# 454 pyrosequencing based transcriptome analysis of *Zygaena filipendulae *with focus on genes involved in biosynthesis of cyanogenic glucosides

**DOI:** 10.1186/1471-2164-10-574

**Published:** 2009-12-02

**Authors:** Mika Zagrobelny, Karsten Scheibye-Alsing, Niels Bjerg Jensen, Birger Lindberg Møller, Jan Gorodkin, Søren Bak

**Affiliations:** 1 Plant Biochemistry Laboratory, Department of Plant Biology and Biotechnology, University of Copenhagen, 40 Thorvaldsensvej, DK-1871 Frederiksberg C, Copenhagen, Denmark; 2 The VKR Research Centre "Proactive Plants", University of Copenhagen, 40 Thorvaldsensvej, DK-1871 Frederiksberg C, Copenhagen, Denmark; 3 Section for Genetics and Bioinformatics (IBHV), Faculty of Life Sciences (LIFE), University of Copenhagen, 3 Grønnegårdsvej, DK-1871 Frederiksberg C, Denmark; 4 Center for Applied Bioinformatics, University of Copenhagen, 40 Thorvaldsensvej, DK-1871 Frederiksberg C, Copenhagen, Denmark

## Abstract

**Background:**

An essential driving component in the co-evolution of plants and insects is the ability to produce and handle bioactive compounds. Plants produce bioactive natural products for defense, but some insects detoxify and/or sequester the compounds, opening up for new niches with fewer competitors. To study the molecular mechanism behind the co-adaption in plant-insect interactions, we have investigated the interactions between *Lotus corniculatus *and *Zygaena filipendulae*. They both contain cyanogenic glucosides which liberate toxic hydrogen cyanide upon breakdown. Moths belonging to the *Zygaena *family are the only insects known, able to carry out both *de novo *biosynthesis and sequestration of the same cyanogenic glucosides as those from their feed plants. The biosynthetic pathway for cyanogenic glucoside biosynthesis in *Z. filipendulae *proceeds using the same intermediates as in the well known pathway from plants, but none of the enzymes responsible have been identified. A genomics strategy founded on 454 pyrosequencing of the *Z. filipendulae *transcriptome was undertaken to identify some of these enzymes in *Z. filipendulae*.

**Results:**

Comparisons of the *Z. filipendulae *transcriptome with the sequenced genomes of *Bombyx mori*, *Drosophila melanogaster*, *Tribolium castaneum*, *Apis mellifera *and *Anopheles gambiae *indicate a high coverage of the *Z. filipendulae *transcriptome. 11% of the *Z. filipendulae *transcriptome sequences were assigned to Gene Ontology categories. Candidate genes for enzymes functioning in the biosynthesis of cyanogenic glucosides (cytochrome P450 and family 1 glycosyltransferases) were identified based on sequence length, number of copies and presence/absence of close homologs in *D. melanogaster*, *B. mori *and the cyanogenic butterfly *Heliconius*. Examination of biased codon usage, GC content and selection on gene candidates support the notion of cyanogenesis as an "old" trait within Ditrysia, as well as its origins being convergent between plants and insects.

**Conclusion:**

Pyrosequencing is an attractive approach to gain access to genes in the biosynthesis of bio-active natural products from insects and other organisms, for which the genome sequence is not known. Based on analysis of the *Z. filipendulae *transcriptome, promising gene candidates for biosynthesis of cyanogenic glucosides was identified, and the suitability of *Z. filipendulae *as a model system for cyanogenesis in insects is evident.

## Background

The advent of new sequencing technologies opens up the opportunity to engage in genomics in non-model organisms, and to study model systems aimed at specific interactions in a biological context. In this paper, we have taken a genomics approach to unravel co-adaptation within a plant-insect interaction at the molecular level, investigating the refined metabolic interactions between the six-spot burnet moth, *Zygaena filipendulae*, and its host plant bird's-foot trefoil, *Lotus corniculatus*.

Plants and insects have co-evolved for the last ~400 million years. A key driving component in these interactions is the ability to produce and handle bioactive natural products. The dogma is that plants produce low molecular mass bioactive compounds as a chemical defense against herbivorous insects. These specialized bioactive compounds are often specific to a particular species. As a countermeasure, herbivorous insects evolve strategies to circumvent the plants chemical defense systems, e.g. by detoxification. The ability to circumvent the chemical defense system of a particular plant species enables the insect to develop an advantageous niche with few competitors. This has resulted in the majority of insects having a restricted diet, feeding only on one plant species.

In some cases, insects are able to *de novo *biosynthesize the same or similar defense compounds as the feed plant, which is why they are immediately able to handle the defense compounds when they encounter the plant. These insects use the compounds in their own defense against predators. If the insects develop an ability to sequester the particular bio-active natural product from the feed plants, the interaction may become highly beneficial for the insects, as they save energy in their own metabolism.

To unravel the molecular mechanism behind co-adaption and balance between sequestration and *de novo *biosynthesis of bioactive compounds in plant-insect interactions, we have taken advantage of the model system of interactions between *L. corniculatus *and *Z. filipendulae *[1]. *L. corniculatus *and *Z. filipendulae *both contain the bioactive compounds cyanogenic glucosides (CNglcs) which can liberate toxic hydrogen cyanide by hydrolysis. While CNglcs are widespread in plants [2], they are in general rare in arthropods [3]. Within insects, particular within butterflies and moths, several species are known to sequester or de novo biosynthesize CNglcs [1,4,5]. Moths belonging to the Zygaena family are the only insects known, able to de novo biosynthesize as well as to sequester the same CNglcs [1,5].

**Figure 1:**
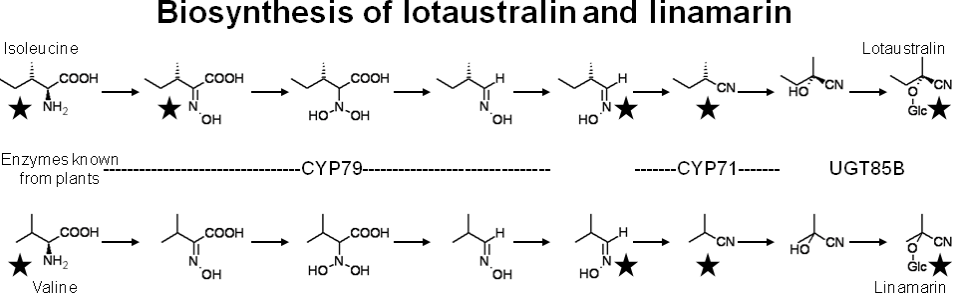
**Proposed biosynthetic pathway for the CNglcs linamarin and lotaustralin**. The types of enzymes catalyzing the equivalent reactions in plants are shown. Intermediates known from insects are indicated with a star.

In plants, the entire pathway for CNglc biosynthesis has been elucidated [6-10]. The precursors for CNglcs are amino acids, and the biosynthetic pathway is genetically relatively simple as only two multifunctional cytochromes P450 (P450) and a family 1 glycosyltransferase (UGT) are involved (Figure 1). In contrast, knowledge on biosynthesis of CNglcs in insects is rudimentary. It has been shown that *Zygaena trifolii *is able to biosynthesize the CNglcs linamarin and lotaustralin using intermediates equivalent to those identified in plants [11-13], and preliminary evidence show that the pathway in *Z. filipendulae *also involves P450 enzymes [14] (Figure 1). It is not known whether the ability to biosynthesize CNglcs in insects is due to horizontal gene transfers, divergence, or convergence.

To identify gene candidates involved in *de novo *biosynthesis of CNglcs in *Z. filipendulae *454 pyrosequencing of the transcriptome was carried out. The transcriptome was compared to the deduced coding sequences of five known insect genomes: *Bombyx mori*, *Drosophila melanogaster*, *Tribolium castaneum*, *Apis mellifera*, and *Anopheles gambiae*. As P450s and UGTs are key enzyme families involved in secondary metabolism in plants as well as in insects [15], a focused analysis of these two multigene families were conducted, to specify gene candidates involved in CNglc biosynthesis.

## Results

### Genome annotation

**Figure 2:**
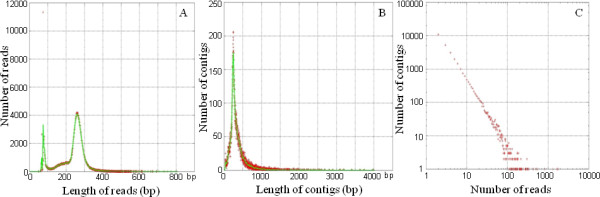
**Distribution of sequence lengths and cluster sizes**. A: Number distribution of lengths of the individual reads. B: Number distribution of lengths of the contigs. Average contig length is indicated by a green line. C: Contig sizes. Contig sizes are scaling invariant as observed by [17].

454 pyrosequencing was carried out on cDNA from a *Z. filipendulae *larva feeding on leaves of acyanogenic *L. corniculatus*. Larvae reared on acyanogenic *L. corniculatus *were previously shown to accumulate the same overall amount of CNglcs as larvae reared on cyanogenic *L. corniculatus *[16]. This balance is achieved by *de novo *biosynthesis of the CNglcs, and imply that the transcript levels for the biosynthetic enzymes are elevated [16]. To achieve depth in the transcriptome by decreasing the number of reads for highly expressed genes, the cDNA library was normalized prior to sequencing. Pyrosequencing resulted in 319,956 reads [NCBI Short Read Archive, accession SRX008323] which were assembled into 29,857 contigs and 42,215 singlets. The average length of reads is 229 nucleotides, while the average contig length is 329 nucleotides, supported on average by 4.4 reads. 3,938 contigs were longer than 500 bp (Figure 2).

The *Z. filipendulae *transcriptome was GO-annotated based on matches to uniprot proteins. The degree of confidence to the assignment of contig or singlet to a particular GO-annotated protein from nr and Uniprot databases is presented as match levels in Table 1[17]. For example, M2 signifies a requirement of the contig to have an identity of at least 85% measured within the matching region, and to cover at least 90% of the matching database sequence. On the basis of a match level 3 (70% identity/70% coverage) or better match, 158 contigs were assigned to a uniprot protein with associated GO-terms, while 335 contigs were associated with GO-terms at match level 4 (60% identity/50% coverage) or better. Upon relaxing the match level criteria to level 5 (20% identity/20%coverage) or better, 3,240 contigs could be associated with one or more GO-terms (~11% of the *Z. filipendulae *transcriptome). Because several of the sequences were characterized by more than one GO term, the total number of GO terms obtained at match level 5 was 8,865. Most of the GO matches based on comparisons at match level 4 and better are obtained with *D. melanogaster *proteins (34%), while at match level 5 and better most matches are obtained with human proteins (41%) and only 17% with proteins from *D. melanogaster*. This is probably due to the much greater number of GO annotated human genes as compared to insect genes.

**Table 1:** Protein BLAST statistics

Match level (identity/coverage)	nr (contigs)	nr (singlets)	Uniprot (contigs)	Uniprot (singlets)
M0 (98%/100%)	33	0	21	0
M1 (95%/95%)	58	2	31	0
M2 (85%/90%)	142	5	49	3
M3 (70%/70%)	495	70	163	16
M4 (60%/50%)	1019	200	326	64
M5 (20%/20%)	6466	2744	4685	1276

Number of contigs and singlets in each match level that match proteins in nr and Uniprot databases. For example M2 means a requirement of the contig to have an identity of at least 85% measured within the matching region and to cover at least 90% of the matching database sequence.

**Figure 3:**
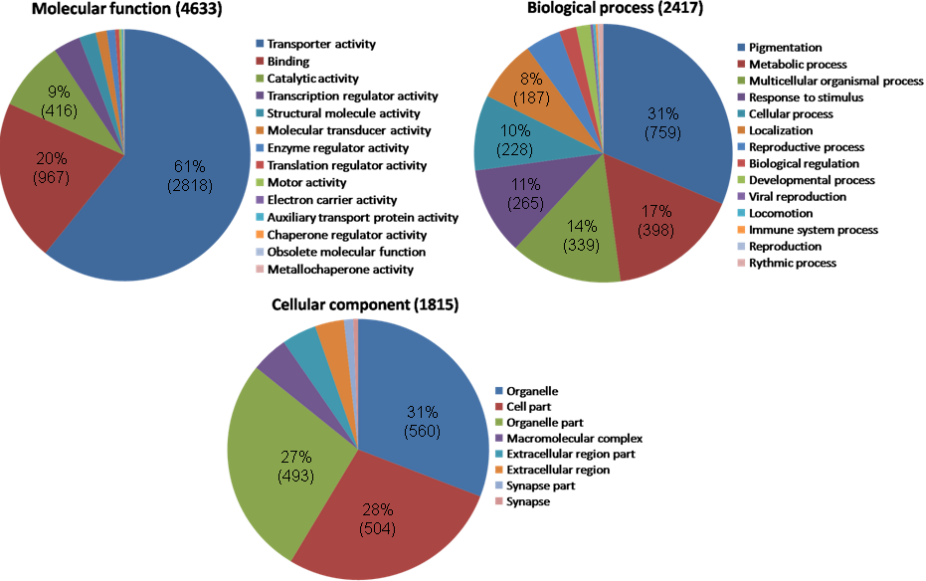
**Gene ontology (GO) -terms for *Z. filipendulae *proteins belonging to match level 5**. The percentage, (total number), and distribution of top-level GO-terms for match level 5 and better in the categories "Molecular function", "Biological Process" and "Cellular component". Total number of *Z. filipendulae *genes in each category is also shown.

To group the proteins with associated GO terms, the top level term for each GO category "Molecular function", "Biological Process" and "Cellular component" was recorded at the different match levels. The distribution of hits in the different categories turned out to be roughly similar for each match level. The distribution of terms for match level 5 or better is shown in Figure 3, and a table listing the different terms and their respective counts for each match level is provided in Additional file 1. The dominant terms for "Molecular function" are clearly transporter activity and binding, while the dominant term for "Biological process" is pigmentation. Within "Cellular component" the dominant terms are evenly divided between organelle, cell part and organelle part.

### Genome comparison

**Figure 4:**
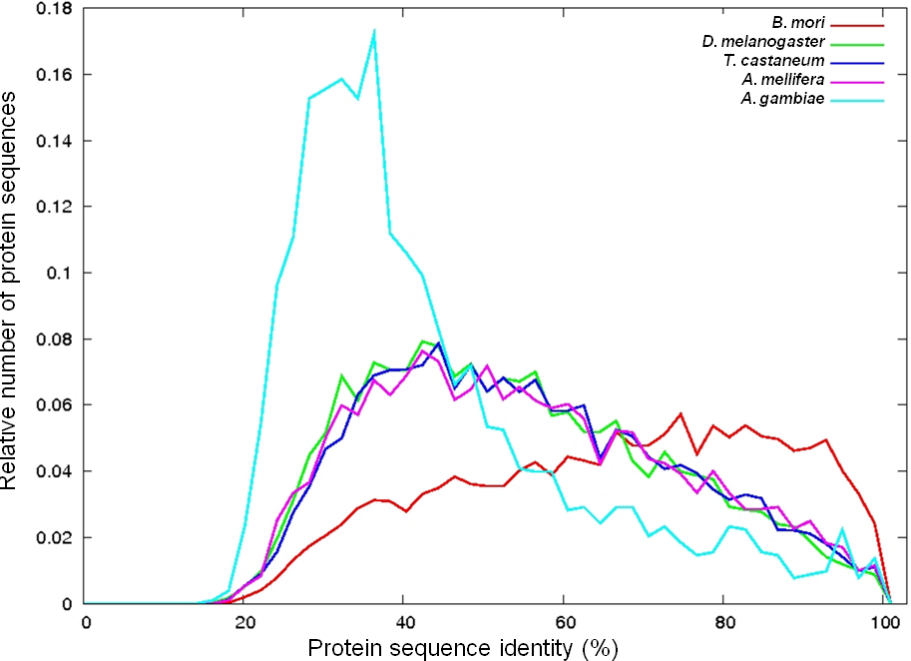
**A comparison of the distribution of sequence identity of *Z. filipendulae *proteins to proteins from five different insect species**. Numerical distribution of protein sequence identity between *Z. filipendulae *protein sequences obtained, and the best BLASTp search hit when compared to available *B. mori*, *D. melanogaster*, *T. castaneum*, *A. mellifera*, and *A. gambiae *protein sequences. All curves are normalized so that the area beneath each curve sums to 1. The drop in matches with less than roughly 25% identity is probably an artifact of the BLAST E-value threshold.

**Figure 5:**
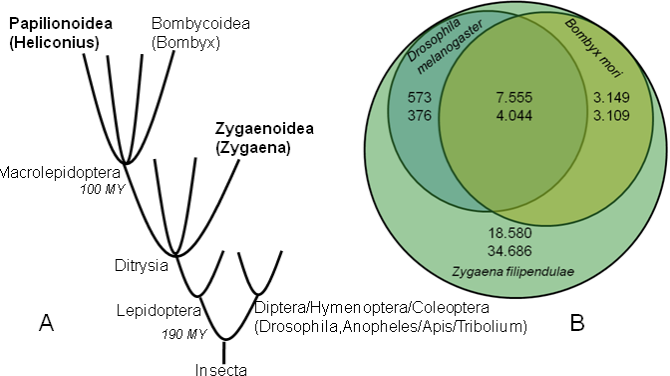
**Phylogenetic relationships and genetic overlap between *Z. filipendulae*, *B. mori *and *D. melanogaster***. A: Simplified evolutionary tree showing the relationships between *Zygaena*, *Heliconius*, *Bombyx*, *Drosophila*, *Anopheles*, *Apis*, and *Tribolium*. Groups comprising cyanogenic species are shown in bold. Time since divergence of the lineages Papilionoidea/Bombycoidea and Lepidoptera/Diptera are shown in italics [20]. B: Venn diagram of the number of contigs (upper number) and singlets (lower number) from *Z. filipendulae *which show matches to *D. melanogaster *(19,389 proteins) and/or *B. mori *(14,623 proteins). Cut-off E-value was 1e^-3^.

Comparisons of the translated *Z. filipendulae *transcriptome to *B. mori*, *D. melanogaster*, *A. mellifera*, *A. gambiae*, and *T. castaneum *protein sequences using BLASTx are shown on Figure 4. The average identity of the protein sequence matches between *B. mori *and *Z. filipendulae *is greater than between *Z. filipendulae *and *D. melanogaster, A. gambiae, A. mellifera*, and *T. castaneum *(Figure 4). This is to be expected, as phylogenetically *B. mori *is closer to *Z. filipendulae *(both belong to ditrysia) than the other species are (belonging to Diptera, Hymenoptera and Coleoptera) (Figure 5A). *D. melanogaster*, *T. castaneum*, and *A. mellifera *have intermediate identities of BLAST hits matching their evolutionary distance from *Z. filipendulae*, whereas *A. gambiae *seem to have more divergent sequences (Figure 4). This could be caused by adaptation of *A. gambiae *to blood feeding and development of insecticide resistance [18], as well as a generally faster divergence in Diptera [19,20]. For sequences with ~90-100% identity, numbers are very similar for *D. melanogaster*, *T. castaneum*, *A. mellifera*, and *A. gambiae*, probably reflecting housekeeping genes (Figure 4). The BLASTx searches of the *Z. filipendulae *sequences against the *D. melanogaster *protein dataset, resulted in 8,128 contigs and 4,780 singlets matching 11,365 and 8,693 *D. melanogaster *proteins, respectively, with a cut-off E-value of 1e^-3^. The BLASTx search of the *Z. filipendulae *sequences against the *B. mori *consensus protein dataset resulted in 10,824 contigs and 7,513 singlets matching 8,195 and 6,993 *B. mori *proteins respectively. Due to overlap, the matches correspond to a total of 9,760 different *B. mori *proteins. The larger number of BLASTx hits between *D. melanogaster *and *Z. filipendulae *compared to the number of hits between *B. mori *and *Z. filipendulae *probably reflects the greater number of recorded *D. melanogaster *proteins compared to *B. mori *proteins (19,389 versus 14,623). Figure 5B presents a Venn diagram of the matches from contigs (upper) and singlets (lower) belonging to the *Z. filipendulae *transcriptome compared to the *D. melanogaster *and *B. mori *protein datasets, respectively (Figure 5). The numbers 18,580 and 34,686 denote the number of contigs and singlets, respectively, from Z. *filipendulae *without matches to either *D. melanogaster *or *B. mori*. The high number of *Z. filipendulae *sequences which do not match either *D. melanogaster *or *B. mori *(Figure 5) probably reflects a mixture of novel, unique genes, poorly conserved genes, and erroneous sequences.

*Heliconius *butterfly species are known to biosynthesize linamarin and often lotaustralin [21,22], and are more closely related to Zygaenidae than *D. melanogaster *and *B. mori *(Figure 5). This prompted a sequence comparison between *Z. filipendulae *and three *Heliconius *species. The *Heliconius *species represented in the sequence databases are *H. melpomene *(4,977 ESTs) and *H. erato *(17,567 ESTs), with the *H. erato *data set containing additional ESTs from the closely related species *H. himera *[23]. These two data sets were assembled separately using the EST sequence assembler geneDistiller (Scheibye-Alsing K *et al*: EST assembly with genedistiller, submitted), resulting in, respectively, 420 and 1,207 contigs and 1,205 and 4,438 singlets. In total, 3,961 *Z. filipendulae *contigs had detectable similarity (E-value 1e^-3^) with sequences in the *Heliconius *data sets (BLASTn), where 1,626 matched *Heliconius *contigs and 2,723 matched *Heliconius *singlets. In addition, 3,138 singlets from *Z. filipendulae *matched *Heliconius *sequences, with 1,117 singlets matching *Heliconius *contigs and 2,345 matching *Heliconius *singlets. In total, 4,035 of the 7,270 conreads (contigs and singlets) of the *Heliconius *datasets could be associated with *Z. filipendulae *(E-value 1e^-3^), corresponding to 55% of the *Heliconius *conreads. On the other hand, only 7,099 of the 72,072 conreads from *Z. filipendulae*, corresponding to 9.8%, are represented by *Heliconius *conreads, which most likely reflects the more extensive sampling of the *Z. filipendulae *transcriptome. Additionally, the fact that the 7,099 *Z. filipendulae *sequences hit only 4,035 different *Heliconius *conreads implies that there is a degree of under clustering in the *Z. filipendulae *dataset, i.e. some reads representing the same gene have not been clustered together in the same contig. This was also experienced in the course of manual merging different *Z. filipendulae *contigs within gene families (see Methods). In addition, many of the *Heliconius *ESTs originate from wing disc cDNA libraries [24] which, unfortunately, may not have a high expression of genes involved in CNglc biosynthesis.

We used BLASTx for the comparisons of *Z. filipendulae *to *D. melanogaster *and *B. mori *because good BLAST hits are more easily obtained with amino acid sequences than with nucleotide sequences, if sequences have diverged much from each other. Because the *Heliconius *dataset consists of nucleotide sequences, BLASTn had to be used for comparisons between *Z. filipendulae *and *Heliconius*. Since it was not directly possible to compare the BLASTx searches to the BLASTn searches, test BLASTn runs were performed between *Z. filipendulae *sequences and the genomes of *B. mori *and *D. melanogaster*. These runs showed that for *D. melanogaster *and to a lesser degree *B. mori*, sequences were so diverged that the number of valid matches obtained was limited. However, using rough estimates we find that around 39% of the *Heliconius *EST sequences show similarity with *B. mori*.

### Candidates for enzymes involved in biosynthesis of cyanogenic glucosides

Our working hypothesis is that the same gene families (P450s and UGTs) are involved in biosynthesis of CNglcs in plants and insects (Figure 1). On account of that, a general search for P450s and UGTs with sequences from both plants [25] and insects was carried out in the *Z. filipendulae *transcriptome. Despite normalization of the cDNA library, transcripts representing enzymes involved in CNglc biosynthesis would be expected to be abundant, because the pathway for CNglc biosynthesis was induced by rearing the *Z. filipendulae *larvae on acyanogenic *L. corniculatus *leaves. Full length or almost full length contigs supported by a high number of pyrosequencing reads would therefore be expected lead candidates for enzymes involved in CNglc biosynthesis. A second important selection criterion is genes which do not have a close homolog in *B. mori *and/or *D. melanogaster*, because these insects are not biosynthesizing CNglcs.

#### Cytochromes P450 (P450)

P450s are heme-thiolate proteins present in all kingdoms involved in primary and secondary metabolism as well as in detoxification [2]. Particularly in plants, they are often recruited for novel biosynthetic pathways, and in plants the first two steps in CNglc biosynthesis are catalyzed by P450s. In insects, P450s play crucial roles in the detoxification of toxic bio-active plant natural products present in their feed plants, as well as in primary metabolism and development. In general, insect genomes harbor ~100 different P450s, which can be divided into four clades http://p450.sophia.inra.fr/[26,27]. For the majority of P450s from plants as well as insects the biochemical function is unknown. However, some functionality can be assigned to each of the four clades based on known functionalities. The CYP6, CYP9, CYP28, CYP308-310, and CYP321 families constitute the first clade, and many of the genes belonging to these families are known to mediate responses to environmental challenges. The families are characterized by a very high diversity, individual members have proliferated by duplication events, show rapid rates of evolution, occur in gene clusters, and show tissue- or developmental-specific expression. The CYP4, CYP311-313, CYP316, and CYP325 families belong to the second clade of highly diverse insect P450s, with some of the gene members being induced by xenobiotics. The third clade is the mitochondrial CYP12 family, comprising many rapidly evolving genes. Some of the slowly evolving genes in this family are involved in moulting. The CYP15, CYP18, and CYP303-307 families constitute the fourth clade of insect P450s. Some members of these families are involved in essential physiological functions like juvenile hormone biosynthesis and moulting.

**Figure 6:**
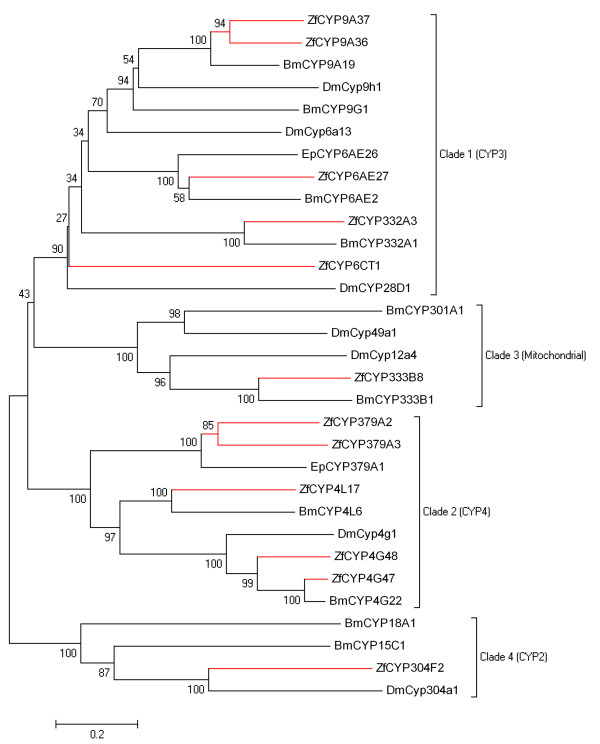
**Neighbor-Joining bootstrap tree of translated full length *Z. filipendulae *P450 sequences**. Sequences from *Z. filipendulae *are marked in red. Clades of insect P450s are according to [26,27]. Bm: *B. mori*, Dm: *D. melanogaster*, EP: *E. postvittana*. Bootstrap values are shown as percentages.

Approximately 120 putative P450 conreads were identified in the *Z. filipendulae *transcriptome (a table of all sequences with length and coverage are shown in Additional file 1). Five *Z. filipendulae *P450 sequences (ZfCYP9A37, ZfCYP304F2, ZfCYP332A3, ZfCYP379A2, ZfCYP379A3) were identified in the dataset as full length sequences. The coding sequence of a P450 is approximately 1500 bp. Eleven P450 sequences longer than 1000 nucleotides were discovered all in all (ZfCYP4G47, ZfCYP4G48, ZfCYP9A37, ZfCYP304F2, ZfCYP332A3, ZfCYP333B8, ZfCYP379A2, ZfCYP379A3, ZfP450-5, ZfP450-13, Zfc3346). To extend these, and other partial sequences, RACE PCR was performed, and this provided further full length sequences of ZfCYP4G47, ZfCYP4G48, ZfCYP4L17, ZfCYP6CT1, ZfCYP6AE27, CYP9A36, and ZfCYP333B8, resulting in twelve full length P450 sequences [GenBank accessions GQ915312-GQ915323]. A Neighbor-Joining tree containing the twelve full length *Z. filipendulae *sequences as well as selected P450s from other species is shown in Figure 6. The selected P450s were most closely related to the *Z. filipendulae *P450s, and were chosen from a much larger tree (Additional file 2) comprising more than 200 P450 genes from many different insect species.

The few P450 sequences from the tree with assigned functions are: DmCYP4G1, DmCYP49A1, DmCYP12A4, and BmCYP15C1. DmCYP4G1 is required for lipid metabolism, DmCYP49A1 and DmCYP12A4 confer insecticide resistance and may therefore be involved in detoxification reactions, while BmCYP15C1 is involved in juvenile hormone biosynthesis. For an overview of known *D. melanogaster *P450 functions see [28]. Based on the known function and its position in the tree, it can be inferred that ZfCYP304F2 may be involved in juvenile hormone biosynthesis, ZfCYP333B8 may be involved in detoxification processes, and ZfCYP4G48 and ZfCYP4G47 could be required for lipid metabolism. However, because the *Z. filipendulae *P450 sequences are not that closely related to the P450s with known functions, these functional assignments of P450s in *Z. filipendulae *are to be considered tentative. Because most of the P450s from *B. mori *and *D. melanogaster *shown in the tree has not been properly characterized, it was not possible to infer functions to most of the full length *Z. filipendulae *P450s obtained.

Based on our criteria for lead candidates, ZfCYP4G47 would appear to be a less likely candidate, because of the close homolog present in *B. mori *(82% sequence identity on the amino acid level). ZfCYP4G47 was the only one of the *Z. filipendulae *P450s with a close homolog in the cyanogenic butterfly *Heliconius*, showing 83% sequence identity over 250 amino acid residues. As the sequence is partial it has been omitted from the tree presented in Figure 6, and due to the close homolog in *B. mori *mentioned earlier, it is not a good candidate for CNglc biosynthesis. The remaining eleven full length *Z. filipendulae *P450s appear sufficiently distant to genes in the other two species, to have been able to acquire functions specific to *Z. filipendulae*. The most divergent *Z. filipendulae *full length P450 is ZfCYP6CT1 (~30% sequence identity on the amino acid level). This may therefore be the best candidate for a P450 involved in the CNglc pathway. The *Z. filipendulae *transcriptome contains two P450s belonging to a new animal P450 family (CYP379 in clade 2) to which a single sequence from *Epiphyas postvittana *(light brown apple moth) has previously been assigned (Nelson, D. personal communication). Like *Z. filipendulae*, *E. postvittana *belong to Ditrysia (Figure 4). Since *E. postvittana *is not cyanogenic, the two putative CYP379 orthologs are not obvious candidates for a P450 involved in CNglc biosynthesis.

#### UDPG-Glucosyltransferases (UGTs)

The third and final step in CNglc biosynthesis in plants is a glucosylation step catalyzed by a Family 1 UDPG-glycosyltransferase [29], and mediating the conversion of a cyanohydrin into the corresponding CNglc [30,31]. Family 1 UGTs are ubiquitously found in plants, animals, fungi, bacteria, and viruses [32]. UGTs generally serve to glycosylate low molecular weight molecules, as a mean to convert reactive and toxic aglycones into more stable and non-reactive storage forms, as well as to increase solubility of hydrophobic metabolites, and as transport cues for intra- and intercellular transport [32]. In addition, UGTs are also involved in hormone homeostasis and detoxification of xenobiotics.

**Figure 7:**
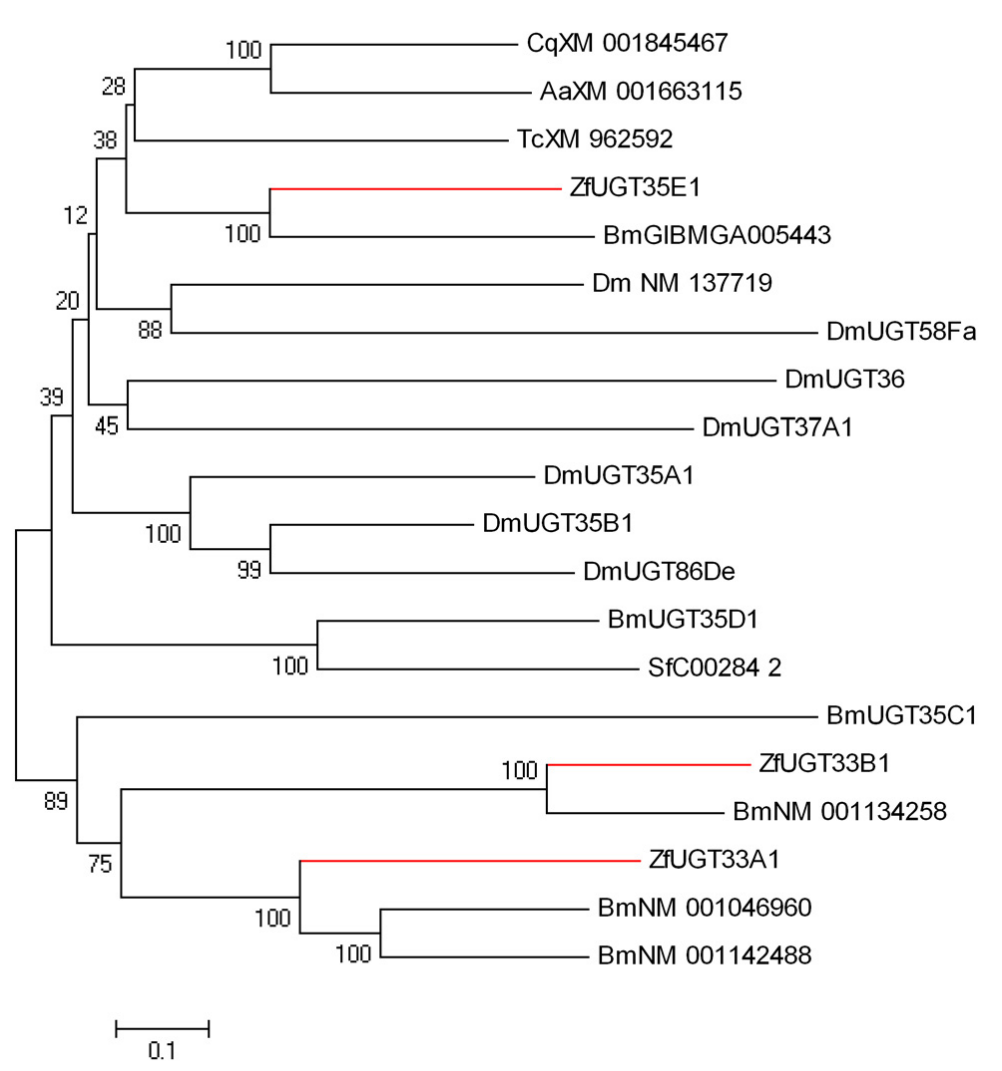
**Neighbor-Joining bootstrap tree of translated full length *Z. filipendulae *UGT sequences **. Sequences from *Z. filipendulae *are marked in red. Aa:*Aedes aegypti*, Bm:*B. mori*, Cq:*Culex quinquefasciatus*, Sf:*S. frugiperda*, Tc:*T. castaneum*. Bootstrap values are shown as percentages.

The pyrosequencing approach resulted in identification of 41 different putative Family 1 UGT conreads in the *Z. filipendulae *transcriptome. Three of these sequences represented full length sequences (ZfUGT33A1, ZfUGT33B1, ZfUGT35E1) [GenBank accessions GQ915324-GQ915326] (Figure 7) (a table of all sequences with length and coverage are presented in Additional file 1). A Neighbor-Joining tree with full length *Z. filipendulae *UGTs as well as UGTs from other insects is shown in Figure 7. The genes most closely related to the *Z. filipendulae *UGTs were selected from a larger tree (Additional file 3) comprising 76 UGT genes from different insect species.

The high abundance of the three full length *Z. filipendulae *UGT transcripts implies that one of these three UGT sequences may be the UGT catalyzing the last step in CNglc biosynthesis. From Figure 7 it is evident that the *Z. filipendulae *UGTs assigned as ZfUGT33A1 and ZfUGT33B1 have fairly close homologs in *B. mori*, while this is not the case for ZfUGT35E1. Accordingly, among the full length sequences obtained, ZfUGT35E1 would therefore be a lead candidate.

### Codon usage and GC content

Genes involved in biosynthesis of CNglcs in *Z. filipendulae *have been hypothesized to be highly expressed in larvae feeding on acyanogenic *L. corniculatus *[16]. Highly expressed genes are generally assumed to possess a more biased codon usage, and a higher GC content in comparison to genes with low expression [33]. Accordingly, to obtain evidence of their expression levels, codon usage and GC content of each *Z. filipendulae *candidate gene encoding P450s or UGTs were examined. Codon usage and GC content of genes encoding P450s and UGTs were similar, and average numbers for each gene family are shown in Table 2. Overall codon usage in *Z. filipendulae *was found to be similar to codon usage in *B. mori *(Table 2). A biased codon usage (ENC) is a measure of how far the codon usage of a gene departs from equal usage of synonymous codons, and is rated from 20 (maximally bias) to 61 (unbiased) [33]. The high ENC numbers signify a low codon usage bias, *i.e*. a somewhat equal usage of synonymous codons within the gene families. *D. melanogaster *has the lowest ENC values, *e.g*. higher codon usage bias compared to *Z. filipendulae *and *B. mori*. Codon usage bias within the P450 genes is significantly different when *D. melanogaster *sequences are compared to sequences from *Z. filipendulae *and *B. mori *using student t-tests (both significant at the 99% level). This was also the case when only 12 *D. melanogaster *P450s were compared to the 12 *Z. filipendulae *P450s (data not shown). Codon usage within the UGTs showed no significant differences between the three species. The high number of P450 genes available for analysis in comparison to the number of UGT genes available, may explain the insignificant codon usage differences observed in the latter group. It has previously been suggested that Lepidoptera exhibits less codon usage bias than other species [34], and this study corroborates with that observation.

**Table 2:** GC content of the transcriptome and within P450s and UGTs in *Z. filipendulae*, *B. mori*, *D. melanogaster*, and *Heliconius *species.

	*Z. filipendulae*			*B. mori*			*D. melanogaster*			*Heliconius *species
	**GC**	**ENC**	**No.**	**GC**	**ENC**	**No.**	**GC**	**ENC**	**No.**	
Transcriptome	39%			48%			54%			43%
P450	43%	56	12	43%	55	70	51%	51	81	
UGT	40%	55	3	42%	56	5	48%	56	7	

Average of groups	42%	56	15	43%	56	75	50%	54	88	

The number of sequences used for the individual analyses are shown in column labeled "No.". *Heliconius *does not offer calculations on individual gene families due to the availability of too few sequences. The GC content calculated for the *D. melanogaster*, *B. mori*, and *A. mellifera *transcriptomes are based on all coding sequences deposited in Genbank, while the GC content of the *Z. filipendulae *transcriptome is based on 29.857 contigs. The GC content of the *Heliconius *transcriptome is derived from 1.627 contigs.

The GC content in the *Z. filipendulae *transcriptome is 38.7% (Table 2). This is lower than measured for *A. gambiae *(55.8%), *D. melanogaster *(53.9%), *B. mori *(48.1%), and *T. castaneum *(47.2%), but more similar to the GC content in *A. mellifera *(43.5%) and *Heliconius *(44%). This is remarkable, because *Z. filipendulae *and *Heliconius *are phylogenetically more closely related to *B. mori *and *D. melanogaster *[35], in comparison to the relationship between *Z. filipendulae *and *A. mellifera*. For unknown reasons, selective pressure within Ditrysia (Figure 4) towards the development of a lower GC content may have occurred. Alternatively, the unexpected high values of GC content may have been obtained due to a prediction bias towards more GC rich transcripts, introduced by the gene annotation methods when applied on entire genomes [36].

### Substitution rate ratios

To test our lead candidates for selection, we calculated the nonsynonymous (*d*_*N*_) to synonymous (*d*_*S*_) substitution rate ratio (ω = *d*_*N*_*/d*_*S*_) on full length sequences of P450s and UGTs from *Z. filipendulae*. ω provides a sensitive measure of selective pressure at the protein level, with ω values < 1, = 1 and > 1 indicating purifying selection, neutral evolution and diversifying selection, respectively. Maximum likelihood models of codon substitution account for variable selective pressures among amino acid sites. By testing different models with more classes of ω, positive selection in specific regions of a gene can be detected [37]. The program codeml from the PAML4.1 package was used for the analysis. Site-models 0, 1, 2, 3, 5, 7, 8 from codeml were tested on the available full length P450 and UGT sequences with likelihood ratio tests [38]. The models used basically implement different rates and classes of ω values, *e.g*. model 0 has one ω value calculated from the data set, model 1 has two ω values, with one value fixed at 1 and the other calculated from the data set, and so on with model 8 operating with 11 classes of ω values all calculated from the data set. The likelihood ratio test was used to calculate which of the possible models offered the best fit to the data set [37]. If positive selection was present in one or more of the sequences tested, residues with positive selection would be identified in the output file.

In our analyses, average ω values range from 0.18 to 0.53, with UGTs having the highest ω value (Table 3). These ω values are quite low and signify purifying selection. Model 3 (three discrete classes of ω values) provides the best fit for P450s while model 1 (two ω values of which one is fixed at 1) offers the best fit for UGTs. This demonstrates a negative purifying selection to maintain the sequence in both gene families. Negative purifying selection pressure is less restrained in some sequence domain areas for P450s as evidenced by the obtained best fit using Model 3 which is based on three discrete classes of ω values. UGTs possess larger sequence domains (46%) where they seem to undergo neutral evolution corresponding to a ω value of 1. This signifies that some areas are prone to accumulate a lot of mutations which could give rise to proteins with new catalytical functions or altered substrate specificities [39,40]. No sites with positive selection were detected in any sequence from any of the gene families (Table 3).

**Table 3:** Nonsynonymous/synonymous substitution rate ratios (ω) in gene families from *Z. filipendulae*.

Enzymes from *Z. filipendulae*	Average ω (*d*_*N*_*/d*_*S*_)	Best-fit model from PAML4.1	
P450	0.18	Model 3:	p: 0.03 0.40 0.57
			ω : 0.00 0.10 0.25$
UGT	0.53	Model 1:	p: 0.54 0.46
			ω : 0.13 1.00$
Average	0.36		

## Discussion

### Characterization of the *Zygaena filipendulae *transcriptome

The *Z. filipendulae *transcriptome was compared to other sequenced insect species, and the results clearly show that *Z. filipendulae *is more closely related to *B. mori *compared to *D. melanogaster*, *A. mellifera*, *A. gambiae*, and *T. castaneum *(Figure 4, Figure 5). The high percentage of *Z. filipendulae *contigs with no match in *Heliconius *and *B. mori *as well as in any other sequenced insects reflects the uniqueness of the species, and corroborates with previous observations of unusually many unique genes in the other Ditrysians *B. mori *and *Manduca sexta *[41]. GO groups could be assigned to ~11% of the *Z. filipendulae *sequences whereas the corresponding number for the *Heliconius *sequences was 25% [24]. The gene sequences encoding the gene families of relevance to cyanogenesis (P450s and UGTs) are not well represented among the genes to which a GO annotation was assigned. For the P450s, only 45 out of 118 sequences (38%) had a BLAST hit (E-value 0.01) with a GO term attached. This demonstrates that the assignment of GO terms is biased towards genes with high overall sequence conservation, and well-known roles in primary metabolism.

In *H. melpomene*, 28.5% of the contigs had GO terms assigned, all within "Molecular function". 15% of those is assigned to the transporter activity category [24], while this category comprises 61% in *Z. filipendulae*. In *H. melpomene*, 20% of GO annotated sequences are assigned to the "binding" category, similar to that observed in *Z. filipendulae*. These terms account for a larger fraction of the overall assignments than expected. This may indicate that genes encoding these functions in general are more conserved among different organisms and thus easier to annotate. Alternatively, this may represent database annotation artifacts. The "structural molecule" category is represented by 30% in *H. melpomene*, but limited to around 2% in *Z. filipendulae*. This may reflect that the original cDNA library from which the *H. melpomene *sequences were obtained was non-normalized, and therefore gave rise to an overrepresentation of ribosomal sequences. The assignments to the other categories within Molecular function are similar in *H. melpomene *and *Z. filipendulae *[24]. Differences in gene assignments between *H. melpomene *and *Z. filipendulae *could signify different selection pressures on the genes in the two species, derive from the fact that many of the *Heliconius *ESTs originate from wing disc cDNA libraries, and not larvae as in *Z. filipendulae*, or, more likely, be artifacts resulting from a low number of GO-annotated genes in both species.

In the "Biological process" group, the term representing the largest gene fraction in *Z. filipendulae *is pigmentation. While "metabolic process" and "multicellular organismal process" are relatively common terms which would be expected to feature prominently in the distribution of terms, "pigmentation" would only be expected to generate few counts among a random set of genes. Hence, the relatively high predominance of genes associated with "pigmentation" was unexpected. This may indicate that genes involved in pigmentation are conserved between insect species or more likely reflect that pigmentation is a frequently studied topic in butterflies and *D. melanogaster *[24]. The distribution of hits in the "Cellular component" category features organelle, cell part and organelle part as dominant terms. These are broad terms, and it seems reasonable that they are widely represented in *Z. filipendulae*. As GO and other public databases are populated with additional data, it will be possible to provide annotation for a higher fraction of the *Z. filipendulae *data set.

### Candidates for enzymes involved in biosynthesis of cyanogenic glucosides

The two multigene families, P450s and UGTs, are found in most or all living organisms. They serve important roles in housekeeping reactions, as well as in the synthesis of bio-active natural products, and in detoxification of xenobiotics and allelochemicals. The pyrosequencing approach was therefore expected to provide a multitude of sequences within both families of which the great majority was not expected to have any function related to cyanogenesis in *Z. filipendulae*. To select genes putatively related to biosynthesis of CNglcs, the presumed high transcriptional activity of the relevant P450 and UGT encoding genes, reflecting the demand for effective *de novo *CNglc biosynthesis in *Z. filipendulae *larvae, was employed. Therefore, P450 and UGT gene sequences providing a high number of reads and long contigs were considered promising gene candidates. Additional priority was given to candidate genes with no close homologs in the genomes of acyanogenic *B. mori *and *D. melanogaster*, because a high sequence identity would probably signify a housekeeping protein. In cases where putative homologs were found in other sequenced insects, sufficient time may have passed since the divergence from a common ancestor for recruitment of the gene to a new function. The shared ability of *Z. filipendulae *and *Heliconius *species to produce the CNglcs linamarin and lotaustralin motivated a search for putative orthologous genes in *Heliconius*. Since the estimated time of divergence between *B. mori *and *H. melpomene *is 103 ± 9 MY [20], the divergence between *Z. filipendulae *and *Heliconius *is probably comparable (Figure 4). However, only very few genes from the *Heliconius *dataset matched *Z. filipendulae *genes belonging to the P450 or UGT multigene families. This is probably due to the fact that the *Z. filipendulae *transcriptome is derived from larval tissue, and *Heliconius *ESTs are mostly derived from wing discs. Furthermore, the *Heliconius *ESTs are biased as biosynthesis of CNglcs in insects in general takes place in the larval stage, and not in wing tissue.

Many of the partial gene sequences obtained by pyrosequencing of the *Z. filipendulae *transcriptome belong to the same gene. The number of gene sequences found in this study predicted to encode P450s or UGTs is therefore an overestimate of the actual number of genes belonging to each of these families in *Z. filipendulae*. Nevertheless, the number of gene sequences found within each gene family appears to closely match the gene numbers within these groups obtained from fully sequenced insect genomes (*A. aegypti, A. gambiae, A. mellifera*, *B. mori*, *C. quinquefasciatus, D. melanogaster*, *T. castaneum*) when overestimation is taken into account. The pyrosequencing approach thus afforded a high coverage of the *Z. filipendulae *transcriptome, and that conclusion was also obtained following pyrosequencing of the transcriptomes of other organisms [42].

### Evolution and selection on enzymes involved in biosynthesis of cyanogenic glucosides

Highly expressed genes are often characterized by a higher codon usage bias and low ENC numbers, in comparison to lowly expressed genes [33]. In the P450 and UGT multigene families investigated here, the codon usage bias is surprisingly low and the ENC numbers high, considering that the full length copies represent a fairly high expression level. High ENC values have previously been found in highly expressed ribosomal proteins from *Spodoptera frugiperda *and *B. mori*, but not in *D. melanogaster *[34], suggesting that genes with an unbiased codon usage may be a common trait in Lepidoptera. This study indicates that this observation is also valid for *Z. filipendulae*. A quantitatively equivalent level of the tRNAs corresponding to each of the codons for the same amino acid would explain the lack of codon usage bias [34].

A recent recruitment of genes related to cyanogenesis in *Z. filipendulae *was expected to be manifested by positive selection. Nevertheless, no positive selection was observed among the two gene families examined, and this would imply that the genes tested have reached a fixed and important function, that needs to be maintained. Biosynthesis of linamarin and lotaustralin was recently shown to be old traits, at least within Zygaenidae, and maybe even in the common ancestor of butterflies and moths [43,44]. Bouts of positive selection present upon recruitment may long since have been masked by a subsequent long period of purifying selection to maintain sequence, once the optimal sequence had been obtained. The fact that large parts of UGTs seem to undergo neutral evolution, fits with the fact that UGTs generally have low sequence conservation albeit conserved secondary and tertiary structures [40]. The N-terminal part of the protein, responsible for binding of the aglycone, is less conserved than the C-terminal part which binds the UDP sugar. Therefore the N-terminal part could be the part of the sequences undergoing neutral evolution.

None of the lead candidate sequences for genes involved in biosynthesis of CNglcs in *Z. filipendulae *show high homology to plant genes involved in CNglc biosynthesis. This supports the notion that the ability to *de novo *biosynthesize CNglcs was developed independently in arthropods and plants [5] by convergent evolution. However, the similarities of the biosynthetic pathways between the two kingdoms indicate that CNglcs may be produced according to an optimal biochemical route affording easy recruitment of the enzyme activities required.

## Conclusion

Pyrosequencing is an attractive approach to gain access to genes in the synthesis of bio-active natural products from insects and other organisms, for which the genome sequence is not known. The transcriptome of *Z. filipendulae *offers a unique opportunity to characterize the biosynthetic pathway of cyanogenesis in insects. Moreover, *Zygaena *species have co-evolved with their cyanogenic feed plants for millions of years [44], offering opportunities for further analyses in this field.

Analysis of the *Z. filipendulae *transcriptome by 454 pyrosequencing has revealed several gene candidates for biosynthesis of CNglcs, and full length sequences of the gene candidates have been generated by RACE PCR. Heterologous expression and biochemical and functional characterization of the recombinant enzymes will serve to identify genes with relevance to cyanogenesis. The obtained transcriptome offers the additional option to design microarrays to study transcript regulation to understand the environmental cues, timing and tissue localization of biosynthesis of CNglcs. A detailed transcriptomics comparison using such microarrays as a comparison between *Z. filipendulae *larvae that are sequestering versus *de novo *biosynthesizing CNglcs will further the understanding of how metabolism is directed by the content and ratio of CNglcs in the host plant.

## Methods

### Insect material, pyrosequencing and assembly

*Z. filipendulae *larvae were collected from a fallow field in Kvistgård north of Copenhagen, Denmark, and reared for a week on *L. corniculatus *plants devoid of CNglcs [16]. The gut was removed from one larva, and total RNA was extracted from the remaining part of the body using RNeasy plant mini kit from Qiagen (Cat. No. 74904). Integrity of the RNA was verified using an Agilent 2100 Bioanalyzer before total RNA was shipped to Eurofins MWG Operon, Inc, Germany http://www.eurofinsdna.com/home.html. Eurofins MWG Operon performed mRNA purification and normalization in order to thin out the highly abundant transcripts before 454 pyrosequencing. cDNA was produced and fragmented by nebulization, and 454 pyrosequencing carried out using a GS FLX system. Sequencing of the *Z. filipendulae *transcriptome resulted in 319,956 reads [NCBI Short Read Archive SRX008323] which were assembled using the "mira" assembler [45] with default settings (minimum 30 bases overlap with 80% identity). A minimum read length of 30 bases was required for assembly.

### Genome comparison

Contigs and singlets from the *Z. filipendulae *transcriptome were BLASTx searched [46] against the protein databases "nr" and "Uniprot" [47], and the results checked manually. Of the 29,857 *Z. filipendulae *contigs, 8,395 matched proteins in "Uniprot" and 10,423 matched proteins in "nr", while the numbers for the 42,215 singlets where 4,830 and 6,715 respectively. The overall statistics of the BLAST-matches are listed in Table 1. The matches at different levels of identity and coverage, i.e. the degree of confidence to the assignment of contig or singlet to a particular protein is as presented in [17]. The *Z. filipendulae *sequences were BLASTx searched against a *D. melanogaster *protein set, downloaded from Flybase (December 2008) http://flybase.org[48], and against the *B. mori *consensus gene dataset downloaded from the silkworm website http://silkworm.genomics.org.cn (November 2008) [49]. Protein sequences from *T. castaneum*, *A. gambiae*, and *A. mellifera *were downloaded from http://www.beetlebase.org[50], http://www.anobase.org[18], and http://www.beebase.org[51] respectively (August 2009). The *Z. filipendulae *sequences were also compared to 22,544 ESTs from *Heliconius *species downloaded from NCBI's EST collection using the keyword "Heliconius". The two species represented in the data set were *H. melpomene *(4,977 ESTs) and *H. erato *(17,567 ESTs) (the *H. erato *set containing 9,394 ESTs from combined *H. erato*/*H. himera *libraries). These two data sets were assembled separately using the EST sequence assembler geneDistiller (Scheibye-Alsing K *et al*: EST assembly with genedistiller, submitted), and the resulting *Heliconius *contigs and singlets are available in additional files 4 and 5 respectively. The *Z. filipendulae *sequences were annotated using the Gene Ontology (GO) terms http://www.geneontology.org) [52] where possible.

### Candidates for enzymes involved in biosynthesis of cyanogenic glucosides

Candidate genes from the multigene families P450s and UGTs were identified in the *Z. filipendulae *sequence dataset by BLAST searches (using BLASTx and BLASTn with default algorithm parameter settings) with selected protein sequences from the gene families from plants and insects. Gene candidates from *Z. filipendulae *were then BLAST searched (using BLASTx and BLASTn with default algorithm parameter settings) against the entire *Z. filipendulae *transcriptome to ensure exhaustive searches. The BLAST searches enabled the assembly of some genes which had initially been assigned to different conreads (33 out of approximately 300 conreads). The sequences of approximately 50 conreads fell completely within another contig. During searches in Butterflybase http://butterflybase.ice.mpg.de/[53], a few contigs from *B. mori *and *Antheraea mylitta *which belonged to the same gene were also identified. The P450 sequences ZfCYP4G47, ZfCYP4G48, ZfCYP4L17, ZfCYP6AE27, ZfCYP6CT1, CYP9A36, ZfCYP9A37, ZfCYP304F2, ZfCYP332A3, ZfCYP333B8, ZfCYP379A2, and ZfCYP379A3 were verified by Race-PCR using the SMART RACE cDNA Amplification Kit from Clontech (cat. 634914), and partial sequences were extended to full length.

### Analyses on candidate genes

Sequences were aligned in MEGA4 [54,55] using CLUSTALW [56] with default settings, and refined manually. Phylogenetic Neighbor-Joining trees were also generated, and bootstrapped with 500 iterations in MEGA4. Codon Usage analysis was performed with CodonW version 1.3 http://codonw.sourceforge.net/, and codon bias was calculated using the "effective number of codons" (ENC) method [33]. GC content for *Z. filipendulae *full length sequences were calculated in MEGA4. Transcriptome GC content from *A. gambiae*, *D. melanogaster*, *B. mori*, *T. castaneum*, and *A. mellifera *are from http://www.kazusa.or.jp/codon/, and for *Heliconius *it was calculated manually. Nonsynonymous to synonymous substitution rate ratios (ω = (*d*_*N*_*/d*_*S*_)) were calculated for full length sequences with the program *codeml *from the PAML package, version 4.1 [57]. Site-models 0, 1, 2, 3, 5, 7, 8 (NSsites) were tested with the settings: model = 0, icode = 4 (invertebrate), cleandata = 0. The rest of the settings were default. Models were tested against each other with likelihood ratio tests [38]. Sequences from other species used for alignments and trees (Figures 6 and 7 and Additional file 2 and 3) were downloaded from Butterflybase and GenBank retaining their respective names.

## Authors' contributions

MZ extracted gene candidates, carried out phylogenetic analyses, and drafted the manuscript. KSA did BLAST searches of the complete transcriptome and assignment of sequences to GO groups. NBJ provided RNA samples for pyrosequencing, and verified and extended P450 sequences by RACE PCR. BLM, JG and SB outlined the project, provided guidance during the work and feed-back on the manuscript. All authors read and approved the final manuscript.

## Supplementary Material

Additional file 1 **Gene ontology terms and putative P450s and UGTs from the *Zygaena filipendulae *transcriptome**. Table with the distribution of different GO-terms in the categories "Molecular function", "Biological Process" and "Cellular component" for match levels 3 and better, match levels 4 and better and match level 5 and better. Tables with putative P450s and UGTs extracted from the *Zygaena filipendulae *454 pyrosequencing. Click here for file

Additional file 2 **P450 tree**. Neighbor-joining bootstrap tree of full length P450s from *Z. filipendulae *as well as P450s from other insects. Bootstrap values are shown as percentages. Click here for file

Additional file 3 **UGT tree**. Neighbor-joining bootstrap tree of full length UGTs from *Z. filipendulae *as well as UGTs from other insects. Bootstrap values are shown as percentages. Click here for file

Additional file 4 ***Heliconius *contigs**. FASTA file of all *Heliconius *contigs. Click here for file

Additional file 5 ***Heliconius *singlets**. FASTA file of all *Heliconius *singlets. Click here for file
