# Supplementary material for: 454 pyrosequencing based transcriptome analysis of Zygaena filipendulae with focus on genes involved in biosynthesis of cyanogenic glucosides
Source: BMC Genomics. 2009 Dec 2;10:574. doi: 10.1186/1471-2164-10-574 (PMC2791780; doi:10.1186/1471-2164-10-574)
Supplement: Additional file 1 — Gene ontology terms and putative P450s and UGTs from the Zygaena filipendulae transcriptome. Table with the distribution of different GO-terms in the categories "Molecular function", "Biological Process" and "Cellular component" for match levels 3 and better, match levels 4 and better and match level 5 and better. Tables with putative P450s and UGTs extracted from the Zygaena filipendulae 454 pyrosequencing. [file 1471-2164-10-574-S1.PDF]

Gene ontology terms and putative P450s and UGTs from the Zygaena transcriptome

Gene ontology terms represented in *Z. filipendulae*

**Molecular function**	**M3**	**M4**	**M5**
Transporter activity	147 (72.8%)	293 (68.8%)	2818 (60.8%)
Binding	32 (15.8%)	69 (16.2%)	967 (20.1%)
Catalytic activity	6 (3.0%)	24 (5.6%)	416 (9.0%)
Transcription regulator activity	12 (5.9%)	23 (5.4%)	161 (3.5%)
Structural molecule activity	2 (1.0%)	8 (1.9%)	100 (2.2%)
Molecular transducer activity	0	0	66 (1.4%)
Enzyme regulator activity	1 (0.5%)	3 (0.7%)	49 (1.1%)
Translation regulator activity	1 (0.5%)	4 (0.9%)	24 (0.5%)
Motor activity	0	1 (0.2%)	15 (0.3%)
Electron carrier activity	1 (0.5%)	1 (0.2%)	1
Auxiliary transport protein activity	0	0	10 (0.2%)
Chaperone regulator activity	0	0	3
Obsolete molecular function	0	0	2
Metallochaperone activity	0	0	1
Overall	202	426	4633
**Biological Processes**	**M3**	**M4**	**M5**
Pigmentation	30 (50.8%)	62 (45.3%)	759 (31.4%)
Metabolic process	9 (15.3%)	25 (18.2%)	398 (16.5%)
Multicellular organismal process	2 (3.4%)	7 (5.1%)	339 (14.0%)
Response to stimulus	4 (6.8%)	10 (7.3%)	265 (11.0%)
Cellular process	4 (6.8%)	8 (5.8%)	228 (9.5%)
Localization	5 (8.5%)	14 (10.2%)	187 (7.7%)
Reproductive process	0	1 (0.7%)	107 (4.4%)
Biological regulation	3 (5.1%)	4 (2.9%)	52 (2.2%)
Developmental process	1 (1.7%)	2 (1.5%)	43 (1.8%)
Viral reproduction	1 (1.7%)	2 (1.5%)	8 (0.3%)
Locomotion	0	1 (0.7%)	8 (0.3%)
Immune system process	0	1 (0.7%)	6 (0.2%)
Reproduction	0	0	4 (0.2%)
Rythmic process	0	0	13 (0.5%)
Overall	59	137	2417
**Cellular component**	**M3**	**M4**	**M5**
Organelle	25 (35.2%)	47 (31.8%)	560 (30.9%)
Cell part	17 (23.9%)	33 (22.3%)	504 (27.8%)
Organelle part	23 (32.4%)	57 (38.5%)	493 (27.2%)
Macromolecular complex	5 (7.0%)	9 (6.1%)	83 (4.6%)
Extracellular region part	0	0	78 (4.3%)
Extracellular region	0	1 (0.7%)	65 (3.6%)

Synapse part	1 (1.4%)	1 (0.7%)	21 (1.2%)
Synapse	0	0	11 (0.6%)
Overall	71	148	1815

Distribution of different GO-terms in the categories “Molecular function”, “Biological Process” and “Cellular component” for matchlevel 3 and better (M3), matchlevel 4 and better (M4) and matchlevel 5 and better (M5).

Putative P450s extracted from the Zygaena 454 pyrosequencing

Contigs	Length (bp)	n	Comment	Verified by PCR	New length (bp)	New n
Zf_c28	1029	117	Pasted together as ZfCYP379A2	Yes	2421	302
Zf_c319	1759	153				
Zf_c68	1930	120	Pasted together as ZfCYP9A37	Yes	2297	160
Zf_c1736	388	13				
Zf_c1079	405	22				
Zf_c353	1197	95	Pasted together as ZfCYP332A3	Yes	1740	132
Zf_c509	704	35				
Zf_c481	2588	220	ZfCYP304F2	Yes		
Zf_c620	258	21				
Zf_c636	1741	153	ZfCYP4G47	Yes		164
E9BX6S102FL4RS	81		Same as Zf_c636			
Zf_c1080	1684	52	Pasted together as ZfCYP379A3	Yes	1705	59
Zf_c19232	143	2				
Zf_c11779	238	2	Same as ZfCYP379A3			
E9BX6S102FM79E	223		Same as ZfCYP379A3			
Zf_c1082	309	21				
Zf_c1293	502	22				
Zf_c1451	675	26				
Zf_c1477	1326	48	Pasted together as ZfP450-5		1611	63
Zf_c2266	567	14				
E9BX6S102JHDZC	213		Same as ZfP450-5			
Zf_c1522	835	30				
Zf_c1560	282	16	Pasted together as ZfCYP4L17	Yes	1677	36
Zf_c12280	226	3				
Zf_c5835	321	7				
Zf_c6985	737	10				
Zf_c1688	989	30				
Zf_c1863	431	14	Same as ZfCYP379A2			
Zf_c1980	510	17				
Zf_c2215	418	10	Same as Zf_c636			
Zf_c2363	1853	55	ZfCYP4G48	Yes		
Zf_c2519	451	11				
Zf_c2900	960	23				
Zf_c3143	593	19	Pasted together as ZfCYP6CT1	Yes	1769	34
Zf_c8955	391	4				
Zf_c7630	511	11				
Zf_c3342	252	10	Pasted together as ZfP450-7		581	15
Zf_c9425	384	5				

Zf_c3346	1240	25				
Zf_c4088	175	18	Same as ZfCYP379A2			
Zf_c4133	955	18	Pasted together as ZfP450-13		1280	22
Zf_c14465	336	4				
Zf_c4443	411	8				
Zf_c4474	362	9				
Zf_c4757	1440	28	ZfCYP333B8	Yes	1575	
Zf_c4873	348	11				
Zf_c5152	263	5	Same as ZfCYP9A37			
Zf_c5251	331	6				
Zf_c5348	307	6				
Zf_c5498	530	10				
Zf_c6025	686	14				
Zf_c6669	787	16	ZfCYP6AE27	Yes	1539	
Zf_c6742	395	14				
Zf_c6744	263	5				
Zf_c6793	533	9				
Zf_c6899	176	6				
Zf_c6974	300	5	Pasted together as ZfP450-8		637	10
E9BX6S102FN6QV	213					
Zf_c19156	194	3				
Zf_c7162	367	6				
Zf_c7429	669	13	ZfCYP9A36	Yes	1590	14
E9BX6S102HFZQ8	261		Same as Zf_c7429			
Zf_c7919	825	14				
Zf_c8045	439	6	Pasted together as ZfP450-9		573	7
E9BX6S102IRAY4	163					
Zf_c8226	528	7				
Zf_c8319	246	4				
Zf_c8409	369	6				
Zf_c8505	459	5				
Zf_c9441	256	6	Pasted together as ZfP450-14		422	7
E9BX6S102F1NRU	241					
Zf_c9556	250	5	Pasted together as ZfP450-11		381	7
Zf_c26845	163	2				
Zf_c10643	352	4				
Zf_c10964	329	6				
Zf_c11779	238	2	Same as ZfCYP379A3			
Zf_c11806	247	3				
Zf_c11879	493	5				
Zf_c12049	395	4	Pasted together as ZfP450-10		592	5
E9BX6S102IDL7L	222					
Zf_c12470	326	5				
Zf_c13218	240	3				
Zf_c13243	227	3				
Zf_c13488	580	6				
Zf_c13529	237	4				
Zf_c14205	433	5				

Zf_c14950	251	3				
Zf_c15136	383	4				
Zf_c15555	323	6				
Zf_c15837	322	3				
Zf_c15910	280	3				
Zf_c16005	429	3				
Zf_c16028	254	3				
Zf_c17720	235	2				
Zf_c17772	339	3				
Zf_c17827	475	4				
Zf_c17899	405	3				
Zf_c18919	251	2				
Zf_c19064	137	3				
Zf_c19156	194	3	Same as ZfP450-8			
Zf_c19273	248	4				
Zf_c19301	264	2				
Zf_c19308	407	4				
Zf_c19387	217	2				
Zf_c19480	235	2	Same as ZfCYP332A3			
Zf_c19699	231	2				
Zf_c20321	256	3				
Zf_c20248	215	2				
Zf_c20568	233	2				
Zf_c20774	648	5				
Zf_c20876	146	3				
Zf_c21051	221	2				
Zf_c21387	232	2				
Zf_c21947	252	3	Pasted together as ZfP450-15		402	4
E9BX6S102IZF1D	231					
Zf_c22545	256	2				
Zf_c23366	171	2				
Zf_c23625	323	2				
Zf_c23857	338	2				
Zf_c24527	185	2				
Zf_c24554	222	3				
Zf_c25199	130	2				
Zf_c25897	303	2				
Zf_c26236	351	2				
Zf_c26307	391	2				
Zf_c27029	329	2				
Zf_c28701	237	2				

The table includes only those singlets that are the same as or part of contigs. CYP-names were assigned to full-length P450s obtained by RACE-PCR by Dr. David Nelson. The full length of the coding region is listed under New Length as compared to the sequence length obtained from 454 sequencing listed under Length. n is the number of reads belonging to the contig.

Putative glycosyl transferases extracted from the Zygaena 454 pyrosequencing

Contigs	Length (bp)	n	Comment	New length (bp)	New n

Zf_c278	1247	64	Pasted together as ZfUGT33A1	1749	91
Zf_c2201	606	21			
Zf_c940	687	23			
Zf_c2042	777	32	Pasted together as ZfUGT33B1	1474	52
Zf_c2187	316	11			
Zf_c8687	528	8			
Zf_c2135	653	24	Pasted together as ZfUGT3	791	26
Zf_c19048	259	2			
Zf_c2688	418	14			
Zf_c2752	865	20	Pasted together as ZfUGT35E1	1794	48
Zf_c2971	1053	28			
Zf_c3352	337	9	Pasted together as ZfUGT5	907	20
Zf_c5719	658	11			
Zf_c4278	602	12			
Zf_c4292	650	14			
Zf_c5066	270	7			
Zf_c5105	250	4	Same as ZfUGT33A1		
Zf_c5170	258	6			
Zf_c5196	594	15			
Zf_c6009	966	13			17
Zf_c6437	234	5			
Zf_c6855	597	7			
Zf_c7972	229	4			
Zf_c10589	448	5	Pasted together as ZfUGT6	680	8
Zf_c12966	260	3			
Zf_c10617	440	8			
Zf_c10844	409	4			
Zf_c11543	552	7			
Zf_c11850	427	4			
Zf_c12814	347	6			
Zf_c13462	247	5			
Zf_c15831	465	4			
Zf_c16328	327	4	Same as Zf_c6009		
Zf_c16473	264	2			
Zf_c16617	347	3			
Zf_c16944	451	4			
Zf_c19478	197	2			
Zf_c19930	320	3			
Zf_c20684	487	4	Pasted together as ZfUGT7	685	6
Zf_c21148	287	2			
Zf_c21364	303	2			
Singletons					
E9BX6S102G9XOX	206		Pasted together as ZfUGT8	488	3
E9BX6S102ICFVF	215				
E9BX6S102I90HF	255				
E9BX6S102IPF1N	227		Same as ZfUGT33B1		
E9BX6S102GBG3U	104		Same as ZfUGT33A1		
E9BX6S102JY74W	94		Same as ZfUGT33A1		
E9BX6S102ITK7C	218				
E9BX6S102FV8U1	228				
E9BX6S102GVRHD	150				

E9BX6S102F5XKF	224				
E9BX6S102GNER2	236				
E9BX6S102F1W96	229				
E9BX6S102HTJOA	247				
E9BX6S102GPIEM	221				
E9BX6S102GFE9V	154				

UGT names were assigned by the UGT Nomenclature Committee. n is the number of reads belonging to the contig. The full length of the coding region is listed under New Length as compared to the sequence length obtained from 454 sequencing listed under Length.
