# Supplementary figures and images for: 454 pyrosequencing based transcriptome analysis of Zygaena filipendulae with focus on genes involved in biosynthesis of cyanogenic glucosides
Source: BMC Genomics. 2009 Dec 2;10:574. doi: 10.1186/1471-2164-10-574 (PMC2791780; doi:10.1186/1471-2164-10-574)

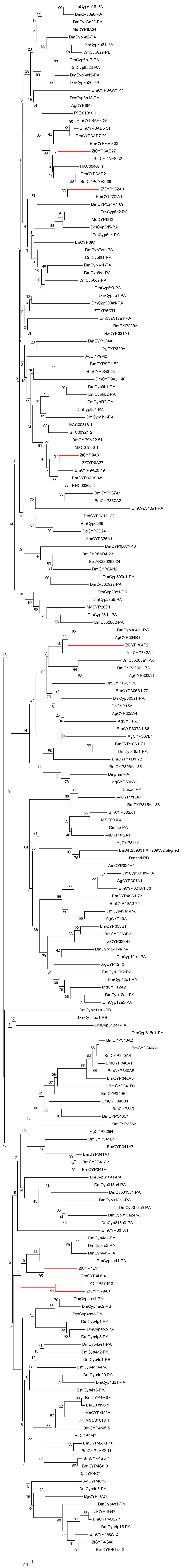

Supplement: Additional file 2 — P450 tree. Neighbor-joining bootstrap tree of full length P450s from Z. filipendulae as well as P450s from other insects. Bootstrap values are shown as percentages. [file 1471-2164-10-574-S2.TIFF]

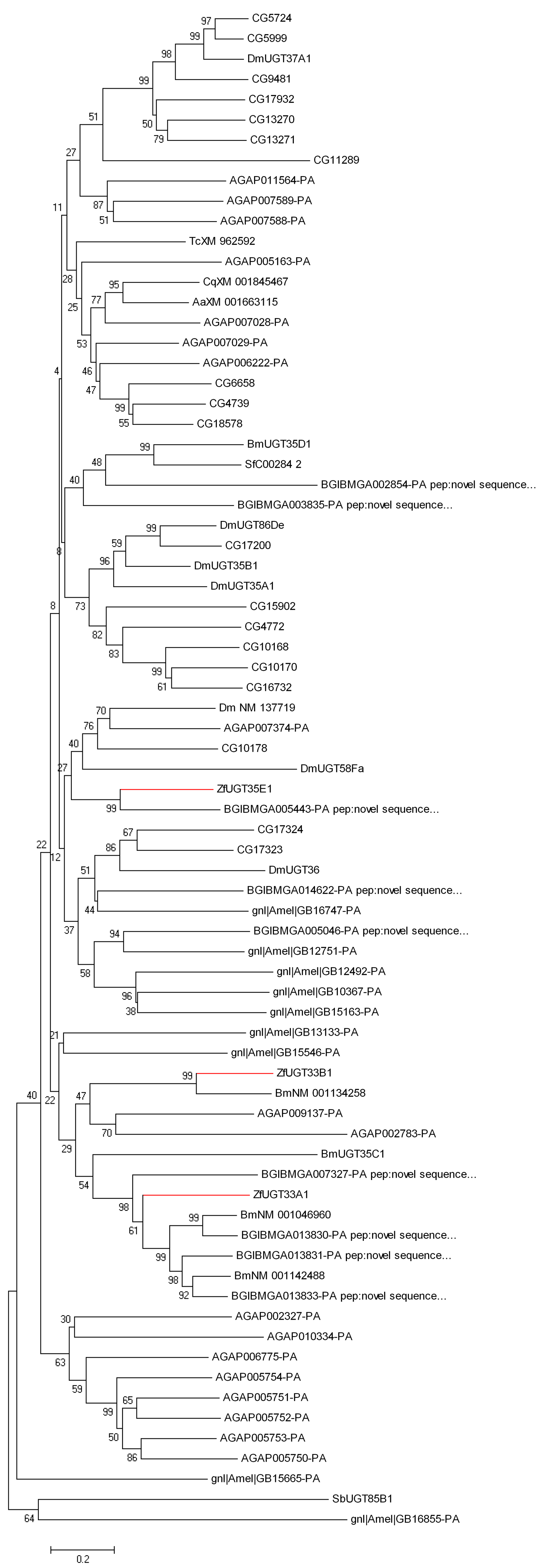

Supplement: Additional file 3 — UGT tree. Neighbor-joining bootstrap tree of full length UGTs from Z. filipendulae as well as UGTs from other insects. Bootstrap values are shown as percentages. [file 1471-2164-10-574-S3.TIFF]
