# Supplementary material for: 454 pyrosequencing based transcriptome analysis of Zygaena filipendulae with focus on genes involved in biosynthesis of cyanogenic glucosides
Source: BMC Genomics. 2009 Dec 2;10:574. doi: 10.1186/1471-2164-10-574 (PMC2791780; doi:10.1186/1471-2164-10-574)
Supplement: Additional file 4 — Heliconius contigs. FASTA file of all Heliconius contigs. [file 1471-2164-10-574-S4.PDF]

>1002049141_1 Heli.1-DT664125.3.5
GCACGAGGCAAGTCCGTCGGTCGGCTGAACTCAAATCGGTTAAAAGTAATGTGACTTGGTGACGGGATACAGG
AGCTTTCTTCGTAAAATGGCGGCGTTACACTTTTTTATGATCTTGCTTGTGGCAAGTAGTTTATCAGCAAAGG
AGGTTGAACAAAAATCTAAAGTCTCCGAGCTCCTCGAGAATGGAATAAAGGATCAGCC
ATCAGGCAAGCTGGCCAAGAGAGGCATCATCTCATCTGGTCTGTCACATGGTATTGGCTTGTCACATGGCATT
GGTGTGTCCCACGGCATCGGCCTTGGTTCAGGAATCGGTATCGGTTCAGGAATCGGTATTGGTTCAGGAATTG
GTATTGGTTCAGGAATCGGTATTGGTCCAGGTATCGGTATTGGTCCAGGAATCGGCAT
CGGTTCAGGGGTTGGTCTTTCTTCGGGAATAGGAATCGGTGGCGGTGCAATTGGTATCGGGGGAGGAGCTATT
GGTATCGGCGGTGGTGCCGTGGGTATCGCTGCCGCACCCGTCGCAGTTGCCGCTGCACCCGTCGCAGTAGCAG
CAGCACCTGCAGTTGCCGTCGCTCCTGCTCAAGTTCAAACTTCTGTATCAGTTCAACC
AAATCCAGTCATTCTCCGCCAAGAAGTACCCAGGTTCATAACTAGAACTATCACCAGGGACGTCCAAGTTCCC
GTTCAAGTCCCTGTACCTGTACCAGTCGACCGCCAAATTCCAGTACCAGTCAGAGTACCAGTCCCAGTTCCCG
TCGACAGACCCGTGCCAGTTATTCACAAAGTACCTGTAGAAGTTACCAGACAAGTACC
TGTGTACTACGAAAGGAAGGTGCCATACCCAGTGAAAGTTCCAGTATCAGTACCTGTGCCTTACCCAGTAACA
GTTGAGAGGCAAGTCCCAGTAGACCGTCCAGTGTACGTTGATCGCCAAGTGCCTGTACCATACCCAGTGCGCG
TAGACCGTCCCGTCAGCGTACCTGTGCCGGTGCACGTCGACAGGCCTGTGCCTGTTCC
GCACCCAGTCGTAGTGGACAGACCTGTGGCTGTACCCCAGCCGGTAGTCGTTGACAGACCTGTAGCAGTGGCT
GCTGCAGTGCCAGTAGCATCAGCAGGCATAAGCATTGGTAGCTCTCTCGGTGGCTCCCTCGGTGGCTCCCTCG
GTGGCTCTCTCGGTGGCTCCCTCGTTGGTTCCCTCGGAAGTTCTCTCGGCGGCTCCCT
TGGTGGCATATCGTATGGAGGAGGATACTCATCCGGCATCATTTCATCTGGAATTGGTCACGGTTTGTCTGCT
GGGATATTAACTGGACACGGATTGTCCTCTGGCATCTCAATTGGTCATGGATTATCCGGCGGCTACTCCAAGA
TCATCAGTAGCCACTAATCTTAATATTATAAGAGCAAAATGTGCAAGTTCATTTATAA
TGGATATTATTATTGAAATAAAATTATAAAACTTTTAGTCTTTATTTTATGTTTATTTTTATTTATACAAAGA
ATATTTACACAAACTTATATATTTCGAGTTTTTTAAATTGAAAATAAAAATCTCAATTATTTCGAAATCGAAA
TCGAATCTATTTATTATTAAATTACTATAAACTGTATATACAAAATACTATTTATAAA
TACTGATAAGATATATTAATTTATGCTGTTAATATTTGAAGACAAATTACAAAGACGGTGTTTACTTGTCTTT
AAATAGAAACTATATATTATGTATTATGTATGTATGTATATATGACTGCTTTTATATAATATAATTACTTTAT
TGTCTTTTCGTTGTTTATAATAAAATGAATGAGT
>1002049142_1 Heli.1-DT668408.3.5
GCACGAGCAGTAAGCATCACTTACTGAGGAAATCGCGTCAAGAAACGGTGATTGGAAACTAGGTAACGTTAGC
TCGACGAAAGAAGAACGATGAACAAACTTATTCTTCTCTCTGTCGTCGTTGCCGTGGCAACGGGGATCAATGT
TTCACCTTCAACTGGCGTCGAGCAGAAGGCACCCTGCCAGACTGCGGAACAGTGGGAG
GGACGTGCCAGTGAATGGGACCATGTTCAGGGTAGAAACAACCGATTTCTGATCAGTTTCGATGGCATAGGTA
TGAGGAAGAGGGTGATTGAGGAGAAGAAGTCGTTTATGCCTGGACGAAGATTTTACGAGTACATCATGGAATA
CAAGACCAACAAAATGTATACCATAAACATGAACTTGGGCACGTGCACCGTCTCAACA
CTGAAGCAACCATGGCAAAACCACACCATTCCTTCCGATGCCACCCTAGAAGACGAATATGAGATGGGTGATC
CTGGTTCTGGCCTCAATGTCCAGGAGTGGTCTGACAGACTCCCCGCCAGGAGAAGCGAGAGCTGGATTGGCAT
CTACACTCCTAAAACAGAGAATGGTGGTTGCTGGCCTGTGGTGGAAGTTTTCACAGAC
GACACTACCGATCCTCCCGTTTCCTTGACAACGCGTTTCTTTGACATCACGCCTGGGATAAAGAACATGTCAG
TCTTCATTCCTCCCCCTTCCTGTAATCAGAACGTGGAGATCGTAGTGGAAAACGTGGAGAATAGTGGCCCCAT
TTATACTTTTTACAACTACATCAAGTCCTGGTTTTAGTTTTGTCAATTTTCATCCCAT
CAACATTCATCAAACAGAAATGACGTACGCATGACC
>1002049143_1 Heli.1-DT668378.3.5
CGAGGCTTAACCGGTCGCGCTGAACGACGACTGATTAATTTATTAAATTATATTTCTACGTTAAGTTCAACAA
AACTCAAACAAAATGCGTGAGTGCATCTCCGTACACGTTGGCCAAGCTGGTGTCCAGATCGGTAACGCCTGCT
GGGAACTCTACTGCCTGGAGCATGGCATCCAACCTGATGGCCAGATGCCCACAGACAA
GACCATCGGCGGTGGAGACGACTCCTTCAACACCTTCTTCAGTGAGACCGGCGCCGGCAAACATGTACCCCGT
GCAGTGTTCGTCGACTTGGAGCCTACAGTAGTTGATGAGGTTCGTACCGGCACATACAGACAGTTGTTTCATC
CAGAACAACTTATCACTGGTAAGGAAGATGCGGCCAACAACTATGCCCGCGGTCACTA
CACCATCGGCAAGGAGATTGTTGACCTAGTCCTCGACCGCATCCGCAAGCTCGCCGACCAGTGCACAGGCCTC
CAGGGCTTCCTCATCTTCCACTCCTTTGGTGGTGGTACCGGATCAGGGTTCACTTCCCTGTTGATGGAGCGTC
TCTCAGTTGACTACGGCAAGAAGTCCAAACTCGAGTTCGCCATCTACCCCGCCCCACA

GGTCTCCACTGCTGTGGTTGAGCCCTACAACTCCATCCTGACCACGCACACCACTCTTGAGCACTCCGACTGC
GCCTTCATGGTCGACAACGAGGCCATCTACGACATCTGCCGCCGCAACTTGGACATCGAACGCCCAACCTATA
CAAACTTGAACCGTCTCATCGGTCAAATCGTGTCCTCCATCACGGCCTCCCTGCGTTT
CGACGGCGCACTCAACGTCGATCTTACAGAGTTCCAGACCAACTTGGTGCCCTACCCACGTATCCACTTCCCC
CTCGTGACCTACGCCCCTGTCATCTCCGCCGAGAAGGCTTACCATGAACAGCTCTCTGTCTCCGAAATCACCA
ACGCGTGCTTCGAGCCCGCCAACCAGATGGTGAAGTGCGACCCACGCCACGGCAAGTA
CATGGCGTGCTGCATGTTGTACCGTGGTGACGTCGTGCCCAAGGACGTGAACGCCGCCATCGCCACCATCAAG
ACCAAGCGCACCATCCAATTCGTGGACTGGTGTCCTACTGGTTTCAAGGTGGGCATCAACTACCAGCCCCCGA
CCGTCGTGCCCGGCGGAGACTTGGCTAAGGTACAGCGCGCCGTGTGCATGTTGTCCAA
CACGACCGCCATCGCTGAAGCGTGGGCGCGTCTCGACCACAAGTTCGACCTGATGTACGCCAAGCGCGCCTTC
GTGCACTGGTACGTCGGTGAAGGTATGGAGGAGGGTGAGTTCTCCGAGGCCCGCGAGGACTTGGCCGCCCTGG
AGAAGGACTACGAAGAGGTCGGCATGGACTCCGCTGAGGGAGAAGGCGAGGGTGCCGA
GGAGTACTAAGCGGCTTAATTAACGTTGAGCACTCGCACCGAATACATTCCGGTGAAATAAAATAAAAAGAAA
TCAAATTTAAAAAAAAAAAAAAA
>1002049144_1 Heli.1-DT668383.3.5
GCACGAGGCGACCACGGACTGTGGTGCGTGCGGAGCAGTAGTGTTAAGTGTATAGTGTTTAAGTAATAATTTT
TATATTAAAATGAGGGAAATCGTACACATTCAAGCCGGGCAATGCGGCAACCAGATCGGAGCTAAATTCTGGG
AGATCATCTCTGACGAGCACGGCATCGACCCCACTGGCGCCTACCACGGTGACTCGGA
TTTGCAGCTCGAGCGCATCAATGTATACTACAATGAGGCCTCCGGCGGCAAGTACGTGCCCCGCGCCATCCTC
GTCGACTTGGAGCCCGGCACCATGGACTCTGTCCGCTCGGGACCTTTCGGACAGATCTTCCGCCCCGACAACT
TCGTGTTCGGACAGTCTGGTGCCGGTAACAACTGGGCCAAGGGACACTACACCGAGGG
AGCTGAACTTGTCGACTCAGTCCTTGATGTAGTTCGCAAGGAATCCGAATCCTGCGATTGCCTACAGGGCTTC
CAACTTACACACTCCCTTGGAGGCGGTACTGGCTCTGGTATGGGAACCCTTCTCATATCGAAAATTCGTGAAG
AATATCCCGACAGAATAATGAACACATACTCAGTCGTCCCCTCGCCCAAAGTATCAGA
TACCGTAGTAGAACCCTACAACGCGACTCTCTCAGTACACCAGCTCGTAGAAAACACAGACGAAACTTATTGT
ATCGACAATGAGGCACTTTACGACATCTGTTTCCGCACACTTAAACTTTCCACACCCACCTATGGAGATCTTA
ACCATTTGGTGTCCCTTACAATGTCCGGTGTCACTACTTGTCTTAGGTTCCCTGGTCA
ACTTAACGCTGATCTTCGCAAGCTGGCCGTCAACATGGTGCCCTTCCCGCGTCTTCACTTCTTCATGCCAGGC
TTCGCCCCCCTTACGTCGCGAGGCAGCCAGCAGTACCGCGCGCTCACCGTTCCCGAGCTTACGCAGCAGATGT
TCGACGCCAAGAACATGATGGCGGCGTGCGATCCGCGCCACGGCCGCTACCTTACAGT
GGCCGCCATCTTCCGAGGCCGCATGTCCATGAAAGAGGTGGACGAGCAGATGCTCAACATCCAGAACAAGAAC
TCGTCCTACTTCGTAGAATGGATCCCCAACAACGTGAAGACCGCCGTGTGCGACATTCCGCCCCGTGGACTCA
AGATGGCCGCCACTTTCATCGGCAACTCCACCGCCATCCAGGAGCTGTTCAAGCGCAT
CTCCGAGCAGTTCACCGCTATGTTCAGGCGCAAGGCTTTCTTGCATTGGTACACCGGCGAGGGCATGGACGAG
ATGGAGTTCACGGAAGCCGAGAGCAACATGAACGACTTGGTCTCAGAGTACCAGCAGTACCAGGAGGCGACCG
CCGACGAGGACGCCGAGTTCGACGAGGAGCAGGAGCACGATGTAGAAGATCACTAAAC
GTTAATTACCGTTGTGTTGCAACATAGTACATGCCATTATTGTCTCGCGTTACTTTAAGTAACTTAATGATTC
CTATACGTACTGTGTATTGAAGACATGCTTGCTAACTTTTAACAGACTGCACGACTAAAAAAGCCCGGCCCTA
CCAAATGTGAAAGCATTTAAATGGCGCTCTCTGGTATGTTTATTGGTTGGTATAAACA
ATCCCTTACGACGTGACCCCTTCATTGACCCACCACCCCCTGCGGTGCTGCGAATACACTGATGACACAAAAG
AAGATCTTTTGTTTGATAAGGCTGAACTTGATTTTTTTAATACCTAAGTTTATTTTTAATCTTTGTAACCAGT
GTGATTGAGTTTATGTTGGCTTTTTGGAAACTAAAAAGCTAATAACAATGTCGTACAA
TTTTTCGAAGTCGATCAAAGTTTTTAATTATTATATTCCAGCTGGTGAATAATAAACTACAATGATTTTAAA
>1002049145_1 Heli.1-DT665882.3.5
GCACGAGGCTTTCTCGGATAATACACGCTATCCGTGGTGAGTGTCAGAAGATTTAAAGTGAAGTGCAAATTGT
TAAGTGAATAGCGAGGGGATAATACACGCTATCCGTGACTAACCAAAAATGGGCAAGGAAAAGATTCACATTA
ACATTGTCGTCATTGGACACGTCGACTCCGGCAAGTCCACCACCACTGGTCACTTGAT
TTACAAATGTGGTGGTATCGACAAACGTACCATCGAGAAGTTCGAGAAGGAAGCCCAGGAAATGGGCAAAGGT
TCCTTCAAATACGCCTGGGTGTTGGACAAACTGAAGGCTGAGCGTGAACGTGGTATCACCATCGATATTGCTC
TGTGGAAGTTCGAGACCGCCAAATACTATGTCACCATCATCGACGCTCCCGGACACAG
AGATTTTATCAAGAACATGATTACCGGTACGTCCCAGGCCGACTGCGCCGTGCTCATCGTCGCCGCCGGTACT
GGTGAGTTTGAAGCCGGTATTTCTAAGAATGGACAGACCCGTGAGCATGCCTTACTTGCCTTCACACTCGGCG
TCAAGCAGCTGATTGTTGGAGTAAACAAAATGGACTCCACTGAGCCCCCATACAATGA

GGCTCGTTTCGAAGAAATCAAGAAAGAAGTATCTTCCTACATTAAGAAGATCGGTTATAACCCAGCTGCCGTC
GCTTTCGTACCCATTTCTGGCTGGCACGGAGACAACATGTTGGAGGCATCCACCAAAATGCCCTGGTTCAAGG
GATGGCAAGTGGAGCGCAAGGAAGGTAAGGCTGACGGTAAATGCCTCATTGAGGCTCT
CGACGCCATCTTGCCCCCAGCGCGTCCCACCGATAAGGCACTCCGTCTTCCCCTACAGGACGTCTACAAAATC
GGTGGTATTGGTACAGTGCCAGTAGGCAGAGTTGAAACTGGTGTCCTGAAACCTGGTACCATTGTTGTCTTCG
CTCCTGCTAACATCACTACTGAAGTAAAGTCCGTTGAAATGCACCACGAAGCTCTCCA
AGAGGCTGTGCCTGGAGACAATGTAGGTTTCAACGTAAAGAACGTGTCCGTCAAGGAATTGCGTCGTGGTTAC
GTCGCCGGTGACTCCAAGAACAACCCACCCAAGGGTGCCGCTGACTTCACCGCACAAGTCATTGTACTCAACC
ACCCTGGTCAAATCTCTAATGGATACACACCTGTGTTGGATTGCCACACAGCTCACAT
TGCTTGCAAGTTCGCCGAAATCAAAGAAAAGGTTGACCGTCGTTCTGGTAAATCCACTGAAGATAATCCTAAA
TCTATCAAATCTGGTGATGCTGCCATCGTCAATCTCCAACCATCCAAACCCCTGTGTGTGGAAGCTTTCCAAG
AATTCCCCCCTCTCGGTCGTTTCGCCGTGCGTGACATGAGGCAGACTGTAGCTGTGGG
TGTCATCAAGGCTGTCAACTTCAAGGAAGGTGCTGGTGGTAAAGTCACCAAAGCCGCTGAGAAGGCCACCAAG
GGCAAAAAGTAGCTAGCGCTGTTAACAGCTCAATTCTTTATCCAACTGCGATACTTCATTCACCGAAAGGTGT
TCAGAAGGAAAAAAGGGCTACAAACTCATTCCTTTTCTATATTTTTTACAAGGCTTAT
ACTGTAACATTATTTTATAATTTATAAGGTTATACATATATCTGAAATATTTTGTTATGACTACAACTAAAAT
GTAAATTCATAGAATAAAAGTAGCCTCCAATAATA
>1002049146_1 Heli.1-DT668782.2.5
GAGGGGAACCACCGCCCGAACTAATAATAACTTAAGAAAATGTTCGCAAAGGCAATCGTCGCGTGCGCGCTCC
TGGTTGTAGCTCAGGGAGGGCTCATTGCGAGCCCTCACGGAGTTGGAGCTATCTCATCTCAGAGTATAGTTCT
TGGACATCCTGCACCTGCCATCGCCGCTCATGCTGTTGCCCCTGCATTGGCTGCTCCT
GCATTGGGACTAGCTCATGGATTGGGACTAGCTCCTGGATTGGGACTAGCTCCTAGACTAGGACTAGCTCCTG
GATTGGGATTAGCTTCTGGATTGGGACTAGGTCACGGTGCCATTGTATCACACGCACCCGTGCTGCACGCGCC
TGTGGCCCACGCCGTTGCCCCTGTTGAGGTTTATGCCCACCCCCGCTACCAATTCAAC
TACGGTGTCACTGACGGTCACACCGGCGATCAAAAGAGCCAATGGGAAGCCCGCGATGGTGATGTCGTAAAGG
GACAATACTCCCTCGTCGAACCCGACGGCACCATCCGTACTGTAAACTACTCTGCTGATGATCACAATGGTTT
CAACGCTGTAGTGAGCAGACAAGGACACGCTGCGCATCCTGCCGTTGCAGCCGTTGCC
CATGCTCCTGTAATCGCCGCGGCTCCTCTCGGCCACGGTTCCCTCCTCCGTGGCTCCCTCCTCCATGGCTAAA
CACTTGAAAATGACATATTTATTACCTTTATTTACTTAGGATAAGACAGAATGCGACAATGTTACTTGAATGC
TTCCTGGTAATGGTACAACGGATGGTCTGCTGCTATAGGCACAGCGTTTCATAAGGTG
TATCTAATATGGGACGACGGCCCAAGACTTCACAACCAGTACCTGTTTTTGATGGGTGCTAACTTTTGTATTA
TAATTTCTTTTAGTTTGTAAGTCTTTCTCCGTTTTCTTACTTAGTTAGGAGCATACATTTTATGAAACGTTAG
ACAATGCCATATGGGTGCTTGACGAAGGCTCCAGATTTTGCTTATATTATGTAATATA
CCTTTCTCATAT
>1002049147_1 Heli.1-ES586659.1.5
CCAGCAGTCTAACAAGAAGTGCAAGATGAGATTTCTGATCGCTTTTGTCGCCATCCTCGGCTATGCTTCGGCT
AGCGCCATCATCTCCCCCCTGGTCTTAGGCGCCAACGCTGGTGACGTGCAAGCCGCCGTCATCGACGCCAATG
TAGCCGCCCGCGACTCCATCCGCGCCCTCGGCGAAGGCCAGGCCCGCGCCGCCGAAGC
CGTCATCCAAGCCAACACTGAAGCAGTCCGCCAAGTCGCTGAGAACAACCGTAACATTCACGAAAACGCTTAC
TGGGGATCGGTCGCCGCCTCCCAAAACCTCGTAGCTGCTGCTCAATCTCAGGTCGCCGCTATCGATGGAGCCG
CCGCCGCCGCCCGTGGTGTCCTCCCCATCGCTCCATTGGCCTACGCTGGTGCCCTCCC
CTATGCCGCCCCTTACGCTCTCGGTCTCGGAGGTCTCGGCCTCCACGGCATCCACGCTTGGTAAATCAACTTG
ACCTGGCACATCACTAAATAGTGATGTACTCGTTTGGGAGTTACCTCGTCCCTGAAGTCAAGGAAACCTCCAA
ACTTAACAACTGGCATTGGCAATGTTCACATTAACAGTGGATATTGCCAAACAGTTGT
TCCAATGGAAACCGATTGCTCAATGGATTTCTACAAAGGTCTCAACGTAACTCTCTTTGTAAATAAGTGAATC
TCATGCCATCACTGCCAAAGTGTAAAATAAACACA
>1002049148_1 Heli.1-EL603934.1.5
AACAGCGCACGCGACAGTCATGTACGACAGTCATGTACACTAAGTTATTTATTTTGTGCTGCGCGTGCGCGTT
GGCGACGGCTGCACCTGGTCTCCTGGATGGTCTGAGCTTAGGAGGGGGGCTGGCCCACTCGTACGCGGCACCG
ATTGCCGTGGCTGCACCCGTTGTTAAGACCATCGCAGCACCTGTTGTCAAGACAGTAC
TCAGAGCTGAACCTATCGACCCGAACCCCGCTTACAGCTACGCATATGGAGTAGCCGACCCTCACACTGGTGA
CCACAAAGACGCCCAGGAGACCCTCCAGAACGGTGTCGTCCATGGCTCCTACAGCCTGGTGGAGCCTGACGGC
CACGTCAGGAAAGTGACTTACACAGCTGACAAAATAAACGGTTTCAACGCAGTCGTTG

AAAGAAGCGGTGCTAGTCACGTTGCGCCTGTAGCACTAGCCCACGCGCCCATCGCCGTTGCCCACGCCCCGGT
CGCCATCGCCGCCCCTATCGCCAAATACTCCCTACCAGCCATCGGACACCCCTGGCGCTAGTCACACCACCCA
CCCGCTTGTCGCATCGCTGACCAACAAAGGATAGGACAAACTTGGAGACAGACTAGCA
AACATTTTTGAAGCTGTCTCCATAGTTTACGAACTGATAGCTCAATAAAGGACGAAGGCTGCCCAAAGCGTCG
TCCAGATAGTCGGAGCTATCAGAAATTAAAAAGGAATTCGACTGTAATATTATAGTTTCCACAACTGTTTTCT
GTGTAGAATAGGTTGGGAAGATGTTTTGTTTATTTTTTTGGTTTTTAATGGAATGTTT
ATATATAGATGATGGCCTGGGATCATTGTGAGATGATGAGATGATGAGATTTGCTTAGTCAGCGATGCAACAA
TATCCACGACCGCCGAGCGCCGAATGAAACTCTCCTATTCTTTAATTTATTTAGTTTTGCTTTGACTGTTGCT
ACGGACTTGATCTTGACGCGTTGTATTACTTACATGTAAATATAATGTGTACTTAGTT
TTTAAATAAAATGCCATTTTTAAGGTGAAAAAAAAA
>1002049149_1 Heli.1-DT669008.3.5
AGGGTTCACGTGAGGTTGTGTGGTCACGGAAATAGGTCACACAATCCTCAGAATTTCGTGAATTATTCTTGTA
AAATATAAAAAAAAATCACAAAATTAATAAAAATCCAAAAAAATAGTAAAATATTTAAAAAATAACGGGAATC
ACTCGCCTTTAAGTTTAGTGTACGTAAGCGTGCAACGTGGTCTCAAGGAATGCGATAG
GTAATCGTAAAGAGAAAGTGAAGCAGCTTTTATTAATTAATGAGTTTTGTTAATCGGTCACAATAAATATAAT
AAAATGAATCCTGGACCGCCAAACTATCCCATGGCTTCGCTATATGTTGGAGATCTACACTCCGATATTACTG
AAGCCATGTTGTTTGAAAAATTTTCTACCGCTGGTGCGGTTCTCTCCATTCGCGTTTG
TCGAGATATGATAACACGCAGATCCTTGGGATATGCTTACGTAAATTTTCAGCAGCCTGCCGATGCGGAGCGA
GCATTAGACTCCATGAACTTTGATATGATCAAGGGCAGGCCTATAAGGATCATGTGGTCCCAACGAGATCCAT
CCCTGCGCAAGTCTGGCGTAGGCAATGTGTTTATTAAAAACCTTGATAAAACCATAGA
TAACAAAGCTATGTATGACACATTTACAGCTTTTGGTAATATTTTGAGTTGTAAAGTAGCCCAAGATGAAAAT
GGAGCATCTAAAGGATATGGTTTTGTTCATTTTGAAACAGAAGAAGCTGCAAATAAGTCTATTGAAAAAGTAA
ATGGCATGCTCTTGAATGGTAAAAAAGTTTACGTAGGCCGGTTTATCCCCCGTAAGGA
ACGTGAAAAGGAACTTGGGGAAAAAGCTAAGTTGTTCACGAATGTATACGTTAAAAACTTTGGCGAAGAATTC
TCTGATGAAATGCTTAGAGACATGTTTGAAAAATATGGCAGAATAACTAGCCATAAGGTTATGTACAAGGATG
ATGGGTCATCTAGAGGTTTTGGATTTGTAGCATTTGAGGATCCTGATAATGCAGAAAG
AGCCTGTATGGAACTTAATGGCAAAGAACTTGTAGAGGGTAAACCTTTATATGTAGGACGTGCTCAGAAAAAG
GCTGAACGTCAAAAGGAATTAAAACGTAAATTTGAGCAGTTGAAATCTGAACGCTTGACACGTTACCAAGGTG
TTAATTTGTATGTTAAGAACTTAGATGACACTATTGATGATGAGAGACTTCGTAAAGA
ATTTGCACCTTTTGGTACTATTACTTCTGCTAAGGTTATGTTAGAAGATGGTCGCAGTAAAGGTTTTGGGTTT
GTATGTTTCTCATCCCCCGAAGAAGCTACTAAAGCAGTAACTGAAATGAATGGTCGGATTGTTGGAACCAAAC
CTTTATATGTTGCTTTGGCTCAACGCAAAGAAGATCGCAAAGCTCACTTGACATCTCA
ATATATGCAGAGAATGGCAAGTATGAGAATGCAACAAATGGGACAAATTTTCCAACCTGGAAGTGCAGGCGGG
TACTTTGTACCTACTATTCCTCCAGCGCAAAGGTACTATGGGCCAGCCCAAATGACTCAGATAAGACCTGCTC
CACGCTGGACTGCCCAACCATCTGTTAGACCAAGCGCTCAATCTGCTGCTTCTGCATA
TCCTAACATGCAACCACAGTTCCGTCCAACACCTCGAGGCCCAACTCA
>1002049150_1 Heli.1-ES588096.1.5
ATTATATTTAATAAAAAAATTTTTAATATTGGGGGATGTTAAAGTATTTTTTAGAGAAAAAATATAATTATTT
ATAATAAAATAGTATTGAAAAAGAAATTTGAAATAAATTTGAAAAATTAATTAATTAAAAAGTAAATTTTAAT
TATTGTATCTTGTGTATCAGAGTTTATTAAAAAATATTTATTAGTTAAAAAAATCTCG
AATTTAAAAGAGTTAATTAATTAAAAAATTTAATGTTGAATAATTATTTTAAATAATTAATTTGAAATGAAAT
GTTAATCGTTTTTAAATATATCTAGTTTTTTTAGAAAAAAATTTAATTTTAAATTTAAAATATTTTTTAATTT
TTTATTTAATTAAAAAATTTTTAAAATTAAATATATTAAGGGATAAGCTTTAATTTAA
ATTTTTATAATATATATATATATATATATATATATATATATATATTAAATTAAATTTAAAATTTTTAAAATTTATAT
TGTTTATAAAATAAAATTTATTATAAAAAATTTTAAAAAGAAAAAATAAAATTTAAAATTATATTTAAATTTT
TATAAAAATATAATTTATAACAGTAAAAAATTAGAAATAATGATAAAATTAGTATATT
AAATTTTTACATATTAAATTTAAATTAAAATTAATTTAAAGGAATTCGGCAAATTTTTATATTCACTTGTTTA
TCAAAAACATGTCTTTTTGATTAATAATTTAAAGTCTAATCTGCCCACTGATTTAAAAATTAAAGGGCTGCAG
TATATTGACTGTACAAAGGTAGCATAATAATTAGTCTTTTAATTGAAGACTTGTATGA
AAGATTTGATGAAATATAAACTGTCTCTAAATTAAAAATTGAAATTAATTTTTTTATTAAAAAGTTAAAATAA
TTTAAAAAGACGAGAAGACCCTATAGAGTTTTATAATTATAATTTTTTAATATTTAATATATAATAATAAATT
TAAATAATTGTAATTATTTTATTGGGGTGATAGAAAAATTAAAATAACTTTTTTTTA

AAAATAAACATAAATAATTGAATAAATGATCCATAAATTATGATTATAAGAAAAAATTACCTTAGGGATAACA
GCGTAATTTTTTTTTTTAGTACAAATAAAAAAAAAAGGTTGCGACCTCGATGTTGGATTAAGATAAAATTTAA
ATGCAAAAGTTTAAAATTTTGATCTGTTCGATCATTAAAATCTTACATGATCTGAGTT
CAAACCGGTGTGAGCCAGGTTGGTTTCTATCTTTTAATAAAAAAAATATTTTAGTACGAAAGGATCAAATATT
ATAAATAATTTAATTTTAAGAATATTATTAATATT
>1002049151_1 Heli.1-DT668352.3.5
GTGAAAAGAGGCCGACAATCGCGCACCTTTGCTGAGTGATCTACTATTTAAGTCTTCTAAGTTACAAGCTAAG
TTACTAATATGGCAAAAGCACCCGCGGTAGGTATCGATTTGGGCACCACGTACTCTTGCGTGGGTGTGTTCCA
ACATGGTAAGGTGGAGATCATCGCCAACGACCAGGGCAACAGGACAACGCCGTCCTAT
GTCGCGTTCACCGACACAGAGCGTCTCATCGGAGATGCCGCTAAGAACCAGGTGGCGATGAACCCTAATAACA
CAATTTTTGATGCCAAACGACTTATCGGTCGTAAATTTGAGGATGCTACTGTACAAGCTGACATGAAGCACTG
GCCATTTGAAGTGGTCAGTGATGGAGGCAAACCAAAGATTCAGGTATCTTACAAAGGT
GAAGACAAAACCTTCTTCCCTGAAGAAGTAAGCTCAATGGTGCTTACAAAGATGAAGGAAACAGCTGAGGCGT
ACCTCGGCAAAACAGTGCAGAATGCAGTCATCACGGTTCCAGCATATTTCAACGACTCACAAAGACAGGCCAC
AAAAGATGCGGGCACCATCTCTGGTCTGAACGTTCTCCGAATTATCAACGAACCCACT
GCTGCTGCGATTGCATACGGTCTTGACAAGAAGGGCACTGGAGAACGTAACGTCCTTATTTTCGATCTCGGTG
GCGGTACCTTCGACGTGTCCATCCTGACCATCGAGGATGGTATCTTCGAGGTCAAGTCCACCGCCGGTGACAC
CCACTTGGGAGGTGAAGATTTCGACAATCGTATGGTCAACCACTTCGTACAGGAGTTC
AAGAGGAAATATAAGAAGGATCTTACCACCAACAAGAGGGCCCTCCGTAGGTTGAGAACAGCCTGTGAGAGGG
CAAAGAGGACTCTGTCTTCTTCCACCCAGGCTAGTATCGAAATTGACTCTCTCTTTGAGGGCATTGACTTCTA
CACGTCCATCACCAGGGCTCGTTTTGAAGAGTTGAACGCCGATCTGTTCAGGTCCACC
ATGGAACCCGTAGAGAAATCACTCCGTGATGCAAAGATGGACAAGTCCCAAATCCACGACATTGTCCTCGTTG
GAGGCTCCACCCGCATCCCTAAGGTGCAGAAGCTTCTGCAGGACTTCTTCAATGGCAAGGAGCTAAACAAGTC
TATCAACCCAGACGAGGCCGTCGCATACGGTGCGGCGGTGCAGGCCGCCATCCTGCAC
GGCGACAAGTCTGAGGAGGTACAGGACTTGCTACTGCTCGACGTGACCCCACTGTCGCTTGGTATTGAGACCG
CCGGAGGCGTGATGACGACGCTCATCAAGCGCAACACCACCATCCCTACGAAACAGACGCAGACGTTCACCAC
GTACTCTGACAACCAACCCGGCGTGCTCATCCAGGTGTACGAGGGCGAGCGTGCCATG
ACCAAGGACAACAACCTATTGGGCAAGTTCGAGCTGACAGGCATTCCTCCCGCGCCTCGCGGCGTACCGCAAA
TTGAGGTGACGTTTGACATCGACGCGAACGGTATCTTAAATGTGTCGGCCGTCGAGAAGTCCACCAACAAGGA
GAATAAGATCACCATCACCAACGACAAAGGCCGTCTCTCCAAAGAGGAGATCGAGCGC
ATGGTCAACGAGGCCGAGAAGTACCGCAACGAGGACGAGAAACAGAAGGAGACCATCCAGGCCAAGAACGCAC
TTGAGTCGTACTGCTTCAACATGAAATCCACCATGGAGGATGAGAAGTTGAAGGAGAAGATCTCGGACGCGGA
CAAGCAGACTATCCTGGACAAGTGCAACGACACCATCAAGTGGCTGGATTCTAATCAG
CTGGCCGACAAGGAGGAGTACGAGCACAAGCAGAAGGAGCTCGAGGGCGTGTGCAACCCTATCATCACCAAAA
TGTACCAGGGCGCGGGCGGTGCACCTGGAGGAATGCCCGGCGGTATGCCCGGTTTCCCCGGTGGCGCACCCGG
CGCGGGAGGTGCGGCGCCCGGAGCTGGCGGTGCCGGCCCCACCATTGAGGAGGTCGAC
TAAACAAACATCCACCTGAACCAAACATTCCACGTTTAACACGAATGCATGCAAATAAAA
>1002049152_1 Heli.1-DT665917.3.5
GCAATCCGAAGTTTAGTTAGCCATCGTAATACGACAGCTCGAGTCGTAGACAGACAGGCTCCAGCCCGTGGTC
GAGACATAAGGCTCTCGCAGACTTTACGCTAGCTAAACGCGCAGTGAACTTCAGTTCAGGCTCCTCTATCTTT
ACGTGTGGTGTTTCGAAGCATGGCCGTCGCAGCTGCCCAAAAGAACCGCGAAATGTTC
GCCATCAAGAAGTCCTACAGCATCGAGAACGGCTACCCATCTCGCCGTCGCTCGCTCGTAGATGACGCTCGCT
TCGAGACACTTGTTGTCAAACAGACCAAACAAAGTGTACTTGAGGAGGCTCGTGCCCGTGCCAATGACTCTGG
CTTGGACTCTGATTTCATCCAAGACGCCGCTCATGTAGGCGAAGACGGGAAGACCGTT
GATGGCGCACAAAATGATGAATGCAAAAACGGTCGCCCTGATGCGGATGCCAACGATGGTAACGCTAATGGGG
ATGAAGATTATACTCTGACCGAAGAAGAGATAATTTTGCAAAATGCCGCCAGCGAGAGCCCTGAAGCCGAACA
GGCGACCCAACAAGCCGCTTTACTTCTTCGTATGCGCGAAGGCATGGGCTCTCTCGCT
CGCATCCTGAAAACAATCGACAACTACAAGGGTTGCGTAGAACATCTCGAAACTCGCCCCTCTCAAGTACCCG
GAAACCAATTCGATGCTCTCGTTAAAGTGAGCATGTCCCGCGTGAACCTGCTACAACTGATCAGATCTCTACG
TCAGTCAACTTCTTTCGTTGGAGTTAATCTCATCTCTGAGAACAACCTGTCTAACAAG
ACTCCATGGTTCCCTCGCCATGCATCTGATCTTGACAACTGTAACCATCTGATGACTAAATATGAACCAGAAC
TCGACATGAACCATCCTGGTTTCGCCGACAAGGAATACAGAGAGCGTAGGAAGCAAATTGCCGAAATCGCTTT
CGCTTACAAATACGGTGACCCGATCCCATCTATCACCTACAGCGAAAGCGAGAACGCT

ACCTGGCAGCGTGTGTTCAACACCGTGCTCGACTTGATGCCCAAACATGCTTGCCGCGAATACAAGGCAGCGT
TTGGCAAGCTGCAATCCGCTGACATTTTTGTACCACACCGCATCCCACAATTGGAGGATGTCAGCAACTTCCT
GCGCAAGCACACCGGCTTCACTCTCCGCCCGGCCGCTGGTCTACTCACTGCTCGCGAT
TTCTTGGCCTCTCTCGCCTTCCGTGTTTTCCAATCTACACAATACGTACGCCACGCCAACTCACCATTCCACA
CACCTGAACCTGATTGCATTCACGAGCTCCTTGGACACATCCCTCTTCTGGCTGACCCGAGCTTCGCTCAGTT
CTCTCAGGAGATCGGATTAGCTTCACTTGGAGCTTCTGACGCCGAAATTGAAAAACTA
TCCACTGTATACTGGTTCACCGTTGAATTTGGCCTGTGCAAAGAAAATCAACAATTGAAAGCTTACGGCGCTG
CTCTACTTTCATCCATTGGAGAACTTCTTCACGCACTTAGCGACAAACCCGAGTTGAGGCCCTTCGAACCGGC
ATCCACATCGGTACAGCCCTACCAAGATCAAGAATACCAGCCGATCTACTACGTCGCC
GAGAGCTTTGAAGATGCCAAAGACAAATTCAGACGCTGGGTTTCGACCATGTCCCGACCATTCGAAGTACGTT
TCAACCCACACACAGAGCGCGTTGAGGTTCTGGACTCTGTGGACAAGCTGGAGACCCTCATCTGGCAGTTGAA
CACAGAAATGCTCCACCTCACAAATGCCATCAAGAAGCTCAAGGACTCGCGCTTCGAG
TGAACCAGTTACCTACATTATTGAACCAGATCAGTGCACGCGGCCTCGCCGTACCTGTATAGCTAGTGATATT
ATAGATGTAAGACTGTTGTCGTGTGGAGCGTGTATGCGAGTGTGGAAACCATCGGATGGTACTTCGTTGAATT
CCTTTTGTATCTTATTATTTATTATGCCGAGTTCATTGCCCGGGTTTCGGAAGGCGCA
CAGAACTCCCGATTGCCCATTTACGGAGGCCGTGCCCGTTCGCGAGACCTTTACTATTATATTAGTGTTAATT
TATAATTTAATGAAAAACACAAACAAATAAAAAATGGAGCACCAAAAAGTAAAAAAAATCAAACAGAAATATA
TGTGTTTGAG
>1002049153_1 Heli.1-EL602937.1.5
GGATTTAATAAAAAAATTTTTAATATTGGGGGATGTTAAAGTATTTTTTATAGAAAAAATATAATTATTTATA
GTAAAATAGTATTGAAAAAGAAATTTGAAATAATTATGAAAATAAATTATTTTAAAAGTAAATTTAAATTATT
GTATCTTGTGTATCAGAGTTTATTAAAAAATATTTATTGATTAATAAAATCTCGAATT
TAAAAGAGTTAATTAATTAAGAAAGTTAATGTTGAATAATTATTTTTAATTATTAATTTGAAATGAAATGTTA
ATCGTTTTTAAATATATCTAGTTTTTTTAGAAAAAAATTTAATTTTGAATTTAAAATATTTTTTAATTTATTT
TTTAATTAAAAAATTTTAAAATTAAATATATTAAGGGATAAGCTTTAATTTAAATTTT
TATAATATATATATATATATATATATATATATATAAATATTTAAAATTTTTAAAATTTATATTGTTTATAAAATA
AAATTTATTATAAAAAATTTTAATAAAAATAAAATAAATTTAAAATTTTAATTTTTTATAAAATAATAATTAA
TTTTTAATTAAAATAAAATTAGAATGATAAAATTAATTAGTATATTAAATTAAAAAAT
AATAAATTTAAAAAAATTAATTTAAAGGAATTCGGCAAATTTTTATATTCACTTGTTTATCAAAAACATGTCT
CTTTTTGAAATTAATTTAAAGTCTAATCTGCCCACTGATTATAAATTAAAGGGCTGCAGTATATTGACTGTAC
AAAGGTAGCATAATAAATAGTCTTTTAATTGAAGACTTGTATGAAAGATTTGATGAAA
TATAAACTGTCTCTAAATTAAAAATTGAAATTAATTTTTTAATTAAAAAGTTAAAATAATTTAAAAAGACGAG
AAGACCCTATAGAGTTTTATAATAATAATTTTTTAATATTTAATATCTAAAAATAAATTTAAATAATTATTAT
TATTTTATTGGGGTGATAGAAAAATTAAAATAACTTTTTTTAAAAAAAATTACATTAAT
AAATGAAATTATGATCCATATTTTATGATTATAAGAAAAAATTACCTTAGGGATAACAGCGTAATTTTTTTTT
TTAGTACAAATAAAAAAAAAAGGTTGCGACCTCGATGTTGGATTAAGATAAAATTTAAATGCAAAAGTTTAAA
ATTTTGATCTGTTCGATCATTAAAATCTTACATGATCTGAGTTCAAACCGGTGTGAGC
CAGGTTGGTTTCTATCTTTTAATAATTAAAATATTTTAGTACGAAAGGATCAAATATTTTAAATAATTTAATT
AAAAGAATATTATTAATAAATTA
>1002049154_1 Heli.1-ES588045.1.5
CGAAAGGGAATCCGGTTCCTATTCCGGAACCCGGCAGCGGAACCGTTTCATTAATCGTTCCCTCGTTTTTGAG
CGAGTGTTCGACGGGGTAACCCAAACTGGCCTGAAGACGCCGCCGAGGGGTCCGGGAAGAGTTTTCTTTTCTG
CCTGAGCGTTCGAGTTCCATGGAATCCTATAGAAGGGAGATATGGTTCGGAACGCGAA
GAGCACCGCATTTGCGGCGGTGTCCGGATACTCTCTGCGGACCTTGAAAATTCAGGTGAGGGATGTACGTGGA
GATGTCGCGCCGGTTCGTACCCATATCCGCAGCAGGTCTCCAAGGTGAAGAGCCTCTAGTCGATAGAATAATG
TAGGTAAGGGAAGTCGGCAAATTGGATCCGTAACTTCGGAATAAGGATTGGCTCTGAG
GACCGGGGCGTGTCGGGTTTGGACGGGAAGCGGATGCGGCCGGTGCCGGGCCTGGTCGATGCTGGTGCGCGTG
TGCGCAGCGTGTTCGCGTCCCTGTAACAGGGGGCGTGCTCCGTTGTCGCAGCGTGTGTCCAGCGGAATCCGGA
CCCGCGTTCCGGCCTTCCGCGGATCTTCCTAGCCGTAAGGCCGTGTCGGTCTCGTCTC
GTGCGCGATCGGCGCGGTTCTGTACGACCGCCGTTCAACGGTCAGCTCAGAACTGGCACGGACAAGGGGAATC
CGACTGTCTAATTAAAACAAAGCATTGCGATGGCCCTCGCGGGTGTTGACGCAATGTGATTTCTGCCCAGTGC
TCTGAATGTCAACGTGAAGAAATTCAAGCAAGCGCGGGTAAACGGCGGGAGTAACTAT

GACTCTCTTAAGGTAGCCAAATGCCTCGTCATCTAATTAGTGACGCGCATGAATGGATTAACGAGATTCCCGC
TGTCCCTATCTACTATCTAGCGAAACCACAGCCAAGGGAACGGGCTTGGGAGAATCAGCGGGGAAAGAAGACC
CTGTTGAGCTTGACTCTAGTCTGGCATTGTAAGGAGACATGAGAGGTGTAGCATAAGT
GGGAGATCGTTCGCGGTCGTCGCTGAAAAACCACTACTTTCATTGTTTCATTACTTACTCGGTTGGGCGGAAG
CGGTGCGCGGTCGATAACAATCGACGGGCGTACGGTGTTTCGTTCCAAGCGTGCAGAGTGGCGGCGTGGCGGC
AACGCTCGTCGCCATACAACTCCCGCGTGATCCGGTTCGAGGACACTGCCAGGCGGGG
AGTTTGACTGGGGCGGTACATCTGTCAAAGAATAACGCAGGTGTCCTAAGGCCAGCTCAGCGAGGACAGAAAC
CTCGCGTGGAGCAAAAGGGCAAAAGCTGGCTTGATCCAGATGTTCAGTACGCATAGGGACTGCGAAAGCACGG
CCTATCGATCCTTTAGTATAAAGAGTTTTTAGCAAGAGGTGCCAGAAAAGTTACCACA
GGGATAACTGGCTTGTGGCGGCCAAGCGTTCATAGCGACGTTGCTTTTTGATCCTTCGATGTCGGCTCTTCCT
ATCATTGCGAAGCAAAATTCGCCAAGCGTTGGATTGTTCACCCATCAAAAGGGAACGTGAGCTGGGTTTAGAC
CGTCGTGAGACAGGTTAGTTTTACCCTACTGATGGCTCGTCGTTGCGATAGTAATACT
GCTCAGTACGAGAGGAACCGCAGTTTCGGACATTTGGTTCATGCACTCGGCCGAGCGGCCGGTGGTGCGAAGC
TACCATCCGCGGGATTATGCCTGAACGCCTCTAAGGCCGAAGCCAGCCTAGCCGAATCCGGCAAGGATACGCT
CACTGTGGAGCCCCGAGAGTCGGGAGGCTCCAAACAATGTGACTTTACTAGTCGCGCT
TTACTCGTAAGGTGCGACGTCGAAGCCCTATTTGGAACGCGGCGATCGGTGCGAGCGGTCTTAACACGTGCAC
CACGGCGCCGAAGTTTCTGATATGACTCAGTTCGATGTCGGGGCTCGGAATAGTCTGTAATTCGGCACGAG
>1002049155_1 Heli.1-DT661904.2.5
GCACGAGGCCTCCGAGGCAGCAGTCTAACAAGAAGTGCAAGATGAGATTTCTGATCGCTTTCGTCGCCATCCT
CGGCTATGCTTCGGCTAGCGCCATCATCTCACCCCTGGTCTTAGGCGCCAACGCTGGTGACGTGCAAGCCGCC
GTCATCGACGCCAATGTAGCCGCCCGCGATTCCATCCGCGCCCTTGGCGAAGGCCAGG
CCCGCGCCGCCGAAGCCGTCATTCAAGCCAACACTGAAGCAGTCCGCCAAGTCGCTGAGAACAACCGTAACAT
CCACGAAAACGCTTACTGGGGATCAGTCGCCGCCTCCCAAAACCTCGTAGCTGCTGCTCAATCTCAGGTCGCC
GCTATCGACGGAGCCGCCGCCGCTGTCCGCGGTGTCCTCGGCGCCCCCATCGTCGCCC
CATTGGCTTTGGCTGGTGGCCTTCCGCTTGCCGCCCCTTACGCTCTCGGAGGTCTCGGTCTCCACGGCATCCA
CGCTTGGTAAATCAAACTGATCCATCACTAAATAGTGATGTACCCGTTTGGGAGTTACCTCGTCCCTAAAGTC
AAGGAAACCTCCAAACTTAACAACTGGCATTGGCAATGTTCACAGCAACAGTGGATAT
TGCCAAACAGTTGTTCCAATGGAAACCGATTGCTCAATGGATTTGTACAAAGGTCTCAACGTAACTCTCTTTG
TAAATAAGTGAATCTCATGCCATCACTGCCAAAGTGTAAAATAAACACAGAAATTTACAAAAAAAAAAAAAAC
TTTCCCGTTCCAATTTTTCTAAATAAATACCTGCTGTGTGAAATTT
>1002049156_1 Heli.1-EE743488.1.5
CGATCCATATCACTTAGTTGACTACAGACCTTGACCCTTAACAGGAGCTATTGGAGTAATAACCTTAGTAACT
GGTTTAGTTAAATGATTTCATAATTTTAATATAAACTTATTATTTATTGGATATATTATCGTATTATTAACCA
TATTCCAATGATGACGAGATATTTGTCGAGAAGGAACATTCCAAGGAAAACATACTAT
TATAGTATCAAAAGGTTTACGATGAGGAATAATTTTATTTATTATTTCTGAAATCTTTTTCTTCATTTCTTTT
TTCTGGGCATTTTTTCATAGAAGTTTATCCCCAAATATTGAAATTGGATCAATTTGACCTCCTACTAATATTT
TAGTATTTAATCCATTTCAAATTCCTTTATTAAATACTATTATTCTTATTACTTCAGG
AATTACAGTAACATGAGCTCATCACGCTTTAATAGAAAATAATTATACTCAAGTTTCACAAAGATTATTTTTA
ACTATTATACTTGGATTTTATTTTACTATTTTACAAGCATATGAATACTTAGAAGCTTCTTTCTCAATTGCTG
ATAGAATTTATGGATCTACTTTTTTTATAGCAACAGGATTTCATGGTTTACATGTAAT
TATTGGAACAATTTTTCTTTTAACATGTTTTATTCGTCATATTAATTTTCATTTCTCAAGAACTCATCATTTC
GGATTTGAAGCAGCAGCATGATATTGACATTTTGTTGATGTAGTTTGATTATTTCTTTATATTTCAATTTATT
GATGAGGAAA
>1002049157_1 Heli.1-DT666816.3.5
CGAGTTGAGGTGTTGTGCAGCAGAACCGAGGCTGTGCAATAGCGATTAATTTATTTATTTAATAGGACTATTT
AAATTAAAACTAATTCACAATGTGCGACGAGGAAGTAGCCGCGTTGGTAGTAGACAATGGCTCCGGTATGTGC
AAGGCCGGGTTCGCCGGGGACGACGCGCCTCGCGCCGTGTTCCCCTCGATCGTGGGCC
GCCCGCGCCACCAGGGCGTGATGGTCGGCATGGGGCAGAAGGACTCCTACGTGGGTGATGAGGCACAGAGCAA
GAGAGGTATCCTCACCCTCAAGTACCCCATCGAGCACGGCATCGTCACCAACTGGGACGATATGGAGAAGATC
TGGCATCACACCTTCTACAACGAGCTGCGTGTGGCGCCCGAGGAGCACCCCGTGCTGC
TCACCGAGGCTCCCCTTAACCCCAAGGCCAACAGAGAGAAGATGACACAGATCATGTTCGAGACCTTCAACAC
GCCCGCCATGTACGTCGCTATCCAGGCCGTGCTCTCGCTGTACGCCTCCGGTCGTACCACCGGTATCGTGCTG
GACTCCGGTGACGGCGTCTCCCACACCGTGCCCATCTATGAGGGCTACGCGCTGCCTC

ACGCCATCCTGCGTCTGGACTTGGCCGGTCGCGATCTCACAGACTACCTGATGAAGATCCTCACCGAGCGTGG
TTACTCTTTCACCACCACCGCCGAAAGGGAAATCGTGCGCGACATCAAGGAGAAGCTCTGCTATGTCGCCCTC
GACTTCGAGCAGGAGATGGCCACCGCCGCCTCCAGCAGCTCCCTCGAGAAGTCCTATG
AACTTCCCGACGGTCAGGTCATCACCATCGGAAACGAAAGATTCCGTTGCCCAGAGGCTCTCTTCCAACCCTC
ATTCTTGGGTATGGAAGCCTGCGGCATCCACGAGACCACTTACAACTCCATCATGAAGTGCGACGTGGACATC
CGTAAGGACTTGTACGCCAACACAGTATTGTCCGGAGGTACCACCATGTACCCCGGCA
TCGCCGACCGCATGCAGAAGGAGATCACCGCCCTCGCGCCCTCCACAATGAAGATCAAGATCATCGCGCCCCC
AGAGAGGAAGTACTCCGTATGGATCGGAGGCTCCATCCTCGCGTCTCTGTCCACCTTCCAACAGATGTGGATC
TCCAAGCAGGAGTACGACGAGTCTGGTCCCTCGATCGTGCACAGGAAGTGCTTCTAAG
CGCGCAGGCGCGCGTTGCTGCCCCCGGAAGCGGCCCGCGGCCCGGTCGGCCCCGCGGCCGGCTGCTCAACTCA
GGCTTGTAATCTCCGTTAATTTAATTGTACGTAAGATATTATTATATGTAATTAAATGGTAACAGTGACGGCA
GGGCGGAGCGCGGCGGGCGCTCCCCGGCCGCCCCCGGGCCGCCGGCCTCGGTACTGCA
CTCGCCAAAGGATTTTGTAATCTCATGAATGATGATTGTATGTGCTGAAACGGATAAGACTACTTTCTTACTA
GGTCATTTATAAATTATAAAATATAACAAAAATACAAGAAAAAAATC
>1002049158_1 Heli.1-DT667044.3.5
GCACGAGGCTTCCTTCTTTTGCGCACGAGGGCCAGGCAGAACTGCGAGAGAACGTGATCGATCGAATTTCAAA
AAAGGCACACTCCTACCCCTGTGCTCCCGAGATCACGGAATTACTTTATATCTCCTGATTGAATAGATCGGGA
GTTCGTGTTTCGTGATTTTGCCATTCCGCGTGTGAACTCGCAGATTCCACCTCGCACA
GTACAGACAGCCCACCTAGTAAAACAAAATGTCGAACCTCGCCGATCCCGTCGCGTTCGCTAAGGACTTCCTT
GCCGGTGGTATCTCTGCTGCCGTCTCCAAGACAGCGGTGGCCCCCATCGAGCGTGTCAAACTGTTGTTGCAAG
TACAGCATGTCAGCAAGCAGATCGCCGAGGATCAGCGCTACAAAGGTATCGTCGACGC
ATTCGTCCGCATTCCCAAGGAACAAGGTCCGCTCTCCTTCTGGCGTGGTAACTTAGCTAACGTGATCAGGTAC
TTCCCCACCCAGGCGCTAAACTTCGCGTTCAAAGACAAGTACAAGCAGGTGTTCCTTGGTGGCGTAGACAAGA
ACACACAGTTCTGGCGCTATTTTGCCGGTAATTTGGCCTCTGGTGGTGCCGCTGGAGC
CACTTCCCTCTGCTTCGTGTACCCTCTCGACTTCGCACGTACCCGCTTGGCGGCCGACGTGGGCAAGGGTGAC
GGCCAGCGCGAGTTCACTGGCCTCGGCAACTGCATCAGCAAGATCTTCAAGTCTGACGGTCTGGCCGGTCTGT
ACAGAGGCTTCGGTGTGTCGGTGCAGGGTATCATCATCTACCGCGCTTCGTACTTCGG
TTTCTACGACACCGCGCGCGGCATGTTGCCCGACCCTAAGAACACCCCCATCGTCATCAGCTGGGCGATCGCG
CAGACTGTCACCACTGTGGCGGGTATCATCTCGTATCCCTTCGACACGGTCCGTAGGCGCATGATGATGCAGT
CCGGCCGCGCTAAGGGCGATATCCTGTACAAGAACACCCTGCACTGCTGGGCCACCAT
CGCTAAATCCGAGGGCGCGTCCGCCTTCTTCAAGGGAGCGTTCTCCAACGTACTCAGAGGCACCGGAGGTGCC
TTCGTACTTGTCTTATATGACGAGATCAAGAAGGTCCTTTAAATCTAATGTAAGAATTATTGTTACCATAATT
TCAATCGTGATTTTGAATCACACTAGCCACTCCCCTGAAATCATGTATTATTTAGACG
CGAAAAACATCCTAGTAACTTATTATTAGTTAAATAAAGTGTTATACAGTGATTCCAGAAAAAAATTGTTTCA
TTAATGTTATGTAATTGTGTACAGAATGATGCTGG
>1002049159_1 Heli.1-DT668400.3.5
TGCGATAGTCGGCAGACATGTCGCACAGGAAATTTTCGGCGCCTCGTCATGGATCTATGGGATTCTATCCCAA
GAAGAGGTCCCGCCGTCATCGTGGTAAAGTGAAGGCTTTCCCTAAAGATGATGCCAGTAAACCGGTGCATCTT
ACTGCCTTCATTGGTTACAAAGCTGGTATGACCCACGTGGTTCGTGAACCTGACCGTC
CTGGTTCAAAAATCAACAAGAAGGAGATCGTGGAAGCTGTTACAGTTATCGAAACGCCTCCTATGGTGTGTGT
CGGTGTTGTCGGCTATATTGAGACACCCCACGGTCTTCGTGCGTTGCTTACTGTTTGGGCAGAGCACATGTCT
GAGGACTGTCGTCGTCGCTTCTACAAGAATTGGTACAAGTGTAAGAAGAAGGCTTTTA
CTAAATCAAGCAAGAAATGGCAGGATGAGCTTGGTCGCAAGTCCATTGAGAAGGACTTCAAAAAGATGATTCG
CTACTGCAGTGTAATTAGGGTTATTGCCCATACACAAATGAAACTGCTTAAGCAGCGTCAAAAGAAGGCCCAT
ATCATGGAGATTCAAGTGAATGGTGGAAATATTGAAGATAAAGTAAAATGGGCGAGGG
AACATCTAGAAAAACCTATCCCTGTTGATTCAGTCTTTGCACAAGATGAGATGATTGATTGTATTGGTGTAAC
TAAAGGAAAGGGATACAAAGGTGTTACTTCACGTTGGCACACCAAGAAACTCCCACGTAAGACACACAAGGGT
CTGCGTAAAGTTGCTTGTATTGGAGCATGGCATCCTTCAAGAGTTTCATTTACTGTTG
CCCGTGCTGGTCAAAAGGGCTACCATCACAGAACTGAAATGAACAAGAAGATTTACAGAATTGGCCAAGGAAT
TCATACCAAGGATGGAAAAGTTATCAAGAACAATGCTTCCACTGAATATGATTTGTCTGAGAAGTCCATCACC
CCCATGGGAGGTTTCCCTCATTATGGTGAAGTAAACAATGACTTTGTTATGATTAAGG
GTTGTTGTATGGGACCTAAGAAACGAGTCATCACATTGAGAAAGTCCCTACGTGTCCACACTAAGAGAGCAGC
TCTTGAGAAGATCAATCTGAAGTTCATTGACACATCATCCAAATTTGGACATGGTCGCTTCCAAACACCTGCT
GATAAGGCTGCTTTCATGGGCACCCTTAAGAAAGATCGTATTCGGGAAGAGGCTGCCG

CATCGGCCGCGCCAGCTGCTGCTCAGTCATAAGTTATAACTTTCCTGAATAAACTGATAT
>1002049160_1 Heli.1-EL596880.1.5
TACAAATCATAAAGATATTGGAACTTTATATTTCATTTTTGGAATTTGAGCAGGTATAGTTGGAACATCTCTT
AGTATTTTAATTCGAATAGAATTAGGTAATCCTGGGTCTCTAATTGGAGATGATCAAATTTATAATACTATTG
TTACAGCCCATGCATTTATTATAATTTTTTTTATAGTTATACCAATCATAATTGGAGG
ATTTGGAAATTGATTAGTACCTTTAATATTAGGAGCCCCTGATATAGCATTTCCCCGAATAAATAATATAAGA
TTTTGACTTCTCCCCCCCTCATTAATTTTATTAATTTCTAGCAGAATCGTAGAAAATGGAGCAGGAACAGGAT
GAACTGTCTATCCCCCCCTTTCCTCTAATATCGCACATGGAGGTTCATCTGTAGATTT
AGCTATTTTTTCCCTTCATTTAGCTGGTATTTCCTCAATTTTAGGAGCAATTAATTTTATTACCACTATTATT
AATATACGAATTAATAATATATCCTTTGATCAATTACCATTATTTGTCTGAGCTGTAGGAATTACAGCTTTAT
TACTTCTTTTATCTTTACCAGTTCTAGCTGGAGCTATTACTATACTTTTAACTGATCG
AAATTTAAATACTTCATTTTTTGACCCTGCAGGAGGAGGTGATCCAATTTTATACCAACATTTATTTTGATTT
TTTGGACATCCAGAAGTTTATATTTTAATTCTCCCAGGATTTGGTATAATTTCTCATATTATTTCTCAAGAAA
GTGGAAAAAAGGAAACTTTTGGATGTTTAGGAATAATTTATGCTATAATAGCAATTGG
ATTGCTAGGATTTATTGTATGAGCTCATCATATATTTACAGTAGGAATAGATATTGATACTCGAGCATATTTT
ACTTCAGCTACTATAATCATTGCAGTTCCAACTGGAATTAAAATTTTTAGTTGATTGGCAACTTTACATGGAA
CACAAATTAATTATAGTCCTTCAATATTATGAAGTTTAGGATTTATTTTCTTATTCAC
AGTAGGAGGATTAACAGGTGTAGTTTTAGCTAATTCCTCAATTGATATTACTCTTCATGATACTTATTATGTA
GTAGCCCATTTTCATTATGTTTTATCTATAGGAGCTGTATTCGCTATTCTAGGAGGTTTTGTTCATTGATATT
CCCTATTTACCGGATTATTATTAAATCCCTATTTATTAAAAATTCAATTTATTTCCAT
ATTTATTGGTGTTAATTTAACATTTTTTCCTCAACATTTTTTAGGATTAGCTGGTATACCCCGTCGTTATTCT
GATTATCCAGATAATTTTATATCATGAAATATTATTTCTTCATTTGGTTCATATATTTCATTATTTTCCATAA
TTTTAATTATTATTATTATTTGAGAATCAATAATTAACCAACGAATAATTCTATTTTC
ATTAAATATACCATCCTCTATTGAATGATATCAAAATTTACCTCCTTCTGAACATTCTTATAATGAACTTCCT
ATTTTAAGAAA
>1002049161_1 Heli.1-DT667413.3.5
GGCTACGCCCGTGTCGCTCCTCTGGTCGCGTGTCTGTTGTTCGCCTGCGTATTAGCTGATGGGCACCGTTGGC
GCCGACAAGCTGATGACTCTAAGAAAGACGAAAGTCTGGAACAAGAGTTATGCAAGGACAAGGACGCCGGCGA
ATGGTTCCGGCTGGTAGCCGGCGAGGGCGACAACTGTCGCGACGTCATCCAGTGTACT
GCCTCGGGTATTCAAGCTATTCGTTGCCCTGCTGGTCTTTATTTTGATATCGAAAAACAAACTTGCGATTGGA
AAGAGGCGGTTAAAAACTGCAAATTAAAAAACAAAGAACGTAAAATAAAACCTCTACTGTTCACTGAAGAACC
TCTCTGTCAAGATGGATTTTTAGCATGTGGTGATACTACGTGCATAGAACGCGGTCTG
TTCTGTAACGGAGAAAAAGACTGCGCCGATGGTTCAGACGAAAACTTATGTGATATTGACAATGACCCTAACA
GGGCTCCTCCTTGTGACACTTCCCAATGTGTATTACCCGATTGCTTCTGCTCAGAAGATGGTACCGTTATACC
AGGTGATCTACCTGCCAGAGAAGTACCTCAAATGATAACTATTACTTTTGATGATGCT
ATCAATAATAACAATATCGATTTATATAAAGAAATTTTCGATGGAAATCGTAAAAACCCGAATGGTTGTGACA
TCAAAGCCACCTACTTTATTTCACATAAATACACGAACTACTCAGCCGTGCAAGAAACCCATAGAAAGGGACA
TGAAATCGCTGTACACTCGATCACACACAATGACGATGAACGGTTTTGGAGCAATGCA
ACCGTCGATGACTGGGGTAAGGAAATGGCTGGCATGCGAGTTATCATTGAAAAATTCGCAAATGTAACCGACA
ACAGCGTTGTTGGTGTACGTGCTCCTTATCTGCGGGTTGGAGGCAACAATCAATTTACTATGATGGAAGAACA
AGCTTTTCTTTATGACAGTACTATCACTGCTCCTCTATCCAATCCTCCTCTATGGCCT
TATACTATGTACTTCCGTATGCCTCACCGCTGTCATGGTAACTTACAAAGCTGCCCCACTAGGAGTCACGCAG
TTTGGGAAATGGTAATGAACGAACTTGACCGTCGTGAAGACCCTACCAATGACGAATACTTACCCGGTTGTGC
CATGGTCGACTCTTGCTCTAACATTTTAACTGGTGACCAATTTTACAATTTCCTTAAT
CACAATTTTGACCGCCACTACGATCAAAACAGAGCTCCCTTAGGACTTTACTTCCACGCTGCCTGGTTAAGAA
ACAATCCAGAGTTTTTAGAAGCATTCTTATATTGGATTGACGAAATCCTTAAGACTCATAATGACGTATACTT
CGTAACGATGACCCAAGTGATTCAATGGGTACAAAATCCACGTACCGTTACCGAGGCA
AAGAACTTCGAGCCATGGAGAGAAAAGTGCGCCGTCGAAGGTAAACCCGCCTGCTGGGTACCTCACTCGTGCA
AGCTGACATCAAAGGAGGTTCCCGGTGAAACGATCAATTTACAAACGTGTGTCAGGTGCCCAGTCAATTACCC
CTGGCTCAATGACCCCACGGGCGACGGTCATTATTAAATGAGATCTCGCGCCTCACGT
GGTGCACATTAGTTGATAAATTGCAATGCAATTGATTGCAAGGACATTATATTCGGAAATGTTATATATAATA
AATAAAAGCCAAGTTTATTCAGTGTTGACTTATTCGATGAGTATGTGTCCGTGTAATTATAAAATGTACTGGG
TCATAATCCCGTCTGTAGTCTAAGTACCTACTTA
>1002049162_1 Heli.1-ES587837.1.5

ACGAGGCATCAACGTAGTCACAACAACATCCTTACAAAATGGTTGCGAAGTTCGTTGTCCTTTTCGCCTTGGT
GGCTGCCGTGAGCGCCGATTTCTCCAGCTTCTCTTATGGAGTAGCTGACCCTTACACCGGTGACTTCAAGAGC
CAAGTCGAGAGCAGAGCTGGTCAAAATGTTTTGGGACAATACTCTTTGTTGGAATCTG
ATGGCACCCGCCGTACCGTTGACTACTCTGCAGGAGCCGAAGGCTTTAACGCTGTTGTAAAGAAAGACCCCGC
TCTCCTCGCCCCCTTAGCTCCCGCTTACCCATATGGTGCCTACCCATATGGTGCTTACCCCTATGGTGCCTAC
CCCTATTCCGCCTACGGATATGCCAAATACGGAGCTCTTCCCTACGGATACCCCTACG
GACGTTACCCAATCGTCTACTAAATAACAACAATCGGCTTTTACCTGCGATTTCAAGTACTTTTGCCATCTTT
GTACTACAAAATTAGGTTATGTAAATAGTGTACATAATAAATTTTCT
>1002049163_1 Heli.1-ES587299.1.5
AACACAGACCACTTACAAAATGTTCAAGCTGGTGGTGTTGTCTTGCGTCCTGGCGGCGACGTCCGCGTCCAGC
CTTTATGGAGGATATGTGGCACCCTTCACCCCATTGGCATACGCTGCACCTTTGTGGGAACCTTCCAACTACC
GTGGACCCCTCTCATTGGCTCCTGGTCAACCTGCCAACATTTTGGGCGCTGATGGCAG
GCCTCTCGACACTTTGGATGTAAACTTGGACCGCTCCGCTCACTTCACAGCCAAAGCTCTCGACAATGGATTC
CACATTCTCAAGAAGCGTTCAGTCGTCTCTCCTTTCATCGCTCCTTATGCTGCACCTTACGTAGCCCCTTACG
CCGCATCATATGTTGCTCCCTACGCTGCGTCTTACGCTGCGCCTTTGGCATACTCTGC
TTCTCTCTTGCAGCCATACAACTACCGCGGACCTCTGTCCCTAGCCCCTGGACAACCCGCCAACATTCTTGGT
GCTGATGGAAGGCCCCTTGACACTTTGGACGTAAACTTGGACCGCTCTGCCCACATCGCCGCTAAAGTTGCTG
CTGGCTCTCACTTCATTAAGAAACGTTCTGCCGCCTTCATCGCCCCCATAAGCACCGT
TGCCGTCGCCAGGTCGCCACTGATCGCCTCATCCTACACATTTGCTGCTCCCCTCGCAGCTGCACGTCTCAGC
CCATTCGCCTACACCCACCCTGTTGCCCGCATCGCACACGTATACTAAATTGACGTCAAAATATCTAAGACTG
ATTGAGATTGAAATAAATGTGTTATAATTTTGAAATTAATAAAATTTTATAAAGTACC
>1002049164_1 Heli.1-DT663049.3.5
GAGGGTCGACTCGACATATTTTCATCAAGGGGAGTAAATAAAAAAAAACCGCCACCATGGACGCGATCAAGAA
GAAGATGCAGGCGATGAAGCTGGAGAAGGACAACGCGCTTGACCGCGCCGCCATGTGCGAGCAGCAGGCCAAG
GACGCTAACCTTCGCGCTGAGAAGGCTGAAGAAGAAGCCAGACAGCTCCAGAAGAAGA
TCCAGACCATTGAAAACGATCTCGACCAGACACAAGAGGGCCTTATGCAAGTCAACGCCAAGCTGGAAGAGAA
GGAGAAGGCTCTACAGAACGCTGAATCCGAAGTTGCTGCGCTCAACCGACGCATCCAGCTCCTCGAAGAAGAC
TTGGAAAGGTCTGAGGAGCGTCTCGCCACCGCCACCGCTAAACTGTCCGAGGCTAGCC
AAGCTGCCGATGAATCGGAACGAATACGCAAGGCGTTGGAGAACCGAACCAACATGGAGGACGACCGCGTCGC
GATACTGGAGGCGCAGCTCGCTCAGGCTAAACTCATCGCTGAGGAGTCCGACAAGAAATATGAGGAGGTGGCT
CGTAAGCTGGCCATGGTTGAAGCTGACTTGGAGCGCGCCGAAGAACGCGCCGAAGCCG
GCGAATCCAAAATCGTCGAGCTTGAAGAGGAACTCCGCGTTGTCGGTAACAACCTGAAATCCCTGGAAGTCTC
TGAGGAAAAGGCGATGGCGTCCCGCGAACACGTCGAGGATAAGATACACAGCCTCTCGCAGAAATTGACGCAA
GCTGAAGCTCGCGCTGAATTCGCTGAGCGCTCTGTCCAGAAACTCCAGAAGGAGGTCG
ACAGGCTTGAAGACGACCTGGTGGGCGAGCGCGAAAAGAGCAAAGTGCTGCAGGAGGAGATGGAGGCCACGCT
CCACGACATACAGAACATGTGAACTAGCACACACACCCCCCACTCCTCCTCCCCCCTACCGCCCTCCCCCGCC
CGCCTCACTACGCCCTGTGAATGTCACGCGCACCTCTAGGGCTGCCAGATCTTGTCTT
ACATGATATTGTAGTTAAATAAAGCTAAAGATGAAAAAAATGCTTAACGATGAATCCCGCATATAGCAGAGTA
AGCAGAAGGGAACTAGTGTTGAGACACTCCGTTCCCAGTGCATAAGTCAATTTTTGACACATTCCATATATTA
CCAGAGCGAGAAAATGAAAATAATTCATAGTATATTTAGGTTACAAATATATATTTAT
ATCATTTATATAGATATTACAAATTAATATATCTAAATGTACACAAATCAAATTACTTCGGTCACGTTGAATG
AATTGAAACTA------------
TCAGTAGAATATCGAGATCTGGCAGCCCCGCGCTGCTCTCCGAGTGACGCTTGTCGAGTG
>1002049165_1 Heli.1-DT666548.3.5
GCGTACGCCATTGCTGGCGTATGGTGGTGTATTATATTAAAAGAAAAATCATAAACCATCCAATATGGTGAAT
TTTACCGTCGACGAGATCCGAGGGATGATGGACAAGAAGCGGAACATCCGCAACATGTCCGTCATCGCCCACG
TCGACCACGGCAAGTCCACTTTGACGGACTCGCTGGTCTCCAAAGCCGGTATCATCGC
CAACGCGAGGGCCGGCGAGACTCGTTTCACCGACACCCGCAAAGATGAACAAGACAGATGTATCACCATTAAA
TCCACCGCCATATCTATGTTCTTCGAGTTGGAAGAGAAGGACTTGGTGTTCATCACAAACCCCGACCAGCGTG
AAAAGAGCGAAAAGGGCTTCTTGATCAACTTAATCGACTCACCTGGTCACGTCGATTT
CTCATCAGAAGTAACGGCCGCCCTCCGTGTAACTGATGGTGCCCTCGTAGTAGTGGACTGCGTATCTGGTGTA
TGTGTACAGACTGAGACAGTGTTGCGTCAGGCCATTGCTGAGCGTATCAAGCCCATCCTTTTCATGAACAAGA
TGGACCGTGCATTGCTTGAATTGCAACTGGAGTCCGAAGAATTGTACCAGACCTTCCA

ACGTATTGTAGAAAATGTCAACGTAATCATTGCCACATATAATGACGATGGTGGCCCTATGGGTGAGGTGCGT
GTCGACCCCAGCAAGGGTTCCGTGGGCTTCGGTTCCGGTCTCCACGGCTGGGCGTTCACCCTGAAACAGTTCG
CCGAGATGTACGCGGACAAGTTCAAGATCGACCTCGTCAAACTTATGAACAGGCTATG
GGGAGAGAACTTCTTCAACGCCAAAACCAAGAAATGGTCAAAACAGAAGGACAATGAGAACAAGCGTTCATTC
TGTATGTACGTCTTGGACCCCATCTACAAGGTGTTCAGCGCCATCATGAACTTCCGTAAGGAGGAAATCGACA
GCCTCCTAAAGAAAATTGGAGTCACCATCAAGCACGAAGATGCTGACAAGGATGGCAA
AGCTCTACTTAAGGTAGTGATGCGTACTTGGCTGCCAGCCGGTGAAGCTCTGCTTCAGATGATTGCCATCCAC
TTGCCTTCCCCCGTGGTCGCGCAAAAGTACCGTATGGAGATGTTGTACGAGGGACCCCATGACGACGAAGCCG
CTATTGGTATCAAGAGTTGTGACCCTGAAGCTCCTCTTATGATGTACGTGAGCAAAAT
GGTACCGACCTCTGACAAGGGTCGTTTCTACGCCTTCGGACGTGTGTTCTCCGGAAAGGTCATCACAGGACAG
AAGGCCCGCATCATGGGACCTAACTTCCAGCCCGGCAAGAAAGAGGATCTGTATGAGAAGACCATCCAGCGTA
CCATCCTCATGATGGGTCGTTATGTAGAGGCCATCGAGGATGTGCCCTGCGGTAACAT
CTGTGGTCTGGTCGGTGTCGACCAGTTCTTAGTCAAGACTGGTACCATCACCACCTTCAAGAATGCACACAAC
ATGAAGGTCATGAAATTCAGTGTATCACCCGTCGTACGTGTAGCCGTGGAGCCCAAGAACCCCGCCGACTTGC
CCAAGCTGGTGGAAGGTCTTAAACGTCTCGCCAAATCTGACCCTATGGTGCAGTGCAT
CAACGAGGAATCCGGCGAGCACATTGTTGCTGGTGCTGGAGAACTCCATCTTGAAATTTGTTTGAAGGATCTT
GAAGAAGACCATGCCTGCATTCCAATCAAGAAGTCCGACCCAGTTGTATCATACCGTGAGACAGTCAGTGAAG
AATCCAACCAAATGTGTCTTTCCAAGTCACCCAACAAGCACAACCGTCTGTTCATGAA
GGCCACCCCCATGCCTGACGGTCTTCCTGAAGATATTGATGAGGGCCGCGTGAACCCACGTGACGACTTCAAG
ACCCGTGCCCGTTATCTTGGAGAGAAATACGAGTACGACCTGACTGAGGCGCGTAAGATCTGGTGCTTCGGTC
CCGAGGGCACAGGACCCAACCTCATGGTCGACTGCTCCAAGGGAGTGCAGTACCTCAA
CGAGATCAAGGACTCAGTCGTCGCCGGCTTCCAGTGGGCCGCCAAGGAGGGAGTCATGGCTGAGGAGAATCTG
AGAGGAGTTAGATTCAATATTTATGATGTCACCCTACATACTGATGCTATCCACAGAGGTGGCGGTCAAATCA
TTCCCACGACGAGAAGATGTCTGTACGCGTGTCTGTTGACCGCCGCGCCCAGGCTCAT
GGAGCCCGTGTACCTTTGTGAAATTCAGTGTCCTGAAGTCGCCGTGGGTGGTATCTACGGTGTGCTCAACAGA
CGGCGTGGTCACGTGTTCGAAGAGTCCCAAGTGGCCGGTACCCCCATGTTCGTGGTGAAGGCCTACTTGCCTG
TAAACGAATCGTTCGGCTTCACCGCTGATCTCCGTTCCAATACTGGAGGCCAGGCCTT
CCCGCAGTGCGTGTTCGACCATTGGCAGGTCCTGCCCGGTGACCCGTGCGAGGCTGGTAGCAAACCCTTCGCT
GTAGTACAGGAAACAAGAAAACGGAAAGGATTGAAGGAAGGTCTCCCAGATCTAGCCCAATACTTGGATAAAT
TGTAAACGCCGTAGCTAATGCAATACTGTAATAT
>1002049166_1 Heli.1-ES586804.1.5
GTCCGAACAACACAGACCAAGATGTTCAAGTTGGTGGTGTTGTGCGCTTTCCTCGCCGCGGCTGCTGCTGAGC
CCGGAACCTTCATCACGCCATTGGCATACTCCAGCAGCGTTGTCGCGCCGGCTGCAACAACTATCACCCATCA
GGCCAGCAGTGTCATCCATCCTTCACCCCTGCTCTACCCACGATTCTACGACCTATCT
TACTCACCGGTTGCCCATCTTATCAAGAAGCGATCCCCTGGAATTTACAATCCCTTCTTTGCACCATCAACTT
ACATTACTTCTGCACCTCTGGCCACGACTTATGCTGCAGCTCCTCTTGCTACTACCTACACTGGAGCTCACTT
GGCTACAACTTATGCTGCACCAATCTACCCTGCTGCTGCGCATTTAATCAAAAAGAGG
TCTGCTCCACTTGTGCCCACAACCTACGTTGCTCCATACTACTCTTCTCCTTTGTTGACGCCGACGTACACAG
CCGCTGCACCCATCTACTCAACACCTTACATCACGAGTGCTCCTCTTACTTACACACATTTGATCAAGAAGCG
TTCCGCCCCTTTGTTCTACGCAGGTTACACCGCTCCCGCTTCATTCTCTCATCAGTCT
CGACTAGACATTAAGAGCTCCCCTGCCACTATTACGTCCTACTCCTACCCTGCTGCTGTATCTTATGCTAGTC
CAGTCGCAATTTCACATGTCTTCTAGATATTTTACAATTGTAAATAAATAAACTGAATTGATATCATTTCA
>1002049167_1 Heli.1-DT667036.3.5
GGAGTTTCTCGGCTGGTGTCGTGTACGACCTGCCTGGTTATATAAATAGGTGAAGAGGGGCAGCAGACACCTC
TTGCACTTTCTTGTAGAGCGCGACGCACCGGCCAGTCTTTGCCGAGCGCCATGGCTCGACACGCGTACCTGTG
TTTAGGGGTATTACTTCTCGCACACTTTGTGATTAGCGAGACACGAGTGAAACGGCAA
GATGAGGATAACGACGATGAAGTGAATCCGGAAAAGCTTTGCGACGGTCGCCCAGCCGACGAGTACTTTAGGT
TATCTGTTGAAGGTGACTGCCGAGATGTCGTCAGGTGTGACAAGGGAGGTGAAAATGGTGTGACTAGGCTAGC
TTCTGTGCGCTGCCCCGGCGGACTGGCTTTTGATATCGACCGACAAACCTGTGATTGG
AAAACACATGTTAAGAATTGCGATAAATTAGAAAAACCAAGAAAAATATTACCCATCTTGAAAACTGATGAAC
CAATATGCCCTGAAGGCAAACTATCTTGCGGAAACGGTGATTGTATCGAAAAAGAATTATTCTGTAATGGAAA
AGGTGATTGTAAGGATGAGTCGGACGAAAACGCCTGCACTGTTGACTTAGACCCTAAT

AGAGCGCCAGATTGTGACACCAATCAATGCAGACTTCCCGATTGTTTCTGTTCCGCTGATGGCACACGGATTC
CAGGAGGAATAGAGCCCAATCAGGTGCCTCAAATGATAACGATTACCTTTAATGGCGCCGTAAACGTTGACAA
CATTGACTTATATGAGCAAATATTTAACGGTAACCGTCATAATCCAAATGGCTGTCAA
ATACGCGGAACTTTCTTTGTCTCACACAAGTACACAAACTACGCAGCCGTTCAAGAATTGCACCGCAAGGGAC
ATGAAATCTCTGTTTTCTCACTAACACACAAAGATGATCCACAATATTGGACAAGTGGAAGCTACGATGATTG
GTTGGCTGAAATGGCCGGTGCAAGACTGATCGTAGAACGATTTGCAAATATAAGTGAT
GCTTCTATTATTGGTGTTCGGGCCCCTTACTTAAGAGTCGGAGGTAACAAGCAATTTGAAATGATGGCAGACC
AATACTTCGTATATGATGCTTCGATAACAGCTCCCTTAGGTCGCGTTCCTATTTGGCCTTATACATTATACTT
CCGAATGCCACACAAATGTAACGGTAATGCTCACAATTGCCCCTCTAGAAGTCACCCT
GTGTGGGAAATGGTAATGAACGAATTGGACAGACGTGATGACCCTACTTTTGACGAATCTTTGCCCGGTTGTC
ATGTAGTCGATTCTTGTTCCAACATTCAAACTGGAGATCAATTCGCGCGTCTTCTTCGCCATAACTTCAACCG
TCACTACTCTACCAACCGCGCTCCTTTAGGTCTTCACTTCCACGCGTCGTGGCTTAAA
TCCAAGAAGGAATTCAGAGATGAACTAGTTAAGTTCATTGAAGAGATGTTAGAAAAGAATGATGTTTACTTTA
ATTCTTTCACACAAGTAATGCAATGGATGCAAAACCCAACAGAATTATCACAACTTAGAGACTTCCAAGAATG
GAAAGAAGACAAATGTGATGTTAAAGGGCAACCTTTCTGCTCTTTGCCAAATGCTTGT
CCATTAACAACTCGTGAGTTACCAGGAGAAACTTTACGTTTATTCACTTGCATGGAATGCCCTAATAACTATC
CTTGGATTCTAGATCCTACGGGAGAGGGCTTCAACGTTAGGAAGTGATCTCGTTAAGTAAAATATTAAAAGAC
TGTACACATGTAAATAATACTCAATAATCTTAATGAATACAATTTATATTGTGACTGT
GGCAGATAAAACCAATGTCAACTATTAAGTGATCGTTAAAATTATTTTTGTTAAGGCTAAAACATTAAAATAT
ATATTTTTTTATTCTTAACGTTGATATTTGGTTCATATTATAATTTAAATGCTGCCGCTCGATGTTTACCAGC
AAAGATAGATAAACTTTCATTACGTAATTTAAATTGTTACAATCTTATCAACAATAAT
AAAAAATATAAA
>1002049168_1 Heli.1-EL599996.1.5
TTACATTTAATAAACGCTGTGTTTCGGTTTTTTGTTTTTTTTGTTAGTTTATTGTTCAGCCAGCTATAAAAAA
AATGATTTGCGGATCCCCAGTGTTTCTGATCTTGATACATCTCAAGGGTCAATAGGAAACATTGGTGGCAACG
CGTTGGGCGGCATTCCTTTGGGTAACTTGGGTCTTGGAAATTTAGGAGGTCTTGGACT
ACCATTATTGGGCGGCGGAGAAAATTCTGTTGGCAATATCGCTGCTATTCTTCAAGCAAATGCGGGTTCCAGC
GACGGCGTAGGCAATGTTGCGAATATAGCTCAAAGCAATGTATCGGGCTCTGGAAATAACAACGGTAACAATG
GTGAAGGCGAAAATGATAACAACAATAATCAAAATGAGCAAAATGGTGCAATATTGTC
TATAGGAAACATTGCCGGCGTTATTCAAGCCAATGTAGCTTCCGGTAACAGTGATCCAAATGGTAACTCAGAA
ACGAATGGTGAAAATATTTCTAACGAAAACAATAATTCCGGTGGCAGTATTGGGAACGTCGCTAATATCGCAC
AAAGCGCCGCTAATACCGCACAAAGCGCCGCAGGAGGAAACTCGCACAATTTGGCTAA
TTTGGGCAACATTCTCGGCAATCTAGGCAATTCTGGCTCAAGCGCTTTGAATAATAACGGTGGTGGCGTAGTC
ACTATCGGTAACGCCGCTGGAGTCACACAAGCCAATCTTGGCAACTTTGGCAGTTTGGCTAATTTGGCCAATA
TACTTAGTGGCTTAGGCAATTCTGGCAATTCCGATCCAAATACTAACAATAATTCCGC
TGGCAGTAATGGTAACATCGCTAATATTGCACAAAGTGCTGTGGGACCAAGCACGATTGGCAACGTCGCTGGT
ATTGCGCAGACCAACGTTGGAAAATCGCAAGGAAATATTGGCAACATAGCGAATATAGCCCAAAGTAATGCTG
CTAGAGGAAACAGCGGCATCAAGGGAAGTTCAGGCATAATAAGTTGGGCTGACGTGAT
TAATTATGTAAACAGTAACCGCCAAAACAATAACAACAATAATGGAGGTGGCATCGGTAACATTGCAAATATC
GCCCAAAGCAATATTGGCGGCGTCGGTGTTGGTAACGCTGCTGGCATTGCCCAGATAAATGGTCTTCTTGGTC
TCATCGGTCTTGGCGGCGTCGGTGTTGGTAACGCTGCTGGCATTGCCCAGATAAATGC
TAATGGTCTTCTTGGTCTCATCGGTCTTATCAATTACAACGAGATCATCGAAAGGCCTTGTCAAGTGTTCGAT
ACATACTGCATAAAGAAGTATTTCGCTGAACACTCTAAATGTAAAGTAGCGCACGAGCCGGTACCAGATCCTT
TGTACAGACATCAGTCCACTATGTTTTTGGGTAGAATAAATGTTACGACGACTGCGAC
TGACGTTTTGTATGGAGGTCTAAATGGGAGAATTGAAGATTTCTACATCAACAGAGAAACTGACAGACTAGTG
CTGACTATTGAATTTAGGAATTTGACGTTCTACTCTAAATACGCTTACTTCAGATTCCACAGACGAGCCAGAG
AACCAATTGTCACCAGGGACTACTTATACTTCAATTACCAGTCTGTGGTATCTACTAT
AATAATACCAAACATAAAGGACCTGCAATTGGAAAAGAGTGAGACTTTCATATATAGTAACGACCGTACGCCA
ACATTCAGTATCGGCCCCAACGGATTTAATAGTCCAGACCCCACAGTCCAGCTCAGCAGGATCCAGTTGCTCG
AAGACGTTACCACCATTGGTCAAGAGGCCCAACTTACCGAGGGCGCATACTACACATC
TACGTACTTACAACATAGTATTTGTGACTTTGGTCTTAAAATACTTTAAAATCACGATTTAAAATATCGAGAA
ATAAAGACATTTTTGAAATAAAA
>1002049169_1 Heli.1-DT665713.3.5

CACGAGGAAGGAAGTGACTATTGCTGTTATACAAAAGGCATTATTTTGAAATCCGACTGAGAATATTTCTATC
AAGCTAATATTTGAGAAAGATTCATCATGCAAATTTTCGTAAAAACCTTAACCGGGAAAACCATCACTTTGGA
AGTGGAAGCTTCTGATACAATCGAGAACGTAAAGGCAAAGATTCAAGACAAGGAAGGC
ATTCCTCCAGATCAACAAAGACTAATTTTCGCTGGAAAGCAACTGGAAGATGGGCGCACACTTTCCGATTATA
ATATCCAGAAGGAATCTACTTTGCACTTGGTGCTGCGTCTTAGGGGTGGAATGCAAATTTTCGTAAAGACACT
GACTGGCAAGACTATCACTCTGGAAGTTGAGCCTTCTGATACAATTGAAAATGTAAAA
GCAAAAATTCAGGATAAAGAAGGAATCCCCCCAGATCAGCAAAGGCTTATTTTTGCTGGAAAGCAGCTTGAAG
ATGGACGCACACTATCAGACTACAACATTCAGAAGGAATCTACTTTGCACTTGGTACTACGCCTGAGAGGTGG
AATGCAAATATTTGTTAAAACATTGACAGGCAAAACTATCACATTGGAAGTGGAAGCT
TCTGATACAATCGAAAATGTAAAGGCAAAGATTCAAGACAAGGAAGGTATTCCTCCAGACCAACAAAGGCTTA
TTTTTGCTGGAAAGCAACTGGAAGATGGGCGCACACTGTCAGACTATAATATCCAGAAGGAATCTACTTTGCA
CTTGGTGCTGCGCCTGAGAGGTGGAATGCAAATTTTTGTAAAGACACTGACTGGCAAG
ACTATCACCTTGGAAGTGGAAGCTTCTGATACAATTGAAAATGTTAAGGCGAAAATTCAAGATAAAGAGGGAA
TTCCCCCAGATCAACAAAGACTTATTTTTGCTGGGAAGCAGCTTGAAGATGGACGCACACTATCTGACTACAA
TATTCAGAAGGAATCTACTTTGCACCTGGTACTACGGCTGAGAGGTGGAATGCAGATA
TTTGTAAAAACTCTTACCGGTAAGACTATCACTCTGGAAGTTGAGCCTTCCGACACAATCGAAAATGTTAAGG
CAAAAATTCAAGATAAGGAAGGCATTCCTCCAGACCAACAAAGGCTTATTTTTGCTGGAAAGCAGCTTGAAGA
TGGACGCACACTCTCCGACTACAATATACAAAAGGAATCTACCTTACATTTAGTACTG
CGTCTGAGAGGTGGGATGCAAATTTTTGTGAAAACACTGACAGGAAAAACTATTACTTTAGAAGTAGAAGCTT
CTGATACTATCGAAAATGTTAAGGCAAAGATTCAAGACAAGGAAGGCATTCCTCCGGACCAACAAAGACTGAT
TTTCGCGGGAAAGCAACTGGAAGATGGGCGCACACTCTCAGACTATAATATCCAGAAG
GAATCTACGTTGCACTTAGTGTTGCGTCTAAGGGGTGGAATGCAAATATTTGTTAAAACATTGACAGGCAAAA
CTATCACATTGGAAGTGGAAGCTTCTGATACAATCGAAAATGTTAAGGCAAAGATTCAAGACAAGGAAGGCAT
TCCTCCAGACCAACAAAGACTGATTTTCGCGGGAAAGCAATTGGAAGATGGGCGCACA
CTCTCAGACTATAATATCCAGAAGGAATCTACGTTGCACTTAGTGTTGCGTCTAAGGGGTGGAATGCAAATAT
TTGTTAAAACATTGACAGGCAAAACTATCACATTGGAAGTGGAAGCTTCTGATACAATCGAAAATGTTAAGGC
AAAGATTCAAGACAAGGAAGGCATTCCTCCGGACCAACAAAGACTGATTTTCGCAGGA
AAGCAACTGGAAGATGGGCGCACACTCTCAGACTATAATATCCAGAAGGAATCTACGTTGCACTTAGTGTTGC
GTCTAAGGGGTGGAATGCAAATATTTGTTAAAACATTGACAGGCAAAACTATCACATTGGAAGTGGAAGCT
>1002049170_1 Heli.1-ES586508.1.5
TCGAGAGGACATACAACCATGTACGCTAAACTGTTCATCGTCTGCATCGTCGCCGTGGTAGCGCTCGCCCGCG
AGTACCCGGCCGGTCTCTACCCTGCTTTGTGCCCCAACTACCCCTTCTGTGACTCTAACACCTTAGCTAGATT
CACCCCAGATGGCATGCCTATCCCTGAATGGGTTTACAACCCCTCCATCCTACCTGTA
GCGCCTGCTGACCCCAATGCCAATATTGCCGCTAAATACCCCGCCAACTTGAACCCCGCGGAATGCCCCAACT
ACCCCTACTGCTGGTGAACAGTTTAGTCGCAGCGTGCCTTCCGGATTTACTTGTTAATGACAAATTCTACAAA
ACATCAACTTGGTCTTTTTTTTCTTCATCTGTGGCAATAGCCTCATGTTTAGCAGAAA
ACTACGCTCATTAACAATGTATTGCCATATTTTTCTGTACCTTATTAAATTTGTACATACACATTTTTGCTCA
GCATCTTGTTGTTTTGTAAAAAATATGTAAAAGCTGTAAATAAATACGGTATAATAAATATTGTTTTTGAAAC
TTCATTCCCTAAATATTTACTAAGTAATTATTTTATGTCAAAATTTTCAAATATATAA
GTATCAATAGAATTTCGCATACAAAAATCCTTAGAAATATATAAAAATAATTTAATATCAATAATACTTAAA
>1002049171_1 Heli.1-DT666684.3.5
AGTCGCACGCTCCTGTTATGCTAAAGTGGTGAACCCACGCTACAAACATGGAGGGGACCATCCCCAGCCAAGT
CATGCCTGAGCCCGACGAGGGTATCTCCACCGACCGCCGCCCGTCCACCGACGACCATGCCGACCTGTCCGAC
ACGGCGTCCGAGGCCGGCTCTGGTGGAGGCAAAGACTCCGGCTGCGAAGTCGCTCCGG
ATCCCCAGGAACCTCCCTACACACCACCCGACGAGGAACTGGCCAACCGCATCGTGTCTCAAGTGGAGTTCTA
TTTTTCGGACGCAAATATCACTAAGGACGCCTTCCTGTTGAAGCATGTCCGTCGTAACAAGGAAGGGTATGTC
TCACTCAAGCTCATATCCAGCTTCAAACGCGTCAAACACCTGACCAAGGATTGGCGAG
TGGTCGCTGAAGCTTTGAAGCGATCGACAAAATTGGAAATAAATGAACCGGGAACGAAGCTTCGTCGAATTGA
CCCCCTGCCCCCGTACGACGAAACTACCCCGTCCAGGACTGTTGTGGCAGTCAAAATGCCCATAGATCGCCCT
TCAGTCGAAAACGTTTCAAGATTATTTGCTAGTTGCGGTGAGATAGCTCTGGTACGTG
TGCTGCGGCCCGGTAACCCAGTCCCAGCGGATGTCCGTCAATTCCTCAACAAAAATCCTAGTTTGGTGAATTG
TGTGTGTGCTTTGGTAGAGTTTACTGAATCTGAAGCTGCGAGAGGTGCCCTTCGTCTTCAAAGTTCTGACGAG
GAAGGTATGCGAGTGTACGAACTAAACGGTGTCCCTCGGGAACCTAAGAGGAAAGTTC

CGGCGCGGCGTCCAGCGACGACAAGACGCCACGAATGCGAGTACTCCTCATGTTGTAGCGGTTCGGAGGCCGA
GTACGACTATAGATACACAGCACCATTTTATAGACGGAACTCGAGTGGATTCTTCACACCACGGTCCCCGGAA
ATACAAACATGGGTGCCACGCAGAACCTCAACTTGCAGTCATAGTTCAGATTCTGGGG
TTTCTTTCTTCTGCGGCTCGCGGAGGGCGTCACAGGCTAGCACCGGAAGTGCGAGCAGCGCCGAGGGCTGGCT
AGCACGGAGGTTGTCCGGTTGTTCAATAACCGGAACCGAATGCGGCGGAAGGAGGTTGTCGTGCACGCCACGA
TTCGAACCCCGCACGCCGGTAGTCCCGGACGGCACGCGCGGCTTCCACGCCGCCGCAC
GCCAACGCAGAATCTCGGACCTCGCGTTGTATTCGCGCTAGAAAGCGATCGCCAACCGTGACACCTTTCTAAT
AACGTTGGATAACCACCTTTCACGTTCTGGCCACTTGGTTTATAATCTACGAGGAAGCGTTTCGTGTTCGCGT
GCTTAGCTCTAAATTTTAATATAAAAATATAATATAGTCGGTATGCTGGAATGAAACT
GTAAGGTAATGAGTTACCTAGTTAGACGCGACGGTGAGTCGACGTAATGTGATCGAAGCTCATTTAGTTAGTC
TAGTTGTTAATTTGTAACAGATATTCCTTTTAATTTATTAGAATATAATTTATTTATTGTTATTGCCGCGTAT
TTATTGCGGACAGGTCGCGCGCTGTGGGGAGTTTTCATCAGAGATTTCATGTCTGAGT
TGTGAGCACAAAACTCAAAAAGCAAAATCGACATCTGAAACTGAACCTGTAGACTCGACATATGGTACGGCGG
CGGCCGGAGACCGCGGCGAGAGCACGTTGCGTAAATAGGATTGACGATTTATATGGATG
>1002049172_1 Heli.1-DT666410.3.5
TATTGAACGAAAATAAAGGCGATGGTGTGGAAGATTATAACATTTTTGGGCATGGCGCTTTTGGCGCTAAGTG
ATGCCAGAGCTCCTATATCAAAACAATCTGTAATAATTGAAAATTATGAAAAGCTTTCACACGTTGAACCTCA
AAAGCCTGAGCATCATGAGGTATACGCTTGGGCGTATCCCTCTTATGAATTTAAGTAT
GAAGTTGCTGACCCTCACACTCATGACTTCAAAGGACACCATGAAACGCGACATGGTGACGAAGTAAATGGAA
AATATTGGCTTATTCAACCAGACGGATTTAAACGAACCGTCAAGTACTACGCTGACAAAAACAGCGGTTTCAA
AGCAGATGTTGACTATAATAAGCTATATCAAAAGTCAGAAGAAAATAACGAAGGAAAT
GAAGAAAATGCAGGTGGTGAGGAAGAAAATACGACCGAGGAAAGTAGTTCTATAGAAAATGAAAATAGTAATA
ATGAGGAGGAAAGTACAAATGAATCACTTAATAGTAATAACGATGATGAGTCCACGAGTGTTGGTGGAGAAAG
TGAAAATGAAAACAATGACATGGAAGACGGAGCTGGAGGTGAAGAATCTAATGATGAA
GGAAATGATAATAGTGAAGAAAACGCACAAGAAACAGAATCCAGTCAACAGAACGGTGAAAGTGAAAACGATG
AAGAAAGTAATGAAAATGAAGGGAGTGATAATGGTAACGAGGAAAGTTCTGCTGTTGAGAATAAAAAAGAGAA
GCATGTCGTGAAACATGAATATCATTTTATAATCCATCATCCAAAATATATTAAAGCT
AATATCTGGGATTGAATAATTTAGCTATGTAAACAGTTACTAATATCCCGCCAAGAGTGTAACTATAGTGTAT
TGTGTTGTAAGATTTAACATTTA
>1002049173_1 Heli.1-EL598039.1.5
TTGATACGGTGGTGTGTAGCGGAACAAGTTAGCGATGTCTCGACTTACGATTCTCTTTGTCATCGTTGCGGCG
TACGAAGTACTTGGAGATGTAAGTAAAGTTGCCGATTCTAGCGCAGCTCAGAGTGGAATAGTTGGATATGGCG
CTGGTATTGGCCTCGGTGTGCCTGCAGCGGGCATCACCAGCGGTGTTATCGGATCACC
TGGCGGAGTCCTCGTGGGATCTGGCCTCTACGGTGGAGGTGGTGGCTTAAATAATGCTGCCGCTGTTGGTGCT
GCTCAGCTTAGCGCTATCCAAAACGCTCAAGCTGTCCAGGCTGCACAGATCGCAAATGCAGCCGCTGCTGCCA
TCCAAGGAGCACGTGTGACTGAAGCGGCCGCTAGGGCAGGCCAAGCTAACGCTATTCA
GAATGCTCAAGCTTTAGATGCAGCTCGTATTGCTAACATACAAAGAGCCCAGGCCGCCGCCTACGAAAATGCT
CGTGCCGCTGAAGCAGCTAGACGTGCTGCTGCTGCTGCAGCTGCTGAAGTTGCTCGTGCTGTTGAAGCTGAAC
GTGTAGCCAATGCCGCTCGTGTCCAGGCTGCTGCTATCGCTAACGCTCGTGCTGTTGA
AGCCGCACGTATTGCTAATGCTGCTAGAGCCCAAGCTGCCGCAGTTGCCACTAGTGCTGCTCAAGCTCAGGCC
GTTGCTGACACCGTAGCAAGAAATGCTCAAGATGGCGCTTTATTACTCGGTGGAGGTGGTGGTGGTGGTTTGT
ACGGCGCAGGTGGTCTCGGTGGTGCAGTGATTGCAGCAGCTCCTGTGGCCGTCGAAAA
AGTCGCGGCAGTTCCCGTCGGCATCGGATACGGACTTGGTGACGGTCTCGGACTCGGTCTTGGTGGCCTTGGC
GGTCTTGGCGGCCTTGGCGGTCTTGGCGGCCTTGGTCTTGGATATGGAAAGAAGATATAGAATAACGATTTCT
GCATATTGTATTAGTGGTGTTCCCCAATAGATAGAGATATGTAATTTCCCCTTCCCTT
TTGACTCCATCCAAGGAAATGGCTAGGGGCATTTTTTTGTTATTTTGATTAAATAAGTAC
>1002049174_1 Heli.1-DT666012.3.5
CACGAGGGTCTTTTTACTCCTCCGACGAGGGGCGGATGGTCTTTACCGCCGCATTTAGTGTTTATTTGGAGGA
ATAACTTCGCTGATATAGCGAAATGAGTCTATCAGTAGCCCGACCTTTAGTGTCGGTATACTCTGAGAAGAGT
GAGAAGGTGGCCGATGCCTCGGTCCCTTTGCCGTTCGTTTTCAAGGCCCCCATCAGGC
CTGACCTGGTCAACAATGTCCACGTTTCTATGTCCAAGAATGCAAGACAACCTTACTCTGTTAGTAAGGAGGC
AGGTCATCAAACCAGTGCTGAGTCATGGGGCACAGGACGTGCTGTAGCGCGTATCCCCCGTGTCCGTGGTGGT
GGTACCCATAGATCAGGCCAGGGTGCGTTCGGCAACATGTGCCGCGGTGGTCGCATGT

TCGCACCAACCAAGCCGTGGCGGCGTTGGCACCGCCGCGTTAACCTACGCCAGCGCAGGGCTGCTGCGGCGGC
CGCTGTTGCCGCAGCTGGAGTGCCAGCGTTGGTACAGGCCAGAGGTCACATCATCGAAAAGATTCCGGAACTT
CCACTTGTGGTCTCCGACAAAGTGCAAGAGATCAGCAAGACTAAAGATGCTGTTATCT
TCTTAAGAAGAGTTAAAGCCTGGTCTGATGTCCTTAAGGTGTACAAGTCACAGCGCCTGCGCGCCGGCAAAGG
AAAAATGCGCAACCGTCGTCGCGTGCAACGCAAGGGACCCCTCATCATATACCACAAGGATAAGGGTCTATCC
AAGGCTTTCCGCAACATCCCCGGCGTAGAAATGCTGAGCGTGAATAAACTGAACCTCC
TGAAGCTGGCTCCAGGTGGTCACGTGGGCAGGTTCATCATCTGGACACAGTCCGCTTTTAAGAGACTAGACAG
CCTGTTTGGTTCATGGAAGACACCATCCAAGCTGAAGAAGAACTTCAACCTGCCTCAGCCCAAGATGGCCAAC
ACCGACCTCTCGCGCCTGCTCCGCTCCGACGAGATCAGGAAGGTGCTGCGTGCCCCCA
ACAAGAGGGTAGTCCGCTCCACCAGGAAATTGAACCCGTTGACCAACTCGCGGGCGATGCTGCGTTTGAACCC
GTACGCCGCTGTGCTCAAGAGGAAAGCTGTGTTGGACCTGGAGCGCAGGAACAACTTGAGAGTCGCGGCCGTT
GCTGAAAAGAGAGGGATCAAACTCCCCGAATCTGACCCAGCAGTGAAGGCACAGAAAC
TGAGAGAGAAGAGAGCCAAGTCAATCAAGTTGGCTCTCTCCAAGAAACCCAAGAAGCCGGCAGCGAAGAAGAC
CGCGCCTCCGCCTAAGAAGGTGAAAAAGGAGAAGGCGGATAAACCGGAGAAGAAAGCGGTGTCTAAAAAGTGA
ATTGAATAAACATTTTAATTGACAAAAAAAAAAA
>1002049175_1 Heli.1-DT664918.3.5
GGGTGGAGCGTTTGCTCGTTGCGGGGTCTAGACTGTGGTTTAGGAAAGTTTAGTTTTATTTTATAAATAAAGA
ACTCAATCAAAATGAGCTGGCAAGATTATGTCGATAAACAGTTAATTGCATCCAGATGTGTTACAAAGGCTGC
TATTGCCGGTCACGATGGTAATGTCTGGGCCAAATCAGAAGGATTCGATATATCAAAA
GAAGAAGTAGGTAAGATAGTGGCCGGTTTCGAAAATGAATCACTCCTCACCAGCGGCGGCGTGACGATAGCGG
GCACGCGGTACATCTACCTTAGTGGAACAGACCTCATCATACGCGCAAAGCTCGGCAAGGTCGGCGTGCACTG
CATGAAGACAAAGCAAGCTGTTGTCATTTCTCTCTACGAAGAACCGATCCAACCCCAG
CAAGCTGCATCTGTAGTGGAGAAGTTAGGAGATTATTTAATTACCTGTGGTTATTAAAGATCGAAGCGCGCCG
TGATCTAAGAATACTATAAGATAATATTTTTTCCACAGGGAAATAAGTAACTGCATTTTGTATATTCTATAAA
AGTGATAAGGACCGTCATTTGTTTTGATATTTGATAAATTATGGCGCCATCAGTGTCT
GATGCGCTCGGCGGCCACGTGCAGGCGTAATTACTTTGCGGATTTTGGACTCTTTTTATACGCTCACCTTTTC
CACAGAGTGATTGTTTCTGTATTGTGGTTTTTATTTGTATTTTAATAAAAAATGCTTTTATCGACCTATACAG
TGAGGCTGAACGGAGCTTTGCGAAGGTCTCGAGGCATGATGTATAGAGATTTTTTTTA
GATTTCTTTTCAAGCTGTCTGACAACTTCACTCCGCGCCACCGCGGTCGCTCTGACCCGGTGAATGGCTCAAC
GTGGAACAAATGATAGCTAAATCAATGAAATCGCACACAGACAAGCTTGATAAGAGATAAAATTAAATAACCA
CGTAATGAAATTACTGTAACAATATACAACAATTATATATGTAATTACAAATAGGCAT
TTGTGTAAGGGCTTGTTACAAAATGAGAGACACTGTAACCCAATTAAGTAAACATGTAACGATTTTAAATGAA
AATCTCTAGTCCCACTGGAACCAATCGTGTGAGAATCTATAACTACAGTGTGCGGGGAACCCCCGCGATCGCC
ATTATTTCGCTATGCTTCCCTTTTTTTTATTACGCCGCGTTTATAATCTTGTGTAGTG
AATAAACATTTTGCATTCTGTTCTACTCTTTATCTCTTAGCTGCAGAAACTAGAAGCGCTCAATTATAATCCT
GTATCCTCAAACAAGGCTTGCAGTAATCGAAGGGTGCATATTGTTTATATTTGATATTACTACTATAGCTTTG
TACATTGTTTCTGATAAGCGATGGGAACTGTGTCTATCTAATAATA
>1002049176_1 Heli.1-DT668678.3.5
CGAGGCTACAACAGAAGTGCAAGATGAGAGTTCTGATTGTTGCCGCCGCCGTCCTCGCCTGCGCCGCAGCTGC
GCCATCTGGCGCTCTGCTGGCTGGTCCCTACGCCTCCGGGCTGCTGGGACATGGTCACGGCCTGGCTTTAGGC
CATGGCGCACCTCTGGCCTTAGCTGGTCCCTTGGCTGTAGGTCACGCTGCGCCTCTTG
CCATCGGCCATGGCGCCGTCGTCCCCACCATTCCTCCAGGTGATCTCCAGGGTGCCGCCATCGACGCTCACGT
CGAAGCCTCCGACCACGCACGCGCCGCCGTCGACGCCGCCCGCGAATACCATGACCAGGCTTCCGAGCTCCAG
GGTCAAGCTGTGAACGCTGCTGAAGACCATGCCTGGCAAGCGGTTGACGCTGCCCAGT
CCCACAAAGCTGCCGTCGACGGTGCTGCTGCTGGCGTCGCCCCCGTGGTTGCCCGCCAGTTGGCTGGACACGT
CGCCCCCGTCGCCTACGCCGGCCACGGTCTCGCCGGTCACGGACTCGCTGTCGCCGGTCATGGTCTCGCTGTA
GCCGGTCATGGTCTCGCCGGCCCCCTCGTCGCCGGACACGGCCTCGTCGGACACGGTC
TCGTCGGACACGGTCTTGCCGGACACGGTGTTGCCCTCGGTGGTAGCCATTCCGTAGCCTCATCCTCCCTGTT
ACATCCCGCTCCCGCTCCCCTCGTGCACGCCCCCGTCGCGTACGCCAGCCATGGTGCCCTCGGCTATGGTGGT
CTCGCTCACGGACACGGTCTCGCGCACGGATGGTAAGCTGTGATAGGTGTTTAGCCAA
AAAATCTTAATACTCATGTTCGGAATGTATATTTAATTGTATAAAATGAAAATTGTACAGACAGGAGGCGAGG
ATGTGTGAATAAACGAATTAACT
>1002049177_1 Heli.1-ES587660.1.5

GTTACGTCGAGAGGATATACAACCATGTACGCTAAACTGTTCATCGTCTGCGTCGTCGCCGTGGTAGCGCTCG
CCCGCGAGTACCCGGCCGGCGTCCACCCCGCTGTGTGCCCCGACTACCCCTTCTGCGACTCTAGTACCTTCGG
CAGGTTCACCCTGGACGGCATGCCTATCCCTGAATGGGTTTACAACCCCTCCATCCTA
CCTGTAGCTCCTGCTGACCCCAACGCCAATATTGCCGCTAAATACCCCGCCAACTTGAGCCCCGCGGAATGCC
CCAACTACCCCTACTGTTGGTGAACAGTTTAGTCGCAGCGCGCCTTCCGGATTTACTTGTTAATGACAGATTC
TACGAAACGTCAACTTGACATTTTTTTTTTTTCGTCATCTATAGCAATAGCCTCATGT
TTAGTAGAAACTATGCTCATTAACAATGTACTGCCATATTTTTCTGTACCTTATTAAATAAGTATAATTATAC
ACAGCTAACTGATGTTTGTTCAGTATCTGGTTCTTTTGTAAGAAATGTGTAAAAATTGTAAATAAATACGGTA
TAGTAAAAAA
>1002049178_1 Heli.1-ES587655.1.5
TAAGTCGAGAGGATATACAACCATGTACGCTAAACTGTTCATCGTCTGCGTTGTCGCCGCGGTAGCGCTCGCT
CGCGAGTACCCGGCCGGCCTCCACCCTGCTGTGTGCCCCAACTACCCCTTCTGCGACACTAACGCCTTCGCTA
GATTCACCCCGGAAGGCATGCCTATTCCTGAATGGGTTTATAACCCCTCCATCCTGCC
TGTAGCTCCTGCTGACCCCAACGCCAATATTGCCGCTAAATACCCCGCCAACTTGAACCCCGCAGAATGCCCC
AACTACCCCTACTGCTGGTGAACAGTTTAGTCGCAGCGTGCCTTCCGGATTTACTTGTTAATGACAGATTCTA
CAAAACGTCGACTCGATTTTTTTTATCTGTGGCAATAGCCTTATATTATTTAGTAGAA
AACTACGCTCATTAACAATGTACTGCCATATTTTTCTGTACCTTATTAAATTTGTACATACACATAACAAGCT
GATTTTTGCTCATCTGGTTCTTTTGTAAAAAATGTGTAAAAATTGTAAATAAATACGGTATAGTAAAAATT
>1002049179_1 Heli.1-ES587430.1.5
AGCACTAAGATGTTCAAATTAGTAGCTCTGTGTTGCTTCCTCGCCGCTGCCTCGGCTACGCCTGGTGTTGTCT
TACCGCTGGCATATTCCTCGAATGTGCTCGCACCGGCACCAGCAGTCGTGTCCAGCTACCCCGGCAGCCTGGC
ATACTCATCTCCTGTCTTCTCTTCCGCGCCATTGGCCTACTCCGGACCATGGGACTAC
CAACACCTCATCAAGAAACGTTCTTTGGCCGTAGGCAGCTACATCGCGCCGTCTTCTTACCTCGCGCCGTCTT
CCTACATCGCACCAGCTGCCTACTCTTACCCCGCTGCTCCTTTAGTCAGCTCTTACTCTGCAGTTGCACCTCT
GGCTAGTTACCCAGGAGCCGGTTACACCGGTGCTGTACATCTGATCAAGAAGAGGAGC
GCTGTACTTTTACCTAACAACTACCTCGCACACGCCGCGTACTCTGCACCCGCACCTTTCGTAGCATCGACTT
ACGCGGCTGCCGCTCCCATTGCACCTGTTTGGTCTAACTCATTGTATCCCGCTGCCCAGTATGCTCACTTCAT
CAAGAAGTAAGCTGCTCGTTTAAATCCGCATTCCGCCCGTTCTACCATTTCTCGTACC
TCAAAATGCGAATTTTATCTTCCATTCCCTAAAAAGTCAATGACTTTATTATCTCTTTGCTGACACTTGTGCC
ATCGTCTTAGGAGGTGTACTTGCCATTTAATTTAAACGCGCATGCGACACCTACATTTATGTTAATTTTCAAA
AATAAAATATTTTGTTTTGGAAAACCCACGAGCACTGTATATAATTATTGTCATTGTA
>1002049180_1 Heli.1-DT665318.3.5
TTTATGAGCATTTATACTATTCAATGTGTATATTTTGAATAATAGTTATTAGTATATCATATATCCCAATTCC
TACCACCTTCCAAGATAGTTTCATTACGTTTTCGATTTAACAGCTGTATTTGTTTAATAATAATAAGTGAATC
CTTCATACCGCAAACAGTTAACGTGATACTTGTTTATCGCAACCGATTCTATATAAAG
GATCCGGCAAGTGTCCATGCAGTATTTGTGAATAAAGTCGTGTGTAGTGTGTTGCTCTTTTTCAACGAAGATT
AATTAAAACAAAATGGCCTCAAGAGAGCCCGCTTCTGATCAACTTGTAGATTACAAAAAATCTGCAAAGGATC
CTATTGGTTATATAACGACTAAATCTGGTGCACCGGTGGGAGTAAAGACTGCGATTCA
AACAGTTGGAAAAAATGGACCTGCTCTTCTGCAAGATGCCCAGTTTCTGGATGAAATGTCCTCTTTTGATAGG
GAGCGTATTCCAGAAAGAGTAGTTCACGCAAAGGGAGCCGGCGCTTTTGGATTTTTCGAAGTAACTCATGATA
TCACTAAATATTCAGCAGCTAAATTATTCGACGGGATTGGTAAAGAACTCCCATTGC
TGTTAGATTCTCTACAGTAGGTGGAGAAAGTGGATCGGCTGATACTGTTCGTGATCCTCGTGGTTTTGCTGTC
AAATTCTACACTGAAGATGGGATCTGGGATCTTGTAGGAAACAATACGCCAATTTTCTTCATTAGGGATCCCA
CACTATTTCCAAGTTTTATTCATACTCAGAAAAGAAACCCTGAAACACATTTAAAAGA
TGCTGATATGTTTTGGGATTTCTTAACTTTAAGACCGGAGTCAATGCATCAGGTGCTTTATTTATTTGGTGAC
AGAGGAATCCCTGACGGTTACAGATTTATGAATGGTTACGGATCCCACACCTTTAAGTTAGTTAATGCACAAG
GAGTTGCGCATTGGGTTAAATTTCATTATAAAACTAATCAAGGCATTAAAAATTTGTC
TGTTGATAGAGCTGCAGAGCTTGCATCGTCTGATCCTGACTACGCGATCAGAGATCTTTATAATGCAATTGCA
AAAGGTGATTGCCCCTCTTGGTCATTTTACATCCAGGTTATGACAATGGCACAAGCCAAAACTGTTAAGTTTA
ATCCATTTGATTTAACGAAGGTTTGGCCTCATTCCGATTATCCTCTTATTCCTGTGGG
CAGGTTTACGTTGGATAGGAACCCTAAAAATTATTTTGCTGAAGTGGAGCAGATTGCCTTTAACCCAGCTAAC
TTAGTGCCTGGTATTGAGCCTTCACCTGATAAAATGTTACAAGGTCGCCTATTTTCATATGGTGATACTCATC
GTCATCGCCTCGGCGCCAACTATCTTCAGATACCGGTTAATTGTCCCTATCGTGTTAC

TGTTTCTAATTATCAGCGCGATGGACCACAAGCCATAAGTAACCAAGACGGTGCTCCCAATTATTTTCCGAAC
TCCTTCTCAGGACCACGAGAATGCCCTCGTGCTCATCGTCTCCAGCCAAGATACAATGTTAGCGGGGACGTTG
ACAGATACGACAGTGGACAAACTGAAGATAACTATACACAGGCTGGCGTTCTGTATAA
AAAAGTATTTAGCGACGCTGAAAAGGACCGTGTTGCTGCTAATTTTGTTGGTCATTTAAAAGATGCCGCAGGT
TTTATACAAGAGCGTGCAATAAAAAATTTCTCCCAAATTCATCCCGATTTAGGAAGCAAGGTAGCTGCTGGGC
TTGCACCATACAAAAAATTCCATGCTAACTTGTA
>1002049181_1 Heli.1-ES588106.1.5
AACAATGGCAGCTAAGTTCTTCGTCGTCCTCTCCCTGGCCGTGGCCGCATCAGCTATCCCATTAGTCCCTGTG
GCTAAGGTAGCATACGCTGAGGCAGAAGCTCCCGCTCACTACGAGTTCCAATACTCCGTTCACGATGAACACA
CTGGTGATATCAAGCAACAGCAAGAGGCCCGTGCTGGAGATGATGTCCACGGCTCATA
CTCTCTGGTACAGCCTGATGGTGTCCACCGTATCGTGGAATACTCTTCTGACAAAGTAAACGGTTTTAACGCT
ATTGTGCGTTACGAGGGTAAACCCATCCCAGCTCCCGTACCAGTAGCTAAGGTTGCATACGCTCCCGTCGCCA
AGGTTGCCTACGCCGCACCCGTCGCCAAGGTTGCCTACTCCGCCCCCGTCGCATACGC
TTCCCCAGTCGCCAAGATTGCCTACTCACCCGTGTCTTATGCCGCCCCTGTCGCCAAGGTAGCCTACGCTGCT
CCCGTTGCCAAGCTCGCCTATGCTGCTCCCGTAGCGAAGGTAGCATACGCCGCTCCTCTCGCCCAAGTCAGCT
ACTCCTCCCCCATCGTCTCCTACCACCACTAAAGGTATTAAGTATTATTAAAGTTGAT
TAATTTATTTATGTGATAAATAGTTACAAATAAATTGTTTGTTTAAGA
>1002049182_1 Heli.1-ES587421.1.5
TCCGAACAACACAGACCAAGATGTTCAAGTTGGTGGTGTTGTGCGCTTTCCTCGCCGCGACTGCAGCGGAGCC
CGGAACTTTCATCACGCCATTGGCATACTCCAGCAGCGTTGTCGCGCCGGCTGCAACAACTATCACCCACCAG
GCCAGCAGTGTCATCCATCCTTCACCCCTGCTCTACCCACGATTCTACGACCTATCTT
ACTCACCGGTTGCCCATCTTATCAAGAAGCGATCCCCTGGAATTTACAATCCCTTCTTTGCACCATCCACTTA
CATTACTTCTGCACCTCTGGCCACTACTTATGCTGCAGCTCCTCTTGCTACTACCTACACTGGAGCTCACTTG
GCTACAACTTATGCTGCACCAATCTACCCTGCTGCTGCGCATTTAATCAAGAAGAGGT
CTGCTCCACTTCTGCCCACAACCTACGTTGCTCCATACTACTCTTCTCCTTTGTTGACGCCGACGTATACAGC
CGCTGCACCCATCTACTCAACACCTTACATCACGAGTGCTCCTCTTACTTACACGCATTTGATCAAGAAGCGT
TCCGCCCCATTGTTCTACGCAGGTTACACCGCCCCCGCTGCGGTTTCTCATCAGTCCC
GAGTAGACATTAAGAGCTCCCCTGCCACTATTACGTCCTACTCCTACCCTGCTGCTGTTTCTTATGCTAGCCC
AGTTGCAATTTCACATGTCTTCTAGATGATAGAATGGATTTTTAAATAAAATGAATCGA
>1002049183_1 Heli.1-ES586308.1.5
TTCGCGCAGTCTAAAAGAAGTGCACGATGAGATTTCTGATTGTAGCCGCCGTCTTCGCTTATGCCGCAGCCGC
ACCTAGTGCGCTGTTGGCCTCACCCTACTCCAATGGCCTCCTCGCCTACGGAGCTTTGCCTGTAGCCTCCTCC
ATTGTGGCACCAGTCAACTCAGGAGACCTCCAAGGCGCTGCTATCGAAGCCCACGCTA
AGGCCGCCGACAACGTCGTCGCCGCTGTCGACGCTGTCCGTGAATACAACGACCAGGCCGCTGAAATCCAAGG
CAAGGCTATCAACGCAGCTGAGGACAATGCTTGGCAAGCAGTTAACGCTGCCCAAGTAGCTGCTGCCCAGATC
GACGGTGCTGCTGCCAGCGTATCCCCCGTTGCCGCTCGTGCTGTAGCCGGAAGCGCAG
TAGTAGCCCCAATTGCTGCCTACTCTGCCCCAGCTGTCGTTGCTCCCGCTATCTCTGCTTACTCTGCCCCCTT
GATCGCCGGCCAATCTCTCGCTTTCCAACGTTTCGCTTCCCCCATCCTATCTTACGGCGCCCACGGTTTCGTC
CACATTTAAATTTAATTTCTAGGCAAAGCAAATCAGGCTAAACGAAGATGTGTAAATA
AAAAATATAAAC
>1002049184_1 Heli.1-ES588057.1.5
GCACGAGGGTTACGTCGAGAGGATATACAACCATGTACGCTAAACTGTTCATCGTCTGCGTCGTCGCCGTGGT
AGCGCTCGCCCGCGAGTACCCGGCCGGCGTCCACCCCGCTGTGTGCCCCGACTACCCCTTCTGCGACTCTAGT
ACCTTCGGCAGGTTCACCCTGGACGGCATGCCTATCCCTGAATGGGTTTACAACCCCT
CCATCCTACCTGTAGCTCCTGCTGACCCCAACGCCAATATTGCCGCTAAATACCCCGCCAACTTGAACCCCGC
GGAATGCCCCAACTACCCCTACTGCTGGTGAACAGTTTAGTCGCAGCGTGCCTTCCGGATTTACTTGTTAATG
ACAGATTCTACAAAACGTCAACTTGACATATTTTTTTTTTTTCGTCATTTGTGGCAAT
AGCCTCATGTTTAGTAGAAACTACGCTCATTAACAATGTACTACCATATTTTTCTGTACCTTATTAAATAATA
TATACACAGCTAACTGATGTTTGTTCAGTATCTGGTTCTTTTGTAAGAAATGTGTAAAAATTGTAAATAAATA
CGGTATAGTA
>1002049185_1 Heli.1-EL602236.1.5
GTCGCGAGCGGAGTATACCACGATCCTTCGGTCTGCCATTTAGTTTGAATATTTGTGAAAAATGTCTCTAATC
TCCGCTCGTTTGGCCTCTTCGGTGGCCAGGCGCTTGCCTAATGCCGCTACACAGGTTTCGAAGGTCGCCCTCC
CCGCGGTGGCAGTGGCTTCCCGCAAGCTCCATGTCTCGTGTCCACAACGGGCTGCCGA

AATCTCCACAATCTTGGAGGAGAGAATCCTCGGTGCCGCACCTAAGGCCGACTTAGAAGAAACTGGTCGTGTG
TTGAGCATCGGTGACGGTATTGCCCGTGTGTATGGTCTTAAGAACATCCAGGCCGAGGAGATGGTGGAATTCT
CCTCTGGCCTTAAGGGTATGGCTCTCAACTTGGAGCCTGACAATGTTGGTGTGGTAGT
ATTCGGTAACGACAAGCTGATCAAGGAAGGAGACATCGTGAAGCGTACCGGTGCTATCGTAGACGTTCCCGTC
GGTGAACAACTTCTTGGACGCGTTGTGGACGCCTTGGGTAACGCCATTGATGGAAAAGGACCCATCGACACAA
AGTCCCGTATGCGTGTCGGTATCAAGGCCCCTGGTATCATCCCACGTGTGTCTGTACG
CGAGCCTATGCAAACTGGTATCAAGGCTGTGGACTCCCTCGTACCCATCGGTCGTGGTCAACGTGAGTTGATC
ATTGGAGACCGTCAGACCGGCAAGACCGCTCTCGCCATCGACACCATCATCAACCAGCAGAGGTTCAACAAGG
GAGAGGATGAGAAGAAGAAGCTGTACTGCATCTACGTCGCCATCGGACAGAAGAGGTC
CACTGTGGCACAGATTGTGAAGAGATTGACTGATGCTGGTGCCATCAACTACACCATCATCGTGTCTGCCACT
GCCTCCGACGCCGCGCCCCTGCAATACTTGGCTCCATACTCCGGCTGCGCCATGGGAGAGTTCTTCCGCGACA
ACGGCAAGCACGCCCTCATCATCTATGATGACTTGTCCAAACAGGCTGTTGCTTATCG
TCAGATGTCTCTGCTGCTGCGTCGTCCCCCAGGTCGTGAGGCCTACCCCGGTGATGTGTTCTACCTCCACTCC
CGTCTGCTCGAGCGTGCGGCTAAGATGTCCGACAAAATGGGCGGCGGTTCCCTCACTGCCCTCCCCGTAATCG
AGACCCAGGCCGGTGACGTGTCTGCCTACATTCCAACCAATGTCATTTCTATCACCGA
TGGACAGATCTTCTTGGAGACTGAATTGTTCTACAAGGGTATCCGGCCCGCCATCAACGTCGGTCTGTCTGTG
TCCCGTGTAGGATCTGCTGCCCAGACCAAGGCCATGAAGCAGGTGGCTGGTTCCATGAAGCTTGAGTTGGCTC
AATACCGTGAGGTCGCCGCCTTCGCCCAGTTCGGTTCTGACTTGGACGCCGCCACCCA
GCAGCTGCTCAACCGTGGTCAACG
>1002049186_1 Heli.1-DT662899.3.5
GGTCGAACGCAGTGTACGTGTTTATCAACGCCTTATAAACCAAATCCCTTAAACAATGGCCTGCTCAGTAGAC
GCCCCCTCCCTCAAGGACTTGCCCAAAGTGGCCACCGACCTCAAGAGTCAGCTCGAAGCTTTCAACCCAAGCT
GTTTACGCGACGTCGACACCAATGAAAAGATCGTCCTGCCTTCCGCTGAAGATGTCGC
CACCGAAAAAACTCAGAAGTCCCTGTTCGACGGCATTGAGAAATTCGATGCTACTAGGCTGAAGCATACGGAA
ACACAGGAAAAGAACCCACTTCCTGACAAAGATGTTGTCGCAGCGGAAAAGGCGCATCAGAACCTTCTAGACG
GCGTTGAACACTTTGACAAGACCCAAATGAAGCACACGACGACGGAAGAGAAAAATTC
TTTGCCTGCCATTGAAGCCATTGAAGCCGAGAAGGAAAAGAACAAATTCCTGAACGGAATTGAAAACTTCGAC
CCAACCAAGTTGAAGCACACTGAGACTTGCGAGAAGAACCCACTGCCCACAAAGGACGTCATTGAGCAGGAGA
AGACCGCTTAAACCATATATAACCGCAACTATGTATCGCTAGTATCGCCGTATTTTAG
TATATAAGTTTCGTATAGTCGGACCGTGGCGTCCGTCCGTTCGAACATGTAATGAAGACTCATATTTTAATCC
CTTTAATCATACACTAAACGTGATTAAATATTCATTAAGCCATTATATCATTCACCTCTAATTTTTATTTGTC
ATCCATTTTCATTCATTCATTTGATATTCACTACAAACTCCCATTTAGAAAAATACCC
AATACTCCCATATCTTCATGGAAA------------------------
AAATATGTCAATTGAATATGGCAAATGGAACGGACGTCGCCGCTCGATCACTATCATTAGAATGTAGGAACTA
AATTTTCTATGTCACTTTTAATATATATCTAATAAGTGTATTTTATGAGCATTTTATTT
>1002049187_1 Heli.1-EL600955.1.5
CGAGGGTCGACATGCGCTCCCTTGTTCTCTTCGCTCTCCTCTCGGTGGCTGCGGCCAAGCCCGCGCCCGGACT
TCTTGGTGGATGGGATGGCGGTCTAGGCCTTGGCCACGGCTCTCTCGCGCTAGGGCACGCGCCCCTCATCCAG
GCCGCGCCGGTGCTCCAAGCGGCGCCCGTCATCCAGGCGGCGCCCGTCGTGCACGCCG
CGCCCGTCGCTATCGCCAAGGCTGCCACTAGCTACTCGTCCATCCAGAGGGTTATTCATCCAGTAGCTCAAGT
GGTGGCCCAGCCCGTGGTCCATGCCGCGCCCGTAGTCCAGGCCGCCCCCGTAGTCCAAGCCGTCGCCCAGCCC
ATCCTGAGCCATGGCATCGGCATCGGTAGCCTGGGCCATGGTATCGGCCTCGGCGGGC
TGGGTCATGGTATCGGCATCGGCGGGCTGGGTCATGGTATCGGCATCAGCAGCCTGGGCCATGGCTGGTAGTT
AACTTATTCGTAATTAATTAATGTTCGTTAGCGCGTTGAGATCATGCCCGATTTGTCGGGTGGTGCAAATAAA
TATTTATATCAGTATTTGTAAA
>1002049188_1 Heli.1-DT664179.3.5
TTAAACTTCGAATTAATTTCCTTTCCTTTCTAATAACGAGGGTCGAGACTGACCCTGCTAAATTAAAATCTCA
AGAAATCAAAATCATCACCAGACAATATTAAAATTTAAATATGTCTGGAAACTGGAACAATAGCCGCGGTGGA
AGCGGTGGTTCCAAATTCGGCGGAGGTGGCAAGTTCGGCGGACATGGAACATCAAGAT
TTGGAAACGGTGGATCTAAATTCGGTGGGGGTGGTGGTGGATATGGCGGAAAGAAAGAGTTTTCAGGTGGTCA
AAATATGAAAAGACCTAACTGGGACTCCATGTCATTGCAACCATTCAACAAAGACTTCTATAACCCACCAGAA
TCAGTTCTGAGTAGATCACCTTATGAAGTAGAAGAATACAGAAATCAACATGAAATTA

CTATCAGTGGAGTAGAGGTCCCTAATCCCATCCAGCACTTTGAGGAAGGGAATTTTCCTGACTATGTCATGCA
ATCTATCAAAGGCATGGGTTACAAAGAACCTACACCCATTCAAGCTCAAGGCTGGCCAATTGCTATGTCTGGC
AAGAACTTAGTTGGTATTGCACAAACTGGCTCTGGTAAAACATTAGCTTACATTTTAC
CTGCTATTGTACACATTAATAACCAGCCTCCAGTCAGACGAGGTGATGGACCTGTTGCCCTGGTCTTAGCACC
TACCAGAGAATTAGCACAGCAGATCCAACAAGTTGCTAGCGACTTTGGCAATGCAGCTTATGTTCGTAACACT
TGTATATTTGGAGGAGCACCCAAAAGAGAACAGGCACGTGATTTAGAAAGGGGTGTTG
AGATTGTAATAGCCACACCAGGAAGATTAATTGACTTTTTAGAAAAAGGAACAACTAATTTGCAAAGGTGTAC
CTATTTAGTTTTGGATGAAGCTGATCGCATGTTAGACATGGGCTTTGAACCGCAGATAAGAAAAATTATAGAA
CAAATTCGCCCTGACAGACAAACTTTGATGTGGTCTGCCACATGGCCTAAGGAAGTGA
GGAAATTGGCGGAAGACTACTTGGGAGACTATGTACAAATTAATATTGGATCCATGCAGCTGTCTGCTAATCA
CAACATTCTACAGATTGTAGATGTGTGCCAAGAACATGAAAAGGAAAACAAATTGAATGTGCTGCTTCAAGAG
ATTGGTCAAAGTCAAGATCCAGGTGCAAAGACTA
>1002049189_1 Heli.1-ES587467.1.5
TCGAGAGGATATACAACCATGTACGCTAAACTGTTCATCGTCTGCGTTGTCGCCGTGGTAGCGCTCGCTCGCG
AGTACCCGGCCGGCCTCCACCCTGCTGTGTGCCCCAACTACCCCTTCTGCGACACTAACGCCTTCGCTAGATT
CACCCCGGAAGGCATGCCTATTCCTGAATGGGTTTACAACCCCTCCATCCTACCTGTA
GCTCCTGCTGACCCCAACGCCAATATTGCCGCTAAATACCCCGCCAACTTGAACCCCGCGGAATGCCCCAACT
ACCCCTACTGCTGGTGAACAGTTTAGTCGCAGCGTGCCTTCCGGATTTACTTGTTAATGACAGATTCTACAAA
ACGTCGACTCGATTTTTTTAATCTATGGCAATAGCCTTATATTATTTAGTAGAAAACT
ATGCTCATTAACAATGTACTGCCATATTTTTCTGTACCTTATTAAATTTGTACATACACATAACAAGCTGATT
TTTGCTCATCGTCTGGTTCTTTTGTAAAAAATGTGTAAAAATTGTAAATAAATACGGTA
>1002049190_1 Heli.1-DT664377.3.5
ACGAGGGGAACATGTCCTATTCACCTGAAACAAGATCGGAGGCTTGGCAGGAGGATAAAAATGGCCCAGCCAA
GGATCAAGGCAACTATGATGGACCTCCGGGCTTGGACTCGGTTGGCGGCGCTCTCGACACGAACTGGCATGAA
GTCGTGGAAAGCTTTGATGACATGAAATTAAAAGAAGAATTATTAAGAGGGATTTACG
CTTATGGTTTTGAGAAACCTTCAGCAATTCAGCAACGCGCGATTATGCCTTGTATCCAAGGTCGCGACGTTAT
AGCGCAAGCTCAGTCAGGAACTGGGAAAACCGCTACGTTCTCTATTTCTATTCTTCAGCAAATTGATACTAGC
ATTCGCGAATGTCAAGCACTCATTTTGGCCCCGACTAGGGAGTTGGCTCAACAGATTC
AGAAGGTCGTAATTGCGCTCGGTGATCACTTGAATGCTAAATGCCATGCCTGCATTGGTGGTACAAATGTGCG
TGAAGATATTCGTCAGTTGGAAAGTGGTGTTCATGTGGTGGTTGGAACCCCTGGCCGTGTATATGATATGATT
ACCCGTCGTGCCCTCCGTGCCAATACAATTAAGCTATTTGTACTTGATGAAGCTGATG
AAATGTTGTCTAGAGGATTTAAAGATCAAATCCACGATGTATTTAAGATGCTGTCTTCTGATGTGCAAGTTAT
TTTGCTGTCAGCTACTATGCCTGACGATGTGTTGGAAGTGTCACGGTGCTTCATGAGAGAACCCGTGCGCATT
CTAGTGCAAAAAGAAGAGCTCACCCTGGAAGGTATTAAGCAGTTCTTTATTTCCATCG
AGATGGAGGACTGGAAATTAGATACTCTTTGTGACTTGTATGACACACTCTCCATTGCCCAGGCTGTCATTTT
CTGCAACACTCGCCGCAAGGTGGACTGGCTCACTGAGTCTATGCACAGCCGCGACTTCACCGTATCGGCTATG
CACGGCGATATGGATCAGCGCGAACGTGAAGTCATCATGAGGCAGTTCCGTACAGGGT
CGTCCCGTGTTCTCATCACCACTGATTTGCTAGCTCGTGGCATTGACGTGCAGCAGGTATCCTGCGTTATCAA
CTACGATCTGCCTACCAACCGTGAGAACTATATTCATCGTATCGGAAGAGGTGGTCGTTTCGGTCGTAAAGGA
ATTGCTATCAACTTTGTGACTGAAGCAGACAAGAGAGCATTGAAGGATATTGAGGAGT
TCTACCATACAACTATTACGGAAATGCCCAATGATGTGGCCAACCTCATCTGAGGCGCCAGCGCATTCCAGTT
TATACTTTGTGTCCTGCATTCTATTGCGATATTTAAACGGTGTGAGTATTAGGGGCAACCTCCTAAGTACCGC
TGTGCTGATTTTTATTGGAACTCATTTTATTTGGACCGTTGTATTGGTTTAGTTTAAT
GAAATAATAATACATTACTATAATATTATTGATATAAGTTTAATATACATAGTCGTAAGCCCGGCTCGGCCGG
ATGGCCCCCGTTCCGCGCGGACCCCGCTGCTACCTGCAGAGGGGTCTCATCGGGTACTGGAGAGGCTATTTCA
ATCACAAAAGTACTAGAAGGAAATAATGTATTGTAGCTTTTTTCCAGTTGTACATAAT
ATGGTAAACTGTTTTTTGTAATACAGTAAATAAATA
>1002049191_1 Heli.1-ES586005.1.5
CACGAGGCTTCAACATAGCGATTCCTGAAAGACACTAGTCTTTCAGCAGCGCTATCATAAACAAAAATCGATT
TATAATCTATATATAACTTTTTCTCTCTTTCACTTCTGTGCTTTACTTCTTATTTCAAAATGAAATCCATGAT
CGTTGTAGCTTGCTTGGCGCTGGCGTGTGGAGCCCATGCTTCCGGATGGGTTGGTCCC
CCAGCCAACATTGCCCTGTCTCAAGATGGTCGCAACATCTTAGACACACCCGAGGTAGCCCAGGCTCGCGCTG
CTCACCTCTCCGCCCTTCAACAAGCCTCACACAACAACCCCAACCCTCAAGATGATGGCTCCTACGACCCCCG
CTGGGACAACGAAGAGTACTGGCAAGGAGCTCAACAGAAATGGAACGGTGCCCCCGCC

CAACAATGGAACGGCGCCCCCGCCCAACAATGGAACGCCGCCCCCGCCCAACAGTGGAGCGACAACTCCGGCA
AATGGAACGGTGACAACGGACAATGGAACGGTGACAACGGTCAATGGAACGGTGACAACGGTCAATGGAACGC
CGCCCCCGCCGCACCCGCATGGAACGCCGGCCCCGCCCCCGTCGCCGAGACCCCCGAA
GTAGCCCAGGCCCGCGCCGCTCACCTCGCCGCCCTCAACGCCGCCCCCGCTCACAACCAGTGGAACGCTCCCG
CTCACAACCAGTGGAACGCCCCCGCCCAGAACCAATGGAACGGTGCCCCCTCATGGCAAGGTCCCCCTGCTCA
GATCAGATTAGCCCACAACGGCGCTGGTATCTTGGAGACCCCTGAAGTCGCCGCCGCG
CGCGCTGCCCACTTAGCCGCCCACGCACAAGTAGGACACAATGCCCCAGCTCACCCCCAACAACGTTGGTGAA
TACCTCCCTCTTCTATTTCTAGGTAGGTTAGATTCCTCTTGCATGTCATTCGCTTTCTCAAAGAGCGCTTGCA
AATGACATCCTGGAGGTGAACTAAGATGCCCTCTTCCTTTCGATCTCCTCTTCCTTTA
GTAATATGTTAAAAGTGCTAGTGCGTCAGACTCTTCCTTTCTTTAGCTTTTGTTACTATTCTCTATAAAATTC
CTACTATACTTTTTGTACATAATAAATAATAATTATATGTATGCGTAAAAGAATTATAT
>1002049192_1 Heli.1-DT663086.3.5
TGCGGATCGGCCACGTATTGTACACAGTGCTACTGAAATTCTTATAAGTGTTCCTCAAAAGTGTCTTTTCGTG
TGTAACTTCTTTTTAATATACTAAGTCAAGAAATTTTTCAAGTGAAAATTTAATATCGCATGGTGAAAATGCG
GTGGTGTCTATTAGCTCTGGCGTGCGTGGTCGCGGCGGTCTACGCCGACGACAAGAAG
GAGAAGGATAAGGATATTGGTACCGTCATCGGTATCGACTTGGGTACCACTTACTCATGTGTGGGTGTATACA
AGAATGGACGTGTTGAAATCATCGCCAACGACCAGGGTAACCGTATCACTCCATCTTACGTTGCCTTCACCGG
CGACGGTGAACGTCTTATTGGAGATGCTGCCAAGAATCAGCTTACGACCAACCCCGAA
AACACGGTGTTCGACGCTAAGCGTCTCATCGGTCGCGAATGGAGTGACACCACTGTCCAACATGATGTTAAAT
TCTTCCCATTCAAGGTGGTAGAGAAGAACAGTAAACCCCATGTCTCAGTAATGACCTCCCAAGGTGACAAGAT
CTTTGCCCCTGAAGAAATTTCCGCTATGGTCCTCACTAAAATGAAGGAAACCGCTGAA
GCATATCTTGGCAAGAAGGTCACTCACGCTGTTGTCACTGTACCAGCCTACTTCAACGACGCCCAACGTCAAG
CCACCAAGGACGCTGGTGTCATCGCTGGCCTTAACGTTATGAGAATCATCAACGAGCCTACTGCTGCCGCTAT
CGCTTACGGTCTTGACAAGAAGGAAGGTGAAAAGAATGTACTCGTTTTCGATTTGGGT
GGTGGTACCTTC
>1002049193_1 Heli.1-CO729703.1.5
GGTCGACCACGCGTCCGATTAAATTATCAAAATAGAGCTTCCCCATTAATAGAACAAATTATCTTTTTCCATG
ACCATACATTAATTATTTTAATTATAATTACAATTTTAGTAGCATATTTAATAATAAATTTATTTTTCAATAA
TTATATTAATCGATTTTTATTAGAAAGTCAAATAATTGAATTAATTTGAACTATTTTA
CCAGCTATTACTTTAATTTTTATTGCACTTCCATCATTACGATTATTATATCTTTTAGATGAACTTAATAATC
CTTTAATTACATTAAAATCAATTGGACATCAATGATATTGAAGATATGAATATTCTGATTTTAATAATATTGA
ATTTGATTCATATATAATTCAATCTAATGAAAATTTAAATAATTTTCGACTTTTAGAT
GTTGATAATCGTATTATTTTACCTATAAATAATCAAATTCGTATTTTAGTTACAGCTACAGACGTAATTCACT
CGTGAACAATTCCGTCATTAGGAGTAAAAATTGACGCTAATCCAGGACGATTAAATCAAACTAGATTTTTTAT
TAATCGTCCTGGAATTTATTATGGTCAATGTTCTGAAATTTGTGGAGCAAATCATAGT
TTTATACCTATTGTTATTGAAAGAATCCCTATAAAAAACTTTATTAATTGAATTAATAATTATTAAAAAAAA
>1002049194_1 Heli.1-DT668469.3.5
AAACGTAGTTTTAAGCAGGAAAGGAACCGTTGCAGTGTTTTTTTACTAAAATACTAAGAAAATGGGTTCGCTG
GGCGTGAGCGTGTTCTTTCTTGTGATGCTAGTGCAGTTTAATTTGATTTATTCCAAGCCATTTTTCTTCGATC
TCATTTGTGACCTGATCTGCGACGACGACGACTCGTCCACATCAACTACCACCAGCGC
CAGCACCGAAGATGACTACTCGGATTTTGATGATTGGTGTTCATGTACTCGCACTACGAGACGGCCACCTGGT
ACCAGAAGGAATCAGGGGAATATGCCGGCAGCTATTAACATGATATTACCACCATCCAACGGCATGAACCTGA
CCATGTTCCAAAGAAACGGTGCATGGACTTTCGATTTGAATGCTGTACCGAATGGTTT
TGGTAATCGACCTGCCGGAGCCAGCCGGCCACCCACTACTGGAGCCCCTACCACGGCAGCTACTACCGCGGCG
GCAGCGGCGACTACTACTGCTGCTAAATAAAAATCTTTAAAATTATTATTGTTTAGAATGAAATCTATGAAAA
TCTGTAACATTCATATAAGAATTGGACGTGACTCCTCTATACAAACTAAGTAGTCTTA
GTTATAAAGTACGTAGACCGTTTCTAAGAGATATTGGGAAATTGTGCTACATTTAATATTTTATATGAAATTT
AGGCAGAGGGAACCTAGTGTTAAGACACTCCGTTCCCCACTTTCTGCCGAATTCTGGAGGCATTGCGTGTTGA
GTGGCAGAACTCAACGCCGCGCTTAACCTCGACACCAGAGCGAAGAAATGGAAATATA
AATTTAAGTAAATATTTCATCTCCTCGAGTGGGGATCGAACCCACAACCAGTCGGTTTTACAGTCACACCTCG
TGCCCCTGCGCCACGACCGGCCT
>1002049195_1 Heli.1-DT665617.3.5

AGAGTTGAGTCCGGCTGAAGTCAGTCAGTTACATAACAGTGCGGCACTGGGGCGCGCGCTTAGTACCTACTAA
ATATTTGTTAATTAATATTATTTATTATTTTAATTTTTTGGTTATGTGCTTAGTTCGTTATTTGTTAATAACA
TGTATCTAGAAAGAATCCTTTTATGGCTGTTGGTTGCAACTTGCGCGTGCGCAGAGCG
TTGGAGCTGGCCAGACGACTCTGTCGGGGAAGACAGTGTACGCATTGACAGTAAATTACACTTCGGTGACCCG
GACACGCAAAGGAATAGTAAAAGAAACAGCAACATACAACAAGATGAATTCCCCTTCCAAGAGGCAACAGACA
CGCAAGGATTCTACAACCGGCCGGCGGACGCGGGCCGCTACCCAATGAGAGTCGAACC
CGGATTCAATCAGGGGATACGGATAAACGGACAGACTGTGTTCTTAAATGGATATGATAGGAATCAAGAATCA
GATGGTACACTAGACTCTCTTCAACACTGTAAATGTGTGTCCCGCCCAGATTGTCAGGTGCAGAACGATTATC
AAAACGCATGTGGCACGAATCAATACTTATGCTGTTATAATCAACCCAGACAGCAAAA
CAGATATCCTTCAGAATATTTGAATGAAATAGACAATGAACGACCCATGATATATCCCAATATACCAAATGCT
GGAGCATTTCCGCCGCCAAATAATGTCAATAAAGGAATATTTAGACCGAATCAAGGAAATGAGCCAGGTGTTC
TGGTTGGACCTGATGGACCTACTGGTAGTATAGGCCCACAAAAGAATCAAGTACTGGT
TGGCCCCGGTGGGCCCACCGGCATTATTGGACCTCAAAACTTAAACAAAAATGTTGGCAATAAAAATATTTTG
GTAGGTCCTAATGGGCCGACTGGTGACATAGGGCCGAGTGAAACCGCACAAAGAGGAGTACTTGTTGGGCCAG
GAGGACCTACAGGCATCATTGGTCCAGCATTCAATCGACCAGTCTTAGTTGGTCCCGG
CGGCCCAACTGGTATAATAGGACCACGTCGTCCCGGCCAAGGAGTCTTAGTTGGTCCTGGAGGACCCACTGGT
ATAATAGGCCCGGCTGGATTCAACCGGCCTTCACAGCGTCCAGTGCTAGTGGGTCCTGGAGGTCCAACAGGGA
TCATTGGCCCAGGTAGACAAATCCTTGTCGGACCTGGAGGCCCCACAGGTCAGATTGG
ACCCAGAAATTACTTCATCTGAGTGTTTTACAGTGATAACTAATAAACTAATTTACTTTATCGGCAATGAATG
TCCGCCACGTGTGTGTTTATGTTTAACCGAGAGCTCGTTGTGATTGCATTAGAGCGTAAATATTTTGGTAGAT
CTAACGAACGCGCTACGTGCTAACGCCATAAACTGCGGCTTTGATCCACTTGTGTATA
TAGCTGTGGAAAATGTGTAAATTTATAGAATTATATGTATATATATAGGCATATATGTATATGATATAGTCAA
AATTATATTTAAATACTTTTTGTACCATTACAGGCTGTTTGCATCACAAGAACAATCGAACAAAATGTAGAAA
AGCAGTCCAACGGCATTAATATTTGTATAAATTCCATGACAATTTTATTTGCCATTTT
AATCAGAATTTTTATGTATATTTTTATTATTTTTCGTGTGTCCTCGAGTTCGAGCCCCTGGCCTCAATAACGG
CAGTGTATATAAAAAAATGACGT
>1002049196_1 Heli.1-DT663138.3.5
TCCAAGTGTGAATGAAGTGGCAGTTTCAGCATTTTGTTAGGGTTCGTCTTCCAAGTTGAATTATTAGTTGTAA
TAAGACCACCGGAACTTGAAGATGCGTGTCTTAATATTTTCGGTAGCGATAGCCCTTCTGGGAGCTGCGCTTG
GAGACGAAATTCCGTCAGAAGACAATGTACTTGTATTAAGTAAAGCCAATTTTGATTC
TGTTGTTTCATCTTCGGACTTCGTGTTAGTGGAATTCTATGCACCATGGTGCGGACACTGCAAATCGCTTGCA
CCAGAGTACGCTAAAGCTGCTACTAAGTTGTTAGAAGAAGAATCTCCTATTAAGTTGGCTAAAGTTGACGCAA
CTCAAGAACAAGAACTCGCCGAATCCTACAAAGTCAAAGGTTATCCCACGCTGATCTT
CTTCAAGAAGGGATCACCTATTGATTATTCTGGTGGTAGACAGGCTGACGACATTGTCGCCTGGTTAAAGAAG
AAGACTGGTCCACCAGCTCTTGAAGTCAGTTCAGCAGAACAGGCCAAGGAACTCATTGCTGCAAACAATGTCA
TTATCTTTGGTTTCTTCCCCGACCAAGACTCAGAAAAAGCTAAGGTCTTCCTTAATGC
AGCTGGATTGGTAGATGACCAAGTATTTGCTATTGTATCTGATGAAAAGTTAGTTGAAGAACTAGAAGCTCAG
GCTGAAGATGTTGTTCTGTTCAAAAACTTTGAAGACCCACGCAACAAGTACGAAGGTGAAGAATTCTCCGAAG
ATGCTCTTAAATCATGGGTGTTTGTACAAAGCATGCCAACCATTGTGGAATTCTCACA
CGAAACTGCCTCCAAGATCTTCGGCGGTCAAATCAAATATCACTTACTCTTGTTCCTGTCCAAGAAAAATGGC
GATTTCGAGAAGTACCTTGATGACTTGAAACCCGTTGCCAAGAACTACCGTGACAAGATCATGTTTGTTGCCA
TCGACACTGATGAAGATGACCACCAGAGAATCTTAGAGTTCTTCGGCATGAAGAAAGA
TGAAGTGCCTTCAGCCCGTCTCATCGCCTTGGAACAAGACATGGCCAAATACAAGCCCGCCAGCAACGAGCTC
AGCGCCAACTCTATTGAAGAATTCGTACAATCCTTCTTCGCCGGTAGCCTGAAACAGCATCTCCTAAGTGAAG
ATTTACCCGAGGACTGGTCCGCCAATCCCGTCAAAGTGCTCGTCGCCTCAAACTTCGA
CGAGGTTGTCTTCGATAACTCCAAGAAGGTCCTCGTTGAATTCTACGCCCCATGGTGCGGTCATTGCAAACAG
TTAGTTCCTATCTACGACAAGTTGGGAGAACACTTTGAGAAGGACGATGACGTCGTCATCGCCAAGATCGACG
CCACCGCCAATGAACTTGAACACACTAAGATCACTTCGTTCCCCACCATTAAACTGTA
CACTAAGGACAATCAGGTGCGTGAATACAATGGCGAGAGGACGCTGGCCGGCCTCACTAAGTTCGTGGAGACA
GAATCCGAGGGAGCCGAACCTGT
>1002049197_1 Heli.1-DT668284.3.5
CCCCTTCCTTTTCTCTTTCCGAATAGACCTCAACCATGGCGGCCGGGGTACTTTACACTTATCCGGAAAATTT
CCGTGCTTATAAGGCGTTGATCGCCGCACAGTACTCCGGAGCCGACGTGAAAGTAGCTCCCAACTTTGTGTTC
GGGGACAGCAATAAGTCCGAGGAGTTCCTAAAGAAGTTCCCGGCCGGCAAAGTGCCAG

CATACGAAAGCGCTGACGGAAAAGTTCTCCTCACTGAGAGCAATGCCATCGCTTACTACGTCGCCAATGCCGC
TCTACGTGGATCAGACGTGGCCTCTCAAGCCGCTATCTATCAATGGGCGTCCTGGGCTGACAGCGAACTGCTA
CCAGCATCATGTGCCTGGGTGTTCCCATACTTGGGCATCATGCAGTTCAATAAACAAA
ATGTGGAGCGTGCAAAGTCAGATCTGCTAGCTGCATTGAAGGTGTTGGATGGCCATCTCCTCACCCGAACCTT
CCTCGTCACAGAGAGAGTCACACTGGCTGACATCATTGTGTTCTCCACTCTGATTCATGCTTTCCAGAATGTT
CTAGAGCCCAGCCTCAGATCATCTCTAGTGAATGTCCAGCGTTGGTTCCTGACCCTCG
CCAACCAGCCTCAAGTGTCCAAAGTAGTGGGTAGCATTGCTCTCTGCCAGGCCGCTCCCGTCTTCGACCCTAA
GAAGTACCAGGAACTGTCTCAGAACAAGAAGGAGGGTGGCAAAAAGGAGAAGAAAGAAAAACCAGAAAAGAAA
GAACAACCTAAAAAGAAGGAGCCAGAACCTGCTCCTGCTGATGACTTTGAGGAGAAGC
CCAAAGAAACCAAGGACCCCTTTGACTCACTACCTAAGGGTACCTTCAACATGGACGACTTCAAGCGCAGTTA
CTCCAACGAAGATGAGGCTGTCTCCATCCCATACTTCTGGCAGAAGTTTGACCCCGAGAATTACTCCATCTGG
TATGCTGAGTACAAGTACCCCGAGGAGCTGTCCAAGGTGTTCATGAGCTGTAATCTTA
TCACTGGCATGTTCCAGCGCCTGGACAAGATGCGCAAGCAGGCGTTCGCGTCGGTGTGCCTGTTCGGGGCCGA
CGACGACTCCTCCATCTCCGGCGTGTGGGTGTGGCGCGGGCAGCAGCTCGCCTTCGCGCTGTCGCCCGACTGG
CAGATCGACTATGAGTCCTATGAGTGGAAGAAACTGGACCCCTCCAGTGAGGACACCA
AGAAGAAGGTACAGGACTACTTCTCGTGGTCAGGAGTCGACTCCAAGGGGCGCAAGTTCAACCAGGGCAAGAT
CTTCAAATAAAATTAATTTATTTCCCCACTGTCCTCCGTCTGTCTGTTTTGATACTAAACACATATTTGTAAT
GTAAAGTCCACAAGTCTGTCAGCGAGCCAGCATGTTCAATATATTGTAAAATATTAAG
TGCATTTACGAGTGCGGCGCACTGGCTCTGTGTTGATTGTAATCAACTTGTATTCAACTGAAAATTGATTTTA
AATTGATTTTT
>1002049198_1 Heli.1-DT665652.3.5
GCACGAGGCTCAACCCTGGAGGACCTGGGATGAAAGAGTGTGTAGTGCTACATTAGGGACATTATATTTTTTT
TTCAAATGTTTCTAATCGAAATTATAAGTACAAAGTAATAGGATAGGGTTTTACCACGGTCGCCGCCATTATG
GCGTCGGACGAACAATTTTCGTTATGTTGGAACAATTTCCACGCGAATATGTCAGCAG
GCTTTCATGGCCTGCTGTCGCGTGGAGATTTAGTTGACGTAACATTGGCTGCCGAAGGCAGATTACTACAGGC
ACACAAATTAGTTTTATCAGTATGTTCTCCCTATTTTCAAGAAATGTTCAAAATGAACCCCACTCAACATCCC
ATAGTATTTTTAAAAGATGTTAGTCATTCGGCGCTTAGGGACTTATTACAGTTTATGT
ACCAAGGGGAAGTTAATGTTAAGCAAGAAGAATTAGCGTCGTTTATTAGTACCGCGGAACAACTTCAAGTTAA
AGGTTTAACCGGTAATCAAAATGAAGAAAGTTCCACGCCATCCAAACCAAAGCCGACGTCGAGGCCAGGCCCG
AGGTCGTCACAACAAAGGCAATCTGTTATGACTAAGTTAGAGACTGATTTAGATTCTA
AGCCCTCCTCAACTCCAGTAGCAATTAAGAGACCAAATAGGCCATCGATAGCATCCAATAACTCGTCATCATC
TCAAAGTGGAAATGCGAAACGGAAATGTGTTGACCCCTTAGAAGCAGGACCCTCAGGCTCTACGAAAGAGGAA
TTTGTTACGATACCCGACGAAGATGAAAACAACGCTGTGGCACCCAAAATGGAACCGG
AATTTGTTAATGAAAGCATGTGGGACGAGGACGACGATGGCACCAATAACGATGAAACTAACTTTGGCGAGGA
CGACTCCAATATGGAGATGTCTGGTTTCGATGGCTCTACGACTGGCGACGGCAATATAACTGGAGGCGGGGAA
GGCGGCGCTG
>1002049199_1 Heli.1-EL601652.1.5
GCACGAGGTAACAGCGTTTCAAGTGTTCTGTGATAATTATACACGTGCGAGTATAAACCACGCTACGGATACG
AAGCAAGAAGTAACTAATACAACTTTCAAGTCAGTTGAAAGGTATATATTTCTTTATTCTATAAATGCCAGAA
GAAAAAATGGAGACTCAATCCGGTGAAGTTGAAACCTTCGCCTTCCAGGCTGAAATTG
CCCAGCTTATGTCATTGATCATCAACACATTCTACTCTAACAAAGAGATCTTTCTTCGTGAGTTGATTTCCAA
CTCTTCAGATGCTTTGGACAAGATCCGATATGAATCTCTCACTGATCCATCAAAGCTCGACAGTGGCAAGGAG
CTGTATATTAAGATAGTGCCCAACAAGAGCGAGGGTACCCTAACCATCATTGATACCG
GTATCGGTATGACAAAGGCCGACTTGGTGAACAACCTCGGTACTATCGCGAAGTCCGGCACAAAGGCGTTCAT
GGAGGCGTTGCAGGCGGGCGCTGACATTAGCATGATCGGTCAGTTCGGTGTAGGTTTTTACTCCTGCTACTTA
GTCGCAGACCGCGTGACTGTACACTCAAAACACAATGACGATGAACAATACATGTGGG
AGTCTGCTGCTGGTGGTTCCTTCACCGTCCGCTCTGACCCCGGTGAACCACTGGGCCGCGGTACAAAGATCGT
ACTCCACGTGAAGGAGGACCTCGCCGAGTTCATGGAGGAACACAAGGTCAAAGAGATCGTCAAGAAGCACTCT
CAATTCATCGGTTATCCAATCAAACTTGTAGTTGAAAAGGAACGCGAAAAGGAGCTCT
CTGATGACGAAGCTGAAGAGGAAAAGAAGGAAGATGAGAAGGAGGACGAGAAACCCAAAATTGAGGACGTAGG
TGAGGATGAGGATGAAGACAGCAAGGACAAGAAGAAGAAGAAGAAAACCATCAAAGAAAAGTACACGGAGGAT
GAGGAACTGAACAAGACCAAGCCCATCTGGACACGCAATGCAGATGACATTACCCAGG

AAGAATATGGTGACTTCTACAAATCTCTAACCAATGACTGGGAAGACCATCTAGCTGTCAAGCACTTCAGTGT
AGAGGGTCAGCTTGAGTTCCGTGCCCTTCTGTTCGTTCCTCGTCGCGCTCCCTTCGACCTCTTTGAGAACAAG
AAGCGCAAGAACAATATCAAATTGTATGTCCGCAGGGTGTTCATCATGGACAACTGTG
AAGACCTTATCCCTGAGTACCTGAATTTCATTAAGGGTGTAGTTGACAGCGAGGACTTGCCCCTGAACATTTC
TCGTGAGATGCTCCAACAGAACAAGATCTTGAAAGTAATTAGGAAGAACTTGGTTAAAAAATGCTTAGAGCTC
TTTGAAGAGCTGGCTGAGGACAAAGAAAACTACAAGAAGTATTATGAACAGTTCAGCA
AGAACTTGAAGCTTGGTATCCACGAGGACTCTCAAAACCGCAACAAGATCGCCGACCTACTCCGCTACCACAC
ATCTGCCTCTGGTGATGAAGTTTGCTCCCTCAAGGAATATGTTTCACGCATGAAGGAGAACCAGAAGCACATT
TACTACATCACTGGTGAAAACAGGGACCAGGTGGCCAACTCATCCTTTGTTGAAAGAG
TTAAGAAACGTGGTTATGAAGTAGTTTACATGACTGAGCCTATTGATGAGTATGTAGTTCAACAAATGAGAGA
ATATGATGGCAAAACTCTGGTCTCTGTAACCAAGGAAGGCTTAGAACTCCCAGAAGATGAGGAAGAGAAGAAG
AAACGTGAGGAAGACAAGGCCAAATTTGAAGGTCTATGCAAAGTAATGAAGAACATTT
TGGATAACAAAGTTGAGAAGGTAGTTGTATCCAACAGGTTAGTCGAATCACCTTGCTGTATTGTCACTGCTCA
GTATGGTTGGACGGCCAATATGGAGCGTATCATGAAGGCTCAGGCTCTCCGTGACACATCCACCATGGGCT
>1002049200_1 Heli.1-ES584678.1.5
TGAATAATTTCATTATTATTTTTTATTTCTATTTTTATTAGTTTTATAATTATAAATTATTTTATTTTTAATT
ATAAAAATAATTTTAATTTTAAAAAAATTAATAAAAATAATTTAAAAAAAAAATTTTGAAAATGATAAATAAT
CTTTTCTCAATCTTTGATCCTTCAACAAATTTATTTAATTTCCCATTTAATTGAATTA
GAACATTTATTGGATTATTATTTATTCCTTTTTCTTTCTGATTTTTTCCTAATCGTCATTATTTATTATGAAA
TTTTATTTCAAATAAACTTCATAATGAATTTAAAACTTTATTAGGACCAAATAGAATTAATGGATCAACTTTT
ATTTTTATTTCATTATTCTTTTTTATTTTATTTAATAATTTTTTAGGTTTATTCCCAT
ATATTTTTACTAGAACAAGTCATCTTAATATATCCCTTTCATTATCATTAACATTATGGTTAAGATTTATAAT
TTATGGATGATTAAATAATACACAACATATATTTATTCATATAATTCCTCAAGGAACACCAACTATTTTAATA
CCTTTTATAGTAATAATTGAGACTATTAGTAATATTATTCGACCTGGAACTTTAGCAG
TTCGATTAACAGCTAATATAATTGCAGGTCATTTACTTTTAACTTTATTAAGTAATACAGGTATTAATATACC
TAATTATTTATTAATTATTTTAATTATTATCCAAATTTTATTGTTAATTCTTGAATCTGCCGTAGCAATTATT
CAATCTTATGTAATTTCTATTTTAAGAACTCTTTATTCTAGAGAAG
>1002049201_1 Heli.1-ES588191.1.5
CATGAGCTAAGACTTGATGGAGACGATCCGAAGATTGATTTTGATACATTATTATATCCTCAAGGTACTGTCC
ATAACGCTTATAGTCATCATCAAGGGAGTGTTAGCCAGTATATTCCAAGTTTAGTACAAGGAGTGTATGAAGT
GGATACTGAACCAAATCCTTTAAGTCCATGTGATCCGCGCTTGCCTGAGGGTAATCCA
CCATTGAAATGTGGTGTCGAACATATGGGTAAGCCTCCTTTAAATTTTCCTAAGAAACATTTAGATTTAGCGT
CAGATGATCTTCAAAGTGTATTACTACAAGCTGTTAAACCTGTTCGTGTTAATATAGGTTTGGTATCTCTTCA
AGATGCCATATGTGGAAATGTTAATGTTAAGGGTTTTGAACCATTGGAATGGAGTTCT
AGTGAAGGGTTTCCCCTAAAATCAATTAGACCACCAAATAAAAAAGGGAAGCGTTGGTTATTTGATTTGGAGG
AAACGTCAGAAGGATTTAAATTAAAAGGTATGCATGGTGAATTACAACGACAATTGAGTGTGTGTCATGCGTT
GCGTAAGAAAGGAATTCGTTGTCCTACAATATTTACTGATTGTTTAAAAGATACATGT
ATAGATATTAACAAGTGCAAAATACCAGGAAAGACGCGTGTATTTTCAATATCGCCTGTACAATTCACCGTAG
CGTTTAAACAATATTTTAATGATTTTCTTGCGTCATACCAAAATGCTAGGATAAACGCTGAGCATGGGATTGG
GATTAATGTAGATTCTTTAGAGTGGACAGAGGTTGCGAATTACATAACTCGATATGGT
TCGTCAATTGTAGCAGGCGATTATAAGAATTATGGGCCTAGCCTGATGCTAAGTTGTGTAGAAAAGGCCTTCG
ATATTATAATGGCTTGGTATGATAGATATGACCCTGATGAGGAGCGTCAATTAGTTCGCCGCGTTTTATTGTC
CGAAATTCTTCATGCTAAACATTTGTGTTTAAATGTAGTATACGGTGTACCTTGTGGT
ATACCGTCTGGTAGTCCTATTACAACCCCATTGAATAGTATAGTTAATTCATTATATCTTCGTTGTGCATGGA
AAGATATCGTAAATGAAAGTTTTGAAATTATGCATAATAATATTAAATTTCTTACATATGGTGATGATGTGTG
TATTAATGTTAGTGATAATTATAAAGATATATATAATACTGAAACTTTAAGTGCGTTT
TTTAAGAAATACAATATTATTTTTACAGATATAGATAAGAGTGATAATATTATAAAATATCGTAATTTAGACA
ATGTCACGTTCTTGAAACGTGGTTTCAAGTTACATCCAAATAGTAAAGCTGTGTTTCTTGCACCAATCGAAGA
GCAGAGTATTAGAAAATGTGTTAACTGGATTACACAAAAAGGTGATCCTGTACAAAAC
ACATTAGAAAATTGTAAACAAGCATGTGAGCTTGCTTTTGGATGGGGACCAGAATATTATAATGCTGTACGAG
AGCGTTTATCACGAGAGTGTTTGACGCGTTGTGGTCAGTCGTTTTCGGCACCTTCATGGTACGAGAAGTCTGA
AATGTGTTATAATATTTAGAGTCTTTTCTTTTAAATTTATATTAATTTTGTGTATATT
TTATTAATATAAGCCTTTCCCGATGTGGAACGCCCATGTGAGCGAGGCCCGGGGAAAATT
>1002049202_1 Heli.1-DT666706.3.5

GACACGGTGTTGCGGTGCGGTTAGAGATAATTCTAAGTGTTTCAAAAGAATTTACCAATTCAATTCAATGATC
ACCGCAAGAGAAAAATTGATACTGTGTCGCAATTTGCTGGCTTAATTACTTTATATCCCTTCAAACAAGGGCA
TCAGTAATATAGTCATCTAAATTCATCAAACATGTCCGTCGACAAGGAGGAATTGGTG
CAACGCGCCAAGCTTGCGGAGCAAGCTGAGCGGTATGACGACATGGCGGCGGCGATGAAAGAAGTCACCGAAA
CCGGAGTCGAGCTCAGCAATGAGGAAAGGAATTTACTTTCCGTTGCCTACAAGAACGTCGTGGGTGCTCGACG
GTCATCATGGCGAGTCATATCTTCCATTGAACAGAAAACCGAAGGATCGGAAAGAAAA
CAACAGATGGCAAAAGAATATAGGGTTAAAGTAGAAAAAGAGCTCAGAGAAATCTGCTACGATGTTTTGGGTT
TACTTGACAAACACCTTATCCCTAAAGCTAGTAATCCAGAAAGTAAAGTATTTTACCTTAAAATGAAGGGAGA
TTACTACAGGTACCTCGCAGAAGTGGCCACTGGAGAAACCAGAAATTCCGTTGTAGAC
GATTCACAGAAAGCGTACCAGGATGCTTTCGAAATCAGCAAGGCGAAAATGCAGCCCACACACCCAATAAGGC
TTGGGCTGGCGTTAAATTTCTCCGTCTTCTATTATGAGATATTAAATTCACCAGACAAGGCGTGTCAGCTCGC
TAAGCAGGCGTTCGACGACGCGATCGCGGAGCTGGACACGCTGAACGAGGACTCGTAC
AAGGACTCCACGCTGATCATGCAGCTGCTGCGGGACAACCTGACGCTGTGGACGTCGGACACGCAGGGCGATG
GCGACGAGCCGGCCGAGGGCGGCGACAACTAACGCGCCCACGCACTTGTTCTCACCCGTGTTTTATAAGAAAA
CGAGTAACATTCGAGAACGTCGCGCCGCCACCGCCGCCGCCGCGAGCCGTCGCTTGTA
AAAAAAAATGTTAAAAATGTCGTTCGCAGCGGACCTTCACGTTCCGTCTAAAATTAAATGTTGTGTCATCTTT
GACGGTTTTGTATTTCTGTATTTACACTGGATTACAGATATCGAGCTGACCTGATGAATATAAATATTTATAA
CTTAAGTTATTAAAATTACGGT
>1002049203_1 Heli.1-ES587373.1.5
AACAACACAGACCAAGATGTTCAAGTTGGTGGTGTTGTGCGCTTTCCTCGCCGCGGCTGCAGCGGAGCCCGGA
ACCTTCATCACGCCATTGACTTACTCCAGCAGCATTATCGCACCGGCTGCAACAACTGTTACGCACCAGGCCA
GCAGTGTCATTCACCCTTCACCCCTGCTCTACCCTCGATTCTACGACCTATCTTACTC
ACCGGTTGCTCATCTTATCAAGAAGCGATCCCCTGGAATTTATAATCCTTTCTCTGCCTTCGGCCCATCAACT
TACATTGCCTCTGCACCTCTGGCCACGACCTATACTGCAGCTCCTCTTGCTACTACCTACACTGGAGCTCACT
TGGCTACAACTTATGCTGCACCAATCTACCCTGCTGCTGCGCATTTAATCAAGAAGAG
GTCTGCTCCACTTGTGCCTACAACCTACGTTGCTCCATACTACTCTTCTCCTTTGTTGACGCCGACGTACACA
GCCGCTGCACCCATCTACTCAACACCTTACATCACGAGTGCTCCTCTTACTTACACACATTTGATCAAGAAGC
GTTCCGCTCCATTGTTCTACGCAGGTTACACCGCCCCCGCTTCATTCTCTCATCAGTC
CCGAGTAGACATTAAGAGCTCCCCTGCCACTATTACGTCCTACTCCTACCCTGCTGCTGTTTCTTATGCTAGT
CCAGTCGCAATTTCACATGTCTTCTAGATGATAGAATGAATTTTTAAATAAAATGAATC
>1002049204_1 Heli.1-EE743462.1.5
ACAGCATCAACATGCATTTCAAAATTGCAGTAGTAAGCATCCTCGTGGCCGTATGCCATGCAGGACTTATTGA
AGAAGGACAAAATCACGCCATTTCATCTCAAAGCATCGTCCGTCATGACCAACCAATTAATGGCCCCACACAC
TATGCACCTCTCGTTGCTCATGCCGCTCCAGTCCTCGCCCATGCTGGACAAATCCTTC
AACATGCTGCTCCCCTGATCCATTCTGCTCCCATCGTCCAACATGTTGCCCCCATAGCCCACGCCGCCCCCAT
CGCCCTCGCTCATGGTGAACAAATTGAAGATCATGCTCCAGCTCACTACGAGTTCTCATACTCAGTTGAAGAT
CCCCACACTGGTGACCACAAGTCTCAACACGAGAGTCGTGAAGGTGACGTAGTCAAGG
GAGAATATTCTCTGGTTCAACCTGATGGTGCTGTAAGAACCGTTGAATACACCGCTGACGCTCACAATGGTTT
CAACGCAATTGTGCATAACTCTGCTCCGTCAGCTCACGCTGCTCCAGCTCCAGCAGTACATTCAGCCCCAATC
CAGGTGCACGCTGCTCCCGTAGTGCATGCCGCTCCATTGGTCCATGCTCCTCTAGTAC
ATGCTGCTCCTTTGGTTCACGGACCTGTGATCCATGCAGGACCTATCCTAGCCCACCATTAGGCTCTAACTTT
TCAAACTATTGTAATTCCTATTAGAAATATATTGTTACTATTTATAA
>1002049205_1 Heli.1-DT668417.3.5
TCTCTCTGGTTCTTTTTCATGGCTGACGTTCGAAGGAGCTAACTGCAGTCATGAAGCTGAACGTCTCCTACCC
GGCAACGGGATGTCAGAAGTTATTCGAAGTTGTCGATGAGCATAAGCTCCGTATCTTCTATGAGAAGCGCATG
GGCGCAGAAGTTGAAGCTGACCAGCTAGGTGATGAATGGAAGGGCTATATCCTCCGTG
TAGCTGGTGGCAACGACAAACAGGGCTTCCCCATGAAACAGGGAGTTCTGACTAACAGTCGTGTCCGTCTGTT
GATGTCAAAGGGTCACTCTTGCTACAGACCACGTCGTGATGGTGAGAGAAAGCGTAAGTCAGTGCGTGGTTGC
ATTGTCGATGCTAACTTGTCTGTACTCGCCCTCGTCATTGTCCGCAAAGGAGCTCAGG
AAATCCCTGGACTCACCGATGGCAATGTCCCCCGTCGTCTAGGACCTAAACGTGCATCCAAAATCCGAAAGCT
GTTCAACTTGACCAAGCAGGATGATGTTCGCCGTTACGTCGTCAAACGCCTCCTGCCCGCCAAGGAAGGCAAG
GAGAATGCTAAACCCAGATATAAGGCCCCCAGGATCCAAAGGCTGGTCACCCCAGTAG

TATTGCAACGCAGACGTCACAGATTGGCCCTCAAGAAGAAGCGCCTCGCGAAACGCAAGGCCTCTGAGGCTGA
TTATGCTAAACTACTTGCGCAAAGAAAGAAGGAATCTAAGGTACGCCGCCAAGAAGAAATCAAACGCAGGCGT
TCAGCTTCAATGCGCGATTCCAAGAGCTCAAACCAGAGTGCGCCGCAAAAGTGAAAGA
CTTTTATCAATAAATTAATAAAATATCTAAAAAAAA
>1002049206_1 Heli.1-DT666464.3.5
CGGGTCGACCACGCGTCCGCGCAAGGCGCACGTATCGTGCTATTTGCGTTTTGTCTTGTCCTTAAATTTTTCG
AAGTCCCTACACACGCAAAGATGGGTAGGGAGGACAAGGCTACTTGGAAAACCAACTATTTCACTAAGATTAT
CCAATTATTAGATGAGTACCCAAAATGTTTCATCGTGGGTGCCGACAACGTTGGCTCC
CAGCAAATGCAGCAGATCCGTATCTCCCTACGAGGAAGCAGCATTGTGCTTATGGGAAAGAACACCATGATGC
GTAAAGCTATTAAAGACCATTTGGAGACCAACCCGGCCCTTGAGAAATTGCTCCCTCATATTAAAGGAAATGT
CGGCTTTGTGTTCACCCGTGGAGATCTCGTTGAGGTCCGTGATAAGCTTCTGGAGAAC
AAAGTGCGTGCTCCAGCCAGGCCTGGTGCCATTGCTCCATTAGCTGTGGTCATCCCAGCTCATAACACCGGTT
TAGGTCCCGAAAAGACTGCTTTCTTCCAGGCTCTGTCCATCCCTACTAAGATTTCCAAGGGTACTATTGAAAT
CATCAACGATGTCCACATCTTGAAGCCTGGTGACAAAGTAGGAGCTTCCGAGGCAACC
CTCCTCAACATGTTGAACATCTCTCCATTCTCATATGGTCTTGTTGTTAAGCAAGTATATGACTCTGGCACCA
TCTTTGCGCCAGCCATTTTGGACATTAAGCCGGAGGATCTCCGTGCCAAGTTCCAAGAGGGTGTGGCCAACGT
AGCTGCCTTGTCATTGGCCATCAGCTACCCAACTGTGGCTTCCGCTCCTCACTCCATT
GCCAATGGATTCAAGAACTTGTTGGCCATTGCTGCTGTCTCGGACGTTGAGTTCAAGGAAGCTAACACTATCA
AGGAGTTTATTAAGGACCCAAGCAAATTCGTCGCGGCTGCTGCTCCCGCCGCCGCAGCCGCTGCACCAGCTGC
CGCCGCCAAGGAGGAAGAGAAGAAACCCGAGAAGGAGGACTCTGAGAGCGACGACGAC
ATGGGCTTCGGTCTCTTTGACTAAAAGCACTATTGAAGTCAAGTTTCCCATGCTTTACCATCTCATTATGGGG
ATAAGTTGTAAAGGGATGTTTGCAATGAAGGTACTCGCGCCGCCGGGTCTGGACGCCCATGTGGCGGCCGT
>1002049207_1 Heli.1-ES588054.1.5
CTTGATCTCCCGAGATGTTCAAGTTTCTTGTTCTGCTGTTGGTCGCGAGCGCTTCGGCGCAGATACCCTCGCT
GGGATGGTGCCCCGATTATCAGCCCATGGCGAACTTCAATATCAATCGCTTCTTAGGTTCCTGGTATGAAGCT
GAGCGTTACTTCACCGTCTCTGAACTGGGAACTCGGTGTGTTCGCACTAAATATGAGA
GCACACCCGAAGGCAGGATCTTGGTCTCTAATGAAATAACTAACTCTTTAACTGGATTGAAACGTGTAATGGA
AGGGTCTATGCAGTTGATTGGTAGAGAGGGAGAAGGTCGCATGATCATCAAATATTCGGCGATCCCGGCGCCC
TACGACAACGAGTACAGCATTCTGGACACTGACTACGAGAACTACGCGGTGATGTGGT
CTTGCAGCGGCATCGGACCTGTGCATATCCAAAATGCTTGGATCCTTACTCGTGAACGTTTGGCAACACCCAT
GGTAATGCAGAAGGCCTACACTGTGCTAGAACGATACAGGATTTCCAGAACATTCTTTGTGAAGACTAACCAA
GCTGATTGCTACATCATACCTAGCCCCGTCGCTGATCCTTTGGACATTAAAAACGATG
TCATTATTGACGCGAAAAACGTCCCATTAGAAGTATCTCTCGAAGAAAAAGCTCCAGCACCTGAAAAGAAAGA
AGACCCCGCACCAAAATCTGAAAACATTCAAGAACGTTCCGGCGAGCTGGATATTTCTGTTGAGGAAAAGCCG
ATGGATATGCCCGAAAAAATGTCTGAAATGAAAGATGAGAAGTTGAAAATATCAGAAG
AAAAAATAGAAGAAGAGATAGAAATGAATAAAGAAAAAATAGTGTAAACTATTAAGAAAATGTAAACAATTAA
GTAATGACTATTATGTAGTTGTTCTTGTGTTCTTAGCTTTTGAAAGCTACTGTACCAAATAAAGCGTTAGAAT
GACATGTCGTTAGACACGTAATAGTCGGTAAACTTTGTCAAAGTTCCATAATAAAATT
>1002049208_1 Heli.1-DT665186.2.5
TGTTCAGTAAAATTGTTACTTTCTGCGCGGTGGTGGCAGTGTCCTCTGTGTGCGCGGTGGTGGCAGTGGCCTC
TGCTGGACTTTTACCTGCAGCAGTACACTACTCACCAGCTGAGGCTGTATCATCTCAAAGCATTGTGCGTCAT
GATCAGCCTCAAGCTATCGCCAAGTTGGCTGTAGCTTCTCCTCTTGCCTACCACGCGG
CTCCTTCCGTCGCCTACCACGCTGCCCCAGCTGCCATCTCCTACCAAGCCGCTCCTGCTCATTACTCTTCTGC
CGCCGCTGTTTCTTCCCAAAACATTGTCCGTCATGATGAATCCGCAGGATTAATAGCCTCAGCCCATAAACTG
GCCATCGCCGCTCCCGTCGCCAAACTTGCCCTGGCCTCTCCCCTTGCCTACCAAGCAG
CTCCAGCCCTTGCATACCACTCTGCCCCCGCTCCAGTCGCCTACCACGCTGCTCCCGCTTCCATCGCCTACCA
TGCTGCTCCCGTAGCAAAGGTTCTTGCCGAGCCTGAAATCATTGCCCACCCAAAATACGAATTCAGCTACTCC
GTAGCTGACGGACACTCCGGAGACAACAAGCAACAACAAGAATCCCGCGACGGTGATG
TCGTAAAGGGCTCCTACTCCTTCGTTGAGGCTGACGGCTCCGTCAGGTCCGTGGAGTACTCCGCTGACGACCA
CAACGGTTTCAACGCCGTCGTACACAACAGCGCCCCCGCTCACGCTCCCCAAGCCGTCCTCAAGGCCGCCCCC
GTCTACGCCGCAGCTGCTCCCCAATACTACCACTAAATTTGTTGATTATTGATCGTGT
CAAATGATTGTTGATAATTTATTTATTGTAATAAACGCATAAGTCGAATAAAAAAAAAAA
>1002049209_1 Heli.1-DT665599.3.5

GCACAGGCAAACGAGCAACTGACTTGGTGTTCAAGTGATTTATTATTTAGTTTTGTTTATTTAAAAACAGTAA
TTTTGGCCAGCAATTAATATTTTAAGCAAAATAAAATCTCAATAGTAGTCATACAGTTTTTAGTTTATAAAAT
AAAGATATCAAAATGCAGATTTTCGTGAAAACACTTACGGGTAAAACCATTACTTTGG
AAGTTGAACCTTCCGACACTATTGAAAATGTAAAAGCCAAAATTCAAGACAAGGAAGGCATTCCACCAGATCA
ACAACGTTTGATTTTCGCTGGAAAGCAATTGGAAGACGGCCGTACTCTTTCTGACTACAATATTCAAAAAGAA
TCTACTCTTCACTTGGTATTGCGTCTTCGTGGTGGAATGCAGATCTTTGTTAAGACTC
TCACAGGAAAAACCATCACGTTAGAGGTAGAACCGTCGGACACAATTGAAAACGTGAAGGCAAAGATCCAAGA
CAAAGAAGGTATTCCCCCAGACCAACAGAGGCTCATTTTTGCTGGAAAACAACTGGAAGATGGCCGTACATTA
TCTGATTATAATATTCAAAAGGAATCTACTTTACATTTAGTATTGCGGCTTCGTGGCG
GTATGCAAATATTTGTAAAGACCCTTACTGGTAAAACAATTACTTTAGAAGTTGAAGCCTCTGACACAATTGA
AAATGTGAAAGCTAAAATTCAGGACAAAGAAGGCATTCCGCCAGATCAACAACGTCTTATTTTCGCGGGCAAG
CAATTGGAAGATGGTCGCACATTGTCAGATTATAACATTCAAAAAGAGTCAACTCTTC
ATCTTGTGCTCCGCCTCAGAGGTGGCATGCAAATCTTTGTTAAAACCCTCACTGGCAAAACCATCACTCTTGA
GGTTGAACCCTCTGATACTATTGAAAATGTAAAGGCTAAAATCCAAGACAAAGAGGGCATCCCACCTGATCAG
CAGCGCCTTATCTTTGCTGGCAAGCAATTGGAAGATGGTCGTACCCTGTCTGACTACA
ATATTCAAAAAGAGTCCACACTTCACCTGGTACTGCGTCTGCGAGGAGGTATGCAAATATTTGTCAAGACACT
AACAGGAAAGACAATTACCCTGGAGGTGGAGCCATCTGATACTATTGAAAATGTAAAGGCTAAAATCCAAGAC
AAAGAGGGCATCCCACCTGATCAGCAGCGCCTTATCTTTGCTGGCA
>1002049210_1 Heli.1-ES586203.1.5
GAGCAGTCTAACAGAAGTGCACGATGAGATTTCTGATTGTAGCCGTCGCCGTCTTCGCCTGCGCCGCAGCCGC
ACCTTCCGGTGCTCTGCTGGCCGCACCCTACTCCGCCGGGCTCCTCGCCCACGGAGCTCACTTGGCATACGCC
GCCGTCCCCGCAGCCCTGCCCGTCAACTCCGGCGACATCCAGGGAGCCGCCATCGATG
CCCACGTGCAGGTAGCCGACCACGTCCGCGCTGTCAACGACCAAGCCGCAGAAATCCAAGGCCGCGCCATCAA
CGCCGCTGAGGATCACGCCTGGTCCGCAGTCAACGCCGCCCAAGTGGCCGCCGCCCAGATCGACGGAGTTGCC
GCTAACGTCTCGCCTGTCGCTGCCCGCCAGCTCGCCGGTCACGGTGTAGTCGCCCCCG
TGGTCGCTGCCCCCGCTGTCGCTGCCTACTCTGCTCCCTGGGTCGCCCCCGCCATCTCTGCCTACTCTGCCCC
CTTGGTCGCCCCCGCCATCTCTGCTTACTCTGCCCCCATTGTCGCTGGCGTCGCTGGATCCCACTCCGTGTCC
GTCCAGAGCCTGTCTCAGTCCCACCCCGCCCCCATCGTCCACGCTCCCCTCGCTCTCG
CCCACGGCCTCGCTCACGCGTGGTAAATTATTAGACCAAATTTAGATTTAATAGACAATGAAGACGTGCGATT
AAAAACATTAA
>1002049211_1 Heli.1-DT666774.3.5
TTTCTGAACAGGCGCAGTCCGAGAGAACTCGCAAAAGGAGTAAAAGGCTCCTACATAACCTGTAACTATCAAT
ATATTGTATGAGTCAGAAGGTAGAAAAACCAGTATTATCGGGTCAACGGATCAAGACCAGAAAAAGAGATGAG
AAAGAGAAGTACGACCCGAACGGGTTCCGCGACGCCCTCGTGGGCGGGCTGGAGCGCG
CCGGCGGCGACCTGGACGCAGCCTACAAGTACCTAGACGCGGCCGGCTCCAAGCTCGACTACAGGCGCTATGG
CGAGGTCATATTCGACGTGCTCATTGCCGGGGGGCTGTTGCTTCCCGGCGGCTCCGTGTCGATGGACGGGGAG
ACCCCCAAGACCAACACGTGTATATTCAACTCGAGCGAGGATATGGAGACTATGCGTA
ACTTTGAACAGGTATTCGTGAAGTTAATGCGACGTTACAAATATCTCGAGAAGATGTTTGAAGAGGAAATGAA
AAAAGTCCTAGTGTACCTGAAGGGCTTTGAACCACACCAACGTTTAAAGCTGGCCCGCATGACCGCACTATGG
ATAGGTAATGGCTGCGTGCCGCCATCAGTGTTGCTAGTGCTGGTGAATGAACACCTTT
TAAAAGATAATTTGGCACTTGAATTCGTTTTGGAGGTATTCTCTACGGTGAAAGCGGAGAGGGGCGTCACTTC
GCTCGTCACCGCCCTTAAGAGAGGACAGTTGGAGGGCAGGTTACTAGAATTCCTACCTCTCAACCGTCGTACA
GAAGAAACCCTGGCCTCCGCGTTCGCAGCGCGCGGCCTCGCGGAGCTCCTACGCCTGC
ACCGCGCTCAGGCCTCTCAAGAGGCCCGCAGAGAGCTCACTCATGCACTCCTGGAACAGTTGGCTGATGCCCG
CAACGTCAGGGACCTCGTGGCCGATACGAGGGACGCTGCTGCGAGACACGCTATACCTGATCATGAAGTTGTT
GCTATTATATGGCAGTGCGTTATGTCTCGCGGAGAGTGGAATAAAAAGGAGGAACTGC
TAGCGGAGCAGGCGGCCAAACATTTAAGACATTACACGCCGTTGTTGGCCGCCTTCGCGCAGTCTGCCAAAGC
AGAGATCGCGCTTTTGACTAAGGTGCAAGAATACTGCTACGAGAACATGAGCTTCATGCGCGCGTTCAGCAAG
CTGGTGCTGATGCTGTACAAGACGAACGTGCTCTCCGAGGAGGTGATCCTCAAGTGGT
ACCGCGAGCCCAACTCCAGCAAGGGGAAGGTCATGTTCCTCGACCAGATGAAGAAGTTTGTGGAGTGGCTCCA
GAGTGCCGAGGAAGAATCGGAGAGTGGCGAAGAAGAAGATTAGAAATAAAGGCAGCGTGAGACTGAACACATA
GCGCCGTCCCACACACACACACACACCCAACAGTAAACCAGAAACCATAACCCAAACT

GCAACCATAACCAACCATAAACCACAACTGAAACCAACAGGAAACCGCATCCAAGTGAGCACATCACTAGCTG
TCAGTGATGGGACGTTTTCTACAATTGTATATGAATCATTTGAAATACATACAACTACTAACTAACATGTTGA
TGAAATACAATATTTTTTATTCAATTTTTTTAGTAAAATTTCAATAAATGGTGGTGAC
>1002049212_1 Heli.1-DT665225.3.5
GCACGAGGGAGAGAGAGAGAGAGAGAGAGAGAGAGAGAGAGAGAGAGAGAGAGAGAGAGAGAGAGAGAGAGAG
AGAGAGAGAGAAAATCATAAAGATATTGGAACTTTATATTTTATTTTTGGAATTTGAGCAGGTATAGTTGGAA
CATCTCTTAGTATTTTAATTCGAATAGAATTAGGTAACCCCGGATCTCTAATTGGGGA
TGATCAAATTTATAATACTATTGTTACAGCTCATGCATTTATTATAATTTTTTTTATAGTTATACCCATCATA
ATTGGAGGATTTGGAAATTGATTAGTACCTTTAATATTGGGAGCCCCTGATATAGCATTCCCCCGGATAAATA
ATATAAGATTTTGACTTCTCCCCCCCTCATTAATTTTATTAATTTCTAGAAGAATCGT
AGAAAATGGAGCCGGAACAGGATGAACTGTCTATCCCCCCCTTTCATCTAATATCGCTCATAGAGGTTCATCT
GTAGATTTAGCTATTTTTTCCCTTCATTTAGCGGGTATTTCCTCAATTTTAGGGGCAATTAATTTTATTACTA
CTATCATTAATATACGGATTAACAGCATATCTTTTGATCAATTACCATTATTTGTCTG
AGCTGTAGGTATTACAGCTTTATTACTTCTTTTATCTTTACCAGTTCTAGCCGGAGCTATTACTATACTTTTA
ACTGATCGAAATTTAAATACCTCATTTTTTGACCCCGCCGGAGGAGGTGATCCAATCTTATATCAACATTTAT
TTTGATTTTTTGGGCACCCAGAAGTTTATATTTTAATTCTCCCAGGATTTGGTATAAT
TTCTCATATTATTTCCCAAGAAAGTGGTAAAAAAGAAACTTTTGGTTGCTTAGGGGATATTATGCTATAATAG
CAATTGGTCTA
>1002049213_1 Heli.1-DT661969.3.5
CGGGTCGACCACGCGTCCGTACTGCTTTTGGAGTAACCGATTCGTCGGATTACGATTATTTTAATAAAATGGG
TTTCGTGAAGGTGGTGAAGAATAAGCAGTACTTCAAGCGTTACCAAGTCAAGTTCAAGAGGCGTAGGGAAGGT
AAAACTGATTACTATGCACGTAAGCGACTGGTAGTGCAGGACAAAAATAAGTACAATA
CACCCAAGTACCGTTTGATTGTCCGACTTTCAAACAAGGATGTAACTTGCCAAGTTGCATACTCCCGTATTGA
AGGAGATCACATTGTTTGCGCGGCTTACTCGCACGAATTACCACGTTATGGTATTAAGGTGGGTTTAACTAAC
TATGCGGCTGCATACAGCACTGGGTTACTCCTTGCTAGACGTCTGCTGCAGCGGTTAG
GTCTTGACTCATTGTATATTGGTGCCACAGAAGTCACTGGTGATGAGTTCAATGTGGAACCAGTTGACAATGG
ACCAGGGGCCTTCAGGTGTTACTTAGATGTTGGTCTTGCTCGTACAACAACTGGGGCTAGAGTTTTCGGAGCA
ATGAAAGGTGCAGTAGATGGTGGCCTTAATGTTCCTCATTCCATCAAGAGATTCCCAG
GATATGATGCGGAGGCAAAGAAATTCAATGCTGAAGTTCATAGAGCACACATCTTCGGTCTTCATGTAGCGGA
ATATATGAGAAACCTGGAGCAAGAAGATGAAGATTCCTTCAAAAGACAATTCGGCAAATATGTCCAACTTGGT
GTTACTGCTGATGCTATTGAAGGCATCTACAAGAAAGCCCATGAAGCAATCCGAGCGG
ATCCATCTCACAAGAAGAAGGAAG
>1002049214_1 Heli.1-DT665541.3.5
GCACGAGGCTGCCTTCAGTGCAACCAACTAAACGCAGCATCATGAAGCTCATGCTCGTGGCCGCCAGTCTGGT
GGCTGTTTTGGCTATCGCTCACGCGGAAGAAGCCAAAGCAGCAGAAAAAGAAGTGGCAGTAACCGATGACAAA
GCGGCAACCGAGGACAAGAAACACGAGAAACGTGGACTATTTGATCTTGGTTACGGTG
GACATGGTGGATTCGGAGGTGGTTACGAAGGTGGTTTAGGCGGTGGCTTCGGAGGTGGCTACGGCGGTTACGG
CGGTTACGGCGGTCATGGCCACGAAGAGGTGCACAAAACCATTACTGTCGTCAAAAATGTCCCAGTACCCTAC
CCAGTAGAGAAACACATTCCCTACCCAGTTGAAAAACACGTACCTGTGCCCGTCAAGG
TGCCAGTACCTCAACCTTACCCTGTTGTCAAACATGTCCCATATACTGTCAAGGAACTCGTCAAAGTGCCCGT
ACATGTTCCTCAACCTTACCCAGTTGAAAAGAAAGTCCCCTACCCAGTTCATGTTCCAGTCGATAGACCAGTC
CCCGTCAAAGTGTACGTGCCAGCTCCTTACCCAGTAGAAAAGAAAATCCCTTATCCAG
TAAAGGTACCAGTGCCAGCACCTTACCCAGTTGAAAAGAAAGTCCCCTACCCAGTGAAGGTACCAGTCCATGT
ACCCGCTCCCTACCCAGTAATTAAGGAAGTACATGTTCCCGTTAAGGTCCCAGTCGACAGGCCCTACCCCGTA
CATATTCCTAAGCCTGTGCCCTACCCTGTAGAGAAACCCGTACCCT
>1002049215_1 Heli.1-DT667171.3.5
TCTGTTATTTACTCGTATGTGGCACTTCTGTTATTTACTATATTCTTAATATTATAATTATTACTATATTCTT
AATATTATTAAAATGCGTCAATTTGACGAAGTGTTGACCGTGCTCGGGGTTACGGTGATGTGCTTTTGCGTGC
ACCGGACTCTCCAACTAGTCCCGTTGCCGGATTTCATCCATCCATGTGGCGAGTTAAC
CGATGACTGCTTCACAAAGGCCACACTCGATGCTATTCCAGGAGTAGTGAAGGGGATTCCTGAAGCTGGCATC
CCACCTCTCGATCCCTTATATCTGGACAGAAACATTACTATGAATCTTCCTGGAAACGTAAAAATGACGTTCC
ATAATGGAAAATTATCAGGATTAAGCACTTGTATACCAGATAAAGTGTCATCACAACG

AGCAAAACGAACATTTATCTTTGACATACATTGTAATTTTACCATAAAAGGGCAATACAGCATCCAAGGCAGA
ATCCTTCTCTTTAACCTCGACACTGACGGCTCCGCCAAAATTAAAATTTGGAACCAACACATAAGGTTGGAAG
TCCGGGAAAAAATTGTCAGAGATGAAAACGGTGAAGGGCATTACAAAATAAACTCATA
CAAATACAAGGCTGATTATGGTTCTGACCTTAAACTAAATCTCACCAACTTATTCAGAGGCAGTCCGCAGATA
AGTGCAAATATATTGGATGTGCTAAATTCGAACTCCCAGCTAGTGGCGCAGGAGTTTGGAGGTCCCATTCTGG
ACTACGCCATAGACTACGCTATGAACGTCACACAAAGATTCTTCAGCACATACACCTA
CGATCAGATCAGCAAAGTACCACT
>1002049216_1 Heli.1-DT663422.3.5
GAACGTTATAGTGTCCACTACTCCCGTGCAAGGACTATAGTTTAACCATGAATTCACGGTGTTTATTAGTGCT
AGCAGCCGCCCTCGGCGTGGCGGTCGCCCAAGAAAGCTTCAAATGTCCAGATGACTTCGGCTTCTACCCTCAC
CACATCTCTTGTGACAAATATTGGAAGTGTGACAACGGAGTCGCTGAACTTAAGACTT
GTGGTAACGGACTCGCCTTCGATGCTTCAGATTCCAAATACTTGACCGAAAACTGCGATTACCTCCATAACGT
TGAGTGCGGCGAAAGGACCCAGCTTGAACCCCCAATCTCCACTCCTCACTGCGCAAGATTGTACGGTATCTTC
CCTGACAGCGCCAAATGCGACGTTTTCTGGAACTGCTGGAGTGGAGAAGCTTCCCGTT
ACCAATGCAGCCCCGGACTTGCTTACGACAGAGAAGCTCGCGTGTGCATGTGGGCTGACCAGGTTCCCGAGTG
CAAAAATGAGGAAGTAGCCAACGGTTTCTCTTGCCCCGCCCCTGGTGAGGTTTCCAACGCTGGATCCTTCAGC
CGTCACGCCCATCCTGAAGATTGCCGCAAATACTACATCTGTCTCGAGGGAGTAGCTC
GTGAATACGGATGCCCAATTGGTACCGTTTTCAAAATTGGTGATGCTGACGGCACTGGCAACTGCGAAGACCC
CGAAGATGTTCCTGGATGCGAGGACTACTACGGTGATTTGGACCTGAAGGCCATCCGCAAGAGCGAGCTGCTC
GCCGGCCTCCACAGCAGCTCGGCCCAATCCGTCCAGCCCAAGATCAAACCACGTCCCC
AAAAAGAAAACTAAATACTCACTTCCCACCCTTACCTTTTAATACTCACTACTTAATCTAGTTTCCACACACC
ACAAGCAAATTTTTTGACGAAAATTCTATAAGAATTCATATAAATATATACTAACGTTGCTTTTGGTGTGAGA
GACACGATACCAGGTTCAAATTCGTATTTTAAGAAAGTGAAGCCAATTGATTTGTATT
CATTTCCATTTGCCAAATACTTTTCTCGTTGTAGCCTTAAACAGTTTCAAAAAGCAGATTAGAAAATTAGGAA
TAATTATTTTATATTCTATTTAAATTGATAGCGTCTTATAAATGTACGATTTTATGAAACATTATGATAAATG
AATATCGGTATGCATTTATGTTTATAATTCTTGACGTAACGATGAAACAATCCAATTC
CAACAGCCAACGACCTTGTAAATAAATATATAATATTTTATATACTAAATTATCGCCGCGACTGAGTGTTGTT
ATAGAAACTGTAAACTTAAGTTTTAATGTTTAGTGATAAAATAAACAAAATACAAAAAT
>1002049217_1 Heli.1-DT665640.3.5
GCACGAGGGTAAACTTCGACAAGCACCGAATTTTTAAAAGAAGAATTTTCTTACACTGTACATTCCCGTAAGC
ACATTCAAAAATCGAACATGTTTTCTACCGTTGGTAGAGCTGGTCTTTTGGCCACGAGAGCGGTAGTTAACAA
TACTTTGGCTGAGAAGACTCCTTTGGTTGCCGGAGCTTTTGCGAACAAACGTGATTAT
GCTGCTAAAGCCGCTGGAAAAGGTCAAGGTAAGGTGGTTGCAGTTATTGGTGCTGTCGTAGACGTACAATTTG
AGGATAGTCTCCCACCCATCCTAAATGCCCTCGAAGTCCAGAACCGGTCCCCTAGGCTCGTCCTGGAGGTAGC
CCAGCACTTGGGAGAAAACACCGTTCGCACCATCGCCATGGACGGTACTGAAGGTCTC
GTGCGTGGACAGCCCGTGCACGACTGCGGCTCGCCCATCCGCATCCCCGTAGGCGCAGAGACGCTCGGCCGCA
TCATCAACGTCATCGGTGAGCCCATCGACGAGCGTGGCCCCATCCCCACTGACAAGACCGCCGCCATCCACGC
CGAAGCGCCAGAGTTTGTGGACATGTCCGTACAGCAGGAGATCCTCGTGACAGGCATC
AAGGTGGTGGATCTGCTCGCGCCCTACGCCAAGGGAGGCAAAATCGGCCTGTTCGGCGGCGCCGGCGTCGGCA
AGACCGTACTTATCATGGAGCTGATCAACAACGTCGCTAAGGCGCACGGTGGTTACTCTGTGTTTGCTGGTGT
GGGAGAACGCACCCGTGAAGGTAATGACTTGTATCACGAGATGATTGAGTCCGGCGTT
ATCTCGCTCAAGGACAAGACCTCCAAGGTGGCGCTTGTGTACGGTCAAATGAATGAGCCCCCTGGCGCGCGCG
CCCGTGTCGCTCTCACTGGACTGACCGTAGCCGAGTACTTCCGTGACCAGGAGGGACAAGATGTACTGCTCTT
CATTGACAACATTTTCCGTTTCACTCAGGCTGGTTCTGAGGTGTCTGCTCTTCTGGGT
CGTATCCCCTCAGCTGTAGGATACCAACCTACACTGGCCACTGACATGGGTACTATGCAGGAACGTATCACCA
CCACAAAGAAAGGTTCCATCACCTCTGTACAGGCTATCTATGTGCCAGCTGATGACTTGACAGATCCTGCCCC
TGCTACCACCTTTGCTCACTTG
>1002049218_1 Heli.1-DT666943.3.5
ACGAGGGTTAAGATGAAAGCGTTCTATGCTGTGCTGTTCCTCGCTGCGGCGACCGCCATGCCGGCGAAGGAAG
ACGGTGTCGTGGACAAGTTCATCTCGAACTTGAGAGATTGCGTGGAAACGGATACGATGCTGTGTTTAAAGGA
AAAAGCAATGAAAGTTACTGAAAACTTGGCCTTCGCGAAGGACTTTAGCGTAATGGAT
GGCATTACCTTCGCAAGAACCGGTTCCCCGAGGTCAGCGCGCAGCTACGAGCCTCTACTTGATGACCCTAAAG
CACGAGAGCTTCAGGTTGAAGAAAGGATTATGGATAATGTCGTGGACTTCCTAGACAGCCACGCTTTGCAACT
AAGGATGCCCAAGTCCTTCACTGAAGACAATTCAGTAGAAGAAGAAGGCCGTGGCAAG

AAGAAGAAGAAGCTGAAGAAACTCCTTCCCATCCTTGCTCTCATCAAATTGAAGTTGGCTGCCTTAGTTCCCC
TGTTCCTCGGTATCATCGCCTTCGCTGTATTCAAGGCTTACCTCCTTGGTAAAGTTGCTTTCATCGCTGCCGC
TATCGGAGCTTTGAAGAAACTTCTGGAATCCAAATCCAAGAGCGGTGGAGGTGGATGG
TCTGAACCAGCTCATGAAGAACATGGCTGGGAGAGCGGTGGTGGCTGGGGACGGTCACAGGATGGGCAAAACA
TGGCTTACGCCGCCCACATCAAGCAAATATAACCTTAGGGTCTAGAAATAATTAATTTATTCTAACTTATTAA
GTTTGACGAGTAAGGTTATTTATTGAATGTTGACCACCGTTGTTTCACGTAATGTTTA
TTTGTTATTTATTCATTTCTATTCTTATTTATATTCTACTTTTATATTCTTTATGAAAACTTTGATATTTATT
TAAAACTGCTATTTATTTGTTTATATTTATATGTATTAGTTCATTATATACATATATTTTCAGTTATGCTATA
TTCATATTTA
>1002049219_1 Heli.1-ES586656.1.5
GGCAAGTGCAAGCTGAGATCACAATGGCAAAACCTAAGGGAGAGAGAAAAGGTAAATCAGCAATAAATGAAGT
TGTGACTCGTGAATACACTGTTAATTTACACAAACGGCTTCATGGAGTTGGATTTAAAAGACGGGCACCACGA
GCGATTAAAGAGATTCGTAAATTCGCTGAAAAACAAATGGGAACCCCTGATGTCCGTG
TTGATACTAGGCTAAACAAATTCTTGTGGTCTAAGGGTGTCAGAAATGTACCTTTCAGAGTGCGTGTGAGGCT
TTCTCGCAGACGCAATGATGATGAGGACTCAGCACACAAACTATTCACCCTTGTGACTTACGTGCCAGTTGCA
TCTATCAAGGGCTTGCAAACAGAAAATGTTGAAGCTAGCCAAGAATAAACAACTAAGT
>1002049220_1 Heli.1-ES585655.1.5
GTCTCATTGGTCAAATCGTGTCCTCCATTACAGCCTCCCTGCGTTTCGACGGCGCACTCAACGTCGATCTTAC
AGAGTTCCAGACCAACTTGGTGCCCTATCCACGTATTCACTTCCCCCTCGTTACCTACGCCCCAGTCATCTCT
GCTGAGAAGGCTTACCATGAACAGCTCTCTGTCTCCGAAATCACCAACGCGTGTTTCG
AGCCTGCTAACCAGATGGTGAAATGCGACCCGCGCCACGGCAAGTACATGGCGTGCTGCATGTTGTACCGTGG
TGACGTCGTGCCCAAGGACGTAAACGCCGCCATCGCCACCATCAAGACCAAGCGCACCATCCAGTTCGTGGAC
TGGTGTCCTACTGGTTTTAAGGTGGGCATCAACTACCAGCCCCCGACCGTCGTGCCAG
GCGGAGATTTGGCCAAGGTACAGCGTGCCGTATGCATGTTGTCCAACACTACCGCCATCGCTGAAGCGTGGGC
ACGTCTCGACCACAAGTTCGACCTGATGTACGCCAAGCGCGCCTTCGTGCACTGGTACGTCGGTGAGGGTATG
GAGGAGGGTGAGTTCTCCGAGGCCCGCGAGGACTTGGCCGCCCTGGAGAAGGATTACG
AAGAGGTCGGCATGGACTCCGCTGAAGGAGAAGGCGAGGGTGCCGAGGAGTACTAAGCGGTTTAATTAACGTT
GAGCACTCGCACCGAATACATTCCGGTGAAATAAAAAGAAATCAAAC
>1002049221_1 Heli.1-ES587380.1.5
CGGGTCGACCACACTTGAAGTTTTAACCCTGTGGTGAAGTGGGGTTATCGCAATCATGGGGAAGGGAAATAAT
ATGATTCCTAATGGCCATTTCCATAAGGATTGGCAAAGATTCGTAAAAACTTGGTTCAATCAGCCTGCTCGGA
AACACCGCAGGAGGCAAAACAGAGTTAAGAAAGCAAAGGCTATTGCACCTCGTCCTGT
AGCTGGACCCCTGCGCCCAGTTGTTCGCTGTCCAACTGTGCGATACCATACAAAAGTTCGCGCCGGGCGCGGA
TTTACTCTTCGTGAGATTAGGGCTGCCGGCCTGAACCCTGCTTTTGCTAGGACTGTTGGAATTTCTGTAGACC
CAAGAAGACGCAACAAGTCTGTGGAATCACTACAGATTAATGTACAAAGATTAAAGGA
ATATCGTGCCCGTCTCATTCTTTTCCCTAAGGGCAAGAAGGTACTGAAGGGTGAAGCGTCCGAGGAGGAACGT
AAAGTAGCAACGCAACTTCGTGGACCTCTCATGCCCGTAGAACAGCCAGCACCTAAATCTTCTGCCCGTGTTA
TCACTGAGGAGGAGAAGGACTTCAAAGCATACCAATACCTCAGAGGAGCTCGTTCTCT
TGCAAGACTTGTTGGAATCCGGGCTAAGAGATTGAAGGATGCAGCCGAAAACCCAGATGATGTCACTAAAGCT
CCGTCTGCAGTTAAGGAAGCTAAAAAGAAGTAATTTCTAAAATAAAC
>1002049222_1 Heli.1-DT665729.3.5
GGGTGCATTGTCTGTTGGTGCTCGCAGTAAAGAGTATCGTGTGCAGTGATTATGAATTATACGAGTACCTTAA
CAGAACTGATCCCGCGACCCGACAAATTTATGAGTTCGCGCTAGAGCATCCGAAACCTATTGGAGTGCATACT
GTAAGCTCAGGGAAATTTGTTGAGATCCGGGAGTCTATTGCATTGAATTCAGATGCAT
TTTCTAATCCTTACCATATTGACCTTTTTTCTCACGTCCATGCTGAAAGAATACCAGAACGAGTTAAGAACGC
CGTAGGAACATCAGCATTCGGTTATTTTAAAGTAACCAATGATGTTTCAAAGTATATAAAAGCGGATTTATTC
AACGGCGTGGGGAAGAAGACTCCTGTAGCTGTTCGATTTTCTACAGCTTTTCAAAACC
TTGGAGGAGGTGAACTGGTTAGAGAATTTAAAGGATTTTCTGTGAAATTTTACACAAAAGAAGGAAATTTTGA
TATTGTCGGAGCTAATTATCCTGTCTACTTCCATAGAGATCCTCTTGACTTTCCACATTTCTTACATGCTTTG
AAGAGAAATCCTAAGACTAACCTATACGATCATACTGCACGTTGGGATTTTGCAACCA
AGAAACCTGAACTTATACATGCAACGTTATGGCAACTTTCTGATTATGGTATACCCAACGGATACAGAAAAAT
GAACGGTTACGCCGTTCACACTTATGAAATTAATAATAAGCATGGTGATATATATTTTGTCAAATTTAACTTC
AGATCCGAACAAGGAATTGAAAATCTTTCGGATTATGAAGCACAAAATATTGCAAAAC

GGGATGCCGCTTACTACACTAGGGATTTGTATGACGCTATTGAGAATAAAACTTATCCTTCATGGAAGTTAGA
AATGGATGTTATGACATTGGATGACATAAAAAAGGTGGATTATAACCCGTTTGAACTCACCAGGTTATGGAAA
AAAGGTACCTATCATACAGTAACTATAGGCCGAC
>1002049223_1 Heli.1-ES587433.1.5
GATCATTTACATAACAACATGTTCTCTAAAATTTTTGGAGTAAGCGCCTTGTTGGCAGTGGCTGCTGCAGGTC
TTCTGCCAGAGCCTCACTATTCCTCAGCTGCTGCTGTTTCCTCTCAAAGCATAGTGCGACATGATCAACCTCA
TGGCCTTGTCGCCTCTCCAGTAGCTTACCACGCTGCACCTGTAGCGTACCAAGCAGCT
CCCGCTCACTATTCATCAGCTGCAGCTGTATCATCTCAATCAATTCAACGCCATGACCAGCCTCGTGCAGCTC
TCTCTGTAGCTCCTGTGGCTCACTATGCTGCCGCTCCCGTTGCCCACTACGCTGCTGCTCCCGTTGCCCACTA
CGCTGCTGCTCCCGTTGCCCACTACGCTGCCGCTCCCGTGGCTCACTACGCCGCTCCA
GTAGCACATTACTCTGCTCCCGCCCACAGCGTTGTGGCCAGCCATGAAGATGTATACTCTCACCCCAAATACG
ACTTCTCTTACTCTGTAGCCGATGGTCACTCTGGTGACAACAAGTCACAGCACGAAAGCCGCGATGGTGATGT
AGTACATGGCGAGTACTCTCTGCTTGAAGCTGACGGTTCCGTACGTACTGTTCAATAC
AGCGCTGATGCTCACAGTGGATTCAACGCTGTGGTCAGCAACTCAGCACCTTCAGTACACGCCGCCCCTGCCC
CAGCCCACATCCTGGCTCATCATTGATTTAGCACAATTTTAACATTTAATTATTATTTATTATGATTACGAAA
ATTGTGATTAAAATAATATTTTAAAAAATTAAAA
>1002049224_1 Heli.1-ES586090.1.5
GGTACGGTCGGAATCCGGGTCGACCACGCGTCCGGTTTCGTTCAAGCAAACGTTTATTTACGGCTTAACACGT
TTCGTGTCTTTACGACTTAAAAAATGCGTTACGTGGCCGCGTATTTGTTAGCCGTGCTCGGAGGCAAAGCTGC
CCCAGCCGCTGCCGACCTCGAAAAGATCCTCAGCTCCGTCGGTATTGAGGCTGATGGA
GAGAAACTGAAGAAAGTGATCGGAGAACTCAATGGCAAATCTGTTGAGGAACTGATCGCGCAAGGTCGTGAAA
AGCTATCATCCATGCCGGCCGGTGGTGCTGCCCCTGCTGCTGCCGCTGCAGCACCAGCAGCTGCTGCAGCTGA
AGAGAAGAAAGAAGAGAAAGAGGCCAAGAAGGAAGAGTCGGAGTCCGACGATGAGGAC
ATGGGCTTCGGTCTTTTTGACTAAACTTTTTAATACGGTGCAGAGCACGCGGTTTTTATTCCATGTATTGGTT
TAAGCGTAAATCGACGGCAAGTTTGTTTCTGTGAAATATATCTTGCA
>1002049225_1 Heli.1-DT662950.3.5
GCACGAGGGTTAACGAGGGTGCAGTGTAGATCGAAAACAGATTCATTTTTAAAATAAATAATTAAATATATAT
ACAATAATTAAATAAAAATGTGTGGGGATATGGGCAAAGCAGTATTGCGGGCTCTGACCGGAGCGCTGCTCTG
TACCCTTGTGTCAGATGCGGCTTATTTAGAAAGATATGAACAATACCCATTCCAATAC
GAGCAAGCAGTATACAGAAAACCCCTAAGAGAGCATGAAAAGCCGCAAGATCTCCGCAATGTACCGGGAACAC
CGGGCGTAGACTACCCCATATATCATGCAGTGCCAGAAACCCGCTTCTCCTGCGCCCACGTGCCAGTCCACCC
GGGAATGTATGCCAACGTTGAAACTGGATGCCAAGCCTACCACGTATGCCACGACGGT
CGTGAAGGTCACCAAGGCGCCGCCTTCCTCTGCACCAACGGCACACTCTTCGATCAGGCCAAGTTCGCCTGTG
ATTGGTGGTATAACGTGGATTGCTCACAGGCCATAGCACACTACAAGTTGAACGCAGATCCCTTGAAAAACCC
CTACGTGCCTAAGCCGAAGCCAGAAGAGTTACAGGAAGCGCCACATGGAGTTTACTAT
AGAAAGATACACGATTAAACATTTGTGTTTTGTCATTTATCTATTACTTTTAAAATCATCTAAAAATTGTGTC
ATCGTCATACATCAATCATATGAATCATTAAATTAGCAACAGCCAACTTTAAACTTTAATATTTAGGCAAAAG
TGTGTATATCCAAAGTGATTAACATGTGAGTATTATTTCAAGACATATTGGATTTAGG
GTATTTGTGAGAAAGAAAGATTTATGAGGGGGTAGAAAGATTTATTAATGCGACGGTTGTGGGTTCGATTCCC
ATTCGTTGAAATGAGCTATCTTCATTTCCCTGCTATATGAAGGATACAGCGTGAACGTAAAAGATAAAATT
>1002049226_1 Heli.1-DT663432.3.5
GCACGAGGCGCGGAGCAGTGCACATTAAAAAAAATGTTCAGGCTCATATTATTAGCCTTTGTGGCGGCGGTCG
CGTGTCAGCAACACGAGGGTGCTAGACGGGTTCCCAAATACGCAGGTGACCCCAAAACAGCTGCCATTGTGCA
AGAAGCTCGGTATCTCAGTGGAAACGGTGCTTTCGGAGCAGCTTATCAGCAAGAAGAT
GGTATCGATTTTAAAGAAGAGACAGATGCTGACGGTAACAGGAAAGGCAGTTACTCATACATTGACCCCACCG
GACAAAGGAAGACCGTGAATTACATTGCGGGTAAAAACGGATTCCAAGCTATCGGAGATCATATCCCCACTGC
ACCTCAGCCCGTGGCACCTACGCCAGGATACACACCTGATCCCCGTTACAACTCTCCA
GACTACAAACAGTACAGTGCTCCTCAGCAATACTCAGCACCAGTCGCGCCTCGCAACTACGGTGGAAAACCTA
GCGCGGACGATGGTCAATACTACCCTGAACTTTACGAACGTGATAACTATCAAGGTCAACAACAATACCAACC
TGCCCCACAGCAACAATACCGATCTGCCCCACAACAACAGTACCAACCTGCCCCACAG
CCACAATACAACACCAACAACATCCCACAATACAACCAACAAGCCCAATACAGCGGGATCCAAAACCAACAAT
ACAGTGCGCCCCAGCAATCCCGCCAAGATTACTACGAACCGACCACACCACCTCCCGCACGTTTCTTCCCACC
AGGCAAGTTCAGTCTGAACCGTGCCCCCGATGGCTACACCTACTCGGCCCCCGATGGC

TACACCTACTCGAAAAGAGGGTTCCTGGCGATTTGTGATGTCTGAAACGTAAAAAACTCGTATCAATTAGTAA
ATCGCTGCGTCACCAGAGGGTAGTTTAGGGTACGTGCCTCCTTGCTTCGGAAGCATTTCTTAATTGTTGTTCG
CTGTGATATTGTGTGCTAATGGACAAAGTATATATACGCTGTTGGTATCGTTACTCAA
>1002049227_1 Heli.1-DT666616.2.5
ACACAGACCAAGATGTTCAAGTTGGTGGTGTTGTGCGCTTTCCTCGCCGCGGCTGTAGCGGAGCCCGGAACCT
TCATCTCGCCGTTGGCGTACTCCAGCAGCTTTATCGCGCCGGCTACAACAACTATCACCAACCAGGCCAGCAG
TGTCATCCACCCATCACCTCTGCTCTACCCTCGATTCTACGACCTACCTTACTCACCA
GTCGCACATCTTATCAAGAAGCGATCCCTTGGAATTTTCAATCCTTTCATTGCACCATCAACTTACATTGCTC
CTGCACCTCTGGCCACTACTTATGCTGCAGCTCCTCTTGCTACTACCTACGCTGGAGCTCACTTGGCTACAAC
TTATGCTGCACCAATCTACCCTGCTGCTGCGCATTTAATCAAGAAGAGGTCTGCTCCA
CTTCTGCCCACAACCTACGTTGCTCCATACTATTCTTCTCCTTTGTTGACGCCGACGTACACAGCCGCTGCAC
CCATCTATTCAACACCTTACATCACCAGTGCTCCTCTTACTTACACGCATTTAATCAAGAAGCGCTCCGCTCC
ATTGTTCTACGCAGGTTACACCGCTCCCGCTGCGGTTTCTCATCAGTCCCGAGTAGAC
ATTAAGAGCTCCCCTGCTACTATTACGTCCTACTCCTACCCTGCTGCTGTTTCTTACGCTAGCCCACTTGCAT
TTTCACATGTCTTCTAGATATTTGACTATTGTATGTAAATAAACTGAATTGATATCATTAAAAAAAAAAAATA
TATCATTCTT
>1002049228_1 Heli.1-CX700745.1.5
TTACAGTTCTTGTTGAAGACTGGAAATATGGCAACCTCTAAGAAGAAGACCAGGAAGTTAAGAGGACATGTGA
GCCATGGTCATGGACGTATTGGAAAGCACCGCAAGCACCCTGGTGGTCGCGGTAACGCTGGTGGAGAACATCA
CCACAGAATTAACATGGACAAGTACCACCCTGGATACTTTGGCAAGCTTGGTATGAGA
AATTTCCATTTAAGAAGAAACAAAATCTTCTGCCCGGTGCTGAACCTTGACAAGTTATGGACACTTGTATCGG
AGCAATCCAGGCTTAAATATGCCAATGCATCTGATGGAAAGGTCCCTGTCATTAACATTGTTAAGGCGGGTTA
CTACAAATTGCTTGGCAAAGGCAAGCTGCCCAAACAGCCAGTCATAGTAAAGGCCAAG
TTCTTCTCTAAAACAGCAGAAAGGAAGATCAAAGAAATTGGTGGGGCATGTGTACTGTCTGCGTAATTTAAGT
TTAATAAATAACATAAAATAAAA
>1002049229_1 Heli.1-ES584621.1.5
GGTTGGTGTTCTTTTTCGGTCACGTATCTGTTCGTGGCCGTTAGATCACTTTATTAAAGTCCATACAAGATGG
TGCAGAAGAAGCCTAAGAAGAAGGTAGGAAAGAAGGTAGCGGCCGCTCCTTTGGTCGTGAAGAAAGTTGAACC
TAAGAAGGTTGTAAACCCTCTCTTCGAGAAGAGGACAAAGAACTTTGCTATTGGTCAG
GATATCCAGCCAACTCGTGATTTATCCCGCTTTGTGAGATGGCCCAAGTACATTCGTATTCAACGTCAAAAGG
CAGTGCTCCAACGCCGCTTGAAGGTGCCACCACCAATCAACCAGTTTACTCAGACCTTAGATAAGACTACAGC
GAAGGGTCTTTTCAAGATCCTAGAGAAGTACAGACCAGAGACTGAAGCAGTCAGGAAG
GAACGCCTCAGGAAAGCTGCTGAAGCTAAGGTTGCAAAGAAAGAAGAGCCTCCAGCGAAGAGGCCAAACACCA
TTAGATCAGGTACCAACACCGTGACCAAGCTGGTGGAGAAGAAGAAGGCGCAGCTGGTTGTCATCGCTCATGA
TGTTGATCCTATTGAGTTGGTTCTGTTCCTGCCAGCCCTATGCCGTAAGATGGGCGTG
CCCTACTGCATCGTCAAGGGCAAGTCCCGCCTCGGTGCCCTAGTACACAGGAAGACTTGCACGTGCTTAGCGC
TCACACATGTCGAGGCCGGTGACAGAGCCTCCTTCTCCAAGGTGGTAGAAGCGATCCGCACCAACTTCAATGA
GCGCTACGAGGAGCTCCGAAGACACTGGGGTGGTGGTGTGTTGGGCAACAAGTCCAAC
GCGCGCATTGCCAAGCTGGAGAAGGCCAAGGCGCGCGAGTTGGCGCAGAAACAGGGCTAACCCTAATAAATA
>1002049230_1 Heli.1-DT663146.3.5
GTGTGTAGGATGGATCGCTAACAACACCAGTAGTTTTTTTTTCATTCCGCCTTCTAACAGTGTTTGTTTATTA
CCTTCCGGCACACTTTCCGTAAATCCTTCTCGACGGTGGTGCTTATTATTTCTTTAGTTTTTTCGGCGTTTGT
TTTTATATTTTCTTAAGCGCCGTTCCGATTTCACGTCGAGAAGGCGGCCGCACAAGTT
TGAAAGCGGCTTTTTGAGAGTAGTAACACCTACTCATATTTTGTTATCGTTATAAGAACACATCAGGCAACAA
TCAGAATGGTGAAGAAGATATCAGAAGAGAGTGTAGACAGCGGCGTCGGCCGCGGCAGTAGTCAGTCGGGTAG
CGAGCGCGAGTCGGACGAGATACCGCGCGGCCCGAGGCGTCCGCGCCGGTGCCCATA
CCCGACAGCGAGGAGCTGCAGCGACGCAAGGAGGAGGCGCGCAGGAAACGCCGCAGGAAGAAGCGCTCCGGCA
GCTCAGTGGTCACGTCATGTTTTCAAGACCTATACAAGCTAACAGGAGAAGTACTGGGCGAGGGCGCATACGC
CTCGGTACAGACGTGTGTAAACATCTACACGGGCCAGGAGTTCGCAGTGAAGATCATA
GACAAAGTGCCGGGGCACGCCCGCGCCCGGGTGTTCCGCGAAGTGGAGACGTTCCACTACTGCCAGGGACACC
CGAATATAATCCAGCTCATTGAATTCTTTGAGGACACGGACAAGTTCTATCTTGTCTTTGAGAAGATCAACGG
CGGCCAGCTCCTATCAAGGATACAGGAGCACCACTACTTTTCCGAGCCGCAAGCGGCA

GAGATAGTGCGCGAGATCGCCAACGCCTTGCACTTCCTACACGGGAAGGGCGTGGCGCACCGCGACCTCAAGC
CCGAGAACATTCTGTGCGTCAACCGCGACAGTCTCTGCCCGGTCAAGATTTGTGATTTCGATCTAGGGAGCGG
CATCAGCTTTACGTCCAGCCTCGCCAGCCCGTTGGCGACGCCGCAGCTCATGACGCCG
GTGGGCAGCGCAGAGTTCATGGCGCCCGAGGTGGTGTCGCTGTTCGCGGGCTCGGCCGCCACGCACTACGACA
AGCGCTGCGACCTGTGGTCGCTGGGCGTCATCGCCTACATCCTGCTGTGCGGGTACCCGCCCTTCCGCGCCGA
CTGCGGCGCCGACTGCGGCTGGGAGAGGGGCGACAACTGTAGAGCCTGCCAGGACCTA
CTCTTCACTTCTATACAGGAAGGCCGCTACTCCTTCCCACAAGAGGAGTGGTCTCATATTTCCACCGAAGCCA
AGGAACTGATCGCCCAGCTGCTAGTACGTGAAGCATCACACCGTATTAGCGCTGAACGAGTGCTGCAGCACCC
CTGGCTGAGACGAGCGGACGCTAATGCGACTAAAGTCAACTACCCACCTTTGCATACA
CCACATAATATTAAACGCAATATGTCAGCTCGTAACTTATCCAACTTCGCGGAATCAGCCATGGCGGTCAACC
GGGTGATACAGCAGCACTTCTCAATGAACTACTCGTACATGGACCGCATCGAGCAACCGCCGGCCGCGCTTCG
AACGAGCAAGTCGTGCCGTATACCCGAGGACGACGACGCTCCCCTAGGCCTATCACCG
CCCACCGACTCGGAACTACTGCGTAGACGTATGCAGAACGGTGTTAAGCCGCCACCGCTGCCTGTGCATTAAC
TTATCCTATCTAAATCCTCATCAAAAAAAAGTAAAAATCCTTCTCCTCTATCCCCTTTCCTTCATATCCATTT
CTGATACGGTATCGATCGAGATAACGAATTAGTTGATATGACGCCAGGACCCTGGTAG
TGATCACGACGGCAGAATGCAATAACCTCCCTCCCCAGTCAGTGATCACAGTAGACGGACGGACAGCGGAGCA
GTGTGCCCTGACACCCGCCGTGCGGCCGTGCCGCAGTACGCAGTACAACTGGACCTACCTCCATGTGATAGCC
TTCGGTTGCAGCCACTCCAGTGATATACACGTACGCCGTGCACCGTAGACGGTACTTA
GAGCTGTAACACTTTTATTTTGAGCCTTTTAAAGATATATTTTTTATGAATAAATAAAAACGACTGAATAGTT
TTTATCGATGTGTAGAGTATTTGTGAGTTTATGTTGTAGGCGGCCGCCACTGTGGTCACGCCGTCGAGACCGA
CGCCGGTCGATAAAATCGTATTATATTATTGAGATAGTTGTGCTGATCTACATACTTT
ATTATAAACTACAAAACAGAGTAAAAATATTGTTATAATATTTTTTAGGATTTATAAAATCCGATTGTCTAAA
ATTGAGCTTATATGTGTGATTGCCTTTTGTACGTGGCCTATCCTTTG
>1002049231_1 Heli.1-DT666998.3.5
GTCCGCCCACGCCGAGGCACGCGTTTAGTCGTAACTCAAATAAACGCAACGATGTCGGGAGGATTAGATATTT
TGGCCCTCAACGAGGAAGATGTAACCAAAATGTTGGCGGCAACAACCCATCTTGGGTCTGAAAATGTCAATTT
TCAGATGGAAACCTACGTATACAAGCGTCGCACTGACGGTACCCACGTGATCAACCTT
CGTCGCACCTGGGAGAAGCTTGTGCTGGCTGCACGTGCAGTTGTAGCTATTGAAAACCCAGCAGACATCTTCG
TCATCTCTTCAAGACCTTTTGGTCAAAGAGCCGTTCTCAAGTTCGCTGCTCACACTGGAGCCACTCCCATAGC
TGGAAGATTTACTCCTGGTGCCTTCACTAATCAGATCCAAGCTGCATTCAGGGAGCCA
CGTCTTTTGATTGTATTGGACCCTGCTCAAGATCATCAGCCAATTACTGAGGCCTCATATGTCAACATTCCAG
TCATTGCTTTCTGCAACACTGACTCTCCTCTGAGATTTGTTGACATTGCCATTCCATGCAATACTAAGTCTTC
CCACTCCATTGGTCTCATGTGGTGGCTCTTGGCTCGTGAAGTTCTGCGTCTGAGAGGT
GCTCTACCACGTGACCAGAGGTGGGATGTCGTAGTAGACCTCTTCTTCTTTAGAGATCCCGAAGAAAGCGAAA
AGGAAGAACAACAAGCTAAGGAACAGGCTGTTGTTGCTGCTAAGCCTGAGGTACCAGCACCACCTGTACATGA
GGAGTATGAGCCCCCAGAGCCTGTTGCTTCCTGGAATGAAGAGGCTGCTGCACCACCA
CCAGCAGCCAGCTTTGGTGCACCTGCTGCTGCTCAAGAAGATTGGGCTGCTCAGGTTCAAGAAGAATGGGGTA
CTGCCGCCCCAGCGCCTGCTCCTGCAGCGGCCCCATCGTGGGGAGGTTCCGCCCAGGACTGGAGCGCCGCGTA
AACCATACATTAAGTTGATTGACGCTAAATAAATATATGAAAATTTGAAAAAAAAAAA
>1002049232_1 Heli.1-DT667883.3.5
GCACGAGGCCCGACTGCAACCACCGTTGAAGAGGCATTATGAAGACGATCTGTTTAGTTGTTCTGCTGGTAGC
AGCAGCCGCTGCCAGCGAGAAGAAATCCACAGAACAAAAAGTTGAAGGCCTCGAAAAGAAGTTGGACAAACGT
GGTCTCTTGAACTTGGGATATGGATACGGTATCAGCGGTTTGGACGTCGGATACCTAG
GCAGTGGCCATGGACTAGGTGGAGTATACGGCTTAGATGAAGGATCTTATGGACACGCCGGATACGGCATCGA
CCTTGGAGGCCGCACCGATGTGACCAGGACTGTCACCCTCGTCAAGGGAGTCCCAGTAGCAGTGCCAGTTGAC
AGACCAGTGCCTTACCCAGTAGAGAAGCACGTCCCTTACCCCGTCAAGGTCCCAGTTC
CTCAACCCTACGAAGTAGTCAAGCATGTGCCCGTCCACGTTAAGGAATACGTGAAGGTCCCCGTACACGTACC
TGCACCATACCCAGTAGAAAAGAAGGTGCCATACCCCGTCCATGTCCCCGTCGACAGGCCCTACCCCGTCAAA
GTTCTCGTACCTCAACCCTACCCCGTTGAGAAACACATCCCTTACCCCGTTAAGGTAC
CAGTTCCCCAGCCCTACCCCGTTGAAAAGCACGTGCCTTACCCCGTCGAAGTCAAAGTACCCGTACCACAGCC
CTACCCCGTTATTAAACACGTTGGTGTACCCGTAAAGGTTCCCGTTGACAGGCCTTACCCCGTCCATGTGCCA
GCACCTTACCCCGTTGAAAAGCCAGTACCAGTCGCTGTCCCAGTTGAGAAACCCGTTC

CTTACCCCGTACACGTGCCCGTAGACAGGCCTTACCCCGTCCACGTTGAGAGGCCCGTCGCAGTGCCCGTCAA
AGTACCCGTGCCAGAGCCCTACCCCGTATACAAACACATTCCCGTTGAAGTAGAAAGGCCAGTAGCCGTGCCC
GTCAAAGTGCCCGTCGACAGGCCTTACCCCGTGCACATCGAGAGACCAGTGCCAGTTG
CCGTTGAGAAACCCTACCCAGTACCAGTCAAGGTACCAGTTCTGGTCAATGAATACGATCATGGCAGCAGCTA
TGGCTACGGAGGCTCATATGGGGGATCTTACCACGGAGGTTCCCTCC
>1002049233_1 Heli.1-DT666846.3.5
TATCGCCAGCTTGGTGCGACGCAAAGTGGGTTTAGGCTCGTCGTATTAAAAATGTCTGAAACTTTACAACTTA
GGGGTAACCTTTACGGGCATAGCGGATGGGTTACCCAAATTGCTACTAATCCGAAATATCCGGATACTATTTT
ATCTTCTTCCCGTGATAAAACACTTATTGTTTGGAAGTTGACCCGTGATGAAACCAAC
TACGGTATACCACAGAAGCGTTTATACGGACACTCCCACTTTATCTCGGATGTGGTACTGTCTAGCGATGGAA
ACTACGCTCTTTCCGGATCTTGGGACAAAACTCTTCGTCTATGGGATCTCGCAGCTGGAAAAACCACCAGGCG
TTTCGAAGACCATACTAAGGATGTCCTCTCGGTAGCATTCTCAGTTGACAACCGTCAG
ATTGTGTCTGGCTCCCGAGACAAGACTATCAAGTTGTGGAATACTTTGGCTGAATGTAAATACACAATCCAAG
ATGACGGTCACTCTGACTGGGTATCATGTGTGCGTTTCTCACCAAATCATGCTAACCCAATTATTGTTTCTGC
TGGTTGGGACCGCACTGTTAAGGTCTGGCACCTTACAAACTGCAAATTGAAGATCAAC
CACTTGGGCCACTCTGGATATCTTAACACCGTGACTGTTTCACCTGATGGCTCCCTATGTGCATCTGGTGGCA
AGGACATGAAGGCTATGCTTTGGGACTTAAATGACGGAAAGCACCTTCACACCTTGGACCACAATGACATTAT
CACAGCTCTATGCTTCTCACCCAACAGATACTGGCTGTGTGCTGCATATGGACCCCTG
ATCAGGATCTGGGATCTTGAAAGCAAGGAAATGGTAGAAGAATTAAAGCCCGATGTCATCAAGGACACCAAGA
CTGATCCTCCTCAATGCCTTTCCTTGGCATGGTCTACTGATGGCCAGACCCTCTTCGCTGGTTACACCGACAA
CATCATCAGAGTCTGGCAGGTTTCAGTCTCTGCACGATAGGACGTAAAATAAGAAAAT
AGTTTCATTTATTTTTGTCCTAAC
>1002049234_1 Heli.1-DT666945.3.5
GCACGAGGCGAATATGAAGGTGGTGTTATTAGTTGTTGCTTTAAGCAAATTTGTGACCGGGGAAGAGTACAAA
ATACCGTCAATTGAAGAACTACCGAAAGATGGCATTGGTTTATACAATGGAGAGATAAAAGAAGACATAAATT
ATTACAATGAATATGACAAAAAATACGAATTGGGCAATCCGGATGATGACCAACAATG
TATAGAAAAACCCTGTAATAACTACGACGCGTTTTGCATACGACGCTTTTTTGCCCAACATTCACATTGTAAA
GAATCCTATGGACCAGTGCCCACTCCATTGTATAGGGCACAAAGTATTTCTTACTTACCTAGAATAAACCTTA
CTGTGGTATCACTTAAGCAAGAATATACTGGAGTTAATGGTCGGGTTGTGGAGTTTTA
CATCAACAAGAAGACTGACAAGTTGGTATTGGCTGTGGAGTTCACAAACTTTGGGCTGGGCTCTAATGAAAAT
TACTTTAAATTTCATCGCCGCGGTCAAGAACCTATAGTCAATATGGGTTCTTTAAACGTCACTTATGCTTCCA
TTGTATCTACAATTGTAATACCAAATTTGAAGGATCTTCAGTATGAAAGAAGCGAAGT
ATTTACATACAATAACGATGTTAATCCACGATTCAGTTTTGGTCTCAGCGCTTTCATTGGTACCGATCCTGTT
CTTCTGGAAACGGCTTTGGCGTTTCTCGCTAGTTATGCTGATAATATAAGAGAGGGCCAAATAACGGACGGAC
CGTTCTTCATAGCGAGTTACATTAAATACTTTATATGTGACTTTGGAATTGAAGTGTT
ATAGATACGGTACAGTAAATGTTAATCATAAATGCAGTTTATAACAAAAAAATAAGCGAATTTAATTAATACA
ATGTATGTGTT
>1002049235_1 Heli.1-DT668064.2.5
AATATGAACAGCAAAATGAACAGCTACATTACTTTCGCTTGCCTATTAGTGGTCGCCGCTAGCGCTTACGGCT
CAGGCCTCGGCCTCGGCCTCGGTTATGGTGGACTCGGTTACGGTGGTTACGGCGGCTACGGGGGCTACGGATC
AATAATAAAGGAACCTATCATCGTCAAGCCTGCCCCCATTATTAGCCCATTGCCCATA
ATTAAGCCCCTCCCCATTATTAATCCTCTTCCCATAATCAAACCTCTTCCCATAATCAAACCTCTTCCCATCA
TAAAGAATGTCGAAATCGCTCCCATACTGACTGAGCCACTCTTAAAATCCGTGCCCGTATACGGTGGGTATGG
ACTTAACAAGGCTCTTGATTATGGTCTTGGTCATGGTGGACTCGGCTATGGTTACGGA
AAAGTCTTCTAAATAACGGGATGGACCCAATGGTTATGCTGCTGCTGCTGGCTGAAATGATATAGTTTTTTAT
ATTTCGTCTATTTCCTTCCTCCTATTTTAATCTTTTATCAATAAAGCAATTTAATTTTAAAAAAAAAAAAA
>1002049236_1 Heli.1-DT666267.3.5
GCACGAGGATTTCATTCTTACTAGCTTCACTTGGACGTTGAAAATACGCGTAGCGGTACCTATTGCATCAGTG
AAAGTTTCGAAAATGTTAGGATCACTTAAAATTTTACTATTTTTAGGTTTAATTTATTTATCTAAAGCCTCTG
AAGAAGATGTACTTGAACTTACTGATGCCGACTTCTCCAGTGCTATATCCCAACATGA
AACCGCTCTCGTCATGTTTTACGCTCCATGGTGTGGTCATTGCAAGAGATTAAAACCAGAATACGCTGATGCG
GCCGGCATTTTGAAAAATGATGATCCTCCAATTACGTTAGCTAAAGTGGACTGTACGGAAGGTGGAAAGAGCT
CTTGTGAACAGCACTCTGTCTCTGGATACCCTACACTTAAGATTTTCCGCAATGGTCA

ACTATCACAAGAGTACAACGGCCCCAGAGAATCCAATGGCATAGTCAAATATATGAGAGCCCAAGTAGGACCA
AGCTCAAAAGACTTGGTAGATGTTGCTAGCTACGAAAAATTCCTAGCCAAGGATGAAGTGGTAGTTGTAGGCT
TCTTTGAAAAAGAAAGTGACTTAAAGGGCAAATTCCTGGTAGCTGCTGATGTGATGAG
AGAACAAGTGGTGTTTGGACACTCCTCGGCTAAAGATGTTTTAGAAAAAGCTGGATTCAAGGACAATGTAGTG
CTGTACCGTCCCAAGCGCCTCCAAAACAAATTCGAAGATTCCTTCGTCGTGTACAATGGTGAACCAGCTGAAC
TCAAGGGCTTCATCAAGGAAAACTACCATGGTCTAGTCGGTGTGCGTCAAAAGGAGAA
CATCCAAGACTTCAGCAACCCCCTCGTAGTTGTCTACTACGATGTTGACTACCAGAAGAATCCTAAGGGAACT
AACTACTGGAGGAACCGTGTACTTAAGGTTGCCAAGGAAATGTCAGAAATATCATTCGCGATCAGCGACAAGG
ATGACTTCATGCACGAGCTGAACGACTACGGCATCGACTTTGCCAAAGGTGACAAGCC
TGTTGTGGCTGGAAAGGACATTGATGGCAACAAATTTGTGATGGGCCAGGAGTTCAGCATTGAAAATCTACTC
GCATTCGCCAAGGACCTGGTAGATGGCAAACTAGAACCATTCATCAAGTCTCAACCGATACCTTCAGAGGCTG
GACCCGTCAAAGTAGCAGTCGGCAAAAACTTCAAGGAGCTAGTGACCGATAGTGGACG
GGATGCTTTAATTGAGTTTTACGCACCATGGTGTGGCCATTGCCAGAAGTTGGTACCAGTTTGGGATGAATTA
GCTGAAAAGATGAAGGATGAAGAAGTTGACATCATCAAACTAGATGCTACCGCCAATGACTGGCCTAAGTCTT
CATACGATGTGTCAGGCTTCCCAACAATCTACTGGAAGCCTAAGGATAGCTCCAAGAA
ACCAGTTAGATACAATGGTGGACGTGCCATTGAAGACTTCATTAAATATGTATCTGAAAATGCCTCAGACAAA
CTCAAAGGTTATGACAGAAAGGGCAATGTTATAAGCAAAGATGAGCTCTAAAGTAGTTGAGAATAAAAAATCC
ATGAAAGATCTGTGAGTGAATGAGTGTATGTATGTATGAAAGTAAC
>1002049237_1 Heli.1-DT664206.3.5
CTCGTCGTGTATTGTATTCTTGTACTCGCAGTGAAAAATATTGTGTGTGTTGATTACGAATTACTTGAATATT
GCAACAGAACAGATCCTGCGACTCGACAGCTTGTAGAGTTCGCGAGAGAGCACCCGAAACCCGTTGGCATACT
AACAACAAGCAGTGGAAAGTTTGTGGAGATTCGAGAAAGTACTACACTAAACTCGGAT
GCGTGGTCTAATCCATATCATGTAGACCTTTTAACGCATACCAACGCTGAAAGAGTTCCTCAAAGAATTGTGC
ACGCTAGAGGTACTGCAGCCATAGGTTATTTTGAAGTAACCCACGATGTTTCTAAGTATACGAAAGCAGATGT
TTTTAATGGAATCGGTAAAAAAACACGAGTTTTGGGTCGTTTCTCGTCACTGGCTCAG
AGTGTAGGAGGAAACGATCTAGCTAGAGAAGTAAAAGCGTTAGCTACCAAGTTTTATACTAGAGAAGGCAATT
TGGACTTTTTAAGCAACCATTTGCCAGTTTTCTTTTTTAGAGATCCACTTGAGTTTCCACATTTATCTCATGT
GCTCAGAAACAATCCTAAAACTAATATATTAGATTATTCTACACGTTGGGATTTTGTG
ACAAAAGTAGCTGATATGTTCCATGCGTTTATGTGGATGTTGTCTGATTTTGGAATACCAAATGGATATAGAA
AAATGGATATATTTCCAATTCATGTCTTTGAAATAAATAACAAAAATGGTGACAAACATTTTGTTAAATTTAA
TTTTAGAACATTACAAGGCTTAGAAAACTTACCGTCTGATGTAGCCGCAGACATTTCA
GCTCGAGACCCCGACTATTATAATCGTGACCTATACAATGCTATCGAAAATAAAAACTATCCAGCATGGGAAT
TGGAAATGGATGTTATGACTCTTGAGGATATTAAGAATCTCAATTATAATCCATTTGAAGTAACTAGACTGTG
GAAAAAAGGGACGTATCATACAGTAATAATAGGCCGTCTAGTATTTAATAAAAACCCA
GACAATATGTTTAGATTTGCTGAGGAAAGCGCCTTTAATCCGTTCAATTTAGTGCCAGGTATTCCCGGACCTA
TAGATACACTATTCCGTAGTAGACGAACATCATATCGACAAGCTCAAGTATTCCGTTTGGGTGTGAATCATAA
CAGGATAAGTGTGAATGCGCCACTCCATTGGAGTGTGTATGACCGTGATGGACTTCCT
CCATTAAAGAGCAACATGCGAGATGCACCAAATTAT
>1002049238_1 Heli.1-DT664402.3.5
CCGCATAAAAAAAATCACAAAAACAACAATGAAAGTACTGGTGGTGTTATCCGCTGCTCTAGCGCTTACAGCC
GCTCTTCCTCAAAGAAGAGTGTCCTTGAAGCCTGAAGGTTTAGAAGACCAACAGGCTGCTGAGCAAAACTACA
ATAACTACCAACAACAGCAAGATTACAGGCAACCACCACAGGAATACAGACAGGCCAA
GCCAATTGAGGATTTCAGACCAAAAGTTCAAATCGACACCACCACCTTCATCCCGATCATTCGTTTCGACAAG
GAACAGGAAACTGATGGCAGCTACAGAACGGCATATGAAACTGGAAACAACATTCAAGCTCAAGAAGAAGGTT
ACTTAAAAACTGTTGGAGAAAACGAAGACAAAGTACCAGCTTTGGTTCAACATGGCTC
CTACACTTATACCGCACCAGATGGTCAAATCATCACTGTAGAGTACACCGCTGACGAACTAGGCTTCAGAGTT
AAGGGCGACCACATCCCAACCCCTCCCCCAGTCTCTCCTGAAATCCAAAAAGGTCTTGATTTGATCTACGCTG
GAATTAAAGCCAACCAGGAGCGTGCCGCCATAGAACTGAAGAACAACCCAGAATCCGC
TAGGTTACTCGAAGACAAGGCAGCTCTTGACTACAAAGGACTTTACTACCAGCAGTAAATTTTATAGTGAACG
CTGACCATAAAGCCAGTGACGTTATGTCAAGCAATAGCGTAAAGTAGTGTCATAGAATATGACTAGTAACCTG
CAAGTGCTCTGTGAGCATGATCAAAGTCATTCGTTTATAGTATAATTAATACAAAACT
AAAACAATATCTCTAGAATCTAATTGGGTTCGCACTTATATGTTTTTTAATTTTATTCATCAAACTGTAGTGC
AAATGCCATCTAAGCCTTATCTTATATTTTAAATATATGTAGGTAGTGTTAAGAATATTCCTTTTATAAGCTG
TAAGTAAACATGATTTCTTCCTTTGGAACAATTAATACTCATTTTATATGTCAGGTAT

CCAAAAGTATAGTTGGTTTTTGTTTAATGTGATATTCAGTTGGTAAAAGCAAGTTTTATTGTACGAACATAAT
TTACTTTTGTACATATTGTTATGTAACTTATCACTAATTTGATATTAAAATGTTAAAAT
>1002049239_1 Heli.1-DT662417.3.5
ACGAGGGTCCCGTGTTGCGTGTGCGCACTCCAGCGAGTGATCCCGCAATAGTTAGAAGAAACGAACCCGACTC
TTATCAAAATGCTCCTACTCGCCAGCTTTTTCCTGCTTTTCGTAAGCACCCTGGGCCCTGTCGCAGCTCAAAG
GATAACGACGATACAGCTCGATGGCGTTCAGTACTTTATATCAAGAATGAATCCCTAC
AGCCCCGAGCTCAACTATTTCCTCGCTTATCAATATTGCAGATCCCTAGGACTACAACTGGCGTCTTTTGAAA
CAAAAGAAAAGGCGGATTCAATTACAACTTATTTGACTAATGCAGGCTACAACAAGTACGATTTCTGGACATC
CGGAAACAATCTCGGTACCGACATGTACTTGTGGATGAGTACAGGGTTGCCTTTCAAC
GCGACGTTTAATTACATGCGTCGCATGACACTCGACGCTCCCAGCCAGCATGCTGACGACAGCATGGACCCCC
TTGACGTGCCTCAAGGTAGCACTGCCCCCCAACGTACTGCCAGGCATGGAACTGAACACGTGATGACAAACGG
GTGTGTGGCATTAAAGGCGCCATCATTCCACTGGGAGCCTCAACACTGTGGAGAAATC
AAAGACTTCATCTGCGAACAAACACGCTGCTACTACTACAACTACGGATCCATCCCCGTCTCCTCGGCGCAGG
GGTAATGCAAGGAAGCCGCTATCGATGACGACCACGACGACTGCACACCCACTAACGTCCCGCCCCCGCGCTC
TGAGCACCTGCCGACTCACTTTACTCTCAACGATTCTCAACGATCTTATCGGCAAGAT
GCGTCCGTCTTC
>1002049240_1 Heli.1-ES584863.1.5
ATAAGGAGTCATCATGCAGATTTTCGTGAAAACCCTAACGGGTAAGACCATCACCCTTGAGGTCGAGCCCTCG
GACACTATCGAGAATGTAAAGGCCAAGATCCAAGACAAAGAGGGAATTCCCCCAGACCAGCAGAGATTGATCT
TTGCCGGTAAACAATTGGAAGATGGCCGAACACTCTCCGACTACAACATTCAAAAGGA
ATCCACACTACACTTAGTGCTCCGACTCCGAGGTGGTGCTAAGAAACGCAAGAAGAAGAATTACTCTACACCC
AAGAAAATTAAGCATAAGAAGAAGAAGGTCAAGCTAGCTGTACTTAGGTTTTACAAGGTTGACGAGAATGGCA
AGATCCATCGTCTGCGCAGAGAATGTACCGGAGAGCAGTGTGGTGCTGGAGTGTTCAT
GGCCGTCATGGAGGACAGGCACTACTGCGGCAAGTGCCACTCCACCATGGTGTTCAAGGATGATGATGAATAG
ATTGGGGTCACTAACTGTTAGTGTCTAATGTTTAAGTCCCAGACTGCTGTGTATGATGGATTTTTAAATAAAT
CTTGACACAGTTGTCTTTAATT
>1002049241_1 Heli.1-DT667716.3.5
GGTGGACGTGTCGTATATTTCTAAGAACCAATTTAATTTATCCGTAAATATGTCGAAGATTGGAATCAACGGA
TTCGGCCGTATTGGTCGTTTGGTGCTCCGTGCATCTATTGAGAAAGGAGCTCAAGTTGTTGCCATTAATGATC
CCTTCATTGGCTTGGATTACATGGTATACCTCTTCAAATACGACTCCACCCATGGCCG
CTTTAAGGGATCTGTTGAAGCTGCTGATGGCAATCTCATTGTGAACGGAAATAAAATCGCCGTATTCTCCGAG
AGGGACCCCAAAGCCATTCCCTGGGGCAAGGCTGGAGCTGAATATGTTGTAGAATCTACTGGTGTTTTCACAA
CCATTGACAAAGCGTCTGCTCATTTGGATGGTGGTGCCAAGAAGGTTATTATTTCTGC
TCCCAGTGCTGACGCACCCATGTTCGTGGTTGGTGTCAATCAAGAATCATACGACCCATCTTTCAAAGTTATT
TCTAACGCTTCTTGCACAACTAACTGCTTGGCTCCTCTTGCCAAGGTCATCCATGACAACTTTGAGATTGTAG
AAGGTCTTATGACAACAGTCCATGCTACAACTGCTACCCAAAAAACTGTTGATGGACC
CTCTGGAAAACTGTGGCGTGATGGCCGTGGTGCCCAACAAAATATCATTCCAGCCTCAACTGGTGCAGCCAAA
GCTGTAGGCAAGGTTATTCCTGCCCTGAATGGAAAACTGACTGGTATGGCATTCCGTGTGCCCGTAGCCAATG
TGTCAGTTGTTGACCTTACAGTACGCCTTGGCAAACCTGCCAGCTA
>1002049242_1 Heli.1-DT667187.3.5
ACTCCTGATCTTTCGAGATGTTCAAGCTTCTTGTTCTGCTGTTGGTCGCGAGCGCTTCGGCGCAGATACCCTC
GCTGGGATGGTGCCCCGACTATCAGCCCATGGCAAACTTCAATATCAATCGCTTCTTGGGTTCCTGGTATGAA
GCTGAACGTTACTTCACCGTCTCTGAACTGGGAACTCGGTGTGTCCGCACTAAATATG
AGAGCACACCCGAAGGCAGAATTTTAGTCTCTAATGAAATAACCAACTCTTTAACTGGATTGAAACGTGTAAT
GGAAGGGTCTATGCAGTTGATTGGTAGAGAGGGAGAAGGTCGCATGATCATCAAATATTCGGCGATCCCAGCG
CCCTACGACAACGAATACAGCATTCTGGATACTGACTACGACAACTACGCTGTAATGT
GGTCTTGCAGTGGTATCGGACCTGTGCATATCCAAAATGCTTGGATCCTCACTCGTGAACGTTTGGCCACACC
CATTGTAATGCAGAAGGCCTACACTGTGCTAGAACGGTACAGGATTTCCAGAACATTCTTCGTAAAGACTAAC
CAAGCTGACTGCTACATCATACCTAGCCCTGTCGCAGATCCTTTGGACATTAAAAATG
ATGTCATTATTGATGCCAAAAACGTCCCAATAGAAGTATCTATCGAAGAAAAAGCTCCAGCACCTGAAAAGAA
AGAAGATCCCGCACCAAAATCTGAAAATATTCAAGAACGTTCAGGTGCTATGGATATTTCTGCTGAGGAAAAG
CCGATGGATATGCCGGAAAAAA
>1002049243_1 Heli.1-DT662283.3.5

ATCGGCACGAGGAGATTATACACGTTTGTTACATTGTGCGCGTGCGCGTTGTACGCCAGCGCGGCGTCCATCC
CCGCGCCGGAGAGGAACATTGATGCTCTAGATAACGTTGACTTAGACAATGTAGACCCAGAGCTGGTCGATCA
GCCTTCGGTCCAAGATCTCGATAACAGTCTTCGTAAGGTACCGGATATTCCCGTTAAA
GTTATCGAAGCGCCAGTCATTCTGGATGAAAATTCAAATGAGTATATACCAGATAAAGATGAAAACATCAACG
TCAAGAGAATTGAAATTGATCTAAATAACCCTGGACCACCTCAGCGCCAGGAACATGAAACGCAAAATCCAGG
GAATTATGGAGAAAAAGAGAGGGCTGTGTATCAAATATTACAAAGGCTCAAGAAGCG
CAGTCTATTTTTACCAAAGGAGTAAACGATATATCTGACAGCTTCTATAACTTAAACAAACATAGTGACGAAA
TTCCAGCGGTTTTGGAAAATATAAGAAAATTAAATGTCTCATTTTCAAATGAAATTATTGAACTTAACAACAC
TATAAGAACTTATCTGGAACCCAAGCCAATACAAGTTGACAATGAACAACAAAATAAT
GTAAAACTTAATTACGTTGAATCAAAATTACAAAATTTACATTCAAACTTTAAAAACGGTGTTGACATTTTAA
ATGAGGGCTTTGAACTTCTTTCAATACTTCAGGAAGAAGATGAAGTTGCCAAATCAGAAACTAAGACATTGGT
AAAAGAAGAAAATTATTCAGTCAAAGATGATGCAGCATCGTCCGCTGCTCCTAGTTCT
TCTACTGCAGCACCTTCACCTATACAGGTGCCTGCAAATGGCTTTAACTCGTTTATAGGATTTTTTACTACAA
CTGTTTCCAACATGCTATCAAAC
>1002049244_1 Heli.1-DT662431.3.5
CACGAGGCCGACGTGTTTATTTCAAAATCGTACTTCATCGCGTTTGGGTGTGTCACGTTTACTAAAAAAATCA
AAATGAGTGGACAGCAATACTACCCGTACAAGTGCACCCCCACGGTGTACCCGGCGGAGCCCTTCGAGCCAGC
TGCCGATGCGGAAACTCTTCGCAAGGCGATGAAAGGCTTCGGAACCGATGAGAAGGCT
ATCATCGATGTTCTCTGCCGCCGTGGAATCGTACAACGCTTGGAAATTGCTGAAACCTTCAAAACTAACTATG
GAAAGGACCTTATAAGCGAGTTGAAAAGCGAACTAAGCGGTAACTTGGAGAACGTGATCGTGGCTCTGATGAC
ACCACTGCCACACTTCTACGCCAAGGAACTCCACGACGCGGTGTCAGGATTGGGCACT
GATGAAGAAGCCATCATCGAAATCCTCTGCACTCTGTCTAACTACGGAATCCGTACCATTTCCGCCTTTTACG
AACAATTATACGGCAAGAGTCTCGAGTCAGATCTCAAAGGGGACACGTCTGGTCACTTCAAACGTCTTTGCGT
ATCATTATGTATGGCCAACCGTGATGAGAACTCGGGAGTGGATGAGGGAGCGGCCAAA
GCTGATGCCGAAGCCTTAGCATCAGCTGGTGAAGGACAGTGGGGAACCGATGAGTCGGTGTTCAATTCCATAC
TTATCACACGCTCGTACCAACAGTTGAGACAGATCTTCGCTGAATTCGAGGCGTTAACCGGTAAAGACATCGA
GGAATCTATTAAGAAGGAATTCTCTGGAAGCGTCGAGAAGGGCATGCTTGCTATTGTA
AAATGCGTAAAGAGCAAAGTTGGCTTCTTCGCTGAGCGCCTCTACTACTCAATGAAAGGTTTGGGAACCAACG
ATAAGACCCTTATCAGAATCGTAGTCAGTCGTTCCGAGATCGACTTGGGAGATATTAAACAAGCATTCTTGGA
CAAATACGGGAAACCCCTAGAAGACTGGATCGCT
>1002049245_1 Heli.1-ES586861.1.5
CGACATCACAATGAGAGTTCTGATTGTATCCGCCTTCTTCGCTTGTGTGGCAGCGGCACCCGCGCCCTCACTG
CTGGCACCACTACCACTGCCTTTCGCCGCAGTGGCCATTCCTCGTCTTGTCGTAGTACCAACTATACCACCTG
GTGACATCCAAGCTGCTGCTATTGATGCTCAGGTAAAAATCGCAGATCAAGCTCAATT
ACTCGCTGACCAAGCTCGTGAATTGGCCGAACAAGCTGCCGAAAACCAAAATGAAGGTGTAGTTGAATCAAAG
AATTTAGTCGAGGCCAAATCTGAGGAAGCCTTTTGGGCCGCAGAAGAGAAGAAATGGCAAGCACTGAACGAAG
CTCAAATCGCTGAAGCTAGAAATGCTGCTTTGTTAGCTGGAAATGCCGAAGCATTAGT
CAAATCTGATGCTGCTATTGTTCCTGGAATTAAGTCAGACTCTGAATCAGGAGTCGCAGTAGAATCTGCATCA
GCTGCTGAAGCTAAACCAGAAGCAGCTGTAAAAAGCGAAGCTATTTCTGAAGATAAAGACGAAAAGGAGGCTA
TTGTAAAATCGGCTGAGGAAAAACCCGCCGAAAGTGAAAGTGAAAAGAAGGAAGATTT
ACTTGCTAGTGCCAAATCTGCACTCGCCGATGCCCTGAAGGCACAAAATTTGGCTATCCAAGCATACAATTCA
GCCATTCTTCCACCTGGTGTAGCTGTAGCTGGAGTGCCTGCTGGTCTGGCAGTGGTACCAGCAGCGGCCATCG
GTTACCACTCGGTAGCACCTTTCTCCTACCAAGCAGCTTACAGATCATTCCCAATTCA
CATATAGTCTAACTGTCGCTAAACAACTGTGGTAATTCGACTATAGTTGTAATAAAAATTATTTAAGAATAA
>1002049246_1 Heli.1-CX700812.1.5
CGGAATTCCGGGTCGACCACGCGTCCGCAGCTTCAAAGCTAAAGGCTAAACCATGGCTCCGAGAAAAAACAAA
GTTGCGAAAGAGGAGGTACAGGTAACTCTCGGACCACAACATTTGGCCGGAGAAACTGTTTTTGGAGTCGCCC
ATATTTTTGCTTCTTTCAATGACACATTTGTTCATGTTACCGACTTGTCTGGCCGGGA
AACTATTGCTAGAGTTACTGGAGGCATGAAGGTCAAAGCTGATCGTGATGAAGCATCACCTTACGCCGCTATG
TTGGCCGCTCAGGATGTTGCTGAAAAATGTAAAACCCTTGGCATCACCGCTCTCCACATCAAGCTCCGAGCAA
CTGGTGGCAACAAGACTAAAACTCCAGGACCCGGTGCTCAGTCTGCTCTCAGAGCGTT
GGCCCGTTCCAACATGAAGATTGGTCGCATTGAAGATGTAACTCCAGTACCGTCAGACTCTACTCGCAGGAAG
GGTGGTAGACGAGGACGCAGGCTGTAAATTTTTAGTATAAGCAACTGTGGGCTGAAATACAAAGGTTTACA
>1002049247_1 Heli.1-ES587952.1.5

AACAACCAAAATGTCCGCTAAGGTAGTGATCGTCCTTGCCTTGGTAGCGGCCGCGTCGGCCCTGCCCGTGCTA
CAGTACGCAGAGCCTGAAGTACCAGCACACTACGAGTTTCAATACTCGGTACACGACAGCCACAGTGGTGATG
TGAAGCAGCAACAAGAGGCCCGCGCTGGAGACGCCGTCCACGGCTCTTACTCCCTCGT
GCAGCCTGATGGAGTCCACCGCATCGTGGACTACAGCGCTGACAAGGAGCACGGATTCAACGCTATCGTGCGT
TACGAGGGCCACCCCATCGTTGCCCCCGCCCCTGCCAAGCTCGCCTACGCCGCCCCCGTCGCCAAGCTCGCCT
ACGCCGCTCCCGTCGCTAAGGTGGCCTACTCCGCCCCCGTCGCCTACGCCGCCCCTGT
CGCCAAGTTGGCCTACTCCGCTCCCGTCGCCTACGCCGCCCCTGTCGCCAAAGTGGCCTACGCCGCTCCTCTC
GCGCACGTCAGCTACTCATCACCCGTCATCTCCTACCAACACTAAGTCAATAGCTACTCCTTAAACTTAATTA
ACTTATTTATGAAATGTGATAACTGTTGTTGCAATTAAATATTTAC
>1002049248_1 Heli.1-DT662116.3.5
TCGCTTTGAGTTTATTGCGTGACGTGCCGTCCCGAATTTTCTTAACGAAATGTGATCCAATAAAAAGATGGCG
ACTTCCGTCCTTCAGCCAGAAGAATGCAGACGTGAACACGAACCCTTACCCGACACCCCGAGACTAACAGACG
ATGAAAGAATTGCCATCCTAGAATCCAAGGAACGGGAAACCGGTGAACTTCCACCGGA
GCTTCGAGAGAGAGCCAGATTGGATATTAGAGAGGAACCGGCCATTCGAGATCATGCGCTTGCCCAGATGCGG
CACTTCATTGACAAACATCCAGCCATCAAGAAATGCCGGACCGATGCACCATTCCTGCTTCGTTTCCTTCGCA
CAAAGAAATACTCCATACCACAAGCGTGTTCAATGCTGGAGAGATATCTAACGATCCG
GCAAATGTATCCCCATTGGTTCCAAAAACTTGACCCCCTGGACCCCAAAATAGCTGCAGTTATAGACGCAGGA
TACTTATTGCCACTACCTAAGAGGGATGCTGAAGGACGGAGAATCGTTTTATCTTGTATGGGCCGTTTCGACC
CCCACGTGTTTGATAGCTGTATTATGGCCCGCGTACACTCAATGATAGTAGAGCTGCT
GCTTGACGAGCCTCGTTCCCAACTGCTTGGGTACACTCACGTGAACGACGAGGCCGGTATGCAAATGCCACAT
GTGAGTCTGTGGTCGCTGACTGATGTACGGGTTATGTTGAATTGTATACAGAATTCCACGCCCATGCGTCATA
AGCGTACACACTTCGTCAACATACCGCACTATGGCGTCAAGTTCTTCGAGTTCGCTGT
GTCATTGCTCAGTGATAAGCTCAAGGATCGTGTTATGTTCCATCGTACATCGGAAGATCTGACAAAATATGTA
GATCCCGCCATATTGCCTAAGGAATATGGCGGTACAGTTCCACTAAAGGACATGATTGATGAACTTAAACGTA
AGCTACTGAAGCATAGAGAAGATTTACTTGCATTGGACAATATGTGTATTGATTTGTA
CGCTCTTGAGAAAAATGATCTTACTCAAGATATTCATTCTACTGCGGGGTCATTCAGGAAATTGGAATTAGAC
TAGAATACTAGACATAAATTAGA
>1002049249_1 Heli.1-ES587346.1.5
TTTTTGAATCTGACCGTCTAATCGATCTCACGGTTCAACAATGGCGGACGCAGCTCCAGCCGGTGGACGTGGG
GGTTTCCGCGGTGGCTTTGGATCACGTGGTGGCGATAGAGGCCGTGGAGGACCTCGTGGCCGTGGGCGCGGTC
GTGGCCGTGGACGCGGACGCGGAAAGGAGGACCAAAAAGAATGGGTTCCCGTAACCAA
ATTGGGCCGTCTCGTAAGAGAAGGCAAGATAGACAAACTTGAGAGCATTTACTTGTTCTCTTTGCCCATCAAA
GAGTTCGAAATCATCGATTTCTTCCTGGGCGCTTCATTGAACGATGAAGTACTCAAGATTATGCCTGTACAGA
AACAGACCCGTGCCGGTCAACGTACCCGTTTCAAGGCCTTCGTCGCTATTGGAGACAA
CAACGGTCATATTGGTCTCGGTGTTAAATGCAGCAAGGAAGTAGCAACCGCTATCCGTGGTGCTATCATCTTG
GCAAAATTATCTGTACTTCCTGTACGCAGAGGTTACTGGGGTAACAAGATTGGCAAGCCTCATACTGTACCAT
GCAAGGTCACTGGCAAGTGTGGTTCAGTAACTGTGCGCTTGATCCCAGCTCCCAGAGG
TACCGGAATTGTGTCAGCTCCTGTCCCTAAGAAGCTTCTTCAGATGGCTGGAGTCCAGGACTGCTACACTTCT
GCCCGGGGCTCCACTGGAACCCTTGGAAACTTTGCTAAGGCCACATATGCTGCCATTGCTAAGACCTATGCCT
ATCTTACACCAGACTTGTGGAGGGATATTCCATTGACCAAGTCACCATACTCTGAATT
CAAAGTCTAAATATAACAATGAATAAATGTTTTTGA
>1002049250_1 Heli.1-DT662541.3.5
GCACGAGGGTGCACACGCTTAGGCGAAATTCTCTAGGCGAGTACTGCTTACAGTAGCGTTTAGTCAGTTCGTT
TGAGATCTCCAGAAATAGTTTAAGAAATATCATCATGCAGTCGATGATCATCCTTGCCGCTTTCTTCTGCCTC
GCGCAAGCATCCTACTACCCCGGCCCTCCAGCGCACATACAGCTCAGCTCTGATGGCA
AATATGTGCTGGACACGCCCGAGGTAGCACACGCCAAGGCCGCCCATCTGGCCGCACATGCACAGGCCTCCAA
CGTTCACGGTGCCTGGGCTCCCGCTGGAGCGTACGTCGCTGGACCCGCCTACGGCGCCGGTGCTCATTACGGA
GCTCCCGCTGCCGGTCTCGTGAAATACGGCCCCGCTCCCCTCGCCCACGACGGACGCG
TCATTGACACCCCCGAGGTCGCCCACCTGAAGGCCGCTCACATCGCCGCGCACTCCGCCGCCGCCGCCAAGGC
CGCGCACGGCGGCTACGCCGGCATCGGTGCTGGCCTCGGCGCCGGGTACGGTGCGGGCTACGGCGCGGGCGCA
GCTTACGGCGCTGGATACGCTGGCGGCTACGGCAAGTGGAACGGACCCCAGGCTCACA
TCCAGCTCACCCACGATGGACAATACGTCGTAGACACCCCTGAGGTACAACACGCCCGTGCCGCTCACTTCTC
TCAGTACGCCCACGCTGCGCAAGCCGCCGCCTCCTCGCCCGAGGAGCCCTGGGACGCCCACAGCGGACACGGC
GCCCACGGCTGGCACTAGATTCTACCTCCGACCTAGCCCAAACACTGCCTGAATATAT

TTTTATACAAAATGTAAAGAAAAAACCAAAAAAATGTTAAACTGTATA
>1002049251_1 Heli.1-EL597759.1.5
CACGAGGCTCGTTTCTAACAATCCTACGACCAACCCTCTATCAAAATGCATAAGCTGATTGTATTCGCAACCC
TCCTGGCTGCTGCTGCCGCAGCCCCCGGTTTGGCACCTCTCGTTTCCTCATGGGGCCATGGCGGACTCGTTGG
TGCACCAGCTTTGGTCGGCGCACCCATCGTCAGCGCTCCCATCATAAAACAAGCCATC
CCCGTAGCTACTTCATATGCCAATGTTAACGCTGTCAGGATTTCCTCTCCTGCTCTTGTCGCAGCTCATGCTC
CTGTTATTGCTGCTCATGGTCCCGTCCTTGGTGCACCTTTAGGTCTCGGTCACGGTCTCGCCCACGGCTGGTA
ACCCGCCTAACAAAAACCGAGCCCTGGTTTCAGTCAAGATACGCAACAGTCAATTTTA
TTTAAATTATAGTCTGATAAGCTATGAATGAAATTCTATTTGGTCATTACTATTAAAAATTTAATTTATATAT
AAAAAAAAAAA
>1002049252_1 Heli.1-DT665131.3.5
GCACGAGGAACACACGATGCTGAATCTAATCAACTAAAGAAACATTTATAGGTAGAATAATACCATGATCTGG
CAACCATTGAATTTGTCAGAAGTCTGTCCTCCTTACACACCTATCACAGCTTGTTCGACATTTGGCTATGTCT
ACCTCAGTCTCCTAGTCCAGATCTATGGAGGTTCTGTTGATACGAGGATACAAAAGCA
ACTTCACATAAGAGATGACGAAAAGGAAAAGCCCAGTGAATATGATTTTGTTGTGGTCGGCGCGGGAGCTGCT
GGTTGTGTCGTTGCCAATCGACTCACAGATAATCCTAAATGGAAGGTATTACTCCTAGAAGCGGGTCCTGAGC
AACCAGACGTGACTTACGTACCGGGTCTATCAACTGCACTCCTGGGATCTAACATAGA
CTGGGCGTATACTACAGAGCCTAATGGGAACAGCTGTCTTGCTCATCCCGGAGGCAGGTGTCCTTGGCCAAGA
GGGAAAATGATGGGTGGTTCTAGTTCAATCAACTCAATGGCGTATATTAGAGGTAACAGGGTGGACTATGACG
GTTGGGCAGCGATGGGCAACAACGGCTGGAGCTATGAAGAAGTTCTTCCATTCTTCAA
AAAGTCAGAACGAAATATAAACATAGAGGCTCTAGATCTAAAATATCACGGAGTCAGTGGGGAACAATATGTA
TCCAGATATCCTTACATTGATAAACCCTCACTTATGTTAACTGAAGCTTTTAACCAAGGAGGGTTACCTTTAA
CTGATTATAATGCCCGATATCAAGAAGGCACAATGCAAGCCCAAGCTTTCTCTTTCAA
TGGCGAAAGAGTATCCACAAACACAGCTTTCATACAACCAATAAGATATAAGAGAAAAAATCTTATCGTCGAA
ATCAATTCTGAAGCCACGAAAATATTGATCGATAAAAATAAAAACGCCTATGGCGTTATTTATATGAAAGATG
GGAAAAAATATACAGTTTACGTTAAAAAAGAAGT
>1002049253_1 Heli.1-CV526129.2.5
TCCGAGCCAATATGGGTATTAGCCGTGATCACTGGCATAAACGAAGGGCCACTGGTGGCAAACGTGCGCCCAT
TCGTAAGAAGAGGAAGTATGAGTTAGGTCGCCCGGCTGCCAACACTAAGCTTGGCTCTCAACGTATTCACTTA
GTGCGAGCTCGTGGAGGTAACATTAAGTACCGTGCTTTACGTCTTGACACTGGAAACT
TCGCATGGGGCTCAGAGTGTGCTGCTCGTAAGACTCGTATTATTGATGTTGTGTACAATGCATCAAACAACGA
GCTGGTTCGTACTAAAACCCTCGTAAAGAATGCCATTGTAGTAGTTGATGCTACACCATTCAGACAGTGGTAT
GAGTCTCATTACCTGCTGCCACTTGGTAGAAAGAAGGGTGCCAAGTTGACTGAAGCTG
AGGAGGCAATTATCAACAAGAAGCGCAGTAAGAAAACAGCAAAGAAATATTTGTCAAGACAGCGCCTGTCCAA
AGTGGAAGCTGCTCTTGAAGAACAATTCCACACTGGTCGCTTATTAGCTTGTGTAGCCAGCAGGCCTGGTCAA
TGTGGCCGTGCTGATGGCTATGTCCTTGAAGGAAAGGAATTGGAGTTCTACCTAAGAA
AGATCAAATCCAAGAGGGCAAAGTAGGGTTTTAATGTATAAATAAAAGTAAAAACAACAAAAAAAAAAAAAA
>1002049254_1 Heli.1-CX700450.1.5
ATGGCGGATCAGACCGAGAAAGCGTTTCAGAAACAGGCTACGGTGTTCCTCAACCGCAAGGGCGGATTGAAGA
AGAGGGATATGCGACATCATAAAAATGTCGGATTAGGTTTCAAGACACCAAGAGAGGCAATCGAAGGCACTTA
TATTGACAAGAAGTGCCCTTTTACCGGCAATGTGTCGATCAGAGGCCGCATTCTTACT
GGTGTCGTACAAAAGATGAAAATGCAACGCACCATTGTCATCAGACGTGATTACTTGCATTATCTACCTAAAT
ACAACAGATTTGAGAAGCGTCATAGGAATATGTCAGTCCATCTGTCACCTTGCTTTAGGGACGTGGAACAAGG
AGATATTGTGACAATCGGTGAATGCAGGCCGCTGTCCAAAACAGTAAGATTCAATGTA
CTGAAAGTCTCCAAGGGCAAGGGATCCAAGAAATCGTTCAAAAAATTCTAAATAAAATTA
>1002049255_1 Heli.1-DT662065.2.5
GCACGAGGCAACGGTCGACATGCGCTCCCTTGTTTTCTTCGCTCTCCTCTCAGTGGCTGCGGCCAAGCCCGCG
CCCGGACTTCTTGGTGGATGGGATGGCGGTCTAGGCCTTGGCCATGGCTCTCTCGCGCTAGGGCACGCGCCCC
TCATCCAGGCCGCGCCGGTGCTCCAAGCGGCGCCCGTCATCCAGGCGGCGCCCGTCGT
GCACGCCGCGCCTGTTGCCATCGCCAAGGCTGCCACTAGCTACTCGTCCATCCAGAGGGTTATTCATCCAGTA
GCTCAAGTGGTGGCCCAACCCGTGGTCCATGCCGCGCCCGTAGTCCAGGCCGCCCCAGTAGTCCAAGCCGTCG
CCCAGCCCATCCTGAGCCATGGCATCGGCATCGGCAGCCTGGGCCATGGTATCGGCCT

CGGCGGGCTGGGTCATGGTATCGGCCTCGGCGGGCTGGGTCATGGTATCGGCATCAGCAGCCTGGGCCATGGC
TGGTAAACTTATTCGTAATTAATTAATGTTCGTTAGCGCGTTGAGATCATGCCCGATTTGTCGGGTGGTGCAA
ATAAATATTTATATCAGTATTT
>1002049256_1 Heli.1-DT661669.3.5
AGCTATCACCGGCCGTCCCCGCCTCAACATGCGAGGGATCTGGAGCCCAGTGATTACCGTGACGGTAATTCTA
CAACTCGCTCTAGTATCAGGAAAGGCGTTAATACCACAAGATCAACAAACTCAGCAGCAGGTGGTTCAAAAAG
ATCAAAATAATGCACAAAAACGAGTCGGATATGACTACCCAGTGCCCCCAAATGATTT
AATCAACCCTTTTCAAGATCACGACAACCTTCATCCGCATGATGATCACCACGAAATTATTGATGACCACGGA
CATGATGTTCATCATTTAGAAGAACATCACGTCGAGGAACATCATGAACATCACGATGATCACCACGACCATC
ATGATCATCACGATCCTGGATACTGGAAAAAGAAGTTGATATGGAAGCCTGGATGGAA
GAAAATTTGGAAACCAGCTAAGAAACAAATCTGGAAGCCATCCTGGAAAAAGATTTGGAAGCCTATCTGGGTA
CCGACAAAAGTACCGGTTTGGAAAGACATAAAGGTTCCAGATTGGAAAAAAATTTACAAACCAGATTGGAAAC
CCATAAAGGTACCTGCATGGAAGGAAGTCAAAGTACCTGACTGGAAAAAGATAACAGT
TCCTGTATGGAAAAACATCGTAGTTCCGGGATGGAAAGATATACAAGTACCAGCATGGAAAAAGTTATGGAAA
CCTGAGTGGGTTAAAGTTGGAATTCCTGGTGAAAAATATCTAGGTAAGGATCATGAAGGTTGGGAATATACAT
CGCATGACCTTTGGAAAAAGAA
>1002049257_1 Heli.1-ES588231.1.5
GGCGGTTGGTAAAAATAAAGGCCTTTCGAAAGGCGGCAAGAAGGGAGTTAAGAAGAAGATTGTAGATCCCTTC
ACAAGGAAAGACTGGTACGATGTTAAGGCACCGTCTATGTTCACTAAGAGGCAGGTGGGAACCACGCTCGTCA
ACCGTACTCAGGGAACCAAAATTGCTTCTGAAGGTCTGAAAGGCCGTGTGTTTGAGGT
TTCTTTGGCTGATCTCCAAGCTGATACTGATGCTGAGAGGTCTTTCCGTAAGTTCCGCTTGATTGCTGAAGAT
GTTCAGGGGCGCAATGTGCTCTGCAACTTCCACGGTATGGACCTCACAACTGATAAACTCAGATGGATGGTCA
AGAAATGGCAAACACTGGTTGAAGCCAACATTGATGTCAAGACCACAGATGGTTACCT
CCTGAGGGTTTTCTGCATTGGTTTCACCAACAAAGACTCGTTGAGTCAGCGCAAGACATGCTATGCTCAACAT
ACTCAGGTACGCGCCATCAGAAAGAAGATGTGCGAGATAATTACCCGTGATGTGGCCAACTCTGAACTGAGGG
AGGTTGTCAACAAGCTGATACCAGACTCCATTGCCAAGGATATCGAGAAGGCTTGCCA
CAGCATCTACCCATTGAGAGATGTGTGCATTAGGAAGGTAAAAGTACTGAAAAGACCCAGGTTCGAGATTTCC
AAGTTGATGGAGCTTCATGGTGAAGGCGGTGGCGGCAAGCGTGGTGAAGTGGGAGACAAGTCTGAGCGTCCCG
AGGGTTACGAGCCGCCCGTGCAGGAGAGCGTTTGAATATATTATTG
>1002049258_1 Heli.1-DT667934.1.5
GCATCAACATGCATTTCAAAATTGCAGTAGTCAGCGTCCTCGTGGCCGTATGTCACGCTGGGCTTATTGAAGA
AGGTCAAAATCACGCCGTTTCATCTCAAAGCATCGTCCGTCATGACCAACCGATCAACGGCGCTACACACTAT
GCACCTATCGTTGCTCATGCTGCTCCAGTCCTCGCCCATGGACCAATCCTTCAGCATG
CTGCCCCCCTGATTCATTCTGCCCCCATTGTCCAACATGTTGCCCCCATTGCCCACGGCGCTCCCATCGCCCT
CGCTCACGGTGAACAAATTGAAGACCATGCTCCAGCTCACTACGAGTTCTCATACTCAGTTGAAGATCCCCAC
ACTGGTGACCACAAGTCTCAACACGAGAGCCGTGAAGGTGACGTAGTCAAGGGAGAAT
ATTCTCTGGTTCAACCCGATGGCGCTGTAAGAACTGTCGAATACACCGCTGACGCTCACAATGGTTTCAACGC
AATTGTGCATAATTCTGCTCCGTCAGCCCACGCTGCTCCAGCTCCTGCAGTGCACTCAGCCCCAATCCAGGTG
CATGCTGCTCCTGTAGTGCACGCCGGTCCATTGGTCCATGCTCCTCTAGTACACGCTG
CTCCTTTGGTTCACGGACCTGTAATCCATGCAGCACCTATCCTAGCCCACCATTAGGCTGGACTTTTCAAACT
ACTGTAATTCCTATTAGAAATATATTGTTATTATTTATAATTAAAAAAAAAAAAAAAAA
>1002049259_1 Heli.1-DT664850.2.5
CATGGCGGTTGGTAAAAATAAAGGCCTTTCGAAGGGCGGCAAGAAGGGAGTCAAGAAGAAGATTGTAGATCCC
TTCACAAGGAAGGACTGGTACGATGTTAAGGCTCCGTCTATGTTCACTAAGAGGCAGGTGGGAACCACCCTTG
TCAACCGTACTCAGGGAACCAAAATTGCCTCAGAAGGTCTGAAAGGCCGTGTGTTTGA
GGTCTCTTTGGCTGATCTCCAAGCTGATACTGATGCTGAGAGGTCTTTCCGCAAGTTCCGTTTGATCGCTGAA
GATGTTCAGGGCCGCAATGTGCTCTGCAACTTCCATGGAATGGACCTCACAACTGATAAACTCAGATGGATGG
TCAAGAAATGGCAGACGCTGGTCGAAGCCAACATTGATGTCAAGACCACAGACGGTTA
CCTCCTGAGAGTTTTCTGCATCGGTTTCACCAACAAAGACTCATTGAGTCAACGCAAGACATGCTACGCTCAA
CACACTCAGGTGCGCGCAATCAGAAAGAAGATGTGTGAGATAATCACCCGTGACGTGGCCAACTCTGAACTGA
GAGAGGTTGTCAACAAGCTGATACCAGACTCAATTGCCAAGGATATCGAGAAGGCCTG
CCACAGCATCTACCCATTGAGAGATGTGTGCATTAGAAAGGTTAAGGTACTGAAAAGACCCAGGTTCGAGATA
TCCAAGTTGATGGAGCTTCATGGTGAAGGCGGCGGCGGCAAGCGTGGTGAGGTGGGAGACAAGTCTGAGCGTC
CCGAGGGTTACGAGCCGCCCGTGCAGGAGAGCGTTTGAATATATTA

>1002049260_1 Heli.1-DT666805.3.5
ACGAGGGGAACAATCTCCAAGAGTTGGTTGTTTCAGTGTAAATTGTGCCGCAGTAGCAGTTCTTTTTGACAAA
AAACGTTACACCCAAACGTAAAATGCGCGCATTCGTGGTTCTCGCCTGTGTGGCCCTGGCCTACGGCCGCCCT
GAGCCTCCAGTAGGATACAGCTACTCTGCTCCTTCTAACAAATATATCCCATCTGAAA
GTTACTCATCCTCTTCTAGTAGTGGTCATTCTAGCGGCCTTTCTTTGGGTGGAATCTCTGGCGGCCACTCTTC
AGGTAGTGGTGGTATCAGTGGTGGATTCGGCGGAGGAATTGGCGGAGGAATTGGAGGAGGAATTGGTGGAGGA
TTTGGCGGCGGAGACGCCGGTTTCGGCTCCGGTGGTTACTCGGGGGCACCTATTGTGC
AGAAACACATCTACGTCCACGTGCCTCCCCCAGAACCCCAGGAACAGAGAATCCCCCGCATCACTCCTATCGC
TGCTCCCCAGAAACATTACAAAATCATCTTCATCAAGGCCCCAACCCCACCCGCACCAGTCGCTCCTATCATC
CCTGTCCAACCTCAAAATGAAGAAAAGACCCTCGTATATGTGTTGGTTAAGAAGCCCG
AAGAACAGCCTGATATTGTTATCCCCACTCCCGCACCCACCCAGCCTGCCAAGCCCGAAGTATACTTCATCAA
GTACCAAACCCAGAAGGAGTCTGGTGGTATCGGAGGCGGTGCTATTGGCGGTGGCTCTTTGGGCGGTGGCGCT
CTCGGTGGTGGTGCTCTCGGTG
>1002049261_1 Heli.1-DT666712.3.5
GCACGAGGCAAAGACTACGCGCGTACAGCTTACAAATATACATTCATAGAAACAACAGTATCGTGATGGAGAA
GGAGCACCAGCCAGACTCTATGGCTACTATAACCATGAAACCGGAGTACCCGCCGTCTGAAGTATATAGTGCT
TCAGAACCTCCGCCGGCATATCGTCAGAGGATGTCATCTTCTGTGCAAATCGCGCGTA
TTGCGGCTCTGACGGTAGTGGCGGCGTCGTTCATCCTGGGTTCCTTCATCTTGGCATCCAGCTGGGTATCAGC
ACGCGCCTCTTGTCATCAATTGGAACAGCTCGATGCCATGCTTGACAAGGAACTGGCATTGGAGGGGAGACCA
TACGGAAATGATGGTTTAATGGCAGATGAGCCATTGCCGATAGCAAATGCTCATGCAC
TCCACGGGTCGCCATCACTTGTAGCAGAAACATCGTCTCCACCCGCGAAATTAAAATCAGTGCCGTCCCCCGA
CAGCCTCTTGAAAGAGGATCCTTTAAGCCACGCTGAATCCAAAGTCGATGAAGATAAATTACAGAAAATCGAC
GACGAAAAGGTAGAAACGGCAAATTCTGGAAGCGATGAGAGTTCCGAGTCAGATAGCT
CCGGTGAAGAAGATGATGATGAGCTCATGCCTAAGTTCACGATGCCTATACGTTTTGACCTCGACGAGCTGGC
GGGGGCTTTCTTAGCAAACAACCAAAAAGGACGTATGAATTGTGTAGTTGAGAGGCGCCATGAAGAAGCCATG
GAGAAGCGCTTCCCATTCAACCTTCTTAGTGCATTGACATCTCGAAGCCCCCAGGCTG
AACGTATTTCCATCATCTGCCACGGTGGTGAGGAACGACCGTTGCCTATGTTCCTTGACCCGCAATCACAAGT
ATTCGCATACCCACTGTCCGGGCCAGTGCGTCTACCGCTGCGCCCAGCGCCTGAGCGACCTGAGCCACAAGGA
CTATTCAAAATATTCAAGAATTTAGAACAAGCGTCCATGATGCAAAGGCCATGGGAAT
CTTCGGAAGAACAACAAATGACCCCTCCGCGTGAGATGCGCATTCACGTTCAACGTATCTTCGGCATCCCCGC
GCCTCACAACATGCCTTCTCCTCTTGAGAACATGGGACAGTTTGCACCACCACCACCACCACCACCCCAAGGT
AACGAGCAGCGTGAACAAGAAGAAATGATGGCGCCAAGACAGATGCGAGTGATTGCAA
TTCCTGCTCCACACAACATGCCACCCCCACCCGAAAACATGGGACCCTTCCAGCCCCCACCGCCACCTCAGGC
TCAAGCGCAAAATGAGCAACCCGCCGTGCTTCATCAGTTTATACCTCAGTCAGCCCCCACACAAGAAGTAGAA
CAGCCTGAGGTACAAAAGATGCAAATGGAAGGTA
>1002049262_1 Heli.1-ES587800.1.5
TGTGTTTTTAATCTTGACAAATCGTAAAAAATACGTGTTTATTTTAATAATTTCTTGATAAATCGTATCCTCT
ACATTGTTACTAACAAGAAGTTTCACTTTGTGTAGGTCACAATGGGGCGCCGTCCAGCGAGATGCTACCGGTA
TTGTAAAAACAAACCGTATCCTAAATCCCGGTTTTGCCGTGGTGTACCAGATCCTAAG
ATTCGTATCTTCGACTTGGGAAAGAAAAGGGCAACAGTAGATGACTTCCCACTATGTGTCCATCTGGTGTCTG
ACGAGTATGAACAGCTCAGCTCCGAGGCTCTGGAAGCAGGCCGTATCTGCTGCAACAAGTATCTTGTAAAGAA
TTGTGGTAAAGATCAGTTCCACATTCGCATGAGGTTGCATCCATTCCATGTTATCCGC
ATCAATAAAATGTTGTCGTGCGCTGGAGCTGATAGGCTCCAGACTGGAATGCGTGGTGCGTTCGGCAAGCCAC
AAGGCACCGTAGCCCGTGTGCGTATCGGCCAACCCATCATGTCGGTGCGTTCCAGCGACAGGTGGAAAGCGCA
AGTGATTGAGGCTCTACGTCGTGCCAAGTTCAAGTTCCCTGGTCGTCAGAAGATCTAC
ATCTCCAAGAAATGGGGTTTCACCAAGTATGAGCGTGAGGAGTTTGAGAAGTTGCGTGAGGATGGCCGTCTCG
CCAATGATGGTTGCAATGTCCGCTACCGTCCTGAACATGGCCCTCTCGACTCCTGGAGGAAAGTGCAGAATGA
AATCTACAGTGTGTAACTGATATCTAATAAACTGTGAAGTTTTTAT
>1002049263_1 Heli.1-CV133753.1.5
GACGTTGTAAGGACGTCATGCCATTCCAGCGCTACGTTGAACCGGGGCGAGTTGCCCTGGTTGCTGAAGGTTC
CCTGAAGGGAAAGTTGGTGAGTGTGGTCGATGTGATCGATCAAACACGAGCACTGGTCGACGGACCCGGTAGC
GGTGTACCCCGACAACAAATTCGTCTTAACCAACTCCATCTTACCAAGTTCCGCCTTA

AGTATCCATTCACTGCACCTACACGTGTTGTCAGGAAAGCTTGGTCAGACGCTAAACTTAACGAAAAATGGGC
AGACAGTCAATGGGCAAAGAATTTGGCCAACAAAGAAAAACGCGCCGATATGTCAGACTTTGATAGATTTAAG
TTAAATGCCGCGCGGGGTAAGAGAAACCGTGCGAGAACAGCAGTGTTCAAGAGTCTGA
AAGTTAAAGCGGCGCGCGGTGGTGTCTTTGGCAAGAGGAAAGTACCCAAAACTCCAGCCAAGAAACAACGCAC
CAAGAAGTCCCCAGCTGCCAAGAAACCAGCAAAGAAGTAATTCTTGTTTTGTGTCTGAAAAAAGTGTAATTTA
TAGGACAAATCAAAATAAAGAAATACTTTAAGCT
>1002049264_1 Heli.1-DT664595.3.5
GCACGAGGGTCGTTCCGCGCTCACACATGTCTCGCGAGTGACAAGTGACACTGACAAGTGACCTACAAAATGA
CCGCCAAAATTTACGTGCTATGTCTGGTGCTTGCCACGGGGATGGCGGCGGCTGCGCGACACCGACCCAGATA
CCAACAACCCGTGGAATATGACTATGAGCCTCAAGCCCAGGAACGAGGAGAACGGGTT
GTATTAGTTGCCCAGGATGCATATGATGGATTATACCAAGCCCAAGATCGTGCAGATGAGGATTACGAACCCA
GAGCATTAGTCAGAGCACCTGCACAAAGGCACAAGCAGTCTCAGTTACAACAAGAAGGACCTAAGCAGCCTCC
AGTGCAGACAATCAGGAATTACAATAAGGTGAACGATGATGGTAGCTTTACATTTGGC
TACGAAGCTGCTGATGGGTCCTTCAAAGAAGAAACTAGGGGCACTGACTGCGTAGTTCGTGGAAAATATGGCT
ACATTGACCCTGACGGTAACAAAAGAGAATTTACTTACGTATCTGGTAACCCTTGTGATCCAAACAAACCAAA
TGAAGACGAAGAACAAGAAGCACCAGTGCCAGAATCTGCAGAAAGAGACGATCCTGAA
CCCAATTATCCGAGCAGACCTGCCTTAAGACCGACAACAGCCCGACCAGCGACCACTTACTTCCAAAACGACT
TTAGAGATGCTGATGAGGAACCTGAAGAAGAGCCTCTTCAAAACATACGACCACGAATTGTTCAACGTCCACA
ACCCGTGAGACCAGCTCCTCGGCCAGCTTACCAAGCTCAACAAATCGCAATTACTCCT
CGTCCAGTACCTATCACTACTGCCAGAGCCTTACCTCCAGCGACTACATTTAGGCCACAATTAATCCAAGTAA
CCCCTAAGCCCCAAATTCAGTATAGTCCTGAGCCTGAATACTCTCCTGCCCCAACACCATCTGCTGTTCTGTC
ATCTGCTAGGCCAGGTCAAATTGATTTCGCTGCTGAATTTGCGAAATTCCATCGTGAT
AGTCAAGGACCTAGCACACCTTCATCGTTGGGTGCATCTGGGAAAGCTACTGCTGCTTCTCCATCTGGTAATC
CACTATACTCAACTGAATTAATATTTGATCCATCCAGTGGTCAATATAATACTCAACTC
>1002049265_1 Heli.1-DT665864.3.5
GTTGATCGTTCGCGGCGTGATAGTGTCGTGCTAGGACATCATGCGGACATTACTGATATGTATGCTGTGTGCA
GTAACAGCTTATGCTCAATATGATTCGCCTACGCCGCAAAGGCTGCAAATTCCTGGGGCGTTTGTAGCAGGAG
CGAGACAGGGTGGATTTAGACAAGGAAGATTACAAGCTGCTCAAATTCCTGTTGGAGT
AGCTAGAATAAGAAGGCCAGGACTCGCCAGACCTTCTTTCAAATCTGTAGATGCAGCCCCACGTCCCAGTCTT
CAATCTCTTGATGAACCATCAAAACCCGTCACAGAAGAACCGGAAGACGAAATTGAAATCAATACTCCCACTG
CATATGTGCCAAACGTTTTCTCTACCGCTGAGCCACAAAATGATTTCTTTGATATTAC
AACCACATCCCAACCAAAGCCACCCGCTGTTTACAATTCTTCACCTTTCCAACAAACCACTCCTTCTGCGCCA
AGACCCGAACCAGTGAGACCTACACAATACCGACCATTGGTACCTCAATCTTTTGTCCCTATAAGAAACGAGC
CTGCTAGACCTCAGCCAGCCAGATTACAACCTTTGTCTAGCAGACCACAGCGACCCTC
CTTCAGACCAGAGCCCAAACCTTTTATTGACGAAGAGGATGACTATATTCAACCTGTTAGACAGTTCTCCCGA
CCTCAAGAACCAATTCGTACACCAGCTAAGCCTCAACAAAAATATACTTCAAGTAACTCCAGAGATAAGAAGC
CAGTAGCTCAGATCATACGTAAATTCAGAGACGAAAACGAAGATGGAAGTATTACATG
>1002049266_1 Heli.1-CX700827.1.5
CGTCCGCAAACGAATTGAAACATGGCTGAGGTAGAAGAAATCAAGAAGAAACGTACCTTCAGGAAGTTTACCT
TCCGAGGAGTAGATCTGGATCAGCTTCTGGATATGCCAAATGAGCAACTCATGGAGTTAATGCATGCTCGTGC
TCGTCGGCGATTCGCTCGTGGCCTGAAGCGCAAGCCGATGGCTCTGGTGAAAAAACTA
CGCCGGGCAAAGAAAGAGGCGCCTCCAAATGAAAAGCCAGAAATCGTCAAGACTCATTTGCGGAACATGATCA
TTGTACCTGAAATGGTTGGATCTATTGTTGGCATATACAACGGAAAGACTTTTAACCAGGTTGAAATCAAGCC
CGAGATGATTGGTCACTACCTAGGTGAATTCTCAGTTACATACAAGCCTGTTAAGCAC
GGTAGGCCAGGTATTGGTGCTACCCACAGCTCCAGGTTTATTCCGCTTAAGTAGGCGGCAACATCTTAGGCTA
AGCAAAAACAAACACAATGATACAATAAAGTATTT
>1002049267_1 Heli.1-DT664265.3.5
GCACGAGGGCAACCCGAAAAATCCGGGTCGGCTGCGGCTAAGCCCGCAGAGAAAAAGCCCGCAACTGCTAAAA
CTCCTGCAGCAGCCCCTAAAGCTGCATCTGCTGCTAGTGCTGCACCAAAACCTGCATCTGCTAAAACACCTGC
ACCTGCTCCCAAGGCGGCTGCATCTAAAGCTGCACCAGCAGCCAAGCCAGCACCGGCT
AAGGCTGCCTCAGCTCCTGCTGCAGCAGCCAAGCCTGCAGAAAAGAAACCAGCGGCAGCACCTTCTGCTAAAC
CTGGATCTGCTAAAGCTGCAGCTCTCAAAACTAAATCTAGTGCAAAACCTTCAGCTAAGAAAGCAGCATCTGC
ATCAAAGCCAGCCAAGCCAAAACCAGCAGCGGCTAAATTGAAGATTGCTCCCAAGCCT

AAGAAGACAGGTATTAAAGGGCAAAAGAAAGTGGTTAAGCCAGTTGTTAAAGCCCTTAAAATCCAGAAAAAGG
TTGTAAAAGGTGAACATGGAAAGCGCGTGCGAAAAATACGCACATCTGTGCACTTCCGCAGGCCCAAAACTTT
TGAACCACCAAGAAATCCTAAATACCCCAGGAAATCACTACCTAAGAGAAATCGTATG
GATGCATACAACATTATCAAGTATCCCTTGACATCTGAAGCTGCAATGAAGAAGATTGAGGACAACAATACAT
TAGTATTCATTGTTCACACAAGCTCCAACAAGCACCACATCAAAGCTGCTGTCAAGAAGTTGTATGACATAAA
TGTTGCAAAAGTAAACACTCTTATAAGACCTGATGGCAAGAAGAAGGCATATGTGCGA
CTCGCAAGAGACTATGATGCGTTAGATGTTGCCAACAAGATTGGAATCATATAAACATATGTACAATATTTTA
TAAGAATAAAAAACATAAATTAC
>1002049268_1 Heli.1-DT662530.3.5
CACGAGGCGTAGGCAAGGTCTAGCCAACGTTCGCTGTATTTTAATAGTATGTCTCGGAAAAAAGGCTTTGAGG
AGACTGATAAATTAACTCGTATAGCGATTGTTAATGCTGATCGTTGTAAGCCTAAAAGATGTAGACAAGAGTG
TAAGAAGAGCTGCCCAGTGGTGCGCATGGGCAAGCTCTGCATAGAAGTTACACCCAAC
GATAAAATCGCAACCATATCAGAAGAGCTTTGCATTGGTTGTGGTATTTGTGTCAAGAAATGTCCTTTTGATG
CTATAACTATTATCAATATTCCATCAAACTTGGAGAAGCACACAACTCACAGATATTCTAAAAACTCTTTCAA
ACTCCATCGGCTGCCTATACCTAGACCTGGTGAAGTACTTGGTCTTGTAGGACAGAAT
GGTATTGGTAAATCTACTGCCCTTAAAATTCTTGCGGGCAAACAAAAGCCTAACTTAGGCAGATATGCTGATC
CACCAGACTGGCAAGAGATTCTCTCTCATTTCCGTGGCTCTGAGCTCCAAAATTATTTTACTAAGATCCTTGA
AGATGACCTAAAGGCACTTATTAAGCCTCAGTATGTGGATCAAATTCCGAAAGCTGTA
AAAGGAACTGTAGGGCAACTCCTTGATAAGAAAGATGAGAGGAAAAACCAAACTCAGATATGTTCTATGCTTG
ATCTCTCGCATATTCGTGATCGTGAAATATCCGCGTTGTCCGGTGGAGAACTCCAACGTTTCGCTTGTGCTAT
GGTTTGCATACAGAATGGTGATATATTTATGTTTGACGAGCCTTCGTCCTACTTGGAT
GTGAAGCAGCGCTTGAACGCTGCTAGGACCATACGATCTCTTATTGACCCCGATAAGTTCATTATAGTGGTAG
AGCATGACTTGTCAGTACTGGACTATTTGTCTGATTTTATTTGCTGT
>1002049269_1 Heli.1-EL603482.1.5
CACGAGGGCAACACAGACCACTTACAAAATGTTCAAGCTGGTGGTGTTGTCTTGCGTCCTGGCGGCGACGTCC
GCGTCCAGCCTTTTTGGAGGATATGTGGCACCCTTCACCCCATTGGCATATGCTGCACCTTTGTGGGAACCTT
CCAACTACCGTGGACCCCTCTCATTGGCTCCTGGTCAACCTGCTAACATTCTGGGCGC
TGATGGCAGGCCTCTTGACACTTTGGACGTAAACTTGGACCGCTCCGCTCACTTCACAGCCAAAGCTCTCGAC
AATGGATTCCATATCCTCAAGAAGCGTTCAGTCGTCTCTCCTTTCATCGCCCCCTATGCTGCACCTTACGTAG
CCCCTTACGCCGCATCATATGTGGCCCCCTACGCCGCGTCTTATGCTGCGCCTTTGGC
ATACTCTGCTCCTCTCTTGCAGCCATACAACTACCGCGGACCTCTCTCCCTAGCCCCTGGACAACCCGCCAAC
ATTCTTGGTGCTGATGGAAGGCCCCTTGACACTTTGGACGTAAACTTGGACCGCTCTGCCCACATCGCCGCTA
AAGTTGCTGCTGGCTCTCACTTCATCAAGAAACGCTCTGCCGCCTTCATCGCCCCCGT
CAGCACTGTTGCCGTCGCCAGGACGCCACTGATCGCCCCATCCTACACATTCACCGCTCCCCTCGCAGCTGCA
CGTCTCAGCCCATTCGCCTACACCCACCCTGTTGCCCGCATCGCACACCTATACTAAACTGAACTTAAAATTA
TGTAAGAAGA
>1002049270_1 Heli.1-DT665707.3.5
GCACAGGTGGTGCGAGGCTTCCTTGCAAACATAGCGTCATAAATTACGACTAATTTGTAATTTTTCTAAACTT
TTTGATCGGTTAACGATCTATAAAATAGTGCTTTAAGTGTTTAAGAAGTTTCACAACAAATAGCAATGAGTTC
CAGCGCGTGCTACAAGTGCAACAGGACAGGGCACTTCGCCCGCGAGTGCACGCAGGGC
GGCGTCGGCGCCCGCGACTCCGGGTTCAACCGGCAGCGCGAGAAGTGCTTCAAGTGCAACCGCACCGGACACT
TCGCGCGCGACTGCAAGGAGGAAGCCGACCGTTGCTACAGGTGTAACGGCACGGGGCACATAGCGCGCGAGTG
CGCGCAGAGCCCCGACGAGCCGTCGTGCTACACGTGCAACAAGACGGGGCACATCGCG
CGCAACTGCCCCGAGGGCGGGCGCGACTCGTCCAACCAGACGTGCTACAACTGCAACAAGGCCGGCCACATCT
CGCGCAACTGCCCCGACGGCACCAAGACGTGCTACGTGTGCGGCAAGCCGGGGCACATCTCGCGCGACTGCGA
CGAGTCGGAGCGCAACTAGCCCCCGCTCCACCACGCATGCACCACGCAAACACCACGC
GAGGACTGTACGTAAGCAAAGGAGGGTGGACGTGCGAGCGCGAGCCGTAGGTGAATGTTTGTTCTTGCACGTG
AACCAGTTGTGTTTTTAAATCATTTAATAATCGTGATTGAACTTTTGATGAGGTAGCGTTACAGTTTTTAGCA
TTACAGAGTATATATTTAATATATTTTGTACAGATTTATTTGTCGAAAGATAAATTGG
ATGCTGGCGTATGATGCGTTTTCATGAGAGGAGTGGGAGTACCCTGTCGGACTCTCTTGTTCGTCTTGTTATT
GTCAGTTACGGTAGACGTCGCTCTCGCCGGTCGAACGCGCCGGCGCCGTTGTCACCGTTTGAGTTTGTTTATC
AATCCTTTGTTTATGAGGTCCTGCTGACTTGTGGAGAATTCAGTGGTTAATTAGAATT
TCATCAATCAGTATATTTTTTATT
>1002049271_1 Heli.1-ES588078.1.5

TTTACCGTATCGGAGGTTATAAAAATGACTCGCAAACGCCGTAATGGAGGACGCGCCAAGCACGGCCGTGGAC
ACGTAAAGGCTGTGAGGTGCACTAACTGCGCCCGATGCGTCCCAAAAGACAAGGCTATTAAAAAGTTTGTGAT
CAGAAACATCGTTGAAGCGGCCGCCGTCAGAGATATCAACGAAGCCTCCGTGTATGCT
TCATTCCAACTACCCAAGCTATATGCAAAGCTCCATTACTGCGTGTCATGTGCAATCCACAGTAAGGTAGTAA
GGAACAGGTCCAAGAAGGACAGGCGTATCCGAACTCCGCCCAAGAGTACCTTCAACAGAGATGCGGCAAGACC
GCAAAATGTACAGCGGAAGTAATTTTAAATATTAAAATTATGTAAA
>1002049272_1 Heli.1-ES584684.1.5
GTTACGTCGAGAGGATATACAACCATGTACGCTAAACTGTTCATCGTCTGCATCGTCGCCGCGGTAGCGCTCG
CCCGCGAGTACCCGGCCGGTCTCTACCCTGCTCTGTGCCCCAACTACCCCTTCTGTGACTCTAACACCTTAGC
TAGATTCACCCCAGATGGCATGCCTATCCCTGAATGGGTTTACAACCCCTCCATCCTA
CCTGTAGCTCCTGCTGACCCCAACGCCAATATTGCCGCTAAATACCCCGCCAACTTGAACCCCGCGGAATGCC
CCAACTACCCCTACTGCTGGTGAACAGTTTAGTCGCAGCGTGCCTTCCGGATTTACTTGTTAATGACAAATTC
TACAAAACATCAACTTGGTCTTTTTTTTCTTCATCTGTGGCAATAGCCTCATGTTTAG
CAGAAAACTACGCTCATTAACAATGTATTGCCATATTTTTCTGTACCTTATTAAATTTGTACATACACATAGC
TGATTTTTGCTCAGCATCTTGTTGTTTTGTAAAAAATATGTAAAAGCTGTAAATAAATA
>1002049273_1 Heli.1-DT664452.3.5
CACTGCAAAGCTTGAGCCATATTTGTCTGTGCTAAAATAGTTCCGTGGAGTGAGTGTGTGCAGATAGTCATCA
CATCACAGTTTTTGGGACCTGTAAATACTCCCACACTTCACATACAAAATGGCCGAAACAAATCCCATCTATG
GACCTTTCTTTGGAGTTATGGGGGCGGCGTCAGCTATCATTTTTAGCGCCCTTGGAGC
GGCTTATGGCACAGCCAAGTCCGGCACAGGTATCGCTGCCATGTCGGTGATGAGGCCTGAGCTGATCATGAAG
TCCATCATCCCCGTTGTCATGGCGGGTATCATTGCCATCTACGGGCTGGTCGTGGCTGTGCTGATTGCTGGGT
CGTTGGAGGCTCCGGCCACTTATCCTCTTTTTAGAGGTTTCATCCACTTGGGAGCGGG
TCTCGCTGTAGGTTTCTCTGGTTTAGCCGCAGGTTTCGCCATAGGCATCGTGGGTGACGCCGGCGTCCGCGGT
ACCGCCCAACAACCAAGGCTGTTCGTCGGAATGATCCTTATTCTCATTTTCGCTGAAGTATTGGGTCTATACG
GTCTCATCGTAGCCATCTATCTGTACACGAAACAACAGTAAATTTAATATTTGACATC
CATTCACCATCTTATATATACCCTCGAAGTTTTTATAAGTTCATATGTCGCGCAAATCAATCGCCGGTAGCTA
TTAAACTTCGAGGGCTAAGATAAAGTATCTAACATCAAGCATTGACTTTGTAGACTTGATGCTGCGTCTACAG
AGATCGTGCTTATACTGTGTATTTAAATGAAATTTAAATTTAAACCTCCTCCAGCTAC
CGAGTGATTGAAATTTCAGCTTTGATTTTTTGGAGTATTAATTATTATTACTTCTACATACTACTACATATTA
TGAGCGCGTTACCTATTGCGAAATATATCACTTAGATTATTTTAGTGTAAGGCGAAACTAATGTACAGTTAGA
GTAGAAAGTT
>1002049274_1 Heli.1-DT666274.3.5
ACGTGACTGTGTCCACTTTCCGCTTGTTTTCAATTCATCGATCGCGCTGGAACATCACTACTGCGCAGTGTAG
CGCAGTGTAAGGTCTCATTTAACGGCGTACCTGGGCGGCTGAACTAGGACAGAGAGTTCGCTACGACATGGGG
GCTAGGTCCCGGGAGTTGCTACTGCTCCTGGCAGTTTGTGCGCTTGCAATCTCAGAGT
CTGTTAACAAAACTGCGAAGAAAGATAACAAGGATTCTGACAATTTCCTGTTTCAATATGAAGACTATGACAC
AGATGAACAAGAAGTATTATTCAATGAGGACAGGCCTTGTCCTAGAGATTGCATATGCACTGTATCTCAAGGA
TACAGAACAGCGAAGTGCAACCGTCTAGAAATGGGAACTCAAAAGTTCGGCGACGATA
TCACGGATCTCGTAATTGACAACGCCGAGACAGAAATTCAACTGGATGATTTTATTTTCAAAAAATTGGGTCT
ACACCAGATTGCTACAATCAAAATAGTAAACAGCACTATAACGTCTGTAGGAGCAAACGCCTTCCATGGTCTC
CACGAACTCTACGCTGTTAACTTGTCTAACAACAAACTAAAGAGCTTACATCCTGAAA
CATTTGCAAATAACAAGAAACTGCTCCTCTTAACACTCTCAAATAATCCGCTCAAATTCCCTGCTCCGGGATC
AGATGAGTATTTCCTTAATGCTTCGTCAGTACAAGAGTTCGATGTTTCTTATTGTAACATGCAATACATTACG
GCTAATACTGTTAAAAATATGCCAGGCGTAATGTACCTAAACTTGGCTGGTAACTATT
TATCAGATATGGAAGCTGACACATTTAGAACCCTTC
>1002049275_1 Heli.1-DT667532.3.5
TCAAACTGTCTATACAAGTTATATATCTCTTAACCAAACTTTTAAATTAATTTTTATCGTAAACTTTCCAATG
GCTTTTACGGGTTCAGCAGACAAGCCCAGCATGCAGCTGGCAGCTGCTGATGCCGGATCTCAACTTCAGCACA
TGTGTTCCTTAGATATTGATTCTAAGGCTTCATATGTGCGTCTATCTGGAATTATCTG
TACCATTGGACCCGCATCCCGCGAGGTGCCAATGCTCGAGAAAATGATGGAAACTGGCATGAATGTGGCACGT
ATGAACTTTTCTCATGGCTCCCATGAATACCACGCGGAGACCATCCGTAACTGTCGTGAAGCTGAAAAGAAAT
ATAGTGCACGTCTTGGAGTGCCATTTTCCTTGGCCATCGCTTTGGACACTAAGGGTCC

TGAGATTAGAACTGGGCTTTTGCAAGGTGGTGGTTCAGCCGAAGTGGAACTAAAGAAAGGGGCAACCATCAAG
TTAACCACCGACCCCGCGTACCAAGAAAGCGGCAACGCCGAGATAGTATTCGTGGACTACAAAAACATAACGA
ACGTCGTGAAGCCCGGAAACCGTATTTTTATTGATGACGGACTCATTTCCGTCATCTG
CGAATCTGCGACGGCTGACGCCTTGATCTGTAACATTGAAAATGGAGGTATGTTGGGATCGAGGAAGGGCGTG
AACCTCCCCGGTCTGCCCGTAGATCTGCCCGCTGTCTCCGAAAAAGACAAGTCGGATTTACTGTTTGGTGTAG
AACAAGGAGTGGACATGATCTTCGCATCATTCATCCGTAACGGCGCGGCGCTGGTGGA
GATTCGCGGCATCCTCGGCGAGAAGGGCAAGAACATCAAGATCATATC
>1002049276_1 Heli.1-CX700804.1.5
GGTCGGAATCCGGGTCGACCACGCGTCCGAGCAACATACCAACATGGTAGCCAAATTCGTAGTTTTCTTCGGA
ATGGTGGCAGCGGCCTCCGCCGTGGCTGTCCCTCTCCTCCCAGTCGCTAAAGTAGCATACGCTGAGGCCGAAG
CCCCAGCACACTACGAGTTCCAATACTCCGTTCACGACGAGCAAAGCGGTGATATTAA
ACAACAACAAGAGTCCCGCGCTGGAGACGCCGTCCACGGCTCCTACTCCCTCGTGCAGCCTGATGGAGTCCAC
CGTATCGTGGACTACAGCTCTGACAAAGTAAACGGATTCAACGCTGTCGTCCGCTACGAGGGCTCCCCTAAGA
TAGCCCCTGCCCCCGTCAAGCTTGCCTACGCCGCACCTGTCGCCAAGCTCGCGTATGC
TGCTCCCGTTGCCAAGCTCGCCTACGCCGCACCAGTTGCCAAGGTTGCCTACTCTGCCCCCATCTCCTATGCC
GCTCCCGTCGCCAAGTTGGCCTACGCCGCTCCCGTCGCCAAGTTGGCCTACGCCGCACCCGTGGCTAAACTAG
CCTACTCTCACGCACCAGTAGCCTACCAAGAGAACCTCGGACACGTCTCCTTCTCCTC
ACCTGCCGTCTCTTACAGCCACTAAATGTTAAACTTATTGTTAAAACTCTATTTATTCTGTTAAATACAAAAC
TTTGCAACTCA
>1002049277_1 Heli.1-EL598133.1.5
ACATTTTGACATTTCTTCCCCGGTTAATATTTTATTACTAATCTTGTAAGAATCTAACTGCAATAATTTTTCG
TCAACATGGATGAAGAGTACGATGCGATCGTTTTAGGCACCGGTCTAAAAGAATGTATTCTAAGTGGAATGCT
GTCTGTGTCTGGAAAAAAGGTTCTGCACATTGATCGTAACAAGTATTACGGTGGTGAA
TCAGCATCAATTACCCCGTTAGAAGAATTGTTCGCAAAGTTCAATGCACCAGCGCCTGATGAAACTTACGGCC
GTGGCCGCGACTGGAATGTGGATTTGATTCCTAAATTTTTAATGGCGAATGGACTTCTTGTTAAGCTGTTAAT
CCATACGGGCGTCACCCGGTACCTTGAGTTTAAATCAGTTGAGGGTAGCTATGTGTAT
AAGGGTGGTAAGATTTCAAAAGTCCCAGTGGATCAAAAGGAAGCTTTAGCATCTGACCTGATGGGTATGTTTG
AAAAGAGGCGTTTCCGTAATTTCTTGATATACGTTCAAGATTTTCAAGAAGAAGATGCTAAGACTTGGAAAGA
TTTTGATCCTAGTCAGGCAAATATGCAATCACTTTATGATAAATTTGGCTTAGACAAG
AACACCCAAGACTTCACAGGTCATGCCTTGGCTCTTTACTTGGATGACAACTATCTCCAACAACCAGCTATTC
AGACCATTCGCCGTATTAAGCTGTATTCTGATTCTCTAGCTCGATATGGTAAGTCTCCCTATCTCTATCCTAT
GTATGGTCTAGGAGAATTGCCTCAAGGATTTGCTAGGCTCTCTGCTATATATGGAGGA
ACTTATATGTTAGACAAGCCAATT
>1002049278_1 Heli.1-DT662615.3.5
ACCACGCGTCCGCGAGGGAAAGCCCCAACATGAAGCAAATAGTTGCTAACCAAAAAGTGAAAATCCCAGAGGG
ATTAACTGTTCATGTGAAATCACGACTAGTGACAGTGAAAGGTCCTCGCGGAGTGTTGAAAAGGAACTTCAAA
CACTTGGCTGTAGACATTCGCATGGTGAACCCTCGGTTATTGAAAGTGGAGAAATGGT
TTGGTTCAAAGAAAGAGCTTGCTGCAGTAAGAACTGTATGCTCACACGTTGAAAATATGATCAAAGGTGTAAC
TAAGGGATTCCAGTACAAAATGCGCGCTGTATATGCCCATTTCCCTATCAATTGTGTCACTACTGAGGGAAAC
ACACTTATTGAAATCCGTAACTTCCTTGGAGAGAAATACATCAGGAGAGTAAAGATGG
CACCAGGTGTGACTGTCACTAACTCTGTTAAGCAAAAGGATGAGCTCATTATTGAAGGTAACTCCCTTGAAGA
TGTTTCCAGCTCAGCAGCTCTCATCCAACAATCTACAACTGTCAAAAACAAGGATATCAGAAAGTTCTTGGAT
GGTCTTTACGTATCTGAGAAAACTACTGTTGTACTGGATGAGATATAAGGACATTAAA
TTTATAAGTTGA
>1002049279_1 Heli.1-DT665656.3.5
TCAGTAGCTCACATCCTGCCACATTTCAGTTTAAATTTAATTTGTAACTGTTTCAACGGTGTGCCTGCGAAAG
TTAAAAAGTAAGCGCTGCTCCGAAACACGATATATTTCGCGAAGCATTTGTATTCGGCGGACGCATTGCATCC
AGCATCCCTGCATTTTGTTTTTCTTTGTTTGGTCCAGTTATTTTCTACATAGCAATGG
AAGTTGAAACTAAATCTACTGAAATTCGTTGCCAAGAGATGTCGAAAGGTGGGCTAGCGTACGAAGTTATCCT
AGCTGAGCCCGTGGGGGTGCCCGTGCCGCGTCGCGCTGATTCCCCCGAGAAAACTCCCTCAGTGGAAGAAATC
CAGGAGAAGCTCAAGGCTGCCGAGGAAAGGAGACGTAGCTTGGAAGCCAGCAAGATGG
CGGCGATCGCACAAAAGATGCATAAAATCGAGGAAGCCTCACGTATTCGCAGCGAACAGACGAATAACTTTAT
TGTGGCCACAAAAGAAGCTCTGGATGCCAAGATGGACTCTCATGAAGAGAAACGCGAGGCGTATATAAATGAA
CTTCGTGCTCGTCTTAAGGATCACCTTGAAGGTGTCGAGAAGACCAGGTTGACCTTGG

AGCAGCAGACTGCCGAAGTATACAAGGCGATCGAAGAAAAGATGAACACCGCCGCCGACAAGCGCGACGAAAA
TATTAAAAAAATGCTTGAACGTCTTCGTGAACATGAGGAGCAAGTGCGCAAGGTGCGCGCCGGCAACCAGGAG
CGCTTCCAGCAGCTGGAGAGCGCCATCCAGGACAAGCTGCAGCACGCCGCCCACCGCC
GCCTGCAGCTCGAGGCCGAGCAGCGCGAGAAGCTGCGCAACCACAATAATAAGCTCGCGGAGGTCCGATCAGT
CATAACAGCCAAGATGGAGGAGATCACCAAGGACATTGAGACCAAACTGACCACCGCCGAACAAAACCGGGAA
AAGGAGATACAGAAGAAACTCGACTTTGTTAAAAAAGAGGAGCGGCGCGCGGAACTAG
TGCGACAGAACAAGAGCGCGCGCGCCGAGAGCGACCCCGTGTCCGGGTAGAGCGGCGAGCTCACTCATACAGC
TCACTCCTTCACTCATACGCGTCTTACCTTTGACTGTTAACATGTATTCTTTTAACTGTTTAGTTCATTCATT
TGTACGCATCTTGCCTTTTAACTGTTAACATGTACTCATTCATTCGTTCGATTCTTAA
TTTGTAGTTGATGTACTTATAGTTTTATTTTAAGGGGGGGTTTTCGCTTTTTAACTTTGTTGGTAGTTATAGT
GTTGTTTAAGGAGAAGTTTGTTTTTTTGTTTAGTGTTCCGTTTAATCGATATGAGGGGTAGATCTATTTTGAT
TTTACATTTTCACAACTATTTCTCGATATTAAATAGGTAATTCGATTTATGTGTATCT
CGCTATTGTTAC
>1002049280_1 Heli.1-EE743520.1.5
GATGTTGATGCCAAAACAAAACCGTGTGTCTATCTATGAATACCTTTTCAAAGAAGGTGTTATGGTAGCCAAA
AAAGACTACCATGCACCCAAACATCCGGATTTGGAGAAGATTCCCAACCTTCAAGTTATTAAGGCAATGCAAT
CTTTAAAATCAAGAGGTTATGTCAAGGAACAATTTGCATGGAGGCATTTCTATTGGTA
TCTTACAAATGAAGGTATTGAGTACCTAAGGATCTTCCTGCACCTCCCACCAGAAATTGTGCCTGCAACACTT
AAGCGCTCTGTTCGCGCTGAAACTGTCCGACGTGGTGCTGTTGGCAGGCCCGATGCCCCAGCTCGCTCTGCTG
AAGACAGATCTATGTACCGTCGTGCCCCCACTACACCTGGAGCACCACACGACAAAAA
GGCAGATGTCGGTCCTGGCTCTTCAGAAGTTGTATTTAGAGGAGGTTTTGGACGTGGTAGACCAACTCCTTAA
CTTAAATTAAGCTTAATAAAAAA
>1002049281_1 Heli.1-CO729949.1.5
GGGTCGACCACGCGTCCGGGAGAGTGATATTTCATATCAACGCTCAACAAAATGGTTGCAGCCAAGAAGCAGA
AAAAGACCATCGAGTCAATCAACTCTCGATTGGCTCTAGTAATGAAATCTGGCAAATACTGCTTAGGATACAA
GCAAACATTAAAAACTTTACGTCAAGGAAAAGCAAAACTGGTCATCATTGCAAAGAAT
GCACCCCCATTGAGGAAATCAGAGATTGAGTACTATGCTCTACTCGCCAAAACTGGTGTCCATCATTATAGCG
GTAACAACATTGAGTTAGGTACTGCTTGCGGAAAGTACTACAGGGTGTGCACATTAGCTATCACAGACCCAGG
AGATTCTGACATCATTACGACCTTGCCTGAGGCAACAGTTTAGTTAATAAATTACTTA
>1002049282_1 Heli.1-ES586627.1.5
GTCTTTCTTGTCGACGGGTTAAGAGTAAGAACCCAACATGGGTAAAATAATGAAACCGGGGAAGGTGGTGCTG
GTCCTCAGCGGGCGGTACGCAGGGCGTAAGGCCATAGTAGTCAAGAACTACGACGAAGGCACATCAGACAAGC
CGTACGGCCACGCTTTCGTCGCCGGCATCGACAGGTACCCACGCAAAGTACACAAGCG
AATGGGCAAAAACAAAATCCACAAGCGCTCCAAAGTGAAGCCTTTTGTTAAGGTTGTAAACTACAATCACCTT
ATGCCAACACGCTACTCAGTTGACTTCAGCTTTGAGAAATTCAGTGCAAAGGACCTTAAAGATCCCGCGAAAC
GCAAGAAGCTCCGTTTCAACACCAGAGTGCGTTTTGAAGAGAGATACAAGAGTGGAAA
GAACAAGTGGTTCTTCCAGAAACTTAGGTTCTAAATACTTTTAAAAATAAACACCTAAAA
>1002049283_1 Heli.1-DT662105.3.5
ACGAGGCGGAGAAGGTAGTGGTGACGTTCAAAATGAAATCCTTATTGTTAGCGTTTGCTAGCTTTTTAGTATT
ATGTTCTGTTAATTGTGATGTATATTTAGATGAGAAATTCCCTGATGATTCATGGGAATCCAAATGGGTGTAC
AGTGAACATCCTGGCAAAGAATTCGGCAAATTTAAGCTGACCGCTGGAAAGTTCTATA
ACGACCCAGAGGAAGATAAAGGTTTACAAACATCGGAAGATGCTAGATTCTACGCATTGTCCCGCAAATTCGA
ACCTTTCTCAAATGAAGGCAAGAACCTCGTGGTTCAGTTCTCAGTCAAGCACGAACAAGATATTGACTGTGGA
GGCGGTTACTTGAAAGTATTCAATTGTGACTTAAACCAGCCTGATATGCACGGAGAGT
CCCCATACGAAATTATGTTTGGACCTGATATTTGCGGTCCCGGCACCAAAAAGGTGCACGTCATCTTCAGCTA
CAAGGGCAAAAATCATTTGATCAAGAAAGAAATTCGTTGCAAGGATGACGTTTACACTCACGTCTACACGCTG
GTCGTCAAACCTGACAACAAATACGAAGTTCTCATTGATAACGAAGTTGTTGAGTCTG
GAGAGCTTGAAGCCGACTGGGACTTCCTTGCACCTAAGAAAATCAAGGACCCCGAAGCCAAGAAACCAGAAGA
CTGGGATGATCGTGCCCAGATTCCTGACCCTGATGACAGTAAACCTGAGGACTGGGACAAGCCTGAGCACATC
CCTGACCCTGATGCTGCCAAGCCTGATGACTGGGATGATGAGATGGACGGTGAATGGG
AACCTCCCATGATTGATAACCCTGAATACAAAGGTGTTTGGGCTCCTAAGCAGATTGACAACCCTGCATACAA
AGGTCCATGGGTCCACCCTGAGATCGACAACCCTGAATATGTTGAAGACAAAAATCTCTACAAACGCGATGAA
CTTTGCGCAGTCGGTCTCGACCTTTGGCAAGTCAAATCTGGCACTATCTTTGATAACA

TCCTTATCACTGATGACCTTGAACTGGCGAAGGAACGAGTGGAGGCAGTCAAGAAGACCATTGAGGGTGAAAA
GAAAATGAAGGGCGAACAAGATGAAGCTGAAAGAGAGAAGGATAAGGCTGAGAAGCCTGAAGAAGAGGATGAT
GAGGACTTGGATGATGAGCTCAAGGAGGGACCACCTGTAGATGACCACGACGAGCTGT
AAGTGTAGTGCC
>1002049284_1 Heli.1-ES587547.1.5
GTCAAGCTAAGGCATCAAGATGACGAACTCCAAGGGTTATCGTCGTGGTACAAGGGACTTGTTCGCCCGCAAG
TTCCGCACACATGGAACTATTCCTCTTTCCACGTATATGAAAGTGTACAAAGTCGGCGACATCGTTGACATTA
GGGGTAATGGAGCAGTCCAAAAGGGTATGCCACACAAAGTCTACCACGGCAAGACTGG
CCGTGTTTACAATGTAACTGCCCATGCTCTCGGAGTCATTGTCAACAAGAGGGTCCGTGGCAGAATTATCCCT
AAGAGAATCAACATTCGCATTGAACATGTCAAGCACTCAAAATGCCGTGAAGATTTCCTTAAAAGAGTAAAGG
AAAATGAAAGGCTTCTTAAGGAAGCTAAGGCGTCTGGTAAAATTGTTAACTTGAAGAG
ACAGCCACAGCCGCCTCGTGCAGCTCACATTGTGAAGGGAGCTGATAAGCCAGTCTTACTTGCACCCATCCCA
TACGAGTTTGTAGCTTAATGTGAAATAAACTATTT
>1002049285_1 Heli.1-DT668132.3.5
GTTTGCAAAAATTTTTGGTTTAAGCGCTGTGTTGGCGGTTGCTGCGGCTGGTCTGCTGCCAGAACCCCATTAC
TCCTCAGCAGCGGCGGTTTCTTCTCAAAGCATTGTACGTCATGACCAGCCTCATGTCGTAGCGGCTCCAATTG
CATACCACGCTGCCCCCGTGGCATACCACGCAGCTCCTGTAGCTTACTCTTCAGCTGC
AGCTGTTTCGTCTCAATCCATCCAACGTCATGACGTAAACCATGCTGCCGTAGCTGTTGCTCCTGTTGCCCAC
TACTCCGTTGCTCCTGTAGCCCATTATGCCGCTCCCGCACGTTATGCATCTGCAGTGTCATCGCAATCTATCC
AGCGTCATGACCAACCTCGTGTTTCTATTTCCGTTGCTCCCGTTGCCCACTACGCTGC
TGCACCCATAGCGCATTACGCCGCCCCTGTGCATTACTCTTCAGCTGCAGCTGTTTCGTCTCAATCCATCCAA
CGTCATGACGTAAACCATGCTGCCGTAGCTGTTGCTCCCGTTGCCCAGTACTCCGTTGCTCCTGTAGCCCATT
ATGCTGCTCCCGCACGTTATGCATCTGCAGTGTCGTCTCAATCTATACAGCGTCATGA
CCAACCTCGTGTTTCTATTTCCGTTGCTCCCGTTGCCCACTACGCTGCCGCGCCCGTAGCCCATTACACCGCC
CCTGTGCATTATTCATCAGCCGCAGCCGTATCTTCCCAATCCATCCAACGTCATGACCAGTCCGGTGCCGCCA
TTGCTGTTGCTCCTGTAGCTCACTACGCTTCTGCTCCCGTTGCACACTACGCCGCCCC
TGTAGCCCATTA
>1002049286_1 Heli.1-ES585173.1.5
TCTCAGAGCTAAGGCCAGTTGGCGACTTGCACTAAATATATTAAAAATGGCATCAAAGGCTGAATTAGCTTGT
GTTTACTCTGCTCTCATCCTCGTTGATGATGATGTCGCCGTTACTGGTGAAAAAATTTCGACCATCTTGAAGG
CGGCCGCAGTAGACGTTGAGCCTTACTGGCCTGGTTTGTTCGCCAAGGCTTTGGAGGG
TGTCAATGTACGTGATTTGATCACCAACATCGGATCCGGCGTCGGTGCTGCCCCCGCTGGAGGCGCCCCTGCC
GCCGCTGCAGCAGCCGCCGCACCCGCCGCTGAAGCCGCCAAGGAAGAAAAGAAGGAGGAAGAGCCTGAAGAAT
CTGATGATGACATGGGCTTCGGTCTTTTCGACTAAAAAGCTTTTTATAAAGGCTTTGG
TATTCCATTGTAAGCTTTATGAAGCTTGTTTAGTTGTAAGGAAACTAAAAATATAAAATT
>1002049287_1 Heli.1-DT668465.2.5
GTTCCTGGGGCTAGGAGCCGTAGTTTCGGTTTTTATTATTTTAATCGCCCGTAATCCTACAAAATGAGAATTT
ATAAGGATATTATTACCGGTGACGAGATGTTCTCGGACACATACAAAATGAAACTGGTTGACGAAGTGATCTA
CGAAGTCACCGGTAAATTGGAAACCAGGGTCCAGGGCGAGGTTAAAATCGATGGATTC
AATCCATCAGCTGAGGAGGCGGACGAGGGCACCGACACCAACGCCGAGAGTGGTGTTGATATAGTCCTCAACC
ACAGGCTCGTTGAGAGCTACGCCTTCGGTGACAAGAAATCCTTCACATTATACCTTAAAGACTACATGAAAAA
ATTAATAGCAAAATTGGAAGAGAAGGCACCTGATCAAGTAGATGTATTCAAAACTAAC
ATGAACAAAGCAATGAAGGATATACTCGGAAGGTTTAAGGAGCTGCAGTTCTTCACTGGCGAGTCCATGGACT
GTGACGGCATGGTCGCCATGTTGGAATACAGGGATATTGATGGAGTATCAACTCCCATCATGATGTTCTTCAA
ACATGGTCTTGAGGAAGAGAAGTTCTGAACAGCATATGTGATACTAACTCATAATATT
CCAGCCAGCCTAATAAATTCCTTTTTTTCTATTTATTTACTTTGTTTTTACCAATCATGTCTGTCCGCTCAGC
AGTACACAGCTTCGGCAACTCCCCGTAAAAGGCTGAACTTTTGTAAAGAATTGATGTTGCATATTATTTAAAA
TAAATAATTT
>1002049288_1 Heli.1-DT662661.3.5
GCACGAGGCTAAGTTCTTCGTCGTCCTCTCCCTGGCCGTGGCCGCATCAGCTATCCCCTTGGTCCCCGTGGCT
AAGGTAGCATACGCTGAGCCCGAAGCTCCCGCTCACTACGAGTTCCAATACTCCGTTCACGATGAACACACTG
GTGACATCAAGCAGCAGCAAGAGGCCCGTGCTGGAGATGATGTCCACGGCTCATACTC

TCTCGTACAACCTGATGGCGTACACCGTATCGTGGAATACTCTTCTGACAAAGTGAACGGTTTTAACGCTATT
GTACGTTACGAGGGTAAACCCATCCCAGCTCCCGTACCAGTCGCTAAGGTTGCATACGCTCCCGTCGCCAAGG
TCGCCTACGCCGCGCCCGTCGCCAAGGTTGCCTACTCTTCCCCCGTCGCCTACGCTTC
GCCAGTTGCCAAGATTGCCTACTCTCCCGTATCTTATGCCGCCCCTGTCGCCAAAGTAGCCTACGCTGCTCCC
GTTGCCAAGCTCGCCTATGCTTCTCCCGTTGCCAAGGTAGCATATGCCTCTCCTCTCGCCCAAGTAAGCTACT
CATCTCCCATCGTCTCCTACCACCACTAAACGTAATTGGTATTTTTAAAGTTGATTAA
TTTATTTATATGATAAATAGTTACAAATAAATTGTTTGTTTAAGATTT
>1002049289_1 Heli.1-ES587428.1.5
AAAATATTCTCTTTGAGAGTACAAAAATGGGTCGTTATTCTCGAGAGCCTGATAATCCAGCGAAGTCATGCAA
GGCACGCGGTTCAAATCTTCGCGTTCACTTCAAGAACACATACGAAACAGCTATGGCTATTAAAAAATTGCCT
CTGCGTCGCGCCGTACGTTACCTTAAGAACGTTGTAGAGCAGAGAGAATGCATACCAT
TCCGTCGTTTCAACGGTGGTGTTGGTAGATGTGCTCAAGCCAAGCAGTTTGGTACCACACAAGGTCGCTGGCC
GAAGAAATCCGCGGAATTCCTTTTACAATTGTTAAGGAATGCCGAATCTAACGCTGACTACAAAGGCTTGGAT
GTTGACAGGCTTGTCATTGATCACATTCAGGTAAACCGTGCCCCCTGTCTCCGCAGAC
GTACATACCGTGCTCACGGTCGCATCAACCCCTACATGTCATCCCCCTGTCACATCGAGGTGTGTCTCAGCGA
GAGGGAAGACGCGGTAGCACGCGCTGCCCCCGCTGACGACGCGCCCGCAAAGAAGAAACTCTCCAAGAAGAAG
CTGGCTCGCCAGAAGGAGAAGATGATGAGGGAATAGACTAATAAATGATTTTAAATAT
>1002049290_1 Heli.1-DT662693.3.5
GGAAGTCTCAGAAGAAAGATCAACATGTTTGCTAAAGTTTTAATTCTTACCATCGCCGCTACTGCGTGCTACA
CCCAGGAACATATTTACAAATACAAGCCTGTCCATCGGGAACAACTAAAGGAGTATAGATATCCATCGGTACA
TAAAGCTTTCAGCCTCATAAACCAGGAACCAGCACCTATCCATGAAGAAGAAGCACAT
GGCGCTCCGGAGGCACAATTGCACGGACACCATGAATCTGCTGTTTCATCTCAAAGCATAGTTCACTATCAAC
CATCAGTTCACGAGGAATCGCAGTCGCAAGAATCTTTTGTAGGGCACAATGAACCATCAGTAGCTTACAAGCA
ATACTATCAAGGCCATGAACAAGTAAAACTTCAACAAGGACACCTTGCCAAGAACAAC
ATTCAAGCTTCGCATTATCCTGTGGTTTCTCACGAAGCACCTCAAGCCCAATCCCATGATGAAGCACAATACA
TCCGCGTCCCCGCCCACAACTACCACTACGCCCAAGAATCTGAACATCACGTACCTGCACATCATGAGGTTGA
GCAACACCGTCAAGTAGAATCCCATTCCCACGATGAGCCTATCGACTATTATGCTTAT
CCCAAATACCAGTATGAATACAAAGTTGAAGATCCTCATACCGGAGACAATAAGTTCCAGCACGAGGTCCGCG
ATGGTGACAGCGTAAAAGGAGTTTACTCTCTTCATGAAGCTGATGGCTCCATAAGAACCGTTGAATACAGCTC
AGACAAACATCACGGATTCAACGCTGTCGTAAAGCATTCAGCCCCTGGTCAACACGTC
CATATTGAGAGTCATCATGAAAAT
>1002049291_1 Heli.1-DT662461.3.5
CTACGAATTTTCTATTTTCTGACAAATAATTCTCTCCAAAAATTATCTTATTGTGAGTGTCTTTTTCGTCAAT
TTTTAAAGCCAAAATGGCTAACATTAATGAAGAGAACGTTAGCAAACTAATCCCCACACTTGGTGATGAAAAA
ATCGATATGATCGCCGCCACAAGTGTTTTACAAATCAGAGCAAGTGAGATTCGTCAAA
GCCAGATTAACTGGCAATCTTATTTGCAATCGCAAATGATAACTCAACGTGATCACGACTTCATTGTGAACTT
AGACCAACGAGGACAGAAAGATTTACCTGATAAAAACCCTGACGGCTGTGCTGATGTATTCCTCAACTTGATT
ACTCATATTAGCAAAGATAACACCATCCAATATGTTCTTGTGTTGATTGACGACATTC
TTTCGGAAGACAAATCCCGTGTGAAAATATTCCGTAATGCCAAACATGGTAATGTTTGGCAGCCATTCCTGAA
CTTACTTAACCGTCAGGATGAGTTTGTTCAGCATATGACTGCTCGTATCATTGCTAAGCTGGCATGCTGGCAC
CCACAACTTATGGAAAAAAGTGATTTGCACTTCTACCTTTCCTGGTTAAAGGACCAGC
TCAAGATGAATACCAACGACTACATCCAGTCTGTAGCGCGGTGCCTGCAAATGATGCTGCGTGTGGACGAATA
CCGCTTCGCCTTCCTCTCGGTCGATGGCATTTCTACATTGCTGTCTATTCTCGCTTCGAGGGTTAACTTCCAG
GTCCAATACCAACTAGTGTTCTGCCTGTGGGTGCTGACATTCAATTCGCTGCTTGCCG
AGAAGATGAACAAGTTTAACGCCATTCCCATTTTAGCTGATATTCTGAGTGACTCTGTCAAAGAGAAGGTCAC
TCGCATCGTCCTGGCCGTGTTCCGCAACTTGATCG
>1002049292_1 Heli.1-ES586094.1.5
TACGGATTGTTGAGGCGGGTTTTAAATCCTAGCCATGGTGCGTATGAACGTATTGAGTGATGCTTTAAAATCA
ATTCACAATGCAGAAAAGAGAGGCAAACGACAAGTGCTTATCCGGCCTTGTTCCAAAGTTATTGTCAAGTTTC
TAACAGTGATGATGAAGCACGGTTATATTGGTGAATTTGAAATAGTTGATGATCACCG
AGCAGGCAAAATCGTTGTGAATCTAACTGGCAGATTGAACAAATGCGGAGTAATATCACCAAGATTCGACGTC
CCTATTAATGATATTGAGAGGTGGACAAATCTACTACCATCCAGGCAGTTTGGATATCTTGTTCTTACAACCA
GTGGGGGTATTATGGACCATGAAGAAGCCAGAAGAAAACATCTGGGAGGAAAAATATT
AGGTTTCTTTTTCTAAGCTAATCCAATACATTTGAA

>1002049293_1 Heli.1-ES585961.1.5
CCGTGTCACGATGAGTTCCTTAAAACTGCAGAAGAGGCTAGCAGCCTCTGTTATGCGATGTGGTAAAAAGAAG
GTGTGGTTAGATCCAAATGAAATCAATGAAATCGCCAACACTAATTCCCGACAGAACATCCGTAAGATGATAA
AGGATGGTCTCGTCATCAAGAAACCAGTTGCGGTGCACTCGCGCGCCCGCGTTCGCAA
GAATACCGAGGCCCGTAGAAAGGGAAGACATTGCGGTTTTGGTAAAAGAAGAGGTACTGCAAATGCCCGTATG
CCACAAAAGGAACTGTGGGTACAAAGACAAAGGGTGCTCCGTAAATTACTTCTCAAGTACAGAACAGCCAAGA
AGATTGACAGACATCTATATCATGCCCTGTACATGAAGGCGAAGGGTAATGTGTTCAA
GAACAAACGTGTCCTCATGGAGTACATCCACAGGAAGAAGGCGGAAAAGGCTAGGACTAAGATGTTGAGCGAT
CAAGCCGAGGCCCGCCGCAACAGAGTGAAAGAAGCCCGCAAACGCCGTGAAGAGCGTATCGCAGCCAAGAAGG
AGGAACTGCTGCAGACGTTCGCGCGCGAGGACGAGGCCGCCGTCACAGCCAAGAAGTA
ATTTATACATAAATATAAATAAAAATATATAAAACT
>1002049294_1 Heli.1-DT665624.2.5
AAGTTTTAACCCTGTGGTGAAGTGGGGTTATCGCAATCATGGGGAAGGGAAATAATATGATTCCTAATGGCCA
TTTCCATAAGGATTGGCAAAGATTTGTAAAAACTTGGTTCAATCAGCCTGCTCGGAAACACCGCAGGAGACAA
AACAGAGTTAAGAAAGCAAAGGCTATTGCACCTCGTCCTGTCGCCGGACCCCTTCGTC
CAGTTGTTCGCTGTCCAACTGTGCGATACCATACAAAAGTTCGCGCTGGGCGCGGATTCACTCTTCGTGAAAT
TAGGGCTGCCGGCCTGAACCCTGCTTTTGCTAGGACTGTTGGAATTTCTGTAGACCCAAGAAGACGCAATAAA
TCTGTGGAATCACTACAGATTAATGTACAAAGATTAAAGGAATATCGTGCCCGTCTCA
TTCTTTTCCCTAAGGGCAAGAAGGTACTGAAGGGCGAAGCATCCGAGGAGGAACGTAAAGTAGCAACACAACT
TCGTGGACCTCTTATGCCTGTAGAGCAGCCAGCACCTAAATCTTCTGCCCGTGTTATCACTGAAGAGGAGAAG
GACTTCAAAGCATACCAATACCTCAGAGGAGCTCGTTCTCTTGCAAGGCTTGTTGGAA
TCCGGGCTAAGAGGTTGAAGGATGCAGCTGAAAACCCAGATGATGTGACTAAAGCTCCATCTGCAGTTAAGGA
AGCTAAAAAGAAGTGATTTCTCTTAAATAAACAAATAAAATCTTAAAAAAAAAAAAAAA
>1002049295_1 Heli.1-DT663883.3.5
TGTCGATAAATATCTTAGTTTTATCGTGATTTCGCTTTGAATAGTCTTAATTTTAATTTTTATCACTCCACAC
CGTAACTAAACACCATAAACAATGGGCGATATTGAGGACACACATTTCGAGACCGGCGACTCCGGAGCCTCTG
CCACCTTCCCTATGCAATGTTCTGCTCTCCGCAAGAACGGTTTTGTCATGCTTAAGGG
ACGTCCATGCAAAATTGTTGAAATGTCCACCTCAAAGACTGGCAAGCACGGACATGCTAAGGTCCACTTGGTT
GGCATTGATATATTTAATGGAAAGAAATATGAGGATATCTGTCCCTCAACCCACAATATGGACGTCCCCCACG
TCAAACGTGAGGACTACCAGCTGACCGACATATCGGATGACGGCTACCTCACCCTGAT
GGCTGACAACGGTGACCTGCGCGAGGACCTCAAGATACCCGACGGCGATCTCGGAACTCAGCTCCGCTCTGAT
TTCGACAGTGGCAAGGAATTGTTGTGTACCGTACTGAAGTCGTGCGGGGAGGAGTGTGTTATAGCGGTGAAGG
CAAACACAGCTCTCGACAAATAAACCAACTAAGAATTTATAGGGATATAAAAGCATAT
AATTTTTTTACAATCAACAACTTACATAAATGTAAAACATAATATTATGTATAATTTAAAGTAAACACGTGCC
GGGTTGTTTGTTTTGTCGCTTAAGTCTTAAAAGTTACTTTGCTAGCCTGAAGTTGTGCGATACCGGTGTGGAA
ACCCCGTAAC
>1002049296_1 Heli.1-DT666661.3.5
TTTATTGATTGTTTAGTGTCCAATTGATGTTAATTAATTAAGAAATATAATTTCAGTGTTAAAATATTGAAAT
AGTTATCCGATTTCTTCAAAAGTGAGTTTTGCCTCGCAGCACAATGGTGGCTTCAACGGACAAAAAGGCTGCG
GCCGACAAGAAGGCTCCGGCTGAGAAGAAGGTAGCGAAAGGCAAGGAGGACAGCAAGA
AGCTGCCAGCGGTCCCCGAGTCAGTACTAAAGCACCGTAAACGCAGAGATGCTCTAAGAGCTCGTCGCATACA
GGTAACAATCAAAAGGCGTTCAGCTGCCATCAAGAAGAGGAAAGAAATCTTCAAGAGGGCTGAACAATATGTT
AAGGAATACCGCATCAAGGAGCGTGATGAGATCAGACTGGCTAGACAAGCACGCAACC
GAGGCAACTACTATGTTCCTGGTGAAGCCAAGCTTGCCTTTGTCATCCGTATCCGTGGTATCAACCAAGTCTC
ACCAAAGGTCCGCAAAGTACTAAAACTGTTCAGACTGCGTCAAATCAACAATGGTGTGTTCATCAAGTTGAAC
AAGGCCACAGTGAACATGCTCCGTATCGCGGAACCCTACATCACATGGGGATACCCGA
ATTTGAAGAGTGTGCGAGAGCTCATCTACAAACGCGGTTTCGCCAAAGTGAAGGGACAACGCGTCGGCATCAC
CTCCAACAAGATCATTGAGGAGAAGCTCGGCAAGAGCGATATCATCTGTGTGGAAGACCTTATCCACGAGATA
TTCACTGTGGGAGACAAGTTCAAGTATGCAAGCAATTTCCTATGGCCATTCAAGCTAA
ACAACCCAACCGGAGGCTGGCGTAAGAAGACCATCCATTATGTGGACGGTGGTGACTTCGGTAACCGTGAGGA
CAAGATCAATGAACTCCTCAGGAGAATGGTCTAAATAAATAATTATA
>1002049297_1 Heli.1-DT665670.3.5

TGGACGGCGTAACCGTAATTATTAAACATGTTGGGGAGATTTTTTCTACTGTGCGGGCTACTGGCCGTCGCAT
CAGCTTCGGTGAAGCTGCAAGAGGTCTACTCTTGGAACATTTTGGACTGGAACTATCCTGACCAGTATCTGAA
ACAGCAAGCCCTTCAAACTGGTGCACTCATCAGACAAAACGCTCTTCCCGTGGGTATC
GAACGCTGGAGGGATAAACTCTTCGTCAGTGTACCTCGATGGCAATCAGGTATCCCAGCAACGCTTAACTACA
TTCCTTTGGATGCACCTTACGAGCCTTCACCAAAATTGACACCCTACCCAAGCTTCAAAGGCAACGAACTAGG
CGATTGTGAAAACGGTCTTACCACAGTTTACAGAATTAAAGCAGATCAATGTGATCGA
CTCTGGGTTCTGGATGTTGGAACTTATGGATATGACCCTAATGTAACAAACGTATGCCCGTATTCTCTAAACG
TATACGATTTGAACACTGATCAACGTATTAGACGGTACGTTTTCCGGCCGGAGGACATTGTCTCTACAACATT
CATTGCAAATATTGCTCTTGACGAAGGCATTTCGTGTGAAGATACTTTCGCATATTTC
TCGGACGAACTCGGATATGGACTCATAGCGTATTCTTGGGAACAAAACAAATCATGGAGATTCAGTCACAGCT
ACTTCATGCCTGATCCTTTAGTTGGTGACTTCAACATCGCTGGCCTTAACTTCCAGTGGGGAGCTGAAGGTAT
ATTTGGAATATCAGCATCACCAATGGGAGCAGATGGTTATAGAACACTCTATTTCAGT
CCTCTTTCAAGTAACACGGAATTCGCCGTATCAACTCGTATTTTAAGAGATGAATCTAAGGTCACTGGATCGT
ACCGTGATTTT
>1002049298_1 Heli.1-DT664482.3.5
GCACGAGGGTGCAGTGAACATTGTTTTGCGTTGTGCGTAGCACGTGCCGGATACTTTTTCTATTTTTTAAATA
ATCTCAAACTTACTCAAACAATGGCAGACGCAGCAGTCGAAAAGAAAGACGCCGTAGCCGAGGAGGTCAGCTC
AACCGAGGCGGCGAAGGAATCTCCAGTAAAGAAATCTCCCGCCAAAAAGGTAGCGGAA
CCCGAAAGTAACGGCAAAGAGGAAAATGGCAGCGGCGACGCACCTGAAGAAACTCCCGCCGAAAACGGTGACG
AAGAGAGCAATGACGCCGTCGAAGCCGTCGAAAATGGAGAAGCGACAGAAGATAAAGAATCTGGTGGAGTCAA
GAGGAAATCTGTAGCAACAGACAACGGTGATGACGCAGCCGAAAAGACCACACCTGAA
AAGAAGGCGAAAGTAGCGGACGAGGCTCCCGCGGCCGAAGAAGAGGCGGCCTAACCTAATAGCAAGCATGCTT
GGTTTACTGCAATGCATTCGTTCATTAGGGCTCAACTCCACCAAGACATATCCTCTATACAGACGCCAAAACT
ATAGTTTTTATACATTGTTACGTATTTCTCTTGTACACGTACGAGTTGGGCCCTTTTG
TGACCACTTATTTTAGGTTGTAATGTAACTTACCCGTCGAGTACCTGTAAAGTGTAAATTATACAGAAATAAA
TGCGTCGCAGTGAATCGATCGAGTCGGACACGTTCAGACATCGCTACGCGCAGACTAAGGGTTTTTGAATTGA
TACAATAACATTTATTCCGCCATTAGGTACTGCCAAAGTGATTCACATGTGTGAAGTC
AATTCACGATCCGTTCTGTGAACGATGCGAAAACAG
>1002049299_1 Heli.1-ES587384.1.5
ATTCAGAAAACATGGCCACGGTCACTTACCTTGCCCTAATATTATCTGCTCAGTTGGCGACTACGTTGGCTAG
GGTGCCACTGAGATGTTTTATTGAAGACGACCGTCCTTTTAAAGAATTTGGTAAAGCCTTGTTTGAACAGGCT
CTAGAGGTACCTAAAATAGACCAACCGGAGCTCGGTAAACTGATTTTAAAAGATTTTT
TAGTAGATAAAGATGGCTTGTTCTTCCAAGCAAAATCCTTTCAAGTTTTGGGTCTTGAAAACGCAATTGTGGA
CGATATTGGAATGAATTTAAATTCTAATTTGAGTCGCGTAACATTCCACACGGACATGTTGATAAGAGGACCC
TACAAGGCTGGTGGAGCTTTGTTTTCCGTGCCTATCGAAGGTGAAGGGGACTGTGAAT
TACAGTACAGAAACATTCAAGTTGATATGGTTTTACCTTTCGAAATTATCAAAGATGCAAATGGTAAAAGATT
TATGAATCTGAAAAGCTACAAATATTGGTACGACATAAAAGAAGGCTCAATGAGATTAAATAACCTCTTCGGT
GGAAACAAGCAATTAAGTGATGCTATGCACTACCTTATCAATCAAAATTGGAAGTTGA
TAACATTCTTATATGGAAAAGATGGAACTGATCAAATCGTTAGTCACGTGTTCAATGTGTTCAAGAGCAACTT
GCTGAGAATGCCGCTTAGACAGTCATGTTAATATAGTGAACATTCACTTACAGTTTTAACACTATCACACTAT
TACATTAAGAAAAGAATAATATACTCTCCGATATAAAAAAAGCTTGTCGGTGAGAACA
TTGTAAGTCATTCTTGTCTATAAAATGTAGCACTATGATTTTTTAAAT
>1002049300_1 Heli.1-DT668809.1.5
TTAACTAAAATCTCTCAAAATGAAGGCTAAAGGAGAGTTGAAGGAATATGAAGTCATCGGCCGCAAGCTGCCG
TCGGACAGTGAGCCGAAACCTCCTCTATATAAAATGAGGATCTTCTCTCCAGACCCAATTGTTGCTAAATCAC
GTTTCTGGTACTTCTTAAGGCAACTGAAGAAGTTCAAGAAGACTACTGGCGAAATTGT
CTCAATTAAGGAAATCCCAGAAAAGAGTCCAGTCAAGATCAAGAACTTTGGCATCTGGCTGCGTTATGAGTCC
AGGTCGGGTGTTCACAACATGTACCGCGAGTACCGTGACCTGAGCGTGGGCGGTGCCGTAACACAGTGCTACC
GTGACATGGGAGCCCGTCATAGAGCTAGAGCTCATTCCATTCAGATCATCAAAGTAGA
AGTAATCAAGGCGGCCGCCTGCCGCCGGCCACAAGTGAAGCAGTTCCACAACAGTTCCATCCGCTTCCCACTG
CCCAAGCGCGTCCACCATTACAAGCGACTCAATACATTCGCTTACAAGAGACCCAGCACTTACTTCTTGTAAT
TTAATGATATAAACGTATAAGA
>1002049301_1 Heli.1-DT666031.3.5

GCACTCGGACACTGTAACTGATATTGAAAAACTGTGTGGTGCAAAAAAAATTGTGCAAAAAAAAAAAATGGAAA
TTAGGTGGAGTTTATTCATAGCGCTGCTTCTCCTCGGAGTCGCTGTCGCGTCTGAAGAGGAAGACGAAGTGCC
TGATGCGGGAGTGGAGGGAGACGAAGAACTGGCTTTAGATCAAGAGGAAACACGCAAC
CTCAGACCACGAGCATATAACCAAGCTGGTTACTCGAACACACTACCTAGCGGTTTCCGACCCACACCTTCGT
TAGCTGAACTGGCAGGTTACCAGAGGCCACAGCAAGAACAGCAGGAATATGAGGTACCAGCCAGACAGCAGAT
CTACGTAGAGGAGCAGAGACCACCAGTTAGAGCACCTCATAGGAAGCCTCAAGAGAAC
CGCCCATCTAAAATCCAGCAGCAACTTTTGGAAGAAGAAGAAGAAGAGAAGGAAGAGCCCGACCGTCTTTCCC
AACTATTGCAGCAATCCAAATTCGATTGTGTGAGCAAGAACACTGGGTACTACGCTGACCAGGAGCTGAACTG
CGAGGTGTTCCATTACTGTCAAGACAATGTTAAGCATTCGTGGATCTGCCCTGACGGA
TTCACTTTCCATCAGGTCCATCTAATTTGCATGCCGCCTACACACGACAACATCTGCCAGAAGTCTCAAAAAT
ATCACTTCGTCAATGATTACCTCTACCGCCCCATCAACGAAGAGGAGGTCCAAAGGAAACCTAACGTGTCCCT
CAAGTACTCCGACAGATTCTACCCCGCTGAAGTGTACAGGGATGACCGTTTTGAACAG
GAAGAAGATGAAGAGGAAGAAGAACCACGTAGAGCTCCCCCCGCGCAACAAGCAAGACCAGCCAGACTTCAAT
ACCAGCCTCAAGTATTTCGATCCGCTGAAGAAGTCAACATACCCTTAGTCCAGAGACGC
>1002049302_1 Heli.1-ES586376.1.5
ACGAGGCAGGAGGTGTTTTAAAAATGGGGAAGGTCAAGTGTTCAGAATTACGCACGAAAGACAAAAAAGAACT
CTTCAAGCAACTTGAAGAGTTAAAGACAGAGTTAACTAACCTTCGAGTTGCTAAGGTTACAGGAGGAGTTGCA
TCTAAACTATCTAAAATCCGTGTTGTGAGGAAAGCAATTGCCCGTGTTTACATTGTGT
ATCATCAGAAAATGAAGGTTAACCTAAGAAACCATTACAAAAATAAGAAATACAAGCCTCTTGACTTGAGGCC
AAAGAAGACCCGCGCTATGCGAAAAGCTCTTACTAAACATGAGGCGAAAATCAAGACCCAAAAGGAGATCAGA
AAAAAATCTCTGTTCCCTCCTAGAGTATATGCTGTGAAAGCGTAAATAAATACTTAAA
>1002049303_1 Heli.1-ES587719.1.5
CCGAATATGGGTGTCGACATCAACCATAAACACGACAGGAAAGTTAGGCGTACCGAAGTCAAATCTCAGGATG
TGTACCTGAGATTGCTCGTCAAATTATACAGATATTTGGCCAGACGTACAGATGCCAAGTTCAACCAGATTAT
CCTTCGCCGTCTCTTTATGAGCCGTATTAACCGGGCGCCTATCTCCCTGTCTCGGTTG
GCTCGTCACATGAAGAAGCCCACTCGTGAGGGTTTGATCGCAGTAGTGGTCGGCACTATTACCAACGATGTGA
GGCTGTACAAGGTTCCCAAACTATCTGTTGCCGCTCTTCATGTTACTGAGAAGGCACGCGCCCGTATTTTGGC
TGCTGGAGGTGAAATCCTAACTTTCGATCAGCTTGCCCTTCGCGCTCCCACTGGCCGT
AAGACAGTTCTCGTGCAAGGCCGCAGGAACGCGCGTGAAGCGGTACGCCACTTCGGGCCCGCTCCAGGCGCCC
CACGCTCGCACACGAAGCCCTACGTACGTTCTAAGGGCCATGAGCGTGCTAGACCTAGCCGTAGGTCTAATGT
GTAATCTTTAATATAAGGAATA
>1002049304_1 Heli.1-EL599205.1.5
TGGAGTACGCGAGACGTCTGCCACAGGCTTCCCAAGATGGGCGCTTACAGATATATTCAAGAATTGTATCGTA
AAAAGCTTAGCGATGTTATGCGCTTCCTTTTACGTGTTAGAGTATGGCAGTACCGCCAATTAACTCGCATGCA
CCGTGCACCTAGGCCAACGAGACCCGACAAAGCTAGGAGACTAGGCTACCGCGCCAAG
CAAGGTTATGTTGTCTTCAGAATCCGTGTTCGCCGTGGTGGCCGCAAGCGCCCGGTTGCCAAGGGTGCTACCT
ACGGCAAGCCCAAGAGCCATGGAGTCAACCAGTTGAAACCCACACGCAACTTACAATCTATTGCCGAGGAACG
CGTTGGACGTCGCTGTGGTGGTTTGAGAGTACTCAACTCCTACTGGGTCGCACAAGAT
TCTTCATACAAATACTTTGAAGTCATCCTCGTCGATCCTTCACACAAGGCCATTCGTCGCGATCCTAAGATCA
ACTGGATAGTAAATGCAGTGCACAAGCACAGAGAAATGCGTGGTCTCACTTCAGCTGGCAAGAGCTCACGAGG
TCTTGGCAAGGGTCACAGGTTCTCGCAAACAAAGGGAGGCTCACGCCGCGCCGCCTGG
ATAAGACGCAACACCCTACAACTGCGTCGCAAACGATAATTTCATACATGCTATTGACTTTCTATTTTTGTAT
TTATTCTTAAATAGAAAGTTTGTAGTTTTAAATAAAAAAAAATGTAA
>1002049305_1 Heli.1-EL600879.1.5
TTTTTACAACTACAATTCTTGAGTTGATGGTGTATTGCGGATCTGTGGGTGGTGTTTTAATATTAGTCATCGC
CTGCATTCTTATTTATTCATTTTATTGTGATAACAATTTTCTTAATTTGTTCTCTCTGCTTACTACAGTTCAA
CCTAATATATAACATAAAGATGTCGTCTCGCAAAACAGCTGGTCGTCGTGGTACCAAC
AAGAAACGCGCCCAGCGGGCTACTTCAAATGTATTTGCCATGTTTGACCAGGCGCAAATTGCGGAATTTAAAG
AGGCTTTCAATATGATTGATCAGAATAGAGATGGTTTTGTAGATAAAGATGATCTACATGATATGCTTGCTTC
TCTAGGAAAGAATCCAACTGAGGATTATCTAGAGGGTATGATGAATGAGGCACCAGGA
CCTATCAATTTCACTATGTTTTTGACACTCTTTGGGGAACGTCTTCAGGGAACTGATCCTGAAGATGTTATTA
AGAATGCTTTTGGATGCTTTGACGAAGAAAACACAGGTGTGATAGCCGAAGAGCGTCTTCGGGAACTCCTTAC
CACGATGGGTGACCGTTTTACCGACGACGATGTCGACGAAATGCTTCGCGAGGCTCCC

ATACGCGACGGGATCTTTGATTATGTGGAATTCACTCGTATATTGAAACATGGCGCCAAGGATAAAGATGAAC
AGTAAAACTTTTTCAACGGCAGGTTGATGATATAACATAATAAACTTTATTTTGGGGGATGTTATGGAGCATG
TTACCAAACT
>1002049306_1 Heli.1-DT668657.3.5
GCACGAGGGTCAGAACGCGCCGTGATTTACCGCTGTGAACAAACGGACATTCTCCTCCTGATATATTCAGTGA
TAGTTTCCTTTCACATTGTATTAAACACAATTTGATCCAATGGAAGCAAAGGAGTTCAAGGATTTCGCCAAGG
CGATGGCTGATTACATCGCGGAGTATTTAGAAAATATACGCGACAGACAAGTAGTACC
ATCTGTTAAGCCAGGTTATCTACGGCCATTGATTCCCGACCAAGCTCCAGACAAGCCCGAACCCTGGACCGCA
GTGATGGATGACATCGAACGCGTAGTCATGTCCGGCGTTACACACTGGCACTCGCCGCGTTTCCATGCGTACT
TTCCTACAGCTAACTCCTATCCTTCTATTGTAGCTGATATGCTGAGCGGTGCTATAGC
ATGCATTGGATTCACTTGGATCGCGAGTCCAGCGTGTACTGAGTTAGAGGTCGTGATGATGGACTGGCTTGGT
CAAATGCTTGACTTGCCTAAGGAGTTCTTGGCTCGTTCAGGCGGAGAGGGTGGCGGCGTAATCCAGGGTACAG
CTAGTGAAGCCACCCTCGTCGCTCTATTGGGTGCTAAGGCCCGCACTATGCAGAGGGT
CAAGGAACAGCACCCTGAGTGGACTGATGTTCAGATACTATCTAAACTTGTTGGATATTGTAACAAACAAGCT
CATTCGTCTGTCGAGCGCGCTGGTCTCTTGGGCGGCGTCAAACTCCGCAGCTTAAAACACGATAACAAGAGAA
GTCTTCGAGGTGAAACTTTACAGGAAGCTATTGATGAAGACATCCGTAATGGACTCAT
TCCGTTCTATGTGGTAGCCACATTAGGAACTACATCGTCTTGCGCTTTCGACGCCCTCGACGAGCTCGGGGAC
GTGTGCCAGGCGCACGACGTGTGGCTGCACGTGGACGCCGCATACGCCGGCTCCGCCTTCATCTGCCCCGAGT
ACCGCTACCT
>1002049307_1 Heli.1-DN172724.1.5
ATTTTTCTTCCAACATTTGTGACTTCAACAAGTGCATCAGTTTCAGAAAATGGGGTTGCTTGCGGCGTTAGCT
GTGAATGTTCTAGGCGGCGCTATAATTTATGGCACCGGGGGGCTTGGAGCCGCGTGGGTGGCCCCCATGATGG
GCTTCGGTAGTTCAGGTATTGCTGCTGGGAGCATTGCCGCAGGTGCTCAGTCGTACTA
CGGCAACCTTGTGGCTGGAAGCATTGTCTCTCAGTTAACATCCGTAGCAATGGCCGCACCCACGCCATAGGTG
TAAAACATCGAAGCATAGCATTTGAAGCTTAGCCTTCATGTAAAGAGAATAAAATTTCTAAAAATAGATGATG
TTTTATTTCCTATTTTAATTAAGCCAATGTTTAATTTTAAGATATAATAAATAAATTG
ATAATTATCAAC
>1002049308_1 Heli.1-EL599528.1.5
GTGTGACGCGACACATTAGCGCGGTAGACGCCTGTAAAAATAAACATTATTGATTTTCCATCACTTAAAAAAT
CAATGAAAATGTACTCCATTGCCGAGGCCACGTTAGCTTTCGCTGTTCTTTATCTATTCTCTTTCCTTTTCGA
AGTGGTAACAGGATTAGTGTCGTAGACGATTAAGTGTTTATTAAAAAAATGTGATATT
TTATTTATTTTAAGCTAGATTTAAGATTATTAGTCAAGTTATCAATGCAATTGTATATAAGTTGTAGTTTTAA
TTAAATTATTTAGGCGCGAAAAAATCCATTCCAATACAAGGACGCTACATTAAAGGTTTTGGTTTTCACACCA
GGTATTCACAAAAAGCCTTACAATAAAAGTTCGAAAATAAAACAGATTCAGTTAGAGC
AAGACAGTGGGAAGTCACTTCATGACATTGAATTAAAAAGGAGTCTAGTGGATTTAAATCGAGCAGGTGCACC
TTTAATTGAACTTGTATTTGAACCTGATCTTGAGGATGGTGAGGAAGCTGCTGCATTGGTAAAGGAACTAGTA
CTAATAGTTCAAAGACTAGGTGCATGTACAGGCCGTATGGAAGAAGGAGCTTTACGAG
TAGATGCTAATGTTTCTATAAGAAAAGTTGGTGAACCACTTTCTACACGGACTGAGATTAAAAATATTGGAT
>1002049309_1 Heli.1-ES585772.1.5
GCGGATACACAGTTTCCACAATGGGTCGTGTCAGGACTAAGACCGTCAAAAAAGCAGCGAAAATCATTATTGA
GAAGTATTACACAAAGTTGACTCTTGATTTTGATACAAACAAGAGAATATGTGAGGAAATAGCAATCATTCCA
ACTAAATCTCTTCGTAACAAAATTGCTGGTTTTGCCACACACTTAATGAGGCGTCTCA
GACATTCACAAGTTCGTGGCATCTCCATCAAGCTACAAGAAGAGGAGCGTGAAAGACGTGACAACTATGTTCC
AGAAGTGTCTGCCCTAGAACATGACATCATTGAAGTAGATCCTGACACTAAGGAAATGTTGAAAATGCTTGAT
TTCAACAACATCAATGGTCTTCAACTCACCCAACCTGCTACTCAAGGAGGTTATGGTG
GCAGACGTAATTAAGGATAATAAA
>1002049310_1 Heli.1-DT664112.3.5
GGGTGAACAAACATGATGCGTGCCACATTTGTCCTATTTCTGGTGGCAGCCGCCCAGGGCATGCCCGCCCTGG
AGAAAGACAACGACCCAGGGCTAGTAACCAGCGTTATAGGAGTAGTCAAGGAGTGCGCTGATGGAGATGTGTC
CTTGTGCCTAAAGGAAAAAGCTCTGAAATATGTGGACAGCCTGTCGTCGGCCAGGGAG
ATGAGTATCACTGAAGGTGTCACTCTCTTGGGTACTGGTTCTCCGAGGTCTGCGAGAGCGTTCGAGCCCTTGG
CTGATGAGCCCAAGGCTAGAGAGGCCCAGGTAGAGTCAAGGCTTGTGGACTCCGCTGCTGACTTCCTGGAGAA
CCACGTGATCCAGTTCCGTCTGCCATCATCAGCTGTAGAAGGCATGAAACGATCTCTC

GAAGAAGCTCGTGGCAAAAAGAAGAAGATCAAGCAGCTCCTCCCTCTGTTGGCCATCGCTAAACTGAAGATCA
TGGCTCTCATTCCTCTGTTCTTGGGTATCATTGCATTCGCTGCCGCCAAGGCTGTCCTCCTAGCCAAGATTTC
ACTCTTGGTCGCTGGTATCATTGCTCTGAAGAAGCTTCTTGCAAGCAAGCATCATGAA
ACAAGTTATGAAGTCGTTGCCCATCCTCACCATGAAGAACATTATGCCAGCAGTGGCCATGGATGGGGAAGAT
CAATCGACGAAGCACAAAACTTAGCCTACTCCGGCCATGCTAAATCTGATTAAGGAGAGGGTCTTACAAGGCT
CGATTATATGACCCAAAGTATTCCTTAGATATACGAACGAAATGAAATTAAGCATGCG
ATTTATTTATTTCTAGATGTAAGAATATTTATTGAATATTTATTGTACCCCTTTGTAAGGGATGGTAAAGTAT
TACCGTTGTTTTGTTTTCCCATAAAGTGTGTTCTAAATTTGTATGATCCCTCTCGTTGTACTACATTAGTATA
TTTTTCACACCTTATACGGTATCCGTATTTGTGTACTTTGTTTTATACAAATATTGTA
TTATTTTCTTTGATACTTTTACTTAGTGTTGTTATTATGTGATTTAGTCCTAGTCCTGTCTTGTAAGTCCTTA
TAGTATATACTTATTTTCATGATATTCTATATAATTACTAGTACTATTCAATATTTCTTATTCTTATTTTATT
TTAACGTATAGTTGTTATGTATATATTGTAAATAATGTATATAAATATTTTTTATATT
TAGATATTCATTCAGTATTTGCTGTATATAGTGCCTCATCTCATATATTATGTATGAATCATTTTATGTACTC
GTATCCAGTCATCATATATTTATTTGACTTATTTACTTTCATATTTTCATAATCATTTTAATTTAATATAATT
ATTTGTATAATGTATTAGGTTTACGTATTTTTTTGTTGTATGTTTCATCATTGTGCTA
TATATTTATTTA
>1002049311_1 Heli.1-ES586748.1.5
CTTTTTCCTTGTACCGCCTTGCGAGACACGTGCGTGTATCGCGCCGAAACCTCTAAAATTATCCAAAATGAGT
GAGGGAACCGCGACAATTCGTACTCGCAAATTCATGACCAACAGGTTGTTGGCGCGCAAGCAGATGGTTTGCG
ATGTCCTCCACCCGGGCAAACCCACTGTTAGCAAAACGGAAATCCGCGAAAAGCTAGC
AAAAATGTATAAAGTGACACCCGATGTAGTCTTCGTGTTCGGCTTCAAAACCAACTTCGGAGGTGGCAAGTCC
ACCGGCTTTGCCTTAATATACGACACCTTGGATTTAGCTAAGAAATTCGAACCTAAACACAGATTAGCTCGCC
ACGGATTGTATGAGAAGAAGAGGCCCACACGCAAACAGCGTAAGGAACGTAAGAACAG
GATGAAGAAGGTGCGTGGTACCAAGAAATCCAAGGTCGGAGCCGCCGCGAAGAAGTGATGTGACGGTTAATAA
TTTGTAAGTAAATAAAAATCCCA
>1002049312_1 Heli.1-DT665400.3.5
GCACGAGGGATTTCTTAGTTTGTCTACAATGGAGAAGCCAATATCTGTAGTGTTATTGCTTATTTTTATACTT
TTTAATTATACCACAGCCAGGGTGCCCGTCGATTATTATAAAAAAAACATTAATAAAATAGTAGGAGGATATA
ACATTACAATTCAAGATGCGCCGTATCAAGTATATTTGTTGCTGCAGATGGGAAATGA
TGACTATCAATGCGGAGGGTCGATTATAAGTGAACGATATGTTTTAACTGCTGCTCACTGCTTAGCTGGAATA
CAAAGGGTATTTGTAAGGGCCGGCAGTACATATGCCGATTATGGAGGCATTCAATACAATTCCACAAGATTCA
GGTCACATCCATTGTACAATCCTGCAACTTTTGACTATGATGTAGGTGTAATAAACAT
TCCTGGAGGAATAGCTCTGGATGGATATAGCACGAGAGCTATATCACTGCCAGCAAGGGGCACTGTTATACCG
AATGATACCAATATTTTCATTTCTGGTTGGGGTGATACCACTGAAGGTGGTCAGGTGAGTGAAAATCTGATGG
CTGTACAAATTCCAACGGTGCCACAAGAGGAATGCCGCGAAACTTACACCACATTAAC
ATCTAGGCAATTTTGTGCTGGCTTAGAAGAAGGTGGAAAGGATTCTTGTCAGGGTGATTCCGGAGGCCCTGCA
GTGTCAACAGATACAGGTCTTCAAATAGGTATCGTTTCATTTGGCTATGGTTGCGCTAGACCTGATACTCCTG
GTGTGTACACAAATGTCGCAAG
>1002049313_1 Heli.1-DT665612.3.5
AGTGTCGATAGGTCAATATTCTGCAGAAATTAGCCAGAATCTTCAGGTATCTACAAAATATACTAAATAACCA
CCCTCGCATGTGAGGGTACTATGATATTCTGCAGAAATTAGCCAGAATCTTCAGGACCGATGGCTGCAATACG
CAAAAAATTAGTGATAGTTGGTGACGGTGCATGTGGTAAAACATGCCTGTTGATCGTG
TTTAGTAAAGATCAGTTCCCGGAAGTGTATGTGCCGACAGTATTTGAAAACTACGTCGCCGACATCGAGGTTG
ACGGTAAACAGGTGGAGCTAGCCTTATGGGATACGGCCGGCCAAGAGGATTATGACAGGCTTCGGCCACTCTC
GTATCCAGACACGGACGTGATCCTCATGTGCTTCTCGGTGGACTCCCCCGACTCGCTC
GAGAACATCCCGGAGAAGTGGACACCGGAGGTGAAACACTTCTGCCCCAACGTGCCCATCATTCTCGTGGGTA
ACAAGAAGGATCTACGCAATGACCCAGCCACAATCAATGAGCTCCGCAAGATGAAACAAGAACCTGTCAAGCC
TCAGGAGGGCCGTGCTATGGCTGAGAAAATTAATGCATTCGCTTACCTCGAATGCTCT
GCCAAGAGCAAGGAGGGAGTCCGCGAAGTATTCGAGACGGCGACTCGCGCCGCGTTACAAGTTAAGAAGAAGA
AGAAGACTAGGTGTTCTCTGCTGTAATTGTGCTGTTGTCGGAAGAACTCTGATTTATGAATCGTTCGCATACT
GCCTGAAAACTTACGTTTGATACGACTCGAACTCTGCAGCAGTCGCGATGTCGGCCAC
ACAGTGCTTCCT
>1002049314_1 Heli.1-ES585056.1.5

CGCGAGGTTATAATTTTCAGTTTGTGAAAAGGTGTTCACAATGGTTGAGGAAAACTGGAACGAAGATGGCGTG
GAAGCGGGCAGCATGGCTATCGACAGCATGCCATTGCCACAACCGGCTGACATTCCCGAAATCAAATTATTCG
GAAGATGGAGCTGTTATGATGTCCAGGTCTCTGATATGTCCTTGCAAGATCATATTTC
TGTTAAGGAAAAATTCGCGAAATATTTGCCACACTCTGCTGGTAGATACGCACACAAGCGATTCCGTAAGGCC
CAATGTCCAATCGTTGAGCGTCTCACTAACTCTCTGATGATGCATGGCCGCAACAATGGCAAAAAGCTGATGG
CTGTGCGTATTGTCAAACACGCTTTCGAGATCATCCATCTCCTGACAGGAGAAAACCC
TCTCCAAGTACTTGTGACAGCTATCATCAATTCTGGCCCTCGTGAAGATTCTACTAGAATCGGCCGTGCTGGT
ACAGTACGTCGTCAAGCCGTGGACGTTTCACCTCTACGTCGTGTGAACCAGGCTATCTGGCTGTTGTGCACAG
GAGCACGAGAAGCAGCCTTCAGAAACATTAAGACCATTGCTGAATGTGTTGCTGATGA
GCTCATTAACGCAGCAAAGGGTTCATCTAATTCTTATGCTATCAAGAAGAAAGACGAGTTGGAACGTGTTGCT
AAATCCAACCGTTAAGTGTTTTTAAGAAAGGGACAAAATAAAAGTGT
>1002049315_1 Heli.1-ES587362.1.5
TCCGCGGACACGTCGTGAGCACGTTGTTTAGTCATTTTTACGTGCAAACTGTAAAAATGTCGTCTCATTTAAA
CTGGATGATAATTAGAAACAACAACGCCTTCCTTGTGAAGAAGCGTAACATCAAAAAGCCTTTCAGTAAGGAG
CCCAACAATGTGACTAACCTCAACGCTTTTAGATACAATGGCTTGATCCACAAGAAGG
CTATCGGCGTGGTCGAGAACCCCGACAGGAAAGGTTTTTCAGTGGTATACAAGAAAGCTAAGGCAACAAACAA
GCCCGCTAAGAACTTAATCCGCCGTCCATTCAAAGCCGGTGCCAGAAGATCACTCCACAAAGTGAAAAGGTTG
ATCAGAGCCAACAACTATCGCACAGACTTGGCTAAAGCATCTCTTCGTCGTGCATCAG
CGATCCTCCGATCCCAAAGGAAAATTAAGGCGAAGAAACCTGCCGCAGCGGCCGCTAAAGTCGACTAAATTTG
TATGTAAAAATAAATAAAAAAAT
>1002049316_1 Heli.1-DT667076.3.5
GCACGAGGCAATCTCCTAACATGTATTCTAAGGTGGTCATCTTCCTCTGTGCCGCGGGCATTGCTTCCGCTGG
TAACGTATTGCATGCGGCCCCCGTGGCTTACGGCGCACCGGCCGCTTCAGTGTCGTACTCCACTACCACCACG
TCTCACTCCGCTCCAGTAGCGATTGCCGCACCAGTAGCCTCGTATGCCGCTCCCGTGG
CATCCTACGCTGCACCTGTTGCCTCATATGCAGCTCCTGTTGCATCATATGCTGCACCTTTACTCGCTAAAGC
AGTAGCTCCCATCGCCTCTTACGCTGCACCTTCTTCAGCTGTTTCATATCAATCAGTGTCTACGCGTTCTAGT
CCAGTAGCTTACGCAGCTGCTGCCCCCGTCATTGCCAAATCATATGCAGCTCCTGTTA
TCGCAAGGGCGTATGCTGCACCAGTAATTAAATCTTACGCAGCTGCACCTTTGGTTACTCGAACATATGCGGC
TGCGGCCCCTGTCATAGCTAGATCTTACTCTGTTCCTGCGATTTCCAGCTACGCTGCGCCCGTTGCGCGTATT
GCTCCCTCATTGTCTTACGCGTCAGTCGCCTCGCACAGTGCACCAGTGGCTATTGCAG
CGCCATCTATTGCGTCTTATGCTGCGGCTCCAGCTGTCTCATACGCTCCTGCAGCGCCAGCTATCTCATACGC
TGCGGCAGCCCCAGCTGTCTCATACGCGGCTGCAGCTCCAGCTCTCTCATACGCTGCTGCAGCCCCAGCTGTC
TCATACACTGCGGCAGCTCCAGCTCTCTCATACGCTGCTCCAGCTC
>1002049317_1 Heli.1-CX700433.1.5
GGCTGCCGCGCAAGAGGCGAGACGCGAACCAGTGCATGCCGTCCAAGTATTCGGACGCAAGAAAACTGCCACT
GCAGTAGCATACTGCAAACGCGGTCATGGAGTGCTTCGTGTAAACGGACGTCCTTTGGATCTAGTGGAGCCCC
GTCTCCTCCAATATAAACTGCAAGAACCTATTCTCTTGCTTGGAAAGGAAAGGTTCTC
CGATGTTGACATCCGTGTGACGGTCAAAGGAGGTGGTCACGTAGCTCAGGTGTACGCTATCAGACAGGCCATT
TCTAAGGCTCTTATTGCCTTTTATCAAAAATTTGTAGATGAAGCATCAAAGAAGGAAATTAAGGACATCCTCG
TCCAATACGACAGGAGTTTGTTAGTAGCGGACCCACGTCGCTGCGAGCCCAAGAAGTT
TGGAGGTCCAGGTGCTCGTGCCAGATACCAGAAATCTTACCGTTAATACATTCACATATGCTATATACATAAT
AAAAAAAGTAT
>1002049318_1 Heli.1-DT666069.3.5
AGAGCGGTGAACATCGAGGAGAGGTCGTGGAGGAATTTACCATCTTTGCCACCATCGCCGTTCGCCAATTACA
CCATGGCTGATACCGAAAAGGCGCCGCAGCCGCAGCCCCAACAGCAGCAGCAACCTGAGCAACAACCCCAACC
CCAGCAAGCAAAGGCAGCTAAACAAAAGCAGGTCATTGCTGAGAAGGTTTCGGGCACA
GTTAAATGGTTTAACGTCAAGAGTGGATATGGTTTCATCAATAGGAATGACACAAAGGAGGATGTATTTGTAC
ATCAAACTGCAATCGCCCGTAACAACCCACGCAAGGCTGTGCGCTCGGTCGGCGACGGCGAGGCGGTGGAGTT
CGCCGTGGTGGCCGGTGAGAAAGGGTATGAAGCTGCCGGAGTGACCGGCCCCGGCGGC
GAGCCGGTCAAGGGCTCGCCCTACGCTGCCGATAAGCGTCGCGGCTTCCCACGCCAATACTACCCTCGGCCAG
GAGGCGGACGTGGCGGCGAGGGCGCTCCACGCAGAGGTGGCATGGGACGTCGCGGGCCCCCGCCCAACCAGGG
GGGCGCGCAGGGGGATGAAGTGCAGGAAGGAGCCGGAGGCCCACCACAGCGTAGTTAC

TTCCGCCGCAATTTCCGCGGTGGACGTCGTGGAGCTGGATCCTCCCGGCCTATGTACCGTGGTGGATACAGAT
ATGTCCGTCTCCGCAACAACGCACAGGGACCAGGCCAGGGTCAGGGACAAGGGCAGCAAGGTCAAGGGCAGAC
TCCGCGTCAAAACGGTCAGGGAGAAGGTGAAGCTCCGGCCCCCGCTCCTGCGGCGCCA
GCACAGCCGAAAGCAAAAGCTAAGCCTGCAAGCACCACCATCGAGACCACCACCAATGAGAGCCAAGCCTAGA
TGGTTGGCACTGTGGCACGCCCCTAGTAGTACATGTCGATTCCAGCCGTGCCGCATGCTATCACAACTTACTG
TCTGTCTATTCAGCTAAAGAGTGTTATGATCAGATCATTGTACACCAATTTTATGTGT
AGAGGTAGCTAAGTCGACGGGGGTGTCGCGAGTGGAACTGCATAGTATCATGGATCAGAGATGTGATATTTTT
ATTATTTTATAATATGTTGTATACCATTCGGCCGCCGGGCGGCGAGCGATAGTATCGAATTATTTTTTACTAT
TTAATAAATATTATAGGTCGCCGTGCGAAACCGCCTCTTAAGAAATTGCATGTTTTAA
TATTTAAAAAAGTTTTTGATGATTATTGGGTATACATGTAAAATTATAAAATGTCCTCTTAAGTGAGATGTTG
TGCAATTTTAAATTATGATATTTATGTATATGTATTGAGAAATGATATGCAGGTTCCGAGGCCCCAAGAGA
>1002049319_1 Heli.1-DT668194.3.5
CATAATGAAGCTTACGGTTGCCCTGCTTCTGCTTGCGGCAGTGACACACGCCGCTAAGCCGGGAAAGAAACAC
AGTGAAAGTGAGAAGACGGAGAAACGAGAGCTGGTTGAAGGGAACAGTGGAGTTGAGAAGAGAGCTCCACTTC
TATCTACTGTATCACCGGCAATATCCACTGGAGGAGTAGAATACGCAAGCACAAAAGA
AAATTTATCTGGAAACTCTCCTGCACAGCAAATCTACGCTACACCAGTGCCTCAAATCGCTAAGATATCAGAT
GTACTCTCTGGACAAGGTCCTCAATTCCAAGCTGCGATTGACAGCCATCTCTACTCACCAATCTCTGTTTACC
AACCCAGGCTTGGGTCACCAACTACATATGAAGTATCAGCACCAGTACCTTCTCAATT
AGCCTACTCTGAGCATAGATTAGCTATACCCTCACCAAATGCCATACAATATGATGCCAAACAACAAAATTTG
GGAAAACCAATAGTTAATTTCGAACAACCTCAGATATTATTCCGACCAAGCCACGAACAAAATTTGGGTCCAG
TATTTCCACAATACTCCAACAACATACAAGCAATTCCTCAGCCAGTTCAATATAGTCA
GCAACAAGTATATCCTGCTCAGCAGTTGCAATTCCTCCAACCATCCCAAAATCCTCAAGGAATTTCATATCAA
CAACCATCATCAATAGTTAAACAAAACGAGCCGAGGGACATCAAGAGCAAATCTGTCCACTCTCAAGATTTGA
TAAAGCAAAT
>1002049320_1 Heli.1-ES588035.1.5
AACTGATCTCTGGTTCAGCGCTGAACAATTTTTAATTGAAAAGTTGTTTATCTCGACCGGGAAATCGCATAAA
CATGGCAGCCGCCGTCGTGTCAGGCAAGGATATCGAGAAGCCCCAGGCTGAGGTCTCACCCATCCACCGTATC
CGTATCACATTGACATCCCGCAATGTACGTTCATTGGAAAAGGTCTGCGCTGATTTGA
TCAACGGTGCTAAGAAACAAAAGTTGCGTGTAAAGGGGCCAGTCCGCATGCCAACCAAGATTCTTCGCATTAC
CACCCGCAAGACACCTTGTGGTGAAGGTTCCAAGACCTGGGACAGGTTCCAGATGAGGATCCACAAGAGAGTG
ATTGACCTTCATTCTCCTTCAGAGATTGTGAAGCAGATCACCTCAATCAATATTGAAC
CAGGTGTAGAAGTTGAAGTTACTATTGCCGATGCGTAGGCACAACTGCCTTAATAAATAT
>1002049321_1 Heli.1-DT668407.3.5
ATGTACGCTAAGAACAACACAAAGATGTACGCTAAGTTGATCGTCGCACTTTGCGCTTTAGGCGTCGCCCACG
GCAGTGGCCTCCTCGCGGCCGCCCCGCTAGCGTACAGCAGCCAAGCTATTGTCGCTCCCGCGGTAGTGTCCAA
GACTGTTGTGTCCCCAGCTGTCTCGTCGGTGTCTTCCTACTCCAGCCACACCGCCCAC
TCCGGCCCCGTCGCCACCTATGCCGCCGCCCCAGCCATCGCCAGCTATGCCGCCCCAGCGATCACCAGCTATG
CCGCCCCAGCGGTCGCTAGCTACGCCGCCGCTCCCATCGTGAGCAAGACCGTCGTGCAGCCAGCCAGTTCTTC
CTACTCTTCTTACTCTAGCAGCGTTTCCCACGGAGCACCAGCAGTCGCATCTTACGCC
GCCGCTCCCGCCGTAGTCGCCGCCCCCGCCGTCGCCGTGTCCCGCGCTGTCCCCGCCGTGTCCGTCGCCGCCG
CCCCCGCCGTAGCCGTCTCTCGCACTATCTCCCCCGCCGTATCTTCTTACTCTTCCTACTCCAGCCAAGTCGC
TCACGGTGTTAAAGCTATTGCTGCCCCAGCGTTAGCTTCCTACGCTGCCCCAGCGGTC
GCCTCCTACGCTGCCCCAGCGGTAGCCTCTTATGCTGCCCCAGCAGTCGCCTCTTACGCCGCCCCAGCCATCG
CCTCTTATGCCGCCCCCGCGGTCGCCTCTTACGCCGCCCCCGCGGTCGCTTCTTACGCCGCCCCCGCCGTCGC
CTCTTACTCTAAGATCTACTCTGCGCCCGCCATCTCCTCTTACTCTAAGATCTACTCT
GCGCCCGCCATCTCCTCTTACTCTAGACTGTACTCCGCCCCCGCAGTT
>1002049322_1 Heli.1-DT666674.3.5
CGTTGTACGCATCGCACGTCTTCTCCGCACATTTAATTTATAATATCTATATATTTATACAGTACCCCGAGTG
ACCTGTGCTCGAACGAACGAAATTTTAACTAATATATATCTAACATAGACCTAATAATTAGTTTTGAGTGTAG
TTTTTAAGTTAAATGGTGTAAGCTGCGGTCAGGATGTCTGAAGGAGACGTGTCCCTTA
TCCAAGACGAGGACGTGCTGCGGCGGATGTGGCAGCAGACTGAAGACTTTTCGCGGAAGAAGGAAATTCGAGC
GCATATGTATAAACTGAGGGAGGAGCGTTTGCGCAACCTTTATTCGCCAGAACCTGGAGTCGAAACTAAAGGT
TGTGAATTCTCATCCACGCAAGGTCATGTGAAGTCATTCGCTGACCAGAGCTTCCAAT

CCATGAAGAGCAAGGAAGTTCGAGACGCGGGCTCCCCGCCCAAGGAGTTCACTTACAGAGGCCAAGATCTCAA
AGAGCTTTCTAATGCTGGATGGAACGTCGAAACTGAGAACAGAACAACTGATGATGGCCATACGCAGATTAAG
TCAGTTCACGCTAACATTGAAGGTCGCTACGATGTTGACGGCGGTCATGGTCAATTTG
CAGCCGTCGATCACAATAAACAAGCTTTCACCGAGTATGACGATGGAAATACTAGTCTTAAGCGTAATGAAAA
TGTTTTTAATACTGCAGCGCATGAACAAGTTATACAAAAAACAAATGATGGTACTAAATTTTCGTCGACAAAG
AGTTCGACTAGTTCTTCATCTAAATTCGAGCAAATTTCTTCTAAACAAGAAAATGTGC
CATATAGTGTGA
>1002049323_1 Heli.1-DT668889.3.5
CAACATGGCTCGTGGACCCAAAAAGCATTTGAAGCGTTTAAACGCGCCCAAAGCATGGATGTTGGACAAACTA
GGAGGTGTGTACGCACCTCGGCCGTCCACTGGACCGCACAAACTACGCGAGTGCTTGCCGCTTGTTATTTTCC
TTCGTAACCGTCTGAAGTACGCTTTGACCGGTAACGAGGTACTCAAAATCGTAAAACA
GAGACTGATCAAAGTAGACGGCAAAGTACGCACCGACCCCACATATCCCGCAGGATTCATGGATGTGATCTCA
ATTGAGAAGACAAATGAGTTGTTCAGGTTAATCTATGATGTCAAGGGTCGCTTCACCATCCACCGCATCACAC
CTGAAGAAGCAAAGTACAAGCTGTGCAAGGTACGCCACGTGGGCACCGGCCCCAAGAA
CGTGCCATACCTGGTGACACACGACGGCCGCACGCTCCGCTACCCTGACCCCCTCATCAAGGTCAACGACTCC
GTTCAACTAGACATCGCCTCCGCTCGCATCATGGACTTCATCAAGTTTGAATCAGGCAACCTGTGCATGATCA
CTGGTGGGAGGAATTTGGGTCGTGTGGGTACCATCGTATCCCGTGAGCGGCACCCGGG
GTCCTTCGACATTGTGCACGTGCGTGATTCAAGTGGACACACGTTCGCCACGAGGCTGCACAACGTGTTCATC
ATCGGTAAGGGCACGAAGCCGTACATCTCGCTACCGCGCGGTAAGGGTATCCGCCTCACCATAGCCGAGGAGC
GCGACAAGCGAATCGCCGCTAAACAAGCTGCCCATTAAGTTAATAA
>1002049324_1 Heli.1-EE743412.1.5
GTGATTTTGTTTGATGTTTCTTTTTCATGTTGACGGCGGTTCTTTCGGAACGTTGAGCCGATAGAATTTATCA
AAATGTCGCTCGTAATCCCCGACAAATTTCAACATATTCTTCGTATTATGAATACGAACATTGATGGCAAACG
AAAAGTTATGTTTGCCATGACTGCTATTAAAGGTGTTGGTCGAAGATATTCCAATATC
GTATTGAAAAAAGCTGACATTGATTTAGACAAACGTGCTGGAGAATGCACTGAGGAAGAGGTAGAGAAAATTA
TTACCATCATGTCAAACCCCAGGCAATATAAAATTCCAGACTGGTTCCTTAACAGACAAAAGGATATTGTTGA
TGGTAAATACAGCCAGCTAACATCTTCTAACTTGGACTCTAAACTCCGTGAAGATTTG
GAAAGGTTGAAGAAGATCCGTGCTCATAGAGGTATGCGTCACTACTGGGGTCTGAGAGTGCGTGGTCAACACA
CCAAGACTACTGGCCGCCGTGGAAGAACTGTTGGTGTGTCTAAGAAGAAGTAAACTGTACAATAAATAATA
>1002049325_1 Heli.1-ES585929.1.5
AACATGTACTCAAAGGTTGTAATATTCTTGTGCGCCGCCGGCATCGCTTCTGCCGGTAACCTCCTTCACGCCG
CACCAGTGGCGTACAGCGCGCCGATCTCTGCCGTGTCCTACTCATCTCACGCCGCTCCTATCGCTTCATACGC
CGCGCCAGTGGTCGCCAAGAGTTTATCTCCAGTGGTCTCTTACCAATCAGTCTCTCAT
GCCGCCCCAGCCATTGCATACTCTTCTCCTTTAGTGGCTAAGAGCTATGCCCCGGCTTTATCTTACTCATCAG
TTGCTCATGCCCCAGCATACGTTGCTGGTCCAGCTGTATCTTACTCCTCACCTATTGTAGCTAAGAGCATATC
CCCCGTGGTGTCCTACTCATCTCCTGTGTCCTATGCTGCTGGTCCAGTAGTTGCCAAA
GCTATTGGTCCCGTTGGTGCCCCAGTACTTAAGTCCGCTATCGCGTATTCTGCTGCCCCAGCGGTTTCCCACG
TCGCTTATTCTGGTTTAGGAGTTAACTACGGATGGTAATCAATGAAATCTAGTCAATTAAATAACTGTATTAT
TGTTATTTATATTATTTTTTTACGAAATAAAAAA
>1002049326_1 Heli.1-DT664972.3.5
GACGCGTGCACTGTGTCAGTTTGAGTTTAAACGAACAGTTGAAATTTGTTTTTTTTTAAACTGTATTACTTTA
TCGAAAAGTGGCCAGTGTTATAACTTTTTTTTTTAATAAAGCAGTGTGCCCAGTGTGGATTGGATACTTGGAC
GCCGTTCAGATTGTTGATTAAGATAATTGATATAATGTACTCGTTTTAAAATGGACCC
TCTCACGTGGTTGTCCCTGGGGCTGCAGCTGGCGGCGTTGTGCTCCATAGTCGGCGCGTTGCTACTCTACTTA
CTGCGTAAGGTCCGAGTGGCTGAGGTCTCACCTGTTGACAGTGCGAAAACTGTTCTTGTCACTTCCGTGGACT
CCGCTCTTGGACTACAGATAGCAACGTACCTAAGCGGCAAGGGTTGGCGAGTAATAGC
AGGATGTCGGCAAGGGGGACTCGGTGCTCGGTTAGCTGAATCATGGCTGCAAGCCCACGTCGCGTCCACCCCT
GAAGACCAGCCACCTCCAAGACTAGCGACCCTGGAGCTGGACGTAGCGAGGGAGGATTTATTGGAGGAGGCGG
CGAGAGCGACTGCCCAACACTTACCTGCAGGAGAACATGGTGTATGGGCAGTAATAAA
CACGGCGGGCAGCAGTGGGCGCGGCGGCGCGGCGTGCTGGGAGAGCGCTCTACGAAGCAATGTCCTCGGTGCC
CTGAGAGTCGCTAGGACCTTCTCACCTTTGCTGGCAGCAGCTGCATCTGACCATCCTTATGCTGGACGACTGT
TTTATATCGGATTAACATCAGACACAGCATGCGAAAGCCTGTCCCGTCTAGATGGGGA
CCTGAGCGAGGGCAGCGCTGGTGCGGCGGCCGTCAGGTGGGGCACCTGGGGGGCGGCCAGGGCTTTACGAACA
TCACTACGCGCGCGGAGATTGAACGTTGTACTTTTACACGCCCCCGA

>1002049327_1 Heli.1-ES588218.1.5
ACCAAGATGTTCAAGTTGGTGGTGTTGTGCGCTTTTCTCGCCGGGGCTGCAGCGGAGCCCGGAACCTTCATCA
CGCCATTGGCTTACTCCAGCAGCATTATCGCACCGGCTGCAACAACTGTCACGCACCAGGCCAGCAGTGTCAT
CCACCCTTCACCCCTGCTCTACCCTCGATTCTACGACCTATCTTACTCACCGGTTGCT
CATCTTATCAAGAAGCGATCCCCTGGAATTTACAATCCTTTCTTTGGCTTCGGCCCATCAACTTACGTTGCTT
CTACACCTCTGGCCACAACTTATGCTGCAGCTCCTCTTGCTACTACCTACACTGGAGCTCACTTAGCTACAAC
TTATGCTGCACCAATCTACCCTGCTGCTGCGCATTTAATCAAGAAGAGGTCTGCTCCA
CTTGTGCCCACAACCTACGTGGCTCCATACTATTCTTCTCCTTTGTTGACGCCGACGTACACAGCCGCTGCAC
CCATCTACTCAACACCTTACATCACGAGTGCTCCTCTTACTTACACACATTTGATCAAGAAGCGTTCCGCCCC
ATTTTTCTACGCAGGTTACACCGCCCCCGCTGCGGTTTCTCATCAGTCCCGAGTAGAC
ATTAAGAGCTCCCCTGCCACTATTACGTCCTACTCCTACCCTGCTGCTGTATCTTATGCTAGTCCAGTTGCAA
TTTCACATGTCTTCTAGATGATAGAATGGATTTTGAAATAAAATGAA
>1002049328_1 Heli.1-DT664957.3.5
CCATTCTACCTACATCGGCTTTATCGAAATGGAAATAGCACTACTTTGTGCAATACTAACAGTCACCGGCGCT
GTGGCGGATGACTGGCAACCAATTATAGCAGATTCACCACAGCTCTATACCACATACTCTAAGGCATCGGAGC
GATCATTAGATGGAAGCCCCCAGCTAAAGATTATCAACCGTAGACCAGTTCTCCAATC
TAAGCCTCCACCTTTCGGCCCTAACAAATTATTCAGCTCCGAGCCAGAGGCTGGTTCAGATGCGTGCAGCGTG
TACAAAGTTTCTATGAACCAAGAACTTTTCTACCAATACGTCGAATATGAAAAAGCTTTGCCAGATCTTAAAG
AGTTCACACTTTGTATGTGGTCAAAGTTCCACAACCACTCCGACGATCATCCACTGTT
CTCATATGCTGTTGGTGATAATCCGAAGGAGATTTCGGCTTGGATATCAAACACTAAAGAGGCAAGCTACTTC
AGCATGGCGATCCACGGACAGACCTACTTCAGGCTCAACTATCCCCTTAAACTTAACACATGGTATCATTCTT
GCCAATCTTGGAATGGTAAAACAGGCGAATGGCAAATTTGGGTGAACGGTGAAAGAGT
TGGCAGAGGATTTCACAACAGGCTCGTGGGTCATATCATAAAAGGCGGTGGAATTGCGACTAGCGGCCAAGAA
CAATCTCTTTTATACAAAAATGACGGCAAAGAACCTGTTTTGAAACAACCCGGCCTGATCGGTGAAATTACTA
TGCTCCAAATGTACCACGTCGCTTTAACTGCCGGAAAAGCGCATAAAGATCACAAGCA
TCATCATGTACATCATTTCAAACATGACGGTACACTCTTCGAGACGTCCTCGGAGGCAGTTACGGAACCACCT
CCACCTCCGCCTACTCCTCTCGGCAATGGCAACTTTTTGATAGGGGGTCAATTACAAAGACCTGCGAGCCTAA
ATCTGGCTCGTCAGCAACAAATGATACCCGTACAGCTTTCTAACGGACTTCAGTTACA
ACAAGAATATGTTAATGGACAATTGGGTAATAGAATAGTCTCCGAGCAACTACTAAATAATGTTCAACCGCTT
GCTAGTAATCAGGGCAGCGGTGGGGTCCAGCGCATACCGTTAGTTGGTTTGTCAGTATCACCAGTTGACCA
>1002049329_1 Heli.1-ES587815.1.5
CACAAAAAACACATACAGAGCAACCATGATCTCTAAAATCGTCACCGTATCAGCTTTGCTAGTGGCAGCAAGT
GCCCAGCAACATTTAAGTGGTCATGCCACGTCATCCCAATCTATCATTCGTCATGACATCTCCCAAAATCATG
GATACCAAGGCCAATTGACTCAAGCGTTTCCCATTTCCTACCACCAAGCTCCTGTCAG
CCACCATGCCATTCCTATTGCTAGCCACGGAAGTCTTCAATCTGCTGCCATCTCACATCACGCATCTCCTATA
ATTTCCCTTCAACACGTTTCTCCTGTAGTCCAGCACACTTCTCCCATCCACTATAACCAATATGAATCTGGAC
ACGGACATCAAGACTATCATGCTCACCCGAAATACGAGTTCGCGTACAATGTAGCCGA
CCACCACACTGGCGACATCAAGTCCCAGCACGAAGCTCGTGATGGTGACCACGTGTCCGGCTCATACAGCCTT
CACGAGGCTGACGGTACCATCCGCACCGTTCACTACAATGTTGATGGACACAGTGGATTCAATGCTCAAGTAG
AGCGTTCCGGCCACTCTCAACACGTCCAGCCCCAACACCAAGCCCCCCACTACGTACT
CGCACATCATTAAGAATTAAACAATGTTCCTAATAGAAAGTTATCATAATATATGTACTCGTATACCCATCAA
TCAAGCTATTGATGAGTCTAGTCTTTTAATATGTGTTCCAATTTTTGTGTATCGATTAAATATAATATTGT
>1002049330_1 Heli.1-ES587914.1.5
AACAATGGTAGCTAAAATCGTCATCGTCCTCGCTCTGGCCGTAGCCGCCTCAGCTATTCCCTTGGTACCAGTT
GCCAAAATCGCCTACGCCGAACCCGAAGCACCCGCCAACTACGAATTCCAGTACTCCGTACACGATGACCACA
GCGGTGATATAAAGCAGCAACAAGAGGCCCGCGCTGGAGACGCCGTCCACGGCTCTTA
CTCGCTCGTGCAGCCTGATGGAGTCCACCGCATCGTGGACTACAGCGCTGACAAGGTGAACGGATTCAACGCC
ATCGTTCGTTATGAAGGACAACCCATCGTCGCCGCTCCCGCTAAGGTCGCCTACGCACCCGTCGCCAAGCTCG
CCTACGCTGCTCCCGTCGCCAAGGTGGCTTACGCTGCCCCTATTGCCAAGGTTGCCTA
CTCCGCACCTGTAGCCTACGCCGCCCCCGTCGCCAAGCTCGCCTACAGTGCCCCCATCGCCAAGGTTGCCTAT
GCCGCTCCCCTTGCCCACGTTAGCTACTCATCCCCCATCATCTCTTACCAACACTAAGTCAAACTGTTGTTAT
TGTTATACTAATTTATTATCCTATGAATAGTTGCAAATAAATAATT
>1002049331_1 Heli.1-ES588189.1.5

TACCACCGCCTGCAAATCCTGCCCTTAAAAAGGATAAGAAGGAGAAGAAGGTTCCCAAAGACCATTCCAAAAA
TGTTATGCGGAATCTGCACATCAGGAAATTGTGCCTGAACATCTGTGTCGGTGAATCTGGTGACAGATTGACA
CGTGCTGCTAAGGTGTTGGAACAGCTTACTGGTCAACAGCCCGTGTTCTCCAAGGCTC
GTTACACTGTCAGATCCTTTGGTATCCGTCGTAATGAAAAAATTGCTGTCCACTGCACTGTTCGTGGAGCTAA
AGCTGAGGAAATTCTTGAAAGGGGATTAAAAGTAAGGGAATATGAATTGAGACGTGACAACTTCTCTGCAACT
GGAAACTTTGGTTTTGGTATCCAGGAACACATTGATTTGGGTATCAAGTATGATCCTT
CCATCGGTATCTATGGTCTTGACTTCTACGTCGTACTTGGAAGGCCAGGATTCAATGTAGCACACAGACGACG
CAAGACTGGCAAAGTTGGCTTCCAACATCGTTTGACGAAGGAAGACGCCATGAAGTGGTTCCAACAGAAATAC
GATGGTATCATCCTTAACAGCAAAAAGTAAATTAAGTAATAAAAAG
>1002049332_1 Heli.1-DT664959.3.5
CGTCTGATAGGCGTCGACTTACTTCTTCTGGCCAGTTTTTTAATTTCTGGCTCAATATCGGCCGTTTTTCGTG
TTAGTGGTGTTATAAAATAACTATCAGCCATGGCGCTCAGCGACGCAGATGTTCAAAAGCAGATCAAGCACAT
GATGGCCTTCATCGAACAAGAGGCCAATGAAAAAGCCGAAGAAATCGATGCAAAGGCC
GAAGAGGAGTTCAATATTGAAAAGGGGCGCCTTGTCCAACAGCAGCGCCTGAAGATTATGGAATATTATGAAA
AGAAAGAAAAACAAGTTGAACTCCAAAAAAAGATCCAATCTTCGAACATGCTGAACCAAGCCCGTCTCAAGGT
ACTCAAAGTGCGTGAAGACCACGTGCGTACTGTACTCGACGAGGCCCGCCAGCGCCTG
GCTGAAGTGCCCGGAGACGTCAAGTTATACTCTGATCTGCTTGTGACCCTCATTGTGCAGGCTCTCTTCCAGC
TGGTAGAGCCCGCAGTAACACTCCGCGTGCGACAGGCCGACAAGAGCTTAGTAGAGTCTCTGCTTGGCCGAGC
CCAGAACGATTACAAGGCCAAGATTAAGAAGGATGTTGTGCTGAGCATTGACCCAGAC
AACTATCTAGCCGCTGACACTTGCGGTGGTATTGAGCTGATTGCAGCTAAGGGCCGCATTAAGATCTGCAATA
CATTGGAGTCCCGTCTGGAGCTGATCGCCCAGCAGCTGCTGCCGGAGATACGTACCGCTCTGTTCGGACGCAA
CCCTAACCGTAAATTCACCGATTAAACAACATACGGTCATTCATATTATTAAAACATT
TATTTAAATTTT
>1002049333_1 Heli.1-ES587811.1.5
ACTCGTTAAACGTGTGTATAACCTACGGCCTCTAAGAATCTCTGATCACCATGGCTGAACAGCGTGAAAGAAC
TTTCATCATGATCAAACCTGACGGCGTGCAACGTGGTCTGGTCGGCGAAATCATCCAAAGGTTCGAGAAGAAA
GGCTTCAAATTAGTAGCTCTCAAATTCGTGTGGCCATCTGAAGAACTTCTGCAAAACC
ACTACAGCGATCTCGCTTCACGCCCCTTCTTCCCTGGCCTAGTGAAATACATGAGTTCCGGCCCCGTTGTACC
CATGGTATGGGAAGGGCTCAACTCAGTGAAAACCGGCCGTCAAATGCTTGGCGCTACCAACCCCGCTGACTCC
CAACCCGGCACAATCCGTGGTGACCTTTGCATTCAAGTCGGCCGCAACATCATCCATG
GCTCTGACAGTGTAGAATCCGCTAAAAAAGAAATTGATCTCTGGTTTTCCGAAAAAGAAGTCGTTGGATGGTC
CCCTGCTAACGAAAACTGGGTTTATGAATAAACTATAATTCCATTCTTTTTTTAAGTTATACAATACATAATG
AAATAAAGCT
>1002049334_1 Heli.1-DN172712.1.5
ATAGTGTCCGGAATGGCAGACGCTGCGCCATCGACAGCGCCCAAGGCGAAGGCGCCTAAAAGCAAGGCACCTA
AGGAAAAGGCTGCGAAAGCGGCCCCAGCGGCACCCGCGGAGCCACCGAAGGCTGTCCGCAAAGTTTCATCCAA
GCCTCGTCACGGAAGATTGTACGCTAAGGCCGTATTCACAGGATACAGACGTGGTTTA
CGCAATCAGCACGAAAACACTGCTCTTTTGAAGATTGAAGGAGCTAAAGGTCGCGACGACGCCATCTTCTACG
CTGGCAAGAAATGTGTATATGTATACAGAGCTAAGAAGAGGACACCCATTCCCGGAGGTCCCCGTGGCAAGAA
GACCAAGTTGAGGGCCATCTGGGGCAAGGTAACCCGCCCCCACGGTAACAACGGCAGT
GTGCGTGCCAGGTTCAAGTCTAACTTGCCTGCTCAAGCAATGGGACACAGGATACGAGTGATGTTGTATCCAT
CCAGGATCTAAGAACCTTTGTAATATAAGACTAAAATAAAACAAAAG
>1002049335_1 Heli.1-ES584628.1.5
CATACAACCAACATGAAAGCTTTTATTATAATCTGCGCCCTTGTGGCTTGCGTTGCCGCTGAGAACGAAAAAC
GTGAAAAGCGTGGTTTCCTTAGCGGCGGCCACTCAGTTGGACTTGGAGGTTACGTCGGATTTGGGGCACATGG
ATACAGCGGGAGCTATGGTGGATACGGCGGACATGGCGGATACGGAGGCGGATATGGC
GGATATGGCTATTCAGCACCTGCCAAGGTCATTTCAGTAAACAAAGTAGTGAATGCTCACAAAATTGTTGAAG
TTCCCCAAATTGTGAAAGTGCGCAAAGTTGTCTCTGTGCCACAAGTGGTTTCGGTGAAGAAGATCGTGTCTGG
GCCTAGCTACGGTGGTGGATATGGGTATGGCGGAGGATACGGATACGGCTCTGGATAT
AGTTCAGATTATGGTTATGGCTTAGGCCATGGCTCGAGCTACGACTTAGGCCATGGTTCCAGCTATGGCTCAG
GATACTCTAGCGGTTGGTGGTAAAAGTGTTTATCTAGTCATTTTAATTAGTGTGTTCTGTTTTCATATTAGTG
AATTTATCTTAAGTCGTTTTTAATATGTATGTATTTGTATATAAATATATAGAAGATG
TAGGTATATAATGTCAAAATAAATATATGTATGCCG
>1002049336_1 Heli.1-DT662631.3.5

GAGAATCTTCGAGAAGTATAAGTTCAGAGCGATCTGAATATTCTGTGAAGTCCACAAATTCATTCCATTTAAC
TTAAAAATGTTGCGTTTGCCTCGTGTCGTCCGCCAAACCGTTTCCTTACAAAAATCTCTTCAATTTTCTCGAT
TTTATGCCAAAGATGTGAGGTTCGGTGCTGATGTAAGAGCCCTGATGCTCCAGGGTGT
GGACGTGCTGGCCGACGCCGTTGCCGTCACAATGGGTCCAAAAGGTAGGAATGTTATTTTAGAACAATCTTGG
GGCTCACCGAAAATAACCAAAGATGGTGTAACAGTTGCGAAAGGTGTAGAGCTAAAAGATAAATTCCAGAATA
TCGGTGCCAAACTTGTACAAAACGTAGCTAATAACACAAACGAAGAAGCCGGTGATGG
CACCACAACAGCCACAGTGCTTGCCAGAGCGATCGCCAAAGAAGGTTTCGAGAAAATTTCAAAAGGTGCAAAT
CCTATTGAAATTCGTCGCGGCGTTATGCTAGCTGTAGATGCCGTGAAAGAGAAGTTGAAGAACATGTCGAAGC
CTGTCACAACCCCTGAGGAGATCGCCCAGGTCGCCACTATCTCAGCAAACGGTGACAC
CGCCATCGGCAAACTCATCGCCGATGCCATGAAGAAGGTAGGGCGCGACGGCGTCATCACAGTAAAAGATGGT
AAAACACTTAACGATGAATTAGAAATTATCGAAGGTATGAAATTCGACAGAGGTTATATTTCGCCATATTTCA
TCAACTCGTCAAAGGGTGCAAAGGTCGAATTCCAAGATGCCCTTGT
>1002049337_1 Heli.1-DT665352.3.5
GCACGAGGTATGGCTCCACCTCAGCCCAAGGCGGTGGCCAAGGCCCCCGCAGCCCCAGCGGGCGGAGTTAAGA
AACCAGCGAAGGTTGGCAAACCAAGGAATTATGACTTAGGAAATGGAATCGTCAGATTCTCCAAGGCTAAAAT
GTTCCACAAGAAGGCTAAGTACAAGTTCATTGGCAAGAAACACCCAAAAGCACCTAAG
CCCAAGAAGCCCTCAGTTGTAGTAAAGCCTATTGGTGGTGAGAAGAATGGAGGAACCCGTAAAGTACTCTTGC
GTCGCAGGAAATCTTTCTACCCCACTCAGGACAAAATCCGCAAAATCCCCCACCACAAGACGTTCAGTAAACA
CGCGAGAAACATTCGCCCCAGCCTCACAATTGGTACAGTGTGCATCCTCCTGGCGGGC
AGACACGCGGGCAAGCGCGTCATCCTGGTGGGAGTACTGCCGAGCGGCTTGCTGCTTGTTACTGGGCCTTTTG
CTTTCAACTCATGCCCACTCCGTCGTATTCCTCAACAATATGTGATCGGTACCAGCACTAAAGTGGATCTCGG
AGACTTCAAGTTACCGGCCCACTTAGACGATGCCTACTTCAAGAAGAACAAGAAGAGC
GTCAAACGTAGTGTCAAGCGTAAAGAGGGAGAAGATATCTTCGCTTCAAAGAAAGAGAAATACGTTCCATCAG
AACAACGCAAGTCGGACCAGAAGTCAGTAGATGAGGCGGTCATCAAGGCGATCGGCAAGCGAGCTGACAAGAA
GATCCTCCGCGGATACCTCAAGTCTGTGTTCGGTCTGCGATCCAGCCAGTTCGCGCAC
CGACTTGTATTTTAGATACATAATATAATAAATAAC
>1002049338_1 Heli.1-CO729595.1.5
ACGGGCTTTAGTAATAAGGCCATAGTAATCGATGGCCGCGGCCACCTTTTGGGCCGTCTGGCTGCAGTGATTG
CAAAAGTACTTCTTGAGGGAAACAAAGTTGTTGTTGTAAGATGTGAACAACTCAACATCTCAGGAAACTTCTT
CAGGAACAAACTGAAGTTTATGTCATTCTTGCGCAAACGCTGCAATGTCAACCCAGCC
CGTGGTCCATTCCACTTCAGAGCACCATCAAAAGTTTTGTGGAAAACTGTCAGAGGCATGATCCCACACAAGA
CTGAAAGGGGAAAGGACGCCTTAAGAAGGCTCCGTGCCTATGATGGTTGTCCACCTCCATATGACAACCGTCG
TCGTGTCGTTGTACCTGCAGCACTCCGTGTTTTCTGCCTGAAGCCTGGACGTAAGTAC
TGTCACGTTGGCCGCCTTTCCCACGAGGTTGGATGGAAGTACCGTGACGTTGTCCGCAAACTGGAGGACAAGA
GGAAGTTCAAGACTATCCATAAAGTTTCCTATGAAAAGAAACTCAAGAAAATCACTAAGGAAGCCGGTGAGAA
AGTAGCGAAGGCAACAGCTCCCTTCAACGCTGTCATGCAATCCTATGGATACAATTAG
GACTAAGGAAATTATATAAAAAATAAACCTTTGGTTGCCCAAAAAAAA
>1002049339_1 Heli.1-DT665985.3.5
CGGCACGAGGCACTACAGTTGTTGGAGTTTGTAATCATGAAGATTGTGGTTTTGGTTGTTTTCTCCTTTATCC
TTGCGGTTAAAACACAAGATGCTTTGGAAGTGAACCCCGAACCTTCACTATTCGGCGACGAATTTGCAGCAAG
CAACCTCTACGACACTATCTTCTCAAAATATTTCGATGAAAAACCACAGAATAACAGG
CAAAATAATAGAAAAGTCCCAAAAATCAAAAATTCTGGACACCAGAGTCAATCGTATAAGGCTAAAGAACCTA
TAAAGATTGAGAAAATTCCTGAGTATATAAAATATAAGAAAAATCCTGAATATAAGGTTGAGTATGAGAAGTT
GGTAGGTCGTAAAGGCCTAGGCTTCGATAAGAGAACCGCCACTACTTACGGTATGAGG
GCACCCATTACCGCGAAGTCACTAGAGGTTGTCGGAAGGAAGTATTACGAAAAGAAAGAAAATAAAGATCTCA
TCATTTTCTAAAAAAATAACTTTTTTTGTTTGAATTTAATAAAGAATTTTCATATTTTGCTCTTTCTTTTCGA
TAACAATGAATACAGTATTTTTAATTTGTAATTAAATTATAACTATAATGTGCTATTA
TAAACACGACGGTTGTGGGTTCGATTCGCACTCGGGCGTTTTTTTGTTTCTTCGCTTTACTAGAAAAGACTCA
GCGTTGAGTCTCAATTTTTAGGAAATTTAGGTAGCGCATAGAAGAAGAGGAGTATTTCTAGTTTCCTTCTGTT
TACTCTATGCTATGCGGGAAAAATCGTGTATGGA
>1002049340_1 Heli.1-DT662779.3.5
GCACGAGGGTCTTTTGAGTTTTCTCCGCTCCTTTGCAATCATGGCGGTGAACAACATTTCCAAAAAGCGAAAA
TTCGTCGGTGACGGTGTTTTCAAAGCTGAATTGAACGAGTTCCTTACTCGGGAGCTTGCTGAGGATGGTTACT
CCGGCGTGGAGGTGCGTGTTACGCCAACCCGTTCAGAGATCATCATCATGGCGACGAG

GACACAGAGCGTGTTGGGAGAGAAGGGCCGCAGGATTCGTGAATTGACCTCTGTAGTTCAAAAGAGGTTCAAC
ATCCCTGAACAGAGTGTTGAACTTTACGCTGAGAAGGTTGCCACGCGAGGATTGTGTGCTATCGCTCAGGCTG
AATCTTTAAGATACAAGCTCATTGGAGGGCTGGCTGTGCGTCGTGCCTGCTATGGTGT
ACTCAGATTCATCATGGAATCTGGAGCCAGGGGATGTGAGGTTGTAGTGTCTGGTAAACTGAGAGGGCAGAGA
GCCAAGTCCATGAAGTTTGTTGATGGTCTCATGATCCACTCTGGAGACCCATGTAATGACTATGTTAACACTG
CTACACGCCATGTACTGCTAAGGCAGGGAGTGCTTGGTATCAAGGTGAAAATCATGTT
GCCATGGGACCAACAAGGCAAGAACGGCCCCAAGAAGCCACAACCGGACCACATCCTGGTGACGGAGCCCAAG
GACGAGCCCGTGCCCTCAGAGCCCACGTCGGACGTAAGGCCCGCGCCCGCGCCCCTTCCGCCGCCCGTCGCAG
CCACTGCTTAGACTGTACTATTATAAACATTGAAAATAAAAAAAAA
>1002049341_1 Heli.1-DT664799.3.5
TTACTCCGCCATTCCCCGATCGTCTTTTCCATCTTTCTTGTCCCCATATAGAATAAGACATTCAAAATGGCGG
CAAAAGCCAATTTGAAGATGATCGTCAATGAGGAGAATGAGGAGAGGTTTGGGTTCGTGTTTGCCGTATCTGG
ACCCGTCGTAACGGCGGAGAAGATGTCCGGATCGGCTATGTACGAGTTGGTGCGCGTC
GGGTACAACGAGCTGGTCGGAGAGATCATTCGACTCGAGGGTGACATGGCAACTATTCAGGTATACGAGGAAA
CATCAGGTGTAACAGTCGGAGATCCCGTACTCCGTACCGGCAAGCCTCTCTCAGTGGAACTTGGACCTGGTAT
CCTGGGCTCCATCTTCGACGGCATCCAGCGACCCCTGAAGGACATCAACGAGCTGACT
CAGTCCATTTATATACCTAAGGGTATCAATGTGCCGTGCTTGGCTAGAGAAACTGATTGGGAATTCAATCCAC
TCAATGTTAAGGTTGGATCACACATAACCGGAGGTGACTTATACGGTATAGTCCATGAGAACACCCTCGTCAA
ACACAAGATGCTGATGCCACCCCGTGCCAAGGGTACAGTCACTTACATTGCACCTTCA
GGAAACTATAAAGTTACTGATATAGTGCTAGAAACTGAATTCGATGGTGAAAAGGCATCATACAGTATGTTGC
AAGTATGGCCAGTACGTCAACCCCGTCCCGTTACAGAGAAGCTCCCAGCCAACCATCCACTATTAACTGGACA
ACGTGTCCTGGACTCCCTATTCCCGTGTGTCCAGGGAGGAACAACAGCCATCCCTGGG
>1002049342_1 Heli.1-DT663271.3.5
GGTTTTTCGGCGACTGTAACACGTGTTCTGTTGCCAAAATGGCAGTAGCAAAGAAACCCGTAGCCAAAAAAGG
CCAGTTACATCAAAAGTCTGGTAAAAAAGGAGTCAAAGGTGGCAAAATCCGCGGCAAAGGCCAAAGAAGGAAG
ATCAGCCTCAAGTTCGCCATTGACTGCACACACCCCGCTGAAGATAGTATTTTGGAAG
TAGGAAACTTCGAAAAATACTTAAAAGAACGTGTCAAAGTTGAGGGCAAAACAAATAACCTAGGCAATCACGT
TGTCATCGCTAGAGACAAGACTAAGATCATCATCAGCGCAGACATTCCTTTCTCCAAGAGGTACCTGAAATAC
TTGACAAAGAGGTATCTTAAGAAGAACAATTTGCGTGACTGGCTCCGAGTGGTGGCTT
CTGCCCACGACTCCTACGAGTTACGCTACTTCAACATCAATGCTGAAAGCGACAATGAGGACAATGAAGATTA
ACCTGTTGCTATAATAAAATAGT
>1002049343_1 Heli.1-EE743450.1.5
GCTCCACCTCAGCCCAAGGCGGTGGCCAAGGCCCCCGCAGCCCCAGCGGGCGGAGTTAAGAAGCCAGCAAAGG
TTGGCAAACCGAGGAATTATGACTTAGGAAATGGAATCGTCAGATTCTCCAAGGCTAAAATGTTCCACAAAAA
GGCTAAGTACAAGTTCATTGGCAAGAAACACCCAAAAGCACCTAAGCCCAAGAAGGCC
TCAGTTGTAGTAAAACCCATTGGTGGTGAGAAGAATGGAGGAACCCGTAAAGTACTCTTGCGTCGCAGGAAAT
CTTTCTACCCCACTCAAGATAAGATCCGCAAAATCGCCCACCACAAGACTTTCAGTAAACATGCGAGAAACAT
TCGTCCAAGCCTCACAGTTGGTACAGTGTGCATCCTCCTGGCTGGCAGACACGCTGGC
AAGCGGGTCATCCTTGTGGGAGTATTGCCAAGCGGTTTACTGCTTGTTACTGGGCCTTTTGCTTTCAACTCAT
GCCCACTGCGTCGTATTCCCCAACAATATGTGATCGGTACCAGCACTAAGGTGGACCTCGGAGACTTCAAGTT
ACCGGCGCACTTGGATGATGCCTACTTCAAAAAGAACAAGAAGAGCGTCAAGCGTAGT
GTCAAGCGTAAAGAGGGAGAAGATATTTTTGCTTCCAAGAAAGAGAAATACGTTCCATCAGAACAACGCAAGT
CTGACCAGAAGTCAGTGGATGAGGCAGTCATCAAGGCAATCGGCAAACGAGATGACAAGAAGATCCTCCGTGG
ATACCTCAAGTCTGTGTTCGGTCTTCGGTCTAGCCAATTCGCTCACCGACTTGTATTT
TAGATACATAATAAATAACAAAAA
>1002049344_1 Heli.1-DT664014.3.5
CTTCTGCTTGCGGCAGTGACACACGCCGCTAAGCCGGGAAAGAAACACAGTGAAAGTGAGAAGACGGAGAAAC
GAGAGCTGGTTGAAGGGAACAGTGGAGTTGAGAAGAGAGCTCCACTTCTATCTACTGTATCACCGGCAATATC
CACTGGAGGAGTGGAATACGCAAGCACAAAAGAAAATTTATCTGGAAGCTCTCCTGCA
CAGCAAATCTACGCTACACCAGTGCCTCAAATCGCTAAGATATCAGATGTACTCTCTGGGCAAGGTCCTCAAT
TCCAAGCTGCGATTGACAGCCATCTCTACTCACCAATCTCTGTTTACCAACCCAGGCTTGGGTCTCCAACTAC
ATATGAAGTATCAGCACCAGTACCATCTCAATTAGCCTACTCTGAGCATAGACTAGCT

ATACCCTCACCAAATGCCATACAATATGATGCCAAACAACAGAATTTGGGAAAACCAATAGTAAATTTCGAAC
AACCTCAGATATTATTCCGACCAAGCCACGAACAAAATTTGGGACCAGTATTTCCACAATACTCCAACAACAT
ACAAGCAATTCCTCAACCAGTTCAATATAGTCAGCAACAAGTATATCCTGCTCAACAG
TTTCAATACATCCAACCATCTCAAAGTCCCCAAGGAATTTCATATCAACAACCATCATCAATAATTAAACAAA
ACGAGCCGAGGGACATCAAGAGCAAATCTGTCCACTCTCAAGATTTGATAAAGCAAATA
>1002049345_1 Heli.1-DT666489.3.5
GCACGAGGCTGAGTTCAACGCGCGGACGTCACGATACATCGACAACATGAGGATTAGTGCACATTTCGCTATT
TTGACCGTTTGCCTTGCCTATGCGCAAGCAGGCCTTCTCCTTCGAAACGCGCCAGCGTGCCCAGAACAGTATG
GAGTTCAGGCTTACGCGCACCCCGAACTCTGCGATCAGTTCTTTTTGTGCACAAATGG
CACCCTCACCGTAGAAACATGTGAAAATGGCCTTCTATTCGACGGCAAGGGCGCCGTGCACAACCACTGCAAC
TACAACTGGGCTGTGGACTGCGGGGAGAGGAAAGCTGATTTGACACCATACTCTACGCCTGGATGTGAATATC
AGTTCGGTATCTACCCTGACAGCGCTGAGTGCTCTACCAGCTACATCAAGTGTGCTTT
CGGAGTGCCACACCAGGAGCCCTGCACACCCGGCCTCGTGTACGACGAGCGTATCCATGGATGCAACTGGCCT
GATTTATTACAACCTTTCTGTAATCCTGAAGCCGTCGTCGGTTTCAAATGCCCAACAAAAGTGCCAGCCCACA
CTCAATCAGCCAAATTCTGGCCTTACCCCCGTTTCCCTGTGCCCGGTGACTGCCACCA
CCTGATCACCTGCGTGGAAGGTAACCCTCGTCTCATCGCCTGCGGTGAAGGCAAAGTCTTCGATGACCAAAAC
CTGACTTGTGAAGACCCTGAACTGGTACCTCATTGCGGACACGCGTAATTTTAAGTCTATCGGCATTATAA
>1002049346_1 Heli.1-EL598034.1.5
ATTTTGACATCCAGCGAATAGTAGGGGACTTCAGTGGATTGAAACGTTTTTACTACATATTACATATATTTGC
TTCAAAAAAAACGAAAATAAAAAATACAGCAAAATGGGGAATATGTTTGCAAATTTATTTAAAGGCCTATTTG
GCAAAAAAGAAATGAGAATATTGATGGTGGGTCTTGATGCAGCTGGTAAAACCACAAT
CTTATACAAACTTAAATTAGGAGAAATCGTAACTACAATTCCAACAATTGGATTTAACGTGGAAACTGTAGAA
TACAAAAACATCAGTTTCACTGTTTGGGATGTAGGTGGTCAAGACAAAATCAGACCACTGTGGAGGCATTATT
TCCAGAACACACAGGGTCTCATCTTTGTAGTAGACAGTAATGATCGGGAACGTATCGG
TGAGGCGCGTGAGGAGCTCATGAGAATGTTAAGTGAAGATGAGTTACGGGATGCAGTACTTTTGATCTTTGCA
AACAAACAGGATCTGCCAAACGCCATGAACGCCGCGGAGATAACGGACAAGCTGGGCCTGCACTCGCTGCGCA
ACCGCAACTGGTACATCCAGGCGACGTGCGCCACCTCCGGCGACGGCCTCTACGAGGG
GCTGGACTGGCTCTCCAACCAGCTCAAGAACGCCAACCGCTAACCTGTGCCCCCACAGACTTATCTGATTACT
GAAAGAGACTATGGTTTAAGATATGTTCACTTTATTTGCTATAAATACAATGTATGTGCGAAATTTATGTACT
ACCGTCACCCGTCGACCGAGCT
>1002049347_1 Heli.1-ES586155.1.5
CACGGAACGTTCCTGTGCAGTTCAGTTTTAAATCCAAAGTAATCTCTTTGGATTTTGTTTAATATCTTAAATA
GATATTAGATTTATTCAGAACAATGCCTCCTAAGTTCGATCCTAACGAAATTAAAATCGTTAACTTGAGATGT
GTTGGAGGAGAAGTTGGTGCTACGTCATCATTGGCTCCTAAAATTGGTCCCTTGGGTC
TGTCCCCCAAGAAAGTTGGTGATGATATAGCTAAAGCTACTGGAGACTGGAAAGGACTGAAGATCACAGTGCA
GCTGATTGTTCAAAACAGACAGGCGCAGATATCTGTAGTGCCCTCTGCTGCAGCACTTATCATTAGAGCTTTG
AAAGAGCCTCCTCGTGACCGTAAGAAGCAGAAAAATATTAAACATAATGGTAACATCA
CATTGGAAGATGTTATTGGCATTGCTAAAGTTATGAGGCCAAGATCAATGGCAAGGTACCTCTCAGGAACAGT
TAAAGAGATCCTTGGTACAGCCCAATCTGTAGGTTGTACTATAGATGGAAGACCACCTCATGACCTTATTGCA
GACATCAACAGTGGTGAATTAACAATTGATGAATAAATTTCATAAG
>1002049348_1 Heli.1-DN172646.1.5
GTCGTGGTTATGCCGCATGGTTTTTAGTGAAAAATTAAGTGTTTAGACTAAAATTTGAGTGTAAAAAGTGCCA
CATTTCTGTTAAAGATGCGTTCAGTCACACTGAAGGATGTTGAACAAGACAAGGTCGTCAAGACCGTGGCTGC
CCATTTGAAAAAAATGGGCAAAGTGAAGGTTCCAGAACACATGGATCTGGTTAAGACT
GGTCGTTTCAAGGAGCTAGCACCCTACGACCCCGACTGGTTCTATGTACGCTGTGCTGCTGTACTCCGTCACA
TTTACATCAGATCTCCAGTTGGAGTAAAGACTGTCACTAAAATCTTCGGAGGCCGCAAACGCAATGGTGTCAC
CCCTTCACATTTTTGCAGATCATCAGGCAGCATTGCTCGTAAAGCACTTCAGGCACTT
GAAGCTCTTAAGTTAGTAGAAAAGATCCAAGATGGTGGCCGCATCCTCACCACCCAAGGCAGACGTGACTTGG
ACAGAATCGCTGCACAGGTCCGTCTAAAGGCCAAACAAGCCGCCAAACAGAGTGTAATAGTTCTGTAATTTTA
AACTTAATAAAAATATTAAACC
>1002049349_1 Heli.1-DT665802.3.5
GGGGCGGCCATTATTCTACGTATAATATTATAATTTTTTTGTGTTTTGCGCTGTACTATATTCGAAACAAAGA
ACTTTACTATATTCTACAAACTCTTGTTTAGCATTATTGTGATCATAAAACTATTATAGTACAAAATGAATCC
AGAATACGATTACCTCTTTAAGCTGCTGTTGATTGGTGACTCTGGTGTGGGGAAATCA

TGCCTTCTACTCAGATTTGCTGATGATACATACACAGAGAGTTATATCAGCACTATCGGAGTGGACTTTAAAA
TTAGGACTTTAGATTTAGATGGCAAAACAATAAAATTACAAATATGGGACACAGCGGGACAAGAGAGATTTAG
AACGATAACATCATCTTACTACAGAGGCGCACATGGAATCATTATTGTTTATGATTGC
ACAGATCAGGATTCATTCAGTAATGTCAAACAATGGCTGGAGGAGATAGATCGTTACGCATGTGACAATGTCA
ACAAGCTGCTAGTGGGCAACAAGTGTGATCTTACCACAAAGAAAGTTGTTGACTACACCACTGCTAAGCAATA
CGCGGAACAGCTGGCCATACCGTTCCTGGAGACATCAGCGAAGAACTCCACGAACGTG
GAGCAAGCGTTCATGACGATGGCGGCGGAGATCAAGGCGCGGGTGGGCCCGCCGTCCGCGGGCGCCGCGCCCG
CCGGCGCCGCCGTCAAGATCGACCAGGGCCGCCCCATCGACACCGGCAAGTCGTCGTGCTGCTGAATGTGTTA
TACGGGTGGCGGCGTGCAGCAGTCGTGGCGGGAACCCGCACCCAGCTGTTAAAATGCA
ATTACCACAAATTCATGATCGCATCCTTATTATAATATAAATAAATAAATAAATGATATATAGTGCCGAGTGG
CGAATGTTAAGCTAACTCCCTTAAGTTGCTATCGTCGAGGTGAAATGGATGGATGTATGTTTATACGTGTT
>1002049350_1 Heli.1-ES584776.1.5
ATGTCTAAGAGAGGACGTGGTGGTTCCGCGGGAGCGAAATTCCGCATCTCACTGGGTCTCCCGGTGGGAGCCG
TAATAAACTGCGCAGATAACACAGGGGCAAAGAATCTGTATGTGATCGCCGTGCAAGGTATCAAGGGTCGCCT
GAACAGACTGCCAGCCGCTGGTTCAGGGGACATGATTGTCGCCACTGTGAAGAAGGGT
AAACCAGAACTTAGGAAAAAGGTAATGCCCGCAGTGGTGATACGGCAGCGGAAACCGTTCAGAAGGCGTGACG
GGGTGTTCATATACTTTGAGGATAATGCAGGCGTGATAGTAAACAACAAAGGTGAAATGAAAGGCTCGGCTAT
CACAGGACCAGTGGCTAAGGAGTGTGCGGATCTGTGGCCCCGTATTGCATCCAATGCT
AGTTCTATAGCTTGAAGTTAATATATGAATAAGGAT
>1002049351_1 Heli.1-ES587044.1.5
ACCGTAAACAGTGCACGGGCGCGCTCGGTTTTTTTATCTTTTTAACATAATATTTTTTATAAATAAATTGTGT
GAAGATGAAGTATCTGATTGTACTCGCCGTCCTATCTTTTGCCTTCGCGGCGGAAGAGGCAAAGGAGGAGTCG
AAGACTTTCAGCCGGTTGATCCCCGCTGATGTTTTAAGAGATTTCCCTGGCCTCTGCT
TCGCGTCAACCCGTTGCGCCACAGTAGAAGTTGGTAACACCTGGGACCTCGCCCCATTCTGTGGCCGCAGCAC
TTGCGTTCTCAGCCAGGATGAGCCTAAGAGGCTTCTTGAGCTGGTCGAAGACTGCGGACCTCTGCCCGTGCCT
AATCCTAAGTGCAAACTCGACTCTGAAAAGACCAACAAGACCGCTCCTTTCCCTGGAT
GCTGCCCAATCTTTACCTGCGAGGACGGCGTTGTCTTAGAATACCCAAAACTCCCAGTAGCCCCCGAAGAGGA
GAACAAAGAAGAAGCTAAAGCCTAAATTTCCAAACAACAATTGAAAACCGACTAAAATTCTAAACCAAATATT
TCATACATTCGGACTATTCCAAAATCTTGATATATTTTTATATTACTCTATACATGAT
TATGTATGACTATTTTAATGGTATTAATAGGATATTTAAATTATTACTTTCGTCGGTTACTTTTTTTTTAGAT
ATCAATTCGACATTACTTTCTAAGTATTTGTTAACAATTACCTATTGTTTGCTTCCATTTCCGTTTTACACAA
AGCCGTAACAGAATAGTAGACCTTTGTCTATTTAATAGGTCATTGTTCGCTTTTTTTA
>1002049352_1 Heli.1-ES585900.1.5
TTTTGCTTCTGACATTTGCGTGATTCGCGTGTTTTACTAGTGTACAAAATGCAGAACGACGCCGGTGAATTTG
TTGACTTGTACTGCCCGAGAAAATGCTCGGCCAGCAACCGCCTCATCCATGCTAAGGACCATGCTTCCGTGCA
GCTGGTGATTGCTGATGTGGACCCTGCCACTGGCCGTGCGGCTGACTCCAGCAAGATG
TACGTGGTCTGCGGAGCCATCAGACGCATGGGAGAGTCCGACGACTGCATTGTTAGACTCACTAAGAAAGATG
GAATATTGACTAAGAACTACTGATGTAGTGGTGTAAGAAGCTTATTTTATAAGAAAAACAATAAAAAAAATTA
AAAACTGAAA
>1002049353_1 Heli.1-DT664911.3.5
GCACGAGGGCGCGGTGCACGTCGTCTGGACTTGTAGTTGTAAAGCCTTATCAAAAACTCTACTAAGCTACGCT
CGTAGCCATGAGCAGAACTTAGAAGTATATATCAAGCGTATATTTATATTAAAGTGATATTTTAAAGTGATAT
TTTTATAGCAATAAGTAGAATATAGTGCAAAACAAATCAATATGTACAATATTTTATT
TTATTTTTCCATTTTATTATCCTACGGATATGCCGAAGTATTAAATTTAGCTGGAGGTGCCAAAAGCAATTAC
ACATTTCCTAAAGGGTTCCTTTTCGGCGTTTCTACTGCAGCTATTCAAATCGAAGGAGCTTGGAATGTGGATG
GAAAATCGGAACATATATGGGATCATTTAGTTCACACGAATCCAAAGTTTACCAAAGA
TGGGTCTACTCCAGAAGTGGCAGCGGATTCTTATCATCAATACAAAAGAGACGCTGAAATGGTCCATCAACTC
GGTGTTGATATTTACAGATTCTCCATGTCCTGGGCTAGAATATTACCAAACGGTTTAGCTAACAAAGTGAATC
CTCTCGGCATTCAGTATTATAAAAATTTAATAAGTGAATTGGAGAGATATAACATTAC
ACCAATGGTCACCATTTATCATTGGGATCTGCCGCAAAAGTTACAAGATATAGGTGGCTGGACGAACGCCCAC
ATTGTGGACTATTACACGGATTACGCGAACGTTCTGTTCGAGAACTTTGCAAATGAAGTAAAGTATTGGGTGA
CATTCAATGAACCGATGCAAACCTGCTTGGAGGGCTACGGGGGCACCTACCGGGCGCC

GGCCCTCAATCGGCATGGTGTTGCTGAATATTTATGCACCCATAATTTACTGAAGGCGCACGCGAGCGTCTAC
CATTTGTTTGCTCAACAGTATCGTCCTATTTATGGAGGTAAAATCGGTATGTCATTAGATTCTAATTGGGCTG
AGCCAAAAACTAATTCTTCGAAGGACCGAGAGGCCGCTGAACGCTACCTACAAACACA
TGTCGGTTGGTATGCACATCCAGTGTATTCTGAGGAAGGAAACTACCCCCCAGAGCTCATTAGGCTTGTAGAT
GAAAAAAGTCGTCTGCAAAACTATACACGCTCACGTTTACCTAAGTTTACTCCGGAAGAAGTTGCGTATATAC
GAGGTACAGCGGATTTCTTCGGCTTAAATCATTATACGACATATCTTCTGAGTATAGC
TGATGGAGAAGT
>1002049354_1 Heli.1-EL601479.1.5
AACATGAAAGCGTTCTTGGTATTAGCAGCCGTTGCGGCGATCGGCTCGTCGGCGAGGCCCGCGGGATACTCCT
ACAATGCGCCCCCCGGTGGCGGTGGTCTCGGTGGTGGGGGCGGCATCATCGGAGGCGGCATCGGAGGCGGCAT
TGGAGGCGGCATCGGTGGCGGCGGTGGCTTCTCCGGCGGTGGCTTCTCCTCCGGTGGC
CTGAGTTCCAGCAGCTTCGGCATCGGTGGCGGATTCGGCGGAGGTATCGGTGGAGGTGTCGGCGGAGGTATCG
GTGGAGGATTCTCCTCCGGCGGAGGTTTCGGCGCCGGAGGCGGCGGCGGCTTCGGAGGTGGCTTCGGAGGAGC
CCCCATAGTCCAGAAACACATCTACGTCCATGTCCCACCCCCAGAACCTGAAGAACAA
CGCCCACAAATCATCTCCGGCGGAGCCATCCCCAGAAACACTACAAGATCATCTTCATCAAGGCCCCAGCAC
CTCCCGCACCTGTTGCCCCCATCATCCCCGCTCAGGCCCAAAACGAAGAGAAGACCCTCGTATACGTGCTGGT
CAAGAAACCCGACGAACAGCCCGACATCACTATTCCCACCGCCGCCCCTACTCAACCC
TCAAAGCCCGAAGTATACTTCATCAAGTACAAGACCCAGAAGGAAGGTGGTTCCATCGGTGGTGGTGCTATCG
GCGGTGGTATTGGCGGTGGTATTGGCGGTGGTATCGGCGGTGGTATCGGCGGTGGACTCGGTGGTTCCGGCAT
TGGTGGCGGTATCTCTGGTGGTAGCATCGGCGGCGGTCTCTCCGGCGGCAGCATTGGT
GGCGGTATCGGAGGTGGCATCGGAGGCGGACACGGCTCCTCGGGCTCCGTCAGCTCCTCGTACGGACCCCCCG
GACACTCCGGCGGTCCATACTAAACCCGCACACCGCGTGCTAACTTGTGATACCTGCTCAGCCGGCGACAAAC
CTAGTATATAAGTAGAGAAAAAATTACGGTGCCTAAATGTATTTATGTAAATAGTAAT
TTTAATGTATAATTTTAGTTGTATTATAAAATCTTTTTTGCATAAAAAACTCGATAAACC
>1002049355_1 Heli.1-ES585426.1.5
AGTTGAAGATCGAATTTGGATTCGGTTGGCGGGCGCGGCCATCTTGGTGTAATAGTGACCTGGCGATTCGGGT
TTAGTTAGTGAAAAATCATTTGTCATCCACGATGTCGGAAAGGGAAGATAATGTGTATAAAGCTAAGTTAGCT
GAGCAGGCCGAGCGCTATGATGAAATGGTGGAGGCAATGAAAAATGTGGCCTCACGAA
ATGTGTCAGATAATGAACTCACAGTTGAAGAGAGGAACCTCCTCTCAGTGGCATACAAGAACGTGATCGGGGC
GCGCCGGGCGTCGTGGAGGATTATCTCTTCAATTGAACAGAAAGAAGAAACAAAAGGCGCGGAGGACAAGCTC
AACATGATCCGCGCGTACCGCAGCCAGGTGGAGAAGGAGCTGCGCGACATATGCTCGG
ACATACTGAGCGTTCTCGACAAATACCTCATACCCAGCTCGCAGACCGGCGAGTCCAAAGTTTTTTATTACAA
AATGAAGGGTGACTACCACCGGTACCTGGCGGAGTTCGCGACGGGCAACGACCGCAAGGAGGCGGCGGAGAAC
TCGCTGGTCGCGTACAAGGCGGCCTCCGACATCGCCATGACGGAGCTGCCACCCACCC
ATCCAATACGCCTCGGCCTCGCGCTTAACTTCTCAGTATTCTACTACGAAATCCTGAACAGCCCGGACCGCGC
GTGCCGGCTGGCGAAGGCGGCGTTCGACGACGCCATCGCCGAGCTGGACACGCTGTCGGAGGAGAGCTACAAG
GACTCCACGCTCATCATGCAGCTGCTGCGCGACAACCTCACGCTGTGGACGTCGGACA
TGCAGGGCGACGGCGAGCCGGGCGAGCCCGAGCCCAAGGAGCCCGCCGCGCCCGACGCCGACGCGGACGACGT
GTCGTAATGCGCCCGCCCGCCCCGCCCACCCGCCGCCGCACAAACACACAGCCCCCTTTACTTGCGAGTATTG
TGATAGAGATTTTTTGTATATT
>1002049356_1 Heli.1-CX700617.1.5
GTTGTCAGATCGGCGATTTTTGTAAAAAGCCGCAACCATGGGTCGTATGCACGCACCTGGTAAGGGTATCTCG
CAGTCGGCGTTACCGTACCGCCGCAGTGTTCCTACATGGTTAAGATTAACCGCAGACGATGTTAAGGAACAAA
TATTCAAGCTCGGCAAAAAGGGTCTTACTCCCTCACAAATTGGCGTCATGCTCAGGGA
TTCACATGGAGTGGCCCAAGTGAGATTTGTCACTGGCAAAAAGATCCTACGTATCATGAAAGCTATGGGCCTT
GCTCCCGATCTACCCGAGGATTTATACTATCTTATCAAGAAGGCAGTAGCCATGAGGAAGCATTTGGAACGTA
ATAGAAAGGACAAGGACAGCAAATTTAGATTGATTCTTGTCGAGTCTAGAATTCACAG
ACTGGCTCGTTACTACAAGACTAAGAGTGTGCTTCCCCCCAACTGGAAATATGAGTCTAGCACCGCCTCTGCT
TTAGTGGCTTAAGTTTAAATAAACTATGTAAAATT
>1002049357_1 Heli.1-DT664480.2.5
GGTCACTTTGTGTCACGATGGGGCGCCGTCCAGCGAGATGCTACCGGTATTGTAAAAACAAACCGTATCCTAA
ATCCCGGTTTTGTCGTGGTGTACCAGATCCTAAGATTCGTATCTTCGACTTGGGAAAGAAGAGGGCTACAGTA
GATGACTTCCCACTATGTGTCCATCTGGTGTCTGACGAATATGAACAGCTTAGCTCTG

AGGCGCTAGAAGCTGGCCGTATTTGCTGCAACAAATATCTCGTAAAGAATTGTGGTAAAGATCAGTTCCACAT
TCGCATGAGGTTGCATCCATTCCATGTTATCCGCATCAATAAAATGTTATCGTGCGCTGGAGCTGATAGGCTC
CAGACTGGAATGCGTGGTGCTTTTGGCAAGCCACAAGGCACCGTGGCCAGAGTGCGTA
TCGGCCAACCCATCATGTCGGTGCGTTCCAGCGACAGGTGGAAGGCGCAAGTGATTGAGGCTTTACGTCGTGC
CAAGTTCAAGTTCCCTGGTCGTCAGAAGATCTACATCTCCAAAAAATGGGGTTTCACCAAGTATGAGCGTGAG
GAGTTTGAGAAGCTGCGTGAGGATGGCCGTCTCGCCAATGACGGTTGCAATGTCCGCT
ACCGTCCTGAACATGGACCTCTTGACACCTGGAGGAAAGTGCAGAATGAAATCTACAGTGTTTAACTGATATC
TAATAAACTGTGAAGTTTTTATC
>1002049358_1 Heli.1-DT668156.1.5
CACGCGTCCGCTCATTGAAAATTGAGGGTTAAGACGTGACAATGGGTCGTGTAATTCGGGCTCAACGTAAAGG
TGCCGGTTCGGTTTTCGTATCACATACAAAGAAGAGGAAAGGAGCACCAAAACTCCGTTCTTTAGATTATGCT
GAACGTCATGGATACATCAAAGGCGTTGTTAAGGATATCATTCACGACCCTGGTCGTG
GAGCTCCTCTAGCTGTGGTCCATTTCCGTGACCCATACAAGTTCAAAACACGCAAGGAGCTCTTTATTGCACC
TGAGGGTCTCTACACAGGGCAGTTTGTATATTGTGGAAAAAAAGCTACCCTAGAAGTTGGAAATGTAATGCCA
GTGGGTGCTATGCCTGAGGGTACTATTGTATGTAACCTAGAAGAAAAGATGGGTGACC
GGGGTCGGCTGGCTCGTGCCTCTGGTAACTTTGCTACTGTCATTGGCCACAACCCTGATGCTAAACGTACAAG
AGTAAAGCTTCCGTCTGGAGCCAAAAAAGTGTTGCCTTCTAGTAATAGAGGAATGGTTGGTATTGTTGCTGGT
GGTGGACGTATTGACAAACCCATCCTTAAGGCTGGTCGTGCATACCACAAATACAAGG
TGAAGCGTAACTGCTGGCCATATGTGCGTGGTGTGTCCATGAACCCCGTTGAGCATCCCCACGGTGGTGGTAA
CCATCAACACATTGGTAAGGCTTCGACTGTCAAGAGAGGAACATCAGCCGGTCGCAAGGTTGGTCTTATTGCT
GCCCGCAGAACTGGAAGAATCCGCGGTGGCAAGACTGAGACTAAGAAGGAGACGTAAA
CATACTTTCAAACAAATAAAAATA
>1002049359_1 Heli.1-DT666773.3.5
GCACGAGGCTAGCGTGACCGTAGGTTGTGTCGCGTTCGTGTCCGGTCCCGTAATGTACAGGTGGTGGTTGGTG
GTGGCGCTGGTGGCGGGCGCGGCGGGTAACCGTCTGCCGCGGACCTCGCCCACGTCTGGCCCTTCGCCCGCGC
CCTCGACTCGTCTGCCGCAACGGAACTTTACTATATCTTCCCGATCCTCCGTCAGAAA
CAGGGAACAATACATTTTTAATGTATTCGAAGTGATCGTTAGCACGCTTGAAACGAAGTTGCAGAGGATTGAA
AACCTTGACAAGGCAGTGGAACATCTCATGAGAAGAGTAGAAGCTTTGGATTCAAGAGTCAATGATAATATTC
ATAAAACCGACGCCATTATATCTAAATTAGGAAATTTAGACTTTAAACTATTTAGCCA
GTTAGGTCAAAGTGAAGAGGATGTTGTAACTGAAAATATCAACAAAAAAAGTGAAAATAAACTTAGTAATGCA
CAATTGCTCGACAAGAAGTTAGAATCTTTAGATCAGAAAGTGTCAGATATAGATTCTAAGCTAGTTGGAATAA
AGACACAAATAGATACAAACTTTCTACCAGTCGATGATATTAATGCGGAAGCAAGTGA
AAAGAAGCCTGTTAATCTAAATGTAATAGAAATTGCAAAAGGGCTAAATTCAGAAGTGGTAAATGAAATCACC
AAAAAAGTTGATCAACTAAGATCATCAATGTCAACAGTCGATCGGAAACTTCAATTTCATATAAATCTTGTAT
CCGAAAATTTAGGAAAAGTCCTTTATATGATGGCAGATGTACATGCTGCTATAGTAGA
GCCCGAATCTGCCTCGATAAATGTTAATAACTTATT
>1002049360_1 Heli.1-ES587935.1.5
GTCGCAACATAAACAATGGCAGCTAAGTTGGTTGTAGTACTCGCCGCCGTGGTCGCTGTGGCCCACTGCTCAG
TGGTGCCAGTGGCGCGAGCAGACGCCGACTACTCAAGCTTCGCGTACGACGTCGCTGATCCCTACAGCGGCGA
CTTCAAGAGCCAAGTGGAGAGCCGCAGCGGTGGCAACGTACAAGGACAGTACTCCCTG
CTCGAGGCCGATGGTTCCAAACGTGTTGTTGACTACGCCGCTGATGATGTGAACGGCTTCAACGCCGTCGTCC
GCAAGGAGCCCGGTGTGGTCGCCCACGTGGCCGCTCCCGCTGTAGTCGCCGCCGCTCCCGCCGCAGTCGCCGC
CGCCCCAGCAGTCATCGCCGCTCGCGCCTACTCCCCTGCCATAGTTGCCTCATCTCCC
GCCGTATACGCCGCGTCTGCCCCCGCAGTCGTCGCAGCCCGCTCTTACGCTCCCGCAGTATATGCTGCATCTG
CCCCAGCTGTCGTTGCTGCCCGCTCGTACGTCGCCCCCTCAGTATACGCCGCGTCTGCCCCAGCCGTAGTCGC
CCCCGCTGTCTACTCTGCTGGTCATTCCGTTGTTGCTGCATCCCCCCTCGCCTACTCC
GCTCCTCTCAAATACTGGTAAACGTTTCATTGAATAGGACGACGGGTAATCTTTCTTGTAAATATGAAATTAA
ATAGATATTTA
>1002049361_1 Heli.1-DT664501.3.5
GATGTTAAATCAGCTATTACATATTACCCAGAGAAGGATGAGCCGAAAGATAGTACAGATGGGCCAAAGCACG
ATAAGCCCACGTACGTGAAAAAACATGAAAAGAATTATATCGATGATAAAACATCAGTTTCTGAGCACTATAC
TCAATACACTGAAGATGACTCTAGTCATTATACAACACTAAAAACATCAGAAGTAACC

AATCTTTCTCAAGGACGTTCTCCTCAACCAAATGGACAGAATGATTATTCTGTTCCTAAAGATGGTAAATCTT
CACGACCTAAATGGGATAAACCTGGAAATAGAGAAGATAATCGTCCAATAAAGAGTGATAAACCAGAAGAAAG
GCCTGAATATAAAACAGAAGAAAAATTGGGTGATAAACCAGAAGAGAAACCTCGTTAT
AGAACTGAGGAGAGGCCGAGTGACAGGCCAGTAGACAAACCAAGTGAGAAGCCAATGTCGGAACCTAATTATC
GCCCCGGAGAGAGACCAAGTGATAAGACTGGGGAAAGGCCTTGGGACACAGCAAATATTAGGCCAAATTTATC
AGAACGACGTCCTGGTGATAAATCTAGACCAGGGGAGCAATCTCGGGATACACAAGGC
AGAAGAGAAGATAATATAAAACCTGAGGAACATTTTGGTCAACCTACTAAACCACAGTGGCAGACAAGCGAGC
GAGCTGGTCCTATTCGCCCGAAGGACAATCTATATCCCGAAGGAAATAAACAATATCCATCATCTGTTAGCAG
GGCTGATTATTCTCAAACCAAGCAAAGTTCTCACTCAGTTATAGAGGAACGACATACC
TCACATGGCTCTAGTCCTGACGCGAGTAAAAGGGTTCACGATACAAATAATTTAACTGATTATAGCAAATATC
ATTCTGCAACAGATATTGACAGCTCTAAACATACGCATTCTGACCATGATGTAAAATCACACATTACTAAAAC
TATATCCGATAAGCACCATGATAGCAGTGTGCAAAAGTCTCATTCAGAGATACGTACT
ACTAAAGTGATGGGAACTCATAATAAAGTAGTTGAAAAGTCTCATATAGTTTCTGGTACTAAAGGTCACGATA
GTCCATCAAATATAAATCGTAATGAATTGCAAAAAACTTCTACTACATCATCATCAAAAGATGTAAGTCAGTC
ATTATCACACGACACTACCGATCGGAATGCAACAGATCGTTCATATAAAAGACAATCA
ACGGAAAGAAATGTTATAAATAAGAGTGATACTACAAATATTCAACATTCATCTTCTACTAATAAAATTATAA
AACAAATAACAGAGAAGAAGTTAATTGCGGGAAAATGGGTAAATGTAACTAGAAATGTTGAAGTCGTGACGAA
TACTGATAAATCAGGAAGTCCAAACTTGAAAAATGTTATCGATGGGCGAACTGAGGTT
GATAAAAACAAAACCAGAAACATCTCAAGCAGCATTGGATCATCTGGACAAGTGTATTTAAGCGATGACAATC
AAATATTCCAAAACACTGCAAACGCCACATATCTAGTGAAAAGGAGAACTGATAATTTAAACCACTCATCTAA
TCATTCAGTCTCTGAACATGTAACAAAAGATATTGGTGAAATATCTTCACAGAAAATT
ATAAATTCTGGACAACATTTAACATCTTCGAACCAAAATATACATACCCAACGTCATGGTCATGTATCCTCAG
ATACCCGTTCCCATGAAAACATACACAATTCCACA
>1002049362_1 Heli.1-EL598992.1.5
CAATGGCATTCAAGTTTGTGGTTCTCGCCTGTTTCGTGGCTGCTGCTAGCGCTGGTATCTTACCGGCTGCTCC
CATCGCCTACGCTGCATCTCCACTCATCCACTCAGCTCCCGTGGCTTACGCCGCCCCCGTAGCCAAATACGCA
GCCGTCGCACCCGTCGCTAAGGTTGCCGTCGAAGAATACGACCCACATCCCCAGTACA
GCTTTGCTTATGATGTACAAGATGGTCTCACTGGTGACTCCAAGAGCCAGCACGAATCTCGCGATGGAGATGT
AGTTCAAGGTTCCTACTCCGTTGTCGACCCTGATGGTGTTAAACGTACCGTCGAATACACCGCCGACCCCCAC
AACGGTTTCAACGCTGTCGTGCACAGGGAGCCTTTGGCAGTTAAGGCAATCGCCCCAG
TCGCTAAAATCGCACCTGTAGTGCACGCTGCCCCCGTGGTACACGCTTCTCCAGCTGTATACTCCTCTGGCCC
CTTGGTGCACGCCGCACCTCTGGCCTACAGCGCCTCCCCTGTCGTCCACGCTGCCCCCATCGCGAAACTTGCG
TACTCTTCCCCTTACTACCACTAAATAATGCCAAATATAAATTAAATCATGAACTGTT
GTGACTACTAGTGATGTTCTAGAGATTTCTCGACATTTTTAGGATAAGTAAATTAAATCGTTTTTTACCGTCA
AAAAAAAAAAA
>1002049363_1 Heli.1-DT663106.3.5
GGCGAGTTGGTCGATTGGAGTAGTTCGTAGTAAATTAATTAAATTGACATAATTTGTGTTTTATCAAATCAGT
GTTTATAAAACGTTTACCATATAATCATTATCATTTAAATTGATTTGGAAAAATGGCACCAGATCCCGATAGA
CATCAAGAAAGTTTCCCGAAACTTAAGTATCCTTTGTACAGAGAAGTAGAACCAAGGA
CCCCGCAATACTGGTTAGCAGCAAAGCGAATACAAGACGGTGCAGACGGCCTTTGGCGGATACACAATGATAT
CTATGATTTCACTGATTTTGTTTCATCTCATCCTGGCGGCTCGCAATGGCTGGAGTTTACTCAGGGTACAGAT
ATAACAGAACAATTCGAAACTCACCATATTAATAGTGCACTAGCCGAAGCACTGTTGT
CCAAATATTTCGTGAAAAAAGCGGAAACACCCAGAAATTCACCTTTCACTTTTAAAGAAGATGGTTTTTACAA
GACCCTCAAAATCAAAATAGCTGATACACTGAAAGGCATACCTAAAGATGTTCGAAAGAAGAGTGATCATGTA
ACTGATTTCCTCTTAATATGTTTGCTACTAGCAAGCCCAATTTGTGGCTGGGTTTGGA
CCAAGAATTTAATATATGGAGCTGTTACAACCTTTATTCTGAGCTTAATATTGAGTGCACTCACTGTTTGCGC
TCACAACTACTTCCATAGAGCAGATAGCTGGCGCATGTATCTGTATAATATTAGCGGATTTTCGTACTCGGAC
TGGCGTATATCTCATGCGATGTCTCATCATATGCACACAAACACGG
>1002049364_1 Heli.1-DT668373.3.5
GCACGAGGACAACACAAAGATGTACGCTAAGTTGATCGTCGCACTTTGCGCTTTAGGCGTTGCCCACGGCAGT
GGCCTCCTCGCGGCCGCCCCGCTAGCGTACAGCAGCCAAGCTATTGTCGCTCCCGCGGTAGTGTCCAAGACTG
TTGTGTCCCCAGCTGTCTCGTCGGTGTCTTCCTACTCCAGCCACACCGCCCACTCCGG

CCCCGTCGCCACCTATGCCGCCGCCCCAGCCATCGCCAGCTATGCCGCCCCAGCGATCACCAGCTATGCCGCC
CCAGCGGTCGCTAGCTACGCCGCCGCTCCCATCGTGAGCAAGACCGTCGTGCAGCCAGCCAGTTCTTCCTACT
CTTCTTACTCTAGCAGCGTTTCCCACGGAGCACCAGCAGTCGCATCTTACGCCGCCGC
TCCCGCCGTAGTCGCCGCCCCCGCCGTCGCCGTGTCCCGCGCTGTCCCCGCCGTGTCCGTCGCCGCCGCCCCC
GCCGTAGCCGTCTCTCGCACTATCTCCCCCGCCGTATCTTCTTACTCTTCCTACTCCAGCCAAGTCGCTCACG
GTGTTAAAGCTATTGCTGCCCCAGCGGTCGCCTCCTACGCTGCCCCAGCGGTAGCCTC
TTATGCTGCCCCAGCAGTCGCCTCTTACGCTGCCCCAGCCATCGCCTCTTATGCCGCCCCCGCGGTCGCCTCT
TACGCCGCCCCCGCGGTCGCTTCTTACGCCCGCCCCGCCGTCGCCTCTTACTCTAAGATCTACTCTGCGCCCG
CCATCTCCTCTTACTCTAAGATCTACTCTGCGCCCGCCATCTCCTCTTACTCTAGACT
GTACTCCGCCCCCGCAGTTGCCTCTTACGCCGCCCCCGCTACTCTACTACGCCGCGGCCGCCCCGTCTACAAT
CAGCTATCTCTACTCGGCTGCCCTGCTGTATCTAC
>1002049365_1 Heli.1-ES588164.1.5
AAGCGCTAAAATGTTTAAATTAATAGTTTTGTGGTGTTTCCTTGCCGCCACCTCGGCTACACCTTGTGGTAGC
GGAGCTCCTTTGTCATATTCCTCAAATGTACTGGCAGCAGCACCAGCTGTCGTGTCAACCTACACTAGCAGTC
TAGGATACTCGTCTCCTGCCATCTCTTCAGTACCATTGACTTACTCCGGATTGTTCAA
CTACCCACATCTTATTAAAAAACGTTCACTGGCTGTAAGCAGCTATATTGCGCCGTCTTCTTACATCGCGCCA
TCTTCCTACATCGCACCTGCTGCCTACACTTACCCCGCTGCTGCTCCTTTAGTCAGCTCTTACTCTGCAGTTG
CGCCTCTGGCTAGCTACTCGGGACCCGTTTATACTGGTGCTGCACATCTAATCAAGAA
GAGGAGCGCTTTACTTGTACCTAACACATACGTGGCACCTGCTGGATACACTGCATCTGCACCTTTTGTAGCG
TCAACTTACGCGGCTGCTTCTCCCATTGCACCTGTTTGGTCTAGCTCGTTGTACCCTGCAGTTCAGTATCCTC
ACTTCATCAAAAAACGATCTGCTCCTTTGGCAATAGCTACATACGCAGCCCCATCTTC
CATCTCTCATACATCAAGATTTGATTTTGAGGCTGTATCTCCTGCTGTTAGCTACACTTCTTTTACGGGCCCT
TCACCATTCGTACATAATGCTCCAGTTTTTTCTCACCTGAGTTAAGCCAGCGTATTTGTCGAATTGTAACGTC
CAACAATGATCGTAAAGGAATCAATTTAAACAATATTGTATATTGTAAAGAATAAATA
>1002049366_1 Heli.1-ES587942.1.5
CATTTCCAAAAAGCGAAAATTCGTCGGTGACGGAGTTTTCAAAGCCGAATTGAACGAGTTCCTTACTCGGGAG
CTTGCTGAGGATGGTTACTCCGGCGTTGAGGTGCGCGTCACGCCAACCCGTTCAGAGATCATCATTATGGCGA
CGAGGACACAGAGCGTGTTGGGAGAGAAGGGCCGCAGAATCCGTGAATTGACCTCTGT
AGTGCAAAAGAGGTTCAACATCCCTGAACAGAGTGTTGAGCTTTACGCTGAGAAGGTCGCCACGCGAGGACTG
TGTGCCATCGCTCAGGCTGAATCTTTGAGATACAAGCTCATAGGAGGACTAGCTGTGCGCCGTGCTTGCTATG
GTGTACTCAGATTCATCATGGAATCCGGAGCCAGGGGATGTGAGGTTGTAGTGTCCGG
TAAACTGAGAGGGCAAAGAGCTAAGTCCATGAAGTTTGTTGATGGTCTTATGATCCACTCTGGAGACCCATGC
AATGACTATGTTAATACTGCTACTCGTCATGTACTGCTTAGACAGGGAGTGCTTGGTATCAAGGTGAAAATCA
TGTTACCATGGGACCAACAAGGCAAGAACGGCCCCAAGAAGCCCCAACCGGACCACAT
CCTGGTGACGGAGCCCAAGGACGAGCCCGTGCCCTCAGAGCCCACGTCCGACGTGAGGCCCGCGCCCGCGCCC
CTCCCGCCGCCCGTCGCAGCCACTGCTTAGACTTTACTATTATAAAC
>1002049367_1 Heli.1-DT664484.3.5
CTGTCGCTACTAGTGTTGTTCTGTTTATTAAATAAAACTTTGTAAAACATCAGAAATGGAGGATCAAGTGCCA
TTTAAAGTGTTTACATATTGGAATGACGATGATAAACCTGAAGTAAGAAGATTTGGTATTGAAAAGAGCGTTG
TGACTAGCTTTCATTATATAAATGCTAAGTTACAAGATGTTTTCCCTGGCTTGAAGAC
CAGACAATATGTGGTTTCCTGGAAAGATGAGGAAGATGATGATGTTACTATATCATCTGATAATGAAGTCATG
ATTGCATTGGTGGCTATGCAGCAACAAAATCTAATAAAACTCAACATTTACTGCAAAGATTCAAAGCCACAAG
ATGATGACTGTGACATCATTGTAACTGCTGTAGCTGATAATGGAGCATGCTCCACCCT
GCCCCATTGTGGTGTAGTTTGTGATGAATGCGACTCACCAGTCGTTGGATTCCGTTACAAGTGTACAACATGT
GATGACTATGATTTGTGTACAAAATGTGAATCTGCAGGTTTACACCCTGAACATTGTATGGTGCGACTACCAA
CTCCTAATATTGCGCGTACTACCATCAAAGCTGCCCTAAGGCGTTCCCGTCGCTTCCT
ACATTCCGTCGCAAGTACAGTTGAAGAAGATTGTAAGAGGCAACGTCGTGAACGTAGCAGTGAAAGGAAACGC
CACGGCCACGGCCACAGCCATGGTCGAAGCCACCGCCACGGTGGAGACGGTGACCACCGACGATCACGTACAA
GTTGGCTGGA
>1002049368_1 Heli.1-DT665053.3.5
GTGTCTATATAGAAATGAAGTTTTTTTCATCTATTGCATGCCTCCTGGCGCTGTCCGTGACAATCAGTGCGGA
TGGACTTGGACTCGATGTATACGCGCCTAAATATTCCTCCGTGGATTACTATTCATATCCCAGTTACGCTTTC
GAATATGCAGTGCGGGATCCACATACTGGTGACAACAAAGCACAGTGGGAAAAACGAG

ACGGAGATGTTGTAACAGGTGCGTATTCGTTGGTGGAGCCTGATGGCAGTGTGCGTGTCGTGGAGTATCGGGC
TGATGATAAGTCAGGATTCAACGCTGTCGTTAAGCGTATTGGCCCCAATCTACACCCAGTCGCTGCTCCCATT
TACAAAGCACCTCTCCCGGTTATTGGTTATAAAGCAGAAATACCAATCTCGATTGCAC
CATTAGCAGGGATTGAGAAGCTTGAAGCTCCACTTTTGAAAGGACCGTATCTAGGTGGTAACGCTATATCATC
TGCTTCTCTATATAAAGCTGCTGCTCCGCCTATTATCAAGGAGGTTGCACCAATAATTCCTGCCCCAATTCTT
TCTGCTCCCATATTAAAAAGTCCCATCATAGCTGAACCGTATCCTCTGTACCAAGCTC
CTGTCCTCGAAAAACCGCTGTTCCAACCTTATGCTAAATCTATATATCCAATATCCTATCCTGGCCTGAAAGT
GCCACTTCCCGAAATTAAATATTCGGCGCCACTTC
>1002049369_1 Heli.1-DT665828.3.5
TCGCCAGAGGGGTCGGACCGCAATCTAGTCATTGTATTTTTAAATATATTTTTAATATCTTAATCAAAAGGTC
ATCATGAAAGTGTTCATCGTGTTGGCAGCAGCCGCCGCCCTTGCGAGCGCCCAATTCAAATGCCCCAACAAGG
ATGGCCAATACGAGGATGACAGACAGTGTGACAAATTCTACGAATGTTCTGATGGTGC
TGCTGTCACCAAATTGTGTCCCGATGGTCTTGTGTTCGACCCCACCATTAGAAAGATCAACAAATGCGACCAG
CCCTTCAATGTAGACTGCGGCAACAGAGTCGAGCTCCAACCCCCCAAGCCCAGCGCTCAATGCCCCCGTCGTA
ACGGGTTCTTCGCTCACCCTGACCCCACCGTCTGCAACATCTTCTTAAACTGCATCGA
AGGTGAAGCTATCGAAGTTAAGTGTACCGCCGGCCTCCACTTCGACGAGTACTCTGGTACCTGCGTGTGGCCC
GATACCGCTGGAAGACAAGGATGCGTCGCCCAAGAAAAGAAGACCAAGGATGGTTTCGAGTGCCCCAAGGAGC
AACAAGTTGATGCCCAAGGCTTGGCCGTCGCTCACCCCAAGTTCCCCCACCCCAATGA
CTGCCAACGTTTCTACGTTTGCTTGAACGGTGTAGAACCCCGTGACCTTGGTTGCCAAGTTGGTGAAGTATAC
AATGAAGAAAGCCAGAAATGCGACGCCCCCGAGAATGTTCGTGGATGCGAGGACTGGTACAAGGACTCCGAAG
ACGCCGCGCC
>1002049370_1 Heli.1-DT663107.3.5
GATCCTGATAATAACCAAACAAAATGAACACCTTCATCACTTTGGCTTGCCTGGTGGCTGCAGTCGCGAGCGT
GCACGGCTCGGGTTATGGTGGATACGGCTATTCTTCAATCATCTCTGTGCCGGTCATCAAAGCCGCTCCCATC
ATTAAAACAGTTGCTGTAGCACCAATTATCCGTAGACCTATCTTGCAAACTGTCCCTG
TATCCAGCTACAGCCTTGCCCTCGGGGGTTACGGTGGACTCGGTTACGGCGGTTACGGACTCGGTTACGGAGG
TTATGGACTCGGAAAACTGGGTTATGGACTCGGATACGGTGGTTACGGACTCGGAAAACTTGGTTACGGTTAC
GGAAAACTCGGTTGAGTGATTCGAGGTTATAAGTCTTCAGACGGAGAAACGCTAGCTA
CTTTATTGGCCAATGTCTTGAATAAAATGTTTACAGTCTTGAATAAAATGTTTACAGTTATATCATTTTGTTT
TTTTTTCTTTTTTTTATATTGTATAATTTCTTTTTAAATATCATATACCATGTTTTAAGAGAAATTGTAAAAA
GACATTTAGAACGGTTTTAACTATTTTACTAAAATTATAAGTTTTCATATGTACAACA
AATGTATAAATTACATTTTGAAAAGTTAAAAAAATATTAAATACTAGCGACCCGCTCGCGGCTTCCCCTTCTT
AAGCTACTGAATTACCATTAGGTAGTGTATTAACACTGAATTACGTTCCTGTTTGAACA
>1002049371_1 Heli.1-ES587628.1.5
GTCAAATGAAGATCGGACTTTGTGCCTACAGTGGTTACAAGATTTACCCCGGCCATGGCAAAACCATGGTTAA
GGTAGATGGCAAGACTTTCACATTTCTGAATTCAAAATGTGAAGCCGCACATTTGATGAGGAGAAATCCTCGT
AAAGTTACATGGACTGTTCTATACAGACGCAAGTTCAAGAAAGGCCAGGAAGAAGAAC
AGGCCAAAAAACGCACCCGGAGGACACAAAAGTTCCAGCGTGCTATTGTGGGAGCTTCACTCAGTGACATCAT
GGCCAAGAGGAATATGAAACCTGAAGTCAGGAAGGCTCAAAGAGAACAGGCTATCAAAGCGGCTAAGGAGCAA
AAGAAGTCCACCAAAGCCGCTAAGAAGGCCACAGCTGCTCCTCCCAAGGCTAAGGCAC
CCCCTAAGGCTAAAGCTGCTAAAGTAAGCCAAAAGGCAGCTCCACGTGTTGGAGGAAAACGATAAGACCTTTG
TAAAATAAACTATTAAGTAAAAC
>1002049372_1 Heli.1-DT664008.3.5
ATCCGCGCCACTGTCTTTTTGTTTCGGCCATTTTTGCCGAAAGGTTGGTGTTCTTTTTCGGTCACGTATCCGT
TCGTGGCCGTTAGATCACTTTATTAAAGTTCATACAAGATGGTGCAGAAGAAGCCTAAGAAGAAGGTAGGAAA
GAAGGTAGCGGCCGCTCCTTTGGTCGTGAAGAAAGTTGAACCTAAGAAGGTTGTAAAC
CCTCTCTTCGAGAAGAGGACAAAGAACTTTGCTATTGGTCAGGATATCCAGCCAACTCGTGACTTATCCCGTT
TCGTGAGATGGCCCAAGTACATTCGTATTCAGCGTCAAAAGGCAGTGCTCCAACGCCGCTTGAAGGTGCCACC
ACCAATCAACCAGTTCACCCAGACTTTAGATAAGACTACAGCCAAGGGTCTTTTCAAG
ATCCTCGAGAAGTACAGACCAGAGACTGAGGCAGTCAGGAAGGAACGCCTCAGGAAAGCTGCTGAAGCTAAGG
TTGCAAAGAAAGAGGAGCCCCCAGCGAAGAGGCCGAATACTATTAGATCAGGTACCAACACTGTGACTAAGCT
GGTGGAGAAGAAGAAGGCACAGCTGGTCGTCATCGCTCATGATGTTGATCCTATTGAG
CTGGTACTGTTCCTGCCAGCCCTTTGCCGTAAGATGGGTGTGCCCTACTGCATCGTCAAGGGCAAGTCCCGCC
TCGGTGCCCTCGTACACAGGAAGACTTGCACGTGCTTAGCGCTCACACATGTTGAGTCCGGTGACAGAGCC

>1002049375_1 Heli.1-DT662680.3.5
ACACGTTATAACGTCAAAATGGCAAAGAAAAAAGGCGATGAGAAAGTGAATCAAACTCCTCAAAGTGATAATG
AGGAGCAAAATTTTGACGAAGAACCTAATTTCGAAGATCCGGAAGGATTCGTGGATGATATTACAGACGAGGA
ACTGTTGGCAGACCTCTTAGAGCAGAAACCTAAGGAATCCGATGGGTACGAAAATGTG
ATCATCGTAGATGGATGCCCGCAGGTCGGGCCTGAACGTTTAGAGAAGCTTCAGAGTGTAATAAACAAAATCT
TTAGTAAATTTGGAAAGATTGTGAACGAATTCTATCCAACCAACGAGAATGGAGTCACGACTGGCTTTATTTA
CTTGGAATATAGTAATCCACAGAATGCTGCTGAGGCCGTACAAGCCACTAACAATTGT
AAATTAGATAAACAGCACACATTTTTAGTAAACCTGTTCACAGACTTCAAAAAGTATTCAGATATCCCCAAAG
AATGGGAGCCTCCAGCACCTCAACCGTTCAAGGTGCAATCAGATCTTCAATGGTATCTCATGGATCCTGATGC
ATATGACCAGTTCCTCGTAGGAATTGGTACTGGTGTGGCACTGCAAGTATGGCAGAAT
ACTCTGCCAGAGCCTATTACATTGCAAGAGAGACCTAATTGGACTGAAACCTACGCTGTGTGGTCTCCACTTG
GCACATATTTGGCCACTTTCCATTGGAGGGGTGTTGCCTTATGGGCGGGACCTAAGTTCAGTCAGTTCCAGAA
ATTCTACCATCCTGAAGCTAGATTTATTTCTTTCTCACCTTGTGAAAATTATATTGTC
ACTTTTTCACCTAACAGCGATAGAGGTGACGATAAGAAACTTATAATTTGGGATATTAGAACAGGCCAAGAAA
AACGTAGTTTCCCTCCACCCGACGAGTATGTAACGTGGCCGATATTCCGCTGGAGTAAGGACGACCGTTTCTT
TGCAAGACTCGGTGCTGATGTTCTCTCTGTATACGAGACGCCCAGC
>1002049376_1 Heli.1-ES585445.1.5
ACGCGTCCGCGTTTCGTTGGTTGATATCTGAAAAGGCCAAAATGGTGAACGTACCAAAACAGCGCAGGACCTA
CTGCAAAAAATGCAAAGTCCACAAGGTACACAAAGTATCACAGTACAAGAAATCTAAGGAAAGGCACGCTGCC
CAAGGTAGAAGACGTTATGACCGCAAACAGCAGGGTTATGGTGGTCAGTCCAAGCCTA
TCTTCAAAAAGAAGGCTAAGACAACCAAGAAAATTGTACTCCGTCTTGAATGTGCTGACTGCAAAGTGAGATC
CCAAGTCGCACTTAAACGCTGCAAGCATTTTGAATTGGGCGGTGACAAGAAGAGAAAGGGACAGATGATCCAG
TTCTAAGCTGGTTAATAAATAAGTTTAATATCTA
>1002049377_1 Heli.1-ES587444.1.5
AAACAACACAAAGATGTACGCTAAGTTGATCGTCGCACTTTGCGCTTTAGGCGTCGCCCACGGCAGTGGCCTC
CTCGCGGCCGCCCCAGTAGCGTACAGCAGCCACGCTATTGCCGCCCCCGCGGTAGTGTCCAAGACTGTTGTGT
CTCCAGCTGTCTCGTCCGTGTCTTCCTACTCCAGCCACACCGCACACTCCGGTCCCGT
CGCCGCTTACGCCGCCGCCCCAGCCATCGCCAGCTATGCCGCTCCAGCCATCGCCAGCTATGCCGCCCCAGCG
ATTGCCAGCTATGCCGCCTCAGCGGTCGCTAGCTACGCCGCCGCCCCAGTTGTCAGCAAGACCATCGTGCAGC
CAGCCAGTTCTTCCTACTCTTCTTACTCTAGCAGCGTTTCCCACGGAGCACCAGCGGT
CGCTTCTTACGCCGCTGCTCCCGCCGTAGTCGCCGCCCCCGCCGTAGCCGTGTCCCGCGCTGTCCCCGCCGTG
TCCGTCGCCGCCGCCCCCGCCGTAGCCGTCTCCCGCACTATCTCCCCCGCCGTATCTTCTTACTCTTCCTACT
CCAGCCAAGTTGCTCACGGTGTTAAAGCTATTGCTGCCCCAGCGGTTGCCTCTTACGC
CGCCCCAGCCATCGCCTCTTACGCCGCCCCCGCGGTCGCCTCTTACGCCGCCCCCGCGGTCGCCTCTTACGCC
GCCCCCGCGGTCGCCTCTTACGCCGCCCCCGCGGTCGCCTCCTATGCCGCCCCCGCCGTCGCCTCTTACTCTA
AGATCTACTCTGCGCCCGCCATCTCCTCTTACTCTAGACTGTACTCCGCCCCCGCAGT
CGCCTCTTACGCCGCCCCCGCATACTCTAGTTATGCCGCGGCCGCCCCCGTCTACTAATCAGCTATCTCCTAC
TCGGCTGCCCC
>1002049378_1 Heli.1-DN172686.1.5
GCCGCCCAAGAAGGACGCGAAATCCTCGGCAAAGCAGCCTCAGAAAACACAGAAGAAGAAGGAAGGATCTGGC
GGCGGCAAAGCCAAGAAGAAGAAGTGGTCCAAGGGAAAAGTACGTGACAAGTTAAATAACCAGGTGTTGTTTG
ATAAGCCCACGTACGAGAAACTGTACAAAGAAGTACCACAGTATAAGCTCATCACTCC
TGCAGTGGTCTCTGAAAGATTGAAGGTCAGAGGATCCCTCGCCAGGAGGGCGCTCATCGAATTAAGAGAAAAA
GGCTTAATTAAGCAAGTAGTTCAACACCACGGTCAAGTGATATACACAAGAGCAACAAAGGGTGATGATCCCG
TCGCTTAATTATGAATAAGTAAATAAAACATAAA
>1002049379_1 Heli.1-DT668453.3.5
CGCTTTTGCGGCGTAGTCTGTGATTTTTTAGTGTAGCTCCAGATAATCCAAGTTAATCTACGATGGCGGATCA
GCTGACAGAAGAACAGATCGCGGAGTTCAAGGAGGCGTTTTCACTGTTCGACAAAGATGGCGACGGCACCATC
ACCACCAAAGAATTGGGCACCGTGATGCGCTCGCTAGGACAGAACCCCACAGAGGCTG
AACTACAGGACATGATCAACGAGGTTGATGCGGACGGCAACGGAACGATAGACTTCCCAGAATTCCTTACAAT
GATGGCGCGCAAGATGAAGGACACAGACAGCGAGGAAGAGATTCGCGAGGCGTTCCGCGTCTTCGACAAGGAC
GGTAACGGGTTCATTTCCGCGGCCGAGCTGCGCCACGTGATGACCAACCTGGGTGAGA

AGCTGACCGACGAGGAGGTCGACGAGATGATACGCGAGGCCGACATCGATGGTGATGGACAGGTCAATTATGA
AGAATTCGTCACCATGATGACGTCGAAGTGAGCCGCCGGCTAGTGTGTGTGTATAAAGCAGAGAATTTTAATA
TACGTTTTGTTTCATGACACACAATATTACCATCGTATTGGACCTGCATTTCTCTAGT
GTTTGCGATAAATTAATATTGTCATCGTTTCAGTTTAATGGTATATCGGCGGCGCTGCGGCGTCGCCCCCCTA
TTTAGACTTTAAAGTATTCCGTTAATTTAAATAAGTAAGGAACACGCCGGCGAACGCCGCCGCCCGACGCCGC
CGCGCCAACACATCTTAATGTAAATTATAAAGTACGTACGTGATGA
>1002049380_1 Heli.1-DT662561.3.5
CGTGCGTTTGGAAAAACTCTGACAATCTGTCAATATTTCGCGCTTAAAATATTTTTTTCAGAATTATAAGTTT
GTCTATGGTATTACGTTTTTTTTTAAGTATATTTTTAAATAAAAAATATATAAATTGTAAAAATAAATAAAGT
GTGTGTTGGGATTCAGTGACTGTGATCTTGTGTGATGGTACTCTGCCGCTTTTTATAG
CTGATAGAGAGCAGCATGAGGGGTTGCGTTTTTCTAATTTTGGCGCATACGGTCGCCGCCATAACTGGACCGA
GGATAGAAAATGACTTTGACAGAAATAGACAGCCATCCCTGGGTCACCCAGGATTCAATGATCATCAAAATAG
ATATGGAGTACAAAATTTCCAACAACAGCAAAGTTTGTTGAGTAGCCCAGCGCCACCC
ATCCAGTTTCCGCAACAGATCTTTCAACAGCCCTACGTACCTCAATTCAACAACTTGTTGCCAACCCAAAATA
TTCCGAATAATGTCCCCATACAAAATAACAACGGATTTCAATTTAGCCAACCGGCAAATGTTAACCCTATAAA
TAACTTACAGCATCAAAATCTCGGTCATATTCAACAACATGCTCAAAATGCACCACTA
CATGCACAAAATGTACCACAACCAATTCCGACTTTAGCAGCATTTCAGAGTAATGGACTACTGCCCACGCAAA
ACTCTCATCCCAGTTTCAACCAAAACCCTAACATTCATAGCTCTATTGATTTGTCTCAACAGAGACCTACTCA
AGCTGCCTTTCTCCCTACCTTAAATTCTGCCCCCATTCAAAATCAGGCTTTTAGTGGA
CCAAATCCCATCACTATTCAAGCTGCGCCAAACTTAGCACCTCAAAATTTAAATAGTCAACAGCTTCCATTTC
AATCTTCTCTCGGCCAGTCTCAATTATACAACAGCCAATCTGCGTCCCAACTACGAGACTACTATGAACAGCA
ATCTAAGGAAAATCTAGAAAAATTAAAAGAACTACAAGAAAGGCAAAGACTCATCCAA
AAACATCAAGAA
>1002049381_1 Heli.1-EL604318.1.5
AATTTTTGTATTTTGTTTATTTTATCACTAAACATTAAAACTTAAGTTTACAGTTTCTATAACAACACTCAGT
CGCGGCGATAATTTAGTATATAAAATATTATATATTTATTTACAAGGTCGTGGCTGTTGGAATTGAATTGTTT
CATCGTTACGTCAAGAATTATAAACATAAATGCATACCGATATTCATTTATCATTAAT
TTTTACAAAATCGTACATTTATAAGACGCTATCAATTTAAATAAAATATAAAATAAATATTCCTAATTTTCTA
ATCTGCTTTTTGAAACTGTTTAAGGCTACAACGAGTAAAGTATTTGGCAAATGGAAATGAATACAAATCAATT
GGCTTCACTTTCTTAAAATACGAATTTGAACCTGGAATCGTGTTTCTCACACCAAAAG
CAACGTTAGTGTATGTTTATATGAAAATTTCCTTTTAAATTCTTATAAAATTTTCGTCAAGGTGTGGTAGGAA
CTTGTGCAAAAGATAGAACAGATTTTATAAAAAATGGCATTTGGAGACGTAAAATCTGCACAAGGCCTCGGCG
AATTGAACAATTTTTTAGCTGAGAAAAGTTATGTGTCAGGTTACACACCTTCTCAAGC
CGATGTTCAAGTCTTTGAACAAGTAGGAAAAGCGCCGGCCGCCAGCTTACCACACGCCTTACGCTGGTATAAC
CACATCGCCTCCTACTCCCCTGCTGAACGTAAAGCATGGGTTGCAGGAGTGAGTCCACTTAAAGCCGGTGCAC
AGCCTACAGCTGCTGCACCAGCCAAGAAAGATGATGATGATGATGAAGTCGACCTCTT
CGACTCAGATGAGGAAGAGGATGAGGAAGCAGCTAAAATCCGTGAGGAGCGTTTGCAGGCATATGCCAGCAAA
AAGTCAAAGAAACCTGCCCTTATTGCTAAATCATCTATATTACTCGATGTCAAGCCTTGGGATGATGAGACAG
ACATGAGTGAAATGGAGAAACAGGTGCGCACTATTGAAATGGAAGGACTTCTGTGGGG
AGCATCAAAACTTGTCCCAGTCGGTTATGGAATTAACAAACTCCAAATTATGTGCGTCATTGAGGACGACAAA
GTCTCCGTTGACCTTCTTACAGAAAAAATTCAAGAATTTGAAGACTTTGTCCAATCTGTAGATATTGCTGCCT
TCAACAAAATCTAAACAATGCCATCTTAATTTATCATCTTTTTCAA
>1002049382_1 Heli.1-ES587202.1.5
CAAGGTTTGCAAAACGTTATAAACCATGGCCGATGTCGATGTTGAAATTCCCAACAACCCCGTCTTGAGCGGT
GGTGCTATGGACGTCAATACTGCACTTCAAGAAGTATTGAAAACTGCCTTAATCCACGGTGGACTCGTCCATG
GTCTTCACGAAGCCGCCAAAGCTCTCGACAAGAGACAAGCTGTACTCTGTGTGTTGGC
AGAAAACTGTGATGAAGCTGCATACAAGAAACTTGTACAAGCCCTATGCAATGAGCACCAGATCCCACTTGTG
AAGGTGGATAACAACAAGAAACTTGGTGAATGGGCTGGTTTATGTAAAATCGACAAAGACGGCAAGGCCAGGA
AGATTGTTGGTTGCTCTTGTGTAGTAATCAAAGACTTTGGTGAGGAGACACCAGCGCT
GGATGTGCTTAAAGACTACTTGAAATCATCGAGCTAATCCTTAGGTGTAATGTCTTAAAATAATAAAAGCCAA
AAAAATTAAAA
>1002049383_1 Heli.1-CO729506.1.5

GGTCGACCACGCCATAGCAACACAGACCACAATGTACAAGCTGGTGGTGTTGTCCGCGGTCCTCGCTTCGGCG
GCTTGCAAGCCCAGTGGCATTGCGCCATTATGGGGTGGACACTTGGCAGCTCCTTGGGCGGCAGCAGTCGCAG
CTCCTTTGGCAGCAGCTCATGTTGCCGCACCCGTTGCTGTGGCTCCTCTTGCTCCTGT
CGGTGCTTACAACTACAGGGGGCCTCTTTCCCTCGCCCCTGGTCAGCCCGCCAACATTTTGGCTCACGACGGT
CGCCCCCTGGATACCCTTAGTGTTAACGTAGATCGCGCCGTTCACTATACTGCAAGAGCTTTGGACCCTGCTG
CTCATGGTGTACATGGTGTACATGGTTTACATGGGGCGCATTTATTAGGTAAAAGATC
AATTGTTGCACCCTGGGCCGCAGCTCCCTTGGCTGTTGCTCCTGCCGCTGCCTGGTCTCATTCCGCCCACGTG
GTCGCACCTGCTGCTGTAGCCGTATCCGCACCTCTTGGTCATTTGGGATGGGGACATGGTGCTGCTTTAGGAC
ATGGACATTGGTGAAGACTACACGAGCTAGGCTAGGATCCTCAAATAATATGATAATA
TACAGAGAGTTA
>1002049384_1 Heli.1-DT663858.3.5
TGTGTTGTGTGTGAATATTAGAATTAACGGATAGCTATTAGTGGATTTCATAAAGAAATTGTAATTGATAAAT
CATCAACATGGGCAAGGATTACTACAAGATTTTGGGTATAGCGAAAGGCGCTACCGATGACGAAATCAAAAAG
GCGTATCGCAAATTAGCCCTGAAATACCATCCGGACAAAAATAAATCCGCTGGTGCTG
AAGAAAGATTTAAGGAAGTTGCAGAAGCTTACGAAGTATTGTCGGATAAGAAGAAACGAGAGATCTATGATGC
CCACGGCGAAGAAGGCTTGAAGGGTGGAATGGGAAGCCACAATGGTCCAGGTGCTGGCCAATCATTCTCTTAC
ACCTTCCACGGAGACCCTAGAGCTACCTTTGCTCAGTTTTTCGGTTCAGCGAGTCCTT
TCCAGGGCTTTTTCGATTTGAACGGAACCCCTGGCAACACATTCTTTGACCGCGACATGGATGTCGATATGGA
TCCTTTCAGTAACATAGGTATGGGTACTGCAAGACCTGGTGGGCCTGGTGGAGCGTTTAGGAGTCACAGCTTC
AACTTCCACGGATCACCCAACCGGAAGGAAAAGACTCAAGATCCGCCTATAGAACACG
ATTTGTATGTCTCACTCGAAGACATTGCGCGTGGTTGTGTCAAGAAAATGAAGATTTCACGAAGAGTAATGCA
GCCAGATGGCGGATCGAAGAAGGAAGATAAAGTGCTAACTATTCATGTTAAACCTGGATGGAAGGCTGGCACT
AAGATCACTT
>1002049385_1 Heli.1-EL597471.1.5
GGTTTAGGGCCAGTTCTAATGATTGTAATTTAATTGGCCGTGAACGCTCAACCGGGTGTGACTCTCGATCGGT
TTTAGTGATACGTAATAGAAGTTTTCAGGCTGGGCAGGGTCTTCAGAATGAAGCTCTCCCTGGTGTTTGAGCT
TCTGTTAATATTCGTACTGCTTGCGTTTGCAGCGTCGAAAAATATCGGTCGTCAGAAA
AACAATAGAAAAGGAATTCAAAAAGCAAGTGACGGCATTCAAATAGAGGACAACAGTATAAATAAATTTTGCA
AATGTTCAGAGGCTCTGTGCAATTGTTGCAGAGATTTTGCGGTGCCTATTATAAATCTTAATGGTCCAGGATG
CGCTTCATTAATGTATTTAAATGGCGACAAAATGTCCGTATCACTCAGCTTCGGTAAC
AAAGTCATTACAAATCGAACCCTTTCAAGCCGCAAACCAAGTCCTGTTTGTATGCCTCTCCCCGGAGGTGTTT
CAAAGTTTTGTGGACGAGTTTACAATATTGCGAGGATTGGAGAAGAATTTCGTGCTTGTTTAGGCTTAGAGCT
TCAAGCTAAAAAATCTGTAGAAGCCGCAGTTAGAGTATCCTGTTTTAAATTTGGTCCC
AGAGGTGTAATTTCTGAACCAGCTGATCCTTTGCCATTAGTGCCACAGGATGAGAAGGATACTGATGACGATG
ATGATGACGATGACGACGACGATGATGATGATGATGTCGATTTTGGACTCGATGCGGAT
>1002049386_1 Heli.1-DT663298.3.5
CATTTTCTGTCTTTTTATGACAATTTCTGGGCTAAAAACTTATTAGTAAGGTAGCCCCAAACGTCCACTAGGA
ATAGTTATAAAAATGGCGTTTTGCCGTTTAGTATTCCCTCTTTTAGTGCTAAGTGTCGTGTCGAGTTATGCCA
ATATACATGTACCAGTATTTCTTTGGGGCGATTTGAAAACATCAATAAAGTCGAATCC
CTTGTCAACCGTGCCTCAAGAAGACTTCGAAGCTATTTTGAAGAAAGAATTAAAAGCCGATCCATTCGTCGTA
ATTTTCGTCGACGAAACTTTGTCTGTTGAAGATTTCTCACTCAAAAACTCCGATGGCGACTCTTCATTCCCCT
ATTTACACGCTAACATTGGTAAAGCTGTATACTTACCTGGCGTGGAGAACGCTTTGCC
CGTCTTGAACAACTTGGCGAACCCTGAACAAGTGGACCGCGTGAAATTGACAGAAGATGGGCTTTCTGCTGAA
TTTGAACCAAGGGATGTAAAAGTACTCTTTATAACGCTTAAAGATGCTCGCGAGGGAGAATCAAGGGCTGAAC
TACTCCGTCGTCATAACGATTTCATGGCTGACATGCTCACTAAACTACAAGAACAATA
TGATAACATTGTAGCTGTATACACTGCAGAGAATCCTTCCTGGACCATTGCTGAAAACCACTCTCGAGTCCGT
CGTCAAGCTGGAGAGAGTCAAAGAGACTATGTAATGGATGGCCTGAGATTGTATGTGAAGAACATTATTCTTT
CCGTTGGTAGTGAAAAAACAAATTTGAATAACATGGCCAGTTTCTCTAGCAAATTCAA
TGAAACAGAAATGTCCACAACAATGAACTTTGGAGAGAACTCCATCAC
>1002049387_1 Heli.1-DT663065.3.5
ACGTCTTGCTGCGTAATTTCTCGAATTCCTACGGCCAGTCCGTGTTTTTGATTGATTCGTTTTGCAAAGGCTT
AAATACGATTTGCCAAGATGACCAAGTTAATACAACAGTTAAATTTTAGTAGTAGTAATTACTACGACGTTAC
TGGTGTCGTAGTTGAACCCCAAACCCCGAAAGCGAAGACCTATCGTGGTGATGGCGCG

TCCTTGAACGCGGCCGGCAGCAAGCGCACCGCTTTGTCGGCGTCCGACGCCGAGTGTTTCTCGCTGTGCCTGG
GCGCCGGGCCTCTGTGGTGGTCCTGATGTCCCTGCTCTCGACTCCGCACCGCCCGGAGGGGTGAACAGCGGGG
CCTCGAGCCCCGCAACCCCGTCCACCCCCGTACACGACGACAACAGCCGTGACTTGTT
GAGCGCCTTGCTATGGGCGAGCGCGAGCTCTCTAAGCACCAGCGCGGAGAGTCTCACCCCCGAGCCCGTGAAC
ACACACCCAGCACTCAACCTCGCTGAGAGACAGACGGTCATCAATAAGATCTTGGAACTCAAGGACAAGCAAC
CGGTTAAGATCGACTTTAAAATCTTCCTTACCGAAAACACTGTTGTGCGTTGGGAGGC
CGTAATCAAGGGTAATACCATGTACCTACGCGTCCCCGGCGTACTGCAAGCTGGTAGCAAGGAAAGTTTTATG
CTGTTGCTTGACTTTGCCGAAGCGAACCTTGGGTGTGCTAACTGCATAATTTGCATATTAAAAACACGCAC
>1002049388_1 Heli.1-DT662581.3.5
AAGATTTGATTGTTGAATCCATTTTAATCCTGAGTTTGTGCTGTTATAATTTTGTGAAAAATGGCCATGTTTC
CAGGAACAGTGGCCTTCGATGAGTTTGGGCGACCATTTATTATTTTAAGGGACCAGGAAAACCAGAAACGGTT
AACCGGTATCGATGCTCTTAAGTCTCACATCCAAGCCGCAAGGCAAATAGCAGGTATC
CTTCGCACGTCCCTTGGTCCAAGAGGCTTAGATAAGATGATGGTATCTTCAGATGGTGAAGTGACTGTCACTA
ATGATGGAGCCACTATCCTTAAACTCATGGATGTGGAACACCAGATTGGTAAACTTATGGTGCAGCTTGCTCA
GAGTCAGGATGATGAAATTGGTGATGGAACTACTGGTGTAGTTGTGCTGGCTGGTGCT
CTCCTGGAACAAGCATCAAACCTATTAGACAAAGGCATACACCCCATCCGTATAGCCGATGGTTTTGAAATGG
CTGCTGCCACTGCCTTAGCCCACTTGGACAGTATTAGCGAGGCTTTTCCAGTAAATAAAGCCACCAGGGAACA
TTTGATCAAAGTGGCCATGACTACACTTGGTAGTAAAGTTGTTGTGAAATGTCATAGA
CAAATGGCAGAGATTGCTGTTGATGCTATCCTATCAGTAGCCGATTTAGAAAAGCGGGATGTAAATTTTGAAC
TAATCAAAGTCGAAGGTAAAGTAGGTGGTCGCATGGAAGACTCAATGTTGGTACGCGGTGTTGTCATCAACAA
GACCATGAGTCACCCCCAGATGCCAAAAGTTTTGAAGAACGTAAAACTAGCAATTCTG
ACCTGTCCATTTGAGCCCCCTAAGCCCAAGACCAAGCATAAGCTCCAAGTTGGCTCCGTCGAGGAGTACAGAG
ATCTTAGGAAGTATGAGCAGGAGAAGTTCCTGGAGATGGTCAAGAGG
>1002049389_1 Heli.1-EL596608.1.5
TTCGGCACGAGGGTTCAAGCAAACGTTTATTTACGGCTTAACACGTTTCGTGTCTTTACGACTTAAAAAATGC
GTTACGTGGCCGCGTATTTGTTAGCCGTGCTCGGAGGCAAAGCTGCCCCAGCCGCTGCCGACCTCGAGAAGAT
CCTTAGCTCCGTCGGTATTGAGGCTGATGGAGAGAAACTGAAGAAGGTGATCGGTGAA
CTCAATGGCAAATCTGTTGAGGAACTGATCGCGCAAGGTCGTGAAAAGCTATCGTCCATGCCGGCGGGTGGTG
CGGCCCCTGCTGCTGCTGCTGCAGCACCAGCGGCTGCCGCAGCTGAGGAGAAGAAAGAAGAGAAAGAGGCCAA
GAAGGAGGAGTCTGAGTCCGACGATGAGGACATGGGCTTCGGTCTTTTTGACTAAACT
TTTTAATACGGTGCAGAGCACGCGGTTTTTATTCCATGTATTGGTTTAAGCGTAAATTGACGGCAAGTTTGTT
TCTGTGAAATATATCTTGCTAAATAAAAAAAAAAA
>1002049390_1 Heli.1-ES585810.1.5
TGCGGTACGGTCCGGAATCCGCGGCCGAGCTGAGGTCCGGTGCAGCAGAACCGAGGCTGTGCAATAGCGATTA
ATTTATTTATTTTAATAGGACTATTTAAATTAAAACTAATTCAAAATGTGTGACGAGGAAGTAGCCGCGTTGG
TAGTAGACAATGGCTCCGGTATGTGCAAGGCCGGGTTCGCGGGAGACGACGCGCCTCG
CGCCGTGTTCCCCTCAATCGTGGGCCGCCCGCGCCACCAGGGCGTGATGGTCGGCATGGGGCAGAAGGACTCC
TATGTAGGAGATGAGGCACAGAGCAAGAGAGGTATCCTCACCCTCAAGTACCCCATCGAGCACGGCATCGTCA
CCAACTGGGACGACATGGAGAAAATCTGGCATCACACTTTCTACAATGAACTGCGTGT
GGCACCCGAGGAGCACCCTGTGCTGCTCACCGAGGCTCCTCTTAACCCTAAGGCCAACAGAGAAAAGATGACA
CAGATCATGTTCGAGACCTTCAACACACCCGCCATGTACGTCGCCATCCAGGCCGTGCTCTCACTGTACGCCT
CTGGTCGTACCACCGGTATCGTGCTAGATTCCGGCGATGGTGTTTCCCACACCGTACC
CATCTACGAGGGCTATGCGCTTCCTCACGCCATCCTGCGTCTGGACTTAGCTGGTCGCGATCTTACCGACTAC
CTGATGAAGATCCTCACAGAACGTGGTTACTCTTTCACCACACTGCCGAAAGAGAAATC
>1002049391_1 Heli.1-DT663234.3.5
TCGTACTACGCGCTATATTATGACAGTAACGTGATGGAGGGCAAAGAATTCGGTGCGGTCCCCGGCTTGCCCA
CCGGCCTGGACTACTTCATGATGCCCCGTGTGAGCCAGCCGGCGCCGCAGTTCGACTTTAGAAAATTAGGCGC
CAGTTTCAGCGGTCGGGATGAAGATAAATGTGAAGAGCGACGAACCCCCGAGTACGTC
CCGGAGCGTCGCGACCCACCGCCCGCGCCGTGGCCTCTCGGGTTGGGGGTTCAATTCGTGAATCCCGCTACGG
GAAAGAAGAGAGTGCAGTGCAATGTTTGCCTTAAGACTTTTTGCGATAAAGGCGCATTAAAAATACATTTTTC
CGCGGTGCACTTAAGAGAGATGCACAAGTGTACAGTGGAGGGGTGTACTATGATGTTT
AGTTCGCGGCGTTCTCGGAACCGGCACAGCGCTAATCCGAATCCTAAGCTGCACTCACCGCACGTTAGACGTA
AGATCTCTGCCCACGATGGTCGATCTGCGCAGCCGTTCCCCCTGCTCCCAGCGTTGGCTCGGTTACCATTGCC
GCCGCCTGCGCTTCTTCCGCCTGAACTAGCAGCTCGTCTTCCGCCACCTCTAACTGCA

CCACCTCCTAATCATCTACCTCCTCGTGTACCTCTCGATGATCTCCGAAACTTCAGTGAAATCGAAAAAATGT
ACAGAAAAATACCTTCTCCAGAAAATCAAACTCGTCACCCATTAGATCTTGTGAAACCTGTGCTACATGAAGA
GGAATCAATTAATTACAGTGAAAACTATGAAGACGGTTCGGATAATGATAATAAGGAC
GAAATTAAATCTCAAATTAAACCTGAAGATCAAAACCGGTTAAACTACGTCGATGAACCCGAAGACCTAAGTG
TAAATAAAAAGAAAGAAGATGATATTAAACCTAAGCAATCTAATCCTAGTGTAAGTACTGACTTGACCACCAC
AGATGATAAATCTCCTTTAATTCCAAACAAACGTAAAAGGAAAAGTGGAAACCCCACG
CGTTGCTCACAAAATAATGAATATAGTGTATCAGATGAAGAGTATAATAGTGATTTATTTAGAAATATGTCAA
CGCCCAGTTCTAGTCGAACAGACGAGGAACCTTTGTCTCTAAAAAAACAAAAGCCAGAAAAATGGGAGCCATT
TCAGTCCAATGAGGAAGCGACTGTGGACTCGGAATCAACAACGAGAGTAAAGGCAGAG
AGTGAGTCCGACGATGAATCGAGTGCGCCCAGCGTGGTTCGGGAAGGATTAAGGTTACGTTCTGATCTCTATA
CACCCAGTGACAGCGGGAGTGATTTACATACCTTAGAAGAGAGACTTGCTCGTGTTAGATCTCCTTCAGCCAG
CAGTGACAGG
>1002049392_1 Heli.1-EL597465.1.5
ACGAGGCTCTTGAGCAGTTCTAGTGAGATACCACCCAACGCCATCAATATGAAATTCTTCGTGTTCTTCTTCT
TGTGCTGTGTTGCTCTGGCATCTGCTGCTCCCCAGTTGCTCACCTGGCCTGGCGTCGTCGCCCCTGGGGTATT
GGGACCTCGGACCATCATAGCCGGACCCACCGTAGTGAACGGAGTGGACCCAGTCGGC
CGGGTCGTTCTCCCCGGGGTCGTCGGGGTCCTCTAGGCGTCAGGCGTCAGCAGCTAGGCTTAAATTTACAACG
ACACAGCTTTACACCTGTCCGGACATCACCGAAGTTATTGGCACCCAAATTATTATGTATCGTATACGAATAA
ACGCATGCTCAATTTAAAAAAA
>1002049393_1 Heli.1-DT662927.3.5
GCACGAGGTGACACTGCAGCGGACGGTAATAATACGCGAAAATGGACGGAGAAACACACAGCCTGGAAGCCAT
TATTAACAACAACCTCACTGGTCGGGATCTGGATGAATTTAACAGAATCTACTACGGCCGAAAGAATCATCTA
GAAATCAAAATTAAAGACTCATCTCTTGCTGCCGCTAAAGACAATGACTTCGATATAG
CAGCATATGCTTTTCCAGCAAAAAAAGAATCCACAAGACCACCCAGAATTGTGAAAGTGGGTGTTATCCAACA
TTCCATTTCCGCGCCGACCGACCGCCCGATCAATGAACAGAAAAATGCAATTTTCGCTAAAGTAAAGAAAATC
ATCGATGTCGCCGGACAAGAGGGCGTCAATATTTTATGTTTCCAAGAGTTATGGAATA
TGCCATTTGCTTTTTGTACACGTGAAAAGACGCCGTGGTGTGAGTTCGCTGAATCTGCTGAGGAAGGGCCCAC
AACACGCTTCCTTCGCGAATTGGCAATTAAATACTCAATGGTAATAGTTTCTTCTATTTTGGAACGCGACGAA
AAACATGCTGATATTCTCTGGAACACTGCGGTCGTTATCAGCGATACTGGCAACGTGA
TCGGCAAACACCGTAAAAACCATATTCCAAGGGTTGGAGATTTCAATGAATCTAATTACTACATGGAAGGTAA
CACGGGACACCCAGTGTTCGCGACGAGATATGGTAAAATAGCTGTTAACATTTGCTTCGGACGTCACCACGTT
CTTAACTGGATGATGTTTGGACAGAATGGAGCTGAAATTGTATTTTATCCATCAGCTA
CGATCGCTGCCGATGCTGGAAGCG
>1002049394_1 Heli.1-ES585557.1.5
GTAGCGGCTCCAGTTGCATATCACGCTGCTCCTGTAGCTTACCATGCCGCCCCCGTGGCCTACCACGCAGCTC
CCGTAGCCTACTCTTCAGCTGCAGCTGTTTCGTCGCAATCCATCCAACGTCATGACGTAAGTCATGCTTCCGT
AGCCGTTGCTCCCGTTGCCCACTACTCCGTTGCTCCCGTAGCTCATTATGCTGCCCCC
GCACGTTACGCATCTGCAGTGTCATCTCAATCTATTCAGCGTCATGACCAACCTCGTGTTTCTATCGCTGTTG
CTCCCGTTGCCCACTACGCTGCTGCGCCCGTTGCCCATTACGCCGCCCCTGTCCACTACTCATCAGCTGCAGC
CGTATCTTCTCAATCCATCCAACGTCATGACCAGTCCCATGCCGCCATCGCCGTCGCT
CCCGTAGCCCACTACGCCGCCGCTCCCATTGCACACTACGCCGCCCCTGTCGCCCATTACGCTGCGCCCGTAC
ACAGCATTGTGGCCAGTCACGATGACGAATACTCTCACCCCAAATACGACTTCTCTTACTCTGTAGCCGACGG
TCACTCCGGCGACAACAAGTCCCAGCACGAGAGCCGCGACGGTGACGTAGTACAAGGC
GAGTACTCTCTGGTTGAAGCTGACGGCTCCATCCGCAACGTCAAGTACAGCGCCGACGCACATAACGGATTCA
ACGCTATTGTCAGCAACTCTGCACCCAAACACGCCATCATTGCTCACCATTAAAATCTAATTTATTAACTTTG
TTATTAGTTATTAATAATTTTTGTTGATGTGAGAATTACAGTGCATTAGTAATAAAAA
>1002049396_1 Heli.1-DT667068.3.5
TTATAGGCGGTTAACACTGCCATGCATAAAATCAAGATTACAAGATCTTGATATGCGGTGATAGTCTCCAGAA
TCAAACCAGTTCAAACAAAAATTAATACAACCTCTTTCCAGGCCAAAAAAAACGCACAAATTACTCACCGTAC
GGGCAAAACTGCACGCCAATAAAAAAATTGGCACAGGCTTATTATTCTCGGCACACCG
AGATAGGTCTTCCAATAAATCATTGTATAGAACGTTTTTTATAAACATTTATTCAAAATGTCTGAAACCAAGG
ACGGTATCGAGGATATATGCAAACGCGTATCCGAATTATCCGTTTTATCGTTCAGAAAAGTGGATGAAATAAA
AAAGTTGGCTAAGCATTTCAAATTTGTGATAAACAATCCGATCACTCAGCAAAATGCT

GTTGTTTTGCGCTACTTGAGGGATTTAGATGAAACCCATCGAAAAATCTTCCTCAAAAGACGGGAAGGTGTTG
AAGTCGAATATATATTTACGACTGAGAAGAAAAGTAACCTCCAAAGACAACGTTTCAACAGTCTTCCCGTTAG
TGAAGTGCCGGAGTTATTGAAAACTGAATTTTTGATAGCCGACACTGCCAAACAAGTC
CACATTTGTACAGACTGTAACGTAGAAATTGAGTTAAACGACGAGATTCCGCTAATGGAGTCGCTTCAAAAGC
ATTTTAACTTGGAGGTGCATTTGCCGAAGTTGAAAAGAGAGAGTATTGACGTTGCAGAACCAATAATCTTACC
TAAAATGTCC
>1002049397_1 Heli.1-ES587780.1.5
ACATGTTCTCTAAAATTTTCGGTCTTAGCGCTGTATTGGCAGTGGCTGCCGCAGGTCTGCTGCCCGAGCCACA
CTACTCCTCAGCGGCTGCAGTGTCTTCTCAAAGCATTGTGCGACATGACCAGCCACACGCTGTAGTCGCGGCC
CCAGTGGCGTACCACGCCGCCCCTGTCGCCTACCACGCCGCTCCTGTCGCATACCACG
CCGCCCCCGTCGCCTACTCCTCGGCCGCAGCTGTCTCTTCTCAGTCTATCCAGCGTCATGACCAGTCACATGC
CGCCATCGCCGTCGCTCCCGTAGCCCACTACGCCGCCGCTCCTATTGCACACTACGCCGCACCTGTTGCCCAT
TACGCTGCACCCGTACACAGCATTGTGGCCAGTCACGATGACGAATACTCTCACCCCA
AATACGACTTCTCTTACTCTGTAGCCGACGGTCACTCCGGCGACAACAAGTCCCAGCACGAGAGCCGCGACGG
TGACGTAGTACAAGGCGAGTACTCTCTGGTCGAAGCTGACGGCTCCATCCGCAACGTCAAGTACAGCGCGGAC
GCACATAACGGATTCAACGCTATTGTCAGCAACTCTGCACCCAAACACGCCATCATTG
CTCATCATTAACATACTATATTTTAAATAAGTTAATAAATTAATTTAAACTTTAAAAAATACAAACACATTA
>1002049398_1 Heli.1-CO729479.1.5
GTCGACCACGCGTCCGCATCAACTCATCAAACAACAATGGCCTTTAAGATCGTGATCTTCTCCTGCCTCGTGG
CTTTGGCCCGCGCCAGTGCATCTGTCGCAGTAGCCGCACCACTTGCAGCGGCTCCTGTTGTCGCAGCACCAGC
AGTTGCAGCCAGACTCGAAGAATTCGATCCTCTTCCGCAGTACAGATTTGGATACAAC
GTTGCCGATGCACTCACTGGCGATTACAAGAGTCAACAAGAACAACGTGATGGTGACTTAGTACAAGGATCCT
ATTCGCTGGTGGACCCTGATGGCACACGCCGCATTGTTGATTATTCCGCTGACTCCGTTAACGGATTCAATGC
GGTCGTGCGCAAAGAACCTTTAGTAGCCGCTGCTCCAGCCGTCGTTGCCGAGCCTGCC
GTGGTACCAGCTCGTATTGCCGCTGCTCCAGTAGCTGCAGCCCCTCTTGCTGCCGCCCCCGTCTTTGCCGCCC
CAGTAGCCAAATACACAGCTGCATACTCCGCCCCATTGGCTTACCCAGCATACAGTGCCCCAGTAGCAAAAGT
AGCGGCATACAGCGCACCATTCGCTTACTCTGCTCCTGTTGCCAGATATGCTGCATAC
TCTTCACCTTTGATAGCTGGCAGATACGCAGCAGCTCCAGTGGTAGCTGTCTAAGTATTTGCTGTACATAATT
TTGACTGATTTCTCTCTATATTTGGCCAATAAATTTTTTATTTGTGA
>1002049399_1 Heli.1-DT668438.3.5
AGCAACCCTCAAGGAGCAGAGAATTATAGTTTTCTAGTGACTGGCCCACGCGAACCGGGCACAAGAACAATCA
AAATGAGCTTCCCTGCCGCCGAAACTGCTCTGGGGAGCAGCACATGGGCAGCAACCAACCTTTTCTACGTATT
GTTGGTGCCAGCGATCATCTTGTGGTACACCTACTGGCGTTTATCCCGGAGGCACTTG
TATGAGCTCGCAGAAGAAATAGCCGGGCCCAAAGGATTACCACTTATAGGAAATGCTCTAGAATTTACTGGGG
GCTCTGCCGAAATTTTCGACAAAATCATACAAAGAAGCGAGGAATTCAAACAGGAAAGTGCTGTGAAAATGTG
GATTGGACCCAGACTTATTGTGTTCTTGTACGATCCTCGCGACGTAGAGCTTATTCTA
AGCAGTCATGTACATATCGATAAAGCCGAAGATTACAGATTCTTCAAGCCTTGGCTTGGAGATGGTCTTCTTA
TCAGCACTGGTCAAAAATGGCGTTCTCATCGCAAATTGATCGCACCTACATTCCATTTGAATGTTCTTAAGAG
TTTCATAGATCTATTCAACGCTAACTCCAGAGCTGTCGTTAACAAACTGAAGAAAGAA
GCTGGAGAATTCGACTGTCACGATTATATGAGTGAATGTACCGTGGAAATCCTTCTTGAAACTGCTATGGGTG
TTAGTAAGAATACTCAAGACCAAAGTGGATTTGAATATGCTATGGCTGTGATGAAAATGTGCGACATCTTA
>1002049401_1 Heli.1-DT662995.3.5
TGTGTTCAACAGTTACCATCGGATATTAGACATTAAAAATGTTCTCATCCTTGCTGGAGACGGCGCGGAACTC
GCCCTTCCGCAGCCCTTTGACTACCGCGAGCTGCGAACCGGCCCTTGTTGATTCACAGGGCATGGCCGCGTCC
GCTCCAGTCCAGGGCGACTCTTGCGAGTTCGGTTCACCCAAATACTTCGCGCTTTGCG
GACTTGGAGGAATTCTTTCTTGCGGTATAACACACACAGCAGTGGTGCCCTTGGACTTAGTAAAATGCCGTCT
ACAGGTGGACCCCGACAAGTACAAGAATGTGGTCAACGGGTTCAAGGTGTCCGTACGGGAGGAGGGCATGAGG
GGCCTCGCCAAGGGCTGGGCACCCACCTTCATTGGATACTCTCTACAGGGACTCTGCA
AATTTGGCTTGTATGAAGTGTTCAAAGTAGGTTACTCTGGCCTCTTAGATGAAGAGATGGCTTACAGCTATCG
TACGTTTGTGTACTTAGCTGCGTCTGCCTCAGCTGAATTCTTTGCTGATATTGCCCTTTCTCCATTGGAAGCT
GCTAAGGTCAGAATCCAAACTATGCCTGGTTTTGCGAACACCCTAAGGGAGGCGTGGC
CCAAGATGGTACAAAATGAAGGTTATGGCACATTCTACAAGGGCTTAGTGCCTCTGTGGGGAAGACAGATACC
ATACACCATGATGAAGTTTGCCTGTTTCGAGAAGACCGTGGAATTGTTGTATAAGTATGTAGTACCCAAGC
>1002049402_1 Heli.1-ES586383.1.5

CTTTCGAGCACATTCGGAAAAGTCTCACAATGCAAATTTTCGTCAAGACCCTTACGGGTAAAACCATTACCCT
CGAGGTGGAGGCCTCCGATACTATAGAAAATGTAAAAGCTAAAATCCAAGATAAGGAAGGTATTCCACCAGAC
CAGCAACGTTTGATCTTTGCTGGTAAACAACTAGAAGATGGACGTACATTGTCTGACT
ACAACATTCAAAAGGAATCCACATTGCATCTTGTGCTTCGACTTCGCGGAGGTACCATTGAACCCTCCCTTCA
CATCTTGGCCTGCAAGTACAATTGTGAAAAAATGATCTGCCGCAAATGCTATGCGCGTCTTCATCCACGTGCA
ACTAACTGCCGCAAGAAGAAATGTGGACACACCAATAACCTCCGCCCCAAGAAGAAGA
TCAAGAACTAAATATTGTTCACATTCTTATTAATGGCATACAGTTAATGGCTTTCAATAATTAAAAAATAATA
AAAACAGTAAA
>1002049403_1 Heli.1-DT665413.3.5
AGTTGAGAAGATTCTCTTGATTCTTTTTGTTATTATTTAAATATATAAAACAGTATTCAAAAATGGCTTTACA
TATACCAAAAGCCCCTGGAGTACCGCAAATGTTAAAAGATGGAGCACGAATGTTTTCTGGCTTAGAAGAAGCG
GTATATCGCAATATAAATGCGTGCAAACAATTTGCCCAAAGTGTTCGTTCAGCTTATG
GTCCTAATGGGATGAACAAAATGATTATCAACCACATCGACAAGCAATTTATTACAAGCGATGCCGGTACTAT
TATTCGTGAATTAGACGTAGAACATCCAGCTGCTAGACTGATGGTACTGGCCAGCCAAATGCAAGATTCTGAA
GTCGGTGATGGAACCAACTTTGTCATAGTTTTATCTGGGGCACTCCTTGAGGCTGCAG
AAGAGTTATTGCGTCTAGGTGTCACTACAAGTGAAATTGCAGAAGGATATGAAAAAGCCCTTGATAAGTGCCT
AGAAATATTACCAGAATTAATTTGCCACGAAATAAAAGATTGCAAAGATATTGAAGCTGTGGTTCATGCTATA
AAACCAGCAATTATGTCAAAACAATATGGAAATGAAGAATTTATTGCAAGTCTTGTAG
CCAAAGCTTGTGTCGCAATCTTACCTGAAAAAACTACATTTAACGTAGATAATGTGAGGATATGCAAAATACT
TGGAGCTGGCCTGTTACAATCTGAAGTGTTATCAGGAATGGTATTCAAAAGGGAAGTTGAAGGAGACATAACA
AGTGCTAATAAAGCTAAAGTAGCAATATATTCATGTCCAATTGATATAACACAAACAG
AAACAAAAGGAA
>1002049404_1 Heli.1-DT665998.3.5
TCTCAGCCGGTTAAAGCATTTTCAACATATTTAAAAGAAATAACATCTAATTTATAATTTTAATGATACTTCC
CTATTAAGTTAGATCAAGTTTATTTCAACCTTACCGCATTTAAACTACACATCAAAAATGAAGCCGCCATACA
AAATTGCTGATGAAAAGTTAGCTGAGCTTGGACGTAAGGAGATCATGTTAGCTGAGAA
AGAAATGCCAGGATTAATGGCTTGCCGTAGAAAGTATGCTCCAGCAAAGATTCTGAAAGGTGCAAGAATAGCT
GGTAGCCTTCACATGACTGTACAAACTGCTGTACTTATTGAGACTCTTATTGAGCTTGGAGCTGAAGTCCAGT
GGTCAAGTAGTAATATTTACAGCACACAAGATGAAGCAGCAGCTGCATTAGTGGCAGT
TGGAATTCCAATCTATGCCTGGAAAGGGGAAACTGAGGAGGAATATGTGTGGTGTATAGAACAAACACTCATC
TTCCCTGATGGAAAGCCCTTAAACATGATATTAGATGATGGAGGTGACTTAACCAATTTAATTCATACCAAAC
ATGCAGAACTTCTAGAAGGTGTTCGAGGTATCTCTGAAGAAACAACTACAGGTGTCCA
CAACTTATACAAAATGTTCCGAGAAGGTCTATTAAAAGTGCCAGCTATCAATGTAAATGATTCTGTCACAAAA
AGTAAGTTTGACAATTTATATGGATGCAGGGAATCACTTCTTGATGGTATTAAGAGGGCAACTGATATAAT
>1002049405_1 Heli.1-DT665501.3.5
CACGAGGCAACACTTGAAAATATGAAGGCTATAGTTTGTGTACTCCTTTTGGTCGCGGCCGTCTACGGTGCAG
AAGAGAAAAAGCAAGAGAAACGTGGCCTGCTAGGTCTCGGGTACGGTGGTGGTCTCTCTCTCGGTGGCCACGG
TCTGGGTCTCTACGGTGGCCACGGTCTGGGTCTGGCCGCTGCACCTATCGTTAGCCAT
AGCGTCGCCATCCCTGCTCCTATCATCAGCCATGGCATCGCCGCTCCCATCCTAGACCATGGTCTGATCGGTG
GTCACGGTCTAATCGGTGGTCACGGTCTGATCGGTGGTCATGGCTTGATTGGTGGTCATGGACTCATCGGGGC
ACCACTGCTAGGCCTTGGTCATGGCGGCTGGCGTTGAGGCTACACTATGTAAATAAAA
AAAAATCAAAATTAATCGTACATCTTGCAAGCACACACGTTGCGTGGGTCTTTCAGTGCCAAATTAGTTTTTT
TGGGTGTTTCTATATTATTAAGGTGAATAAAAATTAAAAACATAATAAAAAAAAAAAAA
>1002049406_1 Heli.1-ES585243.1.5
TCAAAAAGCTTGAAAATGGTACAGCGTCTTACTTTTAGGCGACGGTTGTCGTACAACACCAAGTCAAATCAGA
GGAGAATAGTTAGAACACCAGGTGGACGATTGGTCTATCAATATGTTAAGAAACCCAAGAAGATCCCAAGATG
TGGTCAATGCAAGAGCAAGCTCAGAGGTATCCAACCTGCCAGACCCGCAGAACGTTCT
AGACTTTGCTATCGCAAAAAGACTGTGAAGCGAGTATATGGTGGAGTTCTCTGTCACAAGTGTGTTAAACAAC
GCATTGTCAGAGCTTTCCTCATTGAGGAACAGAAGATCGTAAAGGTCCTTAAGGCGCAACAAGCTACTGCCAA
ACCCAGCAAAAAGGCCGCAAAATGAACTTTAGGGCGTAAATAAATT
>1002049407_1 Heli.1-EL596237.1.5
AACGAGGAGGTTGAAGAAGCCAAGATGTCGAAGGTATCTCGTGATACGCTTTACGAATGTGTAAATGCGGTCC
TTCAATCATCAAAGGACAAAAAACGTAACTTTTTGGAGACAGTGGAACTCCAAATCGGTCTTAAGAACTATGA
CCCACAGAAGGACAAGCGTTTCAGCGGCACCGTCAAGCTAAAGTACATTCCCAGGCCA

AAAATGCAAGTATGTGTTCTTGGTGACCAACAGCATTGTGATGAAGCTAAAAACTTGACTGTACCATGCATGG
ATGCCGAGGCTCTAAAAAAACTGAACAAGAACAAGAAGCTTGTCAAGAAACTAGCAAAGAAGTATGATGCTTT
CCTTGCCTCTGAATCACTCATCAAGCAGATCCCACGTCTGCTGGGTCCTGGTCTCAAC
AAGGCTGGTAAATTCCCTGGTCTCCTCTCTCACCAGGAGTCCATGACACAAAAGATTGATGAAGTTAAGGCTA
CCATCAAGTTCCAAATGAAGAAAGTACTCTGCCTCTCTGTGGCTGTAGGACATGTGGACATGACACCTGATGA
GTTGGCACAGAATGTTCACCTTTCAATCAACTTCTTAGTGTCTCTATTGAAGAAGCAC
TGGCAAAATGTACGATCACTTCATATGAAGTCCACCATGGGTCCACCCCAAAGATTGTACTAAGTCCTTACAA
TAAATTTTGAC
>1002049408_1 Heli.1-EL604074.1.5
ACATTACCAACCAACTATACTACAGTATATTCTTATACGCGGTGACTTCACGGCGAGAACCCGTGTCTGTGAT
TTAATTTATTTATTGTACAAAATAGTTTTAGTTAAAATAACTTACTTAGAAAACATGGCCAACAACAGGGCCG
CCAAATCTGGATTTGCCGCCGAAGCCCAGAGAAAGATTAACAGCAAATACAGTGAAGA
GCTGGCTGAGGAGTGCTTGGAATGGATCCGAGTGATTACCGGCGAGCCGGACAACACATCTGGCGACATGGAC
AACTTCTATGAAGTGCTGAAAGACGGAACTGTACTTTGCAAACTCGCAAATACCTTGAAGCCTGGTATTGTGA
AGAAGATCAACGAGTCTAAAATGGCGTTTAAATGTATGGAAAACATAAACGCTTTCTT
AGAAGCGGCCAAGCAGTTCGGTGTGCCCGCACAGGAAACTTTCCAAACTGTCGACTTGTGGGAGCGACAGAAC
TTGAACTCTGTTGTCATATGTCTACAGTCTTTGGGTAGAAAGGCGGGCAACTACGGCAAACCTTCGATAGGAC
CAAAAGAAGCTGAGAAGAACGTGAGAAACTTCACTGAAGAACAACTCAGAGCTGGTCA
GGGGGTCATTTCCCTTCAATATGGCTCCAATAAAGGTGCCACACAAAGTGGAATAAACTTTGGCAACACAAGA
CATATGTAAAGCTTTACTAATACTTGTCTCTTTCTAATACACTAGATTAATGGTACAGAGAAATGGACAGAAA
TTTATGCTTAAAGTTCAAAATTTATGTAACGTTT
>1002049409_1 Heli.1-ES585799.1.5
CACAGAAGTCACTGGTGACGAGTTCAATGTGGAACCAGTTGACAATGGACCAGGGGCCTTCAGGTGTTACTTA
GATGTTGGTCTTGCCCGTACAACAACTGGGGCTAGAGTTTTCGGAGCAATGAAGGGTGCAGTAGATGGTGGCC
TTAATGTTCCTCATTCCATCAAGAGATTCCCAGGATATGATGCGGAGGCAAAGAAATT
CAATGCTGAAGTTCATAGAGCGCACATCTTTGGTCTTCATGTAGCTGAATATATGAGAAACCTTGAGCAAGAA
GATGAAGATTCCTTCAAAAGACAATTTGGCAAATACATCCAACTTGGTGTTAGTGCTGATGCTATTGAAGGTA
TCTACAAGAAAGCCCATGAAGCAATCCGAGCGGATCCATCTCACAAGAAGAAGGAAGC
AAAGGACTCAGTCAAACAGAAACGCTGGAACAAACGTAAGCTGACACTGGCTGAGAGAAAGAACAGAATTAAG
CAGAAGAAAGAATCTTACATCAAGAAATTACAAGCAGAAGCTTAAAGGCTGACTGACAACCTCGTGCCGAATT
CGGCACGAGGTGACTCCCAAGATGTTCCAATGCTCCAAGCGACCATAGCCAAAATGCC
TGAAGAAGAAGCTGTATACTACATGAAGAGATGTGTAGATTCTGGTCT
>1002049410_1 Heli.1-DT666312.3.5
CCCATCTGAACTAACACAGACCACGATGAACTCGCTGGTGGTGTTGCTTTCCGTGATGGCGCTTGCCACAGCT
AAGCCCAGCCTCTTCAACGCTGGCGTTATCAGCTATTCTGCCCCGGCTATCGCAGTGGCTCCAGCTGCAGTTT
CTCATCAATCACGCGTTGACATTAAATCATCTCCAGCCATAGTCGCAGCTGAGGTTGT
AGCTCCTGTCAGCCGTGCAATCGTGGCGGAACAAGCTGTAGTAGCTCCTGCAGCTTACTCATCACCTGCCGTG
TACGCAGCTCCAATCATTGCCCCTGCTGCAGTTTCATCTCAGTCTCGTGTTGATATTAAATCATCTCCAGCTG
TAGTTAGTAGCGTTTCTGTTGGCCCTGTAGCATACGATGCATCCATTGCTGCTCCTCT
TGCATACGGTGTATCTCTTGCTGCTCCTGTAGCGTATGGAGCATCTTTCGCTGCGCCCGTACCGTACGGTGCA
TCTATCGCTGCACCCGTAGCATATAGTGCATCAGTTGCAGCGCCTGTAGCGTACAGTGCTCCTTTGGCTGGCT
TTTCATCCATCATCTCACCTGCCATCTCTGCAGTTTCTGAGGTCGCCAAACCTGAAGT
TGCTGAATTACCAGTCGCCCCAACTGAAACACCTGAAGTTGCTGCCGCTCGTGCCGCCCATCTTGAAGCCAAA
GCACTTGAAGAGTCACATAAAATTGAGAAACGATCTGTTGCTTTTGTATCAGCTGCTCCACAGGCATCCCTAT
ACGTGGCATCCCCAGTTGCTAGCTACGCTGCACC
>1002049411_1 Heli.1-ES586517.1.5
GAAGTCAAATTTTATCCCTTGAAAATGGCTTTTAAGCTTGTCATTTTTGTGTGCGCTGTAGCCGCAGCCCACG
CCGGCTTGGTCGCTCCCATCTCTTATGCGGCGCATCCCGCGCCGGTCGCATACGCCGCTGCCCCAGTGGTCCA
TGCAGCTCCAGTTGCGTCATACGTCCACTCCGCCCCTGTGGCCTACTCCGCTCCCGTT
GCCAAAGTCGCCGCCGTGGCTCCTGTAGCCAAGGTAGCCGTTGAGGAGTACGACCCACATCCTCAGTACAGCT
TCGCTTATGATGTACAAGATGGTCTCACTGGTGACTCCAAGAGCCAGCACGAATCTCGTGATGGTGACGTCGT
CCACGGTTCCTACTCCGTTGTCGACCCTGACGGCATTAAGCGTACTGTCGAATACACT

GCTGACCCACACAACGGTTTTAATGCTGTTGTACATAAAGAACCAGTAGCAATTAAGGCTGTAGCCCCCGTTG
CTAAAGTAGCTGCTCCTCTAGCATACGCTGCTGCTCCAGTAGTCCACGCCGCACCAGTAGTTCACGCTGGCCC
CGTGGTACACCATGCCCCAATAGTGCACGCTGCCCCCCTAAGCTACTCGGCGCCCCTA
TACCACCACTGAAATGATAATTCAAATATAAACTAGTCATCGAAAAGCAGAAACAGTTAATAATTTATTGTAG
ATATACGATTCTAATAAATGTAT
>1002049412_1 Heli.1-EL601013.1.5
AGTTGATTATAGACCCTGACCTTTAACTGGTGCTATTGGAGTAATAACCTTAGTTACCGGAATAGTAAAATGA
TTTCATAATTTTAATATAAATTTACTATTTATTGGTTATATTATTGTATTATTAACTATATTTCAATGATGAC
GAGATATTTGTCGAGAAGGAACATTCCAAGGTAAACATACTATTATAGTATCTAAAGG
ATTACGATGAGGAATAATTTTATTTATTATTTCTGAAATTTTCTTTTTTGTATCTTTTTTTTGAGCATTTTTT
CATAGAAGTTTATCCCCCAATATTGAAATTGGATCAATTTGACCCCCTACTAGAATTACCCCATTTAACCCTT
TTCAAATTCCTTTATTAAATACAATTATTCTTATTACATCCGGAATTACAGTAACATG
AGCTCATCATGCTTTAATAGAAAATAACTTTACCCAAGTATCTCAAAGATTATTTTTAACTATTTTTTTAGGA
TTTTACTTTACTATTCTTCAAGCATATGAATATTTGGAAGCTCCATTTACTATTGCAGATAGAATTTATGGCT
CTACTTTTTTTATAGCAACCGGATTCCATGGATTACATGTAATTATTGGAACAATCTT
CCTTTTAACATGTTTTATTCGACATATTAATTACCATTTCTCAAGAACTCATCACTTTGGGTTTGAAGCAGCA
GCATGATATTGACATTTTGTTGATGTAGTTTGATTATTTCTTTATAT
>1002049413_1 Heli.1-DT665451.3.5
GATGTTCAAATTAGTAGCTCTGTGTTGCTTCCTCGCCGTAGCCTCGGCTACACCTGGTGTTGTCGTACCACTG
GCATATTCATCGAATGTGCTAGCATCGGCACCAGCAGTCGTGTCAAGCTACCCCAGCAGCCTGGCATACTCAT
CTCCTGTCTTCTCTTCGGCGCCATTGGCCTACCCCGGATGGGACTACCAACAACACCT
CATCAAGAAACGTTCTTTGAGCTACATCGCGCCGTCTTCTTACATCGCACCATCTTCTTACATCGCGCCGTCT
TCCTACATCGCACCCGCTGCCTACAGTTACCCCGCTGCTCCTTTAGTCAGCTCTTACTCTGCAGTTGCACCTC
TGGCTAGTTACCCAGGGCCCGTTTACACCGGTGCTGCACATCTGATCAAGAAGAGGAG
CGCTGTACTCTTACCTAACAACTACGTCGCACATGCCGCATACTCTGCATCCGCACCTATCTTAGCATCAACT
TATGCGGCTGCCTCTCCCATTGCACCTGTTTGGTCTGGCTCTTTGTATCCCGCTGCTCAGTATGCTCAATTCA
TCAAGAAGTAAACTGCTCTTTTAGCTAAATCCACGTTCCGTCCGTTCTACCACTTCTC
GTACCTCAAAATGCGAATTTTATCTTCTATTCCCTAATCATTGACTTTATTATCTCTTTGCGGACACTTGTGC
CATCGTCTTGTGTACTTGCCTTTTAAGTTAATTTGATTTAAGTGTGCATGCTACACATACATTTATATTAA
>1002049414_1 Heli.1-ES585181.1.5
ACCAAAGATTTTGGACTCCGGGGGGAGTATGGTTGCAAAGCTGAAACTTAAAGGAATTGACGGAAGGGCACCA
CCAGGAGTGGAGCCTGCGGCTTAATTTGACTCAACACGGGAAATCTCACCAGGCCCGGACACCGGAAGGATTG
ACAGATTAACAGCTCTTTCTTGATTCGGTGGGTGGTGGTGCATGGCCGTTCTTAGTTG
GTGGAGCGATTTGTCTGGTTAATTCCGGTAACGAACGAGACTCTAGCCTGCTAAATAGGCGTCGTCATTTAGG
TGTGCGCGATTTCGGTCGCGCAACTCACTGGCGACGTATTAAAATTCTTCTTAGAGGGACCGGCGTCTTCGAG
CCGCACGAGATTGAGCAATAACAGGTCTGTGATGCCCTTAGATGTCCTGGGCCGCACG
CGCGCTACACTGAAGGAATCAGCATGTTCTCCCTGGCCTAGAGGCCCGGGCAACCCGTTGAAACTCCTTCGTG
CTGGGGATTGGGGTTTGCAATTATCCCCCATAAACGAGGAATTCCTAGTAAGCGCGAGTCATAAGCTCGCGTT
GATTACGTCCCTGCCCTTTGTACACACCGCCCGTCGCTACTACCGATTGAATGATTTA
GTGAGGTCTTCGGACCGACACGCGGTGGCTTCACGGCCGTCGGCGTTGCTGGGAAGTTGACCAAACTTGATCA
TTTAGAGGAAGTAAAAGTCGTAACAAGGTTTCCGTAGGGGAACCTGCGGAAGGATCATTAACGTGTGTGTGCG
TGTTGTCGTGTTTCTCGCCCTCACCGGCGACGAAGAAGCCCTCATCCACATGACAAAG
ATATGATTTATAAAAAGAGATAAAGGAGAGGCGGGAGCGAAAGCGCGCGCGCGCGCGCATACATTTCGACGAA
GGGACGTGCAACGCGCGAGCACACGACGGTGCATC
>1002049415_1 Heli.1-DT665411.3.5
GCACGAGGCTTTTTCTTGCTGTATATTTATTCACACATAAGATGCAGGCGTGGTTGGTGGTGGCGTGCGCAGC
GGCGCTAGCAGCGTTGGCGTCTGCCCAAAAACCCGGACGGTTCCTCTCCCTACCGATACCGCAGAAATGCGCT
AACAGACCCAAAGAATTTTCTTATCGCGGACACAATTACTTCTACACCGGCCACGTTC
CTGAGCTCGCTAATAGGAAATTCGACTGGCTCGATGGACGCAATATTTGCCGTGAATATTGCATGGATCTTGT
TTCAATGGAAACACAAGAAGAAAACAATCTGATTTTCAAACTAATTCAACAAAGTGATGTCCCGTATATCTGG
ACCTCGGGTCGTCTCTGTGACTTCAAGGGATGCGAGTCGCGTCGAGACCTCGAGCCTA
AAAATATTTTGGGTTGGTTCTGGTCCGCTAATCGCGAAAAGATATCAGCAACTAATCAGATCTCACCCGGTTG
GGGTTACAACCCGTGGTCTCAAACCGGTCACAAAAAGCAACGTCAGCCCGACAACGCCGAGTTCGATATCAAT
GGAACCACCGAATCATGTCTGAGTGTTCTGAACAACGTATACAATGATGGTATAGCAT

GGCACGACGTCGCCTGTTACCACGAAAAGCCAGTTGTATGTGAGGACTCAGAGGAGCTCCTCAATTACGTCGC
GAGCACCAATCCTGGCCTTCGCTTGTAATTGAATAACATATTTAAAT
>1002049416_1 Heli.1-ES587437.1.5
AAGAATTAAATAAGCATTATAATAATAGTAATAGCAACATTTTTAAATTACACATTTAGTAACAAACAACAAT
ATTTATAACAATAAAAATAATTTAGGCTTCGCCAGTCTTCCATAAGTAATAATTAAATTAATTTTCATGATGA
CTCTCAATATGGACGTGTTGACCAGGGGCTGAGTGCTTTACAACAGCGTTGAATCCGT
GGTGTTTGTCCGAGCTGTATTCAACAGTTCTCACGGAGCCATCAGCTTCGTGAAGAGAGTAAACTCCCTTTAC
GCTATCACCATCGCGGACCTCATGCTGGAACTTGTTGTCTCCGGTGTGGGGATCTTCAACTTTGTATTCATAC
TGGTATTTGGGATAAGCATAATAGTCGATAGGCTCATCGTGGGAATGGGATTCTACTT
TATGGTGTTGCCCTCGTGCCGAATTCGGCACGAGGGTGAAAATGACGAAGGGTACATCAAGCTTCGGTAAACG
CCGAAATAAGACACATACACTATGTAGAAGATGTGGAAGGTCGTCATACCACATTCAAAAATCTAAATGTGCC
CAATGTGGATACCCTGCAGCTAAGCTGCGATCTTATCACTGGTCAGTGAAGGCTAAGC
GTAGGAAGACCACTGGAACTGGCCGCATGCGTCACCTCAAGATTGTAAGAAGGCGATTCAGAAACGGTTTTAA
GGAAGGCAAACCTACACCTAAGAAAGCAGTTGCATCCTCATAGACTTATTGCCTAATGTCAATAAAAAATA
>1002049417_1 Heli.1-ES587782.1.5
TTATTAATGGATCTTTAATTGATTTACCTTCTCCATCTAATATTTCTTCATGATGAAATTTTGGATCTTTATT
AGCTTTATGTTTAATAATTCAAATTATTACAGGATTATTTTTAACTATATATTACTCAGCAAATATTGATATT
TCTTTTTATAGAGTAAATTATATTTGTCGAAATGTTAACTATGGATGATTAATTCGTA
CTTTACATGCTAATGGAGCATCTTTTTTTTTTATTTGTATTTATTTACATATTGGACGAGGAATTTATTATGA
ATCTTTCAATTTAAAATTAACTTGACTAGTGGGAGTAATTATTTTATTTTTACTAATAGCTACAGCTTTTATA
GGTTATGTTTTACCTTGAGGTCAAATATCTTTTTGAGGGGCTACAGTAATTACTAATT
TATTATCTGCAATTCCATATTTAGGAAATATATTAGTAAATTGAATTTGAGGAGGATTTGCTGTTGATAATGC
AACTTTAACTCGATTTTATACATTTCATTTCTTATTTCCATTTATTATTTTAATATTAACTATAATTCATTTA
TTATTTCTTCACCAAACTGGATCTAATAACCCTTTAGGAATTAATAGAAATTTAGATA
AAATTCCATTTCATCCATTTTTTACTTTTAAAGATTTAATTGGATTTATTGTATTAATTTTTTTTTTATCTTT
ATTAACTTTAACAAATCCATATTTATTAGGAGATCCTGATAATTTTATTCCAGCTAATCCTTTAATTACCCCA
ATTCATATTCAACCAGAATGATATTTTTTATTTGCTTATGCAATTTTACGATCAATCC
CTAATAAATTAGGAGGTGTAATTGCTTTAGTTTTATCTATTTTAATTTTAATTATTCTACCATTTACTTTTAA
TAAAAAAATTCAAGGTATTCAATTCTATCCTTTAAATCAAATTTTATTTTGATTTTTAATTATTACTATTATT
TTATTAACTTGAATTGGATCACGACCAGTAGAAATACCTTATATTATTACAGGACAAA
TTTTAACTTTAATTTATTTTTCTTATTTTATTATTAACCCAATTTTAAATAAATTTTGAGATAAATTAATTT
>1002049418_1 Heli.1-DT663329.3.5
GAAACGTCTCTATTTATCGGCCAACTTTATTTAACATACGGTTTTAGGTTAATACCGGGAAGATGGGTGTTAA
ACCTGCAATTGGAAGGAAATCTAACAAGAACCCCCCAATTTTTAGCCATGAATTTGTTATCCAGAACCATGCA
GATATTGTTTCATGTGTTGTAATGGTTTTCCTGGTGGGGCTTATGGTACAGGCCACAA
GTCCCGTTGCGAGTTTATTCATCAGCTTGCACCATAATGTTACTGGAGTAGAACCGTCGCGCGATCTTCCTAA
GGGCGAACCTTTCTCGTATGAAGCTGGTTGGAAAGATGCCTGTGCTGTTTTCTTTTACTCACTAGTATGCATT
GTGATGCATGCAATTTTACAAGAGTATTTCTTAGATAAAATATCAAAGAAATTTCACC
TATCCAAGTCAAGATTAAGTGCTCTAAATGAATCAGGCCAGCTTGTAGTATTCCAACTAGTTACATTGCTTTG
GGGAGGAGATGCTATTCTTCGTGAAGGATTTATTTTCAATATTTCTCAGCTATGGGATGGATATCCAAATCAC
CCAATGAGTTTTCTATTGAAGTTATGGTGGGTTGTCCAAGCTTCTTACTGGGTTCACA
CCATTCCTGAGTTATACTTCCAAAGAATTAAGAAAGACGAATGGTCAGGACGCATAAGACATGCTGCAGCTGC
ATTTGCATTTGTAGCTCTAGCTTATGGTTTCAAAT
>1002049419_1 Heli.1-DT663556.3.5
AGTCGGTGAAGTTAGTGGTGGAATTTTTACGTGTATAACATCAAAAAATGGCGTCTGGTGTGACAGTATCGGA
CGCGTGCAAAACAACCTACGAGGAGATCAAAAAGGACAAAAAACACCGCTACGTAGTGTTCTACATTCGTGAT
GAGAAGCAGATCGACGTTGAGACCGTAGGCGAACGCAACGCCGAGTACGACCAGTTCC
TCGAGGATTTGCAGAAGGGCGGCACTGGAGAGTGCAGATATGGGCTCTTCGACTTTGAATACACGCACCAGTG
CCAGGGCACGTCGGAGGCGAGCAAGAAACAGAAGTTGTTCCTAATGTCGTGGTGCCCCGACACCGCCAAGGTC
AAGAAGAAGATGTTGTACTCTAGCTCCTTCGACGCCCTGAAGAAGTCGCTGGTAGGCG
TACAAAAGTACATTCAAGCGACTGATCTCTCTGAAGCTTCGCAAGAGGCCGTCGAGGAAAAACTACGCGCCAC
CGACCGCCAATAAGCATTTACACAGAACGAATAATGATAATGTCGCAATAATGCTAATAAATAACACTTAAGT
TTCTTAAGAACTTTTTTACTGTCCCCGAGCTCAGTCATATTGACTCGTTGTTGTCACA

TTATCAAATTCACAAGCTGTACTGTTTTTTCATCAGTTTCTTTTTATACTGAAAGGGTCTACGCGATGAGCAG
TGATAAGATAAATAAATAATCATTGGTTCTTTAATTACATCTAATAAGCTTTCCTGTCTTGTCGGCATGTATG
TACAGTCACCCGACCTTAGAGTAAGGGATGTTTATGTAATAATTTATATTAATACTAA
TTATTATATATATTTTTCCTATAAACTAAGCGAGTT
>1002049420_1 Heli.1-DT664293.3.5
AAAAAGTGTGACGAGCGTTTGTTTTATCAATTTCTAAAAATCAAATTAATAAAAAATGTCGACTCTTGCTGCA
CCTTTAACTGTTGCGGGGACCAGATATTCTGGAGCCCCAGTCAGAAGTCAGAATGTAATGGCAGCTGCAGCAA
TTGCCAATATTGTCAAAAGTTCTCTGGGTCCTGTGGGTCTGGACAAGATGTTAGTTGA
TGATATTGGAGATGTCACAGTAACTAATGATGGTGCTACTATTCTCAAAATGTTGGAAGTTGAACATCCCGCA
GCAAAAGTATTAGTAGAGCTTGCTCAACTGCAAGACGAGGAAGTTGGTGATGGCACCACATCTGTTGTTATTA
TTGCTGCTGAGTTATTGAAGAATGCTGATGAGCTAGTCAAAAATAAAATTCACCCAAC
CAGTATTATTTCTGGATACCGTCTAGCCTGTAAGGAAGCTGTAAAATATATCCAGGACAACTTGACAGTCTCT
GTAGAGTCTTTGGGCAGGGCTTCCGTCATCAACGCAGCTAAAACCACCATGTCATCTAAATTGATTGGAGCTG
ATGCAGATTTTTTCTCAGAAATGATTGTAGATGCCGCACAAGCTATTAAAATAACTGA
CGTCAAGGGAAATTCATTATATCCTATTAAAGCTGTCAATGTTTTAAAAGCCCATGGGAAAAGTGCAAGGGAA
AGTGTTCTCGTCAAAGGATATGCGTTGAATTGCACAGTAGCATCTCAAGCTATGCCTAAGAAGATTGTGAA
>1002049421_1 Heli.1-ES585279.1.5
CGGAAGGCAAGATGTTGATGCCAAAACAAAACCGTGTGTCTATCTATGAATACCTTTTCAAAGAAGGTGTTAT
GGTAGCCAAAAAAGACTACCATGCACCCAAACATCCGGATTTGGAGAAGATACCCAACCTTCAAGTTATTAAG
GCAATGCAATCTTTAAAATCAAGAGGTTATGTCAAGGAACAATTTGCATGGAGGCATT
TCTATTGGTATCTTACAAATGAAGGTATTGAGTATCTAAGGATCTTCCTGCACCTTCCACCAGAAATTGTGCC
TGCAACACTCAAGCGCTCTGTTCGCGCTGAGACTGTCCGACGTGGTGCTGTTGGTAGGCCCGATGCCCCAGCT
CGCTCTGCTGAAGATAGATCAATGTACCGCCGTGCCCCCACTACACCTGGAGCACCAC
ATGACAAAAAGGCAGATGTTGGCCCTGGCTCTTCAGAAGTTGTATTTAGAGGAGGTTTTGGACGTGGTAGACC
AACTCCTTAACTTAAGTTAAGCTTAATAAAAAAAA
>1002049422_1 Heli.1-ES585387.1.5
CACGATACTCAAAATGCAAGGAATTATCGTATTCACCGCTCTTATGGCTACCGCCATCGCAGCTCCACTCGGC
TTGGAGTACAACGCGTATGTGCCGCTCTCCAAGTCAACTTTCTCATCGAGCAGTCAGGTCGTCAACCATGGAA
GTGCCCACATCGTCCACGCCCCAGTAGTGGCTCATGAAGTCGAATACCACGTGCCTTT
AGCCAAGTCCACTGTGACCAAGAGTAGTCAAGTTGTAGACCATGGATCCACCTTCGTTGCCCATGCTCCAGTC
GCCCACGCTGTGGTCGCGCCAGTGTCCCACGTGGTAGCTCACGCTCCTATTGCCCACGCTCCAGTATACTCTG
TCAGCTCTCTCTACTCTTACCATTGATTGATTGTTAGTTGTTAAATAAATTATTTTGT
>1002049423_1 Heli.1-DT663969.3.5
AGTCCTGCCTTGTCTACTAGCGAGTGCGAAGCGACACCGTGAACTGTGTTTACACTCAAACGTCTCCCAGTCA
GTGTTTGTTTAGTTTCGACAGTTTCCACACGACGCACACGTGAAAATGGCTGACAGTGGTCTGAAGAGAAATA
TTCCTATCAAGCTCGGAGATTTTTCAGTTATCGACACCGAATTCTCAAGCATCAGGGA
AAGATTTGACGCCGAAATGAGAAAAATGGAGGAAGAAATGAGCAAATTCCGATCGGAGCTCATGAACAGAGAG
AGCAATAACTTTTTCAAGAGCTCTACAAGCACCACCACATCGTCACAGCACAGTGACAGCAGACAGCTGGCTG
AGCCCAGCCACTGGGACAGCTTAAACTCTCCTTTAATTCAGGATGAAGGCGATGGCAA
GTCTCTTAAACTCAGATTCGATGTCAGTCAGTACACGCCCGAGGAAATCGTCGTGAAGACAGTCGATAACAAA
TTATTGGTTCACGCCAAACACGAGGAGAAGTCAGAGACGAAGTCTGTATACAGAGAGTACAACCGCGAATTCC
TTTTGCCCAAAGGAACCAATCCTGAGGCTATCAAATCGTCGCTGTCCCGAGACGGCGT
CCTGACTGTGGAGGCGCCGTTGCCGCAGCTCGCGATCACCGACAGGAACATCCCCATTCAGAAACACTGAGGC
GGTTGTCGATTCCTCTCTCGACAGCAGCTATTCCATACTACTGTTAATAATAATTTATTATAATTTACTATAA
GGAGTATTGGATATATTCAGATATTTATTTTGAATCTTAATTATATGGAATCTCATCG
TTTTTAACATGTGTATTGTTAGAGTTTTGAATTATTTTTTTTTATTATCCAATTATACCTGCTCTTGGCAGCG
GAGCACAGTTGCGGTTAGCACTGTACGCATTAAATATTGCTTTTAAGTCAATGCTTTTTCTGTGATCGTTAAA
AATGATGAAAATTATTTCGATTTCACGAAGATCACTTTTTGATATTCAAGCGGCATGC
TGTTTTGCACCGTTTTAAATGTTTACCCACGATTTATATTTGAATTTTAAAGTGTATTCGGCCTACAACCACT
TAGAATGGAGCTTATATGAAGTGTTCGGTGATTTTAGTGCAATTGGT
>1002049424_1 Heli.1-ES587056.1.5
CTTATTCAAAGAATGCATCCTGATTTGGCGGGTAAAATAACTGGAATGCTTTTAGAAATTGATAACTCTGAAC
TGCTTCACATGTTAGAGCACGGAGAGTCTCTTAAAGCAAAGGTTGATGAGGCTGTTGCTGTTTTGCAAGCTCA
CCAAGCTAAACAGCAAGCCACAAAGAAGGATTAAGTTGATAAAATTGACAGAGGGTTC

TTTTTTTGCTATATATATACTTTATATTATGCTTTGAACATCTTTATATATATTAATAAGAGTCCCGGTCTTT
TAAAAAAATATATGAATAAAAATTGAAAAATAAAGAAAAATGTGGATTGCACTAATCCAACTTATACTACTTT
TATAATAAATATTGGGAGTGCATATCTGCAAACAAAAATATAGCAACTATCAACATTT
CTATATATGAAACAGTTATGCTAGTAAGCAATTTTGACTTGCTATATTGACCTTTGATTGAAGAAGTTTAATT
TAAGTTTAGTCTTTAATCTTTTCTTTTATTCTCCCTTCATGGAGTTGGCTTATTTTGTTTTTTGCGGTATCTG
TCTTTCTTATTATTTTCTTATCAGTTATGTGAAGGGCATACCTTGTATTGTAAACTTG
GTCTCTCTAATATGACTGATTATAAGATGTACTATTGTTACACATTATATTCTGAAATAT
>1002049425_1 Heli.1-DT664379.3.5
ATACAAGATGCAACCTCAAATCCTAGTGCTAAAAGAGGGTACAGACCAGTCGCAGGGCAAGCCTCAGCTGGTC
TCCAATATCAATGCCTGTCAACTTGTTGTAGATGCTGTCCGAACAACACTTGGACCACGGGGCATGGACAAGC
TCATTGTGGATCACAATGGGAAAGCAGTCATTTCTAATGATGGAGCTACTATTATGAA
ATTGTTAGATATTGTCCACCCGGCTGCAAAGACATTGGTTGACATTGCCAAATCACAGGATGCTGAGGTTGGT
GATGGCACCACATCGGTAGTGATACTCGCAGGTGAACTCTTGAAGAGATTGAAGCCTTTTGTTGAGGAAGGTG
TTCATCCTCGTATAATAGTGAAGGCAGTAAGAACGGCAAGTAAACTAGCCGTGGACAA
AGTTAAAGAATTATCGGTTAAAATTGAGAATCGATCACCTGAGGAACAGAGAGAGTTGCTAACAAAATGCGCG
GCAACTGCAATGTCATCGAAACTAATTCACAGGCAGAAAGATCATTTCTCCAAAATGGTTGTTGATGCTGTAT
TGTCGTTGGACGCACCATTGCTACCGTTGGATATGATTGGTATTAAGAAAGTGACAGG
CGGTGGTTTAGAAGATTCCTTCCTCGTTGCCGGGGTTGCTTTCAAGAAGACATTTTCCTATGCTGGATTTGAA
ATGCAGCCTAA
>1002049426_1 Heli.1-DT662532.3.5
GCACGAGGCACCAACAGTCCTCCAGTGAACACAAGGAGTTAGAAAACCAAAATGGCAGCGAAAGTGTTGTGTA
TAGCTCTCGCATTATTTGGGGCGGCTTTGGCTCAACCCTCCAAACAAGGATTCTGGAATGGAACTCCTATGGA
TAGCATGGTGGAGGAGATGAGATCTGGATGCGCTGAAGGATCTGATCCCACTGCTTGC
ATCAAATACAAGGTTATGTCCTTGTTAGACAGCATCTTCAAAAAAGAGACTTTCCAGATCTCCGACGCCGTGG
AAGTGACAAAGAACGGTGCAGGTGAATCTTCAGCTCGCTCTAATGGTGATTTTATGGATTCCATTGAAAGCTA
CATTCAATCTCATGATGTTACTTTCCAATTGCCCATCGCTGACACTAAAATAACCGTC
AGCCCCAGAAACCTTGAGAATGATGAACTAAGCCTTAACATCAAGTTCAACAAAGAAGGTGCCCGCTCTGTAG
GTGAGGCTCGCAAGGCTAAGCTCAAGAAGATCATCGTCCCCATCCTCGTGTTTGTCCTCTTAAAGGCCATGAC
CCTTATCCCTCTGGCTATCGGAGTCCTTGGTCTGAAGGCCTGGAACGCCCTTCAGCTG
TCTTTCTTCTCTTTCGTCGTATCCGTCGCTCTGGCCATCTTCCAACTTTGCAAGAAGATTGCTGCGGACAACT
CGCACCCGCAGATCGCTGCCCACGGACCATGGGACCCATGGGACGCCGCCTACGCCGCC
>1002049427_1 Heli.1-DT667427.3.5
GTTTCTCACGAGTCTTGTCGGCAAGGAGATAAAACATGGGTTCCGTGGAACGTGCTGGTGATGCGACATCAGA
GGCAGAGTCTGGAGATGAGGAAAGAATCGTAGGTGGCGAGGAATTGGCAGCACAGTTGATGAAAAGTGGTATC
ACCCGCAATGAAATGCTAGCTGCTATCACAACGCGTATCCACGCCGAGGCGATGGCGT
CCCTACCACCCAATGTTCGTCGAAGAATCCGCGCTTTAAGATCCTTACAGAAAGAATTTGTAGACATTGAAGC
CAAGTTCTATTCAGAAGTGCATGCGTTAGAATGCAAGTACGAGAAACTGTACAAGCCCTTATTTGAAAAGCGT
GCTCTCATAGTGAATGGGGGGTATGAACCAACAGATGATGAATGTCAAAATCCATGGA
GGGATGACACAGAGGAGGAGGAGCTCGCTCGTGCCGTTCAAAGTGCGGCTATCACTGAAGGTGATAAGAAGGA
GGAGGAAAATAAACTTGTTGAACCACCCATGGACCCCAACGTCAAAGGAATTCCCGACTTTTGGTATAATATA
TTCAAGAATGTATCAATGTTATGTGAAATGGTGCAGGAATATGATGAACCTATTATTA
AATCTTTGCAAGACATCAAAGTGCAAATGCATGAAGACCCCAGCGGTTTCACATTAGAATTCCACTTCGCTGC
AAATGACTACTTTACCAACACAATCCTCACTAAGGAATATTCCATGAAGTGTAAACCTGATGAGGAGAACCCC
CTGGAGTTTGAGGGACCCGAGATTTATTCATGCAAGGGTTGTGAAATAAACTGGAAGA
AAGGCAAGAATGTAACGGTGAAGACAATAAAGAAGAAGCAAAAGCACAAGTCGCGTGGCTCCGTGCGCACCGT
CACCAAGTCTG
>1002049428_1 Heli.1-EL603347.1.5
GGTTCATTTTTGTAAAAGTGCAGTTGGAGTCTTATTATTGAACAAAAGTTGAATGGTTTCATTCGAATAAATC
GGTTGATATTTTAGTTAGGATAGTAATATCACAGTAAATCAAAATGGGAATAAAATTCCTTGAAGTTATAAAA
CCGTTTTGCAGTATATTACCGGAGATAGCGAAACCGGAGAGAAAGATCCAATTCAGAG
AAAAAGTATTATGGACAGCAATCACATTATTTATTTTCTTAGTATGCTGCCAGATTCCACTGTTCGGTATAAT
GTCATCAGACAGTGCAGATCCTTTCTACTGGATCCGAGTGATCCTGGCTTCGAATAGAGGAACCCTCATGGAA
CTTGGTATTTCTCCGATTGTAACCTCAGGTCTTATCATGCAATTGCTTGCTGGTGCTA

AGATCATTGAAGTTGGTGACACTCCGAAGGATAGAGCATTGTTTAATGGAGCTCAAAAATTATTCGGCATGGT
GATAACAGTGGGTCAAGCTATTGTATACGTTATGACTGGAATGTACGGAGAACCAAGTGAAATTGGTGCTGGA
GTATGTCTGCTTATTATTATTCAGCTGTTTGTTGCTGGTTTGATTGTGCTGTTGCTTG
ACGAGCTCCTACAAAAAGGTTATGGTCTTGGATCTGGTATTTCTCTCTTCATCGCTACAAACATTTGTGAAAC
AATCGTCTGGAAAGCATTCTCTCCTGCTACCGTTAACACTGGTCGCGGCACAGAATTCGAAGGTGCAGTGATA
GCGCTGTTCCATCTCCTCGCGACCCGCCCTGACAAAGTACGCGCTC
>1002049429_1 Heli.1-DT662503.3.5
GCGCCCGGCTCCGAGCTGAGCGAGCGCAACAAGCGGACCATCGGCATCCTGCGGCAGCTGTTCCCGACCCTCA
CACAGAACGCCATCGCGGATGAGAACCAGCTACTGTCTGGAGCGGAGTCCCAACAAGCTGAGGTGCGAGTCGC
GTTCGGTGACAACCAGGCGGCCGCCAGCGACGACCAGCAAACTGCCGCGCAAACTAAC
TCCGCGTCCATCGACGAGATCACATTAGACGATGCTGATGACAGCGAAGAAAATAGAAATAAGAGGTTCCTTA
ACTTCGGAGGATCAGCTTCAGCATCAGGAGGCGGCTCTGGCAACTTCTTATTTGACATCGTGCGACTCGTAGC
GGGATCTTCGTCCGGAGGCTCCAGCGACGGCGGCGCGGCTGAGGGAGAGGACAACGGC
GCCGCGAAGGGCGACAACCTGACGGAGGGCGTGCCCGGCCCCATCACGCGCCTGTTCATCATCGCCAACCGCG
GCATCGCCAACCTCATCCAGGACCTGATCCTGCGCATCGCGCAAACCTCCGAGAGGATAGTCAACTTCAAAGC
ACGATTGATCACCTCTATCATTTAAATGCGTTCGCGCTTAGAATAATGACGAGAGGCT
TCGGTCCTCGCGCCCTCGTATCCCGACTCGGACATTAAACAAATAAAATAAAGCGCTCTATTTCAAGCAACAT
TTCCTCCGAGTCGTGATCCGAGCGGCGCCGAGTACCGCAGAGTGTTACCGCGGCGAGCACTCCCGCTACTTGA
CTGAACACGAAAATATTGAGTCAGGTGCCTTCAGTCGTTACGGCAGTATCGATTGAAA
GATAAATATCCTCTATCATTAGAGGCCGCAACAAATATTAAGGCCGCG
>1002049430_1 Heli.1-DT662576.1.5
GGGTGTCGACATCAACCATAAACACGACAGGAAAGTTAGGCGTACCGAAGTCAAATCTCAGGATGTGTACCTG
AGATTGCTCGTCAAATTATACAGATACTTGGCCAGACGTACAAATGCCAAGTTCAACCAGATTATCCTTCGCC
GTCTCTTTATGAGCCGTATTAACCGGGCGCCTATCTCCCTGTCGCGGTTGGCTCGTCA
CATGAAGAAGCCCACTCGGGAAGGTCTGATCGCAGTAGTGGTCGGCACTATCACCAACGATGTCAGGCTGTAC
AAGGTTCCCAAACTATCTGTTGCCGCTCTTCATGTTACTGAAAAAGCACGCGCCCGTATCTTGGCTGCTGGAG
GTGAAATCCTAACTTTCGATCAGCTTGCCCTTCGCGCTCCCACTGGCCGTAAGACAGT
TCTCGTCCAAGGCCGCAGGAACGCGCGTGAAGCGGTACGCCACTTCGGGCCCGCACCTGGCGCCCCACGCTCG
CACACGAAGCCCTATGTGCGTTCTAAGGGCCATGAGCGCTCTAGACCTAGCCGTAGGTCTAATGTGTAATCTT
TAATATAAGG
>1002049431_1 Heli.1-DT665515.3.5
GAATTAGTAGATCTTGACAGTTTTAAAACGCACTCTGGAGATATTCCAGCACAATATAAAGAACCACCTACAA
GATATTCGTCAGAAAGCAAAACTACTACAGAAAACACAGACAATTCTTTACTTGCCAATGAACCATCTCATAC
TAAAGAAAGTCAAAGAGAAGGACATAGTTTAGCAAGTGGGATTAGATTCCAAGAAATA
CAAGATGATTCCAAATTGCCATCTGATTACAAACATACAACTACTTATGAACAAGACACTACTGAGTATACAA
CAATGACAGCGATGGAGAAAGTTGCTCTAGACTTGTACGCTTATTTAGCTGGTGAAAATCTAAATAATGAGAT
CAGTCCAGCTGGGGACCTGATGAGTTTCGACGGTTCAACTACCGTAGATGAAGACGCA
TGGACGACGGAGTTGGTCAGCACGACTGAAGAGGAAACTACACCGACAACTACGACCACGACCACCACTACCA
CCACCACCACCACGACTACAACTACGACTCCAGCGCCCACGACGACCGCTGCACCAACCCGTCCTTCAAGATT
CAAGGGCCGCCCCGGATCGAGGGCTCGCGCACCTGCCTCTACCAGCGCTGCCACAGAA
GCACCTCAAGAATCCTCTACAAGAGCTCGAGGTCGTTTTGGTAAGCCCAATGGAATCAAAAAGACAACGGCAG
CTGCTGTAGAATCTTCCACCTCCTCCGCTCCAGTCCAATCACCGCAACCAAAGCAGGCGTTTGGACGGCGCGG
CTTGTTTTCGGGTCGCATACGCCCCAGCTCCACGGCTGCTCCTGCGTCAGAGGCCGTA
AGCAGCGCTCCCAGTGAAGCAGCGGCCCCGAGAGCACGTCTTCGCCCCGGATTAAGGAGACTTCCATCATCTA
CTTCTGCCGCACCGTCTTCTGGAGAAGTCACCGACACGCCAGCGTCTGTTGCTAGTCAAAGTGCAGCATCCGG
AGAAGTCGTAGAAACTACGCCAATTCCTTCTAGAAGTATTGGACGTAAACCAGGGGGT
GTCAGACCAGTTTCACTGCGACCCGGACCTAGACTCAATATAAGACCAAGTCCTCGTCTGAGACCCGGATTGG
CGAGCGTAGCACCTACAGAAGCTGCCTCAGAAGCACCCGCTGAAACATCAGCTCCCGAAGTTCCTGCAACAGA
GGCAGAAAATGAGGGCTCCTCCCCCACGCCTGCACCTGAACAACCTCGTGGTGTGTTA
AAGCTACGTAATAGGCTTCAGGTGTCTCCGTCGCCAAAGCCACGCGTCGCCGTCACTCCGCCATCCCGTCGCC
CTAACCCATTGTTGAAGAAAAGGTTGCCGGCTACTGAGGCCACAACGGAGCCACCAAAGAAGCCGACAGAATC
AGTAGAAGAAGAAGCTAGCGCAGAGAAGAGCGAGGCCGAGGCGGAGACAGAGGCCCAG
CCAGCGGAGACCACCGCCGCGCCGCCCCTGCGCGGCCTTGACGCGCTCATCGCCCGAAGGCGCGCCGCCGGTA
TCGGCGCCCGCCCCGCCCGCCCC

>1002049432_1 Heli.1-CO729504.1.5
CGTAGAGTCAACTTCCAGCGCGTCCCGACAAACACACACAAACTTCAAAATGTGCGACGACGATGTTGCTGCG
CTCGTAGTCGACAATGGCTCCGGCATGTGCAAGGCCGGTTTCGCCGGCGACGACGCGCCCCGCGCCGTCTTCC
CATCCATCGTGGGTCGCCCCCGTCACCAGGGTGTGATGGTCGGTATGGGCCAGAAAGA
CTCCTACGTAGGTGACGAGGCCCAGAGCAAGAGAGGTATCCTCACCCTGAAGTACCCCATCGAGCACGGTATC
ATCACCAACTGGGATGATATGGAAAAGATCTGGCACCACACCTTCTACAACGAGCTGCGCGTCGCCCCCGAAG
AGCACCCCGTTCTGCTCACTGAGGCCCCCCTTAATCCTAAGGCCAACAGGGAAAAGAT
GACCCAAATCATGTTTGAGACCTTCAACTCTCCGGCTATGTACGTCGCCATCCAGGCAGTGCTCTCCCTGTAC
GCCTCCGGTCGTACCACCGGTATCGTCTTGGACTCCGGAGATGGTGTCTCCCACACCGTTCCCATTTATGAAG
GTTACGCCCTTCCCCACGCCATCCTTCGTTTGGACTTGGCCGGCCGTGACTTGACCGA
CTACCTCATGAAGATCCTCACCGAGAGGGGTTACTCTTTCACCACCACCGCTGAGAGGGAAATCGTACGTGAC
ATCAAGGAAAAGCTGTGCTATGTCGCCCTCGACTTCGAGCAGGAAATGGCCACCGCTGCCGCCTCCACATCCC
TCGAGAAGTCCTACGAACTTCCCGACGGTCAGGTCATCACCATTGGAAACGAAAGGTT
CCGTTGCCCTGAAGCCCTCTTCCAGCCTTCCTTCTTGGGTATGGAATCATGCGGTATCCACGAGACTGTGTAC
AACTCCATCATGAAGTGTGATGTAGACATCCGTAAGGACCTGTACGCCAACACTGTCATGTCCGGTGGTAC
>1002049433_1 Heli.1-DT665479.3.5
TGAAAACTATTTAAAATTTTTTTTGGTGATATTCAAGAAAGTGTTAATAAAAATCATGCGATTTTTTATATAT
AGTTGTATTTTGCTATCGCTAACTATAGCAAGCATATCAGCTGACTGGTCTTGGGGAGGAGATGAGAAAAAAA
CAGAAAATGCGGACGGAAAATCGACTGAACTATTACAAGGAGAAGAAATTAGTGAAGC
ACAAGGAAAGAGTTTTAATGCTAACGCAACTGTCCTAGATGATATTGTAGACGAATTAGTTAGCAGTAAGCAG
GGTAGAAGTTTAAGCGGATTTGATGACGTGTATAGTGACCCCACCATTAAAGAAGCGCTCGATTCCGGTGACG
ACTTGGAAGCACGAAATTTGATTAAAGGACGTCTTTGTACATTAGGGCTTATCCAGTG
CGATGAGGAAGATTCACAAGAAAAAAGAACTTATCTTTCACCAGATGAACTAATTTATGCTCAACCCGTTGAC
ATAAAGCCTATTGGTAAACCAGTAGCTTCAATTCCAGTGCGCGGACCACCCAGAGCTTACGGACCACCTAAAC
CAATGCTATACCCTCCCCGCCCGCAAAAACTACCCCCAAAAAGACCAGGATACGGAAA
TCCAAGACCTGGATTTTCAGACAAATATGGCGTAGCCGGAAATAATTATCAGTTTTCTCAAAGCAATGGTATA
TATAATGGGTTTGAAGCTAACTA
>1002049434_1 Heli.1-ES584923.1.5
GAAACCATGCCGCTCGCAGTTGACTTATTGCACCCTACGCCTGCATCTGAAAAGAGGAAGCACAAGCTTAAAA
GGCTTGTGCCACATCCAAACTCTTACTTCATGGATGTAAAGTGCCCAGGATGTTACAGAATCACAACTGTATT
TAGTCACGCGCAAAGAGTTGTCGTATGTGCAGGATGCTCCACAATTCTTTGCCAGCCT
ACTGGTGGTCGTGCCAGGTTAACTGAAGGATGTTCATTTAGAAGAAAACAACACTAAACCTACCTGGAGAGAG
AGCCCTAATTTAAGATTTGCATTTTATTTTCAAATGTTTCAATGAGTGATATTTTTCTTATTCATTGGATTTG
TTAAAGCATTACAAATAAAGGCTTTTTCAAACGG
>1002049435_1 Heli.1-ES586042.1.5
TAGAACTGTCAATCCGGAACCATGGCACCCCGGTTTGAAATCGCAATTGGTCTGCGTAAAGGCCACAAGACCA
CGAAAATATCCGCTGGCAAAAAGGGTATTTCAAACAAAGCAATCAGAATCAGACCAGCTAGGCTAAAGGGTTT
ACAAACCAAACACTCAAAGTTTGTCCGTGACTTGGTCCGCGAAGTTGTTGGTCATGCT
CCATATGAGAAGAGGGCTATGGAATTGCTTAAGGTATCAAAAGACAAGCGCGCCCTGAAGTTCTTGAAGCGTC
GTCTTGGCACACACATCCGCGCCAAGAGGAAGCGTGAGGAGATGAGCAACGTACTGGTGCAGATGAGGAAGGC
GGCCGCCCAGGCTCACCACGCTCACGCCCATTAGTCTTATTAAACATTTAAAATATTA
>1002049436_1 Heli.1-DT667140.3.5
TATTCTCGTCGGAGTCGCGCGGGAAACGCACGCGCAGTCGTTACTTTTTTAATTTTTTTATCGCTGTGGAATC
AAAATCGAGACGAGAGCAAGTGCATTCCGTAGTTAATGGCTGTATAGTGAATTGTGTTTTGTGTTTAAATATA
AGTGTTGCCAGTGTTAAATATATATTTTTAAAGATGGGTCGACGGTGGCCTATGTTCT
GTCTGCCGATTTTACTCGCGGCGTCAGTATGTGGAAGCGACATACTGATGATCACGATGGGAGGCACCAAGTC
CCACAAAATACCATTCTTAGAACTAGCGAGAGGACTTATAAGAAGGGATCACAACATTACATTAATCAGTCCC
TTCCTTCCGGATTTTCACATTGAGGGCTTGGAAGAAATTACTCCCCAAGGTCTGGCCT
CTTTCGTACGAGGATATATGTCCTTCGATCTTGTTGGCGCAAGAATGAGAGGGGAGGAGCCTTTGCCATATAT
GGATATTATAAGATATGGATATGAGGCATGCGATTCTTTTCTCAACGATATCGAGACAAGATCATTTCTACGT
TCAGGGAAAAGCTACGATCTCATAATATTAGATGGGGCATATCCAGAATGCGCTTTGG
GCCTTGTGTATAGAATGAAAGTTCCTTTCATGTACATAAACACTGTAGCCTTTTACGCTTCACCAACAAGTAC
TTCAGGAAGTCCTGCACCATATTCTATAATGGCATTCTTTGCTCGATCATTTACGGATA
>1002049438_1 Heli.1-DT662513.3.5

TTATAGTGTTATAGTGTTAATATAAGCTTATTTGTCATACAAAATACGAAACAAAATGGGGGGTATAAAGACC
CTCATTTTCCTGCAGCTGATCTACATCACCACATGCATGGAACAGCTTGACATCATCTATGAGTGGACTCAAT
TGGACTTCCAATTCCCAACTCCCGAAGCTCGACAACAGGCTATCGACAGTAGAAGTTT
TATACCAGAAAATAATATACCCATGGGCCTTGAGATATTTGGAGATAGACTCTTTGTAACAGTGCCTAGGTGG
AGAACTGGTGTACCTGCTAGTTTGAATTATGTGAATTTAAAAGATAATTCCACCAAATCACCGAAACTGATTC
CATACCCGAGTTGGGCAGCTCACACCCCGGGTCCAGACGGTAAACCAGAAATTGTATC
GCCGTTCAGAATCAGAGCAGACAAATGTGCCCGTCTTTGGGTATTGGACAATGGAAAAATTGGAAATCTAGAA
AATAATACTACCAAATTTCTACCTTCAATCATAATTTATGATTTAAAAACAGATACTCTTCTCAGAAAGTACG
TTTTCCCAGAGGATCAAGTAAAGGAAGAATCTGGTTTTGCCAACATTGCAATTGAAGA
TACAGATTGTGATAAAACATATGCGTATGCCGGTGATTTGGGAAAACCGGCTGTCGTTGTTTATTCTTGGGAG
AAAAACGAATCGTGGAGAATAACTCATCATTTCTTCCATCCCGATCCTTTAGCTTGTGATTTTAGTGTCAAAG
GGCATAATTTTAGCTGGACAGATGCTATATTCGGTATTGGCATTTCAGCACCAAACTC
TGATAACTTCAGTACTTTATATTTCCACCCAATGGCGAGTTACAATGA
>1002049439_1 Heli.1-DT664086.1.5
AAAACTGCAGAAGAGGCTAGCAGCCTCTGTTATGCGATGTGGTAAAAAGAAGGTGTGGTTAGATCCAAATGAA
ATCAATGAAATCGCCAACACTAACTCCCGACAGAACATCCGTAAGATGATAAAGGATGGTCTCGTCATTAAGA
AACCAGTTGCAGTGCACTCGCGCGCCCGCGTCCGCAAGAACACCGAGGCCCGTAGAAA
GGGAAGACATTGCGGTTTTGGTAAAAGAAGAGGTACTGCAAATGCCCGTATGCCACAAAAAGAACTGTGGGTA
CAAAGACAAAGGGTGCTCCGTAAATTACTTCTCAAGTACAGAACAGCCAAGAAGATTGACAGACATCTCTACC
ATGCCCTGTACATGAAGGCGAAGGGTAATGTGTTCAAGAACAAGCGTGTACTCATGGA
GTATATTCACAGGAAGAAGGCCGAGAAGGCTAGGACTAAGATGCTGAGCGATCAAGCCGAGGCCCGCCGTAAC
AGAGTGAAGGAAGCCCGCAAACGCCGTGAAGAACGTATCGCAGCTAAGAAGGAGGAACTGCTGCAAACGTTTG
CGCGTGAAGATGAGGCCGCCGTCACAGCCAAGAAGTGATTTATACATAAATATAAATA
AATAAATATAAA
>1002049440_1 Heli.1-ES585965.1.5
TTCCCATCTGAACTAACACAGACCACGATGAACTCGCTGGTGGTGTTGCTTTCCGTGATGGCGCTTGCCGCAG
CTAAGCCCAGTGGCTTCGAAGCTGGCGTCATCAGCTATTCTGCCCCAGCTATCGCAGTGGCTCCAGCTGCAGT
ATCTCATCAATCACGGGTTGACATTAAATCATCTCCAGCCATAGTCGCCGCTGAAGTT
GTAGCTCCTGTAAGCCGTGCAATCGTGGCCGAACAAGCTGTAGTAGCTGCTCCTGCAGCCTACGCGTCACCTG
CCGTATACGCAGCTCCAATCATTGCCCCTGCTGCAGTTTCATCACAGTCTCGTGTTGATATTAAATCTTCTCC
TGCTGTAGTTAGTAGTGTTTCTGTTGCCCCAGTAGCATACGGGGCATCAATTGCTGCA
CCTGTAGCATACGGTGCATCCATTGCTGCTCCTGTCGCGTACGGTGCATCCATTGGTGCTCCTGTTGCGTACG
GTGCATCCATTGCTGCTCCTGTAGTGTACGGAGCATCTATCGCTGCGCCCGTACCATACGGTGCGCCTATCGC
TGCACCCGTAGCATATAGTGCATCAGTTGTATCGCCTGTAGCGTACAGTGCTCCTTTG
CCTGGTTTCTCATCCATCATCTCACCTGCCATCTCTGCTGATTCTGCGGTCGCCAAAACTGAAGTCGCTGAAT
TGCCAGTCGCCCCTTCCGAAACACCTGAAGTTGCGGCCGCTCGTGCCGCCCATCTTGAAGCAAAAGCACTTGA
AGAATCACACAAAATTGAGAAACGATCTGTTGCTTTTGTATCAGCTGCACCCCAGGCA
TCGCTATACGTGGCATCCCCAGTTGCTAGCTACGCTGCACCTTCAATAGTGTATCCTTCACTCTCCCATGTGG
CCGCCATTTCTAAAGCTGCACCAATCTACTCCAGTGCTGTCAGCATTCATCCATACTGAGCTATCATGGAAGC
GAGATAATAATTTCTCGGTTCAGATGTAATAAAATGATTTACACTAATCTAATTATAT
GAAAATAAAAAA
>1002049441_1 Heli.1-DT664312.2.5
GCACGAGGCAACAACCAAAATGTCCGCTAAGGTAGTAATCGTCCTTGCTCTGGTCGCGGCCGCCTCAGCCCTG
CCCGTGCTGCAGTACGCAGAGCCCGAAGCACCCGCCAACTACGAATTCCAATACTCCGTACATGACAACCAAA
GCGGTGATGTGAAGCAACAACAAGAGGCCCGCGCTGGAGACGCCGTCCACGGCTCCTA
CTCGCTCGTGCAGCCTGATGGAGTCCACCGCATCGTGGACTACAGCGCTGACAAGGTGAACGGATTCAACGCC
GTCGTGCGTTACGAAGGTCAACCCATCGTTTCTGCTCCCGCTAAGATCGCCTACGCTCCCGTCGCCAAGCTCG
CATACGCCGCTCCCGTCGCCAAGGTGGCCTACTCCGCGCCCGTTGCCTATGCCGCTCC
CGTCGCCAAGCTCGCCTACGCTGCTCCCGTAGCCAAGGTTGCCTACTCCGCCCCCGTCGCCTACGCCGCCCCT
GTAGCCAAGCTCGCCTACGCCTCTCCCCTCGCCCACGTCAGCTACTCATCCCCCGTCATCTCCTACCAACACT
AAGTCAATAGCTATAGCTGATATTGTCTTTAAAGTTAATTAATTTATTTATGAAATGT
GATAACAGTTGTTGCAATTAAATATTAACAATCATGTTAAAAAAAAAA
>1002049442_1 Heli.1-ES586406.1.5

CAGAGGGGTCGGACCGCTATCTAGTCATTGTATTTTTAAATATATTTTTAATATCTTAATCAAAAGGTCATCA
TGAAAGTGTTCATCGTGTTGGCAGCAGCTGCCGCCCTTGCGAGCGCCCAATTCAAATGTCCCAACAAGGACGG
CCAATACGAGGATGATAGGCAGTGCGACAAATTCTATGAATGTTCAGATGGTGCTGCA
GTCACCAAGTTATGTCCCGATGGTCTTGTGTTCGACCCCACCATTAGAAAGATCAACAAATGCGACCAGCCCT
TCAACGTAGACTGCGGCAACAGAGTGGAGCTCCAACCCCCCAAGCCGAGTGCCCAATGCCCTCGTCGTAACGG
TTTCTTCGCTCACCCTGACGCCTCTGTCTGCAACATCTTCTTGAACTGCATCGAAGGT
GAAGCTATCGAAGTTAAGTGTACCGCTGGTCTGCACTTCGACGAGTACTCTGGTACCTGCGTGTGGCCCGATA
CCGCTGGAAGACAAGGATGCGCCGCTCAAGATAAGAAAACCAAGGATGGCTTCGAGTGCCCCAAGGAGCAACA
AGTCGATGCCCAAGGCCAGATCGTCGCTCACCCCAAGTTCCCCCACCCCAATGACTGC
CAACGTTTCTACGTTTGCTTAAACGGTATAGAGCCCCGTGACCTTGGCTGCCAAGTTGGTGAAGTATACAATG
AGGAAAGCCAGAAATGCGACGCCCCCGAGAATGTTCGTGGATGCGAGGACTGGTACAAGGACTCCGAAGACGC
CGCGCCAGCCCCCAAGGCGAGGTCCTAAACTAGTGATTAAATAATATTATAGTTACTT
CTAAGACTTGATAAGTTAATATATGATAAGATAAAC
>1002049443_1 Heli.1-DT667041.2.5
ACTTCAATAACAACCACAATGTCCGCCAAGATTGTGATCGTTCTCGCCCTGGTCGCGGCTGCCTCAGCCTTGC
CCGTGCTGCAGTACGCAGAGCCCGAAGCTCCCGCCCACTACGAGTTCCAGTACTCCGTACACGACAACCACAG
CGGTGATGTGAAACAGCAACAAGAGGCCCGCGCTGGAGATGCCGTCCACGGCTCCTAC
TCCCTCGTGCAGCCTGATGGAGTCCACCGCATCGTGGACTACAGCGCTGACAAAGTAAACGGATTCAACGCCA
TCGTGCGTTACGAGGGTCACCCCGTCGCTGCCCCTGCCCCCGCCAAGCTCGCCTACGCTCCCGTCGCCAAGCT
CGCCTACGCCGCTCCCGTCGCCAAGGTGGCTTATTCCGCCCCCGTCGCCTACGCCGCT
CCCGTCGCCAAGGTGGCCTACGCCGCTCCCCTCGCGCATGTCAGCTACTCATCCCCCGTCATCTCTTACCACC
ACTAAGTCAATAGCTATAGCGAATATTCTTATTAAACATGCTTAATTTATTTCTGAATATGTGATAAAAATAC
TTGTAAATAAATATTTACATATAAAAAAAAAAAA
>1002049444_1 Heli.1-EL603456.1.5
GTTCTCTACGTCTGCAGTTTGGTTGTGTACCAGTTCAAAAATCATTTCAAAATTTTTGCCATAAGGAAAAAAG
TTAAGATGACGGACAAGCCGAAGCGTCCTATGTCAGCATATATGCTTTGGCTTAACAGTGCAAGAGAACAAAT
AAAGGCCGATAATCCCGGTCTTAAAGTAACTGAAATAGCCAAAAAAGGAGGTGAAATA
TGGAGAGGCATGAAAGACAAAAGTGTTTGGGAGGAAAAGGCGGCTAAAGCCAAGGAACAATATACAAAAGACT
TAGAGTCATATAACGCTAATGGAGGTGGTGGAGAAGGTGGTGGCAAAAAGGCACAAAAGAGAGGAAAGAAGGG
CAAGAAGGCGGCGCCAGCTAAGTCAAAGAAGAAGAAGGACGAGTCTGATGATGAAGAT
GGTGGTGAGGAGGAAGAGGAAGAAAGCGAATGATCTCCCAATCTATAGGCATTCTGTTCATATTGAATAATTT
ACTTGGACTTAATTTATTGCAAAGTAAAATGGGACTGACTTTCCAAAGTCTGTATTGCATTGTCTTTAATTTT
TATATGAGTATTTTAGAAAAAAATAATTCAAAGAATAATTAATTACACATTTATTATT
GTATTTATAAAGTTTTTGGTCGACACATAATTAAATTTGTTGATTATTTAATGACAGGTGTTATTTACTAATT
TACTTTGTAGTATTGAGTAGTTGATAGGTTAGAAGAGTGCAGAATTTGGAAAGTTTCACCACAGGCTAGGGTC
TCTAGGTTTC
>1002049445_1 Heli.1-DT662733.3.5
CGTATATAGGTAACTTTTCTAAGAATATTACTAACTAGACTGATATTTTTTCCTTAAAAATGAAGAAAGTAAT
ATTTTTATGCGTGTTGTCACTGTCCTTGTGTTTGAGCAAAGCAGCTGAGGACAAAGCCCCACAAGCTCCACCA
GCTTTAAATGCGACAGATGCTAAACTTGCTCCACCAGTAAATGCCACACCGCCTCATG
AAAAATCTCAAGAAGCGCCAGTAAGTAATAAAAATGAAACAACATCTGACAAACCTGCGCCTCCACCCTCACT
GCCACCACATATAAATACAACTAAGCCAGAAGTTGATAATAAGACTGCAGACAAACCAACTCCTAAGGAGGAA
ACTGCTAAGCCAAGTAACAATGAAACAAAAGTAATCCCAGATAAAGAAAGTAAGGATG
TGAAGCCCAATGAGAAGCCTAAAAATGAGACTTCGACCGAGAAGGTCACGGAAAAACCCACTGAAACACCAAA
AGTCACGGAAACACCTAAACCTATGCATATTGTTGAAAAGACTAAAGGTTTTGATGGACCAAGTTTCATTGGA
GGCATCATCCTCACCTTGGGTCTCCTTGCCATAGGATTCATGGGGCTCAAATACTATA
AGAATCAAACTGAAAGAAACTACCACACACTTTAAGTGAAATATTTATACTATTAATTATCTATGTGAGTGCA
CTCTGCTATTTACTTATAATATTATAGCTATATATTTTGGTTTTTCTCTATAAGTTACGAGATTTTTTGTTTT
TTTTTGTGCACTTTACATTTTA
>1002049446_1 Heli.1-EL603452.1.5
TCAACACAATGTTCTCTAAAATTGTATCTCTTTGCGCTGTAGTGGCAGTGGCTTCAGCTGGTCTTTTGCCCGC
TGCTGTGCAGTACTCCCCAGCAGCTGCCGTATCTTCCCAAAGCATTGTGCGCCATGACGAGCCCCAAGCTCTT
GGTGCTAAGATCCTCGCCCCTGTCGCCAAGTTGGCCGTCGCTGCCCCTGTAGCCTACC

ACGCACCTGCCCCCGTCGTGTACCAGGCCGCCCCCGCACCCATCGCCTACCACGCTGCTCCCGCTTCCATCGC
CTACCATTCAGCTCCCGTAGCAAAGGTTCTTGCCCAGCCTGAAATCATTGCTCACCCGAAATACGATTTCAGT
TACTCTGTAGCTGACGGACACTCCGGAGACAACAAGCAACAACAAGAATCCCGCGACG
GTGATGTCGTAAAGGGCTCCTACTCCCTCGTTGAGGCTGACGGCTCTGTCAGGACCGTGGAGTACTCCGCTGA
CGACCACAACGGTTTCAACGCCGTAGTACACAACAGCGCCCCCGTCCACGGCCCAGCTGTCATCAAGGCCGCC
CCCGTCGTCCTCAAGGCTCCCGTGTACGCAGCCCCCGTACAACAATACTACCACTAAA
AATTAAACAATTAGTCACCAAATATGATATAACGACCTGCTATTTATTTATTGCAATATA
>1002049447_1 Heli.1-DT664658.3.5
TCTGTTCTAATTCTTATTATTTCTTAATATTAGTTATTATTATTTTTTATTTAAGTCTTTGAGATTTTATCTT
AATAGTCACTTTTTTATTATTATTGCATAAAACGACTTAAGTGGTACTTTTGCGTTTAGACGGGAGCCATCTT
GCAATCTCTAATCAAAGGTCCTTGGCTCACAGTTTCGTTGTCTAGTTGAAAGGCAGTA
TATTCAATCATAAAATAGTCAAATGGCGTTAAAACGAATTAATAGGGAATTACAAGATCTGGGTAGAGACCCG
CCGGCACAATGTTCAGCAGGACCACACGGTGAAGATCTTTTCCATTGGCAAGCCACAATAATGGGTCCAGTTG
ACAGTCCCTATCAGGGTGGTGTATTTTTCCTCACCATACATTTCCCTACAGACTATCC
ATTCAAACCACCAAAAGTTGCATTCACAACGCGCATCTATCACCCTAATATAAACAGTAATGGTTCCATTTGT
CTTGATATTCTGCGCTCACAATGGTCTCCAGCACTTACCATATCCAAAGTGTTGCTCTCAATCTGCTCACTTC
TATGCGATCCAAATCCCGATGATCCTTTGGTGCCAGAAATTGCTAGGATCTACAAAAC
TGACAGAGAAAAGTACAATGAATTAGCCCGAGAGTGGACTAGGAAGTATGCCATGTGATGAGTCAGATGTGAC
ATCAGTAAACATATACACACAGATCGCCACAGCCCGGGCTTATACCCTTCCTGTTCTCATCCACGCCCAATTC
CTTTTTTGTCTGAAAACCTTACCAATCACTAAAATTACTCAAACTTTGTTCAATCTAC
ATGGTTTTAAAATCTGATTGTTTCTTGTAATTTAAATGCCAATTATTGCGACTAAGTAAAATAATACACATGA
TATGCAAATAGTTTCTGTTCATGTAATTAAGTACCATTTTCTATAAA
>1002049448_1 Heli.1-DT662894.3.5
AATAATATATTTAATAATGGTTAATTATAATATATATATATTAATAGTATTTTTAGTATTCACAGTTTGTGAA
GGGGCTTTAGGGTTATCAATTTTAGTGTCAATAATTCGAACTCATGGTAATGATTATTTTCAAAGTTTTAATT
TAATTTAATGATAAAATATTTATTTATAATAATTTTTATAATACCTATATGTTTGAAA
CGAAATATATTTTGAATGGTTCAATTTATATTTATATTTTTAATGTTTATATTTATAAATATAAGAATTTCAA
TTATGAATTTTTGTAATTTAAGATATATGTTAGGTTGCGATATAATTTCATATGGTTTAATTTTATTAAGAAT
TTGAATTACATTTTTAATAGTTATAGCTAGAGAAAAATTAAATAATAATGATTTTTTT
AAGAATTTATTTTTATTTAATATTATTTTTTTAATAGTAATATTATATCTAACATTTAGAACTATAAATTTAT
TTTTATTTTATTTATTTTTTGAAAGAAGCTTAATTCCCACATTAATATTAATTATTGGTTGGGGGTATCAACC
TGAGCGAATTCAGGCGGGGATATACTTATTATTTTATACATTATTTGCTTCTTTACCT
TTATTAATTGGGATTTTTTATATTTATAAAGAAATAAATTTTATAATTATATATATAATA
>1002049449_1 Heli.1-DT668272.3.5
GTCGGCCGTGTACGCTTACGGAATCGTCCTCATACACTTTATGCGTGATTAATTGAAACGTTTTTCTTAAAAA
ATAACATCGAAATTAAAGTTTAATGTTGAAGAAGCACTGAAGTACAAAATGAACTCGTAAAAACAAATATATT
TAAATTTCACAAAATAAACAGGAATAAAGGAAATTTCTCGAAACAAATTACTAACAAT
GTCAAATGTAGAGGCGAATAATAATATGAATAAAAGTGAAGCTAATAATCAGAAAGTGGGAAAAGACATAGAA
CAGGGGAAATTTGGAGACAGTGCAAGTTCTGGTTTGCCCTGGAGTCGCGTCTTCGTCATGGTGTTTGCAGCGG
CAGTGCTGGTGGCAGCGTGTGCAGTGGCATGGGTGTTGTTTAGCTGGCAGATCAGTCT
TATTGTTTTTGCGGTTCTAGTTATATGCTATACACTAGCTTATTTCTTCAACTGGATATGGATTGCTGTACAA
ACTGCTCCGAGAGACTTAAAAGCTTTATACTGCTATTTCAAGATTTTAAGATTATCCAGTCAATTTACGAAGA
AGAACTGGTCTATGCCAGATATTTTTCATGAAGTTGTAAAGAAACATCCAAAAAAAGC
ATGTTTCTTATTCGAAGATGAAACGTGGACTTTCCAACAGGTTGAGGAGTTCAGTCTGCGGGTTTCTGCAGTA
CTGCAGTCGCGAGGAGTTAAACGCGGCGACACTGTAGCCCTCATGGCTAGCAATTACCCAGAAATGCCGGCTA
TCTGGCTTGGTATCACACGTGTTGGGGCCGTTGCACCACTTATAAATACCAACCAAAC
TGGCAATACTCTACTACACTCTATCAACATTGCCAAATGCGATTATGTTATTTATGGCAACGAATTCGAATCA
GCTATTCAAGACATCAGAAAGGAGATAAAAGAATCTGTTAAATTATTAAAATTTACACGCCGTCCATTAAATA
CATCTAACGATAGCGTCCAAGTAGTCAATTCTGAACATGATTTTACACATTTATTGGA
AACAACCCAACCTGCCCCTTGGTTCTTGTCCGAAGGGGAAGGATTTACTGGGAAATTATTGTACATTTATAC
>1002049450_1 Heli.1-DT666076.3.5
GCACGAGGGTGCGGTGCAGGAGGTGAACTTACTGCCAAGAATTCTCTGAATTCTACATATTTCATTTCAATTA
GTGACTAAATATAAAATACAGAAATGGCTCCCCCTTACTATTCTGATCTCGGAAAGAAGGCCAATGATGTCTT
CGCCAAGGGCTACCATTTTGGAGTCTTCAAACTAGACCTCAAGACCAAGAGCGAGTCT

GGAGTCGAGTTCAGCAGTGGCATCACCTCCAACCAAGAAAGTGGAAAGGTATTCGGTAGCCTTTCCTCTAAAT
ATGCTGTGAAAGACTATGGTCTGACATTCACAGAAAAATGGAACACAGACAACACACTTGCCACAGACATTAC
CATCCAAGACAAGATCGCTGCTGGTCTTAAAGTTACACTTGAAGGAACTTTTGCACCA
CAAACTGGAAGCAAATCTGGAAAGCTGAAGACCCAATTCTCCAATGAAACAGTAGCTGTCAACAGCAACTTGG
ACCTTGACTTGGCCGGACCAATAGTCGATGTAGCCGCTGTCCTCAAGTACCAGGGCTGGCTTGCTGGTGCACA
CACACAGTTTGACACACAAAAGGCCAAGTTCTCTAAGAACAACTTTGCCTTTGGCTAC
CAGACCAATGACTTTGCTTTACATACCAATGTTGACAACGGCAAGGACTTTGGTGGTTCCATCTACCAGAAGG
TTTCAGACAAGTTGGATTGTGGAGTCAACATGAAG
>1002049451_1 Heli.1-DT665436.3.5
AGGGTCGTGTGGCATTATTTAGTTTATTTTATAAAGTGAATTAAAACAATAATTGTGCTTTATCTTAGTGATG
TCAGAACACGTACTCCCGGCTGAAGAGCCTATACGGTTCGTAGCCCCAGTGATTCAAGATAATCCTACAGGAT
GGGGCCCGTGCGAGATGCCGGAGCAATTTAGGGACATGCCCTACCAACCTTTTAGTAA
AGGTGATCGTCTTGGCAAAATAAGTGATTGGACTGTAGTACAAGATAAGAAATACCAGAATAAGTATGCTTCG
CAATTTGGCGCTGGATCGTCATATGCGTACTTCCATGATGAGGACGAAAGTACGTTCCATCTGGTGGACTCGA
CTCGCGAACAGAAACCTCCGTACCAACGAGGTCGTGGTCGTGGTAACCGAGGCAGAGG
AGCAAGAGGCGCTCGCACACCTGGTGGCATGACAACTTTGAGCAAGCAACGTGAACGTAAGCTTGGCAAGAGA
TGGGGCCAGAGAGGTGCTCCTATGAAGATACGAGATGCATCTGTAACTGTCCGACCTACTTGGGTGACAATTG
AAGACATGGACTTCCCACGTCTTGCTAAGTTATCTTTACCTGGAATTAAAGAAGGTGA
AGATATTGTATGTTGTGGTACACTGGAATACTATGAAAAGGCTTATGACCGTGTAAATGTGAAACACGAAAAG
CCCCTTCAGCGCATTGACCGCATCTTCCATACTGTCACAACCACTGATGACCCAGTCATTCGTCGTCTGAG
>1002049452_1 Heli.1-ES584782.1.5
AGCAATGGCAGCTAAGTTGGTTGTAGTACTCGCCGCCGTGGTCGCTGTGGCCCACTGCTCAGTGGTGCCAGTG
GTGCGAGCAGACGCCGACTACTCAAGCTTCGCGTACGACGTCGCTGATCCCTACAGCGGCGACTTCAAGAGCC
AAGTGGAGAGCCGCAGCGGTGGCAACGTACAAGGACAGTACTCTCTGATCGAACCCGA
TGGCTCCAGACGTGTTGTTGACTACGCCGCTGATGATGTGAACGGCTTCAATGCCGTTGTACGCAAAGAACCC
GGTGTGGTCGCCCCCGTCGCCGCCGTAGCTCCCGCCGTGGTCGCCGCACCCTCTGTAGTCGCTGCTGCTCCCT
CTGTAGTCGCCGCCGCCCCAGAAGTTGTCGCCGCCCGTGCCTTCTCCCCTGCCGTTGT
TGCCTCATCTCCCGCCGTAGTGGCCGCCTCTCCCGCGGTATACGCCGCGTCTGCCCCCGCAGTCGTCGCCGCC
CACTCTTACGCTCCTGCAGTATACGCTGCATCAGCTCCAGCTGTCATCGCTGCCCGCTCGTACGCTGCACCCG
CAGTGTACTCCTCTGCCCCAGCTGTTGTAGCTGCCCACTCTTATGCCCCATCCGTATA
CGCCGCAGGTCCCGTCGTATCCTCTCGGTCTTACCTCGCTCCTTCCCTGTACTCGTCCGCGGCCCCATTAGTC
GCTGCCCGTTACGCCGTTAACGCCCCCTACTACTCCGCCTACGGCTACGCCAACCCCATCGCCAATTACTGGT
AAACGAATATATAAAATCTTAGTTTTAGATCGAAGTTGCCATGTAAATAATATTTTTG
TACATTTGCATACATATTAAATTAAACGACTTTTTT
>1002049453_1 Heli.1-EL596932.1.5
GCACGAGGCTTAACCGCCATGGGTCGTTCGTTGCGTTCGCTATCAGTTTACACTCGTCCGTTTGCCCCCATAA
ACTGCCGTCAGGCCACCACAGGATGCCCGTTTTCCAAGCGTCAACGTTCCCAAATAGCTCCAGCTGCTTCCCT
GTCCGAGGAAATTTTTGCAAACGCGAAACCGTACTCCGAAGTTCCTGGACCTAAGCCT
ATCCCTATTTTGGGCAATACGTGGCGGATGGTCCCGGTTATAGGGCAGTTTGATATTTCCGAATTCGCTAAAG
TCACTAAATTATTCCTAGAAAGATATGGCAGGATTGTGAAGTTAGGAGGGCTTATTGGGAGGCCTGATCTGTT
ATTTGTGTATGATGCTGACGAAATCGAAAGAATGTATAGGAGAGAAGGACCCACTCCG
TTTAGGCCAGCGATGCCGTGTCTTGTGAAATATAAATCTGAAGTGCGAAAGGATTTTTTTGGGGAACTGCCAG
GTGTTGTTGGAGTTCATGGAGAGCAGTGGAGACGGTTCCGTTCTAAAGTCCAAAGGCCTATACTCCAACCTCA
AACAGTGAAAAAGTATGTAGCACCAATAGAACTCGTGACCGAGGACTTCATCAGATAC
ATGGAAAACGCAAGAGATGAAAACGGGGACTTACCTCATGAATTTGATAATGACATCCATAGATGGTCCCTT
>1002049454_1 Heli.1-EE743496.1.5
TGATAGAAACCTCAAGATGCAGTTGCACATCAGAGGACAGTCTACGCACGTTCTAGACGTAAATGGAGATGAA
TCCATTGCTCAGATCAAGGAGCGCATCTGTGCCCTTTCTGATCTTGGAAATGAAGAAATAACTTTATCTGCAT
GTGGGTCACCTCTTGAAGATGGTCTCCTTGTATCTGAATTGGCATCAACAGAATTGGA
CCTTACTGTACCATTGCTTGGTGGTAAAGTGCATGGTTCCTTGGCTCGTGCCGGTAAGGTAAAGGGACAAACC
CCAAAGGTTGAGAAACAGCAAAAAAAGAAGAAGAAGACTGGTCGTGCCAAGCGTAGGATCCAGTACAACAGAA
GGTTCGTCAATGTTGTCCAAACGTTCGGCCGTCGTCGTGGACCCAACTCCAACTCATA
GATATGTATTGTAAATAATGTGTTGCAAATATAACATCTGATTTGCCC
>1002049456_1 Heli.1-EL603472.1.5

GACGCACGCTGGCCGAGCTATGTCGTAGGCCCCATGGTGTACCCCGAGGCGTCCCGCTGGGGAGCGCCCGACT
CGCTAGCGCCCCTCTACGATGACGTCTACCGAGATATCTCATCCGCAGCGGACGGTGTGCCAACAGTGAACGT
GGTAGAGGCGTTGCAACGTTTCTGGCAGTCGAAAGCTTCGAGAAATGGCAGCGCCGGG
AACGGCGGCAGCAGTGCACTTGTCATTTACGAGTCAGTACCGGCGGCGCGTCCTCCCTACGTGTGCTATGTGA
CGCTGCCAGGCGGAGCTTGCTTTGGCAGCTTTCAGAATAGCCCTACAAAAGCAGAGGCACGGCGCAGCGCTGC
AAAGATCGCGCTTATGAATAGCGTGTTCAACGAGCACGAGTCGCGGCGTATATCTGAG
CACTTCATCGAGAAAGCAGTAGCAGAAGCGAGAGCGTCCTTCGCTGGAGACGCGGCGCGCCACCAGGACCCTA
ATCCCGGAATAGCTGCTTTTAGATTCATGTTAGAAGCAAACAAGGATCGCACAATGCTAGAGTTCCAGGAGCT
GATGACAGTGTTCCAGCTGTTGCATTGGAACGGGTCGTTAAGAGCGATGCGGGAGCAG
CAATGTTCGAGGCAGGAGGTGGTCGCGCACTACTCGGCTCGGACCTTGGACGATGTGATGCGTGAACAGATGG
CGCGCGAGTGGGCTTCCAGGGAAAGAGAAGCTGTGGCCAAAGGTGGTGGCGTATTACGAAGTGAATTAGCAAG
AGCAGAACTAGAACTACAGACAGCGAGGCTAGCAGCTCGAGAACTACGCTTCCCAAAG
GAGAAACGTGACATCCTCCTCCTGGCGCTACGCCTCGCTCCCCCACAGCAACAACAGTGATCTCACCGCGCGA
CTATTTGTTATTTAAGTATAGTTATAGTATAAGAATATTTTGTGTTTCATTTTCCTTTACCTTGCGTTTGTAT
GCTTTCTTTTTAAGAATCTGTAATGATCTGAATAAGAGCTTTTTTATGTCATAAAAAA
AACAAGTGAATTGAAAATAATTATATCCTTTTTCATTATATGTATAGT
>1002049457_1 Heli.1-EL602474.1.5
GACACCAACATGAAGTACACGGTTGTATTAATAGCCTCGTTGGCGGTGGTGGCCTTTGCCAGTGAGGAGAAAG
GCACACAAAAGGTAGCTGCCGAAGAAAAGAAACAAGATAAACGAGGAATTTACGACATCGGTTCCTACGGAGG
CAGTTACGAAGATGGCGGTCACGGAGGTCATGGTGGATTCAGCTCAGGCCAAGGTAGC
TACGAGAGCTACGGTCACTCAGGTTCCGAATTCGGAGGATCTTCCGGAGACTCGTGGAAACCAATTGCCAGTG
ACCATGGCCATCATCACCACGTGAAGACCATCGAAGTCATCAAAAAAGTGCCTGTCCCGTATACCGTTGAAAA
GCACGTCCCTTACACAGTTGAAAAGAAAGTTCCCTATGAAGTTAAAGTACCTTATCCC
CAACCCTACACCGTTGAGAAGAAGGTGCCAGTCACTGTCAAGGAATACGTCAAATACCCAGTTCACGTACCAG
AACCTTACGTAGTTGAAAAGAAGGTGCCTTATGAAGTCAAGGTCCACGTTGACAAGCCCTACGAAGTGAAGAT
CAAAGTACCCACTCCTTACACTGTCGAAAAGAAAGTACCTTATGAAGTCAAGGTCCCC
GTTCCCCAACCCTACACCGTCGAAAAGAAGGTCCCATACGAAGTAAAATACGAAGTTAAGGTACCCCAACCCT
ATGAAGTCGTCAAGAAGATCCCCTATGAAGTGAAAGTACCCGTCGACAAGCCTTACCACGTATATGTAGAAAA
ACCTTACCCTGTCCCAGTTGAGAAGCCTTACCCAGTAACAGTTCATAAGCCAGTTCCT
TATGAAGTTAAGGTCCCAGTTGACAGACCCTACAAGGTTGAAGTAGAAAAGCCCTACCCAGTCCCCGTTAAAA
TACCAGTACCCAAACCCTATGATGTATACAAGAAAATCCCTTACACCGTCGAAAAGAAAGTGCCTTATGAAGT
CAAGGTACCCATCGACAAGCCCTACCCCGTATACAAAGAGGTTCAAGTACCTCTAGTC
AAAGAGGTCCCCTACCCCGTAAAGGTTCATGTACCTATATACCTGAAAAAGGAGGAGGAACACCATGGCTGGC
ATTAAATTAAA
>1002049458_1 Heli.1-ES587832.1.5
GATAAAAAATGAAATTCTTAATTGTATTCTCATGCCTGGTCCTGTCAGCGTTTGCTGGAGAAAAATATAATGC
TAAATATGATAATTTCGATGTCGAGACCCTTGTAACAAACGAGAGACTGCTCAAATCGTACATCAACTGCTTT
TTAGATAAAGGCAGGTGTACTGCAGAAGGTTCTGACTTCAAAAAAACTTTACCTGAAG
CAGTGGAGACAGTATGCGGTAAATGCACAGAGAAGCAAAAGATTAACATTAAAAAAGTGATCAGAGCTATCCA
AGAGAAGTTCCCCAAGTATTGGGAAGAGCTCGTTCAGAAGAACGATCCCAGCGGCAAACACCGTGAAAACTTC
GATAAATTTATCAAAAGTTAATTTCTTAGTATTATTCTTAATATAGGTGACTAAATTT
AAATTTATTTTTACGCTTTAATCAAAATAAGATAAAAGGAAAGATAGAAAATGGTAGCTTTTTAATAAGATAT
ATAAGTGATACGATGCGTGATATTTGTTAGTCTCTGGAGAGCATTTCGATGTAAATGGCGTAGAGTTATTTAT
TTTCTATTGGTGTTGACTGATTTTAATGAATAAAACTAGTAATTAT
>1002049459_1 Heli.1-DT666827.3.5
GCACGAGGAGAAAACATCTAACACTAAGTTATATATAGTTATATTACATACTTACACGAAAAGTGCTTGGTTT
TAGTGGTACTTGAGTTACAGATTTTGATACTCGGCTACCGGCATCTAGTGACAAGAATCATGACTACGATAGT
GTCCCTACTCTCCGCCGTGGCACTGCTGTCGCTTCAATGTGCAGCATTCGGAAATATA
AATGAAAAAGTCGAAATCGACAATAGTTTGATACCAAATGCAATACCAGCCCCACGCCATAGTCCGGTTCGAA
ATTATGTAAGAATGATGTCACCACCCCCTGAAACTGTGAGGCACATACCTGAAACTTCTCTTGTTTTAATTTG
TCAATTGAGAGGAAATCCGTCACCTGTTGTGAATTGGTTGAAGAATGGTGTACCTATT
ACAGATTTTGAAGAAGACGTGAATGAAATTCTTTCCATACCACCGTTCAGTCCCACTGAAATGACAAGTAAAC
TTGTTGTACGTTCTCCGTCTAACGCCGACATTTTTACTTGCGTAGGATCCGCTGGATTGCTCGAGATCAGTGC
TTCAACTACTGTTCTCATTGTTGGAGAAAATAGGCCTCAACCACTAACTTCAATTATT

CAATCCAAGCCTGTAATCACCACATTTTATAACAATTTATTCCAATTTATTGGTTCCAACGTAATTCTACCCT
GCCGTGTCGACAGTCCTACTAAATCCCAAGTTGTCTGGTCAGTTGAT
>1002049460_1 Heli.1-DT666934.3.5
CGTCATATTATTAAATCAGTTAACAACCTGCACTGCACCACGCATGAAACAATCATCTTAGCAAGATGTTACT
AAGGGTTGTCTGTATAGCAGTGTTTCTAGCAACAAGTGACAGTTTTAGTTCCTTTTCTCAAAGAAAAGCTATA
GAAACCTTGTACCGATGGAAACAAATTGATTTTGCTTTTCCTACACCACAACACAGAC
AACTAGCTATACGAAATGGAGAATTCAATCAGATAAACGTTATACCGCTAGGAATAGAGCGATGGCAGGGTCG
TGTTTTTGTAAGCACACCTCGATGGAAGAAGGGCGTCCCGGCTACCTTATCGTCATTGCCAGTTGCGTCCCAA
GAAGAATCTCCGTTATTGGCACCATATCCTAGTTGGGACTGGCATAATGCAGATAATT
GTACGGGATTTACATCTATATACAGAATGAGTATCGATGATTGCGGTGTCATGTGGGTCCTTGATTCGGGACA
AGTGGAAGCCTTTGAAACTGCTCGTCAGCTCTGCCCACCAACGCTTTTCGCTATTAGCCTAGCGACGGACACT
GTCGTAGGCCGTTTCCCGATTCCCAGCGAGTACGTTCTACAAAATTCTCTAATCACTA
ATCTTGTAGTCGACTCACGTGACGCTCAATGTCGAGATCTACATGTATATATTGCTGATGCATGGAGATTTGG
ATTGATTGTGTTTAGAGATTCTGACACATCCTTCTGGAGGTTTAATCATTATTCATTTTATCCAGAGCCAT
>1002049461_1 Heli.1-DT664833.3.5
AGCGCACACACACACAAGAAAAAATTGGAAGTTATTTAATTGTGTCAGTGCTTACCACCAGCGGCTCATCTTTACT
ATCAAGGAATCCATAAAAAATCAGGATGAAGGTCTTATTACTGTGCATGGCCTTCGCGGCTGTGAGTCTGGCT
ATGCCTGTCGCTGAAGAGAAGCAAGAAGAGCCTATTGCACTTCCATCTGCCCCTGAAA
TCAAATCTGAAGATGTTAAGCCTCAAAGCGATTCAGCGCCAAGTCCTGTAGAAGAAAAAAAAACCAGAACCAGC
ACCTGAAGCAAAAAGCGTTGAAAAACCTGACGAGGTCGCTATTGAAGCCAAGAGTTCAGATGTTGCAATAGAC
GCTTCTCCGGAGGCTAAGGCGGAAGCCAGCCCAGAAGTCAATAAAGAAGAAGTACCTG
CAGCTAAGGCAGCAGACGTAGCAGATTCTGCAGTTGAACAAAAAGCAGACGTTGCTGAACCAGCCAAACCTGA
AGAAAAAGTTCCAGAAGTTAAAGCTGCAGTTGTAGAAGAATCTAAGCCAGATGAAAGTCAAGCTAAGGTTGAA
GAAGTGAGCAATGTACCTTCAGCAAAATCTGCTGTCTCTGAAGATGTAATCGACCTGG
TCAGTGCTATCAAACCTGAAGCAGCCGTTGCCGATGATGTCGTTGACCTTGCCGCGATTAACCCATCTGACTC
TCAGCCAGTAGTTTCTGCTAAAA
>1002049462_1 Heli.1-DT667174.3.5
GCACGAGGTCGACTTGACATATTGAGTGTGGTATTGAGGAATTGGACCTTTGAAGTTGTTCCGAGGGTCAAGA
GATGGCGTTCAGGTCTATAGGAGTGGTGCTCTTGGCGTGCCTTGTGGTATTAGCATCGTCAGCGGTGCCTAAA
GGTGGTAAGAGGAAACCAGCTAACCCACCAGCCCGCGTCGCCGCTGATGCAGAACGAG
ATGCAGAAATAACGAATTCCTGTCCAGATGATGGCTTCTTTGCTGATGCTGAACAGTGTGACAAATATTATGA
ATGCAGAGCGGGTGAAATAATTGAAAAGCTGTGCCCTGATGGTATGGTGTTCAACGACTACAGTGCCCAAGAA
GAAAAATGTGACTTGCCATTCAATATCGACTGCTCTCAAAGGCCCAAACTACAGACAC
CTATCCCTGCCCAGCACTGTCCACGCCAAAACGGTTACTTCTCCCACGAAGATGAGAAGGAGTGTGGCAAGTT
CTACTACTGTGTAGATGGTAAATTCAACATGATCACCTGTCCTGATGGCCTCGTGTACAACGACAAGAGCGGT
ATCTGCACCTGGCCTGATGAAGCTAAGAAGAAGGGATGTGGTGCTGCTGAGGTGTTCC
AATTCGACTGTCCCGCTGTAAATGAAACTTTCGGTCTAACTCACCCACGGTACGCGGACCCTGATGACTGCCA
GTTCTTCTATGTATGCATAAACGGTATCACTCCTCGTCGCTCTGGCT
>1002049463_1 Heli.1-DT665653.3.5
TATTGTGATCGGCTGTGAATTCTGGGAGTGAATAATTTTATTATTTAAAGTGTAATTTCTTTGTCTTCGTTGT
TCGGATTTTAAGACCCAAGGGAAGACAGTGATTATGGGTGCTAAGCAGAGCAAGCGCTCGGTGGACATAAGCG
GCAAGGAGGCCGAGGGCGCGGGCGAAGTGGCCGCGGCCGGCGCGGGCGGGGAGGGCCG
CGTGGAGCAGCTCGCCGACGCCGACGCGCTCAAGCCGCAGCTCAACGGCGACGCACACATCCACGAAACCTCA
GATAAAGAGAAGAACCTCGATAGCGGTACGCCAGAGAATGAGAAAGATGCGACCACAGAGAAGGAATCTAAAG
AACAAGAGGAGGATAACAAAGAAGCTGCACCCGTAACTAATGGTGACGCCGAACAAAA
GGTAGAGAATGGCGAATCTACTCCTTCACCCGAGGACGGCAAGAAACCCAAAAAGGAGAAGGTTAAGAAGAAA
TGGTCTCTGAGATCGATAAGCTTCAGCAGAAAGGATAAACCGAAACAAGAAAAGAAACAAAAAGACGATGAAT
CTAAAACAAACGGAGAACCAGAGAAAGTACCTGAAGAGTCGGCTGAGCAGAGTCCCGA
GTCGGAGAAAAACAATATCGTACCCGAAGAGACTGTCGAAGCTGAGGCTAAAGATGAAAAAACACCAGAGACT
CCGGTGACTGAACCAATTACGAATGGCAGCATGACACCTGAGTCACCGAAAGAAGAATCCCCTCTTTCGGAAG
AACCAGTGTCTAATAATAAGGAAAACGACAGGACGGAGACTCCTAAAGCTGAGGTTGC
ACCAGAAACGTCACCTGTCGTCAATGGCCTCCCCGCAGAGGAACCTAAGAGTGAGACCCCCGAGCCGGTCGTA
ACCGAGCTCGAGACTAGCGAGCCTAAGGAGGCTGTTGTCGAAAAATCGGAGCCCGCTCCCATCATTGAAACCC
CTGTCGAGAAAGAGGAGCCCGCACCAGTCTCTGACAAGCTTGCTGAACTGATTCCTGA

AATTCCTTCAGTGCCAGAAATAAACAATGAATTGGAGGCGCCGAGCGCGGCGGTGGTGGCGGCGGGGAGCGAC
TAGGGCGGCAGCGGGCGGAGGACGGTGGGCGCTAATTTGAAGACGAGCCTTTTATTTATTCCCGAATAGCGAG
TATTCCCGGACGATATCGAACCTAGTTCGATGCCGACATCGAACGTTCACACCACCCA
CGACTAGTGTAGTTTAAGTTAGCGTGCTTTGCTTTTGTAAAATTAAGGTTTTATATATATATTATAACAAAGT
TATGTAGCAGTATACGGTGCGTTAAGTTGGTGCCATGATTACAGGGGTATTAGTTACGTCCCTCCTCGGATAC
TTCGCGAACGCTAGTTACTCCTTTATGATATCATTATTTAGAGAATTATATTATGAAT
>1002049464_1 Heli.1-DT664842.2.5
TTCTGATTGTAGCCGCCGTCTTCGCTTATGCCTCAGCCGCACCTAGTGCGCTGTTGGCCTCACCTTACTCCAA
TGGCCTCCTCGCCTATGGAGCTCTGCCTATAGCCTCCTCCATTGTGGCACCAGTCAACTCAGGAGACCTCCAA
GGCGCTGCTATCGAAGCAAACGCTAAGGCCGCCGACAACGTCGTCGCCGCTGTCGACG
CTGTCCGTGAATACAACGACCAGGCCGCTGAAATCCAAGGCAGAGCTATCAACGCAGCTGAGGACAACGCTTG
GCAAGCAGTTAACGCTGCCCAAGTAGCTGCTGCCAAGATCGACGGCGCTGCTGCCAGCGTATCTCCCGATGCC
GCTCGCGCTGTAGCCGGAAGCGCAGTAGTAGCCCCTATCGCTGCCTACTCTGCCCCAG
CTGTCGTTGCCCCTGCTATCTCTGCCTACTCTTCCCCCTTGATCGCTGGCCCCTCTTTCGCCTACCAACGTTT
TGGTTCCCCCATCCTACCTTACGGCGCCCGTGGTTTCATCCAATATTAAATTTAATTTCTAGGCAAAGCAAAT
CAGGCTAAACGAAGATGTGTAAATAAAATATAAAAACTAAAAAAAA
>1002049465_1 Heli.1-DT662771.3.5
GATAATTATAGTAATTTTGCTGTAATTTAACTTGTTGAAATTAAGTGAAAGTACTTTCCATTGGAAACTGGTT
TAGTCTAAAAGTAATTCATCATGGCAGACTCGCGGAAAAACCATCTTATTATAGATGGGGGTCAACATGCAGA
CAACAGTAACCCTGCCTCTTACAGGCCTATCCCGGGACGTACTTGCTAACTCCGGGCC
TCCAAGGCTAGTCTACACAAGTCCAAGGAGAAGGTATCATCTGACGGCGCTGAAGAGAAGCTGCTTCAGAAGG
AGGACGAGGCGAAGATCACCACGCGCGTGGACATGGCAGATGCTAAATATGTGGTCGGCGACCACAGGAATGG
AGATGCTAAGATCGAGCTAGATGCTAATAAACGGCAATTCACGGGCCTCACAAAGGAG
GAACTGATGAAATACGCAGAGGATCCGTTCTGGGTGCGGCTCAGGTGGTTCATGTTCATACTGTTCTGGGCTC
TCTGGTTTTGTATGCTGGCTGGGGCCATCGCCATCATCATTCGGGCCCCGAAATGCGCGCCGCCACCGCCGCG
GACTTGGTATGAAAAGGGCCCACTAGTAGATATGGCTTCTGTAGAAAATTACAACGAG
GTAGCTTCAGAATTGGACAATCTGGCAAGTTCTCAAGTATCTGGCATGTTTGCCTTCTCTTGTAAGGAGACTT
ACGAGGTGTTGGAAGACCCCTCCTGCCTCGATCAGTTCAAGCAGTTCGTTGCGAAGACCAAGGAAGCGGGTAT
AAAAGTGATCGTAGACCTCACGGCGAATTTCGTATCGAAAACTCACAAGTGGTTCGAA
CTCAGTGAGAATCGATCATCGGTGTACAATGACTACTTCATCTGGGCGAAGGGTCAGGAGTTCGATCCTGAGA
AATCTGTGCCAAAACCACCTAATAACTGGGTATCGAACCTAAACACACCGATGTGGAGCTACAGTGACAAGCG
CAAGGAGTTCTACCTTCACCATTTCAACGAAGATCAACCTGACTTGAACTTCCACAAC
CCTGAGGTTGTTAAACAGTTTGATGCTGTACTCAAGATGTGGATGGACGCTGGAGCTGATGGGATTAGGCTGC
AAAACGCTCGCGATTTACTAGTGAACTCGTCCCTTCCCAACGAGTCCCCCCACGTGGGCGCGGGCAGTGATCC
CGGAGCCGACCACACCCAGCGAGCCTACTGGAAGCGCCAGTACACCAGCGACCAGCCG
CAGCTAGACCAACTATTGGCCCACTGGTCGCATTTAGTTGCTACCGCTAAGCCTGCTGAACAAACAGTATTCA
CGATATCAGAAGCCGGCGGGCGGCCTGAACTGTTCCTGCTACAGCGTAACTTAACCTCGCTGCGACCCGCGTC
CGCCGCGCCG
>1002049466_1 Heli.1-DT663678.3.5
TGGAGTGGCGGCCATATTTAATTTATTGTTTTTTTTTAATTGTGAGGATAGGCTAAGGATGTGTTTTAGTCTG
TAATTTTTTTTGTGTTAAATAATTGTTTTATGTTATTTATTTTGGTGGCAGAAGAATTGTGAGCATTGAAAGA
TAGAAGAGAAGACAGTATACAATGTTGGTTTTCTTCATTATCTTCATTACGGTAGCAA
CCTCTCAAGAGATACCAACCCCGGCCAGCTTGATCGCGCCCAGGAATTTTAACTACCACCCGTATCAGGTTCA
ATTCTTGAAAGAAAATAATATTGAGCCTACTAAACAAGGCCCCGTCCTCTTTCCGAATGACGGCCCCCCACCA
CCTAAACCTCCTCTCGTGGTCACTTCCCGACCCCTCATCGAATCTATCGCCAGAAGTG
ACTTGAGTGACAATACGACCACAGCTGCATCGAATCCCAACAAAATAACCATATATGATCAGAATTTTGCCAA
AAATCCCGAAAATATATATGATCAAACATTATTTGGAAATGATTTTGCTAATAATGTCGAATATCAGGCGTCT
ACGTATAATGGGATCCCGTATGGAGATGTCCTGAAGAATTTAAGAGTGAATGACTTTG
GGAGAAACGATTATAATGATTATGGGGAAAGAACCGTTCTACCTCCAATTTACAAAGCATTAGAAGACCACGC
CAATCAGAATGTCTTAGACTTGCAGAAGAAGCAACTTAAACATTACAACGAACCAAAATATGTTGATTACACA
AACCAATATGAAAACAACGATGACTCACAGCAAAGCAGTGGGTCAG
>1002049467_1 Heli.1-ES585440.1.5

GAGATTTCCCTTTTGTTCAATGGGAACCCTTTCGGCTAAAGCGGCCCAGTTCACAGAGCTCTGAGCGATCCTT
TTTGAAGCCATCCTCGTGCCGAATTCGGCACGAGGGGAGTCTAGCATGTGTGCGAGTCATTGAGTTTATATTG
AAAATAATTCACAAAACTGAAAGGCGCAACGAAAGTGAAGGCGCGCGCTCGTCGCGCG
CTCAGGGAGGATGGAGCGTCGGTCTCGATCGATCTCCCGCACTCCCGAGGCGTCTCGTTTCCAATCCGTGAAT
GCAGGCGCGCTCTGAGCACAGATGCTGGGACCCGAAAGATGGTGAACTATGCCTGGTCAGGTCGAAGTCAGGG
GAAACCCTGATGGAGGACCGTAGCGATTCTGACGTGCAAATCGATCGTCGGAACTGGG
TATAGGGGCGAAAGACTAATCGAACCATCTAGTAGCTGGTTCCGTCCGAAGTTTCCCTCAGGATAGCTGGCGT
CGATTATTAACAGTCCCATCCGGTAAAGCGAATGATTAGAGGCATTGGGGCCGAAACGACCTCAACCTATTCT
CAAACTTTAAATGGGTGAGAACTCCGGCTTACTCGAACGATGAAGCCGGAGATCTGAT
GACGGTGCCAAGTGGGCCAATTTTGGTAAGCAGAACTGGCGCTGTGGGATGAACCAAACGTAGTGTTAAGGCG
CCTAAAAAACGCTCATGGGACACCATGAAAGGCGTTGGTCGCTCATGACAGCAGGACGGTGGCCATGGAAGTC
GGAATCCGCTAAGGAGTGTGCAACGACTCACCTGCCGAAGCAACCAGCCCTGAAAATG
GATGGCGCTGAAGCGTTTTGCCTATACACTACCGTTACGGGCACGTGCGGCGCGCGTTACGCGTGCGTCTGCG
TCATTATGCCGTAACGAGTAGGACGTGCGCGGCGGAGAGCGCAGAAGGGTCTGGGCGTGAGCCCGCTTGGAGC
CTCCGTCGGTGCAGATCTTGGTGGTAGTAGCAAATACTCCAGCGAGGCCCTGGAGGAC
TGACGTGGAGAAGGGTTTCGCGTGGCCACTATATGATCCAGCGCGAGTGGTGGTACTGGCTGTTCACGTACCT
GGAGCGCAACGTGCGCGGGCCAGTGCCGCGCCGCTACGACTGGCCGCTGCCGTGGCCGCGCACCAAGCTATTC
AGCCGGCTCAGCTAGTGACACGCGCTACCGCCCCCGCCGCCCGAGCGCGTATGACGCT
CACCTTCTGCGA
>1002049468_1 Heli.1-DT668739.3.5
GAGGGCGAGGTCTTGATTCGCGTTAAGGCATGTGGTCTAAATTTCCAAGATTTGATCGTACGGCAAGGCGCTA
TCGATTCTCCTCCTAAAACTCCATTCATCCTCGGATTTGAATGTGCCGGTGAGATCGAGCAGGTTGGCGAGGG
TGTCACCAATTTTAAGGTTGGCGATCAAGTTGTCGCTCTGCCTGAGTACAAAGCTTGG
GCTGAACTGGTGGCTGTGCCCGCCCAGTATGTGTACGCGTTACCCGAGGGAATGTCGGCATTGGACGCGGTCG
CTATCACCACCAACTACGTCGTGGCTTACCTTCTCCTCTTCGAGATGGCTAACCTGACCCCTGGCAAGAGCCT
CCTCGTTCATTCCGCTGGAGGTGGCGTCGGACAAGCCGTAGCTCAGCTGGCCAAAACC
GTGGAGGGTGTGACTGTGTTTGGCGTTTGCTCCAAGAGCAAGCATGAAGCTTTGAAGGCAAACAACAATAACA
TCGACCATCTCCTCGAGAGGGGAAGTGACTACACCAGCGAAGTCAGAAAGATTTCTTCTGATGGCGTTGACAT
CGTGCTCGACTGTCTCTGTGGGGAGGAATGCAATCGAGGCTATTCTCTTCTCAAGCCG
ATGGGACGATACATCCTTTATGGATCATCTAACATCGTGACCGGTGAAACCAAGAGTTTCTTCAGCGCAGCGC
GTGCCTGGTGGCAAGTGGACAAGGTGTCGCCCATCAAGCTGTTCGACGAGAACAAGAGCCTGGCGGGCCTCAA
CCTGCGCCACCTGCTGTTCCAGCACGGCCGCGGCGACGCCGTGCGGCGCGCCGTCGAC
GCCGTGTTCGCGCTGTGGGCCGCCGGGAAGGTCAGGCCCATCGTGGACTCCACCTGGGCCTTGGAAGATGTGG
GTGAAGCCATGCAAAAGATGCACGATCGCAAGAACATCGGCAAGCTCGTACTAGACCCTTCATTGGAGCCAAA
GCCTAAACCAGCAACGCCTGCCAAAGGCAAATCTGGCAAAGAGAAGAAACCAGCTAAA
GAGAGCTCTGAAGAAAAGAAGGACAAGGAGCCAAAAGAGGAGGAGAAGAAAGCGGAGAATGGCGACAAGAATG
AGTCAAAGGAGAAAGAGAAGGAGAAGGAGAAAGAATCTAGCTGAATTGTTTCGCCCGAGGACAGGACCAGACA
AACCACCGCCAATTGATTATTTTAAATTTGAAGCTACTCTCGTCTCGTTCGGCTTGTG
CTTACACCGGCCTCATGTTTCCTTTTATAAGTCTGAGTACGGCACTGTCCGATCCCTTCATTTCTTATTGTTT
ACAGCGACGACCTCAGTTCGGACATTTTAATATAGTTTCTATTAATTTTAGTATAACATCACATTCTATCATT
TAGATTTAAAACAAAATATTATTATTATTATTATTCAAGTACCACATTCGAACAGCGA
ATTCGTATTAAGTTAATATGACACACATTCTATTTTGAATGTGGCCTAAGTTAGCTTCCGAACTGTCGAGTCT
TATAGTTTAATGTTTAAGATTTTATTTTTAGGATATTTTAGTCTGCAGTTATTTTGAATGGCTATTGTATATC
TAAGGTGCAGAGATGCTTAATCTTTATGTATGTTAAGCTTTAATATTTTATTAGTAAA
TGTTTAAAATTTGGCCCCTGGTGACCCACCCCCGCCCTCGCGCGCCCCCCGGAACTGCAAACGAAATCCTTGC
GTGTGATATTTTGTACTAGCATGCCTATGCTGGAGGTTCTGCGGTATTATTATGCTGCCTATACATGACGTTC
ATTTTGTTTGGGCTTAAGAAAACGATTGAGTGTTTTATAATTATATTTTTTATACTTA
GAAGTTTTGTAAATTATTTTCTAGTTAAGAGAAAAGTCAAATCAAATACATCGCATCTGCCACTGTCTGGTAT
GACACTAGCAGTGGACTTGCCAGGTATACAAAGCTCGAAGTACCGTCACTCCCTCATCGAGACTAACCAAATA
ATATTTGTAAAATGTTTAAAGTGACTCCTTGAATTTGTGGATCCCGCATTCGTTCAAC
ATTTAAGTATTAATATAAGTCGTATTTTTGTAAATCAAATGTCGACGCGAGATCTCTTTTATAAGTATAAAAA
CTTTTATGTAT
>1002049469_1 Heli.1-CO729579.1.5

CTGCAAGTAGCTCGCCATCATGGTGAACAACAGAGTACCGTCGGTCTTTTCGAAGACTTATGTCACACCGCGT
CGTCCCTTCGAGAAGGCGCGTCTAGACCAGGAGTTAAAGATCATCGGTGAATATGGTCTAAGGAACAAACGTG
AAGTGTGGAGAGTCAAATACACTCTCGCCAGGATCCGGAAAGCCGCTCGTGAGCTGCT
TACCCTGGAGGAAAAAGATCCTAAACGTCTTTTCGAAGGTAATGCACTCCTACGTCGTCTCGTCAGGATTGGA
GTCTTGGATGAGAAGCAAATGAAGCTTGATTATGTGCTTGGCCTCAAGATTGAAGACTTCTTGGAGCGCCGTC
TCCAGACACAGGTGTTCAAGGCTGGTCTTGCTAAGTCCATCCATCATGCTCGCATCTT
GATCAGACAGAGGCATATTCGTGTCCGCAAACAAGTTGTAAACATCCCCTCCTTCATTGTCCGCCTGGACTCC
GGCAAGCACATTGACTTCTCCCTGAAGTCACCATTCGGAGGCGGCAGGCCAGGACGTGTCAAGAGGAAGAACC
TCCGCAAGGGCCAGGGCGGTGGTGCCACCAATGATGAGGAGGAGGATTAGATATTAAA
TGCTTAATATCT
>1002049470_1 Heli.1-ES585196.1.5
CGCTTAGGCGAAATTCTCTAGGCGAGTACTGCTTACAGTAGCGTTTAGTCAGTTCGTTTGAGATCTCCAGAAT
TAGTTTAAGAAATATCATCATGCAGTCGATGATCATCTTTGCCGCTTTCTTCTGCCTCGCGCAAGCATCCTAC
TACCCTGGCCCTCCTGCGCACATACAGCTCAGCTCTGATGGCAAGTATGTGCTGGACA
CGCCCGAGGTAGCACACGCCAAGGCCGCCCATCTGGCCGCACATGCACAGGCCTCCAACGTTCACGGTGCCTG
GGCTCCCGCCGGAGCGTACGTCGCTGGTCCCGCCTATGGTGCTGGTGGCCACTACGGAGCTCCCGCTGCCGGT
CTCGTGAAATACGGCCCAGCTCCCCTCGCCCACGACGGCCGCGTCATTGACACCCCCG
AGGTCGCCCACCTGAAGGCCGCCCACATCGCCGCGCACTCTGCTGCCGCTGCCAAGGCCGCGCACGGCGGCTA
CGCTGGCATCGGTGCCGGGCTCGGTGCTGGGTACGGAGCGGGTTACGGCGCGGGCGCAGCTTACGGGGCGGGA
GCAGCCTACGGCGCTGGGTACGCTGGCGGCTACGGCAAGTGGAACGGACCCCAGGCTC
ACATCCAGCTCACCCACGACGGACAATACGTGGTAGACACCCCTGAGGTCCAACACGCCCGTGCTGCCCACTT
CTCCCAGTACGCCCACGCCGCGCAAGCCGCCGCCTCCTCCCCCGAGGAGCCCTGGGACGCCCACAGCGGACAC
GGCGCCCACGGCTGGCACTAAATCCTACCTCCGACATAGCCCAAACACTGCCTGAATA
TATTTTTATACAAAACGTAAAGAAAAAACCAAAAAAATGTTAAGCTGTATAAAGTTGAATGACCTGTGTATAG
CGATGCTAGAGCGATTTTGTACTTAATGTAATAAAACTCTTATAATG
>1002049471_1 Heli.1-EL602668.1.5
TCATACCGTATTATATCTTGCTTATATTAAATTTGTACACAATTATTGGGTTTTACAGTAGTTCGTATTTCAA
CGCCTATTTAATATCAACCAATAACTCACAATGGTTTCGCTAAACCCTATAAGGATTTTAAAGAATGAGGCTG
AAGAAGAAAAAGCCGAAGTGGCTCGTATGTCCAGTTTTATCGGGGCCATTGCAATTGG
TGATTTAGTAAAAAGTACCCTTGGTCCTAAAGGCATGGATAAAATATTGGTATCTTATGGAAGAAATGCAGGC
CAAGTTGAAGTCACAAATGATGGTGCTACCATTTTAAAATCTGTGGGAGTTGACAATCCAGCCGCTAAAATCT
TAGTTGATATGTCTAAAGTTCAAGACGAAGAAGTAGGTGATGGTACTACATCTGTAAC
AGTTCTAGCTGCCGAGTTGTTAAGAGAGGCAGAAAAGTTGGTTGAACAGAAGCTTCACCCACAAACAATTATT
GCTGGTTGGCGCATTGCTGTGGAAGCAGCCAGGCAGGCTTTAGCTGAAGCTAGTTTTGATCATGAAAAAAACA
TGAATGAAGCAGCTTTAAGGAGTGATCTGGAGAACATTGCTCGTACCACACTTAGCTC
TAAAATCCTTTCTAATCATAAAGAGCATTTTACTAAATTAGCTGTTGATGCAGTCTTAAGGTTAAAAGGTTCT
GGAAATCTTAAAGCCATACAAATCATTAAAATTTCTGGTGGTTTGCTTGAGGAATCTTT
>1002049472_1 Heli.1-DT663193.3.5
GCACGAGGCGCGACGCTTGTCTGTGAGCTCGATCCCTCAGATACGCTGCTGGTTTACGGCGTGCCGTAACACG
ACACGTTTGGAGGGACCTGCTGCTACGTGTGTGCCTGGTGCTCTGTGAACATCGGATACTGTGCTTCTAGTGC
TGTGACAATGCAAATGTGCTGATATTTCAAATTAACATAATCACCTTAAAGGATTTTT
GACGACACAGCCAGTTCCGGATCAGCTCAGCCGATAATCACCATGAAAGTTGTTAGCCTTCAATTGCCATCGG
GACCCAGTATGGAGCGGTTACCGTTACCATTCAGTCCGACGGAGCTATTATGGCGATACCCTTTGCCTTGGGC
ACCACCTCCACCTTCACCTTTAGGTGATACTAAAGCACAGCTGCCAGCTGGTCTTCCA
CCAGAACCTAGATTATGGACTCGTGAAGATGTTGCCATTTTTTTGAAATGGTGCGAAAGGGAATTTGACTTGC
CCAATTTTGATATGGATCTTTTCCAAATGAATGGTAAAGCTCTGTGTCTTTTGACGAAAACTGATTTAGGAGA
GCGTTGCCCCGGTGCTGGAGATGTTTTACATAACGTTCTTCAAATGTTAGTACGGGAT
GCTGCGATACTCGGAAGAGTGCCTTCATCTCCTGTAACACCAACCGCACGTGCTGCACCATACCCACCGTCAC
CACATTCGCATCCACCGACCCCTACTTGGGCAGTAGACGGATTTCATCATTTTCATAGTGCTGCAGCTGCAGC
AGCGGCGCAACCCAACTCTGTCACTTTAAGTCCTGCTCCTTCAGTTGACAGTTCTGGA
AGCCCTCAAAGAGGAGATACTATGACTTACGCCCCTGCTTATGCACAACCAGTTCCTGTAACTACTCAAGCTG
CTAGTTCCGGAAGTAATCATTCTGACTCGGATGAGGAAGCTCAGTTCTCAGCTCCTCGATCGCCAAAAGAAAC
ACCTTTGACCAGTCCAGCGCCACAAACGCATGTTGTACCTCAACATTCACACTATCGC

ACTCAACATCGCGAGTTCTTCCCTAATGATATGCCAGAGTCTAACACAAATGGTAGGCTTTTGTGGGACTTCC
TGCAACAACTTCTTAATGACCCTACACAAAGATACACAAACTATATAGCATGGAAAAAT
>1002049473_1 Heli.1-DT665562.3.5
GAATAGCTCGTGCGTACAGTTGATCTCAGTCGGCTAGTGTGTGATGTGCTCTGATTGGACCACTTAAATCATT
GAGGAGTTGTCCTCTCGTCCTGTGACTCTGGGACCCATTCACGTAAAATGATGCGTCTACTGGTGATACTCAT
AGCAACTACGCTTTGTGGTTTGACTGATTCTGTTCTATCGCCACAGAAATCGCTCAAG
ACATTTACTCTTGAAGAATTGGTGCCTTTGCAACCAGATTTTTTTCCATTCAGAGTATCTGTCAATTGGATAT
CAGATACGGAGTATTTAATTATAGAGCCTGGTTCAATAAATAAATATGATGTCGTTGCTGACAATTTTACTAC
AATTATAAATGAATCGGAATTTAACAATTTAAGCCGATTCTCCGTATCATCATTTTCA
AATGACCAAAAATATTTGCTTTTAACTGCAAATAAGAAGAAGGTGTATCGGTATTCTACTATAGCAGAATACT
CGGTGTATGATCTCGAAAACAAGTCTATATCGAACATTGGCAATGGTTCACTTCAAGTGGTGGTGTGGGGCAA
CGATCATGCGCTCGCGTATGTGCAAGATAATAATGTATACTACGTGCCTGATGTCTCC
AGCCCCGATATAGTTACTCCACTTACTACTGATGGAGTAATAGGCAATATATACCATGGTGTCACTGACTGGA
TCTATGAGGAAGAAGTATTTAATGCAGCAGAAGCGACGTGGTTCTCACCTGACGGAACATATTTAGCAGTAGC
TTCCTTTAACGACTCTCAGGTGGAATCTGCCGTA
>1002049474_1 Heli.1-DT665714.3.5
CGAGGTGTACCTTAATTGTGTGTTGGTTAGCGCTCGGTGGGTTTAAAAATGGCTCTATTCAGGATCACCTCTA
CAAAATTAATTGATATACAGAAAGCATGCCCAACGGCAACTATTCTGTTAAGAAATTTGAGCGCGGAGCAAAC
CAACCTGAAGAGTGTACTTCAGGAGAAAATCCCCAAAGAACAGGAGAAGATCAAGGAA
TTCCGTAAGAAGCATGGCAACGCTAAAGTCGGCGAAGTCACCGTTGACATGATGTACGGTGGTATGCGTGGTA
TCAAAGGTCTAGTATGGGAGACCTCAGTGTTAGACGCCGATGAGGGTATCAGATTCCGTGGTCTCTCCATCCC
GGAGTGCCAACAACAGTTGCCCAAAGCGAAGGGAGGGGAAGAACCTTTACCTGAGGGT
CTCTTCTGGCTTCTAGTTACTGGGGAAATACCCACCGAGGCTCAAGTGAAGGCTTTGTCTAAGGAATGGGCGC
AAAGAGCCGAGCTCCCAGCCCATGTTGTAACAATGCTAAACAACATGCCAAGCAAACTGCACCCGATGTCTCA
GTTCTCAGCAGCCGTAACAGCACTTAACAGTGAGAGCAAGTTCGTCCAGGCCTACACG
GAAGGTGTACACAAGTCTAAGTACTGGGAGTATGTCTACGAGGACTCAATGAACCTGATCGCCAAGTTGCCCG
TCATAGCCGCCACCATCTACCGCAACACCTACCGCGACGGCAAGGGTATCGGCGCCATCGACGACAACAAGGA
CTGGTCCGCCAACTACTGCAAC
>1002049475_1 Heli.1-ES588147.1.5
CAAAGGTTGTAATATTCCTGTGCGCCGCCGGCATCGCTTCTGCCGGTAACCTCCTTCACGCCGCACCAGTGGC
GTACAGCGCGCCGATCTCCGCAGTGTCCTACTCATCTCACGCAGCTCCCATCGCTTCATACGCCGCGCCAGTG
GTCGCCAAGAGTTTATCTCCAGTGGTCTCGTACCAATCAGTCTCTCATGCCGCCCCTG
CCATCGCATACTCTTCTCCTTTAGTGGCTAAGAGCTATGCCCCAGCCTTATCTTACTCATCCGTTGCTCATGC
CCCAGCATACGTTGCTGGCCCGGCTGTATCTTACTCCTCACCTATTGTAGCTAAGAGCATATCCCCCGTGGTA
TCCTACTCATCTCCTGTATCCTATGCTGCTGGTCCAGTAGTTGCCAAGGCTATCGGAC
CCGTAGGTGCCCCAGTACTTAAGTCTGCTATCGCATATTCTGCTGCCCCAGCGGTTTCCCACGTCGCTTACTC
TGGTTTAGGAGTTAACTACGGATGGTAATCAATGAAATCTATCTAGTCAATTAAATAACTGTATTATTGTTAT
TTATATTATTTTTTTACGAAATAAAAAATTATAA
>1002049476_1 Heli.1-DT667803.3.5
GGTGAAATATAATTTTTAGAAAAAATTTCACGTGCTTGAACCAATTATTATTGTTTACTTCATAAATTTTCAT
ACACCAAACCCATAAATCATGGCCGACAATGACGATCTTCTCGACTACGAAGATGAAGAGCAGGCGGATCAGC
AAGCGGCGGACGGAGCGACCGAAGCGGCGCCAAAAAAAGAGGTTAAGGGATCCTATGT
GTCGATTCACAGTTCAGGATTCAGAGATTTCCTTCTAAAACCTGAGATTTTGCGAGCCATCGTCGATTGTGGC
TTCGAACATCCTTCAGAAGTGCAACACGAATGTATACCGCAAGCTGTTCTCGGTATGGACATATTATGTCAAG
CTAAGTCCGGTATGGGGAAAACGGCCGTTTTTGTTTTGGCGACGCTACAACAACTGGA
GCCTTCCGAAGAGCATGTATACGTCCTCGTCATGTGTCACACGAGAGAGCTCGCCTTCCAAATCAGCAAAGAA
TACGAGCGCTTCTCCAAGTATATGGCAGGAGTTAGGGTGTCCGTGTTTTTCGGGGGAATGCCGATTCAGAAAG
ATGAAGAGATATTAAAGACTGCGTGCCCGCATATTGTGGTCGGTACGCCGGGACGTAT
CCTGGCTTTAGTCAACAGTAAGAAATTGAATCTGAAACATTTAAAACATTTCATCCTCGATGAGTGTGACAAG
ATGCTGGAGTCACTGGACATGCGTCGTGACGTACAGGAGATCTTCAGGAGTACTCCCCATGGGAAGCAAGTTA
TGATGTTCTCGGCTACTTTAAG
>1002049477_1 Heli.1-DT668041.3.5

CGTACCCTCAGTCGTGCACACTGCACGCGCGTCGCACTAGCGCAGCTACCACTCCTAACAGATCACAGAGGTA
CGCACGTGCATTACTGTTCTAGTCTGCACGCGCGTCGCACTAGCGCAGCTACCACTCCTAGTGAATTTTAAAG
ATGAGCCGGCACGTATGCCTGGCGGCCGCGGCGCTTCTGGCGTTGCTGTGCGTCGCCG
ATGCCCAGCGGCGCTTGGCCCTGCCTGACCCCAGAAGTTGCGCTAACAGAGTTCGACACTCAACGTACCGTGA
CGCACGCGGTGTCCTTCATTCGTACTTCTTCAGCTGGGAACATGCTCCGACTCGCAGCCTCGAGGTAGATTGG
CTCGATGCAAGAAACATTTGCAGACGACACTGCATGGACGCCGTTTCTTTGGAGACCC
CACAGGAAAATGAATTCGTGAAGCAAAAGATTGCTCGTGGTAATATCCGCTACATTTGGACTTCTGGCCGTAA
ATGTAATTTTGCTGGCTGTGACCGAACTGACCTGCAGCCTCCCAATGTCAACGGCTGGTTCTGGTCCGGATCA
GGTGCTAAGATCGGACCCACCACGCAGCGTAACACTGGAGATTGGTCCTACACTGGCG
GGTACGGCCAGGCCCAACCCGACAACAGGGAAGCTGCCCAAGGCAACGACGAGTCCTGCCTGGCAATATTGAA
TAACTTCTACAACGACGGCGTCAAATGGCACGACGTCGCCTGTCACCACGTCAAGCCTTTCGTGTGCGAGGAC
AGCGACGAGCTCCTCAACTTTGTTCGGTCCCGCAACCCCGGCCTACGTCTCTAAATTT
CTTTCCCACCTATAACTATTAGTGCGGATACAATGCCGTTTTGTGAATATTAAACATAAATGAAATTTAAAAT
AGATGAATTTATACTTAATACATTTTGTCCGCACTTATAAAAGGACTTGTGCTGACTGACTGAGGTAAGACGC
ACGCAGAGCCGTAACCGCTGATGGTAGAAACGTGTAAGTTATTTATATGTTTATTTTC
TGTTTATTGTTTTCCACTAAGAAGAAACATCTTTCAATGAAAGATTACATTGTTATTATAATTTCATATTGGT
CAGTCATGTTTTCTTGTGTAAATCTTTTGCGCTATAGGTTTAATTTATAAATATTAGAATAAATCATTAAT
>1002049478_1 Heli.1-ES584676.1.5
TTACCACGCCATCGGATCACAAATCTGTCACAGTGTAGTTTGTGTGATTCTTCGTGAAATTCAGCTGAACTCT
TTTAAGTTTTGTCTATAGGAATCAATATTTTTACAATCCTAAAATGGCAGATGAAAAAAAGGGAGAAAGCGAA
CACATCAATCTAAAAGTTTTAGGACAAGATAATGCTATTGTCCAATTTAAAATTAAGA
AACACACACCACTCAGAAAATTGATGAATGCCTACTGTGATAGAGCGGGTCTATCAATGCAAGTTGTACGATT
CCGATTTGATGGTCAACCTATCAATGAAAATGACACACCAACGTCTTTGGAAATGGAAGAAGGTGACACAATA
GAGGTGTATCAACAACAAACTGGAGGGGCTTTAGTGTAAATTTAACTTAAGGAACTGT
CCGGGAGAACCACTGCATTTGTGGCCCATCTCAAAAACATTAATTAAGTTTATTATAATACCAACATTTGTAT
CTGTAAAAATTTCCAAGATACAATACAGCACCAAAATCAGATTGTGTCTTGGAAATTTCAAAATAATTAAGAG
AGTAAGACAAACCAAATGTTCTAGGCGACTTCAATTATTCCATTGTGATAATTTTGAG
TTGGCAAACTTAGAAACAGCCGTTCTAAATGCTGTAGCACATAAAACTTTTTCATGTGCATGGGTCAACTTTA
TGGTTATTTATTGGACTTGTGTCCAATCCACATGTCAGATCTGTAATGTTGTACCTAACAGAAATAAAGTA
>1002049479_1 Heli.1-EL602531.1.5
GCACGAGGGTCACTTTTGCTCGATGAGAAGATTGTTGAGTTAGTTTACGCGGCGTCTAATTTAGGATACTTCA
TAAAACATTAAATCATGAATATATTCCGTTTACTTGCGGACTTGTCCCACCTGTTAGCTATTATAATATTATT
ACTTAAAATATGGAAAACCAGATCATGTGCAGGAATATCAGGCAAATCGCAGATACTA
TTTTCTATAGTATATACGACCCGGTATTTGGACTTGGTGACAACGTTCGTGTCTCCATATAACACTATAATGA
AAATAGTGTTCCTGGCTGCATCATACGCGACTGTTTATTTAATGTATGTCAAGTTTAAAGCCACCTACGATCA
CAACCACGACACATTTAGGATTGAATTCCTCTTAATTCCATCTCTTATTCTGGCTTTA
CTGATTAATCGTGAGTTCACAGTAATGGAGATATTATGGACATTCTCTATTTATTTAGAATCGGTTGCAATTC
TTCCACAACTATTTTTGGTTTCTAAGACTGGAGAAGCTGAAAGTATCACATCTCACTACTTGTTTGCATTGGG
TTCCTACAGAGGCCTATATCTTTTGAACTGGATATACCGTTACATTGTTGAAAACCAC
CATGAGCCAATTGCCATTGTTGCTGGCATAGTACAGACAGTTTTGTACTGTGATTTCTTCTACCTCTACATTA
CTAAAGTTCTC
>1002049480_1 Heli.1-DT662891.3.5
TTCTCAAAAATTATTGGTTTGAGTGCTGTGCTGGCGGGTTTGAGCGCTGTGCTGGCTGTGGCTGCGGCAGGTT
TACTACCAGAGCCACACTACTCTTCAGCTGCTGCAGTGTCATCCCAACCCCATGCCGTTGTTGCTGGTCCAGT
TGCATACCATGCAGCACCAGTTGCCTACCAGGCAGCACCAGTTGCCTACCAGGCAGCT
CCAGCTCATTACTCTTCAGCAGCAGCAGTCTCTTCTCAGTCTATCCTACGTCATGACCAACCTCACGCTGCCC
TCGCTGTTGCCCCCGTTGCCCACTACTCCGCTGCTCCTGTTGCCCACTATGCTGCTGCACCTGTTGCTCACTA
CGCTGCTCCTTCCCATTACTCATCCGCCGCAGCAGTATCTTCTCAATCAATTCAACGC
CATGACCAGCAACATGCCGCTATTGCTGTAGCTCCTGTGGCTCATTACAATGCCGCACCTGTAGCTCACTATG
CCGCTCCCGTAGCGCATTATGCTGCCCCCGTCCACGGCGTTGTTGCCAGCCATCAAGAGGAATATTCTCACCC
TAAATACGATTTCTCTTACTCTGTAGCCGATGGTCACTCTGGTGATAATAAGTCCCAG
CACGAGAGCCGCGACGGTGACGCAGTACATGGTGAATACTCTCTGGTCGAAGCTGATGGCTCCATCCGCCATG
TTCAGTACACCGCTGATGCACACAACGGTTTCAACGCCGTAGTCAGCAACTCTGCACCATCAGGACACGTTGC
CCCAGCCCCAGCACATATCCTAGCTCATCATTGATTAATTTATGTAAAATTAATTGTA

AATAATGTCTACTTAAATTAAGAT
>1002049481_1 Heli.1-DT665532.3.5
GACTACGTGTAATTGACCTAGCGATCGTGCAATTTTATCGTTATACATTAACGTAGAGATAGATAATTATTAT
ACTTATCAAAATTATATCGTATTTGTGCTCAGTTCGAGACGTGTGTAATTAGAACGAAGTAAAAGGAAGTGAT
ATTTGAAATAATCTATCTATCGCAGTGGTCTACGCAGTTCGACACAGATTAATCGAGG
TAATTATAAAATACCAAAATGCCTAGAAAAGTTTACGTAGTCGGTGTAGGTATGACGAAATTCGTCAAACCAA
ATAGTGGAAAAGATTATCCAGATCTAGGCAAGGAGGCGGTCGTGGACGCGCTCGCTGATGCTCGTATTAGATA
CGACGATGTCCAACAAGCGGTCTGCGGCTATGTCTTCGGCGATTCCACTTGTGGCCAG
CGAGTACTGTACCAGGTCGGCATGACCGGTATACCCATATACAATGTCAACAACAACTGTTCCACAGGTTCAA
ACGCTCTTTTCCTCGCAAAACAACTCATTGAAGGTGGTGTCTCTGATGTAATACTCGCTGTTGGGTTCGAGAA
AATGACCCCAGGAGCTCTTGGTGGTGGTGCTTATACAGATAGAACAAACCCATTAGAT
AGGCATACTCTTAAAATGGCGGAAATCACAGAACTTACTGCGGCCCCAATGACAGCACAATATTTTGGTAATG
CAGGCATGGAACACATGAAAAAGTATGGCACAACAGAAACTCATTTAGCAAAGATCGCT
>1002049482_1 Heli.1-DT668048.3.5
CACGCGGAGCTGTGGACGTTAAATTGGTACTTTAAAAAAAGTAGTTTGACTTTGACTACTACTTGTTCGTTCT
CGTGTTTTATAATTATTAATTATTTTTTTTTGAAATGGCTGGAAATAATGTCAAGCGGTTATTTAAATATGTT
TCTGATCAAACTATTACATCTACGAGGAATTATGCTTCAAAAAGAGTTGTTGCCTCTA
AGCCTGTTGTGGAAATGGATGGAGATGAAATGACAAGAATCATTTGGGAAAAAATTAAAGAAAAGCTTATATT
TCCTTATGTCAAGTTAGAAAGCTTGTACTTCGATTTGGGCCTCCCGCACCGCGACGCCACTGATGACCAGGTC
ACAATCGACGCGGCGCACGCTATTCTGAAACACAATGTTGGTATCAAGTGCGCTACTA
TTACACCTGATGAACAGCGAGTGGAAGAATTCAAGCTGAAGAAAATGTGGCTTAGTCCAAATGGAACTATAAG
AAATATTCTCGGCGGTACCGTGTTCCGTGAACCCATTTTATGTAAAAGCATTCCTCGCGTAGTTCCTGGATGG
ACGAACGGTATTGTTATCGGTCGTCACGCTCACGGTGACCAGTATAAGGCCCAAGACA
TTGTTGTTTCTAAACCTGGCAAGATGGAGTTAGTATTCACAGCGGAAGACGGTTCTGTGGAGAAGCGACTTTT
GTATGATTACAAGTCACCCGGTGTGGCTATGGGCATGTACAATATTGACGAATCCATTCGTGCCTTCGCACAC
TCTAGCTTCCAGGTAGCATTACAAAAGAAGTGGCCACTTTATTTGTCTACAAAAAATA
CTATTTTGAAAA
>1002049483_1 Heli.1-ES584763.1.5
GCGGTACGGTCGGAATCCCGGGTCGACCACGCGTCCGCGAGCAGTCTACAACAGAAGTGCACGATGAGAGTTC
TGATTGTTGCCGCCGCCTTCCTCGCCTGCGCCGCAGCTGCGCCATCCGGCGCTCTGCTGGCTGGTCCCTACGC
CTCCGGGCTGCTGGGACATGGTCACGGCCTGGCTTTAGGCCATGGCGCACCTCTGGCC
GTAGCTGGTCCCTTGGCTGTAGCTCACGCTGCGCCCCTCGCCATCGGTCATGCTGCCGTCGTCCCCACCATCC
CTCCAGGTGATCTCCACGGTGCCGCCATCGACGCTCACGTCGAGGCCTCCGACCACGCACGCGCCGCCGTCGA
CGCTGCCCGTGAATACCATGACCAGGCTGCCGAACTCCAGGGTCAAGCTGTGAACGCT
GCTGAAGACCATGCCTGGCAAGCGGTTGACGCTGCCCAGTCCCACAAAGCTGCCGTCGACGGTGCTGCTGCTG
GTGTCGCCCCTGTGGTGGCCCGTCAGTTGGCCGGACACGTCGCCCCCGTCGCCTACGCCGGTCATGGTCTCGC
TGGTCACGGACTCGCTGGTCCTCTCGTAGCCGGTCATGGTCTCGCCGCCCCCCTCGTA
GCCGGACACGGTCTCGTCGCCGGACACGGTCTCGTCGGACACGGTCTCGCCGGACACGGTGTTGCCCTCGCTG
GTAGCCATTCC
>1002049484_1 Heli.1-EL597165.1.5
GTTTTGAGTTTTCTGTCAAGGTAGAAATTGGAGAAAAAAAGGCCGTCCCTTTTGGGATTCTCTGGTTTAAGAA
CTCCAATTGATCCAAAAACACCACCAAGATGAGATACTTCTTGACTTACCTGTTTGTGCTGCTGTTGTCGCTT
GCCATACCCATATTTTCGTTATACTACCTACTGACTGGAAAGGGTGAGCAAATAAGTT
TCGGATGGTTCCTCGAGAATACTTCCCCCTACATGTGGGGCACCCTGGGCATAGCGTTTGCTGTGGCTTTCTC
AGTAGTGGGCGCCGCGATGGGCATTCATACAACCGGAGTGAGCATAGTAGGTGGTGGTGTCAAAGCTCCAAGA
ATCAAGACCAAGAATTTAATCTCCGTTATTTTCTGTGAAGCTGTGGCCATCTATGGAT
TAATTACGGCTATCGTATTGTCTGGTATGCTTGAACAGTACAAAGAACCATTCTTGGATATATCAATCAAGCA
ACAAAACTGGATGGCTGGCTATGTGATGTTCGGTGCTGGTCTGGCCGTCGGACTCGTGAACTTGTTTTGCGGT
ATCGCCGTAGGTATTGTGGGTTCAGGTGCCGCTCTCGCTGATGCTGCCAATGCTGCCC
TTTTCGTAAAGATCCTAATTGTAGAAATCTTTGGATCAGCTATAGGGTTGTTCGGCCTTATTGTAGGTATCTA
CATGACATCAAAAGTCAAAATGGGCAACCAGTAAC
>1002049485_1 Heli.1-DT667859.1.5

TGGTGGCCAAATTCGTAGTTTTCTTCGGAATGGTGGCAGCAGCCTCAGCCGTTGCTGTCCCCCTCCTCCCCGT
CGCTAAGGTGGCGTACGCTGAGCCCGAAGCCCCAGCGCACTACGAATTCCAATACTCCGTCCACGACGAACAA
AGTGGTGATGTGAAACAACAACAAGAATCCCGCGCTGGAGACGCCGTCCACGGCTCTT
ACTCTCTCGTGCAGCCTGATGGAGTTCACCGCATCGTGGACTACAGCTCCGACAAAATAAACGGATTTAACGC
CGTCGTCCGCTATGAGGGATCCCCTATCCCAGCCCCCGCACCTGTCGCCAAGCTCGCATATGCTGCTCCCGTT
GCCAAACTCGCCTACGCCGCTCCCGTGGCCAAGGTTGCCTACTCTTCCCCCGTCGCCT
ACGCCGCACCCGTCGCCAAGTTGGCTTACGCCGCGCCCGTCGCCAAGTTGGCTTACGCCGCTCCCGTCGCCAA
GTTGGCCTACGCCCCCGCACCCGTCGCTAAGCTAGCCTACTCTCACGCACCAGTAGCCTACCAAGAAAACCTC
GGCCACGTCTCCTTCTCCTCACCCGTCGTCTCTTACAGCCACTGAATATTAAAATAAT
TGTTAAAACACTATTTATTCTGTTAAATACAAAACT
>1002049486_1 Heli.1-DT664808.3.5
AAGGAAGTGCCATTGTTGACTGTGATCCTGTGTTGTAAATAGTGTACGAAATGTATTCAAAGACATTAACTTT
AATGATATTGTCGATTGGATTTTTGGACTTGGGTGACTGCCAATCTTTACAATGTCCTAAAGTGCGCACCACC
AGTGGATGGTTAGATTTATTCCCAATACCCTGTCGAAAAAGTACAGATTGCAGTGTGA
TGGGAGCCCAGCATCTTTGCTGTAAGGGATTCTGTACGAAAGGAGCAAAAGTGGCTCAACTACGAAACAAGGA
GGCGAGTAGTGTAGTTTCATCGAGTAGTTCAAGCCCCAGTACTAGCACAAGCACAAGCACTAGCACAACCACA
ACAACCACAACAACGACAACAACAACAACTACTACAACGACTCCAAGTACTACCAGTT
CGACGACCACGACGACTACCACGACCCCCAAGCCGACCACCACCACGACGTCAACGGAACCGCCGCGCCCGTT
CCTCCAGTTGCTGCCTATACAAACGACACCTAAAGCTCCCGCAAAAATAGGTAAGAACCAAAAGCTGATATGT
CCAAAGAATATAGCAATTGTGTTGTTCCCAATTCCCTGCGAGACCAACGCTCAGTGCC
GCACGAGCAGCGGGCCCGGACAAGTTTGCTGCCAAGGCCAGTGTGTGAAAGGAGTGCCCGCCCCTCGACCTAC
CGTGTCGGAACAATCACACCAACCTATTCTCGGAGTCCCTCGCGAGTAGTGCCCAGCGGCGCCGCTGGGCGAG
CTGCTGTTCGAGGTGCAGACGTGCAAGTCGGACGCGAACTGCTGGCCGCGCGTGTGCT
>1002049487_1 Heli.1-ES587038.1.5
GTCCGCTAAGATTTTGATCGTCCTCGCCCTGGCCGCGGTTGCCTCCGCCCTGCCAGTTGTGCAGTACGCAGAG
CCCGAAGCGCCCGCCCACTACGAATTCCAATACTCCGTGCACGATGAGCACAGCGCTGACGTGAAGCAGCAAC
AAGAGGCCCGAGCTGGTGACGCCGTCCACGGCTCCTACTCCCTCGTGCAGCCTGATGG
AGTCCACCGCATCGTTGAGTACAGCGCTGACAAGGAGCACGGATTCAACGCCGTAGTACGATATGAAGGACAC
CCCGTCGCCGCCCCCGCCCCTGCCAAACTCGCGTACGCTCCCGTCGCCAAGCTCGCCTACGCCGCTCCCGTCG
CCAAGGTGGCGTACTCCGCCCCCGTCGCCTACGCAGCTCCCGTCGCCAAGGTAGCCTA
CGCTGCTCCCCTCGCACACGTCAGCTACTCATCCCCCGTCATCTCCTACCAACACTAAGTCAATACTTTTAGC
GAATATCCATGTTAAATCTATTTAATTAATTTATATAATAAAAATAT
>1002049488_1 Heli.1-CO729503.1.5
GTCTGCCAGTGAAGACCGGGAGGGAACCGACTAACACCACACAAAATGAGCGACCTCAGCAAGAACGAAGTTG
AAAGGGCATCCTTCGCCTTCTCCATCTACGACTTTGACGGTAGCGGCAAGGTCGATGCCTTCAACCTGGGTGA
TATCTTGAGGGCACTCAACTCCAACCCAACTGTTGCTACCATTGAGAAACTCGGAGGC
ACCAAGAAGAAGGGCGAGAAAATGCTTTCGATTGAAGAGTTCCTTCCCATCTACAGCCAATGCAAGAAAGACA
AAGACCAAGGCTGCTATGAAGACTTCTTGGAATGCCTGAAGCTCTACGACAAGAATGAGAATGGTCTCATGCT
TGGCGCTGAGCTCACACACACACTTCTTGCCTTGGGTGAAAAGCTTGAAGACAGTGAG
GTAGCAGAAATCGTAAAGGACTGCATGGACCCTGAAGATGACGATGGCATGATCCCATATGCATCTTTCCTCA
AAAAAGTAGTTGCGGGTTGGCAGGGCCCTCAGCAAGCGGCGGCAGCGCCGGCGGCGGCGGAAGCGCCCGCGCA
GGGTTAAGCTCCACGGTATAACTTTCGCAGCCGTCGCGACCGCCCCAACTACACACCT
CCGCTCGAACCATCTACGGAGCACCCCTTTTTTACGAAATAACTTCATACATTTTGTACAATCTGTTATAATG
ACTCTATTATGAAATAAGATTAAAGAGAGCACTGTGAACGCACGTTGTACGTGTTTTAAAATTTATTATTATT
ATTTATAAGTACGTTTATAATGACAAAAACACAAACCATAAGTTATTTTATAATAAAA
>1002049489_1 Heli.1-DT665987.3.5
TTGTGGTGTGGTGACCAACGCGGTATTACTATTTTGCCGCCTAGCTCAAGTGTGATATATATCTTTAATTGAG
TTGAACTTTAAATTTTTAACACACATATTAGTTATAGTTTACAGAAATCTCTATCTTTATTAAACTGAACAAA
AACTACCAAGATGTTTTGGGGACTTATAATGGAACCGAACAAGCGGTATTCTCAGCTT
GTGGAGAAACCGTTTCATATATCACAAGCCGCGATGGACATCTCAACGGGTGATAATGACCCATGCCAAGTAA
TGGTTGTAGTTGACGGCAAAAATTTTCTCGTGTGCACGTTACAAAAGAACAAATGCATTCAAGTACCTTTGGA
TCTGTACTTTAAGACTGGAGATTCCATTTCATTTTTAACAAATGGTAAATGCAATGTT

CACTTAACGGGCTACTTGGATCCTGAATTTGAAGAAGAACCTTCTGATGAAGAAGAAGAAGAGGAAGAAGAGG
AGGAAGAAGCACCAGCACTTGTTGCCGCCAAGAATAAAAGGAAATTAGAAAATAGCAATGATGGTGCTGCCAA
CAAAAAGGCAAAGCAAGACAAGAAAGCCAACAAGAAGGCTGCAGCAGCTGATAGCTCT
GATTCTGATGAAGATGGTGAAGATCAATTGCAAAAGTTTTTGGATGGAGAGGACATTGACACGGATGAGAATG
ATGAGTCCTTCAAAGTGAATACCTCGGCTGAAGCTGACAGTGATGACGATGAAGATTCAGATGAAGATGAAGA
GGAGGAGGAAGGAGAAGAAGAAGAAGATGATGATGATGATGAGGAA
>1002049490_1 Heli.1-DT666925.3.5
CGTGCCTTTCGTAATCGGCAAAAATCTAAACAATCCTTTTTCTATTACATATTATATTTAGTCATATTTCGTG
CACAAACATTATATCAAATGGCAGACAGAGACACAGATGAGTTAAACATCGATAACGTAATAAAAAAATTATT
AAAAGTGAGAGGCGAGAAGCCTGGCATGAATGTACAGTTAACGGAAATCGAGATTAAA
GGGCTATGCTTGAAATCGAGAGAGATATTTCTGTCACAACCGATATTACTTGAGTTAGAAGCACCGTTGAAAA
TATGCGGTGACATTCACGGACAATACTATGATTTGTTAAGGTTGTTTGAATATGGTGGTTTTCCACCTGAATC
AAATTATTTGTTCCTCGGAGACTATGTAGACCGCGGCAAGCAGTCGCTGGAGACGATA
TGCCTGTTACTAGCATACAAGATCAAATACCCTGAAAACTTCTTTTTGTTGAGAGGGAACCATGAGTGCGCCA
GCATTAATAGGATTTATGGATTCTATGACGAGTGCAAAAGGAGGTACAACATCAAGTTATGGAAAACGTTCAC
GGACTGCTTTAACTGCTTACCTGTTGCCGCGATAGTTGACGAGAAGATCTTCTGCTGC
CACGGCGGCCTCTCCCCCGACCTGCAGGCGATGGAACAGATACGCAGGATCATGCGGCCCACCGACGTGCCCG
ACCAGGGGCTGCTCTGCGACCTGCTCTGGTCCGACCCTGACAAGGACACGGCCGGATGGGGGAGAACGATCCG
TGGCGTCAGCTTCACTTTTGGCACGGAGGTGGTGGGCAAGTTCCTCTCCAAGCACGAG
TTCGACCTGATCTGCCGCGCGCACCAGGTGGTCGAGGACGGCTACGAGTTCTTCGCGAAACGACAACTTGTCA
CCCTCTTCTCCGCGCCCAACTATTGCGGAGAGTTTGATAATGCTGGCGCCCTTATGTCCGTGGACGAGACACT
GATGTGCTCGTTCCAGATATTGAAGCCCGCGGACAAACGTAAACTGTACTCCGGTCTC
AACATGGGCAGACCCAACACACCGCCTCGTGCACAGCCCAAGAATAAGAAAAATTAGCCATCGCATCGAAGTC
GACGACTTGCATGGATCCCCGCGATACAACACATTGCCTACGTTTTGACAGCTTTTCTTATGAAAAAACAACC
CTTTTCTTACCTATATGTCCACAAATATTCACTTTTTCTCGTAAATGTTTTAAAATAA
ATAGGTTTTGAACTTTAAATTCCGTGATTTTTCGATTCCATACTTATTTTATATCCATATGAATTAATTTGAA
GATGAATTTGATGCGTAACAAATTCAAATTGAAGTTTAAAACCTATATATTTTACTTATTAATTAAATTATGC
CCCGACTCCCATTCATTTTTGTATGTTTTGTATAAAATGTAAGATTAATTCTCAATAG
TTTTAAGTTCAATTCCCGCTCGCATTATCAACAATTGCTGGACAATTGTGTTCAGTGGACATGAGCTTTTGGA
CTCGAAGAACTGTCAAAATTGTGAAAAAAATATAAGCTTAGGCTGTCCTGCGTAAAGCCTTTGCATGTTCCAA
ACAAATGTCAAACGTCAACGTAAACTGTCAGAATAAGAGAAAAATTATAAAGCCTAGG
CTGTATACTTTCAAGTATTTGCTTGTTCGAAAGAAATGTCAACGTCAACGTCAAATGTCAATATCTGTCAAGC
CAGTTGGATGATTTGTAAGCTGCCAGTGCGTTTTA
>1002049491_1 Heli.1-DT664177.3.5
CACAACTCAACTTGTCTTCGATAATGACCGGTGGAAGCTAGTGGTGCCGTGTAGTGTCTGAAAAATTTTTTTG
TGTTGTGTATTTCAGTTTTCATGATGATTTCAAGAGCGCTCCTAGTAGCGCTGTGCATCGTGTACGCGACGTC
GTTGAGCCCATTAAGTGTTCCTGTAGACGACGACTGTGCACTCTACCTCACTGGTCCA
GGAAGATCGTCGGCATATGACTACAGCTTAAACCTCCTTAAAGGAAATTTTGCATACGATGGTCAACACACCT
GCATCACGTATGAGGCAATCAATCAAGCGTATCTTGACGCCAGGTCCAGAATTTTGGTGTCACAACCAAAAGG
CGATTGGAAGGGTGAAGACTTCGCTAGTGTCGGGGAGCTGATTCTGGATATTTCCATT
AGTTTGGCACGCACATACGGTTTAACATATGAAGAAATAGAAAAGGGTCTACCTCTCATAGACACTTCCCGTA
CACTTATCCGCGAGGTGTGTCCACCAGTTTTCTCCCATGTAGAGTGCCGCGCGGGCAAGTACCGCCGCCTCGA
TGGACTGTGCAATAACTTAAGGCATCCCACATGGGGCGCGACTATGGCGCCTTTCCAA
AGATTGATCGGTCCCCTGTTCGCGGACGGCATCAACGCGCCGCGCATATCGCACACCGGACGCGACCTGCCCC
TGTCCCGCGTG
>1002049492_1 Heli.1-ES587354.1.5
TATTAATTGGGGTTTTAATTGGGGTTGCTTTTTTAACTTTATTAGAACGAAAAGTTTTAGGATATATTCAAAT
TCGTAAAGGACCTAATAAATTAGGAATTATAGGAATTTTACAACCTTTTTCTGATGCTATTAAATTATTTTCA
AAAGAACAAGTATATTTAAATTATTCAAATTATTTTTCTTATTATTTTTCTCCTATTA
TTAGATTTATATTATCATTAATTATTTGAATAGTGATTCCCTATTTATTTAATATAATTATGTTTAATTTAGG
TTTATTATTTTTTTTATCTTGCACAAGAGTTGGGGTTTATACTTTATTAATTGCTGGTTGATCTTCAAATAGA
AATTATTCGTTATTAGGAGGTTTACGAGCAGTTGCTCAGACAATTTCTTATGAAGTTA

GATTATCTTTAATTTTAATATCAAGAATTATTATAATTATAGATTTAAATTTAATTAAATTTAGAGAATATCA
ATTTTTAAATTGATTTATTTTTATAATAATTCCATTAAGATTATGTTTATTTTCTTCGATATTAGCTGAAACT
AATCGAACTCCATTTGATTTTGCAGAAGGGGAAAGAGAATTAGTTTCAGGATTTAATA
TTGAATATAGAAGAGGGGGATTTGCTTTAATTTTTTTAGCCGAATATTCAAGAATTTTATTTATAAGAATATT
ATTTATTATTTTTAATATAGGAGGTTATGAATTAAATTTATTATTTTATTTGAAGTTAGTATTTATTTCTTTT
TTTTTTATTTGAGTTCGTGGTACTTTACCTCGTTATCGTTATGATAAATTAATATATT
TATGTTGAAAAAGATATTTACCTATTTCTTTAAATTATTTATTATTTTTTTTAGGTTTAAAAATATTATTAA
>1002049493_1 Heli.1-DN172692.1.5
CTCACACCCAACCAACACAATGGCATTCAAGTTTGTGGTCCTCGCCTGTTTAGTGGCTGCTGCCAGCGCTGGT
ATCGTACCAGCTGCTCCCTTCGCCTACGCTGCATCTCCACTCATCCACTCAGCTCCCGTAGCTTACGCCGCGC
CTGTAGCTAAATACGCAGCCGTCGCCCCAGTCGCTAAGGTCGCCGTCGAAGAATACGA
CCCACACCCCCAGTACAGCTTTGCTTATGACGTACAAGATGGACTTACTGGTGACTCCAAGAGCCAGCACGAA
TCCCGTGATGGAGATGTAGTTCAAGGTTCCTACTCCGTGGTCGACCCTGATGGTGTTAAGCGTACCGTCGAAT
ACAGCGCCGATCCCCATAACGGTTTCAACGCTGTCGTCCACAGGGAACCTTTGGCAGT
TAAAGCAATCGCCCCAGTCGCTAAAATCGCGCCTGTAGTGCACGCTGCCCCCGTGGTACACGCTTCTCCAGCT
GTATACTCATCTGGCCCCTTGGTGCACGCCGCACCTCTGGCCTACAGTGCTGCCCCCGTCGTCCACGCTGCCC
CCATCGCGAAACTTGCATACTCTTCCCCTTACTACCACCACTAAATAATGCCAAATAT
AAACTAAGCCACGAACTGTTGTGACTACTAGTGATGTTCTAGAGATTTCTCGACACTTTTAGGATAAGTAAAT
TAAATTGTTTT
>1002049494_1 Heli.1-DT664781.3.5
AACGCCGCTTGCGATGTTCAACTCCGCTTGGCGTCGTTGCCACTGGATGAGCTGACGCTGATTGATGAGGAGA
CTAGGCGACAGATGTTCGAGTCTTCTCGCAAGCCCCCATCTGACGCCAGCAAGTGGAGAGTAGGTGGTGAGGA
GTTCGAAGCCCTGATGCGTATGTTGGACAACGCTGGGTTCCGCACCGGATACATTTAT
AGACATCCATTGGTCAAGAACGACGTGCCCAAGCCCAAGAACGATGTGGAAGTGCCACCAGCAGTCTCTTCCC
TTGGATATCTTCTTGAAGAGGAAGAGTTGTATAAACAAGGATTATGGAAGAAGGGCACAGGCAAGGCCGGCGA
ACGCACCCGCTGGCTAAACTCCCCCAACGTCTACCAGAAGGTCGAGGTCCTGGAGACC
GGCACCAGCGACCCTAAGAGGATCACCAAGTGGGTGGACGCTAACAACGAAGAGTGGGTGCAAGATGGTTCTC
CGGCGCACTCTAGTACTCCAGTGAAAATTGATACGCTACAATTTGTGCCCAAAAACACAAATCCTAAGGAATT
TAAGCAGCTGCAACAACAGATAAAAGATAACCGTCGCGCTGACAAAATAAGCGCGGGC
CCGCAGTCCCACATACTAGAGGGTGTTACATGGGATGAAGCGTCCAAGCTGGTGGGTGAGGACGCCGTTAATA
CCCACACTGGGGATCATGTGGTCCTGATGGGCGCAGCGTCCAAAGGTATAATCCAGCGCGGCTACCAACACAA
CGCAGCCGTATACAGCGCGCCCTACGCCCGCAACCCGTTCGACCAAGTCACCGACAAC
GAGATAGACGAGTACCGACGCACTGTTGAGAGGAAACAGCGGCACGATTACGACACCGACATCTCCGAGTCGG
AGGCGGTGAGCGCAGCGCAAGTCACGTCACCCAACACCGCGCCTTCTGACACCGAGGATGAGTCACGTGATGA
GCAACGTGTTCTCCGGATAGAGACCAAGCAAGTGCCCGTGCGCAGTCAGCCGGAGGTC
GTGTTGAGTGATGTGGACACGACAGATTTCTTGTCAGCGGAGCGCGAGCACGCCGATAGGACCAGAGGCGAGC
ACACTGTGAACGGCGACCACTCTGATGCCCATCAAAGCACCTTCTCGCACAGCAGTAAAGAGGGTTCTCCATC
CAAGGAGGTATCAACGGAGGAG
>1002049495_1 Heli.1-DT668242.3.5
ATACGATTAGTTTTCCATTTTAATTTTCGTCGTTATCGACGAAAGGGAGTATATTATACTCCCTTATTGTGTG
TAAAGTTTTACTCTGTGTATTCACCGATTATCTAAATATTTTATTTAAGAGGGATATATTTTCTCTTTGTTTT
GTATTATAAACATGGCTTTTTAAAATGTCGGTCCTGAAGGTAAAATCTTCGTCTCCTT
GCGGAGTATCCCGCTCAATTGTTATTGTGGATGTTGGAAGATTCCAGGATGGACAAAAAATCTATAAATCGTT
CTACTATACCACAGTCGAGGGCCGTAGCCATCGACTGTAAGAAAAAATACGGAATTGACACACAAACCTTGGCC
ACGAAATAACAAGAAACGTGAAGGATCTGGCAGTTTACCTAAAAACGAAACTTATCGC
AAAAATGTACCATCACAAAGGGGTAGAGGTCAAATGGATAAGCGCCCGCGCTCTCGTGGTGCCACCAATTTCG
CTGTGGGTGGAGCGCAGAACACCGGGCTAGAAGAAGAGGAAGAGCCTAAAATAGGCTCAGTGTTTATTCCTGG
AAGCAAAAAGCAAAATTTGAACCATTTGCTGAATTTCATGTACCCCTCCCGAGGCAAT
ACAGAACGGAGGGGTCACGCTCCAAGACGTCAAGTCCAACAATGGGTCTCTTATAAAAATGAACATGATCTCT
ACCTTCGAGCCTACTGTCAATTTGTGGTTAAAGAGGATGGAGATTATAAAGTAAATTTAATGGACCCTGACTT
GCCTCTAAAATGGGAGCAAATTGAAGAAATTGTTGTAAGAAGCACTGGTAGATCTGAA
TGTCCCATATGCCTCGGTCCCCCGGTGGCTGGACGAGTAGGTCACTGTGGACATGTTTATTGCTGGGCATGTA
TTTTACATTATTCTGCAGCCCATGAGAAACAACCTCCACCATGTCCAGTTTGTGCTATGTCCTTGCAAGTTGC
TGATATGAAACCTACTCGGATGGTGCAGTGGGAA

>1002049496_1 Heli.1-ES584847.1.5
TGAGTGTTAAGGATCGGCTTCATCATGCCCAGAGAAATCAAAGACATTAAGGACTTTTTACTTAAAGCGCGGA
GGAAAGATGCCAAATCTGTTAAGATAAAGAAGAACCCTGAGAATGTTAAGTTCAAAGTGCGTTGCTCACGTTT
CCTGTACACATTGGTTATCACTGACAAGGAAAAGGCAGAGAAACTAAAACAGAGCCTC
CCTCCAGGTCTCCAGGTTAAAGAGGTGAAGTGATGTGACATAGGGTTAAGAAAGTAAACA
>1002049497_1 Heli.1-DT662735.3.5
GCACGAGGCTCAAGCGTTTCGTTGCAGTTGTAGCTATGGCGTGTTTAAGTTCGAGTAGGCTTTGTGCTCGTGT
TCAGGATAAGTTAATGTGTCAATTCGGACCTAAAGTTAGAAGGCAAACGGTATTTGTGAAACTGAAACCTCCT
GGACCCTCTTCGATGCGGATATTTGGAAAGACCTGTCAGCGAGTAACTACATGTGGTC
CAGCCTGTGGGTCAGCCGGGCCCGGGCCCATCACTGGTGCTGCTCGACGGCTTGCGTCCAGTCTACAGAGCAC
GAGCACAGGCTCTCTCCCAGATTACGGTACACAGATTGTAGACCCATACCGCTTACTAGAGGATGACCTAAAC
GGAATATATGAGGATATAAGATCAGAATTGGAACGCAACACAAACCAGCCCGAGCTGA
ATACCATAGCTACATACTACTTCGACGGACAAGGGAAGGCGCTCAGGCCAATGGTGGCCATACTTATGGCTAA
AGCTGTTAATTACCACGTATATGGAGAAAATAGCGCAATACTGCCATCTCAGAGGCAAGTTGCGATGATCAGC
GAGATGATCCACTCGGCTTCGCTCATCCACGATGACGTCATCGACCAGAGCGACTTCC
GCCGCGGAAAGCCCTCTGTCAACGTGCTCTGGAACCATAAGAAGGTGGCCATGGCCGGCGACTTCATCCTCGC
TGTGGCGTCGATGATGATAGCTCGCCTGCGCAGCGATGAAGTCACTCTTGTACTCAGTCAGGTGGTCACAGAT
TTAGTTCAAGGAGAGTTCATGCAGCTCGGCAGCAAGGAAACAGAAAATGAGCGGTTTG
CACATTACTTAACAAAAACATACAGAAAGACAGCCTCCCTCATCGCTAATTCTGTTAAAGCGGTGGCGCTTCT
AAGTGGTGCAGATGAAACTACATCCGAGTTGGCATTCCAATATGGTCGTAATCTGGGTCTGTCCTTCCAACTG
GTGGATGATTTGTTAGATTTCGTATCATCTGCACAGGCGATGGGAAAACCCACTGCAA
CAGACCTTCGACTTGGACTCGCGACCGCTCCAGTTCTTTTTGCTTGTGAAAAGTATCCAGAACTGAATCCAAT
GATAATGAGGAGGTTCCAAGAGGCCGGCGACGTAGAGAAGGCCTTCGAACTGGTACATAAATCACGTGGCCTC
GAACAGACGAGGTTCTTAGCTAAGAAACACGGGCTCGAAGCCGCGCGCCTCGCCGCCG
AACTCGCGGACTCACCGTACCAGAAGGGACTGGTTGTGACCACCGATTTAGTTCTAAACAGAATTAAATAAAT
TACTAAGAGCTACTACGAACGTA
>1002049498_1 Heli.1-DT662347.3.5
ATATGTCCCTGTATCGCCAACTGTACAATCCTATGTATCCTCCACTCCCGTAACTATCAATGAGAATCCCATT
CTCAAGTATACAATAAAGGCTGCTCCAGTGACTTTAGTTAGCTCTACTTATGCTCCACAGTCTTATAGTCAAC
AAACTGTTCACAAGGATAATTCTCAATTAGATGTTGTATCAGATAACCAATATGTTGC
AGGAATAAAATATGCAACGCCAAAAGCAACCTATTACGCGCAGCCATCTTTGGCTGTTCAATCCACTGTTTCT
ACTTATCAACCCCAAGCTTACAGTCAACAAACTATCCATAAAGTTGAATCAGGTAGAGTGAACGCCTATGATT
CGTCAGCTTTGGGATATGAATATTCACAACCAGCTGTCAGTTATGAAACTTCAAATAT
AGCATCATCAACTGTTTCGCCAGTAATCACCTCAACAAATGGTCCAAAATCATACAGCCAACAAACTATTCAC
AGGGTGGAAAAGCCAAGAATTTCGTCAACACCACTTCCGGCTGTTGAAGTAACTTATCAACAACCGTCTGTTT
CTATTTATGAAAATCCTATATTGAAATACACCCAAAAAGTCACACCAGTCAGCTATAT
TGAACCTACCGCGAGCGTATTTACTCAAACTTATAAACCAATTCAGCCTAAAGTAAGTTATGTTAGTAGGCCA
GTCGAATATGAAAGTTCAGGTCATCAAACGTATGAAGCTGGTTACTCTTACAACCAACCAGAAATTCAAAGAG
AAGTCGAAACACTTAATACTGCAAAATATTCTGACGCGAGTTTAGTTTCTCAGCAATA
TTTCACGTTATCACACAATAGTGCAAACACACAAAGTGAATCTGGCAAAAATGTAGTTTTTGTATCTACAACT
CCATCTTCTCTAGTTTATGAAGACCATTATACTGAGGACCAATCTGCCAAAAGAATAAAAGCATATAAAGCTC
CAGAGTACATACCACCGAAAATTGAGTATCAGCAAGATTTTGTATCTTCAACAGCTTC
AATTCCTATTCTTCAACAAAATTCATTTGTCCAGACACAACATCAATACAACTACAAACCTGCTGAGTATTCC
CAACAATACGAAGTTCAGTCATCTTCACAATACGGAAACAGTCAATCTAATACTGATCAACCACAATTTGTCG
AGTCTATACATTTTTCGTCTTCCCCAGAAGTGTCAGCCAAAACTCAGTATTTCCAAAC
CAATTACCAATCACCTGAAGTTCAAGTAGCCTACAAAGCTGAAGAATATTTACCACCAGTAGTATCAACTGCT
TTGCCTCCAGTATCTTCTACATATAGATCACAATACTCAACGACAGCGTCGAGGGAATATTTGCCCCCAACTT
CAAAATCTAAATACACTGCACCAGAATACTTACCTCCGGTAGGGAAAGCATCTGCGGA
ACATTCTACTCTATCGCCGGTTACGAAATCTTTACAATACTTACCACCATTTGAAAGTGTCGGTTCAAGGGTA
GTCAATTTTGATAGTTTTGGTTATAGCAATGAAGGGGAATCCATAGCCAAATTAAGCACTTACCAAGATTATA
GCGTACCTTCTGTTACACCAATTATAGTGTCATCAACAGCCGCCCCTGTTACAAGAAA
GTCAAATATTGTAGTCGAATTAGCTAAATCTAATTTGCTAGGTTTTGGCACGGTTGGGCCTGATGCAGGTTTA
GTATCACATTATTCTACTCCTGTTACTATTTCATCCAGTCCTGACGATTATTTACCCTCCATTTCATCTACTT
ACGCTCCCGAAGTGTCATTAGTTCCAAGAAAATCGAGACCAAAATATATAAGACCTGC

GGTGAAATCCAGTACTGAGTCTTATGTCGATTCAACTACTGCTTATAGAGCTCCTGAATATTTACCACCATCG
GAGGAGTATGTACAAGATAATGTTAAATCTTTCGAGTACCAAAGTACAGCAGCCCCCCAAATTAAATACAATC
CTTATCAAAAACAAGTACCAACGGTAGTATCAACAATTTCACCTCCTATAAGAAAACA
AAATATTGTAGTAGAAACGGCTAAATCTCACTTACTTGGCTTTGGAAGCGTGGGGCCTGACGCTGGATTAGTT
TCCGAATATAGCACCCCGGTATCTATTGAAATATCGAAATCAGGATCCGCATACTCTACAATCCCCATTAAGC
AAGAAATAGTTGAAGTGGCACAAGTTATAAGAAAATCTAAACCTAAAGTAGCCGTTGT
AACAAAATTAAATGACTTCAATCC
>1002049499_1 Heli.1-DT665452.3.5
TAATGTGGATTTATCTTTACATAGCCCTCATAATATATGTTGATTAGGTTCTTGGGCTTTCCATTTTGTTTTG
ATGCCCCTATTCCATATTTCATTAGTTTACATATTATTAACCCATAAATAGAATAATATTGTACTTAGTTTTC
CAATTATTGATTATATTTGTACTGTTTTGACTGGATGGGGCAATGTTTTTTTATAACT
TTAATAATATTAGAATATATTTTTTAGATCTGATTGAAAGGTTCAAATTCTATTAGAATATTATGTGCAGATA
TTCTTTTCATTTCACTAAAAACAGAACTATTTTATGGGGAAAAATTATTACTGTCCTTTTCACGAGTCTAAAT
TATTAGTGATTATCTTTCAGATCAATATGTATGTTTAAAATAAGTGTGTTAGGTTTAA
ATCTGGTTTGTTAACTGTTGACCATATCGGTATTTACAATGAACTAACACTACTCTCACGGCATTGGCTTTCT
AATACTTTGTCTTAAGCATTTATATGAAGCATTTACAATATGTGGTAATGTTTTCAATAATGTTATTTATAAA
GAGGTTTTTTATTTGAGAGATATGTTATCACTTATAGTTTTATATAAATTGTTATAAG
AATACATAGTAATTTACTTGAAGTAATAGACAAATTAAGTGCTGCATATAAAATATATATGGTCCTAACAGTC
CTTCAGTCTATAATTAAAAAAAATATCTCACTATAATCAATCACATATTTACAATGTGATATTGGTTTTGACG
TATTTGTTTATAAATGAATACTGTAGAATATGTTTTTATTATTACGAAAAATGATAAA
ATAGAGAAGTGTCAGTGAAATTTTTGTAAGAAACTGTAAATACGGTCA
>1002049500_1 Heli.1-DT668658.3.5
GCACGAGGGTGTGGCCGTCTACTTCGCTACATTGTGACTTGACAAGTTAGTGTTCTTCTACGTAACCAGCCGT
ATGCGCGTAACGAACGTTGGCCGTGCAATTTCTCTCGAAAATAATTAGTATGTGAATGCGAATTTTTTATTTG
TTCTGCGTGAAAATTAGTGAAATGCAACCGTTGTTCGCGTACTGACTGACAATCTTCG
CGTCTCTACAATTTCGAACAACCTTCATGAGGAATTGGTTGTTTATATTCTTTTAAGATAATCTGTACCCGTG
GCTCCCAGTTATGATTATAAGAATACCATGGCTACTTCAAATAGTTGCAATAATTACAATAACTATGACATCA
GGTAGTCCGTTGTTTTTGCAGAAACAATCTAGCAGAAATATTAAACCAGTAATAAAAT
GGAGTCCTAAAACGGATACATGGAGTTGGGATGAAACACCTCGCAATGATGAACCTTTTTTCATGCCCTTAAA
TTTTACTCATAAGTTACCTCCATCGGCCAATGAAACAGAACTAATTGCACACATGGCTGCTCTCAAAAGAAAC
TACTCCCGCTTATTGTGGGACAGTGATAAAAGTTCAAATTTACCTCTCTTCGTAGATA
AAGCGCTAAAATTATTAAATTTAAAACAGGTCTATTATGAAGTTGAACATTCTGTTATGTCAAAAGTTTCATG
TACAGCGTGTAAGGCTGGCGCTGGTCTTTTGCAACACTATATGAGACTTGGAAAAAGTAAAGAAGAGATAAAT
AAAATGATATATCAATTTTGTGTTTCATTAAATATTCAATCTGCTAGAGTTTGCGAAG
GCATAACACGACTCTTCGGGAGTGAAGTTGTATATGTCCTGAAACGTATAACGATTGGTGCAGATGAAATATG
CAGTTTTGTAATTGGTGATGCTTGCGGCGACGTTTATAATCCATACCACGAATGGGAAGTAGCATTTCCACCT
GTACCAAAACCAACAGCGCGTGCCCTTGATGCGCCTATAGAAAAAGCTCCTACTTTTA
AAGTTTTACAAATATCTGATACACATTTTGATCCTTACTACGCAGAAGGGGCAAATGCTGAATGTAATGAACC
TCTTTGCTGTCGGGTATCTAGTGGCCCCGTTATTACACCTGGTGGTGGTGCGGGACGATGGGGAGATTACCGA
AAATGTGATACACCGAAACGTACCATCGATCATATGCTGAAACATATTGCAGATACTC
ATACCGATATTGACTATATTTTATGGACAGGAGATTTGCCACCACATGATGTTTGGAATCAGACAAAAGAGGA
AAACCTGAAAGTTCTCCAAGAAACCGTAGCCCAAATGTCAGACATGTTTCCGGGTGTGCCTATCTTCCCTGCA
CTCGGAAATCACGAATCATCGCCGGTGAACAGTTTCCCACCTCCATACATTTCATCAC
CAGAATCTAACA
>1002049501_1 Heli.1-DT665342.3.5
ATTTGAGAATTTGGTTGATTTTCTTTTCTGATAGTTCTCTGAGTTTTGTCTTTTGTATTTAATAAATTAATTT
CAGCAAAATGTACGGACAACAACCAATTATAGTACTTAGCCAAAATACAAAACGTGACTCCGGCCGGAAAGTT
CAGTTGGAGAATATTAATGCTGGAAAGACGATAGCAGACGTTATTCGAACATGCCTCG
GACCACAGGCTATGTTGAAAATGTTGATGGATCCCATGGGTGGGATCGTAATGACAAATGACGGCAATGCTAT
ACTCAGGGAAATCACTGTGCAGCACCCTGCCGCCAAATCCATGATCGAAATCGCAAGAACACAGGATGAAGAG
GTCGGAGACGGCACAACATCTGTAATTGTCTTGGCTGGAGAAATGCTTGCTGTGGCTG
AACCTTTCTTAGCCCAAAACATTCACCCTACAGTCATTATTAGAGAATATAGACAGGCTTTAGAAGACGCTGT
CAAGTTGTTACAAGAAAAGATTTCCACACCTATTGATTTGAATGACAGAGAGAAAGTGAAAGAAGTTATCCGC
TCATGTGTGGGAACTAAATACATTGGTCGCTGGGCCGATCTTGCTGTGGACATTGCTT

TAGATGCAGTGAATACTGTCACCATTAATGACAATGGAAGAGTTGAAGTTGATATTAAAAATTATGCCAAAGT
AGAGAAGATCCCTGGTGGTACAGTGGAAGACTCCAAAGTACTAAATGGTGTGATGTTCAACAAAGATGTCA
>1002049502_1 Heli.1-CO729612.1.5
CAAAACTAACATGTACGCAGTCGTGCTTTTGGGATTTCTGGCGCTGGCCGGTGCCGCGCCACACTACGGCTAT
CACCACCATGATCACTACGAGCATTTTCCACATTCTTTCGAATCGGAACTCGATAGATATTTGAGGCAAAACG
TTTTTGATACCAGAAGATTTTGGGAAGATTTACAAAGAGACATGATGCAGCTCGACAC
GATGTTGGCGGACCTCGGCAAACATTTCCCAAGCAGTGTATCAAGTGAAAAAATTGAAGGTAACGAGTACGTA
ATTACTATACCCTTGACAGGCTATAGAGAAGACGAGATCGCTATTAAAGCGAGGAAGGGATTACTTGTAGTCC
AAGCTGTCCATAAAGTTGATGGTGAATCACAGAGGAACTATCTGGACGTAAGGACTTT
GCCCGATTGTGCTAATGAGGCTGGTAGCTGGACTTTTGATAAGGGAATTCTTAGAATTACATTCCCATTACAT
AAAGATGAAAATACTGATGTAGTGACAGAGGCTGTCGTCACAGATGCACCACAACACAGTCGTGAAGAAATGG
AGCCACATGATGGGAATAGCAAACCAAATAATGATGATAATATCAGAGGAGATATTGA
TGTCAGGGGAGTCCCTAATCCAAGCGACATTGAAGTCAAGACTAAGGTCTTGACTAATGA
>1002049503_1 Heli.1-ES585377.1.5
CAAAATCCAGCATGTTGCACCAGTACATCACTCTGGATCTGTCCATCAAGTACAACCAATATTGCATCATGCA
CCCTCTGTTCACAACAGTGGATCTGTGTACCGTGCTGTTCCAGTAGTACAAAACGTTGCTCCTATCCAACATT
ACACAGTTCAATCTATTCAACACGCCTCACCTATTGTGCAACACATTCCACCTATCCT
AAAACTTTCCAATAATTACCACAACGAGGGATCCCACGACCACCAAGAATATTACGCACACCCTAAATATGAG
TTCAAATACAGCGTAGAGGATCATCACACTGGTGATATCAAATCCCAACAAGAGGCTCGTGATGGTGACCACG
TAAATGGGTACTACAGCTTGCACCAACCCGACGGTTCCGTACGCACTGTACATTATAA
TGCTGACAAGCACAGCGGATTCAACGCTCAAGTACACAACTCTGCCCCATCAGCCCACATACAGCCTGCTCAT
CATGTTGAACCGCATTATGTTCTTTCACACCATTAAGATTTTTTTTAATTATATTAAGTGTAGAGTAGTAAA
ATAAAAAAAACAATTGTTTTAAATTTTAAAATAAAATCTAGTCTTA
>1002049505_1 Heli.1-EL599677.1.5
AATATTTGGTCTTAGCGCTGTATTGGCAGTGGCTGCCGCAGGTCTGCTGCCCGAGCCACACTACTCCTCAGCG
GCTGCAGTTTCTTCTCAAAGCATTGTGCGACATGACCAGCCTCACGCCGTAGTCGCGGCCCCAGTGGCGTACC
ACGCCGCCCCTGTCGCTTACCACGCCGCTCCTGTCGCTTACCACGCCGCCCCCGTCGC
CTACTCCTCAGCCGCAGCCGTGTCTTCTCAGTCTATCCAGCGTCATGACCAGTCCCATGCCGCCATCGCTGTC
GCTCCTGTAGCTCACTACGCCGCCGCTCCCGTTGCACACTACGCCGCCCCTGTAGCCCATTACGCTGCGCCCG
TACACAGCATTGTGGCCAGTCACGATGACGTGTACTCTCACCCCAAATACGACTTCTC
TTACTCTGTAGCCGACGGTCACTCCGGTGACAACAAGTCCCAGCACGAGAGCCGCGACGGTGACGTAGTACAA
GGCGAGTACTCTCTGGTCGAAGCTGACGGCTCCATCCGCAACGTCAAGTACAGCGCCGACGCACATAACGGAT
TCAACGCTATTGTCAGCAACTCTGCACCCAAACACGCCATCATTGCTCACCATTAAAA
TAAAATGTATTTAATTTTATTTGTTTTAGTCATTTTAATTGATATTTAAATACAAACATAGTAATAATTTAT
>1002049506_1 Heli.1-CX700766.1.5
CACTACACAACAACCACAATGTCCGCCAAGATTGTGATCGTCCTCGCCCTGGCCGCAGTTGCCTCCGCTCTGC
CAGTTGTGCAGTATGCAGAGCCGGAAGCGCCCGCCCACTACGAGTTCCAATACTCCGTACACGATGAGCACAG
CGCGGACGTAAAGCAGCAACAAGAGGCTCGTGCTGGAGATGCCGTCCACGGCTCCTAC
TCCCTCGTACAGCCTGATGGTGTCCACCGTATCGTGGAGTACAGCGCTGACAAGGAGCACGGATTCAACGCCG
TAGTGCGATATGAGGGACACCCCGTCGCCGCCCCTGCCCCTGCCAAGCTCGCCTACGCTCCTGTCGCCAAGCT
CGCCTACGCCGCCCCTGTGGCCAAGGTGGCCTATTCTGCCCCCGTCGCCTACTCAGCT
CCCGTCGCCAAGGTGGCCTACGCCGCTCCCCTCGCACACGTCAGCTACTCATCCCCCGTCATCTCCTACCAAC
ACTAAGTCTATAGCGGATATCTTTGTTAAAAATACTTAATTTATTTCTGAATGTGTGATACAAATAGTAATAA
ATAAATGTTT
>1002049507_1 Heli.1-ES586312.1.5
AAGGAAGGTCTTATTCAAAATAAACAATATGTTTGCCTTTAAATCTATCGTCCTAATTGCTGCAGCTTTTACT
GTTGGAGTAGAATGCAGTGTGCAGGCAGCTGCATTGGTTGCACCAGCGCAAGTTGGTTACGCCCATGCTGTAC
CACAAAACATTCCACCATATGCATCTCAAGTGAATATTGAAAGCCGAAGTGCTCTAGT
TGCTGCACCTGCTGTAGCTGCTTATGCTGCCCCTGTATCTGCACCGTTGGCTGCCCCATTTGCTGCACCGTTT
GCCGCACCATTGGCAGCACCACTAGCTGCTCCATTGGCTGCACCATTAGCCGCACCATTGGCTGCACCACTAG
CGGCTAGATACGCATCACCCGCTTTTGCAGCTCCTCTTGGTGCAGCGCCATACCTTGC
TGCACCTGCAGTGCGAGCAGCATATGCAGCGTCACCAATTGTTCCAGCACCTTACGCTGCGGCTTCTTACGCA
CTTCCTGCACCTATTTCCGCGGCTTATCCTTATGGTGCAGCTTCTCCCTTCTTCCGTGCTCCCATCGGCGTTG
CTCCAGCTTTTGTTCGATAGACTTAAAGATAAACTAGTCGACCTTCCTTTATAAAAGA

CTTACTATTTTTTAATTACGAAGAAATAAATATTTT
>1002049508_1 Heli.1-EL599500.1.5
CATGCGTCGCGCGTCTGCCCGCGCTTTTTTTTACGCGCCAATTTTGTGAAAATTTTAAATTATAATCTGTGCT
TACAAACTAATCAACAATAAATAAAATACAGTTTTATTAATATAACATGATTATAAATAATAATTATTTTCCG
TTAAACAAAACGCAGTGATATATTTTCGAACGATACTTTTGATAAAATAAAACCAAAA
TGGAATATAAATCAAATAAAAGGACTAAATGGTGTATGTATAGAAAATTAACGACTTTTAAAACAGTTTATGC
GGGATTGATATTGTTTTATGTGCCAGTTTTATGTGACAAAGATATATCCCGACCACTGACTTATGACTATATC
TACAACAGTACCAACTACGCTCAACTGCAGAATGTAAAGCAAAATCCTAACCTATATA
ATCTTAATAATAATAATCACACCGAAACCAAAGGAACAGAAGAGTTCAAATTTAGCGATAAGTATGAGATAAA
CAAACCCGACGATCTGTTGGTGGGTTTCGCGCATGACCCATTCAGACAGTCCATACCGGTAGCTGATTATATG
AAAGGTGTCAGAACTATGGATCCAAGCAAAATGAGCAAGGATCGTGGGAAGCGGGGTG
TTTTTCATCTATACAACATGTTGGCGTGCGCGACCGGGTGCGACCCCGTCAGCTATAAGGGCTACGGCTGCTA
CTGCGGCTTCC
>1002049509_1 Heli.1-DT662608.3.5
CCCTGTGCATATTAACCAAACAAGCAACAGCAAAATGGTCTTCAAGTTTGTGGTCTTCTCCTGCTTGGTGGCT
GTCGCCTACGGAAGTGCATCCGCCGCCATAGCAGCAGCTCCTGTCGCATATGCAGCACCAGCCGTTGCAGCAA
GATTAGAAGAATTCGACTCTTTCCCTCAGTATAGATTTGGATATGACGTAGCTGACTC
TCTAACCGGTGACTACAAGAGCCAATCAGAACAGCGTGATGGTGATATTGTACAAGGTCAATACTCTCTTGTG
GAACCTGATGGTACCCGCCGTGTTGTTGACTATGCTGCTGACCCTGTTAATGGTTTCAACGCGGTTGTACGTA
AAGAACCCCTCGTGGCTGCTGCCCCAGCTGTAGTCGCAGAGCCTGCAGTCGTCCCTGC
TAGAATCGCCGCCGCTCCAGCCGTGGCAGTTGCCGAGCCAGTAGTAGCCGCCAAGGTCGCCGCCCCTATCGTG
GCCCAACCTGCTCCTGCTGTCCTTGCGGCTCGTTATGCCGTTGCTCCCGCTGCCCGTTATGCTGTAGCTGCAG
CGCCCGTAAACTATGCAGCTGCTGCTCGGTACGTGGCCGCCCCCGCAGCTCAGTACGT
TGCCGCTCCAGCGGCTCAATATGTCGCTGCTCCAGCCGCCCAATACGTCGCCGCCCCTGCTGCTCAGTACGTC
GCCGCCCCTTCTGCTCAATACGTCGCTGCCCCTGCTGCTCAATATGTCGCTGCACCCGCTCGTTATGCCGCCG
GCCCAATCGTAGCAGCTCGTTATGCTGCTCCAGTATCAGCATATGC
>1002049511_1 Heli.1-EL599638.1.5
GCACGAGGCCTCGTCCTATTCGGCATGCCAAAACAAAACCGTGTGTCTATCTATGAATACCTTTTCAAAGAAG
GTGTTATGGTAGCCAAAAAAGACTACCATGCACCTAAACATCCGGACTTGGAGAAGATACCCAACCTTCAAGT
TATTAAGGCAATGCAATCTTTAAAATCAAGAGGTTATGTCAAGGAACAATTTGCATGG
AGGCATTTCTATTGGTATCTTACAAATGAAGGTATTGAGTACCTAAGGATCTTCCTCCACCTCCCACCAGAAA
TTGTGCCCGCAACACTCAAGCGCTCTGTTCGCACTGAGACGGTCAGACGTGGTGCTGTTGGCAGGCCTGATGC
CCCAGCTCGTTCTGCTGAAGACAGATCCATGTACAGGCGTGCACCCACTACACCTGGA
GCACCACACGACAAAAAGGCAGATGTCGGCCCTGGCTCTTCAGAAGTTGTATTCAGAGGAGGTTTTGGACGTG
GTAGACCAGCTCCTTAACTTAAGTTAAGCTAAATAAAAAAAAATACCT
>1002049512_1 Heli.1-ES585057.1.5
ACCAACATGAGAACCTTTATCATCCTTTCCCTTGTGGCAGTAGCCTTCGCCGCACCACAGTTACCTTCGGAGC
CGATCCCAATTTTGCGTCAAGATAGCAACATCAACCCTGACGGTTCCTTCCAATATTCTTATGAAACTGGTAA
TGGAATTAACGCAGCAGCTAATGGAGCTTTAAGAAACATTGGCGCTGAGGAGCCAGCT
CTGCAAATCCAAGGGGAGTTTAGCTACCCCAGTGAAGACGGTGGCAGCATCCAATTAACTTATGTAGCTGATG
AAAATGGATACCAACCCCAAGGTAGCGCTCTACCTACTCCTCCTCCTATCCCAGCTGATATCCAACGCGCTTT
GGACTTCCTCGCCACTGCTCCCCCACAAGCCCAACGCTAAGATGCTAACTAAAAGGAG
TGGCTGTCCACGATAATACTACCTAGAAGGCTATTAGTTATAATTATACCAATAAGGGACTATTTTTCCTAAA
GGCGAGTACACATCAGTTAAAATTTGATTTGAGGCTATGAGTAAAAACATTATTAGAAAAAAAGTATTATTAA
TTATTTATAAATGTAACGTTCAATTGATTTTAATTGTAAATCTTAAGGAAATGTTAAC
TGATGTGTAATCGCTTGCACAGCAACATAAAGATGAGAGGTTGATATCATGTTTGAATTTTTGTACATACCAT
TTTATACTTTATTTAAATAGTCT
>1002049513_1 Heli.1-DT665247.3.5
TCGTTCCGCGGCCCTCGCCACTACACTACAGCTACACTACAGTCGCACACCGCACGATCTGTAGAACGCTCGC
CACAGTGGGACAAAAATGAGTAACGAGTTTTAGTGAGCTAAGCAATCAAGTGATAGTGTTAGTGTTTTGGAAC
TGCCACTTTTTTATATAACCAAAGGAATTGACTTCGACGATGATGGCTCCGGAGACAG
TAGTGACTGTAGACCACAACAAACCAGCACAGCAGCAGGCGCCGCCGCAGCAGCAGCAAGGCGGTGCTCTCGA
TTGGATTAAAATTAATATAAATTATTTCAAAACACCGCCCGGCATATTAAAAGTTATCCAACTGGTCTTGGGT
ATTCTATGTATGTCACTGGGCACACCTTGGGCATCCGCGTGGTATGTCTTCGTGGCAG

TAACAGCTTTTATAACCACTTTAATGTGGAGCTTCGTTTACTTCCTTAGCATCAGAGAAGCCTTGAAGATACC
CATCAACTGGGTACTGTCTGAACTTATTAGCACTAGTTTGGAGACGCTCTTCTACCTGATTGCTTTCATCGTC
ATGTTTGCGTCTGTGTATGGCAACTATGGGCGTAATGTGGCTGCTGCAGTATTTGGTA
TGTTCAACATGTTGGCGTATGGGGCCAGCTCGTACTACTTGTATCAAGAGCACAGGAGTAGCGCAGCGAGCGC
GGCGGCGTAGATATAGTCTGCTCTTGCGCATGCGCAACATAAGAACAATGCTTCTTGTTTTTAATATTATTTC
ACAGAAAACTTTATTATAGTGCCTTGTACAGTCATCTAATGTTTAAATTATATTTTGA
CGAAGATAAATAATAAAATAATTTACATATCTTTTCTATATTATTAAAAAAATAAAAAAA
>1002049514_1 Heli.1-ES587494.1.5
ACCTGATATTTAAACAAAATGAAATATATCTTTCTATTAGTTGTGATTGTTGCTGCAACAGCTGCAGACATCA
GCAGCTTATATCACCAGAACCCACAGTTGCAACAACAGCAGGTGTACACCACGGAACCCATTCCCATCATACG
ACAAGAGCAAATACTCAACCCTGATGGATCTTATAAATGGAATTATGAAACCGGAAAT
GGCATAAGTGCTGAAGAAGCTGGCTTCATAAAAAATGTAGGTATCCCTGAACAAGAATCACAGGTTGCGCAAG
GACAGTACAAATATACAGCGCCCGATGGACAAGTTATCCAGCTGCAATACGTAGCTGATGAAAATGGCTTCCA
GCCGCAAGGTGCTCATCTTCCCACACCACCACCAATCCCTGTTGAAATTCAAAAAGCT
TTAGACCACCTCGCCACTCTTCCCCCACAAGCACCTGAGCAAAATCTATATAAATTATTATAAATCAATATTA
CAAAGAAACATAATTTATTATGACCATAAGAGGCTATAATACAACACCGGCATTAAAGTATAGTAAAATATAT
ATAAGTGTGTGACTTAGTTTTAAGTACAATAATAATTTATTTTATATAGTGGACTTAT
TTGAAAATACATCCAATAAAATAT
>1002049515_1 Heli.1-DT662578.3.5
TACGATCAACTTACATGGAATACCAGTGTGTTAGCGGCTGAATACAGTAAAAAAACAATACTAAACAGTGCGT
CGTGTTTCTATTGACAGGTAGCGCGTGTTAAATACCAGAATGAACACCGAAAAGCTGAAAAAACTGCAGTCAC
AAGTCCGCATAGGCGGCAAGGGAACACCACGGCGCAAGAAGAAGGTCGTCCATGCGAC
CGCCGCCACAGATGACAAGAAATTACAGTCCTCCCTCAAAAAACTATCAGTAAATACCATCCCTGGTATTGAA
GAGGTGAACATGATCAAAGATGACGGCACTGTGATACACTTTAACAACCCTAAGGCGCAGGCATCACTCGCTG
CGAACACTTTCGCTATCACCGGCCATGGTGAGAACAAGCAGATCGCGGAAATGCTGCC
TGGCATTCTCAGCCAGCTCGGTCCTGAAGGATTAAACCAGTTAAAGAAACTCGCCTCCAGCGTGGCCCCGCCG
AAACCTATCGACGAGGACGACGAAGTACCTAACCTCGTCGGTAACTTTGACGAGGCCTCGAAACAAGAGGCTA
AAGAGGTAGCCACTGAGGAAAAAGAGAAGGAAAAGAAACCAGAAATAGAAACCAAAGC
CGCGGACAACAAAGTTGATTAAATAGACCCCCATGATATATAGTTTTTAATTTTGCTCACCTGATGAAGTGAA
TACTATAGTGGGTTTCCAAAGGCATCGAGACTGTGTCTGTGTCTCAAATAATTTTTCTTTTTTTGTATTTTTG
TGTTCCTAAGCGTTCTGACTGTTCGGTTGGTGAT
>1002049516_1 Heli.1-DT665467.3.5
CCTACCACTACCACTACAACTACAACAACCACCACAGTGCGACCACCAAGACCACCGCGACGACGACCACCGA
CCAGATATGATTATTATGATGATGATTATTATTATCCAACTCGAGATGATTATGATTATGAAGATAGAGGAGG
CCGAAGAAGTAGACCAAGGAGACCAGGAAAGCGCAGACCTCATGATGATTATGATGAT
AGATATGAAACTCGGACAAGACCTCGTGATGAACCAGAAGAAGATTATGAAGATAGACGGCCATATGACAGAC
CGAGAAGTAACAAAAGACCATATCCTGATTATAGGAGACCATATGATGACGAAGAAAGAAGGCCATTCAAAGG
TAACAGAAGGCCAAATAGACCTCGAGATAATGATGAAGACAGGTACAAGCCGGATACT
GATGAAGATGATAGACCAAGGGGTGGTAAAAGAGGAGAATCGAGACCTAGAGATAAGCTGAGACCACGAGATG
AAGATGAAAGACTTAGAGAGGATATCAGGCCTAGAGATGAAATCAGGTCTAGAGATGAAATTAGACCTAGAGA
CGAAGTCAGACCTAGAGATGAAATCAAGCCCCGAGATGAAATCAGACCTAGAGATGAA
ATCAGATCTAGAGATGAAATCAGACCTAGAGATGAAATCAAACCTAGAGATGAAATCAGACCAAGAGATGAAA
TCAGGCCTAGAGATGAAATTAGGCCTCGAGATGAAATAAGGCCACGAGATGAAATTAGGCCTAGAACTGAAAC
TAGATCAAGGGACGAAATAAGGTCTAGGGATGAAGTTAAACCCATTAGAGACGATTCT
CGTTTAAGAGATGAAATAAGACCTAGAGATGATTTGAAATCTAGGGACGAGAGAAGGCCGTCTGTAGATGAAC
CTATAGAATCTGAAGGTAAGCAGAGAGAAAGGCCAAGAGATGATCGATATCAACAGAATGAGGGTAGACGTCT
TTATGATCGCCCATACAGAAACCGAGATGAAAGGCCTTATTTAAAACCTGAAAGAAAC
TATGAAGGTGAAGAAAGAATTCAAGAAGAAAAAAGACAAATTCCAACAGCTCCTGAAAGTCAAACGTTGGTTA
AACCAAATGGTCACGGTATATTCAGTCAGCCTCGAAAGCCACCAAAATTTAAACGACCGGTCCCATTAAACGA
GAAAGACAAATATGAGTACGTACCAGTATCAACTACTAAAGCACCCCCAAAACAGGAT
GATGAATATTATGACGATTATGAAGATGAAGATACTAGAAAACCATTGCCTTCTGCGAAAACAAGTACAATAC
AACAAAGACCAAGACCAGATACAATCGAAAAAGAAAAGTTCAAGCCA
>1002049517_1 Heli.1-EL600115.1.5

CCGTTTGGTGTTGCGCTATCCAAATGGGTAAACCCCGTGGAATACGCACGGCGCGTAAGCACGTGAACCACCG
CCGCGAACAGCGATGGGCGGACAAGGAGTACAAAAAAGCCCACATGGGCACAAGGTGGAAGGCTAACCCCTTC
GGTGGTGCATCTCACGCTAAGGGCATCGTCCTGGAGAAAGTTGGTGTAGAAGCCAAAC
AGCCCAACTCCGCCATCCGGAAGTGCGTGCGTGTGCAACTGATCAAGAACGGCAAGAAGGTGACCGCCTTCGT
GCCCCGCGACGGTTGTCTCAACCACATCGAGGAGAACGACGAAGTGCTAGTCGCGGGTTTCGGTAGGAAGGGT
CACGCCGTCGGTGATATTCCTGGAGTCAGATTTAAGGTGGTAAAAGTGGCCAACGTAT
CACTCCTTGCGCTGTACAAAGAAAAGAAGGAAAGACCTAGATCATAGATCTACTTCATTGTGATAAGTGCTGC
TGAGGGAGTCGTCGTTTTAAGTGTAACTTTTCAAAAATGAAAAAATTAAGTATGCAGTAAATTGTGTATGC
>1002049518_1 Heli.1-ES584604.1.5
GCAGTACGGTCCGGAATCCCGGGTAATTGTCTTGTGCGGCGGTGCAGGAGGTGAACTTACTGCCAAGAATTCT
CTGAATTCTACATATTTAATTTCAATTAGTGACTAAATATAAAATACAGAAATGGCTCCCCCTCACTATTCCG
ACCTCGGAAAGAAGGCCAATGATGTCTTCTCTAAGGGCTACCATTTTGGAGTCTTTAA
ACTAGACCTCAAGACCAAGAGCGAGTCTGGAGTTGAGTTCAGCAGCGGCATCACCTCCAACCAAGAAAGTGGA
AAGGTATTTGGTAGCCTTTCATCTAAATATGCTGTGAAAGACTATGGTCTGACATTCACAGAAAAGTGGAATA
CCGACAACACACTGGCCACAGACATTACCATCCAAGACAAGATTGCTGCTGGTCTTAA
AGTTACACTTCAAGGAACTTTTGCACCACAAACTGGAAGCAAATCTGGAAAATTGAAGACCCAATTCGCCAAT
GAAACAATAGCTGTCAACAGCAATTTGGACCTTGACTTGGCCGGACCAATAGTTGACGTAGCCGCTGTGCTTA
AGTACCAGGGCTGGCTTGCCGGTGCACACACACAGTTTGACACACAAAAGGCCAAGTT
CTCTAAGAACAACTTTGCCTTTGGCTACCAGACCAACGACTTTGCTTTACATACCAATGTTGACAATGGCAAG
GACTTTGGTGGTTCCATCTACCAGAAGGTATCAGACAAGTTGGACTGCGGAGTCAACATGAAGTGGACTTCAG
GTTCCTCTGACACATTGTTCGGAGTTGGTGCTAAGTTTGCTTTAGACCAAGATGCGTC
ACTTCATGCTAAGATCAACAACAAGTCCCTTATTGGACTTGGTTACCCACAGAAACTGCGCCCAGGCGTAAC
>1002049519_1 Heli.1-DT664382.3.5
GCACGAGGCACTTATGGCTGCGGCCGCCCGCAACCGCTCGATTTCATTGTTATTATTCAATTCAATTTAATAT
AAAAGAAAAATTGCCAAAAAAATCAATGGTAAAGTTTTTGTAAAAAATAATTGAAACATGCCTGCTGATTCTA
CAATTATAGCAGCGGGGGGAGAGCGGGAGAGTGTAATTTTGTTTCAAAGTCTCTCGGA
GAGATCGAAACATGAAGACTTTCTACGTCGGGCGGCAGATCTGCTGGTGGAAAGAGTGGTTTTCGGTAGGGCG
TCAAGGAGTTCGAAGGTTGTGGAGTGGGCGACCCCTGAGGAAATAAAGAAAACAATTGATTTGAAACCTCGAA
ATGGTCCCGTGTCCCACGAGGAGCTTTTGGCTTTTATGGCTGATATTGCCCGGTACTC
AGTCAATACTGGGCACCCATACTTTGTGAACCAGCTTTTCTCGTCTGTAGATCCTTACGGCCTTGTCGGCCAG
TGGTTGACAGACGCACTAAATCCAAGTGTGTATACCTTCGAAGTAGCTCCCGTGTTTACTTTAATGGAAGAAG
AAGTTCTTCGTGAAATGCGTACAATAGTCGGCTGGCAGGGAGGTGAAGGTGACGGTAT
ATTTTGTCCAGGAGGGTCCATAGCCAATGGATATGCTATTAGTTGTGCAAGATTCTATCTTTATCCAGAAACC
AAGACCAAAGGAGTACACGCTGTGCCAAATCTTGTGTTGTTCACATCAGAACTAGCACATTATTCAACTAGGA
AAATGGCAGCGTTCATGGGTATAGGTAGCGACAATTGTATAGCAGTAAAAACTGATGA
ATTTGGCAAAAT
>1002049520_1 Heli.1-DT662154.3.5
ATACCGCATAAGGTATAAAAGAGCACGTGTCCTTCAGGCAGAGCAATTGTTCTGTTCATCTCTAAGAAGTACG
ACCTGAAATAAAAAATAAATAAAATGGAAAGTCTTTTGAGACACGATGACCACGATCACGATCATGATCACGA
TCATGATGATACAAGCGGAGTGGTTATAGCAAAAGGGGTTGCAATGGCGACCCTCTTC
TGTGCCTCCATGATTTGTGGTATTGTACCACTGTTCCTGGCAAGGCGTTTCAGATGGATCTCCGCTGATGATG
CTGGAAATTTAAAATCGAAAAATAGAGTGGTTATGACTTTGCTCTCGTTCGGTGGAGGTGTGTTGCTGTCCAC
AACATTTCTTCATTTGCTTCCTGAAGTCGATCACAACATTGAAGATTTGCAATCATCG
GGCCTGATGGGAGAATTTGATTTCTCCTTGGCGTCTCTACTGATGATATGCGGCTTCTTCATAATGTATCTTG
TTGAAGAACTGGTTCATATATACATACACCGGAGGGAAAAGAGGAACGGCCGCAATTCACCGCTAGTAAGGAA
CCTCAGCGTTCGTAGGAGTGTATCAAGTAGCGAGAGCAAGGAAACAGGTGATAAGAGC
GTTACCAATTCCACTGCTGATCTCATTGATCCCGCATCTATTATCAAAAGCAAGGACTTGGAAGTAAATCATA
ATCACGCCAACCACGTGCACCATGGCCATAGCCACATGCCCATCAGCAATGCCGACGATGTGACCTCAGCTTT
GCGCGGCCTACTCATCGTACTAGCTCTGTCTATTCATGAACTATTT
>1002049521_1 Heli.1-DT663650.3.5
CGTGGTCCGAAAAAAAAACGATATGAGATCCCTCGTTATATTTCTGAGTGCAGTGGCTGTGGCCAGCGCCGGT
CCTGTGGCCCAAGAAGATTCAGTGATCAAATTGGTTGCCGGAAATTTAGTGAATTGCATGAATAGTGATTTGA
GTTTGTGCTTAAAGGAACATGCGCTCAAAGCGGCTGAAAGACTAGGAACTGTTCGCAA

GCTGAACATCATCGAAGGGGTCACTCTATACAACAATGTACCAAAAGAAAGCAGGAGCTACGAACCTCTTTCT
ACTGAACCAGAACTTAGAAACAAGCAGATCACTGAGAGATTGTGGGAAAGTACCGGTGATCTGCTGCAGAAGA
GCGACTTGGAGCTGAGCTTCAATGGCGGAGAAGAAGAAGAAGAAGAAGAAAGTTCTAG
AGCTATTGGAGATGTTGACGAAAGCCGGGGCAAAAAGAAGAAGCAGCTCAAGAAGAAACTGAAGCTACTCATT
CCTCTGGCTATCTTAGCAAAAGCCAAAGCTGTCGCACTTGTAGTAATTTCCCTTTTGATCATCGCCGTTTCTG
TCTTCAAAATCGCCATTTTAGCCAAAATTGCATTTATCGCTAAGGTTATAGCCATAAT
TAAGGCACTTTTGGCCAAGAAGAATGCCCAAGAAGACCACGGGTGGGTATCTCATGAAGAACATGTACATCC
>1002049522_1 Heli.1-ES586839.1.5
GTGTCGTTTCGGTGAAGACCACCAGTATGAAGTACAACAGGCTGGTAACGTCCTCCAGGAGGAAAAACAGGAA
GAGGCATTTTAGCGCCCCTTCCCACATCAGGAGAGTCTTGATGTCATCTCCACTTTCTAAGGAGTTGAGACAA
AAATTCAACGTTAAATCTATGCCTATCCGCAAAGACGACGAAGTTCAGGTAGTTCGTG
GACATTATAAGGGTCAACAGGTTGGCAAAGTAGTCCAAGTGTACCGTAAGAAATTTGTGGTCTACATTGAGAG
GATCCAAAGGGAGAAGGCCAATGGCGCCAGTGCATATGTCGGCATCCACCCCTCAAAGTGTGTTATTGTAAAA
CTAAAGATGAACAAGGACCGTAAAGACATCTTAGTGAGACGCGCCAAGGGCAGACGTG
CGGCACTCGGCTTGGAGAAGGGCAAATACACTGAGGAGACCGCCACCGCGATGGAAGCCTCGTAAACATAGAT
TTAAGATTAATAAAATTATAAAA
>1002049523_1 Heli.1-EL599180.1.5
TCATAAGATTGGTATTTTTGTTTGGATTAAAAACTTAATTTCGTGGTGTAATTGCAATAGTTTGTGAACACAA
TGGGAAAAGATTATGGAGAACCGATACGATACGATCCAGATTTCAGTGGCCCCACACACAACCGCTCTTGCAC
GGACATCGTCTGGCTAATCATCTTCATCCTCTTTTTAGGAGGATGGGGCTACGTCGGA
TATTACAGTATGACAAAAGGCAACGTTGAAAAACTTTTAGCTCCAATTGATTCAAAAGGTCGCCGTTGTGGTT
TAGACTCCGGATTAGAAGACAAAAAATATTTGCTATTTTTTAATATAGTAAAATGTCTTTCACCTGGGACACC
TATAACAGGGTGCCCAACCACTCAAGTTTGTGTATCCCAATGTCCAAGTAAAACGATA
TTATTTGAAAAAGAATTACGCTTAAATCCAAGTTCATTTGAGCTCATTCGTAAAGACATGGTATGCAGTGACG
AAGTAGAGAACGTGCAAACAATGACTCCGGCTCAAGCTTTACAATACATTCAAGAAGAAAAATGTGCCAGTTT
TATATTACAAAGTCAACCAGTG
>1002049524_1 Heli.1-DT666650.3.5
TAAGAGCTCCGTTCTGTGCTACACACTAAGTGCTTCAAAAGTATTGCAACCATGGCTAACATTCTAGATGTTT
CAACCGATGACAACCTGCAGTACCAATTTTTCCCGGTATCCAGCGGTTCCGTCCAGTTTAAAATAAGAGCACC
TAACGATGCGCATATTGCTCTGACTATGGGTCCCCAAGAATCTGACCCCATGTATGAG
ATATTTATTGGTGGTTGGGGCAACACGAAGAGTGTTATTAGGAGAAACAGAACCAAACCAGATAAAGTAGAAA
TTGAAACACCAAATATTTTGAATGGCGGCGAATTTCGCGGCTTTTGGGTGAGATGGGAAAGCGGCATCGTGTC
AGCGGGCCGTGAAGGAGAGTCTATTCCTTTCATTTCCTGGTCTGATCCAGAACCCTTC
CCTATTGGTTTCGTTGGAGTGTGCACCGGCTGGGGTGCTTCTGGCACATGGAAAATCGAAGATGGAGCCGAGT
TCAATACGCCTGACAAGTTAGAATACAAATTCGGGCCTGCTGCATCTGGTTCTTTGGAACTTGAGTACCGCGG
CCCTCACAATTGTCACGTTTGCCTCACACCTGCACCCGCTGAAGTAGATCCGATGTAT
GAAGTCATTCTTCCCCTTTACGTGGCTAGAGCACAGCATGAAGGCGCTCTGTTACCTGGAAAGCTGGTCTCCT
CTCACGGATGCCCTGGTATAATGAATGCCAACGAGTTTAGGAAGTTCATCTTTGAATGGCGTTGCGGGAGGTT
GTCAGTGCGCGATGGTGTTTCTGGGGCAGTGTTG
>1002049525_1 Heli.1-DT663814.3.5
GGGACTAAGTTTTTAGTTTTGCGATAAGTATTAAAAGTTCATAAGTGCCCTTCATTTACAATAGTGTTTTTTT
TAATAATTAAGTTAAACAAGACTAGATTTGCACGATGTCAGACAACACACAGGATATACTAAAAGAAGCGGCG
ACGGCGGCTGCGGTCGGTGGTTCACTGTTCAGTACTTTTGACATATTCATGCTTGTCG
TTTTATTTGGTGCAGCTGTTTGGTGGCTATATAGTTCTAGAAAAGAAAGTAAAAAGGATGATTTACTTCTTAG
TAATTACGCCATACAGCCTGCAGGTTCTATTCAAGTCACAGAGAATTCTTTTATAAAGAAATTACAAACATCA
GGTAGAAGTCTTGTTGTATTTTATGGCTCACAAACGGGTACAGCAGAGGAATTTGCTG
GCCGTTTAGCAAAAGAGGGCATTCGCTACAAAATGAAAGGGATGGTAGCTGATCCTGAAGAATGTGATATGGA
AGAATTGACTAAACTCAAAGATATTGATAACTCTCTCGCTGTGTTTTGCTTAGCAACCTATGGTGAGGGAGAT
CCCACGGATAATGCCATGGATTTTGTTGAATGGCTTAAAAACGAGCCCGAATTAATTG
GACTAAATTATGCTGTATTTGGCCTGGGGAACAAAACTTATGAACACTATAATTCTGTAGCTATCTTTGTAGA
TAAAAGACTTGAAGAACTTGGAGCCACCAGAGTTCATGAGCTTGGATTGGGGGATGATGATGCAAATATTGAA
GATGACTTCATTACCTGGAAGGAAAAATTTTGGCCATCTGTGTGTGAAAAATTCAATA

TTGAAAGTGCAGGAGAGGAAGAATTAACTCGTCAATTTCATTTGATAACACATGATCCAGAGGAAATTTCTCC
AAATGAAATATTTACTGGTGAAATAGCTAGATTACATTCACTGGAGAGACAGAGGGCGCCTTTTGATGCGAAA
AATCCTTTCTTAGCACAAATTAAAGTAAACAGAGAATTGCATAAAGGTGGTGACAGGT
CATGCTTGCATATTGAATTTGACATATCCGGCTCAAATATGAGATATGAGTCTGGTGATCACTTAGCTGTAT
>1002049527_1 Heli.1-EL601168.1.5
GCACGAGGCCGCGATCCTGTGAGGGTGCAACGAGAGCTAAGATCGTTTTCGTTTACGCGCGAAAAGTGACATA
AATATAATTTTCAAAATGGCGGTTACAAGTGCCAGGAGTATTGTGAATATTAAGGAGAAGGGACATTTCGAAC
ATGAAAAAACAAGGATTGATAAATATACGTTGGCCGACGAGCTGCCAGCTCAGCTGTC
GGAGCTGGCCCTCCGGCCACGGACCATGGCCCCCTCCTACACCCTGCGCAAGTTGACGCCATATGATAAAGAG
CGCGTAATAGAATTTTTAAGAAGGTTTTTCTTCCTCGACGAACCGTTGAACCTGACGATCAACCTATTAGAGA
CTCCCGAATCCCGTTGCGTAGAGCTGGAGGAGTACGCGTCCAGCACTCTCGGTGATGG
CGCGTCACTAGCTGCAGTTGATGAGCACGGAAAATTTGTTGGAATGGTCATCAATGGAGTTGTTAAGAGAGAG
GAAGTAGACTACACAGACAAATCAGAAGACTGTCCGAATCCAAAGTTCCGGCGGATCCTAAAACTCCTGGGTC
ACTTGGACCGTGAAGCCAGGATTTGGGACAAACTGCCGCTAAGTTGCCATACAGTTGT
GGAGATCAGGATAGCATCCACCCACTCTGACTGGAGGGGGCGAGGCCTTATGAGGGTGTTATGTGAAGAGACT
GAACGAATAGC
>1002049528_1 Heli.1-DT667892.3.5
AAATAAATTATATATTGAGTGATGATGAACCATTTTTGGTTTGAAAATGTGATTCAGTCAACTGAATTTTGAA
ATTTTTTTTTCATGGATTTTTTTTATTTGTTTGATATTTGACATTTTGACAAAGTGATTCCGTGGATTTTAGT
GGAGAAGTGATTGTTTTTAAGATGGCGCACACAGTGGTAGCCTTAGTGGCATTGATTG
CCGCGTCTACAACTGCAAAAGAGCAAACGAGATGGTCCCGCCAGATTAGCTCTTACACAGCCGACATAAGCGA
CTGGGTTCCCTTAACAAGCCCAGTGGATAAAGAAGAATTGCCACCAATCAAGAGGCAAGCAGTGGCGGAACCT
AGGATATTATCTGAACCTTTTCCGGGCTTTATAAGACCAACTGGTTTTAGTCAAGACG
GATTTCCATCAAGGGCATTCCTTAACTCTCCTGTTAATCGACAATTATATTTACAATCAGTACCGTCAGCTCC
TCAAAATTATCTCTCAGATCAGGGTTTCAACCAAGGCCTAAGATTTGGCTTGACTCAACCGAATTTCCCTTTA
AGTCAAGGATTTATAACTCCTCAGTTTAACTTTGACAGTGTTCCACAATTAAAATCTC
GACCGCACAGTTCTCTTAATCCGATCAAATTTGAAAACTCTATCAAAACAACTCATCCACTGCCATCTCCAGA
ACCTTATGTCATCAGTCAAACTC
>1002049530_1 Heli.1-DT665417.3.5
ATTAGTCAACATAAGTCTTCCTCCCCCGCTCCCAATGCTACGCAAGTACCTTTATCATCAAAACCTCATGGAT
CAGAAAAAGAAGATAAGTCATCTCTAAATGAGTTTACGAAAGAAAAGTCGGAAACAAATATCCACGAAAAATT
ACCAAGGCCAAAAAGTCCTTCCAAGCTGTCTGTTAAATTACCAACTAAATCGAAAAGT
CCAATGCGTACTGCTAGTCCTGATAAAAAAAGACCTACGTCTCCAACCAAGTCATCGCCAGCAAAACCTAAAT
CGAATCGTTTCAATGAATATGCTTCTGCTTATATGAAAAAGGTGGGACTCAAGGATGAAGAAAAAACAACAGA
GATTTCAAAAGTTAAGAAGATTTCTAATGATGAGCAAAGAATTAAGAAAATGGAAAAA
CATACCTCAGAAGCTAATGCATCGTCGATTATATCCAAAAAGTCCCCTGAACGAACTGTGTCACATGACAATT
TTGAAGTAAACACAACACAAATTAATGGAAAAAGATCACCATCGCCTGAGAATACTATTAATAAAAAAATCTA
TGAATCTGATAAGAATCGCGTTTCTCAAAAGCGTACTTCTCCTGTCCGAAAAACGTAT
AGTCCCGAGAGGATAGCACATAGTCTAGAACGAAACATCCCCGTGGCACGTTCCCCAAGTCCTAAAAGACCCC
AAGAATTAAAGCAAAAATCAGACACGGATGGATCTAAAACACAAACAAAGGAAGTTCTTATAACAACAACAAT
TGATATTGATAAGAAAATTCCTCAAAAACAAAAACAAGAGGAAAAACCATCTTGGGTG
GTGAATAGAAATTTAAAAAAGACATCTGAGACTCGTACGTTTACAACAAAAAAAGTGGAACCCGAGAAACCAA
AGTACCGAGCTGTGAGTCCTTCTAAGGTGATCTCAAAGCCTATCGATGTCATCACTTCGAGCTATGGCCCTGG
ACCCCTGGATGCTGACGGCAGGCCACTCTTTGGTATCAAAGCATTAAGAAACGGAGCT
TCAAATTATCAAGTGAAAGGTACAGTCATTCGTCAAGAGTTCCACTCGCGGAACGGTGGTGAACCAGAGGGGA
CTGTGTCTGTGACCGCCTACTCCACGGAGCCTGAGGACTTGGAAAAATTGTTGAACAGTCAGGGTGAGAAACC
GTCTAGGATTCACGGTCTTGCTGCCATCACAACGACGAAGAAGTTCGGCGGCGACACC
GGCACTACGTTTAGCGAAGTTCATAACAAAGAAGAACGTGCAGTTCTGGACCAGTTCACACACAGCGATCGCC
GCGTGACAGACAGTAGGGTCACCACGAGCAATTTTGAAACATCCGACGACTTCATTCAAAATGAGAAATTAGT
TAATGTAAATAACGTTATACATGTACAAGACAAGAGCGCAGAAGCAGAAACACATGAA
AGCCGTGATCTCAAACAAATGAAATCTGACAAGGAATCTATTAAGCAAACTACCAAAATTCACAGTGAAAAAG
ACAAAAAAGTTGGAATGGAAAGAAGGGACAGAGAAAAGTTTGATAAAAGAGAAGACAAGAAGACAGTGCGACA
GAGTTCGGTGAAATCGCTTACTGAAAAATACATTAAAAGTGCAAGTGAAACATCTAAA

ACCGAACGCCACGTGTACCCCAAGGCTGGTCTCATCCTGCGCACCTCCACTATGAAGGACAACGTATCGAGCG
ATGCTGGCCTCACTCGGACAGACAGTGAACAAAGCCTGGGGTCTGATGAAGAAATAGTGACCACTACGACCAC
AGAACAAGTAAATGACGGTGTGAAGACCACGACCACTACCACCACCAGAACAGGTCAC
TCGCATGGTCAAGAGCGATCCTTCCTCGACAGTTCTACTAAGGTCACCGGGGTACAAGATATTCTTACGAGAA
TGAAGAATGCTGATATAGTAATAGAAGAAGGCGACACCAACGCTGACACAGAGGCGCGTGCTTTATTGAACAA
GTTCCTCGGAGCCACTGTCCTGATGGCGGGCATGCAGAGCTACGTCACGGAGAAACCC
TCCGGCAAAGTCGTGGTTAAACAGGAAACCGTACAGAGCAGTGGTGGCAAGGTGACCAGCAGCCGCCGGGTAG
AGGAGTTCGATATCGACCAGTGCTGGGACGAGCGTGTTTTACGCAAGCTTTTGGACGAGTGCAGTGACTACGA
ACAAAGGCGCCGCCTGCGCGCGCGCATCCGAACCCTCATGGCTGAACAAGAAGCGTGC
ACGAGTGCGGTGACGGAGGCGCTGGCGGCGGCGGGCGCGGCGGACGCGGAGCAGGCCGGCGAGCGTGAAGAGG
AAGTAACTGTAACATCGAGCGTGAGGAGAAACTCGTCCGAGAAAACCGTTAGCAGTAGCACTACCACTATAAA
AAATTCTAAAGTGATCGAGAGCATGACCCGGCCGGCGCCGAAGCCTGTGTCGCCCCTTC
GCCAAGTTTCGTCAGCTCGAGAAACAAAATTCTACTAACAGTCCCAAATCCCCCCAGAGCCCGCAGAGCCCGG
GCTCTCCGTCGCAGCCCTACTTCAAGTTCACGGAC
>1002049531_1 Heli.1-DT661960.2.5
AACATGGCTAAGTGGAGTATTGTCGCCCTCGCTCTCATCGGCTGCGCCGTCGCCGAGCCTCCAGTAGGCTACA
GCTACTCCGCACCCTCTTCCCTCCTGGTTGTCGGTGGCGGACACGGCCACAGTTCTCCTCTCGCCAGCGGAGG
TCACTACTCCTCCGTACCGGTCGGTCATCAGTCCTCAGAGGGCTACCATATTGACCCT
CATCTATTGGAGAAGATCCGTCACATTATCCTAAAGGATGAGATCCAGAACCAGGCCTACCAATCATCTATCG
GAGGCGGACACGGTCACGGTATCTCTTCTCACTACGGTCCCCCTGCCCCCGTCTACGGTGTGCCCCACGACAG
AATCGTAGGCGTTGAGCTCGAATCACTCCAACAAGGCATCCAGGTAGCTCAATACCAC
CAAGCTCAAGAAGACTACTCTGGCGGTTACGCTGGTGGTTACTCTGGTTACTCTGGTGGTTCATCTGGTTACT
CTGGCGGTTACTCAAGCGGTGGTCACGGTTCCATCTCCTACAGCCCGTCGTCCAGCTACAGCACCATCGGCGT
TCCCTCTGGCTCCTATGGTATCCCATCCCCCTCATCATCATACGGCGCCCCCCACCAT
TAAACGAAAATTATAATAGTCATCTCATTTCATTAATGTTATGAAACTCATGTGATCGCTAGACGTTAACTGT
ATTATATTTGTTGTTAATATAAGCTTTCTGTAAATAAATCTATCTTT
>1002049532_1 Heli.1-ES585707.1.5
TTTTTTTCAATAAGGAAGGTCTTATTCAAAATAAACAATATGTTTGCCTTTAAATCTATCGTCCTAATTGCTG
CAGCTTTTACTGTTGGAGCAGAATGCAGTGTGCAGGCAGCTGCATTGGTTGCGCCAGCGCAAGTTGGTTACGC
CCATGCTGTACCACAAAACATTCCACCATATGCATCTCAAGTGAACATTGAAAGCCGA
AGTGCTCTAGTTGCTGCACCTGCTGTAGCTGCTTATGCTGCCCCTGTATCTGCACCGTTGGCTGCTCCGTTTG
CCGCACCGTTTGCTGCACCATTGGCTGCACCACTAGCTGCACCATTGGCTGCACCACTAGCTGCACCGATAGC
GGCTAGATACGCTTCACCTGCTTTTGCAGCTCCTATTGGTGCAGCGCCATACCTTGCT
GCACCTGCAGTGCGAGCAGCATATGCAGCATCACCTATAGTTCCAGCACCTTACGCTGCGGCTTCTTACGCAC
TTCCTGCACCTATTTCTGCGGCTTATCCTTATGCTGCAGCGTCTCCCTTCTTCCGTGCTCCCATCGGCGTTGC
TCCAGCTTTTGTTCGATAGACTTAAAGATAAACTAGTCGACCTTCCTTTATAAAAGAC
TTACTATTTTTTAATTACGAAGAAATAAATATTTTT
>1002049534_1 Heli.1-DT665094.3.5
GCACGAGGCACCACTATACGACTAAAGTGAAAAGAAAATAAAATCGAAAAGATATGAAAATAAATACTTAAAC
TAATTTAATCCTGATATTGTTTGTTTATATAGTGATTGTTTATGTTTAATGTTTACCTAATAACTGTGTGGCC
TTTGATGTGAACAAAATCGATGAAATCGTGTGTTCAGTAATTCCAGACAATATGTGCA
GTGTTGATTGCGATTGACATCTGACGTAAAACAAAAGGAAAACGAAGTTGGAGAACTACTTAGAGGATTAAGG
ATGAGTTCTACTGATACAATTAATGTAACAGCACAACCAATCTCGGATATAACTCACGAGATGTTGACGTCTG
TGTTTTTGGAGACCCGCTCTGCGCAGGTCATTGCGGGCATATTTGTATGGACAGCATT
ATTTATTACTTGCCAACAGATTTACCAGCACCTCCGCTGGTACACGAACCCGTCGGAGCAGCGTTGGATAGTG
CGCATATTGTTTATCGTGCCCATCTATGGATGCTACAGTTGGATCTCGCTCTTGTTTTTCAATGGAAATTCTT
ATTATGTTTACTTCTTCACAGTAAGGGACTGCTATGAAGCCTTCGTGATATACAGCTT
TCTGTCGCTATGCTACGAATATTTGGGCGGGGAAGGGAACATAATGTCGGAGCTGCGAGGACGGCCGGTGCGC
GCCTCCTGCGTCAACGGGACTTGTTGCCTGTCAGGGGCCACGTACACTATCGGATTTTTGAGATTCTGCAAAC
AAGCGACATTGCAGTTCTGTCTCATCAAACCAGTGTGCGCCTTCATCATCATATTCTT
GCAGGCGGCGGGTCACTACCACGACGGTGACTGGAGTCCTGACGGCGGTTACATCTACATAACGATAGTGTAC
AATTTCTCCGTCAGTTTAGCGCT
>1002049535_1 Heli.1-DT664476.3.5

AGACGTGAAAATGGCCAGCGGACGAATTGTCGTATACGGTGGCCGCGGTGCTTTGGGCGCGGCTTGCGTAAAT
CACTTTAAGAAGGCAAACTACTGGGTTGCGAGCGTTGATCTGAATCCTAATGAGAGCGCAGATGTAAACATTA
CAGTCCCAAAGGATGCTTCCTGGGTTCAACAAGAAGAACATGTTGTTAATGAACTAGG
TGAAGCTTTGCAAGGTCAAAAAGTGAATGCAGTAATTTGTGTGGCCGGTGGTTGGGCTGGTGGTAACGCGGCC
AAGGATTTGAGCAAGCAAACTGATCTTATGTGGAGGCAGTCAGTGTGGAGTTCTACAATTGCTGCAACTTTGG
CATCTAAATATTTAAGTCCTGGAGGATTAGTTGCTTTGACAGGTGCTAAAGCTGCATT
GGAAGGGACCCCCGGAATGATTGGTTATGGTTTAGCGAAGGCTGCAGTACATCAGCTTACCAAATCTCTTGGT
GCTAAAGATTCAGGTCTACCAGAAAACTCTCTTGCCGTTGCTATACTACCAGTGACACTGGACACAGAAATGA
ATAGAAAGTGGATGCCTAAAGCAGACTTTGGATCTTGGACACCCCTGTCATTTGTTGC
GCAGCTATTTGATAAATGGCTAAAAGGAGATGAAAGACCTGCAAGTGGAAGCTTAGTTGCGCTTGTGACTAAA
GATAATGTAACAGATCTGATTGTCCAGTAAATCTTGCCAAAATCTCTCACCACCACTAACTTATGTATGATCA
GAGTATAACATTTTCAATAAAATATAATGCAACTATATGTTGGCATTTATAATGAACT
TAGTTCATTAGTCAAATCGCTTTT
>1002049536_1 Heli.1-ES585355.1.5
GTCACTATCATTTCGACAGTCCAAGATGAAGCTTTGCTCAGTTGTATTCAGTCTATTAGTGGTGTGCGCGGTG
AGCTTCGCCGTGGAACATGGCCCGTTGATCAATGTGCTGCAACCCCTTGACTCCCACGACTTAGTCCAGATTG
AACCTGTACAAAATATTAGACAGAAGAGGCATTTACTTCTAGGTGCCGGAGCCCTAGG
TGCTGGTGTAGTAGGAGCTGGAGCTCTTGGAGCTGGTTTATTAGGAGCTGGTTTGATCGGTGCTAAAGCTGGA
CTTATCGGCGGTGCTTTAGTAGGCGGTGCCCTACAAGGCAGAAGTTACGGGGGTGGATACGGATACGGCGGCG
GATACGGTGGCGGATACGGATATGGCGGCGGTTACGGATATGGCGGTGGATACGGGTA
TGGTGGTGGATACGGTGGATACAGTGGACAAGGATACAGACGTTACTACCCAACTACAACATATATTGTAGAA
GAACCCTGGTGCTAATTTCAACATTGCGTCGTAGCCGTTACGTAATCAATTCCAACGTGTGTAGAGATGGGGA
TATCTCATGACTGATACCTGCACGTTTAGTTGTTAAGAATAAATTT
>1002049537_1 Heli.1-DT664368.3.5
TCCGATCACCCGCGAAGCCACGCGGTCTACATTGTGAAAGTGCAGTTTCTCGGTAGTGAGAGTTAGCCGAGCG
CCCACAACGCTAATATAAGTGGTGTGCATTTCTTGTTAACATAACAGAGTTCAATGGAAGCCAGGTTCGTGAC
CTGCCGGAGCTTCCAGCAGATCAAGCCGCCCGAGAAGAGTTTAGCGCCGCCACCGCCG
ACGGTTAAGGGGCTGCTGTCTCGTTTCGGAAGCCAGGCTCAAGCTTTATTCGCGCCGAAGAAGCCAAGATTCG
GGTCGTCAGTTGCTATTCATAAATTACGCGAGTCGAAGTGGTTCAAACATGAAGAGCAGAACATCTTGTGCGC
TGTCCTCGAATCCGGATTCGTACTGGAAGTCCGAGCTGAGGACCCTCTGCCGCCAGAC
TCGACACTGTCCCCTGACTACCATATGTACGCCATTGCCATGTGGAAGAAGGGTAAAGAAAAAACAAAGAACC
CGGAACAGATAGAGGTGTACACGGTGCAACAGAAGGGTGTGCGCAAACGTATTATAAAGACTACTTTGGGTGC
ATTCTGGAAGAAAGACTCTGTCATACGTATCAACAACGTAGACGACAAGCAAGAGACA
CCCAACAGTGAAAAAGACATCAGGGCAAAGCTCGACATGGCTACTAAGTCAAGATTTGAAAGGAACTGGCATA
ACACTCTGCATTTCGTCCACTGGTGCCGATACGGAGAGACTCCCCAAGAGGCTCGTATG
>1002049538_1 Heli.1-ES587350.1.5
GTTCAGAAGCCAACACGGACTTTGTAAGTGACAAAATGTTCAAGTTGATGGTGTTGTCCTGCATCCTGGCGGG
TACGTCTGCTTCTAGCATTCTTGGAGAATATTTGGCACCCATCACCACATTGGCATATCCTACTCCTCTATGG
CAGCCTTCTAACTACCGTGGACCATTGTCGTTAGCCCCTGGACAACCTGCTAACATTT
TAGGCGCTGATGGCAGACCTTTGGACACTTTAGAAGTGAACTTAGATAAATCAGCTCACTTTGCCACCAAATT
ACTCAACAATGAAATACATGTACTCAAGAAACGCTCTGCTCCATTGACCACATCTTTGAAATATGCGACATCC
CTCATCCAACCCTACAACTACCGCGGTCCCCTGTCTCTCGCTCCTGGACAACCAGCCA
ACATCCTCGGCGCTGACAGCAGACCTCTTGATACACTGGACGTCAACTCGGACCGTGCTTATCATTACGCGAA
CAAAGCTCTCAATGGCGTACATTTCTTGAAGAAACGGTCCGCTCCCATCAATGCTATCACCATTGCTAGGACG
CCACTGATCACTCCTGCCTATTCTTATATTACACCAGTCGCTGCTACTCATCTGAATC
CGATAACTTACAATACCGCCTATATTACTCACGTCTTATAATTTAGTATTATAGAAAACCTGAAACTGTGGAA
ATGTAATAAATAAATAATTCATG
>1002049539_1 Heli.1-EL603054.1.5
CGAGGGTTGTTTTTGCAAATGAAAGACAATACTTAAATAAATCATGTTTATGTGCTGTTTTAGAGTTTAATCT
TATATAGCTACTTTTGTTTTTATAGTTTAATTGTTATATGTACAAGTGTGATTACAAAAATGAGTGCCAAAAA
GGGTTTTGAAACAAAAGTTTTCGATGACCAGGAGAGCGATGTTGGTTTCGAGTCCGGA
CCGTTGAGTGAACAGCTGTTGTCTTCCGAGGAAGTGGATATAGAGGAAGATAGTCCAGTGGAAAGTAATGAAC
AAGTGAACAGTGACACAGGGTTAGATAGTGGTTTGTGCTTGTCAGAGCCTCTGTCTAAAGTTAACCTTAGTGA
ATCGCCCGTCGCACAACAAGGGTCATTAGTTAATATTGAAGGTGAAAGACCACAGGAT

ATCCCACCGATTTCGATACTGTTCCAGCAAGACGACGATGGTGATACGCAGCTCCACATTGCAGCTGTGCACG
GATGCCTGAAATCAGTTGGTGTCTTGATAAGAGTGTGTCCAGACAAAGCGTGGCTAGATATACCAAATGACTA
TGGTCATACACCGTTACACCTTGCTGCTATGAGTGGTAATGCAGTTGTTGCCAGAATG
TTGGTCAGAGCAGGAGCCGCCTTAGACGTGAGGGATTCCACAGGAGAAAATCCTCTACACAAAGCAGTTGGT
>1002049540_1 Heli.1-DT663126.2.5
GAGTACGAAAATCATCAAAGCGAACGCTGTGGAGCCCGATGGCTTCGAGACATCAATCTCACAGGCGCTGGTG
GAGTTGGAGACCAACTCTGACCTGAAAGCTCAACTCAGGGAGCTCTATATTACAAAGGCTAAAGAAATTGACC
TGCACAATAAGAAGTCAATCATCATCTATGTGCCGATGCCCAAATTGAAGGCATTCCA
AAAGATCCAGATCAGGCTGGTCCGTGAATTAGAGAAGAAGTTCAGTGGCAAGCATGTTGTGTTCGTTGGTGAC
CGCAAAATCTTGCCTAAGCCAAGCCACAAGACTCGTGTTGCAAACAAACAAAAGAGGCCCAGATCTAGGACCC
TAACCTCAGTATATGATGCTATCTTGGAAGACCTGGTATTCCCAGCTGAAATTGTGGG
CAAACGCATCAGGGTGAAACTGGACGGCTCGCAGCTCATCAAAGTGCACCTCGATAAAAACCAGCAGACCACC
ATTGAACACAAGGTGGACACCTTCCAGTCAGTATACAAGAAGTTGACGGGGCGTGAAGTGACCTTCGAATTCC
CCGAACCCTACTTGTAAACTTAGACTATGACTTTACAATAAAACTAAAACCTTAAAAA
>1002049541_1 Heli.1-EL599998.1.5
GCACGAGGCTTTCTTCTTGTGTGTTGTGCTACTTATGTGGATTATTAGGTGATATTCTTCTTTTTATTGGATT
GTCATCCTAAACCGCAAACATGCCTAAATCGATGAACGTCCGGGTGACGACGATGGACGCGGAGCTGGAGTTC
GCGATCCAGCAGACGACGACCGGCAAACAGCTCTTTGACCAGGTCGTGAAGACCATCG
GCCTCCGCGAGGTCTGGTTCTTTGGACTCCAGTACACCGACTCCAAGGGTGATCTCACATGGATCAAGCTCTA
CAAGAAGGTGATGCAACAAGATGTGAAGAAGGAGAATCCGCTCCAGTTTAAATTCCGGGCCAAATTCTATCCT
GAGGATGTCGCTGACGAACTGATTCAAGAGATCACGCTCAAACTGTTCTACTTACAAG
TTAAAAACGCGATCCTCTCCGACGAGATCTACTGCCCGCCCGAGACGTCGGTGTTGCTCGCGTCGTACGCGGT
GCAGGCGCGCCACGGAGACCACAACCCGTCCATCCATGGACCTGGATTCCTTGCTAATGATCGTCTGTTGCCG
CAACGTGTTACTGATCAGCACAAGATGTCCCGTGAGGAATGGGAGCAGAGTATCACCA
ACTGGTGGCAGGAACACCGCGGGATGCTGCGTGAAGACGCCATGATGGAATATCTCAAAATCGCCCAAGATTT
GGAGATGTATGGTGTCAACTACTTCGAGATCCGCAACAAGAAGAACACCGAGTTGTGGCTCGGAGTCGATGCG
CTTGGACTTAATATTTATGAGAAGGATGATAAACTGACCCCCAAGATCGGGTTCCCGT
GGTCGGAGATCCGCAACATCTCGTTCAACGACCGCAAGTTCATCATCAAGCCGATCGACAAGAAGGCGCCCGA
CTTCGTGTTCTTCGCGCCGCGCGTGCGCGTCAACAAGCGCATCCTGGCGCTGTGCATGGGCAACCACGAGCTC
TACATGCGGCGCCGCAAGCCCGACACCATCGACGTGCAGCAGATGAAGGCGCAGGCGC
GCGAGGAGAAGCTGGCCAAGCAGGCGCAGAGGGAGAAACTGCAGCTCGAGATAGCGGCCCGCGAGCGCGCCGA
GAAGAAGCAGCAGGAGTACGAGGACCGCCTGCGGCAGATTTTTGAGGAGATGGAGCGCTCACAGGCTAATTTG
GTCGAAGCACAAGAGATGATCAGACGGCTGGAGGAGCAACTGCGTCAGCTGCAGGCCG
CCAAGGAGGAGCTGGAACAGCGCCAGAACGAGCTCCAAGCCATGATGCAACGCCTCGAGGAGACTAAGAACAT
GGAGGCAGCTGAACGACAGAAGCTGGAGGAGGAGATAGCGGCGAAACAAGAAGAGGTATCTCGCATCCAACAA
GAGGTGGAGATCAAGGACTCTGAGACGCGCCGCCTCCAGGACGAGGTAGAGGAGGCGC
GCCGCAAGCAGGACGAGGCCGCCGCCCTACTGGCCGCCGCTACCACCCCCACACACCACCACGTGACTGAGGC
CGGCGGTGACGCCGACGGCGACGGCGGTGACGACGGCGAAGGCAGCGGCAGCGAGGCCGGCGGTGGAGAGCTG
GCACGAGGCCCCGACGACCTGGTCGACCCGCTAGAGGAGCGCCGCACACTGGCCGAGC
GCTCGGAGCGGCTCCACAGCCAGCTGCGAGCCCTCAAGCAGGACCTGGCGCAGTCGCGCGACGAGACCAAGGA
GACGCCCATGGACAAGATACACCGCGAGAACGTGCGCCAGGGACGCGACAAGTACAAGACGCTGCGCGAGATC
CGCAAGGGCAACACCAAGCGCCGCGTCGACCAGTTCGAGAACATGTAACCGCCGCCAC
GTCCAACTTGTACACTATTTATGTTAGGGGTCGTTCCTGAACTACGCCACACGTACATGACCATGTTTGTCAA
GGAATGGGTCTTAAATTTGGTGACGTCACATTTCGAAATATAATATACTAACATCATTTTGAATGATTGAAAA
AAAAGATTTATTAAAAATATGTGACGTCACACCAAGGGTTCGAAAATCATGAAATTCG
TGTGACGTAGTTTACGTACGACACCTTAGATGTATGTACGTGTAGCCCGGAACGGTCTCTTCGACCGGTGCTC
TGGTCGGGCGTTGTCGGGACTAAGTGTAAATATTATTGATTATTATTATTACTTAATAG
>1002049542_1 Heli.1-DT663837.3.5
TCGGTTCCGTAAAGGGACTACTGAAGCTAGACTCTGTGTGCATCGACAACAATGTGTTCCGTTTACACTACAA
AGCCACCGTGATCATCCTGATCGCCTTCTCGCTGCTGGTCACCTCTCGCCAGTACATCGGGGACCCCATTGAC
TGCATCGTCGATGAAATCCCATATTCAGTGATGGACACCTACTGTTGGATTTACTCGA
CGTTTACGATCCCCAATCGCCTCGTCGGACGCGTCGGGAAGGACATGCCCGCGCCCGGCGTAGGAAGTCTCAT
TGAAGGAGAGGATGAGGTGAAATACCACAAATACTACCAGTGGGTGTGTTTTGTGCTGTTCTTCCAGGCTATT
CTGTTTTATGTGCCTCGTTATCTGTGGAAGACATGGGAAGGTGGCCGTGTAAAAATGT

TAGTACTGGATTTAAATTCCCCTGTGGTCGATGAAAATTGTAAATCGGACCGTAAGAAGTTACTTGTGGACTA
CTTTCACACAAACCTACATACGCAAAACTTTTATGCATTCCGCTTCTTTATTTGTGAAGTTTTGAATTTTATA
AATGTTGTGGGACAAATTTACTTCATGGACTTTTTCTTGGACGGAGAGTTCTCAACGT
ATGGGCGAGACGTCGTTCGTTTCACCGAGATGGAACCGGAAGAAAGAGAAGACCCTATGGCTCGAGTATTTCC
TAAAGTTACAAAATGCACCTTCCACAAATACGGTCCTTCGGGAACCGTGCAGAAGTTCGACGGGCTGTGTGTA
CTTCCATTGAATATTGTAAATGAGAAGATCTATGTATTCCTATGGTTCTGGTTCATTA
TTCTGTCAATTCTCAGCGGCATCTCCCTTATCTACCGCGCCTGTGTGGTCGCCGGACCGCGTATACGTTTATA
CCTGCTGCGCGCGCGCAGCCGCCTCGCCCCGCAGGATCAAGTCGAAGCTGTAGCCCGCAAGCTACAAATCGGC
GACTGGTTTG
>1002049543_1 Heli.1-DT666641.3.5
AAACATTTTATTTCACTAATAAATTAAATCCAAAATCATCACGATTGTGAAAATAAAAATTCTGATATTTAAA
TAACTAAAGCACTGCAGCTTATATTATAATGGCTCCTAAAGCCGGTGGTGATTCCGCTAAAGTTAATTCAGCT
GTATATAAAGATAAAAGTAAACCTACTGATATTCGCCTTAGTAACATCAATGCCGCTA
AAGCCGTTTCCGATGCTATACGTACGAGCTTGGGTCCTCGCGGGATGGATAAAATGATCCAAGCAGCAAATGG
TGAGGTGACGATTACAAATGATGGAGCTACTATCCTAAAACAAATGAGTGTAGTACACCCAGCTGCTAAAATG
TTAGTGGAGTTGTCTAAGGCCCAGGATATTGAAGCTGGTGATGGCACAACATCAGTGG
TTGTTATCGCTGGGGCTTTACTTGATGCAGCTGAAAAACTATTGCAAAAGGGTATCCATCCCACTGTTATTTC
TGATGGTTTCCAAAAAGCTCTTCAAATGGCACTGCAGGTTGTTGAAGGCATGTCTACACCTGTAGACCTAACA
AATGAGGATGCGCTTCTAAAAGCAGCTGCTACATCTTTAAACTCCAAAGTGGTATCAC
AGCATTCCACTATCCTAGCACCAATAGCAGTGCAAGCTATCCGTGCAGTGATGGAACCAATAGTGGGTGGAGT
AGGTGCGCGTGTAGACTTGAGAGATGTGAAAGTGATTGAGCGTATTGGAGGTACTGTAGAAGACACAGAACTT
GTCAATGGACTGGTCATCCCACACCGTGCCTCAAATGTTAATGGACCCCATACAGTAG
AGAAAGCCAAAGTTGGCCTCATACAATTTTGTATATCACCACCAAAAACTGATATGGATCACAATGTGATTGT
ATCTGATTATGCTGCAATGGATCGTGTCTTAAAAGAAGAGCGTCAGTATATATTGAATATTGTCAAACAAATT
AAAAAAGCTGGCTGCAATGTATTATTAGTACAAAAGTCAATTTTGAGAGATGCACTCA
GTGATTTAGCTATTCACTTCTTGGATAAGATAAAGACAATGGTAATCAAGGATATTGAGCGTGAAGACATTGA
GTTTGTATGTA
>1002049544_1 Heli.1-DT666922.3.5
GAGGCTACAATCGTCACCATATCGGCTTTGCTAGTGGTAGCAAGTGCCCAGCACCATTTAAGTGGTCATGCCA
CGTCATCCCAATCTATAATTCGTCACGACATCGCCCAAAATCATGGTTACCAAGGCCAATTAACTCAAGCTTT
TCCTACTTCCTACCATCAGGCTCCCATCGTCCACCAAGTCCTTCCTATTGCTAGCCAT
GGAAGTCTTCAGTCTGCTGGTATCTCACAACACGCATCTCCTATAGTTTCCCTTCAACACGTTTCTCCTGTTG
TCCAGCGCGTTTCTCCTGTTGTCCAGCACGTTTCTCCCATCCACTATAACCAATATGAATCTGGACATGGACA
TCAAGACTATCATGGTCACCCCAGATACGAGTTCGCGTACAATGTAGCTGATCATCAC
ACCGGTGACATCAAGTCCCAGCACGAAGCTCGTGATGGTGACCATGTGTCTGGTTCATACAGTCTTCACGAAT
CCGACGGTACCATCCGCACCGTTCACTACAACGTTGATGGACACAGCGGATTCAACGCCCAGGTGGAGCGTTC
TGGCCACTCTCAACACGTCCAGCCCCAGCACCGTGCCCCCAACTACGTACTAGCACAT
CACTAAGAATTAAACAATGTTCCTAATAGAAAGTTAGCATATGTATGGACTCGTATACCCATCAATCAAGCTA
ATGACGAGTCTAGTCTTTTAATGTATGTTCCAATTTTTGTGTATCGATTAAATATTATATAATAGTGTAAA
>1002049545_1 Heli.1-CX700763.1.5
CTGCAGTACGGTCGGAATCCGGGTCGACCACGCGTCCGCTCACTCACCAAGGGAACATCTCCAAGAGTTGGTT
GTTTCAGTGTACATTGTGCGGCAGTAGCAGTTCTTTTTGATAAAAAACGTTACGCCCAAACGTAAAATGCGCG
CATTCGTGGTTCTTGCCTGTGTGGCCCTGGCCTACGGCCGCCCTGAGCCTCCTGTAGG
ATACAGCTACTCTGCTCCTTCTAACAAATATATTCCTTCTGAAAGTTACTCATCCTCTTCTAGTAGTGGTCAT
TCTAGCGGTCTTTCTTTGGGTGGAATCTCTGGCGGACACTCTTCTGGTAGTGGTGGTATCAGTGGTGGATTCG
GCGGAGGAATTAGTGGAGGATTTGGCGGCGGAGACGCTGGTTTCGGCTCCGGTGGTTA
CTCGGGCGCACCCATTGTACAGAAACACATCTACGTCCACGTGCCTCCTCCAGAACCCCAGGAACAGAGAATC
CCTCGCATCACTCCTGTTGCTGCTCCCCAGAAACATTACAAAATCATCTTCATCAAGGCCCCAACTCCACCCG
CACCAGTTGCTCCTATCATCCCTGTCCAACCTCAAAACGAAGAAAAGACCCTCGTATA
CGTGTTGGTTAAGAAGCCCGAAGAACAGCCTGATATTGTTATCCCCACTCCCGCACCCAC
>1002049546_1 Heli.1-DT664331.3.5
GCACGAGGCCAAAATGTACGGATATAAGGCAACCTATGTACTTGTATTGTGTATAGCGGCTGTACAAGAAGTC
GCTTTAACATCAAAGGAAAGTAGAACAGGCGGTCTGAATATACCAGAAAAAATTATAGGTGGAGTTATTGAGA
TTGTTCAAAGTCACAAAAACAAACCAGTTCCGGGAGCGCAGCCAGTTCAGCCTGCACC

TTCTTATCCTCCAAATCCTCAATATAATTACCAACAGTATCCGCAGTACCAACAATGGAATTCCCAATCAAGT
TTCCAAAATCAAGGATTTCAGGGTCCACAGTATCCAGTACAGTATCCAAGCCAGGGACAATATAACTACCAAA
ATAATTACAACCAAGCAGGTAATTATCCTCAAGGAAGCTATAGCAATCAAGTCTATCC
TACTGGAACAGGACAGACGTTTACCCAGGGACAAGCTCAACAAGGACAGTATCAAGGAACTTACCAGAACCAA
AATCAATTCAACCAGCCTAATAATTTCCAGAATCAGCAATCGAATTATTATACACAAAATCAGGGTAGTAACC
AATATCAAGGAAGTACCTCTAGCTTCCAAGGACAGACCAGTCAAGCCCAAGGACCAGG
ACAAGGTCAAGGACAAGCGATTGGTGGAAATGGACAATTTACTCAAGGGTTCCTGGTGTCAGGTCAGGGTAAT
GCAGGAGGACAGGGACCGACCTGTGTATGCCAAGCTTGGACCAAACCTCAGCCAAACCAAGAAATACTAGCTG
ATACTCCGGC
>1002049547_1 Heli.1-DT668290.3.5
GTGTTGCTAAAATTTAAAATTTCGAATTTTTATTTTTTTAACCTCGACCGCGAAATGAAGGAGCTCAATGGAA
CTACTGTATGTGAAAAATATATAGGGAGGCGTCGAGCAGTCCCCGTAGTATACGTACAAGGCTCTCACTATGA
AGTCGGCTTCGATGTGGGTCGAAATTTCGCGAGCGTCATCAAAAGCTTTTTATCCTCA
TACGCTAACCTTCGGGACTTCGAAAAGGAATACAAGACGGAAGTAGGCAGGAATGCATACGAAAAAACCCTGT
CCAATATGAAAGAAAGGTTTCCTTACTATGTAAAGGAGATGCAGGGTGTCGCTGACGGCGCCGAGGTCCCATT
CCACCAGCTTTTCCTTTTGCAAATGGATGATATAATTGGAACTATCAACGACAACCAT
ATTCCACGCAACGACACTGGAGGCTGCAGCTCTCTAGCTATTAAAACTCCGAATAGTGCGGTCTTAGGTCACA
CCGAAGATGCTTTTAGTGAGACGCTAAATCACTTCTACATTATGTCGGCACATATTATACCAACCGACGAAGA
CAAGGAACGTGGGGCAATTGAAGAACGTTTTGCTTCGCTGTGCTACGCTGGTCACCTA
CCCGGATACACTATGGGCTACAATGAAAACGGTCTGGTATTTTCCATCAACACTCTCAGCCCCCTGGTACTGA
AACCTGGAAAC
>1002049548_1 Heli.1-DT665869.3.5
TAAGCTGAATACGCGTTCAACCGACTATCAGTTGCGCGCGAGCTTCTTAGTGCTCTATCCGCACATTTGTTAC
CAAATATTATTTTTATCTACAAAAAATAAATACACTGACGAGATTACGCGATTATTTTAATCATAATTTACCT
ATAAATTAAAAATAATAATTTAAAATATATAGTTACTATGTATCGAGTAACGGTCAAG
TGACGTGAGAGATCATATAGTGAAATCTGTGCCAAATTGCCTCATAAGAAAAAATTGCGGTGAAGGGTCTATC
ATAATATTGCTTCAGAAGTGCTTGAGGCTGGATTTGGTTTCTTTTCACACTAGCATCTCGTTGAAAAATATAT
TTTATTTCGTAAAGACTATATTAAATATAACACGTTAATTTATTTTTGGTCCAAATCG
ATTTGCCATAAAAATGGCATTACAATTTGAACCCGATGGCACTCCATTTGTTATGTGGGCAGAACATAAGATT
AAATTGGAAATATCACCAATAACCGAGGATTTTTACGTCGAAAAGGCAAAAACAGAGTTAAGGGAGACACCGG
AAACTATAGAAAACAGTCTTAAAGAATTAAGGCAACTCCTTAAACGAGAGACAAATCT
TGTGATACCAATAGAAGACGACAATTTTCTACTAAAATTTCTCAGACCCTCGAAATTTTACCCGGAGAGTGCA
TTTAAAAAGATTCAAGCGTATTACAAATTTAGGTTGTCACACCTTGATTACTGCAGGGATCTATTTCCTAGTA
ACATTCACTCCGCATTCGACCATTCCATCATTTCCATACTTTCGCC
>1002049550_1 Heli.1-EL600235.1.5
TTTAGTGTTTTATTGAGCGGCCTGCCAAAAGAAAACTGAAACATGGCAAGAAATCTATCCGCTACCGAGGCGA
ATAAAGAACATGCCCTGGTTGTATCCAGGGATTTCATCTCGCAACCCCGTCTTACTTACAAGACAGTATCCGG
AGTAAATGGGCCACTGGTAATCTTAGATGAGGTGAAGTTCCCCAAGTTCTCTGAGATT
GTCCAGCTCAGACTTGCAGATGGCACCCTACGATCTGGTCAGGTACTGGAAGTCAGCGGCTCAAAAGCCGTTG
TCCAGGTGTTCGAGGGTACATCAGGTATTGACGCCAAAAACACTCTTTGCGAGTTCACCGGCGACATCTTGAG
GACTCCAGTGTCTGAAGACATGTTGGGTCGTGTATTCAACGGCTCCGGTAAGCCAATT
GACAAAGGTCCACCAATCTTGGCAGAGGACTTCTTGGACATCCAGGGTCAGCCCATCAATCCGTGGTCTCGTA
TCTACCCCGAGGAGATGATTCAAACTGGTATTTCCGCCATCGACGTGATGAACTCCATCGCCCGTGGTCAGAA
GATCCCCATCTTCTCGGCCGCCGGTCTGCCCCACAATGAGATTGCCGCTCAGATCTGT
AGACAAGCTGGTCTTGTTAAGGTCCCAGGCAAATCAGTATTAGACGACCATGAGGACAACTTCGCCATCGTAT
TCGCAGCCATG
>1002049551_1 Heli.1-EL602529.1.5
CACCGCGACTCGGCTGGGTGAGTCGACAGTTGTAGTGAATACGAATATACATTATTTATTAGTTGTAATATGT
TAGCAACTACTGTGTTTCTATTGGCACTGCAATATGCGGTTGCTGAAATTCCCCCGTATATTAAAATATGCCA
GCGTAATGATCCCAATGTTGACAAGTGCATCATAAACTCCATAGAAGAACTCCGACCA
AAGATGAAAAAGGGTATACCAGAACTCGACGTGCCTGGAATCGAACCTCTAAGCTTAGGTCAAATAGGACTCG
CGCGAGGTCCACAAGGTGCCAAGTTGACTGCAGTTGTGAACGATGTTAAAGTGCGAGGTCCCAGTGATTTTAT
TATAGAAGAGTTGAAATCAGACCTAGATAAAAACAGGTTTGACTTCAAACTATTGCTG

CCGAGGCTCGATTTCGCCGGCAAGTACAAAATGGACATCCAAGTGCTGCTATTGCGGTTGCAAGGACGGGGA
ATATTACTGGATCATTTAAGGATTACGCCTGCAACGTCACGATGAGAGGTCACAAAGAGAAGAGAGGAGACGA
GGAGTACCTCAACTTCGAACCAATGAAAGTAAAACTTCGTGTTGGTGAATCATCCATA
TACTTGACCAACCTCTTCGACGGC
>1002049552_1 Heli.1-EL602549.1.5
AGTTTACCCGCACAGTATCTTATTTCTTATATTCAATTTAAATATTCGAATACACCCTTTAAAATGGCCCGAA
GTGTTACTGCTTTGACTCTGGCCACCCTACTGGCGGTGTTCGAGATTGGTTCGTGCCTAAAATGCTACCAATG
CAACTCCCAATCTGATCCCAACTGTAAAGACCCCTTCGCCGCTAAGGATAAATTAATT
GACTGCATAAGTCAAGATTCGATTAATTACAACCGTAACTACTTGAGAGAGATCCTCCCTCGTGAGCTCGTAG
ACGGCGTTGCTGGAGCACCTCGCTACTGTCACAAGATTGTTATGCAAAACGGCGCCACTGTCCGTACTTGCTT
GGACGCCAACCCCTCTAACCTGAGTGAATCCTGTCGTCTTCTAGAAAACCTGAAGCAA
TCTCCAGCTGACCCCAGCAAGCAGATCAAATCTTGCGCAGTCTGCGACAAGGATGGCTGTAATGGAGCCGGAG
CCATTCAAGTTTCTCTACCACTAGCCGTTATGGCTGTACTTATCTCATACTTGTACAAACAATAATATATACA
AACAAAATATAAACAACGGTCTCTTTGGCCTTGTTGACCATGCTTCATACTTTTGCAG
TATATAACTGAAATAACTAAAACATTTTTCTATGTCTATAACATCCAATCGCTAAAAGCTATTAGGGCCAGTA
TTCTATGAGTAATTAAAATAAAA
>1002049553_1 Heli.1-EL598813.1.5
TTGTTTTAATCTTCATACAATTCTATTATGCCAGTACAGCGGCGTCCAATCGTAATATACGGGAAGGAAAATT
AATAGGTGGTCATGAGTCCAGACCTTACAGCCACCCTTACCTCGTATCTTTACAGATCAGGTTCCTCTGGATC
AGGACGCACGTGTGTGGTGGCAGTATATTAAACGATAAATGGGTTTTAACTGCTGCGC
ATTGTATTAAGGAATCATTTCTATTAAGATGGCTCCCCATGGACGCAGTTGCTGGAACGCACGATGTCAATTA
CTTTAGTCCAAAGGCCCAAATTATATCTGTGGCTGAGCGTATGGCACATCCTAAATATGCTGGAGGCGTAGGT
CCTTACGATATAGCACTTTTGCGAATGAGCAAACCATTTATGTTCACTGATGAAGTTC
TGCCAATACATCTGCCTCATAGCTACGAAGTTACCAATTATTCCCTTACCTTGGCTGGTTGGGGTGCTTTACG
TACAACTTACTTTATACCAGACGTTCCGAGCAAGCTGCAAGAAGTAAATGTCACTTACATCCCATACGAGAAA
TGTCACAATGCTCTCGAACAACTATTGGAAGATGGAGAAAGTAATCCATTGGATAAGA
ATTCGAATATATGCACCGGACCGCTCTCAGGCGGAGTAGCAGCGTGTAGCGGAGACTCAGGTGGACCACTCAT
GCAGTTAGTACCTTTGAGCGTCTACAATCGCGACAAAATAACTGATGAATACGAAGAATACGACGAGAATTAT
AACGAAGAGA
>1002049554_1 Heli.1-DT666681.3.5
TTTATTTTATATCTAAAATAAGGACACATTCAGTCATATTTCAATATCAGCTTCGACGTCTTCTTTTACAATA
TATTTTTTAGTTTGCACTAAGCTATTTAGAAGTGCAAGAAGGTGGTGGGATGTCTTAACGGTTGAAGCTAATT
TTTGTTTGGTGACGTCTAAACGACAAGATGGCGTGGCTTCCTATATGTCTTCTTGCGG
CCTCACTTCTTCTATCGCCGGCATCGGCTGCCGTCTGCCCTAATAGCTGCGTGTGCAGCACCACGCGGGATGG
TCTTCACCGCGTCACTTGCAGTAGTATCGCTGACATCTACAAATATACTATCCGACAGAAGCATCATAACATT
AACATTCTAGATCTTTCCCACAACAATATCACCAAAATCACTCACGAGCTAGATAAAT
TAACAGAAGTCGTCACACTTGATCTATCCACCAATGGACTTACAGAACTAAATAAATTTTTACTTAATGCAAA
AAAATTAGTTCACTTGAACTTAGCACATAATAGAATACAAAAACTCTCATTGACGCACTTGCCCACTAGCATA
AGTTCATTGGACCTAACGGGTAATCTACTGAAAGATGTGCCTTCAGAAATATCACATC
TGCCAAATTTAGAACACTTAGAACTAGATGGAAATCCTCTAGATTGTTCATGTGACAATATAATTGCTCGTGA
CCGGCTTTTACAGGCAAATGTATACATAGATAGTGCCAAATGTCATACTCCAAATTATTTAAAGGAACATTCA
TGGTTGGAAATAAAAACAAAAGATATCTGTAAAGGCTATAAAGCAGATTTTATGGATA
TAATGATGGGTGATCAACCAATGGATGCAGTGCAAGTTGGTGAATAAACTACTGCACTAAAATCTATGCCTTT
AGTAGCTAGTAGTGATTTAGACGACGGTAAAGTAATACATGGTGCGGACGCCGAGGTTGATGATGATAGTTCG
CAATTTATAAAAGTAGGTCACT
>1002049555_1 Heli.1-DT666775.3.5
GCAAAACTTTGGCCTATATGTTACCTGCGGCAGTTCACATTGTTCACCAGCAGAGGATTCAACGTGGTGATGG
TCCCATTGCTCTTGTTTTGGCTCCAACAAGAGAATTAGCTCAACAAATTCAATCTGTAGCTCAAGCATATAGT
GCTCATGGTTGCATCAGAAATACTTGTCTCTTTGGTGGATCCCCTAAAGGTCCACAAG
CTAGGGACTTAGAAAGAGGTGTAGAGATTGTTATTGCTACCCCTGGACGACTGATTGATTTCTTAGAACGTGG
TACCACCAATTTACGACGATGCACTTATTTAGTGTTGGATGAAGCCGATAGGATGTTAGATATGGGATTTGAA
CCACAAATAAGGAAAATAATTGAGCAAATCCGCCCCGATCGTCAAGTCCTTATGTGGT

CTGCTACTTGGCCCAAAGAAATTCAAGCACTTGCAGAAGATTTCTTAACTGATTATGTTAAAGTAAATATTGG
TTCTCTAAATCTTTCGGCTAACAATAACATAAAGCAAATCATTGAAGTCTGTGAAGAACATGAGAAGGAAGTT
AAACTAACTAATCTATTGAAGGAAATTGCTTCAGAGAAAGACAACAAAGTTATTGTAT
TTGTTGAAACCAAGAAAAAGGTGGACGACATAGCTCGTGCAGTGCGACGCAATGGACACAAAGCATTAGCCAT
CCATGGTGACAAGTCTCAACAGGAGCGTGATGCTGTTCTCACAGAGTTTCGTAATGGAGCAACTACCATACTG
ATTGCTACCGATGTGGCTGCTCGTGGCTTGGATGTTGAAGATGTTAAATTTGTTGTTA
ACTTTGACTATCCAAATACTTCAGAAGACTATATTCATAGAATTGGTCGCACTGGTCGCTGTCAACAGTCTGG
CACAGCCTATACCTACTTTACTAGTGGAGATGCAAGACAGGCTCGTTCACTCTTGGCTGTTCTGAGGGAGACT
GGACAGAACCCTCCAGCAAAACTGAATGATATGGCACGAAGCAACAACAACAACAACT
CTAGCCGCAATAGATGGCAACAAAGAAAAGAAAATAATAGCGGTGCAAGTTCCCCACGCCAAAAGAGTAATCA
GTGGAATAACAAAATGGCTAACATGACTAATGAAGAGAATCCTAACAATTATCAAAATAGAAACAACACACAG
CAACAAGCTCCTCGGTTCTCCAATCAGAACCAAAATGCAAACCAGGGATATAGACAAC
AAAATACTTACCAAAAGACAGTACCTTTCCCCAGTCCCAATGTATTTGAGTCTATGGGAAGCTACCAAGGGGG
ATACAATAATGGATATCAAAATGCTTACAGTAACCAAAATGGCTATGGTCAGAGGACCTACAACAGTGAGTAT
AATCGCCCAAATGGCGGTCAGCAATATCACCGTAACACCAACTCTGTGGGCAACAAAT
CTGCGGGATCCGGTTCGTCTTATGGCTCGCCGCCTCCTCATTTCGCTACTCCACCTCATTATCCACAGATGCA
TCCTCACCATCAGGATATGCTTGGAGGCAAGTACTACGGCGGTGGCGTCGGCGGAGGCGGTCCGTGCGGCTAT
CAAGCGGCCGTAGGCGGTGGAGTGCCCTATCCGCATCTGCCACCTGGCCCGTTGTTGT
ACGCGCCAGTCCAACAGTAAATTTATAACATTTTATTCCACATTATTGTGCAACTGTTCAGTCTTTTTGCACG
CTCAATATACTGACTATAATATATATTTAAAAGCACAGAATATGTCGGTATAATATTTAGATTAATATAATAT
TTTGGTTAACCGTATTGCTTAGCAGTGTTCATTTTGACAGACTGAA
>1002049556_1 Heli.1-DT666586.3.5
GTGAAATTTTGTTCAGGTGATAGTGCTAGTGGATTTGTGTAAAAAATGCAAATTCCCAATGAATATGTCTCAA
CTGCAGAAGATGTTGCTGATCAGGTGATAAGAAAAGGCAAGAATATACTACCAACAGTAGCGCGACTGTGCTT
AATATCAACATTTTTAGAAGATGGCCTCAGAATGTGGTTCCAATGGTCTGAACAAAGA
GATTATATGGATATGTCATGGAACTGTGGCAAATTTCTTGCAACTATGTTTGTGATAATCAACTTGGTGGGTC
AGCTGGGTGGCTGCGTTATGGTCCTCGGTAGGCTAAAAGTGGACATCGCGTGCGGAATATTGTTCTTCATCGT
TGTACTACAGACATTCGCCTACAGTATACTATGGGACATGCAATTCTTGCTCCGCAAT
CTGGCCCTCATCGGAGCCCTGTTACTAGTACTAGCGGAGGCCCGAGCGGAAGGGCGAAGTCTCTTCGCCGGAG
TACCGTCCTTAGGTGAAAACAAGCCTAAGACATACTTGCAATTGGCTGGCCGTATTCTATTGGCCTTTATGTT
CATCACGTTACTGAGATTTGAAGTATCTTTCTTACAGATCGTCCAAGACCTCCTCGGC
AGTATCCTCATGGTGCTCGTAACAGTGGGCTATCGTACCAAGCTATCAGCCCTCATGCTGGTACTTGTTCTG
>1002049557_1 Heli.1-ES586223.1.5
ATTTAATGTACCCCTTTGTAAGGGATGGTAAAGTATTACCGTTGTTTTGTTTTCCCATAAAGTGTGTTCTGAA
TTTGTATGATCCCTCTCGTTATACTATATTAGTATATTTTTCACACCCCTATACGGTATCCGTATTTGTGTAC
TTTGTTTTATACAAATATTTTATTATTTTCTTTGATATTTTTACTTAGTGTTGCTATT
ATGTGATTTAGTCCTAGTCCTGTCTTGTAAGTCCTTATAGTATATACTTATTTTCATGATGTTCTATATTAAT
ACTGATACTATTCATTATTTCTTATTCTTATTCTATTTTAATGTATAGTTGTTATGTATATATTGTAAATAAT
GTATATAAATATTTTTTATATCTAGATTTTCATTCAATATTTGCTGTATATTGTGCCT
CATCTAATATATTATGTATGAATCATTTTATATACTCGTATCAAGTGAAATAGTCATTTTATTTATATAATTT
CATATTTTGATAATCATTTTAATTTAATTTTTTTTTTAAATTAATTGTATTATGTTTACGTATTTTTTTGTTG
TATGTTTCATCACTACACTATATATTTATTTATCATCACACTTATAATATATGTATAT
ATGTATACAACATGTAGAATTATATTTCCTATTTATAGTCTGACACATGACTCGTACAAATAAAGAACATTA
>1002049558_1 Heli.1-DT667554.3.5
AATGTCTGTAACAGATACTCTACCCCCTGGATCCCCACGGTCAGCTGGAGGTTCCCCTCCTCAACCGCCACCA
CAGCGCTCTGCTAGCCGGTCTAGCCAACACTCGCCCAATTCATCAGGGTCGAACGGCGGGCGGCGGTCGTCGG
GTTCGCGGCACGTGTCGTCAACGACTTCGGTGTCGTCGACGGAGGCCGGAGAGGGTGC
AGAGGCCGCAGCCCCTGCGCCTGCGCCTCTTCTAAAGTGCACCCTCTGCCAGGAGCGCCTCGAAGACACACAC
TTCGTACAGTGCCCGAGCCAGCCGCACCACAAATTCTGCTTTCCATGCTCCCGTGACTCTATCAAAAGACAGC
AAGGATCTGAGGTATACTGTCCAAGCGGTGACAAATGTCCCTTGGCTAATTCGACGGT
ACCCTGGGCTTTCATGCAGGGCGAGATAGCCACGATTTTAGGGGAAGAATTCAAGCCGAAGAAGGAACGGGAG
ACCTAGGGCAATGTGCCAAAGACCCGACTTTATCTCTTGTCCCTCTCGATGTAAATGAGCACGAGAGGAGTTT
TGCCCGTAAGGGAGCTTTCGATAGCCTTGCGCGTCGAAACTGCGGCTCCGATAGGGAT

ACGCGGCCAGGCCGTGATAGACCGATCACTTGTATATAAATTGTAAATAAATCCATATAACTTTAGTTCGCTC
ATCAAGAGTGGCGATTTGGAATTGGTTCTCCGGATGGTCGGATATAGACCGTATTCAGCGTAATGGGTTCTTT
ACGATTCAGCCGATTGTAACGAAATCGCTGGTTTTTGTTTTAATGAGCTTTGCAATAT
TCTGAATATAGTACATATCTGTTTTTCGAAGATTTTATTATTGTCTTGATTCTTGACGATCCTTTAGAAACAC
GACATCTTTTGATCTGAAGTAACTCTCGATGTACGACGGCGATGTCGCGTATATACATGTTATATGGGATTTC
TTTGGATGCCATTTTCTTAGTTACCTTTAGGTTCCGAACCATAGTTAACTTGGCATTT
ATTTATATTGTGATTCTTATACACTATACGAGTATTATGCGATATATGATCATGACATTTATGTAGTCGAGTA
CTGACTGTAAAAAAGTAGTTTTTCTGATATCGATGGCGCACCGCGCCCTGTACAGATAATTAGCTCGGTGTAC
ATTTGTATTTATTACATACATTCGCATGTGAAAT
>1002049559_1 Heli.1-EL599236.1.5
GAGTGAGAAATTATTTCTTTTAATTCAATAAGAATTGTAATATCTATTTTATTAGACTGAATATCATTATTGT
TTATAATATTTGTATCTTTAATTTCTTCTTCTGTTATTTATTATAGAAAAAGATATATAAGATCAGAGTTAAA
TTTAAATCGATTTATTTATTTAGTTTTAATATTTGTTTTTCAATAATATTATTGATT
ATTAGACCAAATATAATTAGAATTTTATTAGGTTGAGATGGTCTAGGGTTAGTTTCTTATTGTTTAGTGATTT
ATTATCAAAATATAAAATCTTATAATGCTGGGATATTAACTGCTTTATCTAATCGGATTGGTGATGTAATAAT
TTTATTATTAATTTCATGAATAATAAATTATGGGAGTTGAAATTATATTTTTTATTTA
AATTTTATAAGAAATGATTTTTCAATAAAAATTATTGGGTTATTAGTTATTATTGCTGCTATAACTAAAAGAG
CTCAAATTCCTTTTAGATCTTGATTACCTGCAGCTATAGCAGCTCCTACTCCTGTATCTGCTTTAGTTCATTC
TTCTACATTAGTTACAGCTGGAGTTTATTTATTAATTCGATTTAATAATTTATTGATT
>1002049560_1 Heli.1-ES585936.1.5
CAAAAACATGTACGCCAAGTTGATCGTCGCCCTTTGCGCTTTAGGCGTTGCTCATGGCAGTGGCCTCTTAGCC
GCCGCCCCAGTGGCGTACAGCAGTCACAGCATCGCGGCTCCCGCAGTATCTTCTTTATCGTACTCGAGCCAGA
CCTCCCACGTAGCACCTCTGTCCTACACTGCCCCCGCAGTGGCCAGGATTGCCACCCC
AGCCTATGCCTCGTATGCCGCCCCAGTGGTAGCTAAGTCCATCTCTCCCGCCAGCGTGTCCTACTCATCTTAC
TCGAGCAGCACATCGCATGGTGCTCCAGTAGCTTACGAAGCCCGTGCTGTTGCTGCCCCCGCTATTGTAGCCG
CTGCCCCCGCCATAGCTGTATCTAAAACCATTACTCCTGCAGTCTCATCTTACTCTTC
CTATTCTTCACAAACTTCCCACGGCGTAAGAGCTATTGCCGCTCCTGTAGCAACCTACTCTGCTGCCCCAGCT
GTTACATCATACGCTGCCCCAGCGATCGCTTCTTATGCCAAATCATACGCCGCCCCAGCGATTGCCTCTTACA
GATCTTACGCCGCCCCCGCTTTAGCCTCTTATTCCAAGATCTACTCTGCCCCCGCCAT
CTCTTCGTACTCTAGACTGTACTCCGCCCCTGCGGTCGCCTCTTACGCCGCCCCCGCCTACTCTACCTACGCC
GCAGCTGCCCCCGTCCTTAAATCTGTATCCTATTC
>1002049561_1 Heli.1-ES586222.1.5
GAGAAGATCTCGGACGCGGACAAGCAGACCATCCTGGACAAGTGCAACGACACCATCAAGTGGCTGGACTCTA
ATCAGCTGGCCGACAAGGAGGAGTACGAGCACAAACAAAAGGAGCTGGAGGGTGTATGCAACCCTATCATCAC
CAAGATGTACCAGGGCGCTGGTGGCGCACCAGGGGGTATGCCCGGTGGCATGCCCGGC
TTCCCTGGCGGCGCACCAGGCGCGGGAGGTGCGGCGCCCGGAGCTGGCGGCGCCGGCCCCACCATCGAGGAGG
TCGACTAAACAAACATCCACCTGAACCAAACATTCCACGTCGTAACACGAGATCTACTCGTGGTCCTCTAATT
CTGAATTGCAAATAAAACAATT
>1002049562_1 Heli.1-DT665460.3.5
GCACGAGGATGGCCTTTAACTTTACCAGGATGTATTAAATTGTTTTATTAAATTTGTGAGGTGCAGAGACGAT
AGTTAAGATCAAAGATGCAGATCTTCGTAAAATCCCTCACGGGTCACATTGTCCTCGCAGTTGAACCCACTAA
CACCGTAGAGCATGTGAAGTCTAAAATTTACGACAGGGAAGGCATCTTTCATGAATAT
CAAAGATTAATATTTGAAGGCAAACAACTAAATAATGGAAGAACTCTATCTGAATATAACATTCAAAAGGAAT
CGACTCTACACCTTACTCTTTGCCTTCCTGGAGGGGGCAAAGGAGCCATGGTCATACCACCGGACCTAGTAGC
GCTAGCTCAGAAATTCAACGTACGTAAAATGATATGTCGCAAGTGCTACGCGCGCCTC
CACCCTCGCGCCACCAACTGCCGGAAGACGAAATGCGGTCACAACAACGACTTACGACCTAAGAAGGAACTAA
AGAATTAATAATAAATTAGAAATTACATTGTTTACAATATAATAAATAATGTTAATAGAAGATGTTGAGTGAT
GTTTGGTTAGAGGTTGTCGTATCGCAGCGTCTCAGTGTCTGATTGTGAACAATGGTTG
AGAGTTTGATTCTCGGGGGGATTAATTATTTATCCTGCTCTATTAATAAGACAAATCGCAATATTGATAATTG
AGCGGAAAAAGGGAATGTAGCGTTTTTAAGGTTCCGTACCTTTTGAG
>1002049563_1 Heli.1-ES586697.1.5
GCCAGGCGTCTGTTAATGTCAACATGTTCAAATTAGTAGCATTGTGTTGTTTTCTTGCCTCGGTCTCGGCTAC
ACCTGATGTTGTCGTACCTCTGGCTTATTCCTCGAATGTCCTAGCATCGGCACCAGCAGTCGTGTCCAGCTAC
CCCAGCGGCTTGGCATACTCATCTCCTGTGTTCTCTTCCGCGCCATTGGCCTACTCCG

GACCATGGGATTACCAACATCTCATTAAGAAACGTTCTTTGGCCGTAAGCAGCTACATCGCGCCGTCTTCTTA
CATCGCACCGTCTTCCTACTTCGCACCTGCTGCCTACACCTTCCGCGCCGCTGCTCCTTTGGTCAGCTCTTAC
TCTGCAGTTGCACCTCTGGCTAGCTACTCAGGAGCTGTTTACACCGGTGCTGCACATT
TGATCAAGAAGAGGAGCGCTTTACTTGTACCCAACACATACGTGGCATCTGCCGGATACGCTGCACCTGCACC
TTTCGTAGCGTCAACTTACGCGGCTGCTTCTCCCATTGCACCTGTTTGGTCTAACACATTATATCCTGCAGCT
CAGTATGCCCACTTCATCAAAAAACGATCTGCACCTCTGGCCGTAGCTACATACGCTG
CCCCATCTTCTATCTCTCATACATCAAGATTTGATTTCCAAGCTGTTTCTCCCGCCGTCAGCTACACTTCCTT
TACGGGCCCCTCACCAATTATCTACAGTGCACCAACTTATTCTCACCTGGTCTAAGTGATTATGACTTACT
>1002049565_1 Heli.1-CO729614.1.5
GTCGACCACGCGTCCGCTAAGTTGTCCGAGGCCAGCCAAGCTGCCGATGAATCGGAACGCGCAAGGAAGGTTC
TGGAGAACAGGTCGTTGGCCGATGAAGAACGTATGGACGCTCTGGAGAACCAGCTCAAGGAGGCCAGGTTCCT
CGCTGAGGAGGCTGACAAGAAATACGATGAGGTGGCTCGTAAGCTGGCCATGGTTGAA
GCTGACTTGGAGCGTGCGGAAGAACGCGCCGAAGCCGGCGAATCCAAAATCGTCGAGCTTGAAGAGGAACTCC
GCGTTGTCGGTAACAACTTGAAATCCCTGGAAGTCTCTGAGGAAAAGGCCAACCAACGCGAGGAGGAGTACAA
AAACCAAATCAAAACTCTCACGACCCGCCTAAAGGAGGCTGAAGCTCGCGCTGAATTC
GCTGAGCGCTCTGTCCAGAAACTCCAGAAGGAGGTCGACAGGCTTGAAGATGACCTGATCAACGCAAAGGAGC
AGTTCAATGACATCGGTGACGACATCGACGACACAGTCGTGGAGTTGATCCCAGGCGTGGAGATGACGGAGAA
ACAAGTTGAAAATGCGAAGAAAGCCCTCGAGAAAAAAATGAACGCTCCTCCTCCACCA
>1002049566_1 Heli.1-DT666929.3.5
GCACGAGGGTTGCGTCGAACTTACTGCTTTTTGTACATCATAATTCTGTACAAAAATGGCTGATAACAAACTA
TATGAAATATTAGGTGTTTCAAGAAGCTCAAGCGATTCGGAAATAAAAAGAAGTTATCACAAACTCGCAAAAG
AATTCCATCCGGACAAAAATCCAGCAGCTGGCGATAGATTTAAGGAAATAAGCTATGC
ATATGAGGTTTTATCTGATCCTAAAAAAAGACAAACTTATGATAAATATGGATTAAAAGGTTTACAAGAGGGT
GGGCAAGGTGGAGGATTTGCATCTGACGACTTGTTTGGTCATTTCTTTGGTGACATTTTCGGAATGGGAGGTG
GTGGTAGAGGCCGACGACACGCAAGAGGTGAAGATACTATACATCCTTTAAAAGTTTC
ATTAGAGGACATGTATGTTGGCAAAACAGCAAAGCTTCAATTAAGTAAAAATGTTATATGTGGTCCTTGCAAA
GGTATAGGTGGCAAGCCAGGTGCAGTGGTTTCCTGTAAAGATTGTCATGGTCAGGGTATAAAAGTTTCTTATC
AACAAATTGGACCAAACATGACTCGGCAATTTCAATCTCGCTGTCCAACCTGTCAAGG
ACAAGGTGAAACTATCAATGATAAAGAGAAATGCCCAAAATGCAAAGGAAAAAAAGTTCTCAATGAGATTAAG
ATATTGGAAGTTCATGTAGAAAAAGGAATGCGTGAAAACCAGAAAATTTTCTTTAGAGGTGAAGGAGATCAGC
AACCTGACACTCAACCTGGTGATGTCATTATAGT
>1002049568_1 Heli.1-DT665586.3.5
GCACGAGGGCCAAGGTGTGTCTCAGTTAATAATATTTCTTATACATTAAGGGCCAAGTTGATCGTCGCCCTTT
GTTCTTTAGGCGTTGCTCATGGCAGTGGCCTCTTAGCCGCCGCTCCAGTTGCGTACAGCAGTCACAGCATCGC
GGCTCCCGCAGTATCTTCTGTATCATACTCAAGCCAGACTTCCCACGTAGCACCTGTA
GCTGCCTATGCTGCCCCCGCAGTGGCCAGGATTGCCACTCCAGCCTATACTACGTACTCCGCACCCGCCATCA
CCAGTTATGCGGGCCCCGTGGTAGCTAAGTCCATCTCACCCGCCAGCGTGTCCTACTCATCTTACTCGAGCAG
CACATCTCATGGTGCCCCAGTAGCCTATGCGGCCCGTGCTGTTGCTGCCCCCGCCTTA
GCAGTATCTCGTAGCATCGTCGCCGCCCCCACCCTCGCTGTTTCTAAAACCATTGCTCCTGCAGTGTCATCTT
ACTCTTCCTATTCTTCACAAACTTCCCACGGTGTTAGAGCTATTGCCGCTCCTGTAGCAGCCTACTCTGCTGC
CCCAGCTATTGCGTCATACGCTGCCCCAGCGATCGCCTCTTATGGTAGATCCTACGTC
GCCCCAGCGATTGCCTCTTACAGATCATACGCCGCTCCCGCAGTAACCTCTTATGCCGCCCCTGCTGTCGCCT
CTTACTCTAAGGTCTACTCTGCCCCCGTCGTCTCCTCATACTCTAAGCTGTACTCCGCCCCCGCAGTTGCCTC
TTACGCTGCCCCCGCCTACTCTACCTACGCCGCAGCCGCCCCCGTCCTTAAATCCGTA
TCCTATTCGGCGGCACCTGCTGTCTCCACATCTCTTACAGCGCTCACGGTGCTAACTACGCTTGGTAACACAC
TATAAATTGGT
>1002049570_1 Heli.1-DT667359.3.5
GCACGGGGTGTTCTTCGCTCTCGCCGCTGTGGCTTGCGGCTCAATCATCCCCTTGGCGCAGCCCGCGCACCAT
CCGGCTCTCGTGCTGGACCCTCACGGCCGCCCCCTGGACACCGCTGAGGTCATCAACGCTCGTGCCATTCACC
TCCAAGCTAAAGCCCTGGAGCCCCTCGGCCACGCCGCCATCGCTCCCCTCGTGCACTC
CGCTGTTGTAGCCGCCCCCGTCGCTCATGCCGCCATCGTCGCCCCTGCTGTGGTCGCCGCCCCCGCCGCCGTG
TCCCACCAGTCCCGTGTTGACGTACGCACCAGCCCTGCCATCATCGGTCACAGCATCGCCGCTCCTCTGCTGA
GCCACTCCGCCCACGGACTCGCCGGAATCGGACTCGCCGGACACGGACTCGGACTCGC

CGGACACGGACTCGGACTTGCCGGACACGGACTCGCTGGACTCGGACACCTCCTCAAGAAACGCTCTCTCGGC
CACTGGGCATACAGCGCGCCTCTCGCCGTTGCCCCCGCCGCCGTCTCCCACCAGTCCCGTGTGGATGTAGTGT
CCAGCCCCGCCGTGGTATCTCACGCAGTAGCCCCCGTCATCTCTCACGCCGTGGCCGC
CCCAGTGCTCGCGCACGCCATCGCGCCCGCCGCAGTGTCCCACCAGTCCCGCGTTGACGTACGCAGCAGCCCC
GCTGTCATCGCCCACAGCGTAGTCGCTGCCGCTCCCCTCGTCCACGCTGCTCCCCTGGCTCACTCCGCTGCCC
TCCTCCACGCCGCTCCCTTGGCGCATGGTGCTGGTCATTTAGTCCATGGTTGGTGAAG
AGCCCCTCTGACGAACTGTTGATA
>1002049571_1 Heli.1-DT667458.3.5
GTTCTCTGTTAGAATTACTATAACTGCGTTAATTTCCGATAAAAGTTGTGTTTTGTGTGTTAAATTGATCCAG
CAATAACTTAAGAAAATGGCGCGTACCAAGCAGACTGCCCGTAAATCTACTGGAGGTAAAGCTCCTCGTAAAC
AATTGGCAACAAAGGCAGCACGTAAATCGGCCCCTAGCACGGGAGGTGTGAAGAAGCC
CCATCGTTATCGCCCTGGTACCGTGGCGCTCCGAGAAATTCGTCGTTATCAGAAATCTACTGAGTTGTTGATC
CGTAAGCTGCCCTTCCAGCGTCTCGTGAGAGAAATCGCTCAAGATTTCAAAACTGATCTTCGTTTCCAGTCTG
CCGCCATCGGCGCTCTGCAGGAAGCAAGTGAGGCTTACTTGGTAGGTCTGTTTGAAGA
CACAAACTTGTGCGCCATCCACGCTAAGCGTGTAACCATCATGCCTAAAGACATCCAGTTGGCCAGACGGATT
CGAGGAGAACGCGCTTAAATTAGCTTTAATATACCTAATTTTAAATTATTCATAATTCAAATAGACATTAATT
CTATGAAAATTTTTTATGTTGTCCCACATTTAATTTATAGTAATAACATTAAAAATCG
TTTAATGTAAGGAGGCAGCAGTACTGATTTTTTTTATAAATTTCTTATAACATAAGTTAGTATGTTGTGTGTA
CACGGAGCACTGCCACGTCTTGCAGAGATTCTCTCTTGGCCAGCGTCTTGTACAAACAGCGTAGTGTCTATAT
CTTAGTGACTTGTGGTATATCTAGGTAGTACTATAGGCAATATTGT
>1002049574_1 Heli.1-ES587505.1.5
CAGGAAGCTTTCCAGTTATTTGACTCCCGTGGAGATGGCAAAATTCATGTGGCACAAATAGGGGATGCCCTCC
GTGCTTTAGGACAAAACCCAACTGAATCTGATGTTAAGAAGTGCACCTTACACCTCAAGCCTGATGAGAGAAT
ATCATTTGAAGTGTTTCTGCCTATTTATCAGGCAATTTCTAAAGCCAGAAGTGGTGAT
ACTGCAAATGACTTTATAGAGGGTCTGCGCCATTTTGATAAGGATGGTAATGGTTTCATTTCTTCAGCTGAAC
TCCGCCACCTACTTTCCACTCTTGGAGAAAAACTGAGTGATGATGAGGTGGAGCAGTTGTTGCAGGGACAAGA
GGACTCTCAGGGGAATATCAACTATGAGAACTTTGTACACCTCATTATGCAGGGATAG
ACGCGCCCACCTGCAGTCCACTGTTATATAGTATTATGTCATTTTATATTTATTTCTTTTTTATATCTCTTCA
ATTGATAATGCATTTATTAAAATGACTGCGATATGATGGCTCATATATGACACCATTATAATTGTGATTTAAT
CAAAAAGTGAAGAATATTCCGAACTGTACTTTATATATTTGACTAACAATATTTTTAT
TATTTCCATTACTTTACTATAATTTAAGAGCATATTCCAAGATTTTTACATAATTTTAATATATTTTGTGATT
TAGTTAAGTTCAGTTGTACAAACACATGGTTAATAATTAATATTGTAATCCAAAATAAA
>1002049575_1 Heli.1-ES584986.1.5
ACAACAATGTTCTCCAAAATTGTAGCCCTCTGCGCTTTTGTGGCGGTGTCCTCAGCAGGACTCCTGCCTGCAG
AAATCCACTACTCTCCAGCATCTGCTGTTTCTTCCCAAAGCATCGTACGTCACGACCAACACCAAGTGGTTGC
TGCCAAAGTACTCGCCCCCGTAGCTAAATTGGCCGTTGCCGCACCTGTAGCCTACCAC
GCCGCCCCTGTCGCCTACCACGCACCTGCCCCCGTCGTGTACCACGCCGCCCCCGCCCCAGTCGCCTACCACG
CTGCCCCCGCTTCCATCGCGTACCATGCAGCCCCCGTCGCTAAAGTCATCGCTCAACCCGAGGAAATCGCCTA
CCCCAAATACGAGTTCAACTATTCCGTAGCTGACGGACACTCCGGAGACAACAAGCAA
CAACAAGAATCCCGCGACGGTGATGTCGTAAAGGGCTCCTACTCCTTCGTTGAGGCTGACGGCTCCGTCAGGT
CCGTGGAGTACTCCGCTGACGACCACAACGGTTTCAACGCCGTCGTACACAACAGCGCCCCCGCCCACGCCCC
AGCTGTCATCAAGGCCGCCCCCGTCGTCCTCAAGGCTCCCGTATACGCGGCCCCTGTA
CACCAATACTACCACTAAAGTATTTCAATCATTTAAAATAAATGATTGTTAAATATTTAT
>1002049576_1 Heli.1-EL601954.1.5
GCTACAGATAGCCGTGACCACCGAGATAGGGGCCAGTACACCAGTACACTCGGCAGATACAGCCACTCTACGA
GTCGGCTGAATCCGAACAGTGAAGCTGAAGAAGATTATACACGGAGTGCTCAAACACTACCGCGCAAGCTCCA
CGAGCGTAAAGAGAAGGCGGAGAAGCCTAGCTCCAATAAATACTGGCAACGGCTCACT
TCCAACAAACGTTCTACTAGTGCCATTAATATTCCAAACAACGTATCGTCAAACGCAGAAGGAAAAATAAACG
GGCACCCGCCGGGCCCAGCGAAGCCGGCGCGGACCTATAAAGGTCTAAATAGAAGCAAAAGTTTTGCTATGGG
AGCACCCGAACAAACACATCCAAGATACGTCTCAGGAATGAATAGAAACTATTCTACA
ATGTACAAATCAAACCCACACTTGAGTCGGCTCGACGAGACCCCTGAACAACTGAAAAGCCCAGGCATTGTGT
CGATTATAAATAGGAGCCAACGCGATCTAGCCGATGCGGTTTCCCGGGAAACTGAACGCAGACGTGACGGATT
ATTCGGCGCTCGATACGGCAAGACGATAACTAATGGCCACACGAACGGTTACAATAAA

TCCTTCGATGAAACTGATAAAAAGAAAATATTCCTCAAGGGCCTTCGTGAGAGAGCTCCCGAACTGTATCAAA
CTTTACACGACGACGAATCCGACGGTAGTAGATTATCTTCACGATATGGCACCCCGTCGCCTCATTATAAGGA
ACGGATATCGGAGGAGCGCAAAATACCATTGTAC
>1002049577_1 Heli.1-DT665565.3.5
CAGTTTTCTAATTCCTGTTATTTCCCTATAAAATATCATCTATTCATAAGATAGTTGCCATCATGGATACACA
ACTGACAGGAAATGAGATTAGAAAAGCATTTATAGACTTCTTCATCAGTAAAGGTCATAAGTATGTCCATTCC
TCCTCCACAATACCACTAGATGATCCAACACTGCTGTTTGCAAATGCAGGCATGAATC
AATTTAAGCCAATATTCTTGGGATCAGTAGACCCCAACTCAGACATGGCCCAATATATAAGAGTAGTTAATAC
CCAAAAATGTATAAGGGCAGGGGGGAAACACAATGACTTAGATGATGTTGGAAAAGATGTTTATCATCATACT
TTTTTTGAAATGATGGGAAATTGGTCTTTTGGTGATTATTTTAAAAAGGAAATATGTG
CATGGGCTTGGGAACTTCTTACTCAGGTTTATAAATTGTCTGGAGATAGATTGTATGTCACATATTTCGGAGG
TGATCAGTCTTCAGGGTTAGAGCCAGATTTAGAATGTAAAAATATCTGGCTGGACTTGGGAGTTTCAGAATCA
CACATTTTACCTGGTAGCATGAAAGATAATTTTTGGGAGATGGGTGAAACTGGGCCTT
GTGGACCATGTTCCGAATTACACTATGACAGAATTGGTGGACGAAATGCTGCACATCTTGTAAATATGGATGA
TCCCGATGTCCTTGAAATTTGGAACTTGGTGTTCATACAATTTAATAGAGAATCCGATGGATCATTAAAATTG
TTACCGAAAAAACATATTGACTGTGGTTTGGGCTTAGAAAGATTAGTGTCTGTCATTC
AAAATAAAAGAGCTAATTACGACACAGATTTCTTTATGCCTATCTTCCAGGCTATTGAAAATGGTACAAAAAT
AAGACCATACAGTGGAAAAGTTGGTTCAGAAGATAAAGATGGGATCGACATGGCATACCGCGTTCTAGCTGAT
CATGCCCGAACTTTAACCATAGCTTTGTCTGATGGTGGATACCCCGATAACACAGGGA
GAGGATATGTTCTTAGAAGAATTTTAAGAAGGGCTGTTCGTTTTGCTTCAGAAAAACTCAATGCAAAACCCGG
ATTTTTTGGGTCGTTAGTTCGTA
>1002049578_1 Heli.1-EL597915.1.5
GCACGAGGCGGTTGCATTATCACAGTCGGTGGTAGCAAAGTTTGTAAAAATGTGGAAATATCTTGGTTTACTT
GCCGTTTTGGCGGCGGTTCATTGTAACCCAGTGGATCGTGGTTTGGAGGAGAATTTAGTGGGTGCAGTGTCGG
AGTGCATTGATAAGGATACTTCACTATGTTTGAAGGAAAAAGCTTTAAAGTATACCGA
TAGATTGGCCTTCGCCAAAGATATCAGTATCTTTGAGGGCATGAGCCTTATCAATACTGGATCAGCCCGTTCA
TCTCGCAGTTATGAGCAATTGTCTGAGGACCCAAAGACCAGAGAATCTCAGATTGAAGAAAGGATAGCCAGCA
ATGTCGGAGACTTTTTAGACAATCATGTATTACAAATGCGCTTGTCTGAAGACTCAGA
TGAATCTAGGGCCTTAGATGATGAAGAAGGCCGTGGCAAAAAGAAGAAGAAGATCAAGAAGATCCTTCCCCTT
CTCCTTCTCTTGAAGCTCAAGCTTGCTGCTCTAATTCCTCTTTTCCTTGGAATCATCGCATTTGCAGCCATCA
AGGCGGTGTTCCTTGGCAAGATTGCCTTTGCCATTAGTGCTTTTGGCCTAATCAGGCG
ACTTCTATCTAAGAATAGTTCAGGATCCTCTGGAGCTACAATCAGTTATTCTCCCCATCACGCGGAGGAACAC
CCTGGATATTC
>1002049579_1 Heli.1-DT667242.3.5
GCCGTCGCAAACAAGATGACAAAAATTTAGAAATTTTACGTGAACTTATATCTTTGAATGGCAATAAATATTG
CTTAGATTGTAATCAAAGAGGTCCTACTTATGTTAATACTACGATAGGCTCTTTTGTGTGTTCAAAATGTTCG
GGAATGTTGCGTGGCCTTACACCTCCTCATCGTGTAAAATCTATCTCGATGGCTACAT
TTACGCCAGAAGAAATAGAATTCATTAAAGTAAGAGGAAACGATTATTGCAGACGCGTGTGGTTAGGTCTTTA
CGAGGGTGAAAGTGTAAACTTTACTGATGAACAAAGCATTAAAGACTTCATGTCTGATAAGTATGAAAAGAAA
CGATACTACTTGGAGTCATCTTCTAATAATGTGACTATTACAAATGGTAGTACATTCG
CGAAAAGTAAAGTTAAAGATACAAAATCTGGAAGTAGTAATGTGGGTACTACTCCATTAATATCAATTACACC
TCAGGTATCTAAATCAGTCAGCAACAATAATAATAATAATGTGATCAACATAGCTAAAATAGTAAGCAATGAA
ACTAACCATAAATTGCCAAGGCCTGCAAATAATTTTGTTTCACCACAATTGAATAAAA
TACCAACTATTTCAACTGTTTCGAGCCCAGTCCCAGTACCTGATTTTCCTGTTGATTTTACTACAGCAAATAT
TTTCAATAGTGCCCAGTATAATAATACTGTCACCA
>1002049580_1 Heli.1-ES586217.1.5
CTCGTTGCGCAGCACGTGTGTAATTTTTCTTTCGCTCGATATCTGTAATAAAAAATGGATAAACCTAACGTTT
TGGCTCGCGTAATGAAAGTACTGGGCCGCACAGGATCCCAGGGTCAATGTACCCAAGTGAAGGTGGAGTTCCT
CGGGGAAACCAGCCGCCAAATCATCAGAAACGTGAAGGGGCCAGTTCGTGATGGAGAC
ATCTTAACCCTTCTGGAATCTGAGCGTGAAGCTAGGAGATTAAGATAAAATGGGTCAGCTCAAATCTTCATTA
GGTTAAATTTAATTTTTATATGTAACCAATTACTATAATACTGAAACATCAAAACCGTCAAAGATACCATCAA
TGTAATCTGCTTAAATACATAA
>1002049581_1 Heli.1-DT666964.3.5

TATCCTGGCACTATGCGTATGTGCGCATGCGCAGTACGCGGAGGAGCGCGCGCCCCGGTACATCGCCTCGGAG
CCGAAAGTTACATCGACACCCGTGCCAATTCTTAAACAGATTAACAGGCACAACGAAGATGGCTCATATACCT
ATGGATATGAGGCAGCCGATGGGTCTTTCAAGATCGAGACAAAATCTCCTTCTGGAGA
AGTCAAAGGAAAATATGGATATAGAGATGATACTGGCAAGGTACGAGTTATCGAATATGGAGCAAATAAATAC
GGTTTCCAGCCAGCTGGAGAAGGCATCACTGTCGCTCCCCCACATTAGTTGACGAGTCCACACGAGAACCTA
ACAAGCAGGGTGGCCGATCCCAATACCGTGAACAATCAGTCGACTACGACTATGAAGA
GCCTGCACCAGCCCCGCGCCCTCGTCCTACTCCACAGCCCGCCTACCGTGCTCCCCAGCCCCAACAACAATAC
AGACCTGCCCCACAGCCCCAGCAACAATATAGACCTGCCCCACAACCCCAACAGCAGTATAGACCCGCACCTC
AGCCTCAGCAGCAATATAGGCCCGCCCCTCAACAAGCCCCAGCTCCACCTAAGCCTGC
GTTTTTCGCGGGTGCCTCACCAGCGCCAGTAGAAGACAATTTCTTCAACCCAGAACCAGCCCAGCCCCGTCAA
CAGCAGTATAGGCCGAAACAGGACTTCAGGCCTGCCCCACAGCCCCAGCACTTCAGCCCTAAACAGACCCAAG
TCGATTTTAACCAGGACTATAATGCCCCAGCTCCCCAAAGGTTTCCACAAGCGAATCA
AAGGAGTCAATCATTCTCAATGCT
>1002049582_1 Heli.1-ES587363.1.5
CAGTCCTGCCTTGTCTACTAACGAGTGCGAAGCGACACCGTGAACCGTGTTTACACTAAAACGTTTCCCCGTC
ACTCAGTGTTTGTTTAGTTTCGACAGTTTCCACACGACGCACACGTGAAAATGGCTGATAGTGGTCTGAAGAG
AAATATTCCCATCAAACTCGGAGATTTTTCAGTTATCGACACCGAATTCTCAAGCATT
AGGGAGAGATTTGACGCCGAAATGAGAAAAATGGAGGAAGAAATGAGCAAATTCCGATCGGAGCTCATGAACA
GAGAGAGCAATAACTTTTTCAAGAGCTCTACAAGCACCACCACATCGTCACAGCACAGTGACAGCAGACAGTT
GGCTGAGCCCAGCCACTGGGACAGTTTAAACTCTCCTTTAATTCAGGATGAAGGAGAT
GGCAAGTCTCTTAAGCTCAGATTCGACGTCAGTCAATACACGCCCGAGGAAATCGTCGTGAAGACAGTTGATA
ACAAATTATTGGTTCACGCCAAACACGAAGAGAAGTCAGAGACGAAGTCGGTATACAGAGAGTACAACCGCGA
ATTCCTTTTGCCCAAAGGAACCAATCCTGAGGCCATCAAATCGTCGTTGTCCCGGGAC
GGCGTCCTGACTGTGGAAGCGCCGTTGCCGCAGCTCGCCATCACCGACAGGAACATCCCCATTCAGAAACACT
GAGGCGGTTGTCGGTTCCTTTCTCGACAGCAGCTATTCCATACTACTGTTAATAATAATTTATTATAATTTAC
TATAAGGAGTATTGGATATATTCAGATATTTATTTCGAATCTTAATTACTTATATGGA
ATCTCATCGTTTTTAACATGTGTATGTTATTGTTAGAGTTTTGAATTATTTTTTAATTATTCCAATTTATCCT
GCTGTTGGCAGCAGAGCACAGTTGCGGTTAGCACT
>1002049583_1 Heli.1-DT665208.3.5
GACGGAGGCGTGTTCGTGATAATTTCCGGTCAAGTGGGTTTCATTTAATAGTCTCTTAACCTTAATCAAAACA
TTGTATATTTATTACATTATTCATTGCGTTATACGTTCTCCATTAGGAAAGTACCCGTGAACAAGTTGTCGTT
TTCTTAAATAAAAATTAAATATTTGGAAGACATCGAAATGGCTCTATACAGTTTAAAG
AACAATCTTCAGCTGGCTACATTAAAAACTTTGTCCCTGGTCTCCATAAGCGTCATGTCACCACAGCAAAGTG
TGCAAGAGTTATTCCGGACTGCGGACTGCGTATGTTTCGATGTAGACTCTACTGTGATCCAGGATGAGGGTAT
CGATGAGCTGGCCAGATTCTGTGGAAAGGGAGATGAAGTGAAACGACTAACTGCTGAA
GCCATGGGCGGCTCTATGACCTTCCAGGAAGCCCTTAAGAAAAGATTGGACATCATCAGGCCCAGTGTAAACC
AAGTTAGGGAATTCTTAGAAAAGTTCCCCATACATCTTACTCCTGGAATAGCAGAATTAGTGAAGACCTTACA
TGACAGAGGTGTGATCGTGTATCTAGTGTCAGGTGGCTTCAGAAGTCTCATAGAACCA
GTTGCTGTCAAACTAGGCATCCCTATTAGCAATATCTACGCTAACAGACTTAAATTCTTTTTTAATGGAGAAT
ACGCTGGTTTTGATGAAAATGAACCCACATCGAGGTCTGGAGGCAAGAGTTTGGTAGTCAGGCGGCTAAAGGA
ACAGTTTGGTTACCAAAGGGTAGTAATCATTGGAGATGGCGCCACCGACGCTGAGGCG
AGTCCACCAGCTGATGCCTTCATAGGTTTCGGTGGTAACGTTGTTAGAGAAGAAGTCAAAAGGAAGGCGGCGT
GGTACGTAACAGACTTCCAAGAG
>1002049584_1 Heli.1-DT665463.3.5
TATTAGAATAATTTTGAGTATTAGACTATTATTTTAGAACTAAAACTCGCAGAGATGGACGGAATATGTAGTT
ACAAGACATTTATAGCAGCGCATAAGCCGCAACTTTTACTTTCGGGTGTACCAGAGTATTTTTGGCCTACTTT
ATGTAAAAAATTGAAAGATCAAATATTTGATTCTGGTGCTGCATTTCAATTAGTAAAA
ATTGATTATGATGAAGAAGAAAGGAGACCATATGATCCATTATGGAGTGTTATGGCTATAACTGATATAGACA
GAACTGATTCTAGTCATATTTATCTTATAGATCACGCTTGGACTTTCAAAGCTAACAATATAAAAAATAACTT
AAGAAATGTACCAGTGCTTTTAGAGAGAATGTGTAATCTTATGCAGATCACTTCTGAA
AATATTGAAGAACAAATTAACGATGTATCTAAGTGTGTATGGAAATATGCAAACACATACGCAGTTGAAAGTG
AAGAATTATCAGTAGAAGACAGAGTGCCAGTGTGGTATGTTATGGATGAGTTAGGTTCAGGAATTACACATTC
TGATGATCCAAATTTTCGGATTGTTCCATTCATATATATCCCAGAACAAATAACATTC

ACATTGTTGTTTCCAGTTGAAAATGTTGAGGAAGGTGACATAATTACAAGAAATTTTATTGAAGGAAGTTACT
CAAACTCAAAACAAAGAGAAACTATGCTTATTCCATGGCAACATTAT
>1002049585_1 Heli.1-DT666544.2.5
GCACGAGGGTCACACCTACAACCTTACAAAATGGTTGCGAAGTTCGTTGTCCTTTTCGCCTTGGTGGCTGCCG
TCAGCGCCGATTTCTCCAGCTTCTCGTATGGAGTAGCTGACCCTTACACCGGTGACTTCAAAAGCCAAGTCGA
GAGCAGAGCTGGACAAAATGTATTGGGACAATACTCTTTATTGGAATCTGATGGCACC
CGCCGTACCGTAGACTACTCTGCAGGAGCCGAAGGCTTCAACGCTGTTGTAAAGAAAGACCCCGCTCTCCTTG
CCCCCTTAGCTCCCGCTTACTCTGCCTACCCCTATGGTGCCTACCCCTATTCCGCCTACGGATATGCCAGGTA
CGGAGCCCTTCCCTACGGATACCCCTACGGACGTTACCCATTCGTCTACTAAATAACA
ACAATCGGCTTTTACCTGGAATTACAAGTACTTCTGTCACCTTTGTACTACGATATTAGGTTATGTAAATAGT
GTACATAATAAATTTTCTATGATTTAAAAAAAAAA
>1002049586_1 Heli.1-DT663246.3.5
GCACGAGGCGGCGGGGAACTTTCGCTCCTGCTCCGTACAGATACGCTTTAGCTGAACTTTTACGAAAATCGCG
TTCGGAAATTTAAGAACCGGCACCCATTTCCGATTCGTGACAGATTACGATAACCGTTGAAAGTGAAACCGAA
CGATTGGGACGTGATAAGAGTTGTGTAAGGATTTACTTTGGCAGGTAAATTGAGTGAC
TAAGTGGGACGATCTAGTTGAAAAGTGAAATTACACCGGACTGGTTTCGATATCGGCACAGTTTTGCAATAAA
AACTGCGTTTTGAATATTGACACTCGGTGAAGCTGTTTTATTTCCTTATATGGATAATTCACAATCTTGTTAA
TGACAAGACGTGGATTGCTTTTTAAATCTTATACATATGTCGACAGTGGTGTTTTCTT
AAGTGTTGTGAGTTATATCGAATCTTGGACTTTTGTGCAATGAAATGTGAATTTTGTGTTGATGCAGTTTAAA
TCGTTTGTCGTGTGATAACTTTGCCCTGATGATTTATCAAGTGTAACTTTTTTTACTGTTTTCTTAATTGGTT
TACTGATACTCTTTCGCTATGGAGACCGAGGAACTCACTAAACTCGTGGACGGTATAT
ACAAGAACATCTTGGACAAGTTCAACCCCGGCGCCCGGCAGATGATAACAGCTGGCAAAGCCTATTTGAAAGC
GTTACATGGTGCGGCGGCAGCGTCCAGGCTGTATGTGGATGCTGTGGGCAAGCTCGGCCGACAGGCGCAGCAA
GGTACTTGGGGAGGATGCGCCGATATAGGCACTGCCCTTATGAAGGTCGTGGAAGTGT
ACCGGGAAATCCAAGATCAACAAATGAATATCCTGAAAGCGTTCTACGTAGATTTGCTCGTCCCCCTGGAAAC
TAATCTGGAAAAAGATACAAAAGTTGTACAGTCTGAACAGAAGAGATTTTTGCAACAACATAAGCTGCGTTCA
GAAAGCTACAGCAAAGCGGCCGCTACTATTAAGAAACAGAGAAAAAAGAAAACTAATG
TAACAAAAGTTG
>1002049587_1 Heli.1-DT663074.3.5
CACTAAAACTTTCAGTTGTTGATTGCGTGAAATATTTTGTTTTGTGTAGTGATTTATTTTGAAAAAAGAATAT
TGAATATAATTTACCATGGATCCCCTAGCAGTGGACGTCGTACATTTCTACCCACGGGAGAACGCGCAGCCTC
ATAACACTGTAAAATTTGAAGCTGTGAACGATGAAAACCCAACCACTGCGATAGTACG
TCGTGGTCAACCCTTCAACGGCGTCGTACGGTTCACGAGACCGTTCGATGAGAACGAGGACATTGTACAACTC
GTGTTTACACTTGGTGATAAGCCACAGATGGACACGCAAGGTTCCATATTTTTAAGGAGAGACGCCATCCCTG
AGAAGCACTCTTGGTACGCTAAGATCGTTGATATACAAGAGGATACAGTATCTTTTGA
GGTCTCAACACCAGTCAGCCTACCAGTAGGTTGCTGGGCTCTCCGCGTCGTCACCAGACTGAAGAGTTCTCAA
GCGCGAGAAGTTTATGATTACGATCAAGATCTGTACATTTTGTTTAATCCATGGAATCCAGATGACCAAACCT
ATTGGGCAGACTTCGATCTTTTGCAAGAATACGTGATGAACGACGTCGGCAAAGTATG
GGTCGGTCCTATAAACACAACAAGAGGCAAACCCTGGTTCTATGGACAGTTCGACGCGGTTGTTCTACCAGCG
GTCATGTTCAT
>1002049589_1 Heli.1-EL602533.1.5
GCACGAGGGACAACCAAGAATTTGCCAAATAAGGAACTTTACTAGATGAAGACAACAAGATGATAGTAAAAGA
CGAAAGCTACTTCATATAATTAAATTAATATTATAAAGCTTAAAATGAAAAACAAAAGGCTGATAAAATAAAG
ACCAAAAAAATTGAGATCCAAACAATAAAACCCGAAAATGAAGCTTTAATACTAATAA
AACCCAACAAAGATGAAATAAAAACAAAGATAAAAATAAAAACGAAGACTAAAGATACAAGGAATTCAAAACT
TTAATTGATTCCAATGAATATTGATGCCGACGAAAGCCGAAAAATAAGGAGACCGGCGTAGAACAAGATCGAA
GCATACGTCGATCCAAGCAAATGAAGGCCTAAATTAATGAAGGCCGAAACAGAAGAAT
ATCGAAGTAAATGAAGACACTCAAGCAGAAAAGACAGAAGCAGTTGAAACCTTTAATTGATGACGACAAGGAA
GATTGACGACAGAAAGTAGACGAAGACCGAAGCAGATGAAGACCGAAAAAGTTGAATATCGAAACAGATGAAG
ACGAAGCAGATGAAACCCGAATCAGAACAAAATAAAGCTAGAAAAAGATGAGCAGAAT
AGACCTAAGCGTTTAATATTCTAAGCAGATAAAGACTAAACCAGATGAAGACATTAGTAGACGGCCAGCAGA
>1002049590_1 Heli.1-DT664002.3.5

TAAATATAATTTAAATAAAAATTATTGAAAATGATAAATAATTTATTTTCAATTTTTGATCCATCAACTAACT
TATTTAATTTTCCATTTAATTGAATTAGAACATTTATTGGTTTAATATTCATCCCTTTATCTTTTTGATTTTT
CCCTAATCGTCATTTCCTATTATGAAATTTTATCGCAAATAAACTTCACAATGAATTT
AAAACTTTATTAGGACCCAATAGAATAAATGGATCAACTTTTATTTTTATTTCATTATTTTTTTTTATTTTAT
TTAATAATTTTTTAGGATTATTCCCATATATTTTTACTAGAACTAGACACTTAAATATATCTCTTTCCTTATC
ATTGACATTATGATTAAGATTTATAATTTATGGCTGATTAAATAATACCCAACACATA
TTTATTCACATAATCCCCCAAGGAACACCAACAATTTTAATGCCATTTATAGTATTAATTGAAACTATTAGTA
ATATTATTCGACCTGGAACTTTAGCAGTACGATTAACCGCTAATATAATTGCAGGTCATTTACTTCTAACCCT
ATTAAGTAGCACAGGTATTAATATACCTAACTATTTAGTGATTATT
>1002049591_1 Heli.1-ES588021.1.5
GTAATTTGGTCGTCATCGGTGTCCGTCGCCGTCTGCGCGTTGCGTTTACACTATCAGACATCAGTGACATTTG
GTGTTGTCGTGCGTCATCACGCAGCAGCGTTTCGTTAGAACAAAGAGTAGAGACGTTCAATCGTATGTCCATC
CAGAATCTCAACACATTCGACCCATTCGCCGATGCTATCAAAAGCTCGGAAGACGACG
TACAAGATGGACTAGTCCACGTCCGCATACAGCAGCGGAACGGGCGCAAGACGCTGACCACGGTACAGGGGCT
CTCGTCGGAGTATGACCTGAAAAAGATCGTGCGGGCATGCAAGAAGGAGTTCGCGTGCAACGGTACCGTCGTC
GAGCACCCCGAGTACGGCGAGGTGCTGCAGCTGCAAGGCGACCAGCGCGAGAACATTT
GCCAGTGGCTCACCAAATCGGGGCTGGTCAAGCCCGAGCAGCTCAAGGTGCACGGCTTCTAGGCCGTATAACC
ATGTCCGCTGTGACATCATCATAGTTGATATAATATTATGAGAGCGTGCCCGTTATAAACCTATTGAATATGT
ATTTAGAAAGTACCGACGTTTACAGAGAGTATAGGAGAGAGGCTTCGCCTCCCGCGCC
CTGTGCCTTGCTGTCACCCTATAATGTTAATATAAATGTATTTTTGAATATCGACGCCGTCTCCCACATCTCG
GACAGCTTCTGTTGCAATTAACGCCGAACGAAATAGTCAATAATCATTTTTTGTTTTTTTACGACATTATTAT
AAATTGCTGTAATAATTATAATATGATGTGTCTCAATTATTAAACA
>1002049592_1 Heli.1-EL602760.1.5
GCCGTCTGTGTCGTTAACAGTTTTGTGTTTCTCTTGTATATAGTGGTTTAGTTTCGACAACGACATCTAAGTT
TATATAAGCATACGAAATGAAATTCACGGTGATTTTCGTAACTCTGTTGAGTATATCTACATCATGGGCTATA
AAGGCAAAAAAAGAAGAGCTAAAAGATAAAAGGGAAGCTGCTTCTGGAAGTTATTTAC
CTCCACATGGCTCATCAAGTCATAGTTATCAAGGACCTGCCTTAAGTCAAGAAGATGCTAACGCTATAAGTAT
CGGAGCCGGATACAGCATTGGAGGCGCTAAACCTAGTTACAACTTCCTAACTCAAGGTTCAGGTCAACCATCG
TACCAAGTTCAATCAGAGGGTCTTCCAGCCAGTGGTCACGGAACCATTCAACTTGCAC
CTATCACTTTACAACCGAGCCAACCAGGTCTTATTTCTAATGACCTCTCTCAACTCATGAGTCAATTATCTCA
TGGAATTAATTCTGGGGCTATTGCCTTACCTTCATCTGGCCAAGGTGCCATATATCAATTCGCTGGGCAAAGC
GGTCACAGTGGCCAAGACTTGAGTGCACCTCAGTTTAGTTATGGAAGTCCTAAATTAC
AACAGTATAGTTTTGGTGAACAGTCAAGTCAAGCCGGCCAAATAGTCCCCGCTTATGCATTTGGAGCTAAAGG
CCTCAGTAGCTATGGATCTACAGGACCTGTCCTAT
>1002049593_1 Heli.1-DT662253.3.5
AACGTAAAAGATCTGATATTTACCATTTATATATACAGTTTAATTATAAGATTGTATTGTTTGTTTTAAAATG
CTGAGTTCACGCTTATTAGGCGTTATTTTATTAGTATCGGGTACGTATGCCCTTTATGACTCGTCGTCTGATG
TAGTTCAATTGACACCAAGTAATTTTGATAGATTAGTTTCAAATTCCGACGAAGTATG
GATCGTAGAGTTCTTTGCCCCTTGGTGCGGGCATTGTAAGAACCTTGTTCCAGAATATAAGAAAGCCGCCCGC
GCTCTTAAGGGCATTGCTAAAGTAGGAGCAGTAGATGCTGACCAGTATAAAGAACTTGGTCAGAAATATGGAG
TGTCTGGTTTCCCTACCATTAAAATATTCATTGGCAGCAAACACTCTCCTTATCAAGG
CCAAAGAACAGCCGAGTCATTTGTTGATGCCGCACTTAAAGCAGCCAAAGAAAAGGCATATGAGAATCTTGGA
AAGAAATCATCTGGATCTTCTGGAAAGTCGGACGTAATCGAGCTAACGGACAGCAACTTCAAGGAATTAGTTC
TGGACAGCGAGGACCTGTGGTTGGTGGAGTTCTTCGCGCCGTGGTGCGGTCACTGCAA
GAACCTGGAGCCACACTGGGCCAAGGCCGCCACTGAGCTTAAGGGCAAATTAAAACTCGGTGCCGTAGACGCG
ACAGTGCACTCTGTGATGGCCTC
>1002049594_1 Heli.1-DT664486.3.5
TCGCGCCTACAAAATTAGAAGATTCTATGAAAGTGGTAGGACCACTTGGTAGGCGCGGTGCCGACACGACGCT
AAGACGCAGCTGGAGTGACCCTTCAGCTCGAATTCCTGAAAGAAAAATAAACAATACTGATACAGTGTCAGCG
CCAAATCTTTACTCTTTATTTCCACCACCAAGAAAAAGCAGTTCTCCAGAGCCAAGAT
CTCCTCTGGCTTTGGAAATATCCTCGGAAATTGTTACTTCTATAAATGGCCATCGTGATTTGGAAATCAAATT
ATTTTCGACCTCGCCCCCGGCCTCGACCTCGGAACGTCGCTCTTTCTTCGACGATAAAAGTGAAAGTGCCGTT
ACCGATAGATGTGCGAACACAAACACTAAGCTAAAATTAAATCGTGCAGTGAGCAGTG

CAAAAGTTGTGTCGAGTGCAAACAATGATCGTAAATGTGAAAATTTGGATTCGGGGTGCATAAAGGCACTTGT
GGCTGGCGAGAATACGCGCAACATTTGTGATTTAGCCTTAGTTTCTGCCTGAGGTCTTCGCCATGGCCATGTT
TTATACTAATTATATTTGCAAGCGTACGATTACCAGTCCAAGAGCTAGGCTCGCCATT
TGAGCAGGACTCAAATTATTATAGCGAGGCATAAGCCGGAGCGTCTCAACGCGACCCCAATGTAAATGAGATC
TCCGAATCGGTTTCCTTTAGATTCGATAGTTAATTTATTTTTATTAACCATAGCTTAGTAAACTTCAATGGAA
CTTTGTCTGAACGATGTACGTTTTGTGATTCGCCGTTATGACGCTTAAAAAGAAGTTA
AATCATGTCATTCCTTGACGAATGATCTGATATATT
>1002049595_1 Heli.1-DT667031.3.5
GTGTTGTCGTTCTCGACAGAGGCAACAATACAACTTGTACCGTAAATCTTTTCGGTGCTACAGTGGTATCATG
GCGGGTTAATAATCAAGAACAGTTATTTGTAAGTAAGCAAGCTGTGTTCGATGGGAAGAGAGCAATTCGGGGA
GGAATACCGTTTGTATTTCCTCAATTCGGACAATGGGCGTTCGGTCCCCAGCACGGGT
TCGCGCGGGTGGCTCGCTGGCACGTCGAGAAGATGCCGGAGCGACTACCCAGTGGAGACGTGGAAGCTGTCTT
CAGCCTCATGGACGATGACTTTACGCGGTCCATGTGGCATTTCCAATTCAGACTAACATACCGCCTTATTCTA
CGGGAGAAGGAATTGCACTTCAACATAGGAGTGTACAATCCTAGTAAAGAGCTGACGT
TCAGTTGCCAGCTCCTCCTCCATACGTACTTCAAGGTTCCCGATGTGCGACGCTGCCAGATCACAGGCATGCA
TGGCTGCATGTTTATTGATAAGACGCGAGAGGGAGCCGTGTATCAAGAGACGCGCGAGGTGGTCACTATCAAC
GAGTGGACGGATCGCATCTACCAGAACACCATGCAGGAGCACATCATCACCAACGTGG
TCAGCGGACGCAAGATGAGAATACAGAAGTATAATTTCCCTGATACAGTGATCTGGAACCCGTGGTCGGAGTT
CGCGAAGGAGA
>1002049596_1 Heli.1-EL596747.1.5
AACTTGCGGTATATTTAAGTGAAATAAAAATGAAGATTTCCATAATATTGGGAATAATAGCATGCTTTTCAAT
AGCTTATGCTGATCAAGAAACTCTAGTTCTAGTGGACAATTTGAACATTAGAGAGACTCATTCACAGTTTTTT
AAATCTCTTCAAGATCGGGGCTACAATCTTGTCTTTAAGTTAGCTGACGATGCTAACC
TAGTGCTATCTAAGTATGGAGAATATTTGTATAAAAATCTCATTGTCTTTTCACCATCCGTCGTCGAGTTTGG
TGGTCAAATAGACACGGAAGCCATCACAAAGTTTATTGATGATGGTGGAAATGTTCTCATGGCTGGTAACTCT
GCGGCAGGCGATGTCTACAGGGAGATCGCTTCTGAATGTGGATTTGAGATGGATGAAG
AATCTGCAGCTGTAATCGACCATTTCAATTATGACAGTCTGGACGATGGAGACCACACTAGGATAGTAGTGTC
TCCCAAAAATTTAATCAATGCTCCTACAATTGTTGGTTCTCAAAACACACAGCCATTACTCTTTGAAGGTACA
GGTCTAATATTAGATAAGGATAACAGCCTGGTCCTGCCTATACTGACAGCAGATAGTA
CAGCTTACAGCTATAATCCTAAGAGTCAGGTTAAAGAGTACCCTCATGCAGTCGGTCGTAAAACTGTCCTCAT
AGCAGCACTTCAAGCTAGAAACAATGCTAGGATCATATTCAGTGGAT
>1002049597_1 Heli.1-EL600004.1.5
AACGTTTCGGTCAACATTTTAACGTTTTTAACATTTTGCTCAATATTTTAGACCTTGAACACTTATCAAACGT
TTAAAAATTTGTTTGTAAAAGGTAGTTTTAACATAAACAGTTAAAGATGGCAGTCAATGTGTATTCTACAAAT
GTGACGTCGGAAAATCTATCAAGACATGATATGCTGGCGTGGGTGAACGACTGTCTTC
AGTCGAATTTTGCTAAAATCGAAGAGCTTTGTACTGGTGCAGCATATTGCCAATTTATGGATATGTTATTTCC
TGGTAGTGTTCCTATGAAACGAATCAAATTTAAGACAAATCTAGAACATGAATATATTCAAAATTTCAAAATA
CTACAAGCAGGTTTCAAAAAAAATGTCTGTAGACAAAATAGTACCCATTGACAGGCTGG
TGAAGGGTCGGTTCCAAGATAATTTTGAGTTCTTGCAATGGTTCAAGAAGTTTTTCGATGCCAACTACGGAGG
CACGGAGTACGACGCGATGGCGCAGCGTGAGGGGCTCCCCATGGGGCATGGAGCGGCCGGGGCGCCGTCCCGG
GTATCCGCTCCCGTAGCGGTTAAGAAGCCGGCAGCACCAGTTGCCAAAGTCGCCGCTA
GACCCCAAACCATTGTAAAAGCAAATCCGACAGTCAGGACTCCACCAA
>1002049598_1 Heli.1-EL598128.1.5
AGGGTCGTCCCGCCAGCCGCGTCAATAAGTAAATCCGTGTGTGACATCAATTTGTGAATTGTGTGTGAGAGGT
CTATCAAAAATGGGGTGTGGAACGAGTTTTGTGAAATATGTACTGTTTTTCTTCAATCTGATTGTCGCGCTTT
TTGGCCTAGCCGTCATTGGCATAGGTGTGGCCGTGCTCCTGAACTGGGCGGTGATCAA
GGATGAGCTCCAAGGTCACCTGACAGTGGCACCGTGGGTGTTTATCGTGATAGGCGCTATCATGTTCATCATC
GCCTTCTTCGGATGCTGTGGAGCTATACGCGAAAGTCACTGCATGACTGTCACGTACGCGATCTTCCTCCTCG
TGATAATCATAGTGCAAGTGGTACTCGCGGTACTCATGTTCACATATGCTGACAACAT
CAAAGACGCCCTGGTCACCTCCGTGAACAAGCTCTTCGACAAGCGCAGTGTGGACCCAGCGGCGGCTACCGTC
TTCAGCAATATTGAACAACAGCTCGAATGTTGTGGTAAATACAGCGCTACGGACTACTCGGTAATCCTCCCCA
AGTCCTGCTGCTCGAGACTCAGCGCTGTCGGCAAGTTCCTTGGCGACCAGTGCACTTT
GGCCGATGCTAATATAGTCGGTTGCAGTGTCAGAGTTGGCAATTTATATGAAACTTGGAATAAAACTATTGCT
GGTGTCGCCAT

>1002049600_1 Heli.1-DT662521.3.5
GCACGAGGGCCAACCTTATCGTCGCCCTTTGCTCTTTAGGCGTTGCTCATGGCAGTGGTCTCTTAGCCGCCGC
TCCAGTTGCGTACAGCAGTCACAGCATCGCGGCTCCCGCAGTATCTTCTGTATCATACTCAAGCCAGACCTCC
CACGTAGCACCTGTAGCTGCCTATGCTGCCCCTGCAGTGGCCAGGATTGCCACTCCAG
CCTATACTACGTACTCCGCACCCGCCATCACCAGTTATGCCGCCCCCGTGGTAGCTAAGTCCATCTCACCCGC
CAGCGTGTCCTACTCATCTTACTCGAGCAGCACATCTCATGGTGCCCCAGTAGCCTACGCGGCCCGTGCTGTT
GCTGCCCCCGCCTTAGCAGTATCTCGTAGCATCGTCGCCGCCCCCGCCCTCGCTGTTT
CTAAAACCATTGCTCCTGCAGTGTCATCTTACTCTTCCTATTCTTCACAAACTTCCCACGGTGTTAGAGCTAT
TGCCGCTCCTGTAGCAGCCTACTCTGCTGCCCCAGCTATTGCGTCATACGCTGCCCCAGCGATCGCCTCTTAT
GGTAGATCCTACGTCGCCCCAGCGATTGCTTCTTACAGATCATACGCCGCTCCCGCAG
TAACCTCTTACGCCGGCCCTGCTGTTGCCTCTTACTCTAAGGTCTACTCTGCCCCCGTCGTCTCCTCATAGTC
TAAGCTGTACTCCGCCCCCGCAGTTGCCTCTTACGCTGCCCCCGCCTACTCTACCTACGCCGCAGCCGCCCCC
GTCCTTAAATCCGTGTCCTATTCGGCGGCACCTGCTGTCTCCACATCTCTTACAGCGC
TCACGGTGCTAACTACGCTTGGTAAACACACTATAA
>1002049601_1 Heli.1-ES586195.1.5
TTTCAAATTGCCCATCGCCGACACTAAAATAACCGTCAGCCCCAGAAACCTTGAGAATGATGAGCTGAGCCTA
AACATCAAGTTCAACAAAGAAGGTGCCCGCTCTGTAGGTGAGGCTCGCAAGGCTAAGCTCAAGAAGATCATCG
TCCCCATCCTTGTGTTTGTCCTCTTAAAGGCGATGACCATCATCCCTCTGGCTATCGG
AGTCCTTGGTCTGAAGGCCTGGAACGCCCTGCAACTGTCTTTCTTCTCTTTCGTCGTATCCGTCGCTCTGGCC
ATCTTCCAACTTTGCAAGAAGATTGCTGCGGACAACTCGCACCCGCAGATCGCCGCCCACGGACCATGGGACG
CCGCCTACGCCGCCTCCCGCCACAGGAGGGAAGCAGAGCCTCAGGAACTTGCTCAAGA
ACTCGCTTACAACGCATACCACTAAAAAACTATAACGTTAAGAGGGCCAAATAGTACCAACATAACCTCTGTA
GCATTAAGTTAACGACCCTCTCGTCTAGGACTACGAAACGATGACCGACAACCGTCTGCGAGATTCCCTCGAT
CTGGCAAACGGGTACAAACCAAATAGGTTTTTCGGAACCATGTATGTAAATAGTGTGG
AAATTATTTATTTAATTGTGCGCGTGCTTGTGATGAGTGAATGTTATTTATTGTTCAAATATCATTTTGTTA
>1002049602_1 Heli.1-ES586555.1.5
TGAAGCTCCTATTTCTGGCTCAATAATTTTAGCTGCAATTATATTAAAGTTAGGGGGATATGGATTATTACGA
ATATTAATAATTTTACAAAAAATAAATTTAAGATGAAGATATATTTGAGTAGTAATAAGATTAATTGGAGGAT
TTTTTATTAGATTAAAATGTTTTTGTCAAATTGATATAAAATCTTTAATTGCTTATTC
TTCTGTAGCTCATATAAGATTAGTAATTGGGGGATTGATAACAATAAATTATTGAGGATTTTTAGGTTCTTAT
ATTTTAATAATTGGTCATGGATTATGTTCTTCTGGAATATTTTGTTTATCTAATATTAATTATGAACGATTAA
ATAGACGAAGAATATTTATTAATAAAGGGATAATAAATTTTATACCTTCAATAAGATT
GTGATGATTTTTATTAATATCTTCAAATATAGCAGCTCCTCCATCATTAAATTTAATAGGAGAAATTAGTTTA
ATTAATAGATTAGTTAGATGATCTTGGTGAAGAATAATTATACTTATAATAATTTCTTTTTTTAGAGCTGGAT
ATAGATTATACTTATATTCTTTTACTCAACATGGAAAATATAATATAAGAATTTATAG
ATTTTATAGAGGAACTTCACGAGAATATTTAATATT
>1002049603_1 Heli.1-EL600473.1.5
TTTCTTTTTAGTATCTCGTGCCAGTCAGTGTTCTTGAAAGAAAAGAAAAATGAAGATTGTTTTCTTGATACTT
GCTCTCGCTGTGTGCGCTCGCTCTGAAGACGCCAACAAAAAGGTCGAAGTTCAAAATAAGGACAAAAGGCAAA
CCAACGAAGGCACTCCACAACTCGTTTATAGGACGGCTAGAAAACAGGAACAAGTAGC
ACCGGTTGAAGAAAATCAAGAAGATGAAAAGGGCGACAGAGTTCAACCCCAAGAGTACCGGCCCGGCCAAGTG
TTCTCTCTCAACGCGCAAGAGCTGTTAGAGTTGCAGCCGGAAAGGAAAGCACCAATTCCAAGCGGCCAACAAC
TACAACAGCTGTATAGCAACCCTCAACAGGAACACAGGCAAAATTTACAGCAGTTTTA
TTATTTAGAACCACAATTGAGTCGACAGGTAGCACTGCAACCTTCGCATGCAGTGATTGCCCGTCCGCACTTC
ACATCGAATGGTGGTGAAGCTTCCGTTGGAGCAGCATTATCAGTCAGCGATACCGGTGCTAATTCAGCTGAAT
CCCTGGATCAAGAGTTGCTTGCTTTATTGGGTCACCAACTGGCAGGCAGGCAGGACGA
TGTACGACCTCAAACATTTCCACAACAATCACCTCAAATTCAAGCACAAAATATAGCGCCTCAGTATCAGCAG
GTGGATGTGGCGGCATATATCCAAAAACCGAATAA
>1002049604_1 Heli.1-DT664958.3.5
GCACGAGGCTCAAACATGATCGACGTTATCAAACCCCGCGATGGATGTCTCTACGAAATCAGGCAGAGGGGAC
AAAAGGAACCCTGTTACGTGGTCGTGCCTGATACAAAAGATCTGGTTAAGAGCCACTTGATGTTCGCGGTGCG
CGAGGAGGTGGAAGTACTGAAGGAGCGTATCGCAGAACTGATGGAGAGGATCAACCAG

CTTGAGGTAGAGAACAGCTACCTGCGCGCGCACGCCAGCCAGGACACACTGGCGCAGCTACCGGCCGCCGGCG
CCAAGCCGCCGCAGCCGCAGGGCCCGCAGCCCCCGGTGTCGTAGGCCCCCGCCCCCCAGGAGGGACTCGGGAG
GAGTTATGGCTGTGCTATGTTATATCGTACGTTCACGGGAGAGACGACAACGAGTGTC
CCAAAGTGTCCGCGACCCTGCACTATCGTAGTGTCACAGTGTCGCAGTGCCGTAGTGTCGTCCGCGACCGTCG
CGCAGGAGCCGTCGCGAAAAACTACTATGCTTGGATCTATAGATATATTTTTCGTTATTTAATATTTAGGGTG
CAGAATTTCGTGCTAACTGCCATGCTTATGCTACGCGTCTCGACGACGAGCCCGATAG
TGTATCCATGTATTGACTATATTTTATTCAGTTATTCCATTTAAGTATCTCGTAAATCGCGTGTTATTATAAT
ATAATACTACTACATACTGGAGTAACTCTACGCAATGAACGATGACTGTTAATTTATTTTTCATTATTTTAGT
AGATGTCGTAATTTAAGTTATTTATCGAGTTTTATAATGTTGAATACAGATGAAATGA
TATTGTTGTATTTTGTGAAGTCATTCTGCAATTGCTGGATGTTGTTGGATGTGTGGCAGT
>1002049605_1 Heli.1-DT665155.3.5
CTCTAGAGTTATTTTCGCTCTTTCATACGTTTTTTCAGATTCTATTCATCATGCATTATTTCTCGCTTCTCTT
ATAAAATTAAAATATTTATACAGAATAGAGACGAAATTGATTGCTCAAATGTTTATATATATACCTATTTTTA
GGAGAATTTCAGAAAGGAACTATTTGCTATATGTTATTACGGTACGGAGTGAATTTTT
GTATATTGTAAAACGAATTTTTAAATGTTTGTAACGTTTCTACGTACGTTGACTCGACTGCACTAAAACATAA
CTTCCTACCTCAAAACTATGCAAACTATTACATAATATATTGAATATACAATTTATTGGGAAAAGCAGTTTTA
CATGCAATAAGATATAGTGTAATAAGAGTTTTATTAAAAAAAGTATTTACGTATAAAT
GTTTATATTATAAAAAAACAAAAATATAACGCCCTCAGATATATCTATTATATATGTATTGCCTGTTTGGTGC
TTTACGGCCATTTGCATATAAATATTTACCTTGTTGAAGCAATCGATGATATTCTATGTACAGTGAATCTGTT
ATCGGCCCTAGCTGTTGTTCCTTTGTTGCGGCTGGCCTGGTCCTGGTCCTCCAGGTCC
TCCAAAAGTGGTCCTGAAGACATGAGGGCAGATGGGTTAAAGAAAGTAGTAAAAGAGCGATAACGAATGATTT
TCGCATTGAATGAATGATATGAGATAATACTAGTT
>1002049606_1 Heli.1-EL597979.1.5
TAGTGTACTTATTAAGGCACTGTTAAGAATATAAGAGTTGTGTTTTTAGTGTTTTTATTGCTTTTAATATGAT
GCTTCCGTCATCTCTTAATTTGAAGTCAAGTGCTTCTCAACGAGTGTCTTGAACACTACAACTTACAATAATT
TTACTTATTTATTAACAATACATAAGTTTCTTAATGTTAGCACCTAGATATCAGTTTC
TTATCATTTTGCTCGAGATAAAACACACCTAAGTAGCATATCTTTCTATGTTTTATAACAGAATTGTTGTGTT
TTATTGTATTAAGTATTTATTAAGTTTATTATAAAAAGTAAAAATTGTAAAGTTTATTTGCGGCATTTTATCA
AGATGGCAAGTGATCCAGAATTAAAGGATGGATTTACTCTTCCCGTTTGGATGAAAAG
TAAACCAACCAAGGAGTTTGAACCGTTCGCTTCATCCCCACCAGCTCCAGAGGATTCGTTCTTCTACATTCGC
TATCCCAAACCGGACGTCCTGTTTGGCAAAACGAAGTTCAGCAATGACCTTCCTAAAGTCCTGACTGAACAAC
AGGAGTTGTCAGCGACATACAACGTGATCAAAGAGAAGGATTGGTCGAAACACACGAA
GCCGTGCCAAGACGTCTTGCACGGCATTGCGCCTATTAATACTTCTGTTAAATCGGCCAACGTTAAACCCAAG
CCAGCGGGCACAGATGATGGATTCAAAGGGGAGGGTGCGGCGTGCGGCAGTTCCGTCGTCAAGGTGGCCCACC
AAATGATACA
>1002049607_1 Heli.1-EL602725.1.5
GACTATTATTTTTATTTTTCGTCATAATTATATAAATTTTTGTGCATCATGTCTTCACGTAAAAAGGTTCTTC
TAAAAGTTATCATTCTTGGCGACAGTGGTGTTGGGAAAACATCTTTGATGAATCAGTTTGTCAACAAGAAATT
TTCCAACCAATATAAGGCAACAATAGGAGCTGATTTTCTCACGAAAGAGGTAATTGTC
GATGACAGGATCGTCACAATGCAGATCTGGGACACAGCAGGGCAAGAGCGATTCCAATCTTTGGGGGTAGCCT
TTTATCGCGGGGCGGATTGCTGCGTCTTAGTTTTTGACGTAACTGCCCCTAACACGTTCAAGTCCTTGGAGAG
TTGGAGAGACGAATTTTTGATACAGGCGTCACCACGCGATCCTGAAAACTTTCCATTC
GTTATATTAGGTAATAAAGTGGATTTGGATAACCGTGCTGTGTCAGCAAAGCGTGCACAGCAATGGTGTCAAA
GCAAAAACGACATTCCTTATTTTGAAACAAGTGCCAAAGAAGCCGTGAACGTCGAACTTGCTTTCCAGACCAT
AGCACGCAATGCATTGGCTCAAGAGACTGAAGCAGAGCTTTATAATGAATTCCCTGAT
CAAATTAAGCTGAATGCCAATGACAATGGCCGCAACAGGGATGGAGATAACTGTGCTTGCTAAATTCCACATC
ACTTTTAAACA
>1002049608_1 Heli.1-DT661753.3.5
GCACGAGGGAGAGTTCTGATTGTATCCGCCCTCTTCGCTTGTGTGGCAGCGGCACCCGCGCCCTCATTCTTGG
CACCACTGCCTTTCGCCGCAGTGGCCCTTCCTCGTCTTGTCGTAGTACCAACTATACCACCTGGTGACATTCA
AGCTGCTGCTATTGATGCTCAGGTAAAAATCGCAGACCAAGCTCAATTACTCGCTGAC
CAAGCTCGTGAATTGGCCGAACAAGCTGCCGAAAATCAAAATGAAGGCGTAGTGGAATCAATTGATTTGGGCA
AGGAAAAATCTGAGGAAGCCTTTTGGGCCGCAGAAGAGAAGAAATGGCAAGCAGTCAACGAAGCTCAAATCGC
TGAAGCTAGAAATGCTGCATTGTTGGCTGGAAATGCTGAGGCATTAGTCAAATCTGAT

GAAGCTATTGTCCCTGGAATTAAGTCAAACTCTGAATCAGGAGTCGCAGTAGAATCAGCCGCTGAAGCAAAGC
CAGAAGCAGCTGTAAAAAGCGAATCCGAAGTTAAAGTTAAAGAAGAAAAAGAGGCTGTTGTAAAATCGGCTGA
GGAAAAACCTGCCGAAAAGGCTGAAAGTGAAAAGAAGGAAGATTTACTTGCTAGTGCC
AAATCTGCACTCGACGATGCCCTTAAGGCACAAAATTTGGCTATCCAAGCATACAATTCAGCCATTTTCCCAT
CTGGTGTAGCT
>1002049610_1 Heli.1-DT666265.3.5
GTCAAACCATTTTAATTTCACAATTCACAAATTTTAATGCGTTAGTAGCGAAAAGTCGCTCGCGTCCGACCGT
GTACTTTAAAAATAAATTAAACATAAATATATTATCGGAATAATTCAATCAAACGACGGGATAAAAGCTGCGT
TGCAGCGAATCAACCATGAAAGTGTGGGGAGCACATGGAGAGTTCTGCGCGAGACATC
AATGGGAGGTCATAGTAGCCACTTTAGCCTTGTTAGCCTGTGCAGCTAGCGTGGAAAGACATGGCACAGGTAC
CCGAGCAGAGAGATGTGCAGGATGGGCTCGAGCCTGTCCAGGGCTTGAGGCAGAATATCAAGCAGCGGATGCT
GTTATCATGACCTTTGTGAGATGTGCTGCCCTCCTCTACGCGTACTACCAAGTCTCCA
ATCTCCAGAAAATCGCGTCGAAATACCTTCTCATAATAGCCGGGTTGTTCTCAACGTTTGCCAGCTTTATATT
TACTTCAGCACTCGCTAGCTTGTTCTGGAGTGAGCTGGCCAGTATAAAAGATGCCCCTTTTCTGTTTTTGTTA
GTCGCTGATGTAGCAAGGGGCGCAAGAATGGCCAGGGCCGGTTGGAGTGCGGGCGAAG
ATCAGGGGAAGAGAGTGGGGAAAGCGCTTTCGTTACTTGGACCGACAGCGACGTTAGATACACTTCTAGCGGT
CCTACTTGTCGGTGTTGGTGGTTTGTCGGGCGTTCCTCGGTTGGAACATATGTGCACGTTCGCTTGCTTAGCG
TTATTAGTGGACTACCTCGTATTCGTTACTTTCTATCCGGCGTGCTTGTCACTTGTTG
CTGATTTCGCATCCGGAAGAAAGGAAATATCGCCAGATAGCCCATTTTCTGAGGCTGATTTAAAACCAAATCC
AGTTGTTCAAAGAGTTAAAATGATAATGGCTGCTGGCTTGCTGTGTGTTCATTTGACTAGTAGGTGGCCGTGG
ACCAGAGAGAGCGGAATAATGGAAGGATCTTTGACAAACGACTTCAAATCAGGATCAC
ATGATAATGTTTTGTTTCACTCCTATGTTAAATGGTTTTCTGTGAGCGCTGACTATATTGTTATTGCCACTTT
ATTATGCGCTTTAATAATTAAATTTATCTTTTTTGAAGAGCAAAGGAATTGGATCATAGATATGAACGATTTG
ACAGTCAAGGAAGTGGTTAAAGCAAGCGATAAACCTAAATTCTCTCTCGGCGAGGACA
TTAAATCTGAAATGTACACTCAAACTGAAGATATAGGAAATTGTGAAATGTCGGAGTGGTCCTTACTTTCGCC
GAGTTCATCAGCAGCAATGTTGAATTCTAAAAAACGCCCGTTAGCAGAATGCTTAGAAATATATAGATCTGAA
GGCATAGGCACTGCATTGAGCGATGAAGAAATTGTTATGCTAGTTGAACAGTCCCATA
>1002049611_1 Heli.1-DT666965.3.5
GACTGGACAACAAGACGGAGCGAAGTAGGCGAAGAAGTATCGCGCGCGAACTCTATACGCTGCTGGCGATGAC
CGCATTCATATGAGTTTTTATTAAATTGTGAGAGTGCTGTCGTTTACCCCGTCCCGCTAAGTGTTCACGAAAA
TGCTGTATAGGTGAACTTCCAGAAGTAACGTGTTGTGATTGTGTTAACGTACCCTATC
TCTTCTATCTCGCTGTTTCTTGAAGGTTGTAAAAATGCCTTGCTCTGCGGTAACTTTGTCTATAGCGACCATT
ACGGCGATCGTGGCTGCTGCTTTAATGGCAATCGCATTCTCTACAGACAACTGGCTTTATATTGAAGTTAAAC
GGAACAGTATACAGACATACGTAACGGAGAATACGGACATAAACTCTCAGGCCATCCT
CGACAGCCTCAACAGCAAATACTACTTCTACACGCGGACGCGAGGGCTCTTCCGCTATTGCTACCCCAAGGAA
CGTCCACCTACAGTTGAAATATACCTGTCGCCGGTGGAAACTCACTGCAGCAACGTCGATTACTTCATACCCG
ATGAAAACAACGAGACCAGAGGTCTGTCCGAGGATGCGATGAACAGGCTACACATGGC
TCGGTCCACGGTGGCGCTGTTCATCGTGGCGTTCCTGTCTCTATTCATCGCGTTCTGGACCGGCGTGGTGGGC
TGTTGGAAGCGCAGCCCTGGAAACATAACAGCCACCGCCATACTGATGCTTGTTACTTGTTTACTGTCAGCGG
GCGCCATGGCATTATGGCATGGAGTGGAGTTCTACGAGAAGGAAAAAGTTGTTGGCGA
AGAGTATTATCAGCAATGGCCTAATGTATTAAAAGACTACTCGTCGATTTGGTACGATTGGTCGTACATCCTC
GCTTGGTTGTCCGTTGGTGTTTCCTTTGGGAGCTCCATACTTTTCTTCTCGGCGGCCATTTGTCTCAGCAAAG
AGAAGCGTCGTGAGCAACAGAATAATGTGCAGTACATAATGCCAGTGTACCCCCAAAA
GCAACAATACGCGTACGCCGGTTACCCCCCGCCCCAGGCGTACCCCCCCGGCCCCTACTACCACGGCTCCCAG
TACGGACCCTACAACTACTGAGCAAGCGACCAGATGAGGGACATGGTGGCGTACAACGCGCCCGCACCGCAGG
CACCCGCGAGAAACTCCCCACAACAAAACAACCCGTCCATATTAACATACAACTCCCA
CCGGAAAATTAAACGACCGCTTTTAAGCGATAAGAAAGTTTACAGACCGATAAGAAGGCTAACGTTGTCTAGG
ATCGATGAAATACCGCGACCGAGATCGAAGAGTATACAGCATTCCACGGATTTCTCATCAACCAGACCCATGA
TGAGATCAAGGTTCAACGTCTCAGCGGACACTAGCAGATCTGTACCAGTATGCAATCA
AAGTCAGGATATATCGAATCAAATGGAAAGCGTTAGTCCCCAATATGCGGAACCCAAATACGATTCCGGTTCA
TATGAAAGACCAGCCTCCATGATGGATTTACCAATAATGGTTCCGATGGACCCCTTAACCGGGGCGTACATTC
CGTATCCAGAATACGCGTACTATCACGACGATAACTGTAGACTCGGTTGTTATAAGAA
AAAACAATCGAAAAATAATTTGTT
>1002049612_1 Heli.1-DT666601.2.5

GCACGAGGGTTAACCTTCGCTAAAATGATTAAATTAGTAGTTTTGTGTTGTTTCCTTGCCGCTACTTCGGCTA
CACCTGGTGCTATCATAGCCCCTTTGTCATATTCCTCGAATGTGCTAGCACCAGCACCAGCAGTCGTGTCTAG
CTACCACAGCGGCTTGGCATACTCATCTCCTATCTTCTCTTCCGCGCCATTGGCCTAC
TCCGGACCATGGGACTACCAACATCTCATTAAGAAACGTTCTTTGGCCGTAAGCAGCTACATCGCGCCGTCGT
CCTACTTCGCACCTGCTGCCTACACTTACCCCGCCACTGCTCCTTTAGTCAGCTCTTACTCTGCAATTGCGCC
TCTGGCTAGCTACCCAAGTCCTGTTTACACCGGTGCTGCACATCTGATCAAGAAGAGG
AGCGCTGTACTTTTACCTAACACATACGTGGCACCTGCCGGATACGCTGCCCCCGCACCTTTCGTAGCGTCAA
CTTACGCGGCTGCTTCTCCCATTGCACCCATTTGGTCTAACTCATTGTATCCTGCAGCTCAGTATGCTCATTT
CATCAAAAAACGATCTGCCCCTTTGGCGGTAGCTACATACACAGCCCCAAGTTCTTTC
TCTCATACATCAAGATATGATTTCGAGGCTGTATCTCCTGCTCTTAGCTACACTTCCTTCACAGGCCCTTCAC
CATTAGTATATAATACTCCAGTTCTTTCTCGCCTGGTTTAAGCCAGCGTATCTATGTAACTGCAACGGCCAAT
AATGATCGTA
>1002049613_1 Heli.1-EL599350.1.5
GCACGAGGGTCGCGTGGCGCGGCCGGCACTCCCACTCGCGGGTCGCGGTCGCGCTCGTTCACTTTAGTTGTGG
GCCGGCTGCCGCGGCGGATGCACGCCACGAGACACGCGCCTCCGATGTTAGTTTACGATTACGACATCGCGCA
CGAACTCGGACTTTTGCCGTGACATTGAATTTATCTGTGTCGAATATGGGTTAAACGA
TCCTATCGACGAAATGAGTCCGGGCGAGCGCGCCGCCCTGGGGCGTCGCGAAGCCGCTCGCCCTCACTATGGT
GCCACCGCACCGGGTTTCCCGCCACCATACTACCCGCCCTACGGTGTGCCACATCCCAATGCGTGGCTCGGTG
CTCCCCTCATATCGATGGCGGGCCGCACGCCGCAACGCGATAACCCCGTATCCAAACT
GGCGATGCACGCCCAGGGAGCTCCGCTTATTATACACAACAGACACGATAGAAGAAAATATACAACCATACCT
GAGCAGCGCAACCATCGTCCTATGGGCCAAATAGAACATCACACGAAGGATCGATACGATATGTCAGAAAATA
ATAGGCCGAGAAATCAACTTCATCATCATCAACAACAACAACAACAACAACAAAATCG
ACCATCAAGAAGTGGACGTAGTAACAACACTGACGCAGTCAGCGTCGCCAGTGACGAAAGTTCCGGCTCCACC
AATTCGGAGACAATGTTACCGAGAATAATTAAACCACGAAAGCGTCGTAAAAAAGATAGAAAACCGAATAACA
TAGTGCCACATGATTTAACATCCGTTTCACTGGATCTCAGCGATGTTGATCACTGTGC
AGTTACAAATAT
>1002049614_1 Heli.1-DT664423.3.5
GATCTTCCATGAAACAGTGGGAAATTAAAAGATCACGCACTAGGATTAAACGGTCACAAGGAAGCATTTGCTA
TGGAGAATTCGGTTGCTTTGAGGATTCTGGTCCATTTGCATACTTGGAGACGCTACCAAGTCCACCGTCAGAA
GTTGGAACGCATTTTCTCCTCTACTCTACTACGAGCAGAGGAGACCAACCACTTATAG
CTGTCCCGGCGAGCAACATGTCGGCAGCGTGGAGCTGGGCCGCGCGCGCCTTCGACACCGTCCGACCCACACG
CGTCATCGTGCACGGCTTCGGCTCAAACTGTGATAATGTATGGGTGTACGAGATGCGCTCCGCCTTAATGGCA
GTGGAAGAATGCAACGTAATTTGCGTAGACTGGGAAGGCGGTGCTACTATGCCAAATT
ACCTTCGAGCAGCAGCTAATACGAGACTTGTGGGCAAACAGTTGGCAATGCTGCTACAAGGTTTGACTCAACA
TATAGATCTGCGATTTGAGGACGTCCATTTGATTGGGTTTAGCTTGGGAGCGCATGTCGCAGGATTTGCCGGA
TCTGAATTGAGAAATATTAGTCGTATAACAGGATTGGATCCAGCGGGACCTCTATTCG
AATTTCAAGACCCGAGAGCACGGTTGGATAAAACTGATGCAAAGTTTGTAGATGTGATACATTCAAACGGTGA
GACGTTAATACTCGGCGGGCTCGGCGCCGCGCAGCCTCTCGGACACGTCGACTTCTACCCCAACGGCGGCCGA
GTGCAACATGGCTGTTCTAATTTGTTCGTCGGTGCAGTTTCCGATCTTGTTTTGCCAT
GGGCCGGCGCGTCTCCAGAAGGACGGTCATTGTGCAATCATCGTCGCGCGTACAAGTTCTTCACTGATTCTGT
TTCGCCTAAGTGTCACTTTCCAGCGTTTCCCTGCACTGATTATGATACTTTCTTGGAAGGTCGGTGTTTTCCT
TGTGATGGCGACCATCGTTGTGGCAATATGGGTTACTACGCCGACCGTTCTCTCGGAC
GTGGACAATTGTACCTTCTCACGAGAGAGGAAGAGCCTTTCTGTGCACATCAATACCACGTGGCCTTGTGGGG
AGCTAGTGAAGTAGGCCAAAAACCAAACTACGGTCGAGTGACCCTCACTCTACATGGGGATTCAGGGCTAAAT
GAATCTTTTCCTATGACCAAGCGCGAGGCCAGCGACCGCAACGTGCGCTTCGGGCGCG
TGCTGGTGCCGCACCCCGCGCTGGGCGTGCCGCTGCGCGCCTCCGTGCACTACGCCGCCTACAACGGCTGGCT
CAGCGCCGGCGCGCGCGCGCTCCTCGTGCACAAGCTACTCATTACTGACAGCTTTGGGAAGACTTCATCATTC
TGTAAGAACCTACGACTTCTATCCGACGAGTCGGCTCAACTACCATTGCAACCCGGGG
ATTGTCAAATTCAAGAAGTTGAAGAACCGACTAATAGCACTCTATTGGCGCAGGAAGTTATTCATCTAGAAAA
TGTAACTAGCGCTCCTCCAGATGACCCGCACGATAATGAACTACCCGAAGATCCGCAGCAGAGGCCTTTTGTC
GCCGAGTCTTATGACTGGGAGGAAACAGTCGACACTGGTAGAGCATTCGGTATGACCA
ATACAAAAACGGCAGCCAGTGGAG
>1002049615_1 Heli.1-EL600074.1.5

ACAAGCAACACGCACTTTCACCAGTGACGAGTGGCTACCCCGTGGCAGATATCAATAAGTGTTAGTGATTTAG
TGTTTACCCTTTCAAACAAAATAAGCATGCAGCAAAAGGTGACAGGATGGGCATTTCTGGCCCTGATCTGCTC
GTCGACGATTCTTCACACGATGGCGTACCCGCAACATATGCCTCTTGTTTCACAGGTC
AAGAGCAATGTTCGGACTCAAAACCCACAGTATTATAGTAACTCATTGCCAGCACCTCACCGAGAACAAATAG
CAGAAGAACGAAAATTTGCCGAAAAATCCAATGCGCTGAAGAAAGTAGCTCTCGATGATCTAGATGATATCCA
GAACAATTCTATATCAGATGGCGGTTCCTCATGGTCAAACATGTTAGGTATGATAATG
CAAATGATTTTCAATCCTGCGGGCGCCGTAGGACCAAACAAGAGCGACAATCTAGATACAGATTCGGCTGCAC
CATCTCCTTGGACATTCTTATTCTCAATGGGTCTGAAAATCCTGACCGCAATTCTAGGAGGCGCAACCAACAA
CACTGGAATTGACAAAGTGGACAACGAGTCTTCACCAATGCAGTTTATCAACATCGTT
GTAAATCTTCTGGATGCTCTCAAAACCTCGTTCTCTCATCGGTCACTTGCTGCCCGGTCAATGGGAAAGAAGG
ACTCGGTCAGTGACGCCGCTATG
>1002049616_1 Heli.1-EL596523.1.5
ATTCTTCGTGTTTAATAATACTAAAGGATTTTTGTTTATGTTATAACGATGCAACTACTATTAGCTGCTGCTA
CCCTAGTAGCCAGTCTCATAATAGTTGAGGCTCAAGTAAACTCGGACAGATTGGACAGTGTCAACATAGACGA
GGTGCTAGCAAACAAGAGGTTGCTTCAAGCCTATATAAAGTGCACTCTGGACAAGGGA
AGGTGTACGCCGGAAGGGAGAGAGTTAAAAACCCACATAACTGAAGCATTGCAACAGGGTTGCGACGGTTGCA
CAGACGGACAAAGGGAGAGCGTACGAAGGGTTATACGTCATTTAATAAGAAATGAACCGGACTACTGGCAGCA
ACTCGTGGACAAATACGACTCAGAAGGAGTGTTTTCTAAGAAATATGAAGACGAACTT
AATTCTCTTTAGGTAGCGACATCAAATGATTTTAGGAATATAAATTTAATTTTTGCGGCGTTCAATTCCGCCA
CGCAACACGCAGTGCTTGGAAATTCGGTAAAAGTTAAAGAAAATACAGCGTGAAACTGAAAAAAAAGTAAACA
TTTGATTTTAAATGGAAATATTTTCAACGAAATATTGCGTGTAATTTATATAATAGTA
GGTATTTACTATTAGGGTAAAAGTTAATTAATTATACTATTATAAGGT
>1002049617_1 Heli.1-EL601795.1.5
GCACGAGGGATTTCCCCTCGATAAAAAAAAAAAAAAAAAAAGGGTAGACAGAGAGAATCTGAGAAAGTAGTGTT
AAGATACTTTGTTTCCATTAACATATTTCCATTCTGAGGCATAGCGTGGCATCTAATTCCGCCACTCAACACA
CAATGCCTACAGATTTCAAAGGAAAATGGGTAACGGAAGTGTTAAGATCAGTATTAAG
ATACTAGATTTCCAGGGTCCTGAGAGAGCCTTCGGAACACAGCGTCTAATGTGTGGCCAGACCTGGATTTAGT
GAATTAATTAAAGAACACACGCTTTAACGTAGAGGAAAACAAAAAAACAAGAACAACATTGTGATACATGTAT
GTGATAATTTAATTAATATTAATATTAGGTTTTTACATATGAAATTGGTTTTTAGTAT
GGGAGGAACCAAAAGTCGAATACTTGTTTATGCAATATATTTATATGATATATTTAGTTATCTATTTTTTTAA
AATTTAGTTATCTATGAAAAAAATGGTAATCTGTTGGAAAACTGTAATGAGCGACACTGGCACTAGCCCATCA
AACGCTTATAAGTAACTCTTTTTTGGTCAATTTTCGTTTGGGGCAGGATTTGGCTGAC
TTGCTAGGGTCCAGCTTCAGATTACCGTTGAGAAACCACTTGTCATCACAGGTAATGTAAAACCTCGATGGTT
CGATGTAAAAACCTTTCATTATTTTTGCGGTTAAGTAATGTAATGAAACAATTCGCTTTCACTGAATTAAAAC
AGTTGTATCA
>1002049618_1 Heli.1-EL602821.1.5
AATAATATATTTAATAATAGTTGATTATAATATATATATATTAATAGTATTTTTAGTATTTACGGTTTGTGAG
GGGGCTTTAGGTTTATCAATTTTAGTGTCAATAATTCGAACTCATGGTAATGATTATTTTCAAAGTTTTAATT
TAATTTAATGATAAAATATTTATTTATAATAATTTTTATAATACCAATATGTTTGAAA
CGAAATATATTTTGAATGGTTCAATTTATATTTATATTTTTAATATTTATATTTATAAATATAAGAATTTCAA
TTATAAATTTTTGTAATTTAAGATATATATTAGGTTGTGATATAATTTCTTATGGTTTAATTTTATTAAGAAT
TTGAATTACATTTTTAATAATTATGGCTAGAGAAAAATTAAATAATGATGATTTTTTT
AAGAACTTATTTTTATTTAATATTATTTTTTTAATGGTGATATTGTATTTAACATTTAGAATTATAAATTTAT
TTTTATTTTATTTATTTTTTGAAAGAAGATTAATTCCTACATTAATATTAATTATTGGTTGGGGATATCAACC
TGAGCGAATTCAGGCGGGGATATATTTATTATTTTACACATTATTT
>1002049619_1 Heli.1-EL595939.1.5
GTTGCACGCGGGATCTAGTGCGATTTCCCGCCATTTTTAATTAAACTGTTGACTGTGATATTGGATAACTAAA
TTAGATAAAAAAAATGGCGTCTGTGGAGACCAACGTCCAAAAAAATGGTGATATATTATCAAAGGCACCTTGG
ACCGACGAACAAGGCATCGGTATGAAGATTTATCAAGGAGTAGTGTTGGCACTCGAAA
TTATCGTTCTCGTCGTGAAAATGTATGCGACATGGATGTACTGTATTTACAAGTTCTTCGTGCCTCCCGAACC
TAAGAGCGTTAAAGGAGAAATTATTTTAATAACAGGTGCAGGACACGGTATGGGTCGAGAAATGGCACTTAGG
TTCGCGAAGCTGGGGGGCGTTATAGTATGTGTCGATATCAACCCCTCTGGTAATCAAG

AGACAGTAGAACTAATTAAGGAGGCCAAAGGAAAAGCGCATAGTTACCAGTGCGACGTAACAAGTCGGACATC
CATAAACGAAATGGCCGATAAAATACGTAAAGAGGTTGGCGATGTGTCCATTTTGATTAACAACGCCGGCATC
ATGCCTTGCAAGCCGTTACTGCAGACTGGGGAGAAGGAAATACGGACCGCATTTGAAG
TTAACTGCTTGGCGCATTTATGGACCCTACAAGCCTTCCTGCCGGCCATGATGGAAAGAAACCACGGTCACAT
TGTAGCCATGTCCTCGATGGCGGGTGTGATCGGTC
>1002049621_1 Heli.1-DT664236.3.5
GCACGAGGTGGAGTCATATTTTTGCGTAATAAAATCCGATTGTAACACTGTTGGAAATTTATTGAAATTATGT
CATTTGTGTATCTCCTCTCAAATCGTTCGTGGTCAATGCAGAAAAGCTTCGATCGTTTGACAATATCCGCCTG
AGAATTGTTATTGTTAATAAGATTGTCGATCCGGGAGACATCTTGTGTTGAAAAAGTT
ACCAAGTTGTACGACTTGTCCGATTTTGTCTTCACGTGAAAAGTTTGCAAAATTAGATCGGTGCTAGAAAGTA
GGCAAACATGGATGTTGCGTGGGACGAAGATAATTTTGAACCTAAACTTCCGACCACGTTGACATCGTCCAAC
AAATGGGAGGGCGAGGACGAGGAAGAAGCTGTCAAGGACAGCTGGGAGGACGAAGAAG
AAGAAAAGAAGGATGAAGAGAAAAAAGATGTCGCCGCTCCACCACCGAAGCCCAAAAAGAAAATACACGACAA
AATTGCAGAAAAGGAGCGCTTAGAACGTGAGAAAGCAGAACGCATAGCAGAGAAAGAAGAAGAAATGACGCCA
GAAGAAAAACTTGCGGAAAAACTGCGAAGACAGAAATTACAAGAGGAGTCTGATTTAC
GTCTGGCTATGGAAACATTTGGTATATCCGAGAGCGGTGTCGGCAAGTTGGACAGCTTCCTGCCGACGAACAA
AGCGGAGTTCACAGAGTTTGGGGATCTCCTCAGCAAAAAGATCAACTTGTTCAAGGCCAAGGAGGAGTTCCCC
GCCTTTGTTGATGATCTGGTCAAGAATATTATAGTACAAATGGCGTCCGCTGACATAA
AGAGGATAAAAATGACAGTTGACAATTTATACATTG
>1002049622_1 Heli.1-EL599616.1.5
TTTTTAATATATAAGTGAATAATTTGTTGCGCAATTCGAACGCGGGCCGAGGCAAAAGATTCACATTCCAATC
GACGGTACGACAACTGGAAAGGCGACGGTTAGCGGAGAAGCTGTCTAAAGAAGCAGACCTGAAGGAACAGCAG
AGAAGAACCGAGCTGGAGGCCATGAGACGAGTAGAAGAAGAGTTTCAACGTAAGAGAG
CGAGGGAGAAGGCCAACATCCGGCAGCAGCTGAGGCTGGTGACCAGTGGTGCTAGCAGCATGCCGCCCTCTCA
ACATAGCAAGACCAGAGACGAGCCAGACGGTTCCTGCCGGGGCTCCCCCGCGTTAGATCGAACTCGTGACCAT
CGAGTCAAACACAGCAATGCGTCTTCTGAGGGCAGTGGCAGAAGCAAAAGTTCTACAC
CAATTCGTAGTGTAGAGCTATCAGAGTGGCGCACGGAGGGCGGCGCGCGCGTGTACCGCGACTGGGCGGCGGC
CGGCGTGGCGGCGCACGCGCACCCGCCCGCCTGCGCGAGGATGCCCGCCACCGTACACGCTTATGCTGAAGGA
GAAGCCTTCAGCGGTAGTCCTCGCTCAGACAACTATCGCTTAGAGTTTGCTCGTGGTC
GCTCCCCGCGCACGCCGCGACCTCCTCGCCTCTTACACTCCCCCGCCTCGTCTACTACTGGCAGTGATTTGTC
ACTGCGACAGCCTATTAAGATTA
>1002049623_1 Heli.1-DT663391.3.5
GCTAATCAAGGCACCAAAAGAAAAATGAGCAGCTCGGAGGAGGTATCATGGATTTCATGGTTCTGTGGTCTGC
GAGGGAACGAATTCTTCTGTGAAGTCGATGAAGATTACATTAACGATAAATTTAACTTAACGGGGCTTAATGA
GCAGGTGCCACACTACAGACAGGCTTTAGACATGATATTAGATTTAGAACCTGATGAT
GACCTAGATGAAAATCCAAATCAATCAGACTTAGTAGAACAAGCTGCAGAGATACTATATGGCTTAATACATG
CTCGATATATTCTAACAAACCGAGGGATTGGGCAAATGTTAGAAAAATTCCAATCTGGTGATTTTGGCCACTG
TCCACGTGTCTACTGTGAATGCCAGGCAATGCTTCCCATTGGTTTATCAGACGTGCCG
GGTGAGGCGATGGTAAAACTCTACTGTCCTCGTTGTATGGATGTGTACACGCCTAAGTCGTCACGCCACCACC
ACACCGACGGTGCTTTCTTCGGCACTGGCTTTCCTCACATGGTGTTCATGGTGCACCCGGATTATAGGGCCAA
GCGCCCCGCTTCACAGTTTGTGCCTAGACTTTACGGTTTCAAGATCCACCCGCTCGCG
TACCAGATCCAGCAGCAGGCGGCGGCCAACTTCAAAGCGCCGCTCCGGACCCTCTCGTACAACAACGGTAAAC
GTTAGGACGCCCACAAACCAACCCCTTTGATACAGATTCTACCTGTGCGTCTGAAGGCCCACTAGTGATTGAT
CGATTACGTCACGTGACAATGGTTTATATGTTGA
>1002049624_1 Heli.1-DT665593.3.5
ATCCTACAATGGCAGATCCGCGCATCCGGCAAATAAAAATTAAAACCGGCGTCGTAAAACGAATTGCCAAAGA
GAAAGTAGTTTACGAAAAGGAAGCTGAACAACAAAAGAATAGGATACAGAAGATAAAAGACGAAGGGCAGGAC
GAACACAACATAAGGAAGCAAGAGGAGGTCCTGCAGGAGTCACTGATGATGGTCCCTG
ACTGTCAGCGACGATTGGCTAAAGCATTTGCAGATTTGAAAGGCATTCTAGAAACTGAACAAGACCTCAAAGA
AAACGAGGACTACATAGCAGCAGAGCAAGTATTAAAAGACGCTGAAATCCAATTACCAGAATCTGTTTAATTA
TTTTTATTTATATTTTTATACTAAACAATCATGTCGCAAATATTTTCTAACATTTTTA
CATTCCAAAATATGCTTCCTGTGCCCCCACCACCACCGCCACCACCACTGATGACCCAAGAGCTATCAGATCA
AGAATTTGTTAAGTCATTTGAGTCTCGGGTTGAAATATCTAAGAAACGAAAATTGAAAATAAATTGTGGAAGC
ATCTCCCAAACTAAATATTGTTTGGCGAGTTTGGTTTCTGCAATAAGAGATATAAAGG

CACAAGAGAAGTTGCTTGCTGGGAACAATTTAATCAGTGATGAAGAATGGAAATCAGCCATTGAAAGCATAAA
AACGAATAGATATGTTATAGATTCAACTATTAAAAGAGTTACTGGAACATTTATAGAGATTAATAGAAAGGTG
TTGACAAGACGTGCTGCTAAAC
>1002049625_1 Heli.1-EL598902.1.5
GCACGAGGCGATCGTGTCCACCTGACAGAGGTCAATTCCGATACCGGACGGCCTGGTTATGGACCCGATGGCG
CCCACAGCACTTGCAGTTCCAAATCAACTCATGGATCGCATTGGCAGTGGACTGACCATCCTCAGAGATCACG
GCAAAAAGTTCCAAAAGTACCTTCTGAAGAGTAGAAAATTTGAAGTGCATAGAAAATC
TTGGTCTTCAATAGTAAATATAGTGTTGATAGAAGCAAAAGATTTACCTGATGCTCCTTCGAATGGTTCCAGC
GGCCTTTATTGCAAATTCAAACTCGGCAGTGAATCTCACAAATCAAAACAAGTCCAAAAAACGAAACCGATAT
GGCGGGAACGGTTTAATCTGTATTTGTATGAAGACAGTTCCTTAGAGGTCAACGTATG
GCACAAAACGAAACAGAAAAACTTTATGGGCAGATGTGTGGTCGATCTGTCGCAATTAGAAAAAGAGCGAACA
CACGATCTGTGGCAGAAATTAGACTGTGGTTACGGTTCAGTGCACCTACTCATCACACTGAGCGGATCAGATA
GGTGCACCACCGACAACGTCCTCTCAACGACCAACGGCGTCCACAATGATGCTCCTAA
AGACGATAAGTTCCTATGGTACAGACTTAACAATTTAAATGAAGTCGGCCAGCTAACAGTCACCGTGCACGGA
GCCAGAGGTTTAACTGCATTAAATATTAGTGGCAACGTGAATGCTTTTTGCGTGTTAGAACTCGATAATTCAA
GAATACAAACACACACAGTACG
>1002049626_1 Heli.1-ES584652.1.5
GTCGGAATCCCCGGTCGACTTACGCGTCGGGCTGGTGTCACTCACAATACTATTTATTGTAGCAGTGATAATT
CGTAATATATATTTCCATTTTGTTTCAACTCAAAGTATCAATCGCCTTTGTTGGGAGGTCGCCTTCTGCGCCA
GTGAGTGTAAAGGCTTCTCTTTCAACGCATCTATCCTCGCACGCTTATAAAACCACTA
TCTAAAGTCGCAAAAAGCATGATGAAGGCTGTGATCGCTGTCGTCTGCCTCGCGCTGTGCGCTGTATCGGCGG
AGGTTTGCTACAATGAAGTGTCTTTGGAGTGCGGTAGAGCGACCAGTAGTCTATCTTTACCCAGCTGTAACGC
AATATACGGCAACTTTGGACACCAGGGCAACGTGGCCACGGAGCTCCAAGCGTACGCC
AATCTACACCTCAGGAGATCATATGAATATCTCCTTTCTGCTGCATATTACAATAACTATCAAACTAACAGAC
TTGGATTCAGCAAACTGTTCAAGAAACTGTCGGATGACGCTTGGGCCAAAACTATTGATATTATTAAACATGT
CACTTTGAGAGGCGGCAGCATGGATTTCCGCGCCCGTAGTTCTCAGGACCAAGAAACA
AGGAACTCCACCATCGAGCTCCAGGAGCTGGAGTCTCTCGCACACGCCTTGGACGTGGAGAAGGAGATCGCTG
AGCGCGCATTTTTCATACACCAGGAGGCTACAAGGAACAATCACAAGACTCATGATGCTGAGATCGCACAATA
CCTAGAGGAAGAGTTCATAGAATACCATGCCAAGACAGTCCGTGACCTTGCCGGGTAC
ACCACAGATCTCAAGCAGTTCATCACCAAGGACGATGGCAGAGACCTCTCTATATCACTTTACCTCTTTGATG
AATACCTACAA
>1002049627_1 Heli.1-DT667202.3.5
GCGGGCCGCACGTCTACTGGTGGTTAGTGTTCGATGTTCCGTATCTGACACTGGATAGATATGTCTAAAGTCA
AGACGATGTTGAAGATAGGCGGTGGCGCGGCGCTAGTCGCGGCCATAGTAGCCGTTTTCGTAGTGGCTACTCA
AGGGAGGGACCCCGACTTAGAAGCTTTAGAACAAGAAGGTAGAGAATACATTTTGCAT
TTGGACAAAATGGCTGGCCTCAGGAAGAATAAAGCAAGTCTTGCCGAATGGGCGTACACATCGAATATAACAG
AGGAGAATGAGGAGAAACGGATACAAGTCCAGCTAGAATTATCAAAGCAAGAGAAACAAGCGTGGGAAGAAAC
GAAGATGTACAAATGGGAAGATTTTCAAGATTTTTCTTTACGGCGAATGTTTAAAAAG
TACAGTCAACTAGGAGTTTCCGCTTTACCCGATGACAAATATAAAATGTTAATGCAATGTGTTTCTGGAATGG
AATCCAACTATGCCACTTCGAAAATTTGTGATTATAAGAACGCTACCAAATGTGACTTGGCGTTGGAACCAGA
TATCACAGAAATTTTCGCCAAAAGCCAGGATCCAGAAGAGCTAAAACATACTTGGTTG
GAGTGGCACAAGGCGGCCGGCGCTAAGGCGCGAGAGAACTTTACTAAGTACGTAGAGTTGGATAACGAGGCAG
CTAAGCTTAATGGTTTCAAAGACGTAGCAGAGTGGTGGCAATCAGAA
>1002049628_1 Heli.1-DT664435.1.5
ACGTCAAAATGGTGAACGTACCAAAACAGCGCAGGACCTACTGCAAAAAATGCAAAGTCCACAAGGTACACAA
AGTATCACAGTATAAGAAATCCAAGGAAAGGCACGCTGCCCAAGGTAGAAGACGTTATGACCGCAAACAGCAG
GGTTATGGTGGTCAGTCCAAGCCTATCTTCAAAAAGAAGGCCAAGACAACCAAGAAAA
TTGTGCTCCGTCTTGAATGTGCTGACTGTAAAGTGAGATCCCAAGTTGCACTTAAGCGCTGCAAGCACTTTGA
ATTGGGTGGTGACAAGAAGAGGAAGGGACAGATGATCTTTTTCTAAGCTGATTAATAAATAAGTTTAAAATTT
AAAAAAAAAAAAAAAAAACTCGAGACTAGTTCTCATTTTGGATCTCCGCAACGAAACA
GCGATCCTAAGTTAAGGGACAGTCCTAATGGGAGTTTAAGACGTGTGGACTACTCTGACAACGTAATGTCTAC
TAGCGGCCCATCTAGTTTGGAATCAACCAACCATCGAACACCGAGGAAAGATAGCAAAAATGGTTCATCAGAT
TTAAATTCTACCATAAAAAGGAGCTATCAAACAAGTAGCAGCGGTCACTCCAGTCTAG

AGTCGCACATATCAGATCGCACACTAAAACCTTCACCTAGTCATAGTAATGGAACGGGCTTAAGCAGAGCACA
ATCACTTGTCAAAGTTCAAAGCTTACAAAGTTCCCCTAAAAGCTTAC
>1002049629_1 Heli.1-DT662088.3.5
TCCGTCTGGTAAAGAAATTTTAGTGAAGGCAATTAAATTGATTATTCTGAATGAATTAGTATCAAAGTAACTT
TCAATTGCTGGTTTAATTAACCGCTAACCAGCATATGTGATCTTGCGCATTAAAATGGCTACATATGAAGAAT
TTATTCAGCAGAATGAAGATAGGGATGGAATACGGTTTACGTGGAACGTGTGGCCATC
TAGTAGAATTGAAGCTACAAGGCTTGTCGTTCCTTTAGTATCTTTATATCAACCATTAAAAGAACGCCCTGAC
TTACCACCGATTCAATATGAACCAGTATTATGTACACGTAATACATGCCGCGCTGTACTGAACCCTATGTGTC
AAGTTGATTATAGGGCTAAATTATGGGTCTGCAATTTCTGTTTTCAAAGAAATCCTTT
CCCCCCACAATATGCGGCCATATCAGAGCAACATCAGCCAGCTGAGTTAATACCTAACTTTTCTACTATAGAG
TATACTATAACAAGAGCCCAAAGTATGCCACCTATCTTTCTATTAGTTGTAGATACATGTCTTGATGAGGAAG
AATTAGGTGCTCTTAAAGATTCTCTTCAAACATCTCTTAGCTTAATGCCACAAAATGC
CCTTGTTGGTCTTATTACATTTGGGCGTATGGTCCAAATTCATGAGCTAGGTACTGAAGGACTCTACAAGTGT
TATGTTTTCAAAGGGACCAAAGA
>1002049630_1 Heli.1-DT668374.3.5
CACTAAGTTCATAATATATTGTGTCACAAATTAAATATTAGTCATGGTTGCTTAATTTAATTATTTTTTACTT
ATAGTCTTTTGTTTTCACTTTGAAGTAAATTAATAATACAATAATCAAATAATAACAAGATTCAACAATGGGC
GCCGTATTAGGTTTATGTTCAGCAGCTCAGTTGGCTTGCTGCTGTGGTAGTGCAGCAT
GTTCACTTTGCTGTTCTGCATGCCCTTCATGCGCCAATTCGACTTCTACTCGCCTCATGTACACCCTTATGCT
ACTACTAACTGTGGTAGTGGCTTGTATTACTTTAGCTCCTGGATTACATGAAGAAATGAAAAAAGTACCATTT
TGTGAAAATTCTACAGGGATAGTACCAGGAAGCTTTAAAGTAAACTGTGATCAAGCTG
TGGGATATTTGGCTGTTTACAGAATCTGCTTCATAACATGTCTATTTTTCATATTTATGGCATTAATTATGAT
AGGTGTAAAGTCATCAAAGGATCCTAGAGCCGGAATACAAAATGGATTTTGGGGTATTAAATATTTGGTTGTG
ATTGGAGGCATTATCGGTGCTTTCTTCATCCCTGAAGGCAGTTTTGGATATACCTGGA
TGATTTTCGGAATGATAGGAGGCTTCTGTTTTATTGTCATACAACTTATTTTGATTATTGATTTTGCTCATGC
TTGGGCTGAGAAATGGGTCTCAAATTATGAAGAGAGTCAGTCCCGCGGCTGGTATTCGG
>1002049631_1 Heli.1-ES585100.1.5
CTGCACTTCGTGTTCTGTATCTGTTATACGTTAGTTTACTTGTTTTTGCTTAAACAAGATGCCTTGCGGAGGT
TGTGGCGATAGTTGCAAATGCACTACAACGCAGTGCTGTCAGACCTGCAAGTGCGACTCTTCCTGCTCCTGCA
GCTGCAAGCGGTCCACTGACACCACAGCTACCACAAAGAAATGAAACACACACAAATC
AATAACACGTAGAACGTAGAAATATCTCTTTATTCGATTGTTTACAACATGTTATTATATGTAGGAATGCAAT
TAAAAAATACTGTTATAATTTTCCTTATAATAGTCATTTCCGTTGTGAGAAATAATAATTATGTTTAAGTCGC
ATTTAAATATACGTTGAAAATATTCCAATACCAACCAACCTACTACTATGTATCATTT
TCAAGTTAATATCATGTATTTTGAGAATGTTTAAATTAAAAAATAAAGAGATATAATTAA
>1002049633_1 Heli.1-DT664826.3.5
CGCCTAGCCACATCGTGACAAACGTCCGCAATTGTGATCAAACATTTTGTCTTACAGATTTGAAACTTTATTC
TTGATCATAGAGAAAAATCGGAGTAGAAGTCCGTCGATTTATATTTTTTTAATATATTAAGGTAAAAGAAGAT
AAGAAGTAAAGATGATGTCCAGCCCAGCCATGGAATTGGAAATCGAGGAAGCCCCATT
GGCCTTGCGCCGCTTGTGGAATCTTACTCGGGCCCGTCTCGCTGGCACTTCCACTGCTTTGCATGAGGACCCA
GCTTTAGTAGAGACTTTACTTCCTGCTCAGGAACCAGAAGTGACCTCAAAAACCTGCATACCAGTCCCTAGTG
ACTACGAAAGTGATGGTGAAGAAGCTTTTCCAGAACTAGAAGTTCATGAGAAACCTGA
GCTTCCAATTAGATCTTTGTGGAGCGAAGGTGCTTATGAAAAAGAAGTCCTGTTGGATGCCAGCACTCTGGGA
GATGTGCCCGCGGTATACCGCGTACCGGCGCCAGCGCCGCACCAAATGCCCGTCATTGGAGTTTATATCGATC
CTCGAGTCAGGACTGGCTTCAGATACAAAATTAGACCGATGCAGACTTTGGATGCTCC
TCCACTATCGGTGAAACCTCGATACTTGTTCGACGGGAAGGCTCTTGTCCTGCAATCGATTGGCCGTGGATTT
GCCAGAAGACTAACGTTTGAGCCTCATGATTCTACTTTAAATGAAAATAACAATTTCTTTTGGACCGATTCAC
GTCCTGAAGGCTTTCTATTTGAAATTGAAGCTGTTTCTGTTGGAGACAAGTTCACTAT
TTATGATGCCAACCACGAGCCGCAAGGAATATTGGAAGTAGTGCAACAACAGAAGAACCAAATCGAGATTGAT
CAAAAAATCTCAGCAGATGGACTAATTGAAAAGAAAGTGAAAATTAATACACTTTGCAAAGTGGAATGGTATG
AGAATGATGGAATCACGAAATTAGTTCCAGTCAC
>1002049634_1 Heli.1-EL598069.1.5
GTCTCCTTTCTATCCACATAAGTTTTTGTGTCCTACAGATATGGCTAGCCAGACACAGGGGATCCAGCAACTT
TTGGCTGCTGAAAAGCGGGCTGCCGAAAAGGTCTCAGAGGCGAGAAAGCGAAAAGCGAAACGCCTAAAGCAAG
CCAAGGAGGAGGCTCAAGATGAGGTTGAGAAGTACAGACAGGAGCGCGAGAGGCAATT

CAAAGATTTTGAGGCCAAGCACATGGGCACAAGGGAAGGTGTCGCCGCTAAGATTGACGCGGAGACAAAGGTG
AAGATCGAAGAGATGAATAAAATGGTCAAGGCACAGCAGGAGTTGGTGATCACGGACATCCTCAACTTAGTGT
ACGACATCAAGCCCGAGCTGCACGTCAACTACCGCTTAGCATAAGCGCATCTTCGTTA
CGCATAAATAAATTATAAATTTTTTCGCTATTAAATCTTTGCGAAATAATTATAGTTATTATTCTTGTTTATC
TGTAGCCATTAGGGACGCGTGCAAATGTTGCCAATTTTCAAATGCACTATTTATCTTTTAAATATCTATGTAA
CTCGAAATAAATGTTTTTAACATCTAAATTATGATTACCATTCTATTAATAATATCAT
CTTTAATATGTTTTTGTCTTTATTAAATAGTTATTGTCGATTTATTGTGCATTGAAAATGAGTGACAGTGAC
>1002049635_1 Heli.1-DT665861.3.5
AGCGAGCGCTTTGCTAGGCACCTGCCGGCCAATCTTCACGATAACATTCTAGTCACCGCGCAGCAGAAGCTAG
CGCAACGCGGTGTTGGACCGCGCGCGTTCCTACTAGCAGTGATATGTGCAATAGCTCTACTTACAAAATAATT
GTGATAATTTATTTAAATTAGTTATTAGTTATACGCGTTTAGACCGTGATACGGCTGT
GAGTGAAATAAATATCGGATATGTTGTGACTGCCCGACTGGAACTTGTTACCTGTGATCCAAACCAGTGTTAG
TTATTCTTGCGTTGGAGGACCTTTTGTTACCATGGTTGGGAACAACGGCACTATGATGGCGATTCGTATCGTC
CACAGCAAGCTGCGCAAGCGCGAGGAACACTCGGCTTCGGTGCATCCTGCCGAAGCTG
TGCTCCAGACTGCGCCGCAACCAACGGCTGATCCTCTTGCCTTTCGACAGCAGCCCTTGTGGCAGTTCCCACC
GCCGCTGCCTCCGCCTTACGTCTATCCTCACGATCAGGACAACTTGATGCAGCCCATTGGTAACGAGCGAGCA
AGTTTTCGCAGTTTACGTAAGAATATCGGTGGTCGTTGGAAGCGACTCGTTAAAAAGA
AGCCCGAACAGGAAGTGTACACGATCCCGCCTGAGCTCAAGCCTCAACTGAAACAAATATACGTGTACTAGCA
ACTTTCTAAGAACAGCATAGAAGCGGGATACTTCGATGCCACTTCAAGACATGCTCCGAATGGGAGTTCTGTA
GTTATAACTGATAAAGTCATAT
>1002049636_1 Heli.1-ES585811.1.5
TGAAAGTCTTGTCAAAAAACTAAAGAATGAAGTTCGTGATCTTGTGCGTATTCGCTTTAATAGCTGTAGCATC
TAGTCGTCCGGAGAGTAAATACACTGACCGCTTCGATAACATCGATACACAACAAATCTTGGAGAATAGGAGA
CTTCTTGTTGCTTATATATTGTGCATTCTTGAAAAAGGCAAATGTACCAATGAAGGCC
GTGAACTTAAAGCTCACATTAAAGACGCTTTAGAGACCGAATGTTCAAAATGTACGGAAGTCCAACGCAAGGG
CACGAGAACCGTTATCGGTCACTTGATCAACCACGAGGAGGACTACTGGAACCAGCTGGTGGTCAAGTATGAT
CCTGACCGTAAATACGTCGTCAAATACGAGAAGGAACTCAGATCTGTGAAAGCTCACT
AAAAAGGATATCCTAAGTTAAATTATTATAATGTTTTTTTTAATTAAATTAGTTAACAAGACAAAACGAAGAT
ACGAAAAGATTAAAGATAGTTTAAGTTAAAATCCCCAAAATCTTCAAATTAAGTTTGTAATTTTGTAAAGAAA
TTTTGTTGTTTTCAATTTAAGTATTCCTGTAAGAAATGTATATGACATAAAAGAATTA
TGAAAATTAAATTTAGTAGAATTTGGCGTTAAAGAATAATAAAACAGTATGTTAGTCCTTTTGCTATAACATC
TATATGACTTCATATTTTAAACAAAAACAAAACAATTATCATAGATAGATAGAAGTTTCGTAAAAAAAATGTGT
GACGAATAAGTATTATACGTAAATAAATTATATT
>1002049637_1 Heli.1-DT666452.3.5
GCACGAGGGGAAGACGCATCCACTCCGTTGCGTTTAGTGACTTGTGTTTGACATACATACATACATACATACA
TACATATATGTATATATTGCGAGTGTTTTAAGGATAATATTACTTTTAGTTGGAAATCCTCCGGTCTCAGCCA
GACGATTTGACAGTTGCATGACAGACTAGCACACGTGACCAAATAAACATGACTCTTC
CGCTTAGCTTCCGTGTGTTAACTTGCCTGTTGGTGTACGTGTCCTGCGTACACGCAATCAAGGTGACGTTTGG
CACTAAGAATGGGCCGATCGAGCCGCCGACGCCGGAGCCGCTGCCGCCGCCGCAGCCGTACCGCGTGCCCGCT
CCCGTCTGGGAAGAAAGATCTGATGACTCACCAGACCCTAACGCCCATTGGAGACCTC
AACTCTTCAAACCTCAGCCGAGATACACTCAAATTATATTCGCTGCACCAACACCGACGCAACCACCCCAAAA
CAATGCGTTGCGCTTCGTGAACGCGTACAAACCAGCTCATCAACCAATCCAGGCACAACCCATTAAGGAATAT
TTCACTCCATCCCAATTCCTGAGTTCACAAAGTCTACCAGGTATCGGCCTTCGATACT
TCGTCCCAGCCTATATTAATGAACTGCAAGCTAGGAAAGAAGAGAAGAAACAAGAGGATGCTAAGCACAATGA
CATTGAAACAAACGACGTAAACGGAGCTAACAAAGATTCCAGCTCTGATCTTTTATGGAAACATGAAAAGGAT
TCATCGAAAAGATACTTAAGAAATACTTTAGAAGTCACAGCAAGACCAATCTATGTGT
GGCCGGCGTACGTGCAACCTAGGCATTAATAGATATGAATTAGTTTAGTATTTCTCTAGGTTATAGCTTGTGT
AAGCACCACAGAAGGCAATGTGATTTAAAATGGAGAAGCGATAAGAAATGCCAGTATTAGATATTTTATATTT
CCTATATTTC
>1002049639_1 Heli.1-ES587652.1.5
GGTCGGAATTCCGGGTCGACCCCGCGTCCGCTGCTATATCGTTGGCTGTAACAATTCTATTTTAATTTCGTAT
ATTTAATATTTACCAAAATGTCGGACGATGGGTCAACAGCAGTTGAAAAGAAAGGACGTGGTAGACCTAAATC
TAATGGGACACAATCAGAAGCCAAAGGTGATGGTAAGAAAAGAGGAAGACCAGCAGTA

CCAGCTGCTAAATCTAAAGAATCAAAAAATTCTTCTGACGATGAACAAGCACCAATAGCAAAACGAGGAAGAG
GCAGACCCAAAGGCTCTAAGAAAAAGGCAGCTGCACCTAAATCTAAGAGTTCGTCTGGAGAGGGGCGATCTCG
TGGCCGGCCACGCAAAGATGCACCTCCACCTAAAAAAGATGCGGGATCTACTGAAGAA
GAACAAGAAGATGAAGAAGAAGAGGAAGGTTCTGATCAGTAAACACATATGTTTCTCTCACAACTCACTCTTA
GTGTAATTTGATTGTCATCATAACTCCTTAGAGTGTAACTTTTCATAAAAATACTTGTGAAATGTTATATGCT
CTGTATTGGGGTTATCATAACAACAAGTCTCAAATGAAATTGCTCCTAAATTTTAATC
ATGTAAGAAAAATTTTAAATTGAAAATAATTAACACATAAATTAAGAGATCATTTTTCATGATTTTATTGCTT
TTACTGCAGAATGTCTTGCATGGTTAAAATTTTGGACAGATATAGCTCTCCCTAGCAACGTACAAGTCTAAGA
TATAATTTGTAAGATCTTTTTTTTGTAGATTTATTCTATTTCTATTATAATAAGTATT
TTGTAATCTCCTGATTTACATTGTAAGAAATCAAATAATACAATTTAGATTTATTAATTTGACTGAAAAGTCA
AGTATTATTTTCTATAACAATAAATATTTCATTTC
>1002049640_1 Heli.1-EL603044.1.5
CTTCGGTGCTGTTTGTTACTAGAAAACGCAAAAAATAGACGTTACAAATTTTAATAGACACATATCCTTGTAT
TTATTACTACCACGTGACTTAAAATAATCGTTGAGTGAACGTTAAAGTAGTCAGCTAATATTTTTATTTAAAG
TTTATATCACTGCTCTAGCAGTGGGTACATTAAGCGAGTGTGGTAGCGTGGGCATTAG
TCCCACGCTGTCCGAAACAAGACTGGGAGCCGTGGAGATGGTACAAGAGGGGGAGGGCGTCGGCGATGGACTG
ATCGCCCCACGCCGCTTGCCCAACCCTTGTTTAGAGAACCCCGTACGAATGGATTTGCATAGAGAACTAATGT
TCAATCAGAAAATAGGCAAAAATGTTCTCAATCAAAAGAGTGAACTACAAAAAGCACT
TTCGAAACATAAAGAGAAGCAGCTCATGAATCAAATAAAGGAGCACAAGGAAACGCCAGAACTAGAAAGGGCA
ATAGCAGAACGAGCGCGGCGCCTCGAACAGGCGGAGCAAGGCTCGGAGGAAATGGAGCCAGGAACAAATCCTA
CGTTACAAGAGGTCCGCGCCCGATTAAGACACGCGGCACCCGCGGCCTCAGCGCATTA
AGTATCGTTTAACATTTTACATTTTATCATTAAAAACCGATTTATATTTTTCCTAATATG
>1002049641_1 Heli.1-DT663239.3.5
TTATAAGCCAAGAATCATTTTATGAAGATCTACTAGATAAGCCGGCGCCGAAGGGATATTCCCAGCAAGAGAT
TTTCAGAATGACAAAGTCATTTTTCGTACTTTTTCTATATAGAGTCGTTCGTTTAACCCCAGCTTACGCTTTT
GTTATTGGACTGAATGAACTAGCACTAAGATATACGTACGATAATACTGTATTTGAAC
CGGCTATCTTCGATCATATCACTTGCGATCACTATTGGTGGAGAAATTTGCTATATATAAACAATTTGTTCCC
GCAAAAAGACATGTGTATGGTATGGTCTTGGTATATGGCCAACGATACTCAATTCTACGTCGTCGGTATAATA
CTACTATTAATATCAGTCAAACACACGAAGATTGCAATGGCATCTCTGTTTCTGTTAA
TGGTTAGTTCCTGGGGAACCACCATTTACATATCAGTGTGGCATCAATACAAGGCTCGCATACAGGAGCCGTT
CGAAATGTTCGACCCCCTATACGACAAGCCTTGGTCTCGAATTGGCCCATATTTAGTTGGAATGATTATTGGA
TGGTACTTACATAAAACAAAGTGTCAAGTTCGTATACCATATTGGTTAGTTGTGATTG
GTTGGCCCGTGTCACTGGCCATCATCGGCAGCCTGATCTTTGGAATGACTGATGGATATTTTGAGGTTTGGCC
AACTGCGTTCTATGTGAGCATCGGTCATACAGCATGGGGAGTGGCGTTAGCGTGGATAG
>1002049642_1 Heli.1-DT662597.3.5
CTGCTCACATTCGCTCCAAAGAAAAAAACATCGATCGAGTGCTTTCAACGACGATCGTGCCAGAAAATAAACA
TGAGGATTTTACCATTGATTGTATTGGTCGCATGTCTGGTGTACCAAGCGCGAGCGCGACCCCAAGACGAATC
AGAACCGCGTGCTATATCTAACAGGGGACTCTTAAAACGAGGCCTCGTTGGTAAAGCA
AAAACTACAACCACCACACCAGCTCCACAGGAGGCACCAGAATATGAAGATGAAGGTGATTATCCAGAAGAAG
AACCTCAAGAGCTCTCCACTGAAGCGGCACCTTCTTCAACGGAGGGAAAGAAATTAGTAGGAAGTGGAGTTAG
ACCCTTTAGAAGTAACACTGATCTCCTAGAAGCCTTAAAGAGGAGACGACAACAGGTT
GCTGAAGCAAAAGGTTCCGGTTCGCCTGTAGTACAACAACAACAATCTGAAAACCAACCGGAGGCGCCGCCCA
AAGCTAGTTACAGCAAAAAGCGTTTCAACACTGCCACGCGGGAGACTAAAGCTGAAGATGCGCCCGCGCCGGC
AGCTGCGCCCTCCAAGCCAACAAGAGGACGATTCAGTCGACCATCATCGAGGTCCGTC
CAAGAAGCAGAACCTGAAGAACAAAACGACGCTGCTCCACCCGCGAGAACTGGCAGAAATTTCTCTAGACGAG
GAAATTAAATAAATTTATCCAAAAGTGCCCTAATTTCCTTTCCTACATTCTCTCATAAT
>1002049643_1 Heli.1-EL599553.1.5
GCGCCTCGTACTTTGTTACATTACCATTCACGTACTTAATATGTGACTGACTTCATTTTTTCACTTATCTACA
ACAAATAAAATATGATGGTGACGTTTTGTATAAATTAGATTAGTGTCGACTGTTTAATATTTCACTCTGACCC
ACTTCTCAACAGGTGTACGCCGTAATAAGGCTTTGTTTCAATCCGAAAGAAATGAAGC
GACGCGTAACATATTTTTTGATATTTATCCATTTAAAACACTTCAATTATGACAGCAAATGTGAATAAAGACT
TAGCGTTGTGAATATATTTAGATTATTAGCTTTAGAAACTTTTGAAGTTTATTCGTTTAAATGAATACATTTT
GAATTAATTGTCTTGTATATATGTTTATAATTATAAATATACACAATGGGATTGCTCA

TATCGAAAATATGGAGCCTCTTCGGAAATGAAGAACACAAATTAGTGTTAGTGGGCTTGGACAATGCTGGTAA
AACTACAATATTGTACCAGCTGCTGCTCGGCGAAGCTGTTCACACTAGGCCGACCATTGGATCCAATGTGGAG
GAAGTAGTGTGGAGAAACCTTCGCTTTGTGATGTGGGACCTCGGAGGACAACAGAGTC
TACGCTCAGCTTGGAATACTTATTACACTAACAGTGAATTTGTAATAATGGTGATAGACTCGACGGACCGCCA
GCGTCTGGCCA
>1002049644_1 Heli.1-DT666665.3.5
ACATGTCGTCCGTAGTAGCATTAAATTTATTATTATGCACAGTGCTATGCGTTCTAGTGAGTTCCAATTCACA
GACAGTTCACAGGCGTTTCGAATACAAATATTCCTTTAAGCCGCCGTATTTAGCACAAAAAGATGGTTCTGTA
CCTTTCTGGGAGTATGGAGGAAATGCGATAGCGTCAGGCGAAAGTGTAAGACTTGCTC
CATCGCTCAGAAGTCAAAAGGGTGCGATATGGACAAAGACACCCATCAACTTCGACTGGTGGGAGGTGGACAT
CATGTTCAAGGTCACAGGACGGGGGAGGATTGGTGCTGACGGTTTGGCTTTCTGGTACACCTCCCAACGCGGA
GATTACACAGGCGAAGTGTTCGGCTCCTCTGACAGGTGGAACGGTCTCGGTGTGATCT
TTGACTCATTCGACAATGACAATAAGCACAACAATCCATATATCATGGCCGTGCTCAACGATGGCACAAGAAA
CTTTGACCATAAGAGCGATGGTTCCACCCAACTCCTCTCAGGGTGTCTACGAGACTTTAGGAACAAGCCGTTC
CCGACGCGAGCTCGTGTGGAATACTACCTCAATACATTGACAGTGTACTTCCATAATG
GTTTAACAAACAACGAAGCGGACTACGAACTCTGCTTCCGAGCTGAGAACGTTGTTCTTCCTCGTGGTGGTCA
CTTTGGACTGT
>1002049645_1 Heli.1-EL596740.1.5
CAAGATGTCCAAGACCTACTACACCAGAGGTTCCGAAATGTTATCCTGGTAGCTCTGATCTACGTTGTCCGAG
ACCAACAACAACTTCAAAACCTATTTGTTATCCAGGATCCCCAGATCCAAATTGCCCTCAACCTCCAAGACCA
ACAACATTAAATCCTCCAACATATTTACCTCCTACTACTTCTGAACCAAAATGTTTCC
CAGGAAGTAGCGACCCAAGATGTCCAAAGCCCACTACACCAGCACCTCCAAATTGTTTCCCTGGTAACACAGA
TCCTCGCTGTCCGAAACCTACAACGCCCGCTCAACCTCGATGTTATCCAGGAAGTAGCGATCCAAGATGTCCC
AAACCCAGTACACCTGCGCCACCAAATTGTTTCCCCGGTAGCATTGATCCCCGTTGTC
CTAAGCCCACAACGCCCGCCCCACCTAGATGTTATCCAGGTAGTAACGACCCAAGATGTCCAAAACCCACTAC
ACCTGCGCCGCCAAATTGTTTCCCCGGTAGCACTGATTTCCGCTGTCCTAAGCCCACAACGCCCGCCCCACCT
AGGTGCTATCCAGGTAATAACGACCCAAGATGTCCAAAACCTACTACACCTGCGCCGC
CAAATTGTTTCCCCAGTAGCACTGATCCCCGCTGCCCTAAGCCTACAACACCTGCCCCACCTAGATGCTATCC
AGGAAGTAGCGATCCAAGATGTCCAAAACCCACTACACCAGAAGTTCCGAAATGTTATCCTGGTAGCTCTGAT
CTACGTTGTCCAAGACCAACAACACCTTCAAGACCAATATGTTATCCAGGATCACCAG
ATCCAAATTGTCCTCAACCTCCAAGACCAACAACATTAAATCCTCCAACATATTTACCTCCCACTACTTCTGA
ACCAAAATGTTTCCCAGGAAGCATTGATCCAAGATGTCCAAAACCTACTTCTCTAGCACCACCAAATTGTTTC
CATGGTAGCATAGATCCTCGCT
>1002049646_1 Heli.1-CV526012.2.5
GCTGTGTGAATTGTTTACGTGTTTTTAACGATAAATGTTGTGTGTGTTACAATGGCGAATCTTACACTGAGAT
ACCTGGATGAGCTCTCCTATGTCCTGAACAGACGGCACCTGGCGAGCCAAGCGAAGGCATCTTCCGTCGGCGA
TCTTCTCAACACAGAAGAGGAAAGATTTGCACCGTGGATCACGCCAGAAGAATGTGAA
GTGTACTCGAGAGAGAGGAGACCCCCGCTCGTCATACCTCGCATCACGCAGTATAGAGCGCCACGCCCGCGAC
CACAAATAAACTACCCTGAGGATTCCATGCAAGATGTGCCCGCCAAATCTTGGAACTGGTCTCCCACGCCGGA
GCGGAGCCGTCTCGATATCGAGAGGAGCTTCAGCAAAGCAAATTGTGACAGTCCACCG
CTGTGGGTCAGCAAGCGACTGGGCGGTTCAACAGAGAACCTCCTGGCCGCGTCGCGAAGGCCCAACCACCTCC
AACTACCAGCGTCTTCCTCTGGAGACAGCCTGGATGTGGAGGTGCGGGAGCGGGATCTAGGATCGAGGGTCAA
GGAGAGGAAGTCCAGATCGATGGTGGCTCCTAAGCAGAAGCCAAGACCGCCACTCACT
TCCCCCAGGCTTATGATGCATCCGCCTGTTGAGGCAACAGTAATATACGAAGGGCCAATAAAGAGAACCAAGT
TTTCGGAGGGCGGTAAGAAAAGTAAGAAGAATTGGACTGACTGCTTCATGGTGGTGACGCCCACCGCGCTGGT
GTTCTATAAGGACCAAAGGACCTACTTAGCTACGAAAATGCCAAAACCTCTGAACACA
CCACCGAGCCCCACAGCGCCGCGAGCCGAGCTCGTCCTGCCTCTGCTCAATGCGCATATTGTCCAGTGCATA
>1002049647_1 Heli.1-DT666296.3.5
GCACGAGGAACAACCTTGTACTCCAAGGTCGTAATCCTCATGTGCGTCGCTGGCATCGCCTCAGCCGGCAATC
TTCTCCAAGCCGCGCCAGTTGCGTCCGTTGCGTACAGCTCTCCCGTTTCCTCCGTGTCATACTCTTCGCAGAC
AACAGCTCATGGCTCGCCAATCTCCTACGCAAGCCCCGTTGTAGCCAAGACGGTTGCC
TATTCTGCAGGACCAGTGATTGCAAAGAACATTTCCCCGGCTCTATCTTACCAGACCATCTCTCATGCTCCAG
CTATCGCAAGTTACGCAGCGGGACCAGTAGTCGCTAAAGCCATCGCGCCCGCAGTGTCATACTCATCAGTATC
TCGGTCTTCGCCCATAGCGTATGCTGCCAGCCCTGCAGTGAGCTATGGAGCTCCTATC

GTAGCAAAATCTATATCACCTGCTGTATCCTACTCTTCTGTAGCTCATCCTGCATCATTAATACATGCTGCTC
CAGTAGCATATGCTTCTGGTCCAGTCGTTGCTAAAACCTTAGCTGGCCCAGCAGTGTCTTACTCATCTCTGGC
GTCTCCTTCATTATCTTATGCTGCGGCTCCTTCTTACTCCTATGCTGCAGCTCCTTCT
TACTCCTATGCTGCGGCTCCAGTCCTCAAGTCGGCTGTAGCTTACTCCGCCGCACCAGCAGTCTCCCATGTTA
GCTACTCTGGTTTGGGAATCAACTACGGATGGTGATTAAACAAAGACTTAAATATAATTAGGATATGTGCATA
TAATTAATAATTATTGAAATGTATATTAAAAACAGTCAATAAAATTTTAAAAATTAAA
AAAAAAAAAAAA
>1002049648_1 Heli.1-EL596938.1.5
GCACGAGGTGAAACGAGGGTTCAGACCTTAGGTTTTAGTTTATTGTTACTTTAAGTCTAAATATAGTAATAAT
AATAATGGCTAATAACGATAATTTTGCACAAGACGTTACTGAAAATCAAGTGAATGGTAATGCAGAAAACGGT
GGAGGCGATGGCCAAGAACATAACAGTGCCGACGCCCCGGGACGCGATGACGACAGAA
AACTTTTTGTGGGAGGTTTGAGCTGGGAAACAACAGATAAGGAATTACGTGATCACTTCAGTGCTTATGGAGA
AATTGAAAGTATCAATGTTAAGACAGATCCTAACACGGGGCGGTCGCGGGGGTTTGCATTCATTGTTTTTAAG
GCACCCGACTCCATTGACAAGGTGATGGCTGCCGGCGACCACACCATCAATAACAAAA
AAGTAGACCCAAAGAAAGCCAAGGCGAGACATGGGAAAATATTTGTTGGTGGACTCAGCAGTGAAATTTCCGA
TGATGAAATAAAAAACTTCTTCAGTAATTTTGGAGCAGTCATAGACGTGGAGATGCCCTTCGACAAGACAAAG
AACCAAAGGAAAGGTTTCTGTTTCATCACATTTGAATCTGAGCAAGTTGTCAATGAGC
TACTCAAAACTCCCAAACAGACCATCGGCGGAAAGGAGGTGGACGTGAAGCGCGCGACGCCGAAGCCCGATGG
CCCCGGGGGCATGGGAGGGCGAGGGGGCCGCGGCGGTAGAGGCGCGCGCGGGGGCCGCGGCGGCCGCGGGGGC
TACGGCGGGCAGGGCGCGTGGGGCAACCAGGGCTACGGCAGCTACGGCTACGGGCAGG
GCGGCTACGGCG
>1002049649_1 Heli.1-DT664189.3.5
GCACGAGGCTGCGAAGCGCGGGAAAGTTTGACGTTTGCGTTCGGATATTACAAACATGATTTTCAAGGCACTG
TTACTCTGTGCAGTATGCGCGTGCGCATATTCACAGCACCAAGGACCCACCACTACTCCTGTACCGATTCTCA
AACAGATAAACAGACAAAACGACGACGGCTCATACAGCTTTGGGTATGAAGCAGCTGA
CGGATCGTTTAAAATTGAAACAAAATACCCTAATGGCGATGTAGCAGGGAAATACGGATATGTCGACGAGAGC
GGGAAAGTCAGGGAGGTTTCTTATGGTGCGAGCAGCCAGAGAGGATTCGAACCTGAAGGTCCAGGAATTATGG
TGCCACCACCAACGCTTCACGACCCATCATCAACAAATGCTTTGACCGACGGCCAAGA
AGATGATGGCCAATACAGAGAAGATCCTAAAATTTACGAAGACCCGAAATACAATGGTAGAGCACAGGCCCGA
CCTTCAGGCAGCTTCAGGCAGTTCCAGCAACCAGCTCAACAAGCGAGTTTCCAACCGCAACCAAAGTTCCAAT
CGCAACCACAGCAAACGTTCCAGTCAAGTTTCCAATCGCAGCCACAAGCTCAACCACA
ACAGTACCAACAACAATACCAGCCTCAATATCAACAACAACAACAATATCAGCCACAACGGCCTTCATTCCCT
CAACAAACCAACCTCTTCAGTCAGTCACCTCAACAATTCCCTCAAGAGTACAGGCCTCAAAGCCAAGTCTATC
ACCAACAGCCCCAATTCTCCTATCAAGCTTACACTCCACAACCCTACCAAAACTACCA
ATCTCAGAATTACCAAAATCAAAATTACAACCCCTTCCAAGGGCACCCTGCCCAAAACTTCGACCCTAACACT
GGCTCCTACTCAATCAACTTCACTGGCAAATAATTCTTGACTTAATTAGAATATTATAAAATAACTAGCTTTA
TAGTAATACAATTTTTTTAATT
>1002049650_1 Heli.1-EL597986.1.5
TAAGATTATTAAAACTGCGTCTTATACAATACAAATTGTGTGATAAAAAGTCAGTCTAGTGCTAGTAGTGATT
ATAATCAACTTTAATCCAAAGAAAATTTGTTATAATGGCATACGCTTACTTGTTTAAATATATCATCATTGGT
GACACAGGTGTGGGCAAGTCTTGCCTGTTACTTCAGTTTACAGACAAAAGGTTCCAAC
CAGTACATGACTTAACTATTGGTGTGGAGTTTGGGGCCCGCATGATAACCATTGATGGGAAGCAAATAAAACT
GCAAATTTGGGACACTGCTGGACAGGAAGCATTTAGGTCAATCACTAGATCATACTATCGGGGTGCAGCTGGT
GCGTTACTTGTGTATGACATTACTCGACGAGACACCTTCAACCACTTAACTACATGGC
TTGAAGATGCCCGCCAACATTCCAATTCGAACATGGTTATAATGCTTATTGGTAATAAGAGTGACTTAGAGTC
ACGCCGTGAAGTAAAGAAAGAGGAAGGTGAGGCGTTCGCGCGGGAACATGGACTGGTCTTCATGGAAACCTCT
GCCAAGACTGCGGCGAACGTGGAAGAAGCATTCATCAATACTGCTAAGGAGATTTATG
AAAAAATCCAGGAAGGTGTCTTCGATATCAACAATG
>1002049651_1 Heli.1-EL598267.1.5
AGTTAAAATATATTTTAAGAAAACTTTGTATATTTTGCATTTAGTGACATTTTCTCTATTGTGAATTAAAATG
GCAGAAACAGAACCTAACCAGCAAGCTGGAGATACCGGCCCACCGAAAGGCATTCCCGCATTAAAAGCTCATG
TAATAGCAAATAAAATAGATGTAGCATTGTGGGGTATTCGTGTACTCACAGTCCTTTG

TACATTTGGATATGTGTTTCCTCTATTTAATAACCCCGTCTCAGCGTTCTACAAAGCGCTCCTAGCGAACGCC
GCGACGTCCGCGCTAAGACTCCACCAGCGTATACCGGCAAGAGAGATCTCGTTATCAAGAGAGTTTCTAAACA
GATTTTTCCTAGAAGACAGTGCACATTATCTGTTCTATTCGTTGATATTTATGAACGT
GGCGCCCAATTTATTGATATTGACACCCATCTTCCTATTCGCACTTCTCCACGCGGCATCATACTCCCTAACA
ATACTAGACACGTTAGGACAGAACTCATTGTGGGTGGCGCGACTCTTGATCTCGCTGGTGGAGTTCCAGTCCC
GCAACATCCTCCGCGCGGCCGCGCTCGCTGAGATCGTGCTGTTCCCGCTCGTCGTGAT
TATGGCCCTTATTGGGTACTGCGGGCTGATGACGCCGTTCGTGTACTA
>1002049652_1 Heli.1-DT667915.3.5
CGGGTTAGTCGTACGTTTATCTACAATATTAGTACAAACGTCAACGATTGGTGAACAATGCATAAAAATCTTA
TATATTTAACAATATTAGTAGCCGTTGGCACAAATGCAACTAGATCGCAAACACCTTTAATAAAAAATGTATA
TCGTGGTGGAGAGCATTTCGAAGGGCCGCATTTCGCAAAAGACGAGAGTGCATTATAT
TGGGTGGACATTACAAAACAGAATGTTTTAAGGCTCGATATTGACACAGGCAATGTCACCAGTAGGCACATAG
GATATGGTCCAGTCAGTTTAGTTGTAAGGGTAAAAGACTATCCGAAGTTAATATTAGTGACTGTAAGATCGGA
AATATATCTGTTGTCGTGGGATGCACCTGAAGAAGAAAACGCCTTGAAATTATTGAGC
TCGGTAGACATGGGTTTACCTGACAATAGGTGTAATGATGGCAAAGTGGACGCTAAAGGGCGACTATGGTTTG
GAACAATGGGCAAAGAAGTAGGAGATATTATCGATAAGGATCAAGGAACTCTTTATATGGTTAATGAAAATAA
CTATAAGAATCCTGAGATAAAAGTTCGTCCCGTTTCTATATCTAACGGCATCGCTTGG
ACATCCGACAACAAGTTCATGTTCTATATCGACACTTCCACGAGAAATATAGACGTGTTCGATTTTGATTTAG
ACTGTGGCTGTATCANAAACAGAAGAACATTATTTAACTTTCAATCTAACAATGTTACTGGTTATCCTGATGG
TATGACCATAGACAGGGATGGT
>1002049653_1 Heli.1-ES584679.1.5
GACTGAAGCAGATGAAGACCGAAGCAGATGAAGACCGAAACAGATGAAGACCGAAGCAGATGAAGATCAAAGA
AGATGAAGACTAGAACAGATGAAGACCGAAGTGGGTGAAAACTGGAGATTTAAGGAGATGAAGACTAAAGCAA
ATGAAGACTGAAGCAGATGAAGACTGAAGCAGATGAAGACTGAAGCAGATGAAGACTG
AAGCAGATGAAGACCGAAGCAGATGAAGATCACAGCAGCTGAAGACTGAAGCATATGAAGACTGAAGCATATG
AAGACTGAAGCATATGAAGACTGAAGCATATGAAGACCGAAGCAGATGAAGATCACAGCAGATGAAGACCGAA
GCAGATGAAGACCGAAGCAGATGAAGACCGAAGCAGATGAAGACTGAAGCAGATGGAG
ATTTAAGGAGATGAAGACCGAAGCAGATGAAGACCGAAGCAGATGAAGACCGAAGCAGATGAAGACCGAAGCA
GATGAAGATCGAAGCAGATGAAGATCAGAGGAGATGAAGACCGAAACAGATGAAGATCAGAGGAGATGAATAC
TGAAGCAGATGAAGACCGGAGCAGATGAAGACCGGAGCAGATGAAGACCGGAGCAGAT
GAAGACCGGAGCAGATGAAGACCGGAGCAGATGAAGACCGAAGCAGATGAAGACCGAAGC
>1002049654_1 Heli.1-DT668897.3.5
GAAAAAGTTTTCAAAATAATTCCATATGTAATATAGTATATAGATTTATGAAATTATTATTGTATTAACAGTA
GTGATTAAAATTGACTATATGATACTCGAGAAACTTCACCAAAGGTCACAATGAGGCTTTTTATTTACTTAGC
TTTTATAGCTGCTATACTACATTGTTCATTTGCACCCCCTGTTACGCCTGATAAACGT
AAAGAGGAAGAAAAGGATGAAAATGATTTGGCGCAATACACAGAGTACCACAGGTACCTGAAAGAGGTTGTGC
AAGCGCTTGAAAGTGATCCCCAGTTTAGAGATCGATTGGAAAGAGCTAACGAAGAAGATGTTCGGTCGGGTAA
AATAGCCGAGGATCTCGACTTTGTACACCACAATGTAAGAACCCAGTTGGATGAAATA
AAACGGAGGGAGATAGAGCGACTGCGACATCTCGCTACTAAGGAATATGAACTAACAAACGACCTCGACATGC
CCGTGCATGTACCTCCCGTCTACCCAAACTTGGATAGTCAACATTTGGACCACAAAAATCCCCATACGTTTGA
AGTTGAAGATTTAAAAAAGCTAATACAAAAAACCACCGAAGATTTGGAAGCCGTTGAT
AAAAAACGTAGAGAACAATTTAAGGAATATGAAATGCAAAAAGAATTTGAGAAACAACAAAAAATTGAACAAA
TGGACGAATCTCACAAAAAGGAGTACATGGAGACCTTGAAGAAAGAAGACGAAGCTAAGAAACATCACGAACC
GCTTCACCATCCCGGCTCTAAGCAACAGTTAGAGGAAGTATGGGAGAAACAGGACCAC
ATGGACCAACAGTTCGATCCAAAAGCCTTCTTCATGATGCACGATGTCGACGGCAACGGCGCTTGGGACCCAG
ATGAGGTTAAAGCACTTTTCATAAAGGAATTAGACAAGATGTACGGACCGGAAGGGCCCAACAAGGATCTCCA
CGAGCGCGCCGAAGAAATGGAAAGGATGAGGGAGCACTTCTTCAAGGAAAACGATCGC
CACCACGATGGTCTGATTGAATATAGCGAGTTCATG
>1002049655_1 Heli.1-EL597725.1.5
CGTGTGCTATCTTGGCGGTTGGCGCTGTAATATGCGCATCCCAACCGGTGGAGAAGTTACCAGTTCTAAAGGA
ACTTCCTGCGGAAGTCAACTTTAGAGTTCCAAATAAAGAAGATTTGGTCCTGAACTGTGAGACTGTTGACAAA
GATGTTGGAGTCAAATATTCCTGGCTTAAAGACGGTAAACCATTCAGCCTACGATCAG

ACGTAAGCCAAAAGGAAAACGAAGGTACACTCATCTTCAAAAAACCAGTAGATTCTGACGAAGGCAAATACCA
ATGCTTGGCTGAAACCAAATTTGGCGTTGCCAGTTCTCGTATTGTGCTCGTAAAAAAGATTTTCATAGATAAA
CCTCAAGTGTCTCTACAAAAACATAAGCCTGTGAATGGAAAGACTTATAAATTGGAAT
GCGCTATCCCCAAATCTTATCCTAAGCCAGAAATTTCTTGGATAGTGAAGACAGGAAACGAGGCAAAACCTGT
TGCTGGTGCGAAATTTACTATTTCTCCGGAAGGAACTCTATACATAGCAAGCATCTCCTCAGAAGATGTAGGC
AATAAGAAATACGTCTGCCTCGCAAACACTCCAGCTGAAGACCAGCCTATAGAACTGG
CTGAACATGTTCTAGAAGAAGTCATCCAAAATAAAGAACCTGCTGATCATGAAGTTGTAAAACAATACGTCTC
TAACGAAGTGG
>1002049656_1 Heli.1-ES586863.1.5
AATGGTCACAAAATATATTTTAACTTTATTCGCTCTTGCGATTAGCGGCTCTTTTGCACAAAGCTGTCAGAAT
CCTAAAGTAGAAGCTGCGTCTTTCACGAGTTTAGATGCTACCGTCGTCACACAAATAGCTTATATTACAGAAT
TTACATTGAACTGTGATAATCCTGTGGGAGAGAACTATGCTCTGTATGCAGAGGTTGA
AGGAAAAGCTTTGACGGCTGCTCGTATTGCTGAGAACAGATACCAGGTTTCCTGGACCGAAGAACCTTCCAAG
GCAAGATCTGGAGTGCACGAGATCCACATCTTTGATGAAGAAGGCTGGGCATCCCTCCGTAGAGCTCGTCGTA
GTGACCCGTCTGTAGCTGTTGCTCCTTTGTTGGCTATTCAATTACAACACCCTGGCAG
CTACTCCGGACCCTGGGTTAACTCTGAAGTCTTGGCCACCGCAATCTCAGTCATTGTTGCATACACAGCTCTC
AGGAACAAAAGCAAAATCTTAGCTTAGCAGTTAATATAATTGTTTTAATATATTTTTAATTAAATTGTTTA
>1002049657_1 Heli.1-EL596919.1.5
TGAAGGCTCAAGGTATAGAAATAGGCTCTGAAAAAAAAACCGCCGAGACCAGGAACCAGAATTAAGCCAACAAA
ATTAAAAACACAAATGTCACAAGATGCTCCAAGCAGCCCCGCTGAAGAGAAGAAGATTGAATTGGAAATATCA
GATAAAAAGGAAGAACCAAAGAAAGAAGAAGAAAAGAAGGAATCCGATAATGAATCCG
TTAAAGATGCTTGGGACGCTGAGTCATCTGACGAAGAAATTGAACCACCTAAAGATGTTAAGTCTGAACCTAC
CCCACCTACTAATGAATTAAAAAAAGCTGATGATAAACCAAAGGAGAAGGAAGATGAAGAGGAAAGTTCTGAA
GAAGAAGATGATAGCTCTGAAGAAGATTCAAGTTCCGATGAAGACTCGGATGACGAAC
AAATGACAGACGCACAGAAGAAACGTGAAATTGTTTTGAAGAGATTAGAGAAACGTCGCGAGGATAATGAGAA
AAATAAAACAAATAATCCACTCCGTGCCGCCGTCGTCTGTGTGCTGGGACACGTAGATACAGGCAAAACGAAG
ATCCTAGACAAACTAAGACGTACTAACGTGCAAGATGGCGAAGCGGGAGGTATTACAC
AGCAGATTGGGGCCACTAATGTTCCCATTGAGAATA
>1002049658_1 Heli.1-EL596323.1.5
CCGCATCGCTACACGCACGATTCGCGCGCGGCTTTAAGGACTTTATCAAGGTTAATTAAAAATCCACAAAATG
GCTGACTCAGGCCTCGTTAAAATAGAAGATAATCCAAAGATTCTGTCAGGGCCAGTCCTGACGAACTCTCCAA
ACGCAGTATTATCCAAGACCCTGTTGGGTCGCTACAACGACCTGCCGTTGCCCGTCGA
CCGCATCCAGGCCACTTACGTATGGATAGACGGCACTGGCGAACACTTGAGATGCAAGGACCGCACTCTGACC
TTCATTCCCAAGAAACCTAAAGATTTGCCCGAATGGAACTTCGACGGCAGCTCGACTGGCCAGGCCGAGGGGA
ACAACTCTGACACATATCTGATCCCCCGTGCAATCTACAAGGACCCCTTCCGTCGCGG
GAACCACATCCTCGTCATGTGCGATACCTTCAAACACAATCATCAGCCTACAGAGAGCAATCATCGTGTCAAA
TGCGAGGAGGCGTACGAAAATTGCAAGGAGGACGAGCCCTGGTTCGGCATTGAACAGGAGTACATTCTCTTGG
ATTCGGACCTGCGACCCTTCGGCTGGCCGCCCGGCGGCTGTCCCCCACCACAGGGCCC
CTACTACTGCGGTGTGGGCGCCAACAAAGTGTTTGCCAGAGATCTTGTTGAAGCTCACTATAAATGCTGTCTC
TACGCTGGTGTTCCGTTAGCTGG
>1002049659_1 Heli.1-DT663278.3.5
GCACGAGGGTGAACCGGTCGCTGACTGACCTTACGCGGTAAAACCAAATAGTGCACGCCGAAATGAAGGCAAT
TAGCTTTATTATCGCACTGACAGCGGTAATAAGTTGCAAGGCAGCGACCCCACAACTGCGTTTCGCGTGGAAA
GAAGTAGATTTCACATGGGACTCCCCAGAACAGCGGGAAGCTGCTATTAAAGATGGAG
TATTTGTAAAGGCCAATAACCTTCCTTTAGGCTTAGCGAGGTGGAATAACAAAATTTTCGTGACTGTTCCAAG
ATGGAAGAACGGAGTTGCTTCTTCGCTAAATTACATAGACCTAGACGGTGCTCAAGATCAATTATTGAAGCCA
TATCCGTCATTAAAAGAGAACCTTGTAGCTGACAGCGCCAAGGAACTGCCATCAAACA
GCTCAATTATTTCTGTCTTTAGAGTGTTTGTTGATCCTTGCGATAGATTGTGGGTAATGGACTCTGGGTTGGC
GGATATATTAGGATCAGCAAATCAAGTGGTTGGTCCGTCTCTTGTGATATTTGATCTCAATACCAATCAGCTG
TTACATCGATACTTCTTCAAGGTGTCAGATATGAAAGAAGATTCATTCTTCGCTAATA
TCGTAGTGGACGTAAATAAGGATACTTGCGACAACGCGTTTGCATATGTGCCAGATTTAGGTGCATACGGCGT
TGTTGTATACAGCCTTAAGCAGGACGATTCGTGGCGTGTGAGCCATCACTACTTCCACTTTGAGCCATTGGCT
GGGTCTTACAAAGTGGGTGGTGTGGAATTCCACTGGACCGACGGAGTTTTTGCTTTAG

CACTCTCAGAGCCAAGAGAGAACGGATACCGCACGATGTTTTTCCACGCGTTCTCTAGCACCAAGGAGTTCTG
CGTGTCAACGGAGCTGCTTCGCAATTACACACACATAGACAAGAACGAGGCATTCCACGACTTCAAGCTGTTG
GGTGATCGTGGTGAGCGTACACAGTCTTCGGCCAGTTTCTATGATGAAAATACTCACG
TTCTATTTTACACGCAGATAAATCGTAATGGTGTTGGTTGCTGGAATTCTAATAAGCCTTACACTCCACAAAA
CAATCCTCTTATATTCAGTGACCCCGAAATATATGAGTTCCTCAATGACTTNAAGATTGACAACGAAGGCACT
TTATGGTTCCTAAGTGACAAGATGCCACGTTTCATTTTCAAATCTC
>1002049660_1 Heli.1-ES585670.1.5
AATGAACGAACTTGACCGTCGTGAAGACCCTACCAATGACGAATACTTACCCGGTTGTGCCATGGTAGACTCT
TGTTCTAACATTTTAACTGGTGACCAATTTTACAATTTCCTTAATCACAATTTTGATCGTCACTACGATCAAA
ACAGAGCTCCCTTAGGACTTTACTTCCACGCTGCCTGGTTAAGAAATAATCCAGAGTT
TTTGGAAGCATTCTTATATTGGATTGACGAAATCCTTCAGACTCACAATGACGTATACTTCGTAACGATGACA
CAAGTAATTCAATGGGTACAAAATCCACGTACCGTTACCGAGGCAAAGAACTTCGAGCCATGGAGAGAAAAGT
GCGCCGTCGAAGGCAACCCCGCCTGTTGGGTACCTCACTCATGCAAGCTGACCTCAAA
GGAGGTTCCCGGTGAAACTATCAATTTACAAACCTGTGTCAGGTGCCCAGTCAATTACCCCTGGCTCAATGAC
CCCACGGGCGACGGTCATTATTAAATGAGATCTCGCGCCTCACGTGGTGCACGTTAGTTGATAAATTGCAATT
GATTGCAAGGACATTATATTCGGAAATGTTATAATAAATAAAAGCCAAGTTTATTCAG
TGTCGACTTATTCGATGAGTATGTGTCCGTGTAATTATGTACTGGGTCATAATCCCGTCTATAGTCTAAGTAC
CTACCTAAATGTAATATAGACTCACATGATATTTAATAGGTCTGTTTTAATACTATATGTGATTTTTCTACTG
TAAGTCTGTTGGATAAAATTAAAATAAAAATCGT
>1002049661_1 Heli.1-DT662267.3.5
GCACGAGGCTGTACCATTGCATTTCATGGATAGTGTGATTGTGTTTTGCAAGCTTAGTTTGTTAACTAGTTGT
TTATTGTGTTATAAATTACGTCCTTATTCGTATTTTCTAATATATTATTTGGGTTTTATAAAGAGATACTTTC
ACAATTATGCGTCGTAAAAGTGAACGCACCGCCACTAAGAAAAAGTCTTCTCCAGACA
AAAAGGAAAAAGTTGAAAAAGCAAAGCGCAGAGGAACACGTCGGACTCGCAAACATTCTTCATCATCAGAAAG
TGGGTCTGCTGAAGATAATACTCCTAAAGAAACTGGCGAGGTTAGCCAAAAGGAAGCGATCCCGCCAAAATCT
GTCAAAGAGGACAGTCCCGAAGAATCTCAGGAGCAAGTTTGGCATGTTAAAGCAACAG
AAACTTCAGGTGATGTTGGTGAAATTCAAAAGTTAAAAATTTGTCTTGCTCGTCCGCCTTCAACTCCGGAAAG
GGCAGACAGGTCACCTAGAAGTAAAAGAAAGCATTCTCGTGCTACTAGTTCCAGTGACACTCAAAGTGTTGAG
GGAGAGGAAAAAAAGAAATCAAAACATCGTTCGAAACGATTGACAGAATCAAAAGAAG
CACCAGAAACGAGTAAAGAGGATCATGAAGAAGTAGAAGTTCAGCCAGCTTCCTCAGCAGTTGAACCCGAGTC
TACTAATACTACTACTGAAGTAGAGAGCACTGAGCCGAATAAAGTAGTAACAGACGAGACACCCATGTCAACT
ACTAATGAAGAAAACAAAAATGAGAAAGTAGAATCAACTGAATCGACAGAGAGTGAGG
TGAGCCATGAGAATGTACCAAAAG
>1002049662_1 Heli.1-EL600778.1.5
TTATTTCAATATTACAAACATTTTAATTCAAGTTATTATTTGACTACACACAGAAAAACTATTTAGCGCATAT
TTTTATTGCCTCTCGAAACATTATAATTTAAGGCTGCTAAAATGAGTGATTCACCAGATCAAAATGAGCCACC
AAAAGATCCAAAAGAGCTGGAAAAATTAGAGGAAGCAAAATTAAAAGCTAAGTTTCCA
AATGCAGTTCTTGGTCGAGGACCTGGAGGGCATTCAGCTTTTCTTCAGAAGAGATTGGCTAAAGGCCAAAAAT
TCTTTGATTCTGGTGACTATCAAATGGCTAAGCAAAGACCGAGCAACCTGGCTGCCCCATTCAAGGCTCCAGC
GCCCGCTAAGTTGCCTACTGGTGAAGCCATACCCACACCGGACACAGTGCCATTACGG
AAGACCTCCATCATACAGCCCAAGTTCCAGCCCACCCACGGACAGCCATCCTAGCCTTAAATATCCAAACTTT
ATAAATATGCTGTGTAAATAACATGTTGACACTTGCTTTGTTTTATTCATCCATTCCAGCTTATATTCATCAT
GTTGGTCAAGCTTGTTTTGAGTTGCAAAAGTCATCTATGCCTTGCCCTTGTTAAATTG
TACTGAAGAAATGTTATGTATGTATAAAATTTTTAGGTAAATATAATTAAATTGTGAATGAAATTTTGCATGT
GGTACATATTA
>1002049663_1 Heli.1-EL604282.1.5
GATATAACGATATGTATTTTAGGATATTTATAATTTAGATTTAGGCTGAGGCTATTTACTTATTTTTATGTAG
TATTAAGCTTATATTACATATTTATAATATGTCTGGGGAATTGGAGTTTATTACGAGAAGTGGCAAGTTCCGT
TAGAATCTAAAATGGCAGAGTCATCGCCCGGTTGACGCGTAGATCCTTGATTGACCTG
AAAATCAATTCAAGAGAAATACCAAGGATAAAACATAAGAGTCGGTTAAACTCATCGAACATCGGTGGCAGGT
CAATACGTACATTACAATAGACAGACTCCAATACAATTAGCATCACGAATAATGCTTGTTGAATGGTTTGCGC
TGGTACGTTCAGGATAGCCGGTCTCCAGTAGAAACGTCCCCGTTTAGGGATCTTTCTA

GTAACAGTTATTCAGATGTTTAGTAATTAAGAATTGTTTGTTCCTATTTTGGCAGTTAGCATTTAAATGACTG
TTATTAGTTATTATCATTAGTTTAGTATGGATACTAAGCAAGTTTCCTTCGCTATTAAAGCTTTTTGGATATT
AAAGCAAGTTTCCTGCCCCTTTCTTATTACATAAACTTTTGAAATG
>1002049664_1 Heli.1-EL601238.1.5
GCACGAGGAACATCAACTATAATGTCAGCTAAGATTGTGATCGTCCTCGCCCTTGTTGCGGCCGCTTCCGCCC
TGCCCGTCTTACAGTACGCAGAACCCGAATCGCCCGCCCACTATGAGTTCCAATACTCCGTACACGACAACCA
AAGCGGTGATGTAAAACAGCAACAAGAGGCCCGCGCCGGAGACGCCGTCCACGGCTCC
TACTCGCTTGTGCAGCCTGATGGAGTCCACCGCATCGTGGACTACAGCGCTGACAAAGTAAACGGATTTAACG
CTATCGTGCGTTACGAAGGACAACCCATCGTATCTGCTCCCGCTAAAGTCGCCTACGCACCCGTCGCCAAGCT
CGCCTACGCCGCTCCCGTCGCCAAGGTGGCCTACTCCGCTCCCGTCGCCTACGCCGCC
CCTGTCGCCAAGCTCGCCTACGCCGCTCCCCTCGCGCACGTCAGCTACTCATCCCCCGTCATTTCCTACCAAC
ACTAAGTCAATAGCTATAGCAGACATTCTACGTTAAACTTACTATATATATAATATAAATATGTTATTGATTT
TAGGATTATAGCAATAAAAACACTTTTAGTTTCTTCTGTTAAAAGAAACGACATTAAA
TGTTTATTACTACTGTTACAAAACTTCTATATCACAAAATTTTACAATGTACACGTTTCTCATTAAACGGATA
AAATTTAAAAG
>1002049665_1 Heli.1-DT662732.3.5
GCACGAGGGACCGCCGCGATAGCTTGACGCTTTACACCCACGAAAAAATATATAAATAAATATATATAATTGG
CATTTCACATTAGTGCTCCTTTCATTTGACCAACACTCACTTTTCCCCCCTGGAAAGTTGGAGTGGGCAAAAG
ACAGTGTATTCGGAAGTTACTAGTGTTGTTAAAATTTTGTGATTTCTAGGATTGTTGC
GTGTATTAACGGATTGATCGATTAATTATTGTCAACAAAATTGTGGAATCCGATAGATAACGTTTCCACAATC
GCAGGAATTTAATGTTGTGAAGGTACTCAACAATGACATAAGAGTTATAAACAAAATGGCGTTACGACTCAAA
AAAGATATTAAAAAGGCGTCGTACTACGTGTGGTTCCTCGGTGCGCAGGAGTCTCGTG
GCCTGAGGGGTGAGGAGTTCGCTATACCTGCAATACGACTATTAGAAGAGAGGGCGAGGGATTTGGAACCTTT
CAAAGTTACTTTGCAGGTGTCACACAAGGGACTAAAAATAATCCAGAATGTAACAGCAAAAGGCAAACAGCAA
ACGATCAAACACTTCATACCGCACGGCAGCATCACCAGCGCGGTGGTGCAAGGGGACG
TGGTCGCGTGTGTTCTGCTGCTGTACAACCCCATCACCGGCTGTCCCGTCCACGTGCACGCGTACCGATGCGA
CTCAGATCATACGGCGGAGATGCTATACACACATCTACTAGCGTTGATAGAACGTCCAGAAAACCAGAAGAAA
TTTGCTGATATCGAAAGAAAACTTCAAATGCGAGGTGCTTTACCTTCAAAGAAACCAC
CTGACTCTTCCCTCGGAAGTGAGGTTTCCAGAGAATCCGACTCCGGCAGCAACGAGGATAGGAACGTAGCTAA
TCTATACGACAGTCTTGCAGCCGAATTGAAGCAAAAGTTGTCAACAGGTAAAAAGGGTCTAGGCAAAAGTCCA
ATCCTTCTGCCCCCACGTGACTACGACACCGTGCACCGCCAGAAAGGCAACCTAAGCA
ACATAGACACTAGGCGGTGTCTCAATCAGAACATTGTAGGGATCAACGCACGCCGCAAGCTGGAGTCCTCCGG
TGGCAGTTCTGGAATAGGGAGCGACCTCGCACCGTCGCCAGAGAGGAATGAATATCTACGACATGATAACCAT
AGCACTAGCGAGGAAGATTGGGCAGAAAGCACAGCTTACTTAATGCACGAGGCATTTG
ACGCATCTCCACCTCGTCGAGCACACTCTCCACGTCGAGCTCACTCTCCACATCGCTCTTCTCCTCCTCCTCG
TCGTACCTTGTCTCCGCGCCACTTCGAGAGGGCTTACAACGATGACTATAGTGACCAATACCGTGACCAAATG
CCTTCATTCCGTGACCAAGCAACTTTTGAGCGACGCTTCCGTCATGAATCTCTTGAAA
GGAAGGACAAAACAGATAAATTCGATGACGATACGAGCTATAGGCGACGGTATGTCGCAGAAACAAAACCTTC
CAATAGAAGCATCGATAGAGTCGAAGATCGCAGATTGGGTAAGCTTCAAAGGGGTGCAAGCGAAGGAAGCCTC
AAAGTTGTTGACGCCTCCCCAAAAGAACGGTTTAACGTCGCTAAGGAAAAGTTCATGA
ACTTGGAAAGAGAGAGGTTTAATAAAGAACTTGAAGCTCACATGGCCATGAGGAGAAGCATGCTAGAACGGAA
CAGTCGCTCGA
>1002049666_1 Heli.1-EL598818.1.5
GGCCGTATTGTGTCGGGTGAATGGCTCTAAGTCACCACTAAATAACTCATTTTATTAAAATACGTCCATAATA
TAACACAAAAATGAAATTAATCTACCTTACGTTAATAGTTCTGCCTGCGGCGTTACTCTGCTTCCAAAGCGCA
AACACAATCTTTGCTCGTGCTGAAGAAGATGAACTTGATGATGTAGTGGATATTGAGG
GCGAAGACAACCAAGTAGTGGGTGAAGATGCGCTTGACGAAGATGACTCAGTTGTAAAATCATCACAGGATGT
TGATACTACTATTTTGTTCACAAAACCCATAACAAACTATGGTGATGTGGCATTCGATTTACAAGCTGGCTTC
CCTGTCGAGTTCCTTGTTGGTTTCATTAACAAGGGTTCTGAAGATTACATTGTTGAGA
CCATGGAAGCTTCCTTCAGATATCCCATGGATTATACATATTATATTCAGAATTTCACTGCATTGCCCTATTT
TAAGGAGGTTAAGCCTAGAGAGGAAGCTACTTTTGCTTACTCCTTTATTCCCAATGAAGCTTTCGCTGGACGA
CCATTCGGATTGAACATTCAACTCAATTATAGAGATGCCAGTGGAAACTTTTACCAAG
ATGCAGTCTACAACCAAACAGTCAACATTGTTGAAGTATCTGAAGGTTTGGATGGGGAAA
>1002049667_1 Heli.1-EL597590.1.5

GGGCGATGCGCAGTTTGCTGTGATGGCTCTTTATAATACTTACGTATATTTCACTTTTTTATGATATTCCCGT
TCCTTGCACTTCTTCTAGTTTAATTTAGTTCTAATTTATATTTATGCGTCTAAATGCGTTTAAATAAAATGGC
GAATCAATCCCAGAAAATTGTGTATAAGCTGGTACTGACTGGAGGGCCTTGCGGCGGA
AAAACTACGGGACAATCCCGACTTAGCACATTCTTCGAAAACTTGGGATGGAAGGTGTTCCGGGTGCCGGAAA
CGGCTACCGTCCTATTAAGTGGTGGCATCAAGTTTGCAGATCTCAGCCCAGATGAAGCGATAAAGTTCCAGGA
GAACTTGCTGAAGACCATGATTCAAATAGAGAACACTTTCTTTGAACTGGGGCGAACT
TGCCAACGGAACTGCCTCATTATATGCGACCGAGGAGCCATGGACGCTAGTGCATTTATATCCAAGGAGAAAT
GGGAGGCGATGCTGATCGCAAACAACTGGAACAGCGTTGAGTTGCGCGACAATAGATACAATCACATCGTGCA
TATGGTGTCCGCTGCGAATGGAGCTGAAGACTTTTACTCTACTGAAGATCACGCGTGT
CGCTCTGAAGGCGTTGAAATGGCGCGCGAGCTGGACTACAACGCGGCCGCCGCGTGGATAGGACACCCGTACT
TCGATGTGATCGACAATTCCACAGATTTCGATAAAAAGATGAACCGCCTCATAGCTTGTGTGTGTCAACGCGT
CGGTCTCGACACCGGGGACCGGCTCAACGTCAACTCCAAGAAAAGGAAGTTCCTCATC
AAATCCCCCCTACTCCCCGATACAGAGTTTCCGCCGTTCCAAGATTTCGACGTCGTTCACAATTACCTGCAAA
GTGATTTCCGCAAAGCGCAGGTTAGGTTGCGTAAACGCGGACAGAAGGGCCACTGGTCGTATATCCACACCGT
ACGCAAGTTCCATCCGACCAATGGTCAATCGGTGGAAGTACGCACGCAACTAACGCAT
CGGGACTATCTGAATATGCTTCCGCAACGCGACGATGCCCATTTCACGATATTTAAGAAACGCCGTTGCTTCA
TACACAACAATCAGTACTACCAGCTCGATATTTACAGGCAGCCCACGCATCCCAGGTGTCGTGGCTTAGTTTT
GCTTGAGACGTACAGTGCCGCGTACGATCAAAACGCATTATTAGCTTCCTTGCCAAAG
TTCCTAGCTATTGAGAAAGAGGTGACTGGTGATCCAGCGTATTCTATGTACAATTTATCTTTGAAAGAAGATT
GGAAGACCTCCACTAAGTATTAT
>1002049668_1 Heli.1-ES587888.1.5
GTTAGGCAGGTTATATTAAAAATGGCCAAACGTACTAAGAAGGTTGGAATTACTGGAAAATATGGCACACGTT
ATGGTGCCTCACTACGTAAAATGGTCAAGAAGATGGAAGTAACCCAACACGCCAAATACACTTGCTCCTTCTG
TGGAAAGGATGCCATGAAACGTAGCTGTGTCGGCATCTGGTCATGCAAGCGTTGTAAG
AGGACTGTAGCTGGTGGTGCATGGGTATTCTCTACTACTGCTGCTTCATCCTGCAGATCTGCTGTCAGAAGGT
TACGTGAAGTCAAGTAAACATCATAGAATAAGACAATAATAAAAAGA
>1002049669_1 Heli.1-DT667042.3.5
ATCAGGAGCTGATAAAAACAAATCTGACTTCTACAATGTCGCACACGGTCACAATTACGAGGACAACCACTAC
CACATCTGGAGGTGCATTCTTCACGAACACCGGGTACCTTAGCTCTACTCCAGGGTTCCTCAAACTTGTACAA
TTGCTTTTAGGCGCAGCTTGTGTTGGCGTGGTGGGTTACTATTTAGATCCCGGTTATA
GTCGGTACAATGGACAAAAGCCGGAATTATTTTATTTACTAGTCGCGGTGACGTTCCTAATCGGTACCTTCTG
CTTGTTCATATCATGCCTGATGTCCTGGTCAACGGCTTCTGTGATATCAAAAACATTATATGAAGTTATCTAC
CATGGCGTTGCATTTGTTATGTATTTAGCAGCAGGTCTTACACTAATGATCGAAGTAA
ATCATCAAAAAAATAGTTACAGGCGAGATTTCGAGCCATATTTGGCAGCAGCGGTAATGGGACTGGTAATGGC
GGTTCTTTATTTATTTAGTACGTTCATTGCAGTCAGGTCCTATCGCGGTATTTAAGTCGCCGAGAATCTCTAT
AACACTAGCTTTATCAAAATGCATTTCTGTGTTGTTTGTATATACCAAGACGTACTTT
GCTTTCTACTTTTTATAGGCCTTTTAACTATTCCTAGACTCTATATTAACTATGGCGTCGGCATGGAGATATG
TCTACTGACGCTTTTGCAAATATGTATATAAATAACGATGCCGTTGCGACGCTAGGTTAGTTATAGTTATAAT
TGCTTCCCTGTTCAGGTCAGACAGACAACGACCA
>1002049670_1 Heli.1-DT667825.3.5
GCACGAGGCCGCCAGGGGAACCCCCGAGTTTAAGGAACTAGCGGACAGGGTATCCGCAGCGCTGGGCGCCGAA
TTGGGAAGGGTTAAAGGATACCGGGAAGTTGTTCTGGATGGATTTGTACAGAACGAAACTCAATCTGTAATAG
CTGATCTAACACTAAAGACCGTCGAGGACACAACTACGCCAACAACAACAAACACAAC
AGACGAACAGAAGTGGGAGGAGGCGGTGCGGGACATGCTCGCCGCGGGGAGTGTCGGAGCCTTCCAATTGGAC
CCCTTATTCTTTGAGTTCGAAGTCGCCGGTGCCAAGCCAACAAGCCCGGCGCCGCCGCGCGCGGCGGGCGGCG
TGCTGGGCGGCGCGCGCCTGTGGCTGGTGGCGGGCTGCGTGGCCGCGCTGCTGCTGCT
GGCGCTGCTGCAGGCGCTGTGCACGTTGGCGCACTCGAAGGCCAGGAAGGAGCAGCTAATACCACCTACACCG
TGGAAAGAATACACATCGGCCAACACTAACTACGCCTTCGAACCTTTCGAGAACGACGACAAATACCCCCACT
CCCTGCCACGTTCTCCCCCACATTCCCTTCCCCGCTCGCCCCCACAACGACCCGCCGC
TCCCCCTCCCCCACGACCTACACACGCACCTGACAGACAAGAACGGGCCAATAACAGGCATGAACTTGCCAAT
AACAAGCACGAACATGGCAACAACAGGCACGAACATGGCAATAACAGGCACGAGCTTGGCAATAACAGACAAG
AACAGACCCATAACAGGCACGA
>1002049671_1 Heli.1-DT668594.3.5

ATTTAAATTGTCCTGAAAGGCACAAATAAAATGAACTAATGTGTGCACACATGTAATGATGGCATTAAATTTA
GAGAGCTGCGACAGCTTGCCCCCAGATGAAGAGCCTAAACTGAAATATGATCGTATGGGCAATGACGTTCAAA
ATATACTTTTAAAAGACGCTGTAAGCTGTATTTGCGTACATACAAAATTTATTTGCCT
TGGAACACAATGGGGAGTGATACATTTACTAGATCATGAAGGTAACACAGTGCCTATATCTCAAGATAATAAC
CAAAAGGACTTGCAAGCTCATGCCATCGCAGTAAATAAAATCTCAGTGGATATTAATGGGGACTATATTGCCA
GTTGCTCTGATGACGGCAAAGTATTAGTGTATGGCCTCTACACAGATGATAATACCCA
TAATTTGACACTAGGTAGAGTAGTGAAATCAATTGCTTTGGACCCTTTTTACTTTAAATCTGGCTCTGGAAGA
AGGTTTCTTACAGGTGATAACAAACTTATTCTCTACGAGAAAACATTCCTGAACCGTCTGCGCAGTACGGTCC
TTTGTGAGTGTGAAGGCTATGTTCAGGCAATAGCGTGGCATGACAGATTCATAGCGTG
GGCCAGCGAAGTAGGTGTACGCGTCTACGACCTCGTAGCAAGATGTTCATTAGGCTTAATTCAATGGGAAAGA
AATCCTAATAGATCCATTGAAGATTTTCGGTGCAATCTTCTTTGGTCTGCCCCAAAGACCCTTATGATTGGTT
GGGTAGACAC
>1002049673_1 Heli.1-EL597349.1.5
AAATATTTCTGTGTGAATTTTTTTTATAACAGTTTTAGTGTGTGAAGTGCAATGGTTTACTTTTTGTTTTTTA
TTGTTAACATTGATACAAAATCGTCTGCACAATGCAAACCGTTCTAAATTTACATATTATTATTATTATTTTT
TCTACGAGAGTGTGTTATTTGGTTTTACAAACATGTTGACGGGATTTGTTGTTTTTTT
GGTGGTTTTTCTCCATTCACTACAGTTTGCACACTCCCTAAGTCTGTTATGCGGGCACACAAAGGAAGTCGAA
GTGGGCGGCAACGTGGACGCCGGAGCCGCGCTCGCCCTCACCCTGCTCCGGCCGCCCTCCTCCACGTCGACCA
GCGCCTGTCAGTTGCGTCTGACAGCTCCTGATGCTGCTGCTTTCACTGTTCGACTTAT
TGATGTTAAGGAAAATCTATCAGAATGGGAGCGCGGCGTCACCGACATGCAGGCGCCGACCGCTGGAGCCCTA
TCACGCAAGTGGAGCATGAGGAGTGCGCATGCGCAGGACACGGCCCCGGATGAGACCCCGTCGGTTACCAGTT
CAAATTCCACGGACGCTTGTAAATTGCTTGTGTACATCGGCGAGTCCAAGGCGCCGAT
ATGGAGGCTGTCTCTATGCGGCGGCAACGCGGCGGCGGTGGCAGCTCGGGCTGGAACCAAGTTGCTACCACCA
AAAATTCGTATAGTCTGGAATCCACCAACCACACCTTACCATCATACTGAGAAACTGAGATTAGTCGTCACAG
CTGTTAATAGTGGTTCAGTATGCAACAACGACTCCCAATTCACATG
>1002049674_1 Heli.1-EL603032.1.5
CGACTTTTTAGAGCACGGAGTTAAATCTCTAATTTCTTGAATAATCTTGAATCAGATAAATAAATTACAGCAA
AAATGGCAGATGATATGCCCACATTCAAATGTGTGCTGGTAGGAGACGGCGGCACTGGCAAAACAACATTCGT
CAAACGACATTTGACTGGAGAGTTCGAGAAACGATACGTCGCTACACTCGGTGTTGAA
GTACATCCACTAGTTTTCCACACAAACAGAGGCCCTATTAGGTTTAATGTTTGGGATACAGCTGGCCAAGAGA
AGTTCGGAGGACTCCGAGATGGTTATTACATCCAAGGTCAATGTGCGATCATCATGTTTGATGTAACCTCTCG
TGTCACCTACAAAAACGTCCCCAACTGGCACAGAGATTTAGTGCGCGTTTGCGAAGGA
ATCCCAATTGTACTTTGTGGTAACAAAGTGGACATCAAGGACAGAAAAGTCAAAGCTAAAACTATTGTATTCC
ACAGAAAAAAGAATCTTCAGTACTATGACATCTCTGCCAAATCAAATTACAATTTTGAGAAGCCATTCTTGTG
GCTTGCGAGAAAGCTGATTGGTGATGGCAACTTAGAATTTGTTGCTATGCCTGCTCTT
GTACCCCCTGAA
>1002049675_1 Heli.1-DT665373.3.5
GCACGAGGGGATGCGCTTGCGTGATGCCGGGTGCGTGTTATTTTAACTTTTAAAGTGGCGTCGTTTTAGTGCG
AAACTGAATTATTTATTAAAAAGGATAATATATTTTGCATAGCTATAAATTGAAAATGGTCGCCCCGAAGAAA
GGCCGTGGCGCAAAAAATCAAGCGGTGAAGGCGAAAGCTAATCAACCCGAGGAAGTTG
CCGACAACGATATCGCGGAAAATGTAGAGGAAAATAATGGCGAGGCACAGCCGGAATCGGAGCAGGCTGAGCA
GGCTGCTGGGGCGGACGAAGAACATGAAGAACAAGCTGAGGGTGCAGAAAATCAAGAAGGCGATGAAGAAGTG
AAAGGAGAAGAAACAAAAGAAGAAAAAGTCGAAACTGGTAAAATACTAGTAGAGAATC
TACCACCAAGTTACCTATTTGACTATCAAGATAAACTTAAAGAGCTGTTCTCCAAGCATGGAGAAATCATCAG
TGTGAAACGTGGTCCAATCATTGTTACTGAACAGACCACATCTCCAACATTATCGGCTATAGTCGAATTCAAA
AACAAAGATTCCCTGGAGAAGGCATTAACAGAAGATGGTACAGCCCTCGATGGTACCA
CCATATCAGTAGCAGCGGAGTCTAGAGCCGAAACTGCAGTGTTGGTGGGAGTTCCATATGAAGCTAGCACTGA
TTATGTGAAGCTGTTGTTTTCACAGTGTGGAGATGTAGCACATATACATGAGTTTAGCAAGACTAAATTTAAA
ATTTTAAGAGTAACTTTCCTCGAAAAGGAATCTGTGGAGAAAGCCCTCAAGTTAGATC
GTGAGTTACGCA
>1002049676_1 Heli.1-ES587676.1.5
TGTCGATCGCCGCACCTAACTAAAACACAAAAATGTATGCTGCTAGATTGATCGTTCCCGCAGCCAGGTCTGC
TATCTTCAGCAATACAGCTCTAGTGAGGCCGTTGGCGGCGCTCCCATCCCACACCCAAGTAGTACCAGCTGCC
CCCCAGCTGTCCGCAGTCCGTTCTTTCCAGACCACCTCTGTCACTAAGGATATTGACT

CTGCAGCCAAATTCATTGGTGCTGGTGCCGCCACAGTAGGAGTTGCTGGCTCCGGTGCTGGTATCGGAACAGT
GTTCGGTTCCCTCATCATCGGCTATGCCAGGAACCCCTCCCTCAAGCAGCAGCTGTTCTCATACGCCATCTTG
GGTTTCGCCCTCTCTGAGGCCATGGGACTGTTCTGTCTTATGATGGCCTTCTTGCTGC
TCTTCGCCTTCTAAGCTACTTACTTTTAAGAACACTACTGCCATCCCGCGAGGTTTCAGTGTATACTATCTGG
AGTGGACGGCCATGGAATCGAATGTGTGAAACCTCACCCTCATAGTGGTGCGAGGACTTGTGTTAAGTGTTAC
ACTTATCAGTGCATCAAAGCAATTTATTTAATTATGTAGTATAATAAAATCTGCAAAT
AAAATGTTGTACGTAAACTCGACTATTCCTATATCCTGATATTGATTGGTTATATATAATTGTAGATTTGAAC
TGTTATTCAATTTTGTCTATTTCCCAGTGCAAGCGTGTTGGGCGCGTGGTGACTTTTAAGCCAGTTTTAACAC
TTTGCGTGTGAGTATTGTCATCACGTGTCCACTTGTTGTGCTGTGAGGGAATGTCAGC
GTCTGTATTGTACATTTTTAGGGCGATTAAATGATA
>1002049677_1 Heli.1-ES587851.1.5
TACGGTCGGAATTCCGGGTCGACTACGCGTCCGCGCTGGTTCGCAAACGCTAACCAAATATACGGTTACGCAT
AAAAATATAATAAAATGGCTATAAGACCGGTATACAGACCGCAAATCGTCAAAAAGAGGACGAAAAGATTCAT
CAGACACCAATCTGATAGATATGATAAACTTAAACGCAACTGGCGTAAACCTAGAGGT
ATTGACAACAGAGTGCGCAGGCGTTTCAAGGGTCAGTACTTGATGCCCAGCATTGGTTACGGATCAAATAAGA
AGACCCGTCACATGCTGCCCAATGGCTTCCGTAAGGTCCTTGTCCACAATGTAAAGGAACTGGAGATCCTTAT
GATGCAGAACAGGAAGTATTGCGCGGAGATCGCCCACGGAGTGTCATCTAAGAAACGC
AAGAGCATTGTAGAGAGGGCCCAGCAGCTCAGCATTAGGGTGACCAACGCCGCTGCTCGTCTCCGCAGCCAGG
AGAATGAATAAATATAAGAATAAATTTATAAAAAA
>1002049678_1 Heli.1-DT662124.3.5
CGCGCTTCTAAATAGCTTGTGAACTGTGGCTGTTGAACTTTTGTGCTTTGAGTGTGTGTTTGAGCTTATTTCT
TTTTAAGATAAATCGCCGTATTACAAACTGATTTACTGCGCTTGATAAGCTGAAGTACTATTGGACGAAACAT
ATACGCAGCCACAACTACTAAATTGTTGATAATCGAAAACACAGTGTTATAGGTATTC
CCATAGTCACGTCTTACAAAATGTTGTTCCAGCACAGTCTGTTCCAGTCGTGAAATTAAAGTGAAGAACAGTA
AACGATCACCATGGACCATCGGATATTCATCTTTTTGGCAGTATTTGCCCATTTTAGTTGCACATTGGCACAG
AGTCCATTTTATGATGGTGGACCAAAAGCAGCAAACCCAGCGCCAGTGGGTAACTTCT
TAACACAAACTGTTTACGGCTTCCTTGATTTCACCACTACAATAGGCAATACAGTAATGGTTTTCTCACCGCA
GTCCGCCCCACCACCAGAACCACCGTCAACTGAAAAATCTGTACAAGAAAACATTATAGAAACTAAACCACCA
CCAATAAACAATGTAAAACCTGAAGCAATCAAGCCCAGCAAAACTGTGGACAAGGATA
CAACAAATAAAGTGGGAATTCCTATAGGTGCCTCCAGTGCTATTTCAGTTATTAGTTCTCCTCCTAAAATTGA
TATTATAAATATACCAAAATCTGTGGCAAAAGAACAAAAGCAAATTATCACAAAAGTAGAAGTCGTCGCAGGA
CCATCCAAAATCGTAGAAAGTAATGCTGCCAAACAGTTAGCTAAAGCCAACAAACAAT
TACCACAGGTGAAAAAACAGCAGCCGAAGAATCAACCAGTGAAAAGTGTTACTAATATAGTGAGCAGCAAAGT
AGAAGTGAAACAAAACCCCGAACCATCTTCAATAGTTAATTCAAAAGTAAATAGTGTGGTCGAAATCCAATCA
AGTGAAAGCGAAGAGGAGCCGGCAATTCTAGTGTCAAATAACATTAGAGAACCAGAAT
ACGATTACTTATCACGCCAGCCCT
>1002049679_1 Heli.1-DT663055.3.5
GCACGAGGGTTCAACATGAGATATCTAAGTCAGGTGGCCGTGATTATATTTACTTTTTGGTTATGTGAAATAG
TGAAGTGCAATCCACATCGTAGATTAAAAAAAAATCAGAAGAGTGCCCACTTCGCTTTGCAACCTATACCATT
GAAACCTTTTTCGGTTTTATTAAAGAAAATAACACCGTTTGGAGAATATGGAATTAAG
CATTCTATCAAAAAAAGGCAGATTGTCCAACCTAGCGAAATACCCAATATTGAAGGTGGTACACTTGAGGATA
TTATAAAAATACAAAATATTGGAGTAGCAGACAAAGCAGAAGATATTCCACTTGAACCAAATTTAACCCCACA
AGTAATAATAACATCTATCATTAAGCCTATAGAAGATAGTGTGCCTATTTCTATACCT
TTACTTTTGCCAGAGGGTTCAGATTTGCCAATAGACCAGAAAAATATAGAGGATTTGAAGCCTCCGGACGAAC
TTATACATAAAGAATCAGAACTAAAAGTCATAGAAGTAGCATCATTATCAGAAAATGTTATTGAAATTACTCC
AAGGCCAAAGATAGAAGAAATACCGCCAGAGTACCTCGAAATTAAAAATGTAGCCATA
AACACTCCAGTGGAAAGTACAGTACCATCAATATCGAAAATTCCTCTTATAAAAAATCCAAATGGTCCCTTTT
TATTTACTGATATTACTAGACTACCTTTAAATGGTTTTTATCCTGCTCCATCTATTGGTGTTCCTGAATATAT
TGTACCTTCACCACCATTACCACTACCACCGCCTGTAGTTCAACCACATATAAGCCAG
TACTGATACTAC
>1002049681_1 Heli.1-DT662470.3.5
TGGAGAAATTTTATTTAAAAATCAAATAAACATATTTCATCAAAATGGTGTTGGAAAGTACAATGATTTGTGT
AGATAACAGTGACTACATGAGAAATGGAGACTTTCTGCCAACGAGACTGCAGGCACAGCAAGATGCTGTTAAT
TTGGTTTGTCATTCCAAAACAAGATCCAACCCGGAGAATAATGTTGGGTTATTGACTT

TAGCCAATGTGGAGGTACTGGCTACATTAACCAGTGATGTCGGTAGAATATTATCAAAACTTCATCGTGTCCA
GCCCAATGGAGATATCAATATCTTGACTGGTATAAGAATTGCACATTTAGCACTTAAACATCGACAGGGCAAA
AATCATAAGATGCGAATAGTTGTATTCGTGGGGTCGCCAATTAATACAGACGAGAAAG
AATTGGTCAAGCTAGCGAAGAGACTGAAGAAAGAAAAGGTCAATTGTGACGTCGTTTCGTTTGGCGAGGACTC
TGAGAACAACCCTCTTTTAACATCCTTTGTTAACACTCTTAATGGTAAAGACAATACAACTGGCGGAAGCCAC
CTTGTATCCGTGCCAGCTGGTGGATGTGTGGTGCTCTCTGAAGCTTTAATAACCAGTC
CTATTATTGGAGGAGACGGTGCTGGCCCATCAGGATCTGGTTTGTCACCATTCGAATTTGGTGTTGATCCGAA
TGAAGATCCCGAACTTGCTCTTGCTCTTAGAGTATCTATGGAAGAACAAAGGCAGCGTCAAGAAGAGGAGTCT
CGTCGTCAACAAGCTTCCACTGAGGGTGAACAAGGAAAAGCAGAAG
>1002049682_1 Heli.1-DT666443.3.5
GTTCGAATTTTTAAATACGAATATGTGTAACGTTTGCACACGCGATGTCGAGGTTACGCCGGCCCCGGCGAAA
CCTAATAAATCCTTGGAAAGACTTAAGAAGAAGCATAATATACAGACTAACCCTACTGACGAAGAGAAACAGA
GAGAAGTGGAATACCAACCAGATTTATTGGCTGTGTCGACACAATATTCCAAAGTGGC
ATACGATTTGTTCAAGCAAGCTGTTGTTAGGCTGCAGGAGACTAAGGCTTATGCTATAGCAACAAAAACGACA
CAGCCGTTCCACGTAGCGCTCGCTGTGCTAGTAATAGCGGCGTGGCTTACTAAATCTGGGTCCAGTGCCAACT
TGAGGGGCTGGAAGGCGTTGTACGTGGGGGCGGTCGCGACACACCTGGGGGCGCAGAT
ATGGATGACTTTAGTGTCAGGCATAGTGCTGTATTTCTCCCTCCCGCGCCACGAGTTCGGTCGCGTCCAGACG
GTCTTGTTCCCCGTGTATTACGCCTTCAACTCCCTGGTCAGCCTGTTGGCAGCGCTCGCATACTTCCGAACGC
AATGTCTCACCCGCTTCGAGAACACTTCCTGGGTACAACTAGCCCTGCTGTTAGTAGT
ATTCAGTATCGAAGCGTACGTCCGCCTCTGGCTCGTCCGGCCGATGCTGCGTGCGAAACACGTCAAAACACAA
ATGGAGGAAGCCGCCGGCGGTGGACAGGAAGTCGGCCGCCTAGTTCTCGGTGAGCTTGCCCACTGCCCCCGCT
ACCTCCGCGTCCTAAAGACCTTTAGGGCCTACCATTCATCTATAGCGATGGGCACCAT
GATTACTTTAGGGTGCTCCTTCTA
>1002049683_1 Heli.1-DT662784.3.5
CCGATACGAAGTGTGCGTGTGGCCTGGAAGCCTGCCTCACTCACAAGCCTGTCCTGGCTCCTCCGAAATAGCA
CCAGTACCACATTCCCGATTCCAATGTCCTGGGCATGAAGGTTACTACGCTGACCCCGAGAATTGTCGGTGGT
TCTTCGCTTGTCTTGACCATGGAAAATCTCCTTTAGAAGCTTATGAGTTTCGTTGTCC
GTTCGGCCTTGGATTCGACGCTGCGAAGCTCAAGTGTGATTGGCCCTGGCTGGTTCCTAACTGCGGAAATATA
GGAAGATATGAAGCTGAAGCCTTTGGGTTCTCTAGTGCTGCTTTATCTGGCGCTACTGGATTCCATGGGAAGA
CAGCAGATTCAGTGAACATTGCTGCCCACCAAAGCTTAGTATCCGGAGCATCTCTAGA
CAATCTTGTTGGAATACAGAACGGCTACCTATCCAAGGACGACATCCTCGACTCAAATTTCATTGCTTCTCAA
GAAGCTTCCAATCTAGGTTTCGCTGGACCACAAACTTATCAAATTGATGAAGGGTCTATTGACTATGCTTTGG
GTGGTCAAGGAGAATATAGTGGACTTTCCTACAAGTATGTATCAGCTGATAATATTAA
CAAGGGTCTTGTGCAAGCAACGAATTACAATGACGAAGACAAATATACAAGTGGATCCATAATTCTAGATGAT
TATAGATTACCGAGTAAAACGTTAGTTTCTTCCGGAAAATATATTCCTGAAATTTACTCAGGCAAATCATCAT
CTGGTATCGATTCTGGAAAATATAGGGGCAACGGGGCTTATGCCGGTGCTGCAGCAAA
TTACAATGATGGAAAATACAACAGCGGTCAGTATAGAAAAGATAAGTCTGGTGCTTATGTTCATAATCCAGCT
GGAGACAGAGCACAGCCTTACCAACATATTGACGTACCACCAGTACCTTATGAACATTTAGACTCTAAATATA
CACAAAATAC
>1002049684_1 Heli.1-DT668423.3.5
GCTGCACGTGCACCAGTCGGGCTTGCCGTGATCATGAAACTTGTAGTGCTCCTCGCCGCAGCCGTCGTCGCGG
CGGCCCAGCAGCCGCAACGTCGGGAACAGTTCAGAGTCATATATGAATGGCGCACTATAGACTTCCAGTGGGA
GTCACCTGCGGATCGGGAAACTTATGTTAACACCAGTCGATACATCCCGCAAAATGTG
CTCATATCTGGAATCAATTATTATGAAGATAAAATATTTTTGACTCTACCAAGAATGCTCGACGGCGTGCCCG
CCACGCTCGCTTATATACCGGCGCAGCAGAACGACACTACGGCGCCGAAACTGAAGCCGTTCCCGAGCTGGGG
CGACAATACCGTTGGTAACTGCGACGCCCTACAATTTGTGCAAAATATAGAAATTGAC
AGCAACGGGATGATGTGGATTTTAGACAACGGCCGCGTCGGCACTCTGACTCAAAACCCCAGTACTAAGTGTC
CTCCGTCTATTGCATTCATTGATTTAAAAACTGGAGAAAATAGAATTGATCGTATACCATTACCTCCGGACAC
GGTGAATCCTAATACCTCATACCTAAATGACCTAGTGGTCGACAGCCGTGATGGTGGT
TACGCGTACATAACCGACAACAGCGCGGTCGATCCGGGCATCGTCGTATTTCGTCTGAGCGACAAGAAATCAT
GGAAATTGCGTGACTCGAAATCGATGCTGGCCTCACCCGACGCCGCTTTATTCCGCATAAATGGCACAACAGT
GAACCTGCCAGTGAATTTAGATGGTATCGCACTCGGACCACAGTTCCTCACAGAGGAC
GGGAAGGTCGATCGTACCGTCTACTACTGCCCACTCGCAAGCTACCATCTATACGCTATCAACGCTTCTGTTT
TACGAAACGACTCAATCCATGCACAAGACGAGCTGGCCGTTCGCGCGAACGTGGTCGATCTCGGAACGAAA

>1002049685_1 Heli.1-DT667017.3.5
GACCCACAACTGAAGCAACTACTGTACAATATCAATCGTCAGAAGAAGAAGAAGATAAAGAATCTCAAGAATC
CGTATTAGAAAGAGAAATAACTTCACAATCAAATGAACCACAGTACAATTCAATTTTAAGAGCTCGCGCCACT
CTCCCCCCAGAACCAGAATTGAGTAGTCCGGAACCCACTACTGTTTTGTCAGTTCAAA
TTTCTTCCTTATTAAACGAACCAAATTCAGATAATCAAAGTCCCGAACCTGAACCCGCTATACAAACGACTGA
AGCCGAAACGACGACCACATCTACTACCGTAACGACCCTCTCATCGACTACACAAACAACATCAGCTTCATCA
ACTAGACGACCTCTAATAAGACGTCGAGGATCTACAATACAAACGACTAGTACTGAAG
CACCAACAACAAGCACACAGGTACGAGAGCGGCGTCCATTCCTTCGTCGTACCAAAGCCACTGCACCCCCTGA
TGTCACTGACTCCGACATCACCACTCCATCCCTCGAAACAACCACGAACAAGTTCACAAGACGAGGCAACAAC
AGGTTCAAGTTACGGAGAAATGAAAATGATGTCGAGAAAACGGAAAATAAGACATCTT
CAACAGTCGCGCCGCAGCGTAGTGAGAGACCGCGCAACTTCGTACGCCGGCGGCTTGGAGGGGCGAGCTCCAC
TACAGCCGCGCCAACCGTAAGTTCAACTCAAGCTGTCCTACCATCACGTCGTCCGTTCAGAATAGCGAGTCGA
CGTCGTCCCCTTTCCACTACCACTACCACCACAGCCAAACCTACTACTGCACTAGTCG
AGAGTGAAGAGGCTTTACAGGATATTGGAGATATTGATGCCATAGAAGATCCTTCTTTAACACCGAGTACACC
TCGAACGTCTCTCAACGTCCAAAGACGGCGTCCTCTCGTCCAACTCAAGATAGATGACCAGAATCCATCAGCA
ACAAATGAAGAAGAAAAGAAAAGACAGAGTAAGAAGTTTAGCGCCAGCTTCAAACAAA
ATCAATTGGACGAATTGTTAAAACTAAGGGCGAGTGCGGAAGAAATTGATATAACTACGGAAGGAAAAACTAC
TTCAGATGATATAAGTGCTGAAACTGCAGTCGCATTAGCAGCACACCAGCTCTTAGCAGCTCCCATACCAATC
ATACCAGATTACGACGAAGAGTTCTTCACGACGAAGAAAACACCTAAAACTATAGTAG
ATTACAAATTCACGAATCCAGCTTACACTGAAGAATATTTCAAAACACAAAGTTATTCAGACGAAACTCAAAC
TTATAGCACAAGGCAAAGACTAGTGGAAACTTCTACACCTAATTACAGTCCTACTGACACTAATACACCAAGG
TTAGCTGAGGGCAGTTTTACTACATCTTCATTCACCAGTCGATTCTCCAAACCAGATT
CAAATGTGAACCCAACACTTTCCGGAGTAACACTCAGTTTGGGTTCACAAAACGCGGAATCCACAGCTCGCTA
CAACCCTACAGAACCTACACCGTACACAACAGAAAGTTACGAATCCC
>1002049687_1 Heli.1-CO729687.1.5
ACTAACATACAAACCAACCAATCAATCAAAATGATGAAAATCATCCTTGTTATATCCGCTCTGGTCGCCATCA
GCGCTGCCAAGCCTTACGTAGTCAGTGCTCCACTTATCGCCGCCCCAGCACCCGTTGTTACCGCTAGCAGTTC
TCAGTACTACCACCGTATTAACAACGGCTACGCTGCTTACGTCGCCCCGGCAGCTGCT
TATGTTGCCCCTGCAGCATCTTACGTAGCCCCTGCAGCATCTTACGTAGCCCCAGCAGCTTCTTACGTAGCCA
GCCCCTACGTAGCGGCTGCTGCGCCTTACGTATCTGCCCCTGCTGTTGTTGCCGTTTAAATATTCCTAATTAT
GCGCCTTACTCAATATGTGATATTATGAAGTAAATATTTTATTGCA
>1002049688_1 Heli.1-EL599556.1.5
AACGGCGTGTGGTAGTTCCAGTTCGACATGCGTCAACGCTCTGTGTCATGACTGTGACACAACAACGTTCGCC
ATCCACATTGCGCATCAGTCTATCGATAACTGACCCACTCGATTCAAACTGCCAAGTTTCGATTCCATCAGCG
CGCTCACTACTCGAGTAAAATACCAGACGTCTGGACGTCGCATCACGTATCCATTGCA
TACGATGCGGTAGACAGCGTACGATAGTGATGCGCTTTATGTAGATTTGAAATAATAGTAATATACTGGCCTG
CCAAATAAACTATTCTAAATTCAAATTTCTATCAGTTTTGACGGATTGCCGCTCAAGTGTCGTGTAAATAAAT
AATAACCAAAGTGTTTTTTATAGTGTTTTAAAGATTTAGTTGTGTATAAATATATAGT
GCACGATGACTTCGCGATCTAAGGAGAAAGTGGCAGCGGCTTTTCGGAAGCTGTTTCGCTCTCCAGAGAAACT
TGACAACGAGGGAGCCGGCCCGAGCGGGTCGCCAGCTAGACGCGGGCTATTTCGCCGTCACAGAGATGGTGTG
AGTCCGGCGGAAGCCCCTGCGAGTTTACCGTCAAGTCCTGCGGCTTCATCGATTGCTA
AGAAAAAATCTGGAGGATCACACGACGTGCGGGAGAGAGCAGCAGCTGTGCGGTCACGGAGACTCATGAAGG
>1002049689_1 Heli.1-DT662390.3.5
TTACATCGTCGCGAACGTAGCGTAGTCATAATAAAAGTAATTAAATATAATAAAATATGAGCGAAAAAATGAA
ACTATTCACCAGGGAAGAGTTGAAAAGTCACAATTCACGCGACGACGCCTTTATTATTATTGATAATTCAGTA
TACGATGTCAGCAATTTTTTGGATGAGCATCCTGGTGGAGAAGAAGTAATTATGGAAC
TGGCAGGTGAGGACTCCACTGAAGCTTTCGAAGATGTCAGTCACAGTTCTGATGCCAGAGCGTTAATGAAGAA
GTTTAAAATAGGCGAAGTGGTGGAGGCTGACAGGAAACAGACGAAGACCAATCTGCCGGCGCAATGGAACAAT
GACCAACAATTGGAGCAGAGCAAGTGCGTAATCTGGGTGGTGCCGCTGCTGCTGGGCA
TAGCTGCCACCGTCGTCTTCAGATACCTATTCATCTAATACCACGACGAATTACTTTAATTTCACCATAGTTT
AAGAGATTTTCTTACAACTATATAGTTATATAAAGAAATTAAGACTCAATAACGCTATAAAAACAATTTAAAA
CGATTATTTATTAAGCGCAAACTTCAGTGCAGCATTTATAAAATTGAAATCTTCATAT

ACATATATTATATATGTATATAGGAATATTAATTTATAAATAAGTATTTTTCTACATGAAACAGACAGAATTC
CTTTAGTCCATATTGTAATTTAATATCATTAAATGACATTAGTGTCTGTACCTATAGATAATTGTAAATAGCT
TTTCTACTGTATGTTGATAAGAAATAACTAACAAATGCTTGACCTGATATACTTAAGA
ATTTACACTCGGTTACACTATAAC
>1002049690_1 Heli.1-EL602717.1.5
ATTTATTGTGTAGTTAATGTAAAGTTGCAAATATTAAAGTTTTTGTAGAAAAAATACTTTTTTAACTATGGGT
CAAAACCAATCTGGAGGAGGCAGTGGTGGCGACAAAAAAGATGATAAAGATAAGAAAAAGAAATATGAACCTC
CAATCCCTACTAGAGTTGGCAAGAAGAAGCGTAAAGCAAAGGGTCCTGATGCCGCTTT
AAAGTTACCTCAAGTGACACCTCATACGCGATGTAGGTTGAAGTTGCTCAAACTGGAAAGGATTAAGGATTAT
TTGCTTATGGAAGAAGAATTTATTCGTAACCAAGAGAGACTAAAACCTCAGGAAGAAAAAATTGAAGAAGAAA
GATCTAAGGTTGATGATCTTAGAGGGACACCAATGTCAGTGGGAACCCTGGAAGAGAT
TATTGATGATAACCATGCCATAGTTTCTACATCTGTAGGCAGTGAGCATTATGTCAGCATCTTATCCTTTGTT
GATAAGGATCAATTAGAACCTGGTTGCTCAGTGTTGTTGAACCACAAGGTACATGCAGTTGTAGGTGTACTAG
GTGATGATACAGATCCTATGGTTTCTGTAATGAAGTTGGAAAAAGCTCCACAAGAAAC
CTATGCAGATATTGGTGGTCTCGATACCCAGATACAGGAAATCAAGGAGTCAGTAGAGCTGCCACTTACTCAC
CCTGAGTACTACGAAGAGATGGGAATAAAACCCCCTAAAGGTGTTATCTTGTATGGGCCCCCTGGTACTGGCA
AGACTTTGCTGGCCAAAGCTGTTGCCAACCAGACGTCTGCCACATTCCTCAGGGTTGT
TGGATCTGAATTGATCCAGAAATA
>1002049691_1 Heli.1-ES584836.1.5
CTCACAATCCTACGATCAACCCTCTAACAAAATGCATAAGCTGATTGTATTCGCCACCCTCTTGGCTGTTGCT
GCTGCAGCCCCCGGTTTGGCACCTCTTGTATCCTCATGGGGACATGGGGCACTCGTTGGTGCACCAGCTGTGG
TCAGCGCACCTCTCGTCAGTGCTCCCATCATAAAACAAGCCGTCCCCGTAGCTACTTC
CTATGCCAATGTTAACGCTGTCCGGATTTCCTCTCCAGCTCTTGTCGCAGCCCATGCTCCTATCATTGCTGCT
CATGGTCCCGTCCTCGGTGCATCTTTAGGCCACGGTCTCGCCCACGGCTGGTAATCCGCCTAACGAAGACCGA
GCCTTGGTTTCAGTCAAGATACGCAACAGTCAATTTTATTTAAATTATAGTCTGATAA
GCTATTAATGAAATTCTATTTGGTCCTTATTATTAAAAATTTAATTTA
>1002049692_1 Heli.1-EL597981.1.5
CGCCCTAGCCGCCCCGACCGCCCTACCCGCCCTAGCCCGCCACGCCCACGCCCTAGACTAACTGCAACTGCGA
CATAGACTAAACGGTGTCAAATTGACGTCAATGAGAACAGATAATATTTACTCACTATAGGAAATATATGAAT
CTCAAGCGCTGTAGACTCAAGAGCAATAGAATATTATTGTGTAAATGTACACCTGTAA
ATGTAAAGCTTATATTTGCTGTTCAATACACATATGATTTGCTCTAAAGATCAATTAGTACAAACGTTATCTT
ATGATTGAAACTACATGACAATCTCAACTTTCCAGAAATTAAGAGATATTTGATGAATTATGCAAGTATTTTT
CCCATCGCTTTATGTTTAATCACAACACCTTATACTTCTCTGTATATTGAAGCTGTTA
TGTAAAAGCTAAAACGTTAACGCTAGTTCCAAATAATCTAGCAAAAATCGTACAAAATTTTGATCAACTAGCT
TTACTGTAATGTAGAAGTAAAATCAACGCCGAATATAGGGCTGTCCATAGATTTCGTCACACGAATTTCATGA
TTTGACTCACTCCCAAGCCTTGTCAGCTGTGACAATATCATATTTGTGAGACCCTTAC
ATAGTGTGACGTCATATATGTAGCAATTTTACATCTAGGAATTATTGA
>1002049693_1 Heli.1-DT668315.3.5
AGGATGCATGTTTTAAATGTTTACGAGAAGTTTTAATTTATAATTAACATATATCTAGTTATATTTAACGTAT
ATAAAAAGCATGTGAGATTGTGTTTTCAATTGTGATATAAGTATTTGAGGTTGCTAAGATAAGTTATAAGTGC
TTTCATTATTTACGATTGTTTGTATTGAAATACTTCGTCGGTAAGTTTTTAATAGATT
ATTATTAAAATAAAGAAGAGTGATATTAATGCCGTCAATATCCAGAGATAAATATTTTTTATGTTTTTATGAG
GTATATAAAATTACAATTATATTACGTAATGTACGCAGAATGCCTGCCGCCATCTTCGTTAGACGCGTGTATG
GGAATTGTGAAATGAAAGTTAAATAGGTTTGGTGAATTATAGTAACTTATAAATGTAA
TTTCTATATATATTAATAAATATTTAACATGTTACTATAAAAGGAAATATAAATATAATAGTATAATTTATAA
TATGATGTAAAACTATTATATAAAAATGTATATTTACAATTTATAAATATTATATAAATATTATATAAATATC
AGTATGTTTAATGTCCTACGATAAAAGTAATCTTTCAATATGTTTTATATATTACTTT
AATTAAATATACTAGACATTAGACATTAACCAAATATTAATTATTTACGATTTATACGACTGTTAGAGTTCAT
TGACCCTTGTTTTAGTATATATTATATAAATATCATATAAATATGAGTCTGTTTAATGTCCTACAATGAAAGT
AATCTTTCAATGTTTTTTTATATATTACTTTAATTAAATACAATAGACATTAGACATT
AACCAAATATTAATTATTTACGATTTATACGACTGTTAGAGTTCACTGACCCTTGTTTTAGTATTAACTAATA
TTATAGTCAGAACCGACTGCTGCTTGGCTTCGTTGGTACTGGTGTGCTTGGCTAAGTGAATTGCCGTGGCATT
TAATTTTATATATATTATATTTAGATAGTCTCGTGGTGCCTTGTGGTGCGTCAACGTT

AACACTTGGTGTATGATGGATAGGTGTGCGATGCTATTTTATGTCCTGGAGCTCTGGTGTATGTAGTAGTGTC
CGCCCGTCGCATACGGGTGCGCGATGCTATTTTATGTCCTGGAACTCTGGTGTATGTAGTAGTGTCCGCCCGT
CGCATACGGGTGCGCGATGCTATTTTATGTCCTGGAACTCTGGTGTATGTAGTAGTGT
CCGCCTGTCGCATACGGGTGCGCGATGCTATTTTAT
>1002049694_1 Heli.1-ES586722.1.5
GATCCAGTTTTTCATATTTTAAGTAAATTAAAAATAGTTTTAGTTTCTAAATCGTAAAATAAAACATAACATA
CAGTTTAAGTGTAGATTTACTTGAACCTACATAAAAAAAAACACGAATCACCATGAGCTCAGGAGGAACTCGC
GTGTACGTGGGCGGGCTCGTCGAGGGCATTAAGAAGGAGGATTTGGAGCGCGAGTTCG
ATAAATACGGGAAACTCAACTCCGTGTGGGTCGCACTTAACCCCCCAGGTTTTGCGTTCATTGAATTTGAAAA
CATGCAGGAGGCGGAGGATGCTTGTATGGCTTTGAATGGATTCGAGATGCTAGGAGCTACCTTAAAAGTAGAG
TTATCGAGAAAACGAGACGGCCCTCGTCGAGGTGGAGGTAATTTCAGAGGCGGGCGCG
GAGGTAACTTCAGAGGGCGTTCCTTCGGAGGGGGCCCACAGGGCGGCGGCCGATCCTACGGCGGCGGCAGACC
CTTTAACCGCAACAACCAAGGCAACGGGTACGGCTCAAGCTATGGTGGAAACTCCAGATCTAGGTCACCGATC
ACTCGATGTTAATTTAATTCTAGCATTTAGTTAGGTTTAGTGTTATCGTAATCTGAAG
CCTTCCAAGTGTTAAAATTCAATGTCTGTTTAATACTGGGGGTTAATGCTTCAAAGATGGTTAATGAAGTTGA
TGTCCAAGGAATTAGAGTTCCTTGGTATAATTTTAATTTGCATCTATAGAAGTCAAGATATGGATGAACAATC
TTCAGTATATCTTTAACATTTTACCTAAGCACCT
>1002049695_1 Heli.1-ES585792.1.5
TAGATCAACACAACAATTTTAAAACTCAACATGTTTGGAAAGATCGTATTCCTAGCCGCCATCGCCGTAGCCG
TGGCTAAGCCCGGTATACCTGTAGCGGCTCCATTTGTTGCCGCTGCTCCTGTGGTAGCACCAGCTCCCATTGT
TGCTGCATCCAGTTCTCAATATATCTCAAGGAACTACAATGGAGTGGTATCTGCACCT
TTAGTGGCGGCTCCCGTCTATGCTCCAGCAGCTAAGATTGTAGCACCCGCTGCTTCGTATGTGGCTGCTCCTG
CTCCTTACGTGGCCGCTTCACCATATGTGGCTGCTGCTTCCCCATACGTTGCTGCCGCTTCTCCATATGTAGC
AGCTCCTTACTACGCTTCTGCCCCTTTTGTTGCCCTTAAATGATAAGACTGCTACTTC
CTAACGAATAAACAAAATTATACC
>1002049696_1 Heli.1-EL602250.1.5
TGGAGGACGCGCTCACGCTCATCGTTTTAACACTGTTACAACTATGTCGAGCCGAAATATCAAACACGGAATG
GTTCGCCGCAAATTTAATAAACGACCCAAATTACACGCCAAAACCCATCACGGAAATATTTACTACTGATATT
ACATTTATACCGAGAAAAACATCAACGTCGACCCAAGCAACTTCGCGAGAAACACCTT
CTCCAACAATCTCGACCACATTTTCTGGGAGAGAAATGGTTACTCAGGATATGGGTACCGAGCGAGTACCGCA
AGTTATAAACCCTGCAATACAACAATCCCAAGGAAACATGCCACCAAATAATAACATGCCTAGTGCAGTTTTA
GTGTATGATCCAAGCCTTCAAAATAGTCAAATATATCAACAGCCTTCATCTACTCCAG
CAACAGCTGAAGCGGAGTTATCCACAACTTTTGTGACACAAATGTCACCTATGCCTCCTTCTCCACCCTCTCC
TCCGAATTTAATACCAGCTGCTCAAGGCCCTACAATTGTGGTACGGCAGCCCCTAAATCAGAACGGTAATCCT
CCACTAATACCAATTGGCTTAATACCAAATGTACAGAATGGAAATTCTGCTATCCCAA
TGTCAAATATCCCTGTGGTAAACACTTCTTCTGGCTTGATATCGCCTTCATCGGTTATGCCTGACCAACAAT
>1002049697_1 Heli.1-DT667274.3.5
CGCAACATTTTTAGACTGAATATTTTATAATTTTGCATTTCAATGAGAACATTGACATAAATATACCTTATTA
GAACCGAAACAATTGTGAACAAAAGAATAAAGTAACGTATGTGTTTTGTGTTTCAATTTCGTGACGTAACTGG
ATTTCTTGTGAAACGCGAGTAACTGTACTCAACCCGCAAACGAAGGTCGTATCTCTCG
GATGGTTATTACTTTCGTATAGTTCCGTAACGGTACATACACAAAATGTGGGAAGTTGACTCTGACACAGGGT
CCCAAAGTGCGTCGTCGGACGGGCTTCGACGTCGGCAAGGATGGGACCCAGAGGCCGAGAGTTTGGCTTCTCA
AATCGACGAACTCGACGAAGTGCTTGCGGAAGAAGAAGAAGGTTGTCCACTGCCTTCT
ACTCCAGAAGATCAACATTTACTCGACGCAGAAATGGCTGAAGTGTTGAAAGCTGGTGTGCTATCCGACGAAA
TAGACCTCGGTGCATTGGCACATAATGCAGCAGAGCAAGCGGAGGAGTTTGTACGAAAAGTATGGGAAGCGTC
GTGGAACGTGTGCCACTTCAGACACCTGCCGCGTTGGCTACAAGACAACGATTACTTA
CACAAAGGCCACAGACCTCCACTTCCATCTTTCAGCGCCTGCTTTGCTTCTATATTTCGCATCCACACTGAAA
CTGGCAACATTTGGACCCATCTGCTTGGTTGCGTGGCGTTCATCGGTGTAGCTATTTACTTCTTAACTCGCCC
ATCTATTGAAATTCAAATGCAAGAAAAAATGATCTTCGGAGTGTTCTTCGTTGGCGCT
ATTGTATGTCTTGGATTTTCATTTGCATACCATACTTTGTACTGCCATTCTGAAATGGTGGGTAAACTATTTT
CTAAGCTGGATTATTGCGGCATTGCTCTACTTATTATGGGGTCATTTGTTCCTTGGCTGTATTACAGTTTCTA
CTGCCATTATAGGCCGAAAATAATTTACTTATCTGTAGTGGTTGTGCTAGGAATATTA

TCAATAATAGTTTCACTTTGGGATAGATTCTCAGAGCCTCACCTCCGTCCATTGAGAGCTGGGGTGTTCATGG
GTTTCGGTCTGTCTGGAGTAGTACCCGCCATACATTATGGCATAACTGAAGGTTGGTTCAGTCAAGTTAGTAT
GACTTCTCTCTTCTGGTTAGTACTAATGGGTTTGCTATACATATTAGGGGCCATGTTC
TATGCTCTGAGGGTGCCAGAACGCTGGTTCCCAGGTAAATGTGACATTTGGTTCCAGTCTCATCAAATATTCC
ATGTTTTAGTTATAGTGGCAGCATTTGTCCATTATCATGGAATCAGTGAGCTAGCATCC
>1002049698_1 Heli.1-EL600974.1.5
TATTTTTTAAAGTATTTGTTGTATTGTTCTTAAACAAACCTTGCAACTTTAAAAATAGTTAACTAAAGTGACT
AATGTAATTATTTACCTCTTCAGTTGGGTTCTTTTGTTCCTAGTGTAATCGTCAAATATATCGAAAATGCCTA
TTTTATTCAGCGTTATTGCTCGTGGAACCGTAGTTTTAGCTAAATACGCTTCATGTGC
TGGAAACTTCACTGAAGTGACAGAACAAATTTTGTCCAAAATACCACCCCACGATGATAAACTTACCTATTCT
CATGGAAATTATTTATTTCATTATATCGCGGAAAACAAATTAGTTTATTTTTGCATTACTGATGATAAATTTC
AGCGTTCGAGAGCGTTTCTCTTTTTAAATGAAATTAAAAGAAGATTTATCTCAGCATT
TGGGGACACTGCCCAAACTGCTATTCCTTATGCTATGAACAGTGAATTTGCTAGAGTTTTGGCTACTGAAATG
AAGCATTACAGTGAATCCAGAGACTTGGAAACAATATCAAGAGTGCATGGAGAGTTAGATGAGTTAAAGAATA
TTATGGTCAAAAATATTGATAGTATGGCAATGCGTGGTGAGAAATTGGAGCTTCTCGT
AGACAAAGCTGATAATTTAGCTACAAGTTCAGTATCATACCGCACCTCGGCAAGGACTCTACAACGTTCGCTA
TTCTGGAAGAATATAAAAATGTATGTTATTTTGACATTAATAGTTGCGTTCGCCATATATTTAGTTGGCGCAA
TGGCATGTGGCGGTCTCGCTTGGAAGACATGCGTCGGATAAACATAATGTTCCTATCA
>1002049699_1 Heli.1-CV526051.2.5
GGAGTGAGTCGGCACGAGGCTTTAGCTACTGCTATATCGTTGGCTGTAGAAACATTCTATTTTAATTTCGTAC
ATAGCTGTATTTAATATTTACCAAAATGTCGGACGATGGATCAACAGCAGTTGAAAAGAAAGGACGCGGTAGA
CCTAAATCTAATGGGACACAATCAGAAGCCAAAGGTGATGGTAAGAAAAGAGGAAGGC
CAGCAGTACCAGCTGCTAAATCTAAAGAATCAAAAAATTCTTCTGATGATGAACAAGCACCAATAGCAAAACG
AGGAAGAGGCAGACCCAAAGGCTCTAAGAAAAAGGCAGCTGCACCTAAATCTAAGAGTTCTTCTGGAGAGGGA
CGATCTCGTGGCCGGCCACGCAAAGATGCACCTCCACCTAAAAAAGATGCAGGATCTA
CTGAAGAAGAACAAGAAGATGAAGAAGAAGAGGAAGGTTCTGATCAGTAAACACATATGTTTCTCTCACAACT
CACTCTTAGTGTAATTTGATTGTCATCATAACTCCTTACAGTGTAACTTTTCATAAAAAATATTTGTGAAATG
TTATATGCTATGTATAGGGGTTATCATAACAACAAGTCTCAAATGAGATTGCTCCTAA
ATTTTAATCATGTAAGGAAAGTTTTAAATTAAAAGTAATTAACACATAAATTAAGAGATCGTTTTTCATGATT
TTATTGCATTTACTTCGTCTATTTTACTGCAGATGGTCTTACATGGTTAAAATTTTTGGCAGATATAGCTCTC
CCTAGCAACGTACAAGTCTAAGATATAATTTGTAAGATCTTTTTTTTGTAGATTTATT
CTATTTCTATTATAATAAGTATTTTGTAATCTCCTGATTATATTGTAA
>1002049700_1 Heli.1-DT667587.3.5
GCGCGTGGCGTGACTTACGTGATGTTAATGTAACCGTGCTCATTATACTATAGTGTTCCTGTTATAGTATTGT
CGGAGTGACGCCCCGTTTAAGCATGCTGATGGTACAGACTAACGCTCACCACATGAAGACCACTAAAATAAAA
GGACCTCCACCCGTGCCACCGCGGCCTAGTCAGAGCATGGTGGCCGAAGCACTCGCTA
AGACTAGGAAAGCTGTAGCAGATTCGAAAGCCACACTGTCTAAGACCAGAACTTTTACTAAAAATGAAGTTAA
TCGTGTTTCGTCGAAGGCTAAAACATTGGACAGGAACTCTACACCGAGCAAGCCTTCCGTGTCGCCACGACAA
AACGGGCTCGCGCGCGTCAAGTCATTCATTAAAGACGTTATTAGTGATCGTAGCTCTT
CGTCTGATGAAAAGAAATCGAGTGGTCAGAACTCGAGGCGTAGCTCGAGTGATAGCAATTCGATTCACTCTCC
GACATCTAATAAATCAAATGAATCATTGAACTCTAAAGCCGTTAAGACATGTAGGCAAATATTAGTACGGTCA
CTTTCTACTTCTAACAAGTTAGATCAAGATTCTAAATGTAAAGTTTCTAAGTCTTCTA
GCTTTGCTGAAAAAACTTTACCCTTACGTAAAGCCCCACCACCTCCTTTATCGCCAAAGCCCAGATTGAAGCC
AATGAAACCTGTGCCACCTCCTGTACATAGAAGTATAGATGGCAATAAACATACAAAAGAACCTGATATTAGC
ATATACACATTGCCAGTGGATGCTAAGACTCAAATTTTTAGTGCAG
>1002049701_1 Heli.1-DT665212.3.5
GCACGAGGCAGTAATTTACGTCCCTTCCTATCGTGTGCTCCCATTGCCTTAACATGGGACCGGTCTGCCCACA
CTCAGGACATCACCAAGAACATTCAGTACATTACGAACGACAAGATCGATCTGAAGACAGATCCGAAGACCAT
GAAGCTAGACAAACAACAGAATCGTCCAACTTGCGTAATGATCAATTGGCTGCTCGCT
AGGCAGAAGCATGTCATGAAGTATGCCAATCTATATTTGGAACAGGGTTTCGATGTCATATCTGTATCGTGCA
CACCATGGCAACTGATGTGGCCGTTAAAGGGATCTCAGCTGGTAGCAGCTGATCTTATAAAGTTTATGGCGGC
GAATGAAAACGACCAGCCCACTGTAATACATGGCTTCTCTGTCGGTGGCTACTTGTGG

GGAGAAGTGTGCGCGCATGTCATGGATAACAAGGAAGTGTATCAACCAGTAATAGATCGTGTGACGGCACAAG
TATGGGATTCCGCCGCTGATATCACCGAGATAACGATAGGCGTGCCGGCCGCAGTGTTTCCCAAGAACAAGAT
TATGCAGAAAACACTTAAAGCTTATATGGAGTACCACTTAAAAACGTTCCACACGGCG
GCGACGGTGCACTACATCCGCTCGTCGCAGCTGTTCCACACCAACCTGTGCCGCGCGCCCGCGCTGTTCCTGC
TGTCGGCCAGCGACCCCGTCGGCGCCGAGCGCAGCAACCGCAGCGTCTATGACAGTTGGTGTAAAATGGGCGT
CAAGTGCACCTGGCAATGTTGG
>1002049702_1 Heli.1-EL602656.1.5
ATTTGTATTTATTTACATATTGGTCGAGGAATTTATTATGAATCTTTTAATTTAAAATTAACTTGATTAGTGG
GAGTAATTATTTTATTTTTATTAATAGCTACAGCTTTTATGGGTTATGTTTTACCCTGAGGGCAAATATCTTT
TTGAGGAGCTACTGTTATTACTAATTTATTATCTGCTATCCCATATCTAGGAAATATA
TTAGTAAATTGAATTTGAGGTGGATTTGCTGTAGATAATGCCACACTAACTCGATTTTATACATTTCACTTTC
TATTTCCATTTATTATCTTAATATTAACAATAATTCATTTATTATTTTTACATCAAACAGGGTCTAATAATCC
ATTAGGAATTAATAGAAACCTAGATAAAATTCCATTTCACCCATTTTTTACTTTTAAG
>1002049703_1 Heli.1-EL604245.1.5
GAGGCAGAGCGCCGCGTCAAGGCGCTGGAGGCCGAGGCCCTGCAGCATGCCGAGGAATTGGCGGCAGTGGACA
GGGCGAGGAGGCACGCTGAGGCCGAAAGGGATGATAGGGATGACGAGCTGACCGCGACCGGTGCTAAGGTCAC
ATTGCTAGTGGATGAGAAGAAACGTCTAGAAGCGAGAATAGCGGCCCTCGAGGAGGAT
CTCGATGAAGAACAGTCGAACAACGAAATACTTAATGATAGACTTAGAAAGGCACAGAACCAAATCGACCAGC
TCACAATGGAGCTGGGAACAGAGAAGTCCGCCACTCAGAAACTGGAGAGCGGCAAGCTGGTGCTGGAGAGACA
GAACAAGGAGCTGAAGGCTAAGCTGGCTGAACTGGAGACGGCTGGACGGGCTAAGACT
AAGGGCGTCATAACCTCGCTAGAGCTCAAAGTCTCCAACCTGGAGGAACAATTGGAGGCCGAGTCCCGCGAGC
GTCTCGCCCAACAGAAAGCTTCTAGGAAACTCGACAAGAAAATGAAGGAACTGGCCCTCCAATTAGATGAGGA
ACGTCGTCACGCCGACCAGTACAAGGAGCAGATTGAAAAGATGAACGCTCGAGTGAAA
GCTCTGAAACGCGCAGTGGACGAGGCCGAGGAGGAGGTCCAGCGTGAGAAGGTCGGCAAGCGTAAAGCTCAAC
GTGAACTGGAGGATCTGCTGGAGACCCACGAGACTCTGGCCAGAGAGTGTTCCAACTTGCGTAACAAGCTTAG
GCGGCAAGGCGGTCCCATAGGTCTCTCCGGGGCGACGTCCCGCAGTAAACGATCGTCG
CTGGCGCCCGGCGGGGCGGCGTCCGGGGACGAGTCCTTCGACGACGGCACCGACGCCGCTAACTCGCTAGAGT
GACGTCACTAGACACGCAGCCGGCCCAGTGATGTGACGTGACGTGACGTGACGTTGTACTGTCAGATGGCAAT
TGATTGTCATGACAATCATAGATATATCATAGTCGATTGATTGTTAATCGATTGTGAT
AGTCAATTATTGGTCATTGTTAATTGATTCCCAAATGATTGTCAAGTGATAATCAATTGGTAGTCAAGTAATA
GCCAATTGGTTGTCAAATGTCCGACAGACGACGTGACGCCATTGTGATTACGATTATAGCGTTGGTCTTGTAA
TCTTTTTTAATATCTTTTTTCT
>1002049704_1 Heli.1-EL596069.1.5
ACGTGTTACATTCCACGCTAGGTTATTTTCTGGTTTGTCATTGGCTTTTGCTTGTCAAATGTGACAGCCCGCC
CTTAGCTTCGAGCTTCTACGTCAGTTCTATGCACAGATTATACAGTTGCCAAAAGCTACTTTTAAAATATTTT
TAATCTGTAAAAAATTATATAAATCGCTTTAGTCTAGTAAATATAGTAGGCCTATAAT
GTTACGTGTAAATTGTTTGATAGACTCTAATAAATTATGTACACAAGTATATTAGATATTGTAAAAAAAATAT
TAGTTTCAATTTCTCATTTCTATATAAAATAATACGTAATTCAAGTAAAGATTTAAAATGTTTTTGGTTTTCT
TGTAAATTGTCCATAGCAACGTGCGTAGAAGCGTTACTACTTTATTGTTATTGTATTT
ATTACTTAAAATAGTGATTTTTTAATGTAATTTATTTGTAAAGGGTGGTTGTACGGTCTGTAACGTTTAAAGG
GAACGATTTTAGCTTTGCAGTCAATATTGATTGAAAATAACTTTGTGACCAATATTGACTATTAAGTTGATAG
TTTTTAAGACCGCGGTTTTGCGGCCAATGTTGGCCCTGAATGGAACAAACTTTAGCTG
GCCAAAGAGTTCTTATATTGAATCAGTAACTGTTATACTTGGCCAACGTTGGCCAAGGAATGGCTGGAGGACC
AGTCAAAGTTATGTTTTGGAATATTGATTATTATGTTTTTATAATCACTAACACCGGGAATGGGTCATTGTGG
GTGTCAAGGTGTTTAGGTGTAATACTTTGAAACGCTTGTCAAACAAGTGCACAAAGTT
TAGACTTCAGCTAGTGAATAAGCGGTTTTACTGTTGCATATTGACAGTTTTAATTCTGCTTGTATGTATTTAT
TTAAATATTATAACATGCCTACAATAACATAGAGGCGTTTTGTAAATAGTTTGAACGAGAATAAAGCTTAAGT
AAAGGTAAACAATTCCTATACATGGCTCGTGACACGTATTTCTTCAAGTGCTTAACGG
CACTGTGTACAAAGTTAGCGTATA
>1002049705_1 Heli.1-ES588111.1.5
AGTGAGCTTGGCTATCCTGGCATTATGCGCATGTGCGCATGCACAGTACGCGGAGGAGCGCGCGCCGCGGTAC
ATCGCCTCGGAGCCGAAGGTTACGTCGACACCCGTGCCAATTCTTAAACAGATTAACAGGCACAACGAAGATG
GCTCATATACCTACGGATATGAGGCAGCCGATGGGTCTTTCAAGATCGAGACGAAATC

TCCTTCTGGAGAAGTCAAAGGAAAATACGGATACAGAGATGATACTGGCAAGGTACGAGTTATCGAATATGGC
GCAAATAAATATGGTTTCCAGCCAGCTGGAGAAGGCATCACTGTCGCTCCCCCACATTAGTTGACGAGTCCA
CACGGGAACCAAACAAGCAGGGTGGCCGATCCCAATACCGTGAACAATCAGTCGACTA
CGACTATGAAGAGCCTGCACCAGCCCCGCGCCCTCGTCCTACCCCACAGCCCTCCTATCGCGCTCCCCAGCCC
CAGCTCCCCAAAGGTTCCCACAAGCGAATCAAAGGAGTCAATCTTTCTCAATGCTCGATCAACTCCTCAAAGA
GTATTCTCTGCCTCAAGGTGGATCCGCTCCACTCCACGATATCTCATTCGGATCCTAC
TAGTTCGATAATTTGATTACATTTTGTAAATATGTAGTGTATTATTATTAAACGAATTTT
>1002049706_1 Heli.1-DT665810.3.5
TTTTTAGTATTATTTTTCTTATATATATGTGATTCACGATCATCTTATATAGATGAATATAAAAGTTATAAAG
ATTACAAATACTATGTAGTGCGAGGCGATCAAAACGACTTGGCTGAATTGAAGAGGCAGATGCTGGAGAAAGA
TAATTCAGTCATTTACTTGGAGCAAGGGAAAGAGCACCAAGTCTTAATTGCTCCGGAC
TTAAATCCATTCTTTCTTGAAATATCTTCAAAGGAAAAATTAAACGCCACGCTTCTACACGAGGATATTTCTG
AAGTAATACGGTCGGAAAAACCTATAAATTGGCGTTACAAGAATCAAAATTTTACATGGGATGCTTACTACGA
CGTCGATTACATATATAAATACCTTTCAAGTGTGAAAAAATCCTATCCCGAAATAACT
GAGGAAATAATTGGTGGCGAGAGTTACGAGGGTCGCCAGATACGAGGTCTGCGTATTAACACTCCTGCAGAAG
AGGGCGTTGAAAAACCCGTCTTCTTTATCGAATCAGGTATACACGCCCGAGAATGGATCGCACCAGCTACTAC
AACGTACTTCATCAATCAATTGCTCACAAGCTCCGATCCAAATGTGACCAGACTAAGG
GATCAGTTCGATTGGCGCATCTTCCCCACTGTTAATCCTGATGGATATCATTACAGCTATGTATTTGATAGGT
ATTGGAGAAAA
>1002049707_1 Heli.1-DT664141.3.5
GCACGAGGCAGTCCCTTTCAGACTAACGCATAACTAAGTCGTATTTCCCGTTGCATTCGCGCCAAAGTCGGTA
CGAAATCTGCAGTGACATTTGAATTTAGAATTGTGTTTGTGATTGACATTTCTATCCAGCCAGTGGACGTTTT
GGAACTTTTTGGTTTCGGATTACGGCGGAGGCGCGCGAGTAGTTTGGGCCATGTCGGC
AGGCGACAGGGAGGCTGAGGCGCAAGCCAAGAAGGGTGACGATGGATCGTCCACCGAGGACAGCAGGGCCGCC
GCCGCACTCAAGCAGAACTGGTGCATCGGTTTGAGGGTTATTGAGTTGATCCTAGCGGTGATCGCTATTGGCC
TTATAGTGGGCGCTCTGACTGCTCCACAAGTCGTGCAATCGAATGATCGTCACATCGC
ACTTATTTACTCAGCCTATTCGAGTTTCATCATCATCACCGGTGTACTGATAGTCGCAAGATTATTTGGTGAA
TCAGCAGGATGGAGGACCTCTATCGGCTTCTCCATTATTGGCTTCATTATGTTCACTGCTGCAGCTGGTATCA
TTTTCTATGACTGGCACAGATCATACTACACGAACATTCGACCTAATAAGGAGGTTTA
CAACCTTCTTATTTCATCTGGCGTTTTTGCCGTTGTCGACGCCGCTGTGTTCCTTGTTCATGCTTTTCTTACT
TTTAGGAAGGAAGCTGACTACTAAACATGAAATTTAAATAAATATGTATGTATATAATAAAATTTGTCAATAA
TTTAATTATTTTACGAATACAGATTAAAAATATAACATAGTTATTCAGTTATTGTAAA
CATATATCATTTTTATTTACTCCAAAGTATTAAACG
>1002049708_1 Heli.1-EL598831.1.5
TGTGTCTTAAGAAGAGAAACAGTTAAAAATTAACAACTTATTTTAACTTAATTGTTATTGTTAATATAATAAA
TCTGACAAGTGTATTCTTATTCTCAATCAAATATGCCAGTTGCTGATGAAATCCAGTCCTCATGGGCAGATGA
AGTTGAAATTGATCAAGGAGCCCTACCACCTCCTTCTGAAGTAGTTGAAAATGGTCTA
AAAATTGTAACCGAATATAAATACGACAATGACAATAAAAAAGTAAAAATCGTACGGACATACAAGATCGAAA
AAAGAGTTGTATCGAAGAGTATTGCAAAACGCAAGACATGGGCCAAGTTTGGTGATTCTGCAAGTGACAAGCC
TGGACCAAATCCAGCCACCACTAATGTATCTGAAGATGTCTATATGCAGTTTATTACA
AGCAAGGAGGAAGCACAACGTCCAGATGAAGGTGATTTAGAGGCACTTAAGGCTAGACCAACTAAGGCTCTGT
TCAAGTGCCGTACTTGTCATGGGGAGCATTGGACCACAAGTTGTCCATTCCAACACACAGAACTGGCTCAAGC
TAAAGCAAGTGAAGCTGCTAAAGCTGCAGAGGCAAAAGCGGCGACTACGAACAAATAT
GTCGCACCTAATATGCGAGAGGGAGCCAGCCGTGTTCCCGGTCGTGAACCTCCAGGTGCC
>1002049710_1 Heli.1-EL598669.1.5
CACGAGGCGTAGCTGACGTATTTTATATTTATATGAAATAGAAATTGTACAGATTTTTAGTATAAGTGACCTA
TCTGTGTTTATTTTATTATATATTAGTAAATATTGTTAATGACTAAGAAATAATGTCCTTCGAGTACTTGCCC
GTGGCCGAAGATGAAAATGAAGAACCCATAGAACTTCCAATCGAAGAAGATGGGACTT
TAATGTTAACAACAGTGTCTGCGCAATTCCCTGGATGCTGTGGTCTTAAGTATAGACATCCTGAGACAAAAAC
GTTCAGAGGAATCAGATTAAGAGATGGCAGGTTGTATCCACCTCCTGAAGGTTGGGGCAATTATTTATTCATT
TGCAGTTTTCCTAAGGAAAATAAACGCAAGTCAGGCGAAAATTCTGAAACATCTTCCA
TCAAGAGCAAAAGAAATGACAATTTATGCTCTGATTTAATAGTTTTGGGCTTGCCATGGAAAGCTACTGAGCA
AACCGTTCGTGAGTACTTTGAGAAGTTTGGTGAAGTTTTAATGGCACAATTAAAACGCGATCCTAAAACTGGT
ATGTCAAAGGGGTTTGCGTTTATTCGATTTTCATCTTATACATCTCAAATGAGAGTAT

TAGCGCAAAGACATATGATTGATGGTCGTTGGTGTGATGTGCGGATACCTAATTCTAAAGAAGGTTCGGTCGC
TTCTATGCCTTGCAAAGTGTTTGTTGGCCGTTGTA
>1002049711_1 Heli.1-ES587209.1.5
CGGTTGAAGTACCACAGCTGGTGATAGCAAAGTTTGTAAAAATGTGGAAATACCTTGGTTTACTTGCCGTTTT
GGCGGCCGTTCATTGTAATCCAGTGGATCGTGGCTTGGAAGAGAATTTAGTGGGTGCAGTGTCGGAGTGCATT
GATAAGGATACGTCACTATGTTTGAAGGAAAAAGCTTTAAAGTACACCGACAGATTGG
CATTCGCCAAAGATATCAGTATCTTCGAGGGCATGAGTCTTATCAATACTGGATCAGCCCGTTCATCTCGCAG
TTACGAACAACTGTCTGAGGACCCGAAGACCAGAGAATCTCAAATTGAAGAAAGGATAGCCAGCAATGTAGGA
GATTTTCTAGACAACCATGTACTACAATTGCGCTTGTCTGAAGACTCAAATGAATCTA
GGGCCTTAGATGATGAAGAAGGTCGTGGCAAAAAGAAGAAGAAGATCAAGAAGATTCTTCCCCTTCTCCTGCT
CTTGAAGCTCAAGCTTGCTGCTCTAATTCCTCTTTTCCTTGGTATCATCGCATTCGCAGCCATCAAGGCTGTG
TTCCTTGGCAAGATTGCCTTTGCCATTAATGCCTTTGGCCTAATCAGGCGGCTTCTAT
CCAAAAATAGTTCAGGATCCTTTGGAGCTACAATCAGCTATTCTCCCCATCATGCGGAGGAACACCCTGGATA
CTCTTACGAACCAGCTCAGGGTTGGAGCAGAAAAGTCAACGATGCACAAAGCCTGGCCTACTCCGGCCAAATA
GCTCATTGATGTTAGACTTACTTTAGTATGAATGTGTGTGTGAAATATTTATTTTTGT
GTGTTTGTGTTGCCATAGGAGTACCTCAGTTTAATTTATTTAATTTATGAAAAATAAATT
>1002049712_1 Heli.1-DT662893.3.5
CCGAGTCCGATGCGATCGCGAACGCGACGTTTTGTCTCCTAATCTTTTTGTTCTAACAATTCGTTTAAATGGA
AGTATTGAGGACACACCTCATTTCCCTTTGTTGCGATTCTCTTTAACATGCTGTGAATATTCTTTGTTAGTTT
ACAAAAGCAGTGTTAGTTCTACTTTCGTAAAATTTGAATAAAACAAACCCCCCTGTGA
ATATAAATTTGAAATTCTACATCTAGACGAAAATAATTAAAAATAACTTCGAAACATTTGCAGATTCTTTCAG
ATCCAGTGTACTGTTGTGATGAACAATGTATAAACAGAAGCTATTTTTGCTGATGTTCGCGTACAGTGCGCTG
GCTGCATTAGACGGCTACAGTGCCCAGCAAGATCCTGATCAGTTCCATATACAAACAG
ACGAAGACGACGACCGTTATTTCCTTTATCAGACACACAATGGGCAGTACCGTAAGGAGCGGCGCCTCAAAGA
TGGTTCTGTTGTCGGAACCACAGGATGGGTGGGCGCCGACGGATATCTAAGGCTGCAAGATTACATAGCAGAC
GGCCAAGGATATAGAATTTACAAATCAAAAACTGTTTACGTCGGTGAAAATCGGCCTA
TTGGTGAATCTTTAAAAATAGCAAAAACTGCTCCCACAGACTCAGGTTACAATATAACGCCTGCGCCGGCGCC
TCATCCGCCACGAGGCACACCGCGTTTCACTACCACAACGCAATCTCCATTGTCTCATTCTACACCAAAATTG
CACAGCCAATATCCCTTAGATGTATCTATCACACCACCACCGCAAACCTTCTCTTATC
>1002049713_1 Heli.1-EL601889.1.5
AAGCTACGTCTCGTTCGTATCCTCCGTTCCACCGTGATGGTGCGAGTGGGCGGCGGGTGGGTCGCCTTGGACG
AGTTTCTGGTTAAAAACGATCCGTGTCGAGCCAAGGGTCGCACCAACATCGAACTGCGAGAGCAATTTATTCT
GGCCGATGGAGTGTCGCAGAGCATGGCTGCATTCAGACCGCGTACGCCTCGCTCTAAT
ACAAATACACCTCCATCCACCGGACCTATTACCAAGGTAAGAGAGCGTACCGTTCGCTCAGTGCCGATGTCCG
CGGGAGGAGCCGCAGGTCGAGCATCGAGATCATCACTAAGTGCTGGAACACCTGATTCGCTTAGTGACAATGA
AGCTGCAAGTGGTCTTGGTCAAAGATATAGGAAGCCAAGTGTACCAAGATCAACATTA
ACTCCTGGTGGATCACGACCAGGTTCTCGGCCTGGGTCAAGAGCAGGTTCGAAACCACCTTCTAGACATGGAT
CCAATTTGTCTTTGGATAGTACAGATGATGTTCTCACACCATCACGTATACCAATGCGTAAAGTGACGAACAC
TCGCACTTCAATAGCTCGGGCAGCCGCCAATGCCAGTAAGTTAGGAGTGACCACTCCC
AATGGAGGCTCCCGACCAAGAACA
>1002049714_1 Heli.1-DT664019.3.5
ATCACTTCCCGAAGATATTTTCTCAGAGAACACTGAATTGGCTACTCTTCACATTCAAAGAAACCCAATTGAT
TCTGTATATGGACTTCAAATATCCGATTTACTTACTTTAAATGCAGGTCAAACAAATATTAAATTTATCGGAC
CATCCATGTTTAATGGTATGACATACATTGCCAATCTTAACCTTAGCGGAAACAACAT
TGAAAAAATTCACAACCAAGCTTTCCACAAACTGGTGGAACTTAACTACTTGGATCTTTCCTATAACGACTTG
GATTTCATTTCGAGCATTCTTATCAAGGAAAATATAGAATTGGATATTTTCAAAATATCCAACAACCCTCGAT
TAACACATTTGCCTACTGAAGGCTTCAATTGCTCCGCAGAACAATTTAACATATACTT
GTTCGATGCATCAAACTGTGGCCTCGAAGAAATTTATGATGATTCATTAAGAACATTTACTGCTCTCTCACAA
ATTAATCTGTCCGGGAACAAGATAAAAACAATAAGCAACCGAGTTTTTTCGAGAAGTCCTAAACTTATCGAAA
TCAATTTGTCATATAACTTGTTGACAACTTTAGAGGCCAAAGTATTTGAACAAAACAA
AGACCTCGGGAAATTAAACCTCCAAGGAAATCCCCTGAAAGTACTTTCAGCTGAAGTCTTCATCCACACACCA
ATTCTCACCTGGTTGGACATGAGTCATGCTAAACTTACAGCTCTTTGGAAAGTAGAGAAAAATCAATCAAATA
CACTCCTTAACAACTTGAGCTTCCTAAATGTATCACATAACCACAT
>1002049715_1 Heli.1-DT663062.3.5

GCACGAGGCCACGACCTACATCCACACCGCATGTGCCCGCCATCACGGACGGCCACGACCGCCACGACCGCCA
CGACCGCTCCCCGCCGCAGCCGCATAATGAATCTCCTATTTCACCTCAGATGAATGGAAGTCCAGAAAACAAA
GCAATGACACCACAAAAACCAGTCAATTTGCTACCAGAAGTTCCCAGCCAGACTTCTA
GAAAGCTCTCTGATAGAGAACAACACGACTGCGATGTTATTGAACGGTTGATCAAATCATACTTCTACATAGT
GCGTAAATCCATTAAAGATTCAGTTCCGAAGGCAGTTATGCATTTCTTAGTGAACTATGTGAAAGATAATCTG
CAATCTGAGCTTGTGACACACTTGTACAAGTCGGACCAAGCGGAGAGTCTGTTGAATG
AGTCCGAACATATTGCTCAGAGGAGGAAGGAGGCTGCTGACATGCTTAAGGCATTACAACGCGCCGGTCAGAT
TATAAGCGAGATACGTGAGACGCACATGTGGTGATGGTCGTCTGGAGGCTCTAGTCTGTGGAATAGTGAAATC
GAGCTCTGAGCTCTGTAAATAATACTTATTTAGACGAATTATGTAGGCATTTTTACTT
ATTTTAAAATGTTTGTACTTTCAAGTCTGTGTACAGTACGGACGACGAGCCGTGGCAGTCCTGAAGTTAGCTA
TAGATGGCTGCGCATATTTCAATGTTAGTATTTTACTTACTTTGATTTGTTTTTTCTCGAGTGAACAATACGC
AAAATTTTCAGAAACCCTTACTTCAGGGCGGCCATGACTCGTCGACCGTACTGTAAGT
CTCATTTGGACGTCAGCGACAAAACTGTCCTTACTTTTCGTTAAGTAT
>1002049716_1 Heli.1-EL598288.1.5
AATTATATGAAGAAGATTATTTCTTTATTGTATCGCAGTAACACAAAGCAAAATTCCATCAAAATTGTGCGCG
TTCGTGCCCGATGTCTAGTGCTGAAGTCGCCGATAATTCCGTTGACCCCCCGGCAAAAAAGCGGAAGCTAAAT
ACAGGAGAGGCGAGTTGCAAATCCTCAGTAATGGCGAACAATGGAACGCGCGTGGAAG
ACGAAATCGACGAGAGCCTGTACTCCCGGCAGCTCTACGTACTGGGACACGATGCTATGCGCCGGATGGCCAG
CTCTGACGTCCTGATCTCCGGCCTCGGCGGCTTGGGAGTGGAAATTGCTAAAAATGTGATACTCGGCGGCGTT
AAGTCCGTAACCCTTCATGACGACCGGGCATGCACTATAGTTGATTTATCTTCGCAAT
TTTATTTATCTGAGGCTGCCATTGGTAAGAATAGAGCTCTGGCGTCATGCGAGCAGCTTGCGGAGCTCAATCG
TTATGTACCAACAACGGCGCATTCCGGAGTTTTGGATGAGGAATTTCTGAAAAAGTTCCGTGTCGTGGTGCTA
ACTGGAACGTCTTTGGCGGAACAGCAACGCGTAGCTGCCATCACGCATGCCAACAACA
TAGCCTTGGTCATTGCAGACACTAGGGGTCTTTTCTCGCAGGTATTTTGCGATTTTGGGCCGGAATTTACCGT
TTTGGACGTGACTGGCGAGAACCCAGTGTCTGCAATGGTCGCTGGTATCACTCATGAATACGAAGCGGTAGTA
ACTTGCTTGGATGATACCCGCCATGGTCTTGAAGATGGAGATTATGTTACTTTTAGTG
AGATACAAGGAATGTCCGAACTAAATGGCTGTGAACCACGTAAAATTAAAGTGCTAGGCCCATACACTTTTAG
TATTGGAGATACCACCAACTTTTCTAAATACATCAGAGGTGGCATTGTCACTCAAGTTAAAATGCCTAAGAAA
CTACATTTCAAACCCTTAAGCGAATCTATTAAAAGTCCAGAGTATCTTATCACCGATT
TCGGTAAGATGGACTACCCTCAACAGCTTCACGTAGCTTTCTCAGCTCTTCATAAGTTCCAAGATGCAGAGGG
TCGTCTGCCGAAGCCCTGGAGTGATGCAGATGTGGCTAAATTCAATGATTATGTGAAGAATATTGTGGATAGT
GAAGAATTGTTTAAGAATGGTGAAATTGAAATAAATAATGAGCTTATGGAGATTTTTT
GCAAGGTATCATCTGGAGATCTAAACCCCATGAACGCCGCCATTGGTGGTGTAGTTGCTCAAGAAGTGATGAA
AGCCTGTTCTGGAAAATTCCATCCCATAGTCCAGTGGCTGTACTTAGATGCTATTGAGTGCCTTCCCAAAGAC
AGATCTGGCCTCAACGAGGACAACTGTAAGCCCACGGGCTCCAGATACGATGGACAGA
TTGCTGTGTTCGGCAAGGAGTTCCAGAAGAAACTAGGTCAATTGAAGTATTTTATTGTTGGTGCTGGCGCCAT
TGGCTGTGAACTTCTAAAGAACTTCGCGATGATAGGCGTGGGGGCTGATGGGGGACGGGTAACTGTCACCGAC
ATGGATCTGATAGAGAAGTCCAATCTGAATAGGCAATTTCTGTTCCGACCCCATGACG
TACAGAAACCCAAGTCCAGCACAGCTGCTAGGGTAATCAAACAAATGAACCCCCTAATGAATGTAACAGCACA
AGAGAACAGAGTATGCCCTGAAACAGAATCAGTGTACGATGATGCATTCTTCGAGCAGCTGGATGGAGTTGCC
AACGCCCTGGACAATGTTGATGCTAGGATCTACATGGACAGACGGTGTGTGTACTATA
GGAAGCCCCTGTTGGAAAGTGGTACGCTCGGCACTAAGGGGAATACTCAGGTGGTAGTACCATTCCTAACTGA
GTCCTACAGCTCTTCACAAGACCCACCAGAGAAGAGCATACCAATATGCACCCTGAAGAACTTCCCCAACGCC
ATCGAGCACACCCTTCAGTGGGCCCGAGATGAGTTCGAGGGTCTGTTTCGTCAGGCTG
CAGAGCACGCTGCCCAGTATCTCGGAGACCCTCACTTCTTGGAGAGGACCATGAAGCTGCCGGGCAGTCAGCC
CCTGGATGCTTTGGAAAGTGTCAGGAACGCAATCAACGAACGGCCATTAAACATCGACGACTGTGTGACATGG
GCGCGTCTACACTGGGAGGCACAGTATTCGAATCAGATCAAACAACTCCTCTACAATT
TCCCCCCAGACCAGACCACCACCAGTGGGGCCCCGTTCTGGTCTGGACCCAAGAGGTGCCCGTCGCCCTTGGA
CTTTGACCCTGAGGATGAATTGCATATGGACTATGTGGTTGCCGCGGCTAATCTGAGGGCTCAAGTGTATGGT
CTACCGCCGTGTGTGGATAGGGAGAGGATGGCGAAGGTGGCTTGCGAGATTCAGGTAC
CGAAATTCAAACCGAAGTCCGGCGTGAAGATAGCAGTAACAGATGCCCAGCTGCAGCAGAACAACGATGACAT
GGACCAGGACA
>1002049717_1 Heli.1-EL599564.1.5

GCTGACGCCTTGCTATCCACACAAAAATGCCAGTCGACCTATACTACGTTCCCGGCTCAGCGCCCTGCCGGGC
GGTGCTGCTCACCGCGCGTGCTCTCAACTTGAACCTCAATCTGAAACTCGTGGATCTTCATCATGGAGAACAT
CTTAAGCCTGAATATCTTAAGATAAACCCACAACACACAGTTCCTACCCTCGTGGACG
ACGGCCACCCGATCTACGAGTCGCGTGCTATAATAACCTATCTAGTGAACAAGTACGGCAAAGGCAGCGCGCT
GTACCCAGAGGAACCCAAAGCCAGGGCCCTCGTCGACCAACGCTTGTACTTCGATATTGGCACTTTGTATCAG
AGATTTGGTGATTACTTTTATCCACAAATCTTCGGCGGTGCGCCCGCGGACAAGGACA
AGCTAGCCAAAGTAGAGGATGCCCTTAAACTCCTGGATACCTTCCTCGAAGGTCAGAAGTACGTCGCCGGTCC
CAACCTCACTGTGGCCGACCTGAGCATCGTCGCTGGCGTGTCCAGCTTTGAAGCATCGGATATTGACTTCAAG
AAATACGCTAATGTAAAGAGATGGTATGAAACAGTGAAATCGACAGCACCCGGCTACC
AGGAGGCCAACGAAAAAGGATTAG
>1002049718_1 Heli.1-EL604338.1.5
TTCGACACTATCATACCTTATCGAAACGTTTTTATGTGGACTGTAAAGTTAAGACTGTTTCGAAAGTAATATT
TAAGATGGAATTATTAAGGCAAGTGTCGAATACCCAGTTTCTATTTCCTGTGGCAGCTGTAGCGGTTGTTTTA
GTATGCGCTGCGCTCGTATTTATATTCGGATTTCACACAGCAGAACAACCGCAATTCG
ATAAATTACCACTGGTTGCCGATGACAGGAAATCATCCAATAAGAAGAGAAAAAATAAGGAGAAGAAGTCTTC
TCCTAACCGCATTTCTTCCGATGACGCCAAAGCAAAATCAGAGAGCTCTAAGAAATCTCCTTCAAAGGAGAAA
AAAGAGGAGAAAGTAAAGGAAGTTGAAAAGCCCAAGCCTAAAGAGAGAGTTGAACCCA
AAATTGTAAAGAAAGAAGCCCCAGTTGAGGCTAAAAAGGGTAAAAAGAACAAGGGTGTTGCAGAGGCGGAGAA
ACCTGTTGACTATGATGAAGGAGTGTGGGAGGAAGTGCCTAAGAAAAGTGACAAGAAGAAGGTTGTGAAACCA
GAAGAAAAGGAAAAGAAAGAGAGCCCTGTTAAAAAGAATAAGAAGAAGGTGAAAGAAG
CAGATGTTGAAGCTGCTCGCCCGGCCGAGGGCTCAAGTGACAAGATTAAAGTATTGAGTGCTGAGGGGCCAGG
AGTGAGTGAAGATGCAGCTAGAGCTCTACAAGCTCAAGTAGAGGAACTGCAGCGAGTTCTTAAAGAGGCAGAA
CAAAGAGATCAGGGTATATCTGAAGACCTCAATGAAAACGAAATACCTGAAGTAAAGG
ATCTCAGAAGCAACAAGAAGAAGGAAAATAAAGAAAAACAGAGCAAGAAGAAAGCAGTTGAGGCTCCAATACT
AGCCAACAACATCAAAGAAGACAAGGACTCTGAATCTGAGAAAAAGGAAGATAAACCAGCAGGCCCTGTATTC
GATGAGCTTGGAGATACTTGGACAGATGCTAAAGTATCTAAAAAGAGCAAAAAGAAAG
CACGCAAGGATCAGTGAGTGTGACATAAGGCCCGGGTCTTTGCTACCCTCTAGCTCAATGCACTTCACATCAC
TCACAAACTACAATCAATGGGGATAAAGCGAAAAGTGTCACATGTCAATAGTGGCCATCCTTAAATTACGTCA
CACGATTTTCATTATTTTATGACCCCTTTTCACAGCTGATCACATTTGGGGGTCGGTG
AAGTGTGACATACATTCATATAAGAGTAACTCGTATTTATATATACATCAATTGGTCCTGTAGTCAAATTAAA
ACAACTATTCCGAAATTATATTT
>1002049719_1 Heli.1-DT668780.3.5
GCACGAGGGCTAGCCTTTATGCGCGCGCACATAATGTCTAACACCCTCTAGCGCGATCCACCAACTTCTTGTA
CCGACCCTATATCGCTGGCGCGCTGATTGTGCTGATTGCTGTAAAAAGTATGGCAGCCTCGTCGTGAGTGACC
TTCGACCGTTAAGTGCCGATGGCAGAATGACTGAGGAAACTGAGCAGTGCGACTCTTT
CAACTCCTGGGAGCTCAACGGCCTGAACTCCTTGGACCTCTGGGATTATACTGTCGAGCTGGAATGTCTGCAA
GGAACTGAAGATTTGCAGCTCGCGGCTGAACTCGGCAAAACACTCTTGGAGCGAAATAAGGAGCTAGAAACTG
CGTTGCGCCAACACCAAAATGTTATTGAAGACCAGGCACAAGAGATCGAGTACTTAAC
AAAGCAAACGGTAGCATTGCGCGAAGTAAACGACTCAAGACTGAGAATCTACGAGCAGCTGGAAGTGAGCATA
CAAGACTTAGAGCGCGCGAACCACAGGCTCGCGGTCGACCACGCCGCCGACAAGAAACACATTAAGACTTTAT
GCAGCACCATCGAAAGCTTGGAAAACAAGTGTGAGGAGTTCCAAAAGACTGTGGACGA
TCTGAACGCGCAATTAGAAATCGTCAGACGGCGAGCGGAGAGAAAACCTGAAAGCGAAAAACCCAAAGAAAAA
GAACTAAAACCTACAGATACTGCGATACCAAACAACAAAAATGTTAACATCACACCACAAAAATCAGTGGCGC
CTCTTCCGGAACTGACAAAAGAGGACGAGGACTTGCTGAGGTTAAGCGATGAGTTGAG
AGAGAACAAGGT
>1002049720_1 Heli.1-DT662325.3.5
AACACACTGCATCAGTTCTTCAGGGATATTGCTGATGAGGAGTCTTGGATTAAGGAGAAAAAACTCCTCGTCG
CCTCAGACGACTACGGCCGTGACCTCACCGGCGTACAGAATCTCCTTAAAAAACACAAGCGTTTAGAAGCGGA
ACTGGCCAGCCACGAGCCCGCCGTACAAGCGGTGCAAGAAGCGGGCGAACAACTCAAG
GACGTCAGCAATGTAGGCGTAGCGGAAATAGAGCAGCGCTTGAAAGCGCTCGCTCAAGCGTGGGAAGCGCTCG
AAGCACTAGCGGCCGAGCGCGGCACTAAGCTGCAGCAATCATTAGCGTATCAACAATTTTTGGCTAAGCTGGA
TGAGGAGGAAGCTTGGATTAGTGAAAAACAACAACTAGTTGTCGTAAGCGAATGCGGT

GATAGCATGGCGGCTGTACAAGGTCTTCTCAAAAAGCACGAAGCGCTAGAGGCGGAATTGGCGGCGAGAGGAG
AACGTGTGAGAGATCTCTCCGCTGAAGGAGAACAGCTACTTGCAGCTGGAAATCTGCATTCTGAGGCTTTGTC
TCATAGATTGGAACAGTTGAAGGCTAAATTAGAAAAGCTAACAGCACTAGCCGCCCGT
CGCAAAGCTGCTCTCGTAGACAACTCTGCCTACCTCCAACTCCTGTGGAAGGCCGATGTAGTAGAATCCTGGA
TCGCTGATAAGGAGACTCACGTGCGGTCGGATGAGTTTGGACGTGATCTGTCCACTGTCCAGACGTTGCTGAC
TAAACAGGATACTTTTGATGCCGGTCTAGCCGCATTCGAACATGAAGGCATACAAAAC
ATTACCGCCCTCAAGGAGCAGCTAGTGGCAGCTGGCCACGAACAGAGCGCGGCCATATCCCGTCGTCACGGTG
ACGTCATCGCCCGCTGGCAACGCCTCTTGGCCGACTCGGCGGCGCGCAAGCAACGGCTACTGCAGCTTCAAGA
CCAGTTCAGGCAGATTGAGGAGCTATATCTTACGTTTGCTAAAAAGGCATCCGCATTC
AACTCTTGGTTCGAAAACGCTGAAGAAGATCTTACGGACCCTGTCCGTTGTAACTCTATTGAGGAAATTAGAG
CTCTTCGCGATGCGCATGCGCAATTCCAGGCATCACTATCATCTGCTCAAAGCGACTTTGAGGCTCTAGCAGC
ATTAGATGCTCAGATCAAGTCGTTCAACGTCGGCGCTAACCCCTACACCTGGTTCACC
ATGGAGGCCTTGGAGGAGACATGGAGGAATCTGCGCAAGATTATAGCGGAACGCGATGTTGAGCTAACCAAGG
AGGCTCAGCGTCAAGAGGAGAACGACAAGCTGCGTAAGGAGTTTGCTAAGCACGCCAACGCCTTCCACCAATG
GCTTACAGAGACACGTACGTCTATGATGGAGGGCACGGGCTCATTAGAGGCGCAATTG
GCTACGCTGCGTCAACGCGCTACCGAAGTGCGCGCTCGTCGAGCTGACTTGCGTCGTCTGGAAGAGCTTGGCG
CTGCCCTCGAGGAGCACCTGATCTTGGACAACCGCTACACGGAGCACAGCACGGTGGGCCTCGCACAGCAGTG
GGACCAACTCGATCAGCTGTCTATGCGCATGCAG
>1002049721_1 Heli.1-DT667621.3.5
GCACGAGGCCAATATTTTTCATATATTGTTTTGTTAAATTAGGCGGGAATTGAATTTGATCAATTTAGAAGTA
AAAATGAGCGTCGAAGAGGATGTAATGAAAATACAAAAGAAACTAACAAAGATGACATCAGACGATGGCACGG
GTCAAGAAGAAGCATTGGAACTTTTGAAGGTTCTACAAACTATGGCGATAAATTTGGA
CGTCTTGACTAAAACCAGAATAGGTATGACTGTTAATGCACTACGAAAATCTAGTAAGGATGAAGAAGTAATT
TCTCTTTGCAAAACTCTTATTAAAAACTGGAAAAAGTTTCTGTCTACACCAGCAACACCATCTAAAGATTCAG
GCAATTCATCTAAACCTAAAAAGGATTCAAGTAAAGATAAAGAAAAAAAAAGATGACAA
AGAAAAAGATAAAAAATTGCCTGCATCATTCCCACCACAGTCAAACACTACCGATGCTGTCAGACTTAAATGT
CGAGAATTATTAACTCAGGCATTGAAGATTGATGGTGAAAATCCAAATGCTTGTGCTTCTCCTGAAGAACTTG
CCGAAGAATTAGAGGAATGCATTTATGGAGAGTTCAAAAATACAGATATGAGATATAA
AAATAGGGTTCGGTCCAGAGTGGCCAATTTAAAAGATATCAAAAATCCTACTTTAAGGACTAATTTCCTTAAT
GGAGTGATTACTGCATCACGTCTTGCCAAAATGACCCCAGAAGAAATGGCAAGTGACGAAATGAAAAAATTGC
GGGAGAAATTTATTAAAGAAGCTATTGATGATGCTCAACTTGCAACTGTTCAGGGTAC
TAAAACGGAGATGCTTAAATGTGGGAAATGCAAGAAGAAGAATTGTACATACAATCAACTTCAAACAAGAAGC
TCTGATGAACCTATGACTACTTTCGTTCTCTGCAATGAATGTGGAAA
>1002049722_1 Heli.1-ES586166.1.5
ATTTTTTAATAAACAGCCAAGATGTCAGGGAAAGGAAATAAAGCATTTACAAAAGAGGAAGAATTGCTTTTAC
AAGATTTCAGCAGGAATGTTTCAACGAAGTCGTCGGCGCTATTCTATGGAAATGCTTTTATAGTTTCAGCTAT
TCCCATATGGCTATTCTGGAGAGTACATGCTTTAGAAGTTAGTACTTCATTGGCTTGG
TTTGCACTAGTAACTACAGCTAGTACATGGTTACTTGCTTTGGCATACCGAAACACGAAATTCCAATTGAAAC
ACCGTGTTGCGGTTCGCCGGGAAGATGCTGTAGCTCGCGAAATGGCTCGGAAATTAGCTGATGAGAAGAAAAT
GAGCAGAAAAGAAAAAGATGAAAGGATTTTATGGAAGAAGAATGAAGTAGCGGACTAT
GAAGCAACTACTTTCTCAATCTTCTACAACAATGCAATGTTTTTGACAATTGTGATTCTCAGTAGCTTTTACT
TGCTGCGCTCCTTTACTCCTACTGTTAATTACATAGTATCTCTTTCAGTAGCATCTGGTCTTCTGGCTCTGCT
GTCAACTGGTACTAAGTGAAAAGACTGATTTTGTGTTTGTGATAATAATATGAACACA
ATTTTGTACCATAGTGTGTTTGAAGAATATAAATTATTAAGTAAAGTGCTAAAGTTGGTTCAGTTTTGTTTTA
GAAGGAATTTATAACTATAGTCATTAAGAAATAAACACACATACAAA
>1002049723_1 Heli.1-DT668844.3.5
GCACGAGGCAGTTCCTTAAAACTTGATTAATTTGATTAACATTTTATTTTTCAATTCCTATTACATATTTTGG
TTCGAGCTTGACCAGAAATGGCTTTGCTTCCACCTGACCCGGTTTATACAATTCGAAATGTCGACAATTCGCC
TGTGTATTCCTTAGCGTTCAGCTTTCTACCGGGAGGACTCGAAAGGTTACTAGCCGGC
TCGAAAAATGGATATGTTTATGCATACAACCTTCAGACAAACCGGGTCCAGCAAAAAATCAAAGTTGGTCAAG
CTCCCATTCTTCATCTCATCCATACAAGCAGCCAAATGATAACCCAAGAAAAGGGTGGTAAGTTCAAGATTTT
CAACTTAACTAACAGCGGCTATCAAGAAGAACACACAATAGACATAGATTATCCAGGA

TTTTGCCGTTTCGATGCCAACACAAAACTCGAAACACTTTATGTACCTGACTCTGAATCCAAAATATACATAT
ATAACTTTTCTGGAGAAAAATTGGACTGCTTGAAGCCAGAAAACCCAAAACTTGGAGACCCAATGTGTATAAA
ACATATTGAACTGTCTAATGAACAGCCTTGTCTTCTAGTAGGCTACGAAGCGGGGTGG
CTTCTATTGTGGGATCTCAACACTAGTCAATGTATCAGTAAATTGCAAACAAAAGAATGTCCAATGTCAGTAG
ATTTTCATGTGGAACAACAGCGGGGCATGGTGGGCAACGCCTCGGATGTGATACAAATA
>1002049724_1 Heli.1-EL601199.1.5
GGACGTCTCGGCGCAGACAAAATAAATGGAACGGAACCAGTAGTGGTTGTGTTGTGTGTGTCTGTGTTTATTT
TTGTTGTGTGTTTACTCTTGAGACAAGAAATAAAAAATAGTGAACTATAAAAATGCAGGGCCGTGATAAATTG
TGTGCTAAACTGACGAGTGCATTCCTAAGGAAGTGGTGGTTCGTCCTCGCGTCAGCAG
TGGTCCTACTATTGTTAGGCATCATATGCGCTATATTCTTCGGAACGTGGATTCGAATGTTTATAGATCATGA
ACTTGTTCTCCGCCCAGGCTCGATGACATTCGAGTGGTGGGCGCGACCTCCAGTCCGGCCGTTCGTCCGAGTC
TACGTATACAACGTGACGAACGCTGACGAGTTCCTAAATAATGGGTCCAAGCCTATCC
TGGATGAGTTGGGGCCTTATGTGTATTCCGAAGAATGGGAGAAGGTGAATATAACAGACAACGACAACGGCAC
ACTCTCGTTCCACTACAAGAGGACGTACACGTTCGTGCCAGAACTTAGCGCCGGGCCCGACGACGACTCAGTG
GTCGTGCCTAATATACCTATGCTGAGTGCCACATCTCAATCCAAACACGCCGCTCGTT
TCCTCCGTCTGGCTATGGCGTCAATCATGGACATCCTCAAGATAAAGCCTTTCGTTGAAG
>1002049725_1 Heli.1-DT667354.3.5
ACTAGTCGCATGTCATTCGTATAAAAGTGTTGTTGTTAATAATACTTTTTAAGACTTATCCGCGATGTGCCTT
CGGAAGTGACACAGTTCTAGTGTAAAGTGAATTTATAAGCAATCAAAATGCTCGCAGACGGTGAAATAGTTGG
TGACGGATCATGGAACCTCACGATCTACGTGACGGACTTGAACGAGAAGCGTACCATG
GTCGTGAAGGGAGATATGCACATCGGTGGAGTGATGCTGAAGTTGACGGAAAGCTTCGGTAAAGACTTCAAAA
AGGACTGGTCAGATCATGCACTATGGTGGCCGACAAGAAACAAATGGCTATCTCGTCCAAAACACACCCTGGA
CCAATACGGCGTCCACGCAGATGCTGCCCTACATTTTACACCGATGCACAAACCTATC
AGAATTCAATTGCCTGACCTGAGATACATTGATTGTAAAATTGACTTCTCGATTGATACATTCAGTGCCGTAG
TACAACTCTGCAAAAGTCTCGGAATACGGCATCCAGAAGAACTGTCTTTGTGCTATCCTCTCGAACCTTCCCA
CCTAAAACAGAACTACCAAAATTTGAAAGAAGCGAAGAAGGTGAAATCTATTCAGGCG
CCCGATACGAATACGTTTATAGCGGCAACAAGAGGCTCGTCGAATAGCCTGGACAGGTCCTTAGCGTGTCCTG
CGACGCCGCCTCCACCGCGATCGTTGTCAGCCACGCCAGTTGCTTCACAACAGAACGGGACTCTCCGCCGCTA
TGGGGGTCACATCTACAGTACACAGAGCGACGGCTCAAGTGACGGGGGCTACTGCGGA
ACCCCGCCGCGAGCAGCTTCCATGGACGCGTTAGACTCTCTCGCGGAATTTTTTTTGGCAGACTCACCTCTCG
AACCGGACAGCCAGTCTCGGGAG
>1002049726_1 Heli.1-EL602985.1.5
AAGCTCGCCCATTCAGTAGATATTAAACGTTTTTAATTAAATTCAATTATAATCTTATTTTCTTACGCATTTT
TAATATAAAGTGAATTTTAAAATGTCGGCTAAAGTGGCGTTAGCCTTTGTGGCAATGCTAGCATTCGTTAACA
GTCAAAGCTATTTAGACGCGCTCGTGCAGCAGATAATGCCAGAACCCAGTGATCAACC
GAAGAGTAACTGCTTGTGCAAAGGTCCAGCCTGCATATGTTGTGTGGATTTTAATATGACATTCCTTGATCTA
GGAGGTCCAGGGTGCATACACATGAAATACATTTCGCCAGAAGAAGGATTATCGGTAAAAGTTACATATGATA
AAAAATTTCTACAAAGTTCTAAAATAAAAGGACCTCATCCAACTCCTGTTTGCGTGAA
CTTATACGGTGTACATGCCCAAGTTTGTGCCAATTTCAATGATCTTGCTCCCACTGCTGATGGACTACGTGGA
TGCTCAAAATTAGAACTTAAGTTACTTGGAAGTTCACAACTAGAATTCCCAATAGGATGCTTCAAATCGTCTG
CTGGCGTGATGGAAATGGAGGACCCACCAGTTGAATCAGAAAAGGAAGAGACCACAGA
AGAACCTACTGCAGAAGACACCAATATATTCGACCCCGAAGCATTTTTGATAAACATCTATCAGACAGCCGAA
GAAGGTGTCGCTTTTCTTGGTAGCCTTTTAAATAT
>1002049727_1 Heli.1-EL600176.1.5
TGTGTTGTAATTTAATTTTACTTCTTCCATTTACTCAAAAATTCTGTTTAAAATTGCAAGAAAGAAAACTAAG
CTGATAATCAGCATGGGTCTCCTTGCAACAGCACTTGGCGTTCTTTTTATTGTTGCCACGGCTTGGTCTGCGG
ACGCTCCTAAAGGACCTAAGGTCACTCACAAGGTCAAATTTGATATAAGCATTGGCAA
TAACCCGGCAGGGACAGTAGTTATTGGTCTCTTTGGTAAAACTGTACCAAAAACAGTAGAAAACTTCTACCAG
CTTGCTCAAAAACCAGAAGGTGAAGGTTACAAGGGTAGCAAGTTCCACAGAGTTATTGAGAACTTTATGATTC
AAGGTGGAGATTTCACTAAGGGAGATGGAACTGGTGGTCGCAGCATCTATGGTGACAG
ATTTGAAGATGAGAACTTCAAACTAAGCCACTACGGTGCGGGTTGGTTATCTATGGCTAATGCTGGAAAGGAC
ACAAATGGATCTCAATTCTTCATTACTACCACTAAAACACCATGGCTTGATGGCAGACATGTTGTTTTTGGAA
AAGTACTTGAGGGCATGGATGTAATTCGTACTATTGAGAAGTCGCCCACTGGAGCCAA

TGACCGTCCAACAAAGGATGTTGTCATAACCAATACGCATGTTGAAGTTGTAGCTGAACCCTTCAGTGTTACA
AAAGACAGTGCTCAATAAACTTTAGTTTAAAATGTCAATATAACAATAATAATTTTGTTTAAAATGCATTTAT
TATTAAGTTCACTACTAATATTCATTAAGTGATCAAAGTATAAATTTCACCTTTATTA
GTTACCCTTATCCCCAGTTTATGCCAATAATCTTTTTAAATGTCACAGGGTACATAAAAATAGATATGTTTAT
AAAATCATTTTATAATAAGCTATAATTTGCAGTGA
>1002049728_1 Heli.1-DT665077.3.5
GGTGTGGTGTATTTTTGTTGATACTGTTGATACCAAATTTAGAGTTAAAATATTATTGTATGTAATTAATTAA
TTATTTTTTATTATTAACATTCCACTTATTGTTTATCGTTTCTCGATGGATTATTACTTTTATTGTATATGAC
GTTTATTTAATTGATTTTGAATGTAATATAAGATTTATTTACAAGTAAGTTGGCGTAT
GAACAGTTCGTACATTGTTTCTACGGGGCCGATGCGAACCACTGGGTATTTGTTATCGATGTAGTGTAATGAC
AGAAATTAAAACACTCTAGGATATATCTCATTCGAATAAGCTAAAAACCAAAGGTGCCCTTCATAGAAAGATT
ACGTTTTGTTGATAATATTTCAAGTTGAATGATTTTAAATGAAGTTGTATGTAATAAT
CGAATCGATTTTGTTTTAATCTCGATAATATTAGTATATTTAAATGAATTTTCACGACTATCTCAAATTATAT
TTACCTAATGCTTTTTTAGTAATTTTTATAATGTTCAATCACTCGTTAAAGCGCTGGCACACTAGCGTCGGTC
TTACGATCCTTTCTATCTGTATATGGATATTTGCAATAATTGTATAAATAAAATATAG
GGGCCGCCCTCGGTTCCGCAATACAGGAGTGCAATACGCCACTGGCTCCGGCTCGCGCTCGCACCGTGTACCG
CGCACGCGCTGGCTCCCACGGGTCTCTTGTGCCTTGCGGAACTCTTTTCCTAGAACCGCGTATATTCTTTATC
ACATCACTTTTTACGTTTCCTT
>1002049729_1 Heli.1-EL602628.1.5
GCACGAGGCACAGACCACAATGTACAAGCTGGTGGTGTTGTCCGCGGTCCTCGCTTCGGCGGTTTGCAAGCCC
AGTGGCATTGCGCCATTATGGGGTGGACACTTGGCAGCTCCTTGGGCGGCAGCAGTCGCAGCTCCTTGGGCAG
CAGCTCATGTAGCCTCACCCATTGCTGCGGCTCCCCTTGCTCCCGTCGGTGCTTACAA
CTACAGGGGTCCTCTTTCCCTCGCCCCTGGTCAGCCCGCCAACATTTTGGCTCACGACGGCCGCCCTCTGGAT
ACCCTTAGTGTTAACGTAGATCGCGCCGTCCACTATACTGCAAGAGCTTTGGACCCTGCTGCCCATGGTGTAC
ATGGCGCGCATTTATTAGGTAAAAGATCAGTTGTTGGACCCTGGGGCGCAGCTCCCTT
GGCTGTTGCTCCTGCCGCTGCCTGGGCGCATTCCGCCCGTGTGGTCGCACCTGCTGCTGTAGCCGTAGCCGCA
CCTCTTGGTCATTTGGGATGGGGACATGGTGCTGCTTTGGGACATGGACATTGGTGAAGACTACACGACCTAG
ACTAGGATCCTCAAATAATATGATAATATACAGAGAGTTACAAACA
>1002049730_1 Heli.1-DT664847.3.5
AAACATAAACTAGCCACAATGAGTTGCCCATTTAACTTTAACGAAGAACAATTACTGCAGCTCAAGGCGTTTG
TAGACTTATGCAAGTCCCAACCTCAAATTCTTCAACATCCAAAGCTATCTTTCTTCAAAGAATATTTAATATC
ACTCGGGGTAACAATACCTTCAGCTACATTCGAGAATTTTGCACCCTCCGGAGACAGC
GACAGCAACTTCAGTAGTGCTTCTGCTGCTGCTCCTCCTCCTCCTGCTGAAGCCTCATCAGAAGAAGATTCCG
AACCTGAATCAGATGTTGAACTGGATATGGAAGGTGTCATATCTGACTCGTCAGAGGCAAATCAAGATATGGG
CGATGAATCCAAGGAAGTATCTGACGAAGAACGTGATCAGTCGGACGAGAAACGATCT
GAGGCTATGAGGGCGTTCTCGGAACAACAATTTGACGAAGCGATTGTATTGTACACTGATGCTATAAAACTGA
ACCCCCAAAGTGCTTTGCTTTTTGCTAAAAGAGGACAGGTATACCTCAAACAAAATAAGCTACATGCGTGCAT
TAAAGACTGCAGCCGCGCCTTGGAGTTGAATTGTGACAGTGCAGCTGCGTATAAATTT
AGAGGACGCGCTTATAGACTTTTGGGCAAATTTGAAGAGGCGTCCCATGATCTCTGCGAATCTCTCAAGATAG
ATTACGATGATCAAACAAATGAATGGCTCAGTGAGGTGAAACCTAATGCTGAAAAACTT
>1002049731_1 Heli.1-DT668444.3.5
GCACGAGGCAAAATCATATATCTAAGTCAGGTGGCCGTGATTATATTTACTTTTTGGTTATGTGAAATAGTGA
AGTGCAATCCACATCGTAGATTAAAAGAAATTCAGAAGAGTGCCCACTTCGCTTTGCAACCTATACCATTGAA
ACCTTTTATGGTTTTATTAAAGAAAATAACACCGTTTGGAGAATATGGAATTAAGCAT
TCTATCAAAAAAAGGCAGATTGTCCAACCTAACGAAATACCCAATATTGAAGGTGGTACACTTGAGGATATTA
TAAAAATACAAAATATTGGAGTAGCAGACAAAGCAGACGATATTCCACTTGAACCAAATTTAACCCCACAAGT
AATAATAACATCTATCATTAAGCCTATAGAAGATAGTGTGCCTATTTCTATACCTGTA
CCTTTGCCAGAGGGTTCAGATTTGCCAACAGACCAGAAAAATATAGAGGATTTGAAGCCTCCGGACGAACTTA
TACATCAAGAATCAGAACTAAAAATCATAGAAGTAGCATCATCATCAGAAAATGTTATTGAAATAACTCCAAG
GCCAAAGATAGAAGAAATACCGCCAGAGTACCTCGAAATTAAAAATGTAGCCATAAAC
ACTCCAGTGGAAAGTACAGTACCATCAATATCGAAAATTCCTCTTATAAAAAATCCAAATGGGCCCTTTTTAT
TCACTGATATTACTAGACTACCTCTAAATGGTTTTTATCCTGCTCCATCTATTGGTGTTCCTGAATATATTGT
ACCTTCACCACCATTACCACTACCACCGCCTGTAGTTCACCACCAT
>1002049732_1 Heli.1-EL603248.1.5

GCACGAGGGCCGGTGCGGCTGCGGCGCGGAGTCGACTTATACCGCTCAAATAATAATTCGAAGTGCGTGCTGC
CTATAGTGAAAAATTTCAATATAAAGTTTATCGTCAAAACAGTATTAAATAAATTCCACACGCAATTTTTATA
TATTGAACTGTATTTTACCGTTGTGAATGATATTTTACTTATATGTTAGATTATTGTG
TAGATAATGATACTTCATCGATAAGAAATCTGTTCAGGTCCCAGTCATCTTATTCCACGATGTGCTGCGAAAC
TGTTATCATCCGAGTGTTCTGAAGTGGATTATTTATTTAGACAATTTTTTAATATAAATAATAAAATAGTCTA
AAATGGTAAAATTGTTAAGTAAAAATGTATCAGTCTCAGATTTATGGGTATCAACTAA
TGGTAAAATAAGTGATTTTTATTTGACATCATGGCAACGAGGAGATTCGCCGTTGCCCATGCTGCTGGTGAGG
TTGTTCCTGAGTTGTATAGCTATCAGCATTTTCACATGGTCTTTGTGGAGCGGTGCCAGCCCTTACTGGCTGA
TATACCTTACGAACTGGGGCCTATTACTTGTCACATTATTAACACTCAGCGGTTTCCT
AGTGTCCTGTTGCGCTGTTTGCAAAAAACTTCCTGACGGAAGTGAGTTGCCTTGGTACGTGAGTATGTATTGG
CTGTTTTACAACATGACCATCACAGTGGCCATCATGATTACATGTCT
>1002049733_1 Heli.1-DT662130.3.5
GCGCGGGCTACGCGGGCGGCGCGCTGTACGGCGGCGCCTACGCCTACGACGAGCAGCTCATGAGGAGTGGACT
GCCGCACCATATGGGTGGCTACTACGAAGTAGGTTATAGCGCGCGGGAAGGCACGTTCGGTCTGGGAGCGGGA
GAGAGGTTCGGCAGGACCGACGCTGCATCTCCGCAACAGGTGCCAGCAGCGCTACCCC
CCGGCTATGCATACTTCTACCAGCCACCACCCACCACATACCAATATGGCGTTTATCCTACAGCTTACGGCGG
TGGATCAAGTGTAGGCGGCGTAGGCGGTGTAGGCGGAGTGGGAGGCGTAGGTGGCGTAGGTGTGAGTGGAGTC
ACGGGCGGAGGCAAAGTGTCCGCCTACTCGCAGCAACAGCCACCTTACGATGCACAAG
ATTCCTATAAGAGCGCGGGCCCATACACGGCGAACGCTAACAAATCAGCCGGCGGTGCTTCCGCTGATCTCTC
AAACGCAATGTACGCTAAGACACATGTGGCGCTTAATAAAGTTAATAGCTACGAGAAGGCGGGCTTCCACAGC
GGCACGCCGCCCCCCTTCGGCGCGGGCTCGCACCTCTACATACCGGCGCCGCCGCACC
ACCACCCGCACCACCACGCGCCGCAGCACCAGGAAAGCGCCTCTTCGGGCAGATCGGGTGCAGCTAAGCCGGC
CGCCAGCAAGCCCACTTACTCGCAGTCGTACTGGGCGCCTAATTAACTTGCAGGGACCGAACAGACAATATGG
AGACGTGTAAATACAACGCCCGACCTCTACTCAA
>1002049734_1 Heli.1-DT664638.3.5
TAACTCTACTCAACATGATGTAATAAAACGTGAAACTATACCAGAAATTCCTCAATTTGACTTGAATTCTTCA
TTGATATCCCAAGAAAAAAAACTATCAAAAACTAAAATAAAAGAAAAAGGAGTTGAAGCTCTCGATAGCACTC
TTGAAACTAAATATCCATATTACTCAAAAAAAAGTAAAATTATTAACAAAAATTCACC
ACTTAGATATGCGCAAAATTTCGCATTCATCCCTAAAAAATCCAATGGTGGCACTGAATTTTATGATTCACGT
TCTAAATATGTATGTCCAGAGGTAGAAGATGTTGTAGATCCCGTACCGGAGAAATTAAAAAAAGATGGTCATC
CAAATGATAATGAAAATGCAAATAATGATTCAGGAGATAACAGCGATAAGGAAAAATT
CGAAGAAATAGAAAACAAGCAACGATTAAAGGGACTCGGTGATAAAATTGACTGTTTCAAGGCAAAATATTTT
GATTCTAATCCATTAGACAATCCATTTTTTAAAGAAGAAGTAATTGAAGATCCTGAGCCTGTAACAAAGCCAA
ATTTATTTATTTTTGAGACCGGTAAAGCAAGAAGTTACGTTCATCCTAATCAACTAAA
TAAGAATGATGATATTTTTTCGATACCCGCAAATTC
>1002049735_1 Heli.1-DT662316.3.5
TCAATTGAAAAAGCTGATGATGATGCAGCTATCAAAGTCCTCATTGAACTTACTGAATCAGCACCTAAATTCT
TACGTCCACAAGTGGAAACCATATTCCAAGTTTGTATGAAACTAATTGGCGACACTGATCAAGAAGATAACTG
GCGTCAGCTAGCTCTGGAAGCTCTTATAACATTATGTGAGACAGCACCGGCAATGGTA
CGAAAGGTTGTACCAAATGCAATTCGTGTCCTCACTCCACTTATATTGGAGATGATGTGTGAATTAGACGAAG
AACCTGATTGGTCTGTGCAAGATAACGCTGCTGATGATGACAATGAACTGAATTACGTCGCTGCGGAATCAGC
TTTAGACAGAATGTGCTGTGGGCTTGGTGGCAAGATTATGTTGGGGCTTATTGTAGGC
CAAGTACCTGAAATGTTGAGCTCAGAAGACTGGAAACGACGCCATGCTGCATTGATGGCAGTCTCCTCTGCTG
GTGAGGGATGTCACAAACTGATGGAACAAATGCTTGACCAAGTTGTTTCAGCAGTTCTTAACTACTTAACTGA
TCCACATCCACGAGTCCGTTACGCCGCATGTAATGCAGTAGGTCAGATGTCAACTGAC
TTTGCACCAATATTCGAAAAGAAATTCCATGACAAAGTTGTACCTGGGTTATTGATGGTACTTGAAGATAATG
CGAATCCTCGAGTGCAGGCTCATGCAGCAGCTGCTCTGGTAAACTTTAGTGAAGACTGTCCAAAGCCTATCTT
AACTCAGTATTTAGGCCCATTGATGAACAAGCTTGAAGTTATTCTTACTGCTAAATTC
AAAGAGTTAGTGGAAAGTGGCACAAAGCTTGTATTGGAACAAATTGTCACCACAATTGCCTCTGTCGCTGATA
CAGTGGAGAAA
>1002049736_1 Heli.1-DT663687.3.5
GCACGAGGATTCGTATAAAGCTGTATGCGTGTAATGATCGTTCTATTATAAATGACCCAGATGAAGTATATAG
TAGGATCTTGAACGCAAGTAATCGACTATTGGGTATCGCTTAGCTTGTCTTCTACACTCGTCTTTGCTCACAA
AATGTACCAAAGAGTTAGTAGTGTATGGGCAAATGTTTGATAGTTTTATAGTAACGCG

TAACTTAAAAGTGTTTTGGTAATAAAATGTAAACTTCACATAAGCATATCTTTTACTATAATACATATCGTAT
ATTGTTACATATGTAGATCATTACAGTATTGTACGATTCATTAGTTTGTTGGAATTAAACTTCAAACATTTGC
CTGTATTTAGCGACGTTTTAAAATAAAAAGAGTATATAAATTTATAATCTGTTGCGTA
TTTACTCTATGGCATTATTATAGGTGTTGCCAGATAATAAAAATTTTAATAAAACGGCAGCTCTCACTATAGA
CACTGCCACTCCATGCGTCGTATAGTATTATTTTGAAAAGAAAAACAAAGGCTGTTATCGAGGCCGTCGTGGC
ATAAGACGGCAAACGCGACAGTTGTGGGTTCAATTTCCTTTCGTGTGAATGAATTGTT
TTATATTTTCATTTCCTTGTTCTGGTAAGGAGGCAAAGTAGGCGTTGAGTTCCGCCAATCAACACACAATGTG
TCGAAATTTCGGCGGTGCTTGGGAAATGGAATATCTTAACACTAGGCTACCTCTGTCTGCCATGCTGTATGCG
GGATAAAGCACGAATTTTAAAGAAATGTGCCAAAAATGGACCATTCGGATCAAATTGC
TGGAATGGTAATGTCTATAACGTA
>1002049737_1 Heli.1-DT666034.2.5
CGGCACGAGGCAGAGGCGCGTACCTCGCGCTCGTTTCGCTTGCAGCGTCCGTACTCGCCGCACCTGCTCCTCA
TCCACGCGACCGTCTCGGCTTCGAAAGAGAAATAGACACTAGATACAACCAAGTGCCGAAACTCCTCGACTAT
GTTCTCAGTGAGGAATACCTCAGTTCGCAGAAGAACAGGGCGAAGCTCGTCACTAAGC
TGCGGCCGGAGGAAGCTGTGGTCGCTGACAACGTGATTTTAGAATCTAACGTTGTAAAAATTGTGAAGCCGCT
TAGGAAAATAGAAAATGGTGAACTGGTGAGACACCGAAGAGGCTACAACCTTGAAAACGTGATAGTGCCCGCT
AGGTACCCCTACCTTCCCGTGGTGCGTTATAACCGTTTCCGCCGCTGGGGCTAGTGGC
CGTAGTGGGCCCCGATGCGACGGTAAATGTCAAGGTTGCATTCAAGCTAATTTGCAGCGAGAGTCATGTGCGA
ACAGCATCTGTGAGGCGTCTTTATATAGTCTGGACTCTATGTTTACGTTGTTTATATAAGTGTTTGTATAAGT
GGCTGTGACCAAACTCTCAATTTATTGCATTATTTTGGAGATATGCATTAATTTGATT
GACACAGAGTATATGAGACTATTGTTAGTTTTAAATAGTGAAATTTTAAGTGAATTAAGATGTTTACCTATTA
AAAACATATAAATATTGTTAGTGTTTCTATATATTTTGTTCTCTACTATTATTAAATATTTATTAATTATCCT
ATCCTAAAAC
>1002049738_1 Heli.1-DT665798.3.5
CAGCGTGGATAACGTCAAACCAAAAATCTAAATGACGTTACTGTAAAAAAACATTCAAAGAAACTTTTTAAAT
TGTGTGAACATGGATTTCTTCGAAGTAAAGGGGAAAATTGGTTTAATGAAAACGCTCCTAGTTATTTCGTTTT
CGTTTACCTTTGTTGCAACAGCAAACTTAGAATACAGTGTCTACAATAGTCCAGTGAG
TCACAGCAGCAGAATAGCACCAGAGATTATAACATTTGAGAATAGTAGAGTCCGTCGAGATGTCGAGTCAACC
AATGCCTATGCCGTCACCTCAAACTCTTCCAACACCAATCCAGGTGCATCTAACAACACTGCCAACAGCACAA
AAATGACAAATGAGACTGAAATATCGTCTTCCATCGCGTCGACAGTTGTGACTAAAGT
ACCACTATTAGGAGTGAACGGGTCGGGGATTTTGCACGTTAACGTCACTGCCAATTCACCGAAAGTCAGCGAT
CCAATAGTACCAGATGACGTTCCAGATATCAACGATGTGTTCAAAGAGACCCCAGAAGTTATCAAGGCCCAGC
AAAATCTTTCAGAAATTATTACAGACCATCACAGCTTCTACAACAGTTCGTTCATTGG
GAATGTCGACTACTTTAGGGAGTATTGGGCCAATATATCTAAAGTTCCAGCCGATGTACACGAGCTACTTAGC
AACTCACATCGACGTGCTACTACTCTAACCCTTAA
>1002049739_1 Heli.1-DT663061.3.5
GCAGTTGACGTGGCTACTACGTACCGTCCCGTTCGCCCACTCCGGACGCAGCATATACGGTGACGAGGAATAC
GTAACTGGTGAACGAAGTGGGCATGGACGGAATGGTCGAGGAGAACGGCACATCTGGCGCTGCGGGCGTCCCA
GATCCCTTGGCGGGTGCAGGCTCGTCGGCCAGTACCACGCCTCACGTCGTTGTAACCA
GCATTGTGCAACTGACGTTGCCTACACAAGCACCGTCAGCACAGGTCCAGTCAGTCATCCAGCCAAATCAACA
GTCTGTTATTCAAACAGCATCTAATATACAGTCAGTGCAACTTCCTAAAGGAAATGTTATATTGGTTAGCAAA
CCAAGCTCAGTTATACACACTACACAAGGAACATTACAAACATTACAGATTAAACCTG
AACCAAATAGTGTAGTCAGTACACAAGGACAATCTTGCAGTGATGATAGCTGTAGTGATGATGACAGTCCTAA
AAGAAAATATAGAGAAATGTTAACACGGCGTCCATCATATAGAAAAATTCTGAATGACCTTGGAGGAGCAGAA
ATTGCTGTGATTCCAGCTGGAGCCCTGCAAACTGATAGTGGCTTACATACATTGGCAG
TGTCAGGTACCACAGGTGGCGGTGCTATAGTGCAATATGCGACAAGTCAAGATGCACAATTTTATGTTCCGGG
TGAGTATTGTTTATATGACTTTTGTACTATTATTAAACTATGGCAATGATAAAATATTTGTAGAGGGAACGAA
AGGAATATATAAAATGTTTGGAAAATCGAGTTGC
>1002049740_1 Heli.1-DT667394.3.5
TACGATTTTTTAATACCACAATAACTTAAACGATGCATCTCGACCCACCGCACCCCCAGTGACTATAGTGTAA
TACAACATTATCACTTCATAGTGGTATTTGTGATGTTTTGTGCCAAAATCTTCAGTGCCATATATATATTGGG
GAACACTTAAATTTATATCAAAAAAACTGATAACAAGCTGGGTAAGCGATGAGGGGGT

GGTTTGACGCTTTCCGCGAGGAAGGCGGGCCAACCCTATATGCGTACCACAACAGGACCGCAGTCGCTGCTGA
TGTACCGGCACTCGCGCTACTAGTCGCTGCCCTCACATTATACCTCGCGTTCTTAGCCATCTTCCCAGGCATT
AGGAAGGAGAGATTCTCCACTTTTACAATCGTCACGCTTAGTTTATTCGTAGGAACTG
TGATTTTAGTATGTAAGCATGGGTCATCGTGGCATGTCGCGGGGGCACGTGTGGCCCGCGCCGCGTACCGTGC
GTTTTCCACGGACCGCCTGGACTGTTGGCTTGCGGTCCACGTAGGCTTGGGCCACGTTAATGTTACCCTCTCT
GCGTTATCGTGGGGCAACATATCAAGAGGTGATCCTGGCGTGGACTACAATGAACAAT
TCAGGTGGGAAGAAGCGGGGGCGATCCAGGAGTGGTACCGTGCTGGCCTCTTACGAGGACTACCTTATCCCGT
CCTGTCTGTCGCTGAACATTTTGCAGCAGAGCATGAAGGATTCGAATGGGGTGCGAAGTACCGAGCCGCTGGA
TATACCACAACGACATTGCTTTGGACGGCGCTTGCTTTGTGGCTGTTGATGAATCTTC
TCTTAGTGGTTGTGCCAAGATATGGAGCATACGCGATGGCTTCCCTAGGAGTTACATTATGTGCGGCAGCGGG
AGGGTACTGGGCCTCACTGCCTCATGTACCTCTAATAGTTAGGATAGATGGAGCTATGTTATTTTTTTCATTG
GGTTGGTGTTTTTGGCTAGTGTTAATTGCAGGTGGTATTTGCTTAGTCGTTGGGTTAT
TGATAGCTGCGCTAGACCTAGTATGGCCGCATAAGTTCTCGACAGTATTAGAGGTGGACTATGATACACCATA
TGATAGACACGTGCTTATAGTGGACAGTCGGCAGCGAGCCAGACCTCAGACACAAAGTT
>1002049741_1 Heli.1-EL598533.1.5
ACGCGCCAATTTTGTGAAAATTTTAAATTATAATCTGTGCTTACAAATTAATCAATAATAAATAAAATACGAT
TTTATTAATATAAGATGTTTATAAATAATTATTATTTTCTGTTAAACAAAACGCAGTGATATATTTTCGAACG
ATACTTTTAATAAAAGAAAATCAAAATGGAATATAAATCAAATAAAAGGACTAAATGG
TGTATGTATAGAAAATTAACGACTTTTAAAACAGTTTATGCGGGTTTGATATTGCTTTATGTGCCAGTTTTAT
GTGACAAAGATACATCCCGACCACTGACTTACGACTATATCTACAACAGTACCAACTACGCTCAACTGCAGAA
TGTGAAGCAAATTGTTAATTCATATATTCTTAATAATAATAATCACACCGAAACCAAA
GGAACAGAAGAGTTCAAATTTAGCGATAAGTATGAAATAAGCAAACCTGACGATCTGTTGGTGGGATTCGCGC
ATGACCCATTCAGACAGTCCATACCGGTAGCTGATTATATGAAAGGTGTTAGAACTATGGATCCAAGCACAAT
GAACAAGGATCGTGGAAAACGTGGTGTTTTTCATCTATACAACATGCTGACGTGCGCG
ACCGGGTGCGACCCCGTCAGCTATAAGGGCTACGGCTGCTACTGCGGCTTCCTGGGCTCCGGGAGACCCACTG
ATGGGATTGACAACTGTTGCCGTTTACACGACGAATGCTACGAAAACATATACTGTCCTGGCAACACTGTTTA
CTTTCAACCATACTACTGGAAGTGCTTTAACGGAGAACCATATTGTGCCCTGGAGAAC
TATCAAAGAAGAAACAGAGTGTCCAATAGCTGTTCAGGGAGGTTGTGTGAGTGCGACAGGCGCTTCGCGATGT
GTGTCAGGAGGTACCGCTGCCCCAGGGGCAGGAGCTTTTGCACATCCGACCCTTTGAGGCTTATACAGAATAT
ATTGATGTTTAAGTAAATCGATTTTTTTTACATTTTTGGGCACATCGAATTGGTTTCG
CAATCGAATATAATTTGATTTGTATGTGATAGTAGTTTATTTTATTATTATTTTTTATGTTGATTTTTAATTT
TTTTGATTACATTTGTTTACGTATTACTGGAATGCTTTAACTGTGAAACACAATACACTTCAGAAAACTATCA
TAGTAGGAACAGAATGTTGAATTCCTGTTTAGGTAGAAGTGATAAAGGCTTTGCGGTG
TGCGTCAGAATGTCTCATTACCCTAGACCACTGGTTCCCAACCAGTGGTCCGCAGAAGCCTAAATATGGTCC
>1002049742_1 Heli.1-DT664556.3.5
GCACGAGGTTTGGCTTGGCGCCTTGCACGAGGGTTGTTCTCTTGTAGTGATAGTGTGTTACACGTTTCAAAAT
GGCTGGTGGTAAAGCGGGCAAAGATTCTGGCAAAGCCAAGGCGAAAGCTGTATCACGATCGGCTCGAGCAGGC
TTGCAGTTCCCTGTGGGTAGAATTCATAGACATCTCAAAAATAGGACTACGAGTCACG
GCCGTGTCGGCGCCACGGCTGCCGTTTATTCTGCAGCTATTCTCGAATATCTTACAGCCGAGGTTTTAGAGTT
AGCGGGCAATGCATCCAAAGATCTTAAAGTAAAACGTATCACTCCCAGACATTTACAACTAGCTATCAGAGGA
GACGAGGAGCTGGACAGCTTGATCAAAGCCACCATTGCAGGAGGTGGTGTCATCCCAC
ATATTCACAAATCACTCATCGGTAAAAAAGGAGGACCGGGGGCACCTGTTTAATAAGAGTCTTGCTTCAATTC
AACATTCTAACTGCATTTGAACCGGACACTATCAGCGTTGTAAATTCATCAGCCAGGCAGTAAACATAGTTTT
CTACAGGTAGTGATGTGGACAGTGGAGTGACTTCAGACATATTGATCCGGACTAAACT
TATAGTTGTTAAGACTAGTAATATTTTTTAGTATAAGGAGATTTAAATATAATTGAATTAGGATTAAGTTCAT
TTTCTCGTTTTGTTACGGGACATCTTTATTTTATTAGACATGTGTACAATTTATTTCAGCTACTGCTAAAATT
AGTTCATTGTCAGTTCGGCGTGGCCTGAACAACAAGGTTCAGATCGACTGCTGCAGTG
ATTTAATTTTAC
>1002049743_1 Heli.1-ES585817.1.5
GTCACTATCATTTCGTCAGTCCAAGATGAAGCTTTGCTCAGTTATATTCAGTGTATTAGTGGTGTGCGCAGTG
AGCTTCGCCGTGGAACATGGCCCGTTGATCAATGTTCTGCAACCCGTTGAGTCCCACGACTTAGTCCAGATTG
AACCTGTACAAAATATTAGACCGAAGAGGCATTTACTTCTAGGTGCCGGAGCTCTAGG

TGCTGGTGTAGTAGGAGCTGGAGCTCTTGGAGCTGGTTTATTAGGAGCTGGTTTGATCGGTGCTAAAGCTGGA
CTTATCGGCGGTGCTTTAGTGGGCGGTGCCCTACAAGGCAGAAGTTACGGGGGTGGATACGGATACGGCGGCG
GATACGGTGGCGGATACGGATATGGCGGCGGTTACGGATATGGTGGTGGATACGGATA
TGGTGGGGGATACGGCGGATACAGTGGACAAGGATACAGACGTTACTACCCCACTACAACATATATTGTAGAA
GAACCCTGGTGCTAACTTCAACAATGCGTCGTAGTTCTTACGTAATCAATTCCAACGTGTGTAGAGATGGGGA
TATCTCATGACTGATACCTGCACGTTTAGTTGTTAAGAATAAATTT
>1002049744_1 Heli.1-DT662056.3.5
CCGCCAGTGAGGAGCAAAAGGAAAGAAAGACCTCAAAACAAGAAAAAACAAGAGAAAAGGATCACATCATCTG
ATTACACTGCTTGGGAGAAGTTTGATGTGGACAAAGCATGTGAAGAGGTTGATATGGCAGACCTGGGCCCAGT
GTCCCTTGATAGCAAGAAGAGTCAACCTGCGAAACTAGAGAAACTAAGAGAAGAAGCA
CAGTATGAGAAAGAGAGGGGTAATAATTTCGTGAAGCAAGAGAAATGGGATGAAGCAATATCGTGTTACAATC
GTGCAATAGAATTGGTTAAGGATGACGCCATTTATTACGCTAACAGAGGACTTTGTTACTTGAAGAAGGATAG
TTTACACCAAGCGGAAACGGACTGTACCCAAGCGTTAAACTTAGACCCAACCTACGTA
AAAGCGTTACAACGTAGAGCCACGGCCAGAGAGAGACTTGGGTCACTAAGATCCGCTTCACACGATTTAAACG
AAGTACTGAAGCTGGAACCTCATAATGCGGCCGCTAGGAAGCAATTGGAGGCGATTAAAACTAGAATGGGTAC
TAAAGGGTCAAAATCAAAATCATCACCGGCAACCACACCGACAACCGAATCCAAACCG
ATAGTAAAACCAAAAACACAACCAAAAATAGTCGAAATAGAGGAAACTAAGAGAGAAATG
>1002049745_1 Heli.1-DT667981.3.5
TTCCGCCATTTTGATGGAAGTGCTTTGTGAATTCATTTTGTGGTTATATTAATGACACTACGGACAATATCAA
ATTATAATCTAGTGTGGATTAAGTTATAATTTTCAGCCTTGATTGATTTACACATATTAATATGGAGCCGGCT
CTGGATGCGCCATTGGTGGCGCAATCCATCAACTACCATGGGCAACAATTACAAAAGG
CGTGGGAGGCAGAACGAGGAGAAGACGATTTGGGCAAAATTGGTGTAGGACCACTGGACTTTGCAGTTTATCA
GTCCAGACATAAACATCTTACATTCCAAGACCGAGGAAAGCGGTTAAATTTACATCAGTTTATTGCAAAAGAA
GCAAATGCTTTATTTGACGCTTCACTACTAGAAGAAACTCCATCATCTTCCAGTTTAG
GGGCTGATGCAACTGCACCTGAAGATAATTTGTTTGCATTAATGCCGCCATTTGAGACTTTTTTGAATGTGGA
TAAAACTGCAAGATTGCGACATTTTTTTGATAATGTGAAGACTGGTGAACTTATCATTGGGGCAGTGATCAAT
AGAACAGCCTCAGGAATGATGTTGAAGGTTCTATGCACAGCAGCTCCAACTCCAAGAT
ATGTTGCTGATATTAATGTTAAGGCATTTTTGCCAGTTGCAAATATCATACCAGCAGTGGACAAGAAAAATGT
GTCAAGAACCTACCTGATGAATGACACAGTTTGCTGTGAAGTTATAG
>1002049746_1 Heli.1-DT664848.3.5
GACGAAGGACGTTCATTCAACACAAAATTCTTCGACTAATAATATTTTTTATATATATGTGCATAAATGTGAA
TAAAATGTGGACTCTATATATTTGGGCATTATGCCTGGCGGCCATGTGTCAGGCGCAGCTGTCGGACATCGAG
AGATATTTATTGACTATCGCCGAAATGTCACCGTCGCCCCCAGTACAGAGTAACGAAA
AAGCATGTCAAGTTGATAGTTCTACAAACGGTGTTTGTGTGAAACGTACACAATGCAACTTGGACGCGCCGGC
AGTGGATCCGGCTACTACTTTCATACTGCGTGAAGGAGGATCCACGCCTAATGCATCGGATGTGGCGAACGCC
CTAGATACACAGTGCGCATGGCTTCAAATCTGTTGTCCATCTGATAAAGTCCGGGAGG
AAGAACGCCAAGTGAAATTCGAACAGCCACTTGGCTGTGGCTACAGCAACCCCGGCGCCAACGTCTTCAGGGA
ATCTGCTAATGCTTACGGCTATGCAGACTTCGGGGATTTCCCGTGGATGATTGCTTTATTACTGAAAAAAAAC
GACAAAACAAAATTCAATGATCAGTACATAGGGGGTGGAACATTAATTCATCCCTCGG
TCGTGATGACTGTGGCTCATAAAGTAGACACGCTCGAGTCACCTAATGAGTTGACGTGTCGCGCTGGAGAATG
GGATGCAGTAACAGAGGACGAACTCTTTAAACATCAAGACCGAGATGTTAAAAATATTGTCATCCATAAAAAT
TATCTATAAG
>1002049747_1 Heli.1-DT662324.3.5
GTGTCGAGTTATTTCTATGATTCATACGATAAAGTAATGATTCATACGATAAAGTACGTAATATCAAGTAATT
ATTACAACATTACATTCTTCAGTACAACATTACATTCTTCAGTAATTTACTACACTGCACGCTTGAAAAGTGA
AAATGGATGCAACCGAGGTACAATACCGGCCTATACCGTTTAGTGTTCACGTCTGGAG
TGCATTGAGAACATTAATCGATGTGATCACCGCTATTTTTTTTAAATTGTATTATGGCACACAAGGAAATAGG
ATACCGCCAATAAAAGACGACATTCTGAAGCAACCGGCTGTAGAGGTTGCCAGAAGAATAAGGGCTAAACAGA
TAAGCAGTGAAGATGTAGTACAAATATGTATACAACGGATTCGGGACGTAAATCGCGA
GTTAAATTGTTTGGTAGAAGAACGGTTCGAACTGGCTCTGGCTGAAGCTAAAAAGGCTGATGATCTGGTGAGA
AGCGGTTCGAAAACGGAAAATGAATTGGCTAAAGAGAAACCTTTCCTAGGCGTTCCGTTTACGACTAAGGATT
GTATTGGTGTTGAAGGACTTCATCAAACAGCTGGAGTGGTTATGCGAAAGGATTTCAT
AGCGGAGAAGGATGCCGAGTCAATTAAATTATTAAGAGACGCGGGTGCCATTATTCTAGGTCTGACAAATGTT
CCGGAACTTTGCATGTGGTACGAAACTTACAACAAAATTCACGGTAGGACAAATAATCCTTATGATACTAA

>1002049748_1 Heli.1-EL600997.1.5
CGCGGAAGAGGCGCGCCGCCGCGAGCCCGGGTCGCCGGGCGGCTCGCTGGCGTCGCTGTCGTCGGGCGGCTCG
CTGGCGTCGTACAGCGACGGGTCGCGCTCTCCGCGCTCGCCGCACTCTCCGCTGACGCCGCAGTCGCCGCAGT
GCCCGCACTCTCCGCCCTCCTTCGCCTTCCGCCGCGCGCCCTCGCCCGACCCCGAGGA
GGAGCGCCTGCCCGTGTTCAACCGTCTCTCTTCCGCGTTCGGCGACATCGTGATCGCCTAGCTCCAGCGCAGT
GCCCCCTCGACGTCTTCCAGACCGCGCCCTCACCCGCTCAACGAATGATCTCCAGTCAACCTATGCTCTTATT
ACCGCCATGTAATGTTTTAAAGTTAAAGTGTTCTGGCGTCTCGTAAGTTTGCGGTGTG
AATATAAGTAGATATACTTACATGTTTTAGTTATCGGCACGCACTTGTAAATGGCACCCTATGTTCATAGATA
TTTATTGCGTAGCTCGCGTAGCGGACGCCTCCCCGACGCGAGCCTCCGCCGAGCGGTGGTCTTGTAAAGTTAG
CTCATAGTGACGTTTTATTTATTCGTATTGCCTCTCCGCGATCGGTACCGCACCGCGA
GACGGCGCTCGTGATTTATTTTTAAGGATATAAGATAATAATTTATTATTTATTTCGAGTCTCTTTAACTTTA
ATTATTATTATTTTGTAACTTTCGCGATGCCACACCGCACACGGACGAGGTACGCGTCGACGGGACGTGGCTC
GTTGTATCAACCTCCGCTCGGCCGGTCCCTTGATTGACTATATTCTGACAACTTATTG
TGGACGGAGCCGGCCGGGTCGGACCGGCAGTCGAGAGAGAGATGGGCTTAAACACCTCGAAAACATTATTTAT
ACTCTTAATTT
>1002049749_1 Heli.1-EL597152.1.5
GCACGAGGCTCGTCTCAGCGTCAGCTCCACAATTCACAATGAAGGTTTTCGTTTTAGCTGTCCTTATGTCGTC
AAGCGTCTATGCGGGTCTGTTACCTGCGCCACTTGGCTATGCCCGCCCCTACGGCTTGGCTGCTCCACTAGCT
GCTCCCCTGGCAGCACCTCTTGCAATCGCGAGGCCTGCTTTAGCTCCATACGCGGTGG
CTCGGCCAGCTCTAGCGCCCTACGCTGTAGCCAAAGTAGCAGCCCCTGTCGAAGACTACGATCCCAACCCACA
ATATTCGTATGCTTACGACATTCAAGATGCCTTAACTGGTGACTCGAAAAACCAGCAAGAAAGTCGATCTGGG
GATGTAGTCCAAGGTTCTTATTCGTTAATAGATCCAGATGGTACACGCCGTATTGTAG
AATACACAGCTGATCCTCAAAATGGTTTCAACGCAATCGTGCGCAAGGAACCTCTGGGTGGCGTTGTGAAGGC
AGTTGCCCCTATTGCTGCTATTCATTAAAATTAAGCTAATGTAAGCTGATTCAAACACCTCAAGGAAACTTAT
AAAGGAAAGTTACTATAAGGCTCTAAAAACGAAAAATATAATTTATTTTTCGAATTAT
GCCATTGGTTGATATCGTTTCTAATGAGGACTTGAATCTGCTCCAAAGCTTTTATTCACAGTGACCTGCATCT
TTGTTTTTTTATAAATTGTTGTTAAAGTATGTAAG
>1002049750_1 Heli.1-ES587539.1.5
AACGCGGCGATCGGTGCGAGCCGTCTTAACACGTGCACCACGGCGCCGAAGTTTCTGATATGACTCAGTTCGA
TGTCGGGGCTCGGAATAGCCTCGTGCCGAATTCGGCACGAGGCCGACTTGCATCATGAAGACATTCATCGTTC
TCGCCATCTGCTTCGTCGCCGCTCAGGCGCTGACTGACGAACAGAAGGACAAGCTCAA
GAAGCACAAATCGGAATGCCTCGCTGAGACCAAAGTTGACGAAAAATTGGTTGACAAGCTGAAAACTGGTGAC
TTTGAGATAGAAAATGAGCCTTTGAAGAATTACACACTGTGTATGTTAGTCAAATCCGGGCTGATGAGCATGG
ATGGAGAATTCAAGAAGGACGTCGCCCTCGCTAAAGTACCTAATGCAGCGGACAAACA
ACTAGTAGGACAGCTGATTGACTTATGCTTGGCCAACAAAGGAGATGCTCCTGAAGAGACAGCTTGGAACTAC
TCAAAGTGCTACCACCAGAAGGACGCTAAACATTCAATTTTCCAATAAATGACCATTTTTTCCCGTAAAAATC
TGTAAAATTGGCAAAAATTGTGTTTTTTCGTTGCAATTAGATATTTTTTTGTTCGTTC
ACTCTAGAAAATGTAATTACTGTTCTTTAAATAATGTAAACTGATACTGAGCCTGCACGAATTTAATGACGAA
TAAAAAATTTAATGTGAAAAAAAAAAAAAGAAAAA
>1002049751_1 Heli.1-EL599548.1.5
GCACGAGGGTTTTTCCTTTTGCCGCACACGTGCTGAGATTTGTAATATTTTTTACTCAAAAAATTTAGTTTAG
CACGCGCTGTAAAGTTTCTTGAAACTGAGTCTAAATTAATTATATATTCAACAAGATTCTTCAAGGAACATCA
TATTAAAATAGCAGACTACCTAACTTACATGGCAATGGTGTCTGAGGCGAAGGTTAAT
GGCAGTGGAGGTGAAGCACCGAACGAGCTTCTACCAGCACTAGCAGAGAAGCCAGTAGGCCTTCCACCAGGAA
AGTATGTCTTAGTTGGGTGGGACATGGATACAACTGGAAGAAGGCTAATTGATGAAATATGTCAAGTAGCTGC
CTTCACACCAAAGCAAACATATTCCCAATACATTATGCCATATGGGGATTTAAATCCT
GGAGCCCGTAGGCGTCACAATGTACGAGTGGTTACCGTTGGAAGATTTCGTATGTTAAAGGATACTAATACTA
ATAAGATATTAAAAACAAAATCTGAGATATCTGCACTTTCTGACTTCTTAGATTGGTTAGAGAAGGAAAAAGG
AGATGGCAGTGTCATACTCATATACCATGAGCCACGTCGCTTAAGCCCCACTATGCTG
TTAGAAGCTCTAACTCGCTACAAGTTGTTGGACCGTTTTAAATCAATAGTAGCTGGTTTCACAGACAGCTATG
CCCTAGCGGCAGACAAATGTAAATCTACAGTAAAATCTGTTTCTCTACGAGTATTGGCCAGAGTTTTACTCGA
TGCCGACACACTGTCTGTGGAC
>1002049752_1 Heli.1-EL601585.1.5

GCACGAGGGCTATTTAGTGGTGAAATATCGACTGGTTCTTCATTAGAATCATTAACTTGGTTAGGCTTAGATA
ATAATAATATTCATTTTATATCGACTGATGATATACAAAATTTTCCTTCATTAGAATATTTAAATTTGGATTT
TAATAAATTAATCGAATTTCCTAGTAAAGTTAGTAGAACAAGTGGAACGTATAAAATT
AAGGAATTAAGATTATCATATAATTATGTAAGTAGAATAGATTCTGAGTTTTTAGAAAAAATTACGGAATTAC
AAAGCATAGACTTATCTAACAATAGAATGCACAATATCAGTGAACGAAGTTTCTGTAATTTACAGAATCTGGC
TTATTTAAATTTAGCTGGCAACGTTATAGAGTTAATAGCCGAAGAAGCTTTTGTTGAT
CTTCCTAAATTAGAAATATTGGATTTACAGGAAAATAGCTTAATTGAGTTTTCAACAAGATATTTTTGCAACA
TATCAAAGGAAGAAACGAATTTTGCAGTCAATGTTAGTTATAATAAAATTTCGTCCCTTATGGGTAGTTTAAC
AGTTTCAATCAATATCTTAGACTTGTCACATAATATGTTGGAAATAATTCCCTCAGAT
TTTTTCTTTTCTTTAGGTAAACACATTCGACAAATATTTTTATCCTACAATAGAATAACTATTATAGACAATA
CAGCATTTTGGTTATTGGCCTCA
>1002049753_1 Heli.1-EL602762.1.5
GCACGAGGTGGAGACCGGCACGGAATAAAGTATTATATAAAATATTTTATATTTTAACTTTGTAATACATTAA
TCAAATTACCATCATGGGTCGTAAATTCGTTGTTGGAGGTAATTGGAAGATGAATGGAGACAAAATCCAAATA
AATGAAATTGTTAACAACTTAAAGAAGGGTCCCCTCGATCCTAATGCTGAGATCGTAA
TTGGAGTACCCGCTATCTATCTTGCTCAAGTGAAAAGTATTGTTCCTAGCAATGTTGGAGTTGCTGCACAAAA
CTGCTGGAAGGTTCCTAAAGGAGCATTCACAGGTGAAATTTCTCCTGCAATGATTAAAGATGTTGGAGCTGAC
TGGGTCATCCTTGGTCACTCTGAAAGGAGAACCATCTTTGGTGAAAAGGATGATCTGG
TTGCTGAAAAGGTGGCCCATGCCCTTGAATGTGGTTTAAAAGTGATTGCCTGCATAGGTGAAACTTTAGAAGA
GAGGGAGGCGGGTAAAACTGAGGAAGTGGTGTTCAGACAAACTAAGGCTCTATTGCCTGCCATTGGCAACAAC
TGGGCAAATGTTGTGTTGGCTTACGAACCTGTATGGGCTATTGGCACTGGCAAAACTG
CTTCACCACAACAGGCTCAAGATGTCCACGCTGCTTTACGTAACTGGCTATCCAGCAATGCTTCTCCTGACGT
AGCTGATTCTGTTCGCATCCAATATGGTGGCTCAGTGACAGCGGCAAATGCAAAGGAGC
>1002049754_1 Heli.1-EL596856.1.5
GCACGAGGCGTGAATCTTTAAGGGGGTGTTGTAGGGCTGGCCCCGTATCGTCTTGTGATTTCACCAAATAATT
GCTGTTTCCAATATTTAGTGTTAAACAGCATTAAATTTTAAACACCGGTGATCAAGATGAGTGAATATTGGCT
AATCAGTGCCCCTGGCGACAAAACCTGCCAACAGACTTGGGACACCTTGAATAATGCC
ACTAAATCAGGCAGTCTCAGTGTTAACTATAAATTCCCTATACCTGACCTGAAGGTTGGTACATTGGATCAGT
TGGTAGGATTGTCTGATGACCTTGGCAAGCTTGATACTTTTGTCGAAGGTGTCACTAGGAAAGTAGCTCAGTA
TCTTGGTGAGGTATTAGAGGATCAACGTGACAAGCTGCACGAGAATCTAATGGCAAAC
AACAGCGATCTGCCCACGTATCTGACTCGTTTCCAATGGGACATGGCCAAATATCCCATAAAGCAGAGCCTTC
GCAACATCGCCGATATTATCAGCAAACAGGTGGGTCAGATCGACGCCGACTTGAAAGTGAAATCATCAGCTTA
CAACTCTCTCAAGGGGAACTTACAAAATTTAGAGAAGAAACAAACCGGAAGTCTTCTA
ACTCGCAACTTGGCGGACTTAGTGAAGAANGAGCATTTCATTCTGGACAGCGAATATTTGACCACTCTTCTCG
TCATTGTGCCTAAGTCGATGTTT
>1002049755_1 Heli.1-ES588168.1.5
ACGAAGGCTATAAAATATACGATTTTATATTTACCATTTTCAAGAAATTTCATAATATATTTTGTCATCATTT
ATTGACGTCGG--
TAGGTTAAGTACTGAGAGATCCCCTCATAATTTCCCCAAAGCGTAACCATGTGTGAATAA
ATTTTGAGCTAGTAGGGTTGCAGCCACGAGTAAGTCTTCCCTTGTTATTGTGTAGCCAGAATGCCGCAAAACT
TCCATGCCTAAGCGAACTGTTGAGAGTACGTTTCGATTTCTGACTGTGTTAGCCTGGAAGTGCTTGTCCCAAC
CTTGTTTCTGAGCATGAACGCCCGCAAGCCAACATGTTAGTTGAAGCATCAGGGCGAT
TAGCAGCATGATATCAAAACGCTCTGAGCTGCTCGTTCGGCTATGGCGTAGGCCTAGTCCGTAGGCAGGACTT
TTCAAGTCTCGGAAGGTTTCTTCAATCTGCATTCGCTTCGAATAGATATTAACAAGTTGTTTGGGTGTTCGAA
TTTCAACAGGTAAGTTAGTTGCTAGAACCCATGGCTCCTTTGCCGACGCTGAGTAGAT
TTTAGGTGACGGGTGGTGACAATGAGTCCGTGTCGAGCGCTGATTTTTTCGGCCTTTAGAGCGAGATTTATAC
AATAGAATTTGGCATGAGATTGGATTGCTTTTAATCAGCCTCTTATATCCTAAAGTCTTTGAGTGACTATATG
ACATATCATG
>1002049756_1 Heli.1-DT662579.3.5
GCACGAGGCTTGTCCCTCGCACATCCTCGGAACGTGTACGCGAATTAAGGCACTTTTTTTATTGTTTACAGTG
AATGCATTTCTTATTAATTACGCAAAATGATATTAAAATCAAAATATAGATACGGGATTTTGTTTGTAAATGT
ACTTGTCGGCTTCAGGATTGTTTTCGCAGATGTACTTAGGACTGAACTTTCAGAAGAA

AATGAAGTGTACAGCACTGATGATGCCGTGTTTATTAACGTGAATACGGAGCGAGTAAAACGCGAATCAAACA
GCACTCGAGATGGAAAACAACTATTGTTACGATCTAGACAACTTTTAGGCTACCCGGTTGCCGCACCTCCACA
GCAAGAGCCAGACGTTCAATACCTCGGACGGCTGACACCATCATTCCCGATGCCATTA
ACCAAATTACAAATTCCTGCGGGTTTGCCACAATGGTACACCAACTTTTATCGTCAGATACCAGCACAATGGC
CGCCTGCGTTACCGCCGATACCAACACATCCACCAGACCATTCCGCGCCTACTCATCCTCCATCATTAATTGC
AGGTGTAACAAGCCCAAAACCTGTTGAAGGACCTTCAAACACGCCTCCAACTACCACA
TGGGGAACAAAGCCTCCAGTAAGCACTACTAAGCAGACTTGGCCTCCCGTTAACCCAACACAACCAACTAGGC
CCACGGCGCAACCAGGCACAGATTCCTCATGCGGCATCAAAAATGGACCACAGGTATATGGCAGCACATACGA
ATCGAAGGATGAGGAACGTATAGTGGGCGGGCACGACGCCGACCTGAACGAATGGCCA
TGGATAGTCGCG
>1002049757_1 Heli.1-EL599302.1.5
ATTTTCTCTGTCGTCCGTAGGTGCCGTTCGGATACGCTGGTGGCCGCTCGTGGTCACTTCTGACTAAGTCAAT
TTCTTTTAAATAATGCGTTTGCTAGTATTACTTTGCTTTGTGGGGATAGCCAGTTGCTATTCTGTAAACGAAA
GAGCTGGACCACCTGGTCAGGAGAAAGGCATACTGGAATGGATAAACACGTGGTTGAC
AACAACTTCGACTACGAGTAAACCGACAAACGATCCGCCAGAGGAATGCCCAACATGTCAGTGCGGCATAGCT
AGGACTCGCAGGCGAATCGTGGGAGGATATGAAACCAAGAAGCTGGAGATACCATGGATAGTGGTGTTGATGT
ACAACGGCAGGTTCTACTGTGGAGGCTCCCTTATTAACGATCTGTATGTGCTGACTGC
GGCACATTGCACTTCTGGATTCCGAAAAGAAAAAATAACGGTTCGTTTCTTAGAACACGATCGTTCTACTGAC
AACGAGACGAAGACCATTGACAGGAAGGTGGCCAGTATCATCCGTCACCAGCGGTACAACCCCGGCAACTATG
ACAACGACATAGCTATGCTAAAGTTGAATGAAAGGGTAGACCTCAGCACAGCTCTGAA
GAGAGCTCGAGGTGATGAAACAGAGGAAGACAAGGACGTCGGTCTCCGGCCTGTGTGCTTACCGTCGGCTGGA
CTATCCTATAATAACTATACCGCGTTAGTAGCTGGCTGGGGCACTAC
>1002049758_1 Heli.1-DT664214.3.5
CTGTATCCCGCACGTAGCAGGGTGGGCAGAGGGAACCTATTGTTAAGACACTCCGTTCCCCACTTGCCGCCAA
ATTCTGGAGGCATTGCGTGTAGAGTGGCGGAACTCAACACCGCGCTTTTCTTTTTGTGATTTTGTTTTTAATT
TTTAAGAAACATGGCGGCGAAAATACCCTTTCCATATGAATTTAAAAACGTAACGGAG
GAAGAGGATAAAATAGTGAAAAAATATTACAAAGATTACACACGTCCGTTCATCCGCGTGGGCCCGCACGGCT
ACATCTCCATGGCAGGGTACGGAGACCATGCGGCTGATATCTACAATTTGGAAGTTAGACCGGACGATGTATG
GGTGATTGCCTTTTCTAGATCAGGCACTACTTGGCTCCAAGAGCTAGTTTGGCTGGTT
GCGAACGATCTGGACTATAGCGCAGCCTCAGCCACGCCACTGACTAAAAGATACGCCTATATTGAATATCCAA
CGATGGCAGCAGAAACTAAGAAGCATGAACCCCCACCGCCACCACCACCACCAGGTCGACCTCACCACCGAGC
CACCTTCGACGACTTCAGAACCGTCCACACGTTGCCTTCTCCGCGTTTCATCAAAACT
CACTTACCATTATCTCTCTTACCGCCGAAACTTTTGGACACTGCAAAGATTCTTTACGTAGCACGAGATCCCC
GCGACGTGGCCGTTTCATTCCACTTCATGCACAAATTGTTCAGATACTTCGACGGAGGGGTGGAGTTTAAAGA
ATTTTGGGATCTCTTTAAGAGAGATTTAATAATGCACACACCTATATTTCCACACATC
AAAAAGTTCTGGAACGTCCGTGATCATCCCAACTTGACATTTTTGTTTTATGAAGAAATGCTAATGAATCTT
>1002049759_1 Heli.1-DT662342.3.5
TCTCCACCTCCACAAGTGCGAGATGGTAGCATCGACAGCCGCGAGGGCTCCATAAGTCCATCGCGACGTCGGA
AGAAAGCCCGTCGTACATCCACCGAGAGCACCCCCGTACCAGCGCCCGAGCCCGAGGACCTGCCACTGAAGGA
GGAAGTATCACGAGATGGCGTGGAGGATTTGACGTTAGATGAAGAAGCAGAGCCGTCT
GTTACACACAATGATGTTGTACGCAATTTTCAATGGCATATGGAAAGATCTCAAGATGAAATGATGAATTCAA
ATGATAGTGTTCGAGAGACTCAAGAGGTGCGCAAGGAGGCCCTGCCGTCGGCCTTGCTCTCGCTGCTGTCGGC
GCAGCGCGCGCAGGCGCTCGTGCCGCGCCTCCTCCCCCTCCCCGACCTCCCCGAGCCG
CTGCAGGCGCGCCTGCTGCAGCGCGCCTTCCTCGACAACAACAACGACGAGCCCGACGAGCACTCGGGGCCCG
ACTGCGACGACACGGACGACATCGTGCTGCCCGACGACGAGGCGCGCCGCGACGACGACGCCAGCCGCCAGTT
CGAGTGCCGCCACTGCGGCAAGCGCTACCGCTGGAAGTCCACGCTGCGCCGCCACGAG
AACGTGGAGTGCGGCGGCAAGGCGCCCAGCCACCAGTGCCCCTACTGCTCCTACCGCGCCAAGCAGCGCGGCA
ACCTGGGCGTGCACATCCGCAAGCACCACAACACCGAGTGGTACATATACGCCAGCAACAAGGTGCGCCGCAA
CAAGAAGACGCCCGCCTAACAC
>1002049760_1 Heli.1-DN172647.1.5
GCACGAGAGAGCTCATCTTACTCTATCGGAAAATATTAACTTGATCCGAAAAGCAACGAAATATTAAATAGTA
AAAGCCTTTAGGCTTGAAGTTATAAAGCATGAGAGCAAAGTGGCGTAAGAAGCGTATGCGAAGGCTAAAGCGT
AAACGCAGGAAGATGCGTGCGAGGTCTAAATAAAGAGGTACATGTGTGAGTGATGAAA
ATTTTATTCTGAACTTCATGTGTATTCAATAAAAAT

>1002049761_1 Heli.1-DT663662.3.5
GCTCTTGTATTTTGGCGTCTGTCGAAGCAAATTTCATCTCATTACATCTGGAATTATCCAGATATTAAAGATT
AATTGTAACAAGTGCCCGTGAATATATAATCTAAGATGTCTATCGGTGTGCCTATTAAAGTATTACACGAAGC
GGAAGGCCATGTAGTGACCTGCGAAACAAATACCGGCGAAGTTTACCGCGGGAAGCTT
ATAGAGGCCGAGGATAATATGAATTGTCAGATGACCCAAGTAACAGTCACTTATAGAGATGGAAGAGTCGCGC
AATTAGAGAATGTATACATCAGAGGATCTAAAATCCGTTTCCTTATATTACCTGATATGTTGAAAAATGCGCC
CATGTTCAAACGACAGGGGAATAAACCCACGGCTGGGCGTGGGAAAAGTGCTATACTT
CGTGCACAAGCTGCTGGTCGAGGCAGAGCAGGAGGTAGAGGTGGAGGGCATCGTGGAGGCTGGCAGGGTGGCT
CAGGACCTTCGAGACGATAAACCTACAGTTAACTTAAGTTCAGTTTGTTTAATACCTATGATGTCCAAACACA
TTTGTCAGATAAGGATAATTATTTAATATTAAGATGTGTTTTGATATTGAGACATGAT
TGAACTCAGTGTGGTTATTAAAACAGCATTTATATCAAAATGTAGCTTTATTTGAATTTTATTTAGGACATGG
ACAACAAACTGTAAATATATAAGTAACTGTTCTTTATCATTTTTAACCTATGAATATTTGTCTTTGCCAGGGT
GGTTAGGAAGCTCCCCAACTGTCCCTACATTTATTAGATATATCAAAACAAAATACAA
TCTTTTACCACAACTAATTAAAATATAAATGTTTTG
>1002049762_1 Heli.1-ES584602.1.5
ATGGAAAAAATAGATGAAAAAGTAAATATGTAATTAAAATGTATTTCCAACGCCCATAAATGGCAAAAAATCG
CAACTTATCGAGCTAGTAAGTATATCAATGGCAGTGGCAAGTGGAAACAAATCAATATAATTAAGAGTAATAA
TTAAATGGTAAAAAATATTAATATCAAATTACATTGATACTAAAAAATGTACAATTAT
AGCGACAATAAGGTCAAAGCAAGAATTGAAATTCCTTAATTATATCAGCAGTCATATAAAAGGATTTGCAGAA
GAAAATTGCTGCTATATAAGAAAATATATATTAAAATCAAAAGATTTAAACAACAACAACAAACATAATTATA
TCAGATCACAACAAAATTAGTTTTTTAATAAATCAGTTTGTATGATTATAATCTGAAG
TTGAATGTATTATATAGATTGAGCTAAAAACTTGCTAATAATATAATGTACAATTATAAATGATAAAGACCCC
CAAAAAGAATGGTAACAATATAAACGTGAAAAAAGAACTTTAAATTAAGTGCCGTGCAGTTACGGTTGCCAGT
CATTTAATTTAAAGTGACTGGCAAGAAGACTCTAAATGACTGGCGAGAAGTTTCTAAA
TGAAGAACTTTATTAGATGATAACGAAATAAGATGACAGTTAAAGACGGCAGCTACGTAATAAAATAAAAAAT
AATATTATAAAACTTAAAATCAAAACCGAAAGGCTGAAAAAATGTAGACCAAAAAAGATGAAGGTCCGAACAA
TAAAACCCGAAAATGAAGCATTTAAATGAAGCCCAACAAAGATGAAGATGTACGTAAT
GAACACTGAAGAAGAAGACTGATAAATATAAAGGCA
>1002049763_1 Heli.1-DT667253.3.5
GCACGAGGGTTCACCCTGGCAGGAGATCAGGCGCAATTTTACCAATTACTAAATACAATATTGTCTATAGATA
ATGACATCAGATCCCAAGCAGAAAAAGTATACAATGATATACCAACAGAAACAAAGGTAGTCCATTTAGTCGG
TGCTATACAAAATGCGGATCTTGGACAAGACTGTAGGGAAACAGCTGCCGTACTCTTA
CGGCGGCTGCTTAGTGCAGAATTCTTTGAATTCTTCCCAAAACTTCCTTTTGAACAACAAGCCATGCTCAGAG
AACAACTTCTCCTTACACTGCAAATGGATGTTAACCAAAGTTTAAGACGAAAGATATGTGATGTAGTATCAGA
GTTAGCAAGAAATCATATTGATGATGATGGAGTCAATCAATGGCCTGAATTTCTTCAA
TTTATGTTTAATTGTGCTAGTGCACAAGATCCTAATATTAAAGAAGCTGGCATTAGGATGTTTACGTCAGTAC
CAGGAGTTTTTGGTAATCGACAAAATGAAAATTTAGATGTCATTAAGAGAATGTTGCTCTCAGCACTACAGCC
AACAGAATCTGAAGCATTGCGCATGCAAGCGGTCAAGGCAGTGGGGGCTTTCATCTTG
CTGCACGATAAAGAGCCAGCTATTCAGAAACATTTTAGTGACTTGCTCTTGCCTTTTATGCAGGTTGTAGTTC
AATCAATTGAAAAAGCTGATGATGATGCAGCTATCAAAGTCCTCATTGAACTTACTGAATCAGCACCTAAATT
CTTACGTCCACAAGTGGAAACCATATTCCAAGTTTGTATGAAACTAATTGGCGACACT
>1002049764_1 Heli.1-EL601015.1.5
GCACGAGGGATCAGACCTCTCTCCAGGAATGCTAAGAAAATCTAGATTTCAAAAATTTCTAGACTTCTGCGCC
ATGCAGCCAAATTCCCGAATAGCAAGAAATTGGAGTATCAAAGAAAGGACCAGATCTCTAAATTTGGAGGCAG
ACCAACTTGAGCTGCAGTCTAGGACAACATCCGGGCATGAGGGTGTACAGTGGTGCAC
TCGAGTATTACGAGACATGCAGATCGGTCGGTAGGGAAGCGAGCTGTTGAAATCTAACCAGACGTCAACAATT
CCTGGCCGGATGAGAAATTTATTAATGAAATGTAGAGTTTAATCGATGTATTCTAGTTTTAAGGGTGGATGTA
ACCAAATGAAATGCTGAACATATGGGGACCCAACACCTGTGTCTCGAGACGACGGGAG
CGGAATAGTGCAAGTCATAAAGTTACTCTGTAATATGAAGTTACTCTGTAACTAAGTCACCTTGTTACATGAA
GTCACTCTGTAAAATGAAGTTACCCTGTAACGTANAGTTACACTGTAACACGAAGTCACCTTGTTACTGGATA
TTAATCAGTAACATAAATCACTCTGTAACATGATGTTACCCTGTAACATGCACTTACC
CTGTAACATTATGATGGCACTGTCTGCTACGCTCGTCGACTCATACTTAAGCGTATTATCTCTTCATATACT
>1002049765_1 Heli.1-ES585888.1.5

GCACGAGGGTCGCCCCATGCAACGCGCTCCTCGCGCTGTCCGCCGCGCTGCTCGCCGCCGCCGCCGCCGCCGC
CCCCTCCGCCGCGCACCACCTGCGCCCGCGCCGCCAGCTCGCCGACGACAACACTCTACAGCCGCTCGACGAC
GCGTCGAGCGACGACCAGGCCCGGGAGAAGCGAAAGCTCGAGGGCGTCACGCAGGCTA
AGTTTGGTATCAAGAACGCCGTTCTGGGCTTCGTATTCGGGAAAATCAATTCGTTGATCGACTCAAAGACGAG
GCTCGTGGAGTCGCTGGATCGCCAAAACATCGAGTTGAATAAACTTTATGGCATCGAAGCGCCGAAAGGAGGA
GGCCTGAGCTCCCTGAGCGGCATCGTGGGGCAGGTGATTGGGCCCAAGCTGCAGTTCC
TGGGCCCCAAGATCCAAGCGGTGACAGGGCTGCTGGGCGGCGCGTCCGGCGGCGGTGGTTCAGGTGGGGGCTC
CGGTCTGGGCTCCATCTTGTCGCTCGTAACATCCTTGTCGGGATCCTCGTCAGGGGGAGCCGCGGCGGGCGCT
GCCGTCGAGACCGTTGACTCCTCCGAGGAGGACGACTCTTAAACGCTTTCAATTCCCT
TCTTTCTCCCGCTCCGACTTTTTGCGTCACGTTGATTCCTAGTTCTTGCCGATATCTCGTGAATCACAAAACG
AGGCCGTCGTCGTTTCGGTAACTTCGTAAGTAGTAAGTAGCGACCGCCAGTGACTCCTTGCCAGTGCCGCAAA
TCGCCGAAGCGGGCGCACAGTGTACAGTCCCAGTGGCGTTCCTCTCTTAACGCTTCTG
ACCCTCCACGCGCTCTCAGCGCGTTGTATTTATATGTAAATAGATTTAGGTGAGTGTTGGTGATATATATAAT
AATTAATAATATATGTTTTTTTT
>1002049766_1 Heli.1-EL597438.1.5
GTCACATCTGTACAGTGCTCCCGCGAATTTTCAAACAAGTGAACTATTGTGTGTCAAAATGCCGAAGAATATT
TTAGCCGAGATGGGCTCGCAAGTGGGACTGACCCCCGCCCGACAAAATAAAAAGGAAGCGGAAGAATGGGCCC
GCAGACAGAGCGGCCCTATGGTCAATCCTTCCTTCGAGCCCGACGATTTTCTGCCACA
GAGTTTATCACCCGTCAAAGATGAAAAGAAAAGGCCGGAAGACAAAGGCATCTTCCTCGTTAACATGAAAGAA
AAGAACGTGCAAGAAGTAGAAGAATACGAGCCTTACGACAACAGAGTTGTGGAACATCCTACCACAAACACAG
AAACCCTCTTGCATTTGTTAAAAGGCAGTTTAGGTACGGGTATTTTAGCAATGCCTCA
CGCCTTCTCTAAATCGGGGTACGTAGTGGGTACTATCGGAACCATTGTCATCGGGGTGCTGTGCACATATTGC
ATACATGTGCTCTTGGACTCTTGTTACGTGTTATGCAAGCGGCGGAAAGTGCCCTCCCTCACGTACACTGCTG
CGGCCGAGGCAGCGCTGTCTGAAGGCCCTGACTGGTGTAAAGCCTGTGCACCTTACGC
CGCGCACGTGGTGAACGCTTTCCTCCTTATTTATCAGATCGGCACCTGCTGCGTGTACGTAGTTTTTGTGTCC
GAAAATATACACTACGTTCTGATAGAACACTTCAAGCTGTCTGTAACCGTCACAGAAGTGATGTTATACATCC
TTTTGCCTCTGGTTCTTATCAACTGGGTTAAGGATCTCAAGTACCTGGCGCCGTTCTC
GGCTATAGCTAA
>1002049767_1 Heli.1-EL597851.1.5
GCACGAGGCAACACTCTACATCGTGGCTGCCATGGAGTTTGCCCGTAGCCGTCGGGCTGAGTGTGACGGGTGA
CGTGGTCCTAGTATCGCGCCACCTTACCGTCCAATCCACGGGTCGCTTGCCGCATTGCCTTCCACTTCACTTT
GAGCTCTAAACGCTACCCAAACCAATTGCATATATCGATACCGCGTTGAAACACGTCT
CGCAGTGCTGTCTCTTATACTATAAAACACGTGAATAATTTTATATTTTTAATTCTTGTCTCAAACGTGACAG
GCCGCCGGCGGATTATCTAAATAAACCTTGCATATGATAAGTGTGTGAATATTGTCCTATTGTTGTTTCGTTT
TTGAAATTAATCAATAGACAGTGCTGTAGCCTTAATATATTATTTGTAAATTATTGAA
TAAAAAAAATATAATAAAATGGCACCTAATTCCGGAGATGCAAATGGAGTTCTATTCGAATCAGATGCCGCAA
CACCTGATATGGCTTTACCTATAACTCCTATACAGCAAGCTGATCAATATCCACGAAAATTAGTTTGGAGGAA
TATAATTGCTTTTGCATATTTACACTTAGCTGCTTTATATGGTGGATTTTTGTTTCTG
TTTTCAGCTAAATGGCAAACAGATATTTTTGCATACATCTTGTATGTGATGTCTGGACTTGGAATTACAGCAG
GTGCCCATCGTTTATGGGCTCACAAATCTTATAAAGCAAAATGGCCACTTCGAGTAATCCTTATTATGTTCAA
CACCTTGGCATTCCAGGATTCCGCAATTGACTGGGCCCGCGATCATCGCATGCATCAC
AAGTACTCGGAAACTGATGCCGACCCACACAATGCTACTAGAGGCTTCTTCTTCTCTCAT
>1002049768_1 Heli.1-ES588224.1.5
CTAAAGTAAAGTGCCTCCGACATGAAGGTGTTTGTGTTATTTATGTGTGCGGTTGCCTCGGCTGCGGCTGGTG
AGAGTGTCGCATGGCCGGGTGCGATACCAATCACAGCATCACAAGTTAAAACCATAACACCAGGACAAATCAA
AGGATTTGCCTATTCTACGAGTCAATACATAAATGCGATACCGTCTGAGCCGTTTGGA
TATCAGCCGCAATTCGGATACCCAATATCAATTCCAAGTTATCGTCCAGTTCAGTCAGTAGTAGGCGCTTCAT
ATTTCCCTCTACCGATTGCTCCATTGGTCCCTGCTTCCCCAATTCTTCCTGCTGTTCCAATTGCACCTATTCA
ACCACCACTTCAAAACGTACCATCTCAACCGGCAGAACAACCAGCGGGAGACGAAGAC
TCTGCAGTAATTGAATCTGCAGATTCTTTTAATCAACAAAGCTCCCAATCGCCTCAACCTTCTTTAGATACAA
AGAATATGCCTGGTTATCCTCAAGTTCCCCAAGGATCTCCGCAAATCCCGCTCTTTCCTCAAAGGCCTGAGGG
TCCACATATACCATTGTTCCCTCCATTCTCTCAGGTACCTCAACAAAACCCATCAGCT

CCTCAAATTCCTCAAGGGCCTCAGCAAAATCCATTAGTTCCTCAATTTCCCCAGGGATCTCAGCAAATTCCAT
CAATTCCTCAATTTCCACAGGCATCTCAAGTAATCCCATTGTTTCCTCAAAATTCTCAGAGACCATCGTTCCC
ACAGTTTCCCCTCAGTCCCTCACAAATCTCAGCG
>1002049770_1 Heli.1-EL601087.1.5
GCGAGTGATAGTGAGGTGCACGTTATCGATATAGACACAGATACAGAAACACCGGTGATATTTAAACTAGTGG
ACATCACTATGAACGGAAACGGAACATTATCAAACTCCAAACCAAAGCCAGTTAACATACCGGAGGTTGTACA
AAAAGCCCATGACACATACAACAGCGGTTTAACGAGGCCAACAGAATGGAGGAAGAAA
CAACTAAAGAACCTTATGAGGATGTATGAAGAGAACAGGGGGGCTATGATCGACGCCCTAGTAAAGGATCTAC
GGAGAAGTAAAACTGAGGCCATATTACTTGAAGTGGACTACTTGGTTAATGATCTTAAGAATGTATTAAGTAA
ATTCGACGAATGGGTCCAGCCTGAACGGCCACCAAAAGGATTAGTGAACATTCTAGAC
GACGTGGTCATATTCAACGATCCCTATGGAGTCGTTCTCATCATTGGAGCTTGGAACTACCCATTACAACTTC
TACTACTACCAATGGCTGCAGCCCTCGCCGCTGGTAACGTGGTCATACTGAAGCCAAGTGAATTGGCAGTGGC
CTCCGCGCAGTTCATAGCTGAAACGTTGCCTAAATATTTGGATAATGATGCCTTAGTA
ATAGTGGAAGGTGGTCCGGAAGAAACAACAGAGCTGTTGAAACAAAAGTTCGACTATATCTTCTATACTGGTG
GCACAAATGTAGGCAGGATCGTGTATGAAGCGGCG
>1002049771_1 Heli.1-EL603536.1.5
GCACGAGGGTGCGTGCGAGGCGACGGGCGCGTAAATTGCGGCGTATAAGCGGCTCGACAAGAGTCCCCACGTT
GCAAGTAGCTATAGGTACTTCTTATCATTAGTTAAGAGGTCACGAATAGTTCGTAACTTCATTACCAACTACA
TTTAGGAACGACGGACCGGAGATCAATTTAGTCCGGTGCTGATGAAATCAACTATTAT
TCTCGAAGAAGTAGTGAATAAATTAGACCATCTCGCTTCACCGTTACCTGCCAGGATGCCTAACATTAAAGTT
TTCAGTGGTAGCTCCCATCCCGATCTGGCACAAAAGATCGTGGACCGACTCGGAATTGATCTCGGCAAAGTGG
TAACCAAGAAATTTAGCAACATGGAAACGTGCGTTGAAATCGGAGAATCCGTCCGCGG
CGAAGATGTTTACATCGTTCAAAGTGGTAGTGGTGAAATTAATGACAACCTAATGGAATTACTTATTATGATC
AACGCTTGTAAAATAGCCTCAGCGTCTCGTGTTACCGCTGTCATCCCGTGTTTCCCGTATGCAAGACAGGACA
AAAAAGATAAAAGCAGAGCGCCCATCACTGCTAAACTTGTTGCTAATATTTTATCTGT
TTCTGGAGCTGATCATATAATAACCATGGACCTTCACGCGTCGCAAATCCAGGGTTTCTTTGACATCCCCGTG
GATAATCTGTTTGCGGAACCAGC
>1002049772_1 Heli.1-DT665376.3.5
AGTATATACCGACTTCGACTTCGAGCTTTGTCCATCATATTTAGAATGAAGTGTTATCTGTTTCCCCTTATAA
CGTATTATTAAGCATACATAGTGTCAGTGATAGTGTGCAAGTACACTAGCCAGTGTGTTTTTGTCGTTGAACC
GTCGACGTTAACGGTTAAGTTGTGTCAATATCCTTAATTTAACTAAGTTCGTGACAGT
GTTTTTATCAGTGGGATGTGTTCGAGTATTTAGAATCTTTTGAAAAATACAGGTACAAGTCATGTAATTTCTT
CTGTTTTTAATTGAAGCGGCTTGGCGCGGCGTAGCGGGCGGGCCGCGCCATGGGTAACAAGTGCTGCAGCCGC
CGTCACGACCCCGACAGACCCTTGGTATATCCAGCGTACAAGAAGAACGAGGCGGGCT
TCACAACATGTCCCTCGCTGGAGACGCGGTACACCGGCGAGCCGAACAACCGCGCCGTGTCGCGCCGTCCCGC
GGACATCGTGCGCACACGCAACCCTGGGGCGTGTATATCAAACGGCGGGCGGCGTGTGGTAAAAGCACTATGC
GCGTACACAGCCCGCGCGGAATCCGATATCTCCTTCCGCAAAGGCGACCGCATGGAGG
TCCTCAGCGATGCGGAGACGGACTGGTGGCGCGTCCTCCACCTCACCACGCGGCGAGAGGGTCTGGTGCCAGC
CAACTTTGTGGCCGAGGAGAGTTCCGTTGAATGTGAAGATTGGTTCTTCCCGCACGTATCACGCAAAGAAGCC
GACAAATTACTTTTAGCAGAAATCAATCCCCGAGGAACTTTCCTAGTTAGACCTGCCG
AGCATAACCCGCACGGGTTCAGCCTCAGTGTTAAAGATTGGGAAGAAGGAAGGGGTTACCATGTTAAACATTA
TAAGATAAAGCCACTGGACAACGGCGGTTTTTATATCGCCACGAATCAAACATTTCCTAGTCTACCTGCGTTG
GTCATGTCGTATACAAAGAACGCGTTAGGCCTCTGCCACGTGTTAGCCAGGCCTTGTC
CAAAACCTGAGCCGCAAATGTGGGACCTGGGGCCAGAACTGCGGGACAAATGGGAGATACCCAGGAGTCAGAT
ACAGTTGATCAAGAAACTTGGACAGGGGAACTTTGGCGAAGTGTATTATGGCAAATGGTGTAATAATATTGAG
GTCGCCGTGAAAACTTTAAGAGAAGGCACGATGTCCAAACAAGCCTTCCTTCAAGAGG
CGGCCATTATGAAGAAATTCCGTCACAAACGCTTAGTCGCCCTCTACGCGGTCTGTTCCCAACAAGAGCCCGT
GTACATCGTACAAGAGTACATGTGCAAGGGATCTTTGTTAGAATTTCTAAGGAACGGCGAGGGGAAGTCCCTG
CATTTCGAAGATTTAGTATACGTTGCTGCGCAAGTAGCTTCAGGCATGGAGTATTTGG
AATCAAAACTTCTCATCCACAGAGATTTGGCGGCCCGTAACGTCTTGATAGGTGAGAACAATGTCGCCAAAAT
ATGCGATTTCGGTCTAGCAAGAGTGATAGAAGACAATGAATACTGTCCAAAGCAAGGCTCCAGATTCCCAGTG
AAATGGACAGCGCCGGAAGCGATCATTTACGGACGGTTCTCCATAAAGAGTGACGTGT
GGTCCTACGGGATATTACTCATGGAGTTGTTCACATATGGTCAAATACCTTATCCAGGATTACATGGAAAGGA
AGTGATAGAACAAGTGGAGAGAGGGTACAGAATGCCAAAACCGGTGGGACATTACCTACCGGACGACATAT

>1002049773_1 Heli.1-ES588238.1.5
TGCAGTACGGTCCGGAATCCGGGTCGACCACGCGTCCGCGACAACTTGACATATTGAGTGTTGGATAGGAGAA
TTAGACCTTTGAAGTAGTTCCAAGGGTCACGAGATGGCGTTCAGGTCTATAGGAGTGGTGTTCTTGGCGTGCC
TTGTAGTATTAGCATCGTCAGCGGTGCCTAAGGGTGGTAAAAGGAAACCAGCCAACCC
ACCAGCCCGCGTCGCTGCTGATGCAGAACGAGATGCAAAAATCACAAACTCCTGTCCAGATGATGGCTTCTTT
GCGGATGCTGAACAGTGCGACAAATATTACGAATGCAGAGCGGGTGAAATAATTGAAAAACTGTGCCCAGATG
GTATGGTGTTTAACGACTACAGTCCTCAAGAAGAAAAATGTGACTTGCCATTCAATAT
CGACTGCTCCCAAAGGCCCAAACTACAGACACCTATCCCTGCCCAGCACTGTCCACGTCAAAACGGTTATTTC
TCCCACGAAGATGAGAAGGAGTGCGGCAAGTTCTACTACTGTGTAGATGGTAAATTCAACATGATCACCTGCC
CTGATGGCCTCGTGTACAACGACAAGAGCGGTATCTGTACTTGGCCTGATGAAGCTAA
GAAGAAGGGATGCGGTGCTGCTGA
>1002049774_1 Heli.1-EL596824.1.5
TACAGGTCATCCAAAACCCCGTGTAATGTGGCTTATTAATGGAAATACAATTGTTAACGGCTCTAGATATAAG
CTAACATATGATGGAATGTATCATCTTGATATACCAAAAACTAGACAATATGATACTGGTAAAATTGAAGTAA
TTGCCCGAAGCTCAGTAGGAGAGGCATTAGCAACGACCGAACTTAAAGTTATCCCGAG
ACACGACGATTATAGAGGTGTTCTGAAAAATGCACCAAGACCTTGGTATGATGAAACAACTCAATATCAACGC
AATGAATTTTAATTAGAGAAAACATTTGAAGAAAGACAAACTATGCAACGGCAAGGAGTCATTGATCATAGAC
CTGAATTAAAATCAAAGGTAATTAAAGATTCTGAAACTGAATGGCAACAGACAGTAAA
GAAGAAGAAGAGTGAAGAATACTATAATAAGCTACAGGAGCTAGAGAATGAACAAATTGTTAAAGAAAGTAGA
TTAAGAGAATCATCACATCAATATGCCATCCCTGGAGAGAAAGTAACTAGCTCTTCTGTGGCCAGAAGTATGG
CGCAGAAATATGAAGATAATTTGGAGACAGAAGAAGTCGTTGAAAAGCAAATCCAAAG
TAAAGTACAAAAGCCTAGAATTCCTGGACCTTCAGAATCAACTGTTCATGGCAAAGAAGTGCATGTTGCAAAA
CAGAAACAAGTTCAAAAAGAAGTAGTCGGGGACACTGAAATAACACGCAAAATAACATCTACAGAAACTACTG
AAGTAGAACATAAGGCACAAACTCAAGAAAGGGTGGTCGAAGGTCCAGTTAAGCCTAG
TACACCACCTGTGTTTACCAAAAAAATGCAACCCTGTCGCGCTTTTGAACATGAACAAGCAAGATTTGAAGTA
GAATTTGACGGAGATCCATTACCAACAATAAAATGGTATCGTGAGAATTTCCCAATTAAAAACTCACCTGATT
TCCAAATTCACACGTTCAGTACCAAGTCTATATTAATTATTAGACAAGTCTTCATTGA
AGATTCTGCTGTCTTTGCTGCTGTAGCTGAAAACAGAGGAGGAACTGCTAAATGCAGCGCTAATTTAGTCGTA
GAAGAACGAAGACGACAGGGTAGAGGTGGCGTTATTCCACCTAGCTTTACGTTAACAGCTCAAAATGTTAACG
TTACTGCAGGACAACTGGTAAGATTTGACACCAAAGTTACTGGAACTAAGGCTATTGA
TGTCTATTGGCTAAAGAATGGAAAGAAAGTTCAACCAGATATTCGAAACAAGATTCTGGAAGAAGATGGTACA
TACACTCTCCTCATTTTAGAAGCCTTTACTCAAGATTCTGGTAAATACGAATGCGTTGCAATCAATAGTGCTG
GTGAAGCGCGTTGCGATGCTGAATGTGTCGTGAATGCTCCTGCTACTAAAGATAAACC
GAAACCTCAAACTAAGGCTGCTAACTCTCCGCCAGAAATTATTGAACCTTTGAAGGACAAGGTAGTAACTGAG
GGCCAGGCTATTGAATTCAGTTG
>1002049775_1 Heli.1-EL603552.1.5
GCACGAGGCCACGTCTCTCTAGCCCTGGAACTTGCTCGGTCGTCAGTGAATTTTATGTTCTTATTCATTTACC
ACGTTGTCTGAATAATTTGTATCAAAGATGAGTGCTCCAGCACCAACGCCTTCCTATAAAATTGGTATCGTGA
AGCAGGTACTATCAGGAGACACGGTGATCATTAGAAAACAGCCTCAAGGTGGACCACC
ACCAGAGAAAGTGATTGCTCTATCTGGCATCACCGCGCCGAAACTAGCTCGTCAAAGAACTGCCAACAATGAC
ACTGAGACAAAAGATGAACCTTTTGCCTGGGAGGCTCGAGAGTTTCTTAGAAAGAAGTTAATTGGTAAAGAAG
TTATATTCACTGCAGAGAAACCACCTAACTCTGCAACCAGAGAATATGGGTCTGTTTG
GGCTGGAAAAGATCCCTTAAAGGATGAAAATATGACAGAAGCCCTCTTGTCTGAAGGGCTTGTTAAAGTGAGG
GAGGGAGGTCGTAATATTCCTCAATTGAAACGTCTTGTTGAAATTGAAGAAGTCGCTCGTTCTCAGGGAAAGG
GAATCTGGGGATCTGAATTGCAGAGCCATGTGAGAGATATCAAGTGGACAGTGGAGAA
TACAAAACAATATGTGAACAAGTTCCATGGACAACCAATAAAAGCAGTTATTGAGTATGTCAGAGATGGATCT
ACAGTCAGACTGTGCTTGTTGCCTGATTACACACCTATAACAATGATGCTGTCTGGAATTCGATGCCCTGCAG
TAAAGCAAGATGGTGATTCAGAACCTTATGCGGAAGAAGCCCGTTTCTTTTTGGAATC
GAAATTGCTGCAAAGAGATGTGGAAGTGATCTTAGAATCTGTAAACAATAACAATTTTGTTGGTACAATTTTA
CACCCACAAGGCAATATTGCAGAAGCTTTGCTTAAACAAGGTTTTGCAAGATGTGTTGATTGGTCACTAGCTG
TAATGAAATCAGGTGCTTCTTCACTAAGGGCGGCAGAACGTGCAGCAAAAGATGCTAA
ACTACGAATCTGGACTAACTATGTCAGCAATGCCCCTGTTATTGCTGCTAAGGACAAAGAGTTCTCGGCAATT
GTAATGGAAGTTGTAAACGGAGATGCACTTGTTGTTAAACAAATTCCCACAAATATCCAGAAAAAAGTCTTTT
TGGCTAGTATTAGACCACCACGTGAAAAGAACAGTCCAGATGAAGAAGGCAAACAGTC

ACCTAGGCCTAAAGGCTTCAAACCCCTGTATGATGTTCCCTGGATGTATGAAGCAAGAGAATTTTTGAGGAAA
AAACTGATTGGAAAGAAAGTAAATGTAACCGTTGATTACATTCAACCAGCTAAAGACAACTTCCCTGAAAAGA
CCTGTTGCACTGTTGTCACTGGTGGAACTAATATTGCTGAAGCATTAGTAAATCTGGG
ATATGCCACTGTGGTCAGATATAGAAATGACAATGACCAGAGAAGTTCCCACTATGACAAATTGCTTGAAGCT
GAACTCAAGGCCCAAAAGTCTGCTATTGGTGTCCATGCTAAAAAAGATATTCCAACTCATCGCATCCAAGATA
CTAGTGGAGATTCAGCTAAGGCAAAGAAATTTTTCCCATTCTTGAAGAGAGCTCAAAA
GACAGAAGCTGTTGTAGAATTCGTCGCTAGTGGATCTCGTATGAGAATCTACATCCCCAAGGAATCAGTTCTC
GTCACGTTCTTGCTTGCGGGTGTCAACTGTCCGCGGGGCGCGCGCCC
>1002049776_1 Heli.1-ES584766.1.5
AATAAAAAAAAAAAAAAAAAAAAACGCAAACAAACAACTATCAAAATGAAAGCCTTCGCTTGCATCGTCCTCT
TAGTAGCAGCAGTGTCAGCCGCACCAGAAGGCAAGCGCGACAAACGTGGCTTCCTCCACGGTGGCTTCAGCAG
CGGCTATGAACTGGGCCACAGCTCCTACGGCCACGGCTCCCTGGACCTCGGCCTGAGC
CATGGCCCCATTGTAGAGCATGTGTACTCCGCCCCAGCCAAGATCGTGTCTGTTAACAAAGTTGTTGAAGTAC
CTAGAGTTGTCGAAGTCAAGAAAATCGTCTCAGTACCCAAAGTAGTATCCGTGCCACAAATAGTGAAGGTATC
TAAGATCGTGGAAGAACCCCATTACGGACACGTTTCCTCTGGTTGGTGGTGAAGAGTC
AATCAGCCTTAGCATAATGTCGGCATTCCACCGCCCCGTGTTAGCTATTTCTCATAATATATTAAAAACAACC
TCCAGAACCAGAGCAGAAAATATCTCATCAAAATCTCTCTCGTCAAAAAGAATATAATAGACCAAAATCATTA
ACTGATGAAACAAGTTTAAGCTAACAAACAAACATGCCGACTTACGAGGCTTTCTACA
ACCAAAATTAGTTTTAATAAAATCTATTTTTTTTACTAAAAAAAAAAA
>1002049777_1 Heli.1-DT666598.3.5
AACTCAACGACAAGAACGAGCAATTGGCCAAATTCATAATGACTTACGTGTGAAAACAAATCTTATTATTGAA
AATTTAGACATGGTAGAGAAGCGTTTAAAAAAACAAGAAGATGATGTGGCAACTCTTGCACAGCGACCAGTTC
CAGCTGAACTGCTTTTGGATCCCACAATAGACAGACTTGTGGAATATGCACCCAACAG
ATACAGAGTGGATGAGCCTTCAACGGACCCTATTACCACAGTTGCCTACACTACTCCTGCGACTTCAACTGCT
AATAACAGCCCGAGCATCCCGAGCGTCCCGAGCAATTCGAGTACAAGTCCAACGAGTGCTACCGTGACAGTGA
CGCCAACAAGTACGGGCAGCAGTGCGGGTCCTACCAGCCCGCGCCCCACGAGCCGCAA
GGGCGGCATCATCTTCCCCAGTGTCAAGAACAAACCCATAATCGGCAACAATACCTTTGCATCGGAGATTGTC
GCCAACTATAAAGATGTTAAAGGTTACTCTTGTGTTGATCTGTCGAACGCCGGCATGCGAGAATCTGGTGTGT
ACTATTTGCAGATACGAGGCACCACTTACTGGTTCCTGAAAGTGTACTGCGAACAAAA
CGTCGCCGACGGTGGATGGACGGTAATTCATCGTCGTGATGACTATGGGATACCAGCGGAAAATTTCAATAGA
GACTGGAGTGACTACAAAAACGGATTTGGTGACCCCAGCAAAGAATTCTGGTTGGGCAATGAGAACATTTATA
TGTTGACTAATAATGATGACTATATGTTAAGAGTTGAGCTGGAAGACTTTGATGGTAA
TAAAAGGTATGCACAGTATTCGCACTTCAAAATATATTCAGAAGCGGAATATTACAAATTGGAAATAGATGGA
TACGAAGGCAACGCAGGCGACTCTTTGAATGATCCTTGGTACGGGTCTAACAATAGTCCTTTTTCGACTTATA
ACAGAGACAACGACAGATCTTCGTTGAATTGCGCGTCTATGTTAAAAGGCGGTTGGTG
GTGGAAATCATGTGGACGCGGTTTAAACGGTCTATATCTGCACGACCCTCAAGATCTTACAGCACGACAAGGT
ATAGTGTGGTTCCGCTGGCGTGGCTGGGACTATACACTAAAAAGAGCTTCTATGATGATCAAACCTAAGGGAC
TGCAGCCAAACACATGAAAATAAAGAAACAACTTCTACAATTTAATCTTATTTGGCCT
CTGTTCAATTGTTACGTGGATTTTGGACATAAAAAACTTATGAATAATATAACAGTATTCACTTTTTTTATCA
GCAAAAAGATTTAAATAATAAGCACTGAATATTTCACAAAATAACATTCGCAGATATTTTAACAATTGGTACA
ATCGTATGCATATTACAATAAATAAAGAAAAAATAATGAAGATTCAAATCCGTTCTAC
ATAACCATTAGATCTTAACGTTTAAAATAACTTAACTTTTACACTTTAATAATTATTTACCAACGAGTTATAG
CTGTCTTAGCTTCTTGTGCTATATATTTAAAACTATAAGAAATTTGGTACTGAATCCACGTAATGACCTAG
>1002049778_1 Heli.1-DT665487.3.5
GCACGAGGATTTTACTTTATTAAATATTAGACAGTATAATCTTATGTAGACGTTTCTTACAAAGATCTATCGT
TAAAGTAATTCCAATACTATGCCTCTTTTTGGGAAATCTCAGAAGAGCCCCGCGGAGTTAGTAAGGTCTTTGA
AAGATGCTGTCACAGCGTTAGAAAGAGGTGATAAGAAAGCTGAAAAGGCCCAAGAAGA
TGTTAGCAAAAATTTGGTCTTAATCAAGAACATGCTCTATGGTACCTCTGATGCAGAACCACAAACTGATATC
ATTGTTGCCCAACTTGCACAAGAGCTTTATAATACAAATCTGCTGCTGCTATTAATCCAAAATTTAAATCGTA
TTGATTTTGAAGGCAAGAAAGATGTAGCCCAAGTTTTCAACAATGTTCTTAGACGTCA
AATTGGCACTCGCTCACCTACAGTTGAATATATTTGTACTAAACCAGAAATATTGTTCACTTTGATGTCTGGA
TATGAACACCAGGAAATTGCTTCCAATTGTGGTACTATGTTACGAGAGTGTGCTAGATATGAAGCCCTAGCCA
AAATAATGTTGTATTCAGATGACTTTTACAATTTTTTCCGTTATGTAGAAGTATCCAC

TTTTGATATTGCCTCTGATGCCTTTTCTACTTTTAAGGAATTATTAACTCGACATAAAATATTATGTGCCGAG
TTTTTAGAAGCAAATTATGATAAAGTGTTTAGTCACTACCAGCGTCTATTAAATTCCGAAAATTATGTCACTC
GACGGCAGAGTTTAAAACTACTTGGGGAACTACT
>1002049779_1 Heli.1-DT667252.3.5
GTCGGCATATTGTTTCAGATAGGGGGCGCTGGCTGGTTCACGGGGAGGTATAGCTTTCAGTGCCAGCCGGTTG
ATCACTCGAAAAGTCCACAAACAATGAGGATGGTTCATGTTTGTTGGTGGTACTACTTCTCAAAATTCACAGA
ATTCTTTGATACGATCTTCTTTATCCTAAGAAAGAAATTCGATCACGTATCAACTTTG
CACGTAATTCACCACGGAGTGATGCCGATGTCTGTTTGGTTCGGTGTTAAGTTCACTCCTGGCGGCCATTCCA
CATTTTTCGGTCTTTTGAACACTTTTGTACACATCATTATGTACTCGTACTATATGTTGGCGGCGATGGGACC
GCAAGTTAGGAGGTATCTGTGGTGGAAGAAATACCTCACTGCCCTACAAATGGCCCAA
TTCGTCGGTATAATGGTGCACGCCTTCCAGCTGTTGTTCATCGAGTGCGACTACCCGCGCGCCTTCGTGTGGT
GGATCGGAATGCACGCCGTCATGTTCTTCTTCCTATTCAAAGACTTCTACAATCAATCATACTCCAAACCCAA
GGTCCGCGCGAAATCTCCACAACCTGTAACAACGGAGATCGAAGACGACGTCAAGCTC
TACAAAAACGGCTCCGTCAAGAACGGCTACACGAACGGGCACGCGAACGGGCTGGTGCGTGCGCGCACCGTGC
TGCCCGCCGGCAACTGACCGCGGACGCGCGCTCGCCGCCCGTCGCCACACTTCGTTACCTAATGGGCACAAGT
CAAAAATTATACAGCCTGCGCACAATGTTGTGTTTCATGAAAAAAGTACTGGCCATAA
GTAAAACCTTTTATGACGAATTTATATACTCGCGCGCTCGAACTTGCAATGCAAACCTCAATATGTTGTGAGT
TAATTTCGTTTAGAAAAGTTTTGTATAAAATGAGAAATAATGTTAGACGAATGACACGTTAAAATATGAAATA
ATAAATTTTTGACTTGTTGTTT
>1002049780_1 Heli.1-DT663779.3.5
CTCCTATAAGCCCGAGGGTAAAACTCACGCAAACACAAAACATGTTTTCGTCGCGACTGTTGATTATTTTCGC
ATTTGCAGCACTAACGGCTGCCTATCCCGCCGATAACGGGCCGTTCCCGCGGCCTATTACAACTATCACAGAA
ACTGAATTCGATGATCCGTCACCATGGTTCAGATTCCCGGCGTTTGGTAATATTTTTG
CACCGCTAACTAGATTGTTCTCTAGTTTCGCTGATATTGGTCCCAGGATCGAATCTGATGACGAGAAATTCAG
AGTTATAGTCAACGTTAAAGATTATAGCTTGAGTGATTTGAAAGTTAAGGTGAAAGGCGACTTTATCCTCGTG
CAAGGATCCCACGAAGCCAAACAAGACGATCACGATCTGTTTGCCAGCCAATTTGTAC
ATACATATTCTTTGCCGATCAACGCTAGCGCGTCGGATGTAACAGCGAAACTAACATCTGATAAATATTTAAT
CATCACAGCTCCACTTAACGGTGCGGGTGCTGATGACGGAAAAGCAGTAGACAGAGAAGTACCTATTGTAGAA
AGTAAAGAAGCATGGAAGAGTGAAGACAAAGAAGATAAAGACAAAAAGCCAGAAGATG
TGCCAGTTCAGGCCAGCGGCGTATTGCCAGTGGAAATTGATGAAGCCAAGGGCACTACTGATCTTCCAGCGAC
GGCAGCTGAGGAACTTACTACCCCGTCCAACCGTGAAGAAGTAACGTCCGTGATGTCCTAACGAAGTGACC
>1002049781_1 Heli.1-CX700533.1.5
CAGTACGGTCCGGAATTCCGGGTCGACCTAGCGTCCGCACGAAACAACAATCAACACAATGTTCTCTAAAATT
GTATCTCTTTGCGCTGTAGTGGCAGTGGCTTCAGCTGGTCTTGTGCCCGCTGCTGTTCACTACTCCCCGGCAG
CTGCCGTATCTTCCCAAAGCATTGTGCGCCATGACGAGCCCCAAGCTATTGGTGCTAA
GATCCTCGCCCCTGTCGCCAAACTGGCCGTCGCTGCCCCTGTAGCCTACCACGCACCTGCCCCCGTCGTGTAC
CACGCCGCCCCTGCCCCCATCGCCTACCACGCTGCCCCCGCTTCCATCGCCTACCACGCTGCCCCCGTCGCTA
AGGTCATCGCTCAACCCGAGGAAATTGCCTACCCCAAATACGAGTTCAACTACTCCGT
AGCTGACGGACACTCTGGAGACAACAAGCAACAACAAGAATCCCGCGACGGTGATGTCGTAGAGGGCTCCTAC
TCCTTCGTTGAGGCTGACGGCTCCGTCAGGTCCGTGGAGTACTCCGCTGACGACCACAACGGTTTCAACGCCG
TCGTACACAAGAGCGCCCCCGCCCACGCCCCATTTGTCATCAAGGC
>1002049782_1 Heli.1-DT667365.3.5
TTCGGCACGAGGCCAACCCCGGTCATAGCGTCCTAAACTCATAAAAAGGACGCGGGCGGAACCTCTTCCGCTG
GAGCCTGAAGGACTCGTCAGTGAAAAGTCAAAGATACGAGATGAGGTCGTTGTTTTTATTAGCGGCGTTGTGC
TGCTTAGTCTTGTCGGCTTATGCTGGCCCGGTTTTCCGATCTGATGAAAGCCAGAAGG
AAGTAGGCAGGCAATCTGCTGCACCGGCTGCGCCCGCGCCGGCTGCTTCTGATGACGACGACGACGACGATGA
TGATGATGATGATGACGATGACGTCCTCGATGACCCATTATCAGATGATGACGACGACGACGATGATGATGAT
GATGAAGCAGATGAGGAATCCGATGATGATGATGAAGATTATTTAGAAAGATTCTTTG
ATGACATCTTAGGAGGTGATGACGATGATGACGACGACGACGAATCTACAGGTGTTCAATCCGTAGTGGCTGC
TGCCGACACCGTCGCTGCCGCACCAGCACCTGTAGCTCCGGCTGAAGAAGCTGCAGCATCTTTGGAAGAAGGA
AATGCAGCTGACGCTGCTGAAGAAGTTGCTGAGGGCGATGAGGCTGAAGCTGGACAAA
AACAACCCGCTAGCGCCGAAGTAGAATCACCTGTTGCCACGAGTTCAGAAGTAGCTGCTGCTTCTGACTCAGA
TGATGAAGAAGAAGATGATGATATTGCTGATGCTCTCGACCCCGAAGACGATGATGATGAAGAGGACGATGAC
AAGGAGGATGATGACGACGATGATGATGACCTAA

>1002049783_1 Heli.1-DT663097.3.5
TCTTGCCAGAAACTTACATACTAGTTTAACCATGAATGACAAACTTTTAAATGGAAATTTATGGGACACAGAT
CCTGAATTGTTTGATATTATTAAAAAAGAAAAGCAAAGACAAGCAAGTGGATTAGAATTAATTGCTTCCGAAA
ATTTTACTTCAATACCAGTCCTACAATGCCTTAGCTCATGTCTTCATAACAAATACTC
AGAGGGTATGCCACATCAAAGGTATTACGGTGGTAATGAATTTATTGATGAAATAGAAATTTTGACTCAGCAA
AGATCCCTGGAAGCATATAGACTGAAACCTGAAGAATGGGGAGTAAATGTTCAGCCATATTCAGGATCACCAG
CAAATTTTGCAGTATATACAGGAGTTGTAGAACCACATGGTAGGATTATGGGATTAGA
TTTACCAGATGGCGGTCATCTTACTCATGGCTTCTTCACTCCCACTAAGAAAATATCAGCAACATCAATTTTC
TTTGAAAGCATGCCGTATAAGGTGGACCCCCAATCAGGTTTAATAGATTATGACAAATTAGCAGAAACAGCCA
AATTGTTTAAGCCAAGATTAATTATAGCTGGTGTTAGTTGCTATTCTCGTTGTCTCGA
CTATAAGCGCTTCAGACAGATCGCAGACGAAAACGGCGCCATTTTGATGGCGGATATGGCACATATATCTGGA
CTCGTTGCTGCAGGTGTTATTCCAAGTCCATTTGAATACTGTGACAT
>1002049784_1 Heli.1-DT664348.3.5
TTGAATTGACCTTATTTACTCAATCCGCTGTTCAAGTGAGGTCGATAACCTTTCGCTTATTAGTTACTTAGTT
TTTACATATCAGTTCGCACCGAGAGACGGCCCCGGCTCGTTTGTGTTTATGGCGATACACTGACTCCGACACC
CGTGACAATGGTCGAAGTGAAGATAACGGAAGACGTTAAGGTTGGCGGCAACAATCCC
TGCTTTATCATAGCTGAGGTTGGGCAAAATCATCAAGGTGACATTGGAATAGCAAAAAAGTTGATCCGGGCTG
CTAAGGAAGCCGGCGCGAGTTGTGTTAAATTTCAAAAGACATGCCTAAAAGAGAAATTCACAAAGAAATATTT
GGAACGGCCATATAACAATCCAAACTCTTGGGGTGAAACGTATGGCGATCACAAGAAG
CATTTAGAATTTAGTGAGAGCCAATATAGAGAACTTTTCAAATATGCACAAGAAGTTGGCATTCTTTTCACTG
CATCGGCCATGGATATGGTTTCTTTTGACTTCTTGGTGAACATAAAAGTGCCGTTTATTAAAATTGGTTCCGG
GGATTCAAACAATTTATTATTCTTGAAATATGCCGCGTCGAAGAACGTCCCTCTAATC
ATATCGACCGGGATGGTTGACAAGGAAGCAGTAAAAACTATTTATGACATAATATCGGCTCATCACAAACAAT
TTTGTTTATTGCACTGTATATCGGCGTATCCTGTGCCTTTTGAAGACTGCAATCTTACTGTATTACAAGACTA
TAAAAACACATTCGATATCCCTGTTGGGTATTCTGGGCAGGAAGTTGGTACTGCAGTG
GCTTTAGGTGCAGTTGCGTTGGGA
>1002049785_1 Heli.1-DT665875.3.5
GCACGAGGGACGCGTCTTACCTACAATATAAATAGTAGAACAAAAAGATATTTATCTTTTATTATATTTTCTT
AGTCTTTAAAAACTTCGAAAACGTTGCGTTCAAGTGAGAGTGTTACCGTCGTAGAGAGTTTGGCGAGTGATTT
GATATTCCATTACAAAGAACCAGAACCCAAGATGACACTCGAACAGCCATCAGGAGAG
ATGTGGAAGAAAATTCGCGAAGAATTAAACGAAAACGCTGACACAAAAGATCAAGACCTGGCTCATATCAAGG
AATGGCTGAAGAAAGAGCCCCATCTACCTGATGAATTTGACGATCAACGTATAATGACCTTTCTCAGAGGGTG
CAAGTTTTCCCTTGAGAAGTGCAAGCGTAAGCTGGACATGTACTTCACCATGCGTTCG
GCTGTACCAGAGTTCTTCAACGACCGAGATGTCAACCGCCCCGAGCTACAGGAGATTATCAAGATTGTCCAAA
TGCCACCAATGCCAGGTCTCACCCCTGATGGTCGCCGAGTCATCCTTATGAAAGGTATTGAAAAAGAAGTCCA
AACTCCAAATGTTGCCAACGCTTTCAAGCTGGCTCTTATGCTTGGTGACGTTAGATTG
ATGGAAGAGAAGGAAGGTGTAGCTGGAGATATTTACATCTTGGATGCTTCCGTCGCTACTCCTACTCATTTTG
CCAAATTTACACCAACTTTGGTCAAGAAGTTCTTAGTTTGTGTCCAGGAGGCGTACCCAGTAAAATTGAAGCA
AGTCCATGTCATAAATATATCA
>1002049786_1 Heli.1-DT662606.3.5
CACTTGGTTCAGTGGTGCATATTTATACGGCCATGAAGCTGTTTTGGTGCTGCTTGGCAGTAGCGTTACTGAC
GATTACTCAAGCTGCTGTAGATGTTGAGAAAACAGTACAACAAGTTCAAAGTATTTTGAAAACCAATGCACTT
CTACCTAGACTAACCAGGGAAGAAATTATACAATTATTAAATGACATAAGAGCAGAGG
ATGCAAAAAGTACCTTACCCAATAATAAAATCAACAATGTAAGAACAACTACAAAGGGTCCCACCAAAAATGT
TTCAAAGAAAGTAACTGAATCCTTAAAGTACAATAACTTACAAGATAATGAAATTGCGACTCAACTTTCACAT
GTAGAAGTAAGTAGCGACAGCCTTGTTAAAGATAACGATGATACAGTATCAGCTTCTT
CAAGCCCGATAGAATCTACATCAAAAGCAAGTCCAACGTTAACTGTGGTTTTGCCTTATACGCCTAGAGACGG
ATCGTCCTTGCAAGAACTGTACACAAAGCCTCCACGTCTTCAGATCGTTACTGACCAAGTCACTCCGAAACCA
ATCAAGCAAACGAAATTAAAAAATGACATTAAGCAAGTACCGAAAATAAAAAATACAA
AGAAGCCAGAACTAGACCTTCCAGCTGAATTACAGGCATTTTTAGAAGCACATGGCTTAAGAGACAAACCAGG
AAAAGACAACTTCTTGTTACCACTGGAAGGTTTCAAGCCATTACCACCACCAAGGATTGTTGATGGCACGGTA
CAGTTACCAGAAAATTTATTACTTACTTATGATC
>1002049787_1 Heli.1-EL599856.1.5

TCGTTCCTATAATAAATATATCATTTAATTTTTAAAATCAGTTCTTTTCCAACATGAGAAGTTTAATCATCGC
GTTCGCTTTTTGCGCCTTAGTTTCAGCACAGGACTTCGATTGTCCAGAGAAGAGTGGTTTCTACGCGGACCCG
TACCAATGTGACCTCTATTACAAATGCAGCAAAGGTAAATCAGAAGCCAAACTTTGCC
CAGACGGTCTTGTATTCGCTGACGAGAACCCCCACAAGGAGCTCTGTGACATCCCCTCAAATGTAGACTGCGG
TGACAGAAAGGAGCTTCAGGAACCCAAGCCTACTAAAGGTTGCCCACGACTAAATGGATATTTCAAGCATCCC
GATCCTCAGGCATGTGACAAATTCTACTACTGCTCAGATGGCGTCCCCAATGAGTTAC
CTTGCCCTCCTGGCTTATATTTTAACGAAGAGACCTCGAACTGCGACTGGAAGGAGTCAGTACAAAGACAATG
TGACCAAATCACTAAAGATACACTAGACGATGGTTTCTCGTGCCCCGATGGTGAAGTAATGGGACCTAACGGA
CGTGCTCTTCCTCACCCAACCTTCCCACACCCTGAGGACTGCCAGAAGTTCTACATTT
GCCGTAATGGCTTCCAACCTCAAAAAGGCAGCTGTCCATCCGGCAAGGTCTATAATGAAGACACTTTCATGT
>1002049788_1 Heli.1-DT668361.3.5
GCACGAGGCGGACGTTTTATAAAGTGACGCAAGCTAATGCTTTACGGCATGCGTTTATCTAACATTTCGATAC
TAGAAATTGTTGATCAATTACTGTCGCTATCAATAAACACATGACCAGTTAGGGGTGTATTCATATATAAATA
ACAAGAAATGGCTCGTGAGTTTGATCACCTCTTCAAACTACTGATTATCGGTGACAGT
GGTGTAGGTAAAAGCTGTCTCCTACTGCGGTTTGCCGACAACACGTTTTCCGGTAGTTACATTACAACCATTG
GTGTTGATTTCAAAATAAGAACTTTAGAGATAAACGGTGAACGAGTTAAACTGCAGATATGGGATACCGCGGG
CCAAGAGAGGTTCAGGACTATAACAAGCACGTATTACAGAGGGACTCACGGCGTCATC
GTTGTGTACGATGTCACTAATGGGGAGTCCTTTGCCAATGTCAAACGGTGGTTACACGAGATTGAACAGAACT
GTGATGTCGTTAACAAAGTTCTAGTTGGTAATAAAAATGACTGTCCATCGAGAAAAGTGGTTGTAACAGAGGA
TGCACAACGGTTTGCCAGTCAAATGAACATACCATTATTTGAAACCAGTGCAAAAGAG
AATATAAATGTTGAAGAAATGTTTCTCACTATCACAAAAATGGTATTAAGATCTAAATTAGAAATGAAAGAGA
GACAAAATGTAACAGCAAATGACACAGTACACTTAAAGAAGACCAACAAAACAAAAAAGCGATGTTGCTAGTG
ACCACAGACAATGGCACTCGGCCAGGGATGTCACTTTAACAATGCTCAAATATTGAGA
GACAGTTGCACAACCCTAGCAGGACACTATGGGACCTCAGTCATATGTAATGTCAGCTAAGAATTGATAAGTT
GTAAATTCTTTAATGGACAGATA
>1002049789_1 Heli.1-DT667652.3.5
CGGTTGGATCTTCGGGATGGAATCGAAAGAAATATTAGACAGACTTCGCTTTTTATTAGAATTCTTGGATCTG
CCGAGTGAAAACAGAATGGTTAAGAATTTGAGTGGTGGTCAACAACGTCGTGTGTCATTTGCTGTGGCTCTCA
TGCATGACCCCGAGCTGCTCATCCTCGATGAACCCACAGTGGGCGTGGACCCGTTACT
CAGACAGTCCATATGGACCCATCTCGTTCGCATCACCAGTTCCGGCGATAAGACTGTTATCATAACAACACAT
TACATTGAAGAGGCGAGGCAGGCGCATTGTATCGGTTTGATGAGGAGTGGACGCCTATTAGCTGAAGAATCAC
CACAAGCCCTCCTCGCCATGTACAGCTGTATTTCGCTGGAAGACGTCTTCTTAAAGCT
GTCAAGGAGACAAGGTCAAGCCAACCAAGTAGTAGAATTAAACGTATCGGGCGGAAGCCTAGGGCTCAACAAG
ATGTCCAAACGCGAGGAGGCGCCGTACGCGGCGGAAGACAACCAAGTAGTCGGGCTGAATTTCCATCAAAGCA
AGGAAGTGTTGATCGTAGAACACGCGGCGGGTTCTAATGGGGATCTTCCAGGCAAAAT
GACTGAAGCTGTGAAGTCGGAGTGCGAAGATTGCGGGAACTTTTTCAACCTAACATCCAAAGGCAAGATTAAG
GCTCTCTTACAAAAGAACATACTCCGGATGTGGAGGAACATCGGCGTCATGTTGTTCATCTTTGCACTTCCTG
TCATGCAAGTGATACTGTTCTGTCTTGCCATCGGTAGAGATCCTAGCGGGCTGAGGTT
GGCGATAGTGAACGACGACGTCAACATCATCGACGGTAACTGCCCATACAATGCATCGTGTTCAATGAAAAAC
CTATCATGTCGTTATTTAGCACATTTCAATAATAAATCAATAGTAAAAGAGTACTACGCGACGGTGCCGGACG
CCATCGCGGCCGTGCGGAGCGGCGACGCGTGGGGCGCCGTCTACTTCAACGACAACTA
TACTGATTCACTTGTTGCTAGGTTGGCTTTGGCTGATACAGCGGACAACGAAACTATATCGTCGTCCGAGGTG
CAAGTGTGGCTCGACATGTCCAACCAACAGATCGGACTCATGTTGAACAGGGATATACAGTTCTCATATCGGG
ATTTCGCTAAGGATCTTCTTTCCACCTGCAATTACAATCCCAAAGTGGGTGACATCCC
CATAGACTTCCAGGACCCCATTTATGGTGACAGTAACCCATCGTTCACTGATTTCGTCGCACCTGGAGGTATT
CTTACGATCGTGTTCTTCCTCGC
>1002049790_1 Heli.1-DT664297.3.5
GCCGCCGATACTCGATAGTTGGATTTTGTGAGGAGTAATCCGTGTATTTGTTTTTGTTTGTGACTGAAATACT
GCTTTAAAATGTCATCCAAATCTAAAAAGACTGTAACATCGTCAACCAATATAAGTGTTGTGTCATCAGTGCA
AAGCCCTCAGCAATCTAGCACTCCGGTTGGTAGCCGCCCTTCAAGTTCTGCCGGTCGA
CCTAACAGCCCATTAAGCCCAACAAGACATACACGTCTTCAAGAAAAAGATGCTTTACAAAATCTTAATGACC
GTCTCGCAGCGTATATTGATAAAGTTCGTCAGCTTGAGAGTGAAAACTCTGGACTTCGTCGTGAAATTCAAAC
AACACAGGAGGTGGTCACACGTGAAGTCTCTAACATTAAGGGAATGTATGAAAATGAA

TTACAAGATGCAAGAAAGTTACTAGATGATACATCAAGGGAAAAAGCAAAACTTGAAATTGACTTAAAGAGGT
TGTACGAAGAAAATGACGATCTTAAGAAACGCTTGGACAAGAAAACTAAGGATTGTCAGCAAGCCGAGAACTT
GGCGCGTCATTACGAAACTCGTTACACCGAGGAAAGTAACAAATACAACACTGCCCTT
GCTGACAAGAAGAAGGCTCAAGATGAGGCCAGGGAACTGGCCAAAGAGCTTGAGAAGTTGCGTAAAGTATACG
CCGATACCCGCAAGACCCTGGAAGAGGAGATGCTGTGTCGCATCGACATGGAGAATACCGTGCAAAGCCTGCG
CGAGGAGCTCTCCTTCAAGGATCAGGTGTTCCAGCAAGAGTTGCAGGAGACCCGCACT
AGACGACAGGTTGAGATATCTGAAATTGATGGTCGTCTGGCTCAGCAGTATGAAGCTAAGCTACAGCAAAGCC
TACAGGAGCTGCGTGAGCAGCAAGAGGCTAATATCAAGGCCAATAGGGATGAAATTGAAGCCCTTTATGAGAA
TAAGATGAAGAACTTGCAATCAGCGGCGACTCGCAACAGCAGCGCCGCCACCGTGGCC
GTGGAGGAACTGCGCACCATGCGCACGCGCATTGATAGTCTCAACGCTACACTTAATGATTTGGAGAACAAGA
ATGCTTCTCTTAGTAACCGGTGCCGTGAATTAGAACGTCAGTTAGAATCCGAGCGCGCCCGTCATGCCGAAGA
TCTGGCTTCTATGGAGCAGGAGTTGGCCCGACTTCGTGATGAAATGGCGGCTCAGCTG
CGTGAATACGCCACGCTTATGGACATCAAGATATCCCTGGACCATGAGATCGCTACGTATAGAGCGCTTATTG
AAGGCGAGGAGGACAGGTTGAACCTAACATCCCAATCGCCCGGGCGCGAGTCGCGCGCGTCCGTGAGCGCGGC
CAGCGCCAGCGGCGGTCGCGCCACGCCTGGCCGCCGCGCCACGCCGCTGCGCGCCGCG
CGCAAGCGCACGCTGCTGGACGAGAGCGAGGAGCGCAGCCTGCAGGACTTCAGCGTCACGTCCAGCGCCAAGG
GGGACCTGGAG
>1002049791_1 Heli.1-CX700604.1.5
CTGCAGGTACGGTCCCGGAATTCCGGGTGACCACGCGTCCGCATAATTTGACCAAAAAATCCCATAAAAAAAC
AATCAAAATGTTGGGTCGTTTTGCACCTGGAGTCGCCCAGGTAACCATCATGGTCAACCAGCAACAGAACCGT
AACATGGCCACTCTGAAAGCAATTTCTATCCGCTTGAAGTCTGTCAAGAACATTCAAA
AGATCACCCAATCTATGAAAATGGTGTCAGCTGCTAAGTACACTCGCGCTGAGCGCGAATTGAAGGCTGCCCG
TCCCTATGGCGAGGGTGCTGTGCAGTTTTATGAGAAGGCTGAGGTCGCCGCCCCCGAGGATGAGCCAGCACAG
CTGTATATCGCTATGACCTCAGACAGAGGTCTCTGCGGCGCTGTGCACACTGGAGTGT
CCAAGGTGATCCGCAACCGCCTCAGTGAGCCCGGAGCCGAAAACATCAAGGTGATCTGCGTCGGGGACAAGTC
GCGCGGCATCCTGCAGCGCTTGTACGGGAAGCACATTATTAGTGTGGCCAATGAGATTGGTCGTCTCCCCCCG
ACTTTCTTAGACGCTGCTAAATTGGCAAACGCCATTATGACTTCTGGTTACGATTTCG
GCTCTGGCAAGATCATTTATAACAAGTTCAAATCTGTGGTGTCTTATGCTCAGTCTGATTTGCCTTTGTTCA
>1002049793_1 Heli.1-EL599786.1.5
TCCACAACGCAAAAGGCACCGGTGGCAGCGGCACAACCTACCCGGCCCTCCTGCGGCCCGACGAGCAGTTCCT
GGTGCCGGACCGGCTGCTGGTGGTGCTCGACATGGACGAGGGCACGCTGGCCTTCTGCGCGGACGGCCGCTAC
CTGGGCGTGGCGGCGCGCGGCCTGCGCGGCAAGACGCTGTACCCCATCGTGTCCGCCG
TGTGGGGGCACGCGGAGATCACTATGAAGTATATCGGCGGGCTCGATCCGGAACCTCTCCCTCTAATGGAGCT
GTGCAGACGCGTGATCAGACAGCGCGTGGGGCGCGCGCGCCTGCGGGCCGCCGCCTCGCGCCTCGCGCTGCCG
CCCGCCCTCACCGCCTACCTGCTGTACCGCGCGCCCTAGCCGCCCCGCCCGCCCTAGC
CGCCCCAACCGCCCTGCCCGCCCTAGCCCGCCACCCACGCCCTAGACTAACTGCAACTGCGACATAGACTAAA
CGGTGTCAAATTGACGTCAATGAGAACAGATAATATTTACTCACTATAGGAAATATATGAATCTCAAGCGCTG
TAGACTCAAGAGCAATAGAATATTATTGTGTAAATGTACACCTGTAAATGTAAAGCTT
ATATTTGCTGTTCATTACACATATGATTTGCTCTAAAGATCAATTAGTACAAACGTTATCTTATGATTGTAAC
TACATGACAATCTCAACTTTCCAGAAATTAAGAGATATTTGATGAATTATGCAAGTATTTTTCCCATCGCCTT
TATGTTTAATCACAACACCTTATACTTCTCTGTATATTGAAGCTGTTATGTAAAAGCT
AAAACGTTAACGCTAGTTCCAGATAATCTAGCAAAAATCGTACAAAATTTTGATCAACTAGCTTTACTGTAAT
GTAGAAGTAAAATCAACGCTGAATATAGGGCTGTCCATAGATTTCGTCACACGTATTTCATGATTTGACTCAC
TCCCAAGCCTTGTCACAGTTGGTCATATTTGTGAGACCTTTACATGGTGTGACGTCAT
ATATGTAGCAATTTTACATCTAGAAATTATTGAATTCAAACAGCAATTCTGAAATTATGCTTGTATATGTTTT
GAAGTATTCACGTTAAGAGACGTGTGACGTAATTTATGGGATGACCCATTGCAGCTAATATTTTTCTTTTTTT
TTTTACTATTGTCTTCTTCTCTTCAGTGTACTTGTTTGACAGGTACGTACACACATTG
TTCCAATGAGGATACATGTTTTATTTTTATTTTTACACTGTCTGTGATATGTGTTTTTTTATGCATCACCATT
CTCAAATATTATTCATCAGTTGCATTTTAAATCATATTATAATAATTATTATGAGAACGGTTATAAATGAT
>1002049795_1 Heli.1-DT663701.2.5
GCACGAGGTTTACTTCCTCAAGGGGTATACGCACAATTTTTAACACAACTAACAGTTACTACATTTTTTAATA
AACAGCAAAGATGTCAGGGAAAGCAAATAAAGCATTTACAAAAGAGGAAGAATTGCTTTTACAAGATTTCAGC
AGGAATGTTTCAACGAAGTCATCAGCGCTATTCTATGGAAATGCTTTTATAGTTTCAG

CTATTCCCATATGGCTATTCTGGAGAGTACATGCTTTAGAAGTAAGTACTTCATTGGCTTGGTTTGCATTGGT
AACTACAGCTAGTACATGGTTACTTGCTTTGGCATACCGAAACACGAAATTCCAACTGAAGCACCGTGTTGCG
GTTCGCCGGGAAGATGCTGTAGCTCGTGAAATGGCACGGAAATTAGCTGATGAGAAGA
AAATGAGCAGAAAAGAAAAAGATGAAAGGATTTTATGGAAGAAGAATGAAGTAGCTGACTATGAAGCAACTAC
TTTCTCAATCTTCTACAACAATGCATTGTTTTTGACAATTGTGATTCTCAGTAGCTTCTACTTGCTGCGCTCC
TTCACTCCTACTGTTAATTACATAGTATCTCTTTCAGTAGCATCTGGTCTTCTGGCTC
TGCTGTCAACTGGTACTAAGTGAAAAGACTGATTTTGTGTTTGTGATGATAATATGAACACAATTTTGTACTG
CAGTGTGTTTGTACTCAAAAATTAAATTATTAAGTAAAGTGCTAAAGTTGATTCAGTTTTGTTTTAAAAGGAA
TTTATTACTATAGTCATTAAAAAATAAAATACACATACAAAAAAAAAAAAAAAAAACT
>1002049796_1 Heli.1-DT667054.3.5
ACGCGCCTCTCGCGCAATCGATAGGAATGCCCAGTGATACCTTTACCTAGTATGAAGTGCTTTGGAAAATAAA
AATCTTAAAAGCGAAACTATTGACCATAGATAAAAGGAGGAGCGCGGATGAGTCTGCAGCGACCGACGGGCAG
CTGAGGGCCGGCCCGGCCTGATGGCGAACGTCGCGGTGTACGCACTGCTCGTTACGGC
CGTCACGGCCACCCTTGCGGAGGCTGGTGCGGCGCGCTTCGGCGACCGCCTGGCCCCGCGAGTGCCCGCGCCG
TACGCGCCTCGCAGTGCACCCGCGTCGCGACACTACGTCGAAAATGAAGAATATGATGCTTATGAACAAGATT
ATGAAGAAGATGAAATACCAATTGAGACCAAGCCACCACCTCGAAGACCTACACGTAC
AGAAGCTCATCGTTTAGAAATGAGCACGGAATATTTTATTATTCCCGAAAGACCTTCGTCGGCACCGTCCACC
TCTTCAACATACCGAGTATTTACTGTATCGACATTAAAGCCATTAAATGTGACGCCCGTTCATATAATACCAT
CCCCAAAGTTCAATATACTTAGAACAGAATCCTGGGCAGTGCCAATTTTAGCGTTATC
TAGCGCAAGCATGATCATCATAGCGGGTTTTGAAGCATTCATAGTGTGGGGAGCAAGTAAGAAAGCTCCGAGT
CAGCGACACCTCCTTCTTGGTCAAAGTTTACTTTTTGGACTTTTTAC
>1002049798_1 Heli.1-EL601888.1.5
GCACGAGGCTCGAGTTTCATGTCTTTACTGAACAACAATAATTAATTATTTTATGTTACCAGCTATAAAACGT
GATAGTTCGTGAATATAGTGCATTATGGATTCTTCGGAGTTTCGCGAATGTGCAAAGGCCACTGTCGATTTGC
TTATCAATTATATAGATAATATAAGAGACAGGAATGTTTTACCATCAGTGGAACCTGG
ATATTTAATGAAAATGCTACCAGAAAGCGCTCCAGAGGAACCTGAAGATTGGAAAACTCTACTTAAAGATTTT
AATGAAGTAATTATGCCTGGAATGACCCACTGGCAGTCACCGTACTTCCATGCTTACTTCCCTACTGGGACAT
CATATGCCAGTATGATTGGGAATCTCCTGAGTGACGGTTTAGGAATCGTAGGATTTAC
ATGGATGGCCAGTCCAGCATGCACGGAGCTAGAGGTAGTGACTATGAACTGGTTAGGAAAACTACTTGGTTTG
CCCGAGGAGTTTCTCAATTGTTCAGCAGGTCCTGGGGGCGGTGTTATTCAGGGATCTGCAAGTGAAGCAACAC
TAGTGGCATTATTAGCAGCGAAGGATAAAAAAATTCGAAAGATGAAAGAAATAGATCC
AGAGCTAGATGAAAGCATAACAAAAACAAAATTTGTGGCATACACATCTGAACAATGTAATTCTTCTGTAGAG
AAAGCAGGAGTACTTGGTTCGATGAAAATGAGGCTTCTAAAAAGCGATCAAAATGGTAG
>1002049799_1 Heli.1-DT664192.3.5
TTTAAATTTATATATTTTTGCTCTTTTTGTGAGTTTGTTTTTAATTTTTAAGAAACATGGCGACCAAAAAACA
CTTTCCATATGAATTTAAAAATGTAACGGAGGAAGAGGATAAAATAGTGAAAAAATATTACAAAGATTACACA
CGCCCGTTCATCCGCGTGGGCCCGCACGGCTACATCTCTATGGCAGGGTACGGAGACC
ACGCAGCTGATATCTACAATTTGGAAGTTAGACCGGACGATGTGTGGGTGATTGCCTTTTCTAGATCAGGCAC
TACTTGGCTCCAAGAGTTAGTGTGGCTGGTTGCGAACGATCTGGACTATAGCGCAGCCTCAGCCACTCCGCTG
ACTAAAAGATACGCCTATATTGAATATCCAACGATGGCAGCAGAAACTAAGAAGCACG
AACCCCCACCGCCACCACCACCACCAGGTCGACCCCACCACCGAGCCACCTTCGACGACTTCAGAACCGTCCA
CACGTTGCCTTCTCCGCGTTTCATCAAAACTCATTTACCACTATCGCTTTTACCGCCGAAACTTTTGGACACT
GCAAAGATTCTTTACGTAGCACGAGATCCCCGCGACGTGGCCGTTTCATTCCACTTCA
TGCACAAATTGTTCAGATACTTCGACGGAGGGGTGGAGTTTAAAGAATTTTGGGATCTCTTTAAGAGAGATTT
AATAATGCACACACCTATATTTCCACACATCAAAGAGTTTTGGAACGTCCGTGATCATCCCAACTTAACATTT
TTGTTTTATGAAGAAATGTTAATGAACCTTCGCAGCGTTGTAGACAAAGTATCGGCCT
TCCTCGGCAAGA
>1002049800_1 Heli.1-EL599260.1.5
ATCAGCAATCCGCAATTTTACACTATTGGCTATAAAGTAGAACTTTGGACTGCAAGAGGGGGCGGAAAATAAG
GAAGAACCGGTCCAGACATTATATTTAATCTCTTGTCCACATTTGTTCACTGGCATATATCGGAACAGGGACT
CACCAATTTACAATCACCGGTTACTTGTTATAACCAACATAGTATCACAGTACAGTAT
CAACGTAGTTATCTAAAACACATAAAATGGCTGCACCTATCAAAGTGGTTGTGACTGGAGCCGCTGGCCAAAT
TGCTTACTCCCTTTTGTATCAAATTGCATCTGGTGCAGTTTTTGGACCGGAGCAGCCAGTATACCTTCACCTT
CTCGATATAGCTCCTATGATGGGAGTATTAGAAGGTGTTGTTATGGAATTGGCAGATT

GTGCTTTACCACTGCTTGTTGGAGTTTTGCCCACCGCCAGTCCCGAGGAAGCTTTTAAAGACGTAGCTGCTGC
CTTCCTTGTGGGTGCTATGCCAAGAAGAGAAGGTATGGAAAGGAAAGACCTCCTCTCTGCTAATGTACGCATA
TTTAAAGAGCAGGGCCAAGCTCTAGACAAAGTAGCACGCAAGGATGTTAAAGTGCTAG
TTGTTGGTAATCCTGCTAACACTAATGCGTTTATTTGCTCCAAATATGCACCTTCAATTCCTAAAGAAAACTT
CTCAGCTATGACTAGGTTAGATCAAAATCGCGCACAATCTCAACTAGCGGGAAAACTCGGAGTGCCGGTACAG
GATGTCAAAAATGTTATTATCTGGGGCAACCACTTCCTCCACACAGTTCCCAGAACCA
TCTAACGCTGTAGTCAAAATAGGTGGAGTTGAAAAA
>1002049801_1 Heli.1-ES585367.1.5
AAATAAAACTATTAAACAAACCAACATGCAGAAATTCGTAGTATTCGCTGCTTTCTTGGCCTGCGCTGCTGCC
GCACCCGGTCTCTTAGTAGCCCACGAAGCCCCAGTGGTCGCGGTCCACTCTGCACCAGTTGTCCACGCTGTAC
CAGTGGTAGCTTCCAAGTCCACCATCACTAAGAGCAGTCAAGTTGTCAACCATGGATC
CCCTGTGGTCTCCGTACCAGTTGTGAAAGCCGCTGTTGTCGCTCCCGTAGTACATTCCGCTGCTGTAGTCCAC
TCTGCCCCAGTTGTCCATGCCGTACACGCCCCAGTAGTCCATGCCGTACACGCCCCAGTAGTCCATGCCGTAC
GTGCCCCAATAGTCCACGCTGTACATGCTGCCCCAGTCGTCCACTCGGTCCACGCTGT
AGCCCCAGTCGTCGTCAAGTCTGCCCCATCCGCCGTCTCTCACAGCAGCTCAGTCGTCCACCACTCCTCAATC
AAAGCTATCCCCGTTGTAGCCATCCACTAAATGCTTTACTTAAAATCTAGATGTGTTGATTATAATATATTAC
CTCCGAATGT
>1002049802_1 Heli.1-EL602165.1.5
GCACGAGGCCTCGTGCCGAATTCGGCACGAGGCGGACCGTGTACACCCGGTGCGATGTGAAGGCACACCTCAC
TCCTCTGTCTCCGACGGCAGCTTTTGTTGCTTGTGCCGCGTGTGTGGTGAATTTATTAGCTGTTCACGGCTGA
ATCCGTTCAAGTTTTATTCAATGTGTATGGCTGTGGAGCTGGGCAACTGTTCTGTGGC
GACATTGATGGAGTTCCGCTTGGACAGCGCGGAGTACTGCCAGGCCCAACACAAATAAGTTCACGGGCTTCCG
TGGAGACGAGGTAGCGGGCCACGACTAGACCCATGCTGCCGGGAGCGCGCGACTCACGCACCCCAAGCACTTT
GCGATATACACCCACCCATTTTACCTTAAAATCGGATCCCACGTTTAATTGGACCCTA
TAGTTTGCAGGACGCGTCGTCTTATGTTCGGTCGCATAATTTATTAGTTTCGTTTCCCAATTCGGAACCTTAG
TTTGTTAGTCGTCGAATGTGCTCGAGTGGTTGAAATCGTCATCCATGCGGGTGGTTCTAATTATATAATTGGA
CCCTTTTTAAACAGAAGCCACGGAACTGTAGTGTGTTGTGTGGAAATAGAGTGACAAT
TTGTCTTAGACTCTGACGAATCGTTACTTGACGCGTGTGAATTTGCCATCTTGGTGCGAGAGTTTAACTGTGT
ATTGTGCGTCAGTGAAGTAAAGTGGTCATCATGAAGCAGAAAGACATCAGGCCGGCGCCGTCTCTCATTGAGT
ACAAGAACATGCGCTTCCTCATCACCGACCGACCATCGGATGTCACCATCCAAGGCTA
CTTACAGGAGCTAAGGAAGCACAACGTGTGCACCGTGGTGCGCGTCTGCGAGCCGAGCTACGACACGGCGCCG
CTGAAGGCGGAGAGCATCGAGGTGCGCGACCTCGCCTACGACGACGGCACCTTCCCGCCGGCCAACGTTGTTG
ATGACTGGTTCGAGATTTTGCGCGACAGGGCCGCCAACAAGCCGGAGGCGGCGGTGGC
GGTGCACTGCGTGGGCGGGGCTGG
>1002049803_1 Heli.1-EL596112.1.5
AGTCTGTGCAGAGACGGTACAGAAGACGCTATAAACTTATATAAACATATATAAAAATATTTAATCGGCAAAA
CAAGCGGTCGGTCTGAAGGAAGGCATTGTGTTATAATGGTAATGAAATTGAATATTAATTCGGATTAAATAAT
AATTTTAATTAATTGAAGTTCATATGGATTTTATAAAGGTGTCTTCTAATTTGTGTAC
ATCGTAAATTAGTTCTGAAACCTGAAAACGTAAAAGGAAACATAAGCAAGAGGTGACAGCTTGCGGAGTGCAA
ATTGTTGCCGTGACAAGAAAGTGACTGGAAAGTAAAAATAAAGTGTTTAAGTGTATGTGTGTAAACTTAAGTG
TAGAAGTGTTAACTTGTCAAAATGTCGGGCTTCGTTAAATTCGCCATAGATGACTATT
ATTCAGACAATAACTGGACAGAAATCATCAATAAGTATTGGATGCTGGCGGAACGAGTTTCAGATCCTCGTGT
ACAAGGATGGTTTCTCTTCGATACTCCTATAGCGACACTCGCGATGGTCGTAGCGTACCTAAGCTTCGTCATG
GTAGTGGGCCCACTGTGGATGGCAAACAGAAAGCCTTTCAAAATTAAGAATATATTAG
TCGGTTACAACGCAGCGCAAGTTTTACTTTCGTCATATATGTTTTATGAGCACTTAATGTCAGGATGGTGGTA
CGACTACAGCCTCACTTGCCAACCGGTCGATTACAGTGACAGTGATAAGGCGAGACGGATGCTACACTTATGC
TGGGTTTACTATTTCTCGAAGCTTTCGGAGTTCGCGGACACTGTGTTCTTCGTTCTTA
GGAAGAAGAAAAGTCAAATCACGTGGTTACATCTCTATCATCACTCCTTGACGCCATTTGAAGCTTGGCTTTT
ACTTAAGTTCATTGCACGTGGCCACGGAACGTTTTCTATCTTTATCAACAATTTAGTACATGTTATAATGTAC
AGCTACTACATGATGGCTGCCATGGGGCCTCAGTACCAACAATATTTGTGGTGGAAGA
AACATTTGACGACTTTGCAATTGATACAATTCTTTATGGTGCTGTTCCATTCAATTAGCGCGCTTGTATACGA
CTGTGGCTATCCAAAAATCATCGCATCCGGCCTCATCCTACACTCGACGATATTCATTATCCTTTTCACCAAT
TTCTATATTC
>1002049804_1 Heli.1-DT667334.3.5

TTTTTAAAAATGTCCAATAACGGTGCTCCGGACTCTTGGGAAAGTCAAGCTGATGTTATAGTTTTGAACGGAT
GTGGAGACGGCGCTGAAAGTGGCGATGGAAGAGCGGCTTCCGCGTCTCCCCGCGTAGAAGAGCCACCTCCTGT
CCCTCCAGCTGTTGCTACCGCCCCACTGCCTGTACCTCCAGACGTAAGCCCTACTGCT
GATAGCTGGGAAGCGGAAGCAGATGACGCCCTTTTAACTCCTGAAGACAACAACGAAGCTGAGGAAGAAGAAT
TAGAACAACAGGATGATGGAGAAGCTGCAAAAAAAAATACCTAAAAAGAAGCCACCTAGGGTAGAGGACACTCG
TAGCAAGAAGGAGCATGTTAATGTTGTATTTATTGGTCATGTGGATGCTGGAAAATCG
ACTATTGGGGGTCAGATTATGTCATTAACGGGTATGGTTGATAAACGAACATTAGAAAAATATGAACGAGAAG
CAAGAGAAAAGTCTAGAGAATCTTGGTATTTATCTTGGGCACTTGACACTAACCAAGAAGAAAGAGATAAGGG
CAAAACTGTGGAAGTTGGCAGGGCATACTTTGAGACTGATAAAAAGCATTTCACAATT
CTTGATGCACCAGGGCATAAAAGTTTTGTGCCGAACATGATTGGTGGTGCCGCACAAGCTGACCTTGCTGTTC
TTGTGATATCTGCGAGGAAAGGAGAATTCGAAACAGGGTTTGACAGAGGGGGACAAACTCGTGAGCATGCTAT
GTTGGCAAAAACTGCCGGCGTTAAACATTTAGTAGCTCTTGTAAACAAAATGGATGAC
CCCACAGTAAACTGGGATGAAAAAAGATACAATGAATGTCGTGACAAAATTATGCCATATCTCAAGAAGCTGG
GCTTTAACACTGCTAAAGACTTGTCATTTTTACCAGTTTCTGGCCAAACCGGTCAAGGTTTATTGGAAAGAGT
AACAGAGGAAATCTGCCCCTGGTATCGTGGTCCATCATTCATCCAGTTGATCGATGAG
CTCCCATCCCTCAATCGCAAGATGGACGGGCCCTTCATCATGCCAGTTGTTGATAAGTACAAGGATATGGGCA
CTGTCCTAATG
>1002049805_1 Heli.1-CX700527.1.5
CTGCGGTACGGTCGGAATCCGGGTCGACCACGCGTCCGGTCAATCATTCCCACGACGAGAAGATGTCTGTACG
CGTGTCTGTTGACCGCCGCACCCAGGCTCATGGAGCCCGTCTATCTTTGTGAAATTCAGTGTCCCGAAGTCGC
CGTGGGTGGTATCTACGGTGTGCTCAACAGACGGCGTGGTCACGTGTTCGAGGAGTCC
CAAGTGGCCGGTACCCCCATGTTTGTAGTGAAGGCCTACTTGCCTGTAAACGAGTCCTTCGGTTTCACTGCTG
ATCTCCGTTCCAACACTGGAGGCCAGGCCTTCCCGCAGTGCGTGTTTGACCACTGGCAGGTCCTGCCCGGTGA
CCCGTGTGAGGCTGGTAGCAAACCCTTCGCTGTAGTACAGGAAACAAGAAAACGGAAA
GGATTGAAGGAGGGTCTCCCAGATCTAGCCCAATACTTGGATAAATTGTAAACGCCGTAGCTAATGCAATATA
TAATGTAATATAACTATATATGCGCCTCACGCACTCAGTTTTGGTTCAACCATTTGCCTGATGACGTCATCAA
GGGATACATTCAATACAATAAATACCAATACAAATAGATATTTGAGAGACGCGTCGTC
AATAAACAAACAAATGTGATACCCGGCTGTATCATACATTCTATGTCCCCATTATCATTTTTGTAGTCGGGAA
CCTGGACACTTTAATTATACTGTAATTAATTTTTAAATTATAATTTTTCTATTATAAAA
>1002049806_1 Heli.1-DT665558.3.5
GACGAGGCAACGCCTTCTGGATGTGCCGCTAGCTGCCCCGCACACTGTGAACAGCGAGGAGACTTACCGTGTA
GTGTTATGATGCTAACTTGTGTTGATCTATGGACGGGGTGATATATTATAATAGAACTTGTGATAAACACGAA
CAAAAGGATCCAGTGCAGTGTTAATTAGCGAGTAAACATGCCGAAGATATTCCTTATA
AAGAAACGGCTGCACGAGCAGCAACTCGGGCTACAGGAGGGTCAGGAGCTTCTAGGCAAAGGTGACCCGCTGT
GTCCTGGCTCTCCTCTGGACGATGGTCCCATACCCCTTCTCTCTAAGAAGGATAGAGATTGTCTTGATCGCGA
TGTATTTGGAGAGTGCAACACCAAAACTGCACAAGAAAGACGTACTAAAGAACCTAGG
AGATTTATTTCATCAATACTCGGTGGCGAAATCCCATACGGAAGCCATAGAGGCATACATGTGCTTACTCAAG
CAGAACGAAAGCAATATTTACCGGTTGTAGCAGAAGATAAAAAAAATGATGATAAACCCCTTATCAAAGAAGA
AAAAGACTTATTAGAAACTGAGCCAGTAGTTTTACCATCTAGAACTTCACCTTCACCT
GAAACTGCTCCATCTCCTGGTATCGATAGTAATGTCACCTGCCAAAAAGTTTCCGTAATACAAAGAACACCTT
CTCAATCACAATTTCGCGATAATAAAAAAGAATTATCAGATGTAACTATTCCACCTACTTTGCCTTCTTCTGA
ACCGGAACAAGATCAACCCATTGATTACGTTATTGCTAAAAAAAGGGGTGAATCTGAA
GACGAAGAGACTGAAAAGAAAATAAGAGAACAAAGAAGGACTAGCAGCTCTAAAATTGCTAATGGCATTTTAG
CCAGACCTGTTTTAGTATTACGAAATCGTCCTGGGGGTAAAGTTCCCGGTATTATCAATGCTGCAGCTGGCCA
CGGGCGTTCAACAAGCGGAAATAGTTCAGGTGGAAATACGCAGAATAGTGGAAGTTCA
GGAAGCTTCAGTTCTAGCTCTGGGAGTGCAGCTCCATCATCAGGTGGAGGAGGAGCTTTAGGAGGAAGATCTG
GTAGTGGTGGAACGGTCGTGATGGACGTCAAATAT
>1002049807_1 Heli.1-ES585609.1.5
CAGTTTGGTTGTGTACCAGTTCAAAAATCATTTCAAAATTTTTGCCATAAGGAAAAAAGTTAAGATGACGGAC
AAGCCGAAGCGTCCTATGTCAGCATATATGCTTTGGCTTAACAGTGCAAGAGAACAAATAAAGGCAGATAATC
CCGGTCTTAAAGTAACTGAAATAGCCAAAAAAGGAGGTGAAATATGGAGAGGCATGAA
AGACAAAAGTGTTTGGGAGGAAAAGGCGGCTAAAGCCAAGGAACAATATACAAAAGACTTAGAGTCATATAAC
GCTAATGGAGGAGGTGGAGAAGGTGGTGGCAAAAAGGCACAAAAGAGAGGAAAGAAAGGCAAAAAGGCGGCAC
CAGCTAAGTCAAAGAAGAAGAAGGATGAGTCTGATGATGAAGATGGTGGTGAAGAGGA

AGAAGAAGAAAGCGAATGATCTCCCAATCTATAGGCATTCTGTTCATATTGAATAATTTACTTGGACTTAATT
TATTGCAAAGTAAAATGGGACTTACTTTCCAAAGTCTGTATTGCATTGTCTTTAATTTTTATATAATTATTAG
AAAAATTTAATTCAATGAATAATTAATTACACATTTATTATTGTATTTATATAGAATT
TTTGTCGACATGTAAAGAAATTTTTGATTATTTAATAGCAGGTGTTATTAACTAATTTACTTTGTAGTATTGA
GTAGTTGATAGGTTAGAAGAGTGCAGAATTTGGAAAGTTTCACCACAGGCTAGGGTCTCTAGGTTTCTAAGCC
TTGGAAAAAAAAATAATGTTTAAGTGTTAGCATGTATTCACTGGGGAATTGTACAGTT
AGCCCTCCCTCGTTAAGTTCGCTGTGACTACTGGATGCTCACCTTTGAAATGTACCTCAAATGGTTTTATCAA
AACTGTATGTCTACCACATTATTTTGCCGTAAAGCTGTGCTTTAAATTTGATATGCCATAAGTTTAATTTACG
CTAATTATAATTTGCTCTATGGACATGATAGTTTTGATGGGATCGTGTCAACATGTAA
AATTAGAGACAATTTCATGAATGATGTGACGGCTTATTGTTAATTAAACCTACCTCTCATTGTAAATTTCAAT
GCTTATTAATGGTACCAAACTTTAAGCTAAGAATGGACAGCTTGGGTCTTAAGCCCGTGCTGAGCAAATTAAA
TTTAGGAAATTCATGTGTATGTTGTATTTTTAGTTAGTTATAGGTAGGTTTTCAATGT
GATTAGTCTCTCAAATTTTACATTTGTGTTATTTTGTAAATGAGAATTAGCATTCCTCATGTCAATACGGATT
TTACATGTTCAATAAAGTAATTT
>1002049808_1 Heli.1-EL599919.1.5
GTGCGCGCTCACAACCAACCGACCCCTCCGACCACAATTCAAAAGGAATTTTCAATTATAAATTAAATAACTT
GTGGTTGTATTGGTAAATATCTGTCGCAATTGTGCGGTGTTCGGTGTTAAACGGCGGAACAGTGTCAAAATGA
AGACGGATTATCCTATGGATGAGATGCATAAGAGGACGAGCTCAATCATCGGTTTGGC
CGATGTAACAGACAACAAGCTGATCTGGAGGCAGCTGACGGCGGAGCTGATCGGCACGTTCCTGCTTACCTCC
ATCGGTGTCGCTTCGTGTATTGCCATATCAGATGCAGCAGTACCACACGTCGCTAGTATTGCGTTGTGCTTCG
GTCTTCTCGTTGCGTCTATTGTTCAGGCTATAGGCCACGTGTCCGGCGGACATATAAA
TCCAGCAGTGACATGCGGACTCTTCGCGGCTGGCGACGTGAAGCTGTTGAAGGCGATATTCTACATCGTGGTA
CAATGCTTGGGAGCTATAGCTGGAGCAGCGTTCATCAGGTTGGCTGTACCTGAAGATAGAGTTATGTCTTTTG
GAATGACTCTACCTGCATCTAATGTCACCGAACCACAGGCACTGCTAATAGAGGCGCT
GATCACGTTCGTGCTGGTGATAGT
>1002049809_1 Heli.1-EL604196.1.5
GACGGACGAGTCACGCAGTGTTTGTCGTTGTTGCTAATCTATTCAGTTTACTTGTGGTTCGAATAATCGAACA
CAAGTGAGACAATTATCATATTGATTACTATTTTGTTATAATAAAGTTACAATTTATTTGAGAAGCAGCCAAT
TTACTTAACTAATTAAAAATGTCGGTTATAGGCAAATTTGTTGTGCCTAACGTTGCGG
CCCGGAATTTTACTAAAATCGGTGCCGCGCTACAAACTGTTAAATCGAACAGAACTTATTTTACTTATACACA
AGAATTATCGCAGCCATTGGATAGGAAACCTGAATTCCTCAGTGCCAAGCAAGCATTTGAAAAATGTTTAAAA
TCAGGTCAGACAGTGTTCTCGCAAGGCGCCGCGGCCACGCCCGTGCCGCTGCTGGACG
CTATGACAGAAGTGGGCAAGGCTGGCTCATTGAGAGACATCAAAGTCGTGCATATGCACACCGAAAAGGATGC
CGCGTACGTTGCACCGGAATGCAAGGATATATTTAGATCCGTATCTCTATTCATGGCGGCAAACGTAAGGAAG
TCAGTAGCGGAGGGTCGCTCGGACGCGATCCCTATCTTCTTACAAGACATTCCCAAGT
TGTTCCACAGGAAGATCATCCAGCCCGACATTGCTCTTATCCAGGTTTCTCCCCCGGACCAACATGGATATTG
CAGCTTGGGTACTTCAGTGGACT
>1002049810_1 Heli.1-DT666583.3.5
TATTTGAACGTTCTTTTAATAATTGAAAAAAAAATAAAGTGCGTGTTGCCAGTCGTAATATAAATAATATAAA
CCACAAAATGTCAGTCGACCGGTGTCGCTCCTCTCTCATAGTTCTTATCGCCAGTTTAGTGGTCGCGTCTTGC
GCACAAATCGAAGATCCATGCAAAACAAAATCTCGCATTGTGGGCGACGACAAGTACT
GTGACAAATACTGGGAGTGCGACGGCGGTCAGTCTGTTCAGTATGATTGTCCCAATGGCCTCGTGTTCGCGGG
GAAGCACCGGGGAGTGACTGAGGGATGCGACTACCCCTGGAGATCGAATTACTGTGAATACCCTAAAGTACAG
ATAAACCCACCAATCGGTACAGAACATTGCGACTGGCTGTACGGCATCTTCGGACACG
AGACATCCTGCACTCGCTACTGGACCTGCTGGAACGGAACAGCCACTGAACAACTGTGTATCGGTGGACTCCT
CTACAATGAAAACGCTCATTCCTGTGACTGGCCCGAAAATGTAGATGGCTGTCAGAAACATCCTCTTTGTAAC
GAAGATCCCAACGGCAACGTGCCCCTCGGCAAGTCCTGCAACAGATACTGGCAGTGCC
AAGGTGGATATCCTCGTCTCCAGCGATGCCCAGCGATGTTGGTGTTCGACAGACGCTCGCTAAGATGCGTAGT
GCCACCAACTGAGGAATGCGATGTTCCGACTACCACGCCAGCTCCTGTTGATGCTGAAGAGGAACTGAGACAG
AGCCCCCCACATCAATCAAAGAGGCCTTCCAACAACGAATATCATGAATCTCAAGGCC
AAAGTCGTCAGAGGACTAGAAACTAGAATAACATATACATATTATATAGTATAAGTATGTCTATATATAATAA
TATAGTACTTCATGGGCATAATCTAAGATTACCAA
>1002049811_1 Heli.1-EL602332.1.5

GCACGAGGGCAACATGTTTATGTACGAGCCACCCACTAAGCTAAAACTTACTTCAGTGGAAGAGTTCCAAGAA
GCATTCCAGCTTTTTGACTCCCGTGGGGATGGGAAAATTCATGTGGCACAAATAGGAGATGCACTCCGTGCTT
TAGGACAAAATCCAACTGAATCTGATGTCAAAAAGTGCACCTTACACCTCAAGCCTGA
TGAGAGAATATCATTTGAAGTGTTTCTGCCTATTTACCAGGCAATTTCTAAAGCTAGAAGTGGTGATACTGCA
AATGACTTTATAGAGGGCCTTCGCCATTTTGATAAGGATGGCAATGGTTTCATTTCTTCAGCTGAACTACGTC
ATCTACTTTCCACTCTTGGAGAAAAGCTGAGTGATGATGAGGTGGAGCAGTTGTTGCA
GGGACAAGAGGACTCTCAGGGGAACATCAACTATGAGAACTTTGTACACCTCATTATGCAGGGATAGACGGAG
GCGGACACACCTCCGGCCACTGTTTTATAGTATTATGTAGTACTTTTTCTTTATCTCTTCAATTGATAATGCA
TTTATTTAAATGGCTGCAGTATGGTGGCTATACTATATAATATAACACCATGATAACT
GTGATGTATTATAATCAAAAAGAGGAGAATATTCCAAACTCTACTTCTTTATGTATTTGACTAACAATATTTT
TATTATTTCCGTTGCTATAATTTAAGAGCATATTC
>1002049812_1 Heli.1-DT663150.3.5
GCACGAGGCCCATCTTGGACTTCGTTGCCGTCTGTTACTGTTGATTATCTTTCACTATTGCTTCCCTATTTTA
TGACACTCCTATCCATATCTTTATCTACTCACCACGTCGCAATTACAATTATCACTGAGTGTGAGTTCAATGA
TCCGTCTCCTAGTTTCATATTCCCGTGTATATCGCTGCTCTTTTGCTGCGCACATTAA
ATTGTTGTCGAATTTCGCTGATTATGCCCCCACTATCCAAACTGATAATGAGAACCTCGGAGATGTAACCAAA
GTTGGAGATGTATCGCTTGAGTGATTTGAAAGTTAAGGTGAAAGGCGACTTTATCCTCGTGCAAGGATCCCAC
GAAGCCAAACAAGACGATCACGATCTGTTTGCCAGCCAATTTGTACATACATATTCTT
TGCCGATCAACGCTAGCGCGTCGGATGTAACAGCGAAACTAACATCAGATAAATATTTAATCATAACAGCTCC
ACTTAACGGTGCGGGTGCTGATGACGGAAAGATAGTAGACAGAGAAGTACCTATTGTAGAAAGTAAAGAAGCA
TGGAAGAGTGAAGACAAAGAAGATAAAGACAAGAAGCCAGAAGATGTGCCGGTTCAGG
CCAGCGGCGTATTGCCAGTGGAAATTGATGAAGCCAAAGGCACCACTGATCTTCCAGCGACAGTAGCTGAGGA
AGAGGACAGAAAAGAACTTACTACCCCGTCCAACCGTGAAGCAGTAACCGTCCGTGATGTACCTAACGAAGTG
AACGAAATCAGCAGCTCTAATGAAGTCCAGCCTTAAGTGAATATTTTAGTATGTATGT
ATATAATATATATAAAACAGTATTGTAATAAATATTTTTAATACTAAACAGTGTTTTGTTTGCGTGTGGGTAT
GCGCTTAGTGCGTTCATTGTTAGCGACCTGAATACAAATCACGTATGGCTAAACAAAGATTTCAATTATGTTT
TTTATCAATTGCACGCATCTCTTGAATTTTGCATGGTTGTTAGAGAAACTAGAACGCA
CAACATTCACATACTTATATTGAATATTTTATTTGAAGAANAGGAAAACATACAACCTGGTATATTAATTTTT
TTTTAAATATATTATATATAAGTATATATGTATGTATAATTTAGCATATAAATCGATGCGTTTTGGTAAATAC
TATCCAAATGCGCCGCAGTGAC
>1002049813_1 Heli.1-EL599315.1.5
ATAACTTCACGAAAATTAACCTGTATGGATCGCTAAAATGTGAATTGGTGAAGGTCACCGTCACGTCATACGT
CGCTGCCAAAGCCAATTGCGATTTGCGTATTTTTCCGCGTCGCGCTGAGACTCGAGCCGCGAGCCGCGAGCCC
TGCAGCAGTGAGTGAGCGTCGCGCCCCTTTACGTAACGAGCCTACGACAACTAGAACA
TAATACGTTACCACCACTTGAGATAATCTCCGCTCGCAGGACAAGAAAAACAAAATGATGTCTACAATATGTG
CGAGGGCCGCGCTCCGCGCCCGCTCCATGGAGCGCTGCGCGTCGAGCGTCGTCGAGCTGCCCAAGTTCCAGGA
CTTCGGCATCTACAACGAGCCTGTGCTGGGGTACCGCGCCGGCAGCCGTGAGCGTTCC
GAACTGATGGACGAGCTGAAGCGCACCTCCGCCGTCACCGAGGAGGTGCCTATCGTTATCGGGAGTGAGAACA
TCAAAGAAGGCCCGCCGCAGTACTAGGTGATGCCGCACGACCACGGCCGCAAGCTCGCCAAGTACTACTATGC
CAGCGAGAAGACAATACAGAAAGCGATCCAGGTGTCGAGCGAGGCACAACGCCGCTGG
GACCGCACCCCTCTAGATGAACGTATCCGCATCTGGCAGCGCGCGGCGGACCTTATGGCCGGCGAGCACCGCC
AGCGGCTCAACGCCGCTACCATGCTGGGACAGTCCAAGACCGTCGTGCAGGCTGAGATAGACGCCGCCGCTGA
ACTCATCGACTTCTTTCGCTTCAACGTCTTCTTTTTGAAGGAGAATGCTAAATATCAG
CCAATCTCCGAAAATCCCTCCGTCACGCGAAATTCGCTCAGGTTTAGAGGCCTGGATGGCTTCATAGCTGCTA
TCAGCCCATTCAACTTCACAGCCATTGGAGGTAATTTGGCATACACCCCAGCTCTGATGGGTAATGGAGTAGT
GTGGAAGCCTTCTGACACTGCTGTCCTATCAAATTGGCGTATCTTTAACATTATGAGG
GAGGCAGGTCTACCTGACGGAATTGTTAATTTTGTGCCAGCAGATGGCCCTACATTTGGTCGCACTATCACTA
AATCCCCGAACTTGTCAGGTATTAATTTCACTGGATCTGTGCCTACTTTTAACTGGCTCTGGAATGAGGTTGG
TAAAAACCTTGATATGTACAAGAACTACCCCCGGCTCATTGGCGAGTGCGGTGGCAAG
AATTACCACTTCATCCACCCATCAGCTGATGTACAATCAGTTGTAACATCTACAATTCGCTCTGCTTTTGAAT
ATTGTGGACAGAAGTGCTCCGCATGCTCAAGAATATATGTTCCTAAG
>1002049814_1 Heli.1-DT663667.3.5

CACGGCTGCCCCCGGCACAGGATACGTCCAGCAAGGCGAGCTCATCGAGTACGCGGGGGGTGCGGCAGGTGGC
CCGGCAGGCCACCACCAAGCCTACCGACGACATCCCCGTGTTCGACCGCAACAAGGTCAGCCTGGACTTTCCG
GGGAGCCTGTTCGGGCCATCCGTGTCGCTCTTCATCCGTACCACTAAAATTATTGGCG
ACGTTATTCAGAACTCGGCAGTACGCTATCAGTCATTCTTACGATTGTTCCGCCCCCTGTTCCGCGGTCCCTT
CGAAATCAAGGGCCTGGACCCTCCGACCACCACCACCACGCGCCGCACCACCACGACGAGGGTCACCACGACC
ACGACGCTCGCACCACCCGCTGACAATGAAATCAATCGTAGAAGATAAACCTAATTTT
TTGCCATTCCAATATGCAAACTTTAAAATATTGAATAAGATGAGTCATTCATTGACTTTAATACTCATATTTT
ACATTAGTTGTTAATGTTATAAAAAAACCGGCCAAGCGCGAGTCGGGCTCGTTCACCGAGGGTTCCGTACAGA
AATTAGAAGTCTCAAGCTTTTTCTGAAATTGGGGCTTACTTTAGCCTAGCCAGAGCTC
TCTGATACCTCACTAGATCTAGCGGCATTAATACCAAATTTTGTCCCCCTTTTAAGGTTATTATTTTTTAGGA
TTTAACCATAAATGTATAGTTTTCATATTTTTTTCTGAATTAGTAATCCCTGTAGCTGCTCACTGCCAAATTT
CATAACTATATAATCATATAATCAACCCGAAGTACCCTCTTAATTTTGATACCCTTTA
TAGTATCGAAATACGTGTTGTTTTGGTATATAAACTACCCTACTATAT
>1002049815_1 Heli.1-CO729537.1.5
CGACCACGCGTCCGCTTGAAGTATAGTGAAACAGATCAATATGTTCAAGTTAGTGGTGTTATCGGTCGTGGTG
GCTTTGGCTGCAGCTAAGCCCAGCCTTGTGGCTCCTCTGTGGGGAGGAGGAATCGTATCTCCATTGGTAGCTG
GTGCATGGGGTGCCGTAGCCCCAGTACCTGTTGGTATTGGCAACTACCGTGGACCGTT
GTCTCTTGCACCTGGACAGCCCGCTAACATCATTGCTGCTGACGGCAGGCCTCTAGATACTCTTGATGTCAAC
GTTGACCGCGCTGTCCACCTCACTGCGAAGGCTCTGGACCCTGCTGGAATTCATCTTCTGAAGAAAAGGTCCA
TTGTTGCACCTTTGGCCCCAATCGGTGTACCTCTCGCTGGTACCGTCATTGCTCCCGG
TCACATCGCCGTGGCCGCCCATGCTCCTGTTGTAGCTGCAGCACCAGTAGTTGGTTTGGGTCACGGTATCGGT
TGGGGACACCTCTGGTGAAAACACTAGTATTTAAGCTTCGATCTAAAAAATCTAATGAAATAAAGAGAGTT
>1002049816_1 Heli.1-DT665109.3.5
GGCGTTTTGTGTTTTGTGTAATTTTATTTTATTTTTAACTAATTGAACCGTAACCCGTGATTTGTTTGTTTGT
GTGTTTTATTTACAAAGAAAATGACACGTAAAAGCCAAACACAACGCAGCCGTTCGTACGAATCAGACGAGCA
CGAGAGGGGTGCTTGCGAATGGTGTGAATATGCCTTGATTATCACACTAGCTACGATC
CTCCTGATAGGAGTGTCGGCTCTCACTATATTCTGGACCTTCTTCTATAGAGAGGGTTATGGATGGGCCGACG
ATATACCCGAGAAGCAGTTCAATTTACATCCAACGCTTATGGTCGCCGGCTTTATTACTTTTAGTGGATTCTC
GGTACTCCTCTACCGTATCTGCCGGTGCTTCAGACGCATCTACGTGAAACTATTACAC
GCTATATTCCACGCGTTGGCGTTTCCTTGTATTGTCATCGGTTTCCTTGCTGTCCTCGACTTCCATAATAAGA
AGGGCATCAACAACTTCTACTCGCTTCACAGCTGGATCGGTTTAGTGGCTATGGGACTGTTTGGAATTCAGTA
TGCGGTCGGCTTCTTCAGCTTCCTTTTGCTATTGATCTGTAATAAGGGAACAGCCAGT
TTCCGCGCATCTTTGGTGCCTATCCACGCCGCGTTTGGCATCTTGACTTTTGTTCTCGGAGCCAGCGCCTGCC
TCACCGGACTCACTGAGAAAGCTCTCTTTACACTTGGCCCGGAACGGTACAGCGGTCTCCCGGACGAGGGTGT
GATCCTGAACGCGATCGGCGTCACCATCGTTGCCATACTGGCCGTCGTCATATACATC
CTGTCCACGGACAAGTTCAAACGA
>1002049817_1 Heli.1-DT663000.2.5
CAAAATGTACAAATACCCTTTGATATTCGCTCTTTTGAATCTTGCCATTTGTGAAGAGAGATCACATAAAACA
CACTATGTTGATCCCTTCGCGCTGATTGATAAGCATTTGTCACACTCCCTTACTTATCACTATCTGTGGCCTT
GGGGACAATTGGTGAAAGCAGCAGCGGCTTTGGACTTGGAAGAAAGCTTGGAAGATCC
AGAAATTATATCTGATTCTGAGAAATATCAGATCAGCTTGAATGTGAGGCGGTTCAAGCCTGATGAGCTGAAG
ATTAAAGTGAAGAACCGGTACATTATTGTGGAGGGAAGACATAAAGTAGATAGTGACAACAAGTTCATGGCAA
ATCACTTCGCTCAGCGTTTCGTTCTGCCACCGGGTACCAAACAGGAGGAAGTCAAGGC
TGTATTAAATGAGAAAAGTATTTTGATCATCTCAGCACCAAAACACGAACTTCCACCGCCGATTCCAGAACGA
GAAGTGCCTATTGAAGTTATTTTGCCCGAAAAGAAAGTTGAGGAGCCTACAGAAAAAGAGGACGGCAAGGAAC
AAGCATCATCAGTTGCTCCTATTGAGCAGATCGAAACAGAAGCTACTACCCATGTAGG
CAAAATAAGGAAAAAGGAATTGAAAACGACCACTAAAACTACTAAGGATAACGAAGTTACGAAAGGTGTGGAT
GGAAATGGGCTGGACTACGCTTTGATTGAAGCTGAGGAATGATTGTAAGATATTTTATGGAAAAGACTGAATA
AGCTTTAAAG
>1002049818_1 Heli.1-DT668508.2.5
GCACGAGGGGCAAAATGGCTTTTAAGTTCGTCGTACTCGCATGTTTCGTGGCGGCCGCCAGCGCCGGTCTCGT
TCCGGCTCCCGTCGTCTACTCTGCCCCGGCATACCACGCCGCCCCCGTGGCGTACGCCGCCGCACCCGTCGCC
AAGCTCGCCTACGCCGCCCCGGTCGCCAAAATTGAGGAATACGACGCCCACCCTAAAT

ACAGCTTCGCTTACGACGTCCAGGACTCCCTTAGCGGAGACTCCAAGAGCCAGCACGAGTCCCGCGACGGAGA
TGTAGTACAGGGCTCCTACTCTCTTGTTGAACCTGACGGTTCCAAGCGCATCGTCGACTACTCTGCCGATCCC
CACAACGGATTTAACGCGGTCGTACGCAAAGAAGCCGGTGTAAAGGTTGTTTCTAAAG
TAGTAGCACCCGTAGCTTACGCCGCTCCCATCGCTAAGGTCGCTGCCGTCTCCTATGCTGCCCCTGTGGTCCA
CGCCGCCCCCGTGGTCCATGCTGCCCCCGTGGCGTACGCAGCTGCCCCAGTAGTGCACTCTGCCCCATTAGTC
CATGCACCACTTGCCTACTCCTCCCCCATCTACCACCATTGAGTTCATTATTAGTTTT
AAAATAAACATATTTATTTAAAAATATATGTATATTATTCATCATCCTAATTAGAAATTA
>1002049819_1 Heli.1-EL597724.1.5
GCACGAGGCCTCGACTTACGTAGTATAGAATTACATCCGTGAGTGGAAAAACTTTTAATATTAACAAAAAAAA
ATGTGCACATAGTTTTTTTAAACATAACACAAAAATGGGTTCTAAAATAGAGTACGTTGATTCATCTCATTTA
TACGCCACAAACTATGTCCGGAATTCGAAAGCTATCGGAGTGCTTTGGGCTATATTCA
CGATTTGTTACGCGATTATAAGCGTTGTGGCGTTCGTTACACCGGAGTGGATAGGTGACTTAGAGACGGAGTA
TCCCAGGAAGTTCGGCCTCTTCCAAATATGTAGGACGGATGACTCCATCGAGGACTGTAAAGGCAGGTTGGAT
GACTTCCTGTCGATCAATGGTTTTGTGTTCAAAGTGGCCACCGTGCTGGTGGGAGCGG
CGGTAGCGCTGGCCTTGTTTACCATCTGTGCCATACTTTTGTTTTTCTTCTGCCAGTCGACCACTGTGTTCCA
TATATGTGGATGGTTGCAGCTGATTTCGGCTATGTTCATGATCGGTGGTGTGGCGGTGTACCCCGCGGCGTGG
AGCGAGACCGCCGTGCAGGAGACCTGCGGCCCGACCGCCGACCAGTACAATATTGGTC
GCTGCCACATCCGCTGGGCATATCTTCTGGCGATCATCGGATGTTTGGACGGCGTGGTGCTGGCAGCCCTCGC
CTTCATACTGGCCACGAGACATGTGAGACTACAGCCCGATACCTCGTACCCTGCCGATTTGTATAAGGGTGAA
GTAAACAACGCATACGTAACAGATGCCACCTCGGTCTCAGGTTCCCGCAAGTCCCTGG
CCCTCCAGCCGGTACTCTTGATGCATCCTCACGCTCCTCTCGACGTCGACACCTACTCGCATTACTCGGGTCG
TACTGCAAGATCTAAACATGGTATTTATGCAAATACAATGCATAATTATCAGTTGTAGAATTATTATTTTTTA
TTTTTTAATTAGATTAAATTTAAATGCGCGGCGTTGAACTCCGCCGCTTAACACACAA
TGTTTTGATTTGGTGAAGAACTCAGTAGTTAATACTGATGCACTTATATTATATTACTCCGACCGCATTACGA
GGTCTAACATGGTATTTATTTTTGTGGTAATATAATGCATAATAATCGGTCGCAATATTGAATTCACAATGTT
ATTATTATTT
>1002049820_1 Heli.1-DT666080.3.5
AGCAACAGGAAAAAGTAGAAAAATTACATGAAGAGTTTATAAATAAACAAGCAACAAAATCCATACCAACTTA
TACAACGACGGAAAGTTACGAAGAGTTCATTAAACGAACTCAAGAAAAGCGAAGACCTTTACATCCACATGAA
ACAGATTTATTTAGAAAAGCTGTTGAACTTTATGAAAAAGATCACCCGACTACAACGA
CTACGACGACGACCACCACAACTACAACAAAACGCCCGACCACTGCAAGATACAGAACTCGTTCACCAACGCG
AACCAGACCTCCTCCTCAAGATAGGAACAAACAAAAACTTTATAATGAAATAAAAAATCTTCTCGAAGAAAGT
GACACTAAAGGTTTTGATGATAACTTAAGAGCAAAGAGTGTAGCACTTTTGCAGAAAC
CAGACATATTAAAGCAACTTAAAGTAGCTTTGGCGGAAAATGCAGAAGATTTTAATGAGAAAAATTTTACATC
TCGTGAAATTTCTTTAAACGGACAGAAATTTGAAGTAATAAGAACCACAAATCCAAATCTTATTCCACAGGGA
GCGATTGCAGCAGATAGTCCTGACTTAGGTAAACTTATAGCTGCTACTCAAGAAACAC
CTAAGGAAAGTCGCATTTCTTTCGATGATTTGACAAAAGGTGTCCTCCCACCTGGTGCCAACTTCGAATTAAT
CAAACAGGCAGATAATGGAAAATTGGAAGAAGTATCTAAAATACCAAATACATTACAAAACAAAAAGAAAGTT
ACTTTCGTTTTCCTTGAAGAACAAGACGATGGTTCATTTAAAGTAAAAGGTGTGAAAG
CCAATGGGCAACAAACGGAAGAGGGTCCTGAAGTGGAAGATATTTTAAGCAAAATCAAGAAAGGTGAAATACA
ATTGCCCGGGCCAACAAAAATTTCCAACAGTGGATATACAACTAGCACTCCAAGTCCATCCACTTATACAACT
GATTATATAGCGGAATCATCACATCCTCCTTCAACATACACAAGTTTCGTGACAACCT
CTAATTACGGGGATTCTGGACACACTAACCCTATGCAAACAACCCCTGCACCGACATATCGCACGATAATACC
ATCGACAAAACCTACTCCTCTGGCTCAAACATTCCCCTCTAGCACACGAGGAACTACAAGTGAAAGATATTAC
ATAAAATCTTCTACACCAAACTATATTAAAAATAGAAATGGTTACAGTTCCCCGTCAA
GAGGCCACGTCCAAAGTAGTACAGTATTAAATACAACACCTGTTTACTCAACATCTTCATTCCGAACCACACC
TCAAGACATAGTCGTTATAGGCTCGAGCGCCGCCCCTAATTTTGAAGAGTCTACCATCAATTTAAGCTACGCC
AAGACTGGCAATCCAGATCTTATAGATATTTTGAAAGAAAACGGTCTGTTTGCAACCG
CTAAATATCTGAAACAGTCAGGTTTGGATGCGATATTAAATGAAACTGGGCCGTACACAATATTTGCACCGAC
AGACAAAGCATTTAGAACGTTGCTAGTTCAATTGGGTGGTCCCGATAGAGCAGAAGAGAAGTTCCGCGATAAC
CCAAGGCTATTGAGTGGGCTTCTCCTCCACCACGTGATCCCTGGTGCTTTCGACATCG
GCTCGTTACAAGATGAGATGACAGGGGTGTCCCTCGCGGGCACCCAGCTCAGGGTCAACC
>1002049822_1 Heli.1-DT665209.2.5

GCACGAGGTGTCACTATTATTTCGACAGTCCAAGATGAAGCTTTGCTCAGTTGTATTCAGTCTATTAGTGGTG
TGCGCGGTGAGCTTCGCCGTGGAACATGGCCCGTTGATCAATGTACTGCAACCCGTTGACTCCCACGACTTAG
TCCAAATTGAACCTGTACAAAATATTAGACCGAAGAGGCATTTACTTCTAGGTGCCGG
AGCCCTAGGTGCTGGTTTAGTAGGAGCTGGAGCCCTTGGAGCCGGTTTATTAGGAGCTGGTGTGATCGGTGCT
AAAGCTGGACTTATTGGCGGTGCTTTAGTAGGCGGTGCCCTGCAAGGCAGAAGCTACGGAGGTGGATACGGAT
ACGGCGGCGGATACGGATATGGCGGCGGTTATGGATATGGCGGTGGATACGGATATGG
CGGTGGATACGGTGGACACGGATACAGACGTTACTACCCTACTACAACATATATCGTAGAAGAACCCTGGTGC
TAATTTCAACGTTGCGTCGTAGTTGTTACGTAATCAATTCCAACGTGTGTAGAGATGGGGATATCTCGTGACT
GATACCTGCACGTTTAGTTGTTAAGAATAAAATTTTATAAAATAAAAAAAAAAAAAA
AGGTATTTGAAACCTCTCTATTTC
>1002049825_1 Heli.1-EL602296.1.5
GAAGGATGCATGTTTTAAATGTTTACGAGAAGTTTTAATTTATAATTAACATAAATCTAGTTATATTTAATGT
ATATAAAAAGCATGTGAGATTGTGTTTTCAATTGTGATATAAGTATTTGAGGTTGCTAAGATAAGTTATAAGT
GCTTTCACTATTTACGATTGTTTGTATTGAAATACTTCGTCGGTAAGTTTTTAATAGA
TTATTATTAAAATAAAGAAGAGTGATATTAATGCATACAATGCCGTCAATATCCAGAGATAAATATTATTTAT
ATAGTAAATGGAAATAAATACATGAAGATGTTTTATGACGTATATAAAATTACAATTATATTAATGTACGCAG
AATGCCTGCCGCCATCTTCGTTAGAAGCGTGTATGGGAATTGTGAAATGAAAGTTAAA
TAGGTTTGGTGAATTATAGTAACTTATAAATGTAATTTCTATATATATTAATAAATATTTAACATGTTACTAT
AAAAGGAAATATACATATAATAGTATAATTTATAATATGATATAAAACTATTAAATCAAAATATATATTTACA
ATTTATAAATATTATATAAATATCAGTCTGTTTAATGTCCTACGATAAAAGTAATCTT
TCAATATGTTTTATATATTACTTTAATTAAATATACTAGACATTAGACATTAACCAAATATTAATTATTTAC
>1002049826_1 Heli.1-DT663601.3.5
GCACGAGGCTCAAACTTAAAGAGGACCAAGAAAAGATAGAAAAAGAAAAGGTAGAGAAAGAAAAAAAAGAAAA
ACTGAGAAAAGAGCGAATTGAAAGAGGAGAAGATGTTGAAGATGAAGTGGAAAGCGATACTGAGAACAAAAAA
GCTGAAGAGCTTGCTAATGATAAGAAACTGAAAGAAGAGAAAAAACAACGCCGAAAAC
GTCGAAATGAGGATGATGATGACTCTTGGGATGGTGAGTCATCCAGTGAATCTGAAGAGGATGGTCTCAGTGA
TGATGAAACTGTGGTTATTAAAGACAGAGATGATGTTATAGACGATGTCAAAGACCCAATTAATATGGTGATT
AAAGGTATTGCTGGTAAAAACGATAGCTACTTTGACTGTTCATTAGGTCAATTTTTTA
TAAACATAGGTATTAATTTAGTGCAAGAATATGTCCAAAGTGATTTGCTAAAAACACAAAACAGAAAACTTTA
TAAAGAAAAGAAATCTGGTCGTAGTACTAGAGCTACTGAAACTGCTATAGCCTCTTTGACTCAAAATCTAGCC
TACAGTAAAAAACTAAATGCTCCTTATGCTCTTGAGCAAAAACGATGTGATTTTTGTA
GTTTTAAAACTGAATCTATTTTAGTTATGGCTCATCATTTGGAGGCGCCTCATATGAAAAACAATATATATAA
ATGTAACACATGTGCATTTGAAATACGAAGTCCTCATGATATTCTGT
>1002049827_1 Heli.1-DT662629.3.5
GCACGAGGTTGGCTCCTTGCATTGCTCATTAAAACAAGTAGAGTAGAAGCGTTCCTTTCGTTTATTATTGTGA
TTCACTTTGGTTTATTCTACTTTTAGTGTAACTGTCTACATTTGTTTATAAATTAAATAAAATAAAATGCGCA
AAAGGAAGTCCCTTAAGAAAGCAGACGACGAATCCGACGCTTCGGAACCTGGAGCCGA
ACCAGAAGAATTTCAGGACTCTGGCGAAGATTGGACGCCGGATGCCGATTCGAATGAACAGCCAACCCGTGGT
GGCAGAAAAAGGACCTCAAGAACATCATTAAACAATTCGAAAAAGAAACGCAAAAATTCATCATCCGAAGAAA
GTGAAGGTGAAGCTGAGGAAGATGAAGAGGGAGAAGACGAAGAAGCTGAATTAGATGA
AGATGAAGGTTCCGATGATGAGAAAAATGGCTCTGATGGAAATAAGTCTGACTCCAGTCAGTCAAAAGACATA
CCAAAGCATTTTCATTCTGGAAATTTTGTTTTGTTAAAATCTGACGTGAAATGGGATGGGGACACAGTCATTT
CAAAGTTGGATGAATTAAATCTGTGGAAAATAGATGGAAAAGCTTTACTACAGAAATT
CATTCCTATGGAATCAAATGGAAAAATTTTACATAAATGTACATGTGTGTACTCTGGATGGAATGTTGACAAC
CGGGACAATTACTACCCTATAACTGATATATTGGACAGAAATCCACGTACAGACTCTAAAGAAATTTGCGTAG
CTTTAGATCTTAATGATCTTATTAAAGTAAGAGA
>1002049828_1 Heli.1-DT662299.3.5
GCACGAGGTGACACTGTCGAGCGGAAGGTCGTCGACTCAGCGGCTGAAACTCGTGAATTACAAACATTACTTC
AATACCGCACTATCTCGTTGTTACATCATATTGCAATCGAAATCAGTAATTAGACTCCAAGAGTCCTACATAG
GCGGTCGTGTTGTGCATTAAATAAATAAAAATGGGTTCACGTTTCCAAGTCGTCGAAC
AAGGTCCACCTATTGAAGTTTTCCAACTTAACAAACTTTGTACTGAAGATACATTTAAAAATAAAGTAAACCT
TGGTGTGGGGGCCTACAGGGATGAAAATGGCAAGCCCTGGGTTCTGCCTGTTGTCCAGAAAATGGAGAAGCAA
ATGGCAGCTGATGCGAGCCTGCTCCATGAATATCTGCCAGTACTTGGCTTAGAACAAT

TCAGCAAGGCTTCAGTGTCTATGCTTCTGGGAGAAGACAACCCTGTGATTGCCGCTGGCCAAACTTTCGGAGT
CCAAACACTGTCGGGAACAGGTGGTCTCCGAGTCGGCGCTGAGCTTCTAAGCAAACACTTGAAATACTCCACA
TTCTACTATTCAAATCCAACATGGGAAAACCACCATCTAGTATTCGTAAATTCCGGCT
TCACCCAGCCGAAGACCTACCGATACTGGGACGCCAAGAACCGCGCAATAGACTTCGAGGGTATGATCGAGGA
CCTGAAGAATGCTCCAGAGAATTCCGTGATAATACTCCACGCGTGCGCTCACAACCCCACCGGTATCGACCCC
ACTCAGGAGC
>1002049829_1 Heli.1-DT667570.3.5
GCACGAGGCCTTTCAATTTCTTGAGTACGATAATTTATTACGAACCTGTAACAGTTGTTACTTATAAATAAGT
AAAAAGTGTGTATTAATTAATAATTATGGTAGTTTTGGAAGGAGATAATGGGGACGCCCAGCTATTAACTGCA
GATAATAAACAAGATGGGAAAGTAGCCAATCCTTTATCATTAGAAATATTTCGTATAA
CTAAGGATGGTCAACAGCAGCATGGTCTTCGTCATGGAGATTATCAACGTTACCGCGGCTACTGCTCGCGGCG
TATTCGCCGTTTGCGCAAAGTGCTTAAAATTCCACAGGGAGACAGACGTCACTATCGTCGGCGTGACATAACA
TCATCTCATCTTACCGGCACCAATGCTGAGAACCGTTTGCTTTGCGTACCCTTACTCC
AGGCTGAGAGGGCCTGGGCCCATGCAATGCAATTACGGCAAGAGGCCAACACGGAGCCCAGAAAAAAGTTCCA
TCTTGTATCCCGCTTGAAGAAAGCTTGCGCTCACGCTCATATGCTTTTGCAACTTTGTGAGGAAAGCGGGGCC
TGCAACGCTCGCACACAACTGGAGGCGGGCGCGTACTCGGCGTGGTTAAACGGCGTTT
TATTACTAGAACTGCAGCAATGGCGTCCGGCCGCTGAGAGCTTGCAGAGGGCTCAATTAGTGTTGGAGAAACT
GAGTGCCGCTTTGCCTGAAGAAGAAAGAGTCATTT
>1002049830_1 Heli.1-DT668826.3.5
GCACGAGGGGCCCATACTAAACGCTATGACGGCGCCAGACGACGAAATGTTCATGGACCCTAGAAGCATGAAC
GATATGGTTTCTATTGATACTGGTCTACCTCTTGTAAAAAGAAGAAAAATTGAACAAGAACAACTACAACAGG
CTCTGAGAGGCGCCGGTCACGTCATCATCAAACGAGACCCGGCACAAGGCGACCATTT
GAGTGATTATGCTTTCATGGAACCAGACCACATTGGCAAAAGGTTCAGGCGGAAAAGATCGACCGAGTCCCAT
AGCAGACAGAGGCGTGAAGCACCATATGTGATATACCCAGAAATTCTTGTGATAGTAGACTATGATGGTTACA
GATTGCACGGAGGAGATAATTTACAAATTAAAAGATACTTCGTTTCCTTCTGGAATGG
CGTCGATCTGAGATATAAACTATTGAAAGGACCCAGAATAAGGATATCAATTGCCGGTATTATTATATCTCGG
GGTCGAGACGCGACACCGTATCTAGAAAGGAATCGTGTAGGACGAGACGCGATAGACTCAGCGGCCGCGCTAA
CCGACATGGGCAAATATCTATTCAGGGAAAGAAGGTTGCCCGTTTACGATATAGCTGT
CGCTATTACTAAACTAGACATGTGCAGAAGACAGTATGCAAACGATGCGTGCAATAGAGGGACTGCAGGATTC
GCGTACGTCGGAGGTGCATGCGTGGTGAACAAACGATTGGAAAAAGTCAACTCAGTTGCTATTATTGAAGACA
CTGGTGGTTTTTCTGGTATCATTGTGGCCGCACATGAAGTTGGACATTTGTTAGGAGC
AGTACACGATGGCAGTCCCCCACCCTCATATCTAGGAGAACAAGAGCTCTCTACTCTACGTAGTTTAGTCATT
GTATAATCTGTCATTTAGATCACAAAATCATAAACCTCAAAATCTATTTCTGGAGACCTTCGACTTTATGACT
CACATGCCCAACATTACCAATTGGTACTTTCGTAAAGCATGCGATGGATTCTTTACTT
ATCAACTAAAACAAACGTAGCATTTAAAGTGTTGCTTTATATATTACC
>1002049831_1 Heli.1-EL599535.1.5
TAACTTCAGACATTTAATATCTGATAACAAAGTATAGTGAGATTATAATAGATAAAAAGTATTGTGTAATAGT
AAATAATGCATCTATTTATCGCGATAGCGACAGTTGTGGCCGTTTTACTGCCGGTGGACGACGCGCTTGCAAC
AGACATGGCTAAAAATCAAAAAGTACTCGTATCGTGCATCGATTTTCCAGAAAAAGCA
TTGAATTTACTTGGAGAACATTTCACAGTACTCCAAAGTAACTACGTAAATGTCGAGAAAGGCATCGTCACGG
TAAACAATGAAGATCTACTGAAAAAACTACCTGGAGTATCCGCGTTGATCTGGGCGTCCCATCTTCCTATAAC
TAAAGATATATTGGATACAGCAGGTCCTCAATTAAAAATCGTAAGTACAGCATCAGCG
GGCTACAATCACTGCAACTTACCAGAACTGAAAGCGCGAGGAATCAAACTGTCGAACACACCCAACGTGCTCA
GTGCTGCTGTCGCTGAGGTGGTCGTCGGTTTGCTGCTCGGTGCGGCGAGACGGTTCACTGAGAACCTGGAGAA
AGTTCGTAGTGGCGAATGGGAAATTGGATTTGGTAAAATGTTGGGGCAAGATGTTCGC
GGCAGTACAGTGGGCATTGTTGGCTTCGGGGGCATCGGGCAAGCCGCCGCGAAGAGACTGTCCGGGTTCGAGG
TCGGCAGGTTC
>1002049832_1 Heli.1-DT661662.3.5
GCACGAGGTCGAACCCTGAGATCGGCGCTGCGCCGCTTAATTCGTATTGCCGTTGCCACCCACTCCGCTCGCT
TGCTTCAGCGCCTCCAGTCATTCAGTCCCGACGCGATCGCCGTTTTAGTCACACGTGGTCCTCGCTTTCTCTA
CGTGCAGTCCAGTGTTCAAGGCGAAACTGTTTCGACACGGCATTCGGTTGCGAAAGTC
TCGCGATGATATACAAGTGAGAGCTACCATCGGACGCCAGGGTAACGCTCATCGCGCACGGTGAGCCAGTTTG
TTACACGAGAGACATTCAAAACTTGCCAACCAAGGGACGGGGGGCGCACGACGCCGTCGGGTCGCACACGCGG
GAGCAACGATTCCTACGCATACTTACTGTTAGAGAGCCACGGAAACCAATTTTTAATG

TGTCAAGTCAGTATAGCTTATTGCTGCTACGTCAGATCGCAGAACGCCTCGAGTAGGAATTACAAGGTTATGC
AAATGAGTTCTGAACGCTTCTACACTAGGGGTAATCAAAAGACCATTCATAATTTTAATAAACTTTAACGTTC
GGTCCCCATACTGTGTACTGAGTGCTTATCTGTATCGTATTTCTATCCTTCATTATCG
TTCGGTGTCGCGATCGCTAATTGCTAATTACTTACCTGGAGTCGAGTGATAAGGCTCGAGATCGGTGGCCGAG
TGTACGACGTACGCAGATCAGCACCAGCACGCGGCGGCGATGGCGGCCTTGCAAGAGGAGACCCTGCTCTC
>1002049833_1 Heli.1-DT668172.3.5
TGGCGAGCAGGTCGAAAGTGAAGTGAAAGAGCAACTAGAAAATGTGTTTAAGAAAATGCAAGAGATTGTGGAT
CGCAAATCAAACAAAATCAGTAGTAGAGTTCGTTTCATGATCCAAGATGTCATTGAACTCAAGAAAAGGAAAT
GGGTGGCTAAAAATGTTGTAGATTCCCAACCTAAAATGATGGATCAGATTCAAAAAGA
AGCTGAACAACAGCAAAGACATATTGAGTTGATGAATGCGTCACCAATGGGCGGTGGAGGTTTCCGACGTGAG
GATGGTGGTCGCGGCAAGCGTGGAGGTGAGAGACGCCAAGGGTCGAACTCCTTTATGGACAACCAGTGGAAGA
CCTCGCGCAGTAATTACGTTGTTGACACTTCGAAATTAAAGGCTGTCACACAAAAGAG
TCTTAGTAACATTAAATTGGCCCCAACAAACTTTGCCTGGAATCAAGGCTCGGGTACAAAGACTCCAGCTCAA
ATAAGCAGCAACTCAATGATTAGCATTTCCAAAAATATGTATAGTGTGCTTGAAAATGTCCAAGCTGATCCTA
CATCTCTCAGAGCAAACAAGGATCTAACACCAAGTTATCAACATTCAAAGTCGATTGA
GAGATCGACATTCAATTCCAGAGGTGATTTCAATAGTGGCAGTGGAAGTCGCTCAGGGTCTATGGGACAGACG
CGCTCCAACTCAAGTAGCAGAAGTTCGAATGCTACACCGGCACCTGCCCCTGAACCAGTGCCCTCTGCACCCG
TGGCTCAAGAGCCTCTCCCTGAGGCCAAGAAGAGGATTGTTAAAATCATGATCATGGA
AATTATTGTTAATCCTAATGATGAAGAATTGGTTATAGAAGTCAAACAAACCTTCCCTGCTCAGTACCATGCA
GCAGTGGTCACTGAAGTACTTAATGTTGCCCTTGAAAAGTCAGCAAAGGAAACTATGGCAATTGCGAAAGCAC
TTCACCACCTTGTTTCAACTGGGACAATATCATCTGACAATTTATTGGCAGGATTAAA
TGAAATCTTGGAGTTTGCGCCAGATTTATATATAGATATTCCTATGTTATATGATTACTTAGGAAAGTTTATA
TCACCTCATATTGAAAAGAGGCATATCACATTTGTACAGATATTTAGGTTGTGTGAACAAATCGTCAAAGCAA
ATCATGGACATATGCTCCTAAAAGCCATTATTAAAGAATTGAAAGAAAGCATGGGACC
TTTCTTTGTTAAAACAAAGTGGCAGGAATCCAACTTGGAATTCAAACAATGGATGGATGAAGAACAGGTTCCT
AAATGGATAGAAGACAATAAATTAGAATTTATGGAGGGAGGTGACGCCATCGAAGATACGAAAAAGATTATGA
CCCCAAGTGAAACACAAAGCAAATTACTCCAACTCATGAATGCTGATGAGAGCTGTGA
CTGTATCCGAGGATGGGTGCAGGATAACCTCGGCGCATCGTCCAACGAGGACTGGTTCATGCGTGCACATATT
CAAGCGATATGTGAGCACGCGCTCTACGGCAACGACGGCCGCGATGTGCTGCACTTCAGCCACGACCGCATGA
ATAAATATTCGTCGCTGATCAACGAGTTCGGCGAGACCCGCGAGCAGCGCGAGGCCAG
CTGCCTCTTCGGCATACAGCAGCTAATACATAGACTGGAACATCCACAAGGATTAACACTAGATATATTCCAA
TACTTGCATGAACAATATATAATTAGTGTTGAAGGATTCATAGCGTGGGAAGTATCTGAAAAGGAGCCCGAGG
GAAAAGCCGTTATGCTAAAGGCGCTGACGTCGTTCTTCACGAACAT
>1002049834_1 Heli.1-ES584880.1.5
CTGCAGTACGGTCGGAATCCGGGTCGACCACGCGTCCGTGATTAAAAAATGATTGACAAGATATCTGTAATAA
TAGCCTGCATGGCTATTTGTAAAGCTGGAGTTGACGCCCAGGGAATTTACTTATCTCCTGGGCGGAATCAGTC
AATCATCACTCAATAGAATTCAAGTTTCCAAAGAACCTGCACCTATATATGAACCAGA
AGACGTGGAAACGAAGACCCAAGCTGATGTAGTTCAACAGTCTATTTACCTCAACAGATACAATAATGCCATA
TTATCCGCTGTTCCTAAAATTTCAGCACAAAATGGCCAATCACCTGTCTATAATACTTTATACTCTGTGCCAC
AGTCTAATATAAATGGTTATCCTAACACTATAGCATTGTTAAATTCGTATTCAAATCA
AAAAGCTTTATCGCAGCCCTACGCAGGTGTACTTCCCAGTAAGGGGGTTGGGGTTCCTTTAGTACAAAATGTC
AAAACAACTATCTCTTACTCTGACGCCCCAGCCGTATCTCACACTACATTTACCGGCCTCGGTACGACTTACA
CCTGGTAAATATTTGAGATTCTAAGTTAGTTTTAAGATTTCATGGTATGCGCGACTGA
CGGTATAATTAAGCTTAATTTATTACACTACTTTAAGAGTTTAATTGGATTATTAAGTTATTAATTATTTAAA
CATTATCATTA
>1002049835_1 Heli.1-ES587572.1.5
AAAAGATCAAAATGCCAGAACTTACAGAATTGGACAAGGCCACCGCCTCAATGACTGAGAAACGCAAGGAAGA
GACTGCATCATCAGACAGTGACTCTGATGACACTATCCCAGAATTAGAAGATGCAGGAGCGCCAGGTGCAGGT
GGTATCACCAACCCAATTGGTGGCATTGACATTGTATCCAAAGCTAAACAATCACGTG
GCGAAAAGAAAGCCCGCAAGATTATGAGCAAACTTGGACTTAAACCGGTGCAAGGTGTAAACAGGGTAACAAT
AAGAAAATCAAAGAACATTCTCTTTGTGATAAACTCCCCTGATGTGTACAAGAACCCCCATTCGGACACTTAC
ATAGTGTTTGGTGAAGCCAAGATTGAGGATCTGTCACAACAAGCAACCATGGCTGCCG

CTGAACGTTTCAAGGCTCCAGAGACGGCAGCGGCTGGCAATGATGCTGTGACGGCTGGCACTACCGTCGCACC
CATAGCAGAGGAAGAAGACGAGGAGGGAGTAGATGAGACAGAAGTGGACGAGAAGGATGTAGAAATCGTCATG
ACCCAAGCAAACGTGTCGCGAGCGAAAGCAGTCCGCGCCCTCAAGAATAACCAGTCGG
ATATTGTTAATGCTATAATGGAGCTGACAATGTAAACATTGTAGTCACAAGTGTTATTTTATGTTCCTAGCTT
TAAGTCGGTTAAACGGACATATGTCTGTCCTTACATCAATATATTTCAAAATCCAAAGTCAATCTCAGTGTTG
AGTGGAATTCATGATGGATAATGGGATTGTATATAGACATATTTACTTCAATAAAGTA
TAACTGGTAACT
>1002049836_1 Heli.1-EL597307.1.5
GGCGGCACTACAAAGTTTACCTTGGCTCTTTGTGATTTTTGCGGTGAATTTGGCTGTACCAACTCCAGGAAGA
ATACCGAAGGTGTACAACGCTTTGATCACGTCTAATCAAAACTTGGAACCAAGCAAGGCTTTTCCTGTATACC
AACCGGTGTTGCAGAATGCCTTTCCATTTCCGCTGCAAACGGTCGTCTACGGAGATTA
TCCATTAACCAATGGTCTGGTCCCAGTTCCTGCCGTGCCTCCAAAGGATGTGAATGCAAAACCTGACGAGACT
CCTTCATCTACTGCAGCAGTTCCATCTGAGAAGGCAGCCACAGAAAAAGCGCCTTCACCTTCTCCTGAGCCAA
GTTCTGCTCCAGAGAGTGAAGCGACACCTTCACCACCACCAGTTCCAAAAACTGAGTC
GCCAATACCTCTTAACGAATTTGGTTTACCTCCGCAAATATTACCTCTCGGTCGTATAAATCCAGGGTACCAG
TACACTCAGTTAGCCACATATCCTTACACTTATCCAGGACTGAGGTTTTACGATCCTTATGATCCCTTTGGAT
TTCATACCTATGCAAACTGGCCTCTGTACCAACCACTTACAAATTTCATAGGACAAAC
>1002049837_1 Heli.1-DT667094.3.5
CTCTTTTATGATAAATACAACATGAAGTACGCGGCGCTTTTTGCGCTGCTGGCGGTCGCCTGCGCCAGGGACA
GCGATCCTACAGCGGTTGACAATTTACGCAATAAGTTTTACGCGCTGGAAAAACAATTATGGTTGAACGTGAC
GAACCCTGAGTGGAGTCTGGGAGGCCTTGGAGGAGACGTCGAGCTAACAAAGGCCTTT
GTGGCTTTCGATGAGCAAATACAAGCGGTGCCGCCTCCGCCGCGAGTACCGTTCGAGACCTGGCTTTGGGCGA
AGGCGACGGAGAAGTTGCGGGTCATCGAGGGGTACTACAAGAATTTCATTATATTCGTGAGGCGTCAAGCCCA
GCCCGGCATCGTGCCCGCGCCAGTGCGCGAATGGCTGGACCTCGCCCAGCAAATGATG
GATGAGAAGTCACCTGTTGTGCAGGCCACGAAAAAGATCACCGATATGCTAGAATTGGGCGACATGTTCCGTG
GTGTTTTGCAGGAGGAAAATTCAGACCTATGTGATCTTCAGCTGTCGCCGCATCAGTTGATCTACGATATGTA
CAACACCATCTCCCTGACGGAGATAAAGGGGTACGCCATGACGCAGTTCTCGTGGATG
CTGCTCAGGATTTACGGCAAAGGAAATTACACGCAAGAAGCAAGTTTGACGCGTCGAAGGTATGGAGAAAGAA
CGACTAGGACGGCGGCCGCTGCTCGATCTGCTTTGGCTATGGCCAATAGAGGTTTATACCGGTGCGACCCGCC
TCAGCATAAACAAGGAGAGACA
>1002049838_1 Heli.1-DT666777.3.5
AAAACATGTAATATTTTGACTAACACTATCTAAAACTAGCATTAATAAGTATTGCTGCAATATTCTAGAAAGA
TTTGTAATTAAGTAAGATTTTGTGAGAAATATTGTGTGAAAATAAACTATATATAGGTTATAAAGTGCAATGA
ATAACAATTGGAATCGTAAACCGCGCGGGGCGTATAGAGGAACGAGAGGATTTGGTAG
AGGACGAGGTTCTGGAGGCTTCTTCCCTAATAGAGGCTTTAGAGGAGGCTTTTCCCGTTTTGATAGAGGAAGA
GGAGGATATCATTACGATGGATATGTCAATAATCAATGGACACCTAAGCCTAAACCAGAAATGCCCTCTAAAA
GGCTTTCTGAACAAGATATTTATGTTACGGAGTTCATCAGCGAACATGAAGGTTTCAA
TGGCATTATTAAATCAAGATTTTCAGATTTTCAAGTGTCAGAAATAAATTTAAAAGGTGAAGTAGCCAAGTTA
ACCGATACTTCCCCACCTGAAGTTGTTAAAGAGGAATCAGATGTTGAAGATGATGAGGACTTGCTATTAAAGA
AATATAACCTTGAGATCTTGCCAATGGAGACTTGGGATAGGATCAATAAATTGTCATT
GTCTCCTAAGTCGCAAGCATGGCAGAAAATAGAAATAGATGTAACAGGTATGTCAAAGGAAGACAGAACTAAA
ATACATGATGCTGTCAAAAAGGCATTTGGAGAAAGCATTGTTGGTAGCACAGTCAGTGTGGATGATAAGAAGT
ATGTTACATT
>1002049839_1 Heli.1-EL602648.1.5
TATTTCGAAACTTTAAAACAATTATTTATTACGTTGTGGAGTTTCTTTTTTCATTTATTTTATAATATAAATA
CTTAAGTGGGTCGTGTAAAGTGTTTTTAAGTGAAAATATTTTTACTGGTAATAGTTAGTGTAGAAATCAACGA
GTTTTGCGACGGACCGGTCTTTATAAATATACACTATGTTGCCGAGGAAAAGACACAA
TATGGGCATCATACTTATGACGGTACTAATGTTTGCCAAATACTCACATCAGTATGGATCTGGTGCTCCACCT
ATGGCATGTTTAGACCAAACACCAAGGCACAGTGAGATCCCCGCACAGTCATCAGTCCCACCATACAGTCTCA
TCCCATCATCATCGCAAGTCAGGCAGGGTGATGTCTTAAATATCACGATCGGCAGTCC
TTTTGGTGCTCCCACACCCATTGGTGGTTTTATATTACAAGCTAGAGAAATACAGGATATGGAAAAGATCGTG
GGCAAATTTACAAAAGTACCCAGTTCTGATTTGGCACAAATCAAGAGCTGTAGAGGTCAAAACGACTCGGTAA
CTCATACGAATCCGGAAGATAAACAACCTTTGACCTTCACTTGGAGAGCGCCTGACGA
TTTTTTGGGAGGCGTTGCATTTAGAGCAACGGTCGCACAAAGCTATGC

>1002049840_1 Heli.1-DT667224.3.5
AACATCTCGGNCCCGGGCTGANGAATCGGCCGAGGTGAAGAGTGCCTGCTCGTACAAAATACATTCTATATAA
TAATATAATGTTTAAAATAAAGATTACTTTGATATCAGCGCTTTTGGCCTTAGCGTCATGTGGTCGTCTGGAA
CCTCAGTACCTACCACCGAGGCCTGGCGGAGGTAACGCAGGATTGGGAGGACTGGGAT
CTGGAGGAGCTGGAGGTGGAGGAGGTAACGGAGGACTTGGCGGAGGATCTTTTGGCGGCGGAAGCGGCTTCGG
TGGCGGTAGCGGTTTTGGAGGCGGATCCGGAGGACCAAATATACCCATACTCCGATATGAAAACGAAAACAAC
GGCGACGGCACTTACAAATTCAGCTACGAAACCGGTAACGGCATATCAGCACAAGAAA
GCGGAGCGCCGCGCGCCCCCGGCCCCGAAGGACCAGCCGTCACAGCCGAAGGCGCGTTCTCCTACCGCGCTCC
CGATGGCCAACAAATCTCCCTAACCTATACAGCTGATGAAAACGGCTTCCATCCCGTTGGTTCCCATCTTCCT
ACACCTCCCCCCATCCCGGAAGCTATCTTGCAATCAATCGAATTTAATAGACGCAATC
CTTCATCTGAGGGGTCGTACAACGGTGGATCAGGCGGCAGCAATTTCGGAGGTTCCAGCAGTGGGTTTGGATC
TGGTGGAAGCGGTTTCGGGGGCTCCGGTTCTGGTGGTGGTGGCGCGGGCGGCTACCATTACTGATCTCATTTA
TAGAAGTATCTAAGATACGTCACTGAATCGAAGTTTTCATGGATGCAATGTCCTAAGC
AACAGATACTTTAAATTTCTTAGATATTTTTTATTT
>1002049841_1 Heli.1-ES586440.1.5
GCCGTTCCAGCGCACACACACAAGAAAATTGGAAATTATTAAATTGTGTCAGTGGTTCATCATACCACCAGCG
GCTCATCTACATTGCCAAGGAATCCATAAAAAATCAGGATGAAGGTCTTATTACTGTGTATGGCCTTCGCGGC
TGTGAGTTTGGCTATGCCTGTCGCTGAAGAGAAGCAAGAAGAGCCTATTGCACTTCCA
TCTGTCCCTGAAATCAAATCTGAAGATGTTAAGGCCCAAAGCGATTCGGCGCCAAGTCCTGTAGAAGAAAAAA
AACCAGAGCTAGTACCTGAAGCAAAAAGCGTCGAAAAACCTGACGAGGTCGCTATTGAAGCCAAGAGTTCAGA
TGTAGCAATAGATGCTTCTCCAGAGTCTAAGGCGGAAGAAAGCCCAGAAGTCAAAAAA
GAAGAAGTACCTGAATCTAAGGCAGCAGACGTAGCAGGTTCTGAAGTTGAACAAAAAGCAGACGTTGCTGAAC
CAGCCAAACCTGAAGAAAAAAGTCCAGAAGTTAAAGCTGCCGTTGTAGAAGAATCTAAGCCAGAAGAAAGTCA
AGCTAAGGTTGAAGAAGTAAGTAATTTACCTTCAGCAAAATCTGCTGTCTCTGAAGAT
GTAATCGACCTGGCCAGTGCTATCAAACCTGAAGCAGCCGTTGCCGATGATGTCATTGATCTTGCCGCTATTA
ACCCATCTGAGCCAGTAGTTTCTGCTAAAAGTGCTGCGCCTGAGGAAAAGAAAGAAGCTGATAGCGAATCAGT
TTCAGAAAAAGTTGACAAACCTGAGGAAAAAAAGGAATCGGTAGAA
>1002049842_1 Heli.1-DT664663.3.5
TATACCAACAATTCAAGCGTCTGGCATCACAATGGCGGCAGCGGGTGACTCAGTCATCTTAGAGTGCAGAGTG
GAAGCGCTGCCCAAACCTACCATCGCGTTCTGGAGAGACCCTAATGGCAGAACACCGGTTATAGATGGACCAA
ATTATACGATACAACTGCTTCCTGATCCTGAGGAACAAACCAAATACACAATGCGCCT
CGTAATAAAGAAGATCACGGAAGCTGACGAGGGGGATTACTTCTGTCATGCGGAGAACGCTTTCGGCAAGACA
CTGAGGCCGGTCTCTGTACGTTTGAGGTCGAATAGTCCTCACCACAACGTCACTGAATGCTGTACTCAGATGA
ACGTATCATCCGCGTGCATCGACGCCTGCAGCTTCCACTTGGACATGGACAACATCAT
GGATCGTCCGGAGTGTATGAACGATTTTGACAAACTCATGAAATGTGCTGCTGATGGTTCAGATCATCGAAGC
TGCTGTGCATCATGGGGAGTTCCCCGTAACTGCCTAGAACTTTGCCGCGGCGGCCCCGTCTCCAGGACTTGTG
CCCTACAGCACGCAAGACGTGCACTGGCCTGCTTTAGAGATTCTGGGGCCAGAATGCC
TGGACCACCCAGAAACTTGAGGGCTCATGCAGCACCTACACCACATGCTGTTTTATTAAGTTGGGAGCCACCG
CGCAAGAACCCCGATACTGTTTACCTATATAGAGTGTTCTGGCGAGCTTACGGTGCTAAGGTACCAGAGAAAT
TGGACACTAGCGAAACAAGCGTTGTTCTAACTGGATTACAGGATGATGTCAGATATGA
ATGTGTCGTGAAATCTGCTAATGATGTCGGCACATCTTCCCTAAGCCAGCCAATAATGTTCACGACCGCTGGT
CAAGAGACAGGTGCGGCGGCTGCTCCTGTATCGACTTCGGGAACAGCTTCAGCAGTCGGAGTAGCCGTGGCCT
GTATATTAGTGGCCGCTATTCTCTTAGCTGCCGGATTTTATTATAGGCATAGAAAGAA
TCTTAGACTTAAAGCACAAGGAGGTGTTGCTTTCGAAAACCCAAGCTACCTTCGAGAACCAAATCCTGACAAC
GCTGTTAATGGCTCAGTACCAAACGGTACTTCTGGAGAAAACGGCCTAGCCAACGGCATATCAAACAGCGCGG
CCTGGAGGCAAGAGACCCAGAACCCAACAACCACTGGGCCCAGGGAAGTTGATCCCAC
ACTGTACGAGGAGCTGAAGCTCGGTCACGATGGAGCGGGCTTCAAGAGACTGAAACCATAGCCCGAGGTCCGC
CGCTCGCCGCTCCGCTCGCCGCGACTGTCGGCGCGCGTGGCGCCGTACTGAGCGGCTTTACTCGCGTTGTCGG
CTCTTTTACTGGTGTAGGGTTGTCGAAGCGTATTATAAAATATATCGGTGTTTATAGT
CTGTGGTATATTTTCTATCTATAGTTTTATCTGTAGTGATATATATTCTATTCTTTGGCAGTAATGACAGCCA
AGCCATTACCTAAGTTTATATAATTTACTTTGGGCCATAGTTGTGGCTTGACTTTCTTTACCACCAAAGCAAC
GATATCTATATTGATATCTAAAATATTTTTATGTACGTATGTCAGTATTTGTAAGGTA
AAGTTACCATGTTATTGAAATTAG
>1002049843_1 Heli.1-EL603159.1.5

ACGAGGCCTCCTTTAAGAATAATTTTACCAAAATTGGTGATTTTCATAATATTAACACTAGAAATAAGCATAA
TCTTGGTGCACCTGTAACTCGGTTGCATCGAATCCTTAATTCTTTTATGGGACAATGTGTACGCTTTTACAAT
AGGATCCCAGAAAACGTCAGAGAATTTTCTATATCACGATTTAAAAAAATTGTGAAAA
GACGTTTGTGTGTTAAAGGTTATTACCAAATTAATGATTTTGTAAATGATAACACGCCTTGGGAATGAAATGC
TCGCTCCAGTCTGTTTCAAAGGTATCATTGTCACAAATAATTAAATATGTATTTGTTTAAAATTGATTTTAAA
AAATAGGTCCCGCTGAGTTTCTTGCGCCGGTTCTTCTCAGGTCTGAGGCATTAATTTT
AGTGAACTGGTGGTAAATTTGACGTTCAAAAAATGAAGCATGTAACTTCTTATTTTGAATAAAATTATTTGAA
TTTGAATTTGAATTTAAAACTTGTACATTTTCATTTTATTCTTTGCTATTCTTATTTTATACAAAGATATTTT
TCATATAACATTCAAGTACAACAAGCAATAATGCTATATATAGAAAACTCCACGTTAA
ATAAATTAAATATAAATAAAAAAATACAATAAAGTTTGAGAAACAGAAATTTTTTGTTTACATTTTTTGTCAT
GCCATAATGAATCGAGTATAGATACACTTGCCTCGTTGTTTTTAGGAAAAGGACACAAAGATTACTTCATAAG
TTACATAACTTAGTTGTATTAAATGTTAAGCTAACTTAATAATATAAATAATTTTTAA
AAAATGTTTGCAAAAATTATTATTATGAAATTAACTACAAATATACATTTCTGAAAGTTTTATTGTAATATAA
ATATAAAAAATTGACATTTTTCGGAGATTTTTCCCTTATTTTACTTTATGTTCTCTCACTTCTTGTCNAATTT
TATAATTGTAAGTCTACAGGAAATATTCTATAAGATTTTGCGACAAATTTTGATACTA
AATGCAGCAAAAACTATACACATAAATTACGTAAATTCTTATATCGTT
>1002049844_1 Heli.1-EL599439.1.5
GCACGAGGGTCGATACTAGTGAATGAAGACTTAGTAAAATTTTCAAATTCTCTCATAAGAGAGATACTTATTC
AAGCGTTTACGACCACGTAGGTACTACCTACAAAGAAACGAACATAAAACAGCACTATGCAGTTTAACGCCTT
CCAAAATCATCATCCAATCTATAGACCTCGACTAGACCGGAATGAACAATTCGGTAAA
ATGCCTTTCATCAATAGTCTGCATGATTTCGGTGGGCCAAAAAACTTTCAGCCAAGAAATGGGATGGGTAATA
AGAACTTTAGGCCTCGAAATGATTTCAATGGGATAAGAAATGATTTTAACAATATGAAAAATGAAAATGGAAA
TCCTAGTGATTTTGGCGGACCGAAAGAATACAGACCCAGAAATAATTATAATAATCAA
ACTCAAAAAAAGAATGATTATGATGGTGAAAAAAATTCAGGAGGCAATATGCAGTTTTATAATGCAAAAGGTG
ATTTTGGTGGACCTAAACAACAAAACTTCCAAAAGGGTTTTGCACCCAAGAATTTTAACAATCAAAATGGGGG
CAGCAATGGGCAACAGTCATTTGTTCCTAAAAAAATATTTAACAACAGTTCATCCCAG
CCATCTTCAGATTTCAATGATCGCAAATTACAAAGCCGAAAAGCAAAGTATCCTGGTGATGGATTGATAAAAC
CAGTATGGGACATGGCTAATCTG
>1002049845_1 Heli.1-EL602431.1.5
GCACGAGGGTTTACTATTTGGCTCCACAACTTTTCGTAAGTTCAAAATGTCGTTTCGCTGTATACTCGCGTTG
CTGGCTCTCGCCGGAGCCACGCTGGCCGGGCCCGAGGTCACCGAACTGAAGGTAGAGGTGGTCAGCGTGCCAG
AGGGCTGCACGGCCAAGTCCAAGCACGGCGACATGCTGACCATGCACTACACGGGCAA
GCTCGAAAACGGGCACAAATTCGATTCCAGCTTAGATCGCGACCAACCCTTCACCTTCCAAATCGGCGTTGGA
CAAGTTATCAAAGGATGGGACCAAGGTCTCTTAGACATGTGCGTTGGTGAGAAACGTAAGCTGGTGATCCCGC
CGTCCCTCGGCTACGGCGAGCGTGGAGCCGGTAACGTAATCCCCTCTAAGGCCGTCCT
CTTTTTCGACGTCGAACTTATCAACATCGGTGACTCCCCTCCATCCACCAATGTCTTCAAAGAGATCGATTCT
GACAAGGACAATATGCTGTCCAGAGAAGAAGTAAGTGAATACCTTAAGAAGCAAATGGTGCCATCCGACGGCG
CTGAGATGAGCGAGGATATCAAGCAGATGCTGGAGAGCCACGACAAACTGGTGGAGGA
GATCTTCCAGCATGAGGATAAAGGACAGAATGGCTTCATCAGCCACGAGGAAGTCTCTGG
>1002049846_1 Heli.1-DN048385.1.5
GTAAGGGGACGTTGCGATTAGTCTACATCGTTGGGATTTTCCTACGTTGATCAGTATTTAAAATTATTTGAGT
AAAACACAGCTAGAATGGGTGTTCCTGCAGGAAATGCTGAAAATGGAAAAAAAATCTTTGTGCAAAGGTGTGC
TCAGTGTCATACCGTTGAAGCAGGTGGAAAACACAAAGTAGGACCTAACCTTCATGGA
TTCTATGGACGTAAAACTGGTCAGGCTCCTGGCTTCTCTTACTCAGAAGCTAACAAGGCTAAAGGTATCACCT
GGGGTGATGACACACTTTTTGAATACTTGGAGAACCCCAAGAAGTACATCCCTGGAACCAAAATGGTGTTTGC
TGGCTTAAAGAAGGCCAATGAACGTGCAGATCTTATTGCATACCTTAAGGAAGCTACT
AAGTAACTTTAATGACATTAGGAAAAGTAAAATGAAATAAGAAGAATATCCCATTATATGGGAGATATTTCTT
ATTTGTTAGCGCAATCAGTATCATTATTATTTATATATTTATTTTGAGTTAACATTTCTGGACAATTGTGAAC
TTGTTACTCATTTATACCATCTATACTATTACAAAAGAGGAATGTGTGAATTGGATGA
ATTGTAAATTAT
>1002049847_1 Heli.1-DT668252.3.5
GCACGAGGAAACCAACATCCGCTACTGCGCTTAATACGTCAACAGATGATGATGATATAATAATTGATGTTCA
ACAAGCAAAATCTTCACGTGAGAACTCGCCAGATCGCATTTGTCCAACCCCAGTAGGATTTTCGGAAGATGTA
GGAACACCAAGATTTCCAGATGAAGTGAATGAACCAGACGATGAATATCAAAAAAGAG

CTCATCAAATCATACACGAAGCTGAAACTATTGTAGATGATATTGTGGAAATTGATGAAGATGACGAACTATT
CGTGAGAAAAACAGATTTCAGTGAATATGTAGAAGTAGATGAAAGCGTAAATAAAGTTTCGAAGACTACAAAT
AAATATGAAGCTACGTCACGTTTCAAAGATACTGAAAAAAAAGATCACTCTGACTTTT
TGGATGAAAATCTAAAATCAGATGATTGCTTATTATCAGTTTCTGAAAAAGTTAATAAGTTTGCCAAGGGACC
TACTAACACAGAAGTCAGAAAGAGCCCATCTCGAAATATTATTGAAGAGTTTGACAAGCATACATCATATCAA
GATGATTATACTAAACTAAGTGTGAATGATAAAGCTCACTTATTCATAGAAACAGCAG
AAAATGTAAAAGCAACAAAATCTAAGCCTAAACAAAAAGTAGAGCGCCCTAGTTTTAACGATATAGATGAAAC
ATTAAAAAGTGACGATTGCTTACTTAGTGTTTCTGACAAAGTCAACAAATTTGTAAAAACAGCTGAACAATTT
TTAAATGAAACCCATGAAGTTGAAGAGAAAGAAAAGAAAATTGTTGAGGAACATGAAA
AGATTATGAAAAAAATTGTAGGCAATATTGACGATACCACTATAAAATCACATGACGAAAATTATGCTTGCAA
TAATAATACTTCTAGTAGGAATACCGATAATGATGATAGGCGAAGCTCTTTAACAAAAGAAACAGTTTCATCT
CATGCGAAAATCAAGGACCCGGTACACCCTAGTATTAAAACTAGTGAAAAATCTGCAG
TAAGAATAACAACGTTACGAAGTAGTGATGGAGTGAAAAAAGCGAAAGCATTATTTGAAAACATTTCATCTAC
CACCCCACAAAAAGTTGCCGATATATCTCACACTAAAATCTCTAAATCAACAGGTACACCCAAAAAAATAACA
AAAAATAATCCTAAAAATATAAATTCCTTAGAAAACAATTCTCGGGCTATTTAAGAAA
CGGAAAAAATAAAAGTTTCTACTT
>1002049848_1 Heli.1-DT665055.3.5
GCACGAGGTGAAGACGAAGTAAAAACTGAAGCTCATGTACCAAAGATGACTAACACAACAAGTGAAGTCGCTA
TCAGTGATGAAATATTGAGACAAGATTTTTCAATAGACGATGAATGCGGCTGGAATAATGTAGGAACATATAT
ATTTCCCAATAAAACTAAGGACCCGCAAATTTACGCCAATTATGGAGAATATAGATGG
ATGATTGCACTTATCAAGAAAAGAAAGAATGTAACCGTCTGGGTGTCCGACGACTATATTGGTGGAGGTTCGT
TAATACATCCATCTGTAGTAATAACTGCTGCACATAAAATACATAACATCAAACCTCGTGAGCTTAAGTGCCG
AGGCGGTGAATGGGATACACAGACGGAGTTAGAAAAATATAAATTTCAAGAAAGAGAC
GCCAGAAAAATTATGATCCATCCAGATTACTTTAGAACGTCTTTGTACTACGATGCGGCTCTAATATTCCTCA
AGAAACCTTTTGATTTACAAAGTGCCCCTCACATGGGCTTAGCTTGTCTGAACAAGATACTGCCGGAGCCCGA
TACTGACTGTTTTACAATGGGATGGGGAAGGTTATTTAATGAGAAGAATAAATATGCC
GTGGTATTGAAAAAGACAAAGCTATCTTTAGTGAGTTCACAAGCGTGCGAGACCAAGTTACAAGCAACAAGAC
TAGGCCCGTACTTCAGGATCCACTCGTCTCTGACTTGCGCTGGAGGGCAGGAGGGTGTCGACGCGTGTGACAA
AGATGGCGGGTCCTCTTTAGTGTGTCCTATAAAGAAAAATGGTGAAAACGTTAGATAC
GCGATGGTGGGAATGGTAGCGTACGGCATTGGTTGCGGGATCAAAGACCGTCCCGGGGTCTACGTGAATGTAC
CAGCTATTTACCCCTGGGTGGATAAAGTTATGACAGATGAGAAATATGGCAACGTCACTTATACTGTATAAAA
AATGTACTTCGTTATATAAATAACTATAGTTGTAGCTTTACATAAAATAAAAAGGGGC
>1002049849_1 Heli.1-DT665135.3.5
GCACGAGGCGACCCCTGCGCAGCTGGTGTGCGGTGCGGCTGCCGCTGTTGCCACTCGTATTACTTCCAACGAT
TTAAACATCACTAATTTACGTATCCCGAATAATATGACGTAAAACTTACGTGACCAGAAGCCATCGAACGCGC
GGCCGTGTTTGTGTTGTGATCCGGGGGACGTGCGCGATTTAGGAAATCTCGACGGGTG
AAAGAAATTACAGTTACTTTTGAAAAGAGGAGAAGAACTAATCAAAAATGGCGGTGTTCGGAATGGCCTCCGC
GGTGGCGGGCTTTGTCAAAGTCCGCTACCTGATCGACAAATTTGTCATCGACAATATGGTATTTAGGATGCAC
TACAGAATTACTTCTGCTATTATGTTCCTGTGCTGCATCCTCGTTACTGCTAACAACC
TAATTGGTGACCCGATCTCGTGCATCAGCGATGGAGCTGTACCTGCACATATATTGAACACTTATTGCTGGAT
CACTTACACCTTCACGCTTCCCTACAGCGGGGCTAAAGGCATCGCCCACCCAGGTCTCGGCAACGATTACGAA
GAAGAGAAACGCATTCATTCATACTATCAATGGGTGCCTTTCATGCTGTTCTTTCAGG
GCGTCCTTTTCTACGTCCCTCATTGGATCTGGAAGAATTGGGAAGAAGGCAAGGTTCGCATGATCTCCGACGG
TATGCGTGGAACTACAGCCATAATTGCCGATGACAAAACTAACCGTCAGAATCGCTTGGTTCAATATCTGCTT
GATACACTTCATATGCACAACACCTATTCTTTTGGATATTTCTTCTGCGAAGTTCTCA
ATTTTATTAATGTCGTTGGCAACATATTCTTTTTGGACACGTTCCTCGGAGGTGCATTCTTGTCCTATGGAAC
TGATGTAGTTAGGTTCTCAAACATGAATCAAGAACAACGTACTGATCCTATGATTGAGGTATTCCCTCGTATA
ACTAAGTGTACGTTCCACAAGTTCGGAGCTTCTGGTACGATCCAGAAACACGACGCCC
TGTGTGTTCTTGCATTGAATATTCTCAACGAGAAGATTTTCATCTTCCTCTGGTTCTGGTTCATCATCCTATC
TGTCGTTTCTGGCTTGGCTATCGTGTACTCGGCTGCAGTCATACTTCTGCCTAGCACTCGCGAGACTATACTT
AAAAGGCGATTCCGTTTTGGATCTCCCAATGGAGTTGAAGCTCTCGTTAGAAAAACTC
AGGTTGGGGATTTCCTTCTTTTGCACTTGCTTGGCCAAAACATCTCCCTTCGTGTGTTCGGCGAAGTATTGGA
TGAGCTAGGACGTCGACTTCACCTCGGTTCTAACCCACCGTCAGCACCGTCTACTCTAGAAATGGCACCCATT
TACCCCGACATCGACAAGTTCTCCAAGGAAACTGAGACGTAAATTGAAAAATATAAGC

AGTATACGAGTATTCTGTAATCACTTTCAAAATAGCTCAAACACATTTTTTAAGATCTCTTACACTAGTTAAA
ACTTTCATTCATGGCTGTTTCTTAAAATTTGTGTTTGACCTAATATTGTTTCAATTTAAAAAAAACGTTCCCG
CCCCTATATCTCAATATTTTCTTTATTATTACTTTAGTTATAGCGTGTTTGTGTATGT
TAATAGGTTCCAGTATGGGAATTTGAAGTTAATGACATTCCTTATTTT
>1002049850_1 Heli.1-ES587370.1.5
GTTGGTGCAGCGTTCGAGTGGTCGACAACATGACGCGATTAATTTTTATTTGCTTATCAGCTCTGGTAGTGGC
CATTTCGGCGGAGGAAATCGATGAAGTACAGGTTACGCGTCTTCTTCATGATGCCAGCAATAAATTCACATCT
AGAATGTTTAACGAAGTAGCACACGCCAATGAAGGTAAAAGCTTTGTGCTCTCAGCAT
TTTCAGTTATGCCCCCACTTGGTCAGCTAGCTCTAGCGTCTGTGGGCGAATCTCATGATGAACTCCTTCACAA
TTTAGATATACCAAATGACAATATGACTATAGCTGTTTTCTCATATTCAAATGCGTTAATACAATCAGTAGAA
GATGTAACGCTTAATATGGCAGCTAAAGTATATATCGCCAAAAATTACAAAATAAACA
AGAACTTTGGTGAAGTTTCAAGAAATGCATTCCAATCTGAATTTCAAAATATTGACTTTGATAATAATCAAGC
TGCTGCAAACGAAATCAACCAATGGGTTGAAGCGCAAACAAATAACAAAATCAAGGACTTAGTAGATCCTAAC
AGCATTTCAGGCGCCACCAGAGCTATTTTAGCAAACGCAATTTATTTCAAGGGTAATT
GGAAAGATCAATTCTCCAAATACGCTACACAAGAAAGAGATTTCCATGTGAGCAAAGATAAGAAAAACAAAGT
CCAAATGATGAACAGGAAAGGACACTATAACTACGTAGAAAGTAGTGATCTTAATGCTCAGGTACTGGAAATT
CCTTATAAAGGAGACCAATTATCTCTCCTCGTTGTCCTACCACGTGACATTGACGGTC
TGCCTGCATTAGAAGAAAAATTGAAAGATCCTTCGGCTTTAGACAAGGCAGTCAGTTCTATGTCTAATATAGA
AGTAGATGTTTTCCTACAAAAATTTAAAATTGAAACTAAAACTGAAC
>1002049851_1 Heli.1-EL601178.1.5
ATCGTATTTTAAAATATGAGAAAGTAATTGTTACAGAAGTTACTCCAGAAGGGACATTCTATGCTCAAAACAT
TGAACTGGGTAACAAATTAGAAACACTCATGGACAAAATTCACCAAGAGTTTAAAGCTAACGCCCCCCTCCCT
GGATCTTACGTGCCAAGGAAAGGTCATATTTGTGCAGCACGCTTTTCTTTAGATGATC
AATGGTACAGAGCAAAAGTTGAGAAGCTTTTGGAAGATAAACAAGCCCAGATTCTCTATATCGACTATGGGAA
CAGAGAGGTAGTGAATCAGTCTCGACTAGCACAACTGCCCTCTGGCACGGAGTCGGAGCCACCTTACGTGACC
GAATACGCGCTGGCGTGCGTCAAGTTCTCCAGCGACGCAGACGACAAGCTGGAGGCCG
TGCGCGCCCTCTCGCTAGACACGCTCAATAAGAAGCTGCTGCTCAACGTGGAGGTCTTCGGCGCCCCGCCCGC
CGTCACGCTCGTCGACCCCACCGCCAGCACCGACATTGGCAAGAACCTCATCAAGGAGGGGCTCGTGTTGCTG
GAGCACAGCCGCCAGCACCGGCTGAGCGCGCTGATGGCGGAGTACCGCGCGGCGCAGG
AGCACGCCAAGAGCTCGCGCCTCAACCTGTGGCGGCACGGCGACATCACCGACGACGACGCCGTGGAGTTCGG
CGCGCGCCGCTAGCGCGAGCTGCCCTGGTACGACGCCACCGCCTCGCCCTCGCACAAGCTGCACTGCGCCACG
TGCACCTCCCCGCACGTGGAGTCCATCATATACACGGTCATATACGACTTCCTGTGAA
GGCACTGCGGCGCGCGGCACGGCAGCGTACCCGTGAACCGATTTAGGCGTGTTGTTTGATATATCCAAGTTTC
GGATCTGCCTTCCCAAAAATCAATTAACTTCCTTTACGAGTTCGATACATTTTTAAATATTGTGAATGTTATT
TATGTAACAGAGCGGCGGCGGCCGTCCGAACGCGCTCTCGTGTGGCTATTCTTGTGTC
CAAACTCTTGTAGTGTAACGATGAGTGGCACCTTTTGCCCTTTGTACCTACTATGATACAATAATCACTTTAG
ATATTGTCTACATATTTTTATATTTCGCTAAGACAAACTGAATTATTAAATATAATAGTGAATTATACATAGA
TAATTGTAGAGACGAGGAGCTCCTCCCTACTCCTCTGATTTAATTTATGGCATCATAT
TAAATATTAATA
>1002049852_1 Heli.1-DT665299.3.5
GCACGAGGCGCCGTTTTCAGTTTCGTCCTCAATCCTCTGACTCCAGTCCAAGAAACGCGTTGCCTGCTGCAAA
ATGATACACAGGCTAACAACCCTATGGCCATACCACAGCGGTCGGTGAACACTTACTATATGGTGAAGTTACA
AGTGAGATCAGAAAATTTATGCTTGAGGCCTTGGGCATTTGAAAGAGTACCTGGCAAA
GCGCTTCGAGGGTTCGACAACAGTATAATATATACTTCTACTAAAGAGGCCTGCCTTGCTTCTTGTCTCAGCG
AGAAAAAAATTCGCGTGCCGGTCCGCCGAGTACGAGTACGGGAGCATGAGGTGCTCGCTTAGTGATTCTGATAG
GAGGACCTCTCAGCATATTGCGCCGCTCGTGGATACGCCCGGCACTGATTATTTTGAG
AATCTATGCCTGAAGGGGACCCAAGCGTGCAAGGGCCAGAGAGTATTTACTGCCCCGCGTATAGGAGTCGCCG
AGGACAAAGTGGCGCAGTACGCTGGTCTACATTATTACACAGATAAGGAGCTGCAGGTGACATCAGAGTCAGC
GTGTCGACTGGCTTGCGAGATCGAATCCGAATTCCTTTGCCGTTCTTTCTTGTATATG
GGCGCGCCACACTCCGCCACGTACAACTGTCGATTGTATCACCTCGACCACCACACCCTGCCCGATGGACCAT
CAGCATATTTGAACGCTGAACGACCGTTGATCGACGATGGTGAACCAATTGGGAAATATTTTGAAAACTTCTG
CGAAAAACCACCTGCCAGTCCCAGTGGGGAACTGCCCGTGTCTATAGATCACCAACAA
GATACGAACATGTCCAGCAACTTAACGAGGAACGACGCCAACTGCGACCAGACGGGAACT
>1002049853_1 Heli.1-ES587037.1.5

TACGGTCGGAATCCGCGGTCGACCTAGCGTCCGGAACATCAAACATGTACTCCAAGGTCGTAATCTTTATGTG
CGTCGCTGGCATCGCCTCAGCCGGCAATCTTCTCCATGCCGCGCCCGTTTCGTCCGTGGCCTACAGCTCTCCA
GTTTCCTCCGTGTCATACTCTTCTCAGACAACAGCTCATGGCTCGCCAATCTCCTACG
GAAGCCCCGTCGCCTATTCTGCAGGACCAGTGATTGCAAAGAGCATTTCCCCGGCTCTATCATACCAGACCAT
CTCTCATGCTCCAGCTATCGCAAGTTACGCAGCGGGACCAGTTGTCGCTAAAGCTATCGCACCGGCAGTGTCA
TACTCATCGGTGTCTCGGTCTTCTCCCATCGCGTATGCTGCCAGCCCTGCAGTGAGCT
ATGGAGCTCCTGTCGTAGCAAAGGCTTTATCACCCGCTGTATCCTACTCTTCTGTAGCTCACCCTGCATCATT
GTTACATGCTGCGCCAGTAGCATATGCTGCCGGTCCGGTCGTTGCCAAAAGCTTAGCTGGCCAAGCAGTGTCA
TATTCATCTCTAGCGTCTCCTTCAGTATCTTATGCTGCGGCTCCCTATTCCTATGCTG
CGGCTCCTTCTTACTCCTATGCTGCGGCTCCAGTCCTCAAGTCGGCAGTAGCTTACTCCGCAGCACCAGCAGT
CTCCCATGTTAGCTACTCTGGTT
>1002049854_1 Heli.1-EL600409.1.5
GCACGAGGGTGATTTTCAATCTTCTTGTTACCCACATTCCAAATCTCGTGCTTGTTTTGTACATTCCTTCACT
GTACTGAACAATTATAAGTTAACAACAACTCGATAATACCTTAAATTTCATACTAAATAATTTAAAGTATAAA
ACTATAAACATGTCTGAAGTAAATGAGCAAGACAATGGTGAAACAGAACAAAGACCTA
ACGGCGGTGGTGATGAAGGCGACGCGTGGGGAAGTCATGAATCTTCTTCGATTCGTCACTATGCTAAAATGCC
TGTGGAAAGAAACGCTCCATATTATAATATGAACCATAAAAATCGTGGTATCGCGGTTATATTTAACCATGAG
CATTTTGATATCCACAACTTGAAGTCCCGCACAGGAACTAATGTGGACTGCGACAATT
TATCAAAAGTATTAAAAACTTTGGGATTTCGTGTAACAATATTAAATAATTTGAAATTTGAAGATGTTAACAG
GTACTTGCAACAAGTGGCTGAAATGGATCATACAGAAAATGATTGTTTGCTCATGGCAGTATTGAGCCATGGA
GAAATGGGTATGCTGTATGCAAAGGATACACATTATAAGCCTGACACCCTCTGGTATT
ATTTTACTGCTGATAAATGTCCTACTCTAGCTGGAAAACCAAAGCTTTTCTTTATTCAGG
>1002049855_1 Heli.1-DT668631.3.5
TTCGGCACGAGGCGGTGTCATTACGCGTGGTTAAGAGGCGAAACTGGTTGTGTTTTTGTGATCGAAAAATTCT
CTGAAAGAAAGTTGTGATTATTGTGTTTATGGTAATTTAACAAAGAATACCAGAAAAGCTGTGAAATATGAAC
ATAATATGGTACAGCGTAGCAATACTGGTTTTACTGGAAAAAGGCGTCAACGGTCATG
GGAGACTCATTGAACCGCCTTCTCGAGCATCGGCGTGGCGCTACGGATTCGACACACCACATAACTACAATGA
TCACGAACTGTACTGCGGAGGATTTTCCAGACAATGGAATAAGAACAATGGGAAATGTGGTGTCTGTGGTGAC
GCCTGGGATGCACCTAAGCCTCGGCCTCACGAACTTGGAGGCAGATTTGGTCAAGGTG
TTATCGTCCGACGATACGCGCCCAAAGATGTCATAGTTATAAAGGTAGAGTTAACGGCGAGCCACAACGGATT
CTTTGAATTTAGAGTATGTGATGAACCGAAATCTACGAATCAGGACTGCATGGACAAACATGTGTTACGGTTG
GAGGGGAAGGATAACACAAAATACTACCCACGTGAAGGAAACAAAATATATGAAATGA
AGTACGAACTACCAGAGGGTTTAGAATGCGCCCATTGTGTGTTACAGTGGCGATATATAGCCGGGAACAACTG
GGGTACGTGTGCGGACGGAACAGGAGCCGTTGGCTGTGGACCCCAGGAGGAGTTCAGAGCGTGCGCTGATATA
GCTATAGGTAGCAAATTTACTACAACAACGAAAC
>1002049856_1 Heli.1-EL600392.1.5
GTCTCCCGTGTGGATGGTTCTCTTATTACTCCCAATCCTCGGGGGAGCAGAAAGATTTAACTGTCGGGGACGC
ATATTGGGTGCATATTATGCTGACGCCAAATCTGGATGCAAGGCTTTCCATGTCTGTGTAAGAGTGGCTGGCG
GTGGTATTCGAGACTTCAGGTTTTTCTGTCCACCCGGAACCTTATTCCACCAGGAAGC
TCCTCTCGCTTGCGACTGGGGCGATGACGATCCTCTCGCTTGCCCTGCCGATATCTACGATGGGTTCGACACA
AAGAAGTTGTCGTCTTCAGGGAATCGAGAAGAAGAACCTGAATTTGGTCTACAAAGAGCAGAAACTGGTGACC
GACGTCTTTCAGCAAATAATAATGGAGGAACATCAGACCTTCGAGCAGCGCACTCTTC
AGATTTCTTCACCGGCCAACGTGATAGAGGACGCGACGAAGCTGCAGTTCAAACCCAGTCACCGATATCCCAT
GCACCTGCTAGGCAATCTTTCAGGCGACCCACAACAACACGTGCTCCTTACACAACTACACAAAATTCTTCTC
CACCTTCACCAGTATCACAATCGTCAGGTCATTCTCAGTACGATCACTCAAAACGGAA
ACTTGTCAGGAAACGTCCAGTCTATACACCTACACCTCCACCCACTACACCAGCTCAAACAGTCCCTCCTCA
>1002049857_1 Heli.1-EL599300.1.5
GTTAGTGCTATTTATGTGTGCGGTTGCCACTGCTGCGGCTGGCGAGAGTGTAGCATGGCCGGGTGCGATACCA
ATCACAGCATCGCAACTTAAAACCATAACACCCGGGCAAATCAAAGGATTTGCCTACTCTACCAGTCAATACA
TAAATGCGATCCCATCTGCGCCATTTGGATATCAGCCCCAATTCAGCTACCAAATAGC
AATTCCAAGTTATCCAGCTCAGTCAATAGCAAGCGCTTCATATTTCCCTCTACCGATTGTTCCATTGGCTCCT
GCTGCCCCCATTCTTCCTGTTGCTCCGATTGCACCTATTCAACCACCGCTTCAAAACATACCATCTCAACCGG
CAGAGCAACCAGCGGGAGACGAAGACTCAGCAGTAATTGAATCTGCAGATTCTTTTAA

TCAGCAAAGCTCGCAATCATCTCAACCTTCTTTAGATACAAAGAATATGCCCGGATTTCCTCAAGTTCCTCAA
GGAGCTCCGCAAATCCCGCCATTTCCTCAAAGGCCTGAGGGTCCACACATTCCACTCTTCCCTCCATTCTCTC
AGGTACCTCAACAAAACCCACCAGTTCCTCAATTTCCTCAGGAACCTCAGCAAAATCC
ATCAAGTTCAAATTTTCCACAAGGATTTCAGCAAATTCCATCAATCCCTCAATTTCCTCAGGGATCTCAAGTA
ATCCCATTGTTTCCGCAAAATTCTCAGGGACCAGCGTTCCCTCAGTTTCCTCAAGTTCCACAACAAATCCC
>1002049859_1 Heli.1-EL598810.1.5
GCACGAGGCACACCCCTTTAAGGGCTGTGGCGGCTGGTGATACAGAGTTCAAACCAAATTTAATCTATTAATA
TTATAAATAATACTTTTTATCTTATTTAATCACATTAAAACACAAAAATGAGGGAAATTGTGCACCTGCAAGC
CGGACAGTGCGGAAACCAGATTGGCGCCAAATTCTGGGAAATAATATCTGAGGAGCAC
GGCATCGACCCCACCGGCGTGTACCGCGGCACCAGCGACTTGCAGCTAGAACGCATCTCCGTATACTACAATG
AGGCCTCTGTTGCGACGGCGGAGAGCGGCGGCAAGTACGTCCCCCGCGCCATCCTGCTCGACCTGGAGCCCGG
CACCATGGACGCGGTGCGCTCCGGCGGCTACGGCCAGCTGTTCCGCCCCGACAACTTC
GTGTTCGGGCAGTCCGGCGCCGGCAACAACTGGGCCAAGGGCCACTACACCGAGGGCGCCGAGCTCGTGGACG
CCGTGCTAGACGTGGTGCGCAAGGAGTGCGAGAACTGCGACTGCCTGCAGGGCTTCCAGCTCACGCACTCGTT
GGGCGGCGGCACCGGCTCCGGCATGGGCACGCTGCTCATCTCCAAGATCCGCGAGGAG
TATCCCGACCGCATCATGAACACCTACTCCGTCGTGCCCTCCCCCAAAGTGTCCGACACCGTCGTCGAACCCT
ACAACGCCGTGCTCTCCATACACCAGCTCGTCGAGAACACCGACGAGACTTACTGCATAGACAACGAAGCCCT
CTATGACATT
>1002049860_1 Heli.1-DT663887.3.5
CCCTTCCTCGATTACCGTACGTACGCTATGAAAATATTGTTCCCTAATATCGACGATCACGTCGTCCTCCAGT
GGGAACGCCCAGAGCTTATTAGAAAAGAAAAGGGTCTGAGAATGTTCGGACAGTTAATTATGAACAAAACATT
CCTTCTATTATTTATTCGTACTTTAGAAAGCAATAGGTATTTTTCAATGCGCGATCGT
GTTAATGTAGCTTCACTTATAATGGTAACTTTACAAAGTAAGATGGAGTACTGTACGGACGTTTTAAAGACGC
TCCTAGCGGAGCTTATAGAAAAGTGTATGGAGAGTAAGAGCCATCCTAAATTATTGCTTAGGCGTACTGAAAG
CGTCGCCGAGAAAATGTTGAGCGCCTGGTTTACTTTCCTCTTATACAAATTCCTTCGC
GAATGCGCCGGCGAGCCGCTTTACCTTTTGTTTAGGTCAATGAAAGGTCAAGTAGACAAAGGGCCGGTAGACG
TTATAACCTCCGAGGCGCGATACTCTCTGAGCGAAGAGAAACTAATTAGGCAGTCGATCGATTTCAAAGCAAT
GACCGTGAGCGTGTCTATATCGCAGCAAACCATCTTCGTGAGCGGCTTAGAGGCTACG
ACGGAAAACGTGCAAGTAAAAGTTCTCGACTGTGATACGATTAGCCAAGTAAAAGAAAAATGTTTAGACGCTA
TTTACCGCGCCACGCCGTACTCGCAGCGGCCGAGTCGTGACGAATTAGATTTAGAGTGGCGCACGGGCGCTTG
CCGCCGTTTGATACTGTACGACGAGGACAGCACCACTAAATGCGAAGGCGAATGGCGC
AAGCTTAACACCCTCAATCACTACCGCGTCCCCGACGGCGCTTGCTTGAGTTTAGTCGCGAAACAGAGCTCGG
TTTATAACCTTTCCAATATGGAAAAGAACGATAAATCGCACACTTAC
>1002049861_1 Heli.1-EL599892.1.5
CGTCCGGTGAACGGGTGAACACTTAGGAGTTTATAAACTTTAAAGTGTGCGATTCGCGTACTTTCTTTTTCAA
GTTTATTCTGCAAGTTAGTAATTAAAAGGAGAAATGTCTCTGCTACCCTATTTGTTCGACGATTACTCTTTCA
GACGACCGCGTCGTCTCATGGACCAGGATTTCGGCTTGGGATTGACACCAGATGATCT
TCTTACGGTTGCAGCGGGACCTCTTCTTACAAGAGAATATTACAGACCCTGGCGTCACATGGCAGCCGCGGCA
CGAGATCTAGGCTCCAATATCAAATCTGATGCAGACAAATTCCAAATCAACTTGGACGTCCAGCATTTCTCAC
CTGAAGAAATATCCGTTAAAACAGTCGACGGTTTCGTTGTAGTGGAGGGTAAGCATGA
AGAGAAACAAGACCAACATGGCTACGTTTCCCGTCAATTTGTAAGGAGATATGCTCTACCTGAGGGTACATTA
CCAGAAACCGTCGAATCTCGTTTGTCTTCTGATGGTGTCCTAACAATAACTGCGCCCAAGAAAGTTCCAGAAG
CTATAAAAGGAGAGAGGAAGGTGCCTATAGCCCAAACCGGTCCAGTTCGTAAAGAAAT
CAAGGATCAAAACGAGGGAACCAGTGATAAGCAATAATAAAAAAACATTCCAAAGAGGCAGTGCCATTTAAGA
CTGATAACGTGTGTATACTTCGA
>1002049862_1 Heli.1-DT663031.3.5
GGAAAATATACATTTTTGTTTTATAAAAAAACAAGTTGTGCGTGCGACAGTGTTCTACCGGTTTCTCAAAGTG
CCAAATGACGTAACCTCAAAATGAAGGATCTCGGGTTGAAAGTACCTACGCGTAATGTCCTCACTGGCGCCGT
GGTCAACTGGGACGACGACGAAATGGCAGGACTTGGAGATTCTCCAGAAAACCTAACG
CTATCTTGGAAAGACCTATCAGTATATAGAAAAAAGAAAATTCAAACAAGTATATGGAGGTCACCGACTTATG
AAGAAGTTAAAGTTTTACATGGAGTGAGCGGAGTCGTTTCGTCAGGCAACTTAGTGGCTTTAATGGGTTCAAG
TGGAGCTGGAAAAACAACTCTCCTAGCTGCAATCAGTAGGCGGGACAAAAGCGCTATT

TCCGGTTATTTAATGTTGAATGGCCGCCTCGCTGGAGCCGACCTGATCGCCAGGATATCGGGATTTGTGCCTC
AAGAGGATTTGTCGATTGATGATCTGACGGTCGCTGAGCATATGGAGTTTATGGCTCGTCTAATGATGGACAA
GAGAGCAACCACAGCGGTGCGCGCGAGACGCGTGACGCAGCTGTTAGCCGATTTAGGA
GTAATGTCGTGTACTAGTACAAAACTTAAAGCTCTGTCTGGAGGAGAGAGGAAACGAGTTGCGTTGGCTGTAC
AGCTACTCAACGATCCACCTATATTGTTCTGCGATGAGCCCACAACGGGTTTGGACAGTTCAGCTGCAAGTGC
AGTTGTATCACGTTTAAGAAGGCTGGCCATAGGTGGTAAACTAGTCATCTGTTCCGTG
>1002049863_1 Heli.1-CX700710.1.5
CACGCGTCCGGTCGGGTGAAGGCTCTAAGTCACCACTAAATAACTCATTTTATTAAAATACGTCCATAATATA
ACACAAAAATGAAGTTAATCTACCTTATGTTAATAGTTCTGCCCGCGGCATTACTCTGCTTCCAAAGCGCCAA
CACAATCTTTGCTCGTGCTGAAAAAGATGAGCTTGATGATGTGGTGGATATTGAGGGT
GAAGACAACCAGGTAGTGGGTGAAGATGCACTTGACGAAGATGACTCAGTTGTAAAATCATCACAGGATGTTG
ACACTACTATTTTGTTCACTAAGCCCATAACAAATTATGGTGATGTGGCATTCGATTTACAAGCTGGCTTCCC
TGTCGAGTTCCTCGTTGGTTTCATTAACAAGGGTTCAGAAGATTACATTGTTGAGACC
ATGGAAGCTTCCTTCAGATATCCCATGGATTATACATATTATATTCAAAATTTCACTGCATTGCCCTATTTTA
AGGAAGTTAAGCCCAGAGAGGAAGCTACTTTTGCTTATTCCTTTATTCCTAATGAAGCTTTCGCTGGACGGCC
ATTCGGATTGAACATTCAACTCAATTATAGAGATGCCAGTGGAAACTTTTACCAAGAT
GCAGTCTACAACCAAACAGTCAACATTGTTGAAGTATCGGAGGGTTTGGATGGGGAAACCTTCTTCCTCTACA
TATTCCTTGGGGCTGCTTGTGTTTTAGCTCTAGTTTTGGGTCAACAAGGCCTGTCTTCCCTTGCACGTCGCAG
GTCACCCAGATCTGCTCCGAAACCTTTGGAGACAGGAACAGCAAAT
>1002049864_1 Heli.1-DT667452.3.5
GCACGAGGGCGCGTCGAGTCGCGATATGCGTCGGTGGTGAAGTGTAGTGCAGTGTCGTGTACAGTGAATTAAG
ATGAAGTCCCTCAGCGCTACCGCGCTTTTCGCACTAGCTGTTATAGCAGAATGTCTCGGAGCACAGGACCGCG
GATCACGGCAGCGCTCCGGCCCTATACGAAGCGTTCCCGTCGCGGCTGATATAAAGAG
GGAAGTTGATTTTGACTGTCCTGAAGAATTTGGATACTACCCTCATCCCACCGATTGCACATTGTATTACGTT
TGCGTATTCGGCGGCGCGTTACTCGAATCATGCACTGGTGGCCTAATGTACAGCCACGAGCTCCAAACATGTG
ATTGGCCACGTAATGTGGGCTGCGACGCCACTGGTGCTGTGGTAGCAGAAGACTTGGA
ACGATTAAATGAGCGCGAGCCTCCACCCCGTCCCCCACCACGTCGTAATCCTCCTCCACCACCGAGGGCACAG
CCGAATCCTGTTATCACTTCTCGAGGTCAACCTAAATTTAATCGACAAGAATACGAAAAGCAACAACAGTTGT
ATGCGGAAGTAGATGATTTACCACCAGTAGAAGAACTCGAGAATGACCGACAACAACG
AGTATACAGAGGTCAGCCTTCCACGATTGGGCAAGTTCAGAAGGACAGAGATGGATACGGGTCTCAATCCATA
AGTTCTGGAAGAACACTCAATTCAAATATTATACCTTCCTCGATTAACCAGAATAGTAAAATTGGTTCATTCT
CATTTGGAACTCAAGTTGACGACAGGAGAACAGCCACTGTAACACCAGCGCCTCAAAC
TTATAGCTTGTATCCGACTGAGAGCATTACTCCGTCTTCATATTCAGCTAGCCCGCGGCCGAAGATGCTGTAC
AATGGCTCTCAAGCCTATAGTCCCGACCAATACGATCCTTATTACGCCGTTTATGACGAGGATGGAGAATTAT
ACAAGGATACAGTCCAGCAGACGTACCGCGGCACACCTCCTCCAGCCCGCCGTCCCGT
CGAAACATATACACCCAGGCCTTCTCTGGATGACTACGAGGATACTCTCATTCAAGGACAGATAAGCAACCAG
AACCAATATCAAACACCAATTCGTCAGCCATCGAGGGGCGAAGGTAACGAATTGGGCTATGATCATATTCCAA
GCAGCGTGAGGACGACTGTATACGAAGCTACTTCCCTTCGCACAACTCCTCCTACTAC
TAGCACAAGCACGACCACTACCACCACTACTACTACTACCACTACCACAACTACACGACGTCCAACAACTGCA
CCTTACACCGAAGCTATGACCCCGTCTCGACAAACTTTAAGGCCATCCATTACAAGAGGACGTGGTTCAGCTC
ATTATTCCACTTCAGGTGCATCGGGGTCTTCACAACAGACTCCTAATAGAGGAACACC
TCCAACTCGCAGCCGTCCTACTTTAAAGCCCTCTACAGCAATTGTATCAAAAGTATCTGAGGTGGATATCTAC
ACAAATCCTCCTAATCGGCCAGCACCTGTCTATCCTCAACCAACTCCCGACAAGACAGC
>1002049865_1 Heli.1-DT666521.3.5
GCACGAGGTACTATTCTTTATAAGAATTAATTAATATTTTATTAGAAAAGTGCGTGAAATGTTTCGTAAGTGT
ATCATATTGGCGGTGTTAGGGTGCGCTGTCGCAGCCCCACAGTATCAAATTCAGCAACAACAAGATGAAGGCA
TTCCTCCGCACCTACTGAGACAGTACATCGGTCAACAACAAGCACAGCATATACCGCG
ACCTGCACCTCAACCAGCGCAAGCACCTGCCAGACTTCCTTACAATGTACAAGAAGAGCAATACCAGCCCAGA
GCCCAATACCAGCCCCAGCCCCAAGCGCAAGCGCAGCCCCAGTACAGGCCACAACAACGCGAACCTGAAGATT
ATGACCCTAATCCTTCCTACCAATTCGGTTTCGACGTGAACGACGATCAATACACGAA
CTACCAGAACCGTAAGGAGCAAAGAGACGGTGATGTCATCAAGGGCTCTTACTCTGTAGTCGACAGCGATGGT
TTCGTACGAACTGTTACTTACACTGCTGACCCTAAAGAAGGTTTTAGGGCTGAGGTTTCACGTAAACCCACCG
ACATCGTAGTAAAGATCCCCACACCCAAACCTCAAATTCAGCAACAACAGCCCCAACC

TCAGATTCAGCATCAACCCCAAATTCAGCAACAACTCGCTCAGAGACAACAACAGCAACAGCCTCAGCAATAC
TATCGTTATGAATAAATAATGCGAATATAATATCCACTCTTACATATTAGGACCAAAATCTAAATATTTATCC
TCCATTTGTCGATTTTTCTTACTACATCTTTATTAAAAACGAAATGTTTTAACATTTT
CTCTTCATTATCTTTGTCAACTTCGTTTAGAAAAAAAAAGATAAAAAATACGATTAATTTTCAAACATTTTA
>1002049866_1 Heli.1-DT667018.3.5
GCACGAGGATTGCCCCTAGATCAAGAAAGCAAGAAGATAACGAAAATACAGAAAAAAGTACAGAAGTTCCAAG
TACGACTGACGGATGCAGATCAAATAGTGGATATATAGAAGACAACCTTCAACCGACCCTTCAAGTAATTTCT
TCGAAAATATAGTTGTTGATGAACAACGTGCAGGAAAATTAAAAATATTTTCTTATAA
CATTTCATTAGAATTAATGGAGTGAAGAGATGTCAATTATTTTATACTATTTAAAAAAAGCCATAATACCGCC
CATCGCCATTTTTAAAAGAAAAGGAGGTTATCAATTCGACTGTATTTTTTTGTGTTTGTTACCTCAGAACTTT
TGATAGGGTGAACCGATTTCGATTATTCTTTTTTTATTTGAAAGCTAGTGATTCCCGT
GTGGTCCCGTTTTAATTCTTTCTTGTCCTTTCTTGTCAAGGGTATCCATGAGAAAATTACATAAGTTTTAAGT
TTGCATTAGGTATGTGCGCGACAAATGGACGAATAACTCATTACGCTAAATGATTGCGAAAATTCCTCTTTTG
ATTGAAAAGGATACACGTTAGTGGAAATTTGATGAAAGTTTGATTAGTTTATGGGATC
CGGGTAAAATTGAAGAAGTCGGTCTATTTTTGTTAAAAATTATAAATATGATTTCACTATGACATTACCGTCT
AAACGCTTAGTGTAGACACGCCTAGCAACATGCTA
>1002049867_1 Heli.1-CV525911.2.5
CGGCACGAGGGAAATACTCATACAATACTATTTTTATTTCCTTCTTCTATGTATATACGTACAAATTAAAGTT
AATTGATTGGTTATTACTAAACAAAGTAATAAGTGCGTGTGCTGTATTCATAAGAAGATTTATTGTTTGTTTT
TGTTTGCTTAAAATACATCAATAAAATGAGAAATCATGTTGCTGTTGTAAGATGGCCG
TTTCGTAGACTATGCGAGCGTAGTGTTGCCACTTCCGCTAGTGTGAACCAAGCAACTGAGGGATCAGATCTAG
GTCATTTATCACGGCCTCAATCGTGTCCAGTGAAGAATCATCGTGCCAGGTCTACACATGCCGTAGCATCGGT
ATTTGAAACTCTATCGCCTTCAGTAAAACCGTGGGAGGAGGTTCCAGGTCCCAAACCC
TTGCCACTACTAGGAAATACTTGGAGATTCATTCCATATATTGGTGGATATTCAGTAGAGCATATAGATAAAA
TTTGCTTATCACTGAGAAAACAATATGGCAAGTGTGTCAAAATGGCAGGTCTCCTGGGAAGACCGGATATGTT
GTTCGTGTTTGATGCTGGCGAAGTTGAGAGGGTCTTCCGAGGAGAGGACAGTGCTCCT
CATAGGCCATCTATGCCATCTTTAAATTACTATAAGCATACACTAAGGAAGGATTTTTTTGGTGCAGAAGAGA
ACTGTGCTGGTGTGATAGCAGTTCACGGCGATTCATGGGCGGCGTTTAGAACCAAAGTATCTCGAGTAGCGCT
CAGTACTGGCGCAGCAGCGCAATACACAGAACAAGTAGGGGAAGTGGCAGAGGCATTC
TTTAATAGGATACGAAAAATCAGAAATCAACAATCT
>1002049868_1 Heli.1-EL601005.1.5
GCACGAGGGAAAAGATCTGAACTCACCACCCAATACCACCCCTAAACCTACAACTACTTCTACGACTTCTACT
ACGACAACTACAACAACAACTACTACTACTACTACACCAGAACCTCCAACAACAACACCATTTATTACAACAA
CAACAACAACAGAAAAGCCAACTACCACGTTAGCTACAGAAAAACCCACAACAACAAC
AGTGCCAGAAGGAGAATACGAATATTACGATGAAGAAGATATAGAATACGAATCCAAAGATAAAAAGGAACCG
ACTACTACCGTAATAATTAAACCAGCTCAAATTAAAGAAGTACCTACTACTACAGTGTCATTCTCCGAAGAAC
TAACTACAGAAAACAAGGCAAGAGACTTAGCAACAAAAGTTTTGAGAAATTTCAATGA
TAACTACGAAGCTATTAAAGAACAGCTCGCATCTACGCTGACACCAGGGGATTATCTAAAACCATATTTAGGT
CAAACAATAAATAACAAGAAAGCGGAACCACAACCGGAAATATCAAAAGGCTATACAGCAACTGGAAAACCGT
TACAAATACCTGACATAAAACAAAATTTGGCAGATTCTTTAGAAAACGACTACGATGT
TAGACTAAATGAAGCATTAAGCCCTAAAATAAGGATACCCAGTGGTTTTATAGTTCCTGATAGAGATTATACG
TTTTCGAGATTCAGAAATAACGTGCATTCCGAACCGCAGTTTGCTGCTTCAGAAATTAATCATTACCAAGTTA
GGAAACGACCCCAAATTTCTGGAGTTTCGATCAGAACTCCTTCATCATACTACATTCA
ACCCCAAAGAGTGGTCTACGAAGACCTTGTGAGACCAGCTCGGCACTTAGTGTACAGGGAGTTTGGTAATATA
TTTTAATATAGATAAGGGTAACCTTTGAGAGATTCAAAAAACTTTGAAAGTCACCTATAGACTTTTGGAGTTT
TTAAATATATGAGATATACATATATATATATTAGACTGACATACAATATATAGAAATT
TAAATGATATATTATGTCTTAAATAATAAATTCAAGTATTTAAATATAAATTAAAACGTC
>1002049869_1 Heli.1-DT663440.3.5
GCACGAGGGGTGATTCGATATTGGGAGTAAAATCCTGAATAGGGATAAAAAGGCTTATATTTTCCTAAATCAA
TATGTCTGGAAAAGATAAATTGCCCATTTTCCCTTCTCGGGGAGCTCAGATGTTAATGAAAGCCCGTCTGGCT
GGAGCACAGAAAGGCCATGGTCTCTTGAAAAAGAAAGCTGATGCTTTACAAGTGAGAT
TTCGTATGATCCTCAGCAAAATTATCGAGACTAAAACCCTTATGGGTGAAGTTATGAAAGAAGCTGCATTTTC
CTTGGCCGAAGCAAAGTTTACTACAGGAGACTTCAACCAGGTTGTTCTTCAGAATGTTACCAAGGCTCAAATA
AAGATCCGCTCCAAGAAAGATAATGTTGCTGGTGTAACCCTCCCAATTTTCGAGTCTT

ACCAAGATGGCTCTGATACCTATGAGCTCGCTGGTCTTGCTCGCGGTGGACAACAACTGTCCAAGCTCAAGAA
GAACTTCCAAAGTGCTGTCAAATTACTTGTCGAATTGGCCTCCTTGCAAACCTCATTTGTGACTTTAGATGAG
GTCATCAAGATTACTAACAGGCGTGTTAATGCTATTGAGCATGTGATCATCCCCCGCC
TGGAGCGTACCCTGGCTTACATCATTTCGGAGTTGGATGAGCTTGAGCGTGAAGAGTTTTACCGGCTGAAGAA
GATCCAGGATAAGAAGAAGATCATCAAGGACAAGGCGGAGGCTAAAAAGCAAGCAATGCTGGCAGCGAGCAAA
GAGAGAGATGTGCGCGACATCGCCAATCTATTGGACGAGGGTGACGAGGATTTACTTT
TCTAAATATCCCATCTGGATTAAA
>1002049870_1 Heli.1-DT664400.3.5
GCACGAGGGTGAACTTCATCAAAGATTAATGACCAATATGGAAATATACAAAAACAAACAACAATAGGTTACT
CCACGGAAACACTACCTCAAAAAGTGACGCAAATGCAAGGAGGAAAATCTGTGGAATGTTCGCAGAGTGGATT
TGTGGGTGACCCAAATGATTGTTCTAAATTTTATAGGTGTGTCGATAGCGGTAACGGG
AAATTTATGAAATATGAATTTTCTTGTAGCCAAGGCACGGCGTGGGACAGTACAGCCGAAGCTTGCAACCATG
CGTGGGCTGTAAAGGAATGTGGTGGACTGTCACCAATTAATAGTGTTTCCCCTAGCACTACTCTAACAACATC
TAGTGATAGTTTTGTTTCTAGTAGCAGTTCAACGACTTATAAGATTACTACAATTAGC
ACTACTAACGATGGTTTAATTAATGAAAATAATGGTAATGATTACCTTAATCCAATAAAGGTTCAAGAAGAAA
CAACCAAGCCCACTACTGTATCACTTATTTCTAGTACAACATCTACAACAGAGTCTGTTAAAGAATGTAAGGA
TACTAGCTTTGTTGGAGATCTAAATGATTGCCAAATATTTTACCGCTGTGTTGATAAT
GGCAACGGAGGTTATACCAAGTTTGAGTACAAATGTGGAGAAGGTACTTTATGGGATCAGGATATCGAAGCGT
GTAACCATGCGTGGGCTGTAAAATCTTGTGGGTCTAAGTCAACTACAAATATAACAAAAGTCGAAAAAGAAAC
AACTAAAGTATCAGATAAATTTACCACTAACTCATATGTCCAAGCCACGACAATGAGC
GTTATACCAAGTAATAATTATAACACAGAATATAATGCACAAGAGACACAAACTAAGAGACCTGATAAAAAGG
AAATAAACGAGGTTTCAAAAGAACCGGAAACGAATAAAAATATATGTCAAAATGTTGGTTTTATCGGTGATTC
TAATGATTGTAGGAAGTTTTATAGATGTGTAGATAATGGAAACAGA
>1002049871_1 Heli.1-EL598633.1.5
CGGCACGAGGGTCGGCCGTGTCGAGAGGTCCGCTGTGTAGATTGTATTTGAATTAGAATTTGAAACCTCCAGC
GTAAAAGACAAAATGTTCTCCCGCGCAGCAAAACCAGTAGCCACAGTCGCCCAAAATGGCATCAAAAACTTCT
CTACAACATCACAGAAAAACTTCAAAGTTGTGGTGGCGGGAGCTGCTGGAGGTATCGG
TCAGCCCTTAGCCCTATTATTAAAGCAAAATAAGCTTGTAACTAGGCTGGCTTTATACGATATCGCTCCAGTA
ACCCCTGGAGTAGCCGTGGACCTCTCGCACATGGACACCGCAGCCAAGGTATCTGGACACCAGGGCCCAGACC
AACTGGGTGATGCCATTAAAGGCTCCGACGTAGTAGTAATCCCAGCCGGCGTACCCCG
CAAGCCTGGCATGACCCGCGACGACCTGTTCAACACAAACGCCTCAATTGTCAGAGACATTGCTGCCAGTATT
GCCAAGAACTCTCCGAAGGCCATCGTGGCCATTATAACAAACCCAGTTAACTCGATGGTGCCTATTGCTTCTG
AAGTGTTGAAGAAGGCAGGAGTATATGACGCAAACAGGGTCCTCGGTGTCACCACCCT
AGATGTAGTCCGGGCTGCCGCCTTCATTGGAGAGATCAATGGGGTCGACCCCCAGTCTGTGTCCATCCCGGTG
ATTGGAGGTCACTCCGGGGTCACCATTATCCCTGTCTTGAGCCAGAGCAAGCCAGCGGTCAAGTTGTCAGAGC
AAGCCAAGATTGATGCGTTGAC
>1002049872_1 Heli.1-DT664225.3.5
TGGAAAAAAACGATTGCAGAAGACTTGGTTGTTACCAAGTATAAATTAGCAGGGCAAATTGTTAACCGTGTTTT
AGAGCAAGTTATAGCTAAATGCGTACCGGACGCTTCAGCGAGAGAAATATGTGAATTCGGTGATAATTTGCTA
TTAGAGGAAACGTCGAAAGTTTTTAAAAAAGAAAAGGATTCCAAGAAAGGAATCGCTT
TTTCGACATGCGTTTCCGTAAATAATTGTATATGTCACTTCTCTCCAATACCTAGTGAGGCTGACTATGTATT
GAAACTAGGGGATCTTGCCAAAATTGACTTGGGTGCCCATATTGATGGTTTCATAGCAGTTGTAGCTCACACA
GTAGTTGTTGGTGGTGGGGAGGCATCCGGTAGAGCTGCAGATGTCCTTCTTGCTGCAC
ATAATGCTAGCGAGGCCGCCTTGAGATTATTAAGACCAGGCAATGAGAACTATGCAGTAACAGATGTTGTTCA
AAAAATTAGTGCTGAATATGGCTGTAAGCCCATAGAAGGCATGCTTTCTCATCAATTGAAGCAGTTCCGTATT
GATGGTGAAAAAAGCATAATTCAAAACCCATCAGAAGCCCAAAGGAAAGAGCATGAGA
AGGCCTCTTTTGAAACTTATGAAGTCTATGCCATGGATGTACTTATTTCTACTGGAGAAGGTGTTGGAAGGGA
AATGGATACAAGGTGTACAATATACAAAAAAACTGATGAAATTTACCAGCTTAAATTGAAAGCATCTAGAATG
TTCTACAGTGAGGTCCGCAACAAGCATGGATCAATGCCTTTCAACCTGCGCAGTTTCG
ACAAAGAAACTAGTGCAAGACTTGGTGTTGTTGAATGTGTAAATCATAAACTCATTGAGCCTTTCCAGGTATT
GTATGAAAGGCCGGGTGAAATTGTAGCACAGTTCAAATTTACAGTACTGTTGTTACCAAGTGGCACTCATCGT
ATCACAGGACTACCATTTGACAAGAGTCAGTGTAAAACTGAACGCATAATCAAAGATC
CTGAATTGAATGCCCTTCTGAACTCGTCCGCAAAGTCAAATAAGAAGAAGAAAAAGAAAACTGGTGCTGAAGA
ACCAATGGAAGTTGAAACGGCAGCCTAAAAAAAATTCACAAAATATCTGTGGGCACTCA

>1002049873_1 Heli.1-DT666567.3.5
GCACGAGGAGATTTCTTAGGGGGTAGAAAGATTTATAAATGCGACGGTTGTGGATTCGATTCCCACTCATTGA
AATGAGCTATCTTCATTTCCCTGCTATATGAAGGATACAGCGTGAACGTAAAAGATAATATTTAAAAAAATTA
AAGAATTTTAAAAACAAATAGCTTGTGATATATTCTCTTTGAAAGTAAATAGATACTT
AATAGATTTTTAACCAACTTCTGTAAAAAAAGGAGGTTATCAATTCGACAGATTTTTTTAAATGTTTTTTACT
TCAGAATTTTGAACTGGTTGAATCGTAATATTTTTTATTTGAAAGCTGGTGCTTCTCGTGTGGTCCTATTTTA
ATTTAGTTAATCTATGACAATGTCATTCATGAGAAAATCATATAAGTCTTAAATTTGC
ATTAAGTATGTGCGCGACAAAATGGATGAATAACTCAATATTCTGTCAACCGATGATTCTTTTTTTATTAGAA
AGGATAGATGATTATGAGATCCGGTTGAAATTGAGGAGTCAGTTTTTTTGTTATTTTAAATATATACTTTTTT
GTTTAATTTTTAAAAGATAAGACAGTAACAAAATTTTCGTTTTCCAAAACCAGGTATA
AAATAAAATATTCAAAAATAAATCAAAACACAAAGCTGTAACAGAAATTTCATAGTCAAAGATAAATTCTTGA
TACTAAATTTTGATTCGTAGAAATGAAATCTTTGAAGCTCACATTAATAGGTCACTTAGAAATTACAAGTTCA
AAGTTGGCTCTTAGCAACTCATTTATCATTCATAATATTCATTTTTCATGAATCCACT
GTATATTTATTTTGATTGAATATAAATCGTTATCATTACTACTGTGCTTATATCACTCGCTACTTTATCCCAA
TCGCTTAGTTCTTGGATTTAAATTTTTGTCTAGGTATAGTATGGTGATAACAACATAATTTACAGATTTTGAT
ATATACCTATTAATGACATATTACATTCAGGCCTTTGTGGCGTAGGGGCTCACGCGTG
TTACTGCAACGCGACAGTTTTGGTTTCGATTCCTACTGGAAGGAATGTATTATTATTTATTAATATTTTCATT
TCTTCACTCTG
>1002049874_1 Heli.1-CO729753.1.5
CGTCCGCTGATAGAAAACAGACCAATATGCTGAAGCTGGTGGTTTTATCTGTCGTGGTCGCGTTGGTGGCCGC
CGAACCCATCGCTCCTCTTTGGGGCGGTCACCTCGCTGCTCCACTTGTTGCGGGCCCATTGGCAGCCGTCGCA
GCACCCTTAGCCGCTGCTGCTGTCGCTCCAGTACCCATCGGTCTTGGAAACTACCGAG
GACCACTGTCTCTCGCACCTGGCCAGCCCGCTAATGTCTTAGCTGTAGACGGCAGACCTCTCGACACTCTCGA
TGTTAACTTAGACCGTGCTGCTCACTACACTGCGAAGGCTTGGAATCCTGCTGGTCTTCATTTGTTGAAGAAA
AGATCATTGGTAGCACCTTGGGCCGCCCCTGTAGGCTTGCCCCTGGCTGCTCGTGTGA
TCGCCCCCGCACCTATCGCTGTAGCCGCTGCCCACGGTGCTGTTGTAGCTGCTGGTCCAGTGGCCGCCTGGGG
CTTGGGGCATGGTGCCCGATTGGGACACCTTTGGTGTACACTATGTATATATATTTCGTAAGGCTATAATA
>1002049875_1 Heli.1-EL603885.1.5
GCACGAGGGAAGCAACAGACGAAGATTGAAGCAGGAAAACCGAAGCAGAAGAAGACCCATGCAGATGAAGACC
GAAACATATGATGATTGAAGCAGGTGAAGACCGAAGCAGATGAAGACCGAACGAAGCAGATGAAGACCGAACG
AAGCAGATGAAGACCGAAGCAGATGAAGACCGAAGCAGATGAAGACCGAAGCAGATGA
AGACGAAGCAGACGAAGACCGAAGGAGATGAAGATGAAGCACATGAAGACAACAGACGAAGATTGAAGCAGGA
AAACCGAAGCAGATGAAGATTCAAGCAGTAGAAGACCCAGGAAGATGAAGACCCAGGCAGATGAAGACCCAGG
CAGTTAAAGACCCAGGCAGATGAAGACCCAGGCAGGTGAAGACCCAGGCAGATGAAGA
CCCAGGCAGATGAAGACCCAGGCAGATGAAGACCCAGGCAGATGAAGACCCAGGCAGATGAAGACCCCGGCAG
ATGAAGACCGAAGAAGATGAAAACCGAAGCAGATGGAGACAGAAGCAGATGAAGGCCAAAGCAGATGAAGACC
GAAGCAGATGAAGACCGAAGCAGATGAAGACCGAAGCAGATGAAGACTGAAGCAGATA
AAGACCGAATCAGATGAAGACCGAATCAGATGAAGACCGAATCAGATGAAGACCGAATCAGATGAAGACCGAA
CCAGATGAAGACCGAATCAGATGAAGACCGAATCAGATGAAGACATATGAAGACCCAGGCATATGAAGACCCA
GGCAGATGAAGACCCAGGCAGATCAAGACCCAGGCAGATGAAGACCCAGGCAGATGAA
>1002049876_1 Heli.1-EL597623.1.5
TCATACAAAGATTGGTGGATTAAAGATCTTACAATAATGGCTCTCCCACGAGTTTTTATGGATGTCACCGCCG
ATGGTTCACCTTTGGGTAGAATTGTGATTGAGTTGAAAGCCGATGTAACTCCCAAGACTTGTGAGAACTTCCG
TGCTCTCTGTACTGGAGAAAAAGGCTTCGGATATAGAGGCTCTACCTTCCATCGCGTG
ATCCCTAACTTTATGTTGCAAGGGGGTGACTTCACAAACCACAATGGCACTGGTGGCAAGTCTATCTATGGTG
AGAAATTCGCTGACGAGAACTTCGTCCTGAAGCACACTGGCCCCGGAGTTTTGTCTATGGCCAACGCTGGCCC
TAACACCAATGGCTCTCAATTCTTTGTCACCACTGTAAAAACTTCCTGGTTGGATGGA
AGACACGTCGTTTTCGGCAATGTTATTGAAGGCATGGATGTTGTTAAACAAGTGGAAGCACTAGGCTCCCAGT
CTGGCAAACCATCAAAAAAGGTGGTCATCGCTGACTGTGGACAGCTTTCTTAAGATAATGTTGTAATATTATA
AAATGAAATGCGATAATTTAAGTTTTAACACGATGTCCTCCCAAGTAATACCTTCCCA
GTCATAAAATTTACCAATATATTTGAAGTTTACTGAGGGTCATTCCAACTATTTCCACTATGTTATATAAATT
TATAACTATGC
>1002049877_1 Heli.1-DT666676.3.5

CTGCTGATGGCGGCCGTGTTCCTGGTGGTGGCGGTGCTGGTGGCCGTGCTGGTGCAGCGCCTGGTGGAGCCGC
GCCCGTGACGCGCGCTGGCGGCGGCGCGGCCGCGCGCCCTGGCCCGCCTGTAGCCGCCCGAGGCGATGGAGTG
CAATTATTCGATTTATATAAACCTTCGACGAGACGCCGCCCCGCGGGAGAGAATCTGA
AATATTTATATGTATATAGTAGATAATAAAATAATCAAATTGTACGCCGCCGCCATTAAATGACGGGACGCGC
GAGGTAATCGCTGTCTTTAATCGCTTTATTTTTAATATATTTTGATTTCGGTTAATGAATATTCAGATGAACT
AGACGATGAGTTTATTATATCTCCAATATTTTAATCGACTGTACTAAACGAAAGGCCT
AGGGAACGTCGAGTTTAACGAGTTTTTTTAGATTTCACGTATATGTATTTTTAGTGAATTTACAATTATGAAT
TTGCTATTTTGATGCGAATGTATATGTAGGAAGTGTTAACTTTGCCTAATCCAATCTGTTTCGCAGATCTTCT
AAGTTCTAGTCTATGCTTTTAAAATTTCATATCTTCTGCTGAATCGTCTATTTTACGT
AAATTATATGAAACATAAAAGTTGTATTTATTGTGATATAAAATATACTTCTCGATTATAAAATCACCCTGCC
GCTTGTATTTCCCATACATTTCTATATTTTTATTAATAAAAAAGATCTGTTGCATGTTTTATCATATTTAAGT
ATAGCAAGTTAGCGACCAAGGTGCTCTAATATTAATGTTCTGTTACGAGGGACAAGGC
CCCTCGCTCTCCAACGAGTTTCCAGTGTGTGTGCTGTGATCACTTCCTTATGCGAATGAGACTGATTTTAAAA
ATATATGCTTCGTGTCTTAGGATTGTTTTGACAATGCTGTCGTTCATTTTGATGAAAACCGAAGTGTTTTATG
ATTTCCCGAGAACTTAGCAACCCGCCAGCTGAGGCGCGGCGACCGACGCGCACGTCTC
TTTTACCCTCTAATGTACCTTCTG
>1002049878_1 Heli.1-EL603369.1.5
GCGGAAGGCGCACGAGAGCGAGGTGCGCCGCGAGGTCGATAAGTTCAAGACTGAGTTCCTGGCGAGGGGGAGC
CCTGATCTCGGACAGCTCAGCTCGAGGCACCAGCAAGAAATGGAGGAGATAAAGCGTGAGATACTCTCGTTAT
CCGAGAAGTATTCAGTTAAGTGTGTGGAGTCGGCGTCGCTGGAGGAGCGCCTCGCAGC
GGCCAGTTCACAGCTTGCGCACGCGCACAATCACATCATGCAATTGGATGCCAGGAACAAGCAGCTCCGAGCT
CACATAATGTCTGAAGCTAACGACATGAAGAACTCTGAAGCGTCCACACTTGCTCAACTCATTGAAGACACGG
CACCGGGGAAATCCCCAAGGTCGAGCGGGATGGAGCGAGCACGTGCATCCGGCCTGCA
GTTCTCGAAGACGGAGTCTCCCAGAAAGCAGTTGTCTCCCGTTCAGAACTAGAGACGGAATCTAGCAGCGTGC
TCGCAGGCGTCGTCGCAGAACGGAAAAAACAATTTGAAACATAACGACGCTATTTGATTGTATTTTACGAGTA
CCCGCCATGTCTATATATAAGTATGGCGGGTCCCGCACTTACCGGGTCACAAGCTTCC
CACCACACCGGCACCTGGCCACTTAGCTTCTAGCTATAAATGGATATAAAATATTAATATAACATTAAATTAA
ATACCTGTTAGGTATAAGCTAAG
>1002049879_1 Heli.1-DT667901.3.5
GGCGCCGGGCTGAGGAATCGGCACGCGGCTCGAGTAGATTTGATTTGAATTTTAATTTTTTATAACATAGAAA
ATATTTTACTATAATCATATTATAATAATTAAGTCGTTTCCGACAATACCAAAAGACAATTACTTTAGCATTC
AGTATGAGTAGTGAACCTGTAGAGGAACGGTCCAAAATGAGTGCTTTAGATCAACTGA
AACAATTTTCTACTGTTGTAGCCGATACAGGTGATTTTGAAGCTATGAAAGCTTATAAGCCCACTGATGCAAC
AACAAATCCAAGCCTTATTTTATCTGCTGCTGGAATGGAACAGTACCAACACTTGCTGGACAAAGCCATTAAA
TATGGCAAGGATTGTGGTGGAAATGTAGATGAACAACTGTCAGAAACATTAGACATGC
TCAGTGTACTATTTGGCTGCGAAATCTTGAAGATAATTCCTGGAAGAGTATCAGTTGAGGTGGACGCCAGGTT
ATCTTTTGATAAAGATGCTAGTATGGCAAAGGCTATTAAGCTTATTACTATGTTTGCAGAGCATGGTATTAAA
AAAGAAAGGATTTTAATAAAATTGGCCTCAACTTGGGAGGGAATTCAAGCTGCAAAGG
AACTGGAGAAGAAGCATGGTATACACTGTAATTTGACGTTACTTTTCTCGTTATACCAAGCGATAGCATGTGC
GGAGGCGAACGTCACTTTGATTTCACCGTTTGTTGGTAGAATTTTAGATTGGTACATTGAGCATACTAAGAAT
GCGTATGAGGCTAAAGATGATCCAGGTGTGCTATCAGTGACTCGTGTATATAATTATT
ACAAGAAGTTTGGATACAAAACCCAGGTGATGGGTGCGTCTTTCCGTAACACTGGAGAAATAAGGGAGCTTGC
TGGTTGCGACTTACTAACAATCAGTCCAAAACTTTTACAAGAGCTTGCTGGTAGTGGAGAACCATTAAGAAGC
ATTAGACGCC
>1002049880_1 Heli.1-DT668481.3.5
GTGAATATTCTTAAATTAGATCCAATTGATTCTACAAAAGATTTCATCGACAATGAAAACGTAGATTCGTCAT
TTTTATTACAAAAAATTAACAAAAATACAGACTCTGAGAAAATAGTATTAGAACCCTTTGAGGAAAAAAGTAA
AAAGGATGGTGATGAAAATACAGAGAGACATTCAGAAGAAAATGTAAAAATGATTAAA
GCGGCCAATAAAGGACTCTGCGATGAAAATAAACAGGAAAAGTTTCCGTCTAAGTCATCAGAAGAACCACTAA
ATTCACCATATAAATTTGAAGATGATAAATCATCAGATGTACTACATGTGAAGTCTACAGATATGCCAAAAAT
CATACCTATCGATGGCAAGAAACATATTACAAAAGAAATTATAGAAGAACCAGATGAT
ATACAAGAAATTGAATTAGAGCCGAAATTGACCTCTTTCGTCGGAGAGCATAAAACAGAATTAAATAGAGCAA
CACCTTTGCAAAATGTTCAATTTATTGTAAACCAAAATATAATTTCAGAAACAGAGGAACCAATAGATGAGAA
CATTTTAAGAACAAGTGTCACGAGACAGGTAGATTCACAACATTCTCCACAATCAAAA

AAAACTAATGAAAAATTATCAGAAATATCTTTTAGTCCAGTTAAGCAGTCAATGGAAGCTGAGAGTTTTGATG
ATCTACCACCATCGTTTAAAAGTATTACGGAACAATTTTTATCTCAA
>1002049881_1 Heli.1-DT664596.3.5
GCACGAGGCGCAGCGCGTGCGAGATGACGCGCCGGTGAATTTTGTATTTTAGAAATTGACGTCATTTTACTGT
GTACGCTCTATTTATAAGGTGAATTCTTTTATTATGAGATGACGCGCCGGTGAATTTTGTATTTTAGAAATTG
ACGTCATTTTACTGTGTACGCTCTATTTATAAGATTTCACAATGGCCGATCAAGTAGA
TTCAATGTCAGACGCGGAGCTCCGCACTAAACTGGCCGAGCATGGGTTCCCTGTTATGCCAATTACAGCATCT
ACAAGAAAGTTGTTAGTGAAAAAGTTAAAAATGGTATTGGACAACAAAACCAAACCCAGAAACAGTGTGGACG
CCAAAGTCGAGAGCAGACGTTCTTTAGCCAGGTATTCCAGTGGTGAAGAATCTGATTT
AGAATCTAATAATGCGAAAGAAAGACGAGGTCGGCGAGTTACAACTGGCGGGGCTATGCTACCTCCTGTGACC
AACAAAGCTAGACGAACCCCAATAAAAAAAGATTCACCTATCAAACGTGATGCTGATAAAGGCTCTGAGTCCG
AGGATGAAAAATCACCAGTCCGCACTCATGAAGAAGTTGAAACTGTTACAACAAGAAG
AATAACACACACCTATGCAACAAATGACCAAGATGACTATGAAACTGGTTCAGATAGTGATGTTGACAATGAT
AAGAAAACATCCAGTCCATTTAGAAGTAGCTCTAGGTCTAGTGATTACATTTCTAGCACTCTACCCACTAATG
ACGCGTCAACAAGCCCACCCAAAACTTCTATTTTTTCCCGCCCATCTTTATCTCAGTC
AAACTTTTCATCATTATCATCTTCAGACCAATTGAATTCAATAAGATCTAGACTCGGGCTAACTTCATCTTTG
GCTGACAGGCCATCTCTTAATAGTTACACACCAAG
>1002049883_1 Heli.1-EL595937.1.5
GCACGAGGACAAATTCTAAATTGTAGGGTATTTTGTTTTGTTAGTCTTCATAGAACCGACGACGAAAGTGGAC
AGTGGATTTTAAACGTTAACAATGACAAACGCATTTGCAGTTTGTCTTATTACAATATCATTCTTTTGTTGTA
CAAGTTTGTCAACTGCTCGACAAATTCCAAAAGAATGTTCTAAGGGACCAGAATATTG
GTGTGAAAATTTGAAGCGGGCTGCAGACTGCGGCGCAGTGCAACACTGCATCGGTACAGTATGGGAACAAGAA
CGAATCGAACTCAAATCCAATGAAGTGTCCGACAAGATCGTCCGTCTCTTCAGACAACTTAAGGATGTCAAGG
ATATGATTAATGATGAATATTTAGGAACTCGTGTGAGCTCCGCTTGCCGCGATGTGCC
GTCGCCGGCCATCACGGCCGTGTGCAAGGAGAACACAGCGGAACTACAACAGTACATGAGCCACCTCCTCCAA
TCCGACACTACTCCTGAGACCATGTGCAAGATAATCGGCATGTGTAACAACCCTAAAATTGATCGTTTGATCT
CGAAAAAGAAGACTGAAGAACCTACCAAACAAAAGCCACATCTATTGGGAGCTACGAA
ATGTACCTGGGGACCTTCTTATTGGTGCAGCAATTTCAGCACTGGTCGTGAATGCAAAATGACGCATCATTGT
GTGCAACGTGTGTGGTCCAAAATGACGTTCCCACAAGACGATGACGG
>1002049884_1 Heli.1-DT662505.3.5
GCACGAGGGACACGCATTAAAAGTAAGAAATAATCATAAGTTTGACCTTTTAGCGTTGTAACAAAAATGGCAC
CATCAACTGATTGGGATGAAATCAAACGTTTAGCAGCTGACTTCCAAAAAGCTCAACTAAGTTCAACTTCACA
ACGATTATCAGAACGAAATTGTGTTGAAATTGTATCAAAATTAATAGAATTAAAACTA
TTAGACGTCATTTTTACAAATGATGGAAAAGAATATCTAACCCCACAACAACTTATAAAGGAAATGAAAGATG
AATTATATGTAAGAGGAGGAAGGGTAAATACTGTAGATTTGGCAAAGGAGCTTAATGTGGATTTTAATCATAT
AAATGTCCATGTAGCTGAAATATTAAAAGGAAAAGATGTACAATTAGTTTCAGGATCT
TTAATTGCAAACTATTATTTGGAAAAAATTGCTAGAGAAATTAATGAGAAACTTCAGCTACAAGGACAAATTA
CTGTTGGGGACCTGATATTGCAATATGACCTACCAGCAGATCTTCTACAACATGGTATTATGGAAAAAAATCT
TGGTAAGATTATCAATGGAAACCAAGATCCATATGACCCGAGGATATTTTATACTGCA
GAGTATATTATGAGAATGAAAGCAAAAATTAGAGGGGCTTTAATGGGTCTGCTTAAACCTACACCTGTAAGCT
TAATCATTTCTCATTGTAACATAGGTGAAAGACTATTCATGTATCTTTTTGATAAGTTAAATGCACCAGGCGT
TCTCACTGGAAGACAAGCTGGAGCTCAATATGTACCTTCATGTTACACAAATCCCAAA
ATGAGTGGGTTCTAAGCTTTTACAACAAAACATTAT
>1002049885_1 Heli.1-DT666571.3.5
GCACGAGGGTTAAACATAAGTGAAATTTAAATTATAATTCATAATAAATACAGTTAAAAACAAATAAGTCAAT
ATGATAAATGCGGTCAACGAGTTCCTAGATGACCTGCGTGGCGGCAGAATGATTGATTCACCGGTCGTGAGAG
TTGAAGAGGGTGAATTACAGGGGAAACTGGTGAACTCACCGAGTGGCAAGGCGTTCTA
TAGCTTCCAAGGCATACCCTATGCTAAACCACCTATTGGATCTCTAAGATTTAGAGCGCCACAACCACCAGAA
CCATGGGAAGGTATACGAGATGCCACATGCGAAGGCAACATTTCTGCTCAAATACCACCTTTTGAAAACCCAT
CATATTCGGGAGACGAGAATTGCTTATTTTTAAACATCTATACACCAAATCTCGACGG
TGAATTCCTCCCAGTTATGTTTTTCATCCATGGAGGAGGATATCGTTTTGGATCTGGCAGTGCCAGTCTTTAT
GGTGGCGATTATTTAGTTGAAAAAGATGTAGTCGTTGTAACCATCAACTATAGAGTTGGGCCATTGGGTTTCT
TAAGTCTCAATATTCCGGAAGTGCCAGGAAATGCTGGAATAAAAGATATGGTTCAAGC

TTTACGATGGGTTCACCAAAACATAAAGAATTTTGGTGGTAATTCGGGAAATTTGACCGTTTTCGGACACAGT
TGTGGTGGCGCGGCCGTATCTTTGCTCACAGCGAGTCCATTGTCGAAGAATTTGATAAACAAAGCTATTGTCC
AGTCCGGCTGTTCGTTAAGCTGTTGGGCAATGCAAAAAACACCGATCGAAGATGCCAG
ACGCTTAGCGGAAGAATTGGGCTGTGAATCATCAGATGTTTCTGATATTTTAGAATTCCTATCAACAACACCA
GTGATCGATATCGTCGAAGCTACCGAGAGAGTTTTCCCATTTAATGATAACATTAANAAATTCACAAATTTCT
TCTGTCCGGTCGTTGAGAAAGAGTTTCCTGGAGTCGAAGCCTTCATGACGGAGCCTTT
>1002049886_1 Heli.1-EL603568.1.5
GCACGAGGCTTGAACTTTACAAAGCTAAATTTGTCGATCTTATTTACTGTAAAACCACCCATTCACCCATTTT
ACATAGCTATTTCAAATTAAATAAAATTTAATAATCATATTCACATCTCCAATCACTAACAATGCCACGCGGA
AAATATACCAACCACAAAGGGCGTAACCGCAAATTCACTAGTCCTGAAGAGTTAGAAG
AACAAAGGAAACAGGAAGAGCTAAAACAAAAATGGAGAAAGGAACATGGTGATGTGAGTTCTAGTGAAGAAGA
CTCTGAGGGAAACAAATCTGGTTCAGATAACAGTGACGATAGCGATTCAGATGAAGATCATCCTACAAAAGCA
AAGGGAGTGTCAGGTCTCATTGAAGTAGAAAACCCAAATCGAGTAGTAAAGAAGAATA
AAAAATTATCTAATCTAAATACCCTTGGAGAAGCAGAGAAACCTCAACTCTCAAGGCGTGAACGTGAAGAAAT
AGAGCGTCAGCGAGCAGCCGCTGCGTACCAGAAGGCCCACGCAGAGGGTAAGACCGACCAGGCGCGGGCCGAC
CTCGCGCGCCTCGCCATCATCAGGCAGCAGAGGGAAGAGGCCGCCAAGCGACGCGAGG
CCGACAAGAAGGCTAAGGAGGAGACTACTAAGAAGAGGTAGATAAATACTCATCAATTTTCGTCGATGACGAG
ATGCCATTCTGAATTTCGGCGCTTCAAATCCATTGTCTCAGTTTCTTTTGTGATATTTGTTTTGAATAAATAC
AAATTTTTTT
>1002049887_1 Heli.1-DT666966.3.5
GAAATTGTTAAATACAATCCGTGTGGTGAAGATAACGATTACTATAACTACTCTTTCGATACGAAAGTGCAGG
TGCTCAATAGATTCGTTTTCAATTTGGCGGTGGCGTTCTCCAGATGCATACCTAGTGTCACGCTAGCCAACTT
CGGCTTCATCGCCGTGACATCTGGGCTGTTCGCTATTTTTAATATGTCTTACCTGGTC
GTATATCCGTTTTGCAGATTGGTATTTGGGACTCTCTACCCAGCATACGCGTCATACAAGGCTGTACGGACAA
AGAACCTCAAAGAATACGTAAAATGGATGATGTATTGGATTGTTTTCGCACTATTCACATGCACAGAGACGTT
CACTGACGTTTTTCTGTCCTGGTTCCCCTTTTACTACGAAGTGAAGATAGTACTCGTT
TTGTGGCTGCTATCGCCTGCTACGAAAGGCTCGTCCATTCTCTACAGAAAGTTTGTTCACCCGGCGTTATGCC
GGCGAGAACAGGAGATCGATGAGTATATAGCTAAAGCCAAAGACCAAGGTTACCATACAGTACTGAATTTAGG
AACTAAAGGCGTCAACTATGCAACTACTGTTATTATGCAAACAGCTATCAAAAATTTG
AATCTGCCGGGTCCCGAAGGCAATGTTCGTGCTATAGCGGACCGTGACGTCACCGATTACCATGATTACGAAG
AAATGGAGTTCCAAAGAGAAAATACACCTAGAGTAGTAGAAATTGTCGAATTAGATGAGAACGCTGGGGATAG
TAATGATCCGGATTACGAACCGACGTCGGCTAGAAGTCGTGGGGAT
>1002049888_1 Heli.1-DT662493.3.5
GCACGAGGGTCACTCACAATACTATTTATTGTAGCAGTGATAATTGCTAATATATATTTCCATTTTGTTTCAA
CTTAAAGTTTATCAATCACCTTTGTTGGAAGGTCGCCTTCTGCGCCAGTGAGTGTAAAGGCTTCTCTTTCAAC
GCATCTATCCTAGCACGCTTGTAAAACAAGTACATAACGTCGCAAAAAGCAAAATGAG
GGCTGTGATCGCTGTAGTCTGCCTCGTCCTGTCCGCAGTGTCTGCGGAGGTTTGCTACAATGAAGTGTCTATG
GAGTGTGGTAGAGCTACTAGTAGTTTAGCTTTACCCAGCTGTAACGCAGTGTACGGCAACTTTGGCCACCAAG
GCAACGTGGCCACGGAGCTCCAAGCGTACGCCAATCTACATCTAAGAAGATCATACGA
ATATCTCCTTTCTGCGGCATATTATAATAACTATCAAACAAACAGAATTGGATTCAGCAAGTTGTTCAAGAAA
CTGTCGGATGACGCTTGGGCCAAGACTATTGATATCATTAAACATGTTACTTTGAGAGGCGGCAGCATGGACT
TCCGCGCCCGTAGTTCCCAGGACCAAGAAACAAAGAACTCCACTATCGAGCTGCAGGA
GCTGGAGTCTCTTGCGCACGCCCTGGACGTGGAGAAGGAGATCGCTGAGCGTGCATTCTTCATACACCAGGAG
GCTACAAGGAACAACCACAAGACTCATGATGCTGAGATCGCACAATATTTATAGAGGTTTTGGAGATTTTGGC
GTTCTTGGGCCTGGAAACTGGAAGTGGAGGAGTTCTGGGAAGAAGAAGAGAGTACAAC
TAACTACAAGATATAAAAAATCAGCTTCACGCTGCATACCGCACACAACAGGGTAGGCAGTGTGAATACACTC
AGTTGAGTGGCGGAATTCAACGCTGCGCTTTGCCTTAACGCCAGAGTAAAGAAATGGAAATATAAATGAAAAT
TAAGTAAATATCAAATATTTAT
>1002049889_1 Heli.1-EL596364.1.5
GTATAGAATTAATTCTCAATTATAAAATTATTGCTTTAATTGTTTACAAACGATGTTATATGTTTCACTTAGT
GGCGCGATTAGGTTATATTTTTAGTCTATTTTAAACTATAAGCATTCAAGTTGAATTAACGGTTCAATTAAGA
GGATCATTAGAAAAAAAAAACCAAACAAAATGAAACTTTAAAGTGACGAAAATTTATT

GTTACGTCCGTGTTATTGTCCAAATTGATATATTTAGTGCTAAAAATGTTAAATTTAAATGTATTTATAAATA
ACTTTCTCGGCTGTATTCAAACGGCCCGGGACTCCCCCTGGCTGCGGTGGCTGCTTCAGCTGCTTGCTATTTC
ACATGCGATCTCGCCGGTAGGATGGCCTGCTTCTTATCCTCTGAAAGATGGAGACACA
TTCGACTTTATAGTGATAGGTGCAGGATCTGGTGGTGCGACAGTGGCGGCCAGACTGAGCGAAATTCATCACT
GGAAAGTTCTTCTTTTGGAAGCAGGGGGAGATCCTCCGCCAGCCAGTGTGATACCCAGTTTGTTTGCAGTTAT
GGCACATACTGAGAATGACTGGGATTACCACGCTAAACTGGATGAGGGAGTAGGGCAG
GCGCACCCCGGGGGCATCATATACATGACACGAGGAAAAATGTTAGGAGGGTCATCGTCTATAAATTACGAGA
TTTACGCAAGAGGCGTTCCAGAAGATTACGACGACTGGAACACTGTAGCTCCAGGGTGGGACTGGGGGACAGT
TCTTCATTACTTTAAAAAACTTGAACATATGATAGATGCAACGATATTTAAAAATCCA
TATAATGCCTATTTACATTCACAATCTGGTCCAGTAGCCATATCCAGACCAAAAGTTAATGCATACTTCAAAA
GTGTAAATGAAGAAGTTTTAAGTTCCTATGAGGAAATAGGTATAAAAAGAGTTTTAGAGAACAACGGTCCAGA
GCATTTTGGCGCTTCGACACCTCATTTTACTTTT
>1002049890_1 Heli.1-EL600440.1.5
GCACGAGGCTTTCAGGCAGTTGCGTCACCCACATGGTCTTGGTCAAGTGAAATAAAACGAAATAATTCATAAA
GTTAGTTAAAAACGCAATAAATATGCTCACAGCAACGCGGGTGATAAGCCGAAAGGCTTTGGAATGCACTGGT
ATATCAGATTTTTCCACACATAGAAATTTCTCGTCGATCCTAAAAAGTACAGCAGCCC
CCACAGTGCCTATTTATCAGCGCCATGGACTCCAGTACAGACAGAAGTCCGAAGGTGTTCGTGGCGCCGTCAT
TGGTATCGACTTGGGAACGACAAACTCCTGTGTGGCAGTTATGGAGGGAAAAACACCTAAGGTGGTGGAAAAC
AGCGAAGGTTCCAGAACGACGCCGTCCCACGTGGCTTTCTCTAAAGAAGGTGAACGTC
TCGTAGGAATGCCAGCCAAGAGACAAGCTGTCACTAACAGTGGAAATACATTCTATGCGACGAAAAGATTAAT
AGGTCGCAGGTTCGAGGACCCGGAGGTGCAGAAAGATATGAAGAACCTGTCGTATAAGGTTGTGAAAGCATCT
AATGGCGATGCTTGGGTGTCAGGTACTGATGGTAAAGTTTATTCACCAAGTCAAATAG
GTGCTTTTGTTCTTATTAAAATGAAGGAAACGGCCGAGGCCTACCTAAATACAAGTGTTAAGAATGCAGTGGT
GACAGTTCCGGCATACTTCAATGATTCACAGAGGCAGGCTACTAAGGATGCAGGTCAAATCGCGGGTTTGAAC
GTGTTACGTGTGATCAACGAGCCGACAGCTGCTGCACTGGCTTACG
>1002049891_1 Heli.1-CV133783.1.5
CGTCCGAATAGAGAAATATCTTGTAAGCCGGGGTAGTGGCAGTCAAGGGATTTCACATTTTTATTGTTTGCCA
AGTGCGTGCGGGGTCGCATCCACCTGACCGGCACTCTGTATTTGGCACAGTTTCTGATTTAAATAATAAGTAG
ATTTAGTATAGACGTTGGATTCATAAATTGGAGTTTTTAGATGTAATCGTTTGTCAGT
CGGTTAGAAGTGGTTATCCCTGATAATAGTTACCATTTTCGTTACGTTAGGTTGTGCTCACTACTCTGGTACT
TGCCACCTGTGGGAAGATAATAAAAATAAACAAAC
>1002049892_1 Heli.1-DT667404.3.5
AATCCGATCAATTTTCTACAAATCTAGCAACAGATTAGTGTGAACGTATCAAAACTTATTGTTTCGGTTTTAT
TGATTATTTTGTGTGATCTAAATAAATTAAAGGACAACCATCTGCGTACACCGTTGCCAACTGTCGAGTCGTC
GATATTTTGCGACCGAATAATGATCCTTGGTTTCGTGCGAGTGTAACTTTGCTAAACA
TTACTGTTTTTTCGAAAATAATATTGTAAAACCAGCAAAAATGGCATCGTTTCAGCAGTTGATAGCTTTTCGC
AGAGAGCTTTTGATTACAGACAAATATAGAATTACAAGAAGAATCGGAGGTGGTTCTTTTGGAGATATTTACT
TGGGGATAAACATCGCTAATGGCGAGGAAGTAGCAGTGAAGGTTGAGTCTATAAAAGC
GAGACATCCCCAACTTCTCTATGAAAGCCGTGTATATAAAATGCTCCAAGGGGGCATCGGCATACCACATATA
CGATGGTATGGCTTCGAGAAGGAACACAACATCCTCGTGATGGACCTCCTGGGTCCATCACTAGAGGACCTGT
TCAACTTTTGTTCGCGTCAGTTCACCATCAAGACAGTGCTCATGCTTGCAGATCAAAT
GCTCGGGCGTGTGGAGTTCGTCCACTGCAAATGTTTCATCCATCGTGATATAAAACCTGACAACTTTTTAATG
GGCATCGGCCGCCATTGCAACAAATTATACATGATCGACTTCGGCCTCGCTAAGAAATTTAGAGATGTACGTA
CCAGAGCTCACATCACCTATCGGGAGGATAAGAATCTTACTGGCACTGCCCGTTACGC
GTCCATCAACGCTCACCTCGGCATCGAGCAGTCCCGACGTGACGACATGGAGTCACTTGGCTACGTACTTATG
TATTTTAATAGAGGATCTTTGCCGTGGCAAGGATTGAAAGCAATAACGAAGAAACAAAAATACGAACGTATTA
GTGAAAAGAAAATGTCCACGCCTGTCGAAGTTTTATGCAAGGGATTCCCAGCGGAATT
TTCTATGTATTTAAATTACTGCCGCGGTCTCGGTTTCGACGAAGCACCAGACTATATGTATCTTAGACAGCTG
TTCCGCATCCTATTCCGCACGATGAACTACCAGTACGACTACACGTTCGACTGGACCATCCTGCGCCAGCGCA
AGCAGCAGGCCATGGACGCCATCGTGCAGCGCCGCTCGCCCGCCGCGCAGCGCCGCGC
GCAGGCGCCCTC
>1002049893_1 Heli.1-DT665734.3.5

GCACGAGGATCTTGTCTTGCAACATGTTTATGTACGAGCCACCTACCAAGCTAAAACTTACTTCAGTGGAAGA
GTTCCAAGAAGCATTCCAGCTTTTTGACTCCCGTGGGGATGGGAAAATTCATGTGGCACAAATAGGAGATGCG
CTCCGTGCTTTAGGACAAAACCCAACTGAATCTGATGTCAAGAAGTGCACCTTACACC
TCAAGCCTGATGAGAGAATATCATTTGAAGTGTTTCTGCCTATTTACCAGGCAATTTCTAAAGCTAGAAGTGG
TGATACTGCAAATGACTTTATAGAGGGCCTTCGCCATTTTGATAAGGATGGTAATGGTTTCATTTCTTCGGCT
GAACTCCGCCATCTACTTTCCACTCTTGGAGAAAAGCTGAGTGATGATGAGGTGGAGC
AGTTGTTGCAGGGACAAGAGGACTCTCAGGGGAATATCAACTATGAGAACTTTGTACACCTCATTATGCAGGG
ATAGACGGAGACGGACACACCTCCGGCCACTGTTTTATAGTATTATGTAGTTTCTTTATCTCTTCAATTGATA
ATGCATTTATTAAAATGGCTGCAGTATGGTGGCTATACTATATAATATGACACCATGA
TAACTGTGATGTATTATAAACAAAAAGAGAAGAATATTCCAAACTCTATTTCTTTATGTATTTGACTAACAAT
CTTTTTATTATTTCCATTGCTATAATTTAAGAGCATATTCCAAGATTTTAACATAATTT
>1002049894_1 Heli.1-EL596336.1.5
TCAAAATCATTACGTGAGAAATTGTTCAAAGACCGACTTAAATCTATACAGACAAGATGTGTGACCGCAAAGC
GGTGATCAAGAATGCTGACATGAGTGAGGAGATGCAGCAGGATGCTGTGGACTGCGCGACGCAGGCTCTTGAG
AAATTTAACATTGAAAAGGACATTGCAGCGTTCATCAAAAAAGAATTTGACAAGAAGT
ACAACCCAACCTGGCATTGCATCGTGGGCCGTAACTTCGGTTCATATGTAACACACGAGACCCGCCACTTTAT
CTACTTCTACCTGGGCCAGGTGGCAATACTGCTCTTCAAGAGCGGCTAATCGAAATCTGTCTAGCAAAACCTC
TTTTCGGACATTCCATTGATCCAACTAGGGTTGCCAACATTATTTTTGACCATAATGA
TAAAATAATACAGCAAATTTAGTATATTTTAGGTATATTACAGTGAAATTGACGGATTTTTTTAAATTTTGGG
TGCTGAAATGGCATCAAGCTAAAAACTTATAGTATTTTTGCATATTGTATATTTCTTGTACACAGACAGACAA
GCATACATACACTATTCATTACAGTACAGTTGGCAACACTAGCTCGAACCCACATTCC
TATCCCAAAGGTATACTAATGGTGTCAGATTTTAATTAAAAAAAAACCTGAAGATTACCTTATTTATTTGATT
TTTGAGTTACTTTTACATTCAAGATCTTGGTAAACAGATAGTGCAGG
>1002049895_1 Heli.1-DT665230.3.5
GCACGAGGGATTGTCCTTTTAAACATCTAGTTTGTAAATATTCGTATTGCTAAAAGAATTGATATAAATAGGA
ATATTAAATTCCGGAATTCAATAAGGAAAATCTTGTATATTGTCAATATAATTTATCTTTTTCTTTAACTATG
GAGAATAAATGTGACTTTGAGTATCCTCATACTTTTGTACTTCTCGGAGCATCAGGTG
ATCTTGCTAAGAAGAAAATTTATCCAACACTTTGGTATTTATATCGTGATAGCCTTTTGCCAAAAAATACCAC
ATTTATTGGTTATTCAAGATCGAAGTTAACTATAGAAGATATAAAAGAAAGATCAAAAAAATATATTAAAGTA
CGTCCAGGCGATGAAAAAAAACTTGAAAAGTTTTGGGAAGTTAACGATTACGTTGCAG
GTTCTTATGATAAAAGAATTGATTATGAACTTCTTAATCAACACATATCCAGACATGAAAAGGGTGCAAAGTG
TAACAGAATATTTTATTTAGCAGTTCCACCTAATGTATTTGAAGATGTAAGTGTGAATATTAGAAATGCTTGT
TTAGCAATTAAAGGATATACGCGAGTTATAATTGAGAAACCTTTTGGTAGAGATTCAG
AGAGTTCCGATAAATTAAGCAATCATTTAGCTAGCTTGTTTAAAGAAGAGCAAATTTATAGAATTGACCATTA
CCTTGGAAAAGAAATGGTACAAAATTTGCTGACTATTAGATTTGCTAATCAAATTTTTAGCCCATCATGGAAT
AGAGAAAACATTGCTTCAGTTCTAATTTCATTTAAAGAACCTTTTGGCACAGAGGGAA
GGGGTGGTTATTTTGATAATTTTGGTATTATAAGAGATGTGATGCAAAACCACCTTTTACAAATATTATCACT
TGTTGCAATGGAAAAGCCAGTAACTTTAAACCCTAATGATATTAGAGATGAAAAAGTAAAAGTCCTTAGACAT
ATAAAACCTATAGAGCTCAAAGATTTGGTTGTTGGGCAATATGTGGGTAACCCAAATG
GGCAAGGGGAAGAGAAACTTGGTTACCTTGATGACCCTACTGTGCCTCCAAATTCTATCACTCCAACATATGC
TTTAGCTGCTATGTATATTAACAATACCAGATGGCAGGGTGTGCCATTTATACTGAGATGTGGAAAAGCGCTT
AATGAAAGGAAAGCTGAAGTAAGATTTCATTTAAAGATGTACCAGGTGATATTTTTTG
GCACACAAAGAG
>1002049896_1 Heli.1-DT667630.3.5
GCACGAGGGTCGGAGCGGTTTGCTGCAGAATAGATACGTCAGAGAATTCTAGAAGATTGTACATAATGACTCT
GGGATGTCTGTATCCTTATAGAAGATTAGGTATTGGTACTCTAATGGTGGAACATGTGCTCAAGTATGTTGAA
CAGGATGGCAACTTTGACAGCATTTTTTTGCATGTGCAAGTGAATAATGAAAGTGCAA
TAGATTTTTACAAAAAATTTGGCTTCGAAATTGTTGAAACAAAAGAATTTTACTATAAGAGAATAGAACCAGC
CGATGCCCATGTATTACAGAAGACCATTAGACAGCCACAGCCAGCATTAGTTAACGGGAATGTTCCTCATGCA
AAAACCAACGGCCACGATTGAAATAACAATAATTAGAATTGTCTCAACTCTATAATAG
TGAAAAGGAGCAAGTGATAAATAACTTAAGAATAATAATCAAGTCACTTTTATATTTACATGTTTGATTGATT
AAGATGAGAGATTATGAATGATACTTAACATTATTGTAGTGGGTTGTGGTAAAGGAGATTCTATCTTCTCTAG
TGCCCCTTGTAATCTGACATGTGTAACTCTATTCATTGTATATAAATATTTTTGGCAA

GCCTTAATGGTATCTTATCCTTATATTTTTTCAGTTAAATTTGATTGTATAAATGTATGTAGCAGCCTAGAAG
GCATTCAGTTGTTCAATGTTTATTAATTGTGTTGTATTTACATGCCCAAAAGCAAAAGCATAAAACTTATAAT
TAAAAGAAAATGATGTTTTGGATCGCATAAATTGTAATGTATCCTTCAGTATAGCAAT
TAACATGGTGTTTTATATAATTTG
>1002049897_1 Heli.1-DT663879.3.5
GCACGAGGGTTTGTGAAGGCTTTTTAGACACGGACCCTTACCGTCCTTGACAATTTGTCTTTAATGAACACTA
AAGTCATAGGAGCCATTAACAACGTAGATTAAGTTTAAATTAAGGTCGATTTAGCTTAGTTACTTGCGTATTG
TACCTACGTTATGTTTTTAATTTTAAACCAATTTTGTTTTCTTTAGCGTTTTGTACAG
TCAAACGAGAGTATATTTTATTTCTGAACAATCAAAGAATAATATTTTTATTTATTATACAGATATATAAATT
ATTTACATAAATAAATATAAATAAAATACCTATTTATTTAAACTACTTTTTCGTATGAAATATCTAAAGGCAG
TGATTTCGAACAAAAACTGTTATTGTAGTTTTACATTATCGAAACTGCCACTAGAGCT
CTTTAAGTTACCGACTCTACATACAAAGATATACATTTAATTATATTTATAATACATATTTTAATTTCACTAG
TTGCACACAATTTCAAATCATTCACTATAGTTTTTGTGATAACCCTCGCTTGTAATATAACTAATCTATTAAC
AAGATGTACTCTTTGAACTTTAACTTTTTATGTTGGATAGACTCATGCATGACATGTG
ACTTCAAAACTTATTAGTTTTTCCGAATGTGATTAACATTTTTTTTTAAATTAACTATCAAAATCTCTGAATG
GCGATAAAATGAATAAATGATTCAGTGATTACAAGCAAAAGGGACCAAATACAAATTGAATTATATTACAAAC
AACACAATCGTAATATTTTAATTATTAACAAAAC
>1002049898_1 Heli.1-ES587385.1.5
AGTCCTGCAGTACGGTCGGAATCCGGGTCGACCACGCGTCCGAGCAATCATGTTCATGGATTTAATCGTGTAG
TGGAAATATATTTTAAGAAAACTTTGTATATTTTTCATTTAGTGATATATTCTCTGTTGTGAGTTAAAATGGC
AGAAACAGAACCTAACCAGCAAGCTGGAGACACCGGCCCACCGAAAGGCATTCCCGCA
TTAAAAGCTCATGTAATAGCAAATAAAATAGATGTAGCATTGTGGGGTATTCGTGTACTCACAGTCCTTTGTA
CATTTGGATATGTATTTCCTCTATTTAATAACCCCGTCTCAGCGTTCTACAAAGCGCTCCTAGCGAACGCCGC
GACGTCAGCGCTAAGACTCCACCAGCGTATACCGGCAAGAGAGATATCGTTATCAAGG
GAATTTCTAAACAGATTCTTCCTAGAAGACAGTGCACATTATCTATTCTATTCGTTGATATTTATGAACGTGG
CGCCCAATTTGTTGATATTGACACCCATCTTCCTATTCGCTCTACTCCACGCGGCGTCATACTCCCTAACAAT
ACTAGACACGTTAGGACAGAACTCAATGTGGGTGGCGCGACTCCTGATCTCACTGGTG
GAGTTTCAGTCCCGCAACATCCTGCGCGCGGCCGCTCTCGCTGAGATCGTGTTGTTCCCGCTCGTCGTGCTTA
TGGCCCTTATTGGGTACTGCGGTCTGATGACGCCGTTCGTGTACTACTACTTCGTGACGTGGCGCTACACGTC
GCGCCGCAACCCCTACACGCGCAACACGTTCCGCGAGCTGCGCGTGTTCGCGGAGAGC
GCGGCCGCGCGCCCGGCACTCCCCGCACCCCTGCGCGCCGCCCTGCTCGCTGCCGTGCGCCTCGCATGCCGCG
CCGCGCCGCCG
>1002049899_1 Heli.1-DT668995.3.5
GCACGAGGGCCTGTGAGTGTTTTCTTTACTAAAGTTAATTTTAAACTTTGTTTAAACTTTATTTGTGTAAAGT
GATACTTGTGTTGCTCTGTGAAACTTAGTGTCTATATATTTGTGTCAATAATTGTGCTAATAAATATATAACA
CGATGAATAAAACTTGTGCGAGGTGCGAAAAAACAGTTTATCCTACTGAGGAATTGAA
ATGTTTGGACAAGGTGTGGCACAAAGGCTGTTTCAAATGCCAGGAATGCGGAATGACACTCAACATGAGGACG
TACAAGGGCTACGGCAAGCTGCCGTACTGTGAAGCGCACGTGCCAAAAGCGAAGCACACCACAATGGCCGAAA
CGCCAGAACTGAAACGGATAGCGGAAAACACAAAACTACAGAGCAACGTGAAGTATCA
CGCCGACTTCGAGAAGAGCAAAGGCAAGTTTACACAGGTCGCAGATGATCCAGAAACACTCAGAATTAAAGCA
AACACAAAAATTATAAGCAATGTTGCCTACCACGGAGACTTGGAGAAGAAAGCTCAAATGGAGAAACAGAGAC
AAATTAACGAGAACGGAGAGATAGTGGACGTAGCGACGAACGATAACTACCATCAACA
AATAGAGAACTACGCGACGGAAATGCTACCACCAAACGTACCACCACCAAACTTGCCACCCAAAAATCATTAT
CAGACACCCGTCGTCGCCCAAAGTCATCGTCAGTATCCGGAATATCGTCAACAAGAGGAATATTATCAACAGC
CAAAGTACGAGGAATATCCTAAAAATGCGAATTTTCATTACGGAAACCAAAAGATTGG
CAGGATTCAAGATTATGATCCCCTAATCGATGCTCCGAGAGCTCCCTTAAATGTTCAAAGGGCTTCGGCTAC
>1002049900_1 Heli.1-ES585486.1.5
GGATTCCGGGTCGACCACGCGTCCGGAAATAATCGTGACTCTTAATTATATTTTTAGTTACTGCCATTTAATT
TTATTTATAAGTAAACATGAGTTTCATTGCCAGAACGAGAAATCCTATTTTGCGAGGACTTTCTAGTATTAGA
ATGTATTCTGGAGAACCAGGATCTGGTGCTGGCAAGGGTGGTGGCGCAGGTGGTTCCA
TCCGTGAAGCAGGTGGTGCATTCGGCAAGATGGAGGCTGCTAGAGAAGATGAATATTTCTATAAAAAGCAAAA
AGAACAGTTGGCCAATATCAAGGGTCATTTGGACAAAGAAATCTCCTTCCATCAAGAACAGATCAAACGCCAC
GAGGATGCCATCAGACGTCACAAGGAACAAATGGCTGACATGAATGAGAACAAATAGA

TTTTTTTACCTTAATTATTAAATTAGCATCAAGTCCTGTTCATAACGAATACTATGCTGCTGTTTAACAAAAG
TGAAACTTAAAGTCAAGATGTCAATTTTTTTTGCTTTTATTTTATTTAGGAATTAGTCTTGGACATCAAATAT
ATTGTACAGG
>1002049901_1 Heli.1-EL598392.1.5
GCACGAGGGTAACTACCGTTGAATCAACTACCATGCAGTACCTTATTTTCTTGGGACTTTTCGCCCTTGCGGC
TGCTTATCCTGGTATCATTCACCAGGAAGCCCCACAACTCTCTGAGGGTTATGATCATCAAGGCCTCATTGGC
GAATCAGGACATCATCATCAAGTTCAACACGAACATGCTAAGTCACATCAATCAATCA
AATTCGAGCACTTCCATCCAGTACCTGTATACGTTAAGAAAGAACATAGCCATCTGCTGAAACATCCTCTTGA
GAAGGGCCATTCTGAACAAAACTTGAAACAAATTCACCCTGAGACCCAACACAGCCATGGTGGCGGCCTTGTC
CTGGAGGACCACAGGTTAGACACTGAACAGTTCGCCGCCAGTCTCGGCCACGGTGGTC
TTGAACACGGCCTCGAACAAGGATCACTTGAACAAGGTGGATTGGAACACGCAGGTATTCAGCAAGGTTATGA
ACATTATGCTGGGAGCTACGAAGGTGGAGAAGGATTAAAATCATACGCTGAATCTCAGCCTGAAATTCAGGGA
CACTACTACTCCGAACACGCGCAAGCCCTTTCAGGGGACAGCGGAGAGGGTTACAAAT
TCGAGCATTATTAATCGCATTTAGCTTAAGTCCGACGGGCATATCGGTGTTATAACACCGTTCTCTCATATAT
TTATAAGATATTTTTCTTGCCTGTTAAGTACTGTAAATGTACAGTATGTAAGTATCTTAACTGACGCTACGCG
GTTGATAATATATCGAAATTTTAATATAAAATGTACCTGATATATAAGTTAGAAACAA
ATTCATTTAGACAACCCCGTGAATTCTTTTAGTGGTGTAAATGTTGCGAATCTTGTGGCACTTAAATTATAAA
CATGTATTTCATTCCTTCATGTGTGTCCTTGCTTGTTTCTTATAGTATTATTGTGATTATGATGAGGACGTGT
GACGAGTCCCAGTGAAATTGTGATATTAATAAATGTTGAATTTTAA
>1002049902_1 Heli.1-EL599570.1.5
CCCGGAGCTTAATCGCACGAAGGCGCTGGTATATACAATTTGACCAAAAAATCTCACAAAAAAACAATCAAAA
TGTTGGGTCGTTTTGCACCTGGAGTCGCCCAGGTGACCATCATGGTCAACCAGCAGCAGAGCCGTAACATGGC
CACTCTGAAAGCCATTTCAATCCGTTTGAAGTCTGTCAAGAATATTCAAAAGATCACC
CAATCTATGAAAATGGTGTCAGCTGCTAAGTATACTCGCGCTGAGCGTGAATTGAAAGCTGCCCGTCCCTATG
GCGAGGGTGCTGTGCAGTTTTATGAGAAGGCAGAGGTTGCTGTCCCTGAGGATGAGCCAGCGCAGCTGTATAT
CGCCATGACTTCAGACAGAGGTCTCTGCGGCGCTGTGCACACGGGCGTGTCCAAGGTG
ATCCGCAACCGCCTCAACGAGCCCGGAGCGGAGAACATCAAGGTCATCTGCGTCGGGGACAAGTCGCGCGGCA
TCCTGCAGCGCTTGTACGCCAAGCACATTATTAGTGTCGCCAATGAGATTGGTCGTCTCCCCCCGACTTTCTT
AGACGCTGCTAAATTGGCAAACGCCATTATGACTTCTGGTTACGATTTCGGATCTGGC
AAGATCATTTATAACAAGTTCAAATCTGTGGTGTCTTATGCTCAGTCTGATTTGCCTTTGTTCAGTAAGAAGG
CTATTGAGTCAGCACCAAAGCTAGCAGTCTACGATTCCTTGGACTCGGACGTTCTTCAATCCTACATGGAGTT
CTCTCTCGCCTCCATGTTGTTCTACGCTTTAAAGGAAGGCGCTTGTTCCGAACAATCC
TCGAGAATGTCGGCCATGGACAACGCATCGAAGAATGCAGGAGAAATGATTGACAAACTAACACTCACATTCA
ATAGGACCCGCCAAGCCGTCATCACTAGAGAGCTTATCGAAATTATCTCTGGTGCTGCCGCTTTGGATTAATA
ATGTTATGCCTTTGTCTGAAAAATGGCGCCGTTTTATGTTTCAATGTGGATTAATAAA
GTTATACCCGACTTACAAAAAAAAAGTTGGCGCCATTTTATCTGTCAATGTCATTTTGTTATATAGCCCGAGA
TGTTTATGTAGAATCATGTATTTATATGTTTAAATTATATTTTTTTTATTTCGATTTTCGATCATATTATTAC
AAAAAAAACTGCCATTTGCAATAGTAATAATGTATCTAACATTATGCTGACATTCCTT
TATAAGTTTGAATAAGTATTGATCTGTTTATAAACCTCACACGCGACACAAACTGGCTTTTAACATCTTTATA
AATATATCGATGCTTATTTTGATTTGTGGCTGTTCTAACTGAATTTC
>1002049903_1 Heli.1-DT663347.3.5
CACGAGGGTTCGAGCAGCAAGTGCGAGACTTACGACGACTTCATTTTAAAGAAGCCGGCTAGGTCGAGTCCTC
GCTACGTCTCGTTAAAATTTATAAGTTAATATAATTATATTAGATTTATAAAAATCATTTAACAGTATAGTAC
GTACTATTAAAGCGTAATTAATAATTATACAAAAAAAATCTATTATAATCTTCCTCAA
AATATTCAACAAAAGTACTCGCACCACGTGTATTTAAAAGTATTAAAGTGAAATAAGTCCTCCAAGAAACTTT
TTGACTAGATACTATTCAGAAAGCATTGTTCTAATCTAAAGAAATCGAATACTGTAATAAACTTGTGCTTAAC
AAAATAATTTTTGCACGTGTTAGTTTTTACAACAATAATCATGTCAAACGCAGAAGGT
GACTTACGGGATGGACTCTCGGCAAAAGAAATTTTTGCTAACAGTGAAGGACTAACTTACAATGATTTTCTTC
TTCTTCCTGGATACATTGACTTTACCGCGGAGGAGGTTGACTTGACATCACCTCTCACCAAGAAAATCAACCT
GAAAGCACCTCTAGTGTCTACACCTATGGATACTGTGACCGAAGCTGATATGGCTATC
GCTATGGCTTTATGCGGAGGTATTGGCATTATACATCATAATTGCACTGCTGAATACCAAGCAAATGAAGTCC
ACAAGGTAAAAAAGTACAAACACGGTTTCATTCGTGATCCTGTTTGCATGGGTCCCAAAAATACTGTAGCAGA
TGTCATAGAAGCCAAAAAGAAAAATGGATTTACAGGTTATCCTATCACTGAGAATGGC

AAACTTGGAGGACGCTTGATTGGTATTGTGACGTCTAGAGACATTGACTTTAGGGAAGGTGACCCACATCTCA
GCTTAAAAGAAGTTATGACCCCTATTGACGAAATGATCACTGCACAGTCTGGTGTTACTTTACAAGATGCTAA
CTATATCCTTGAAAAGAGTAAGAAAGGAAAATTACCTATTGTAAATGGTGCAGGTGAG
CTTGTGGCTTTAATTGCAAGAACAGATTTAAAGAAGGCTCGTAGTTATCCCAATGCCTCAAAAGATTCAAACA
AACAGTTGCTGGTAGGTGCTGCTATTGGTACAAGAGACGCAGATAAAGAGCGTCTTAAGCTTCTGGTGAGCAA
TGGTGTGGAT
>1002049904_1 Heli.1-EL603226.1.5
GCACGAGGGCAACGCTCTCGTTGTGCTCTACAGTGATTTAGGTTAAATATTATTTGTGTCTATATTTCATACG
TCATAAGCAATGCCCCAAACCAACGCGATCTCTCTCAACGGACAAAATAATGGGAGCTTGAAGAATGTCGATC
AGTCCTCCGAACACCAAAATGGATACCACACGCCGCACCGGAAGACATTTGACGACGA
AGAGTTCCATCTTAACAATACGGGAGAAGTGGACAAAAACTGTGTTAGGTATCCCGATGGCAGTGTACGTCTT
CGCAATCCCAATATCGAACTTATGGACCAAGATATTCTCTATCATTTGGCGCTGGGCAGTGGTTCTCATGACC
TTGTTGAGATGTTTGGAGACGTTAAGTTCGTATGTATGGGAGGGACACCGAAGCGTAT
GGAGCAGTTCGCATACACTATCATGGCAGAGATTGGTCACAAGTTGCCTTGCGGAACCACGTTGCAGGACATT
AGTCAATTCTCGTATAGATACTCCATGTTTAAAGTCGGACCCGTCCTGTGCATCAGTCATGGCATGGGCATAC
CTTCTGTCGGGATCCTACTTCACGAGGTGATAAAGCTGATGTACCACGCTAAAGTGCG
AGACCCAGTATTCTTTAGAATTGGCACATGTGGAGGCATCGGTTATGAGGGTGGTACCGTTGTCATATCTGAA
GAGGCGGTTGACGGCGCTCTTAAAAATGTTCTTGAACTGTGCGTCCTTGGTAAAATATACAACGACCGGCA
>1002049905_1 Heli.1-DT662026.3.5
GCACGAGGGGATTATCTATCTTGATCTGTTAAATTTTGCCTACGAATAACAATATTTAATACAAAATGCCGTT
CCAAGTATTAACTATAGAGCCGCAAAATGAACTGAAGTTCAAAGTTGACTTTTCAGGATTGTTTGAGCAGGGC
TACACAACCTACATGAAGCTGACGAATCCCTCAGAACATACCGTCTTGTTTAAGATCA
AGACCACAGCCCCAAAGAAATATTGCGTTCGTCCCAACTCTGGTGTGCTCGATCCTAATTCTAAAGTTGATAT
AGCTATTACGCCACAGGCCGTTCACGTCGACTCCAATGAGAAACACAAACACAAATTTATGGTGCAAAGTGTT
ATTGCTCCAGAAGGAAAAACTAATATAGATCAAGTGTGGAAGGAAATTAGTCAGGATC
AGCTAATGGACTACAAGCTGAAATGTGTATTTGAAACACCTAGGGGAACAAATCTTAACGATGCCGGTGATAA
TGTCGCTCAGAATGAAATCTCCAAGAAACGAGTAGCAGTCGCCGCAGCGGAAGACTCAAAGTCACTATCAAAG
GATGCTGTAGAAGGTCTCCAGAATGATGCTAAGAGGAAGGATGATGACCCAGCACCGG
GAGCTTTAAATGCAAAAACGACCGCTTTCCCAAAATCTGAAAACCTCGAAAGCGATTTGCAAAAGGCGACGAA
TGAGGTCATTCATTTGAGGGAGGAAGAAAGCAAATTAAGACATGAAAATCTGCGATTAAAAGAGGAGTTACTT
CGCCTGAACCAGACGGCGGGCGAGGGCCGCGTGC
>1002049906_1 Heli.1-EL604060.1.5
GCAGAGGATTAAATAGGCGCTTATTATTTTTGATATTTTAGGTGTTATCGCAATTTGTAAACCAAAATGTATA
TTCCATCTCTTATATTCACTGTACTAGTCACTGTTATTAACGGTATTTATTGTAAAAGTGTAGAACTTACATT
TGAGTTACCTGATAACGCCGTAGAATGTTTTTATCACGAGATTGACCAAGATGTATCT
GCCTCGTTGGAATACCAGGTTATAACTGGAGGTCAGTATGACGTTGATGTAAAGATTGAAGGTCCAAATAATC
AAATTTTGTATCAACAGCAAAAGATGCAATATGACTCACATCAATTTACAACCCAACATAAAGGAGTTTATAA
AATATGTTTCAGTAATGAATTCAGTACATTTACACATAAATTAGTATATATGGAATTA
AATGTTGGTCCAGAAGAACCTCTCCCTGGCATTGGTGAACATGCTACAGTTTTAACACAGTTGGAAACTTCAG
CGGAAGAAATCCATTCATCTCTTAATAAGATTATTGACCATCAAACCCACCACAGGCTGAGAGAGGCTCAAAG
TAGGAAAAGGGCAGAAGATCTGAATGAAAGAGTATTCTGGTGGTCGACGGGTGAAACC
TTGGCTATTGTTTGTGTTGCAATCGCACAAGTTATGATTTTGAAGAATTTCTTTAGTGATCGACCTACATTAT
ATAAAATGTAATAGCAAATTAAATAGTGTATTTATTAATATATTTTT
>1002049907_1 Heli.1-DT665270.3.5
ATATGAAATTGTTCATAGTTGCCGCTGTACTTGGTGTCTGCTCTGCAGCCCGCCTTGACAATGCCTACCTCCC
TCCTAGAGGAGGAGCTGGTGCAGGCTATGGCGCTGGTTCCGGACCTTCGGGCTTCGGATCTGGCTTAGGAAGA
GGAGCAGTTGCAGGATTTGGAGGCAGTGGAGCTTACAGCGGGTCAGGATCAGGATCTG
GATCCTTTGGAAGTGGATCAGGTTTTGGAGGAGTTGGTGCTGGTGCAGGAGCTGGAAACTATCAGGGAAGTTT
CGGAGGCAGAAGCAACTCAGCTGACGCTAACGCTCAAATCCTCCGACTGAACAGTGACGTTACCGCAGAAGGT
TTCTCTTACGACTTCGAAACATCCAACGGAATAAGGGCTGATGCATCTGGTGTTGCTA
CCAATGGCGTTCAATCTCAAGGCAGCTTCTCTTACAAGGGTGATGATGGCCAAGACTACAGCATCCAGTACAC
AGCTGACGAAAATGGTTACCAACCTCGTGGTGCTCATTTGCCCACTCCTCCCCCAATCCCTGAAGCTATCTTG
AAATCCTTGGAACAAAATGCCCGTGATGAGGCTGCTGGCATCATTGATGATGGCACAT

ACCGCGGAGAAGGCGCAGGTGCGGGCGCTGGCGCATACTCCGGCCAAGGTGGATATTCTGGGCCAGGTGGATA
CTCTGGACCTTCTAACCAATATGGTGCTCCGGCCAGAGGATTTGCAGGTGCTGGTGCCG
>1002049908_1 Heli.1-EL596245.1.5
GCACGAGGGTACGCGTGTCAGCTTTGATTGACCGCGTGTGCCAGGTCGTTGAACGGGACGGGTCACCCCAGGA
AATTTGTCGCGCTTACCTCCGCAAAATTGACCATCTTTACTATAAATTTGACCCACGCGCCGTCAAGAAAGAC
CTTCCTGCGGGTGAAGAGACAACCATTAAGAAAATGGAACGTCTCTGCAAATATATTT
ATGCGAATGATGACAGCGATCGTCTGAGAACACGCGCCATACTGTCTCATATATATCACCACGCGTTACACGA
CAACTGGTTCCAAGCCAGAGATCTCTTGCTTATGTCTCACTTGCAAGAAAACGTTCAGCATTCGGATCCTAGC
ACTCAGATTCTGTACAATCGTACTATGGCCAATTTGGGATTGTGCGCTTTCCGCCGGG
GCAACGTGAAGGAAGCCCACGGATGCTTGGCGGAGCTGATGATGACAGGAAAGCCCAAGGAGTTGCTCGCACA
AGGTCTTTTGCCGCAACGGCAACACGAAAGGTCTAAAGAACAAGAAAAGATTGAAAAGCAACGTCAAATGCCA
TTCCATATGCATATAAACTTGGAGTTGTTGGAGTGTGTCTACTTGGTTTCCGCGATGT
TAATTGAGATCCCATACATGGCTGCCCACGAGTTCGACGCCCGCCGTCGTATGATCAGTAAGACGTTCTATCA
GAACTTGCGCGCGAGTGAGCGGCAGGCGCTGGTGGGCCCGCCGGAGTCCATGCGGGAGCACGCGGTGGCCGCC
GCGAGGGCCATGCGGCGCGGGGACTGGCGCGCCTGCCTCAACTTCATTGTGAATGAAA
AAATGAATGCTAAGGTTTGGGACCTAATGGTTGGTGCTGATAATGTGCGCGCAATGCTGGGACGTCTTATCAG
AGAGGAATCCCTCCGCACATATCTCTTCACTTACGCCCACGTGTACGCGTCTCTATCCCTACGTTCTTTGGCG
GACATGTTTGAGATGCCACGACAACGCGTACATTCCCTCGTATCTAAGATGATTATAA
ATGAAGAACTCTTGGCGTCTTTGGATGACCCGAGCGAGTGCGCCATCT
>1002049909_1 Heli.1-DN172713.1.5
TGCGGTACGGTCCGGAATTCCCGGTCGACCTCGCGTCCGATACCGTGAGATAAATATTTATATTTCAATTTCT
TTGGGGTTGAAAAGTTCATAACTTCATATTGCTTTTCATTTCAAAATGGTGAAATTCGCAATAGATTGTGAAA
TGGTCGGCAGCGGTAACAGAAGTTTGCTAGCGCGTGTCTCTATTGTTAACCAATATGG
AACCACCATCTTGGACAAATTTGTAAAACCCACTGCTGTTATCACAGATTACAGGGACTTTGTAAGTGGGATT
AGAAAGAGAGATTTGGACAATGGCTGCGATTTTAATTCTGTCAGAAACCAAGTGGCAAATCTAATGAATGGAA
GCATTTTAATCGGTCATTCCTTGAAATTTGATCTGGAAGCACTACATTTATCACATCC
AGAAAGAAATCAGCGTGATCTGGCGAGGTTCCCGCCTTTAATGAAAAATGGTATGCCCGTCGCTCTGCAAACT
CTCGCTCGAGTACATCTAGGAAAAGTAATCCAGACCGGCGAACACGATTCTGTAGAAGATGCGAGGGCGTGCA
TGGAATTGTATCAAAAATTCGCATCTGATTGGGAGAGAAGCTTTTAATGAGTAATTTT
AAATTTAGGATCAGTGTTGATTATTTTTTATGAGTAAAGTTAAATGTATGTGTATTAGCAAACATTTGACGTT
GCCAATATAAAAGGTTATGTTCACTTAATATATTAAACAATAAAACACAATTTACTTGC
>1002049910_1 Heli.1-ES584685.1.5
CTTTAAACCTTAATATTTACGCAAGAGTTTGGATATTCCCAGATATATTGGATTTTATGGGCTTAGGGTCTGG
AAAAAAGATTTATGAGCCCGTCGCGGCAAAGCGCGATTGTGGGTTCGATTCCCACTTGAAGTAACGAATTATT
TTAATTTCCCCGCTCTTGAAAGAAAACAGCCTACCTTGCTGAATCCTGTAGGGATACA
ACCAGAAAGTTTCGATAGATTCTCTTTTATACGTAAAGAGATATCCAATAACTTATTTTGAATATCTTTAAAA
TGCACAGCATGGACAATAACAACACTTTCTTGTCCCAAATCCAGGAATTCCATAAAATATTCTATAATAATAA
TCAAAACATAAAGCTTTAACAGAAATTTCATAGTCAAAGATAAATTCTAGATAGTAAA
TTTTGATTCGTAGAAATGAAACCTTTGAAGCTCACATTAATAGGTCACTTAGAAATTACAAGTTCAGAGTTGG
CTCTTAGCTACTCATTTATCATTCATTATATTCATTTTTCATGAATCAACTGTATATTTATTTTGATTGAATA
TAAATCGTTATATCATTTTTGTGTTTACATAAATCGTTAGTTTCGTTAGTTTTTCGGT
CAAGAAGTTGCCGAATGAGTCACAGTTTGTGGCTTTTTTGTCTCCATAAAGTATGAATTATGCTTACAATGTT
ATAAACTTAATATAAAAAAATCTAGAATAAAATAATTACTTTAAAATTATATAAATGTAATATTACAACAAAA
TTGATAAGATATTGAAAAAGCAACGGTTTTTGTAAGCACATTATTGTTAATTTCATGT
TCATATTCATACAACCATTATCATTCATTTTCATTCTTTGTATAAGATAAAACAAATTAGAATAATTTTCATA
TTGGGTAAGCTTAAATTGTAATATTTTATAAGAATGCCATTAAATAG
>1002049911_1 Heli.1-DT668896.3.5
CACGCAGTGCAGCGGACCACCGGCGAATTTGATATTTGACGTTTCGTAAATATAAGTGGGAACTTTTGTTACG
AATCTCAAAAATAAATCGTGTTTTCAATTTCAGTTAAAAAGTGTAGTGAAAAATTTATCAACAATGATTACCC
TAGTGTTTGTTTTGGGAGGTCTCCTCGTAGCGACAGAGGGTTGCGGAGTTGGCAAGCA
GATGCTAAGATCGGGAGGGTTGACAGCATATGAAAAGCAAACTATAGTCGATGCACACAATCGTCTTCGGCAG
TCCGTGGCTTTGGGACAAGTGTCTAGTCAACCTCCAGCGGCTAATATGATGGAAATGGTGTGGGATGACGAGT
TAGCAGCAACTGCTCAACGTTGGGCTGACCAATGCACCACTGCCCACGATCGCGCCGC

CCAGCGTGACGTAGGACGCTTTCCTGTAGGGCAAAACCTTGCAGCCACATGGACCACTCGCCCACCATCTGAA
CAAGCGGATTCCGAGCCTGACTTCATGAAACAAATCAACGCTTGGTTCGATGAGGTCCGCATCTACGGATTCA
AGCCGATCAGCGGTGGACACGGAACAGGACACTATTCTCAGCTGGTATGGGGAGAAAC
TTCACACGTCGGTTGTGGATATACTTTCTACTATGATAACACCCGAGGATACACCAAACTGTACGTCTGCAAT
TACGGCCCTGGTGGAAACGTCATCGGATCAAATCCATATGAGAAAGGAAGTCCATCTTGCAGCGCCTATGGGC
TAGCTGACTCCAGAAAATACTCTGGACTTTGTTCTGCCAGTTTTAGCGCGACATCGAG
CTACCAAACGGTGGACAACGTCAATAACAACGGATACATCTCGAACGTCATTCCTGACAGCACTGTGGAGGCT
CCTGGCTTCAATTTCCAATTCTACAAACAATTTGACAATCAATATCAATATCAGTATCAACAGCCTGAAACCT
TCTACCAAGCGGAAACGTCGACCTTCAGGCCACTCATAAGCATATTCAAAACTGCCTC
AAATTTGTTCGCAAAACCAAAATCATCGGGCTTCCGAATTGGAACCGTCTACGTTTAGGCCATTTGCAATAGC
ATAAAAATTTGTAACAGTATTTTATCACTATTTAGACAGAGTGACTTGACTGATTAATTATTCGAGCCAGAGT
GATGCGTTCGGAACATTGTAGAGAATATTCCTTGGTTTTACAAGGAGCTTTAGAACTA
AACCAGTTGCGTATATGAATTTTGAACCGATTGAAGGTCTGATAGTTTTTTCCAAGAAAACAATGTGCTGTTT
GTGAATGACCTTCTAGTATTAGCTTAGCGTGAAGTATGTTGTTATTTAGATAATTTATATTATAATAATAAAC
ATTTACATATTAATTTATTTTTTATTCATTGTTATTAGTTATAATTGTCACATAAAAT
TTTAATAACCAATACAAAATTTGT
>1002049912_1 Heli.1-EL599267.1.5
GCACGAGGAACAGCCATCCCTGGGGCTTTCGGTTGTGGAAAGACTGTGATTTCTCAAGCCTTGTCCAAATATT
CCAACTCAGATGTTATTATCTATGTGGGTTGCGGAGAAAGAGGTAACGAGATGTCAGAAGTACTGCGGGACTT
CCCCGAGCTGACGGTGGAAATCGAAGGTGTGACCGAGTCTATCATGAAGCGTACCGCG
CTCGTCGCCAACACCTCCAACATGCCGGTAGCTGCCCGTGAAGCTTCCATTTACACTGGTATCACACTTTCCG
AGTACTTCAGAGACATGGGCTACAACGTGTCCATGATGGCTGACTCCACATCCCGTTGGGCTGAGGCTCTTCG
TGAAATCTCCGGTCGTTTGGCTGAGATGCCTGCGGACTCCGGCTACCCTGCCTACCTC
GGCGCCAGGCTGGCCTCCTTCTACGAGAGGGCTGGACGTGTCAAGTGCTTGGGCAACCCTGACAGAGAAGGTT
CAGTATCAATCGTGGGTGCCGTATCGCCCCCTGGAGGTGACTTCTCGGACCCCGTGACCGCCGCCACTTTGGG
TATCGTCCAGGTGTTCTGGGGCTTGGACAAGAAACTGGCACAAAGGAAGCACTTCCCG
TCTATCAATTGGTTGATTTCTTACAGCAAGTACATGCGCGCTCTAGATGACTTCTATGAGAAGAACTACACTG
AATTCGTACCCCTCAGAACTAAGGTCAAGGAGATCCTCCAAGAAGAAGAAGATCTATCAGAAATCGTGCAGCT
CGTGGGTAAGGCGTCGCTGGCCGAGACCGACAAGATCACGCTGGAGGTGGCCAAGCTG
CTGAAGGACGACTTCCTGCAGCAGAACAGCTACTCGTCGTACGACCGCTTCTGCCCCTTCTACAAGACGGTGG
GCATGTTGCGCAACATCATCGCCTTCTACGACATGTCGCGCCACGCCGTGGAATCCACC
>1002049913_1 Heli.1-ES585305.1.5
GCACGAGGGGTGAGTGGAGTGCCTTGTCATCAGTCCTTTAAGTTTTTGGATGATTTTCTTTGGAATTCATATT
CAAAGTGGAGTAACTATTCATTGTGATGCCGGTGGATGCTGACCTCAACCGCACAGACGTGGTGTCGCCTGGT
TGTGAGCTGAAGGGTCTTCGTACTTGGAAAGACATTTGTCTCGTCCGCAACTCTCTTT
TTCAACATCAGGGCCCCTTGCGATCCTGACATAGAAAGAAGTAGCGTCTCCACGGCTATCAGAAGGCCGCTGT
TCGCATACCGTGCGACCCCCACCCTACGACTAAAATAACGTCTACAAGTGCATTGTACTGCGACCATTGTGAA
CTTTGTGTTAAAAACAAGTGCAATATACTGATAGTGCAATCGTACTCTTAACTTTTGT
TGTTGTTGTTAAAGTGAAATTATCGTGGAACTATGGAGAGAGAGTCGAACCCCATGGAGGCTCTGTGCCGATC
AGGATGCGGGTTTTACGGAAATCCCTCCACGGACGGACTATGTTCAGTTTGCTTTAAGGAAGCGTTAAAAAAG
AAGCAGCAGCCGCCGGCGACGACGCCGTCGCCGGTGTCCACGTCGTTCGTGGCCGCCA
CGCCGCTCGCCGCCGCCACGCCCACAGTGCCCGCGCTGCCGGTGCAGCCGCAGCTACACACAGACAAACTGGA
AGAGGAGAGTGGGGCCGGCACAAGCGGCGCAAGCACCGGTGCATCAGACACCGACGCCGACGAACAGGACAGT
AAAGACAAAA
>1002049914_1 Heli.1-DT668381.3.5
GCACGAGGCTCGCCACCGCTCAACCACACGCGAGTGTTACCCTTTCTCTCTACGTGATCGTATACGTGATTAC
CGGAATTAAACTCGCCACCGCTCAACCACACGCGAGTGTTACCCTTTCTCTCTACGTGATCGTATACGTGATT
ACCGGAATTAAACACCAAAGGAACAACTTTCGGACCTGTCTCAATTTATATCAGGGGC
AAAACGAGAACACGGTATGACTGCGACGCACGGTGGCGGCTGTGAAGACCACCGGCCGGACAAGGACTCTGTG
TACACTGTGAAAACTGGGTTCAAAAACGATGGCTATCAACATGATAGAGATTCCAATGATGACATCGTCAAAC
CACCACCTCTGCCTACCGAGGATGACTCTTATCCTTCAGGCAAAGTGAAATTATCCAG
AAATGAAAAATGGAGAATATTGAAGAATGTAGCGGCCGTCAGTGCAGCCTTCATGGTGCAGTTTACAGCGTTT
CAAGGAACTGCCAATCTTCAATCTTCAATAAACGCCGCCGACGGTCTTGGAACCGTTTCCCTTAGCTCTATTT
ACGCGGCTCTCGTCGTCTCTTGTATATTCGTGCCTACTTTCCTTATAAAGAGGCTAAC

AGTGAAATGGACCTTATGCCTTTCTATGCTTTGTTACGCGCCATATATTGGCGCTCAATTTTACCCTGCATTC
TATACCCTGGTCCCTGCTGGAGTCATAGTTGGCTTAGGGGCAGCACCTATGTGGACTTCCAAAGCCACTTACT
TGACTCANGCTGGAAGTGTGTACGCTAAGCTGACAGATCAAGCAGTCGATGGTATCAT
CGTTAGATTCTTCGGCTTCTTCTTTCTAGCGTGGCAAACTGCTGAGCTATGGGGGAATCTTATTTCTAGTTTA
GTATTTTCTCG
>1002049915_1 Heli.1-EL596180.1.5
GCACGAGGTAAAATGTGTTTTCTTGTAGATACAGGATCCGATCTTTGTGTGTATCCACTGCGAGCTCTTCGTG
AAACACGCGCCGCACGAGGCTATGACCTGTTTGCTGCAAACGGAACGCAGATAGCTACCTACGGCTGGATCCA
TCTGCAACTGAACATTGGCCTGCGACGCACATATAGGTGGAGGTTTGTCGTAGCTGAT
ATAACAAAGCCAATTATAGGTGCTGATTTTCTCAGATTTTATAACCTGCTTGTAGATCTGAGGAACCACCGCT
TAGTAGACGGCCTTACTTTACTGTCTACGCCAGCTCGTCCAGCAGGAGAAAAAGCAGCAGAGGTCGCTTCAGT
CAAGGCTGTGTCGGGTGAAAGCAAGTATCACCAGCTACTTCGTGAGTATCCTGACATC
ACTCGACCAGCAGGGACCCAACGTGAGGTAAAACATAATACCACTCATCATATACGCATTACTCCTGGACCAC
CTGTATCCTCACGGCCACGTCGACTTCCACCAGATAAGTTGAAAATTGCCCAAAAAGAATTTGAGGATATGCT
GATGAATGGTACTGCACGGAGATCAGACAGTGCCTGGTCATCGCCCCTACACCTGGTT
CCCAAGAAAGAGAACGGTTGGAGACCTTGCGGGGATTACAGGGCTTTGAACTCACGGACGATACCAGATAAGT
ACCCAATCAGACACATCCAAGACTTCGCACATCAACTCGCCGGCAGT
>1002049916_1 Heli.1-EL596064.1.5
GCACGAGGCCACCCTCTAACGATGAAGATGAACATCATGCAGACTATGTTACTGTTACTCCTGAACCTTTATA
CATACCAGTGCAAACAACAACTCTGAGAGCTATTATAAAGCCAGAATCAGAAATTTATCATACTGACAGTAAT
GTTAAAGTCACATTCGGTAATGATCATCACCATTATGGCAATAGACGGGCAGAAAGTC
CACAAGGAGAGCAAAGAGAAGAAACTGCGCCAAGACCCGCAATTGCTTTGCCAAATCACAATTTAAATGAACC
ACCACAATCAGAACGTTCGGTATCACCTAGACCGCAGTATAATGATCATGAAATAAAATTAAATCAACACAAT
CCCGCAATTTTTCAACAAGAAAAACATGCTCAAAGCCGTATACAATTCCAGCAACCAT
TCCTTAGCCACGAACCACAGTTACCAAGGCAAGGGCCTTCATTAATTCCTTTTAGAGCTCAACCACATCAAAA
TAATTTTGGCCCATCGCCTTCGTTAAGCCCTCGTCCTTCATTTTTCAATGTTTCTCCAAGCCCTAGTAACCAT
TTCGCTAACATTGCCCCTACGCTTCCAGGTCCGATTGGTCCAAGTTTTGCAACATTTT
CAAGACCAAATGGACCGCCACAATCTTTTAATTCTCGCCCAAGTAATACACACCCTTTTGGAACAAGGCAGCA
TTTCCCACCCAGGGCAAGTTTCCATTCTGGACCTCAGAGACCGTTTCATCAACCTCAAAATTACTTTGATGAA
GCTAAATATTTACAAGAAAATTATCACACAATAACTACCGATCAAGTTGATCCGCCAA
AGGTTCAGTTGTTACCAAATAAACAGATTCAAAATTTTAATATTGGTCCTAAGCCTGGTACAAATCCTACTTC
ACATTTTGCTGATCATCAGGGCCGTCCACAACAAATAATTCCTTTCAACCAGCAATTACCTCAGCAAAGAACA
CCACAACAATTTTTAGATTCCAATAAAAATAAGC
>1002049917_1 Heli.1-DT665079.3.5
GCACGAGGGTCGACGTAGTTCTTTCGACAGATAAAAAATAAGACGCGGTCACTCAAGGACGGTCAACAACTAT
CGAGTGGTATTTCCCGTTTAACGGGCACCAGAGTGAAACACAAATGACGCGCAAACGACACCAGTCATTAACG
CCGCTAATGTATGTGGCTTTAAGGCTGGTAGGTCGCGGGCCACACTCCGGCGAAGCAT
TCTTGCAGTATAGAGTGTGCAACGTGAGGGGCCTCTAGCGCGTTTCCCAAGTTATCGGCACTGGAAAAACACG
CTACATTTTATCACATACAGTGTGTTCGCTATCAGCTGTTGCGAGCGATGCGGTGTGCGGTCGCCAACACGTC
ATGGTACGAAAATACGAAAGAATGAGTGGTCGCCAATCTTGGAGCGAAGAAGAAATGG
CAAGGGCCGTGGCCGCCGTGGTATCTGGTAAAATGGGCTATAAGCTCGCTGCTAGAACTTTTCACATACCACG
ATCAACTCTCCAAAGACGTGCAAGCAAGGTGCGATATCAGCAACCAGATGAACCAAAGCCTTTGATGGGACAC
TATCGACGTGTGTTCACAGAAAGTCAAGAGAAAGATTTGGTAGGTTATATTAAAAGCA
TGGAAAAATATTTTATGGGAGTTTCGAGAAGAGATATAAGAGAACTTGCTTTCCAATACGCAGAGGATAATAA
CCTCAATCATCCATTCGACGTCAACACTCGGATGGCTGGTGAAGATTGGGTGAGGAATTTTTTGAAACGTAAC
CCCGAATTGCTCCACAGGTCGGATAAAGAATATGAACTGGAACCTGTTAATTTCGATC
AATTTTATCACTTTATGTGTCAATCATCATGATTGTAATTGTAAACTCCTTAGACATATTGAAGGTCGTATGC
AATGAAGCCCACCTGACGGAGCCTCAAATATTGTATAATATGGTATATACTGTATGGAACGATAGATAGTAAT
TCTACTGAAGTTGTTTGCAGTT
>1002049918_1 Heli.1-EL603900.1.5
GCACGAGGCAGATACACCCGGCCTGTAATATAGTACAAAATCGGCCAGTTTGTGATATAACTTCGCGATGGCT
CAAGCTCTTAAAAAACTATCTGCGCAAGCTCTGAAAAATAATTATGCAGAGGCTTTTACAACCGGTATTATAT
CCAGAGCAAGCAGCTCGTGGTGGAGTAATGTTCAAATGGGTCCACCTGATGTCATCTT

AGGTATAACAGAGGCCTATAAAAGAGATGCGGATCCAAAGAAAGTAAATTTAGGAGTTGGGGCCTACAGAGAT
GATCAAGGCAAACCTTTTATTTTGCCATCAGTTAGAAAGGCTGAAGAAATAATCTACAATAAAGGATTAAACC
ATGAATATGCCCCTATTGGTGGAGAAGCAGCATATACTGATGCAGTAGCGAAGCTTGC
ATTTGGAGAAGACAGCCCAGTGCTTAAAAACAAAAGTAACTGCACAGTCCAAACCTTGTCTGGTACTGGTGCC
CTTCGTCTGGGTTTGGAATTCATCACAAAGCATTATGCCAAGAATAAAGAGATCTGGCTTTCGACTCCTACTT
GGGGTAACCACCCACAAATCTGCAACATGATCAATCTCCCACACAAGAAATACAGATA
CTTTGATGCTAAGACCAACGGCTTTGACTTGAAAGGAGCTATTGAAGATATTAGTAAAATCCCGGAAGGTTCA
ATTATTTTGTTGCACGCGTGCGCACACAACCCCACAGGCGTTGACCCGAGGCCAGAGGAATGGAAACAATTAT
CACAGGTAATAAAAGACAGGAAGCTGTTCCCATTCTTTGATATGGCGTACCAAGGTTT
CGCCACTGGCAGTGTAGACAATGATGCCTTCGCTGTCCGCTTGTTCGTAAAAGAGGGACACCAGGTCATGCTC
GCTCAGAGCTTTGCTAAGAATATGGGATTGTACGGCGAGCGTGCAGGAGCTCTCACTTTCCTTTGTGGTGACG
AAGAATCTGCGGCTCGCGTGATGTCGCAAGTTAAGATTATGATCCGTACTATGTACTC
CAATCCGCCATTGTTCGGCGCACGTCTTGTCCAGGAAATACTTAATAACCCACAGCTTAAATCACAGTGGTTA
AATGACGTTAAGCTCATGGCCGACCGTATCATCTCAATGCGCCAGCAGTTGCGCGCCGG
>1002049919_1 Heli.1-DT665290.3.5
GCACGAGGCAACAGTCAACAATTAACGAGGAAACGCCTAGATATATAAAGTATTATGACCTATAAGCGCAAAA
AGTACCATCACGGTGATACTCATCTGAAGAAACGGTGGAGAGTACGGAATAGAAAGAAGGATCTCGACCAAAT
CGACGACGACATAAAAGAAGAAAATGCTGATAAATTGCTAAACCAAGAAGTAGATCTC
GACGTACCAGGCGCCGCACAACATTATTGTTTGCACTGCGCACGATATTTCATTGATAATCATGCACTTTTAG
AACATTTTAAAACTAAAGTGCATAAAAGAAGGTTAAAAGCTCTAGAATTAGAACCATACACCGTAGAAGAATC
TGAAAGGGCAGCAGGACACGGTAATTTTAAACTTCCTAATAAAAGGAAGATAGTCACA
CAAAACACAGAAAGTGCAGATATTACTGACAGAGATAGTGAAGCTTTAGAAGTGAGTCCTAATAAAAAGAAAA
AGGTTGAGGATAATGCTACGTAAAATATGTTATAATTTTTTTTGTAAAAAGATAATACTAAGGCGATATTCTG
CATTGTTTATGAATGGTCCTAAAGCTACTGAAAATTTTCTTTTTGTTACACCATACAT
TGATTTTCATGAGCAAATAAAGAACAAAGAAGTCATAAAGACCGAACTGCATAAGAGACAAATAGATTTAGAT
TTACAGAAATTGGAGAATCTTTGGGATGTATATGACACTTTAAAGAATAAAAGAAATGAACTTGATTTAAAGA
AGTCAACTATAGGTAAAGAATTAGTGGAGCTACAACAGAGTAAGGAAGAGAGTGATCA
TAAAGAAAAATTGAAAATACAATTAAATTTAGTCAAAGAAACATTCAAAAATTAAAGTACCGTTGTGGTCTA
>1002049920_1 Heli.1-EL598220.1.5
GTCGACCGTTTTCGTCCACATTTATTTTAACGTGAAAAATGACGAGGACTTAAAAGTAAATAACAACTAAGAT
AAATTATAATATATAAGTGAAAGTGTTCAATATTAACGTCAAGAAATCAGCTTTGGATTTCTTGCTTACGTTA
TACAAGATGGCGATATTTGGTTTCGTAATTTTGAGCATTTTACTAATTCAAGCAAGCT
CAAAAGCTGCTCCAAGTTCAGACGTAGCTAAAAGTTTAGACGAAATTAAAAACATTGAAGACGGCCCTAAACT
AGACGATGAACCATATGGTACCGCTGAAGATATAAAAACTATAAACGAAAGCGAAAATAACGGACGACAGAAG
AGATATTACGATTATGTAGAGTTTGGCCTTCCACCAATGTTTGAATCAAATTTCCCCT
TTCCTAGCCGATTTAACTACAATAAGAGAGATGAATCCCAAAATACAGGTGTATATGGCAGTGAAGATTCGTT
ACTTTCCATATTAAAGGCTCTACAAAGAATTCTTAATGATGCAAGGCGGCAACAATCAACGCCCCAGCCGCCT
CGCGTTCACATACCTACCTATATACCAATATTTTACCTACCTCAAGCAGCATGCGAGT
GCAATAATAATGTACCTCAGGTACCCATAAATAATGGTAATAATAAAGAGACCAATAATAAAGATATAAGCCA
TAATAATAATA
>1002049921_1 Heli.1-DT665906.3.5
GCACGAGGCGCATTTCTGTGCTAGATTTTTCAGTTTAGCAATTCAATTAATGATTTCATTCGTATAAGAGTGT
AATTAATGAAATTATCCGAATCACGGCGACCTTGCGAGGATTTGTCGCCCAGTAAAAAGTGTGTGACGGCGAT
ATAATAACAGGGAGGGCGATGGCAACAAACCGAAGTGGTGCGGCTCAGAGGCCCAACG
GCGCGCCCCAGACTAAAGTATGTCAGTTCAAATTGGTACTTCTGGGCGAGTCGGCGGTGGGTAAATCTTCCCT
CGTGTTGCGCTTCGTCAAGGGCCAGTTCCATGAATATCAAGAGAGCACCATAGGGGCAGCTTTCCTAACTCAA
ACGCTTTGTTTAGATGATACTACTGTTAAGTTCGAGATTTGGGACACGGCGGGGCAGG
AGAGGTATCACAGCTTAGCACCAATGTACTATAGGGGCGCGCAAGCGGCAATCGTAGTCTACGATATAACTAA
TCAGGACACATTCGGCCGTGCGAAGAACTGGGTCAAGGAGCTCCAAAGGCAGGCATCACCGTCCATCGTCATC
GCGCTCGCCGGCAATAAGAGCGACTTGGCCGCCAAACGGATGGTGGAATTTGAAGAGG
CTCAGGCCTACGCGGACGAGAACGGACTGCTCTTTATGGAGACCAGCGCCAAGACGGCCATGAACGTGAACGA
TATATTCTTAGCTATCGCCAACAAGTTACCAAAAAGCGAGAGCGTGGGCGCGGCCGGCACGCGCGTGCTCAAC
AACGCGGACGCGCCGCGCGCCGCCTCCTCCTGCTGCAAGTGATACCCGCAGACCGTGT

CGCTACAGAGTGAGGGCGCGCGCGCCGCCTAGCCGCGCCGCGGGACGCCACACACACACACACACCACAAACA
CACACATACATTCAGTGTCTGACATTCATGTACTTACATGTATCCATTCTCATACCGACATACAAACATTAAA
CAACACCCACACATACAACCCTACACCCACACCCACACATACAACCCTACACCCACAC
ACATACACCCCTACACCTACACACCCTTCTATACACAAATACATGAAAGTGCGCACCTCCATGCAACAACACT
TAGCCTTACACACACATCTACACGTCCACACAATCATAATACAATACACACATTCACGTAAAGTCTACTAC
>1002049922_1 Heli.1-EL602030.1.5
CTAAGAGATTTTAAGTTTTAAGTAAATTACTATATACTAAATTAATAACCAATTCCACATAGTGGATTCATAA
ATAAACTGGGTTTCATTGAAAAAATTATCATAAAGATGAGCAAGTTGATTCCCTCTGCAGCCAAGCTGTTTGG
TGCAACAGTGACGAAAGCAGCCGCCCCAGTAGTCACCACGAATGTGAAATATAGTACA
AAATCTGAGGCATCTTTCGAAATTAAACCTTTCAAACTCCACAGGTTGGATAAAGGTCCGGCTACAACAGCCA
CTTTAACATCTGAAGATGCAATCCAAATGTATGAAAAACTTGCATTACTTAGAAGGATTGAGACCGCAGCAGG
CAATTTGTACAAGGAAAAGATTATTCGTGGTTTCTGCCATTTATATTCAGGCCAAGAA
GCGGTAGCTGTGGGTATGCGTATGGCAATGCGCGATGCTGACTCGGTGATCACTGCCTATCGTTGTCATGGTT
GGACCCACCTCATGGGGGTCGATGTGGTGGGCGTCCTAGCTGAACTTACGGGCAAGAAAACCGGGTGCTCCAG
GGGCAAGGGAGGTTCTATGCATTTGTATGGACGTAACTTCTACGGTGGTAATGGGATT
GTAGGTGCTCAGGTACCACTCGGTGCAGGTATAGCTTTTGCTCACAAATATAATGGGGACGGTGGAGTCAATT
TTGCCTTGTATGGAGACGGCGCCGCCAATCAAGGACAGCTTTTTGAAGCGTACAATATGGCGAAACTATGGGA
TTTGCCTTGTGTGTTTGTTTGCGAGAACAATGGATATGGTATGGGCACGAGCGTGGAG
CGCGCGTCGGCCAGCACGGACTACTACTCGCGCGGCGACTACATCCCGGGCGTGTGGGTGGACGGCATGGACG
TGCTCGCCACGCGCGAGGCCACCAGGTTCGCGATCGACTACTGCACC
>1002049923_1 Heli.1-EL603045.1.5
GCACGAGGACGTTATCGAGAAAAATGAATCTTAAACAATTTATATTTCTGTTCTTATCACAATTATTTAATAT
TTCAACAGCTGTTTCCCCGATACCATTAAAGTGTTGGTTATGGGACTCCCCATGCACGACTGTAAAAGCTCAA
GCAATATTTTCGATCCTCGCGGCTGGAGTTCCTGATTTGGATATACAACCACTGGACA
CTATATGGATAGACTTGATGCATGTTGACCAAGATGGATTAAGAGCAGACTTTAGAAGTATGAGCATTAAAGG
AATGACTAATGCAATTATTGATAATATGAGCATGGATATGCCTTCGAAGATAGTTAATTTAGTATTCCACACG
GATTTCGTCACTAAAAGTCGATATATAAACAACGGTACTTTACTTTCAACTCCCATTT
ACGGTGAGGGCGATTACTTTATGAAACTTGAAAACGTTCGTATTACAATGAACATACCTTTTGATATCATAAA
AGACGCTCATGAAATGGATATAATGATGTTAAAGTCATTTAACTACACCTATGATGTAGCGAATGGTGCCCAA
TTTTATTTTGAAAATCTTTACAACGGTGATAAGTATTTAAGTGACAATATGCATAGTA
AAATGCATCAAAATTGGAAATATTTGACAATTGCTTATGGAAAACATTTGTTTGATCCAGTAACTAAAAAGGT
GTTTGGTTCTTTCCGAAACTATATCCAATCTGATCCACTGAGAACGGTCGCTTTGTAAACTTTTCCAACTATT
TTGAAAATTAAAGTATATTGAATTATAATATGCATAATAAAAGAAT
>1002049924_1 Heli.1-EL600832.1.5
AGATCACCTTCGCCGTCGGATTGCATGAGTCCGGATACCATTAACCCTCCGTTGTCGCCTGCCGACTCCACCT
TCTCCATGGCGTCGTCAGGCCGGTCAGGCCGGGACAGGACACGTGCTTTCTCCGACGAGGAACTGAAGCCACA
GCCCATGATCAAGAAGTCCAGGAAACAGGTAAATGAATTCGTGCCCGATGACCTGAAA
GACGACAAGTACTGGGCACGCCGACGGAAGAACAATATGGCAGCCAAGAGGTCACGTGACGCGCGCCGCATGA
AAGAAAACCAAATCGCGCTTCGAGCTGGCTACCTAGAAAAGGAGAACATGGGCCTACGACAAGAAGTGGAACT
GCTGAAGAAAGAGAACCACATTTTGCGCGAGAAGCTGTCCAAATACGCGGACGTATAA
GCGAGTTCCGCTCAGCACAAACACGCCACATCATATGTTGCGGGCATCGTCGCGCGTTGCGCCGTCTCGGGCA
CACCGCTCCTTCTATTTGTAACGCTTTTCAAATGTTCGGTTATAATGTACTGTACTATACAATACTGACTAAA
CTGTTGTTAAATGTAATGCTCTTCACCACCCACCACACTCGAACTACCGATTCGCTGT
TCCCAAAGTACTTATGTTGTAACC
>1002049925_1 Heli.1-DT666173.2.5
GCACGAGGCACACATCAACCACAATGTCCGCCAAGATTGTGATCGTCCTCGCCCTGGCCGCGGCCGCCTACGC
TCTGCCAGTTGTGCAGTACGCAGAGCCCGAATCGCCCGCCCACTACGAGTTCCAATACTCCGTACACGACAGT
TACAGCGGTGATGTGAAGCAGCAAGAAGAGGCCCGCGCTGGAGACGTCGTCCACGGCT
CCTACTCGCTCGTGCAGCCTGATGGAGTCCACCGCATCGTGGACTACAGTGCTGACAAGGTGAACGGATTCAA
CGCCGTCGTGCGTTACGAGGGGCACCCTGTCGCCGCCCCCGCCAAGCTCGCGTACGCCGCTCCAGTCGCCAAG
CTCGCGTACGCCGCTCCAGTCGCCAAGCTCGCCTACGCCGCTCCCGTCGCCAAGGTAG
CCTACTCCGCCCCCGTCGCCTACGCCGCCCCTGTCGCCAAGCTTGCCTACGCCTCTCCCCTTGCGCACGTCAG
CTACTCATCTCCCGTCATCTCCTACCAACACTAAGTTAATAACTATAGCGGATATACTTATTAAACATACTTA
ATTTATTTTTGAATATGTGATAGCAATGTTTGTAAATAAATATTTACATATTATTTAA

AAAAAAAAAAAA
>1002049926_1 Heli.1-EL603538.1.5
GCACGAGGCAACAGTCGGGTGTGAGTGAAAACTCCAAATTTTTTATAATTGAAAAAAATATATATTCGAAAAT
CGTGCTCCTAGTGTAATTTATTATTTTTAATAAATCTGAATTATTAAATCACTACGATGGGAGAACTCGGTAA
AAAGCTTGGTTTGGACGAATTGTCGGGAGGTGCAGCTGGTATCAGCAAAGCATTGCTC
GCGGAATTTGTCGGAAATCTACTGCTAAACTTGTTTGGTTGTGGAGCTGTCATCAATATTGCGCAAGGTTCTG
AAGGTGCCCAGGACATTGTACTCATTGCCCTGGCTTTTGGTCTCGCTGTCTTCGCCATTGTTTCTGCCATAGG
TCACGTATCAGGTGGTCACGTGAACCCTGCTGTAACAGCTGGTATGGCCGCCACCGGC
AAAATCAAACCGATTCGAGGTATCCTCTATGTGATAGCGCAATGTGCTGGTGCAGCTGCAGGATCTGGACTGC
TTAAGGCGTTCACACCAGAACAAGTGGCCGGTACCCTCGGTACTACGGGACTTGGCAAAAATGTCACGCAATT
ACAAGGTTTCGGGATTGAGTTCTTCCTGGGATTCGTCCTCGTGTTCGTTGTTTGCGGG
GTTTGCGACGCTAACAAACCTGACAGCAAATCAACGGCGCCCCTCGCCATCGGCCTGACGGTGACACTGGGGC
ACTTACTGGCGGTGGACTACACCGGCTCCGCCATGAACCCCGCGAGGTCCTTTGGCTCTGCCCTCGTCGCCAG
CACCTGGGATGACCACTGGGTATACTGGGCGGGACCAATAGCTGGCGGTGTGGCCGCG
GCACTACTCTATGTTCACGGCTTCGCAGCACCTCCACCGGATACCAACTCGCTCTCACCTCGGTACCGCCCCG
TCGCCTCCGATGAAAAAGAGTTGAAGCGCCTGGACGGTAAAGCCGACGACATGGCCTGAAAGCGAGTCCCGCG
GCTCCGGCCGCACTCGCCGCTCTCCGTCCCGCAAGACACGCTCCCACGCTCCTACACC
CACCACTCACAA
>1002049927_1 Heli.1-EL601619.1.5
GCACGAGGGGAGGCAGCTCTGACTGGACGACACGCTCGTCGGGAAATAATACACGATGAGGAGTTACTTCCTG
CTGCTGGCCTTATTGGTCGCAGCAATTTGCTCCACTGAAACAGTCACGAAACGGCGTCGTCACCGGCGCCTGC
GCACCACCACGACGACGGAGCCGGCCATGCTGGCGGCCGCGCGCCTCTTTCCCCCCGC
CGAGGCCGAGCACATCGAACTCGGCATGGCGGAGCGCCTCGACGAGAAACGCTTCCAGGAAGCGGAACGGGCT
CGCGTCAATGAATTGATGAACGAAGTAGACGAGGAGAGCGGCAACGAGGTGTTCATGGACGACCCGTGCCTGA
AGGTGCACTGCAGCGCGGGCCGCGTGTGCGAGATCGACGAGCACGGCGACGCGGTCTG
CAACTGCATCAAGGAGTGCCCCTACGAGACCGACTCGCGCCGCAAGGTGTGCACGCACCGCAACGAGACCTGG
TCCTCGGACTGCGAGGTGTACCGCCAGCGCTGCCTGTGCCTGGACGGCAGCGAGCTGTGCCGCGGGCCCGAGT
ACCACCACGTGCAGATCGAGTACTACGGCGCGTGCCGCGAGATGCCGGCCTGCTCCGA
CAGCGAGATGTCGGACTTCCCGCGGCGCATGCGCGACTGGCTCTTCAACATCATGCGCGACATGGCGGAGCGC
CGCGAGCTGTCGCCGCACTACCTGCGCATGGAGCGCGAGGCCGAGTCCAACCTCACGCGGCGCTGGACCAACG
CCGCCATCTGGAAGTGGTGCGACCTGGACGCGCACGACAACGACCGCTCCGTGTCGCG
CCACGAGCTGTTCCCCATCCGCGCGCCGCTCATGGCGCTGGAGCACTGCATCGCGCCCTTCCTGGACCGCTGC
GACGAGGACGACGACCACCGCGTCACGCTGGCCGAGTGGGGNCAGTGCCTGCAGCTCGACGAGTACGAGCTGG
AGGACCGCTGCGACCAGCTGTC
>1002049928_1 Heli.1-ES585469.1.5
CGTCCGCAATCACAATTTCACAACCTTCTCTTGTAAAAGTAACTTTTAGGCCATAATTTAACTGAAATAGCAA
TTTAAAAGTTAGTACAATCCAAAAATGGGTAACGCGTGTGCGGCGGGAATTGTGAACGTACTGGGAAGTATTG
TACTCTGTACGGGGAAAGGCGCCTGGCTTTTATTGAAGCTAGGATTTTCTGCAACTGG
AGTGGTGGCAGGCAGCTTGGCAGCTCTGGCCATGTCATTTTTTGGCAACGTAGAAGCTGGCAGTGTTATATCA
CGGGCTACGTCTATGGGAATGAAGTCTTAAATCATTTTGTTCCTGCACCGAAAGATGAATAAATCAATACA
>1002049929_1 Heli.1-ES587901.1.5
TCGGGGACAGTGGGCAAAGACCCAGTCAATATCAGATCTGATGATAGCGCTTCGCCCCGATCTCTAAGCTAGT
CCACAGTGCTTTTAGCGGTTTTTAATAAATAATATTAATAGTAATAACGTGTTGTACAAATAATAATATAGGA
AAACGTGATATTTTGTTAGTAACAGTAGTCGTCGGTTCCGATCGAGGAACAATTTTGA
TAAAGTAAAAACAAAGTGTACAATATAAGTCATAAAAATTTGGAAAATATGGAGATACTGCCGGTCACGAGTC
AGTTTAACGTCGGAGTCGGATTCAGTAGTGAAAAAGCGTGGAATGGTCCGACGTGGAGAGAGGCACCGATATC
TGTACCAACAGAGGCAGCATTGGCCCAGAGGCTCGAAAGGGAACTTCGAGCAGCCAAG
GGAGCCAGTGAGTTAGCCACAGCGGAAGTCCTGGTGCCAGCGGAACTGCTGGCCAGGGCTTCGAGACAGACCC
TGGCTCTTGCTGAAGGCGAACCCTGCGGGTCGAGGGGAGCAGCCGTGATAGTGGACGTGGCTGGAAGACGCTT
AGCCGCATTCAAAATAGACCCAAACACATTAACCACCCATGAAATACACCTCCACTTG
GAACATGATGCGACCAATTGGACTAGCTTACTGCCGCAATTCTTAAAAAACCTAACGAGAGGCGGCACCATCA
TAATCAGCCCACAATTCACGATA
>1002049930_1 Heli.1-ES587459.1.5

CAAGATAGCTTGTGCGTGTTTGGCTTAAAAGCCTTTAATTTTAAGATTATTGACAAATATCACTGTTTTTACA
AGAAGAATGGCGCCCAAACAGAGAATGCGTATTGCAAATGAAATTGCAAGCAAAAACATCACCATGAGGGGAA
ATGTTCCTAAAACCACTAAGGAAAAGGACGATCAATACCCCGTAGCACCATGGTTACT
AGCTCTTTTCATTTTTGTAGTTTGTGGCTCGGCCGTCTTCCAAATCATTCAATCTATTAGGTTAGCCTAAACC
TGTGATTCCCCGACTGATAATTTTAGTATCACGAAATTGTGGCTAAGTATTCTTCCTTTCATCTTTCTTTTAC
TTTCCATCCTATCAATTAAACATATCCCTTCTAATATATTTTATAATTCTATTTGCCA
AATGTAAATATTTTTTAAATAAATGTTCAGAGAAGGGCCTTAATATTATTTGATTTAATTATGCATTATTTTA
ATAGAAAAAATAAATTTATAAATACTAATTGTTTTTAACATACTTTTGCCACAATTTCTAGAAAAAATTGTAA
CATCTTCATTACTTTGTATGAATTAAATATACACATGTATATATATTTGATTGATATA
AAGCCTATCCATTTTTCCTGTAGCTGCAAATGCAATCTGTGTTTAGACTAAGCTCAGGTAATCTCTTTGTATG
TACTATATTCTTGTATATTGTAAAATAAATATAAGCTTGGCCCGCACTTGAGAATTTTTATAGTACCTTATTA
TTATTATATTAAATAATAGTTAATTTAATTATATGTTTTTAATATATAATTATATTTG
ATTGTCAAGTGTGGTCTCAGCTTTACTTTTAAGGTGCATTTCGACCAAGAAATTTAATTTTCATTCCTTAATT
TTTTTATTATTAAATTAAGTTCAACGCACAAATCATAATTTTGTTTCTGCAAAGTTATGTTGACTGTAATTTT
TTTATGTTGAATATGTTTCTGTCCCAATGTTATGAAATTATTTGTCGGTACTATTTAT
AATTTTAAATGT
>1002049931_1 Heli.1-DT664552.3.5
GCACGAGGTTGACACCCACGTCCCGAAATGTAATATTATTTAAATATACATAATAATACATCGATTTTGTGTT
GTGCTGTGAAATCTTAATAAATAAGATGAAGCCGGTCCTGCTGGTTCTATTCTTGTCGGTGGTGTACGCCGAC
GAGCTCCCACTGTCGTGCAATAGACCTGTCTATTGTAAAAGCAATTTGCTACACTATG
TTCAAATGTCTAGAATCTTCTCAGATTCTAAAACTTTCGTGGATCTTCATATGAAAAAAGATGAAAATAGTAC
CATAACAGCTTTTGATGAGTTGCTCAAAAATACCAACAATATCCCAACAAACGAACAAATTAAAGAATTTTTA
GATAATTATTTTGATTCATCAAGTGAATTAGAGGATTGGACGCCTCTTGATTACAGCC
CTAATCCACCTTTCCTATCTACTATTCGAGATGAAACATTAAGAAATTTTGGAAAGAATATAAACGACATATG
GCCTACTCTAGGAAGAAGAGTAAATCAAAAATTATTTGAAAATCCTGATCAATACAGTTTAATACCGGTAGAT
AACGGGTTCATTATTCCTGGAGGTCGATTTAAAGAATTGTACTATTGGGATACGTATT
GGATTATGGAAGGTCTCCTTGTTAGTGGTATGCGAGATACCGTCAAAGGTGTAATTGCAAATTTAATACAACT
GTTGAAAAAACTTGGTCACATTCCCAATGGCAGCAGATGGTACTATGAACAAAGAAGTCAACCACCTCTTCTA
TCAGCTATGGTATCTTTATACGTTCGAGAATCGAAAGACATAGACTTTCTTAAGCAAA
ATATAAATGCTCTTGAAGAAGAATTAGAATATTGGTTAGATACTCAATTAATTACATTCAATGTGAATGATAG
AGCGTACACGTTGCTAAGATATTATGCGCCAAGTGAAGGTCCAAGACCTGAGTCTTATTATGAAGATTATAAA
GATGCTCAAATATTCGACAACCCTGACCGTAAGCAGGAATTTTATACAGACATAAAAA
GCGCTGCTGAAAGTGGTTGGGACTTTTCGTCTCGTTGGTTTATTGGTAATGATGGTAATAATAAGGGTAATTT
ATCAACAATTCACGCTAGTAAGATTATACCAGTTGATTTAAATGCGATATTTGCAAATGCTCTTCAAAATATG
GCATATTTTT
>1002049932_1 Heli.1-CX700733.1.5
AATGGTCCTGCAGTACGGTCGGAATCCGGGTCGACCACGCGTCCGGGGAATTCGCACTTAGGTTTTTTTATGA
AAAGGCTTATTTCTTAATCGCCAGTTCTACTAATAATAGTGTTATATAAATATATCGATTTAACTATTGCCTG
TCGTTGCTATAGTAATTTGCATACAACAATAAAATGGATGCTGGGAAAGGTATAATTT
TGGGTGTCGGCGTTGTGGTGGGACTGGTGACGCTTTTCGTCGAGTTGCTCAGATATAGGAACGTTCCAAGACC
ACAAGCTGCATATGGGAGACGACCTGAAAGGAATAATCAATAATATGTATATCGCCAGAGTGATGAAAATTGC
TTCAAAAAGCAACCAATATTATGCTTATGCAGTACAATTTTTCCTTTAAATTAATATT
TTATGTTTTAAAGTCTGTAATGTATTTCTTTTTATATATTATTAATCTGTATTCCATATATCCATATAAATAT
AATTTTGCTAA
>1002049933_1 Heli.1-EL600358.1.5
CGGCACGAGGCATTGTCCTAAAAGCCATGGGATTGTTCATTTAAACGCGTATTGTGTGTTTCCTAGTTATTTT
TTATTTCTTTTATTCAGTAGTCACTTAATCATACAATAAAAATATTTTCATATCAGTGCTTACTTTTTATAAA
GTTCGTCGTATATGAGAAACAGTTGTGGCCGGATCAATCGGTAATTTACTGTATTTTT
CGCCCAGTGCAATGCTAGTGATGTACCCGAAAGGTGACTGTTGATAATAAGTAGAAATAAATTTATCTTCATT
TCGCAGGTATCTTACTCGTATGCAAGCGCCCCGCGTACGTGCCTCAATAAACCGCGCCGCCTAGCCGCCTGAT
ATATTGGCGGCGCGGCCTAGCGTGAGGTGTTAAAGCGCACCCTGGAGCCGCATCGAGG
AATTTTGGTGCGGTGGGCGCGGCGCCACAATGGACGACGGAGGCAGCAACAAGCAACGCCAAGCAACGCGAGT
CTTCAAGAAGAGCTCGCCAAATGGCAAGATAACAGTGTATTTAGGGAAAAGAGACTTTGTAGACCATATCACA
CATGTAGACCCGATTGACGGGGTCGTGCTAATCGACCCGGAGTACGTGAAGGACAGGA

AGGTGTTTGGGCACGTGCTCGCCGCCTTCCGATATGGGCGCGAGGATTTGGACGTCCTCGGCCTGACGTTCAG
AAAGGACCTTTATTTGGCTGCTGAACAGATATACCCAGCAACGGGTACCCCAAAGCGGCCGCTGACGCGACTC
CAAGAGCGTCTCGTCCGCAAGCTCGGGCCCGCCGCCCATCCGTTTTACTTCGAACTGC
CACCGCATTGTCCCGCCTCAGTGACTCTACAACCGGCCCCCGGTGACACAGGAAAACCTTGTGGCGTTGATTA
CGAATTGAAGGCTTTTGTTGCTGATTCTCAAGATGATAAGCCACATAAGAGAAATTCAGTTCGGCTTGCAATC
AGAAAGATTATGTACGCGCCAAGCAAGCAAGGAGAGCAGCCATCCGTCGAAGTGTCCA
AAGAGTTTATGATGAGCCCTAACAAACTGTATCTGGAAGCCTCTTTAGACAAGGAACTATACCACCACGGCGA
GAACATAGCAGTGAACGTGCACATAGCAAATAATTCCAACAGATCGGTTAAACGCATCAAAGTGTCAGTGCGC
CAGTTCGCTG
>1002049934_1 Heli.1-DT665168.3.5
GCACGAGGCACCCCTCGAGCCGGCCCCCGCCCTCGCCCTCGCCCCCGCCCCCGCGCCCGTCGACTTCATACAA
GAACATTCCAGGCCGCGTACAACCCACGGACCACCCCAAATCCCATCACCACCGTCGGTCAGCAGCAACGGTA
GTAGCGGCAGTAGCGGTAGTGCCCCCCGAGCAGGTACCCGTGAGGTCCACAACAAATT
AGAGAAAAACCGTCGAGCTCATCTGAAAGAGTGCTTCGAGCTCCTCAAAAGACAGCTGCCAGTCACACCTGAT
GATAAGAAGACGTCGAATCTGTCGATACTTGGGTCCGCAATACGGTATATTCAGGTGCTCCGCAGAAAGGAGC
GCGAGTGCGAGCACGAAATGGAGCGTCTGGCGCGCGAGAAGATAGCGGCCCAACAGCG
CCTCGCCACTCTCAGGAGGGAGGTGACTGTGAGGGCGGCGGTGCGGCCGCGCACGATCGATGCCGTTTCTGAA
GAGAAAGATAGGGACGATAGTGTCAATGGGCAAGTGTTAGGGATACCTATAAGTATTACCTCTTCGCCGCCAC
GGTCGCAAGTGAGAATGGAATCCCCGCCCCGCACTCTAAATCTAAGCACGAAGCTGCG
CACGCTGCCCATACAGATAACGCCCACCACCTCGCAGGTCGTGCGGACGTCATATGGCACAAGGCATAACACG
TTAACGCTTACAACCATTGCTCCCGCGGAACCCACTGCACAGAACGGTCAAGAAAATGGTATCAATACATTGA
GTAATTTAGTACATCCGGCTCAAATTCACCTTCTCTTGTCACAAGTGGTGAGCAGTGG
TGGTCTCGTGGTGAGTCCCGCCGCGTTACAGCTGCTCTCGACTAGCGG
>1002049935_1 Heli.1-EL596088.1.5
GCACGAGGGCGCCTTCCAGTCTTGAGTAGTATGGCCTGGTTCCAGTTAACGTAATTATCCCCATTTTCTGCAA
GAACCGTCAAATCTGTTTTTTGCCAGATCTTTCACTCGTGTTCTGCAAGGCGCTAGTCACCGTGGTGCTCACT
TGCCAGTCTGCAGCCTGCCGGTCGCGCAGTGTATCCCGCTACCCGGTTGCAGTCGACA
CAACCAGCCCTGTGTTTTGCCAGACTTTGGGCCGGGCAGTGTGACCTGGTTCCAGTCGCCGCAACCAGCCCAG
TCGCTCCTAGTGCTTGGGACGCGAAGTGTGTCCTAGTCCAGTTAACACAGCCACGTACATCAGACACACACAG
ATACTTCACTTCTGGCCTCTGTGGTCAATTCTGCGTGGTCTTAGTGACCGTGCTGCCT
GCCCGCCTGCCTACCAGTCTTCGGGCCAGCCAGTGTGACCTGCTTCCAGTCGGCGCAAGCAGCCCTGTTTCCG
GGCCAGGCAGTGTGACCTGCTTCCAGTCGGCGCAAGCAGCCCTGTTTCCGGGCCAGGCAGTGTGACCTGCTTC
CAGTCGGCGCAAGCAGCCCTACCTCCGGGTCAGGCAGTATGACCTGCTTCCAGTCGGC
GCAAGCAGCCCTGTTTCCGGGCCAGGCAGTGTGACCTGCTTCCAGTCGGCGCAAGCAGCCCGATCTCCGGGTC
AGGCAGTGTGACCTGCTTCCAGTCGGCGCAAGCAGCCCTGTCTCCGGGTCAGGCAGTGTGACCTGCTTCCAGT
TGGCGCAAGCAGCCCCGTCTCTACAAGTCTCAGGCCGGTCTGTTCGACACAGCTCGCC
>1002049936_1 Heli.1-EL600133.1.5
CGCCGGCGAGCTGGCTGGTCGGTGTTGGTAACAGAAAACAAAATGAATTGCGCGCGAATTCTAACGAACACAT
TCGAAGTTTAAAAACGATTTGACAGTTACAATAAATTTTTTGACACATCGATTTAAACTAGATTTATTTATTT
CAAACTAATAATAAATCATTTAAGAAAAAGTGTTGTTTTTTTGGTGTTGTGTGAAGTG
TTTATTATTGAAAATGTTGTTGTGAGGCAATGCTTGTGGGGGAGCAATCGCCGGCACCGCGGGTGGTGGCCGG
CTTGCGGAAACTTAGACCTGAACTGACACCAGGACCTGTGCCTACGCCAGCTGATCCTAATCTAAGGATATCA
GGTGTTCTACGCCAATACTACAATGACGATGACCAGTCGTTCGCGTGGGTGCGGGCGG
CGGTACGAGGGGGCTGCTTGCTGGCGTGGCGTGACGGCACCCTGCCCCGGCGGTCAGCTGCCCGTCTGCCCCT
CAGACACCTGCACCTTCGGGCGGCCGCCACCCTGCCCAATGCGTTCCAGTTGTCCAGGTTGCGGGATGATGCC
GCTGTTGCCACGTTTCAGGCTTGCAATGCTACGGAATACGCCCGGTGGGTGCGAGCGC
TTTGCGTTGAGATACTCGGCCAGACACCATTGCCACAAGTCAGATTTTTGGATGTCCTACCGGCCGCGGACAC
GTCCAGCAAAGAACCAAAGAAGGTACCCATTCAGGAGACGCCTGTATGCCCTCCCAGACCACCTCCCCGAGCG
AGACGAAGGTTGCTAACCGCTCCGGAGACCCAGCTTCCTAGAAGAGACCATTCCCCTG
CCGCTACGGACGAGGGCATCGTCGTAGAAGACGACGATTATGATTCATCGTCCGATCGCAGCCTCGATCTGTC
TCTGTCACTAGACGCACTGAAGACAATTGATGTGGTCGACGCGCCAGTGCGGAAAGCTGAGGTCGTGAAGTGT
GATAACTGCAGCAAACTAAACAAAAGCCCTCAACATCACACTCTTCCACGAGCGAGGA
>1002049937_1 Heli.1-DT664844.3.5

GCACGAGGAAGCTGTCTTTGAGTGTGGTACACCATTGTTGGCCGCCACACTGGCTGGACGCCAAAGACTGGAT
TCTCGCGGATCTGTGGATGAAGAAGCTCTCGGTGTAGCTCACCTGGCCCACAATGGATTAACCGTAACGGCTG
TTCGCCCTCCCCCGCTTTATCGTAAACTCGATTCTCCTACAGATTCGGCCTCGTCCTG
CGGAAACGAAAAGCATGAAAGAATTGTCGGCCCCGGTTCAGGCTGCTCGAGTCCACGATCATCTTTAGAGGAA
CACTTGGAAGACAGGCGGCCGCCTGCTCTCGCAGACGACATGCCAGTTTTAAAGCGCGCGCTAGAGGCGCCGC
CGATAACACGCCCGCCCTTTGATGTGCCACGTCCTCGCTCTCTGATGGAGGAAGCGTA
CAACCGTCATAAAAAGTTCCGCGCCATGCGCCGCGACACGGGCGAGGCGGAGGCGCGCGCAGTGCCGCTCACA
CCGTCGCCGCAGCCCCCGCAACACCCGCACCCCGCCAGCCCCGCGCACCCGGCGCACTCGCCGCGGCCGCTGC
GCGCCTCGCTGTCGTCCACGCACTCGGTGCTGGCCAAGAGCCTGATGGAGGGCCCGCG
CATGACGCCGGAGCAGCTCAAGCGCACCGACCTCATCCAGCAGTACATGAGACGCGGCGAGGCGGCGGGCGAG
GGCTGCCCCATGCGCGCCGGCGCGCTGCTGGCGTGCTACCGCGGTGCGTCGCCGGCGCCGGAGCCCGCGCCCG
TGCTGGAGCTGCAGGTGGACGTGGCGGATGCGCCGCTCAACCTCTCCAAGAAGTCGCC
GTCGCCGCCGCGCTCCTACATGCCGCGCATGCTGGAGGCGTGAGGCCGGCCCGCGGAGCCCTGCGTCTCCGCT
ACCCACCCCGACCTACTTTAAATTGAATAAACTGTAGTTAATGATCGTTCGCAAATCGGTTTAATATATTATA
TGTGATAATTCGTTTAAGACGCGTGCGTGAGTGGCCGGTGCCGGACGAGGAGAGCGCA
ATAGACTGAAACATAATTTAAGACTATATAAGTATAATTTATAATGCAATGCAGCGCCGGTCGAGGTGTGTGT
GTGTGTGTATTGTATGTGCGTATGTGAGCGTGCTCAGTCATTTGTTTGTTAATGTAATGACAGGTTGTCGAAT
TAAAATAAAACAAACTTGTAAA
>1002049938_1 Heli.1-DT664009.3.5
TTAAAGAAGTCACACATAAGCCACCTTAATTTTGTTCAGCCGGTGTTTATTGAATTTTATCAAGATGTCTATC
AAGTCAATTAAAGCGCGTCAAATCTTCGATTCCCGCGGTAACCCTACTGTGGAAGTAGATTTAGTAACCGAGC
TTGGACTTTTCCGTGCAGCAGTACCATCTGGAGCGTCTACTGGTGTTCATGAAGCTCT
AGAGTTAAGAGATAATGTTAAAGGTGAATACCATGGCAAAGGTGTCCTCACCGCTATTAAAAATATTAACTCT
ATAATTGCACCAGAATTGTTAAAACAGAATCTAGAAGTAACTCAACAAAAAGAAATTGATCAATTTATGCTGG
GTCTGGATGGTACAGAAAACAAATCTAAATTGGGAGCAAATGCAATTCTTGGTGTATC
TTTGGCTGTAGCTAAGGCTGGTGCTGCTAAAAAGGGTGTCCCTCTTTACAAACACTTAGCTGACTTGGCCGGC
AATAATGATATTGTATTGCCTGTACCTGCTTTCAATGTAATCAATGGTGGTTCACATGCTGGAAATAAACTTG
CCATGCAAGAATTTATGATCTTGCCCACAGGTGCTGCTTCATTCAGTGAGGCAATGCG
TATGGGATCTGAAGTATATCATCACCTAAAGAAAATCATTAAGGATAAGTTTGGCTTAGATTCCACAGCTGTT
GGTGATGAAGGAGGTTTTGCACC
>1002049939_1 Heli.1-DT664040.3.5
GCACGAGGACACTCTTAAGTTGTCAGTGTCACTTTCAGTTGTTTCGAATTTATCTGGAGTTTGGATATCATCA
TTAATAAATAAATTACTAAGTTAGATATCACCGTTAAAACTCCGAACAAATACATTTTACTAATGTTTATTGA
TATTCTAAAAATATATCTATTTTTAAACCTCTGATACTTGTGCTTTAATTTGTAAACC
ATTAACTAAATTATAATTTATATAATTTTAAGCCGGTTAAATTTATAAATAAGTGGTACATCATGGCATGTGA
TATTATGACTCAATTTTATTCTTGCTTGAATTTACCACTACAAGAAAGAATTTTGGAATTGACTAGGCTTATT
GATCGAGCTAGTCCTAATAAAGATTTGCAAGCAATATTTCCTCAGCTTGTTAGTAATA
TTTTTGCACCCTACAATCAAAATGGATGGAACCTAAGACAAGTAACATTTGAAACAAACAGATATGAATATCA
GACTTTACTGAGTTTTTTAGAACCACTCGGACCGATGTTTCGTCTTTGCTATAAACTATTGTCTGATACACAA
CTGAAGTACAATCTTCCATTAAACACCCTTCCTATAGACCTACAGATAACCCTTGAAA
GAGGGAGATGTCCACAATTTTATGCAGACCTGCTAGCTACAGACTCCCAATCAATGAATGTAGTGGCTCTTGC
TTTAAATCCTTTTGATTATTATATATTTCACTTTGCATTGTACTTAGTGAATAATAATCAAAATAAAGCCTTG
TGGGAGAATTGGAATAGTGTATATTTTGCATTAGCTTGTGACTATCTGTTGATTTTTT
TACCTTCGGATCCAAATATTCCTGTTTTACCACATATCCCACATTACACTGGAAAGGTACCAATGGCTGCAC
>1002049940_1 Heli.1-DT662739.3.5
GCACGAGGCGAGTTCTTTTTTTTTTTTTTTATATAGTAAATGTTAAGTTTATTTTATTTTCAAATACAGATCA
AACTGAATTGGAATGAATCACATTTTGGGGCATGAACATGCTAAGCTGTAGGATATGCATATACATATATAAT
TTATAAATAAAAAAATTAGGACAGGGGAAATTACAACTTCTTAACCTTTAGCTAATCC
TCACTAAAATATCACAGTAAAAATTAAATTAACAACAGAGTTCACTCAGCTTCAAATAGATTGAAAATCAGAA
ATAAATACAAGTTCAAGATATTTTAATGTATCAGTGATTAACTATTTCTCTAACATCAACAAAGAGACACATA
GCTCTTCTCATTCATACACTTTAAGTATTCCCATGAGGATTTGCTTAAATCTTATAAA
TTAAATTATACTATTCAATATTCGTTAAATCTCTTGTGTCCGCCTCCGCCATGTCCATTGCCCCTGCCAAAGT
TTGATCCTCTCTTGAATGAGCCACCTCCTCCGCGGCCACCTCCATACCTGTTTCTATTCCATCCACCGCCACC
ACCACCACAACGGTCAGCCATTCCTTGTAGTTGAGGACTAATCACCTGGTTAGCTTCC

TGTAATACTGATACAAGGTCCTTAGCCTGTCGCGAATTTGAAGGGGTGAAGAAGGCATATGATGTTCCTTTTG
ATTTTGACCGTCCAGTTCTACCAATGCGGTGAATATAATCCTCAGATGAATTAGGATAATCAAAGTTTATAAC
ATATTTAATTCCATCCACATCTAGTCCTCTAGCTGCAACATCTGTTGCAACAAGTATA
CTAGCACGACCTTGCTTAAATTGATATAAAACATCATCCCGTTCGTGCTGTGTTTTTACACCGTGCCGGACGC
GTGGTCGACCC
>1002049941_1 Heli.1-ES585641.1.5
ATTAGCAATGCTAATTCCGGGTCGATATGCGGGAGAAAAAGCCAAATTTGGTCAGCCCGAGATCAACATCGGT
ACCATTCCTGGAGCCGGCGGCACCCAGCGTTTACCCCGTTACGTGGGGAAATCTAAGGCAATGGAGATCGTTT
TAACTGGCAACTTTATTGACGCTCAGGAGGCTGAGAAGATGGGTCTTGTAAGCCGCGT
CTTCCCAGTAGAGAAACTTCTAGAAGAAACAATCAAACTGGCTGAAAGGATTGGAACTCACTCCCCCCTCATT
GTTAAGATGGCGAAACAAGCAGTCAACCAAGCATATGAAACAACTTTGGTTTCTGGTCTGCTTTATGAGAAAT
CACTGTTCTATGGAACTTTTGGCACTGAGGACCGCAAAGAAGGCATGTCAGCATTTAT
TGAAAAGAGAGCACCCAATTTCAAGAATAATTAAAAAACATGAGAATGTAGTTAGAATTTGATCAAAAATTGG
TAATTGAAGATGAACCACCATTATCAAACTCATCAGTGATTTTTTTAGCAATATAGAATATAAGGGAAAATGA
ATTTATACCTGTGCTTTTTTGATTGTTGTTATATTTGATATATGTATATTTTACAGAC
AATATCTTTCTA
>1002049943_1 Heli.1-DT664845.3.5
GGGAGGACGAGGACGCAGAATGGCCGAGGTACATCGCTTTTGTAGTTGTTTTCAGCAATATCATGGCTAATGT
GCTATTTTAATTAGTAAATTAGTAGAACGTGCCTCAAAAAAGTTAATTACACGTACAGTGCAAAGACTATTTG
ATATTTTAGACGATTATATGATTACATGCTCTCATTTGACAAATTGCGCTAGACCGCA
TAAATAAAATGGCAGCCCTACCACGTAGAATAATCAAAGAGACACAGCGGTTGATGCAAGAGCCGGTGCCGGG
AATCAGCGCGGTGCCGAGTGAGAACAATGCGCGCTACTTCCACGTAATCGTCACCGGCCCCGAGGACTCGCCG
TTCGAGGGAGGTCTATTCAAATTAGAACTATTCCTTCCAGAGGACTACCCTATGTCAG
CGCCTAAGGTTAGGTTTATTACTAAAATTTATCACCCGAATATAGATCGACTGGGTCGCATATGCCTTGATAT
TTTGAAAGACAAATGGAGTCCCGCACTGCAGATCCGCACGGTGCTCCTGTCGATCCAGGCGTTACTATCGGCC
CCCAACCCTGACGACCCGCTGGCCAACGACGTCGCAGAGCTATGGAAAGTGAACGAAA
GCGAAGCTATCCGAAACGCCAAGGAGTGGACCAGGAGATACGCCATGGACAACTGAGCGCCTCCCGTCGCAGC
CAGTGCCTCCCCCACGCGCTACACTAAGTGGACGTATCTAACATATATATCATGATTTTAAGAGTGCAAGTTA
TGTTTTACAACCTAGATCGAGTAACTTGTGAGGAGACAATTGTGGATAAATTTTTATT
ATCGGCGGTAAAAGCCGCCGCGGCGCACCGGCCTCGCGCCCGCTCCGGTCAACCTCTTGC
>1002049944_1 Heli.1-DT666813.3.5
CATTGTTTTCCAGTGCGCAGTAGTTTCTCTGTCGTGAAATCGGGATTCTGTTTTAGTATTATATCTATTCTGC
CATTTTGAAAATAGTGGCGTGTATGGATTAGACCTAGTGTTTTTTATGTGACAAATATGTCCGGGAGCAATGA
TAAAGTGTCGACTACGCAGCGTATGATCCAAAGTGTCGCATTCCCTCCTAGCCACAAA
CTTACTGTGTCTGAAGTATTTGATGAAAAAACTGGAAAACCTCTACCAGATGTACTGAAGCAACACTTTATCT
TAGAGGGTCGAATTGAAGAAAATGCAGCTTTACGAATCATCCAAGATGGTGCCACCTTATTACGATCTGAAAA
AACTATGATCGAGATTGATTCACCGGTGACCGTGTGCGGAGACGTCCATGGCCAGTTT
TACGATTTGATGAAATTATTTGAAGTGGGAGGCTCTCCTTCCTGCACCAAGTATTTATTCTTAGGTGATTATG
TCGACCGAGGTTATTTTAGTATAGAATGTGTTCTCTACCTCTGGGCATTGAAATTGTGCTATCCGAAGACATT
ATTTCTATTACGCGGCAATCATGAATGCCGACATTTGACAGAATATTTTACTTTCAAG
CAAGAATGCAAAATTAAGTATTCTGAAAAAGTGTATGATTCATGTATGGATGCTTTTGATTGCTTACCTCTTG
CTGCTCTTATGAACCAGCAGTTCCTATGTGTACATGGAGGACTTTCTCCGGAGATTAACAGCTTAGATGATAT
TCGTAAACTAGATAGATTCAAAGAACCACCTGCATTTGGGGCTATGTGTGATCTGCTG
TGGTCTGATCCTCTTGAAGATTTTGGAAATGAAAAAAATGCAGAGCATTTTTCGCACAACTCTGTTAGAGGCT
GCTCGTATTTTTACAGCTATGCTGCTTGTTGTGACTTTCTACAGAGGAACAATTTATTGTCAATCATCCGTGC
TCATGAAGCTCAAGATGCTGGTTATCGAATGTATCGTAAAAGCCAAACTACTGGGTTC
CCAAGTCTTATTACTATCTTTTCTGCTCCCAATTACTTGGATGTGTACAACAATAAAGCTGCAGTCTTAAAGT
ATGAGAACAATGTCATGAATATTCGGCAGTTCAACTGTTCTCCCCATCCATATTGGCTGCCAAACTTTATGGA
TGTTTTTACTTGGTCACTTCCATTTGTGGGGGAAAAGGTTACAGAGATGCTTGTTAAT
GTCCTAAATATTTGCTCTGATGATGAACTTATGACAGAAGGAGAGGATGCTTTGGAGGAAGCCAATCTCAGAA
AAGAAGTGATTCGTAACAAAATCCGCGCTATTGGAAAAATGGCCCATGTCTTTTCAGTACTTCGTGAGGAAAG
TGAATCAGTTTTGCAGCTTAAGGGGCTCACTCCAACTGGTGCTCTACCTTTGGGTGCA
TTATCAGGTGGTAAAACCTCTTTAAAAAATGCTCTTCAAGGATTCTCGCCCAACCACAAGATTACCTCGTTTG
CTGAAGCAAAAGGCTTAGATGCTATTAATGAACGGATGCCACCACGCAGAGATGGGCAGCGTACTCCAGAT

>1002049945_1 Heli.1-EL596844.1.5
GTCGTGCGCCACGTGCTGGCGCTGCGCGCGGCGCGCTGGGGCCTCGCCGACCAGGAGCCCGACTCCAGGGTGC
TGAGCGAGCGGCGCCGGGTCAACACGGTGACAAACCACGTGCCCCCGCCACCCGCTGAAGGGGAACTGGTGGG
GTACCTGGCAGATGGACAGACGCTCACTGTGGAGGAGTGCGACTTCTTGCAAAACAAC
CTGCCCACTAAACCTGCTGCATTGGACGAGGATATACTGGAGGAGCTGGAGAACGACGCGTGGGAGACGGACA
TGGACGCGGAGATGCAGGCGGGCTTCCTCGAGTTCCTCAAGATGTCCAACCAGATCAAGCGATAACCGCGGCT
CTACGAGCAAAAACTATACAAAAAAAATATAAAATTTTATAAAACGATAAAAAGATAT
CAGTGAACGCTTTTCGATCGCTCTATAAATATGATCATGACGTACTCCGCTACTTAAACACATTGCCTATACT
CTCCCGTTACAATAGTACACGTGTCCTAAATTAAGCAATATTTTATAGAATTATAGTAACATCAGTACCATTT
AAACGCAATAATTCATATTAAGTAAAGAAAAATATATCTAGATATTATCGACGGTCCC
TACGTATAACGGACATGTCAAAAGAGCACGTTTACGGGAGAATGGAATAAAAGTTCGTCTTGCTCTTAAAAAT
TTAAATAAAATTATAAATATACAGTATACTGTTCTAATACTATACAGTTATGTTTATAATTTATTTTATTTTT
TTAAGAGCAAAATGAACCTAAAATCGTAAACCTTCTCTTTTAGCATGTCCGATGGCAC
GTACGAACCGTCGTTCTCTTAGATACGGTTAAGTATTGAACGTTGAAGGTTGTTCGTCTAGTTGAATGATAT
>1002049946_1 Heli.1-ES586998.1.5
GTCTGTTTGTAAGCAATTTACCTATAAATTGATGTTTTTGTCAGTAGTGACGTTGTTCTTACCGACTTCAACT
GTTCTTCTCCGTTTCTCTCCTCATCTGTGCTGTCCGGATTTTGAGGGATGTCACTGTTTAATGTACATGTTTA
ATCTGTTGACAGATTTTTGTATAACCTTTTTTTTTGCTGATGGTACTTTTTTAATGTT
AGGTGATTTTTTTATATTATATGTATACTTATTTTTTAGTTGTAATTTTTAAATATGTAATAAACCTACTTAA
TTTGGCACTTTTCCGTGCAGCGTTTAGGAACGAATTGGTTTGTAATTTTTTTGTTGAATTTAAAAAAATGTTT
CTAAACGTGACTTTTTACATTACTTACATTTGTTAATTGTTTACGTTACGTTTGAGTT
TTACTCAATAACCGTCCAAGTAAATTCCCCGTTAATTTTATATAGTTTTAAGATCTGAAATAAATATCGGCCC
AAATGCTATTTTTTTCATTCATTTCATATTTTATAACCATAAAATAGCTTTCTTTTTAATGATTGATTTTTTA
TATAATTTTTACTTTGTATTTTTAATTGCGGTATTTTAACTGTATACTTATTAGGTAA
CTTCTCTGATGTATGGATTATTATATTTTAGCTAAAAATAAAGTTGGT
>1002049947_1 Heli.1-EL599354.1.5
GCACGAGGAACACTCGTCACGCGCCGCGACTCGCTTACTAGTTAGTGCTCGTGTTAAGTGTCTCTTACGATGA
AAGCCATAACGGCGGTGTGCGCAACCGGAGCGTCCGTACCGGCAATCGCCAGCGGACGGGTGCAGAGGCACCG
GGACGGCGAAAACGCTGAAATTCAAATGTATCTCTCCAAATTACAAGACTTGGTGCCG
TTTATGCCAAAGAATAGGCGGATCTCAAAACTGGAAGTGATCCAGCACGTCATCGATTACATCTGCGATCTAC
AGTCAGCATTGGAAAATCATCCTGCTGTGGGGCAGTTCGAAGCGGAGGCCGCACTGGCGCCTGCATGCGCTTC
ACCACCCAGGCCACGTCGCCGACCTCTTGGACCTCGCCCAGCACCCAATACCATTCTC
CCTGCTGATAGAACTCTATCTACAACACATCACTCTAATCACATGACTCCTGAAAAGCAAGATCAACCAGATA
GGCCCACGTCGTGCTAGGTGATCTTTAGAGTTAAGGACAGTTGTATTAATGGTGAATATCAAATATACCTCGA
GATGCAAGGGCCGCGCTCCGTAGGAATGAACCTGGTACGAGAAGGTGCCTCTGCACCG
GGAGCCTCCTGCATCAGTTCCAGTGAACTTCATCTCACGATGATATCCTATGCACGATCTCGTTAAGATCAAT
TATTTAATTATTTGGTGGCATTA
>1002049948_1 Heli.1-DT664021.3.5
GCACGAGGGTAATTCTATAGCGTTCTTCTGTGTAGTAAATATCTTTTTTCTACATAAAATTAACAACCATTCT
AAAACAACGAAATAATGGCTCCTGTTGAGCAAGATGTGGAAATGAAAAATGTAGACAGTCCAACCGCTGCGAG
TGATGTAGAAACTGGTGAAGTGAAGAAAGATGCCGATGTCCTAGCTGTTCACGATTTA
AGGGAACATGTTAGACAAATTGACAAGGCTGTAACGTCCAAGGAACCGAGGTTTGCAATGCGCGTTCTTAGAT
CTATTCCAAATACACGAAGAAAACTAAATGGTAATGTTTTACGTGCTATTATTAATCAGCTTTATCCTGCAAG
CACAGACAAAGAAGCTTTGATCGCGTTTGTTGAGAACCCACTGCCAGGTGCAGTTGAA
ATAGAGGCTCCACGTTCCCGTAGTGTTCCGAAAACGCCAGCGCCAGAAGTTGATGCATATATGCACCTGTTAG
TGTTGCTTCGTTTATTAGATACTAATAAACTTGAAGAGGCAGTAGAATGTTCTCAGCAGCTGATGAATAAGGT
AACAGCTCAAAACCGTCGAACTCTAGATCTAATTGCAGCCAAATGCTATTTCTATCAT
TCACGAGTGTTTGAACTTACCAATAAGTTAGACCTCATTAGAGGGTTGCTGCATGCTCGTCTGCGAACTTCGA
CTTTACGAAATGATTACGAAGGCCAGGCAGTGTTAATAAACTGTCTTTTACGAAACTATCTACATTATGCACT
GTATGACCAGGGCAGACACATTGGGTTACCAATCAGTGTTCGCTGGAAGTGCCAGTAT
TATTGATGGGCACCCTTTTGTCGAGCGTTGGCAAATGAAGGGGTAATTCAGTAGGGATCCCCAAACTTATCCA
CTAGTGGCCCT
>1002049949_1 Heli.1-EL599054.1.5

AAGCCTGCACACGCGGTGAAAAAAAATAAACTGTCAAGTTTCCCGCGCTCACAGATTAAATCGGATTTTCGCT
TTACAATTATTTAAATCATTGATTTATTATTATAGTAAATTATTTATTATTATCAGTTCTTAAAAATAAAGAT
ATAATGTCGGCGGACGTGTTAAGTGACGATTTTAAATCGCGTCTCCACTTGACAACTA
AAACGGTAGGGCAGGAGAGGATACGGAGGGCCCGGGAAAACAATGAGGACGTCGCCATATTGTCCATGTTCCT
CCCAAATGCGACAGCTGAGCGGGAAAATAAGAAGTTCTGTTGTGCCATGAAAGACCTTTGCTTGCTCGGCGAT
CCGAGGAAAACAGTCGCAGGAACCAAGAAAATGGAGTACAAAGCAAGGCAATGGCATG
AGAAGTGTTTCTGTTGCGTTGTCTGCAAGAACCCGATCGGCACCAAGAGCTTCATCCCGCGCGAGCAGGAGAT
CTACTGCGCCGGCTGCTACGAGGACAAGTTCGCCACACGCTGTGTCAAGTGTAACAAGATCATCACGCAGGGC
GGCGTGACGTACAAGAACGAGCCGTGGCACCGCGAGTGCTTCACGTGCAGCAACTGCG
CGCTGTCGCTGGCGGGGCAGCGCTTCACGTCGCGCGACGAGCGCCCCTACTGCGCCGACTGCTTCGGGGAGCT
CTTCGCCAAGCGCTGCACCTCCTGCACCAAGCCCATCACTGGCATAGGTGGTACCCGCTTCATCTCCTTCGAG
GACCGCCACTGGCACAACGACTGCTTCATCTGCGCGCAGTGCCGCA
>1002049950_1 Heli.1-EL599620.1.5
TGAACGCGACTTATCTGACTCTCAGTCAATTACCGGTCGTATGCGAGAAGGTCAATTCGAGTGATATATGCGA
GTCCCTTACGCTCAATGGAACGAAATACTGCGAATGGGGCTACAATGGTTTGGGGATCGATTACCAAGTGCTG
GCAGGGCCGGCCTTCATGGCCGTGTTCACTGTTGTCGGCGTTATATTGGGTGTAGCTG
CTGATAGATATAACCGAGCAAAAATCCTAGGTGTATGCACCTTAGTGTTCGTGGTGGCGATGCTCTTGATGGG
CACAGTGACCGAATATTGGCACCTAGTGTTACTAAGAATGATTATGGCAGCAGGCGAGTCCGGGTGCAATCCA
CTAGCTACTGGCATCCTGACTGATCTGTTTCCGGAACATCAGAGGGCTTTGGTCCTGT
CCATCTTCAACTGGGGTATCTATGGAGGTTACGGGATTGCGTTCCCGGTGGGAAGATATATACCTGCTATAAA
TGCTTGGGGTTTGAGCTGGAGAGTGTGTTACTATGGCGCCGGTATCATTGGTATAGTCATCATTATCTTGACG
TTCCTCACTCTTCGGGAACCTGAGAGGACTACTATTGGAGAAGAAGGAAACGCGAAAG
CAGGAGATGCTGCTCTAGAGGCCGGTAAGAAAATGCCTCAAGTCGCCATCTGGCATATAATCGGACAGCCGAG
GATCCTGCTGCTGTGCTTGGCCGCCTCTATCAGACATTGTGGTGGTATGACATTCGCATATAATGCAGATTTG
TACTACCGCGATTACTTCCCCGACGTGGACCTCGGCTGGTGGCTGTTCGCGGTCACTG
TGGGCATCGGCTCCGTGGGCGTAGTGGTTGGCGGAGTTATTTCTGATAAATTTGTATCAAAAATGGGTATCAG
ATCGAGAGTACTAGTACTTGCATTATCACAATTGATAGCGACATTGCCAGCGTTCGGCTCCGTCGTGTTCGGA
CCACTGTGGGCCATGATCACGCTTGCCTTCTCTTATTTCTTCGCTGAAATGTGGTTCG
GAATAGTATTCGCTATCCTCGTAGAAATAGTGCCTGTCGCTGTCCGCTCGACCACCGTGGGTGTGTTTCTCTT
CGTAATGAACAATATTGGTGGTAACTTGCCCATTCTGGTCGACCCCGTGTCGAAGGCTATAGGATATAGGGAG
TCTATAATGATATTCTACGCTGGTTTCTACGGTATTAGTAGCATACTGTTCTTCTTAA
CAATGTTCTTAATGGACGGTCCAGTAGAAAATAAAGATAAAGAAGAGAGTGACAAACGCAGCGGCCTAGACAA
CAGAGCTTATTCACACGACGAGGTTGCAAGAGATAGAATCGATAATATTAGATTATAAATGAAATCAAAATTA
TAAGACAAATATCCATATATTT
>1002049951_1 Heli.1-DT664941.3.5
AAATAAATTTTATTTTTTATAAGGCGGTTTCAAACTTACTACCACCACGGAAGCATCCGGGTACTAGGGCCTC
CGCGCCCGGTCGCTCAAACCGTCGGGTCGACGAGCAGTGGGGGGATCGGTGCGGGCATTAACACTGTTTGCTG
GTTGCTCGAGCTATAGCTTTAGGTGCCGATTCACCCTCCGCTCGCGCACAAAGCCCGA
TCATGTTGAGGTCGGGCGCGAGGCACCCGGATGCTGACCAGGGAGCGTCCACGTCCACCGGAAGTGCTGTGGA
CTTGAGACAGCTGTATACCACTCTAAACGAGTACCTGCAGCTGGAGCCCAAGTTCCAGCCGAGATTGTGCTTG
CCGAGTGAAAGTGAGAATGGGGAAGTTACGACGGGCGCCCGTGACGGCGCTGCCCACG
TGCTCCGCTGTCTCAAGGTGTGGTTCGATCTACCCGCAGATGTTCTAATAAGTGCTATTAATCTGTTTGATCG
TTTCCTAACCAAAATGAAAGTGCGTCCGTGTCACGTGCCCTGTATAACCGTGTCGTGCATGAACATTGCGATC
GACGAACACGCTGATATTACTAAAAAACCGAGGAATGTCAGTGTGGAGGAGCTAGTGT
CAGTATCACAATCAGCGTGCACCTCGGGCGACGTAACCCGCATGTCGCACATCATAACCAGCAAGCTGGCGCT
CGCCCAGCGGT
>1002049952_1 Heli.1-EL596358.1.5
TTCGCCTACGTCACATCGACGAAGGAGACATCATCGCGACGAGTCTTCGCCACGTTCACCTCCTGAACAGAGC
TCCCGGGCACGCTCCTCCGTCAAGGACGACAAGGACGGACACCTGCTGTACTGGCCCGGATATGTCATGGGAG
CGAGATACAAAATCATCGAGACTCTCGGTGAGGGCACCTTCGGCAAGGTGGTCGAGGT
GAAGGACTTGGAAATGGAGCACAGAATGGCTCTGAAGATAATAAAAAATGTTGAGAAGTACAGAGAGGCTGCA
AAACTAGAAATTAATGTTTTAGAAAAGTTAGCTGATATTGACCCAGATTGTAAAAATTTATGTGTTAAGATGT
TAGACTGGTTCGAGTACCACGGACACATGTGTATCGCGTTTGAAATGCTCGGACAAAG

TGTATTCGACTTCCTGAAGGACAACAACTACCAGCCGTACCCGCTAGAGCAAGTGCGGCACATCTCCTACCAG
CTCATATACAGCGTGCTCTTCTTACACGACAACAAGCTGACCCACACCGACCTTAAGCCCGAGAACATATTGT
TTGTGGACAGCGACTATGAAGTGCTCAGTGTTTATAATAGTTCTAAGAAGAAACATGA
CTTGAGACGGGTGAAACGTAGCGACGTTCGTCTCATAGACTTCGGCAGCGCCACCTTCGACCACGAACATCAC
AGCACAATTGT
>1002049953_1 Heli.1-DT667437.3.5
AATAAACCTAAGTCGTTTACTGAAGAGTTACCGTCAGAGAATATTAATATACAAGTTGGGAGCTCTACTAAAA
TTAAGGAACCTCGAGACCGTGAAATTGTACATAAAATTAAAGAAGACCATTATAAATTTATTGATGAAGAAAC
AAAAATGTTCTCCATAACAGACATTCATGATAATAAATTAACAGACACTGTCAGAACT
CATAATAAATATCCCAAAAATCGTCCGTCCAGCCCTGACAAAACTTCAAATTTTTCAACTAAAAATAGCCCTA
ATAGGGAAATATCAAACAAAAATAAACCATCAAAATTGATTGAAAATATTCCCGAATCTCGCGTATCCCCAAC
AAGAAATATATACCCAACCAATCATGCAACAGACACAAGTATTACTTCTTCTTCAATG
ATTTCAAAAGATGTATTAAGATCACACAAAAGCCCATCACCGACAATACAAAGACCATCTTCGACTGAACCTC
GTAATGAATTTCGTAAGTCTTCATCTCCTACAAGAGAGATCTCAAAAAGAAATAAAGAAATTCTTGAAGACAC
AACCGCCTTCATTCCAAAAGAAGAAAGTAATACTATCCCTGATAATACCCCTCTTCGT
AAACGTACTCCATCACCAATAAAGAACATAAAAAATCTTAACAATAAACAAATAACTTCAGAAAATAGTGTCA
CTTTTAGTTCATCGAAAACGATAGAAAGAACTGAAAATATAGAAGAAGAATCCAGTAATATCACAATTAATAA
TGATAAACAACCAAGAAAAAATGATTTTCCTAAGAGGGAGAGTTCACCGCTTAAAACA
AAATCCATTAACAGTATAAAGGATAATATACACTTTACAGATACACTGATACATCAGTATACTGACAAAAAGG
ATACACAGATAGATAGATCCCCCAATGATTGTAGTCAACAACCTTTAAATAAATCCCCCCGAAGTAGCGTGTC
TCCAACTGAATCAATTGTTCGTGAAACTAAATTTAAACAGATGTCTGATTTCATTGAT
ACTGAAAAAAAATAATGAAGAAATTAATAAGCGCTCTTTAAAAGATCGGCCTAGACAATTAATTACACCTTCTA
CAAGTCCGACACGGAAACCTAAATACATTGAAAATACAATATCTTCAGGGCAAAGTTCTCCAACAACATCTGT
TAGTGGATTTGAATATTTTCCAGCCTGTAAAGACAAAAAAGTTGTTACTGACTTAGAT
GAAAAAGAATCATACACAAAAGATATAGAAAGCTCCAATAATGAAAGAGTTAAGCAAGTAAATAATCAAATTT
CAATCAAAAATCCTTGTAGATCGCCATCACCAGAAAAACGTCCAACAAAGGAAACTCTTCCAAGAAAAAGTTC
ATTAAAGAAAACTTCTAATAGCCAGATGTCCCCTACAGAAAAACCACCTTCTAGTTTT
CTTATTTCCCCTAATGTAGAAACAAAAGAATTTACCGATCATAAAGTCACTATCCAAGATCATCCTATAAAAG
AGACTGAAAAACCCATAAAATCAAAGCCTCCATTTGAGAGACGTGAAACTTATGAAGAAAGATGCCGCAAAAT
TCTTGGTATGATTGAGACAGACACAATGGAATCGGAAGATACAATAGATTACACTATA
AAGAGATCCAATATACCGACAAGTGAACGCAGTTCAGCCAAGTGTATCACCTTGCCGTAGTCCGTCTCCCAAT
GAAAAAGCATCTAATAATGAAATTAATAAAGAAAATACAGATAAAGAATATAAGGATGATTACAAAAATACAT
CTAACAACCGCAACGTCAGTAAAATTCCAACAAGGGAACCATCACCTACTAAGCCCCA
TGATTCGGTTGAATATAAACTGGAATCTCATACGACTGCATTGAAAGATACCTCTTTAGTTACATCAAACAT
>1002049954_1 Heli.1-EL603503.1.5
GCACGAGGGTTAACTTTGACACAACATGAAATCCTTCGTATCCATCCTCGCCCTTGTGGCCGTCGCTGCTGCT
GACGTCGCCCATCTCCGCTCCGTTGACGCTGACGCGGTCATCGTAAGGCAAGACGCTGACGTCCTCCCCGACC
AATACCAATTCGCCTTTGAAACCAGCAACGGAATAAGCGCCCAAGAAAGCGGAGTCTT
GAAGAACGCTGGCCGCGAGGACGAAGCTATCGAAGTTCAAGGTGCCAATGCCTACTCCGGCCCTGATGGTCAA
AGATACGAAGTCAGATACGTTGCCAACGAATTGGGATACCAACCTGAGGGAAGCCATCTCCCCACCCCCGTGC
CAATCCCAGACTACATCGTCAGGGCCCTCGAGTACATCAGAAGCCACCCACCCAAGGT
TGAGGCCATCAGGAACTAAATTTAGTATATAGAATATGTAATATCTCTAAACACTGCCATATATTTGTTACTT
TACCTCTAGTCGCGTTTGTGATGAAGCAATAAAATTCCTTACCAACTTAAAAAAAAAAA
>1002049955_1 Heli.1-EL601756.1.5
GCACGAGGATTTGTTTCCTTTCGTGCTATATATTGCGTTAAGATGGGTTTCTAGATTTTTAAACGTTTTGCAC
ACCTGCGTGTGCTTTCACAAAATAATAAAAATGCTTGCCAACATGGCGCCTTCAACCGATACCTTCGTCCCTC
GACCACTATCATCAGCATTAACGGATTTCGATCAATACCGATACCTAATTATTCCTTT
GAGTGCAATTAAAACTGACATCAAACATGAGCTTGAATCAGTGGAGTTTTGCACCGGCTTCGGCCGCGGGCCT
CTTAACTGAAAATGAATGCTCTATGCTGCTCGACGTCGACGTCTTTAACACCGATGAAGATCTATTCAAGTCG
TTCTCCGGCGCTGCACCGAAGATTGAAACGGGCAAATTTTTACAAGATGACATATCAA
ACACCCTCTATCCACCATCTCCGCCCGAGTTCAAGCCCAGCCCAGCTGAGCTTGCCAACGATCTTCTGCAGCA
GCTGGAGACGCAATGCAAACGAGAAAACATATTTCCTAACTGGTTAGAAGAGAAAGTGGAACTTTCAATATTC
GAGAACATCCCCCAAGTGTCCGAGCGCGCGGAGGTGCCGCTGGTGCCCCTGCCGGTGG
CGCAGCTGGCGCGGGCGGCGTACCCGCAGACCGCGCAGCCGACCGACGAGGAGCTCCTGC

>1002049956_1 Heli.1-DT662870.3.5
GCACGAGGCCGCACTCTGCACACGATTCGACTTATAGTTATTGAAAGAGTAACCAAAAACTTAAAACATATTC
ATTATTTGAATGTAGCGTGTATATATGAATTAAATTCTTCGCTGCACAGGTATTTATTATAAAGGAAAACATG
CCTACAATATCAGTTAAACGCGATTTACTTTTCGATGCACTTGGAAAAAGTTATAGTG
ATGAAGAATTTCAAAATTTATGTTTTGAATTTGGCTTAGAATTGGATGAAGTGACTACAGAGAAACAGATGTT
AATGAAAGAGCAAGGAGATCAAGCTGGTGCTGGTGTATCAGAAGATATACTATATCGTATTGATATTCCCGCT
AATAGATATGACCTGCTGTGCCTTGAGGGACTTGTAAATGGGCTATTGGTTTTCCAGG
GAAAAAAGGATCCTCCAATATACCACCTCAAGAAATATGAAGATTGTTATTCCCTTCATTTAACACCAGCAAC
ATCTCAAATTAGACCTTATGCAGTAGCGGCTGTACTCAAAGGAGTTTCCTTCACTAAGGATAGCTATGATAGC
TTCATTGATTTACAGGACAAACTTCATCAAAATATCTGTAGGAAAAGAACACTAGTAG
CAATTGGGACTCATGACCTAGATACAATCCAGGGACCATTTGTATATGATGCTTTACCGCCAAATGAAATAAA
GTTCAAAGCGTTAAATCAGAGTAAAGAGATGACAGCTCCTGAATTAATGGAATTGTATTCGAGTCATGCCCAA
TTGAAACAATATCTGGGTATCATTAAGGACAGTCCAGTGTATCCAGTTATTAAAGACA
AAAATGGTATAGTTTTGTCCATGC
>1002049958_1 Heli.1-EL600478.1.5
TGTCATCATGAGGGAGGAAAAGCCACCCCTTTTACCCCCCGCGCCTGACTACCGCGGGGACGACGCCCCCTAC
CGCCCCCCGCCGCCCTTCGCCGCCTCCCGCACGCCGCCCCGCCCCAAAGCTACCCCCACTTATAGGAAACCAC
CACCATATGTGCCCCCTTAATTACTTTACCAGTACAATCTAATTATAACAAACTTTGT
AGTGGTAACGTCTTAGAACTGACGATTTTATGATATTATGTAAAGTATATCACATTCAGTCCTGCATTTTTAA
ACCAGGTGATAATATAATTTTACCTAATTTTGGAAGGTTATTTGAACATTTCATGTCAACGGTATTAGAAAAT
CGTCAATTCTACAATACGGCCACGACAATTATACTAATACAGATTATGTAAACGTTGG
ATAGTGAAGACGCAATTTTATATTTTAAAATTATTTTGCTTGATATATTAAGATTTTTGCTTTTACTTTTTAT
ATGGAAATGTGCACTGTTTGTGTGAAATTACGTCTTCAGTATTCTAACTATATTATATTATGGGAAATTTAAA
TAGAATTTTCTAAAATCAAGTCAAGGCGAATACTTTGAAAAAATCCAATTAATATTAT
ATATTTAATGCATGTCAAGATATCTTAATTTGGATTTCTTAAAGTATTTGTGTTCGTGTATAATAAACTCGTA
CATATATACGT
>1002049959_1 Heli.1-ES587478.1.5
CTTCATCAGTCAACTTCGTTTGGATAAAAATTACGACAATAACAAACACGAATATATTTCAATTTATTATTTT
TATTGTTCAACTTAGACTTAAAATATTAGAATCAAATTCAAAATATTTTTTCTTTATTGATATCGCTTTCTTT
CATATTCAATTATCTAGGAGGTAGATAGAATTTAGTGTTAAGACACTCCATTCTCCAC
TAAGTGCATCTTTAGTAACATAACAATTGCTTAATAGAAATTGGTCCTTCGAGTGTGATGACGTTATTGTCTA
TAATTGGCTTCTAGGAACTTTTTCAATATAAAAAATAATACACGTGTTACCGATATATATTTAATAATTTTTA
TTTTTTTATTTGTTACATATCGTAGACTGCCAATTGTATACAAATTGTAGTGTAAACG
TAGTTTAGTTAGATGTGTTATTGCGAACTATTAGCATAGGATTTCGATTCTAAGTCCCACTGAATGTAAAATG
ATTTAAATAAATTTTTTATTATT
>1002049960_1 Heli.1-DT664364.3.5
AACAGAAACTTGTTATAGCGCTAGACCCCACAACATCCAAACCAATCTCTAATATCAAAAATGACATGCCAAG
TGAAAATTCAGATGTGACTTTTGACACTATCAATATGCTTTATAATCGGTCCTCTAAAGCCATAACAAACGAA
GAAAACAATAGTAATGATGTAACAGAATCGAATAATTTTAAAGATATGACTGAAACTA
CAACAGACTCTGACTGGCTTTCTGAATCAGTGACAGAAATAAATTTAGATGATGCTATGAAAAAAGATCAAGT
TGAGACTACAGAAACACCGTCTTCTAAAATAGATGAAATAATGGGTAGTGGAGTTAGTAAAGATGATTTTGAG
CCCGACTATTTAAATAATATGGAATCTAACACGAAAAAGATGGACCAAAGTGACGAAA
TGTATAGTATGTCCCATGACTATGATAATGATGAAGCTAGAGTAAAACGAGTGAACAGTCAAGTGGAAAATTC
TGACGAAAATGCAAGCAACAGCGTGCATGATGTTCCAGCATTAAATTCTAATATAAATAAGCTCGAAACAACA
ACTGTAAGTCAATATATATACAAGATCGCAGAGAAAAATGTAAATGAAATGAATGTTA
ACACTGAAAGCTCAGCACATCCAGCTCCTGTCTGGGAAGATATTGAACCAGAAAATGTAGTTCCAATAACTAA
CGAACCTAGAATGGAAGAAGAGCTAAAAGTAAATGATAACGTGATTACTTCAACGACAATGCCTCCAACAACC
GTGTCTAGTGTAGAAGAAAATGATCAAAAAAATCCATCTGAAATCAACACAAACACCC
AAAATTCGACACAAATAAATAATCTAAACGTAACGATATACGAAATATCTAGTCCTAACGACAATGAATCTTT
TATCACTTCTAAACCTGCAAGCGTTCAAGATTTTGAAGACCACGAAACAGAGATGAATCCTTTCTTACCAGAA
GTGGAAAATAATAAAAGCCTCG
>1002049961_1 Heli.1-DT662966.3.5

TCGGAATCGTGTCAAGACGGCAATTGTCACGGAAATAATGAATTTGGGCCTGATGAATCACCTACGAAGCTTA
TCGTCAATTATATTCCTGAAGTTATGACCCAGGATATGATGTTCTCGCTGTTTTCAACAATGGGTAAATTAGA
AAGTTGCAAATTGATAGCGAACAGAGGGTACGGTTTCGTCGAGTACTCTCGCCCAGAG
GATGCGGTCAAAGCACGCAAGGCCTTTAATGGGTTGCTAATGCAGAATAAGACTTTAAAAGTTTCACATGCCT
TACTTAATCCAGAAATGAAGCCACCCTCAAAACCGGAAGCAGACTGGAACCTCTACGTATGTAACCTGCCTAA
TGAACTGACCTTGCAAGATTTACATGGACTATTCGCACAATTTGGTAAAATAGTAAAT
TCTCGTATTGCCTCAGGCATAGCTTTTGTTCTATACGAACATCATTATGAAGCAGAGAGAGCCATTCACAATG
TAAATGGCACTACTCCACCTGGTTTTCTACACCCATTAACTGTTAAGTATGCTAATAAGAGTAATCCCAATAA
ACATAAAAACAATAATAATAACTTTTCAAAGAATTCTCTTGTGAAGCCTTATCAATGG
ATTAATCATGTGGGTGCTATAGGTGACCATAATTCTCCTAGTACATGGTCTATATATATTTATAATATTGCTC
CTGAAGTAGAAGAATTGACTCTATGGCAGTTATTCGGTCCATATGGTGCTATTGTATCAGTTAAGATAATTAA
AGATCATCAAACTAATAAGAGT
>1002049963_1 Heli.1-DT662035.3.5
GTGTTTATCGAAATAATTATGTTAGTTTAAGATAAACAGCTTTTATAATGAGTAAACTATACGTGCTTTTCGA
GCACAGCGCGGGCTTCGCCCTTTTCCGCGTGGCGGAGTTTGAGGAGCTCGCAGCATTTCTACCTCAAGTGGAG
GAATCAGTGACAGACTTGCAAAGATTCAATTCAGTGGTCACACTTATTGCGTTCCAGC
CTTTCAAATCTGCCGTACTAGCTTTAGAGAACATTAATGCTATATCTGAAGGTATTCTACCAGAAGATCTAAA
TCTATTCCTTGAAGGAGCATTGCCAAAACGCAAGAAACGCAGCAAATGTACCCTTGGAGTGTCTGATCCCAAA
CTCGGTGCTGCTATCAGCGAAGCCTTAGAGATACCATGCTCTCATACAGGAGCTGTCC
CGGAGGTCCTCAGAGGTATAAGACACCATTTCCATGCTCTCATCAAAGGTCTTACACTCAAAGCGTGTAGTGT
AGCTCAACTCGGGCTCGGCCATTCATATTCGAGGGCTCGAGTCAAGTTCAACGTACACAGAGTGGATAATATG
ATAATACAGTCAATAGCACTGTTGGATCAGTTGGATAAAGATGTGAACACATTTTCTA
TGAGGATTAGAGAATGGTATTCCTACCACTTCCCAGAGCTCGTGAGCATTGTTCCAGACAACAACTTATATGC
AAAATGTGCTG
>1002049964_1 Heli.1-DT668551.3.5
TATATTTAATTAAATTTCAAAGTTCACTATTCGTATTGAATGTAAAAGTTGACATCGCTCTCAAGCGCTTTTG
TTAAGCTATTTTTTTATTTTCCCATAAAGAGTAACTAACGTCTTCTGTGTTACCTGCAAAAAGATACTTTTTG
TAAGGTCTACTGCTGTGACAGTTTCTTGCCAAGGAGCCTAGAAGGTTAAAATTTCCGT
AGGGACTCTCGAAGAGTTCAAAGAATTCTATTCAAAATGGTTGCTCAAAAGATCACCGTCTGTCCGCGTCGAG
GAGACGATATCGGAAATCAGTTTAAGTCGAACACCGAAAGACATGGTTTTTTCGCAAACGAATGTCAATACAC
GACAACTTTTGAAACTGGTAGAGATAAATCGCTTAAACTCCGAAAAGAATCAACAGGT
GAACGGTCCAACCGGAGTGGTTATCGGCGGCATTCTCGACTCTCCTTCTCCAACGCCCGCTCCCAGCACGAGC
TTTTCAGAGGAATTTCTCAAACCGGAGCCTCTATTTCCGGAAACAATGTTACACGATTCGGACGGCGAATTCT
TCCAAGACCTGGTGCAATGGTGCGCCGCGCCCGCCGAGGAGCCACTAGTACAGTTCAT
TGATCCATTAAAAGTAAGTGAAGAAAGGGCACACAAAACAGACACACTAAACACTCAATTCTCACCGCAAGGT
TGGGAAATATTCGACGCTAACTCACCGCCGGCGCCCGCCGCCCACGCCTGCCCGGCCAC
>1002049965_1 Heli.1-DT664132.3.5
GCACGAGGCTTCGCTGAACATCATACAAAGCGACAATGAGGCTCTTGAGATCTTCCTTCATCCTGGCCGTGGC
TATAAGCCTGGCCACGGCAGAGGAAAAGGCGGAGAAGGATTCAAAGGCTGAACCTAAACAGGTTGAAGAAAAT
AAAAAACAAGACAAAAGAGGTCTCTCGGATTATTATGGTGATCACGGATTTGGAGAGA
GCAGCGGGTATGGTGACCATGGTGGACTTGGAGGAAGTTATGGTGGATACGGTGGACACGAAGAACATGAAAA
GACCATCACTGTGATCAAGAAGGTTCCTGTACCTTATCCAGTAGAGAAGCACATTCCCTATCCTGTGGAGAAA
CATGTGCCAGTACCAATCAAAGTACCCGTGCCACAGCCATACCCAGTAGTAAAGACTG
TACACTTCCCAGTCAAGGAAACAATCAAAGTACCTGAATATATCCCTAAGCCTTACCCTGTGACCAAACATGT
GCCCTATCCAGTGAAAGTACATGTGGACAGGCCAGTTCCAGTCAAAGTATACGAACATGTGCCCTACCCAGTG
GAGAAGCACATCCCTGTGCCCGTGAAAGTCCACGTACCTCATCCTTACCCAGTCGAAA
AGGAAGTTCCTTTCCCAGTCAAGGTACCAGTGAAAGTACATGTACCGTACCCAGTCGAGAAGATCATCCATTA
CCCTGTTAAAGTGCCAGTTGACAACCCTGTGCCAGTCCATGTTGAGAAGCCAGTCCCAGTCCACGTAGAAAAA
CCAGTGCCCTACCCAGTGGAGAAACCCGTGCCCTACCCAGTGAAGGTTCACATTGACA
ACCCAGTGCCAGTACATGTGGAGAAGCCCGTGCCCTACCCAGTCAAGGTTCCAGTACCAGCACCTTATCCAGT
GGAGAAAGTGATCCCTTACCCAGTGGAGAAAAAAGTACCTATCCCTGTACACATTCCCGTGGATAGACCTATC
GCTGTCCCAATTGAGAAACATGTGGCGTACCCAGTGGAAAAGCATGTACCTTTACCCG
TGAAGGTGCCAG
>1002049966_1 Heli.1-EL602755.1.5

GCACAGGGTCAATTTCGTCAACATTGTTTTCAATACTATTTACTATATTTTTCAATAATTTCAATGTAAAAAA
ACTAATTTTATTTTGTAAATTCTGAACATTAAGTCATTTACAATATTTAAATTTAGTTTTTTTGTTTGTACAC
AAGGTAACTGTGCCATTTCCGTATTTGTGGAACAAAGAAGTCAAATATAGACATTTTT
TATTTTTATTTAAAATACTTTAGATTGATTCAGTAATATAGAATGGATTGTGTTACTACGCTTAAGATATTAG
TGATCGGAGAAAGTGGAGTTGGAAAATCTAGCATTATCTTAGCGTTTACAACTGGGGATTATAATTCGTCATT
CCCAGCAACAATCGGAGTAGATTACAAATGTAAGGTTATGGATGTGAATGGTTTGAAA
GTCAAACTTGGTATATGGGACACAGCCGGTCAAGAGCGGTACAGAACATTGACAAACAGTTTTTATAGAGACG
CACACGGTGCAATTTTAGTTTACGACGTGTCCGAACCGAAAACACTGCAGAAGCTCAACGAGTGGGTTGAAGA
GTTACAAGTGTATTCAACTAAGAAGAATATAGTTTGTCTTGTAGTTGGCAACAAAATT
GACAAACCACGCGCCGTATCAAGGGAGTCGGGACAGGCGTTCGCACAAAAACACAGAATGCTCTTTATTGAAA
GCAGCGCGAAAACACAAGAAGGCATCAATTTGGCATTCGAAGAACTTGTACAGAAGATAATAGAAACACCTGG
CCTTTGGGAGTCAACCGGTTCATCGTCCAACATT
>1002049968_1 Heli.1-DT668473.3.5
ACTTCCACATCGTTCAACGTTGGTATCGGTGACGGTTCGAGACTGTCTAGCAGGGTGCTCCGCCCCCCTGGTG
GTGGTCATACGGACATCTTTGGAGGTGAGCCTGAGCCCCCGCGGGGACGTCGTCAAGCGCCTTCGGCTGCTGC
TAATCTTATACAAGGTCAATCCAACGAGCCATCACAACCAACAAATGGTGATACCCCT
ACCCAAAATGGTCAAGCAAGCGAACCTGCTCCTATCATCCAAGCAGAGCCTGCCGCTCCCCAGTCCACACCAG
AGCCAAGAAGCCCCGAGCCATCGACTCCTGACTCTGCATCCGCGTCCGCATCACCCAAGGCTGTCACCCCCAC
AGAGAGGAACGATGCTCCAAAACGTGTGAGGGTACCTCCGGGTGGTTTCTCGTCGGGT
CTGTGGTAATGCCATGTGATGGATGGATGATTGGTATGAAATATGATGTTTAGTAATGTGTATATGGAATTGT
AGTCTTTTTTTATATATGTGTAATTTCAATTGGAGTTTTCTTTTTGATAATTTGTTCAGGTCAATCGAAGTCT
CCATAGCTCTCTCCATATCTGAGTCGAATTCTTTTCGTATATGGAGGGAACATTATGA
TAAGACATTTCATTTCCCACGCACAGCACAATTATTTGGGACATCACATGTTGAATGGTGGAGCTTAAGCGCT
TTCTCTTTTGTTTAGAGCTAGCCACGCAAAAATAAAAAAATGAATTATATAAATTAGAGACCGTCTAGCGGCA
CAGCGTCTGAGTGTAAGCGTGACGATTGTGAGTTTGATTCTCATTCAAAGATAACAAG
TCAAAGCGCGGCATTAAGTTCCGCCATTCATCTCGAAATGTCTCGAAAATTGCACGAAGATACAGCCGGAAGC
TAATAATAAAAAAAGTCTTACTCCTTATTCTAATTGAATTTTTATTTCGTATGTATATTATTAAATAAATAAA
TATATTTTATTACAATACATCCTCGATCCAAGGTAGTCGCTTCTTTTTGGTACTTTTA
AATCCTCGCAAA
>1002049969_1 Heli.1-ES588149.1.5
GGGNCGACCACGCGNCCGAATTCTTTCGTATAATCACTTGTGAGAAGTCACCGAATCAAGCAAAATGATCGCC
AAATTCACCATCATCTTTGCTCTCACCATCGCTGCTGTCCAATGTGGCGTAGTACCCTTAGCTGCAGCGCCTG
CTGCTCTGGTGTCTCCTGCTGTAGTTCCTGCAGCCTACGCCGTGGCTCCTTACGCTAG
CTCCTATTCCGCACACGCTGTCAATCATGCAGTAGCTGCACCCGTCGTAGCCTCTGCCCCAGTTGTAGCTGCT
GCTTCCTATGTTGCACCATCTCCTTATGTTGCCGCCGCCTCACCATACGTTGCAGCACCATCCCCCTACGTCG
CCGCAGCTTCCCCTTACGTAGCTGCTAAATACATTTCCTCTCCTTACTTCCTATAAGA
ATTTTCGGCGATATCATTGCCTGGTGACTTGCTCAAAAATGCTGTGTTATATTAATATGTATATGTATATGTA
ATATCTTTATAAATAGAGAATAAATATGTTTTGTT
>1002049970_1 Heli.1-DT668410.3.5
GCACGAGGGTCGCGTCGGCTGAACCTGTATTTTTGGGTACACCTTGTATTCTATTTATTTATGTTAAAAATGC
ACTTTTTATATTTATAACTGTAGTTAAAATTATAAATAAAAATGGATTTGTTGAGTGAAGCAAGTAACGGTCC
AAGATTATTAGCGGCTCGTCATATAAGCGCTGACCCACCACTCCAAGAAGTAAACAAT
GCAATCAATACAACGCTGTTGTGCCATACATATAGCCTTCAACCGGATTTCGCCACATACTTTGCAGTTCTCA
AAAGTAACGCGTGGGTAATTTGGTCATGCGGTCTCGTGGTTCTGCTGACGTTATGTGTTCTCTACATAATCAC
GCTGCGGTCGGCTCTACGATATTGGAAGGAGTCTGGTATCAGCATAGCGATAGTTCTA
GCTGTATATCCTGTGGTAGCAGCTACGGCTTTACTCACCACAGTGTTACCTCGAGTTCGAATCTTATCTGAGG
CAATTGCCCAGGAGGCTGTGATGGTAGCCATGTATCACTTCTACTTTATTATATTAGGGGAATGCGGTGGAAC
TAATCAACTTATAAGACGATCCGAGGGCTCTCAGATGGAAACAAGAGTTTTGCCATGC
TGCTGTTGGCCTTGCTGCATCCTGCCCAGACCTCAAGTTCAAAAAAAAAAGCTTAACTTGGTTGCGGTACCTGG
TGCTACAGATGCCTATAATCCAAGCTATACTCTACTTGATAATTCTCATACTCTGGTCAGAAGACATGATGTT
ATATATAAACAGCTTTACTTACATCCAGCCATTCATAGTGATATCTATATTATCTGGC
GTTTGGGGTATAATAAATGTGTGT
>1002049972_1 Heli.1-DT665921.3.5

GCACGAGGCCGATTCCTATTTAGATATGTAACAAATTCCATTCGTATTCCAATGGATTGGAATAATGAAATAA
CAATAATGAGATTATCTGTGTAAATTGTGATAAATATATTTACAAACTATTTTGATAGTGTATTAATCTAATA
AACTATGCCTGAGCATTTGGAATTACAACACCTTGTGGATCTCTCAGTCGGTAGCGGC
GACGAATCTATACCAAATTTGCTGGAGAACAAAAGGCCACTTATCAAGAGATGTCCTTACCTTGGATTGATAC
TAGCGACGTTATCGTCGTTATTTTTCTCATTATGTTCAGTTATCGTCAAAAGTTTAGTGAATATTGATCCAAT
GCAACTCGCAATGTTCAGGTTCATAGGAGTACTACTACCGACAATACCTATTGTGATA
TACACAGAGCAACCGATATTCCCGCAAGGTAAACGTGTTTTACTAATATTGCGTTCTTTTGTAGGAACAGTCG
GCTTAATGCTAAGTTTCTATGCCGGCAGAAACATGCCTCTAGCCGATGCATCTGTTATTGTTTTCTCTGTACC
AGTGTTTGTTGCACTTTTCGCAAGAGTTTTTTTAAGAGAACCATGTGGTATTTGGAAT
ACGATTTCCATTATTCTTACTTTGATTGGTGTTATATTAATCACTCATCCTCCATTTATATTTGGTGATACAC
CATCTGAGACTAATCAGAATTATAATAGCTTAAGAGGTGCTATAGCCGCCTTCGTTTCTACAATCTTTGGGGC
AAATGCCTATGTTTTGCTAAGAGTGCTAAAGGGATTACATTTTTCTGTAATCATGACC
AATTTTGGAGCCATTGCAATTGTCCAAACACTTTTCTACTCTTTTATATTTGGTGTTTTGTGTATGCCAAATT
GTGGTACAGAAAGATTTTTAGTTGTGTGCCTCGCCTTATTTAGTTATTTAGGTCAGATTTTACTTACAATGTC
TTTACAGATGGAGCAGGCTGGACCTGTCGCCATTGCCAGATCAGCAGACATTGTTTTT
GCTTTCCTATGGCAAGTCATGTTCTTTGATGAGATACCTAGTAAATTTTCAATATGTGGTGCAATATTGGTTA
TGAGCTCTGTACTTCTAGTAGGTTTACGTAAATGGGCATTAGCGTTGCCCCCAGATTCA
>1002049973_1 Heli.1-ES587761.1.5
TCAGATCAAAACAACAAACTTAAAACAATCAACATGTTCGGAAAGATTATCTTCTTTGCCGCTATCGCCGTAG
CCGTCGCTAAGCCTGGTATACCTGTAGCGGCACCATTAGTAGCCGCCGCCCCTGTGGTAGCTCCAGCTCCCAT
TGTTACAGCATCTAGCTCTCAATACATCTCAAGGAATTACAATGGAGTGGTATCTGCC
CCTCTAGTGGCGGCACCTGTCTATGCTCCAGCGGCTAAAATCGTAGCGCCTGCTGCTCCATATGTAGCCGCTG
CTGCTCCTTACGTGGCCTCTTCACCATACGTGGCTGCTGCTTCCCCATACGTTGCAGCCGCGTCCCCATACGT
TGCAGCCGCGTCCCCATATGTTGCAGCTGCCTCCCCATATGTTGCGGCTGCCTCCCCA
TATGTTGCGGCTCCTTACTATGCCTCTGCCCCCATTGTTGCCCTTAAGTGAAAAGACTGTTTATAGTATCTGA
ACAAAATAAATGAATTGATATAT
>1002049974_1 Heli.1-EL599263.1.5
GCACGAGGATTGCACATTACGTAAATCAGCGAGCAGAGTTCTATAAAGTGCCAGGAAAAGAGAAAAATGCTGA
AAGTAGCGAGGCTTATAACACAGCACGGCAATAATGTTCGTGCCCTGGCAACAGCGGCGGCCTACAAGCAAGC
CCTAGTCAACATTCCCCCAACTCAACTTACAATTCTCGACAATGGGGTTCGCGTTGCC
TCTGAGGACTCCGGTGCTGCTACAGCCACCGTAGGTCTCTGGATAGACGCTGGCTCAAGGTACGAGAACTCCA
AAAACAATGGAGTCGCCCACTTCTTAGAACACATGGCTTTTAAGGGAACAAGCAAAAGATCACAAACTGACTT
AGAATTACTCGTTGAAAACATGGGTGCTCACTTAAACGCTTACACATCCCGGGAGCAA
ACTGTGTTCTATGCTAAATGCCTCGCCAACGACGTACCTGCCGCTGTAGAAATCCTTGCTGATATTATTCAAA
ACTCATCACTCGCAGAACCCGAAATCGAACGAGAACGTGGAGTTATTCTACGTGAAATGCAAGATGTCGAAAG
CAACTTGCAAGAGGTTGTATTCGACCACCTTCATGCCACAGCATTTCAGGGTACTCCC
TTAGGACAAACCATACTTGGACCCACTAAAAACATCAAGAAGATCTCTAAGGCTGATCTCCAACAATACATTA
AGACTCACTATCAACCAGGCCGTATTGTCCTTTCTGGAGCTGGTGGCATTGAACATGGCAAGCTTGTAGACTT
AGCCAACAAGCATTTAGGTGGA
>1002049975_1 Heli.1-EL597889.1.5
GCAGTACTCGCAGGAGTCGCAGCCGCGCGGCTCGCTGGGCCGCCGCGAGCGCGTGCTGGTGGCCTCGCGCCAC
CTGCGCAACGACTCCAAGACCATCCAGCTGGAGTGCGCCGCGCCCGCGCCCGCCGCGCGCGACCTCAGCCTGG
ACCAGCTCAAGCAGCTCGCGCTCAAGAGCATGGAGGGGCTGGAGCTGCCGGCCTGCAA
GCGCGACGACGAGACCAAGCGCCTGCTGGAGGCGGGCGGCGTGCTGCGCCACCGCCGCACGGGCTCGCGCGAC
CTCAAGCCGCCCGCCGGCCGCCACAAGCGCACCTCCTCGCACCACATCACCATGGAGCCGCACGAGCTGAGCC
TGCAGCTGCAGAAGGGCCGCAGCGTGGACCAGCTGGCCGCCCCGAGCGTGTGAGCGCA
CCCGAATCTCATTGTGCACTGGACAGTTGACACTGTGTTGGGAGGAATAGAAAGAATAATTTATGCAATTGTC
GCTTCGGACGGATGATGATAACACATTAGGCTAAACGAATTATTTCTTCTATTACGCTTAAGCGACCGTGAGT
GATTTCGAACCATATCTTAAATGTAGTTTAATTAACGCTTATCGCGATTGAGATCAAA
CGTTCTCATTGCGTCTCTTACTGTAGCTATATATTTAAATAATCAAAACGGTAAGTTTTTATTTTGTGTTCAT
AGTCAAAATGGTCGTAATTTAATAGGTTTAATACTCACATCACTTTTGCTGTCACTGTGCATATTTCGCTTAT
AATTTTGGCCTAACTATGCCTAGGTAATTTCCTTAGATTAAAAAATGCAAATTGCCAT

AAAAAAAATACTATATTTTATTCTAAACTATTTCTGGAATAAAACCAGAGCTGTACTTAAATTTTACAAATAC
AGGAAAAAAATTATGTCATTGTCATAACATAACTTTTTAACATTAATGGAAATTTTATCAAAACAAGCACTAA
AGAACTCTTAAGTAAGATATAAAGCTATAAATCTCGTAATAATACCAAATATCCTTTA
CAATAATAGCTAAATATGTGCAATTTTAAATAAATAACTACAGTCTCACATATTTAAAGATCTATTAGTATAT
TGGACTGTGCAAAGCAATATTTTTATAAAAATTAAACTTTTAATAGTAATGTAATTTATCATTAAATGAAAGA
AAATAAATGTTTAATTGCTAAAAATCATTATTTTGGAAAAAATATTCCTTTTTTATTA
AATTTAGCATCTCACCTAAACCAATATAAAATAACTGAAAATTATGGAAGGCAGGCAGGAAGAACCATCTCAT
ATGAGAATTCAGCTGAAAAAGTGTATTCTAGTGAACAATAAAGTATA
>1002049976_1 Heli.1-DT666919.3.5
GCACGAGGGGTAGATTGCTTTAAAACAACAAAGAAAAATAATGCATAATGACGAACGCATAATAAAGTTAGTA
CTGAAATTTCCTCAAATGTTCATGTGAGTGACTAATGTAATAAACTCTGATTAGTTCTCGCGCTTCGCTAGTA
AAAATTGTCCTTGAATATATTACCTTGGACCACGTAATGTCGTCCTCGGAAGAAAATG
AAAACTTACCCTCAAATTTCGATGATAAAACCAACACAGCAGCAAGAGATGATCATCAGGAAAGAGAGCAAGG
TCAGTCGTCATGGTCAGCACCCAATACCTCGACGCTAATTCAAGTAGAAGAAAAAATGCGACGTAAATTACAA
TTTTTTTTCATGAATCCAATTGAGAAATGGCGCGCCAAGAGAAAATTTCCGTACAAGT
TTGTAGTACAAGTTATCAAGATTGTTTTGGTGACACTACAACTTTGTCTGTTTGCACATAACAGATATAATCA
TGTGAACTATACCTGGGACAACAGAATCAGTTTCTCCCATTTGTTTTTGCTTGGCTGGGATTCTACAAGGGAA
ATTAATGCTTACCCACCAGGTGCAGGACCTTTAGCAGTATATAAGGTAGATGAATTTT
ATAATGCCTTGGACTATGCCTACACAGGATATTCAAATTTAAGCAATGCCATTGGACCATATTCTTACAACAA
TGAAGATAACAATAAAACAGATCCCATGTTTTGCACATACTATTACAAAAAGGGAATAATTCATGGTTTTAAT
GAAAGCTATGAATTTAATTCAGAAATAGTATACAGCTGTGTTAATTTACAAATGAAGA
AACAAACTTTTTAAATCAAAGGAATTTATAAAAATGTCGGCATAGAAGTCATTTTGCAGC
>1002049977_1 Heli.1-EL598768.1.5
GCACGAGGCCTCGTGCCGAATCGGCACGAGGGCCGTGTTGTGTTCAAACAAAGCTACCAAGGAAAAAAATATG
CAGTCCTCAACAATCATCGTTCTGGCTTGTCTCGTAGCCGTTGTTGCAGCGAGCGTGCATTATGAGTACCCAG
AAAATACGCAAGACGTACAAAATATCCACGAGTCACTGCACGAAACACCAGTGCACGA
AAGAGGCGCAAGAACGAAAAGAGGACTGCTACTTCTGAAGAAGAAACTTATCCTTGGAGCTTTAGGTTTGAAG
GCAGTGAAAGTAGGTGCTGTGGGTGCCGGAGTAGTGGGCGCTATAGCACTCAAGAAAAAACACTGGTCCGGTT
GAGGCGCTAAAGATTTCTGTTTAAGCATCCTGTGCGTTAAGAAATCAAGAAAGCATCG
TTAAGTAATATTAGAAATAGTTAAAAGCTGAAGCCAATGGGTATTTTTTAATATGAACTAGATAATAAGAGTA
AATACAGGCTATTAAGTGAAATCAATTATAAAATTCGAAGGACAACATTGCTACATCGAAAAAGAAACGGAAT
ATACTCTGCACATTATTATTAAAAATATTACACTCATGTCAGTTACGAACCTCTGGTG
ATAACATTGAAAAACAAGTAAAAAATGTAATAAATAGTCTGTAAATTGCCATAAATACAAATCTTAATACTTA
AAAGTCAGATAAAACGCAACTGATTTTGTTTATACAATAATAAATATGTACATAACATAAATATCACTTTATA
GAACATGTAT
>1002049978_1 Heli.1-DT668349.3.5
GCACGAGGCGCGCGCCCACTTAGGTACCAAACGGGACGCCGGTTGAACGCCATAGTTGCTCCAATTGCAAGAT
ATCTTTCGCTTCGCTTCGCTTGGATAGGAAAGAAAGAGTGCAGCGAGTTACAATGCGGTGATCGCTGACATTT
GTACTGTGTTTAAGTGTCAATATCAAGTGCATAGCTTTCAAAGGTGTCCCAACATGTT
TATTTTAAAGGTTGCACTTTTATTTTTCATGGTCATCCTCGTGGTGGCTGAGAAAAAAATTGAGTTGCAGGAC
ATAGAAGAAGACAATTTAAGAAGTGAAAAGGAAAAGCAATTATGAAAGTCAGAGGCCCGATCTCAAGAACCAA
ATTCAGTTAGTGCTCCCGATTTACCACTGGATTTATTAAAAAATGGATTTTTCCAATA
TTTCAAGGAACCGGTCCAGACGCCTCAACAACAACGTTTTGTACATCAATATGCTGTTACAGAACAGCCAGAG
AGACCACCTCCACTGCCGACCCCTCAATATGGACCACCAGCTCCACAGCAAGCTATGGTTGGATATTTATCAA
ACGTGCCAATGCAAATATACTTAGTGCCACAATACTATAATGAACCTTCTGAACAAGG
GATCCACCACCAATCACAAAATGGTGTACAGCAACAGACCTTGGCTCAAGTATCAGGCTATGAAAATAGACAG
CAAATACAACAGCAACCCGCATACTTTGAAGTGCCGACATACATTACACCAACAGGCAAGACACTCATTCAGC
AATATACTCCTCCAGTATCTTACGTGACTTACGCTCATCCGACCATGTCGCCAATACA
ATCCACAGTTACACCGGTGCTAGCATATCAAATGCCCGTCGTTCGCTATCCTACAGCAATCTCCGCCCCTCCA
GTCAAGGGCTATTACCAAAGTACACATTATCCAGAGACCAATGTTGTCGATGAAGATCAAGAAACTGAAGTGA
ATATACCTAAGCCATATCCTAGTCACAATGATATACATTCTTCANAGGCCTTAGGCTT
GGGTTATCCGAGATATTATAATTCCCGGACCCCAGTAACAGAAGGATATAGATCTGCACCCATTGAGCTTCCT
CATCCCAACCCTCTTCTGTTAAAAGCACCCCCATCTCACCTGGCACA
>1002049979_1 Heli.1-EL600991.1.5

CGGCACGAGGGGCACTACGGCTCTCCGGCCCTCCCTCGAACTTTCACCTCCCGCGATATCTTCATCTGTGATA
AACACCAAGTGAAATTGAATGTGCGTTCCTAGAAGAGATTTCTGCGACGGCACGCGGACGAACGGCCGCCGGC
GAAGTCGATTTCTGCAAAATAATTTCTCCGGTGCATTTCGCTTCCACGAAACAGCTTG
TGTAGAGTTTACCGAGAAGCGAAGCGATAGAGTGGATTTTATGAAATATCAGTAATTATAAGTGAACTGTGTT
TCCGTGTTCTGTTGTTAGTTACGGCGTTTACATTGGATCCCAACCAGATCGCACCCCCAATGGTGGTTAAACT
ACCAGATGCATGCCTTGAAGTGGGGGTTCCACTCAACAGTTGGTCAGCGGGAGTTGCT
TGCTGTCCAAACTGCAGGCATGAGCTCCGGGTGTCCCTGGCATGCCCCAAGAACCACCATGTTCCCGTGACAC
CACTCACTCCTACTTTCACAGTCCAGCCAACATACTTGCCACAGATCCCTTTATATAATACACCTTCATCACA
ATTTATTACATCTCCTTTAAATGATGAGTTGAAACGGTTAGCCGAGACTCTTAGAGCA
TTAAGGTTATCAGGCTGGTACTATGGAAATTTAGATTGGCAGGGTGCTCGGAATCTATTGAAGGATGCAAGCG
TTGGTGCATTTGTGATCAGAGATTCAGGAGATAGGAACTTTATATTTTCTCTGTCAGTACAAACGGAGAGGGG
ACCAACTTCTGTGAGATTGCATTATGAACAAGGCTGCTTTAGGTTAGACTGTGATAGG
CCACTAGCAAGGTACATGCCTCGGTTCCGCTGCGTT
>1002049980_1 Heli.1-DT668401.3.5
GCACGAGGGGTAAAACGTCTTTGGTAGATGTTGATTATTCAGATCAAGCTAACCACCAATCACAATCTTACAG
CTCTAATGTTATTCATGAGAGTCAAGGAGTTTCTCAGCCTGCAGTTTCTATTACTCATGTTGGAACTGACGCA
CAAAATATTGACGGATATAGTTATGTCACTACTCCTACTTCATCTGGTATTCCAACTA
CCGCAAATCCTTTGCCAGTAACATACAAAACAACATATGTACCAGAAGCGCCTAAGAGCAGTATTAAGCAAGT
ATTTGGTTACACTCAGCCCGCTGTTACCTACGTTCAACCGACATTAATTCCCGTCAAAGTAACTCCACAAATT
CAAGTTACTAATTATAATCAAGGTCTAAACTATCAGTACGAAAATAAGGACTATGCGC
TTTATAATTCAGGATCAGCTAGTAATGCTGAGTCAGGATATGACTACTCAAAGCCTGCAACAAAATTTGTAAG
CACTATTACTTACACTACTCCTACTCCAACAGCTGCTGTTGTCTCTTATAAGCCACAAGGATTTGGTCACAGT
CATCAAACTATTCATAAAGTGGAATCAGTTGGATTTGAATATTCTACTCCTACCCCTA
TTATTGAGGAACCATTTAAAAAAATAGTAGCATATACAACTGGTCCATCTGTTTCTACTTACGTACAGCCAAC
AGCTCAGTCGTTCAGCCATCAAACAATACATAAAGTTGAATCCGGGAATGTTTACACTCCATCAGAAGAAGTA
ACTGGAAGCCAATCTCTTTACGAAGTTCCACAAACTGTCGTGCAATATGCAACATCGA
AACCAATTTCAGTACAAAAGGTGGCAATATCGACAAATGGACTGCAATCAGTGAGTCAAGGTCAATCAGAAGA
CAGTTATGAATACAAGCGGCCATTGGTACAGTTTGAAGAAGCGCCAAAAATAATTCAATATTCCACACCCGCG
CCTACGGCATACAAATCTCCAAGTTATAGCTCACAATCTATAACCAGATATAATACTA
AACAATATGTCCCTATATCGCCAACTGTACAATCCTATGTATCCTCCACTCCCGTAACTATCAATGAGAATCC
CATTCTCAAGTATACAATAAAGTCTGCTCCAGTGACTTTAGTTAGCTCTACTTATGCTCCACAGTCTTATAGT
CAACAAACTGTTCACAAGGATA
>1002049981_1 Heli.1-DT664699.3.5
GCACGAGGGCGCAGCGTATAAGGTTACCCGGTTGCAGTCGACACAACCAGCCCTGTGTTTTGCCAGACTTTGG
GCCGGGCAGTGTGACCTGATTCCAGTCGACGCAACCAGCCCAGTCGCTCCTAGTGCTTGGGACGCGAAATGTG
ACCTAGTCCAGTTAACACAGCCACGTACATCAGACACACACAGATACTTCACTTCTGG
CCTCTGTGGTCAATTCTGCGTGGTCTTAGTGACCGTGCTGCCTGCCTGCCCGCCTGCCTACCAGTCTTCGGGC
CAGCCAGTGTGACCTGCTTCCAGTCGGCGCAAGCAGCCCTGTTTCTGGGCCAGGCAGTGTGACCTGCTTCCAG
TCGGCGCAAGCAGCCCTGTTTCCGGGCCAGGCAGTGTGACCTGGTTCCAGTCGACGCA
ACCAGCCCAGTCGCTCCTAGTGTTCGGGACGCGAAGTGTGACCTAGTCCAGTCAACACAGCCACGTACACCAG
ACACACACAGATACTTCACTTCTGGCCTCTGTGGCCAATTCTGCGTGGTCTTAGTGACCGTGCTGCCTGCCCG
CCTGTCTACCAGTCTTCGGGCCAGCCAGTGTGACCTGCTTCCAGTCGGCGCAAGCAGC
CCTGTTTCCGGGCCATGCAGTGTGACCTGCTTCCAGTCGGCGCAAGCAGCCCTGTCTCCGGGTCAGGCAGTAT
GACCTGCTTTCAGTCGGCGCAAGCAGTCCTGTCTCGGGGTCAGGCAGTGTGACCTGCTTCCAATTGGCGCAAG
CAGCCTCGTCTCTACAAATCTCATGCCGGGCTGTTCGACACAGCTCGCCTCGTTTCTG
CCAGTCTGCAGGCTGAACAGTGTGTGACCTGGTTCCAGTCGACGCAACCAAACCCGTATCTGGCCAACTTCAC
ATCGGTATGACGTAGTTCCTGCCTCTGTCAATCTTCGGGCCGGGCAGTGCGACCTGGTTCCAGTCAACGCCAT
CAGCTCCGTCACTGCAGTCTTCGGGTTGCGTAGTGCCACCTGGTTCCAGTTGATGCAA
ACAGCCTTTGCCTCTGTGGTGTACCCTGCATGGTCTTGGTGACCGTGTTGCCTACCAGCCAGTTCTTCAGGCC
GTGCAGTGGCACCTGGTTCCTTTCGACGCGATCCACCCTGTTTCTGCCCGTCTCTGGAGCTGGCAGTGTGACC
TGGTTCCGGCCAACGCAACCTG
>1002049982_1 Heli.1-EL598396.1.5

GCACGAGGGTCGTCCTTTAAGGGTGTTAATTGTAATCGTGAATTTGTGTATTTTGATTTAAATCGTAGGGCGA
CCCGATACCTCACCTGTCTTAAGTACTTAAACCAGACAAAATGTTACACCTTAAGAATATAGCAAAATCGGTT
GTGCCGCCCCTCAAAAATAACATCCAAAATGAGGCAGTCAATAATATGCTAAAGATCA
CGCCTGCAGCTGTAAACGTTTGTTGCCGTACCTACGCAAGTCATGAGATCCCAGACAGGCTCAAAGACATACC
GACCAGTGCTAATCCCAGATTTTTTGACATGGTGGAGTATTTTTTCCACCGGTCGTGTCAAGTAATCGAAGAC
AAATTAGTTGAGGACATGAAATCCAGAGTTTCAATTGAAGAGAAAAAGAAGAAGGTGG
CCGGTATCCTCAAATTAATGCAGCCATGTGACCACATTCTCGAAATTCAATTCCCACTGAGACGCGATTCCGG
CGATTATGAAATGATTTTAGGATATCGGGCACAGCATTCTTCGCACAGAACCCCAACAAAAGGAGGTATTCGA
TTCTCAACCGACGTGACAAGAGACGAAGTAAAAGCTTTGTCCGCCTTAATGACATTCA
AGTGTGCGTGCGTGGATGTGCCCTTCGGCGGCGCCAAGGCCGGAATCAAGATCAATCCTAAGGAGTACTCTGA
ACACGAGCTCGAGAAGATCACAAGACGATTCACTTTGGAATTGGCCAAAAAGGGATTCATTGAGCCCGGTGTG
GACGTGCCTGCCCCAGATATGGGTACGGGTGAGCGCGAGATGTCTTGGATCGCGGACA
>1002049983_1 Heli.1-DT667393.3.5
GCACGAGGCTACACACCTCCTCAGGATGGACAAGATGGCCTCGCTTCTTATTGGGAACATAGACACATACACA
ATGAATATGGTCTATGGCACATTAGAGGTACGCACGAGGGAATGCTCTCAAGGGCCAACGGCAAATATAGGCC
TTTCATACTAACGAGATCTGCTTTCTCTGGTACACAACGTTACGCAGCAGTGTGGACG
GGTGATAATGCAGCGGAATGGGGTTTCCTACAGGCGTCAGTTCCAATGTGTATTTCCTTAGCCGCCGCCGGTA
TTAGCTTCTGTGGATCAGATGTTGGTGGTTTCTTCAAGTATCCCGAAGCTGAAATGATGACCAGGTGGTATCA
GGCGGGCGCGTACCAAGCGTTCTTCCGCGCGCACTCCCACATAGAGACCAAGCGCCGC
GAGCCGTGGCTGTACGACGCGAGCACCACCGCGCGCATCCGCGACGCCGTGCGCCGCCGGTACGCGCTGCTCG
ACTTCTGGTACACGTTATTCTACGAACATACGCAAGATGGCCTCCCCGTTACCAGACCTCTATTCCAGGAGTT
CCCCAACGAGGAAGAAACTTTCACTATTGATAATAGTTATTTGCTTGGTGACAAGTTG
CTAATCCGCCCAGTGTTAGAAGCAGGTGTGTCAAATGTCAAAGTGTACCTCCCAGGAAGAGAATCTCATACCA
TCTGGTATGATGTAGACTCCTACCAGGCATACCCAGCTAATGGATATATGAATGTTGATGTTAATATTGCTAA
GATTCCAGTATACCAACGCGGCGGTACAATAGTACCGCGGTAAGAGCGAGTCCGTCGT
CCTTCAGCCTCTAATGGTCAAGGATCCTTACACACTCGTCGAGGCCTT
>1002049984_1 Heli.1-DT667141.3.5
GCACGAGGCAAGCCTCAAGTCTTCGGTGAATGTTTGACGTTTGTGAAGTATATTTTTAACTCTTTACAAGTTT
ACAAGCTTACAATACGTGCTTAATTAAAAGTATTAAATACAAAGAAGAACAAGTAAAATTATCGTAATGGAAA
TACATATAATACCTGACACATAACGTCGATATAACCATTTAATTGTTTAAATTGTGAG
GTTGTAACTATACAGCAGGTGTATTTTTGCTATTATTTAATACAAACAATTTGATAATGACGGAAAAACAAAA
AACGCTCGTTTGTGGTGATGTAAACGGAAATTTTAACGCACTATTTTCTCGTGTTGAGAGCATTACAAAAAAA
TCTGGGCCTTTTGATGTATTGCTCTGCGCGGGAAACTTCTTTGGTTCAAACAATGCTC
AATTAGACCCATATCGTTCAGGCGCAAAAAAAGTTCCAGTCACAACTTACATATTTGGTCCATCTAATAGTGA
TCATGTTCCATATTACTGTGAAGAAGGTGCTGAGATAGCTCCTAATATTATTTATATGGGTACAAAAGGTATT
TTTACAACTAGTGCTGATGTTAAGATTGCTTATTTAACTGGCCTGTCGAGACGTGAGC
TGGGCAAAGATATACCAATGTGTACATTTGAACCTAGTCATTGTAGTGCTGTGAGAGACGCATGTTTTAGAGG
ACAAAGTGAATATAGAGGTGTTGATATCTTAATTACTACCGTGTGGCCTGTTGGTATACAGCAAGACAATAAT
CAAAAGGCAGATGTGGAACCAGACTGTCTATCAGACTTAATATCTTGGTTATCAATAC
ACATTAAACCTAGGTATCATTTTGTGCCTGCAAACAATAAATATTATGAGAGGCAGCCTT
>1002049985_1 Heli.1-EL598900.1.5
TTAATTGGTGTTTTAATTGGGGTTGCTTTTTTAACTTTATTAGAGCGAAAAGTTTTAGGATATATCCAAATTC
GAAAAGGGCCTAATAAATTAGGATTAATAGGAATTTTACAACCATTTTCTGATGCTATTAAATTATTTTCTAA
GGAACAAGTTTATTTAAATTATTCCAATTATTTTTCTTATTATTTTTCTCCAATTATT
AGATTTATATTATCTTTAATTATTTGAATGATAATTCCATATTTATTTAATATAATTGTATTTAATTTAGGGT
TATTATTTTTTATATCTTGTACTAGAGTGGGGGTGTATACTTTATTAATTGCTGGGTGATCTTCAAATAGAAA
TTATTCGTTATTAGGGGGTTTACGGGCTGTTGCTCAAACAATTTCTTATGAGGTAAGA
TTATCTTTAATTTTAATGTCAAGAGTAATTATAATTATAGATTTAAATTTAATAAAATTTAGAGAGTATCAAT
TTTTAATTTGATTTATATTTATAATAATTCCTTTAAGAATATGTTTTTTTTCTTCTATATTAGCTGAAACTAA
TCGAACTCCTTTTGATTTTGCTGAAGGTGAGAGAGAGTTAGTTTCTGGGTTTAATATT
GAGTATAGAAGAGGGGGATTTGCTTTAATTTTTTTAGCAGAATATTCGAGAATTTTATTT
>1002049986_1 Heli.1-DT662881.3.5

GCACGAGGCCGCGGCGTCTCCGCGCACGCCGCTTGTCCATAGAGAGTTCGCACGCCGCACACGCACCGGCCTA
TGTTAAATAACTGTTATTTCGGTCTTTAGTGGAAATGTTCCGCATTCTGAACTCAATAAACTCTCATCTCGAT
TTTATTATTACATCACCACAGATCTTAAGTGGTTAGGCGCTGTTTTTCGTTAACATTT
AGTGAAGATGTTTTTACTCATTTTTTATTGACGATGAAAGTAAATGTTTTATGAATGAGCGGATGCCGAAACA
GGTTTATTAAATGGATGTGTAGTGTTATGGATTGGTGCAGAAGATTTTAAACATGGGAACTGATCAAAAATTT
TTTACAATGCTGGACACACGGAAGCCGCCCAGACGCTCTATGTCGCTCGTCGGTCCTT
ACTTGAAAAATCGTGTAAATAGAAGTGACGCTGAGGATAAAAGTCTTTCAAATGATGAAACACCGATGCCATC
CTGTCCTCGACAACCACCCGACGGTGTTGAGACGCCTTCAGATGTTGTCGAAAAAACCCCAGAGTTGCCTAGT
AGGTTACCTCCTCGACTTTCAACAGGATGGCCTGGCACACGTTCTCGTTTTTCATATT
CTTCAAGAACTCAAGATGCAAGAAGTGAAGCGAGCGTTAAAGATAAAATTGCTATGTTTTCTGTTGATTTGGG
GAAATCCACCGATCGACTCGATAAATGTGGAACTTTACCAGTTAGGTCACATTCAACTTTAAGAAAAACTCCT
AATATATACTCAAATAATTATAGCGAAGTTTCATCTTTAGATAGGAGGGTAACGAAAT
>1002049987_1 Heli.1-ES586820.1.5
TCGTAACGGTTTCTTCGTGCCGAATTCGGCACGAGGACAAATTTCAGAGCTCGTATCTTTTTTTTCGGAGCCT
ATTACGATAAATTCAAGATGTTAGCATCACGACCCGTCATCTCCCGTGTTATGCAGCAATTTGCAAGGCGCTA
CCACGGCGAGCATGGCCACTTCAAGCCCCCAAGCATGGATGAGCTGCCCGTGCCAAAA
GGTTCCTGGCAGGCGCAGTATGATGCCAACCAACGACGATACAACTCTATACTTTTGTTCGGCATTGCTTTTG
CCGCTGGAACAATCATCATTGGTAAATCTACCGGACTCATTTACTTGAACTACTCCCCACCAAAGTCTCTGGA
TTAAATTTGGAGTACCTGCTTAATATTGTGAATTAATATTTAGTTTATTTAAATATAG
TTTGAAAAACTA
>1002049988_1 Heli.1-DT665485.3.5
GCACGAGGCAGCCATCTTTTGTGGAAATATCATTTGTTTCGTGTCGTCAGGGTAAATTTCATAATTGTAATTA
ATGTCAGAGCCACAGTCCTCGCTTTTATTAAAAAAGCAATTAGCAGAACTAAACAAAAATCCAGTAGAAGGTT
TTTCTGCTGGGTTGATAGACGACAATGACATATACAGATGGGAGGTCCTCATCATTGG
CCCTCCAGACACATTATATGAAGGTGGTTTTTTTAAGGCACATTTACACTTCCCTAAAGAGTACCCTTTGAGA
CCGCCTAGGATGAAATTTGTTACTGAAATTTGGCATCCAAATATTGAAAAGAATGGAGATGTCTGTATATCAA
TATTACATGAGCCAGGTGATGATAAATGGGGTTATGAGAAAGCCTCTGAAAGATGGCT
GCCTGTACACACTGTTGAGACTATATTGATAAGTGTTATTTCTATGTTGGCTGATCCTAATGATGAAAGTCCT
GCTAATGTTGATGCTGCAAAAGAATGGAGAGAGAGGTATTCGGATTTCAAAAAGAAAGTTGCCAGATGTGTTA
GGAAAAGTCAAGAAGATTGTTTTTAGGATCAAAGGGAATTACACAGCAGAATGTCATG
TCGAAAAAGGGCCACGTGACACCATTGACATATATAATTTAACATTAGGTTGCACACCCACCGGCGCCAAACC
AAATTTATAGTAGTAGCTGTGAGCTCATTCTCGTTCTTTTTGCAGAAAGAATGGAGAGAATCATATTCGGAGT
TTAAAAGGAAAGTAGCGCAGTGTGTGAGAAAGAGCCAGGAGGATTGTTCATAAATAAC
AGTGCCCTTTTTCATTTATTTATTTGGATTGTGAGTGTTGGGTGACAGTTTGGAGGTCTCGCCACAGTGTGAA
GACTCGCCACAGTGTTTTTTCTAACACCGGGCCAGAGACAATATTGAAATTGATTTAATAAATAATTATTGTA
ATTAAATGGTATCGCTGCCGTATTTTATAAATAATAAATTAAATGTAGAACTCGAGGT
CTTTTTCATCTTTTTTAAGACATGGTAGTTAGCTATTGTTATAATTAAAATGGTGTACGATATTTTTTCCACA
GAAAATAAATCTGCATTTGTTCTTTTGACAGATCTCTTTTTAGTGAAACCAAATTTGTAATGCAATAAAAACA
TTATTTATTAGTCGCTAAAATGTATAGTCATGGCTGAGACAAATCATCGAAAATTGTA
TCTGGCAATTTTTGTATTTGTAAATTTTAGTTACCATAGTTAATGCAAGGAATCATTGATAGGCTTGTGTGGT
CAGATTCTTAAATTACTGTTTTGTTTGTTATGCTATCTTAGTAGTGAAATTAAAACTTATTATTTTAATGAAT
AAATGCAAATTTTTTAATATTATCTTTTTATATCAATATACCTTGCCCATACTACACA
CTAGCTACTTATAGCACCAAGTCGTCTTTCATATTGACAAAGTCAAGTTACTGTTGCAGCGTAACCATTGCTT
CTAATAATTTACTGTTAAAAACCTGCCTTTTAATGATATCTAAAAATCAATGGTTCACT
>1002049989_1 Heli.1-EL596877.1.5
AGCTGCTCTGAAGAAGGATCTGACCCCGCAGTCCATTTGTCACATCAACGGACAGTGCAACTATCTATACCAT
TCACACAATTTGTACACATTTGATGATGAAACTAAGGATCTAAAGGCTTCTGATGACATTCCATGTGAATTCT
GCGAACAAGTGATCCAACACTTACGCGACACGCTTATAGCGAATACCACGGAATTAGA
GTTTCACAAAGTACTCGTTGGTTTATGCAAGCAAACGGGCGAATTCAAATCCGAATGTCTCCATCTCGTCGAG
CAATATTCTTCAACAATCTATAACTTCTTAGTATCGGAATTAAAAGGTGCAGAATTATGCAGTTTGATTGGAA
TCTGCACTAACAAAACTGAAGTAACTATTGCACCTCTCATCCCTAAGGAGTTGGCTGT
GAAAGCTGTTAAAATGTCAACCGAACTTATTGGTCAAGATGAAGCCAATAGCTACATTACAGAGAACACCCAA
GCAAAGAAACATGAGAACGTACAAATATTGGGCACTGAAGCGAGCCAGGTCCCCGTGTTGCCCATAGAGAGGA
TGTTCGTGTCCGCGCCGCGCTCCGACGCCGTGTGCTCCTTCTGCCAGTACTTCCTGCA

CTACATACAGGTGGAGCTCAGCGACGACAACACTGAGGAGGCCATCAAGAAGGCGGTGGAGGAGGCGTGCGAC
GTGCTGCCGGCGTCCATCGACGACGAGTGCCGCCAGTTCGTGTCGCAGTACGGGCCCGCCGTCATCGCGCTGC
TCGTGCAGGAGATCGACCCGGCCAGCGTATGTCCAGCTTTGGACTTGTGTCCTAAAAC
CGAGGAAGTACGTCGTGTTGATATAAATGCAGAGAAATCGAACTGCCCCCTTTGTCTGTTTGCTGTAGAGCAA
CTTGAAACCATGCTGAAGAATAATCGTAGTGAGGAAAGTATCCGTAAAGCATTAGACGGACTGTGCAACCACC
TGTCAGCGAAACTGCGCACTGAATGCGTCGATTTCGTGGACAATTACACCAACCAATT
GGTAGAAATGCT
>1002049990_1 Heli.1-EL596488.1.5
GATACACTTAAAACACTCTTAAAACAAAATATTGACAAAACAATAAAGATGCTGATTTATAGCACTAAGACTC
AGTCCGTGAGGGAAGTTATGATAACACCTAGTGCAAATTGGGGTGGACAAGGCCTTCTAGGTGTCAGTATAAG
GTTTTGTTCTTTTGAAGGTGCAAATGAAAATGTGTGGCATGTACTTGAAGTACATCCA
TCATCACCAGCAGAATTGGCTGGATTTGTAGTATTTGTTATTTATCTCTTGTTAATAATGAGTTAACTGCACT
TATATTAACTTATTATTAATTATTAGCTTCACGCTATATCCCGCACACAGCAGTTAGGCAGAGGGAACCTAGT
GTTAAGACACTCCGTTCCCCACTTTCTGCCGAATTCTGGAGGCATTGCGTGTTGAGTG
GCGGAACTCAGCGCCTCGCTTTGCCTCGACACCAGAAGCGAAGAAATGGAAATATAAATTTAAGTAAATATTT
CATCTCCTCGAGTGGGGATCGAACCCACAACCAGTCGGTTTTACAGTCACACCTCGTGCCGCTGCGCCACG
>1002049991_1 Heli.1-DT668794.3.5
AAACCTCGTAATGTTGTTTTTGTGGACTTTGGTCATAGCTCTCTTCAGGTTGCTGCATGTGCATTTAATAAGG
GTAAATTGAGAGTGCTGGCCACTTCCTCAGACCCCTTCTGTGGTGGGAGAGATATAGATATGGCAATGGCAGA
GTATTTCTGTCAAGATTTTGTAACACGATACAAACTTGATGCTAAAAAAAATCAAAGA
GCTTTCTTGCGTCTTTTGCAAGAGGTGGAGAAACTTAAGAAGCAAATGTCAGCAAATAGCACCCGCCTACCAT
TAAATGTTGAATGCTTCATGGAAGAACGTGATGTATCTGGAGACCTACAACGCTCGCAGATGGAACAAATTTG
TGGCGACACATTCAACAGAATTGAAAGGACTTTGAAAGGAATTTTGTATAATGCTAAA
TTGAGACCTGAAGACATAAGCTCAGTAGAAATTGTCGGAGGTTCTACTAGAATACCGGCAGTCAAGGGTCTCA
TTGAACAAGTATTTGGCAAACAGGCTTCTACAACATTGAATCAGGACGAGGCGGTGTCGCGCGGGTGCGCGCT
GCAGTGCGCCACGCTGAGCCCGGCCGTGCGCGTGCGCGAGTTCAACGTCACGGACGCG
CAGCCCTACGCCGTGCGCCTCGCCTGGGACGCCGCGCGCGGCGAGGACGGCGACATGGAGGTGTTCCCGCCGT
TCCATGCCGCGCCATTTTCCAAAATGCTTACTTTCTACAGAAAAGAACCATTCACTGTTCGCGCTTATTACTC
TGATCAAGCAATCTATCCCGATAGTTTCATTGGACAATGGAACATAAGAGACGTGCAG
CCAACCGCCGAAGGAGAATCGCAAAAGGTTAAACTGAAAGTCCGCGTAAACATCCACGGTATCGTGACCGTCG
CGTCCGCGTCCCTGGTGGAGAAGAAGCAGGACGCGGCGCAGAGCGAGAACGTGGAGATGGAGAACTCGAACGA
GGCGAGCCAG
>1002049992_1 Heli.1-DT665609.3.5
GCACGAGGAAAGATTCTCTGTTGTCTTGTTAAGACACCGCTTCAAATTAATATATAAACAAAAAATAAAATTG
GATATATAGTGTGTCGTAACCGAATATATATGAAAATTTAAATAATTACGTATAATATAAAGCCATAAGATGA
CTGCCGCTGATAATCAATATCCTAAGGCATCTAATGCCACCTTAATAGGAGCATCAAT
AACGTACGTAGCAGGACTGTTTTTATTATTATCATTTGCTGGACCGTATTGGATCGAATCGTATCCTGAAATG
TTTTCTTCGTTCAAACACATGGGCTTGTGGGAGTATTGTTTTGATCGCTTCTGATTTCCATCGTTTCAATATG
ATAAATATTTCAATGGATGTCATTACATATTTGGTGAAGAACTTTATGTCATTCGAGA
ATGGCTTCTACCTGGTTGGTTAATGGCTGTGCAAACATTTGTGACTCTTGGTCTAATGTTGTCATTTACTGCA
CAGACCCTTCTTGCTTGTGTTATTGTTCGGTGGCCTTTAAGAACTGTGTTAAGATATGAATGGATATTTGTAT
CTACATCTTTTGTTATGGTTTCACTATCAAGTGTTTTCCTATTTCTGGCTGTAGCTAT
ATTTGGAGGAAATTGTTATCGCAGGGACTGGTTACTATACCCTTCATTTAATGTATTATCCTGGTCTTATGCA
TTTGCTGTAATTGCCTTTATTTTATTTGGTCTCGCAGCAATATTATTATTCTTGGAATCACGTAAGTTATACG
AATTGCGTTC
>1002049993_1 Heli.1-DT665416.3.5
GCACGAGGGCCCCCTGCGCATCATCTGGCAGCCATGGGCTAAACCGACCTTACCAGATTGACACCGTTACCAA
AAGCCTCTAGCTCGAAAGTGACCTCTTTCCCACTGTTTTGAACTAAATATGTTGGACTCGAGTGTTTGATCAA
AATCCTAGCGTGACAGTGATTGTAAAAAGTGGTCAGTTTCATAAAATATTCGGAAAAG
TGCAAAGGATTTAAACACAAAATGATTGAATATTCACAAAATCACTTGTTGTTGGCGACAGTGTTTATGACTA
TTGTCCATGTCACACAAGGACAGTGTCCATGGAAAGATTTTCCTGATTTACAGGCAACTTGTGTGTGCGCCTT
AAATCTAGCAAGGCAACTATCAGTGCAATGTGATCAGGTTGATTTTCCAATCTTATTG

TCTACATTGAACATTAGTGCAAGAAAAAATACAATAGATCTCTTATATATAAATAATTCAACAATCCCTGTAT
TAAGTAATGAAATGTTCGCTAACTTAGCGATACACAATTTACAAATATCTGGATGCAAAGTGAACAAGATAGA
GGACAACGCATTTAGAGGTCAAGGACAGTATTTAAAAAATTTAAACTTGCAAGACAAT
GAGTTGACTGAAGTACCTGTTAGAGCATTGAGAGTATTGACAAGTCTATCCCTATTGGATATTTCTAAGAATA
AAATAACGTATATTGATAATTACTCTTTTACGACTTTACAAGAATTAACAACTTTAAAGTTGTCAGATAATAA
CATTACGTTAGCGCCACAAGCACTGTCTGGTTTAGAGAGCTCACTCAAAAATCTTAAT
TTAAAAGGAACTAAGCAAAAAACAGTGCCTCAATGTATCAAGGGACTCCACAGTCTTGCTTTTCTGGACCTGT
CACAAAACAGTATAAGAGAACTTCCAGGACCTGATGGCGCGCTTACCTTTGATGGATTAGATTCGTTAACTGC
ACTGAATTTAGAAAGAAATCTATTAGTTAATTTAAGAAAAGATGCATTCTTTGGGATT
>1002049994_1 Heli.1-EE743391.1.5
GCACGAGGGGAATGTGCGATTCCTGGGAAAAAAGGAACTCCATGGGAAGGAGGTCTTTACAAATTAAGAATGA
TTTTCAAAGATGACTACCCATCAAGCCCACCAAAGTGCAAGTTTGAACCACCACTATTCCATCCCAATGTATA
CCCATCTGGCACAGTATGCTTGTCACTACTTGATGAAGAAAAAGATTGGCGTCCAGCA
ATCACTATTAAACAGATTTTACTTGGTATTCAGGATCTGCTAAATGAACCCAATGTAAAAGACCCTGCACAAG
CTGAAGCATACACAATTTACTGCCAAAATCGATTAGAGTATGACAAAAGAGTCAGGGCACAGGCACGTGCTAT
GGCGGCAACAGAGTGAAAAAGTACATTCCTGACATAAATGTGAATGTAGGGATCATCT
ATGTAGCAAATGGCAGTTTTATTTTGTGTACAGTCATATTGGCATTTTACAAATCATTAATTTAATAACAGTC
TTGCTACAGAGTTGATCAACCAAGAAGATTAAAGTTTAAGTAAGGTTTTACAATCTATTGTAATAAAAGATAG
TAAATTAACAAAACAAACTTTAAGAAATTGCTTAATTAAAATACCACTTATGTCCAAT
TAAAGATATGGAGCATTTGTATTACATATTAAGTGATAGGTTTTAACTTATTAAAAGTGCTTGGAAATCTTTG
ATCAAAATTAATTTAATTTAAGAAAATTTAAATGTATGATGTATACTCGCTTAAAAAATATTAGATAGTATTG
CAGTCTCATGTGTAAACTTTTCCATTTAATTTTAATTAGTTTCATATAACTCGATTGG
ATCTTTATTATGTTAAAGACAGGAATACTACAATTTAATTTTCNACTATTTTTTGTATTTTTATGTTAGATCT
ACCCACAATAAATAATAAAAAAT
>1002049995_1 Heli.1-DT663059.3.5
GCACGAGGCGCCCCCCTCTCTGCTCCTCGGACCTCGACCCGTGGACTCCAGCTGCTCCTATAGCTCGCGAAGA
GTTGTGATGTGCCTCGTGAGCTGATCATGTGCAGAGTGTATACAGTGCGGGAGTGCGCGAGCGACGAGCGCCG
GTGGCCCCCGCGGCCGTAACCTGCCATGTGGACGCTCCTGATGCCGCTGCTGGCACTC
CTCACCTCGGGTGGCGGTGACTCTGTCTCCTCCCACAGAGAACTTCTAGAATGTGCTAGCCAGATCCTGTATA
TGGGCCACCATTCATATGAACTTCCAACTAACTACAGTAGAAACATTCCACCCGGTAAGAATACGACGGTTTA
CATCGGAGTGAACGTCAACAGAGTGTCCGGAGTAGACGAGAATAGAGAGGAGATCACA
TTGGACGCTTTCCTGCAAGTTTCATGGGAAGACTTGCGTCTCAAAACGCCTCATCACATGCCCTACATTGACT
TACCCTGGGAGTTTCGACAACTGATCTGGACTCCAGATCTCTACATATGGCAGTTACAGACAATGAGGATCAT
GTCAGTCCTCCAAGAAATGGCTTCGTTACGATTATATGCTAATCGTACTGTCTCTGTC
AGCATAGGTGCTACAATAACTATAAAATGCGAAATGGATTTCGTTCTATATCCATTGGACGTACAAAATTGCG
CTATAGACTTTAGCAGTTACAAATACACAACAGAAGATGTACGTTTCGAGTGGCGGGAAGTAGCGCCGTGGTT
CGGCCTCGCCGGCGGTCAACGCCGCGAGTTCCGCCTACCGAAATACGTGGTCACATTT
GTAACGGACAAAAGTGAACATTCTGCAGCCAGATTGCGAATCAAGCTTTCCAGGGAGTTGCGGAGCTACCTCC
TTGAGAGCTACTTGCCATCATCTCTCTTCGTCATCATATCGTGGGGCAGCTTCTGCGTCATACCCGAGATAGT
GCCGGGTCGTATGGTGCTACTTGTGACCACACTGCTTTCATTGGTCACCATGTTCGAT
ACAGTCAGCACTCATTCCCCAGATGCATTAGAACTAAAATGCATCGAAGTCTGGCTGATCTCTTGTACGATAT
TCGTATTCCTGGCGTTACTGGAATACTTCGTGGTGCTGTTCGGCATCCGCTACGACAAGAGCTGGAGCAGGCG
ACGGCAGGAC
>1002049996_1 Heli.1-EL600985.1.5
GCACGAGGGAAAGGATAGTTGAGTGCGTGTTATATTTTAAAATAAATCCCCAAAAATGGCTGAGCCTGTAACT
GAATTACCTATAAATATAGAAGAAACAGTAAAAGAAGTTGTACAAAATGTTACCGACAAACCCCCGACAAGTA
TCGAAGGAGTCGCAATTGCGTACTTAAGTTTAGTGATAATGGCTATTTTACCCATATT
CTTCGGATCATTTCGATCTGTTAAATACTTAAAAGAACAAAAGGAATCAGGTGAAAGAGCTGAAACTATGTCT
AATAAGGATGCTCTGATGTTCCCGTTGATAGCATCCTGTGCCTTATTTGCTCTGTATATATATTTTTCCAATTTT
TCTCTAAAGAGTACATAAATCTGCTTCTTACTGGATACTTTTTCTTCCTTGGAGTTTT
AGCACTGAGCCATCTTTTAAGCCCAATAATATCTTTAATAGTACCAGCTTCAGTCCCAAATGTACCGTACCAC
ATTTTGTTTACACGCGGAGAGCGCGACGGCCGCTCGGACATCGTGAACTATAAGTTCACCTCGTACGACGTCA
TTTGCCTGATCATATCACTTATTATGGGGAGGGTGGTATTTACTGAAGAAGCATTGGA
TCGCAAATAATTTATTCGGTATCG

>1002049997_1 Heli.1-DT663425.3.5
AAAAAAAACACACCGGGCTGAGATCGGCCGAGGGTCGACTGAAGACATTGCTGTGTTCGGTGTTGCTCGCGGT
CGCGGGCACGTTGGCTCATCATCATCATCATGTCCATGAACATCCCTCTGGAACAGAGTTACCGTCGTCCCCT
GTCACCGTGTCCCCATCAGGGGAGTTCCGCGGCGGGTACAACGTGACGCGTCTCGGGC
GCCGTTTCGAGACCTACCGCGGCATCCGATACATCGAGCCGCCCATTGGGGAATTGCGCTTCCAACCTCCCAA
ACTGATCTTGTCGTACGCGTCGCCAGTGGATGCAAGTGAGGACGGACCCGCGTGCCCACAGCCCGCACCGCCC
GGATATCCCATCGACGAGGATTGTGTCACAATCAATATTTATACGCCACTCAAGAAAG
ATAGGGCAAGTAATGCTCCTCTGCCTGTAATATTTTATATGCACTCGGGCGGGTATTATTCGATGTCAGGTCG
CAGTGACCTCGCAGGGCCGCACTACCTTCTTGACCGTGACATCGTCCTCGTTACTATCAATTACCGACTCGGT
TCTCTTGGATTTCTAAGTACGGGAGATAAGCTCGCGCCAGGTAACAATGGGTTCAAGG
ACCAAGTGGCGGCTTTGAAGTGGGTGCAACGCAACATTGCGGCATTTGGAGGAGACCCCAACAGCGTCACAAT
TGCGGGATGCAGCGCCGGTTCTTCCAGCGTTATACTGCATATGATCTCTCCTATGTCTAAAGGACTCTTCATC
CGCGGTATTTCAATAAGCGGGTCTCCTGTCACGCCTCGCCCGACGCCCGATAATATGT
ATAGTCGCGCGGTGAAGCAGGCGCAGTTGCTGGACTGTCCCACCACCAACTCTAAGGCGATCATCGATTGCTT
GAAGACTAAACCTTGGAGGCAGTTGGGAGATTCCCTTATGAGACTCTATGAATTTGGATTTGATCCAATTTTG
ATATGGGAACCTGTAGTCGAGAAGGATTTCGGCCAAGAAAGATTTTTACCTATAGATC
CAACTGAAGCAATCCGGCAGGGGAAAATGCATGCAGTTCCGTACATCATCAGTCAGACAAAAGATGAATTATT
CTGGATGGCACTCACGGTCCTTCGCAACCAGACGCTGCGCGATAGGATGAATGCGGAGTGGGAGTCCATTGCG
CCCATCTCATTTCTGCTTCCGCATGAGAACACCGTGCAGGCAACCCGTACACTGAGCC
CGCAGTATCTGC
>1002049999_1 Heli.1-DT666757.3.5
GCAGAGGATTCACGCATGCAGACGCTGTTATAGTGTCGCTGCCATTGTAATTTTTATTTTTTTTGTTATTAAC
AAGTTATCTCTTAGATAATTTACATTAAGTAGTGATTTTTAATAACTACTGTCGATAAACAGTAGTAGGACAT
TTTTATATTGTGTAAATCTTTGTGATTGATCGGACATTTTGACTGCCATTCTTTATCC
CTAACGGGATAATAGCTTGGTTCTGAGCCTTTGTTCATAAAAAGTGTCTAAAGCAGAGTGCATGCATCTTTCC
CTGCAATACACAGGATTTTTGACCGACGACTGTGATGATACGATTGTTGAGCTATCAAAACTATCGATCGGTG
ACTCTGATCACGAGGTCACTGTTTTAGAAAAATTTCATGACACCACGAGCCGTGTAGC
GACCGCGCGGGACTACGTCTTGCGACGTTGCAATCAAACAGAAGTTGTACTGTTTGATGAATGCTATCCAGAT
ACAGCCCTGAAAAACTGCCGCAAGATCGGCGAGGGTGTCTACGGGGAAGTTTACCTCTGGCGCGCTCGAGACG
GCAGAGCCCGTGTGCTGAAGATAGTGCCCATAGCCGGCCACATCAAAGTAAATGGGGA
AGATCAGAAGGACTACCATGAAATCATATCTGAGATTGTGATTGCTATGGAATTGAGCGCACTACGCGCTCCT
ATAGCAGAAATAGAACAACATTTAGATGAAGGCAAGAGTGTTGAAACTTTGGATCTACATTCTGTAATGAATG
CTTCTGATGTCTTCAATGAGGTTCTAGCAGTCAGATGCGTACAAGG
>1002050000_1 Heli.1-EL603302.1.5
GCACGAGGCGCAAGAGTAGTAGTAATACGAAAAATACTGTCATTGTTGTGTAATTGGAAAATTGGAAATTAGC
GACATGCTTCGCTACACTCGTTTGGTTCTTTGCTTACTTTTAGTGTTTTTAAGTGTTGATGTGATATTATCAA
AACGTATTTCTGGAAGACACTCTTATCCAAAACCTCATGGCGGTGGTCTCTCTGGTGG
GGGAAGTCATTCGGGAGGCCTATCAGGCGGTGGACATGGATATCCTTCGTCGGGAGGCCATATAACCCCTTCG
AATACTGGCGGAAGCCACGCATACCCCTCGCCTGGAGGAAGCCATACATATCCATCGTCTGGAGGAAGCCACA
CATATCCATCGCCTGGAGGAAGTCACACATATCCTTCTAATAACGGAGGTAGCCATGG
ATATCCACAGGGTAAAGGATTATCAGGTAACTCACCAAGCCAGAGTCATGCCACACACACTACTACAAATATT
CATCACCACTATAATTATAATCCTCCTCAACAAATAAGATATGCACCTGTACATGGTGGGCCTCCAGTGAGCT
ACCCAGTTTATCATGGTACCCCTCCAACTTATGTCTATCAGTATAAAGATTCTGGAAG
CAAGTATGGTACCCTACTTGCTGGCTTAGCCCTTCTTAATCTTGGCACTCTTGGAGTCAGTGCCTATGCTGCA
AGTCAGAGTCACAGCCATAGTCA
>1002050001_1 Heli.1-EC091315.1.5
TGACGGTAAAGCGTGGTAAGCCCCATATACTCACTTTTGCCACGCTTACGCGGGCAAGCTCTCCGGTGGGATG
AAACAAAAACTCGGTCTGGCCTGTACCCTGGTGGGCGAACCGAAAGTGTTGCTGCTCGATGAACCCGGCGTCG
GCGTTGACCCTATCTCACGGCGCGAACTGTGGCAGATGGTGCATGAGCTGGCGGGCGA
AGGGATGTTAATCCTCTGGAGTACCTCGTATCTCGACGAAGCCGAGCAGTGCCGTGACGTGTTACTGATGAAC
GAAGGCGAGTTGCTGTATCAGGGAGAACCAAAAGCCCTGACACAAACCATGGCCGGACGCAGCTTTCTGATGA
CCAGTCCACACGAGGGCAACCGCAAACTGTTGCAACGCGCCTTGAAACTGCCGCAGGT

CAGCGACGGCATGATTCAGGGGAAATCGGTACGTCTGATCCTCAAAAAAGAGGCCACACCAGACGATATTCGC
CATGCCGACGGGATGCCGGAAATCAACATCAACGAAACTACGCCGCGTTTTGAAGATGCGTTTATTGATTTGC
TGGGCGGTGCCGGAACCTCGGAATCGCCGCTGGGCGCAATATTACATACGGTAGAAGG
CACACCCGGCGAGACGGTGATCGAAGCGAAAGAACTGACCAAGAAATTTGGGGATTTTGCCGCCACCGATCAC
GTCAACTTTGCCGTTAAACGTGGGGAGATTTTTGGTTTGCTGGGGCCAAACGGCGCGGGTAAATCGACCACCT
TTAAGATGATGTGCGGTTTGCTGGTGCCGACTTCCGGCCAGGCGCTGGTGCTGGGGAT
GGATCTGAAAGAGAGTTCCGGTAAAGCGCGCCAGCATCTCGGCTATATGGCGCAAAAATTTTCGCTCTACGGT
AACCTGACGGTCGAACAGAATTTACGCTTTTTCTCTGGTGTGTATGGCTTACGCGGTCGGGCGCAGAACGAAG
AAATCTCCCGCATGAGCGAGGCGTTCGGCCTGAA
>1002050002_1 Heli.1-EL599133.1.5
CATAGTCGAGCATCGGCCGAGGCCCTGCGCAGTTGGTGTCGCGCGGCATTAGTTTTCCAGCCACGGAAAAATT
GTGTCGCGAGTGTCGGACACTATATACCTGTGCTAGAGCAGTGTCTGACTGTGTGTCATGGATTATGTGTTTG
GCTCCTAAGTGTCGTTGTGATTTTCTTTACTGCAGCACTCCAGGCCCACCCCTTCGCT
GCTGACTTCCTTAACACTTTCAACATGGAAGCGACACGACAAGTGTCGGCCCACTGGCTTTCAGCTATAGTTC
TTCTCTGCTCATTTTGTTCTTCATATTCTTTTAATGATAAGATTCACGAGCACATGACACCAGATGAGTTACG
GAGCGTCTTCCACGTAGAGCATCACAGCAACGTACCAAAATATCACTTAGTTCATCTC
ACTCACCATCTATCCAGGCGGAATATCGCAACATCACACTCATCTAATTCCCACTCCCGTACAACCAGCAGTG
GGAAAATACCCCACGAGAAGATCTCGAAACCAGCTCCATCGCTTCTAAATGATCAAATGTACATGAAAGCAAA
GGAACATATCAAGGATGTTGATATAATAGGTGATCTAAATAATACAGTGAATTTGCAA
TATAACGTGTCGAGTTCTAGTGATAGTGTAAGTGAAGGTGATAAGGAGCACGCGGTGGAAGATCAAGAAGATG
ATGTACATAGAATTGAGTTGGAAGCTTTTGGGAAACAGCTTAAGTTGGTACTGAGAAAGCAGGAGGGATTGGT
GAAGAAAGATGGACTGAAGATGTGGCGAGCGCTGACAAATGAGTCGCAGCCTCACGGT
GTTGAATACGAGGAGATGCAAACTGATGACAGCGAAGAGATTGGAGATCTTTACCATGATGACGAAAACGGGG
CCGCCCTTCTGATTAGAAGACAT
>1002050003_1 Heli.1-CV525799.2.5
CCCGGGGCTCGGGATACGACGAGGTTAAACTGATCCATTGTTATCGAAAGCGGTTTCAAGCCAATAATACGTG
AGCCATTAATGGCTGAAGAAGACAAAATAGCAGAATATGATGGCAATAGTGCAAATAGACGTGAGGATACAGA
CATAGAAGAAGAATATGAGGAAGGACCGCAATATATTAATGGAATCAATCACGCATAT
CCGAGTGATAAGATAACGGAAACTTTTGAGCCTATGTTTATTCCGTCTCCTCCAGATCATTTGCTGTCAAGTA
ACGATAGAACCAAAGAAATCTTTCCAAGCAACCATGCTAAAGAAGATAGACCACATCCGGTATACGTGAAAAC
TGAAAACGAACTAAATGCTTTGTTTGGAAAGAAAAATATGGAACGAGAAGCGCCTTCT
GATATGATAATGGAATCGGATAAAGTTAGTCCACAATACTTACCTCCCGTTACAAAGCTATCAAAAGAACATT
TACAAAAATTGTCATCTGATCAAACGTTTACAACTTACGATGGTAAAACTATATCCGGTGCAACTTTGACCAG
CGTGCCGGAAGTAAAATCAACAAAAATATTCTCTTCAAAATTACCAGCACATACTGAA
CTACTTCTAAAAACACCTCAATTTGGTCCTTTTAAAGGCGAAATCCCTCCAGTAGTAGCGGTACATGTTCAAA
CAGATACGCCAAATCCTTCGGATAAAAGAACTACTCGTTCGAAATTAGTGAATTCTTTACATGAACTCAAACG
TCTATAATAATTATTAGCGCAAACGTGTGAGAAGACGGCAAGTTAAGACATCCGGATT
GTTGNGAAGAGATCGTAAAGATAA
>1002050004_1 Heli.1-ES587018.1.5
TTTTAAATGAACAGACATTTTTATATTTATATTTAGTATAATAACATATATTCTAATAATATGAAAATTTATT
ATTATTATAATAATTGTGATTTATAATATAAATTATATAGGACTTATAAAGATCCTATACAACTAAATAAAAT
TTATATTTTTTCTTCATAATCGAACAACACGAAGATGTTGTTCGCTGTAAGCTGTCTT
CTTTTAATTATACAAGTACATGATGGCACCGCTCAAAACCAGGGTGAAAATTGTGTTATAAGTCATACTAAGT
CTCAAGGAGTTTGTACACAAGCGTCTAAATGCGTATCAGCCAGGCAGGATTTTCAAAATAACAATATTGCCCC
AACATTTTGTAAGCACTCTGGCAACGCATTGCTAGTTTGCTGTAGGGACGGGCTTTCG
ATCGTCAATACGGCGCCTGCATATGATCGCAGACCAGTTTGGAGTTCATTTAGCACGAATACAAACTCAAATA
CAAATAAACGTTTAAGTGAACGAAAATGTGAAGAATACAGCAAGAGTGTGATCCAAAGAGTGGATTTCATACC
TCTGCTGACGGAACCCGAAACAATGTCAATAACTGCACCTAAATGCGACTATACAGGC
ATCCAACTTATTGTGGGCGGAGAANATGCTACACAAGGAGAGTTTCCTCATATGGCCGCCATTGGTTGGATTG
ACTTCGATGATAAATACGCTTTCCAATGTGGAGGT
>1002050005_1 Heli.1-EL598160.1.5
GAAAACGATACAAAAATTGTTCATTACTATTGTTTAACATAAGACACAATGGCTGCTAAATTATTATCGTTGA
TTCTCATTGTCGCTGCAGCTGTAACGGCAGACGAGGAACCATCAGTTGCAAGATTATTAGTATCAAAGCAAGT
TCTAAACAAATATTTAGTAGAAAATATGGATATTTTAATTAAATACACACTCTATAAC

GTTGGAACAGCACCTGCTGTGGATGTAAAACTCGTAGACAATGGCTTCCACCCTGAAGTATTTACAGTGGTTG
GTGGACAGTTAACTGCTGAGATTGATAGAATAGCCCCTCAAACAAATGTGTCTCATGTAGTCACACTCAGATC
AAACAGATATGGCTACTTCAACTTTACATCTGCTGAAGTTAGATACAAAGCCAGTGAA
GATGCTGTTGAGAGCCAATTTTCAATCAGCAGCGCCCCAGGTGAAGGCACAATTGTTGCATTCAAAGACTATG
ACCGCAAGTTCTCGTCCCACATTTTAGATTGGGCAGCTTTTGCAGTCATGACCTTACCGTCCTTGGCCATCCC
CTTCGGCCTCTGGTACTCCTCAAAGAGTAAATATGAGAAGCTGGCGAAACCCAAAAAG
ACTCATTAGGTAGCTTAGGTTTAGACATGTCATTGCTAATGTTGTTCTTTATCAATTTTGTTTGATGAAAGTT
AACTTATTAAATGCCTGATTTAATTAAAAGAACACACAATTTGAATAAAATCAATATATGATTGATATCAGTT
ATCAGTTTTCTCAATTTAGTCAAATTAGTAGTAATTTTTCCTATATTTCTTCAAGTAT
GATTTTTATTTAAATAGAAATTTTGATTTTTGATAT
>1002050006_1 Heli.1-DT666018.3.5
GCACGAGGCTGCAGCCGTACTCTGCATCTGCAACAAGAAATACGTACTCTATATAAAATTTATAGAAAATAAA
ATTTGATCTGCAATTCTACAGTTGTCACTTTTCAAATATTATCACGTCGACCCTTTCTTCAGAAAATATTACT
GACATACTACAAAATAAATGTGACATAAAACTGTAACTGTACATATAAATCACCATTT
CATTATAAATATAAAATTAACAATGCCTGCAATTGAGAAGGTTACTTCTGATGAAGATGAAAATTACATTATA
TCAAGAGCCAAAGAAGCTCAGAAACAGAATATATATTCTGCTAAAGCTTGGATGTTAACTGCTAAAACTTTAT
TTCCCACTAATTTCAAAATTCAATTTGAAGCCTATTTAATGGAAAAGCAATCCGGAAA
TGTTCAAGAAGCTGCAGAATGTTTCAGTTCGCTAATGTTGGTACCACAAAATCTATCAAGTTTATTGTCTGAA
ATATTTGCTATTGCTAATGCTCTTAAATCAAATGAATTAAACTTTTTGAGTCAAATGTTTGATCATATTAGCC
CTGACATTCAGTTAACAATACTTAAAACAAGTGTTGAAAATAGTGATGACACTATGGA
ACATTGTAGATTATTAGTATTATTATTAAAAAAGTTTCCTCAATTAGGTGTTGATAGCCTTGTCAAAACTCTA
ATAAATGCAGAGAAATTTTTCTATGACAACCGATATGCTCGATTGCTTGTAGTAGAAACTTTACCTTTACTTA
ATTCACTAGAATCCCCTAGATT
>1002050007_1 Heli.1-EL597857.1.5
GCACGAGGGTTCGATCGCTTGGCCACTTTCAACGTCATCTTTTTAGACATCTAATGTCTGATTTTCGCATCCC
ACATGCTTAGGGGTAACCGTATTAAGAAGAAATCCAAGAATCCCCGAACAAGGAGAAAAGTACGAGTGAGAGT
TGCAAAGATAAATGTGAACTAGAAAGTGAGTGAGCGAAGTCGCGAGGTTTCGACGTCC
GGTTCGATCTGGATTTGACCAGTTAAAACCGATTTGCGGGCTGCTTTCCCTCTTGAATATTTTTGTGTATACT
GTGTTTTGTATAACAATATCTTAATTTTGTTATTTGGAACTTTATTTTGGTCAGTCCGTAACAAATCTTAGCG
AAAATGTCTGAGGAATCGTCAGCGGATCGCAATGTCGAGATATGGAAGATCAAGAAGC
TTATCAAGAGCTTGGAAATGGCTAGGGGGAATGGTACATCAATGATATCTCTCATAATACCACCCAAAGATCA
AATCTCCCGTGTATCTAAGATGTTGGCGGATGAATTTGGTACAGCATCCAATATTAAGTCACGTGTCAACCGT
CTGTCTGTACTAGGCGCTATAACTTCTGTACAACATCGACTCAAATTGTATACAAAAG
TACCGCCGAACGGCCTTGTAATATACTGCGGTACAATCGTAACGGAGGAGGGCAAGGAGAAGAAGGTCAATAT
TGACTTTGAACCATTCAAACCCATAAACACCTCTCTGTATCTGTGCGACAACAAGTTTCACACTGAAGCACTA
ACCGCTTTACTCGCGGATGATAATAAATTTGGTTTCATCGTCATGGACGGTAATGGCG
CCCTCTTCGGTACGCTACAGGGAAATACTCGTGAGGTTCTCCACAAGTTCACCGTCGATCTGCCCAAGAAGCA
CGGCCGCGGTGGTCAGTCGGCGCTGCGTTTTGCGCGTCTCCGTATGGAGAAGCGTCACAACTACGTGCGCAAA
GTGGCCGAAGTTGCCACCCAGCTGTTCATCAGCGCCGACCGCCCTAACGTGGCCGGAC
TCATTTTGGCCGGTTCGGCCGATTTCAAGACTGAACTGTCGCAGTCTGATATGTTTGATC
>1002050008_1 Heli.1-ES587725.1.5
GCACGAGCCCTAAACAACCAACCATGAACAGCAAGATTGTAGTATTCATCTGCATGGTAGGCGTAGCCTCCGC
TAGCATCGTAGCACCAGTACCAGTCGCCCGCGTTGATCCTCTCCCTCAGTACTCCTATGGCTACGACGTTCAA
GATGCCTTAACCGGTGATTACAAGGGCCACCAGGAACACAGGAATGGTGATATCGTCA
CAGGGTCCTACAGCGTTGTAGACCCTGACGGTACCAGAAGGATTGTGGACTATTCCGCTGACCCTCTTAACGG
ATTCAACGCTGTTGTCCGTCGCGAACCCCTTGTATTAGCTGCTCCTGCTAAGGTCATCGCTCCGGCTCCCGTC
GTAGCCCCTGTGGCTCCAGTCGTTGCTAGGGCACCCATCTTTGCACCAGCACCTGCAC
CCATCTTAGCGCCAAGATTACCATACTACGTGTAGATTAATTACTACAATTTTGTTGTGTTGATGTATATACC
TATTAAAAAATAAGGCTTTTTATAATTGTTATTTATGTTATTAAATGATGATTTCAAATAGAAAAAAAAAA
>1002050009_1 Heli.1-DT667639.3.5
GCACGAGGTGAAACTTAGGAGTCTTTATTGCAAAAAATTAAGGAACAAGGAGATCTTGTGAGAAAATTAAAAG
CTGCCAAGGAACCCAATGCAAAGATTCAAGAAGAGGTATCAAAGTTATTGGCACTGAAGGCCCAACTGAATAC
TGAAGATGCAGCTCCTCAGAAATTTACTCTTAAGACTCCAAAAGGGACAAGGGATTAC

AATCCACAGCAGATGACTATAAGAAACAATGTATTGCAAAAAATTGTTACAGTATTTAAAAGACATGGAGCTG
AATGTATTGATACTCCTGTATTTGAATTGAAGGATGTCTTGACAGGAAAATATGGTGAAGATTCTAAGCTTAT
TTATGACCTAAAAGATCAAGGTGGGGAAATTCTTTCTTTAAGATATGATTTAACTGTA
CCATTAGCCAGATACTTGGCGATGTATAAAATAAACAATTTGAAAAGATATCATATAGCTAAAGTATATAGAC
GAGACAACCCGGCTATGACAAGAGGAAGATACAGAGAGTTTTACCAATGTGATTTTGATATTGCTGGCCAATA
TGACCTTATGGTGCCTGATGCAGAATGCCTTAAAGTTGTTACTGAAATATTGGATGCC
TTAGACATAGGTAAATATATATTAAAGGTGAATCACAGGAGATTACTTGATGGCATATTTGAAGCTTGTGGTG
TTCCCGCAGACAAGTTCCGCTCCACTTGTTCCACTGTGGATAAACTTGATAAGTCGCCATGGGAAGAAGTTCG
TACGGAAATGATAAATGAGAAGGGGGTCTCACCAGATGCAGCTGACCGTATAGGAGAA
TACGTCAGGCTTAACGGCAGTATAGAATTGGCTGATAAACTACTTAAGGATGAGAAACTATGCAAGTCCAAGG
GAGCAGTGGAAGGATTGGAAGGAATTAAATTACTGTTAGATTATTGCGAACTATTAGGTATAAAGGATAAGAT
TCTATTTGATCTGAGCCTTGCCAGAGGCTTGGATTATTATACTGGTGTTATTTATGAA
GCTGTTCTTACACAACCTATAAAAATCGGCAATGAA
>1002050010_1 Heli.1-EL600998.1.5
GCACGAGGTTTTGCTTGGTCGGGAGTGAACGTAAATGTATTTCTATGTATTCTAATGTGGCAAATAGTTATTT
AGTTTAAAAATTAGTGTTTCAATACACTAATGGCTTGCTTAAATACACTTAAGTTAGAAATAAAGACTTTAGA
ACAAGTGTTTCCAAAAAACCATGAACGGTTTCAGATAATGTCAGCTAGTGTCGACGAA
CTGACCTGCAGATTCGTTGGCAAAAATGGAAAGAAATACGAAATCCACGCAAATATCACTGAGACGTACCCCA
ACACTCCTCCGGTGTGGTTCGCTGACAGCGAAGATCCCATTGTAACAAATGCGGTGCAAATCCTCACCAATAC
ACAAGGGAGGGATAATCACGTTATAAATCAGGTGGGTATACTATTGAGGGAGCTGTGC
AAACTACATGGTGTTCCCGAACCGCCAGATTTGGACTCGTTATCACTACCCCTCCACCCTGTACCGCAGCTAA
GAGTACCAAGCGTGACGTCAAACGGTGCGGAGTCCGGCACCGAGGAGGATGAGGAGATGGCGGCAGAGGAGGA
TGAATCTGAGGGAGAGGACGACTTGCCACTGGAAATGGTTGACGATGCTGGACGTAGC
AACAAGGACGACATGGAGACAGAGCACCTGGCGACGCTGGAGCGGCTCAGACAGAACCAGCGACAGGACTACC
TGTCGGGCAGCGTGTCGGGCCTCCTTGCAGGACAACGGACCGCTCAT
>1002050011_1 Heli.1-DT668500.3.5
GCACGAGGTATAAATTCATCTTTATTCGCTGTTGTTAACTGTTTGTAACATAAAAAGTTTCACAAAAATGGAT
ATAGTACATTTAGAATCTTCTGCTTATGCTAAGATAATACTACATGCAGCTAAATACCCATACTGTGCTGTAA
ATGGGGTACTTCTTGCCGATAACATATCAGCTCGGTCTGTAAATAAAAATCATGACTT
GGATATCCTGGATGCCGTGCCACTGTTCCATCATAGCCATTATTTATCCCCAATGGCTGAAGTTGCGTTGACA
CAGATTGAGACCATGGCACAAGCTGATAATAGAGTCATAGTAGGATATTATGCAGCTTGTGAGAATTTTAGAG
ACAATACAGTTGAGAAGTGCCCTGGTTTAAAAATTGCAGAAAAGATTGTGGAACATTT
TCCTTCAGCCGTTTTTATAGTTGTGGATCACTCCAGTGTAATGGACATGGCACAAATGCTGTATGCTTTAAAA
GTACACAAATACAGTGAGGGCAAGTGGAAACCAAAAGATGCATCTAGTGTTGTATTCCGAAGCCCCTATATAC
TAAAGACAGTAGATGAATTACTAAATAGAGGTGTTCAAAAGGAGTTAATAGATTTTGA
CAATTATTTGGATGATCTATCACAGGATTGGACTAACTTAGGTATAGAAAAACTTATAGCCAGTGTTAACGCA
TCTAACTCAATTGATGATGATTACGAAGATAGAGATTGAAAAAAATACCTAAATGTATAATAATTATCACATT
TTTTTTTTTT
>1002050012_1 Heli.1-EL598624.1.5
GCAGTACGGTCCGGAATCCGGGTCGACCACGCGTCCGACATTTGACAGTTAATGGCGTCTATGTGAAATAATT
TTCCCGCTAACGAAATACCAAAAATTAATCTGTAATTTCGTTTATAATCATTTTGTAAACATGACTATCGGAA
AGAATAATAAAATGCAACAGCATATAAATTACCGCGTAAGGGTAATTTTACAAGATTC
ACGTACATTTATTGGAACTTTTAAGGCTTTTGACAAACATATGAATTTAATTTTGGGTGACTGTGAAGAATTT
AGAAAAATCAAATCGAAAAACAGCAAAACTGCGGATCGTGAAGAAAAACGTACTCTAGGATTTGTGCTTCTTC
GTGGAGAAAACATTGTTTCTCTGACGATTGAAGGCCCACCCCCTCCAGAAGAGGGTTT
ACCCCGAGTTCCTCTGCCCGGCGCTATGGGTGGACCAGGTGCTGGTAGAGCAGCAGGACGCGGTGCAGGAATC
GGTGGTGCTCCACCAGGTCTACAGGGCCCTGCGAGAGGCGTTGGTGGACCTTCACAACAGCATATGGCTCCAG
GAGCCAGAGGTCAACTATCAGCACCTCCACAAATGCGAGGCGGACCCATGATGGGAGG
TCCACCTCCCGGCATGATGGGTGCACCACCAGGAATGGGAAGAGGTGGACCCGGAGGAAGAGGCCCTCCTGGA
ATGCGTCGCTATTAGTGTATTTGTGTAGATTTATTAAGTGAGTTAAACCACAAGTTTGTAAAGTTGTGATGTA
ATATTCCATTAACGTGTTTCACAAGTTATACTTTGTACTGATTGATTACTAAATATTG
TAATATTAAATGTAGTTTGTCATTTTATATTAAAAGGAAATTTACACTACAGGATGAGAGCTTTACAAATAAT
ATAAGATAAGTATTTTTCATGCAGAAGATAATTGAGCTCAGGCATTATGCCTTACTAAAAATATGTTTTCAAG
AGTATCTGGAATTGATTAAACTTTTAATACACCGAAAAAAAAAAAA

>1002050013_1 Heli.1-DT663605.3.5
GCACGAGGAATTGTGATTATGCTTGTGGTCTGTACTGGACTCATAAAATATCAATATTTATAGCGGTTTTTGT
AATATCAGAAATAATAATTGGAACAGGCAAATAAAAATACGCAAAAATGGCTTTAAGAACTTACGGCGACAAG
CCGATAAGTTTTCAGATAGAAGAAGGCGGTGATTTTTACTGTGTTGGATCTGAGGTTG
GTAATTATTTACGGCTATTCCGTGGTTCTCTTTATAAAAAGTACCCTGGCATGGCCAGGAGAACGTTGACTAA
TGAAGAAAGAAAACGTTTAGTCGATAATGGCTTAGGCCCTCACGTTCTGTCAAGCTCTGTATCTTTACTAAAA
GCTTCTGAAGTAGAAGATATAATTGAAGGAAATGATGAAAAATATAAAGCTGTATCAG
TCAGTCAGGAACTAGCGACACCCCGTGAAGGCAAAGGTAAAAAGCCACACAATCCATCATGGATGCCCGCTAT
GCCAAATTCATCTCATTTAGATGCTGTACCGCAAGCTACTCCTATTAGTAGAACTAGGGTTCATAATAAAAAG
GTGAGAACATTCCCATTATGTTTTGATGATACGGACATGACTGCCATGTTGGAAAATT
CATCACAGAAGCAAATACTAGTGCCAATAAGATTAGACATGGAGATAGAGGGGCAGAAATTAAGAGATACATT
CACTTGGAATAAGAATGAATCAATAATAACACCGGAACAATTCGCGGAGGTGCTGTGCGACGACCTGGAGCTG
AACACGAGCACCTTCATACCTGCCATAGCGACGTCCATCCGCCAGCAGATAGACGCGT
ACCCGAGCGAGCCGCCCGCCATACTGGAGGAGCAGAGCGACCAGCGCGTCATCATTAAGCTGAATATACACGT
TGGGAACACCTCCTTGGTGGATCAGGTTGAATGGGACATGGCGGAAAAAGAGAACAATCCTGAACAATTTGCG
ATGAAACTTTGCGCGGAACTGGGCCTCGGTGGTGAATTTGTTACGGGCATCGCTTACA
GTGTGAGAGGGCAGCTTAGCTGGCATCAAAGGACTTACGCCTTCAGCGAGGCCCCGTTGCCTGGTGTTGAGAC
ACCCTATCGTCAGCCATCCGAAG
>1002050014_1 Heli.1-EL596188.1.5
GCACGAGGTAAACCTTATGGACTAACTTAAAAATTAAAATTTGTTTTTGATTATAATAAACGCTCATTGTATA
CAACTTAGTTTAATGGGAAATTTGAACAGTAAGAACAGTGAATATAGAAATGATAAGAGATTAGTGCAGTCCA
CTCGCAACGGTCAAGCGTTTACGAGTCAGTTTAGTTTATCTCATTTCGTCGGCAACCT
CAGCGGGCGCTCCTTCGCCAGTGTTGTTAGCAGCCAGTCAGTATACAGCGCATCTCGGCCTTGGTCTCGGGTT
TCTAGACGAAGATGGAATGACTCAACTTTGAACAATCCACTTGATGCAAGTAAGACAGCTTGGCCAGTAGCAC
ATAAAGAGTCAATATTTCTGCCTGAATTCCCCATAACAACTGATTTATTGCAAAAAGA
TTTTGAAATAATTGAAACTGTAGCAAAAGGAGCTTTTGGAGAAGTGTACAAAGTAAAGAAGATCAGTGAAGAC
AAGGAATATGCACTAAAAGTTTTAAATAAATCCCAGATAGTGAGCGAAAATGCTGTACGTCAAGTGAAAGAGG
AGGCGCGTATACAAGCTGCGGTCGGACACCACTCGTGTATAGCTGGTTCTGTATCGAA
GTGGCAGACCAAGAAAAGACTGTATATTGTTTCAGAATACATTCCTGGAGGAGAATTGCTAGCTTTATTAGAT
AAGTACGGAAAGTTACCAGAAGAACTTGTGAAGATATTTGTTGCTGAGATTGCATTAGCTATAGATTTCCTGC
ACAACGCTGGCGTGATATATCGTGATCTCAAACCAGAAAATATACTCTTAGATTCAGA
CTATCACATTCAGTTAGTTGATTTTGGACTGTCCAAGTGGTTGTCCATANGCTCTCGCACCACCACACTTTGC
GGCACTCTCAAATATATGGTGTGGGACTTGACCCGTCGCTTACCTTGTAAATGGGACAG
>1002050015_1 Heli.1-EL598477.1.5
GCACGACGGTCGGATGCCTCTGAAGTAATGGATTTACTTTTAAAAACACATACAGAGGGAGAACAATTACCAG
CTGATGATCCACAGACATCTTACCTTATTTCAGCATGGTCTAGAATTTGCAGAATAATGGGCAAAAAATTCGC
TCAGTACCTGCCCATGGTTATGGAACCAGTAATGCGTACTGCTGCTATGAAACCAGAG
GTGGCTTTGTTAGACAATGATGATCTTGAGGTAATTGAGGGAGATCTTGACTGGCATTTTGTCACATTGGGTG
AACAGCAAAACTTTGGCATCAAAACTGCAGGTTTGGAAGACAAGGCCTCTGCCTGTGACATGTTAGTATGCTA
TGCGCGCGAACTTAAAGAGGCGTTTGCAGAATATGCTGAAGATGTAGTAAAACTAATG
GTGCCAATGCTCAAATTCTATTTCCACGACAATGTGCGTACGGCGGCCGCCGAGTCCCTGCCCTATCTGCTGG
AGTGTGCCCGCATTCGCGGCCCACAGTACATACAAGGAATGTGGGCATACATACTCCCAGAGCTGTTGAAAGC
CATCGACTCTGAACCGGAGCAAGAAGTGCAAGTGGAGCTACTCAACAGTTTAGCTAAA
TGTATAGAGTTGTTGGGTGGTGGATGCTTATCAGATGAAATGATGGCTGAAGTTCTTCGTATCTTAAAGAAAC
TTCTCACAGAACATTTCGAGCGTGCCACTGAACGTCGCCAGAAACGAGCTGATGAAGACTATGATGAGGTAGT
GGAAGAACAACTAGCAGACGAAGACAATGAAGATGTATATGGATTATCCCGTGTGGCC
GACGTTCTTCACGCCCTTATGTCTGCATACCACGAAAGTTTCTTCCCACACCTTGATTCACTACTACCACACC
TTGTACAGCTGCTAGGCCCTGGACGCCCCTATGCTGACCGACAATGGGCTATTTGCATTTTCGATGATGTCAT
TGAGTTTGGAGGTCCTGCATGTGTAAAATATCAAGACATATTTCTGGAACCTATGCTT
AACGGACTACGCGAACCTGAACCTGAAGTAAGACAAGCTGCCGCCTATGGATGTGGAGTACTCGCTCAGTTTG
GTGGACCACAGTTTGCGAGTGCTTGCGCTAGGGCAGTGCCATTATTGGCGGCCCTCATCGCAGAGCCTGACTC
CCGTTCTGTTGAAAATCTAAATGCTACCGAAAATGCCATCTCCGCTGTTACCAAGATT

ATTAAATATAACCACTCTCAAATTAATAGGGACGAAATTATTAGGCATTGGTTGACATGGCTACCAGTAGTTG
AAGATACAGAAGAAGCGCCTCATGTTTACTCTTTACTCTGTGAGTTGGTTGCAAGTGGTCACGAAGCTCTTGC
GACTCCAGATGCACCTCAAAGAGTTATAGCTACAATAGCAGAGGCTTTCTTACAAGAT
GCTGTTCCCGAAGATAATCCAGTTTTTGCGCAAATGGTGGCCCTCGTCAGACAAATACAAAGTAATAACGAGC
TGTTTAATTCATGTGTGATGCCGCTGAGCAATGACCATAAGGAAGCCCTCAAATTGGCGCTGTCCACCTAATG
CCAATGTAACTTGGTAACTCTATTTATTCATCAAACGCCCCTCGCTCTCGCTCGCACC
GCCTCCTTTGAC
>1002050016_1 Heli.1-EE743433.1.5
AGAAACCCGAGGCTGCAGAATCGGCCGAGGCGCGACAAGTCGGATTCAATCGTTTTAAAAGTGGTGTTCATTG
ATTAAATTAAAAGGAATAAAGATAACATGAGTGCCGTACCCGTGGGTCCCAAGCCCACCACTTTGGTGTGTCC
TTCCTGCAGATCCACTATCGTCAGCAGAGTGGACTTCAACTCTACCACAAGAACACAC
ATTATAGCCTGTCTTTTGTTTTTATTTTTATGTTGGCCTTGCATTTGCGTGCCTTATTGTATGGACTCGTGCA
AAAACGCCGACCACTATTGTCCGAGCTGTAACGCGTATTTAGGCACATACCAAGGATAACTTCCGTACTGGCA
CTTCCGTATAATATAATTTTATATCGATCTCCTATTTATCTATTTCAATTAGATAATG
AACTGAGATTAACAATAATTATTCTTTTATTATGGCACCTATGCTGTTTATTTATTGTTAATACCGTTTAAAT
ATTTTGTAATTATTATTTATACGTTCAATATGTTTTTTGTTTATTATGTTATAGTATACGAGTATAGATCTTA
CTACTAAAGTATTTATATCTATACTTTATTTACTTTTATTACTATTTATATCTATATT
TAAATTAAATCTTAACCCATCGGTATCTGTACATGGAACTTGTATAAGAAAAAATGTTATTTGTGGCATTTAA
TAACGTAATATAATCAAAAACAATGATTTTTTTTGTCTCTTCGTTTGTTTACGCTAATATCAGAAACAGCTTA
ACATGGTATCCGGTATAAAAAATTATAAGACTGTAAGTGCGCCTGTTGTAGAGGTGAA
CCTCAAATAATGTATAAATGAAATAATTATTTCATTTCTTCATTTAGAGAACAATCTTCTTAACATTGCGTTA
ATAAGTAAAGA
>1002050017_1 Heli.1-ES586365.1.5
CTTTGTCAAAGCAAACAAAGAAAACAAACAACCCTTAAAAATGTTCAAACAGGTAATCTTCGCTGCCCTCCTC
GCCGTCGTGGCTGCTAAGCCTGGCATCCACTCCATAGCGTACTCATCACCTATTGTTGCTGCACCTGCTGTAG
CCGCATATAGTGCTCCTGCTGTTGCTGCATACGCTGCACCGGTAGCTGCTTATAGCGC
AGCATACAGTGCACCTCTAGCTGCAGCTTATTCTGCACCCGTTGCTGCTGCCTACTCTGCGTACTCGCCCTTT
GCGTACTCCGCTCCAGTAGTTGCTGCAGCTTACAAAGCTCCAGTTTTCCTTAAGTGAGAAGGTGTGAAGATCT
CTTTGTGATATGAACATTTAATTAAACGACTGTA
>1002050018_1 Heli.1-DT667069.3.5
AAAACTGTGTTTAAGACTGTTTTTGGACATAAATAGACATGGGACGTTGTTCAAAACGGCGCAATAACTCAGA
ACTACGATGACTAAAAGTTTTAGTTGGGGAATAAAAAGTTGTCCGAATGCACTCGGCTACAAAAAAAATTATG
TGCTGGTAATAGTGATGGCCTGATGGGATCGCAGTGCGACTGTTCGTGAACCGTGTAG
CTAGTGATGTGCTGTGATATAAATTATAAGCAATCGCGTAGATGATTGCCATTTTTGTGTGTGATAATATTAG
TTTATTAGTTACCAAAGATTCCTGATGATCTTGTACATTAGTTTGTGCTTCATATTGGTAGTTTTAGCGGACC
CCTTGCCCGGCAAACATGTACCACTCGAAAGCTACGAGAGTGAATTGGAGCGAGAGCA
CGCGCTGCCCACGTCCGTCCCGAACGCAGATATACTCAAGACTAACAACAATCCCACCTATCATAAGCCCAGG
CCTCCAGCCCACCACGGCGCTGCGTTACTGGCGGGTGTGGGCGCGGTGCCGTCGTACGCGCCGCCCGGACGAG
CATTCTTCACGCCGCCCTTGCCGCCTGAGTATCGAAACCCCTTCGCTGATAAGCCGAC
ACTCAGAGGCACAAACACAGATGGGAACAGTTATCTCAATCGGCGGCCGATACCACCTCCGTCGCTCGGACCT
GGACATGAGAGGATACCGATTCGACCTCCGGGACAGGAAACATCCTCCCCACAAGTGCCGGCGCCACCGCCCT
TACCGCCGCCGGGAATTGAACCACAAAAGAAAAAAGCTCTTAACACGCCGAGCCATAA
GCCTGACGAACTAGACGACAAGCACGGTACGGACCAAGCGAACTTCACAGACTTTGTGCCAAATTCTCTTAAC
ATACCAACTATATCAAGAATACTATCAGGATCAAATGGAAGAAAAGAAGATATTCCCGACGTACTTCTCAGGA
CAGTCACGGCTAAACCTCAACATAATCAAGAAAAGACTTCTCGAAGTGACACGCATGC
AACAAAAGATGCTGTAACGCTAAGTGATTCGAGAGATTCTTCTACAGAAGGTGTTTTAGCCGTACTAGACCCT
GAAATGAACAATTTAGATAGACCACTCACTGTAGAACAAAGCGAAACGAATACTCGAAGAAATCTACAATATG
CTACAAACAAGCAGACAGACAAGAATGATTCTAGAAAAGACGAAACTTATACACCGGC
TACTACTGCCGGATTCGAACTGAGTGACCCAGAAATGTCTACAGAAAGATATCACAATTTGGCTAACGTCAAT
TTAGATAAGAAAGTGTCTCACGATAGTAAAAATGTAGGTCCAATACAAGGTCCGGATGAAACTTGGCCAGTTT
TGTGGAAATGCCATAACTGCTTAATGGTCACCATGTTCACTGCTCTCGCCGTATACTC
GATTGTTAGAATTATCCGCTATGATAAATTTACTCA
>1002050019_1 Heli.1-EC091423.1.5

CTTACCAGAGCTTAGCTCACCGAAAATATCATCTGCCTCTGCGGCCGCTTTTGCTGCAGCGGCTTTATCAGCT
GCAGCTTTTGCCGCTGCCGCTTTTTTGTCTGCGGCGGCTTTTTCTGCCGCTGCTTTCTTATCAGCTGCTGCCT
TTTCAGCAGCCGCTTTTTCTGCTGCTTTTTTGTCGGCTGCAGCTTTCTCTGCTGCCGC
TTTCTTATCAGCTGCAGCCTTTTCAGCAGCCGCTTTCTTCTCAGCTTCTGCTTTGGCTTTTTCAGCAGCTTCA
GTTGCCGCTTTCTTTCTTGCTTCAGCTGCAGCTGCTTCTGCCGCTTCCGCTTTCTTCTTCAGTGCCGCAGCGG
CTGCCTCGGCTTTTTTCTGCGCTTCGGCTGCGGCTTTGGCGGCTTCTGCTTCTGCTTT
TTTCTTTGCGTCTGCAGCCGCTTTCTTCGCTGCTTCTTCCGCAGCTTTAGCATCTGCTTCGGCCTTCGCTTTA
GCATCTGCCGCCGCTTTCGCTGCCGCCTCTTCAGCTTGCTTCTGCTTTAACTCGGCCTGTTTTGCGGCTTCTT
CAGCCTGCTTTTTCTGCTCCTGAGCCGCTAACCGCTCTTTCTCAAGTTGCTTCAGGCG
TTCCTGTTCAGCCGCTTGTTTCTCACGCAGTTCTTCAGCAGCCTGCTGTTCCTTCATCTTGCGCTGTTCATCA
GAACGCTTCGCGCTTGATTCCTGGCTTTGCATGCGTTTGTACTGCTCAACTACCGCACCTGAATCAACCATGA
CAGCGTCGATGGACGAACCACCGCCGCCTCCGGCTGAAGCTTCTATATTCTCATCGAA
CGAACTCCAGATCAGCGCCGCAAATAAGATGACATGCAGCACTGCTGAAATAATTATCGCCCGCTTGAGCTTG
TCGTTTTGTTCGGTTGCCTTTGACACTCTCGGTTTCCAAAAACTGTTCGCCTGTTACCCGCTCTCTTTCAAGC
AAGGGAAACGCAGATGTTTAGATAGGCTGCGTCATTAAACCAACCGATTTCACACCCG
CACTATGTAACAAGTTCAGTGCTTTAATTATTTCATCGTAAGGCACATCTTTTGCGCCACCGATCAGAAAGAC
CGTTTTCGGGTTGGCCTTGAAACGGCTGGACACTTCCGCCACCACCTGCTCTGGTGGTAAACGCTCCAGGCGA
TCTTTCTCAA
>1002050020_1 Heli.1-DT666520.3.5
TGCGGTACGGTCCGGAATTCCGGGTCGACCACGCGTCCGCGGGAAACTTGTTTTTTAGTTCTTATTATTGAAA
GTGACATATAAAAGCTATAGAAAAAGAATGAAGCTCTTTTGTATTTGGGCTATAGCCCTAACAACAGTGTACG
CGAGGACGGCGACCAAACCACAGAATATATCACCAGTGATAACGTCTTTATCTGATAA
GGATGCCAAGCTGAAAGCAAAAGTTCATCCAGCATTTGCAAATGCAGGACAAAAAGCTGGACTTGAAATTTGG
CGTATTAAGGACTTCGAGCCAGAAGCTGTTCCGAAGGCTGACATCGGAAAATTTTACAAAGGGGATTCTTACA
TCGTTCTAAAGACTACATCAGATAAGAAAAACAACCTATCCTGGGACATCCACTATTG
GATAGGAGCTGAGACCAGCCAGGATGAGGCCGGAGCAGCCGCTATACTGACTGTTGGCTTAGATGACAAGTTT
GACGGAAAAGCTGTGCAACATAGAGAAAGCATGGGTTATGAGAGCTCGCAATTTTTGGGTTATTTCCCTGGAA
AAGCTGTACGCTATGTAGATGGTGGTAACGCATCTGGCTTCAATCACGTCCAAACAAA
TCCCGGTGCTGAAAAACGACTTTTCCAAGTTAAAGGAAAGAAGAACATTAGGGTGAGACAGGTGGACCCTCTC
ATTTCTTCAATGAACAAAGGCGATGTTTTCATTTTGGACATAAACAGTGACATATTTGTTTACGTCGGAGAGA
AAGCGAAGAATGTGGAGAAATTGAAGGCTATCTCTGTGGCAAATCA
>1002050021_1 Heli.1-ES588177.1.5
TTTATATGAAAGTTTAAGCTATGATTTCAATGAATTTAATCCCGCTTGTTTGCTTACTGGGCGCCGCTTTAGC
CCAAAGACTGACCGACGTGAGTGACCCAAGCTACATCAGACGAGGCGGGGGCTATGGACTGCTGAGACCAGGA
TATAACAACTATGGTAGCGGTTACGGAGGATATGGGAGTGGATATGAGGGCTATGGGA
CAGGATACGGTGGTTATCCTGGACAATATCCTAATTACGGAGGATACGGTGGTTATCAGGGATTTGGATCGGG
ATACCAAGGAAATGGGGGCTACCGCCCTGGATACAGGCCTGGCTACGACTACCGCCCTGGATACGATTACCAG
GGATCAAATTATCCCGGGTACAGACCGGGTTATCAATCCAGACCTGGATACGATCGCT
ATCCGGGATACAGTAGTAATTATGGATATGACGGTTATGATAACTTTAGATCGTTGACGCGTAGAGCTGGCGA
AGTGAAGAAAGATAGTATCTGAGGAAAAGTTTAGAATTAACTTATAAATATGTTATTTAATTTTATTTTTATA
AGATTATTTTTGGTATAGAAGAATATTTTGTCTAATATATATGTATATGTTCCAGTTT
CCTAAACTACTGTTCTATTAATTTAACAAATTTTAAACGATTATTGCTGTTGTGTTAATACAAAACAAATTTT
AAACAATTATAAAAAACGTATCGGCAAGATCTACTTATTTTTATCAGATACGTATATTTATTTCTGTATATTT
AAGTTAATAGTATTTAAATTTCTTCTAATTGTAATGAATATATTACGTAATTAAGATT
TTCATTTAAGATTCATGACAATTTATTATAGCTTTTTCTCAATTAGTTGTATATTAGGAATTAAAAAGCTTT
>1002050022_1 Heli.1-EL604403.1.5
GCACGAGGGGTGTCTGCTATGCATTTTCATATTAAATTATTTTTTATTTCTAGTTATGCTTAAGTAATATGTA
CAGTTGTAAATTATAGTATTGTAAACAAATTTCGAGAATTTATGAATCATCCTTAGGATTAAAAAGTAAACAG
TTATATTTATAACCTATATTAGTCATCTATTGAAGATAAATGAAGAGGTGACAAACGT
TGTCTGGAATTGCATTAGTATAATATATTAGAAATTTATATTAACAATGCTACAATTAAGTACCTACTTTATC
GCGAAAGTTTATACGCGTATTATATTTTGTATTCAATCGTAGATCCTATACAAGTTTAAAAGGGAATCCGTTT
CCTATACAACCATTTAGGACAGTTCTCTATATATATAATTAGTTGATAAGAGTATTAT

ATCCAAAGATGGAGAGTATCTACGGTTGGTACAAGGATCTCATGGACAACAGAGGCGATCCACGCGTGAAGGA
TTGGCCTATGATGTCTTCACTGTGGCCAACGTTATTCGCGTGTATTTGCTACGCAATATGCGCAAGGAAAATT
GGTCCAGCGCTGATGGCCAACCGGAAACCCTTCGAGCTGAGAAATATACTCATAGTGT
ATAATCTAGCGCAAACAATATTCAGTGCCTGGATATTTTACGAGTATTTGGCAAGTGGTTGGTGGGGTCACTA
CGACTTTAGGTGTCAACTCGTAGATTATTCTCGAAGCCCTAAAGCGATGAGGATGGCGAACACTTGTTGGTGG
TATTACTTCAGCAAGTTTACGGAATTTTTCGATACACTGTTCTTTGTGCTGCGAAAGA
AGAACGAGCATGTGTCAACCCTCCACGTAATACATCATGGAATTATGCCAATGTCCGTCTGGTTTGGACTTAA
ATTTGCTCCAGGTGGTCACAGTACCTTCTTCGCTC
>1002050023_1 Heli.1-DT665243.3.5
GCACGAGGGTAGGCACGAGGATTATTCAGGATTATTTGTAATAACATAAAATAAATTGAAATATTAAGTAATT
TGTTAATTTCATCAGTTCTTTTTAAACATAACAATTGAAAATGGTAGACAGAATTGATATGGCTTTAGACGAT
ATTATAAAAGCAAGTAAAAAAGGAAGAACAGGAGGCGGTGGAGCTGGAAGAAAATTTG
ACGCAAAAAAATCTAGTCGCGGTGGAGGTGGTTTTCGAAATGCCCGCTCAGGTGCTGTGCTACGAGGTCGTAA
CCGCGGAGGGGTATCAAAACCTGCTAATTACACAAGGGGTGATGTAAACAGCACATGGAAACATGACATGTTC
AATGACTTCAATGATAGGAAGATACAGAGGAGTGCACCAATCACAACTGGGCCAACCA
AACTCCTTGTTTCAAACTTGGATTTTGGTGTCTCAGACTCTGATATTCAGGAGCTGTTCTCAGAGTTTGGCAT
TTTAAAAAGTGCAGCAGTGCACTATGACAGATCAGGAAGGTCATTAGGTACAGCAGATGTAGTTTTTGAAAGA
AGAGCAGATGCATTGAAAGCAATGAAACAATATAATGGGGTACCACTGGATGGGCGTG
CAATGAATATACAATTAGCAACTTCTGAAATTAGTACATTTAGAAATGAAGAGAGAAGTAGGCCAGTAGTAGG
AGGACCAGTTCGAAGAAATTCAAATAGAGGTGGAGGAAATAGACCTCAAACTGGAGGTCCTCGTGGAGGAAGT
GGTGCAAGAAAAGGTGGTCGTG
>1002050024_1 Heli.1-DT668567.3.5
GCACGAGGCAGGCATTGAGAATAATATTTTAAGTACTTATTACCTATTATTCGGCGATTCCTGTCCTTTATAA
AGTAAATTCCTGATTGTTGATGGAGTATCTGTTCAGTATTAGGAAGCAAATATAATAGTGAAATGTGCTTAAG
AATTGAAATAATAGCATTTTTTTAAATTACTTATTATTTTATGAAAGTACCCATGCAA
GATGCTCTACTTAAACATTTTGAAGCAGTCAACTATAGTTCACTTATGTTTTGCTATATCATATTTTACTTCA
GGCTTGATATTAAGCCTTATCCAGGCAATATTATTTTCTAGCCTTAAGCCTTTCAATAAAGTGTTATATAGAA
AAATAAACTATTACCTATCATACTCATTTTATTGTCAATTAGTGTTTATGTCTGAATG
GTGGTCAGGGACAAAATTGTCAATATATATAAAAAAAGATGAATATGATAAGTATTATGGAAAGGAACATGGC
TATCTAATTATGAACCATAGTTATGAAATAGACTGGTTAATGGGTTGGCATTTTTGTGATGGCATACAAGTTT
TAGGAAATTGCAAAGCCTACGCGAAGAAATCCATACAGTATTTACCACCTATAGGTTG
GATGTGGAAATTTTCAGAATTTGTATTTTTAGAAAGATCATTTGACAAAGATAAGGAAATAATCAAAAGGCAG
ATATCAGAAATTTGTGATTATCC
>1002050025_1 Heli.1-DT663209.3.5
GCACGAGGGGGCCCCTTGTCTAATTAATCAAATCTAATGACAAAGTGAAAATAACGAAAAATGAAGTGTCTTT
GCCTCCTCCTGGCTTCCGCGGCCCTCGTACACAGTTACGCTATCCAGGATAATGAGATCGAGACCTCTCGATT
GAACACAAGTGACGATCTTTTGAACAGTGTGATTAGTGATTGTTTTGAGGCTGAAAAC
CCAACGTCATGCCTTAAAGTGAAAGTCCTATCGTTCTTAGATACTAAGCTCGGAGTGACATCGGAGTCTGCGA
GAGCTTTTGATGACAAGAACATCGACAAAGTAATCTTCGATCGCGTAGGAAGAGTTCTAAACGACAATGAGAT
CAGGGTACAGTTGCCCGAATTCATCTTCCAGAATGCCGAAATCTCGTACCGCGCCGAT
AGAGGATTTGATGTAGATTTTCCTGAAACCAACGCTGAAAATGGTGAAGCTCGCGGCATTTTAAAGAAGAAAC
TTTTACTGCCAGTTCTTCTCCTTCTCAAGTTGAAAATGAAGGCTCTTATGCCTATCCTCTTAGCTATCGTCGG
TATCAAGGCTGTGAAGGCACTGATTCTTAGCAAGCTGGCCATTACTCTTGTTGTTGGA
TTCCTCGTCTACAACTTAATTATGAAGAAAGGAGCTATGCCAATGATGATGGCTCCCACAGAAGCACCTGCAC
CCGCTCCTCAGTACGGTCCACCATCTTCTCAATATGGACCTGCTTCCACAGCTGCTCCACAAGACTCCTACAG
TGCGCAATGGGAACCTGCCGGTTCTGGACCTTACGCTAGGGTATGGGATCCCACTCAA
CTGGGCTACAGCAGCTACTACCCC
>1002050026_1 Heli.1-EL600977.1.5
GCACGACGATGTAGTCATGTCATTGAGTGAATTTTGTTTATATTATTTTCTTATATCAATATAAAAAAAATAC
CTAAACTTTATTACAAATAACATATGATCTTAAACTAAAGCTCATCATGGATAAACACAACAGTTCTGAATTA
TTAAATTCAGATTTTTCATTATCGAAGAATGCTAGTTCCGATTCATTGGATTCAGATT
CTAAATCAAGTTCATTGAATTCCAAACACGGACAAGATTCCACTCATGTTCTGGGAGATATTATGTTATTGGA
TGGGGAAAGGGTGATGGGAGTTGCTCGAGATGTTACATACTTATGTCCCTACAGTGGCCCATCTCGTGGAGTG
CTTAAAGTTACAAATTATCAACTACATTTCCGTCCAACGGAAGCAACATCTCTTCAGA

CAACTCTTAGTGTGCCTCTTGGAGTAGTTTCTCGTATTGAAAAGGTAGGAGGAGCTTCATCTAAAGGGGAGAA
TTCATATGGTATTGAAGTTTTTTGTAAGGATATGAGAAATTTACGTTTTGCTCACAAACAAGAAAATCATTCC
CGACGGGGAATATTTGAAAAGTTGCAACAACTAGCATTCCCGTCATCACATAGACAGC
CCTTATTTGCTTTTAGTTACTCGGAGAGTTTTCCTGATGATGGATGGAATGTATATGAACCTATCGCTGAATT
AAAAAGAATGGGTGTGAACAATGATATGTGGAGGA
>1002050027_1 Heli.1-EL596921.1.5
GCACGAGGGGACAGATGACATTGACTGTCAAAAGCATACTGAATATAAAAACTAAGAACATGATAATTACGAA
CTTGAGTCTGCAAATAAAACAAATATGACTGTGTTAAACTCTTAATATATAAATCTATTTGGACTAAAATATG
GCGCGTCACAGAAATGTTAGAAATCGAAATTATTCGGATGAATACGACTACGATGATG
TGTATGGACATTCTGTTGAAGAAGACAGCTGCTTATCGCCAAGCGATGCAGCTCAGTGGTTGTATGATCGTTC
TGGTAACCAAACTGCTATATCAAATTTTTTAGACAGTCGTAACGATATTCAAGAAGAGAATGAAGATCCCGAT
ACCACAGTTCCAGTTTTGGAACGACGGGAGTCTATGGATCTACGCGGCCTAAATTTAA
GTGAAGATGATGAAGCAAAACTGATGTCATGTCTCGATGAGCTAAGAAATATTTTAGGAGATACAATACCAGA
AAATATTTTAGTGCAATCTGTTTTGAATCATAACTTTAATTACAACAATGCATTAGATGAAATATTGAATAAT
AATAAAGGTAAAAATTCAGAGGTAAGTAGTACCAAAACAGCATTACAGCCTGTACCTG
TAATAGTTGAGCCTAGTAAAGTTCCCAATGTAGTAGTTGACACTACACCTAAAGTTATTACCACAAAAGTAGA
ACCAAGTAAAATTGGGGTTATCAAAGGCTTTAAATTAGGAGATGAGAATCTTAGAAGTCAGCCTGGAACACCA
AGAGATCAGTCTCCAGCCTCTAGAGAAAGACTCC
>1002050028_1 Heli.1-DT668871.3.5
CCTCGTGCCGATTCGGCTCTTAGGATTTGGAAGGGAGAGATCGGAGTTGTGTCGAATAATCTGAAATAATTTC
AGAAATCTTTATAGTTATTAATGTATTAAGAGTTATTTTAAAATTTTAGTGTTATTTTTCGATAAGATATACT
AATATTTTTCGAGTTTTGTCCTATTTATGAGTAACTTAAAATTAGTTGTATAAAATCA
GTAAAGGACGTTCGTTGGGCGTAAAAAGAAAATGGCGTGTGCTACACTTAAAAGAAATTTGGATTGGGATGGC
ATGGCACAGATGCCTTCTAAAAGACGAAGATGTGCTCCACTTACTCCAAGCACAAGCACAAGTCCTGGTTTAA
AAATGGCTGAAGCTAAAAGTTGTGTATTTGGAGAGACCGTTACTGCGTCTGCAAAAAT
GACCCCAGAGCGCATGATACAAGAAATATACGATCAGATAAAGCGGCTTCAGCGTCGTCGTCAACTGCGGATG
GCGTCGGGCGTCACCGCGTCGTGCTCGTCGTCCAGCGGCAGCGAGGGCGACACCTCCCCGTCACAGCGCTCCA
CGCATTCAAGGAAGAAAGCGCAGCACAGGGCCTTGTTCACTTTTAAACAGGTGCGTAT
GATATGTGAGCGTCTGCTGCGCGATCAGGAGGCGGCGTTGCGCGCCGAGTACGAGCTGGTGCTCAACAACAAG
CTGTCCGAGCAGTACGAGGCTTTTGTGCGCTTTAACATTGATCAGGTGCAGCGCCGGCCGCCTCCCAACACGT
GCATGCCGCTGGGCATGGAGGCTGAGCACCACAT
>1002050029_1 Heli.1-DT665766.3.5
GATGAAGACGAAGCAGATGAAATCCGAATCAGAACAAAATAAAGCTAGAAAAAGATGAGCAGAATAGACCTAA
GCGGATAATATTCTAAGCAGATAAAGACCAAACCAGATGAAAACATTAGTAGACGGCCAGCAGATAAAACACA
AATAGATGAACAGCAGATGAAGATACAAATAGATGAACACTTAAATAAGTTAAGAACT
AATTACATAATGACCACCCTAGCAAAAGATCGAAGCTAATGAAGACTGAAGCAGATGAAGATTGAAGAAGATG
AAGACTGAAGCTGATGACGACTGAAGTAGATGAAGACCGAAGCAGATAACGACTGAAGCAGATGACGACTGAA
GTTGATGACGACTGAAGTAGATGAAGACTGAAGCAGATGAAGACTAAACAAATGAAGA
CCGAAGCAGATGAAGACCCAAGCAGATGAAGACCGAAACATATGAAGATTGAAGCAGGTGAAGACCGAAGCAG
ATGAAGACCGAACGAAGCAGATGAAGACCGAAGCAGATGAAGACCGAAGCAGATGAAGACCCAGGCAGATGAC
CGAAGCAGATGATGAAGACCCAGGCAGATGAAGACCCAGGCAGATGAAGACCCAGGCA
GAAGATTGAAGCAGGTGAAGACCGAAGCGGATGAAGAAAACTGAAGCAAGATGAAGACCGAAGCAGATGAAGA
CCGAAGCAGATGAAGACCGAAGCAGATGAAGACGAAGCAGACGAAGACCGAAGGAGATGAAGACAAAGCACAT
GAAGACAACAGACGAAGATTGAAGCAGGAAAACCGAAGCAGATGAAGACCCAAGCAGT
AGAATACCCAGGAAAATGAAGACCCAGGCAGTTAAAGACCCAGGCAGATGAAGACCCAGTCAGATGAAGACCG
AAGAAGATGAAAACTGAAGCAGATGAAGACCGAAGCAGATGGAGACA
>1002050030_1 Heli.1-EL603801.1.5
GCACGAGGGATGAACAATTCGAATTTACACTAAATTGCACCATATGTTAATATAATTTTTAATTTAATTAAAC
TAACGTCAAAAACGATGTCTTAATGTACTTGTAATATTATATAAATGCATGAATTGACAAGTCTTAAAAGCGA
AAATGTCGAAAATCAAAAAGTTAACAGGCTCATTCCGCTATACGCAGTGGGATCCTTG
TCTTATTATATCACAAATAGTGGCAATGCAGTTTGTTTTGTATCTAACACTTAGTCTTTTGATGGCTATAATG
CAAGATTTAACTTCTTCGACTCGAACTTTGGATCACTTGTTTGAATACCATGAAATTCATGTGAAAGATAATG
AAGGTAGAGCTGTTATATTAGCTTTTGTGTTAAATTCTGTTGTTGGAGCCTTCATGTT

GTGGATGTTGGTGGGAAGGACCAAATTGTGCTTGGACTTTAGTTGTACATTCTACGTCATACACTTACTTTTT
TGCTGGGTGTACAACGGTCGGTTTCCAACTACATTTTCATGGTGGGCTTTAAATGTTGCCTGTACTGCAATAA
CTTGTGTTTCAGCAGAATTTCTGTGTTTGAGAACAGAATTACAGGCCATTCCATTAAA
TATTGGTGCTAAAGTGGACTTATGAAAGTTAAAAGTTTCATCTATTATTGCTTAGTGTTTATTTTTTGACGGT
GAATCAAAATGTGAAGTGATTCTCGTTACATTTATTATATAATATACATTTATTATATAATATAATGTACAGT
TGACAAGTTGTGGTCGTCCTGAAGTAAGCTGTAGACAGATAGTTGCACAGATTTCATT
TTTAGGATTTTTCTTAAATTGATA
>1002050031_1 Heli.1-DT667464.3.5
GCACGAGGCTGACCCCTGACAAAATGCGGGTACTACTATGCACGTTGGCAGTCTTGGCTGTATTTACATCAGT
GAAATCGGACAGCAAAGCGCGTATAGTATGCTACTTCAGCAACTGGGCGGTGTACCGGCCGGGCGTCGGGAGG
TATGGCATCGAGGACATACCAGTAGACATGTGCACTCACATCATATATTCTTTCATTG
GAGTTACAGAGAAATCGAGTGAGGTTCTTGTCATTGATCCAGAGCTGGACGTTGAGAAGAGTGGGTTCAGTAA
CTTTACGTCTCTGAAGAAATCTCATCCAAACGTCAAATTCTTGGTGGCAGTGGGAGGTTGGGCCGAGGGAGGG
TCGAAATACTCGCACATGGTGGCTCAAAAGTCTTCCAGAACGTCCTTTATCAGAAGTG
TCGTTGATTTCCTCAACAAATACAACTTCGATGGTCTAGACCTCGACTGGGAATATCCTGGAGCAGCTGACCG
CGGAGGTTCCTTTTCTGATAAGGACAGATTCTTATACCTGGTCCAAGAGCTAAGAAGAGCCTTTATCAGAGCT
GGTAAGGGATGGGAACTCACTGCTGCCGTACCTTTGGCCAATTTCCGATTGATGGAGG
GATACCATGTGCCTGATCTTTGCCAGGAGTTAGATGCTATCCACGTAATGTCCTATGACCTGCGAGGGAACTG
GGCGGGCTTCGCTGACGTTCACTCTCCTTTGTACAAGCGTCCACACGACCAGTGGGCGTATGAAAAACTTAAT
GTGAACGATGGTCTCAATCTATGGGAAGAAAAGGGTTGTCCGTCAAACAAACTCGTCG
TGGGTGTTCCTTTCTACGGAAGATCCTTCACGCTGTCAGCTGGAAACACTAACTACGATCTTGGAACGTATAT
AAATAAGGAAGCTGGTGGTGGCAACCCTGCACCTTACACAAATGCTACAGGATTTTGGTCTTACTATGAGATT
TGCATGGATGTTGACAAGCCAAAATCTGGCTGGACCAAGAAATGGGATGACCATGGCA
AGTGTCCTTACGCGTACAAAGGAACCCAATGGGTTG
>1002050032_1 Heli.1-EL598332.1.5
GCACGAGGTATAGTGACACAGTCACCGCGATATATCTTGAGTATTCTTTGTGAAACTTACTAGTCAGAAATAT
GTCTGAAGCTTTCTTTGATGAATACGACCACTACAACTTCGACCACGACAAGCACATCTTCTCCAGCCACAGC
GGCAAACAGCGCACCAAAAAGGAAGTAAGCGAACACACCAACCACTTCGATCCATCGG
GCCATTCCAGAAAGATCGTCACCAAGCTTGCCAACACAGAAAATAATAAGAAAGGGGCTAACAAACATTAAGT
TGGCTTATAAACAATAGATGACTATGGCGTGTAAACGGAATATTGAGTTTTTGCCGACGGAACGGAGGACGTT
ATGGCGAATACCCGAAGAAAGCACGGGTCGCTCGTCTCTGAGATCAGTGGAACAGCCG
AGAAGGTTTTTGCTAACGCAGAGTTTTTACTATGCACACCACAATGCTAGAACACGGTGGCGCAGCCGGCGCG
CCTCGTGAGTCGTGACCCACCGGTGCATGCCGATGGATATCTATTTTGAAGAACACCGTTCCAACGACTGATC
TCATCTCTGCTGGGCTTGTTGTAAAGCTCAATGTCGCTGTGTTATTAACTTCTAGGTA
GTTATTATGTTGTTGTATCTGTACGTGGTACGTTCATGACTGTAATAATTTAAGATAAATAAAGTTAATTTA
>1002050033_1 Heli.1-ES585783.1.5
TAACTCCAGTCTAGTGACTTTGAACTTCATATAACAATACAAAAATGGCTCAAGACAAACCTATTTTTGAATA
CCACGAGGAAAGCCAGGGCGAAAGATTAGCGCGCAAAACTAAAGAATCTCCTTTCATGGTAGTTGGATTGGCT
GGCCTGGCAACTGCTGTCGGCTATGGAGCCTACGCATACAAGAACAGAGGAAAGATGA
GCACCAGTGTCTACCTCATGCAGTTCCGTGTCATCTCACAGGGCCTCGTAGTGGGCGCGCTCACCGCCGGCAT
GGCTTACACTCTCTATAACAACCATTTCAACAAACCCAAAGCAATTAGCAGTAGTGTCCTACCCAGTAACGAG
CACTAAGTCATATGTTTTAGAATTACTTTTAGAGTAAAGCTGTATTATTAGATTATTT
CCAAAGCTTAATTAATTGGAATGATTTGCGTTTATTGAACTATACAAGATAATTTCATGTAAGGCACAACGGC
ACCGTCCTGAGCACAGTTAAAGGCAGAAGAAATAAACGCTTTTACGTGTTACACGTGATGTAGAAGTCAGTTG
GAGATACTAAAGAAACATCTTAGAAATTAACACTTAAATTTTAAATGATACAAGAAGT
GCAGAATGAGAT
>1002050035_1 Heli.1-DT667084.3.5
GACGAGGCACGAATTACATTCAGCCGAGGCTCGCCTCTGCAACACGCGCTCCACTTCCATACATCAATGGTGT
TGTGTCTTACAGGATCTCGACTTCAGCACACCGGGAACTTCTAGCTCTGTCGCCATGGAAAAGGAGCACGCTC
TTGAGGAGATGATGGGGAAACTGGGGGACTTCGGGCGTTATCAAGGATTTCAATTTTT
TCTACATGTGTTGGCAGCCATGACAGCTGGTATACATATGTTATCCTTGGTGACTGTGGCTGCAGTACCGGAA
CATAGGTGCTGGATAGACGGAGTAGATACAAATGAAGCCGCAGCACTTTGGAACTCATCAGAAATATTAGCAT
CCATACCTCTAACAGCATCTGGCGGCCTCGATAACTGTCACATGTACAATGAGGACAA

TGTATCAGTCACGTGTGACAAGTGGGTTTTCGATACCCGATACAGAACATCATCACGCGCCATCGAATGGAAT
CTCGTCTGCAATCAACGATGGAAGGGTGCTGTGGCTCAAACTGTTTTCATGCTGGGAGTCTTCACTGGCGCGG
TATTTCTCGGAGGTTTAGCCGACAAAGTAGGTAGGAAGACTGTTTTCTGCTGGTCAGG
TGTATTGCAGTTAATTTTAGGAGTCGTTGTATCGTTTATTCCTGAATATTGGTCTTTTTTGGTTGTTATGTTC
TTTTATGCAATTTTTGGTTCTGCTCCATATATAGCTGGATTTGTCCTTACTATGGAGCTAGTTGGGCCAAGCC
GTCGGACAGTATGTGGAGTGATGTTTCAAGTAATGTTTGCTTTCGGCATAATACTCTT
>1002050036_1 Heli.1-DT667312.3.5
GCACGAGGGCAAAGTCTCGGGGCTCGACCGGTTGAAGCACCCCGGCTTAAATAAGGGTATGGCATTTTCCATT
GAAGAACGCCAGGCTCTGGGTATCCACGGACTTTTGCCCCCTAGAGTGAAGAGCCAAGAGGAACAGGTTCAAC
TATGCAAGCTCTCTATCGAAAGATACGAAGATCCTCTCAATAAATACATCTACCTTAT
GGGACTTCTTGATCGCAACGAGCACTTGTTCTACCGTTTCGTCGGCGAGAACGTAGCGGAGTGTATGCCCATC
GTGTACACGCCGACCGTGGGCCTCGCGTGCCAGAAGTACGGCCTCGTGTACCGCAGACCGCGCGGCCTCTTCA
TCACGATCCACGATAAAGGACACATTTATGATATTTTGAAAAACTGGCCCGAGACAGA
CGTGCGCGCCATCGTGGTGACGGACGGCGAGCGCATCCTGGGGCTGGGCGACCTGGGCGCGTGCGGCATGGGC
ATCCCCGTGGGCAAGCTGGCGCTCTACACCGCGCTGGCCGGCATCAAGCCGCACCAGACGCTGCCTATCACGC
TGGATGTCGGTACGAACAACCAGACAATGCTGGACGACCCTCTCTACATCGGCCTCCG
CCAGAACAGAGTGCGCGGCGCCGCCTACGACGAGTTCATCGACGAGTTCATGCAAGCGGTTGTAAGGAGATTT
GGACAGAACTGCCTCATTCAGTTCGAAGACTTCGCCAATGCCAACGCTTTCAGATTGTTGGCGAAGTACAGGG
GAAAATACTGTACATTCAACGATGACATCCAAGGAACAGCTTCAGTCGCAGTGGCGGG
ACTCCTCGCCTCTTTACGCATTACTGGGAAAAGGCTGTCCGACAATGTTATAGTGTTCCAGGGAGCTGGAGAG
GCTTCTCTAGGTATTGCGGAGCTTTGCGTAATGGCGATGAAGAAGGAGGGCACCTCCGACGAGGACGCGCGCG
CTCGCATCTACCTCGTGGACTCGAAGGGGCTCGTGGTCAAGAACAGGCCCGAAGGAGG
ACTCAACGAACATAAGCTGCACTTCGCTAAGGACTGTCCCCCCGTCAGGACTTTGGCTGAAGTGGTCACTCTG
ACGAAACCTACTGTTTTGATTGGCGCGGCGGCCATCGGCGGCGCGTTCACGGCGGACATCCTGCGCGCGATGG
GCGCCACCACGAGCGCCCCGTGGTGTTCGCGCTGTCTAACCCCACCAGCAAGGCCGAG
TGCACCGCCGAG
>1002050037_1 Heli.1-DT668231.3.5
GCACGAGGGTACGTGATATCTTTCGACATTTACATTATAAATTGCATTTCTATTGCACTTTTGCAGATTGTAC
TTTGTCCTCTAGTCGATTTTGTATTATATTTTAAACATAACAAGTATATTCTAAATTAGGAAGTGCAACAGTT
TATTGTCGATATTAAAGATGGCGACAGATGTTGGATCAGCACCTGGCATGGCTCAGCT
TCAATCAGTACAGAAAGTTATGGCATTGCCAACAATAGAAGCAGCAGTTGGCCATGTAGGAGCTCTGTACACA
AGAGTAAAGGGAGCACATTCATTGCTTGAATGGGCACTATCCACAGCAGAGACTAGCGTAACTTTAGCTGCGG
CTACTGCGGCTCCGTTTGTTGCTGCCCCACTTGCAGCAGGTGATGCTAAAGTGGCAGC
AGTAATTGATGAACTAGAACGCAGAGTTCCACTCGTAACTGAACAACCAAAAGTGATAGTAGAAACTACTAAA
CAAGCAGTGCTTGCTAAAATAGCACCACAATTAAATAAAGTGTACGGCGCTAAGGACGTAGCGGAACAACGTG
TGAAGTCTCTCAAAGAGCTGACATGGGCTAAGGCCAATGTTCTTCTCACGACTGCTTA
CGGACAGAAGGCCATGACCAGCGTTGATACTGGTGCCTCCTATGCTATGCAATTGTTGAATCACTATCTGCCG
CCTACGCAGGAAGAAACTAATGCGGTGGGTGCCGAAATAGTAGCAGCCGAGAGTGACCCAGCACTCCACACCG
TGCAGACAGTGGGGCGGCTGAGCGCGGTGGCGGCGCGCCGCGTGTGGCCAAACCTCGC
>1002050038_1 Heli.1-ES586346.1.5
GAGAAGCGAAGGCTTCCCCAATTAAGATATTATAATATTAGTTTTTGAATTGTTTTTATTTTGTGAAACACAC
ATTTTACGAACGTACAAACAAGTATACGTAATTAGTGTAAGTTGTAGGAAAATCTATGTATTGAAAATTGAAA
AATGGACGATCCGAATAGAATGATGGCGCATAGCGGTGGTCTCATGGGACCTCAAGGC
TACGGTCTTCCTGGAGGCGACGGTGCTCCTGCCACGGGCGATGGAGAAGCTCGCAAACAAGACATCGGAGAAA
TATTGCAGCAAATTATGAATATAACCGATCAGAGTCTCGACGAAGCTCAAGCGAGAAAACACACATTGAACTG
CCATAGAATGAAGCCTGCCCTATTTTCCGTGTTATGTGAAATTAAAGAGAAAACAGTT
CTGTCCCTTCGTAACACACAAGAGGAAGAGCCCCCAGATCCCCAATTAATGCGGTTAGACAACATGCTGATCG
CCGAAGGTGTTGCTGGCCCGGAAAAGGGTGGCGGTGCAGGCGCTGCAGCGTCCGCGTCAGCCGCGGCGGGCGA
ATGGGATAACGCGATCGAGCACTCCGACTACCGGGCCAAGCTGGCGCAGATCAGACAG
ATTTACCACCAGGAGCTGGACAAGTATGAGAACGCGTGCAACGAGTTCACTACCCATGTTATGAATTTTGTG
>1002050039_1 Heli.1-DT664890.3.5
GCACGAGGGTTGTCCCGTTATTTTGTCAAACAAGACAAAATATAAAAGTTAAATTATAAAATTTCTTATTGCA
ATAATTATTTATATGATCAGTCAATTGCTTAAAAATCTATCTACTTGTCCGCTTCATTATTTTAACCATTTTC
CATAAGAGGTTATGACACAATAATATCGCTATTTTAATAACGCTATGGATTCATTGCG

TTCTTGAGTGAATAAATTAGTTTTCAAGTGAAAAACATTTAGAACAATTAATACTTATGATTTCATTTATTGG
GAAAATTATTATATCGAATGTGCGCCAATTTATAAATCGGCGTGCCATAAATACAATTTTTTTTCAATGATGT
CATCTCTCACCCTGACCACGACACTGACCAATGGACAGCTTAATGGAAAATTGGAGAA
CGGCACGAAAGGTTACCGGAAGAATGGTTACGTCAAATCAGAAATACCGGGCTCAAGTAGCTCGTTACGGACA
GCAGCCCCCTTCAGCACGGACTTCGCGGCGGTGGCGGAGCGGGATAGGTGCGCGTACGGCCGCATACCCAGGG
CGCTGGCGATAGCGGGCACGGCATCCCGCAAAGTGCGAGTGTACTCGGATGGCATATA
CGATATGTTCCACCAGGGCCATGCGAGACAGCTGCAGCAAGCCAAGACAGTATTTCCCAATGTCTATCTCATA
GTTGGAGTATGCAACGACAACCTCACACACAGCCGTAAGGGGCGCACGGTGATGACAGAAGACGAGAGATACG
AGGCCGTCCGACACTGCAGATATGTTGACGAGGTTGTACGAGACGCGCCGTGGGAGTA
>1002050040_1 Heli.1-ES584634.1.5
GCACGAGGCTATTTTATTACCTTACGACGGTGGCAGATACGCTTTATTGCTATTGGTACCACGGGCCCGAGAC
GGTTTAAGACGGTTGATAGCTGATCTGCCCGCCACATCAGTGTCGGAAATCCAAGAAAGTTTGAACGAAGAAG
AATTACAATTCTCAATGCCAATATTCTATGTCGAAACTACTACCAAACCTGTTACTGC
ATTGGCGAAGTTTGGAGTTAGCAGTATATTCGGTAAGGAAGCAGATCTGACCGGCATATCTGCAAAGGAAGGC
TTATTTGTTCAAGAATTGGTACAAAATGTCGCCGTTCGCGTTGATAATAATGATTCTTCGTCCACTTTAGTTG
GAGCGGCCGTAGCTGTAGAAGACGAACTGGCATCTTTGAAGAATCTCCCGTTATACGA
TAGCAAGGAATCCCGTAAATTATGTGTGGATCATCCATTTATTTTCTACATCCTTGACCATTTAGACAACTTA
GTTGTTGCTTCTGGTAAGGTCATTGACCCTCAATTGCCGACTGACATTTTGACATAAATTTTTAAATTTAGAA
AGACAAATAATACTTATAAGATTTTTTTAAAACTATTTATACAAGAAATATGACAATC
TTATTTCATTATATTTACAATTCTTAACATTTTCATTTTTAAATAGTTTTTTATTATAATTTATAAAATGTAC
TTAATTTCTTTCCCACCCCACCCAAATGTAATTTTAATTATATAACTTTCGCTGTGGGGTTGTCTTGAAATAA
TGTTTTTTATCCAGATGCATTTGATAATGAAAAAGTACACAGCGTAGTTTAGTTTAGT
TGTAAGTTTATTTATTTATTTTTTTACTGTTTGTGATATTTTTAAGAATAAACAAAAATA
>1002050041_1 Heli.1-DT666584.3.5
AGTCGGCGGCGGAATATACTTGTCCTAATAAAATGGCAGATATAACTAAGGAAGATCTCAAAAAGGAAATAAA
GAATGTCCTGAAAAACGCAAATCTGGCAAAGACTTCAACTAAAAAGGTCATTCAGCAATTAGAGAAAGTGTTT
GACACAGACTTGAGTGAAAAAAAAAAAGATTATAGACCAGTTAGTGATGGATTATGTTA
CAAGCAAGGAAAAGGAGAAGGAGGATGATGATGATGACGATGATGAAGAAGACGAGGAAGAAGAAGAAGAGGA
AGAAAAGAAACCAGCCAAACGATCAGCTCCACCAGCCAAGTCGTCATCTAAGAAGTCTAAAAAAGAAAGCTCT
GATGAAGATGAAGATGATGATGATGATGCAAGTGAAGATAGTGAATCTGAGGAGGAGA
GAAAGAAGCCAGCAGCCAAGAAAGGCAAAAAGAAGAAGGGATCAGAGGACTCTGACAGTGACTGGGGAAAGAA
GAAGGAAAAAGCGTCTAAAGCTAAAAAGGCAGCAGGCGGTCGTGGCAAAGGCGGCGGCTACACCCGAGCATAC
AAACTATCCCCGGCCCTAGCCGAGCTGATGGGCCAAGAAGAAATGCCCCGGCACGAAG
TAGTCAAGCGTGTTTGGACTATAATCAAAGAGAAGAACCTATACGACCCTAATAATAAGCAATTTGCCATCTG
CGACGATGCCTTGTATAAAGTTATAGGTACTAAACGATTCCGTACGTTTGGTATGATGAAATACTTGAAGACG
CATTTTCTTGATGAGTAAATTGTGTTTTTTTGTATATATTTAATAAATATAAACTTAA
ACTTTTATGGGATTTCTGCTTTATATGTGTGTGTATGTTTGTTTGAATGATATAATGTGGTAATAAAGAAAGA
TTTGTTTCTAGGTTCTCTAATAAAAAAAATTAATTAATTAGGTGTCTTTATACTTGTATAACATTGGATATAC
GATTTTGGGTACTATTACATAATAATATAAAATTAAATATATTTTTTTCATCATTTTC
CTTATTAATGGTATGAAAGTGCTTTTTATTTGTTATTAACAAATTATTAGTTACTATTGGTTGATTTTATCAT
CATGAGCAAACGTATTTCTTTATAAATATATATAAAGTAGAAATGTTATAATATAAATA
>1002050042_1 Heli.1-EL597490.1.5
ATCCGTTGATCGTGACTTTCAATAATTTAAAAAGTAGCAATAATAAATTTTCTTTAATATTATACTATTTACT
ATCATTATTAAATAATAATCATGCCAAAAGTCTCAGTAAAAGTAAAATGGGGAAAGGAGACATACCCTGATGT
AGAAGTAAATACGGATGACGAGCCTGTATTATTCAAAGCACAAATATTTGCTTTAACT
GGAGTTCAACCTGAAAGACAAAAGGTAGTTTGTCGAGGGGTAACATTAAAAGATGACAGCTGGGCAAATTTTA
AATTAACAAATAATGCAATTGTGCTTGTAATGGGAAGTAAAGAAGAAGATGTACCAGCTGCTCCTGTTGAGCA
AACCCGTTTTGTTGAGGATATGAATGAATCAGAACTAGCTACTGCTCTTGATCTTCCA
GAGGGCCTAATTAACTTGGGTAACACTTGTTACATGAATGCTACAGTGCAATGTCTTAAAACTGTTCCTGAAT
TGAAAAATGCTCTGCTCAAATATGACCAATCATCTGGCGGTGGAACAGCTGGTGGTCTCACATCTGCGTTAAG
TGAAACTATGAGAGCTTTAGAGAATGGTGGTGCCGGAGCCTGTGCTGCAGCTGCTGCC
AGGCTTCTCCATGCGCTGCATGCGGCTGCTCCCCGCTTAGCAGAGAGAGGAGCTGGAGGACAATTGGCTCAGC
AAGATGCTTCAGAATGCTGGACTGAGATGGTTCGTGCTTTGAGGATGAGACTGCCAATAACACCTGAAGCTGA
CAGNCAATCCGTAGTAGAACAGTACTTTGGTGGGACACTCGACGTGGAGTTAGTATGC

AGCGAGGCAGACGAACCTCCGACACGCTCCTCCGAGTCGTTCCTACAGCTGTCCTGCTTCATATCCCAGGATG
TCAAATATTTGCAATCCGGGCTTAGATCTAAAATGCACGAGGAAATCACTAAAATGTCACAAACATTGGGAAG
AGATGCTGTTTATACGAAAACTAGCAAAATAAGTCGTCTGCCGGCGTATTTAACAGTG
CAATTTGTACGTTTTTACTACAAAGAGAAGGAGTCCATCAATGCGAAGATCCTCAAAGATGTTAAGTTTCCTT
TGGACCTGGATGTATATGAACTGTGCACGCCGGAGCTGCAAGAACGTTTGACGCCTATGAGAAATAAATTTAA
AGAACTCGAAGACGCAACGGTGGAATCATCACTGAGTTCAAAAAACAAAAGTCACGGC
GACAGCAAAAAA
>1002050043_1 Heli.1-CV526214.2.5
AACGTCGCAATACGAATGCCCCCAGTTATCCCTATTAATCATTACCTCGGAGTTCTGAAAACCAACAAAATAG
AACCGAGATCATATTCTATTATTCCATGCACGAAATATTCAAGCGGCATTTTGAGCCCGCTTTGAGCACTCTA
ATTTGTTCAAAGTAAAATTGTCGGCCCATCTCGACACTCACCGAAGAGAACCGCGATA
GGATTTTGATATTGAACCGGCGTTTTACCGCCGGCTCACCGACGATATGCTCCGCAGACGTGTCAGTATCACC
GCGGATGCGGTGCACCGACAGCGCGGCGCACAAATGCAACTACGAGCTTTTTAACCGCAACAATTTTAGTATA
CGCTATTGGAGCTGGAATTACCGCGGCTGCTGGCACCAGACTTGCCCTCCAATTGTTC
CTCGTTAAAATATTTAAAGTGTACTCATTCCGATTACGAGGCCTCGAAAGAGTCCCGTATCGTTATTTTTCGT
CACTACCTCCCCGTGCCGGGAGTGGGTAATTTGCGCGCCTGCTGCCTTCCTTGGATGTGGTAGCCGTTTCTCA
GGCTCCCTCTCCGGAATCGAACCCTGATTCCCCGTTACCCGTGACAACCATGGTAGTC
GCAGAAACTACCATCGAAAGTTGATAAGGCAGACATTTGAAAGATGCGTCGCCGGTACTTGACCATGCGATCG
GCAAAAGTTATCCAGATTCATCAAAATTAACGACTTCGGACGCGAAGCCCTCCGTCGATTGGTTATGATCTAA
TAAAAAGCACTCATCCCATCACTGGTCAGAGTTCTGATAGCATGTATTAGCTCTAGAA
TTACCACAGTTATCCCAGTAACTGAGTCAGAACTAAGGAACCAAAACTGATGTATTGAGCCATTCGCGGTATC
GCCTCAATTCGGTTGCACTTATACCTGCTTGGTTAAGTTTTGAGACAAGACTATACCATCTAGAGGGGCCTCA
GTTAAAGTTGACCACTAAGTAATGATTGAAAGGGAGAATCTCGTATTCCTGGTGACAC
>1002050044_1 Heli.1-DT664780.3.5
GCACGAGGGCAAGGTCAAGTGCGAGAGTCATTGCTCTGCGCATCCTATCCTATGTGTATTAGTTGTCTTCACG
AGGTACAAAATGGGACACGGAATACTTTTGTTCACTCTGCTGAGCCTCGCGGCGTTGGCGCTGGCTGACAGCG
ACGACAAATTCTTCAAAAAATATGCTATGATGAAAATATACGAAAGTTGTTTCGGACC
AGACGTAGTAAAACAAGTGCGACAGGAAATGAAGGAAGCGTACGCTAAATGTTCATCACCGCCATCTGTTGGC
GAGGGGCTAATTCAACGCGTTCCTTCACTTATACTTGCTAATCTACCACAAGGGTTTGAATCTGATCCGGCCA
CTCAAACTATAACTAAGCCGACAGATGGCGCTGTTCCAGAAAATGTACCACAGATTTT
GTCACATAAACAAATGTTCGATAATATGTTAAAACAGCGGCCATTCCAAGCGCCAGCAGTACCAAACCCATAT
CCAATGCCGAATCAATTCAGGCCGTACCCTCAACCGTACTACCAGTTCCCCCTCAACCCTATGTACTACCCGC
CGGGAGTCCAATACAACCCTTACACGTATCAGCAACAGCAGTACCAACAGGGATTCTA
TCAGCCACCTTATTTCCAGCAGAACAATAGAATATCTCGTGACATCAACATCCGTGACCGTTTCGAGATGCTG
TCTCACATGAGTAACCCTGGCCGAACCCGCAACATTACCTGCGTGGTTCAAGAACTGGGCTATCTGGACCACA
ACATGGAACCTAACTATGAACAAATAGCTCAAAGAATCAACAACCTGCCTGTGTCTAG
TCAATTGAAGGGCGATATACAAGATGGTGTGCATTTCTGTCAAAAGTTCTCGCAATGTGTACCAGAGATCGAA
CGTAACGCGCCGCTGTCTCAAGAACTCATCAAGCCTATGTTCTTTTTCCGCTGTTATAAGCACAAGAAACTTG
AAGCGTGTATTATGAAAGATATACGTGAACGTTTCGCCGCTGACGATGACCTCGATAC
TGACACGGACTTCAGATCGCTATCCAGAGCAGCAAGATCGGTCCGCGAAGAGACAATCTCAGATCCACGTCTT
GAAGCTTTAGATGAAATGTCTGTCTACTTCTACGACTACCTCAGCGGCGGCACCGGCTTAGATTTCGATTTAT
ATCTATAAAA
>1002050045_1 Heli.1-DT664332.3.5
ATGAAACCGTTATTCCTAGTTCTCTTGGCCGTGGGACTGACAAGTGCAAGATGGGTGCGCCGTCAGCGTTCTA
ACGAACCCAGAGAGACTAGTTTTGTTGGCAAAGCTACTAACGAACTTTCTACAACTATTCTACAGGGATACAT
TGATGATGAAAAGAACATTGCCTTCTCGCCTCTTGGCTATACAGCTATTCTAGCTATA
CTCGCAGAAGGTGCTATGGGTGAAACTAGGAATCAACTGGTGTCAGCATTACACTTACCAGAAGATCAAAATC
TAACAAGAAAAACGTATAAATATATAATGGAACGTCTTAAATATAGAAATGAATATAAATATAATCAACCTGA
ATTGAAAAACTTCTTCTATATATACAAAAATTACACTATCAATGATGATTATAAGAAG
ATTCTTGAAGATTACTATTTAACAGATGTTAGATCTGTTGAAAGGAGTTACGACCACGAGCTAGATACTAAAA
ACGACGAAGACGACGAAAGCGATGTAAAATCAGATGAACCCCTACCATCTGACAATAATGAAAAATTAATTTC
ATATGCTGTTGAAGATATTCCAGAAAAGATCGATGTGACTCATGCTGAATATAAACCA

GCGAAAAATATAAAAGAACAGATAAAATTAGTAAAGACAAAAGAAGAAGTCGGTGATGATGAAGAGACGATGG
TTGCTGTTGAAGCAAGGAATCACGCGCGCAGCCTTAATGTACTCAATGATAAAAATGACGTTGCAACCAGTAT
TTCAGTAAACAGCGTTGGAAAGAAATCTAGCAAATCTACAAATTCTTTGATGCTTATC
>1002050046_1 Heli.1-DT667633.3.5
GCACGAGGACGGCACGAGGGTTTGTATTGATATGTAATTTATTGAGTTTTTCAGCATTATTGTTTACCACTCG
AAATATTTGTTATTTAAGATTTTTCCACAATGATTGGTAGTGATTACACTTTGGAATTGTGCAATGTTTTTCA
CTCAGGGCAAGTCGAGCCAGGCAATTTTTTCCAACGATTAACAGGTGGGGTAAAGACA
GGAATAATCTTAAAAGATGTCTCTTTTCTTACTCACAGTGGAGAGGTCACCGCTATCTTGGGCTCTAAAGGTA
GTGGTAAAAGAGCGCTTCTGGATGTCATTAGCCGACGAGTACCCTGCAAAGGTCATATTCTTCTGGAAGGAGT
GCCTTTAGAAGAGGATCAGTTTAGAAATACTTGTGCATTAGTGCGTCATTCCACTAAA
CTTCTGCCTGGACTTAGCGTTCAACAAACATTAGCTCTATCGTTGACAAAGATATCCGGATACCTGAAATCGT
CGAAAGTCAAACAAGTTATGGCTGACTTGGCTCTATCTCAGGTCGCACATAAATGTGTTACCAGTTTGACGAA
AAGTGAATACCGAAGATTAGTAATCGGAGTTCAACTCATACGAGATCCAATTATTCTT
CTTCTTGATGAGCCTACTTGGGATTTGGATCCTCTGAACACATATTTAGTAATTTCTATTCTTTCCAATGCTG
CTAAAAAGTATGGAACAACTATTATACTCACCATGGAGAAACCAAGATCAGACGTGTTCCCGTTCCTCGACCG
TGTGGTATATCTATGCCTGGGTGACGTCGTTTACGCTGGTCCTACAAGAAATCTTTTA
GACTACTTCGGCAGCATTGGCTTCCTTGCCCTCAGTTGGAGAATCCTC
>1002050047_1 Heli.1-DT664925.3.5
GCACGAGGCTGAACCCTCCCAAAGCAGGCAAACTACGAATAATCCTTTGCTTAGATTTAGAAGACCACCGGTC
GTTCGACAGCCAGGAACAGCGATAACTCCAAGGTCTACAACACCTTCCTCAAGGCGAAGCTTAACAACGCGTG
GTAGAACCACGACAACAAGACGATCAACTACACGTTCCACCTCAAATCCTCTACTTGC
ATTACGTAGACAAAGACCAACAAATTCTCTTTTCCCCAGAAGAAATCTTTTTAAGCAACCAGAACCAGAGCCA
GAAGAAGAACTAAGAGAAAATGAAGAAAATGAAGAAGATGAGGAATTATTAGATGAAGAAGAATTCGAAGAAG
ATAATGATTATGAATCATCAGACAGAAAAGAACAACAGGTGGCTTCCCCTATAGCTTC
ATCAACAAAAAGAATCCAAAACGCCGTGCAGATACGACCTTTTGCATTTAAAAGACGAGTTAAGCGTCAGGTT
GATTATGGTAACAGAAAATATAATAATTTTAGAAGACCTGGAGTCAAATCAGCAAGTACTCGCAGACCCGAAC
CTGTACCTGAAACAGAACCTCCTACACCTGTTACTCCAAAACAAAAATCTGGACGTTA
TAACTCACGTGCCGGAAGTAGCAGAACTACAACTGCATCAGCTCCGAAGGTTTCATCCAGGCAACCGTTTATT
GTAAGAGGAGATAGCAGTAGAACAACAACTACTTCGGCACCAGCATACAGAAGGGGAAAAACGAGAACTAATT
CGAGAACTACAACGCTTTCATCAAGACCTAAAGCACCACGCTTAACTAGTGGCAAGTC
AACACAATCATCAAGATCTAGTCCTAGGACGACAAGCTCCAGATCAAGATCAAGAACGACAACTAGCAGGGCC
TCTAGCAGACAAAGAAATTCCTTTGAAAATTTAAGTGGAGAGAGATATAATAAATATGATAATATTTTAAATG
ATGGCAAAATTACCATAACACATCAGATACCGTTTGAAGTAACAATACCGGTGGTAAA
TGGAAAGATTACAGAATATAAAAATCTTTTGACAGCTAAACCTAGCGCAGAAACTTTATTACTTAATCAGGTA
TCGACATCACTTAGTCCTATTGGTGCTCAACAGTTAGTTCTTGTAGCTGAGTCTACAGAATTGGCAGAAAATG
GTGCAACAAGAATAACTAAGTATGTTTTACATGAAAGCCCTACAGCCACAATCATCTT
TAACTCCAATCAACAATAAGAGGTCGCAAAACATCTTTTTCACATGTTGGGCCCTAGTACTGTGTACACTGTC
GAGCCCGTCGGGCAATCTATAGCGCCTTGAACTGAGTGCTCATGTGCCTTTTTGGTTACTGTGCTTTGGTCCC
AACCTTTTTTTCAGGGACACCGACCGGTCTGGGATAGATCGGTACCTGGCTCTTCAGG
>1002050048_1 Heli.1-ES587630.1.5
ACCAAAACAAACAAAATGTTCAAATTTGTGATTTTCTTCGCTCTCTTGGCTGTGGCCTTCGCCAAGCCCTTAG
TGTACACTGCGCCATTTGCCGCAGCGTACACGGCTCCCGTAGCTGCAGCGTACACTGCGCCCGTAGCGTACTC
GTCGTACAGCGCGTACTCTCCCGTCGCTTACTCCGCCTACTCATACGCATCTCCTTAT
TCTTACTACCTTTGAGAAATAAAACGACTAACTTAAACGGACTCTCGGAAATATCAGCAAAATGGATTATTTA
ATATATTTATTATGTTTGAACGCAATAAATGACAA
>1002050049_1 Heli.1-CX700582.1.5
GCAGTACGGTCCGGAATCCGGGTCGACCACGCGTCCGTTTGGCTTGGCGCCTTTCCTGTTGGTTGTTCTCTTG
TAGTGATAGTGTGTTACACGTTTCAAAATGGCTGGTGGTAAAGCGGGCAAAGATTCTGGCAAAGCCAAGGCGA
AAGCTGTCTCACGATCGGCTCGGGCGGGCTTGCAGTTCCCTGTGGGTAGAATTCATAG
ACATCTCAAAAATAGGACTACGAGTCACGGCCGTGTCGGCGCAACGGCTGCCGTTTATTCTGCGGCTATTCTT
GAATATCTCACAGCCGAGGTTTTAGAGTTAGCGGGCAATGCATCCAAAGATCTTAAAGTAAAACGTATCACTC
CCAGACATTTACAACTAGCTATCAGAGGAGACGAGGAGCTGGACAGCTTGATCAAAGC

CACCATTGCCGGAGGTGGTGTCATCCCACATATTCACAAATCACTCATCGGTAAAAAAGGAGGACCGGGGGCA
CCTGTTTAATAAGAGTCTTGCTTCAATTCAACATTCTAACTGCATTTGAACCGGAGACTATCAGCATTGTAAG
TTCATCAGCCAGGCAGTAAACATAGTTTTCTACAGGTAGTGATGTGGACAGTGGGAGT
GACTTCAGACATATTGATCCGGACTAAACTTATAGTTAAGACTAGTAATATTTTTAGTTTAAGGAGATTTAAA
TATAATTGAATTAGAATTAAGTTAATTTTGTCGTTTTGTTATGGGACATGTTTATTTTATGAGGCATGTGTAC
AATTTATGTCAGCTACTACTAAAATTAGTTCACT
>1002050050_1 Heli.1-EL597145.1.5
GGGCTGCGATCGGCACGCGTCAGTAGCTTGTGCGTGTTGGCTTAAAGCGCCTTTAATTTTAAGATTATTGACA
AATATCACTGTTTTTACAAGAAAAATGGCACCCAAACAGAGAATGCGTATTGCAAATGAAATTGCAAGCAAAA
ACATCACCATGAGAGGGAATGTTCCTAAAACCACTAAGGAAAAGGATGATCAATACCC
CGTAGCGCCATGGTTACTAGCTCTGTTCATATTTGTAGTCTGTGGCTCGGCCGTCTTCCAAATCATTCAATCT
ATTAGGTTAGCCTAAACCTGTGATTCCCCGACTGATAATTTTAGTATCACGAAATTGTGGCTAAGTATTCTTC
TTTTAATTTTACTCTTACTTTTCATCCTATCACTTATACATATCCCTTCTAATATATT
TTATAATTCTATTTGCCAAATGTAAATATTTTTTAAAGAAATGTTTCAGAAGGGCCTTAATATTATTTTATTT
TATTATGGTTTATTTTAAATAAAAAAATAAGTTTATAGATAACTAATTATTTAATTTTTAACATACTTTTGCC
ACGATTTCTAGAAAAAAATTGTAACATCTTCATTACTTTGCATGAATTAAATATACAT
ATATGTATATTTGATTGATATAAAGCCTATCCATTTTCCTGTAGCTGCAAATGCAATTTGTGTTTAGACTAAG
CTCAGGTAATCTCTTTGTATGTACTATATTCTTGTATATTGTAAAATAAATATAAGCTCGGCCCGCACTTGAA
TTTTTTTTATAGTACCTTATTATTATTATTATATTAAATAATATTTTTGTTCATTTTT
ATGTTTTTAATA
>1002050051_1 Heli.1-DT666707.3.5
GATTCCGGGTCGACCACGCGTCCGCAAATTTATACATGTAGACCTTTGGGGCCTCATCTTTTAAAATAAAGCA
GCATAATAAATAAAAGTGAAGTATTAATTCAATTCTAGGTGCAAACAAATAAACATATTGCAATGGTTCCTTA
AAATATTTAGAAATATTGATTTAAAAGCTCCCGTGTCATGAGACTCTAAGCTTATGTA
ACATATTTTTTCTCAAAATAACTGTACGATCAGTGATAACTAGTGTTTACTATCGTAAAGAGTGAAAACGAAA
AACAATGTTGCGACGCTGCTCTAAACACTTGCAGACGATATACCGGAGGCAGAGCCAAGGCCTCCGGTTTAGG
TCTACAGAGGTGCCCAAGATCTGTGGCGCTGTGGCTCCAGGGACCAAACTCCTGAAGC
CCAGAGTCCTGGCCGCCCACCATCAGGTCGCCACCATCCACTTTACCAACCCTCTTCAAGCAGAACAGGATGT
AATGACTCCGGGATTCCCCGATTCCGTCTCAGAAGGAGATGTCAAATTGGACAAAAAAGTTGGAGACGCTGTA
GCAGCTGACGAAGTAGTGTTAGAAATAGAAACAGACAAAACAGCCATACCCGTAATGG
CGCCTGATAATGGTATTATTAAAGAACTGTATGTCCAAGATGGTGACACAGTGAAGGCTGGTCAAAAGTTGTT
CAGACTTGAGATAACTAGTGGTGCTCCACCAAAAAAAGAAGCGCCAGTTGAGAAGAAAGCTGATGCTCCACCA
CCAGCTGCAGCTGCGCCACCTCCACCTCCACCCCCTCCACCATCAGCTGCTGCTCCAT
CTCCACCTCCTCTCCCACAAGCTACTCCTCCACCTCAAGCTCCCCCCAAACCTGCATCTCCACCCCCTGCAGC
TCCAGTCTCCTCAATACCCATAGCTGCTATCCGACACGCGCAAGCAATCGAAACTGCCTCAGTGAAAGTCCCT
CCAGCGGATTATAGCAAAGAAATAGTGGGGCACCCGCAGTGAACAGCGAGTCAAAATG
AATCGTATGCAACAACGCATCGCCGAAAGGCTAAAGGAAGCTCAAAACACCAACGCCCTT
>1002050052_1 Heli.1-EL599258.1.5
CGGCACGAGGGCCTTATACCTAACTTGTCTATTTCTAGTAATTAACACAAAATCAGAACAATCATGAGCTCCA
ATGAAAATAATAGTGAAGTGGCAGTAGACAAGGTGGGCGAGGAGAAGTCCGGAGTTGCTAAAAGTGACCTAAA
AGGAGCCAAAAGGGCAGCAGAAGAAAAAGCAACAGAAGCAAAAAAGGCGCGAAAGGAG
GAAAATGGCGGCGGTGGGGAAGACGACGCTCACAGTGAGGAAGATGACTTGGAAGGATATGAAGTGATAGGCG
AAGGCGAAGAGGACGACGACGAGGAAGTAGAGGGAGAAGAGGGCGAGGAAGACGAGGAAGAGTTCGAAGAGGG
AGAGGATGATCTTGAAGATGAGGACGTCGAGGAGGAATTAGGGGAAGAGGAAGAGGAC
GACGCGTAATACGGCCGAGCCTCTATAAGGACAATGTCACAGTGTTAGTGCGCTGTGTGTGTAGTGTGTGTGA
CGCCCCGGCCGCAGTTGAACGCAACAACGGGTGCACTATGAATGACAACGTAATTAAGTCTAATCCGAAGAAA
ATTACATTTTTTAATTGATTACATTTAAGTTTGATACTCATGTATCAATTTCTTTTTC
GAGCAATCTAATTTAATTTTAAGACTAGTAGAATACAGTTGAATTTTGTCATGAAATGTACAAATGTGTTTTC
GGAAACTTTGTTCTAAGTGAACATCCACTCTGTATTTAAGTTAACCTTTAATATATGCCCGAATGGTGAAGTT
GTGATATTTTTCTATATAAACAGTAGTATAATTCACATTGTAATGAAATATAGACAAT
>1002050053_1 Heli.1-DT664662.3.5
CGTCAGCTCAACTCTTAAACACAGAACTTTATAATCAAAACCAAAGACCTAATCCGATAGTTCAAAATCTTGC
TGGAGAATTCCAAAGACCAGCGATTAAATCACAAGATACATTTAACTCTCAAGAATTTCTTGCAAGCTATAAC
AACCCTCTACTCGACATGGATCTTTTTCCTAAATTTTCAACAAATAATTTACCATTTC

CTACGACTGCATCAACAACGTCTAAGCCAAAAAAGCTGAAGCCTCATCAGCCACCATTAGCTATTTTTCTTAC
CCAAGAAGACAAAAAAGAAGACGAAGTCAAAGTCGGAGATGTACTACATTCTTTAAAGAGTGCTGATACAGTT
GCTGTGCTGGATTCTGTCAATCCATTAAATGCTCCCAAAGTATTTATTGGTCCTTCGT
CATTGATTCCACCACAAAATTACGTGAAATTTGAGCTTCCATACTTATCAAACATAGAAAATGTTGATAAAAA
TTTAAGACAATTGCCATTTTTTGTAGCACCACTTAGCTACAATACACCACAAGGTTTTGCTAAAATACCTTTT
CCATCCCCACATGTGGGATCTGTCGTCATTAACTCTTTTATTAAAGATACATCTTCGC
ATCGTACGACTACAACACCTGCAAATGTTTATTCTAACTCTTATACTAAAGAACCAGCCCCGTACAAACAGGA
ACAAAAATATGTCACACAAAAACCTAAAATTAGTTATTATTCTACCACTTCTCCAAATGTAAATTCACCAAGT
TATGAACAAAATTACTACTCCATTGAACCTCAGTCAGTAAATACTATACGGCCCTTGA
AAGAATCCAATTTCAATAATATGCGCCAGCCGACTCCCATTAAAACTGAATCATATTTTTTAAGCAACGCTGG
AAATCAGTACAGCGTGCCTCAATTTTTCCCAAAACCCGAGGAGCCTCATAAATCCGTAGATGTCTTCAAACCT
GAAACTACAACCATACTGAGTACATCATCTACTACTACAAAACCACCTTCGACATATC
CTAGTCAATTACTAGAGACACACAATCCCTATTCTATTAATCAAGCTTTTCACTTTAGTACACCCTTGGATTA
CCACAACTTCTTCGATGAGTATAAAGAGCCTTATGTTGAAGAAGCAGTAAAACAATCTGTGCATCCTGTTACA
CCTGCATCTACACCAACAACAAAAATTGAATCTACATCAACATTACCATCAAATCAGT
CAAGTCGGCAGTCATCACTAAATTATTTACAGAGCTATAGTCCTGAAATTCATCATGAAAATGAAAATCATAA
TACAAGATATCCGGTTTTAGAAACAAACGATTACTCCACAAAGACAAACTCGGCGCCACTAACTGATGTAACT
AATTTCAATTTAGCCAATTCAAATGATAATAGTAAAAAAATATATAATACAAATATAT
CTGAAGAAAAATATTTAGAACTTCAATCACCTATAT
>1002050054_1 Heli.1-DT667804.3.5
GCACGAGGATTTAGAGTAGATATTATAAAGTCTTATTAGATAAGTTAATTGAATTAAATGAAGTGATTTATTA
TTAGTAAAATAGTGCTGAGCCTAACAGAGTCAACATGCGTATGATAATATTTTCCATAGCATTTTTGCTATGT
AGTGTCTATATGACTCGGGCTTTTGAGGACACTACACTAGAGGACGATGATTTTGCTG
AATTTGAACAATTTGAAGCAGATGAAGATGAGCAGACATCAGATGGAGCATTTAGTGAAGAAGAAGCTCCAAT
ATTGAAGCCAAAATTAAATGCTGCCAATAAAGATCAATTTGATGTTACTGTTGAAATAGAGGATGATATAATT
ACTGAGGAGGAGGATAATGAATTCGAGCATTTCCAAGATCCTGAAGAGTTTGAGGGCT
TCCAAGAAACCACCCCACGGACTGTTGAGCAACCAAAGATTACAATTTCAAAGGTTCCTATAATGGTGCGTCC
ACGCTGGGATGCATACTGGTTAGAGGCGATCCTCTGCTGTCTACTCGCTTCATATGCATTGGCATATGCGATC
GGTCGCGCAAAGAATACTTCGATAGCTGTTAATTTCTTGAAATTACATAAGCCATTGT
TAGATGAGAATTTTACTTTGGTCGGTGAAACGGGCCTAGACGTAGTAGCGGCGGACGAGCGCGGCTGGCGGCG
CGAGGCGGAGCACTGCTTCACCATGTGGTGCAGCGGCCGCCAGTGCTGCGAGGGGATGCTGCTCACTTTGAAA
CTTATTAAGCGTCAAGATCTAGTCCACGTGTTGCTAGGTGTAGTCA
>1002050055_1 Heli.1-DT665309.3.5
GCACGAGGCCAATACCTTACCGCCATGACTGTCTTTACGCGAATATTTCTATTTTGTGCTATACTTGTGAATG
TTAATTGCCAGAAAGAAAAGGAAGGAGAGGAATTCGTTTGTCCCGAAGGTACTCAAGGCAACGGCAATTTTGC
TGATCCGGCCACTTGCCGGCGCTTCTACCAATGCGTAGACGGATACCCATACCTGAAC
AGATGTCCTTCTGGCTTGTACTTCGATGATATCAGCAAGTACTGCACTTTTAAAGCTGAGGCAAGGTGCGGCC
CGATAGCGACTACACAAGCTCCAATAACAGAGGCGCCCCATGATCTGGCAACGAAGTGTGATACGGCAGAATG
TCAGTTGCCGTACTGTTTTTGCTCCAAGGATGGCACACTCATACCAGGCGGTCTGGAC
CCGGAGGACACGCCGCAAATGATAATGCTCACATTTGATGGTGCCGTTAATTTGAACAACTTCGACTTGTACA
AGAAAGTTTTTAATGGGAAAATACGTAATCCAAACGGCTGCCCAATTCGGGGCACTTTCTTCTTATCTCACGA
GTACAGTAATTACGTAATGGTGCAGTCCCTCGCACACGACGGTCACGAGATTGCGACC
GGTACCATATCTCAGCAACAAGGACTCCAGGACAAAGGATATGAAGAGTGGGCGGGCGAGATGATCGGTATGC
GCGAGATACTCAAGATATTTTCAAACGTTTCTCGGTCTGAAGTCGTGGGCGCGCGAGCTCCTTTCCTTAAACC
CGGCAGGAACACTCAATTTAAGGTGTTAGAAGACTTCGGCTACATCTACGATAGTTCG
GTGGGCGTGCCT
>1002050056_1 Heli.1-ES585142.1.5
ATCAGACACACCATATCATCATGAAAGGACTACTAGTATTGTCGGTACTCGCTGTTGCGCACGCGCAGTTCCC
CAACGGACGTATCCTGGAGCCCCCGGTACCTGCACAATGTGCACAGCGCGTAATACACGAGAGGTTTGCCGAT
AATAAAGGCTACTTCTTTTCGTGGAGAGACCCGGCGTTACGTGGAGTAGAAGAAGACT
GGTTGAGCGCCAGAAACTACTGCCGCCAACGCTGCATGGACCTCGTGTCGTTGGAAACAAGTGACGAAAACGA
ATGGGTGAAAGCCCGCATTGTACAGGATAAAGTAAAATACATTTGGACGTCCGGGCGTTTGTGCGATTTCAAA
GGATGTAATCGCCCAGACTTGCTGCCCAACGAAATCAATGGCTGGTTCTGGACCGCTG

AATTGCAAAAGCTGGCTCCTAGCACTAACCGTCAACAAAACGACTGGTCCGAGGGTGGTGGCATCGGAAAACC
TCAACCAGACAATCGGGAATTGATCCAGGGCGGAGCTGCGGAACACTGTGTGGCTATCTTGAACAATTTCTAC
AACGACGGTGTCCATTGGCATGACGTCGCCTGTCACCACCGCAAGCCCTTCGTGTGCG
AAGAAAACGATGCTCTTCTCAAATACGTTAGATATACGAACCCTAACTTGAGAGTATAACAAAATAACAATCA
AAATTCACAATTAAGTAAATAAAGAAAATCTTAAAATCTATGCTATCGACAAAAGTATC
>1002050057_1 Heli.1-DT665706.3.5
GCACGAGGAAGCCCCTTCAAAGCTGAGCATCATTTTTCGTCATTGGCTTTTCAAACATCAATTAAAAGTATTT
CGTTTTGCTAAGAATGCCTTAAATATACAAAGTAAACATGGAGTTTGATGAAGTTGCGGTGAAGGAGAGCCCG
GGGCTTTGCCGGTGCTGTCTATCCGAAGGTTGTTACAAAGACTTGGGTACTGAGTACG
TTTGGATGGATGAGACCGAAATTTACGCCGATATGCTGTTAGAATGTTTTGATATTAGTATATCACAACACAT
AGAAGGGCCAAATGGTCCAAACCGTTTCATCTGTGAAGTCTGCATAACTCGTCTCCGAGATGCATGCAACTTT
AAAAAGCAAGTGCTGGAATCTGAAAAGAAGTTCATTGATATGCTTGGCAGGGGGGAAT
TCAAGTCAAAAGTGAAAGCATATCAAGATCAAATGAAAGCTGAGGAGGTTCAATCTGGTATGGACACGGAAGT
AGAATTTCTTGAGGATGATATTGATTATGATGATGGGCCAGGCAATGACATAACGGAACCAAGTATATCTGAA
GATATAACTGTAGAGACTTTACCCATTAAAAGCAAAAAGGGAAGACCCCGTAAAAATG
CAGTCAAAACGGAAAAGAAAAAAACTAAAATCGAAGAGAAACCTAAGGCTAAAATTGTGAGAGATGAAGTCCC
GTACGATGTCTGCCCCTCTGAGCGCATACGAAAAAACTTGCAAATACTGTTCAACAACACAACAATAATACAC
GGAAATGTTGTGCCATTCCTCTGGACACAAGATGCTTCAGATGCTTCTACTGTCATAA
CAAATAAAGGAT
>1002050058_1 Heli.1-EL603150.1.5
AGGGGGAATCGCAACGAGGTAATGAAAAGTCGTTAAAATTATTGTTTCCTTGTTATTTATTGTTTTAAATAAA
CATTTGTATACAAAATGTCGTTTGATTTAAACGCAATAAGTTCAGCGCTTAATGAACCGAACAGAACACTTAG
CGGTGTGGATTTTTCCGGAAAATCCTTAAAATTGGATACTGAGCTAGATGCTCAACCC
ATAGTAGATGCAATCAACAGTTGTCCAAACCTCCAATATCTTACTCTCACCGGCAACACATTGGGTGTAGCAG
CGGCACAAGCAATTGCCAAAGCCCTGTCCAAACACCCGGAACTTAAATCTGCAAGATTCTCTGATATGTTCAC
TGGAAGAATGAAAACTGAGATACCACCAGCTTTGAGTGCACTTGGTGATGGTATGATA
GAGGCCGGAGCTCGTCTTTCCATACTTGATCTGAGTGATAATGCCTTTGGCCCCATAGGAGTGGAGGGACTAG
CAAAGTTGTTACAAAGTCATGTCTGTTCAGAATTGGAGGAATTATACTTAAACAACAATGGCTTAGGTATAAC
CGGGGGTAGGTTACTTGCTAAAGCACTTTCAGCAAGTCCCAGAAAACTGAAGATCTTC
ATAGCAGGACGTAATAGATTGGATAATGATGGGGCTAAGGCACTTGCTGCTGTATTTCAGGCAATGGGTACAT
TAGAAGAACTAGCCATGCCTCAAAATGGGATATACCACGTGGGTATA
>1002050059_1 Heli.1-DT668884.3.5
TGGCACGAGGGTCACCATACAAGCAGCCGGCCCAACGATGTGACGTGACGTGACGTTGTACTGTCAGATGGCA
ATTGATTGTCATGACAATCATAGATATATCATAGTCGATTGATTGTTAATCGATTGTGGTAGTCAATTATTGG
TCATTGTTAATTGATTGCCAAATGATTGTCAAGTGATAATCAATTGGTAGTCAAGTAA
TAGTCAATTGGTTGTCAAACGTCCGACAGACGACGTGACGCCATTGTGATTACGATTATAGTGTTGGTCTTGT
AATCTTTTTTAATATTTTTTTCTTTTTGTCGTTATAACTTTTTTTTTAATCTTTTCCATATTTGTTATGAAAT
ATTGTCAAGTGCCAATTTTTAGCTGTATTTTTTTTTCTGGTTTGATTTTTAATATTAA
TTATTAATGCTATAATCGTAATCGAACACATCTAGTGGCCCTTCAGTATGCTGCGTTATCAAAAGGCTATAGG
TAAACGATTCACTTTGATATTTCGTCATAATTAAAGATATTAAAAATAATTATACAACATGTTATTCTAATAT
TCTCATGTTCTTTTTTAGAATAAATAAAGTACAAGTAACAAAATTTTGCTTTGAATAA
TAATATCGACTGAATCGTATATCTACCGCCTTACAATTTTGATGCGGACTAATATGAAATATTATAATTTTGT
TTTGTCCATCTGTCCTGCATCTATATATTTGTTTTCAATCTATATGTAAGCGAAGTAGGAAGCCTCGTTCTGT
ATTTACTATCGCCATTGTTTTAACTGTTATATTGTACATTCAACTATTGCGTATCAAG
TTAATTTTTCACTGCATATACGATTGTATATACGTAATGAGGTCTTTAATGTCATTATTAATATTTTGTTTTT
TTTTAATTTTTGGATTTTTGCACCGAAATAATAATGAAAGTAATAAAAGTGAATAATAATTCAGAACTGACTG
CGCTCATACAATTGGTGCTCATGTTTGATTGCATTTATTGTGATGTAAAAATGATTGA
AATGTCGTTTTTTTTTAATCTTTTCATCTAAAACATTTAATCTTTTTATATATATATAGTCTGTAATTTGTCT
ACGTCTATGTTATTGTCTATAATCTATGGTTTTCAAGTTACATCACGGTACAAAATTTCCGTTTGGTTTTTTT
CCCACCAGTATTAATTATTAATCAATTTAATAAA
>1002050060_1 Heli.1-DN172743.1.5
GCACGAGGGTCATGTCGAAAAGAACACTTCCCATTCGACACATGCCAATGTTATACAAACTTCAGGACAGAGA
CATATTACAGGAGATCATATTCATAGAAAGCAAATAATTACAGAGTCCCCAATTCATCACATGACTTCATCTT
GTCATACAGACGATCAACATCATCGTGGTACTCGTTCAAGCGAGCGTCAAGTCCAACG

ATCGTCCACTGGAACATCATCTTCAGGAAGTCACGTTGAGCAAAATATTTCTAAAGTATCATCAGTGCATCAA
TCACATAGCGTAAACAGAAATCAAATAAGTGACAGTGTAACAAAAACGAGTATGGAATTTCAGAGAGCTATGC
ACGGTGGAGGAGAAGCCCCAATTATATCAAAAGACCAGGCTTCGCGCAGTGGACACCA
TAAACGTACATCCGATTTGGTATCTGATTTGTCAAGTACAAATGCAGTACTTCATAGAAAAGGTGTATCTTCG
ACAACTGAAGCCCATCATTCTATCAGTTCCACTGCAGCCGCACAAAGAAAGAGCATAACGAACCTCAATGAGC
GTGGAGAATATATAAGTAACTCAACACATAGTGCTGACAGAAAGAGTATAAGTTCAAT
GCATCGTTCTGGTCGCGATCATACATCTGTAATTTCTGAACAAAATGAAAACAGCGACTTCCGCCGTCAACAA
AAACGTGACGGTCAATCAACACTGGCTGTAGATCGACAGCCGCGTGTTGTTGTTCGAGATAATCTTCGTGTCG
GTGGAGAGTTTTATGGTCAATCTGAGGCTCGCTCTTACGGCAGCTTCTCAAGATCTAA
TCACAGTGAAAAATCTGAAAGGAATGAACGTGTTGAAAAAGTGGAACGTCGAGTAAGGCATGGTACTACGTCA
AATTTCGTTTTGGGAGACGGAGGCACGAGTTATAAGCGAGAATATACTACTCACGTCCATGGAACTTGCCCAG
CAACGTTAATCGAGTCCCCGCGGACACCATTTAAGCATACTCGCGACTCAAGAGAGCA
CAAATTTTATACGGCAAAAACAAATCAGCAGAAATCTTAAATTTGTTGTATGTAACAACTACTAACCGATAGC
CGTATCATTCTTTATGTACTCCAGCTTTGCATCTGATTAAGATTTTTATATAAATTTAATATTTTATACAAGC
TAACATTGCTAGTCTAAAAAAAAATTGTGCCTTTAATTTTAAAGATTATGTATGCATAA
TATCACTTCATTTAATATTGTTTAACTTAATTTTTTAAATATATTTATAACTTATCAATTATTGCCATGTAAT
AAAAAAATACT
>1002050061_1 Heli.1-DT663599.3.5
GCACGAGGCTTCAGTAAAACTTACATAACATTCTTCAGTGAAGTGTTCGACTTCTCATTATAATACAGAGGAT
ACACAAATATACAAATAAAATGATCATTATGTGGATTTTTTATTACGTTGGACAGAAAGGTTTCTTCGTAACA
AACTTTCTCCTGTCCTGAGATTAAAAGGATTTTAATTCATAGATAATTGAATACATAT
GGATGTGAAAACATGGATGTGAAAACTGTTTAAAGGTATGCACAAATTTGTACAGTTTTGTAATGAACGTCAT
GTGGTCTTAATCTATGTAGATTGAAAACTTCTAACAATATTGCAGCTTCTTAGAATGTAAGATATAACTAATA
AACATATTTCAGATATAAGAATGCGCAGTAGGTGCATGTTTTATACTTTTGGTAGCCA
TGAGTTTATAAAAGTTTATTATAATTGAAGAATATCAGAAGTCTTATCATACACGTAAATGATCCTGCCGTAC
TGTTTGTGACTGATATACAGGAAGGTTTAAAATGTATATTTTCTAGTATATATCAAAGAAATAGCTAATTTAA
TAAGCAATGCCGAACAACGTTTTTTATGATAGATCATTTCAAATATGCAGCATGTTCC
TTCAATAATATTGTTGGTACGGTTTGTAAATAACCTGCAGTTCATTTCAAAATTTAAATAATATATCTAGAGT
GCTTCATGCCTGCTAAGCTTATATATGAGAACGATAGTGTGATGTGCGATTACAATTTATACAAGTAAATAGT
AATAGTATTATTGTATTACGTTTTTTATGTGGAAAGTATACAATACTTAAGATACGTA
AGATCGCATGTGATAACCATCTCAAAAGATTGTAGTTAGCACATTTTTCTTCCTAATTTTTCTTTCTCAGCTC
TGCATCCATGACTGCGCTCAATGGACTTAACAATTAAAATCGTGTTTGGGGTCATTGCCTGGTAGCATCTAGC
ATGCAAGTCATGACTAGCGCCAACATGTTTTGCCTAATGAAGCTAATATCTAAAAGAA
ACCTAAATGTATCGATGAATGGCAATTATTTAAAAAAATAGGAGAATCCAGCCTATGTCAGCAGTCTTTGCAG
GAAATGCAACGAATAACGTATACCTAGTTAACCAGTTTTTTCGGCAGGCGACGCGTGATTTCTGCAGCATCTG
TGGACAATTGTACAAGTTACTGGTAACCCGTCAGTCAGCACCATCTACCGCCTGAACT
TCTACTCCTGGTCTACGAGGCTCGGACGAGCGTGCTTCGCGCCTTCCAGTCTTGAGCAGTATGGCCTGGTTCC
AGTTAACGTAATTATCCCCATTTTCTGCAAGAACAGTGTATCCCGTTACCCGGTTGCAGTCGACACAACCATC
CCTGTGTTTTGCCAGACTTTGGGCCGGGCAGTGTGACCTGCTTCCAGTCGGCGCAAGC
AGCCCTAACTCCGGGTCAGGCAGTATGACCTGCTTCCAGTCGGCGCAAGCAGCCCTGTCTCCAGGTCAGGCAG
TGTGACCTGCTTTCAGTTGGCGCAAGCAGCCCTGT
>1002050062_1 Heli.1-DT667788.3.5
GCACGAGGAAGCATTTATGAACATTTTAAAGTGGGTATAGTGAGCGTCGGCGTTCTTAAATAGTGTCTTTCGC
AGCGTGGTGAATATTATAAAAGTTATAACATACTATAAAGCTGAAGTAAAATAAATTAAAAATGTCTTCTGAT
AATATAGAGAAATTGTATCAAAACTACGGCATTCTAGCTGATGCCAAGGATGACATTT
CTAAGCATGAAAAAGAATATCTGGAAATACTTGCTGCCGTTAAAGGTTCCGAAAAAGAAAAACGTTTAGCATC
ACAGTTTATTGCAAAATTCTTTAATAGTTTTCCAAAGTTGGCTGATCAAGCGATTGAAGCTCAGTTTGATCTT
TGTGAAGATGATGACGTGGCGATTCGTAAACAAGCTATAAAAGATTTACCTTCAATGT
GTAAAAATAATAAAGAACACACAACTAGGATTGCTGATATATTAGCCCAATTATTACAGTCTGAAGATGTAAC
TGAAATTAACATTGTCACAAACTCTTTAGTGACAATCCTTAAAAATGACCCCAAAGGTACTTTAACAGGTCTA
TTTTCTCAAATTCACCAAAGTACTGATAGTGAAGTGCCTAATGAAGTGGTCAGAGAAA
GATGTATAAAGTTTTTAGCCACAAAAGTCAAAATATTGGGAAGAGAAATCATTGATAAAGAAGCTGAAGACCT
TATTATAGCTGAAGGGAAGAAAATTCTGCAGGATTCTGTTGCTGAAGAATTTGAACATATAATGGATTTATTG
ACATGGTCAAGATTGGGAAAAACTCCAGCAGGAAAGAAGGAGCTAACTCAACTCATAG

CAGCATTGGCGTTTACACCAGAAGATTGGCATCCAGAGGATCCAGAATATGTTGACAGATTAATTCAATGCAC
ACAACATGCTTTGCCATTTTTTACGGCTCAAGTGGATTCCACACAATTTGTAAATATATTCTGTGACCATGTT
CTCCAAAAATGGAGTGACATTGCGGCAACAGGTGGGGGAAGTGATCTAAAACTGGGAC
TTTTAAAGACTTTTGCTGAAATTA
>1002050063_1 Heli.1-DT664302.3.5
GCATGAGGCGGACACTACGCGTGTGTAGTGACAGAGTGTTGCGTGCAAACTGGATGATTTTTATATATGAAAA
TTTGTGTTCGTAAGGATTTATGTATTTGAATTTAGAATTTTTTGGATTGCATTTTGGAAGTGGGTTGGTGAAT
GAACTGTGAATTCTACCAAATGTGTTCAAATGCAATACTGCCTTCCATATTATATATT
AATATTTTAAGAAAAGTAATGGCGTGGAGTTGAACCTTGGACGACGTACGACTGTTCGCGAGCTGCGATAGAC
TAGCATAGTGTACGCAGTGGTTGTTGGTGCGGCGCTGCCTCTATGCCGCATCGACGCAGGTTCGACATACACC
GGGACGTCGCCAGGTCATTACTGAAGATAACCGGAGAGCTGGGTGTCAGATAACCTGG
CCGGAGCCAGCAGCGACAATACAAAACAATGTGTTCCATTAAGTACCTAATAATAACATAATATATACAAAAT
ACATCGAATAATGTACGGTCCGTCCATAAACTGCCAAACTTTTGAAACGAATTTATTAATTTAGTGTTTATAT
TCGTTGTGTGAGTGTAACGCCATCTGGCGTGATCGAGGCGAGGTGGCGGGGTCGTCGT
CTGTGCCTTTGGCGATGCCATTTATCGAAGACGATTGGTGGCCGGAGAATGAGGGCAGGATGGTCGATCTCTC
CAATTGCCTTCAGGACGCGGTAACTGGATCTGGGCAACAGCGACACAGTTACAGTTACAAATGGCGACGTCTC
TGGGGGAAATGTCCCAAGCTGA
>1002050064_1 Heli.1-EL601315.1.5
CTGTGGTCAAAGTCATTCGGTAACAAACGTTCGTCTTGTATAGTTGTGTAAAAGTTGGTCGTTGCGTGTATAG
GCGGCTTTATTTTGTTTTAAATTTAATTTTCTTTTTAGTTGTTATTTGTATTTTTTATCTGTGATTGTGTTTT
TATGAATCTATAGAAAAGAAAGAAAAATGTGGCGGTTCTTTGTACCAACACTAGCACT
GATCGCGGGGGTCTCATCCATCATACCGAGACCCATATCAGAACCCTACTCAGCAGCATCTGCAGCTCAAATT
AGGTTCGCCATCGAGAACAGTTACGACAACACCGGTCCAGGAGCTAAGGAACCCGAATATGCTTATCACACTT
ACAAAAATGTGGATGACGCTCTCATCAGTTACTTAGACGACGCTGATACTAAATTGCC
AGAATATGAAAGGGAAAGAGCCATATCAGTTTTGCAAGGCAACCCCTACACACCACCTCCTCGTCAGCTTCCC
AAGTCAGTAGAAGAATACACAGGATACACTGAAAGACCAAATCCCATCAACCAATTTAACTATGGCGAACAGG
TTCTATATGATCCTAAAACCAATTACAAATACACAGATTTTAGAGGCTACGAAAATAA
TCTGGCACAAAAGAATTATGACTATAAAGTGAATGAAGACAATTTCTACAATTTGCAATCAGTTCAAAGTCCA
ACTGCGTCGTCGTATGTTAATAATTATGATTTGAGCTCAGCCCATGACCAGTTCGATCCTCACCCAAAATTAC
AGCTTCTCTTATGGAGTACACGATAAAAGTACAG
>1002050065_1 Heli.1-ES586230.1.5
CAGCTACCCAACTTTGGCTTCTGTCCCTCACTCCATCGCCAATGGATTCAAGAACCTGTTGGCCATTGCTGCT
GTCTCGGACGTTGAGTTCAAGGAAGCTAACACTATTAAGGAGTTTATTAAGGACCCAAGCAAATTTGTCGCTG
CCGCTGCACCCGCCGCCGCAGCCGCTGCTCCAGCGGCCGCCGCCAAGGAAGAAGAGAA
GAAACCCGAGAAGGAGGACTCTGAGAGCGACGACGACATGGGCTTCGGTCTCTTTGACTAAAACTACCATTGA
AGTCAAGTTATTCATGCTTTACCATCTCATTATGGTGACAAGTTGTAAGGGGATGTTTGCAATGAAGGTACTC
GCGCCGCCGGGTCTGGACACCCATATGGCGGCCATTTTGTATCTCTCATTGTGAATAT
ATACACATTTAC
>1002050066_1 Heli.1-EL600226.1.5
GTTACGGCCCTGGTGGTTGGAGAGGAAGAGATGAAACATTACAAGGTTCAATTGATTGTAAAAGAGCAGGTTT
ATTCAGACATCCTAAACAGTGTAACAAATTCTACGCTTGTAGATGGGACTGCACAAAACAAAGGTTCACCCTT
CATGTATTTAACTGCCCAGTTCAACTCAGTTTCGACCCTAATCTAGGTGCTTGTAATT
GGCCCAGCCAAGGACCAGCTTGTCAAGGCGACACTCTCCTAACAAATGCTCTTTAACATGTATTATAACTGAA
AGCTTGCGAATCTCAACGTGCCATCAAAATTGTGACAAGCGTCACGAATGTCCTACGAGCTTACAAATTTCGA
ACGTATATAAATAAGTTGAGTCTCTCTCTTTTAATTTTACTAGGCTTATTTATTTGTT
TGATTGTTTTAATTTTAATTCATAGTTATTTATTACTCATGCTCTATTAAAGCTTGTGATTGCAAAGCATTAC
TTTCGGCATTGATTGAACATAAGTTACCATCGTCTAGTTCATTTAGAATATATGTTTTGTGAAGTTGTGGAGT
CATTTTTATATGTCGATAAACGCTGTAATGTAAATATATCATTTAT
>1002050067_1 Heli.1-DT666536.3.5
GCACGAGGAAGACCCTTATAATTGTTTTAGTTTAATTTATATAAAAAAAATAATAACTTTCAAAATGGCTAAC
GTCGTTGAAAGCCTTCAACTGTTGTTTGATCGTCCTAATGAACCCATGGTCTCACTTAAGGGCGACAAAAAAG
CATTATTCCAGCTTACTGAAAAGCATTTATCAAACGACTACAAAAGAAATGGAATTGA

AATCAACAACCGTTTTGGAAAGCAAGCAAACGAAGTTATACCGGTGAAAGAACTTCAAAAAGTGCCTCGGTTC
AATAAAGCTAAGCGCCTACGACACGATCAGGAATTCTCCATATTACTACCTGCGCACCAGGAGATGGCTGACG
AAGTCATCGACGAACTGCTAGCTGTTCCTGAAAATCAGCTTCAAGACTTCCTATCCAC
GTGTATCTTCGCAAGAGTAAACTTGAACCCACAACTATTCAACTACTGCTACTCTGTTGCTTTAATGCACCGA
AACGACACTAAGAATGTGCCTATTCAAAACTTTGCTGAAACTTTCCCTTCTAAGTTTGTCAATTCGCAAGTGT
TCGCGCAGGCTCGCGAAACTGCCGCAGTAGCTGCTCAGGGTGCTAAGCGTACACCCAT
CATAATACCAAGAGACTTCACTGCCACGGACCTCGACATCGAGCATCGTCTGGCGTACTGGAGGGAAGACATC
GGAGTCAACCTCCACCACTGGCACTGGCATCTCGTATACCCTTTCACTTCTAACCAGCGCCAAATCGTTGCAA
AAGACCGCCGGCGGGGAGCTGTTCTTTTATATGCATCAACAACTTATGCTAGATATAA
CGGAGAGCGTCTAAAGCACGCATTAGCCAGGGTGAAGAAATTCAGTGA
>1002050068_1 Heli.1-EL600633.1.5
TTGTCACTGCCCGAAGGGAAGACTTTGAAGGATGTCTCTTGGTTTTCTGTTTGGTGTGATGAATATTCGATAA
ACTTCGGCTCAGTAGAGATCCCTCGTAACCTTGAGATCCCCAAGCCTTTGAAAGTCGGAGCCCTGAACGGAGT
GCATTTTTTATCCTCAGAACCCATCGTCATCGTCGACGCACAGACACTATTGATACCT
AACTTTTCATACGATGGAGAAGCGCCAGATGCAAAGTTCTGGGTGGGTCGTGGAGCATCCCCCTCCCCGCAGG
GCATCCGTATACCCGACGAGAACGGCAAGGAGACGCCTCTACGCAAATACGACAAGAAGACCATCGTGTTGAC
TCTACCAGGGGAACTAACAGTATTCGACATCGGCTACTTCGCTATATGGTGTGAGGCC
TTCACCGTGAACTTCGGGCACGTAACTCTGCCCCGCGCCACTACCCTCAATATACCGCCCAGCTTGAAAATGT
TGGGAGTTTCTCCACAGTCCAAATTGAACTGCGAATCATTAGATGATGAACTGGCGTTCGAAGTGCGATGGGC
TATCGCCGGCGACAGCATCGTGCTACAACTAGTTGCTAAGCTTGATGAAGGTGAATAC
ATGTCTTTCGGTGTCTCTGGTAGCCTCGAGCGTTCCCAAATGGTAGAAGGCGACGTAGCGGTCGCGGGCTACA
ATGTCACCACAATGAAGGGATTCGCAGACGATTATTACTTGGACGCTAAGAGTCAGTGCGCTGGGAAACATGG
GTCATGCCCCGATGACAAAATTGTTAAAAACACCAACTCAATTCGTCTGTTAAACGCC
GCACGCGTGAACGGGTACTCCATAGTAACGTACCAG
>1002050069_1 Heli.1-EL602452.1.5
GCACGAGGCAGATTGATATTTCGTCGTCAAGAATATCGTCAATTTTTGAGACGAAACAAATTGCAAATAACAG
CAAAATAGTGTGATAAACCTCTTTTAGTGTTAGTTAATCTAGTACAATTTTCATAAATTCAAAATGTTTATTT
GGGATTGGTTCACTGGCGTATTGGGATTCTTGGGTCTATATAAGAAATCTGGGAAACT
TTTGTTTCTGGGCTTGGACAATGCTGGCAAGACTACACTCCTGCACATGTTGAAGGATGACAGACTGGCTCAA
CATGTACCTACATTGCATCCCACATCAGAGGAACTCTCAATAGGTAGCATGAGATTTACTACCTTTGACTTGG
GCGGGCATCAGCAGGCCAGACGTGTATGGCGTGATTACTTCCCTGCGGTAGATGCCAT
CGTGTTCCTTGTGGACGCATGTGATCGCCCCCGACTGCCAGAATCTAAGAAGGAACTAGACTCCCTCCTAACA
GATGAAACTCTAAGCAATTGCCCTGTTTTAATTCTTGGAAACAAAATAGACAAACCTGGTGCTGCCAGTGAAG
ATGAACTAAGACAGTTCTTCAACTTGTATCAGCAGACCACTGGAAAGGGTAAGGTATC
CCGGTCGGAGCTTCCCGGACGGCCGCTTGAGCTGTTCATGTGTTCGGTGTTGAAGCGCCAGGGCTACGGCGAG
GGTTTCCGTTGGCTCGCACAGTATATTGACTGAAC
>1002050070_1 Heli.1-EL604033.1.5
CGGCACGAGGCCGTTTTGCTGGAACAAGCGTGCGTCATTGGACTGTGAGGCGTCGTTGCCGGCGCCGCGCGTT
TCTAAACCCCGCCCCAATAAAAAGCGAACCACGTACACTTTCGGCGAACTCAAAAACACCGTCCCCAACTCCA
CCGACGCGTCCGCCGTCGATGGTATTCACATCTCGCCGGAGGATTTCCTAAAAATAAT
CGACAACTAATCGTTTGTCGTGTCGCGGAAGTGGAACTTTAGTTAGTCGTTTTAACGAGTTATTATATTCGAC
GTGTATATTACGATTTGCAGATGTATCTATTGGCGGCTGATGTAAATAGTGTAATGTGCTTCTAATTTTAGGT
ATACGCCACTGAAACAGGCCATTGCTCAGTTTCATTGACTGCTGAGGTATTTAGGGGT
AATAAACATATATGGACATTGTTTCGCTATATGTAATATTAGACTAGTGGTGGATCTTCCTCTTGCATAGTAT
ATGAAAATATTATCATTGGTTTTTAGTTACTAAGTATTATTCTATTGGTTTATCTTCGTTTTAATTGATTTCG
ATATTGTCTTATAATTGTAGTTAGTTAATACTGATGTCATATTAATTTAACAATTCGC
TGGCAATGGTTTAGTTTCACTGAACGATCTCTATAAATGAATCTCTATTTTTTCTACATACTATGTACCTATT
GTACAAATTTACTCATACTTATGTAAAGTGTTCAT
>1002050071_1 Heli.1-EL597200.1.5
GCAGTACCGGTCGGAATTCCGGGCTGACCACGCGTCCGATTTACTTTAATCTTATCAGATTTAACCTCCTCAT
TTTCTTTTTAATTCAGTTATAATTCCTATTAGTTTACAATAATGTCTTCTCCCGAGGAAAGCTCTCAACTCAA
AGCCGGTCATCCCCCCGCGGTCAAGGCTGGTGGCATGAGAATTACACAACACAAAGTT

CCACATGCAAAAGATGCAAAGGAGGTACCAAATGAAGATCTGACTGGTCTTGCTGGACCATCTCCCGTTCCGT
CAAACCCAGTATCAATTTCAGGAGCCCCTAATAGAGGTAATGCGGATTTCACACCTCAGGCTGCCCAAATTGC
TCATAGCCCTAAGCCGCCTGCACATATAAATGTTAAACCTAGCCCAAATATCCAACAG
CCAAGAAAGTAGACTGAAATGATAGGTTGTGACAGGCATATCAACATCTTGTGTGTTTTGCTAGGTGTTTTAT
GACATTTAATTGATATTAGAATGCTTTTATTCACATGCATAATTTAAACAAATATATTACCTATTTGTAATTG
TTAACTAAATTACAAAAAACTGAACAGAAATAACAAATTATATGTTGAAAACTTTAAA
CCAGAGGTCTTTATCCTATTGATTAAACAAAAAGCAACAATAATATTAATCATTGTTTAGTTAAGTGTTCTTG
CAATTGTTTAAACAAAAATTTACTAAAATGAAAATACCTAGCAGCAA
>1002050072_1 Heli.1-ES584674.1.5
GCACGAGGAGGAGAGGCCCCGCCGCGCGCGTGCACGCGGCGCACCGGCGCTCCTTGGAAAACGACTCCGAG
AGCGCGTGCGGCGCGCTGGGCCCGCTGGCGGAGGCGCGCGGCGCGCAGTGGCGGCCCGCCGTCACCGACACCG
ACCTCGTGCGCGCCATCGAGGCCGTCGAGGACGTGCAACCAAAAACAAAAGACAGCAG
GCTGCCTTGGCGAAAAACAGAGGAAAACGAAGAGATGCGACGTCTCAGGAGACAGCGATCGCGTCAACAAATC
GACTCGCCTCAACCACTTGTATCCATTACGGAAGAAGAAAAACGAAAACTACCCGACATCACCGTTACCAGTG
TTAAAGATAACAAAACAGAAAAATCTGATCGAGTCGAAATAGATTCTGATAACATCGA
AACGCCTCCCGTACAAAGAAAAATATTCAACCCACCGCCAGAAAGAGAGCCTTGTAGGAAATTCCAATCCTCA
ATACCAGAAAAATCTTCCATCGAAATGAAGGACTTGTGTCAGGAGATATTAGGTGACGGACAGTTCGACAGAT
TCTCAGCTGCTAGAAGAACGAGAAGGTACAAGAGAACACCAGAATCGAGCTCACCCGA
GGAAGAGAAGAAACCGGAAAGTACTGAATTAATAGCAGAAACCCAAGTGTTGAGACCGTCTAAATTGGAAATT
CAAGCTTCGTATCCAATTGATACTCCAAAAGAAACGAAACCAGTAGAAGAAACAATAAAAGACGAGAAAGAAG
TTAGGTTAAAACGTTGGCAGGATAGACTAAAAGGTCAAGGTGGGCCAGAAAAACCAAC
CAAAGATAATAAATCAGTGCCATATTCCAGGATGAGGCGGCAGACATCCATCAACCAGGAGGACGTTCAGAAA
GCCATAAGAGAACTAAAGTCACCGACACAAACACCTAACTGGTGTCTGGTCAAGGAATGCTTATAGGAAATCT
ATGAATGCTAAAATAGATAANATTCCTACAGAAAGGACGATTTCTCCTAGAATTCCGA
AAGTAAAAGCGAACACGAGCTCAACGATGAAGGATTCGAAGAGACGCAAAGTCTTAACTCCGAAAGCGCCTCC
CAGGGAGCGTC
>1002050073_1 Heli.1-EL597440.1.5
GCACGAGGGTTTTAGCGGCAAAGGTTGAACGTTTGTATTGCGGCGCGCTTATTACAATATACAAAGTATAATT
ATTTTGTTTAAATATTGTAGTTGTATATATAGTAGTAGTAGTTTTGTACTCATCTGTGTTACTAAAAGTCTAC
TTAGTGAATTTATATTTTTTTTCTGTGAAAACACGAATATTATAAGTGTATTTCAAAA
CGTTGTGTAAATAATAAAAGTATATTATTTTTACCAAAAAAGTATATTATAAGTTCAAAAATGAGTTTAAGCA
GGTTGAGATCTGCAGTGAGATATGATTTAGTGAAAGCTTGTCCTTCAGGCGCCCTAGTTGCGAAGCTCTGGGG
GGCTCACATTGGTAGGGAAGTGGCCACAAGTAGCTCTCACCGGGCCGTTGCACAACAT
AACACGAAGCCAGACTGGAACAGGGCAGTCAGCGAGGCCGAGAAGATCGTCGGTTACCCAACATCTTTTCTTA
GCTTACGATGGGTATTGAGCGACGAAATAGCAAACGTTGCTCTCCATTTGCGAAAACTCGTTGGTAGTAACCA
TCCATTACTTAAAACTGCAAAAAATCTCATTTACAATGGAAAAAACAATATGCAAGCG
TGGGGCCTCATCGTGCTGTTGGTGTCCAAAGCGGCGGGACACAGTCCGGAAATACCCGACATGGAACAAGACA
AGGCGGCCGGAGTGTTACATAGCCAACGCGCGTTAGCCGAAGTAACAGAAATGATTCGGACTTCCCACCTCGT
GCATAAAGGCCTGGTCAACATGAACGCTCGACAAGCGGTCGCGGGGGAGCCCACGACA
TGATGTTTGGCATAAGATCGCTTTACTAAGCGGTGA
>1002050074_1 Heli.1-DT664386.3.5
GCACGAGGAAACACATATTATACATAGCCGCGTAAGAGCTAAAAGAAAACAAGTTATAAAACATTCATGTTTT
ATATTCAAAGTAATAGTTGATTTAATTTAAAGTATACCATTTTTTATTATTATTTTAAAATTAATATATTTAT
AACAGAATTAATTCTACAAATGATAAAACCTAATGTCGTTGGCATCAAGTAAAATGTA
GCAATTTACTTCAAAATATTAACTTTCTTATATCATCTGTCTATATATATGTATACGTATAATTAATTGCTGT
TTAGTACTTCAAATCTATGAGTAAAAGTGCTTCAAATGTATTGCGTCGTAGTATTCTCACAAGTTTCTTACGT
AGGTATTTCATAATGTTAAATTATTATATCTAATTAATTGTATGTATATGATACAGTA
AATTTCTCGAATGTTCACGAATTTATTGTATGGTTTACATTAGAATTACGTCTGATCGTAAACATGTTGATTG
TGGACACGTTCCCCATTCGGTGGAATGATTATGTTTAAGTTATTTCATTTACTTGCTCTGGAAAGACAAAGCG
CAGCGTTAAAATTCGCCACATATCATGTAACGTCTAGTTATAGGGCAAAAATCTGAGA
ACGAAGTTAATACTTGGTTCTCTGCCTACTACGGTCCGTGAAAAATAGCGAACAGGAGAAAAATGTACTAGAA
ATTAGAAAATGATTTTGAAGTAAAGTTGATAAATGTGTATTAAGTACTTATAGGGCTTCTAAAGGCTGAATGT
ATGATAACGAATTTCAGAGTAATGCGAAATTGAAAAGCAAGCAATTTATAATTGCTAT

CAAAGTTTTGTCTATTACTCTTTTAAGATAATTATATTTTCTTCTTATTTATTTTTATAAAGACTGGATCTTT
TTAAGTTATATATTTTAAAATGTAATTTAATATTTGACAAATAAAGA
>1002050075_1 Heli.1-EL604164.1.5
GCACGAGGAAATGAACGGTCAATATTAGATAGTGGCAATGATTTACGATTCCATGCATTTACCTGAAACTATT
AGTAATTACCTGAAATACGACCAGTGGTGGTACAGTGACAATTGGTGTGATAGTAATTCCCCGGGTTTCGAAC
ACCAGCAGAGGTAACAGGAAGTTTCTGGACCTTGTGAACTTAATAGTTGCTGACACTC
TACATTAGATATCAGCTGTAAATATACTTTAAAACCTTGAAAAGAAATTGCCTTGAATTGAAGTATAGTAGAA
GTGGAAATTGAATAAAATGGCTACCGTACAGAGCCCACCTGAACTTAGCCTGTCGGCACCCCTTGCCGTGCAA
CAGGATACTGACATAGATTTTGTGGCGTTAACAATGGCCGCTCTACGTTTCGTTAGTC
CTTGGTCAGTGGAGACCAAATCTAACAAATGGTCGCCAAAGATTGAACCCGGGACTCCAGAAGCTAAAGATCG
CGTCATTTCCCTGGCAGAAGTATCTCTACATGATACGCAAAAAGATTGCTGGGTTGTGATATACGATCGCGTA
TACGACATCACGGATTTTCTTTATGAACATCCCGGTGGAGATGAGATCATGTTAGAAT
ACGCAGGGCGCGACGCGAGCACCGCCTTCAGAAGCTCGGGCCACTCTAAAATGGCGACTAAAGCCTTAGACAG
GTTCCTTGTGGGAGAACTGCCGATGCACGAGCGCATGTACCGACGACCAGGAGGCATTCGTCTTAGTGACATA
CCAGAATAATTATATCATCATTGATGTAATCTGGTTATTTAAAATTAATAATATGTTA
AACTTAATTTAGCTTTAATCAAATGTTTTCTAATGTACTTAATTTTCTAATTAAAATTAGCTCAATTTTATTA
GCACATTGTCGCAGGTCTCATATCATGTAATCTTTGTTTATTGTAAGTAAAGCAGTTATTTAAAACAATTCCA
CTGTAGCTAGGCAAATTATCACTAACGTTCTTTATCTTTATGTTAAACTTTGTATTTT
CTGTATAAATGTGCTTTTCGGTATTCATATAATTTA
>1002050076_1 Heli.1-ES586637.1.5
CAGGCTTGAAGACGACCTGGTGGGCGAGCGTGAAAAGAGCAAAGTGCTGCAGGAGGAGATGGAGGCCACGCTC
CACGACATACAGAACATGTGAACTAGTACACCCCATCCCTCACTCCCTCTCCCCCTACCGCCCTCCCCCGCCC
GCCACACTACGCCCTGTGAATGTCACACGCACCTCTGGGGCTGCCAGATCTTGTCTTA
ATAATGTAATTAAATAGAGATAAAGATTAAAAAAAATTTGCTAAATGATGTATCCTGCATATAGCAGGGTAAG
CGGAAGGGACTTCCGTTCCCAATGCATAACCCAATTGTAGAGACATTGCGTATTTTCTTACCAGAGCGAGGAA
ATGGTAATAATTCACATTATATTTTAGACGAGAGATTTAGAGAGATCTGACAACCCCG
GGCTGCTCTCCGAATGACGCTTGTCAAATGTGACAGCTGTCAATGCGACAATACGGCTTAGGCGCGCTTTTTG
TGTCATTTTTTTTATTTATTAACTATGCTTTGATTCTTATTTTTTGATACGTATATTTGTATTAGCCGTGTAG
TGAAGCGGGCGGGCGACTAGATGTTACTAAATGTAAAGTAATTGCCAGCTGTCTTTTT
TATTTTAATATGTAACGTCATATCGAGTGTTCGCTACGACACACTGCCATGTGCAATTTTTTTTATTTAAATC
TGCTACACAAATTACTATTTTTTATGTCCCGAACGAGAGTTTAAGTTTATTTCTTTATTTCTTAGTATTGTCA
AAATGAAAGATTAGTCGCGATTAATCGTATCTATGTTTTATTAATGTTAGGGATTTTC
TACATTCCACCATTGGGACCAAAAATGAGTCGTTAATATTATATTAATCAAATTAAAATT
>1002050077_1 Heli.1-DT664109.3.5
GCACGAGGCTCACCCTTGGCACCAACGCCCTGAACTGTATACGCGTCCGGGAGCTCGACTCTCGTAGCGACTC
GACGAACAGGATAAGTTAAGCGAGCACAATGCCTTTCAAACCCGCAGATAACCCTAAGTGTCCGAAATGCGGC
AAGTCCGTATACGCGGCTGAAGAACGTGTCGCCGGTGGACTCAAATGGCACAAGATGT
GCTTCAAGTGCGGTCTGTGCCAGAAATTGCTGGACTCTACTAACTGCTCAGAACACGAAGGTGAACTTTACTG
CAAAGTTTGCCACGCACGCAAGTTCGGACCTAAAGGCTACGGATTCGGCGGTGGTGCTGGTTGTCTTTCCATG
GACACCGGTGACCACTTAAAAGAGAACGCGTAAGTACAACATTTATTCGAACTCCCGC
CGCCCGCGCCACTGAGGGTCTTTCCTTATCTAAGGTCGCTACGACGACATCGCCAATGTACTTACATTAAATT
ATTTTAGTTAGGATTATTTATTCGCTATCGCTTAATTTATTTTGTTTATCGGTCTATATATTTATAATTTATA
AAAAAATGTAATAAAACAAGTCGACTACCAATTCCGAGTTGTTAAATGGGAATGTGGT
TGAGTTCAATGTGAGTTCAAAGCTTGTCGGCAGCGAATTGCATTGGCTCTTCACGTGACCCCCTAGATCCTTT
ACTTCCTGATCAGGGTGATCCGAAAACAACAGAATTACGGTGAGAACCCAATTTAAAGCCTAGTCTATGCACG
GCTTAGTAACGCTCAAGCCGCACCCTAAACGCATGCCAAAAATTTGTCATTCTTATTA
TTCGTAACTTTC
>1002050078_1 Heli.1-DT663625.3.5
GACGAGGATTAAAGGCTGACCTACTTTTAGTGCAAGAAACATTGTACCTTTAATTCTATACTTTCTAGTGTTT
TCTTATATTTATTCAAATATTGGTTCTTGTGTAGTTATATAAATATTAATACTTAAATATGGCTACAAATTAT
CCTCCGAACACTTCATCTATTCCAATTGGAAATCCAAATGATCTTCCTCCACCATACT
CCGCTGTCGTAGGCAACCCTCAATACGGTTTCGTAGCTCCTGCTGGTGAACCGTATCCTGGTAATGAAGGAGC
TTTCCCACAGCCGAAACAATTTACTGCGACCGGAGTTTATCCACACCCCACAATTGTTACTACTTCTCAACCT
CAAACAGGTGACATTCCACCTCCTCCTCCTGGCATGTCAGTACCTGTAGGAGTGATAA

TACCCCCTGCTGTTGGAACTGAGCCTACAACTGTCACATGTTATAACTGTGGGAAAGTGGTCACAACAAGAGT
TACTTACACAACTGCTTGGCATACTCATTTTGTGGCTGGATCTATTTGTGTGATAACAATGGTGTGTTCACTT
TGCTGTCTTGGACTTGTACCTTACTGCTTCGATACATTTAAGGATGCTGAGCATTATT
GTCCAAATTGCAGTACATTTATAGGCAAAAGTAACAAATGTTAATGACCCATAATGACTATAAAAATGTCTCT
GTTAAAATGTCTCCAATAAATATTTATTACATAAGAAGGTATATAATTACATTTATTGTTCAAAAATGTAATG
TTCATCTTGAATTATTTTTAAT
>1002050079_1 Heli.1-ES588008.1.5
CTCGTTCCTATAATAAATATATCATTTAATTTTTAAAATCAGTTCTTTTCCAACATGAGAAGTTTAATCATCG
CGTTCGCTTTTTGTGCGTTAGCTTCAGCACAGGACTTCGATTGTCCAGAGAAGAGCGGTTTCTATGCAGACCC
GTACCAATGTGACCTCTATTACAAATGCAGCAGAGGTAAATCAGAAGCCAAACTTTGC
CCAGATGGTCTTGTATTCGCTGACGAAAACCCCCACAAGGAGCTCTGTGATATTCCCTCAAATGTAGACTGCG
GTGATAGAAAGGAGCTACAGGAACCCAAACCTACTAAAGGCTGCCCAAGACTGAATGGATATTTCAAGCATCC
CGATCCTCAGGCATGTGATAAATTCTACTACTGCTCAGATGGTGTCCCTAATGAATTG
CCTTGCCCTCCTGGTTTATACTTTAACGAAGAAAGCTCAAACTGCGACTGGAAAGAGTCGGTACAAAGACAAT
GTGACCAAATTACTAAAGACACACTAGACGATGGTTTCTCGTGCCCCGATGGTGAAGTCATGGGACCTAATGG
ACGTGCTCTTCCTCACCCAACCTTCCCCCACCCTGAGGACTGCCAGAAGTTCTACATT
TGCCGTAATGGCTTCCAACCCCAAAAAGGCAGCTGTCCATCCGGCAAGGTCTATAATGAAGACACTTTCATGT
GCGACGCACCAGAAAAAGTACCCGGGTGTGAAAACTACTATGAAGGCCAGCCACTAGAGAAAAACAAGTTACC
GAAGAAGGGGTAATAATTAGAATATATAAGAATTATTTTTAGTTTTAAAATTTAAATA
AATAGTGACATA
>1002050080_1 Heli.1-EL601703.1.5
GCACGAGGGATTTCTGCTGTTATCAAGAGCTGTGAATGTAAAGATCGCTGAATTCATTGCATTTAAATCGTAA
TTGCTTCGTATCTAATTAAGAAAAACCAAAAATCGTAAGTAAGTTCACCAAAAATGAATAGCAAAAGTGGTTA
CAATGAAGATCCATTTGATTATATTAAAGTCAGTGAAGATGAAGATGATTCTAATGCG
GTAGAAATACCCTGTGAAACAGACGGAACTCTTCTACTATCTACGCTTGTTGCTCAATTCCCTGGAGCATGTG
GACTTAAATACCGTCATCCCGATAGTAAAGTTATCCGTGGAATACGTCTTAGTGATGGTAAACTTCATCCTCC
GAATGAAGCTGACTGGGGTAAACTCTTATATATTTGTGTTTTTCCTAAAGAAAATAAG
CGAAAGATGGAAGATGTATCCTCAGAAAACTCCGCTGCAAAAACAAAGCGCTTGGAGAAAAAACTCACTTGCT
CGGACTTAATATGCCTGGGCTTACCATGGAAATCAACTGAAGAATCTATCAAACAGTATTTTGAACAATTTGG
AGAGGTGGTTATGGTTCAATTAAAACGAGATAAGAATGGATCATTTAAAGGCTTTGGA
TTTATTAGATTTGCTACATATGCTTCCCAAATGCGAGCCTTGGCTCAAAGACACAACATAGATGGTCGTTGGG
TTGATGTTCGTATACCTAATTCCAAAGAAGGAGTGGTGCCGCAAATGCCATGTAAAGTTTTTGTTGGGCGA
>1002050081_1 Heli.1-DT666941.2.5
CGGACGAGGTGTAAGCTCCCTACTTGCACCTGATATTTAAACAAAATGAAATATATCTTTCTACTAGTTGTGA
TAGTTGCTGCAACAGCTGCAGACATTAGCAGCTTGCAGCCGTTCTTATATCATCAGCAACCACAATTACAACA
GCAACAACAGCAGGTGTCCACGACGGAACCCATTCCCATCATAAGACAAGAACAAATA
CTCAACCCTGATGGATCTTATAAATGGAATTATGAAACCGGAAATGGTATAAGTGCTGAAGAAACTGGCTTCA
TAAAAAATTTGGGTATTCCTGAACAAGAATCAGCGGTTGCACAAGGACAGTACAAATATACAGCACCCGATGG
ACAAGTTATTCAGCTACAATACGTAGCTGATGAAAATGGCTTCCAGCCGCAAGGTGCT
CATCTTCCTACACCACCACCAATCCCTGTTGAAATTCAAAAAGCTTTAGACCATCTCGCCACACTTCCCCCAC
AGGCGCCTGAGCAAAATCTATATAAATTATTATAAATCAATATCACAAAGAAACATAATTTATTATGACCATA
AGAGGCTACAATACAAACACAGGCATTAAAGTGTAGTAAAATATATATAAATATGTGA
CTTAGTTTTAAGTACAATAATAATTTATTTTATATAGTGGAGCTATTTAAAATTACATCGAATAAACAATTTG
ATGTTAAAAAAAAAAAGATCCATCTATTTAGACATTAATTAATAAAGCATTTCTGTAATAATATTACAAAGTA
CCCGTCGTTTTATTGGTTAACCCTATTGTCATTTTTATACGAATAGAACGTCAGCTGA
CTAGTGTAATAA
>1002050082_1 Heli.1-CO729799.1.5
CACATAGGATATCTTGCACATCCTTAGTACTTGGTTTGGAAACATGATGAAAATGAGATTACTTTTGTTTCTT
GCTGTGCTAGTTATCGCAGCAGCAGCGCCACAAGAAAAAGTCATAGCTATTCTTAAGCAAGAGTTTGATCAAC
AAGCTGATGGATCTTATGTATACAGCTATGAAACTGAAAATGGCATTAAGGCGGATGA
AACTGGCAGTTTGAAGAAGGCATCAAGTCCTGACAGCAATGATGTAATAGTCGCACAAGGCGCGTTTAGTTAC
ACGGCCCCAGATGGCACTGTTATCAACTTAAGTTATACCGCTGATGACGAGAATGGCTTCAGACCTGAGGGTG
CTCATTTGCCAACTCCTCCGCCTATACCTCCAGCAATTCAGAAGGCGTTGGACTACAT

CGCATCTCTGCCTCCTCCACCACCGTCTAGAAACTGAAGAGACACTAGCACCGACTCCTCAATGACCTCATGC
AGACCAATCTTTTTTGTCAAAGG
>1002050083_1 Heli.1-DT666273.3.5
AGCCCGTAGCGTGCTAGTGTTGTGTCACAGCGGTGGGCCAGGTCGGTTTCGAGATTTATTCGTTCCGACTCAA
AAATGGCTCTCAACTCGGACGGTGAACAGAAATCGAACCTGGTCCTCCGCGTAGACCAGGATTCTGACTCGGT
TTTGCAGTCATTATTCGACACAGTGCTCAAGCCCGACTCTAAACGACCGCTACAGGTG
CCTCTGCGTATGCGACAGCTTCCAAAGTCGTTCTTTAACCCGCCGTCGACCGGTTCCAAGTCGCCATCTGTTT
CCCACTCGCGAGAAAATTCGGCCGATTCGGCTTTTGGATCTTCTTCTGCCACCGGAGCTGCTCCAGTATCACA
TTCTCGAGCACACTCTTCTCCTGCTAGCTTGCAACAGACTTATGCAGCTGGTCAACAA
ACACAGCAACCACCTCTACCACATCAACATGCGAAGCAGAGGTCCTATGATGTGGGAACGCATATCCCGGACG
ATCTTGGCCCTTTACCAGCCGGCTGGGAACAAGCACGTACTCCAGAAGGACAAATATATTATCTCAACCACAT
TACAAAAACAACGACATGGGAGGATCCCCGCAAGACGTTAGCTGCGCAAACCGTAACA
AGCAGTGTTCAGCATCAGAGTGCAGAGACGCTGCTCACACCAGCGGCCGCGCCGGCTATAGCTGCTGTAACGA
CAGCGAAGAGTACAAGCACTAATACGACGACAGATCCTCTCGGGCCGTTGCCGGACGGCTGGGAACAGGCAAC
GACACCGGAGGGAGAAATATACTTTATCAATCACGCGGCTCGGACGCATCCTGGTTCG
ATCCAAGGATACCGCAACACTTGCAACGTACTCCAGCGGGGAGCGGCGCCGCGGGCGGAGGTTGGGCCAATGC
CTCAATACAGGCTTGCCAGCAAAAATTAAGATTGCAGTCACTACAGTTAGAACGAGAAAGACTTAAACAACGC
CAGCAGGAGATACTACTCCAGTCAGAGTTAATGGCGCGGCAATCCTCGCCTATCGTGG
TGGCGCTGCCGGCGGGCGGCGGGGGCGCGGGCGCGAGCACGGAGCTGCCGCTGGACCCCTTCCTGTCGGGCCT
GACCGACCACCAGCGCCAGGAGTCGGCGGACAGCGGACTGGGCATGGCCGTCACGCAGTCCTACTCCATGCCG
CACACGCCCGAGGACTTCCTCTCCGGCATGGACGACCGCATGGACTGCACCAGCGAGG
CCGGCGCCGCGCTCGACTCCGCCGACATCACCGACGACCTGTTGGGAGAATTTACAAATGACATACTACTGGA
CGACGTGCAGTCACTGATCAACTCGACGCCCAGCAAGCCCGACAACGTACTCACCTGGCTGTAGAGGACAAAA
AACACAAATTTTTTAAATTAAGTATTATATCGTAACTATAGAGTACTTACCGCATCAG
AATATCGACTTCGTACAGGAGTGTCGGTGGACGGTAATGGTGTATATT
>1002050084_1 Heli.1-DT668428.3.5
CGTCTGAGCGTAGTCGACTCCACGGAGATTATGTTGTATTCGTACAAACAATACCAATACACATAAAAATTAT
TGTACAGTGTCGGTCGACGGGCGCGCCTTGTGCGTTGTTCGTATTACAATTTTAGATACATTTTGTTTTCATC
ATGTATTCTTATATTGTTCAGCGATCGTTCGAATTTATGTGTGCGCGTCCCTTGCGGG
CGATATATCTTTAAAATTGGCCACGATCTCTCCAACACCGGGCGATGATTTGTATACCACATGTTCACGCTCA
TGTTAAACGATATTATGAGAGTGTGTTGTGTGTGTTATTGCGACCGTTGTCGCTGCACACGCATTAAAGATCT
CGTCAGAGAACGTTCAGTACGGTGACCGTGCATCGCGTTCGTTGTCGTTTGTTTTTTG
ACAGGCGGCGACGACTGCGCTGCGGCGTCGTTGTAATAAATATAGAGTATATCAGAACGGAGAAAGTTTGCGC
GAACCGACACACACACACGCACATACATGCGTTTGTGTTATCATGTTGAGCTTTCGAAGTTTTCGAAATTTAA
ATATTTTTTTAAAGTCATCGCGACGAGTCTTTCGACCCCCGCGTACGACCTTTAAGAG
AAAATATAAAACAACGCGAGAGTTAGCGATGTTATTATTGTTAGCTCCCTGGTTGATCCTGCCAGTAGTTATA
TGCTTGTCTCAAAGATTAAGCCATGCATGTCTCAG
>1002050085_1 Heli.1-EL597378.1.5
GCACGAGGCCGCGTTGTTAATAAGTGCCCTCTCTCTGGCCACCGCGCGTGTTTACGAACGGTGCGAGCTGGCA
CGCGATCTTGTAAAACTCGGTGTCAGTAGGGACGATGTGGCTACGTGGGTGTGTATCGCGTTCCATGAGTCCA
GGTTCGATACTGCGGCAAGGAATCCCTTCAGTGGAGACCATGGACTGCTACAAATAAG
TGAACTGTACTGGTGTGGTCCCGGAAAAGCGTGTGGTGCACCGTGCTCCGCTTTCCGGGATGAAGATATATCT
GATGATGTCAGATGTGCCCTCCAGATTCATGAAGAGCACACTCGCTTGCAAGGGAATGGATTTCTAGCGTGGG
TAGTATATCCACAACATTGTAAACACAACGCAAAGAAATACTTAGTGGACTGTGAGGC
CACAAAATCGGTTTCAAAATCTAATGAAGCTCGATCCCTCAAAATATTTGAAAAGAACATTACATCGCAGCCT
CAGCTGGCATATAATTATGCAGAAAGTAAGAAACCAAATTTTTTGGCCATCAGCGATTTATTTAAAGCACATA
TTGCTCGCGATTTCGAACAAAACTATTACGAAAACAAAAAGCAACCTAACTGGTTAAA
CGTTAAAATAGATAACGTAAATGACGTTAAAACTCCGGTCGCGGGACGAAATCTATTAATAGAATTTACTACT
CCTCGTGCTGTCGCTACAACGATAGATCCTACTTTAAAAGGTGTAAAGCCGCCATCACCAAGAAAAATAGAGA
CGAATCAATTTCGTCGACGTATGTTGAATTTTGATGTTCCTGTTAAATATGATTATAT
TTTGAATGAGCAAACAAAAACTACTAAAGCACCGAGTATTGACAATGATTTTTTATCTCACAATATTTTTATT
AACCGAGCAAGAGCATTATCAAATTTTAACTTGAACTATATAGAAAATAATACACAAAAACCGACAACAACTG
TGGAAGTGAAAAACGATTAAAATAAATGTATCAACCCAAAAACCAACACCAACAACCA
CGACAGTGCCAACTCATATTTCTA

>1002050086_1 Heli.1-EL596641.1.5
ATGTTGATGCGAGAAATCGGAAAAAAATAATTATTTATTAAAGTTATTAATACATAAGAATTAATGAAATATCT
AAAATATGAAATAGTGAAATCGTAATTGTGTAAATCGAAAAGACACACATTAAATGAACTAAGAGATCATTTC
TTTAAATCATACGTGTACATCATCGGTCGTAAATATATTATATATGTAAAAATTGATT
ACATTTATTTATAAATCACTAGAAAATCTATCGAAATGAGGTAATTTAACGTTTTACTTCTACAATAAATTAT
ATAATAAACTAAACTCTAACGTAAATACAGTTTGTTACAATAATAAGTGCAACGATAGTTTTTCTTCATGATT
TGGGACTATGTGTTTCGCACCAAGCACATTATGCAAGAGTTCGAAGATATAGAAGTCC
ACAAAACCAGCGTATCGGTGTTCGTCGCACTATTTTACTTGGAAATGCTGTATATATTGGTAATGACATTATC
AATTTTAAATCGTATTAATCAATCATCTTCATAAATTAACTAATAATTAAACAAAAATGATTGGAGTTAAGCA
AAACATTATAAGTGAATGTTTATATCTACAAAGGGAAGAGAAAAATGAAGTGTTTTTA
AATGATTGTCAACTTTTAAAACAGCATCAAATAGATGGATTGCGCTTT
>1002050087_1 Heli.1-EL601861.1.5
TTTTGAGGCGAGTTTGTTCTTCGTACTTCGTTAATACTGTTTAAGCAGACATTGCTTAAACATATTAAAATTT
GTGATATTTACTCAATTATCCCCCTAATAGATATAATAATCTAGTAGTTATAAGGTATAAACACCAGTTACCT
GAAATAGAAGTACTCGTAAAATTTTGTCTGTGACAGATCGATTTAGCGTACTTTGTGA
ACTTACAGGTTATATACGCTTTATATTACTTTTTGACTTTGTGCTATTCTTAATTAATAAAAATGGAGAGCAT
GGACGTCGCGCAAGAGAGCACTTCTGCAGCATCTACGCCAGCAACTGAAAATGGACCAGACAAAAACGTGACA
GCAGAAGAAATGACCTCACGAGACTATTATTTCGACTCATACGCACATTTTGGTATAC
ACGAGGAGATGTTAAAAGATGAAGTCCGAACTCTTACTTACAGAAATGCTATGTATCACAATAGGCATCTCTT
CAAAGGAAAAACAGTATTAGACATAGGTTGCGGTACTGGCATCCTCTCCATGTTTGCTGCTAAAGCTGGTGCT
GCAAAAGTAATAGCAGTAGAATGCTCGAACATTGTAGACTATGCTCGGAAAATAGTTG
AGGCAAACAGACTCGATGATATTATTGAAATTGTTAAAGGAAAGGTAGAAGAAGTAGAACTGCCAGTGGAGAA
AGTTGACATCATAATATCACAATGGATGGGCTACTGCCTCTTCTATGAGAGCATGCTCGACACTGTTCTGTAT
GCCCGTGACATATGGCTGAAGTCAGATGGCATGCTGTTTCCTGATAGATGCACCCTGT
TCATTTGTGGCATAGAAGACCGTCAGTACAAGGATGAAAAGATCAACTGGTGGGACGATGTATACGGTTTCGA
CATGTCCTCAA
>1002050088_1 Heli.1-DT666915.3.5
CCAGGCGTCGTGCTTTAGATACCAGACATGCTCTACAGGAATTACAGAGCGAAGTTTCAAGTCTTGAGGAAAC
CAAAGACGATTTGCTTGAACACGCAGATGTTGTAATAACATTATTAACACCACACTCTAAAGAGGCTGCCTCT
GAAACTGAAAGGAATGTAAAAGAACTGGTGGAGGCTTATGAGAAATTGCGACAGACTA
TCGCGGCGAGACTCGCGGAAATCGACGAAATCGTTTCCGAATTCGATCGTGTGTCGGAGAAAATTGAGCAGCT
TCGACAGCAGATTGAAGTTTACATAGCTAAAGTTGGAACCTTCTACGTGTTTGGAGAGGATAACGAAGGGGAC
GTTCGTGATTTGACTAAGCAGGTGTCCGATTTAGTAAGACGGACAAAGACTTTTACCG
AAGACAGTAGGAAGCGGTACGGTGGCTCTGCGCCTGCAGACGTGGCGCAGGAGCTATCCTCTCTTGAACTGTC
AGCGGAAGCTCTAGGAGCAGCGATGGAAGAAAAAGAAAGGGAATGGAAGCGCGCGCGCACCTCTCGCAGTGAA
TATGCGAGTGATGTTGAAAGCGTGCAGGCTTGGGTCCGTGGGGCTGAATTAACTGCTC
GTGACAGAACATTACCGCCAGAACCTTACAAAGAACGACTTGTCGCCATGCGATCTGAAATACCCGCGATATC
TGATAGAATCGAGCGGCTAACTCGTAACGCCCGCGCGATAGTTGAGGGTAGCCGCGATTCTGGTGAACGTCAA
CTTATTCAATCCACTGTATCAGCTTTAACGGAACAATTTTCAGCAGTTTGCAGTGAAC
TTGAAGCCAGACAGGCAGCAGTAGAGGATGCCTGTGATTCAGTATCTAGATTTTTGACTTTATTGGAGAAAGT
TTTACTTTGGGTGGAAACACAGCGATCTTTTCTTGCGCGGCCACTTCCACTAGCTGACCTGCAAGAGGCCCAA
CAAAAACAGACTGAATATGGGAATGCACTTAAGAGTTGTAAGCAGCAAGCTAAAAACT
TAGCAGATATGACCAAGGAATTAGAGGCAATAGAACGCGTAACTAGTCCTGGAGATTTGCTTTCCAGGCTCGA
AGCTGCTGAAAACTCAACAGTTGATGTCGAAAAACGCTTGGCAAAAACAAATGGCTTACTGCAAGAATTAGCT
GAAGAATGGGAGAGATGTGAAAAAAAATTAAAAGATGTCGGCCATTGGCTTGAAGCCA
CAACAAGAACCATTGAGACACCTCAAAATGCGAAAAAACCATTGCGAGATAGATTGGCTTTAAGAGAGAAATT
AATCAACGATATATCGACACAGAAAACAAAGATATCATATGCGGTTGAAAAGTTGAATGTACATTTCGGTCCT
GACGGCGTAGCGTCGCAGTCTCGGTCTCCAGAGGGCGTGCAAAGTGCTGCGCGTTCGT
TAACAGCGGCGCTGGATGCTCTAGCAGCGACCACTA
>1002050089_1 Heli.1-DT664604.3.5
AAAATCTCAGTATTCTCTTAATATCGACAGTGTTATTGTACACGGAAATTCTCTTTCCGCGGAAGTGGTCACA
GTTGACACCGATGGTGATTTTTTTAATATATTGTGGCACTATGCACTAAGGCTATCTGCCATAGCCGATGGTA
CATTCCGTGTGGAGATAGATGAAGCTGATCCTCTGTATCCAAGATATAGGACACAACT

GGCCTTGAATGGTGAACCAAAGGAGGATGGATTAAAACTAGTATCAAATGAAAATGGAAAAATAACTTTAATC
AACACCCAAGGTCATAAAGTGTTTATAACAGCGGAACCATTAAAATTAGAGTTTGTTGACCTCAATGGGGAAA
TTTCAGTAATACTTAATGATAACAACCAGTTGTTTGTGGAACCTTTGAGGGTAAGAAG
GGAGAAAATTGGTGATGATGATGACGCAAATGTGGTGGAAATTGAAGAAGAAGGCACATGGAGTGAAAACTTC
AAATCTCACATGGACAGCAAGCCCAGAGGGAATGAAGCTATTTCCTTGGACGTGTCCTTCCCAGATGCCGAAC
ATGTTTATGGTATACCGGAACACACAGACAATTTCTATCTAAAAACAACAACATCGGG
CGAACCCTACAGGCTGTACAATTTGGATGTTTTTGAATACGAATTGGACAGTCGTATGGCTATTTATGGAGCA
GTACCAGTGTTATATTCTCATAGTGCTAAACGCAGTGCAGGTGTGTTTTGGCACAATTCAGCTGAAACTTGGG
TGGATGTTGTCAACTACGCCGATGGTACAGTGGTCTCTTCCCTTGTTAACTTGGTGAC
>1002050090_1 Heli.1-EL603605.1.5
GCACGAGGATTTTTACTAATTCAGTGCTTTAAATAATATTTATTGGAAAAAATATTATAGTTATTTTGGATCA
TTTAAAATATTATAAAATGTATCCATTTTATATTAAAGATATGAAAAGTTTTATAAAACATTTGCTCATTTAT
AATCTTGCCTTTAAACTTTGCCATTTTTTTTAAATGCAAATCCAGATAATGTTTATTC
AATTAGTACTCCTTTTATTCAAATTTAATAATTAATAATTTCTTTAGCGTTTGAAATATTTTTGCCTGATTTT
ATTTGACTTTTATGTAAAATTGATTATTTGAATCTATAAGTAAATTTGTAATTCCACACTTTTGTCAATGTAT
TCATTAGGTTGGGGTCTTTTTTTTCTCGTAATAGTTGTATTATGTTGTCTAAACAAAT
AACGATGCGTTAAAAAAACAAAAAACAATGTTTACGTCCTTAGCCCCCGTATGTAACTCCAAGTGTTAAGATT
CTATGAGAAAAACCGAGAGTTGCATTTGTCCTTCAAGTTTATATCATTTCTTTCTTCACTTACTTAAAAAAAA
CTAATATAAGAATATACCAGATAAAAATAATTAAAGTGAAGCCAATGAAGCCAATAAA
CAAACCTATAATATTATATTAGCTATGAGTATATTGAACAGCAATACAAGTTAATTTGTTTCCAGTAATGGGA
AAAATATAGATTCATAGTAATACTTATATAATAGGTAACTGTAATTATTAAATGGAGATAAAAGTAAGTAGGA
AAAATAAGAAGACTGAATTATGTGATAAAAGTTCAACTTGCTTTGAAAGTCCAGACGA
TGTAATTAAAAAAATAGACAGAATTTAAAAACCAAAATGAGCGAAGTCTCTTTCTCACTCTCAAACACAATGT
ATACATCGTGT
>1002050091_1 Heli.1-DT667524.3.5
CTTGGTATCAGCTCACGACGCGAACTCCTCGCTGACCATCGACAAATATTACACGGAGAACATGGAGGAGCTT
GACTTCGACCCCAACAACCCTAACGGGGCTGACTCCATTTGGAACTACCACGTGTGGAAGGATGTTTGGATGG
CGCGACCTGATCTGCCGCCAGGTTACGGAGGCTGGCAAGCGATAGACGCAACACCCCA
AGAAACTTCATCAGGAATGTACCAATGTGGTCCAGCACCTTTGGAAGCTATCAAGCAAGGTGTGATCGGTTTG
AATTACGATGTAGAGTTCATGCTAGCGTCCGTCAACGCTGACCTGATGAGGTGGAGACGAGACCCGGAAAGTG
AATCTGGATATTCCGTGGTTAAAACGAATAACTATCACATCGGTCGTATGATCCTCAC
AAAGAAACCCTTCGTGTTCGACCCCTTGGGTGATGAAGATAGGCAGGATGTGATAAGCGAATATAAGCACAGG
GAGGGCACAGCCTCCGAACGTATGGCTTTGATGAATGGAGTGCGGTACTCTGAGAGGGCGAAGAGATATTACG
CTGTGGCCGCAAACGTATCGGATGACATATCGTTCAAGCTCCGCGACATCGACACTGT
GTCCATCGGCAAAGAGTTCAGAGTCATCGTCGATATCGACAACAATTCCAGCGAGGGTCGCAACATTAAGGCA
GCTTTAACAGCAACCAGTGTCTTCTACAATGGAGTAAGAGCTGAAGTCGTTAAGAAAATAGAAGGAAAACTTT
TCGTCGGTCCGCAGAAACATGAACAAATCAGCATCCAAGTCCAACCAGACGACTACTT
GCCCAAATTGGTCGAGTACTGTAACATGAAGATCTCCGCTATGGCCATCGTCGACGAGACCAAGCAGTCGTGG
GCTGATGATGACGACTTCCAAATATTGAAGCCTAATATAAATATTAAGTTCAACGAGGACCTAATAATCGGAC
AACCGGCAACAGCGGTTTTATCCTTCTTGAATCCTCTAGAACGTTCTCTTACCGGATG
CGAGTTCAGAGTGACCTCGTCGGGTATAGCTGGTAGGACGTTGCGTTTTGGGGCCCCCGATGCCGCCCCGAAG
GGTATGCTGAG
>1002050092_1 Heli.1-EL602857.1.5
GCACGAGGATTTTATTGCTGGTTGAAGAACGGAGGGAGAAGCGAAGGCTTCCCCAACTAAGATATTATAATAT
TAATTTTTGGATTGTTTTTATTTTGTGAAACACACATTATACGAACGTACAAACAAGTATACGTAATTAGTGT
AAGTTTTAGGAAAATTTATGTATTGAAAATTGAAAAATGGACGATCCCAATAGAATGA
TGGCGCATAGCGGTGGTCTCATGGGACCTCAAGGCTACGGTCTTCCTGGAGGCGACGGAGCACCTGCCACGGG
CGATGGAGAAGCTCGCAAACAAGACATCGGAGAAATATTGCAGCAAATCATGAATATAACCGACCAGAGTCTC
GACGAAGCTCAAGCGAGAAAACACACATTGAACTGCCATAGAATGAAGCCTGCCCTAT
TTTCCGTGTTATGTGAAATTAAAGAGAAAACAGTCCTGTCCCTCCGCAACACACAAGAGGAAGAGCCCCCAGA
TCCCCAGCTCATGCGATTAGACAACATGTTGATCGCCGAAGGTGTCGCTGGCCCAGAGAAGGGTGGCGGTGCA
GGCGCTGCAGCGTCCGCGTCAGCCGCGGCGGGCGAATGGGGCACTGTGGCCCAACCAG

ATAACGCGATCGAGCACTCCGACTACCGGGCCAAGCTGGCGCAGATCAGGCAGATATACCACCAGGAGCTCGA
CAAGTATGAGAACGCGTGCAACGAGTTCACTACCCACGTCATGAATTTGTTGAGGGAGCAGAGTCGCACTAGG
CCTATCACGC
>1002050093_1 Heli.1-EL596111.1.5
GCACGAGGGGCAAACGACGCGAGTAGACGTACGGTCATGCGAGAAGCGGCTGGTAGGATGGCCGAACTAGAGT
TCGAGGCGCCACTTGCTCAAATCGAGGAGGCGGACGACTGCCTCTCCACATCAGATTATCCGCAGAAGACCAT
AAATGGCAACGAGATACGCCTCAACAACATCAACAATCTCAAAAATGATCTGGTCAAA
ATGCAAACCACTGATTCCAATAAAATTAATGACAACGACGAACCGAGACCGGTGAAGTGTAAACAGGACAAGA
TGCAAAATTGTGAAGGCGACAATTTCACGGAGACTTTCTCCGGGTCACTGGAAGATTTAGTGAATACGTTCGA
CGAGAAGATCACGAAGTGCTTTGGAAATTACGAAGAAAGCGTGGAGAAGCTGGCCCCG
GTGCAGGTTCGATCTCAAGAGGAGATTATGAACGAGTGCCAAATGTGGTGGACAATTACTGGCAACTTCGGGA
ACATTCTGCCGATAGACTGGACTAAGTCCTTCTCAAGAAAGATGCACATTCCCACATTAAACCTGAGCGACAA
AAAGGGTGCCATGACTCCAGACGATGAGATACATAGCTCTGAGGACGAGGCCGTCGCG
TCTGATTTGGACATGCACGCCCTAATCTTGGGAGGGTTGCACCAGGATCACGAATGTCCAGTTAAGACTGCTG
ATGAGGTGATACAAGANATAGATGACATGATGCAAGAGACGCCGTCCTCGGAAGGGGACCTGCTCGAGTATAA
TGAGGCGTTG
>1002050094_1 Heli.1-EL599422.1.5
GCACGAGGATACAGCACAAACGGTCACGGCAGCGTTGCTTCAATGAACACAACAGCCACAACTTTAACCGGTT
TATCAGATGCCAGTACAATAACAAGATCTCCATTAAGATCCTCATTAAAAAAACCCAAGCCAAAAGAACCTGG
CGCGTTGGGAATACAGAATCCAGGTTATTCCGGACATTCACCACCTTTGAACAGAAAT
GGTAGTCAAAAGAAGGTACGCATACAGACACACAGTACCGAAGTTTAAGGTTTATTTATTTACTTATTGAATT
TGGTTTTGACAAGATTTTTTTTCTAAGTTATAGATTTTTGTTATTGAGAATTTTTTATACTTATTAGAAAAGT
TAGTAAAAGTTCTGACTGATGTTATATGTATACACTGACAGGTTATTCATAACGTAGA
GAAAAATCGATAATAATGTATGATCGATTTTATACATTTATTTTATTAAAATCATTAAGTTGTTTGCTAAAAG
ATTTCTAATTGCTATTAAGCACTACGTTATTCTGTTTGATTATGCAAATAACAGTTTTATTTTATTGTAAATT
TTTATAAATTGAACGTAGGCACAATAATATACAAATATCTTTTGTATATATAAAAATC
ATGTATTGGTATTTTTATTGTTTTAATATTATGAAAAATACCATTTAATACATTATACCTTGCTGTATCTCAT
ATATATAGCAGATGTAGAAGGGAAGGTAAAGGGAACCTAGTGCCAGTACACTATTCCCGCACGCACCGCTTAA
CTTTCGAAATTATTTTTTTCATTGCTTGTTGAGAGGCAGAACACGACGCCGCATTTGT
CTTGTTACCGAGCGAGGAAATGAAAATTATTCAATCCTCCAAATGACAATCGAACCCGCAATCGTCACGCTTA
CAGTCACGTACGCTATGCCGCGGCGATAATTCTACTAAAGTTTGTTTCTACAGAATCTCATATTTCTGTCAAA
TAATCTAGATTCATATAAAAGTGTATAAATAGTGTTAATGTGTTTTAGTTTTATAAAA
ACATAGTCATTAGTAGTACTATAAATTATATTTAAGTTTAATATAAGTTCGTATAAAAGATCTATTTACGTAT
ATCAATTAGCACAGAATTAGTTTTAGTTTTTTTTT
>1002050095_1 Heli.1-EL603112.1.5
GCACGAGGGCCATGTTATACACATTTCTACGCTCCCTATTATATTACTTTATTTAACGAGATGAGTCTAGGCA
TCAAATCAGTCCTAGTAGTAGATGGGGTCGGTGCAAACTGTGCCGAGATACTTAAATCTCACAATATTAACGT
AACGACAAAGGCAAAGATCAGCAAAGAGGAACTTTTAAAAGAAATTCCTAACCATGAA
GCTTTAGTGGTACGATCGGCATCACAAGTGACTAAAGAGGTATTGGAAGCTGGATCTAAACTCAAAGTTGTAG
GCCGAGCTGGAGCAGGTGTGGACAACATCGACGTGGCTGCTGCTGGACAAAAGGGCATCGGAGTTATCAATGC
ACCGGGCGCCAACGCTCTCAGCGCATGTGAATTGACTTGCGGATTGACCCTGGCTTTG
TCCCGTCACATCGTCCCTGCTGCCACTGCTCTGAAAGCCGGTCGTTGGGAGCGCACACAGCATACAGGAACCG
AGGTTAATGGGAAAACCCTGGCTATCCTGGGACTCGGCAGGGTCGGACGCGAAGTAGCAGTACGCATGAACGC
GTTTGGCATGAAGATCATTGGATTTGATCCTTTTGTAACGGCAGAGCAGTGCTCCCAA
TTTCACGCCACAAAGATGGAGCTGGACGAGATCTGGCCCCTTGCCGATTATATCACACTTCATACACCACTTA
TTGAATCCACCAGAAACTTTATCAACGCCAATGTC
>1002050096_1 Heli.1-EL599187.1.5
GCACGAGGGAGGACGCATGTGCTCGCGTTATTCGCGGTGGTAGTGTTTACATCTGTGCAGGCCGCACTCCAGC
GTCACGATATAATCCCCATATATAACTATCAGACAGTACATAAAATAAATACAAATGATATACCTTTCACAAA
GAATAAAGGTGTGAACATACCAAAGCCAGTTAATGAATTATATCCTTCTGCGGTGCTG
TTCGGCAGAACTTGCGGCGGCACCATCATCAGCCCCAAATGGGTTCTTAGTGCAGGTCATTGCACGTTGTTCA
CTGGCGGTCGAGAGGTCCTCGCAGGTACCAACAACACGGCGGACGGTACCGGCGTAAAGCGGAAGGTCAAGCG
CCTGATCATCCACCCTCGTTTCTCAGTGGGACCGTACTGGCTTGATGCTCAACAATTT

AATATTAAGCAGGTGGGAGCAAGATTTGATTTTCTCCTTGCGGAGTTAGAAGAACCTTTACCTTTGGACAATG
TGAAAATTGCGGCGGCGCAACTTGAGACTCGTGAGAGACTACCCAAAGGTGAAGCGGTCGGCTATGGAGGCTT
CGGCGCAGAAAGACACGGTGAAACAATGCGAGAAGATATGCATGGCATGGACCTAGAA
ATATTGGATAATTCAGAATGCTCCTCCCTCGAGGAGTTCGACGCCCAAGACATGGTGTGTCACGGTGAAACAA
TGCGAGAAGATATGCATGGCATGAACCTAGAAATATTGGATAATTCAGAATGCTCCTCCCTCGAGGAGTTCGA
CGCCCAAGACATGGTGTGTACGAGAGGAAGAGCACCTCGCTTTGACTCCGCCCTGTAC
GGTGACAGTGGCAGTGGACTTATT
>1002050097_1 Heli.1-EL597627.1.5
CCGACTTGCATCATGAAGACATTGATCGTTCTCGCTATCTGCTTCGTCGCGGCTCAGGCGCTCACTGATGAAC
AGAAGGACAAACTCAAGAAGCACAAATCCGAATGCCTCGCTGAGACCAAAGCTGACGAAGAATTGGTCAACAA
ACTGAAATCAGGCGACTTTAAGAATGAAAATGAGGATTTGAAGAAATACTCCCTGTGC
ATGTTGATCAAATCCGACCTGATGACCAAGGATGGCGAATTCAAGAAGGACGTCGCCCTCGCCAAAGTACCTA
ACGCAGCGGACAAAGTACTAGTAGGAAAGCTGATTGACTCATGCTTGGCCAACAAAGGAAATGCTCCTGAACA
GGCCGCTTGGAACTACTCCAAGTGTTACCACCAGAAGGACGCTAAACATTCGATTTTC
CAATAAATCACAACTTTTTCCCCAAAAACATTGGTAAAATTATCAAAAAAATGTGTTTTTTGTTGCATTTATG
TATTTTTTCGTTCTAAAAACGTATTTACCATGCACCATTTTGATTTATAAATTTATATTCGTTTGACTTAATG
TAAACTGATACTGTCTGCACGAATTTAATTGCGAATAAAACCTTTT
>1002050098_1 Heli.1-EL603319.1.5
GCACGAGGGTACACCCTTCCCTAAATGGCAGTTAGCCATCATTCTAGGAGCTCCTTTAGCTATTGGTTTAGGT
TACCTTTATCTAAGAAATAGACTAGAAGATCCGGAAAAGAAGAAAGTAGCTGAGTTGAAGGCTAAAACAACTA
TTTCTTTAGATAATGAAGATAACGCGAAAGCCGCCGAAAGCGCTATTGATCGAGCGAT
GAAATTAAAAGGAGCCGGAAACCGAGCTTTTCATGCTGGTGAATATGATAAAGCTATTTCCCTGTATAACGAA
GCAATTGAAGCATGTCCACCTGATCGTCCTATTGATTTAGCTACGTTTTACCAGAACCGGTCAGCATGCTATG
AGAAGCGTGAAATGTGGGAACAAGTTAAGGAGGATTGCACATTTGCTCTTAAACTAAA
CGAAAAATATGTTAAAGCATTTCTGAGACGATCTCGAGCCGCTGAAAAAAGTGGTGATCTCGTTTTAGCTCTT
GAAGATGTAACTTCTGCTTGTATTTTAGAAAGATTCCAAGTTCAGAGCTCATTAGTTAATGCTGATCGCATTC
TTAAAGCCCTTGGTAGGCAACATGCAAGGGAGGCACTTGCGATGAGACAACCTGTAAT
GCCATCTAAACATTTTATTAAAACATACTTCTCAGCCTTTTCTGAAGATCCCATTAACAAGATTGAAGTCGA
>1002050099_1 Heli.1-EL602626.1.5
CGGCACGAGGCCTCGTGCCGCACGTGTTTAATAAAAAACATAAAAAATAACATCAAATAACCAGTGATAAATA
GACATCAATACAATTACATGTGATATATTAAATAAGTTAAACAAATAAATATAAAACGCAGTTATGACGGAGG
TAATGCAGTCCGGCACCGAAGTGTTTTCAGAGAAGAAAAAGAAGAAAAATAAGGAAGG
AGTAAGCTTGGGCGCCTTTCAGAAGATCGGCGAATTCAAAATAGAGCCGTCTGAGAGTGTTCATAAGTTAGAT
ACGGCGTATTGGCCTCTGTTATTGAAGAACTTCGATCGTCTCAATGTGCGCACTAACCATTACACACCCCTGC
CTTTTGGTAGTTCACCGTTGAAACGCCCAATTACAGAATATGTGAAGTCAGGATTCAT
CAACGTAGATAAACCTAGTAACCCTAGCTCTCACGAAGTTGTATCTTGGATAAAAAGAATATTGAAAGTCGAA
AAGACTGGTCATTCAGGCACCCTCGATCCCAAGGTAACTGGTTGCTTAATAGTCTGCATCGACAGAGCTACAA
GATTAGTCAAATCTCAACAGAACGCTGGAAAAGAGTATGTCGCGGTGTTCAATTTACA
TTCTGCAGTGGAAAACATTCAGAAAGTTACGCAGGGGTTGGAGAAATTGAGAGGAGCACTGTTCCAACGACCT
CCTTTAATATCTGCTGTAAAACGTCAACTACGTGTTAGATCAGTGTATGATAGTAAGCTATTGGATTATGACC
CAGAACGTAACATTGGGCGTTTTTTGGGTCAGCTGCGAGGCAGGGTCGTATATACGTA
CCATGTGTGTACATTAAGCCTCAT
>1002050100_1 Heli.1-EL597121.1.5
GCACGAGGCCGGCGCGTACGCACGTCCTTGAAACAAAAAGTTATTTCGATCAACAAAACTAACATAAAAAATT
ACAAATAATTAAAAAAATACATATTAGTTGTTAACTTTAATATAGATAATAAAATAAAAAGTACGCGGTTTGT
GTAACTTAAGTTTTTGTTGTTAAAAGTGTTATTTTAGAAGTGAAATCGGCTTTATTTT
GTCAGAATGTCCACCTTGCTGTTCTGGCAGGGCAAAGGCAAGGCGAACGGCACGCCCAACGGCAGGAACTGGA
TACACGCGCCCGATGCGCTGGTTAAGGGACACGTTGCGTACTTGGTGAAGTTCCTAGGCTGTACACAAGTGGA
CCAGCCAAAAGGTATCGAAGTCGTAAAGGATGCTATCAAAAAACTACAATTCACACAA
CAACTAAGGAAATCTGAGACCAAGGATGGAGCGAAATGCAAGAAAGTTGAAATCACTGTGTCTGTCGATGGAG
TTGCTATACAGGAACCAAGATCCAACAACGTGATGTATCAGTTCCCGTTACATCGGATATCCTACTGTGCTGA
CGATAAGGGAGCAAAAAAATACTTCTCTTTTATTGCGAAAGGTGGCAGCACCGTCAAT
GTCAATGGGGTAAACGGCCACGATAGTGGAAATGTGGAGAAGCACGAATGTTTTGTGTTCATATCCACTAAAT
TGGCTTCCGAGATCACTTTAACT

>1002050101_1 Heli.1-EL603613.1.5
GCACGAGGCGGTGTCGTTCAGTTGTGAACATGAAGGTCCTCTTACCGCTGCTGGGCCTCTTCGCCCTCGCTGC
TGCCACTCCCGCCGTCTCTCAGAGCAAGGTGGTCTGCTACTACAACAGCAAGAGTTATGTCAGGGAATCTCAA
GCACGCATGCTGCCCATGGACCTGGATCCAGCTCTGTCATTCTGCACCCACTTAGTCT
ACGGATATGCTGGGATCCAGTCTGACACCTACAAAATGGTGTCCTTGAACCCCAACTTGGACGTGGACCGATC
ACACGCTAACTACAGAGCCCTTACCAACTTCAAGACCAAATACCCTGGTCTGAAAGTGTTGCTGTCTGTTGGC
GGTGATGTCGATATTGAAGAGAAAGAGAAATATAATTTGCTGCTCGAATCACCACAAG
CTCGTGTAGCTTTCGTCAACTCAGGTGTGCTATTGGCTGAACAGCACGGCTTTGATGGTATCGACCTCGCTTG
GCAATTCCCCAGAATCAAGCCTAAGAAGATCCGTTCCACTTTTGGATCCATCTGGCATGGTATTAAGAAGACA
TTCGGCACTACACCAGTCGATGACAAAGAATCTGAACACCGTGAAGGATTCACTGCTC
TCGTACGCGAAATGAAGTCCGCTTTGAACCTGAAGCCCAACATGCAGCTCTCCGTCACCGTTCTACCTAATGT
CAATGCTAGCATTTACTTCGACGTTCCAGCCATCATTAACTTAGTAGACATTGTTAATGTAAACGCATATGAC
TACTATACTCCTGAAAGAATCCTAAAGAAGCTGA
>1002050102_1 Heli.1-DT662931.3.5
GCACGAGGAATTTACATTACAGATACAGGGACAGGCCAAGGTTATCGGAAAGATATAATAAAAAACCTGAGAC
GGAAACAGAAGACCAAGTAAGTGAAGATCAAGATTCTAACTATTCTATAAATGTACCAAAATATGCCGCACCA
GAATCTGAACGCTGGTCACCCAAAATTTCAAAAGATTCCTTTAAGCCATATAATCCAA
ATAACATTGCCGACGACACAAAAAGTGATACAGTTAGTGAAAAGTTAAAGGATGATGAATTAGATATCATTAC
AGCGAAAAATGAATATGAAGATATACTTATTTCTGTAACACCGTCTTCAAATAACAGACAACAGAAAAAAATA
CCTGATATTCCGCCAACGCTAGAGGCTTTGGTAGAACAAAGTAAAGTGACCAACAGTG
ATTCTAGTGACAATATGTCCACTTTTGAAAGCATGCTCGAAGAAGTGATGAAAAGTCTCGAGGAGCAAGATGA
AAATGAATATACAGGAAACGTAATGAAACATAAAGGTGGTGAAATTGGAGAAATTCCTCCGGAAAAAATCATA
TCATCGGGAGAAAATTATTCTATTAGATCCACAACGCCATTACAAGAAGAAAATTCAA
CACTTAGAATTGAAAATACATCTTCCCCTATCAACACGGAGGAGGTGACTACGCAGCCTAACCGCCGCCGCGG
TTTTTGGAAACGAGTCAGAAAAGTGCGGCCAACGTCATCGTCCGAAGAGATTGAGGTAGCTGAGTCACAGTAT
TATTCAAATGTTGTGAATAGGCTTGGAGAAACAGTAAATAAGGAAGCTTTAGAAAAAA
CTGGAAAAGGAAATACTAAAATAGTGGTAACAACTTACAAACCAAACTATCAATTCCTAAAAGATTTCTTCGA
AACAGATGATGATGACGATCAAGTCGACGTGATACCAAATATAGAAATACCTAAAATAT
>1002050103_1 Heli.1-EL603198.1.5
CACGAGGGGAAAGGATAGTTGAGTGCGTGTTATATTTTAAAATAAATCCCCAAAAATGGCTGAGCCTGTAACA
GAATTACCTATAAATATAGAAGAAACAGTAAAAGAAGTTGTACAAAATGTTACCGACAAACCCCCTACAAGTA
TCGAAGGAGTCGCAATCGCGTACTTAAGTTTAGTGATAATGGCTATTTTACCCATATT
CTTCGGATCATTTCGATCTGTTAAATACTTAAAAGAACAAAAGGAATCTGGTGAAAGAGCTGAAACTATGTCT
AATAAGGATGCTCTGATGTTCCCGTTGATAGCATCCTGTGCCTTATTTGCTCTGTATATATATTTTTCCAATTTT
TCTCTAAAGAGTACATAAATCTGCTTCTTACTGGATACTTTTTCTTCCTTGGAGTTTT
AGCACTGAGCCATCTTTTAAGCCCAATAATATCTTTAATAGTACCAGCTTCGGTCCCAAATGTACCGTACCAC
ATTTTGTTTACTCGCGGAGAGCGCGACGGCCGCTCCGACATCGTAAACTACAAGTTCACCTCGTACGACGTCA
TTTGCCTGATCATATCACTTATTATGGGAGGATGGTATTTACTGAAGAAGCATTGGAT
CGCAAACAACTTATTCGGTATCGCCTTCGCAATCAACGGTGTTGAGCTCCTCCACTTGAACAATGTGGTCACG
GGATGTATTTTACTCTGTGGACT
>1002050104_1 Heli.1-DT663079.3.5
GCACGAGGCCGTGGTGGGCGCCAACACCGTCATCGAGCAGGACGGCCGCCGCATTCGCGGACGCAAGTACCCT
TGGGGCATTGCTGAAGTGGAAAATCTGGAACATTGCGACTTCCTGGCGCTCCGCAACATGGTGATCCGGACGC
ACCTACAGGACTTGAAGGACGTGACGAGCTCCGTGCACTATGAGAACTACCGGTGCCG
CAAGCTGGCCGGCCTCTCGCACGACGGGAAACCGCACAGGATCAATTCTAACAAGAATCCATTGGCCCAAATG
GAAGAAGAGAAAAGAGAACACGACCTTAAAATGAAGAAGATGGAGAGTGAAATGGAACAGGTATTCGAAACAA
AAGTGCGCGAGAAGCGCGCTAAGCTGAAGGAGTCCGAGGCGGAACTGGCGCGGCGCCA
CGAGGCCACGCGGCGCGCGCTCGAGGCGCAGGCGCGCGAGCTCGAGGAGCGCCAGCGCGCGCTGCTCGCCGAG
CAGGCCGCCTGGGAGCGCGAGACCGGCCTGTCGCTCGACGACCTGCGCCGACGCTCGCTCGAGGCCAACAGCA
AGGAGACGGTCGATGGCAAGGATAAAAAGGATAAGAAAAAGAAAGGTTTGTTTTAATC
GCCATTGTTCGCGTCGGCTTCGCACCCACCGTTACTGTTGCGTGACTAACATTGGAGACCTACAGTCCTACAT
ACATGCTTCTTTCAGGACTCTATTTTGGTCGTATATATTTTGATTTAACATATGCTAATAATGTTACATTTAT
AAATGAAACTCTTTAACCGGATTAATTACGTATATGTACTTAGAATTATACATTTTTG

GAAACCCGACGCTTGGAACACTCGGCAGCAGTAAGTGGTCACGGGTACGGACCAAAAAAGTGTATTTCTAAAT
ATATTTCTATTAATCCGCCTAAATAGTTTTGTTTAAAATTTGTAAAAATCGCGAAAATCAAAGATATTAAAAA
ATTTATTTTTTTCTATATACGAATATAGACAGTTTAGAGTTGAAGTGTTATTTTGTGT
TTAATACGTAAATAAAAAAAATATTTGTCTATTGTCACTATATTTTTCTGTCCTTTCACACTGTAATGTGTTC
TTTCAAGTTCTTTTACTATGTACTTACTAAGCAAAAAAAGTGTTAATCTTAAGGCAATTAGACAGATTAAACC
TTTCACTATTTTGTATTGACTGAAATTGTAAAAAAATAATAATAACATAATCTTGGAC
TCTAAACTGATTCAAAAAGCGAAATTCGAAACAAAAAAATCATTCGAATTATCGCAAACGACATTTACATTGT
AAATTAATTTAATATTTTTACATGTATTTATTAATAGCTGTAACACACTGACGGGAGCATGTAAAGTGCTTCA
TCACAATGGCTAAAGCAACCATCGTCGGCTACCATGTTTGTACTGTGTACTCATTTTA
ATGAAACTTTTATTTATACTTTCAAGGTATTTACTCTGCATATCGTAATGTATTGAAGAAGCGTACTTTGTAA
CCGTGCCGTCACATGCACTGGCCTGAGATTTTACGAGATTTACTTATTTATAGTGCCATTTCGATTAATACAT
TTTGTGAACACGTTTACTTATGTATGTTAATAATACATACTTGTTCAACTAACACGTG
GGCTTCTGTTCTACGCCTGCTCTTATTGACGTTTACTAAAACATGTCT
>1002050105_1 Heli.1-CO729566.1.5
GTCGGATTCCCGGGTCGACCACGCGTCCGCTCTGACAATGTGGGGTCTTCTCGTTTTGGTTACTCTGGTTTTA
GGGTGTGAGGCGAAAAATTATAAAGCGGAACTTCTCGATCCAGAATGGCAGGATCTGGTCGCCGCTGGTGGAA
TGCACATCGGACACGGACGCGCTAAACGGTTCGTGGAGCTCAACGACAATCAGCCTAA
CGTACCGCACCAAGCCTGCCTGCTACCAGATGGCAAGCCGGGGCACTGTCGCCACTTGCACTACTGCATCCAG
GAGGACTTCCAAAAGGACTTCATGAAGTTCATGGACTATCTTTGCATTATTAAACATTCAGCAATCGGTGTCT
GCTGTCCAGACAACTTAGGCGAAGGCGCCATAGATGCTCTTGCCGGTGACCTCCCCGC
GACCGCACCTAAGGATGAAAACGAAATTGTGTTCAAAATTGAACGCGCCGAAAACCGAGGCTGCGGTCTCAGT
ACTCGGGCTCAAGGTAGGGTTCTGGGCTCCCGGCCAGCAAGTCCAAGGGAATGGCCATGGATGGCATCCGTCA
CTCCTGAAGGCTTCGAACAGTACTGCGGAGGCGCTCTTATCACTGATCGCCATGTTCT
CACTGCTGCCCACTGTACTCGCAGGTGGAAAGCCGAAGAATTATACGTACGACTCGGCGAGTACGACCTGAAG
CGTACAAACGACTCCCGCTCCTACAACTTCCGCGTGATAGAAGTACGCCAGCACGAGCT
>1002050106_1 Heli.1-EL603956.1.5
GCACGAGGGTAGCACTGGGAGACAGAAAAAGTCGATCCTGGCGTCGCTACTATAAGTTATCAGGTTGATTAGC
TACAGTACATAAGTACTGTAACGACTTGGCGCGGACAAGGACTGCTGGAGGGAAAGAAAATGATGCATCAGCA
ATCCATGATGGTTGGAGATATGATTCCTGTACACTCTGAAATACCCGTGCATTCTGAA
GAAATGAATAATATTGGATATGATAATAGCAATTCATATACTTATGATGACCTTTTTCCGGCCCTACCGCATG
CTCAACCACCTGTGCAGCGCAATAATATTCAACAAGTAACTAATAAACTACGTGTTGGCTCTTCATTGCATAC
TCAGGTTTTCCATGTGCCATATGAAGAAAGAAAATTAGATAATGCTAATACTTTTGGT
GAAGGTGAATCTTTGAGAACATGTCAATCTATCACTAAAGATACTGGGGCCCATATTGAGATATCTACAAGCA
AGGATGGAAGCTTGACTTTCCTTATTACTGGAAAGCATAGTGCTGTTCTTGACGCTAGAAGACTCATTTTAAC
TCAATTTCAACAACAGGCAAGTAAGCAAGTTGCAATTCCCAAAGAACATCATCGTTGC
ATTCTGGGTAGACAAGGTCAGAAACTGAAAGACTTGGAGAAGGTTACAGCCACAAAAATAAGTGTACCCAGTA
TTGCTGACAATAGTGAAACAATTACCATTACTGGA
>1002050107_1 Heli.1-DT668626.3.5
CGGCACAGGGAAATATCGGCACATGTGACCGTAGAGACCACGGGCTTGCCTCCAACGCCGTTACTCCCCCCAC
AAGTACTTAATGCACCAGCACAACCACGAGCGTGCCCATCACTAGGCGCATCGGTTTCACATGATTTGCTCCT
GGGAAGGTGGCGATTATCACTGGACCTATTCGGACGTGTGTTCACTGAAGATGTGGGA
CTCGAGCCAGGATCAGTAGTAGCAGAATTAGGCGGGTTTCCTGTGAAAGAAGTTAAATTTAGACGTGAGATGG
AGAAATTAAGAAATTCACAACAGAAGGATTTGACATTACACAAGATGGAACGCGATCGTGCAAAGCTTCTCCA
GCAGACGTTTGCGGAGTTAAATACAACATTTTCAGGGCAAAGTCGGCGCGCTCACAAT
TCGGTTCAACCACCGTTAGCTGTAAATCGGGTAAAAGTTACATTCCGAGACGAGCCCGGTGAGGGCAGTGGAG
TTGCGCGTTCTTTTTATACCAGTGTAGCCGAAGCTTTGCTGGCAAATGAGAAACTCCCTCCATTAGAATCAAC
GACAGGAAGTGGTAGTAATAACAGTGCAACCAATGGTACCTCGGGCTCTTCGGGCACG
GCTGCCAGTGGAGCTAGCGGCACCAGCGGGGCGCGAAGTGGCGCTGGAAGAGCTCGGGCGAAGGACAATGCAA
GGCGTGCGCCAGGGCGACCGGCGCCGCGACCTCCAGCGGTACGCGAACCTCGCAGAGTACTTAGTGTTGATGC
GCGACCTTACTCGCCACAGGCAGCTCCTGGCACAGAAGGTCCAGGTTACAGCGGAGAT
AGACCAGGAGGTCACAATGAGCACCTCACATTACACCAAGCCCAACTTGGTGAAAGGCTATATCCAAAAGTTC
ATTCATTACATCCAACATTTGCGGGTAAAATCACGGGAATGCTATTGGAATTAACACCAGCTCAACTGCTTGT
ACTGTTAGCCAGTGAAGATGCTCTCAGGCAGAAAGTACGTGAAGCAATGGACCTCATA

GTTTTACACCCCTCAGAAGCAATTTTAGATCTGGACGTGTTCTCGCTGTCAGAGCGCGGCGGCGGAGGGG
GCGCGAGCAGCGGGGCCGGCAACGGTAGTAGTAGCGGCGCCAGCTCCTCAACGTGACCGCCGACGACGCGGCG
CCTCTCTTCT
>1002050108_1 Heli.1-EL599006.1.5
GCACGAGGGCCAACTTCTCGTTGCCCCGTTACTTATAACTTACGATACTTACGCGCCCACATTCTTTTTACTA
GTGAAGGAAATATAGAGCAACTCAATATCAATGACAAACACAAGTTTTTAGTTCTTTTTTTTTATTTGTGGTC
GAACATTTAAAGATGAATCGGTGGACCATATACAACATCTTACTACTTTTTAATATCA
TCACGCTTAAGGGGATATGTGGACAATTTCAATCTGACCCAAGTTATACTTCAGATGACGTATCAACCAGCCT
AAGTGAAGTAGAGTCACGCGACGCGGGCCCCGAATTGGAATTCAAATACCATGACCACGAGGAGTTAACGAGG
TACCTGCGCGCGGTGTCCGCACGCTATCCTGCTCTCACCGCTCTGTACTCTATAGGAA
AATCTGTACAAGGGCGTGATTTGTGGGTGATGGTTGTATCGGCATCCCCATATGAACACATGATCGGAAAGCC
CGATGTCAAATATGTGGCTAATATACACGGGAACGAAGCTGTTGGCCGCGAGCTTTTGCTTCACTTAATACAG
TACTTAGTAACTGCTTACGAGACGGATTCATATATAAAATGGCTGTTAGACAACACAA
GGATTCATTTAATGCCATCTATGAATCCTGATGGTTTTCTCATTTCCCGTGAAGGACAGTGTGACACCATCCA
TGGCAGACACAACGCACGCCGGCACGATTTGAACCGCAATTTCCCGGACTTCTTCAAGC
>1002050109_1 Heli.1-DT666744.3.5
GCACGAGGGAAGTGTATTATCTCGCTTAGATTTTTGCTTTACGACTGTTGATATTTCTAAGCTAGCATTTTAA
ATTTAACAAAATTATTTTAAAAACTCTAAGATTTTACATTTGTATACTACAATTTTAAAAAAATATATGGATA
TATAAGTGTATACAATGGAAGCTTACGAAAGTGTGTTACTTGTTAAAAATGAAGTATT
TGTTTTTAAAATACCCCCTAAAACAACTAATAGAGGGTACAGGGCGGCTGACTGGAATCTTCAAGAACCACAA
TGGACGGGGCGTATGCGTCTCGTTGCAAAAGGTGACGAATTGATTATGAAGTTGGAAGACAAACAAAGTGGAG
AGCTCTTTGCGAAATGCCCCATCGATAAATACCCTGGAGTGGCCCTGGAGGCTGTTAC
TGATAGCTCAAGATATTTTGTTGTCAGAATACAAGATGATAATGGAAGAAGTGCTTATATTGGTTTGGGTTTT
GGAGATAGATCAGATTCATTTGATTTAAATGTAGCCCTACAAGATCATTTTAAGTGGTTGCGAAAGGAACAGG
AAGGTGAACAAGGACAGCAACAACAACTTGATTTGAGTTTTAAAGAAGGGGAAACCAT
TAAAATAAACATGAAAATAACTAAAAAGGATGGAAGTGAAGGCAGTAGGCCTAGGAGAGCTGGTGGGGTTAAT
GTTGGGGGATTTGCTCCCTCCACCACCAGGGGGTTCAAAGCTGCCTCCACCACCATCACCACAACATGTTCCA
ACACCAATTACACCAGCTGTGCCAAACACAGAATGGGGAGACTTTAATTCCGCTAGTG
>1002050110_1 Heli.1-DT667758.3.5
CACGAGGCCGCGCTCGTTGGACTCTAACCCGTGACAATAATTTCGTATCGAGAAGCACTCTACGGGCGCTCGC
GCTCGGCTCGCGACCTATGCCTGCCAACGTCTATGCGCCTCTCGGACGCCGTTAGAATGAAGTACCACTATCG
CTGCATTTGGAATAAGGAGATAGTGTTCAACTCACAAAAACTTGTAAAGAGACGTTAG
GTTCCCGTCGCCGGTTCCTGACACCCAACGACGACTATCGCTCGCACCACGAGGATTCCAGTTTCTGCAATAC
CACTGCCTATTTTAACGTGTAGAAGTAAAATTTAGTACTGTATTGTAGCAGTAAGAACATTTTATAGAGGACA
ATTATATTGTTAATATATTTTGGAATTCGTGATGGCACTTGCAGATCTGTATGCTGAA
AACATATTTCCTAACTGGTTAGAAGAGAAAGTGGAACTTTCAATATTCGAAAACATCCCCCAAGTGTCAGAGC
GCGCGGAGGTGCCGCTGGTGCCCCTGCCGGTGGCGCAGCTGGCGCCGGCGGCGTACCCGCAGACCGCGCAGCC
GACCGACGAGGAGCTCCTGCGCGAGTTCGAGACCGTGTACGACGCCGTCGAGCTCACG
CACCTCACGCCGCCGCAGAGCCCGCCCGGCCCCGCCACGCAGCTGCTGCTCACCTACGCGCAGCAGGCGCAGT
GCGCGCCCGCGCCCGCCGCCCACTGGCCCGTCGCCGCGCCCGCGCCCGCCCCCGCGCCCGCCGCCCCCTACGA
CTACGACGCGGCGTCGCTCGAGGAGCTCATCCGGCAGGGCGCCGCGCAGCTGGGCGCG
CCCTCGCCGTGCGCGTCGGCGCGCTCGTCGCCCTCGTGCTCGCCGCGCTCCAGCACCGACGAGGAGTGGGGCG
CGCCGGCGCGCGGCAAGCCCTACGCGCGCGGCGACGACCGCCGCTCGCGCAAGAAGGAGCAGAACAAGAACGC
CGCCACGCGCTACCGCCAGAAGAAGAAGGCGGAGGTCGAGGTGCTGCTCAGCGAGGAG
ACGGAGCTGCGCAAGCGGCACGCCGACCTGGGCGAGAAGTGCTCCGACGTGCAGCGCGAG
>1002050111_1 Heli.1-DT665133.3.5
GCACGAGGCAATGTCTATGCGTTTTATTAGGTTATGGGCCCAGACCATAATTCGCGATAAAATTGCAGTTTAT
ATTTCGTAAATATTTACTTTTCGTGGACTATCAGTGTACCGTTTGATATTAAAATATAAAATCGCGGTGGAAA
TCGCTCCTTTGTGGACCTATATCCAGATTGACTGTCGGCTACCTCGTGGCGTAGTAAA
TTTTTTGCAAGATGGCTGGAAGTTACATAGTGAATGTGCCTAAACTAAAAGGACGAGAAAATTATGATGAATG
GGCGTTTGCAGCGGAAAATTTTCTAATGTTAGATGGTGTGGATATCCATAAACAGGAATCTACTGATGCCTTT
TCCGTTGATGAAAAAAAGGCAAAAGCGAAACTCATCATGACAATTGATTCATCTATTT

ATGTTCACATCAAAAACGAGCAAACAGTTCAAGGTGTGTGGAAACGTTTGAAGTGTCTTTATGACGACTCTGG
TTTTACGCGTCGCATCAGCTTACTTCGTCAGCTAATTTCTATTCGATTGGAAGATTGCGAATCAATGGGGACA
TACGTTACACAAATGGTGGATACTGCCCAGAAATTAAAAGGTACCGGTTTCCAGATCA
CTGAAGAGTGGATTGGTTCGATGCTATTGGCGGGGCTAACAGAAAGGTTTGCTCCCATGATAATGGCTATAGA
ACATTCTGGCATGATTATCAGTACGGACATTATCAAGTCAAAATTATTAGACATGAGTTCGGAAGTTGATACC
AACGTACCAAACAACGCTTTTTTGTCGAAAGGTT
>1002050112_1 Heli.1-EL599744.1.5
GCACGAGGAAACAATCCGCCATCTTGTTCCAGTGACAATTTAGTATTTAATATAGTTTTTAACCCATATTTTT
TCAATAAATCCTTCATCGAAAGTGATATTTTGTGAGCTGTGCAGTGTTTGAAAAGCCTGTGATATTTTTGTGT
AATACTATTTTGTTTTCGATTTGCGTATCTTGACGTTATTAGTTGACCGATTTTGACG
TCGAACGGACCGCTAGATAATCAAAATGGGTACAAGAGAGGACGAGTACGATTATTTGTTCAAAGTTGTTCTG
ATTGGTGACTCAGGGGTGGGTAAAAGTAGTTTACTCTCACGTTTCACTAGAAATGAATTCAACTTAGAATCAA
AATCTACAATAGGAGTGGAGTTTGCAACAAGAAGTATAGAGGTGGACGGTAAAACCAT
AAAAGCTCAAATATGGGACACCGCCGGCCAGGAGAGATATCGCGCTATAACGTCGGCGTACTACCGCGGCGCG
GTGGGCGCGCTCCTCGTCTACGACATCGCGAAGCACCTCTCGTACGAGAACGTGGAGAGATGGCTGCGCGAGC
TGCGCGACCACGCCGACCAGAACATACTGATCATGTTAGTCGGTAACAAGAGTGATCT
TAGGCACCTTAGATCTATCCCAACAGAGGAGGCGAAGGCGTTCGCGGAAAAGAACGGGCTCAGTTTTATTGAA
ACGTCCGCACTCGACTCTACCAACGTGGAGCCGGCATTCCAGAATATATTAACCGAGATCTACAGGATCGTGT
CGCAGAAGCAGATGCGCGACCCGCCCGAGGGCGACGTGATCCGCGCCGACGTGGAGCC
CGCCGACGTGCGCCCCTCCGGCGC
>1002050113_1 Heli.1-EL601561.1.5
GCACGAGGCGATTGCCTTTAAGTTGGTAATATTATTTGATAAACTACAAAAGTAGTGCCTACATAATACATAT
TTATTGTACTAGTGAAATTCACTAAGTTCCCCAAACATGGATGACAACATCAATAACGAAGTTGGAGTTGATC
AGCAATTTAATAATGCTGATCAGCAATTTAATGAAAGTGATCAGCAATTCAATGACAC
TGATCAGCAATTTGATAACTCGGATCAGCAATTCAATAATTCTGATCAGCAATTTAATAACGCTGGTGGTGAT
TTTAACTGTGATCAAGGTGGAGAAGATTATAATGAGCAAGATAATCAAGATTATGATAATCTAGATGAAAACA
ATCAAGATGAAGATTATCAGGACCAAAATAATGACCAACCTAATGATGGTGGATATTT
TGGCCCAGCTAGGAAATATCGGAATAACTTTAGGAATGATCGCTACAACAATGCTTATGGAAATTATAGCGGA
GGTTATGGTCCACCGGGTGGATATAACAACCGTAATTTTAGAGGCAGAGGTAACTTTTACCCACCAATGAATT
ATGGACCTCCACCAATGTTCCAACAACCTCCTGGTAATTTCTCTTGGGGACAGGGCTT
TGGGCCCGGTGGACCCCGAAGAAAAAATACAAAGGAAGACAAAACCATTAGATATTTGCTACGATGTGGTGTT
GGGAAAGACAATGTTAAAAACTTGCCCCGTGATTTGATATCATTGATTGAACCTGAGTATTGCGGGATCTGTG
CTCAGGGTTTGGATTCTTTTGCTATATGTAAGTAATAAAATTTTGAATAAAAAAGAAA
AAAGTTTTTTTGTAATGCTTGATCATTAATTGCACT
>1002050114_1 Heli.1-DT664128.3.5
GCACGAGGCAGTTCTAAAAATACTAATAGTGTGGTACATTGGACTAATTCTGTTGAAAATAGAGTTTGTTGTC
CAGAAACAGTGTTATACGGCGCGGTCTACGGGCTAAAGTGTTTGAAATAGTTGAATAGTTTAAACAAATCGAC
AATTTAGTGCAAAAGTGAATGAAATAGTGCGGCTCACGGCGTTAAACTCTGAACTTTG
TAAGTGAAGTGAGGTGACCCCCGCCATTGCCTCACACGGTTCGTGTCGAACGCTTAACCCTACACTGCCTAGC
TCTTGTAACTTCGATATAGATTTAAAATACGCTTAAAATCGAGTGTTGCATTTAAATTGTATTACAATGTCGA
TTAGTGTCGGTATACAATAAGCTTGTACATCGAATTACTATTGCCATTAGGTGTATTC
CCTACAAAGGGCGCGAGGTATTGCGCCCTTTGAACAGAATACTATTACGTTAACGACTCATGTTCCTAACTAC
TCGATGGTAGTCGCGTTTGGGGCAATGGCGGTCGCGGCGGCTAAAATGATCTCAGTAAATAAATAAAGACTAA
CTAACGTATGTTATACAGACAACTAGTGGAATTGTGCGAGTTTTACTGTTTTCTATGT
ATTATACATTTTAAGGACGTTGATTCGAGCGTGTCGATGTTAGTGCCTCCTAAGTCTCGTTAGTGATAGGAAT
CCTCGACCACTGTTTAAGGGAACTCTTTGAACATTTTACCGAAACGACTTGTAATTTCGAGAGGTGAATTTTT
TGTGTACTGCCTTCCAAGCGCTTTGAACATTCGTAGAATCTTGTACCGCTTTATACAA
GATTTTAGCTGAATTTCAATGGAGTTTCGCTTTCTTTCGTTCGTGGTAGTTATCGCAATATTTGATATAGTTG
TAATTGAAAAAAAAATTCGCTCTCGAAACCCCCTAGGTTAAGCTATTGTGTGTGACTAATAACGAATTTCTAC
AGATCAACTGTCATTTTTGTTTTAATTTTATTCGACGTAAGCGTCTCGTATAAGTTAA
TCCTCCTCAAGTGATATAGAAATGTGAATGACTTGAGCATTCTTAAAGGACGTAAGTTTAGTTAGTCGCAAGG
CCTTTTTTATCTAGTTAGGGACT
>1002050115_1 Heli.1-EL601303.1.5

GAAGAGGGAAAGTCCCTTAAAGTGTGTTGTGTTTGTGCGGTGGGTTCGATTCCGATTCGGGAAAAAGAAAATA
TTTTAATTACCTCGCTCTGGTAATGAAGCAAAGCACAGCATTGAGTTCCACCAATCAACACGCAATGCCTCAA
GAATTCTGCAGATATTGGGAAACGGAAGTGTGTTAATGAGAATGGAGTGTCTTAACAC
TAGGTTAACAGGTTCCCTCTGGCTACACTGTCATGTGTGGTATACAGCTGAATGCCACAAATACGAAGATTGA
TACCATTTAAACAGCCTTAATTTAACACGCTACACCACAGTCTTGTGTTAAACACATAATAACTGGCTTACTA
TAATTTAATGAAAAAAAGTATTGTCTAAAACTGACTACTTATAGATTATTGTATAATT
ACACCTTCCTCTTAAGTAAGTCTAAAATGATAGATTACTTGGAGTTTCAATATAAAATCTGATAGCTATATTG
AATCTTCGATTTGTTTATCGTGTTTTTATTAAGATTATTAATAATAAGAAATGTTAAAAGATATGTATAATTT
ATATTATGTATAACGTGTATGTTATTTCTGAATTATATGAAATTTTGTGGTTAGTCTC
ACTGCTCTCTAATTTCTAACAAAACAAATATTTTGTATTGACGGATCTTAACGTTGCATTTTGTTTTGAAAGA
TGAGAGAACCAATTTTACTTCAATAAATATTTTTCGGAATGCAAATTTAATGTTAAAAC
>1002050116_1 Heli.1-EL602900.1.5
GCACGAGGGGAGAATCCAGATGCTATTACATATGAACCCATAGATATTGTCACAGGTATTACCGATGAACAAG
TATCACGTGTAGTCAACAAGATTGGACTCGGTGAACAAGCTAATGAAGCCTGTGATATGATTAAAAGAATGTA
TGACCTCTTCCAAAAGAAAGACGCCTTGTTGATTGAAGTCAATCCCTACGCTGAAGAT
GCTGTTACAGGGCAATTTTTCTGCTTGGACGCTAAATTTCGCTTCGACGACAACGCCCAATTTCGACAAAAGG
AACTATTTCAACTGCGGGACATTACCCAAGAGGATCCGAAGGAAATTGAGGCTGCAAAGTTTGATCTGAACTA
CATTGCCCTAGATGGTAACATTGGGTGCATGGTGAACGGCGCCGGGCTGGCCATGGCC
ACCATGGACATCATCAAGCTGTACGGCGGCGACCCCGCTAACTTCCTCGACGTTGGCGGCGGTGCTACCGCAC
AAGCTGTCTCCGAAGCCTTCAAGATTATCTTATCAGACCCGAAGGTGACAGCGATCTTGGTGAACATCTTTGG
AGGCATCATGCGATGTGACGTCATCGCTGAAGGCATCATCAACGCCGCCAAGAACCTC
AACATTCAGATACCAGTTATTGTTCGTCTACAGGGTACAAAAGTGAACGAAGCCAGAAAGCTGATAGCCGACT
CCGGCCTGCGCATAGTGCCGCGCGACGACCTGGACGAGGCCGCCCAGCTGGTGGTGCAGCTGTCGGAGATCGT
GGCCCTCGCCAAGAACGCCGGTGTCGAGGTCAAGTTCGACATACCT
>1002050117_1 Heli.1-EL603244.1.5
GCACGAGGCTTCACTAATATAATTATTTTAAATGCACTATACGAGTACATTGTAAATGGGTCGCAAATTCGAG
TTTGAGTTGAGACAAAATATTTTCTATTATTTTTCTTATAATACATATTATAAGAATCTAAAATCTCGTGTTT
TATTGATCGGAGATTTAAGTTTCCTTTATTTGTTTTTTTGGTAATATTCAGCTGTATC
ATATAGCAGGTTAGGCAGTGGGAACCTAGTGTTAACACACTCCGTTCTCCACTTTCCGCTGAATTTTTCGAAA
CATTGCGTGTAGAGTGGCGGAACTCAACCCTAGGCTAATGCGTTGATACCAGAGCGAAGAAATCAATATATTA
ATAAATAATAATTCATTCTCCCGAGTGGAGATCGAACCCACAACATTCTTTAACGTAT
TGAATAAAAATGTCTCATAACTCGAACTCGAACTGGCTACCTTCGCCTCAGTGCTCGACATATTATAAATTAG
ATATAAAATATACACAAAAAAGTCTCTAAGCGTATAGATTAATACCATATCGAAAGTTTCTACATTGGTACTC
GATGGCTATAAGTTCGATTCCCACTCGACGAAATGATTATATTTCAAGTATTTTCATT
TCTCCGCTCTGCTAATACAGAGCGCGGCGTTGAGCTCAGCCACGCAATACGCAATGTCTCGAAATTGGGCAT
>1002050119_1 Heli.1-EL603053.1.5
GCACGAGGGTCCGTAGAGAGGCGCGTTTATTTCTAACTTACACATATTTTTATACTTTAACGTTTAAATACAC
CTATTATGGAAGCCTTTATAGAAGGCGTTCGGCTTCAGCCCTGTTTATGGAATCCCTTACATCCGGACTACCG
CGAGATACATGTCAAGGATGAAGCATGGCAGTGCGTCGTCGATCATTATAAAAGCAGT
TCCATTCCAAATATACAGGTAGCGAAATCAGAATGGAAAAAACTGCGAGACAACCATAGAGATGCGCTGAAAA
GAGCTAAACTGGGTAAGAATAAATTGTTACCAGCTCAAATTACAACATGGAAATACGCTAAAGCGATGCAGTT
CCTTGAACCTCATATGAAGTACAGAATAACAGAGAACATTGAAATCGACCCGCCGCCA
AGTTCGAATAGTAACATAAAGAGTTCAGAACACGACGTTTCTACCACGGAGAGTGCCGATGAGCAGTTTGCAG
CACCACAATCCAAACGACGGTGTCCCGAACGCAAAACCGATGGCCGCCAAGAGACTGATGCTCTGGAGTCATT
TTTTAATTGTATATTGCAAAGCACTAAGACGATGCCCCATTGGATGCAAACACAAGTT
AAGAAAAAAATATTTGCAGTTATGATTGACGCAGAGGAATATCTGTCG
>1002050120_1 Heli.1-EL600017.1.5
GCACGAGGATTGTTCTTTATACTATTTACTATATTTTTCAATAATTTCAACGTAAAAAAACTAATTTTATTTC
GTAAATTCTGAAAATAATGTCATTTACAATATTTAAATTTAGTTTTTTTGTTTGTACACAAGGTAACTGTGCC
ATTTCCGTATTTGTGGAACAAAGAAGTCAAATATAGACATTTTTTATTTTTATTTAAA
ATACTTTAGATTGATTCAGTAATATAGAATGGATTGTGTTACTACGCTTAAGATATTAGTGATCGGAGAGAGT
GGAGTTGGAAAATCTAGGTAAGTTTTTATAGTTAATTATAATTTGCATGACTCACACAGGTAAATTACAGTAC
ATGATAGATATTCCACACTAAATGCAGAATTTATCAAAAATTACTTAAAATAACATAT

ATACAAATATATATATACCAAAAGAAAATTATTATTTTCTACAATTGTTGATAAGAAACAAGAAACTGATTCA
AAATTAGCTCTTCCAAACATATTGGTTTTGTCAAATAACATTATCTGTATAATATAATATTGCATGACTGTAC
AAATGTATCAGACCTTGACTTATTTAAATGTATCTTACCAACAAATTATTTGGTTATT
AAATTATATTTGTAGCAGTCATGATGTAGCAGCACAGAATATGAATGTAAATGATAGAATTCTTGGATGGAAC
GATTATATCCAAATTTAACTTTAATTGAGGTCTGCCACTTAACATAC
>1002050121_1 Heli.1-EL600424.1.5
TCCTGCAGTACGGTCGGAATCCGGGTCGACTACGCGTCCGATAACATTCGTTGTTTAGTGCTGTTTTGCTATA
AACTGTTAATAATGGAAGAAATTGGAATTATTCTACCTGAAAAGGATGATCAAGCCCCGGACACCAAGGGACT
TGCATATGTAGGTCCACAAACATTTGATGAATTAGAATCAGAAGATCTTTACACAAAA
TATAAGAAGTTACAAAGAATGCTTGAATTCCTTGAAGTACAAGAAGAATATATTAAAGATGAACAAAGAAATT
TGAAAAAAGAATACCTCCATGCTCAAGAAGAGGTTAAAAGGATTCAATCAGTACCACTTGTTATTGGTCAATT
CCTTGAAGCTGTTGACCAGAACACAGGAATAGTCGGCAGTACAACAGGCTCCAATTAC
TATGTGCGCATCTTGTCCACCATAGACAGGGAGTTGCTAAAGCCGTCAGCGTCTGTCGCTTTACATAAGCATT
CTAATGCATTAGTCGATGTGTTACCACCAGAGGCTGACAGCTCTATATCTATGTTACAAGCAGATGAGAAACC
TGATGTCCAATACTCAGATATAGGAGGTATGGACACACAGAAGCAAGAGATTAGGGAA
GCTGTAGAACTGCCGCTCACACATGTTGAATTGTATCGTCAGATTGGTATTGAGCCTCCGAGAGGCGTGCTAA
TGTATGGCCCACCAGGTTGCGGCAAAACAATGTTGGCTAAAGCTGTCGCTCATCATACTACAGCGGCATTC
>1002050122_1 Heli.1-EL601845.1.5
CAACCTCGTCATACAGTACGCGTCGCAGGGCCGCTACGAGGTGGCCGTGCCGCTCTGCAAGCAGGCGCTCGAG
GACCTGGAGAAGACCTCCGGGCACGACCACCCCGACGTGGCCACCATGTTGAACATACTGGCTTTGGTCTATA
GAGACCAAAATAAATACAAGGAAGCGGCCAACCTCTTGAACGATGCTCTCTCGATCCG
TGAAAAGACACTCGGCGAGAACCATCCCGCCGTCGCCGCCACGCTCAACAACCTCGCCGTGCTCTATGGGAAA
CGAGGCAAGTACCGCGAGGCGGAGCCGCTGTGCAAGCGCGCGCTGTGCATCCGCGAGGCCGTGCTGGGCCGCG
ACCACCCCGACGTCGCCAAGCAGCTCAACAACCTCGCGCTGCTGTGCCAGAACCAGAA
TAAGTACGAGGAAGTGGAACAGTACTACCAGCGCGCGCTAGAAATATACGAAAGCAAACTAGGACCTGATGAC
CCAAATGTCGCTAAAACCAAGAACAATCTCGCCTCTTGCTATCTCAAACAGGGCAAATACAAGGAAGCTGAAA
CTCTATACAAGCAAGTGTTGACGAGAGCGCACGAACGTGAATTTGGCACTATTGATGG
TGACAATAAACCAATCTGGCAAGT
>1002050123_1 Heli.1-ES586856.1.5
CAATCGCCGGTAGCTATTAAACTTCGAGGGTTAAGATTAAAGTATAACATCAAGCGTTGACTTTGTAGACTTG
ATGCTGCGTCTACAGAGATCATCCTTATACTGTATATTTAAATGAAATTTAAATTTAAACCTCCATCAGCTAC
CGAGTGATTGAAATTTCAGCTTTGATTTTTTGGAGTATTAATTATTATTACTTCTACA
TACTACTACATACTATGAGCGCGTTACCTATTGCGAAATATATCACTTAGATTATTTTAGTGTAAGGCGAAAC
TAATGTACAGTTAGAGTAGAAAGTTAAGTCCAAACGAAAAAAATATTTATGAACCGTATCCCCAGCGGTACTG
TGTACCGCATAATGTATCGTTCAATACCTGGCTTTTAATTATATTGTTGTAATAATTG
CTACGTTTCATGGGTGTGCCTGTCCCGGTGCTCTCGTCTGTATGCTGTGACCATACTTAAATTATTATATATT
TATTTATATATATGTATTAAATAAATTATCAGTCTGTCTGTTCGAAAGTGAGTCACAAACCAATGTATTTGCC
AGGGATCAGTTAATAAACATGATTTGTACATTCCATATTATTTTGTTTCTTACTTGAT
TGTTTTTTGTGTGTTTTATTTTTCCTCGACATTATTATTATTATTTTTAATAATAATATTAATAACTAGACAT
TGCACTTTTAATATATATATTAATTGAACAATGGAATTTTTAAAAAAAAAAAATATTTTTATTATGTATAATA
CCTCATAGACAAATGATGTTTGAATGATATATAAAATTGGTAAAAATAAAATAAAAAT
ACAAATAGGTGAAATTGTATTATTTTAGGTTTTCATGTTAGAGAATGTTATAATCTTGGTGTTATTGAATGAA
TTGTGTTAATAAAATATTTTGAAAACTACTTGTAA
>1002050125_1 Heli.1-DT667812.3.5
ATTTGGGAATTTTTTCGAAGCATATGGTCCCAAGGATGTGGTCTTGTTCTTGAATAAATGGTTCTAAGTGAAT
GCCTTAAAGCATAGTGTTGTGAGATTTGTGGCTAAAAATAAGTTTAAAAAGACTGAACAATGTGGAGATTGAT
GATGATGGCGGCGCTGGTCGCGACCGCGCGCGCCAAGCAGATACCTTCTAAAACCGAA
CTAGCTACAACCTCAGTAGCGACGGCAACAGAACCGGTCACTGAGTGGCCAGCACCCCTCGCGCCCCTCGACA
CCCTCGCGGTCGCGTGCGAAAAGGACAACATGCATGTCACCATCTCTCTCGCACACACTCGGCCTCATCCTAA
TAGTATATACGACTCATTCAATGGGATTGTCTACCCCGCGGGTCTCGGCAGTAACTCT
TCGTGTCTGCGAGAATATGTTTCTGCGAGGGGCGACTTGCAATACACGCTGCCGTTGAAAGGATGCAATACTA
TGTCTAATGATAATGACGACGGTACAGTGGAATACTACAACAACATAATAGTCCAGCCTCACCTCCGCCTGGT
GACGGGGCAGGGGCGCGGCTACCACGTGCGCTGCAGGTACCGGCGACGCGACCTAACG

CTGTACCACCTGCACCGGCCCCACGCTGATAGGTTGACTTCGAATGGGAATTACGATAGCGACGAATTCGACG
AGGAGTCAGGACTGCTGCCATCCGTCACTATGAAAATTTTCAAGGGGGATCCTGAAGATAAAGAGGTGGCATC
AAACGTGCGTATCGGCGACAACCTCACCCTCGTCGTGTCTTTGGAGAAGCAGAGGCAA
TTCGGTCTATTAGTCTCTGAATGTTCGGTCAGAGATGGCTTGGGCTGGGCTGAACAGAGCCTGATTGCTGATG
ATGGATGCCCCCTGGACGGTGAAATTATGGGGCTGTTCCAATACTCAAAAGACAAGCAAGAGGCCCGTGTGTC
GTTCCCGGCACACAAGTTTCCCTACACTGCCAGTGTTTATTACACATGCGAAGTTAAA
CTTTGCGATTTAAATCATCCTACGGATTGCGAGCCATGTTCCCATAAGAAGAACCGTGTGCGTCGTCAATCGG
ACGAGGGCTCCCCGGCCACAGTGGAGGTGTTCTCCGGACTCTACGTCAATGAAGCGGACTCGCCGGAGAACGA
CGAAGTCACTAGCGTTAAGACTGAAGACGAAATC
>1002050126_1 Heli.1-DT665403.3.5
GCACGAGGGCCCCCATATACTACTCTCTTCATTTTAATGTACTTTATATTTTATAGAATAAATAAAATAAACA
CATTTCACCGTCTTTCAAATCCTATTAAGTTTTTATAGAAGTTTTAATCTTGTGGGCTGTCTGATATTGGCCT
CGCTGTAAGCAGCCTGTTTTGTCTTAATGCTCTCGGTTGTTATACGAAGCATTACATT
ATTTACAATTTTATAGTAACTTTTAGTAACAATCGAATATATATCATAGCATTGTTTCGTTTAACAGCAGCAT
CTGGCTACATTTTCATTTATGTACAGAGTTATGTTGACTCGCTAGCTAGTAATATAGCATCCATTAAGGCTCA
TTTTTATATATACAAATTATATTTTTTTTATTATAGATTTGTTCTTATTTCTAATTAT
AGTTTTTTCCATTAAATTTATAACGAAATGTTCCCCAATGTTCACAAACTGCAGTATCTATTGTTTGTTAAAG
TATGTAATGTAGCTGATTAATTGTAACAAAACTTTGTTCGATTATAACTTAAATCTTCAATGTAATGTACTTA
CATACAATATAAGAGTACAAAAAATATACTGATAATATTTTTCTGTTGGGAACGAGCC
AGACGTAACTAGTTAGCGAGCTGAAATATGAATGTGGTCAAAATAACTTCGCCTCAAATAGCTTGCAATGTAT
CATTGTGTGAAGCTCTCGCGGCCTTCGAACCCGCATCTACCGTGCGTGTAATGATTATATATATATATATATA
TATATATGTATATATATATATATATATATATATATATAAAATGATTTT
>1002050127_1 Heli.1-DT661668.3.5
GGTCGGCGTACTAAGGAGATGCAGATCTTCGCTGAACTGACTGCCGCGCTGCCAGCCAAGAAGGAGGACGTGG
AACAGCTCGACAAGGCGTCCGTTATGCGCCTCGCCATCTCCTACTTGCGCGTTCGTGATGTCGTCTCGATGTT
GCCCGAAGACAAAGAAGCACCAAAACTGCAAAGTCCAAAAGGCTTAGAAGAAGTCCAG
TCTGAGCTCTCCTATATGAAAGCTTTAGATGGCTTCGTCTTGGTGTTGTCGCAACAAGGCGACATCGTATACT
GCAGTGAAAACATCTCAGAACTCCTTGGAGTATCACAGATGGAGATCATGGGCCAGAGCGTCTTCGAGTTCAG
TCATCCTTGCGATCACGACGAGATACGAGAAGCACTTCGCCCAGGGAAAAACGGCAAA
CGCGATTTACTTCTACGCCTCAAATGCACCCTTACCAGCAAAGGAAGGAATGTGCATCTGAAATCTGCATCTT
ATAAGGTAATTCACGTTACCGGTCACATGCTGAACTGCGAGGAAACTAAAGAACAAGAAAAGAACGTAGAGTC
TGAAGATGAAAATAAAGATGGTGGCATGAAGATGTTCACCAAAGGAGCCCTAGTAGCT
GTAGGGAGGCCGATCCCGCACCCTTCTAACATCGAAATTCCACTGAACAGCATGACCTTCCTTACGAAACATA
ACTTGGATATGAAATTTACTTATTCCGATGAATGTCTATTGAACAGTCTCGGGTACGATTCAGAGGATTTGGC
AGGTCGCTCCTTATACGACTACCACCACGCCGCCGACAGTGCAGCTTTGGCTCAGCAG
TTCAAATCATTGTTCTCAAAAGGACAATGCGAAACAGGACAGTACCGCTTTCTGGCCAAGAGTGGTGGTTACG
CCTGGGTACAAACCCAAGCCACAGTCATCACGGACAAACAGCAGAAGCCGATCAGCGTCATCTGCGTCAACTA
CGTCATCAGCGGTATCGAGTGCAAAGATGAAGTATTTGCTGCACATCAAGTGCAGCAC
GCAGACTTAAAGCCAATAGTGGCATCAGCTGCGTCATTGCCATTGCAAATTGTTGTGCCCGCTGAACTAGCCG
CTGAACCTGCTAACGGTGCCATAGTGGCCACTATTCTTCCGGAAGAAGTAAATTCTGAAGAACGTCCTATACC
TGTAACAGAATTGATATTTGCACCCAGAAAGAAAGAAATGAACAAGGGATTTCTCATG
TTCTCTCCACAAGAGGGACTTACAATGCTTAAAGACGAGCCGGAAGATCTGACTCATTTGGCACCCACTGCCG
GTGATGCTTGT
>1002050128_1 Heli.1-ES585730.1.5
GCACGAGGCGGAAGCTTGCGGATAAAATTACGGTGGTAGCGCTGAGTTAAAATTAGTGCGACATTTAAAAATA
TTGTGTAGACCGTTCATCTGTGAGCTAATTTATTATTTGTGATATTTTAAATAACAGGAAAAATGTTCAGCGG
AATCGCAGGGGCGTTCCGAAGCATCGGCGAGAAGACTGCATCCCTCTTCGATAGGAAG
AAGAAGGATGCCGAGCAACTTGCCGCTGAGAAAGTATCTGAAGCTGCCCACGTTGTGGAAGACCAGGTGAAGA
AGGCAGGTGAACTGGTCAGTGGGGCGGAGAAGACTGCGGCAGATGCTGCTAATTCTGCTTCCAACGCCAAGCA
TTCAGCGATTGACGCGCTGGACAAGGAATTGGCGAAGGTATCAGTTGCTGCAGGAAAA
GCTGAAGATGCTGCCAACAAAGCCGTTGCAGATGGCAAGAAAGTCGTCGACACAACTAAAGATACTATTGCTA
CAACTGTAGATAATACGAAAAAGGCCGCAGAATCTGCAATTAACACAGCTAAAGATACGATCAACGCGACTGT
AGATAATACAAAGAAGACAGCTGAAGAGGCAAAGGCTCAAGCGTTGAAGGCCGCTGAA

GACGCTAAAGAGAAAGCCCGAGTCGCGGATAAGTTTTTGAGCGAGAAACGTGATGCGTTGGTTTCAAACTTCT
CTCATTCGGCTCACGATGGCGCTGAGGGAGCCGCCGGGCTTCTTAGCAAGGGCCTTGCTGCCTTCCCTAAGTA
AATATTCAGTCATTACTGGCATTTGGGAACCATCATCAAACAGAAAAAATATTTTTAG
TACTCATCAGGAAATGGTCAGAATAAAAAGTTTTTT
>1002050129_1 Heli.1-EL599213.1.5
GCACGAGGGAAATTTTGAAACCCGTCGCATTGCATCGGCGCGGCGGCGGTTAATGGCCGTCTTTATTAGATTA
TCTACAAATGAGTTACAAATCTTAGGATATTGTAGCAAAGAATAACAGCGACATCTAATAAGATAAGGTTTAA
TTGATTACAAAATCTGATACGCCTTCGCGATCCAGATACCTCGAGATCGCGGAGCCGC
GACCGTTGTGACACACGGCGCGAGAGGGCGCGAGAAGGCGCTGGAGGACGCGGAAAGACCACGCGCACGCGCG
GCCAGAGGACCGACCGTAGTAGTAGTACATGTATGTCGATGATATCATTAAAGTCCCAGTACTTAGTAATATA
CGGGATTTCAATTCAAAAAGTAATCAATTCTATGCACGAGCTCGAACTCCACGGTAAT
CATTTAGGTATCACTCTGAGAATCTAAATATGTATATTAATTGAATGCTAGAACAAGGTACATTTACGATATA
AGTGGTTATAACTGATAACAAGACAAAGTGTTATAAGTAGGCATTGCATGAAAATGTAATACGTATATATACA
TATATATAGTAAGTAATTAAGGTCTCAAAACGTCTAANATAATTTGTCATTCACATCC
GTCTTGAAACTTGAATTGTGACAAAAAAATATACGTAAGTACGTAAAAACGACAACGTAT
>1002050130_1 Heli.1-EL598600.1.5
GCACGAGGGATATAGTCATGATGAAGTCGGTTTGTAAGTTCTTGTTAATCTTCATATTGGTTGGTGGAGTTTG
TAGTAGACGTGTGCCTGATGTGAATAGTACGAGCCATGACGATAATATGGAGGAAATGGCCAAAAAACCAGTA
TGTCACTACAGCGACCCAAACATAGCGGACTGTATCAAGCGTGTGGCGGAACAAGCGA
GGCACCTTCTCGCTCACGGCATACCCTCGTTCAATATACAACCCCTGGAACCCCTAAAAGTGCCCAGCATCAG
GTTAAGGCAACACAATATGCCACAGGCTCACTTCAAATACGACGCCTGGCTGACCGATTTGATTTTAAACGGA
CTCACAAATTATACTTTTAATAAATTGGACGTTTATCCCGAAGATTTAAAGGTGACTG
GAAACATAAGTCTGCCACATCTAACAATGTCCGGTGAATATGTTATTATCGGAGAATTCCAGATGCTTCCCGT
GGAATCTACTGGAATGATCACGGCTAATTTTACTGATTGTAGTGCCGCCCTCGATGCTGTTGGAGCTAAAGTC
CATAAAAGGATGGTGATACGCGATGCAAATGTCAAACTACGCTGCCTTGGACCGTTGA
GGGCTAATTTAAAGGAAGCTCACTCAACTACAGGAGAAATGGAAATGATAACCGACCACATAGTCCACATGCA
CGCTTCAGAACTCGCTAAGGAAGTTCAACCGGCTGTCGAAACCGCTCTTGGAATGGTACTTGAGGACATCGCT
AATAAATTCCTTAAGCACATCCCAACTAATATGGTATTCCCAAATTAGAAGCACCAGG
AAGCTCTCTCTAGTCAAATCCCACGTAAATACGTTGGGAGTGGCCCCTTAACGTTACGTCAGTTATGTAAGTC
AAGACTTAGGTAATCTATTGTAATTATTTAATTATTAGTTATTTAAT
>1002050132_1 Heli.1-EL596883.1.5
GCACGAGGGCGACTTTTAGTGATTTCTAATCGTAGGCTTTCTTTTCTCATTTATATTTAGTAGTATTTAAACT
TTTTAATTTAGAATTAGCTTTAATTTAAGAGGAACTTTAGTTTTTAAGTAGGCATGGCTGACTTTTTGGAATC
TGAAGCAGAAGAAAGCGAGGTTGATTCTGAAGATGATCAACCAGTAGAACGCAAAAAA
CCCAAACGTAAAGCGGCTGTGCAGAGCGACGACGAAGATGATGAAGAAGAAGATGATGAAGAGCGTCTTCGCG
AAGAACTAAAAGATTTAATTGATGATGCACCTATAGAAGAGTCTGGTAGTGATGGAGAGGATTCAGACGCAAG
TGCAGGTCCTAAGAAAAGAAAGAAAAGCGATGATGAATTAGATGATAGATTGGAAGAT
GAGGACTATGACCTCATTGAAGAAAATTTAGGTGTGAAAGTAGCTAGGAACAAGTTCAAAAGATTACGTCGGT
TAGAAGATGATGATAGTGACAATGAAGGTAATGATGATCCGGATTTGGAGAGAGAGGTTATTGCAGAAAAACT
ATTTGTTGGTGGATCTGACGAGGAAGATGAAAACAGATCAGAATCAGCCGCACCCCGA
GAAGTTGACTATGATGATGAAAATGATGATATGGAATCTGATGCAGATGACTTTATAGTAGATGGTGACGGT
>1002050133_1 Heli.1-EL601908.1.5
GCACGAGGGGAGATCGGGCGGGTCGACGCAGGGCGCGCAGTCCAAAGATGATAAGACGAGCCCCTTCGTTTCT
ACTATACACTCGCATCCGGGCTTCCAACCGCAGAAGATTGGGAAAGGATCTGGTGGTGGTGCAGGGCTTCCAA
AGCCACCAAAGCCCCCAGAAAAGCCACTCATGCCATACATGCGGTACTCCCGCAGAGT
ATGGGACAGTGTGAAGGCGGCAAACCCTGATCTAAAATTATGGGAAATTGGACGTATCATAGGTGGCATGTGG
AGAGATTTGCCACAAACTGAAAAATATGCATTTGTTGATGAATATGAGGCTGATTTTTTGGAATATGAGAAAA
GTTTGAAAACCTATCACAACTCGCCCGCCTATCTGGCGTACGTCGCGGCCAAGAACAA
GGCAGTTGTCGGTAACTTGGAAGAAGAAAGCTCAAGTAAAAAGGGTAGTTCTCAAAAGGAGCAACAACAGCAA
GACAGAAGAATTGATATACAACCTGCTGAAGATGAAGAAGACCAAGACGAGGGTCTATCAGTGAAGCACGTGG
CCTACGCGCGTTATCTGCGCAACCATCGGCTTATCAATGAAATATTCTCGGACACAGT
AGTGCCTGATGTTCGATCTGTTGTTACTACTGCTAGGATGCAGATCTTGAAGAAACAAGTACAATCACTTACA
ATGCACCAAAAGAAATTAGAAGATGAGTTGCAGCAAATCGAAGAGAAGTTCGAAGCAAAGAAACGCAAGTTTA
TAGAAAGCAGTGAGGCATTCCAGGAAGAATTAAAGAAGCACTGTAAACCAGCGGTAGA

CGATGACACGTTTTCTCGTATGGTGGAGCGCGCCTTAGAGCAGCTGAGACGGGGCGTGGCCTCGCAGCCACAT
AGGGATAGGCCAAATGAGCCAATGGAACAAGATCAAAACAATTCAATTAAAACTGAGTCCGGTCCCAACGCTC
CCACTCAAGTGGACAAAGTATCCACAACGGAAGTTAAACCTGATACTAACAACACTAC
TGAACTTAACAAACCTGCTGCTGTAGAAGCTAAAGAATGTGTCCCTGAGACCAGTGGAAATCAGGAGAATGAA
GGAGAACGCAAAGATGACAAGCCACCTGAAGTGAAACCAGAAAATAGGGAATTGCCCGCAGCCCCCGTACACC
ACAACCACAGATTAATTCACCT
>1002050134_1 Heli.1-DT664462.3.5
GCACGAGGGAAGCATTTTATTAAGGTGTAAAAACATTACAATTTTGAACAGTCCTTGAAAAAATGTCGTCAAA
AACAATTGCTACACCACTTATTCGTCACGTAACGGCCAGAAACTACGCACAAGTTGCTCCAGCATTGAAAAAG
GATGTGAGAATTCAGTCTAGTGTTTTGCCCAATAAAACATTTGTGGCGGCATTGGATA
ATGGTTCCCCAGTAGCACGAGTGACTATTGCCTTTAAGGCTGGATCACGCTATGAACCACAAGGAGAATTGGG
CTTAGCCCACCTCTTACGTTCTGCTGCAGGACTTACTACAAAAAATGCTAGTGCTTTTATCATCCGTCGTCAA
CTTGCACAGATTGGTGCATCTTTCAATGCATCAGGAGACCGTGAATTCATCTACTATA
CTCTTGAGGCTACCCAAGACAACTTACAGCAAGCTGTGGAGTACTTGAATAATATTGTATCTAACCAGGAGTT
TCGGCCTTGGGAATTAAGTGACAACCTTCCCCGTCTGAAGTATGATCTTGCAGCACTGCCTCCTCAGGTTCGT
GCAGTAGATTTGCTTCACAAAGCCGCATACCGCCGTGGTCTAGGCAATTCTCTGTTCA
TTGCCCCTAAGAGAATTGGTAAAATCAGCTCAGAGTCCCTTCAACACTTTGCACGCAGTACTTTGACTCCTGG
TCGATGTGCTGTTGCAGTTATTGGTAATAGCCAGGACAATGCCAGTCTAGTAGCTCAGGCCCTTCAGTTGT
>1002050135_1 Heli.1-EL600000.1.5
GCACGAGGGTCTCGCGTTTTTTTTTTTTTTTTTGACACTGACACTGACAGCCGTGCCACTGCCAGACCGCCAC
TGCCAGGCGCCTACAGACTACAGTAGTGTTCTATCCGTATATAATCATAACTTACATTATATATTATTTACAG
TTCTATTTCTATTAAAATGAGCTGACGTGGGTCCAGTACAAAGTTTATAACTTATGCA
TTCCAGAATTCGTATCGTTAAGTAAAGTTGTGATAATGGCAGAAGTGCCGGTTAAGTTAATCGTGTCCTTATG
TGTTGTTTTTTATGCTATTTTCTATAGAAAAGGTAACTTAGAAGATGTGAGACTTTCCGATGTAAAGGAAAAC
CTTTTGTACCTGGAGACTCAAAATAAGGTAGCTTCGGGAATTTATCAACCAAAAGTGG
CTCTCGGCTACGGCGCCTGCCATGATCTCTTTGTAAACGCAACGTCACTCTTGAACCCGGAAGAGTTACAAGG
CAACCCGGAACATTTCAACGAAATATCTAACATGGAAGAATTTTTGAAGAGTTTTACTTATTTTTTCAAACAT
GGAGCTGCTGCAGAGAGATTTATGTCAAACAGTGATCTATATGATGAGCTTGTGAAAC
TTTCATTAGAATTACCTGACTCAAGATGGGCGATTGGTGGGAATGCTCCACTTATGGCAAAAAGATTTTTTAT
GGAAGGTTGGAAAGTTTTGCTTGGAGCTAAGATGAGTAAAAAACTAAAAGAATACATTCCAAAAGATATCCAA
ATAGTAGGAGATGAAGAAAATGAACAAGTAAAGGATGATGTTCACATGATTTTAGAAT
ACAGAGCAGATGAAAAGTTTGGTGCTTATAAGTCTCCTCGAGCTAACAGATACATAATGCATAATGATGAAAA
TAACCCTCTCTTGACATCTTTAGAAAAACTGGGGGAACATCTGCCTGTCTTCAGCCCCAATTTGCTAGTCATC
AGTGGACTACAGATGATGGACAATTTTCCTTTTAATCAAAATGATCTAAGAGCAGAAA
GACTAGAATTAGTTAAACAGCAGATATTGTCACAACCACTACACACATTGGCTCACTTTGAAATGGCAAGCTA
CGTAGATCTAGAACTCCTTCTACATTTGACAACTAAAGTATTGCCTTATGTAGACTCAGTTGGTATGAATGAA
CAGGAACTATCAAATCTAAACAGTATTTTGGAATTTGGTAAGGTTATGGTGGTTGCTG
ATTCTAACCCAAGGATTGCTACTGCACTGGACCAAATGCGTAAAACTTTCCAGCTTATAAGGCAGAAAAATAA
AGAAGTAGGCAGCTTAAGAAGACTAACACGAATCCACGTGCACACACTTGCATACCAAGCCATTTTGACAGTC
AAAGATTCAAGTTGGAAACGAACCAAAGCAGCCGCCGCAAAAGCCTCACTAACTGCAC
ACAGATATGTCTGTAACAGTCCAGATATATCTTTAGGCAAATGTAAACTTCTGCTAGATGATAGTTTCTCAAC
TACCACAGACGATAACAACAATAGCAGAGTTTATTTCAAATCTACTGAACCTGTGGCCTGTTGGGACGAGGTT
CTAGATAATGATGAAGTTAATATTTGTGTTGCGCCTGTCCTCATAT
>1002050136_1 Heli.1-DT669007.3.5
TTTTTTTTTAATTTCTATATCCTTTAATTAAAATTTATAAATAAACAACAACACAGATAAACAACATAAAATA
TATAGATGCTCATTTTGTGTGGGATACATACTAGATACATTACAAATTATTACAGTAGGTATAGATAAGCAAC
AAATGAAATATCTATAAAAAATGCTTTATTAATATGTTTATAATTATGTTGTGTGCTT
ATTTTACCAGTAATATAAAAAAACTATAAATTTGTATATAAAGTGATAAAAAATAATTGTAATAAATCTTTAA
ATAAATTATTTGTTGTCGTGCGGTAGCACTCTAATAGTTACACACTATGTTACACGTAAAATTGTATTATTGT
CATGTTTTAATATAAATAAGCTCATAAATTCGTAATAATTATTGTTAAATAAAATACT
TCTTACGCATAATTCTAACGTACTTTTTGATACATATTTTTTCACAACAACTACTCACATTAATAGCTATATG
CAGTGGCAAGATCAACGACTTTATATAGAACTTCAACAGTAGTTACAAGACAGTATATAATTTGGTTAAATAG
ATAGGTATATATTAAATATCTCTAAGTTAAGTCTCTAAGTTACACTCAGAGAACTCAG

TTAGTGTTAATATGAAATCAAACCATTACATACATACATTTTTTTACCCGTTAAACTTTAACGTCAATTTACT
CTTATTAAATTTTCATAGAGCAGTATTATTATAGTAAAGTAACGATTTTGCTAACCATTCATATTATGTTTAT
GCATCACATTCATACATCATCGTATAATGACGTTTTGATGATATTATTATAAAATTTG
TCGCCTTAGACTTTGGCAACATATATATAAGGATATATAAGGAATAGTATAGTATACACATGGTTTTTTTCTA
CATTTAAACATGAATGAAACGTACACGCGAAGCCTGTATGATCTAAAGCAAATTTTCTACGATTATTAATTTT
CAGCTAAGACTTCCTCAGCAGATTCGTTAATTTCCTTAAAGGAACTGTGAATGTCGCT
TTCTTCCGTGTAACGGGAACAAATTGCGAGTCTAAGGAAATATACATCATCTACTTTAGAAGGTACAAGGTGA
TTTTTCCACGACCGTTGATACGTCTAATAGTGTCTCGAGCCTCGGCCGATCCTCGCCCC
>1002050137_1 Heli.1-DT666767.3.5
GCACGAGGTGACAGCCTTAGCGCTTTTAAATTTAAGGAGGAATTTGTGTAGATTTGCCTTAAAATATTATTCA
CATACGTATTTTCTCACTGATAACGAAATAATAAATTTAAAAGCAAAATGAGTGCATTGGCAAGGAAATTAAT
TGATCAGCTGAAAAGCAAAGAATTTAGAGATTATTTAATGAGCACTCATTTCTGGGGT
CCTGTAGCAAATTGGGGAATACCATTGGCTGCCATTGCTGACACCAAGAAGGATCCTAGCTTTATTAGTGGCA
AAATGACTATTGCCTTGTCGCTATATTCTCTCATGTTCATGAGATTCGCCTGGAGAGTCAAACCAAGGAACAT
GCTGCTATTCGCATGCCATTTCACCAACGAATGTGCACAAATAACACAGGGTGTTCGA
TTTGTCAACTATTACTATATACAGGGCGAGGAAAAAAAAGAAGTTAAGCCTTAAGTTAAATAGATATTAAAGG
GTAGTATAATATTTAATAGGTACAGTCAAAATGACCACAACGCTACAATTCCGTGTTTATTATCTAATTAGTA
TGACACTATATCTAAGCACTTTTACATTATATTGTCTGTGCAATATGACTTTTTACTA
TTTTGAATCTGTGCAATATTGTGACATAAAAAATCTTAAATGGTCTAATACATACTTAGTGTAGATTGTGCAA
TAATATTGAACGATGTCTTTATGATATCAAACAGTAATCATATATTTTGTAATATATTTTCAATCTGTATTGA
GTGTGTATGGGCCGCTTTATATTCTATATTAGATTATATAGAGTTGAATACTATATTG
>1002050138_1 Heli.1-EL598683.1.5
CGGCACGAGGGCACGACGGAGGGGACACACGCCCGGTCCACCGAGTAGCGATAGTTACTATATTAATATTTAC
ATATCTTCTTACTCTTCCTCACCAGGCAGTGGAGACGTGGAACGTGCTATAGTGGACGACAAATTGTCCCAAT
CATCTCGTTGAGCATAAACAAAATCGATCGATAAAATGAAGCCTCCATTATGTATACT
GTGTACATTGGCTATGTTTGTAATATATGTTAACTCCGCTACCATACCAACTGAAGATCAGACTATCGTAGTG
GCCAAAGCTGATGATGAAGTTAGCGAGGCTACAGCTACAGCGGAAATGGCTACAGAAGAACCAAATGCGCCTA
TAAAATTGACAGAAATGCTAGTACAGTCAGAGGTGTCCCTCCGATACGCCCACACTGC
TGTGGTAACGCACGTGAGGAACGCAGCGAGTCGGTCGCAGCAAGCATCGTTCCACGTACTATTACCTGACACA
GCGTTTATTACTGGCTTTGTTATGACATTGAGTGGTAAATCATACAAAGCTTATGTAAAAGAAAAGGAAGAAG
CAAAGAAAATTTACTCTGAAGCTGTTTCTCAAGGAATTGGAGCTGCTCACGTAGCTGC
AAAAGCACGTAATTCTAACCACTTCACGGTAACAGTAAACGTAGAACCAAATAGTAAGGCGACTTTCAATTTG
ACCTATGAAGAGCTTCTAGTAAGACGCAATGGAGTCTACAACCATGCCATTAACCTACATCCCGGCGCTCTGG
TACCTCACATGGAAGTTCGTGTACATATTAAAGAATCGCAAAAGATAAAGCTGCTTCG
TGTGCCTGAACTTAGAACTGGAAATGAAATTGATGCCACGGAGAACGATGCTCAAAACACAGTCGCTGTTATC
AAAAAATCCGATGATAAAAAAGAAGCTACTATAACATTTACTCCAGATCTAGAGGAACAAAAACGACTCGCCC
GAGTATTTGCAGATAATCTTAAAAAAACACAATCTCACCATTGGTACTCCAATATCGA
CGAAGACAACAACAACGAGGGTGCTATCGGGCAGTTTGTAGTACAGTATGACGTTGAACGACCCGATAAAGGA
GAAATTTTAGTAAATGACGGGTATTTTGTCCATTTCTTCGCACCACAATCCCTGAGTCCATTGAGCAAGTACG
TGATATTTGTTTTGGACACGTCGGGGTCTATGGGCGGGCGCAAGAT
>1002050139_1 Heli.1-EL596733.1.5
GCACGAGGCCTCGTGCCGATTCGGCACGAGGGTTTATCCAAGGATATAATTATAAGGTTGTTGTTAATAAAAT
CTACAGATACACTAAATCCTGATCCGGAGGTGCTTAAATCTGCTAATGGTTCAAAGAAATCTGTAGTTATTGA
GGAAAAGCCTTCGCCCAGGCATCATCACGACCTTCGAAGATTGGGTTTACTGGACCGA
TTGGGACAAGACCGCTGTGTACCGAGCTAACAAGTTCAATGGGAAAGATGTTGAAGCTATTACATCTACACAT
ACGCTACAAAATCCGATGGTGATCCACGTGTACCACCCGTACCGTCAGCCCGATGGGGTCAACTACTGTGCGG
CGGTGAACGGTCACTGCTCCCACCTGTGCCTGCCCGCGCCCAGGATAGGCTCGAACTC
GCCGCGCGTCTCCTGCGCCTGCCCTAACGGCCTTCGACTGCTGCCAGATAACCAGATGTGCGTCGAAGACAAT
ACAATAAATCCTGATCCGGAGGTGCTTAAAACTGCTAATGGTTCAAAGAAATCTGTAGTTATTGAGGAAAAGC
CTTCGCCCAGTGTACCTGAAACTCCTAAGGCCGGAGCTGGTCTCCCGACTGCAGGACG
CGACGCCAGTGTCGTAGCTGGCATCGTCGTTGCTGCGTTCTCAGTTATTATTATAGTTGCTGCTGTGATCGCT
GTTGTAATGTACCGGCATTACGTGCACCGTAACGTCACTTCTATGAACTTTGACAACCCCGTGTACCGCANAA
CAACAGAAAATCAATTCGCATTAGAACAAAATGGCTACGCACCGGGCAGCAAATTGTA
CCCCAGTACCGT

>1002050140_1 Heli.1-DT662295.3.5
ATACAGTGAAGATTTACTAAGAGTATTATTTATACGAGTCTACTTTACTAAGAGTGTTATTTATACGAGTCTT
TATAAGGAAGTAAATCAATATGCCAGCTATTGGAATTGATCTTGGCACCACATATTCCTGTGTTGGTGTGTGG
CAACATGGCAATGTGGAAATCATTGCCAATGACCAAGGTAACCGTACAACACCTTCAT
ACGTTGCTTTTACTGACACCGAGCGTTTGATTGGTGATGCGGCGAAAAGCCAAGTGGCTTTGAACCCCAGCAA
CACGGTTTTCGACGCGAAAAGGCTGATCGGACGCAAATTCGACGACCAAAAGATTCAACAGGACATGAAACAT
TGGCCATTTAAGGTAATTAATGATGGCAGTAAACCAAAGATTCAGGTTGAATTCAAAG
GCGAGGCCAAACGATTTGCCCCTGAAGAGATCAGCAGCATGGTTTTGACAAAGATGAAGGAAACCGCAGAGGC
ATATCTTGGCACATCAGTTCGCGATGCTGTCATCACAGTTCCCGCTTACTTCAACGACTCTCAGCGACAGGCC
ACGAAGGATGCTGGAGCGATCGCTGGCTTAAACGTCCTAAGAATCATCAACGAACCCA
CCGCTGCAGCTTTGGCATACGGTTTGGACAAGAACT
>1002050141_1 Heli.1-EL603654.1.5
GCACGAGGGTGCAGCGTGCCAGTGCCGGTGCCAGTGCCACTCTGTTCCGTTGACCTGCGCGGACCACGTGCTC
GTGACCCCGTCGCTCGACCACACTGTAGAGCACAGTGTAGAGACACTGCGGTCGAAGCGATCTTTTGGGTGGT
GAACATTAATATATTAAAGTGACGCGTCGGACGGCGATGGTGTATCGGACACTGGGCT
AGTGACAGTTGTACGTGTGCGGTGCGTCGTGCGTGTGATGTGCCGGGAGCGGTGACGGGCAGCCGGGGCAGCC
GGGGCAGGCAGCGAGGGCCGGCCGCGAGCTGGGGGCCAGCGAGGTGGAGGTAGCGGTGGGCGGGCGGTGGCGC
GCGCGGGCGCTGGATGCGGCAGGAGCGGCGGCGGCGGGCGCGGGCCCCGGCGCCGTGG
CGATGACGGTGCAGCGCGACGAGCGCGACGCGCGCGCTCCACGCACGCGCTTCATGATCACCGACATCCTCGA
CGCTGCGCCCAGAGACCTCAGCGCGCACAGGGATTCCGATTCGGACCGATCAGCCACGGATTCACCAGGTGTA
AAAGATGATTCAGACGACGTATCGAGCAAATCATGCGGTGGCGACGCGTCAGGACTCG
CGAAAAAGCAGAGAAAGGCACGAACAGCCTTCACGGATCACCAGTTGCAGACATTAGAAAAGTCTTTTGAAAG
ACAAAAGTACCTCAGTGTGCAAGATCGTATGGAGCTTGCAGCAAAACTGGGCCTTACAGATACGCAAGTGAAG
ACGTGGTATCAAAATCGACGAACGAAATGGAAACGTCAAACAGCCGTCGGCCTAGAAC
TTTTGGCTGAAGCTGGCAACTATGCGGCTTTCCAGCGGTTGTACGGTGGGTACTGGGCGGGAGTACCCGCTTA
CCCAGCACAGCCGGCGCCGACCGCTGACCTGTACTACAGGCAAGCCGCGGCCACAGCTGCCGCCGCAGCATCT
GCTTCTGCAAACACATTGCAAAAACCTCTGCCTTACCGATTATATCCAGGTGCTCCGT
TAGCGGGCGTACCACCTTTAGGATTGGGTCTACCGGGTCCATCGGCTCACCTGGGTTCCCTTGGAGGCCCAG
>1002050142_1 Heli.1-DT666397.3.5
GGCCGCGGGCTGCAGAATCGATACACGCTCGATCGGATACCGACTGACAAAAGAGTGTAAAAGTTATCAATAT
TGTTAAATATAAATGTGTATTTACATTTAATACAATATGTTTGTATATTATTTGTAATAGATTAAACATTTGT
ATTGAATTACTTATTATTATAAAAATAAGGAGTAAGTAAGGTCAATGTCAGGCAAACC
TGATTTTACAAATCCACAATCGCAATGATACATACATCTAAATAAATCAAAATGAAAATTTTAATGAGTTTGG
TCATTTCATTATTGATATTTGAAGTGGAAAGTGCAAATGATTCTGGATGGTCGTTTGTTGGTAATCCTTTAGG
TTTCGCTCGTCCGTGTAATGGTTCTGATGATATAACCTCGACTTTCGAGGGCAACAGA
CCTCCAGAACTAGAGAACTCTTACAGCGTATATATACAAAAAAATTTTCCGGGTGCAACTAAAATCAAGTTGC
AATTTGATGCTGATGCCAGCGTTGAGTTACACAACAAATCATATGCAAGAATATCCTATGAACAAAATGTAAA
TGCATTTCTAATACGTTTTTTTAGGGCAAACGATCTTATCGCATTTGATGTGAAGGGA
CTGACGCCTGGAGTCGTTCCGTATTTTAAAAGTTTAATTATAAATAATGTGGAATACTGTCAAGATCCTGAAG
TGGGTTATCTA
>1002050143_1 Heli.1-EL597624.1.5
GCACGAGGGTCAGTCCTCGCTACGCAGCTAACGCGCCCCGCTGGCGGTAGTGAACAAATTATATATTAGACAA
AAAAAACTCTTGTGTGTGAATAAAGGACTTGTGTTGAACACCCCGTCTGCATAATGGCTACAGAAGTTATAAA
CACCGTGCCGGAGAATACGGAGAAAGAGAAGGAATTGAAAGAGGACGCGCCAGACAAT
GAGCAGACAAAAGAGGAGCCGCCGGCATCCGAAAAAACCGAGGTGAAGGTGCCGGAGGAGAAAAAAGCAGAGC
CGGCCCCGCCCAAGCCCAATGTCCACAAGATTAATTTCGAAAAAGACGTTGTATATTTGTACCAGTTCTCGAG
AACTCCTTTGTTGCCGTCGACTTCGCCGTACTGCCTGAAGGTGGAGACATGGCTGCGG
CTCGCCGGCATCAAGTACGAGAACGTCGATCACAAAACCAAGTTCCGCTCCAAGAAGGGACAACTGCCGTTCG
TAGAGCTCAACGGCGAGGAGATCGCGGACAGCGCCTTCATCATCAAGGAGCTCTCGGAGAAATACAACAAAGA
TCTAGATGCAGGTCTGACGCCCGACCAGCGGGTGGTGACCCACGCCATGGTCTCCATG
ATAGAGAACCATCTCTCCTGGGTGATCCTCTGGTGGCGCGCCAAGTATCCGGACAGTGTCATCAAGGGCTACC
AAGTGAATCTCCAGAACGCGCTCAACACCCGCCTGCCCAACCCAATATTGAACTTCTGCTACAAATTCACGTC
TGGACGCAAGGGTATGAAGAAAGCGAAAGCTCACGGTATCGGTGTGCATAGCCAGGAG
GAGATCATCGAGTTCGGCAAAAACGATCTTCGTGTGCTCTCTGACCTG

>1002050144_1 Heli.1-DT665116.3.5
TTTCATCCTCCTTTCCCTTTTGCTCAGGAGGCTGTTGTACGGCCCGGGAACGCTCGAGCTCGGCCCTCATCGA
CGACATCTCCTTGCGCAACTCCGACAGCTCCTGTGACAAGCGACTGTTCACCTCCCGCAACTGCGTGACCTCC
TCGCTGGCCGATTTATTTACTAAAATCATCACGGCTGCGTTGATGGCGCCAGCGGCTT
CCTTAAGGGCTTTCGTAAAAGTGCCCTTAAGGTTGCCGGATCTGCAGGCCACTTTGTTGATAATGGCCAAGCT
GTCCGCGACCTGGAGCTGTTTTTCCTGCACCAGGGGGCCATCCACCTTAGGTCCCTCCAAATCTGAGAGCCTG
GAGTTTGCCCTCAGTTGGACCGTCTCAGCCAAGCTTATCACTTCCTCCTCCGCTTGGA
GCCGAAGTTCTCTTCTCTTCGCCGTGGTGAGCTTCTTTGGCTTCTCTTGCCCTTCGTTATCGGAAGAGCCGGA
GTTGTTGTTTTTACTCCTCGTTCTCTTCCGATATTTGGAGCTCTCTTGCACCGCTGCACTTTGAGACTCCATC
GAGACACAGCTGTCCGTCTCGGATTCAGATGCCGCCCGGAGACGACTTCGTGAAGACG
TAGAGCACGACCTGGGCCTCCCGCTGATTTCGGAGGCGATTTTCGCCAAGTCCAGCCGCTCTACTCTCGCGTC
TTTGACGCTTCCGCCCGCTACGGGCACCACAAACCCCTCATCATCTTTTTTAAAATTTTTATTGATCTCCATA
TTGTTCCCACGAGTATGGGGGAAAAACGGTCCATCCAAGCAGTGCCCCCTGTACTTGG
ATAAGCCTAATACGCCACGGGTGTCGGCCGGTACCCTGGCGGGACCAGCAGTAGCGACTGATCATCCCATCAG
CCACGCACCCCTCGCGCTGCTTCGCCGCTTGGGTTGGGGTAATTTTTTATAGAGGTTTTCATCCTCGCAGCCC
GGCCGGTAAAGGCAAGACCCTCAGTTCCACTCGTGATTACGAGACATGACCGCAACCA
CGAGTGGAGTTCCGATATAGTCGGCGCTGGCACATATAAATGCACCTGCGTGGAGGTATTGTGAGTACACACC
ACGCGCGCCCTCCCAGTTCCTCGCACTGTGTCCTCTGCTAATCAGAGGAACACGCGGAAGAACGCCGTCGCGT
CGGATTATTCGCCGACGAGCAATCATTACCCAAGGACGGATCCTGTGGGCAATAATTA
CTCGTCGCCCAGGGTGCACCGGTACACTATACTGCTCGTCCTCCCCGAATGACAAGCATTCGAAAAGGGCCAA
CAGCAAAGGCACACACGTGGAGGTTATCCGACTTCGCACCCCACCCT
>1002050145_1 Heli.1-DT664488.3.5
GCACGAGGCACTGAGCGCGCGTCTACCGTAGACACCCAGTCGAGCTTATTTGAAAGTTAAAAGTTTGTTCAAG
CTTATATTATGAATTTTGTGTTTATGTGATTTATTTGTGGATTTTTGTTTTGTGTCATTTAATATATTTATAT
TTTTGCTGCTTAGAAATTAGTTGGCTCTTATAGCTGCAGTGAATGAGACGAGTGAGGA
TGACATTCGCGATGCTTTGAAAGTGCAGACGGACCATCAGCTCGCTTTGGTACCTCGGGAGCCTACCCTCGTG
AAGGACACCGTTGCCTACAACACTACTATTACCACTGATGACACGGAGGAGCTGCGAGCACTGCGTCCCGATG
GCACGATAGTGACGGAGACGCGTCACACGAAAGAACAAGAACGACTTTGTGACGAGGA
ACTGTCTAAGGAAGAGGCAAAATCTCTAGCAAGTAACGAGAGTTTGGTAGAAGAAACGGGAGGAACGGAGCGA
CGCAAGCGAGTGGACCTCGAACACTCAACGGACCATATGGCGGGTGGACTGCGGGTAGCGACACAAGTTCACT
CACGGACACGGACAGAAGAAGTCGAAAGAGAAGGCCAGCCAAACATGGAAGACGATTG
GGAAAGCTTGTCAGTGAGAATGCGACGCCAGCGCCGGCTACGGCAAAGTGATCACGAAAAAGATTACGCGAAA
CGACCTACCAACTTTGACATTGAAGAGGAGACACGTAAAAAAGAAACTTCTAAGTGGTTAGAAAGTCACTTCG
GCAGCGACTCCCGTTCTTCCCACGACTCCATTGCGGAGGAAATCGAAAGGCGTCCAGA
AGCTGCATATTATGTGAAAAATAAACCGGAAGACGAGGGCTACTACGGGAAAACAACTTCTTCTATACCACCA
CCGGTGTACGGAGATCGAGTTCTACGGAAAACTAAATCGTCCACACCATCTGACGAACGTCCTGTAGTTTCAG
GAAATAGAACTGGTGGGTACTTTAAAGGAGTAGCTGATTGGTCACAACGGCGTGCTAG
TAAGCCGAGAATGCCGGAGCCAAGAAGCTCCTCGCCGTCCCCACCGGCTGTGCGTTCTCATATGCTAGCAAC
>1002050146_1 Heli.1-EL601335.1.5
CGTCCGCGCATCTTACTGTGAAAACGAAAGTGACTGATACTGATACTGATACTGATACCCACCAGACTGTGAT
ACTGTGTTTGTGTGACTGGAACAGTGATCGGTAGCTCGCTGTGTTATCCTGAAACTGTTTCCACGGCCCTTTA
CCACATTGAAAGCACACCTGAAAAGGTTTTCTCAAAAATTTGGAGACTTTATGCCACC
ACCAAATTCTTCTACTGCCGACTGACCGGACCGAGACTATGAGGAGGATCAGATTGCTGCTGCTGTTCACGAC
GCTCCTTTTACAGGACGCGATATGCGAACCACCCGTCGACTCGGCATCTCTACCTCTAGAACACAGAGGCGGC
GCCTATGGCCCGCCCCTACCTCCAGCACACACAGACGACCCCTGGCCACTCGCCACAC
CAGACAGTCCAAAAATTAAACATCTTCAAGTACAATGTGAAAAGACTCATATGCGAGTCAATATAGAATTCGA
TCGACCATTTTACGGTATGATATTTTCAAAAGGATTTTATAGTGACCCTCACTGTATGCATTTGAAACCAGGT
ACGGGACACTTAAGTGCGACATTTGAAATATTCCTAAACAGTTGTGGTATGGCATCCT
CTGCCAATCACAACGTTGCAGCTTACGGCAGCCCTACGCCTAGTGGATCGTACGTCGAAAATACAATTATAGT
TCAATACGATCCTTATGTTCAAGAAGTATGGGATCAAGCGCGAAAACTAAGATGTACGTGGTACGACTATTAC
GAAAAAGCAGTAACATTCAGACCTTTCCAAGTCGATATGTTGCACGCTGTAACAGCTA
>1002050147_1 Heli.1-DT661878.3.5

GCACGAGGCGGGGGCACACGTGTGCACAGTTAGTGTCGATGTTTAGTGTTCAAACAATTGGTTGAAAAAGTTT
TACAGAGCTTTGTGATTAATATGGACGGATAAAATTAATTATTACTGGCGCGAGCATCAATGGCCTGAAAGGA
TGTTCAGAACGGCGTCAGTCGAGTCCGGGCCGCGGACGCCGCCGGCGTACGCCTCGCA
CCCGCCCTACGTGCCACCCCGTTTCTTTCCACCCCGACTACGACCACCGCCCCCACAGTACTGCTTCGACGCC
GCCCGCTTCGCCTGTGATGCATCTAAACCTAGATACCTGGACGTCGAGAGGGAACGGCTCGTCACTAGATGGC
TTGCTGAAGCCAACGAAGCTATGGTCAGACGAGACCGGCCAAGGTACAAACCAAAAAG
TACCGAGAAAAGGCCAGAATTCCCTGAACGGAGACACGAATGGTCGGATAATTTTCTGCTAGGACGGAGAAAT
GGTTCCGTTGAACCGGAAGAGGAGAGTAGTGATGTGATGACGCTTAAAGAGTTTGTCGATAAGTTTTCTTTGC
CTCGTGTAGTGAGGTTCGAGGGAGAGGATAGGGCTGTGCTATTATATCGAGTGCTGGA
AGCTCATAGACGTGTAGAAGCTGTTCCGCTGTCTGGGAAGAAAGGGAAATTGGTTGGCAAGCCGCTTTATATC
CCGGATTCATATGACGGTTGGTTTTCAGCGTGTGGTTGTCGAGGTGGAGCTCTTGCAACTGCTCGGGGCTCCA
TAGCCGCCCTCCTACGAAGTCGAGCCTTCGCCCTCGTGTCTCCACGTGCCATCTCTGC
TTATCGAGCCAGGCCTGCTTCTGAAAGTGGCCGAGTGGGCGCGCAATACGAGCGTGCCGCAGCGCGACCGGGG
ACTCCCCTACG
>1002050148_1 Heli.1-EL604304.1.5
GTACGGTCGGAATTCCGGGTCGACTCAGCGTCCGAACAACGACTGAACAAATAAAATTACAGGTTTTGGTGGT
GAATAACATTTCTCAATAACTTTAATAATTATAAGGTTTTATTAAATGATGGCCTCGCCTTTAGAACAATTTG
TTAATAATGTGCGAACAACTTCTTCCTCAGGCAATTTTCGTGAACTTTGTGATATTAT
CGGAAAATCAGATGAAGTTTTACAACGGAACAGTGCCCATCTTAATACGGTTTTGGAAACGTTGGATATTCAA
CAACATTCTCTGGGTGTATTAGCAGTTCTGGTTGCTAAATTTTCTCTTCCACAAGGAAATATAGATTTTGATC
GATCTACTATGTTCCAAGAAGTACATGAGTTCATAACCAATTGTAATGGAGAACAAGT
ACGATTCTGTCCTGAGTTGTATGCCGATTTATGCCATTTATTAACTGAACATCTTGTTGAACTCAAACAACCA
ATTCGAGGAATCGAAATTTTAAAAAAAGCTATTAGAAAAATTCAACTGTTTGATTCGCAACTAACATCAATTC
ATGCAGATTTATGTCAATTATGTCTTTTGTCAAAGTGTATGAAGCCAGTACTAGAATT
TTTGGAAACAGATGTTACAAGTATTGGATCAGAGCTTGGTGGTGTAAATGACTCAAAGCATTTCCTGTTATAT
TATTATTATGGAGGGATGATCTACACAGCCATGAAGAATTATGATAGAGCATTATATTTTTTCGAAGTTGTTG
TTACAGTTCCTGCTTTGGTTGTTTCCCATATTATGTTGGAAGCATATAAAAAATATAT
TTTAGTGTCACT
>1002050149_1 Heli.1-CO729608.1.5
GGGTTCTGATCCCGGTTGAGAAAACCGTTGCAAGACAGCGTGGTGTCCGACGTACGTAGTAAAAAATAGTATA
CAATAACATTCTTTTAATTCGTTTCTTTCAATCGTTCATTCAATTATTAATTCATTCATTCATTCTATATAGA
AAGGGACTTTGTAAGGTATTTATGTAGGTAACTAATGGTTTTGCTCAAAGTTGGCAAC
ATTAGTTGGTCGTTCCTTCATTTTTTATGTATAGATAATAAATATTCATTTAAGACATTATTATAGATGTTTG
TAGGTTTTTAACTGATTTATTTTAAGAAATTTTATTAAATGAAAAAGTTAGAAAAACTCTATTGTTTTATTAA
TATTAAACATACTAAAATATGGTACTTGCTTAGTTCGGTGTAACAAAGTGATATAATA
TTTTTTAAAACAGGCGTAAAGTAAACAAAAAACACACTCCTAACTCAAACACATAACTAAATCTAAATTCGAT
TAATATCCTTATTTTACTAAAATAATAATTAATAGATAATTAAAAAAATAAAATATTTTTGCTCTAAAATCGA
TCTTCTCTAATAATTGATCAGTTAAAAATCTATAAAGCCAATTTAATAGATATAATAA
ATAATCAATTAAATCATTTAATTTCAATTATTATCATAATTTCAACTTTACGTGTGTAAAATTGAATTATCTG
TAGATTTTTTTATTTTTACTTCATAAGCATGTATTAGCAGATGGCTATGTGACACTTAATTTAAAAAAAAACA
ATTGCTTTGTAAAAAGTCTCGATTCGCTTAAAACTAAGACTAAAATGTATAATTATCT
AAAGAGAAACTATAGAAATGTGATGTAATTTAATAGATTTTTAAATGA
>1002050150_1 Heli.1-DT667065.3.5
GCACGAGGCAGCGGCGGCGCGGCCTCCGCGCACAGGGATGAGCTGGAGCTGTCTCTGTCTAAGGCGCTGCAAG
GCCGGAGCACCAGCACTGGTTCCAAGCTGTCTAAAGTCACTCAGCTGACAGGGTTCTCGGATCCAGTATACGC
AGAGGCTTTAGTGTCGGTACATCAATACGATGTAGTGCTAGATGTGCTAGTAGTGAAC
CAAACGGACGACACGCTGCAACATTGTACCCTGGAACTGGCCACTTTGGGAGAGCTCCGGCTGGTGGAGAGGC
CGGCGGGGGTAGTGCTTGCGCCTCGTGATTTTACCACTATAAGAGCCCATGTTAAAGTTGCGTCAACTGAAAA
TGGGATCATATTTGGGAATATCGTGTATGAAGTGTCAGGTGCTTCAATGGATCGCGGC
GTGGTGGTGTTAAACGATATACATATAGACATTGTGGACTACATACAGCCCGCGGCCTGTAGTGATGCTGACT
TCAGACATATGTGGGCTGAGTTCGAGTGGGAGAATAAGGTATCAGTCAACACAAACATCACGGACTTGCGCGA
CTATTTGCAGCATCTTCTCGCCTCTACCAACATGAAGTGTCTCACGCCAGATAAGGCG

CTGTCCGGTCAGTGCGGGTTCATGGCGGCCAACCTGTACGCGCGCAGCATCTTCGGCGAAGACGCCCTCGCCA
ACCTCAGCATAGAGACGCCCCTGCAGCGCCCCGGCGCGCCCGTCGTCGGACACGTGCGCATACGCGCCAAGAC
ACAGGGCATGGCGTTAAGTCTAGGGGACAAAATTAATATGATGCACAAGTCTCCGCCG
CAAAAACCTCCAGCTGAACCAATTGGCGCCGCTGCATAGGTACTATAAAATAATTATAAATGTTATAATATTC
CTTGTTTTGATACTATATTTATATTTTAAAAATGTATTCATTATTTAAATAAAAAAAATCTAGAATATCTCCT
TTTTCATTGTCGGTAAATAAATTTGCATTTTGGCTTAATTTATTTTTATCTATGCAAT
TATCTTGAGTTACTTTTTTCCGTTGGAGCACTAAAATTTTATTTTATTTTATTATATCCAAACATATTTAAGA
AAATTTTTCTTTTTAATTATCAAAACAAGGATCTTTAGTATAAAGATGTAGTTTCGTAATATCCATTATAATA
TTATAATAGTTGTTATATTTAGTTTTTAACTATATTTGTGGCTGTATTATAAATTTCA
AATGAGAATTACTTTATAATCTGTATTCATTAACTTAGTATGGACAGTAAACATAAATTATAAATAATATCCT
CACATCCGGTCATCCTGCCACAGTAGAACAGCATCTGACTGTAATTGTGGCAATTGTGGGTTCGATTCTCACT
CAGAGGTGATTGTATTGACATT
>1002050151_1 Heli.1-EL597451.1.5
GCACGAGGGGAAATAGTACTTGTTATTCTCACGTCGTCAAAATTCTTTATTTTACCATTCTATCCAAATTGCA
CTTAAGTTCCAATTAAAAACGACAAATATCAAAAAAATCCAATCAAATTTATATAATGTGAAACGTCTCACAC
CGTTTAAAGATTCAAAGATTATTTCTTTTCATAAATAGAAGAATAAGTGAAGGTAAAA
ATGTCGCTGTGGGCAAGAGCTCAGCAGCTGCCGCCTGAAAGCTTGCAGAAGGTGCGAGCGATATATGGGGAAC
ATTTTCCCATAGAGGTCCGCCACTGTCTCGCTACTTGGATCGAGAATAGGATATGGACACCGGAGCCAGAGGA
ACAACAAAGGTTCTTCGTAGACGAGCTAGTGCAAGAGATCCACACGCACACGGAGCTG
ATGCTGTCTCCAGAGATGTTTGTTACTAAGATGAAACTGATTGAGGCTGCCAAGTTATTTAGGATGCAGTATA
GCCACGCCCCTCACGAGCTATACACATACATGCGGCGTTGCTTGGCTCTCGAGATGGAAGTTATACAGAGTGC
GATGGGCACACCCTATATCGCGCAACCTCAGACCGAAAGGAAGTATAGTGAACTCATA
ACCGGCCTACAAACTGTGCGTCAGAAGGTCAACATGGCGAGCGAGGAAATAAGATCACTT
>1002050152_1 Heli.1-EL603792.1.5
GCACGAGGCGGGGACGCTGTGTGGCAGACTCAAGTCAATGGATTATACGGAGTACTTTTCAGGCGTAACAAGG
AGGCAGCTTTCTCTAACTACAGGCTTTGGGAGAGCGCCGGTTTTGTGGTGGCTTACGCCTACTCTACACATCT
TTGCGCCAGAATGAAGCTCTATGTGCTGATGGTAGCGTTGCTGATCGGTGTAGCCGGT
TACATCGTTGTCGAGATCCTGCATATGAGAAAGGCTCGTCGTATGAAAGCTATTGCAGAGAACCCAGTGGCAG
CAGCTGAAGCCGCCAAGCAACCTCCAGAAGAAGATGATGAAAAGGATGAGATTGATGACGATATCATCATAAC
TCATCTCTGAGCGCAGGGTATACCTGTATTAAAGCGCACTAGCGCATTTGCCATAAAC
ATTTTAGGTACAAAACCACGAGCAATGTCATCTCGAATTTAATATATTTATGTATCCAATAGTTAAGAAAATT
ATTGAAGTACAAAAATTGCTTGATCAACTTCTATCACTAAACTGAAATTTGTGTACGGGTAGTTTTGTATGCA
TATTATTTCTGTAATATTTCCAACTTTTATACTATAAATATCGTTATTTGGAACAGAA
AATTGCCTGTGAAATGAGATGTAGGTTTTTGCTTTTTTTGACCATGTTAGGAGAAAGGAAAATAGTGAATTTC
TCAATAACATCGCCTGATGTACTGTTAGAGTGTTGTCGAAATCATTTGCTAAAAACTATAAAATAGCTTCCAT
AGATTTTTGAAAGCGAAGATATTTCTAAAATTCGTGCAAATAATGCTTTTTGTCAATC
CTAGACTAAAGAGTCGTTCTTATATTGAGTGCATGCCAAGCTTCGGTCTGAAAGTACCGTTACTGACAAGACA
TAATCGACACACGTTTTTTATAAAAGTTTCTAACAGGTTTGTTCGTAGTATGCATGATATGCTATTTTTAGAA
ACCTAAAAACCGACTTCATAGTAGAGGCAATTATTTTGTACCAACTTTATAATGTTAC
AACTAATATGCATACAATTGATTACCTCATACTTAAATTATTATGAAA
>1002050153_1 Heli.1-ES586558.1.5
GCACGAGGGATACTCCTTGAACAAAGTCGAATCATTCAGGATGTCGGAGACTCCCGCTAGACCCATGAAATAT
CCTTACACCTTCAGTGCCAAGATAGCACAGTTCCCCTTCAAATATTACGTCCAAAACCAATGGATCTGGCGCT
ACTGGATGATCGGAATTGCGGTTGCTACTCCCGTGTTCTACAAGATTCACAAATTGGC
CAACTCCCCTGAGAATGTGAGCAAATGGGCTGAAATGAGGAAGAAGGAAGCTGCGGGACACCATTAATATTCT
AACTTATTTTGCTCTGCAGTTAATAAATGTTATATTTATAATAAGAATTAAATCATGTC
>1002050154_1 Heli.1-ES584993.1.5
TTTGTTTATTAAATATAGTTGTTAAGTTAATTAAGCTGTTTGTAATAATGAATGTTGGGTATTGCTGACCTTT
TTATAAACGAGTTTTCATACAAAATTTTATAGTTTATCTTTAAGTCAGTTTCAATATAATAAGTTCAAATATT
TACATCACATTTTTATAAAAGAAAGAATATTCAAATTGCATTCATTGAAAGTAGTTAT
CTAGAAAATTAAAGAACTCGGCAATATACAATATTTTTTACAATTCTATTGAAATTATTGAAAAAAAAAACAA
AGTATTTTCTTAATCTTGTAATTTGTACGTCCAATGTTTATATAATATTATATGATATAAAAGTGTTAAAATT
TTATAATCTCAATGTATATAGCTATAAATATCGCACTAATGCCATGAGTCCATCAAAA

CGAAACATTTTCGGGGAATTACCTATTTGTTTTTTCTTAAAACATTAATATATTCTACTTTCCCCTATTGACA
CTTTAACTTTCCTAGAAGTTTTAATGAAAAAATAATAGGAAATTATTTTTATTTATTTGACTAAAATCCGGAA
ATAAAACTATTTTTTTCTATAACATGCGTTTTTTATAAGATACGACGACGACGATATA
AGAAAAACAAGTATTGTGACACTCATAGTGCGATATGTTGGGAGCAAATGTCTATTGAATCTCTTGTGCATTA
CCGATACTGCTTTTGTGGATGATTTAACATTGTATGATTAAATAAATCTTAATCGGATAAAAAAAAAAAAA
>1002050155_1 Heli.1-DT668520.3.5
GGCACCACCACCGAGCCAAGGCAAAAAATTTAAAAATAGAAACTCTTCTCAATCTCAACGAACTCAAAGTGAA
AGTTCATCACCTTCGTTTTCAAATAGACGAAAGAGCAATCGTAACTCTGGACACAAAACAACTGTAACACCAT
CAAGCAACACTGAAAATCAATCGAAGAGGGGATTCAAACCTCGTATACAACCAAGTTC
AGTCGAACAAGTGACTCCGGCTGCTACAAGTGTTTATAAATTCAAGCTTAATAGACAACAGGGCTCTGGACGC
TGGCAGTATAAAACTAGTCCTAAGCCAAAAGTAACTATACGGAAAATGAATGACGATGATCAAACAACCCCAA
TGAGTGCTGAAGACCAACCCTCTAATGAAATATCTCCTCAAGCAAGATCAGATAACGA
CTTAGAACTATCTGGCTCCCAAAGTGGTCCGGGAACGTTACTTGACAATGAAACTGAAGACAATTCTATCGAG
AAGCCACCGCCGGTCGAAACACTCAAAGTAGAAATTTCCACTCCGGCTGATTTCCGAGATGTTTATTACGAAA
TTGCAACTCTTAAGTCTCCGTATACTTTCCAGGTTGGACGCGTGAAAAACACTCGTAT
TATAACAGTAACTACGACTATAGAAAAGCGAATTGAGCCAACAGCTGTGCCTCTGAATTCTCAAATTCAGCTG
AACGAGCCTTT
>1002050156_1 Heli.1-EL597671.1.5
TTCGGCACGAGGCGCGAGCCCTGTCACATTCTCTCTAACAAATAGTATATTCTTATTTCATTTCAAAAATGGA
ATTCGTTGGCAAGAAATACAAAATGGTCTCCTCCGAGAACTTCGATGAGTTCATGAAGACTATTGGTGTTGGT
CTCATCACCCGCAAAGCCGCCAACGCAGTGACACCAACTGTGGAACTGCGCCAGGACG
GCGACAGCTGCGTGCTGGTTACCTCATCCACTTTCAAGACCACCGAAATGAAGTTCAAGCCCGGTGAAGAGTT
CGATGAGGAACGCGCTGATGGTGCTAAGGTTAAATCGGTGTGCACATTCGAGGGCAACACGCTGAAGCAGGTG
CAGAAGGCGACAGATGGCCTCGAGGTCTCCTATATAAGGGAATTTGGCCCTGAAGAGA
TGAAGGCTGTGATGACCGCCAAGGACGTAACCTGCACCAGAGTCTACAAAGTACAGTAAAACCAACAACCCTC
CAAGACTGCAGGGCGGTGCCCTACACTATCTCCTAATATCGTATTATTATTTTATATTCCCTTAGCACAACTA
TGTATTTACATATTATTTAGGCTATGAATGTGAATTTAAAGAACTAAATGAGATTGTA
AACGATCTGTTCGTACTGCCTTCATATGGTGTGGAGTTAAAATGGACCTAAAACATACAGTTGTATAAGTTTT
TCAGTGCATATTTAAATAAAGTTTTCTTAAACTCTAAAAAAAAAAAA
>1002050157_1 Heli.1-CX700528.1.5
TGCAGTACGGTCAGGAATCCCCGGTCGACGTTAGCGTCCGCAGGTTTAGAAGAAGGTAGTCGAAGCCGAAAGA
CCTTATTTTCCATAATTTTCATTACACTGTGTGATCAAGATTAAAAATGGCTTTAGCTCTGCGTAATGTACTA
CGAGGAGTAGCCAGGCGGGTTCAAGCTCGTGGGTATGCTGATGCTCCCAAAGAAGGAG
AAATGGCTTTTACTTTTGCAGCTGGAAATAAAGTATTTTTTGATAAGCAGATTGTAAAACAGATTGATGTGCC
ATCATTCAGTGGAGCTTTTGGTATCCTGCCTAAACATGTACCTACATTAGCTGTCCTTAGGCCTGGCGTAGTT
ACAGTGACAGAAAATGATGGTAAACAAAGCAAGATTTTTGTTTCTTCTGGTACCGTCA
CTGTGAATGATGATTCTTCAGTCCAGGTCCTCGCTGAAGAGGCCCATCCACTGGAGAACTTGGACCGTAGCGC
TGCTCAGGAAGCACTCAGCAAGGCGCAATCTGAACTTAATTCTGCTGCTAATGATCAGGCTAAAGCTGAAGCT
GCCATTGCTGTGGAGGTTGCTGAAGAAATCGTAAAAGCTGCATCAGCGTAACTCCAGT
GAATAATCGAATGATTCCAGCATGTTAGTTTAAAAACTATTTAATTTAATAGTCATCAACAATAATAAACACT
ATTAACTGTCA
>1002050158_1 Heli.1-EL601968.1.5
GCACGAGGAACGTGCGATACGCGTGCTTTAGTGCTTGTTTGTGCGTCGATATAGGATGAGATCTAAATTGGCG
GCTATAACATTTTAACGGAATTTAATAGTAAATTAGTTATATTTAGCAATTACAAATTTGAACCATGACTCCA
CCTAATGAAGAAGGCTTGGAACCACCTCATATTGTTGTTTTGGACGGTGGATTCTCAA
CGCAACTTTCTTGTCACGTAGGTCACGTCATTGACGGTGACCCTCTTTGGAGCGCCCGCTTCTTGCATACACA
CCCCAATGAGATCGTGAATACTCATCTTGACTTCCTTAGAGCTGGTGCAGATTTAATAATTACAAATACTTAT
CAAGCCTCGGTCGATGGATTCGTTGAACATCTAGATGTCACACCTGAACAAGCTATGC
AGCTCATAACCCGCGCCGTAGAACTTGCAAAGCAGGCACGTACCTTATACCTTGAAGAGTATCAAGATTATAT
ACAAAATGATGACGTCCCATTAGTGGTGGGATCAGTAGGACCGTATGGTGCTCATTTGCACGATGGGTCGGAA
TATGACGGCAGTTACGCTGACACTACATCTGTTCAAACAATGAGAGAGTGGCACAGAC
CCCGTATACAGGCGCTAGTAGAAGCAGGAGTCGATTTATTGGCATTGG
>1002050159_1 Heli.1-CO729966.1.5

CAGTACGGTCCGGAATCCGGGTCGACTCAGCGTCCGATCTTTAGATCAAAACAACAAATTTAAAACAATCAAC
ATGTTCGGAAAGATCATCTTCTTTGCCGCTATCGCCGTAGCCGTCGCTAAGCCTGGCATACCTGTAGCGGCAC
CATTGGTAGCCGCTGCCCCTGTGGTGGCTCCAGCTCCCATTGTTACAGCATCCAGCTC
TCAATACATCTCAAGGAATTACAATGGAGTAGTATCTGCACCTCTAGTCGCGGCACCCGTCTATGCTCCAGCG
GCTAAAATTGTAGCACCTGCTGCTCCATATGTAGCCGCTGCTGCTCCTTATGTGGCCGCTTCACCATACGTGG
CTGCTGCTTCCCCATACGTTGCAGCCGCGTCCCCATATGTTGCAGCTGCCTCCCCATA
TGTTGCGGCTGCCTCCCCATATGTTGCGGCTCCTTACTATGCCTCTGCCCCCATTGTTGCCCTTAAGTGAAAA
GACTGTTTATAGTATCTGAACAAAATAAATGAATT
>1002050160_1 Heli.1-EL599968.1.5
GCACGAGGGTTATGTTGTGACTGGAAGAGAACTGGGACTAAGCTGTGATTAATTGCTATTTGCAATTGAATTT
ATAGCCAATACTTTGTACATAACAAAAAACGAACGATAAATAAGTAAAATACTATTCAATAATCTAATATACG
GAGAGCATGGCTAATCTAAAGTCAGATTTAGACGAGTATTTACTCCAAAACGAGAACC
GTCGCAGCTACAAGATTAGTTTACCATTCTCTACTCCTAGTTTCTTTTCACGAAGTAATGACGAAACTCCCAC
GAGCTCTAACAGCTCTACCGGAACTTGGTTTGATAATGTACAGAAAGAATATTTTACTTTGACTCGAACACAA
CGATTTATGGGATTTGGTATATGCCTAGCTTTAGGTATATTGTGTTTCTTTTTGTCAT
TCCTTTACATACCAGTTTTAGTATTGAAGGCTAAAAAATTCTCTCTTCTATTCACACTTGGCAGTGTCTTCTT
TATATTTAGTTTTAGTTTTCTATATGGTCCATGGGCACATATGAAATCGCTGTTCTCCAAAGAGAAAGTACTT
AACACACTACTATATGGTTTTACATTAATAGCTACATTATACTGTGCTTTATATTTAG
AAAGTACACTTTGGACTGTGCTATGTGCAATAGCACAAGTTATGGCATTAATGTGGATGATGTTGGGTTCCAT
TCCTGGGGGGTCCACAGGAGCAA
>1002050161_1 Heli.1-DT662586.3.5
GCACGAGGGTGACTTTAAGCTTAGGTTAGGCCTGAACAGTGAACATGATTTTTAAACCATAGTTTATTGTGCG
TGTCATATATAAACAGAATTTCTTTATGAGGAATAATATTTAAAATTTTACAACCATGGGGAAGAAAACAAAA
GTAGGAAAACAACGAAAAGACAAATATTATCAACTTGCAAAAGAAACAGGTTATCGTT
CTCGAGCTGCTTTTAAGCTTATTCAATTAAATAGAAAGTTTGGATTTCTTCAAAAATCGAGAGTATGTATCGA
CTTGTGTGCAGCACCTGGTGGATGGATGCAGGTGGCGCATCAGAATATGCCCGTTTCCAGCGTCGTAATAGGT
GTAGATTTGTTTCCAATAAAACCTGTACCTGGTTGTATAAGTCTGACAGAAGATATTA
CGACAGAAAAATGTAAAACCGCTATAAAGAAAGAAATAAAAACTTGGAAAGCTGATGTTGTTTTAAACGATGG
TGCACCAAATGTCGGTCTTAACTGGATTCATGATGCATATCAACAGGCATCGCTTACTTTAAGTGCTATGAAA
TTAGCTTCTCATTTTTTGAGACAAGGTGGTTGGTTCGTAACAAAAGTGTTCAGATCGA
AAGACTATCATGCTTTGCTCTGGGTTTTGAAACAATTTTTTAAAAAAGTTCATGCAACAAAACCACAAGCTTC
TCGTAATGAGTCTTCTGAAATTTTTGTTGTATGCCAAGGATACATTGCTCCTGATAGTA
>1002050162_1 Heli.1-EL598144.1.5
GCACGAGGGGAGCATCTAACACCGACTCGCAGTTAGTATCATATCACATTAAATTTACTTTGGATTTACTGTG
TAGATATATTAATTTAGTTACTATTTTTCCTTATTATAATCTTAAAAAAGTTGTGCTGGTAAGGTTGATTTAC
AAATGGACGAAATGTGGTAGAAGAGGACTGAAGCTCTAACATCTTTACGGCCACAAGA
GAATTCAAGCTTTAGTTTCAGTAAGGGAGTGGAACAGGTCGTGGTCGGGGCAGTGGAGCAGTTGAGAGCAAAC
CAGAGTCAAAAGAAAATAAGCCAAATCCTAAAATGTCGAAGGCGCTTGGTACGTCAGCTAAGAGGATCCAGAA
GGAATTGGCAGAAATTACACTAGACCCACCTCCTAATTGCAGTGCAGGGCCAAAAGGA
GATAATCTATATGAATGGGTATCCACAATACTTGGACCGCCTGGGTCAGTGTACGAAGGTGGAGTTTTCTTCT
TAGACATACACTTCTCTGCAGAATACCCTTTTAAACCTCCTAAGGTAACATTCCGAACTAGAATATACCATTG
CAATATAAACAGTCAAGGTGTTATATGCCTTGATATTCTTAAAGACAACTGGTCGCCA
GCATTGACCATATCTAAAGTGCTACTGTCCATATGCTCACTTTTGACTGATTGTAATCCAGCTGACCCTCTAG
TAGGGAGCATCGCTACACAGTATCTCCAGAATCGCGAGGAGCACGATCGCATCGCGCGCCTCTGGACAAGCGC
TATGCGACATGATTGCCTTCATTCTCCCTTACCC
>1002050163_1 Heli.1-DT662912.3.5
GGCTCTTTTCTGGTGTACCCTATTAATATCCATTATTGGTTCTCATGCTGCTGGCGAATTCAGCATCTTGCAC
AGTCCACCATCCTTGAAATTTACTGGATCTAGCAAAACATTGGAAAGTCTTCTGAAAGAAATATTCTCTGCAT
CTCTTGGGCTGTCCGTTGAAGAGAGTTCTGATTGGAATGGCTTATCCATCGTGGATCC
GTTTAATACTCCCGAAGCAGTTGTTGAGGTGTACATTGATGGTGTATCTTCTCTAGGAGATAATGCTGGACTC
AAAAGTAAAAACTTTCCATTGATTGTTGATGAATATGAACCAGACACCTATGATGCAGTTAGACACAGAATTC
GACAGAGGTTCACAAGTGGTGGAAACAAACTTGTTAATATAAGGTTATCTGAACCAGA

TGATCTCATATCAGCTTCAAATGTATTTGGAGAAATTGTACCACCAAAAGTATCTAAGCAAACATTACAACAC
TTGAAATATGACACTGTTGAAGAAGATTACCAATTTATTTATGAATTAGAAGTATTGAAAGCTTTAACTGCCA
AGATCAGATCTGGTGCAGTAACTGCTGATAACATTGTTGATTTCTACAACATTAGATT
CCGTTCCCTACATGCACTTTCAGACTACCATGGACCAAATTCCCTGCAAACTAAAGAAGCTAAAAAGTTATTG
GGTAATGCTCTTGAAGACCTAAGCAATGCGTTGGTTAAGGCCTATGATGGTTCAGTACTTGTTACTGCGGTAA
CCACAGATGTAGCTCACACCAGACGTGCCGTTCGTGCTGCACCTGA
>1002050164_1 Heli.1-EL600620.1.5
GCACGAGGCTCTTACACCTGTTGAAGTACCGTTCGTTTAACTTTTAGTTTTTGTTCCGTTAGTGCTATTGTGC
TGTGTACGTATTTAGATTGGCACAGGTTGTGTTTGATTGTGAACATGGTTATGTAGATCTTGATTTCTAAAAG
AGAAACGAAATTACGATCTACGGCGTTCTTATCCAAGTCACAAAACTTAACCAATCGA
ATATAACAAAGCCATATCTGTGAATTTCCAGTAAAAAACTCAAAATGCACAAGTGACAAGTGTCAAGTGTGAA
CGTCGAAGTGACAAAGTGTTGTGAAGTGAAGTGTTTGTATAATTATGATGTAATAGCCCCTAAACCATAGCCA
TGTATCGGCCTAATTTCTACGAGAGCACCTGCTTCAGATGCAACGAGATCGTTTACCA
GGTGGACAGAGTGGGTCCGCTAAAGGACTTCACCTTTTTCCATAGTGGATGCTTTAAGTGCGCTGTGTGTGGA
ACGAAACTTACATTGAAGACCTACTACAACAACCAACATTGGACAGAAGACAAAGAAGTGTACTGCTCCAGTC
ACGTCCCAAAGATCGGCCCAGGCCATCTGGACAACTCCTCAGTGGGCATACGTAGCGC
TCTTAATGTGCCGAAGAGTAACAACTATGTGAACGAACAGATACGGGGCCACGCCAGGAACAGCCATGAAACA
GAATTGTCACCCACCCGCGCCACGAACGGCGGCAGCGTGCACGCGTCGCCCGCGCGGGAGCTGTCGCGCGACA
CTCCGGACTACCAGTATGGCAGGTTCGATGCCAGCGCGTTACACATCGCGCACGCGCT
CTTACAGACCGAGCTGCAGAAAGCATATCACAGACCGAGGGAGAAACCCATCGATTGTTATTTGGATCGAGAT
GAACAAACCAAACTGGAAATGAAACACAGACAAGAAGAAGATGACTTATATAGAAAATTCTCGAAGCACAGAG
AAGAGGAAAACAGAAGAATACGAGAAGAAATACAGGATGAATGGGAAAGAGAACTGGA
GCGGCTAACAAACAGGTTCCAGCAAGAAATGCAAGTGAAGAAGAGAAGACCAGACGCTGAAATAGGTGCTCTT
ACTCTTCGACACCAGCAAGAGAGAGCGGATTTGGAAAAGAATATGACCTTACGGAGAGACAAGAAAAAAGAGA
GCTTGACGAGAAAAATGTTGGAACATGAGAGAGCTGCAACAGCAGCCCTTGTAGAAAA
ACAAAGCCACGAGATGATGGAACTCATCCAAGAGAGGAGATCGGAGTACATGGCAGCTTCCTCCTTGTTCCTG
GACGGCGAAGAGGCACCTCCCTACCCAGCACGAGCACCGCCCCCGCAACCACCGCTTGTATCAAAGTTCCATA
TCTATACCGACCCTGCGGAGTTCACAGATGTCGATAAGATTGCTATTTCTGTAGCTCA
AGAAGACCAGAAAACCTTCACGGACCTGGTGAGACAGCTGGTAGGCAGATGTGCGAGCGACGTCGAGAAGGCC
CGCACCATTTTCAGATGGATAACAGTCAAAAATCTAAATAACATTCAATTTGATGACAATTTGAGAGGAGATT
CTCCTTTAGGATTATTAAGAGGAATAAAACATGGAACGGAGAGTTACCACGTTCTGTT
TAANAGATTGTGCAGCTACGCGGGCCTTCACTGCGTAGTAATAAAGGGCTACAGCAAATCAGCGGGCTACCAA
CCAGGAGTCAG
>1002050165_1 Heli.1-DT666937.3.5
GCACGAGGCAAAACGTTTCCATTTTGCTGCTAAATGCGTAAAATTTTACAAAAAATTAACGTACTAAATTGTG
GTTGGATTTGCAAAACGTGATTATTTGGTGAAATTATTAAACGCAGTGATTAACAGTTAGAATGGCAGCCAGC
GATTCAGGGACGGATGACTCACTTTATCCCATTGCGGTACTGATTGATGAATTGAAAA
ATGAAGATGTACAACTAAGATTGAATTCCATTAAAAAGTTGTCGACTATTGCACTTGCTCTGGGCGTGGAAAG
GACAAGATCTGAACTGATTCCGTTCTTAACAGAAACTATATATGATGAGGATGAAGTACTACTTGCACTCGCT
GAGCAACTTGGAAACTTCATAAACCTGGTTGGAGGAGGTGAATTTGCCCACTGCTTGC
TTCCACCACTAGAATCTCTTGCCACTGTAGAAGAAACTGTAGTAAGGGATAAAGCAGTGGCTTCGTTGAGGGC
TGTTGCAGCTCATCATTCGCCTCAAGCCTTGGAACAGCACTTTGTGCCTCTTGTCCAACGTTTGGCAGGAGGA
GATTGGTTTACTTCAAGAGCCTCAGCATGTGGATTGTTCAGCGTCTGTTACCCCCGTG
TCTCAGCGCCAGTGAAAGCGGAACTCCGTGGCCATTTCCGCACCCTTTGCCAAGACGACACGCCGATGGTTCG
TCGCGCTGCCGCATACAAGTTGGGTGAATTTGCCAGAGTCGTTGAAGTTGAATATGTGAAAAGCGATCTTATA
CCTATGTTCGTTTTTGTGGCTC
>1002050166_1 Heli.1-EL602780.1.5
GCACGAGGGCTATTTAAAACTTTATATTAAGCGGTGTTATAAGATTTTTCAATGAATATGGGATACACAGAAG
CCGGCCAGAAAATGAATGCCTATATTCTAGCTGGATTTACTATTGTCTTGTGTGTAATATTACACTTGATATA
CAAGACCATCTTCGGTAATAAGAAATGTGAAGTACAAAATCATGAAGCGGAGAGCGTA
ACAGAATCATCCGCCGACGCCCCTACTACAACCGCGGCCGAGTCGCGTGCAGTTGTTAAAAGTAAGAAACGAG
CACCATGGAAAGGAAAAACGGACTTCAGCCACCCCTGGCTGTTAAAGAATCTCAAAGGTCATCCTGGGACTGT
ACTCCTCATGGATTTCTCTGCTAACGGCAAATTTATGGCCACTACCTGTGATGATGGT

TCAGTGATGCTTTGGGACATACGTGATCTAAACGTAAAAGAACACAAGACATTGCGGGTGAATATTGAATTCG
ATCATGCGACACATGTTGCTTGGAGTCCGGATTCAAAAGCTTTCGTTTTACATACAGCGAGGGAAAATAATAT
AGTTGTATACAAGATTGAGAAGAAAAAAGATGGTACCATTGGTTCAGCCACACCAGTC
ATTAATTTTGATAAGGCTCACGAAGACAATATCGTCGGATTTGGAATATCAAGCAATGGAAAATTAATGATGT
CCTGCTCATGCATAACCGATATGATAATCTGGGAC
>1002050167_1 Heli.1-EL604263.1.5
GCACGAGGGAATTAAGCGCCAATAAGGTTATCATGGTGTCCGCGATAGGCAACGATGGACCGTTGTATGGAAC
ACTAAATAACCCAGCGGATCAAATGGACGTCATCGGCGTGGGGGGTATAGGGTTCGACGATCGGATCGCCAAG
TTCTCTTCCAGAGGCATGACTACATGGGAACTGCCAGATGGCTATGGTCGTATGAAGC
CCGACATCGTGACGTATGGGAGTGGCGTTCGCGGCTCCAGTGTGAACGGCGGGTGCAGATCCCTTAGTGGTAC
TTCAGTAGCTTCACCAGTGGTGGCCGGCGCTATAGCACTTTTAGCTAGCGGCGTGCCCAGACAGAACCTTACA
CCTGCGGCGGTTAAACAGGCCCTTTGTATAACAGCAAGGCGACTGTCCGGCCCTAACA
TGTTTGAGCAGGGCCACGGGAAATTAGATCTTATCAGTGCTTATCAGTTTCTCCGCGAGTACGAACCCCAAGC
TAGTCTCAGTCCGCCGTATATAGATCTAACCGAATGCCAGTATATGTGGCCCTACTGTACCCAGCCACTGTAC
TATAGTGCACAACCCACTATAGCCAATGTCACAGTAATCAATGGACTTGGCGTTGCGG
GCGAAGTGATAAAAGTAACATGGCATCCACATTTACCTCACGGTGTAATACTGTCGGTGTCAGCAGATTACAG
TCAAATACTATGGCCGTGGTCCGGTTGGCTTGCACTGAGTTTCAATGTTTTGGAAACGG
>1002050168_1 Heli.1-EL601662.1.5
GGGCACGAGGCAGACGCTTGCGCGGGAATAAAAAAATATAAGGATTAAACATTATCTATAGTCACATTTAAAT
TTCGAATAGCGGGAAAATTATATAGTTTTTATTATTCAAGATGGAGAGATATCAAGTTTTATTTTTCGTTTTT
ATAGCCGTACTTGCGCCGAGCTCTTCAAATTCAAATATATTCGGGGGTCAGTGCTCGA
AAAGTGATGAAGAAGTTGATCGGTGCTTGTTAGTACGATTTAACAATTTTATCAACTATCTTAAAAGTGGATC
ACCTGAATTGGACATTGAACAGCAGTCGGACTCGATAGTAATAGACGAGCTGTCGATCGCGCTGGGCGGCGGC
CCCGACGGGTACAGGGCCACCTTCAAAGACATCAACGCGAGCGGCGTCGACGACGTCA
CCATCACCAACGTTAGGTCGGACCTGGACACGCACCAGTTCCAGCTGACGCTGTCCATCCCGCACATCAGCGC
CACGGCGCGCTACCGCTCGTCCGGCGTGCTGCTGCTGGTGCGCGCGTCGGGCGGCGGCGACTACTGGGGCGAG
TACGAGAACGTAAAGGCCAAAGTGTATTTCCGCGGTGAGCCCTACAAGCGCAAGGGCA
AGACCTACCTCAAGCTCAAGCAACTCAAGCTGGACTTCTCCGTCAAGAACATCAAGATGGGAGTGGAGAACCT
GGAAAATAGCAACAGTGTGCTGCAAGCCGCATTAAATCTCTTCATCAACACGAACTCGC
>1002050169_1 Heli.1-DT662271.3.5
GTATTCCCCCAGATCAACAACGTCTTATCTTTGCTGGCAAGCAGCTAGAAGATGGTCGCACCCTGTCTGACTA
CAACATCCAAAAAGAGTCTACCCTTCATTTAGTACTGCGTCTGCGAGGTGGTATGCAAATATTTGTCAAAACA
TTGACTGGAAAGACCATTACCCTGGAAGTAGAACCATCTGACACAATCGAGAATGTCA
AAGCTAAAATACAGGATAAGGAGGGCATCCCCCCAGACCAACAACGTCTCATTTTTGCTGGAAAGCAATTGGA
AGATGGTCGCACTTTGTCTGATTACAATATTCAGAAAGAGTCAACCCTGCACTTAGTGCTTCGTCTCCGTGGT
GGAATGCAAATATTTGTCAAAACACTTACTGGAAAAACAATAACTTTGGAAGTTGAGC
CATCAGACACCATTGAAAATGTTAAAGCTAAAATTCAAGATAAGGAAGGAATTCCTCCAGATCAACAACGTCT
TATCTTCGCTGGAAAACAACTGGAAGATGGCCGCACTCTATCTGACTACAATATCCAGAAAGAGTCCACCCTC
CACTTGGTACTTCGTTTGCGTGGAGGTATGCAGATCTTTGTAAAGACCCTCACAGGGA
AAACTATCACCTTAGAAGTTGAACCCTCAGACACTATTGAAAATGTTAAAGCCAAGATCCAAGACAAAGAGGG
TATTCCCCCAGATCAACAACGTCTAATTTTCGCTGGCAAGCAGCTGGAGGATGGTCGTACTCTCTCTGACTAC
AATATTCAGAAAGAATCCACTTTGCACTTGGTACTACGTCTACGAGGAGGTATGCAGA
TATTTGTTAAAACACTAACTGGGAAGACCATCACCTTGGAGGTGGAGCCGTCCGAAAAAATCGAAAATGTTAA
GGCTAAGATCCAAGACAAGGAGGGCATTCCCCCAGACCAACAGCGTCTCATCTTTGCTGGCAAACAGTTGGAG
GATGGCCGCACTCTGTCGGACTACAACATCCAAAAAGAATTTACTTTGCATTTAGTAC
TTCGTCTTCGTGGTGGAATCTAAGCGCACGGCAAATTGGAATAAAATTAAAGCTGTTTTGATTTCAGTATTA
>1002050170_1 Heli.1-EL603308.1.5
GCACGAGGGGGCCCCGCCGCCGCCGCAGCACTCGCCGCCCGCGCCGCCCTCTCAGCAGGAGATCATGCAGGGC
TATTCCGTACAAGAGCGAGCGGCGCAACTACAAAGTTTTGCTGATCAGGTCCACCTTAAAAACCAAGATGTGC
ATAAGAACCTTCGGCGGAATAAGCCAGGGATTCGTTTAAAAGAGGTGACAAAACCTCC
AGAAGAATATCAGCCACCTCCTGGATTCAGTCTAGTTCCTTCTATCATAAGAGGTCAGCCATCACAAGTAGAA
CAAGATGCCAGTAGACTTTCCAGAGAACCAGATAACAGAAATGAATACGACGAGGACAAACCTTTACAAAATA
ATCGCAACCGTCAAAACCTTCCCTGTAGTAGGCAGTGTAGTGGCCTAGTCAGTAATAA

AATAATAGAGCTAGTTAAACAAACTCAGAAAGCCGATATCCCACCGCGTCCGGTGTACAGTCCAGCGTACGAC
CCCGAGTCGGACCTTCGGAAGATCCTTCGCAATTCACCAGAGATGCTCGCAACGCCTGTCTTTAGTTCAGATG
ACGATTACACAGATGGACCTTTTAGGTTTAAGCAACTACTTCGACCAACATTAGGCCC
AACAGAGAGTCTCCGAAAACGCAAAGTACGCAATTCGTCCGGTCGCAGCCCATGGATGTCGGACAGTTCTCAG
GACAGCAGTAGTTCTAAAGAAGC
>1002050171_1 Heli.1-ES587244.1.5
AAATTTGATCAATTTAGTATATTTTGATTTCATTCATGATAAATTCCTAATAATAGTATTAAAATAAAAAAAA
AACTTATTTTAAATCAAGATAAAAAATTTACTAAATAAAATGTTGGGATAATTGGAAAAATTAATGCAATTTC
TACATCAAAAATTAAAAAAATTATTGTGATTAAAAAGAAATGTAAAGAAAATGGAATT
CGAGCTAAAGATTTAGGGTCAAATCCACATTCAAATGGTGAACATTTTTCTCGATCTAGAAATGATTTTTTTG
AGATTAATAGTGAAATTAATATGAAAATATTAGCAATTGCTATTATGAGTCTGTTTAGAATTTTTTATATTAC
CAACGAATGA
>1002050172_1 Heli.1-DT665721.3.5
CGGCACGAGGGTGCGGTCTTCCTTTAAAGTTTTGTAATAAGATTTATAGCTATATAATACAATATAAAATACA
AAACGTAACCAAAAACAGCAAATTAAGTTTATTTGTAATTGAATCTTGTTATCGTGTAGATCTTCTTTTTTTA
TTATTTATTTCTGAAGTAAGAAAACTGGTTTTTGTGCCCAAGTATTTGTGAACGAGTT
TATTGTATATTATTAATTGAGGAAAAGACCAATTACCACATGTGAAAAATAAAATATGTCACTTAAATACTTT
AATTTCTGCATTTTTCTATTGTTGAATTCCGTATATTCTGGAGAGGTTGGTGATAGATGTTCTACACAAGATG
GAACTTCTAATGATGGAACCTGCAAATTAGCCTATGAATGTCCTGTTGCTATAAGGCT
CATTCAAGATTTAGGAAATCATCCATTTCGCAGATGTGGTTTCCGTAGTTTAACAGAAATAATCTGCTGTCCA
TCAAAACCATATGAAGTTAAATCTACTACAAAAAAACCAACACCTATTCCTACAGATAAATTTGGTGAAACAC
CGATTGAGTCTTTGCGAATTGCAGATAAAGAATGCCAAAAGATTGTGAGCACAAATCA
ACCACCTTTGAATTTACATATAATTGGCGGTGACACGGTTTCTGCTGGGGAATTTCCACATATGGTCGCACTC
GGCTATGAGCGTACAGATGGATACGAGTTCCTTTGCGGCGGGTCCTTGGTGTCGGACACTTACGTCCTTACCG
CTGCTCATTGCGTGGACACCTTAGATCAAGTGAAGCCGAGTATCGCGCGTATGGGTGT
CATTGAAATCGGCGGGCGAAATTTCTCTAAGGAGTCCGACGTGAGGATAGATCAAATAATAACGCACCCGTCA
TACATTAGGCGCACAAAGTATCACGATTTGGCCTTGCTCAAGTTGGAAGCCCCAGTGACGTTTTCGATGGAAA
TAAAACCGATATGTTTATATACGAAAGATGATAACCCTACAAAGCCGTTAATTATTAC
GGGATGGGGTAAAACCAGCAATACACGCGACGTGAAAAGTACGCTGCTGCTGAAGGCGAACGTGTCGGTGGTG
GCGCGCCGCAAGTGCAGCGAGTCCTACACCAACTGGCGCAAGCTGCCCGACGGCATCGCCGACACGCAGATCT
GCGCCGGTGACCCGCTCGGCCT
>1002050173_1 Heli.1-DT668474.3.5
ATTATTTATTTGTTTATTATTATTAATTCTGTGTTCGTAACAATTAAAAGATTTACAAATAATGGGTTTTTTA
GAAGAATTCGAATTTAAGTAATGACCGGACATACCCAGAATATTATAAGGGAAGACTCTTTTTATAACAGGCG
GTTCAGGTTTCATGGGCAAAGTACTGATAGAAAAACTGCTATACTCATGTCCAGACCT
AGAAAAAAATCTATATGTTGTTAAGAAGTAAAAAAGGGGTCAATCCAGAAGATCGATTATCAGCTATATATTCT
TCTAGATGTTTCGATCGTCTCAAGAAAGAAAGGCCGGATATATTCAAGAAAAAAGTTTTCTTCATCAGTGGTG
ACGTCAGTGAGATAGGATTAGGTATTTCAGATGAAGATAAAACGTTGTTAATTAAAAC
TACTAACATTATTTTTCACGTAGCTGCTAGTGTTAGATTTGACGATTCTCTTAAAATAGCTGCAAACTTGAAC
TTACGTGGAACAAGGGAGCTGCTAGAACTTGCTAAGAATATAAGAAACTTAGAAGTTTTAGTCCACGTTTCGA
CATCATACGCTAATACGAACAGACAATGTATAGACGAAGTTATATA
>1002050174_1 Heli.1-DT663459.3.5
GCACGAGGGTTAATGTTATGAAAATTTCTATTTAAGAAATTAAAACCAATGTTTTCACATTACATTAAAATTA
TAAATTAATTTTAATATAACCATTAAAGTCTAATACAATAATGCCAAGTCCATATGAAGAGGAACGTGTCTGG
GAGGGACCAAATCACACACAATACGAAGAAGAAGGCACAGAAGATGTTATTGATAATC
CAGAAGAAGTGCTGCAAGAATGCCTTGAAAAATTTAAAACCCCCGACTATATTATGGAGCCTGGTATTTTTGG
CCAATTAAAAAGATATTTTCAAGCTGGAGGAAATCCCGAACAAGTGATTGAACAACTTTCAATTAATTATAAT
GCAGTTGCACAAATGGCTAACTTACTTGCTGAGTGGCTAATTCTTGGAGGAGTTAAAG
TTACTGAAGTTCAGGCTATGGTGGAAAATCATTTAAAGGATATGATATTAAAAACATTTGATCCCAAAAAAGC
TGACACAATTTTTACAGAAGAGGGTGAAACACCGGCTTGGCTTACAGAAATGATTGAACATCCAACATGGCGT
TCACTGATTTACAGATTAGCAGAAGAATACCCTGACTGTCTAATGTTAAATTTTACTA
TTAAGCTTATATCAGATGCTGGATTCCAAGGAGAAATAACTAGCATATCTACAGCTGCTCAACAAATTGAAGT
CTTCTCTAGAGTGTTAAAGTCATCAATAGTTGGATTTTTACAAAGCTCTGATGAATGGCAGGATAGTGTTACA
GAATGTGCTAAAATGGTTTGTCATGGTGCTCATACATATGTGTACAGCCAAGTTATAG

TTCACATATTGT
>1002050175_1 Heli.1-DT668100.3.5
GACGAGGCGATGGCCCCTGTACCGGCCGGCGTCATATAAACATATTTAATACGATATTTAGACAACTACTAC
TACTTCTAGTGTTTACTATGACTTTTTGTAAATAAAACGAATTTCTTTTATAGTGTCCAATGTATAACAAACA
GCATGTGTGATTAAAATTGGGAATTCTTTGAAAATTTGTGAATCGAAGACATTTTACG
AAAATGGAAACGAAGAAAGAAATTAATCTAGAGTCAATCCTAGAAAGTCTGGGTCCCTACGGTCGATACAATC
TACTTAATTTTGTTCTTCTACTTTACCCTGTGCTTTTAAGCAGCTTTTTCGAGTGTGGATTTATATTTGAAGC
TCAGGAACAAAGTTATAGGTGCAAAATCTATGGATGTGAAGGAAGGTACAACGACACG
TCATGGCTCGAATACGCTATACCCACCAACGCCGAGAGGAACAGACCAGAGTCGTGTAAAAGATATGCTTATT
ACAACGTTTCGAGTTTCCAATGTTTGCCTGAAGAGTTTTCCAATACCACTGTGCCTTGTGAAAGCTATGTTTA
CGAGGAAGGCGAATCATTCGTGAAAGAGTTTGACCTGGGTTGTCAAGAATGGAAGCGA
GCACTAGTAGGAACTGTTCACAATTCTGGAATGTTTGCCGCCATGTTGGTCACTGGTGCTATATCAGATAGAT
TTGGACGACGAGTAGCAGTGGGCATGGCAGCAACAGCTAGCTTCATATTTGGTGTTGCTCGAGCTTTTTCACC
AGGATACCTGACCTATCTGACCATGCATTTCTTGGAGCGGGCCTTG
>1002050176_1 Heli.1-EL596933.1.5
GCACGAGGCCATGGCGTGCCTTCCGAGCCTGGCCGTGTGTTGAATATTTTACTCGACGTCAACTATAGGCTGG
ACCAAATAATGACGGGATCGAACCCTCCAAGCCAGGCAGCTGTATGCGTGCACTGCTCGGCCGGCATTGGCAG
GACCGGCACGTTTATAGTCATCGACATGATACTTGACCAAATAAGGAAAGAAGGGTTC
GACTGCGAGATAGACATACACCGCACGGTGCAAATGGTGCGCGACCAGCGATCTGGGATGGTGCAAAACGAGG
CCCAATACAAGTTCATATACATGGCCCTGTTGGAGTACATCGAGACGGAGAAGCAGCGCCTCAGCTGCGTGTC
GGCGCCGGCGCAGGACTCCCCGCGCATATGATCCGAGCAGAACAAAAAGTTTCGCGAC
AGGAAGAAAAGTGTCCTGAAGTGCTTCCCGAGTCGCAAACACAAGGCCCGCGCCGACTTCCAATGTCTCCCCA
AACGGTAATAAGGCTTGTTAGTCAAATCAGCAGTTTATTCGTTTGATTATTACATATTATAGATTTATTGTAC
TGCAATATTTTATAAAGAAAATAATATTTTTCACTAAATGTTATGGAGTAAGTAAATT
TTTAACTTTTAAGTAACATTTACAAAAGACGCAATTTAAAATCCATTATAAATATACATACATATTTATCACG
TAGCTATGTTTATGTGCATATTATAATTTTCTATATTAATGTCACGACCGTAATCACTCTATGTATTCACA
>1002050177_1 Heli.1-EL601018.1.5
GCACGAGGCCACCGCGCACTCGCACGAGCAGTGCGCGCGCGCCGCCCTGCGCGCGCTCTCCGTCATGGGGCTG
GACGCGCCCGCCACCGCCGGGATGCAGAGCAGCCTCCCGGCCGGCGCCAAGTCGAACGTGATCACCAACGGCA
TCGAGTGAGGGCGCCGCACAGCAACACGGGAATATCTCACGGCGCGAAATAAATAGTT
CGGGAGTCCTCGCATCAGGCAACATCGTTTATTTCGTTACCCACCAGCCATGACGTATTCACCGCCGCCCTCC
CTCCTCCGCCCTCCTGATCTCGGGTCTTGCAACAAATTGTAGTCTCTTTATCTTTCTAAACTTTAACCTAATT
ATATTACAAATACAAATATGTATATTTCTTTTGTTGTACTTTGCTTTTTCTGTTGTCA
CTTTATCGAACTGAACGCGAAACCATAAAATCTATCTATATATAAGCGATGTATTTATTGAAATATTATTATA
TTATTGTGTAATTGCAAAGTAAATATATACAAAAACTCGACGAGTCGAATCTGAATCAACACAAGTAAGTCAA
CGTAGATTTAAAATTAGGATAATAATATCGTTATAAAATGCAGCTATCTCGTTTTTAT
ATAGCTATCGAATAGACACTGGTATCATATAATATGTAGAAACGAACG
>1002050178_1 Heli.1-ES587588.1.5
GCACGAGGCGGTTTTGAAGTGATCTAGTTCGTCAGACAAACACAGACCACGTTCGCAATGCAATCGCTGGTGG
TGTTCTTCGCTCTCGCCGCTGTGGCTTGCGGCTCTATCATCCCCTTGGCGCAGCCCGCGCACCATCCAGCCCT
CGTGCTGGACCCCCATGGCCGTCCCCTGGACACCGCTGAGGTTATCAACGCTCGCGCC
ATCCACCTGCAAGCTAAGGCTCTGGAGCCCCTCGGCCATGCCGCCATCGCTCCCCTCGTACACTCCGCCGTCG
TAGCCGCCCCCGTCGCTCACGCTGCCGTCGTCGCACCCGCTGTGATCGCCGCCCCCGCCGCCGTGTCTCACCA
ATCCCGCGTGGATGTACACTCCAGCCCCGCCGTCGTCGCCCACGGCCTCGCCGCTCCC
CTATTGAGCTACTCCGCCCACGGACTCGCCGGACACGGACTCGCTGGACACGGACTCGTCGGACACGGACTCG
CTGGACTCGGTCTCGCTGGACACGGACATCTCCTCAAGAAGCGCTCCCTCGGCCACTGGGCGTACAGCGCACC
TCTCGCCGTCGCTCCCGCCGCCGTCTCCCACCAGTCCCGTGTGGATGTAGTGTCCAGC
CCCGCTGTCGTCTCTCACGCAGTGGCCCCCATCGTCTCTCACGCCGTGGCCGCCCCCGTGCTCGCGCACGCCG
GACACCTCCTCAAGAAGCGCTCCCTCGGCCACTGGGCATACAGCGCGCCTCTCGCCGTCGCCCCCGCCGCCGT
CTCCCACAAGTCCCGTGTGGATGTAGTGTCAAGC
>1002050179_1 Heli.1-DT667966.3.5
GCACGAGGCTTTGGCCCTCTTGTTTTTATTGACTTTGTTTTAATAATTCAATAATTAAATAAATGTAGCACTG
AAATACCTTTATTTAATTTATTATTTATAAAGTATCATTGACTTTTTATTTAGAAATGCGTTAACTAGAAAAT
AAGATGCCTGGCATAAGAAAATACCGACGTGACACAACTGAAGTCAGCTGTTGTTTGA

AATATGTAATATTTGGAGTTAATGTATTATTTTGGTTTCTTGGCCTTATTGTGTTGGCCATAGGTGTGTGGGC
TTGGTCAGAGAAAGATACATTCAATAATCTTTCACGCTTGACAAACATTGCTTTGGATCCTGCTTTCATTTTA
ATATGTGTTGGTACGATAACATTTATAATCGGTTTTACTGGATGCGTCGGCGCCTTGC
GTGAGAACACGTGTTTGTTAGCCTGCTATGCAGTATTCCTAGCTCTTCTACTGCTGGCTGAGATGACGGCTGG
AATACTATTCTTCGTTTTTAAAGACTGGATAAAACAACAAGCAACAACCGGCTTCCAATCTTTCATCACTAAC
TATAGAGCAGATCCCGACCAACAGAATTTGATAGATTGGATACAAGAAGATTGGTTAC
AATGTTGCGGTGTAGAAGGCCCCCAGGATTGGGACAGGAACGACTACTTCAACTGCTCGAGCGGCGCCGTCGG
CTCGCGCGAGGCGTGCGGCGTGCCTTTTAGCTGCT
>1002050180_1 Heli.1-CO729580.1.5
GGTCGGAATTCCGGGTCGACCACGCGTCCGACAAGATGGCGGATAAGGAAAAGAAGATCAAGAAGAAGAAGGC
GAAAGAAGACGCGCCCGCTGAGGAGGCCCCAGCAGCGCCCGCGCCCGCCGCCGCCGCCGGTGGTGAACGTCAA
TCCTCCCGCGGCAGCCGCAAGGCTAAGCGCACTGGCTCCAATGTCTTCTCCATGTTCT
CCCAGAAGCAGGTCGCCGAATTCAAGGAGGCCTTCCAGCTAATGGACCACGACAAGGACGGTATCATCGGCAA
GAACGACCTTCGCGCCACCTTCGACTCTCTCGGCAGGCTCGCCTCCGAGAAGGAACTCGACGAGATGGTCGGT
GAGGCGCCCGGCCCCATCAACTTTACCCAGCTCCTCACCCTTTTCGCCAGCCGCATGT
CCGGTGGCTCTGATGATGACGACGTCGTCATCAACGCCTTCAAGTCCTTCGACAACGAGGGCAAGATCGACTC
TGAGAGGCTGAGGCACGCCCTCATGACCTGGGGCGACAAGTTTTCCGCCGACGAGGTCGACGAGGCATACGAC
CAGATGGATATTGATGAGAAGGGCTTCATTGATACCAACAAGCTGATCACCATGCTCA
CAGCCAGCGCCGAGGACGAGGAGGGCGGAGAGGCCGCGTAAATGCTCACTAATATCCAAGGAGTCGAATAGAC
GGTGGCTACCA
>1002050181_1 Heli.1-CO729577.1.5
TACGGTCGGAATTCCGGGTCGACCACGCGTCCGGCCAAAGATCTAAGGAACTACCACACTGGCTTCCCAGTAA
GGCTACTTTTGCCTAAAGGTCTTGTCGGTGGAATGAAGATGATGCTTTACGTTATCGTAACACCACTGAAACC
AATTGACAACATTGACATATCCATTCTGGACATAAACCGCAAAGATTTATTGGTAGAT
TTTAGGTCTACAGTCCTTCTCGACAAAATGCCTCTTGGTTTCCCATTCGACCGTTACATTGATTACACCAAGT
TCTTTACTCCCAACATGAAATTCTTGGATGTCATGATCTTCCACAAAACTCGCGTTTGCGACATGAAGACTCG
CTGGAATCGCTTCGTGCTCAAAGATTACGGCTACTTCAACCAAACGCCAATGGTAAAT
AACTATTTCACTGACCTTGACATCAACACCAAAATCGACACAGACGTCGTAGGTACTTTTGATGTCTAAACGA
TAGATTATTTTTAACCAAAAATGCTGCCACAGACGAAAAAATTATCCAAGTTTAAATGTAACTTATTTGAATC
TACATAAGCTTGTACAGCCATAATAATGATTAATGAGTAAATAAATAAATATTTATGA
>1002050182_1 Heli.1-EL603753.1.5
GCACGAGGCGAGAGTCCATCTTATCTTAATACATTATTTTATTATCGATAAATATATTATAATCTTTTCGTTT
GATCAAGAAAATGAAATCTAAAAAGCGTCATCACGACAGTTCTGATGAGTCTGAAGAATCAAATGATAGTGTA
AAATCAAGCAGTTCTTCAGATAGCGAAAGTGATGATTCTTCAGTATCTGAGCCCCGGC
ATAAGAAAAAAAGAAAACGTGCTGATTCTTCCGATTCTGATAGTAACACGGATGATCAAAAACGCTCAAAAAA
ACGTAAGCATAAGAAAAAGAAAAAGAAGCACAATAAGAGAAAACGACGATCGGAGTCATACGACGACATATCT
GATGATGCGATTTCGATAAGTGAAGGCGAAATATCTCCTAAAAAGAAGAGAAAACATA
AACATAAGCATTCTAAACGAGCTCAATCAGCTAGTGGAGATGAAAATTATGAAAGAGAATCAGAGCGTCGGTT
ACACAGTGTTGTTAAAGAACGCAGACCATCTTCAGAAGAAGGCAATCCACCAACCCGCACATATTATCAGAAG
GAATATCGTCGTTCGGAATCAGTAGAAGAATATGATCGTGGAAGAGAAAGCCAAAGAT
ACTATCAAGAACCTAACAGGCGACATCAAGAACCCCCAGTTAGTTATA
>1002050183_1 Heli.1-EL597439.1.5
GCACGAGGGTCATTACTCTGTGTTCATACAAAGTTTATAGAATATAAAAATTCTTTGGTTCATAACTTCATTC
ATATACTTCATTGTTCATTAGCCATAGCCTCCCCCTCCTATGTTATTTGTTCTGTAGTGTGGTTATTATTTCA
TTGTTAATAAAAAGTTTCTACGTGAAAATTCTTAGTTCTGGTTTTTCCTTATAATATA
TTCTTGTTTGTGTGTTAGTTTTTGTTTGTGACTTGTGAGTGACTTGGTACAATTTTTTAAATTAAAAGGCGTA
ATCTTATTTAACAATGTCGGGTCATAGATACTTTCAGGAAGGCAAAGACTTATTTGATGACGAAGTGGATGAT
GATACTTTCGTTAACAGCTATAGATCAAGGATTCCACCACCACAACCAAAACCATCGC
CCTACATTGCTGATTTGGAGAGGCAAAAACAAACAATGTTACAAAAACAAAAAGAAATTGAACAACGCACTGT
GGAATCTTCGATACGAAGTATAGGTTTATTACGAGACTCAGAACAAATTGGCATTGCAACTGCTGAAGAACTC
ACCCGTCAACGTGAACAGTTACAAAGCACTAGTAGGCGACTAGATGAAATAAATACCA
ACTTAAATTACAGTCAAAAACATCTAAATGGCATAAAGTCAGTATTTTATGGATTCAAAA
>1002050184_1 Heli.1-EL603360.1.5

GCACGAGGAGAAGGCGAAAACTTTCCTTATTCGAAATGGAACCAAAAGTTAATTTAAAAGAAGACTCGGCGTT
TAAAAAACTACAAGAATTCTACAATGTAAACGCTGAAAAAATAAATATTCAGCAATTATTCCAACAGGATCCG
GCCCGATTTAATAAATTCAGCCTTCGCATTCCAACACCTAATGATGGAGATATTTTAC
TGGACTACTCCAAAAACCGAGTGGATGATGCTGCCTTCCAACTCCTTTTGCAACTGGCAAAGAGTCGTAATGT
AGAACAAGCGAGAGATGCTATGTTTTCAGGTCAAAAGATCTTTTTTACTGAAGATAGAGCAGTACTTCACATA
GCATTACGCAACAGACAGAACAAGCCTATACTGGTTAATGGGAAAGATGTAACACCGG
ATGTGAATGCAGTACTCGCTCATATGAAGGAATTCTCCCAACAAGTTGTTAGTGGGAATTGGAAAGGATATAC
AGGCAAGCCAATAACTGATGTTATAAATATTGGCATTGGTGGCTCCGACTTGGGCCCTCTTATGGTCACTGAA
GCACTTAAGCCATATGCTAATCATCTTAAGGTACATTTCGTGTCAAACATAGATGGCA
CCCATTTAGCGGAAGTCCTCAAACGCTTGAACCCCGAGACGGCGTTGTTTATAATTGCGTCTAAGACATTTAC
AACTTCAGAAACTATTACTAACGCGACTTCAGCGAAGAATTGGTTTTTGGAACTGGCTAAAGATCCGGTCAGC
AGTATCAAAG
>1002050185_1 Heli.1-EL602923.1.5
ATTGATTCCTAATAGATAATGTAAATTCTCATCTGAACCCCGAACCACCTACGGAAAAACATATGGATTTCCA
TATAAATCTCACATTTCAGAGATTACAAGTTTTCTGATAAAAAATAAAACATTGGCCTCCGTAGCTGTGATGG
GATCGTACTTCAAAGATACCAGCAGACGAAAAATGTCTACAGTGGAGGGATATAAATG
ATATACTGCTGACTATTTTTATTTCGTCGCTTATTTTGTGATAACTGTATTGTTGATCGTCGATCGACGTATT
TTATGTGCGCGTGACGGTGACATGAGCACAAACAAGAGACGCGGCGCCAGCAGTTCCAGTTGTGCTGTCCCGG
GGGTGCCCGCGCTTGTCAACGTCCCGACCGCAATGTCTGCCGACCTTGCCTCGCTGTT
CGAGTGTCCTGTGTGTTTCGACTATGTCTTACCACCCATCCTGCAGTGTCAGAGCGGGCACCTCGTGTGTTCG
AGCTGCAGACCTAAGCTTTCCTGCTGCCCGACGTGCCGTGGCCCTCTCGGCAACATACGCAACCTGGCCATGG
AAAAAGTCGCCAGCAACGTGATGTTCCCATGCAAACATTCCAATACGGGCTGCACCGT
TACTTTAGTTCACACGGAGAAGGCGGAGCACGAGGAGGCGTGCGAGTTCAGACCTTACTCCTGTCCCTGTCCT
GGAGCATCGTGCAAGTGGCAAGGAGGCCTTGACCAAGTCATGCCCCACTTGATGATGTCTCATAAGAGTATCA
CAACATTACAGGGAGAAGACATTGTTTTCTTGGCGACAGATATAAACTTGCCTGGTGC
AGTGGATTGGGTGATGATGCAGTCATGTTTCAATCATCATTTCATGTT
>1002050186_1 Heli.1-DT661968.3.5
GGAATATGGAACAGCACTATGTGCGACACTTATTACTGCAACAGCAGGAACGTGCTACGGTTGGCCGTCACCG
ACTCTATCCTACCTACAATCAAAGGAGAGTTCGATACCAACATCATCAGCCGAGGGGTCATGGATAGTTTCTA
TAATGATCCTATGTTCAGCACTAACCCCTGTCCCCTCGGCGTATATGGCTGATCGCTT
TGGAAGAAAAACCACACTTCTTTTCGGAGCAATACCCTTCATCATCGGCTGGGTGCTGGTCATCGTAGCAAAC
TCAGTTCCCGTTCTTTACGTGGCCAGAATGTTCTCTGGTCTCGGCTACGGTATCGTGTACACCGTCGCCCCCA
TGTACACAGGAGAGATTGCCACAAATGAAGTTAGAGGAGCCCTCTCTACTCTTATTAC
ATTAATGAACAAGGTCGGTATACTTGCCCAGTATTGCATCGGGCCCTTTGTTTCGATGCGAATCTTAGCCGCC
ATCAACTTGATCCTGCCCATTTCGTTCGTAATTACCTTCATCTTTCTACCGGAATCGCCTTACTACTATCTAA
AGTTTGAACGTAGCGAAAGAGCGGAAAGATCACTCCGGAAATTGCGAACTGGTGATAT
AAGGTTAGAACTTAAGAACATAGAAGTTAGCGTTCAAGAAGATATGAAAAATCGAGGCACTTGGATGGATCTA
ATTTCCGAAGCGACGAACAGGAAAGCTCTTTGGATAAGCCTTGGGGTGTTTACTATACAACAACTTTGTGGGA
GCGCTGCCGTGGTCGCGTACGCCCAAGTTATCTTTGAAGTCACAAACAGTCGCATCGA
GCCGTATCAGGAATCAATAATCCTGGGTTGCGTACAGGTGCTAACATGCTGCGTGTCGGTGATGCTGGTGGAC
CGGCTTGGAAGAAACCTTTATTGTTGTTGTCTGCACTCGGAGTTGGGTTTATGAATGGCATCATTGGCACATA
TTTCTACTTC
>1002050187_1 Heli.1-ES586608.1.5
AATTTAATTTATTAAAGATATTTATTATTTATGAGCGAGTGGTGGCGGGGCCGACCCATCCGCCCCCCCCCTA
CCAAAAACAATCTTTCTGAGGGGCGCCCAAGAAACCACCCTTACCGGGGGGGGGATGGAACCCACCCTGTCCT
AGTTTGAAGGATGGGTCAAAGAACTATTGATTGACCTTCTCACTACCCGCTCACTTAG
GGGGGACACGGACGGACTTTCCGAATGGAATGACTCCTTTCTTACGTCCCACTACAACCTCCCTTATTCCTAA
GGAATTGTTTGTTTACTTCACCAAAAGGAAGCAACCCCCGCAGGGGAGGGAAGAACTTCACAAACCAAACTCA
TCCCCCCGCCGGCCGAAGTAGGCAGAATGATTCTGTCTTAACTCCTAACCAAAAAACT
AATTTCAAAATGCCGCAGGTTTCTTTCTACTACTTTCCCCTGAAGGCTCTGGGTGAGTCCGGAAGGTTGCTTC
TGGCTTATGGTGGCCAGGAGTTCGAAGACAAACGCGTTTCCATGGAAGATTGGCCTGCGTTTAAACCATCAAC
CCCATTTGGTCAGATGCCAATACTTGAGATCGATGGCAAGAAGTACGCACAGAGTATG

GCCATAGCTCGCTACCTCGGCCGCAAGTACGGAGTGGCCGGTGCAGATATCGAGGAGGACTTCGAGATCGACC
AGAATGTGGAATTTGTCAATGATATCAGAGCTAAAGCAGCAAGCGTTCACTACGAGGCCGACGAAGAACTCAA
AGCAAAGAAGCACGAGGACCTCAACAAAAACATATACCCAGTATTACTGAGCAAGCTG
GATGAGCTTATAAAGAAGAATAACGGACATATTGCAGCTGGCAAGCTCACATGGGCAGATTTCGTTTTCGCCG
GCATCTTTGATTACTTGAAAGTAATGCTTCAAATGCCCGACCTGGAGAAGAAGTATCCCAGCTTCAAGCAGGT
CGTCGATGCAGTGTACGCCGACGATAAGGTGAAGGCCTACAGCGAGAATATACCTAAA
TCAGAATATAACTTCTAATTTAAAGAGGCTTATTTTTGTATGGAAAAA
>1002050188_1 Heli.1-DT665830.3.5
GCACGAGGATGACCCTTGAAGATGAAGACCGAAGAAGATGAAAACTGAAGCAGATGAAGACCGAAGCACATGA
AGACTGAAGCAGATGACGACTGAAGCAGATGGAGACCGAAGCAGGTGAAGACCGAAGCAGATGAAGACCGAAG
CAGACAAAGACTGAAGAAGCAGATGAAAACTACAAGCAGATGAACACTGAAACAGATG
AAGACCGAAGCAGATGAAGACGAAGCACATGAAGACAACAGACGAAGATTGAGGCAGGAAAACCGAAGCAGAT
GATGACCGAAGCAGGTGAAGACCGAAGCAGGTGAAGATTTTTGCAGAGGAAGACCGAAGGAGATGAAGACGAA
GCACATGAAGACAACAGACGAAGATTGAAGCAGGAAAACCGAAGCAGATGAAGATCCA
AGCAGAAGAAGACCCAGAAAGATGAAGACCCAGGCATATGTAGACCCAGGCAGATGAAGACCCAGGCAGATCA
AGACCGAGGCAGATGAAGACCCAGGCAGATGAAGACCGAAGAAGATGAAAACTAAAGCAGATGAAGACCGAAG
CAGATGAAGACCGAAGCAGATGAAGACCGAAGCAGATGAAGACCGAAGCAGGTGAAGA
CCGAAGCAGATGAAGACCGAAGCAGATGAAGACCGAAGCAGATGAAGACCGAAGCAGATGTAGATCGAAGCAG
CAGATGAAAACTAGAAGCAGATGAAAACTAGAAGCAGATGAATACTGAAGCAGGTGAAGACCGAAGCAGACGA
AGACCGAAGAAGATGAAAACTGAAGCAGATGAAGACCGAAGCAGATGGAGACCGAAGC
AGGTAAAGACCGAAGCAGATGAAGACCGAAGCAGATGAAGACCGAAGCAGATGAAGACCGAAGCAGATGAAGA
GCGAAGTANATGATGACCAAAAAAATTGAGATCCAAACAATAAAACCCGAAAATGAAGCTTTAATACTAATAA
AACCCAACAAAGATGAAATAAAAACAAAGATAAAAATAAAAACGAAGACGAAAGATAC
AAGGAATTCAAAACTTTAATTGGATTCCATGAATAT
>1002050189_1 Heli.1-CO729575.1.5
ATTTTAATAATTTATTTTTCATTTATTTTACATATAAATTTTAGTGAATAATTTTAATTATTTTAAAAATAAT
TAATAATGTTTTGTAGTAATTAATATTAAAAATTTAAGAAATTAAGATTTAGTAATAAAAAAATTAATAACTA
ATTTTGTGCCAGCAGTTGCGGTTAAACAAAAGTTAAATTAAATTAATTCAGTAAATAA
TAAATAATAATATTATTTTAAAAAATATTAAATTATAAGGTGAAATTTTAATTTAATTAAAATTTATATAAAA
ATTATGATTTAGTAAATTTTATTATAAACTAGGATTAGATACCCTATTATTAAAAATTTAATTTTAAATACTA
AAATAGTAAATAATAAATATTGAAACTTAAATAAAATGGCGGTATTTTAGTTCATTTA
GAGGAATCTGTCTAGTAATTGATAATCCACGAATAAATTTACTTAATAAAAAATTTTGTATATCATTGTTAAA
AAAATATTTTTATATAAAAATAATATTTTAAAATTTTAAATATAAATTAATTCAGATCAAGATGCAGATTATA
ATTAAGATTAAGATGGATTACAATAAATTTATTTAAACGAAAATAAATTTTTTAATAT
AAAATTGAAGGTGGATTTAAATGTAATTTTAATTAGTTTGTTAAAATGATTAATAATTGAATATGTACATATT
GCCCGTCACTTTCATTTAAAAAT
>1002050190_1 Heli.1-EL600227.1.5
GCACGAGGCGAAGTAGTAAGCAATGGGTTAAACTTAATGTGGGTGGAACTTATTTTTTAACTACAAAGACTAC
TTTATGTAGGGACCCCAACTCATTTCTTTATCGATTAGTTCAAGAGGACAGTGACTTAATTTCAGATAGGGAC
GAAACAGGCGCTTATTTAATAGATAGAGATCCTACGTATTTCTCACCAGTGCTCAATT
ACCTTCGACATGGTAAACTTGTAATAAATAATGATATAGCAGAGGAAGGAGTTTTAGAAGAAGCTGAATTTTA
TAATATTACAGAGCTTATAAGATTAGTAAAAGAGAGAATATGTTTAAGAGAAAGAAGGCCTCTCAAAGATTCC
AAAAAACATGTTTATAGAGTATTACAATTTCATGAAGAAGAGCTGACACAAATGGTAT
CCACAATGTCAGATGGCTGGAAGTTTGAGCAATTAATAAATATTGGGTCTCAATATAACTATGGCACTGAAGA
ACATGCAGAATTTCTTTGTGTTGTTAGTAGAGAATGCGGCAGCTCCTTAAACAGTAATGACATAGAACCAACA
GATCGTGCAAAAGTATTACAACAAAAGGGATCGAGGATGTGAGGTTACCTCAAACATT
ATATCTGATGTTTTTTGAGACTTTGTGTGAAATGTGTATATACATATTTTGTAGTGTGAAGTTTGTTACTCTG
TTCTATAATTCCAAGTTAAATGTAATTTTTTAAATCCACTCATTTTAAAATTAGTCCTGTTAGGCCAAAATGT
TGGAAATCGATATTTTAATATT
>1002050191_1 Heli.1-ES584749.1.5
CTCGAAACGGCCAGTCGTATCGGAATCAGCCAATAGTCAATATCGTTATCTAATCTTACTTTACCTGGATTTA
TATTCAGTTTAGTTAAATAAAAAAATCGTAAAATGTTGGGCACCGTAAGCAGAACTACTCTTATGGGAAGGAA
TGTGATGAAGAGCTTCATCAGGAATGGCTCTCACGGAGGAATCCCGGGGGAGAACCTG

CCTTTCGATATTCACAACCGCACAAAAATTACCTTGCATTTCCTATGGTATGGTGTGACAGGAATTTCGTCCC
CATTCCTCATCTTGCTGTACCAGCTTTACAAAAAGTAAATGTAAATGGAATTTCAACGTAAATAAAACAGCAT
AAAAAATATTTGTAGTAGTTTAATTATAATTAATTGAATATAAACG
>1002050192_1 Heli.1-EL601751.1.5
AAGTGTAAAGTTTCAATTTACTAAGTATAATAAAATTATTACAAAAAATCGTTTTATGTATTATAAACTTTGG
TATATCTTTGTGAAAATGCATATTTGTATTAGTTTAACAAAAAATATGTGACTGATATAATAAAATATAAAAA
TCGAAGTAAATTACAAAAATAATAGATAACAGTTAGGCTGTATTTCAAACGAGTCTTC
TTTTCACGATTCTTTTTCATTTTCATTAGTTACATTAAATTCTAACGAAAGTACTTAATGTAATAATAGGGTA
CTAAAACTTTGTCACTTGCGATCTTGTATTTATTGAAATATATGTCAAACTGACAGCACAACAACATTTAACA
TTTTAATTTAAAAATATTAAAACTGTGATATATTCATCTACAAATGTAGCTAAAAAAT
GTATAACTATTGAAAATTTGTATAAAATAGTTAAACAAAGTCAAGCCAAAAATAAAATTGTTGACAATATGTG
TTTTCTTAGAACTCTACCTCTTAATTGAAGAATGAAACATAGCCACTCCATAGTTCGTTCCTTTCATGTAGGG
TTTCTAAAATAAGTGTTAACCATTACCTTTATCTATATGTTGAGTAAAACTTATAAAA
ATCTAACGTGAAAGTCGGGAACAAGGGCAAGTTTCATACTCGCCTATGGAATGTATTCTTTATATTAACATT
>1002050193_1 Heli.1-ES585055.1.5
CAAAGTCGCGAGGTATCGACGTCCGGTTCGATCTGGATTTGACCAGTTAAAACCGATTTGCGGGCTGCTTTCC
CCCTTGAATCTTTTTGTTTATAGTGTGTTTTGTATAACAATAGCTTAATTTTGTTATTTGAAACTTTATTTTC
GTCAGTCCGTAACGAATTTTAACGAAAATGTCTGAGGAATCGTCAGCGGATCGCAATG
TCGAGATATGGAAGATCAAGAAGCTTATCAAGAGCTTGGAAATGGCTAGGGGGAACGGTACATCAATGATATC
TCTCATAATACCGCCCAAAGATCAAATCTCCCGCGTGTCTAAGATGTTGGCGGATGAATTCGGCACAGCATCC
AATATTAAGTCACGTGTCAACCGTCTGTCTGTACTGGGCGCTATCACATCGGTACAAC
ACCGACTCAAATTGTATACGAAAGTACCGCCGAACGGCCTTGTAATATACTGCGGTACAATCGTGACCGAGGA
GGGCAAAGAGAAGAAAGTCAATATTGACTTTGAACCATTCAAACCCATAAACACCTCCCTGTATTTGTGTGAC
AATAAGTTTCACACTGAAGCCCTGACTGCATTACTCGCGGATGATAATAAATTCGGAT
TCATCGTTATGGACGGTAACGGCGCCCTGTTTGGTACTCTACAAGGAAATACTCGTGAGGTTCTCCACAAGTT
TACCGTCGATTTACCCAAAAAGCACGGCCGCGGTGGTCAGTCGGCCCTACGTTTTGCGCGTCTGCGTATGGAG
AAACGTCACAATTACGTGCGCAAAGTGGCCGAGGTCGCTACCCAGCTGTTCATCAGTG
CCGACCGCCCAAATGTGGCTGGACTTATATTGGCCGGTTCAGCCGATTTCAAAACTGAGCTGTCGCAGTCTGA
TATGTTTGATC
>1002050194_1 Heli.1-CO729588.1.5
ATTTTGACAGTTCAGTACCAATTTGGGAGTGCTATAGTTCTGATTAGAATTCACAAATAAAATCCACAATGAC
CACATACTTCCAATACCCCACACCTGAACTCCAGGAGGAGCTTAGAAAGATTGCTCAGGCAATTGTTGCTCCT
GGCAAGGGTATTTTAGCTGCTGATGAGTCCACAGGAACCATGGGCAAGCGTCTCCAAG
ACATTGGTGTTGAGAACATTGAAGAGAATCGTCGTAAGTACCGTCAGCTGCTCTTCAGTACTGATCCAGCCAT
CTCCGAGAACATCTCAGGTGTAATCCTGTTCCACGAAACCTTGTACCAGAAAAGTGATGATGGCACTCCCCTG
GTGTCTCTTTTGGAGAAACGTGGAATCATTCCCGGCATTAAGGTCGACAAAGGTGTTG
TACCACTCTTTGGATCTGAAGATGAATGCACTACTCAGGGCTTAGACGATCTCGCCCAGCGTTGCGCCCAATA
CAAAAAAGACGGATGCCATTTCGCAAAATGGCGTTGTGTCCTCAAGATCGGTCGCAACACACCCTCATACCAG
GCCATCTTGGAGAACGCTAACGTTTTAGCGCGTTACGCTTCGATATGCCAGAGCCAGA
GGATCGTTCCTATTGTGGAACCTGAAGTGTTGCCTGATGGTGAACACGATTTGGACCGTGCCCAGAAGGTGAC
AGAGACCGTATTGGCTGCCGTCTACAAGGCGTTGAATGACCACCACGTCTACCTTGAGGGTACTCTTCTGA
>1002050195_1 Heli.1-CX700793.1.5
CTGCAGGTACGGTCGGAATCCCGGGTCGACCACGCGTCCGCGTTCACGCGACACAAAGTATTCTTCTAAATGT
GCATAAATGTAAATAAAATGTGGACTCAATATATTTGGGCGTTATGCCTGGCGGTCATGTGTCAGGCCCAGCT
GTCGGACATCGAGAGATATTTATTGACTATAGCCGAAATGTCACCGTCGCCCCCTCTG
CAGAGTAACGAAAAGGCATGTCAAGTTGACAGTTCTACAAACGGTGTTTGCGTGAAACGTACACAATGCAACT
TGGACGCGCCGGCAGTGGATCCCGCAACTACCTTCATACTGCGTGAAGGAGGATCCGCGTCTACCGCATCTGA
TGTGGAGAACGCATTGGATACACAGTGCGCATGGCTTCAGATCTGTTGTCCATCTGAT
AAAGTCCGGGAGGAAGAACGCCAAGTTAAATTCGAGCAGCCGCTTGGTTGTGGCTACAGCAACCCCGGCGCTA
ACGTCTTCAGGGAATCAGCTAATGCTTACGGCTATGCGGACTTCGGGGATTTCCCGTGGATGATTGCTTTATT
ACAGACAAAAAACGATAAGACAAAATTCAATGACCAGTATATAGGGGGTGGAACACTA
ATTCATCCTTCGGTCGTGCTGACGGTGGCGCATAAAGTAGATAAGCTCGAGTCACCTAGTGAGTTGACGTGTC
GTGCTGGAGAATGGGATGCAGCAACAGAGGACGAACTCTTTTCAACATCAGACAGAAATGTTAAAAATATTGT
CATCCATAAAAATTATCTAAAA

>1002050196_1 Heli.1-EL599289.1.5
GCACGAGGTCGTCGTTGTGAACAGACGGTTTCTTTGTTTATTTAAAAAAATTAACTTGGATCATAAGAGTTTG
AGAAAGATTTATAAACAAAGTATATTGGATATATTTGTAAAATAACATATACATTGAATGAAAAGCAGCAAGT
TTTGAAGTTTTCAAAATGTTCTCCCCTACCAAGAAGATCTTCGAGCAAATTTACAAAG
ACGTTGCACGTCGGCATATCAGCAGGACTACCAGCGTAACTAATTCCGACTCGATGGCTCCTAGACTCGACCA
GCAAAATATCCAAAGTGATGATGCTCTACCTAAGAGGTTGCTGAAGTTGCAGAAATATCACAGATTCCTGTCG
ACTCTGACCCCTCAGCAAATGCCACTGGCAGCCCGTGGACTGGCCGTCTCCAAAGACC
AGTCCCGGGAGTTCATGGCTTGCTTCCCGGACATCGTGAGGGACCTGACTGAGTCTGGCAAGCATATTGACGT
GCCTGACGCCAGCAAGTGGTTGGCCAAGCTTCTCCAATACAACGTACCAAACGGCAAGAAAAACCGCGGGCTA
GCAACGATACTTGCTTACAAAATGGTGGAAAAGCAACAAAATCTGACTCCAGAAAATA
TACATTTAGCTAACATTATGGGATGGTGTATTGAAATGTTCCACGCGCACCAACTAATTCTGAAGGATATAAT
GGAGGGAGCGGAAATGCGTCGGGGTGTACCCTGCTGGTACCGTCAGC
>1002050197_1 Heli.1-CV525802.2.5
GGGAAAAAAGGAAAAAAAAAAAGAAGGCCCGTGGTCGAGGAATCGGCACGAGGCCGCATGTCGGTGCGTGGCT
TTCTAGTGTTAGTTGCATTCGCGACCACGCACGCGTTGGTTGACGAAGCTGTCGACGTTATCAAACTAGGCAT
TGATATTGGTGAGGAAGTGCTCAACTCCTGGGATGTTATCAGTAAACCTTTCAATGTC
TCCGGTGGAGTGGAGCTGCCTTTCATTAGGCGAAGAGAAAGGCAAATTTTGGCGAAATTGGCCCAGATTTCGC
GCAGCATAACCAGACTTGAGTTGGGCATAGAAAGAAATGGTGCAATCGCAATGCTTCTAGCCAAGCGCAGTGG
TCATAGCGCCAGACTGGAATTAAAATTACACGAAATGGCTGACCTTTTGAATCGAGTG
GCATCAGCTGACAGGCAGATGCGCGAATATATGAGGATACAGCAAGACCTGGAGAGGAGTACATTGGAAGACT
TTGCAGAGTGGTGTGTGTCACACGACGCGGGAGCGTTGCCCAGCCTTCTGGAGCGGGTGCACTCGTTGATAGT
ACCACCACATAAGAATATCTTGGGAACTAATATTTTGCAGTTGCTTTTAGAGGACTTG
CAGGAGGAAAATTCAGATCTATGTGATCTTCAGCTGTCACCGCATCAGTTGATCTACGATATGTACAACACCA
TCTCCCTGACGGAGATAAAAGGTTACGCCATGACGCAGTTCTCGTGGATGCTGCTCAGGATTTACGGCAAAGG
ATATTACCCGCAAGAAGCAGTTTGACGCGCGACGGTTGGAGAAAGGACACTAGAACGG
GGCGATGTCCGATTGGTTGGTATTGCCCATAAAGGTTTTACGGGGAACGCCTCTGATACCAGGTAACTTGAG
>1002050198_1 Heli.1-EL602197.1.5
GCACGAGGCGTAAACCCTTAAAGGTGCGGAGTGTTTTTTGTTTTTTTTTTATATTTAAATAAATATACTTTGG
TGATCCCATTTATATATAAATAAGATTTTTTAAAATTAATTTTAAATAAATAAATCAAAATGGAGCGAAAATT
TTTAAACGTAATTTTACTTGGTTTCGGATTTATGTTTGTGTTTACGGCATTCCAAACT
ATGGGGAACATAGAGAAAACCATTTTGGATAGTATAACACAAGATGATCCCGAGTTCACCGGTGATGGGTACA
CGTCGCTGGCGATCATCTACGCGACCCTCGCCATCTGCAACTGGATGGCTCCTTCCATTATCAGCGTCACAGG
CCCCAGGGGTGCCATGCTTATTGGTGCATTGACGTATTTATTCTTCATAATAACGTTC
CTGTTCCCGCGCACATGGCTACTGTACGTCGCGAGCGTCCTGATAGGCGCCGGCGCAGCTGCCATATGGACTG
GGCAAGGCAACTATCTCACGTTGAACAGTGACGCTGAGACCATATCTAGGAACTCTGGAATATTCTGGGCTAT
GTTACAATGCAGCTTGTTCTTTGGAAATCTCTTCGTATTCATCAAGTTTCAAGGGAAA
AGTCACATAGACACGGAGACGCGCAATGTAGTCTTCGGCGCGTTGACAGCAGTATGTGCTGTCGGCATCGTCT
TCTTGCTGTCGTTGAGGCCGACCAGACGAGCTCCAGCCATTGATGATGTAAAGAGTGAAATGGCTGTGTCCGA
GTCTGAGGGTCCTATGGAGGCCTTCAAAGGGGCCATTCGTTTATTCTGTACCAGCGAT
ATGCTGTTGCTGAGTGCCGCTTTCATTTATACTGGTGTGGAGCTATCATTCTTCATCGGAGTATACAGTCCTA
GTATCTGTTTCACTCTCTTTATGTGTGATAACGCAAAGCAACTTGTTGGTCTTTCTGGTGTCTTCATTGGAAT
GGGAGAAGTT
>1002050199_1 Heli.1-ES588034.1.5
GTTTTAGACGCGTTTTTTTATAGAGCAGCTCTATGTGGGCTATATTTTTTTTGTAGATGAGGTGCCTTACGAT
AATGCATGTTACGGTGCAACGATTGCATTTTTAGACTAGTTGTACGATCGTTTGTTTTTATTTATTTTAATTA
TTATTATGTACATTATTGTAATAGTGTGTATCTTGTTTACAATTTTTTACGTACTAAA
TTTCATGTGATATTATTGTTCTCTAAGGCTATTATTCGATGTTTCCTATTGATGAAATGTGTCATTCCGACGG
GCGTCTTTTGAATCTCTTAGTGCCAAACTGCATTTTGATCGATGTTTGTTGTTTTAATTCTTGTAATTTTCGA
ATAAATGTTATCGAGATAATATAACAAATGTGAAATACATTGAAATATTATTGGATAT
CGCTTTTAAATTAAAAAACACATCTTGACACACGGAGACAGACAGACTGACAACAAAGCGGTTCTACAAAGGT
TTCGTTTTTTCTTTTTGGGGGGTACGAGACCCCTAACTATAAATGGACATTGCCGAATTTGCTTTTTTTTTAA
TAATTATATATGAATAATTTGTTTAATGTCAGATTGTAGTAGTAAAATATACGCGTAT
AGTTGTTTTTTATTTATGTAACTATCGCAAACCATATATTCGTGCAGCTCGACGCTAGTTTTCTTATAGTATA
TTTGCTCGTGGCTTTTACGTTTTGGCATGTGTCGATATCCCAGGACATTAAAAATTGTA

>1002050200_1 Heli.1-DT663994.3.5
GCACGAGGTTGAAATTGAAAATCACGATAGATTTGTTGCGGGCCGCTTGCCACTTTGAGTTTCCTAAATTTCT
TTCATTGTGAACTTGAAATTTGTTGAATGCATGTATTTTAATGTATAAAAACTTATAATAGTCGTTAATTTAA
GAAGATGGCTATTTATTTTCGGCGATTGTTTACAAGTGCCTTGCTGGTGCTTTTAATT
GCATCCGCGGTAGCTGATAATGACGAATCTGATGATGGAGTCACTGTTGAGACAGTAGAAGAAGAACTATACC
AAAGCCCCACAGTGGACCCAAAGAAAATATATTTTGCGGAACATTTTGATGATGAAGCTGCATTTAAGAAGAA
ATGGATTAAATCTGAGGCCAAGAAGCAAGGTGTTGATGAAAACATTGCAAAATATGAT
GGCAAGTGGGAGATTCAATCACCGTCGAGGCGTATCTTGAAAAATGACCTAGGTTTAGTTTTGACGACTGAAG
CTAAGCACGCGGCGATATCAGCTCTGTTGGACCGCCCCTTCGAATTCAAGGAGAAGCCGCTTGTAGTACAGTA
TGAAGTTACTATGCAGGAAGGCCAAAACTGCGGCGGTGCTTACCTCAAACTGCTGTCC
CGCGGCATGAACACGAAGGCGGACCTGCGCCAGTTCCACGACCAGACGCCCTACACCATCATGTTCGGGCCCG
ACAAGTGCGGGAATGACAACAAGTTGCATTTCATATTCAGACACAAGAACCCCAACAACGGCACTATCGAAGA
GAAACACAGCAAGAAGCCCACTCAGCGTCTCGACGACATATACAAAGATAAAGAACCT
CACCTCTACACTTTAATACTGCGTCCAGATAACACGTTTTCCATACTA
>1002050202_1 Heli.1-DT663196.3.5
GCACGAGGGTGAATAATGAGAAGAAAAAAATAAATACTTTCTAATTGCATAAATTAATAAAAGTGACATTTGT
TTTTAAGTGAAGGGACTCATATAATTAACAAAATGGTTCTAATGACAATGATCGCCCGTGTTGTAGACGGGCT
GCCTTTAGCCGCTACTATGCAAGAAGACGATCAGAGCGGCTGTAATATATTGGAGTAT
CAAAACCAAGCAAAAATGCTGTTTAGAAAATTAAGTCCTCAATCGCCAATCCGCTGTTCCATCGAAACTGGAT
CCTATCTCTTCCACTATTTGATAGAGAATGAGATCTGTTATCTAGTTTTGTGTGAAAGAAACTACAGTAAACG
TCTTGCCTTCAGTTACTTAGAAGAGATTGCGCAGGAATTCTATCAGCAATACGGTAAA
AGAGTGAATACAGTAACTCGGCCATACACGTTCATAGAGTTCGACACGTGGATGCAGCGCGCTCGCAAGCAGT
ACGCGGAGGGCGGGGCGAGGGCGCGCCGGGCGACCTCCGCCCTCGGGGGGCAGCTGGGCGACGTGCAGAGGAT
TATGATGCAGAATATTGATGATGTGCTGCAGAGGGGCGCTGTTTTGTCAGAATTGGAC
ACAAAAACCCAAAAGCTGTCGATGATGTCGCAAAAATACAAAAAGGACGCCACTTACCTAAATACTAAATCAA
TGTTAGTGAAAGCGACCGCCGGAGCCGTCATATTGCTTGTATTTGTGCTGTACTTCTGGGTGCTGTAGACTTT
TGGGACTTTAGAAGTTATAAAAAATTGTTATGGACATCCTCATATGTTTAACGTATTG
TTTAATTTTCTTTTTGATTATAGGCTATTTTTATTTTTTACTTGTCTG
>1002050203_1 Heli.1-ES587002.1.5
CACAACCGATATGGACCGTAACGTGAACGGTAAGATTGTGGCTGTTACTGGCGCCGCCCAGGGCTTGGGCCTG
GCGATGGTCAACAGTTTCCTTCAAAATGGAGCTAAGCTAGCGATCATTCTTGATATTGACGAGGAAGCGGGTC
AACAAGCTATAGCGGGCTTACAAAGTAACTATCCAAACAAGGCTGTCTTCTATAAATG
CAATGTTGTGACAGATTTAAACATGATTTACGATTTGATCATCAAAAATCATCAAAAAGTTGATATTTTAATA
AACAATGCTGGAATCCTCGATGAATTGAATATCAAGAGAACTATAGATATTAATACGACTGCTGTTATGGAAT
GGTCCATGAAATTTTATGAGCATATGAGATTTGATAAAGGAGGCAGTGGTGGTACTAT
TATAAATGTGTCTTCGATCTTCGGCTACAGGATTACGGCTCACCTACCGTACTATCATGCTGCTAAGTTTGCT
GTCCTTGGATTTTCTAAGTCTTTAGGACATGAAACCAATTTTAAAAAGACCGGAGTGCGAGTAGCGACCCTCT
GTCCTGGATTGGTTCTAACAAATATTAGTGCAAATCCTGTGATCAGAGAAAAAGATTT
GTTAGCTGAGTTTTTGGCAGACCTTAAAACTTATGAATGGCAAGAAAG
>1002050204_1 Heli.1-DT664428.3.5
GCACGAGGCTACAGTACACAGAGCGACGGCTCAAGTGACGGGGGCTACTGCGGAACCCCGCCGCGGGCAGCTT
CCATGGACGCGTTAGACTCTCTCGCGGAACTCTCCTTGGCAGACTCACCTCTCGAACCGGACAGCCAGTCTCG
AGAGTCGCTTTTGACTCCGAAATCCTTGATGGAACGCGCTAGGATGAATGTTGGTTGG
CTAGATTCATCGCTCTCAGTAATGGAGCAATACGTACGCGAATGGGATACACTACAACTTCGCTTCAAGTTCT
ATTCGTTCTTCGACCTGACTCCGAGAGCTCAGGACGCGCCGAGATTGAACCAGCTGTACCAGCAGGCTCGTTG
GCAGATACTGAACCAAGAGGTGCACTGTACTGAGGAGGAAATGCTGCTTTTTGCTGCT
TTACAGCTACAAATCGAACTCCAGACCCTAGCCGGTGGCGGAGTAGATGCTAACGACGGCTCAGCATCTGCCG
CTGCACCAGAAGACGAGATAGACGCAGCCCTCAGTGAGCTGCAAGTGCAGCTGGAGGGAGGGCCGCCCGCGCG
CCCCGACATCACCCACGTGCCGGAGCTGGCTGGCTACTTGAAGTACAGAGGTCGTCAA
CGTTTTACTCTACGCGCCTACAAACGCGGTTGGGTGTCGTGTCGCGACGGAGTGTTGCGCATACACTCGTCTC
AAGAGGCCGCCGCCAAAGGCGATACGCCGTCTTACGCTGTTGAACTACGGGGTGCTGAGGTGACCCCGGACGC
ACACCCTGCTTCAGGCAGATACGGCATCAAGCTGGAGGTGCCTTCTGAAGACACTATG

CACGAAATGTGGCTTAAATGTGAAAATGAAGACGAATATGCAGAATGGGTGGCGGCGTGTCGTTTGGGCGCTA
GAGGGCGCTCTCTCGCAGACGCGGCCTTCGCGGCCGAGGCGGCCGGCGTGCGAGCCTTACTCGCTTTACAACG
ACCACAACCAGCCGCCGCTTTAAATCAACATAGTTTGCCCCATTTGGACCATTTGCAG
CCCGAGAATTATCTTGCGCCGAGGTTCTTGAAGAAACTGAAGGGAAAATTCACCCAAAGAGTATTGGAGAGCC
ACGCCAACGTCAAGGACCTTCCTCTACTAGAAGCAAAACTCCAATACATAAAAACATGGCAGAATCTACCAGA
CTACGGGCAAACCTTGTTCGTGGTGCGTTTCATGGGACACCGGANAGATGAAATTATT
AGTATCGCAAACAACCGCATCATGCGACTGGACCCTAATACCGGAGACCATATTAAGACATGGCGTTATAGT
>1002050206_1 Heli.1-CV133754.1.5
CGGGTCGACGCCGCGATGGAGAAGTTCGGCCGCGTGGACGTACTGGTCAACAACGCCGGCGTCGGAACCGCTG
TTCCGGCAACCCGTGAGACCCCGGACCAGTTCCGCGCCGTTATCGACATCAACCTCAATGGGTCCTACTGGGC
AGCGCAGGCC
>1002050207_1 Heli.1-EL604011.1.5
GCACGAGGAGGCAGCGCTAGAACGTGAAAAGACCCACATAGCAGACTTCGCGCCGGAAGTAGCGTGGGTGACG
CACTCGGGCTCCTCCGAGCTGGCGGAGCACATCGCCATCAGGCCCACCTCGGAGACCGTCATGTACCCGGCGT
ACGCCAAGTGGATACAGAGCCACAGGGACCTGCCGTACAAGCTGAACCAGTGGAACAA
TGTTGTGAGGTGGGAGTTCAAGCAGCCGCAGCCGTTCTTGCGGACTCGTGAGTTCCTCTGGCAGGAGGGACAC
ACAGCCTTCCGTACGAAAGAGGAAGCGGATGAGGAAGTCCTGCAGATATTAGAGCTATACGCCCGAGTATACG
AGGAGCTGCTGGCGGTGCCCGTGATCAAGGGGCGGAAGACGGAGAAGGAGAAGTTCGC
GGGCGGCGACTACACCACCACCGTGGAGGCCTACATACCCGCCAGCGGGAGGGGCATACAGGGTGCGACCAGC
CATCACCTCGGTATGAACTTCTCTAAAATGTTTGAAATAGTGTACGACGATCCCGACACGCAGGAGAAGAAGT
ACGTCTTCCAGAACTCATGGGGTCTAACCACTAGAACTATAGGTGTGATGGTCCTCGT
CCACGGTGACAACCGCGGCCTCGTCCTTCCCCCCAGAGTCGCAGACAT
>1002050208_1 Heli.1-DT668276.3.5
CGGCACGAGGAAATCGGATAATCGGGCCGTCGCTCAAGTGTTGAACTGTGATAAGTTTATTTTAAGGTTGTAG
TGATATATCTGATGTGCATTTAAGTGAAATATAATAATTAATCAAGATGCTTATCAAAGAATATCGTGTTACA
CTTCCTTTAACAGTTGAAGAGTACCAAGTCGCGCAATTATACTGCGTTGCGGAAGTTT
CCAAAAATGAAACCGGAGGCGGTGAAGGCATTGAAGTCATCAAGAACGAACCCTTCAAAGACTATCCCTTGCT
TGGTGGGAAATATTCTTCTGGCCAATACACCTACAAGATCTACCATCTCGCGTCGAAGGTGCCAGCTTTCATC
AGGCTCCTGGCGCCCAAAGGGTCCCTAGAAGTCCACGAAGAGGCCTGGAATGCGTACC
CATACTGCCGGACTGTATTAACGAATCCCGGTTATATGAAAGAGAACTTCGTCATCTGTATAGAGTCCCTGCA
CTTACCTGACGCCGGCGACCAGCAAAACGTACACGAACTGCCACCAGAAAAGCTGAAGAACCGTGAAGTGGTC
AACATAGACATCGCCAATGATCCTTTATCCTCGGCCGATTACAAATCTGAGACGGATC
CAACCAAATTCAAGTCGGCCAAGACTAACCGTGGCCCCCTCGTGGGACCCAACTGGAAGAACGACGTGAAACC
TGTCATGACCTGCTATAAGTTGGTAACAGCTGAGTTCAAGTGGTTCGGTTTGCAGAGCAAAGTCGAAAATGTG
ATTCAGAAGTCGGAACGACGGCTGTTTACCATATTCCACAGGCAAGTATTCTGTTCCA
TGGACGACTGGTACGGGATGACAATGGCGGACATCCGCGCCATCGAGGACCGCACCAAGGAGGAGCTCGAGAA
GCTGCGCCGCGAGGGAGAGGTGCGCGGGATGCGCGCCGACAACGAGTAGGGGGCCTCCCCGACCCCACGCCCA
CGTTAACCCTTTAAGGTCCTTGATCATTTTTTCTCAAAAAATGAATTTGAAAATTATT
AATTTTAAACATTTTTTTTTATACTTAGCGATTTTGTTATTTAGTTAAGATAGTTAATTT
>1002050209_1 Heli.1-CX700861.1.5
GCGGTACCGGTCGGAATTCCGGGTCGACCACGCGTCCGGACATGCCGGTGCTTGCAAGTTGCATTGCATGTGG
TCTATAATAGAAACACGTGTTTTATAATACTAACACTTTTTCTCATATTTATATAATTTAAGTCATTAATTAA
TAAGAACAATTTGCCCTATACTCTGATTAGATACAGTGTAGAACTTTGAAGCATTAAT
ATTAATAAAGTGCGCCTTAATTTTTGCACGCGTGCATATACACATCTCAATCAAGTACAAATTTTCTAAAAAT
GACGGAAGAAAAAGAAGTAGTAGCAACAGAGCAAAACAATGGGCAGGAGAACGACAAGGAAAATACTGTTGAA
GAAAAAACTGACCTGGATAACGCTATTATACGGCAGTTGGAGTATTACTTCAGTGACA
TCAATTTGCCAAGAGACAAGTTCCTTAGTCAGCAAGTGACGCTGGACAACGGTTGGGTGCCATTGGACATACT
CACAAGGTTCAACAGACTTGCCAAATTATCTACAGATACTGATATTATTGCTGAAGCAATTAAAAAGTCATTG
TCAGGATTACTAGAGATATCAGAAGACAACAAGAAAGTGAGACGAAATCCAGAACAGC
CAATACCAGAAATGAACGAAGAGAGGCGCAAAGAGCTATCTTCTAGAACAATCTATGCCAAGGGCTTCCCAAA
AGACTCTGTTTTAGATGACCTAC
>1002050210_1 Heli.1-DT665606.3.5

CCACGAGGGATTAGTGGTTTTCATCTTTATTTTATTTGTGATAGTGGTTCACCAAACTGAGCTTACATGATTG
GATTACCGCGTAGCGATAGAGATAGAGATCGTATAACGGTGAAAATACGTAACATGAAGAGAAGTACTTCTCG
AGATAGTATGCCGCGTTCCAAAAGAACGCGCAGTAGCATAGGAAGGTATGACGACAGC
TCCGATGAGCGCGTCACCCCTGAGAGGGTGCGCCGCCGAGTGCGCTCGCCGAGTCCCCGCGGTCGGTATGTGT
CTCCTCACCGTGACGACTACGTACGTTCACGAGAGGTACGGGAGGAATCCTCGCGTGCCTACTATAAAGTTTT
ATGTGTAAGTGCTTTGCATCCCAAGGCTTCTGACGAAATTGTGAAGGATACACTTTAC
AGGGAGTATAAAAAATTTGGCGATTTTAGTATCAAAATTTCTCATGAGTTGGACGAACGCGTCGCATACATTT
GTTTCCGAAGTATTGAAGATGCCAGAGATGCCAAACATGCTAAACCAAGGATTATTCTATATGATAAAGTAGC
TATCGTCGATCCAGTGTATGAACCGATGCGCTCAGAGTATAGGAGCAGACCTAGAAGT
ATAAGTCCTCCTGATTATGATCGCCCTTACTCTTATGCATGGTCTCCAGTTCCCGAGAGACGTAGGCCACCAC
CAGATGAGAGGGCTTATGGAATACCTCCTACTGTTCCTCCTCACCATAGAGATTTTCGTCCTCCAATGCATGA
GTATCCAATGGCTGGGACCCACGGTCCTCCAATGCATCATCGTCCACCTATGCACCCA
CCCCCACATCCTCATTATATGCCCAGGCCTTATATG
>1002050211_1 Heli.1-EL597096.1.5
GCACGAGGCCAACATACACATGAGAATCGGAGGACGTATAATTTATAATCACGATCAGAGCGGTGAAAAAGGA
ATTGTTTTAAATTGTTTTCTCAAATATTTAATTATTATAGAAAAAAATGTATAAACATAAAATAATATTATTA
ATTTATTTATTTGTTTTATTTTGTAAAGGTTCTCCAAAAGTGTCACCGGACTACATTC
AGCCATGTCCGGGATTAAAGCCAGACTGTCTCAAGAGGAACTTCCAGGAGACAATTCCTCAATTCGTGAAAGG
TATACCAAGTTTAGGTATACCGTCTATGGATCCATTGAATCAGGACCAAGATAAAATTAGTTTAGATTTACCG
GGAAACTTCAAAATAGAAATGACTGATGGCACAGTCAGCGGCTTTAGAAAATGTATTG
TAGAAGATGTTAGATTTGAAGGATTGAGCGCAGACCTGGAACTTCGTTGTAATTTAACATTAAAATCAAAATA
TAAAGCCAATGGGAGAATACTGATTGTATCTATTAATGGTGATGGAGATGCAAAGATCAAAACACCCAATGTG
GTTTTTAAAGCAAAAATAAACTTAGAGGATAAAACGCGTGATGGTGTTGTTTACCGTG
AAGTAAAGAATTACAAAGTTACTTATAAATTTGGTGAGACTGGTAGCTTCGTCTTCACCACTTATTCAAAGGG
AACGCTGAACTTAGTCAAACAGTATTAGCATTCCTTAATCAAACTGG
>1002050212_1 Heli.1-EL598159.1.5
GCACGAGGCAGGCCCTTCCAGCCCCACACCCTATGGCGAGCAATATCCACGCCAGACGGGGTCCTTTGCTAGC
GAGCCCTACTATCGGGAATACTTCGTTGGTGACAGCTACCAGCAGAGGGCTGCACCACCTTACAGTGACGCCG
AGTCAGCTTCAGCATCGGCTTTCGGGGATCGGTACTCGGCCCCTCGCTACCATAGCAA
GAGTGTAATTGCTGCTGCTGGTCTAACTGTGGATCTGCCGTCTCCAGACAGCGGCATCGGAGCTGATGCTGTT
ACACCCCGTGATCACACCACCGTTCAGCAGTCGTTCGAGTACACAGTGATATGCCAGCAACCTGGCGTCGGTG
AGGATGGACGAGGCACCCCCTCGGCGCACCACTCTCCTGCACAACCACCTCGTACGCG
CCCTTGGCACGACTTCGGCCGCCAGAATGATGCAGATAAAATTCAAATCGCTAAATTGTTTTCACCGTATGGA
TTCAAATATCACCTGGAAACGGCGAGTAGCAGTTCACAAAGACGTGAAGACGATCGTATCACTTATATCAATA
AGGGTCAGTTCTATGGCATCACTTTGGAGTACATTCATGATCCGGACAAACCACTTAA
GAATCAAACTGTTAAGAGCGTTGTAATGCTCATGTTCCGGGAAGAGAAATCACCAGAAGATGAAATAAAAGCA
TGGCAGTTCTGGCACGGAAGACAGCATTCTGTAAAGCAAAGGATACTTGATGCTGACACTAAGAACAGCATCG
GACTGGCGGGGTGCATTGAAGAAGTAGCTCACAACGCCATAGCTGTCTATTGGAATCC
ACTTGAGAGCGCTGCCAAGATTAATATAGCAGTACAGTGCTTGAGCACAGACTTCAGCAGCCAGAAGGGTGTC
AAGGGTTTGCCGCTTCATATTCAAATTGATACATTTGAAGATCCACGAGATACACAAGTTTATCATAGGGGAT
ATTGTCAAATAAAAGTGTTTTGTGATAAGGGAGCTGAGAGAAAGACAAGAGACGAGGA
AAGAAGAGCAGCAAAGAGAAAAATGTCAGCTACAAATAGAAAAAAATTAGATGAGATTTATCATCCTGTTACG
GAAAGGAGTGAATTTTATTCTATGGCTGATGTAGCAAAACCACCAGTTTTATTTAGCCCCGCCGAGGACATCG
ATAAACTAGCAGGAATGGATATACAGGGATTCTACGGACATGACGAAGCAGCCCTAGC
TGAAGCTCACCTAAAAGGAGCTTCGCCTTTCCTTTTACATGCTGCCAAGCCGCAGGCACCTGCTCTGAAGTTT
CATAATCATTTTCCACCTGATGCCCCAGCGTATCGATCGGACGTAGGTGGATTATCACCGTACAGTGACAGGA
AGGACTCTTTGGAGTTAGAAGGCGTGCTGGGGAAGCGCGCGCGCACGTCCACGCCGCC
GCTGAGCGAGCGCGTCATGCTGTACGTGCGCCAGGACACGGACGACGTCTACACGCCCCTGCACGTGGTGCCT
CCCCACACGCAAGGCCTCCTGCACGCCGATTACGG
>1002050213_1 Heli.1-DT663052.3.5
CAAGGACTTGGAGCTGGTGTACAACAAGGACTAGGAGTTGGAGTATCTCAAGGATTTGGACAAGGTATCAGAC
AAGGCCAAGGTCAATTTGTATCACAAGGAGAAGGAATTGGTGTGTCTCAAGGCTTCGGTTCTGGTCTACGACA
AGGGCAAGGACTCGGTGTGATTCAAGGCCAGGGCCAAATTGTGTCTGAGGGACAAGGG

TTAGACGTATCACAAGGATTTGGACAAGGAATCCGCCAAGGACAGGGTCAATTTCTATCTCAAGGCCAAGGTG
AACTAGTGAGCCAGGGCGAAGGAGCTTACGTATCGCAAGGATTTGGTCAAGGCATTCGTCAAGGTCAAGGACA
ATTAGTTAGCCAAGGACAAGGACAATATGTTACACAGGGACAAGGCAGAAAAGTCATC
CAAGGTTTTGGCCAAGCTATCCGCCAGGGCAGTGGTGTTGGAGTCGAAAACGAATACAGCGAATCAGTCAACA
GAGTATTCTTACAACAGTACAACGGCGGAGGACAGTGCGGATTGCTGAACGGTCAGAGGCCATCCGGAAACCG
TAGGGACCTGGAGGTAGACTTCGCTGAGATCCCCTGGCAGGCCATGGTCTTGTTGCAG
ACTAACAGAAGTCTTCTTTGCGGTGGTGTCATTACTAGGCCAGATGTTGTTGTTACATCTGCCGCATGTGTAG
AAGGCCTTGACGCCAAGAACGTGCTCATCAAAGGTGGTGAATGGAAACTTGGAATCGACGATGAACCTCTACC
ATTCCAAATTGTCCAAGTCAAGACTATCCTTCGTCATCCTCTGTACAAGCCTGGCAGC
CTCCTGTACGACGCCGCTATCCTTGTCCTGACTGAAAACTTAAGATTGGCGAAGAACATCTATCCCATTTGCT
TGCCAGGAGCGAATGAGAATTTGGATGCTTTCTACAGTGGTGCAGGACAGTGTATAGTTACGGGATGGGGC
>1002050214_1 Heli.1-DT662248.3.5
GCACGAGGAACAAGTTTCGCGCATATTAGTGCACGTGTTCTGTTTATAACCATATAAAATATATAATTTATAT
TATATATACTAATTTCTAAGTCTAATATAAATCATATAGAATATAAGTTTTCAATAAAGTAAAACATACAAGA
CTTAAAGATGTAAGATATTAGTTGTGCAATGTCTAAAGAAGGTTGTAGTGCCGAAAAG
AAAGAAAGTGACAGTAAAGTAGTATTAGAAAAGTTACAATCCTATTTGAAAGATAAAAGAAGTATAGAATTAT
CAGAGTCTAACATTGGCTACAGTAAGCCAAGCACGAGTTTCGAGCAAAGTCCTCACTCAACAATCACTGACGA
CAACAGCAACGTATTGAGAGAAGTATTGAATCACAAAAAAGCAGAGTTGCTTCGGCAG
CCCGAAATAGTGGAATTTCTAAAAACTCTGACATCAGATATTAAGAAGAGTATATCACTAGAACAGAAATAAT
GGCTGTCAGTGCTCATAAAAGTGTAGCATCTTTGATGCGTAAAACTCTTGATCGCCAAGCAGCTATTACTCGA
ATGAAAATTAACTTTAAAAGAAACCAAATCGAGCAAATTTTGCTAACTTATGTAAGAG
ATGGTAATTCTGCATCATATAATGAATTGCTAGCAGTCTTGAAGGATTGCCAGTTAAATGATGATAACTTTAG
AATTTTAATAGAAGATTGTCTGACCTGTGTAGTGCTGCTGGGCAGGGAATTAAAGCATTTGTAAATACTTTGT
GTTCTTTAGA
>1002050215_1 Heli.1-DT662976.3.5
GCACGAGGGGAATACATTCGTGAACCAAAAAACATCCTTTTTAAAGTCCATATTTAATAAGGTATTTATTTTT
CCTGTCTATTAAGATGCAACGTTTCATTCCTTCATTTATATTTTATACCAACAATATAAATAATTTCTTGCTT
TAATATCTTTTATTTAAAAATAAAATAGTGATTTATAACAAAAATGTAAAGAGTCCAA
TTAAAAAGTAATTATGAACTGTCACTTTAGTAGAACATAGTAGACCAAGGTTCTTAGAGCGTAGTGTATTGTA
TAATTTCAATGAGATTTTCCGGTATGCCTTAAATATTAGCATAATTAATTCATTTGTTCTTACCTGATACAAA
ATTCGAAGAATTATCGATTGACTCGGTGTATAATATCCTCTTTGCATTTTTCTGTTTT
AATCGTATACGGATGTTGAAGGTTTATAAGATACGTAACGAATATAGATTCGATTACTTGGAGAATTAAAATA
TATAAATCAGTTTTACGCTCCACACGTAGCAGGATAGGTAGAGGGAATCTAGTGTTAAGACACTCCGTTCGCC
ATTTTCTGCCAAATTCTGGAGGCATTGTTGAGTGGCGGAACTTAACGCCTCGACATTT
GGAGAAGTTAATGTATATTAAAGCTCACTTGCTTCTCCTATACCTACATAAAAAAATACTGAAAGGTAACTAA
AACAGTCATTTTTTATTGTTTAATGTAAATAATATTAAAAGTCAAATATTTAAATGAACTAAGATTGTCATCT
ATTAAAATGGATCATTTTATATAAAATATGCTAA
>1002050216_1 Heli.1-EL596843.1.5
GCACGAGGCTTCGATATGTTTGTTAAATTTGTGCGAAAATCTGTTTTTGGTGATGCGGAGTTATTATCTTTTG
GTGAACTGGCATATGAAGAATGAGGAATCTTTACCAAATTCGGGTAATGTGTTTGATATAGAGAAGAATGTTA
GTAGAGAATATATTAAGCAGAAGTTAAAAGCTGGTTTGATAAGAATTTGGCATGACGT
TCAAGCCAAAGTATCAACGTTTCTCAAAAGTTCAGGATTAGAGGAGTTTCCTTTCGAAAAGTTCATCCAAATG
TTGGGCATTTTGAGAAAATTGACGCAAGTTGCGGAAATTTTTTGTGGTGATAAGTCAGATTTTCTACAGGATT
TTATAAAAACGCAAAGTGTTTCGTATATCAAGAATTATCATAGAGGAAGGATGGAGGA
GTTGAAATTATTTTTGGAAAACGAGGGTTGGGAACAATGTCCTGTGAAATCTTCTTTTAATGTATTGAATTTA
CAGGAGTTTAAACAATTTAAAAAATATCTAAACCCATCGAAATCAGATTTGACCAAAACACAATCTGATGCGA
CATCATCAGCCCCTTCTCAAGACGACAGTGTGTATATATCAAAATATTTCACGCAAAA
GACATCTCGGACACCTTTTGAGATATTTAGAAATGAAAACGTGACAAATGATGACATTTT
>1002050217_1 Heli.1-EL602195.1.5
CGGCACGAGGAAACGCACGTAATGATAAACAAAAGCCTACCTACTGTTCTCTTAAACTAAAATACTTATACGG
AAGAAATTATGAACAAAAGACTACCTACTATTCTATTAATCAAAATAGACGAAACTAAATATATTAAACTCCA
ATTATCCAATACGTACAATCGCATCTACATCTACTTTTCTACAGTACAGAGGCAGGCG

GGCGCGGCGGACGCGCTTCTATAAGCAAAACGTCGCGGGGCGCGGGGTACGCATGGCGCGTGGCGAGCCGTTT
CGCAGCAAGGATTTTTTGATATTGTAACAACTTCTAAAGTACTTATTAAAATGAAAAACAGTAAAAGACAGTT
ATAATCTACATTAAAAGGCCAACGTAACTAATATACTTATTTATATGCTTTATGAGTA
AGGGATAAACGAAACATGAGCCAAATCTGCGGTTTATAGCCGGCGCGATGTGTCAACAATTAAATGCGTTTTT
CTCTAAAACTATTCGTAATTTCCAATTAGTATAAAGGACAAATTTTATCTTCGTTTAACGTAGAAAATTAATA
AAGTATTTAAAAGGATAAGGATTGATACCATTATGGACAAACATCCCTCACAATAAAA
AATATATTCAAATTACTATTTTTAGACAGAAATATGCTCACTGTATGTTATCCTTAGAAGATACAAATATGCT
GTCGCTGACTTTTTTGTAGATCTTATTAAGAGGAATAATACTCTTGTACATTGTTTTCATGTAGGACTTACCA
TTAAAGCAGCGCATTCGTGAAAGTTTCTCAACGGGTAGGACTTTATGCACATTTATCA
TATTTGTGTCAAATTACATTAAAATATACATATAATAAACTATATATAAACCTTCCCTGAGAAATACTCTAC
>1002050218_1 Heli.1-EL603042.1.5
GCACGAGGGATAAGGAGGGATCTCTCGTGTATGACATACAGATAGAGGATATAAGTGTCACAGATCCAAAAAT
CACTTTGGTGGAGGAACAAGGGAAACGTCATTCGCAGGTGGAGATTTTGGACACGAGGATAGGGGTTTTGGCG
AGGCCAAGCGCTCGTATTTTCCCACCGCTGTACGAAGATCAACTTGCGGTCCATATTG
GTTCTGATACGGGTCCTCCAATGCTGAGAGGTCGCTTGTTGTCGCGACCACTGCCGGACACAGCGAGTGCAGG
TCCAGCTCTACTCCGTCGTACGGATGCACGGATGCCACCATCCATCAATCCCGTTGCTGGTCTTGTGTGGCTT
TCAGTTGATGCCTTATGTGGTATAAACTATGAGGTAGTACTAAATGGATATTCCAGTA
CGTGGTCAGCGTGGCTAGACACGCACACTTCCAAGGGACCGAGAGTTCTAGCTGGTTCTGAAGGGTCAGTTCT
AGAACCTTCTGCAGGCGAACTGACGGCGTTAAATGTGGGCACCGCAGTCCTTAAAGTGAGGACATCAGACAAT
GATACGGAATTTCTGCGCGCGCGATTACCACAGATAAGCGTGCCACCCTCGTGCCTTT
CCTCGGTACCTG
>1002050219_1 Heli.1-DT666306.3.5
GCACGAGGCTTTTCCTTCATAAATTTCCTTAGAATAAGGAACACTTATCAGTCAAAATGCCAGAACTTACAGA
ATTGGACAAGGCCACTGCGTCAATGACTGAGAAACGCAAGGAGGAGACTGCATCATCAGACAGTGACTCTGAC
GACACTATCCCAGAATTGGAAGATGCAGGAGCGCCAGGTGCAGGTGGTATCACCAACC
CAATTGGTGGCATCGACATTGTGTCCAAAGCAAAACAATCACGTGGTGAGAAGAAAGCTCGTAAGATTATGAG
CAAACTTGGACTTAAACCGGTGCAAGGTGTAAACAGGGTGACAATAAGAAAGTCAAAGAACATTCTTTTCGTG
ATAAACTCCCCTGATGTGTACAAGAACCCCCATTCAGACACTTATATAGTGTTTGGTG
AAGCCAAGATTGAGGATCTGTCACAGCAGGCCACCATGGCTGCTGCAGAACGTTTCAAGGCTCCAGAGACGGC
GGCAGCTGGCAATGATGCTGTGACAGCTGGCACTACAGTGGCACCCATAGCAGAGGAAGAAGACGAAGAAGGA
GTAGATGAGACAGAAGTGGACGAGAAGGATGTGGAAATCGTCATGACCCAAGCGAACG
TGTCCCGAGCGAAGGCAGTCCGCGCCCTCAAGAATAACCAGTCTGATATCGTTAATGCTATAATGGAGCTGAC
AATGTAAACGACGTAGTCACAAGTGTTATTTTATGTTCCTAGCTTTAAGTCGGTTAAACAGACATACGTCTGT
CCTTACATCAGTATATTTCAAAATCCAAAGTCAATCTCAGTGTTGAGTGGTATCATGA
TGGATAATGGGATTGTATATAGACATATTTACTTCAATAAAGTATAAC
>1002050220_1 Heli.1-EL595895.1.5
GCACGAGGCCCGGCACGATGTTCACCGGACATCTACATTTTTAACGAAACGATCGACATGAAAAAAATAGAAT
ACTTACAAAGGAAGCTTTGGCCTTACAGAGTTGTTAATTATGAAACTACAATTTCAGTGCGTGAATATCGTGT
CAAGGCGGCCTCTGTCGGTTGTTTCTTCGATCGAACGACTATATAACGTTCACTGTAT
ATATAGCCTGTCTATCTCATTTAAAATGTCCGTTTTCCTGTTTCCATCTAACTCGTAGACAGGAGAGCGGGCT
CAATGTAAAATCGCAAAATAAATCGATAGCCATGGCAAATCGCGACTAGCAATAATAATAGATTATATATATA
TAAAAAACCCTTGGATGAATGGAATGGTCATGGACTTGTGGTACATAAAGGCTATATA
AATGCTAACATATCGATTTGTAAAGGGGATGTATTTAAAAGATTATTTTAGTTCAATGTTTTCTTTTTTTTAG
CGATTTTATATTGATGTAGGAACGTTTGGTGGAATACTGATGTTTGTACTTTGTAATTAAAATTTATATGACG
GGGGTTTTTTCACAATGATGTTTATTATTGACTTTCTGTCATACCATCCATCTCTCGG
CCAGTTTATTTATTTATATATGGAATGGTATATTAGTTATTTGTAATCGTGAAAATTATCGATCAACAGTAT
>1002050221_1 Heli.1-ES586736.1.5
TATTCTCCGTGTTAAGTAATCAAGACAATATGAAATTTAAGCAATTAATATTTCTACTTTTAGTACAATTATT
TACTACTTCAACAGCTGTTTCCCCGATACCAGTTAAATGTTGGTTATGGGATTCCCCGTGCTTGACTGTAAAA
ACTCAAGCAATATTTTCGATACTTGCGGCTGGGGTTCCTGATTTGGATATACAACCAT
TGGACACCATACATATAGACTTAATACATGTTAACAAAGACGGTTATAGGGAAGACTTGAGATGTATTAAAGT
TAAAGGAATGACTGATGCAGTAGTCGATAGTGTCAGCGTGGATATGCCTTCGAAAATGGTTAATTTAGTATTC
CACACGGATTACGTGACTAGAAGTCGATATATAAACAACGGTACTTTACTTTCAACAC

CCATTGAAGGCGATGGTGACTTCTATATGAAACTTGAGAACGTTCAAATAGCAATGAGCATACCTTTTGATAT
CATAAAAGATGCTCAAAACAATGATGTAATGACCTTCAAGTCATTTGACTACACCTTCGACGTTATAAATGGC
GCTCAATTCCATTTGGAAAATCTTTACAACGGTGATAAGTACTTGAGTGACTATATGC
ATCATAAGCTGCATAAAAATTGGAAATATTTGACAATTATATACGCAAACTATTTGTTGGATCCAGTAACTAC
AAAAGTGTTTACTTCTCTCCGAAACTATATTCGTTCTGATCCACTGGAAACTGTCGCTTTGTATACTTTCCAA
TAAAGGAATCCACATAAACTTTTTTGGATATTGAGCTGAATTGAATCGTTGCATGTAA
AATAAAGAATTT
>1002050222_1 Heli.1-DT662110.3.5
GCACGAGGATTTAAAGTAGGTACTATATATGAGTTTAAAAAATATTGGCATAACTTTATTTTATTGTAGTTCT
GTATTTGTATTTTTTTAATTGGTAATAATTTATTATTTTTATATAGTTTTATTTATTTATATAGTATTATTTT
TTCGCTCTATTTAACAAACATTCTGAATAACCCCATAAAACATAGTTTGGAATTTGAA
AGAAATTAAGAACTTAATACTTTATTGGTCAACACAGTCTGCACTAGAATATGACTTATATTGGAATAGACAG
CTTATATTGAGAGAACAAAACATACTTTATCAAAAAATTTTTAATGACATATCTTAACTTGGTAAAAAAATTG
AAACTTTTAAATTATACAAACGAAAAACAAAAAAAAAATTAATCCGTACACAATGCAG
TGTTGGTATCAACGTTGTTATACGGCAGCGAAACGTGGGAATTACAGAAGAAGAATGAAAGAAAGATGAATGC
AGTGGAAAAGCAATCTCTTCATTGGATATGCGGAGTCAGCTTAGCTGATCGAATCCGCAATGAAGAGATACAC
AGAATGGCAGGTACTAGCGAGGATGTCATGGTGAGAATGAAGAAGACCGTGCTCAGCT
GGTTTGGGCACGTCGAACGAATGAGTGATGAAAGAATGGCAAAAAAGATTTATGATGGATAAGTGAGTGGTAA
GAGTGGTAGGGGGAGACCTCGGTTGACCGTCGAAAACGTTAACAAATAAAAATTAAATTAATTTTGCCAATAA
AAATCGGAACTAATCGTGGTGCAGAAGCACAACCATGGGACTGTAAGTTCGACAGGTT
GTGGGTTCAATGCCAGGTTAAAAAAAACTTCATTTCCTTATTCTGGGAATGACGCATTGAGTTTTATGGCGCG
AAATTGAGTTTTAACATTCAACACATAATGGTTCAAAAATTCAATGGAATGTAGGAAACGGAAGTGTCTTAAC
GGGAATGAAGTGTCTTAACACTTTCCCAGATTTCGTATGCGTACCCTGCTATGTGCAG
GGTACAGTGTGAAGCTAAAAAAAA
>1002050223_1 Heli.1-DT662618.3.5
GCACGAGGACCCTGCGCACGCGCTCTCGCAACGAAAAGCTCAGCTTAACGCTTCGAGCACTGCGATTTTTTTA
AACGAATCAACTTTTTAGTTATTTAAGTGTTATATTAAGGATATAGGAAGCATGGCTTCGCAAGCCATATGTC
TGTTGGTGATTCTTTGCGCGGCATCCGTTACAAATTCGCTCACACTGGACGAAGATGC
ATCAAGCGATGTAATAAATACAGCAGGCTTATCTGGTGACGGTCAGATCGCAATCCTAGTCAGAGGACCCTCC
AGAGTAAGTTCGAGTCGTGAAGAACTGCTGCATGCAAAAGGCGCTGAAGATAATTCTATTTCATTGTATTATA
ACAGTAAAACTAATAAAGTTTCTCTAGAAAGTTTAAATGGAGGTCATATCAAATCAGT
TTCATGGGGTCTGGGTGCTCATCCAAGGGGTACTATATTGTTGGTTGTGAACCATTCTCGGGTGAAAATCGTG
GTCAGCTGCAAGCCATTACACTGGCATTCGATGTCCAATAGACACGACATATTCAAGTTATTGACCAATCAGA
AATTAAAATTGTATCATGAAGAAAATGCACCAGTAGAAGTATATAGCAACGAGAAATC
AGCACTAGATTCTATAAGTTGTAACCATCAGGATGAACTAAAACCACCAACTCTAGTCACAGTGGATTCCGAT
GTAGATGAAGTAAAAGATTTTATAAAGAAAGAAGAGAGAATGAAGATGGAGGAAGAAATGCAAGGAGATTACC
CTAATAATAACTATATAGATCCAAATATATATCC
>1002050224_1 Heli.1-EL599480.1.5
GAGAGCAGCAAGGCCGCCTTACCCGGGCTGAGCACTGTCAAGCGCGTTAATCTGCACTATAACAAACCCCAAG
AAGTAAATTTTGAAGTAGTAAAGTACAACCATGGAAAGAAGACATGATTTTTGATCCGTCTTTAAAGAAAAAA
AAGAAAAAGAAGACGGGTTTTGATATAGACGCTGCCCTCGCTGGTGACCAAGGAGATA
CCACGAGTGTCGGAAGCGCCTGCCGAGTCCGGTGACGTAGACATACCTGAAGATGACAACCTCGACTTAGATA
ACTTCGGTAAAAAAAAGAAGAAAACTAAAAAGAGATTCTTTAATTTGCAAGAAATGGAAAATGCTCTCCCTGA
AGTGACCCAGAATGAAAATACTCCTGTCGAGGAACCTGAGCCTCAGGAAGAGGAGGTT
ATTGACGATTTAGACTTAGATATAGATTTCTCTAGGACTAAGAAGAAGAAAAAGAAGAAAAATATTGATGAGT
TTGCAATTGAAGATGATGTGAAGGGAGAAGATCAAGATAAAGAGGAAGATCAGCATGGAGATTGGATTGGCAG
TGATCGTGATTACACTTATGATGAGTTGTTAGAGCGTGTGTTTGATATTATGAGAGAA
AAGAATCCCAGTATGGTTTCTGGTAAAAAACAAAAGTTCATCATGAGACCCCCTCAGGTTGTAAGAATTGGGA
CAAAGAAAACATCATTTGCAAACTTTACAGAAATCTGTAAAACCCTCCATCGTCAGCCTAAACATTTGTTAGA
CTTTTTACTTGCTGAATTGGGTACAAGTGGTTCAGTAGATGGTAATAGCCAACTTATT
ATCAAGGGTCGTTTCCAACAAAAACAAATAGAAAATGTTTTACGTCGATACATAAAGGAATATGTTACCTGTC
ATACATGTCGTTCCCCTGATACTATTTTACAAAAAGATACCAGATTATTCTTCCTTCAATGTGAAACTTGTGG
TTCTCGTTGTTCTGTTGCAAGTATCAAATCTGGTTTCCAGGCTGTTACTGGTAAAAGA
GCTGCCATGCGTGCAAAGACTGCA

>1002050225_1 Heli.1-DT665510.3.5
GCACGAGGCCGCGTTCACTCACGTGTCCTCCTTCCTGTCTATCGTCTCCCGCGAGACATTGTTACGGTGTAAA
AATATATAAAGCCGCCGACAGACGGAGTCTCGAGTTAAATTTAAATGATACGGGTTTCGCCATGAGACTAAGC
CGGGGCATATTGTGCGAGTGCGCGGGCGCCAGCGCGTGTCCCGACGGCGCCGCCAACG
GCACCTGCACCACTCAGCCCGGCGGGTACTGCTTCGTCGCTGTCGAGAAGGTCTTGGATGAGAACGAGCTGGT
CGTCTTGGAGAGGACCGCTGGATGCTTGCCGCCTGATGAGTCGGGTTTTATGCAGTGCAAAAGTTCACAAGTA
CCACACCAACACCCGAAAGTTATAGAGTGCTGCGAAAAGGATCTTTGCAATCGTCGGC
TCCAGCCACAACTGCCGGAGCCCCCGCCCGACGTGACGGAGGCGCCGGGACTGCGCCCCACCACGCCTGCAAG
CCCGACTATATTAATGGCCGCCGCGCTGTGTGTCGCGCTGGTCGCCTTCCTCGCCGCCTTCTGGCTGCTATTC
AGGAAACGTAGACGAGGATGCAAGAGACCGCCTTCTCCTCCAGCACCCTCGCACCACG
CTGAAATATCATCAGGGTCAGGTTCTGGCCTGCCTTTACTCGTGCAAAGGACAGTCGCGAAACAAATACAAAT
GGTGGAATCTATAGGTAAAGGGCGTTACGGAGAAGTGTGGCTCGCGAGGTGGCGAGGTGAGAAGGTGGCGGTC
AAAGTGTTCTTCACGACGGAAGAGGCTTCGTGGTTCCGAGAGACGGAGATATACCAGA
CCGTGCTGATGCGGCATGAGAATATACTTGGGTTCATTGCAGCGGATATCAAAGGAACTGGCTCGTGGACTCA
GATGCTTCTAATAACGGATTACCATGAGAACGGCTCGCTCCATGATTACTTACAAACAGTTGTACTGGACCCG
CACATGTTGCTGACGATGGCGTATTCGATAGTGAGCGGTCTCGCGCACTTACATATGG
ACATATTTGGTACAAAGGGCAAACCGGCGATAGCTC
>1002050226_1 Heli.1-DT661756.3.5
GCACGAGGTATTATTCTGTGGATGCGAGTTGAACCTACGTAACGTAATTGCTATCGGCGAAACGTCTGTGATT
AGTGTAGTGTTCGAATGGTTCGTACATCGTTTATAAATCACCAAAAGGAAGATATGAAATTGATTTTACCCTT
TGAAAACAGTGACAATTATTCCTATGAATGTTAAATTTAATACTCAAGCATATTGATT
ACGTTTAGAGGTGATTTAGTGCAGTGGTAACCGTTCGAGATTGGTCCGGAGGCCGTCGTTGTGATAATCGCCC
CTTGTTTAGTGTTAACAGAAATACTGAAACGCAGCAATGCAGGCGATCAAGTGTGTCGTTGTAGGTGACGGTG
CCGTCGGTAAAACATGTCTGCTCATCAGTTACACGACGAATGCCTTCCCCGGCGAGTA
TATACCAACAGTATTCGACAACTATTCAGCTAATGTAATGGTAGATGGGAAGCCCATCAACCTGGGCCTATGG
GATACGGCGGGGCAGGAGGACTATGATCGGCTGCGACCCCTGTCCTACCCACAGACTGACGTGTTCCTCATAT
GCTTCTCGCTCGTCAACCCTGCCTCCTTCGAGAACGTTCGCGCGAAGTGGTACCCAGA
AGTTCGGCATCACTGCCCGTCCACGCCCATCATCCTGGTCGGTACAAAGTTAGATCTTCGCGAAGACAAGGAC
ACCATTGAGAATTTGAAAGAGAAGAAACTTGCCCC
>1002050227_1 Heli.1-EL600307.1.5
GCACGAGGCACGGTAGTGGTAGCCATTTTATTCTTGTGAATGTGACCAAATATTTTTTGTGATTTCTTGTACT
CGAACTGATCGTGTCAATGTGTGCCCACAACAAGTGTCTTTTGTGACGTGCATTCGTGCTTAAAATTTGATGA
TGTACGTGTTTCAAATCGGATTGTGTTGACCTGCCCGTTTCTTACACGGACGTACAGG
CAGGCATCGATTAGTGCAGAAGGCAACCTCAATAACGGGGAATCAAGACACTAGAACAGTTTGTAGAGTGAGC
CATGGGCAACAATGCTGCCACCGCCAACAAAAAGGTGGATGCCGCCGAAAGCGTCAAGGAATTCCTTGACCAG
GCCAAAGAAGATTTTGAAGAGAAATGGAAGAAGAATCCTACCAACACCGCCGGATTGA
ACGACTTTGAACGAATAAAAACGCTCGGGACGGGCTCGTTCGGCCGAGTTATGATAGTACAACACAAACCCAC
TAAAGAATATTATGCGATGAAAATATTAGATAAGCAGAAGGTCGTAAAACTAAAGCAAGTAGAGCATACGTTA
AACGAAAAACGTATATTACAGGCAATAAATTTTCCTTTCTTGGTCAGTCTCAAGTTCC
ACTTCAAGGACAACTCTAATCTATACATGGTTCTCGAGTATGTTCCCGGAGGTGAGATGTTCTCGCACCTTCG
TAAGGTCGGAC
>1002050228_1 Heli.1-EL603091.1.5
GCACGAGGGTTCGATACAGTCAGCACTCATTCCCCAGATGCATTAGAACTAAAATGTATCGAAGTCTGGCTGA
TCTCTTGTACGATATTCGTATTCCTGGCTTTACTGGAATACTTCGTGGTGCTGTTCGGCATCCGTTACGACAA
GAGCTGGAGCAGGCGACGGCAGGACCTACGGCGCGCCGCCTCAGCCGCCCAACTTGCT
CCACCTCTACATATTAACCACTCGCAGGACTCGCCAATATTAGAGACGATGGCAGCAGGTGATTCGTCTGGAA
GTTTCTACGTTAGGTTCGCCGCTATGGTCGACAGGGGCGTCATGTTTTGTGGCGCCCAGGATGGCGCTCTCGA
CAAATTCGCTCTCATATTGTTTCCCTTCTGTTTCCTACTCTTCACCATTATTTACTGG
ACCACATATCTCAGTGAAGCGCATCGGGCGATGCACGAGTGACTTTTTTGAGTGGCTGATAGCCACTAAGACA
TTTATTTACATGTAAATACCTACATAAGATAAGTTACTAGATATTATTAAAAATGTTTGAACTATATTTGACA
CATAGTTAAATTTTAAATAATTGTTTGCTAGATTTTCATAGTTTATTGATATGTTATG
CATTGAAATGTTTATTTATCTTTTTTTGTTAAATTACATTGTTTTAATATTCAGTAGTTGGGTCCTATACAAT
TTTTAATATATGATGACATATTTTAACGAAGTATATTTTTTATTTTTACTATATTATTAATTATTACCATGCA
GATAATACATAGAATACTAAAAATATAAATAATATGTAATGCATCATATTAAATAAAA

TATTTAAGTTTTTTTAAATGTACCTATTCTTATCTCTGTAATATTTCATCCGTCCTTCGTCTAATCTTGATAC
AAGTAATTATTTCTGTAGCTGTA
>1002050229_1 Heli.1-CO729796.1.5
AGAGTGCTTGTCAAAGTGAGTTTTACATTATTCGTTCTGACGAGCATTATTTGTATTGCACTCATCTGAACTT
ATACTTACGTAGTTAAATAGAATTGAATGAGACTTCAAGGAGTTTAGGCGTATGTAGTCAAATTAAGAGTTTT
CGTCTTAAATTTAAGTGTCACGTTCAACGAAATGACTGAAAATGTCATGTGTGGAGTG
CGCATCGAATTTCATTTTCTTTTATTCTCAGTGTTTCAATCATTTCGTTGAACGCAGTTGTGTGGGTTATGTG
CTATTTATATGAGATGAAATTTATATAATAAATAATAATTTACGTGAAGTACAGACTTTTCTTTAAAATCCTA
TTAAACACAACGCTACATTTCTGCCGAGACTTTGTTCTCATATAATTTTTTTGTATTA
ATGACATTGGTTTGTATTGATGCTACTTTCGATTATTTCACTATTTCTATTATATTTTTCTACTGTTAAGTCG
TACGTTCTATAAGTCGTGCCGCCCTTAAGTTAGCTATAGAACGCTAT
>1002050230_1 Heli.1-EL603180.1.5
GCATGGTCTTGGTGACCGTGTTGCCTACCAGCCAGATCTTCAGGCCGTGCAGTGGCACCTGGTTCCTTTCGAC
GCGATCAACCCTGTTTCTGCCTGTCTCTGGAGCTGGCAGTGTGACCTGGTTCCAGCCAACGCAACCTGCTCCG
TCTCCGTCAGTCTTTGGACTGGGCAGTGTGACCTGGTGCCATGTAGTTCTCTACGTCC
AACTCTTCGCGTCTCTGGTGACCGTGCTGCATGCCTGCTGACCTTCACTTCGGGCAAAGTGATGGTGCGTTTC
TTATCTTGAATAATTACTTTACTTATGTGGGTAAGAATTTGATAGCAATGATTTAGATAATAAGAAATAATTT
TCAGTTGTTTACAAGGATTACTTAAATTATATATAAAAACACGGTAACGATGGTTTAA
ATTATCAACAAGTCATAGAAAATTTTTTGATTTAACAACTGAGTAGCAATATATGGTAAGTTCTGCATTATAT
TCGTATTTGCTCCTAGATTCGTAGTTAAGATTTTCAGGGGCAAATAATTGAAGTAATTGTAGCTACCCATTGA
GGCAACCAATGCAATTGGACAAATTATAAAGATTAAGAATATCGCCAGCTGGTTCATA
ATTGGATTTCATTTTCTACGTAATATAAGATGAAAAGATTTATTTTTATATGGAATAAGGTTAATGGCATTTA
TTACAGGTTCTTAAATGTAAAAATAGAAAACTTTAAACTGCATAATTTTGACATTTTTTTACAGATATGAT
>1002050231_1 Heli.1-DT667622.3.5
GCACGAGGCGCGCTTGGCGAGATGCAGCTGACCGGAGGTCCGTTTGGCGCGGAGTGGAAGCCGCCTTGCACCT
TAGGAGGCCAGCGCCCGTGTTATTTGCTTCGTTGGCGAGACGTGGAGTCAGGAAACTACAGGTTTTGTCGCTG
AGACGGGGGTTAAGAGACGCGGTCGCTGCTTTACCTGGATTAGAATCTCTTTCCTTGA
GTGGATGCTACAGTGTCACAGATGCCGCTCTAGCCAGCGCGTTTGCCACAGAATTGCCGGCGCTCCGACGATT
GGATCTTTCACTATGTAAACAGGTGACAGACTCTTCCCTCGGTAGAATAGCACAGTCGCTTAAAAATTTAGAA
GAATTAGAATTAGGTGGATGTTGTAATGTAACAGATACCGGACTTTTATTAATTGCTT
GGGGATTAAGAAAGTTGCGTCGTTTAAATTTACGGTCGTGCTGGCACGTCAATGACGATGGAATAGCACACTT
ATGCGGCGGAGGTGAGGCTAGAGGAACCCCCGAACTAGAACATTTGGGCTTACAGGACTGCCAAAGATTGACA
GACGAGGCTTTAAAACATGCTGCCACTGGTCTTCCTAAGCTAAAGTCGATAAATCTTT
CCTTCTGTGTTGCGGTAACGGATGCCGGGCTGAGGCATCTCGCTAGGCTTCCACATTTAGAAGATGTCAATCT
GAGAGCCTGCGACGGTGTTTCGGACGCCGGCGTCGCCCATTTAGCGGAGAGCGGGCGATTAAGAGCTTTAGAT
GTTTCATTTTGTGATAAAGTTGGCGATGAGGCAT
>1002050232_1 Heli.1-DT663154.3.5
GCACGAGGGTCGTCCTTGCCGCTCGCGTTACATCAGTTAGAGCCCACGCGGCTGAACTGATTCGGACGCCAAA
ACGAAACAAATAAACAACATATATAATAAATATATATATTATTAATATAATAATTTTATTATATATAAAATAT
AACAGAATCGCGTATCAAAAATCCCTAAGGGAGTCGAACGTGATACTGTATTTTATTC
GTATTGGAAGTTATTGGATTTTAAGTTTTCGAAGTACAGACGTGGTCAAGACATCATTAAAGAGGCAGGACTA
GACACGCTTCCCCTGCTTTACGCGAGGCATTGCGATTGTTGCGAGACGCGTGGCCTGAGCCTCGGTCGTTGAA
AGAACGCTGCAAAGCGACTAGTGAAGAAGAATCGTGTGGCACGAGCGAAAAGAACGTA
CGAAATATGTGGGGAAACGATGTGCCAGCGCGCGCCGCAGCCTCCCCCGGCCTCCTGCGCCGCCGCTACTCAG
TGCCCGAGACCATTATGAGAAAGTATCGGTTGGCTCAACAACGATCTGAAGGGGAAAGCGATGAAACGGGTCG
CTGGAGTGCATCCGGCGCAGCGGCGGGCACGCGCAACTCATGCGCATGGTGCGGTGCA
AAGTCTGGCGCAAGGCGGGAAAGAGAACATATGAGAGAATCTGCATTATTACGCCTTTGGGGCAGAGCTGGAG
CCTCTCCAGCTCGCACTTGCCCTTGTGGTCCATGTCCACCGTGTGGA
>1002050233_1 Heli.1-DT666014.3.5
GCACGAGGGCCATTCTTTCAAGATGGTACACAATGGTATCGAATATGGAGACATGCAGCTCATTACTGAAGCA
TATCATCTCATGAAAGATGTTCTTGGAATGGAACAGGACGAAATGGCACAAGTATTTGAAGAGTGGAATAAAG
GAGAACTCGACTCTTTCCTCATTGAAATCACTCGTGATATTCTTAAGTTTAAGGACTC

TGACGGTAAATATTTATTGCCAAAGATTCGTGATGCGGCTGGGCAGAAGGGTACAGGGAAGTGGACAGGAATT
AGCGCATTAGAGTACGGAGTGCCAGTCACTCTAATTGGTGAAGCTGTATTTGCACGATGCCTATCTGCCATTA
AAGATGAACGTTTAACAGCAAGCAAGAGTCTACCAGGTTCCGATTTGAAGTTTACAGG
CAATAAAACCGAATTCCTGGAACATCTCCGTAAAGCTCTCTTTGCAAGCAAAATTATTTCTTATGCACAAGGA
TTCATGTTACTGAGAGAAGCTGCTAAGGTTAACAACTGGAACTTGAACTATGGAAGTATAGCTCTCATGTGGC
GCGGAGGATGCATCATCAGAAGTGTATTCCTTGGAAACATTAAAGATGCGTATACAAA
GAACAGTCAGCTATCCAACTTGCTGTTAGACCCGTATTTCCGCGACCGTATCAGCGTGTGCCAGCCGTCGCTC
AGGCAGGTGGTGGCCCAGTCGGCGTTGGTGGGTGTTCCCGCGCCGGCTTTCAGCGCCGCCCTCGCCTTCTACG
ACGGATACCGCTCTGACATGCTTCCGGCTAATTTGTTACAGGCACAAAGAGACTACTT
TGGCGCTCATAC
>1002050234_1 Heli.1-DT662343.3.5
GCACGAGGAAAAAGCCTGAGAGACAGTTTTAAAACTCGATCTTTTAATTTAACCAAAAATTAAAATGGAAGAT
AATAAAATAACACAGTCTTTGGAGTATGAAAAATCAGTTGATGATAAAAACCCTAAGAAAAAAAATAAGAACA
AAAAAAAACGTAGTGGAGGAGATGGCGATAATCGCGGTTCGGTCTCTAGCGATGGAAT
TGCTATTCCTAATTCCGGAGATGTTCATCAGAATATTTCTTTTAAGGATTTGAAAATGGCAATGGAAGTTCTT
AATTTACAACAGAAACCCGCAAAAACTACTGAGGAAGCTTTGCATAAATCTTATCAATTTTGGTCGACACAAC
CAGTTCCTAAATGGGATGAAAAAATTGTCACAAATGAATCAATAGAACCCCCTAAATC
TCGTGATGAAATACGATCAGAATCGTATACTCTACCAGATGGTTTTCAATGGGATACGCTTAATTTAAACGAA
CCTTTGGTTCTTAAAGAATTATACACTTTGTTAAATGAAAATTATGTTGAAGATGATGATTGCATGTTTCGTT
TTGACTACCAAACCGATTTTTTAAAATGGGCCCTACAACCTCCTGGGTGGAGAATGGA
GTGGCATTGTGGAGTGCGTGTAGTGAAATCAGGTAGATTGGTTGGTTTTATATCTGCTATACCAGCCACACTC
AGAATTTATGACCACGTCCAAACTGTAGTTGAAATTAATTTTTTGTGTGTTCATAAGAAGCTTCGTTCAAAAC
GCGTTGCACCAGTATTAATAAGAGAAATAACTAGAAGAGTGAACTTGACTGGAATTTT
TCAAGGTGTTTATACAGCAGGAATTGTATTACCAAAACCTATTGGTACTTGTCGTTACTGGCACAGATCACTT
AATCCCAAGAAATTGATTGACATTAAATTTAGTCATTTATCACGCAACATGACTATGCAAAGAACTCTAAAGC
TATTTAAACTACCTGATTTACCAAAAACTCCGGGATTTCGCAAAATGGAGCCTAAAGA
TTCTGAAAAAGTTGTTAAGTTACTAAATGACTATTTACAAAAATTTGACTTGGTGCCAATATTTTCAGAAGAG
GATTTTAAACATTGGTTTGTCCCACAAGTTGGTATAATTGATGGTTACATAGTAGAAGCAGCTGACGGTTCTA
TTACTGACTTTGTTAGTTATTATACCCTGCCATCAACGGTTGTGTATCATCCTATACA
TAAAACCTTAAAAGCTGCTTATTCATTCTATAATGTTTCTACCAAAACTCCATGGATTGACTTAATGTTAGAT
GCTCTGATTACAGCAAAAAATTCTGGATTTGATGTGTTCAATGCTTT
>1002050235_1 Heli.1-EL603703.1.5
GCACGAGGCATCTCCTAAACGATGCAAACGGACACTAGCCTTCTTTCCTTCATTTTACATCTTCACGCTTCAA
CGTAAAAAGAATAAACGTATATAAAATACATACATATCAATAAAGAGAATGTATTAGTTCGCGGGACGGCGAT
GATAAATTTGTACGATCAAAACACTAATTCGGTTTCCAACCAATGTTTTGCTATTTTT
AACTCTGTGATTTACTTTATGTATTTTCTAAATTATAAGAATCTAGTGGAGACTGATATCGCATAGATTAGTT
TTAGCAGCGCTAGCTTCTCTAACTTTTTTAATTTTTAGAACTGGGCATAATTTTAGTGAAGGAAAACAATACG
TACATATTTTTGATAATTTCCTTATCTGTGGAGATCGGGATATCTCTTCGCCATCTTC
AGCGATCGCTTATAAAATTAGGTAACAGTGGTTTTTTACGAATTTATAGTTGCAAAGCCGATAACGCCCGATG
TCCACAGGAAAGCGCGTGCAAACTCAATTTGTATAAAAAAAATCGCATAAACTGTCAATTCATTGAAAATGGT
GTCAGTTAATAAGCGGGGTAATGTTTCCGAAATGAATATTACCTCAGTCATTTTAAGA
TATAGATATAAATCTTCTAAAATATAAAGTTTCAGATAACGAGAATAATATTTGTAGATATTCCGTGTCCCTA
AAATATATTTCGTAATAAGGGATCTATTGTGCTGATAAAAAGAAAAAATGCTTTACTTATATTATATTCTTAT
AAATATATTGTGTAAAAATAAAACTCGATGTAATAAGTAGTGTGTGTCTATGAAAATC
CAATTATTTTTAAAGAAATGCGTAAATATATGTTATCTATACATAAATCTTAACGTGAATCTATGTAACTGTC
TTGTGCGTTGTAGGCGATATATA
>1002050236_1 Heli.1-EL603516.1.5
GCACGAGGACTTACGCGGATATTATATTACACACGACTGAATACACATCATTATGTAGGGATGACAAGAAACT
TGTACCTTTTAACGTAATATTCAAATAATATAAATAATAATTGTTAAATGACGTCGGTGCCTATTTGTACGCC
CGCTGCTAGGTTACGTGTTGTAGTGTCCGCGCCTACCGCTCCTCTACTCTCTCACTTG
ATCGTTCCTATGTAAGTTAGGTTTAATTTGTTTCGATTCATTCCTCGTTGAGTCCGTCGTTGGTAAGTTCGCT
TCCACACCCCCCCCACTGTACCTCGCGTACTTCCCCCCTGTACCCCGGTGTACCCTCCGGTACCCCTTCACCT
CCTCCCGCTATCGTTTCCGCGCACCCGCGTATCGGGTTTATCATTTACTCGCCGTAGG

TTAATACACAACCACCGATAAGTGACGAATGTCCTCACGTCTGTTTAATGTTAGGAAACGATGCCTTAAACAA
AGCGTTAAGTGAATATAGAGATGATTTATATTATGTTATTTTCAGTGATATTGAATTAGCACTAAGGTGTTTT
AGACGCGGGCTCCGAACCCGCACCCACGAGCCTTCGGTAACAATTTTTGTATGAAGTT
AGTTTGTAATCAACCATCGTGGCAATCTTCTAAGTAACATAATAAAAGAGTATAATTTATTTAAATAAAGTAT
AATATGTATTAGAAATTTTAGATTTACTGAACAATTATTAATTAAAACTTAGTGCAAATTATTGTGAGAAATC
AATTGATAAAACTATTTATATGAGTTTTTGATTATTATTCTTATTATTATTATTATTA
TATAGGAGAAGAGACATAAAAATATTGGATGAATTTATTAATTAAAAATAGTTAGCAAAATACATAATACGGT
AAGACTCGTTTAGAGGCTCCGTCTGTCAAATGTGGTCGTCGGGCGTCGGGCCGTGACGTCACTGTGCGGCGTC
GCGCGCGAGGTGTATGTAACAAACACTACACATACTACTGTATTTTTAATATTATAAT
ATAAATAAATAATGTTTATTGATAATATTTGATCATGGCTTAAATGTAGTACTGTTGTGCTGTTTGTAGTTTA
CAATTTCGACTCTTGTTTGTATTTTTACTTTATATAAATGATAGTGTACGCGGTTTTTTATATGTGTATGTAT
ATTCAAGTAGTAACATGTTTGTGTAAAAGTGTATTTTTTGGTATATTATTATTAATAT
TAATTAATATAATAATAATAGTGAACAACAGGCGCGCGCGGTCGACTG
>1002050237_1 Heli.1-EL596096.1.5
GCACGAGGTTTTTGCTTAACGGTCATTTGTATAGTATTTATTTATTATTTAAATATATTAAGTGAATATGAGA
ATATTTATTGTTTTGCTCTGTTTGGGGAGTCATGCGGCTTTGGACACTGGATATCCAGCAGGGCTAATGGCAG
AATGTCCCGGGATGACCAAAAATACAACTTTATCGCAAAAAACAAGGGACTCACTAAC
AGTAGTAGCGCTGAAACCCAGTGATAGCTGGCTCGGCACCAAGGAAAAAATTTGTCAACTGAGCCACGGTGGT
GTAGCCTGTGTAGCAAAGCATTTGAATTTTAGAAAAAGGAATTCTTTGGTATTTATAAGCGGATATTTGGACG
CGTCGTTTTCGCCTATAGTGCGATCTACAGCAATGCCGTATTTGAAGCGTGATTATAA
TGTAATTATTATGGAAATATACCCAATACTTCACAGGAGTTACCCAATAGCAGCGCGCCTTACAAAGCCGTTG
GGTATAATGCTGGGAGACTTTTTAGCACTCTTAACACATAGAGGGCTAAAACCCGACAAGCTAGAGCTACTCG
GGGGGAGCCTGGGGGCGCATATAGCTTATTTTGCAGCAACCAGGTATCGCATATTAAC
GCACTTCAAGCCATCACGGCTTACGGGGTTAGATCCAGCGGGTCCATGTTTCCGGACACTACCCCGCGCCGAA
CGGTTTAACTCCGACGCGGCACATAAAGTGGACGCCGTTCACACAAACATTGATGGATTTGGTTTAGCTGACC
CGGTGGCTGATATCGATTTCTACGTAAATGGAGGTGAATTCCAGCAAGCGATGTTATA
CGACCACCTCCTCCCTTGCTTACAGCTTTGCAGCCACGTCAGATCAGCCATATATTGGTTAATATCACATTAC
GAACCACAAAAATTCTTAGCAGTCCGATGTCCATCGCTGGCTTCAGCGAGACATGGGGAGTGCTTCAGTGGGA
CTCCAGAAGTCAACGTTCTTGG
>1002050238_1 Heli.1-DT668370.3.5
GCACGAGGGCACACCTTTAAAGACGCGCCACACCCACTGTCCCAGCAACCCGTTTATAATAAAACAATAAATT
AATTATTGAGAAAATGTCCTACCCGAAGGAAGTTGGCGGCTACAAACTCGGTAACCTTCTTATTGAGGGTAAA
ACCAAGCAGGTATTCGATTTACCAGAAAATCCCGGAAACTGTCTTCTCTTGAATAAGG
ACAGAATAACTGCTGGCGATGGCGTCAAGGCACACGACATGGAAGGCAAAGCAGCTATTTCAAACCAGACAAA
CGCTAAAGTCTTTGAAATTCTCAAAGCTGCTGGTGTTAAAACTTCCTTTGTAAAGATCGCTTCACCTACTGCG
TTTATTTCAAAGAAGTGTGATATGGTCCCCATTGAATGGGTAACAAGGCGTTTAGCAA
CAGGATCCTTTTTAAAACGTAACCCTGGAGTACCTGAGGGACACCGTTTTACTCCACCAAAGCAAGAGACCTT
CTTCAAAGATGATGCTAACCACGACCCGCAATGGTCAGAGGAACAAATCATCTCTGCTAACTTCAAATTTAAC
GGTCTTCTTATTGGTCAAGATGAAGTAGACTATATGCGTAAAGTTACAATATTGGTCT
TTGAAATTTTGGAGAAAGCTTGGGCCCTTCGTGATTGTGCTCTTATCGATATGAAGATCGAGTTCGGTGTTGA
CTTTGACGGCAACATCCTCTTAGCTGACGTCATTGACTCAAATTCCTGGAGATTATGGCCATCCGGTGACAAG
AGACTGATGGTAGACAAACAGGTGTACAGAAACCTCGCAAATGTAACAGCAGCCGACC
TTGATAACGTAAAACGTAACTTTG
>1002050239_1 Heli.1-DT661905.3.5
GCACGAGGATATATATACAAGTATTATATGAAAAAATATTAATTTCAATCCATTTATTACTTGATCGCTTTCA
ATAAAGAACATGGGATAGCCTCGTTGTGAGAATATTTAAGTAGCGTTACCCCGTGAGTGTTGTAGAACGACAT
CGAATTTATTGTTGTATTTAGCGCTTCTTGTTGTGGTTTTTCAATAATTGGAATTATC
AGGGTTTCGATGTTTTCGTTTGAAATTAATATTTGTAAAAAGATCTTTTGTAAATTGTACTTTACACACCCTT
AGATGTTTCAAAATTGTTTTTCCAATAGTTTGCTTGGGTCGATAGGCTTCTCCCCTCTTAGCCTTTTAAAATC
TGTCAAGGTATTTTAAAATGTAAATAAATAAGTATTGAAACTGTTTATAATAATGTAA
AAGTCAAAATAAAGCGATACTGTGACAAAAAATTAGAGGATATATATAATACGCGTCTCAAATCATGAAATAA
ATATCATCCGAAGACGTAGTTAGTTGTTGTAGGTTATGTATGTAATTATAATAAAATTGTTGTGTAATACATT
GTATCGAAATTTTCTATATAACTCTGCGTATATGATTTTATTCCTTTTTGGGTGAAAA

AAGGAGAAAAATTTGATATGCACAGTTTACTCTTCCGTTCTAATCTTTAATTCTGATACTGTTGTTGACAAAT
TGTGCTAACGATCCCTCATTTAGATGTAAAAATAACGATTTCTTGTATTTGTTAGATGTAAAATTGACTACAT
TCAGAGATGTGATCTCATTCAAACCAAAAATAAAGCAATAAGTTAAGATAACAGTACA
GAATAAAAGATTGATAACACTTCGCCTGACTCTGTGCTTACAAAATCGCTTGTCACACAAGTAGATTGATGTT
TATAATATATGTACGTACGTANGTTATGTATGTTTAACATTATACATAATGTAATCGTATAAAGTGTTTCG
>1002050240_1 Heli.1-DT666411.3.5
GCACGAGGGTCGCGCCCGTGCGCATTTCTTTCGTGATCTTTAAAGTCAATCATTAATTCTAAATAAAAAGTAT
TTCATTTAGTAACATTAACGATTGAATTTTATATTTATACAAAGTTTTGTGATATCGGAAGCTATTTTAAGTC
AATATTATCAAGATGAAGTTATCTATAGTATTCTTAGCGATACTATATTATATTGTTG
GAATACAAGAGGTCAGTACAAGAACAATTCACGAGAATGATGGTGAAAAGCATCGCCACCGTCGAAGATCATA
TTATGATTATGATAATAAATATTATGGAAGAATCGATTATGGTCATCAACATCAGGAGTTGCTTACTGAAATA
ACGCACTTATTACAAGAACTATCGCTATATATAAGACGTCCACCAAGCCCTCCACCAC
CACCGCAAATTATTTATGTGCCATATCCAGTTACCTTGCCAGTGCGCGTTACATGTAAGGGGAATAATACAAG
TTTAGAAAATAGGTTTAAAGTGGAGGATGAACGCCAGATTTGGAATTTAATACCTGGTGATAATGGACTTGAC
ATAGATAATGGCAACGATGGTGCGAGGCCTATATCTTTCGACATCTTTGAACGACCAG
AGTCAGTTCCAGAGATGCCACCAGTTGAGCACGGGAGCAGTCAAGCTGGGATAACAACTACAACAACACAGGC
ACCTCCATCAAAATCTCGCATATGCCAGGCCGCCATTATAATATGTT
>1002050241_1 Heli.1-EL598509.1.5
TTATGACCCTCGATTATTAGATGGAACACCAAAGTTCAGTATGTATGGAAAAGGCCCGGATGCAAAACCGAGT
GATACTCCTGGTCCAAACTCTTATGACCCTCGATTATTAGATGGAACACCAAAGTTCAGTATGTATGGAAAAG
GCCCGGATGCAAAACCGAGTGATACTCCTGGTCCAAATTCTTATGACCCTCGATTATT
AGATGGAACACCAAAGTTCAGTATGTATGGTAAAGGCCCGGATGCAAAACCGAGTGATACTCCTGGTCCAAAC
TCTTATGACCCTCGGTTGTTAGATAGTACACCTAAGTTTAGTATGTACGGTAAAGGTCCAGACGCGAAACCAA
ATGACACTCCAGCACCTAATGTATACGATCCACGAATATTGGACGGAACTCCGAAATT
CACCATCAGTGGTAAAGGGCAAGTTGAGAAACTATTCAACGTGCCAGCTCCCAATGCTTATGATCCTCATCTA
CCGAATGACTCGCCGAAATTTACGCTATCCGGTAAAGGTAAAGATGGAAAACCTTCGGATACTCCAGGTCCTA
CCAATTATGATCCACATCTTCCCGTAAACTCTCCAAAATATACCATGGGTGGTAAAGG
TCCGGATGGTAAAACTTCAGACGTTCCCGCACCGAATGCTTATGATCCTAACTTACCTAACGGAAGTCCTAGA
TTCACTATTTCAAGTAAAGGCCCATCACCTAAAATAGAGCAAACACCCGGGCCAACGGCTTATAATCCTAATT
TACCACAAAGTAGTCCAAAATTTACAATAAGTGGGAAAGGGCCTGACTCTAAACTGCC
AGACGTTCCGGCACCAAATGCCTATGATCCGCATATTCCGGATGGGGCACCAAAGTTTACATTATCAGGGAAA
GGGGTACCTGGGAAAGTTCCAAATAATCCAGCTCCAAATGCGTATACGCCAGAGAAAGCCGATAGAGTTATGC
ACGAAAACTCACCAGCGTATACGTTTAGGCCTAAAATTGAAAGTGGAAAAATTCCTGA
TACACCGGCTCCTAACTACTATAACCCACAAAAGGCAGATAAAGTTATATTAGAACATACACCAGCGTACACG
TTATCGCCTAAGGGTAAAGATGCTAAAATCAATGATACGCCAGCTCCCAATGTTTATAA
>1002050242_1 Heli.1-EL599647.1.5
TTCGGCACGAGGCTTCTGCGGCATTCAAATAGTTGAAGTACTGAAGCTTATTTTTAAATATATGATATTTATT
AGCATTTGGATATCTTAATTTTAAAAAATGGATGTATCAAGTTCTCAAAAATTTATAGAGCGAGGTTCTCATA
AAGGGAAGGGCCTCGCAGTTTTTACAAGTGGTGGTGATTCTCAGGGAATGAATGCAGC
TGTAAGATCTGTAGTTCGAATGGGTATTTATTTAGGGTGTAAAGTATATTTTATTCGTGAGGGATACCAGGGG
ATGGTCGATGGAGGAGACAACATTGAAGAAGCCAATTGGTCATCGGTTAGTTCTATTATCCACAAAGGTGGCA
CAATAATTGGATCAGCTAGATGCATGGATTTCATGAAGCATGAAGGTCGTCTTAAAGC
TGCTTATAACTTAGTAACAAGGGGTATAACTAATTTGGTTGTAATTGGAGGTGATGGCTCCCTTACTGGTGCA
AACTTATTTCGGCAGGAGTGGTCAAGTTTGCTTGATGAATTGTTAGAAAATAATAAAATTACAAAGGATCAGA
GGGAAAAGTACAAATACTTGCATATTGCTGGAATGGTTGGTTCCATTGATAATGATTT
CTGTGGTACTGATATGACAATAGGTACAGATTCTGCTTTGCATCGAATTATTGAAGCTATTGATGCTATTGTC
AGTACTGCATACTCTCATCAAAGAACTTTTATCATGGAAGTTATGGGAAGGCATTGTGGTTATCTGGCATTAG
TGGCCGCTCTGGCGAGCGAGGCTGATCAAGTTTTTATACCTGAAGACCCTGTTCCCAA
TAATTGGGTTGAAAAACTTTGCAAACGCCTTGCGCAGGAGCGTAAATCCGGTCAGCGTCTGAATATCATAATA
GTAGCGGAAGGTGCAATAGATCGTGAGGGCAAGCCGATAACTGCAGA
>1002050243_1 Heli.1-EL598395.1.5
CGGACGAGGGCGGAATTGTCAAGTTTCAAGTATACACATAACCCAATTTCAAACAGAATTGGGTTTTGGTGTT
AGTTTATCATTGCTAACATAAATCACTCAAATTAATTTATAATATGTTCGCGTAAAAATATTTCCAATTATAT
AAAATAATGAAGTATATAAAATTATAACATACTTTTTACTATTACATTACGTTATTTT

TATAAAATTTACGTTGAAAGCTACTCCTTATTGAAGCCAACATGGAAAATATGAAAAAATCTGATGACGTGGA
TAGTCCAACTGCTGACGGAAATTTATTGCAACCACCAAAACACACTAGCATTCCAACACGAAATCCTCTGATG
AAAGTGCGTACTTATGTGTTGGCACTTCGACCATGGTCTCTAAGTGGAAGCCTATTAC
CGACGTTACTGGGTGCTGCACTAGCGTATCGTCTTCCTGGAGATAGTGGATTCAGCTGGGTTACACTACTATT
GACACTGTGTACAGTTGTTCCAGTTCATGGAGCTGGAAACGTTGTAAATACGTACTTCGATTTTGTGAAAGGA
ATAGACAATCGAAAATCAGATGACAGAACGCTAGTTGATCACATTTTAAGCATTGATG
AAGTAGTATCTTTGGGAGCTTTATTATATATGGCTGGATGTGCATTTTTTGTGTTGCTTGATTATAATGTCAC
CAGCTATAATGCAGCATCTAGCATTAGTATAATAAGCGGTCTGTCCTCATCATTACTGTATACAGGAGGACAT
GGCTAAAAATATATTGCCCTTG
>1002050244_1 Heli.1-CX700545.1.5
ATGTCCTGCGGTACGGTCCGGAATCCGGGTCGACCACGCGTCCGCGATATCTCGAAGTAATATTGCGGCATTG
TATTTCATTTATCGTCAGAATTGTTTCAAACAGTACGTTATATATATTTAGGGATCGTCTTCTATTTCAACAG
TGAATTCTTAAAGTCCGTCATGAGTCTCCAGTGGACTATCATCGCCACATTTCTGTAT
TCAGAAATAGCATTCGTGCTGTTATTGACTTTGCCGATCGCCAGCCCGGCGAGATGGAACAAATTCTTCAAAT
CGAAGTTTTTAGCTTATATAAGTGGGCAGGCTTCGATATACTTCGTGATCTTGATCGGGGTGCTGGTTCTGTG
CCTGCTTGATGCAATTCGCGAAATGCAAAAATACTCCAGCATTGAGTCCTCGGATCAT
CAACATTTGGATGCGGAAATGCAAGGTAACATGCGCTTATTCAGGGCACAAAGGAATTTTTATATCTCCGGAA
TCGCCTTATTCCTTCTTGTTGTTATTCGCCGTCTGATTCAAATGATTTGTGAGTTAGCTGGGTTATACGCTCA
ATCTGAAGCCAACTTCCGTCAAGCGCAGAGTGCTTCTGCTGCTGCGAGAACTCTCCTT
GAGAAACAAGGTGCAGGTGATGAAGTTGCAAAGAAAGAGGTTGAAGAGTTGAGAGGACAAATATCAATCCTAG
AAAGAGAGTTAGCTAAAGAGAAAAAAGACAAGGAAGCCGTTAAATCTCAAGCGGAAAGCCTTAATCGTGAATA
TGACAGGCTTTCAGAAGAACACAGCAAATTACAAAAGAAAATCACAATTGCTGGAGGT
GATAAGAAAGATGATTAAATTCCTATTTTAAAATAATGTCTAACATAAAAAAACAGTCATGGTAAATATGTTT
TTTAAAATCTATGGTAATCATTATTCAATTATCAAATCAAACTTAAACTTTGTTCACTATAATGATGTAATGC
TTTATGATTCAACTTAGGCCTTGAAATATTTGTTTATCTTGAATCTATTATAATAAGA
CATTAAGCACATTAAAAATGTTTATATTATATCTATAGTAAAGATATTTTTTATAAGTGA
>1002050245_1 Heli.1-EL601859.1.5
CGGCACGAGGGTCGTACTGGCCGCTCGCGTTACATCAGTTAGAGCCCACGCGGCTGAACTGATTCGGACGCCA
AAACGTAACAAATATACAAAATATATAATAAATATATATTATTATTAATATAATAATAATTATTATATATAAA
ATATAACAGAATCGCGTATCAAAAATCCCTAAGGGAGTCGAACGTGATACTGTATTTT
ATTCGTATTGGAAGTTATTGGATTTTAAGTTTTCGAAGTACAGACGTGGTCAAGACATCATTAAAGAGGCAGG
ACTAGACACGCGTCTCCTGCGTTACGTGAGGCCTTGCGGTTGTTGCGAGACGCGTGGCCTGAGCCTCGGTCGT
TGAAAGAACGCTGCAAAGCGACTAGTGAAGAAGAATCGTGTGGCACGAGCGAAAAGAA
CGTACGAAATATGTGGGGAAACGATGTGCCAGCGCGCGCCCGCAGCCTCCCCCGGCCTCCTGCGCCGCCGCTAC
TCAGTGCCCGAGACCATTATGAGAAAGTATCGGTTGGCTCAACAACGATCTGAAGGGGAAAGCGATGAAACGG
GTCGCTGGAGTGCATCCGGCGCAGCGGCGGGTACGCGCAACTCATGCGCATGGTGCGG
TGCAAAGTCTGGCGCAAGGGCGGGAAAGAGAACATATGAGAAAATCTGCATTATTACGCCTTTGGGGCAGAGC
TGGAGCCTCTCCAGCTCGCACTT
>1002050246_1 Heli.1-EL598213.1.5
GCACGAGGAAACAATTTTGTAACGAGAGAAAAATTTGCTCCTTCGATTTCGACACGTTCTGTGAACCAAAATA
AAATTAGTTAACGACATAAGTGTAATTATTTTAAGCACATTCTATCTATTAAGTGTATAAATAAATATTTCTT
TGAGACAATACAAATAAAAACATTACATAATGCCACTGTTGAGGTTGACTGCTGGCAG
TTTTGGGCAGCTGCGTACCTTCGCAACGTCATCTGCCCTTGCTAAACGGGTAAAGACCTTCGCCGTTTACCGC
TGGAACCCCGACGAGCCGGATAAGAAGCCGCACACGCAAAACTTTGAAGTGGACCTCGACGATTGCGCTCCTA
TGGTCTTAGATGCTTTACTAAAAATAAAGAACGAAGTCGATCCCACTTTAACTTTCAG
ACGATCATGCCGCGAGGGTGTCTGCGGCTCTTGCGCTATGAATATTGATGGCACCAACACTCTTGCCTGCATC
AGCCATATCGATCAAGATCTGTCTAAGCCATCGAAAATATACCCTCTACCTCATATGTATGTAGTGAAGGACT
TAGTTCCTGACTTGACTAACTTTTACCGTCAGTACCAATCTATTGAGCCTTGGTTACA
GCGTGACAAGGAACCTGGTAAAGGTAAGGAGGTACAATTGCTCCAAGATGTAGAAGATAGAGCAAAACTTGAT
GGTTTATATGAATGTGTACTATGTGCCTGTTGTTC
>1002050247_1 Heli.1-EL599466.1.5
GCACGAGGGTTTTAACTTTGCGCCCGGCGCCTAGAAAAATACCTATCGAGGAAATAATAACTAACGTTGAGGA
CTGTTTATATAAAAACCAAATTTTGAAGGAAGATTCTGAAGTTATTCGCCAAGATATATCATCTTTACTGCGC
CGTACTAAACCACCTAAATCAAATCTTTCTCGTGATGAATCTCGAGCTCTTAGTAAAC

TACGAAAAAACGAGGATATAACAGTCCTCACAGCCGATAAAGGTAACGCGACGGTTATACTATACACCGACGA
GTATATACAAAAAATGTCAATATTGCTATCGGATCAAAAAACTTATAAAATTGTTGATAAAGACCCTACAACA
AAAATACTTAAAAATACATCCGATTTGATCAAAAAACACGCGAAAACATTACATCTTG
ACATTAAATCTCTCGTGCCTAGCTGTGTAAAACCTCCAAAGTTGTATGGGTTACCAAAAATACACAAAAAAGA
TGTACCTTTTCGACCAATAGTCAGCCAAATCGATACGCCGACGTATAAACTGGCTCAACATGTCGCCAAAGTA
CTTTCTCCACTGCGTGGCCACACAACATCTTATGTCAGGGACTCTTACCATTTTGTTA
GCGGACTGAAAGATCTTAAGTTAGCTAACAATGAAACCATGGTCAGTTTTGATGTCCAGTCGCTGTTCACCAG
TATTCCCGTCAAGGACTGCATCAAGATTGTCAAGAGGAAGCTAGAGGATAACAATATGCCTATAGAATATCTT
GAACTTTTAGAACATTGCCTCACATCTGGATACCTTATGTTTCAATCAGAAATCTATT
TACAAATAGATGGCATTGCTATGG
>1002050248_1 Heli.1-DT665724.3.5
GCACGAGGCCGGCTCGCGCTGCGGCTGCGGGAAGTCTCCGCTACTATTAACCATCAACCATGTATTGCGAGGG
AGAGAAGCGCCACCGCCGTGTCCTCTGGAAGATGTGCAAGTGGAGAACGAACCAGATGCCGCAGTAATACCTG
CTCCTGAAGATCCAATAGAACCCGAGGAAGAACCTTCTATACCCCTGGCAGACACCGA
AATCTCAAATGAGGGTGCATCTTATGAAGGTCAGTCTTGTTCCGGTATAACGTTCCGGGAAGAAGAATCAAAC
TGTGCCAAGTATTATTTATGTATAAACGGCCAGTACATGCAGCTCGAGTGTCCTGCTCCATTAGTCTGGAATC
AGAATCATTGCGATTTTCCTAATAAATCAAAATGCAAGGGCAAGAACAGCTTGAGGAT
AGTTGACGACAGCAATGAAGTCGAACCCGAATTAAAACCTATTATTGCATGCTATTTCACGAATTGGGCTTAT
TACAGACATGGCAATGGCAGTTTCGGACCCGAACAAATAGATCCCACTCTATGTTCTCATATCATTTACTCTT
GGGCACATCTGGACGGTGAAAAAAATACAGTCGTCGCTGGCAATGCAGAATTGGATAT
AAGCAATGATTTCTATGGCAAAATAACAGAATTGAAACTGAAAGGCGTAAAAGTGATCTTAGGTGTTGGTGGT
ATCGAAAACTCCATAGATGGGAAATGGAGCCGCATGGCCGCAAAATCGGAACAAAGGAGGATATTCGTACGAT
CGGTCTTGAA
>1002050249_1 Heli.1-EL603106.1.5
CGGCACGAGGGCTGCACATGTTCATCGATTTTCGTATAAATTTAAAAAATAGTGAAATAAATTAAATAAAATG
GGTGCTATAGCCAGCAGATATCGTGTTTCTAACTCGGCACTCAGTAGCGCAGATGCACAAAACATAGAAAACG
CTAACAAACGCGAAAATCCCGGGACATTGGATGAACTACATAAAAAGACCAAAGAGGT
CATGCCAGTGAACTTTGAGGGTGGCAAACTTATGGTTAATAAAGGAATTTCCAATCACTTCCAGATGTCACAC
ACCCTCACAATGAGTTCAATGCAAAATGGCTACAAATTAGGAGCAACGTACATCGGCACTAAACAAATTTCAC
CCACCGAAGCTTTCCCTGTTGTTCTTGGAGATGTTGATCCAGCAGGGAATGTAAACTT
TAATCTTATTCATCAGCTTACACCAGAAATTAGAGTTAAGGGTGCTGCACAGGTCCAAGAATGCAAGTTAACT
GCAACACAAGGCACGTTAGAGTACAAAGGCTCGGACTATACACTAGCACTAGCTGTAGGTAAACCAGACTGCA
GTGAGAAATCATCTGTTTTTGTTGGACATTATTTACAGGCAGTAACGCGGCGATTAAC
GCTGGGCGCGGAGCTCGTGTATCAGTCAAGTCCGCGCATAGCGGGAGGAGAGGTGGCTGTAGCGTCTGCGGCG
GCAGATATACT
>1002050250_1 Heli.1-CO729556.1.5
TCCGGGTCGACCACGCGTCCGCACAGTCACCGCGATATATCCCGAGTACTTTTTGTGAATCTTACTAGTCATA
AATATGTCTGAAGCTTTCTTTGATGAATACGACCACTACAACTTCGAGCACGACAAGCACATCTTCTCCAGCC
ACAGCGGCAAGCAGCGCACCAAGAAGGAAGTGAGCCAGCACACCAACCACTTCGATCC
GTCGGGCCATTCCAGAAAGATCGTCACCAAGCTCGCCAATACAGAAAACAACAAGAAAGGCGATAACAAACAT
TAAATAAATTTATAAACGACAGACGACTATGGCGTGTAAAAGGAATATTGAATTTTTGCCGACGGAACGGAGG
ACGTTATGGCGAATACCCGAAGAAAGCACGGGTCGCTCGTCTCTGAGATCAGCGGAAC
AGCCGAGAAGGTTTTTGGTAACGCAGAGTTTTTATTATGCACACCACAATGCTAGAACACGGTGGCGCAGCCG
ACGCGCCTCGTGACTCGTGCCCCCACCGGTGCATGCCGATGGATATCTATTTTGAAGAACACCGTTCCAATGA
CTGATCTCATCTCTACTGGGCTTGTTGTAAAGCTCAATGTCGCTGTGTTATTAACTTG
TAGGTAGTTATTATGTTGTTGTACGTGGTACGTTACGTTAATGACTGTAATAATTTAAGA
>1002050251_1 Heli.1-DT662186.3.5
GNACGAGGGATTAGCTTTAACAGATAAAATCCACAATGACCACATATTTTCAATACCCCACACCTGAGCTCCA
GGAGGAGCTTAAGAAGATTGCTCAGGCAATTGTTGCTCCTGGCAAGGGTATTTTAGCCGCTGATGAGTCCACA
GGAACCATGGGCAAGCGTCTCCAAGACATTGGTGTTGAGAACAACGAAGAGAATCGCC
GCAAGTACCGTCAGCTGCTATTCAGTACTGACCCAGCCATCTCCGAGAACATCTCTGGTGTAATCCTTTTCCA
CGAGACCTTGTACCAGAAGACCGATGATGGCACTCCCCTAGTGTCTCTATTGGAGAAACGTGGAATCATTCCC
GGCATCAAGGTCGACAAAGGTGTTGTACCACTTTTTGGATCTGAAGATGAATGCACTA

CTCAGGGCTTGGATGATCTTGCCCAGCGTTGCGCCCAATACAAAAAGGATGGGTGCCATTTCGCAAAATGGCG
TTGTGTGCTCAAGATCGGTCGCAACACACCCTCTTACCAGTCCATCTTGGAAAACGCTAACGTTTTAGCCCGT
TACGCTTCCATATGCCAGAGCCAGAGAATCGTACCCATTGTGGAGCCTGAAGTGTTGC
CTGATGGTGAACACGACTTGGACCGTGCCCAGAAGGTGACCGAGACCGTATTGGCTGCTGTCTACAAGGCGTT
GAATGACCACCACGTCTTCCTTGAGGGTACTCTTCTGAAGCCTAACATGGTGACTGCTGGACAGTCCTGCAAG
AAGACATACACTCCAATGGACA
>1002050252_1 Heli.1-EL595934.1.5
CGGCACGAGGGTATTTTGTTGTGAATTGATCGAAGAGGTTGAATTTTATTCTTCCTTGTATTTGTGTGTTTGT
TTTAAATTATTCTTACTGCTGAAAATGGCGTCGCCGCGTACCCGTCGCAAACTAAACTTCATACGTACTCAAG
ATGAAAATCACAAATGTTTCGAGTGTGGTACACTAAATCCGCAATGGGTATCGGTGAC
CTACGGTATTTGGATTTGCCTGGAATGTTCAGGCGTTCATCGCAGCCTTGGAGTCCACTTATCATTTGTGCGT
TCTGTTACAATGGATAAATGGAAGGACATTGAATTAGAAAAGATGATGGTAGGGGGAAATCTGAAGGCTCGTA
CATTTTTTGAAAGTCAACCTGACTATAGGTCTGATATGAAGATACAACAAAAATATAA
CACTAAAGCTGCCTCAATGTACCGCCAGAAGATCACAGCCCTTGCAGAGGGTAATGATTGGAGTCCTTCCGAT
TACAAGCCAGAAGTAATAGAAAGACCTTCTGACTGGAGTCAATCCCAAACTTTTTACTCTTCAGGGGATAACT
TTTTCCCGACTAGTGGTTCCGATAATAACATCAGCTACCACAGTGAGTATGGAAGTGG
ACGGTATACAGGATTTGGAAATTCACCCAAGCAATCTCAGTCGACACCAATGTCCCCAATTCACAGTGGCAAT
GAAATTGTTGACAACACACTTGCTTCACTAGCAACGGGCTGGTCTATGTTTGCTTCATCTGTATCAAAAGCCG
CCCGCTCGGCTACAGAGAGTGCTGTGAAATATGGAGGAATTGCTACCCAGAAAGTATC
AGAGATGGCCTCCACTGTTACTGACAAGGTGAACAACCGCGGCGGGTGGAGTTCCCTGGGCGGCACTAGTGAA
CTACGCCGCGCCAGCAACTCGAGCACCCTCTTCCAGTCACCGAGCTATGGCGCCATGAAGAATTCTACATCTG
AGCCGTACTCTAAATGGAACGAACAGACTCAACCCGGAATAGTGCCATCGGTGGCGGC
GCGTAACAACTTAGACTCGCGCGATCTATCTCAAGTCGGCGTATCGAGCCCTCCCCCGGATATCAAGAGCATC
ACCATCAAGAAGAAGCGCGATGATGACTCGTGGGATTGGCTAAATAACTAAGGACAATTGACAACAGAAAAGG
ACAAATGCAACAAAATTTTTGCTGATGTGCAATTATCAGTAGATCATAGATTTTGACT
TTATAAATGTTTCATTTCCTTCATATACGTATGTGTAGTACAAAATAT
>1002050253_1 Heli.1-DT667396.3.5
CTGGTCACTTGTGTTCTAGGAAAGGTTTTTTATTGGAAATTAAAAACATTTGAAGCAGCCATGATAGTAGCAG
AGGCGTGGAAAGCCAACTATCTCTTCTGGATATTACCAGCGGTCTTCGCCCTCGGTCTTCTCGCTGTTTTGGG
GCTGATCGGTCTTTTCTTCGCGGTACGTTTATACGCCAAGTTCACCTGTGGCAGGTTT
AGAGAGAAGACCAGGATGGATGGGAAGACCGTGATTGTGACTGGATGTACTAGTGGAATCGGAAAGGAGACTG
CGAAGGAACTGGCCAAGAGAGGCGCTAGGGTTATCATGGCGTGTAGGAGTATGGAGAAAGCTGAGAAAGTGCA
AGATGAAATTATGCAGTCCACAAAGAACGAGAACGTACTAGTGAAGAAGTTGGACTTA
AGCTCCTTCTCATCAATCCGGACCTTCGCCGAAGACATAATAAAGACTGAAAAGAAACTTGACGTGTTAATCC
ACAATGCTGGTTACGCTGAAACCTTCAGAAAGAACATTTCGGAAGACGGCTTGGAACTTACTATGGCTACAAA
TCATTATGGCCCATTCCTTCTGACCCATCTTTTGATAGATTTACTTAAGAAAAGTTCG
CCGTCACGTATAGTTATCGTGGCCTCTTCATTGTATCGTTTGGGATCAGTCAATTTGGACAATCCCAACCCAG
TTAACACAATGCCTGGATACTTGTACTACGTTTCAAAGGAAGCTAATATTCTCTTCACTAAGGAATTAGCGAG
GCGTTTGGAAGGCACTGGAGTGACTGCTAACTGC
>1002050254_1 Heli.1-DT665159.3.5
GCACGAGGCAACTTCCTCAGCCTCATGGAGGGCGGCACCGGCCTCACGCCCGTGCACCCGCACCCGCACATGC
TCGCCCACGCCCACGCGCATCCCCACGCTCACGCCCACTGCGCGCAACAGCAGCGCGCCGCCCCCGACCTCGC
CTCGCCCGACCACGGCAGCCTCGTCAGCCTCTGAGACCTCCGCGCCGCCTGCCGCCTC
TTCGAGTTCGACCTCGACCTCGGCCTCGACCTCGACCTCGACGAGCAGTAGCGCGGCCGCCCGCAGCGTCCCA
CGTGAGACGTGGCGGCGCACCGAACATAGTTCGCCTCCTGTATAGACTACTGCGCTATTTATTCTTCTTCTTC
TGTCGCGGCGCCTCTCGCGTCGCCTGTTCGTCTATCCTCTGTCCCCCGTCCTCTGTCC
CCTGTCCCCCGTCCCCCGTCCGGGCCCCGCAGGCTATCTCTTCTATTCATTGGGGCCCGCAACGTACTTGCTC
ACTGCGCGCTTTGTATTTATTGATATATCTCGTATAGTATCGTAGCGTAGTGTAGCGCCGGCGAGGCGACGCC
GGATCGAGAGTCAATTGATGAGCTGTTCTAGTTTTCTAAAGGATAATATTTTTTATAC
CTAGATTGTATTACGTCGTAAATTATAATCGTGATGTAAAATAAAACAGAGTTTTATTTGAAGCATTTGAGGG
TTCAATTCCGAGTCGTGCTCGATCTAGCGAGGCTAGACTAGTTGTATGTCGGTACGTAC
>1002050255_1 Heli.1-EL602811.1.5

GCACGAGGGTTCTTCTACAAATAAGCAGTATTTTACATCCACCCTTTTTTTCAATTATCAGTTACTTATTCAC
AGAAATGTCCTCTAAAAGTGAAAAAGACCGAGCCAAACAGATCCAGGATCGATGTCAAAATATATTGATACAA
TTACTAAAAGATGAAGACAACAAATACTGTGTTGATTGCGACGCCAAGGGACCTCGCT
GGGCGTCATGGAATCTTGGTATTTTCTTATGCATCCGCTGTGCCGGTATCCACCGGAATCTGGGGGTCCATAT
TTCTAAAGTGAAGAGCGTCAACCTCGACTCCTGGACTGCAGAGCAAGTAGTGTACCTCCAACAAATGGGGAAT
TCTCGTGCTCGTGCCGTATACGAAGCGAATCTACCAGATTCGTTCCGACGGCCGCAGA
ATGATTCCTCTTTAGAATCCTTCATCAGAGCCAAGTACGAACAGAAGAAATATATAGCCAAGGAGTGGGTGCC
GCCTACTATGCCTAAAGTTAATTGGGATAAAGAGATAGACGAAGAGCTGGAGAAACAAAAACGTAAGAAGAGG
GCCACTACTTCAGGATTGGGACCACTGCCAGCACCCTCTGCAGATAAGAAGTATAATA
AATCGGATGTAATACCGAGCCTACCGAAACCCAAGTCGTCAGTCAGTCCCAAATTGGGTAGGAACACACCAAC
GAGCCAAGCAGAAACTAGCAAGACATCAAACGGTTCAGCTGATCTTC
>1002050256_1 Heli.1-ES587699.1.5
GCACGAGGTTTTTACTTTTAATTTAGATATGAGTGATATTAATTTATGAATTTAGTTTTCAATTTTCGGCTGT
GTCTGGTGACCTGCTTCTCCGGTCCCGGTGTAAGGCAGCCTTTCATCAGATTATCTTTGTTGTTTTATAGATT
TATTGATATTATAGAAAAATTATTTCGTTAATATTGAAGTTGAAGGTTAGCGATCTTA
GAGATGCGGGCATTGGACGTGAGAGTTATGTTTAAATATTTTTGAAGTTGTAGTGGATCGGAAGCACCATTGC
TTTGTTTATATTCTATAACTAATGTGCCTATAAATGTATTTTATATATAGGTCTCGGTATAAGTGACGGAAAT
TGGTTAATTTGAAAATTCTAGTTTTCTGTAAAGTTTATTGGCGAATGTTTTCAAAAAA
CATATCGTCTATTTATCACATGTGTCCTAGTAACGTATTATGTGTTTATTATGTTTTAGAGCTGGCCTAAAAC
ATAATATCAACCTTTGCCGAGTAGGTAATAAGTGTCCAAGTTCAATATAAGGACTCCATTGCAATCACACTTT
GCTCAAGGAGCGAACTTAAACAAGCCCAGTGGATTATTATTTATTTTTGCACACAGTA
ATTAATATTATGGATAAGTAGAAGTTTCAAAATTATCGGCGAATTGGCGTGATCTCCCTACTTGAGGCTTTAA
CTTCAAAAATAATGATAGGTCTCGAAAACAATGTAATGTAAAGCGATGGTAGAAGCATTGTAGTCCTCGCAAC
AATAGTGCAATTGCTATATTATAATTATATAAAATATTGAAAATTATATGCAGGTTTT
GCATTGTACAATTTAATTTTTTAGCTATGTTAACAATGTCAAATAATATTCATTAATAAATATTACTATTTAA
AGTAAGAGCTTTATTTTTGTTTTATTTTCTTTTAGATATACAGATTGTAATTTTTTTATGATATACACAGGTA
AATAAATGTATGTAGTGAAACAATGTATGTGTTATAATCTATTTTCCATATTTCACTT
CAAAATTTCTTAAATAGCTGTAGATTATCATATTGCTTTTTCATGTTTAGTCTTCACTTTTCTAATTGTTTGT
AAAATTATTAGATAATTCATATAATATGTTGCGAGATAGATTTTGAGATTTTCCTGTTTGTATAACACTGTAT
TATGATAATCTTCTCTTTTTTCATCTACTATAAAACATAGTTTCAAAGTGATGTTGAT
TTTAAACTATCTAGATATTAAAAATTTTGTTGTACTTTCTCTCATATGTACAATTTTCTGAAATATAAAATT
>1002050257_1 Heli.1-DT662524.3.5
GCACGAGGAATACTTCTATAAACAATAACAACTACTTTTATAATTTTTTTTAATTTATTTATTAAAAAAAACA
ATAACATTCTATGCTTCTTTTACACATTTGACTTTTAAAAACTTTATCCAGCGATACTAAATTTAAAATTCGT
CTAGACATATCAAACCGGCAACCACTAAAGATAGACATGTGAATGAGTCACTGGATTT
CTTTATTATAAATCCATATACCAGTATCCGGTTTGTCGGCAACAGCAAGAAGCTATTCGACAAATAAGTATTA
AAACACCACATCCACTCCCACGCTGCAATTATCATCACTTTTTCCGCTAAAAATCTTTAGGGTTCCGTACATC
TTAAAGATAAATAACCCTTGAAACCCTTATAAGATCACTTTGTTGTCCGTCCGTCTGT
CTGTCTCTCAAGAGCCTTTATCTCAGGAACGTTTGGAGATATCGAGTTGAAATTCAAACCATATACTCAGGTT
TACGGTTCCTTGATTTTCTAAAAAAAATCGTCTTTTTCTATTTATTAAAAAAAAGATGCCGCTGTTTACATAT
ATCACGAAAACGAATTTCGAGACTATTAAGGTAATCAAAATTAATAGGGTACTTCCTG
TTGACCTAGAATTATGAAATTTGGCAGCTAGCAAGCTAGCTAGCTGAAAACTATACATTTGTGGTTAAATCCT
AAAAATAATAAGCCTTAAAAGGGGAACAAATTTTGGTTTTAAAGCGTCTAGCTCAAGTGAGGTACCAGATGAA
AGGGCTCCAATTTTTGTCCCAGACATCCAATTTTTGTACGGAACCCTCGGTGGGAGAG
CCCGACTCGCGCTTGTCCGGTTTTTATTAAACATTTACTTGACCGACA
>1002050258_1 Heli.1-EL599160.1.5
GCACGAGGCACCAACACCTCCTCATGTCTTATATGACGGCTGGAAACCAATTCCAGGAGTATCAAAACCTTTG
ATTGAAAACATTCAAAATGTTCATACAATACAATCTATTCAAAATCTCGGCCACAGTATTGGTCATATTGAAA
CATTAGATGAATATGGACCTCCACCTCCTGTAGTCCAAGAAGTCAGTATAAACTTAGA
TGGACATTCTTTACCAAATATTAATCAGCATTCACAGTTTTCTGGCGCCCAAATTTCATCTGGTTATTCTTCT
GTTCATCAAGATGCATTGGCCAATATCGATCTTAACGCTATTGTAGGAGGCGATTCGAATAATATCGATCAAT
CTAAAACAGTATTCGAAGCTCACTACACCGAAAATAATGTCCCTCAAGCCGACATCGC

CTTCATAGGTGGTATTCCAGAAAAACCTATAGACTCCTACGGTGCACCTCCTTTGGATTCCCTATCAAATGGT
CCTTATCCACCATCAATTAGACAACAAGGAGCTAAAGGATTAATTCCACCTTCAGGATTATATGGGTTACCAC
CTACAAATCAATATGGCGTCCCTAAAATTGCACCATCTAACTTACCGATTCCATACGG
ATCTGTTTCCGGGGGTGGCCATTCTTTACACACAGGAGGACATTCACCAAGACATCCCATAAAATTCAGAGAT
TCCGTACCAGAAGGTTTGATCCAGCAAATTGGGAAAGTTTCACATCATAAGGATATTCACGGGTTAGATCATG
TGAAACAAGGCCCAGCATATCTTCCTCCACCAATCAGAGAAGTTAAAGACATAAATCA
ATTTTCAGCTAACCATGGTTCCAATTTTAATAGTCTATCATTGTCTATTGAACCTACGAATCTCTACAGTTTA
CCTCATGCCGGACAACCTTTAAACTTCCAACAAAATCAAAACCAGCCCAGTAATTTATATGGCTCACCCATAG
ATTCTTATTCTGTTCCTCTCTTAACTGTTGGCGATCACACAGCTTCTGGTTCCAGCAG
TAACGTAGTCGCTGCAACAATTGATGGAACAATTTTAGCTAACCTTAGTAATTTGGAAGCGGCCGCAATTTTG
AAACATTGTCCCTATCATGAAGCTATATTGAAAGCTGCTAGATTAGGGGAAAAAATACCTTCAGAATTAGCTT
CAAGTTACGTTGCCAGCTTAAGTTCTTTAGGATCTACTTTGCAAAAGAATCATCAAAC
TTTAATTTCACCACAAAATGATTTTTATACACCAGTATCTGGAAGTGAATCCGTATCATACAATACAAAGGAT
TTGTCGACAGCTTCCCATACATTAGTACAAACCATACTACCAAATCAAGTAGAAAATGAAAAATCTATAAAAT
ACAGCGCACAAAAAGGCAAATCTTTAAATCCAAAAGATCTATATAA
>1002050259_1 Heli.1-EL603921.1.5
GCACGAGGAGTTTATATATTTTTGTTCTTTTTGTGATTTTGTTTTTAATTTTTAAGAAACATGGCGGCGAAAA
TACCCTTTCCATATGAATTTAAAAATGTAACGGAGGAAGAGGATAAAATAGTGAAAAAATATTACAAAGATTA
CACATGTCCTTTCATCCGCGTGGGCCCGCACGGCTACATCTCTATGGCAGGGTACGGA
GACCACGCAGCTGATATATACAATTTGGAAGTTAGACCAGACGATGTATGGGTGATTGCCTTTTCTAGATCAG
GTATTTTTCTTTTCTTATTTTATAATAATATTAATGTTAGTTTACAATGAAAAATATTTAAATAAGTTGTTCG
CCGTAACATTTTGTGCTCCGTTCTCAATGGGAGAACCCGTTATGGAGTAAAGCTAAAA
AACGAGGAAAGAATAAATAAATTGTTGTTGTGGATTCGAATTCTAACGACGGTTTTTTTTTTCAAAATTATGG
TGTTCTTTTTAACCGACTTCTGTAAGAAAAGGAGGTTATCAATTTGACTGTATTTTTTTATTGTGTTTGTTAA
CTTTTGCGTTGGTGAACCGATTTTGATGATTCTTTTTTTTAATTGA
>1002050261_1 Heli.1-CX700755.1.5
GCAGTACGGTCGGAATCCCGGGTCGACTACGCGTCCGCGGGAAGGCAAACATGTCGGCCCACAAGACATTTAT
TATCAAGCGCAAGCTCGCTAAAAAGCTAAAACAAAATCGCCCAATTCCGCAATGGGTTAGAATGCGCACTGGA
AATACTATTAGGTACAACGCTAAGAGGCGTCACTGGAGGAGAACTAAGCTTAAGCTGT
AAGCTTGATGTCTTGTATCTGTTAAGTGAAATAAAC
>1002050262_1 Heli.1-EL603298.1.5
GCACGAGGCTGAAGTCATAAAGGTGAAATTTAGTACACTACACTACAAAGATTAATAGTGGTAGTTAACGAAG
TTAAATAATAATGTTGAATCTATTAGCAGATAAGCAAATATCGCATAAACTTACCTCCTGATAATAGTGTTGT
TCAAGTTAGTTGTAAAAAACTAATACGTAACTAACGTTATAGAATAATAGTGAAAAAA
ATATTAATGTTCAGTAATGGATAACGAATTCCTGGATATATCAACAAGCTTAAAGCAGCAACTTGCATATGGA
ATTGGGCATGTACTAAACGATGTGTGTGCAAGTCTGTGGTTTATATATTTTTTGGTATTTTTCCATCTTGTGT
TGGAGTTTTCTGGCGCGGAAGCGGGATATTTAATGTTAATAGGTCAAGTGGTTGACGC
CTTATCAACGCCTTTTATCGGTTACCATTCTGATCATACAAACAACTATTTGAGTACAAAATATGGAAGAAGA
AAACTATGGCATTTGTTTGGAACTTTATGTGTTTTGTCATCGTTTCCTTTTATATTTATGAAATGTATTCAGT
GTGAACTTGCACATAAATGGGCACAGATGTTCTACTTTGCTGCATTTATAACAATTTT
TCAAATTGGCTGGGCTTCAGTACAAATATCACACCTTAGTCTTATTCCTGAGCTTGCAGAAGACCCTCATGTT
AGAACACATCTGACTGCTATAAGATATGGATTTACTGTGTTTTCAAATATATTTGTATA
>1002050263_1 Heli.1-EL599678.1.5
AAACGAACGCGCAGCTGCCGCAGAAAGCGGATGCGTATCTTCAAGGATCCCTTTCGCCATAGACATTGGCAGG
AATGGAATCTGAGCACTTTCAATAAATTCACGAAGCTGTTCATCAGCTTGTGAATACGCCGCGCCTTTGCCAA
GGATAATTAATGGCCGTTCAGCTTTTGCTAAAAGCGAAATTGCGCTAGTGACTGACTT
CGGGCATGGCAATAATGCTGGCGACGGATTTTCAACTTTAACAATCGTGGTTAACGCTTCGTCTTTTTCCATC
GTCGCGGCCAGGACATTTGCTGGCAAATCAAGATAAACTCCGCCAGGGCGACCCGATACAGAGACCCGGATAG
CGCGTGCCAATGCAATGCCAAGATCCTGCGGCTGATTAACGCGAAATGCTGCTTTGGC
ATACGGTTTTGCCGCATTCATTTGGTCCAGCTCTTCATAATCACCTTGCTGTAGGTCGACGATCGCGCGGTCG
CTGGAGCCGCTAATCATGATCATCGGAAAACCATTTACCGTTGCGTTGGCCAATGCGGTCAAACCATTGAGGA
ATCCTGGCGCAGAAACTGTCAGGCAGATCCCCGGTTTTTGGGTAAGAAAACCGCTTGC

CGCAGCGGCATAGCCTGCCGACTGCTCATGACGAAAACCAATATAACGAATGCCTTCCGCCTGGGCATGGCGT
GCCATATCCGTCACAGGAATACCTACAACACCATAAATAGTGTCAATATTATTCTGTTTTAATGCTTCAACGA
TGATATGCATACCATCTGTCATTTGAAGTTGATCTGACATGATTAATAACCTCGAAAT
>1002050264_1 Heli.1-ES586560.1.5
GCGCGAGCACGTCTTACTTCTAACTAAAAGTATATTCTTATTTCATTTCAAAAATGGAATTCGTTGGCAAGAA
ATACAAAATGGTCTCCTCCGAGAACTTCGATGAGTTCATGAAGACTATTGGTGTTGGTCTGATCACCCGTAAA
GCCGCCAACGCTGTGACACCAACCGTGGAACTGCGTCAGGACGGCGACAACTGTGTAC
TGGTCACCTCCTCCACCTTCAAGACCACCGAAATGAAGTTCAAGCCCGGTGAAGAGTTCGATGAAGAACGCGC
TGATGGTGCTAAGGTTAAATCGGTGTGCACATTCGAGGGGAACACGCTGAAGCAGGTGCAGAAGGCGACAGAT
GGCCTCGAGGTCACCTATGTGAGGGAATTCGGCCCTGAGGAGATGAAAGCTGTAATGA
CCGCCAAGGACGTAACCTGCACCCGAGTGTACAAAGTACAGTAAGACCACCAACCCTCCAAGACTGCAGGGCG
GCGCCCTACACTATTTCCTAATATCGTATTATTACTTTATATACCTTTAGCACAACTATATATTTACGTATTA
TTTAGGCTATGATTGTGAATTGTAAGAACTAGATGAGATTGTAAACGACTTGTTCGTA
CTGCCTTCATTCATATGGTGTGGAAAAAATGAACCTAAAACATACAGTTGTATAAGTTTTTCAGTGCATATTT
AAATAAAGTTT
>1002050265_1 Heli.1-DT666760.3.5
GGGGGGAGAGGGCCGGGCCTGAGAATCCGCCGAGGTATCTATATCTTTCTCAGTTAAGATTTGATTGAAAATT
ATTTTATTTTGAACAACGCACTGGTAACAGGACTCATACAGACATGGTTGTTAAAGTGTATATAAGTGGTATT
TCAGGAAACAAAGAGGTAAAAAAGAGACAGCAGCGTGTTTTAATGATACTTGATTCCA
AAAACATTAAATATGAAATAATTGACATAACGGAACCTGGTAGAGAAGATGATAAGGACTTCATGCAAAATAA
TTCAAAATCGAATGGAGGCACCGTAAGTGACCCTAATCCTCGAACACCTTTACCTCCCCAAGTGTTCAACGAC
GTCGAGTACTGTGGGGACTATGATCAATTTGACTTAGCTAATGAAGTAGATACACTGG
AACAATTTCTGAAGGTAGAAGTCCCTACTGAAGAACCTCAATTAAACAATAAGAGTAATGCTGTTAATGGAGT
GCTTCAAAATAATGAGGATGAAAAGGAGTCAAAAGAAATATCCCCAGAACAAGGTTTAGCTGAACATTCAGAC
AAATCTAAAGACACAAATGAAGTTTCTGCTGACGATGGCCATTCAAAAGAAAATTCTC
CAGAGATTGCAGCAGCATCAGTAGAAAAAGAATCCTCACCTGCAAGAGACCCTTCGCATGACAAGGAAGAATC
TGCTGAAGACGATCAGAAACCTAAGAACGAAGCTGAATCCAAACCAATTGCAAAAGTAAGCTTCTTGAATTTC
CAACATGGGACGTCCTGACCTCGACAGAATGACAATCACATGCAGGAGCTCAAATTTT
CCAGACGGGCTA
>1002050266_1 Heli.1-EL601369.1.5
TAATAAAACAGAAATGATTTTTAAAGTTATACTTAGTTTTTTAATGTTTTACTTGTGTGAAGTATCTGCAAAA
GCTCCATGCAACGAGTGTATTTTACTTCGATCGTGTCAGGGAGCGATAGAATATGCGTCCAAAAACAGAAACA
ATGCTGAAAGTCAAAGTAAACTTAGAGAATCATTTTGTGGAACAGAATTTGTTAATGG
TATCAAAGCACCAAAGGTGTGCTGTTCTGATTTTAAAGAATATGATAATCGCTGGGCCGATCCAGGCATCGAG
TCTAATGGTGATAATTTGAACAACGAGATTAAAATGCATGAAAATTTAAATCTCCTTACCGAGTCATGTGGAA
ATATCGACGGAAACAGAATAGTTGGAGGCAGGATTGCTAATCTCCATGAGTTCCCCTG
GATGGCACTCATTTCTCACAAAACACGTGCTAGTTCAGGTAATCATAACCTTCAATTTAAATGTGGGGGTTCA
ATTATCAACTCAAGATATATTTTGACCGCTGCCCATTGTGTCGTCAATAAAAAGATTGCCGGAGTTCGCATCG
GTGAATTCGATATTCGCTCGAGGGAAGATTGTCAAGGAGAATATCCGTACACAGTGTG
TGAATCTCATATTCAGGATATATC
>1002050267_1 Heli.1-ES587903.1.5
AAGCATTATGGCGTAGACGTGATATTCTTATACATACTGAATTAGATTTAGAATACGTCAAACCTGGGTGCAT
TCATTGTGAAAAGAAGTTACCTGTTAATGCGCTTTTGCCATCCGAAGCAATTTCTTCTTTAAAAGATTTTCAT
CATTTGAAATTTAAGTATACTTTCGATGTTAAAAACCCATCATGCACATATTTACCTG
AGGGTAGATATTTAAAGTATGATGAACTTCTCGTTCTTCTTAAAGATTTATTTAAGAAAAACAGAGAGGCTGA
GAATATTAAGTTTGCACGTCGTGTAGAACAATGCAATGAAGTTGTAGGAGATCGACATTCTATTGTTGGAAAT
GTAGAAAATTTGGAACATTTATGGAATGATGCTATCATGAAAAGAAAAGCAGCAGCTG
ATCTTGTAAAAAAATTCTACATTTTCCACGATATGTAAGAGTTTTGCTTCGAATGCGCTAGAAAAAATGGGAACA
AGGAAAACATCATGTTCTTAAAACTATCGCTGGAATTATTAAACCTAATAATAATAAGTATTTATTAAAGAAT
GATTTATGTGAGGAGTGTAATAGAATTAAGTATCAATGTATTTCATGCAGAATTGCTA
TGGAAACTGTAATGTTGTGTAATTCTACGCCCTCAACGTCTCAGGGTTCTACGTCTATAGAAGTTTTGCTTGA
AGATGATGATAAAATGGGGTATCAAGCAGATAATGATGGTGAAATTTATACGCCTGAATTCCATCATGATATC
ATCTACCATC
>1002050268_1 Heli.1-DT664004.3.5

GCACGAGGGGAGTGCGCTAGGTCGAACTTTCGGCACAAATTTATTTAAAAATAACATCACAGCACCATACCTC
GTAAAAAATGTAAATTTCTCTGTAGGCAAAGCCCTTAATGGCGATCATGGGCACAGAGTTATGGCAATGCAGC
CATCAAGATGGCAATGGCACAAATTCAAGGATATGTTCCACTATTATCTAATGGTTGG
ATTAATTCCTGTAGGTGCTATTATTTTTTACACAAATGTGTTTATTGGACCTGCTCAGTTGACACCAATTCCA
GATGGTTACGAGCCCAAGCACTGGGAATATCATCGTCACCCAATAACAAGATTTATAGCTAGATACATTCATA
ACAACCCACAGCAGGATTATGAAAAATTTATGCATTACATTGATGAAGAAGCCCAAAG
GGGAAAATTGAGAGCTTTAGAAAAGGAAGTGCAAAAGAAAATGGCTGAAAGGCTCGACTACCAAGCATATTAC
TACAGGCCAATGGTTAACAAATACCTTAGAATCAACAAGAAGGTTGGTGACGAGATGTTTGACCGTCATGGCG
ATAACCACGAGGAATAAAGAAGTTTTTATCTAAATTTAGTTATTTAGAAATCAAGTGT
ATAGTAATCCACATTATGTAGAACAAGTGAACAATATTCTTTTGAATTTAGATTGTTGCTTTTATCCTGCCT
>1002050269_1 Heli.1-EL599984.1.5
GCACGAGGGTTTATTGGAATATTTATTATTATAAAAGAACCATATTATTATTATTATTAATGTGAAGACCCAA
CTAAGTTATTCCTTAAAATGCCAGCTACAATACATAACGATGTTCTAGTGTACAAAGGCAGCAACTTTTTTCG
TCAGAGGTTGTTATTATCGACGTTAAGTGGTCGTTCTATTAAAATAGAGGAGATTCGC
AGTACACACGATGATCCTGGCCTCAGAGAATACGAAGTAAACTTAGTACGTTTATTAGATAAGATAACAAATG
GTTCTAGAGTAGAGTTAAATGAAACTGGTACATCAATATATTACCAACCAGGTATTTTGATTGGGGGACAAGT
TACACATTCTTGTTGCACACAAAGAGGAATAGGTTACTATTTAGAAGTGCTCCTAGCA
TTAGGACCATTTTGTAAAGAACCATTAAATGCAGTCCTTCAAGGTGTTACACATCATGAATTAGATGTATCAG
TCGATAAAATAAAGAGTACAGCTCTGCCAATACTATTGAAGTTTATTCTAGTTGATGATGGTTTAGAATTGAA
GGTTGTAAGAAGGGGAGCCCCACCACTTGGTGGAGGTGAAATAGTATTCAAATGTCCG
GTAAGAAGACACTTAAAGCCCTTGGCATGGAGACAATGGGGGCTCATCAAGAGAATACGAGGAGTAGTGTACG
CTCTACGAGTGTCACCCACTATGGCTAATAGAGTT
>1002050270_1 Heli.1-EL596706.1.5
CGGCCGAGGCGATGATTGGCGCCCGGCCGAACGTCCTGATGTAAAGAAACCCAAGGATAATCTCAAGCCTGAA
GGGGAAATGGAAGTTATTCGTAGAACTGATTACTCTGTAACTAAAGGAGATAGAGCTGATGTTGTTAGACATG
AAGACCACTTGAAAATGGAAGGAAGTATTGATGTTCGTCGCTCAAGAGACGATTACAA
ACGTATTGATAAAACTGAAAAAATAATATTACAGAGACATGAAGACAATTTACGAACAGAAGGTGAATTTATT
GATTTACATGTCCGAAATGATTATAATGCTACTAAAGGTGAACGAGCACCAGTAGTAAAACCTCAAGATAACT
TAAAACCTGATGGTAAATTTTATAAACCAGAAGTAGTGCCATCCCAGCCAGCTGAAAA
ACGCAAACTTATAAAGCATGTTGATAATATAACAATTGAAAGAGATTTGGATATACGTAAAAATGTCGATAGA
GTTGATATAATTCGAAGAGAAGACAACTTAAAGATAGAAGGTGAATTTATAGATATGAAGCAAAGAAATGACT
ACAATGTAGTTACAGGTGACAGAAGAACTATCGTTAAACATGAGGATAACCTTAAGAT
GGAAGGTACGATGGAAAATATTCGCTCTTCTGATACATATCGTGTTGTAAAAGGCCAAAGAGTTAAATTAACT
CGTAGAGAGGATAATCTTAAAATAGAGGGTGTTTTTGAAGACCATACTCGCAGGGATGACTATAAAATTAAAA
AAGATGTTAAATCAGCTATTACATATTACCCAGAGAAGGATGAGCCGAAAGATAGTAC
AGATGGGCCAAAACACGATAAGCCCACGTACGTGAAAAACATGGAAAGATTATATCGATG
>1002050271_1 Heli.1-EL602527.1.5
GCACGAGGCCAATTCTAGCAAACGAGTCAGTTGGATAGATGTTGCTTCCGTGTTTGTACTAATCTAGATTAAA
CTGAATACAATATTACCACGATGCAGTCTAAGTTGTTTTGCCACTCGTGTAACTAAAATGGCGTCCCTGTTTA
TGATTTAGTTACCATTCTTTATGTGAACATCTCCTAGTGATCGCATCCGAGTGAGTCA
TTATCCAACATAGCAATGACATTTTTATAGCACAAAGTGAGAATACGTCTCTGAAACGTTTATAGAAGACTGG
CAAGTGTGATTAATATAGCACAATAATGCGCGAATTCAAAGTGGTCGTGCTAGGCTCGGGTGGGGTCGGGAAG
AGTGCTTTGACTGTGCAGTTTGTGTCTGGGTGTTTTATGGAAAAATATGACCCCACGA
TAGAGGATTTCTACAGGAAAGAAATAGAAGTAGACAATTCCCCATGTGTTCTAGAAATATTGGATACTGCTGG
AACAGAACAATTCGCCTCCATGCGAGACCTGTACATAAAGAATGGTCAAGGATTTGTAGTTGTGTACTCGTTA
ACAAATCATCAAACTTTTCAAGATATCAAACCTATGAAGGAATTGATAACTCGCGTCA
AAGGATCAGAACGTGTACCTATTTTGTTAGTAGGCAACAAAGCCGATCTGGAACACCAGCGCGAGGTTTCACA
AGGAGAAGGCAATGCGCTCGCGCAAATGTGGGGCTGCCCATTCGTGGAAGCATCGGCCA
>1002050272_1 Heli.1-EL600542.1.5
GCACGAGGCCCTGTTCGGTACGCTACAGGGAAATACTCGTGAGGTTCTCCACAAGTTCACCGTGGATCTGCCC
AAGAAGCACGGCCGCGGTGGTCAGTCGGCGCTGCGTTTTGCGCGTCTGCGTATGGAGAAGCGTCACAACTACG
TGCGCAAAGTGGCCGAAGTTGCCACCCAGCTGTTCATCAGCGCCGATCGCCCGAACGT

GGCCGGACTCATATTGGCCGGTTCGGCCGATTTCAAGACTGAACTGTCGCAGTCTGATATGTTTGATCCGCGT
CTACAATCAAAAATTATCAAACTGGTAGACGTCTCGTATGGAGGTGAAAACGGGTTCAATCAGGCCATTGAAT
TAGCAGCGGAGAGCTTACAGAACGTCAAGTTCATACAAGAGAAGAAATTGATCGGAAG
ATACTTTGATGAAATATCTCAGGATACTGGCAAATATTGCTTTGGTGTAGACGATACTCTGCGAGCTCTGGAA
CTTGGAGCCGTTGAAACACTTATATGTTGGGAGAATCTTGATATACAGAGATACGTACTCAAATCTCATGCGA
CCAATCAAGAAACTATTTTGCATCTCACTCCTGAGCAAGAAAAGGACAAGTCACATTT
CACTGATAAAGAAAGTGGCGTAGAACTCGAATTGGTAGAATGTCAGCCGTTGTTAGAGTGGCTGGCGAACAAT
TACAAGTCGTTTGGTGCGACTCTCGAAATTATAACGGACAAGAGTCAGGAGGGGAGCCAGTTCGTGCGCGGAT
TTGGTGGCATTGGCGGTCTACTGCGTTACAAAGTGGACTTCCAGTCGATGCAGTTGGA
CGACGAAGAGATCGACAATCTATACGATATCGATGATTATTAAACAATTTACTGTTAAACTGTGGAGCTTTTG
CAATACAATATGTTATTAAAAATGAGTTTTCCTAATTATTTATTTTATTTCATCTTTTTTTATCTGTCTATAC
TTAGTCGTTTTTTTTTGTGATCTATGTCCTGAAAAAAGTAGCGGTTCCTATTTTTTAT
TTGTTTTTCAAAAGCTAAACTGATAATTTAAAAAATCGGGGATTTGTTTCATTATTTAAAATATATTAAATAT
ACATAACTGAA
>1002050273_1 Heli.1-EL599216.1.5
GTCCCATCAACGAGCCGACGTTCAGGGAAGTACAGTGCGCTAAGTTCAACAACATGACTTACCACAATGAAAC
TATCACAGAATGGATACCCTACATCAATCAAGATAAGCCATGTGACCTACAATGCGTGCCTCGCGACGGCAAG
GACATAGAGGGAATCGGCGCGTTCGTGGCCGACGGAACCCCTTGCAGGCAGAGCCTCG
GCAGTCGAGATATGTGTATATCTGGTGTTTGCTATAAAGTAGGCTGTGACTGGATTGTCAATTCCGACGTCGA
AGAAGACGAGTGCGGTGTTTGCGGTGGAAATGGATCGGCGTGTAAAACTGTACAAGGCATTTACAATAAGGAC
ACTACCAGGCAATCTGGATTTAGTGAGGTTGCTGTTATACCGGCCGGTTCAAGGAACG
TAAGAATTCAGGAGAAAGTTAGTCCAGGAAACTTTATTTCTATAGGTAGTGCGAAAACGAGGAAAATATATTT
AAGCGGTGCAAGAAACGCAACGGTAACGGAACACTTTGTAGCGGGGACGCAAGCAATCTACGAACGGGATAGA
GACTGGGAGAAAGTCCGAATTAGTGGACCTCTCGCTGAAGATATAAAAGTTTATCAAC
GTATATTCCGCGGGCGCCACCGCAACCCGGGCGTGACGTACCAGTACACGGTGGACCAGCCGCGGCGCCGCCA
CTCCTACCGCCTGTCCGACTGGTCGCCGTGCTCCGCCACGTGCGGCGTCGGCCGCATGCGCCGCCACTACGTG
TGCGTCGACGAACACCAGCGTCAAGTGGAGTTGTCGGCGTGCTACCACACGGAGCCGC
CGCGCCACGAGGCGCTGCTGCAGCAGTGCCGGCAGCCCGCCTGCACGCACTGGTGGGTGGGCGCGTGGCGGCC
CTGCGAGCCCTGCCACATGCCGG
>1002050274_1 Heli.1-DT664049.3.5
GCACGAGGCTTGCCCTATGCTCTTATATTATCTAAATAGTATGAGAAACACGGAAAATATTAATATATAGTGA
ATTATTTTCATTTTGAAACATAGTTAGTTACGTTGTTAGTGATCCGTATGTTATATTTGCTCAGCTGTACGTT
CTCAACTGGATGTGGTCTGGTCTATTCAGTTCATATGAAGAGAGGACAGTCGCAAGTC
CAAGTTCAGAAGTCAATAAATTATAAAATCTCGATTTTTGATTACACAGAGGTGTTTTTTTTCGCGAAATGGC
AAACCTGGTGTTGTGAATATTTTTTTTCGAAGTGTTAATTATAGTAATCTGTGTCGTTTTCACACATTTTCAT
GATGCAACACATACTAGTTTTTATAACAATATGGATATTTATGAGAGGTATACAGTCG
AAATGCCCGGCAACCCAACCAGAGACCTTCACTCGGTGGTCGGGCATGGTCCCACAGCATGCCACACCGGTTT
TCATGTATTCTGCTAGTGGGGGCGAGGAGTCCTTAACTGGTGCCTGCTTGGCCAGATGTCGCGAACTTGAAGA
CTGTGCTGCTGTCATAGTGTCGTACGGCAAAGGAACCTGTCAGGGCATCGCCGGTGGT
GAAACAACTAAGTTCTACTCTGACAATGATGCCGTTTTTTTCCACAGAATTTGTTTGAAACTGCCAGATACTT
GCAGTAAACGCTGGTGGTCCTTAGAAAGCACACCCGGATATTACCTCAATTCTGAGGGTACTAATATAAAAGT
CATAGTAAATACAACCATCGAAGAGTGCTACAACGCTATTTTCTCTAACAATGAGAAA
>1002050276_1 Heli.1-EL602861.1.5
GCACGAGGGAGAATTGTAATTGTTTTGCGCATTTCTTATTACAATAAATCAATTTATCCATTAATAAATTTAT
TTTGTTTAATGCCGTGAAGTTTCGTAAGAAAAACTGTTTTAGTCCATATTAATACGAATGTGTTGTGAGAGAA
TAAACTAAATTGTGAATTCAATAAGTGTATTGTAAAAATATATGTTTTTTGTGACGTA
ATATTACAAAAAAAAGTAAAACAATAAGTAGTACAATGGCAGAAACAACGGAAGCGAAGGCGAGCGCCTCAGC
TCCCGGCGGTGACGCTGGTGGTGAAGAGGAGAGGCGCGATGAACGTATGCTCGAATGCAATATTTGCCTCGAC
ACAGCGCGAGATGCAGTTGTTAGTATGTGCGGACATCTTTTCTGCTGGCCATGTCTTC
ATCAGTGGTTGGAGACCAGACCAAATCGTCAATCATGTCCAGTTTGTAAAGCTGCTATTAGCAAGGAAAAAGT
TATTCCATTATATGGTAGAGGAAATACTAAACAAGAAGACCCTAGGAATAAGGTACCACCAAGACCAGCAGGC
CAGCGCACGGAGCCTGAGTCGAACAGCGGGTTCCCCGGTTTCGGTTTTGGTGAAGGGT

TCCACATGTCGTTCGGTATAGGCGCGTTCCCCTTTGGCGTGTTCACTTCCACATTAAATTTTGGTGATCCTAG
GCCTAGTGCTGCCCCCCGCGACACCGCCCACTACCAAAAGGAGCATTTTCTATCACAAATCTTCTTGTGGGTG
GCCATTTTATTTGACATCTGGCTCGTTTTTGCCTGACATGGACTGTTTAACGAACCCC
GGCTGCGAATGGAAACTTTCTCATGACTAAGTGAATCTACTTAAAATATATATATTATTATTTAAATATTTTA
TTTTGAAGCAAAAAGTGCCCAATAATAATTTGTTAACAGATAAAGCGTTTCTGTATGATAAGGAAGTAAACAT
GGCCGTAATTAACATTGTTCTTCACATAATACTGTTAAATATAAATAAATCAGATTAT
AATATTTTACATACTATAACATCAAAATATCTTTGGATATTGATTTAACTGTATACGAAGCTTATGATTTACT
TGGTTGTTTTT
>1002050277_1 Heli.1-DT667926.3.5
GCACGAGGTGTGTAAAAATAACACTCGATGTAATAAGTAATGTGTGTCTATGAAAATCCAATTATTTTTAGAG
AAATGCGTAAATATATGTTATCTATACATAATCTTAACGTGAATCTACACGAGGCTTGTGCGCTGTAGGCGAT
ATATATTGTAATATTATATTGATAATACTTAAATAAAAAAAGAAGAAATAAATTTGTG
CCTAAAGAATTACTTGAAAAATTTTCATACATGTATATTTTAGTGATGCTCAATATATCTTTGTAAGCGGGTA
GTGGTTAGAAGCTGTTGAACGTCAAACCGTATTTTCGGACGCCAGTCATGCATTTTGTGTTGATTTTCCTTCA
TTTAAGGCTTTGCTTCTCTGCTAAGTAAAAGATACCACTTTTGCGTCTTCATTAATAT
CGTCTGTACTACCTTACTTATCGGGCTTACTGTACGTAATTGCTAAGCAAATCTTGATTAAACTTTCACTTTT
TCATTAAGATGATCCAATTTTAATAGAAATTTGAACGTAATGGTAACCTTTTTGAGAGATAAGTGCTTAACGA
TTTTCCCACAGTTATTACAACTTTAGAATTAAAAAAAGAAAACAGTGCAGACGATATT
ATAAGTTCACTAGAATAAGATGTTTTGGTAATATCGCCATTTTGAAATATCGTTATCACCATTCCTTTTGTAT
TTGTGAATATAGCAATAATCGGGTAACATTCATCTCATAACAAGCTAACGATTCGTTTTGTGCAAGGTTTTTG
TTTTGACACATTGATGTTGTATTATTTTTTGCGCTCTCGCTAGGTAATTTTGAGAAGC
AATATTATTATTGTGACATGAACGCTCAAATTAGACCTAAATGTCATTTCTGTAGAAGTTAATTATGCATACA
TACATATATAT
>1002050278_1 Heli.1-DT668124.3.5
GCACGAGGGGTGACCATGCTCCTGAGATTTGTAGAGGAAATGTTACATGCAGATTAGATCAGTTCCAATGCAA
GGATCACAGCTGTATCCCCGGGGCCCTCTACTGTAATGGAGAAAAGGATTGTCCTGATGGAAGCGATGAATTC
AATTGTACTCGACCCAAACCTGTGTGCGATAAAAAAACCGAATTCGATTGCGGTGCTG
GTATGTGCATACCGTTATCTAAGGTTTGCGATAAGCATCCGGACTGTCCCAACTTCGAAGATGAACCCAGAGA
CCGGTGTGGGGAGGACGAGTGTGCTATAAACAATGGAGGCTGTACACAAAAATGTATCGACACCCCCGTCGGA
TACTACTGCGATTGTGAAAATGGATATAAGCTGACAGATAATCGCACTTGTGAAGATA
TCGACGAGTGTACAGACCCCGGTGCATGTTCCCAAATATGCATAAACGAGAAAGGCACCTTCAAATGCGAGTG
CCACACAGGGTATGCCAGAGATCCAAGAGACAGAACACGGTGTAAGGCAACAGAGGGCCACCCATCACTGCTC
TTTGCCAGGCGTTTTGATATCAGAAAGATCAGTTTGGACCATCATGAGATGGTAGCTA
TAGTAAATGATACGAAGTCAGCCACAGCCCTCGATTATGTGTTCAGAACTGGCATGATATTTTGGAGTGATGT
CACCGATGAAAAGATATATAAGGCACCAATAGACGAAGGTAGTCAACGTACGGTAGTGATAGGCGATCAGTTG
ATCACATCAGACGGCTTGGCAGTGGACTGGATATACAACCACCTCTACTGGACGGACA
CTGGCAAGAATCACATAGAACTGTCCGATCTTCAGGGCAATATGAGAAAAATCCTCATTAGAGACAAACTTGA
AGAACCTAGAGCTATCGCTTTAAATCCTCTTGACGGTTGGATGTACTGGACAGACTGGGGTCAAATACCGAAN
ATCGAACGCGCCGGTATGGATGGTTCACACAGACAGACAATAGTCTCCTACGACGTGA
AGTGGCCAAACGGCCTCACTTTGGATTTAGTAAGGAAACGCGTATATTGGGTCGATGCAAAACTGAACACAAT
ATCATCCCTGCACTACGATGGAA
>1002050279_1 Heli.1-EL602167.1.5
GCACGAGGAATTGCCTTCAAGCAATTGGTACCTACGCCATTTTGTAAGTGTCTGCCATAGTCAGTATTTCTAG
GCACAGTGTAAAATACAAAATTCGCATGGTTTTGTGTACATTAAATAGATAAAAATATCTTTAAACTAAGTTA
ACGTCTAGTGCGTTAATTCACCGACACAAATTATGGAGGACAAGGCGTCATTAAAAGA
ACTGGATCAATGGATAGAACAGCTAAATGAATGCAAGCAATTGACAGAAAACCAAGTGAAAACGTTATGCGAT
AAGGCAAAAGAGATTCTAAGCAAAGAGTCCAATGTGCAAGAAGTGAAATGTCCAGTGACCGTATGCGGCGATG
TGCACGGTCAGTTCCACGACCTGATGGAGCTGTTCCGCATCGGCGGCCGCTCGCCCGA
CACCAACTATCTGTTCATGGGCGACTACGTGGACCGCGGCTACTACAGTGTGGAGACAGTCACATTGCTAGTT
GCACTTAAGGTTAGGTACCGCGAGAGAATAACAATCCTCCGCGGCAACCACGAGTCACGTCAAATCACCCAAG
TTTACGGTTTCTACGACGAGTGTCTCCGTAAATACGGCAACGCGTCCGTGTGGAAGCA
TTTCACGGATCTCTTCGACTTCCTGCCACTCACTGCTTTAGTAGATGGACAGATATTCTGCCTTCACGGTGGG
CTCAGTCCTTCTATAGATACTTTGGATCACATCCGCGCACTGGATCGCGTCCAAGAAGTGCCCCATGAGGGAC
CCATGTGCGATCTGCTGTGGAGTGACCCTGATGACAGAGGCGGATGGGGTATATCGCC

CCGCGGCGCCGGCTACACCTTCGGGCAGGACATCTC
>1002050280_1 Heli.1-EL603262.1.5
GCACGAGGGATAAAACGAGACGGAACGAAATATAAACCTTGTAAAAAAAACGAATAATACGTGATTTATGCCA
ATATTTGGTGTTAGAGAGCATATTACGTTATACAATTAAAGTGACCTTAATCATTATTTATACTACTACGATT
TCAAAATGAGCAGCGTTCCAGAAAACGCGCCACATCATTGCCCTGGGACTCAAAGCGA
GGACGCAGGCAAGGCCTCAGCTTGTGCTGGTTGCCCCAATCAAAATATTTGTGCATCAGGTATCCCAGCTGGA
CCTGATCCTGCTATAGAAATCATAAAAAGCCGCCTAGCAAATGTTAAACACAAAATACTGATTCTCTCCGGAA
AAGGAGGTGTTGGTAAAAGCACAGTCACATCACTACTTGGACATGGGCTTGCCAAACT
TAATCCAGATTTAAATATAGGTATCTTGGACGCGGACATCTGTGGGCCGAGTCAGCCTCGCGTGCTCGGCGTC
AGGGGTGAGCAGGTGCACAAATCCGGCTCGGGGTGGTCGCCTGTTTATGTGACAGACAATCTATCACTTATGT
CCATCGGTTTTCTATTAGGAAGTCCGGATGACGCAGTTATATGGAGAGGCCCCAAGAA
GAACGGTATGATCAAGCAGTTCCTGAGCGAAGTGGACTGGGGGGATTTGGATTAATTTGCTAATAGATACTCC
GCCAGGTACATCAGACAAACATCTATCGCTAGTCC
>1002050281_1 Heli.1-DT665616.3.5
GCACGAGGCTTTTATCTACAGCTCCAATAAGCACGAATTTGCCTAAAGGTCCTCATTATGTGCAATCTGGTTC
TACACCTAATTTAAGATATGTTCAAACGTATGGTCCGGAGAACCAACTGTCGACGGTTTCCCAAGTGTCTGCC
AATCAGCAGCTCACTGCTCAGATATTGCAGTCTTTGTCTCAGCCTAAGTTAATGCTGC
AAAGTCCAACACCGATACATACAACAGTTAACATCACCCCTGCTCCGATTGTTGAGGAACCAGAGATTAAACC
CATTAATCAAAAGAGGATTGTATTCGGTGACAAAACAACACTTGTATTAACCGATGATATCATTGGTGTGGAG
GAACGGCCAAACCTATCAGAGGAGTTGCGTCGCTATTCATATCAAGCACTAGATCTTA
TTATGCTAGACCATACATACGCGTTACCCGCACAGAAACAACATGATAGTCCTCCAACAACGACAGTGGCGGC
TATATCTCCAG
>1002050282_1 Heli.1-EL598715.1.5
GCACGAGGGATGCATAACAACGCTACGTAAATAAAAAAAAAACGATTTTAATCTGCTAATTCTACTTGTATTT
AATTTAGTTTCTGTCCAGTGGATTTTGCAAGTCCTACTTTAACTTTGTGGACCTCTTATTTGTTACTGTGTTT
ATAAACGAGGACGTGCGGAGAACGAAGCTAAGATGCTGAAGCTGGGGGTCGTTTTCTT
CCTTGCTTTGTCGACGACCGTTCACAGTCAGTGTTTTTACAAAAACGATGGCGAGAAAAAACTAAATCCTGCA
GCTCGTACTAGCTTGTACAGAAATCAATTAGATTTTACACTAAATCTCTTCAATATAATAAACAAAGCTGTTC
CGGATGACAATATATTCTTTTCACCATTCTCGGTGTACCATGCGCTACTGCTCGCTTA
TTTTGCTGCAGGTGGACAAACTGAGAAGTCATTAAAGCAGTCATTAAGAATTTCTGATGAACTGGATAAAGTA
AACCTGTTGGCTGTTTACAAGGTGGACAAGAGAGAGCGCGAGTTTAACAACAACAGCGACAGCTATGAGTTCA
CGAACGCTAACAAGATGTTCGTGGACAGCGAGTTACACTTGCGTCAGTGTATGCTTGA
CCTTCTTTCTGGGGAATTGGAAGCTCTGAATTTTCACGACAATCCCGATAAATCTCGTCTTATAATAAACGAT
TGGGTGTCTCGCGTCACTAAAAACAACATCAAAGATCTAATCCCAGTCGACGGCATCAGTCAAGCCACTAAAC
TAGTTTTGGCAAATGCTGCATACTTCAAAGGAGTATGGGCCTCCAAATTCCCTGCAGA
AAGAACGAAGAAACAACCGTTCTACGTATCAGAATCTCGTCAAACATTTGTACCGTTTATGCGACAGAAGGGG
ATATTTCATTACATGGTAAGTGACGAATTGGGTGCAAAAATTTTGGAATTACCGTACAAGGGCCATGACATGA
GCATGTTCATTTTACTTCCGCCGTACGCCTTAGAAGAAGGCCTATCCAACATTATTGC
TAAGCTCACCCCTGAACGCTTGGCGGCCGTAGTGGAGGAAAGCTACATGGGTCGCGAGGTGATAGTAGAAATA
CCTAAATTTACAGTCGAGAGAAGTCTTCCTCTCAGAAATATTCTGGAAAAGCTTGGCGTAGGAGATTTATTCA
ACGCCACCTCTGACTTCAGCACTCTCTCAGATGACCACGGAATCGTTTTTGACGATGC
TGTACACAAAGCCAAAATACAAGTAGATGAAGAAGGTACTGTAGCCGCGGCTGCCACTGCCATATTCGGATTC
CGTTCTTCCCGCCCCGCTGAAGCAAGCGTCTTCATCGCTAACTTCCCTTTCGTCTACGTCATATATGAGCGTC
CCACGAATTC
>1002050283_1 Heli.1-DT665024.3.5
GAAGAAGAAGAAGGGAGCGGGAACTGAAAATGTGAATGTTGAAAATAATGCTGTGGATTCGGAAAACCCTGAG
CCTCCTCAGGTTGATGAAACTACCGGAGAAGCAGATGAGAAGAAAAAAAAGAAGAAAAATAAAAATAAAAACA
AAGGCACTGGCAAAGTGCAAACTAATCCTCCAACAATCCCAATTGCAGAACTTTTCCC
TAATGGTAATTTCCCTGAGGGTCAAATAATGGAACATGGACCAGCGGAAGGTATTGACGAGAGAACAGCAAAA
GATCGCTTTTCCAGTGAGGAAAAAAGAGCTTTAGACAGAATGCACAATTATATATATCAAGAAATACGACAAG
CTGCAGAAGCCCACAGACAGACTCGTCAATATATGCGTGACTGGATAAAACCTGGTAT
GACTATGATACAAATCTGTGAAGAACTAGAATCCACTGCAAGGCGTCTTATAGGAGAGGATGGCTTAAAGGCA
GGATTAGCTTTTCCTACTGGCTGCAGTCGTAATCATTGCGCTGCCCACTATACACCAAATACAGGTGATAATA
CAGTACTGGAGTATGATGATGTAGTCAAAATAGATTTTGGCACTCATATAAACGGCCG

TATCATTGACTGTGCCTTCACATTGCATTTTAACCCTCGATATGATCCTCTGGTAAAAGGTGTACAAGAAGCT
ACAGAGGCTGGGATCAAAGCATCTGGTGTAGACGTGAGGCTGTGTGA
>1002050284_1 Heli.1-EL600412.1.5
GCACGAGGCGTGTCTCCACTCTCCGGTCGAGGAGGAACGCCCTGGACACGGCGTGGACCGCCCGCGCCAGGAT
ATATGAACAGCATTTGGACGCTCTACTGTTCTTGAGAGATGCTGATGCTCTGGACCAATGGATTGCTGCCAGG
GTACCTCTGGTCCGCGACGGCAAGTACGGAGAGACCCTCGCTCAAACTGAGGAACTGA
TCAACCGACACCGAGACTTAGAAGAGACCATCGACGCCCAACGAGACAAGTTCTTAGCTCTAAAGAGGATCAC
ATTGATTGAAAAAGCCTTCAAACATCAACAGAACGAGGAGCAAGAAGAGCGTAAGAGGCAAGCGGAAAAGCAA
GAGGCCGACCGTCTTCAGCAAGTGAAGAGGCGGGAGATGGAGAGGATCACCGAGGAGA
GGCGACGGGAGACGGAGTTCCAGGAGAACCGAGGAGTTGGAGTACTGGAGATGCAGGAGCAGAGACCGACTAT
GGTGGAACGTCAACACAGCGTAGCCTCCTCAGAGGAGATCATAAACCCGGCGCCGCAGTTCGAGCGTCTTCCG
AAATCGGACACCAACGTACAGAGAGCGGAGAGTATGAGTGTCGTAAAAACACCAAAAC
GTACACCGTCGTTCACTACCCGGCGTCGAACCCAGAGCTTCCGCAGACACAGACGACCCGACGACCTACCCCC
TGTTTGAATAGAAAGTCATCTAGAACGTAAACAAGAAGCGGGTTGCG
>1002050285_1 Heli.1-EL600894.1.5
GCACGAGGGTCTCGTCCAGAATTTTAAAGAATTCTAAATAACTTCTATTCTGGTTTTTAGCTTGTGCAAGCAC
ACGTACTGTCGGATACAATATTTTCAACTGCAAATTATTTTAAAGATACAAAAATGGAGGCGCTCGGCGATGT
CCTGCAACCTTATAAGGAGCCAGTAGCCATAATCACTCGTCTAGTGACTATGGGACAG
ATGTTCTCTGGGGCTTTCATTTGCTATGATATTTATAAACAGGGCAGCACTAAAGGGATAGGAATTATGCCTT
TTTTGGGCGGTCTTATAATGGGAGTTCTCAACTTGAAGTTCGGCTTCATGCTCCGCGACGACACAATGATCCA
AGTCAACTTTGTGGGTATATCCCTAAGTATTATATACATGATGGTGTACTACATGTAC
TCTAGGGACAAAGTAAACGAAGTGTGGGTTCAAACCGGTGCAGCGTTAGCTGTCTGTGTCGGTCTACTCGCAT
ACGCTGAAATGGAAGATCCTAAACTAATTGAAAACCGATTTGGGAGTATTATAACTGCTTTCATGTTCTATTT
GATAGCTTCTCCTTTATTGAATTTGAAACATATTGTTAAAAATAAGAGCACAGAAGGT
ATGCCGTTCCCGATCATCTTTTCTGGTACAATCGTCACTTTCATGTGGCTGCTATACGGCATCATATTGAAGA
ACAAGTTTATTGTGCTTCAAAACGTAGTTGCGTTTGCTCTATGTGGGTTCCAACTAAGTCTCTTCGTGGTGTA
CCCATCGAAGTCCAAGGGCAAGGACAAGGCAAAGGCCAAAGCAAAGAAATCCAACTAA
TTAGGTTATTTAAAAAAAACCTATTTTATAATATTAAATGTATATGTATCATATAAAAAT
>1002050286_1 Heli.1-DT667097.2.5
TTTTTTTTTTTTTTTTTATATCACAATATAATTTATCACATATGAACTACACGAGCGGGTAGGAGGCGGAGGA
GGCGATGCCGCAGTGGTTGTTGCGGCCGCGCGCCATCTTGATGTACCCGAGCTCGCCCCACGAGCGGCCCCAC
GAGTTCTTCACCAGCCAGTAGTCGCCGCCCTCCTCGTCCGTGCCGTACCCCACCACCA
GCACCCCGTGGTCCAGGTTCTCGCTGGAGCAGTCCTCGTCGAAGTAGACGCCGGCGGAGTAGAGCTGGAAGGA
CTCGTGGCTGGCGTCGATGGCGACGGCGACGGGCCCCACCGTGGCGACGGCCAGGCGCAGCGCGTCCTCGTCG
CCGGCCGGCACGTCCACGAAGCCCACGTCCTCCGCGCCCGCGTTCTTGGGGTTGTACC
TGCACTTATCGTCCACACCCTCATAGGGATAGCTCTTCTCGGTGTCGATGCCGCCGTTGTCCTTGATGTATTT
GAAGGCGTTGTCCATGAGGCCGCCGTTGCAGCCGTTGTTGCCGTACGCTGACGAGCAGTCGATCAGGTTCTGC
TCCGACAGGGATACCAGGAAACCCTTCTGACGGAAGTGCTGGCCCTCGAGGGCTCCCG
TGGTGCTGAAGGACCAGCAGGAGCCGCACTTGCCCTGGTCCTTGACGTCGGTGACGGCGCCCTGCTGGCGCCA
GTCCACGCGCGCCGGCGCCGCCACGTTGGCGGCCGCGATGTACGCCGCGCCGCGCTGCCCCGCGCCCTTGC
>1002050287_1 Heli.1-DT665161.3.5
GCACGAGGTGACTTTCGTGGCTTAGTCGGTATTCGATGTATTATGGTTTTCTTATCTTCGTAAACTAAATAAA
TAAGCAAAAAATGTCCGACGTAATTGTACCTAAAGGTCAACCTGTTCCTGGTGAACCAGAAAAAAAGGGCTGG
CTTTTTAAATGGACAAACTACCTCAAAGGATACCAACGAAGATGGTTCGTGTTGTCAA
ATGGATTATTATCATATTACAGAAATCAAGCAGAGATGGCGCACACATGTCGCGGCACAATATCATTATTAGG
TGCTTTGATTCATACAGCAGATTCCTGCACATTTGTAATAAGTAATGGAGGAACTCAAACTTTTCACATAAGA
GCACACGATGAAGTGGAACGGCAAAGCTGGGTCACTGCATTAGAATTAGCCAAAGCAA
AGGCAATACGTGCACAAGAATCAGATGATGATGAAGATCAATTGTCATCTGGACAAGCTGTTGTTGGGGGTAC
TGATGGTGAAGGTGAAGATGCAGGAGGTATAGCCCGAGAGCTGGCAGCTAGATTCCATGACTTAAGAACATGT
TCAGAACTTGTAGCAAGACATGGGGCAGCTTTACAAAGGTCCCTGGTAGACTTGGAGA
TACCCCCAACTGACACAACTAAACAAGTTTCCGAAAGAGCAACTTTGTTCAGGATATCATGTAATGCTATGAT
GAATGCATGTTCAGAGTTCATGAGTGCAGCGGCAGCGTCGAGTGCGCGTATGAATCGTGCGTTACAACACGAA
CGCGATCAGAAAATGAGATTACAAGAGGTAGTGGAACAGCTCGCAC
>1002050288_1 Heli.1-DT662587.3.5

GCACGAGGCTTGTTCGTATGGTGTTCTACGGAGTTTGGGGAAAAAATGAATTGAAGTTTCCTCCACACTTTAA
GTTCGGGGCAGCGACTTCAGCTTACCAAATAGAAGGCGGATGGAACGCTGATGATAAAGCTCCTAGTGTATGG
GATGACTATTTCCACACCTATCCAAATTCTGCAGATGATGGCGGTAATGGAGATGTGG
CATGTGATTCGTATCACAGATGGCAAGATGATATAAAAATCGCTTCAGATTTAGAATTAGATTTTTACAGATT
TTCAATATCATGGTCGAGAATTTTACCTACAGGTACACCAGAATATGTGAGCAAATCTGGTTCTAAATATTAT
AACAATCTAATTAACGGCTTACTGAAAAGAAACATTGAACCAGTAGTCACTATTCATC
ATTTTGATCTACCACAAAGACTTGAAAACTTAGGTGGATGGGCAAACCCTCTAATCACCGATTGGCTTGCTGA
CTACGCCAGGATCCTATTCTCTTTATATGGTGATAAAGTGAAAACTTGGATATCTATTAACGAACCTTGCATC
ATGTGCAACCTAGCTTACAATTCAAATATAGGTCCGGGTATAATAGATTATGATATTG
GTTACTTTCAGTGTGTAAAAAACGCTATGGTAGCTCATGGGAAGGTTGCGAAGGTATATAGGGAAGAATTTGA
ACACATTTATAAGGGTCGTATTGGAATTGTAAACCAGTTATTTAATTTTATACCAGCGACGAAAGACGATGAG
GGGCTTACAGAACTGGCAGCTGAAATATATTCGGGATTGGTCTTATATCCTATATACT
CAAAAACTGGTGGATGGCCGCCAC
>1002050289_1 Heli.1-DT662382.3.5
GCACGAGGGCTGCGCAAGTTTTCATGCGCAATGATAACTTCCTCCAACCTCCTGGTCGATTGCGGTGTTCCAT
TTAGTTTTTCAGCTGATCACGCTTTCCACGGGTGCCTTGCAGACGCGTCGGCGTGTGTCATGTGCGGATCTGT
CGCAGCGTAGTAAGACGAGTGCCTGTGTACCGTGTAGTTGCTAGCTAGTGGCTGTGAA
AATGTTTTCAGGACTTACTAACCAGGTCAGTTCTTGGATGGGAGCGGCTAAGGGCGAGCCCCAGGATGAAGAA
GTGCCCACCCCCACAAAGGAAGCGACGGGTGAAGCTACGGCCGAGGGCGAAAAACAAAGTCCAACAAAAGGTG
GCAGCAAGCTTGATCTTATAACAAATGTCAAGTCACAGATGACTGGATGGCTTGGATC
AGGAATCCCAATTCCTGGACTTAGAAAAAATGAGGCAGCTCCATCTGAAGCAACTGAACCAGTTCCAGAACCT
GCTGAAATTGCAGAACCCAAGCCCGAAACTAAAGATGATGATGATAACTCTAGTGCCACCGGTGGAGCGGATA
GTAGGCCCGCGTCGACCGGCGGCACTCCCACTGAGGATCAACCGGCTGGGGTAGGAAA
TGGTCAACAGAATAAAAAAACGTTCACAGTAGATGCCATCGAAGTGCCAGATATGCAAGAGTTGACCACGAAA
GCGGTGGCCGGCGCCAAGTCGCTCGGCAACTTCCTCTACTCGGCGGTGAACAAGGCCGGCGCTAAAGTGAGCG
AGGCCAGCGCCAAGATAAAGAAGACCGTTGAAGAGAACAGCATCCTCGGCGAGTTCAA
CCGTGAGCAGGACGCGTTCATCAAGGGCCAGGACAAGGGCAACGGCGCCGCCGCCCCGCCCTGGATGGGGGCG
CCCAACGAGGCGGCGCTCAAGGAGGAGTGCCTGTCGTTGTCCACTGACCGAAGGAACTTCGTGCGCGCCCCTC
CCGCCGGCGTGGACTTCGACTTCGACTACAACAAGATGTACCCGGTGGCGGTCGCCAT
>1002050290_1 Heli.1-EL599903.1.5
GCACGAGGGTATATTGCATAGCATTGCGTCGTGGTACGCTTCAATTATTTAATATTACAAGTTAATGTAGTGT
TTATGAATAAATAACTGGTTCAATAACATATATAACTATGAGTCGGTTTTTTGCAACTGGTACGGACTCTGAA
TCCGAAAGTTCATCTGAAGAGGAGCAAGTAGTACGGGCACCAGCGCCCGTCTATACGT
TTAGTGATGATGAAGAAGAAACTAAACGTGTTGTGCGTTCTATGAAAGAGAAGCGATATGAAGAGTTAGAAGC
AATAATCCATTCTCTTCGTAACCACCGTAAGATCAAAGATTTCTCATCGGCGTTAGCGTCTTTTGAAGAACTC
CAGCGAGCTTATACTAGAGCGGCTCCTGTAGTTCAAAAAGAGGAAAATGGAATTGCCC
CAAGGTTCTTTATCAGAGCCTTAACTGAATTAGATGATTGGGTATCCGGTGCATGGAATGATCGTGATAGTCG
GAAAGCTCTGTCAAAGGGTAATAGTAAAGCCCTAACATCTTTAAGGCAAAAATTAAGGAAATATGCTAAAGAT
TTTGATGCTGAAGTTTCCAAATTCAGAGAAAATCCTGATTTGCCTGATGAAGATGATG
AACGCAAGGACACTTCATCCTCCGACGAATCTGACGACGAGGACAAAGTGAAGGAGAAACCTAGAATCGCTCA
TTCACCTGAACCGCCACGCAGGCCCATACCGCAAGATGACGAGTCATCAGATTCTATGGATTGGGGCTCTAGT
TCTTCGGACTCAAGTTCCAGCTCCGACGACGAAAACCGTGGTGCTGCAACACTAAGGG
AGAAGTTCTTGAAACGTACCACCGAAAGAGATGAAGAAGAAGAAAGGGACAGACGTCGTGTTAGACGCGATAG
ACGCGAACGGGGCGGCAAAATTAGTAAGAAAGATCAAGCGGACGATGGTGGCGAATGGGAAACAGTACGTAAA
GGAGCCGCCACCTCGGATAAACCTAAGATGTTTGCTAAGGATAGTGACATTGATGCAG
CGTCAGTTGAAAAAAACTGGGTGAGATAAGTGCTGCGCGGGGCCGCAAAAAACTGACCGC
>1002050291_1 Heli.1-ES585703.1.5
GTCTTGGAGTGCGCTAGGTCGAACTTTCGGCACAAATTTATTTAAAAACAACATCACAGCCCCATACCTCGTA
AAAAATGTAAATTTCTCTGTAGGCAAAGTCCTTAATGGAGATCATGGACACAGAGTTATGGCAATGCAGCCAT
CAAGATGGCAATGGCACAAATTCAAGGATATGTTCCATTTCTATCTAATGGTTGGATT
AATTCCTGTTGGTGCTATTATTTTTTACACAAATGTGTTTATTGGACCTGCTCAGTTGACACCAATTCCAGAA
GGTTACGAGCCCAAGCACTGGGAATATCATCGTCACCCAATAACAAGATTTATTGCTAGATACGTTCATAACA
ACCCACAGCAGGATTATGAGAAATTTATGCATTACATTGATGAAGAGGCCCAAAAGGG

AAAGCTGAGGGCTTTAGAAAAGGAAGTACAAAAGAAAATGGCTGAACGGCTCGACTACCAAGCATATTACTAC
AGGCCAATGGTTAACAAATACCTCAGAATCAACAAGAAAGTTGGTGATGACATGTTTGACCGGCACGGTGATA
ACTTTGAGGAATAAACAAGTTTTTATCTAAATTTAGTTATTTAGAAATCAAGTGTATA
GTAATCCACATTATGTAGAACAAGTAAACAATATTCTTTTGAATTTAGATTGTTACTTTTATCCTGCCTTATA
ATTTTTGTTGGAATAAACAATTT
>1002050292_1 Heli.1-DT666032.3.5
GCACGAGGTTTTTTTTTTTAAGCCGACACGATATCGCTGTCGACTGTTCATTGCGTAATAATTCTAATTATAT
TACATGAAAGTTGAACATTTTCATTGAGGATTTCGCCGGCTTTTGTATATCCTAAAAGAACCGCAACGACATC
CACAGAAACAGCACGTCAAAATGTTCGCCACGCGCAGACTTGGGAATTTAATCGTAGG
GGCATCTCGTCAAAAACATTCCCTTCCTGATCTGCCTTATGAGTACAGTGCTTTGGAGCCTGTTATCAGTCGA
GAGATTATGAGCTTGCATCACAGCAAGCATCATGCTACTTATATAAACAACTTGAATGCTGCTGAAGAGAAGT
TAGCACAAGCTCAAGCCAAAGGTGACATACAGACAGTTATCAACTTGGCCCCAGCTCT
TAAGTTCAATGGAGGAGGTCACATTAACCATACAATCTTTTGGCAGAACCTATCCCCCAAAGGTGGCAAACCA
TCTGATGTCCTCTCCAAAGCTATTGAGAAAGATTTTGGTTCATTAGAAAACATGAAAAATCAACTGGCTGCTG
CATCAGTAGGTGTACAAGGATCAGGCTGGGGCTGGCTCGGTTATAATAAGTCAATGAA
GAAACTACAGATTGCTACATGTCAAAACCAAGATCCTCTTGAGGCAACTACTGGTATTGTTCCTCTCTTCGGA
ATAGATGTGTGGGAACATGCTTACTATTTGCAATACAAAAATGTTCGGGCTGACTATGTGAAGGCAATATTTG
ATGTTGCCAACTGGGCAGATATTTCTGCGAGATATGACAAGGCTTTAAATTAAAACTT
GTAAACCATTTGAAAATCTCTTTGTAGCGTTGGCTTGGAAAGCAATGATCAACAATTATGTTTTAGGCATTAG
ACTGTACTTTGTGATGCACCCATAAGTTTTAAATCTTAATATAATGT
>1002050294_1 Heli.1-ES586299.1.5
GCAGTACGGTCGGAATTCCGGGTCGACCACGCGTCCGATCGCGGCGACTGACCGTTGTGCTACAGTATACAAT
ATTTATTAATAAATATTAAATAACAAATTTGTTTTATAACTTTGAAATTTTTAACGCAAGTGAAAATAAATAG
AATTTGAAATGCCAATTGAAACTTTTGTATTCTTCGATCTGGAAGCCACAGGCCTACC
TGATGAAGAAAGGAACCAGACCAAGATAACAGAGTTGACTTGCTTGGCAGTGTCACGCAAAGATATCATCGCG
GCCAAGGTTGGGGAAATACCGCCGATGCGCAAACTTACTTTTCTCGTCAACCCGCAGAGAAAGCTCCGACCGA
ATGCACAGTTGATGACCGGCCTCACGGAGGAAATTTTAAGCACACATCCCGTTTTCAA
AGACAAAATTGATTGTATCAACGAATTCCTGGATTTGCCCAAGCCAGTATGTCTTGTTGCACATTGTGGATGT
AAGTTCGACTTTCCGCTGTTGAAAACTGAATATAAAGAAGCTGGAGTATCATTACCTGACGATTTGTATTATA
TTGACTCAATGGGAGGTTTTAAATACGCATTAAGACAGATTAGTCTCTATGCAAACGG
TTTGTCTTTGAAAAATTTATATAAAGTGTTAGTGAAAAAGGAGATGAAAGGTATACACCGAGCTGAAGCCGAC
TGTCTAATGGTGTTGGAATGTGTGATTTGCACTAAAACACATTTTTTTAAATTTTCTGA
>1002050295_1 Heli.1-DT664859.3.5
GCACGAGGCAGATACTTCTAATCACTTATTTTGGAAGCATGGCACTGAAAGTGTTTTGTGTAAAAAGTGTCTA
CAGAAAAAGTGGCCTCATGTTTATCATTTCTGTACACCACCAGCCGTGTTTGTTTGCGATGAATGCACTTTGC
AATTTACGAGAGCAGTTTGTTTAAAAGTTCATAAACGATTTCACTCTGAAGAGTTTCC
ACATACGTGTAGTGAAGAAGGGTGTACAGAAAAAATTTGTATCTAAAAAACTCCTTAATAAGCATTTAGAAGTA
CATAACAAAAAATTAATTTCAGAAAAATTAGATGAAGAAAAACCTGTTTTAAATGTATCTATTTCAGAAAGTT
CTAAGGAAATTCCTATTATTGATCTAGTTGACGATAAGACAAAAGAGGGAGCTGGAAC
TCCCGAAAATAAAACTGAGGCTGAAAGTGAACAAAAATCTAAAAGAAGTAAAAAGAAAAAGCTGAAAGACAAA
GATGCTTTATTATTAGATGTTAATCTGCCGGCATTAAATTTCTTTGAGAGCGACAGTAGTGATGAGTCTGATA
GTGGTATTCCGCCGATAAATAAGGAAGAAACAACTCAGGACAAGCCTGATAATGTTCA
AGAAACTAATCCTACATCTAATGTTGAAAAAGATGTGAAAACAGTAGACTCAGAAATGCCACCAATTAACGAA
GAAGAAAAAACAGATTTGAAAGAAGACGATAAACCTAGTGACCAAAAAATTCTAGAAATATGGGATAATTTTA
ATAAATATCAAGCTAAAGTTGAAAAACAAAAAGAAAAGACGCCTCCTTTGGTACCTAT
ACGAAAACATGTATGCTAGTCAGACCACGATTACTGCGTTATACCTGCTGAAATTAACGGAGACGATGATGTA
TTTGGTTCTGATTCGAAAAAAAGTAAAAAATCTCCAAAGAAAAGGCACGGTAATCTATCAGTAAGTTCAAGCA
GTAGTAGTAGTGACAGCGACTCAAGCTGCTCCTGCGGATCTAACTGTAGTTGTTCATC
TTCAAGTGGATCTTCTTCATCAAGTTCATCCGATTCTGATTCTTCAGAAGAATCAGGCAATGAAAAGGATAAG
AAAAAGAGCAAACAATTTAAAAAAACCCTTCCTAATAGGAGATTAAGTAATGGTTCAAATGTTGATGTAATGG
GTATGTCGGAGACCCCTATTTTAGTACCAGAACAAAGTGAACCAACAATTGCTGAAAG
TGACTTAGAAACTGAAGAAAGCGAAACTGATGAAGAGTTTTATGATGAACATCCTCAACAAATTGCAAATAAA
ATGCATAATGA
>1002050296_1 Heli.1-ES585979.1.5

CCTAAACAGAGCGGGCGGCGCCAACTACGGCACCGGCGTCAGGGACTTATGAATTACCTAACACAAATACATG
CTTGCGTTTTAACCTAGCAATACTATTGTCTATATCTAGAGAAAAGACAATAATGTTCTACATATATTATTGT
AATAATCAACCAAAGCTTAATAGTAAATGAATGGAGAAAACAACATCTTTTCAGTATT
GCTTCGTTAAAATATTTGTGGTATAAGGTATTTTAAATTCAAGTCAAAAGTGGCATATTCAGAAAACTTTTTG
TAGAATAAAAGTATTTGTAACGGATTTAAAGAAATTTTTGTAATGTTTTTTATATTTAAAATATAAATTAGAA
TTTCGATTCGTATTTTGTAATGATATATTGTGTGACAATATATTTTAGTGATGTGCTC
TTGGCATTATACATGTGGACTTTTGCCTTTATTATTGGAACGCTGCATCTTGCCCTTTTAGTATTTTTAAGAA
GTCATTTCAATTTATTTTCTTATCTTTTTTTTTAATGTTTTTCGTGTTAAAAAATTGTATTTATTTTTTGATG
TCAAAGATTTATTTATTTTAGGATTAATTTGTGGTCTCTTTGGGGATTTTCTAAAATA
TTCGTCGTGGCAGGGTGCAATGATCGCGTGACGTATTATTGCCATATATAGGCGCAAAGGTGCATTTATTTTT
GTCTACATTTGGATTTTTATTGCTTTTATATACATTACTCTAGTATTATATCACCCATACATTATTGACTGGT
AATACAATAA
>1002050297_1 Heli.1-EL597631.1.5
GCACGAGGAGCGGTTGTCTCGTGTGGTGCAGTGCATTTATTTTATAGTGTCGATTTTGATTGTGTTTCCTTTG
ATTTTATAGTTCTAGTTCATATTATTTTGTGTTGAAACTGGTGTGTGACATTTCGCTATCGTCGGATGCAATT
TTTGAATAATATGCTGGCGAATACAAAATTATGAATTGATGCAATTCAGTCATTCCTC
ATGTGTGAAAACAGCTTAAGGAGACAAATATATCGATGATCGAGCGTCGTAATCGATACCAAAAGACAATCGA
GGTACCTCAGAACTAGGTACCTCAAAAACCGATGTCTGAAACTGCTATCAAGACAGAGACGCAAGATAACGAT
GGGGTAGTGAAAACGGAGAAAGAGGTCAAGGAGAAAGACGGAAAACAAAGATCTGTAT
CATTTAATAGGGACGTACACGTCAAACGTTTCGGTAAACCGCGTGAGCGGGGTACCTCTGTTCAGCCGCCGAT
AATACGAAAAGAACCTTTTACTCATTTATCAGAACAAGAACTTATCGAAGAAGCGAACAAAGTAATCGCTCAA
GCTCAGAGTGTAACCTGTACTGCAGATCATCCTCCTGAAAAATTCTTTTCGCTTCCAC
ACAGAAGAAAATTAAAAGACGACAGATCTGGCAGAAGAAATAGTGACGACGGAAGCACTCCAGAAGAAAACGA
AAAAACTCCTTTAGGAAGATCTACTAGTGATGTAAACCCGAAAAGAAGAAAGGAAAGAACCTCTTTGTCAACT
CTCTTTAGACGTGCTCAAAAAAGTATAAGTCCTGATGCACCAGTTGTAATTTCTACAA
AGCCAACCGTTGTTGTAAAACGAAGTAAGAGTGACGTATCAGATTTAAAATCTAATACCGATCTTACCAAAAG
AAAACCTATTCGAAAAAGATCTGGGAGTGAAACAGAAGAATTTCTTAGATCATTGCGAAACAAAAAATCCCAA
TTGTCTCCTATTATTGAGAGTTCTCCAAGAGAAGATTGTGTTGCAAAAACTACTCCGT
TGGACTTGCTTCAACATAATAAATCTTCTGAAAAGCAATTTAAGCAATCCGATGTCCATTATCACTAGAAGTA
AATAGTTCATCATAGAAGAACTCCAAGTATACACCGATAGTACGAAAACCAGTACCAAA
>1002050298_1 Heli.1-ES585113.1.5
GGGACGCGGAATCGGCCGAGGGAAAACTTTGATCTCATATTACATCTCGAAAAAAAGTAATATTGTATTTTAA
CAATTGTAATATGACTAAAATATATCAAAATGAGTTTTGGACTACCTTCATTAAAAGTAAAACCAGAGCATGC
CGGAAATATCCCAGTACAAGAAGGCCCTTTCGGGATACCTAGCCCAATGGTTGCAGGA
CTTGCGGCCACAAAAAACAAATTAGGCATGTCTCATCCTCTTCAGGCTTCTGAACAGAATTACCATCTTAATG
AAGAAAAAAATGAATATGGCTATGCTACGAAACATCCAAGGACTCCATGCGCCTTTAAAACTTTCAATGGAGCG
AAAGTTTGCTAGCAAGGTTGGCCATCTTCCTTTCCTCACCAGCTCAAATTTGCAACAT
GATGTATTTACTGGGAGATATCTTGATATTGGCTTCGAAGATATTCTCAATACTCCTGAATTCATTGAAGTCA
ACGGGCAACCCCATGCAGTTGTTGAAAAATCTTTGGGTGTACTTTAATGCACTTATAAATTAAATGTATAATT
ATTTTGTAATAAATATCTCAGTAAGAATTAAACATTTTAAAAACAA
>1002050299_1 Heli.1-EL604087.1.5
GCACGAGGCTTCGGTAGAGTAGTACAGTGCAATGTCAATTTTCTAAAAGTAAACTGTGTGGTGTGCGACAATG
TCATCTTTAAAATTTTGTATATTAAGCATCATCTTACTGTCATCGTGTATATCAGCCCGAAACACAATCTTGG
AAGGTATCGCCGGCTTCCTTAGAGACACAGCGGAACTTTACAATGGAGAACCACAAGA
TGCTGAAAGAATATTTGAAGAGTATGATTTTGTTATCGTCGGAGCAGGCACTGCAGGATGCGTCCTTAGCAAC
AGACTGACTGAAATCAACAAATTCAAGGTGTTACTTATAGAAGCTGGTGACTCCGAACAAGTGTTCATGGATA
TACCAATTCTGGCTACTCTCCTACAATTCACAAATGCCAATTGGAATTACCACACAGA
ACCGCAAAAGGCTGGCTGTAAAGGCATGCGGGCTGGACGTTGTTCGTGGCCAAGGGGGAAAGTAGTAGGGGGA
TCTTCCGTATTACATTCCATGATGCACACGCGAGGAAACAGGAAGGATTACGACAGATGGGCCGCAAATGGAA
ATCCCGATTGGGATTACAGCAATGCTTTGAAATATTTCAAGAAATCTGAAAACATTCA
GATACCTGAGCTAGCAAAAGATACCAAATATCATACAACGACGGGACCTATGACCATTCAATATACAAATTGG
CGTACGCCATTAGCCGATGCATTTTTACAGGCTGG
>1002050300_1 Heli.1-DT665205.3.5

TGGGAAATGAAGTGGATTCTAACACAACCCTTTTAGATTTCATTAGAAGCAAATTAAACCTTCGCGGTACAAA
GTACATGTGTTTGGAGGGAGGTTGTGGAGCATGCATCGTTAGTGCATCTACTTCTCTGGGGCAACCTCCGCAT
AGTGTTAACTCGTGCATGGTGTCCATAACATCATGTCAAGATTGGGATATAACAACCG
TTGAGAAGATTGGCAATCGTTTAGACGGCTACCATCCCATACAGACGACGCTCGCTGAAAACAATGGCAGTCA
ATGTGGATACTGTAGTCCGGGATGGGTCATGGCCATGTACAGCCTTATGAAAACAAAGCGTATGACTATGCTT
GAAATAGAACAGTCGTTTGGTAGCAACGTATGCAGGTGTACAGGATATAGACCAATAT
TAGACGCTTTCAAAAAATTCGCATCAGATTCACCAAATCCCGTTGAAATTCCTGACATTGAAGATTTGAAAAT
ATGTGATAAAACCGGTGAAGCATGTTCAAAAACAAAATGTGAAGAAGCCGATTGGTGTTTTGTAAGCAAGGAC
GAAGTTTTTAATGAAGTCAAATGTTTGTACTTAAAAGATAATAAAATTTGGTATAGAG
TTGAGACACTATCTGATATTTTTGTTATATGGGCAGAGAGAGGTGTCCAGTCTTATACTTTGGTGGCTGGTAA
CACTGGAAAAGGTGTTTATCCAATTATCGAATATCCAAGGATCCTTATAGATATTTCAGGACTAACAGAATTG
AAAGGCTTCTACATCGACCAGAATTTGGTACTTGGTGCAGGAACGACTCTTACAGATA
CTATGAACATTTTCGAAAAAGTTTCAAACTATGATTACTTTAGTTATCTAAAAACATTTAATGAACACTTAAG
ATTAGTAGCGCATATTCCAATTAAAAATATAGCGACGTTGGCTGGAAATTTAATGCTCANAAATCGAAATCCT
GAATTCAAATCTGATATATTCTTACTTTTAGAAACAGTTGGAGCTCAACTTGTTATAT
TAATAGCTCCAG
>1002050301_1 Heli.1-DT667640.3.5
GCACGAGGCTTACACTATTATTCAATTTAGTCAGCGGTAGTCAGGTGTCTATTTGAAAAAAGTGCTTTTTTAA
TTAATAAGTTTATATATTTTATAAAGATGTCTTGCGACCAAAACGTAGAAAAGTTTAAACAAATAGGTTTAAG
TGAGCAAAAAGCAAAGGAAACTTTAAAAAATGCCAACGTTACCAAATTTTTGCTTGCC
GCTATACAGGAGGTTAATCCTTCAAGTTTACCAAGCGGCGCTGGTTTGTTGGTATACCATCTGGCTACTAAAA
TAAAGCCTCAAATTTCTGATAAAATACCCTTTCTTTGTAAATATATCGCTGAAGGCAAACTAGATTCCTCACT
GAGGATTGATGCTGCCTTAGAGTATCTTTTAAGTTGTGTTAATGAAACAAACTTAAAT
GTTAAAGAATTTGAAAGCACCTGTGGTGTTGGAGTAGTTGTTACTCCTGAGCAAGTGGAACAAGCTGTAGAAA
AGCATATGGCTAAATTTAAGCAAGAATTATTAGAGAAAAGATATCGCTTTAATTCAGGTATTATTATGCAGGC
AGTTAGATCAGATTTAAAGTGGGCAGATGGAAAAGCAATTAAGAATGAAGTTGATGTT
CAGATTTTAGATTTATTAGGTGCAAAAACAGAGGCTGACTTAGCACCACCCCCTAAAGCTGAAAAAAAAGTTA
AAAATGTTGAGGTTAATAAGAAAATTAAAAAAGGAGAAAGCCCAGGTAATAATAAATGTGAAACTGTTGAGTC
AAGTGAAGAAGTCAGTGGACCTTTATCTATGCCAGAATTAATGAGAAAGCTACCTTTT
>1002050302_1 Heli.1-ES587846.1.5
CTCGCTGCCGCGGTCCGCGCCTGCGCCCAACTACTTCTACTTACGTCTACGAAAAATATTCGCAATGTATACT
AAATCATTAATTTTTGTTGGTGTTCTGTGCGTCGCTTTGATTGACGCACGTCCTAAATGGCAGACTCTACCAG
AAGTTCCCGGTTACGTACCAGTTTACATTCGAAACGGCGACACGCCGCTATATGAAAT
CAATCCGGATTTAGCAGAAGCTTTCCACGCTTTGCCTATAGGCCGAAGCGTTAATAAAGAAGTGGAAGCCTCA
TCAGATATAGAGCTAGAACCGCCACAGAGTGTTCCAGAGGTCATACCCATTGACGATCGTCGCATCTATGAAA
AGAAACTTTTGGACAAAAAGAAGAAGGCTAGCTACGATATCCTTAAACCTGTTGAACG
TAGATAAATTGTGTACTTATCTATTAAAAATACTTATTCAATTTTAGCGATAGGTTGTTATTTAGATTGACTA
CACTTAATTTTTAAATATCGTGTGGTCTAAGCCATTTAGTATTTAATGATAAAAAGTGAGATTTTGGGTGGAA
CACCCGTATGTTTCAGTTCGATGAACTCTACTTGCCACTTAGCATACTAAGTATTTGT
AAGAAATGAAATATAACAATTTACATATTATATTCTAATTTTTGTACTCATTATGTTATATAAATATAAATT
>1002050303_1 Heli.1-EL597756.1.5
GCACGAGGCCCGCGTTTACTTGCCATACCGATACCTAGTGCCCGGACGTTTTGGTTTCAATAACACAAAAACA
ACCTCAAACTTACACATTTATTCGTTTAAAACAACACCTGAATTATATTTGTAAATAGATCAACCAAAAAGAT
ATCGACTTAAGAATATATTTCAAGTTAATAGTGTGAATGCCACGGTCTACAGCCTACG
CAGCCGGCGGCGTTCCCCTCCTCTGTTCATTCAAGGACCAGGCGTTGAACTCATGTTGTCACAGATATATCAA
CGCAAACTTCACGATTTGGAAATGTACATAGAAACGGTACAAAGACCGGTATGAAGCCTTGATAGAGCTGATT
GACCATTAGCGATTCGGTGTCAACGACAATGTCAATCAGACAAAATACGGGCGAATTT
ACTAAACACAAACAACAAGTAGGTAAACAGGGCCCGGAACCGGTCAACTTTCCACCGCGTTATCATCCGCCGC
CACCTGCCGCAGCGACTTCTAAACTGGGCACTGTGGATTCAACCTCTAAAGAGTTCAAACCTCCTAACGCTGT
AAAACATATTGACCCTAATAAAATTCCCTACTCTCTGGGGGCGCAAGGATCTATGCCA
CTACTCTATCCCGGTGCACCATATCCACAAGTAAAATACCTACAGGGTTATCCCCCGGGCTACCCTGTGCCTC
CAGCTGCGCGTCGAGTACTCAGACCTGCAGGTGAA
>1002050304_1 Heli.1-DT666585.3.5

GCACGAGGTGCCGCGATCGGATGAAAAATTTATAAATAACAAAATTTTTCTCGAAAATCATTAATCATTTAGA
TTAATTTTGTGTTAAATATTTTTATATAAAGGAGTTTAGTTTATATTTGTGTTGTGTCTCGCAAAATGAGTGT
ATATATTTCAGAAGTACAGGAAAAGGAAAGTTGCTGTTTTCAGAATTCAAAATGGCCA
ACGATCCCTTGAAGTTTTTAGCTATTTTTCCTAGCTTACTAAAAATGAGAAGAAAAAATTGTAAGCCCCCTTT
ACTATTTAAGAGTTCAAAAGTAAAGTACAAACTTCTGATACTTAGAAAATTAAAATAGTAGTCTAATTGAGAC
TGGTAAGTTATATTTCAGCATATAAGCTATTACATAGCTACTGAATTTTATTAGAGAT
TTTCTATATGATATTTTGACAAAATATTATACATTAAAAATGTCAACACTCAAGGTTATTTCGAAAATGTCTT
TTAAAACATTCTATTTAACATGGGCAAGGTTAAGCCTTACACTTATTAAGTGACTTTACTGTACATTATATAC
AGACATAGATCTAAAATGTTTATGATAACTATAAAAAAGTAATAGTAAAGCTTATTAT
TCTATATATAATTACTTGCATTACAGATTGTAATAATAACATGTACCTATGTAAATACAGATGGTAATAATAA
CATGTACCTATGTAAATAATAGTTTGAAGCATAGCTGCAATTACATTGTTTTAAAATTGTAACTTTTATTGAA
AAGAGCTGTTTTTTAGTTCTTAGGCAACAGAACTGCTCCTCGGGCCGGTGGTGGCTCT
ATATTCATTTGACAATAACTGTCTATTTAATACTGAATTGCAGTATAATTTGAATTTAACTTTATTTGTAACA
ACCACAACAACATTTTGTGTTAA
>1002050305_1 Heli.1-EL601849.1.5
GCACGAGGGTTCCGTCCCCAGATTTCCCTCCAAGTGGAACCGGAGGTTATGGACAAGAACCGGATTATGTTGA
AGAAGATCAGGTCCTTGGACGAGATACGTGCCGCTATGGAGGCAAGATATATATGTCAGCGCAACAAATCCCA
AGAGATGACCCTTGTGACTTCTGTTTCTGCTTTAGGAGCGACATTATTTGTTTACAAC
AAAGTTGCCCGCCACCAATTCATGGCTGTCACGAAGAACCAATTCAAGGTTTCTGTTGCCCCCGCTATGAATG
TCCTGTATCCATGGCAACTACTCTTAATATGACCACTACTACTACGACAACGACAACAACATTACCTCCACAT
TTCCTCCCTCATGCTTATAAGGGTGCAGCCCAACGAAGAGGATGTCAAATAAAAGGTC
ACACCTATAAAGTTGGAGAAGTAGTTCGAGCGTCTTCAGGACCATGTTTGCATTGCACATGTGGCGGTGATGG
TCAAATGAAATGTGATCCTAAAGCCTGCACTCCGGAACCTATGTTGAGACAAATGATTGCAGCAGCTGTTTCT
GCGAAAAGACGGAGGTGAAACACCTCCGAGGTGTCCTTGACAGCATGAAGTACATCAT
CACGAATGTTAACATCTAAGTGATAACCAATTTCATGTATTTTCTATGACCCTACGTACGACGGGACTTTGCA
ATTCTCTTTTAATAATATGGATACTCGCATCTCTCCTATATATATTTTTTTATTTATAAGAAAAATATAACAT
TAAGTAAGAACTTCCCTTACATTTAATCATTTTTTATATCTGCGCAAGTAGATATGTA
ACATACTATTTACCACTTTCACTTAATATACTATTTCCAGCATTGGTAATACAATTAACA
>1002050306_1 Heli.1-EL600691.1.5
GCACGAGGGTGGAACCCACAAATTGGTTATTTATATGATAAATAGCTAGAAAACAATACAAAATGAGTTTGTA
CAATTTCAAAAAGATAGCCGTCGTCCCTACGGCGAAGGACTTCATTGATATTATTTTATCAAAAACACAAAGG
AAAACTCCAACAGTTGTCCACAAACATTATAAAATAACAAGGATACGTGCCTTTTACA
TAAGAAAAGTGAAATTTACTCAACAGAATTTTCACGATAGGTTATCACGGATTATACAGGAATTTCCAAAATT
AGATGATGTCCATCCCTTCTATGCTGATCTTATGAATGTTCTGTATGACAAAGACCACTACAAGCTTGGTCTA
GGTCAACTAAACACTGCCAGACACCTCATTGATAATGTAGCCAAGGACTATGTACGTC
TTCTTAAGTTTGGTGATTCATTATATCGATGCAAGCAGTTAAAAAGAGCGGCGCTCGGGCGAATGGCCACCAT
CATGAAGAGACAGGGAGCCAACCTAACTTATCTTGAACAGGTTCGACAACATTTAGCCCGTTTGCCGTCAATA
GACCCCTATACTAGGACTATCATCATATGCGGTTTCCCCAATGTGGGCAAGTCCAGCT
TTATAAATAAGATTACACGCGCAGATGTAGAAGTACAGCCGTACGCGTTCACTACAAAGAGTTTGTACGTCGG
ACACACAGATTATAAATACTTGAGATGGCAAGTAATCGACACACCCG
>1002050307_1 Heli.1-DT665615.3.5
TCAGACTACTCTTCACCAGGCCCCTAGAGGATGGTGTTTTGATAAACACATCCAAATGTGTCTTCGGGCAGCG
AAGGTAAAATTTCTTGGTTACCTAGTGTCAGGTTCTGGGACTCAACCTTTAGAAACGAACGTAAAGAGCATAC
AGGAGTGCAGCGTGCCTAAAACTGCTAAGGAATTAAGACGTTTCCTTGGTATGTTTAA
CTTTTATCGTCGTTTTATTCCCGGCGCCGCAAACATTCAAGCACCATTAGACTCTGCGCTTTTAGGACCCAAG
ATGAAAGGAGCTAGGCCGATCACAATGACACCTGAGATGATGGAAGCTTTTTTTTTTTGTAAGACTTGTTTAT
CTCAAGCCACGTTATTAGCTCATCCAGATTCATCTGCAGAGCTAGCTATTGCCACTGA
TGCATCTGATGTCGGTATTGGAGCAGTACTTCAGCAACATGTTAACAACATATGGCAACCTCTTGGTTTCTTT
TCTAAGAAATTAACATCTGCACAAAAAAAATATAGTCCTTACGACCGAGAGCTTTTAGCTATTTATGAAGCCA
TTAAATATTTTCGACACATGGTTGAGGCTAGAAACTTCGTGATTCACACTGATCATAA
ACCTCTCACCTATGCATTCAACATTAATAGGGAGAAATGCTCACCGCGCCAATATCGGTACTTTGACTATATT
TCACAATTCACAACCAGCATTCAATACTTACCAGGACGTTTGAACGTAGTGGCTGATACACTGTCACGACTAG
AAGAGGTGAGTCGTTCTATTGATTACCAGGCTCTGGCACTTGCTCAAGATACTGATCC

TGAACTGCTAGGTCTGCTTCAAAAGGGCTCATCGTTAAAACTGGTTATGGTAGATTTGCCAGACTCTAACCAG
AAGATCTACTGTGACGTATCAATGTCTTCTCCACGCCCGTTCATTACTGAGAAATTTCGCCGACAAGTTTTTG
AAGCGTTACACAGCTTGAGCCATCCTGGTAGGACAGCTACAGTGCGGCTGGTAACACA
GAGATTCGTATGGCCAGGGATTCGCAAGGACTGTAGAGAGTGGACACGAGAGTGTATACTTTGTCAAAAGAGC
AAAGTTCACCGTCATACCCTTGCTCCCTTATCCTCAATCCCAACTCCTACGTCGAGGTTTGCACACATCCACC
TCGACATCGTCGGACCTTTAACCATTTCGTCTGATTTCAGGTATTGCCTTACAGTCAT
CGATCGTTTCACACGATGGCCTGAGGCATACCCACTCAAAGACATCACAGCTGATTCCTGTGCTGCCGCCCTT
CTCTCTGGTTGGATAGCAAGATTTGGTTGCCCTCTTCGAATTACAACCGATAGGGGACGTCAATTCGAGTCGC
ATCTCTTCAAGGCACTCTCAGCCATGATTGGTGC
>1002050308_1 Heli.1-ES586362.1.5
ATTTGAAGTTCATGTAACTTAACTGAAATGTGAAGATTTTGGTTTTGACCGGTTTTGTGTAATTCATAATTTT
TGTCAGAAAAAGTTTTTTTTAAAAATATGTATGTTAATAAAAGATGTTATTTCCATCTTATACATATTTTGAC
ACATTTGTTGAATAGTAACTGAGTGTGATAAATATTCTGTGCAAAAATATTTAGGGAA
GTTTTATAAAATGGTGTCATAGTCTAAAATTTACATCTGTTCTATGTAAATGGTATTAAGCCTTTACGAGGCT
TTAGAAGTTGTATATGAAAATTTTATCTTTTTTACAGAACAAAATTTCAAATGTCTAATACAATTGTTAAAGG
CTATGAAACCGAGTGCTTTATTCATTATAAATACTCTTCCCTGTAAGTAAAAATATGT
TATTTTATGTAAATGTTAAAAACTGAAGCAAAATTAAATTATTCAAGGAAAATAAAATATAAGGGAGTTGCCT
TTAAATTGTACCTAATTGGCCTATCAGAAAGAAATGTTCAATTTATTAATTAAAATTTATTTTGGTCAATTGC
TCCCAGAATGAATACAAATTACAACTGATGTTAAACAAGGACATAATGAAGTTTAAAA
TTTAAAAACATCGAGTAGAAGCAAATTAAATGTTTC
>1002050309_1 Heli.1-EL604185.1.5
GATGTTAGATTAACAAGAGCTTTACACTTAGCGCCTGCACCTACAGATAAAATTGCTACTCTTAAAGACTTAG
ATGGCGTCGAAGAAGATTTACGAGAATGTGCTGCATTAGTCGATGAGGCTGATCGCCTCTCAGTATCTGTAAG
CAGTATACCCGGAGTCTCGGACATGGTGGGCGAGTACCGCGCCTTGTTCGCTGACGTT
AGAACTAGATTAGACATAGCAAAGGCTGAAGTGGTTGGTGATGTGGATACTGCTGTACAGGTTGACACCTTAA
AATGGGAGACCGATGCATCATTACAAGTAGACACTCTAACATCCAAAGAAACATACCGAAGTGAATTAGCGAC
CGCCGTTAAAGAAACATCAGATTCCATCGAGGCTCTCAGAACAGCACTACTACTAGAG
ATCAAAGAAGATGCCAAAAGCGATGAATTGGCAACTGCTGCCAAAGAAATAGCTAAGGCCGGAACAAAACCTG
AGCAAACCTTAGAACTAGCAAAACACTTATCAGAGTTGCTTTTAACAGAATGTGATGCGACAGAAGATGAAGC
TATGCTTAAAGAAGTAGAGTCCTTGTCTTTGAGATACGAAGACCTTCTGACACAAGCT
AAGAAACGTGAATTACAAATTAATAACCTGAGGTTGCCCCACTCCGCATCGTACGCTCTCACGTGCGAACATG
AATCTGGACGTCTAACATGTCCACTTTGCTCGGACCGTAACTGGAAACAACTTGATAATGATCTATGGCGACT
AGAACAATGGTTACAATTCGCTGAAGCAACAGACGAAGCGAGGATAGATCCTCCAGAA
CAGTATGATGCTCTGGAAGATGCTATACAGGACCATCGCGAGTTTCTTCTTGATCTAGAC
>1002050310_1 Heli.1-DT665160.3.5
GCACGAGGACCTACATCTGGAGCAGGCATGTGGGCGTCACAAATCCGTGTGGTGGACGTGAGCGCGGCGGGCG
GGCAGCCGGACACGGTGTTCAAGCTGCCGCTGGAGCAGAACGAGGCGGCCGTGTCGCTGTGCGCGCTGCGCTG
GGCGGCGCACGCCGAGCACGCCGTCGCGCACCTCGTCGTGGGCGTCGCTAAGGACGCG
CTGCTCTCGCCGCGCTCCTGCACCGAGGGCAGCTTACATGTGTATAAGATCTACAACACGGGCAAATTAGAAC
TAGTACACAAAACACCAGTTGATGAGTATCCAGGTGCTCTCGCAGCATTCAATGGGAAATTACTGGCCGGAGT
TGGTCGCATGTTACGACTATATGACATCGGTCGAAGAAAACTACTACGCAAATGTGAA
AACAGACACATTCCGAACCTCATTGCAGACATAAAAACTGTTAGACAAAGGATTTTCGTCTCAGACGTACAGG
AATCTATATTCTGCGTTAAATATAAGAAACGTGAGAATCAGTTGATCATATTTGCTGATGATACTAATCCAAG
ATGGATAACAAACAGTTGTGTACTAGATTACGATACGGTCGCCGTGTCTGATAAATTT
GGTAATATATCTATAATGAGGCTACCCCAATCTGTTACTGATGATGTTGATGAAGATCCGACAGGAAATAAAG
CACTCTGGGATAGAGGTTTATTAAATGGAGCATCACAAAAAGGTGAT
>1002050311_1 Heli.1-EL600973.1.5
GCACGAGGACCGACATTGACAGTGCTCGCCGGACCGCCACACCGAGCAGACGCGTCGCGGCCTCACTCCAAGT
GGCAAGTGCTTCACGCGACCAAATCTCATCAATTCCGGAAAATCGGCAATTTATTCCATCATTTGTGTATGCT
CTGCTTCATAAGACGACTGTGCACCATGAAACCACCGACCAACGCTGCGTTACTCCAT
GATGTGGATATACCGACGAAGAAGGCAACATTTGGTATGGGTTGTTTCTGGTCGAACGACTCCTTATTCGGTG
CTACGCCGGGCATCGTACGGACAAGAGTGGGCTACGCGGGTGGCACTACAAAAAATCCACACTACAGAAACTT
AGGCGACCATACAGAAGTAATCGAGGTGGACTACGACCCGAAGACCGTAACATATGAA

GACCTTCTTGACATGTTTTGGGCTAACCACGAATATGGACTCACTACAAAAATAAAGAGACAGTACCAATCAT
TGATTCTGTACCATGATGAAGAGCAACGTAAAACAGCCGAGACTTCGTACAAAACCATGCAAGTCCGATGCAA
TGAACAACTGAGGACAGAGATTGCACCCGCTGGCACATTTTACCCGGCTGAAGACTAT
CATCAGAAATACAGATTACAAGGACACAAGGATCTCTGCAGAAGTTTGGGCCTGGACGCTTCCAAGTTACAAA
CATCACATTTGGCGGCGCGGCTCAACGGCTACCTGGTGGGGAGTTGGTGGGAAAGCACAATTTGATCAAAA
>1002050312_1 Heli.1-DT664083.3.5
TCAATATTGATTACACCATAAAGTTCTACTTAGAAAATGGTGCAGATCCTGATAAGCTAGTGCTTGGTATACC
AACGTATGGAAGATCATACACACTCTTCAATCCTGATGCTGTAGAGATTGGTTCTCCTGCTGACGGTCCAGGT
GAACAGGGGGATGCTACTAGGGAGAAGGGATATTTGGCGTATTATGAGATCTGTGAAG
CTCTCAAACCTAAAAGTAGGAAAAGAGCCATTGCCTCAGAAGAGTATTCTGAAGAAGATTCAGAAGAAGAGGA
AGAAATTGAAGAAGAATGGACTATAATGCATCCGAATAAGAAAGCAATGGGTCCTGTTGCTTATAAGGGCAAC
CAGTGGGTCGGATATGATGATGTGGAAATCGTTAAGAAGAAAGCTGAATATGTTGCTG
AAAATGGTCTTGGAGGTATTATGTTCTGGTCTATCGACAATGATGATTTCCGAGGCACCTGCCACGGCAAGCC
CTACCCCCTCTTAGAGGCTGCTAAGGAATCTTATATTATGAAACTTGGATCATCCGAAATTACTGTTGAAAAG
GAATCAGAACCGATAGCTAAAGCAAAGTCTAGCAGCAGCCGCAGAAGGAACCGCCCGC
GGCTCAGCTCCACTACCACTACGACGACCACTACTACTACTGCTAAACCTAAGATCAAAAGTAACAAGCGCAA
GAGCAGTACAACTGTCAGCACAACTCCTGCATGGAATATCATCACACCGGAACCTCCCACCACTCCTGACCCA
GGATCAGATTTCAAGTGTAAAGACGAAGGTTTCTTCCCCCATCCTCGTGACTGCAAGA
AGTACTTTTGGTGCTTGGACTCCGGGCCATCAGATCTTGGTATTGTGGCACATCAGTTCACTTGTCCTTCTGG
TCTTTACTTCAACAAAGCGGCCGACTCCTGCGACTTCGCACGTAACG
>1002050313_1 Heli.1-ES584806.1.5
AACAATTAAAATATGTTTATTAGTATTTAGTACAGAAAATTAATTATAATAAATATATCTTCCGGCAGTTGCA
CGGCGATGCGCAGGCTCAAGGCGGCGCGCGCTTGCAACTGGTGGAAAATGCGTCTGAATGGATGAGCCCACAC
CATGGAGTTAATATAGTTCCTTAGAGAACTATCAGTTAGTGCCTTAAAGCTTTAGGGT
TGGAGGCGCCGTATGACCAGCCAGGAAGATCCCATCCGCCATTTCCACGCGCATAAGCGTTGGCATTAGCGTT
ATCATTAGCATCTCCTCCCCCAGAATAGGCATTTGCGTTGGCATTGGCATTAGCGTCTGCGCCTGAATACGCT
GGGTACGGCGCTACCGACCGGCGCAGCCTACGGAGCGGCACCGGGGCTGAAGCGTGAC
CTTCCGCATCAATCACATAGTACGCACGAGGCACGTAGTATTGGCCCTCTGCCAGTGCCACGGCTGCGATGGC
TGCGAGTACGAGCAAAATAGCACGCTGCATGTCGACGTTGTACCTCCGCCTCGTGCCGAATTCGGCACGAGGT
AATAATACGATGATGACGAGATATTTGTCGAGAAGGAACATTCCAAGGAAAACATACT
ATTATAGTATCAAAAGGTTTACGA
>1002050314_1 Heli.1-DT664090.3.5
GCACGAGGCAAGAATGGACGTGTGTTTGTATTTTAAATTGTTATAAATCAAAATTTACAAAAACAATGTAATA
TTATGAAAAGATAGTTATTATATTTTATGTTTTAGTGTTTTAGTTTGTTAAAATGAAAATTGTGGAGGCGAAA
ATTGTTGTTTTGGGCTCGCAAGGCGTGGGCAAAACGAGCCTGGTGGTGCGTTACATCG
GGAAAATGTTCTCCAAGCATATATCACCCACCATAGGCGCGTCTTTCTTCACCTGCAACATTAATTTAGATGA
CGCTAGGATTAAATTACAAGTATGGGACACTGCAGGCCAGGAGAGGTTCAGGTCAATGGCCCCCATGTACTAC
AGAAACGCTAACGCCGCCCTCCTAGTGTTCGACATCACTAGCGCTAACAGCTTTGTGT
CCATGAAGAGCTGGGTCAAAGAACTAGAGAACAACGTGCCAGAAGCGATGATCCTCTCGTTGGTGGGCAACAA
GAGTGACCTGGAAGAGCACCGCGCGGTCACGGCGCTGGAGGCGGCGCAGTACGCCGCGTCCATCGGCGCCTCC
TACTGCGAGACCTCCGCGCTGCACGACCAGGGTATTGACCAGGTATTTCTAAACACTG
CAACAGAACTGCTCAAAATGTCATCTTCTAACCTCATGTCCTCACTAAGATCCTACGACTCAAACGAAGATGA
CAAATTGCCAATAGGTATTCCTTCCGAAGAAAAAGCGACTCACACAGGGGAAATAGAGCTGCCGGCGCTTAAC
TCCAACGTGAAGCACGGAGAGTTACAGAGAAACATGTGTTGTTAGC
>1002050315_1 Heli.1-DT667686.3.5
GCACGAGGGAAAACTCTTTATTTAATTAGTTTTGCCAATTAAAATACATAAAGTAATTTTGATATAACTTTGT
AAATACATAGTGACATTTTTTTTACATATTAGTTATGTGCGTTTAATCAAACAAATTATTGAAAAGTGAAGTG
ACGGTGACGCTATACGCTATTGTGTCGGTGATGTAATTTAAGTGATTGGGTGGCTTCC
TAGTTGTAGATACTACAATGGAAGAATCCTCTTTTACTATTCCAGCTGACGACAATGGTGACACTGAGCTCGA
TCCTAGAATACAGGTCAGTGAGTGAAAAATTATGTATTGAGTAACAAATCGTTGCCTTCGTAACAGCAAAGAT
GAGTGTCAACTTTGTAGATTATTTCGCCAACGAGCATAATGGATACGATATTTTATAT
CAAAACATGAAATACGGACTGATAGCTAGCAAGGAGCTCTCAGAGTATCTCAGAGAGAGGTCGAACATTGAAG
AATCCAATTCGAAACTCCTAGCAAAGTTGGCTAAACAAGCAAATAGTAACTGCGCCCAAGGTACATTTGCGCC
TTTTTGGGGCGTACTAAAATGTTCAGCAGAGAAATTATCTAATTTACATCAGCATATG

TTTCAAAAAGTCAGCGAGCTTGTTAAAGATGTTGCTCGCTATGCTGAAGAGCTTCATAAGAAACATAAAGCAG
TTAAAGATTCTGAATCAAGTACACTTGAAGTAGTATTACTCATACAAAACACTAAACAAGCTTTACAGAAAGC
TAAAGATGTGTACACATCAAAATCAGCTGAACTAGAAAAACTTAGAAAAGAAAATGCT
TCAGCAAAAGACATAGAGAAAGCTGAAGTCAAAATA
>1002050316_1 Heli.1-EL600696.1.5
CGGCACGAGGATGCTTTGGGCGAGCATTTTCGCGTAAACTTAATTGATTTCTTCTAATTAAATTATTGTGAAT
CACTGTGTTGGCTATGAGAATGTGATATGAGCCATGCGAAAAAATATTGTGTTAATATATATGAAAGTGACTT
TAAAATAGGGGTGTAACTTGCAAAATAAAAATGGCCTCAGGAGATAGTGATCATATTG
AGGTGATGGATATTTCTGTGATGAAGAAAGAAGCAGCAGGACAGTCGGGCTCCGAAGATGATGATCGCGATCG
AGCGTCTTTGGCTGAAAAAAATATTCCCTATGACCCAGCGATCGGTCCCATGGGTGCTATCAGCAAAGTCCAC
AAGTCGGACAGGAAGATCGGTCACCGCAGAGTGGGCGAGGGTGGCGAGATCACATACA
AGAAGATCCAGTCGTCGCAGATCATGGGATCTATACAGCTTGGTATCCAACACGCGATTGGTGGTCTAGCATC
CAAGCCGGAGAGAGATCTTCTCATGCAGGACTTCATGACGGTGGAGACTACCAACTTTCCGTCCGAAGGCTCG
AACCACACGCCCGCGCATCACTACTCCGAGTTCAAGTTCAAAACGTACGCGCCCATAG
CCTTCAGGTACTTTAGAGACCTGTTTGGAATACAGCCGGATGATTTCCTTATGTCAATGTGCAGCGCCCCGTT
ACGCGAGTTAA
>1002050317_1 Heli.1-ES586665.1.5
TTGGAACGTACGCTGCGAGTTTTATTCTGGAAAGACAATCCGCTGGAACCTTCAACCAATTTTACGAAGCTGG
ACGTCGTGGACGTTCAGGAGCAGACTGCCGCCAAATCTATCTTGAATGTAACGAAGTTTAATTAAGTTTTTTT
CCAAATTTAAATTTTAAATGCCACAAATACATTTTTTTTTATAATAGACAGTAATGCG
TTAAAACATTTGTGTAAAATATCCGTTAGAATTTTTTCGAAATCCGTAAAAAATATTTCAAATCACTAATAAT
CTTGAATGTTTGCTTTGAACAAACTGGCAGTTTATGCCATTGCACTGTGTTATGGATAAATTAAGGTATTATT
TATTTAACTTATTTATTTATTTAACTTATTCTAGAACTTATCGCTTAGACCCCAATAA
AATAGCGCGAAATGTAGTTTTCCATTTTCAAAACGATTATGTAAAACAGATCTTTTGAATTTTTACAAATTGG
CATCGAATTTATTTTTTAATTCTGCCAATAATATGTAATTAACGACTGATGTTTGGAGTGATATGAATAAATT
AATGTCACATAATATAGTTAGTAATAATATTGTGTATATTTTTGTGATCGAGTGATTG
AGGACGTCCTTGTGTGATTGTGATTGTAAAATAAAAAAAAATGTTAAA
>1002050318_1 Heli.1-ES586356.1.5
CCTATATCCACCATCTCCGCCCGAGATCAAGCCCAGCCCAGCTGAGCTCGCCAACGATCTTCTGCAGCAGCTG
GAGACGCAATGCAAACGAGAAAACATATTTCCTAACTGGTTAGAAGAGAAAGTGGAACTTTCTATATTCGAGA
ACATCCCCCAAGTGCCCGAGCGCGTAGAGGTGCCACTGGTGCCCCTGCCGCCGGCGGC
CTACCCGCCGCAGACGGCGCAGCCGACCGAGGAGGAGCTCCTGCGGGAGTTCGAGAACGTGTACGACGCGGTC
GAGCTCACGCACCTCACGCCGCCTCAGAGCCCGCCCGGCCCCGCTACGCAGTTGCTGCTCACCTACGCGCAGC
AGGCGCAGTGCGCGCCCGTCGACCACTGGCCCGCACCCCTCGCCGCCGCCCCCGCGTC
CTCTCCCGTGCCCGCCGCTCCCTACGACTACGAGGCGGCGGCGTCGCTCGAGGAGCTCATCCGGCAGGGCGCC
GCGCAGTTGGGCTCGCCCTCGCCGTGCGCGTCGTCGCGCTCGTCGCCGTCGTGCTCGCCGCGTTCCAGCACCG
ACGAGGAGTGGGGCGCGCCCGCCCGTGGCAAGCCCTACGCGCGTGGTGACGACCGGCG
CTCGCGCAAAAAGGAGCAGAACAAGAACGCCGCCACGCGCTACCGCCAGAAGAAGAAGGCGGAGGTCGAGGTG
CTGCTCAACGAGGAGGCGGAGCTGCGCAAGCGGCACGCGGACCTCGGCGAGAAGTGCTCCGACGTGCAGCGCG
AGATACGCTACATCAAGGGGCTCATGCGCGACCTGTTCAAGGCGAAAGGCCTCATAAA
>1002050319_1 Heli.1-ES586943.1.5
AAGCAGACTGCCCGTAAATCTACTGGAGGTAAAGCACCTCGTAAACAATTGGCTACAAAGGCAGCCCGGAAAT
CGGCTCCTAGCAAGGGAGGTGTGAAGAACCCCCATCGTTATCGCCCTGGTACCGTGGCGCTCCGAGAAATTCG
TCGTTATCACAAATCTACCGAGTTGTTGATCCGTAAGCTGCCCTTCCAGCGTCTCGTG
AGAGAAATCGCTCAGGATTTCCAAACTGATCTTCGTTTCCAGTCTGCCGCCATCGGCGCTCTGCACGAAGCAA
GCGAGGCTTACTTGGTAGGTCTCTTTGAAGACACAAACTTGTGCGCCATCCACGCTAGGCGTGTAACCATCAT
GCCTAAAGACATTCACTTGGCCAGACGGATTCGAGGAGAACACGCATAAATTATGTTT
AATATACCTAATTTTAAATTATTCATAATTCAAATGGACATTATTTCTATGAAAATTTTCTATGTTGTCTCAT
ATTTAATTTATAGTAATAACATTAAAAATCGTGTAATGTAACGAGGTAGCAGTACTGATTTTTTTTTATACAT
TTCTTATAACATAAGTTACTATGTTGTGTGTACACAGAGCACTGCCATGTCTTGCGGA
GATTCGCTCTTGGCTAGCGTCTTGTACAAACAGCGCAGAGTCTATATCTTAGTGACTTGTGGTATATCTAGGT
AGTACTATAGGCAATATTATAACTACATAAGCCCCCGTGTATGTAATTTGATGCACTATTGGTATGTGTAATG
ACAAGTTTTGGAGTTAATAAAT
>1002050320_1 Heli.1-EL603851.1.5

GCACGAGGTCGATTTTCCCAGAGAAAACGTCAGGCACCATTCAACATGGGCTTGAAATACGGTATCGATAGCT
AGTTATAGCTGAAATATTGCTCTAATCGTAGTGTTATTGTTATTTTAGTTACGTTTTATATGTACATTGTGTA
ACTCATAAATTAGTCGAAAATGGATACGTTTATCTTAGGATACTTCGGTTATGGCGTT
TGTTGATCACTAAATTATTTTGTTCGGTTGATAATTTACTTTCCGTTTGATGTCTGCTTTTCGTTTCTAAACG
TCTGTTTTGTTCGTGTTTACGTGTTTTATCGAAATGGTCAAGGAGAAGCCCAATTCGATACGGATATACGACG
ACGAAGGGTTTAGGCGCCGTGCGGCTTGTATTTGCGTGCGGTCGGATGCCGAAACGGA
GGTGTTGCTTGTGACGTCCTCGCGTCGGCCTGACAACTGGATCGTGCCGGGCGGCGGCGTAGAACCGGAGGAG
GAACCGTCGGTGACGGCCATGCGTGAGGTCCTAGAAGAGGCCGGCGTCATCGGCAAGCTGGGCAGATGCCTCG
GGGTCTTTGAGAACCGAGAGCACAAACACAGAACAGAAGTATATGTTATGACTGTCAC
CCAAGAGCTGGCCGAGTGGGAAGACTCTCGTCTCATGGGCCGTAAGCG
>1002050321_1 Heli.1-DT665723.3.5
GCACGAGGGTTTATTTCTAATAAAATACGCAACCCTAAACCCCGCGAAATACCAACGAAATTGGGAAGAAAAT
TATAAATCAACAATACAAAGGTTCCCTAAACAGAGGTCCATTATCGAGAGCTTTCCGAACATTACGTATTCCC
ACACCACGCTCCATGTAAATTGAGACTCGATGATATACCTCATTGTGTAGACCATTGA
ATTTTAGAATAAATAAACCCAAATATTTACGCTTTTTGGTAAAATTTATCAATATATATGTATTTAGTGAAAA
TTAGGATCGATTTAACCATTTTCGTCAAAACATGAAGCCGTACTGCTGCTGTTGTGGCGCTTTGAGATAGCGT
CTATAAACGCTATGGCTGTATGTTCGATTCCCTCTTGGGGAGGAATTATATTTAGATT
AAATTTGTTTAATCGCTCTGGTAGATAAGCGCGGTGTTGTCTTTAAATTTACAATGTTACGAAAATTCAACGA
ATAGAAAATTTTAAAACTGACCTCTGCCTATCCTGTTATGCGGGAAAAATTGTGAATTTGGAATCTTAGTGTC
CGCTTTTATATCAACTCTTAGCATGCCGTGCCGTCTCACTACTGGTTGTACTTTCTAT
TTTCTAAACTTATCTTATTAACATTATTTTAATATAATTCGCCGAATGGAGATCGAACCCACACGTCTACCGT
TACGGTTAAACGTTCCACTGCGCCACGACGGCCTTAACAACATATTT
>1002050322_1 Heli.1-ES586448.1.5
TTCGGCACGAGGGCCAGTTCATTTTATTAATATTGAAATATAATAATTAAAACATCAATATGCAAAGCTTGAA
GATTATTTTTGCGCTACTAGTATGTGCTATCACTGTAAATGCTAAACCCGCGGAGCACTACACTGACAAATAC
GATGGCATTGAAATAAAGGAAATTATAAACAACCGTCGATTACTACTTCCTTATCTGA
AGTGCTTACTTGACCAGGGCAAGTGTTCCCCGGAAGGAAAGGAACTAAAATCTCACATCCAAGAAGCTCTGGA
AACCTATTGTGCAAAATGCACAGAAGCACAACGTAAGGGTACTCGTCAAGTTATCGCACATTTAATAAACAAC
GAAGGTGACTATTGGAACGAGCTGACTGCGAAGTATGATCCTAAGAAGCAATACGTCA
AGAAATATGAAAATGAACTGAAAACTGTCAAATCTTAATTGTTATTAATTTTAATTATATTTAATGTTAAAAA
TTAAATTAAATTTATATTTTATAGTATGTACTTCATTGTATATAAAAATAGATAAATAAATTTATTTTATT
>1002050323_1 Heli.1-DT668155.2.5
GCACGAGGGAAAAAGATAATACTATGTGGTTAAAGATTAATGTGTTAAAGAAAACTGTTATAAATTTAATTTT
GTCTCTATTTAAACATAAATATCCAAAAAATATTAACAAAATAATGCTTGCAGTTATAATATAATATTGGTAA
AAAAATGCTAACTTTAATGTTGATCGTACTTATTTATTATAGGATTTTATATTCTATG
TGCTCTCTGCCTACCCTGGTATGTCCGGGATATAGCGTAACGCTAAAAAAAAAAATTCTATGTGCATATCAAT
TCTTTTACTTTTCAATTTATGATAAAAATAAATGATGCCAAAAAAAAAGAAAGTCTCGTAGCAATTCAGAAAT
TAACATTGTTTTGTAATAATTTAAAAAAATCAGTTTAGAAAGTTGTATCGAGTATAAT
TTGTCCAATACAATATTACTGCTTTAAAATTTTATGTTACAGTTTGATTTACGAAGAAAAGGCGTATTGGTGA
TGATGATAGCAGCGAGTGCCAGACCAGCCGTGGGACACAGCTTTGCAGGATGGCCGAGTGTTAAACAGCACGA
CCAAGACCATATGGAATATTTCTATCTATTATCATATACTTATTTTAGTACATTAAAA
GAGTATGTTATTATCAATTTAATTATACATAATAGTATTGGCACTAATTGGCAAATTGAGTAGATTCATTAAC
TTTATATATTTAATAAGTTTAACATATAAGAAGTACTGTTAAAAATCACTAGCAACACTGTATAGAATTATTC
AATTTCCTTTTGCGTAACTTTTATTTTAAGACCTATAAACAATGTTATTCTCCATTCA
ATTATAGCTAATAATTAATTACACATTGAATTATATAGTATATGGATAAATACATTATGTAAAAGAGCCACTG
TTGAGTTTCTTGCCGGTTCTTCTCAGCAGAACTGCCTTCCGAACCGGTGGTAGCTTCTTTTTTGTTTCCATGT
TAATTCGACGATTCAAAAGTGCTTATAAGAGTTTATTTGAATAAGTAATATTTTGAAT
TTGAATACTTTAATGATTGTATAAATTTATCATATAAAAGTTGCTCATTTGACAGTATGTCCAAATATATATA
AATAGACAGTTTCGTTAGCCATGATCAGTCGAATAAACTAAACAGAATGAGAGAAAAAA
>1002050324_1 Heli.1-DT663806.3.5
GCACGAGGGTTTATTTCATTATTTGTGTAGTGGCGCGCACGAGGACTTGCTCTTAAAAGAGGAATATATTCTC
TATAGAGCTGAAAGTCTTATTAAATAAATCAAGAAACAAACTGAACTTATATTAGAAATTTATGTTATTAATT
ATAAGAAGAATCTTAATGGGTAACGTAGTATCTTAACTGGGGGTTCCCTCTGCCTGCC

ATGCTGTATGCGGGATACAGCGTGAATTTTAAAAGGGATGAACTAATAATAGAACAATAAAAAATTCTAAAAT
GAGCCTTACAATTGAAAATAATTGTTTACTGTTTAAAGATCCAAAAACATTCCCCTTAACCAAAATTGTAGCA
GTGGAATTGACACTTCAACAAACTATTTCAAAATTTAAAGACCTAGTTAGTGATTTTG
AAGAGTTACATTTAGAAAATGAAAATTATAGAACTTTCATATCACAAAAAGAGCAAAATGGGTACATTTGTAA
TAATTGTGAAAATTTAAAAAAGGAAGTGATCAACAAGGAAAATTTATTTAAATCTATTGTTTCAGCTCTTAAT
AAGTTAAATGAAACACAAGACTTCAAGATTGTAGACAAAATCTACAACATTTTGAATA
TACAATCACCTAAAAAACAGTGTATAAAAAAGGAAAAATTAGAACCACAAGTAAATTTTGAAAGTCCAAATAA
AGAAATTTTAGAGAATAAAGATGAATCTATATCAGAAATTGAAACTACTCCGTTGGGTAGAAAGAGTCCAATA
ATATTTACTAAAAAGAAAACTAGCACACATTCCTTAAGTTTAAGAAGATAAAAGAAAT
>1002050325_1 Heli.1-DT663808.3.5
GCACGAGGTCTGCGTTTGAGAAACCCCTGGGGAAACGAAGCGGAGTGGAACGGAGCCTGGAGCGATAAGTCAC
CAGAATGGCGCTTTATCCCTGAATCGGAGAAAGAAGAATTAGGTCTAACATTCGATGACGATGGAGAATTTTG
GATGTCATTCAAAGATTTTACAAGTCATTTTGATAGAGTTGAAATTTGTAACTTGAAC
CCCGACTCATTGGACCCCGAAGAATGTCCCGAAGGTTGCACTAAGAAGTGGGAGATGTCTGTGTTTGAAGGGG
AGTGGGTGAGAGGTGTAACAGCGGGTGGTTGCAGAAACTACTTAGAAACGTTCTGGAAGAATCCTCAGTACAC
TGTGACACTTAAAGATCCGGATGAAGATGACGCTGAGAACAAGTGTACTATTATCGTG
GCACTTATGCAGAAGAACCGTCGTTCTCAACGTCATCAAGGCTTAGAGTGTTTGACCATTGGATTTGCGGTAT
ACCGTCTACCCGACTACGGACATGTACCAAAACCTTTGGATGTCAACTACTTCAAATACAATGCTTCTGTTGG
ACGTTCTCAAGCTTTTATTAACTTGAGAGAAGTTAGTGCTAGATTCAAATTCGAACCT
GGTTCTTATGTAATAGTGCCTTCAACTTTCGAGCCCGACGAGGAAGGTGAATTCTTACTGCGTGTCTTCTCTG
AGAAGAGCAATAATATGGCTGAAAATGATGAAGAAATGGGAATGGGTGATGTAGATGATAGGGTAAAAGAAAT
AGCCCCGAATCCAGAACCGGCAGATCCGGTGCGTGAGTTCTTCACTCGACTCGCCGGA
TCAGATGGAGAA
>1002050326_1 Heli.1-EL603550.1.5
GCACGAGGGTCACCTTAAAGTGATAAAATGGCAGAAGGCCAAGATGAACCTAAAAAGATTACAATTACAGTTA
AAACTCCAAAAGAAACGCAGCAAGTGGAAATAGAGGAGGATGCCGATATCAAAAAACTTAAAGAAGTTCTGTC
CCCTAAATTCAATGCAGAACCGGAACAATTATGTCTAATTTTTGCTGGAAAAAATCATG
AATGATTCCGACTCACTGAAGCAGCATAACATCAAAGATGGATTGACAGTTCATCTTGTGATAAAAACTCCAC
CAAGACCTGAGCCAGAAGGAGCAACTCGACGTCCTTTTTCTGATATTGGTGCAACACCATTTGGATTAAACTC
TCTTGGTGGCCTTGCTGGACTTGAAAGCTTGGGCTTAGGTCAAAGTACATTTATGGAT
CTTCAGGCCCGTATGCAACAAGAACTATTATCAAACCCTGAAATGCTAAGACAAGTACTTGACAATCCCTTGG
TACAACAAATGATGAATGATCCAGAAAATATGAGATCACTTATAACTTCTAATCCACAAATGCAAGACTTAAT
GGCTAGAAACCCAGAAATAAGTCATATGTTAAACAATCCTGAATTGTTACGGCAAACT
ATGGAATTGGCTAGGAACCCTGCCATGTTGCAAGAGTTGATGAGGTCCCATGATAGAGCTTTGTCCAATTTGG
AAAGCATACCTGGTGGATATAATGCTTTACAAAGAATGTATCGTGATATTCAAGAGCCAATGCTTAATGTAGC
CAGCAGTATGGCGGGGAATCCATTTTCTGGACTTGTTGATAACTCAGATGGTACCAAC
CCACAACAAGGTTCTGAAAACAGACAGCCATTACCAAACCCTTGGAATAGAGGTGGTTCCAACACTTCAGGGA
CAGGTGCAGGAGCAGGGATAGGACCAATTACAGGATCTGGTCCAGGCCTCATCAATACACCTGGCATGCAGTC
CCTCCTCCAACAAATGTCGGAGAACCCTCGACTGGTACAGTCCATGCTGTCAGCTCCT
TATACCAATAATATGCTACAAGCATTAGCTGCTGACCCGGAAGTGGCGTCACAATTGATTAACCAGAACCCCA
TGTTTGCCAACAACCCGCAGCTGCAAGAACAGATGCGTGTCATGATGCCACAGTTGCTCACTCAATTACAGAA
TCCTGATATGCAGCAAATGATGTCTAATCCCACAGCACTAAACGCGCTACTGCAAATA
>1002050327_1 Heli.1-EL604395.1.5
GCACGAGGAAAGTTATAAGCGATGAAAAACTGAAAAATGGACGACCGATAGATTTTCCTTCGCACGTGAACTT
CATAGTTAGTTTTTATTTGTTTATAAAGAGCGGTTTGCCGTCAAGCTGGGTGCCAAAACCGTGGAGCGCTTCT
TGAACCTCGCCGACGACAGCTTCGTCCAGGATGTCAACGACGACTTAAAATGTTTTCT
AGCCGAAAAAAATGTTGACAGTGTCCTGATATTCCCGCCGGTGAGCAACTACCGTCGTTTCCTCATCCACCGC
TGCGTGGAGCAGCTGTGCCGACCGGAGCTGACCACCTTCTCTATAGGGCTGAGCGACGAGAGGCGCACCGTCG
TCTGCTATAGAGATAAATTGTTGAGTGATGAGGCTTGCGCACGACCAGCCATGGGCTT
GACTCAGGAGAGGAACGATGAAGCCGGCGCTGAGAATGGCGCAGACGCAAGTTGCAGGGCGGCGCGCGCGCAG
GAAAAGATCAGATCCGGGTCCACTAAACCTCGGCGGCCAGACCGCGCCGTTTACGTACCGCGAGCTCTACGGT
CGAACGAGAACGCGGAAGAAACCAAAACGCCAGATAATAAAAAGACCAATTCTGACGC

GAAATGCCCCAAGTCTCCAGCATCCAAAGATAAATCAGCTCACGGCACCCCCAAGCGCTCTCTAGACAAGCAC
TTCTGCAACGTGTACACACCGCCGCACCTGCGCGGCCGAGTGCCTCCCGGCGACGACAAGCCTGTGCCTTCCG
TGCCAAATAGTGATAGTGCAATATCTTATAGTGATAGTAGTTTTAACGACGTAGAAGT
AACTGAGAATTTTTATATAGGAGATTATTTTGCCAACGGCCAACTAGGGTTCTATAGCCCCGCGTACTACGAC
GAGTGCTGGAGGAATGAATCTGGTGACATGAACATGGCGCAAGAAACTCAAAGCAATATTATTGATAATGATT
ATTTATATAATAAGGACGAGGATGTCGAACAGAACGAGCTGAAGCG
>1002050328_1 Heli.1-EL603020.1.5
TGAGTTTAGTTAATTTTGCCAAAGTAGAATTTGTTTTTCTATGTTTGTGTTGGGGTCCCCTGATTTTGCTGTA
GCCGGCTTCCCCAAAATTCGACGAGCTTTGTGCCGTTGGAAACGAACTCGTAGCCAGTGAAGTAGTATCAGTC
CGCGTGACCGCCGGCATACACCGCTACCCGCCCGCATAGGATGATAGAAACTGACCCT
GGGCCTAAGAAACCTCACAATTGTGATGTAAAAAGTGACCACCAACTAAACGGACCAGGTTCCAAAAATATTG
ATCTTGGCGTCGTGCCGTCGCTGAAGACACCAGCAAAGCTCGACCCAGAAAAAGGATCGTCGGAAAAGGCTGA
CTTCGAGCGGGCTATCGAACAAGCTGGCTATGGACGCTTCCACTACCTCCTGCTGGCC
GTGTGCGGCCTCGTCAGCACCTCAGAGGAGATGGATGTCATATCCATGTCCTTCATCTTGCCGTCAGCGCAGT
GCGACCTAAACCTCACCACACAAACCAAAGGATGGCTAAACAGCATAATCTTCATAGGAATGATGGTGGGCGC
GTACGCGTGGGGCTCCGTGGCGGACTCTCTGGGCCGCAAGCGGGTGCTGATCGCGATC
TCCATCATCAACGCGCTCGCCATCGTCGCGTCGTCGTTCAGCCAAAATTATGAACTTTTTATGCTCTTCCGCT
TCATAAATGGTGCTGCTTTGGGAGGATCAGGTCCAGTGATCTGGTCATATTTCGCCGAGTTTCAACCTAAAAA
GAAGCGAGGTGCTATGTTAAGTTTCATGGCTGCCTTCTGGACTCTTGGTAACCTTTTC
GTCGCCGGTCTCGCTTGGGTCATCATTCCAAGTGAAATCGGAGGAATGACTGGTGGATTCGTTTACAACTCAT
GGCGTATATTCTTACTAGTGATGTCACTGCCTTCTTTCATAGTGGCCGCACTATTATTCCTACTGCCGGAATC
TCCCAAGTTTTTAATATCAACGGGCCGTCAAGAAGAAGCTTTAGAAGTATTCCGTGGT
ATTTACATGATGAATACCGGGAAA
>1002050329_1 Heli.1-EL597375.1.5
GCACGAGGGATGTCTGAAAAAAATTGTGATGATTTCTTTGGTTACATAGCCGAAAACGAAATACTAATATATA
ACTTATTTGTAAAAAGTCCCAACGTACAATATATTTTGTAATATATTTATTATTTCCTAATCAAAATGTCAGA
TAACGATGATGATTATATGTGTGAGGAGGAAGAGGATTATGGACTGGAATATTCGGAA
GATAGTAATACAGAACCAGATGTCGATCTAGAAAACCAATACTATAACAGCAAAGCTTTGAAAGAAGACGAAC
CTCAAGCTGCGTTGTTAAGCTTTCAGAAGGTTTTAGAGCTGGAAGGTGGAGACAAAGGAGAATGGGGTTTCAA
AGCACTGAAGCAAATGATAAAAATTAATTTCAAATTGAGTAATTTCACTGAAATGATG
TCTAGATACAAACAACTATTGACATATATTAAAAGTGCGGTCACAAGAAATCATTCAGAAAAATCTATCAACT
CTATTCTTGATTACATATCAACATCTAGAAATATGGAATTACTACAGGATTTTTATGAAACAACATTGGAAGC
TTTAAAAGATGCAAAAAATGACAGATTATGGTTTAAGACTAACACAAAACTAGGCAAA
TTGTACTATGATAGAGGGGACTTCAACAAACTGGCCAAGATACTAAAACAATTACATCAGAGTTGTCAAACT
>1002050330_1 Heli.1-DT665321.3.5
GCACGAGGGGGATTTGACTATATTAGAAAAAAGAAGAAAATTATAAGGCGAGAAAATAATATGCCTAAAGTTC
GCCGTGCAGCACCTAAACCGAGCCCTGAGTCTGTCCAAGATATACAAAAAGAGATTAAAAGTTGGTTTATTAA
CAAAAGCATAGGCGAAACACATTTGCACCGTGCTGCAAGACTTGGTTTTACCGACTGC
GTAGCTTACTGTCTGGAGAAAATGGAAGCCGACCCTACAGCGAAAGATAATGCGGGTTTCACTCCGTTACATG
TGGCATCGGCTAAAGGTCACGTAAGAATTGCGAAGCTTCTCCTTCAATATGGCGCCAATGTCTCAGCTGCAGC
TCAGGGTGGAACAAGACCACTTCACGAAGCTTGTGAAAACTGTCACGTAGAAATTATA
AGACTATTATTAGCCTATGGGGCCGATCCTCTACTAGGAACATATGCCGGTCAAACTCCTGAAGAATTGGCGG
AAGGTATAGCTGCAAAGTTGTTGCGGCTTCATATACTTGATGTACAAGGACGGGCTATAGAACCTTGGAGATT
TCCATCACTAGCAGAAATCGTCGATCGCGAAGAACTCGGCTGTGACCCATTATCGTCC
CCGCCGCCTGCGTCGCCGCCGCCGCCGCCAGACTCCATGATAGAAATTCAATGCACTGAGACGCCACTGCCCC
CTTTCTATAGCCTGCGGACCGCGCCTGGACAGCCAGCAGATGGGCTGTGGTGCTTACTGCAGGATGTTACTAA
TATTTTACAGATAAAATCAAAAGACAGCCTTTTAAAGCAAATCCACTGTGGTTCTGGA
TCACCCAAGGAGTTGCTCCGCGAAATACGAACACAAGAGTTCCTAGAGAGAGCCCAATGCCACCAATTGCTAT
GCGCGGGTGAAAAAGTGAACGTGCGCGCGTCCAAAGTGTCACTTATACGGGTTACCGATAAACTGAGGCAATT
ACTAAAGATAGAGACCGTCCTTGTCAGCTGACATTAGTTGAAAAATAATACCCACCTG
CAATGTTACTAGGCTTGTCTTAAACACATCCTGATATTATTGCAGGTGAATTTTTTTATCTGAAAAACATTTA
CTCAACACATATTGTTATTTATTATTTTTTACTCCTGTGTAAATATTTCTATAATGTGGTATGAATCTTATAC
CATATATACAGTGTTGTACTAACACAACTCTTATATGTAAAGGTCAACCATATTAGAA

CGAAGCGGGCTTTAAATCTACTATGACTGATTTATTGTAAAAAGGATGTATCTCATTTAGGAACAAAATAACA
TATTTGTTTTATGAGACTGTGTAATTTTAGGCAGCCATTACATGTTTTGTGTCTGAGCCACACATGTGTTGTG
TTCGAATGTGCGCTCTTTTGAAGAACTTCTGTATATAGTGTAAATAATTTTGCAAGTG
TAAATAATAAATGTTGCTTTAATTGTATTATATTCACATGGGTGCTATTTAATGATGGTGTATGGGCTTTAT
>1002050331_1 Heli.1-DT667339.3.5
GCACGAGGGACGCCCCTTCTACTGTTTTACTTATTTTGAATAAGAATTTTGTATTTTTACAAACAAAATACAC
AGGTTGTAATACGAAAAATGTCGGCATATGACTCTCACGCTTACTTTACAGGGAATGGGACGTACAAGTCTAT
AAATCCGTTAATTTCTAGAGTGGGTGACTTCACACCGTTCTATATAACTATAGCAATC
TGCTCCGTCATCCTCGGCTCTCTGCTCATCTTGAATTTCGTCTGCTGCTGTTCAAAGTACTCTGAGTACTGGC
TTGATAGACACACTGGGAACCGCTGGATCGTGTCAGTGTGGTCATCAACTCCTCACAAGCAGCCTCCCCTCGA
CTTTGCTGAGCTCGAAAGCCAGTTCCAGCACCAATACATCCCTTACATCAGTGAAGAG
TACCAGGAGGTCCACCACCACTCCGAAATCCCCTCGTCTGTATCTCGTCAGCCACTGACCTCTTCCCAGGAGT
ATCTTGAACTACATAAGAGGGAAAGTGACATTTAAAATGGCTTTCCTAATGATATTTCCTGCCGTAGCCATTC
TTTCTTTATTTGTCCTCATGGTTATTATATTAATTCTGCATTTCGGAGCTCGCTGGTG
CAAGCTCCGGCATTCTACATTTGTAGAGACGGAGATTTGGAACGAGGATGCTGCCTATGAGCACAAAGTTTCA
TATGCCTAATTATTTTTATATTATGTAACCCTTCCGTCCTGTTTTTATTTTACTTTTTTTTAAGATACTTTAT
TTTTACTTTCTATATTAGGAAACGTCTATGTTACTTCTAAAGTATATAATGTAAGTGT
CTAAGAAACATATATACATATGTTTAAATATAAATGTTGTAGGAACCTTTATGTCCCAACTTTGTTTTAGTCC
CTAGCTTTCATAGCAAGATGGGTCCAATGCTGGTACTAAGACTGTGGACAAAAAGAAAGCAATAGATGCATTT
ACTAAAAACTTGAATCTCAAGTCAATTTGTATTGACTTGAGTGGTGTGCAGTTTATTT
AATGTTTTATATTGTATAACAATTATATTTCTGGCGTATATTGCATAAAATATATACTTTTTACTTTTACTTT
TAGTATATGACAATAACAAATTGACTATTTTTTATAAGAAGAGTGAACTGCACACTACTTATGTACTTTAGTT
CGGCGACCCCTCCGTCCATTGATTGTCTGATTTATTTTTTTGTAATTTTAAGTTAGAT
GTAAGGATTATTTTATTTTAAATATAAACTCAGTTAAAGTGCACAACATCCCCCTCTCCTGTGTTCTTATCAT
TGTTAGTGTTTTACTGATAATATATTGAATGTATTGATGTCTATATATCGATGTATCGCCTACGGAATACAGT
GTTGTAGTCTAGTAATATTTACCAGTTGATTGAATAAACCCTCGTG
>1002050332_1 Heli.1-DT666146.3.5
GCACGAGGTGTGAACGTCACGCGCACCTCTAGGGCTGCCAGATCTTGTCTTACATGATATTGTATAATAGTTT
AAAGCTAAAGATGAAAACATGCTTTACGATGAATCCCGCATATAGAAGAGTAAGCAGATGGAAACTAGTGTTA
AGACACTCCGTTCCCATTGCATAACTCAATTTTTAACACATTGCATATATTACCAGAG
CGAGAAAATGAAAATAATTCACAGTATATTTAGGTTACAAATATATATGTATATCATTTATATAGATATATTA
CAAATTGGTAAATGAACACAAAATATTTCGCCAATGTCACGCTGAATGAATGAAACTCTATTAAAAAATATCG
AGATCTGGCAGCCCCGAGTGACGCTTGTCGAGTGTGACAGCTGTCAATGCGACAATAC
TATGGCTTAGGCGCGCTTTTTGTGTCATTTTTTTTTATTTATTAACTATGCTTTGTTTCTTATTTTTTTGATA
CGTATATTTTGTATTAGCCGTGTAGTGAAGCGGGCGGGCGACTAGATGTTACTAAATGTAAAGTAATTGCCAG
CTGTTTTTTTTTTAATATGTAACGTCATATCGAGTGTTCGCTACGACACACTGCCAAG
TGCAATTTTTTTTTTAAATCTTACACAAAGTACTAATATTTATGTTCCGAACGAGAGTTTAAGTTAATTTCTT
TATTTCTTTATATTGTCAAAATGATTTTTTATTAATGTTAGGGATTCTCTACATTCCACCATTGGGACCAAAT
ATGAAGTTGTTAATTTTATATTAATCTAAATTAATTGCACTGAAAAGACAATGCGGAG
AAACGACATTCCTGTAACTATTCCGATTTGAGTGAATATTTACTGTTAATGTATAAGAATGTGCACTGTTTGC
GGTTTATTATGAACTTATATACATATTTACTTAACGATAGGAAAGAGATTAGTTACATAATTTTATTTAATGA
GTGATGGTAACACGTATTTATGAGTATATGCTTGATATAGATTTGATATATTTCTATA
TGTTTTGTGTCCTGTGTCATAATTTCCGACGGTAATTATTTATATAAACAGAAAATTATAAATAATGACAACG
TTTCCTTATAAAGCTGGCAATTAAATCCCTGATACATTATATCGGGCTATTCGCGTGATGTTACGAAAAACAA
AAATTGTAATTTAAAATCGATCGAGGGTAATGCGTCTTTTTTTTAAATGTTATCGATT
AATGAATTGATAGATAAGTACGTGAGAAATAAATTAATGATTTTTAAGTATATTCTAACACAAGTGTTTATC
>1002050333_1 Heli.1-EL602825.1.5
CAAAAATGATTGATAAGATATCTGTAATAATAGCATGCATGGCTATTTGTAAAGCGGGAGTTCACGCCGATGG
AGTTTACTTATCTCCTGTCAGAATCAGTCAATCACAAATTAATAGAATTCAAGTTTCTAAGGAACCTGCACCT
ATATTCGAACCAGACGTGGAAAAGAAGACTCAATCTGATGTAATTCAACAGTCTATAT
ATCTCAAGAACAATTACAATAATGCCATATTGTCCGCTGGTCCCAAAATTTCGCCACAAAATGTCCAATCACC
CATCTACAATACTTTATACTCTGTGGCACAGCCCAGTATAAATGGTTATCCTAATACTTTAGCATTGTTAAAT
TCGTATTCACATAAAAATGCTGTATCGCAGCCCTATGCAGGTATACTTGCGAATAAGG

AAGTTGAGGTTCCTTTAGTACAAAATGTTAAAACAGGGATAGTCCCTTATTCTGTTTCTCACACTACATTTAC
CGGCCTCGGTACAAGTTACACTTGGTAAATATTTAAAATTCCAAGTTAGTTTTAAGATTTCATGGTATGCGCG
ACTGACGGTATAATTAAGCTTAATTTATTGCACTACTTTAAGAGTTTAATTGGATTAT
TAAGTTATTAATTATTTAAACATT
>1002050334_1 Heli.1-DT662896.3.5
GCACGAGGACGAACACTGGGAACCCCTTGTCGGGGCTATACTGCAGGACATGTCTACATCTCCTATACTGCAT
GTCGAACAAAAAGAAGGTGGAACGCAGCTTAAGCTTATCATCGATTATCCTAACGGCGTCCAAGCACTGTTTA
AACCAATGAGATTCGCGCGGGATGTTCAAACATTGCCGAACCACTTCTACTTCTCGGA
CTACGAGAGGCACAACGCCGAGATTGCGGCTTTTCATCTAGATAGGATTCTCGGTTTTCGCCGGGCGATGCCG
GTCGTGGGTCGAGTTCTCAATATGACAACTGAAATATACGACGTGACAGAAGGTGACATCCTAAAGACATTCT
TCGTATCTCCAGCGAATAATTTCTGTTTCCACGGGAAATGTTCGTATTACTGTGACAC
TGGACACGCTATTTGTGGCAACCCTGACATGCTGGAGGGCAGCTTCGCCGGCTTCCTGCCGACCGCAGATTTG
GCAGAACGAAAGGTTTGGAGACATCCATGGAGAAGATCGTATCACAAACGAAGAAAAGCGCAATGGGAACTAC
AATCCGATTATTGTGATACAGTCCGTTCGACACCTCCGTACGACTCTGGGCGGCGACT
TTTGGACTTAATGGACATGTCCATATTCGATTTTCTAACTGGCAACATGGATAGACATCACTATGAAACGTTC
AAAATGTTCGGTAACGACACCTTCACGCTGCACCTGGACCAGGGCCGCGCGTTCGGGAAGGCCTTCCACAATG
AGCTAAGTATCCTGGCGCCGCTGCTGCAGTGCTGCCTCGTCAGACACACCACGCTCCT
CGCTCTGCTCAA
>1002050335_1 Heli.1-EL602968.1.5
TTTGCACGCAACGGAAGTCGTAACAAGACATAATAATATTAAGTTGATGGTTAATATTAAATAAAAAAAAAAA
CATAGCTGTCAAATAAAAAAAAAAGATTTCTATTTCTTAAATAAAATTTGTGTTTTAAGTGAGTAAAAATAAT
AAATGTGTTAGTAATTGATTTAATTTGAATTTTAAAATAAGTTTAAAGTGATTTTTAA
ACAGAGTGATATATTAAGTTGTTCAAAATGAAAATACATCTGTCTCCTTTGATCCGTCAATATGCACTAGTAG
TTACAGTTAACTTGGCTGTCCTCACAACCGGAGCTAATGTGTCTTGGTCTTCGCCCGCACTAGTAAAATTACG
GAATGCCACAGAGACACCTCTCCCAAGACCTATTACTGAGGAAGAGGGTTCGTGGATT
GTGTCTGGAGGGTACTTGGTAGCAACATTTACTACCATAATTGGTGGTATGTTCATTGATACAATAGGACGGA
AGAACTGTGTTTTGATGGCAGCAGTACCAAAACTTGTAATGGCAATAGTATTAATATTTGCTAACGAAGTGTG
GATTTTTATTCTATGCAGAGTAATTATGACTGTAGCTGACGCTTTAGTGTTAGCTGTT
GTACCGATATACGCTTCTGAGATAGCCAGTAAAGAACATCGAGGCTTTCTTGGAACATTTCTTCAGTTGTTTT
CTTCGTTAGGCATATTGTTTACACTCTCCGTGGGT
>1002050336_1 Heli.1-EL596285.1.5
GCACGAGGCTACGATGCTTATTTTGAACGATATATTAAATCGTTTCTTGTTATGAAGACAAAAACATCTGATT
TGTTGTTACCTTCCAATGGAGAATTTTAACCAAAACATTTAGAACACACACAAAATAATTCCAAACTACTTAG
CTGTAATCGTTAAAAATGTCGAAGAATGGCGAAGTGTCGTCAAGCCAAGGGCCAAGCA
GACCGAATAATACAAAACCGGAACTAAATTTATTGGGTACAGAGAAACGTAAAGTTTTAAACCTTCAATTTGG
GGGGCCTTCAGGTGACAATGCCGCATTTATGCCTTTTTCTTCTAACACACCGTCACAGAAGTCCAGAATACCA
TCACGGACAATCAGAGATGTATTGCCTGATAACACTAGAGACAGATGTAGGATTTATC
CATCTATGCAGTCTTCAGGAAAGTTACAACTATCTGCCACAGAGATATATGATTTTACTGCTGATGATTTACA
TGATCTTGGCGAAATAGGCAGAGGTGCATTTGGAGCTGTCAATAAAATGGTTCATAGAAAAACTAGCAAGGTG
ATGGCTGTGAAGCGAATTCGTTCAACAGTAGATGAAAAAGAACAAAAACAATTATTAA
TGGACCTGGAGGTGGTTATGAAAAGTAATGAGTGTCCATTCATTGTGCAGTTTTATGGAGCACTCTTTAAAGA
AGGTGACTGCTGGATTTGTATGGAGTTAATGGATACCTCGTTAGACAAATTTTATAAGTTTATTTGTGAAAGG
ATGCAGACTCGGATACCTGAAAATATTATGGCTAAAATAACATTAGCAACTGTCAAAG
CTTTAAATTATTTAAAAGAAAAATTAAAAATTATACACAGAGATGTCAAGCCTTTCTATATATTATTAGACAG
GAGAGGTAATA
>1002050337_1 Heli.1-DT666819.3.5
GCACGAGGGTCGTTCGCTCGATAAGCTTAAAAAAATAAAATGTCACTTTTTCCCGCCTTTACGTCAAGGTCAA
TGCCCCTGTACGTGTTTTGATTTTGAAAAATGCGCACGAATCATTTGTCACTGTTTCTATACACTGAAATATA
ATTATTTCTTAAATTTTTATTCTTATTCGTTTCTGAAGTGAGTTTTTTGAAAAAAAAT
AAAAAAAGATGGCTTTAAGTTTTCTTTTGTTTATACTTTTGTTCTTTGGAGTGGAGTGCTTAGATACTAGACA
GGAAGAAGGCTATCCCAGAGGATTTATGTCAGTATGTCCAGGATCGAACAAGCCGGCCTCAATACCTCGAAAT
CAGCTGAAATACTTATATTTCGTGGTGCAAGGGAAAGGGCAAACGCGACATAAATACA

ATTATTGGAACGCGAAAGATATCGCCACAGATCCCAGGATTGATTTTCGTAGGAAAACAATAATTGTGGCTAT
AGGATACCTGGACAGCACCAGCTTCCCGATAGCCGCCATGTTCGCTAACGAGTACGAAGCGAGGGACTACAAT
GTTATATTACTGGACAATCAGCGTTTTGCCACCGTTCATTATCATTTAGCAACTCGCC
TAATGCGACCAGTGGGCAAACACGTAGCAGAGATATTAGTACAGCTAACACACTTAGGTCTAGATCCTTCTAG
AACTGAGTTAATAGGGTTCAGCTTAAGCGGACAAACTGTCAGTTATATCGCTCGAAACTATCAAATGATGACC
GGAACTAATATATCCAGAAATTACGCTTTAGAACCTTCGGGGCATG
>1002050338_1 Heli.1-DT662171.3.5
GCACGAGGAATTGACACTGTTTTGAATTTCACGATGGTGTTTTTACGAAACAAAGTTAAAAACAATTAGTGAT
ATAATGATGACAACGATTTAAAAGATAATTTATCAATTCAAAATGAGGCGCAGTTTAGCGCCGAGCCAAATAG
GCTCTAGCGACACAGTATTTAAAAGTCCGCTGCTAGAATCTGCGAAAAGAAAGAAGCG
GGAATGTACAAAAATTCCCCTCACACAAAAATCGCTTTCTCAAGCAATGTCCACTTCGGATCACGAGAATCTC
ATCAAACAGATATTGAGTAAACCATTCAAGGTACCAATATCTAATTATGTTTCGTCATATTGCAGCAAAAGCT
TAGGATTAAAACGAACTCAACTAAGAAGAGCTTTGCACGATCCAGACGAACCAAACGC
ACTTGTTCTGTATCGACCACCGTATATTCCAGAACATGAAAAGATGAAAATGAATGCCAATGATATAAAAGTT
GCGGTTGTAGTGGATCCTATTCTAGGGAACATATTGAGGCCGCATCAAAGAGATGGTGTTAAATTTATGTATG
ACTGTGTCACTGGGATGCAGATTGAAAATGCATATGGATGTATTATGGCAGACGATAT
GGGTTTGGGTAAAACTCTGCAGTGTATTACGTTGCTCTATACTCTTTTAAGGCAGGGTCCAGATTGTAAGCCA
ACTATCAGCAAAGCTATTATTGTATGTCCTAGTAGTTTAGTGAAGAACTGGTACTATGAAATACAAAAATGCT
TAGGACAAAGGATAAATGCTTTGCCAATGGATGGAGGTTCCAAAGC
>1002050339_1 Heli.1-EL603261.1.5
GCACGAGGATTACCACAATCATACTTACGTATAAATCCATGTAAATTTTAAACAGGTTTAATTATAAAATCAC
AACGTGATGGCTGAAAATAGCGAAATAAATCCCAGTAATGCTGACACAACACCTAAACAGGAAGAGAAATCAT
CAGGCAACTGGTGGGACTCATGGATCTCATCAGCAAAATCCAAGTCAGCAGAAGTTTA
CACAATGGTAAAGAAAGATCTAGATGAAATAGGTACAGCAGTTAAAAGTGAAGCCAGTCATGTGTTCAGTTCT
ACGTCTAATGTAATCGGTAAAACTTTGAAGTTGGACTCTCCTGAATCAACTGCAAATGTAATGAAGAAAAGTT
TGAGCAGTTTCATAGGTCAAGTAAGTACAGTTCTAAACCCGGAGCCAGATGATGAAGA
TGACACTGAAGTGATCCTATCATCTGGTGATACAACGATGCTGTCCACGTACAAGAAAGAATTAGAATCTCTC
CAGCGCGTAGACGCTACCTACATTGTGCCAGCATATAGTCCAGAATTCGAAGCCTGGAAAGGTAGTCTCGAGG
ACTCGGAGACAGTAATCACTTTAGCCTCAGTGGTGCGTCGTCTGGACTCGAACCCGAT
ACTAAAGTCACAGTATGACAAGCTGGTGCCAGACGCAGTGTCTCATGAGGAGTTTTGGGAAAGATACCTGTTC
CGCGTGGCGCTGCTGCAGGACCGGCTGGCGGCGGCGTCGCGCCACGGGGAGGACGCGCCCCCCGCCGACCCCG
TGCTGGCGCA
>1002050340_1 Heli.1-EL598130.1.5
GCACGAGGCTGCTGCCCGCCGAGGGCGCCGTGCAGGTGTCGGACGGCAGCGTGAACGTGCTCGTGAGCCTCAC
GTCGGCCGTGATGCGCGAGCCCGCGGAGGGAGGCGACATAAAGCGAGACGACAAGGATGTGCTCCCGCGGCAG
AAGTGTTTGGACGCGTTGGCCGCCCTTCGGCACGCCAAGTGGTTCCAGGCTAGGGCCG
CCAGCCTGCAGTCGTGCGTCATCATCATCCGCATCATGAGGGATCTGTGCCGGCGGATACCCAACTGGACGCC
GCTCGATCCTTATGCGATGGAGCTGCTGGTGTCGGGCGTGATGCAGTCGGCGGGCGGCGCGCTGTCGCCGGGC
GAGGCGCTGCGGCGCGTGACGGAGGCGGTGGCGGGCGGCCTGCTGCTGGACTCGGGGC
CCGGCCTGCGCGACCCCTGCGAGAAGGAGCTCGTGGACGCCCTCGGCAACTTGCAACCGCAGAAGCGTGAAGA
CTTAACTGCGTCAGCTCAGCAGTTCCTGAGACAAATTGCGTTTAGGCAAATACATAAAGTATTGGACATGGAG
CCTCTACCGAAGTTGAAGCACACAGCCGGCAACTGGAAGTTCCCGCGCAAGCGCAGGC
GCTCCAACACCGACCACGACCCCGACACGCCCAACGGTGAAGGTAAAG
>1002050341_1 Heli.1-ES585993.1.5
CCAAAACAAACAAAATGTTCAAATTTGTGATTTTCTTCGCCCTCTTGGCTGTGGCCTTCGCCAAGCCCTTAGT
GTACACTGCGCCATTTGCCGCGGCGTACACGGCTCCCGTAGCTGCAGCGTACACTGCGCCCGTAGCGTACTCA
TCGTACAGCGCGTACTCTCCCGTCGCTTACTCCGCCTACTCATACGCATCTCCTTATT
CTTACTACCTTTGAGAAATAAAACGACGCCTTTAAACTCTCGGAAATATCAGCAAAATGGATTATTTAATATA
TTTATTATGTTTGAACGCAATAAATGACAATTAAA
>1002050343_1 Heli.1-DT666545.3.5
GCACGAGGGTGAACCCTCGACTGCTGACTTCCCCTTGCATTTGCTCCACCCTACGTCATTGCTATATACGATA
GCTACAATTTAAAAACCTAATTACTTATAAATAATCTATTTATTCTCTCATGAACGTATAGAATTGAAATTTA
AATAAATATTTACATGTAATATGGAATACATATTTGGTGTTTTGTTTATGTGATTTGT

GACTGAAACGTATGGAATATACTGTTTGATGGTGTAAAGTCGTCAAAATTAGCTCTTACGCGGAACTAGGTGC
GCGTGCGTCGTCCCATGGGGCGCGCCGCCGTCAGTTCATCATGGGTTGGAAGAAAACCATGGGGGTCATATCT
AAAACGTTCGGTGTCGTTTTCAGTCTCCTCTTCGAACAGGAGATCATGAGCTACGTGC
CGGCGTCAGGTTGCGCGACCGCGGAGCCCTCGCCCGTGGCGTCGCCCGCCGCGCGCTCCACCAGGCAGCCAGC
GCCGCAGCGACGCGACTTTGAAGCTAAGCTGCGAGCTTTCTATAGGAAATTGGAAAGCAAGGGATATGGACAA
GGTCCTGGGAAGTTAAAATTACACATCCGTCGCGACCATCTATTAGAAGACGCGTTCA
GACGCATAATGTCCTGTACCAAAAAGGAGTTACAAAAGGGGAAACTTTGTGTACTTTGGGACGGTGAAGAGGG
CCTGGACTATGGGGGGCCCAGCAGAGAGTTCTTCTTCCTACTGTCTAGGGAACTGTTCAACCCATACTATGGG
TTATTCGAGTATTCAGCTAACGACACGTATACGGTGCACGTGTCACCTATGTCGGCGT
TCGTGGACAATCATCACGAATGGTTCAGATTTTCCGGTCGAGTGCTGGGCCTGGCTCTAGTCCACGGGTATCT
TCTAGAGGCGTGGTTCACTCGTGCTCTGTACCGCGCACTGCTACGTCTGCCCCCAGCAT
>1002050344_1 Heli.1-DT666790.3.5
GCACGAGGGGCGGACATAGTCTCGTTAATCTGACATACTTTGGCTTAGTTTAGAGTCACCGGTAGAGACAAAA
ATGTATAAACATTATGATGCAAATCTTTTCATAAAATTGTTGCTTTTTACAATATTTGCACGATTGTGTATTT
GCGAAGAAAAAGTCTGTGATCGCCGACCACTCGGCACTAAAACCGAGCCCTTGCCTCC
TGATAACAGATTTCAAATAGACATTATTGGGATCATCGACAACCAATATATTCCTAACAATAAATATAAAGTA
CGATTGTTTTCGGCGGACAATGTATCCACATTCATCGCTTATACAATCTCAGCCCGAGGTGACTCTCTTTTTA
AGGATCGAAATCCTCGAAAAGAAACAATTCTTAACGCTGGCAGGATAGAAGTTCCAGA
ACATTCTAGGGACGCAATGAACGCGCCGATATGTACTAACTCTGTTATTCAAACTGATATTACACCAAAGACA
TCTGTAGAGGTGATTTGGAAAGCGCCACCCAAGAACAATAAGTGCGTTACAATATTCGCAGTTTTGGCTGTGA
GACCGGACGTGTGGTACAACTTTGACGGGCCGCTCTCGAAGCGCGTGTGTGAAGATCG
ACGTAACATGGAGGATATGCAACCCATTGAAAACGATAATTGTATCACTTGTGAAGATGCTAGATATTTGCTT
ACATTCGACGGCATCTGGTCATATAACACACATCCACAAATGTTCCCCACAACGAGAGAGCTTGCCCGCTTCA
GTGACGTGGTTGGGGCTTCCCACAACAACAATTTTCAGCTATACAG
>1002050345_1 Heli.1-DT668693.3.5
CTACCGCCGCTAGGCGCCCTACCCCGCGCCGCCCTACCGCCGCTAGGCGCCCTACCCCGCGCCGCCCTACTGC
CACTAGGCACCCTACCCTGCGCCGCCCCCGCCTCGCGGAACTGATCACGCTTCGCATATCGCTTCTTTTCGTT
AAGTAGTTAGATAGTGAATTAATTTTATGTAAGGTGCTCCTCTCGCCCTTCATTGTTT
CCTCACGAAAATTCTTGTATGTACTTGTATGTCGCGTTCGACCCGCGGTCGCCCGCCGCGGCGCCTCGCCGTC
GCCGGTGGGCTCAACTCTAGGTTAGGTTTTTAGAGTATTTTTTTTTTTTCAAAACTCAACGGAGAAAAGTAAA
AGATGACTTGTCCGCGGAGGCCGACCGCCGGTCGCCGCCCCCGTCCCGTTGGACTCCT
GTAATTATTATATAAGTTTATATTAAGCGGTTAAAATTTAATTTTTGTCTATAGCTAGCGCTCGTGACGTGTA
CAGCCTTTAATTTCTTTAGATATTCCTATTTCCTGTAATTTACTTTTAAAGCATAAAAGTGTTATGCGTCACT
TATAATATAATAATTATCGTCGTCAATGCGTCCGGGCGCCCGTTTCCATTTTGTAAAT
GTGTGAGATATAAGGACTAGTGCTGAAGCCGGCCTGTCCTCGTCAGAGTGCGTCAGCGGAAGCGAAGTCTTAT
TATACAACATAAACTCTTGCAGTGCAGGCCTTCAGCTTTTTGTATATTTAAGTTGTACACTGCAGTTCGTGTC
TCGGTGTTTGACGCGTAACACAACCGCGTGTGACCGATATTACATACGGTGATGCCCG
CGTCGCCCTGGTCCGCTGACTTTGTGAAAAGCGAGTAGATTTTATGCAATCACTTCTCATATTGTTTACCTAC
TGTGCTACTACATAGAACTTTGTTGATCAAAACACTAGCATTCTAGTAATGATATATGTCATTTAAAATTTGT
ATGTAGATGTGGAAGATTACAATGTAAACTGGGACTTACATTTAGGTATTTTGATTTA
AAAATGTTAAGATACATTATATTTTGGCTGCGCCTTGTCCTGTACTTAGGTACGCAATTGACATTTGTCGTAT
ATCTTCTTATAGATTATAAAAGTCTTTTGATGTCATTGTTAGGAGTCTATGTCGTCGTTATATTGGATGCTCC
AAAGCATTCATAAGTCGGTCGTGTCTCTGGTAGATATTACATACATATATGGCAATAT
TCAATATATAATTATTTTCAAATCTAGAAATATAATAATTCCTTATCATGTAAATCAACGAGTGATAACATTT
CATAATATTCTGTATATTAGTTTTAATGTATTTTTACGTGACGTAAATAAGTTGGCAATATTGACATATTTGA
ACCGGCTACTGTAATTTTACTTTAAATAGCTATGCATTTAATTTCATATTACACTAAA
TAATAATTGAACAAATCGGTCTGCTTACTTACGTCATACAAAAATGTCATTATTTTGAGTTAACCAATACTTA
TTAATTATTGTATTTGTAGAGTATGTTCAAATAGAAATTATACGTGT
>1002050346_1 Heli.1-EL603018.1.5
GCACGAGGTAAAGCTAGTTTTACAAAAAAAGTGATAAAACATAATTATTATATCGAATACATATATAAGTTAC
ATTCTTAAATATGAGCCAACAGGCCGATGCTGCTCCAAAAGAGCGATATATTCCTAATGGACTTAAATTTGCT
TTCGGAGGTCTTGCCGGTATGGCAGCCACATGTGTAGTACAACCGTTAGATTTAATCA

AAACTCGAATGCAGTTGAGTGGAGGTGGAAGAACCTCTTTTGCTGTAGCTGGTGAAATTGTTGCAAGAGAAGG
TTTCTTCTCTTTATATACAGGACTATCAGCAGGGCTTCTACGACAAGCAACATACACAACTACACGTCTTGGA
ATATACAATTTGTTATTTGATGCTTACAAGGAGCGTAATGCAGGTGCTGCACCTGGTT
TCGGCACGAAGACGTTCCTCGGCATAACTGCGGGGTCGATCGGTGCTTTTGTTGGCACTCCTGCTGAGGTAGC
TCTCATCCGTATGACCGCTGATGGTAGACTACCAAAGGAACAACGAAGAAATTACAAGAATGTTCTAGATGCA
CTTCTCANGATAATACGCGAAGAAGGTGTATTGAAGCTGTGGCGCGGCGCCACGCCCA
CTGTTGGGCGCGCCATGGTGGGTCACGCGGCGCAGCTTAGTACTTACT
>1002050347_1 Heli.1-EL597949.1.5
AATCTGTGGTCACAAAATAAAATTATTTAGTAAATCATATTACACTTAAGCGGACAAATTAAAATAATATTTA
ATATATTATATGTATATTTTAATTATTTTTTTATTTAAATTATTTTTATAACCAAGGTTTAATTTTTATACAT
TCCATTTTTTTATAATTACTTTATGAGTTTTATTGACTCATTTTTTGTACAATATTTT
AAAATACAAGCATTCTAAATTTTCCTTAATTAAAAATATTATACTAAAACTATTTTCAACAGATAATAAATTC
AGAAGAGTTTATATTTTGACGTTCAAAAGATAATATATACATATTATTTGTTAAAAGCAGTTTTAGTATGAAA
ATATGAAAAATATAAAAAGTATACATACATTTTCCAGATTATAATTAAGTTTTTTTAA
CAATAACGCACAAGGGTGGTGGGTCACAAGAGCATTGTAACGCTTCCATTTATTTTGTTAATAAAATTTTTA
>1002050348_1 Heli.1-EL603493.1.5
GCACGAGGACGCGTCGGCCATTTTTTATTTTACGTTCTCTCTTCAAAATTCGCGCCAAATAAAAATTGTGCTG
TGAAAAAAATTGGTTTTCGTTTATGGATTTGTGTATGTGATTTAAAAGAAAAAAAGCAAAATGCCGGCATACG
ACAATGAAGGTGCCACAATATCCATGGAGGATGTCCGAGATCCTGAGCTGACGATCAA
TACTATAGGAGAAGAATTAGCTGCTAGAAGAAACCGGATGTTGAAACCGCAGAAGTCCACATTAGCTTCAAGA
AGACAGCAAGCAGTATGTGTGAGACGAGCTCATAAGAAATACGGCCCAACAAAGAATCCTAATGTCATATTGG
ACGGATTGAACATGACTGTACCTAAAGGCGCCATATATGGTCTTCTGGGAGCGTCAGG
ATGTGGAAAAACTACATTACTCTCTTGTATAGTTGGTCGTCGTCGTCTCAATGCTGGTGAAATATGGGTCCTT
GGTGGAAGACCTGGTGGACCTGGTAGTGGTGTTCCTGGACCCAGGATTGGTTATATGCCTCAGGAAATCGCGC
TATTCGGAGAGTTTTCTATACGAGAAACAATGATTTACTTTGGATGGATAGCGGGCAT
GACTACTAAAGAAGTGGACGAGAAAGTCGATTTTTTAGTATCACTGCTACAGTTACCTAATCCTACTAGACAA
GTCAAAAATCTATCTGGTGGTCAACAGCGTAGAGTGTCTTTAGCAGCTGCTTTATTACATGATCCCGAGCTAC
TTATCCTGGATGAGCCTACTGTTGGAGTGGATCCAGTACTACGACAGAGCATATGGGA
TCACTTAGTGGATATTAGTAAAGGCGGAAGAACAACAGTCATCATCACAACACATTATATCGACGAAACGAAA
CAAGCTAATATTATTGGCCTCATGCGAGGAGGCAGGTTCCTAGCTGAGGAGTCACCAACTGAGCTGATAACTA
GGTACAACGCGGAGAGCCTTGAAGATGTCTTCCTAAAACTATCTGTACTCCAAAACAT
GGGGAAACGACGTCGATCAAGTATCCTCGCTGATGTTGTTGAACGTGTGGAAATTCCTTCAATACCTTTTTGC
TACACCAAAAGGCCGCTAAATCT
>1002050349_1 Heli.1-EL601787.1.5
GCACGAGGGTTTGCCTGCGTACTAAGAGCGCGTCCACAGAGTACAAGCGCGTCGTTGACTCGTTGCTTTGCAA
ATCGTAGTCGAGCTCATAGTCTTGTCGCGGAAACAGGATTGTCATATGTGCGATAAAAAACTAAAGACCGTCT
GCAATGCGGCTTTGTTTTGTTGTGATAGTTAACTGTGCCGGATCAAAGGAATCTATAT
TTTTAAGTGTTTTACAGTGTTTATCATGATCATATGATGTTAATTATTTGTCATATTAATGCACAAATTAGCT
TCGTTTTCATATATGAGAAATAAAATGAAAAAGTGAGTTTATTAAAAGTGACAAGAAGTCTATTAAGAAAGGA
AGCAAAATTATATTGAATATATTACAGAAATTTTTCCTTATTTAAAAACATGACCGTT
ACGTACACCGGTGAAGTTGCGACTTGTAGAGGTTTTGGAACATTTTTGAAGGTTTTAAACAGATGGCGAGGAA
GCATATACAAGTTAATATGGCTAGATTTATTAGTATTTCTTCTATTATATTATATATTAAATTTAATTTATCG
ACTACTTCTAGATGAAGATTCTAAAAAGACATTTGAAGGGATAGTTAATTATTGTAGT
TTCCACGGAAATGTAATTCCGTTGTCTTTTGTGCTAGGTTTTTATGTAACTGTGGTAATGAATCGTTGGTGGA
ATCAATATACAACTATTCCGTGGCCAGACTCCATCGCCGTGTTCGTGTCAGCAACTATACATGGACAAGACGA
GCGAGGGCGGCTGATGCGTCGT
>1002050350_1 Heli.1-ES584811.1.5
GTCAACTTAAGCACTTCAAAACTTGATGTTTCTTACATTATGAATTGTATTTAATGAAAATTACATAATTTAC
TCTAAAAGTAAACAATAACCAAGAAAATGCTTAAATTTGGAATTAAGTCTGCCATACTTGGGTCAGCTGTATA
TTATACAGTTGATAAAGGAGTGTGGAAAGATAGTGCTACAACAAGTGAGCTTTATGAA
GAACTCGAAAAAGGAATATCTCCATATGTAGGCGAATTTAAAAGTCAAATTCCTTACGAGCTTCCTCCTTTGC
CATCAAACGATAAATTAACATATTTATGTAAATATTATTGGAACAGTGGAGTCAAAGCTACTTTCAAATTTTT
AGTAGATCTTCCATCACACGCTTCAAAGGCAGCAACTAAAACATATGACTTCATTTCT

GCCTCGTTAGATCCTATTGAACCCAGTGCAGATCACAGTCAAGAAAAAACAAAATAATTCATATAATAATAGA
TTAGTGTAATAACTGAACTAACTTATTAAATCTGT
>1002050351_1 Heli.1-CO729780.1.5
GTAAAAAAGTCGCTTGCAAATTTTGTTTTGTACAACACAAAAATACTAAAAAGTTATATTATAAATAGATAAC
CAATACTTATAACATTTAGTGCCGTTTATTAGGAAAAGAATAAAATGAGATCAACCTTTTGTTTATCCGCCAT
TTTATTGGAGGAGGATGATGAATTTGAAGAATTCCCCGCTGAACATTGGGGAACTGAA
GACGCTGATGATGAGGATGTATCAGTGTGGGAAGACAACTGGGAGGATGACATAGTACAAGATGATTTTAGTC
AGCAACTGCGTCAACAACTGGAGAAACTCCGAGAGCAAAACAAGTCTTAACTAGTTTGTAAATAATCTCTTTC
ATTTATGATTACTTTTTAAATAAAATAATACAAA
>1002050352_1 Heli.1-DT664583.3.5
GCACGAGGGTCCCTACAACTAGATTATGCGAAGTTAATAAAATACTAAAATGTGTATTTTAAAATATACGATT
GATGTCTTCTTCCTTCTATTCTTACTGCTAACTCCTATTTTAGCAGAATGGAATACGAAGGATTATATACGAC
GAGAACATTCATTAACAAAACCTTACCAAGGTAGTGGTATGTCCGTACCATACTGGGA
CTTTTTAGGTAACACCATAGTTACTACAAATTACGTTCGTTTAACACCGGATTTGCAGTCAAAGTCCGGCGCT
ATTTGGAATACCGCGCCATGTTATACAAGGAACTGGGAGTTGCAAGTTCAATTCAAAGTGCATGGACGAGGCA
AAGATTTATTCGGAGATGGTTTTGCCATTTGGTATGTCCGTGATAGGATGCAAACTGG
ACCGGTCTTTGGCAGTAAAGACTATTTCCAAGGACTCGCAATCATATTGGATACCTATAGCAACCATAATGGC
GCTCATAATCATCAACATCCATACATATCGGCGATGATAAGCAACGGCTCTCTACACTACGACCACGACCGGG
ATGGAACCCACACTCAGCTCTCAGGTTGCGAAGCCAAGTTCCGAAACTATAACCATGA
TACTCATTTGTCTATTATATATAAGGATGATACACTTGTTGTATCAACAGACTTAGAAGGCAAAAACGCATGG
AAAGAATGTCTAAGAGTAGAGAATGTATTGCTACCAACCGGCTACTACTTCGGTGCAACTGCAACTACTGGTG
ACTTGAGCGATAACCATGACATTATTGCTATTAA
>1002050353_1 Heli.1-EL601148.1.5
GCACGAGGAAACGTTTCAAAATGGCAGCTAAGTTCTTCGTCGTCGCTTTGTTGGTAGCAGCCGCTCAAGGCAG
CGCCATCCACGGTGCTGACTACACCAGCTTCTCTTATGGAGTTGCGGACCCACACACCGGTGACGTGAAGAGC
CAACACGAAACCCGAGTTGGAGACAATGTCGTCGGCCAGTACTCCTTGTTGGAATCTG
ATGGAAGCCGCCGCACCGTGGACTATGCTGCTGATGCCCATTCTGGATTCAACGCCATCGTACGCAAAGACCC
AGCTCTTATTGCCCACGCCGCTCCCGCCGTCGTTGCCGCACCCCTTGCCCACGCTGCCCCTGCAGTTGTAGCC
GCTCCTCTGGCCCATGCCGCTCCCCTCGCATACGCTGCCCGTGTAGCTCCCATCGCTC
ACGCTGCTCCCGCCGTCATCGCTGGACCCGCTGTATCTTACTCTGCATCTTCAAGCTCAGTAGCTCATGGAGG
TTATGCCGCCCCATTGGCCCATGGTGCCCTTGCCGCTCCCTTAGCCCACGGTGCCCTTGCCGCTCCCTGGGGC
CATGGTGCCCTTGCCGCTCCCTGTCTCGCTCACGCCCTTGCTGCTCGCGCTCACGGTC
TCGGACACTACTAAATAATGATATAAGAATTTATTTATGTACCTACCTAATTAATAATTATGACGATGGTTAT
GAATGATGAATGATGAACTTGCTGTGATCAATTAAAAAACTTTATAC
>1002050354_1 Heli.1-CO729534.1.5
GTCGGAATTCCGGGTCGACCACGCGTCCGCGAGGATAGGGAAGATTTTGTGACATGGCTGGAAATAATGCTGA
AGAAGTTGAGGGTTCAGCAGGCGTTGAGAGCAAAGCCAACACTAACACAGAAATTCCCCCAGAATTCTTGATA
AAACATCCACTTCAGAACAATTGGAGTCTCTGGTTCTATGATAATGATAGAACAAAAT
CATGGGAGGAGAATCAAATTGAACTAACATCGTTTGACACAGTTGAAGATTTCTGGAGACTCTACCATCATAT
TAAACTACCCTCTGAGCTTCGTCAAGGCCATGACTATGCGGTATTTAAACAAGGCATACGCCCCATGTGGGAG
GACGACGCCAACAAAATGGGGGGGCGCTGGTTAATAAGTCTCGAGAAGAAACAAAGAA
ATTCGGACTTGGATCGCTTCTGGCTTGATGTTGTGCTTCTTCTGATTGGTGAAAACTTCGAGCACTCTGAGGA
AATATGCGGCGCAGTCGTAAATATTAGAGCAAAAATTGATAAAATCGCTATATGGACAGCTGATACATCGAAG
CAACAAGCTAATTTGGAAATAGGCAGAAAATTAAAAGAGCAACTCGGCATCCATGGGA
AAATTGGTTTCCAACTCCATAGAGACACTATGGTCAAGCACAGTTCTGCCACTAAGAATCTGTACACCGTCTA
GATCATACTTATTAATTATTTGTAAACTAAACACATAATAATATGACATTATATTAAACTATTACATTATAAA
ATTACATTTCTATATGGTCCAA
>1002050355_1 Heli.1-DT667710.3.5
GCACGAGGTCTGCGCTCGTGTGTGCCGCAGGAGTGGCCCGAACTGTCATTGTTCATGTAGTTTTCTGTGATAA
GAATTACTGTGAAATTATAGTTTAGCGAATTAATTAATTATAACAATCACTATGTCTACGTCGGGGGAATTTG
GTAATCCTCTACGCAAGTTTAAACTTGTGTTCCTTGGTGAACAAAGTGTGGGAAAAAC
GTCGCTCATCACTAGATTCATGTATGACAGTTTTGACAACACTTACCAGGCTACAATTGGTATAGACTTTCTG
TCTAAGACCATGTACTTAGAGGACCGTACAGTGAGACTGCAGTTGTGGGATACAGCAGGACAGGAGCGTTTCC
GCTCCCTTATTCCTTCTTACATCAGAGACTCCACTGTGGCTGTTGTTGTTTATGATAT

AACTAATGCAAATTCTTTCCATCAAACATCCAAATGGATCGACGACGTGCGCACCGAGCGTGGGTCGGACGTC
ATCATCATGCTGGTGGGCAACAAGACGGATCTCTCCGACAAGAGGCAGGTGTCCACAGAGGACGGCGACCGCA
AAGCCAAGGAGCTCAACGTTATGTTCATAGAGACGAGTGCCAAGGCTGGATATAATGT
GAAACAGTTATTCCGAAGAGTAGCCGCCGCGCTGCCCGGCATGGACTCGGCTGAGAGCAAGCCACCGGAAGAC
ACCGTTGATCTCCAAACTCAGTCTCCAATGGGCTCCGAGCAGGAAGGCAACGAGAGCGGATGCGTATGCTGAA
TAATACCACCCCCCTCTTTACATACCCTTGCCCCCCCCCATTCCCCCGCACCGCTTC
ACGGATACTTACGTGCCTGGCGTCATACAAAACATTATTATATAGGCTAGTGTTGCTAGATTTAATTTATTAC
GGGAATTAATGAATAGTTGAATCGAAATGTTTATC
>1002050356_1 Heli.1-EL596201.1.5
CGGCACGAGGGTCTTATTGAGAAGTCAGTTTACCAAAAATAAGGAGAAGAATTTGAGACGAGACGACCGCACC
AACAGATTTGACAGAGAGAGACCAAGAACTGAAAAAGAGTTCCGCGATAAATACAGAAACGAAAGAGAACGCA
GATATTCCGAAAAAGATGATAAAAATAAGAATATTGAACATGATAAAGACACAAAACG
TTATCAAGATAAAGATAACATTGAAACAGATAAAGATCGTAGAGACATAAGAGACAGGAATGAAAGATCTAAG
GAAAGAAACGAGAGATCTGTTGATAGATCAAATGAGTTTAAAGAAAGGAAATTATCTGAAACGGATCGATCTA
GGGAGACCGATGATAGAAAAGAACGTGAAAAATCCAAAGAACACGATGAAAAAAATAT
ACTAGATAAAGATAGATCGCCAGTAAGAGAAGAAAAACGTAATAAATCTAAGGAAAAGGAAGATAAAGCAAAT
ACTGAAGAAAAAGATACTTCCAAAGAAAGTGATACTAGAAAAGATAAATCTGATAAAGATACAAGGAGATACT
CTCGGGATAGAGAAAGATCTAGAGGTAGAGATAGCAGAAGAGATAGATATCAGAGGGA
CAGATCGCGAGATAGGCGCGACAGGTCTCGAGATAGGCACGATAGATCTAAAGATAGGATTGATAAACGGAAT
AAAGAGGACACCCAAGATATATC
>1002050357_1 Heli.1-EL601454.1.5
GCACGAGGACACGTTAAGGGGGTTGACGGAGTGTGAGATTGTGTGACAACACATTGTGGGTGTCAATGTGTGA
CAGTAAATTGTGGAGGGGGTCTGAAATTTCGTGGTCACATTTGAAAACATAATATGCAAACCTAATTTCTGAA
CTGTGTGACGTAGTTGCCGGGCGGCGGCGGTGCGTGGTTGGCGCAGAGCCGAGCATTA
TTCGACGATAACTTGCAAATTGAGCATTTTGTTGCATATAAAAGCATACTTGTAAATTTTTTATAACTAGCGT
AAACTGATCTCTCATGATAATATTAATATTTAATGATATTTCAGACTATTATTGTATTTGTATAAACAATTCG
AAAAATATAAATTTTAGATTTTACTACATTATAAATATTCGTTTATTTGGGCAACTCT
TAGTAATTTTGTGACGCGTTGTACTTGTACCTCACCTTCTTATCCAGCGCTGAGTAAATGTAACGTATTTTAT
AAGCACTGTCGGTGTCCTATTGTAATGTCATAGATTTTTCGATAATATTGTATTCTATATTCGATAAATATTT
CTGCTATGTAAAAACAACTTCACACATTAATATAGTAGACATGTATGAAGTTAGTTAC
AGAACTATGTTAAAATAATTAAACTCATTGTCATAGTTTTAAGTGTTTTGGGCAATTTCGTTTCGCACTTCAC
TTGACATTTAATTTTTATGAGAACGTTGTCGTTCGCCTCTCGCAATTCGGAGGACTTGC
>1002050358_1 Heli.1-EL598454.1.5
GCACGAGGGCGCTGTATAATTTATTTTTCGTATTTGTTTCACTGTATTATATAGAGCAATGTCAGGTATTGCA
AGTGCACGTTTAGCTGAAGAAAGAAAAGCCTGGCGGAAGGACCATCCTTTTGTAAGTGGCTGTATGATAGCTA
TGAAAAATATTTTTTAATTTCTACAAGTAAATAAAACATTCGATTTTTTAGGGTTTCG
TTGCCAGACCAATGAAAAATCCTGATGGGTCATTAAATCTCATGACTTGGGAATGTGCGATTCCTGGCAAAAA
AGGAACGCCATGGGAAGGTGGTCTTTATAAATTAAGAATGATTTTCAAAGACGACTACCCATCAAGCCCACCA
AAGTGCAAGTTTGAACCACCACTATTCCATCCGAATGTATACCCATCTGGGACAGTAT
GCTTGTCACTACTTGATGAAGAAAAAGATTGGCGTCCAGCAATCACTATTAAACAAATTCTACTTGGTATTCA
GGATCTGCTAAATGAACCCAACGTAAAAGACCCCGCACAAGCTGAAGCATACACAATTTACTGCCAAAATAGA
TTAGAGTATGACAAAAGAGTCAGGGCACAGGCACGAGCTATGGCGGCAACAGAGTGAA
AAAGTACATTCCTGACATTTATGTGAATGTAGGGATCATCTTTGTAGCAAATGGCAATATTATTTTGTGTACA
GTTGGCGTGTTACAAATCATTAATTTAATAACAGTCTTGCTACAGAGTTGATCAACCAA
>1002050359_1 Heli.1-DT663230.3.5
ATTATAGACATCACACAATGGATCTGGAGTTTCGAAACTTGACGTACACCGTCTACAATAAAATAGTTAGAGG
ACCCGGAAAGCAAGTCGTTAAAGGCGTAAGCGGCAAGTTCATGTCCGGGCAGCTTATAGCTATAATGGGACCT
TCGGGTGCAGGAAAAAGCTCTCTTCTCAATGTCATATCTGGTTATAAATCTGCTGGAG
TATCAGGTGAATTGTTAGTAAATGGTCAACCCCGTGACGAGCATAATTTCAAAAGGTCCTCATGCTACATCAC
GCAGGAGGATCTTCACCAACCACTACTTACAGTGCGAGAAGCTATGGACGTGGCAGCTAAATTTAAACTGCCT
AAAGGAGCTAAGATACCTTCTGAAGATATTTTACAACAGTTGGGTTTACTTGAACACC
AACATACTAGAACAGATCAACTATCCGGCGGACAAAAGAAAAGATTATCTATAGCTCTAGAGCTTGTGAACAA
TCCACCAGTTTTCTTCCTCGATGAGCCAACGAGTGGCCTTGATACAGTCACAACTATGCAATGTGTGAAGTTG
TTGAGGCAATTGGCACGACAGGGTCGGACAGTGGTGTGTACCATACATCAGCCTACTG

CATCGCTCTTTGAAATGTTTGATCAGGTTTACATAGTAGCTGATGGTCGATGCATTTACCAAGGCGATACAGA
TGCAATGGTCCCGTTTCTGAACAGTATAGGTTTAACGTGTCCACGTCATCATAATCCTGCAGATTTTATAATA
GAAGTAACAGAATCCCCACATAACATAAATATGCTATGCAACGAAATGCTGAACGGTA
TGGTATACAAATCCGCGAAAATGGAGTCTACAGTTAACGAGCCCTTAAACAACTTGGAATTGAAGATCTGCTA
CCAAATAGAGGACGAATCTGGAGAAACACAGATTTTATTATCAAATGACAAATGTACATATAAAGTGAAACAG
TCGAACGAGGCTAGTAACTCTACTCTGTCTCTAAGTCAGATATCTCAGGACGACTCAT
CTTTGGCGTGGATGAAGATGCAGGATAATGTTAATCAATACCCAACAACGTATTTCGAGCAGTTCACAATACT
ATTATGTAGAATGTTGCTGCAAATATCCAGAAATAGACAAGCGCTATGGATCCAAACAATACATCATGTGATG
TGTTCGGTTCTTATAGGGTTATGTTTCTTCGATACGGCCAATAACGGCAAGGAAATGT
TCAACCATTTAAAATTGTGCGTCGGCCTCGTTATATTCTTCGCGTACACGCACATCATGGTGCCGGTGTTAGT
ATATCCGCAAGAAGTTAAGCTGGTTAAAAAAGAGCATTTTAACAGTTGGTATAGTTTGTTTCCGTACTATGCT
GCACTAACAGTATCAAAGTTGCCAGTGCAAATAACTTTGAATTTGGTTTTTTGCACCA
TCGTGTTCTTTATGGTTGGCGTGCCATTCAATGTTGCCAGATTTATTGTATTCTGTTTTATTGGCAACATTGT
TTCTTTGGTGTCTGAGGGGATTGGCATGGCGATCGGATCTCTGTTTAGTGTTAGGAATGGATGCGCCATTGGC
CCAGCAGCCATTGCGCCGTTCCTCGGTCTTGCTA
>1002050360_1 Heli.1-DT664280.3.5
GCACGAGGTTGTGCTTGTTTGTATCAAGAAAAAGCTATGATGGCTGAAGATACGACGGCAGATGCGACCAGAC
GCCTAAACGTTAAGAAACAGACGCTCGACGACGCGTACGCGGCTCCAGCGAACTTCCTAGAAATTGACGTGGT
AAATCCAGTTACGATGGGAGTTGGGAAAAAACGCTACACGGATTACGAAGTTCGCATG
AGGACCAACCTTCCCGTATTTAAAGTAAAAGAATCCAGTGTGAGACGGCGGTACAGTGACTTTGAATGGCTCA
GGACTGAACTAGAAAGGGATAGTAAAATTGTAGTACCTCCACTCCCTGGTAAAGCTTTAAAGAGACAACTACC
TTTCCGAGGGGATGATGGCATTTTTGAGGAAGAATTCATAGAAGACCGTAGAAAAGGA
CTAGAAGTATTCATAAACAAAATAGCTGGACACCCCCTAGCTCAGAACGAGCGTTGCCTCCACATGTTCTTAC
AAGACCCAACCATTGATAAAAACTATGTGCCGGGAAAGATAAGGAACACATAACCGATTCACTTCGAACTGTA
AGATGATTCTCGACTCGCCATTTTGTTTGTGTCAGTTATGTCAGTCAAGTCAGTTATG
TCAGTCGATATTTAGGTAAAAAGTTCTAAAGTTCGTGAAATTAATTGTTAGATTAATTAACTCATTGAGGACT
GTAGGTACTAAATTAGTATTTTTTTGGGGAGTTTTTTTTTGTCATAACATTTGGTTTTTTTAATTTTTAATGT
TATATGTACTATGTAAATAGATTAGCATGAGATTTTAGCATTTTTTGCATACTATTTT
TTCAGTAACGAA
>1002050361_1 Heli.1-DV501305.2.5
GCACGAGGTTTACCTTCTTTATTAAATCAACGCAGACACAAAACAAATTCAATAGAATCAATAAAACCGGTGT
GGAAATTACATACATAAAAGCTAAAATGGTAAAGTACTATTTAAACTTCTTCAGCAATATCTGTAATATGTGT
ATAAAAAAAATATTAAGTGTACACAAATATGTTTGATATGATGAAAATATGACTTGCT
ACAGCAATGAGGTGGAAGACATGAAATTAAATAAAGAAAACGTAGGCTAAACATGACATTATATGTATACCAA
ATACATATGTATACTTATGAATACAAGAAAAATATGTTCTTTTCAATTAAATTACTATTGATAAGGCATATGG
TATCATAGATATTAGACGACAAGTCGACAATTTATTTAGATAAGTTGCATAACAAAAA
TATATTTTTGAAAAATCAATATGTAAGGCGTTTTTTTCTTTCTAATAAATACATATTATTTTGCTTGAAAAGA
ATAATCTGTATAATCACTAACATCGTAACTATTTCTATTCAAAATATATCATTCATAATAAAGTTTTTTTTT
TCGTTTGATTTATTGGTATTATACATGTCATCAATTCTTTGTTTATGTTAAACATTAG
CACCATGTTCGGTTGATCCTATTGAAGTCAGTGGGATTGTATTATTAATTTTACAAAGTAAACCATGACTAAG
CACACACCTGAAAATGAATAAACTATATTTACACAGAACATATATATTGCACTTAAAATTGAACATGAAAAGT
GATGGTTCAGAAGTGGATATTTGCATGTTCCTTACTAAACTGTATGGACCTTGAAGAA
CTTTCAACTTAT
>1002050362_1 Heli.1-DT663381.3.5
GCACGAGGCAACCCCCTGCTTCTATATTGTGATTTTTAAGTGTGTTTGTTATATTTGAGTACATATATAAGCG
TCTATTTATTTACCAAGTACCAAACGAAATCAGAAGCTACAGGTGCTATGCATATTATATGAAAAAAATTAAT
ACATTGAAAGTAAATTCATTAATTTAGCTATATTGCTTTAAAAAATAGAATTATACAC
TAAAGAAAATGCAAACAATCAAATGTGTTGTGGTTGGTGATGGTGCAGTGGGTAAAACCTGCTTGCTCATCAG
TTATACCACTAATAAATTTCCTTCCGAATATGTGCCTACTGTGTTTGACAATTATGCTGTCACAGTTATGATA
GGTGGTGAACCTTATACCCTGGGCTTGTTTGACACTGCAGGTCAGGAAGATTATGACA
GGCTCAGACCATTGAGTTACCCACAAACTGATGTTTTCCTTGTTTGCTTTAGTGTTGTGAGTCCCAGTTCTTT
TGAAAATGTCAAGGAAAAGTGGGTTCCGGAAATAACACACCATCAGCAAAAAACACCTTTCCTTTTAGTTGGC
ACTCAAATATATCTACGAGATGATCCTGCTACTATGGAGAAGTTAGCTAAAATAAAGC

AAAAACCAGTTTCTCTGGAACAAGGTGAAAAACTGGCCAAGGAGCTGAAAGCTGTCAAATATGTGGAGTGCTC
TGCTCTCACTCAGAAAGGTTTGAAGAATGTGTTTGATGAAGCAATCCTAGCAGCTCTGGAGCCGCCGGAGCCA
GTTAAGAGGAGGAAATGTGTTTTGTTGTAATGAGTGTTATATGCAATCTGGCAGACTG
CTGAGCTGATTCATACAACTTCACAATTTTCTTAAGCTATTCATATATTGTTCATTTTTTTAAAATAATTATA
ACTATGAAATATGCCATACTTTAAAATAGCATCCTGAGAGTAAGTCTTGTGCTACTTTATATTATACTATTTT
ATACCACCATAGTAATATTTTGTCAGCTTATAATTTTATATTTATAAGTCACTATACA
AGACCATTTTGATACCTAACAACTTATATGAAGTACTATAGATTTTATGATTATAAAAATATTATATGGCTAT
ATCCTTCGAAGAGACTCATGTATTCTACTTTATTTATTATCCTTATGTGTTGTATGCATATTTATAACGTAAG
TGTTCTTAAGAGGTGGAGATATTCTATAAATTGTGATAATTCATTACTCGAGAAGTGT
AAAATGACCCATAGTAGAAACAAATATATTGTGTCTATTAGTTTAAGAAATGATTTTAATTTGTCTATCTACC
ATTTATAAGAATATTTTGTAATGGTGTTATAAACAAAGCACAAACAACATAAACATTTCTGATTTATACTGCT
CCAAGCGATCACAGATTTTATAATATTTATCAACGTTTAAAAATGAGTTTCATAAATT
GGTCTGAATATTCCGCCTCTTAATAATAATTGATATAATAATTTTATAAATACGTTTTCCAACGTATTCCTAG
TTTTAACCCTATTAACATCTTAAATTAGAATATTAGAGCTGATGATATTAATGATGCTCTAAATTTAACGCAT
CACTAATGTCACCATGGGTTTGTTATACAGAGTATATAATAACGGTGTGAATATCAAT
TACTAGTATTTAACTTTAGTGGAAAGTAGCATTTTTATCACGCTATATATAGCTATGTGAATTTTGACTGAAT
TAAAAGTATTCATTCCATAGGATGTCAATGCATTTTTTTTTTTAATTAATATACGCGCTGCGGTTTTTTAATG
TATTATATATGATCGCTTGCGTTCATACTACGGCGTGACTTGCTTGTGTCCCAATTTT
ATACTCTGTATACGCTGCTGCCTC
>1002050363_1 Heli.1-EL603119.1.5
AAACTACCATACAGTGGGAGAACAGCTTTGTATCGGTCTACTCAAAGGATAATCCCAACTTACTGTTCAACAT
GGCAGGATTTGAATGCAGAATATTGCCTAAATGTCGAAGCCAACACGAGGAGCTATCTCATCGAGATGGAGTG
TGGAATCTCCAAAACGAAGTGACAAAAGAACGAACAGCACAATGCTATTTACGTGTGG
ACGATGAATCCCTAGCGCGTTTCCATAATAGAGTGCGACAGATTTTAATGGCGTCAGGCTCCACTACATTTAC
TAAGATCGTTAATAAATGGAATACTTCGCTTATTGGTCTTATGACATACTTCCGTGAAGCAGTTGTTAACACA
CAAGAGTTGTTAGATCTCCTCGTCAAATGTGAGAATAAGATACAAACTCGTATTAAAA
TCGGTTTGAATTCAAAAATGCCCTCCCGTTTCCCGCCCGTCGTGTTCTATACGCCTAAGGAGTTGGGTGGTTT
GGGAATGTTGTCTATGGGCCATGTTTTGATTCCACAGTCCGATCTTCGGTGGTCGAAACAAACAGACGTTGGC
ATCACCCATTTCCGCTCAGGAATGTCTCATGATGAAGACCAGCTTATTCCCAATTTAT
ACCGTTACATCCAGCCCTGGGAAGCTGAATTTGTTGACTCACAAAGAGTATGGGCAGAATATGCATTGAAGAG
ACAGGAGGCTAATGCTCAGAATAGGCGTCTAACATTGGAAGATTTGGAAGATTCGTGGGACAGAGGTATACCG
AGAATCAATACGCTGTTCCAAAAAGACAGACACACGCTCGCCTATGATAAAGGATGGC
GTATACGAACTGAATTTAAGCAGTATCAGGTTCTAAAACAAAACCCCTTCTGGTGGACTCACCAACGTCACGA
CGGTAAACTGTGGAACTTGAACAACTACCGTACCGACATGATACAAGCACTCGGAGGAGTGGAGGGCATTCTC
GAACATACACTGTTCAAGGGCA
>1002050364_1 Heli.1-DT668615.3.5
GGTGGAGAACCAGTCGGGCTTTGCATGCTTAGCATTGGCGAGGATTGAAGTGTCGCCGGATAGGTTAGAAAAT
TGTCCTTTTTAATTCTTTGATATTTTCAAAGCATTTTGGTCGAAAGATGGCCCAAAGTAAATTAAATGATATG
GCGGGCAAGTTTGCTAAAGGGGGCCCGCCTGGACTTGGTATTGGATTAAAAGTAGCTG
CTGTACTAGGCGCAGGGGTCTACGGTATTTCACAGTCCTTATTTACTGTCGAGGGTGGTCATCGTGCAATTAT
GTTTAACAGAATAGGAGGAGTGCAACAGCATGTTATGTCTGAAGGTCTTCATTTTCGCGTTCCATGGTTCCAG
TATCCTATCATTTATGATATCAGGTCACGACCTCGTAAAATATCTTCTCCAACCGGTT
CAAAAGATTTACAAATGGTAAACATTTCGTTGAGAGTGTTATCTCGTCCTGATTCTAGCAATTTATCTACTAT
GTACAGACAATTGGGTACTGATTATGATGAAAAAGTCCTTCCTTCAATTTGTAATGAAGTGTTGAAATCGGTT
GTAGCCAAGTTTAATGCATCCCAACTGATCACTCAACGTCAGCAAGTATCATTATTAA
TAAGAAGAGAATTGGTTGAGAGGGCAGCAGATTTTAACATAATCTTAGATGATGTATCTCTAACAGAATTGAG
TTTTGGTAAAGAATATACAGCTGCAGTTGAAGCTAAACAAGTAGCCCAACAAGAAGCTCAGCGTGCTGCTTTC
GTTGTTGAAAGGGCTAAGCAAGAGCTCCAGCAGAAAATTGTACAAGCTGAAGGAGAAG
CTGAAGCAGCAGAGATGTTAGGTAAATCTATGGGAATGAATCCTGGGTACTTAAAGTTGCGCAAAATCCGTGC
AGCTCAAAGTATTTCTAGGATGATTGCTCAGTCACAAAACAGAGTCTTCTTGCCTGGCAATAGTATAATGATT
AACCTGCAAGACCCATCCTTTGATGACTTATCAGAGAAACTCACAAAGAAGAAGTAAC
ATACGGTAGAGGAAGAGTATGAAAGAAGCCTGGAGCTGGTGACTACTCTTTCTGAAAATAATGAAACTGCAGT
TATTTTTAGTGGCGCTGCTTCAGTTGCTATATAAGTAATTATTTCTTTAGGCATATTGTGGTGTTTCACATAC
ACTTAACATTTGAAAGAATATATTATGCATTTTATGTTAAATACTTTGTAGGTTTAGT

ATGTATGGAGATCAGTTGGAATAAATTTTTTAAGACATTAAAAAAAAA
>1002050365_1 Heli.1-DT663946.3.5
GTTGAGGTAGTGAGGTCGAGCCTACTGCAGCAGCTCACAGTCCTGCAGCTGACCTCGAAGCACTCATCGAGCG
GCTTGAGCGTGTGACGTCACGCTTAGAGCGCTTGCCCGTCCTGCGCGAGAGCACTCCTACCCCGGCGAGTTCC
CCGGTGCTACCGCCTGTCAGTGATAGCGCATCCACTCCACAGACCATAAGTTTTATAG
AAACTGACAATATGAGTGTCAACGGATACCAGGACATTGTGCAGGGACCCCTGCTTACCTATCTACAGCTGTC
GAAGCAGCTTGGCGGTGACATTGCCACGCACGCTAACCTCGTTAACGAAGCTTTTCAACAACAGCTTCGTTAT
ATTCAACTTGCGACTTCGCGGTCTAAGCCGTCGCAGACTGAGGAGGTACAGCTCCTAG
CGCCAACCAGCGAAAAGATCTCATCTATCCAACAATACCGCGAGAAGAACAGAGCATCGCCTTTCTTCAACCA
CCTCTCCGCTATATCGGAGAGTATCCCCGCTTTGGGATGGGTCGCCGTGTCTCCCACACCGGCGCCGTATGTC
AAGGAAATGAACGACGCCGGCCAGTTTTACACGAACCGTGTCCTAAAAGAGTGGAAGG
AGAAAGACAAGACCCACGTTGAGTGGTGTCGCGCTTGGGTGCAGCTGCTATCTGACCTGCAGGCGTACGTGAA
ACAGTATCACACTACTGGATTAGTATGGTCTGGCAAGGGCGCCGGGGCCCCGCCGCCGCCGCCCGGTGGCATG
CCCTTTTTTTCGCCCGCGCTGCCCGACGTGGACTTCGCGAGCATGCCGGCCGACGACC
GCAGCGCTCTCTTCGCGGAAATCAACAAGGGAGAAGCTATCACTAGCAGTCTTCGTAAGGTGACATCGGACAT
GCAAACCCACAAGAACCCTCAGCTGCGCGAGGGTCCGGCGCCCTTCAAGGCGGCGGCTCCCAAAGTGCCTACC
AAGGCGCTACCCACGCCCGGCGCCGGGGTCTTACCCGATAAACCACCTGTGTTTTCTA
GAGACGGCAAGAAATGGATTATTGAATATCAAAAGGGCAACCCTGGCCTTGTAGTGGAGAACGCGGAGATGAA
CAACGTGGTGTACATGTTCCGTTGCCGTGACTCTGCCTTGACAGTGCGCGGGAAGATCAACGGCGTGGTGCTC
GACTCCTGCACCAAATGCTCCGTGGTCTTCGATAATCTGGTCTCCAGTGTGGAGTTTG
TTAATTGCCAGTCCGTGCAAATGCAGGTACTGGGTAAAGTGCCGACTATTTCGATTGACAAGACGGACGGCTG
CCAGATCTACCTGTCGGAGGAGTCGCTGGCCGTAGAGATCGTCTCCTCCAAGTCTTCGGAAATGAACGTGCTC
GTTCCAAAGGGCAATGGTGACTATTCGGAGCTCCCTATCCCCGAGCAATTCAAGACTG
>1002050366_1 Heli.1-DT661699.3.5
GCACGAGGCTTATTTTCTGTTTTTCAAGGTCCACTTTTGTCACGTCGCGGCTATAATCTTTGAGTGCTGCGCA
AATCGATAGTGCATAGCGAATCGCGTGTGGAAGCTGCGATTTATTGCTTATTGTGCGAAGTAGTTACGCCGGT
ATTGGATTTGCGGTCGTTATACCTATTTTATTTACTTTAATAATTATTTTTACAATTT
TTTAGCTTTTTGAATAATTTATTGATAAGAATTTGCGTTTATTTGAAAACAATATAGAATTTTTTAAACTCTA
TTGGTACAAATTTAAATTATTTACGTGTCAATGACGTCAATTAAAATGTAGTTGTATAAGAAACGTTATCTGT
TATCAATAATTATATGAGGAGTATAGTTTCAAAAAGGGCCAACCCTATTTTTGACTAA
AAACATTGTTTCGTTTGAATATGAAGCGTCATCATTCATTGTTTCTTTTAGTTTAAGAATTATTAACAAATGT
CGCTAAAATCTTAACAAATCAGCTTACAAAAGTAACAGTTTGTTGCAAACGCCGCTAAAATATTTCTAAAGCG
TTTCAAATTCCGCCAAAACAAAATCGACCAATCACAGCAAGGCGTTTGCATCACGTGA
CGCAACAAACACTATGGCGATTTAGTTACAAACGTGAAGAAGTGCTGTTAGTTTTTTCTGTTTATTTTAAAGA
ACAAATGGATGACTTACCACAATTTGTTACTAAATGTGATCCTAAACAGGCCAGAAAGAGAAAAAGTGATAGA
TCAACATGGAAACGCACTGCAGAGCAAAAAAAAAAGGTACGTAAAATTTTGTATCAAAC
TACATTTCAAAGTAAAGGGGATGGTGCTATGCATAATGTTATCAAAATATTTTTAGTTTTATAAAAGTAGTTC
GTCAGTTTATTAGCTGTATATTTAAACTATTAGAGGGGTTTATTTTTTGTTTAATTTGTCGTAATGAAGATTT
TTTGTAGGCATTTAAAAGTACCTACTTATTGCTTATATTATGTTGTAATATCCAAGTA
TTAAAAAATGCTTAATTATCTACTAATATAGTGCCAAAAAAAATACTTTTTATATTAACCATAAGTCAATATA
ATGCTACGTTTCCATTGAGGTAAAGCGGAGCGGAGCACAATACGCGAAATCTTAATCTTGCACTTCCGTTTGG
TTGGTTAAATGTAAAAATATTATTTCAGGTATGAAAGTAAAGGTTTACCGAAGTTCCC
>1002050367_1 Heli.1-DT666716.3.5
GCACGAGGGAAGAACTTAAAAAGAAAAGCGTGCGTAGATCAAAATGGAGTCGGCCGGTGATTGGTGTTTAATT
GAAAGTGATCCTGGTGTATTTACACAATTAATCAAGAAATTTGGTGTAAATGGTGTTCAAGTTGAAGAGCTGT
GGTCTATTAATGAAGAAGGAGTCTTCGACAATTTGAGACCTGTACATGGCTTAATATT
CTTATTCAAATATCAACAACATGATGAACCAGCACAACCTGTTGTAAAAGATAAACAGCGACTTGAAAAAATT
TTCTTTGCAAAACAGGTCATAAATAATGCGTGTGCAACTCAAGCAGTAATTAGTTTACTGTTGAACTGCAACC
ATCCAGATGTAGATTTAGGTCCAGAGTTAACAAAATTAAAAGAATTTAGTATGTCATT
TGATCCTAAAATGCGAGGCCTAACTCTCAGCAATTCTCAGACAATACGATCTGCACATAATTCTATGTCACAG
CAAACACTTTTTGAATTTGACAACAAGGCTCCAAGTAATGATGAAGATGCATATCATTTCATTGGCTACATGC
CAATAGACGGGCGACTGTATGAACTTGATGGGCTACGAGATGGACCAATTGATCATGG

TGCTGTGGCCCAGAATCAGGATTGGCTTGATGTCATTCGTCCTATTATTTTAAAGCGAATAAATGAGTATAAG
GATGGTGAAATTCACTTCAATCTTATGGCACTCGTATCTGATAGAAAAATGATTTACGAGCGGCAAATCCAAG
AATTAATAAATGAGACGAGAAT
>1002050368_1 Heli.1-DT662315.3.5
GCACGAGGACGCCTCTGCCCACACACTACTGTGCTGACGTGACCATTTCAGTTAAACTTTTGCCCATTCCACA
GTTCTGTGTTACGCGTAAAACCTATAACCACAACTAGGAAGAGACATAACGGAAAAAAATCGATAAATTATTA
TAAATTTTGGATTTTTGCTTGATACAATAAGTTTAAAAATTGGGATTTTAGTTGATTT
TGAAGTTTTTTCTTTGAAAAAAAGGAGTGTGTGAAGGAAAGTGAGACCGAGGCATGCGCCTGCGCTGATTTTC
ACTTTCTACTCGACGGGACATATAGCGATGGCTCCCGTAAGCCAAATGCGGATATGTGCCATCCTTTGCCTGT
TCTGTGCAATCAGCATTAGAGCAGACCCTAGTGAAAATGCTACAACAACAAAGACGAT
ACGAACAACAATCTTACCATCAAGGACCACGCGGACTGAACTGAAGGTCTCCAGAAGTCTGAACCCGCACGAA
ACTCAGACCCTAAATGTGAGGACCAAGTCAGGTCATCTGACTCAACTAATAGTTAAGAAAAAGGACGCAAAGT
CAACCACGTACAGCAGTACGACGCCTGCGCCTACGACAATTGCAGTCAGAACGACGCT
TACAACTGCTAGACTCAACAATACGGATGATAGCAACAAAAACAAAACTGATAGCAGAGATCAGCGTANATTA
CATTTTGGTTCAATGAGTAGCGATCTAGCTGGTTTCGATTATTATTTAAATAGAAACAAACACGATGATTTCG
CTGATGTTAAAGCTGAATATGGAAACTGGTCACCAGTATCTGTTTA
>1002050369_1 Heli.1-EL601708.1.5
GTATAACAAATTATGAAATATGAATTACATGTGTTCGTAGTGCGCGGTTTAAGTTATTTGTGAAATTTTAATA
TTCATTCGGAAAGCGCTTTGAATATGTTTTAATTTTACGCACAAAATGAACTATACTGTAGATATTTCACTAA
TATTACGATGAAGGGTGGGCTGTGTGTAATTTACACGTCTTTACTGACTATATCAGTG
CTTTCTAGTTGTGCTCTCTTCATATTGGTGGTGTCTGAAGCTCTTCAAACTGAGGATGGGAACAACAACGTCG
ATCTATCTGGTCTCCACAATGTTTCTCAGAAATCTCCAGATATTGAGAAAGAACCATCAGTGGACCCTGGTGT
TCAAGATGCTCAAGAATTACCCGAAGAGACGAAAAGTGAAGAAATCGATAACCAGATG
ACATTCCCTGAAGCTTTGTCTCTTAACACAGTGACTATTGATGGGTTACCTGACACGGGACAACCTCCTATAC
TCATCTCTTTGGCGGAGAACGCGAAGTTGGACTTGAGACCTCCAGATGACTTATTGTACGTTAACGACTCGGA
ACAACACAATGAAACAACAAGTTTTGCCATCGTCGACAATGCAGCT
>1002050370_1 Heli.1-EL600683.1.5
GCACGAGGGATAGGTCGTCGTACGAATTAAATGTTTTCAATATCTTTACAAACATCACTTAAAAATGTATATA
TTCTACAAATAATGCAAATTACTCTAAGAGTTCTTGCTCTCTTCTGATATCTTTATATACTTTTCGTTATTAC
TTACTGTGTAAGGTTACATACCGCTAGTGAAGTACACGTATGCAGTGAATCTGGTGTA
TAACGTGTTTTTTTTGTAATATTTCGGCTGAAATGACGTGAGATCAGTATTAAACTATTAATTAAAATTTTGT
TACAATGCTGCCACTTACACAAAAAGATACGCGAATGTCAAGGGGCCCAATTACTGGGATCCTAGAAAAATTT
CAAAATTGGCTTAGGTTAATTTTTTCCGATAAAAATTCCAAAAATCTATTCCTTTTCC
TTCTTCTTAATTTATCCTTCGCATTTGTTGAGTTATTTTATGGTGTTTGGACGAACAGCTTAGGTCTTATTTC
TGATGCATTCCATATGTTCTTTGATTGCACTGGATTGGTAGCTGGACTGGCTGCATCATTAGTGTCTAAATGG
AGAGCCAATGAACGATACTCTTATGGATATGCAAGAGCTGAAGTGTTAGCTGGCTTTG
TGAATGGACTTTTCTTACTATTTATATCATTTTTTATATTAAAAGAAGCAGTGGAAAGAGCCATAGAACCACC
AGAAGTAAGTTAATTTGGTTCTATTAAATATATTTTGTTAGTAAAGGAGAATATATCAAGTAAATTAATATCA
TATATAAAAAAATCATTCATTGAGTAAGTGACTGACAGACAATGTACAGCCAAAAAAA
CATGCAGCATTGATAACTTTAACATGTAGATTTCTTGATGTGTTTTTTGAGAGTACTGAGAAAGGATTTTTAA
AATATGCTACAGGAAATGAAGAAAATTGTATTTTAACTTTGGTTGGTAGGTTAACTAAGGTGTAACAACACTG
ACATATGTTATATTTATAAATACATTATTTAGATGTGCACCATTTTTGAAAATGGCTG
GCAGAAATAGAGCAACTGAGCTCCTAATCAGACTATTAACAACTACTTATAGCTAGTTTATTATGTGTTAAAG
ATGCTGCTATATTTATAAAATATCTTATCTAAGTTGCTGTGTCTCTA
>1002050371_1 Heli.1-DT666469.3.5
GCACGAGGCTGAGTTATACTTCCAAAGAATTAAGAAAGACGAATGGTCAGGACGCATAAGACATGCTGCAGCT
GCATTTGCATTTGTAGCTCTAGCTTATGGTTTCAAATTCCAAAGGGTGGGAGTATGCTTAGTGGTTTTACATT
CTTTAGCGGAATTTGTAGCTCATAGCTACCGTCTTAACACTATCCTTAGAGGAGAAAG
GGAAGATTATTTGGACAAATTTTTCAGCTTATTAAATGGTGTAGTGTTTGTGACTGTACGCCTCTGTTCACTG
GTGCTTGGTGTGTTGACATTCTACTTTGGAGTGGCGGGTGCTGCCCCATTGTTACTCAGAGTGGCGGCCCTAT
CAGTCCTTGTATCATTCCAGGTTTACCTTATGTTCAACTTTATTAATGAAGCTATTAA
GCAACGTCAAGAAGCACGTCAACTCGCTCTGGCCAAACCCAAAAAGGAAAAGAAAGAGAAGCCTAAGAAAGAA
AAAGTGAAGAAAGTCTACAATGAAGACTCTGACCTGCCAGAGGTGGACCAGAATACAAACAAAACGCTGCGAC
AGCGACAAGCCGCCAAGACTAAATAAACGCACATGACAAACAACCTATCAAAATTTAA

ATGTAACCTTAATCTCCTGTTTGTTACTTTCTTCCTTAAATGTCAATGCACAAAAGGAAATGTAATGCTTAGA
CATCGCATTATATGAAATATATTAAAATACGACAATTTATTTTACACTCAAAATCTATTATACTGCTATTATC
TTCTTCGACGTGTAATACGATGTCTGATATTTATAAACATTTAAATTTTAGTTCTTTA
TAGAAAAATAAATGGAAATTTTCGTTTGTTCGAGATTGTTATTGATGTTATACGTATGTGTGAATTGATGGCG
GTACGTTCCACCCGACATTTAGTATGAAACGCACTATTGTCATTACGTATAGTGGATACAGGGGTGTAAAAAT
ATCTTTTTAAAATATTTAATGAAAAACTTAAAATTATAGGTTTAAATGTTGTTTATAT
>1002050372_1 Heli.1-ES586721.1.5
GCTTCATCTGCGCGCAGTGCCGCACCTCGCTCGTGGGCAAGGGGTTCATCACCGACGCACAAGACATCCTGTG
CCCAGAGTGTGCTAAGCAAAAACTACTGTAAATCCGTTTAAATACATATAAAAAAATGCATATAAACATACAC
ACATACAGATAAATGCGTATAGATAAACATAAATGCATATAAATAAATGGTATTTATA
TACTAACACCACTACAGCTATAATTTAGAGCTTTTTAAATGTTAAGGTTTTGTTATTTTATACAAAACCCTAT
TTAACCCAGCCTACACTTAAAAACTGCTAAATCCGTAGCTACGGTGTCGTTAATACATAAATACATGCATGTA
AACATACGTAAATGCATATAAATACTAATAAAAAAAATTAAATAAAAACATACGCAAA
TCTCTCGCATTTTGCAATCTAACTATTTAAAACTTACGTAACTTACTTTTAAATTACTTATGATAATAAATAA
AATTATTTTAAATTGATAAAATTTTGTCATGTTAAGCTTGTGCTTCAAATCTGTTAACGCTTTGTTTGAGTGC
GACTTAACTAATGTGTTGCCAGATTATGAGATTATGTAATTAATAGAGGCAAAATAAC
ATATTGTTTATTAAGTAAGATCTTTTACTAAGAATACTCGGAGTTTTAGGCTTTCTTTTAATGGAAAGTAAAA
AATCGTTATAAAGAACTAAGTTTTCTCTGAATCAGAAAACAACTTAGTTTCATCAAGTTACTTAGTTATACAG
CGTAAAAGTATTTTAAACCTGGCAACACTAAAAGTGACTAAATCTTATTTTGTACTGT
GCAGCGATAAAACGATCCTTCCGTTAGTCGCAAAAGCGAGAGACTGGTGCAATTTACGTCGAATATCCCACG
>1002050374_1 Heli.1-EL597919.1.5
GCACGAGGTTATGACCTAGTGGCTTGGAGTGGAACAAAGAATATTAGCTAGTTTACTTCTTGGCATACCTTTG
TCTGCGGGAGCAGCGTTACTCGCTTTGCTCGATTACCTGACCGGCTATTGGCGCACGTGGGCTCGCCTGGCTT
ATCCACCTTCATTTCTTCTCTTGCTCTACCCCTGGGTTTTACCAGAGAGTATCAGATG
GCTCCTTGCTGGTGGAAAAGTAACTGAGGCCGTACGCGTAATTAAGCAAGCTGCGAAAGCCAACAAAGTCGCC
ATTCCAGAAGACACGTTGGATAAAATGCTGTCGAGAGACCTTGTCTCGGAGAAAGTGGAACCTATAGTTGAGG
AAGAAAGTCTGTTCAGGGCGTTTATCAAATACGGAGCTTTACGCCGCCGCCTCCTCGT
CTGCTTCGCGTGGTGGTCTTGCGCAGTATTCGTTTTCTACGGCCTAGCTGTCCGATCTCACGCGTTGGCTGGC
TCAGTGCATGCTAACTACGCACTACTGGCCGCGGCGGAGTTGCCCGCACTGGCGCTCAACACGCTGCTACTGG
ACCGCGCTGGACGCCGCCCGCTACTCACCGCCGCCTTCCTGATGACCTCTGTTGCGCT
TATTGCTATACCCTGTTTGCCTAACCAAGACAGCGGTGTAGGCATAGTTCTGTTCCTAGCGGGCAAGATGGGT
GCCACCATGGCGTTGAACGCCCTGTACATATATACAGCCGAGCTGTTCCCGACGCGCGCGCGACAGCGGCTAC
TGGCCGCGTGCTCCACTTGCGGACGCATTGGTGCTATACTGGCACCCCAGACGCCTTT
GCTGGCTGCATACGGCGCTTGGGTGCCCACAACGCTTCTAGGCGCATTACCACTAGTCAGTGCTGCGCTTACA
CGACTAGTACCAGATACGCTCGGACGTCGACTTCCAGATTCCTTCGCAGATCTGGACTCATCAGCCTCCGCTG
AATCCATCTCCACCACTGTGCCGTGACCCTATACGTTAATATATTATAAGGATCTCAC
ATAGAATAGTTTACTAGCTTATAGATAATATGAGGTTAAAGGGGATCACGATCTGTTGGA
>1002050375_1 Heli.1-DT666771.3.5
GCACGAGGCTCGAGTTTTTTTTTTTTTTTTTTTTACTTTTTGGCAATGCCAAGCATTTATTACTTACGTATAA
CTTGTAATTTTCTACACATTTCGTTCTATTTATTATAACAGGTACATCAACTCGAAATTAATACACAACATTT
ATCGATTCACAAAATAAAAAAAAATACGTTTTTAACAAATAAAAAAAATTAAAAAAAA
AGAGTTAGTTCTAATATAACTAATCGATCGTGAACACCGAAGTGGCTTATCCAGAGATCTGGCGTATCCACTA
TTCGCTTTGATAAGTATTTCGATGTTCACGATCGTTAGTTTTTGCTATTCTTAGATTTATATATCACAATACA
GCGTCTGCTTCCTGCAGCCATTAGTAGCTACCATGATGACCATACTCGTGACCGTGAC
TGATGACAACCGGGACTGGTACCTTGACGGGGTAGGGCACGGGTTTCTCCACGTGGACTGGCACATGCTTTTC
AATATGGACGGGGTAGGGCCTGTCTACTGGCACTTTTACGGGGAACGGCACTGGTTTCTCTACTGGGTAGGGA
ATGTGCTTCTCGACGGGGTATGGCGCTGGTACATGCACCTTTACTGGGTAAGGCACGG
GTTTCTCAACATGTACGGGCACTGGGCGGTCGACGTGGACCTTGACGGGGTAGGGCACGGGCTTTTCAACTGG
GTAGGGTACGGGTTTCTCTACAGGGTAGGGCACAGGTTTAGGAATATGTACGGGGTAGGGCCTATCGACTGGG
ACCTTAACGGGAACATGTACTTCCTTAATTACTGGGTAGGGAGCGGGTACATGGACTG
GTACTTAACTGGGTAGGGGACTTTCTTTCAACTGGGTAAGTGCTGGTA
>1002050376_1 Heli.1-DT666607.3.5

GCACGAGGACCTATTCTACTTAGATCTTCAGCGCAAATCTTAATTTGTGGTATAAAACAAACAGATAACAATG
TTAAGATTCCGGCCAAAGCTGGGTACTGAGCGCAGTAAAGAATGGCGGTGTTGTTTTTGTCTTCATGTTCGTA
CAGGAACAATCTTGTTGGGTACTTGGCATTTGCTGCTCCACATGATGGCATTAGGATT
TCTGGCAACAATAGTGCGTAATCCAAACTTTCTTGATGAATTGGAACGTGAATCAACACCTGTTTCTGATTGG
GGTGATATAGGTGCCATATCAACTCCACTGTCTAATGTGGAGACCCGGCCAGGCATACAGCATTCACCAAATC
ATCCCCGTGATCATAGTCTGATATATCATGATGTGGATGTGGGTGCACTGGTTACAGT
GTGTACACTGGCCATCACACTGATGCTGTTATACGGTGCGTCAAGAGGCAAGCCTGCGCACCTGCTGCCCTTC
TTCTGCTTGCAGATATTTGATTTTGCTATTACTGTGCTAACAGCGACGGGCTACCTGTGCTACATCAGCTCCA
TCCACCGGCTGGTGGAGGAGGCGCGGCGCGTGCCGTGGCGCGAGCAGCTGCTGGCGTT
ACACGCGCCCGCCCTCGCCTTCATTGTTATTACTACGTTCCTACTGGCTGTGTTGCTTAAGGGTTACTGCATC
AGCGTAGTATGGCGGTGCTACAAATACCTCACAATGCGCACCCACGCGCTGCACTCGCTGACCCCCTTCGTGA
TCTCGACCGAAGGGGGTGGTGACCCCCGCGCCCCCCTACTCCGCCG
>1002050377_1 Heli.1-DT664684.3.5
TTTACCTTTTAGTCTATAGTGGTAATTTAATGTGATTGTTATTTTTAGAATTTATGAAAACGATTTATAACAC
TTTAATTTCGTTTCAATAGCAATATATTATTTATTGCTACTTCAAGATGGATGTGCTTTAAATATCATTCGCA
ATAATTATCACGTTAAAATGTGGAAATATGTATCACGTCAAATTAGAGATACGTTTGA
GAGGAGTGCTAACCATTTTGATAAACGCGGTTCTACTGTGATTAATTCCTCGAATAGCAACATTACTGAAGAA
AAAAATAGAAATATCAATTCAACTTGTTGTTGGTTTAGTGCAAGGAAATGTTGGAATTCTTTTGAAAGTGGAA
ATGATACGAATAACAAGAGATGGAACTTTGAGCAATTAAACCGTTCTTGGATTGGTGC
CATAACATGGAGTAGTGCCCTTGTATTTGGTTGGTATACAAGTCAGCTAATACATTTTAGCTATAAAAAGCAA
GGTATTGAAAAAAAGAAAAATGGATTCTACAGAAACAGTTTTTTGTTGGAATGTTTACCATTCTTGGCTTCTA
TTGGTAAAAGAGACTTAAAACCTCTTAATATACAATCCACATTTGAAGATCCTTTATT
CAATAACCCTTCAACTGTTCATTTGATCTCAAATGATCAAAACAGAGGAAATGATACAAATTCAAATACAAGC
ACCAGCGCAAATAGTTCACAAAAATCTGAAAATGATTTAGGTGAAGTTCTCAATTCCATTGAAAATAAATTAG
GCCTAGCTGCCATTGAAAATGGACAACATCAAGATGGGCTCAATTTACTTAGATCTGC
AGCACATCGTAACCATGCACCAGCTCTATATAACTTAGGTCTGTGCTATGAGTTAGGACT
>1002050378_1 Heli.1-CX700558.1.5
CCTGCGGTACCGGTCGGAATCCGGGTCGACCACGCGTCCGGCATTTTATTTCAAATAAATTATTAAATCTAGG
CTTATATTTTCTTGTGCGTCTTATAATTCAAATCTATTTAAATTTGAAGTTTTAACAAAAATGCCTGTCGATC
AGATACAGTATTCGGAAAGATACAACGATGACGTCTACGAGTACAGACATGTCATCCT
CCCACCAGATATAGCTAGATTAGTTCCAAAATCCCACCTCATGACGGAAACCGAATGGAGAAATCTTGGTGTT
CAGCAAAGCCCAGGATGGTTACATTTCATGGTGCATAACCCCGAACCCCATGTCCTATTATTTAGACGACCTA
GAACTAATATACCTGCTCCGATCAATGGCTTAGATTCAAGCAGCTCCTCAACCGTTAA
AGTATGATAAAAGTTTAGACAATGAAGGCCACATGTAAGATATTTATTTGATATGTTTATAGCGGCTTAAAAT
TTATATTGCTTAAATAAAAAAAAAATTATATGAATTTTGTAACTTTGTAACAAAAAATAGAACAACCAATGAA
TAGTGTAATTTTGTTTAAATAAATTTGTAAGGATTAATTACCGTCAACTTAATGTAAA
TGAAAATGAGTCCTTCAAACTATTATTTATGGTGTTAAAGATAAAAAATCATAATAATAAATATTAAACATAG
TTGTTAATAAAAACTATATAAAA
>1002050379_1 Heli.1-DT664223.3.5
GCACGAGGAATATCTTTTGACGTGAGCGATGTGTACCTATTTTATTTCTATTGAAAGGAAAATTTTGGTCTAT
TTTTTCATTAATATATAGTGCATATCCAATCTTTTCATTAAAAATTAAGGCAAAATAATTTATTCTCACTTGA
TAACGTAAATTTAACAAATGTTTGTGAAGCATTCCTTGCAAAATTTTGTGAAGCTTAT
GTGAAATTATTATTATAAATGTCATGGTTCTCGGACCTCGCTGGCAAGGCTGAAAGCCTTCTCAACAACCTTG
ACGAACAGACAGGTGCAGTTCTACGTAATCATAATGGTGTAAAAGAAACTAAAAATGATTTTATCCTTCATCC
TGATGGTACATGGGGGCAAAAGAAAAAGACAACCCCTCGTAGCGTTAAGAAAAATAGT
TCAATTTCTGAAACGAAATCTAATGGTACAGCTGCATACAAAAGTCCGCCTACAAGACAAGCCAGATCCACTA
CAAAACATTATGATAACATGAGAAATGGGGTTGAAAATGTTAGGAGACAATCACCTTTGCGGAAAACCCCCTA
CACTAATAAAAATGGCCCTAAAACTCCTAGTAGTAAAGACTATAATGATGAATCTATT
AACCAATTCGGACTAAGACATAGAAGATATAGTTTGCCATCTGACTTGGAATTTATAAATACTGAAAAACTAA
CATATAATATGCAAAATCTAGAAGTTGAAAATGCTATGTTGAAAAATGAACTTAATGTTGTAAACAGAGAAGT
TTCAGAGTTGCTAGATAGATTA
>1002050380_1 Heli.1-EL599672.1.5

GCACGAGGGTTATCCATCGCGGCCATGAAAATGAATCCTTGGCTCTCATGCAGTCTTATTTGCCAAAAGAAGC
TGGTCCTTCTTCAGGCTACTCGGAGGGTGGGGGTCTCTATGCCTTGGGTTTGATACATGCAAATCATGGTGCC
AATATCATTGATTATCTTTTAACTCAACTAAAAGATGCTCAAAATGAGATGGTACGTC
ACGGTGGGTGTCTCGGTTTGGGCCTGGCCGCTATGGGTACCCACAGGCAAGATGTCTACGAACAGTTAAAATT
CAATCTATATCAAGATGATGCCGTTACTGGTGAAGCTGCCGGTATCGCTATGGGTATGGTGATGTTAGGCTCT
AGACACGCGGCAGCCATTGAAGACATGGTCGCATATGCTCAAGAAACTCAGCACGAGA
AAATCTTACGAGGATTGGCTGTTGGTATCGCATTTACGATGTATGGACGCTTGGAAGAAGCCGACGCTCTCGT
CCAGCAATTGTTAAGAGACAAGGATCCCTTACTGCGGCGCGCCGGGTGCTACACCATTGCCACAGCGTACTGC
GGCACCGGCAACAACGACTCCATCCGCACGTTGCTGCACGTGGCGGTGTCCGACGTCA
ACGACGACGTGCGCCGCGCTGCCGTCACTGCACTTGGTTTCCTGCTGTTCAGGACACCAGAACAATGCCCCTC
TGTGGTGTCTCTTTTGGCTGAATCTTATAACCCTC
>1002050381_1 Heli.1-ES585778.1.5
TTCCGCACGAGGTGACGCCTTGGTTGTTACCTAACTCTCTGTGTATTACCGGTGGGTTCCAGCACCCGTTGTG
GCATTTACGACTGCCCAATGACATCATCCTCCCTTGTTCCTTACAATCGTCCTATAACATATAATTCTAACAG
ATATCTCCTTTAAGGTCAGTTTGGGTGTTGGTTCAGCCACTCGGGATAACCGAATGAG
TGGGTGGATAATACAACCAAGGTTGCTCGATGGTTGGGCGATAGAAGCCTGGCCACGAGACATGGAATTGTAG
ATGGTCTAGAGTGCGACCGCCCGCCACAGTGGTACATCACTGGACTGTTAAAACAATTTCTGTGTTTGATATA
TGAAGGGTCCTGTAGGAGGCACCCTGAGACAAATAACTGTCTTATAGCCTCTTAAAAT
ACTGTCTTAATAGCCTCTTAAAAA
>1002050382_1 Heli.1-EL600124.1.5
GCACGAGGGTAAAATAGAGTTAGTGGTGTTTTCCTGCAATCGTGTTGTAATACCAAATTATGGATAGATTTTA
TGATGAAAACAATGATGTTTTAGCAATATTTTTTGATTTAGATAATACTTTAATACAAACCAGGAAGGGAGAC
AGCAAGGCTTGTAATAAGCTCGTTGACATACTAGACCACAAATATGGCATACCCCATG
AACTGGCAGCAGATAGTGCCTCCACTTTCCTTCGTGCATTTAGGGCCAGACCTGACGACGAATCGTACGCAAT
AGATGAATGGCCTACGCATCTGTGGCGAAACTCCTTGCCGAAAAACTATAGGCACATTGCCAAAAATGTAGCA
ACCGAATGGTTAAAATTAAGGTTTCAATATTTAGCATTGACGCCAGATGTGATACACC
TTTTAGAAACTTTGAGGTACGTTGACCTTCTGGGTTTGATCACGAACGGGCCGTCCCGAGCACAGTGGCAGAA
GATCGAGCGTCTGGGCCTCCGCAAGTACTTCGACTGCGTCCTCGTGTCCGGGGACCTGCCCTGGGAGAAGCCT
GACCAGAACATCTTCTTGGAGGCCTGTAAGCTTCTCAACGTGGAAGCGAGGACTTGCA
TCATGGTTGGGGATAAATTAGAAACGGATATTAAGGGTGGCAAAGAAGCAGAATTAGGTGGTACAGTATGGAT
TCCGTTGCGATATGATGAAATGGACTCTGATTTACCAGATTTCAAAATACAAAAGGTGACAGAGTTACCGGAA
GTTCTGCCTAACTCACCAAAACTTAAAAAGAAAACAGCACAACCGTGTATGTGTTGAT
GGTGGTATGCATTTTGACAAACCATATACTATTTAAATATGTGTTTAACAGTATCAATTAGATCTCTATCAGT
ATAAATTCAAAAATCTCTCAAGTCGCTACACTAGTAGTTCTCACTTTGCTTATATTTTCTAGATTTAGATAAT
CTTTTCTCTGCAACTAAAATATTTGAGATTAAATTTAAGGGTTTTGGTATTTCACATG
AAATTATAAAATATAGAATGATTTACAAGCAATTATTACTTATTCACTTTGCTAAATAATAGCAATATTAGTT
GACATTCCTAACAGGAACTTGTTGTATTTTTTAAAATTTTTAGAGCT
>1002050383_1 Heli.1-DV501175.2.5
GCACGAGGGGTACCCTGAGGGAACCTAGTGTTAAGACACTCCGTTCCCCACTTTCTGCCGAATTCTGGAGACA
TTGCGTGTTGAATGACAGAACTCAACGCCGCGCTTATCCTCGACACCAGAACGAAGAAATGGAAACATAAATT
TAAGTAAATGTTTTATATCCTCGAGTGAGGATCGCACCCACAACCAGTCAGTTTTACA
ATCACACTTTGCGCCGCTGCGCCACGACTGGCCTCAACTCACTTTGTTATGTTTCAATGAATACTATGTTTTT
TTTCTATCAACAGCGCGGCTATCTGACCCGCCGTGTCCGCCATAGAGGCGCATCACACGAACAGTTGTGAAAG
TTAAACGTTTACTCTACGGACGTTACGTGCGTGTGGGAATGCCTTAACTTAATTCATC
ATTATTTTTTTAGTTTTTAAGGCCGGAATTTATTTGGGAGCTATATGTATAGCCGTTGATAAATGAAGTGTTT
CTTCTCTATAAATGTTTTTTTTTTTATTTATATAGAATATAGCGAAGTAAGAATAGAAAGAAGAAGGGAACTA
GGTTCGGTCACTCCGATCTCCGTGCGCTGCCCAATTTTTAAGATATTACGTGTTGAGT
AGCGGAACTCAACACCGCTTTGTCTTTTATTTAGTGAGAATCGAACCCACAATCGTCGCGTTGACAGTCAAAC
GTTGCTGTCGACGGTCTTCTTGTTTAGATGTGTATGTATTTTATTTTTTATGTACAAATTCTTTTATTTTGTT
TTATTTCAAATTGTTTTATTGTAAAAATTTTTATTCTTTCTATTTTAACTTACATATT
TTTATAGATTTGATAATGCACTTCATTTTTCAATATATAATTTGAATTTAAAATAAAGTTTTTGTTTACCTAC
TTCTATAAGGAATCAAAATGGATGAGGAACATATTTGTTAATCGTTCTCCGAGCGTCATTTAATTTAAATATT
ATTTTACGTTTTACTTTTATTTAAAATTATTTTTCTTTTTAACACGTTAAAGCATATG

GATATTAAATTAATTAAAAATAAAATTTTATATAATTCGAAAATAATTTTAAGGTTCGATGCTAATTTTTCAT
AAAAGTGTGGTACACAAAAATAATATTTAATATAGTATTTGAACTGATTTTCAATGTTTATTATTACATCGTA
GTTACCATGG
>1002050384_1 Heli.1-DT663958.3.5
GCACGAGGGGCCCCCCTATCGACACCGGCAAGTCGTCGTGCTGCTGAATGTGTTATACGGGTGGCGGCGTGCA
GCAGTCGTGGCGGGAACCCGCACCCAGCTGTTAAAATGCAATTACCACAAATTCATGATCGCATCCTTATTAT
AATATAAATAAATAAATAAATTATATATAGTGCCGAGTGGCGAATGTTAAGCTAACTC
CCTTAAGTTGCTATTGTCGAGGTGAAATGGATGGATGTATGTTTATACGTGTTTATATATATATATATATAAT
GAATAATATTGAATATTGTAGATTAATTTTGTGCTGTACTCCAAAATAACATCTAAATTTGGATACCAAGTTT
TATATTGTATTGTATGTTGGTAGTATGGCGGCCCGTGTCGGTTTAATAATGTAGTTTG
TAATTCACTTAAATTATTCTGTATTTGTTATGATTTTTTTCGCATTAATATGAAGTCTTAAGAGATTATTTCA
TGGTTATCTCAAACAGCAGGCACTATTTGCAAACGAAACAAACAGTCGAAAGTAAGGAAAGTCGGGCCGCCAT
TTTATAATACGGATTGCTAACTCATAATTTCAGTCCATTTCAAGTAGGCTAATTTCTT
TATTAGTTTGCTTTTAGCGTTTTACTTTCAAACGTCACCGAGGTACTAATTCGTTCTAATATTCGTCTGCACT
GGGTGTTATTCCATATTATATTGATATATTATTATTTTTTTTAATTTTAACGCGTACTGGGGTTGTGCTGTTC
GAAATTTTGTGTTGAAACTTCTCATTGATTGTATCTCGTAGTTTTTTAACACAAAATT
GTAGATATAGCAACTTGGCCTTAGTGTTTAGACAGATGTATAGAGTAATGTAGTCGGTTCAGATCAAGCGGGA
ACATAGAGTATTAAAATATTTGTGTTGTTTGACGTCGGATTGTCACTGTCACTTTGACATAAGCACAAGATAA
TAAATAAATAAGATGTAGGTATTGTTATGGTGAAGATGTATTTTAACTGCATCTCTTT
TTAACGTGCAGTCTCCTATTGTGTAATATTATCTCTTTTTCACCGTACAGTTTATATATCGTGTATTCTCTAT
TTATACGTGAT
>1002050385_1 Heli.1-DT668890.3.5
GCACGAGGCGCTTCTTTAGTCCAACTGAAGAGAACAAAAAAATAACCCGTGATGTGGTGGAAAACGGTGATTG
TAGGCACCTTAATAGGGGCCTACGTTGTTCAATGTGCCAAATTTCCACCTGGTTTCCGATTTGGTGCGGCAAC
ATCTTCCTACCAAATAGAAGGAGGCTGGAACGCAAGCGACAAGTCAGGAAACGTTTGG
GATCATTTCGTTCACTCAAAACCTGATAGAATTATAGATTTGGCAAATGGAGATATTGCGTGTGATACCTACA
ACAATTGGAAGAGAGATATAGAAATAGCTGAAGAATTGGGCCTTCATTTTTATAGATTTTCTCTTTCTTGGAC
ACGAATATTGCCAACTGGATATCCATATGAGATAAGTAAAGATGGAAAGGAATTCTAT
AATAATCTCATAAATGGTTTACTTGCAAAAAATATAGAGCCTGTGATAACTCTGTATCATTGGGATCTACCAC
AGAACTTACAGGATTTGGGAGGATGGGCCAACCCTTTAATAGTAGATTGGTTTGCCGACTACGCAAGAGTTGT
GTACAATCTGTATGCTGATCGTGTCAAGATCCTGTTGACGTTGAATGAACCTACAGCG
TTCTGTGACTTTTCTTTCAATATGGGTCTTCATGCACCAGGAATAGTTAGCACTGGAATTGGAAGCTACTTAT
GTAACAAGCACGTTTTATTGGCTCATGCAAAGGCATGGAGAATATATGATAAAGAATTTAGACAAAATATAAT
GGTAAAATTGGTATACCAATGCCCTCTTATGGTT
>1002050386_1 Heli.1-ES587746.1.5
CCCAGTTGACAGGCCCTACAAAGTTGAAGTAGAAAAACCCTACCCGGTCCCCGTTAAAATACCAGTACCCAAG
CCCTATGATGTATACAAGAAAATCCCTTACACTGTCGAAAAGAAAGTGCCTTATGAAGTCAAGGTACCCATCG
ACAAGCCCTACCCCGTATACAAGGAAGTTCAAGTACCTCTAGTCAAAGAGGTCCCATA
CCCCGTAAAGGTTCATGTACCCATATACCTGAAAAAGGAGGAGGAACACCATGGCTGGCATTAAATTAAACCT
AAGAATAGGTTTCACAGCAGTACCTTATAGGCTAAGTTCGAAATGTGATCATTTAGTATTCGTAGTGTAATAA
TAAATTTTAAATTGAGGTAGCTGAGGTACCAACAGTTAGTGAGATGTGAGAAACGTTT
CGCCTATCCCAAGTTTCCGACCAGCTCACGTTGCTCTGCAACGCCATTCGAGTATACGACACCTAATGATTGA
TCTGTTATTACTTGTTTATCTTTTGTATAATGTGCTTTGTTTTATATAATTGCGAAAGTACGCCTTTTGCAAT
CTTGTGTTGAGGCTGATAAAAATAAATATGTTAT
>1002050387_1 Heli.1-EL599453.1.5
GCACGAGGCTATACCCCTGTCCCTGCTGGAGTCATAGTTGGCTTAGGGGCAGCACCTATGTGGACTTCCAAAG
CCACTTACTTGACTCAGGCTGGAAGTGTGTACGCTAAGCTGACAGATCAAGCAGTCGATGGTATCATCGTTAG
ATTCTTCGGCTTCTTCTTTCTAGCGTGGCAAACTGCTGAGCTATGGGGAAATCTTATT
TCTAGTTTAGTATTTTCCTCGGGAGTCCATAGTGGAAATACATCCAAAGAGAATACCAGTAACACGTTCCTCA
CATGCGGAGCTAACTTCTGCATGATTGGAGGGGGTCATCATGACAACCAGAACTTACACCGGCCGCCTGACAG
CGAGATTTATGAAATTAGTGCTATATACCTCGCTTGCGTGGTAGTGGCCGTGCTGATG
GTGGCCTTACTCGTCGACCCTTTGTCTAGGTACGGCGAGAAACAGAGAACTGCCGATACTGCCAAGTTGTCAG
GGATCCAACTTCTATCGGCCACTGCATACCAGTTAAAGAAACCTAACCAACAACTGCTCATACCAATCACACT
GTGGATAGGTATGGAGCAGGCATTTATCGGTGCTGATTATACACAGGCGTATGTATCT

TGCGCTCTTGGAATCCGATCAATCGGTTACGTGATGATTTGCTTTGGAGTTGTGAATGCGATATGTTCGCTTC
TCTTCGGTACAGCTATGAAATATATAGGACGATTCCCAATATTAGTAATGGGAGCGGCACTCCATCTTGGTCT
CATCGTGTGGCTGCTCATCTGGAGGCCAAACCCTGAAACACCTACC
>1002050388_1 Heli.1-EL599808.1.5
GCACGAGGCAAATACTTGACGAGACCTTTGTTGTTCTGAATTTTATGTGGAGAAAATGGTTGACCCAATATTT
GATTATCAATTTAGGCTTATTCTAATCGGTGATAGCACGGTAGGCAAGAGCTCACTGCTAAAATACTTCACAG
ATGGAAAATTTGCAGAGCTTTCGGATCCTACGGTGGGAGTTGATTTTTTCGCACGGAT
AATAGAAGTACAAGATGGTACGAGAATTAAATTGCAACTATGGGACACAGCTGGACAAGAAAGATTTAGATCT
ATCACAAAATCGTACTACAGAAATTCGGTTGGGGCACTCTTAGTATACGATGTGTGCAACAGATCAAGTTTCG
AACATATACCGCTTTGGATGATGGAAGCTAAAAGGCATATAGAACCACACAGGCCTGT
CTTTGCTTTGGTGGGCTGTAAAGTAGACCTGGTTGGTACTGATAATAAAAACGGAGCCCGAAGAGAAGTTTCT
TGTGAAGAGGCTAGAATGTTTGCAGAGGAAAATGGCCTCCATCATGTAGAAACCTCTGCCAAAACGGGATTAA
ACGTAGAACAGGCTTTCATACTTGTTGCTCAAGAAGTATACAATCGTATACAAACAGG
TGAATATAAAGTAGAAGATGGCTGGGACGGTATCAAAACTGGCTTCAACCGACCAAATGGGATGGACTTCAAT
CTTCTTGAAGCTGAAACAGTTCAGTCGACATGTTGTTAAATGAAACAGGCTGATATATGGTGAAAAGTAAACG
TATTTTTCATATATTCTTTTAATATATGTATAGA
>1002050389_1 Heli.1-EL598410.1.5
GCACGAGGGCAACGCGCGTCTTAATGTCACAGATTAAATAAATGCTAAATAAAATTATTTAAAAGTACTAAAT
TAAATTAAAATATATTTAATTAAAAAAATATGAATATTTTTTTGTGTTTTTAAAAAATATATTTTTTTACAAT
GTTGATATTTTTTGGAAGAATGTGATTGATTCACCTTATTGTTTTTGAAATAAAAAAA
CATATTAAATTAAACAGTGAATGTACTATTTTGTTCCAGCTTTAACTTTGAAAAATGCCTTTTCTAAAGCATA
CAACGCTCATCCCTATACTGCTGGTGCTACTCGGTCCCGCTTGCATCAGTAGCTTCGAGCCACGACCTGAAAT
AGGATATCCTGCTGGACTTATACCTGAATGTCCAGGAGTAACAAAAAATGCTACAATA
ACCCCAGTTATGATGAAAATGCTTCAGATCACTGTACATAGGATGACTGCTGCCGGACAGGTTATAAGGAAAG
ACTTTCCTATTCTATCTGCACATAAAAATATAGGGAAGGATAAAAGTATTGATTTTAAGAACAAAAAGACGGT
TCTGTACGCAGTTGGATTTTTAGATAGTTCAGTGTGGCCTCAAAGTCAAGCTATTGGC
>1002050390_1 Heli.1-DT662500.3.5
GCACGAGGGTTGTGTGTACATTTTGCAGTAATTGGATTTTGTTAATCTAAGTTTCATTTGTGCTTAATATGAT
AATTTTTAAATGTGCGCTTATTGTATTATCATCAACTTATATTTATCCCTTTGTTCTCAAATTGTTCATGAA
GTAAACTTTTTTGATACCGGCCTGGCCTTTATGTATATTGGGAGTGGTGCTTTAGCTG
GCAAAATCATATATGACAGATACCAGCAAATTCATATACTAGGATCATTAACTCAAAATCCAAATGTGACTTC
CTTTGCCAATAGGATATCTTGTTTCTCAGCCTCATTCTTGTCATTGGGTTTCCTTGTCTACTACTTTTTGTTT
CTATTTAAGGTAGACGAACAATGCCTGCGAGCACTAAATATATTTATATGTGGGTTTG
GAACTTTGTACATGTGGGTTCAGTGCATCATAACCACATATATCTGCACACTGTTCTATGACAAAAGATTAAC
GATACTTAGGCAATGCCTTGCAAACGTGAGCTTCCCCGTTTTAATCGTAATGGCGATATTTGGCACAGTGTCT
TCATTTTTGCCTGAAGATGGCTCGAGAGGCGTCTATGTGTGTCATGTCGTCAGTTCCC
TTTGTAGCTATATCCTTACGGGAATATTCTGTGTATTTATATTATCATTTGAAAAGGATTACGAATATTTTTC
TGAGGGCTTGCGGTCCGAATTATTACTCGACGACGCTTGTTCTTTGAACAACGCGTCACTTGCCGACGTCGAT
AGTATATTGAATCACGAGCTTATATCGAACACTATAGTTATAACTGGAAACATCTCTT
GTGGCAAGATCTCCTGACCCGAGGCCGTGCCTATCAAAGGCACGGTACACGCAAGCTACTGAAGCTTTGACAA
AACAAAATCTACTTATAATGAAA
>1002050392_1 Heli.1-EL602479.1.5
GCACGAGGCTCGTTGCTGGGGTTGCTTTCAAGAAGACATTTTCCTATGCAGGATTTGAAATGCAGCCTAAAAG
TTACACTGATTGTAAAATTGCACTATTGAACATTGAGTTGGAGTTAAAAGCCGAAAGGGATAACGCTGAGGTA
AGAGTGGACAACGTAAAGGAATACCAAAAGGTGGTAGATGCTGAATGGCAAATATTAT
ATGAAAAATTGGCCATCTTGCACGCTAGTGGTGCCAACGTGGTGCTGAGTAAGCTGCCCATAGGAGACGTTGC
TACACAGTATTTTGCTGATCGTGACATGTTCTGCGCCGGCCGCGTAACAGAGGAGGACCTTAGGCGCACACAG
CGCGCCTGCGGGGGCGCGGTGTTGAGCTCTGTGCGAGACCTGAGCGCCGACGCACTGG
GGCACTGCGCGGAGTTTATCGAGAGACAGGTCGGCGGGGAGAGATATAATATATTTACTGGATGTCCGGCCGC
TAAGACCTGTACTCTAATCCTTCGTGGGGGTGCCGAGCAGTTCCTTGAGGAGACAGAGAGGTCACTCCACGAT
GCAATCATGATTGTGAGACGTACAATCAAGAATGATGCTGTAGTTGCAGGCGGCGGCG
CAATAGACATGGAAGTGTGCAAGTACCTCCGTGACCACTCCAAGTCGATAGCCGGCAAGGAGCAGCTCCTCAT
AGGCGCAGTGGCGCGCGCCTACGAGGCCATCCCACGGCAGCTCTGCGATAACGCAGGGTTCGACGCCACCAAC
TTGCTGAACAAGTTACGGCAGAAGCATCATCAAGGTGATATATGGTACGGCGTTGACA

TCCAGAAAGAGGACATTGCTGACAACTTTGCGGCTTGCGTGTGGGAACCTGCGGTGGTCAAGATCAATGCTAT
TATTGCTGCTAGTGAAGCTGCTGCACAGATCCTCTCAATCGATGAAACAATAAAGAATGCAAAGAGCGGTGGC
GGTGACATGCCGGCGCCTGGACGCGGTATGGGACGACCGCGGATGGGATAATTTTGTA
TACATACATATAAATAAATGCATATACAGTACAATCTCGGAGTCGTGGCCACTCTGAAATCTTGGTTTTCTGA
TCTTTTACTTTACATTGGTCATTTGCGCTCGGGAACAAAATATTCAAAAGTAAGAATAAACCCAAAATCAAAT
CGGCGCAGCCATCTCCAGCTAATTTCAGGGCGGCCACGACTCGTTGACCATACTTTAT
ATAGATGGAAGCCTTAATTTGTTTTAGTTGTGCTATTTAATGCGGAGGAAAAGTTGACTAGTTTTGACTGTAC
TTGAAAATTATTTAGGAATAAAAATACAAATCAAGTGCGAGTCGGTCTCGCTCACCGAGAGTTCCGTATCAAT
TTAATGATTGTAGGTCAATAGGTTATTTTTTTCTGATTCCCCATAGATCTTTATATTT
>1002050393_1 Heli.1-EL604353.1.5
GGCACGAGGCAAAACTGCCGGACGGCTTTTAAAATAAATAAAAGGTCTATTTAGAAAACTTGAAATATGTTCC
GTAACCAATATGACAGTGACGTCACAGTATGGAGTCCTCAAGGACGTCTTCACCAGGTAGAATATGCGATGGA
AGCAGTAAAATTAGGGTCAGCAACAGTTGGCTTAAAGAATAAGCAATTTGCTGTTTTA
ATAGCACTTAAGAGGGCTGCCAGTGAATTATCTGCGTATCAGAAGAAAATTATACCCATTGATGAACATATTG
GTATTTCAATTTCGGGTTTAACAGCTGATGCTAGAATGCTAAGCCGTTTCATGCGTACAGAGTGTTTGAACCA
TCGTTATGCTCATGACTCGCCAATGCCTGTTGGCCGCCTCATTGCCTTGGTTGGCAAC
AAAATGCAAATCTGTACTCAGAGATATGATAAGAGACCTCTTGGTGTTGGACTATTGGTGGCTGGTTATGATG
ATCAAGGACCTCACATCTATCAAACATGTCCATCTGCTAACTTCTTTGATTGTCGAGCTATGGCAATTGGTGC
AAGGTCTCAGTCGGCCAGGACTTACTTGGAGAAACATCTTAGTACTTTCATAGATTGT
GACCTAAATGAATTAGTTGCACATGGACTTAGGGCTTTGAGAGATACCCTTACAAATGAAGTTGATTTGAATA
ATAAGAATGTATCAATCGCCATAGTTGGTCCTAAAACACCACTTCAAGTGTCCGAAGAAGCTGAACTTGCT
>1002050394_1 Heli.1-EL603790.1.5
GCACGAGGACAGAGACACACCAGTTTTTCGTGATGATGTTTGTTTCTTTATTTCCCAATCTGGAGAAACTGCA
GATACGCTGATGGCCCTTCGTTACTGCAAACGTCATGGAGCTCTTATTGTTGGAATAACAAATACTGTGGGCA
GTTCTATTTGTCGAGAGTCACATTGCGGAGTACATATTAATGCTGGTCCTGAGATTGG
TGTTGCTTCCACAAAAGCTTATACGTCACAGTTTGTGTCTCTGGTGATGTTTGCACTAGTTATAAGTGAAGAT
CGCATCTCCCTACAAAAACGTAGAGCAGATATTATTGAAGGATTACACCATTTACATGAGAAAATCCGACAAG
TACTGGCTTTGGACGGTAAAGTGAAGGCCTTAGCTGAAGATTTATATCGTCAGCGCTC
GCTACTGATTATGGGCCGTGGGTATAATTTCGCGACGTGTCTAGAAGGAGCGTTAAAAGTAAAGGAACTGACT
TACATGCACAGTGAGGGTATTATGGCAGGAGAGTTAAAGCACGGACCGCTGGCTCTGATTGATGACTCCATGC
CTGTTATGATGATAGTTATGCGTGATCCTGTTTACACAAAATGTATGAACGCATTACA
GCAGGTAACGGCTCGTCAAGGCCAACCTATTGTTATTTGTGAAGAAGGAGACACCGAAACGATAAGTCTCGC
>1002050395_1 Heli.1-DT666085.3.5
CACGAGGTAAATATGACATCAGAATTGAACGTCAGACTCAGGATGAATCCGCGTGTAATCGCGACCTGTTATC
AGGAGCGGACGAGGCGGATGGCGCGGCGGACACGCACTCCCGCTTCCTAAAAGGCCAGAACGCAAGCTACAGC
AGTATGAACAACAAGTTACTGCGTCGGCAGGTCGTGAGTTACGGTGGTACAAACAAAG
ACGGTTTAACGCTTAGTTGTACGGGTGATATTGTGGATGTGGATGTGCCAGACGGTGTAGAGCGGACTTCTGA
CAAGAATGAATGTGAACATGATGTGCAAACGCAGACGCCGACCAATTTCTGCACTTTGCCCAGGAGGCCGCGA
GCACCGACTCACACGTATCATACTATTACGTTTGAGAAAGGACACGGAAAGAAACCAT
TAGGCTTTACTATTGTGGGAGGACGCGACTCTCCTCGAGGTCCTTTAGGGATATTCATTAAGAGCATACTTCC
ACAAGGCCAAGCTATTGATGATGGAAGACTTAAAGCAGGTGACGAAGTGTTAGCAGTGAATGGACAAGCGTGT
CATGAGCTAGCGCATGTAGAGGCACTGGCTCTCTTCAAAGCAGTGCGGAATGGTTCTA
TCGAACTAAGAGTCTGTCGCAGAGTTAAAAATCAATCTACCAAAGCAAAATCCTGTACTGATCTACTAAACGA
CGATGACTGAAAGTATGCTGTCAAAATCATCATCGTGTTAAAATATAAAAAAAGCAACAATGACGTCACCGTC
AGCAAGAGATCATTTCGATTTAAATTTGGAAGTCACAGACGATAAAATGAAGCAATTC
AGAATAAAAATAGTGCATGCAACATTAATTTTTATATTATTTAATACGTAAAAATGTACATTAATAGTGAGTG
CCATTCACTTCGTATTATACAGCACGCTGTCTTCAAAGATGTCTTTTGTTCTTACACTCCAAGCATTATTTTA
TAATTCAGTACCTGACTTTCGTTTTCATTTATTAGGGAAGCTCAATTTGCACAATAAA
AGTAATAATTGTCCCAAAGATAATGTTAGGTAACTATTCTGTAATCTCTTTAAATAAATAGGGAATGTATCAA
TGTACCGCTAACGGTAGTGTTAGTTCTGTATTTTTCTTTAGGACTTTATAAGACTGCTATGTTTCAGTAGTAG
AGTTCGAGGATTCGCACTTTTAAAGTCACCTTAATATAAGTTACAAATTAGTAGTATG
TTTTAAATATATATAAGTATACGTTTCAAACAAAAGAATTAAGTTTATTTCAATAGTCAA
>1002050396_1 Heli.1-EL599124.1.5

GCACGAGGACGGTTTAATGTCTAACGAGTTTTAAACATTTATTAAAATGAGAATTATTTTAGTTTTTGTGGTG
TATTTCTTACCCAATAGCAATGGTTATAAAGGATTTGCAGGACCAGACCACGACAAAATTGTAGCATGTTACT
ACACAAACTGGGCTGTGTACAGACCTGGGGCGGGAAGTTTTGATATCTTTGATATAGA
ACCGTCTCTCTGTTCTCACTTGATTTACTCCTTTATGGGTCTCGATGAAAACTCTTTTAAAATTAAGAGTATT
GACCCTTGGCAAGACTTTGAAGAAAACGGTGGTAAAGGAGGGCTGAAAAGATTTGTAGCTTTAAAAGAGAAAC
ACAAACATCTAAAAACCACTGTAGCTATCGGAGGATGGAACGAAGGCTCTTCAAAATA
TTCTAAAATGGCAGGTGATCCAAAGTTGAGAAATATATTCGTTAAAAGCGTTGTTCATTTTTTAACTAAACAT
AAACTCGATGGTCTTGACTTATTTTGGGATTATCCAACAAAACGAGGTGGAAAATTAGAAGATAGAAAAAATT
ATGTCTCTTTAGTAAAGGAACTGTCAGAAGCGTTCGAGCCTAATAATTTCCTTTTGAC
GGGATCTCTTCGATGTGGTTCAGACGAAATGGACTCGATTTACGATTTGGCTGAAGTCAATAATTATTTGGAC
TTTATGCATATAATGGCGTATGACTATCATGGGGCATGGAATGATGTCATAGGAGCTAGTGCACCGCTATATG
GACTGAGTGAAAATGATACATTAAGTGTGGAATATTCTATAAGACAACTTTTAAAACA
TGAAGTGACACCAAGTAAAATAATTCTCGATATATCAACGTTGGGACGTACTTCCTTCTTAATCAATCCTAAA
GAAAATATTGTTTTCGGGGAAACCACTACAAAGAGTGAAGGGTTCACTGGACCATTTACTAATATTCCAGGAC
TTATAGGATATAATGAAATTTGCTTAGAAATGTCAAATCAATCATCACAATGGGTATA
CGAGTGGAACGAGCAGACTAATACACCGTATATAAGAGACGATGGAAAATTCATCTCATATGATAATCCAAGA
TCTATCGCCAATAAAGTTAAATTTGCTATGGACCATAGTCTTTGGTGGTTTTGCCGTTTGGAGTATAACAACA
GATGACTTTAGAGGTGCATGCA
>1002050397_1 Heli.1-EL599614.1.5
GCACGAGGGTTTTTTCTCACTTCTCAGTGAATTTAAAGAAAAAAGAAAACTTAGCTTTATTGAAGGCATTCGC
TGTTGAATTTAGTCTGACTAGATAAATTAATATCTTTAACCAATAAGAGAAATTACTTGACATATTTATTTAA
TTAATTTATACTAAAGACTGAATTCTTGTATACCTCTATTATCAAAATGAGCGGTGTT
CAAACAGCTGAAGAGGCGATGGCTGGTGCGGGAGCCGGCGATGCGACCCCTGACGAGTTAGCTGGGCTAACTC
CGGAGCAGGCAGAACAGCTTCGCGCCGAGTGGAGCCGGGAACTAGCTCGTGTTGAAGACGAGATTGCTACACT
TCGGACGGTTTTGCAGAGCAAAATGAGGCAGAGTTCCGATTTGAAAAGAAAACTTGGC
ATCACAGTTTGGAAGGAAATAACCGAAGATGTGAATCAAGGTTTGAAAAACGTTAAAGAGAGCCATGTATACC
AAAAAACTGAGTCTGTGATAAAATCGACCGCCGAGAAGACGACGTCGATATTCGGCGGCATCACCGCCGGCGT
GTCCACCAAGCTCGGACAGATGCGCAACTCCGAGTCGTTCCGCTCCATTGAGGAGAGG
GTCGGCTCTGCATATGAAAATGTTAAGGGTAAAGTGGCGTCGAGGTCGAATTCGACCCAGAGCTTCGACGAGG
CGCTGCGCGACGCGAGCCGCGCGGCCAGCGGCGCCGCCTCGCCCACCATACCCGAGAACAAGCCCTTGCCTTA
AACGACAACACCCTCTAGTGGCGGATTTACACTGGGGTCAACCATGTTACTATTGCCC
ACTTAAGGAATTGCGAGTTACTAAATATTTTTTTAATTTAACGTATCAGTGGAAATTATATTAAGAAAATATA
ATAATATAAAA
>1002050398_1 Heli.1-ES587147.1.5
TTTTGGTTGTTGCATCATCGATTTAGAAATAATGTCTGGAAAAGTGATCCCTGTGGTCAGGGCTGAGGAAGAG
GAGGAAGAACTCGTTGATCCCCAGACACAATTACGAGAACAATGCAGCCAGAAACAAGATGCCCAGAACCTGT
GGTCTAAATTTCAAGAGTGCAATGACCGAGTGAACTCCAGATCCAACACAGCAGAAAC
TTGTGAAGAGGAGCTGAGGGACTACTTGCATGTCCTTGATCATTGTGTTACTAAAGATCTTTTTAAAAGTCTT
AAGTAGATTTATTGTTTAGCAATATTGTACCCGCCTTCATTAAACACAAAGAACCCAATTGTTTGAATGCATA
TTTTCTTTGGAGGCAAGTAGGTTGTTAGTAAATTATTTTACCTTAAAAACAGAAAAGA
TGAGTGCTGGCATTGTTGTAAATAAATAATCACTTT
>1002050399_1 Heli.1-DT665701.3.5
GCACGAGGTTCATCCCCTTAGACGTGGCGCGGTAGCTGGATTTTATAAAATAATAACAAATACTATTACAGTT
TAGCTGTAGGAAAATGGATAATAAGCGGTAAAACACAGTGCATTACTTAACGTCGTAATAATAATTGTTACGA
GTCGTGTCTCAAAGAAATATTGTATAGTCGCGTAGTTTTTGCGAATCATTTCATATTG
ATTTTTATTCTTCGCCGGTTTTGTAACTTTGTTTTAGTTAAAATCATATCTTGGTAAAGTATTATATCGTCTA
ATATAATAATTTTGTGCTACTAATAAGTTTGTTATTGCAAAGTGAATAATTTAAAAACTTTCGACCCTGCATT
GTGTTGTAACGTGGGAAAAATACAAGATGAAAAAGATAGAGTCGCCACTGGACGTGCT
GTCACGTGCTGCCACAATGGTGCAGCCGTGCCCGGCTTATCCTGCCAGCGATTCAGACGGCTTTGAGACTCCG
AGTACAGCATCGAGCGGAGAATCAGAGGGCGACCGCACTCTATCTCCTGAGGCGCCGCGCGCGCAGTCCAGGG
AGCTTACCGGCGGGAAATGGAGAAGGGAACGACGTTCTATGCGCCTGCCGGAGTATAG
ACCGAAAGAGAACGGTAAAATGGCGCTACGGTCCCAGTCGTTCAACGAGCGGCGTAGCGTCGCCTCAGTGACG
CGCGCGCAGCCTCACAGCGACGGGGAGCTGTCCGACGAATTGGATGATGGACCCGCTAACTTAACTATGAA
>1002050400_1 Heli.1-DT664505.3.5

GCACGAGGACTACTCATGCGTTAAAGTCGACCGCTACTCTAAAGTAAAGAAAGACAAAAACGAGATCAAATAC
TCTGGAATGTATTACAACTTTGAAATATTCCATATGAGAGAGAAGGAGATTCCGGTGGATTCTGTGGAAATAA
AGGAGCCTATACAAGCGTTTGCTTGGGAGCCGATAGGTTCTAAATTTGGCATTATACA
TGGTGATGCGGCGAATATATGTATTAGTTTCTATCAAGTGAATACTGGACAGGCTCCCACTTTATTGAAGAAA
TTTGATAGAACTTTCAACCATTTGTTCTGGTCGCCTATGGGACAGTTCATAGTGTTGGCCAATTTTGGTTTGA
CCGGTGGAGCGCTGGCCTTTGTTGACACCAATGATTTTACTATTATGAACATTTCTGA
TCATTATCAGATGTCAGGCATTGAATGGGATCCAACTGGTCGCTACGTAGTGACCGGTGTCTCCTCGCTCAAA
TGTAAAATGGATTGTGGATACTATTTGTGGTCATTCCAAGGCAAGATCTTGAGAAGAGTAATGAAAGAAGGTT
TCGCACAATTCCTATGGAGACCGCGTCCCCCCACACTGTTGTCGGAGAAACAGCAGAA
GGAGATAAAGAAGAACCTGAAGAAATATTACTCTCAGTTCGAATCTAAGGATCGTATGCGCAGCAGTAAAGCC
AGCAAGGAATTGGTCGCCAAGCGTACGGAGCAAATGAAGAAGTACATAGAGTACCGCGAGCAGAAGACGCTCG
AGTGGGCCGAGCAGAAGCCGCGACGTATCGAGCTTCGGGATTATGTAGACACGGACGG
TCTCGACACCGACCAAACAAATACAGTGGAGGAAGTTGTAGAATTCTTTGTCAAAGAGGAACAATCGGTCATC
GAGTAGTCGCCATAAATAACCACTTGTCGCCGGTTGTAACCGCCTGTATCGGCTTATAACCGGCCAAAACCGG
TCGAACTGGTACATACCGGTCGAAACCGGTTATTAAAGGATAACTTTTTAATAAACAA
>1002050401_1 Heli.1-ES586706.1.5
AGTGTGGCAGAGTGGCCTGACGCGCCGTGGCCTGGCGCCACGAGGCCATGGCTCGGCCTGGCCTGGCATGGTC
TGGGCTGGCCTGGTCTGGCCCGCCATGCCTATAACGTGCCATTGTGCGTGTGATAGTTTTATACAATGAACAA
ATCCGGCCTCAGGCCTTGTAAATATAAACTTATGCTAGTGAGATGTTTTACCAATTAA
TGATTAAAAAAAAACAAAGACAATTATAGTAAATGCTAATGGGAATTTTTCGGTGGAGTATTATTATTCATGA
AAATTTTACATTGTTATCGTTTCGTTCAGCGTTTCGAGGTTTGTTTTGTATTATATTACGTGGCGGGCGATTA
TGAACGATTATGAAATGGTTATGAAATCAATTTATTACTATGGATATTTCAAAATGAA
TTCTGAACTTATTATGAATTATTTCTTTGCTTCTTTCTAAACTTGGGATCCTTTAGAAAATGTTCCCGGAATC
TTTTTTCTTGATCAATTATGGAAATTATGAGTCATTATGGAATGGTTCATAATCGTTCGTACTGTATATTATG
TATAGCGTGATTTGTGTTGCAGTGAGAAGCTCTATGACGTACATATCTGTTCCGTAGC
TTGTATTATATATAATTTGATCTTTTTCATCTTGATAATAAAGTTGATTTGTTTCTAAAT
>1002050402_1 Heli.1-EL599503.1.5
GCACGAGGGCTGTGCTATAATGGATAACTGTCAAACTCAGAACTATAAACGAATTATAAAGTATTTAAACGAA
ATATGTAACTTTTTAAATATAATTTCTCATCGATTCGCTCAACATAGGTTTAAGTATCATTAATGAACGACTA
TGGTGACCTAAAATAATGAACAATGAAAGAAATAAACGTTTTTTATATCTTTTCAAAA
TTTACAAATAATAATGGTGTAGTATAAGTAAATATAATAGAAGTAAATTAATATTAATATAACATTTTAAATT
GGCTCAAAGTGAGTTAAGAAATTTAGATTCAATTTCAAGGTCATTTTTGAATGAGATGAATGAATGTTTTGTG
CATTAAACAATATTAGTCTATTTTTTTTAATTGTTTATATTTGAAGAAAATTAAAATT
AAGTTGCAATTTTTTTACTAAATTAAGGATATTTCTGAATGTAATGGATGACTTAACAAATGTTTTGTGGATTA
AAACAATGACTTTCTAATTTTAATAATTTATAAAAATAAATGTGTTGAGTGAAATATAGGAAGTGTCATGTAG
TTTTAAAGACTTCAAGTCTTGATTCAATACATTTCATACAAAAATCATGATGTACATA
TAAATGGTGTACTCATCTGTCACTAGATGATTAATAATGGAATATTTAATATAATAATTATAACTAGGACTTT
TTTCTATATAT
>1002050403_1 Heli.1-DT665938.3.5
TTGCAGCGAGACGGCAGAGGCTAGTGAACGGTCGCTGACTGACTTACGCGGTAAAACCAAATAGTGCACACCG
AAATGAAGGCAATTAGCTTTATTATCGCACTGACAGCTGTAATAAGTTGCAAGGCAGCGACCCCACAACTGCG
TTTCGCGGTGGAAAGAAAGTAGATTTTACATGGGACTCTCCAGAACAGCGGGAAACCG
CTATTAAAGATGGAGTGTTTGTTAAGGCCAATAACCTTCCTTTAGGCTTAGCGAGGTGGAATAACAAAATCTT
CGTGACTGTTCCAAGATGGAAGAACGGAATTGTGTCTTCTTTTTTTTACATAGACCTAGACGGTGCCCAAGAC
CAGTTAGTGAAGCCATATCCGTCATTAAAAGAAAACCTTGTAGCTGACAGCGCCAAGG
AACTGCCATCAAACAGCTCAATTATTTCTGTCTTTAGAGTGTTTGTTGATCCTTGCGATAGATTGTGGGTAAT
GGACTCTGGGTTGGCGGATATATTAGGATCAGCAAATCAAGTGGTTGGTCCGTCTCTTG
>1002050404_1 Heli.1-DT667429.3.5
AGTACCACGAGCCGCCTCGGCCTGAAACTCCGTCCAGACAGCAGCAGAAGCAGCCATCACCGCATCTGAGACA
GCCCCCAGCACCGCCGCAACAACAACCACCACAACCCCAACAAAAACAACAGCAGCAACAACACTATCAACAA
CAAGAACAACAACATCAATCTCAACAAAACCAAGCCCCGCCACCTCCTCCTCAGCCTT
CGGCCAATGACCCCATCACATTGATCCTTAGCATTCAAACTGACGTCCTCAACCTAATGACAGACGTTGAAAA
CTTCACCGGCGCGAAGAATGACAAAAGATATCTTTACCTAGACGAAATGTTAACCAGAAATCTGATTAAGTTA
GATAATATTGAAACAGATGGCAAAGAAAATATTCGACAGGCGAGGAAGGAGGCTATCA

AGTGTATTCAAAAGTGTATAGCTGTTCTTGAAGCAAAGGCGGAGAGCAATAAGGCGAATGCGGCACAACCCCA
AGATGTCGAAATGCAGAATGAGAATGCGAAACAGGCAGCTGAGCAAAATAGCGTTCAAAATGGCGACGTAGAG
ATGAAAGAAACCAAAGAAGATAAAGTAGAATCGCAACCAGAAGTACAAGCGCAGGAAC
CACCTGCGCCGGCGCAGAGTGGAGAAGTGAAAGAAAAGCCCGATGAAGTAAACAAAGATGAACCAAAGCCACA
GGAAAAAGAACCAGAAAACCTGGAGGTAGCTGACACGCGACCAGAACAACCTAAAACCTCAGAACAGTCGGAA
TTGAAGCCCGAAGAAAAATCTGAAAAGAAACAGAGCCCCAAAAAAGTTGTCAAGAAAC
GTGACAAAAGCAAAGATAAGAAGGACGTTGCCAACGAAAACAAAAAGGAAGAAGTAGAGAAACAGGAAGAGAC
AAAGAAAGAAGACGAAAAAAAAGATTTAGATAAAACTATAGCAAACGAAAATGTAAACAAAGACGACGTCACA
GATAAAAAAGATAAAGTAGATAATAAAGAGAAAATAGAAGATGTGATGCAAACAGATG
CGAGCGCGAAAGGAAACGTGATGGAGGTCGACGGTGCTGCTAGTCAATAAATATAGTTTGGATTAGTGAATTA
TAGAGCACGGATAGTCCGCACGAGGCCTTCCACTACTTTATAACTACTTGTGCCTGTGTTTATCTGTACCAGA
CTTGGATAAG
>1002050405_1 Heli.1-DT668670.3.5
GCACGAGGGCAGTATACGACTTGGGCGGTGGTACCTTCGATATATCTGTACTGGAAATCCAAAAGGGCGTCTT
CGAAGTTAAATCTACAAACGGTGACACGCTGCTCGGTGGTGAAGATTTTGATAATGTCATCGTTAACTTCTTA
GTTGACGAGTTTAAGAGGGATCAAGGCTTAGACATTCGCAAGGACGCCATGGCGATGC
AACGTCTCAAGGAAGCAGCGGAGAAAGCCAAAATAGAACTTTCTGGATCACTTCAAACTGATATCAACTTGCC
CTACCTCACCATGGACTCGTCTGGACCTAAACATATGAATTTGAAGATGACCAGATCGAAACTAGAATCTTTA
GTAGGCGATCTAATAAAGAGGACAGTGGCGCCGTGTCAGCGAGCGCTGCAAGACGCTG
AAGTCAGTCGCACTGATGTAGGAGAGGTGCTGTTGGTTGGTGGAATGACCAGAATGCCTAAGGTTCAGTCAAC
TGTACAAGAGATCTTCGGCCGAGCGCCCTCCAGATCAGTCAATCCTGATGAAGCAGTCGCAGTCGGAGCGGCC
GTCCAGGGAGGAGTTTTGGCCGGCGATGTGACAGACATCCTGCTTCTGGACGTGACCC
CACTGTCCCTGGGTATTGAGACCCTGGGAGGAGTGTTCACTAAGCTGATCACTAGAAACACTACAATACCCAC
TAAGAAAAGTCAGGTGTTTTCTACTGCCGCCGACGGACAAACGCAGGTTGAAATCAAAGTACACCAAGGCGAA
CGTGAAATGGCGTCCGACAACAAGCTATTAGGACAGTTCAGTTTAATTGGGATTCCCC
CGGCACCTCGGGGAGTCCCCCAAATCGAAGTGACCTTTGATATTGACGCCAACGGCATTGTACATGTGTCCGC
AAGGGACAAGGGAACTGGAAAGGAACAACAGATCGTAATCCAATCGTCCGGTGGTCTATCGAAGGACGAAATC
GAAAACATGGTGAAGGCGGCGGAGCAGTTCGCGGCCGCGGACAAGAACCGGCGCGAGC
GCGTGGAGGCGGCCAACCAGGCCGAGGGCGTGCTGCACGATACTGACACCAAGCTGGATGAATACAAGGCCCA
TTTGCCACAGGATGAGTGTGATAAGCTACGCGAAGAAATGGGTAAACTACGTGAACTGTTAGCCCAAAAGGAA
ACTGCAGACCCAGAAGCCATTAAGTCAGCCACCAGTACCCTACAGCAGGCCAGTTTGA
AACTATTCGAACAGGCCTATAAGAAGATGGCAGCAGAACGTGAAGGATCATCACAACAATCCCAGTCTCAAC
>1002050406_1 Heli.1-ES586610.1.5
CTTAATCATCGCCGTTTCTGTCTTCAAAATAGCCATTTTAGCTAAAATTGCGTTTATTGCTAAGGTTATAGCC
ATAATTAAGGCACTTCTGGCCAAGAAGAACGCCCAAGAAGACCACGGATGGGTATCTCATGAAGAACATGTAC
ATCCAAGCCCTGGCTGGGAAGGTGGATGGTCCCGTTCTAGGAACGAAGCGAATAATTT
AGCTTATTCAGCCTATACTCAACAGTAATTGAGTTATTTATTCATAAAAGGTTAAAGATTAATTTATTTTTGA
ATGATTAATTTATTTGTGTGAATGTGTGATACGATTTTGATACGACATTTCTTTTCGGTCATTTTATTTATTT
TATTTGTTGCTCTCTTTTCTTTAATTTGACTGAAAAGGATTTTTTTGTTATTTTTATA
ATATATTATTATGTATACGAATATAAAATTATATTTCATTAATTTATGTATTAGTATGTGATATTTAACTTAT
ATTTTATCATTCTTTTATTTTTTATATAAAATATGTTTATTAAACATTTTACTACTTAATTTTACTTAATATG
TGTATGTATATCTTTTATAATTATTTTTTTTATGTAATGAGCAATTATTTTGACTATA
TTTTTATAAATATTTATTAATATTTTTTATTGCTTATGTATATTTTTGTATATTTATTATAATTCATTTATTA
TTATTATTATTTTAAAAAATATATATTTAATCTCGTTACCTTTTATGATCTCTTTTTATGTTTTATTATTAGT
TCATTTTTATTTTTACTTTCTTATTTTGTATTGTATCGTGTATCACGAATCGCAAGTG
ACTTATTTATATGAGTGTATGTGTTTCCATATAATTTAATAGTCATAGATTAAATAAAAA
>1002050407_1 Heli.1-EL596204.1.5
GCACGAGGCGAGTGTTCTGTGCGAAAGCCCTGGTTGCAAATCCCTGGCTATGCTACAATGCCCAACGTGCATT
AAGCTGGGTATACAGGGCTCGTTTTTCTGCAACCAGGAATGCTTTAAAAAGACCTGGAAATCTCATAAAATCA
TACATACATTAGCTAAAGGAGAGAGTACAGAAGGATCTGGCTTGGGTTATAATCCTTG
GCCGTCGTATAACTATACGGGGAAACTGAGACCGGCTCCGCTTGGGGCTACGCGTACTGTGCCACCTCATATA
GGCCGCCCGGATTATGCCGATCATCCTACTGGATTTCCGGCTTCTGAAAATGCAGCTAAAGGTTCTGGCCAAA
TCAAAGTGCTAGATGATGAGGAAATAGAGGGCATGCGTGTAGCTTGCCGTCTAGGACG

AGAAGTACTGGATGAAGCAGCAAAAGTGTGTGACATTGGTGTAACAACTGACGAAATTGATAGAGTTGTACAC
GAGGCTTGTATTGAGAGAGAATGTTATCCAAGTCCATTAAACTATCACAATTTCCCCAAAAGTTGCTGTACTT
CTGTCAACGAAGTTATTTGCCATGGTATTCCTGATTTGCGTCCATTAAAGGATGGTGA
TCTATGCAATGTAGATGTAACAGTCTACCACAGAGGTTTCCATGGTGACCTCAATGAAAC
>1002050408_1 Heli.1-EL600512.1.5
GCACGAGGGTGCGGATGTGCTTAACGCTGGTTTAAACAAAAATGTCTAAGAAAAATGGTTTAGAAGTAGCTGC
GTCGAGTAAATCGGACGCTACGGAAACGACACCTCTCGTGTCCAAGAATGAAGAAGGTGGTAACGGCGGAGAG
CCCGGTAAGAATGGAAACGGAGGAGGACTGTCCACCTATCAAGCAGCTTTCCTGATAG
CTGGTGAAATGGCCGGTAGTGGAGTCCTGGCGCTACCACGGGCACTCGTTAAAACTGGTTGGGTAGGAGTACC
CATCATAGTTTTGATGTGTGCCATAGCAGCGTTCAGTGGGAAACGATTGGGTGACTGCTGGACCATAATAGAA
GGTCGTGACCCTGAGATGAGGACCAGAAAACGAAATCCATATGCAATCATTGCTGAAC
AGTCTTTGGGCAAGATGTGGAGTGCGGCGGTGTCCCTGGCAATGATTTTAACGCAGTTCGGTATAGCCGTCGT
GTATTTACTGCTGGCAGCTCAGATCATAGAACAGATCTTCATGTCGATAATGCCGACTGTCACGATCTGTATC
TGGTACCTGGTGGTGGTGGGCGCCATGACACCACTTATGCTATTTGGATCACCTAAGG
ATTTCTCCTTCCTAGGAGTGATCGCGTTCTTCTCCACCGTAGTGGCTTGCCTCTTGTACTTCATACAAATGA
>1002050409_1 Heli.1-DT663979.3.5
CGGCACGAGGCTTAAGTAAATCGAAATAAAAAATGTTTTATATTGAACTAATAAAATTTAAAATAAATAACAA
TATAAATATTAAATTCGCTGTTAAATGTTGATTGTTTGATATTTACAAAAAACATCGGCCATTTTGTTTATTT
TTTAATAAATATAGTAAAACATATATAATATTATGAATATATATATTAATTTTATAAC
ATGTTTGTGATATATCGACGGCAACAGCAGGGCTCGGTTGTTTTGTACAGGTAATGTTTAAATAACGTCAATC
AATTCAATTTTTATATCAATCTCATATCAATCATTTAACGCGTTTTGCGCAAGTAAATCTTTTTACATTAGCC
TTTGGATACTTTAAACTATAGTACTTAACTAGGTATTGAAAATAAGGTATAGCTATCT
CTTTCTGGCAAATTTAATCTGCCATATAATAGAGGAGATAGCAAATACTAAATTAAAAATAAATAAAAGTAGG
AATTGATTTCGGCTTCATGAATTTATTCATCAATTCTAGCTACAATTGAAACGTTGACAATAAAATTACTATA
ATAATAAATATTTTTAGAGTAACGTCCTTGAGAGTAACGTCGTGTGATTGACAATAAG
GTAAACGTAAGGTAACTACTAACGTGTATGTACAATTTTATCCACAGTATTTCTAGTTAATAACAAGATTTTT
TAAAAATAAAATGGAATGGTAAGATTTGACAATAATTTGACAATAATGTCTCAATAGGTCTAATTGAAATCAA
ATAATGTTATAATACTTATATACACATATATATGCTTATATACATTTTTATATAATAT
AATATTTTTCAAATATATATCTAATATATAGACATATATAAAAGAGGCAATAAAATTCCCAAAGTTTACTTTT
TTTATCAATACTTAAAATAACATATAAACAATAATATTCAAGAAAATCAAAAGTGAATTAATTAAAAAAAATA
CAAAATCTATATAATCTTTAATAACATAAAGACAATAAATAATTTTAAATTCATTATA
CAAATTTATATTTAAAAATGATTTAAATTTTTTGCATATTTTTAACTTTAAATGTAAGCT
>1002050410_1 Heli.1-EL601044.1.5
CTCCTAAAGAGGCGGGCTGCAGGAATTCGGCACGAGGGGCGAGTCCTAAAAGGCCCAACGCAAATGTAACCGG
TCAAACGCGGGAAATAGTTGCGTTTAAAAACTTTTGAATTAAATTTAAAACAAATACTAACTTTAAAATGGGT
GTCAGTGCAAGGATTGCTGTCCTGTTTCTATTGATTTCTGCGACGTATGCGGTTCCTA
CAGGAGATATAATGAGGACTCCAAAGATCGATGATGACACAGACCTGACGGCAGAATCATCAGAAATTATATC
AGAAGCAGTGGCACCACCCCTCCACATTCACCGTCCAGACGACGCTGAAGCAGCCGCAGACCATTCCATCGAA
GTTATTGGCGACTCACAAACATTCTTTCCTACTTTCGCCAATTTATTTGAACCACGGC
AAGCCAATAACTTTCGACTAGGATTTGGAAGCCAAGAGGCTGCATACAGCCAGCGTCCGAATTCTTATAGGTC
ATTGTTAAACTATCCGTTATTTGGCGGTTATAAATCTGTCGAGAACTTTGGCAAACAGAACGGTCAGGCTTCT
GAGCCCCTAGTAGATGCAAGCTCTAGCGTTCTCGGCTCTGGAAATTTTGGCATTATAA
GAGGAGGTACTTTCTTCGCCCAAAATGACGAGGACGGAGATTACAGCGACAATTATAATTCCTACTATAATAA
CGGCCATGGCAGACCATCCCTAAACGTTGGTTACATAGCAAACCCACGCCCCAACTACAATCAAGACCAGTTT
GCTAATTTTAGAGACTTTGCCGATATAAACGCGCCTTCCAATACTGCTTACTCTCACT
>1002050411_1 Heli.1-DT667624.3.5
AGGTTTCCCTTTACACGTGTTTCATGGTTTTTAAGAATTCACCCCCTAAGGGTTCAAATAAGGGGTTGAAAGT
TTATATGGAAAAATCTTTAAATTGCCACCGATTTTTATAAAAATTTAAATTCAGACTGTTAAAAATATTTTAT
TGAGCACGTGTTTCGTGGTTTTTTTAGAATTCACTCCTAATAAGCGGGCGAAGCCGCG
CGCGCCCTCTAGTGTAAGTATATGTACGTATGGACGTTTGATTATGACTCGATCGACGGTTCAAAGTTGGCCT
GTGAGTAGGGTACCTCAATGCCTATCCTTTTCGCCCCATGTATGTTTAGGGATTTTTACTGTTTTTTTGCAAA
TTTGCAAAATGGCGTCTTTTTGAAATAAGTCAGATTTTTGAAGAAACTTAAAAATACA

AACACAATGTCAAAACATTTTCACTCTTTAAATATTGTCCTACTACTATAGTTGAGTAGTAATAAATATAAAT
AGTTAGGTAAATCAGAATAAATATATTTCAAGGTAATCAATTCTTGTAACCCAATAAGATTTACTGTTTCTTA
ACTTGAGTACATTTTTTATCTTGTTGTCATTAAGCTAGCCTTTCAGTCAGGATACTTT
CAGAGATTATTCCACACAAATGCCAGTAGTTGGTGGATACATTACAGTTTCTTCCGCTACAGCCCTTGAAAGT
TATAGGGAATA
>1002050412_1 Heli.1-ES585052.1.5
AGCTACGAGAAGGCGGGTTTCCACAGTGGCACGCCGCCCCCCTTCGGCGCGGGTTCGCACCTCTACATCACGG
CGCCACCGCACCACCACCCGCACCACCACGCGCCACAGCACCAGATGGATGGTCGGGTGAACAGTAGTCATAA
TAGGCGGGAAAGCGCCTCTTCCGGCCGATCGGGTGCAGCTAAGCCGGCCGCAAGCAAG
CCCACTTACTCGCAGTCGTACTGGGCGCCTAATTAACTTGGGACCGAACAGACAATATGGAGACGTGTAAATA
CAACGCCCGACCTCTACTCAACTGCCGACGGTCGCCGCGTCATATACGACTTCCTCTGAACCACCCACTGTCA
TTGTCATAAACACGTATAAATACACATAAACACATATAAATACACATAAACAAGTATG
AACACATACATATGTGTTTGTGTAGCCGGCGCGTTAATCGTGTCAATTTGTATGGAAAATATTAATAAAAGTA
TTTGATAAAAATAATTAATTGATAGTGTGTATAGTTGTCGATTTTGTAAATGCTCTACTGAATACTTATTAAT
TAATATCAACGCGCGGGCGACGCCATTTTGTTTGCACTAGTGTTGTGTTTTATGTGTG
TTTGTATGTTTGTAACTTTTTTTTTTAGTTGTTTTAGCGACACACTGGCCCCCCTATGTAATTTTTTTCTTAT
TTTTTTATTTCATTTAGCAATTAATATATATTATAGTACATATATACATAAACAGTACGTGTATCTATTAAAA
AACGGAACTGATATTGTAAAATAAATAAAATAAAAGGTAATTGAATGTTTAAAAGCTA
TGTTAATAAAAGTGTATCATGTGTTTTTTTATTATTATTATTAATATTAAATTCGTAGTGTATTTCCGACGCG
TCGTATCGCAGCGCTCTTTAATT
>1002050413_1 Heli.1-DT664230.3.5
GNAGAGGAGCCGCCCTTTAAATTCACTTTATTAATGTCGTGTACTCTGCTCCTAGAATTAATAAGTTTTTTAC
GTAAACATTTTTTTATCTTTAACCGTTATGGTTGAAAAAGCTGAAATTAAGGCAGAATCATTACTGAATTTAG
ACGACTTACTACAATGCCCTGTTTGCTACGAGATTCCAACAGGACAAATATTTCAATG
CAACGAAGGTCATCATGTCTGCGGCAGGTGCAAGATGCGTCTGGATGTGTGCCCTGTCTGCCGGGCTCTTTTT
TTTGGGACACGCAATTATGCAATGGAAGAATTAATAGCCAATTTCAGAAAACTGCGTGCCTTCAAAACAAGTG
CCAAAGCACAAACTGGTTCAGGCTCTTCAGAAAGCAGTGCTCCAGCTAGAGAAACAAC
CCCTGGGGAAGGTGAAAATGAAAACAATGAAGATGATGAAGAAAACAATCAAAATATTATAAATCAATCTGCT
CAAGGACCGCCGCAAGCATGTAAAGGTCTCTTTCGGTGTCTCTGTTGTAAAAATGGCAATGGAGAGAGATTAC
CAGCTGCTAGATTACTCAACCATCTTCGTTATTTTCATTCACCAGATCTACTTGAGGG
TCGATCTGAAAGTGGAGAGTATTTACAAGCATGGCAATTCTCCACTACACCCGGCAAGATCGTGACGGCGGTG
AGAATTGCTGACATGGGCATATTTTTCTTAACTATAGAATTAAATAATGATGCTGTATGTGCATGGCTGACTA
TGGCTGCATCACCGTGGGTAGCTCACGCTTTTCATTATACTGTTACCATTTGTGGTAA
TGACCGTGAAGCAATTTTTTTCTGATTGTGTTTGGTCGGTAAGGTCTTGCGAAGGATCTTT
>1002050414_1 Heli.1-DT666930.3.5
GCACGAGGAAAACCTTTCGTTTCGTTGTAGAATATTCTGTGGAGGTTAATGAATCAGCAACGCATATAGTGCC
GTTGGTGTTGAAACATGTCCGATTAGTTAATTGCATTATTCTTTTAAACAATTTCTTTATTTCATTGTGTATT
ACGTATAAACGTTTGGCCACCACGACCGCGACAATAAAATGGCGACTGACCAAATGAA
ACACCGCATGCATGTTTTCAAGAATACTGGCAAAGATGTCGATGAAATGCGCAGGCGGAGGAACGAAGTGACA
GTCGAGCTGAGAAAGAATAAACGGGAAGAAACTCTACAATAACGACGCAATGTGCCCGTTAGTTATTCTACAG
ACGAAGATGACGCAGACAGGACCTTGGCCACGACAGACCTCCAGGAGTTGGTGATGAA
GGCCGCTAATGTGGAAAACCCTGAGGATCAACTGTCAGCCGTGCAGCAATGTAGAAAGTTATTATCCTCAGAT
AAGAATCCGCCAATAGATGACTTGATAAAGACTGGAATACTACCTATACTCGTTCAGTGCCTTTCGAGGGCGG
ACAACCCGACGTTACAGTTCGAGGCGGCATGGGCCCTCACGAACATAGCGTCAGGAAC
ATCCGCACAGACTAACAAAGTGGTTCACGCGGGAGCTGTGCCACTGTTCCTCTCCTTGCTCATGTCGCCGCAT
GAGAACGTGTGCGAGCAAGCAGTGTGGGCCCTCGGTAACATAATTGGAGATGGTCCCGTATTGCGAGACTTTG
TGGTAGAACTGGGGGTTGTTAA
>1002050415_1 Heli.1-DT661768.3.5
GCACGAGGGTTGAATCTATATTTTTATATATTGTTAAAAGCATAATAAAACAATTGGACACTTAGAGGAATAC
ATTAAAAACTAATAAAATGTCCAATTTAAAAATCAAAGTTATAAGCCGAAATCCAGAAGATTATTTACGCGCA
ACTAAGAGGGATATACATAAAATACCTAGAAATTATGATCCTTCATTGCATCCAATGG
AAGGGCCTCGAGAATATGTAAGAGCTTTAAACGCAGTTAAACTTGAAAGGGTATTTGCAAAGCCATTTGTAGG
TAGTTTAGATGGACATACAGATGGAGTATCAAGTCTTGCTAAACATCCAAGCAGATTGTCATGTCTTGCAAGT
GGAGCATTTGATGGTGAGATAAGATTATGGGACTTGGCAAGTCGAAATTGTACCAGAC

ATTTTGTTGCTCATGAGGGTTGGGTCCGCTCTATTTGTTTTACACCAAATGGCCAGCAGTTTACTAGTGTTGG
TGATGATAAAACTATTAAAACATGGAAAACCGAAGTACAAAACCCAGATGATGAGGATCCTGTAAATACACTT
TTAAGTATGTCAATTGTCTCTGGTATTACTCATCATAGATCTAAACCAATATTTGCAA
CGTGTGGTGAACATTGCCAACTGTGGGAGAATACAAGGAATGAACCTATTAAAGTATTCAAATGGGGTGTTGA
TAGTCTTCATCATGTTGCTTTTAACCAGGTGGAGACCAATTTACTTGCATCTTGTGCCAGTGATAGAAGTATT
ATTTTATATGATTTTCGTGAAT
>1002050416_1 Heli.1-EL603496.1.5
GCACGAGGGATGACTTAGCGATTATACAAAATTACAAAGAAAGTGTAGCTGTTGTTGTGTCATTTTAATATAC
ACTTTTTAAATAGTTAACATATACAGTATGGCCGGTGCAATGTTATTTGAGAGGTCACGAGTTTCATCGTCAA
ACAGAGACGAAGACGTCCGCATGACTGACAAAATGGTTAGCACTGGAGAGCTCCCCGA
AGACGACGAGGAAAATATCAGGGCCAAGGAGCAAGGAATATTAAACCTCGGCGAAAAGTACAAGAAAGAAGGT
AAAGCTAAAGAACTTGCTGAATTAATAAAAGCAACCAGACCTTTCCTTAGTCTGATAAGTAAGGCTAAGGCTG
CGAAATTGGTGCGATCCCTAGTAGACTTCTTCTTGGATTTGGAAGCTGGCATTGGAAT
TGAGGTCCAGCTGTGCAAAGAATGTATAGAATGGGCCAAAGAAGAGAGGCGCACGTTCTTAAGACAGTCCCTC
GAGGCAAGACTTATCGCGCTCTACTTCGACACCGGTATGTACACAGAGGCCTTAGATTTAGCCACCGCACTGT
TAAAAGAGTTAAAGAAGTTAGATGATAAAAATTTATTAGTTGAAGTACTGCTTTTAGA
GAGTAAAACTTATCATGCTCTTAGTAATTTACCGAAGGCCCGTGCATCGTTGACGTCAGCAAGAACTACAGCA
AATGCTATTTATTGCCCTCCTAAAATGCAAGCGGCTCTCGATTTGCAGTCTGGTATTCT
>1002050417_1 Heli.1-DT666768.3.5
GCACGAGGGACACGCCTTAAAAGAACGCGAGCCGACTTATTTTTGATAGTTTTTTTCTTTAAGTGGACGCAAA
AACTGTGACTTAGCCGAACACGTTATTATCGTTCGTATCGAAACTCAGGTGTTTGAAATTTTTGTTGAAATTA
TTGTGTAATTAATTAGATAAATGCTATATTTATTACGTTGAAAGAGAAGAGTGATGAT
TTGAAATTATTTCTGTCTTGATATTTTCCGTCGGATGTATCTACATATGTATATGTACGTGAGAGCGTGACTT
CGGGTCGGTTCTGTTGCACCGAACTACTAAAGTGCATTTCGTGTTTGTGTGGCCTTTGTCCTCACCCACTGCT
CGCCGCTCCGAGTACGGCAGCAAAGGCCACACTTGACACGGACCCAAGGCCGCGAATT
ACTCCAGACTCATGTGGACATGTGCCGTGGTGCGTTGGGAAGCAGTAGACTGAGGCCGTGAACTCTTGACAGT
TCATTTAGACGATAATGAACAAACTGTAATTAAATACGTCGTAAGTACTTAAATTAACCGATGAACTTTCATT
TGTTCCGCATCCTCTGCTCCAGGGCGGCGCTGTAATTGGGTGTCAATTAAACAGCCTT
ATCGACAGTCATGTGTGGAAGTCTCACCATTTGGGATTTGGGAATTTAAACAAAAAAAACGTGATTAAACATT
AAAGTACTTAAGCATCTAAAAATCGGAATTGTTATACAAACTAAATCTGTCCGAGAAACCGGTGGCCGTCTCG
TGAGCGCTAATCTTTTAACAATATTTAACGATTAAGAACAATAATTTAGGAATAATGC
ATACATTATTATATATCTATGAACTAAATAAACATGGAATAATTTCTTACGGTATTTTACTACTATAGTTAAT
ACAGTTCCTAATATATAATTAGTTAAATCGTAAGTAACAAATGACAATTTATTTGCAGTATTTAATTCTAGTT
TTTTACAAGCGCATTCGAGACGGCCAACGGTCATACTCAAGTAGTGAAGCGAAATAGA
TAAAGATATTAAAAAACTTTAAAAGTTCATTGAGAAATGCAATGAGATTTGAGACATCGAAAATATTAATTAA
AATGTTCAAAACAATAGATAAAGTGAACCCGACCTCCCTAAGAGAGTTCTAGTCAAACCGATTCTACGTCATC
GTGCGGGAGTGTGGAGTGCGGCGAGGCGTAGCAACGGTGTGCCCTCTCGTTAGTGACG
TCGGGTTGCGAGCAGCTCGCGTGAAATGGGACTTCGGGGGTTGCGGTCGACGCTAACTGCGATACGTTTTTAG
CTGCTGCGGGC
>1002050418_1 Heli.1-DT665756.3.5
GCACGAGGAATAATCCTTTAATGACCTAAAAGTGTATACAATTAAATAAAATACGATATATATTTTTTTAACA
TAAAATATATGTTATTATTTATTTGGCCTACAAAGAAATGTTAAAAAGAATGAAATTTACACAACAAGGGCAT
CATACTTAAAAAAAAAATGAATGATAAGTTTACTCATTTTGCCTAAAAAAATATATTAA
AAATGTATATTGATAAGAGCATGCATAAGTTCTGGAGACGAAATTAATTTTGTATACTTTCTACATAAAAATG
TATCATGAAGGATTATATTCGATTTTTTAAGTATAGTTGTAACTAACGTAAACAAATGAGTGTCTCCGAAATA
TAAAGGAACTGTTTATAGTGAATTTTATGGATAGTTGTGTGTATTGTATTTTTGGTTG
TTCTTCCCTCCAAACTTGAAGCAGCAATCTGTTGAAAGGCATCCACAGTTCACCGCCTTATTAATTTACATTC
GACGAACTCTTCCGAGTACTTTCTTGTTCTTACACCTGAACTTCCAAGTACTTTGGAAGATATAATGTATCAA
TGATAAATATTTAATTCGTTAATGTTTCGAACGAAACACAGAATTTTGGATACCTTTT
AGAAGGTAGAGTGACGTTAAAATATATTTGAAATAAGTATGTATGAATTCTGTTATGATTGAGAAACGTTACG
TGCGTCCTTAGGCTACTGTGTATTCGTGTAAAATATTTTATCTTTAAAATAACCTTAGATATTCTGTCAAGTG
CCCAAACCCAAATGTTATTTGATATCATGGTTAAATGTAAACTGATCACTTGACACCA

AATTCGTATGTCATTAGTACGTGACTTTAAATTTTATTTATTTTATTAATAACATGTTTTATCTTACATTATT
CTTTCCCACACATATGTGACCTGATTTATTTTAAGTTTTCTTTGAATATTACGTTCATAGTAAACTATATTCT
ATTCGTCTAACTATTCGATTACTTTGACTAGCCACTGTTTATAGATAAGCGTGTACGT
AACCGTAAACACCCGTTACCAAATAGGGGCCTTCCGTAAATTACGTCACACGTGGAGGGGAGTGGCCCCTCAC
CTTCCTTTGCGTGTGACGACGAAGTGTGACATTATACGACCTTATATATATATAAATGGGTGGGTAGGGGTCG
TAAATTCGTGACATCACACTTTAAAATGTACTGTTGTTTTAATTTA
>1002050419_1 Heli.1-DT665820.3.5
GCACGAGGGTTCGCCGGCAATAGCGTTGACGTCTGCAGTAAACAAGTTATTCCCCCATTATCGTCACTATTCA
ACAGTAGAGAAAGAAGTATCCAAGAAGATGGCAGCCGATAAAATGTTGCCTCAAATCTTTAAGTTCGTGGATC
AGAACAAAGAATCCTATAAAGGGCTTTTAAAGGAGGCAGTAGCAATTCCATCAGTATC
GAGCGATATAAAGCATCGCGATGATTGTATTCGAATGGTGCAATGGATGCAAGATAAATTAAAGGAAGTTGGT
GCATCGACGGAATTAAGGGATGTTGGGTTTCAAGTTTTAGAAGGAGTAGAAGTAAAATTACCGCCTGTTCTAG
TTGGCACATTAGGAAATGATTCGAAGAAAAATACTATTTGCATTTATGGACATTTAGA
TGTTCAACCAGCTTTAAAATCAGATGGCTGGCTATCTGAACCTTTTGAGTTGGTAGAAAGAGATGATAAACTT
TATGGACGAGGATCTACTGATGATAAAGGGCCTGTTCTAGGCTGGCTTCATGCTATTAATGCATATAAGGGTA
TTGGAGAAGAACTGCCAGTGAATTTGAAATTCATTTTTGAATGTATGGAAGAATCTGG
ATCAGAAGGCCTAGATGAGTTGCTTATGGAAAAACTAAAACCAGAAGGTTTCTTTGACTCAGTGGACTATGTA
TGTATATCTGACAATTATTGGTTGGGAACAACAAAACCATGCATTACATATGGATTAAGAGGATTTAGCTACT
ATTTCTTGGAGGTTGAATGTGC
>1002050420_1 Heli.1-EL604148.1.5
GCACGAGGGGCGCTGCCGCTGGTGGCGGGCATGCGCGTGGCGGCGCCCGCGCTGCCGCCCGCGCCCGGCGAGC
CCGCCTTCACGTGCCCCGACTGCGGCCGCGTGTACAAGCTCAAGTCCTCGCTGCGCAACCACCAGAAGTGGGA
GTGCGGCAAGGAGCCGCAGTTCCAGTGCCCCTACTGCGTGTACCGCGCCAAGCAGAAG
ATGCACATCGCGCGCCACATGGAGCGCATGCACCGCGAGGTGCACATGAAGCCCGAGCAGTACATCAAGCAGG
ACAACGTCGACGCATGCACCTAGGCGCCGCGTGGCGCCCCGACGCTCCGACTCTCGCTTATATACCTATTCAG
GCTCCTCAAGATTATTGCCGATTGTGATAAATGTGATGAAATCTCTCTTGTGATAGTG
ACTCGGGACGGTCACGTTAGATGTAGTCGCCTGGCTCGTTACCGCTATACATACTCTATGAATTTAATCTCGC
GTCGAATTTCTCTTAGATTGACACTGGTTGCAAATTTTGCAGCGGTGTGAGCGACTGTTGCATTTATCCAGTA
CGTTAAGGAAAGTGATTTGTATAGTTACGCTTTATATATTTTTATGTACAGCATTTAT
CGTGTAAATTATCATTCCTGGTGTTCGTCGACGTCGGGCGTGTAGCTTTGCTAGATCGTTGCTTAGAAAAGTT
TACTTCTTGTTACCAGTTACAGTTACTACGGACTAAATTTTAACATTGATTACGAGATTATTATAAACGTTTG
GCAATATATTTTACCTACTTACTCATTATCGAGT
>1002050421_1 Heli.1-DT662991.3.5
GGATGCGGATTCGGCACGACGTCGCGATATATACTAAGTTACTTACTTTTGTGACATTCCTTGTCTATTTTAC
TAATGCATCAATACAAGTGCCGAAAATAAAGTTGAACGATGGGAATGAAATGCCGGCTATCGCACTGGGGACG
TGGCTTGGGAATGCAACTTTTGGTCGTCTTCCACCGGAATCACATGAAGTGGAACAAG
CAGTGAAATGGGCATTGGACGCCGGTTACAGGCATATAGACACCGCTTGGATATACAAGGTGGAGGATCAGGT
TGGGAGGGGACTGAAGGGCAGCGAGGTTAAGAGAGAAGATATCTTTATCACTACTAAGCTCTGGAATGACCGT
CATGCCGGAGATGATGTAGTGCCTGCTTTACGAGAATCCCTTAAAAATCTGCAGCTCG
ATTACGTCGACTTGTACCTTATACACTGGCCTTGTGGACAATTTTCAAACTCTACCTTCGACATAACGGACTA
TCTGGACACATGGCGTGGCATGATAGAGGCCAAAAAGCTTGGGCTCACCAAGTCCATAGGATTGTCCAACTTT
AATCAAGAACAGATTCAACGGATCCTGGATCATGGACTGGAGAAACCTGCTGCTTTGC
AAGTTGAGATAAACTTGAACCTACAACAGCCAGAACTCCTTGCTTTCTGCAAATCACACAACATCGTGGTGCA
AGGCTACACTCCGTTTGGCTCGTTGTTCTACAGCAAGGCCGCGCCTGGCGCTCCCCTACCGGTAGATGATCCA
ACCTGGTGCGAATAGCTATAAGTTTAGGTAACGTGCCTAGATGGCCTAAGTATTGGCT
CACTAGGGTGGTCACTTCCATTTGGGTAAGCTTGNTTAAAAACTGACTTTTGTGAACTGTGATCGAAGGAATG
AATTACATCGTATACCCCATAAGGGGTCCTTTCTAACGAGTGAGAGGCGCCGGTCGGAATACACATAGAGT
>1002050422_1 Heli.1-DT665370.3.5
GCACGAGGGTTCCTTCAAATAAGTTTTTCTTTTTTAAAATTTAAAGTATTTAACTATTTATATGTATATGAAT
TTGCCATAAAATTATTATCACATAACGATACATTATTTTTTCTTCAATATATATAAAAAAGTCTTAACCAAAA
TGGATGTTGGGGAATTACTATCTTTAAAACCTGTAGCTACTCCAAAACGCCCCAATGA
GGATGAGATAGAACACACAGACGATGATAATAGTCGTTCTTCCAAAATGCGCCGATTAAATAAAGCTGCTATA
GAGCGAATGTCTCAGTCTACAATTATTTCTCTGCCTAAAGAACCAGCAATATCAGATAAAGAAAGAGAAGATA
TACTTAAATTTGTTGAAACGGAAGCCACCGAGGGAGAAATTTTAGATGATACAGCAGT

TAAAAAATTAGTGCTCAATTTTGAGAAGAAAGCATTAAAAAATAGGGAAATGAGAATAAAATTTCCAGACCAA
CCAGAAAAATTCATGGAAAGCGAAATTGATTTGTTTGAGGCATTACAAGATTTGAGTGCAGTGGCAACAGTTC
CTGACCAGTACCCTTTATTAGTCGACCTAAAATGTATTAATTCTATACTGGAGTTACT
TTCACATGACAATACTGATGTATCTACTAAAGTTGTGAACCTTTTACAAGAGTTAACTGATGTGGATATATTA
CACGAAAGCGAAGAAGGTGCAGAGGAATTGATTAATGCTTTGGCAGAAGCAGAATGTCCTTCATTACTTTTAC
ATAACTTGGCTCGTTTAGATGAGCAAGTACCAGATGAAAGAGATGCTGTTCATAATAC
ATTAGGTATTGTTGAAAATATAACGGAATTTAGACCAGAATTATGTATTGAAGTAGCTAAACAGGGTTTCATT
CAGTGGATATTGAAAAGATTAAAAATGAAAGTTCCATTTGATGGCAACAAATTGTATGCAACAGAAATTTTAT
CAATATTACTGCAAAGCACTCCAGAGAATAGGAAATTTCTTGGTGAACTTGATGGTAT
TGATGTCCTGCTTCAACAGTTGGCTTTTTATAAACGTCACGATCCCAG
>1002050423_1 Heli.1-DT665596.3.5
TACATGCATTTAACATAGAGACACAATAAGTATTTTGGTCATTTCAAAGCGCCAAGGAATGGTAACTTCTTTC
TTTTCTTAGCAACAATAAGTGTATTCTTTGTGATCGTTTTCGATTATTTTTATAATGTAAATTTCACATAATT
CTAGTTAACTACTGGAGTTTCTTTGTACGTAAAGGGGTTCTAAGGGCTAAAGGTAGGA
ACGTCAATTGGTACAAAATATAATTATAAAAACAAACATGTATGTATTTGTGTTGTTTTAGAAGCCCATTACT
GTCCAAAGAGTAAAAATCTGTGATATTTCATTACTATAACCTGTCGTTGCGTTGGTCAATTGTTAATACATAA
GAACACTTCCTTTGGTCGGAGAGTGTAAAACTAGACAAAAATATATTTTTTGACTATT
TGAGCGAATTTCATGAATGTTTGTGACTACAAAGCTTGCAAGTCGTTCACAAAGTATATGATAGGTGTAAGAA
TATTTTATTGTTATTCAATTTAGTTTTCAGTTAACCGTGAGGTGACGAAATGTACCAGTTCGTGGGACGTAAA
CGTTATTATCTAGGATTCCCGTCATTAAGTGCATCTCAAAGATACGAAATGATAACTC
GCTCTGATAGATTAAAGGGAAGGGGTGAACAACGTTGTGTACATATATGATCGCGGATCGATGTATCGCAGGT
AGGTACCTCCTGTTTCATGTACAAAGTACTGCACCCACCTCTACTAAACTAAACAACTGTGATAATAACACTA
AGTAGCGTAGCAGGCGATTTATATACTAGTTACGTCCAAGAGAAGGAATGACATCATA
TCTGACATACTGTATTGTGATAATAAATATTATATCGACACGTGTTCGTTATCGGCGCGTATTGATAAAACTT
TTTCTCTAAATAAGACTGATTATAATTATTTCTAATTGAGTATTTTATACAAGTTTCACTTTGACTGAATGAA
TTAAGATGTCTGTATTTATATCGGTAGTATTCTTGTGCTGTGTGTTTTCACCAAAATT
TATTATTCACTATGTTGGATATGAGTCAAAGCGCCTTTCTTATGCGGTCACCTGGTGAAGATGATGATATTCA
ACTCTTCGTAAGTTGATTTCTACTTTCTTTGTTGTGATGGTAAAATGTTGTTGACGAATTGTGGCAGCGACAG
TATGAGAACTAACGTTATAAAATGTATGTTATTATTTGTTGTTAATTTGCTAAATTTT
ATTTATTCAACCAGATTATCTCATAGACGATCGTAGTTTCATGCTTTACTATATACTATTTAAGGATTTATTT
TCTAAAATTGTAGGTTTGTGAAAAATATACATAGATTTTTAAGTATGTGGCAATGCAGATCTTCATAAATATT
GATTCTATAACAAAGTACATAG
>1002050425_1 Heli.1-DT665995.3.5
GCACGAGGGTCTCGCCGGTCACGCGTATATTCCTGTCTTTCGGCCGTTCCTCGACACGTGCTATTATCTATTC
ACTCGACCTTCCGAGAACTCGATAATGGGCCGATAACGGCTAATTGTTCCTAGTTTGCTATCTCGTGGCAGAT
CGCGTGTGCAGTGTGCAGTAGGGGTGGGATGGCCGAAAATCGTGCGTTAATACCTGAA
CCTATCACCATGTATACTCAATCGGAATCGTTGAAAGATTCAAAGGACTTATCTAAGAATTTAATGAGTGGAG
AGTTTGAACGGCAATATCCAAGAGGAGAGAATAAAAGGCCTCGAGGAGGCTGCATCATCCTAGGAATTGCTGT
TGGAGCTTTGGCAGTATGGCTTTTGGTGTTGACTATTCTGCGAGAAGTGCAGATTGTT
CGCCTGCAAGCTCAGGTAGAGGAGCTTTCTGCAAATATGATGGAAATGACCGTAAATGTAGTGTCGTTGAACA
CGAGACTAACTAACAACAGATTATTCAATGAATTCAAAACTTTAGAGGACACGATATACGCAGATGAAGATCA
AGACAGCAATTTAGTTGAAGAAGACAAAGATAAGAGCAAGGAAAAGCAAAGTTTCGAG
TCTCTTGAGAAGATTAAAGGTCTTACAGTGTTAGAAGATGATAACCAGTTCGGAGACGACGAGGATATGTACG
ACGGGGACGAAGACGCAGAGAGCGGGGATTGGTATCCAGACTATGACAGGCGTAGGGAGAAGATTGGTACGTC
AAATATGGTGCACTTGAAACCTTGAGACTTCACA
>1002050426_1 Heli.1-EL597498.1.5
GCACGAGGCAGACTCTTATAAATCACACGCCAGTTATTTACTTTTTATAATATTAGATTTTACATTAAACATA
GCAATCATTATATTGAATAACACTATTTATTTTCTAAAAGTTATTATAGAACACATCTTCGATGTTAAAATGA
CAGAAAAAGTCAAGAAACGTAAAAGAGACCAAAATAAAATATCTAACCAAACTGTTTA
TAAAAGAGGCGCGATAGATGCAAATTGGCAAAAACTATTGTCTAGTTCTCTAATGGAAGAGAAAAAAGAGAAA
GTAGAGGAAACAACAAATTACACAGGCACATTTCGTAGAGCGCGTAAGAAAAATAAAGATTTAACAAACAATG
TCAATTTAACAACTTTAAACATAAGTGACAATAATAAAAGTGAGTGTAGTAAAGCTGG

AATCGAGGAAAAAGACAGAAAAAAGAATAAACTAACTAAATTCCTAGCAATGGACTGTGAAATGGTTGGAGTA
GGGTACGATGGTAATGATCACATGTTGGCAAGAGTATCAATAGTAAATAAATTCGGGGATTGCATTTTTGACA
AATTTGTGAAAGCTAGAGAAGAGGTAGTAGATTATAGAACGGAAATAAGTGGTATACG
GAAAGAGGATTTGTTGAAAGGAGAGGATTTCAACGTAGTACAAAAAGAAGTGTCAGAATTAATAAAAGGCAGG
ATTCTTGTCGGACATTCGCTTAA
>1002050427_1 Heli.1-DT666673.3.5
GCACGAGGCCGACGTAGATCATAACAAAGGTAGTCTAAGTGGAGCTGATTGTGTCGCAGAGTTAGGACTAGCC
ATGGCTTCGAAAGTTCTTGTACCAACCGCACCATTTACTAAATTACAGGTGAAGAAATCTCCTCCAGAATCGA
ACGGCAGTGGATTCATAATTGGTAACGATGCAGCTGCCCGCGTCGCCCATTTGGAGCA
CAGTGTACGCTTCCTACAAGAGCAGCACCGACTTATGCTTAGCGGACTTCACACTGAGATCGAAGCTTTGCGA
GATAGAAATAGAGACTTGCAATTTCAACTTATATTTAACAAAGAGTCCTCGGTGAAGCCAGCTCAGCCCGCAA
CCGATGACACAGCTGAAAGCAATGAGGAAAAACTAGGAAAAGAAGTGAGCCGCTTAGA
GAAGGAAGCGGCGGCGGCGCGCGGCGAGGCGCGGGCGGCGGAGGCCCGCGCTTTACAACTACAGCGTATTGTC
GACGCACAGGCTGAAAAATTGCGTGAACTCGAACTGCGCCCTGAGGCACCAGCGACAGTGAGCGCAGGCGGCG
CGGGCGCGGGTGGGGGCGGGGAGGCCGAGGAGAGCCGCGCGGAGCTGCGCCTGCGGCT
GGCGGAGGCGGAGCGGCTGGTGCGGCGCCTGCGCGCGGACGCCGAGCGCCAGCGCAGAGAGTTGCAGTGTATG
AAGAATTCGCTGCATGCGAGTCTTCGTGCGAGTGGTCTTGACGCTTATGGATATCAAAATAATTACCACTTCC
CTCCAGTACATACTCCTGACTTCTGGCGAGAGCC
>1002050428_1 Heli.1-DT661665.3.5
GAGAGGCACGGGCCCTACGGGCGGCTCCGGCAACGCGTCGCGCCGCGAGAGCCACGTCAACGTCAACGTCACG
CCCACCGGCCACGACCTCTCCTCGGACACGCCCGAGATACGTAAATACAAAAAGAGGTTCAACTCGGAAATTT
TGTGCGCGGCTCTTTGGGGTGTGAATCTCCTTATTGGAACTGAAAATGGTTTGATGCT
GCTGGATCGTTCTGGCCAAGGCAAAGTTTATCAATTGATCTCAAGACGCCGCTTCCAACAAATGGAAGTGTTA
GAGGGACAAAATATACTTATAACTGTTTCGGGTAAAAAGAATCGCGTACGAGTATATTATCTGAGCTGGTTGA
AGTCTAAGATATTACGGACAGATGGGTTAAGTGACCAAGTAGAAAGGAGAAACGGTTG
GATAAATGTAGGAGAATTACAGGGTGCTGTTCATTTTCGTATAGTCAAGTATGAGCGTATCAAGTTTTTGGTT
ATCGCTCTTAAGGATTCTATCGAAATTTATGCTTGGGCTCCAAAACCATATCACAAATTTATGGCCTTCAAAA
GTTTTGGGGATTTACAGCATAGACCATTGTTAGTTGATTTGACTATAGAAGAAGGGAC
AAGACTGAAAGTAATCCATGGATCTGCAGATGGTTTCCACGCTGTTGATCTTGATACTGCCAGTGTATATGAT
ATTTATATTCCAAAACATACCCAAGGAGCTATTGTTCCTCACTGTATTGTACCACTGCCGAATTCTAATGGAG
TGCAGCTATTATTATGCTATGATAATGAAGGTGTTTATGTCAACACCTATGGAAGGGT
CAGCAAAATATT
>1002050429_1 Heli.1-DT665007.3.5
GCACGAGGACGCCTACGGCTCATACTAGAGACGCCATTTTGATTTCTTCTATTCCATGAAATTGGCATTTGGC
GTTTATGACTTCTGTTATATATATTATTAGATAATCCTATAATTAAGACTATACTTTGCCGGTTAATATTAGT
ATCTCGCTTAAAATAACGCACGGCGATGTCGGAACGTAAGCGCTTGTTAGAAGACCTC
CGTGTAATAGATCTACGTGCAGAGTTAGAAAAACGCAATTTGGATAAAAGCGGTGTTCGCAATGTGCTAATAC
AACGTTTGTCTAAGCATTTAGAAGAGCAAGGAATTGATCCAGCAACATTCACATTCGAGTTGAGTACAAGTGA
AGCAAAAACACCTTCTAAAAAGACAAGACGTACTGAAAGTTCAGCAGGGACAACAGAG
TCGGAAGATACTCCAGCTATGGAAGATATGATTGTTCGGGATACTGCAGGAGAAGAAGAAGAAGCTGAAAACT
CAGTGGAGAAAGAAAGTAATATAGCTAAAGAATCCCCAAAAAAGCCTGATGAAAATATGGAAGTAGATGAAAG
TGAAAAATCTATCACTAGAAAGCGTGAAAGCAAAGAAGAAACTGAGGCTACTGAGGCA
AAAAAACAATGTCTAGACAAAGATGTAAAAAATGAAGAAGATCATAAAACAGAGAATAACACTGATGCTGAAG
ACAGTATTAATTTAGACATTGGTGATGATGAACTTTTAAATGAAGAGACTGACAACACAGCTAAGAATAAAAA
AGGTAAGTGCTTAGCTAATAATTTACACAATTTTTATAATTCTATTAAACATTCTATT
TTTGTAAAAAGGAATATGTAAACGAAGTAATGGTTTCAATTTTAATTGTAATAATAAAATTTTCAAAAATGAC
AATTTTTATAAAAATTGATATTAAAATATTTTGTGAAGTGACTTTGTTTCTGGAATGGATAACTATGAAGAAA
AGTAATGGTAACAAAGCCTTATAGAGACAAACCAGCCAAAGACCACAGTGGAGAAATA
TCAAGGATATTCTAATTGAGAGGAGGAAACATTAGTGACGGATTACGTTTTCAGCACTCTAATATTGCTATTT
GAATTCATCTGTCTTCAATAAATAAATTGTTACAGTGACTGTTTTAATTTAGTCATTTGTGTGAGTTTTCATG
ACTCAGGTATTCATCAGCAATAGTATACCCATAAATGGCACAGGAATATACTCCATGT
GTTACACTGGCACTGGTGTTAATGTCAAATCAAGTT
>1002050430_1 Heli.1-DT668915.3.5

ATTAGCTTGGACCAAGTCGTTAGAACATTGTTTGTGACTAATAACACCTGTTTTCTAAACGCGTATTGGTTAT
TTAGTCATTTATCGGCTATTTATAGTTTTTTTTAAAAAAGATTTAGTGAGTAATACAAAAAGAAGACAGTTTA
TTAAAAATGCAATTTATATAATTTTTAAATTATAAAACACATTTAATTTTGTGCTATA
TATGTAATTAAATAACTAGCTAATATAGAATCAAGTTTTTAATGTGCTATCTGTATAGTGTTTTCTTATAACT
GGAAGATACATCTATATAATATAAAGATCCTCATCTTCTGGACATCTACAAGGCCGAAATGGGGTACGGTGTC
GAGAAATTTTTCCTACTATCGTACTGCTTGGCGTGTTGTTGGCCTGGTCTTGCGGCCA
AAAATAACCTTAGGATCGTCAAACAATGGGCAGAAATGAACTTCGTGTTCCCCAGCGATTCTGCCCGTGAAGC
CGCTATAAGCAATCGATACTACGTTCCTGGAAATTCGGTGCCAATTGATGTGGACGTTCAACATCGACAAGGA
CCAGAAAAATCACGAATCTTCGTCACGATACCGAGATTTGACGAAGGTCGTCCTATCA
CTCTGGGGACTGTCAACGACCAGGGTCTAATCGTCGCCTATCCTGATTACAGTTGGCACGACAATCAAGGACA
CAATTGCGATGGATTAACATCTGTATTCAGAGTCGCTATTGACAAATGCAACAGATTATGGGTGATAGACACA
GGAAAAATTGGAGACAACGCTGTTTGTCCGCCGCAACTCCTCGCGTTCGATCTAAACA
CAGACCAGCTCATCTATCGGCATCGACCAGACCCTTCGACTTATGTTGCAACCTCATTGTTTATTACACCAGT
TGTCGACGTGC
>1002050431_1 Heli.1-DT665324.3.5
GACGAGGCGCACCCCGACCTTTACACGCAACGCACGGTCGCCACGCACGCGCGCCACCTTTTCTCCGCCCTCG
ACTGGCTGCACTCTAATAACGTCGCGCATCTGGATGTTCGGCCGGAAAACATTCTCGTTGAATTGAGTGGAGC
ACAGCCGCAACTGAAGCTAGTGGATTTGGGTTCGGCCGTGGAACTGGGTGCGGATGGC
CAGGGTGGTGCGGAAGGCACAGCGGTACTGCCACCGGCGCCGGCACAGCTCGAGTTCGCGCCGCCGGAATGCG
TGCTCGGTCGGCCGCCCGCACCGGCATGGGACGCTTGGGCTGCTGGCGTTTTCCTATACGTTTTTCTAACAGG
GTTGTCACCGTTCCTAGACGAGTCTATCGAGGAGACGACAGCGAATATAATAAAATGT
GACTACTGCTTCCCCCCGGAGCACTGGGCGGGCGTGGGTGAGCGCGCGCAGGCGCTCATCCGACGTCTGCTGG
AGCCCGCGCCCGCGCGCCGCCTGCTGCCGCGAGATGCACTCAGAGACCCCTGGTTTGAAGAGGCGAGCAATAC
AACGCTGTCGGCCAGCCAGCTCAAGACGTTCCTGGAAAGGCGGCGTCCCTCCGGCCTT
GGCAACGCGCTGCACTCCCTGCGCTCCCCCAACGACACCTGATGTGATGACTTCTTATATGCCAACTGACTAC
CTTTACCCGAACGACTTGTGTCATACGTACTTTATAGTCCACAGCGACTGTGACTCTGATCTTTCTAGAACGA
ATGTAATACAATTTAACTCTGTCCACGCGAGTGTAAATTTAGTACGTCACAATATTTT
TAATAAAGGTGGAATTATTTACGATGGACAGCTTTGCTTAGATAGCACTGTTTATTAAAATTGTGGTTTTACG
ATTTAGAAAAAAATTGAACAAAACACGTAAGAAGGTGAATGGTGAAAGTGGATGGCCGGTCACTGGATCACTG
GTTGCACTCACTCTCACCGTGAGGATATATAAAGCTTTAATTAGTTTAGGTATAGCAT
CGCATAGCATCGTAATATAGTCATTGTGCCCACCTATGTATGACGTCGATTCCTGTAACAGTCCCGACGTGGT
ACTCGTTTTCACTCATACTGTATTGTTGAAACTTT
>1002050432_1 Heli.1-EL597670.1.5
GCACCAGGAAATAGAATCTCAGTCTTATTTATAAGAAAAATATAACAAATTGTAAAAAATTATAATATATTTC
GATTATACTTGGATTTATATTTCAAAAATATAAAAATACCGTAGTCGAGTGAAACATGATTTTATATAGTTTA
TTTAAAAAAATCTTTCATAATAAAAAAAATACCATTTTATATAAATGAAATAGAAATA
ATTTTTATATGTTTAACCGAGAGGACAATAATTTTGTCTCTTTTTGCACAAAACATTATAAAATGGTGTTTCG
AGAACTTTCTCCCTGTCATTTCGTTGTTGACGTTTGTATCGACGAGCTTCCGTCTCTTTCTATTATACGAGAG
TGAAAGAGATAGCAAATACCCAAGGGTAACGCAAATATGTTAGAAGAAATTTAAAAGA
AATTAACTTATTTTATTCTTTTTTAACATGAGATTGAGTCGAAAATTAATAAATATGAAGTATAACGTTTGTT
TTTATTGATAAATATATATCTGTCTCTTTCGTGCGTATGAAAGGGAATGAGTGTTAGAAAGAGACGGAATGCT
TGTAGTTCGATTACATAGGTGCAAAACTGTTGTATATTGTAGTGAAGTTAGTTATAAT
CGTTATAGAGCCGTCGTCGTCGCTTTTTTTTATTTAAAAAATTTATTGTATTATTATTTTTATAATTTTGATA
TATGTTTATCT
>1002050433_1 Heli.1-DT666120.3.5
GCACGAGGGCGAAGCGCTCCACCCGCACGCTAGCTCGACGCTTTACGGCGTATTCGTATATATAACTTTTATT
CACATAAATCATATTTTAAAATTGTTAACGATATTTTTTTTGGATTGTTGAATGTCGCACATCAGTTGTGATC
ATGGACTTTTGGATGCTAATTTTATGCGTTTTTCCAACAATTGTATTACCTATCACGG
TATCATCAAATGGAAATAGTCCAGAAACAAAAATGATCGGTGGAATGAGAGTCGACACCAGCATAACAGAAGA
TATGAATTCAAAAATAATTATGAACAACACTGGAAATCAAGCATACAATATTCCAAAACTAGCTATTAATGAA
AGGTATCGAAGATTAATACCTTATATGACTTTTTATTACGCTAATGACGTAGTTCCTC
CAACAACTAACGGAAATGTGCGTAATGCCGAAAATGTCGAAGTAGATAAAGCAGTAATATTAGAGTCCTCTTC
AGACAGACCCAGTGATCGTGAACCTAAGCTTATTTATAAACCACATAGATACAAGAATATACCGAGGTACAAT
GGGAATAGATTAACCCAATTTAATATAGCGTCGTCCAATCCTACCAAAATATTCTATA

AAGAAATACCTGTGCTGTACCAGAATTCACCTAAGGCCGTCGCTCATTACAATAATCACCGATTGGAGGACGA
GCAATTTGATAATAGAGACGTTCCATATGATCATTATGTTCCCAAACAAATAAAAGCTCCGAGTACACCCTTC
ACAGTACCTGAGAGGAAACCTGTATTAAATATTTACCAACACGAAGATTTTTCCCCAA
>1002050434_1 Heli.1-DT662625.3.5
GCACGAGGAAAGAACCGTCAAACAATTAGGAGTACTATGTATTGTTATAAATACTATTTACATTATAAAACTA
TTAACAACATTCTTTATCTATTCTTTTGTAATTTTATTTTTGCTTAAAATGTGGTCTATAATAATTTTGTTAC
TGTTATCAGTAATAGCTTATTTCATAACTGATGAATTAATACCTAAATTGAAAAATCT
TTTTATTACTGCTGGTTTATTCGGAATTGATCTTTGTAAAGTAACAAAAGAAAAAATACCAGAAGCCATAGGC
GTAATATCAGGATGTGTGTTTTTGGTGACAATATTTTTATTTATACCAATAGCATTTGGAAATGGACTTATGG
ACAGAGGAAATTTCCCACATAATGAGGTAATTATAGTTTAAATATATCAATATATTTC
ATCTTATTTTAAAGATGTTTATATAAGCAATGTAACAATATAATACCACTACAAGGTTTTAAGTTTTTTTTAT
TGAAGTTTTTGTCACTATAGCATTGAAAATTATGTAGAAAAGATTAATTTTAAGTTATAATTTTTAAGTTTGC
AGAGTTGTTGGCAGCATTATTATCAATATGTTGCATGCTACTATTGGGATTTGCAGAT
GATGTGTTAAATCTAAAATGGAGATATAAATTGTTGCTTCCAACTATAGCATCTTTACCTTTACTAGTTGTTT
ATTATGTAAATTTTAACTCAACTACCTTTATTATCCCTATACCATTACGGCAGTTTTTTGGAGGTTCTGTTAA
TATTGGATTTTTATATTATATTTACATGGGAATGTTAGCAGTCTTTTGTACTAATGCT
ATCAATATTTTGGCTGGTATCAAT
>1002050435_1 Heli.1-EL604127.1.5
GCACGAGGATTCCAGGGTGAACACGGCATCGAGGAGGATGTGTTCCTTTCCTTGCCCTGCGTTCTTGGCAGTA
ACGGAGTGTCTGACGTCATCCGTCAACCTCTCACCGATAAGGAACTTGGACAACTTCGCAAGTCAGCTCACCT
CATGTCGCAAGTCCAAGATGGTATTAAGTTTTAATTAGGTCTTAATTTGAAACTTTTG
TATCTCTAATTTAAAAGGGATATAAGTAAGAAAATAATCAGGATTGAAAAATACGTTTTTAGAGAACGTATAA
TAATTTGAACATGGCGATTACTAAGCGCGCATTTCCAAAGCTTCGAATGCTGCTACGTTTTTTGAAAAATCAT
ATAATAATAATAATACTAAAGTGTTAACTTAATAATATACATAATTCCGAAATTGATC
AATAGATTTTTTTAACTATTAACTTTTTTTTATAATATCGAACTTATTATCCCTTTTAATTTGGGCACTATTT
TTTTCTGTAACTTTTAGGCTTTTCAATACGCTTCTTTAAAATCTTTATGGTATTCAAAACAAATTATTAATAT
TTAAAATTTGAATATTAGTCATAACCTATTCTTCTGGAAAACAAAATAATAAAAAAAT
AATTAGTCATAACCTATTCTTCTGGAAAACAAAATAATAAAGAAATAATTAAACTTAAAATATTATTAAACTG
GTTTCCCGGTTGAAACTTTATAA
>1002050436_1 Heli.1-DT665232.3.5
GCACGAGGCTTTCTCGAAATCTAACAAAAATCTACTTAAAATGTCTGGCCGTGATGGAGGTAAAAAGAAGCCA
CTGAAGGCACCCAAGAAGGCATCAAGAGAACTTGACGATGATGACCTGGCCCTAAAACAAAAGTTAAAAGAAC
AACAAAAGGCACTAGATGAAGCAAAGGCGAAGGCCTCCCAAAAAGGTCCACTAGTTAG
TGGTGGCATAAAGAAATCAGGCAAAAAGTGACAGAATTTCCAAAATATAAATTTAATTTTAGGATAAAATTAT
TGTAAGTCAACATTCGGGATCTTTTTGTTTGTAATTTCGTTCATTCTGTGCTATGTATTTTTTTAAATAATTTT
TAATTCAATGAATGAAGGTGATATGAATAAATTTTTACTCATATTTTTGGTGTCGGAG
CAGCAGTTTTTTTTATTGTAAAGAGACAGAAGAAACTGTGTGTAAGGCAATGATGTCAATACTTTAAAAGGGT
ACTGAGATTGTATTGTTTCTTTGATAAGCTTATATACCATTGCAACACTAGCTAGAGGGACACTTCAATTATT
ATTATTTTTTATTTTTGTTCTACAAGTTGCAATTCAAGGCAAACTACTACCACTGACG
TACAATAGTGTCTTGATTTTCATCAACATGATGTGTGGGTTCGATTTCCACTTGGTTCCATTCCACACACAGT
ATATTGTGGAGAGTGGACTATTTAACTACCCCTAT
>1002050437_1 Heli.1-EL596874.1.5
CGGCACGAGGGAGCTAGTGTCAGTATCACAATCAGCGTGCACCTCAGGCGACGTAACCCGCATGTCGCACATT
ATAACCAGCAAGCTGGCGCTCGCCCAGCGGTGTTCAGTTACGCAACTCCACTGGGTCCGCGTTGTACGGGAGC
TTGTGTTGGTGGTCGCCGAGTCGCTCGGTGTCACGTGTTCGTTGCCGGAATCTGAACT
AATCAATCTGTTGGAGATTGCCGTGTGCGATTCGGGCTGTGTTAACTCGAGGGCCAGTGAACTAGCGCTAGTA
TTGGTGTTTCAGCAGTTAGAAAAAGAACTGGTGGAATGGAGTCGTTTAGCCACAGAAGGTGGTTCTCCGGTGC
CCGAGGATGCATATTATTTGTATGAGTTTGCAGCTCAGTTGCAGGCGCATTGTAATAT
GTCAGACGCGTCGCTGGCGGCGGTGCGTGCGGTGGTGTGCGACGTGCGCGCGCGGTACGAGGCGCGCGCGGCG
GCCCCCCAGCGGCAGCGCCTCGTGTGGAGACTGTCGGAGAGGACGCTCAAGGTACTTCTTCCCACCGATCGTT
TAACATCTCTTCTACCAACCATCGAGGAACAACACTTGGCTGTTGATTCAACGCCCAA
ACGAAATAGATCCGGAAGCGAAAGCAGCGACAGCGAAGAGACCTCCGACTGGCCGAGAAGTCCAGTGCCGCCA
GTTTACTGCGATAATTGAACAAATAAACACGTGAAGGCTGAATGCACGCTACCTTTGCCCCTTGCAAACAAAT
CCATGAGAAAAATATCTATTTTTGTTACTAAAAAAAAATAAGAAAATTTTTCTATTAC

AAACAAATTACTTTTTTGTTAGGCTCCACGAAGTAAGGTCTTTGTTTAAAATTGTGAGGTATTTTCTGCCTTG
AAAAATTTTGGCTGAATAAAATTTGGCTTTATTTTAGTTTTTTTTAATTAAATATATATAAAGAAACGGAAGT
GCAATAGTATATTTCTAAAAGGTCTTTGTCTAACAAATATAAAAAA
>1002050438_1 Heli.1-EL596835.1.5
GNACGAGGATTGAATATTTTAATATTGAAAATTCTTATCTACCTTAAATATGGAATTGATGAAAATCTAATCT
TAATTAGATAACAAATAATTAATGGCCTAGTAGTGCAGTGCAGGGGAGTAAGTTGTGAGTTTGATTTCCACTC
AAGTTTCAGTTCAAATCAAAATGGTAGTATCTGAAAATCAACCTCCACCAGCTGAAGG
AGAAGCAGCTTCAAGTAAGAAAGCTGCAAAGAAAGCAGCGAAAGCCGCTGAGAAGCAACAGAAAAAGGCTGAT
AACAAGGCAGTCTCATCACAAAACGAAGCCCCCGAGCCTGATATATCAGAAGGCAAATATGGTAACTTAAAGA
TGATACAATCATCCGGTGAGAATAGAGAGAGAGTGTACACTGATGTCAAAGATCTTAA
TGTAGAGCTACATGGGAAGAATGTGTGGGTTAGAGCTCGCCTCCAAACGTCAAGAGCCAAAGGTAAACAGTGT
TTTGCCGTCCTCCGTCAGAGTTCAAGCACAGTACAACTACTGGTCAGTGTCAACGAAAATGTTAGCAAGCAAA
TGGTTAAATTTACTGGCAACATAACAAAAGAATCAATAATCGACGTAAACGCGACAGT
TGTTAAGACAAATTCCCCAGTGGAGGCTTGCACCATACAAAATGTGGAGCTATCAGGGCTCCAAGTGTGGCTC
GTGTCGGGGGCGAAGTCCCAATTGCCTTTGCAAGTGGAGGATGCGGCTAGGCCTGAAACTGACGATCCAGAGG
CGCTAAAAATTCGTGTGAATCAAGACACGAGGTTGGATAATCGTGTCCTAGATCTGAG
AACGCCGGCCAACCAGGCCATATTTAGAATAGAAGCCGGGGTGTGTCGCCTGTTTAGAGATCTGCTTACGCAG
CGTGGTTTTGTAGAAATACACACTCCTAAAATTATCTCGGCGGCTTCTGAGGGAGGCGCTAATGTGTTCACTG
TATCGTATTTTAAATCGTCTGCATATTTGGCTCAATCACCACAATTGTACAAGCAAAT
GGCGATAGCTGCTGATTTTGATAAGGTATTTACAGTAGGTGGTGTATTTCGCGCAGAGGATTCGAACACGCAC
CGACATTTGACAGAGTTCGTCGGCCTCGACCTCGAGATGTCCTTTAAGAATCATTACCACGAATTTTTGGAGA
CTATAGGACAAACATTCACCGATATGTTTAGAGG
>1002050439_1 Heli.1-ES584976.1.5
GTAACCTTAATCTCCTATTACTTTCTTCCTTAAATGTCAATGCACAAAAGGAAATGTAATGCTTAGACATCGC
ATTATATGAAACATATTATTAAAAAATACGACAATTTATTTTACACTCAAAATCTATTATATTGCTATTCTCT
TCTTCGACATGTAATACGATGTCTGATATTTATAAACACTTAAATTTTAGTTCTTTAT
AGAAAAATAAATGGAAATTTTCGTTTGTTCGAGATTGTTATTGATGTTATATGTATGTGTGAATTGATGGCGG
TACGTTCCACCCGATATTTAGTATGAAACGCACTATTGTCATTACGTATAGTGGATACAGGGTTGTAAAAATA
TCTTTTTAAAATATTAAATGAAAAACTTAAAATTATAGGTTTAAATGTTGTTTATATG
AATATATTGATAAGAATTTTACAGTACATTGTGTTCGTACACTAATAAAAAAATTCCATGTAATTTTGTGCAT
TTCGATAGCCGTAACGTGCAATTCCAAGCTTTTTTTGAATTTATATTTTATTTTTATAGACTTTATACTTCAA
TTTGACCATATCTATTTTTGTATGACAATAGACATTTTCTATTGCACTTCATGATGTA
TTTCGGTGCTTTTCATCTCTATACTACTATCTTACTATACACTATTTATATACACGTAGTCAACGATTCAGGA
CCTAATTATAATAAAAATATAATGTATAATATATAAGCAGCAACTCCAAGAGGATTTAATTTTTAACAATTTG
CGAGAATGAAGCCATGATTGTTGTTATACTCGTATCGGTATTAGACGTTAATATAATT
ATTATTCTTCCTAAAATGAAAATGTGCTCATAAATTATTATATAATTTTCATGAGATATGTCAATTTTTAAAG
CTTGATGTTATTTTTATTTCTGAATAAAATTTTGT
>1002050440_1 Heli.1-EL602908.1.5
GCACGAGGCGCACGTCTCTTACACCTGTTGAAGTACCGTTCGTTTAACTTTTAGTTTTTGTTCCGTTAGTGCT
ATTGTGCTGTGTACGTATTTAGATTGGCACAGGTTGTGTTTGATTGTGAACATGGTTATGTAGATCTTGATCG
TTCTTATCCAAGTGACAAATTTCAACCAATCGAATATAACAAAGAAACCATATCTGTG
AATTTCCAGAAT
>1002050441_1 Heli.1-EL601772.1.5
CGGCACGAGGGTCCTGCGTTCACAAAGTCGCCGATTTTGAATGCATGCGGTCAACGCATAGCGTGCACCAGTA
AACATTTATAAAACGTATTAACCACCGTACCGTGAAAAGCAGGCTTCTTAACAAACACTTAGAATTTGTGCAA
TATACAATGAGAAAGAACTCGTTTCAATTTACATTTACATAAGGAGTACTAACGTAGG
AAGGTCATAGCGCCTGCAGGTTTCGGAACAACTGACGCATTTCGGTTGTTTAGTTTTGGATTAGTGTGATCTA
TCGCGATGTGTGAAAGACATGTTTTAGTTGTTTCCTTGGTGGCGATAGTGCTGGCCTTCGGGAGCAGCTCCTC
GGCCCGCCTCGCGCCTGACTTCATCCATCCGTGCAATAACACAGAATCATCCTGTCTA
ATACAATCTACACAAGATGCTATCCCAGACTTCTCTAAAGGCATACCACTCCTCGGAGTACCACCATTAGACC
CATTTGTCATTGATGAACTTCCGATCCAGTTGCCAGGAGTCAAGGTGACCTTTTTAGATGGAAAAGTGACGGG
ACTTCGGAAATGTCAAGTGCTAAATGTCGAAGCTTACCTGGAAAGAAATATATTTATT

TTAGAAATAAGATGCAACATAACCATAAAAGGAAAATACACGGCTGTTGGAAGGTTACTTTTATTCCCGATAA
ATGGTGAAGGTGATTCAAAGATTAAAATAGTGAACTCTGTCATAAAACTGAACATAAAAACCAAGTATTTTAA
AGATACGGAAGGACGAGACCACTTTGGTATAAGAAATTATAGATATACATTCGACTAC
GGCGACAGAGTCCATTATACCATCAACAACCTATTCAAAGGGAACCCGGAATTGAGTAACACAGTCTTACAAT
TCTTAAATGAAAATTGGCGGGTGGTGACAGAAGAGTTGGGCAAACAGTAGTAGACTACGCTATGAATGTCA
>1002050442_1 Heli.1-EL602860.1.5
TTCGGCACGAGGCGCAATTCTACCGCGCGTGCTTCCCATACGCAATACCGGTTATGTCGGACTATACCTATAC
GATTCGATTTATTTAATTAAACAACAGTAATCGTATCCTCTACAGAGATATTATAAATTTTATTATTCTCTAC
TGAAGAGAAGACGTTATGGATCATAAAAGAAGCATAAAGATCGGTATAATCGGTGGGT
CAGGTTTCGACGATCCTGAAATATTAGAAAACGCGGTCGAGAGGGATGTGACGACGCCTTTTGGGAAACCTTC
TGATGTGTTGATAGAAGGCACTATCAGAGGTGCTGATTGTGTACTGTTGGCCAGGCATGGGAGGAAACACCAG
TTTCAACCAAGTGATGTCAACTATAGAGCTAATATTTGGGCTCTGAAGCAAGTAGGAT
GTACGCACGTGCTGGCCACCACCGCGACGGGTTCGCTCGTGAAGCACTACAAGCCCGGCGACCTGGTGGTCTT
GGATGATTTCATTGACAGGACATGGGGTCGCAAATGCACGTTCTACGACAACACGCCAGGCGGGCCCAAGGGC
GTGTGCCACATCCCGATGCGGCCCGCCTTCTGCGAGCGCTCGCGCCAGGCGCTGATGG
TGGCGGCGCGCGCGCAGCGGCACACCTGCCACAGCCGCGGCACTGCGGTCACCATACAGGGGCCTAGGTTCTC
AAGCTTTGCCGAAAGTTTAATGCACCAGCAGTGGGGAGGCCACGTCGTCAACATGACTACTGTACCAGAGGTG
GTTTTAGCGAAAGAAGCCGGGCTCAGCTACGCGGCGATAGCCCTCGTCACCGACTACG
>1002050443_1 Heli.1-DT664641.3.5
GCACGAGGAAGAAACCAAACAAAATGAACACCTTCATCACTGTGAGTAAATTTAAATGTATTTTTTGTATCTC
CTTTCATTAGCGTCATCGTTTATTGTTCAAAGAAAAATATATTTAATCTTTATTTTAAAATGAAATTATCTTT
ATATTTAATCTTTTCTTTATTTTAAAATGAAATTATCAATTCAATTTCAACTTGGGAG
AATGAACTGGCAGATAAATCGCGGCGTGAATTCTTCCACTGAAAGAGGATTTTTTCGGCGGGATGTGCATAAT
AGAATGTTTTAACACTTCGTTCCCTCTGCCTGCCCTACCATAGGCAGGACAAAGCATGAAGCTAAAAAAAATA
TATAATTATGTATGTATTACGCATCACACTCACAATAAATAATCAGAAAATTGAGAGC
AAAGTGTGTGCGGCTTACCTTTCATAACCTGACGATGTGAAAATTTGGAACAAGAATTATAATGTATATGTAG
ATCTGTTTATTATCTACATAAAAAAGTTTGGCATTTAGGATCCGTGGAGAGTACAGAGAGGAAAGAGGATTTT
TTAAAATGCCTACAACAATGGGAAAAGGGAGTTTCCATTTTACGTCTCCTAAAATATA
ACTGCACATAGTTCTACACAATATCTTTAAGTTAAGATGTTGGGAGGAAATTAGACAAATTATATTTTTATTT
AATTATTTCTTAATACATAACAAATAATATAATTATTGTACTTTTTG
>1002050444_1 Heli.1-EL602537.1.5
GCACGAGGGAGTCTTAATATGCTTGCTGTAACGTAATGTAAACTTAAAATAAAAATTTATAAAATTAGTGGGA
AAACCATTATTATATTAGAAATATACTCCAGATAAAAATACATCAATAACTATGAAAATTTGGACATCAGAAC
ATACTTTTAACCATCCTTGGGAAACTGTGGCTCAAGCAGCATGGAGGAAATATCCTAA
TCCTATGAATCCAGCAGTTATAGGAATTGATGTAGTTGACAGAAAAGTTGTAAATGGAGTTTTACATACTCAC
AGACTAGTCAGCTCTAAATGGTTCTTTCCAAGATGGGCACAAGCAGTTATTGGCACAGCTAAGATTTGCTATG
CTAGCGAAAAGTCTGAAGTGAACCCCAATGAACGTCAAATGACACTAAAGACGAGTAA
CTTAACATTTTGTCACTATATTGCTGTAGATGAAACTGTTAAATACACCCCTCACCCATCTGACCCTACAAAG
ACACTTCTAAGGCAAGAAGCTGTGGTTACTGTACAAGGAGTACCACTAAGCAGCTATATGGAAGATTTACTGA
CCAACAAAATTTCATTAAATGCTGGCAAAGGACGCCAAGCAATTGAATGGGTAATAGG
GAAGATAGACTCAATAAGCAGCTGTTGGAAGATTACTGAACACAAACTTCATAATGCTGGCAAGGGGCACACA
ATGAATGGACTGGGAACATGCCCGAGATAAGATACTATAGATGTAAGTCGATATCGTTAGAAAGCTGAATCAG
CGTAAACGTAACCTTAGAGGTGGATCACCAGAGTTAATAGTCTAGGACATTTATGTTT
>1002050445_1 Heli.1-DT663077.3.5
GACATTATACACGCATGACTATCCAGTACACTTCACTTATGATACATCGGTATAATGATTGTTTGTTTTTTTT
TTTCGGTTATATATTTTACAGTTAGTACCAGTTGCATACCTACGGTGCGATGGTATAATTTGAGACGACGATC
ACCAATTCTACCTATTTCTGTAAGTTGCACTATCCCTTAATTTATTAAAAAATATATC
TCATTTAAATCATCATGAGTTTGGAATGTTACGTGTGCCGTGCCGTGCAACCACTGGGATACATTATTTAATA
AAGTACTACACTGCAGAATAGGTATTTTTCAGTTTGTATTAATTATATAAATTTACTATTAATAATATCTCGT
TTGAATATTGTACTGTCTATTGGTGTGTATGTTTGAGTTTGTCGCTTTGGCTATAATT
ATGTGGTCTTAGTGTGTTTGCCCAAAACTAGGTCGAGTATCATCAAATTTGGCATGATGTGCTTAAAAAATCC
AGAATGAAATATTTAATATGGAATAAGTATACGTCTATCCAGAAGTTCACACCAGGTGCTTACTACTAGAGAA
TCTGAGTAAACTTATTGTTGCTACCAGCCAATGTCAAAAATATCATTGCGAACAAACA

AGAACGATTTAAATTCGGATGAAAGCAGAATTTTGTGTCGTATGTACTGCTCATTTTTGAATTTTTATATGTT
AAATTTTATCACAAGGTGGAATTTTACTTTTCGTTTGGCTAATATGT
>1002050446_1 Heli.1-DT666658.3.5
GCACGAGGGGTCTATAGTATGTGTATTATGTCAAATCGAAGCAAATAGTACTCGTGCATGTAACAAAATAAAA
ACGCCATGTATCATATGAAAAATATCATATCTACTAATGAAGAAGTAATCTTGCCTAAGTGCATGTATCGATT
GAAGAATTTAACCGACATATACCAAAACGATATAGAAATCAATATAAGCGATCAGAAA
ATGCCTACTAATCAGCTTGTTGACTTAGAAAACAGGCAGGACAAACTGCTAATAAAATTAGATATTCTTTATG
AAAGAATAAAAAAGATAAGCTCATTGTGTGTTTTAAATCCATATGTAAAACAAGAATTGACAACCAATGTTGT
TGATACTACAGAGCAACAAGAAATAGTAATTATTTTAAATACTGAAAATCTCCCTTGG
TTTTTAAATATATTTTTAAAACATAAAACATTAAGTGTGTCATGGCACATTCATTCTTCCATTTCAAATGCAA
AAGTTGCAAAAGTAAAAGCATTTTTTAAAAAATTTCAAGACTTGTACCAATTAAAACCTGAATCCAAAATAAA
CCTCAGACTCATATTTAAATCTGAGTCAGCAAACCCAGAACTGAAAATTTCTTCTTTA
GATGTCCCAATTCTTGGCAATGTGAACATCATAAGATACTTATGTCTTGTATATAGTGATGTAGTACCTTACG
ACTACCAAAACCATCAATTAGACAGTTTCCTTGACATATGTCATCAACTTGAAACTGCACCAGAAAAAAGAAA
GGAAAATTATATCAACAATATATTTGCAAATAATAGTTCTAGCTGCTGGATCTATAAT
AATAATTTCTCT
>1002050447_1 Heli.1-DT667316.3.5
GCACGAGGGGACATTGTACCTGTAGCGCTAGACCAGTCGCGTCGTTACCTAAACCTATAACCGGTATGGGGCA
AAAGGCGTTGGCGTTTGCGGTCGCTGCCCTCCTCGGGCTGGCGGAGTGTGGACCTCCAGGGAAACCTTCATTA
GGATGGGGTGAGAGGACTTTTGCGATTGTGGAGGTCAATCAAGCAGCAACGGCGTACA
ACCAGCTCGTCACAAGAAAAGAAGCGGCTGATGTTTCCGTTTCCTGGAATGTTTGGTCTGGAGATGCAGCGAA
GAGTGCTAGAGTGTTGTTTGATGGTGAAGAGGTTTGGTCTGGTGCATCTGGAGCGGCTTCGTCTGCCACTTTT
CCCATTTCGAAAGGTGGAAGATACCAAATGACAGTGGAACTTTGCAATGAAGACGGCT
GCAGTTCCAGCGATCCCACCGAAATTGTCGTGGCCGATACCGATGGAAGTCATTTACCTCCTCTAGAGTATAC
ATTGGGGGAAAAAAATAAGCCGTTCAAGCAAACGTCTGGAAAAGTTGTAGGTGCTTACTTCGTAGAATGGGGT
GTTTATCCTAGGAAATTCCCCGTGGATCGTATACCTATACCTAATCTTACTCATCTGC
TCTATGGCTTTATACCAATTTGCGGCGGTGACGGTATCAATGACAGCTTAAAAGAGATCGAAGGCAGGTTCCA
AGCACTACAAAGATCCTGTAGCGGTCGCGAAGGACTTCAAAGTCTCA
>1002050448_1 Heli.1-CO729874.1.5
GTCGGAATTCCGGGTCGACCACGCGTCCGAGAAAATGAACAGCTACATCACTTTTGCCTGCCTATTAGCGGTC
GCCGCTAGCGCGTACGGTTCTGGCCTCGGCCTCGGCTATGGCGGACTCGGTTACGGTGGTCACGGCGGCTACG
GCTACGGGGGCTACGGGGGCTACGGATCAATCATAAAGGAACCCATTATCGTCAAGCC
TGCTCCTATTATAAGTCCACTGCCGATTATTAAACCTTACCCCATTATTAACCCGCTGCCTATTATTAAGCCT
CTCCCCATAATCAAACCTCTTCCCATCATAAAGAATGTGGAAATTGCTCCCATACTGTCTGAGCCTATTTTAA
AATCTGTGCCCGTATACGGTGGGTATGGACTTAATAAGGGACTTGGTTATGGACTTGG
TCTTGGTTATGG
>1002050449_1 Heli.1-EL598508.1.5
GCACGAGGAAAAAGGTTATACACTGAAAAAGAGGGAATAGGCTGATTATGATTAAATTATTGTAAAAATAAAT
AGAATATAATAAAAGTGTTCGAGATTTTCTTAATTTGCAAAATATAAACATTGAATTGTAACTTTGTACATAG
CCTGAGAGTAAAAAAGAGCTACGTGTCCTAGGTTAGGCCGTTAGGGAATCATGTTAGT
ATTGGCGTAACATGACTGAAAGAAAAATGTGCAAATTCTATAATTTGAATGTGCCTGACCGCCGATCCGGGGA
GAGTGGGATCCACAACCACGTGAACAATAACGATGACGACGATCGTAACCAAGACGATCCTCTACAACCTTAT
GTGCCCCCACACGAGCACAAGCTTGAATATAGTTATTGGATGTGGTTTTCAAGGAGGC
CTCCGGCTAGAGAGCTCTCAGCAACCACTACTGGCTATGGACAGGCTCTACGTCTGGTGGGTCGCGTGGGGTC
TGTTGAACAGTGGTGGGGTCTTTACACGCATTTAGCGCGGCCCTCAGAGCTTCCTCCGCTGTCAGACCTGCAC
CTGTTCAAGTTAGGTATCAAACCAATGTGGGAGGATCCAGCCAATGTCAACGGTGGAA
AATGGGTGGTGAGGCTGCGTAAGTCGCAGACAGGTAGAGCGTGGGAGGACCTATGTATGGCGATGCTTGGGGA
ACAGTTCATGGTGGGCCCCGAGCTGTGCGGCGTTGTGCTTTCTGTTCGTTTTCAGGAGGACCATCTAGCAGTG
TGGCACAGAACGGCGTCCGACATAAACGCAGCGGGCCGAGTGCGAGACGCCCTACGCC
GTATACTACAACTGCCCGCCTCCATACCAATTGAATACAAAGCCCACGGCGACTGTCTACGTACCGCCAATGC
CAGTGCCAGTGCTAGTAGTGCCAGCACTAGTGCCAGTGCCAGCGCTAGTGCTAGTAGTGCCAGTACCAGTGCC
AGTGGTAGTGGTAACACTCGCAATGACGATGAACGCTCGTAGCGAAACGCCCCCCCGC

GTCCACGCCCGCCGAGACGCCTCTGATATGCCTTTTTGTAATTAATTAACAGAACTGAATTTGTAATTAACGC
AACTATTGTACATTTTGAAATTGATTAAACTTTTTAGTAGAATTTTGGTTTTCTGTATAATTTTTTTTGATTA
GAGTTTTGACATTAATCCTTTT
>1002050450_1 Heli.1-DT664624.3.5
GCACGAGGCGGTGTCGAGGCGCACCAGGGAGCGGGGCGAGCTTCCAGCATCCTTGAGGAAATCTTACACATCT
CTTGTGGCTCATTTGGTGCAACAACGACGTAATTGCACTTTTTTATATTATATCATTTAAAAAAAATAGTTAT
ATATTTCAATATATGCGGAAAATATATTCACAAACCTTGATTTTGTCGCAATATTACA
AAAAAAATGTTAATATGTCCTTTTAGCACCAAATGGGCGCTATGTCGAGAGTCGGTCGTATGCTATTTAGTGA
TAGTACATTTCGTGTTGGCCATCGGGAAATTTATTGCATAATAGATGTTTTCGGCGAGTAATCGTGTGATACT
GTAGCGTAATTGTCGATAAAGCTTGAGCTGTCGTAGTCGGTAAGAAGGGTAAATCCGC
CGTAGATTAAACCGTCGCAATGTGATTACTTTGTACTTTTGTAAGCTCAAATCCTTCGTCGTGTACATACCTT
AACGCAGTTTTTAAAATACATTAAAAGTCCGTACCGATTTAGATCGCTGTATTTTCAAAAGCCATGTCGTAAA
TTTACCCTTCGCAGTGAGGGGCCGTCAATAAACTACGTCACACGTTAAGGGGATTGAC
GGAGTGTGAAATTGTGTGACAACACATTGTGGGTGTCAATGTGTGACAATAAATTGTGGAGGGGGTCTAAAAT
TTCGTGGTCACATTTGAAAACGTAATATGCAAACCTAATTTCTGAACTGTGTGACGTAGTTGCCGGGCGGCGG
CGGTGCGTGGTTGGGCGAAAGC
>1002050451_1 Heli.1-DT665372.3.5
GCACGAGGCTTTTCTTCAAGTAGCTACTAGCCATTATTCATGAAGATCACAGATTTGATCTCGGTCTCGGACG
GATATATTGTGATCGTTAGATTCATAATAAATATTTTAATTTTTTTTGCTTCATCACTGTTCCATCATCAAAG
TACTTTCCTACTTTAAAGGGAAGCAAAATGTGATATATTTTTGTTTTTATATATTCAT
TATAAATTAATATTAATAATTAATAAGAACTGCCAAAAACACACTGAATATTAATACTTGTGGTATATTTCTT
GAAGCAGAATTTTTCTTCAGAGATTGTTGGGCATATGTTTGTAATAATCCGCAATCAACAGATTTTCGCATGT
TAGAAGAAGGAGTATGTCTTATAACATAACATTTCATTCCTGGAGTCACACTTACTGG
TGCACCTTTTACATCCAGTGCATTTACAGTAGTAGTATTTGTAGTTGTTATTGTTGTAACTGAAGATGTCTTA
GAGGCATCTGATGACGGATTCTGGCTTTGAAGTGCAGTTGTGTTATCTGCTGTTACATTCACAGGAACAAGAG
TGCCATTCGGCAACATTTGATATAAGACATTTTGTGGTGGTGGTGGTGGTGGAGATTC
TCCTGGTCCTGTTGCATTAATAGTTGTCACATTTGTCACAGTTGTTGTAACCACAGTGGAGTTCATGCCTGTA
TTATGAGATTTAGCTTCTTCTTGGAATATGAACCTTGGAATTAAGTT
>1002050452_1 Heli.1-EL602906.1.5
GCAGAGGCTATTTTGGGCGCAAAGTGAAAATTAATGTTATTTAAATCTTATATACATAAAATCACTTTTATTA
TAACAATTAGCGTAGAAGGTTTAAATTTTTACAATCCGTACAATAAATCAAAAAGCTAAAGACTAAGAATATA
AATAAAAATGACCGACTCCGTCAATATGTCACTTGATGACATAATTAAACAGAACCAA
CAAAATAGGACTAGTCGTGCCCGCGGCAGAGGTGGTGGTAACATTCGAGGACGCGGAGGTGGCCGTGGTGGCC
GAAGAGGCCGTGGTGGTCGAGGCGGGGTAAATACACGTGGACGATCTCAATCTAGAGCTGATAGTAACAGTAG
GCAAGGCAGATCACGTTCCCGTTCTCGAGGTCCTAGAGGACGATCACAATCACGTGCC
CGTTCCCAGTCTAGAAATAGAGCCAGATCTCAACAAAGACGATTTTCTTATCCTAGAACAAGATCTGCATCTA
GATCTAGATCTCAATTAAGAAGCTTGACTCCTAAAGGAAGGGCTGGTCTTGAAAATGCAATAAATGATATGCC
ACCTTTAATTCGATCTACCAGAGGAAATAGAGGTGGTCTTCAAACAAGGTTCCGGCGG
TCTAATTCTAATCAGCAAATAAGAGGTGGTGTACATAGTAGACTTGGAATGAATACACGAAGAGGTGGGGCT
>1002050453_1 Heli.1-EL600506.1.5
GCACGAGGGGTCGATATGGATCCTCCCAGCATCCGTCAGTGCCCGCCAGACTGCCGGTGACACCTGCAGTACC
ACATGGCTCTCCAGTTGGTTCCTCGCCCTTCTCCGGTATTTGATCTTTGCCCTGTAATCGTCATCCACGATAT
GGTCCACCAGGTGAGAGTTTTGAACCTTTATTGAGTTCAAAATGTCCTCGTCCGAGTT
ATACTCTAGTACGTCCTTTATTATGACCGTTAAGTAGATCCTTGTTCCTTTTGTCCTCCACTGTTAGGTCCGG
CGGTCCTGTATTTATCCTTTCCTTTACCCTCTCGAGGTCCTCCCTGTTCGTACACCTTATGACGACATTCTTC
CTGGCTTTCCTGACCCGGTCCACCCTGATGCCTGTCGACTTGGCCTCGAGAGCAGTGC
GGATCTTATTTATAACATCCTCACTGCTGTCCTTGGCGTTTGTCGACGATACCACGATGGAGTGGATCGAGAG
GTCGGCTGGACCCCTGATCGGCATAGGGTTGCACTTCTGGGCGGCTTTCTCCGCGTAGGTGGGTGCTCCTTTC
TCGCGCTGTTTTCTTACCTCTTCCCTTGTTTCTTCTGCCCTAGCCTTTAGTTGGTGGA
GTCTGCTATTGAGCTGGAGTCTATTCTCGCTCAGTTTGAGAATGATCTCGTACATCCTTCCCAAATTCTCTAT
TACTGTCTCCTTTATCGTGGTTTTAATGTTCCCCGACTGTTCCAGATGCAGCTTGGCCTCTTGGAAGAGATGG
TTCGCAACCACTCTGAGGTTTTCAGGTTCTTCTTCTGAGGTCTGAGGATGTGGTATCT

TTTATATCAAAAACCAAAATTTTAGATACTAAATCTATTCCTGATTTACTTATGATCTGTGGAGTAACGAGCC
AACAGCCAGTCTATTTTAATGATTATGTCCAGATATATTTGTATATATATGTATATATAATACTTGTCCAAAG
TATATGTTACACAAAGTAAAATTAAGAGCAAATATAATAAATTGAATAAAAAGTGTAT
CTAGGGTCAAGTATGTCTCTACTCCGTGGCAGAGCAACGGTACTGGGTCACCATATGTTATTCCTGTGGGTAT
GGCCACATCGG
>1002050454_1 Heli.1-EL596601.1.5
GCACGAGGGTCGCTCTTGCTGGAAGATAAATAATTTAATATACGTTAATATACTTAATTTATAGTCTACTGTA
ACAAATTTATATTTTGTAGTATTAAATATGGCAGATATTGATAAGTTGTCCATAGGAGAAATTTATACTTCTG
ATAAGAATGGCAGTGATGAGAATGGCGACGGATCCGCAGAAAAGCCCTTTAAAACAAT
CTTGCAGGCCATGCGCCATGCCGGCAAAGAACCTTTCCCTGCAATTTATGTAGACTCTAAAGAAGAAGGAAAA
GATTATGATGTAGCAGCAAAATCTCAATTAAAAAAGATTCAAAAAATCTGGGTGCGTGAAGTTCATAAGGCAG
CTGAGAAGACGAAGGCTGAGGAGGAAAACTTGGAAAAAAGGCAACAAAACCTTGAAGA
TGCCAAAAAGATAATTATACAAGAAGATCCTTCACTTCCTAAGGCAGTAACTGTAAAAATTCTCAATGCTGAG
GAACATCGTGGGAAAAGAATATGTGTGAAAGGATGGGTACATCGTCTTAGGAGGCAGGGCAAAGCTTTAGCGT
TCCTCATGCTTAGAGATGGCACTGGCTTCCTACAGTGCGTCTTACACGGGGAGCTCTG
TCAGACTTATGATGCCCTGGTACTGTCCACTGAGTCGTCCGTGACATTGTATGGACAACTTGAAGTGGTGCCT
GAAGGGAAGACGGCGCCTGGAGGTCACGAATTGAATGTCGACTATTGGGAATTAATAGGACTCGCTCCCCCAG
GAGGAGCGGA
>1002050455_1 Heli.1-EL597393.1.5
GCACGAGGCTGAGGCGTCGTGCTAGTATCAAATATTAAAAAGTGAAAAATTATACATTATTGTATTTAAACGT
TATATTTTACTGCAGTTACATCGTGATAAGTGCATTTTGAATTGTTATCATGTTGGTGTTGTTTGAAACACCG
GCGGGATACGCTATATTTAAGTTACTTGATGAATCCAAATTATCTCAAATAGATGATT
TGTATCAAGAGTTCAATACACCAGAAGGAGCAGCATCTGTAGTAAAATTAAAGAACTTTATTAAATTTGAAGA
TACCACAGAAGCATTAGCTGCTACTACCGCAGCTATTGAAGGAAAATTATCTAAAACATTAAAGAAAGCTCTT
AAAAAACATGTTTGTAAGGAAATTCAAGATCAGTTACTTGTTGGAGATGCTAAGCTTG
GGAGTGCAATTAAAGAAAAGTTTGATTTACAATGTGTTTCAAATTCAAATGTGCAAGAGTTATTGAGATGTAT
ACGGTCGCAAATGGACAGTTTACTAACAGGGCTACCTAAAAAAGAAATGACTGCCATGGCACTTGGTCTAGCC
CATTCGCTGTCTAGATATAAACTAAAGTTTTCACCTGACAAAATTGACACTATGATTG
TTCAAGCCCAATGTCTTCTTGATGACTTAGACAAAGAGTTAAATAATTATGTAATGAGATGCCGAGAGTGGTA
CGGCTGGCATTTTCCGGAACTCGGCAAAATTATAACTGATAATACGTTATTTGTCAAAATAGTTAAACTTATG
GGTACAAGAGATAATGCGGCTC
>1002050456_1 Heli.1-EL599108.1.5
GCACGAGGCTTCCCTTAACGCGCTACGATTTTCGATTGTGTGTTTGGAAATAAACAGTTCGGCGAATTTAATT
TATGATATTTTTGGTTATATGAGGAGTTTAAAGTCTGAAAAATGTTTGTGTGTGGTGAAATTAGTGGAACAAG
AGAAATGCAAAGCTTCTGATAACACCGATCCTCGCTCACCGAAGTGACGCCATTTTAC
GGGAATGACCAGCCCGCGGCCGCCTCTCATTAGGTCTGATGTAAGGAGCGCCCTATACAAACGGATGCATGGA
AACAAACCTCTCATATTGCACTCGTACTGGACGTACGATTTCAGAGAAAACTATGGAAGATGGTAATATTGTC
AATACTAAAGACGATAAAATCGCTTCAGCATCTTTAGCAAATTTGTATAGTGATCGAT
CTTGTAAATTGGCAACAAATGGACACGATTTTGCTGGCGAGCCGTCCAGTCCCACAGACAGGCACTACGAGCC
GCGTCGTCACAGCGCTGACACACTGCAGGAAGATAAGTTTCGAAAACGCATAAACCCGCTAAGAATACACCTG
GGTAAAAGACCCTTTGTGGATAACTCTGTACCATATAATAGTGTTTCAAAGAAACGAA
AACCTTTTGTGTCAAAACCTAGTGATGTCAGTGTACCCAAAGCAAATACTCCCCGAGTACCCAAGTTAGTGCC
CCAACGTGTAGATCATAGTCAATTTCATAATGTTCCAACACATTTGGATAGTTCTCTTT
>1002050457_1 Heli.1-DT666568.3.5
GCACGAGGGTTTATTCGTAAAACAGAGGATTCCATTTAAATGACGCCAACCAAGACTACAAAAACTGAAGTAG
AAATGGTGATACGAAATAGAAAAACTGTTATGAAAGACGGGATAATGACAATGTCGGGCATCGGTGAGCCTTC
GGTTTTCCACGCTTTGGTGGTAATATTTTTGGAATTTTTCGCGTGGGGGCTTCTCACC
ATGCCGATAATATCGGTTCTCAATGCCACGTTCCCGGATCACACGTTTCTGATGAACGGCCTCATAATGGGTA
TAAAGGGAATCTTGTCGTTCCTTTCTGCGCCGCTGATTGGAGCTTTATCCGACGTGTGGGGGAGGAAGTTCTT
CCTACTAGTGACCGTGTTCTTCACGTGCGCTCCTATCCCCATGATGACCTTCAACACC
TGGTGGTTCTTCGCGATGATAAGTATTAGCGGTGTTTTTGCCGTGACCTTCTCCATCGTGTTCGCTTACGTGG
CTGATGTAACAACAGAAGCCGAGAGGTCACGCGCCTACGGACTCGTGTCAGCGACATTCGCTGCCAGCATGGT
GATATCGCCTGCACTAGGCGCATATCTAATGGACTTATACGGAGAAGGCCTTGTTGTC

GCCGCTGCTACTGCCGTGGCGGTTCTCGATGTCTTCTTCATTATGGTTGCAGTTCCTGAAAGTCTTCCTGAGA
AGGTTCGCCCCAGTGGTTGGGGTCCAGCTATTAGTTGGGAGCAAGCT
>1002050458_1 Heli.1-EL597501.1.5
GCACGAGGATTGAGCAGTTACGAGCGGCCATGCGCGCCATTCTCGCCGATCTCAACCCCAATGATTACTTCAG
CATTGTTGAATTTAATTCTGATGTTCAGGTGCATGAACTTAAAGAAGCTGATGAACCAGAGAAACCACGCACT
TATTCATACCACTCATGGAGGTCAGAGTCCAAGCCAGTCGTACTAGTGCCACCGTCCC
ACGCGTCACCCGAGAACATCGCCAAAGCAAAGATAATTGTATCGAGGCTTGCTGCATCTGGAGGTACCAACAT
CTATGATGCTCTTAAAGTAGCTCTGGATCTAATCCATAAGGCTTATGCAAACAATCATTCTGACTCCAGCAAC
AAGGAGATACAAAGCGCGGATATAAGTGATTTGGAACCTACCATCATTTTCTTGACGG
ACGGCGATCCGACCGTTGGCGAGACCAGCACTCAACGCATCATCTCTCACATGACTGAAAAGAACTCTGGTCA
GAAAAAGGCTGCCATCTTCTCACTCGCTTTTGGCGAAGACGCGGACCGGACTTTCCTGCGTAAGCTGTCTCTC
CGCAACGACGGCTTCATGAGGCACATCTACGAGGCGGCCGACGCTGAGCTACAGCTGC
GCCACTTCTACCAGCAGGTGTCGTCGCCCCTGCTCGCCGGCGTGCACTTCGTCTATCCACCTAATCAGGTTAA
AGAAGGCTCAGTGACTAAAAATAGGTACAAGGCCATATACGATGGATCGGAAGTGATAGTGGCGGGACAGCTG
GTGTTAGGCGTCAGCGAGTTGGAACCGACTGTACAGGCGTTCTGCGCCGATCCCAACG
ACCATCGCAAGAAACCTTACGAAGTGCGTCCAGCTTTGACGCCAGGACGCGCCGGCGAGTTCCTACCACTGGA
GCGCATGTGGGCGTACCTCAGCGTGAAGCAGATGCTGGACGAGCGGGAGGTGGCGGACAGCCCCAAAGAGGTC
GAGAAACGCGCACTGGATATAGCGCTCAAGTATGCCTTCGTGACACCGCTGACTTCTC
TGGTAGTAGTAAAGCCCAATGCGACCGATGCAGTCAACGCAGAGTCTGTTGATAACAACAACAAACCTTCTTT
CGGTTCGTTCCTACAAAGTCCATCATTCGCTTCGTACCCTGGGGGGGGTTGCCTTGAGTGAAGCAGCACAGTC
GTTCGTGGTGGATGAAGAGGAAGACTACGCTGACCGATTTTCTCCTGATTCATACGTT
>1002050459_1 Heli.1-DT665865.3.5
GCACGAGGAAAATGGCTGACGCGTCGTCGTCGGTGCAAGTGAAAAAAATACCCGACAATTTATACGAGGTGTG
CTTAACTAATTTAGTGAACTATCTGCAGAAATCCAAGTGCGAACGGAATGATTTACGATCTCTACCGGACAGC
ATCCTCATGGATGTTTATTATAAGATGCTCCAGGAGAAGCGTTTATGCATCCTGGGTG
CGGAGCTATCAGAATTGGACATCTTCGAACGCCTGCTGCGTTTCTCCGGTGCTCAGTTACGACTTTTACAGTG
TTTTCAGGCGGTGATAGAACATGGCTCCAAACTATCAGCAGAGTTGGCCAGCGGATACTTGGCGAGGTGTGAC
GCCAATACAAAATCTCAAAACTCACTCATACAGCTTGGTTTAAGACTTGGTGGTTTCC
TAAACGAGGCCGGGTGGTACGCGGACGCGCAGCGCGTCTTACTGAGATGCCGAGACCTCTGTCAAGCGCAGCC
GCAGACCACGCACTATAAACGACTAACTTTGGAATGCTGTCACAGATTACTCAACACACAATCAGCCTACTGC
TGTTTCCCAGCTGCAGCTGAGACCTACGCGCTGGCACTGAAATTACTAGGAATAGAAG
AGGAGAGCACCAGCAAGTCACCCGGCGATGCTTTCTCAGAGAAATACTGTCTAAACGCCTTGGAGGATGAGGA
GATGAAAAAATCGTCGTGCTCAG
>1002050460_1 Heli.1-DT662211.3.5
GCACGAGGTAGACGTGGAGGTTCAGTAACAGAAGAAATAATGTTACCTGGACCTAAAGTGGGTCTTATCATTG
GCAAAAATGGTAAAACCATCAAGCAGTTGCAAGAGCAATCTGGCGCTAAAATGGTTGTCATTCAAGAGGGACC
CAATACAGAATATGAAAAACCTCTGCGGATATCCGGTGATCAATCCAAGGTGGAGCAC
GCCAAGCAGTTAGTATACGAACTGCTCGCCGACAAGGACATGCAACAAGGCGGCTCTAGGCCTTACGATGATG
GATACTCACAAGAGCAGGGAAATGGACTCGCTACTAATTCTACTGAGGTGCTGGTACCAAAAGTAGCCGTGGG
TGTTGTCATTGGCCGCGGCGGTGACATGATAAAGAAGATTCAAGCGGAGACTGGCTGC
AGGGTTCAGTTCCACCAGGAGAGGGATGATGGGCCTGGGGATAAGAGATGTTACTTGCAAGGCAAGCCACATC
AAGTGGATCAGGCCAGACAAATGATCGAAGATTTGATTGCCAGCGTAAATATGGCGATCCGCTCGGGCCGCGG
CCGCAACCCGAACAGCGGGGGCGCGGGTAGCGGGCCGCCGGGCCAACGCAATGGCAGC
GAGCGCGACTTCCAGCAGTGGCCCGAGCAGCAGGGCCAGGAGATACGCGTCACCTTCACCGTGTCCAACGTCA
AGTGCGGACTCATCATTGGGAGAGGTGGTGAAGTAATCAAACAAATAAACGCCCAATCCGGCGCCCATTGCGA
GCTAGACCGTCGCGCGCAGAACAACGATCGCAACAACCGTACGTTCTATATACGCGGA
CACCCCGAGGCCGTGGAGCATTGCAAACGAATTATTATGGAGAAAGTTAGCATGCCGGTGAACTTCATCCCAG
ATAATGGCAATGGGAACGGTAACGGCAATGGCAACGGCAATGGTAATGGTAACGGCAACGGTAACGGGAATGG
TAACGACGGGACCGATTACTATAGGGGAGGTGGGGGTCCAGCACCCGGTCCTCCGGCC
TGGGGCTATAATCCTCATTGGCATCATCAACAACAGCATCAACCACAGCAACAAGTCCAAATAAATCCAGCAA
CAGGCCAACCAGACTACTCTCAACAGTGGATAGACTATTACCGCTCCTTAGGACTGATGAGGGAAGCCGAGGC
AGTTGAACAGCANGGGCTAACACAGCAGCAAGCGGGCGCCCCAGCACCCAGCACGCCG
ACGCCGGCCGCGCCCGCCGCGCAGTCCGCGTCGTCCGCGCCCGCCGCCGACTACAGCGCG
>1002050461_1 Heli.1-EL600497.1.5

GCACGAGGTCACACCCTTAACTGTACAGTCACTCTGATGGCAGTAGAACTGACTATAGTAGTTCACATTATGA
TAATTACAAAATAACCAAGGAAAATGACTTAGCTGACTTCGATAGTGTCTCATCAATATTGAAAAACGAGTCC
ACTCGGCTCAATCGCTCTCCTAAAGATAAGAGATTACTGGAAACTTTAAGCACTGCTG
ATTGCGTTGATGGCTCAACACAATTTTGTGTAGAAGAACATAATGTTAAAGAGGATTTATTTCGTGAGCGAAC
AAATTCAAGTATTAGTGCTGCTTCATTTCATGGTGACGGAAGTGATCCTACAGATAAAAAACACAACTTGTTA
AGTGCAAGCGAATTAAGTGATCTAATTGTTGGAAGAGGCGTTTATCCCAAAAATCAAT
CTGTAAGTGATACTTTTGATTCCGTCTCTGACTACGTAAGACTACCTTTACCATTTTCTGGTGACAGTTACCT
TCAAGGACATGAAGATACGGCTCCTTCAGATGACAACTATCCAAGTAATTCTTATTTTGATAGACCCCCAACA
CCACCAACAAGAATAGACAGCCGCAAGGGGCTAAATTTATCCTTGCCAAATATACTTG
ATTTAGATGAAAACGTGCCCGCCGTACCAAGATATCCAGATAAACCTCCACCACCGTATGAATACAAACATCT
AACTCTCTCATCTTGTCATGGTACTCCAAAACCACCGCCAGCATATCCCGGAACATCACCGTCAGTTCCTTCA
AAACACACACTCGAAAAAGAAGAAGTAGCGGCTAGAGTTGTGACAACAAAACCAATGA
TTACAATTCTAAAAGCGGAAGCAGGAGAAGTTAGCACGTCTGGTGAAAGGACATTCGCTAGTCCGATGGTCAT
GGAACATCGTTTTCAAAATCAAA
>1002050462_1 Heli.1-EL602852.1.5
GCACGAGGGTCCTTCAGAAATATAGGCCGTGGTCGCCTCGCATATGAAATCGAAATTTATCTATAGATTAGTT
CGATTTTATACGTAGATTTAGTATTTTTCCTTTGAATAACGCCTTTTGATTTTTGAAATTTATTTTTTTATAT
ATACAGTCTGTGGATTTTGTGCACATTTTTGTGTTATCTGGCAAGCAGACGGCAACAA
CGGTGCCATGGCGGACGTTGGGATTTCACAATCAGTGCTAGTGGAGCCTAGAGGTGATAATGGGAGGAAACTA
CCCCAGTATATTGCTGCTTTGTCAGCGACTCTCGGCGCCCTGGCAGCAGGTTCGATGCTCGGTTGGTCATCTC
CGGTAGTCTTCAAAATAACGCAAAGCAACAGTACTGATTACGATTTTGAAGTTTCTGG
ATCACAAGGGGACTGGGTGTCCTCCCTTATTAATTTAGGTGCGGCTGCCGTTTGCTTTCCCATAGGTCTCATC
ATGGACGCCATAGGAAGAAAGACCACTATGCTTTTGCTAGTTATTCCGTTCACTTTAGGATGGCTCTTGATAA
CGTTTGCTACGAATGTCGGTATGTTAATGGCGGGAAGAATTATCACTGGAGTAGCTGG
AGGTGCGTTTTGTGTGACAGCCCCGGCTTATACAAGTGAGATAGCCCAAGATTCTATACGAGGAACTCTTGG
>1002050463_1 Heli.1-EL600343.1.5
GCACGAGGCTCCATTGAGTGTGCACGCGTTAGTTGGTCGCTACATGCCGCAAACTTTACACGAGCTCACTCAT
TAACAACATTCCGATGTTTTCTTAAACTGTTGTAAACTATTTTAATACAGCGACGCGACACATTATTTGTAAA
TTATTACCGCATTCGGCCACGTAAATTTCGTTGAGCCTTTTTCATTCGTTAGGTCGTA
AATCCTTTTCAAAGTGACAGTTGAAGTGGACAAGCAATTTTCGGTTAAGTGTCTAAATTGTAACTGTGATATT
GTTTTTGTTGTGAATCAAAAGGACACTCGGAATTAAGTTTTAGTTGAGAGTGCTTCAGTGAACAACGAGAACC
CTACAGGATAATTACTGCCGTGTGATGCTCGAGTGACGAGCTCAATATGATTCGTGAT
CCGCCGGCCATGCACAAACCCGACAATTCTAATAATCAACAAGAAGAAAACAAATATGCACTGAGCGGTGGTG
TGGTTAACAACCCAGGTGGAGCGAGGCTATGTGCGCAATGTGGAAAGGTCATCACTGAGAGATTCTTATTGAA
AGCTATGGAACGCTTCTGGCACGAGGATTGTCTCAAGTGCGGTTGCTGTGACTGTCGT
CTGGGAGAAGTCGGTTCCAAATTGTACTATAAAGCCGACCTTATGCTTTGCAAAAGAGATATCTTAGGCTATT
TGAAGCTACAGGCAACTGTGTGG
>1002050464_1 Heli.1-DT664347.3.5
GCACGAGGCTACCCCTTCCAATCGAATGTCTCCTTTTTGTATTGAGTCTCTTTTGAATGTGCTCATATTTTTT
TTTTATCTTCCAAAATTCAAAAAAATATTTTTTTAATGTTGTCGTGGCGCATTGGCACAGCTTCTGAGTGTAA
ACTGAGGGTTCGATTCCCACTCGGGGGAAATAAATTTTGTTTTCGTTCAAAAGACAGA
GCGCGGTGTTAAGTATCGTCGCTCAGCAAGCATTGTTTCAAAAATTGGACGGTGCGTGGGAGTGTCTTAACAC
TGCCATGTGCAGAATACAACGTGAAGCTAAAACAATCATTTGTATTTTCAAAGAGAATAATGGTTTTATTGTC
TCCATACTGTATATTATAATATAAACTTTTAAAAATTGAGTACAATAGTCCAGTTATA
AAAGGTCTAGTCGTTTCCGGAATCCTATCAGTACTTATCTGTGCAAAACTGCGAATGCAATAATTTTTTTTAT
ATGTTATGGTATGTATATAAAACAATCAGTGTTTAGAGCTTTTGGTAGCCTCTATTTTACTGGGTTCTTATTG
ATTGTATGAAAATTGTCAGGTTTTGTTTCATGTCATTGTATTCAAAAGCAAGATATAC
ATTTTATTTTTACATTTATTTCCTTTTCATTGATTTTAAATAAAAAATGGATTTTGTGTCAGTTATAAATTGC
TGAATTTTATAGTATTACTTGAAATGAGTCGACTTATGTTTCTAATTTTTGGTCAGATTTTAGGATATTTTTT
TCTTTTTATCAAGTCTCAGAGCAAGATTTACAAT
>1002050465_1 Heli.1-DT663729.3.5
GCACGAGGATTTGCATTGTAATTTAGTATTATAAATAATCAATTCATCATGTTCATAAATTTTATGTTTTTAA
ATAATTTTTTTGGTTAATAGGTATTGTTTATTGTTCATAAAATATAAATTGTGGTTCTCATATTTTCTTTGGA
AAATTACATTAAATTTTAATTTTCTCTTTTCCATTATTCTATATTTCCAATTAAATCC

TATGAAATAACGATTACACAAATTTTATATGAAAACGTAATTAAATATTTCTTAAGATTTATTATTTAGGTAT
ATATATGTATATATGTCGCATCGCGAAATTGTATAAAATTGGTAAATAAACCCGTCTAAGCTGTGATAGTAAC
AAAGATTTTAGTGAAGAAATTTGAATCTGTACCTCTAGGAAAGCTCGAGCTAACTAAT
CAATACATTCTTTTCTAACTATATCGAATTTGTTATACACTTGCTACAAACAAAATATATTAAATTTAAATAT
TTTTTTTATGTTGTTAGTTTTGTATGTAACATAATTATAATTATTAACATTTATATTTTCATTTCTTCGCTTT
GGTGTCGAGGCAAAACGCGGCGTTGAGTTCTGCTATTCAACACGCAATGTCTCCAGAA
CTTAGCGGAAAGTGGGGAACAGAGTGTCTTAACACTAGGTTCCCTCTGATAATGTATTATTATGGCATTTTAA
AATTAAATTTAAACAGTGGATTTTATTTAGAATGTAAACAGGCCTCTTAGACCTACTACAACTGGCTTGTATT
GTTATATTATAGTCAAAATTATACTAAATATTTTCATAAATGTGAGTCTGTGTGTTAT
TCTGATATATAATGTCTAAACTATATAACTGTTCTGTATTTATTTTTAATAAGTGTGTTCTGTAGTTTTGGTA
CGAAAGTGAAACAAACATCCATCTTTATAAGCTTACACTTGTATATAAAATGATAAAACCAAAGTACAATCAC
AGTGTGGTCGACGAATCGTGACCACCATGAGCTGTGTTAGCGCAGATTTGGTTTTAGG
ATGTTTCTTACTTTTGATTATTGT
>1002050466_1 Heli.1-DT665595.3.5
GCACGAGGGTAAAACTCTTGCAAGTAGAGCATACGTTAAACGAAAAACGTATATTACAGGCAATAAATTTTCC
TTTCTTGGTCAGTCTCAAGTTCCACTTCAAGGACAACTCTAATCTATACATGGTTCTCGAGTATGTTCCCGGA
GGTGAGATGTTCTCGCACCTTCGTAAGGTCGGACGGTTCTCCGAGCCGCATTCGCGAT
TCTATGCAGCTCAAATAGTACTCGCGTTTGAATACCTCCATTACCTGGACCTTATTTATCGAGATCTTAAACC
TGAAAATTTGTTGATCGACTCTCAGGGCTACCTCAAAGTGACTGATTTTGGTTTCGCGAAGCGAGTCAAAGGC
CGTACGTGGACGCTTTGCGGGACCCCCGAATACCTTGCACCTGAAATCATCCTCTCGA
AAGGATACAACAAAGCCGTGGACTGGTGGGCATTGGGCGTTCTCGTCTACGAGATGGCGGCTGGGTATCCTCC
TTTCTTCGCAGATCAACCGATACAGATTTACGAGAAAATAGTATCAGGTAAGGTCCGTTTCCCATCGCACTTC
GGCTCCGACCTGAAAGATCTTCTGCGCAACCTCCTGCAGGTTGACTTGACTAAGCGCT
ACGGTAACTTGAAAGCGGGAGTCAACGATATCAAGGGGCATAAATGGTTTGCGAGCACGGATTGAATAGCCGT
GTTCCAAAAAAAGATCGAGGCGCCTTTCATCCCGCGCTGCAAGGGCCCTGGCGACACGAGCAACTTCGACGAC
TACGAGGAGGAGGCTCTGCGTATCTCATCCACGGAGAAGTGTGCGAAGGAGTTTGCAG
AGTTCTGAGCGTGTACTTGGAGCTTGCAACTTTGTATTAGTGAATTTTCCACAACTAAGTGGTTGTGGGAAGG
AATTGAGTGCGTCTCTTGTGGGCAGCTTACGGTGTACACAGACATTGAGTGCCCCGAGCTGACACCTCAGGTG
TATCAAATTTTGAGTATAGGCGAAATGGTAGAACTGATTCTGATATTTTTGTGGCATT
TGTTTTCTTAGTGCCCATTCAATTTAAATATAAACAGACATGGTGTGTAGCTCCCATTACGGTGATATTCTCA
TAAGCTACTGCAGACATAGTAGAGTGTAAGCCGACCTGTCGTCGGTACTAAGTGTTTTTGGTACTCTGTAGTT
TGCCTTTAATAGCATTGGGATGTGGTAAGCTTTAGTCCTTTTTTAATTGTTTTTATTA
TGGAACTCATACAAATTGTATATTAATCAGGTTTTCTTGTCGCTTATTTAGACTTGTAAGTCTTCACTTCATG
TTTATACATCTNCATTTTAATAAACTGAAATTGTATCACTTTATTATATTATGGATTCAAACACTTATTTAGT
CAGTTAATAAAAATAATATTTTATTATGCTGCTAATATTGTAAAGAACATGCCTTGAA
>1002050467_1 Heli.1-DT667612.3.5
GCACGAGGCAGACCTTTATTACGAAAGACAACCAGCCTCTGCATTAGTTGAACCAGTTGTAGAAAGTGGATTT
AAACCTATTTTTAAAAATCCCCCCGAAGTCACAAAAAGCCCCATCTATACTTCGTCTGTTCAACAAGACGAAT
TGTCGTATCATAACTATTATATTGAGAATCAAAAGAATCCAGTCGATGATTCAACTTT
AGATGTGCCAAGAGATAATTCGTATAAGCAACAATATATTATTGAACAACCTACCGTACCTGTATCTACACAG
AAACAGGAATCTTCAAATTCTGTTACATTGGCAGAACTTTTAAATTCTTTACAAATTAATAAATCTATACCAA
AACCAATCACAAGAGAAAATGTAGGCGATTCAATAAGAACATTACTTAATGTATTGAG
TACATTAAAAGCTGCTCCATTACAAAATGAAGGAGTGGCAGTTGTCAGTACGCCAAAACCGTTTGAACCGCCT
GAAATTTCACATGTCACACCTCTCAATTCGGTATCATCGACAACTCCTAAGGCCGAGGAACCAGATGAGGAAT
CGTATCTTGCACCAATACAAACACCTTCGCAATATCTAGACGATTATCCAACTAGCAG
TGGAAGTACGCAAAGATTTCCTCTTCCCGTGACCTCGGACGAGGAAGGTGGGACTCCTGGCCGCCCTGGCGTT
GATTACCCTATATTGACTGTCATCCCTACAACGAATTCAACTGCAAACACAACGTTATAAAGGTTTCTTTGCA
GATACTGGAACAAGATGTCAAG
>1002050468_1 Heli.1-ES585857.1.5
GTTTAATATACTTTTAAAGAACTATGTATAAAGGAAAATAAAACATTTAGAAACATCATCGGCGTTGGTTTCC
GCCACTCAATATCTCCAGAATTCAGCTGAAAATAGGCTAGGGCAGTGACTTAATAAAACAAAGTGTGTCAACT
CTAAGTTCCCAGGTTTCCTCTCATTCTTCACTTAAAAATAAGTACAAGTTTTTTAGCT

TCATGCTGTATCCTGCACATAGCAGGCAGCAGGGTTGCTGCCAGTAAGAAAGCTTATACTTATTTTATGGTGT
AAGAATCTCTGTTTGGAAAAAGTCATAAATTTCAAATTCCCAAAGAATATTCCTTAAAATTGATCATTTGGTT
GAAAGTAAGCTGTATAAAGCATACATATAAGAATACAGAGTGCTTTGATGTTTTACGA
AAGGCACAAGTTTGAGGCCTGCTTCTTTAAATTATTTGTTTCTAAATGTATGGATAATAAATTTATATTTATA
ATTTACTTAATAAATATTTGTTTTAAAGCATTTTTTTATGGCGGTATGTAAGACAGCTTTGTAATATTTATCA
AGAGATCTGTAATGTAATTATAAAAACAAATATTCTTTTTTATTTAATTTATTAATTT
TAAATGAATTACAAGAAATTATAGTAGAATATTAAATGTATGTTACTACTTTAGAACTTTTTTTATTAATAGT
AAGTTCTTTATATTGTAAATCGTGATTTATAATTT
>1002050469_1 Heli.1-DT662835.3.5
GCACGAGGGAAATACTGGTGACTCGTATAGCGGCTGCCTTTGCATAGTGGACGTTACTTAATTATTGCTAAAA
TTACTAAACAATATTCTAAAAACATGTACAAGTAAAAAAAATCAAAAAGAATTCCCAATAACATTACAAATAT
TTTTAAAACTGTGATTTACTATGTAACGAGAGTGTTCTCACTGACAAATATTAGCCGA
ATAAAGTCCCGACGCACTCGACGACCAATCTTCGACGTGTTCGATACCGTACGTCAAAACGGGCCTTCGTGCT
ACCAATTTTGTTTACTGCGTTCGAGTATCTCGATAGTCGAACGTAACACGATACAAAAAATGGTTCGTGCTGG
CATGAGTTAGTTCCCTTTACGCACGGTAGGGGCGCTGTTCAATTCCTATACAAATTGC
TCTCGATGACGATACGAATACTTTTTCGATAGTTTTTCGTGCTTGAGCTACTGAATGACAAACTTAATTTTGG
AAAAAGACAAATATGTTTACGACCTATATATGTAAACAGAAAGAGATAGTATTACGTATCTGTCGAGTAACAC
GAAGTTATCAGAAGAAAGTGGATCTCGTCATTTTGTATACAACGATATTTTACATTTT
TTCTTATTTTAATGTCGCTAATTGAATTTATTTATTACCAAATTTTCGGTAGTACATATGTTGAAAAAGTGTT
CATTATTAGTTATATTTTTAATTTTGAAGTAACTTAATATTTTAATGTACTTAACAGTTACTTAGCTATGTAT
GTATTGGTATCAAGAACTTTATCATTTATTTGTAAGAATAATTTATGTTAAAACGTCG
GTTTAAGAGTGTAAACCCTATAAT
>1002050470_1 Heli.1-EL601275.1.5
GCACGAGGCGGAAACCTTCAATCTGCAGGCGTTTGCATTTTATACTTGCATTCATCTCTTAATAATAAAATAT
TACTAAAACTATCCACAATGACAGTTTTAACAAAGCCCAGTGCTATAATAAAAAAGCCAGAGATAACAAATAC
GCCTTTAGTCGAGAAAGGCGTTAATGATGAGGAAAAGATTGAAATACATGCAGCAGAG
TTTCCGAAAAGATCCTCAAAGGGGATGGTATGTCTGTCCCTCACAGCTTTCCTGCTGGCCATCATAGGATTCT
CAACAGGCCTGGTTCTGTGCAAGCACATGCTGAGGAATACTAACCGCCGCTACCAAGGATTCTGCTCTATTGT
CATTCCTAATGATATAAGGGAGCTTGAAAACAACCACGTGGAACCCCATTTCCGAGTT
CTCCCACTCCAATGGTCCCACGACGTTGAAGTCATTGCCACTGGCGACGAGGACTCGAGCGATGAGTTCGTGA
ACGCCCTACGCGAGGAGCTGGATATAGGTGACATGGTGGAGAAGATATCTGTTCTGAATAATGGACATCGGGT
GGATTTTGTGCACGATTTCAATGATAATACTACTTCTATAATCGACATAACACGTTGT
TTTGTAATGGACCTGGCACCAGCTCTGGTCCTCACTCCAATAGAACTGGGCGAGGAACTACAACGTGGTGCTG
CCTTCGATGTTTCTCGTGTTCGTACTGAACTGCGCGCGTCGTTAAACCCCGTGAACACACACGGTACGGAA
>1002050471_1 Heli.1-DT663988.1.5
GCACGAGGATCGACCCGTACAGCCCGCACCTGTCGTCCCCGACCCTACGCCAGTGTTGACCCCCGCTCCGTCG
GCGCCTCCAGTAGCGTCTGCTCCTGTTATCGAGTCTAAGCCCGAGCCGAAACCCGAAAGCATGGCACCGCCCA
AGCCAGCACCTTCGCCAGTACCGCAAGTCCAGCTGCCGCCCGCCCCAGTGCCCGCGCC
TGTGCCGGAGCCCGAGGCCGTGAAGCCGGAGCGGAAGGCACCCAGCCCCGAACCCGCTCCAATCGACAGCAAG
CCGCAAGCGGAGAAGCGCACCGCCGTCGCACATGTGATCGAAGTGTAGGTCATCTTCTGCCAGATGGGACTCG
TGGAGTCGCTGATCTACTGGCGGTCGGCGGCGCGGTCGGGCGCGGCGCTGGGCGCGGG
CCTGGCGCTGCTGGTGGCCCTCGCGTGCTGCTCCGTGGTGTCCGTGCTGGCGTACAGTTCGCTGCTGGCCCTT
TCCGTGGCTGTGGCCTTCCGCATATATAAGAACGTGCTGCAAGCAGTCCAGAAGACCAACGAGGGGCATCCCT
TCAAATGGCTGCTGGAGAAAGACATCAGTGTGCCGGCAGAGCGCGCGCAGTCGCTGGC
GGCCGCCGCCACCGCGCACCTCAACGCCGCGCTCGCTGAGCTCCGCAGGCTGTTCCTGGTGGAGGACCTGGTG
GACTCGCTGAAGTTCGGCGTGCTGCTGTGGTGCCTCACGTACGTTGGCGCCTGCTTCAACGGCATCACGCTTA
TCATACTTGCGTGGATAGCATTGTTCACATTGCCCAAGGCGTACGAGATGAACAAGGC
GCAAGTGGACGGCAACCTGGAGCTCGCGCGCGCCAAGATCAACGAGATAACCGCTAAAGTGCGAGCGGCCGTC
CCCATCGGCAGGAAGGCGGAGAGCGAGAAGGACAAGTAGTGTGCGAGCGAGACAGCACTCACACTCAGCGACA
CCACCACGCAGTTGTTTTAGCGCTCACTGTTTGTTATATTTATTATTAATACGTATTT
AATGAACATACAACATACACATACTTTACGTGTAAAGCTTTATGAGACGAGTCTCGCGGAGATTATTGTATTT
GCTTTTTTGAAATTTTAATTTAAATACTGTCGAAC
>1002050472_1 Heli.1-DT667240.3.5

GCACGAGGCTTCGCATGAGGAAGCAAATCTCCTACCAACAGTTGAACCAAAACCGGAATCTGAATCTGCTGAA
GTACAACCAGAACCAACAATAGAACCTCAGCCTGAACCAGAACCTGGATCTGAAAATGAACCAAAATCTGAAC
CTCAACCTGAACCTGAACCAGCTGCTGAACCACAACCAGAACCTACACCCGCTGCAGA
ACCACAACCTGAACCTACGCCAAGTGTACCACAATCGGAACCTGAATTAATACCTGCTGAACCAAAACCAGAA
CCCGAACCTACATCTGAACCTGAAGCTTCAGAAGAGGCAACAGCAGTACCTGAGCCAAAACCAGAACCCCAAC
CAACAGCAGAGTCAAATTCTGATCCCGAGCTAATAGCAGAACCTAAACCCGAACCTGA
ACCAACAGCTGTAGCAAGTTCCGAACCGGAATCCACAGCTGAACCAAGACCAGAAATGGAAGGAATGCCAAGC
TCTGAAATACAGAAGCCTGAAACTAAAGAAGAATCTGGGGAAGTTTTTATAGCTCCACAGCTAACAACAATTA
ATCCAGACATACAACAGACAACTATGCCCAGTCCTGAATTAATGTCTCAAGGAGAAGA
GGGAAAGCAAGACGAAATGACTACAACTCCCAAACCTATATTCGATCATCCGACATTTGACTTGAACAGTTTA
ATGGGCATTCACACTACTTCAGAATCTTCCCAAGCTTCACAAACTGAGAGTGACACACAAAAAAGTGATAT
>1002050473_1 Heli.1-DT662993.3.5
GCACGAGGCCCGACTCGATATCCGGGGAAAAACCTAAAGCCTTTATAGTATCTCAGCCCGGGGTAGAACTCAG
TGAGAAAGAAATAATAGATTACGTCAATAACCAAGTGGCGCCGTTTAAGAAGATAAAGGAAGTCCAGTTTGTT
GATGCGATACCTAAGAATCCTACCGGGAAGATACTGAGACGGGTTTTGAAAGAAAAAT
ATTGTTGATTTTTATTTTGTTTTTTCGTGTGTATAATATTTTTTTACATTATTATGTATGTATGACAGTATTA
TGTGTATTGAATTTAGTCGAAGTCAAATTTCATTTATTTTATTCAAAACTAGCGACCCGCTCGCGGCTTCGCC
AGCTTGCAGTTGTTTGCTTGTGTGTACTATATAATTATTTATTTACTAGCCTACATCT
AAATTGGTTAAGTAATTTCCGAGTTTAACGTATATAAACGCGCAAACACAGAGACCAACCGACAAACAGACAA
GAAATAAAAAAATGTTCGTTTTGTGTTTTATTACATTGACTAACATACACCCAATGGCAGATTTTCTCATATA
TTTCCTGCAAACAAACAGCGACAAGTTACAATTTTATTATGTATATAGATAAGAAGTT
ACATGTTGAATTTTTTTAATCAATATTTTACAGTTTTTAATTATTGTTTATTAATGGACAATGTTATCTTTTA
AATGAAGGTGGAAGTATACAATTTATCCCCAATTCGTGTTATATATAAAGTTGTTTTATGATCGATTATATAC
TAAATCATTTTTTTTTGTTTTAGAATAAAAAAAATATGTATTGTTG
>1002050474_1 Heli.1-ES585808.1.5
CCTGCAGTACGGTCGGAATTCCGGGTCGACCACGCGTCCGCTGCCTTAATTTTATTCTTACTGTGTAAAATTC
GGAGCAAATCCGAATAACCATGGCATTCGGTGATTATCCCAAAGAATACAACCCCGCGGTTCACGGGCCTTAC
GATCCAGCTCGGTACTATGGAAAACCTGATACTCCCTTCGGACAACTGAAGTTGAATG
AAATTGGATCGTGGCTCGGACGTAGGAACAAAACCCCTTCAGCAGTAGCTGGAGCTGTCAGCCGAGCTTGGTG
GAGGTGGCAACATAAGTATGTGCAGCCCAAGAGAGTTGGAATTGCACCATTCTTCCAACTGCTGGTTGGATCA
ATGACTTTCTTCTATGTTATCAACTACGGCAAAACTAAGCACCACAGGAACTACAAGT
ACCACTAATCTGATCAAGATGATGCATGCAATGTTATAATTATTTATCTGTGTTAAATATCTGCAGGAAACCT
TTCTAGATTGCATTCAATTAATAAATGAATATTATTAGTTCAAATAT
>1002050475_1 Heli.1-ES586889.1.5
CCGCCATTTTGGACGTATTTTATACGTTTACTCGGTGTAAAAGTAATAACAAATATGGGACACGCAAATATCT
GGTATTCCCACCCCCGTAGATACGGACAGGGATCCCGTTCATGCCGCGCCTGCTCCAATAGACACGGTCTGAT
TCGTAAATACGGCCTGAACATCTGCAGACAGTGCTTCAGAGAATACGCACATGACATT
GGTTTCAAGAAGCTGGACTAAAATGTGTTGGAGTACTTAGGTTAATAAAAATTTAATCCC
>1002050476_1 Heli.1-EL597827.1.5
GCACGAGGCTTGAAGTTTCAACTTTAGTTCAAGTTTGTTACTTGAATTGAATAATTGTTGTTTGTTAACTTAA
TTTTGAATTGTAAATAATTTTTAAAAATAAGTTTTATCCCACTTAACAAGTTAATTCAAAATGAGTTTAGTTG
CAAATACGGGGAAATTAGCGGGTCGTACGTTATTTATAACTGGTGCATCGCGAGGCAT
AGGAAAAGCTATAGCCCTAAAAGCGGCCAAGGATGGAGCAAATGTTGTCGTGGCTGCTAAAACAGCAGAACCT
CATCCTAAATTACCAGGCACGATTTATACCGCTGCAGAAGAAATTGAAGCTCTTGGAGGCAAAGCTTTACCTT
GCATAGTGGATGTAAGGGATGAAAAACAGGTTCAGAATGCGATTGATGAAGCAGTTAA
AAAGTTCAACGGCATTGATATCTTAGTCAACAATGCTTCTGCTATCTCTTTGACTGGAACTGCACAAACTGAC
ATGAAGAGATATGACCTTATGCATAACATTAACACAAGAGGAACCTTCTTAGTATCAAAATTGTGCTTACCGC
TATTGCAAAACAGTGATCATGCACACATCCTTAATCTGTCACCTCCGCTCAACATGAA
TCCGTATTGGTTTTCCATCCACGTAGCGTATACAATGGCCAAGTATGGCATGTCGATGTGTGTTCTCGGTATG
AGCGAAGAGTTCAAACCGCTTAATATAGGAGTAAATGCGTTATGGCCAAGAACAGCAATAGCCACCGCGGCTA
TAGAGATGTTGACAGGCGATACGTCAACGAGCCGCAAACCTGATATAGTGTCTGATGC
CGCTTACGTCAT
>1002050477_1 Heli.1-EL598623.1.5

CAAAACTATCCCGTCACGCTTATGGAGAGAACAAGTACTGTTGATATTGAAGCCATCACAGAACCATCTGTTA
AAAGTATTTCCACAAATAATATAGAAATATCGAAAAATTTCGATACAGAAGATGCATACAGTAATGACCAAAT
TTCTTATACGGAACCCGTAAATAAAGAACTTAAGAGACCACAACAATATACGACGCCT
GCAGATATCGTATCAGAAACCTCTTATACATCTAAATTTTCTTCTTCTAACGAAGAATACTTGTATGTACCAC
AAAATAGTGGATCTCACGAAATAAAAGACAATACTGGTCCAGCACAAACTTCCCTTGAAAATAAAAAGGAACT
ACAAGACGATATGTTCTTTACCAGACCCGCCAGCCCTATAACAGAACAAAATGTGCCC
ATTTACTCAATTAATGACGAATTTCAGCCAATCAATCATGAAGAAATAGTAACACAATCAGAACCAGAAGAGA
CTGGAAATATTAGCCATGATGTTCATCAAGAACAAGAACCCCTTTAAGAAGAAAATGTTGTTGAAACAACTCC
TTCCTATAACAGAGTTAGAGTCCGCCCTGGCGTTATAAGGCAATATCACAAAGCGTCC
ACTGAATCTACAAAATTGAAAAATGAAAGAAGAAAACCTCTACAAACTATTACATACAGACCAGCTTTTGATA
AGCGTCGCACTACTATGAGAATTGAAGAAATTGAAGCAGATTTGAAAACAAAGCAAGTTCATACTAGACCGGA
ATTTCAAGATTACCAGCAGCCTGTTTACAAACCTGAACCTACAACAGAGGCAATTCCT
ACATCTTCTATTTCAGAAAGTTCTCAAAGACGTAGTCAATTCAGACGCAGAAGACCGATATACAGCTCCACTA
CAGAAGGAACTGTTACTAGAAGAACTTTTGAAGTTAAAAATCGATTCAGAGGACGCCGACCCACGGAAAAACC
CACTGATAAACCTGATATACAAGCAGATACCACTACAACAACAATAAAGAGTAATTTA
TATAACAGATACAGGGACAGGCCAAGGTTATCGGAAAGATATCATAAAAAACCTGAGACGGAAACAGAAGATC
CAGTAAGTGAAGATCAAGATTCT
>1002050478_1 Heli.1-DT668617.3.5
GCACGAGGCGCCGCCTCTCGACACTCGATAACTTATCAAAAATATAATTGCATGAAGTCATCGATTAAATTAA
GTGCCGTGGAACCTTTCCGTTGCCATTATATTTATGCCGCACGAAGGATAGTTAGAGTTGCCAAATTGTCCAT
GTTCAAGTTTGACAGTGAACTCTACAAGTGTCCGTGAAAAAGTTTGGTGCGATCCGAT
TGGCTGATTTTGTTTTGATTTTTGTGTAACTTAAGGATGAACGGGACGCCAGCCAGAAGGCCGAAGAGCGCCT
TTTCCGAAACGGGCGTCCGCATAAAGGAGGTGTCAGAAGGCAGGCCGTGTGACAACTGTCCTGAGCTTTGTCC
CGGCTTCATTTCACACCCTTGGAGGAAAACCTGCAAGTCGTGTGGCTGCGACCGCCTC
CAGCACTCAGTGTATCACGAAGAGCTGGGCTCGGTGCGGGACCGCCTCGGTCTGCGGGACACGCGCACCCACC
ACTCCTACGATAACAGCCCACCTGGACTTACTGCTAAACAGACAGAACTATATTGGTCAGCCTTATCAGAACG
AGCACCGAACGCGGTAGGCGGCGCCACCGGCACCGCGGCGTGGCGGGCGTGGAGGACC
CGAGCCTTGGCTGCCCAGTTACCAAAACAGGATCTGTCTTTAGCACACTGCAGGAACATTGAAGAATCCTATC
GTAAACAGTTTGAAGATTTTGTGGCGGCTAGAAATGAGATTGCTTTGGATATCGGACTGGCATGTCAACATCA
CGGCTCCTCGATCGAGTGTATGGGATGCAATAAAACGATCCGCAATGGTAATATTGCT
ATTCAGGCCCCAAGGATCGGTGAAGAGGCGTTCTTTCACCCAGCGTGTTTCACATGCTCGGAATGCGACGAGC
TCCTCGTGGAGCTCGCGTACTGCGCCTGTGATGGACGACTCTACTGCGTACGACATTATGGGGAACGGGTGAA
TCCGAGGTGTCACGCTTGCGATGAGCTTATTTC
>1002050479_1 Heli.1-EL601860.1.5
AAGTGAAAATGTCTGTGGAAGGCATGGAATACGACACAATCGTCCAGAATGGGCCTAAAAAAGTTACCATGAC
AACGGGACTGGACCGCGACACGATACGCGCCGCGTACGAGGATGTGAGAGCCGACAGTTCACCAACTGAGTGG
GCGGTGTTCAAGTTCGACGGCGCCCGCATCGTGTGCTCGGCGCGCGGCAGCGACTTCA
CCGAGTTCCGTACGCAGTTCGCGGACGAAGAGCGCGCTTTTGGTTACCTCAGATTACAAATGGGTGACGAGAT
GTCAAAACGTCGGAAGTTTTTATTTGTCACGTGGGTGGGGCCCAACGTGTCGGTCATCAACCGCGCCAAGATG
TCCACCGACAAGGCGATCATCAAGGATATTATATCTAACTTTGCTGTGGAACTCCCAC
TGGAGAGTCAAGCTGAAATAGATATCGATCAATTCAAAGACGCCCTGAACAGAGCGGGCGGCGCCAACTACGG
CACCGGCGTCAGGGACTTATGAA
>1002050480_1 Heli.1-EL596506.1.5
GCACGAGGGCAAGAGCCATCATGCCAGTCAGATCTCATGGAAACTACTTACTTTGTACGATTTTGCTGAGTAA
CGTTGCTGTGAACAGCACTTTTACTGTTATTTTGGATGATTTGACGTCAGGCTTAGTTGCTGTAATAGGTTCC
ACGCTAGCTATAGTGTTTATAGCCGAAATCACGCCTCAAGCTATTTGTGCAAGACACG
GACTGTTTATTGGTGCTAAGAGTATTTGGATAATGAAGATTGTTATGGGTATCTGTGCTCCACTCGCATGGCC
GACCAGCAAACTTCTGGACTACTTCTTGGGAGAGGAAATCGGTACACATTACAATAGGGAGAGGCTAAAGGAG
TTAGTGAAGATCACGAACCACGTGAACGACTTGGACAAAGAAGAAGTAAACATCATAT
CAGGTGCTCTCGACCTTCGCAAGAAGACTGTCGGGGATGTTATGACCAAACTGAAGGACTGCTATATGCTGCC
TATCAATAGTGTATTGGATTTTGAAACTATGACAGAAATTGTTAAATCTGGTTACTCCCGTATTCCGGTATAC
GAGGGCAACCGCGGCAACATAGTGACGGTGCTGTTCATCAAGGACCTGGCCTTCGTGG

ACCCCGACGACAACACGCCGCTGCGCACTCTCTGCCAGTACTACCAGAACCCGTGCAACTTTGTGTTCGAAGA
TATCACTCTGGACATCATGTTGAAACACTTCAAGGAAGGTCACAAAGGCCACATGGCGTTCGTGCAACGCATA
GAGGAAGGTGACGGCGACCCTGTGTACGAGACGGTGGGACTGGTGA
>1002050481_1 Heli.1-EL600573.1.5
GCACGAGGAGGAAACAGGGAAAAGATTGTCATTACAAACGACCAGAACAGATTAACACCTGAAGATATTGAAA
GAATGATCAAGGACGCTGAGAAGTTCGCTGACGATGACAAGAAATTGAAGGAGCGTGTAGAGTCCAGAAACGA
ATTAGAAAGCTACGCCTATTCAATTAAGAACCAACTCCAAGATAAAGAGAAACTTGGA
TCTAAGATCAGCGATGATGAAAAGGCAAAAATGGAGGAAGTAATCGATGCCGCCATCAAATGGCTCGAAGACA
ACCAAGACGCAGAAGCTGAAGAGTACAAGAAACAAAAGAAATCAGTCGAAGATATTGTCCAGCCCATCATTGC
CAAGTTGTACCAAGGCCAAGGTGGTGTTCCACCCCCAGGAGCTGGAGGAGAAGAAGAC
GACTTCAAAGATGAGTTGTAACCAAACAGACTGAAAAGTGATGATAGCCAAATGTGAAAGTAACGTATGAAGA
ACGGATACGCAGTGTTTTAGCTAGTGTTGTGTATATTTATTGAGTGCGAGGAGTGCGGCGGGTTCTCACCTCT
CTGTCAGTCACTGTCAGTGGCTGTCAGTCAGCCGCCGCGCGCGCGTGTATGGGCGTGT
GCTATGTAATGTATAAAATGATGCATTTTCTCTGTAATATGTAAAGAGAACTGTCGAGGACCTTTTGGTTTCT
AGGTAGATCCCGATTAGTCTCTTGTCACTCGAGACTACCTCAATTGATGATTGTTTGTTCATTCCTGTATGTA
ATTGTTTTGAGACTGCCGTGTTGTTAGGTCTTAATTAGAATAACTAATAAATACATAG
TTATAATTCTTTGGTTGAGTACGGAGGAGTACATAAATAAAAATTTTC
>1002050482_1 Heli.1-EL604350.1.5
AAATTATTAAAAAAATTACTATAAGTAATATTCATAAAATAAATCTAAATAAATAAATTTTAAAATTATTTAT
TTGATAAATATAATAAATTAAAGAATTATTTTTAATAATATTAAATAATCCTGGACCTCTATATAATTCTCTT
CATCCTAAATCAATATTTTTATACAATATTTGACCTATTTTTAAATAAAAATAATTTA
AACCATAAGTTGATAAACTAGGTAAAAATCACATAGTAACAAAAAAATATCTTAATTCATAAAAATATAAAAA
TTTATTTAAAGAATAAATTTTTATATTTCTAATTAAATATCCTAATAATATTCCTAATAAAGTAACATAAATT
ACTATTATCTTTAAATTAAAAGGTAAATAAATTATATAAGGGTAATAAAAAATTATTC
ATCTTAAGGCTCTCCCGGAAATTAATCTTATAAATAATAAAATTATTATTCTTTTTAATATAGTATAATCTTC
ATCATATAAATTATAAATAGATAATAAATTAAAATCATTAATTATTAAATATATTAATAAACGAATAGTATAA
AACATTGTTAAACCCGTTGAAA
>1002050483_1 Heli.1-EL597412.1.5
GCACGACGCTGGGTTCCAGAATATGTAAAAGAAAAACGTTTTGGTAATTGGTTAAAAGATGCCAGAGATTGGG
CTATAAGTAGAAACAGATATTGGGGCACGCCTATTCCTTTGTGGGTTTCCAGTGATAAGCAGGAAGTTGTTTG
TGTGGGAAGTATTGCAGAACTAAGTTCTTTGACCGGCAAAGAAATTACAGATTTGCAC
AGGGAAAATATTGATCATTTAGAAATTCCTTCTGCAAGGCCAGGGCATCCACCACTTCGAAGAGTCCCAGAAG
TATTTGATTGTTGGTTTGAGTCAGGATCTATGCCTTATGCTCAAAATCATTATCCCTTTGAAAATAAAAAAGA
ATTTGATGAAATATTCCCAGCAAACTTTGTAGCTGAAGGTATTGATCAGACTAGAGGT
TGGTTTTACACATTAATTGTTTTATCTACAGCATTGTTTAATAAACCACCATTTAAAAATCTAATAGCAAATG
GTTTAATTCTGGCATCAGATGGGCAGAAAATGTCCAAAAGAAAGAAAAACTATCCAGATCCTTTGGATGTCGT
TGCAAAGTATGGTGCTGATGCTTTAAGACTCTATCTTGTTAACTCACCAGTGGTTAAA
GCAGAAAATCTACGATTCAAAGAAGAAGGTGTTAGAGATGTTATTAAAGATGTTTTCCCTGCATGGTATAATG
CCTTTAGATTTTTAATGCAAAAT
>1002050484_1 Heli.1-DT668651.3.5
ACACGAAACCGAAAATAGCGAAGAACTTCTCACATCAAAATCACTTCAAACAAACCTCGATAGTTATCTTGAT
ACTAACAACAGAATGATCAGCGTATATCATGTAGCCGAAGAAAATATTAAAACAAGAGCTGTCCTTGACTTAT
GCAAAGTAAATAACAACGATTGTGAATATAAATGCGAATTCGACGATTCCACTCTCGA
TTTCGCTTGCATTTGTCCTCACGGACAAGTTTTGGACCCAAATAATCCTAAGAAGTGTGTTTTATTGCTAGCT
AATGGCACTATTCCCGAAACAAATCAAGCTGATAACTCCAGACAAAATGAATTTGATTGGAAAGAAAAAGCAC
ATAACACTCCGGAAACGACGACTGAGAGTGACCCTGATATGAATTTCTCCCATATCTT
TGGTCATTCACATGCGGATGACTCGAAACCTGACCCTGAACCTGAACCCCAACCTGAACCTCAACCTGAACCT
CAACCTGAACCCCAACCTGAACCCCAACCTGAACCCCAACCTGAACCCCAACCTGAACCCGAACCTGAACCCC
AACCTGAACCACAACCTGAACCCCAACCTGAACCCCAACCTGAACCTCAACCTGAACC
CCAGCCAGAACCTGCACCTGAACCACAACCGGAACCTGTATCTAGTGAACCTACTGAAATGCCACAATCTTCG
GAAGAGGAAGCAAAGCTCCAACCAACAGTTAAACCAAAACCGGAATC
>1002050485_1 Heli.1-CX700792.1.5

CAGTACGGTCGGAATCCGGGTCGACCACGCGTCCGGACGCTTAATGTAAAGATCGCAACAAATATTTTGTATT
TATTTTTTTAAAACCGGTATGGGGCAAAAGGCGTTGGCGTTTGCGGTCGCTGCCCTCCTCGGGCTGGCAGACT
GTGGACCTCCAGGGAAACCTAATTTGGGATGGGGTGAGAGGACCTTTGCGATTGTGGA
GGTGAACCAAGCGGCAACGGCGTACAACCAGCTCGTCACAAGAAAAGAGGCGGCTGATGTTTCCGTTTCCTGG
AATGTTTGGTCTGGAGATGCAGCGAATAATGCTAGAGTGTTGTTAGATGGTAAAGAGGTCTGGTCTGGTGCAT
CTGGGGCGGCTTCGTCTGCCACTTTTCCCGTTTCGAAAGGTGGAAGATACCAAATGAC
AGTGGAACTTTGCAATGACGACGGCTGCAGTTCAAGTGATCCCACCGAAATTGTCGTGGCCGATACCGATGGA
AGTCATTTACCTCCTCTGGAATATACATTGGGAGAAAAAAATAAGCCGTTCAAGCAAACATCTGGAAAAGTTG
TGGGTGCCTACTTCGTAGAATGGGGTGTCTATCCTAGGAAATTCCCCGTGGATCGTAT
ACCTATACCTAATCTTACTCATTTGCTCTACGGCTTTATCCCAATTTGCGGCGGTGACGGCATCAATGATAGT
TTAAAAGAGATCGAAGGCAGTTTCCAAGCACTACAGCGGTCCTGTAGCGGCCGTGAGGACTTCAAAGTCTCAA
TCCACGATCCATGGGCAGCATTACAAAAACCACAAAAAGGATTGTCTTCATGGAACGA
GCCTTACAAAGGCAACTTTGGCCAATTAATGTCACTAAAACAAGCCCGCCCTGAATTGAAGATTCTTCCCTCT
ATTGGTGGGTGGACGCTTGCCGATCCTTTCTTCTTTTTAGTTGACAAATCTAAGAGAACACGATTTGTGCAGT
CAGTAAAGGAGTTTCTTCTAACATGGAAATTCTTCGATGGTGTTGATATCGATTGGGA
GTTCCCTGGTGGCAAAGGTGCTAACCCTGACCTCGGTAGCCCAGAAGATGGTGATTGTTATGTAAGTTTGAT
>1002050486_1 Heli.1-DT668928.3.5
TTTTTTTTTGTTTATATTCATCGGACAACATTTTTATTTATTTAAAATGCTTTCTTCTTTACAGATTACTATA
ATATATATTTAAGTTATATTTAAGACTGATGAGTGTGAAATTTATATAAAATGTTCACTACTAACGCGGTGCA
AGCCGGGTCTGTCCCAACTCTTCAATACCAAGAGCACCCTCAAAAATCTCAAGGCAAA
AATATAGAAAATGTTCAACAAAAGACTCAACAAACCCAACAAACACAACAGGAGTTCCCTACATTTTGTTATA
CAACAAATGTTAACATGATTGGTAAAATTGGTACTGGTGCGACTGGTAGTGCAGGAGGTGTTACAGGCGGTGT
TAATATAGCACAACTGACCACTAGTGACGATAAAACATGTTATATAGCGCAACCATTC
TCATACAACTATGCTTTGGTTAATCAAATGCAAATAGCACCAAATGGACTACAGAATACAATATCAAATATTA
GTTTTAAATGTGATGTCTGTGGACTTATGTTTGGTCATCTTACATTACTTAATGCTCATAAAAGGATACATTC
TCAAGACACAGATAACAATATAACTGTAGTAGCGACAGGAGTAAGTTCAGCAAATGAA
GTAGCCATGCCACCTCATATACAAATTCTATCATCAGATCCTAGTGATCAACAACAACATCATGTACAAATCC
AAGAAACTAAACCAGTAGTTCTTGACAAAGTACAGAAATGTATAACATGCGGTGGTCCTATAACTAACAATCC
AAAAAGGAAAGGCCCGAAACTTATAAGATGTGAAAACTGTATAGCTCAAGACTCAGTG
GAACAAAGAAACAATCAAATAAATTCAACGCAAATATTTGTAGCCACAGAAAATAATGTGAAATTTGAAGTTG
GTGGAGTGAGTGGGGTGCAGAATACATCGGATCCTTTAACATCTACAACAAACAATCAGCAGCAACCAACACA
ACAGAAACCTTTACCCGGACATCATCCTGTGAAAAAAAGAAATTTAGCATCTGTTACC
AAGTGTCAAAAC
>1002050487_1 Heli.1-EL596961.1.5
GCACGAGGGTTGTCTATTGTCAGCTTGTAATTGAAACTCGGTAATATTAAGTAATAAATAACGATCAGTTACC
ATTTTGGTGCTAATTTGTCTTTAGCGCTGATATTTCGTGTTTTACAGTTATTATTTTAACGTATCATCAGTTG
ATTATTTAAAGGAAGATATAATGTTTACGGGTTGATCTATTGGGCCAGTCGCCATCTT
GTTAAGGTATTATTTTTGGCCCTAAGCTACAAATATAATTTTAAGAATATTGTTATAAAAATTGTCATAATGA
CCGACAACACGTCTAAAGAACCTCCATTGCAAACAATGAGTGGCATGCCACCGAATCACAACCCGTGGATGTA
TGGTCTTTATCATCAATACAATGGGTATCACGGTGGCATGTTCCATCAATTTTACAAC
CATCAGTACTTCAATCAAATTGGGAATAACGGAGGATTTCATAACGACGGCCACCATTTACAACAAAATAAGG
ACGCTCAGTTTTCCAAACCGCCTCCGCCTTTGTTGGGAATGTCTCCACTCGATACAAATCGTCCATTTAATAA
TCAATCTCCTATTAGGTTTAATCTTTCTGGTAACAGAAAATCAGCTCCCATTCCCCCT
AATGAAAACCCTTTATTAAATAATTATAATGTAAAGAAAAAACGAAAGAAAGGAGGCAAAGCTTGTGATGATT
CAGAGACGATTTTAATCCCGCCTCTACCTGATCAT
>1002050488_1 Heli.1-DT667589.3.5
GCACGAGGATCACCTGGCGCTGCTGTCGCTGTGTCGCCATACGTACACACATGCATACATGTTGTTAGATGTG
TGAGTGGATATTTCGCTTCAAAGTACGGCGGTTGATCGGTGGGTAGTTAAATATCGGGTTACTTGTTTCCGTT
TTGCCTTACTCCGTTTAGTTAGTATCGGATCGAGTGCGGTGGGTGACCAACGTGGCAC
GTCCAGTCTGTGGCACGCGTGCGCGTGTCCATTTAACTTTGTACATAAATTTAAAAAAATACCTTTAGATTTT
ATTGTCATATGTTTGACATATGTTTAATTTTATTCATCTAAGAAAGTACAGATGGCTGTTGTTATTTTTATAT
TATTTGGGTAGAATTATATTATATATATATTATTATTTTTTTGTAAATTACCTCTGTA

TTTATCGTTTCTAAGCTTTGTAAGCCGTAGGGACATAGATTAATTAAATATACGAGTTACGAACATTGTGATT
GATCTAATTACATTGGTTTTGTATTTACTTATAATTTCGTTCTGCGTATAGGATTTTACTTCGTTTCGACAGA
TATATCTAATAATTTTTTGTTACTTCAGAGTGCAATATGTACAAATATTTACATATAA
AATATTAATTGTACAGTAATTTTTTTTTTAGTTTTTAATGTGTCAACGCAATATCCATCGCTGTTCATTCGTT
GAATGTACACGGGAAGCATAATACTTGTATCACAGGTGGGCCCAGAG
>1002050489_1 Heli.1-EL603756.1.5
CGGCACGAGGGTTTGGCTAGCTGATTGTGGATTCGTATTTTGTTCGAGTGTGTTATCATATACATTGGCGATT
AATTTTTATATTTAGAATATCACTATAAAAATGGCAATCGGAGCAGGAATGTCGTGTGTGAAGTACCTGTTAT
TTTCTTTCAACCTTTTGTTTGCTATAACAGGATTGATAATATTAATAGTTGGAGCAAA
ATCTGAGATCAATGCCCAGCCATATGTGAGTTTGACTGATGAAAGCTTCTACACATCAGCCCCAGTGATCCTG
ATTATAGTTGGATTGATTGTTTTTGTTGTCGCCTTCTTTGGATGCTGCGGAGCTGTGAAGGAGAACCATTGCA
TGATTGTTACATTTTCAGTATTCCTGCTATTAATCTTCATAGCGGAACTGGCAGTGGG
CATCGCAGGCTATGTGAAGCACACGGACCTAGAAACGTCCATCGTTCGCACTCTCAACGAATCGATCACCCGG
TACTCCTCAGATCCTGATATTAAGAAGAATTATGATATCATCCAGACTGATCTCCAATGCTGTGGTGTGAATG
GAGTAAACGATTGGATTTCCCACGGCCTCCCCATACCGTCAACATGTTGTGCCGGACA
GGAGATTTCCAACTTCAAGACCGTGGCCTGCACCGTGGACTCCCCCTCCCTCCACACGCAGGGCTGTCTCCCA
CTTATCCTGGCTCGTTTGAAAGATGTGGCTTTAGTTCTGGGCGGTGTGGGACTGGGCATTGCGTTTGTACA
>1002050490_1 Heli.1-DT666729.3.5
GCACGAGGGATTCCTTCCTACTTCGAAGACGCTCGATTCATTACACGAACTGGCGTGTGAAAGGGTGCCGAAT
AAAAATTCACCTTCAATATCCTCTAGTGGATATGGTTCCCAAGCAGTATCATCGACAAATCTCACTAACGACG
ATACTCTCTCAATCCGAAGTATGTCAGTTGACGATACACCGGACTTTGACAAGACAAT
GGACTACACACAACTTAGCCGGACAAAAACCTCCATGGCGGGCCTCAGGAACGAAATAACTGAACTTAGAAAC
GACATAAGTGAACTGAAGAATGAAATTGTTGAATCAAAGAATGAACTGGAAGAGGCGACTTTTGAGAAAGCCA
AAACTCCAGTTTTGACGCCAGGATCTCGGAATAGAATTAATCCTTTCTTAAGGGATTG
TGAAGAGATATCTAACAAGGAAGCACCCTTGGTGGACCCCCTTGGAGCTATTCCGATAGAGAACGCGGTGTTG
TCTAGTCAAATAAATAAAGATAATGGACAAGTGAATGGTGAAAACGCTCATGAGACCTCTTTTAGTGAAGCAG
ACGTAGAGTCGGTGAGCTCCGAGCAGTCGAGTCACGAGACGCACAGCCGCGAGGCGTC
CTCCGTGGGGGAGAGCGACTCGCCCGGACCCGACCCGGGCCTCAACACGTCGCTGCCGGCAGGGAAGGTGGTA
CGTCGTCGTGCGGGCGCCTCAGCGCGGAACGCGTCGCGCGCGTCCTACCCGGCCGCGCGGCCCAAGTCCGCGC
ACGACCCCATCACCGCCGACAGGAGTGGGGATGAAGAGTCGCACGACAGCCGCGCGCC
GGCCAGCTGCGCGGTGCCGGAGTGGCTGTCGCTGGGCGAGAGCGTGCAGCTGCGGCTGAGCAGCAGCACGGGC
GTGGTGGCGTACGTGGGGCCCACGCACTTCGCGCACGGCGCCTGGGTCGGCGTGGAGCTGGACGCGCCCACCG
GTAAGAACGACGGCTCGGTGGGCGGCACGCGCTACTTCTCGTGCCGGGCGCGTCACGG
CATCTTCGTGCGCGCCGACAAGCTGGCGCAGGACCGGCGCGGCCGCTCCGCGCGCGCCTTCCGCGACGCAGAG
CTGCGCCGCGCCGCCAGCAAGGGTGAAGGACTACACAACCTGCACAGATCAAGGAGCCGCGGGGACAGTATAA
ACGCGGTGGGCACCAAGAGCCGCGCCAAGTAGACCGCGGCGGGCCTCAGCCACGTCCA
TTCACGTTTATTTTAAGTTGCGATCCTGTAACGCGTATGGTGCTTATAATTTATCATATTTTATCATGTTTTA
CCCTTTTGACGAAGCAATATACTTTATTTATATCGTTGCGAGTGTTTTGGGGAAACTCGATACTCCTCCCG
>1002050491_1 Heli.1-EL598645.1.5
GCACGAGGTGAAGAATCATTAAAAGTTTGGACGAAATAACTTACCGAAATAAAGAAACAATGTCGAACGTACA
TACTGGTGAATTATTAAATTTTCAAGACTATTCTCCGGAGCATAATGAAAGAAGTGCTAGAATCGACGTAGAA
GCTGTTCACACAAATCAATATAATAATCCTATGAGCAACTCAAGCTATTCCTATGAAG
AACCTCAAGTAGTTGAGGAACCGCAGACTGCTTCACAAACTAATCATAACTTCTGGACAATAGAATATTATCA
GAAGTATTTTGATGTACGGACAAGTGAAGTGGTGGAACGAATTATTTCGTCGGTGTTGCCTCAAAAGGTGTCA
CGCAATTATTTTGATGAAAGGATAAAGGGAAAACCTGACCTGTATGGACCAATATGGA
TTTCGGTGACACTGATTTTCACCATTGCTGTTAGTGGAAATATTGCAAATTACCTACAAAGTATAAACAAAGC
TGTAGTATGGAGGTATAATTACCACTTAGTTTCATATGCAGCTACTGCAATCATATGTTATGTATGGCTTGTA
CCTTTGGCATTATGGGCAGCTCTTAAATGGACTACTGTGCCCGATGGACAAGATGATA
TTGAGACTCAGTCATCAACTTCACCAACAATGATATCATTATTTTGCCTATATGGATACTCACTATCTATATA
CATTCCTGTTGCAATACTATGGACCATACAAATCTCTTGGCTCCAAT
>1002050492_1 Heli.1-EL600816.1.5
GCACGAGGCCTCTGCTTGACATGATGGAGCGCCTCGGCGGATGGCCGGCGCTGCAAGTCAGTTCATGGGACGA
AGCTTCCTTTTCCTGGGAAGAATCTGTGTACCGCTTCCGCAGAGCGGGCTATTCCGTGGACTACTTCCTTGAT
TTTTCTATCAGCGTGGATGTAAAGAACTCTACTAAGCGTATTATAGATTTGGACCAAG

CCTCCCTTGGATTAAGTCGGGAATATTTGAACCGTGGATTCAGCGACAAATTAGTGCTTGCTTACTACGACTA
CATGGTGGACATCGCTACTCTCCTAGGAGCTGATCGTGCTCGAGCTGAAGTGGAACTCAAGGATTCTCTACAG
TTCGAAATGAAACTAGCCAATATATCACTACCGTTAGAAAAACGTCGTAATGCCACCA
GTTTATACAACCCAATGACAATAGGCGAACTCCAACGTAAATTTCCAAGGATTCCATGGCTGGCTTACATAAA
TCGTCTATTATCACCTCACGTGCAAGTTGGGTTGGATGAGATAACCATTGTGAATGTCCCCAAATATATTAGC
GATCTCGAGGACCTCTTGGATAAAACACCAAAACGGGTTCAAGCCAACTACGTTATGT
GGCGAGTCGCAGGCGCATCCATATCTTACTTGACTGACGATCTCAGGAGGCGTCAACTGGCGTACGTCACTGC
CCTCTCTGGGAAAACCGAGCGCGAAAGCCGTTGGAAGGAGTGCGCTGACACTACTAGCGTCAGTATGTCTATA
GCTGTGGGAGCTCTGTACATCAGGAAATACTTTAATGAAAATTCAAAAGCAAACGCCT
TAGAAATGGTTAATGATATACGACAACAGTTCCGTAAGACACTTACTGAAGTGGATTGGATGGACGAGCATA
>1002050493_1 Heli.1-DT665710.3.5
GCACGAGGAGCACGATGAAGGCACACGTAATTATCATCCTCCTAATAACCTTCCAAATGGCAACAGCTACGGT
AACATTAGACAAAATCACGGAAAAAGTTCAAGCCGGATTAAAAACTTTAAAGGAGAAAACGGTCACTAAGCTC
CATAAACTGAAAGAGAGTATTGATTTGAGAAAACTGCATCAATTGGACGTCCTACATT
TATTACCTTATGCGCATAAACACTATGTACACGAGCACTACTCTAAGAAGACGCCACTAGTTATAGATCAACA
TCAGGTTGAAAGGTTGAAGAAATATTTCGAGCCACACAAGCCAGCTGTACATTACCACCATTTAGAGCATTAC
GGGCATTTTTAGATCATTTTGGCCCTTAATTGAGATGAAACATTCTTTAATTCTTTAT
AAATTAAGATTTATTTATTAAGCTTGTATTTTGCTGACAAAGATCTGTTTTAATAAATGTATAAAGTTTCGTT
ATCTGAAATTATATCTAGCGTTTTGCGTGATACCTATTCATATAGACTTTCAACTTAAAATAATAAATCACTT
TTATGATTGTTTACGTGTTTGGCTGAAAAATAAGGGTGTTTGGTATAATACCAGGAAA
AAATTGCACACTTAGTGTAGAAGTGATTTATCTCAATATGTGATTTTAATTAAAAATATAAATTAAAATCATT
TTCTTTCTCGTGAGTTATTTTGATGGGTACAATGTGTAATTTGTTGGCACAATATATTTTTTGGGTTAAAGAT
GTATAAAATAAAAAATAATGAA
>1002050494_1 Heli.1-DT662702.3.5
CCCCTATAAGCCCGGGCTGCAGGAATTCGGCCGAGGAGTTTACCCTGAAAGGCCGTCTGCGCCGCCCGGTAGG
AGGTACCATGGACGCGGACAGTGCACGAACACACGACTCTATCACGGACGCTTCGACAGTCATCGTGGAAAAC
GGAGAAGAAATTGTAAAAGTTAGCAAAATGCAAGTACCTAATGGCATAGATAGTAAAA
CATACTTAAATGGACCTGACCAGATTGTACCAGCCGACAGCTATGTATGTTGTACACCAGGAGGTCATGTAAA
ACTCCGCATGGGGTCGCGAGGACCTGCAGCGGAACCTCCTCTGTCAGTGCCTGGTCTGCTTAATAGGATGGTT
TTTCGTTATCCTAACGCCGCCGCCCTAGTCACAAAGAAACAAGACGGAAAATGGCATA
CACTTACATATAAGCAATACCAGGACAACGTACGCACAATAGCTAAAGGTTTTCTAAAATTAGGTCTTGAACG
ACACCACTCAGTATGCATTTTGGGATTTAATTCCGAACAGTGGTACATCTCCGACCTTGCGGCTATTCATGCT
GGAGGTTATGCAGCGGGTATATATACAACTAATTCGGCAGAAGCGTGTTTCCACTGTC
TTGAAACATCAAGGGCTAATATTTGTACTGTTCAAGACAAAAAACAGCTAGAAAAGATACTCTCCATACAAAG
TCGACTGAAACATCTTAAAGCTATCGTACAGTGGGAGGGCCCTGTAGACACCTCCGTACCTGGAGTGTATAGT
TGGGAACAGCTAATGGAAATGGGTTCTAAGGAACCTGACACACAACTTGATAACGTTT
>1002050495_1 Heli.1-DT666798.3.5
GGCACGAGGCAGAGTACATTTTAATATACACGGCGTCGACACTTTTTAAAATAATTTTAAACTGCAGAAATAC
TTGTAATTGATAAATTACGTATTAATTAAGAAATAATTACTAAATAGACTTGTTATTTAATAAAATTGATCCT
AAATATAGTTCATTTTACCTATATTACGTGTCTTGAAACTTCGACATGGCTACAGACG
ACAACGCTGAGATTGATTGGGGAAACGAAAGATTAATTCGAGCTCAAAGGGCAGTGGAAGCTAACATTTATGA
TGTAGACTCATGGTCTCTTTTGATCCGTGAAGCTCAGACAAGGCCTATCAATGAGGTTAGGACTATGTATGAG
AAGCTGATTGTTGCTTTTCCTACAACAGGCAGATACTGGAAAATTTATATAGAACAGG
AGATGAAGGCGAGAAATTTCGAAAAAGTAGAAAAGTTGTTTCAGAGGTGCCTAATGAAGATCCTTAACATAGA
ACTGTGGAGACTGTATTTGAACTACGTTAAAGAGACAAAGTGCATGTTGCCGACGTACAAAGAAAAAATGGCC
CAAGCCTACGATTTTGCGCTAGACAAAATAGGTCTAGACATCCACGCCTATCCTATAT
GGAATGATTATGTTACGTTTCTCAAGAGCGTAGACGCTGTGGGCTCCTACGCTGAGAACCAGAAAATATCTGC
TGTTAGAAAGGTCTACCAAAGAGCAGTTATAACACCCATTATTGGAATAGAAACACTATGGAAAGATTATATT
GCGTTTGAACAAGGAATAAACACTATAATTGCTGAACGTATGGCAATGGAGAGGTCCA
GAGAGTACATGAATGCAAGGAGGGTGGCTAAAGAATTAGAAACTGTTACTCGAGGCCTGAACAGGAACATGCC
GGCCACCCCGCCTAACGTGGACAGGGAAGAAATGAAACAGGTGGAATTATGGAAGAAATACATAACATGGGAG
CGATCCCACCCACTTCGTTCAGAAGATACTGCGT
>1002050496_1 Heli.1-EL603959.1.5

GCACGAGGCTTTGGTGTAGCGCTGTTACCTTTTGTTCGTATTTATAAGGTTTTAATTTATTTACTTTTAAAAT
AATACCTATAATAATCAGTAAGTTTTGGGTTCTGTACAATATTGCACGGAGAAGAGGTTGAACAAGATGATGG
ATGGAGTAGTACACCCTCATCCTGTCGTCCATGGTGGTGTACCAGGGGTCGTCCATGG
TATGCCTGTACATGGAGCTGGACCAGATGGGGAACATGGACCTCATGGTCCTCGTCGTCGCTACCAAAACAGA
CCTAAACCTCAAAACAATTTAGAGCGTCTGCCACTTGATGAAGCGGCCAAGAGGGAAATGGAATCCCTCCCAA
TGAAAACACCAGTGTCGGTACTGCAGGAGCTTCTCGCCAGGCGCGGTACGGTACCCAA
GTATGAACTGGTTCAAATCGAGGGCATGATACATGAGCCCACCTTCCGTTACCGTGTCACTGTTGCTGATTTA
GTATCAATGGGCACTGGAAGATCAAAGAAGGAAGCAAAACATTCAGCTGCTAAAGCATTGCTAGATAAATTGA
CTGGAAATGCTCCGGCCGATCAATCTACTAATGGAACAGTACCAGAAACCAGTGGCAC
TGTAGTATCATCGTTTGAAGATAAGCTGATGGGGAACCCAGTGGGATGGTTACAAGAGTTGTGCATGTCTCGC
TTCTGGCCACCCCCTTTGTACCATGCTGAAAACGATGATAATGTTGCTAGACGACTGCCTCATGAACGTCAAT
TTACGATCGTGTGCACTCTTCTCAAACGTCGCGA
>1002050497_1 Heli.1-DT665381.3.5
GCACGAGGATTTACTATGGTTCAGTAATCATAATAAATTGTCACATCTTTATTTGAGTGTTATTTGAAAATTT
ACACATCTTGACTAATAAGAAACAATTAATTTTCCAAAATATTGATACAATGAATAGAATCTTTAATAAATGT
CAAAAACAATTTATATGAAATTATCTTTATTTATGGATATAGTAAATTTTTGTATGAT
TTATTAGACACAAATTCAAATTTCACCACATCATAACAATTTTTTTGTTGGAAATTTAGTTATCTTATGATTT
TTTTTTGTTTCTACATATTTACACCATTGTACATGTACACTAGATTATGATAAATTAATTACAATTTGAATAT
CCACGTAATAAAGGTGTAAATAAATTATATTAGTGTCACGATTTTTATTAGCACTTGT
ATTCAAAAATAATTCTGCTTTGAGTCTGTATTATTTATATAATACTTATGTACAAAGTAATTTTATTTGTTTT
TTTTTTAATTAAAACTACTTTTAAATGGATTACAATGACATATTAGTAGCAACTTTCCTATCATTTGCTTATT
ATAATAACCACAGCGTGTTAGGATTTCGAAGAAAACAGGCGCGAGTCAAACACTCATG
TCGAGTCGTGCACAGAGTCGTGCATAATATTTAATATGCGGTCGAACGCATCACTTTGCGTCACATAGAAAAC
AATCTTAGTAAATAATAGTTATTTACGTTTTAATAGTAACATTATAATAATATTTGTGTGTTAGTCTTAAGAA
ACACAAAATC
>1002050498_1 Heli.1-EL598291.1.5
ATATAAAATATTATTAAATAGCGTATTTCACAAAAACCGCTATATTGGTGACCCCGAAATTAGTGAACACTAC
ATTAAATATCTACGTTTTCTGCTGGCAAGAAGATTAAAAACGGCAAGAGGCCATATTGACGACCTTTGCAAGG
TGACCTCTAGTACACGATGGAGGACGCGACTACATCCAACGTTTGTAGGATTGGCGTG
CGTATTCCACCATTCTGGCCGGAAGAACCAGCCCTTTGGTTTTCACAAATTGAAGGTCAGTTTGCTTTATCTA
ATATAACAAGTGATGCTACAAAATTTTATTACGTTGCCGCTCAGTTGGACAATCAATTTGCTGTCGAGGTGAA
GGATATAATAATTTCTCCTCCCGAGACGGATAAATACATCACTCTTAAAACGCAGCTG
ATTAAAAGATTATCTGCGTCTCAAGAAAAGAAGGTGATGCAGTTGCTGAGGCATGAGCAGCTAGGTGATCGCA
AGCCATCGCAGTTTCTGCGATACATACAAGATCTTGCGGGTTTGTCTGTCCCCAAAGATTTTTTAAAAACATT
ATGGTCTAGTAGATTACCGCAAAATATTCAAACTGTTATAGCTTCGCAGCCTGATCTA
CCTATAGAAAAATTAGCTGACCTGGCAGACAAGGTGCACGAAATAGCACCCTCATCACCACAAGTCGCAAGCA
CGTCTGCGATGGCCACACCCCCTGCACCCTCCATGTTGGAAGAAATGGCCAAGCAGATCCAACTCCTGACCAA
GCAGGTATCTGAACTCTCGACCCAGGTACGTCATAATTCGGTACCATTCAAGTTCAGA
GAGCGTTCCCGGAGCCATTCCAGGCATCGTCGGAAGTTAACTTCCCACCAAAAATGTTGGTACCATTTTAAAT
TTGGTTCCAACGCAAAGAAATGTGTACAGCCATGTACTTACGGAGAGTCGGAAAACTCTCTGGGCAGCCGGAA
GTAGCGGCCAACGTCTGCCCTTTACCTACAGGCCGCCTTTTTATTACCGACCGTATTT
CAAAAATGCTTTTCCTCATAGATACTGGTTCTGATCTCTGCGTTTTTCCACGCTTGGCTACCAAGGCGCCCTG
TACCAAGACGAAGTATGAACTTATGGCAGCCAACGGTACAGTCATTCACACTTATGGTCCTACGTTATTACCT
TTGGAGCTAGACTGAGACGTACTTTCACCTGGAAATTTACCATCGCTGATGTTTTAAA
GCCGATTATTGGCGTAGATTTTCTGATATTTTACAATCTTCTTGTAGATTGCAGATATCAGCGCCTCGTCGAT
GGAACAACTACACTGTCAGTGCAAGCACTACAAGAAAATATTTCCGATGACCTTGCGTCAGTCAAAGCCATCT
CAGGAGAATCAAAATTTTTATCAGCTGCTTCATGAGTTCCCAGAGGTAACGAGACCAT
CAGGTACGTCTTCCCTACCTAAGCATAATACTGTACATTACATCAAAACCATGCCCGGACCACCAGTTACCAG
CAGACCTCGTCGTTTAGACCCCGTTAGACTGAAAGCAGCCAAAAAAGAATTTGATGATATGCTCTCACAGGGA
ACTGCCCGCTCGTCTAACAGTTCGTGGTCTTCCGCCCTGCACTTAGTTAAGAATAAAG
ACGACGGATGGAGACCATGTGGTGATTATAGAGCACTCAATGCTCGCACAGTTCCTGACAGGTATCCTATACG
CCACATCCAAGATTTTTCCTACCAACTCTCTGGTTGTTCAGTATTTTCAACTATCGACTTGGTTAAGGCGTAC
AATCAGATACCGGTACACGTCGATGATATCCACAAAACAGCCATTACAACTCCTTTTG

GACTTTTTGAGTTCCCGTACATGACCTTCGGACTGCGCAATGCAGCACAAACCTTTCAAAGGTTCATGGACGA
AGTACTACGTG
>1002050499_1 Heli.1-ES586040.1.5
CCTGCGGTACGGTCGGAATTCCGGGTCGACCACGCGTCCGATCTCTTATTAATATTACGTGTTTATCACGAAA
CTACTTTTACTAACTTTTTAAATACTACACTACTTAATTTGAGTCACAATGTCCGGTGAACCTGAAGTAGCTC
GTACCCCCGAAGAGGAAGAGACTGAAGAAGAAGAAATCAAGTCCTCGTACAGACCTCC
TCCAGAAAAGACAATCGAAGAGATCTTAGCAGCCGACCAGGAAGATGAATCACTAAGGAAGTACAAGGAGGCG
CTGCTGGGTCAGGCACAAGCGGGTCCACTTATAGTTGATCCAAACGATCCAAGAAAAGTCATTGTCAAGAAGT
TGGCGCTGTGCGTGACGGACCGGGAGGACCTGGAGTTGGATCTGTCGGGAGATCTGAC
GGACTTAAAGAAACAGGTGTTCATAATAAAAGAGGGCGTGCAATACAGAATAAGGATCGATTTCATAGTGCAA
CGTGAGATCGTGCACGGCTTGAAATATGTGCAGAAAACCTATCGCGTCGGTGTGCCAGTGGACAAAATGACAC
ATATGGTGGGCTCGTATCCGCCAAAAACGGAAATCCACTCGTACACTACCCCGCCAGA
GGACGCCCCGTCAGGCATGATGGCGAGAGGCGCCTACTCTGTCAACAGCCTGTTCACTGATGACGATAAAAAC
GTGCACCTACAATGGGAATGGAGCTTCGAAATCAAAAAGAACTGGAAAGACTAAAAAAACCAAAAAAAAACCA
GATGGGATAAGTATAAATAAAATGGGATGAATGGCTGGGAACCATTTGGCACGTTACA
TCTTATAAAAAGAGGAAATTGATATTTGAATTTGACATTGACATTTGGTTTGTGACAGTCTGTGCCATATCTG
TCTTGTCTATACTGTCTGTGGAAAGTACTTCGCAC
>1002050500_1 Heli.1-CO729590.1.5
GCACGAGGGTGCTGAATACTTGTTCGCCGTGTCCTCCAAGGAGCTGATGCGGGAATGGGTCGCCAAGCTCGGC
TTCCACGCGCAGCTGCCCCCGCAGCTGCAGCTAACGCCCTACCGCGCGCCCGGGGACTCCCCCAATGCGGACG
TCAAGAGGAGGTTGCAGCAAAATGCCTCATCATCATCATCAGCAGCGTCATCTCCAGA
ACCTCAGCGACGTACTCGTACCCAAGCGGAAATACTACAGGAACATCGTGACAGCCAACGAGCTTCCTATACA
ATTGAACAGTCGAATTCCGGGTCGACCACGCGTCCGATAGAATCGTCAGTGCTACCATCTCTACCTCCGCGAC
AACCACCACCAGTATTAGAAGACGCTGCTGAAGTTGTGCTCAGGTCGGAGCAGGCGCA
CACATGGGGACGATCGCGATTCTCCAACGGAAGAGACATCAATGCTGACTTCATACGGTCACAGAAAGACGCG
TATGGAGGCAGCAGCGGTAGCAGCACTCGTCCCGCCTCCGTAGCGGGCAGTGGTGGATCGCCTGCTTTAGATC
AGAGACCGGCCTCGAGATCGTCAGGGGAATCAGAGCTGTCAGTGGGTTCCGATAGGAA
CTCGAATGTTCCAGAAAGGAACTCGAACATTCACGAACGTAACGATCGAACTAACAATAACGATCGAAGTTCT
TCCGAGAGAAGTGAGAAATCAGTGACAGCGCTAGTTAATAGCTATCA
>1002050501_1 Heli.1-DT665213.3.5
GCACGAGGGTCCGATACCAATAGTAGCGCATAAAATAAATATGGCGAAAGTAGAGATGTCGTGTCTAGCTCGG
ATCCTGATAAATACCAAAGACTTGTTATTGTTGATTATTTTGGGTTAGTAAATTAAAGTGCAAGTGTCAGTTT
TATTATCACAATATTCTAGTTTTAGTGTTGTGTATATTTTTTATTTTAATAAAATGGA
TGATAATAACATATCAGCAATAAAAGATGGCGATCCAACCCGATCTCTTGTGTTAGTTGAAGCGCCATCTATG
TTGGAAGTGCCAAAACATACAGTGGAAGGAAGTTTTTTAAAGAAGAGGAGGCGCGGAAAAACAAAAAGAAAAG
TCTTGAAACCTTTTTCTAAGATGCCTTGGCAAGATAGGCGCAAACTTGATAATACCAA
AAGAAATAACCGTTTTCGAAAAGTAGTTTTATCGAAAACACAGGCTCCTTTTAATAATAATCAATTCTTAATG
GAAATTCATAAGCCAGAGCCAGAAAATAATTTTCACCTTTTCAGAACACCCTCAGCTCGGACAAGAGATTCAA
GTTTTAGTGTTGATTCTGAAGAAAATTATTTTTTCTCTCTCCCAGAAGACGAAGAAGA
GTACTTGACTAAAGAGTTTTCAAGTGTGTATGAAGATGCTCAAAGCGAACGTCTGTCTAATATGTCTAAAAAT
GAATTAATACAAGAATACCTTTTGCTCGAAGCTAAATATGATAATCTTATTAAGAGAAATGAGCGTAATAAAA
TAAAAGATTATGATGACGATAAAAATTCTACTGGGAAAGATATATCTCTAATTGACAA
GGATGAAATGTCAACTGACAAAGATACTTCCGGTATTTCTATTGTATCTGGATTTATAGAAGAACCTTCTTTG
TCGGATATAATGTTACGATTAAAGGAACAAGAACAACAAATCGCAGAACTTCAATTAGCCAATGAAAAACTTT
TACTTGAAAATAACCATTTAAGGACAAGAAATCAGGGCTCAAGTGAAGATTCGGAGAG
TGATAGTTCATCTACTTCAGACAGCTGCAGTAGTTCTAGTTGTTCTAGTCCAGAGCATGAATCAGTAATGGTA
AATGGAAACATTACTGATCCTGCACTTGTAAATGGTGATTGTCACAGTGAAAATGGACCCCAACCCCTAGTTA
ACGGGTTCCATAGCCCAAAGGAATAAATAAAATTGTGATTAAATGTGTTTCTGAAAAT
GGACAAATTTGTTGAGTTATCTTGTTAAATGACATAGTATTCTAACATGTAATAAGACAATGTGTTTCTTGCC
TCTGGTTTGTGTTTGTTCTATTCCATGGTATGACTTGGGAGACTGGTAGAATGTAATTGTGAGATCTCACTTT
AGGTTTAGTGTACCAACTGTGATATCCATGTGGA
>1002050502_1 Heli.1-DT665482.3.5

GCACGAGGAGAACCTTTAGAGTGTCAAGTTAAATAATTTAAGAACAATGAAAAAATTTGAATTTTCATCAAAA
GAGAAAGAATTTCCAATATTTTCTGGAAAAATGTTCTATGATGTATATAAAGATTCAGAATTTTTGAGACATA
GTGACAATATTAAGACTATATTATCAAAGGGGTATGAATTAAGGCAATCCCTTAAAGA
TGTGAAGCCTGGTGAAGTAATAGCACTTAAAGAAATGTATCTGGAAAACAAAACACCTTTGCTGGTGAAAAAA
ACTGGACCAAATGTTTTAATTGAGAACTTTGAATTCACTGCGAATCGACTCTCTGGCTTAGTAGCGGCCTATG
CTTTTGAGAACCGCCACAGATTTCCCACTTTGCAATCATCCGAAGCCCTCACATTGGG
ACTCACTTGGAATAATGAAAACGAACAAAGATGTAAATTATATCTATCAGCTGTGACTGGGACAGAACATTTT
TATGAACAATTCTTATATTGGCCCTTAGTTTGTGCCATAAGAAAATTTCAGCTTAAGAAAATGCCTTTAGAAC
CAGTTGTCAAAATAGCTAAAGTGAAAAACCCCGAAGGTATTCAACTTGCTAAAGTTAT
GATGAGAAATTTGGATTCAGTGAGAATTATGTGGAGTTTGTTCCCCGGATCTAACACTGAGGAACTGCAACAG
TTACTGGCAGCCGCACCCCAACAAGCTCAAACTTATTTTCTTTGCTAAATGAAAATTTGAAAACAAATGCA
>1002050503_1 Heli.1-DT665492.3.5
GCACGAGGGTCGCCGGCGTCGGGCGCGCTGCCACCGCCGCGGTTGCTGCACACGATCCCGTCGGCGGCGGAGA
CGGAGGCGCCGATGGAGGACGCGGACGTGCGCTGAGGCCGCTCGGAGTCATCATTCATCACCGGTCGGAGGCC
GGAATCAGTGTCTTATCATACCGTCCATTAACAGTCGGCCTGCAACTCTCGCGCGCCG
AGAGGTCAGCAGCCTTACAATGTTTCCTCCATAAATTGCAGTTCACAAACGATCCACCGATACCACTATACAA
ATGTAACTTCGATCGTATAGCCAGTTGTCCTCAGGTCTTCTCATAGCCGAATATGTTCAGTCTCTTTTGTACA
GTATTAAAGAAACCATGACAATTTTAGTGATAGAAAATAGTGTAACGAAGATACAATA
TTTATTTATAAAAACAACATTTTATTTGATAAGGCATAAAGTTCTAAATTGATGTAATCATAGCTAGATCGTT
CATATAAATGTGCGTGTTTATTTACAAAGTATATAGATTATTAGCGTTAATAGTTGAAAGTGTACATAAATGT
ATGTATCGTTGAAGCTACGGCCGCGCGCGTTGTTGACTTGTAGCGCCGGCTGAAGCCT
TACGTCCGAAAGGACCGAAGCAGTATTCGACTACAGTATCTATTAATTAATTATTATAAATAATGTTACGTTG
ATGGTAAGTTAGATTTTATATGACATAGAGTTTATAATGGCTTTGCTGAGGTGTCACCGTCATGTCGTACATG
TTATAATGTAATTAATGCCTAAATACTACAAGTGAACAGTACCTCG
>1002050504_1 Heli.1-EL602732.1.5
GCACGAGGGAACACTTGGTGGTTTTTTGTGTACCTGCTTAATTTGTTTTGTAAAAACATTGCCTTAGAAATTC
AGCAAATAACGAATGTTTTTCTGGTGCCTTCGTGTTAAATATATTAGATGATTAGAAGTTGTTAAGTATTAAT
ATTCATTTATTTCTATGAACTCAAAAAGCGAAACTGACGAAGCTTCAAGGGGGAGCGA
AGCATCATCGTCAAATAACAATTCAACCCCAAGCAATTCTAAGGATCATTCTGCGCCAGGTTTATGCGCCGAT
TCAGGTTCAAGCAATACTCAAGACATTATGAAGCGGGCTGCGGCGAGACAATTGATTGAAAGATACTTTTATC
AGTTGTTGGATGGTTGTGGTAACCCTAACTGTGACAACCAATACTGTGCATCAAGTGG
AGAGGCAAGAAATTTAACCCCAAATGAAGCAGCAGCTGAAGCAATAAAACTTTTTTATAAAGAAGCACGCCTT
TGTGATACTTTACCTAACAAGGTGCCAAGAACAGAAGCAAGTACTTGTGATTCTAGTGCAAGCACAACTTGTA
CAACTTCTAGCCCAAAAGATAAATCAAATGAAACGGAGAAAAAATCAGAGTCTGCATC
ATGCAGTACATCACCAACAGGGAATAAAATGGAAGCAATTCCTGAAGCTAGTCAAAATGTATCTCAAGAGGAT
TATAAAACTCTAAGTCATAATAATGGTGTTCCACTAACAGAAGAACGTATATACGAGTTGTGCAATGAATGTC
TTCAAACAAAAAAACCACAGTC
>1002050505_1 Heli.1-EL598708.1.5
GCACGAGGGAGCAACATGGCGGCACTACAAAGTTTACCTGTAAGTTAACTATTCAAAATTTTACTAGTATACA
AACAACTTTCATAAATATTATCTTTTTGATAGTTGCTGATTTAAATTTTGATATCATAATTTTATCGTATGCT
ATATAGTACTCGATTAAATAATAATATTTTATAGAATTATACTTAATAAACTTAATAA
GATTAAATCAACATCACTTTAAATCTTAGTAACGATTGAGTTGCCGACTTAAGAACGTTATGCAAGACGTTGG
GAATATGTGCCTTATGATAATACTTCTTGACAAATCATAAATTTTTCTAATGTAATGTTATCTATCTATTACT
TAAGATGAAAACGTAAAAAAATATTATTTTGAATATAAATGTATAGTAAATTAAACAT
TCTTTTTAGGTACTAAAATTTGGATACTGTATTTATTATTTCTTATTTATCATATTCAACATACATCTTAAAA
ACAATAAAACACTATAAAAAAATTACTAACAGCTATCAATTTCTCATAATATATTATACGAATAAGAAAATAA
AAAAATCTAAACTTAGATGATTTTTTTATAATCAATTATTTTTTAAAAATCAAAACTT
AGATAACAAGACTTAGAATATTTTTTAGACATTTTTTAATAATGTGACGTTTATGGTATTGAGAATGAGTAG
>1002050506_1 Heli.1-DT665218.3.5
GTTGGATTTGATTGTTTGAGCGTTATGATTTTTTTACTGTTTTATTTTAAAGTGACGTTCGTGAATCATTACT
TGTGATGAGAATTGAGGTGTAATAAACATTTTTTGTTTTGTACGTAAATTTAAATGTACTTGCAACAGCATAG
ACTTAGTATGGTTTATAACACTATAATTTTGTTAAACAATGTTAAACTTAGATAAAAT

CTGGTTCCAAACGGCTTCGCACCCCGCCCGATGAACGAAATGATCCGTCCGATGTCGTTAGCTCCCCGTGTCG
TCGCCGTGGCCATGGAGGGTGGACTTATCTTTATATCTATAATAGCAAATGTATATAATTACAGCGTGAAGTA
AGCGTAATTTAATTAATTTTTTTTTTTTAAGCTATAAACAATTGAACTTACATACCTGA
GTTACTTATATTTTTAAAAAATTATAGCGATGGATGGAAAATATAATCTAGTGAGCTATCACTCTGTTTCTAA
AAACTCATAAAAAAATCCTTATAAAACTCATGTATAGACACTCCATATTGTCCAACCAGAACAAAACTAATAA
TTTCTCAGAATCCATCCAATAAAGTACTAGTACCTTTATACAGCTGTGCCAGTTTCAG
TGATTGTGGTCTAATTTTGTTTTAATTGCCACGAGTCAATGACTTCGAAGCTCGAGCTTAGCAGCTAATGTTA
GTTACGTTTAAATATTTTTTTCTATATTTCCAAAGACCGTTACTGTACTTATATATAATGTATCAATCAACGT
TCGCCACTCTGGTACTTAGGCAAAAAGCGCGGTGTTGAGTTCCGTCTCTCAACACGCA
ATGCCTCGAAAATTCGATAACACTAGATACACTCTGCCTACCCTGCTACACGAGGGATACTTAAAAAAAAAGA
AATCAACCAACTTTTTTGGTTGTGTCAATCTGTCAGTGCGGAGTTTTTACCACAGGTGTTTTCTGTTGTTTCC
CTTTATATGAGTACGAATAGGAGTAGTCCTTTGCATCCATCTATGTCATTGTGTCTTT
GGGGTGATTAGAAATTCTTTTATCTAANAACAAAAATAGTAATAACCTTTGGAATGAAAGACTTAATTTAAGA
ACTATGCAAAA
>1002050507_1 Heli.1-EL596632.1.5
CAGTGCCATCTGAAATGTGAGTGAATTTTGTGCTTCGGATACTAGTGTTTTGGGTAGGTTGTGTTTTTTTAAA
GAATTGAGAATAATAAAAATGGGGCACAAACAGTACTTGGCCGTGGGGCAGTCGGCGCGCAACAGGCTGTTCG
GTATACCGAATAAAAAGCCCGTACCACTGAACATGATGATATCACAACAAGGCAAATT
AAACCATGGTCGCCTCGCAGTGATAACTTTCAGCATATGCACAGTGGTGTTGGGCGTCATACTGTCCTTCGTG
CCGTGGCTCGATTATATTATATTTAGGGAACTTAGATTATGGAATGGCTCAATAAGCTACAGCTACTGGCATC
GTCCCGGCGTTATAAGATTGACAAAGGTGTACATATTCAATATGACCAACCCGCAGGG
GTTCTTGGAAAACGGGGAAAAACCTAAATTGGTTGAAGTCGGGCCCTTCGTGTATAGAGAAGACATGGAGAAA
GTGAACATCAAGTTCCACGACAATGACACAGTGACCTTCCAACATAATAAAATATTAAGATTTGTGCCTGAAC
TCTCCGTCGATAAGACACAGAAACTGGTTGTACCTAATATACCTTTATTGACTGTCAC
>1002050508_1 Heli.1-ES587006.1.5
CAGATCTCGTGCCTTCGTTTTGTATTGTTCGTGTGCGAGTGAATTACGTTAAAGGGACGTCTCATTGTGATAT
GTAATTATTATTATATCTAAATTAAGTGTTTATCGTGACGAAGAATGAACGAGTGCCTAACGGCTTATTCCTA
AGTTTTAGATAGTTAGTTATCTAATGTAATTTGTTTGTTATCGAACGTTTGCTCGATG
CGATATTGTATAAAGCAATAATGAACTTTTACTATTAGTATTAAGAGATCAATTATTGGAATTGGTTAGCGAC
AATATTGTAATTTTTACTAATTGATACATGTGTCTACATTTTTTATACCGACGAGATCGAGTCGATTATAGTT
AATATTTTCTGAATATATTTAC
>1002050509_1 Heli.1-DT668196.3.5
GCGGTACGGTCCGGAACAAGTCTATTATCGAAACAGGCTCGCCGTCCAAATGGATACGTTAAATATAAAACCG
TTAAGTCAGGACTTGGAGTGGTCTCCGGAGGCGCCCGTCGGCGTAGGAGCCACTCGGGACGTGGTGGACCACG
CTCCCACAGACATGGCGTCGACATCGAGCGAGGAGAGGGCCATACGCCGCGTCACTGG
CCGCTGGGGTGCCGAGTGGTTGAGCTGACTCGGCTCAGGCTATATCAAACCCCTCGGGCCCCAGTGGCCTGCA
GACCACCCCCTACCAGCGCCACGGTGGGGGGCCCCGCGCCCAGCTTTACACTTGTCTCTCTCCAAATACGAGG
ACTTGGTCGCTAAAGCTGAGGCGATATTGAGCCGACTCGTCGTCGCGGAAAACTATGA
CTCCGTTAACAACTTCCTCACTCTCTATGAAACCTATATGGCATCACCTGATGATACTTTGAAGGAGTTCTTC
CCTAAGTACAATCCGCCGATTCGCGCTCACAAGCACACCTGTGTGGGCTTGGGTATGGAGGTCATGAAGCGTT
TGAAGGTCCTGGAGAACGATTTCCCTGGTATCACCAAGTCCATGATGCTAGTATCATG
TGATGAAAACATCGAAGATCTTTTAGACTACACAACATCATTTCCTGGACCGCAAGGCTTCTTGATAGAGACG
GAGAAAGATCACGTCATGATGGCCATCCACGTCAGGGTCGAGGGAAGACCTGGGATATTTTTATCAGATTTAG
GTTATCACATTTCTCGTGTCGTCACCGTTATGGCTGAACGTTGCTACCACATACCGGC
TGGTCATTCATC
>1002050510_1 Heli.1-EL596225.1.5
GCACGAGGCAGAGCCTTTAAAGAGTCGCCCATAGTGCTGTTATTTTAGCGTGCTTCAGCTTTTTGTGTTTAAC
AAAAGGAGTGAGCACGTTTGTAAACAGTGATGGGTTGAGCAACTACACGTTTCCAAATGATTTTATGTTTGGG
GTCGCGACGGCTGCATATCAAATAGAAGGTGGTTGGAACGAAGATGGAAAAGGCGAAA
GTATGTGGGACACTTATTTGCACAAACATCCAAAATTCACTGTAGACCGCTCTAACGGAGATGTCGCCGCTGA
TTCTTATCATAGATATAAGGAAGATGTCATAATGGTACAGTCTCTCGGAGTTAAATACTACCGTTTGTCATTG
TCATGGTCGAGGATATTGCCTTACGGAACTGACAATTACATAAACAAAAAAGGTGTCA

AATACTATCGAAGGCTTTTCGAAGAGCTGTTAAGAGTCAACATCACGCCCGTAGTCACGCTTTTCCACTGGGA
TTTGCCTACACCCCTAATGGATTTAGGCGGGTGGAGTAATCCAAAAATGGTAGATTACTTTGAAGATTACGCG
AGAGTAGTCTTTAAGTTGTATGGTGATATTATAAAGACTTGGACTTCTATGAATGAGC
TACACCAACACTGTGCTAATGGTTATGGCAGCAAATTATTTGTTCCTGCAATAAACGCACATGGTATTGGAGT
TTATTTATGTTCACACTACATGCTTTTGGGTCACGCCCGAGCTTATCATTTATATAATAAAGAATTTAAAGAG
AAACAAAAAGGTAGAGTTGGAA
>1002050511_1 Heli.1-EE743505.1.5
GCACGAGGCGTGTGTTGTCCTTGCTTTGTTCCTGGTGGTGGCTTTTGTGGCAGTTGCCCAAAGCTCCGAGGCC
AATGAAGATGTACCTAAAATTCACAATTTGCTGAAACGGCAAATTAAGCCGATTGGCTCGATGTACCCTCGTT
GCGCGCTCTGGGAATGCAATTCCAACTGCATACGACGCGGGTTTAAAGGAGGTTACTG
TGCCTTCACTGGATGCCAATGCTACTGAATATATTAAATATTGTATTTATATGAGTATTAGTATTAAAATGTA
TTGCCTATTTAGATTATAAGAATAAATATTTTTTTATGATAATTTATTTCTTTAATTTGTTTAGCTCGTGCTG
TAAAAATATATTAATTAAAATA
>1002050512_1 Heli.1-ES587553.1.5
GTATTATGATTTTGGTAAGTTGAAGTGTGGTGTGTGTGTCAGTGATGTAGAAGACAGTGAGAATGCTAAACGC
CACGTGGTGTCGCCGCGGCACAGGGAGAACATGGGGGCCAGTTATGTTAATGTTACTATGGATGTGTCAGCTA
TGGAGTAAGGTGATGAATATGGACTTAACTTATACCTTAAATAAATTAATAAATATTT
TATTATAAGAAATCTGCCACTTATGACCTGAGAATCTTCAAAATACTGAATATATATTTTTAAATCATTCAAG
TTGTATGTATGTTACGTCGACCGTTGCTCGTTCGAGTTGTTATAGTTTCCCAGATCGGAAGAAATTTTACAAG
ATTGAATTTAATAAATGAATTAATTTTTTAATTT
>1002050513_1 Heli.1-EL602641.1.5
GCACGAGGAGGATACTAACCTTGTTCAGGCTTATTCGGAATTAAACAAATTCTGCCAAAGCGGAGACTATGAG
AGAGCTCTAAAAGCTGCAGGGAAAGTCCTGCAACTTGCACCAAATGAGCAAAAAGCATTCCATTGTAAAGTGG
TTTGCTTCATTCAGCTTCACAATTTTAAGGAGGCCCTGTCCATTCTGTGCAACACCAA
AAATGCATCTTTGGCAGCAGACTTACAATTTGAAAAAGCCTATGTTCAATACAGGCTCAACTGTCCAAAAGAA
GCCTTAGAGACAGTTGATAGTGTTTCAGAACTAACACCAGCTTTAAAGGAACTGAGGGCTCAAATTTTGTACC
GTCTAGAACAATACCAAGACTGCTATAACATTTATCGAGACATTGTAAAAAATACCAC
TGATGATTATGAAGATGAGAGGAAAGCTAATATGTCCGCTGTGGTTGCTAATTTATCTGCTCTTAATCCGTCA
GCAGATCTACCAACATTTGAAGAAAGTACCTACGAATTATCATACAATGCTGGTACAACACTCGCCATGCGTG
GTAAATATAATGAAGCTTTGCCAGTTTTAAAGAAAGCTGAACAGGTTTGCTCAGAGAG
TGTCATTGACGATGGTGGCACTGAAGAGGAGGCTAAGGAAGAAGCTGCGATTATCAGAGTGCAACAAGCATAC
TGCCAACAGCAAATGGGGAAAGAGAAAGAGGCTGCAGCCCTTTACCAGAATGTATTGAAAGATAAGCCATCTG
ACCAAGCACTAATTTCTATTGCGAGTAACAATCTTGTTGTCATTAATAGAGATACAAA
TGTTTTCGACTCCCGCAAACGTATGAAGGCGGCGACCCAAGATGGATTAGAACATAAACTGAACTCCCGCCAA
CGCGCCGCCATTGCTTACAACCAGGCGGTACTTGCTATATATTCTAATCACCCGGACTTCTGTAAAACATG
>1002050514_1 Heli.1-EL600583.1.5
GCACGAGGATTTAATTGCGCTTCGTACGCCTTGAAGCGTTTTTTTGTATAAAATAAATAGTTTATAGTTTAAA
ATAATAATACCATAATGGATGTCTTTCTAATGATTAGGCGAAAAAAATTGACCATCTTTACAGATGCAAAGGA
TACTACAACTGTTTTAGAATTGAAGAAAATGATAGAGGGTATCCTAAAGGTGGCACCA
CCAAGCCAGATGCTATTTAACAAAGACAGCCAGCTTATGGAGGATGACAAGACCCTTGCTGAATATGGCCTCA
CATCTGCTACAGCTAAAGCTCAATGCCCTGCACCGATTGGACTTGCATTAAGAAAAGACAATGGCGAATTTGA
AACACTGGAATTAACACCGTACTCCACCCCACCAGATCTACCAGATGTCATGAAGTCT
CAGGAAACCAATGGACAGGAACAGATGGATCAGCACTAACCGACTCGGCCACGACACGAGATACGACCACCAG
TATAATATCTCGATACGATTTTTTAGTTCTTTGTTTTGAACTACTTTGCCGCCCGCCATTGTTGCGGGCATTT
ACTAATTTTACATATATTTTGTTAACGATTTATC
>1002050515_1 Heli.1-DT668750.3.5
TTTTTATGTAGTCGTACCAACATTTAGCAAATGAGGTTAAAGAACTTTTTACCAGTGAACTATTACGAGTGAA
TATAAATATATTCACATTATCAATGCAATAAATAAAAAAAAAACTTTTTATTAACATCTAGTTCCTATAAAGA
ACTTATTTACTTTTTATAGTACGTACCAACATTTAGCAAATGAGGTTCTTTATACTGT
TACAAGTGAATAAAAATCTTTTCACGTTATCAATGCAATAAATAAAAAAAATATTTTATTAACATCTAATTCC
TATAAGGAACTTATTTACTCTTTATCGTATAATTTAACTATGTACTATAAAGAACCTCAAGGTGGTCGCCTTC
ATTTCAATACGTTTATTTAAGACTGATTTTATGTAACCAACAAGTATTATAGTCTTCA

TAATCTGACATTTAACTTTCGAATATGTAAGCCGTGTATCTGTTTAGAATCTCCCGTTGACTTAGAAAATGTT
CGACAACGATTCGTGTAGAGTAGGAGAGTAGCAGGTTCAAATCTCAATATCAATTAATCGTATATAGTTAGGG
TACGCGGTGATTGCGAAGACTTGAAAGAGAAAAAAAATATAAGAAATGCTATTTTTGT
CACAGAATTGATTAAGGTAATTTGATTGATTATTAT
>1002050517_1 Heli.1-DT662948.3.5
GCACGAGGGCCAACTCTACGCGAGTGTTGAGGCTTAAAACAGGTAAACGCTGGTAGAGAAGAATGTAGAGTCA
CTGATGTTTCTTTGAGCTGAGTAAAAGGGGCCATCCGTAAATTATGTTTACAGTTTTTTTATTTTTTTTTGT
TTCTTCGTCCCTTTCTTGTCACATTTTCGAAGTATGTTATGCAATTTTACGTCTAGGA
ACTGTTAAATTGAAACAGCAATTTCAAAATGTCAGACTATTATAATTTGGAATGGGATATTCCGAAATTTTGG
GTCACTTCCCACCACTTGTCACTCAAGGTCACATTACGTCGACCCCCCCTCTCGTTTTGAAATTAGACGTAAT
TTATAGATGACCCCTAATTATATTAATAGATTTAATAAAGAATTATTCAACATTAATC
TGTTGTTGAATTTTTTGTTTCATAATTTCAGTTTGTAGTTTTTGAAACACTCTGTGTAATGTTTGATGTATAT
TGTAAATGTTGATATGTAGCGTCAATTGATTTGGATGGTTTGTTTTCTATTGAAGAAAATATATTATAAAGTT
CATATTTCATATTAGAAAAATATTATGTATTGTAAAGAGATTGTTATATGAAATATAT
GAGTAACTTAAAATTGTAATAAATAAGTACACAAATACATGAATGTACAATGGCCTTAGTACAATCTTTAATT
GTTCTTCTTTACCAGCGCTAAATAATTAACTTCCAATTTACAACATATCTCATCTATTCTTGATAGTATAAAA
AACCTATATCGGGGTAATTCAGAAATATCGTTTTGACGTTTTAACAGATAAAAAAATA
>1002050518_1 Heli.1-EL603850.1.5
GCACGAGGCCACCACCGAGGGTGTCCAGTGAAGAGTACAAGGCGGTAACTAACGTACCAAAGCCAGCGAGGCC
CCCGCCACCGAAGTCTGTTGCTTTACAGAAAAAACAAAAACAACAAGCCTGGGCGGTGGTGCCTAAACAAGAT
GATAACATTTATGCCAATTTAGGAGAGATCAGATCAGCGCTCGCTCCACGCAAACCAG
AACGTACGGCCAGTATGCGCGAACGGGAATCACATTTAGAACTGCTTAAGCGTCGCACACCATCCGACGACGA
AAAAGACGAAGACGAGCCTCAGGAACTAGTGCAAGCGACGAACGAGACAGTTATACACAATTACGACGACGTA
TACGCGACGAAATACGACGAAGAGAATTGTGACTCAAAGAACAGTGACAGTGACTACG
AATACGTACACCACGCGAGGTCTAGTTCACCAGAATGTGATTCAGCAATAAAACCGAGAGAAGAGAGAACAGA
ACGGAAACCAGAGGAATTTCCAAAGTACAACGGTTTTCGGACATCTCTGCCCTACTGTGGAAGCGAAACGGAA
TCCGAAATATATTCTCCATACAGTTTTTACAGTTCAAACGACAACATGGATAGTCCTT
CCAACGACGATTATCAGTCAGATATATCGAAAACGACGAACAAATTGCGCGTGAGAAAAGGAAGGAGCGTAGT
CCACAAGAACTTGGAGGATAATTACGGGGCGGTCGTTATCGCGAATCACGAAGCGCTAGCTCAGGTTTTGGAA
CAGGTACAACAAAGCGCGACAATGCAGCCTTTATTGCGATTTTTTTTAGCGTGTACCA
ACTTGCGCTGGTCAGATTTCTCCATTCAAAACAAAAGCTATACGAAAGCCGGCAAACGTTTCTTCTACCCGGC
CCTGTGGAACTCCAGCGGCGGCCAAGTTAACGTGACGCTGATGCTGTGCAAGGAGCAGACCGCCTCGCAGCTG
GTCTCTCAGTCGGTGCAGCCCAGTACTTTGAATTTGAATGCCATCACGGAATTTTGTG
ATCTAGTTCCGCTGCAGCAGTTTG
>1002050519_1 Heli.1-EL604343.1.5
GCACGAGGCGCGCGCCTCTCCCGCTCCTTTCGTTATACATACGGTACATAGCACTTAATAGATACTGATCGAA
ACTGGAAAATGAAAAAAAAAATAATAAAAAAGATTCAGAGTTATAATAGTATGTTAGTTTTAATTATATTTCT
GATATTGATTGATGTTTTGCTAAAAGATAATTTATCTGTGCGTTACGTTTGATGTCGC
GGAGCTCGCTCTGCCGTCGCAGCCTCCGCCTCCGCGTCGAGTTGTTCCTCACTTTAGCAATAGAATAGCCGGT
AGCGGCCTCACCGGTTATATTCACATAGTCATTATTGCGTATTGATTTTTTTACTAATTTTATAGATCACAGT
AATATACGTTGTTTCATGTACTCATATATTTTTATCGTGATTGATTTTTTTACATTTT
ATGTCGCGGTAGGTTTGTTTCAAGGCACTAACGGCTGTTCGCCGCGGGTTATCCGAACGTGTCGGCGTATATC
CCTACAGGCTTCTTCACTCACCGATCGCCGCCACCATGTGATACACCTCTAAATTCATATATTACATTTAAGC
AATACCAGTACCTGAAATGGATAAGTAAAACGGAAACAGTGGTCGGGTTCCGGCCGGC
CGGCAGCAGTTCGCAGTCGTTACTTAGCGGGACAGCGGTAGGGTAAGTGTCTGTACGTCGGACGCCCGCGCGG
GCGTCGTGTTGACGCAATTATGTATAGGTCGGGGAGTGTGATATCACGAGAGCGGGGTGAAGTGAACGAATAC
TGCCAATCGATTGTAGTTTATATTAAATAATGGTAAATGATTTGTAGGGTTAACGTTT
CGAAATATTATGTCCTAAATATTTCATATTGTATGTATAGTATCGGCGCGTATCGCGTGCCAGTGTTTCATTA
GAGCGAGGACGGTGGTGGATACGAACTGTGTTCCGTAGGGCGTCGTCTAGTTAGGCATGCTGATTTTAAAATA
CATAGAACGCAATACAATTTGTGGGGAGGTGCGGTAGGTAGTACGGTGGTACGGTGGT
>1002050520_1 Heli.1-ES585158.1.5
GCAGTACGGTCGGAATCCGGGTCGACCACGCGTCCGAAGAAGTCTCTACCTATATAAAAATACTTTACCTTTA
ACGGTAGTTATCGTTTTATTACGGACGGCGTTGGAGTAGCGCGTTTAATTTTTGATTTAATATACATTTTGTT
AAGCGTCATTTTGTTTATTAAATATAGTTGTTAAGTTAGTTAAGCTGTTTGTCATAAT

GAATGTTGGGTATTGCTGATCTTTTGATAAACGAGTTTTCATACAAAATTTTATAGTTTATCTATCTTTAATT
CAGTTTCAATATAATATGTTCAAATATTCACATCACATTTTTATAAAGAATGAATATTCAAATCGCATTCAT
TGAAAGTAGTTATCTAGAAAATTAAAGAACTCGGCAATATACAATATTTTTTACAATT
CTATTGAAATTATTGAAAATAAAAAAAACAAAGTATTTTCTTAATCTTGTAATTTGTACGTCCAATGTTTATA
TAATATTATATGATATAAAAGTGTTAAAATTTTATAATCTCCAATGTATATAGCTATAAATATCGCACTATTG
CCATGAGTCCGTCAAAACAAAGCATTTTCGGGGAATTACCTACTGTTTTTTCTTAAAA
CACTAATATATTCTACTTTCCCCTATTGACCTTAACTTTCCTAGAAGTTTTAATGAAAAAATAATAAGATATA
ATTTTTATTTATTTGACGAAAATCCGGAAATAAAACTATTTTTTTCTATAACATTATGCGTTTTTTATAAAAT
ACGACGACCACGACATAAGAAAAATAGATAAGTATTGTGACACTCATAGTGCGATATG
TTGGGAGCAAATGTCTATTGAATCTCTTGTGCATTACCGATACTGCTTTTGTGGATGATTTACATTGTATGAT
TAAATAAATCT
>1002050521_1 Heli.1-EL604335.1.5
GCTTTAGCACTGAATAGATTACCGATTGAATGTAAGACTGACTGTTACAATAACGAAATGAATGACTGAATCA
CTTACTGACTTAATAGAGCTGACTTACTGACGGGTAGTGTACAAACTAAATGTTTGTGATAACAATTAGGCTT
AAAGCTTCTCGACAACTATTAAATTGTACTTACTACGAGATTTATATATATCACGGTT
TATAATCATCCAATAAATCCAAATGTTTACATACAAAACGAGAAAAAGAGTTAAAAAACATTAGTATTAACAT
ATTTCATAATGCTAATCACACTTTTTTTTATTTCGATATTTTTTCAATTTCCTGCAAAAATAACTTTGTACAT
GGAGTTTTAA
>1002050522_1 Heli.1-EL597863.1.5
GCACGAGGCGCGGCCGCATTCGCGCCGAAACTTTCAAAACATTTGACAAGTTCACTTTTTAAATTTAAAACAA
AAATTTTATAGCAAAAACAGTTCTGTATACAGCCAAGTGTGTATAAAAAAAACTATTTATATGTGATTGGATT
GTAACATATTTATGTTAATGTGATGTCAATATCTTCTGTGATCTGACGGGCCTGACCG
GCCGACGATGTTGAACATGTTCGACATGTGGAACTCTGTGACCACCAAGCTGGAGGCTCAGAGCAACGTCCAG
CAAAGCCAGCAGCCACACACCTCAGCGCAAATCGAGATAATACCATGCAAGGTGTGCGGGGACAAGTCGTCGG
GCGTGCACTACGGGGTGATCACGTGTGAGGGGTGCAAGGGGTTCTTCAGACGTTCCCA
GAGTACAGTGGTGAACTACCAATGCCCGAGAAACAAGGCGTGTGTGGTGGACCGCGTCAACCGGAACAGATGT
CAGTACTGTCGTCTACAGAAGTGTCTTAAGTTGGGAATGAGCCGTGATGCGGTAAAATTCGGACGTATGTCAA
AAAAGCAGAGGGAGAAAGTGGAAGATGAGGTCAGATACTTTCAAGAGAAGATGCGTTC
CCAAGCGGATGCTGCACCCGACTCAGTGTACGACACTCAGCAACAGACGCCAAGCTCAAGCGATCAGTATCAT
GGACATTATAGTTTCCCGAACTA
>1002050523_1 Heli.1-ES587740.1.5
ATGAAACAGTCAAACTAAGAAAAAATAAAACAATTCAGAAACTTGAATAAGTGACAAAATATGAAATTGGTGT
TGAATAATATAATAGTGTAATAGATTTTTTACAAGTGTTTGTGTAAATGTTTATAAAGACAACTGATATATTG
TGTAGTTTTTCCTTGCTTTTACTTTGCTATAAAACCTGTAAAATAAAATCCGATGATA
AATCTTAAGATATCTTAACTTAAATCTTAAGATATGTTTTAAGATTTGTCTTGTTTCTTGATCTTTACTATAT
AAATGTACAAAATGTAACTATGAGTTAAATTAAAAGGCACTTATCCTATTGAGTTTAGTGTAAATAATTTCTT
TGTAAGCTGATATATTTTCATATGAAAACGAAATTATGTGTTAGATTTTCTATTTTTG
TTAATGTAATTTGCTTAACTGATATTTTTTTAAAGTATTTGTAAAACAATACATATTAGAAAATATTTAACTG
TTTTAATGTGGATTTTTAATAGATTTATAACAAATAAACAAAAGTTTATTTACATATATATACATGTAAGCAG
TGTTTAAGTATGACTTTTGTAAGTTATGTAGATAGAAGTTGCGTTGTAATATGTTGAA
GCTCGGCGGGCGCCCCCGTTGCCATACACCCGCTCTCCTGTACAGTGGACTGCACGAAATAAACTATCAGACT
TTTCATATTTTGAATTTTCACTTTTATTTATCACCTTTATTTACAGGTACTTTATATATTAACGTTATATTAT
ATCTTTTTTTATATATGTCCGATATATTATTGCT
>1002050524_1 Heli.1-DT668994.3.5
GCACGAGGCAAAGAGGAAATGCAAAAGAAGGGACTCTATCAAATCTCAAATGCGATCTCCTCCTAGTCTAGCA
CCACCGCCATATTATTCATGCTCGGGGTTGGAGAACAAGGCTCTAGAAAATTCCATGGACATACCTCTAACGA
AAGAAGACTCTAAAAGTGCAGTATACGAAACGCAAGGCGGCTACGGGTATCATATCGC
TCGCCACGTGCCGCCCCTCGCCGCGCAGAATATGACAAATTCAGATTGGGCGAACATGGGGTACTCCGAGAAC
AGCTACGCGAACAGCAACAACGGCGGCAGTGTCAACTCGCACGACTCCATGTGGCATATGAAGTTGGCGGTGG
CCAACAACAACCCACCGGGCATCCCCACTCATCAAATGATGGATCACCATCAAGGCAA
TATGGGGTACGACCTGATCCCGCACGGCGGCTACGGCGCGCTGGACGACTACGCGCCGTTCCCGCCGCTGCCG
CACGGCGCGCACGGCTCGCACGCGCCGCTCGCGCCGCACCCGCCGCTGTCCGACTACGGCCTGCGCAACTCGC
AGAACCCCTCCAGGCAAGACTACTGTTCGGACTCCTACGCTTCTGTGCACAAACCGAA

GAAACGTATGGATCAACATATAGAATCTCCATATCATGAAGTCAGCGGGCTCCAGGACGCCTTCGAGGAAGCG
GGCGGCGATGATAAACCACCGCACCTCTCACTTGGCTACGATGAGTCCCTCGAGTCCGGTTACTCCACGCCCA
ACTCACGCGGACGACGCGTGTTGCGGGAGATTATCGTGTGAATGCTTACACTATCTGA
TATTAATACCTTTAAAATGAATTTAACTCGAAACATAATCAAAAAGGGTTTTGCAGGATAAGCAAACTAAAAC
TGCATTAGCTTCTAATGATTTTTTTAAGATGTTTATTTTGCTTTTCATCCGGAATCCTAACCATCCTCTAACC
CTTTATGGTAAGAGAGTTATGGGGATTACAACCCTTAACAAAATGGTCCATTTTCTAG
TTATTTATTCAAGCTATTATGAAACTTTATTTGAACTGTGACGTATTGTGACGTATTCATAACCTCAATATAT
ATTTTTTCCTGTTTTTTTTTTTATAAATTGTACAAAATGAGAAGTTAAGTTTTTTAGGGCGGCATTATATACCT
CCACGTGAAATCACAACTACTAAAATAATATATATTAATCATATAGTAGTTTCATTTT
TGTATTATATCTCAAAACAATAAAAGGAATTATATTATGTTATGCATGTATCTAATTTATTCACATAAACGTT
AACAGAAAACAACGCCTAACGCAATTATAATAATTATATAACAATATCTTTGTTTGACATAAATCCATTTTAT
TTTCATTTTGCAAAATGTTAAAACAATATGATTTCGTCCCATTGTTTTTTTTAAATAA
CATAATGTATGATACTATATTTAGAAAACTGTATAAAACAAAAAAAAAACTAAACTAGGATTTTATAAAAAGT
TTTAAAATTTTTGTTGAACGTTACTTATTACACAAAATTATATTATTAGTTATCTGAGGTATTGCATTGTATT
ATGCTTCGCCGCCATTGCCCACCCCTCGACTTCTTGACGTGTGTAACGTTGATAGACT
ATATTTGGTGAT
>1002050525_1 Heli.1-DT666006.3.5
GCACGAGGAAACTCCTCACATCACCACAAAAAAGTATTCTAACTGCACAACAATGGAATGTTCAAACAAGATA
TTATTCAAGTCTCCCTTCCCATACAATGGTAAATCTACCAGCACTATCACCAACTATGGAGAATGGCTCCATA
GTCAGTTGGGAAAAGAAAGAAGGAGATAAATTAAATGAAGGTGATTTACTATGTGAAA
TTGAGACAGATAAGGCAACAATGGGCTTTGAGACTCCTGAAGAGGGCTATCTTGCAAAAATTATAATTCAAGC
TGGAACCAAAGGTGTTCCAGTTGGAAAATTACTTTGTGTCATTGTTGAAAATGAAGCAGATGTCGCAGCATTT
AAAGATTTCAAAGATGACTCTTCAGGAGCTGCTGCACCAGCACCATCGAAACCAAAAG
CCGCAGCTGCCCCACCACCTGCCGCCGCTCCTTCAGCCCCAGCAGCTCCAGCACCTTCAGCCCCAGCGCCAGC
AGCTCCTGCCCCAGCTTCTGCTGTACCTGCCCCAGATCAGGGTAGGGTCTACGCGAGTCCAATGGCCAGACGA
CTGGCTGAACTGAAGAATATAAGGCTGGGTGGGCACGGCTCAGGCGTATACGGATCTA
TAAAGAGTGGGGATGTTCCCGCAGCGGCTGCCCCCGCGGCGGTCAGCCCCGCTGCCCCTGCACCAGCTCCAGG
AGCTTCCTACGTCGACATACCTC
>1002050526_1 Heli.1-EL596879.1.5
CCTGTACTCACTTATCTGGACAGCCATTGCGAATGTACTGAAGGTTGGCTTGAACCTTTATTAGACAGAATAG
CGCGTAACAAAACTACAGTTGTATGCCCTGTCATTGATGTTATTGATGATAATACATTAGAATATCACTACAG
AGACTCCTCATCCGTCAATGTTGGCGGTTTCGATTGGAATCTTCAGTTTAATTGGCAC
CCAGTACCATCTAGGGAAAGGGCCAGACATAAGCATACTGCTGAACCAGTATGGTCTCCAACTATGGCTGGAG
GCCTTTTTGCAATTGATAAGGAATTTTTCGAAAGACTCGGCACATATGATAGCGGATTTGATATTTGGGGTGG
GGAAAATTTGGAATTGTCTTTTAAGACGTGGATGTGCGGTGGAACACTTGAAATAGTT
CCTTGTTCTCATGTTGGGCATATATTTAGAAAGAGATCACCATATAAATGGAGGACTGGAGTAAATGTATTGA
AAAAGAATTCTGTGAGATTGGCTGAGGTATGGTTAGATGATTACGCCAAGTACTATTATCAACGTGTAGGTAA
CGATAAAGGAGACTATGGCGATATAAGCAGTCGTAGAAACCTCAGACAAAAGCTCGAG
TGTAAATCATTCGAATGGTACTTGAAGAATATATATCCCGAGTTGTTTATCCCCGGAGAGTCTGTAGCTCACG
GCGAGATTCGAAATATCGCCTTC
>1002050527_1 Heli.1-CV526195.2.5
TGGGATCGGCCGAGGGTTGGCTCATTTCCCAAGTGATGTAAAATTGACGCAGGTAGAAACGTATGGGTAATAT
CGGATCGTATGCCGGTATTCTTAGAATCAGAATTGAATTACGGTGATGTTAATTTCAGAATATACGTAGCCCC
TATGGAAAAACTTATCCAAGGAACGGTTTGTGAAGTTGAATCACCAATAATAACAACA
CCTAGATCACTTTCAGTACACAGTCCACGACTTCAAACTATTTCTCAAATTCCATCTAAAATCTTTTTACCAG
GAACTTCGCCTAGTCCTTTAACGTCTTCCTACAATCCTTTTATATCTCAAAATGTAATTTCTAATATTCCAAA
CCAGCCTATGTACACTAGGGAACCATCCAGATCATACTTCTTTCCACCATCACAAAAC
AACAATCGTCAGTGGTGGTTAAACAATAACTAAACGTTTCACCTATAAAATTGATTGTGTAAATTTAGATATA
TTTCAAATTGCAGAAAGTATCTAAAATTCAATAAGCAATCGAGTATTTAATCGAAAATACTCATTTTAATGTT
GTACTCAGGCTATAAAAACATATAGCTAATTAGTTACCACATTCCACATGATGTATAC
AGGTATAGTTAAGGACTAAGGTTATATAAGGACTTGGCTTAATACTGGCTTTGCTCAGCGTTACATTAAAAAA
TACATATAACTAAAAAAATTACGAATATAAAGTAATTACTCCTTATTATAAATTTCGTTTGTCAATTTTGCAA
TCCAATAAATTAGCAGGATTTGCGCTATATCACGCTAGTTAACTAT
>1002050528_1 Heli.1-CV526261.2.5

AAGTGTCATGTGATAGTGTAGTGTTAAAAATGGAATCGACGCCGATCTGTCGGTGTCGCGTGTTGTACCTCGG
CTCGGCGGTGCCTCAGCAGAGCAAGGACGGCCTACAGGGTATTCAGGAGCCTTTGCGCGAGCTGTATCCGGAA
AAGGGGGCGACTACCGGCGGTATAGACTCGTGGCTGTCGGTGTGGTCGAATGGAATCT
TGCTGGAGAACGTGGACGAGAGTGGGTCGAGGGTATCGAGATTTTTCCCCATTTCTAGCTTGCACTACTGTGC
CGCGGTAAGGCGTGTGAGTGTCGAGGGTGCGCCCAGATTCTTACCTTTGGACTCTCCATTTGCAAGAGCGCCG
GCGCCGAGACGGCCTCCTTTATTTGCAGCTGTGTTGCGTCGTACACAAGGTATAAAGG
TGTTGGAATGTCACGCCTTTATCTGTCGTCGTGAAGCGGCCGCAAATGCCCTTGTGCGGTGTTGTTTTCACGC
GTACGCCGATAGTTCATATGCAAAACGATTGGAAGCAGAAAGGTCACCGTCACAGCTGCCGCCGGAAGGTGAC
GAGGAGGTTGAAGTATTCGATGGAGATGAAAATCATAAAGTATGGGTTGGGGAAATAG
AGCGCGACGATGCGAGCGATGACATTCCTCATCCAGCTCGAGCACCTCGACCTCGGCAAATTACACGACCCGC
GTCGGTACCACCACCACCTCCACCGCCTGAGGAACCAAAAAAGAAATTAACAACCAAGAAATCAAAAAAGAAA
TCATCAGCGTCAGCAGACGAATTATACGCGACGATGCCTGGTCCCGCTGTGATGAATG
GAAGATCGTTAGGACGCGCACCACCCGCCTGGGCGGCGGCGGGTCATCCGATGGTTCTGGTCGCGCATCCGGC
TGCTACTCTTCCACACGCTAGACCAGCAACGATGGGCCACAGAGGTCGCGTGCCCGCAGCTTTGGCACCTGGC
CCTTTTCCACCTGGGCCCCCTCCTGGACGAGGTCGAATACAATACGCTACAGTTGACC
CGAGACGTTCTAAGCCACCTCCATCAGCAATGAAAGCCGCTCAAAGTATGACAGGCCTGGAAGCTGTCGAGGA
CTCGGGCGGAA
>1002050529_1 Heli.1-CO729664.1.5
CGGAATTCGGGGCGACCACNCGTCCGATCTTATAGAATATTTTTTGCCACCTTACATTAATTATGTACTACAA
ACACGGTAAACTGCAAACGCTGTCAATCCGTTTACTAACACGTGTTTCTGATCAAATGTAAAATTCAATTATG
TAAACATCTGTCTGTTTATGTTTTCCACTATCAAAAATTTGTAAAATGGTCGAATGAG
CTTGATCGATTGATGCAAAGTCACTGTGATAATTTACAGAATATTTGTGTATGATAAAAATAAAACAATGATA
ACATCAACCTTAACTTTTGCAATGCACGTTCTAGTTTCATCTTATAAAGTACTGGCTCCAGTCGCCCAGATAT
TGGAATACATTTTACCGTATAGAAACATGGCTTCTAACGCAATAAAGGGAAAAATCAA
CTTTAACTCGAAACACTTAAAGAGAGGTGATGCTCTCCCTCCGTACAATGGCAAATTACGTATATACAATATG
CGCTATTGTCCCTACGCACAACGTACCATTTTGGCCCTCAACGCGAAGCAGATCGACTATGAAATTGTGAACA
TTGACCTGGTAGAAAAACCAGAATGGCTAACTAGAAGAAGCCCTCTAGCTAAAGTGCC
ATCATTAGAAGTAAAAGAAGATGTGGCTATATACGAAAGTCTTATAACAGTTGAATATTTAGATGAACAGTAT
CCACAAAGACC
>1002050530_1 Heli.1-CO729865.1.5
CGGGTCGACCACGCGTCCGGTAGTTTTTGTACTGCACACTGTTTGGTGATATAAAATGAAATATTTAATAATT
CTGTGTTTCCTTGCGCTCGTGGGATGGACCCTTGCTGGCACAGATTTGCATCCTAGAGGCTGTATTTATGCTT
TGGGACGTTGTGTAAGAGACTGTGAAGAAGGCACCCACGCATTCACCACTGGCTGTGG
GGTCCTAACTCCTGAGGCCACCTGCGACGAACCCAACCCTAAGCCTGATAACAGAGGTCTGATATGCGATTAC
TCAGCATGCTACTGCGACCCTCCCACAGTGAGGGACACTGTGAGCAAGAAGTGCGTAGCCGTGGAGGACTGCC
CGAAATGAGGGTTTATTGACAGGATATAATATACTTAAATGGCTATAAGCTTCGTTTT
TTGTTATTGTGTTTTATTAATAAA
>1002050532_1 Heli.1-EL600932.1.5
GCACGAGCGACGAGTATTGCCACCAAATGTGCCAGACGATGGTGTCCTTAAACAGAAAAGTGTAGAAAATGGC
TCTGTCATTCTAGAGAAAAAAGCAACTGACGAACCTGATAAACCTGTAGCTCAAATAAAACACGAAAATGAAA
CCGAACAGAAAAAGCCCGTAGTAGCCAAACGCAGGGAGAAAATGGCGCCTCCCATACC
AAATGCTAGAGTCAAATCCACAGATTCAGAAATAAATATCAGCCAAGAAACAACAAAATCTGATATCTCTCAA
TCGTTCACTAGAACAGAATTGAATTCTAGTGGAATCAAAAGAGATGAAAATGGCATACCTCAAGAATTACCTC
AACATATGTTCGACGCTGCCAAAGCCGCAAGAAATAATAGAAAAAGCTCAGCAGAGAT
ACAAGACAAAGATAAAATAGATAAAAAAGAAGAAGTACCAAAACCAAAAAAATCTAAAGGCAAAGCTCCTTCT
CCTCCAGAGCCAGAGAAAAATAAAAGCGATGATAGTATTCTAGAACAAATGAACAATTTACATGATTTCTTGA
TAAATGAGAAACATCATTCAAGTTTATTACATGATAGCATTAAATCTACGTTTAGTAT
AAACACATCTACACCAAAAGTGAATAAAACCAAAACACGTCACGAGGAATCCATTACTTTTAGCCAAGATGAC
ATAGATGATATCGTTTCAAAGCCCTTTAGAAGAGAAAGTGATTCTTTAAATAACTATTTCGAAGATTCTAAAT
CTAATCTATCATCCAATCAAGATGTACATTCAGTTGTCTCATTAAATAATAGTGATAA
AAGTGATAAGGGATCTACTACTAT
>1002050533_1 Heli.1-EL599204.1.5

GCACGAGGCTCGAACTAGTATAGTTACATAGGATTGAAAGCGACGACAAATTATCGAACACTTCTGTAATACT
GCTGAATTCTGCATTAATTTAACTAAACATAAGTGTAATAGAGCATAGAACACTTTGTTTGTAAGTAGTTAAT
ACAGAAAATAATAATATAGTGCGCTAATCTTAGAATCCTTGAACATTAGATCCGAAAA
ACGCGGATCATTCGGCACCTCGTGTTAACGTTTTGTGGTGAAAATGGCCAAATGGGGAGAAGGAGACCCTCGC
TGGATAGTCGAGGAACGTCCTGACGCAACTAACGTAAATAATTGGCACTGGACGGAGAAAAACGCTGGCCCTT
GGTCGAAAGATCGGCTAAAAGAATTATTGACTAATTTAAAAATAGTTCAAAATGGAAT
AGATTGTAAGATTAGCGAAGTAGAAAAAATAGATGGTGAAGCTACAGCGAATAATAGAAAAGGAAAACTAATA
TTTTTCTATGAGTGGGATATTAAATTGAAGTGGGAGGGTATGTTGGCCGGCGGTAAGGACAAGGTGAATGGAG
AAATACATATTCCAAATCTATCAGAGGAAAATGATGTCAGTGAAGTTGATATGACTGT
CACCATAAAAGGCACTGGTGATGAAGCCCAAAGAGTAAAAGCATTCATGCATAACAAGGGCAAAGACGAAATC
CGTAAACAATTAAATGAATATATAAGAAGTCTTAAGGAAGAATTCTC
>1002050534_1 Heli.1-ES587261.1.5
ATGCGCTGTTGCAGTAGTATGTATGGCGCAAACACAAAGGCCCGAGGTGTCTGACACAGCTTTAGATGACGCA
CTAAACGACAAAAGATTCATACAAAGACAATTAAAATGCGCTCTCGGAGAAGGACCCTGCGATCCTATTGGTA
AACGCTTGAAAACTCTCGCCCCGCTAGTGCTTCGAGGCGCTTGTCCGCAATGTACACC
GCAAGAAACGAATCAAATACAGCGAACACTTTCTTATGTCCAGAGAAACTATCCCCAACAGTGGACTAAAATA
GTCCGTCAATATTCCGGGTGAAAACTAGATATTAATTTAAGATGTAAATAGATTATAATCAAAAATTCCAAGA
GTATTATAGTATTTGTTATATAAGAATTTTAATAAGATATTTTACAACTTAATCCAGC
AAAATGATGCAATAGCAACCTGCTTTCAGTCGTCAAGGCAAGACAAATTTATTCCATGACAAAAATGTAAATA
TATTAAAAATAAGTTATTTAATATTGTCTACTGTAGATTAGTATAATATATATATATATTATTGTAATAAAAA
AAAAGAACAA
>1002050535_1 Heli.1-EL599906.1.5
GGCACGAGGATTTGGTTCAACTATCAAAGTGGACTCTATGGCTAAAATTGCTGAATTAGAGGTTGCTGAGAAG
GAAAAAATGAAGGATAAAGTGAACAAAATCCTGGGTCACAAATGCAATGTATTTATAAATAGGCAATTGATTT
ACAATTATCCTGAGCAATTGTTTGCTGATGCTGGTGTGATGGCTATAGAACATGCTGA
CTTTGATGGTATTGAACGTCTCGCTCTTGTCACTGGAGGCGAAATTGTATCTACATTTGATTCACCCGATAAA
GTCAAACTTGGCCACTGCAAGGTCATTGAGCAGATACTTATTGGTGATGAATGTTTGATCCGTTTCTCTGGAG
TAGAACTTGGTGCTGCCTGCACTATCGTAATTCGTGGAGCTACTCAGCAAGTGATTGA
TGAGGCTGAACGTTCCCTACACGATGCTCTTTGCGTGCTGGCCGCGACTGTAAAGGAGCCTAAAGTGGTGTTT
GGTGGTGGTGCCAGCGAGATGCTGATGGCGGAGGCGGCGTCGCGGGCGGCGGCGCGTACGGCCGGCAAGCAGG
CGGCGGCGGCGGAGGCCTTCGCGACGGCGCTGCGCCGCCTGCCCGCCGCCGTGGCCGA
CAACGCCGGCTACGACAGCGCCGACCTGGTGGCCCGTCTGCTTTTTTTCCACGCCCAGGGGGAGAACACGATG
GGATTAGATATGGAAAATGGTCGTGTGGGAGACATGAAGAAACTCGGTATTACTGAATCGTACGTAGTGAAGC
GGCAAGTGCTCCTGTCAGCATCTGAAGCCGCTGAAGTCATACTTCGTGTGGATAACAT
TTTGAAGGCAGCACCACGTAGACGCGGTCCAGATCACCGTCCATGCTAACAAAATTCAACTTCAACTACCATA
AAGGGCTATCCATAAATTGCATTACACGAATATCATGATTTTTTGACCCTGCCCCCACTCTTGTCACATTTAC
TGTACCTAAAACTCAAATGTTAGACTTGGGTAATATAACGTTTAGGAATTATTAAATT
TAAACAGTTCCCAAAATGTGTGTATATTTTATTTAG
>1002050536_1 Heli.1-EL600382.1.5
GCACGAGGTAGACTTGCAGTGTCGAAAAAAAAAACAATCGCTTGACAGCGCGCTACGTCGAAAATAGCGCGGG
AAAAGTGAGGTGATCAGTTGTCAGTGTTGAATTTTGGTGGTTTTCTTGTGAAAATTTTGACGTAAACAATGGG
TTCTATACCACGTGTGTCAGTGGTCTCTGGCGGCCGGGTCCCAGTGCCGGCGGCGCCG
CTCACCAAGCACTTGGAACGTATGGGCGCCGCTGATAGGACAGCTTTAATATATTCAGATGAAACAAGTAATG
CACGGGTAAGCTACGCAGAGTTGGACGGCCGCAGCAACGCTATGGCCAGAGCTATAGCTGCGCACGCGCGGCC
CACCGGCCCCAATAGCGATCAGGACTATGTGATCGCTGTATGTATGAAGCCGACACAC
AATACCATAATAACCCTCCTGTCCACCTGGAAAGCGGGAGCTGCGTACGTACCTATGGAACCAAGTTTCCCTC
AAGGAAGAATAAGCCACATTTTAAAGGACTCCCAACCTTCACTGGTTATATATGATGATACAGCAAATCCTGC
AATGTTCAACGGTAGCGGTGTACCAGCTATATCATTTGAGGAGTTGTCTCTCGAAGCC
AGTGCTTTACCAGCAGACAGGTTTTTCGAAGACGAAGTGCTCGTGAAAACTGATGCAGATACTATTGCCATTG
TGTTATATACTTCTGGCAGTACTGGAATACCTAAAGGTGTACGTCTGCCATACTCGGCGATATGCAATCGTCT
CTGGTGGCAGTTCCGAAACTTCCCGTTCTCAGATACAGAGAGTGTGTGCGTTTGGAAG
ACGGCACTGACGTTTGTGGACTCCGTATGCGAAGTATGGGGGCCGCTGTTACATGGCAGGACCTTGTTGATAC
TATCCAGAGAGACTACCAGAGATCCGCAAAAGTTGGTGCACATTTTGGCGGATTATCAGATCGAACGGTTAGT
GCTAGTACCGACTCTTCTAAAATCAATATTGATGTATTTATCATTGAGCCCATCTGAA

AGGCCGTTACAGCATCTCAAATTGTGGGTATGCTCGGGAGAAACGCTCAGCAAGGAACTG
>1002050537_1 Heli.1-EL604036.1.5
GCACGAGGGCAGAAAAGAAAATTATTTTTTCTCAAGTTGCTGTCATATCTGGTGATTCAACAATAATTACAAG
TGATAATAAGACCTTAGTTGAAGAGCCATCTTCTTCAAGTACTGCACTATCTACAACGACTACGGAGGAGTCT
ATTATTAGCCCTACTCCTGAAGTAATAGAGAGTTCACTTGCTGAATCTGAAATAGCAA
CTGAATCACAATTTGAAACAGAAGAAGACGAAGAAGATGAATCTAATGCTAAAAAGAAAAGTCGGTTGACATT
TACAACTAAAAAACCTTCATTCACTCCTGTTATTATTCCATTTGCTTCGAGGAATAGACCAACATTTAGTCCT
AAGCGTGTAGGTACGCCAAACAGTGCCACCACAATTACTCGAGCAGATTCTACTCCAA
CGATCACTGCTACTCCTACGTTAAAATCAGTAAGACCTACAGGTGCTTATAACGGTAATCGGAAACAAGCTGC
TGTGGCTGGATCAGCGAGCGCTGGAAGGAGATTCCCCGGACGTAATAGTCCAACAATAAATTTATCAGCAAAT
ATTCCTGCCTCACGTGCTGGAGGATTTTCTAGAGGAGGAAGTATTTCTCCAACTTCAA
GGAGAACAGGATTTAGATCGAGTTCACTTAGAAGCAACAGCTTAGATTACGCAAGTCGATCTGCAATTCCTAG
GATACGTCCTACAGCTTCATCTAGATTACGTGGAAGTAGACAATCAAGTATAATAAGCTATCCTCCAGCAGAT
CAAAATGAATCTGAAACTTATGCAACTCAACAAGAAGAANATGCCACAGAATCTCTAG
ATGAATTTACTGAAGCATCCCAAAGCAGGCAAACTACGAATAATCCTTTGCTTAGATTTAGAAGACCACCGGT
CGTTCGACAGCCAGGTACAGCGATAACTCCAAGGTCTACAACACCTT
>1002050538_1 Heli.1-EL603833.1.5
GCACGAGGTTAGTTTGTACGTTCAATTTTTCATTATAAATTCACATTAAAACCTATTGTAATGAAGATTTTAT
AGCCATAAATTACTCAGTGATGTGACGTTATAAGTCAAGTGGGTTTATAAGTGATTATTTCATAAGTTCCGCA
CACAGTTTTAAGAAATCAAAATGACTACTACAACTATACAAAATGCGATAAAAACTAA
CAATGCTGCCCCCGCTAAACCACAAAAAACGTACGGTAAGGTGGTTTTGACCCTGCACAATTGTTTGCTGCCA
GAATCTACGTTTAAAGAAACACCATCTCAGGCCGATGGCTTGGACATTGATACTGAGACAGACTTGCGCATCC
TCGGTTGTGAAATGATACAAACGGCTGGTATTTTATTAAAATTGCCTCAAGTCGCTAT
GGCTACTGGACAAATGTATCTTCAGAGATTCTACTACTCAAAATCATTTGTCAGATATCCTATGGAAACTACG
GCCATGGGAAGTATTTACTTAGCTTCCAAGGTCGAAGAAAAACCTTGCAGAATCCGTGACGTAATCAATGTTT
TCCATCACATAAAGCAAGTTAGAGCTCAAAAAACAATATCCCCATTGATAGTAGATCA
AAATTATATTGAACTAAAAAATCAAGTAATTAAAGCTGAGAGAAGGATTTTAAAAGAACTAGGTTTTTGTGTC
CATGTCAAACACCCGCATAAGCTTATTGTAGTGTATCTACAAGTACTACAATATGAAAAGAATAAACAGCTTA
TGCAAATGGCCTGGAATTACAT
>1002050539_1 Heli.1-DT668084.3.5
GCACGAGGTTTTGGTATGCATTTCACATCCGGTCATCAATGAGCAGCAGCGTAGATTTTTTCGAAAGTCTCAT
CGACGAAGATGGAAGATTTGCTGAGCTGAATTGCCAGGAATCAGCGCAGCGAGCGCAAAGGCGACGAGTCAAT
ACTAATACTGTGCAACGACCGAGACCGTTAATTCAAGCGGAAAATATAAACAATGACG
GTGGTTTGTCGTTGTCTCCGCCGCACACGGTATCACAGCGTGAGACTGGAACACAAGTTTCTCAATGCTACAG
CGGACCACCATCACCATGTGGCTCGACAAGTTCCACAACATCGCCCACAGTGTCCCCGGCGCCGCCTCCGGGA
ACTTTGACGATAGATCCTAATTGGCAAGCGACTAAACCTACTGTGAGAGAACGTAATG
CGGCTATGTTCAATAATCAATTAATGGCTGATATTACTTTTATAGTTGGTGCATCCGGGCACACACAAGTCAT
ACCTGCACACAAATATGTCTTAGCAACTGGCAGTTCAGTATTCTATGCAATGTTTTATGGAGGCTTAGCTGAA
TGTAAACAAGAAATTGAAGTTCCTGATGTTGAACCATCAGCTTTCCTTAGCTTACTTA
AGTATTTATATTGTGATGAAATTCACTTGGAAGCAGATACAGTACTTTCAACATTATATGTTGCCAAAAAATA
TATAGTTCCACACTTAGCAAGGAGCTTGTGTTACCTATTTACAAACTAGTCTGACTGCA
>1002050540_1 Heli.1-EE743504.1.5
GGTCGGATTCCGGGTCGACCACGCGTCCGCTTGTTTCGTAACAGGTAGAATTTTAAGCCTCGTTTTATAAAAT
CATATATCTTCACTTTTGCACAATGTATCACCAACTAGGCAAAATTTCTGGCAGGCTTGTAGCCTCTGCTAGT
CTTCAAAGCCCGCTGCTCCCAATTGCACCGATTGCTTTGCAAAGAGTAGAGCCTCCGT
CTATCCAATACAATACAGCTCATATGTCAAGTGTGAGCGAGGCTTGCCCAGCACCTGGCCCCAAGAAGCTTAT
CCCTGGTACCAACGTACCATACGCACCTCTTAACGAAAAGTTGAAGCAACGCCAGCAACTCTTCCAGAAAGAG
AATGATGTCCCTGTTTTCCTGAAAGGTGGTCCGTTCGACGCTGTCTTGTACCGTCTCA
CCATGTTTCTTTGTGTTGTTGGTCTAGTTGGTATTGGACACACTGTCTATATCCACGCTGTACCTAAACAATA
AAAACTGAAATAGATTATTTCCTAAGTTATGTATTAGATTCTGTGTTAATAGTACTCTATTATTGAAAATAAA
GAACTACAGA
>1002050541_1 Heli.1-ES586781.1.5

TCTACACGTATCTACTATAACGTAGATATATGAACTACATATACACTCCAAGCTCCTTAGGTCAGATTAGATC
GCCGAAATCTTGACCATTTTACATAATATGATAAACAAAATTTTTTACCCATATAAGTTTAGTCAATTCTTTA
CGTTTACATGATTTTATATACGAGTGTGTAAGTTCTAACATCGTGTAAACTTAGAGAC
GTTTAAGAATTTCAGATGTTGTAAATGCCTTACTTAATTTGTAATAATTTATATATTTTAGATGTATAGTACT
TAAGCCATGTTTTGAGACCAGTGACTACTTATTATAATTTAGGAGTAAACCGAGGCCCTTTCCGTGATTTTGA
AAAATTTCAGTCGATGCGTCGAATAATGACTGAGCCTCGGCCGACATTTTTCCTTTCT
TTCACATCTTACGGAAAACGACATATATATATTGACCTTGTTTTCTTTAATTTGTGAGAGTATAATACTAGCA
CTTGTTTAGTAATAGAGGCCGGACGTAAAATTGTTTTGGAAAGAAATTTACAATGTAGCTAATGGTGAAAGCA
TATTGTAGAGTCTTTTGTAACTTTTGATACAAAAAGTGCTGGTAGATTAATAATAATT
AAAACCGAGATTAAGGTTGGTACGAAAGTTTCGTGGCTATTGTAATTGTTATTATAATTATTGATAAAATTAA
TATACGATTACATTAATTCGTACCGCCCTTGTCTGTATAATGAGGTTGTAGTTATAGCTCCTTATTTATTTAA
AGTTAATAGCAATAGTAATACATATTTGAATTAACTGGTGATAAATGTATTGTTTCTA
AAGTTGTCCATGGTTTATGACAGTTCAGTATAAAATGTAATGTCAATTATATATTTTTTT
>1002050542_1 Heli.1-EL597175.1.5
GCACGAGGACGACGTGGCCACGTGTTGGCCGAGCTTTTGCAGACTGAGAGGGTTTATGTTCAAGAACTTGGCT
CTATATTAAATGGTTATAAAGAAGAGATAGAAAAGCCCGAAAACCAGCACGTACTGCCGTCAGCTCTGGTCGG
TCAAGCTGATGTGCTCTTCGGCAACTTACACGAGCTTTTTACATTCCACCAAGAAGTG
TTCCTCAAAGACTTGGAGAAATCAATTTCAGCAACAGAACTCGTCGCACTTTGCTTCGTTGAAAAGAGAGAAA
CCTTCTTCCGCCTTTACAGCTACTATTGCCAGAATATTCCACGGTCGGAAAGGCTAAGAGAGACTCTCGTAGA
TACGCATCTATTCCTACAGGCTTGTCAGCAACGTTTAGGCCACAAGCTACCGCTCGCC
GCATACCTTCTGAAACCTGTGCAAAGGATAACTAAATACCAACTACTTCTTAAGGATCTTCTACGTTATAGCG
AGTGTGGCTCTATGAGTTCAGGTCTACAGCGGGCATTGGACTGCATGCTAGTTGTACTCAAATGTGTCAACGA
TTCTATGCATCAAATAGCTATCACGGGAGTTCCTGTGGATTTGAGCCAGCAAGGAGAG
TTGTTGTTACAAAGTTCATTCCTCGTGGTGTCAGAAAACAAACGTGACCTCCGTTTAAGGATACGGCCGAGAC
GTCGGCACATCTTCTTATACGAGAAGGCAATGCTGTTCTGCAAATCTGCGGCTAAGAACAGCCATAATAAAGC
TACCTACCATTTTAAACATGATCTCCAAATGTCACAAATCGGCCTCACAGAATCAGTA
AAGGGCGACGCTCGTAAATTCGAGGTATGGCGGCAAGGGCGGTCCGAGGTGCACACCATAACGGCCCCCAGCG
TGGACGTGAAGCGCACGTGGGTCGAGCGCATCAAG
>1002050543_1 Heli.1-ES586380.1.5
GGTTAATTTAACTGCATCTTTGTCGATTTTGAAGAAAAAATAATAAGTATTTGCGATTAACATCGTTTTCTCA
AGACAAATTATTCTGTTTCGATAACATTATATATACAAGAAAGTCAAGATGCTGATTAAAGTTAAGACACTCA
CCGGCAAAGAAATCGAAATAGACATAGAACCCACTGATAAAGTGGAGCGTATTAAAGA
AAGAGTTGAAGAGAAAGAAGGTATTCCTCCACAACAACAAAGACTCATATTTTCTGGAAAACAAATGAATGAT
GAAAAAACTGCCCAAGACTACAAAGTTCAAGGAGGATCTGTTCTGCATTTAGTACTAGCATTGAGAGGTGGCA
AGTGATCTGTTGCAACAATCATCTTAATGTAACAACACTTATTTAATTTTATGGTTCT
TATAAAATATAACTGATTAAAGTAATGTACAACACTTATATTTAATCATAAAAAAACTTG
>1002050544_1 Heli.1-ES587366.1.5
AGATCTATCTTTATTCACAATGGAGAAGTACGAAGCAATTACCAAATACAAGACGTCGTATTATACTTTCCAA
ATGCCGGTTAGCTTGGCGCTGCTTATGAGCGGAGTCGACGATCCTGAAACACACAGACAAGCGAAAACTATAT
TATTAGAAATGGGAGAATTCTTCCAGATTCAGGACGATTTTCTCGATTGTTTCGGTGA
TCCAGCTGTTATTGGTAAAAACGGAACAGATATACAAGATGGTAAATGCACTTGGCTCGCAGTAGTTGCTTTA
CAGAGGGCGAACCCGTCTCAACGTCAATTAATGGAGAACCACTACGGGAGTTCCAACTTCGAAGATGTTCAGA
AAATAAAGGATCTCTATGAAGAACTGCAATTGCCTCACACATATTCCGTATATGAAGA
AGCCACGTACGATCTCCTTAGGACTCAAATACAGCAAGCCACCAGAGGCCTTCCACATGACTTGTTCTTTAAA
ATACTTGACAATATATTTAGGCGCAGTATTTAAATATTTTTTATGGTATTAGGATAGGAATGTTATCAAGGAA
GTTCTAACAGTGCAAGTTCATTAGTTCATGTCCTATAAATATTAAGAACTGTTTATAT
ATGAGATGATTTATTTATAAGAAAGGCTTATTTAATGACTAGCTTTAGAACATGAAGTTAGGGGGAGAATTCG
TTATTGACTTAATATAAGTTAAGTAATTAAGAAAAATGTAGGAAGGTTACTTTTTATCTTGTTTGTCAATGAT
AAAATATAACATAAATAATATTACTGTTTTTATAAAGAATCATCACAAAAAATGTATA
AGACCCCTGGATAAGGGTGTGAGGTTGTGTTGGTTGTGTTTGTAATAT
>1002050545_1 Heli.1-DT663968.3.5
GCACGAGGGTGTTGACTGCCCAGAGTCCACGAATTCGTTTCTAGTTTCATCTTCCTAAAGTTGTTTATCGGGA
TTTGTTTATCAGATATTGAAAATGTCTCAAACGCTTACGTTCGGTGACTCATGCGCCCCGATAACTTGTCATG
CGTGGAATAAAGAGAGAAATCAAATAGCCTTTTCACCCAACAACAATGAAGTCCACAT

TTACCAAAAAGAAGGTAGCGAGTGGAAACAGACAAACAATCTTGTCGAACATGACATGAGAGTGATGGGCATT
GACTGGGCACCTAATACAAACCGAATAGTCTCTTGTTCAGTTGACCGTAATGCTTACGTCTGGACACAGGATG
AAGATGGGAAGTGGAATACAACTTTAGTATTGCTACGTATTAATAGGGCTGCCACATG
TGTTAAATGGTCACCCATGGAGAACAAATTTGCTGTCGGTTCTGGAGCTAGATTGATATCTATCTGTTATTTT
GAGAAAGAAAATAATTGGTGGGTCTCCAAGCATATTAAGAAACCGATACGTTCCACTATAACCACTTTGGATT
GGCATCCTAACAACATACTTTTGGTTGCCGGTTCAACCGATTTCAAAGTGCGAGTCTT
TTCAGCTTATATTAAAGATATTGAAGATCAGCCCGGGCCTAATGTATGGNGATCTAAGCTACCTCTTGGGCAA
TTGTTAGCCGAGTTTCCATCGACTGGCAGTGGGTGGGTCCATAGTGTTTCTTTTTCAGCCAGTGGTCATAAAG
TGCCGTGGGTTGGCCATGACAG
>1002050546_1 Heli.1-EL599893.1.5
GCACGAGGGTACGCGACGCANATGGTTTTACATTTGCCTATTAATTATGTTTGTTAACGTTGAAACGGAAACA
GGAAATTATTTCGATTCGTGATTTTTATTCTTATTAAAACAGTGTGTGCGGTAGACATAGTAGGCTTAGTGAT
GTTTTCTCATAGCAATTAAATTACGGCACGATTGTCGATTTTACGTTTTTTTTCTTAT
AATTTTGGTGACACAAATACAATTCGATTGTAATTTTTTGGTATCGTGTGTTTAAAATTATTGTAGGTGTTAA
AATTGTTCATTTTTGTTTTAAATAATTCGTAATATACACTTACTGCCTTATGAATATTTGGAAATTTGGAATA
ATTCGACGTAATTATTGTAGCTTTATGGAAGCTATAGGACGATAAAGGTTTAACTGTC
ACAATATTTAACCGTGTACTTAATCATTCCAAAATTAATAATGTACTTACAAATACTATTCGATTATTAAATA
TAATAGACGATTAATTATTATTATTATTATTATTGTTGTAAATTTTTAATCACAATTTAATTATTTTATTGTA
AGATTTCATAAAGTTTTTTAATATTTAAATTGATATTTTATTAATTAAACATCATTCT
CACAATAACATGCAAATTTTAATATCATTATCTAGATAAGATAATTGCTAACTCCGTTTTTTAATGTTTATGT
TTTTAAGGGATAGTTTCCCACTTTACGCTCAATTTTAAAAAATTTAA
>1002050547_1 Heli.1-DT665195.3.5
CGGCACGAGGGTTCTTTTAAAAGCTATTCAATTGTGAATGATCATTATGTTATAAAAATATTAAGGATGTACT
TTGGATTTACTAGATCTATTGGCTAGAGGAGATAAATGTTAGCGACAACGAGATTCAGTCATTAGACTGCATC
CTGCGACTGCCACGATTACGCGCAGTTATAGCAGCTCGAAATCTTATTACAAATTTCG
GGACTAGTAAGACAGCGCAAATGGGTCCTGGAGAGGAAAACAGAGGCGAGTACCGCGCGCCGCTGACAAACGT
CGATCTTCGTTACAACAGATTAAAAGGAAGCATTTTTTTTTGGTAATTATGAACATCTCGTCTCGCTCGATGTG
TCTCACAATGCTGTGGAGGTGCTCGTGGTGTCGGCGCTACGAGGCCTCCGCGAACTTT
ACGCGGCCCACAACAATATACAGCATCTGGCATTACACGGCGCCTCGCTCAGAATATTACATGTGCCTTATAA
CCAAATGGATAGCTTAACGACGATGGTGCCACCAATAAATTTAGCGGAAATAAATGTTTCACATAACAAACTC
ACAGCACTACCTCAATGGCTCAGTGGGTGTTCAGATCTGACCAAGCTCTACGCTAGCA
ATAACCAATTAACTTCACTCCCAGAACATCTTTTCTGTAGTGAACTGTCAAGCTTAAGTCATCTTCATTTAGC
TCATAACAAAATAACGAACTTCCCGTCCATGCCTAGACTAANGTCGCCGTTGAAAGAACTGTTATTACACGAT
AATTGTATACAAGCACTTCCAGAAACTTTTTCTCTATATGTGATAGATTATGTGTGTT
>1002050548_1 Heli.1-DT663559.3.5
CGGCACGAGGAAACAACTCGTTAATGATCTATGGCGACTAGAACAATGGTTACAATTCGCTGAAGCAACAGAC
GAAGCGAGGATAGATCCTCCAGAACAGTATGATGCTTTGGAAGATGCTATACAGGACCATCGCGAGTTCCTTC
TTGATCTAGACTCTCACAAAGCGCTGGTAGTATCTCTCAACGTGGTGGGCGCCCACGT
GACGCGCCATGCACGGTCTGCCTCCGATGCGCGCAGAGTGCAAGAACGGCTAGCAGCAGCCAACACTAGGTGG
GACAACGCATGTGCGCGCGCCGCCGCCTGGCAAGGCAGGTTGCAGATTTTTTTCGTTAATAATAGAGAATTCC
ATGATATTGTCGTCGAACTAGTATCACAACTGGCAGCAGCCGAGAAGTCCGTAAGAGC
ACGAGAGCCGCTGCGTTTAACTCGGCCTGCGCCGGAACTCCGCAAAGACTTCCGCCGCTTCAGCGAACTCCGT
GACGAATTGAGCAGAGCTGAACCGCGTGTACTCGCGCTTAGGGATGCTGCACAATTATTGACAAATGACGCGC
AGGATGTTTGTCGACGGCTGGGCGAGCTCCGCCTCCGTCTCCAGTCGCTGCGCAAGCT
GTCGGGCGTGTACGCGCTGAAGCT
>1002050549_1 Heli.1-EL596582.1.5
GCACGAGGCGCGATCCATAGAAGCAGCGGCCACGACAGGTGAGCCACAGCCGACACGCGGTCACTCAGCACGA
ACCGCGACTTATCCTTAACCCGACTACACTTCCTCCGACCCCATGAGTGGGGACGCCGAATACATTACCACAA
ACTACAACATGCGTAACAACGTGAAGCCGTCGTTTTGGGCGCAAAGGACGAGAATGGA
AAAGAGGTTACTACTAGCCGTTGGGGTGATTTCTGTGATCGCAGTTGCTTTCCTCGCGGCGTTCCTTGCCACT
GTGCCCGTCAAGACATCAGCTGATATAAATGACATGCCACAGACTAGCGAACTAAGACTTACCCCGTCCATGC
CGCCAGCCGTCATCGCCAGGACCGACAGCGACAGCAAAATGTGCAACGCACCTGGATG

TATCCATACAGCGTCAAAATTGCTCTCCAATATGGACGAAAAAGTGGACCCCTGTGACGATTTCTACGACTTT
TCCTGTGGATCTTTCCTGAAAAACACGCGGATACCTGACGACAAGACTTCTGTGAATACCTTCTCCATCATCA
CCGACCAACTACAAGAACAAATCCGTACACTTCTTGACGAGCCGATTGCTGAAGACGA
ACCTCGTCCTTTCGCACTCGCAAAGACGTTGTATCAAGCGTGTATGAATAGAACGGCCATAGAAAGTCGTGGT
GTCAAACCTCTGCTTGACATGATGGAGCGCCTAGGCGGATGGCCGGCGCTGCAAGGCAGCTCATGGGACGAAG
CTTCCTTTTCCTGGGAAGAATCTGTATACCGCTTCCGCAGAGCGGGTTATTCCGTGGA
CTACTTCCTTGACTTTTCTATAAGCGTGGATGTAAAGAACTCTACTAAGCGTATTATAGATTTGGACCAAGCC
TCCCTTGGATTGAGTCGAGAATATTTAAACCGTGGATTCAGCGACAAACTAGTGCTTGCTTACTACGACTACA
TGGTGGACATCGCTACTCTCCTAGGTGCTGATCGTGCTCGAGCTGAAGTGGAACTCAA
GGATTCTTTACAGTTTGAAATGAAACTAGCCAATATATCGTTGCCATTAGAAAAACGTCGTAATGCCACCAGT
TTATACAACCCAATGACAATAGGAGAACTCCAACGTANATTTCCAAGGATTCCATGGCTGGCTTACATAAATC
GTCTATTATC
>1002050550_1 Heli.1-ES586206.1.5
ATTAATATCAGTTGTGTGTAAATATGAGTCAAAGATTACTTGTAAGTTCTGACTTGAAGAGTACATGAAAGAT
GAAGTCAGGGCCTGTTGTGTAAGGGCAACATGTCGAAGAAACATTCGCATCGCACTTGAGAGCATTGCGAAGA
AATGTGCGTCCGATGCGATTGTGTTCTACTTCTATGGTACAAATTGTTACGATTTTTG
TTCTTGTGGAAAGCTTTAATAGGTTATGAAACACTACAGTAACGAGTGCTTCAACTATTTTCGGTTTTTTATT
ATTAAAAGTAATAAGTAAATTGCAATTATGGAATCCCAATTTACAGTCGATCCATCTCGTTTGCTGTCACAAT
CCAGAATATCAGATTTCTTTCTCAATTGGATCGAAGATGTTGTTATTAAATGAAGCTA
TAACGAGGCTATAATAAATTAGATCGTTCGTGATTGCGAATTCTCTCGCCTCCTGAAATCATATCTACGAGTT
ACCATTAAATAAGTCTCTAACTTGTTTCCATTTCAAATACTATTAATAATGAGTGGCTGTCTATTAAACTTAT
TGATGAACTATAAATTATTAGTATTAAGACTGCCATAATTATTATTAAATTAATCGAT
CACAACGATTCGTCTAATATTTTTTTTTTTAAAATATATAAAGTATCAAAAAATTCTCGATATTTACGAGATT
CTTTACCCACTGAATACTTTGTTATGTTGATTTGTGTGTATTGTTAAGTTTATATTTAGTTGTAAGTTTTTAG
ATGATATAGCGGAACCAATGAAATAAAGAATAAGATTTTTCTTCAT
>1002050551_1 Heli.1-CO729680.1.5
GCACGAGGATTTCTGTCAACTACCGCGTGTCGTGCCGGTTTTCAGCGTTTACACTTGTAATTTTTGCAGCTAA
CTTCAAAATGGCAATCCACATCAAGGACTCCGATGACCTCAAGAACAGGCTTTCTGAGGCCGGTGAGAAGCTC
GTCGTGATCGACTTCATGGCGACCTGGTGCGGCCCTTGCAAGATGATCGGGCCCAAGC
TAGAGGAACTGGCCAATGAAATGGCGGACTCCATTGTTGTACTTAAGGTCGACGTAGACGAGTGCGAGGACAT
CGCCACCGAGTACAACGTAAGCGCGATGCCAACCTTCGTCTTCGTGAAAAGTTCTAAGAAACTTGAAGAGTTC
TCTGGAGCCAACTACGACAAACTAAAGAACACCGTGTTGAAACTAAAGTAAAAATATA
AACAATGTATATGTAACAGATGAGGCAAAGGAAATAACTTGCTTTACGTGTATTTTCGTATATTATTCATAAA
AAAAATGTATTTATAATATTTAAAGCATAATAAAAAGAGCGTAAATGATTATTTGAATCTCGTTTTCTACTGT
AATTTCTCTTAAAATGGATTTTCTTGACACCATTGGGCTTTTATTGTATGGCGGTTAG
TGCGGGTTCGATTTCGATTTTTTTTTATTTATATATGAAATGTTGGCAAGGAGCAATCTAATATTTTTATATA
ATGGCCGTAAAGTGTCTTAAATAATTTGTATTTTAGTAACACGTAGCGGATGCACAAGTACTGGCCTACTG
>1002050552_1 Heli.1-DT668429.3.5
GCACGAGGCTACACCTTATTAATAAAGGTCGTCGACACTTCCACCAAGTATAACTTTATTTGACAATTGTAAA
TTAAACCCATTTTTTTTTATTTAAACTTGTGTTGTAAACGTTTTAGAATAAAAAAGAAATAATAATTTCAATT
TGTAATATTATCTTTTTTTTTAATTTTTGTTTTATAGTTTATACTTATGGTTATTGTA
TATTTAAATATATAAATTTGTTAATTTAACTGCGCTGCTATCTTTAGTTAGCAGCGAGAAATTAACATTGAAA
AAATATTACTGATTGTTATTTACATTGATATTCAAAATTAAAAAGGAAAACGAATAAAACAGAAATAGATATG
GACGTAATATACCTAGAGTATCAATTTATTAAAGTTTTAGTTTATATATATTTTGGTATA
AATTTTAAAATGTATTATCACGTAACATAAACCTTTTGAACAAATCAATATATCCATAATTGAGGAGTATAAA
AGTTAAAGCGCTATATTTCTCATTTCGTATATGTTACTAGCAATCTGTATAAATCGATGTTCTATACAATACC
TGGACAGTGTATAAAAAACCCGGGAAGTCCAGGCAATGTATGAAATATTATGATGGTA
TAGAGTACAGAAATTATACAAAGTGCCCGTAACATATATTTATAATAGTAATTTTAATTTATTTTGATTCATA
TTAGAGCCAACATGTGATTGTAGAACTAATTATTTTTCTATCCGAAAAGAAGAAATTCCCAGTTTACTCTGTT
TCAGTATTTTCTTACTGAAGTGCCTTCGCTTTTATAGTCCTCAATTAGTACGCTGGTA
GGTTAAGTAAGTGAACCATTTATATATTTACTTAATATTTAATTATAACGCCGCAGACTCCATTTTGTTCGCT
TTGTCTTGATGTTTAATAAAAAAAAATATAACTTTGCAAAAACACTTACATCGATATTAATATCTTGTATTAG
GTAATTATATAATTATTCACAGCAATTTTAATACAGATATATATTTAATATTTCCTTT

TTCTAGATTTCAAAATTTGTTTCGGTGTTTGTTTCCTTATTGAGTATTTAAAAAAAATGTCTGAAGAAAATAT
TATGTTGTAAACAATAAAGTATGTTCGTGAATAAACTTATCTATTTAGAACAAGGATATGCAAGCATAAACAG
CCATAGCTGCATACGCTTAATAACCAGTTTTCCTCCTTACTTTATTTGTGAAGACGAG
TTAAATATAATATATAGGTAAAAAATGTATATATACTGTACAAAGAACAGTCTACTTCGACATATATTTATTT
TATTATTATACATTGACTAAATAATGCATAAAATAAACAATATTTACCTAACATCGTTTACTATATGGCATAT
CATTTAGTGGTCTAGGTTTATATTTCCATTTCTTCGCTCTGGTGTCGAGGTTAAGCGC
GGCGTTGAGTTCTGCCACTCATGCCTTAATGCCTCCAGAATTCAGCAGAAAGTGGGGAAC
>1002050553_1 Heli.1-DT667894.3.5
GCACGAGGTGAATTTTAATTTAAATCCACGAACGTGGGCGGTGGGATACATCTTAGGTTATAGGTTGTAGTAA
ATATAATATTTTGTATTTTAAATATATTAAGAAGAAATGGCTGAGGCAAACGAAAAAATTTCAAAGAACGAAC
TCAAACGGAGGCTAAAAGCCGAACAAAAACTAAAAGAAAAAGCTGAGAAGGTAGCTCA
ACAGCCAACTGTTATTGCCGAAAAAAAACCAACAAAAGCAGAAGAGGAAATTAGCCCTAACGAGTATTACAAA
CTTCGATCAGGAGCAGTAGCAGCTCTTAAACAAGGAGCTAAAGAAGATCATCCATATCCTCATAAATTTATCG
TTACTATTTCTTTAGAAGAATTTCTTGAAAAATATAGTAATTTGAGCAATGGAGATGT
GTTGGAAAACATTTCCGTATCTGTAGCTGGCAGAATTCATTCAATAAGAGAGTCTGGAGCAAAACTTATATTT
TATGACTTAAGGGGCGAAGGAGTAAAACTACAGGTAATGGCCAATGCAAAACTATATGAATCTGAGGACAAAT
TTGCTAGTGACACTGATAAACTTAGACGTGGGGATATAATTGGTTGTATTGGAAATCC
TGGTAAGACCAAAAAAGGAGAACTTTCAATAATACCAAAAACAATAAAATTGCTGTCTCCCTGTTTACATATG
CTTCCGCATCTACACTTTCGGCCTTAAAGACAAGGAAACCAGATTTAGGCCGAGATATTTGGATCTAATTTTA
AATGATAGAGTCAGGCAAACTTTTTACATAAGGGCAAAAATAATTT
>1002050554_1 Heli.1-ES585966.1.5
GTACGCTGCAGCCATATCCTGGTAAAGCATGCTGAAAGTAGACGTCCATCGTCTTGGCGTGAAGAAAATATCA
CTAGAAGTAAGGAGGAAGCAATGGAAATGCTTAAGAATTATCGGAAACAGATTGTAGCCAACGATGCTGCATT
TACTGATATTGCAATGAAATTTTCTGACTGCTCATCTGCTAAGCGAGGTGGGGATTTA
GGGATGTTTGGACGTGGTCAGATGCAAAGGGCTTTTGAGGAGGAAGCTTTCAAACTCAAGGTCGGGCAGCTGA
GTAAGCCAGTGGAGACTGACTCAGGAGTCCATATCATATTGAGGACTGTCTAATTTGTTCCTTTTTCATGCAT
TTATGTTTTGTTTGGTTATAGGCATATCTTTGTCATTGTTAAATTTATACTAACTACA
TGTTTTATATTTCTTGATTAGTGTGGATAATGAGTTTTCTTAAAATATCAAATGATTGTAAAATTGACCTTTT
AATCAAATAAACTATAAAAATAT
>1002050555_1 Heli.1-ES585786.1.5
TGAAAATGGAAAAGTTAAAGGATTGAATGAAGATGCTTTGAAAATGTTGGTCCAGTTCTATTTGAATACTCCA
GAGGAAAGAAATTATGATATGAAACCTTATTTAGGCAAAGAACAAATAGTTGCAGATATAGATGATGACAATA
GACGTTGCTGGTTAGAATCAACTTATAAACATATTGTTTCAAATCGTCCAAAGCATGA
CTTATTACCAGAAATCTATCTGTGGGAAAAGATATATAAAATAAAGTTCAACACACGGTTCTTTGAAGCAAAG
AGACGACCATTTGAGTGGGGAATTAATCCATTTAATAGACGATTGGATGAACATTTGCCTCCTTATATACCTA
AAGTTTTGAGGCCTTATCCCAGATCTAAGAAGAAGTTTGAAACTACTTACTATCCAAA
AGTGTAATATGTACAAATTAAATTAAGTAATGTAGTGGTAAACTTTAATAAATTAGTGAT
>1002050556_1 Heli.1-DT663454.3.5
GCACGAGGCCGCTGTCAGTTCTATACAGCTGGAGAATGCTATTATAAAAAACCTAATACCAGGCATGAAAAAG
TATAACAATTCAAAGCGTCTAACAGATTTACTAAATGAGACAGAAACTTATACATATACATCAGAATGTCCAG
CTGGAGGATGTATATGTATAACTGATGATACCACCAGTTTGAAACAAGCCATGAATTT
GATGTCTAATTACGGGTCCAGCAATGATTTTACGTATAAATTATGGGATATTTTAAGAGACTGTGAAACAGCT
GAAGAAATGATAACATTATTAATTCAAGCTCTCAAGTTCATTTCGTCAGGCAAGATAAGGCCTTTTATCGACG
TAAACAACAAGACGTATCTATCAAAATTGGTGCTGAAATTGTCTCGTGGACATTCTCA
AACTGTTAAAGTATTGAAAAATCTACGTTCCAGTCCGCCCCAAGCTTTGTCTTTGGTGGCTCAAGTGGGGTTT
GAGAAATCTGTATGGGAATATACTAGGATAATGTCGTTGTTTGTACATTCCTTCTTTGTTGCTGGCATTTGGA
ACGCGGATTCTAGATCCCAGGAATCAATAGAGCAAATAAACCAAACCATCCAAGATAT
GACAATGGGTGGTGATTTCACGCTAAACCCCTTCGAAACTATAACCACATCTGATCACTCCATACGTTTTGAA
ACTGAATCGATTTGCTTGGATGATGGCAATGAACTGACAGTTGATGATTTTGCATCTTTGAAAAAACATGGCT
TAGTTAGTGAGAAGAAAGATGCTAATGAGGTACCGTTAATAACAGATGAGATAGACAT
AGGTCCGTGGAA
>1002050557_1 Heli.1-EL599864.1.5

GCACGAGGCATGGACTGATTGACTTTTGATTTCTACAAATGAACTAGAAAAAAGCTTCGGTCATCAAATAATA
TTTATTTGGATTACAGAGAAAAATAACTTGAACTTTTGCCTTGTTGAACATCGGTGCTACAATAAACGTTTTC
TTTCAAAATGTCGAAAATAAAGGCCGGACCCGTTGTTGACATCCTCGGCGATGAAATG
ACTAGAATTATTTGGGATTTAATCAAGGATAAATTAATTTTGCCATTTTTGGACATTGAATTACATGTTTACG
ATTTGGGAATGGAAAATCGTGACAAAACTGATGATCAAGTAACAATTGATTGCGCAAATGCGGTTAAAAAATA
TAATGTCGGCATTAAATGCGCTACCATCACTCCTGACGAAAAAAGAGTTGAAGAATTC
AATTTGAAAAAAATGTGGAAGAGCCCTAATGGAACTATTCGTAACATTCTTGGGGGAACTGTTTTTAGGGAAG
CCATTATTTGTAAAAATATTCCCCGATTGGTGACTGGATGGGATAAGCCAATTATAATTGGTCGTCACGCGCA
TGCTGATCAGTACAAAGCTACAGATTTTGTAGTGCCTGGCGCAGGAAAATTGGAACTT
GTGTTTACACCTCCATCTGGAGAGCCAATTAGACATGTCGTAAACGAGTATAAAGGCGCAGGTGTTGCTCTCG
GTATGTTCAATACTGATGAGTCTATTATTGATTTTGCACACTCATCTTTCAAATACGCT
>1002050558_1 Heli.1-EC091318.1.5
TTTGGCCCGAGCTACGAGCTTTTGCCCCTCGAGTTTCGCGATATCATCTACCGACACATTATTTTGCTTTGCC
AGTTGTTGGTAACTCGCTTTACGTTCGGCATTAATGTCGGCTACTAATTTCTCTGTTTCAGCATCCGTTTTCA
GCGCAACCAGATAACCGTAAAATGTTTCACCTACCCGCCCCTGAGTTCTGGCTTCATC
CAGAGTCAATGCCATTACATTGCTGCTTACCAGCCCAACAAGAAACGCACAAAGAAGTAATGTTTTCTTCATC
ATCACCTCAGAAAAGATCGCTACGAGTTTCAAGCAGCTCTTCGACATCTTTGTCTGCCTTGATGATGATCTCA
TGCTCAATTTTAACGTTCATATTGATAGTGATCGGTTCCTTAGGTGCAGCGACTTCAA
TGCGAGGCGTACAGCCAACCAGCATAAAAGATGACGTCAACGCAGCCAGTAAAATTTTCATTGTTTTTCCTCA
CACTCTTTTCCTTGCGGACAGTCATTTCCCGGCAGACGTGCGTTCTGCTCCAGCCATGCCTGGAGATTATCGC
CAAAGCGTAAACTGCGCCACAGCGTAAAAATATTCTCTTCATGATGGTAATTAAGGTT
TACCGTACCGCTTTTACCATCAACGCGACTGGTACCTGTAATGTTGGCCTGCATGGTTAATAAACCGAGATTA
TCTAAATTAATTTTTGTCGATGAACGGCTAATTTCCATATAGCGCAACCAGTTAATTGCTGAACCCGCAGTCA
TATTGTCTTTTACCACCGCATCCGCAGTGTCTTTATCCAGTCGCAATGTCATCGGCCC
GCTATTCGGCAGCCAGCCATCTTTCACAATCCATTTTTCATTGTTCAGCCATAACGGCAGTGCACCACTAAA
>1002050559_1 Heli.1-EL602796.1.5
GCACGAGGCCTCGTGCCGATTCGGCACGAGGAAACAGTTTATTTGTTTCGGAAATTAATTAATAATTTCTTGT
AGATAACAGTGTCTGAAGATAATATTGCAAAAATTAAGCAAGTTCTTGTTGTGTCTATAGTGTACAGAATGAT
TCAACCGGATTACGCCTCTGCAATGGGCTTACTAGCTCATCTAAATTCAGATGAATTA
AAGGAAATGCTCAATGATGATACAAAATTCGATTCTGTCCTTAAAGATTTTAAACAGGTTAAGGACTGGGAAA
CTGAAAAAGAAATGATTATTGCTAGTAATCGATCACTAGCAGAATTCAATTTAAGTAAGGAGCCAGAATTAGA
AGAATTAAAGGCGCAAGTTCAAGAAAAATCTAAAGATGGTGAAGAAATGTGCACTCGC
ATACAAGACCTGTTAAATGAATATAAATCAAAGTCAGCCGGAATATCTCCTGACACAACATTAGCGTTACTAC
AAACTGCAGCAGCTGAAGCAGAAGAAGAATCAGAAAATATATCACAAGATTTTCTTTCTGGTAAAATAGATGT
AGACAAATTTCTTGAAGATTTTGAACCAATTCGTAAGACAATGCACTTGAGAAAATTC
AAATCTGATAAAATGAATGAACTTTTAAGAACAAATCAAAATTCTTATGGCAATGGTATTGCTAAGCCATATT
TACCGTATCCAAGTTATGGCCAAACTCCAGGTGTACCAAATATACCTTATCCTGTAGGCCCTCTTAACATGCC
TATGCCAGGTATGTATGGAAACCACTTTTGAAAACCCCCATATGATCTTATGAATATA
TATATTGAGCTATTACGTAAATATTATTTTAATCATAACAAAAATTCA
>1002050560_1 Heli.1-ES585958.1.5
TGTGTCTCTTATACGTTAGAGGCCTGTTAAGAGTTTATAAACATATCCTGTAATTCTATATAATAATTACAGA
TGTATAGATGTTGAATTTATCAGCGCCAAGAGGCGCTGGTTCCAATTCAAATAAGATTAAAGATTTGAAAGTG
TTCTTTTTAGATTAGGCGTTTATTTAACATATAATTAAATGTTATATTGTTCTCGTAT
AAGAAAACACTAGAATGACGTTGATTTTCTTAATTCATACATTTTTATATAAATAAGTACATATTTATAATGA
TCAACTTGCTGCTATGTCTTTCTAAATATTTCAAAATTCTATCAACTTAGATAAAAAAAAAACGTAAAATTAA
ATTAAACAATAAATTAAATTTTGGAATAATTTTGTAGCAACTGGGTTTGTGGGCGGTT
AAAAAAACGCTTTTATATGTCTCGAAAGAATTTGATTATATACATACATTACAATAGTGAAATAATAGATTTC
ACAGTTATATATTTCTATTTTGTCCCTATCTGCTCATGTCTTGTATTTAGATTCAGACGTAGAATAAATTTGT
AATACCCACTCCGCGGCAACAGGATACTTAAATAGGATTAAATACGTGGTATATCCCA
CTTACGACTTAAGTATAATATTAAAATTATTACGATTCAAAATCGATATGTAACAATTATTTTAATATTTAGA
ATAAAATATTAAAAACTAAACGCTAAAACTAAGTAAGTATTTGTATATACAGAGGCCCTCAGAAAGTCCGTCC
ACTTGTTTGTAAGAATATGTTAAGAAATATTTTACATAAGGAATTTGATTAATATTGT
AATTAATACTGCTCACACATTTATTGTGTAACGTGAGAATATTGTTATTAATAACATGGTATGATACTTTACT
AATATATTATA

>1002050561_1 Heli.1-DT664612.3.5
GCACAGGTATGTGTTATAATATTAATATTTACAATTTTATCACACGTTGGTGAGTCAACTTCGACATCAGCGG
CGCCATGGTACGAAAACCTTCCCGCAGTCACGATGGACTACAAAGTGCACATCGACGCCGGCAAGGAAGACTG
TTACTTCCAATACGTCCAACCGGGCGCTACATTTTACGCGAGTTATCAAGTGCTAAAA
GGCGGTGATGGAATGTGTGGGTTCGCTGTGCGGCATCCAAATGGTCAAATAGTGCACCCTTATGAGTGGCGTC
AGAGCGCAGAGTATGCAGACCAGTCCAGTTCTGGTGGCTATTATGCAGTGTGCATTGATAATCAGTTCTCAAG
ATTTGCAGGGAAGCTAGTCAATTTGTACCTGTCTGTTATTAGATATGATAAGTGGGAA
CAATACGCAAAGGAAGTGGAAGAGCTAGATATGAATATTAAAAATTTCACTAACTCAATCCAGTTTGTGGAGC
GTAATGTAAATGACATTTTGCAATATCAGTATCATTCTAGGGCAAGAGAGTCTCGGGATTATAATTTATTACT
TGACAATAATACTTATGTATTAAGGTGGTCTCTTGTTCAAATCCTAGCCGTAGCAGCA
ACTGGCACACTACAAGTATATTTTGTAAGAAAGCTATTTGAAGTTAAGGATAATTCTTCTAAGACAAGAATTT
GATTGAATTCAGAATTTAAAAGTTAAAATCACTACTGACTGAATATAATCATTCCAGGACAAATTTCTCAAAG
TACACATTCTGATCTCTAATTTTTTTAACCATTTTTTCTAATCTCAAATAACGTTTTA
TATTTAGGTTTTTTACATTCTGTTAACGAATTAGTCCTCAATGTTATATCAACCTATAATAATAAAAAACCTT
TTTTTTCTAACAAAGCTTTAGGATAATGCATTTTTTTACTTTTTATAAATGATAGTCAAACCTTTCCCATA
>1002050562_1 Heli.1-EL597726.1.5
GCACGAGGCTTCAATTCTTGCGATTTCGTCATCAACCACGCGAGCAGAGTTTACCGGTTAACAGATGTGTTAT
ATATAAGCCGAGTGTTGTTCATTTATTTAAATGAGACACATTTTACCATTAGTTTATAATTGTAGTGCTTTTA
GTATAAAATTACCGGGTCTCTTCGCTCAACTTAAATTTGCACATAATCTTCCGAACTG
GGGAAGTAATAAAGTTCATCGAAAAATTAAAATACTAAATCCATTTCGAGATATAATTATGGCTGATCCAAAA
ATCGAAGAAATTCTGGCGCCACTTCGCGCTAGTGTTAAAGAACAAGGCGATTTGGTTAGAAAATTAAAAGAAG
AGAAAGCCCCTGAGATAGATATAAAAAAAGCTGTCGCAGAATTAAAGACCAGAAAGAA
AGTACTAGAAGATAAAGAGCTTAGCTTAACCCCAGCTGAAGAATTATTTGATCGTGCGAAAATGGAGGATTTG
ATTAAGAGAAGATTTTTCTATGACCAATCTTTTGCTATATATGGTGGTATTACAGGTCAATTTGACTTTGGAC
CAATGGGCTGTGCTTTAAAAAGTAATATGATTCAACTATGGAGAAAATACTTCATTCT
TCAGGAACAAATGCTGGAAGTTGATTGCTCAATTTTAACCCCTGAACCAGTGCTTAAGGCATCTGGTCATGTA
GAAAGATTTGCTGATCTCATGACAAAAGATGTTAAAAGTGGTGAATGTTTCAGGTTGGATCATTTTATAAAAG
CTCATCTAGAAAAAATTAAAAGTGAAAAAATACAAAAGCTGAACTCA
>1002050563_1 Heli.1-EL597690.1.5
AGAGTTAAATATTTAAAAAAACTCTTAGTAATCTCATGTACTCACTATTAAAAGTACGATTTATACTATTTTA
TACAGATTAGTCATAGACCAAAGTCGAATTACAAAACAAAACAACACTATAGAAAAACATATTTTTAGCGGCG
TTAAGTTTTGCCACTGAACACCGAGTGTCTCGAATATAGACGGATAGAGGAAAACGGA
GTGTCTTGACACTAAGTTCCCTTTAACTTCCCCGCTATATGTGGGATAAAGAAATTAAGAAGTATCTTTTTTA
AACTAAAGGCAGCATTGAGTTCTGCCATTTAACATGCAATGTAGTTTCTATAATATAAAAAAGTAACACATTT
TGATGCGACTTTGCTCTAAAATTACTAAAAACGTGTGCTTTATAATGATATTCGTAAA
TTATAGATATGATTATCTAATAAGCCTATTTAAGTACTTTTTAAGACTTTTTATTATTATACGTATAAAACTA
TCCGTCCGTTAAAATCGGACTAACAATTTATATTATTATTTAGTTATTTTAGTTACTTTTGAGTTATATTATA
AATATTTTTGTAAAACTCGACTACGAGCTATAAATTGACTGACATTTAATCAGTAAAA
AATAATCTAAACAAAAAACAACAAAAAGAAAAAACTACTAAACTAACATAAAAAAACAATTTACTTAAATTGA
TAGCTTGCTAGCTTTATATTTAAAATTTAAATTTCAATAAAAATTGCTTAACATAGTCGAGTAAAACAGGGTT
CTTCTGGAGGCTTTATTATGTAGAAGTCCCTTTATTCACTGTTTTATATTTTTTAAGG
TACAAAATATGTTATTGTCGGCTGTATATTGATATTATATGAACAAGAAAATAATATGAAATATTTGTTCGAT
AGGTTCTATTAAGTTATTCAAATGACTTTTAAACTATTCTTATTATA
>1002050564_1 Heli.1-ES587232.1.5
TGTCTTCGTGCTTTTATCTCCTCGAAATTACTACATAAATAGTTAATTACTTAGTAATATATTCGCTACTTGT
GTGTTCCAAAGCATCTTTTGAGGCTACGCCCTATTTTGCACGGACTCCGTTGTTCTTTTAGCCTTCAACATAG
GCCTATCAGACACTGCCGTAATGGATATGATGGTATCAACTTTACAACAGCAACGAGC
TGTTACTGAGCAACTTCGAAGGGAAGCCGCGATAATGCGTATTCCTGTTTCTGTAGCTGTGGCAGATATTGTA
CGATATATAAATGAACACGAACAAGAAGATTGTTTGCTTGTCGGCTTTTCAAGTCAGAAAGTAAATCCTTTCA
GGGAGAAAAGTTCTTGCACTGTTCTTTAAACTAAAACATGATATTGGCGTTATTTTTG
TGGTAATTGAAGCACAGGATCTTATTTGTAACATGTGAAATTTATATTTTATTATAACAACATAATATAATAA
ACTGTATTTGCTAACACTACATTTATATTATGTTCTCATAAATGTAATATTTTTAAGTCCCCACACTATCTGT
ATTTATTAAAGTAACTTCTCT
>1002050565_1 Heli.1-DT661925.3.5

GCACGAGGGTGATGCCCACAGTAGGGGCCCGGGCTGGGGACAGTTCATGGAGGGTCAGATATATGGTGGCCGA
TAAATTTGTAGAACTCCAACAAGCAGTTGGTCCTGAATTGGCTCGTTCAGATCTGGCTCAGATCTTCCAAGCT
CTGCTCAAAGATACAGAGGCTGAAGTGCGAGCTGCAGCTGCTGGGAAGGTAAAGGACT
TCTGCATGAACCTGGACCAGGCGCACCAAGAACACATCATCATGACAATGATCCTCCCCCAAATCAAGGATTT
GGTCTGTGATCCCAACCAACATGTGAAATCTGCTTTAGCCTCTGTCATCATGGGATTAAGCCCAATTGTAGGA
AGACAGAATACTATAGAACACTTACTACCATTGTTCCTAACACAACTTAAAGATGAAT
GCCCTGAAGTCAGACTTAATATAATATCAAACTTGGAGTGTGTGAACGAAGTAATTGGAATACAGCAACTTGT
ACAATCTCTTTTGCCCGCTATAATGGAACTAGCAGAAGACACAAAATGGAGGGTCAGACTAGCCATAATTGAA
CACATGCCGCTGTTAGCCGGACAACTCGGCCAGGAGTTCTTCGATGACAAGCTCACAG
GACTTTGTATGCATTGGCTTTTTGATCATGTCTTTGCTATACGTGAAGCTGCCACTCTAAACTTGAAGAAACT
AGTTGAACAATATGGCGCTGCATGGGCTGAGAGCTTTGTCATACCCAAAGTGCTGGCCATGTCCCGAGAACAG
AACTATTTACACAGAATGACTTATTTATTCTGTATTAACGTTTTATCTGAAGTATGCG
GTAAAGATATTACAACTAGAGTTCTTCTACCAACAGTATTATCTATGGCCGACGACAATGTTGCCAACGTTAG
GTTCAATGTTGCCAAAACGTTACAGAAAATGGCGCCTTTCCTCGATCCGGCGGTCATTCAGCCACAAGTCAAA
CCTGTTTTGGAGAAATTAAATGTAGATGAGGATGTGGATGTCAAATACTTCGCGTCTG
AAGCCATTGCTGGTATCGCCGGTTAATATTTTGTTAGTCCGTAATTTTTTCTAGTTTGTATTTATTCTGTAAA
AGCCCTTTTTATTACCGTTTTCATTCCAGTGCAGTAAAATAGAACACTGAACGGCATTTATCTTGCACTTTAT
AACAATTGTAAAACAATCTAAT
>1002050566_1 Heli.1-DT661863.1.5
GCACGAGGCAGAATCCTAAACAGAGGCCTAAACCCAAAAAGAAACTGTAAACATGTACTCTAAGGTATTGTGT
TTAGCTGCTTTAGTGGCAGTGGCCCTAGCGTCGGATGGCCATGGTCAGGCCTACTCCTCTCAACATATCAACC
GCCACGACGGCAAACCGGAAGTAGTGCATGTAGGACATGGACATGGTGACCAGCACGG
ACATGGCCACGTAGTTGATTATCATGCTCATCCGAAATATGATTTTGAATACAAAGTAGAAGACCCTCACACC
GGAGACTTCAAGTCCCAGCACGAGAGTCGCGACGGTGATGTAGTGAAGGGATACTACTCCCTGAAGCAGCCCG
ATGGCTCTGAGCGCGAAGTGCACTACGAAAGTGACGACCATACCGGTTTCCACGCCAA
CGTGAAATTCGGAACACACCATGTCGTCCCTCATCATCATTGAGCTTATAACCAAATGTAGAATTACCGAAAC
ATTATAGATATTGTGTTTTTATACGTTTTTTATAAATATAACAATTTATTCACAATTTT
>1002050567_1 Heli.1-EE743521.1.5
GCACGAGGTAACCTTTAAATTTTTAGATATTGCATGTGTGTTCCGTAATAAATACCAAGGAACAAAATCAACA
ACCTATAAGCCGGATGGTAAAAAAATACATATTCAATACGGAACAGGTTCTATGGAAGGATTCATTTCATTGG
ATACCGTTTGTATTGCTAATATTTGTGTTACGGGACAACCATTTGCTGAATCAACCTC
AGAACCTGGTGCAACATTTGTTATGGCTAATTTTGATGGTATTTTGGGTATGGCATTCCCGGAAATTTCAGTT
CTTGGATTAAATCCAGTTTTTCATACAATGATTAGCCAGAAAGTTGTCCATCAACCAGTGTTTGCTTTCTGGC
TTGATAGAAATCCAAGTGATAAAATTGGTGGTGAAATTACATTCGGCGGCATTGATGC
AAATAGATTTGTATCACCGATCACATACACTCCCGTGTCAAGACATGGGTACTGGCAATTTAAAATGGATAGA
GTGCTGGGCAGAGGTAAAGCAATTGGTTGTGGTAATGGATGTCAGGCTATCGCAGATACAGGAACAAGCTTAA
TTGCTGGTCCAAAGAGTCAGATCGATAAAATTCA
>1002050568_1 Heli.1-DT669010.3.5
GCACGAGGAATATACGCGGACGTTTTTTGTTTATTTATAATATACTATTATAATAATATTTTTAAAAAATCTA
TAAAAAAAATTATGAAATTTTAACATTGAATACATTTTGAGTGATGTTCTAAGTGAAAAATTGAGTTAATCAG
CTATATATTCCGAATTTGCTGAGAAAAAAACTAAACTAAAATAAAAACTATTATAAAC
ATATAATTATGTGTTCATATTCCGCTTTGGATGTAACGTAAAGTGGCGTTCAAACATGGTTTTTTGATTGAAC
AAAAAAGATGGAATTTTCGTGGCAAATGGAGGACGCTGGGAGCCAAGAGACTAACGGGAGGGCGAACAGAAAC
CAGGGGAGAAGTCAAGGTCCGGGACTTATAGACGAGTTGGAGGCGTGCATGGCCTCTG
TCGGCGATGGATGCTATCCGCCGCAATCCATCGACGCACGCTGTATTGGGGGTCACCAATTACCAGAAAGTCC
ACCAGATTCGGGCTCGGAAAACCCATATAGTCCAGACACTCAAGTCTCCCACACAATTGCTGTATCACAAACT
GTATTAGGAACTGATTACATGTTAGTCCCGGACCATATCACATCACATGAACTACTAC
AACAAAATGGTGACTATATCTACGAAGAGTTGAAAACAGATAACATAAACCATGAAGTCTTGAGAAGTAATCT
CAATGATGTAGTAGTATTACCACAAGATCCTAGTATTGTAGAATTAGGATTGAGATCAGTTCGCCATGATTTG
GGCTTATCTGATCCAATTTCTTATCAAAATCGATATAATCAAATGAGGATAGATTTGC
CGGAATTAGAGCAAGGGATGATAAACCCCCAATTGGTAGCTCTAGGACATGAAACTTTAACACCAGTGTACA
>1002050569_1 Heli.1-DT667419.3.5

GCACGAGGATTTGCCTTATAATACTATAATTATTCATCATATATTAATTCTAATGGCTATACAAATTATCGTA
CTATTAGAAAAGAACTGAAAAGGTACCTGTAGAATGAAAAATATGTATAACTAAACAAGCTAAGAAGTTGACT
GTGTGTTGACGACCTAAACTGTCCATTTGATTTAAGTATAAAAAAACATAAATTATAT
TTAACAAAAAAATTAAGTTTTGTAGGTAGAGAGAAAGAACCCAGTGTCAAGACATTCCGTTTTCCACTTTCCT
CCAAAACTCAAGACGACATCTCTTGAGTTTTGGAGGAAGAGTGATTCTTGAGTCTTGAGTTTTGGTGTTGGGT
AGAAGATCTCAACATATACTTTGTATCACCAAAGCGTGAAAATCGAAACTACTGCCTT
CGCGCCAATGCGATCCCACTCCGCCGCGATGACCTCTATAATAATTTTTAATAATAATATTCGAGTCCTGTAA
TTACATTTCTGTATCATTTTAGGTACATTTTTTTAAATCGGAAATCGTTAATTTATTATCTTTGGTTAAGATA
AGTTTAAAATATCTATTTGACGTAAAATTAATTTCTTAGAACTGAAGTTTGTAAGAAA
TAAGTATTTTATTTTTATTTACAAAAAAAATAAGTATCTATTAATTATTGTTAAGCATCTATCAATTATACAT
TAGACCTTGATAAATAAATAAATATATCTAACATTAATATAATTATGTACGTAGTAATGTAAGATCAGCAAAA
TGTCATCTATAGATTTAATAAAATTATCCTTAATTAAATATCCTATAAACAAGGTTAT
ATTGTTAGTTTATGAAACTAACAAAAAGATATATATATTATTTCAGATAATTTAATATAAAATATTGAATGTA
GCAGAATTTCCGAATCTTTTTAAGGAACGGTCTACGAGAGGCCGCCT
>1002050570_1 Heli.1-EL598298.1.5
GCACGAGGGCTTTACCTTCTGGTGGACTTTCCGGGACTTCCGCCCGACGTCGCTGGAGCGCGGGCAGGAGGCG
TGCCGGCTGCGCGTGTACATGGTGTACGCGTGGGGCGGGCCGCTGGCCGTGGCGGGCGCTGCAGCGCTGCTCG
ACCGCTTGCCGCCCGCCGCCGCGCCCGGCCTGCTGCGCCCGCGCTTCGCCGTGCAGCG
CTGCTGGTTCTACGGCGACATGGAGATCCTCGTCTACTTCTTCGGGCCCGTCGGCGTGCTGCTCTTCATCAAT
CTCGCGCTCTTCATCTCCACTACGCGCCAGCTCACCTGCGGTTTCTGGCGGCGCGACGAGGTCAAGTCCACAT
CCGAGAGGGCGGCACTGGGGCGTGTGTGTGCCAAGCTTGTGGTGGTGATGGGCGTTAC
GTGGAGCGCGGACGTGGTATCGTGGGCGGCGGGCGGGCCAGAGTACGTGTGGTACGCCACGGACCTGCTCAAC
GCGCTGCAAGGCGTGTTCATCTTCCTGGTGGTGGGCTGCCAGCCACATGCGTGGGCGGCGCTCAAGCGCGCGG
CCGCCGCGTGGTGCGCGCGCGCTCCCGGCGCCGGGCGCGCATTCCTCTTCACACTTGC
CCTCCTGCGGGGAGTCCCTCACGCACACCACCGCCGCGCCCGCGCCGG
>1002050571_1 Heli.1-EL604037.1.5
CATTTTATTATAATATAGATTTTTTTTATATAGTACTGTCAAGTTTATTTTTTTATTATGAAATTAATAGATT
CATTACGATTTTCGTTTTATCTTTCATATAAACTTAATATCGGCTCTTCATATTTTAATTTAATATTTTTAAA
TTTAAATAATTCAGTGAATGTACATAATGTAAATATTATTCATATATAAATTGTCAAT
TAAATAATGTATGTTATTATAAATGTGTGAAAACAACGAGTAGTAACACCGGCCTAATAGTTAGTTTACGAAG
ACTTATTGCAGTCTCTTTTATAATAATAATAATAATAATATGATAAATATTTATTAAGTATCATTTTGTTC
>1002050572_1 Heli.1-ES586276.1.5
AAACCAGTGGCGGTGCCGGTCGACAGGCCTGTGCCAGTGCCCGTGCCTCATGCTGTTCCTGTACCAATAGTGA
AACAGGTCGGTGTACCTGTACCCGCACCGTACCCAGTACCAGTAGCCAGACCAGTAGCAGTACCAGTAGCACA
ACCCGTTGGCGTACCGGTCATCAAATAGATAATTGGTGGTGGATTAGCCTCACGAATT
GGAGGAGGTATTGTATCCGGATACTATGGAGGTGGACTCCACTATGGCTACCATGGACTTGGACACATTCACT
CCTGGATCTGGAAAAAATATGTTCAGAAGACGTTCCGTTTTTAAAAAAATCTTTAATAAGCATATATCCTTGC
AAATGAAATATGGTAAAGATGTACCATCATTTGAGTTGCAAGACTGTAATAGTGGAAG
GTCCGTAGACATTTCCCCAAAGAAATCTGAAAAGAAGATTTTCTAGTAACCGAATGTCGAAGAAGTAAGGCTT
TATTTAGTCACGGTTGGTATTATGTGATTTAGTATAGGTTAGTTTTAACGTATGTATGTTTTGGATCTAAGAT
GGAAATAGTAAGCGCTATTGCCAAATAGAAGCTACTAATGTAATAAATTTTATATTTT
TATACTAATAAAAAAGTATGGTAAAAATAAGATAGTTCGAAATAAAAGTTTTGTAAAAGAAATTTTCAAGAAA
AAGGCTGAATTGTTGTTTTTTATATTGTATTGCTTCATTGGGTTCGGGGTGTATTGAAATATATATATGTATG
TATATACTTGCTATTAGTTACTTTAGGTTTTTCTTGATCTCCTATGTATACGCATGGA
GGCAAGTCTTCTAACGTATGTACCTGGTTTTTTTACTTTTGTATATAACTCCGTAGTGTAGTTCTGATCAAAT
GAGTTTATTTCGCTCGCAGTCGACTAAACACTATTGTTAATGGACCTTTTGCAATAAAA
>1002050573_1 Heli.1-DT665759.3.5
GCACGAGGAATACAAATAAGCTACAGACACTTGATGATGAAAAAGATGAAAAATCCTCTGAAATAACCACAGA
AAAATCTTTGACAGAAGAACTTCATCCTACTACGTACGAACCTACTCGGATGGAGGCTAGTACTGAACATATT
CCTAAACTGCAAACAGAGGTAGACACACAAGAAAAATATATTGATGAAAACCAAGAAG
AAAATACTAGAATACCCGAAATTACCGAAACCGAAAGAATATCAACAACAGAGAGTATTTTACCTTCCCAAGA
CAAAGAACCAAAATTACCGGAGCAATCATTCAATGCTGATCATACCCTTTCATCAGAAGTAACTGAAAAATTA
GATGAAAATAGAGATAAATTGCCTGAGTATGAGGAATCTACGCAATTTGAAATATCAC

CGACAGTATTAACACCATCAAAAGATGATGTCGTTACAGAAACACCACATGCTCTTGTTACGAATATTATTCA
AACTGGAAGCTTTGATGAAATTGAAACGAAAATAGAAACAACAACTAAAGTAGACGAACGAATGCCATCCTCT
GATGACGTATTTATCACACATTCTCAAGAACAAGCTTCTCAAACTCCAGAATATGCAG
ATCATCATAAAATCCCTGAAATACAGAAAGAAATAAGTCAGGATAGTAGTCTTACTTCAGCCAATCCAGAAAC
TGTACTCTCAGAACAATCATATACAACAACTTTATCGCCTTTACATGAAGTAGATGTAGAAGAACAAAAACAA
GAAATACAACCAACAGAGCAACAAAGTGATATATCGGAAAATATAGCACCTACAAATG
TTCTTCCAGTTTCTCAACAAAGTGAATCATTTACTGAAAATGTATTTGCCCCAACTTCTTCTAAAAAAGACGA
TGAAACATTATATGAAGAAAAAATGAAACCTGTGACTGAATCTATTAATTACGAGAAAACTCCTGAACATTCA
CCTGAAGATTATATTCCTGTATCTGTACAGGTTACTGAATCGACCCATTTAGAATTAA
GTACGGATACAGCAAGTATTATTGATGTCGCAAAAGACCAAGAATCTAAGGTCACTGAATTCACATCGACTAA
GCCTCAAAAACAATTTACTTATGAGACAATTGCTACTGACTCTGTTACGGAAGTATATGAAAAAGAAAGATCT
TCAGAAAAAATTGATACAGATATTACTACAGAAATTTCAAAGATCCCTGATGAAGTTT
TGGTTAATATGCCAGTCAGTACTACGTATCCAGAGAAGTCAACAGAAATGATAGATTTACAGGAACCTGAAAT
GTCTGAACCAGAACGTACTGAAATTAATGTAACAGAAAAAGAAGAATCGTCAACTCCTTCAGTATTTGAAACA
ATACCAGAAGAAAAAGAACACTTAAGCCTATCTC
>1002050574_1 Heli.1-EL596078.1.5
ACAATCATGGATGAAAGAAATCAAAATCGACAAGAGAGATTACGACCGAGTTTTGCACCATGGGTCAAGAGCA
ATCGTACATCGCTCACAACAGCGTTGCGTCAGTGAAGAAATATCAGGACAAATACGAAGACTCTATACAATAT
AGAGTATCATCTGTCGATTATCGAACGAGCGACTACGACAGGAAAGATTATAGGCGTC
ACAGAGCCAGAAGAAGTGCGGAGAGCTTCAAAAGCGATAATTCTTCAAACGAAAGTAGCGGGTACCGAAGCGG
GTCAAGCGCCTGCGAGAGTCGATCTGACAGATCGTCCAATAGTGATTACTATTGCACATCCTTATATAAGAAC
AATCACTACAAGGATATAAACAAAGAAATATATAAACCGATAAAAATTCCCAAAGATA
AACGCTACAAACAGATATTAGACGATAAGGACGATATAAAAAGTGGTAAATTGAAAGGTTACCTTAGTGAAAC
TGAAAAAGCAAAATAAAAGCAGG
>1002050575_1 Heli.1-DT667599.3.5
GCACGAGGCGAAACCTAACTATTCACAAAAATTGTTCTACATTTTATTTAACTCTTAATTTTTGCTTGATTTT
CAATACAATCAAGATGCCCTCAATGAAAGTTGAGGGGGACAGCGATGGTGATGGAGATATGTCTGGTGGAGAC
ACTGAAAGGTCTGTGGGATCGTCAAGACCTGATAGAGACTCAAGTGCTGAAGAAGCTG
ATGAAGCGGACTCTGATGACTCCTCGGAGCTAGATGAGAGTGAATGTGAGAGACGACGCAACGAATGCTTGGA
CAATCTAGCGAACCTGGAGCGTCAATTCACAGTTCTTCGAGAACAACTGTACAACGAAAGAGTGAAGCATGTC
AGATTGCAGCATGCTGAAGTACAGAGCGGGAGATCTCAAGAATATTTGGTGCCGTTGC
ATCAATTGCAAGAGAATATGAAAGTTAGAATCGAGGTAGCAGGTATCTTAAAGCAGATGCGTTTGGAGAACAT
CAAGCACAAGTATGAAGCGGAGGAGCAGGCTGCCCATCAGAATTTTCGAAGCGAAAAATGGCTAGCGTTCGAG
AAGCTAAAAGAAGAAATACTAGAGAAAATAAGAAAAATAGAAGAAGAACGACACACAG
TCGACCTATGGTCGTGTGGAGCTGAGTGGGGCCGCAAGAGGAGGAGGAGGCAGGTGTCTGTATCACCACCTTA
CGTGGTCTATATGCTGCCTGATTCTGACATCATGGAGGACTGGAGGTTAGTTCGTAAGCTGTTGGAAAGAGCC
GACTGATTGTGTGACATTGTATTGATCGTGATTTTGTGTATGACTT
>1002050576_1 Heli.1-EL601204.1.5
GACGAGGCTCTTACAGCTGAAACGGGTATTACAGGAGCCCGGGGAGGAGCATCATTCGATGAGTAGTGAAACT
CTCGACCCGTTGCTGGCGGCGTGCGCGGAGAGCCCCGCGCGCGGCAACGGGCGCCGCCCCCCCGCGTCTCCGC
TGCACCCCGACGCCTGCGCCGAGATGCGCCGTCAGATTGTCTACCTGCAGGGTCAGCT
GGAGGAGCGCGACCGGCTTGTGCGGGTGCTGCAGCAGCAGATGCTGCGCATGGCGGAGGGCCACGAGCCGCGC
GCTGACGACACCTGCAACGTCGCGACGCAGACTGACCGGTTGCGGCCGCCGATCGGCACAACGCTAACGAGTT
CTGAAAATTCCGGATTAGTGAGTTGGAATGAACAAGCTAGCGGGAGGAAGCTGCCGTA
CAGTGATGCACAAAAGACAAAGAGGCCTGTCACCAACGGGACCGTGAAATGTCAGTGCGGTGCGAAAGTGAAC
GGACTTATTGACAGACCGACAGACAAACGAACGGACTCGGTTCAGCTTAAGTCAAATAGTCGAGTCGGATCTA
AATACCTTCAAGCCTTAGCCAGCAATACGGAAAACAGAAGATACATCAAACGTGATTC
TTCTATCGGTGAGAAATCGGAACGAAGAAACTTATATTCGGCTAGATCGCGATCTATAGAAAATTTCCACAAC
CAACACTACCTGTCGACCGAACCGCTTCTCTCAAAGTCAGCGGTTATCGAAGAAAGCATTAAACATCATACAT
CGTACGGTGACTTGTATAATGGCAGGATCAGCAG
>1002050577_1 Heli.1-DT667485.3.5
GCACGAGGAAGCCGCATTGCATGCTCTAAATTACAATCGAGTTTTGGTTTAATTATAAATTCTGAATTAAATT
ATACGTTTGATTAATAAGTATACAAAATTTTAACAAGTTTTATCCAAAACGTATCGTGTATGGTGCGACGTAA
CTAATGTTATCTTCACGTGCCGTAAGAGTAAAAGAAAGTTGGTAACTTCCGCAGTAGG

TCAATGGTACAATAATTGCGTGCCGGTAACGTTGGGGACTGTCGAGTCACTCGCTAGTACTACACACTCGTCA
CCACACATACTTGAGGCTAGTGAAGCACCGAGCGGAGTCTCGTTCGAGACGCGGCAGACTACAGTTGTTCGTT
TGTATTCTTTCGTCTAAATAACGTGTTCACCGTGACCGTGAAAGTTATAATCGTGTCA
TATTACCGTAATCATGGCCAACAAAGTAGAAAATGTTGGCATACTTGCCATGGAATTATATATTCCTTCTCAA
TATGTGGATCAAGAGGAATTGGAAAAATTCGATGAAGTGGCCGCTGGAAAATACACTATAGGATTAGGACAAA
GTAAAATGGGATTTTGCTCAGATCGTGAGGATATTAATTCTTTGTGTCTCACTGCTCT
ACACAATTTGATTGAGAAGAATAATATAAACCTCCATGACATTGGTAGGCTTGAAGTTGGCACAGAAACAATT
ATTGATAAAAGTAAGAGTGTGAAAACTTTTCTTATGACTTTATTCGCCAAAGAAGGAGCAACAGATATAGAAG
GTATTGATACAACTAATGCTTGCTATGGTGGAACTGCTGCTCTTTTCAATGCAATTAA
CTGGGTGGAATCATCATCATGGGATGGCAGGAAGGCCATAGTTGTAGCTGGAGATATAGCTGTATATGGAAAA
GGTCCAGCTAGACCTACTGGAGGTGCAGGGGCAGTAGCTATGCTCATAGGACCAGATGCACCATTGGTTTTCG
ATTGTGGAGTCCGTGCTTCGTATATGACTCACGCATATGATTTTTACAAGCCTGACTT
AGCATCTGAATTTCCTTATGTTGATGGGAAATTATCAATTCACTGTTACCTTAGTGCTTTGGATAATTGTTAC
AATTTATTTTGTAAAAAATGAGG
>1002050578_1 Heli.1-EL601276.1.5
GCACGAGGCAACTGCGGAGGATTGAAGTCAGGCGTTTGTTGTTATAAATTGTTCAGTGTTGTAATTATTAAAT
TGGATTAGAAAGTGAGACTATCAGGAGATCTACAGAATAAATTGTGATTTTATAATGCGAGGACAAAGCTGAC
GTTATGAGGCATCGACTAATTCGATTACATTAGCCAAGTTATGATTCCCTTCCACGAA
TCTTGAAATAATTTCGTAACTTTAAATGGCAAAAAATTAATACGTTTAAAAAATGGATGGAATAAATGGTTCT
GATTTACCGCCCGTTAAGGAAGAGACATTTTGTAAATACGTCTTATACTATGAAATGAACAACTCAAGTGTTC
GTATATGGGATTTGATTATATTGATACCTAACGCTCTATTCGTGCTGTTCTTAGTGGT
TAGATTTAACAAAGCGCAATTAAAGTTACGAGCAACGAGTAGTCCTATATTTTTAACGTTCTACTCGCTTGTA
TGGGGCAATGTGATCATCAGCTTAATACGATGTGCAGTCTCAATGACGTTAAATGCGGCAGTGCCAATTGGTG
GGCTCGTCGATAAAGTGTTATGGGTCACTGTAAGATTTTTTTACTTGCCACAGAACT
GAGTGTTGTGATATTTGGATTGGCTTTTGGTCACATGGACAGTCGAAGTAGTATCAGATATGTGCTATTAGCT
ACATCACTTATTTCTCTAGGGTT
>1002050579_1 Heli.1-DT661706.3.5
GCACGAGGCGTGACCTTGAACGGGTGTTCGAATTATGTTTATGTGATAGACAGACGAAAATATATATATTTTT
AATATGAATTTTATATCTTTTTTGTGTGTTTTAGTGTTAAGTTGTTATGTTGATTGTAGTGTATTGAGAAATA
AATTGTTGAAGTTGGATATAATACATTATAATGATTTTCATGCGAGGTTTGAAGAAAC
AACTGTCTCCTATCCAGTATGTACATCAAACGACACATCATGCTTAGGGGGCTTCCCCCGTCTTTACCATGAA
ATTAAAACGTTACTTAAAGAAAAACCCCGCGCTCTAGTGCTGAATGCTGGAGATACGTTCCAGGGAACGTACT
GGTATACGCTGTTGAAGTGGAACATCACTCACAAATTCATCAATTTATTACCTAATGA
TGCTCATACAATAGGCAACCATGAATTCGATGATGGAATAGCGGGCCTCGCACCATACCTTGCGGCACTCCAA
GCTCCTGTTGTCGTTGCAAATATCAACACAACCCTTGAACCTAGTCTGAATGGTTTATACAAACCTCATATTG
TGATACAAAGATACGGAAGAAAAATAGGAATAATTGGTCTTATTACGACAGAAACAAA
CACATCTTCAAATGCAGAAAGAGTGAAATTCTTAGACCCAATAAAAGCAGCACAAAAGGAAGCTCAGTTACTG
ACGGATAATGGAGTCGACATAATAATAGTTCTGTCACACTGCGGTCTTGACGTTGACAAAAAATAGCCGCTGA
AGTAGGTGAAAATATCGATGTGATTGTCGGTGGACATTCGCACAGT
>1002050580_1 Heli.1-EL596854.1.5
ATTTACCCTCAATTATTTCATATCTTGTGTTTAACGCCACCAGCGTTCGCCCGCAGCAGCGGAAACCAGCCAG
TCCCGTCGCGCAGAGTCAAATTGCGACCGCATGGACCAAACATACCTAGCACGACTTTTAATAGAGCAAAGTA
CAAATTTCAATAAAATAAGATGCATTAGTGCTCCGGCGCATGTTTGTCCACCGTTGTC
AATTACTCTAATATTTGAATTGTGATTCGGTAGCTAATGAGAGAGACTCTATGTTCTTAAATGTAGTGTACAT
ATTATATTTGGAGTTGGGCATTATGGCGCGGTGGT
>1002050581_1 Heli.1-EL601020.1.5
GCACGAGGATGATGAACCTGCTGACAATACATGTAATGAAAATATTGATGCGCCGGAAACAAAACATTCTGGA
CATTCTAAAGATGATCAAGAAAATTCATTAAATGTAGAAGAGGGAGAATGTACATCTGTGGATTCTACAATAG
GTGAAGAACAGTCTCAGAAAGCAAGCAAAAGTGGAGATGAAGGTGCACCACGTCGATT
GAGCAGAGTTATTAAAAAGAAGGTAATATTCGATCCTGATAACCCAGATACATTTACAAAAGGTAAAACAACA
AATAAAATAAAAGATTCTTCACTTGGCAAAGAACAAACACCATCAAAAAAGATTAAACTTGAACAGATAACAC
AAAGATCAAAATCTAAATCTCCACTGTCAAAATTACAATGGAAGAAGCCTACTCCTAA

ACATAATAAACAAAATAAAAGATTAACAGAAGTTGATAAACTTTTAATGGATGAAGGTGCAGTAAATATGATA
TATCAACTCACTCCTGAAGCCCAAAAGGGTAAAAAAAACATAAAAACCAAAGCTGAATTTATAAAAAAATTAC
AAAGCTCTACTCCTGAGGGGAAAGAAATGAATTTTAGAGAAAGAAAGAAGGAGTCTTC
GAAATATGAAGATGGAGAAGCTAAACGAATCCTTATTGGTAAGCAGAGAACATCTCTAAGCAGTTCTGTGAAA
TCTCCATCTGTTTGTGAAGATTTTGAAACTCACAGTGCTGATGATTCTATTATTTATAGACGACACTCATCAA
GTTCATATTCAAGTAGCTGTATGAGCCCTAAACG
>1002050582_1 Heli.1-ES584637.1.5
GCACGAGGTAAAACCATATTCCAAGGGTTGGAGATTTCAACGAATCTAACTACTACATGGAAGGTAATACAGG
ACACCCTGTGTTTGCTACGAGGTACGGTAAAATAGCTGTAAACATTTGCTTCGGACGTCACCACGTTCTCAAC
TGGATGATGTTTGGGCAGAATGGCGCTGAAATTGTATTCAATCCATCAGCTACGATTG
CTGCTGAAGCCGGAAGCGAATATATGTGGAATATCC
>1002050583_1 Heli.1-DT667233.3.5
GCACGAGGCTACGCATATGATCGAGATGAGCCTGATCTGTATGGAGAACAATATGCACCGTACCCGAGAAATA
CGCGAACGTTAACTCGCGAAGACTCCGGCTCAGCAGCATCATCTCCTGAAAGAGAGCCTGCCTACAGAAGTGA
TCGTGACGACGATGACCTAGGTGAGGCGTGGGCGCGTGGCGTGTCAGCTCGCGGCGGC
CGCGTGGTGCGCGTCACGTACCCACGCACCGCCAACAATGACAAGGAGCTCACGGTCGTACGCGGGGAGTACC
TCGAGGTGTTGGACGACTCCCGCAAGTGGTGGAAGGCGCGCAACCGACGCGGCGTGACGGCGCACGTCCCACA
CACGATCGTGGCGCCCACAGTGTCGCCGCCCTCGTCGCCGCACTTATATCCTAACCCC
ATATATACACATTATCAGGAGGGCGGCAGAGGCTCCGGCGGCAGCAGCCCCACCAACATGCCCGGTAGGGGAG
GCGCGGGCGCGGGCGGGGGTGCAGGCGCGGGCGCGGGCGGGGGCGGCGCGGGGGGCGCGGATTGGGTGCGCCG
CGAGCGCCTCGGCAAGAAGGGCGAGTTCCGATACTTCTAGCGCCAACTATCGCAGCAT
TACGCGCCTTCCCCATACCGGATCACCTACCAAAGCGCTTACCGCACGACGTCCGCCGGACACGGGACACCGG
ACACCGCTTAGTCATAATTTATGTATCCGTTAAGATATTTATTTCACATCATTCGACACGCAGTAGTTGCATT
GTCTTGGGCTCGACAGATGGCGCTAGTATCGGAACGCGTCGCTCGCGTCGCGTGTGTG
TGCGTGTTGTCGGTCTCCTTGTTGACTGTCGCCGGCGGAAAATCCCACTGGCAGGGCGCGGTTAATCCCCGCA
GTCAAAGCTTGATGTATTGTGTATTGTGTAATATGTATTCCGCTGTGTCGTGCGTTGAATGTAACTTTCTAAT
CACATTTACCTGTAAACGTAGTGAAACGATATACTCACGCTGTAGATGTACGCGTATC
CCGATAGTAACGAGCAGATAGTGCTATGTGTTCAGTTTAAAATTCTCTATTATACGTACATGTTTATTAATTA
ACAGAGTCTATATTTATATTCATTATGAATAACTAGTACATCTCATTTATATTATAGATGTTTTACACGCAGT
ACGTTGTATCATGTACGGGAGCGGGCGCAGACAGTCGCACAGTCTACCCAAATATACA
AAANAAAAACAAAAATGTACATAATGTTACTAACATGTTACTAAAGGTACTGTACAGTTG
>1002050584_1 Heli.1-EL603705.1.5
GCACGAGGCCATCCCTTTCTACTCAGGATGAAAAAGAGCTAATAAGAACCCTTTCCTCGGCGATGGATGGTCC
CGAGCCGCTAGTAGAAGCAGACGAGTACTTGCAGCCAAAGTTTAAGTCCAATGCTGGGACTGCCACTACTAAC
ACTACATGTACCGTCGGTGCTGAGCCACAGGCCATCAAGGCCTGCGCCACGTGGACCA
ATAACACTACCGGCCAGGAAGGCATTATAATAGACAATTCAAATAGATCAGGCTCCTGGGATCGTGACCTTTT
AAGATACAACAACTCCGGTAATCAAGAAGCCATTGAACTGCGCCATTATTATAACCACGGCGTCAGCGCCTCG
GAGAGTTCGAGCTCGAGATATTGCAGCGACCCTATGAGAATGAGGCCGGATTGCATAG
ATGTAAATATGTCTAAAGCAAAGGAGGCGCACGTAGGAAATTTAAAATTAAATTTGCCTCTTGACGAAGACGA
CTATCTCATGCCCTCACCTCAGCATACCCAAAACGCCTCGGCCTATATGGACCTCATAGGCGAGAGCACCGAG
GGGCAGGATCCTAAGGATCTTTGTTACACCGGATATCTGGCATCGAAGAGATGCGTCG
ATAACCCGGAATATCTCATGTCGGAGCAAAATATTCCTTCACAAACGCTCGGTATCCCTACAGAGCCCGAGAC
ACCGGACACAATACATTCGGCTGAGGCAAGTGGCGTGAGCGTCGGAAGCGGGCCCAGCGCGAGTGAGGCCTGT
CCCGGCGCGCCACCCGAGCTGGTGTGCAGCAGCCGTTACCTGCCGCACCGCTCCGTGG
AGGAGGAGTCCATGTCCGACCACGAGTACTACAACGACTTGCAGCGCGAGTTGCAGCCGCTGCGCCGCAACGA
GACGACCGTGTAGGCAAGCGTGTACGTCGCGTGTGTACGCCGCGCGGGAAGCTCACCGTGTGTTATCCTCGTG
CCATTATGCAATGGCTAGTGTTAGTTGTTGTGAAATCTTAAAACTAGATTGTTTATAA
TTATGAACCAGTTTTGTTATTTTATTTACTTAATTATTGTAAAAACTGTTAAAACTATAATTTGTATAAATTT
TTTCACACGAGACGCTTGATCAAAGAAGCGCCTCGCAAATTATAAGTTATTTATTAATTTAGTATAAGAAAGG
CTATGTTTATGTTTTTCGAATGTATAATTATTATAAGCAAATGTTATTTTAATTTTAT
AACTGTGATAATTAGTTTAATTACATTTAAATATATTTCGGTTATAGACGTTTTATCTCCAGATGTTTAACAA
TCGCGCTGTACTTTACATATGTATGTATGCTTTGATTTAACCTCAAGTGAACCTGTCACCACACCTAGCAGGT
GCTTAAGAGTTTACTGACCAAAAGGGAACAACTATTCACTATTGTAATAACATTAGAT
>1002050585_1 Heli.1-EL599253.1.5

GCACGAGGTCTGTAAATAACCTCTAAATTTTTTAAATCTGAATAAGGATTACAAAATTTGTAAATTGTCTGTC
TTTTATTATTTTCCACTGAAGAAATAGAAAACAATCTATAGCTAAGCCAGAAAATACGGATACAAATAAAATA
AAATACAGAGCACTGTCAAGAATATGAAATGTAAATAAATTGAGGTCTCAAGGGTGCG
ACCATTTATGCGATGGTTGTGAGATCGATTCCCACTTGGGGGAATGAATTATTTTCGATCTGGACCCTTACCG
TGTTGTGCGGGATAATGCGTGAAGGTAAAACGAGATATAGATTTTCTCATGTGTCAATGTAATTTGTCATCTC
TTTCTCTCACACGCCCAATCTAATGAACATTATTTATACTTACATATTCATACATATT
AATTTGCATTCGGATACTCGTACTTAACATATTTGACAGCTTAATATATATTTGTTTTATATTATCTGTGTAC
GTTTGAATATTTGTCAAAATTTTACTCGCATTTCGTATAAATTTTTGTCTGTACAAAGTTTAATATGGTTATT
AAAACATCCTTTTTGTTTATCTATATGGTTTATATTATTATTTTCTTAAATCTGAAAA
GCTGTAGTGTTATTCATACTTGAAAATGCCAATATCGTCGCTACGAAGTGAAAACCTAGTGATTGATGATTTT
TTTTCTGATATATTTAGTATAAGAAGTACGTTGTTAAGGCCATTGTACAATATATGAATAATAATAATAATAT
TAATAGACATATTACAAAGCTATCGTTGAATAAGTTGAAAAAAATGAAGTTGCATTTA
CTAGGTTTTTATTGCGTATCGCCAATTTAAATTCCATTTTCTTAATCTTTCTCTTATTAA
>1002050586_1 Heli.1-DT667787.3.5
GCACGAGGTAGGAGCGTTATTTCATTTTAATTTTATTTATGTTTTAGTATCTTTTTTATACATCATGAATTCC
ATTTTAAATCCTGAAGCACCTGAGTTTTATCCGCATCTAACTTCAATGACACAGGATGGCTATACAACCGTTA
AAAAAACAATTAAATCATCAAATCAATTTCCTGTTGGTACATCATTTAACATATTCAA
GACTTTTACCGATTTCAACGTAGTGACATGCAAGCTAAGCAAAAATGTTCTTATACAAACAAACGATATTATT
ATAACAGAACTGCCTTCCGTGAAATTGAAGCTCCAAGACGACGAGTACAACATGGACAAAATAATGGACGTCA
ACGACCACTGGCTGGACAGGGTCAATATTAACATTGATAGCGTTATCAAGCCGGAACC
CTCGGAACAGACGGTGTGGACCAATCAGCCCGTTATTACTAAGATTAAGGTTGGATCGACCATGTTCATCGGC
GCCAAGAACATACCGCGGCCGCAGCTCAATTTCAAAGACACGATCGACAACACGGAGAACCTGTGGGTGCCCA
GGATATCGGATAAACCTAACAACATAAAGCCGCTGGCTTTGAATATTATTTACAATGA
CGAAGGGGAGGCTGTTGGCTACGAGCATCCATACAAAGTAGAGCTGGACCTCTACGAACCTCCCCAGCACTTC
ATAGAGCCTGACCCTCACCCCCCGACGTTTCCCCCGACCTTGGAGGAAACTAAGCTAAACTTCATAGAGAC
>1002050587_1 Heli.1-EL602599.1.5
GCACGAGGGCTAAGTGTGCTTTTCATTATACATGATTAATTTTATTATTCGAAAAATAACCTACTGGTTATAA
TATAGGAAATATCAATATTTCTTTCATTTATAAACTATAAAAAGTTACGTCCATAATCTATTTCGATCAGCCG
TTTTTCAAGACTGCCATCAGGTTATTATGTTACTCAACAAAAGCAGACTGGTTGGGTT
TACAAATCGTTCAAGCAAGTGCAATATGGAGAACAATTCCACGTCAGACGTAATAATTTTAAGTGGTAATTCG
CACCCTGAATTGGCTGACCTAATTGCGAATCGCTTGGGTGTACGCAAAGGAGGATGTTCAGTTTATCATAAAA
CTAATAGAGAAACTATGGTAGAAATAGCAGATTCAATTCGTGGAAAAAAATATCTATAT
TGTACAAACTGGAACAAAGGATGTTAACAACAATATCATGGAACTATTGATAATGGCTTATGCTTGTAAGACT
TCATCAGCTCACTCTATTGTAGGGGTCATTCCATATTTGCCATACAGCAAGCAATGCAAAATGAGGAAGCGTG
GGTGCATCGTCACAAAATTATTAGCCAAAATGATGTGCAAATCTGGTCTCACACACAT
TATTACTATGGATTTACATCAGAAAGAAATTCAAGGATTTTTCGATTGTCCTGTTGATAATTTGAGGGCATCA
CCCTTTTTGTTACAATACATACAAGAAAGTATACCAGATTATCGCAACTCCGTGATAGTGGCGCGCAACCCCG
GCTCTGCTAAGAAAGCCACTTC
>1002050588_1 Heli.1-EL598407.1.5
GCACGAGGGGACGCTCGGCGGCCCGGCGCTGTACCCCGTGCGTGTGCCCGCACCCGGCCAGTCTGTGCATGCC
GGTCGTAGCGCGCGGTTAGTCGCGCTGCAGCGCGGCAACATGTCGCGCCTCCCCGCGGTGCTCTCGCTTCTGT
CTCTAATTGCCTTTGTGAACACCGAACACAGCAATGGAGTTTGCCAATTGAATTATTG
CGAGTGCGTTCCAGCGAAACACCCGTCCCGAATGGATGTTAACTGCACTGTCCCTCACCATCAGAACCTGAGT
GTTCTAGAAGGGGACCTACCAGAAACTACGGACGATTTAACTATTACCGGAGCGGAATCACTAATGATAGAAG
CAAACTCGCTTTCTCGATTGAATGATGCTCGATATCTACACTTCGTAAACAATACACT
TATCTACATCAGGACCTCAGCAGCAGTCAGTCTGAATATAATTAATCTTTTCCTTGAAATTGACAATTGCGAT
GTGCTGAGAATTGAAGAAAAGGCATTCAACGATATCAAAGGCCCACTATCTGTGACAATACGTAACTGCGACT
ACGTCTCCTTGCAAGGCGAAGCATTTTCATGGCTTTTATTTATTCACATTGAAAACGT
TCGACACCTGGAACTTGGTGTTGGTACTTTCGTACTCGATCCAACTGCAGCAAATGTTGGGGAACACGGACCA
GGGATGTCGATTCGTATGAAGAATGTTACAATAACAGAATTCCCTGAGCAAACTTTCGGATCGTCAGCAGCGC
TGATCTCTCTCGAACATGTTAA
>1002050589_1 Heli.1-DT663942.1.5

GCACGAGGCCTCGCCCTTAATCGGCACAGGAACATGAACACCTTTATTACTTTTGCCTGTCTGTTGGCGGTAG
CCGCTGGCGTACATGGATCAGGCCTCGGGCTCGGCTACGGTGGATACGAAGTATCAGCACCCATCATCTACAA
AGCTCCAATCTTAGCGCCGGCAGTTTCCACTGTAAATTTCTATAAAGCGCCCCCAATT
CCCATTATAAGGAAAGTAGTTGCCCCCATAATACCTGCCCCAGTAATTCCTGTCCCAGTTATTCCTGCCCCAG
TCGTTAAGGTACCTGTAGTCAATTATGGCTTAGGTCTTGGAAAAGGTCTAGATTATCTGAATCTTGGCATAGG
TCTTAACAAACTTGCTTACGGAGGACTCGGATACGGTGGACTCGGCCACGGTATACAT
TAGATGCTATAGGAAATGGAGAAGATTAATTTTTTTTTGGAAATTACTTGGCACTAATAAAGTATATCGATTT
GGGTTCTGTATATTTTTCTCTTTATTTTATTCTATATAATATTGTTCATTTTGTTACATTATAATTGATGATA
AATCTGTTTTTAAATGCCACTATGTTTATGATGAATTTATCGATTTTTAACATTTTTT
ACATGTTATTCCACATTTGGTTTTGTTTCTTTCGAACCATATTGTTTTAGAGAATGTAACTTGGTAACAGTAG
TACTAGCTTAGTATTCGGTGCAC
>1002050590_1 Heli.1-EL600154.1.5
GCACGAGGGCAACCCCTCTATGGAAAAACTGAGGATGACAGCCTCCAGTTCTAATGGAAGTGCCTATGATCCT
TTGTCGTCTCTTAAAAATAAAGATGCGGATCTCACCAACCCTAACTTATACCACTGTATTGATGAAGACAACA
AGCTGGATCATGTCTATGATGAAATTAAACACAAAGAAGGATATGAAATGGAATATGA
TCACCTGAATTATACACCTCCATCCAACACTTGGAAGCCACACTACCAAAGGATGAATAATATGAATAACGGT
GTACCTGCGGGGTCTCCGGGGAGCGACTTGAGTACTTCTACGCCCCCTATCCCCCCTCTACCTAAACTGCACG
GACCTCCCCCCGCGCCCCTTACACCGCCTGCGCCACCCAAAAGAGAAAACCCACCTGA
AGAAAATAACTCCACTACTAGCCCTTAATGTAACATGATTTAAACAAGTTCGATAAAAGTATACGACTAGAAA
AGAGCTGATAATCTTAAAACGATTAAACTTATTTTCTTGAATCATAGCTAAGATCACGCTTGATTAATTCACC
TTTCAAACGAATAACCGAAATTAAAATCAGTTCTTTCCTTAGCTACGATACATACACA
CGTGCACAGACACGTCAAACGTATAACACCCCTCTGTTTTTGTCGGAGGTTAGAAATATACTAGTATCATCAT
TATTTTTGTGAAAATTTTAAAAATTTATTATATTTTGATATAAATTTAAAAAAACCTTTTTGGGTACCTAATT
AATGGAAACGAGTTGTTCTTTGTATGATTAAGGATTATTCAAGTCTTTAATTGTACAA
TATTAATAGTAAATTCAGTCAAATGTGTAGGGTTAGTTTATATGTTAAAATCTCATTTGTTTTTTGTAATTTT
AATTTTTTAACTTTTATGTGTGTCATTAAAAATATTTATTACTGATATTTGTAAAGTTAGTGATTTATGGACA
GCAATAATGATATTTAAATATAATATTATATTTAACGATTAATTTAAAATTAAACCTT
TTCTAAATGTATAGAAATTAATTTTATATTTACATTTATTAGTGCGCATGTATTTTATGCGATCTCGAGGCAA
TCGTTTAAACATATGTTTTTTTTAAATACTTTAACAAATTATTTTGTCATATACACTTT
>1002050591_1 Heli.1-DT663398.3.5
GCACGAGGAACAAACTTCAAGCATGTTCTTCATCATTGTTTTTTTATAACTAACGATAAACTTTAATTTGCTT
ATATTTGAAACTTAAATAAGTTTTGTTACATTAAACAATTATAATAAATTTTTAACCACCTTAAATAGATTTT
ATTATAAAAAACTTAAAATGCCTTCGAATATTGAGGAAAGTTCTTTGAGATATCGGAG
AGCACAGAGTGTTACTAAAGCAGAAGAAATAACGGAAAAAGAGAAAAAAGCAAGAAATTCTCAACTTGATAAA
CCATGTCACAAGCCCAGAGATTCTTTATTTTCATGGAGTTCAGAGTTCACAAATTTTACTGGACTTGTCAATT
GGGGTTTTTTGATGTTAACTATTGGAGGGCTTCGCCTCTGCCTTGAAAACTTTTTAAA
ATATGGTATAAGAGTGAACCCATTTGAATGGATTATTGTCCTCACTGGCTACAATGAAGGCTACAGTCATCAG
TATCCCTCAGTTATTTTATTAATTTGTAAGTCATGTTATTTTTAATTATAAAAGTATATAAAAAAGTGAGGTT
TCATAAAATTGATGAATTAAAATATCTGCGCCATGGTCCCAAAATTTTTAAAAAGTCA
TAATAGAATAGATAAAGTTGTAGGTACCATAACTATACCACTTTAAAAGCTGTTGATGCACTTATAGCCTCTT
ATCTGTTGTTCCATGCAGTTTTGGGCAGCAGTGATTACCTAACCCATCAGTCGAGCAGTGACTTAAAATCATT
TGCCTTGTCTTCCCTACATAAAAAAATGATTACTATAAATATTAAATACCCAACACAT
>1002050592_1 Heli.1-DT668756.3.5
GCACGAGGTGATGACCTGCTCGGATTTGATATATTCAAAAGTAAAATTACAATATAAAAAAGGATTATTTATT
TGTATACCTTAACGTGATATAATTATAGAAACATATTTAATAAAAAAAATGTCCGCGAATAAAGAAGACACAT
TGCGTCAATTTTGTGATGTTACCGGCGCTGATGAAAACCGTAGTAGATTCTTTTTAGA
ATCATCCAATTGGCAACTAGAAGTGGCCCTGTCTAGTTTCTATGAGCATGGGGGTAATGTAGAAGATCTACCG
GCAGCAGCGAATGTACCGCCCATGTCGGACAGTGATATGGATTCGCCACCTAGGTCTCCAAGTAGGCCTCAAA
AGAAAGACAAGAAGAAAGGCAATTCTCAATTTGCCACTTTGGATTCACTTCAGCAAGA
GAGTTCCAGTGAGGATGAAGAAGGCCAAGCTTTCTATGCAGGTGGCTCTGAAAGATCTGGCCAACAGATACTT
GGGCCCCCTGGCAAAGGACGACAAGATATTGTCACAGAGATGTTCAAAAGTGTTCGAGAACGTGGCGCCGTAG
TGTTTGAAGATGGACCAGCTTCAACAAGTCGGGGGCGGGGCGGTGTTTTCAGCGGAGT

TGGCTATAGATTAGGTCAAACCGCTGACGACCATGAACAAGTGACTCCAGGGAAGACAAAACAGGACAATCAG
CCGCGGTCTGTCCGTCTACAATTATACAGAGAAGGATTTACAGTGGACAGTGGACCTTTAAGACATTATACGG
ATCCCAACAACGCTGAGTTCCTTAGTTGCATTCGTAGAGGTGAAATACCAGCGGAGTT
GTCATCAGGCGGCGCTGAGGTCCGTCTTAGTTTGGA
>1002050593_1 Heli.1-CX700785.1.5
GCGGTACGGTCGGAATCCGGGTCGACCACGCGTCCGATTAAATTTTTGCGCAAATCAAATTATTATAATACCA
AAAATGAGTAAAGTTCCCTTGGAGGGATATTTCACCTGTCTTACAGGCATTTAGAAATTTTCTCCTTGGTAGG
AAACATACAAATGCCCTGAGATTCGAGCCTCTTGTAGCCGCCAGGACTCAACCTCCAC
CTCAAATTCCCGATGGAGCTACCCATAAGCATGCTCACAATTACTACTATACTCGAGATGGTCGACGTGAAGT
CATCCCGCCTGTGTCAGTGACACAGCAACAGCTTGCAGACAGCAGTGCTGATAAAGATACTCCAAAATCAGCA
GCAAATGTGAGACCAACTCCAGGAAAATTATATGAATGGGACAAACATTATTAATTTG
TCAATTGTTATAAATTATGTTTAAATTAAAAAATAAATAATAATAGAT
>1002050594_1 Heli.1-DT661838.3.5
GCACGAGGAATGGCTTCTTTGGTGAATGTTTTGCTGCGAAAAAGCCCAAATTTGGGTCAATCTAAGGCTCTTT
TATTTAATTTACCTGCCGTTTACAATGTGAACTCTCAACGTAAAGCTCATAGATGGATGCCCGATGAAACATT
CGTGAAACAATTTGAAGGGGCTGTTATGTACCCTGAGGGTGTCACTTCTCTCATGAAG
CATCCACCTTATAATAGCATTGTAGCCCCAACCGAAAAGCAAGTTAAGAACATGATATTGAACTTTGGACCAC
AACATCCTGCTGCTCATGGGGTGTTGCGTTTAGTTCTTGAATTGGAGGGAGAGATAGTCCGCGGGGCTGACCC
CCACATCGGTCTTCTACACCGTGGTACGGAAAAGCTAATTGAATACAAGACGTATACT
CAAGCTTTACCATACTTTGACCGTCTCGATTATGTGTCAATGATGTGCAACGAACAGTGCTACAGCTTAGCTG
TTGAGAAGCTGCTTAATATTGATATTCCGCTGCGTGCCAAGTATATTAGGACCCTCTTCGCGGAAATAACCCG
CATATTAAACCACATAATGGCGGTCGGTACTCATGCCTTAGACGTGGGAGCCCTAACG
CCTTTCTTCTGGCTGTTCGAGGAGAGGGAGAAGATGATGGAGTTCTACGAGAGAGTGAGTGGGGCGCGGATGC
ACGCAGCGTATATACGGCCGGGTGGTGTTTCTCTGGATATGCCCCTCGGTCTGATGGACGACATATACGAATT
CGCCAGTAAATTCAGCGAGAGACTCGACGAGGTGGAGGATGTGCTAACCACTAACAGG
ATATGGGTGCAAAGAACTAAAGATGTCGGTGTGGTGACCGCTCAAGACTCCTTGAATTATGGGTTTAGCGGCG
TTATGCTCCGCGGCTCAGGCATTAAATGGGATCTCCGCAAGAGTCAGCCGTACGATGCATATCACTTGGTTGA
CTTCGATGTTCCCATAGGAACTAACGGGGATTGTTATGATAGATATCTTATCCGTGTA
GAGGAAATGCGTCAATCACTACGCATAATAGACCAGTGTTTGAACCCAATGCCCTCCGGAGAGGTTAAAACTG
ATGACGCTAAGCTCACGCCGCCT
>1002050595_1 Heli.1-DT667548.3.5
GCACGAGGATTAACTCTAGGGGATATTGACGTGTTATTTTTGTGAGCTAACTCTAAGTTCACAATGCCGGAAA
CCTCGAAAATGAATGGATACGCTAAAACAAATGGCCACAGTTATGAAATGGAAGATGGATCTGTGTTTCTATT
CACATCGGAGTCAGTCGGCGAGGGTCATCCAGATAAAATGTGTGATCAAATAAGCGAT
GCTATCCTTGACGCACATTTGAAACAAGATCCCAATGCCAAAGTAGCTTGCGAAACCGTTACAAAGACAGGAA
TGATCCTGTTATGCGGTGAAATAACGTCGAAAGCCAATGTTGATTATCAAAAAGTTGTCAGAGAAACAGTCCA
GCACATCGGCTACGACGATTCCTCTAAAGGCTTCGATTACAAGACATGCAGTGTGATG
CTGGCTCTCGATCAGCAGTCTCCCAACATAGCTGCGGGAGTGCATGAGAACAGGAATGAAGAGGAAGTCGGTG
CTGGAGATCAGGGCTTGATGTTTGGTTACGCAACTGATGAAACAGAAGAATGTATGCCCCTCACTGTGCTACT
TGCACACAGACTAAATCAGAAAATCGCAGAACTAAGGCGCAATGGTGAATTTTGGTGG
GCACGACCTGATTCGAAGACACAGGTGACATGCGAATACATATTCGCGGGAGGTGCGACTGTTCCACAGAGAG
TACACACCGTGGTTGTCTCACTTCAACACTCTGAAAAGATTGAACTGGAAACACTCCGTGAAGAGATCAGAAA
CAAAGTGATCAACCAAGTGATCCCCGCACACTACTTGGACGAAAGGACCGTAGTACAC
ATCAATCCATGTGGAAACTTTGTCATCGGTGGACCTCAGTCGGACGCGGGGCTGACGGGGCGCAAGATCATCG
TGGACACGTACGGCGGCTGGGGCGCGCACGGCGGCGGCGCCTTCTCGGGCAAGGACTTCACGAAGGTGGACCG
CTCGGCCGCCTACGCGGCGCGCTGGGTGGCCAAGTCGCTGGTGCGCGCCGGCCTGTGC
CGCCGCTGCATGGTGCAGGTGGCCTACGCCATCGGCGTGGCCGAGCCGCTCTCCGTCGCCGTCTTCGACTACG
GCACCTCCCGCAAGACGCAGCAGCAGCTGCTCGCCATCGTGCAGAACAACTTCGACCTGCGCCCCGGCAAGAT
TGTTAAGGAACTAAACCTCAGAGCACCCATCTATCAAAAAACAAGTACCTACGGCCAC
TTCGGGCGAGAGGGCTTCCCGTGGGAGAACCCCAAACCACTGGTCGTGGATTGACTCAAAACAATCATTATAT
AGCATATTGTACATAATTTACTTTTAAAACTTACTGCGTTTCTGTAGAGCGGACAAACTGGCGGAAGTCTCTT
AAAAAAACGATTTTTATTATTGAAATCTAATAAAATATCATGTATTTTTGAAAATACC
>1002050596_1 Heli.1-EL603857.1.5

CCCTGATAAGGCCGGGCTGCAGGAATCGGCACGAGGCTTTACCCATCAAGGGGGTTTTTGTGATAAAATATCT
TTAAAATTGCGAACTAAAGTTCGCGAATAGCATTAATATGGGTGATAAAGGAGATGGAGAAACATTTGACGAT
GCGGTCGAAGAGAGAGTTATTAACGAAGAGTATAAAATATGGAAAAAGAATACCCCAT
TCCTGTACGACCTGGTCATGACACACGCACTGGAATGGCCCTCTTTGACGGCGCAGTGGCTTCCAGATGTCAC
CAGACCCGAAGGAAAGGATTATTCTGTTCATAGATTAATATTGGGAACACATACATCAGATGAACAAAATCAT
CTTTTTTTTGCAAGTGTTCAACTTCCTAATGAAGATGCACAATTTGATGCCAGTCATT
ATGATAATGATAAGGGTGAATTTGGTGGTTTTGGATCAGTTTCTGGTAAAATAGACATAGAGATTAAAATAAA
TCACGAGGGTGAGGTGAACAGAGCAAGATACATGCCACAGAATCCTTGTGTCATAGCAACAAAGACACCTTCC
TCTGATGTACTAGTGTTTGATTACACCAAGCATCCCTCTAAACCTGAGCCTTCTGGAG
AATGTCACCCTGACCTCAGATTGCGTGGTCACCAAAAAGAAGGCTATGGTTTATCATGGAATCCCAACCTAAA
TGGTTATCTTCTATCTGCAAGCGATGACCACACAATCTGCCTGTGTGACATCAACGCCACGCCCAAAGAGGGC
CGCGTCATAAAGGCGAAGTCGG
>1002050597_1 Heli.1-ES585552.1.5
GCTAGACTACCTTATTCAGCCACATTCTAGCGGCTTCGTGTCCCTAGTCAAATTAAATTATAGAATTGTATAT
GGCATGACACATTCTATGTGTTCGAAGTAACTGACCTCAGGTACATTGGAAATTGACGCAGATAAATTGAGAT
GATTAACTCGATGTAGCCTTCCTCGATGAATGGATTATCCAACAAAGAGTTTTGTTTA
ATTGGTACCAGAGATAGTGCGTAGTACATTAGAACATTAGATGAGTGCGTTCTTACAAACTCTTCAGCTTTAA
GTATAAACATTTACTTAACAAGTCGTTATGTATTCACTTTTACAATAAGTCTGGCTAAAGAGTCATTAGAGGC
TCTTTTTTTTTAATTATGTTTTTTTTAAGTAAATATTGTCGCAATTTTTAGAAATAAT
TTTTATGCCATTAAGTAATAAAATAATATTTGTATGACTTTCTGTCTGTGAATTAAAGAATTTTAGAATTAGA
TATTGTTTGTG
>1002050598_1 Heli.1-CO729886.1.5
AAGAATGACAAGGCTGTCACTTACATGGTCATCCTATTATACGAGACTGCTTTATTGTCCTCTGGCTTCACCC
TTGATGAGCCTCAAGTGCACGCTTCTCGCATCTACCGCATGATCAAGCTTGGTCTCGGAATTGATGAGGAAGA
GCCAATTCAAGTTGAAGAAGCCAGCGCTGGTGATGTTCCCCCTCTAGAAGGAGACGCT
GATGATGCGTCCCGCATGGAGGAAGTAGATTAAGTTCCCCGCCTACTCCTAGCCATGTTATAATGGTATCTAT
GTGTATTGCAAGTTTTTTTTGTGCACTGGTGTACAGCCAAAGACTGATTTTAGTTCAAATTCCATTTAATTAA
TAAAGGATTACATTTAATGTAA
>1002050599_1 Heli.1-ES584796.1.5
TAAAAATAGAGAAAAATGATGTTACAAATTACGTGTTGATTGTTGCCCAAAGCTTTATAAGATATGGTGAAGC
TGTAGACATGTTGCAGTACCGTCTTTGCTCTTACACTATACAGTACTACTATACAGAGCGTTTTTGTCAGCAC
CACTTTAAATGACACCACAGTTTTAACTACTCTATAAAAGCCTAATACAATTTTTAAA
AGAAGTCGGTTAAAAAATGTCCAAAAATGAAAGTTTCTTTTTGAAACAACATAAATAATAAAATTATCGCTG
>1002050600_1 Heli.1-EL599496.1.5
GGTCCGGATTCCCGGGTCGACCACGCGTCCGCACACAAGGCGCACGGCAACATGAAGATCGCTATATTGTTAT
TGTTTTTCGTGACGTGTTACGCGGAGATCGATTTTAGCGGGAGAGCGAGGAATTTTAGCATAGAACTGCTATA
TCATACACAATTGGAGACAGATGGCCACGTTGTTATATCACCTTTTGGCATATGGACT
CTGATGACTGGCGTCACGCTGGGAGCTAGCGGTAACAGCCGGAAACAACTGTCACGGGCATTACTTCTACCAA
GTAGTGACGACTCAATCATCAATGGATACACAAATTTGACGAAGACCGTCCTCGACCCGCGGACCGAGGGCGT
CAAGTTGAAAAGTAAAAATTTTGTGTTCCTCGACAAAGGTTTCAATATATTTCCCGAA
TTCAAAAAATTGTTGACAACTGAATTCGATTCTAAAGTGATGTCTTTAGATTTTAAAGATCCCACAGCAGCCA
GCGTAGCAAACGCTTTAATAGAGAACTCTGGCGCCACCGTCTCCAATGTACTAAGGTCAGACGATTTCGCCGT
TTCAAGAATGATTTTGACAAATGTTATATCGTTCAAGGGTCTCTGGGCGTTACCCTTC
AACGCATCAGACACACAGGTTGAGCCATTTTACAATGAAAATAAAATGGAAATAGGTTCAGTGAAAATGATGT
ATCAAAGGGCTCCATTTCCATTTTCAAATGTGCAAAGCCTTCAAGGTCACGTGCTCGAACTGCCCTACGGAAA
CGACAACAAGTATTCTATGTTAATCATATTACCATATCCGAGAATGAAGGTCGGCGAC
GTCTACAGACGATTATCAAAAGTA
>1002050601_1 Heli.1-ES587462.1.5
AAAAGAGGTCTGCTACTTCTGAAGAAGAAACTTATCCTTGGGGCTTTAGGTTTGAAGGCAGTGAAAGTAGGTG
CTGTAGGTGCCGGAGTTGTAGGCGCTATAGCACTCAAGAAAAAACATCATGCGCCAAAATACGTCGAAGTTGA
GAGCTGGTCCGGTTGAGGTGCCTAAGATTTCTGTTTAAGCATCATGTGCGTTAAGAAA
TCAAGAGAGCATCGTTAAGTAATTTTAGAAATAGAATAGTTAAAAGCTGAAGCCAATGGGTATTTTTTAATAT
GAACTAGATAATAAGAGTAAATACAGGCTATTAAGTGACGTGAATTATAGAATTTCCAAAAGACAACAAACAT
TTCTACATAGAAAAAGAAACGAAATATAATTTGCATCATTATTATTGAAAAAAGTACG

CTCATGTTAAATACTCTCTGGTGATAACTTTGAAAGACAAGAAAACACTAATAAATAGTCTGTAAATTGCCAT
AAATACAAATCTTAATACTTAAAAGTCAGATAAATCGAAACTGATATCTTAAAATATTTTTATACAATAATAA
ATATGTATTGTAATGTAAATTGAGTTAAAAACAATATTTATAACCTTAAAGTCAATTT
TAGTTTCAGTACTAACTATATAAATGAGGTCAAAATTTGTCTTATATTTGTTCAATAACATTTTGTAAAAACC
GATGGTCATATTAATATAATACCGGCGTTAATGTT
>1002050602_1 Heli.1-ES587871.1.5
GCGTATTTCCTTCAGATCATTCGTATTTAAAACATCATTTAGTCTGTGCTAAAGTTTAATTTAATTTGTAAAA
ATGTTCTTAAAAGTTTTAATTTTATTATCCATTTTAATGTGCTACGAGTGTATTTTGTATTCAAATTTTTGGG
CGCCATTGTTTAATCCGAGGATTTTAAATTATAAAGTGGACGTGCCTTTTAATCCGGA
CGCTGGTAAAAACGCAAGAGAAGATTTCCAAAAGAAATATGGAGTTAGAGGAGAAAAATTAATTGCTGATTTG
GGAAATGGAAAAGGACCTGAAGGCTACTCGGAAAATAAGAACTTTGGTGACGTAAAGTTCTCATACACATACA
GCGGAGCTGAGAAAAATGCAAAGTATGAATATTAAAGCATAATTATATTATTTTAGTA
TAATATTTTATATTATTTTAGTTATAAGTTTATGTTTCATATCAAGAAATAAACTGCTAT
>1002050603_1 Heli.1-ES587357.1.5
GGAAAATGGCTTTTGTGTCGGTTTTTAGTAAATAAAAAAAAATGTTTTTGAGTTGGGACATTTTATTTTGGGC
GTGAAATTAGGCGATAGCAAATTTGATAATTAATAAAATAAGCAATACATTTTAGTATATGTGGTATTTTAAC
GCTCTATTGAGTGTTACTATTTAGTTTAAAATTATATATATAATATGTAAGTATATTC
TGATTTCTGTATATTCTGGATCTTTGTATTGCTTTAAATATTTTTGTAAATTTTATAATTTTTATATGCATTT
TACTATGAAATAAAGTATATTTA
>1002050604_1 Heli.1-EL596986.1.5
GCACGAGGGCACTGCCCTGTTATTTGGTACACCCACCTGTATTAGACACCCTTCTTTAAGTTATATTTTAAGG
TATATCAATGATTCTTATCAGCATAACTTAGTATAGTTGATATATTTTTAATTGTTATTTTCTGAATTACATA
AAGCAATGTATTTTACAAACAAACGGAAGAACAATAATTATAATGTAAGTTTAACAGT
TAGTGAATTTAAGTGTGCTATTTTCCATTCCAGAACTCAACGATATCAATATGTTCTCGTATGACTTATGTTC
GGGTTTTAAAAAAAAAAGAAAAGAAAGAAGATGTTTTATTATTATCTATATAATTAATTATATAACTTACTTG
AACCTTATGTGATTGTAATGCAATGTTTAGAAGTATTTTTTATTATTTTTTACCATTT
AAAATAATAGTTAGTAGAGATCTATGTAAAGCTGGCTGTAACTAATTAGCGAGTTGCATATAAGTAGAAATAT
TTTTAATATAAAAAAAAAACATTTGGAGAAAGTTTTATGTGGACGACCGGCCAATTGTCTGTTGTACGGTTAA
AGGTGTGCTATTTGAGTATAGTGGTATGTGGTATGTGGTAGTGGTAAGTGGTGATCGT
GAACTAGCGCCGCATGTGCGCACAAGTACTGTGTACGTACACCTCGACTCTCACTGCACAGCACATGTAGTTT
TTAATATTAATAATTGGTTATTACGCCAAAATAACATTTATTATTATATAGGCAATAATGTTTAACAAAAGTC
CCTATACAATCGAACTGACGACAGAAACGCTGTTATATTTGTGGCC
>1002050605_1 Heli.1-DT662163.3.5
AACAAAGCGGGGAAGACCCCTAACCACTCAGAAACGCCCAACAACGTTGTCGTGCAGAACAACACCAGCGCAA
ACAACACTATTGACAAGAAAAATATAAGTGCACCCATAGCTGTTACAAATGGCACAGCACCAACACCTAAATC
TGCTAAAACGTCCCCTGCAAAGGATAGCCTCGGCAAAGACAATCCCTTCGAAGAAGAA
GAGTGGGACGAGGAGTCGGGGGGAGCCCTCACCGACACGGGCGAGCCCGGCGTGCCCGTGCGCGCGCTCTACG
ACTACTTTTTCTCCGAGAGCGACGAGCTCAGCTTTAGACGAGGCGATTTATTCGAAAAACTAGAAGACGAGGA
CGAGCAGGGCTGGTGCAAGGGGCGCAAGGACGGCCGCGTGGGGCTCTACCCCGCCAAC
TACGTGGAGCCCGTCGGACACTAGCCGCGGCGGCGCAACGTCACTGCTACTCACAACAATCGAACATTTTACT
TTTTGTTTGTAAAAAAATATTTTTATTTACTTTTAGTTCATTAAAAAAAGTAGATTTAAAATCGCTGTTTTAG
AAAATTTTTAGTCTAGTCCTCTAGATGTCAGTTGCACTGAGGCCGTCGTGGAACAGCG
GCGCAGCGTCTGACTGTAAATGTGACGGTTGTGGGTTCGATTCCCACTCGGGGAATGAATTATTTTCATTTCC
TCGCTCTGGTAACAAGACAAACGCGGCGTTGAGTTCCGCCACTTTGAAAATATGGTGATGCGTGGGGATCGAA
TTGTTTTAACACTAAGTTCCCTGTGCCCATCCTGATGTGTGCGGGATACAGCGTGAAC
CTATAAATAAAAATATTATTCCAAAGTACATACGATAAAACATTGTATAAAGGCAATAAATATATCACATAAA
ATTAAGTTCATTTTATTATTATTATTATAATAGTAGTTTTTTTTAATTGATTCAGAAATAGCCATTTATGAGT
ATGCCATTTCGAAATGTAAAAAAATAACGAAAAATCGAAATCTTTTGCCTTTTTATGT
GACTTTGTACTTCTGCGAATAACTGTTAGATGTTTTCCGAATTTCCTTGTGGTTTTAGAAAGTGTATAGAGTA
AGAGAGCGGGACTAGTATACTAA
>1002050606_1 Heli.1-DT665331.3.5
GCACGAGGAGAATCCCATGCGGCCGCCATATTGGAAGAGCAGAGAAAGAAATTGGCTAAAGATATCGAGGCGT
TGCATTTGCAACTCGATGAAGTTAGACAGGCCAATGATAAATTGGACAAGAGCAAGAAGAAAATTCAAGAAGA
GTTGGAAGATACCAATATAGAGTTGAGCGCTCAACGCGCCAAGGTCCTCGAACTGGAG

AAGAAACAGAAGAACTTTGACAAGGTGTTATCAGAAGAACGTGCCGTGGCTGAAAGGAATGCGGCCGAACGAG
ATGCTGCAGAGAGAGATGCTCGTGATAAGGAGACTAGAGTGCTATCACTCACTAGAGATTTGGATATCGCGGC
TGAGAAGGTCGAAGAACTAGAACGTTCCAAACGTCTCCTCCAATCCGAACTGGACGAA
TTGGCCAACACTCAAGGTACAGCGGACAAGAACGTCCACGAACTATAGAAAGCCAAGCGAGCCCTGGAGTCAC
AGCTAGCTGAGCTCAAAGCACAGAACGAGGAGATAGAAGATGACCTCCAGTTGACGGAAGACGCCAAGTTGCG
GTTGGAGGTTAACATGCAGGCGATGAGGGCGCAGTTTGAGAGGGATTTGCAGGCTAAA
GAAGAAGCCGGCGAGGAGAAACCC
>1002050607_1 Heli.1-ES585728.1.5
AGATAAAAAAATATGGTTTCGACGACCAAAACCGCCGAAACTTGAATAAAGTTCCAAATACCCATTTTACATC
GGATTTATATTAGTTTTGTTTATTATAATCGATCATTAGATTAAGTAACATGACTGTGGCTGTGGTCTATGAA
TGTATACAGATGAAAAATCATAGACATGGCCGCAAATTGTAATTTTAAAATTATTTCT
ATGTCAGCATTTGCCTCATAAAATGTATGCCTATACAAAGTTATCTCTGATTTCACTAATACAGTTTGTTTTA
AACAAAACAAACAAACCTATTGTTATATTTTTTAGAATATTGTAGACCGCACCCAGTCTACGAAGAGCTTTAC
TAAAACAGTTCTGGTCATTTTGGATCCTCTTTGAGTATATTTGTATATATGGAAACTA
TTTTGGTGTATTTGTTGATATAGAACGTATAATAAATATTTTTTATGACTAAACCTAACATACGAAAATCCCT
CTGTTAGTGGAACCCACATATAATATTTTAAATTCTCTACCCAGAAATTTTCCCAGAAACTGTGTCTGTCAAT
TAATCAGTTATTAATATATATTTTTTTTATTTTATATATATATAAATGATGACTATGG
TTTTAGACTGAAAATTAATGCTGTGAACAAATATACGCCACAGTGCCAATATTAGATGTAACCTGCTTACTTT
ATTTTATTTATTTAAAAATTATAATGCAAGCAGGAATTATCTTTCAATAGAGTTTATTACGTCCGCAGCAAAT
TCGTATTAAATATAATACCTTTGTTGTTTAAAAAAAAAAAATAAAAACCTCGTGCCGAA
>1002050608_1 Heli.1-EL603062.1.5
GCACGAGGTGACGATCCAATCCACGTTATATCAATGCCAGACGAGGAGACGATGCAGCAGAAACCTCCAGACT
ACGACACGGTGGCGGGCGCGCCGCCGACGTACGACGACGCCATCAAGCTGAACCCGGCGCGCCTGCTGCCGGC
CAGCAGCAGCGCCCGCAGCGCGGACGCGCCCCCGCCGCCGCCGCACGCCGACGTCATC
CCGGGCCAGCTGGACGAGGCGCCCGTGCCCGTGCACGCGCCGCTCGCGCCCTCGCCCGTGCCCAAGACGCCGC
CGCCGCCCTACCGGGCGCCGCAAGCCATCAGATGAAAGGTGGGCGCTCCCGGCGCGACCCATGTGTTACTTTA
AATCGACTGCTGTTTCATGTGATGTGACTATGACTTGTAAATACACCTTGCGCCGTGA
GCGGTTGACCCCGGAGCTCCTCGGATGCATCGTCCTTATGCGGTATTATTTCATTGTATCATAATATGGATCC
ACTGTCCACACATGGAACGACTTTACTTTTTTTAATCTTAGTAGAACGTCGACTGTAGAGACGAAGCGTACTT
ATGTGATATTAAAGAGTGAATATTAATAGATACTTAATTACCTACGAGCTGTATATAT
TTGTTATAAAAATTTTGTAAATTAAACACAATAAGCGAACTTAGAAATACCTCTTCCAAATAGCCGGAATTTC
TCTAACTTTAGAGAATTAAGAAGTGATCTAATTACCTACATTTAAAAATTGCTTAAACTGAATAAAAAATACA
AAAAAAAATTAGAGTAGCGCTGTGCCATTATTAATTAATTAATTATGATAGTTATGTG
TAATCATCACATTTGGTACAGTATTATATTAACGTCCGTGGCTGGTTTGGTTCTTGATTTTTTTACTATATAT
TTATTGCTCAG
>1002050609_1 Heli.1-EL602863.1.5
GCACGAGGCCTTGCCGTAGTCCGTCTCCCAATGAAAAAGCATCTAATAATGAAATTAAAAAAGAAAATACAGA
TAAAAAATATAAGGATGATTACAAAAATACATCTAACAACCGCAACGTCAGTAAAATTCCAACAAGGGAACCA
TCACCTACTAAGCCCCATGATTCTGTTGAATATAAACTGGAATCACATACGACTGCAT
TGAAAGATACATCTTTAGTTACATCAAACATAAAAAAAGAAGATATAGACAAATCTCATGAGGAATCTATACA
TATCTACAAGATTATCGATAAAATTGATGAAGCAGATAACAGCCGTAAACCTACTGAAAAGCCTAAAGTTAAT
AATGTAAAAGAATCTGTAAAAATTATTCCGGATAAACATCTAAGCAGTCCAAGAGATC
AACTTAAAGTAATTAGTACAAAAAAATCTGAAGTTAATCCTATAATTATACCGTCAACGGAGCATGATGTACA
GCATGCCACTGAAAGTATAATAGAGTATAACATTGATGAAAATACTGATGATCAAATATCATGTGATAAAACT
TTATTGTCTCCACAACATAAAACTTCAGTAATACCAAAGGAAATCAATAATTTGAATA
AAAACAAGAAAATATCCACTTTAACTGATGAATTTATTGTATCAGAAAAAGAAAAGGAAGTACTGAATAGGGT
TCAAAAATCTTTACGAAAGCTTTCACCTGATCGAAAAGAAAAATCTCCCATTCGTGAATATAGCCCTTCTAAG
TCAGCTACTCTCCGTGATTTAGAAATAATTTCAAAGTCTGATGAAATTGAAGATGTTA
CAATGGAAGAAGTATCAGAAACATTTAAAACATCTAAAAATAATATTAATGAAAATGTAAACTTAATAGTAAA
ACAAAAATATGATGGAAAGGTTAAAGAACAAAAAACTTTAAGTAAGCCTCCATCACGAAACGTATCATCGACG
AAAAAACATCATAGCGCATCTCCAATACAGTCTAGAAGTATTTCCCCAAAAAAACCAA
TTTCACCAACAGAACGACCTCAATCTCCATTAACGCCTAAAGTAAGTGGCATTAAGCCAAGAGAAACTTTGTC
TTCTCATATTCGCAAGCCCTCACCAAGTACATCATTGCACACAAAATTAGAACCTTCATATGTAGATAGTAAA
AAGAACGCTACAGTTGCTAAGCAAACAAATCACTCGAAGACTGTGCCCAATAAATTGC

CATCATCTAAAACCATTCAAGCAACTAATAATAAATCAATAGACCAAGAGTCAAAACGAACTGCCATAACAAA
AACTAAAGATATCATTCGTAAAGATACTGAATCAAAAGTATCTCGAAGTATAAGTGATATGACAACAAAATCG
AAAAAAACTTCCCCTCAAAGAGTTAAATCGAAACCCGAAATTCAAGTAAATGATGTTA
CAACTGGGAAAGTAACCAAGCAGATCAATACTAATGCAAAAAATCAAAAGATTTCTCCTAATCAACCTCACAC
AAAATTACCAACAAGTAAACCAAAATCCGCTACTGCGCTTAATACGTCAACAGATGATGATGATATAATAA
>1002050610_1 Heli.1-DV501199.2.5
GCACGAGGATTAAAGCATTCTTTCAACAGTTTCAAACATTTTCGTTAAACGCTATCATGTGACGTTAAGTCAC
GCGGTCGACATAGTAGAGTTGAATTGCATTTGAAGAGTTATTATTTACTGTCATTTAAGATAATTCATTTAAG
AGAACTTGTCGATTAGTAAATCTAAATATTTGAATCGAATATATTATTTTAAAAGTAT
ATTATAATATTTATAAAATTACAAAATTCGGCAGAAAGTGAGGAACGGAGTGTCTTAACACTAGATTCTCTCT
GCGTACAATTTTTAAGCTATTTTTTTTATAAATCTTTTAAATATCATCAAAATCATTTATTTCAATAGATAAC
ATATGAAAATACCATATATGTATATTCAAACTTGGTTGTACAAGCGATGGTAAAAACA
ATTATATAGTCGGAAGAGGGAAATTAAGGCCGGTAAGTGTGCAGCTAGCGCGAGTGAGCAGACGGCAGCCGGC
GCGGCCGGCGCGGCTCCCCCTCATCATGTTGAACTGCACCGACCTGGAATGCATCCTGCACCACCACCATTAT
TATGCCCATCTACACCACATTCATCACTTGCAAAATGCCCAGGATTCAGCAATGTCGG
CCGACAGGTCGACGACAGATATCCATGGCTTACTTAACGACACTGAATCGGATCTCACCGGCTTATTACATGT
TGATGCCCCGAATGATGCGAATTACATGATCGGAGGTGTAGTGATTGCCATGGCCCTCGTGGGGCTCATTATA
GTGCTGCTGGCTCTCACAATCAACAAGCTGCGCAAGCGCGAGGAACACTCGGCTTCGG
TGCATCCTGCCG
>1002050611_1 Heli.1-EL598531.1.5
GCACGAGGGGGCTTCCTCACGGAGTACGTCGCGACGCGCTGGTATCGCGCACCCGAGATTATGCTGAATTCTA
AGGGTTACACGAAATCCATCGATATCTGGTCAGTGGGTTGCATTTTGGCTGAGATGTTATCAAATCGGCCAAT
CTTCCCCGGCAAGCACTACCTCGACCAACTGAACCACATCCTGGGCGTGTTGGGATCA
CCCAGCCAGGAGGACTTGGACTGCATTATCAATGAAAAGGCACGCAGTTACCTAGAGTCATTGCCGTACAAGC
CCCGCGTCCCATGGACGGATCTGTTCCCCGGGGCGGACACCAGGGCGCTCGACCTCTTGCATCGCATGCTGAC
CTTCAACCCCAACAAGCGTATCACGGTAGAGGAAGCCCTGGCACATCCTTATCTGGAA
CAGTACTATGATCCTAATGACGAGCCCGTCGCCGAGGAACCGTTCCGATTCACAATGGAACTGGACAATTTGC
CGAAGGAGACACTCAAGAAGTTCATATTCGAAGAGACGCTGCTGTTCAAACAGTTAAATGAAGACGCGTAGAT
GTCCGTACGGTTCACGTCACCAACATGGCGGATGCATGCAAGCTCAATTCAGAATTTC
AATAAAATTTAATATTTCCCCATAGGTTGTTACGAGTTAACTTAGCGTTGCAGTCATCCTTGTTAATGTGAAT
GTATTTCGTATTGTCATATCTTATCTTATCAGCTCGTTCACTGGAAATCGAACGACGGACACTCAATAGAAAT
GTTTATTTAAAAGACGTAAAAGCAAGAGTTTGCATGCTTTTTATCCTTATTTCAAGTT
TCAGTCTCCCACCACGTCGCGGTCAACCGTATGGCTAACTAGGTATTATTAATTCTTCGAGTATTTTCAGTGC
GGTTTTATTCGCTATTTTCGTATTTTATATATAATGTTACTTTTAAGATTCGTACTTCTATAGTTATTGGCAT
GTTAGCTACCACCTACTTAAAAACATGCTCGCTTTATAATATGTATATTATATCAGTT
CAAATATGAATGTTTTACAACGATTTTATATTAATTCATTTCTTTATCTTGACATTAAGTATTTTTTTAAATC
TGTTTTAAGTATATAAATTAAGTGTATTAATTAAATAATATGAATAGTAAACGTTCGACTGGATGTAGGATTT
TGGAACAGTAGATAAATAGATGATTATAGATAGGAGTTTTCTCAATTTTATCAACGTT
ACACCTAGTCGTGCGGGTTCGGAACGATGTGTGGTATTGTGTGATTTTACAGTATTACAGCGAATTGGTTTAG
AGGCGACAGGCGTAGGAAGGTTTACAGGAAGAACATTATTCCTGGATGATACATACTCA
>1002050612_1 Heli.1-DT661978.3.5
TGAGTTCTCCTGCACGCCGGACGTGTCGGACATGCTGAGCGAGATCGGCGTGGCGGGCGACTGGGACGACCAC
ACGTTCTCGTCGTACCTCACGTCGTCTTAAAGCGGTGCTCGGGTCGCCACGGCCGCTGGCCGGTACCACTGAT
CTCATACTCGAGCCGCCGCATGGGGGCCGACGACGCGGCGACCACTCGCCGCCCCGCC
TAGAGAGTGCCTACATCCAATTGACGTGTCGACATTTGGATTTAATGTACAAAGTTATTTAATAAGATTCATG
TTTGGAATTTGGTTTCCAAGTTAATTGGCCACATGGTCGCGTTCATTTTGGTGTTCTTTTTTCGGTTTATTTT
CAATGCGAACTATTTGGAAACGGCTCCTCGGCTTTGCCGGGTCAAAACATAGGCTGTG
AGTTGGTTCTCGTTTTGTGGTATTGCTTTTTTTTTTTAAGATGTTTTTTTTTCAATCGGGGTGATTTCCAAAC
GGCACGTCTCAGACGCATCTCAGTAAATAATCCGTCTATACTCTCCGTCGCTGTTAGCAAGCCACGGTCCAGT
CGCCGTATGCTCGCACCGCGTTGTCGCGTTCGTTTCTGTATTCGTATGTACTAGTTAC
TACATTAATCAGCATGGCTTCTATAGTAAGTTCTCAAATACTTTATAATTAATAATTTTACTTTTACAATTTT
ATTCTAAGAAACGTTCGTTTAAAAATATTGAGTGAATATCATTTATACAGTTAAATAAAAATGTTAAGTGCGT
ATCTTTTAATATTGATAGAAATATAAATAACACT
>1002050613_1 Heli.1-DT668978.3.5

GCACGAGGCTTCCTTTAATTCTAACACATCTTTAATATACTAATTAATACAAAATGATGGACTCAGTGGATGA
CAACAACCATGATCCCCTATCAGTACCTCCAATATCCCCACCGTCAAGTGGAGAAATTAAACCTGATAAGAAG
AAACCTAGTGAGAATATTTCCTTAGCTGACAACAGCTTGCTGGTTGACATATCAGATG
CACTTAGTGAAAAAGAAAAAGTGAAATTCACAGTCCACACAAAGACAACTTTGCCAGAATTTCAGAAATCTGA
GTTCTTTGTTGTTAGACAACACGAGGAGTTTGTTTGGCTACATGATAGATATGAAGAGAATGAAGAGTATGCT
GGTTATATTATTCCACCCGCACCCCCAAGGCCAGATTTTGATGCATCTCGAGAGAAGT
TGCAAAGACTGGGAGAAGGTGAAGGTGCTCTAACTAAGGAGGAGTTTCTTAAAATGAAAGAGGAATTGGAAGA
GGAATATCTAGCCACTTTCAAGAAAACGGTGGCCATGCATGAGGTATTCTTGCAACGGTTAGCGGCACACCCT
GTATTCAGAAATGATGCTCATTTAAGAGTATTTCTGGAATACGAACAAGATTTATGCG
CGAAACCTAGAGGCAAGATGGATTTGATTGGAGGATTGGTCCGTTCAATGACCACAACAACTGATGAAATATA
TTTAGGCGCAACAGTTCGTGATGTGACCGCTTTCTTTGAGCAAGAAACGGCATTTTTGCAAGAATACTATTCG
CATTTGAAGGAAGCCGTAGCTAAAGTGGATAGAATGACGTCGAAGCATAAAGACGTAG
CAGATGCTCACATAAAATTATCATCCTGTATAACGCAATTGGCGACTCGAGAACAACCGCCAACTGAGAGATT
TTTGACTAGAGCCGCTGAAACTTTTGATAAGTGTCGGAAAATCGAGGCCGCATGGCGTCTGACCAGGATCTGA
AACTGGCAGACACGCTTCGCTACTACATGCGTGACGCCCACGCAGCAAAAGCAGTACT
TGGTAGACGATTGCGATGCCTCGCCGCGTATGAAGC
>1002050614_1 Heli.1-EL602643.1.5
GCACGAGGGCGGGGAAGAACGTGTTGCCCGTTCGCCCGTCCGCTTCCTCCCTGACGAAAATCTCGGTAGCTAG
TGTCGTTCCCCTATTTGTTTTCGAATACGTGACCTTCGAACGCTTTCCCTATGGATTGGTTCAAATTTAACTG
TGAATAAATATCGTATAGGTGAATTTTTTTTATACTTTAAAAAACACAAATTATATTG
GAAAATAAGGAAAAAAACAACTTCTTATATTGCAAGTTTTGGATGTTACGCTCACTGGATCATCATGATCAGC
TAGATGTGTCGAAGTAAATATATGCAAAGTATGAGGTATCACACCGCCGAAACAATGCGGGACTCACCACATA
GTACCGACTAGCTGACATTTGGTACACGCTATCTGTTAAATTGAGATGCGACGGCAGG
CTCGGACCTCAGCGGGCGGCGCGCTTGTTGTGCGTTCCGCCTCTGCTTTACAAGTATGGTTGTGGTTGCTGCT
TTTTGTTTTTCAAAATGAATTTCGGATATCAACGTCCATTCAACCAGGATATTTGGACTTTGATAATCTTCCC
GAAACAAATTTTACCTGCGCCGGGAAGGTAATCGGCGGTTATTACGCAGACCTCGAGA
CTTCCTGTCAGATGTTCCACGTTTGCACTGTTGGACAGCAGGATGAGCCTATGGATATTAAGTTCCTTTGCCT
TAATGGCACCGTTTTTGATCAGGAAACACGAGTTTGCGAAAGAGTGGATGAAGTGGACT
>1002050615_1 Heli.1-EL601957.1.5
AGTCTGTGGACGCGGTAGATATCGTCGACGGTCGAAGTCGATTCTGCCAACGGGCCGACAGTTGCGAGTTTCG
CGCTCATGAAAATGTGTCGTAGCATACTTCATTGCTGGATATTGTGATTTTTAATGGATTAAAAGTAAAAGTG
GGTTTATAACTGTTGAAACATTGGAGTTAAAAGTGCTTATATGATCAAGGATTTTGTG
GACTACCAATGGATTCACTAAAAGTATCTGCAAATGCACAACCGGCTTCGGGAATGTTGGCACAATTTTTGAA
AAATCATCCACTGGTGCTAACACCTCCGGGATGTGCTTCCAACACAGATACACCCTCGACCTCGCCCACGCCC
GTGTCACCTTCGCCTTCGTACGACAAGCCCGACAACCGCGAGCGCGACGTGTTCGCCT
TCTGCCCGGACGCGCAGGACATGCGACGCGAAGACGAACGGTTAAAGACATTTACCGGATGGCCTGTCGCATT
TTTATCACCCGAATCACTCGCAAGGAATGGTTTCTATTATTTAGGCCGCGGTGATGAAGTACGTTGCGCGTTT
TGTAAAGTAGAAATTATGAAATGGGTCCTGGGCGACGATCCCGCCAAGGACCACCAGC
GATGGGCGCCCCAGTGCCCCTTCCTGCGGCGGCAGGCAGGCTCCGGCAACGTGCCGCGCGAGCCCGCGCCCGC
GCCCGCCGGCCGCGACGAGTGCG
>1002050616_1 Heli.1-ES584771.1.5
CTGCGGTACGGTCCGGAATCCGGGTCGACCACGCGTCCGGGCGCGCGGGCCCGCGGTGACGTGTAGAAAGGCG
ACGGCGTGGCGGCTCCGGCACACCGCCTCACTCTGCCGCCATGGCGCGCCCCAAGCAGTCGCCTCGCCACGCA
GCCTCCTACGTCGAACTGCCGCCGCGAGGACCAGTGGAGTCGCTGATCTACTGGCGGT
CGGCGGCGCGGTCGGGCGCGGCGCTGGGCGCGGGCCTGGCGCTGCTGGTGGCGCTCGCGTGCTGCTCCGTGGT
GTCCGTGCTGGCGTATAGCTCGCTACTGGCCCTGTCCGTGGCTGTGGCTTTCCGCATATACAAGAACGTTCTG
CAAGCAGTCCAGAAGACCAACGAGGGGCATCCCTTCAAATGGCTGCTGGAGAAAGACA
TCAGCGTGCCGGCGGAGCGCGCACAGTCGCTGGCGGCCGCCGCCACCGCGCACCTCAACGCCGCGCTCGCTGA
GCTACGCAGGCTGTTCCTGGTGGAGGACCTGGTGGACTCGCTGAAGTTCGGCGTGCTACTGTGGTGCCTCACG
TACGTCGGCGCCTGCTTCAACGGCATCACGCTCATCATACTTGCGTGGATAGCGTTGT
TCACATTGCCCAAGGCGTACGAGATGAACAAGGCGCAAGTCGACGGCAACCTGGAGCTCGCGCGCGCCAAGAT
CAACGAGATAACCGCCAAAGTGCGAGCGGCCGTCCCCATCGGCAGGAAGGTGGAGAGCGAGAAGGACAAGTAG
TGTGCGAGGCGAGACGGCACACACACTCAGCGACACTACCACGCAGTTGTTTTAGCGC

TCACCGTTTGTTATATTTATTATTAATACGTATTTAATGAACATACAACATACACATTTACGTGTAAAGCTTT
ATGAGACGAGTCTCGCGGAGATTATTGTATTTGCTTTTTTGAAATTT
>1002050617_1 Heli.1-DT664490.3.5
GCACGAGGCCGGTACGGTCGTTTGCCTTTGCTTGTTTTCTTTTCCATAAAAAAAGGCTGTTTGCTTATTATAT
TTATATTGAAAATTTCACGGCCATGCCTACTGTTTATTTGACATAAATAACATTATATTTAATATTAAAATCA
AGCAGAACTTCAATTAACATAATTTTTAATATAAAGTCGGTCGCTGCGTAGCGCGGCA
TTGTCTTACTGCGCAATCAATGAGCGATGGCAACAACAATCGAGGCGTCCCAATCCCTCATCCAATCAGAATC
TAGAGTTCACTCATACCAGGAGGTGACGGAGACGCGGCAGGTTAAGAAGAAGACCAAGTCGAGGAGGCACAAG
GATGAAGGTTCTATATCTGTGTCCAAAAGCTCAGAAAAGCTGTACAAAAAAATGAAAG
CTGACAATGAAAACCCAACGTGCGCCAAATGTGCCCGCCCAGTGTACGCCATGGAGCGCGTCAAGGCGGATAA
GAGAGCGTGGCACAAGGAGTGTTTCAGATGTGTGCAGTGTAATAAACAGCTGACAGTGGAAACGTATCAAAGC
GATCATACCACGTTGTATTGTAAGCCGCATTTCAAGCAGCTATTCGAACCTAAACCCG
TTGACGACGATGATGAAATTGATGCTGCACCCAAAAAGCATCAGATGATCATTTGCGAGAGCCAGCCGGTTGA
ACTACCACCAGATGTCGTTAGAGCGTCCGACAAACCAGATTTAGGTCTCGAAGAGTTAGCCTCTCTAGACGTC
AAGTCTAGGTTCGAAGTGTTCGAGCGCAAGGCCAAAGGGGAAGATGAACCTGTTACCC
TCGAACCGAGAGTGCCTAGAGAGAAGAGTACCGCGTTATTGTCAAAGTTGGCCAAGTTCAAGGCGAAGGGCAT
GGATATCGGCGTGTCGGACGAATATTTGAACGGAGTACCCGTAGAGCACAGCTCCAGCG
>1002050618_1 Heli.1-ES587883.1.5
GAAGTTTACCATTTTTTACTTAATTTATGAAATACTAAACGGAAATTAGTATTGTATAAAACATGCAACGAAT
AAGATCATTTATCATATCATTTAAAATTAAATTATTAACATGACAACTATATTTAATTTTTTTAAATATCAGT
ATTTCTAAAATACATTCCCGTTGCATTTAAAAAAAAAGTTTGCCTGTGAATGGGTTTG
ATTGATCATTATTGTTTTTTTTAAATGTTAATTTAATACTATTCTATATATATTTAAAATAATAAAGGTTAAT
ATAATACTATTAGAGAAAACAAAGCAATAGAATGAAATACAAAATGTGGATATTTTCATATTTTAAATTAAAG
ATTTTAAAAATCCTAAATGTTTTGAATAATCACTTTCTTCACAAATTTGAATGAAATA
TAATGTTTTTTAATTAAGTTGTAGTATTTATTTGTGTTGAGAGGTCGCGTATTGTAATGTTAACAGAATTGAA
ATAATAGAGGA
>1002050619_1 Heli.1-ES587646.1.5
GTTTTTTTTTTAACACTATTGCATATTGAAGTGAGCATGGATTTGTTTTGTATTTTAAAATCACTTTAAAATA
ATATTTACAAAGTAGAAATTAAGTTACATCAAAGCATTTTCCACTTTTCGAATAAATTATGTCTAATTATTTA
TTACATCTTTGTTATCATGTGATAATAGATACAATATTATAATATTATCATAGGCAAT
GTTTAATATAGCCATATCAGATTACTAAACGACAATTAAAAAAAATGTTGAAGAGATAGCAAAAAAAGTAACT
GCAAATACTGACAATAAATTGGAGGTACTGTCGTATATTTTTGTAATTTTGTGAAAAATATTTACATCGTATA
ATATATTTTGATTGTATATTATTATATGTAAAAATAGTTCTGTTATTCTTACTCTTTT
AATAAAAATATAAAAGAGTTATAAAAACTTTCTTATTTTTATTAGGAGAGGATGCACAAGAAGATGTTGTGTT
AGCTTTATTTATTAAATAAAAGGCAGTGTTTGACT
>1002050620_1 Heli.1-EL602295.1.5
GCACGAGGCCTCGTGCCGAATCGGCACGAGGGGACGCAAACGGTCACATCTTGGATTTATTTAAAAATATATT
TATATTAAAAATAAAAATCTAGATATTTATATTATTTTTGGAATTTATGTGTGATTCTTTTTACTTTTAATAT
TGAATAAATTAATATATAACTTATGAAATAAAGCCTAGTGTTATAATGTTGGGAAAAA
ACGCAAAATTGTTTTTTGGTGTATCTGTGGGTGTTTTGATTGGGGCCGTCGTCCTAGCTGCGTGGGGATTCCC
GAAAATTATCCAGGATCAAATACATAAGAAATTACAATTGTCTAACACATCTGCGATGTTCGACAAATGGAGA
GAGCTGCCCATACCGCTGACGTTCAAAATATACGTATTCAATGTGACCAATACTGAGG
AGGTGAATGAAGGATCGAAACCTGAGGTGAAAGAAATAGGACCTTATGTTTACAAAGAATATAGAGTGAAACG
TATACTTGAATACGGAGAGAATGACACTATTAAATATATGTTGAAGAAAACTTTCTACTTCGATCAAGAAGCT
TCGGGAGGTCTTAAAGAAGATGATGTACTTACAGTCATCAATTATTCATATTTGGCAG
CTATGTTGTCAGTGCAGGAACTGATGCCGTCTGCAGCCAGCCTTATAAGCAAAGCACTAGAATCCTTCTTCCC
TAATTTGATCG
>1002050621_1 Heli.1-EL596643.1.5
GCACGAGGGCAGCCTTGTGATTAATAATTTCAAACAAACAAGATTCATGAAAATGTTGAATTAGAACTGTAAA
AATGAGATTCTTCTTGCGTTGGCAGGAGTTCTTCTCTGCTGGTACAGCAGTAGTGCGTCAAGGTGACCGTGGT
GATAAATTTTATTTAATCCGTGGCGGTACAGTGGTGGTAAGTAAACGTGAGGAAGATG
GCGAAGATAGACGTGTTGGTGCTCTTCGGAGAGGAGATTACTTCGGAGAACAGGCACTACTTCACGAAGATAG
ACGTCTTGCAACTGTTACAGCGCTGCCTCCTGGCGTCGAATGCTTAACATTGGAACGCGGACCATTTACAGAG
TTGTTGGGCAATTTAGAAGAACTGAAGAATGTAAGACACTCAGATCCACGGCCTTCCC

AACACAAAAAGACCTCAGTTATTAAAAGCGAGTACCAATTCGTGGAGTTAAAGGATCTAGAGATAGTTGGCAC
TATGGGAGTCGGTGGATTCGGGCGCGTGGAGCTGGTTCAGTACAAGCGAAACCCATCCCTTACATTTGCCCTT
AAATGCTTAAAGAAAGTGGAGATGGTACAGCAACAACAACAAGAACATGCCTTTAATG
AGAAAAACATTATGATGATCTGCAATAGTAGATTTATTTGCAGACTCTATAGTACGTTCAAAGATAATAAATA
TATTTATTTCTTGATGGAGCCTGTGTTAGGTGGAGACGTGTGGACAATACTCCAAAAACAGCGATATTTTCCA
GAAAATATAGCTCGCTTTATGGTCGCTTGTGTGGTCGAGGCTTTTCAGTATTTACACT
CCAAAGATATTATTTATAGAGATCTTAAACCTGAAAATCTTATGCTGGATAAACAAGGTTATATTAAATTAGT
TGATTTTGGATTTGCGAAAAGGTTAAGTGCAAACAGTAAAACATGGACATTTGCGGGCACTCCTGAAATAT
>1002050622_1 Heli.1-CX700649.1.5
GCGGTACGGTCGGAATTCCGGGTCGACCACGCGTCCGGGAAAAATTTATCGAACTACTTGCTGTGATTAGAAC
CACCTTGATTTTACGACTTGAATTTCCAATAGAAAGATGGCTGATAAGGCAGTAGCAAATATCGCCAAGCCTG
AGATGCGAGGTCTGCTCTTCAACGCGATCAAACGCAATTTAATTGTTGCTTTTGTCCT
CGCTGGCACAAGTGGGTTCGCTTACAAATTTCTGGTTGGAAATGAGCGCAAGAGGAAGTATGCTGAATTTTAC
AGAACATATGATGCCGAAAAAGAATTCGAAGAAATGCGCAAAAAAGGACTTTTCCAATCCTGCTAATTTATCA
TTTGGTTATGTAGTTGTATTATTGCTGTGTATAATGTAATTTAAAAAAATAAAAATCC
>1002050623_1 Heli.1-DT668997.3.5
GCACGAGGCCCTCCCTCGACTCCGCCTCCGCCTGCCAAACCGAAACCACCTGTACCCCGCAAACCGCAAACGA
AGCGAGCTTCCCCACCTCAATCTGGTAATTTTGTGAAAGCATCGGTAAGCGATAAGAAAAAGTTTTTCGAAAA
CGCAATGGAAGAAAGCCATAAATCATCACCGAAGCCAGAAAAAGTGTTCACATTCCTA
TCAGCGGATGAGGTGGAGAAATTGAAGCAGGAAGAGGAAAGGAAGATGGCGTCTCTGTCGCGCTCCGAGCTCG
CGTGGTCCCCCGGGGAGGACCGCAGCGGAGAGAGCGAGCCTGACGACGAGCCCTATGAGAATGGACACAGCGT
ATCGCTGGGCGGCGTGAGTCCGTCGGCCAAGTCGCGCCGCCGCCGCGCCGACGCCGGC
TCGGAGGAGGACGCGGAGGCGCGCGCCGCCCGCCGCGCCGCCTGGCGCGCCGCGCGCCTGCGCTCGCTCGAGA
AGAATATACCTGGATTCTAGACCCGGAATCTTCACTCGAGAAAGAGGTGGAGACAACACAAGAGAAGATAATA
TCACTGGAACTGACAAGCAGCCCGAGCAGCGAGACGGCGCCCGAGCTGGACGCGAGCC
TGGAGGCGGCGGAGGCCGGCGAGCACTCGCTGCAGCCCGACATCGTGCAGGTCGTGAACCCGCTGCTCGACGG
CGGCGCGGGGCTCGTCACCGCCATACCCGTGGGGCCCGCGCACAGGCTCACCTACAACGAACCCAAGTAGATT
GTCTACGTTTCAATTTGGAAACCCCTTCGAAATTATAGCCCGACCAGATACTTTATGA
ACTTTGTAAACACATGCAGTCGTTTGCATAATATTCGATATGCGGGATTCGCAGCAATTTGTGTCGTATATAG
ACTGCCACAGTAAATGACAGTTAGCGTCGCGCTCTACTGTACTTTCGCTTAAGTGTTGGGATTGGGCTCAAGG
GGTAAGGGCGAGGACTGCAATGTGAGCGAGGGCATCACTATGAAGGTACCTTAAATAT
GCATTCAGCGCAAGTCTCCGCGCGTATCTCTGAAAATTCGTAAAGTTCTTGTCGGGCTATACAGTATATTATT
GCTGACATTGCTTTGGGATGTTTTTTTTTTCTATAGTTGGCTCTTTGTGACAGTGACTTGTGAATTCTTATTC
CGTCTTCTCTCGTCAGAGTTCATGGTTATTTTAATTTAATTTATTGTTTAGAATCGAG
AATGTTCTTTTTATTATAAGATTTTCTTTGATTTGA
>1002050624_1 Heli.1-EL599880.1.5
TCGGGACGATTTGTTTGAGGAGCTTACTAGGCACGCACCGGGGCTCGTTGAAGTTATTATATATAGTTCGCCC
GATGATAAGAAGAAAAATAGAGGGTTTTGTTTTTTGGAATACGAGTCTCACAAGGCCGCGTCGCTGGCCAAGC
GCAGACTTGGGACCGGTAGAATAAAGGTATGGGGCTGTGATATTATAGTGGATTGGGC
TGATCCTCAAGAAGAGCCCGACGAGCAAACTATGAGTAAAGTTAAGGTGTTATACGTCCGAAATCTGACCCAA
GACATATCAGAAGAGGCGCTTAAGGAGGAGTTTGAGCGCTACGGCAATGTGGAGCGCGTTAAGAAGATCAAGG
ATTACGCTTTCGTCCATTTTGAAGACCGAGATTGTGCTGTAAAGGCAATGCAGGAGCT
GGACGGCAAGGAGCTGGGCGGCGCGCGCCTCGAGGTGTCGCTGGCCAAGCCGCCCTCCGACAAGAAGAAGAAG
GAGGAGATCCTGCGCGCGCGCGAGCGCCGCATGACGCAGATGATCTACGGACGTGGCGGCTGCTCGCCGGTGC
ACGGCGCGCTGCGCGGCCGCACGCCGCAGCCGCAGCCGCGCCCGCCGCAGGCGCGCGG
CGACTACGGCGGCTGGGTCGTGTGGGACGGGCGCGCGATGGGCGCGGCGCGGGGCCGCGGCTGGGGCCCGTGG
TGGTGCGCGACGCGCCGCTCGTGGCCGCCGCACGCCCAGCGCCATGCGTGGCAACCCGCGCGCCAAGCCAAGT
TTACCAGGTAAACGTAAGCACG
>1002050625_1 Heli.1-ES586257.1.5
ATACAATGCAAATAAAATATTTATTGCTGAGCGAAATTCTAGTTTGAAAAATTTGATTTGACATTCAAAATGT
ACCGACATACAAGGCAAAGCCTTCGGTTGAACACCCTGTATATAGTCACCGTCCCTTAGTTCATCCGTTGGCT
GAGCAACGAAGTTAGTTTTAAACATTTTTTTCTATCTCATCGTACCCTACCACCGTTA
CTCGTTATTAGTGCACAATGTTAATGTTGTTCGTATATTATTAATAATTTACTGCGACTGTTATGTTTATGTA
TAAAATAGCTTTTTATTTTTTTATAATAAAAATAT

>1002050626_1 Heli.1-EL600698.1.5
GCAGAGGCGAAGTGATAAGTGTTGTGCGCAACTGTTATGTACTCTTAAGTTATAAAAGACAATGGATGTTGCA
GTTAAAATATTAAAAATATGTTTCTTTTACAATATTAGTATTCTGTCCCTGTGGGATATTTGATTTTTGAAAG
ATGCTCGATTGGTATAAAATTCAATAACCCTCAAGTTAGAAAAACGACATGGCGGACT
AATAATCAACCAATGAGAAGCGAGCATTAAGCGTTTCAGAATTGCATGCACCTATTCACATTTCGCGTCATTA
CGTTTTGGTGCCTTGTTTCTTAGATAATAATCCAAATCTGTTAAGTGGTAAATTATGTTCTGTATCTTTTATT
AACGATATCGTGGAAATAATCGCATTTTTAGGCAGTGTTAAACTCTCTTCCTTTGAAG
CGTCTCAAAAGCTGGGACCATGAATGCCTTAGATTTAGGATGAACCTAAATACGCTTATGTACATCGTTTTGT
TTTTGTAAACAATAATGTGTTTTCAATACACGTGCAAATTTGTGCAAAATTGACAGTTGTTTATAATAATGAC
GGGAAGGGGGGCAAAAAAACGCGGGAGGCCCCCGAAATCGGGAAGTTTTGAAAAGAAT
AGAAAATTTCAATACCATTTATTAAAAAAACCAAAATATTTACAAACACAAAATGTGGGATCTAGTTCCCTCG
CAAGTACTCCATCGGCTTCTAGAGCATCATCACCACAAGGAAGTGATATTAGCAGGCGGAGTACAAGAAAACA
GAGAGGTGGCAAGTCTCATAAAAGTAAAAGAGGAGGTTTGTCAAATGCTTATTCTCGG
AGAGGATACAATCCACATGCTGCCGACTATCATGAGTCGGAGTATCAT
>1002050627_1 Heli.1-EL603554.1.5
GCACGAGGGATTCCCTTAAAAGATTGGTTTTTACAAAGAAATTAAATTTACAATTTACAAATTTTTGATAAAA
AAAAGAAAAATCGAAAACTCCTGGAAGACTGTTTTTTCATGTTATTGTGTATTCATAGTCGGTAACATTTTGA
TATTGTATTAATTGTTAAGGTTTAAGCCGTATTGGATTTTGATAATTATGTCGTCCTT
ATATAGTATGCATTATCGCCGTCGTCGCGCCTCCAGCGAACCCCCCGCGGAGTTGTTCCCATCCCCCTTTCCC
ATCCACCTGGTGGACCCCCCCAGTGTGCTCGACCCGCTGGAATCCCTGCGCGTACCCTCCATACCCCACGTAC
CCCATGCACCCCACGTACCCCCGGTACTCCACGGGCCGCACGTGCTGCACGTGGAAGT
GTCGGACCCCGCGGGCGGCGGGGGGCACGCGCAGGTCCTGCACTTGCACTACCACTACGACCCTGCTGCCCCC
CACCACCATCCTCCATCTGCGCCTCGCCCGCACCTGCACACACTGCACACAGTCCACCTCATCAACAACACCA
TCGTCACCATATTGGTTCTCCTCATTATCGTCATGGTGTTCACAACCATCGTCAAAGT
CGTCCCTTTCTTCATCAAATTTGGAAGACACTTGTAAACCATCTGTTGTTAAGTATCAACTAACATCACGGTG
TTTCGAATCAAATGTATAATATTATAATTATTTATCACTGAAACAGGCTTAACATGTAAAATATTATGTAGAT
AATGATAGAGATCCCCGTTTTCGGTCTTCACCCATTTTTTGCCTTATCAGTAAACGCG
TTCTGGCTTTTTCTGTTTAAGATTTGCGAAGGTCAGTCGTTTACGAACCCGCCTCACGGCGGGATGAGAGTGT
TTCACCTGCATGACGTACAAGCATACACAGTAGCTCGTTTATTGGTAGGGTCCTTTAGTTGGAGCAGTCGTCA
GACCTGTATAGCAGTTTCGGGGTGTAGAAACGTTTTCACCGACGTCCAACGTCAAGTT
TGGACGCCGGCTTGAAATAACTGTCGTGATTACATAAAGGATCTGCAGTTCAGAGGCGGCGTTGCAAACTTTG
GTCTCGGGCGACTTTTTACTTTTTTGTATCCTCGCAATGTGGGGTATTTGACGCTGTTCGGAACGCAATGGAC
GATGAGGACCAGTCCTGGTTGCTTCAACATTCTGCATTGGAGAGTGACGTTACTGTTT
TTACTGTCAGTCGTCAGTCAAATGCCCCCGATATCCGAGACTACGATCGCGGTCCTGGTCTCACGCTTCGTCC
CCTCATAGAGCGGTCGTTCGCTAGGTGCGACTCTGTAGTTCCACATCTAATCTCGAACCTTTATAATTAATGT
TAGGATAGTAGAATAATGTATATTTGACAGATGATCAATGATGTTCATAAAGAGTAGA
TATCATACTAAGTGAACTCGAGACGCCGACACGAGCGCGGTTCACTAAATGATATTAGATATAGGTGGTAGCG
TAACTTAGATAGCCGCGGCCGCCCGCCTCGCCGCGGCTGTAAGCTCCGGCCCGTCGCCGCTGGGGGGCCGAAC
TACAAGTGGATAAGTTCTGGAT
>1002050628_1 Heli.1-DT668056.3.5
GCACGAGGAGAACATTTTAATGAAGTTTTAACACTTGGGAGGGATTTAGTAAGGCTTCTACAAAATGTTGCCC
GCATTCCTGAATTTAACCAATTATGGCAAGATATATTAATAAATCCTAAAACACTTTCTCCCACATTTATAAA
TGTTATGCAACTACTCCAGACTAGAACGTCAAGACGATATTTACAATCAAGATTGACA
CCTGATATGGAGAGAAAATTGGTATTTCTAACTTCCCAAGTTAGGTTTGGTCATCATAAAAAATATCAAGAGT
GGTTCCAGAGGCAATACTTAGCTACACCTGAATCTCAATCTCTCAGAAGTGACATGATTCGCTTTATAGTAGG
AGTTATACATCCAACAAATGAATTGCTATGTTCTGATATAATTCCAAGATGGGCAGTG
ATTGGATGGCTATTGACTACTTGCACATCCAATGTAGCCGCATCAAATGCTAAATTGGCATTATTTTATGATT
GGCTGTTTTATGACCCTGAAAAGGACAATATTATGAATATTGAACCTGCTATCCTTGTGATGCATCATTCAAT
GAGATCACATCCGGCTGTCACAGCCACTTTATTGGATTTTCTTTGTAGAATCATTCCA
AATTTTTATCCACAATTTGCAGATAAAGTGAAACAAGGTATCTTTAATTCCCTTCAACAGATCATAGAGAAAA
GGGTGTTATCTAATTTACAACCATTATTTGATTCACCTAAACTGGATAGGGAACTAAGAACAACTGTGAGGGA
GACTTTTAAAGAATTTTGTAGTAATGGTAATGGAGAAGGTGTATCCGGGAATAGTAGA

TGAAGGTAGCGAGGATTTGCCAAGAGGAGGGCCTGATGAACCAGCATTTTCAGACGACGAAGATGATCCGCTA
CCGACTATTGCAGAGGATACTGACGATGATGATCTACCGTTGTCAGAAGTACGTGCTCGGGAAAGACCTGAAT
TAACGGCCGC
>1002050629_1 Heli.1-ES584817.1.5
AACAAAGTCAATTGCAAAGAGATAGAGCAGCAGCAAGAGCTCAGGCACAAAGACTTGCCATTTCTCAGGCTCA
GGCACAACCTCCACAACGTCAAGCCCAGTCACCTCAATACTACTATGTACCACGAGGAGAAAATCCTTCTTCA
GGTCAAATTGATGCGTTTCTGAGAGGTCACGGCATTCAGCTTTAGATGAATTTTTGAG
TGAGTATCGATCGAGTTCGAAGAGCCATAGTACAAGTCATACACCGGTCGATAACAAAGTGAATCTAGTTAGG
ACAGTGGCGTGTAGTGGCCGCGAGTGTACATAATGCTGTGTCTCAGTACCTTAGTTAGACGTGGTTAAGCGGA
AATGACTCAACTAATAAAAGAATCGAAAATAAACTACTGTCCTATACATATCTCCTAT
TTGAACTTCGACTGTATTCACTTGAATTATTGTATGAGAATAAACTTTAGTGAATAAATTTAATATATAAAAA
CAGATTAAAAAAATATTTAAAAGTAAACTAATTAATTGATTTACAATAAATTATGACACATAATGTCAAAGGC
TTCTAAAATAGGCTAACGATAGGAGTCCTTTCCGCTTAGCTACTTCTAGTTATTAGTG
TTAAGGTTTAATTATAAATAACCACTTTCCTACACGTTCCATATGCGAACGATGTAATATCGAAATACCAATT
TTGTACTATTTTGTTTTATAATAAATTATATTTTT
>1002050630_1 Heli.1-ES586571.1.5
AGGAAGTGCCTGTAATCAAGCACGTGCCTGTGCCTGTAGTGCAGCACATTCCTGTACCTGTAATTAAGAAGGT
GGAGGTGGAAAAGCAGGTGTTCGTACCTGTGCATCACGAGGAGGAACATCATCATCATGGTTGGGAAAGCGAG
TCGCTGTCGCACGGCTGGCACTGATTTACAACACTACTCTAAAATAATCTTAGGTACA
AATTAAGTAGTAAATACGTTTATAATACATATATTGGAAGCTGTTAGTATATTTTTTTAATTTTTTGTGGCAA
TCGTTCATTTGTTTTTGTGAAATACCCTTCGTAGTATGAGCTAGTCATTAACTTTATCCATCTTTACTGTCTG
TGTCGAAAAAAATTAAAATTGAAATTGATTATCTGTATTATATAATTATTTTAAAAAG
TTACCATGACATACATATATGTATAAGCCAAAGAAAAATAAATGCGAA
>1002050631_1 Heli.1-CO729763.1.5
GCCAGAGATTGGTTATAGCACACTAAAGACTGGAGTAGTTCGTGAAGCGATTTTCTTCAGTATTTTTGGTATA
AGACTGGAGTAGTTCGTGAAGCGATTTTCTTCAGTATTTTTGGTTTAAGCACACAAGCCTAACAACAAAAATG
GCAATCACAAGCTTGGACCCCGGACCAGGTGCTGCCGCTCTTCAGGCATGGGGTGGTC
AGATGGCAGCTTTCGGCAGCAACGAAACAGTTGTCGACAAAGTACTACCAGATATGCTCCACCTCATTGACGC
TCATTGGCATCAGTTCCCACCCATGAACCCACTATGGCACGGCTTACTTGGTTTTGTCATCGGCGTTCTTGGT
TTCATCTCCGTCACAGGCAACGGCATGGTCGTCTACATTTTCACAACCACCAAGAGTC
TCAAAACACCATCAAACATTCTCGTTGTCAATTTAGCATTCTCTGACTTCCTCATGATGTTCATGATGGCACC
GCCAATGGTGATAAATTGTTACAACGAAACATGGGTATTCGGACCGTTAGCGTGCCAGTTGTACGCGTGTGCA
GGTTCGCTCTACGGATGCGTTTCCATCTGGACAATGACGATGATCGCTTTCGACCGCT
ACAATGTGATCGTAAAGGGTATCGCCGCCAAACCAA
>1002050632_1 Heli.1-EL602981.1.5
GCACGAGGCACGCACCGAGGTCTCCGCATGTCCCTCTCTGTCGCGGCCCTTATCAAAACAGTCCCGCATTGGG
ATCGATCTGAACGTCTACGGTGCGTACCGTGATAACGTAGGGGCCCCGAACGACGTCGCGCCAGTGTCCCGTT
TAGTGTTGTGACTTACGTATGCTCTGTGAACGCTTATTGGATTATTGTTTACGATTTT
TTTGTTTGTTTTGCTACGTCACATTGCAAGCTTACCATCGAGCGTCGCCTGCACACGCCGCCGCTGTCCGTCT
GCTCCATGAGCATTCCTGGTATGAGAGCAGGGTGTGCAGTACAAGCGCAATGGTTGCGTGATCGGTCACCATG
CGCCTCTTCCGCGGTGCGAAGCGCAGGGAAGTGACCGGGCCGCAGGCCGCGCCGCTCG
GGGACCTCGCCGCGAACACCCCCCACAGTTACACGCTCGTCACTGCACTCCCCGCCATGGTCATGGAGGATGA
TGTCAATGATATGAAAAAAGTGTTTGCAACATGGGGTCGCCGCATGGGGAAAAAATTAGATATGTTGAAAAAA
AATGAATCTAAAGAACTTTCCGAAGAAACTACAAAAAGCGAGAACTTAAACGATGAAA
ATTCTACAATTGTTACGGGACAATATAAGAAAAATGGAAAACAAAATTGGAAAATGGGAAGAAGCAGTTCCGA
TTCTTCTTCTATGAAAAAAGATAATGACTCTGATTCTATTAGAAGTGGCTCAAGAGATAGGTCGCCTAGTCCA
TTTAAGTCCTTCTTCCATCGAATGGGTTCTACTGGAATGTTAAATAATTCTAAAACAC
ACTCTCTTAATACACCAAAAACTCAAGAAAATTATTCTCTGTCTAATGGTCCTTCTCTTTATCGTAGTTGTTC
TACGTCACATTTGTCTACGTATGTAAAAGCTGATGATCCTTCTGATGATATAGACTTACAAAATACAAGTG
>1002050633_1 Heli.1-DN172703.1.5
CCTTCTGGTATATTAACTGGAAAGCCCTAGAAGAAAATAGGAAGAACCCCGTTTCATATAATCAACGACCCAG
CGTCTTCACTGATCCAGGTTCACCCAGTGCCAGTCAAAGTGCACCAAAATCCGATATTAGTTCGAGATTTAAT
AATGACATACAGAACGTTAACAGGGCACAGGCTCTACGTCAAAACTATAGAAATTCTC

AATTAAGATATTAATTTTTATTATGTTTTTTTTTATTATAATTTTTAATTAAGTATTAATGTAAATCCGAATT
GTTCATTGAAATATAACTTGAGAAGCATTATTTTTTTTACAAAATTTAATAGGATTTAATTTTTTCGTATTTT
TTATGTAATGTATCACTTATTGGTACAGTAATCTTTGACAATTTAACTCTACGCAAAA
GACTGTTAATTGGTATTCGTATATAAATATAAGAAAGAAAAACAAACATGCAATCATTTCTTTTATACTTGTA
CTATAAACGAA
>1002050634_1 Heli.1-EL599551.1.5
GCACGAGGGCCGCCCGACTCGTGCCAAATACATTTCCATACACCCCTGACTTTTTTACCAAAATGACTTCCAA
ATTACAGGAATATAAAGACACTATAGAGCGCCACTTGAATGACAAAAACAGTCCATGGGCAAAATACTTTGAA
GTAGCTGAAAAGAAGAGCGGTGTGAGCAGGGTATATATATTTCTCGGGTTGGTCGCAT
TCACCGGTTTATATTTAGTGTTTGGTTTCGGCGCGGAGTTGATATGCAATTCTATTGGATTCGTGTACCCAGC
GTACATGTCGATGAAGGCGTTAGAATCGCCTGTAAAAGATGATGATACAAAGTGGCTAACATACTGGGTGGTA
TACGCTTGCTTCTCTATCGTCGAGTACTTTTCCGATTTTATCGTCGGCTGGTTCCCAT
TGTACTGGCTGATAAAGTGTATCTTCATAATCTGGTGCTACTTGCCCACGGACTACAACGGATCTCTTATCAT
CTATCATCGTATCATTCGTCCCTACTATCAAAAACATCACAGCCGTATCGATGATATTGCCAACTCAGGTAAA
CAAGAATAAAGCTGTGCCAATATGAATATCATCTCGTTTGGTGACAGACGCTATCAGG
AAGAACAATTAAATTCCATTGAGATTTCTACTTGCCAAGAAATCTATAAGTACAAAAATTTGCTTCAGTACAC
GCTTCTATGCACTCATTGGTGAAAAAAAAGAAAAATCATAAACAAAAAAAAATATTTTT
>1002050635_1 Heli.1-DT666209.3.5
GCACGAGGAAAAATTCTTCTGAAAACAATGAAAATGCTGATACTGACTTAAAAACAAATATGGAAACTGTGCA
GATTAGCGTCGAAGAAGGAAACCTACTCCCAGAAAACAATACAACAGAAACGAGCATTGCATCTTCGTCTGAC
GAGCAAGGTCTTCATACAAGCAAGGAAGAATCTGCTACTGAAATTTCTGCTACAGAAA
CCGAAATAATGAGTGAAACGACTACCACAAGTACAACTCAATTAACTTCGCCATCACCAAGGTCAAGGTCTCC
ATATAGGCCACCTAAAAGACTCTTCACATCCACAACTGAGTCTACACCGTCAAGTAGTAGAACCTTTAGTAGA
AAATATAATCCCGGAGCATACACAAGCCCTTCTACGGTTGAAAGGCCTGGTATATTTA
GCCGTGGAACTACAAAAAGACCTTTATTTTCTAGAACTTTTACAAGACGAACTTATCCTCCTGCACGAACGAC
TCCAAGGCAACCAGTAGACGAGGAAGAAGAATATTCTGATGAAGAAATTCTTGAAGAAGAACCTGAAAGTCCA
TTTTCTTTCGTCCCACCGAATAAATTATTCACCAGAAAACCAGATTCTGAAGAATATG
AGGACTTAGATGTAGAGGAAGAAGTCGGTAATGAAGATGAAGAAGAAATACCCGAGGAAGAAATATATGAAGA
AGAGGAACGTCCAGTGAGATTCAAACCTACATCTAAGAAACCGTCATTCAAACCACATGTGGTTAATTCAAAT
ACTTTTAGAACTTCTACATCCACAACTGAAATACCAAGGCATCTCATATCATTTGGAA
ATAGTCAAAATAAAACTATCACATATAACAGATTTTCTGGAAATAAAGTTGTTAATGATACAAAGAAACGAGT
CCAAAATGTACCTATTGGATATAATATACTAAAATCGTCACTAAATGGGAGTTCTAAGAACTCAACAAAGCAA
TTTAAAAATAATGAAACTAAAGAACTAGAAAGCAACACTACTTTAGCTCCCATTTCAA
AAATAAACGACGAATCAACCACACAAGATGATGACTACCTTTCAATGTCCGAAGTAACTACAACTGTTTCTTC
AGATATTTCCA
>1002050636_1 Heli.1-CO729663.1.5
CGATAACGACATGCAAGAGGTGAAAACAATAGTGAACAGCCTTGCAGCAGCTGAGGCCGAGGTAGAGCAACCA
GAGCCCCAGGCGCCGCGTGCGCCTCGCACTCTCCTATTGCCGCAGGATATGCCTGTTGATCAGCGCCCGCATT
ATATGCAACCACGTTCAGTGCGCAGTGTAGACTCATTGTTGCCTGAAAAGCGTGTAAA
GCGCTGCGCATGCAGCTGCGCCTAGACGCGGCAAGTGCCTCTCGTACAAACGCCGCGCTTCAGCGCTGCAATC
CGTCCATTCATTCATTCATTCATTATCCATAGTACGGTTTCGGTTTTGTGTAAATAAAGCAATCCAATCATTT
ATATGCTCTTTATTTTGTATCATGTATTATATTATTAAAAATTAAATTAAATCATATT
ATATTATAATGTTAATTTAATCTATTTTTTATTTTTTTATTTAATCTGTTTATACATTCGCTCATAATCATAA
TAAAGTTGCGAGCGATGTAAGTTTATTTTTTAAGCATTTGTTATCTACAGAGATTTCGTTGGAGTTTGTTTTA
ATGGACCGTACTGTATCTATAATATAAAAAATCCATCGGGTGTTATGGTCGTAACCTA
TCATTATAATAAACTTTACCTACTTTTTGTGATCCTAAATTAAATTTTAATTAGTTTTAA
>1002050637_1 Heli.1-DT666604.3.5
GCACGAGGTATGGCGCTAAAGACACCTGAGGAATTTACAAAACATGGTTAGAGTCAGCTGGTTCTGCCCTCCG
TCCATCTTTGTTAGCTGAACACCCAGTTGACTCAGCTCGACAGAACTTATCAGCTACATTTGTAAATGCATTT
GTGAATGCTGGCTTTGGGAGAGATAAGTTAGTTACAACTGAAGATGGAAACAAATGGA
TGTATAAAAACAAAGATCACGGTATGCTATCAGCTGCAGCATCTCTTGGTATGATACATTTGTGGGATGTGGA
TGGTGGTTTAACTCCCATTGACAAATATTTGTATACTGTTTAGGAACATATAAAAGCAGGAGCGTTGTTAGCA
CTTGGCCTTGTCAACTGCGGTGTAAGAAATGACTGTGATCCAGCCCTTGCCTTGCTAT

CTGATTATGTTTTACATTCAAGTGCTAATTTGCGAATTGGTAGTGTGTTAGGTCTAGGCATAGCATACGCCGG
TACACAACGTCAAGAGGTACTGTCTCACCTCTTGCCCGTGTTGGCCGACACAACGGCCCCGCCAGAGATCTGT
GCGCTGGCAGCTGTCGCCTGCGGCCTCATAGCTGTGGGATCTTGTAATGGAGACGTAT
CTTGTGCCATTATTCAGCGGCTCATTGAGGATAACAAAGAACTTCACTCTTCAACTTATGCTAGATTCCTCCA
CTTGGGCCTTGGATTGTGTTTCTTAGGCTGCAAAGAACGCACAGAAGCCACAATGGCCGCCCTAGAAGTACTC
CCGGAGCCCCAACAGTCCCTATGCCAGACGACGCTGTCGATGTGCGCGTACGCGGCCA
CCGGCGACGTGCTCGTCGTCCAACAGATGTTACATATATGCTCTAAACATTATGAAACTGAGAATGAGCAATC
TTCAGCTGAAGACACAGCTTTCAAGAAACAAGACAAGAAAGAATCCAAGGAAAGTAGCAGCGGTAACAGTAGC
AGCTCTGCTGGTGGATCAAAGGATGACAAAAACAAAAGCAAGTCCAAAGAGAGCAAGA
GTAAGGACAAGGAAAAGGAAAAAGAGAAGGAAGCCAACAAGGAGCTGTCGTCAGTGCAAGCGGTGGCCACGTT
AGGCGTTGCTGTCATTGCGCTGGGCGAGGAGACCGGTGCGGAAATGTGCACACGTATATTTGGACAACTTGGT
CGTTACGGAGAGCCAGCAGTGCGACGCGCGGTGCCGCTCGCTATCGCCCTCTGCTCCA
TCTCGAACCCGCAGCTGTCGGTCATCGACGTGCTGAACAAGTACTCGCACGACTCGGACAACGATGTGGCCTA
CAACGCCATCTTTGCTATGGGCCTAGTTGGCGCTGGAACTAATAATGCTAGACTGGCAACAATGCTCCGCGCG
CTTGCGCTGTACCACGGCAAGTCGCCCGTGCACCTGTTCATGGTGCGGCTGGCGCAGG
GGTTGTGTCACGCAGGTAAGGGCACGGTCACGCTGAGCCCCGCGCACGCTGACCGCCGCCTCATCAACCAGCC
CGCGCTCGCCGGCCTGCTTGTCGTGCTTACTGCGTTCTTGGATTGCAAGAATATTATTTTGGGCAAATCTCAC
TATCTGTTATATGTGTTAGCAACAGCCATGCAACCACGTTGGCTTGTAACTCTTGATG
AACACCTACAGCCACTTAATGTGAGCGTGCGCGTTGGACAGGCTGTGGGATGTATTGGTAAGGCTGGTACTC
>1002050638_1 Heli.1-EL598896.1.5
GAGGCGGAGGATTCAAAGGAGGCAAACAAGTTATTATTGAACCACATAGGCATGCAGGAGTTTTCATTGCTCG
GGGTAAAGAAGATGCGTTAGTAACAAAGAATTTAGTGCCGGGGTCGGAAGTGTATGGGGAGAAGAGAATATCT
GTTGAGACAGAAGGAGATAAAATTGAATATAGAGTATGGAATCCATTCAGATCAAAGT
TAGCGGCAGCTATCATGGGTGGTGTAGATGCTATTCACATGCCACCAGGCTCGAGGGTCTTGTATCTCGGAGC
TGCGAGTGGAACTACTGTCAGTCATGTTTCTGATATTGTGGGACCTGAAGGTTTAGTGTATGCAGTTGAATTC
TCACACAGATCGGGCAGAGATTTAATAAATGTGGCCAAGAAAAGGACTAATATTATAC
CCATTATTGAAGATGCGAGACATCCATTGAAATATAGAATGTTAGTAGGTATGGTTGATACAATATTCGCTGA
CGTGGCTCAGCCCGACCAAGCTAGGATTGTCAGTTTGAACGCACAACATTTTCTCAAGAATGGTGGACATTTT
GTTATTTCTATTAAGGCCTCTTGTATAGATTCCACGGCACAACCCGAAGCAGTGTTTG
CAGCGGAAGTGAAAAAATTACAAGCAGACAAGTTAAAACCTCTAGAGCAGTTGACGTTGGAGCCCTATGAAAG
AGATCATGCTGTAGTTGTGGGTGTATTTAGGCCTCCACCGAAGAATGCATAGACAATAA
>1002050639_1 Heli.1-EL601892.1.5
GCACGAGGGTTTTCTTAAAATGTAAAAAGAAAATAGGAGTGAAATGCAGAACGCGTATTATACATTGTGCAAT
ATAAAATTACAACATTTTTATACGGAAACTTTCTGTTGGACATTCAAACATCGGCTAACTGTTGTTGTAAGGA
TGACATCAACATGTAGGTGGCGCGAGCTATAAAACGCGTTTCCTACCACTTTGTGTGT
ATAGAACCTTTCGAAATTGCTATCCTCCTTAAATTATGGCTTAATTCACATCTCCTCTCTGTAGCAGTGATAT
CCGATCTGTATTTTGCAATAACGGTACGGCGCTGCCCACTTTTAAAAGTCATGGTCCTCGTCGAAAACATTTT
CGCTTAATGACAGTGGATGGGGTCGCATCAAGCTTATATAATTTATTGTGGTTGTTTG
CGAATAATTTTTTAAAGATCATTTTAATTTGTATGCCCTTTATTTTGTTAAGAAATGTTGACAAAAAGCTCTG
GCATAAAGAACTGTCGAGTTCTTAGTAAGCATAATATTTTGCAAGGTGCTTAATCTATTCTTGGCTTAGCCAT
TTCACGTTTATTACGAGTAAGCAATGTTTGCTTTTTTGTTTACATAAATTATGTTAAT
TATTAGTAAACGCATATTTTATTCCAAAGTCGAATCCTCGAAAACGCTCAGATACATCTGTGAGTCATTTTAA
TAAAAGCATCATAAATAGTCGACATCAGAAAGGAACAATACACAGTAGTCGAGGACTGGCAGAGAAACAAACA
CATACTGTAATATGTATACATGAACGTGACTCGATTTACCAAATCAAAAGAGACGCGA
GGCTTAAGGTAACATTTTACATTCGCTATGTAAATTTTGTTTATAATTTCGCGTTATTACTATAAGATTTTAT
TCTAATGCATTTTACTCGGTTAATTCTCAGGCCATTAATAAAGTGCCATCGTCTCTATAGTTTTAAGTACAAG
AGGTTGATATTCAAAGCATCGATTAGGATTTAGGTTCATTTGCACTTCCATACTCATC
TCACCCAAATCATCAATCACTCACTCTAAGTAAAATATTGGCGGAACGTAACTGTTGTTAAGTTATCTGGTG
>1002050640_1 Heli.1-EL597052.1.5
CACTGTCGTGTCGTATTGGCCGTATATTTAATATATGTTTATTTTTGTGATCGCGCGCGGTCGCGGTTGCCTG
TTTGCGGCCGCGCGTCGGGTTGCGACCCGTCGACTGTCTGTTTTTTTTAATACATGTTATTCGATATATTTTA
TTCCTGCGAACGCATTTTGCGATGTGATGTGTATTTTAGCAGTAATTATTATATTATA

AGGAGTTTGACTGTTGTTTTTTGACATACTTACAACTTAGGTTACACGAGAATGATTTAAATGGAATGAATTG
ACCTCGACGACTCTGTCAATTACGGCTAAAATAAAAACACCTGTAATCGATAACATTTCCCAACTGTGTTCCT
GCTTTAGTTTTTTTGCTTTATTTTTATTACATAT
>1002050641_1 Heli.1-CX700655.1.5
CGAGTGTAAAGGGCACTAGTGAACTGAATTGTATGAAATGTCATATATTTTTGACCTGAACTGCGTTGGTTAA
GTGTTGCATGGAGTTCCATGGAACTGCCATAAAATTGTGATCAGTTTGAATACTTTTATATTGCTGAAGAATG
AAATGTTATAATAATGTTAAGTGTAGACTTGCTAGAGTATAACCCTGCGGATCGTTGA
TTGGAAAAAGACTCGTCTACGCCACCCATTGGTTGTTTATGTTACTCAAATTTTATATTTACAATTCATAATA
ACTTCAAAGTCTTTCTTTATTTTTGTATAGTTCTGGTGGCTTTATCCAATCAATTCTAAAATTATCGCAGCAA
ATTTCTTTGTTAAAATTTTGTTTTCGACACAATCATTGATTTGAACTATCATTTGAAA
TACAGATGTTTG
>1002050642_1 Heli.1-EL603547.1.5
GCACGAGGGTTCAGAATGTTTTTGCGCGTGTTATTATTTATTTTTATTACTTCTACCACAGCATTTAAGGTGG
ATAAAGCTTCTCCGCCGCAATGGAGTAATGTGTATACAGTTAAGGGATTGCTAAATATTCCTTATGCAGAACT
CCACGAACCGTTCTACGCTTGGTTCGACAGCAAGAATGGTAAATCCCGCATCGACTAT
TATGGCACAATGGTCAAAACCTATCAACTCTCAGCCTCTGTATATCCAGAGTTTGGCACGTCCATAAAGATAG
CGCCAATAACGACAGAACAAATATTAAATCGTGATACATGTTTGCAAGTGAACGGAACGAAAGACGAGAGCAT
CGGCATACAGTCAGTTTTGCCTGACATGACTGACTTCAAATTTGTTGGTACAGAGACT
ATGCAAGACTCTGACACTTCAAAATGGCGGATGGTCATGAAAATTGGTGATAAAGTGAATAAATATACAATGT
GGGTGAAATACAGGAAGAGTTTGGGGGGAGATCCCGTTGCTATACCGGTCAGATATGAAATGAAAGGATTCAA
TTCCTTACTAGGTTCCCACTATGATCACTATTACATAGATTATAGGGACTACGATGTG
GATGATATTGATCAAGACGTGTTTAAAGTTGATCCAGTGTTCAAGTGCACTTCCTTCCCCGGACCGGGATCAA
AACACTTTGCAACATTCAATCCTATGAAGGAATTTGTCCATCCGATCCACGATGGTCATGTCGACAGTGAATT
TGAAAGATTCAAAATGAAACACAACAAGCAATATTCGTCTGACATTGAACACGCAAAA
AGGATTAATATCTTCAGGCAGAATCTTAGATTCATCCACTCTAACAACCGGGCGCGTCTTGGTTTCACTATGT
CGGTGAACCATCTTGCCGACCGCACTGACGATGAGCTCTCCGCTCTC
>1002050643_1 Heli.1-EL598256.1.5
GGAAAAACAAAACACAGTTCATCCGGTGCTGAGGATCGGCCGAGGTGTACAGGTCCGTACAAATTCACATATC
GCTAGTATTTACAATGCCAGCTAACGGTATTGGCAAGAATGGAAGAATGGCAACCCTTGAGCAATTGTTCCAG
CCGGTCCAGCCTAAGTCTGACGACAATGGGAACAAAGTGACTGTCGTCGGTACTGGTC
AAGTTGGTATGGCAGCTGTGTTCTCTATGTTAACTCAGGGTGTCACTAACAACATAGCATTGGTCGATGTAAT
GGAAGATAAGTTAAAGGGAGAAATGATGGACTTGCAACATGGATCCGCTTTCATGAAAAACGCCAAAATACAA
GCTAGTACTGATTACGCTATCTCCGCTGGTTCAAAAATTTGCGTAGTGACAGCTGGAG
TGAGACAACGCGAAGGCGAATCCCGATTGGACCTCGTGCAGAGAAATACTGATGTACTAAAGATTATCATTCC
GCAGCTAGTCAAATACAGTCCAGAAGCTGTATTCATAATCGCGTCAAACCCTGTTGATATCCTAACTTACGTT
ACTTGGAAGATCAGTGGGCTACCCAAGCATCGTGTTATTGGATCTGGTACCAACCTCG
ACTCGGCCAGATTCCGCTATCTCTTGTCTGAGAAACTTTGCGTCGCAGCCACTTCCTGCCATGGATATATTAT
AGGAGAACATGGTGATAGCAGTGTACCAGTATGGTCGGGAGTAAACGTAGCCGGTGTACGCCTAAGCGACTTA
AACTCTAAAG
>1002050644_1 Heli.1-EL596355.1.5
GCACGATGCCTGCACCGTGCGACGCCTGTAAAGGCACGTGTGGCAACGGCGGCTCCGCCGCTTGCGGCAGCGG
CTGCACTTGCGGCCCGAGCTGCCCCTGCGGAGATGGCAAGCCCTCCGGCGGCAAACCATGCTGCCAGGGCGGC
AAATAAACTATCCCTTTCCTCTAAATCCCAACACGTAGCCCAAATGGAGACCTATACA
ATGTAATTTAGTTTAATTTAATTTAATTCAATTAACTATATATATCATGATATATATAACCGATATAATCGCA
TATATAACTGTAGCTACATAACATATAAACTGACGTGGATCCGTTGTTATAATTACTGTACTTTAAGTGGGCT
AATTTATAAGTTAATATTAAGCATAATATAATATAACAACATAATACTAACTATAAAG
GTATGTTGTGTCGTCATGAAATAAATGTTATTAGAATAATTATGCTTCAATTTTTAAGAAAAAAGTTTCTGTG
TTAGCATATAAGCATTTAAGTATATATTATTCGATTATAGTAGTTATTAATACATTATATTATATCAATATTG
CTTATCGATATCGAATTACCGATTATTACGTAAATATGTGTGTAAGTTTAAGTGTAGT
TTTGTCACATTCAGTGTATTTAAAAGTTTTTTCTCTTTTTAAATTATAAGATGGATCTGTAGTACTACAATAC
TCATTAGATTTAAGATTGTGTAGTGCACGCACACATGCATATTAACCGTGTAGTGGGTGTAGATGTAGATAAA
TAAAGTAAGTAAACCAAAAAAA
>1002050645_1 Heli.1-ES587532.1.5

CGGATTCCCGGGTCGACCACGCGTCCGGGCAGCTGATATTTGTTTGACACAAGATTTTTGCTAAAAATCAGCT
TCTATAATCCCAAAATGGGTGCCTCATTTGTTCCCATCACAATTTTCACCGTTTTCTGGGGTGTTATTGGTAT
TGTGTGCCCCTTCTTTGCTCCTAAAGGTCCAAACAGAGGGATAATCCAAGTGGTATTG
ATGCTAACTGCTGCGACGTGTTGGTTGTTCTGGCTGTGTGCATACATGGCACAAATGAATCCTCTCATTGGCC
CCAGACTTTCTAATGAGACTCTCATCTGGATTTCTGTAGCTTGGGGAAACAACACAAAGTGATATTTCAAACC
GTAACATATTAATTTAATTTTAATAAAAAATCAATTGTATTACAATAAATTTATTATT
TTTCTCCGCAATTTAAAAAAAAATAAATGAAATCCATTCCAAGTTAAATGAAATGTAATTATTGTGTAAGTAT
ATAGTGATGTGCTCGATGGGTCGATGAATTGTATTAATATATTATTATAGCGAATATCTATTTATATGTTACA
TTTCAATAAA
>1002050646_1 Heli.1-EL598696.1.5
GCACGAGGTAAAACTAGGCCAATTTAGGGAAAAAAAATTACCTAAACAACTTAACTTAAGCTAAATGACTTGT
ATAAATAAATATTTGGTATATATATCGATATGTATGTTTAATATTAGTGTTTTGGTTGTTTACCACCCACTAG
AATTAGGAAAACGTTAGGGATCTAATGAAATAATAATGAACATTGACTGAACGTGATT
TAAATATTTTTTTAATTTCCCGACGTTCGTAATGGGAGCAATTCTGTAATAATAATATAAATCGTAATTGTAA
TGATGATTATTTTTTATTTCCTTTTTTTTCTTATATGAATTATTAGTTTTTTATTGCAATATTTAAGTTTATA
TAATGAATAAATAAATAAAATAAAATACTTTTAATGAATTACTACCTGCCAAATTGTC
GACGAGCAAGAGAGGACGGTGTATTGTTGTAAGTGACGACGCTCAAGCACTTAGGATAGAGAGGCGCCGCACC
GTTCGCGCGGACGTGTACATACTAGCTACTCTTAACTAAAATATAATATTTCTATTGTAAACTTAATTTATGT
GAATCGCCAAACACACGCGGCGCCCGCCCGCCCCCCGGCCCGTGTGAGTTATTATTTA
AATTAACAATTTTTAGATTAAATTTAATGTTAGTCCTTTTTACTGTACTATATATCGATCAATCCGTTGACCG
ATCGACTGATT
>1002050647_1 Heli.1-EL601110.1.5
GCACGAGGGGTTAGGCTAGGATAGATTTTTCGGCTTATAACGTTTGCTGCACCCTCTCTATTATGGAATAATT
TTTTTTTAATTTGCAAATTCAAGATTTTTTTCTTTTTTTAAATAAATAATCAAAGACTGCTGCAGTGACTTGT
GTCGTGTTGTGTTTATGATTTAGAATTTTGTTTGGACTCTAGTGACTTTTTTGGTTCG
TTTTGGTTTTGATTCGGTGTGATCGAAGCTCTGGATAGATATCACATGACTCGATAATTGAAGACTACAAGAT
AACAAGATGGTGGACACACAGCACTTTTGTCTCCGATGGAATAATTATCAGAGTAGCATAACAAGCGCCTTCG
AGAACCTGAGGGATGATGAGGACTTCGTGGATGTCACCCTCGCGTGTGATGGGAAGAG
TTTGAAGGCACACAGGGTGGTACTGTCAGCATGCAGCCCTTATTTCAGGGAACTGCTTAAGTCGACACCATGC
AAACACCCTGTCATCGTGCTCCAGGACGTCGCTTTCACGGATCTCCTGGCCCTGGTGGAGTTCATCTACCACG
GGGAAGTGAATGTCCATAGACAGAGCCTACCATCATTTTTGAAGACCGCCGAAGTACT
ACGCGTGTCTGGACTGACACAGAACGATGATGCCCAGGGGCCTCTCGTGCAGAACATCGCTCGAGCAGCTGCA
GCTGCCGCGTCTTCCCACACACTCCTCCCCACCCTGCACACACTCCTCAAACTCCTCACACTCCAAATTATAC
CGAGAAGCTAGAGGAAGCTCTC
>1002050648_1 Heli.1-DT662113.3.5
GCACGAGGTTTTTGTTACACGCTACATGGTTCAAAGTCCGACGCAACTACTATAGCAAAAAATACTTGATTTT
AATTATTATCCGATATGAATCCTCTTACCCAAATTGCAAGGGTCACACGCCCCATCTATCGTCAATTGTCTAC
GACAAAGGCACTTGCATCAGCACCAAGACCAATTCCACCTACTGGTTTATGTTTTGAG
CTCAGTGATGAACAGAAGGCACTACAGGATCTGGCCCGCAAGTTTACCAAGGAGGAGATCACACCTGTCGCGG
CGCAATACGACAAGACCGGGGAATACCCGTGGCCCATTATAAAGAAGGCCTGGGAGATTGGTCTAATGAACGG
ACATGTTCCCGAACATTGCGGTGGTTTGGACATGGGTGTCTTTGATGGGTGTATGGTT
GCTGAAGAGTTGGCCTTCGGCTGTACTGGCATCATGACAGCAATGGAAGCCAGTGGCCTAGGCCAAACCCCAG
TTATAATCGCTGGGAACAAAGAACAACAAAAGAAATATTTGGGCAGATTGATCGAAGAACCGCTTGTCGCTGC
ATATTGTGTGACTGAGCCCGGTGCTGGTTCTGACGTGGCTGGAGTCAAAACGAGAGCG
GAAAAGAAAGGCGACGAGTGGATCCTGAATGGACAAAAGATGTGGATCACCAACGGCGGGGTTGCTAACTGGT
ATTTCGTACTAGCTAGAACGAACCCCGACCCCAAGTGCCCGGCGAGCAAGGCTTTCACTGGGTTCATAGTGAA
GAGAGATTGGCAGGGCGTCATCCCTGGACGCAAAGAATGAATATGGGACAACGCGCAT
CTGAACACGAGG
>1002050649_1 Heli.1-EL597260.1.5
GCACGAGGGAAGGACCTCCAGCGACATCGTCAACTGGGCCTTGGATAAAGTAGCTGAGAATGTACCGGCTCCG
GAAATCATACAGGTAGTGAACGAAGAAACGATGAAGGCGTGTAGTGACAAACCTCTCTGCGTAGTGTCCATTT
TGCCTCACATATTGGACTGCGACGCGGCGTGCCGTAATGGTTATATAGCTGTTTTGGC

CCGTTTGGGAGAGAAATATAAGGCCAAGATGTGGGGTTGGATTTGGTCGGAGGCCGGCGCTCAGCCGTCATTA
GAGGAGTCGCTGGAACTGGGCGGCTTCGGTTACCCGGCGATGGCGGTCGTAAATGCTAAGAAATTGAAGTTCT
CGACTCTTAGAGGATCTTTCTCTGAGAGCGGAATTAATGAATTTTTGAGAGACCTTTC
CTTCGGTCGCGGACAAACAGCACCCGTACGAGGAGCTGAAATGCCCAAGATTCAATCCACTGAGCCCTGGGAT
GGCAAAGATGGTGAACTGCCCCCAGAAGAGGATATTGACCTCTCTGACATAGACTTGGAGAAAGATGAACTGT
AAACTAGTGATGTGAAAGTGATATGAAACTTTTTTTCTTTACAAAAAGCTGTCTAAGA
TTGCAATGCTATGTATGATGTGGATTTTTAATGTGTTTTGTAATTGGTAGATATAAAATCTTTTTTAATGTAT
TTTGATATTTTTTGTGATGCCTATATATATTTTTAGTCAATATCTCAACTGTGCATATCCAAAACTATCACAT
AACTTATCAATAGTTTTCCTTATACTTTACAGTCATACTCAAGAACGTTAATTACCGT
TGAATTGTCATTAAACTTACAATAATAAATATAGATTTGTACCTATTTAAAATGAATGTTACTTATTAATGTT
TCTGAATTAAAAATAAGTGTATATATATATTTATTTTGCCATACATTAAGATAACTTATATTTATAAAAATCA
TCAACAATTAGTTATATGTTCAAAAGTTATTTTGTTTACTAAAGACTGGTTTTAAAAT
AAAAAATAGCTTTTTTTAAATTTTTTTAGTAATAATTTGTCAGTCAGAATCATCCATTTGTTATATTTTTTTA
AATTTTATTATAAATCCATTGTGTATGAATAGTTTAGTTAACAGTTACATATATTGGATATTTTATAATTTAT
ATATTGTAATAATAGAAAATATTGTTCGCTGCATTTTTTCCATAATATTAATATTTTG
AGGTATTTATTGTAGTCACTGACATTTTTAAGATAAATAAATTTTTTA
>1002050650_1 Heli.1-EL600751.1.5
GCACGAGGCGTGTGGTCGCGTTAGCGCTGCACTGTTGTTCTTGTTTGTTATTTTAATCGTTTTAAAATACACA
TACGGTTTGAAATCGATTTCTACGCAGTTCAGTGAAGTGTTTATGTGCCAAAATGATTGAGCCAATGAACGCG
TCCGCGGTAGATGAACTCGAATCCTGGGTGGAGTTAAATTCCGGTGGCATACTGAGCG
GCGCTGAAAACGAATACATCAGGCTGCTCCGGGAGGCTCAGAGAGAGAGCAGAGATTCCTCGGTGCGGCACTC
CCGGGCCTCCAGCGTTAAGGGCAGCCCAAAATCTCCACCGAACAGTCCGAATTTGGAGCCGTCGACTGAAGAC
GAATTGAAAGGAGTTTACATCAACTGTTGGAGAGATGACTCCAACGATTGGGTGTGGG
AGTGGAGCAGTCGGCCTGATCAAGTGCCGCCAAAGGATTGGCGTTTTAAACATCCGGCGGGCGCTCGCGGTCC
TCCATCCGCTACTTCTTCTATAGAAGTACTAGAGCAACTGGCTCCTGCACAGCAGGCGGCGGCGGGGCTGTCG
GTGCGGCGCGAATTTGAGACTCGAAAACGGAATGTCTTAACACTAGTTATTGTCTATT
CTCTATTCTCTATTGTCTATTTTATTTACAGAGTATGGCTGAGCAAAAAGGGCATGCTGCCGCCCAGACTCAT
CATCTTGAACTAGATTTAAGACTGAGTTGGCATTGATTATCTGCTTTGCTATGGATTTAAGAAAAAAATAAAAG
ACAAAAAGTACACTTAGGATTTTATACCTATGCCTTATTTGATAAATGTTGGTTACGG
ATTAAATACATTTTCTTCAATTTGACAGTTAAATATGAAATATTTTTTTCCTAAATAATAATTAGAAATAATA
AATAGTAATTAGCAGTTGGGATGCACCGTTTTATACCTTTCCCTTATTTGAACATTTATGATATACATGTTTT
ATGTTTCACT
>1002050651_1 Heli.1-EL601981.1.5
GTATGTACGAAGTTTATAGCTAATATAACAAATGGATTGAATAACTACATCATGTCGACTAACGCAGTAATAT
ACACATTTCCATTTTCTTGAGTTTTTAATTTAAACATTTAGCAAAATTATTTTATTATTTAAATCACATAATT
TTAATATTATTGCCACGTCATTGCCTTTTATTTATATTCAATATATTTCTAAATTATA
CTAAATGTTCTATTGAAATTTCAAATGTTTCAAATA
>1002050652_1 Heli.1-EL599045.1.5
GCGGCGCGCCGCTAACGCTCATAACGCGCTGCCACGCCGGCCTGCGCACGCGCACTCTCAGCTTTAAGCTCCC
TCCGTGGAAAGGATATTCCATGTGCTGAGCTACTCGCGAGGATATTTGCACTTTTTTTTTTATAAAGTTTGAA
CGTACTGCTTACTGAAAACTATTTGTGTTATTAACATTTATATTTAAATAACGATTTT
TTTTTGGACTTTTAATTAGTTTTTTGTGTTTTAAATTTGAGTTGAAAATAAAACGGAGGAACATCTTCAAGTT
TTTTTTACTAAGCAGAGTACAAAAAACGCCGCCAAACTGTCTACCGCAAATGAGATTATACCCAGCTTCCACG
TCCAAGCGCTAATAGGTCGGTGGCGCACGAGCCCGATGAGCGCGTGCGCGGCGGCCGC
CATGCGCGAAAAGCACGCGGCGCCGCGCCGCCTAGCCTCGCCGCCCTCCTCGCCTCTCGAGCGACATCCCAGC
CCTCTCGGCTCTCCCCGCGCTGACGAACCTCTGGACCTCAGGGTCACACACAAGCGTCCGCCACGATTAGAAG
ATGAAAACTGTAACCTTATCACTCAGTCGCCGCCGCCGCCGCCGCATCCCACACACCC
AGCTCATCCAGCGCATCCAGCGTTACTACAGTTTTGCCGCCGATTGCCGCTGGCACTACCAGCGTCGTTTGGA
CGCTATCCCTTCTTGCCCGCCGCGGCTGCAACATTACTAGCTCCTGGTGCGCCGAGAGCACCGCCGGTTCCCC
AAAACCCTGGAGTAAACCGAGC
>1002050653_1 Heli.1-EL598545.1.5
GCACGAGGTTTTTTTATGTATGCAAAAATATTTAATAAGCAACAGATTAGTAAATACAAATGGCGACTTACA
GACTATTAAGTACAAAAACAGCAAGCTTTAATGTTATTGACAAATGCTTAACAAAATAAAAAAAAAAACAAAT
ATAACTTAAAATCACAAATAGCGAAATGATAAACTAAACAAAGTAAAGAAAAAACAAC

GTACATTGAGGGAACCTTATTAACTAAACACATTCACGTGCTAAGACACCCTGAAATCAATTCAACACCCCAT
CAATATGAGAATTTTACATTTTAAAATAGTTTTAAACAATTAAATAAAAAGCTTTTTCTTTTTATTAATATGT
AAAATAAAATTATTGATGGATAGAAGTATTGACCCTGCTACTGTAAGAACACAAACCA
AGCAGTGACCTCTATTATATTGAATTATCGATGAAAATGATTTTAATATACTTAATTATATAATTACTTTTTA
AAATTGACAAACATTTAAAAAAATGTAATTTCAACCAATGATATTTGCTTGCAAGACTCATTCATATTATTAT
CACATATTCATAAACCAATGTTAGAAACAATAATAAATATTGATTAATTCGTTTATTT
TAAAACAGTTTTAACAATATTCGATATTTCGTTGCGAGGTCAGCACGGCGGCGGTGCCTACTTACAGTTTGG
>1002050654_1 Heli.1-DT667108.3.5
GCACGAGGGTTGCTTTGCAGGTATCAATCCAATGGTCCCGCGCCGAAGTTCAGCTGTCCCTCTCAGTGGAGGA
CCAAACGCTGTACCTCTGTATAGAGTTCTCGGACGATCATCCTTTAACGCCGCCGCGGGTCACAACGCCGCAC
TCGCTGACCTCGCACGCGTCCCCCTCGCGCGGTCCCTTACATGGAACGGACACGCAGT
GGATTGTCATGTATTTGGCTTATCAGAACGGCTCACTCCTAAACGCCTCCAAGATGTGGGTGAGCGCGCTCAG
CACGCGCGTGGCGGCCGCGCCCGTGTGCTACATCTGCTACTGCCGCCTGCTGCCGGCGCCGCGCGCGCGCCTG
CCCGCCGCCTGCCACACCTGCCGCAACGAGTTCCACGCGCAGTGCCTGCGCAAATGGT
TTGCCACCAGCTACAGGTCCAACTGCCCAGTTTGTCGTTCCAAGTTCTGATACACAACGCTTCTAACGAGATG
CAACAAGAACATAATAGAACTAAAGAATAATATATAAATCAATTGAAATATAAAAATCTATTGACAACTTTCT
TATTAAAATTAATATTTTCTAAATAAAGATGTTGTTTGAAAAACTAAATCGTACTTGA
TTTTATTGAAATGCTCTCATGTTAAAAGTACTGATGATATTTTTTTTATATTAAATTTGGTTTTGTTTTTACA
ACTATCCATATGTAAACTCGTTTGACTTAACTATCAGGCATTTCAATTCATACAAGACTTGAAAACATAAGCT
AAATAATCCATGTTTTTAACTAAATTCTAATTACATCATAATAGTA
>1002050655_1 Heli.1-ES585701.1.5
GCACGAGGCCTCGTGCCGCACCAACTTACAATGAAATTCCTGATCTTATTAGCCGTTGTGGCCTACGCCTCGG
CAGATGTAGCCCACATCGTCGGTAAGGAGGCTAGTGCCCAAATCCTAAAGCAGGAATTGGATGTGGGAGTCGA
CGGCCAGTACCAATGGGCGTATGAAACCGAGAACGGTATCGCTGCGAACGAGCAAGGT
GCCCTTAAGAATGTTCCTGGAGCCGAAGCCCCAGCTCAAGTAGTCCAAGGTCAAGCAAGATGGACTGCTCCTG
ACGGTCAAGTCATCGAATTACAATACTCGGCTGACGAAAATGGTTTCCAAGCTCAAGGTCCCCATATCCCCAC
ACCACCCCCGATTCCCGAAGCAATCATCCGCGCAATAGAATTCATCAAGGCAAATCCA
CCAAAAGAAGCTTAAATATTATAAATAGAAAACATCCTGTAAATATGACTAAATTGAAAGAAAGTATATTTCA
AACAAAATTTAACCATAAATATGTTTAAATTTTTATATGAGACTTGAGAATGTGACTAGTCTGATTATTATTT
CAATTTTATCATATTAAAAAAATACTCATATTTGTAAAAAAAATAAAACTAATATATA
AAAACAACAACAACAATCTGTGATTTCTATGAAATCAGGAAACATTTTTATACTTGTGATAAAATAAAATAA
>1002050656_1 Heli.1-ES587035.1.5
CGGATACTCTCTGCGGACCTTGAAAATTCAGGTGAGGGATGTACGTGGAGATGTCGCGCCGGTTCGTACCCAT
ATCCGCAGCTGGTCTATGCTGCCCACGCCGCTCCTCTCGCCATCGCTGCCCACGCTCCCTCCGTGTCCTACTC
CGCATCATCCAGCTCAGTGAACCATGGAGGCTATGCTGCCCTGTCCGCTCCCCTCGTG
CACGCCGCTCCCTACGCCGCCCACGTCTCTCCCCTCGCCTACAACGCATGGTCCGCTCCCTTGGCCCATGGAG
CTATCGCCGCTCCTCTTGCCCACGGCTCCCTCTATGGCGCCCACGGATACGCCGCTGGTCTCGGATGGTAAAT
AATACACAAACTGTGATCCTAGTTAGATAAAAATGGCAAGAAGATGAATGCACGTCAT
GTATACTTCAATGTAGAATAACGTCCCGAATGTGTAATACTCATACAAGTAAGAGCCTCATGTAAATATTATT
TAACTCATCAATAAAATATTTAA
>1002050657_1 Heli.1-EL596255.1.5
GCACGAGGGAAACATGGCGGCCATTGACGTTGAGCCGACTAAGATCGAGCACGCGATGCGTACGCTTAAGTTG
TCAGGGCATGTCGGTTTCGACAGCCTGCCTGATCAGCTCGTGAATAAAAGTGTGCAAAATGGATTCGTATTCA
ACATACTATGTATTGGGGAGACTGGCTTGGGTAAATCAACACTTATGGATTCTCTGTT
TAACACAAACTTTGAATCAACACCCAGTCCTCACAACTTGCCCACTGTTAAATTGAAGGCACATACATATGAA
CTTCAAGAGAGCAATGTTAGATTGAAGCTAACAATCTGTGACACAGTGGGTTATGGGGATCAAGTCAACAAAG
AAGATAGTTTCAAGGCAGTTGTCGACTACATAGATGCTCAGTTTGAAGCCTATCTACA
AGAGGAACTCAAGATAAAGCGAGCCTTGCCTACATATCATGATAGTCGTCTGCATGTCTGCTTATACTTCATT
TGTCCTACTGGACACGGCCTTAAGTCCATCGATCTGGTATGCATGAAGAAGTTGGACACTAAAGTGAACATCA
TCCCTATCATTGCCAAAGCTGATACTATTTCTAAGACTGAGTTGCAAAAATTTAAGGT
AAATTGTATGAATGCTTACATTGCCTTTTTGTTTTGATTAGGGATGAATAGGTAAGTTATTATGTAATTTTGA
CATTTTTCTACTTTTATATTAATCGTTTTTTTTTAATGATGCTGATTATAGATAAAAATGCTATATTGATTTT
TTAAAATGTATTTTTTAGAATCAAAATGTTATTG
>1002050658_1 Heli.1-CO729955.1.5

CTACGCTATAGTGAAGCGCTTGCCACGCAAATTCAACGAATAAAATAACATCGTTTATACTAAAAATAATTAA
TCTACACAAAATGGCCTTACTACCTTTGTTAATAGACTACGAAATGAGACCTAGGCGTTTAAGCGACATGTAC
GGTTTGGATTTATCTCCTGAAGACTTCATAACCGATATCTTCGACAGACCATCCCGCA
ACTATTTACGTTCCTGGAGAAACCTTGCTTCTTTAACAAAAGATGTAGGGTCTACTATCAAAACCGACAAAGA
TAAGTTCCAAGTAAGCTTAGACGTCCAGCATTTTGCTCCAGAGGAAATAAGCGTAAAAACTGTCGACAACTAC
ATAGTGGTTGAAGGAAAGCATGAAGAAAGGAAAGACGAGCACGGGTTCATATCCAGGC
AATTTAAACGCCGGTACGCTATACCAGAAGGCTTTAACCCTGAGATGGTAGAGTCCAAACTTTCTTCTGATGG
TGTATTAACTATATCTGCGCCTAAAAAATCTGAAAAAACAGGTGAAAAAATAGTACCTATTGTCCAAACTGGT
CCGGTGAGAAGGAAACTCCAAGATACAGACGATCAAATTGCTGGACAGGTTGGACAGG
ACCACCAAAATGCTCAGCCTGAAGTTAAAACACCTCAAAAGAAGAAACGACGCTGATTTTGTGGTTAATGATT
TCTAGGTTTAATTTTGGTAATTAAATGTTAAGATTTAAGACTGATTTTTAATCTAAAATATTATTATATGAAT
AAATAAGTAT
>1002050659_1 Heli.1-DT666282.3.5
GCACGAGGGAGGACGCTGTCGAGACCATCAGAGACCAGCGTCGCGGCGCCATCAACGCGAAGCAGCTCTCGTA
CTTGGAGAAGTACAGGCCGAAGTCGCGCCTCAAGAAGAACGGCCACAAGAACTCGTGTTGCGTGCAGTAGGCC
CCGTAGGCGAATCGAGCTCTGCTTTCACACACACACTCACACATACACACGACTAACT
AGTTGTAACTTCCCTCAAAATTCCCCTCCCTTACTTTACTCTCCCCCCCACTGTTTGCTAAAGATCATGACCA
AAAGTTATTACTATATTACATTATATATTGGTTTTGTTTATCAATGCAATGTTTATTCCCTATTTGTACTCTT
AGATAACTTTCTGAACTTGCTTTAGTATTGCATTGCAACAGTCTAACGTCCATTTAAT
GCAAAAGCTGATTTATTTACAGAACGTCCACTCGGTTATGATCTTTAATGAAAATGTTTTTGAATCGTTCGGT
GTTTTCTATTGTACAAGTATCTATCTATGTAATTTGAGAAGTAATGTAATTTCAAGGTATATACCGTCGTGCG
GTGGGACAGGCGTAGACGCGAGCCACTATTTGTGTTCATAGTCGACAGTTACTTGGTT
TTTATAAAAAACATCATAATAAGTTAATGTCCATAATAAACTGACAACTTACCGCTTATTTTTAATTGTGTGT
TGAAATCATTATATATTTTCTTTGTAGACACAGTATTGTAATTGAGATTGTAATTGAGACATAATAGAAGAAA
TGGTTTTTGTGTTTATGGTGCGGCTTTGTGCTGACCAATTTGTATAATTTTAATTTGA
ATTCTTATTTTATAGATAAGAACGTGCAATTCCTATAAGCTATTATTATTATGATTATATCACAAATACTTTA
CAAACCTTTGACTAAAATATAAATTCTACTTGTATCCTGACACAAAAGATAACTTGACTCACTGAATGTTCCA
ATGAAAATAGCTCTTTGATATTATTTTTCTATCTCTAATTTTATGAAGATAATCAAAA
TATATGATTGTGTAACCGCGGAACAGTCACATTCATAAATAAAAATAT
>1002050660_1 Heli.1-ES586764.1.5
GCGCGGCGGTGGTGGTGGCGGGGAGCGACTAGGCCGGCAGCGAGCGGAGGACGGTGGGCGCTAATCTGAAGAC
GAGCCTTTTATTTATTCCCGAATAGCGAGTACTCCCGGACGATATCGAACCTAGTTCAAACCACCCACGACTA
GTGTAGTTTAAGTTAGCGTGCTTTGCTTTTGTAAAATTAAGGTTTTATATATATATAT
ATATATTATAACAAAGTTATGTAGCAGTATACGGTGCGTTAAGTTGGTGCCATGATTACAGGGGTATTAGTTA
CGTCCCTCCTCGGATACTTCGCGAGCGCTAGTTACTCCTTTATGATATCATTATTTAGAGAATTATATTATGA
ATTTACCAATTTTTCATTTTTCTGGACGAGGAAGTTGGTTTTGCGAGCGTGCAGTATA
TACAACGGGGGGCCGGCTGCCGGCTCCCATTATATAGGAATATTGTAAAACTAAATTCGATTAAGATGTAGGA
AATAATTTGCGGATATGCATTACGAGTCGCGGCCACTGTTGCGGTACTGCTCCATCGGTTACGAGCTGGTAGC
GACGACTGTGGTCCGCTAAGAAACATTTGTCACTCAAAAGTTATAAAATTAATATAGG
TTTTAATGATGAGACTATTATTATGTATATACGAATAATTACGTTTTCACAAAACATATTTTTTTCTTAAATT
CTGTTGTCGAATAAAAGTTTAAAATTGCATTTGTATATTGTTATAAGTGATATGAAAATAATTAATTTCATTT
AATTTTTTAAGTATATTTTATGTAGTTGTGTTATTTTTACACCTTATAATTATTAATT
ATAATATTAATATAAATAAATTTTGGATTCGAGCTCTCTTTCTAACGTGAATTTTGGAATATTTTATAGACAA
GCGCAAATCGCCCCGTGTCGATTGCCTATTTTGATATATTTTGTATTTGATAGTGTCGCTTCGCCCATCACCC
GCCAAATTTGAGCGAAGTAAACAGAATTTAGTCGACATGTCTCTTAAAATATATTTCA
TTGAATGAATTGTCTTCAATGTGAATTGGACTGGTTACTAGCGGCCAAGTTTTCGATTAATCTATACAATTTT
TATGCATATACAAACAACAATCTATTATAATCTGTACTATTTAGATGTGTAGTGTTTGTAACGACCAACGAGT
ATAAGAGGTGGCGGAGGTGTCTGTAACCTCGTCGCTCGTTGGACTGTACCTATGTAAG
ATTTGATATCTCTATACAATTATTGTCGTACTCTATAATCGTGTCCGATGAAATTATTTTCCATTCCGTATCC
ATTTTTGTAATTTTTTACATTTTTTATGAAATTTTTGAAGTGACACATCTGCATAACAGCGGGGGTATTACTC
CCAAGTTAAATTAGCAACACATCAATACGAGTGAGACATTCCATATCAAATGCGAATA
TCCAAAACGTATTCATTCCCTTTTTTACATTTATACATTTGAATGTTGAGAGTGATGTCACGCTATGGAGTGA
ACCAGCCTGCTGATACTTTGTGATCTTATTGGTTAAGCTGTTAGGTTTGTAGCTTTTATATAAAATAACTTCT
AACCATTTAGTTCTTAAGACTTGTATTCGCTCAAAATTTTTATACTTTTATAGGATTT

TTTTAAAATTAACCAGGAGCACAACTATTGAAAGTTCTTACCAATTGTCTCTGTTTCGTTCAAATATCATATA
AGTGCAGAAGTGCCAATTCGAAGTATGTATCTTTTTATATTATTATATATTGCAAAAATATTAATTTGATATA
TTTTACTTTGACTGCTCACGTTACTACAAACATAATAGTAATAATGGTTGTTTAAATT
CCGCGATTGAACTATTTTATATATTTATCACTGTTCAATCTTGCTGAAATTTGATGTTGATGTGTGAATTTCT
CTAATTAATAAATTTTAAAATACAAAAAAAATTTAAAAGGCTCGCCT
>1002050661_1 Heli.1-DT665590.3.5
GCACGAGGGAAAAAGGTCTAGCAGAAAAGGCAACAGAACTTGAAAAAGCAATGGAGTTTAATGAAAGGGAACC
ACCAGCAGGCACGCTAGCAGCGGTTCCAGTACCGTCCTGCGATCGTCTCAAATGCCATAAAATATCTAGTTGG
AGCACCGGTGACGATTGTCCTGACTTCAATCTAAAAGATGTACCCTTGTATACTAGGG
TGCACAGTTTGAATACCAATTTTGTCTATATAAACCTGATCCTCGACACAACCCACATGCCGCCGCTGCACCG
GCGCTGGATCCCGCTGCTGCTGGACGCGCTGGGCGAGTGCGGCGTGAGGCGCGGCGACCTGCTGGTGCCGCAC
GAGGAGGTCATCTCCGCCACCGAGAGACTCACTGTCACCTTCTCCAAGGACATCGGCT
TTAATAGACCCGGCAACTTCTCGGTGGGGCCGTTTGGGAACTATATACACGTCGAGACAAGGTGTGAACCGTC
AGATTATGAAAAAGTTGTTGAACATCTATACGACGTGATCTTCATGTCGGTGATAACTAAAGAGAGACTGCTG
GTGTTTGCTCAAAGGCTGATCAATGATGTGGCACAGGTAAGAAGAAACGCTCACAAAA
TGTCATACGATATTCTCCGGGATTCACTCTACAGTAAAAACAGCAACACGCACTGGTGCTCCGTCCTCCGTCA
ACAGAAGTTCCTCAAGGAAATAATAGAGAGACTGACCAAAAACGATGAATCAGACGGAGAAATTATCAATGAA
GCCTTACAAAGCTTAAAGGATTTGACACAAAAGCCCTGGCTTCATTTAGCCACGGACT
TAGAACGGTATAAGTTGAGTGCTGAACCGTGGCAAAAATATGCAAGGGATGATGAAGTGCCAAAAGAGCCGCA
GCTGTACCTGGACGCGGAGCTGATCGCTGCGGGCGCGGGCGGCGTGGCCGTGGGCGTGGGCGGCGTGGAGTCC
TCGTTCGTGGTGCAGACGGGCGCCGGCCCGCGCGGGTACGCGCCGCACCACGTGGCGC
CGCTCACCGTCGCCTTGAACTACTTTACGCAACTGGAGGGCCCCATGTGGCGCCTGATCCGCGGCTGCGGGCT
GGCGTACAGCTACTGCGTGCGCGCGGCGCCGGGCG
>1002050663_1 Heli.1-ES586248.1.5
ATTGGATTGGTTAGCTTTAATGTTGTTTAGAAATCAATACGTTCGCGGTAATTAACATTCCTGTTATAAAAAG
ACGCGCCCCGCGGCTTCTAATATAAATAAATATATGATATAATCGTATTACTATTCGTCATATTTTTTACAAC
TAAGACGGTAATTAGAATTTTTTGTTCACTATTAATAATAAGAATTTCGATTTTAATA
AGACTGTTCTGATTTTTGAAATCATAATTTGAACTGCGAAAAACTGTGAAATGGTCTAAAAATCTGGTAATTT
CCTTCAAATCGTATATTCCATGTAACTGTAGTAAAATAACAAAATTATCTCTATACTTAGCTGGCACTAAATG
TACTTAAAATAATATAAATTTTAAATTCATATAAGCTAAAGCATTTTAATCAGGATTC
TATGTGATAGCCACTTAGTTTTTCGTAGTTTGTACTTTGATGAGTATTGTTAAGTATTCATAATTTTAACGAC
CTAATGAAATGTTTGTGACATATTGTGTTAGAATGTTTGAGAGTTACGTACAAGTTAATGTTCTATTTATATG
AAGCATTTTTTTATAAATAAAGTGTATGTATAAA
>1002050664_1 Heli.1-ES587771.1.5
GNTCGGAATTCCGGGTCGACCACGCGTCCGAAATCGTTAATCGACATATTGATAGAATTTTCCTGTCCTCCTA
ATACTTTCAGACGATTGCGGATTACTTGTTAATAAAGACTGTAACAATCAATAATGCAGGGCCTCAGTTTCTC
CAGCTTAAAGAAGCACAAAGCGTTGATCCCGTTGTACGTGTGCGTCGGTCTGGGATGC
GCCGGATCGGTGTTCTACCTGGCCCGCCTCGCGCTGCGCTCTCCTGACGTCTCCTGGAACAAACGCACCAACC
CCGAGCCCTGGGAGGAGTACAGGAACAAGCAGTACAAGTTCTACTCACCCACCAGGGATTACTCAAAGGAGGA
TGGTGCACCCAAGTTCTAAGATTTCTGCATTCAAGTTATCATCATTATGTACATTAGT
AAATTTTATTTAATAAATAGTGGT
>1002050665_1 Heli.1-EL595911.1.5
AAATTGTAGATAGTATAATGACTCCAATAGAAACGCCTATAGAGATTCAAAAACCTGTAGTTGATATCGATAA
AACCATAGAACCTATAGTAACAGTTGTTAGTGATAAGAAAATGATAGCGAAAGCGGAAATTATTGAAAAGATT
AATGAGACGCTAGTATTTTTTGGACCAGAAGTGGAAGTGCAGAGTTTAGATGTGTGGA
CGCCTGGTGTAGACAAAATCACATTGCAAAGTAAATCTGCAAAAGGCAGCGCTTCAGATAAATCGCATAGCAA
TGGTGGATTTCCCCAAAGCACGAGTACCACACCAAAACCACCACCAAGAAGTAATCGCGATCAGAAATCCAAG
AGCCAAAGTCCTCCTGTCGAACCTATTGAAACCGTCGGAACTCCTAAACCAAATATAT
GTTCACGATTAATGGGCAAATGCAAGGCAAAATGTTGTCCTTGTTGCACCAAGTCACCAGATGAAGAAGAAGA
AGAAAGGAAAGAAAGAGAAGATGAAGTTACGGAGTCAGTAGAAAAGAAAAAAAATATGGCACAAAATGAATTGT
TTCAAAAAGAAGATTCCGGAAGAAGATATTGAAATCGCTGCGGGGAAAGGAACAACAA
TAGAATTCGAAACTGAAACAAAACGAAAACGTAAGCTCCGAGATGTATTATGCGCGTGTTGTCGGAAACGTAA
AGTAGCTGATATAGAGCCCCCTCAACGTGTTGAGGCTTCACCACCAGTGATGAGTGAAGACGTGGTGAAGGAG
ACCGGATGCTGTGGGAAGAAGAAGGAGATTGAAAGGCGGGACAGTATATTGAGTGATC

AGGCGCCTACTACCTGCTGCAACAATCGCCTCGGTCGCTGGATCCGCGGCGCGTGTCGACGTCAGTCGGAGCA
GTCGTCGAGTAGACGAACTAGCTTATTTTCAAAGAATAAGAGTCTCTCTCCTACTTTACCACCTGAGGACACT
CGTAAAAAGCTAGATCCATCTCTGATCGAGCACACGAGCGTGATGCGCGGCGCCATCC
CGGTGCTGCCAGCGGCATTAGCGTACTTCTGTTTACTGTGTAACATTGTTGTTCCGGGACTTGTGACGATCTT
CAGCGGTATGTTTTGTCTTTGCTTCGGAATCCCCCGCTTCGGTGTGCACGATGGAGCTAAACATCGGATAGGT
TCTTTCATAATCAATCTCCTTGTGGGTGCCGGGCAGCTGTTCACGGTTCTCTTCTGCC
TCGTGGGGTGGGGCTGGTCCATTTGGTGGGGTGTTATTATGGTTAAGACATCACGCAAATACGCGAAACTGAA
AGCTGAGGCGGCGGCGGAGGAAGCTGAAGCGCCTCCCGTCACCTCGAATAACCACACGCGAGCGTAACAATGA
CTTACTGATTGTTTATAAATATATTTATATGTATGTATATAACAACATTTAAGAGAAA
AACGTAACAAATAAACGGTAAGGCGTACAAAATGAT
>1002050666_1 Heli.1-CO729971.1.5
CGGTCGGTATTCCCGGGTCGACCACGCGTCCGTGAAGATGAGCAAATCAACTCTTTAATGCGCAAATTCAAGT
TTGATTTGTCCAAGCAATTACAGACAATAAGAAAAATAATTATTAATTTATTGAGTTTAATTCGCTGTGGATT
GTAAACCTACGGCATTTAAAATGGATCTTTTAAACTTTTGGCAGGCTATTGATGTTTT
AATCCATAAGCCTCATGTTATTAATAAAAACCTGTGGGGATCAATAGTGTTAAAAAGATATTACATTAAAACA
GAAATAAATAATTGGAATGAGTATCTTGCTGACTATAGACTAAATACAATGGTATTTTTAAATATAGATCAAT
ACTTAGCGGAATTATCACAAATGTTCCACTTTCAAATTGTTAATGAAGAAACGCAATA
TGAAATAATGTTCATAGAACTCCTCCCTAAAATTTATATTGTACAGCGTGCGATTCAAATGATTTGTATAAAT
AGGATTTTAAAACAAGTAACTTTTTATGATATCACACCAGCTAAACAAATAGTGTGCACTACATTTCCATATA
CTTTTTTCTTCATAGATAACAAAATTACTGTAAAATGTGGTGATTCTAGAAGCTATCA
ATGGTTAAAAAATTCTGTTTTACCTCGACTTAATAAATGGAGTACTGAAGAAATCAACAAACAAGTTCAGATT
TGCAATGAATC
>1002050667_1 Heli.1-DT662801.3.5
ATCCTGAGACCCGTCAAAGGATGACCGACTGCGCGGTGCACTTGGCGAGGCACGTCGGGTATGAGAACGCGGG
CACTGTGGAGTTCCTACTTGACGATAAAGGCAACTTCTACTTCATTGAAGTTAATGCTAGGCTGCAAGTAGAA
CACACAATAACAGAAGAAGTGACCGGTATAGACTTAGTGCAGTCACAAATTAGAGTTG
CCGAGGGAATGACTCTACCGGAAATGGGATTGACGCAGGATAATATCAAAACTCAAGGGTACGCGATACAATG
TAGGGTGACTACTGAAGATCCGGCTAACAACTTCCAGCCCAGTACTGGCAGGATTGAAGTGTTTAGATCCGGT
GAAGGCATGGGTATCCGACTTGACTCCGCTTCAACTTATGCAGGCGCTATTATATCAC
CCTACTATGATTCTTTACTTGTGAAAGTAATATCCCACGCTCAAGATTTATCAGCATCAGCAGCTAAAATGAA
CAGAGCGCTGAGGGAGTTCCGTATACGTGGCGTTAAAACCAACATACCGTTCTTACTCAATGTATTGGAGAAT
CAGAAGTTTTTGAATGGTGCTGTGGATACTTACTTCATAGATGAACATCCACAACTAT
TCATGTTCAAAGCATCGCAGACCAGAGCTCAGAAAATATTGAACTATTTAGGACAGGTTCTAGTAAATGGCCC
CGCTACACCTTTAGCTACTAAAATACCACCATCTGATGTCAAACCGTATATACCGCCAGTGCCTTTGGACCTT
TCCCCAGAGGCCGTTAAAAGGCAAGAAATGACTGGAGAAAACACAGCGGTAGAACCTC
CGAAAGGTTTTAGACAGATTCTCAAAGACGGTGGTCCAGAGGCTTTTGCGAAAGCAGTAAGAAACCACAAAGG
TCTCTTACTGATGGACACAACCTTCAGAGATGCGCACCAGTCCCTCCTGGCGACGCGCGTGAGGACGCACGAC
CTGGTCTCGGTGTCGCCATATGTAGCGCACAATTTGAATAATTTGTATTCATTGGAAA
ATTGGGGAGGAGCTACTTTTGATGTTGCCTTGAGATTTCTACACGAATGTCCATGGGAGAGGCTAGAGGATAT
GCGCAGGTTAATACCGAACATTCCTTTCCAAATGTTACTGAGGGGCGCCAACGCTGTGGGCTATACCAACTAC
CCTGATAACGTTGTTTTTGAATTTTGCGATATGGCGGTGAAAACCGGTATGGATATAT
TCAGAGTGTTCGACTCTCTAAACTACTTGCCCAACCTAATATTGGGTATGGAGGCTGCAGGCAAAGCCGGAGG
CGTGGTCGAAGCTGCCATTTCTTATACCGGTGATGTATCAGATCCCAGTAAAAAGAAATATGATCTGAAGTAT
TATTTGAATTTAGCTGATGAGCTCGTGAAGGCAGGGACACATGTTT
>1002050668_1 Heli.1-ES586393.1.5
GCACCCTCTCAGCCCGCTGCTGAAGCGAAGAGTGATGACAATGTTGAAGTCAAAAATGATGATAAAGTTGAAG
CGAAGAGCGACGCTAACGTAGAAGCCAAAAGCGACGCTAAGGTTGAAGCCATGAATGACGCACCAGCCAAAGA
AAAGAGCTCCGAAGAAAGCAATGAGAGCGCAGACAAAGATTCATCAGAAGAAAGTGGT
GAAAAATCAGATAGCGCTAAACCTTAAAATTGTTAAAATAGTCTAAGCGGCAGGCGTGTGTCGACTCACGCCT
CGACACGCGCCTCCCGCTGTCGCAGCGCAGTGCGTTTGTGTTGTCACGCAGCGGACCGAACCCGTCCTCGACG
TGACCTTCGACTATTCACTAGTTATAAAGACTTATTTAAATAGTGGAACAGAAAAATT
ACCTACGTTGTAATTATTTTTCAAAGTAAATTATCGAATTCACGGTACTGTAAGTAACAATAAATACGTTTT
>1002050669_1 Heli.1-ES587275.1.5

AACGACTTATTGTATTATGATAGTTTATTTTTACGGAAGTTAACTAGATAGAGTAAGCTGTTATGTATTTAAC
ACTCGAAACCGTTCGGAGCGACCTTGTCGGATGGAGTCGTGACGACTTGCATTTAAAGTTGACTTCTCATGAC
GACCCGAGATTTAATTTAGGTATATGTATTACTGACATGGTGTCATTGAAGTAACGTG
CGATTGTGCTCAGAACTCTTCGGTGGTACTCTGCTCTTACACAATTAATTATAACTTAGCATTTTTATAATAA
TTTAGAAGAAATTATTAAACACAATTTAAGGATATGATTATGTGGCGTGTGGTGGAGATTTTTTTTTAATTAA
TTTTTATGAAAATCAAGATGATTCAATGGAAAACGAAATGTGACATTCCGGTTTGTCT
TAATGCTGTATTATGGAATAAAAT
>1002050670_1 Heli.1-DT664489.3.5
GCACGAGGCTCATCATGACATTTTAGTTAAAAATATCTTTATCAGCGATTTCTTACGGTACCTGCTATTTACA
CAGCAGCAGCACACTCGTCGCCGGCACAAATGACGAACCAGGCATCCCCTGTCGAAGAACAGGAGAAGCTTCT
TGATGAAGCGTTAAATGTCGTCAAAGTTCAGGCATTTCAAATGAAAAGATGCCTGGAC
AAATCAAAACTAATGGATGCCTTAAAGCATGCTTCAACTATGCTTGGAGAACTAAGGACTTCTCTTCTATCAC
CAAAGAGTTACTATGAGCTGTATATGGCAATAACAGATGAGCTCCGTCACTTAGAGCTCTATCTTCTAGAGGA
ATTCCAAAAGGGTCGTAAAGTGGCAGATCTGTATGAACTGGTACAATATGCTGGCAAC
ATTGTACCGAGGCTGTACCTGTTGATAACAGTGGGCTTGGTTTATATAAAGACAAATTCTAATTTGCGTAGGG
ATTTATTGAAGGATTTAGTTGAAATGTGTCGTGGAGTACAACATCCTTTAAGAGGCCTTTTCTTGAGAAACTA
TTTGTTGCAATGTACAAGGAATGTGTTGCCAGATACAATTGAAGCACAGAATGAAAAT
GAAGGCACTGTTCGCGACGCAATAGATTTTGTTCTCATGAATTTTGCTGAAATGAACAAACTATGGGTTAGAA
TGCAACACCAAGGGCATTCTAGGGACAAAGAAAGGAGAGAACGTGAACGTTCAGAACTACGTATCTTGGTCGG
GACGAATTTAGTCCGTCTGTCCCAATTAGAGTCCGTAGGAGAACAGGACTACAGGAGA
CTGGTACTTCCCGGGATACTGGAACAAGTGGTCAGCTGTAGAGACGCTATAGCCCAGGAGTATCTCATGGAAT
GTATTATTCAGGTCTTCCCCGATGAATTCCACTTGGCGAATCTCCAGCCATTTTTGAAGTCATGTGCGGAGTT
ACAACCCGGTGTTAATATAAAGAATATCATAATTGCTTTAATAGAAAGATTGGCAGCT
TTTAGTCAGAGGAACGAAGGCAATGTGAATTTAAGTGTGGTTCTAGAAGATGGTAAAGAACAAGAAGTACAAT
TGTTTGAAGTTTTTTCAGATCAAGTGGCAGCTATTACTCAGAGTCGCACAGACATGCCACCAGAGGACATGCT
TGCCCTGCAG
>1002050671_1 Heli.1-ES588113.1.5
CCCTGCGAGAGGCCTTCTACAGGCAGAACCTGCCCAACTTGATGAACCTTCTGGCCACTGTTCTAGTCTTTGC
TATTGTTATATACTTTCAGGGCTTCAGAGTGGATCTACCCATCAAGTCCGCCCGTTACCGCGGTCAATACTCA
TCATACCCAATCAAATTGTTCTACACTTCAAATATACCTATCATTCTACAATCTGCGT
TGGTCTCCAATCTTTATGTTATTTCTCAGATGTTAGCAGTAAAATTCAGTGGCAATTTCTTAGTAAATCTCCT
CGGTGTATGGGCAGACGTTGGCGGTGGTGGTCCCGCTCGTGCTTATCCAGTCGGTGGACTATGCTACTACTTC
AGCCCACCCGAATCCCTAGCCCATATAGCCCATGATCCCGTGCACGCGGTCCTCTATA
TTATATTCATGTTGGGCTCATGTGCTTTCTTCTCCAAGACTTGGATCGATGTGTCAGGGTCGTCAGCTAAGGA
TGTAGCGAAACAGCTGAAAGAACAGCAAATGGTGATGCGTGGACACAGAGACAACTCAATGATCCATGAGCTC
AACCGTTACATACCAACGGCAGCTGCCTTCGGAGGTCTATGTATCGGAGCCCTGTCTG
TTTTAGCAGATT
>1002050672_1 Heli.1-ES587269.1.5
CCTATTTTTCGGGCCAACTCATCGGTCTATTTGACATAGGATAATATGTTTATTTTCAAAGAGTTATGAATTG
TAACTTATTTATTGTTGGATAATATAAAATAGATAATAATAAAAACATGGAGGTTCAATATCTCGTCTTATTT
TTGAGTATTATCTGGCATGGAGCCGACGCTATTATGTGGAGTTTGGCTCCTAACACGC
AAAAATGCTTGAAAGAAGAACTACACGCGAATGTGCTCGTTGCCGGTGAATATGAAATAACAGAAGCAGATGG
CCAGAGAATTGACTATATTGTACGTGATTCAAAGGGACACATTCTAGCCCAAAAAGATGGTATCACCAAAGGA
AAGTTTACATTTGTAACAGAAACATATGACACATTTGAAGTTTGTTTCATATCAAAAG
TGCCTCAAGAGCGCCGTGGTGTAGCACAAGATGTGACACTAGACATTAAAGTTGGCATTGAAGCTAAGAATTA
CGAAGGGATTGGTGAAGCAGCAAAACTGAAGCCCATGGAACTTGAATTGAAACGGTTAGAAGATTTATCTGAA
GCAATCGTTCAAGACTTTACTCTAATGAGAAAGAGGGAAGAAGAAATGAGAGATACTA
ATGAATCTACAAACAACAGAGTGTTGTTTTTCAGTGTATTCTCTATGGTGTGCCTACTAGGGCTGGCCACGTG
GCAGGTGTTATACCTACGCAGATACTTCAAAGCCAAAAAACTCATTGAATAACATTTCATTTTTTTATATATT
TAATACACAGATCTAGTCACACTGGATTTTTTAAATTTTTAGTAGCACAAATGCTTAA
GTTTTTTTTTCTTTTTTTTTTGGTTTTTCATTAGAATTTTTGTATAATAAATGCGTTTAGTAAATCAATTTATGA
TGTATTAAGTGTTGGATACGATTAAAAGAAAAATAATATTTTGTCGTAAAAATATTTTTATAGCAAGTTCCTT
GATAGCTTTTTTAATTTTCATCACAATAATCCAGTGTGATTAAATCAGTTCAGATCTC

GTGCACAGGCACGTATTATAAAAAATAAATAAATACAATTTTTCGTTTACTTAACAAATTTATTATTTAAATA
AATGTTTGTATGTATATGAATGTGTTTCGTAGCTAAGGGTGTAAAGAAATGATTTATTAAAAAACAATCAT
>1002050673_1 Heli.1-EL597390.1.5
TTCGGCACGAGGGGAAGTTTTTGTTATCTTAGTTTGCAGTTAACCGAGTGACTGACTGGTGATTTCGATGGGC
ACTGTCTGTTTTTAAATGTGATAAAGATACATTCAATGAAATATAAGGTAATCGTGGCAGTTGTTGAATTCTA
AAGATACAGGTAGGCTAATTAGATAATATTAAAACTTGCTCATAAACTTTTGCCGTGT
CTTTACGTCGTTTTTGCAGTTACCGCCTGCACGTCTCCTTATCTCGTCGCGTCGTGTTGCGGATACAAAAGAT
TGAAGCACAATTATGAAAGGTATATTTAGTTATATTTTCCGGTTTATCCAAAGATGGCAGTAGTAACGTTCTT
GATAATAATAATTTCAAAAAGGAAAATGTAGAAATAATAACTAATGGTTAGCAGAGTA
GGGTAAGTTTTATGGCATGTAAATAGTTTTCCGTGTCCTAATGTAATTTTTACAGTTCCTGGCGTCGTTTCAT
GCGTTAAATGAAGTCCAGTGGATAGTATTTAGCAACCAGTAGTAGATCAACGGAGTTTTTTGCAGTTACAAAG
TTCTACTTTGTGAACTATAATGTGGCGGCCATAGATAATGATATGACGTAAAAAAAAA
TAAACAGACCGCGAATATAGGCGTAACATACACCAGGTAAACTATATGTAAAACCATAACATCGTATTATAAC
TAAATAAATAGTTTGTAAGTATATTATAAATTATTATTTGCTTGTCCCCGTGTTGTTTGATCTCCTTACAGTT
GATGGTTTCTGCAGCTATTCTTCTAACCTTCATTAATTTTAAAATT
>1002050674_1 Heli.1-DT668036.3.5
GCACGAGGCCGCGTCATTCGAACGCCGTGAACGCGCGTGCTTCGATCCCGATCGGGCAACGGTTCAATGACAT
TTTGAGATCGAGATGAGATTATGTTCAGTTGATTTTGATATTTGGTAGCTTCGATATATATTCTAGGAACAGC
ATAATATAGAAAGAAAAGAAATAATATTATTGTATTTTTTTAGTATCTTAAAATAATA
AATAATTAGACATTATAAGTTATTTTCGACAATAGAACAAATGTGATACTATAACTGATGTGATTCAAAAGTA
ACCGCCAAAACAAAACATAAAAAACAATATTGTTCATAATACAACAAATTCAAAGCGTTAGATTGATTTGAGT
TTTTTACAGATGATATAAACCTATAAAATAATCGAAGATTTAAAAACAGTGAGCAAAA
TGCAAGCATTCACGTATCTGGTTGTTGCCATTGTGCTGTTTACTCCTGGATCGTTGGCGAAAACCAAGAGGTT
TTCAAGGCTGCAGACAGATCCATTATCAAAGACTCGCTGTGATTTGATATGCGTCGATGCTGCCAAGGAAACT
AGATCGCAGTGTCGTTCATCATGCCGATCAGAAGAACGGAAGCCAGGCACGTGTCCGG
AAGGTGAATTTCCTAGATGGATGGCAGCGTGTGTAGAAGCCTGTAATCAAGACTCGCAGTGTACCGGCACTCA
GCGGTGCTGCAAACTTGGCTGCAGCTCTATGTGCAGCGAACCCATTGATCTACTTACAGTGCCAGGTTTGCCA
GCCTTGCCTGTCGTGGAAGAAGCTAAGGAGAGACGGCGAGCTGTGCAAATAAAGTGGT
CGGATGGGGTGGGTGATGCAGCGAGGGCTGTCCCGGGTCGAATTCTTTACATACTGGAAGAGCAACATCATTT
AGGACCAAAGTATGAGGAAGCACGACTTGGAGACTGGAACTTAATATTAAGAACCAATAAGACTAAAGTATCT
CTTAAAGATCTACTGAAGCCAGGACGGTGGTATCGTTTTCGCGTAGCCGCTATTAGTG
CGTCTGGTACAAGGGGATATTCTGCACCAAGCTCCCCATTTACACCAAGAAAAGGACCGAGGCCACCACCATC
ACCCAAGAAATTGAAAGTTTTGCCAATAGGATCAAATAATGACACAGTAACTGTTCGCCTGGAATGGAAAGAA
CCGAAATCAGATTTGCCGGTGATGCGCTACAAAGTTTTTTGGAGCAGAAGAGTCCGCG
GTTTAGGGGGTGAATTAGATTCTGTTCTTGTGAATCATCAAACAGTTCCTAAGGAACAAACCTACGTCGAAAT
AAGCGATCTACAGCCAAATTCGATGTACTTTTTACAAGTCCAGACAATCAGTATATTCGGCTTGGGGAAACTG
CGCAGCGAAAAAGCCGCTATTTTTTATAATACAACCAGCATTGCGGAAGCAAATGCTC
CAGAGGCTCTCACGCGGAGGAAGGACGAGAAACTGATAAAAGGTGTTAAGTTAAATAAATTCATATGGTTGGA
TCATAAGATAAAAGCAAAAATTTCATGGGAGCCAATGTCAGCACCTAAAGGACAACGTANAAGATATTACGTT
CATTGGAAAACGATATCATGCCCATATCCAGTTAAAGATTTAAAAGAATTGTCCGCTA
TAACTGAGCAAAACTCATTTGAAA
>1002050675_1 Heli.1-EL597629.1.5
GCACGAGGGGAAAAGGTAAATGAAGAGAAAAGCTTAGGAAAGCTTCAATTAAGAATTAAAGATGAACCAATGG
ACACTGATTTACCAGAAGAAGATGATGAACCTCCTGCTTTCGGTCCCGCTGCACTGGGCCTTCATAGAGTTGG
AACTAAATTGCCGCCAAAACCAGCACCTAGTGAACCCGTCCAGAAGTTAAATGCAAGA
GGTATGCCAGCT
>1002050676_1 Heli.1-EL598490.1.5
GCACGAGGGTGCACGTCACTTTTGCGGTACACGCTACTATGGCTCTGGTACGGCGGCTTGCCCTGAACGCGCC
TAGAGGGATCCGGGCCCTGTCCACACCCTCCCGCGACGACCTGGACCTCTCCTTCAACAGTCCGAAAGATGCT
TTCAAATCGAAGAAAACCAGTGAGCTGGTCCGGGCGTACCTCGTCTACCAAATATGCT
CAATCAATTGGATTGTTGAGAACAACGATATGCTCATGAAGAGGCTGCGCCAGGTGGTGGGCAACCGTCTCTT
CGAAGTCATCATGAAAGCGACCTTCTATGGGCAGTTCGTAGCCGGCGAAGACCAGAACAAAATCAAGCCCACT
CTTGAGAGATTACGTTCTTTTGGAGTAAAGTCGATTCTTGACTACTCCGTGGAAGAAG

ATTTGTCTCAAGAGGAAGCCGAGAAGCGGGAAGTAAGCGCGTCGATTTCAACGTTTGGGGACCAGCAGGAGGA
AGGTCAGCTCAAGCAGTACCACGTGGAGCAGAGGTTCGCCGACCGCCGGTACAAAGTGACCAGCGCCAGAACT
TACTTCTATTTGAATGAAGCTGCCTGCGAGAAGAATATGGAGGCCTTTTTGAGGAGTA
TCGACGCTGTGGCTGAAAACACAAAAAGTACGGGTTTGATGGCAGTTAAACTGACAGCCCTGGGCAGACCTCA
GCTACTTCTCCAATTATCCGAAGTGATAATGAGGGCGCGTAGCTACATGCACCAAATAGCTGGTGGGACCGGA
AACGTACTCACACATCACAAGACTATCGAAGACCTCCAACGGTACCTCGGCGATCTGA
GCTCCAAACCGGAAGTTCAGGATTTCATGAAGAAAATCACTTCGGACACGGAAGGCATCGTCCACCTGTTCCC
ATGGTCGAATATTCTAGACAAAGACATGGGTCTGTCTGATTCTTTCCGGGTGCCAGACCCGAAGACCGGCCAG
ATGAGGCGTCTCATCTCCCAGA
>1002050677_1 Heli.1-ES586216.1.5
GTTAAGTGCGGTTGTTTCATTTCAGTTAATTGCCGATCCATAACAAACATGTCTCTCCAAGAGCAATTTGATC
AAGCTGCCGCTAATGTAAAGAAGCTAAAGTCTCTGCCCAGTGACTCTGACCTTCTTGAGCTGTATGCTTACTT
CAAGCAAGCAACCGTTGGTGATGCAGATCCTGCAAACAAACCTGGCATGTTCGACCTC
AAGGGCAAAGCTAAGTTCGAAGCGTGGAGCGGCAAGAGTGGTGTCTCAAAGGAGGACGCACAAAAAGCCTACA
TAGCCAAAGTTGACAGCCTTGTTGCTTCAATTGGCCTTCAATAAACTAATCTAATCACTAATATTATACTATA
ACATGACAATTATTTATAACCAAACTTAGAAAAATCTATGAAGATCGTGTAAACGCTA
ATTGATATTAAGTCCTATTAAATTCTATACAAAAATGTCGTAGTATAATGTTATTGTTATGTTATTTATAGTT
TTGGTGGATAGTAACGATAGCACAAACTAACTCTAACTCAACTAACTCTTCCTTTACGTTTTGTATATTAAAA
TTATTTACAATATACATAAAGACTTATAGATAATAAGGATAAAACTGTTCTTTTTTTG
TTACAAGAAATCATTTTTTTTGAAGACTTGAGTACTAGTTTAACTATTTATTCCTTTGGTCTAGGTTTTCTAT
ACATCACATTTATATTGTACCTAGAAACGGTGTTCACATCTGTTATATAGTCATGAACTTTGTTATATTTTTT
ATATCGGCTTGAAATAAATCAA
>1002050678_1 Heli.1-EL601648.1.5
CGGCACGAGGCATGCGCGCTCGTGAGATGGGTCCACGGTTTTCTGAATCTGGTAGCTTTGAGCACGAGTATGG
AACCAGGTGGAAGCAGCTGCACGAACTACATCGCCAGAAGGAGGAGGCACTGAAGAAGGAGTTGGCCGCCGAA
GAGGAGAAACTGGAGGCCCAGATGGAATACGCCAAGTACGAGCACGAGACGGAGCTGC
TGCGCGAGCAGCTGCGCCAGCGCGAGCAGGACCGCGAGCGCCAGAAGCGCAGCTGGGAGATGGCGGAGCGCGC
CGCCGACGAGCGCCGCGACGCCGAGCGCGCGCAGCTGCACCGCACCGAGGAGGAGCTGTCCGCGCGCATGCGC
CTGCAGGACGACGAGCTGCGGCGCCGCCAGCAGGAGAACACGCTCTTCGTGCAGGCGC
AGCGGCTCAACTCCATGCTCGATCGGCAGGAGCAGGGCATGTTTGATCACCAGCAGCCGATGGACGGAGGCTT
CAGAGACCAGTACGACATGCCACGTGGAGGCTTTGACGACATGCCGCAGAATCGCGGTGGTTGGGAAGGACCG
CGCCAAATGGATGATTATCCTAATAAACGCCGGCGCTTTTAAATAACGCGCGACCGCG
GCTCTGTAACACCTGGGATAGGTTCTACGCTGCCCTGGATAATTCAGTCTTTGGTTACTTACTGTCACAGGAC
AGGCATTTATTTTAAAATATTTTATTGTTCATCCATTCTTTAATTCAAGTCAAAGTGCAATCCCAATTTGAAG
TAACCAAAGGTTGCGTTACAATGAGATGAGATGCGTCGTGGCTTGTGGATGAATTCAT
TTATGTACTAAATAGTACAAGAAGTTATAGAAATGTAAACGCGAAATAAATTATATATTAATCATAAATGTTA
TAAAAAAGACAAAACAAATTGTTTATCACTTCTAACTCGAAATGATTTCATCTCGGGCTTGCTGCACTCATAT
TTTATATATATAAGGTTGGTAGAGTATACATAGTATATATAAGTAAGCACATAGTTTC
TGCATATGTGTGTTGTAGACCAATTTTTAATATTGGACGGACTACATGTCAGATTTAAAAATAATTTTATTAT
AGTTAGCATGGATTCTCTCAAATTGCGTTTTATCCGCATGACACCAAGTAATACTGTGTAACATGAAGACTGG
TTCACGGCGAAGTCTCGCAAGCTGACGCGAAGTCACAAAATGTTATCATATGTATAAC
AATAGTTTAACTGAGATTTGTTTTTCTTTGCTTCAATTATTTTTTTATTGTAAAAATAAGAAGAAACTAAATA
GCTTTATTTCTTTTATTAATACTAGCAAAATACTTTTCTAAAATATTTATTGTTTATAAATTTTATTTAATAT
ACCTATCTGTCTTTCTAGTATACGTTGATATAATTACTTTTTTTTTGTCTCGTCGGGA
CATCCGGACAATCGGGACATATTCGGCGGAGATTTCTTAAATGCTATTTCTTGGTTTTTGAAAATTACTAATA
ATAACATTTAATATTTCACATAATAGATTGTTTGT
>1002050679_1 Heli.1-ES587598.1.5
GTCATCGAGAAATGAACGCACTTATTGTATTTTATATCATTTTAATGTAAAAATGTAATGTATGAGTGTATAT
TGAACGAATGTATAGTGTGAATTTATTATGTGAACAATTAATTATTAAGCTATTATTATATTTTTGTACGGGA
ACTTTATTATATAATAATATTTATGACAATATACGAACTCTGAGAATTGAAAATGGTA
ACTAAAGTCAGATAAAATGTAGAATACTCAGGAATTCGTGCGTGGTCTATATCCATCACAGCTGATACCACAA
TTAGTGATTACATTAACAATAATATTTTACAAAGAATAAATTAACAT
>1002050680_1 Heli.1-DT668402.3.5

CGCACGAGGGGTCCACTTAATAGTCAATGTCTAATTTTTGTACTATTTCAGTCTTTCTTGATTTAAATATTCT
TATTCGTATTGCAACATTTTGTAACGAAATTTAAATATGTATTTTATTTCACAAATGTAATAATCGAGTCAAC
GTTGGAATAAGCGATCAAAGGTATAAACATATGGCAGACAAAAAATAAGATGGCTGAT
AAGCACACTTTGAATTATGATGTGAGGGAGTTGAAATTAGGATCAAGTTTCACAAACAATAAATCATCACAAT
ATCATACAATTAAATATGATTTTAAACCAGCGTCGGTGGATGTTAACAAAATGGCGACGGTTGATGTTGGGAC
TAATAATCAAGTGACCGTTACAGTGCCGCATTTAGATGGAGCAGGAGTACCCCAAACA
GTATTTAAAGGAAGCCAACGGCCTTATACAAAAGAATGTGTCCTAATTATAGATAGAGTTACAGGAGAAATCA
CACTGGAAAAACTTTCTAGTAATATACAAGTAAAGAAAACAAGACAAGAAACCAACCAAAAGCCCAGACCATT
GACGCCTGTTACAACAGATTTTACGAATACAACACAGCGTTCAACTTCGCGTACAAGA
GTTGCGACAAACAAACGTACTAATAATACAGGTCCAAGCGGTGTACAAGTGAATACACAAAATCGGTTCAACA
ATAATGCACCTAAATCACAGAATATGGTCAGATCTCCTACTAGAGTAAAAACGTCGCCACCGCAAGCGCCATG
GTCCGGAGGCGGCAACAGTACTCTAGCGTCCCTCCCGATGATTGGCTTTGACGAAGCA
CCTCCCCGCTCTCCCCCAGCTCCACCACCATATTCCCATAATAATCAAAATAACCATAATACCCATAATACTC
ATAATACCCATAATACCCATAATGCCCACAATACCCATAATGCCCATAATGCACATAATCCCCATAATACCCA
TAATACTCACCCTCCACAAAGACAAGCGCCTCCTCAACCTTCCCTTCCACCTGTGGCT
CAAACACCGCGCCAAGAGGACAGCTCGTCCAGCTCGTCCAGCGACAGCGACAGCGACGACAGCGGCAGCGACA
GCGACTACTGCACCCCCCCGCCGCCGCACGCCGCGCTGCCCGCGCTCTCCAACGGTGTGTCAAACGCAGCATT
ATCGGCCGACGTGCTCAACGACGACCTGTGCCTGTCCGAGTCCGGCAGCGACTCCGAC
TGACCCCCGCACACACATGCAGACACAAAAACACACAAACTCACAAACACACACACATACACAAATACACAAA
TACATATACAATTCCTAGCGAACGATCACTTGGCAAGTGTCCTACCTCTTTCTGTTTTTCATCAAATAGCCTT
ATTGTGTGATAATAGCTTAATAAAGATTAGTGTGATGGATCATTCTAGAACTACAAAA
TAATGTGTAGTTTTTAACTATTATTTTTAATGCACGTGCACGTGCACGGTTTTGACTTTTGCCGTGTACGTAT
CATTTTTTTTTATAAACTTGGGC
>1002050681_1 Heli.1-DT668686.3.5
GCAAGGGTTTAGTTGAGTTTATTATTGTTGAAGTATAACTTTATTATCGAGATATACTGAAACTCTAGAAAGT
GCTAAGACAAAGAAATATGGAAAAACCTACAGTCACGGAACAACCCCCTCCTTATTCCGCTTCTGTGCCACCG
GGTCAACCACAACCACATTCATATCCGCCGCCACCTCCTCAACCGCCGTCAGCGTTTC
CACCCCCGCCCCCCGGCTACACCCCCTACGGTAATACCCCTCAAGCTACAGCGTTCGTGCCAAACTATGGAGC
TACCAACATTATAATACCTCCACCTATAATCGCGGTAGGAGCATGTCCAGCTTGCAGGGTTGGTATCCTTGAA
GATGATTTCACATGTCTAGGCATTTTGTGCGCTATCCTCTTCTTTCCTATTGGAATTT
TGTGCTGTCTCGCCCTGAAAAACCGAAGATGCTCGAATTGTGGCGCTATGTTCGGATAGAAGTGGTTTTAAGT
GGGCTCCATACTTCATTTCTCGTTAGTGTTGCCAGGTTCTGTATCATTCTATATTTTTTTCGAGTGAATAGTA
AAGGTAAAAAAAGTCGATAACTATTTATTGATTTTGTAAACTAATAACATGGCAATAT
TAATGGGATTATAATATAAAATAAAGCAATGAAAGATGGTACCGTCCAGCATTTCACATTTTTGTATACTAGA
GGACTTTATAATTATCGTAAACTCCAATCTCTGAAACAGACAATGGAGCCTTAAATTGCTGAATGTCAATTTA
TTAGATGAAAAGAGTTCAATTTAAAAATTAATTGACGTATGACGTCATCAGTGACGTT
TTAGTCGTATTAATTGCACCAAAA
>1002050682_1 Heli.1-EE743490.1.5
GCACGAGGCACCCGCCGAATTTAAGCATATTAGTAAGCGGAGGAAAAGAAACTAACCAGGATTTCCTTAGTAG
CGGCGAGCGAACAGGAAATAGCCCAGCACTGAATCCCGCGGTTGTAACGATCGCGGGAGATGTGGTGTTCGGG
AGGTCCCGCTTTCTCGTCGTCGCCACTCCTGTCCAAGTTCGTCTTGAACGGGGCCGTT
TTCCCGTAGAGGGTGTCATTTAATGTCAGGCAAAGAAAATAAGTAATTATATAGAATGATTTGGTCTCAGTTG
TAAATAGTGATAAAATACGTGTAAAATATATTTATTTAGTAAGGTGATTGTTTTGAACTAAATGCTATTACTG
AAGGCTTCAAATATTGTTGTCTTTTGTGTAAATTTATTTTGTTATTTAATTTTTAAAA
TCATAAATAAAC
>1002050683_1 Heli.1-ES584807.1.5
AAGGGTTTGTCGGAAATGGACAATATAGCCAAGGATTTTTGGTATCAGGTCAGGGTAATTCGGGAGGACAGGG
CCCAACCTGTGTATGCCAAGCTTGGACGAAACCTCAACAAAACCAAGAGATATTGGCCGATGCTCCGGCAACA
GAAAAGACTGAGAAGAAAGAAGAAACATCTAAGTAGATTAAAGATTTACTTTTGTGGT
GTTTATTTAGTTTATAATTAATTTAAGGATATTTAATAAATTATTTATTTATTGTACGAGTTAAATTTTAAAA
TAGTTATGTTACTTAAAGCATTATTTTTCAATGCAGCCATATATTGAGGCAATCGTGGAGAAGTGGAACCGCG
TCTTATTGTAAACGCGATGGTTGCGGGTTCCATCCTTAGTCCGGGAAACGAACAATTT

CCATTTCCCCGCTCCGATACGAAAATACGCGGCGTTGAGTTCCGTTACTCGATACGTAATAGCTAGGCAATGT
ATTTTAAAAGGTTGATAAAGTTGAAATTTAAAAGAAAAATAATATTACATGCTACCATCTAGAAATAATCCTA
GCATATACATGTATGTAATTTTTTGCCTTTAAAAACAGACAGGCGTATAGTATTTAGG
TTTCGATCTCTTCTTATTTATTGCATTATATTCATCGCTTAGACACTTTGCTATAATTATTTTTTTCATATGC
TCATTAAATTTTGTTTATTGAATAAAAAATAGCTA
>1002050684_1 Heli.1-EL602234.1.5
GCACGAGGGTCGTACCTAGTGCAACAGCAAAGCTATAACGTTCCGTCTTCACCACCAACAACCATACAATAGG
TGTAAATTCTTACTGGTATTCATCATTTTGGGTACATGTCAACGCTTTTGTATTATCTGTGGAGTGACATTGA
CAGCTGTCACTGTCACTTGTCTGTGACATTGACGTTTGGATAGGGAACACGTTTACAT
TTTATATTTGGGTGCTTTTTCTAATGCGATCTTATTACAAACATTCCTACATTGTTGCTTCAACCGTACGCCA
TTTTGTTTACTGTTTGACACTTGATTAGAATTATTAATATTAGAAATTGTTTGAACAGCGTCGGTTAATGTGG
AATTGTTAATTTTTTTTTGTGTTTACACAGAGATTATATTTTTCGATTTATTATTGAT
TATATTTTTGTTGTAGTCGTAGATGTTGATGCACAAATTCTGTTTTTAAGATCTTTATTAGAAGTTATAAATT
ATATAAAAATTTTAGACAATTGAATCATGTTGTTACAAATTATAATTGAAATATATAAACATAATCAAATTAA
TATATTGAAATATATGGCCTAAAATACACAATGTATGATTTGCTATTACTTACATGTA
TACAAAACTAGACCGCCATTGAAATACAAGTATAGTACGGTCAACAAGTTGTGGCCTCCCTGAAGTTAGATTT
TCTAAACTTTTGCGCGTTGTTCACTTGGGAACAAAATAATCAAACGTAAAAAAATCCTAAAATCAAATCCACC
ACACAGCCATCTACAGCTAATTTCATGACAGCCACATGTAGACCGTACTGTACCTTAA
TATTTTTCATATATAATATATTCATTTATTAATAATAAATAATGCATGTGCTGTTCTTATGATAAAAGAAGAA
TTATTAGTGAAATAAAATTATTAAATAATTGCCACTATCAATTTACATATTAATTATGAGCGATAATTTTTTT
GCTTGTGAGAATACCAAATATATTCAATTCGGTACTTTCTCCATATTACTACCAAGAT
AATAATACTTTTTTTACTAGATTTCCATAATATGTTATCGAAGGATGGCTTTTAATTTAAATATGAAATGCTC
ATGCAAATTATTAATAATATTAAATATCTTAAATACTGTTTTCAAAGTAAAAATATATAACATAACTTTAATT
TATTTGCAAAACAAATTATATAAAACGTCCATTTCATAAAACAGTCATAAATATTCTA
TAAAGAAAGAGTTATTAAAACTTATTTATAAAAAAATATCATTAGGTACATTGATTTTTATATTCAAAATCTA
AAGTATTATTGTTGTTATGTTTCATTAAACTACAATTTCTTTCTGGC
>1002050685_1 Heli.1-DT668367.3.5
GCACGAGGCTCAAATCAGAACACCAACCAAAATCAAAACCAAAATCAGAACCAAAATCAGAATCAAAACCAAA
ATCAAAACTCGAATCAGAGCTGGAACCAGTGGGCCGGTGGTTGGGGAGGATGGGGCAACTGGAATAATCAGAA
TCAAGGCTGGAATAACTGGAACAACTGGGGTTGGGGCAACCAGGGCAGCGGCAGCGGT
GGCAACCAAGGCGGCGGAGGCGGCAACCAGGGCGGCGGCGGCGGCAAGCAGCAGTGGCCCAACTACTCGGCGC
AGCAGTGGTACCAGTGGCAGCAGTGGCAGCAGCAGCAGCAGGGCTGGCAAGGGTATAACCAGCAAGGTCAAGG
AGGCGGCGGTAACCAGCAACAAGGAGGCACCAATGCAGCTGATGCCGCCCAAGCCTGG
GCTCAATATTACCAGAACTACGGCGGCGCCAACGCTAACAACTCCAACTCAAACAACTCCGCCATCGACAAGA
AGTGATGACTGACACACTCTGACACACCGACGGCGGCGACGGTCACTGACTGACAGTGCTCCAGTTACCTTTA
CCTTACTTACTTAACTTACTTCGACTTGATAAGGTACTACAAGCCTGGTTTAAACTTG
CTCATAGTATTTTACAATACAGTAGAGCGTCGATTATCCTAACACATTGTATACAATATTATATCTTTAACG
>1002050686_1 Heli.1-DT664983.3.5
CTATATATTTCACTAAATAATATTTTATTATTTTAATATTCTATATTTCCAATTAAACCCAATGACCTAACGT
TTATACTAATTTTATATGAAAACATAATTGAATATTTCTTAAGTTTTATTATTTAAGTATATATATGTATATA
TGTCGCATCGCGAAATTGTATAAAATTGGTAAATAAACCCATCTAAGCTGTGATAGTA
ACAAAGATTTTAGTTTACCAAAAGTATTTTTAAATTGTTTATACGTTTTGTATCATTTGTTGTGACACCTTTG
TTAAATACTTTTTTTTTGCTTCGTTTGAGGTTATTTAAAAAAAAAAACAATTAGTTTGTATTACCATTAAAAA
ATAATATAATCATTTCGCTCTGGTGTTGAGGCAAAGCGCGGCGTTGAGTTCCGCCACC
>1002050687_1 Heli.1-DV501205.2.5
GCACAGGATATTTAGGTATTACGAGTGCGGTTTGCGTGGCGCGAGCGCGGTGTTTACACCGACGTGCATCTTA
GTGAACTTATATATTTTATGTACTATTATTGTATCTTAAGAAGATATATTCTTATTTTTCATGGTCCAAGTTT
TAAATGTTTTATCGACTATACAAAAATATGAATAATCATTATTATATTCTTGTCGACT
TTAAAAATTATAACCCACAAAAAAATCCTCAAAAAAAATTGATATCTTCACATTTGTGACATTTTTTGGTGTT
CAATCGGAGTATATGAATAAGGAGGAAATGGAAAAAATGTATACGCTCTCACAGAATAAATTTTAGGTAAAAT
ATTTGGATATAAAATTATCATACGATTATTAGTAGGTTACGTGTTAATGTGCGCCTCA
TTGAAATTAAAAAGTCTTTGTATCGTGTATGTAATTAAAATATACATATATTATATACAAGTTAAATTAAAAA
ATTATTATCGTACATTTTTCTTCGGAGACCATGAGTAAGAGAAAATAGGTCCGAGTCTGACTTCGACATTTCG
ACATATAATATCATTCGTTCGTGGGCCCGAAATAGCTATATATTTAAAAAATAAATAA

ATAAATAAATGTTTTATATACACTGTAAGTAAAATTGTCATACATATCCTTTGCCGAGATGTCCAACGCACAC
TCTGTGAAAAGAAAAAAAAAGAAAAATTTTTATATTCCAAATTTAAACTACTCGATAGCATGTTTCATTGTTT
CCTTTCCCTG
>1002050688_1 Heli.1-EL595925.1.5
GCACGAGGCTCTGCTGGGACTCGGAGTGCGACGTGTCTTTACTAGTGGGCACGTACAAGCACGGCTACGAGAA
CTACCTGGCGATGCGCTCCGACCCACAGCTTTGCTACGTGGACAAGTTGGGCCCCCCGACGATGCTGAATGC
ACCGATATAAAAAGTGAAGAGAGGAAGGTGCTGGGCAGCGTGGCCGGCGACGAGGAGT
GCACGGACGACGTGTCCAGCGGCGCCGGCGCGGCCTCGCCCGCCGCCTCGCCCGCCTCGCCGCGCGACGACGA
CGGCACGCACCTGTGGCCCTCCATGCAGGACCTCAACACGCGCCTGCGACGCCTCATCACCGCCTACCAGAGG
AACTACAAGCGCGAGGAGCTCAAGCTGCAGCAGCGGGCTAAGATGGAACGGCGTGAGC
GCATGGATCAACTTGCTCGAGATGAAATTTCTCAATGGCGTTGGTCGCGTGCGGACGAAGAAGCTTTTAGAAG
AACAGTGGCATCTTATGGAGTCGAGTTTGATCCAGCAACTAAGAGCTTACGCTGGAGTCGGTTCAGATCATTG
GCGAGACTCGACTCAAAAAGTGATGATGCTTTAACAGATTACCTCAAAGCATTTATGG
CGATGTGTAAACGACAGTGTGGCCTCGCTGTGGGAGAAGCAGAGCTTCCTACTCGAGCTGATTTGAAAGCAGA
ACCAATCGGTGAAGAAAGAGCTATTGCTACTTTAGAAAGAATTGACCTGCTTCGCGTATTACGGGAGGAGGTG
GCGCCCCATCCAGCTTTGGAAGCTCGCTTGGCACTATGCGAGAGATCGCTAGATGCAC
CGCCGTGGTGGCAGCCTGCGAGGCACGATAAACTATTAGTTCTGGGCGTATGCAAGCATGGATTAGGAGACAC
ATACAATAAATTATTTTGTGATCCAAAAATGCCTTATGCGGATGCTGCGAGAAAATGGCAAGGAAAATATAAA
ACTACAACAAAGACTGATACAC
>1002050689_1 Heli.1-ES586335.1.5
TGTCACTGGATTTGGTCTTTTGGGGCATGCCCAAAATCTTGCATCACACCAAAAGAATGAAGTATCTTTTGTT
ATCCATAATTTACCAGTAATTGCTAAAATGGCAGCTGTTGCAAAAGCATGTGGAAACATGTTCCAGCTTTTAC
AAGGACACGCTCCTGAGACTTCTGGTGGACTTTTAATTTGCTTACCCCGTGAGCAAGC
TGCAGCATATTGTAAAGATATTGAGAAACAAGAAGGCTATCAAGCCTGGATTATCGGAATTGTTGAGAAGGGT
AACCGCACTGCTAGAATTATTGACAAACCCCGAGTTATTGAAGTGCCTGCTAAAGATTAAGTTGTTTTTTATA
CAAATAAGACATTTACAATGAATACATTAGACATTAATGTTATTGCAGAGCTGGACAA
ATCTTGAAATTTTAATTGGAATATCAATATTTTAACTCCATTTTAGTGTTAATGTTTTATAGTTAAGTGGCTA
GAAGGAATTTAAACAATATTTACTAGCTATCTTGTTTTAAAGTTGGTGTTCTAGGTGTACAATAAGATATCAC
TATTAACTTTAAGAGAAATTATTCCATTAGATACCCTTTCTCAACATTGTTCATGGCT
TGTGGATTGATTCTGCAATTTGTGGAACTTTTTAAATTAGAATAAAATTATTTTTCATTC
>1002050690_1 Heli.1-ES586831.1.5
GATATTAGTGTGACGTAATTTATGGATGGCCCCGAACGTTTAATATAAAAATATAAATATCTTAAGACGACGT
AGTTGCTTACTACGGATGCTAATTCGATGATAAATTAAATAAAATGGTATCGCATCGTCGCGTGGCACCACTG
TGCCGGTGTACTTAGTTAGCGTTTAACAATATATAGTCAAGAATTTCAAATTAATAAC
TTTGAGTGCTCGTTAATCCAATGTGTAGATATTTATTTTTGTTATCGTAATGTATCCGAAATTTTGCTATATT
TATCGAATCGTAATTTTAATGGTTACGTAATTTAATAATAATAAAAAAATCGTAAATCGTAATTCATTAGTAA
GAAGTGAATGTTTCCAACGGAAAATATATAATTTGTATGAGGATTCCCATCAAGCGAA
GCATTTAGCTGTGTGCATTATATAGTGTAAGTTTGGTATCGCGATATAGGAACGCACATGTGAGGTCGCCGGT
GGACTGTGAGCGTTACATGGCATGTAATAAGAAAGTGCTAATCTATAATAATCAAATATTTAAATAGTTAAGT
TATGGTTCTCCAATATTGTGGAAGATGTATAATATTTTTTACTAATAAATTTAATATT
>1002050691_1 Heli.1-EL602503.1.5
GCACGAGGACCACCAGGACCTCAAGGATACCTACCAGGAGTTGACGCTGGCGCGCCAGCAGCTGCAGGACGAC
TACGAGAAGCTGAAGCGCGAGGAGGCCGACAAGTCCGCCAAGCTCAAGGAACTAATCCAAAGTGTGGAAAGAC
GCGAGCAAGCCCGCTCCGATCTCAAGGGCCTGGAAGACACTGTGGCTAAGGAGTTGCA
GACTCTACACAACTTGCGAAAACTATTCGTACAAGATTTGCAGGCTAGAATAAAGAAATCTACGAACTCTGAA
GAAGGAGCAGAAGAGGAGGGAGGATCTCTCGCACAGAAGCAGAAGATCTCCTTCCTGGAGAACAACCTGGAGC
AGCTGACGAAGGTTCACAAGCAGCTGGTGCGAGACAATGCCGACTTGCGCTGCGAACT
GCCCAAGCTCGAGAAGCGCCTGCGCGCCACTATGGAGCGCGTTAAAGCGCTTGAAACTGCACTCAAGGAAGCT
AAAGAAGGTGCAATGCGTGACCGTAAGAGATACCAATTCGAAGTCGACAGAATCAAGGAAGCAGTACGCGCTA
AGAATCTTGCCCGAAGAGGAGTGCAGGCACAAATCGCCAAGCCAATCCGCGCCGGCGG
CGGGCACCTGGTGGGCGGCGGCGCCGTGGGCGTGGTGCGGCCCGCCAACGCGCCCGCGCAGGACATCAAGCGG
AAGTCCATCATTGTCGGCGCGCGCGATGAAAGTTGAAGAGACAGCCGTTGCATAATAGAGAATCTAAATGAAA
CTTTATAGATGTATCTAGAATAAGGCGGCAAAAATCATTCTTGGAAAATATATCCATA

ATATAATAATTCTACTTATATGTTTATGAAAAGCCTAATATAAGCTACAACAAAATGAAAAAAAAAATAAAGTA
GTTCTCTTAGGCTTAACGTTTCGAGCTGTCTAATCTTAAAGTCAATAAGTGCACATGTATTTAAAAACGTTAT
AGCTTCTAGCGATCTTTGTCGTTCTGTCTGTGTCTATACTGGTTCGCTTCAATGTAAC
TGTATAAGTTTGTCATGTTACGTATAGGCACAGGCTCTTCAACCTTTTACTGTTTAGCCATTAGATGTCCACA
GAGTATTAAATTCATACAATTTCTAAATTCTATCC
>1002050692_1 Heli.1-DT665289.3.5
GCACGAGGATCTCGCTATCTGTGGGCTCCCCGCGATATGTTTACCAAAATTCGTTCATTGTTATTGTGATTCG
GGACTAGCTGAAATGCCTCGTGTGTACGTGGACTGCGAGAAGTGCCCCACAGTGTTATGTCAATAATCTACCT
GTAGATAGCTTATTCAATTTTCTATCGATAGTGAAAAATAATTGTGAAAAATGTCTAA
TACGGTACCTACCAGTTCAGCTTTGAGGGCCTTAGCCTTAGAGTATAAGAGTCTTCAAGAGGAACCCGTGGAA
GGTTTTCGTGTAAAGTTATCGGGTGAGGACAATTTATTTGAATGGGAAGTGGCGATTTTTGGACCTCCAGACA
CGCTTTATCAAGGCGGATACTTCAAGGCACACATGAAATTCCCTCCTGATTATCCATA
TTCACCACCATCTATTAGGTTCTTAACTAAAGTATGGCATCCAAATGTCTATGAGAATGGCGATCTCTGTATA
TCGATCCTGCACCCACCCGTGGACGACCCGCAGTCAGGTGAACTGCCCTGCGAGCGTTGGAACCCCACGCAGT
CGGTCCGCACGGTGTTACTGTCGGTCATATCGCTACTCAACGAACCGAACACATTCAG
TCCGGCGAACGTGGACGCCAGCGTTATGTACCGCCGCTGGCGCGACTCCAAGGGCAAGGATAAGGAGTATGAA
AATATTATAAGAAAACAAGCCCAAGTAGCACGTATGGAAGCGGAAAAGGAAGGAATAGTAGTACCCCTCACAT
TAGAAGACTATTGCATTAAAACACAAGTTAAATCTACGACACAAGAACCACAGCTGGA
CATGACAGATTTCTATGACGACGACTACGACGATCTCGACACGGACTCCGAAGGTGAGTTGGGAGGTGAGGAC
GGCGACGACAGCGGCAATGGCGAATCGTGATACCTCACGAAGAACAGATCTCACGCGACGGTCGGACCACGCG
TTCGTTGTAGTTGGCATTTTGTAACTTCATATATTGACCGCCATTTTGGATGTGCGTT
CGGTTTGACGATCAATCCAAATTCAATTTTAAAATATCGTTCTATCTTTATTTTGTGAGTTAAAAGGAAATAA
TTGATGTTTAATATTATTTGTATCGTGATCTAATGTTGTTTTTGTCAATTTTATAATTTACAGACTATAGAGA
TGTCTATGGTCATAGACTAGTGTCAATAAATAATCATATATTTAAGTTGCAATCGTCA
AACAGAACGTAACAACAAATAAAAGTAATAATCAATATAACTATTTACTTTCCTATATATCATGTGCTTATGA
TATCTGAACAATATACTAAATAAAAAAAATCTAATCAAAAACAAATTGCTTGACCAATAAAAAATAACTTACT
GGGATTTTTAATCCCAAACTTTAAGCTTTACAAG
>1002050693_1 Heli.1-EL601379.1.5
ATAGAAAACATTGAATAATTTATAGCGCTTAACCAAAAACTAGGAACATCACTCGTCTTCTATACAATTCTAA
TGACAAGTAGTCCAGAATTAGGTATTAGGAAAATTTTAGAACACCAATTTATTATTTGTATGAAGCTAGAATT
TCTTTGGAAATGTTTAAATAATGTGGAGTGTACAAGATACAAACAAATATAACAAGGG
TTTTTAAATATATAATTGTTTATATATAATCATAAAATTTGTATATATACAGCAGTGACAGCTATTATAAAAT
GGTAATATAATATTTAGTGGATATTCAAAACTTTATCCTTATAGTCAAAGTCAGATGTGTTGAGAATGTTTTT
CTATCTTGTTATTTGTAATTTGTTTGTTTAAAGTGACTATAGCAAGAATAATATTTAA
AGAAAGTTGATTTAAGTATAAAACGCGGCGTTGACTTCCGCTACTCAACACACAATTGTCTCGAAAATTAGAC
GAAAAGTGGGGATGGAATGTCTTAGCACTCCGTTCCTTATGCCTACCGTGCTATATGCGGGATAAGCTAAAAA
GGAGATGACTTAAATGGCCAAAAATAACCGATCAAGACCTAATTTTTTATTACAAATT
TGTATGTACATGAATATTATACATAAAATTTGTTGTAGATTTGGCTAGGCGCATTTCAATTATAAATAAATTC
TTGTTATATCATGCTGAATTTAAATAATTTAATCTGTCTTAAAATTTTTAATAGTAAGTACATGCCATAACAA
AAGTAAATATAATTTTTAAAATGCATCTAGCTTTATCACTTTTGTTTAAACGGCCTTA
ATATTGAAATTTAATGATCGTTTGTATTATAAAGTAGGCGGCCAAAAGTTGTATTTGTGAATCAGTTATATTA
TTGTTGCAGTTTGATTTGCATGTCTTTATTCGGGTTAATTTATTTCAAGGTATAAATTAAGAGTTTCTTGAAC
TGTTGCCTATATTGATGCTGTGTTGATGAAGATCACACAACAAAATCGTAATACAATA
TAACATTTTGACATAATTTTTATAAAAAAACATATATTCTAAGGCGGTTAGGGGTTACTAAGATGTGCAGAT
>1002050694_1 Heli.1-EE743473.1.5
GCACGAGGTTCTAATATATTATGTTATATTATGTATTATTTAGAAACAATCGTTTTTTTTTTTGTATTTTTTT
AATATACATTTTATAAGTAACAACGAAATATATGTATATATTGGTAGAAATAAGATTTTTTCTAAGCATCTGG
AATTGAATCTGTTAACTTTGTGGAATTCTGGTCCTTTATAAATCTCTTATATGTTGTT
TTATTTTTATCTGTAAGTGTTCATTTTGAAGTACATATTTAGTTTTGCTTAAAAAGTAGTTATATTAAAAAC
CTTTTTGTTAATTTCAAGAAAAACATTGAGCTCTGTGATTTGTTTTTTTTTTTATATTAAGACAAGCAAATAT
TTAAAAAGCT
>1002050695_1 Heli.1-ES584919.1.5

CCCCTATTTAGGGTACATCAAATGTAAATATCAGTGAGGTGCCACTGAACGCTTAGTTGCCATAAAGATTTTA
ATCCTAGTTTAAGTAATGTTAGTTCAGGCTTCGCTATGTATCACACTTACTGGTCTTGGCTCGGCGATGTACT
TACCGGATTTGGAAGCGGTTTTCTTTTTTTTTTTTAATAGTTGGTCCTTTTTTTATTC
ATTAGACTGTGTTTTAGGTAAACATGTAAACGATCATTTGTATTCGACATTTTTTTTAATCAGTCGGCTTTAG
TAATGTACGCGCTAGCCGATATTCTATTCTATTCTATTTATTCTATTCTAGAAGTAATAACTAAGAACTAGT
AATAAATTGTGGCCTTGGGTTTCGTACGTCTAACGTGGAAGTGTGGCTTCTATACATT
TTATACTATCTTACTTACTACCAATATTTTTTACCCCGAGTTTTGTTATTTGTAATGACCTCTTTTTTTATGA
ATAATAATATAAAATTAAATGTAAGTATACCATTGATACATATTTTACATAAATATTATATTTAATTATTAAC
AATATTGATGATTGTAAAATTGGCCATAGGTTGTAACGAGTTGTCGGTTGGTATGCCA
ATTAAACAATTTTTCATGATTTTT
>1002050696_1 Heli.1-EL603855.1.5
CCTGCCCTGAGGAACGTGCCGGAACTCGCGCCCGCATTGTCGCGGCGAGATCGCAAACCAATTAGATGTCTAG
TCCGTTCTGGTGAAATGACACAACTGCTCTGGAAGACCGACGAGCTCAAGCTGACATTCGGAAAGAAGTTCCA
CAAAGTGCCGTTACATCTGTTCTTGTTCAACGATCACCTTGTTATTACTAAGAAAAAA
GGCGAGGAGAGCTACGTAGTGGTGGATCACTGCCCGCGGTCACTAGTCGAAGTGTGCTCCAGTGACGCGGCGG
CGGGCGCCAAGCACACGCTGTTGTTGACGCTGCTAGAGAACCACGAGGGAAGAACTATCGAGATGATGATGTC
GTGCCCCACCGAGACGGACCTGCGGCGCTGGTCGGAGGCCCTGGCGCCGCCCGCCGCC
GCCGACGGGGAGACGCTGTACGCGGGCTGGGACTGCCCGCAGGTGGCCGCGCTCTACGCCTACGCGCCGCGCC
AGCCCGACGAGCTCGCGCTGGCGCAGGGCGACGTCGTCAACGTCACCAGGAAGACCAGCGAGGGCTGGTACTA
CGGCGAGCGCACGCGCGACGGCGAGGCGGGCTGGTTCCCCGGCGCCTACGCCACCGAG
ATCGCGTCCCCGCACGTGCGCGCGCGCAACCTGCGCCAGCGGTACCGCCTGCTGGCGCTGTCCGCCACCTACT
TGGGACAGAAGCGGAAACCGGCCGCGTCACACTGACAGAACGACAGCGCCATTGATCTGATAGATTGTAACTG
GATATTTTACATCTTTGATACTGAATAAAATACTTTTGTATTATCTGTATGAAATTTT
GGAACTAACAACTCTATATATTATATATATTGAACT
>1002050697_1 Heli.1-EL603543.1.5
CCTATTTTATTATTCTTTAATAATGACTTTGAAAATGTGTTATTAAAGGCTAATATCTATTCCCTTTCTCACT
TTTTTTCTTTTTCTTATTAAAGAATATTATTATTAATTTTAGGTAAAGATTTTTTTAGCTTCACGCTATATCC
CATATTTTGCAGAGGGAACGTAGTGTTAAGACGCTCGGTTCTCCATACGCCGCACAAT
TTTCAATTTATT
>1002050698_1 Heli.1-EL601671.1.5
GCACGAGGCTCGCTCAAAGCGCCGCGCCGTGTCAACAACAACAATAATAACAAACGCAAGAGGAGCCCACAGC
GAATCGACGTTAAGAGAGTTGTTGAGATACAAATTGCTGGACAGACAGACGTTATTAATGATAAACCTCCAGC
AGCACCGGAAATAATTCCCAAGATATCGGACAAGCCCCCCAAAGGCCGCGCCCCCTCG
CCTGAAACTACTGAGCCCGCCGAAAACATTACTGTAGACGAAGCACCTTCCTGGCGCAGATCCGCTTCATTTA
GGAATAGGCAACAAGATAATAATACACGTGAAAATAAAAAAGATAATAATAACTTGGATAATGACACCATATT
GAGAAGAACACACAGCTTCGAAACCGACAAAACTTTCTATCAACGCTACCGCGACGTG
ACGGCGCGTATAAAGGCGTCGTCGTGTCCGTCCATACCGCCGGCGACCGTGCCCCCCGCCACCGTAGCGAGGA
AAACAACGGAGATTAACACCACTACTGAGAATACTAACGAGGACGTTCACACCAGAATTAGTCCAGCAGAGAG
ACGGGAGAGAGAGGCGAACACATGGAACGCTCCTTCATCTACAACTACTACAACAATT
CCCACAAACACAACTACACAAACTATTAGAAGATCGTTCGTGCCTCCAGTTCGTGATGAAGAGAGTGAAACAC
AAAGGAAAGCTCACGCAAAACGTGTTCGCGAGACGAGAAGGTCTACTCAAGGGGTAACTTTGGATGAAATAAA
ATCCGCTGAGCAGTTAGTAAAGAAGAAATCCGGCAATGGTGCCGCAGAAATACCACCA
CCGACCAGTGTCAAGAAGGAAGATCCTCCTGTGCCTGCTAATACAGATAACGCATTCGAGCTGGAGGACGCGG
GACGGGCGTCCAGGACGAGTGCCCCCGCCGCGGAGACCACCGTCACTCTGCCGCTTCGACGAGCGTCTGCTAA
CAACACAAACACAACCACAGCCACAAACAACGCA
>1002050699_1 Heli.1-DT662033.3.5
GCACGAGGCGATAAAAATCGTCTTATAATTGGTGTTCCAATGATAACAACAATAAGATTGTATCCAAAAGTGT
TACTTATCTATACTTATGCTAAAATTAAATTATCACTGTTCTAAAGTAGCTGTGACACGTGATTCGTGCTTTG
GCTGAAATCTGAAAAATTTATCTAACACATAAATTATGTTTTAAATGAAATGTTATTT
AATACAAATTGTACTATCAAGAATAAGTAGTCATACAAAAGTCAATTATTTGTTTTGAAATTTATACAAATAC
GTCACACTTAAAATTATATAATGTTTTATTATAATATGATTGTATTTTTTTTTTCACAGTAAGTATGTATGTG
ACCCACGCCTGCAATCAAACTTAAAACAAAAAAAAATAAAAAAATACTTATAAGAATA

GAGCTTTGTATATAATTAATTTAAAAATATTTCTATGAAGGGCTCAGCAAGCAAACCAATTACGTGGCTCGAT
TTATGGAAATTTAATATACCTATATACAAAGTCGGTCAAAGGTCGGAACCTTGTAAATCCTTAAGTAACTCTA
ATTAAACAGAGGTGAGAACAAATGATGGGTACTTCTAATGTACAATTCTTAGATTACT
AAAATTCTCTTAACGGCAACCATCATTGTCAGTCTAGACGCAAAGTAATAAATGAATTTAACTAAAATTTAAA
TTAGAAAAAAGCACAAACAACATGTACAATACAAATATTTTAAATGTATTTATTTATAT
>1002050700_1 Heli.1-EL601510.1.5
GCACGAGGTGTAGTGATGCACGCCACGTCTGACAGTATATCATCTGTATCTATGAATGATATGAGCAATTGGT
ATAATGAAACACAAACAACCAAGCACAACCAAACACAATTGAACACACAAAGTATATGTGCAACGGAAAAACG
CTTCGCCGCTGACTTACAAACAGTGGAGACCAGCAACCACAAGATGAGAGCGTTAAAC
GCCGATCTCGATACGGTGTTGGATAGATTACAACAGATATTAACACATAATTTTGCAATGGATGATTCATGTT
TCGACAACACCCAGTTACGTGAAACAGCAAACGAAATGGAAGGTCTTTTGGGTACATTAATACGAGGTGTGGA
ACAACGCGCTACTTTAAACGCTAGTAAAGGACTCGTCTAGGTTATAGATATGGAAAAA
AAACTTAAATATATTGTTAATTCATCCATCCATCTTATATGACGTCATTAAACCGCTCTTACAGTGTTGTTGA
GATTGTTGTTGTTATTAAGATTTCTTGTTAAATCAATAGATGAAAATCAATATTGATCTATTGTAATATTAAT
TTCTTAATAGCTATAAAATTCTTTCTACCTTCATTCCAAGTTTACAAAGGGCGCAGAT
TCAAATTCGGCTATGTTAGATTTTTTATGCGTTTCTTAAATAAATATTCGTCTATCCTAACTGTGGAATAACA
TTTAAAAAATATCAAAAATAGCTATATAATTAATAATTAAAAACATTACAACACTGCCGCCACTTAAAGCAAT
TAGGGGCTAG
>1002050701_1 Heli.1-ES586962.1.5
CTATGGAGGAGACGGTAACAGAGTGTGGCGTGGCGAGCGCGAGTGCCGGTGCGGCCGTCGACTCCGACGGTGA
CTCCTGTTCCGACAGCGACTCGCACAATGACTCCTCTGATGAGGAGGATGAGGTGGTTTCCGCTAAGCAGCAC
AAATATAGGAACATCAGCCAGCTAGAGTGGGATAACAGCACGATGTAAATTGTATCGC
CGTCTCGCTCGCACGCGCCCACACCACATACACCCCTCTCGCTCGCTCTCACCCCCTTTAAATAGGTTACTAG
TAAAACCACTGGACCTGGTTATATATAACTTGCTTATATATAAATAATTGTATTTAATAGTTAATTAAATATA
AATTTATAATGGCATTTAAAACGTACAAATATTATATGATGTTGTGGGAATCAGATTT
TTTTTGGTTCATTTTTTTGTCGATTTTCTATATACATTTATTTTTATCATAGCTAGCCCGTTTCTCCCGTTTC
AACTCGCATTTTTTTTTAATAGGCGATAGGGCTACAAGCACAAACTAATTTTTAAAAGTAACTTATTTAAATT
TATCATATACTTACGTCATAGTAAGCGAGTTTTTCACTGTTTATAATAAGGAATTGAT
TGCGTCACAAGTTTTGAAAAGCACAAACTTCCGTTTCTGGTTTGGAAATTAATAATAAATAATTATTGAGAAA
ATAAAGAAATAAAAAATATCGCGGGGGAATCCTAT
>1002050702_1 Heli.1-EE743460.1.5
GGTCCGGAATCCGGGTCGACTACGCGTCCGTTTTTTTAATCTTAATATGTAGCTAGTGTCCGAGAGTTTCTTA
TAGGGACATGACTCATTGCAATAAGTAATGTAAAAAAATATCTGACATAAATTTTTCTCTTAACACTCTCGTC
GAATGACACCACTATTTTTTATTGTTAACTATTTCTATCGCTGTTGGTGAGTTTAACT
TGAATTTTGAATTTTTATTTCCAATTGTTTTCTTTGAATTGTGCTGAGTTATTTATTTTTAGACGTCTGTACT
TAGGGCCTAATAATACTTTGTAAAGTGTACATCAAAAATTGTATAGATTTTAATAAACTATAATTTTACATTC
ATTGATAAGAGAGCGTTAGATATAAAATATATTTTGGGCTTCGTATTAATAGATTTAA
ACATAATGGACGGTTAGTAAAGAGTTCTTAACGAAGATGCAGACTTATCATGTTAGTATCATTGTATCATATA
AAGTTTATAGGCTCTAAAACATGAATGTATAATATAAATATTTTTTTAAAATCAATAAAGAGTGATATTAA
>1002050704_1 Heli.1-EL601658.1.5
GCACGAGGAAATATCTTACAAATTTAATTTTTATATTGCGCTTATAAGTTATAAGTAACGTAACGAAATGAAA
GTGTGCCAAAACAGAATAAAAAGTGATTTGTTATAGTTCTTATACAAAGTGGCGGCAATATGGTCGTCGATCT
CACCGCCGTGCCGTTGTCTGCACTCTCAAATGAATCTCGTAATATATTATCTTCTCGA
TTAAATCCTAAAAAAGTTATACCAATTGTTGGGCCCGATCAACTACCTAGACACAGAGATTGGCGAGGGCTAG
CGTCACTGGCCCAAATATCAACAGAAGTAGCAGCTAGTATAAATGACTATCCAGATAAAATGGCCAGAGTTTT
GGAAATATGGTCAAAAACTTCAGATGGTGCTGCAACAATTGCTAAACTTCTAGATTTT
TTACAATTAATTGACAGATTTGATGTCTGCGAAGATTTAATAGACCTTCATAAAGAAAATAAACTTATAGTTC
ACCCAAGCAATGGTGACCATCAAATTGTAACATGGGATGAAGAAGACAAAGATATAATAACATATGATGACAA
ACTATTAGGATTTCCACAAAAATATCATGCATATGTGTTACATGCCAAGGAGGATAAG
GATTTTGTCGATGAGCTGGTCAACCGGATGAGGAGCCAAGGTCTGAAGCTGTGTACAGAAGAGGATTTGCTAA
TAGGGCACCCCACGCGAGTGGAGCCGGTGTCCCGGCTTATACTACATAGGGTGTAGATATACAGTCCTTATAT
ACTCCTCGGACTTCTGGAAAATTCAAACATTATCTTTTACACTAAT
>1002050705_1 Heli.1-ES586510.1.5

ATGTGTTTTAAGAATGAAAGTGGCAGATTGGCAATGCTTAGTAGTATTTGTGTAGTGATAGTGGTTACGTTTT
TGGTATCTATTCGTAAATGCATCACACATAATAAGGTATGGATAAAAATTGTGAAATGAAAAAAAAAAACCTG
TGAAATGAAAAAAAAAACTATTGACAAATCTTGATAAAATTTTTTAGGTATGGGTTTT
AAATATTTTATTCTATATGCTGCACCTGTAAAAGGAGCGCAGACGATTCCACCTGGTATAATTATTATTTGAA
ATATACTTTTTTAAAGTGAACCTATTAGGCCTGTGTGATATATACTATAGATTATGAATACTGTAAATCCTGG
TGTACAATCAAAATAGGGATTTTGATTTTTTGATAATTAAAAGGCACTAGTCATTCAA
GTTTTACTCTTTTTATATATAACATATATTGTATTTATTTCAATTTAAAAAGAATTTGATATATTTGAAAAAA
AGGGAAGTAATTTTGAAGTTGCTATATTTAAAGGACAAAGAACAAAAAAATGCAATAAATATGTTTTTTAAAT
TGGCTTTATTATCAAAAAATTATCTTAATTAAAGCATAACAGAAAGAACTTCTTTGGC
CAGTGATAATTTTTTCCTTTGTTGCCTTTTTTCTGGTAGAGAAAATAAAAATATTTCATTCCTTTCATATCGT
TCTGTCTAATCAAAACACTACGTCCCAAATTTGGACTGAAAGTGTATTGATGTGTCTTAAATTAGGTTCACTC
TGCCTGCTATATGCCAGATATAGCGTGGTTCCAAAATATTACATTAGTTTAACCCAAG
CGGACAGAACCATGAGTGAATACAAGTCACAATTAATTTTCTTTCTGAGAAAAAAAAAGAGACAAAATATCAG
ATGTATTTTTACCTCTTTCAATAATTTCAATACATGTTATCTGTATTTAATATACTAAAAAAAGTTATCACTG
GCTTTGTATGAATATTCTAAATCAATTATTAGGAAATTTTTTGGTCATATAAACTAAA
ATACAATCTATTGTGTTACTTCCATCATTTGCGGTGCATCTCCAGAACCGTCGTTAAACATAAACTCATCTTG
ATATTCTTTCAAAAACTGCTCAGACCGATGCTCATCGAGAAATAGTGAATATACTTTCTTAACGTCTTCCATG
CTCACCTCGGTGGCCTTCCTGAACTTTTGCGCATTCTACGCTCGGG
>1002050706_1 Heli.1-DT662204.3.5
ACCACATTAAATGAAACCACAGTTTTAACTACTCTATAAAAGCCTAATATAAATCAGAAAAAAATAAATTTCC
AAAAATGAAAAAGTTATTTTTTCAAACAAAATAAATAATAAAATTGTCATTTTAAAACAAAACGCCATTTGTA
AGGATGACGCACGCACACATAAACCTATCAAAGGAACATCGCCGCACACGCCTCATTT
CGAAATCGAGTCGTAGGTATTGGCGATAAAGTTCAAAGTTGTTTAACCTTTAGTAGTATTTTTGTAGTAGTAA
GTGTTTATGTTATTGGGATCTAT------------
CACACGAAATACAGGTGTGGATAAACATCGTGAAATGAAAAAAAAATCCTACGTCTGAACTGTTGAGGTATC
>1002050707_1 Heli.1-ES585267.1.5
TAATTATTATAAGCGCATGTGACAGCACTTGTATGAAAATAGATAGATTAATCTTTTTATTTGTAAATTTTTA
ACCTTTTTTATTAAATCGTTATAGTTACGGCAACGTTTTTAAAATAATCTATCTGTTTTCATTGTATTTTTCA
TTGTATTGTTTTCATTGTATTGTGTACAATCGTTAAAATGGTGTGAAGTGGAAGTATT
GTAAAGAGTAACTTCTCTTTGTAATTTTAAGTTTATATATTTAGATAAAATGATAATAAAATCGCTATTAGT
>1002050708_1 Heli.1-ES587475.1.5
CGAGATCGTCAGAGACCTTATTAGTCTTAAATTAGTTTACGATTAATATAATATTACCTATTTTAATTGTATA
CATTCAAAATTATGCTATATTCATTTTTAAAACAATGATTGACGTATCAATACTTACTGACATTAATTCCGTT
TTTGAGACAGATATTTGTTCGTGACATAGAATGTTATTTTTTGTTACCTTTGTAATTA
TATGAGTCAAACTATTTCAATAGGCTAAAATAGTGTTGAAACTTCTAGTGGTATGTGAAAGAATTTATCAATA
AAATAAATACTTTGTGTGAGTATTACAGATATCTAACTTTAAGCTTTCTATTAAATTGACAAAATCCCTTT
>1002050709_1 Heli.1-CX700458.1.5
TTAGCAGACGAGTAGCGCGATGGTCAACTACAAGCGTGTTGTATGCGAGGTGGCGGTGCTGGTGGTGGTGGCG
TCGATGGCCGTCGCGGCCCCCACACCCTGACTGCCCTCATGCGCATATTGATCAAAGACAGAACGGTTCAGAA
AATTATCGCTTAAGTATCGACGGTGTCGTCATAGCAGTAGCGCCAGCGGAAACTCTTC
TTGACGCAGCGTCTGATTTAGGGGATCTATTCGATCAATCAGATATTGAATTTTTGCTTAAACCACCATCTGA
ATCATCATCGAAGCCCGATGTGTCCAAACCTGAGGAGCCCAGCCCTGAAAAACCTGAAGCCGAGGGCCCTATT
GACAAGCCTCTAAGCGATGTTAGTTTAAATTCTGATTTATCCGCACAGAAGAAGGATG
CCTCTCTGAAGAAACAAGAGAAAGCTCAAAGACTGAAAAACAGGTTGGCGAACGTTTTGATACCTTTGCTACG
AAGGACGCGCCACCACTAGCGTAATTGATTGATGATACACGGCCATACAGCCGCGAGTGATGCATTTACTATT
TATTATATTTCGTTTTTAAGTTGTATGATTGTATGTAGCATTGTATGCCATTGTTTTG
TTGAAAATAAATGATAATTATTATTGAAAAATCCTTACTACGTCTTTTATTAATTTGTACTAAAAATAAAAAT
AAAAGATATCT
>1002050710_1 Heli.1-ES586034.1.5
CTTATATCCGAACGAAAGTAAATCGAGAGGAAATCGACGGTACGATAATACGACTCGAGCAAATGACGAATAT
TACGTAAAAATTAACAAACTTACCGACGAAACGTGACCGAGCAATAACTTTGTAAATGAAAAATCCAAATATA
TTTAAAATTTATTTTTCATAATTACTAAATTAGTATGAAAGTAAAGTATTTATTGAAA

TCTGGATATGATTGTACAAGTATAATTATTTTAATATCAAAACTGTTAAATTAAAACTAATTTACGGTAAAAA
CAAAAATTTGGAACCTTTTTGCATAAATGAAATGATGACATTGTTTTAAGTGTTAGGCTTAAGTTACGTTATA
ATTGTGATGATAGAAAGATTATTTATTTATAGAATTAAGACAATTCAGAGTAACATAA
GAAAAATGGCTAAAACAGGTATTAAAAGGTGACTTATATGAGACTGGACAATTGGAATCATTCATCCGATGGA
GGATCATCAATGTATGTATCGCTGGTCCCGAGTCAAAGGCAATCAAAGTATACGATGTCAGTAACAAACCAAA
ACACAACAAGAAATTAGCCATAATGCGTTTTTAAAAAATACGGCTTTTCTCATTTCGT
TACCATTTCATTGCGTTCTTATTCCATCATGTATTCATTTTCTTTATATTATTATATCCAAAGTTCTTTTAAG
ATTATTTATGAAAGAATTAAGCTAATATAATACTTACTGTATTTATTTAAATTATTATAGACGCGCTAAGTTG
TAAATACGCTTGGAAATAATGTAAATTATTTATTTTATTTAATAAAATTAATATTTAA
>1002050711_1 Heli.1-DT668553.3.5
GCACGAGGGGACCATAAAAGATTTGTCATAAATGTGACAAAGCTATAACCAGGGGTCCGTTTATCACCGCTTT
GGGTCGCATTTGGTCTCCAGAACATTTTGTTTGCGTTAACACTACTTGCCGACGACAACTACAGGACATTGGC
TTCGTGGAAGAGAATGGCCAACTCTACTGTGAATACTGTTTCGAACAATACATCGCGC
CTGCTTGCAACAAGTGCCATGCCAAGATTAAGGGGGATTGCCTAAACGCTATCGGCAAGCATTTCCATCCAGA
ATGTTTTAGCTGCGTCTATTGTGGCAAGTTGTTTTTAAACAACCCATTCTTTTTAGAAGATGGCTTGCCTTAC
TGTGAAGCGGATTGGAACGAGTTGTTCACAACGAAGTGCTTCGCGTGCGGATTCCCGG
TGGAGGCGGGCGACAGGTGGGTGGAGGCGCTCAACAATAACTACCACAGTCAGTGCTTCAACTGCACGGTGTG
CAAAAAGAATCTCGAAGGACAGAGTTTCTTCGCCAAGGGAGGACGACCTTTCTGCAAGATACACGCCCGCTAG
ATCACCTCAACAACTAATGTACAATTCTGACACTGTTTATACGACAACCACTTTTAGA
CCAGTTTCCATCTCTCCGTCCACGGTTCCTGCGAGCACTCCCTCTCCACAAAGAACTAGAAAATTAGAAACGC
CCCTCCACTTTGATGGAGGGCTGTTTTCTGATATACGAAGCGCAGATGGTAAGTTACTGAGCAAAGTTACCGT
GGATCGAGTTCACAGTTCCTCTCGCCACGATGTCATCGACTCTCCACATTTGTATGAA
GACATAGACCTGAATGGCAATAAG
>1002050712_1 Heli.1-EL602047.1.5
CATATACAATGGAAGTTTATTTTTTTATCACATTAGTTTATTTTTTTTGTATATAGATAGTTAGTAATATAAA
GGTGTATTTAGTCTAAACTGGACTAGACTAAAACAGCAATTTATTAGAGAAAAATGATGTTACAATGATTTTG
TGAATGATTTAGTTAAATAATTATGTTTATTTGACATCTCACGTACTAGCCGTCAAAC
CCAAGATCCCTAATTAAATGTTTGACGAATTGAAAAAGTTATAAAATATGGAATTATTTTAAATAAAGTAAAT
TTGTCTTTAAATAACTAAATGTAATTAACCAAAAACGGTTTTAGCGACATTTATTATTATTATTAATAATAAT
GATATTTACTTAATAATAGAATATTTTTGTACCCGCTTCATTGTAGTGTTAGTGTAAG
TGTTTAATATTATCTGTTTACCGTTATGTGGGGGTGATTATCGATTTTACTAGCTATATCGGTAATTTAATTC
ATTTGGATAGGTACAATCGTATGTGATTGTCGATTATTTTTTTTTCTTCCCGCTTAACGTCGTATGCTGCATA
TAGCAAGGAAGGCAAAGGGAACCTAGTGTTAAGACACTCCATCCCCACTTTCCACTGA
ATTTTTGTGTTGGGTGTTTATTAGTGGAACAATAAATTGGTATATACAAATAACAATTTATTCCACCAATTGG
GAATCGAAACCACTATCTTCACGTTTACAGTCACACGCTTGTGCTACTGCACCTCGACCGTCCGTTGGCGCAA
GTTATTAATTAACAAAGAGGTCATGGTGATAACTCTGTCCAAAAAATAATTTTGGACA
TTTCAAAGTATCAATTTCAAATATTTATGGTCGGAA
>1002050713_1 Heli.1-CV525695.2.5
AGTACGGTCGGAATTCCGGGTCGACCACGCGTCCGCTTGCTTGATTGATGCTCATTTTGTTCAGTAAACTATT
ATAATTTAAACAGCTATTTTAAGTTAAAAAATAATTAAATTCATCAAAGATGTTCCTAACTCGTTCCGAATAT
GACAGAGGTGTTAATACCTTCAGCCCAGAAGGAAGGCTTTTCCAGGTGGAATATGCCA
TAGAGGCCATAAAACTGGGATCCACAGCTATTGGCATTGCTACTTCGGAAGGTGTTGTGTTGGCCGTGGAAAA
AAGAATCACTTCGCCTCTAATGGAACCAACTACTATAGAAAAGATTGTAGAAGTAGACAGACATATTGCTTGT
GCTGTATCTGGTTTGATGGCTGATTCTAGAACTTTGATAGAAAGAGCTCGTGTAGAGT
GCCAGAACCACTGGTTTGTATACAATGAGCGCATGACAGTGGAGTCTTGTGCTCAGGCTGTTTCTAACTTGGC
TATACAATTTGGGGACAGTGATGATGACAGTGGCACTGCTATGTCAAGACCATTTGGTGTGGCTGTTATGTTT
GCAGGTATTGATGAGAAAGGTCCTCAATTGTTCCACATGGACCCCAGCGGTACATTTG
TACAGTATGATGCAAAAGCTATTGGTTCAGGAAGTGAAGGAGCTCAGCAAAGCTTGAAGGAAGTCTACCACAA
ATCTATGACACTCAAAGAAGCTATCAAGTCTGCTCTAACAATTTTGAAACAAGTAATGGAAGAGAAATTGTCC
GAGAACAATGTAGAAGTTGTAACAATGACTCCACAATCATTGTTCCACATGTTCACGA
GGGAACAACTCGCTGAGGTCATTAAAGATATACCTTAATTTGATTATTTTTCTAAATGGTTGGATTTTTTTTA
CAAATATTTATATATGTATGTATAATCGATTTTTAAAATATTTTTTTACTTCTGTATTTGTAATTTTTTTGTC
TTTATTCAAAGGTAAAATGGATGATTAAAGTCTAATAATGATGACAAATTCTGTTTGT

CTTTGTTTTAGTTTTGTATTTATAAAAATCCAACCATGTGTGTCACAAGTAGTAATATTTTAAGTAAATTGTA
TTTTATTGTATCATTTAGTGTAGTGTAAACAGTAAATAAATATTTAGCTGGCATAATTGGGGCCATCCATAAA
TTATGTAAAATTTAATGTCTCATGTTACGATATTATATCTAAGTAAAACTCGGAATTT
TGTTGAACAAACGTAATAGTTCCTGAACTACTTTCTTCAATAATTAAAATAATCTTGCTA
>1002050714_1 Heli.1-EL596102.1.5
GCACGAGGGCATTGCTTTCAATTTTCATTGTGTATTTTATTATATTGTTTGAACTTAGTTGCTGTTCATTGGA
AAAGAGAACATAATATGATTATTATATTTTCAAAACCTTAATTCTTATATAAGACATTGATATAAAACTTTTG
ATTACATTTGTTTATAAATGCTATTAGTATTTCAAGGAAAAAACAAAAAATAAATAGC
AAATTGTCTCTTTGTAAAAATGGAGAACGCAGAAAGCAGTGGAAATTCAAGAATGAGTCAGGAGATGCCTACA
TCTATGTGCCATCCAGCACACAAAGTGCATAGATTTGTAGCACTGATATTAATGTGTTTTCTTTGTTTTGGAT
CATATTTTTGTTATGATACACCTGGAGCATTAGCTGATAACTTTAAAGAAGATTCACA
CTTAAATACATCACAGTTTGCATTACTATATTCCATATATTCATGGCCAAATGTAATATTATGTTTTATTGGT
GGTTATCTTATTGATAGATGTTTTGGAGTAAGGTTGGGAACAATTATATACATGACCATTGTATTTATTGGTG
CAGTACTTTTCGCCTTTGGAGTGTACATTAATGCATATTGGCTAATGATTCTGGGCAG
ATTTGTATTTGGTATTGGAGGTGAATCATTGCAAGTAGCTGTAAATAACTATGTAGTGTTGTGGTTTAAAGGC
AAGGAGCTGAACATGGTCTTTGGACTTCAGTTATCCTTCTCCAGATTTGGCAGTACAGTCAACTTCTGGGTGA
TGGAACCTATCTACAGATGGGTGGCTAATTTTTATGGAGGATATGAGAGACTTGGTGT
AGCTTTGTTTATAGCTTCAATGACTTGCTTGGGATCACTTTTGTGTGGTGTGATACTGGGATGGATGGACCAT
AGAGCTGAGAGGATATTGGGAAGGCAGGAGGAGCAAGCAAAAGATGAACCATTCCATCTTTTGGATATTCTAC
ATTTTAAACCTGTATTTTGGCTTGTTACAATCAT
>1002050715_1 Heli.1-EL595936.1.5
GCACGAGGTTAAATTATAAAAAAAATATATAAAATATTTTTGTGGCAAATGATACAATATATAATTGTGGTGT
TTTTGTGAAATAGAACAAAAATATAGAATATATTTAAATAAAAAATTATGTGGTGTTGAAAATTGAGATAAAA
GAGAAACATATAACGTTGATTTGATAAAATATATATTTTTTATTAAGGTACATGAAAA
AAAAAACAAAATGCGTTGCTCTATATATTCTATTTCGCATACACTTATGTACATTTCGCTGCTCATATCCGGA
TCATCTCTAGAGACGTATATTTTTTTTTATATTTGGACCAACCTATTTCGACCATTACCCCGAAGTATAAACG
AAGGATTTGTACCAGATTACACACCACAGGACGGTCAAGTTTATGACTTCATTATAGT
CGGGGCTGGGTCATCGGGTTGCGTCTTGTCTAATAGACTTTCAGAAATATCACAATGGAAAGTCTTACACTTA
AAAGCTGGCGGTAACAAAAATTTCTTTTCAGATATACCCATATTCTCACCCTTCCTTTC
>1002050716_1 Heli.1-EL599261.1.5
ATTAAATTTAAATTTAAAGCATCTTTTTTAAATGTTTGCTCAAACATTCTGCCTACTAACCGGTAACTCTTAT
CGATTCACACTAGCCGAATTATAAATTCTGAGAGCGCTCCCCTGCCATCCGTGGTCCATTCATTACTACCATT
ACGATTTATTATATAATTATGTATATTACAATAAAATTTGTAACCTATAGAACGAAAT
TTCCTAAAAAGGAAAAGTTAACGGTCGCGAAATCCGCGCAATCGACTAAATATTCAATAACCAATTCAAATAT
TTTTTTTAAATTTAAAAACATCTGGAATAATATGACATAAGTAAATGACTACAATTTAGCTACATACATCCTT
TATGATAAAACTTTTGTCATTAAATTGGCGGACTCGGCGAACGTTAAAAGACGAAATA
AATAAGTTAAAAACTACAGTAATATTTACAAAAGCAAGAAATAGAAAAAAGAAAATCGCTTTGAGAATTTCAC
CTACCTATATAAAAATACGATTAAAACTTTAGTTTTATTTTTTTAATATATAAACTTTATTAATCACATAAA
ACGGGCACTGGTTTGTTCGGAGTTCGGACTGAGATCGTTCTTAGTTCGTACCGATTCG
AAACACAACACTGGGCAACGTAACACACGATCGGTATAAGTTAGTTAGTTGTATTAAGGTGTGTTTCGGTTCA
CTTCCCGCGTTAAGTCGGTCCCGCACTTAGCACCCATTTTCTTTACATTCGATTCTCTTAAGGCTTTACTAAC
CCTTTACAGTTAGACGAAACGCGCAGACCGATATACATACATAGAACGAGTCTTACTC
GCTAAAAAATCGATATTTAATGTTTTTTTATATAAAAATATTGGAAAATCGGAATAATAACTGTCACTGTGGG
ACTGCGCGTTCCGTTTTGGACGCACCTGTCGATTCTTCAATCAACCACTCTAATATACTCTGTAGCTGTAAAC
TCAACGGGAACTAGGCTAGCTTAAACCTGACCTTTTAACGACTATCTAGTTAAAAGTA
TGATTCATTAACATTGGACCACAAATCGTCCTCGTG
>1002050717_1 Heli.1-ES586298.1.5
GGCAAACCTGCCAGCTATGAAGCAATCAAACAAAAGGTTAAGGAAGCTGCAAATGGACCATTGAAGGGCATTC
TTGGATACACTGAAGACCAAGTTGTATCAACTGATTTCATTGGGGACTCTCACTCATCCATCTTTGATGCTGC
TGCTGGTATCTCTCTAAATGACAACTTTGTAAAACTGATCAGCTGGTATGACAATGAA
TATGGATACTCCAGCCGTGTCATCGATCTCATTAAGTACATCCAGAGCAAGGATTAAATTGTTGATTTTAACG
AAACATAGATGATTGTAATGTTGAGATTACCAATGCTTATTTAACTATTAAGTTTTAAGTGTAGCTGTGTATT
ATTCTAAATTATTAAACTACTTCGACAGTAGTGATGTGCTTGTGTTATCTGTTTAATC
AGTAACTGTATACAAGAATTAAAATAATTTAAAAATAAAAAATCACTGCGAACAATAAATCTGTTCTATAAT

>1002050718_1 Heli.1-EC091342.1.5
CGATCTTTTCGTCCATGCGGAAAACGCCCCTTTCGTCCAGAGTCTGCTTGAAAACGCGGCACGGGCCATTGCC
ACCAGCATTAATCTGTTCGATCCCGATGCGGTGATCCTGGGCGGTGGCGTGATGGATATGCCCGCCTTCCCAC
GCGAGACTCTCGTTGCCATGACCCAAAAGTACCTGCGCCGTCCACTGCCGCATCAGGT
CGTGCGCTTTATTGCCGCCTCATCTTCTGACTTTAATGGCGCTCAGGGTGCAGCAATATTGGCGCATCAACGT
TTTTTGCCACAGTTCTGTGCTAAAGCCCCATGAGTAAAACGTTGGCACAGTAGCGCAATGTCCCGCTGTGCCA
ACGACAGGTACAATATATATAATAAACACATAAAACGACTCATATTATTTTCTGCAAT
GATAGTTTTACTGTAATTTTCCCTCTTCAGCACAAATGCCTAATAAAATGGCGCGTTCCGCTCACACATCCAC
TTATTAAAGTCGCAATTTCCCAATTATGATAACGCCAGCCGTTCCGGACGTATTTTGCATGGATTACATATTT
ACGACATAAATGATAAATGGAGATTTATATGAATAACTCTCGGTTATTCCGTTTGAGC
AGGATTGTTATTGCGTTAACTGCCGCCAGCGGCATGATGGTAAATACCGCTAACGCGAAAGAGGAAGCGAAAG
CCGCCACTCAATATACCCAACAGGTTAATCAGAATTACGCCAAATCATTACCGTTTAGCGATCGTCAGGATTT
TGACGATGCCCAGCGTGGATTTATCGCCCCGCTGCTGGATGAAGGTATTCTGCGTGAT
GCGAACGGTAAAGTTTACTACCGCGCGGACGATTACAAATTTGATATTAATGCCGCAGCGCCGGAAACCGTAA
ACCCCAGCCTGTGGCGTCAGTCGCAAATCAACGGTATTTCTGGCCTG
>1002050719_1 Heli.1-CX700434.1.5
AACAGTATGCCATGGATGTGTATGAACTGAACCGAAGAGTTAATGCTTCTGAAAACATTGTGGGATGGTGGGC
AACCGGTACTGAAGTAACAAACCATTCCTCAGTAATCCACGAGTATTACTCTCGTGAATGCCGTGAACCTGTC
CATGTCACTTTAGACACTTCCCTTGCTGGAGCTCGAATGGGATTACGGGCTTATGTGT
GTGTAGCTCTGGGAGTGCCACGGGGGAAACAGGGGTGCATGTTCACACCAATTGATGTTGGACTAACATATTA
TGAACCTGAGATAGTAGGTTTGCAACTGTGCCAAAAGACAATGGGTCCAGGTGGACGCTCGCGTCAAGTACAA
CCCATGGTGGACTTAGCGCAGGTCGCTGAGGCCGCTTCTAAACTATCAGGATTATTGG
ATCCGCTAACACCTCTTCCACATACTGTACCCAACAATGAGGTTGGACGTCAGCTGCTGGAATTAGTCAGTGC
GTTGCCAGACCTCGCATCTAATTCATTCGCTGACTCCTTTGCGTCTAGTGTTAAGGATTTGCTTATGGTGGTA
ACACTAGCACAGCTCATCAAGACCCAACTGCAGTTGAATGAGAAACTTACATTACTTA
CATCGCAGTAATAATACACTATTGTATAATAATGTATGGAGAACACCTTTAAAAAACCATAACTACTAATAAA
TTACAATTTTGTACACAAATACTTTTTATTACTGAAATAAGATTATAATTTATGTCCACAATAAAGATAAA
>1002050720_1 Heli.1-EL602379.1.5
CCCCTATAAGCCCGGGCTGCAGGAATTCGGCACGAGGGTTAAACCCTTAAAGGCCTTCCAAGCCTTCAAGGAC
CGTTTGCAGAAGAAACTTGATATACCAGATAAAGAATGGGAAAAGTACAATTTTGCTGTAGTTACAAACGGCA
GACCTAATTATATAAGCGAAGGGGCAACAATTAATATATTTGACTTCAGGCCAAATAG
CAATACAAACGCGACGGGGCGGCCCTGGCTCGGGCTCGAGCACATAAACAAGACGCCCAAGCGCTCGCGCGTC
AACTACCTGGAGAAGGCCATCAAGATATACAACTGAGTCGAGGCTCCCGCGCCGCCGCCCCCCTTATAGCACA
CCCTTTCCCCTACTCATACTATATGCAAAGCTACTGGACTCACCTCTAATAATGCCTT
TTCTAATGTACTTGCTAGATTATATTTGTATGTTATATTTGTTTATATTTTTTTCATTCATTCTTATGAGTGT
ATGTTTCATTCGTTCGTTGTCTTTGTTTCTTGATAATAATATGTAAATATTATAATATTATAATATGATTTAC
TATTTGTGTATCATCTTATTCTTTTTTCAAACAATATTAAATTTCAAATAAAACTAGT
TTTATATTCAAAATACTATTGCTTTTTAAAGACTTAACGTGCATTTTGATTGAGCTAGACAGATTTGCATTTT
ATATATAGAATGTGTTATTTAAAAGTTGTTATTACATTTATACATTCATAGAATCGAGGCTGCTCAATTTTGT
TTATATTATATTCACATACCGATAAAAGAAATTGAGAAATGTGCATTATGATATACCA
AAATTTAATTTATTATAGTAAAGTCCAGTAGCGATGATATATTTTTCT
>1002050721_1 Heli.1-EL600076.1.5
GCACGAGGCGGAAATGTATTTTTCTACGGCGGGATGTATGGAGGGGTTTTCCCATTAGTTTTAGACTAATATC
ATTATTTTTAATGTCTAACATAACGCAACAGTATTTTAGGGAATGTAATCTAAATTTAAGTTCTCTCATAGTA
TATCATGATCACGCTATAGACTGTCGGTTATAATATCGTATATTAAAATAATTTTTTA
TATGTATGTATTTAGCGGAGGTTCGTCGTCGCGGGTTTGTGGGTGTTAGGGTCGGTGGATGCTTTGCCCGGAA
TTAAATAGTTCTAACACTGGATCTGTTTGATACTGTGTTACATACAACACGTTTAAATCCGAGACACAATATA
GCGGTATAGACAATTCCAGTGTTTTAACTCTGAATACCTAAAATCAGATCTAGTCCAC
GTTTAAATTTAAGTACACAAATTCTTATGATCTCAAAAGGAGCACAATAAAACCAATAGTTATCGTTAGGAAG
TGCCTCGAGCGAAGTCGTTTTATATTTTTTTAAACGTAGGATGAGGCGCCGGCCCTCGGCGTCGACACAAACT
GCTTCTGTGATGTTCGCTCTAGTGATATCGATTACTCGGTTAGGCACAAATTATAACG
ATACGATGTAGGGTTAATCGAGTCGGCGCCCGGTTATCGATTTTAATCTATAATCTTTAATAAAATAGGCTTT
GCCGATTGGTGGACGGGTGCGGCACCGATCGGGTGCGGGAGAAGAGGTGACGAGTGATGGAGATATAAAATTT
AGATGTATGTATGTTTTCCGGCGGTCGGTCCGTCGGTCCGTCGGGCGGTCGTCTGTAT

ATATGTATCTGCTTGGCGGCGGGCGAGCTTGCTGCTTCACTCTATGTTAGCAACGACTAAGAATTAGGTGTAA
GTGAGCTTCAATAGGTGAAGACGTGAAAACTTCTGACCAATTTGCCTTGGGTATTGTGTAGCCTTTTAGACTC
GATCGATACGAATCATGTATAGCGACGCCAGTATAGCTTTCTGATATTTCTAATTATT
TTTTTACGTTCTCATGTGTTAATTTTTAGTGTTCTACGGTGTTTATGTTTATTTTATTTTTTTTATATTTTAT
GTATTAGTATTAATTATATTAATTATTAATATGTGCTTGAGTTTGTGAGGTATAAGAAATTGCTGTTTGAAGT
GCAGGCAACTTTTTGTAGTTTCATTGTCGTATTGATTAAAAAGATA
>1002050722_1 Heli.1-CO729677.1.5
GTCGGAATTCCGGGTCGACCACGCGTCCGCATCAGTTCGCGATGCTGTCCTTTAGTACCCGCTTACTTCAACG
ACTCCCAGCGACAGGCAACCAAGGATGCTGGAGCCATAGCTGGCTTAAACGTCCTAAGAATCATCAACGAACC
CACCGCTGCAGCTTTGGCATACGGCTTGGACAAGAACTTAAAAGGCGAAAGAAATGTA
CTAATCTTCGATCTTGGAGGAGGTACTTTTGACGTGTCTATTTTAACAATCGACGAAGGTTCCCTTTTCGAAG
TCAAGGCAACCGCTGGAGATACTCACCTTGGAGGCGAGGACTTCGACAACAGGCTCGTAAATCACCTAGCCGA
GGAATTCCAGCGTAAATATAAAAAAGATTTGAGAACTAATCCACGCGCGTTGAGACGC
CTCCGCACCGCCGCCGAGCGTGCCAAACGCACTTTATCATCGAGCACTGAGGCGTCTATTGAAATTGACGCAT
TATATGAAGGAATTGACTTTTACACAAGAGTATCACGGGCTCGTTTTGAAGAGCTTAACTCCGACCTTTTCCG
TGGTACTTTGGAACCGGTGGAAAAAGCTTTGAAAGATGCGAAAATGGATAAAAGTCAA
ATTCATGACGTTGTGCTCGTGGGCGGGTCTACTCGTATACCGAAAGTTCAGAACCTACTGCAGAACTTCTTCG
GCGGCAAGAAACTTAACCTTTCAATCAATCCTGACGAAGCCGTAGCGTATGGAGCGGCTGTACAAGCGGCCAT
TTTGAGTGGCGAAACTGATTCAAAAATCCAAGACGTCCTCCTCGTCGACGTGGCTCCA
CTATCCTTGGGTATCGAGACCGCGGGCGGAGTGATGACAAAGATCGTTGAACGCAACAGCAAAATTCCGTGCA
AGCAATCGCAAACCTTTACTACCTACTCCGACAACCAGCCGGCCGTGACCATTCAAGTTTACGAGGGCGAGCG
AGCCATGACCAAGGACAACAACCTGCTTGGTACGTTCGACTTGACTGGCATTCCACCA
GCACCTCGTGGAGTGCCAAAGATTGACGTGACATTTGATCTTGACGCGAATGGCATCCTTAATGTATCGGCTA
AAGAGAACAGCACCGGTCGTAGCAAGAACATTGTCATAAAGAACGACAAAGGTCGACTTTCTCAGGCTGATAT
TGACCGAATGTTATCTGAAGCAGAGCGTTATAAGGAAGAAGATGAGAAGCAGCGTCAG
CGAGTAGCAGCG
>1002050723_1 Heli.1-DT664962.3.5
GCACGAGGATACTAGATTACTAGAATCAATTGTAATTTGTATTTTAGTTGCTTAGGCGTATCCTGTGAATTTA
AGCGCAATATTTATTTAATTCTTACGTAACTATCAACTTTCGTTATCTTGTGAACACAATAAGTAAATATTTA
ACATAGAAATGGCGACACCGCCGATATACAGTCCGGCGATACCATGTGTGCATCCTAT
ACCAGGGGGCCTGTTCCCCGGACGCATGATCAGATTTCAGGGCAGCGTGCCACCAGGCGCCCAAAGGTTCGCG
ATTAATCTCCAATGTGGTCCCAATACTGACCCCCGGGACGACATCGCCCTCCATCTTAACTTCCGTTTCATAG
AGATGTGTGTAGTTCGTAACCATCTCACGACGATGAACTGGGGCGTCGAAGAGACCAG
CGGAGGGATGCCCCTGGTCCGGGGGGAGTCTTTTGAGGCCCTTGTCCTTTGTGAGCATCAGTCGATTAAGGTG
GCGCTAAACGGCGTTCATTTCTGCGAGTTCCCCCACCGTGTGCCATTCCAGCGTATCTCCCATTTCACTATCG
ATGGCGATGTTATGATACAATTTGTAGGCTTTGAAGGTGCCCAGCCACCACCCAGCCA
GATGTACATGTCTGAACCTCCGAACTATGGGGCATATGGGGCACCACCGTCCTACGGAGCCCCAGGATATGGG
GCACCTCAGGGCTTCTATTAAACTGCTNCAGCGTAAGCGGTATTCGATACATGTTTATACGTGCATTGTGAAT
CGACGTCGAAACATTATTTATTATTAAGCCTAAAATACTTTTAGCATTTTTCTTCTTT
ACAAAATCTATT
>1002050724_1 Heli.1-DT665896.3.5
GCACGAGGCGGACTCCGGTCACCTGGCGTTCAACGAAGGAGAGAGGCTGCGGCTCATACTGGAAGTCGACGAC
CAATACTTATTGTGCTGTAGAGGAGAACAGAAGGGTTTAGTTCCGCGGGATGCGGTGCTGTTGGAGGATTTCT
GATATTTGCAGGCATAGCCGGACTGGCAGCCGCGCTGGCGACCCCTCCGCGTGGCTGC
CAACGTCGGCAAACATTAGTGCAAAACACTCGAATGTATGTCGGTACGGATTTACTCAGAAACTAAAGATACT
CTTGGTGATTAAGTGAAAGTGTGAACTTAACGTGTCGGTTAGTGAAAAGGGATATTGTATTGTAAATAGAAAC
TGGATCGTGGATATTATAGTAAATATTTGATGTTACCCAAATATCATCTAATAGTAAG
GTCCTCGGCCAGGGATTTTCGTGACTGTGATTTTTTTTATTTATTTACTCGTTTACCCTTTCATAAGTTTTAC
CTTTATGACATACATTTAATAAGTATTAAGTATTTATTTTTTTTATTTAAAAAAAATCTTTGACTAATCTAGT
ACCTGTCATGGCAATCTCTGCCCGACGGCCGCATTTGACATCACTAATTCGCACATTT
CTAACTCAGTTGTAAAATCTAGTCTTACGTTTAAGGCACGACTCGGAACTGTATATATGATACCTTGTTATAA
GAACACTACTTTCGGGAGTATTTGCTAACGAACGTACATACATACACTTAAACCAACGTTCGTCGGAGATACT
CTCGCAATGGCATGTGATATATTTACTTGTGTTAAGAAATCTCGTTAAAGAACAAACT

CTGTTTTATGAAACGCTCGCGACGGCGGACGGGAAGGCGCATAGTAAATTCTAAGTAAATCTATTGGAATTGA
TGTCTAAATATATCTAATCGAAT
>1002050725_1 Heli.1-CV525817.2.5
CCACGAAACCCCGGGCGCGGATCGGCCGAGGCACAGTGACGTCGAAGATGGAGATCAGCAAAGAAAGGCTATA
TCTCTGATGATGAACTTGATGTAAAACCAGTCAAAAAGTCACAAAAAAAAGCTAAGAAAAAAGGTGGTGATAT
ATCTGATGATGAGCATGATGACAATAAGTCCATTGTTAGTAATGCTACAAGTACACAG
TCTAAGACTTCAAAACAAAATAAAAAAAAGAAAGGAAAGGGAAAAAAAGATGATGATTGGTCTGAAGAAAACA
GTGATATTGAAATAAAAGAAGTTTCTGATGAAGAAATAGCAAAACCAGTTAGTAAAAAA
>1002050726_1 Heli.1-EL598333.1.5
GCACGAGGATTCGTTGATTTTTATCGGAAAACATCCAAAGACTTGCTTAGTTCACAAGATAGTGCTGTTTTCG
TGTAGTATTCTTTAAGTAAATATTATAAAAGTTAACTTAAAAGGAATATCTATGCTGTATTTGAATTATATTG
CGATGTTGGCGAAATTGTAGCAGCCTTCACGGATTATAAATAATTGGAACAAATTGGC
AATGCTTCATTGACACCGTGTAAAATGTATATAGTTAACATGATTGTTGTGTTGTTTGTGTTGGTGAGTGTTG
TTTGTGGGGACGTGAACTCGACGGAGGTTAAAGGGTCCCGCATGGCGTGGATGAAGAAACTGGTGGATCACCA
CGACTGGTTGAACATTTTTAATGGAAATAACACAATACCGCAAGAGTGTGAAAGAGAT
TTGAAGCTGTATATCAATGCTTTGAATCAAGGTCAGCTGTGGGCTTCTAAAATATTCGACGCGTCCGGCCAAT
ATATTCCCAACATCCTGTTCGGCAATGACTACTGGATGGGCTCATGGAACGCGTGTAAGGATCTACAGCTCAN
AAAGTACTATGCAGAAACACCACCTTTCTCCACGGCGTTCTACGTAGCGAGAGTTAAC
CTTACCGTTGACGGGGATCATATACAGCCGAGACAAATGCTAGTCGGGCAGTGTCTCCCAGCGTCGTGCAGC
>1002050727_1 Heli.1-DT664215.3.5
GCACGAGGACTTTACTATAATTAGAAATTGTTTACGCGTAAACAATTGAATTAAAAATATTTTAATTAATAAT
ATAATAATGAATATCTCTATAGAAAAGAAAAGATCCGATTGTTCTGCATTGCCAAAAGGTTGGCAAAGAGAAG
AAATAATAAGAAAATCTGGATTGTCTGCAGGCAAAGTTGATGTTTATTACTATAGCCC
AACAGGTAAAAAGTTTCGCAGTAAACCCGAGCTAGTGCGTTATCTTGGTGATAGTGTGGACTTGTCGTGTTTT
GACTATCAACAAGGTCAAATTAACACAATGCTCCTGTGCAAGGCCAAGAAAGCTCGAGCTCAATTTGATTACA
GGGGTGTCCGTTCAGACACTTCACTTGTGCCCCCGATACGACAAACTGCCTCTATATT
TAAGCAACCAGTCACTGTTTACAAGACTCAAGAAAGTAAAGTAAAAACTGACCTCAAACAAGGCACACAAGAA
AAGCCGAAACAACTCTTTTGGGAAAAACGTCTAGAAGGACTGACTGCTTGTGATTCCAATGGAGTAGTTGGTA
CAACATCTCTACCAAAATATATTAAACCTCTGGGACCATATACTTCAGATGCTACAAC
AATACAGTCCTTAGCTACAGCTTTACATGTTTCATCCCAACCCATAACAGGTCAAACTGGATCAAAACAAACG
ATAATAGAGAGCCCGGGAGTTTTTTTAAATCCGGACCAGCCATTGATAGCTGCGGTGACCATAACAAAGGATG
ACCTGCGTCGGCAAGAGGAGCGCGTGAAGCGCGCGCGTTTGAGGCTCCACGAGGCCCT
AGCGGCCGCATAGGGGTGCGCTGGCGGGGTCACCTCCGCCGCAACACAGGAAGAGCCTGTCGAACTGATGCTC
CCAGACTCTACTGCTGATTCACCTAAGCCCTCTAATTCGGAATGTATTGCTGATAGTACTAATTAGGTTTTTC
TTAGAATAGGTCTCANAGACTTAAAAGTATTTTTTCTTTTTTCTCTTAACTGTCATCA
GTCGATAACGACAAAAATAAGTTTGAATGCATTATTCATGTCGTTTGGGTTTTATAGGCTATTGTCTCAGCTT
ATTTTTTTTTTATCAGCTTCACG
>1002050728_1 Heli.1-EL603118.1.5
GCACGAGGGCACTGCCGACCTGGCCGCTTCACTTTTACGTTAAAAACGTATTTATTATTTTTTTTCTGTGATT
GTGGTTTGAATTTTGAGGATTTTTGTGTGTTTTGTGTCGTTTGATTAAAGAGATTGTAAAGGTGCCCATCAGT
GAACGTGACTGTGATGTGCAACAAACTGCCAACAATCGATTGATTGAAATTAATCGAT
TAACTGTTAAACGAATAATTAATCGAACCAGTATCTCGTCATTACAAAATGTGTTTACGCGGTTGCTAGTCGT
GGAACTAAACGCGTAAAAAAAGTTTATTTTTTAGCAAGAAATTGAATTGATTTGTGACTTAGTGAAGTGAAAT
ATACTATTGGGCGGAGCTCAATCTCGTCGTGTTTTTTCTTGATGTCAGTCCGCTCCAA
AAGTAATGGATAGCCACAGTAGATCGGCGTCCCCCAGGGAGCCAGCGCGGCCGCAGCACCGTCCTCCGTCCCC
ACCTGCCTCGGGCTTCGACAACAGAGCCTACCAGCACGATGAGGCTGACCCAAACCACAACGACTCCTTCACT
TCTAACGGACCACACCAACAACAAAATGGCCACAGCAAAGAACCTAACGGTGACACAA
AAACGCTGGAAGCAGTAAATTTAGAACTGATCAACCTTACACCTAAGAATGGATCGAAGAAGAAGGATGTGGA
AGTCGATATGAATGCGACCAACCCGTACGATGAGTACTTCGTTCCGGTGAACGAGCACAGGAAGTATATGAGA
GGAGAAAAGCTCTACGTGACAGCTGACAAACGCGGTGAAAAGGGCGGTTGTAAGCGGC
CTTTGTGCTGGACATTATTAGGACTAATCGTAGCGGCCATCGTCGCGCTCATTGTCTTAGCAGCAACTGGCAT
CCTCTTTAGTAACTCGCCTACACCTCTCGAACAGTACAACGCGTCGGTAAGCTCCGCGCGCGCGTTTGGCGGT
ATCGCAAGTGGTCATCACCACGACCACGACCACGACCACCACCACGACGACCACGACC

ACAGCGATCAGACCACGCCCCCGCAGTACATGCCGGAAACACAAAACGATGAAGCACGGAACCCTTCGCTCGT
GTCTGACGAATCTGATGATATGTCTATGTATGTAC
>1002050729_1 Heli.1-DT665140.3.5
GCACGAGGTACAGCCGTGAGTTGATTTATAAGTTGTGTCATATAAAGATAATATATAGACAAAAACTTTAATT
TTATCCTAAATCAATCAATTAGATGTAGATTGAAATTAATGTATGTATCTTATAATTTATCAGAATATGATTT
GTAATGGTACAAATGTCAAAAAAATATCGCTAAATAAAAATGTTTATATTAATAATAA
TATAAATAACTTAGTACACTACAATAATATAGTAAATTTTCGATTCATATATCCCTGCGGACTGAGTGTTCAA
AGGGAAGTAATACAAGACTCATTCTAAGTTCGTTAACTCTAACCACAGCATAGGTGCAGAGGGCTACATCGAA
ACTATTAACTTATTTTTCAATTAGAACCATAGGTTCCAAACTTATCGCGGTCAATCAA
ATAAACCTTTCAGCTTTTTAATATTGTATTTAGATTAGTATAAATTCCAAGGCAACGTTATTTTACATTTCAT
CCATGCGTGGCCCCGAAATATTCAAATAATCTTTTTTTTATAGAACGTATTTGTCAGGCAATAGCGTTTTCAA
ACATAAAACGTAATTTATAGGTTATTTAAACATTTCAATGTCGTTCTCAACGTCGAAT
TATAAATTGAACCACTCCTTTGAGTGCAAGAATTCGTTGCGTTGTTAAATTACCAAATTAACATTGAAATTAT
ACTGATATTAATATAAGGCGAGTGTCCACTTAAAAGTATTAAATAAGACGTTATGATTTTCGTTATAAAGATT
GGGCAATAGAAAAATGTGTATTGTGGGTCGATGCCATTTTAGGAAATCCATATTTAAA
TTAATTCCTCTC
>1002050730_1 Heli.1-ES587278.1.5
AAGAAATATCCAAGTACACTCAAATGTCAAAAAATCTTATAACTGAAAGGAAAACGCGTTTCTTTACAAAATG
TTAATCTAAAAATGCTCAACTATTGAACGAACCCTAGATATAAACACATAATATAAACTTCATTTGTAGATAA
TATTCACGACTGCGCCAATTCTCATACGTTTTATAAAATCCCTCTAATTTATTATTTT
ATGTATGTAGTAGGTTCAATTCATACCCCGGACAAAATATATTTTTGCGATGAGAAACTTTCCATTTCTTTCT
ATTAAAATATGTAGATATGTATGTATGTTTATCCCTATAAAATAGACTTTTTTAATAATGAGGCACCAGCTTC
TTTAGGTTGTCTAAATTTTTTATTTATTGTTTTAATATTGTATAATATTACTTGCCGT
TTAGTATTCTAATTAGACATAAGCATAGACTATTTGGGATATAGTTACCTATTATAGCTTCGACATTGCCAAT
CGACTTGTAATATATTGCTAAGGGTTAAATAAGATGTATTTTAAGATTATTTTTATACAATAAACTTTTTC
>1002050731_1 Heli.1-DT666886.3.5
TCTTTGCCAGAGATAATCTATCAAATTTTATTGAATGGTGCCGCAGAGCCTTGGGCATCCTCGATTTCGTATT
ATTCGAAACTGATGATTTATGCCTGAGAAAGAACGAAAAACACGTCGTGCTATGTCTCCTAGAAGTTGCAAGA
AAAGGCGCCGTATTGGGTATGCCAGCCCCGTTGCTTGTTCAAATGGAGAAGCAAATTG
AGAGGGAATTGGCTGGAGAAGAATTACGCCCAGATGATTCTGCTTTGGGCTTGGTACCGTCCGGACCACAGCC
TCAGTTGGTGACCAATGATTTGAGGAGTTTGGACGAGAGAGTCAGAGATCTGGTAGAGAGATGCTCTTGCCCC
ACACAATTCCCCATGGTCAGAGTTTCTGAGGGAAAATACAGGATCGGTGACACTAGAA
TGCTGATATTTGTCAGAATCCTTCGTTCCCACGTTATGGTGCGAGTTGGTGGAGGATGGGATACCCTCGCTCA
TTATCTGGACAAACATGACCCTTGCCGTTGCAAGGCTCAGCATCGCACTTCCTTATCAGCCCGTCTTGCGCGA
CCTAAACACGACCTCGCCGGCGCCACTGTCACTTACGAAAGACCCGACCCTGGTCCGA
CTTCCCTCCCATACAAAGATTACAAAGAACCCAAAACATATGAGCCAATGTCATTGCAATACAATTCAAAGCT
TTATGCTGACGATCCAAAATCAGTACACTATCAATCGAATAACAAATTAGATGGCAATAGCTTGCCATATTTG
GGTGACGTGGACAGGGCTGCTTCTCCAAACAGGAAATACTTGACACCTAGAAACAACA
GCCCTGGAAGACATTCTTCCTCACCAGATCGTAGGACCAAGATTATCACTACGAACCATTTGGTTCCATCGCC
TCATGCTGTGAGGAATAAGAGTCCAAGGCCAGTGTCTCCTGCCCCAGCGACGGANAGTGCTTCAGATAATGGG
TCCGAAGTGTCAGATGAAGGTTACAGAAGCCTGGGGGTGGTAGCCGGATCCACTCAGG
GCTCACCATCAACGAAAACGACGAACAGATACTCGCTGCATAGTCAGAATTCTATGGACGACGCTGATTTTAG
TGAGCGTCTCGACCATGACGACAGCTGTCACCTAGATAAGAACGACCGCGTCGATGACTATGTCAGTTTGAAC
ACTGGGATACGCAAAACTGATTTCTCTGATACAT
>1002050732_1 Heli.1-DT668599.3.5
ACTGAGTCCGACGTCTTCCCCAAATGGCGCTACAACCATATTGCATGGTGGAAAATTAATACCTCATCCACCA
GGTATACCAGGTCCAAATACGCCTGGTTTATATATGGGGAATCCGAGTTCACAAATAAAAACTAATGATAGCT
ATAAAAATACAATAAAGACTACATATGAGAACCTTCAAGAAAATATATCGTATAATAT
ACACTCAGCTAAAGGATCTTCAGTTATAAACTACCACAGAATTCTTCATACCAGAAGTCCAAATATGAAATCA
CATCCAGATATAGAAAGATTTTTTTCTCAAAAGAATTATGGTCCGGAAGTTCAGAACAATTCTGCTGTTCCAA
TTATTAGGATAAAACACGTAGATGAGCCTATTTCAGGGAAACCCTTTTTAATAAATAA
TACAAGGCCGACGGTGCGTAGTGATGGAAGTGTTAAACGAAATGCTGAAAGTCTATCCAGAACCCCGGTTTTA
AAAGAGAATACGAATTATTTGTCAAGTAAAGCTAAAAATAAAGATGCTAATTTAAATCTTTCAGCCAAAATTG
TAGATATTAAATCTGAAAATGAAATAAAAAACTTTAATTTCCAAAATTTAATAACTAA

ATCTGAGATCTATAATAATCAAATTCAAAGTTCTACGGAGGTCCAAAAAAATTGTAATGAACAAGAAATGGCA
ACTTCTGTCTCCATTCAAAATGAGAGATCTGAGACATCTTATTTTCAAAAAAATGTAGCCTCAAAGCCTGTTA
CAGAAGAACGGAAACCAAAATTTTTGCGACCTTCTACTCTGCCACTGAAGCCAGGAAC
TTTCACTCCAAAGAGGCATCACGGGATCACGCCGAATGCAAACACAATGCCTTTAGTATCTCCAGAAACGCCC
CGTCCGGCTAAAGCTTACGGTCAACTTTACTTAAATGGCAATGCATATACCTATTTAGGTCTCAAATGCTCAA
CGAAAGTTTTTTACTGTACAATAAATCGGCCACAGCCGACATATGTGCCGAATCAACA
TTTTCTGTCCATGTACAGTAATTGGCAGTTATTATCTGAGCTGACGCCAGACCCGCTGGGATTGTCAGCATCG
TCTGCTATGTCTTTATATGACTCACGTCATCGACCGCAAAACGTGGCCGTCGCTGTGATTAAACAGGATCTCA
TATTGACTCA
>1002050733_1 Heli.1-DT664796.3.5
CAAATTATTAACAAGCTTGGTTTTAGTATATTTTGTGTTTATTTTGAATTGTTACTTATTACTTTTGAAATGG
CTCCTATCAACAATAAAGAAAATTTGTACGATGGATTAAGTAATGTGCATATTAAATCCCCTGTCAAGAATGT
GCTCTCGGCACGGAATGATCCTAATGGTGATACTGACGATAAAATGGACATAGTCAAA
GAAAATAAGGAACAAACGTTTGACCCACAACTTGAGCCCCTGCTGAGGGAAAACCCTAGACGTTTCGTTATAT
TCCCAATCCAATATCCAGACATATGGCAGATGTATAAAAAAGCCGAAGCATCGTTTTGGACTGCGGAAGAAGT
AGACTTATCCAAGGACTTAGCAGATTGGGAGACTTTAAAAGATTGCGAGAAACATTTT
ATCAAGCATGTCTTAGCATTCTTTGCGGCTTCTGATGGTATTGTTAATGAGAACTTGGTGGAGCGTTTTTCAC
AAGAAGTTCAAGTAACTGAAGCTCGATGCTTCTATGGTTTCCAAATTGCTATGGAAAATGTTCATTCAGAGAT
GTACTCTTTATTAATTGACACATATATAAGGGATCCTAAGGAGAGGGATTTCTTATTT
AATGCAATTGAGACTCTACCGTGTGTTAAGAAGAAGGCAGACTGGGCATTACAGTGGATCGGGCAGCAGACTG
CAACATTTGGAGAACGTATTATTGCTTTTGCCGCAGTTGAAGGAATATTTTTCTCAGGCAGCTTTGCTTCAAT
ATTTTGGTTGAAAAAACGAGGCCTGATGCCCGGATTGACTTTCAGCAATGAACTTATT
TCTAGGGATGAGGGACTACACACTGACTTCGCATGTTTGATGTTCAAACATCTGGTGCTTTTTTTTAGTCCGG
CGCGTGTTTTGTCGATCATTAAGGATGCGGTAGCCATCGAGCAGGAGTTCTTAACAGACGCGCTGCCAGTGCG
CCTGCTTGGCATGAACTGCGACCTCATGTCCAATTACATAGAATTTGTGGCTGATCGC
TTGCTTGTTGAACTTATTGGTGAAAAGCACTACAACACGCAGAATCCATTTGACTTTATGAATCTCATCTCAT
TAGAAGGCAAAACAAACTTCTTTGAAAAGAAAGTGGGAGAATACCAGAAGTGGGGAGTGATGGCACAACCTAT
GGACAATGTGTTCACTTTAGACGCTGAATTTTAAATTTAAGTTTAATTTATCAACTTA
ATTTTGAATATGTATATGAATCTACTTATAGGTTAG
>1002050734_1 Heli.1-EL597163.1.5
CGGCACGAGGGGCTTTCAAAGAAAGGCAGCGTAAAAGAGCTATTTAACGTTAAAAAACTATTTTATTAAGTGA
AAGTTAGTTAGTTTAGTTTGTGATAAGTGTATTGTACATAATGTTGGACTTAGAAATCGTTCCGGAACGTTCC
CTGGGCTGCGATGCCTGGGAGTTTGTACTAGGCATGCACTTTTCTCAAGCAGTTTCAA
TAATACAGAGTCAAGTGGGGACGATACGAGGGGTACAAGTTTTATATAGTGATCAGAATCCCTTATCAGTAGA
CTTAGTAATAAACATGCCGCAGGACGGCATACGGTTAATATTCGACCCGGTCGCGCAACGGCTCAAGATTATA
GAAATATACAACATGAAATTAGTTAAGCTTAGGTATAGTGGTATGTGTTTCAATTCGC
CGGAAATAACGCCGTCTATAGAGCAAGTGGAGCACTGTTTCGGCGCGACGCACCCCGGCCTCTACGACAGCCA
ACGTCATTTGTTCGCGCTGAACTTCCGTGGCCTGTCATTTTATTTTCCTGTTGATAGTAAATTTGAGCCTGGA
TACGCTCACGGTCTCGGCTCCTTGCAGTTCCCCAATGGCGGTTCACCCGTCGTGTCGC
GAACAACGATATATTACGGCTCTC
>1002050735_1 Heli.1-EL601160.1.5
GCACGAGGCTAAACCTTAATCTTGTTGCGGTTTTCCTGAAAATCGGCGTAGATTTTGTTTTTTTTTTATATTC
TCACATGTATTATCTGAAATATAGAATAAATGTTCCTCGATGCGAGCGCCACTGCCTCGGCCTCCGCGGTCGT
CGCGGAGGTCGCGGATACAGTTCTCGCATCACACAGTAATTGCACAGCGCACTTTATT
AGTCCATAGGTTAAACTAATTTGTGTACTTATAAGAAACAAATGTCGAAACCTAAGTCATTGTTTATAAAAAA
AAAACTATTAAGTTTTAGTGATTTAAGTATATTCTCCATTTCTTGGTTGGGAGTACTGTGACAATATAAACGT
CATTAGGCAACATAGGATTCGATATTGCAAGTTGTTTTTAACTTATTTATTTTAATAC
AGCCCACGTTGTTTATACTGATTGAGTTCATGAGTTTTTACGTGTTCTACAGCGAACACGTGAGAGCTCGTCG
ACTAGTCAGCTCTATATTAAATTGCAGTGTTATAAAAAAAAATGTTTGTAGATATTACTATATAAGTGGTAAG
CGTTACCTCTTATGTTGCCTTTTGTACATACATTAGAGGTTTAAATCGACAAAATTGT
AAAATTTATATCAAAATATGAATAGACTGATGATCAATAATAATGTTCTTGTATTTAATTTTGTAAGGTGAC
>1002050736_1 Heli.1-CV526138.2.5

AAACCCAANTTGGACCGAGGTCTGCAGGATCGGCCGAGGTGGGCGTCATCTTGAAATTTGCTGAATTTGTGCC
GGTTGTGTGATAAAAGTGTAATTTGTATATTAGACCCCCAGTTAATTGTTATTTTTCAAGAAGTTTATGTATG
CTTATACTATTTATTGTTTAAAATAGGATAATCTACAGGAATGTGTTAGTATCATTAC
TCAAGACATATGCGATTTCCGGTAAGAGTGTCGTCGACAAGACCTTAGGTTGGCAAAGATATAATACGAGGTC
AGTACCTAGTAGAGCTCGTGTATATGCCGATGTTAACTCTCAAAAGCCTAGAGAATATAGGGATTATAAGAGC
TATGCTGTGGATTGGGGTACCCAGGAAAACTATCAGTTGTTCGGAAGCTAGGGCGCGG
AAGGATACTGTGAGGGTATTTGAAAGCATTTAATGTCGTAAAGCGATGAGAAATGTGTAGTTACAATATGGAA
GCCTGTCAGGGCGAAGAAGATTTCGCGAGAGATATACCTCTTAGAGAACCCAAAGAGGAGGCACCATCGTTCT
TACGTTACAAGCGGTTGCTGCCGAATCCGTATCTCTTCCCCCAGTGTTTGGTTTTACG
AGGATTGTTGGTTGATGGTGGGCCAAACGCCTTTTTTTTTCCACCGGTGTTCGCGTATGGATCCTAGATCCCG
CCCGCTGTTATCTATCGCGGCGCGCGGAAATTAGAGCCAGTTTTGGGGGGCATTTTTNA
>1002050737_1 Heli.1-EL601984.1.5
GCACGAGGCACGTCTGGGGTGATCGCTATATACAATATATTAATCTCAATGGCAACCCGTTATAGTTGCCGCA
ATCCAACCGTGAATTTGTTTTTAAAATTGTAAAGTCACTCTTTTTACGTAGTTTGATTTAGAATTTTTGAACA
GTGTAAAGTGATTACAATAGGAGCTACAAACCAGTGAAACCTTGAGATCCTGACTGAC
AATGTTCGGTTATAGTTATTCAAATCTTGAAGAAATGTACCCGTGTTAAAATTTCAACTAATTCTGGTATTGG
ACGCTAAGGGCGGAAATGTATGCCATGTCTACGGCGTCCACGACGACGTCCGGCTCACGTGCCGATATGACAG
CGGGCATGATGGAGCGGCGCGTGCGAGTCGTCCGCCTCTTGCGTCCTCGCAAGGCTGC
ACCTGCTTTCGGCTTTTCGCTACGAGGTGGCCGTGAATATGCTACTGGATTTTTTATTTCAAAAGTTGAACTT
AATTCTGAAGCTCATCTTCAGGGACTGAAGGTTGGCGATCAGGTGATAAGTGTGAACGGCTATCGGGTGGATG
ATGCGGTTCACGCTGAACTAGTCCATTATATCACCTCACAATCTAGGATAAAAATTAA
AGTTAGACATGTCGGAATGGTTCCAGTCAAAGACAAAGAACATGAATCTTTATCATGGCAATTTGTTTCGGAA
CGCGCCTCCTTGGCTTCGGATGTCTCCTCGTTTTTTTTTTTATGCCACAATACGGATCATTTCTCAGATCTAC
AATTATCTATATTAGTACCCCCAAGGGCCAAACTTGGGTGCGGAATTTGTAAAGGACC
CGACTGGATACCGGGAATCTTTGTACTATTCGTAAGGGAAGGTGGCATAGCCCGGGAAGCTGGATTGAGGCCG
GGGGATCAAAT
>1002050738_1 Heli.1-EL601111.1.5
GCACGAGGGGTGATGCTTGTTCGTAGTCTAAAAACGTATTTGTATATGTCGGTATAAAATGGCTGACACTGGT
CTACGTATGAATTTTGCCGCATTAAAGAGGGCAGATCCATATGCGAGAGAAATAATTGACAGTGCTACCCACG
TCGCACTTTATACATTTGAAGAAAACGAATGGGAAAAAACGAACATCGAAGGTGCTTT
GTTTGTGTACAGTAGAAATGGTGAACCTTACCACAGTTTGGTTATTATGAACAGATTAAATACGAATAATCTT
ATTGAACCTGTTTCTAAAGGAATTGAATTGCAATTAAAGGAGCCGTTTCTATTATATAGGAACGCCAAGTGTC
GCATTTACGGCATATGGTTCTACGATAAAGACGAGTGCGTTAGAGTTGCCACAAAACT
GAATTCTATAGTTAAGGATTCCATCAAAGCGCCAAACGACATGTCACAAAACCCAGTGTACCCTCCGGCAGCC
AAACCTAGCTCGTCTGTTGATATATTTAGCATGCTGAGCAAAGCTCAAGATGACTTCAATTCTAATAAGGGTT
TGACATCTAATAAGAACGAATCGACTCCGTCGCGAGCTCCTGATATGGCATCGCAGAG
TGTGATGGACTTCTTTGCAAAGGCCGGCAGTGGTGCAGCAGCTCAGATGCCGGCCGTATCTTCACTTCCATCA
CCGGGCATGTTCGGGCCGCGGCCGACTGACTCCCGAGAGGGGCCGTTACTGTTGCAGCGACTGATGAGCAACC
CTGCTCACTCTGTGGAGCACATTGAAAAACAACAACGATCTGTCACTCCACAGGATGC
GCAAACATCTAATGGAAATATATCTTTAGATCCCAGTATGAGGCAAAACAACTCCTTCCCACTTAAATCCACT
CCAATCGACAAAAATCAGCAAAGGATAAACATGAAGAAAGTGCAACATTTAGAGAATGTGGACAGTAATGGTC
CCAATCCGTTAGAAAGTGAGTTAAATTTGATGCATATTTCGTCACCTAAGCCTACTTC
TCCTTTAGCAGCTTATTTAAACCAATCTCAAGATATTTCTCACCAAGTGAACTCATTCAATGGAGGAAAGCTA
GAAGATATAGGAGGTATTTTTCCAAATTTAAATACTTTTGCGAATCAGCAAAAACCTGCTCTCATGCCTCCTA
CAATGTTTACGGCAACTCCGGGCAGCGACATCCATCAGTCACCAGAGCCTCTCACCCG
AAACCAGCTTCTGCAAGCCTTTAATTATCTTTCTCAGCACGACACTGACTTTGTTAACAAATTGCATGAAGCA
TATGTGAAATC
>1002050739_1 Heli.1-EC091406.1.5
GAATGCATTGCCTTCAGATTTTATCCGGTGTAAGCAGCGGGACATCAAGCTGAACGACTGTTTGAAAACCGCC
GTGCCGGACGCCTTACGGAAGATGAAAAGTGGCATCCCACAACTAGGAGTGCCGCCGCTGGAGCCACTGGCAG
TGTCCGAAATCAATATAGATTCAGGCATCGGTCCGGTCACTATAACGCAGAACTATAG
AAATATTAAGATATTTTGTTTGACTGACTCCATATTGACGAGTTACAAAGCTGATCTCAAGCATTACCGTCTA
AGAACTGATTCTTTAACACCTAAGATGGAATTTTTAGCTGACTATGTGATGAAAGGGAGGATTTTAGTATTAC
CTATATATGGGAAAGGCATCGCTAATATTACTATGGTGGACTTGGTGGTGAAACACGA

TTTAATAGGAGAGCCTGTGGTCAGGGATGGAGTCACATACATGCACATGAAGGACTACAAGATTAAATTCATT
CCAAAGAAGGTTTATTTGCAGTTCTCTAATTTATTCAATGGAGATAA
>1002050740_1 Heli.1-EC091307.1.5
ACGCTGCACATCTTGCTTTGCTACAACGGCCTGAACTGGGCATTACTTTCACAAAAATCCACTGCTCCAATCT
CATACAGTATGAAAAATGCGTCTTCCTTGACGCTGACACATTGGTTGTTCAAAACTGCGACGAATTGTTCGAG
CGCGAGGAGTTGTCCGCTGCACCCGACGTCGGCTGGCCCGACTGTTTCAACTCAGGAG
TATTTGTATTCATGCCCTCCGGAGACACATTCAGCAAACTCGTCAAATTCGCCTCAGAACGCGGCAGCTTTAA
TGGTGGTGACCATTTTTTTTCTAAATTCGTACTTTTCGGATTGGTTTTAGGGCGACATTAACAAGCATCTACC
ATTCCTCTATAATGTCACATCAGCTGAATTTTATTCTTATTTACCTGCCCTTAAACAT
TATGGTTAAAAC
>1002050741_1 Heli.1-EC091324.1.5
TCCCGCCAGATATATGGTCTGCGGCATCCTTCTGGCGTGCCTGAACAAGCATTGGCGGCGAGAGATCTAAGGC
CGTCACCTGCGCGTGACGTTCCCGCCAGTGGCGGCTCATCCAGCCAGGTCCACAACCCGCGTCCAGTACGTGG
GTGTATTTACGCTGTGGAAGCATTGCCAGTAAGGCGTCAGCACTCTGGCGCTGTAGAT
CTGCATGTTGCTCATAGTGTGCGGCTGCCCGACCAAATGCCGCTGCAATGGCTTGTTTATTAACCGTTGCCAT
GCAGCACCTCCAGCAGACGGTCGATATCCTGCATTTCATGCGCAGCGGTTAGCGTTAAGCGCAGTCGCGCAGT
ACCAGCGGGTACGGTTGGCGGGCGAATCGCCGTGACCCAGCAGCCTTGCTGACGCAGT
TTTTCTGCCAGTTGTAACGCACGGCTGTTATCACCGACAATCAATGGCTGGATGGCGCTGCATGAATCAGCAA
GCGTAAACGGCAAATCCTGTACTCCGGCACGAAAACGCGTAATGAGTGCCGCCAGTTTTTCGCGCCGTGCATC
ACCCTCATCACTGCGAATGACCGCCAGCGACGCACGTAATGCCTGCGCCTGAGCGGGC
GGCATACTGGTGCTGTAGATAAGGTGGCGGGCGAATTGCAGCAGATAATCCGCCACCGTACTGGAGCAAAGCA
CCGCTGCCCCGCTGACGCCAAATCCTTTGCCAAAAGTCACTACCAGCAATTCTGGTTTTACCTTTTGCAGCCA
GCAGCTGCCGCGCCCCTGCTCCCCGATAACGCCCGTGCCGTGGGCATCATCGACCATC
AACCAGCCATTGTGCTGTTGCGTTACCTGCTGGATTTCCGCCAGTGGCGCACTATCGCCGTCCATGCTGAACA
CGCCTTCTGTCACCACCATTTGCTGCCCCGGACAGGGGGAAGCAAGCACCATCAAGCTGAGTGTGTAGCGGGT
TAATTCGGCGTTTAAGCCCTGGCGACTGCTGGCATTGAGCACGCCGAGCAGGGTTTCC
AGCCCGACGCGCAGGTTGGCTTCGCTACCGCCAAAAACCGCCAGCGTCGAGCTGGGGTTCATATCAATAATAC
TGTTGAGTGAGACGTGTAGCGCATCGGCATCACAATGCCCCTGGTGAGCGAGTTGTTGCACCAGGCGTGCCGA
CTGACAAATACCGGCCAGGGCGAGGGTGATGTCATAGTAATTCTTTGCCACGTTCACT
GCTTCCTTGTTTTAAGTAAAGATAATAATCAGACCGGCAGCGGCAGACGCTGCTCAATAATACCGCCACCGAG
GCACACTTCACCGTTATAGAAGACGGCAGACTGGCCCGGCGTCACGGCGGCAACCGGTTCATCGAAAATCACT
TTCATGCGAT
>1002050742_1 Heli.1-DT664748.3.5
GCACAGGGCAAACCGTGATGAATACATAAAAGATAGGAAATCTCTTACTGAGGAGTCAGTGGAACCTTTGACA
GAGATACTCGGTGACTCGGAGAAGGCACAAGCAATAATAGATGCGTCCAAAATGTCCATGGGCATGGATATTT
CACCTGTTGACTTGATTAATATACAAATGTTTGCTGGGCGTGTGGTGGCTTTGAGTAA
TTATAGAAAACAAATAGCCGAATATCTCCATACAAAAATGACCTCCGTAGCACCGAATCTGACCACGCTAGTT
GGTGACCAGGTCGGAGCTCGTCTCATATCAAAGGCCGGTTCGTTGACCAGCCTTGCTAAGTATCCGGCTTCTA
CTTTACAGATTTTAGGTGCCGAAAAAGCTCTTTTCAGAGCGCTAAAAACAAGATCTAA
GACACCCAAATATGGTCTCTTATACCACTCGACGTTCATTGGGCGAGCTGGACTCAAGAATAAAGGCAGGATC
AGTAGATATTTGGCAAACAAATGCTCCATTGCATCCCGGATTGATTGTTTCTCAGAAGTCCAAACTTCAATAT
TTGGTGAAAAACTCCGTCAGCAAGTCGAGGACAGGCTTAAATTCTATGAAACGGGTGA
CATTCCTATGAAGAACATTGATGTAATGAAACAAGCTATAGAAGAAATGGAAAAGGATAAGCCGAGTGATGTC
AGTCTTACTAACAGTAGTAAGAAAAAGAAGAATATGAATAAGGAAGTCGCTATGGACTTAGATTAAATGTTTT
AATATGAATTATATGTTTGGATTCAAATGGTGTCTTATTATTTGTAGTTAAGTATGTT
CTTATTTAGATTTTTTAAATATACGATTTAATGGCGGTTAAACACATCTTTGTGTAGTGTGAGATTTTTAAAA
ATTTTTTAAAGTATTATTCCACTCTTAGGTAAGAAAAATACTTATTTAGATTTTTTTTAAATTTGAGTTTTCT
AAATAAGTATATTTCTTCGATTTGTCACGATAACAACAACAATATGTTTCCAAAAACA
AAATAGCATTTCATAATTCATATATTGTTTAACTTTTTAAAAAATTAAGTAGATTGAGATCAATCTTCAGATG
CCGATTTATACGTAAATTACATATCTATAAATGAAAACGGCTTTAAACAAAAGTAAGAAGAACAATAAGGAAA
GGAATTTGCTGCTGGCTTTGATATTTGAATATGAATTATGTATGAATTAAAATTTGTT
TTATTTCTTAGT
>1002052286_2 Heli.2-DT664125.3.5

GCACGAGGCAAGTCCGTCGGTCGGCTGAACTCAAATCGGTTAAAAGTAATGTGACTTGGTGACGGGATACAGG
AGCTTTCTTCGTAAAATGGCGGCGTTACACTTTTTTATGATCTTGCTTGTGGCAAGTAGTTTATCAGCAAAGG
AGGTTGAACAAAAATCTAAAGTCTCCGAGCTCCTCGAGAATGGAATAAAGGATCAGCC
ATCAGGCAAGCTGGCCAAGAGAGGCATCATCTCATCTGGTCTGTCACATGGTATTGGCTTGTCACATGGCATT
GGTGTGTCCCACGGCATCGGCCTTGGTTCAGGAATCGGTATCGGTTCAGGAATCGGTATTGGTTCAGGAATTG
GTATTGGTTCAGGAATCGGTATTGGTCCAGGTATCGGTATTGGTCCAGGAATCGGCAT
CGGTTCAGGGGTTGGTCTTTCTTCGGGAATAGGAATCGGTGGCGGTGCAATTGGTATCGGGGGAGGAGCTATT
GGTATCGGCGGTGGTGCCGTGGGTATCGCTGCCGCACCCGTCGCAGTTGCCGCTGCACCCGTCGCAGTAGCAG
CAGCACCTGCAGTTGCCGTCGCTCCTGCTCAAGTTCAAACTTCTGTATCAGTTCAACC
AAATCCAGTCATTCTCCGCCAAGAAGTACCCAGGTTCATAACTAGAACTATCACCAGGGACGTCCAAGTTCCC
GTTCAAGTCCCTGTACCTGTACCAGTCGACCGCCAAATTCCAGTACCAGTCAGAGTACCAGTCCCAGTTCCCG
TCGACAGACCCGTGCCAGTTATTCACAAAGTACCTGTAGAAGTTACCAGACAAGTACC
TGTGTACTACGAAAGGAAGGTGCCATACCCAGTGAAAGTTCCAGTATCAGTACCTGTGCCTTACCCAGTAACA
GTTGAGAGGCAAGTCCCAGTAGACCGTCCAGTGTACGTTGATCGCCAAGTGCCTGTACCATACCCAGTGCGCG
TAGACCGTCCCGTCAGCGTACCTGTGCCGGTGCACGTCGACAGGCCTGTGCCTGTTCC
GCACCCAGTCGTAGTGGACAGACCTGTGGCTGTACCCCAGCCGGTAGTCGTTGACAGACCTGTAGCAGTGGCT
GCTGCAGTGCCAGTAGCATCAGCAGGCATAAGCATTGGTAGCTCTCTCGGTGGCTCCCTCGGTGGCTCCCTCG
GTGGCTCTCTCGGTGGCTCCCTCGTTGGTTCCCTCGGAAGTTCTCTCGGCGGCTCCCT
TGGTGGCATATCGTATGGAGGAGGATACTCATCCGGCATCATTTCATCTGGAATTGGTCACGGTTTGTCTGCT
GGGATATTAACTGGACACGGATTGTCCTCTGGCATCTCAATTGGTCATGGATTATCCGGCGGCTACTCCAAGA
TCATCAGTAGCCACTAATCTTAATATTATAAGAGCAAAATGTGCAAGTTCATTTATAA
TGGATATTATTATTGAAATAAAATTATAAAACTTTTAGTCTTTATTTTATGTTTATTTTTATTTATACAAAGA
ATATTTACACAAACTTATATATTTCGAGTTTTTTAAATTGAAAATAAAAATCTCAATTATTTCGAAATCGAAA
TCGAATCTATTTATTATTAAATTACTATAAACTGTATATACAAAATACTATTTATAAA
TACTGATAAGATATATTAATTTATGCTGTTAATATTTGAAGACAAATTACAAAGACGGTGTTTACTTGTCTTT
AAATAGAAACTATATATTATGTATTATGTATGTATGTATATATGACTGCTTTTATATAATATAATTACTTTAT
TGTCTTTTCGTTGTTTATAATAAAATGAATGAGT
>1002052287_2 Heli.2-DT668408.3.5
GCACGAGCAGTAAGCATCACTTACTGAGGAAATCGCGTCAAGAAACGGTGATTGGAAACTAGGTAACGTTAGC
TCGACGAAAGAAGAACGATGAACAAACTTATTCTTCTCTCTGTCGTCGTTGCCGTGGCAACGGGGATCAATGT
TTCACCTTCAACTGGCGTCGAGCAGAAGGCACCCTGCCAGACTGCGGAACAGTGGGAG
GGACGTGCCAGTGAATGGGACCATGTTCAGGGTAGAAACAACCGATTTCTGATCAGTTTCGATGGCATAGGTA
TGAGGAAGAGGGTGATTGAGGAGAAGAAGTCGTTTATGCCTGGACGAAGATTTTACGAGTACATCATGGAATA
CAAGACCAACAAAATGTATACCATAAACATGAACTTGGGCACGTGCACCGTCTCAACA
CTGAAGCAACCATGGCAAAACCACACCATTCCTTCCGATGCCACCCTAGAAGACGAATATGAGATGGGTGATC
CTGGTTCTGGCCTCAATGTCCAGGAGTGGTCTGACAGACTCCCCGCCAGGAGAAGCGAGAGCTGGATTGGCAT
CTACACTCCTAAAACAGAGAATGGTGGTTGCTGGCCTGTGGTGGAAGTTTTCACAGAC
GACACTACCGATCCTCCCGTTTCCTTGACAACGCGTTTCTTTGACATCACGCCTGGGATAAAGAACATGTCAG
TCTTCATTCCTCCCCCTTCCTGTAATCAGAACGTGGAGATCGTAGTGGAAAACGTGGAGAATAGTGGCCCCAT
TTATACTTTTTACAACTACATCAAGTCCTGGTTTTAGTTTTGTCAATTTTCATCCCAT
CAACATTCATCAAACAGAAATGACGTACGCATGACC
>1002052288_2 Heli.2-DT668378.3.5
CGAGGCTTAACCGGTCGCGCTGAACGACGACTGATTAATTTATTAAATTATATTTCTACGTTAAGTTCAACAA
AACTCAAACAAAATGCGTGAGTGCATCTCCGTACACGTTGGCCAAGCTGGTGTCCAGATCGGTAACGCCTGCT
GGGAACTCTACTGCCTGGAGCATGGCATCCAACCTGATGGCCAGATGCCCACAGACAA
GACCATCGGCGGTGGAGACGACTCCTTCAACACCTTCTTCAGTGAGACCGGCGCCGGCAAACATGTACCCCGT
GCAGTGTTCGTCGACTTGGAGCCTACAGTAGTTGATGAGGTTCGTACCGGCACATACAGACAGTTGTTTCATC
CAGAACAACTTATCACTGGTAAGGAAGATGCGGCCAACAACTATGCCCGCGGTCACTA
CACCATCGGCAAGGAGATTGTTGACCTAGTCCTCGACCGCATCCGCAAGCTCGCCGACCAGTGCACAGGCCTC
CAGGGCTTCCTCATCTTCCACTCCTTTGGTGGTGGTACCGGATCAGGGTTCACTTCCCTGTTGATGGAGCGTC
TCTCAGTTGACTACGGCAAGAAGTCCAAACTCGAGTTCGCCATCTACCCCGCCCCACA
GGTCTCCACTGCTGTGGTTGAGCCCTACAACTCCATCCTGACCACGCACACCACTCTTGAGCACTCCGACTGC
GCCTTCATGGTCGACAACGAGGCCATCTACGACATCTGCCGCCGCAACTTGGACATCGAACGCCCAACCTATA
CAAACTTGAACCGTCTCATCGGTCAAATCGTGTCCTCCATCACGGCCTCCCTGCGTTT

CGACGGCGCACTCAACGTCGATCTTACAGAGTTCCAGACCAACTTGGTGCCCTACCCACGTATCCACTTCCCC
CTCGTGACCTACGCCCCTGTCATCTCCGCCGAGAAGGCTTACCATGAACAGCTCTCTGTCTCCGAAATCACCA
ACGCGTGCTTCGAGCCCGCCAACCAGATGGTGAAGTGCGACCCACGCCACGGCAAGTA
CATGGCGTGCTGCATGTTGTACCGTGGTGACGTCGTGCCCAAGGACGTGAACGCCGCCATCGCCACCATCAAG
ACCAAGCGCACCATCCAATTCGTGGACTGGTGTCCTACTGGTTTCAAGGTGGGCATCAACTACCAGCCCCCGA
CCGTCGTGCCCGGCGGAGACTTGGCTAAGGTACAGCGCGCCGTGTGCATGTTGTCCAA
CACGACCGCCATCGCTGAAGCGTGGGCGCGTCTCGACCACAAGTTCGACCTGATGTACGCCAAGCGCGCCTTC
GTGCACTGGTACGTCGGTGAAGGTATGGAGGAGGGTGAGTTCTCCGAGGCCCGCGAGGACTTGGCCGCCCTGG
AGAAGGACTACGAAGAGGTCGGCATGGACTCCGCTGAGGGAGAAGGCGAGGGTGCCGA
GGAGTACTAAGCGGCTTAATTAACGTTGAGCACTCGCACCGAATACATTCCGGTGAAATAAAATAAAAAGAAA
TCAAATTTAAAAAAAAAAAAAAA
>1002052289_2 Heli.2-DT668383.3.5
GCACGAGGCGACAGGGGACTGTGGTGCGTGCGGAGCAGTAGTGTTAAGTGTATAGTGTTTAAGTAATAATTTT
TATATTAAAATGAGGGAAATCGTACACATTCAAGCCGGGCAATGCGGCAACCAGATCGGAGCTAAATTCTGGG
AGATCATCTCTGACGAGCACGGCATCGACCCCACTGGCGCCTACCACGGTGACTCGGA
TTTGCAGCTCGAGCGCATCAATGTATACTACAATGAGGCCTCCGGCGGCAAGTACGTGCCCCGCGCCATCCTC
GTCGACTTGGAGCCCGGCACCATGGACTCTGTCCGCTCGGGACCTTTCGGACAGATCTTCCGCCCCGACAACT
TCGTGTTCGGACAGTCTGGTGCCGGTAACAACTGGGCCAAGGGACACTACACCGAGGG
AGCTGAACTTGTCGACTCAGTCCTTGATGTAGTTCGCAAGGAATCCGAATCCTGCGATTGCCTACAGGGCTTC
CAACTTACACACTCCCTTGGAGGCGGTACTGGCTCTGGTATGGGAACCCTTCTCATATCGAAAATTCGTGAAG
AATATCCCGACAGAATAATGAACACATACTCAGTCGTCCCCTCGCCCAAAGTATCAGA
TACCGTAGTAGAACCCTACAACGCGACTCTCTCAGTACACCAGCTCGTAGAAAACACAGACGAAACTTATTGT
ATCGACAATGAGGCACTTTACGACATCTGTTTCCGCACACTTAAACTTTCCACACCCACCTATGGAGATCTTA
ACCATTTGGTGTCCCTTACAATGTCCGGTGTCACTACTTGTCTTAGGTTCCCTGGTCA
ACTTAACGCTGATCTTCGCAAGCTGGCCGTCAACATGGTGCCCTTCCCGCGTCTTCACTTCTTCATGCCAGGC
TTCGCCCCCCTTACGTCGCGAGGCAGCCAGCAGTACCGCGCGCTCACCGTTCCCGAGCTTACGCAGCAGATGT
TCGACGCCAAGAACATGATGGCGGCGTGCGATCCGCGCCACGGCCGCTACCTTACAGT
GGCCGCCATCTTCCGAGGCCGCATGTCCATGAAAGAGGTGGACGAGCAGATGCTCAACATCCAGAACAAGAAC
TCGTCCTACTTCGTAGAATGGATCCCCAACAACGTGAAGACCGCCGTGTGCGACATTCCGCCCCGTGGACTCA
AGATGGCCGCCACTTTCATCGGCAACTCCACCGCCATCCAGGAGCTGTTCAAGCGCAT
CTCCGAGCAGTTCACCGCTATGTTCAGGCGCAAGGCTTTCTTGCATTGGTACACCGGCGAGGGCATGGACGAG
ATGGAGTTCACGGAAGCCGAGAGCAACATGAACGACTTGGTCTCAGAGTACCAGCAGTACCAGGAGGCGACCG
CCGACGAGGACGCCGAGTTCGACGAGGAGCAGGAGCAGGAGATCGAAGATCACTAAAC
GTTAATTACCGTTGTGTTGCAACACACCACCCCTTTCCCCGTCCCATTCGGGACATTCTCGTTTTCGCTTCAG
TAGTTTTTTACAAGTTTCTAACAGTTCTGGTAGTTCCCAACATAGAGGACGTTCTGATGTCGCGCATAGTATT
TACATTCCCAGTGTTATCGACGGAAGGGCGCTCTCTGGTATGTTTATTGGTATAAACA
GTTGATCCCTTACGACGTGACCCCTTCACTGACCACCCCCCGCGGTGCTGCGAATACACTGATGACACAAAAG
AAGATCTTTTGTTTGATAAGGCTGAACTTGATTTTTTTAATACCTAAGTTTATTTTTAATCTTTGTAACCAGT
GTGATTGAGTTTATGTTGGCTTTTTGGAAACTAAAAAGCTAATAACAATGTCGTACAA
TTTTTCGAAGTCGATCAAAGTTTTTAATTATTATATTCCAGCTGGTGAATAATAAACTAC
>1002052290_2 Heli.2-DT665882.3.5
GCACGAGGCTTTCTCGGATAATACACGCTATCCGTGGTGAGTGTCAGAAGATTTAAAGTGAAGTGCAAATTGT
TAAGTGAATAGCGAGGGGATAATACACGCTATCCGTGACTAACCAAAAATGGGCAAGGAAAAGATTCACATTA
ACATTGTCGTCATTGGACACGTCGACTCCGGCAAGTCCACCACCACTGGTCACTTGAT
TTACAAATGTGGTGGTATCGACAAACGTACCATCGAGAAGTTCGAGAAGGAAGCCCAGGAAATGGGCAAAGGT
TCCTTCAAATACGCCTGGGTGTTGGACAAACTGAAGGCTGAGCGTGAACGTGGTATCACCATCGATATTGCTC
TGTGGAAGTTCGAGACCGCCAAATACTATGTCACCATCATCGACGCTCCCGGACACAG
AGATTTTATCAAGAACATGATTACCGGTACGTCCCAGGCCGACTGCGCCGTGCTCATCGTCGCCGCCGGTACT
GGTGAGTTTGAAGCCGGTATTTCTAAGAATGGACAGACCCGTGAGCATGCCTTACTTGCCTTCACACTCGGCG
TCAAGCAGCTGATTGTTGGAGTAAACAAAATGGACTCCACTGAGCCCCCATACAATGA
GGCTCGTTTCGAAGAAATCAAGAAAGAAGTATCTTCCTACATTAAGAAGATCGGTTATAACCCAGCTGCCGTC
GCTTTCGTACCCATTTCTGGCTGGCACGGAGACAACATGTTGGAGGCATCCACCAAAATGCCCTGGTTCAAGG
GATGGCAAGTGGAGCGCAAGGAAGGTAAGGCTGACGGTAAATGCCTCATTGAGGCTCT

CGACGCCATCTTGCCCCCAGCGCGTCCCACCGATAAGGCACTCCGTCTTCCCCTACAGGACGTCTACAAAATC
GGTGGTATTGGTACAGTGCCAGTAGGCAGAGTTGAAACTGGTGTCCTGAAACCTGGTACCATTGTTGTCTTCG
CTCCTGCTAACATCACTACTGAAGTAAAGTCCGTTGAAATGCACCACGAAGCTCTCCA
AGAGGCTGTGCCCGGAGACAATGTAGGTTTCAACGTAAAGAACGTGTCCGTCAAGGAATTGCGTCGTGGTTAC
GTCGCCGGTGACTCCAAGAACAACCCACCCAAGGGTGCCGCTGACTTCACCGCACAAGTCATTGTACTCAACC
ACCCTGGTCAAATCTCTAATGGATACACACCTGTGTTGGATTGCCACACAGCTCACAT
TGCTTGCAAGTTCGCCGAAATCAAAGAAAAGGTTGACCGTCGTTCTGGTAAATCCACTGAAGATAATCCTAAA
TCTATCAAATCTGGTGATGCTGCTATCGTCAACCTCCAACCATCCAAACCCCTGTGTGTGGAAGCTTTCCAAG
AATTCCCTCCCCTCGGTCGTTTTGCCGTGCGTGACATGAGGCAGACAGTAGCTGTGGG
TGTCATCAAGGCTGTCAACTTCAAGGAAGGTGCTGGTGGTAAAGTCACCAAAGCCGCTGAGAAGGCCACCAAG
GGCAAAAAGTAGCTAGCGCTGTTAACAGCTAAATTCTTTATCCAACTGCGATACTTCATTCACCGAAAGGTGT
TCAGAAGGAAAAAAGGGCTACAAACTCATTCCTTTTCTATATTTTTTACAAGGCTTAT
ACTGTAACATTATTTTATAATTTATAAGGTTATACATATATCTGAAATATTTTGTTATGACTACAACTAAAGT
GTAAATTCATAGAATAAAAGTAG
>1002052291_2 Heli.2-DT668782.2.5
GAGGGGAACCACCGCCCGAACTAATAATAACTTAAGAAAATGTTCGCAAAGGCAATCGTCGCGTGCGCGCTCC
TGGTTGTAGCTCAGGGAGGGCTCATTGCGAGCCCTCACGGAGTTGGAGCTATCTCATCTCAGAGTATAGTTCT
TGGACATCCTGCACCTGCCATCGCCGCTCATGCTGTTGCCCCTGCATTGGCTGCTCCT
GCATTGGGACTAGCTCATGGATTGGGACTAGCTCCTGGATTGGGACTAGCTCCTAGACTAGGACTAGCTCCTG
GATTGGGATTAGCTTCTGGATTGGGACTAGGTCACGGTGCCATTGTATCACACGCACCCGTGCTGCACGCGCC
TGTGGCCCACGCCGTTGCCCCTGTTGAGGTTTATGCCCACCCCCGCTACCAATTCAAC
TACGGTGTCACTGACGGTCACACCGGCGATCAAAAGAGCCAATGGGAAGCCCGCGATGGTGATGTCGTAAAGG
GACAATACTCCCTCGTCGAACCCGACGGCACCATCCGTACTGTAAACTACTCTGCTGATGATCACAATGGTTT
CAACGCTGTAGTGAGCAGACAAGGACACGCTGCGCATCCTGCCGTTGCAGCCGTTGCC
CATGCTCCTGTAATCGCCGCGGCTCCTCTCGGCCACGGTTCCCTCCTCCGTGGCTCCCTCCTCCATGGCTAAA
CACTTGAAAATGACATATTTATTACCTTTATTTACTTAGGATAAGACAGAATGCGACAATGTTACTTGAATGC
TTCCTGGTAATGGTACAACGGATGGTCTGCTGCTATAGGCACAGCGTTTCATAAGGTG
TATCTAATATGGGACGACGGCCCAAGACTTCACAACCAGTACCTGTTTTTGATGGGTGCTAACTTTTGTATTA
TAATTTCTTTTAGTTTGTAAGTCTTTCTCCGTTTTCTTACTTAGTTAGGAGCATACATTTTATGAAACGTTAG
ACAATGCCATATGGGTGCTTGACGAAGGCTCCAGATTTTGCTTATATTATGTAATATA
CCTTTCTCATAT
>1002052292_2 Heli.2-EL603934.1.5
GCACGAGGCACGAACAGCGCACGCGACAGTCATGTACACTAAGTTATTTATTTTGTGCTGCGCGTGCGCGTTG
GCGACGGCTGCGCCTGGTCTCCTGGATGGTCTGAGCTTAGGAGGGGGGCTGGCCCACTCGTACGCGGCACCGA
TTGCCGTGGCTGCACCCGTTATTAAGACCATCGCAGCACCTGTTGTCAAGACAGTAGT
CAGAGCTGAACCTATCGACCCGAACCCCGCTTACAGCTACGCATATGGAGTAGCCGACCCTCACACTGGTGAC
CACAAAGACGCCCAGGAGACCCTCCAGAACGGTGTCGTCCATGGCTCCTACAGCCTGGTGGAGCCTGACGGCC
ACGTCAGGAAAGTGACTTACACAGCTGACAAAATAAACGGTTTCAACGCAGTCGTTGA
AAGAAGCGGTGCTAGTCACGTTGCGCCTGTAGCACTAGCCCACGCGCCCATAGCCGTTGCCCACGCCCCGGTC
GCCATCGCCGCCCCTATCGCCAAATACTCCCTACCAGCCATCGGACACCCCTGGCGCTAGTCACACCACCCAC
CCGCTTGTCGCATCGCTGACCAACAAAGGATAGGACAAACTTGGAGACAGACTAGCAA
ACATTTTTGAAGCTGTCTCCATAGTTTACGAACTGATAGCTCAATAAAGGACGAAGGCTGCCCAAAGCGTCGT
CCAGATAGTCGGAGCTATCAGAAATTAAAAAGGAATTCGACTGTAATATTATAGTTTCCACAACTGTTTTCTA
CTGTGTAGAATAGGTTGGGAAGATGTTTTGTTTATTTTTTTGGTTTTTAATGGAATGT
TTATATATAGATGATGGCCTGGGATCATTGTGAGATGATGAGATGATGAGATTTGCTTAGTCAGCGATGCAAC
AATATCCACGACCGCCGAGCGCCGAATGAAACTCTCCTATTCTTTAATTTATTTAGTTTTGCTTTGACTGTTG
CTACGGACTTGATCTTGACGCGTTGTATTACTTACATGTAAATATAATGTGTACTTAG
TTTTTAAATAAAATGCCGTTTTTAAGGTGAAAAAAA
>1002052293_2 Heli.2-DT669008.3.5
GGTTCACGTGAGGTTGTGTGGTCACGGAAATAGGTCACACAATCCTCAGAATTTCGTGAATTATTCTTGTAAA
ATATAAAAAAAAATCACAAAATTAATAAAAATCCAAAAAAATAGTAAAATATTTAAAAAATAACGGGAATCAC
TCGCCTTTAAGTTTAGTGTACGTAAGCGTGCAACGTGGTCTCAAGGAATGCGATAGGT

AATCGTAAAGAGAAAGTGAAGCAGCTTTTATTAATTAATGAGTTTTGTTAATCGGTCACAATAAATATAATAA
AATGAATCCTGGACCGCCAAACTATCCCATGGCTTCGCTATATGTTGGAGATCTACACTCCGATATTACTGAA
GCCATGTTGTTTGAAAAATTTTCTACCGCTGGTGCGGTTCTCTCCATTCGCGTTTGTC
GAGATATGATAACACGCAGATCCTTGGGATATGCTTACGTAAATTTTCAGCAGCCTGCCGATGCGGAGCGAGC
ATTAGACTCCATGAACTTTGATATGATCAAGGGCAGGCCTATAAGGATCATGTGGTCCCAACGAGATCCATCC
CTGCGCAAGTCTGGCGTAGGCAATGTGTTTATTAAAAACCTTGATAAAACCATAGATA
ACAAAGCTATGTATGACACATTTACAGCTTTTGGTAATATTTTGAGTTGTAAAGTAGCCCAAGATGAAAATGG
AGCATCTAAAGGATATGGTTTTGTTCATTTTGAAACAGAAGAAGCTGCAAATAAGTCTATTGAAAAAGTAAAT
GGCATGCTCTTGAATGGTAAAAAAGTTTACGTAGGCCGGTTTATCCCCCGTAAGGAAC
GTGAAAAGGAACTTGGGGAAAAAGCTAAGTTGTTCACGAATGTATACGTTAAAAACTTTGGCGAAGAATTCTC
TGATGAAATGCTTAGAGACATGTTTGAAAAATATGGCAGAATAACTAGCCATAAGGTTATGTACAAGGATGAT
GGGTCATCTAGAGGTTTTGGATTTGTAGCATTTGAGGATCCTGATAATGCAGAAAGAG
CCTGTATGGAACTTAATGGCAAAGAACTTGTAGAGGGTAAACCTTTATATGTAGGACGTGCTCAGAAAAAGGC
TGAACGTCAAAAGGAATTAAAACGTAAATTTGAGCAGTTGAAATCTGAACGCTTGACACGTTACCAAGGTGTT
AATTTGTATGTTAAGAACTTAGATGACACTATTGATGATGAGAGACTTCGTAAAGAAT
TTGCACCTTTTGGTACTATTACTTCTGCTAAGGTTATGTTAGAAGATGGTCGCAGTAAAGGTTTTGGGTTTGT
ATGTTTCTCATCCCCCGAAGAAGCTACTAAAGCAGTAACTGAAATGAATGGTCGGATTGTTGGAACCAAACCT
TTATATGTTGCTTTGGCTCAACGCAAAGAAGATCGCAAAGCTCACTTGACATCTCAAT
ATATGCAGAGAATGGCAAGTATGAGAATGCAACAAATGGGACAAATTTTCCAACCTGGAAGTGCAGGCGGGTA
CTTTGTACCTACTATTCCTCCAGCGCAAAGGTACTATGGGCCAGCCCAAATGACTCAGATAAGACCTGCTCCA
CGCTGGACTGCCCAACCATCTGTTAGACCAAGCGCTCAATCTGCTGCTTCTGCATATC
CTAACATGCAACCACAGTTCCGTC
>1002052294_2 Heli.2-DT668352.3.5
GTGAAAAGAGGCAGGCAATCGCGCACCTTTGCTGAGTGATCTACTATTTAAGTCTTCTAAGTTACAAGCTAAG
TTACTAATATGGCAAAAGCACCCGCGGTAGGTATCGATTTGGGCACCACGTACTCTTGCGTGGGTGTGTTCCA
ACATGGTAAGGTGGAGATCATCGCCAACGACCAGGGCAACAGGACAACGCCGTCCTAT
GTCGCGTTCACCGACACAGAGCGTCTCATCGGAGATGCCGCTAAGAACCAGGTGGCGATGAACCCTAATAACA
CAATTTTTGATGCCAAACGACTTATCGGTCGTAAATTTGAGGATGCTACTGTACAAGCTGACATGAAGCACTG
GCCATTTGAAGTGGTCAGTGATGGAGGCAAACCAAAGATTCAGGTATCTTACAAAGGT
GAAGACAAAACCTTCTTCCCTGAAGAAGTAAGCTCAATGGTGCTTACAAAGATGAAGGAAACAGCTGAGGCGT
ACCTCGGCAAAACAGTGCAGAATGCAGTCATCACGGTTCCAGCATATTTCAACGACTCACAAAGACAGGCCAC
AAAAGATGCGGGCACCATCTCTGGTCTGAACGTTCTCCGAATTATCAACGAACCCACT
GCTGCTGCGATTGCATACGGTCTTGACAAGAAGGGCACTGGAGAACGTAACGTCCTTATTTTCGATCTCGGTG
GCGGTACCTTCGACGTGTCCATCCTGACCATCGAGGATGGTATCTTCGAGGTCAAGTCCACCGCCGGTGACAC
CCACTTGGGAGGTGAAGATTTCGACAATCGTATGGTCAACCACTTCGTACAGGAGTTC
AAGAGGAAATATAAGAAGGATCTTACCACCAACAAGAGGGCCCTCCGTAGGTTGAGAACAGCCTGTGAGAGGG
CAAAGAGGACTCTGTCTTCTTCCACCCAGGCTAGTATCGAAATTGACTCTCTCTTTGAGGGCATTGACTTCTA
CACGTCCATCACCAGGGCTCGTTTTGAAGAGTTGAACGCCGATCTGTTCAGGTCCACC
ATGGAACCCGTAGAGAAATCACTCCGTGATGCAAAGATGGACAAGTCCCAAATCCACGACATTGTCCTCGTTG
GAGGCTCCACCCGCATCCCTAAGGTGCAGAAGCTTCTGCAGGACTTCTTCAATGGCAAGGAGCTAAACAAGTC
TATCAACCCAGACGAGGCCGTCGCATACGGTGCGGCGGTGCAGGCCGCCATCCTGCAC
GGCGACAAGTCTGAGGAGGTACAGGACTTGCTACTGCTCGACGTGACCCCACTGTCGCTTGGTATTGAGACCG
CCGGAGGCGTGATGACGACGCTCATCAAGCGCAACACCACCATCCCTACGAAACAGACGCAGACGTTCACCAC
GTACTCTGACAACCAACCCGGCGTGCTCATCCAGGTGTACGAGGGCGAGCGTGCCATG
ACCAAGGACAACAACCTATTGGGCAAGTTCGAGCTGACAGGCATTCCTCCCGCGCCTCGCGGCGTACCGCAAA
TTGAGGTGACGTTTGACATCGACGCGAACGGTATCTTAAATGTGTCGGCCGTCGAGAAGTCCACCAACAAGGA
GAATAAGATCACCATCACCAACGACAAAGGCCGTCTCTCCAAAGAGGAGATCGAGCGC
ATGGTCAACGAGGCCGAGAAGTACCGCAACGAGGACGAGAAACAGAAGGAGACCATCCAGGCCAAGAACGCAC
TTGAGTCGTACTGCTTCAACATGAAATCCACCATGGAGGATGAGAAGTTGAAGGAGAAGATCTCGGACGCGGA
CAAGCAGACTATCCTGGACAAGTGCAACGACACCATCAAGTGGCTGGATTCTAATCAG
CTGGCCGACAAGGAGGAGTACGAGCACAAGCAGAAGGAGCTCGAGGGCGTGTGCAACCCTATCATCACCAAAA
TGTACCAGGGCGCGGGCGGTGCACCTGGAGGAATGCCCGGCGGTATGCCCGGTTTCCCCGGTGGCGCACCCGG
CGCGGGAGGTGCGGCGCCCGGAGCTGGCGGTGCCGGCCCCACCATTGAGGAGGTCGAC
TAAACAAACATCCACCTGAACCAAACATTCCACGTTTAACACGAATGCATGCAAATAAAA

```
>1002052295_2 Heli.2-DT665917.3.5
GCAATCCGAAGTTTAGTTAGCCATCGTAATACGACAGCTCGAGTCGTAGACAGACAGGCTCCAGCCCGTGGTC
GAGACATAAGGCTCTCGCAGACTTTACGCTAGCTAAACGCGCAGTGAACTTCAGTTCAGGCTCCTCTATCTTT
ACGTGTGGTGTTTCGAAGCATGGCCGTCGCAGCTGCCCAAAAGAACCGCGAAATGTTC
GCCATCAAGAAGTCCTACAGCATCGAGAACGGCTACCCATCTCGCCGTCGCTCGCTCGTAGATGACGCTCGCT
TCGAGACACTTGTTGTCAAACAGACCAAACAAAGTGTACTTGAGGAGGCTCGTGCCCGTGCCAATGACTCTGG
CTTGGACTCTGATTTCATCCAAGACGCCGCTCATGTAGGCGAAGACGGGAAGACCGTT
GATGGCGCACAAAATGATGAATGCAAAAACGGTCGCCCTGATGCGGATGCCAACGATGGTAACGCTAATGGGG
ATGAAGATTATACTCTGACCGAAGAAGAGATAATTTTGCAAAATGCCGCCAGCGAGAGCCCTGAAGCCGAACA
GGCGACCCAACAAGCCGCTTTACTTCTTCGTATGCGCGAAGGCATGGGCTCTCTCGCT
CGCATCCTGAAAACAATCGACAACTACAAGGGTTGCGTAGAACATCTCGAAACTCGCCCCTCTCAAGTACCCG
GAAACCAATTCGATGCTCTCGTTAAAGTGAGCATGTCCCGCGTGAACCTGCTACAACTGATCAGATCTCTACG
TCAGTCAACTTCTTTCGTTGGAGTTAATCTCATCTCTGAGAACAACCTGTCTAACAAG
ACTCCATGGTTCCCTCGCCATGCATCTGATCTTGACAACTGTAACCATCTGATGACTAAATATGAACCAGAAC
TCGACATGAACCATCCTGGTTTCGCCGACAAGGAATACAGAGAGCGTAGGAAGCAAATTGCCGAAATCGCTTT
CGCTTACAAATACGGTGACCCGATCCCATCTATCACCTACAGCGAAAGCGAGAACGCT
ACCTGGCAGCGTGTGTTCAACACCGTGCTCGACTTGATGCCCAAACATGCTTGCCGCGAATACAAGGCAGCGT
TTGGCAAGCTGCAATCCGCTGACATTTTTGTACCACACCGCATCCCACAATTGGAGGATGTCAGCAACTTCCT
GCGCAAGCACACCGGCTTCACTCTCCGCCCGGCCGCTGGTCTACTCACTGCTCGCGAT
TTCTTGGCCTCTCTCGCCTTCCGTGTTTTCCAATCTACACAATACGTACGCCACGCCAACTCACCATTCCACA
CACCTGAACCTGATTGCATTCACGAGCTCCTTGGACACATCCCTCTTCTGGCTGACCCGAGCTTCGCTCAGTT
CTCTCAGGAGATCGGATTAGCTTCACTTGGAGCTTCTGACGCCGAAATTGAAAAACTA
TCCACTGTATACTGGTTCACCGTTGAATTTGGCCTGTGCAAAGAAAATCAACAATTGAAAGCTTACGGCGCTG
CTCTACTTTCATCCATTGGAGAACTTCTTCACGCACTTAGCGACAAACCCGAGTTGAGGCCCTTCGAACCAGC
ATCCACATCGGTACAGCCCTACCAAGATCAAGAATACCAGCCGATCTACTACGTCGCC
GAGAGCTTTGAAGATGCCAAAGACAAATTCAGACGCTGGGTTTCGACCATGTCCCGACCATTCGAAGTACGTT
TCAACCCACACACAGAGCGCGTTGAGGTTCTGGACTCTGTGGACAAGCTGGAGACCCTCATTTGGCAGCTGAA
CACAGAAATGCTCCACCTCACCAATGCCATCAAGAAGCTCAAGGACTCACGCTTCGAA
TGAACCAGTTACCTACATTATTGAACCAGATCAGTGCACGCGGCCTCGCCGTACCTGTATAGCTAGTGATATT
ATAGATGTAAGACTGTTGTCGTGTGGAGCGTGTATGCGAGTGTGGAAACCATCGGATGGTACTTCGTTGAATT
CCTTTTGTATTTTATTATTTATTATGCCGAGTTCATTGCCCGGGTTTCGGAAGGCGCA
CAGAACTCCCGATTGCCCATTTAAGGAGGCTGTGCACGTTCGCGAGACCTTTACTATTATATTAGTGTTAATT
TATAATTTAATGAAAAACACAAACAAATAAAAAATGGAGCACCAAAAAGTAAAAAAAATCAAACAGAAATATA
TGTGTTTGAG
>1002052296_2 Heli.2-EL600230.1.5
GGCACGAGGATTTAATAAAAAAATTTTTAATATTGGGGGATGTTAAAGTATTTTTTATAGAAAAAATATAATT
ATTTATAGTAAAATAGTATTGAAAAAGAAATTTGAAATAATTATGAAAATAAATTATTTTAAAAGTAAATTTA
AATTATTGTATCTTGTGTATCAGAGTTTATTAAAAAATATTTATTGATTAATAAAATC
TCGAATTTAAAAGAGTTAATTAATTAAGAAAGTTAATGTTGAATAATTATTTTTAATTATTAATTTGAAATGA
AATGTTAATCGTTTTTAAATATATCTAGTTTTTTTAGAAAAAAATTTAATTTTGAATTTAAAATATTTTTTAA
TTTATTTTTTAATTAAAAAATTTTAAAATTAAATATATTAAGGGATAAGCTTTAATTT
AAATTTTTATAATATATATATATATATATATATATATATATAAATTTAAAAAATTTTTAAAATTTATATTGTTTA
TAAAATAAAATTTATTATAAAAAATTTTAATAAAAAATAAAATTTAAAATTATATTTAATTTTTTATAAAATAA
ATAATAATTTTTAATTAAAATAAAATTAGTAATAATGATAAAATTAGTATATTAAATT
AAAAAATAATAAATTTAAATTAAAATTAATTTAAAGGAATTCGGCAAATTTTTATATTCACTTGTTTATCAAA
AACATGTCTTTTTGAAATAATAATTTAAAGTCTAATCTGCCCACTGATTATAAATTAAAGGGCTGCAGTATAT
TGACTGTACAAAGGTAGCATAATAAATAGTCTTTTAATTGAAGACTTGTATGAAAGAT
TTGATGAAATATAAACTGTCTCTAATTTAAAAATTGAAATTAATTTTTTAATTAAAAAGTTAAAATAATTTAA
AAAGACGAGAAGACCCTATAGAGTTTTATAATAATAATTTTTTAATATTTAATATCTAAAAATAAATTTAAAT
AATTATTATTATTTTATTGGGGTGATAGAAAAATTAAAATAACTTTTTTTAAAAAAAT
TACATTAATAAATGAAATTATGATATATTTTATGATTATAAGAAAAAATTACCTTAGGGATAACAGCGTAATT
TTTTTTTTTAGTACAAATAAAAAAAAAAGGTTGCGACCTCGATGTTGGATTAAGATAAAATTTAAATGCAAAA
GTTTAAAATTTTGATCTGTTCGATCATTAAAATCTTACATGATCTGAGTTCAAACCGG
```

TGTGAGCCAGGTTGGTTTCTATCTTTTAGTAATTAAAATATTTTAGTACGAAAGGATCAAATATTTTAAATAA
TTTAATTAAAAGAATATTATTAATAAATTATATAA
>1002052297_2 Heli.2-DT661904.2.5
GCACGAGGCCTCCGAGGCAGCAGTCTAACAAGAAGTGCAAGATGAGATTTCTGATCGCTTTCGTCGCCATCCT
CGGCTATGCTTCGGCTAGCGCCATCATCTCACCCCTGGTCTTAGGCGCCAACGCTGGTGACGTGCAAGCCGCC
GTCATCGACGCCAATGTAGCCGCCCGCGATTCCATCCGCGCCCTTGGCGAAGGCCAGG
CCCGCGCCGCCGAAGCCGTCATTCAAGCCAACACTGAAGCAGTCCGCCAAGTCGCTGAGAACAACCGTAACAT
CCACGAAAACGCTTACTGGGGATCAGTCGCCGCCTCCCAAAACCTCGTAGCTGCTGCTCAATCTCAGGTCGCC
GCTATCGACGGAGCCGCCGCCGCTGTCCGCGGTGTCCTCGGCGCCCCCATCGTCGCCC
CATTGGCTTTGGCTGGTGGCCTTCCGCTTGCCGCCCCTTACGCTCTCGGAGGTCTCGGTCTCCACGGCATCCA
CGCTTGGTAAATCAAACTGATCCATCACTAAATAGTGATGTACCCGTTTGGGAGTTACCTCGTCCCTAAAGTC
AAGGAAACCTCCAAACTTAACAACTGGCATTGGCAATGTTCACAGCAACAGTGGATAT
TGCCAAACAGTTGTTCCAATGGAAACCGATTGCTCAATGGATTTGTACAAAGGTCTCAACGTAACTCTCTTTG
TAAATAAGTGAATCTCATGCCATCACTGCCAAAGTGTAAAATAAACACAGAAATTTACAAAAAAAAAAAAAAC
TTTCCCGTTCCAATTTTTCTAAATAAATACCTGCTGTGTGAAATTT
>1002052298_2 Heli.2-DT666816.3.5
CGAGTTGAGGTGTTGTGCAGCAGAACCGAGGCTGTGCAATAGCGATTAATTTATTTATTTAATAGGACTATTT
AAATTAAAACTAATTCACAATGTGCGACGAGGAAGTAGCCGCGTTGGTAGTAGACAATGGCTCCGGTATGTGC
AAGGCCGGGTTCGCCGGGGACGACGCGCCTCGCGCCGTGTTCCCCTCGATCGTGGGCC
GCCCGCGCCACCAGGGCGTGATGGTCGGCATGGGGCAGAAGGACTCCTACGTGGGTGATGAGGCACAGAGCAA
GAGAGGTATCCTCACCCTCAAGTACCCCATCGAGCACGGCATCGTCACCAACTGGGACGATATGGAGAAGATC
TGGCATCACACCTTCTACAACGAGCTGCGTGTGGCGCCCGAGGAGCACCCCGTGCTGC
TCACCGAGGCTCCCCTTAACCCCAAGGCCAACAGAGAGAAGATGACACAGATCATGTTCGAGACCTTCAACAC
GCCCGCCATGTACGTCGCTATCCAGGCCGTGCTCTCGCTGTACGCCTCCGGTCGTACCACCGGTATCGTGCTG
GACTCCGGTGACGGCGTCTCCCACACCGTGCCCATCTATGAGGGCTACGCGCTGCCTC
ACGCCATCCTGCGTCTGGACTTGGCCGGTCGCGATCTCACAGACTACCTGATGAAGATCCTCACCGAGCGTGG
TTACTCTTTCACCACCACCGCCGAAAGGGAAATCGTGCGCGACATCAAGGAGAAGCTCTGCTATGTCGCCCTC
GACTTCGAGCAGGAGATGGCCACCGCCGCCTCCAGCAGCTCCCTCGAGAAGTCCTATG
AACTTCCCGACGGTCAGGTCATCACCATCGGAAACGAAAGATTCCGTTGCCCAGAGGCTCTCTTCCAACCCTC
ATTCTTGGGTATGGAAGCCTGCGGCATCCACGAGACCACTTACAACTCCATCATGAAGTGCGACGTGGACATC
CGTAAGGACTTGTACGCCAACACAGTATTGTCCGGAGGTACCACCATGTACCCCGGCA
TCGCCGACCGCATGCAGAAGGAGATCACCGCCCTCGCGCCCTCCACAATGAAGATCAAGATCATCGCACCCCC
AGAGAGGAAGTACTCCGTATGGATCGGAGGCTCCATCCTCGCGTCTCTGTCCACCTTCCAACAGATGTGGATC
TCCAAGCAGGAGTACGACGAGTCTGGTCCCTCGATCGTGCACAGGAAGTGCTTCTAAG
CGCGCAGGCGCGCGTTGCTGCCCCCGGAAGCGGCCCGCGGCCCGGTCGGCCCCGCGGCCGGCTGCTCAACTCA
GGCTTGTAATCTCCGTTAATTTAATTGTACGTAAGATATTATTATATGTAATTAAATGGTAACAGTGACGGCA
GGGCGGAGCGCGGCGGGCGCTCCCCGGCCGCCCCCGGGCCGCCGGCCTCGGTACTGCA
CTCGCCAAAGGATTTTGTAATCTCATGAATGATGATTGTATGTGCTGAAACGGATAAGACTACTTTCTTACTA
GGTCATTTATAAATTATAAAATATAACAAAAATACAAGAAAAAAATC
>1002052299_2 Heli.2-DT667044.3.5
GCACGAGGCTTCCTTCTTTTGCGCACGAGGGCCAGGCAGAACTGCGAGAGAACGTGATCGATCGAATTTCAAA
AAAGGCACACTCCTACCCCTGTGCTCCCGAGATCACGGAATTACTTTATATCTCCTGATTGAATAGATCGGGA
GTTCGTGTTTCGTGATTTTGCCATTCCGCGTGTGAACTCGCAGATTCCACCTCGCACA
GTACAGACAGCCCACCTAGTAAAACAAAATGTCGAACCTCGCCGATCCCGTCGCGTTCGCTAAGGACTTCCTT
GCCGGTGGTATCTCTGCTGCCGTCTCCAAGACAGCGGTGGCCCCCATCGAGCGTGTCAAACTGTTGTTGCAAG
TACAGCATGTCAGCAAGCAGATCGCCGAGGATCAGCGCTACAAAGGTATCGTCGACGC
ATTCGTCCGCATTCCCAAGGAACAAGGTCCGCTCTCCTTCTGGCGTGGTAACTTAGCTAACGTGATCAGGTAC
TTCCCCACCCAGGCGCTAAACTTCGCGTTCAAAGACAAGTACAAGCAGGTGTTCCTTGGTGGCGTAGACAAGA
ACACACAGTTCTGGCGCTATTTTGCCGGTAATTTGGCCTCTGGTGGTGCCGCTGGAGC
CACTTCCCTCTGCTTCGTGTACCCTCTCGACTTCGCACGTACCCGCTTGGCGGCCGACGTGGGCAAGGGTGAC
GGCCAGCGCGAGTTCACTGGCCTCGGCAACTGCATCAGCAAGATCTTCAAGTCTGACGGTCTGGCCGGTCTGT
ACAGAGGCTTCGGTGTGTCGGTGCAGGGTATCATCATCTACCGCGCTTCGTACTTCGG

TTTCTACGACACCGCGCGCGGCATGTTGCCCGACCCTAAGAACACCCCCATCGTCATCAGCTGGGCGATCGCG
CAGACTGTCACCACTGTGGCGGGTATCATCTCGTATCCCTTCGACACGGTCCGTAGGCGCATGATGATGCAGT
CCGGCCGCGCTAAGGGCGATATCCTGTACAAGAACACCCTTCACTGCTGGGCCACCAT
CGCTAAATCCGAGGGCGCGTCCGCCTTCTTCAAGGGAGCGTTCTCCAACGTACTCAGAGGCACCGGAGGTGCC
TTCGTACTTGTCTTATATGACGAGATCAAGAAGGTCCTTTAAATCTAATGTAAGAATTATTGTTACCATAATT
TCAATCGTGATTTTGAATCACACTAGCCACTCCCCTGAAATCATGTATTATTTAGACG
CGAAAAACATCCTAGTAACTTATTATTAGTTAAATAAAGTGTTATACAGTGATTCCAGAAAAAAATTGTTTCA
TTAATGTTATGTAATTGTGTACAGAATGATGCTGG
>1002052300_2 Heli.2-EL596880.1.5
GGCTTTTTTCTACAAATCATAAAGATATTGGAACTTTATATTTCATTTTTGGAATTTGAGCAGGTATAGTTGG
AACATCTCTTAGTATTTTAATTCGAATAGAATTAGGTAATCCTGGGTCTCTAATTGGAGATGATCAAATTTAT
AATACTATTGTTACAGCCCATGCATTTATTATAATTTTTTTTATAGTTATACCAATCA
TAATTGGAGGATTTGGAAATTGATTAGTACCTTTAATATTAGGAGCCCCTGATATAGCATTTCCCCGAATAAA
TAATATAAGATTTTGACTTCTCCCCCCCTCATTAATTTTATTAATTTCTAGCAGAATCGTAGAAAATGGAGCA
GGAACAGGATGAACTGTCTATCCCCCCCTTTCCTCTAATATCGCACATGGAGGTTCAT
CTGTAGATTTAGCTATTTTTTCCCTTCATTTAGCTGGTATTTCCTCAATTTTAGGAGCAATTAATTTTATTAC
CACTATTATTAATATACGAATTAACAATATGTCCTTTGATCAATTACCATTATTTGTCTGAGCTGTAGGAATT
ACAGCTTTATTACTTCTTTTATCTTTACCAGTTCTAGCTGGAGCTATTACTATACTTT
TAACTGATCGAAATTTAAATACTTCATTTTTTGACCCTGCAGGAGGAGGTGATCCAATTTTATACCAACATTT
ATTTTGATTTTTTGGACATCCAGAAGTTTATATTTTAATTCTCCCAGGATTTGGTATAATTTCTCATATTATT
TCCCAAGAAAGTGGTAAAAAGGAAACTTTTGGTTGTTTAGGAATAATTTATGCTATAA
TAGCAATTGGTT
>1002052301_2 Heli.2-DT667397.3.5
TGCGATAGTCGGCAGACATGTCGCACAGGAAATTTTCGGCGCCTCGTCATGGATCTATGGGATTCTATCCCAA
GAAGAGGTCCCGCCGTCATCGTGGTAAAGTGAAGGCTTTCCCTAAAGATGATGCCAGTAAACCGGTGCATCTT
ACTGCCTTCATTGGTTACAAAGCTGGTATGACCCACGTGGTTCGTGAACCTGACCGTC
CTGGTTCAAAAATCAACAAGAAGGAGATCGTGGAAGCTGTTACAGTTATCGAAACGCCTCCTATGGTGTGTGT
CGGTGTTGTCGGCTATATTGAGACACCCCACGGTCTTCGTGCGTTGCTTACTGTTTGGGCAGAGCACATGTCT
GAGGACTGTCGTCGTCGCTTCTACAAGAATTGGTACAAGTGTAAGAAGAAGGCTTTTA
CTAAATCAAGCAAGAAATGGCAGGATGAGCTTGGTCGCAAGTCCATTGAGAAGGACTTCAAAAAGATGATTCG
CTACTGCAGTGTAATTAGGGTTATTGCCCATACACAAATGAAACTGCTTAAGCAGCGTCAAAAGAAGGCCCAT
ATCATGGAGATTCAAGTGAATGGTGGAAATATTGAAGATAAAGTAAAATGGGCGAGGG
AACATCTAGAAAAAACCTATCCCTGTTGATTCAGTCTTTGCACAAGATGAGATGATTGATTGTATTGGTGTAAC
TAAAGGAAAGGGATACAAAGGTGTAACTTCACGTTGGCACACCAAGAAACTCCCACGTAAGACACACAAGGGT
CTGCGTAAAGTTGCTTGTATTGGAGCATGGCATCCTTCAAGAGTTTCATTTACTGTTG
CCCGTGCTGGTCAAAAGGGCTACCATCACAGAACTGAAATGAACAAGAAGATTTACAGAATTGGCCAAGGAAT
TCATACCAAGGATGGAAAAGTTATCAAGAACAATGCTTCCACTGAATATGATTTGTCTGAGAAGTCCATCACC
CCCATGGGAGGTTTCCCTCATTATGGTGAAGTAAACAATGACTTTGTTATGATTAAGG
GTTGTTGTATGGGACCTAAGAAACGTGTCATCACATTGAGAAAGTCCCTTCGTGTCCACACTAAGAGAGCAGC
TCTTGAGAAGATCAATCTGAAGTTCATTGACACATCATCCAAATTTGGACATGGTCGCTTCCAGACACCTGCT
GATAAGGCTGCTTTCATGGGCACCCTTAAGAAAGATCGTATTCGGGAAGAGGCTGCCG
CATCGGCCGCGCCAGCTGCTGCTCAGTCTTAAGTTATAACTCCTGAAT
>1002052302_2 Heli.2-DT667413.3.5
GGCTACGCCCGTGTCGCTCCTCTGGTCGCGTGTCTGTTGTTCGCCTGCGTATTAGCTGATGGGCACCGTTGGC
GCCGACAAGCTGATGACTCTAAGAAAGACGAAAGTCTGGAACAAGAGTTATGCAAGGACAAGGACGCCGGCGA
ATGGTTCCGGCTGGTAGCCGGCGAGGGCGACAACTGTCGCGACGTCATCCAGTGTACT
GCCTCGGGTATTCAAGCTATTCGTTGCCCTGCTGGTCTTTATTTTGATATCGAAAAACAAACTTGCGATTGGA
AAGAGGCGGTTAAAAACTGCAAATTAAAAAACAAAGAACGTAAAATAAAACCTCTACTGTTCACTGAAGAACC
TCTCTGTCAAGATGGATTTTTAGCATGTGGTGATACTACGTGCATAGAACGCGGTCTG
TTCTGTAACGGAGAAAAAGACTGCGCCGATGGTTCAGACGAAAACTTATGTGATATTGACAATGACCCTAACA
GGGCTCCTCCTTGTGACACTTCCCAATGTGTATTACCCGATTGCTTCTGCTCAGAAGATGGTACCGTTATACC
AGGTGATCTACCTGCCAGAGAAGTACCTCAAATGATAACTATTACTTTTGATGATGCT

ATCAATAATAACAATATCGATTTATATAAAGAAATTTTCGATGGAAATCGTAAAAACCCGAATGGTTGTGACA
TCAAAGCCACCTACTTTATTTCACATAAATACACGAACTACTCAGCCGTGCAAGAAACCCATAGAAAGGGACA
TGAAATCGCTGTACACTCGATCACACACAATGACGATGAACGGTTTTGGAGCAATGCA
ACCGTCGATGACTGGGGTAAGGAAATGGCTGGCATGCGAGTTATCATTGAAAAATTCGCAAATGTAACCGACA
ACAGCGTTGTTGGTGTACGTGCTCCTTATCTGCGGGTTGGAGGCAACAATCAATTTACTATGATGGAAGAACA
AGCTTTTCTTTATGACAGTACTATCACTGCTCCTCTATCCAATCCTCCTCTATGGCCT
TATACTATGTACTTCCGTATGCCTCACCGCTGTCATGGTAACTTACAAAGCTGCCCCACTAGGAGTCACGCAG
TTTGGGAAATGGTAATGAACGAACTTGACCGTCGTGAAGACCCTACCAATGACGAATACTTACCCGGTTGTGC
CATGGTCGACTCTTGCTCTAACATTTTAACTGGTGACCAATTTTACAATTTCCTTAAT
CACAATTTTGACCGCCACTACGATCAAAACAGAGCTCCCTTAGGACTTTACTTCCACGCTGCCTGGTTAAGAA
ACAATCCAGAGTTTTTAGAAGCATTCTTATATTGGATTGACGAAATCCTTAAGACTCATAATGACGTATACTT
CGTAACGATGACCCAAGTGATTCAATGGGTACAAAATCCACGTACCGTTACCGAGGCA
AAGAACTTCGAGCCATGGAGAGAAAAGTGCGCCGTCGAAGGTAAACCCGCCTGCTGGGTACCTCACTCGTGCA
AGCTGACATCAAAGGAGGTTCCCGGTGAAACGATCAATTTACAAACGTGTGTCAGGTGCCCAGTCAATTACCC
CTGGCTCAATGACCCCACGGGCGACGGTCATTATTAAATGAGATCTCGCGCCTCACGT
GGTGCACATTAGTTGATAAATTGCAATGCAATTGATTGCAAGGACATTATATTCGGAAATGTTATATATAATA
AATAAAAGCCAAGTTTATTCAGTGTTGACTTATTCGATGAGTATGTGTCCGTGTAATTATAAAATGTACTGGG
TCATAATCCCGTCTGTAGTCTAAGTACCTACTTA
>1002052303_2 Heli.2-DT663049.3.5
GAGGGTCGACTCGACATATTTTCATCAAGGGGAGTAAATAAAAAAAAACCGCCACCATGGACGCGATCAAGAA
GAAGATGCAGGCGATGAAGCTGGAGAAGGACAACGCGCTTGACCGCGCCGCCATGTGCGAGCAGCAGGCCAAG
GACGCTAACCTTCGCGCTGAGAAGGCTGAAGAAGAGGCCAGACAGCTCCAGAAGAAGA
TCCAGACCATTGAAAACGATCTCGACCAGACACAAGAGGGCCTTATGCAAGTCAACGCCAAGCTGGAAGAGAA
GGAGAAGGCTCTACAGAACGCTGAATCCGAAGTTGCTGCGCTCAACCGACGCATCCAGCTCCTCGAAGAAGAC
TTGGAAAGGTCTGAGGAGCGTCTCGCCACCGCCACCGCTAAACTGTCCGAGGCTAGCC
AAGCTGCCGATGAATCGGAACGAATACGCAAGGCGTTGGAGAACCGAACCAACATGGAGGACGACCGCGTCGC
GATACTGGAGGCGCAGCTCGCTCAGGCTAAACTCATCGCTGAGGAGTCCGACAAGAAATATGAGGAGGTGGCT
CGTAAGCTGGCCATGGTTGAAGCTGACTTGGAGCGCGCCGAAGAACGCGCCGAAGCCG
GCGAATCCAAAATCGTCGAGCTTGAAGAGGAACTCCGCGTTGTCGGTAACAACCTGAAATCCCTGGAAGTCTC
TGAGGAAAAGGCGATGGCGTCCCGCGAACACGTCGAGGATAAGATACACAGCCTCTCGCAGAAATTGACGCAA
GCTGAAGCTCGCGCTGAATTCGCTGAGCGCTCTGTCCAGAAACTCCAGAAGGAGGTCG
ACAGGCTTGAAGACGACCTGGTGGGCGAGCGCGAAAAGAGCAAAGTGCTGCAGGAGGAGATGGAGGCCACGCT
CCACGACATACAGAACATGTGAACTAGCACACACACCCCCCACTCCTCCTCCCCCCTACCGCCCTCCCCCGCC
CGCCTCACTACGCCCTGTGAATGTCACGCGCACCTCTAGGGCTGCCAGATCTTGTCTT
ACATGATATTGTAGTTAAATAAAGCTAAAGATGAAAAAAATGCTTAACGATGAATCCCGCATATAGCAGAGTA
AGCAGAAGGGAACTAGTGTTGAGACACTCCGTTCCCAGTGCATAAGTCAATTTTTGACACATTCCATATATTA
CCAGAGCGAGAAAATGAAAATAATTCATAGTATATTTAGGTTACAAATATATATTTAT
ATCATTTATATAGATATTACAAATTAATATATCTAAATGTACACAAATCAAATTACTTCGGTCACGTTGAATG
AATTGAAACTA------------
TCAGTAGAATATCGAGATCTGGCAGCCCCGCGCTGCTCTCCGAGTGACGCTTGTCGAGTG
>1002052304_2 Heli.2-DT666548.3.5
GCGTACGCCATTGCTGGCGTATGGTGGTGTATTATATTAAAAGAAAAATCATAAACCATCCAATATGGTGAAT
TTTACCGTCGACGAGATCCGAGGGATGATGGACAAGAAGCGGAACATCCGCAACATGTCCGTCATCGCCCACG
TCGACCACGGCAAGTCCACTTTGACGGACTCGCTGGTCTCCAAAGCCGGTATCATCGC
CAACGCGAGGGCCGGCGAGACTCGTTTCACCGACACCCGCAAAGATGAACAAGACAGATGTATCACCATTAAA
TCCACCGCCATATCTATGTTCTTCGAGTTGGAAGAGAAGGACTTGGTGTTCATCACAAACCCCGACCAGCGTG
AAAAGAGCGAAAAGGGCTTCTTGATCAACTTAATCGACTCACCTGGTCACGTCGATTT
CTCATCAGAAGTAACGGCCGCCCTCCGTGTAACTGATGGTGCCCTCGTAGTAGTGGACTGCGTATCTGGTGTA
TGTGTACAGACTGAGACAGTGTTGCGTCAGGCCATTGCTGAGCGTATCAAGCCCATCCTTTTCATGAACAAGA
TGGACCGTGCATTGCTTGAATTGCAACTGGAGTCCGAAGAATTGTACCAGACCTTCCA
ACGTATTGTAGAAAATGTCAACGTAATCATTGCCACATATAATGACGATGGTGGCCCTATGGGTGAGGTGCGT
GTCGACCCCAGCAAGGGTTCCGTGGGCTTCGGTTCCGGTCTCCACGGCTGGGCGTTCACCCTGAAACAGTTCG
CCGAGATGTACGCGGACAAGTTCAAGATCGACCTCGTCAAACTTATGAACAGGCTATG

GGGAGAGAACTTCTTCAACGCCAAAACCAAGAAATGGTCAAAACAGAAGGACAATGAGAACAAGCGTTCATTC
TGTATGTACGTCTTGGACCCCATCTACAAGGTGTTCAGCGCCATCATGAACTTCCGTAAGGAGGAAATCGACA
GCCTCCTAAAGAAAATTGGAGTCACCATCAAGCACGAAGATGCTGACAAGGATGGCAA
AGCTCTACTTAAGGTAGTGATGCGTACTTGGCTGCCAGCCGGTGAAGCTCTGCTTCAGATGATTGCCATCCAC
TTGCCTTCCCCCGTGGTCGCGCAAAAGTACCGTATGGAGATGTTGTACGAGGGACCCCATGACGACGAAGCCG
CTATTGGTATCAAGAGTTGTGACCCTGAAGCTCCTCTTATGATGTACGTGAGCAAAAT
GGTACCGACCTCTGACAAGGGTCGTTTCTACGCCTTCGGACGTGTGTTCTCCGGAAAGGTCATCACAGGACAG
AAGGCCCGCATCATGGGACCTAACTTCCAGCCCGGCAAGAAAGAGGATCTGTATGAGAAGACCATCCAGCGTA
CCATCCTCATGATGGGTCGTTATGTAGAGGCCATCGAGGATGTGCCCTGCGGTAACAT
CTGTGGTCTGGTCGGTGTCGACCAGTTCTTAGTCAAGACTGGTACCATCACCACCTTCAAGAATGCACACAAC
ATGAAGGTCATGAAATTCAGTGTATCACCCGTCGTACGTGTAGCCGTGGAGCCCAAGAACCCCGCCGACTTGC
CCAAGCTGGTGGAAGGTCTTAAACGTCTCGCCAAATCTGACCCTATGGTGCAGTGCAT
CAACGAGGAATCCGGCGAGCACATTGTTGCTGGTGCTGGAGAACTCCATCTTGAAATTTGTTTGAAGGATCTT
GAAGAAGACCATGCCTGCATTCCAATCAAGAAGTCCGACCCAGTTGTATCATACCGTGAGACAGTCAGTGAAG
AATCCAACCAAATGTGTCTTTCCAAGTCACCCAACAAGCACAACCGTCTGTTCATGAA
GGCCACCCCCATGCCTGACGGTCTTCCTGAAGATATTGATGAGGGCCGCGTGAACCCACGTGACGACTTCAAG
ACCCGTGCCCGTTATCTTGGAGAGAAATACGAGTACGACCTGACTGAGGCGCGTAAGATCTGGTGCTTCGGTC
CCGAGGGCACAGGACCCAACCTCATGGTCGACTGCTCCAAGGGAGTGCAGTACCTCAA
CGAGATCAAGGACTCAGTCGTCGCCGGCTTCCAGTGGGCCGCCAAGGAGGGAGTCATGGCTGAGGAGAATCTG
AGAGGAGTTAGATTCAATATTTATGATGTCACCCTACATACTGATGCTATCCACAGAGGTGGCGGTCAAATCA
TTCCCACGACGAGAAGATGTCTGTACGCGTGTCTGTTGACCGCCGCGCCCAGGCTCAT
GGAGCCCGTGTACCTTTGTGAAATTCAGTGTCCTGAAGTCGCCGTGGGTGGTATCTACGGTGTGCTCAACAGA
CGGCGTGGTCACGTGTTCGAAGAGTCCCAAGTGGCCGGTACCCCCATGTTCGTGGTGAAGGCCTACTTGCCTG
TAAACGAATCGTTCGGCTTCACCGCTGATCTCCGTTCCAATACTGGAGGCCAGGCCTT
CCCGCAGTGCGTGTTCGACCATTGGCAGGTCCTGCCCGGTGACCCGTGCGAGGCTGGTAGCAAACCCTTCGCT
GTAGTACAGGAAACAAGAAAACGGAAAGGATTGAAGGAAGGTCTCCCAGATCTAGCCCAATACTTGGATAAAT
TGTAAACGCCGTAGCTAATGCAATACTGTAATAT
>1002052305_2 Heli.2-DT667036.3.5
GGAGTTTCTCGGCTGGTGTCGTGTACGACCTGCCTGGTTATATAAATAGGTGAAGAGGGGCAGCAGACACCTC
TTGCACTTTCTTGTAGAGCGCGACGCACCGGCCAGTCTTTGCCGAGCGCCATGGCTCGACACGCGTACCTGTG
TTTAGGGGTATTACTTCTCGCACACTTTGTGATTAGCGAGACACGAGTGAAACGGCAA
GATGAGGATAACGACGATGAAGTGAATCCGGAAAAGCTTTGCGACGGTCGCCCAGCCGACGAGTACTTTAGGT
TATCTGTTGAAGGTGACTGCCGAGATGTCGTCAGGTGTGACAAGGGAGGTGAAAATGGTGTGACTAGGCTAGC
TTCTGTGCGCTGCCCCGGCGGACTGGCTTTTGATATCGACCGACAAACCTGTGATTGG
AAAACACATGTTAAGAATTGCGATAAATTAGAAAAACCAAGAAAAATATTACCCATCTTGAAAACTGATGAAC
CAATATGCCCTGAAGGCAAACTATCTTGCGGAAACGGTGATTGTATCGAAAAAGAATTATTCTGTAATGGAAA
AGGTGATTGTAAGGATGAGTCGGACGAAAACGCCTGCACTGTTGACTTAGACCCTAAT
AGAGCGCCAGATTGTGACACCAATCAATGCAGACTTCCCGATTGTTTCTGTTCCGCTGATGGCACACGGATTC
CAGGAGGAATAGAGCCCAATCAGGTGCCTCAAATGATAACGATTACCTTTAATGGCGCCGTAAACGTTGACAA
CATTGACTTATATGAGCAAATATTTAACGGTAACCGTCATAATCCAAATGGCTGTCAA
ATACGCGGAACTTTCTTTGTCTCACACAAGTACACAAACTACGCAGCCGTTCAAGAATTGCACCGCAAGGGAC
ATGAAATCTCTGTTTTCTCACTAACACACAAAGATGATCCACAATATTGGACAAGTGGAAGCTACGATGATTG
GTTGGCTGAAATGGCCGGTGCAAGACTGATCGTAGAACGATTTGCAAATATAAGTGAT
GCTTCTATTATTGGTGTTCGGGCCCCTTACTTAAGAGTCGGAGGTAACAAGCAATTTGAAATGATGGCAGACC
AATACTTCGTATATGATGCTTCGATAACAGCTCCCTTAGGTCGCGTTCCTATTTGGCCTTATACATTATACTT
CCGAATGCCACACAAATGTAACGGTAATGCTCACAATTGCCCCTCTAGAAGTCACCCT
GTGTGGGAAATGGTAATGAACGAATTGGACAGACGTGATGACCCTACTTTTGACGAATCTTTGCCCGGTTGTC
ATGTAGTCGATTCTTGTTCCAACATTCAAACTGGAGATCAATTCGCGCGTCTTCTTCGCCATAACTTCAACCG
TCACTACTCTACCAACCGCGCTCCTTTAGGTCTTCACTTCCACGCGTCGTGGCTTAAA
TCCAAGAAGGAATTCAGAGATGAACTGGTTAAGTTCATTGAAGAGATGTTAGAAAAGAATGATGTTTACTTTA
CCTCTTACACACAAGTAATGCAATGGATGCAAAACCCAACAGAATTATCACAACTTAGAGACTTCCAAGAATG
GAAACAAGAAAAATGTGATGTTAAAGGGCAACCTTTCTGCTCTTTGCCAAATGCTTGT

CCATTAACAACTCGTGAGTTACCAGGAGAAACTTTACGTTTATTCACTTGCATGGAATGCCCTAATAACTATC
CTTGGATTCTAGATCCTACGGGAGAGGGCTTCAACGTTAGGAAGTGATCTCGTTAAGTAAAATATTAAAAGAC
TGTACACATGTAAATAATACTCAATAATGTTAATGAATACAATTTATATTGTGACTGT
GGCAGATTAAACCAATGTCAACTATTAAGTGATCGTTAAAATTATTTTTGTTAAGGCTAAAACATTAAGATAT
ATATTTTTTTATTCTTAACGTTGATGTTTGGTTTACATTATATTTTAAAAGCTGCCGCTCGATGTTTACCAGC
AGAGATAGATAAATTTTCATTA
>1002052306_2 Heli.2-EL599996.1.5
TTACATTTAATAAACGCTGTGTTTCGGTTTTTTGTTTTTTTTGTTAGTTTATTGTTCAGCCAGCTATAAAAAA
AATGATTTGCGGATCCCCAGTGTTTCTGATCTTGATACATCTCAAGGGTCAATAGGAAACATTGGTGGCAACG
CGTTGGGCGGCATTCCTTTGGGTAACTTGGGTCTTGGAAATTTAGGAGGTCTTGGACT
ACCATTATTGGGCGGCGGAGAAAATTCTGTTGGCAATATCGCTGCTATTCTTCAAGCAAATGCGGGTTCCAGC
GACGGCGTAGGCAATGTTGCGAATATAGCTCAAAGCAATGTATCGGGCTCTGGAAATAACAACGGTAACAATG
GTGAAGGCGAAAATGATAACAACAATAATCAAAATGAGCAAAATGGTGCAATATTGTC
TATAGGAAACATTGCCGGCGTTATTCAAGCCAATGTAGCTTCCGGTAACAGTGATCCAAATGGTAACTCAGAA
ACGAATGGTGAAAATATTTCTAACGAAAACAATAATTCCGGTGGCAGTATTGGGAACGTCGCTAATATCGCAC
AAAGCGCCGCTAATACCGCACAAAGCGCCGCAGGAGGAAACTCGCACAATTTGGCTAA
TTTGGGCAACATTCTCGGCAATCTAGGCAATTCTGGCTCAAGCGCTTTGAATAATAACGGTGGTGGCGTAGTC
ACTATCGGTAACGCCGCTGGAGTCACACAAGCCAATCTTGGCAACTTTGGCAGTTTGGCTAATTTGGCCAATA
TACTTAGTGGCTTAGGCAATTCTGGCAATTCCGATCCAAATACTAACAATAATTCCGC
TGGCAGTAATGGTAACATCGCTAATATTGCACAAAGTGCTGTGGGACCAAGCACGATTGGCAACGTCGCTGGT
ATTGCGCAGACCAACGTTGGAAAATCGCAAGGAAATATTGGCAACATAGCGAATATAGCCCAAAGTAATGCTG
CTAGAGGAAACAGCGGCATCAAGGGAAGTTCAGGCATAATAAGTTGGGCTGACGTGAT
TAATTATGTAAACAGTAACCGCCAAAACAATAACAACAATAATGGAGGTGGCATCGGTAACATTGCAAATATC
GCCCAAAGCAATATTGGCGGCGTCGGTGTTGGTAACGCTGCTGGCATTGCCCAGATAAATGGTCTTCTTGGTC
TCATCGGTCTTGGCGGCGTCGGTGTTGGTAACGCTGCTGGCATTGCCCAGATAAATGC
TAATGGTCTTCTTGGTCTCATCGGTCTTATCAATTACAACGAGATCATCGAAAGGCCTTGTCAAGTGTTCGAT
ACATACTGCATAAAGAAGTATTTCGCTGAACACTCTAAATGTAAAGTAGCGCACGAGCCGGTACCAGATCCTT
TGTACAGACATCAGTCCACTATGTTTTTGGGTAGAATAAATGTTACGACGACTGCGAC
TGACGTTTTGTATGGAGGTCTAAATGGGAGAATTGAAGATTTCTACATCAACAGAGAAACTGACAGACTAGTG
CTGACTATTGAATTTAGGAATTTGACGTTCTACTCTAAATACGCTTACTTCAGATTCCACAGACGAGCCAGAG
AACCAATTGTCACCAGGGACTACTTATACTTCAATTACCAGTCTGTGGTATCTACTAT
AATAATACCAAACATAAAGGACCTGCAATTGGAAAAGAGTGAGACTTTCATATATAGTAACGACCGTACGCCA
ACATTCAGTATCGGCCCCAACGGATTTAATAGTCCAGACCCCACAGTCCAGCTCAGCAGGATCCAGTTGCTCG
AAGACGTTACCACCATTGGTCAAGAGGCCCAACTTACCGAGGGCGCATACTACACATC
TACGTACTTACAACATAGTATTTGTGACTTTGGTCTTAAAATACTTTAAAATCACGATTTAAAATATCGAGAA
ATAAAGACATTTTTGAAATAAAA
>1002052307_2 Heli.2-DT665713.3.5
GCACGAGGGTTACACGAGGAAGGAAGTGACTATTGCTGTTATACAAAAGGCATTATTTTGAAATCCGACTGAG
AATATTTCTATCAAGCTAATATTTGAGAAAGATTCATCATGCAAATTTTCGTAAAAACCTTAACCGGGAAAAC
CATCACTTTGGAAGTGGAAGCTTCTGATACAATCGAGAACGTAAAGGCAAAGATTCAA
GACAAGGAAGGCATTCCTCCAGATCAACAAAGACTAATTTTCGCTGGAAAGCAACTGGAAGATGGGCGCACAC
TTTCCGATTATAATATCCAGAAGGAATCTACTTTGCACTTGGTGCTGCGTCTTAGGGGTGGAATGCAAATTTT
CGTAAAGACACTGACTGGCAAGACTATCACTCTGGAAGTTGAGCCTTCTGATACAATT
GAAAATGTAAAAGCAAAAATTCAGGATAAAGAAGGAATCCCCCCAGATCAGCAAAGGCTTATTTTTGCTGGAA
AGCAGCTTGAAGATGGACGCACACTATCAGACTACAACATTCAGAAGGAATCTACTTTGCACTTGGTACTACG
CCTGAGAGGTGGAATGCAAATATTTGTTAAAACATTGACAGGCAAAACTATCACATTG
GAAGTGGAAGCTTCTGATACAATCGAAAATGTAAAGGCAAAGATTCAAGACAAGGAAGGTATTCCTCCAGACC
AACAAAGGCTTATTTTTGCTGGAAAGCAACTGGAAGATGGGCGCACACTGTCAGACTATAATATCCAGAAGGA
ATCTACTTTGCACTTGGTGCTGCGCCTGAGAGGTGGAATGCAAATTTTTGTAAAGACA
CTGACTGGCAAGACTATCACCTTGGAAGTGGAAGCTTCTGATACAATTGAAAATGTTAAGGCGAAAATTCAAG
ATAAAGAGGGAATTCCCCCAGATCAACAAAGACTTATTTTTGCTGGGAAGCAGCTTGAAGATGGACGCACACT
ATCTGACTACAATATTCAGAAGGAATCTACTTTGCACCTGGTACTACGGCTGAGAGGT

GGAATGCAGATATTTGTAAAAACTCTTACCGGTAAGACTATCACTCTGGAAGTTGAGCCTTCCGACACAATCG
AAAATGTTAAGGCAAAAATTCAAGATAAGGAAGGCATTCCTCCAGACCAACAAAGGCTTATTTTTGCTGGAAA
GCAGCTTGAAGATGGACGCACACTCTCCGATTACAATATACAAAAGGAATCTACCTTA
CATTTAGTACTGCGTCTGAGAGGTGGGATGCAAATTTTTGTGAAAACACTGACTGGAAAAACTATTACTTTAG
AAGTAGAAGCTTCTGATACTATCGAAAATGTTAAGGCGAAGATTCAAGACAAGGAAGGC
>1002052308_2 Heli.2-DT666410.3.5
TATTGAACGAAAATAAAGGCGATGGTGTGGAAGATTATAACATTTTTGGGCATGGCGCTTTTGGCGCTAAGTG
ATGCCAGAGCTCCTATATCAAAACAATCTGTAATAATTGAAAATTATGAAAAGCTTTCACACGTTGAACCTCA
AAAGCCTGAGCATCATGAGGTATACGCTTGGGCGTATCCCTCTTATGAATTTAAGTAT
GAAGTTGCTGACCCTCACACTCATGACTTCAAAGGACACCATGAAACGCGACATGGTGACGAAGTAAATGGAA
AATATTGGCTTATTCAACCAGACGGATTTAAACGAACCGTCAAGTACTACGCTGACAAAAACAGCGGTTTCAA
AGCAGATGTTGACTATAATAAGCTATATCAAAAGTCAGAAGAAAATAACGAAGGAAAT
GAAGAAAATGCAGGTGGTGAGGAAGAAAATACGACCGAGGAAAGTAGTTCTATAGAAAATGAAAATAGTAATA
ATGAGGAGGAAAGTACAAATGAATCACTTAATAGTAATAACGATGATGAGTCCACGAGTGTTGGTGGAGAAAG
TGAAAATGAAAACAATGACATGGAAGACGGAGCTGGAGGTGAAGAATCTAATGATGAA
GGAAATGATAATAGTGAAGAAAACGCACAAGAAACAGAATCCAGTCAACAGAACGGTGAAAGTGAAAACGATG
AAGAAAGTAATGAAAATGAAGGGAGTGATAATGGTAACGAGGAAAGTTCTGCTGTTGAGAATAAAAAAGAGAA
GCATGTCGTGAAACATGAATATCATTTTATAATCCATCATCCAAAATATATTAAAGCT
AATATCTGGGATTGAATAATTTAGCTATGTAAACAGTTACTAATATCCCGCCAAGAGTGTAACTATAGTGTAT
TGTGTTGTAAGATTTAACATTTA
>1002052309_2 Heli.2-DT666684.3.5
AGTCGCACGCTCCTGTTATGCTAAAGTGGTGAACCCACGCTACAAACATGGAGGGGACCATCCCCAGCCAAGT
CATGCCTGAGCCCGACGAGGGTATCTCCACCGACCGCCGCCCGTCCACCGACGACCATGCCGACCTGTCCGAC
ACGGCGTCCGAGGCCGGCTCTGGTGGAGGCAAAGACTCCGGCTGCGAAGTCGCTCCGG
ATCCCCAGGAACCTCCCTACACACCACCCGACGAGGAACTGGCCAACCGCATCGTGTCTCAAGTGGAGTTCTA
TTTTTCGGACGCAAATATCACTAAGGACGCCTTCCTGTTGAAGCATGTCCGTCGTAACAAGGAAGGGTATGTC
TCACTCAAGCTCATATCCAGCTTCAAACGCGTCAAACACCTGACCAAGGATTGGCGAG
TGGTCGCTGAAGCTTTGAAGCGATCGACAAAATTGGAAATAAATGAACCGGGAACGAAGCTTCGTCGAATTGA
CCCCCTGCCCCCGTACGACGAAACTACCCCGTCCAGGACTGTTGTGGCAGTCAAAATGCCCATAGATCGCCCT
TCAGTCGAAAACGTTTCAAGATTATTTGCTAGTTGCGGTGAGATAGCTCTGGTACGTG
TGCTGCGGCCCGGTAACCCAGTCCCAGCGGATGTCCGTCAATTCCTCAACAAAAATCCTAGTTTGGTGAATTG
TGTGTGTGCTTTGGTAGAGTTTACTGAATCTGAAGCTGCGAGAGGTGCCCTTCGTCTTCAAAGTTCTGACGAG
GAAGGTATGCGAGTGTACGAACTAAACGGTGTCCCTCGGGAACCTAAGAGGAAAGTTC
CGGCGCGGCGTCCAGCGACGACAAGACGCCACGAATGCGAGTACTCCTCATGTTGTAGCGGTTCGGAGGCCGA
GTACGACTATAGATACACAGCACCATTTTATAGACGGAACTCGAGTGGATTCTTCACACCACGGTCCCCGGAA
ATACAAACATGGGTGCCACGCAGAACCTCAACTTGCAGTCATAGTTCAGATTCTGGGG
TTTCTTTCTTCTGCGGCTCGCGGAGGGCGTCACAGGCTAGCACCGGAAGTGCGAGCAGCGCCGAGGGCTGGCT
AGCACGGAGGTTGTCCGGTTGTTCAATAACCGGAACCGAATGCGGCGGAAGGAGGTTGTCGTGCACGCCACGA
TTCGAACCCCGCACGCCGGTAGTCCCGGACGGCACGCGCGGCTTCCACGCCGCCGCAC
GCCAACGCAGAATCTCGGACCTCGCGTTGTATTCGCGCTAGAAAGCGATCGCCAACCGTGACACCTTTCTAAT
AACGTTGGATAACCACCTTTCACGTTCTGGCCACTTGGTTTATAATCTACGAGGAAGCGTTTCGTGTTCGCGT
GCTTAGCTCTAAATTTTAATATAAAAATATAATATAGTCGGTATGCTGGAATGAAACT
GTAAGGTAATGAGTTACCTAGTTAGACGCGACGGTGAGTCGACGTAATGTGATCGAAGCTCATTTAGTTAGTC
TAGTTGTTAATTTGTAACAGATATTCCTTTTAATTTATTAGAATATAATTTATTTATTGTTATTGCCGCGTAT
TTATTGCGGACAGGTCGCGCGCTGTGGGGAGTTTTCATCAGAGATTTCATGTCTGAGT
TGTGAGCACAAAACTCAAAAAGCAAAATCGACATCTGAAACTGAACCTGTAGACTCGACATATGGTACGGCGG
CGGCCGGAGACCGCGGCGAGAGCACGTTGCGTAAATAGGATTGACGATTTATATGGATG
>1002052310_2 Heli.2-EL598039.1.5
TTGATACGGTGGTGTGTAGCGGAACAAGTTAGCGATGTCTCGACTTACGATTCTCTTTGTCATCGTTGCGGCG
TACGAAGTACTTGGAGATGTAAGTAAAGTTGCCGATTCTAGCGCAGCTCAGAGTGGAATAGTTGGATATGGCG
CTGGTATTGGCCTCGGTGTGCCTGCAGCGGGCATCACCAGCGGTGTTATCGGATCACC
TGGCGGAGTCCTCGTGGGATCTGGCCTCTACGGTGGAGGTGGTGGCTTAAATAATGCTGCCGCTGTTGGTGCT
GCTCAGCTTAGCGCTATCCAAAACGCTCAAGCTGTCCAGGCTGCACAGATCGCAAATGCAGCCGCTGCTGCCA
TCCAAGGAGCACGTGTGACTGAAGCGGCCGCTAGGGCAGGCCAAGCTAACGCTATTCA

GAATGCTCAAGCTTTAGATGCAGCTCGTATTGCTAACATACAAAGAGCCCAGGCCGCCGCCTACGAAAATGCT
CGTGCCGCTGAAGCAGCTAGACGTGCTGCTGCTGCTGCAGCTGCTGAAGTTGCTCGTGCTGTTGAAGCTGAAC
GTGTAGCCAATGCCGCTCGTGTCCAGGCTGCTGCTATCGCTAACGCTCGTGCTGTTGA
AGCCGCACGTATTGCTAATGCTGCTAGAGCCCAAGCTGCCGCAGTTGCCACTAGTGCTGCTCAAGCTCAGGCC
GTTGCTGACACCGTAGCAAGAAATGCTCAAGATGGCGCTTTATTACTCGGTGGAGGTGGTGGTGGTGGTTTGT
ACGGCGCAGGTGGTCTCGGTGGTGCAGTGATTGCAGCAGCTCCTGTGGCCGTCGAAAA
AGTCGCGGCAGTTCCCGTCGGCATCGGATACGGACTTGGTGACGGTCTCGGACTCGGTCTTGGTGGCCTTGGC
GGTCTTGGCGGCCTTGGCGGTCTTGGCGGCCTTGGTCTTGGATATGGAAAGAAGATATAGAATAACGATTTCT
GCATATTGTATTAGTGGTGTTCCCCAATAGATAGAGATATGTAATTTCCCCTTCCCTT
TTGACTCCATCCAAGGAAATGGCTAGGGGCATTTTTTTGTTATTTTGATTAAATAAGTAC
>1002052311_2 Heli.2-DT666012.3.5
TCCTCCGTGGCATGGCGGATGGTCTTTACCGCCGCATTTAGTGTTTATTTGGAGGAATAACTTCGCTGATATA
GCGAAATGAGTCTATCAGTAGCCCGACCTTTAGTGTCGGTATACTCTGAGAAGAGTGAGAAGGTGGCCGATGC
CTCGGTCCCTTTGCCGTTCGTTTTCAAGGCCCCCATCAGGCCTGACCTGGTCAACAAT
GTCCACGTTTCTATGTCCAAGAATGCAAGACAACCTTACTCTGTTAGTAAGGAGGCAGGTCATCAAACCAGTG
CTGAGTCATGGGGCACAGGACGTGCTGTAGCGCGTATCCCCCGTGTCCGTGGTGGTGGTACCCATAGATCAGG
CCAGGGTGCGTTCGGCAACATGTGCCGCGGTGGTCGCATGTTCGCACCAACCAAGCCG
TGGCGGCGTTGGCACCGCCGCGTTAACCTACGCCAGCGCAGGGCTGCTGCGGCGGCCGCTGTTGCCGCAGCTG
GAGTGCCAGCGTTGGTACAGGCCAGAGGTCACATCATCGAAAAGATTCCGGAACTTCCACTTGTGGTCTCCGA
CAAAGTGCAAGAGATCAGCAAGACTAAAGATGCTGTTATCTTCTTAAGAAGAGTTAAA
GCCTGGTCTGATGTCCTTAAGGTGTACAAGTCACAGCGCCTGCGCGCCGGCAAAGGAAAAATGCGCAACCGTC
GTCGCGTGCAACGCAAGGGACCCCTCATCATATACCACAAGGATAAGGGTCTATCCAAGGCTTTCCGCAACAT
CCCCGGCGTAGAAATGCTGAGCGTGAATAAACTGAACCTCCTGAAGCTGGCTCCAGGT
GGTCACGTGGGCAGGTTCATCATCTGGACACAGTCCGCTTTTAAGAGACTAGACAGCCTGTTTGGTTCATGGA
AGACACCATCCAAGCTGAAGAAGAACTTCAACCTGCCTCAGCCCAAGATGGCCAACACCGACCTCTCGCGCCT
GCTCCGCTCCGACGAGATCAGGAAGGTGCTGCGTGCCCCCAACAAGAGGGTAGTCCGC
TCCACCAGGAAATTGAACCCGTTGACCAACTCGCGGGCGATGTTGCGTTTGAACCCGTACGCCGCTGTGCTCA
AGAGGAAAGCTGTGTTGGACCTGGAGCGCAGGAACAACTTGAGAGTCGCGGCCGTTGCTGAAAAGAGAGGGAT
CAAACTCCCCGAATCTGACCCAGCAGTGAAGGCACAGAAACTGAGAGAGAAGAGAGCC
AAGTCAATCAAGTTGGCTCTCTCCAAGAAACCCAAGAAGCCGGCAGCGAAGAAGACCGCGCCTCCGCCTAAGA
AGGTGAAAAAGGAGAAGGCGGATAAACCGGAGAAGAAAGCGGTGTCTAAAAAGTGAATTGAATAAACATTTTA
ATTGACAAAAAAAAAAAAAAAA
>1002052312_2 Heli.2-DT668678.3.5
CACGAGGGCAGTACGAGGCAGAAGTGCAAGATGAGAGTTCTGATTGTTGCCGCCGCCGTCCTCGCCTGCGCCG
CAGCTGCGCCATCTGGCGCTCTGCTGGCTGGTCCCTACGCCTCCGGGCTGCTGGGACATGGTCACGGCCTGGC
TTTAGGCCATGGCGCACCTCTGGCCTTAGCTGGTCCCTTGGCTGTAGGTCACGCTGCG
CCTCTTGCCATCGGCCATGGCGCCGTCGTCCCCACCATTCCTCCAGGTGATCTCCAGGGTGCCGCCATCGACG
CTCACGTCGAAGCCTCCGACCACGCACGCGCCGCCGTCGACGCCGCCCGCGAATACCATGACCAGGCTTCCGA
GCTCCAGGGTCAAGCTGTGAACGCTGCTGAAGACCATGCCTGGCAAGCGGTTGACGCT
GCCCAGTCCCACAAAGCTGCCGTCGACGGTGCTGCTGCTGGCGTCGCCCCCGTGGTTGCCCGCCAGTTGGCTG
GACACGTCGCCCCCGTCGCCTACGCCGGCCACGGTCTCGCCGGTCACGGACTCGCTGTCGCCGGTCATGGTCT
CGCTGTAGCCGGTCATGGTCTCGCCGGCCCCCTCGTCGCCGGACACGGCCTCGTCGGA
CACGGTCTCGTCGGACACGGTCTTGCCGGACACGGTGTTGCCCTCGGTGGTAGCCATTCCGTAGCCTCATCCT
CCCTGTCCCAGCCCGCTCCCCTCGTGCACGCCCCCGCCCCCGTCGCGTACGCCAGCCATGGTGCCCTCGGCTA
TGGTGGTCTCGCTCACGGACACGGTCTCGCGCACGGATGGTAAGCTGTGATAGGTGTT
TAGCCAAAAAATCTTAATACTCATGTTCGGAATGTATATTTAATTGTATAAAATGAAAATTGTACAGACAGGA
GGCGAGGATGTGTGAATAAACGA
>1002052313_2 Heli.2-DT665318.3.5
TTTATGAGCATTTATACTATTCAATGTGTATATTTTGAATAATAGTTATTAGTATATCATATATCCCAATTCC
TACCACCTTCCAAGATAGTTTCATTACGTTTTCGATTTAACAGCTGTATTTGTTTAATAATAATAAGTGAATC
CTTCATACCGCAAACAGTTAACGTGATACTTGTTTATCGCAACCGATTCTATATAAAG
GATCCGGCAAGTGTCCATGCAGTATTTGTGAATAAAGTCGTGTGTAGTGTGTTGCTCTTTTTCAACGAAGATT
AATTAAAACAAAATGGCCTCAAGAGAGCCCGCTTCTGATCAACTTGTAGATTACAAAAAATCTGCAAAGGATC
CTATTGGTTATATAACGACTAAATCTGGTGCACCGGTGGGAGTAAAGACTGCGATTCA

AACAGTTGGAAAAAATGGACCTGCTCTTCTGCAAGATGCCCAGTTTCTGGATGAAATGTCCTCTTTTGATAGG
GAGCGTATTCCAGAAAGAGTAGTTCACGCAAAGGGAGCCGGCGCTTTTGGATTTTTCGAAGTAACTCATGATA
TCACTAAATATTCAGCAGCTAAATTATTCGACGGGATTGGTAAAAGAACTCCCATTGC
TGTTAGATTCTCTACAGTAGGTGGAGAAAGTGGATCGGCTGATACTGTTCGTGATCCTCGTGGTTTTGCTGTC
AAATTCTACACTGAAGATGGGATCTGGGATCTTGTAGGAAACAATACGCCAATTTTCTTCATTAGGGATCCCA
CACTATTTCCAAGTTTTATTCATACTCAGAAAAGAAACCCTGAAACACATTTAAAAGA
TGCTGATATGTTTTGGGATTTCTTAACTTTAAGACCGGAGTCAATGCATCAGGTGCTTTATTTATTTGGTGAC
AGAGGAATCCCTGACGGTTACAGATTTATGAATGGTTACGGATCCCACACCTTTAAGTTAGTTAATGCACAAG
GAGTTGCGCATTGGGTTAAATTTCATTATAAAACTAATCAAGGCATTAAAAATTTGTC
TGTTGATAGAGCTGCAGAGCTTGCATCGTCTGATCCTGACTACGCGATCAGAGATCTTTATAATGCAATTGCA
AAAGGTGATTGCCCCTCTTGGTCATTTTACATCCAGGTTATGACAATGGCACAAGCCAAAACTGTTAAGTTTA
ATCCATTTGATTTAACGAAGGTTTGGCCTCATTCCGATTATCCTCTTATTCCTGTGGG
CAGGTTTACGTTGGATAGGAACCCTAAAAATTATTTTGCTGAAGTGGAGCAGATTGCCTTTAACCCAGCTAAC
TTAGTGCCTGGTATTGAGCCTTCACCTGATAAAATGTTACAAGGTCGCCTATTTTCATATGGTGATACTCATC
GTCATCGCCTCGGCGCCAACTATCTTCAGATACCGGTTAATTGTCCCTATCGTGTTAC
TGTTTCTAATTATCAGCGCGATGGACCACAAGCCATAAGTAACCAAGACGGTGCTCCCAATTATTTTCCGAAC
TCCTTCTCAGGACCACGAGAATGCCCTCGTGCTCATCGTCTCCAGCCAAGATACAATGTTAGCGGGGACGTTG
ACAGATACGACAGTGGACAAACTGAAGATAACTATACACAGGCTGGCGTTCTGTATAA
AAAAGTATTTAGCGACGCTGAAAAGGACCGTGTTGCTGCTAATTTTGTTGGTCATTTAAAAGATGCCGCAGGT
TTTATACAAGAGCGTGCAATAAAAAATTTCTCCCAAATTCATCCCGATTTAGGAAGCAAGGTAGCTGCTGGGC
TTGCACCATACAAAAAATTCCATGCTAACTTGTA
>1002052314_2 Heli.2-DT664918.3.5
CGAGGGTTTGCTCGTTGCGGGGTCTAGACTGTGGTTTAGGAAAGTTTAGTTTTATTTTATAAATAAAGAACTC
AATCAAAATGAGCTGGCAAGATTATGTCGATAAACAGTTAATTGCATCCAGATGTGTTACAAAGGCTGCTATT
GCCGGTCACGATGGTAATGTCTGGGCCAAATCAGAAGGATTCGATATATCAAAAGAAG
AAGTAGGTAAGATAGTGGCCGGTTTCGAAAATGAATCACTCCTCACCAGCGGCGGCGTGACGATAGCGGGCAC
GCGGTACATCTACCTTAGTGGAACAGACCTCATCATACGCGCAAAGCTCGGCAAGGTCGGCGTGCACTGCATG
AAGACAAAGCAAGCTGTTGTCATTTCTCTCTACGAAGAACCGATCCAACCCCAGCAAG
CTGCATCTGTAGTGGAGAAGTTAGGAGATTATTTAATTACCTGTGGTTATTAAAGATCGAAGCGCGCCGTGAT
CTAAGAATACTATAAGATAATATTTTTTCCACAGGGAAATAAGTAACTGCATTTTGTATATTCTATAAAAGTG
ATAAGGACCGTCATTTGTTTTGATATTTGATAAATTATGGCGCCATCAGTGTCTGATG
CGCTCGGCGGCCACGTGCAGGCGTAATTACTTTGCGGATTTTGGACTCTTTTTATACGCTCACCTTTTCCACA
GAGTGATTGTTTCTGTATTGTGGTTTTTATTTGTATTTTAATAAAAAATGCTTTTATCGACCTATACAGTGAG
GCTGAACGGAGCTTTGCGAAGGTCTCGAGGCATGATGTATAGAGATTTTTTTTAGATT
CTTTTCAAGCTGGTCTGACAACTTCACTCCGCGCCACCGCGGTCGCTCTGACCCGGTGAATGGCTCAACGTGG
AACAAATGATAGCTAAATCAATGAAATCGCACACAGACAAGCTTGATAAGAGATAAAATTAAATAACCACGTA
ATGAAATTACTGTAACAATATACAACAATTATATATGTAATTACAAATAGGCATTTGT
GTAAGGGCTTGTTACAAAATGAGAGACACTGTAACCCAATTAAGTAAACATGTAACGATTTTAAATGAAAATC
TCTAGTCCCACTGGAACCATCGTGTGAGAATCTATAACGACAGTGTGCAGGGGAACCCCCGCGATCGCCATTA
TTTCGCTATGCTTCCCTTTTTTTTATTACGCCGCGTTTATAATCTT
>1002052315_2 Heli.2-DT662899.3.5
CGAGGGTCGAACGCAGTGTACGTGTTTATCAACGCCTTATAAACCAAATCCCTTAAACAATGGCCTGCTCAGT
AGACGCCCCCTCCCTCAAGGACTTGCCCAAAGTGGCCACCGACCTCAAGAGTCAGCTCGAAGCTTTCAACCCA
AGCTGTTTACGCGACGTCGACACCAATGAAAAGATCGTCCTGCCTTCCGCTGAAGATG
TCGCCACCGAAAAAACTCAGAAGTCCCTGTTCGACGGCATTGAGAAATTCGATGCTACTAGGCTGAAGCATAC
GGAAACACAGGAAAAGAACCCACTTCCTGACAAAGATGTTGTCGCAGCGGAAAAGGCGCATCAGAACCTTCTA
GACGGCGTTGAACACTTTGACAAGACCCAAATGAAGCACACGACGACGGAAGAGAAAA
ATTCTTTGCCTGCCATTGAAGCCATTGAAGCCGAGAAGGAAAAGAACAAATTCCTGAACGGAATTGAAAACTT
CGACCCAACCAAGTTGAAGCACACTGAGACTTGCGAGAAGAACCCACTGCCCACAAAGGACGTCATTGAGCAG
GAGAAGACCGCTTAAACCATATATAACCGCAACTATGTATCGCTAGTATCGCCGTATT
TTAGTATATAAGTTTCGTATAGTCGGACCGTGGCGTCCGTCCGTTCGAACATGTAATGAAGACTCATATTTTA
ATCCCTTTAATCATACACTAAACGTGATTAAATATTCATTCATATATTATATCATTCACCTCTAATTTTTATT
TGTCATCCATTTTCATTCATTCATTTGATATTCACTACAAACTCCCATTTAGAAAATA
GAAAAATACTCCCATACGTTTTTC

>1002052316_2 Heli.2-EL602236.1.5
GTCGCGAGCGGAGTATACCACGATCCTTCGGTCTGCCATTTAGTTTGAATATTTGTGAAAAATGTCTCTAATC
TCCGCTCGTTTGGCCTCTTCGGTGGCCAGGCGCTTGCCTAATGCCGCTACACAGGTTTCGAAGGTCGCCCTCC
CCGCGGTGGCAGTGGCTTCCCGCAAGCTCCATGTCTCGTGTCCACAACGGGCTGCCGA
AATCTCCACAATCTTGGAGGAGAGAATCCTCGGTGCCGCACCTAAGGCCGACTTAGAAGAAACTGGTCGTGTG
TTGAGCATCGGTGACGGTATTGCCCGTGTGTATGGTCTTAAGAACATCCAGGCCGAGGAGATGGTGGAATTCT
CCTCTGGCCTTAAGGGTATGGCTCTCAACTTGGAGCCTGACAATGTTGGTGTGGTAGT
ATTCGGTAACGACAAGCTGATCAAGGAAGGAGACATCGTGAAGCGTACCGGTGCTATCGTAGACGTTCCCGTC
GGTGAACAACTTCTTGGACGCGTTGTGGACGCCTTGGGTAACGCCATTGATGGAAAAGGACCCATCGACACAA
AGTCCCGTATGCGTGTCGGTATCAAGGCCCCTGGTATCATCCCACGTGTGTCTGTACG
CGAGCCTATGCAAACTGGTATCAAGGCTGTGGACTCCCTCGTACCCATCGGTCGTGGTCAACGTGAGTTGATC
ATTGGAGACCGTCAGACCGGCAAGACCGCTCTCGCCATCGACACCATCATCAACCAGCAGAGGTTCAACAAGG
GAGAGGATGAGAAGAAGAAGCTGTACTGCATCTACGTCGCCATCGGACAGAAGAGGTC
CACTGTGGCACAGATTGTGAAGAGATTGACTGATGCTGGTGCCATCAACTACACCATCATCGTGTCTGCCACT
GCCTCCGACGCCGCGCCCCTGCAATACTTGGCTCCATACTCCGGCTGCGCCATGGGAGAGTTCTTCCGCGACA
ACGGCAAGCACGCCCTCATCATCTATGATGACTTGTCCAAACAGGCTGTTGCTTATCG
TCAGATGTCTCTGCTGCTGCGTCGTCCCCCAGGTCGTGAGGCCTACCCCGGTGATGTGTTCTACCTCCACTCC
CGTCTGCTCGAGCGTGCGGCTAAGATGTCCGACAAAATGGGCGGCGGTTCCCTCACTGCCCTCCCCGTAATCG
AGACCCAGGCCGGTGACGTGTCTGCCTACATTCCAACCAATGTCATTTCTATCACCGA
TGGACAGATCTTCTTGGAGACTGAATTGTTCTACAAGGGTATCCGGCCCGCCATCAACGTCGGTCTGTCTGTG
TCCCGTGTAGGATCTGCTGCCCAGACCAAGGCCATGAAGCAGGTGGCTGGTTCCATGAAGCTTGAGTTGGCTC
AATACCGTGAGGTCGCCGCCTTCGCCCAGTTCGGTTCTGACTTGGACGCCGCCACCCA
GCAGCTGCTCAACCGTGGTCAACG
>1002052317_2 Heli.2-DT664179.3.5
TTAAACTTCGAATTAATTTCCTTTCCTTTCTAATAACGAGGGTCGAGACTGACCCTGCTAAATTAAAATCTCA
AGAAATCAAAATCATCACCAGACAATATTAAAATTTAAATATGTCTGGAAACTGGAACAATAGCCGCGGTGGA
AGCGGTGGTTCCAAATTCGGCGGAGGTGGCAAGTTCGGCGGACATGGAACATCAAGAT
TTGGAAACGGTGGATCTAAATTCGGTGGGGGTGGTGGTGGATATGGCGGAAAGAAAGAGTTTTCAGGTGGTCA
AAATATGAAAAGACCTAACTGGGACTCCATGTCATTGCAACCATTCAACAAAGACTTCTATAACCCACCAGAA
TCAGTTCTGAGTAGATCACCTTATGAAGTAGAAGAATACAGAAATCAACATGAAATTA
CTATCAGTGGAGTAGAGGTCCCTAATCCCATCCAGCACTTTGAGGAAGGGAATTTTCCTGACTATGTCATGCA
ATCTATCAAAGGCATGGGTTACAAAGAACCTACACCCATTCAAGCTCAAGGCTGGCCAATTGCTATGTCTGGC
AAGAACTTAGTTGGTATTGCACAAACTGGCTCTGGTAAAACATTAGCTTACATTTTAC
CTGCTATTGTACACATTAATAACCAGCCTCCAGTCAGACGAGGTGATGGACCTGTTGCCCTGGTCTTAGCACC
TACCAGAGAATTAGCACAGCAGATCCAACAAGTTGCTAGCGACTTTGGCAATGCAGCTTATGTTCGTAACACT
TGTATATTTGGAGGAGCACCCAAAAGAGAACAGGCACGTGATTTAGAAAGGGGTGTTG
AGATTGTAATAGCCACACCAGGAAGATTAATTGACTTTTTAGAAAAAGGAACAACTAATTTGCAAAGGTGTAC
CTATTTAGTTTTGGATGAAGCTGATCGCATGTTAGACATGGGCTTTGAACCGCAGATAAGAAAAATTATAGAA
CAAATTCGCCCTGACAGACAAACTTTGATGTGGTCTGCCACATGGCCTAAGGAAGTGA
GGAAATTGGCGGAAGACTACTTGGGAGACTATGTACAAATTAATATTGGATCCATGCAGCTGTCTGCTAATCA
CAACATTCTACAGATTGTAGATGTGTGCCAAGAACATGAAAAGGAAAACAAATTGAATGTGCTGCTTCAAGAG
ATTGGTCAAAGTCAAGATCCAGGTGCAAAGACTA
>1002052318_2 Heli.2-EL600955.1.5
CGAGGGTCGACATGCGCTCCCTTGTTCTCTTCGCTCTCCTCTCGGTGGCTGCGGCCAAGCCCGCGCCCGGACT
TCTTGGTGGATGGGATGGCGGTCTAGGCCTTGGCCACGGCTCTCTCGCGCTAGGGCACGCGCCCCTCATCCAG
GCCGCGCCGGTGCTCCAAGCGGCGCCCGTCATCCAGGCGGCGCCCGTCGTGCACGCCG
CGCCCGTCGCTATCGCCAAGGCTGCCACTAGCTACTCGTCCATCCAGAGGGTTATTCATCCAGTAGCTCAAGT
GGTGGCCCAGCCCGTGGTCCATGCCGCGCCCGTAGTCCAGGCCGCCCCCGTAGTCCAAGCCGTCGCCCAGCCC
ATCCTGAGCCATGGCATCGGCATCGGTAGCCTGGGCCATGGTATCGGCCTCGGCGGGC
TGGGTCATGGTATCGGCATCGGCGGGCTGGGTCATGGTATCGGCATCAGCAGCCTGGGCCATGGCTGGTAGTT
AACTTATTCGTAATTAATTAATGTTCGTTAGCGCGTTGAGATCATGCCCGATTTGTCGGGTGGTGCAAATAAA
TATTTATATCAGTATTTGTAAA
>1002052319_2 Heli.2-DT664377.3.5

GCACGAGGCGCGATTGGAACATGTCCTATTCACCTGAAACAAGATCGGAGGCTTGGCAGGAGGATAAAAATGG
CCCAGCCAAGGATCAAGGCAACTATGATGGACCTCCGGGCTTGGACTCGGTTGGCGGCGCTCTCGACACGAAC
TGGCATGAAGTCGTGGAAAGCTTTGATGACATGAAATTAAAAGAAGAATTATTAAGAG
GGATTTACGCTTATGGTTTTGAGAAACCTTCAGCAATTCAGCAACGCGCGATTATGCCTTGTATCCAAGGTCG
CGACGTTATAGCGCAAGCTCAGTCAGGAACTGGGAAAACCGCTACGTTCTCTATTTCTATTCTTCAGCAAATT
GATACTAGCATTCGCGAATGTCAAGCACTCATTTTGGCCCCGACTAGGGAGTTGGCTC
AACAGATTCAGAAGGTCGTAATTGCGCTCGGTGATCACTTGAATGCTAAATGCCATGCCTGCATTGGTGGTAC
AAATGTGCGTGAAGATATTCGTCAGTTGGAAAGTGGTGTTCATGTGGTGGTTGGAACCCCTGGCCGTGTATAT
GATATGATTACCCGTCGTGCCCTCCGTGCCAATACAATTAAGCTATTTGTACTTGATG
AAGCTGATGAAATGTTGTCTAGAGGATTTAAAGATCAAATCCACGATGTATTTAAGATGCTGTCTTCTGATGT
GCAAGTTATTTTGCTGTCAGCTACTATGCCTGACGATGTGTTGGAAGTGTCACGGTGCTTCATGAGAGAACCC
GTGCGCATTCTAGTGCAAAAAGAAGAGCTCACCCTGGAAGGTATTAAGCAGTTCTTTA
TTTCCATCGAGATGGAGGACTGGAAATTAGATACTCTTTGTGACTTGTATGACACACTCTCCATTGCCCAGGC
TGTCATTTTCTGCAACACTCGCCGCAAGGTGGACTGGCTCACTGAGTCTATGCACAGCCGCGACTTCACCGTA
TCGGCTATGCACGGCGATATGGATCAGCGCGAACGTGAAGTCATCATGAGGCAGTTCC
GTACAGGGTCGTCCCGTGTTCTCATCACCACTGATTTGCTAGCTCGTGGCATTGACGTGCAGCAGGTATCCTG
CGTTATCAACTACGATCTGCCTACCAACCGTGAGAACTATATTCATCGTATCGGAAGAGGTGGTCGTTTCGGT
CGTAAAGGAATTGCTATCAACTTTGTGACTGAAGCAGACAAGAGAGCATTGAAGGATA
TTGAGGAGTTCTACCATACAACTATTACGGAAATGCCCAATGATGTGGCCAACCTCATCTGAGGCGCCAGCGC
ATTCCAGTTTATACTTTGTGTCCTGCATTCTATTGCGATATTTAAACGGTGTGAGTATTAGGGGCAACCTCCT
AAGTACCGCTGTGCTGATTTTTATTGGAACTCATTTTATTTGGACCGTTGTATTGGTT
TAGTTTAATGAAATAATAATACATTACTATAATATTATTGATATAAGTTTAATATACATAGTCGTAAGCCCGG
CTCGGCCGGATGGCCCCCGTTCCGCGCGGACCCCGCTGCTACCTGCAGAGGGGTCTCATCGGGTACTGGAGAG
GCTATTTCAATCACAAAAGTACTAGAAGGAAATAATGTATTGTAGCTTTTTTCCAGTT
GTACATAATATGGTAAACTGTTTTTTGTAATACAGTAAATAAATATGA
>1002052320_2 Heli.2-DT663086.3.5
TGCGGATCGGCCACGTATTGTACACAGTGCTACTGAAATTCTTATAAGTGTTCCTCAAAAGTGTCTTTTCGTG
TGTAACTTCTTTTTAATATACTAAGTCAAGAAATTTTTCAAGTGAAAATTTAATATCGCATGGTGAAAATGCG
GTGGTGTCTATTAGCTCTGGCGTGCGTGGTCGCGGCGGTCTACGCCGACGACAAGAAG
GAGAAGGATAAGGATATTGGTACCGTCATCGGTATCGACTTGGGTACCACTTACTCATGTGTGGGTGTATACA
AGAATGGACGTGTTGAAATCATCGCCAACGACCAGGGTAACCGTATCACTCCATCTTACGTTGCCTTCACCGG
CGACGGTGAACGTCTTATTGGAGATGCTGCCAAGAATCAGCTTACGACCAACCCCGAA
AACACGGTGTTCGACGCTAAGCGTCTCATCGGTCGCGAATGGAGTGACACCACTGTCCAACATGATGTTAAAT
TCTTCCCATTCAAGGTGGTAGAGAAGAACAGTAAACCCCATGTCTCAGTAATGACCTCCCAAGGTGACAAGAT
CTTTGCCCCTGAAGAAATTTCCGCTATGGTCCTCACTAAAATGAAGGAAACCGCTGAA
GCATATCTTGGCAAGAAGGTCACTCACGCTGTTGTCACTGTACCAGCCTACTTCAACGACGCCCAACGTCAAG
CCACCAAGGACGCTGGTGTCATCGCTGGCCTTAACGTTATGAGAATCATCAACGAGCCTACTGCTGCCGCTAT
CGCTTACGGTCTTGACAAGAAGGAAGGTGAAAAGAATGTACTCGTTTTCGATTTGGGT
GGTGGTACCTTC
>1002052321_2 Heli.2-DT668469.3.5
AAACGTAGTTTTAAGCAGGAAAGGAACCGTTGCAGTGTTTTTTTACTAAAATACTAAGAAAATGGGTTCGCTG
GGCGTGAGCGTGTTCTTTCTTGTGATGCTAGTGCAGTTTAATTTGATTTATTCCAAGCCATTTTTCTTCGATC
TCATTTGTGACCTGATCTGCGACGACGACGACTCGTCCACATCAACTACCACCAGCGC
CAGCACCGAAGATGACTACTCGGATTTTGATGATTGGTGTTCATGTACTCGCACTACGAGACGGCCACCTGGT
ACCAGAAGGAATCAGGGGAATATGCCGGCAGCTATTAACATGATATTACCACCATCCAACGGCATGAACCTGA
CCATGTTCCAAAGAAACGGTGCATGGACTTTCGATTTGAATGCTGTACCGAATGGTTT
TGGTAATCGACCTGCCGGAGCCAGCCGGCCACCCACTACTGGAGCCCCTACCACGGCAGCTACTACCGCGGCG
GCAGCGGCGACTACTACTGCTGCTAAATAAAAATCTTTAAAATTATTATTGTTTAGAATGAAATCTATGAAAA
TCTGTAACATTCATATAAGAATTGGACGTGACTCCTCTATACAAACTAAGTAGTCTTA
GTTATAAAGTACGTAGACCGTTTCTAAGAGATATTGGGAAATTGTGCTACATTTAATATTTTATATGAAATTT
AGGCAGAGGGAACCTAGTGTTAAGACACTCCGTTCCCCACTTTCTGCCGAATTCTGGAGGCATTGCGTGTTGA
GTGGCAGAACTCAACGCCGCGCTTAACCTCGACACCAGAGCGAAGAAATGGAAATATA
AATTTAAGTAAATATTTCATCTCCTCGAGTGGGGATCGAACCCACAACCAGTCGGTTTTACAGTCACACCTCG
TGCCCCTGCGCCACGACCGGCCT

>1002052322_2 Heli.2-DT665617.3.5
AGAGTTGAGTCCGGCTGAAGTCAGTCAGTTACATAACAGTGCGGCACTGGGGCGCGCGCTTAGTACCTACTAA
ATATTTGTTAATTAATATTATTTATTATTTTAATTTTTTGGTTATGTGCTTAGTTCGTTATTTGTTAATAACA
TGTATCTAGAAAGAATCCTTTTATGGCTGTTGGTTGCAACTTGCGCGTGCGCAGAGCG
TTGGAGCTGGCCAGACGACTCTGTCGGGGAAGACAGTGTACGCATTGACAGTAAATTACACTTCGGTGACCCG
GACACGCAAAGGAATAGTAAAAGAAACAGCAACATACAACAAGATGAATTCCCCTTCCAAGAGGCAACAGACA
CGCAAGGATTCTACAACCGGCCGGCGGACGCGGGCCGCTACCCAATGAGAGTCGAACC
CGGATTCAATCAGGGGATACGGATAAACGGACAGACTGTGTTCTTAAATGGATATGATAGGAATCAAGAATCA
GATGGTACACTAGACTCTCTTCAACACTGTAAATGTGTGTCCCGCCCAGATTGTCAGGTGCAGAACGATTATC
AAAACGCATGTGGCACGAATCAATACTTATGCTGTTATAATCAACCCAGACAGCAAAA
CAGATATCCTTCAGAATATTTGAATGAAATAGACAATGAACGACCCATGATATATCCCAATATACCAAATGCT
GGAGCATTTCCGCCGCCAAATAATGTCAATAAAGGAATATTTAGACCGAATCAAGGAAATGAGCCAGGTGTTC
TGGTTGGACCTGATGGACCTACTGGTAGTATAGGCCCACAAAAGAATCAAGTACTGGT
TGGCCCCGGTGGGCCCACCGGCATTATTGGACCTCAAAACTTAAACAAAAATGTTGGCAATAAAAATATTTTG
GTAGGTCCTAATGGGCCGACTGGTGACATAGGGCCGAGTGAAACCGCACAAAGAGGAGTACTTGTTGGGCCAG
GAGGACCTACAGGCATCATTGGTCCAGCATTCAATCGACCAGTCTTAGTTGGTCCCGG
CGGCCCAACTGGTATAATAGGACCACGTCGTCCCGGCCAAGGAGTCTTAGTTGGTCCTGGAGGACCCACTGGT
ATAATAGGCCCGGCTGGATTCAACCGGCCTTCACAGCGTCCAGTGCTAGTGGGTCCTGGAGGTCCAACAGGGA
TCATTGGCCCAGGTAGACAAATCCTTGTCGGACCTGGAGGCCCCACAGGTCAGATTGG
ACCCAGAAATTACTTCATCTGAGTGTTTTACAGTGATAACTAATAAACTAATTTACTTTATCGGCAATGAATG
TCCGCCACGTGTGTGTTTATGTTTAACCGAGAGCTCGTTGTGATTGCATTAGAGCGTAAATATTTTGGTAGAT
CTAACGAACGCGCTACGTGCTAACGCCATAAACTGCGGCTTTGATCCACTTGTGTATA
TAGCTGTGGAAAATGTGTAAATTTATAGAATTATATGTATATATATAGGCATATATGTATATGATATAGTCAA
AATTATATTTAAATACTTCTTGTACCATTACAGGCTGTTTGCATCACAAGAACAATCGAACAAAATGTAGAAA
AGCAGTCCAACGGCATTAATATTTGTATAAATTCCATGACAATTTTATTTGCCATTTT
AATCAGAATTTTTATGTATATTTTTATTATTTTTCGTGTGTCCTCGAGTTCGAGCCCCTGGCCTCAATAACGG
CAGTGTATATAAAAAAATGACGT
>1002052323_2 Heli.2-DT663138.3.5
TCCAAGTGTGAATGAAGTGGCAGTTTCAGCATTTTGTTAGGGTTCGTCTTCCAAGTTGAATTATTAGTTGTAA
TAAGACCACCGGAACTTGAAGATGCGTGTCTTAATATTTTCGGTAGCGATAGCCCTTCTGGGAGCTGCGCTTG
GAGACGAAATTCCGTCAGAAGACAATGTACTTGTATTAAGTAAAGCCAATTTTGATTC
TGTTGTTTCATCTTCGGACTTCGTGTTAGTGGAATTCTATGCACCATGGTGCGGACACTGCAAATCGCTTGCA
CCAGAGTACGCTAAAGCTGCTACTAAGTTGTTAGAAGAAGAATCTCCTATTAAGTTGGCTAAAGTTGACGCAA
CTCAAGAACAAGAACTCGCCGAATCCTACAAAGTCAAAGGTTATCCCACGCTGATCTT
CTTCAAGAAGGGATCACCTATTGATTATTCTGGTGGTAGACAGGCTGACGACATTGTCGCCTGGTTAAAGAAG
AAGACTGGTCCACCAGCTCTTGAAGTCAGTTCAGCAGAACAGGCCAAGGAACTCATTGCTGCAAACAATGTCA
TTATCTTTGGTTTCTTCCCCGACCAAGACTCAGAAAAAGCTAAGGTCTTCCTTAATGC
AGCTGGATTGGTAGATGACCAAGTATTTGCTATTGTATCTGATGAAAAATTAGTTGAAGAACTAGAAGCTCAG
GCTGAAGATGTTGTTCTGTTCAAAAACTTTGAAGACCCACGCAACAAGTACGAAGGTGAAGAATTCTCCGAAG
ATGCTCTTAAATCATGGGTGTTTGTACAAAGCATGCCAACCATTGTGGAATTCTCACA
CGAAACTGCCTCCAAGATCTTCGGCGGTCAAATCAAATATCACTTACTCTTGTTCCTGTCCAAGAAAAATGGC
GATTTCGAGAAGTACCTTGATGACTTGAAACCCGTTGCCAAGAACTACCGTGACAAGATCATGTTTGTTGCCA
TCGACACTGATGAAGATGACCACCAGAGAATCTTAGAGTTCTTCGGCATGAAGAAAGA
TGAAGTGCCTTCAGCCCGTCTCATCGCCTTGGAACAAGACATGGCCAAATACAAGCCCGCCAGCAACGAGCTC
AGCGCCAACTCTATTGAAGAATTCGTACAATCCTTCTTCGCCGGTAGCCTGAAACAGCATCTCCTAAGTGAAG
ATTTACCCGAGGACTGGTCCGCCAATCCCGTCAAAGTGCTCGTCGCCTCAAACTTCGA
CGAGGTTGTCTTCGATAACTCCAAGAAGGTCCTCGTTGAATTCTACGCCCCATGGTGCGGTCATTGCAAACAG
TTAGTTCCTATCTACGACAAGTTGGGAGAACACTTTGAGAAGGACGATGACGTCGTCATCGCCAAGATCGACG
CCACCGCCAATGAACTTGAACACACTAAGATCACTTCGTTCCCCACCATTAAACTGTA
CACTAAGGACAATCAGGTGCGTGAATACAATGGCGAGAGGACGCTGGCCGGCCTCACTAAGTTCGTGGAGACA
GAATCCGAGGGAGCCGAACCTGT
>1002052324_2 Heli.2-EL601652.1.5

ACAGCGTTTCAAGTGTTCTGTGATAATTATACACGTGCGAGTATAAACCACGCTACGGATACGAAGCAAGAAG
TAACTAATACAACTTTCAAGTCAGTTGAAAGGTATATATTTCTTTATTCTATAAATGCCAGAAGAAAAAATGG
AGACTCAATCCGGTGAAGTTGAAACCTTCGCCTTCCAGGCTGAAATTGCCCAGCTTAT
GTCATTGATCATCAACACATTCTACTCTAACAAAGAGATCTTTCTTCGTGAGTTGATTTCCAACTCTTCAGAT
GCTTTGGACAAGATCCGATATGAATCTCTCACTGATCCATCAAAGCTCGACAGTGGCAAGGAGCTGTATATTA
AGATAGTGCCCAACAAGAGCGAGGGTACCCTAACCATCATTGATACCGGTATCGGTAT
GACAAAGGCCGACTTGGTGAACAACCTCGGTACTATCGCGAAGTCCGGCACAAAGGCGTTCATGGAGGCGTTG
CAGGCGGGCGCTGACATTAGCATGATCGGTCAGTTCGGTGTAGGTTTTTACTCCTGCTACTTAGTCGCAGACC
GCGTGACTGTACACTCAAAACACAATGACGATGAACAATACATGTGGGAGTCTGCTGC
TGGTGGTTCCTTCACCGTCCGCTCTGACCCCGGTGAACCACTGGGCCGCGGTACAAAGATCGTACTCCACGTG
AAGGAGGACCTCGCCGAGTTCATGGAGGAACACAAGGTCAAAGAGATCGTCAAGAAGCACTCTCAATTCATCG
GTTATCCAATCAAACTTGTAGTTGAAAAGGAACGCGAAAAGGAGCTCTCTGATGACGA
AGCTGAAGAGGAAAAGAAGGAAGATGAGAAGGAGGACGAGAAACCCAAAATTGAGGACGTAGGTGAGGATGAG
GATGAAGACAGCAAGGACAAGAAGAAGAAGAAGAAAACCATCAAAGAAAAGTACACGGAGGATGAGGAACTGA
ACAAGACCAAGCCCATCTGGACACGCAATGCAGATGACATTACCCAGGAAGAATATGG
TGACTTCTACAAATCTCTAACCAATGACTGGGAAGACCATCTAGCTGTCAAGCACTTCAGTGTAGAGGGTCAG
CTTGAGTTCCGTGCCCTTCTGTTCGTTCCTCGTCGCGCTCCCTTCGACCTCTTTGAGAACAAGAAGCGCAAGA
ACAATATCAAATTGTATGTCCGCAGGGTGTTCATCATGGACAACTGTGAAGACCTTAT
CCCTGAGTACCTGAATTTCATTAAGGGTGTAGTTGACAGCGAGGACTTGCCCCTGAACATTTCTCGTGAGATG
CTCCAACAGAACAAGATCTTGAAAGTAATTAGGAAGAACTTGGTTAAAAAATGCTTAGAGCTCTTTGAAGAGC
TGGCTGAGGACAAAGAAAACTACAAGAAGTATTATGAACAGTTCAGCAAGAACTTGAA
GCTTGGTATCCACGAGGACTCTCAAAACCGCAACAAGATCGCCGACCTACTCCGCTACCACACATCTGCCTCT
GGTGATGAAGTTTGCTCCCTCAAGGAATATGTTTCACGCATGAAGGAGAACCAGAAGCACATTTACTACATCA
CTGGTGAAAACAGGGACCAGGTGGCCAACTCATCCTTTGTTGAAAGAGTTAAGAAACG
TGGTTATGAAGTAGTTTACATGACTGAGCCTATTGATGAGTATGTAGTTCAACAAATGAGAGAATATGATGGC
AAAACTCTGGTCTCTGTAACCAAGGAAGGCTTAGAACTCCCAGAAGATGAGGAAGAGAAGAAGAAACGTGAGG
AAGACAAGGCCAAATTTGAAGGTCTATGCAAAGTAATGAAGAACATTTTGGATAACAA
AGTTGAGAAGGTAGTTGTATCCAACAGGTTAGTCGAATCACCTTGCTGTATTGTCACTGCTCAGTATGGTTGG
ACGGCCAATATGGAGCGTATCATGAAGGCTCAGGCTCTCCGTGACACATCCACCATGGGCTACATGGCAGC
>1002052325_2 Heli.2-DT665652.3.5
GCACGAGGCTCAACCCTGGAGGACCTGGGATGAAAGAGTGTGTAGTGCTACATTAGGGACATTATATTTTTTT
TTCAAATGTTTCTAATCGAAATTATAAGTACAAAGTAATAGGATAGGGTTTTACCACGGTCGCCGCCATTATG
GCGTCGGACGAACAATTTTCGTTATGTTGGAACAATTTCCACGCGAATATGTCAGCAG
GCTTTCATGGCCTGCTGTCGCGTGGAGATTTAGTTGACGTAACATTGGCTGCCGAAGGCAGATTACTACAGGC
ACACAAATTAGTTTTATCAGTATGTTCTCCCTATTTTCAAGAAATGTTCAAAATGAACCCCACTCAACATCCC
ATAGTATTTTTAAAAGATGTTAGTCATTCGGCGCTTAGGGACTTATTACAGTTTATGT
ACCAAGGGGAAGTTAATGTTAAGCAAGAAGAATTAGCGTCGTTTATTAGTACCGCGGAACAACTTCAAGTTAA
AGGTTTAACCGGTAATCAAAATGAAGAAAGTTCCACGCCATCCAAACCAAAGCCGACGTCGAGGCCAGGCCCG
AGGTCGTCACAACAAAGGCAATCTGTTATGACTAAGTTAGAGACTGATTTAGATTCTA
AGCCCTCCTCAACTCCAGTAGCAATTAAGAGACCAAATAGGCCATCGATAGCATCCAATAACTCGTCATCATC
TCAAAGTGGAAATGCGAAACGGAAATGTGTTGACCCCTTAGAAGCAGGACCCTCAGGCTCTACGAAAGAGGAA
TTTGTTACGATACCCGACGAAGATGAAAACAACGCTGTGGCACCCAAAATGGAACCGG
AATTTGTTAATGAAAGCATGTGGGACGAGGACGACGATGGCACCAATAACGATGAAACTAACTTTGGCGAGGA
CGACTCCAATATGGAGATGTCTGGTTTCGATGGCTCTACGACTGGCGACGGCAATATAACTGGAGGCGGGGAA
GGCGGCGCTG
>1002052326_2 Heli.2-DT668284.3.5
CCCCTTCCTTTTCTCTTTCCGAATAGACCTCAACCATGGCGGCCGGGGTACTTTACACTTATCCGGAAAATTT
CCGTGCTTATAAGGCGTTGATCGCCGCACAGTACTCCGGAGCCGACGTGAAAGTAGCTCCCAACTTTGTGTTC
GGGGACAGCAATAAGTCCGAGGAGTTCCTAAAGAAGTTCCCGGCCGGCAAAGTGCCAG
CATACGAAAGCGCTGACGGAAAAGTTCTCCTCACTGAGAGCAACGCCATCGCTTACTACGTCGCCAATGCCGC
TCTACGTGGATCAGACGTGGCCTCTCAAGCCGCTATCTATCAATGGGCGTCCTGGGCTGACAGCGAACTGCTA
CCAGCATCATGCGCCTGGGTGTTCCCATACTTGGGCATCATGCAGTTCAATAAACAAA

ATGTGGAGCGTGCAAAGTCAGATCTGCTAGCTGCATTGAAGGTGTTGGATGGCCATCTCCTCACCCGAACCTT
CCTCGTCACAGAGAGAGTCACACTGGCTGACATCATTGTGTTCTCCACTCTGATTCATGCTTTCCAGAATGTT
CTAGAGCCCAGCCTCAGATCATCTCTAGTGAATGTCCAGCGTTGGTTCCTGACCCTCG
CCAACCAGCCTCAAGTGTCCAAAGTAGTGGGTAGCATTGCTCTCTGCCAGGCCGCTCCCGTCTTCGACCCTAA
GAAGTACCAGGAACTGTCTCAGAACAAGAAGGAGGGTGGCAAAAAGGAGAAGAAAGAAAAACCAGAAAAGAAA
GAACAACCTAAAAAGAAGGAGCCAGAACCTGCTCCTGCTGATGACTTTGAGGAGAAGC
CCAAAGAAACCAAGGACCCCTTTGACTCACTACCTAAGGGTACCTTCAACATGGACGACTTCAAGCGCAGTTA
CTCCAACGAAGATGAGGCTGTCTCCATCCCATACTTCTGGCAGAAGTTTGACCCCGAGAATTACTCCATCTGG
TATGCTGAGTACAAGTACCCCGAGGAGCTGTCCAAGGTGTTCATGAGCTGTAATCTTA
TCACTGGCATGTTCCAGCGCCTGGACAAGATGCGCAAGCAGGCGTTCGCGTCGGTGTGCCTGTTCGGGGCCGA
CGACGACTCCTCCATCTCCGGCGTGTGGGTGTGGCGCGGGCAGCAGCTCGCCTTCGCGCTGTCGCCCGACTGG
CAGATCGACTATGAGTCCTATGAGTGGAAGAAACTGGACCCTTCCAGTGAGGACACCA
AGAAGAAGGTACAGGACTACTTCTCGTGGTCAGGAGTCGACTCCAAGGGGCGCAAGTTCAACCAGGGCAAGAT
CTTCAAATAAAATTAATGTATTTCCCCACTGTCCTCCGTCTGTCTGTTTTGATACTAAACACATATTTGTAAT
GTAAAGTCCACAAGTCTGTCAGCGAGCCAGCATGTTCAATATATTGTAAAATATTAAA
TGCATTTACGAGTGCGGCGCACTGTGCACTACTGTGCTCTGTGTTGATTGTATTCAACTGTAATAAAGTGAAA
AATTGATTTTT
>1002052327_2 Heli.2-EL604180.1.5
ACATAGCGATTCCTGAAAGACACTAGTCTTTCAGCAGCGCTATCATAAACAAAAATCGATTTATAATCTATAT
ATAACTTTTTCTCTCTTTCACTTCTGTGCTTTACTTCTTATTTCAAAATGAAATCCATGATCGTTGTAGCTTG
TTTGGCGCTGGCGTGTGGAGCCCATGCTTCCGGATGGGTGGGTCCCCCAGCCAACATT
GCCCTGTCTCAAGATGGTCGCAACATCTTAGACACACCCGAGGTAGCCCAGGCTCGCGCTGCTCACCTCTCCG
CCCTTCAACAAGCCTCACACAACAACCCCAACCCTCAAGATGATGGCTCCTACGACCCCCGCTGGGACAACGA
AGAGTACTGGCAAGGAGCTCAACAGAAATGGAACGGTGCCCCCGCCCAACAATGGAAC
GGCGCCCCCGCCCAACAATGGAACGCCGCCCCCGCCCAACAGTGGAGCGACAACTCCGGCAAATGGAACGGTG
ACAACGGACAATGGAACGGTGACAACGGTCAATGGAACGGTGACAACGGTCAATGGAACGCCGCCCCCGCCGC
ACCCGCATGGAACGCCGGCCCCGCCCCCGTCGCCGAGACCCCCGAAGTAGCCCAGGCC
CGCGCCGCTCACCTCGCCGCCCTCAACGCCGCCCCCGCTCACAACCAGTGGAACGCTCCCGCTCACAACCAGT
GGAACGCCCCAGCCCAAAACCAATGGAACGGTGCCCCCTCATGGCAAGGTCCCCCTGCTCAGATCAGATTAGC
CCAAAACGGTGCTGGTATCTTGGAGACCCCTGAAGTCGCCGCCGCGCGCGCTGCCCAC
TTAGCCGCCCACGCACAAGTAGGACACAATGCCCCAGCTCACCCCCAACAACGTTGGTGAATACCTCCTTCTT
CTACTTCTAGGTAGGTTAGATTCCTCTTGCATGTCATTCGCTTTCTCAAAGAGTGCTTGCAAATGACATCCTG
GAGGTGAACTAAGATGCCCTCTTCCTTTCGATCTCCTCTTCCTTTAGTAATATGTTAA
AAGTGCTAGTGCGTCAGACTCTTCCTTTCTTTAGCTTTTGTTACTATTCTCTATAAAATTCCTACTATACTTT
TTGTACATAATAAATAATAATTATATGTATGCGTAAACGAAATATAT
>1002052328_2 Heli.2-DT666706.3.5
TGTTGCGGTGCGGTTAGAGATAATTCTAAGTGTTTCAAAAGAATTTACCAATTCAATTCATCGTTCACCGCAA
GAGAAAAATTGATACTGTGTCGCAATTTGCTGGCTTAATTACTTTATATCCCTTCAAACAAGGGCATCAGTAA
TATAGTCATCTAAATTCATCAAACATGTCCGTCGACAAGGAGGAATTGGTGCAACGCG
CCAAGCTTGCGGAGCAAGCTGAGCGGTATGACGACATGGCGGCGGCGATGAAAGAAGTCACCGAAACCGGAGT
CGAGCTCAGCAATGAGGAAAGGAATTTACTTTCCGTTGCCTACAAGAACGTCGTGGGTGCTCGACGGTCATCA
TGGCGAGTCATATCTTCCATTGAACAGAAAACCGAAGGATCGGAAAGAAAACAACAGA
TGGCAAAAGAATATAGGGTTAAAGTAGAAAAAGAGCTCAGAGAAATCTGCTACGATGTTTTGGGTTTACTTGA
CAAACACCTTATCCCTAAAGCTAGTAATCCAGAAAGTAAAGTATTTTACCTTAAAATGAAGGGAGATTACTAC
AGGTACCTCGCAGAAGTGGCCACTGGAGAAACCAGAAATTCCGTTGTAGACGATTCAC
AGAAAGCGTACCAGGATGCTTTCGAAATCAGCAAGGCGAAAATGCAGCCCACACACCCAATAAGGCTTGGGCT
GGCGTTAAATTTCTCCGTCTTCTATTATGAGATATTAAATTCACCAGACAAGGCGTGTCAGCTCGCTAAGCAG
GCGTTCGACGACGCGATCGCGGAGCTGGACACGCTGAACGAGGACTCGTACAAGGACT
CCACGCTGATCATGCAGCTGCTGCGGGACAACCTGACGCTGTGGACGTCGGACACGCAGGGCGACGGCGACGA
GCCGGCCGAGGGCGGCGACAACTAACGCGCCGCACGCACTTGTTCTCATCCGTGTTTTATAAGAAAACGAGTA
ACATTCGAGAACGTCGCGCCACCGCCGCGAGCCGTCGCTTGTAAAAAAAATGTTAAAA
ATGTCGTTCGCAGCGGACCTTCACGTTCCGTCTAAAATTAAATGTTGTGTCATCTTTGACGGTTTTGTATTTC
TGTATTTACACTGGATTACAGATATCGAGCTGACCTGATGAATATAAATATTTATAACT
>1002052329_2 Heli.2-DT666464.3.5

GAGGCACGTATCGTGCTATTTGCGTTTTGTCTTGTCCTTAAATTTTTCGAAGTCCCTACACACGCAAAGATGG
GTAGGGAGGACAAGGCTACTTGGAAAACCAACTATTTCACTAAGATTATCCAATTATTAGATGAGTACCCAAA
ATGTTTCATCGTGGGTGCCGACAACGTTGGCTCCCAGCAAATGCAGCAGATCCGTATC
TCCCTACGAGGAAGCAGCATTGTGCTTATGGGAAAGAACACCATGATGCGTAAAGCTATTAAAGACCATTTGG
AGACCAACCCGGCCCTTGAGAAATTGCTCCCTCATATTAAAGGAAATGTCGGCTTTGTGTTCACCCGTGGAGA
TCTCGTTGAGGTCCGTGATAAGCTTCTGGAGAACAAAGTGCGTGCTCCAGCCAGGCCT
GGTGCCATTGCTCCATTAGCTGTGGTCATCCCAGCTCATAACACCGGTTTAGGTCCCGAAAAGACTGCTTTCT
TCCAGGCTCTGTCCATCCCTACTAAGATTTCCAAGGGTACTATTGAAATCATCAACGATGTCCACATCTTGAA
GCCTGGTGACAAAGTAGGAGCTTCCGAGGCAACCCTCCTCAACATGTTGAACATCTCT
CCATTCTCATATGGTCTTGTTGTTAAGCAAGTATATGACTCTGGCACCATCTTTGCGCCAGCCATTTTGGACA
TTAAGCCGGAGGATCTCCGTGCCAAGTTCCAAGAGGGTGTGGCCAACGTAGCTGCCTTGTCATTGGCCATCAG
CTACCCAACTGTGGCTTCCGCTCCTCACTCCATTGCCAATGGATTCAAGAACTTGTTG
GCCATTGCTGCTGTCTCGGACGTTGAGTTCAAGGAAGCTAACACTATCAAGGAGTTTATTAAGGACCCAAGCA
AATTCGTCGCGGCTGCTGCTCCCGCCGCCGCAGCCGCTGCACCAGCTGCCGCCGCCAAGGAGGAAGAGAAGAA
ACCCGAGAAGGAGGACTCTGAGAGCGACGACGACATGGGCTTCGGTCTCTTTGACTAA
AAGCACTATTGAAGTCAAGTTTCCCATGCTTTACCATCTCATTATGGGGATAAGTTGTAAAGGGATGTTTGCA
ATGAAGGTACTCGCGCCGCCGGGTCTGGACGCCCATGTGGCGGCCGT
>1002052330_2 Heli.2-DT665599.3.5
GCACAGGCAAACGAGCAACTGACTTGGTGTTCAAGTGATTTATTATTTAGTTTTGTTTATTTAAAAACAGTAA
TTTTGGCCAGCAATTAATATTTTAAGCAAAATAAAATCTCAATAGTAGTCATACAGTTTTTAGTTTATAAAAT
AAAGATATCAAAATGCAGATTTTCGTGAAAACACTTACGGGTAAAACCATTACTTTGG
AAGTTGAACCTTCCGACACTATTGAAAATGTAAAAGCCAAAATTCAAGACAAGGAAGGCATTCCACCAGATCA
ACAACGTTTGATTTTCGCTGGAAAGCAATTGGAAGACGGCCGTACTCTTTCTGACTACAATATTCAAAAAGAA
TCTACTCTTCACTTGGTATTGCGTCTTCGTGGTGGAATGCAGATCTTTGTTAAGACTC
TCACAGGAAAAACCATCACGTTAGAGGTAGAACCGTCGGACACAATTGAAAACGTGAAGGCAAAGATCCAAGA
CAAAGAAGGTATTCCCCCAGACCAACAGAGGCTCATTTTTGCTGGAAAACAACTGGAAGATGGCCGTACATTA
TCTGATTATAATATTCAAAAGGAATCTACTTTACATTTAGTATTGCGGCTTCGTGGCG
GTATGCAAATATTTGTAAAGACCCTTACTGGTAAAACAATTACTTTAGAAGTTGAAGCCTCTGACACAATTGA
AAATGTGAAAGCTAAAATTCAGGACAAAGAAGGCATTCCGCCAGATCAACAACGTCTTATTTTCGCGGGCAAG
CAATTGGAAGATGGTCGCACATTGTCAGATTATAACATTCAAAAAGAGTCAACTCTTC
ATCTTGTGCTCCGCCTCAGAGGTGGCATGCAAATCTTTGTTAAAACCCTCACTGGCAAAACCATCACTCTTGA
GGTTGAACCCTCTGATACTATTGAAAATGTAAAGGCTAAAATCCAAGACAAAGAGGGCATCCCACCTGATCAG
CAGCGCCTTATCTTTGCTGGCAAGCAATTGGAAGATGGTCGTACCCTGTCTGACTACA
ATATTCAAAAAGAGTCCACACTTCACCTGGTACTGCGTCTGCGAGGAGGTATGCAAATATTTGTCAAGACACT
AACAGGAAAGACAATTACCCTGGAGGTGGAGCCATCTGATACTATTGAAAATGTAAAGGCTAAAATCCAAGAC
AAAGAGGGCATCCCACCTGATCAGCAGCGCCTTATCTTTGCTGGCA
>1002052331_2 Heli.2-DT662665.3.5
GCACGAGGGAGAGAGAGAGAGAGAGAGAGAGAGAGAGAGAGAGAGAGAGAGAGAGAGAGAGAGAGAGAGAGAG
AGAGAGAGAGAAAATCATAAAGATATTGGAACTTTATATTTTATTTTTGGAATTTGAGCAGGTATAGTTGGAA
CATCTCTTAGTATTTTAATTCGAATAGAATTAGGTAACCCCGGATCTCTAATTGGGGA
TGATCAAATTTATAATACTATTGTTACAGCTCATGCATTTATTATAATTTTTTTTATAGTTATACCCATCATA
ATTGGAGGATTTGGAAATTGATTAGTACCTTTAATATTGGGAGCCCCTGATATAGCATTCCCCCGGATAAATA
ATATAAGATTTTGACTTCTCCCCCCCTCATTAATTTTATTAATTTCTAGAAGAATCGT
AGAAAATGGAGCCGGAACAGGATGAACTGTCTATCCCCCCCTTTCATCTAATATCGCTCATAGAGGTTCATCT
GTAGATTTAGCTATTTTTTCCCTTCATTTAGCGGGTATTTCCTCAATTTTAGGGGCAATTAATTTTATTACTA
CTATCATTAATATACGGATTAACAGCATATCTTTTGATCAATTACCATTATTTGTCTG
AGCTGTAGGTATTACAGCTTTATTACTTCTTTTATCTTTACCAGTTCTAGCCGGAGCTATTACTATACTTTTA
ACTGATCGAAATTTAAATACCTCATTTTTTGACCCCGCCGGAGGAGGTGATCCAATCTTATATCAACATTTAT
TTTGATTTTTTGGGCACCCAGAAGTTTATATTTTAATTCTCCCAGGATTTGGTATAAT
TTCTCATATTATTTCCCAAGAAAGTGGTAAAAAAGAAACTTTTGGTTGCTTAGGGATAATTATGCTATAATAG
CAATTGGTCTA
>1002052332_2 Heli.2-DT667171.3.5

TCTGTTATTTACTCGTATGTGGCACTTCTGTTATTTACTATATTCTTAATATTATAATTATTACTATATTCTT
AATATTATTAAAATGCGTCAATTTGACGAAGTGTTGACCGTGCTCGGGGTTACGGTGATGTGCTTTTGCGTGC
ACCGGACTCTCCAACTAGTCCCGTTGCCGGATTTCATCCATCCATGTGGCGAGTTAAC
CGATGACTGCTTCACAAAGGCCACACTCGATGCTATTCCAGGAGTAGTGAAGGGGATTCCTGAAGCTGGCATC
CCACCTCTCGATCCCTTATATCTGGACAGAAACATTACTATGAATCTTCCTGGAAACGTAAAAATGACGTTCC
ATAATGGAAAATTATCAGGATTAAGCACTTGTATACCAGATAAAGTGTCATCACAACG
AGCAAAACGAACATTTATCTTTGACATACATTGTAATTTTACCATAAAAGGGCAATACAGCATCCAAGGCAGA
ATCCTTCTCTTTAACCTCGACACTGACGGCTCCGCCAAAATTAAAATTTGGAACCAACACATAAGGTTGGAAG
TCCGGGAAAAAATTGTCAGAGATGAAAACGGTGAAGGGCATTACAAAATAAACTCATA
CAAATACAAGGCTGATTATGGTTCTGACCTTAAACTAAATCTCACCAACTTATTCAGAGGCAGTCCGCAGATA
AGTGCAAATATATTGGATGTGCTAAATTCGAACTCCCAGCTAGTGGCGCAGGAGTTTGGAGGTCCCATTCTGG
ACTACGCCATAGACTACGCTATGAACGTCACACAAAGATTCTTCAGCACATACACCTA
CGATCAGATCAGCAAAGTACCACT
>1002052333_2 Heli.2-DT666774.3.5
TTTCTGAACAGGCGCAGTCCGAGAGAACTCGCAAAAGGAGTAAAAGGCTCCTACATAACCTGTAACTATCAAT
ATATTGTATGAGTCAGAAGGTAGAAAAACCAGTATTATCGGGTCAACGGATCAAGACCAGAAAAAGAGATGAG
AAAGAGAAGTACGACCCGAACGGGTTCCGCGACGCCCTCGTGGGCGGGCTGGAGCGCG
CCGGCGGCGACCTGGACGCAGCCTACAAGTACCTAGACGCGGCCGGCTCCAAGCTCGACTACAGGCGCTATGG
CGAGGTCATATTCGACGTGCTCATTGCCGGGGGGCTGTTGCTTCCCGGCGGCTCCGTGTCGATGGACGGGGAG
ACCCCCAAGACCAACACGTGTATATTCAACTCGAGCGAGGATATGGAGACTATGCGTA
ACTTTGAACAGGTATTCGTGAAGTTAATGCGACGTTACAAATATCTCGAGAAGATGTTTGAAGAGGAAATGAA
AAAAGTCCTAGTGTACCTGAAGGGCTTTGAACCACACCAACGTTTAAAGCTGGCCCGCATGACCGCACTATGG
ATAGGTAATGGCTGTGTGCCGCCATCAGTGTTGCTAGTGCTGGTGAATGAACACCTTT
TAAAAGATAATTTGGCACTTGAATTCGTTTTGGAGGTATTCTCTACGGTGAAAGCGGAGAGGGGCGTCACTTC
GCTCGTCACCGCCCTTAAGAGAGGACAGTTGGAGGGCAGGTTACTAGAATTCCTACCTCTCAACCGTCGTACA
GAAGAAACCCTGGCCTCCGCGTTCGCAGCGCGCGGCCTCGCGGAGCTCCTACGCCTGC
ACCGCGCTCAGGCCTCTCAAGAGGCCCGCAGAGAGCTCACTCATGCACTCCTGGAACAGTTGGCTGATGCCCG
CAACGTCAGGGACCTCGTGGCCGATACGAGGGACGCTGCTGCGAGACACGCTATACCTGATCATGAAGTTGTT
GCTATTATATGGCAGTGCGTTATGTCTCGCGGAGAGTGGAATAAAAAGGAGGAACTGC
TAGCGGAGCAGGCGGCCAAACATTTAAGACATTACACGCCGTTGTTGGCCGCCTTCGCGCAGTCTGCCAAAGC
AGAGATCGCGCTTTTGACTAAGGTGCAAGAATACTGCTACGAGAACATGAGCTTCATGCGCGCGTTCAGCAAG
CTGGTGCTGATGCTGTACAAGACGAACGTGCTCTCCGAGGAGGTGATCCTCAAGTGGT
ACCGCGAGCCCAACTCCAGCAAGGGGAAGGTCATGTTCCTCGACCAGATGAAGAAGTTTGTGGAGTGGCTCCA
GAGTGCCGAGGAAGAATCGGAGAGTGGCGAAGAAGAAGATTAGAAATAAAGGCAGCGTGAGACTGAACACATA
GCGCCGTCCCACACACACACACACACCCAACAGTAAACCAGAAACCATAACCCAAACT
GCAACCATAACCAACCATAAACCACAACTGAAACCAACAGGAAACCGCATCCAAGTGAGCACATCACTAGCTG
TCAGTGATGGGACGTTTTCTACAATTGTATATGAATCATTTGAAATACATACAACTACTAACTAACATGTTGA
TGAAATACAATATTTTTTATTCAATTTTTTTAGTAAAATTTCAATAAATGGTGGTGAC
>1002052334_2 Heli.2-DT665541.3.5
GCACGAGGCTGCCTTCAGTGCAACCAACTAAACGCAGCATCATGAAGCTCATGCTCGTGGCCGCCAGTCTGGT
GGCTGTTTTGGCTATCGCTCACGCGGAAGAAGCCAAAGCAGCAGAAAAAGAAGTGGCAGTAACCGATGACAAA
GCGGCAACCGAGGACAAGAAACACGAGAAACGTGGACTATTTGATCTTGGTTACGGTG
GACATGGTGGATTCGGAGGTGGTTACGAAGGTGGTTTAGGCGGTGGCTTCGGAGGTGGCTACGGCGGTTACGG
CGGTTACGGCGGTCATGGCCACGAAGAGGTGCACAAAACCATTACTGTCGTCAAAAATGTCCCAGTACCCTAC
CCAGTAGAGAAACACATTCCCTACCCAGTTGAAAAACACGTACCTGTGCCCGTCAAGG
TGCCAGTACCTCAACCTTACCCTGTTGTCAAACATGTCCCATATACTGTCAAGGAACTCGTCAAAGTGCCCGT
ACATGTTCCTCAACCTTACCCAGTTGAAAAGAAAGTCCCCTACCCAGTTCATGTTCCAGTCGATAGACCAGTC
CCCGTCAAAGTGTACGTGCCAGCACCTTACCCAGTAGAAAAGAAAATCCCTTATCCAG
TAAAGGTACCAGTGCCAGCACCTTACCCAGTTGAAAAGAAAGTCCCCTACCCAGTGAAGGTACCAGTCCATGT
ACCCGCTCCCTACCCAGTAATTAAGGAAGTACATGTTCCCGTTAAGGTCCCAGTCGACAGGCCCTACCCCGTA
CATATTCCTAAACCTGTGCCCTACCCTGTAGAGAAACCCGTACCCT
>1002052335_2 Heli.2-DT665640.3.5

GCACGAGGGTAAGCACGAGGAAGCACCGAATTTTTAAAAGAAGAATTTTCTTACACTGTACATTCCCGTAAGC
ACATTCAAAAATCGAACATGTTTTCTACCGTTGGTAGAGCTGGTCTTTTGGCCACGAGAGCGGTAGTTAACAA
TACTTTGGCTGAGAAGACTCCTTTGGTTGCCGGAGCTTTTGCGAACAAACGTGATTAT
GCTGCTAAAGCCGCTGGAAAAGGTCAAGGTAAGGTGGTTGCAGTTATTGGTGCTGTCGTAGACGTACAATTTG
AGGATAGTCTCCCACCCATCCTAAATGCCCTCGAAGTCCAGAACCGGTCCCCTAGGCTCGTCCTGGAGGTAGC
CCAGCACTTGGGAGAAAACACCGTTCGCACCATCGCCATGGACGGTACTGAAGGTCTC
GTGCGTGGACAGCCCGTGCACGACTGCGGCTCGCCCATCCGCATCCCCGTAGGCGCAGAGACGCTCGGCCGCA
TCATCAACGTCATCGGTGAGCCCATCGACGAGCGTGGCCCCATCCCCACTGACAAGACCGCCGCCATCCACGC
CGAAGCGCCAGAGTTTGTGGACATGTCCGTACAGCAGGAGATCCTCGTGACAGGCATC
AAGGTGGTGGATCTGCTCGCGCCCTACGCCAAGGGAGGCAAAATCGGCCTGTTCGGCGGCGCCGGCGTCGGCA
AGACCGTACTTATCATGGAGCTGATCAACAACGTCGCTAAGGCGCACGGTGGTTACTCTGTGTTTGCTGGTGT
GGGAGAACGCACCCGTGAAGGTAATGACTTGTATCACGAGATGATTGAGTCCGGCGTT
ATCTCGCTCAAGGACAAGACCTCCAAGGTGGCGCTTGTGTACGGTCAAATGAATGAGCCCCCTGGCGCGCGCG
CCCGTGTCGCTCTCACTGGACTGACCGTAGCCGAGTACTTCCGTGACCAGGAGGGACAAGATGTACTGCTCTT
CATTGACAACATTTTCCGTTTCACTCAGGCTGGTTCTGAGGTGTCTGCTCTTCTGGGT
CGTATCCCCTCAGCTGTAGGATACCAACCTACACTGGCCACTGACATGGGTACTATGCAGGAACGTATCACCA
CCACAAAGAAAGGTTCCATCACCTCTGTACAGGCTATCTATGTGCCAGCTGATGACTTGACAGATCCTGCCCC
TGCTACCACCTTTGCTCACTTG
>1002052336_2 Heli.2-DT663422.3.5
GAACGTTATAGTGTCCACTACTCCCGTGCAAGGACTATAGTTTAACCATGAATTCACGGTGTTTATTAGTGCT
AGCAGCCGCCCTCGGCGTGGCGGTCGCCCAAGAAAGCTTCAAATGTCCAGATGACTTCGGCTTCTACCCTCAC
CACATCTCTTGTGACAAATATTGGAAGTGTGACAACGGAGTCGCTGAACTTAAGACTT
GTGGTAACGGACTCGCCTTCGATGCTTCAGATTCCAAATACTTGACCGAAAACTGCGATTACCTCCATAACGT
TGAGTGCGGCGAAAGGACCCAGCTTGAACCCCCAATCTCCACTCCTCACTGCGCAAGATTGTACGGTATCTTC
CCTGACAGCGCCAAATGCGACGTTTTCTGGAACTGCTGGAGTGGAGAAGCTTCCCGTT
ACCAATGCAGCCCCGGACTTGCTTACGACAGAGAAGCTCGCGTGTGCATGTGGGCTGACCAGGTTCCCGAGTG
CAAAAATGAGGAAGTAGCCAACGGTTTCTCTTGCCCCGCCCCTGGTGAGGTTTCCAACGCTGGATCCTTCAGC
CGTCACGCCCATCCTGAAGATTGCCGCAAATACTACATCTGTCTCGAGGGAGTAGCTC
GTGAATACGGATGCCCAATTGGTACCGTTTTCAAAATTGGTGATGCTGACGGCACTGGCAACTGCGAAGACCC
CGAAGATGTTCCTGGATGCGAGGACTACTACGGTGATTTGGACCTGAAGGCCATCCGCAAGAGCGAGCTGCTC
GCCGGCCTCCACAGCAGCTCGGCCCAATCCGTCCAGCCCAAGATCAAACCACGTCCCC
AAAAAGAAAACTAAATACTCACTTCCCACCCCTTACCTTTTAATACTCACTACTTAATCTGTTTCCACACACC
CACAAGCAAATTTTTGACGAAAATTCTATAAGAATTCATATAAATATATACTAACGTTGCTTTTGGTGTGAGA
GACACGATACCAGGTTCAAATTCGTATTTTAAGAAAGTGAAGCCAATTGATTTGTATT
CATTTCCATTTGCCAAATACTTTTCTCGTTGTAGCCTTAAACAGTTTCAAAAAGCAGTTTAGAAAATTAGGAA
TATTTATTTTTATATTCTATTTAAATTGGTACCGTCTTATTTGGTACTATAATATAAAAATTCATTATAAATG
AATATCGGTATGCATTTATGTTTATAATTCTTGACAAAACGTTTTGAAACGATGAAAC
AGGACACGACATATACAACATTTCAATTACAACAATCTTGTAAATATATAATATTTTTATATACTAAATTAT
>1002052337_2 Heli.2-DT665729.3.5
GGGTGCATTGTCTGTTGGTGCTCGCAGTAAAGAGTATCGTGTGCAGTGATTATGAATTATACGAGTACCTTAA
CAGAACTGATCCCGCGACCCGACAAATTTATGAGTTCGCGCTAGAGCATCCGAAACCTATTGGAGTGCATACT
GTAAGCTCAGGGAAATTTGTTGAGATCCGGGAGTCTATTGCATTGAATTCAGATGCAT
TTTCTAATCCTTACCATATTGACCTTTTTTCTCACGTCCATGCTGAAAGAATACCAGAACGAGTTAAGAACGC
CGTAGGAACATCAGCATTCGGTTATTTTAAAGTAACCAATGATGTTTCAAAGTATATAAAAGCGGATTTATTC
AACGGCGTGGGGAAGAAGACTCCTGTAGCTGTTCGATTTTCTACAGCTTTTCAAAACC
TTGGAGGAGGTGAACTGGTTAGAGAATTTAAAGGATTTTCTGTGAAATTTTACACAAAAGAAGGAAATTTTGA
TATTGTCGGAGCTAATTATCCTGTCTACTTCCATAGAGATCCTCTTGACTTTCCACATTTCTTACATGCTTTG
AAGAGAAATCCTAAGACTAACCTATACGATCATACTGCACGTTGGGATTTTGCAACCA
AGAAACCTGAACTTATACATGCAACGTTATGGCAACTTTCTGATTATGGTATACCCAACGGATACAGAAAAAT
GAACGGTTACGCCGTTCACACTTATGAAATTAATAATAAGCATGGTGATATATATTTTGTCAAATTTAACTTC
AGATCCGAACAAGGAATTGAAAATCTTTCGGATTATGAAGCACAAAATATTGCAAAAC
GGGATGCCGCTTACTACACTAGGGATTTGTATGACGCTATTGAGAATAAAACTTATCCTTCATGGAAGTTAGA
AATGGATGTTATGACATTGGATGACATAAAAAAGGTGGATTATAACCCGTTTGAACTCACCAGGTTATGGAAA
AAAGGTACCTATCATACAGTAACTATAGGCCGAC

>1002052338_2 Heli.2-DT661969.3.5
CCGATTCGTCGGATTACGATTATTTTAATAAAATGGGTTTCGTGAAGGTGGTGAAGAATAAGCAGTACTTCAA
GCGTTACCAAGTCAAGTTCAAGAGGCGTAGGGAAGGTAAAACTGATTACTATGCACGTAAGCGACTGGTAGTG
CAGGACAAAAATAAGTACAATACACCCAAGTACCGTTTGATTGTCCGACTTTCAAACA
AGGATGTAACTTGCCAAGTTGCATACTCCCGTATTGAAGGAGATCACATTGTTTGCGCGGCTTACTCGCACGA
ATTACCACGTTATGGTATTAAGGTGGGTTTAACTAACTATGCGGCTGCATACAGCACTGGGTTACTCCTTGCT
AGACGTCTGCTGCAGCGGTTAGGTCTTGACTCATTGTATATTGGTGCCACAGAAGTCA
CTGGTGATGAGTTCAATGTGGAACCAGTTGACAATGGACCAGGGGCCTTCAGGTGTTACTTAGATGTTGGTCT
TGCTCGTACAACAACTGGGGCTAGAGTTTTCGGAGCAATGAAAGGTGCAGTAGATGGTGGCCTTAATGTTCCT
CATTCCATCAAGAGATTCCCAGGATATGATGCGGAGGCAAAGAAATTCAATGCTGAAG
TTCATAGAGCACACATCTTCGGTCTTCATGTAGCGGAATATATGAGAAACCTGGAGCAAGAAGATGAAGATTC
CTTCAAAAGACAATTCGGCAAATATGTCCAACTTGGTGTTACTGCTGATGCTATTGAAGGCATCTACAAGAAA
GCCCATGAAGCAATCCGAGCGGATCCATCTCACAAGAAGAAGGAAG
>1002052339_2 Heli.2-EL599135.1.5
ACGAGGGTTAAGATGAAAGCGTTCTATGCTGTGCTGTTCCTCGCTGCGGCGACCGCCATGCCGGCGAAGGAAG
ACGGTGTCGTGGACAAGTTCATCTCGAACTTGAGAGATTGCGTGGAAACGGATACGATGCTGTGTTTAAAGGA
AAAAGCAATGAAAGTTACTGAAAACTTGGCCTTCGCGAAGGACTTTAGCGTAATGGAT
GGCATTACCTTCGCAAGAACCGGTTCCCCGAGGTCAGCGCGCAGCTACGAGCCTCTACTTGATGACCCTAAAG
CACGAGAGCTTCAGGTTGAAGAAAGGATTATGGATAATGTCGTGGACTTCCTAGACAGCCACGCTTTGCAACT
AAGGATGCCCAAGTCCTTCACTGAAGACAATTCAGTAGAAGAAGAAGGCCGTGGCAAG
AAGAAGAAGAAGCTGAAGAAACTCCTTCCCATCCTTGCTCTCATCAAATTGAAGTTGGCTGCCTTAGTTCCCC
TGTTCCTCGGTATCATCGCCTTCGCTGTATTCAAGGCTTACCTCCTTGGTAAAGTTGCTTTCATCGCTGCCGC
TATCGGAGCTTTGAAGAAACTTCTGGAATCCAAATCCAAGAGCGGTGGAGGTGGATGG
TCTGAACCAGCTCATGAAGAACATGGCTGGGAGAGCGGTGGTGGCTGGGGACGGTCACAGGATGGGCAAAACA
TGGCTTACGCCGCCCACATCAAGCAAATATAACCTTAGGGTCTAGAAATAATTAATTTATTCTAACTTATT
>1002052340_2 Heli.2-DT662950.3.5
GCACGAGGGTTAACGAGGGTGCAGTGTAGATCGAAAACAGATTCATTTTTAAAATAAATAATTAAATATATAT
ACAATAATTAAATAAAAATGTGTGGGGATATGGGCAAAGCAGTATTGCGGGCTCTGACCGGAGCGCTGCTCTG
TACCCTTGTGTCAGATGCGGCTTATTTAGAAAGATATGAACAATACCCATTCCAATAC
GAGCAAGCAGTATACAGAAAACCCCTAAGAGAGCATGAAAAGCCGCAAGATCTCCGCAATGTACCGGGAACAC
CGGGCGTAGACTACCCCATATATCATGCAGTGCCAGAAACCCGCTTCTCCTGCGCCCACGTGCCAGTCCACCC
GGGAATGTATGCCAACGTTGAAACTGGATGCCAAGCCTACCACGTATGCCACGACGGT
CGTGAAGGTCACCAAGGCGCCGCCTTCCTCTGCACCAACGGCACACTCTTCGATCAGGCCAAGTTCGCCTGTG
ATTGGTGGTATAACGTGGATTGCTCACAGGCCATAGCACACTACAAGTTGAACGCAGATCCCTTGAAAAACCC
CTACGTGCCTAAGCCGAAGCCAGAAGAGTTACAGGAAGCGCCACATGGAGTTTACTAT
AGAAAGATACACGATTAAACATTTGTGTTTTGTCATTTATCTATTACTTTTAAAATCATCTAAAAATTGTGTC
ATCGTCATACATCAATCATATGAATCATTAAATTAGCAACAGCCAACTTTAAACTTTAATATTTAGGCAAAAG
TGTGTATATCCAAAGTGATTAACATGTGAGTATTATTTCAAGACATATTGGATTTAGG
GTATTTGTGAGAAAGAAAGATTTATGAGGGGGTAGAAAGATTTATTAATGCGACGGTTGTGGGTTCGATTCCC
ATTCGTTGAAATGAGCTATCTTCATTTCCCTGCTATATGAAGGATACAGCGTGAACGTAAAAGATAAAATT
>1002052341_2 Heli.2-DT666616.2.5
ACACAGACCAAGATGTTCAAGTTGGTGGTGTTGTGCGCTTTCCTCGCCGCGGCTGTAGCGGAGCCCGGAACCT
TCATCTCGCCGTTGGCGTACTCCAGCAGCTTTATCGCGCCGGCTACAACAACTATCACCAACCAGGCCAGCAG
TGTCATCCACCCATCACCTCTGCTCTACCCTCGATTCTACGACCTACCTTACTCACCA
GTCGCACATCTTATCAAGAAGCGATCCCTTGGAATTTTCAATCCTTTCATTGCACCATCAACTTACATTGCTC
CTGCACCTCTGGCCACTACTTATGCTGCAGCTCCTCTTGCTACTACCTACGCTGGAGCTCACTTGGCTACAAC
TTATGCTGCACCAATCTACCCTGCTGCTGCGCATTTAATCAAGAAGAGGTCTGCTCCA
CTTCTGCCCCACAACCTACGTTGCTCCATACTATTCTTCTCCTTTGTTGACGCCGACGTACACAGCCGCTGCAC
CCATCTATTCAACACCTTACATCACCAGTGCTCCTCTTACTTACACGCATTTAATCAAGAAGCGCTCCGCTCC
ATTGTTCTACGCAGGTTACACCGCTCCCGCTGCGGTTTCTCATCAGTCCCGAGTAGAC
ATTAAGAGCTCCCCTGCTACTATTACGTCCTACTCCTACCCTGCTGCTGTTTCTTACGCTAGCCCACTTGCAT
TTTCACATGTCTTCTAGATATTTGACTATTGTATGTAAATAAACTGAATTGATATCATTAAAAAAAAAAAATA
TATCATTCTT
>1002052342_2 Heli.2-DT663146.3.5

GTGTGTAGGATGGATCGCTAACAACACCAGTAGTTTTTTTTTCATTCCGCCTTCTAACAGTGTTTGTTTATTA
CCTTCCGGCACACTTTCCGTAAATCCTTCTCGACGGTGGTGCTTATTATTTCTTTAGTTTTTTCGGCGTTTGT
TTTTATATTTTCTTAAGCGCCGTTCCGATTTCACGTCGAGAAGGCGGCCGCACAAGTT
TGAAAGCGGCTTTTTGAGAGTAGTAACACCTACTCATATTTTGTTATCGTTATAAGAACACATCAGGCAACAA
TCAGAATGGTGAAGAAGATATCAGAAGAGAGTGTAGACAGCGGCGTCGGCCGCGGCAGTAGTCAGTCGGGTAG
CGAGCGCGAGTCGGACGAGATACCGCGCGGGCCCGAGGCGTCCGCGCCGGTGCCCATA
CCCGACAGCGAGGAGCTGCAGCGACGCAAGGAGGAGGCGCGCAGGAAACGCCGCAGGAAGAAGCGCTCCGGCA
GCTCAGTGGTCACGTCATGTTTTCAAGACCTATACAAGCTAACAGGAGAAGTACTGGGCGAGGGCGCATACGC
CTCGGTACAGACGTGTGTAAACATCTACACGGGCCAGGAGTTCGCAGTGAAGATCATA
GACAAAGTGCCGGGGCACGCCCGCGCCCGGGTGTTCCGCGAAGTGGAGACGTTCCACTACTGCCAGGGACACC
CGAATATAATCCAGCTCATTGAATTCTTTGAGGACACGGACAAGTTCTATCTTGTCTTTGAGAAGATCAACGG
CGGCCAGCTCCTATCAAGGATACAGGAGCACCACTACTTTTCCGAGCCGCAAGCGGCA
GAGATAGTGCGCGAGATCGCCAACGCCTTGCACTTCCTACACGGGAAGGGCGTGGCGCACCGCGACCTCAAGC
CCGAGAACATTCTGTGCGTCAACCGCGACAGTCTCTGCCCGGTCAAGATTTGTGATTTCGATCTAGGGAGCGG
CATCAGCTTTACGTCCAGCCTCGCCAGCCCGTTGGCGACGCCGCAGCTCATGACGCCG
GTGGGCAGCGCAGAGTTCATGGCGCCCGAGGTGGTGTCGCTGTTCGCGGGCTCGGCCGCCACGCACTACGACA
AGCGCTGCGACCTGTGGTCGCTGGGCGTCATCGCCTACATCCTGCTGTGCGGGTACCCGCCCTTCCGCGCCGA
CTGCGGCGCCGACTGCGGCTGGGAGAGGGGCGACAACTGTAGAGCCTGCCAGGACCTA
CTCTTCACTTCTATACAGGAAGGCCGCTACTCCTTCCCACAAGAGGAGTGGTCTCATATTTCCACCGAAGCCA
AGGAACTGATCGCCCAGCTGCTAGTACGTGAAGCATCACACCGTATTAGCGCTGAACGAGTGCTGCAGCACCC
CTGGCTGAGACGAGCGGACGCTAATGCGACTAAAGTCAACTACCCACCTTTGCATACA
CCACATAATATTAAACGCAATATGTCAGCTCGTAACTTATCCAACTTCGCGGAATCAGCCATGGCGGTCAACC
GGGTGATACAGCAGCACTTCTCAATGAACTACTCGTACATGGACCGCATCGAGCAACCGCCGGCCGCGCTTCG
AACGAGCAAGTCGTGCCGTATACCCGAGGACGACGACGCTCCCCTAGGCCTATCACCG
CCCACCGACTCGGAACTACTGCGTAGACGTATGCAGAACGGTGTTAAGCCGCCACCGCTGCCTGTGCATTAAC
TTATCCTATCTAAATCCTCATCAAAAAAAAGTAAAAATCCTTCTCCTCTATCCCCTTTCCTTCATATCCATTT
CTGATACGGTATCGATCGAGATAACGAATTAGTTGATATGACGCCAGGACCCTGGTAG
TGATCACGACGGCAGAATGCAATAACCTCCCTCCCCAGTCAGTGATCACAGTAGACGGACGGACAGCGGAGCA
GTGTGCCCTGACACCCGCCGTGCGGCCGTGCCGCAGTACGCAGTACAACTGGACCTACCTCCATGTGATAGCC
TTCGGTTGCAGCCACTCCAGTGATATACACGTACGCCGTGCACCGTAGACGGTACTTA
GAGCTGTAACACTTTTATTTTGAGCCTTTTAAAGATATATTTTTTATGAATAAATAAAAACGACTGAATAGTT
TTAATCGATGTGTAGAGTATTTGTGAGTTTATGTTGTAGGCGGCCGCCACTGTGGTCACGCCGTCGAGACCGA
CGCCGGTCGATAAAATCGTATTATATTATTGAGATAGTTGTGCTGATCTACATACTTT
ATTATAAACTACAAAACAGAGTAA
>1002052343_2 Heli.2-DT662417.3.5
ACGAGGGTCCCGTGTTGCGTGTGCGCACTCCAGCGAGTGATCCCGCAATAGTTAGAAGAAACGAACCCGACTC
TTATCAAAATGCTCCTACTCGCCAGCTTTTTCCTGCTTTTCGTAAGCACCCTGGGCCCTGTCGCAGCTCAAAG
GATAACGACGATACAGCTCGATGGCGTTCAGTACTTTATATCAAGAATGAATCCCTAC
AGCCCCGAGCTCAACTATTTCCTCGCTTATCAATATTGCAGATCCCTAGGACTACAACTGGCGTCTTTTGAAA
CAAAAGAAAAGGCGGATTCAATTACAACTTATTTGACTAATGCAGGCTACAACAAGTACGATTTCTGGACATC
CGGAAACAATCTCGGTACCGACATGTACTTGTGGATGAGTACAGGGTTGCCTTTCAAC
GCGACGTTTAATTACATGCGTCGCATGACACTCGACGCTCCCAGCCAGCATGCTGACGACAGCATGGACCCCC
TTGACGTGCCTCAAGGTAGCACTGCCCCCCAACGTACTGCCAGGCATGGAACTGAACACGTGATGACAAACGG
GTGTGTGGCATTAAAGGCGCCATCATTCCACTGGGAGCCTCAACACTGTGGAGAAATC
AAAGACTTCATCTGCGAACAAACACGCTGCTACTACTACAACTACGGATCCATCCCCGTCTCCTCGGCGCAGG
GGTAATGCAAGGAAGCCGCTATCGATGACGACCACGACGACTGCACACCCACTAACGTCGTCGTCGCCACCTC
TGAGCACCTGTGAGCACCTGCCGACTCACTTTACTCTCAACGATCTTATCGGCAAGAT
GCGTCCGTCTTC
>1002052344_2 Heli.2-DT666945.3.5
GCACGAGGCGAATATGAAGGTGGTGTTATTAGTTGTTGCTTTAAGCAAATTTGTGACCGGGGAAGAGTACAAA
ATACCGTCAATTGAAGAACTACCGAAAGATGGCATTGGTTTATACAATGGAGAGATAAAAGAAGACATAAATT
ATTACAATGAATATGACAAAAAATACGAATTGGGCAATCCGGATGATGACCAACAATG

TATAGAAAAACCCTGTAATAACTACGACGCGTTTTGCATACGACGCTTTTTTGCCCAACATTCACATTGTAAA
GAATCCTATGGACCAGTGCCCACTCCATTGTATAGGGCACAAAGTATTTCTTACTTACCTAGAATAAACCTTA
CTGTGGTATCACTTAAGCAAGAATATACTGGAGTTAATGGTCGGGTTGTGGAGTTTTA
CATCAACAAGAAGACTGACAAGTTGGTATTGGCTGTGGAGTTCACAAACTTTGGGCTGGGCTCTAATGAAAAT
TACTTTAAATTTCATCGCCGCGGTCAAGAACCTATAGTCAATATGGGTTCTTTAAACGTCACTTATGCTTCCA
TTGTATCTACAATTGTAATACCAAATTTGAAGGATCTTCAGTATGAAAGAAGCGAAGT
ATTTACATACAATAACGATGTTAATCCACGATTCAGTTTTGGTCTCAGCGCTTTCATTGGTACCGATCCTGTT
CTTCTGGAAACGGCTTTGGCGTTTCTCGCTAGTTATGCTGATAATATAAGAGAGGGCCAAATAACGGACGGAC
CGTTCTTCATAGCGAGTTACATTAAATACTTTATATGTGACTTTGGAATTGAAGTGTT
ATAGATACGGTACAGTAAATGTTAATCATAAATGCAGTTTATAACAAAAAAATAAGCGAATTTAATTAATACA
ATGTATGTGTT
>1002052345_2 Heli.2-DT666267.3.5
GCACGAGGATTTCATTCTTACTAGCTTCACTTGGACGTTGAAAATACGCGTAGCGGTACCTATTGCATCAGTG
AAAGTTTCGAAAATGTTAGGATCACTTAAAATTTTACTATTTTTAGGTTTAATTTATTTATCTAAAGCCTCTG
AAGAAGATGTACTTGAACTTACTGATGCCGACTTCTCCAGTGCTATATCCCAACATGA
AACCGCTCTCGTCATGTTTTACGCTCCATGGTGTGGTCATTGCAAGAGATTAAAACCAGAATACGCTGATGCG
GCCGGCATTTTGAAAAATGATGATCCTCCAATTACGTTAGCTAAAGTGGACTGTACGGAAGGTGGAAAGAGCT
CTTGTGAACAGCACTCTGTCTCTGGATACCCTACACTTAAGATTTTCCGCAATGGTCA
ACTATCACAAGAGTACAACGGCCCCAGAGAATCCAATGGCATAGTCAAATATATGAGAGCCCAAGTAGGACCA
AGCTCAAAAGACTTGGTAGATGTTGCTAGCTACGAAAAATTCCTAGCCAAGGATGAAGTGGTAGTTGTAGGCT
TCTTTGAAAAAGAAAGTGACTTAAAGGGCAAATTCCTGGTAGCTGCTGATGTGATGAG
AGAACAAGTGGTGTTTGGACACTCCTCGGCTAAAGATGTTTTAGAAAAAGCTGGATTCAAGGACAATGTAGTG
CTGTACCGTCCCAAGCGCCTCCAAAACAAATTCGAAGATTCCTTCGTCGTGTACAATGGTGAACCAGCTGAAC
TCAAGGGCTTCATCAAGGAAAACTACCATGGTCTAGTCGGTGTGCGTCAAAAGGAGAA
CATCCAAGACTTCAGCAACCCCCTCGTAGTTGTCTACTACGATGTTGACTACCAGAAGAATCCTAAGGGAACT
AACTACTGGAGGAACCGTGTACTTAAGGTTGCCAAGGAAATGTCAGAAATATCATTCGCGATCAGCGACAAGG
ATGACTTCATGCACGAGCTGAACGACTACGGCATCGACTTTGCCAAAGGTGACAAGCC
TGTTGTGGCTGGAAAGGACATTGATGGCAACAAATTTGTGATGGGCCAGGAGTTCAGCATTGAAAATCTACTC
GCATTCGCCAAGGACCTGGTAGATGGCAAACTAGAACCATTCATCAAGTCTCAACCGATACCTTCAGAGGCTG
GACCCGTCAAAGTAGCAGTCGGCAAAAACTTCAAGGAGCTAGTGACCGATAGTGGACG
>1002052346_2 Heli.2-DT668064.2.5
AATATGAACAGCAAAATGAACAGCTACATTACTTTCGCTTGCCTATTAGTGGTCGCCGCTAGCGCTTACGGCT
CAGGCCTCGGCCTCGGCCTCGGTTATGGTGGACTCGGTTACGGTGGTTACGGCGGCTACGGGGGCTACGGATC
AATAATAAAGGAACCTATCATCGTCAAGCCTGCCCCCATTATTAGCCCATTGCCCATA
ATTAAGCCCCTCCCCATTATTAATCCTCTTCCCATAATCAAACCTCTTCCCATAATCAAACCTCTTCCCATCA
TAAAGAATGTCGAAATCGCTCCCATACTGACTGAGCCACTCTTAAAATCCGTGCCCGTATACGGTGGGTATGG
ACTTAACAAGGCTCTTGATTATGGTCTTGGTCATGGTGGACTCGGCTATGGTTACGGA
AAAGTCTTCTAAATAACGGGATGGACCCAATGGTTATGCTGCTGCTGCTGGCTGAAATGATATAGTTTTTTAT
ATTTCGTCTATTTCCTTCCTCCTATTTTAATCTTTTATCAATAAAGCAATTTAATTTTAAAAAAAAAAAAA
>1002052347_2 Heli.2-DT664206.3.5
CTCGTCGTGTATTGTATTCTTGTACTCGCAGTGAAAAATATTGTGTGTGTTGATTACGAATTACTTGAATATT
GCAACAGAACAGATCCTGCGACTCGACAGCTTGTAGAGTTCGCGAGAGAGCACCCGAAACCCGTTGGCATACT
AACAACAAGCAGTGGAAAGTTTGTGGAGATTCGAGAAAGTACTACACTAAACTCGGAT
GCGTGGTCTAATCCATATCATGTAGACCTTTTAACGCATACCAACGCTGAAAGAGTTCCTCAAAGAATTGTGC
ACGCTAGAGGTACTGCAGCCATAGGTTATTTTGAAGTAACCCACGATGTTTCTAAGTATACGAAAGCAGATGT
TTTTAATGGAATCGGTAAAAAAACACGAGTTTTGGGTCGTTTCTCGTCACTGGCTCAG
AGTGTAGGAGGAAACGATCTAGCTAGAGAAGTAAAAGCGTTAGCTACCAAGTTTTATACTAGAGAAGGCAATT
TGGACTTTTTAAGCAACCATTTGCCAGTTTTCTTTTTTAGAGATCCACTTGAGTTTCCACATTTATCTCATGT
GCTCAGAAACAATCCTAAAACTAATATATTAGATTATTCTACACGTTGGGATTTTGTG
ACAAAAGTAGCTGATATGTTCCATGCGTTTATGTGGATGTTGTCTGATTTTGGAATACCAAATGGATATAGAA
AAATGGATATATTTCCAATTCATGTCTTTGAAATAAATAACAAAAATGGTGACAAACATTTTGTTAAATTTAA
TTTTAGAACATTACAAGGCTTAGAAAACTTACCGTCTGATGTAGCCGCAGACATTTCA

GCTCGAGACCCCGACTATTATAATCGTGACCTATACAATGCTATCGAAAATAAAAACTATCCAGCATGGGAAT
TGGAAATGGATGTTATGACTCTTGAGGATATTAAGAATCTCAATTATAATCCATTTGAAGTAACTAGACTGTG
GAAAAAAGGGACGTATCATACAGTAATAATAGGCCGTCTAGTATTTAATAAAAACCCA
GACAATATGTTTAGATTTGCTGAGGAAAGCGCCTTTAATCCGTTCAATTTAGTGCCAGGTATTCCCGGACCTA
TAGATACACTATTCCGTAGTAGACGAACATCATATCGACAAGCTCAAGTATTCCGTTTGGGTGTGAATCATAA
CAGGATAAGTGTGAATGCGCCACTCCATTGGAGTGTGTATGACCGTGATGGACTTCCT
CCATTAAAGAGCAACATGCGAGATGCACCAAATTAT
>1002052348_2 Heli.2-DT663432.3.5
GCACGAGGCGCGCACGAGGCACATTTAAAAAAAATGTTCAGGCTCATATTATTAGCCTTTGTGGCGGCGGTCG
CGTGTCAGCAACACGAGGGTGCTAGACGGGTTCCCAAATACGCAGGTGACCCCAAAACAGCTGCCATTGTGCA
AGAAGCTCGGTATCTCAGTGGAAACGGTGCTTTCGGAGCAGCTTATCAGCAAGAAGAT
GGTATCGATTTTAAAGAAGAGACAGATGCTGACGGTAACAGGAAAGGCAGTTACTCATACATTGACCCCACCG
GACAAAGGAAGACCGTGAATTACATTGCGGGTAAAAACGGATTCCAAGCTATCGGAGATCATATCCCCACTGC
ACCTCAGCCCGTGGCACCTACGCCAGGATACACACCTGATCCCCGTTACAACTCTCCA
GACTACAAACAGTACAGTGCTCCTCAGCAATACTCAGCACCAGTCGCGCCTCGCAACTACGGTGGAAAACCTA
GCGCGGACGATGGTCAATACTACCCTGAACTTTACGAACGTGATAACTATCAAGGTCAACAACAATACCAACC
TGCCCCACAGCAACAATACCGATCTGCCCCACAACAACAGTACCAACCTGCCCCACAG
CCACAATACAACACCAACAACATCTACCCAGGAGTACAACAAGCCCAATACAGCGGGATCCAAAACCAACAAT
ACAGTGCGCCCCAGCAATCCCGCCAAGATTACTACGAACCGACCACACCACCTCCCGCACGTTTCTTCCCACC
AGGCAAGTTCAGTCTGAACCGTGCCCCCGATGGCTACACCTACTCGTTCCACAAAGTA
TAAATCTCACTTAAAAGAGGGTTCCTGGCGATTTGTGATGTCTGAAACGTAAAAAACTCGTATCAATTAGTAA
ATCGCTGCGTCACCAGAGGGTAGTTTAGGGTACGTGCCTCCTTGCTTCGGAAGCATTTCTTAATTGTTGTTCG
CTGTGATATTGTGTGCTAATGGACAAAGTATATATACGCTGTTGGTATCGTTACTCAA
>1002052349_2 Heli.2-DT666998.3.5
CACGAGGGTTTAGTCGTAACTCAAATAAACGCAACGATGTCGGGAGGATTAGATATTTTGGCCCTCAACGAGG
AAGATGTAACCAAAATGTTGGCGGCAACAACCCATCTTGGGTCTGAAAATGTCAATTTTCAGATGGAAACCTA
CGTATACAAGCGTCGCACTGACGGTACCCACGTGATCAACCTTCGTCGCACCTGGGAG
AAGCTTGTGCTGGCTGCACGTGCAGTTGTAGCTATTGAAAACCCAGCAGACATCTTCGTCATCTCTTCAAGAC
CTTTTGGTCAAAGAGCCGTTCTCAAGTTCGCTGCTCACACTGGAGCCACTCCCATAGCTGGAAGATTTACTCC
TGGTGCCTTCACTAATCAGATCCAAGCTGCATTCAGGGAGCCACGTCTTTTGATTGTA
TTGGACCCTGCTCAAGATCATCAGCCAATTACTGAGGCCTCATATGTCAACATTCCAGTCATTGCTTTCTGCA
ACACTGACTCTCCTCTGAGATTTGTTGACATTGCCATTCCATGCAATACTAAGTCTTCCCACTCCATTGGTCT
CATGTGGTGGCTCTTGGCTCGTGAAGTTCTGCGTCTGAGAGGTGCTCTACCACGTGAC
CAGAGGTGGGATGTCGTAGTAGACCTCTTCTTCTTTAGAGATCCCGAAGAAAGCGAAAAGGAAGAACAACAAG
CTAAGGAACAGGCTGTTGTTGCTGCTAAGCCTGAGGTACCAGCACCACCTGTACATGAGGAGTATGAGCCCCC
AGAGCCTGTTGCTTCCTGGAATGAAGAGGCTGCTGCACCACCACCAGCAGCCAGCTTT
GGTGCACCTGCTGCTGCTCAAGAAGATTGGGCTGCTCAGGTTCAAGAAGAATGGGGTACTGCCGCCCCAGCGC
CTGCTCCTGCAGCGGCCCCATCGTGGGGAGGTTCCGCCCAGGACTGGAGCGCCGCGTAAACCATACATTAAGT
TGATTGACGC
>1002052350_2 Heli.2-DT664402.3.5
CCGCATAAAAAAAATCACAAAAACAACAATGAAAGTACTGGTGGTGTTATCCGCTGCTCTAGCGCTTACAGCC
GCTCTTCCTCAAAGAAGAGTGTCCTTGAAGCCTGAAGGTTTAGAAGACCAACAGGCTGCTGAGCAAAACTACA
ATAACTACCAACAACAGCAAGATTACAGGCAACCACCACAGGAATACAGACAGGCCAA
GCCAATTGAGGATTTCAGACCAAAAGTTCAAATCGACACCACCACCTTCATCCCGATCATTCGTTTCGACAAG
GAACAGGAAACTGATGGCAGCTACAGAACGGCATATGAAACTGGAAACAACATTCAAGCTCAAGAAGAAGGTT
ACTTAAAAACTGTTGGAGAAAACGAAGACAAAGTACCAGCTTTGGTTCAACATGGCTC
CTACACTTATACCGCACCAGATGGTCAAATCATCACTGTAGAGTACACCGCTGACGAACTAGGCTTCAGAGTT
AAGGGCGACCACATCCCAACCCCTCCCCCAGTCTCTCCTGAAATCCAAAAAGGTCTTGATTTGATCTACGCTG
GAATTAAAGCCAACCAGGAGCGTGCCGCCATAGAACTGAAGAACAACCCAGAATCCGC
TAGGTTACTCGAAGACAAGGCAGCTCTTGACTACAAAGGACTTTACTACCAGCAGTAAATTTTATAGTGAACG
CTGACCATAAAGCCAGTGACGTTATGTCAAGCAATAGCGTAAAGTAGTGTCATAGAATATGACTAATAACCTG
CAAGTGCTCTGTGAGCATGATCAAAGTCATTCGTTTATAGTATAATTAATACAAAACT

AAAACAATATTTAGAATCTAATTGGGTTCGCACTTATATGTTTTTAATTTTATTGATCAGACTGTAGTGCAA
ATGCCATCTAAGCCTTATCTTATATTTTAAATATATGTAGGTAGTGTTAAGAATATTCCTTCTATAAGCTGTA
AGTAAACATGATTTCTTCCTTTGAAATAATTAATACTCATTTTATATGTCAGATATCC
GAAAGTATTGTTGATTTTTGTTTAATGTGATATTCACTTGGTAAAAGT
>1002052351_2 Heli.2-DT666495.3.5
GCACGAGGCCCGACTGCAACCACCGTTGAAGAGGCATTATGAAGACGATCTGTTTAGTTGTTCTGCTGGTAGC
AGCAGCCGCTGCCAGCGAGAAGAAATCCACAGAACAAAAAGTTGAAGGCCTCGAAAAGAAGTTGGACAAACGT
GGTCTCTTGAACTTGGGATATGGATACGGTATCAGCGGTTTGGACGTCGGATACCTAG
GCAGTGGCCATGGACTAGGTGGAGTATACGGCTTAGATGAAGGATCTTATGGACACGCCGGATACGGCATCGA
CCTTGGAGGCCGCACCGATGTGACCAGGACTGTCACCCTCGTCAAGGGAGTCCCAGTAGCAGTGCCAGTTGAC
AGACCAGTGCCTTACCCAGTAGAGAAGCACGTCCCTTACCCCGTCAAGGTCCCAGTTC
CTCAACCCTACGAAGTAGTCAAGCATGTGCCCGTCCACGTTAAGGAATACGTGAAGGTCCCCGTACACGTACC
TGCACCATACCCAGTAGAAAAGAAGGTGCCATACCCCGTCCATGTCCCCGTCGACAGGCCCTACCCCGTCAAA
GTTCTCGTACCTCAACCCTACCCCGTTGAGAAACACATCCCTTACCCCGTTAAGGTAC
CAGTTCCCCAGCCCTACCCCGTTGAAAAGCACGTGCCTTACCCCGTCGAAGTCAAAGTACCCGTACCACAGCC
CTACCCCGTTATTAAACACGTTGGTGTACCCGTAAAGGTTCCCGTTGACAGGCCTTACCCCGTCCATGTGCCA
GCACCTTACCCCGTTGAAAAGCCAGTGCCAGTCGCTGTCCCAGTTGAGAAACCCGTTC
CTTACCCCGTACACGTGCCCGTAGACAGGCCTTACCCCGTCCACGTTGAGAGGCCCGTCGCAGTGCCCGTCAA
AGTACCCGTGCCAGAGCCCTACCCCGTATACAAACACATTCCCGTTGAAGTAGAAAGGCCAGTAGCCGTGCCC
GTCAAAGTGCCCGTCGACAGGCCTTACCCCGTGCACGTCGAGAGACCAGTGCCAGTTG
CCGTTGAGAGACCCTACCCAGTACCAGTCAAGGTACCAGTTCTGGTCAATGAATACGATCATGGCAGCAGCTA
TGGCTACGGAGGCTCATATGGGGGATCTTACCACGGAGGTTCCCTCC
>1002052352_2 Heli.2-DT662431.3.5
CACGAGGCCGACGTGTTTATTTCAAAATCGTACTTCATCGCGTTTGGGTGTGTCACGTTTACTAAAAAAATCA
AAATGAGTGGACAGCAATACTACCCGTACAAGTGCACCCCCACGGTGTACCCGGCGGAGCCCTTCGAGCCAGC
TGCCGATGCGGAAACTCTTCGCAAGGCGATGAAAGGCTTCGGAACCGATGAGAAGGCT
ATCATCGATGTTCTCTGCCGCCGTGGAATCGTACAACGCTTGGAAATTGCTGAAACCTTCAAAACTAACTATG
GAAAGGACCTTATAAGCGAGTTGAAAAGCGAACTAAGCGGTAACTTGGAGAACGTGATCGTGGCTCTGATGAC
ACCACTGCCACACTTCTACGCCAAGGAACTCCACGACGCGGTGTCAGGATTGGGCACT
GATGAAGAAGCCATCATCGAAATCCTCTGCACTCTGTCTAACTACGGAATCCGTACCATTTCCGCCTTTTACG
AACAATTATACGGCAAGAGTCTCGAGTCAGATCTCAAAGGGGACACGTCTGGTCACTTCAAACGTCTTTGCGT
ATCATTATGTATGGCCAACCGTGATGAGAACTCGGGAGTGGATGAGGGAGCGGCCAAA
GCTGATGCCGAAGCCTTAGCATCAGCTGGTGAAGGACAGTGGGGAACCGATGAGTCGGTGTTCAATTCCATAC
TTATCACACGCTCGTACCAACAGTTGAGACAGATCTTCGCTGAATTCGAGGCGTTAACCGGTAAAGACATCGA
GGAATCTATTAAGAAGGAATTCTCTGGAAGCGTCGAGAAGGGCATGCTTGCTATTGTA
AAATGCGTAAAGAGCAAAGTTGGCTTCTTCGCTGAGCGCCTCTACTACTCAATGAAAGGTTTGGGAACCAACG
ATAAGACCCTTATCAGAATCGTAGTCAGTCGTTCCGAGATCGACTTGGGAGATATTAAACAAGCATTCTTGGA
CAAATACGGGAAACCCCTAGAAGACTGGATCGCT
>1002052353_2 Heli.2-DT667716.3.5
GGTGGACGTGTCGTATATTTCTAAGAACCAATTTAATTTATCCGTAAATATGTCGAAGATTGGAATCAACGGA
TTCGGCCGTATTGGTCGTTTGGTGCTCCGTGCATCTATTGAGAAAGGAGCTCAAGTTGTTGCCATTAATGATC
CCTTCATTGGCTTGGATTACATGGTATACCTCTTCAAATACGACTCCACCCATGGCCG
CTTTAAGGGATCTGTTGAAGCTGCTGATGGCAATCTCATTGTGAACGGAAATAAAATCGCCGTATTCTCCGAG
AGGGACCCCAAAGCCATTCCCTGGGGCAAGGCTGGAGCTGAATATGTTGTAGAATCTACTGGTGTTTTCACAA
CCATTGACAAAGCGTCTGCTCATTTGGATGGTGGTGCCAAGAAGGTTATTATTTCTGC
TCCCAGTGCTGACGCACCCATGTTCGTGGTTGGTGTCAATCAAGAATCATACGACCCATCTTTCAAAGTTATT
TCTAACGCTTCTTGCACAACTAACTGCTTGGCTCCTCTTGCCAAGGTCATCCATGACAACTTTGAGATTGTAG
AAGGTCTTATGACAACAGTCCATGCTACAACTGCTACCCAAAAAACTGTTGATGGACC
CTCTGGAAAACTGTGGCGTGATGGCCGTGGTGCCCAACAAAATATCATTCCAGCCTCAACTGGTGCAGCCAAA
GCTGTAGGCAAGGTTATTCCTGCCCTGAATGGAAAACTGACTGGTATGGCATTCCGTGTGCCCGTAGCCAATG
TGTCAGTTGTTGACCTTACAGTACGCCTTGGCAAACCTGCCAGCTA
>1002052354_2 Heli.2-DT662283.3.5

CCATGAGATTATACACGTTTGTTACATTGTGCGCGTGCGCGTTGTACGCCAGCGCGGCGTCCATCCCCGCGCC
GGAGAGGAACATTGATGCTCTAGATAACGTTGACTTAGACAATGTAGACCCAGAGCTGGTCGATCAGCCTTCG
GTCCAAGATCTCGATAACAGTCTTCGTAAGGTACCGGATATTCCCGTTAAAGTTATCG
AAGCGCCAGTCATTCTGGATGAAAATTCAAATGAGTATATACCAGATAAAGATGAAAACATCAACGTCAAGAG
AATTGAAATTGATCTAAATAACCCTGGACCACCTCAGCGCCAGGAACATGAAACGCAAAATCCAGGGAATTAT
GGAGAAAAAGAGAGGGCTGTGTATCAAATATTACAAAAGGCTCAAGAAGCGCAGTCTA
TTTTTACCAAAGGAGTAAACGATATATCTGACAGCTTCTATAACTTAAACAAACATAGTGACGAAATTCCAGC
GGTTTTGGAAAATATAAGAAAATTAAATGTCTCATTTTCAAATGAAATTATTGAACTTAACAACACTATAAGA
ACTTATCTGGAACCCAAGCCAATACAAGTTGACAATGAACAACAAAATAATGTAAAAC
TTAATTACGTTGAATCAAAATTACAAAATTTACATTCAAACTTTAAAAACGGTGTTGACATTTTAAATGAGGG
CTTTGAACTTCTTTCAATACTTCAGGAAGAAGATGAAGTTGCCAAATCAGAAACTAAGACATTGGTAAAAGAA
GAAAATTATTCAGTCAAAGATGATGCAGCATCGTCCGCTGCTCCTAGTTCTTCTACTG
CAGCACCTTCACCTATACAGGTGCCTGCAAATGGCTTTAACTCGTTTATAGGATTTTTTACTACAACTGTTTC
CAACATGCTAT
>1002052355_2 Heli.2-DT665131.3.5
GCACGAGGAACACACGATGCTGAATCTAATCAACTAAAGAAACATTTATAGGTAGAATAATACCATGATCTGG
CAACCATTGAATTTGTCAGAAGTCTGTCCTCCTTACACACCTATCACAGCTTGTTCGACATTTGGCTATGTCT
ACCTCAGTCTCCTAGTCCAGATCTATGGAGGTTCTGTTGATACGAGGATACAAAAGCA
ACTTCACATAAGAGATGACGAAAAGGAAAAGCCCAGTGAATATGATTTTGTTGTGGTCGGCGCGGGAGCTGCT
GGTTGTGTCGTTGCCAATCGACTCACAGATAATCCTAAATGGAAGGTATTACTCCTAGAAGCGGGTCCTGAGC
AACCAGACGTGACTTACGTACCGGGTCTATCAACTGCACTCCTGGGATCTAACATAGA
CTGGGCGTATACTACAGAGCCTAATGGGAACAGCTGTCTTGCTCATCCCGGAGGCAGGTGTCCTTGGCCAAGA
GGGAAAATGATGGGTGGTTCTAGTTCAATCAACTCAATGGCGTATATTAGAGGTAACAGGGTGGACTATGACG
GTTGGGCAGCGATGGGCAACAACGGCTGGAGCTATGAAGAAGTTCTTCCATTCTTCAA
AAAGTCAGAACGAAATATAAACATAGAGGCTCTAGATCTAAAATATCACGGAGTCAGTGGGGAACAATATGTA
TCCAGATATCCTTACATTGATAAACCCTCACTTATGTTAACTGAAGCTTTTAACCAAGGAGGGTTACCTTTAA
CTGATTATAATGCCCGATATCAAGAAGGCACAATGCAAGCCCAAGCTTTCTCTTTCAA
TGGCGAAAGAGTATCCACAAACACAGCTTTCATACAACCAATAAGATATAAGAGAAAAAATCTTATCGTCGAA
ATCAATTCTGAAGCCACGAAAATATTGATCGATAAAAATAAAAACGCCTATGGCGTTATTTATATGAAAGATG
GGAAAAAATATACAGTTTACGTTAAAAAGAAGT
>1002052356_2 Heli.2-DT662541.3.5
GCACGAGGGTGCACACGCTTAGGCGAAATTCTCTAGGCGAGTACTGCTTACAGTAGCGTTTAGTCAGTTCGTT
TGAGATCTCCAGAAATAGTTTAAGAAATATCATCATGCAGTCGATGATCATCCTTGCCGCTTTCTTCTGCCTC
GCGCAAGCATCCTACTACCCCGGCCCTCCAGCGCACATACAGCTCAGCTCTGATGGCA
AATATGTGCTGGACACGCCCGAGGTAGCACACGCCAAGGCCGCCCATCTGGCCGCACATGCACAGGCCTCCAA
CGTTCACGGTGCCTGGGCTCCCGCTGGAGCGTACGTCGCTGGACCCGCCTACGGCGCCGGTGCTCATTACGGA
GCTCCCGCTGCCGGTCTCGTGAAATACGGCCCCGCTCCCCTCGCCCACGACGGACGCG
TCATTGACACCCCCGAGGTCGCCCACCTGAAGGCCGCTCACATCGCCGCGCACTCCGCCGCCGCCGCCAAGGC
CGCGCACGGCGGCTACGCCGGCATCGGTGCTGGCCTCGGCGCCGGGTACGGTGCGGGCTACGGCGCGGGCGCA
GCTTACGGCGCTGGATACGCTGGCGGCTACGGCAAGTGGAACGGACCCCAGGCTCACA
TCCAGCTCACCCACGATGGACAATACGTCGTAGACACCCCTGAGGTACAACACGCCCGTGCCGCTCACTTCTC
TCAGTACGCCCACGCTGCGCAAGCCGCCGCCTCCTCGCCCGAGGAGCCCTGGGACGCCCACAGCGGACACGGC
GCCCACGGCTGGCACTAGATTCTACCTCCGACCTAGCCCAAACACTGCCTGAATATAT
TTTTATACAAAATGTAAAGAAAAAACCAAAAAAATGTTAAACTGTATA
>1002052357_2 Heli.2-DT662116.3.5
TCGCTTTGAGTTTATTGCGTGACGTGCCGTCCCGAATTTTCTTAACGAAATGTGATCCAATAAAAAGATGGCG
ACTTCCGTCCTTCAGCCAGAAGAATGCAGACGTGAACACGAACCCTTACCCGACACCCCGAGACTAACAGACG
ATGAAAGAATTGCCATCCTAGAATCCAAGGAACGGGAAACCGGTGAACTTCCACCGGA
GCTTCGAGAGAGAGCCAGATTGGATATTAGAGAGGAACCGGCCATTCGAGATCATGCGCTTGCCCAGATGCGG
CACTTCATTGACAAACATCCAGCCATCAAGAAATGCCGGACCGATGCACCATTCCTGCTTCGTTTCCTTCGCA
CAAAGAAATACTCCATACCACAAGCGTGTTCAATGCTGGAGAGATATCTAACGATCCG
GCAAATGTATCCCCATTGGTTCCAAAAACTTGACCCCCTGGACCCCAAAATAGCTGCAGTTATAGACGCAGGA
TACTTATTGCCACTACCTAAGAGGGATGCTGAAGGACGGAGAATCGTTTTATCTTGTATGGGCCGTTTCGACC
CCCACGTGTTTGATAGCTGTATTATGGCCCGCGTACACTCAATGATAGTAGAGCTGCT

GCTTGACGAGCCTCGTTCCCAACTGCTTGGGTACACTCACGTGAACGACGAGGCCGGTATGCAAATGCCACAT
GTGAGTCTGTGGTCGCTGACTGATGTACGGGTTATGTTGAATTGTATACAGAATTCCACGCCCATGCGTCATA
AGCGTACACACTTCGTCAACATACCGCACTATGGCGTCAAGTTCTTCGAGTTCGCTGT
GTCATTGCTCAGTGATAAGCTCAAGGATCGTGTTATGTTCCATCGTACATCGGAAGATCTGACAAAATATGTA
GATCCCGCCATATTGCCTAAGGAATATGGCGGTACAGTTCCACTAAAGGACATGATTGATGAACTTAAACGTA
AGCTACTGAAGCATAGAGAAGATTTACTTGCATTGGACAATATGTGTATTGATTTGTA
CGCTCTTGAGAAAAATGATCTTACTCAAGATATTCATTCTACTGCGGGGTCATTCAGGAAATTGGAATTAGAC
TAGAATACTAGACATAAATTAGA
>1002052358_2 Heli.2-EL597759.1.5
CACGAGGCTCGTTTCTAACAATCCTACGACCAACCCTCTATCAAAATGCATAAGCTGATTGTATTCGCAACCC
TCCTGGCTGCTGCTGCCGCAGCCCCCGGTTTGGCACCTCTCGTTTCCTCATGGGGCCATGGCGGACTCGTTGG
TGCACCAGCTTTGGTCGGCGCACCCATCGTCAGCGCTCCCATCATAAAACAAGCCATC
CCCGTAGCTACTTCATATGCCAATGTTAACGCTGTCAGGATTTCCTCTCCTGCTCTTGTCGCAGCTCATGCTC
CTGTTATTGCTGCTCATGGTCCCGTCCTTGGTGCACCTTTAGGTCTCGGTCACGGTCTCGCCCACGGCTGGTA
ACCCGCCTAACAAAAACCGAGCCCTGGTTTCAGTCAAGATACGCAACAGTCAATTTTA
TTTAAATTATAGTCTGATAAGCTATGAATGAAATTCTATTTGGTCATTACTATTAAAAATTTAATTTATATAT
AAAAAAAAAAA
>1002052359_2 Heli.2-DT666846.3.5
CTTTCTTTTATCGCCAGCTTGGTGCGACGCAAAGTGGGTTTAGGCTCGTCGTATTAAAAATGTCTGAAACTTT
ACAACTTAGGGGTAACCTTTACGGGCATAGCGGATGGGTTACCCAAATTGCTACTAATCCGAAATATCCGGAT
ACTATTTTATCTTCTTCCCGTGATAAAACACTTATTGTTTGGAAGTTGACCCGTGATG
AAACCAACTACGGTATACCACAGAAGCGTTTATACGGACACTCCCACTTTATCTCGGATGTGGTACTGTCTAG
CGATGGAAACTACGCTCTTTCCGGATCTTGGGACAAAACTCTTCGTCTATGGGATCTCGCAGCTGGAAAAACC
ACCAGGCGTTTCGAAGACCATACTAAGGATGTCCTCTCGGTAGCATTCTCAGTTGACA
ACCGTCAGATTGTGTCTGGCTCCCGAGACAAGACTATCAAGTTGTGGAATACTTTGGCTGAATGTAAATACAC
AATCCAAGATGACGGTCACTCTGACTGGGTATCATGTGTGCGTTTCTCACCAAATCATGCTAACCCAATTATT
GTTTCTGCTGGTTGGGACCGCACTGTTAAGGTCTGGCACCTTACAAACTGCAAATTGA
AGATCAACCACTTGGGCCACTCTGGATATCTTAACACCGTGACTGTTTCACCTGATGGCTCCCTATGTGCATC
TGGTGGCAAGGACATGAAGGCTATGCTTTGGGACTTAAATGACGGAAAGCACCTTCACACCTTGGACCACAAT
GACATTATCACAGCTCTATGCTTCTCACCCAACAGATACTGGCTGTGTGCTGCATATG
GACCCCTGATCAGGATCTGGGATCTTGAAAGCAAGGAAATGGTAGAAGAATTAAAGCCCGATGTCATCAAGGA
CACCAAGACTGATCCTCCTCAATGCCTTTCCTTGGCATGGTCTACTGATGGCCAGACCCTCTTCGCTGGTT
>1002052360_2 Heli.2-DT661669.3.5
AGCTATCACCGGCCGTCCCCGCCTCAACATGCGAGGGATCTGGAGCCCAGTGATTACCGTGACGGTAATTCTA
CAACTCGCTCTAGTATCAGGAAAGGCGTTAATACCACAAGATCAACAAACTCAGCAGCAGGTGGTTCAAAAAG
ATCAAAATAATGCACAAAAACGAGTCGGATATGACTACCCAGTGCCCCCAAATGATTT
AATCAACCCTTTTCAAGATCACGACAACCTTCATCCGCATGATGATCACCACGAAATTATTGATGACCACGGA
CATGATGTTCATCATTTAGAAGAACATCACGTCGAGGAACATCATGAACATCACGATGATCACCACGACCATC
ATGATCATCACGATCCTGGATACTGGAAAAAGAAGTTGATATGGAAGCCTGGATGGAA
GAAAATTTGGAAACCAGCTAAGAAACAAATCTGGAAGCCATCCTGGAAAAAGATTTGGAAGCCTATCTGGGTA
CCGACAAAAGTACCGGTTTGGAAAGACATAAAGGTTCCAGATTGGAAAAAAATTTACAAACCAGATTGGAAAC
CCATAAAGGTACCTGCATGGAAGGAAGTCAAAGTACCTGACTGGAAAAAGATAACAGT
TCCTGTATGGAAAAACATCGTAGTTCCGGGATGGAAAGATATACAAGTACCAGCATGGAAAAAGTTATGGAAA
CCTGAGTGGGTTAAAGTTGGAATTCCTGGTGAAAAATATCTAGGTAAGGATCATGAAGGTTGGGAATATACAT
CGCATGACCTTTGGAAAAAGAA
>1002052361_2 Heli.2-DT662065.2.5
GCACGAGGCAACGGTCGACATGCGCTCCCTTGTTTTCTTCGCTCTCCTCTCAGTGGCTGCGGCCAAGCCCGCG
CCCGGACTTCTTGGTGGATGGGATGGCGGTCTAGGCCTTGGCCATGGCTCTCTCGCGCTAGGGCACGCGCCCC
TCATCCAGGCCGCGCCGGTGCTCCAAGCGGCGCCCGTCATCCAGGCGGCGCCCGTCGT
GCACGCCGCGCCTGTTGCCATCGCCAAGGCTGCCACTAGCTACTCGTCCATCCAGAGGGTTATTCATCCAGTA
GCTCAAGTGGTGGCCCAACCCGTGGTCCATGCCGCGCCCGTAGTCCAGGCCGCCCCAGTAGTCCAAGCCGTCG
CCCAGCCCATCCTGAGCCATGGCATCGGCATCGGCAGCCTGGGCCATGGTATCGGCCT

CGGCGGGCTGGGTCATGGTATCGGCCTCGGCGGGCTGGGTCATGGTATCGGCATCAGCAGCCTGGGCCATGGC
TGGTAAACTTATTCGTAATTAATTAATGTTCGTTAGCGCGTTGAGATCATGCCCGATTTGTCGGGTGGTGCAA
ATAAATATTTATATCAGTATTT
>1002052362_2 Heli.2-DT665186.2.5
GCACGAGGGTTCAGTAAAATCGTTACTTTGTGCGCGGTGGTGGCAGTGGCCTCTGCTGGACTTTTACCTGCAG
CAGTACACTACTCACCAGCTGAGGCTGTATCATCTCAAAGCATTGTGCGTCATGATCAGCCTCAAGCTATCGC
CAAGTTGGCTGTAGCTTCTCCTCTTGCCTACCACGCGGCTCCTTCCGTCGCCTACCAC
GCTGCCCCAGCTGCCATCTCCTACCAAGCCGCTCCTGCTCATTACTCTTCTGCCGCCGCTGTTTCTTCCCAAA
ACATTGTCCGTCATGATGAATCCGCAGGATTAATAGCCTCAGCCCCTAAACTGGCCATCGCCGCTCCCGTCGC
CAAACTTGCCCTGGCCTCTCCCCTTGCCTACCAAGCAGCTCCAGCCCTTGCATACCAC
TCTGCCCCCGCTCCAGTCGCCTACCACGCTGCTCCCGCTTCCATCGCCTACCATTCTGCTCCCGTAGCAAAGG
TTCTTGCCCAGCCTGAAATCATTGCTCACCCGAAATACGATTTCAGTTACTCTGTAGCTGACGGACACTCCGG
AGACAACAAGCAACAACAAGAATCCCGCGACGGTGATGTCGTAAAGGGCTCCTACTCC
CTCGTTGAGGCTGACGGCTCCGTCAGGACCGTGGAGTACTCCGCTGACGACCACAACGGTTTCAACGCCGTCG
TACACAACAGCGCTCCCGCCCACGCTCCCCAAGCCATCATCAAGGCCGCCCCCGTCTACGCCGCAGCTGCTCC
CCAATACTACGTACAACAATACTACCACTAAAAATTAAACAATTAGTCACCAAATATG
ATATAACGACCTGCTATTTATTTATTGCAATATAAA
>1002052363_2 Heli.2-DT666712.3.5
GCACGAGGCAAAGACTACGCGCGTACAGCTTACAAATATACATTCATAGAAACAACAGTATCGTGATGGAGAA
GGAGCACCAGCCAGACTCTATGGCTACTATAACCATGAAACCGGAGTACCCGCCGTCTGAAGTATATAGTGCT
TCAGAACCTCCGCCGGCATATCGTCAGAGGATGTCATCTTCTGTGCAAATCGCGCGTA
TTGCGGCTCTGACGGTAGTGGCGGCGTCGTTCATCCTGGGTTCCTTCATCTTGGCATCCAGCTGGGTATCAGC
ACGCGCCTCTTGTCATCAATTGGAACAGCTCGATGCCATGCTTGACAAGGAACTGGCATTGGAGGGGAGACCA
TACGGAAATGATGGTTTAATGGCAGATGAGCCATTGCCGATAGCAAATGCTCATGCAC
TCCACGGGTCGCCATCACTTGTAGCAGAAACATCGTCTCCACCCGCGAAATTAAAATCAGTGCCGTCCCCCGA
CAGCCTCTTGAAAGAGGATCCTTTAAGCCACGCTGAATCCAAAGTCGATGAAGATAAATTACAGAAAATCGAC
GACGAAAAGGTAGAAACGGCAAATTCTGGAAGCGATGAGAGTTCCGAGTCAGATAGCT
CCGGTGAAGAAGATGATGATGAGCTCATGCCTAAGTTCACGATGCCTATACGTTTTGACCTCGACGAGCTGGC
GGGGGCTTTCTTAGCAAACAACCAAAAAGGACGTATGAATTGTGTAGTTGAGAGGCGCCATGAAGAAGCCATG
GAGAAGCGCTTCCCATTCAACCTTCTTAGTGCATTGACATCTCGAAGCCCCCAGGCTG
AACGTATTTCCATCATCTGCCACGGTGGTGAGGAACGACCGTTGCCTATGTTCCTTGACCCGCAATCACAAGT
ATTCGCATACCCACTGTCCGGGCCAGTGCGTCTACCGCTGCGCCCAGCGCCTGAGCGACCTGAGCCACAAGGA
CTATTCAAAATATTCAAGAATTTAGAACAAGCGTCCATGATGCAAAGGCCATGGGAAT
CTTCGGAAGAACAACAAATGACCCCTCCGCGTGAGATGCGCATTCACGTTCAACGTATCTTCGGCATCCCCGC
GCCTCACAACATGCCTTCTCCTCTTGAGAACATGGGACAGTTTGCACCACCACCACCGCCACCACCCCAAGAA
CGTAACGAGCGTGAACAAGAAGAAATGATGGCGCCAAGACAGATGCGAGTGATTGCAA
TTCCTGCTCCACACAACATGCCACCCCCACCCGAAAACATGGGACCCTTCCAGCCCCCACCGCCACCTCAGGC
TCAAGCGCAAAATGAGCAACCCGCCGTGCTTCATCAGTTTATACCTCAGTCAGCCCCCACACAAGAAGTAGAA
CAGCCTGAGGTACAAAAGATGC
>1002052364_2 Heli.2-DT663066.3.5
GCACGAGGAAATCATCATGATATTGGAACTTTATATTTCATTTTTGGAATTTGAGCAGGTATAGTTGGGACAT
CTCTCAGTATTTTAATTCGGATAGAATTAGGTAATCCTGGGTCTCTAATTGGAGATGATCAAATTTATAATAC
TATTGTTACAGCTCATGCATTTATTATAATTTTTTTTATAGTTATACCCATCATAATT
GGGGGATTTGGAAATTGATTAGTACCTTTAATACTAGGAGCCCCTGATATAGCATTTCCCCGAATAAATAATA
TAAGATTTTGACTTCTCCCCCCCTCATTAATTTTATTAATTTCTAGTAGAATTGTAGAAAATGGAGCAGGAAC
AGGATGAACTGTCTATCCCCCCCTTTCATCTAATATCGCCCATGGAGGTTCATCTGTA
GATTTAGCTATTTTTTCTCTTCATTTAGCGGGTATTTCCTCAATTTTAGGGGCAATTAATTTTATTACTACTA
TCATTAATATACGAATTAACAATATATCTTTTGATCAATTACCATTATTTGTCTGAGCTGTAGGAATTACAGC
TTTATTACTTCTTTTATCTTTACCAGTTCTAGCTGGAGCTATTACTATACTTTTAACT
GATCGAAATTTAAATACTTCATTTTTTGATCCTGCAGGAGGAGGTGATCCAATTTTATACCAACATTTATTTT
GATTTTTCGGACATCCAGAAGTTTATATTTTAATTCTCCCAGGATTTGGTATAATTTCTCATATTATTTCCCA
AGAAAGTGGTTAAAAGGAAACTTTTGGTTGCTTAGGAATAATTTAT
>1002052365_2 Heli.2-DT664595.3.5

GCACGAGGGTCGTTCCGCGCTCACACATGTCTCGCGAGTGACAAGTGACACTGACAAGTGACCTACAAAATGA
CCGCCAAAATTTACGTGCTATGTCTGGTGCTTGCCACGGGGATGGCGGCGGCTGCGCGACACCGACCCAGATA
CCAACAACCCGTGGAATATGACTATGAGCCTCAAGCCCAGGAACGAGGAGAACGGGTT
GTATTAGTTGCCCAGGATGCATATGATGGATTATACCAAGCCCAAGATCGTGCAGATGAGGATTACGAACCCA
GAGCATTAGTCAGAGCACCTGCACAAAGGCACAAGCAGTCTCAGTTACAACAAGAAGGACCTAAGCAGCCTCC
AGTGCAGACAATCAGGAATTACAATAAGGTGAACGATGATGGTAGCTTTACATTTGGC
TACGAAGCTGCTGATGGGTCCTTCAAAGAAGAAACTAGGGGCACTGACTGCGTAGTTCGTGGAAAATATGGCT
ACATTGACCCTGACGGTAACAAAAGAGAATTTACTTACGTATCTGGTAACCCTTGTGATCCAAACAAACCAAA
TGAAGACGAAGAACAAGAAGCACCAGTGCCAGAATCTGCAGAAAGAGACGATCCTGAA
CCCAATTATCCGAGCAGACCTGCCTTAAGACCGACAACAGCCCGACCAGCGACCACTTACTTCCAAAACGACT
TTAGAGATGCTGATGAGGAACCTGAAGAAGAGCCTCTTCAAAACATACGACCACGAATTGTTCAACGTCCACA
ACCCGTGAGACCAGCTCCTCGGCCAGCTTACCAAGCTCAACAAATCGCAATTACTCCT
CGTCCAGTACCTATCACTACTGCCAGAGCCTTACCTCCAGCGACTACATTTAGGCCACAATTAATCCAAGTAA
CCCCTAAGCCCCAAATTCAGTATAGTCCTGAGCCTGAATACTCTCCTGCCCCAACACCATCTGCTGTTCTGTC
ATCTGCTAGGCCAGGTCAAATTGATTTCGCTGCTGAATTTGCGAAATTCCATCGTGAT
AGTCAAGGACCTAGCACACCTTCATCGTTGGGTGCATCTGGGAAAGCTACTGCTGCTTCTCCATCTGGTAATC
CACTATACTCAACTGAATTAATATTTGATCCATCCAGTGGTCAATATAATACTCAACTC
>1002052366_2 Heli.2-DT663023.3.5
ACGAGGGGAACAATCTCCAAGAGTTGGTTGTTTCAGTGTAAATTGTGCCGCAGTAGCAGTTCTTTTTGACAAA
AAACGTTACACCCAAACGTAAAATGCGCGCATTCGTGGTTCTCGCCTGTGTGGCCCTGGCCTACGGCCGCCCT
GAGCCTCCAGTAGGATACAGCTACTCTGCTCCTTCTAACAAATATATCCCATCTGAAA
GTTACTCATCCTCTTCTAGTAGTGGTCATTCTAGCGGCCTTTCTTTGGGTGGAATCTCTGGCGGCCACTCTTC
AGGTAGTGGTGGTATCAGTGGTGGATTCGGCGGAGGAATTGGCGGAGGAATTGGAGGAGGAATTGGTGGAGGA
TTTGGCGGCGGAGACGCCGGTTTCGGCTCCGGTGGTTACTCGGGGGCACCTATTGTGC
AGAAACACATCTACGTCCACGTGCCTCCCCCAGAACCCCAGGAACAGAGAATCCCCCGCATCACTCCTATCGC
TGCTCCCCAGAAACATTACAAAATCATCTTCATCAAGGCCCCAACCCCACCCGCACCAGTCGCTCCTATCATC
CCTGTCCAACCTCAAAATGAAGAAAAGACCCTCGTATATGTGTTGGTTAAGAAGCCCG
AAGAACAGCCTGATATTGTTATCCCCACTCCCGCACCCACCCAGCCTGCCAAGCCCGAAGTATACTTCATCAA
GTACCAAACCCAGAAGGAGTCTGGTGGTATCGGAGGCGGTGCTATTGGCGGTGGCTCTTTGGGCGGTGGCGCT
CTCGGTGGTGGTGCTCTCGGTG
>1002052367_2 Heli.2-DT662530.3.5
CACGAGGCGTAGGCAAGGTCTAGCCAACGTTCGCTGTATTTTAATAGTATGTCTCGGAAAAAAGGCTTTGAGG
AGACTGATAAATTAACTCGTATAGCGATTGTTAATGCTGATCGTTGTAAGCCTAAAAGATGTAGACAAGAGTG
TAAGAAGAGCTGCCCAGTGGTGCGCATGGGCAAGCTCTGCATAGAAGTTACACCCAAC
GATAAAATCGCAACCATATCAGAAGAGCTTTGCATTGGTTGTGGTATTTGTGTCAAGAAATGTCCTTTTGATG
CTATAACTATTATCAATATTCCATCAAACTTGGAGAAGCACACAACTCACAGATATTCTAAAAACTCTTTCAA
ACTCCATCGGCTGCCTATACCTAGACCTGGTGAAGTACTTGGTCTTGTAGGACAGAAT
GGTATTGGTAAATCTACTGCCCTTAAAATTCTTGCGGGCAAACAAAAGCCTAACTTAGGCAGATATGCTGATC
CACCAGACTGGCAAGAGATTCTCTCTCATTTCCGTGGCTCTGAGCTCCAAAATTATTTTACTAAGATCCTTGA
AGATGACCTAAAGGCACTTATTAAGCCTCAGTATGTGGATCAAATTCCGAAAGCTGTA
AAAGGAACTGTAGGGCAACTCCTTGATAAGAAAGATGAGAGGAAAAACCAAACTCAGATATGTTCTATGCTTG
ATCTCTCGCATATTCGTGATCGTGAAATATCCGCGTTGTCCGGTGGAGAACTCCAACGTTTCGCTTGTGCTAT
GGTTTGCATACAGAATGGTGATATATTTATGTTTGACGAGCCTTCGTCCTACTTGGAT
GTGAAGCAGCGCTTGAACGCTGCTAGGACCATACGATCTCTTATTGACCCCGATAAGTTCATTATAGTGGTAG
AGCATGACTTGTCAGTACTGGACTATTTGTCTGATTTTATTTGCTGT
>1002052368_2 Heli.2-DT665864.3.5
GTTGATCGTTCGCGGCGTGATAGTGTCGTGCTAGGACATCATGCGGACATTACTGATATGTATGCTGTGTGCA
GTAACAGCTTATGCTCAATATGATTCGCCTACGCCGCAAAGGCTGCAAATTCCTGGGGCGTTTGTAGCAGGAG
CGAGACAGGGTGGATTTAGACAAGGAAGATTACAAGCTGCTCAAATTCCTGTTGGAGT
AGCTAGAATAAGAAGGCCAGGACTCGCCAGACCTTCTTTCAAATCTGTAGATGCAGCCCCACGTCCCAGTCTT
CAATCTCTTGATGAACCATCAAAACCCGTCACAGAAGAACCGGAAGACGAAATTGAAATCAATACTCCCACTG
CATATGTGCCAAACGTTTTCTCTACCGCTGAGCCACAAAATGATTTCTTTGATATTAC

AACCACATCCCAACCAAAGCCACCCGCTGTTTACAATTCTTCACCTTTCCAACAAACCACTCCTTCTGCGCCA
AGACCCGAACCAGTGAGACCTACACAATACCGACCATTGGTACCTCAATCTTTTGTCCCTATAAGAAACGAGC
CTGCTAGACCTCAGCCAGCCAGATTACAACCTTTGTCTAGCAGACCACAGCGACCCTC
CTTCAGACCAGAGCCCAAACCTTTTATTGACGAAGAGGATGACTATATTCAACCTGTTAGACAGTTCTCCCGA
CCTCAAGAACCAATTCGTACACCAGCTAAGCCTCAACAAAAATATACTTCAAGTAACTCCAGAGATAAGAAGC
CAGTAGCTCAGATCATACGTAAATTCAGAGACGAAAACGAAGATGGAAGTATTACATG
>1002052369_2 Heli.2-DT667187.3.5
ACTCCTGATCTTTCGAGATGTTCAAGCTTCTTGTTCTGCTGTTGGTCGCGAGCGCTTCGGCGCAGATACCCTC
GCTGGGATGGTGCCCCGACTATCAGCCCATGGCAAACTTCAATATCAATCGCTTCTTGGGTTCCTGGTATGAA
GCTGAACGTTACTTCACCGTCTCTGAACTGGGAACTCGGTGTGTCCGCACTAAATATG
AGAGCACACCCGAAGGCAGAATTTTAGTCTCTAATGAAATAACCAACTCTTTAACTGGATTGAAACGTGTAAT
GGAAGGGTCTATGCAGTTGATTGGTAGAGAGGGAGAAGGTCGCATGATCATCAAATATTCGGCGATCCCAGCG
CCCTACGACAACGAATACAGCATTCTGGATACTGACTACGACAACTACGCTGTAATGT
GGTCTTGCAGTGGTATCGGACCTGTGCATATCCAAAATGCTTGGATCCTCACTCGTGAACGTTTGGCCACACC
CATTGTAATGCAGAAGGCCTACACTGTGCTAGAACGGTACAGGATTTCCAGAACATTCTTCGTAAAGACTAAC
CAAGCTGACTGCTACATCATACCTAGCCCTGTCGCAGATCCTTTGGACATTAAAAATG
ATGTCATTATTGATGCCAAAAACGTCCCAATAGAAGTATCTATCGAAGAAAAAGCTCCAGCACCTGAAAAGAA
AGAAGATCCCGCACCAAAATCTGAAAATATTCAAGAACGTTCAGGTGCTATGGATATTTCTGCTGAGGAAAAG
CCGATGGATATGCCGGAAAAAA
>1002052370_2 Heli.2-DT666274.3.5
ACGTGACTGTGTCCACTTTCCGCTTGTTTTCAATTCATCGATCGCGCTGGAACATCACTACTGCGCAGTGTAG
CGCAGTGTAAGGTCTCATTTAACGGCGTACCTGGGCGGCTGAACTAGGACAGAGAGTTCGCTACGACATGGGG
GCTAGGTCCCGGGAGTTGCTACTGCTCCTGGCAGTTTGTGCGCTTGCAATCTCAGAGT
CTGTTAACAAAACTGCGAAGAAAGATAACAAGGATTCTGACAATTTCCTGTTTCAATATGAAGACTATGACAC
AGATGAACAAGAAGTATTATTCAATGAGGACAGGCCTTGTCCTAGAGATTGCATATGCACTGTATCTCAAGGA
TACAGAACAGCGAAGTGCAACCGTCTAGAAATGGGAACTCAAAAGTTCGGCGACGATA
TCACGGATCTCGTAATTGACAACGCCGAGACAGAAATTCAACTGGATGATTTTATTTTCAAAAAATTGGGTCT
ACACCAGATTGCTACAATCAAAATAGTAAACAGCACTATAACGTCTGTAGGAGCAAACGCCTTCCATGGTCTC
CACGAACTCTACGCTGTTAACTTGTCTAACAACAAACTAAAGAGCTTACATCCTGAAA
CATTTGCAAATAACAAGAAACTGCTCCTCTTAACACTCTCAAATAATCCGCTCAAATTCCCTGCTCCGGGATC
AGATGAGTATTTCCTTAATGCTTCGTCAGTACAAGAGTTCGATGTTTCTTATTGTAACATGCAATACATTACG
GCTAATACTGTTAAAAATATGCCAGGCGTAATGTACCTAAACTTGGCTGGTAACTATT
TATCAGATATGGAAGCTGACACATTTAGAACCCTTC
>1002052371_2 Heli.2-DT664452.3.5
CACTGCAAAGCTTGAGCCATATTTGTCTGTGCTAAAATAGTTCCGTGGAGTGAGTGTGTGCAGATAGTCATCA
CATCACAGTTTTTGGGACCTGTAAATACTCCCACACTTCACATACAAAATGGCCGAAACAAATCCCATCTATG
GACCTTTCTTTGGAGTTATGGGGGCGGCGTCAGCTATCATTTTTAGCGCCCTTGGAGC
GGCTTATGGCACAGCCAAGTCCGGCACAGGTATCGCTGCCATGTCGGTGATGAGGCCTGAGCTGATCATGAAG
TCCATCATCCCCGTTGTCATGGCGGGTATCATTGCCATCTACGGGCTGGTCGTGGCTGTGCTGATTGCTGGGT
CGTTGGAGGCTCCGGCCACTTATCCTCTTTTTAGAGGTTTCATCCACTTGGGAGCGGG
TCTCGCTGTAGGTTTCTCTGGTTTAGCCGCAGGTTTCGCCATAGGCATCGTGGGTGACGCCGGCGTCCGCGGT
ACCGCCCAACAACCAAGGCTGTTCGTCGGAATGATCCTTATTCTCATTTTCGCTGAAGTATTGGGTCTATACG
GTCTCATCGTAGCCATCTATCTGTACACGAAACAACAGTAAATTTAATATTTGACATC
CATTCACCATCTTATATATACCCTCGAAGTTTTTATAAGTTCATATGTCGCGCAAATCAATCGCCGGTAGCTA
TTAAACTTCGAGGGCTAAGATAAAGTATCTAACATCAAGCATTGACTTTGTAGACTTGATGCTGCGTCTACAG
AGATCGTGCTTATACTGTGTATTTAAATGAAATTTAAATTTAAACCTCCTCCAGCTAC
CGAGTGATTGAAATTTCAGCTTTGATTTTTTGGAGTATTAATTATTATTACTTCTACATACTACTACATATTA
TGAGCGCGTTACCTATTGCGAAATATATCACTTAGATTATTTTAGTGTAAGGCGAAACTAATGTACAGTTAGA
GTAGAAAGTT
>1002052372_2 Heli.2-DT665190.3.5
GCATCAACATGCATTTCAAAATTGCAGTAGTCAGCGTCCTCGTGGCCGTATGTCACGCTGGGCTTATTGAAGA
AGGTCAAAATCACGCCGTTTCATCTCAAAGCATCGTCCGTCATGACCAACCGATCAACGGCGCTACACACTAT
GCACCTATCGTTGCTCATGCTGCTCCAGTCCTCGCCCATGGACCAATCCTTCAGCATG

CTGCCCCCCTGATTCATTCTGCCCCCATTGTCCAACATGTTGCCCCCATTGCCCACGGCGCTCCCATCGCCCT
CGCTCACGGTGAACAAATTGAAGACCATGCTCCAGCTCACTACGAGTTCTCATACTCAGTTGAAGATCCCCAC
ACTGGTGACCACAAGTCTCAACACGAGAGCCGTGAAGGTGACGTAGTCAAGGGAGAAT
ATTCTCTGGTTCAACCCGATGGCGCTGTAAGAACTGTCGAATACACCGCTGACGCTCACAATGGTTTCAACGC
AATTGTGCATAATTCTGCTCCGTCAGCCCACGCTGCTCCAGCTCCTGCAGTGCACTCAGCCCCAATCCAGGTG
CATGCTGCTCCTGTAGTGCACGCCGGTCCATTGGTCCATGCTCCTCTAGTACACGCTG
CTCCTTTGGTTCACGGACCTGTAATCCATGCAGCACCTATCCTAGCCCACCATTAGGCTGGACTTTTCAAACT
ACTGTAATTCCTATTAGAAATATATTGTTATTATTTATAATTAAAAAAAAAAAAAAAA
>1002052373_2 Heli.2-EL598133.1.5
ACATTTTGACATTTCTTCCCCGGTTAATATTTTATTACTAATCTTGTAAGAATCTAACTGCAATAATTTTTCG
TCAACATGGATGAAGAGTACGATGCGATCGTTTTAGGCACCGGTCTAAAAGAATGTATTCTAAGTGGAATGCT
GTCTGTGTCTGGAAAAAAGGTTCTGCACATTGATCGTAACAAGTATTACGGTGGTGAA
TCAGCATCAATTACCCCGTTAGAAGAATTGTTCGCAAAGTTCAATGCACCAGCGCCTGATGAAACTTACGGCC
GTGGCCGCGACTGGAATGTGGATTTGATTCCTAAATTTTTAATGGCGAATGGACTTCTTGTTAAGCTGTTAAT
CCATACGGGCGTCACCCGGTACCTTGAGTTTAAATCAGTTGAGGGTAGCTATGTGTAT
AAGGGTGGTAAGATTTCAAAAGTCCCAGTGGATCAAAAGGAAGCTTTAGCATCTGACCTGATGGGTATGTTTG
AAAAGAGGCGTTTCCGTAATTTCTTGATATACGTTCAAGATTTTCAAGAAGAAGATGCTAAGACTTGGAAAGA
TTTTGATCCTAGTCAGGCAAATATGCAATCACTTTATGATAAATTTGGCTTAGACAAG
AACACCCAAGACTTCACAGGTCATGCCTTGGCTCTTTACTTGGATGACAACTATCTCCAACAACCAGCTATTC
AGACCATTCGCCGTATTAAGCTGTATTCTGATTCTCTAGCTCGATATGGTAAGTCTCCCTATCTCTATCCTAT
GTATGGTCTAGGAGAATTGCCTCAAGGATTTGCTAGGCTCTCTGCTATATATGGAGGA
ACTTATATGTTAGACAAGCCAATT
>1002052374_2 Heli.2-DT667532.3.5
GCACGAGGGTCAAACTGTCTATACAAGTTATATATCTCTTAACCAAACTTTTAAATTAATTTTTATCGTAAAC
TTTCCAATGGCTTTTACGGGTTCAGCAGACAAGCCCAGCATGCAGCTGGCAGCTGCTGATGCCGGATCTCAAC
TTCAGCACATGTGTTCCTTAGATATTGATTCTAAGGCTTCATATGTGCGTCTATCTGG
AATTATCTGTACCATTGGACCCGCATCCCGCGAGGTGCCAATGCTCGAGAAAATGATGGAAACTGGCATGAAT
GTGGCACGTATGAACTTTTCTCATGGCTCCCATGAATACCACGCGGAGACCATCCGTAACTGTCGTGAAGCTG
AAAAGAAATATAGTGCACGTCTTGGAGTGCCATTTTCCTTGGCCATCGCTTTGGACAC
TAAGGGTCCTGAGATTAGAACTGGGCTTTTGCAAGGTGGTGGTTCAGCCGAAGTGGAACTAAAGAAAGGGGCA
ACCATCAAGTTAACCACCGACCCCGCGTACCAAGAAAGCGGCAACGCCGAGATAGTATTCGTGGACTACAAAA
ACATAACGAACGTCGTGAAGCCCGGAAACCGTATTTTTATTGATGACGGACTCATTTC
CGTCATCTGCGAATCTGCGACGGCTGACGCCTTGATCTGTAACATTGAAAATGGAGGTATGTTGGGATCGAGG
AAGGGCGTGAACCTCCCCGGTCTGCCCGTAGATCTGCCCGCTGTCTCCGAAAAAGACAAGTCGGATTTACTGT
TTGGTGTAGAACAAGGAGTGGACATGATCTTCGCATCATTCATCCGTAACGGCGCGGC
GCTGGTGGAGATTCGCGGCATCCTCGGCGAGAAGGGCAAGAACATCAAGATCATATCTAA
>1002052375_2 Heli.2-DT665656.3.5
TCAGTAGCTCACATCCTGCCACATTTCAGTTTAAATTTAATTTGTAACTGTTTCAACGGTGTGCCTGCGAAAG
TTAAAAAGTAAGCGCTGCTCCGAAACACGATATATTTCGCGAAGCATTTGTATTCGGCGGACGCATTGCATCC
AGCATCCCTGCATTTTGTTTTTCTTTGTTTGGTCCAGTTATTTTCTACATAGCAATGG
AAGTTGAAACTAAATCTACTGAAATTCGTTGCCAAGAGATGTCGAAAGGTGGGCTAGCGTACGAAGTTATCCT
AGCTGAGCCCGTGGGGGTGCCCGTGCCGCGTCGCGCTGATTCCCCCGAGAAAACTCCCTCAGTGGAAGAAATC
CAGGAGAAGCTCAAGGCTGCCGAGGAAAGGAGACGTAGCTTGGAAGCCAGCAAGATGG
CGGCGATCGCACAAAAGATGCATAAAATCGAGGAAGCCTCACGTATTCGCAGCGAACAGACGAATAACTTTAT
TGTGGCCACAAAAGAAGCTCTGGATGCCAAGATGGACTCTCATGAAGAGAAACGCGAGGCGTATATAAATGAA
CTTCGTGCTCGTCTTAAGGATCACCTTGAAGGTGTCGAGAAGACCAGGTTGACCTTGG
AGCAGCAGACTGCCGAAGTATACAAGGCGATCGAAGAAAAGATGAACACCGCCGCCGACAAGCGCGACGAAAA
TATTAAAAAAATGCTTGAACGTCTTCGTGAACATGAGGAGCAAGTGCGCAAGGTGCGCGCCGGCAACCAGGAG
CGCTTCCAGCAGCTGGAGAGCGCCATCCAGGACAAGCTGCAGCACGCCGCCCACCGCC
GCCTGCAGCTCGAGGCCGAGCAGCGCGAGAAGCTGCGCAACCACAATAATAAGCTCGCGGAGGTCCGATCAGT
CATAACAGCCAAGATGGAGGAGATCACCAAGGACATTGAGACCAAACTGACCACCGCCGAACAAAACCGGGAA
AAGGAGATACAGAAGAAACTCGACTTTGTTAAAAAAGAGGAGCGGCGCGCGGAACTAG

TGCGACAGAACAAGAGCGCGCGCGCCGAGAGCGACCCCGTGTCCGGGTAGAGCGGCGAGCTCACTCATACAGC
TCACTCCTTCACTCATACGCGTCTTACCTTTGACTGTTAACATGTATTCTTTTAACTGTTTAGTTCATTCATT
TGTACGCATCTTGCCTTTTAACTGTTAACATGTACTCATTCATTCGTTCGATTCTTAA
TTTGTAGTTGATGTACTTATAGTTTTATTTTAAGGGGGGGTTTTCGCTTTTTAACTTTGTTGGTAGTTATAGT
GTTGTTTAAGGAGAAGTTTGTTTTTTTGTTTAGTGTTCCGTTTAATCGATATGAGGGGTAGATCTATTTTGAT
TTTACATTTTCACAACTATTTCTCGATATTAAATAGGTAATTCGATTTATGTGTATCT
CGCTATTGTTAC
>1002052376_2 Heli.2-DT667568.2.5
TTTTTGAATCTGACCGTCTAATCGATCTCACGGTTCAACAATGGCGGACGCAGCTCCAGCCGGTGGACGTGGG
GGTTTCCGCGGTGGCTTTGGATCACGTGGTGGCGATAGAGGCCGTGGAGGACCTCGTGGCCGTGGGCGCGGTC
GTGGCCGTGGACGCGGACGCGGAAAGGAGGACCAAAAAGAATGGGTTCCCGTAACCAA
ATTGGGCCGTCTCGTAAGAGAAGGCAAGATAGACAAACTTGAGAGCATTTACTTGTTCTCTTTGCCCATCAAA
GAGTTCGAAATCATCGATTTCTTCCTGGGCGCTTCATTGAACGATGAAGTACTCAAGATTATGCCTGTACAGA
AACAGACCCGTGCCGGTCAACGTACCCGTTTCAAGGCCTTCGTCGCTATTGGAGACAA
CAACGGTCATATTGGTCTCGGTGTTAAATGCAGCAAGGAAGTAGCAACCGCTATCCGTGGTGCTATCATCTTG
GCAAAATTATCTGTACTTCCTGTACGCAGAGGTTACTGGGGTAACAAGATTGGCAAGCCTCATACTGTACCAT
GCAAGGTCACTGGCAAGTGTGGTTCAGTAACTGTGCGCTTGATCCCAGCTCCCAGAGG
TACCGGAATTGTGTCAGCTCCTGTCCCTAAGAAGCTTCTTCAGATGGCTGGAGTCCAGGACTGCTACACTTCT
GCCCGGGGCTCCACTGGAACCCTTGGAAACTTTGCTAAGGCCACATATGCTGCCATTGCTAAGACCTATGCCT
ATCTTACACCAGACTTGTGGAGGGATATTCCATTGACTAAGTCACCATACTCTGAATT
CAAAGTCTAAAT
>1002052377_2 Heli.2-DT662105.3.5
ACGAGGCGGAGAAGGTAGTGGTGACGTTCAAAATGAAATCCTTATTGTTAGCGTTTGCTAGCTTTTTAGTATT
ATGTTCTGTTAATTGTGATGTATATTTAGATGAGAAATTCCCTGATGATTCATGGGAATCCAAATGGGTGTAC
AGTGAACATCCTGGCAAAGAATTCGGCAAATTTAAGCTGACCGCTGGAAAGTTCTATA
ACGACCCAGAGGAAGATAAAGGTTTACAAACATCGGAAGATGCTAGATTCTACGCATTGTCCCGCAAATTCGA
ACCTTTCTCAAATGAAGGCAAGAACCTCGTGGTTCAGTTCTCAGTCAAGCACGAACAAGATATTGACTGTGGA
GGCGGTTACTTGAAAGTATTCAATTGTGACTTAAACCAGCCTGATATGCACGGAGAGT
CCCCATACGAAATTATGTTTGGACCTGATATTTGCGGTCCCGGCACCAAAAAGGTGCACGTCATCTTCAGCTA
CAAGGGCAAAAATCATTTGATCAAGAAAGAAATTCGTTGCAAGGATGACGTTTACACTCACGTCTACACGCTG
GTCGTCAAACCTGACAACAAATACGAAGTTCTCATTGATAACGAAGTTGTTGAGTCTG
GAGAGCTTGAAGCCGACTGGGACTTCCTTGCACCTAAGAAAATCAAGGACCCCGAAGCCAAGAAACCAGAAGA
CTGGGATGATCGTGCCCAGATTCCTGACCCTGATGACAGTAAACCTGAGGACTGGGACAAGCCTGAGCACATC
CCTGACCCTGATGCTGCCAAGCCTGATGACTGGG
>1002052378_2 Heli.2-DT668132.3.5
GTTTGCAAAAATTTTTGGTTTAAGCGCTGTGTTGGCGGTTGCTGCGGCTGGTCTGCTGCCAGAACCCCATTAC
TCCTCAGCAGCGGCGGTTTCTTCTCAAAGCATTGTACGTCATGACCAGCCTCATGTCGTAGCGGCTCCAATTG
CATACCACGCTGCCCCCGTGGCATACCACGCAGCTCCTGTAGCTTACTCTTCAGCTGC
AGCTGTTTCGTCTCAATCCATCCAACGTCATGACGTAAACCATGCTGCCGTAGCTGTTGCTCCTGTTGCCCAC
TACTCCGTTGCTCCTGTAGCCCATTATGCCGCTCCCGCACGTTATGCATCTGCAGTGTCATCGCAATCTATCC
AGCGTCATGACCAACCTCGTGTTTCTATTTCCGTTGCTCCCGTTGCCCACTACGCTGC
TGCACCCATAGCGCATTACGCCGCCCCTGTGCATTACTCTTCAGCTGCAGCTGTTTCGTCTCAATCCATCCAA
CGTCATGACGTAAACCATGCTGCCGTAGCTGTTGCTCCCGTTGCCCAGTACTCCGTTGCTCCTGTAGCCCATT
ATGCTGCTCCCGCACGTTATGCATCTGCAGTGTCGTCTCAATCTATACAGCGTCATGA
CCAACCTCGTGTTTCTATTTCCGTTGCTCCCGTTGCCCACTACGCTGCCGCGCCCGTAGCCCATTACACCGCC
CCTGTGCATTATTCATCAGCCGCAGCCGTATCTTCCCAATCCATCCAACGTCATGACCAGTCCGGTGCCGCCA
TTGCTGTTGCTCCTGTAGCTCACTACGCTTCTGCTCCCGTTGCACACTACGCCGCCCC
TGTAGCCCATTA
>1002052379_2 Heli.2-DT662461.3.5
CTACGAATTTTCTATTTTCTGACAAATAATTCTCTCCAAAAATTATCTTATTGTGAGTGTCTTTTTCGTCAAT
TTTTAAAGCCAAAATGGCTAACATTAATGAAGAGAACGTTAGCAAACTAATCCCCACACTTGGTGATGAAAAA
ATCGATATGATCGCCGCCACAAGTGTTTTACAAATCAGAGCAAGTGAGATTCGTCAAA

GCCAGATTAACTGGCAATCTTATTTGCAATCGCAAATGATAACTCAACGTGATCACGACTTCATTGTGAACTT
AGACCAACGAGGACAGAAAGATTTACCTGATAAAAACCCTGACGGCTGTGCTGATGTATTCCTCAACTTGATT
ACTCATATTAGCAAAGATAACACCATCCAATATGTTCTTGTGTTGATTGACGACATTC
TTTCGGAAGACAAATCCCGTGTGAAAATATTCCGTAATGCCAAACATGGTAATGTTTGGCAGCCATTCCTGAA
CTTACTTAACCGTCAGGATGAGTTTGTTCAGCATATGACTGCTCGTATCATTGCTAAGCTGGCATGCTGGCAC
CCACAACTTATGGAAAAAAGTGATTTGCACTTCTACCTTTCCTGGTTAAAGGACCAGC
TCAAGATGAATACCAACGACTACATCCAGTCTGTAGCGCGGTGCCTGCAAATGATGCTGCGTGTGGACGAATA
CCGCTTCGCCTTCCTCTCGGTCGATGGCATTTCTACATTGCTGTCTATTCTCGCTTCGAGGGTTAACTTCCAG
GTCCAATACCAACTAGTGTTCTGCCTGTGGGTGCTGACATTCAATTCGCTGCTTGCCG
AGAAGATGAACAAGTTTAACGCCATTCCCATTTTAGCTGATATTCTGAGTGACTCTGTCAAAGAGAAGGTCAC
TCGCATCGTCCTGGCCGTGTTCCGCAACTTGATCG
>1002052380_2 Heli.2-DT664850.2.5
CATGGCGGTTGGTAAAAATAAAGGCCTTTCGAAGGGCGGCAAGAAGGGAGTCAAGAAGAAGATTGTAGATCCC
TTCACAAGGAAGGACTGGTACGATGTTAAGGCTCCGTCTATGTTCACTAAGAGGCAGGTGGGAACCACCCTTG
TCAACCGTACTCAGGGAACCAAAATTGCCTCAGAAGGTCTGAAAGGCCGTGTGTTTGA
GGTCTCTTTGGCTGATCTCCAAGCTGATACTGATGCTGAGAGGTCTTTCCGCAAGTTCCGTTTGATCGCTGAA
GATGTTCAGGGCCGCAATGTGCTCTGCAACTTCCATGGAATGGACCTCACAACTGATAAACTCAGATGGATGG
TCAAGAAATGGCAGACGCTGGTCGAAGCCAACATTGATGTCAAGACCACAGACGGTTA
CCTCCTGAGAGTTTTCTGCATCGGTTTCACCAACAAAGACTCATTGAGTCAACGCAAGACATGCTACGCTCAA
CACACTCAGGTGCGCGCAATCAGAAAGAAGATGTGTGAGATAATCACCCGTGACGTGGCCAACTCTGAACTGA
GAGAGGTTGTCAACAAGCTGATACCAGACTCAATTGCCAAGGATATCGAGAAGGCCTG
CCACAGCATCTACCCATTGAGAGATGTGTGCATTAGAAAGGTTAAGGTACTGAAAAGACCCAGGTTCGAGATA
TCCAAGTTGATGGAGCTTCATGGTGAAGGCGGCGGCGGCAAGCGTGGTGAGGTGGGAGACAAGTCTGAGCGTC
CCGAGGGTTACGAGCCGCCCGTGCAGGAGAGCGTTTGAATATATTA
>1002052381_2 Heli.2-DT663883.3.5
ATCTTAGTTTTATCGTGATTTCGCTTTGAATAGTCTTAATTTTAATTTATATCACTCCACACCGTAACTAAAC
ACCATAAACAATGGGCGATATTGAGGACACACATTTCGAGACCGGCGACTCCGGAGCCTCTGCCACCTTCCCT
ATGCAATGTTCTGCTCTCCGCAAGAACGGTTTTGTCATGCTTAAGGGACGTCCATGCA
AAATTGTTGAAATGTCCACCTCAAAGACTGGCAAGCACGGACATGCTAAGGTCCACTTGGTTGGCATTGATAT
ATTTAATGGAAAGAAATATGAGGATATCTGTCCCTCAACCCACAATATGGACGTCCCCCACGTCAAACGTGAG
GACTACCAGCTGACCGACATATCGGATGACGGCTACCTCACCCTGATGGCTGACAACG
GTGACCTGCGCGAGGACCTCAAGATACCCGACGGCGATCTCGGAACTCAGCTCCGCTCTGATTTCGACAGTGG
CAAGGAATTGTTGTGTACCGTACTGAAGTCGTGCGGGGAGGAGTGTGTTATAGCGGTGAAGGCAAACACAGCT
CTCGACAAATAAACCAACTAAGAATTTATAGGGATATAAAAGCATATAATTTTTTTAC
AATCAACAACTTACATAAATGTAAAACATAATATTATGTATAATTTAAAGTAAACACGTGCCGGGTTGTTTGT
TTTGTCGCTTAAGTCTTAAAAGTTACTTTGCTAGCCTGAAGTTGTGCGATACCGGTGTGGAAACCCCGTAA
>1002052382_2 Heli.2-DT665707.3.5
GCACAGGTGGTGCGAGGCTTCCTTGCAAACATAGCGTCATAAATTACGACTAATTTGTAATTTTTCTAAACTT
TTTGATCGGTTAACGATCTATAAAATAGTGCTTTAAGTGTTTAAGAAGTTTCACAACAAATAGCAATGAGTTC
CAGCGCGTGCTACAAGTGCAACAGGACAGGGCACTTCGCCCGCGAGTGCACGCAGGGC
GGCGTCGGCGCCCGCGACTCCGGGTTCAACCGGCAGCGCGAGAAGTGCTTCAAGTGCAACCGCACCGGACACT
TCGCGCGCGACTGCAAGGAGGAGGCCGACCGTTGCTACAGGTGTAACGGCACGGGGCACATAGCGCGCGAGTG
CGCGCAGAGCCCCGACGAGCCGTCGTGCTACACGTGCAACAAGACGGGGCACATCGCG
CGCAACTGCCCCGAGGGCGGGCGCGACTCGTCCAACCAGACGTGCTACAACTGCAACAAGGCCGGCCACATCT
CGCGCAACTGCCCCGACGGCACCAAGACGTGCTACGTGTGCGGCAAGCCGGGGCACATCTCGCGCGACTGCGA
CGAGTCGGAGCGCAACTAGCCCCCGCTCCACCACGCATGCACCACGCAAACACCACGC
GAGGACTGTACGTAAGCAAAGGAGGGTGGACGTGCGAGCGCGAGCCGTAGGTGAATGTTTGTTCTTGCACGTG
AACCAGTTGTGTTTTTAAATCATTTAATAATCGTGATTGAACTTTTGATGAGGTAGCGTTACAGTTTTTAGCA
TTACAGAGTATATATTTAATATATTTTGTACAGATTTATTTGTCGAAAGATAAATTGG
ATGCTGGCGTATGATGCGTTTTCATGAGAGGAGTGGGAGTACCCTGTCGGACTCTCTTGTTCGTCTTGTTATT
GTCAGTTACGGTAGACGTCGCTCTCGCCGGTCGAACGCGCCGGCGCCGTTGTCACCGTTTGAGTTTGTTTATC
AATCCTTTGTTTATGAGGTCCTGCTGACTTGTGGAGAATTCAGTGG
>1002052383_2 Heli.2-DT665670.3.5

TGGACGGCGTAACCGTAATTATTAAACATGTTGGGGAGATTTTTTCTACTGTGCGGGCTACTGGCCGTCGCAT
CAGCTTCGGTGAAGCTGCAAGAGGTCTACTCTTGGAACATTTTGGACTGGAACTATCCTGACCAGTATCTGAA
ACAGCAAGCCCTTCAAACTGGTGCACTCATCAGACAAAACGCTCTTCCCGTGGGTATC
GAACGCTGGAGGGATAAACTCTTCGTCAGTGTACCTCGATGGCAATCAGGTATCCCAGCAACGCTTAACTACA
TTCCTTTGGATGCACCTTACGAGCCTTCACCAAAATTGACACCCTACCCAAGCTTCAAAGGCAACGAACTAGG
CGATTGTGAAAACGGTCTTACCACAGTTTACAGAATTAAAGCAGATCAATGTGATCGA
CTCTGGGTTCTGGATGTTGGAACTTATGGATATGACCCTAATGTAACAAACGTATGCCCGTATTCTCTAAACG
TATACGATTTGAACACTGATCAACGTATTAGACGGTACGTTTTCCGGCCGGAGGACATTGTCTCTACAACATT
CATTGCAAATATTGCTCTTGACGAAGGCATTTCGTGTGAAGATACTTTCGCATATTTC
TCGGACGAACTCGGATATGGACTCATAGCGTATTCTTGGGAACAAAACAAATCATGGAGATTCAGTCACAGCT
ACTTCATGCCTGATCCTTTAGTTGGTGACTTCAACATCGCTGGCCTTAACTTCCAGTGGGGAGCTGAAGGTAT
ATTTGGAATATCAGCATCACCAATGGGAGCAGATGGTTATAGAACACTCTATTTCAGT
CCTCTTTCAAGTAACACGGAATTCGCCGTATCAACTCGTATTTTAAGAGATGAATCTAAGGTCACTGGATCGT
ACCGTGATTTT
>1002052384_2 Heli.2-DT662693.3.5
GGAAGTCTCAGAAGAAAGATCAACATGTTTGCTAAAGTTTTAATTCTTACCATCGCCGCTACTGCGTGCTACA
CCCAGGAACATATTTACAAATACAAGCCTGTCCATCGGGAACAACTAAAGGAGTATAGATATCCATCGGTACA
TAAAGCTTTCAGCCTCATAAACCAGGAACCAGCACCTATCCATGAAGAAGAAGCACAT
GGCGCTCCGGAGGCACAATTGCACGGACACCATGAATCTGCTGTTTCATCTCAAAGCATAGTTCACTATCAAC
CATCAGTTCACGAGGAATCGCAGTCGCAAGAATCTTTTGTAGGGCACAATGAACCATCAGTAGCTTACAAGCA
ATACTATCAAGGCCATGAACAAGTAAAACTTCAACAAGGACACCTTGCCAAGAACAAC
ATTCAAGCTTCGCATTATCCTGTGGTTTCTCACGAAGCACCTCAAGCCCAATCCCATGATGAAGCACAATACA
TCCGCGTCCCCGCCCACAACTACCACTACGCCCAAGAATCTGAACATCACGTACCTGCACATCATGAGGTTGA
GCAACACCGTCAAGTAGAATCCCATTCCCACGATGAGCCTATCGACTATTATGCTTAT
CCCAAATACCAGTATGAATACAAAGTTGAAGATCCTCATACCGGAGACAATAAGTTCCAGCACGAGGTCCGCG
ATGGTGACAGCGTAAAAGGAGTTTACTCTCTTCATGAAGCTGATGGCTCCATAAGAACCGTTGAATACAGCTC
AGACAAACATCACGGATTCAACGCTGTCGTAAAGCATTCAGCCCCTGGTCAACACGTC
CATATTGAGAGTCATCATGAAAAT
>1002052385_2 Heli.2-DT666031.3.5
GCACTCGGACACTGTAACTGATATTGAAAAACTGTGTGGTGCAAAAAAAATTGTGCAAAAAAAAAAATGGAAA
TTAGGTGGAGTTTATTCATAGCGCTGCTTCTCCTCGGAGTCGCTGTCGCGTCTGAAGAGGAAGACGAAGTGCC
TGATGCGGGAGTGGAGGGAGACGAAGAACTGGCTTTAGATCAAGAGGAAACACGCAAC
CTCAGACCACGAGCATATAACCAAGCTGGTTACTCGAACACACTACCTAGCGGTTTCCGACCCACACCTTCGT
TAGCTGAACTGGCAGGTTACCAGAGGCCACAGCAAGAACAGCAGGAATATGAGGTACCAGCCAGACAGCAGAT
CTACGTAGAGGAGCAGAGACCACCAGTTAGAGCACCTCATAGGAAGCCTCAAGAGAAC
CGCCCATCTAAAATCCAGCAGCAACTTTTGGAAGAAGAAGAAGAAGAGAAGGAAGAGCCCGACCGTCTTTCCC
AACTATTGCAGCAATCCAAATTCGATTGTGTGAGCAAGAACACTGGGTACTACGCTGACCAGGAGCTGAACTG
CGAGGTGTTCCATTACTGTCAAGACAATGTTAAGCATTCGTGGATCTGCCCTGACGGA
TTCACTTTCCATCAGGTCCATCTAATTTGCATGCCGCCTACACACGACAACATCTGCCAGAAGTCTCAAAAAT
ATCACTTCGTCAATGATTACCTCTACCGCCCCATCAACGAAGAGGAGGTCCAAAGGAAACCTAACGTGTCCCT
CAAGTACTCCGACAGATTCTACCCCGCTGAAGTGTACAGGGATGAC
>1002052386_2 Heli.2-EL600879.1.5
TTTTTACAACTACAATTCTTGAGTTGATGGTGTATTGCGGATCTGTGGGTGGTGTTTTAATATTAGTCATCGC
CTGCATTCTTATTTATTCATTTTATTGTGATAACAATTTTCTTAATTTGTTCTCTCTGCTTACTACAGTTCAA
CCTAATATATAACATAAAGATGTCGTCTCGCAAAACAGCTGGTCGTCGTGGTACCAAC
AAGAAACGCGCCCAGCGGGCTACTTCAAATGTATTTGCCATGTTTGACCAGGCGCAAATTGCGGAATTTAAAG
AGGCTTTCAATATGATTGATCAGAATAGAGATGGTTTTGTAGATAAAGATGATCTACATGATATGCTTGCTTC
TCTAGGAAAGAATCCAACTGAGGATTATCTAGAGGGTATGATGAATGAGGCACCAGGA
CCTATCAATTTCACTATGTTTTTGACACTCTTTGGGGAACGTCTTCAGGGAACTGATCCTGAAGATGTTATTA
AGAATGCTTTTGGATGCTTTGACGAAGAAAACACAGGTGTGATAGCCGAAGAGCGTCTTCGGGAACTCCTTAC
CACGATGGGTGACCGTTTTACCGACGACGATGTCGACGAAATGCTTCGCGAGGCTCCC
ATACGCGACGGGATCTTTGATTATGTGGAATTCACTCGTATATTGAAACATGGCGCCAAGGATAAAGATGAAC
AGTAAAACTTTTTCAACGGCAGGTTGATGATATAACATAATAAACTTTATTTTGGGGGATGTTATGGAGCATG
TTACCAAACT

>1002052387_2 Heli.2-DT668657.3.5
GCACGAGGGTCAGAACGCGCCGTGATTTACCGCTGTGAACAAACGGACATTCTCCTCCTGATATATTCAGTGA
TAGTTTCCTTTCACATTGTATTAAACACAATTTGATCCAATGGAAGCAAAGGAGTTCAAGGATTTCGCCAAGG
CGATGGCTGATTACATCGCGGAGTATTTAGAAAATATACGCGACAGACAAGTAGTACC
ATCTGTTAAGCCAGGTTATCTACGGCCATTGATTCCCGACCAAGCTCCAGACAAGCCCGAACCCTGGACCGCA
GTGATGGATGACATCGAACGCGTAGTCATGTCCGGCGTTACACACTGGCACTCGCCGCGTTTCCATGCGTACT
TTCCTACAGCTAACTCCTATCCTTCTATTGTAGCTGATATGCTGAGCGGTGCTATAGC
ATGCATTGGATTCACTTGGATCGCGAGTCCAGCGTGTACTGAGTTAGAGGTCGTGATGATGGACTGGCTTGGT
CAAATGCTTGACTTGCCTAAGGAGTTCTTGGCTCGTTCAGGCGGAGAGGGTGGCGGCGTAATCCAGGGTACAG
CTAGTGAAGCCACCCTCGTCGCTCTATTGGGTGCTAAGGCCCGCACTATGCAGAGGGT
CAAGGAACAGCACCCTGAGTGGACTGATGTTCAGATACTATCTAAACTTGTTGGATATTGTAACAAACAAGCT
CATTCGTCTGTCGAGCGCGCTGGTCTCTTGGGCGGCGTCAAACTCCGCAGCTTAAAACACGATAACAAGAGAA
GTCTTCGAGGTGAAACTTTACAGGAAGCTATTGATGAAGACATCCGTAATGGACTCAT
TCCGTTCTATGTGGTAGCCACATTAGGAACTACATCGTCTTGCGCTTTCGACGCCCTCGACGAGCTCGGGGAC
GTGTGCCAGGCGCACGACGTGTGGCTGCACGTGGACGCCGCATACGCCGGCTCCGCCTTCATCTGCCCCGAGT
ACCGCTACCT
>1002052388_2 Heli.2-EL599528.1.5
GTGTGACGCGACACATTAGCGCGGTAGACGCCTGTAAAAATAAACATTATTGATTTTCCATCACTTAAAAAAT
CAATGAAAATGTACTCCATTGCCGAGGCCACGTTAGCTTTCGCTGTTCTTTATCTATTCTCTTTCCTTTTCGA
AGTGGTAACAGGATTAGTGTCGTAGACGATTAAGTGTTTATTAAAAAAATGTGATATT
TTATTTATTTTAAGCTAGATTTAAGATTATTAGTCAAGTTATCAATGCAATTGTATATAAGTTGTAGTTTTAA
TTAAATTATTTAGGCGCGAAAAAATCCATTCCAATACAAGGACGCTACATTAAAGGTTTTGGTTTTCACACCA
GGTATTCACAAAAAGCCTTACAATAAAAGTTCGAAAATAAAACAGATTCAGTTAGAGC
AAGACAGTGGGAAGTCACTTCATGACATTGAATTAAAAAGGAGTCTAGTGGATTTAAATCGAGCAGGTGCACC
TTTAATTGAACTTGTATTTGAACCTGATCTTGAGGATGGTGAGGAAGCTGCTGCATTGGTAAAGGAACTAGTA
CTAATAGTTCAAAGACTAGGTGCATGTACAGGCCGTATGGAAGAAGGAGCTTTACGAG
TAGATGCTAATGTTTCTATAAGAAAAGTTGGTGAACCACTTTCTACACGGACTGAGATTAAAAATATTGGAT
>1002052389_2 Heli.2-DT664482.3.5
GCACGAGGGTGCAGTGAACATTGTTTTGCGTTGTGCGTAGCACGTGCCGGATACTTTTTCTATTTTTTAAATA
ATCTCAAACTTACTCAAACAATGGCAGACGCAGCAGTCGAAAAGAAAGACGCCGTAGCCGAGGAGGTCAGCTC
AACCGAGGCGGCGAAGGAATCTCCAGTAAAGAAATCTCCCGCCAAAAAGGTAGCGGAA
CCCGAAAGTAACGGCAAAGAGGAAAATGGCAGCGGCGACGCACCTGAAGAAACTCCCGCCGAAAACGGTGACG
AAGAGAGCAATGACGCCGTCGAAGCCGTCGAAAATGGAGAAGCGACAGAAGATAAAGAATCTGGTGGAGTCAA
GAGGAAATCTGTAGCAACAGACAACGGTGATGACGCAGCCGAAAAGACCACACCTGAA
AAGAAGGCGAAAGTAGCGGACGAGGCTCCCGCGGCCGAAGAAGAGGCGGCCTAACCTAATAGCAAGCATGCTT
GGTTTACTGCAATGCATTCGTTCATTAGGGCTCAACTCCACCAAGACATATCCTCTATACAGACGCCAAAACT
ATAGTTTTTATACATTGTTACGTATTTCTCTTGTACACGTACGAGTTGGGCCCTTTTG
TGACCACTTATTTTAGGTTGTAATGTAACTTACCCGTCGAGTACCTGTAAAGTGTAAATTATACAGAAATAAA
TGCGTCGCAGTGAATCGATCGAGTCGGACACGTTCAGACATCGCTACGCGCAGACTAAGGGTTTTTGAATTGA
TACAATAACATTTATTCCGCCATTAGGTACTGCCAAAGTGATTCACATGTGTGAAGTC
AATTCACGATCCGTTCTGTGAACGATGCGAAAACAG
>1002052390_2 Heli.2-DT665400.3.5
GCACGAGGGATTTCTTAGTTTGTCTACAATGGAGAAGCCAATATCTGTAGTGTTATTGCTTATTTTTATACTT
TTTAATTATACCACAGCCAGGGTGCCCGTCGATTATTATAAAAAAAACATTAATAAAATAGTAGGAGGATATA
ACATTACAATTCAAGATGCGCCGTATCAAGTATATTTGTTGCTGCAGATGGGAAATGA
TGACTATCAATGCGGAGGGTCGATTATAAGTGAACGATATGTTTTAACTGCTGCTCACTGCTTAGCTGGAATA
CAAAGGGTATTTGTAAGGGCCGGCAGTACATATGCCGATTATGGAGGCATTCAATACAATTCCACAAGATTCA
GGTCACATCCATTGTACAATCCTGCAACTTTTGACTATGATGTAGGTGTAATAAACAT
TCCTGGAGGAATAGCTCTGGATGGATATAGCACGAGAGCTATATCACTGCCAGCAAGGGGCACTGTTATACCG
AATGATACCAATATTTTCATTTCTGGTTGGGGTGATACCACTGAAGGTGGTCAGGTGAGTGAAAATCTGATGG
CTGTACAAATTCCAACGGTGCCACAAGAGGAATGCCGCGAAACTTACACCACATTAAC
ATCTAGGCAATTTTGTGCTGGCTTAGAAGAAGGTGGAAAGGATTCTTGTCAGGGTGATTCCGGAGGCCCTGCA
GTGTCAACAGATACAGGTCTTCAAATAGGTATCGTTTCATTTGGCTATGGTTGCGCTAGACCTGATACTCCTG
GTGTGTACACAAATGTCGCAAG

>1002052391_2 Heli.2-DT662661.3.5
AGCTAAGTTCTTCGTCGTCCTCTCCCTGGCCGTGGCCGCATCAGCTATCCCCTTGGTCCCCGTGGCTAAGGTA
GCATACGCTGAGCCCGAAGCTCCCGCTCACTACGAGTTCCAATACTCCGTTCACGATGAACACACTGGTGACA
TCAAGCAGCAGCAAGAGGCCCGTGCTGGAGATGATGTCCACGGCTCATACTCTCTCGT
ACAACCTGATGGCGTACACCGTATCGTGGAATACTCTTCTGACAAAGTGAACGGTTTTAACGCTATTGTACGT
TACGAGGGTAAACCCATCCCAGCTCCCGTACCAGTCGCTAAGGTTGCATACGCTCCCGTCGCCAAGGTCGCCT
ACGCCGCGCCCGTCGCCAAGGTTGCCTACTCTTCCCCCGTCGCCTACGCTTCGCCAGT
TGCCAAGATTGCCTACTCTCCCGTATCTTATGCCGCCCCTGTCGCCAAAGTAGCCTACGCTGCTCCCGTTGCC
AAGCTCGCCTATGCTTCTCCCGTTGCCAAGGTAGCATATGCCTCTCCTCTCGCCCAAGTAAGCTACTCATCTC
CCATCGTCTCCTACCACCACTAAACGTAATTGGTATTTTTAAAGTTGATTAATTTATT
TATATGATAAATAGTTACAAATAAATTGTTTGTTTAAGATTTAAAAAA
>1002052392_2 Heli.2-DT665612.3.5
AGTGTCGATAGGTCAATATTCTGCAGAAATTAGCCAGAATCTTCAGGTATCTACAAAATATACTAAATAACCA
CCCTCGCATGTGAGGGTACTATGATATTCTGCAGAAATTAGCCAGAATCTTCAGGACCGATGGCTGCAATACG
CAAAAAATTAGTGATAGTTGGTGACGGTGCATGTGGTAAAACATGCCTGTTGATCGTG
TTTAGTAAAGATCAGTTCCCGGAAGTGTATGTGCCGACAGTATTTGAAAACTACGTCGCCGACATCGAGGTTG
ACGGTAAACAGGTGGAGCTAGCCTTATGGGATACGGCCGGCCAAGAGGATTATGACAGGCTTCGGCCACTCTC
GTATCCAGACACGGACGTGATCCTCATGTGCTTCTCGGTGGACTCCCCCGACTCGCTC
GAGAACATCCCGGAGAAGTGGACACCGGAGGTGAAACACTTCTGCCCCAACGTGCCCATCATTCTCGTGGGTA
ACAAGAAGGATCTACGCAATGACCCAGCCACAATCAATGAGCTCCGCAAGATGAAACAAGAACCTGTCAAGCC
TCAGGAGGGCCGTGCTATGGCTGAGAAAATTAATGCATTCGCTTACCTCGAATGCTCT
GCCAAGAGCAAGGAGGGAGTCCGCGAAGTATTCGAGACGGCGACTCGCGCCGCGTTACAAGTTAAGAAGAAGA
AGAAGACTAGGTGTTCTCTGCTGTAATTGTGCTGTTGTCGGAAGAACTCTGATTTATGAATCGTTCGCATACT
GCCTGAAAACTTACGTTTGATACGACTCGAACTCTGCAGCAGTCGCGATGTCGGCCAC
ACAGTGCTTCCT
>1002052393_2 Heli.2-DT664112.3.5
GGGTGAACAAACATGATGCGTGCCACATTTGTCCTATTTCTGGTGGCAGCCGCCCAGGGCATGCCCGCCCTGG
AGAAAGACAACGACCCAGGGCTAGTAACCAGCGTTATAGGAGTAGTCAAGGAGTGCGCTGATGGAGATGTGTC
CTTGTGCCTAAAGGAAAAAGCTCTGAAATATGTGGACAGCCTGTCGTCGGCCAGGGAG
ATGAGTATCACTGAAGGTGTCACTCTCTTGGGTACTGGTTCTCCGAGGTCTGCGAGAGCGTTCGAGCCCTTGG
CTGATGAGCCCAAGGCTAGAGAGGCCCAGGTAGAGTCAAGGCTTGTGGACTCCGCTGCTGACTTCCTGGAGAA
CCACGTGATCCAGTTCCGTCTGCCATCATCAGCTGTAGAAGGCATGAAACGATCTCTC
GAAGAAGCTCGTGGCAAAAAGAAGAAGATCAAGCAGCTCCTCCCTCTGTTGGCCATCGCTAAACTGAAGATCA
TGGCTCTCATTCCTCTGTTCTTGGGTATCATTGCATTCGCTGCCGCCAAGGCTGTCCTCCTAGCCAAGATTTC
ACTCTTGGTCGCTGGTATCATTGCTCTGAAGAAGCTTCTTGCAAGCAAGCATCATGAA
ACAAGTTATGAAGTCGTTGCCCATCCTCACCATGAAGAACATTATGCCAGCAGTGGCCATGGATGGGGAAGAT
CAATCGACGAAGCACAAAACTTAGCCTACTCCGGCCATGCTAAATCTGATTAAGGAGAGGGTCTTACAAGGCT
CGATTATATGACCCAAAGTATTCCTTAGATATACGAACGAAATGAAATTAAGCATGCG
ATTTATTTATTTCTAGATGTAAGAATATTTATTGAATATTTATTGTACCCCTTTGTAAGGGATGGTAAAGTAT
TACCGTTGTTTTGTTTTCCCATAAAGTGTGTTCTAAATTTGTATGATCCCTCTCGTTGTACTACATTAGTATA
TTTTTCACACCTTATACGGTATCCGTATTTGTGTACTTTGTTTTATACAAATATTGTA
TTATTTTCTTTGATACTTTTACTTAGTGTTGTTATTATGTGATTTAGTCCTAGTCCTGTCTTGTAAGTCCTTA
TAGTATATACTTATTTTCATGATATTCTATATAATTACTAGTACTATTCAATATTTCTTATTCTTATTTTATT
TTAACGTATAGTTGTTATGTATATATTGTAAATAATGTATATAAATATTTTTTATATT
TAGATATTCATTCAGTATTTGCTGTATATAGTGCCTCATCTCATATATTATGTATGAATCATTTTATGTACTC
GTATCCAGTCATCATATATTTATTTGACTTATTTACTTTCATATTTTCATAATCATTTTAATTTAATATAATT
ATTTGTATAATGTATTAGGTTTACGTATTTTTTTGTTGTATGTTTCATCATTGTGCTA
TATATTTATTTA
>1002052394_2 Heli.2-DT667076.3.5
GAGGCTAACATGTATTCTAAGGTGGTCATCTTCCTCTGTGCCGCGGGCATTGCTTCCGCTGGTAACGTATTGC
ATGCGGCCCCCGTGGCTTACGGCGCACCGGCCGCTTCAGTGTCGTACTCCACTACCACCACGTCTCACTCCGC
TCCAGTAGCGATTGCCGCACCAGTAGCCTCGTATGCCGCTCCCGTGGCATCCTACGCT

GCACCTGTAGCCTCATATGCAGCTCCTGTTGCATCATATGCTGCACCTTTACTCGCTAAAGCAGTAGCTCCCA
TCGCCTCTTACGCTGCACCTTCTTCAGCTGTTTCATATCAATCAGTGTCTACGCGTTCTAGTCCAGTAGCTTA
CGCAGCTGCTGCCCCCGTCATTGCCAAATCATATGCAGCTCCTGTTATCGCAAGGGCG
TATGCTGCACCAGTAATTAAATCTTACGCAGCTGCACCTTTGGTTACTCGAACATATGCGGCTGCGGCCCCTG
TCATAGCTAGATCTTACTCTGTTCCTGCGATTTCCAGCTACGCTGCGCCCGTTGCGCGTATTGCTCCCTCATT
GTCTTACGCGTCAGTCGCCTCGCACAGTGCACCAGTGGCTATTGCAGCGCCATCTATT
GCGTCTTATGCTGCGGCTCCAGCTGTCTCATACGCTCCTGCAGCGCCAGCTATCTCATACGCTGCGGCAGCCC
CAGCTGTCTCATACGCGGCTGCAGCTCCAGCTCTCTCATACGCTGCTGCAGCCCCAGCTGTCTCATACACTGC
GGCAGCTCCAGCTCTCTCATACGCTGCTCCAGCT
>1002052395_2 Heli.2-EL597855.1.5
TCTCTCTGGTTCTTTTTCATGGCTGACGTTCGAAGGAGCTAACTGCAGTCATGAAGCTGAACGTCTCCTACCC
GGCAACGGGATGTCAGAAGTTATTCGAAGTTGTAGATGAGCATAAGCTCCGTATCTTCTATGAGAAGCGCATG
GGCGCTGAAGTTGAAGCTGACCAGCTAGGTGATGAATGGAAGGGCTATATCCTTCGTG
TAGCTGGTGGCAACGACAAACAGGGCTTCCCTATGAAACAGGGAGTTCTGACTAACAGTCGTGTCCGTCTGTT
GATGTCAAAGGGTCACTCTTGCTACAGACCACGTCGTGATGGTGAGAGGAAGCGTAAGTCAGTGCGTGGTTGC
ATTGTCGATGCTAACTTGTCTGTACTCGCCCTCGTCATTGTCCGCAAAGGAGCTCAGG
AAATCCCTGGACTCACCGACGGCAATGTCCCTCGTCGTCTAGGACCTAAACGTGCGTCCAAAATCCGCAAGCT
GTTCAACTTGACCAAGCAGGATGATGTTCGCCGTTACGTCGTCAAACGCCTCCTGCCCGCTAAGGAAGGCAAG
GAGAATGCTAAACCCAGATATAAGGCCCCCAGGATCCAAAGGCTGGTCACCCCAGTAG
TGTTGCAACGCAGACGTCACAGATTGGCCCTCAAGAAGAAGCGCCTCGCCAAACGCAAGGCCTCTGAGGCTGA
TTATGCTAAACTACTTGCGCAAAGAAAGAAGGAATCTAAGGTACGCCGCCAAGAAGAAATCAAACGCAGGCGT
TCAGCTTCAATGCGCGATTCCAAGAGCTCAAACCAGAGTGCGCCGCAAAAGTGAAAGA
CTTTTATCAATAAAAATTAATAAAATCTAAAAAAAA
>1002052396_2 Heli.2-DT668194.3.5
CATAATGAAGCTTACGGTTGCCCTGCTTCTGCTTGCGGCAGTGACACACGCCGCTAAGCCGGGAAAGAAACAC
AGTGAAAGTGAGAAGACGGAGAAACGAGAGCTGGTTGAAGGGAACAGTGGAGTTGAGAAGAGAGCTCCACTTC
TATCTACTGTATCACCGGCAATATCCACTGGAGGAGTAGAATACGCAAGCACAAAAGA
AAATTTATCTGGAAACTCTCCTGCACAGCAAATCTACGCTACACCAGTGCCTCAAATCGCTAAGATATCAGAT
GTACTCTCTGGACAAGGTCCTCAATTCCAAGCTGCGATTGACAGCCATCTCTACTCACCAATCTCTGTTTACC
AACCCAGGCTTGGGTCACCAACTACATATGAAGTATCAGCACCAGTACCTTCTCAATT
AGCCTACTCTGAGCATAGATTAGCTATACCCTCACCAAATGCCATACAATATGATGCCAAACAACAAAATTTG
GGAAAACCAATAGTTAATTTCGAACAACCTCAGATATTATTCCGACCAAGCCACGAACAAAATTTGGGTCCAG
TATTTCCACAATACTCCAACAACATACAAGCAATTCCTCAGCCAGTTCAATATAGTCA
GCAACAAGTATATCCTGCTCAGCAGTTGCAATTCCTCCAACCATCCCAAAATCCTCAAGGAATTTCATATCAA
CAACCATCATCAATAGTTAAACAAAACGAGCCGAGGGACATCAAGAGCAAATCTGTCCACTCTCAAGATTTGA
TAAAGCAAAT
>1002052397_2 Heli.2-DT666674.3.5
CGTTGTACGCATCGCACGTCTTCTCCGCACATTTAATTTATAATATCTATATATTTATACAGTACCCCGAGTG
ACCTGTGCTCGAACGAACGAAATTTTAACTAATATATATCTAACATAGACCTAATAATTAGTTTTGAGTGTAG
TTTTTAAGTTAAATGGTGTAAGCTGCGGTCAGGATGTCTGAAGGAGACGTGTCCCTTA
TCCAAGACGAGGACGTGCTGCGGCGGATGTGGCAGCAGACTGAAGACTTTTCGCGGAAGAAGGAAATTCGAGC
GCATATGTATAAACTGAGGGAGGAGCGTTTGCGCAACCTTTATTCGCCAGAACCTGGAGTCGAAACTAAAGGT
TGTGAATTCTCATCCACGCAAGGTCATGTGAAGTCATTCGCTGACCAGAGCTTCCAAT
CCATGAAGAGCAAGGAAGTTCGAGACGCGGGCTCCCCGCCCAAGGAGTTCACTTACAGAGGCCAAGATCTCAA
AGAGCTTTCTAATGCTGGATGGAACGTCGAAACTGAGAACAGAACAACTGATGATGGCCATACGCAGATTAAG
TCAGTTCACGCTAACATTGAAGGTCGCTACGATGTTGACGGCGGTCATGGTCAATTTG
CAGCCGTCGATCACAATAAACAAGCTTTCACCGAGTATGACGATGGAAATACTAGTCTTAAGCGTAATGAAAA
TGTTTTTAATACTGCAGCGCATGAACAAGTTATACAAAAAACAAATGATGGTACTAAATTTTCGTCGACAAAG
AGTTCGACTAGTTCTTCATCTAAATTCGAGCAAATTTCTTCTAAACAAGAAAATGTGC
CATATAGTGTGA
>1002052398_2 Heli.2-DT664265.3.5
CTCCTAAGAAGCAACCCGAAAAATCCGGGTCGGCTGCGGCTAAGCCCGCAGAGAAAAAGCCCGCAACTGCTAA
AACTCCTGCAGCAGCCCCTAAAGCTGCATCTGCTGCTAGTGCTGCACCAAAACCTGCATCTGCTAAAACACCT
GCACCTGCTCCCAAGGCGGCTGCATCTAAAGCTGCACCAGCAGCCAAGCCAGCACCGG

CTAAGGCTGCCTCAGCTCCTGCTGCAGCAGCCAAGCCAGCAGAAAAGAAACCAGCGGCAGCACCTTCTGCTAA
ACCTGGATCTGCTAAAGCTGCAGCTCTCAAAGCGAAGTCTAGTGCAAAACCTTCAGCTAAGAAAGCAGCATCT
GCATCAAAGCCAGCCAAGCCAAAACCAGCAGCGGCTAAATTGAAGATTGCTCCCAAGC
CTAAGAAGACAGGTATTAAAGGGCAAAAGAAAGTGGTTAAGCCAGTTGTTAAAGCCCTTAAAATCCAGAAAAA
GGTTGTAAAAGGTGAACATGGAAAGCGCGTGCGAAAAATACGCACATCTGTGCACTTCCGCAGGCCCAAAACT
TTTGAACCACCAAGAAATCCTAAATACCCCAGGAAATCACTACCTAAGAGAAATCGCA
TGGATGCATACAACATTATCAAGTATCCCTTGACATCTGAAGCTGCAATGAAGAAGATTGAGGACAACAATAC
TTTAGTATTCATTGTTCACACAAGCTCCAACAAGCACCACATCAAGGCTGCTGTCAAGAAGCTGTATGACATT
AATGTTGCAAAAGTAAACACTC
>1002052399_2 Heli.2-DT664972.3.5
GACGCGTGCACTGTGTCAGTTTGAGTTTAAACGAACAGTTGAAATTTGTTTTTTTTTAAACTGTATTACTTTA
TCGAAAAGTGGCCAGTGTTATAACTTTTTTTTTTAATAAAGCAGTGTGCCCAGTGTGGATTGGATACTTGGAC
GCCGTTCAGATTGTTGATTAAGATAATTGATATAATGTACTCGTTTTAAAATGGACCC
TCTCACGTGGTTGTCCCTGGGGCTGCAGCTGGCGGCGTTGTGCTCCATAGTCGGCGCGTTGCTACTCTACTTA
CTGCGTAAGGTCCGAGTGGCTGAGGTCTCACCTGTTGACAGTGCGAAAACTGTTCTTGTCACTTCCGTGGACT
CCGCTCTTGGACTACAGATAGCAACGTACCTAAGCGGCAAGGGTTGGCGAGTAATAGC
AGGATGTCGGCAAGGGGGACTCGGTGCTCGGTTAGCTGAATCATGGCTGCAAGCCCACGTCGCGTCCACCCCT
GAAGACCAGCCACCTCCAAGACTAGCGACCCTGGAGCTGGACGTAGCGAGGGAGGATTTATTGGAGGAGGCGG
CGAGAGCGACTGCCCAACACTTACCTGCAGGAGAACATGGTGTATGGGCAGTAATAAA
CACGGCGGGCAGCAGTGGGCGCGGCGGCGCGGCGTGCTGGGAGAGCGCTCTACGAAGCAATGTCCTCGGTGCC
CTGAGAGTCGCTAGGACCTTCTCACCTTTGCTGGCAGCAGCTGCATCTGACCATCCTTATGCTGGACGACTGT
TTTATATCGGATTAACATCAGACACAGCATGCGAAAGCCTGTCCCGTCTAGATGGGGA
CCTGAGCGAGGGCAGCGCTGGTGCGGCGGCCGTCAGGTGGGGCACCTGGGGGGCGGCCAGGGCTTTACGAACA
TCACTACGCGCGCGGAGATTGAACGTTGTACTTTTACACGCCCCCGA
>1002052400_2 Heli.2-DT664957.3.5
CCATTCTACCTACATCGGCTTTATCGAAATGGAAATAGCACTACTTTGTGCAATACTAACAGTCACCGGCGCT
GTGGCGGATGACTGGCAACCAATTATAGCAGATTCACCACAGCTCTATACCACATACTCTAAGGCATCGGAGC
GATCATTAGATGGAAGCCCCCAGCTAAAGATTATCAACCGTAGACCAGTTCTCCAATC
TAAGCCTCCACCTTTCGGCCCTAACAAATTATTCAGCTCCGAGCCAGAGGCTGGTTCAGATGCGTGCAGCGTG
TACAAAGTTTCTATGAACCAAGAACTTTTCTACCAATACGTCGAATATGAAAAAGCTTTGCCAGATCTTAAAG
AGTTCACACTTTGTATGTGGTCAAAGTTCCACAACCACTCCGACGATCATCCACTGTT
CTCATATGCTGTTGGTGATAATCCGAAGGAGATTTCGGCTTGGATATCAAACACTAAAGAGGCAAGCTACTTC
AGCATGGCGATCCACGGACAGACCTACTTCAGGCTCAACTATCCCCTTAAACTTAACACATGGTATCATTCTT
GCCAATCTTGGAATGGTAAAACAGGCGAATGGCAAATTTGGGTGAACGGTGAAAGAGT
TGGCAGAGGATTTCACAACAGGCTCGTGGGTCATATCATAAAAGGCGGTGGAATTGCGACTAGCGGCCAAGAA
CAATCTCTTTTATACAAAAATGACGGCAAAGAACCTGTTTTGAAACAACCCGGCCTGATCGGTGAAATTACTA
TGCTCCAAATGTACCACGTCGCTTTAACTGCCGGAAAAGCGCATAAAGATCACAAGCA
TCATCATGTACATCATTTCAAACATGACGGTACACTCTTCGAGACGTCCTCGGAGGCAGTTACGGAACCACCT
CCACCTCCGCCTACTCCTCTCGGCAATGGCAACTTTTTGATAGGGGGTCAATTACAAAGACCTGCGAGCCTAA
ATCTGGCTCGTCAGCAACAAATGATACCCGTACAGCTTTCTAACGGACTTCAGTTACA
ACAAGAATATGTTAATGGACAATTGGGTAATAGAATAGTCTCCGAGCAACTACTAAATAATGTTCAACCGCTT
GCTAGTAATCAGGGCAGCGGTGGGGTCCAGCGCATACCGTTAGTTGGTTTGTCAGTATCACCAGTTGACCA
>1002052401_2 Heli.2-DT664959.3.5
CGTCTGATAGGCGTCGACTTACTTCTTCTGGCCAGTTTTTTAATTTCTGGCTCAATATCGGCCGTTTTTCGTG
TTAGTGGTGTTATAAAATAACTATCAGCCATGGCGCTCAGCGACGCAGATGTTCAAAAGCAGATCAAGCACAT
GATGGCCTTCATCGAACAAGAGGCCAATGAAAAAGCCGAAGAAATCGATGCAAAGGCC
GAAGAGGAGTTCAATATTGAAAAGGGGCGCCTTGTCCAACAGCAGCGCCTGAAGATTATGGAATATTATGAAA
AGAAAGAAAAACAAGTTGAACTCCAAAAAAAGATCCAATCTTCGAACATGCTGAACCAAGCCCGTCTCAAGGT
ACTCAAAGTGCGTGAAGACCACGTGCGTACTGTACTCGACGAGGCCCGCCAGCGCCTG
GCTGAAGTGCCCGGAGACGTCAAGTTATACTCTGATCTGCTTGTGACCCTCATTGTGCAGGCTCTCTTCCAGC
TGGTAGAGCCCGCAGTAACACTCCGCGTGCGACAGGCCGACAAGAGCTTAGTAGAGTCTCTGCTTGGCCGAGC
CCAGAACGATTACAAGGCCAAGATTAAGAAGGATGTTGTGCTGAGCATTGACCCAGAC

AACTATCTAGCCGCTGACACTTGCGGTGGTATTGAGCTGATTGCAGCTAAGGGCCGCATTAAGATCTGCAATA
CATTGGAGTCCCGTCTGGAGCTGATCGCCCAGCAGCTGCTGCCGGAGATACGTACCGCTCTGTTCGGACGCAA
CCCTAACCGTAAATTCACCGATTAAACAACATACGGTCATTCATATTATTAAAACATT
TATTTAAATTTT
>1002052402_2 Heli.2-DT668407.3.5
ATGTACGCTAAGAACAACACAAAGATGTACGCTAAGTTGATCGTCGCACTTTGCGCTTTAGGCGTCGCCCACG
GCAGTGGCCTCCTCGCGGCCGCCCCGCTAGCGTACAGCAGCCAAGCTATTGTCGCTCCCGCGGTAGTGTCCAA
GACTGTTGTGTCCCCAGCTGTCTCGTCGGTGTCTTCCTACTCCAGCCACACCGCCCAC
TCCGGCCCCGTCGCCACCTATGCCGCCGCCCCAGCCATCGCCAGCTATGCCGCCCCAGCGATCACCAGCTATG
CCGCCCCAGCGGTCGCTAGCTACGCCGCCGCTCCCATCGTGAGCAAGACCGTCGTGCAGCCAGCCAGTTCTTC
CTACTCTTCTTACTCTAGCAGCGTTTCCCACGGAGCACCAGCAGTCGCATCTTACGCC
GCCGCTCCCGCCGTAGTCGCCGCCCCCGCCGTCGCCGTGTCCCGCGCTGTCCCCGCCGTGTCCGTCGCCGCCG
CCCCCGCCGTAGCCGTCTCTCGCACTATCTCCCCCGCCGTATCTTCTTACTCTTCCTACTCCAGCCAAGTCGC
TCACGGTGTTAAAGCTATTGCTGCCCCAGCGTTAGCTTCCTACGCTGCCCCAGCGGTC
GCCTCCTACGCTGCCCCAGCGGTAGCCTCTTATGCTGCCCCAGCAGTCGCCTCTTACGCCGCCCCAGCCATCG
CCTCTTATGCCGCCCCCGCGGTCGCCTCTTACGCCGCCCCCGCGGTCGCTTCTTACGCCGCCCCCGCCGTCGC
CTCTTACTCTAAGATCTACTCTGCGCCCGCCATCTCCTCTTACTCTAAGATCTACTCT
GCGCCCGCCATCTCCTCTTACTCTAGACTGTACTCCGCCCCCGCAGTTGCCTCTTACGCC
>1002052403_2 Heli.2-DT662631.3.5
GAGAATCTTCGAGAAGTATAAGTTCAGAGCGATCTGAATATTCTGTGAAGTCCACAAATTCATTCCATTTAAC
TTAAAAATGTTGCGTTTGCCTCGTGTCGTCCGCCAAACCGTTTCCTTACAAAAATCTCTTCAATTTTCTCGAT
TTTATGCCAAAGATGTGAGGTTCGGTGCTGATGTAAGAGCCCTGATGCTCCAGGGTGT
GGACGTGCTGGCCGACGCCGTTGCCGTCACAATGGGTCCAAAAGGTAGGAATGTTATTTTAGAACAATCTTGG
GGCTCACCGAAAATAACCAAAGATGGTGTAACAGTTGCGAAAGGTGTAGAGCTAAAAGATAAATTCCAGAATA
TCGGTGCCAAACTTGTACAAAACGTAGCTAATAACACAAACGAAGAAGCCGGTGATGG
CACCACAACAGCCACAGTGCTTGCCAGAGCGATCGCCAAAGAAGGTTTCGAGAAAATTTCAAAAGGTGCAAAT
CCTATTGAAATTCGTCGCGGCGTTATGCTAGCTGTAGATGCCGTGAAAGAGAAGTTGAAGAACATGTCGAAGC
CTGTCACAACCCCTGAGGAGATCGCCCAGGTCGCCACTATCTCAGCAAACGGTGACAC
CGCCATCGGCAAACTCATCGCCGATGCCATGAAGAAGGTAGGGCGCGACGGCGTCATCACAGTAAAAGATGGT
AAAACACTTAACGATGAATTAGAAATTATCGAAGGTATGAAATTCGACAGAGGTTATATTTCGCCATATTTCA
TCAACTCGTCAAAGGGTGCAAAGGTCGAATTCCAAGATGCCCTTGT
>1002052404_2 Heli.2-DT666069.3.5
AGAGCGGTGAACATCGAGGAGAGGTCGTGGAGGAATTTACCATCTTTGCCACCATCGCCGTTCGCCAATTACA
CCATGGCTGATACCGAAAAGGCGCCGCAGCCGCAGCCCCAACAGCAGCAGCAACCTGAGCAACAACCCCAACC
CCAGCAAGCAAAGGCAGCTAAACAAAAGCAGGTCATTGCTGAGAAGGTTTCGGGCACA
GTTAAATGGTTTAACGTCAAGAGTGGATATGGTTTCATCAATAGGAATGACACAAAGGAGGATGTATTTGTAC
ATCAAACTGCAATCGCCCGTAACAACCCACGCAAGGCTGTGCGCTCGGTCGGCGACGGCGAGGCGGTGGAGTT
CGCCGTGGTGGCCGGTGAGAAAGGGTATGAAGCTGCCGGAGTGACCGGCCCCGGCGGC
GAGCCGGTCAAGGGCTCGCCCTACGCTGCCGATAAGCGTCGCGGCTTCCCACGCCAATACTACCCTCGGCCAG
GAGGCGGACGTGGCGGCGAGGGCGCTCCACGCAGAGGTGGCATGGGACGTCGCGGGCCCCCGCCCAACCAGGG
GGGCGCGCAGGGGGATGAAGTGCAGGAAGGAGCCGGAGGCCCACCACAGCGTAGTTAC
TTCCGCCGCAATTTCCGCGGTGGACGTCGTGGAGCTGGATCCTCCCGGCCTATGTACCGTGGTGGATACAGAT
ATGTCCGTCTCCGCAACAACGCACAGGGACCAGGCCAGGGTCAGGGACAAGGGCAGCAAGGTCAAGGGCAGAC
TCCGCGTCAAAACGGTCAGGGAGAAGGTGAAGCTCCGGCCCCCGCTCCTGCGGCGCCA
GCACAGCCGAAAGCAAAAGCTAAGCCTGCAAGCACTACCATCGAGACCACCACCAATGAGAGCCAAGCCTAGA
TGGTTGGCACTGTGGCACGCCCCTAGTAGTACATGTCGATTCCAGCCGTGCCGCATGCTATCACAACTTACTG
TCTGTCTATTCAGCTAAAGAGTGTTATGATCAGATCATTGTACACCAATTTTATGTGT
AGAGGTAGCTAAGTCGACGGGGGTGTCGCGAGTGGAACTGCATAGTATCATGGATCAGAGATGTGATATTTTT
ATTATTTTATAATATGTTGTATACCATTCGGCCGCCGGGCGGCGAGCGATAGTATCGAATTATTTTTTACTAT
TTAATAAATATTATAGGTCGCCGTGCGAAACCGCCTCTTAAGAAATTGCATGTTTTAA
TATTTAAAAAAG
>1002052405_2 Heli.2-DT665985.3.5

CGGCACGAGGCACTACAGTTGTTGGAGTTTGTAATCATGAAGATTGTGGTTTTGGTTGTTTTCTCCTTTATCC
TTGCGGTTAAAACACAAGATGCTTTGGAAGTGAACCCCGAACCTTCACTATTCGGCGACGAATTTGCAGCAAG
CAACCTCTACGACACTATCTTCTCAAAATATTTCGATGAAAAACCACAGAATAACAGG
CAAAATAATAGAAAAGTCCCAAAAATCAAAAATTCTGGACACCAGAGTCAATCGTATAAGGCTAAAGAACCTA
TAAAGATTGAGAAAATTCCTGAGTATATAAAATATAAGAAAAATCCTGAATATAAGGTTGAGTATGAGAAGTT
GGTAGGTCGTAAAGGCCTAGGCTTCGATAAGAGAACCGCCACTACTTACGGTATGAGG
GCACCCATTACCGCGAAGTCACTAGAGGTTGTCGGAAGGAAGTATTACGAAAAGAAAGAAAATAAAGATCTCA
TCATTTTCTAAAAAAATAACTTTTTTTGTTTGAATTTAATAAAGAATTTTCATATTTTGCTCTTTCTTTTCGA
TAACAATGAATACAGTATTTTTAATTTGTAATTAAATTATAACTATAATGTGCTATTA
TAAACACGACGGTTGTGGGTTCGATTCGCACTCGGGCGTTTTTTTGTTTCTTCGCTTTACTAGAAAAGACTCA
GCGTTGAGTCTCAATTTTTAGGAAATTTAGGTAGCGCATAGAAGAAGAGGAGTATTTCTAGTTTCCTTCTGTT
TACTCTATGCTATGCGGGAAAAATCGTGTATGGA
>1002052406_2 Heli.2-DT664799.3.5
TTACTCCGCCATTCCCCGATCGTCTTTTCCATCTTTCTTGTCCCCATATAGAATAAGACATTCAAAATGGCGG
CAAAAGCCAATTTGAAGATGATCGTCAATGAGGAGAATGAGGAGAGGTTTGGGTTCGTGTTTGCCGTATCTGG
ACCCGTCGTAACGGCGGAGAAGATGTCCGGATCGGCTATGTACGAGTTGGTGCGCGTC
GGGTACAACGAGCTGGTCGGAGAGATCATTCGACTCGAGGGTGACATGGCAACTATTCAGGTATACGAGGAAA
CATCAGGTGTAACAGTCGGAGATCCCGTACTCCGTACCGGCAAGCCTCTCTCAGTGGAACTTGGACCTGGTAT
CCTGGGCTCCATCTTCGACGGCATCCAGCGACCCCTGAAGGACATCAACGAGCTGACT
CAGTCCATTTATATACCTAAGGGTATCAATGTGCCGTGCTTGGCTAGAGAAACTGATTGGGAATTCAATCCAC
TCAATGTTAAGGTTGGATCACACATAACCGGAGGTGACTTATACGGTATAGTCCATGAGAACACCCTCGTCAA
ACACAAGATGCTGATGCCACCCCGTGCCAAGGGTACAGTCACTTACATTGCACCTTCA
GGAAACTATAAAGTTACTGATATAGTGCTAGAAACTGAATTCGATGGTGAAAAGGCATCATACAGTATGTTGC
AAGTATGGCCAGTACGTCAACCCCGTCCCGTTACAGAGAAGCTCCCAGCCAACCATCCACTATTAACTGGACA
ACGTGTCCTGGACTCCCTATTCCCGTGTGTCCAGGGAGGAACAACAGCCATCCCTGGG
>1002052407_2 Heli.2-DT664014.3.5
CTTCTGCTTGCGGCAGTGACACACGCCGCTAAGCCGGGAAAGAAACACAGTGAAAGTGAGAAGACGGAGAAAC
GAGAGCTGGTTGAAGGGAACAGTGGAGTTGAGAAGAGAGCTCCACTTCTATCTACTGTATCACCGGCAATATC
CACTGGAGGAGTGGAATACGCAAGCACAAAAGAAAATTTATCTGGAAGCTCTCCTGCA
CAGCAAATCTACGCTACACCAGTGCCTCAAATCGCTAAGATATCAGATGTACTCTCTGGGCAAGGTCCTCAAT
TCCAAGCTGCGATTGACAGCCATCTCTACTCACCAATCTCTGTTTACCAACCCAGGCTTGGGTCTCCAACTAC
ATATGAAGTATCAGCACCAGTACCATCTCAATTAGCCTACTCTGAGCATAGACTAGCT
ATACCCTCACCAAATGCCATACAATATGATGCCAAACAACAGAATTTGGGAAAACCAATAGTAAATTTCGAAC
AACCTCAGATATTATTCCGACCAAGCCACGAACAAAATTTGGGACCAGTATTTCCACAATACTCCAACAACAT
ACAAGCAATTCCTCAACCAGTTCAATATAGTCAGCAACAAGTATATCCTGCTCAACAG
TTTCAATACATCCAACCATCTCAAAGTCCCCAAGGAATTTCATATCAACAACCATCATCAATAATTAAACAAA
ACGAGCCGAGGGACATCAAGAGCAAATCTGTCCACTCTCAAGATTTGATAAAGCAAATA
>1002052408_2 Heli.2-DT666489.3.5
GCACGAGGCTGAGTTCAACGCGCGGACGTCACGATACATCGACAACATGAGGATTAGTGCACATTTCGCTATT
TTGACCGTTTGCCTTGCCTATGCGCAAGCAGGCCTTCTCCTTCGAAACGCGCCAGCGTGCCCAGAACAGTATG
GAGTTCAGGCTTACGCGCACCCCGAACTCTGCGATCAGTTCTTTTTGTGCACAAATGG
CACCCTCACCGTAGAAACATGTGAAAATGGCCTTCTATTCGACGGCAAGGGCGCCGTGCACAACCACTGCAAC
TACAACTGGGCTGTGGACTGCGGGGAGAGGAAAGCTGATTTGACACCATACTCTACGCCTGGATGTGAATATC
AGTTCGGTATCTACCCTGACAGCGCTGAGTGCTCTACCAGCTACATCAAGTGTGCTTT
CGGAGTGCCACACCAGGAGCCCTGCACACCCGGCCTCGTGTACGACGAGCGTATCCATGGATGCAACTGGCCT
GATTTATTACAACCTTTCTGTAATCCTGAAGCCGTCGTCGGTTTCAAATGCCCAACAAAAGTGCCAGCCCACA
CTCAATCAGCCAAATTCTGGCCTTACCCCCGTTTCCCTGTGCCCGGTGACTGCCACCA
CCTGATCACCTGCGTGGAAGGTAACCCTCGTCTCATCGCCTGCGGTGAAGGCAAAGTCTTCGATGACCAAAAC
CTGACTTGTGAAGACCCTGAACTGGTACCTCATTGCGGACACGCGTAATTTTAAGTCTATCGGCATTATAA
>1002052409_2 Heli.2-DT667283.2.5
CACGAGGGCAACACAGACCACTTACAAAATGTTCAAGCTGGTGGTGTTGTCTTGCGTCCTGGCGGCGACGTCC
GCGTCCAGCCTTTTTGGAGGATATGTGGCACCCTTCACCCCATTGGCATATGCTGCACCTTTGTGGGAACCTT
CCAACTACCGTGGACCCCTCTCATTGGCTCCTGGTCAACCTGCTAACATTCTGGGCGC

TGATGGCAGGCCTCTTGACACTTTGGACGTAAACTTGGACCGCTCCGCTCACTTCACAGCCAAAGCTCTCGAC
AATGGATTCCATATCCTCAAGAAGCGTTCAGTCGTCTCTCCTTTCATCGCCCCCTATGCTGCACCTTACGTAG
CCCCTTACGCCGCATCATATGTGGCCCCCTACGCCGCGTCTTATGCTGCGCCTTTGGC
ATACTCTGCTCCTCTCTTGCAGCCATACAACTACCGCGGACCTCTCTCCCTAGCCCCTGGACAACCCGCCAAC
ATTCTTGGTGCTGATGGAAGGCCCCTTGACACTTTGGACGTAAACTTGGACCGCTCTGCCCACATCGCCGCTA
AAGTTGCTGCTGGCTCTCACTTCATTAAGAAGCGCTCTGCCGCCTTCATCGCCCCCGT
CAGCACTGTTGCCGTCGCCAGGACGCCACTGATCGCCCCATCCTACACATTCACCGCTCCCCTCGCAGCTGCA
CGTCTCAGCCCATTCGCCTACACCCACCCTGTTGCCCGCATCGCACACCTATACTAAACTGACGTCAAAAT
>1002052410_2 Heli.2-DT665802.3.5
GGGGCGGCCATTATTCTACGTATAATATTATAATTTTTTTGTGTTTTGCGCTGTACTATATTCGAAACAAAGA
ACTTTACTATATTCTACAAACTCTTGTTTAGCATTATTGTGATCATAAAACTATTATAGTACAAAATGAATCC
AGAATACGATTACCTCTTTAAGCTGCTGTTGATTGGTGACTCTGGTGTGGGGAAATCA
TGCCTTCTACTCAGATTTGCTGATGATACATACACAGAGAGTTATATCAGCACTATCGGAGTGGACTTTAAAA
TTAGGACTTTAGATTTAGATGGCAAAACAATAAAATTACAAATATGGGACACAGCGGGACAAGAGAGATTTAG
AACGATAACATCATCTTACTACAGAGGCGCACATGGAATCATTATTGTTTATGATTGC
ACAGATCAGGATTCATTCAGTAATGTCAAACAATGGCTGGAGGAGATAGATCGTTACGCATGTGACAATGTCA
ACAAGCTGCTAGTGGGCAACAAGTGTGATCTTACCACAAAGAAAGTTGTTGACTACACCACTGCTAAGCAATA
CGCGGAACAGCTGGCCATACCGTTCCTGGAGACATCAGCGAAGAACTCCACGAACGTG
GAGCAAGCGTTCATGACGATGGCGGCGGAGATCAAGGCGCGGGTGGGCCCGCCGTCCGCGGGCGCCGCGCCCG
CCGGCGCCGCCGTCAAGATCGACCAGGGCCGCCCCATCGACACCGGCAAGTCGTCGTGCTGCTGAATGTGTTA
TACGGGTGGCGGCGTGCAGCAGTCGTGGCGGGAACCCGCACCCAGCTGTTAAAATGCA
ATTACCACAAATTCATGATCGCATCCTTATTATAATATAAATAAATAAATAAATGATATATAGTGCCGAGTGG
CGAATGTTAAGCTAACTCCCTTAAGTTGCTATCGTCGAGGTGAAATGGATGGATGTATGTTTATACGTGTT
>1002052411_2 Heli.2-DT664911.3.5
GCACGAGGGCGCGGTGCACGTCGTCTGGACTTGTAGTTGTAAAGCCTTATCAAAAACTCTACTAAGCTACGCT
CGTAGCCATGAGCAGAACTTAGAAGTATATATCAAGCGTATATTTATATTAAAGTGATATTTTAAAGTGATAT
TTTTATAGCAATAAGTAGAATATAGTGCAAAACAAATCAATATGTACAATATTTTATT
TTATTTTTCCATTTTATTATCCTACGGATATGCCGAAGTATTAAATTTAGCTGGAGGTGCCAAAAGCAATTAC
ACATTTCCTAAAGGGTTCCTTTTCGGCGTTTCTACTGCAGCTATTCAAATCGAAGGAGCTTGGAATGTGGATG
GAAAATCGGAACATATATGGGATCATTTAGTTCACACGAATCCAAAGTTTACCAAAGA
TGGGTCTACTCCAGAAGTGGCAGCGGATTCTTATCATCAATACAAAAGAGACGCTGAAATGGTCCATCAACTC
GGTGTTGATATTTACAGATTCTCCATGTCCTGGGCTAGAATATTACCAAACGGTTTAGCTAACAAAGTGAATC
CTCTCGGCATTCAGTATTATAAAAATTTAATAAGTGAATTGGAGAGATATAACATTAC
ACCAATGGTCACCATTTATCATTGGGATCTGCCGCAAAAGTTACAAGATATAGGTGGCTGGACGAACGCCCAC
ATTGTGGACTATTACACGGATTACGCGAACGTTCTGTTCGAGAACTTTGCAAATGAAGTAAAGTATTGGGTGA
CATTCAATGAACCGATGCAAACCTGCTTGGAGGGCTACGGGGGCACCTACCGGGCGCC
GGCCCTCAATCGGCATGGTGTTGCTGAATATTTATGCACCCATAATTTACTGAAGGCGCACGCGAGCGTCTAC
CATTTGTTTGCTCAACAGTATCGTCCTATTTATGGAGGTAAAATCGGTATGTCATTAGATTCTAATTGGGCTG
AGCCAAAAACTAATTCTTCGAAGGACCGAGAGGCCGCTGAACGCTACCTACAAACACA
TGTCGGTTGGTATGCACATCCAGTGTATTCTGAGGAAGGAAACTACCCCCCAGAGCTCATTAGGCTTGTAGAT
GAAAAAAGTCGTCTGCAAAACTATACACGCTCACGTTTACCTAAGTTTACTCCGGAAGAAGTTGCGTATATAC
GAGGTACAGCGGATTTCTTCGGCTTAAATCATTATACGACATATCTTCTGAGTATAGC
TGATGGAGAAGT
>1002052412_2 Heli.2-DT664501.3.5
GATGTTAAATCAGCTATTACATATTACCCAGAGAAGGATGAGCCGAAAGATAGTACAGATGGGCCAAAGCACG
ATAAGCCCACGTACGTGAAAAAACATGAAAAGAATTATATCGATGATAAAACATCAGTTTCTGAGCACTATAC
TCAATACACTGAAGATGACTCTAGTCATTATACAACACTAAAAACATCAGAAGTAACC
AATCTTTCTCAAGGACGTTCTCCTCAACCAAATGGACAGAATGATTATTCTGTTCCTAAAGATGGTAAATCTT
CACGACCTAAATGGGATAAACCTGGAAATAGAGAAGATAATCGTCCAATAAAGAGTGATAAACCAGAAGAAAG
GCCTGAATATAAAACAGAAGAAAAATTGGGTGATAAACCAGAAGAGAAACCTCGTTAT
AGAACTGAGGAGAGGCCGAGTGACAGGCCAGTAGACAAACCAAGTGAGAAGCCAATGTCGGAACCTAATTATC
GCCCCGGAGAGAGACCAAGTGATAAGACTGGGGAAAGGCCTTGGGACACAGCAAATATTAGGCCAAATTTATC
AGAACGACGTCCTGGTGATAAATCTAGACCAGGGGAGCAATCTCGGGATACACAAGGC

AGAAGAGAAGATAATATAAAACCTGAGGAACATTTTGGTCAACCTACTAAACCACAGTGGCAGACAAGCGAGC
GAGCTGGTCCTATTCGCCCGAAGGACAATCTATATCCCGAAGGAAATAAACAATATCCATCATCTGTTAGCAG
GGCTGATTATTCTCAAACCAAGCAAAGTTCTCACTCAGTTATAGAGGAACGACATACC
TCACATGGCTCTAGTCCTGACGCGAGTAAAAGGGTTCACGATACAAATAATTTAACTGATTATAGCAAATATC
ATTCTGCAACAGATATTGACAGCTCTAAACATACGCATTCTGACCATGATGTAAAATCACACATTACTAAAAC
TATATCCGATAAGCACCATGATAGCAGTGTGCAAAAGTCTCATTCAGAGATACGTACT
ACTAAAGTGATGGGAACTCATAATAAAGTAGTTGAAAAGTCTCATATAGTTTCTGGTACTAAAGGTCACGATA
GTCCATCAAATATAAATCGTAATGAATTGCAAAAAACTTCTACTACATCATCATCAAAAGATGTAAGTCAGTC
ATTATCACACGACACTACCGATCGGAATGCAACAGATCGTTCATATAAAAGACAATCA
ACGGAAAGAAATGTTATAAATAAGAGTGATACTACAAATATTCAACATTCATCTTCTACTAATAAAATTATAA
AACAAATAACAGAGAAGAAGTTAATTGCGGGAAAATGGGTAAATGTAACTAGAAATGTTGAAGTCGTGACGAA
TACTGATAAATCAGGAAGTCCAAACTTGAAAAATGTTATCGATGGGCGAACTGAGGTT
GATAAAAACAAAACCAGAAACATCTCAAGCAGCATTGGATCATCTGGACAAGTGTATTTAAGCGATGACAATC
AAATATTCCAAAACACTGCAAACGCCACATATCTAGTGAAAAGGAGAACTGATAATTTAAACCACTCATCTAA
TCATTCAGTCTCTGAACATGTAACAAAAGATATTGGTGAAATATCTTCACAGAAAATT
ATAAATTCTGGACAACATTTAACATCTTCGAACCAAAATATACATACCCAACGTCATGGTCATGTATCCTCAG
ATACCCGTTCCCATGAAAACATACACAATTCCACA
>1002052413_2 Heli.2-DT666773.3.5
GCACGAGGCTAGCGTGACCGTAGGTTGTGTCGCGTTCGTGTCCGGTCCCGTAATGTACAGGTGGTGGTTGGTG
GTGGCGCTGGTGGCGGGCGCGGCGGGTAACCGTCTGCCGCGGACCTCGCCCACGTCTGGCCCTTCGCCCGCGC
CCTCGACTCGTCTGCCGCAACGGAACTTTACTATATCTTCCCGATCCTCCGTCAGAAA
CAGGGAACAATACATTTTTAATGTATTCGAAGTGATCGTTAGCACGCTTGAAACGAAGTTGCAGAGGATTGAA
AACCTTGACAAGGCAGTGGAACATCTCATGAGAAGAGTAGAAGCTTTGGATTCAAGAGTCAATGATAATATTC
ATAAAACCGACGCCATTATATCTAAATTAGGAAATTTAGACTTTAAACTATTTAGCCA
GTTAGGTCAAAGTGAAGAGGATGTTGTAACTGAAAATATCAACAAAAAAAGTGAAAATAAACTTAGTAATGCA
CAATTGCTCGACAAGAAGTTAGAATCTTTAGATCAGAAAGTGTCAGATATAGATTCTAAGCTAGTTGGAATAA
AGACACAAATAGATACAAACTTTCTACCAGTCGATGATATTAATGCGGAAGCAAGTGA
AAAGAAGCCTGTTAATCTAAATGTAATAGAAATTGCAAAAGGGCTAAATTCAGAAGTGGTAAATGAAATCACC
AAAAAAGTTGATCAACTAAGATCATCAATGTCAACAGTCGATCGGAAACTTCAATTTCATATAAATCTTGTAT
CCGAAAATTTAGGAAAAGTCCTTTATATGATGGCAGATGTACATGCTGCTATAGTAGA
GCCCGAATCTGCCTCGATAAATGTTAATAACTTATT
>1002052414_2 Heli.2-DT663106.3.5
GGCGAGTTGGTCGATTGGAGTAGTTCGTAGTAAATTAATTAAATTGACATAATTTGTGTTTTATCAAATCAGT
GTTTATAAAACGTTTACCATATAATCATTATCATTTAAATTGATTTGGAAAAATGGCACCAGATCCCGATAGA
CATCAAGAAAGTTTCCCGAAACTTAAGTATCCTTTGTACAGAGAAGTAGAACCAAGGA
CCCCGCAATACTGGTTAGCAGCAAAGCGAATACAAGACGGTGCAGACGGCCTTTGGCGGATACACAATGATAT
CTATGATTTCACTGATTTTGTTTCATCTCATCCTGGCGGCTCGCAATGGCTGGAGTTTACTCAGGGTACAGAT
ATAACAGAACAATTCGAAACTCACCATATTAATAGTGCACTAGCCGAAGCACTGTTGT
CCAAATATTTCGTGAAAAAAGCGGAAACACCCAGAAATTCACCTTTCACTTTTAAAGAAGATGGTTTTTACAA
GACCCTCAAAATCAAAATAGCTGATACACTGAAAGGCATACCTAAAGATGTTCGAAAGAAGAGTGATCATGTA
ACTGATTTCCTCTTAATATGTTTGCTACTAGCAAGCCCAATTTGTGGCTGGGTTTGGA
CCAAGAATTTAATATATGGAGCTGTTACAACCTTTATTCTGAGCTTAATATTGAGTGCACTCACTGTTTGCGC
TCACAACTACTTCCATAGAGCAGATAGCTGGCGCATGTATCTGTATAATATTAGCGGATTTTCGTACTCGGAC
TGGCGTATATCTCATGCGATGTCTCATCATATGCACACAAACACGG
>1002052415_2 Heli.2-EL598034.1.5
GACATCCAGCGAAATAGTAGGGACTTCAGTGGATTGAAACGTTTTTACTACATATTACATATATTTGCTTCAA
AAAAAACGAAAATAAAAAATACAGCAAAATGGGGAATATGTTTGCAAATTTATTTAAAGGCCTATTTGGCAAA
AAAGAAATGAGAATATTGATGGTGGGTCTTGATGCAGCTGGTAAAACCACAATCTTAT
ACAAACTTAAATTAGGAGAAATCGTAACTACAATTCCAACAATTGGATTTAACGTGGAAACTGTAGAATACAA
AAACATCAGTTTCACTGTTTGGGATGTAGGTGGTCAAGACAAAATCAGACCACTGTGGAGGCATTATTTCCAG
AACACACAGGGTCTCATCTTTGTAGTAGACAGTAATGATCGGGAACGTATCGGTGAGG
CGCGTGAGGAGCTCATGAGAATGTTAAGTGAAGATGAGTTACGGGATGCAGTACTTTTGATCTTTGCAAACAA
ACAGGATCTGCCAAACGCCATGAACGCCGCGGAGATAACGGACAAGCTGGGCCTGCACTCGCTGCGCAACCGC
AACTGGTACATCCAGGCGACGTGCGCCACCTCCGGCGACGGCCTCTACGAGGGGCTGG

ACTGGCTCTCCAACCAGCTCAAGAACGCCAACCGCTAACCTGTGCCCCCACAGACTTATCTGATTACTGAAAG
AGACTATGGTTTAAGATATGTTCACTTTATTTGCTATAAATACAATG
>1002052416_2 Heli.2-DT664484.3.5
CTGTCGCTACTAGTGTTGTTCTGTTTATTAAATAAAACTTTGTAAAACATCAGAAATGGAGGATCAAGTGCCA
TTTAAAGTGTTTACATATTGGAATGACGATGATAAACCTGAAGTAAGAAGATTTGGTATTGAAAAGAGCGTTG
TGACTAGCTTTCATTATATAAATGCTAAGTTACAAGATGTTTTCCCTGGCTTGAAGAC
CAGACAATATGTGGTTTCCTGGAAAGATGAGGAAGATGATGATGTTACTATATCATCTGATAATGAAGTCATG
ATTGCATTGGTGGCTATGCAGCAACAAAATCTAATAAAACTCAACATTTACTGCAAAGATTCAAAGCCACAAG
ATGATGACTGTGACATCATTGTAACTGCTGTAGCTGATAATGGAGCATGCTCCACCCT
GCCCCATTGTGGTGTAGTTTGTGATGAATGCGACTCACCAGTCGTTGGATTCCGTTACAAGTGTACAACATGT
GATGACTATGATTTGTGTACAAAATGTGAATCTGCAGGTTTACACCCTGAACATTGTATGGTGCGACTACCAA
CTCCTAATATTGCGCGTACTACCATCAAAGCTGCCCTAAGGCGTTCCCGTCGCTTCCT
ACATTCCGTCGCAAGTACAGTTGAAGAAGATTGTAAGAGGCAACGTCGTGAACGTAGCAGTGAAAGGAAACGC
CACGGCCACGGCCACAGCCATGGTCGAAGCCACCGCCACGGTGGAGACGGTGACCACCGACGATCACGTACAA
GTTGGCTGGA
>1002052417_2 Heli.2-EL602411.1.5
TGCCTCGCGAACTCGCCACGCCACACTATACTCTAAACTTTCCTCCATTCTCTACATCTCTATTTGTCGTCAT
CATGAGGGAATGCATCTCAGTTCACATTGGCCAAGCTGGTGTCCAGATTGGTAATGCCTGCTGGGAGCTGTAC
TGTTTGGAGCACGGCATCCAGCCTGATGGTCAGATGCCCTCAGACAAGACCCTGGGAG
GAGGAGATGACTCGTTCAACACCTTCTTCAGTGAAACTGGTGCTGGGAAGCATGTTCCTAGAGCCGTTTTTGT
TGATTTGGAACCTACTGTAGTTGATGAAGTCCGCACGGGCACGTACCGGCAGCTGTTTCATCCAGAACAACTT
ATTACTGGTAAGGAGGACGCGGCGAACAACTACGCGCGCGGACACTACACCATCGGCA
AAGAAATCGTCGATGTCGTCCTCGACAGACTCAGGAAGCTAGCTGACCAGTGCACTGGTCTTCAGGGTTTCCT
GGTGTTCCACTCGTTCGGCGGCGGCACCGGCTCCGGTTTCACTTCCTTGTTAATGGAGCGTCTCTCCGTCGAC
TACGGCAAGAAGTCCAAGCTCGAGTTCTCCATCTATCCTGCTCCCCAGGTATCAACAG
CGGTAGTAGAGCCATACAACTCCATCCTGACCACGCACACCACGCTCGAGCACTCCGACTGCGCCTTCATGGT
CGACAACGAGGCCATCTACGACATCTGCCGCAGGAACTTGGACATCGAACGTCCGACTTACACTAATCTTAAT
AGGCTTATTGGACAGATCGTCTCCTCCATAACCGCGTCGCTCCGTTTCGACGGCGCGC
TCAACGTGGACCTGACAGAGTTCCAGACCAACTTGGTGCCGTACCCGCGCATCCACTTTCCGTTGGCGACGTA
CGCGCCCGTCA
>1002052418_2 Heli.2-DT665053.3.5
GTGTCTATATAGAAATGAAGTTTTTTTCATCTATTGCATGCCTCCTGGCGCTGTCCGTGACAATCAGTGCGGA
TGGACTTGGACTCGATGTATACGCGCCTAAATATTCCTCCGTGGATTACTATTCATATCCCAGTTACGCTTTC
GAATATGCAGTGCGGGATCCACATACTGGTGACAACAAAGCACAGTGGGAAAAACGAG
ACGGAGATGTTGTAACAGGTGCGTATTCGTTGGTGGAGCCTGATGGCAGTGTGCGTGTCGTGGAGTATCGGGC
TGATGATAAGTCAGGATTCAACGCTGTCGTTAAGCGTATTGGCCCCAATCTACACCCAGTCGCTGCTCCCATT
TACAAAGCACCTCTCCCGGTTATTGGTTATAAAGCAGAAATACCAATCTCGATTGCAC
CATTAGCAGGGATTGAGAAGCTTGAAGCTCCACTTTTGAAAGGACCGTATCTAGGTGGTAACGCTATATCATC
TGCTTCTCTATATAAAGCTGCTGCTCCGCCTATTATCAAGGAGGTTGCACCAATAATTCCTGCCCCAATTCTT
TCTGCTCCCATATTAAAAAGTCCCATCATAGCTGAACCGTATCCTCTGTACCAAGCTC
CTGTCCTCGAAAAACCGCTGTTCCAACCTTATGCTAAATCTATATATCCAATATCCTATCCTGGCCTGAAAGT
GCCACTTCCCGAAATTAAATATTCGGCGCCACTTC
>1002052419_2 Heli.2-DT663107.3.5
GATCCTGATAATAACCAAACAAAATGAACACCTTCATCACTTTGGCTTGCCTGGTGGCTGCAGTCGCGAGCGT
GCACGGCTCGGGTTATGGTGGATACGGCTATTCTTCAATCATCTCTGTGCCGGTCATCAAAGCCGCTCCCATC
ATTAAAACAGTTGCTGTAGCACCAATTATCCGTAGACCTATCTTGCAAACTGTCCCTG
TATCCAGCTACAGCCTTGCCCTCGGGGGTTACGGTGGACTCGGTTACGGCGGTTACGGACTCGGTTACGGAGG
TTATGGACTCGGAAAACTGGGTTATGGACTCGGATACGGTGGTTACGGACTCGGAAAACTTGGTTACGGTTAC
GGAAAACTCGGTTGAGTGATTCGAGGTTATAAGTCTTCAGACGGAGAAACGCTAGCTA
CTTTATTGGCCAATGTCTTGAATAAAATGTTTACAGTCTTGAATAAAATGTTTACAGTTATATCATTTTGTTT
TTTTTTCTTTTTTTTATATTGTATAATTTCTTTTTAAATATCATATACCATGTTTTAAGAGAAATTGTAAAAA
GACATTTAGAACGGTTTTAACTATTTTACTAAAATTATAAGTTTTCATATGTACAACA
AATGTATAAATTACATTTTGAAAAGTTAAAAAAATATTAAATACTAGCGACCCGCTCGCGGCTTCCCCTTCTT
AAGCTACTGAATTACCATTAGGTAGTGTATTAACACTGAATTACGTTCCTGTTTGAACA

>1002052422_2 Heli.2-DT662680.3.5
ACACGTTATAACGTCAAAATGGCAAAGAAAAAAGGCGATGAGAAAGTGAATCAAACTCCTCAAAGTGATAATG
AGGAGCAAAATTTTGACGAAGAACCTAATTTCGAAGATCCGGAAGGATTCGTGGATGATATTACAGACGAGGA
ACTGTTGGCAGACCTCTTAGAGCAGAAACCTAAGGAATCCGATGGGTACGAAAATGTG
ATCATCGTAGATGGATGCCCGCAGGTCGGGCCTGAACGTTTAGAGAAGCTTCAGAGTGTAATAAACAAAATCT
TTAGTAAATTTGGAAAGATTGTGAACGAATTCTATCCAACCAACGAGAATGGAGTCACGACTGGCTTTATTTA
CTTGGAATATAGTAATCCACAGAATGCTGCTGAGGCCGTACAAGCCACTAACAATTGT
AAATTAGATAAACAGCACACATTTTTAGTAAACCTGTTCACAGACTTCAAAAAGTATTCAGATATCCCCAAAG
AATGGGAGCCTCCAGCACCTCAACCGTTCAAGGTGCAATCAGATCTTCAATGGTATCTCATGGATCCTGATGC
ATATGACCAGTTCCTCGTAGGAATTGGTACTGGTGTGGCACTGCAAGTATGGCAGAAT
ACTCTGCCAGAGCCTATTACATTGCAAGAGAGACCTAATTGGACTGAAACCTACGCTGTGTGGTCTCCACTTG
GCACATATTTGGCCACTTTCCATTGGAGGGGTGTTGCCTTATGGGCGGGACCTAAGTTCAGTCAGTTCCAGAA
ATTCTACCATCCTGAAGCTAGATTTATTTCTTTCTCACCTTGTGAAAATTATATTGTC
ACTTTTTCACCTAACAGCGATAGAGGTGACGATAAGAAACTTATAATTTGGGATATTAGAACAGGCCAAGAAA
AACGTAGTTTCCCTCCACCCGACGAGTATGTAACGTGGCCGATATTCCGCTGGAGTAAGGACGACCGTTTCTT
TGCAAGACTCGGTGCTGATGTTCTCTCTGTATACGAGACGCCCAGC
>1002052423_2 Heli.2-DT662561.3.5
CGTGCGTTTGGAAAAACTCTGACAATCTGTCAATATTTCGCGCTTAAAATATTTTTTTCAGAATTATAAGTTT
GTCTATGGTATTACGTTTTTTTTTAAGTATATTTTTAAATAAAAAATATATAAATTGTAAAAATAAATAAAGT
GTGTGTTGGGATTCAGTGACTGTGATCTTGTGTGATGGTACTCTGCCGCTTTTTATAG
CTGATAGAGAGCAGCATGAGGGGTTGCGTTTTTCTAATTTTGGCGCATACGGTCGCCGCCATAACTGGACCGA
GGATAGAAAATGACTTTGACAGAAATAGACAGCCATCCCTGGGTCACCCAGGATTCAATGATCATCAAAATAG
ATATGGAGTACAAAATTTCCAACAACAGCAAAGTTTGTTGAGTAGCCCAGCGCCACCC
ATCCAGTTTCCGCAACAGATCTTTCAACAGCCCTACGTACCTCAATTCAACAACTTGTTGCCAACCCAAAATA
TTCCGAATAATGTCCCCATACAAAATAACAACGGATTTCAATTTAGCCAACCGGCAAATGTTAACCCTATAAA
TAACTTACAGCATCAAAATCTCGGTCATATTCAACAACATGCTCAAAATGCACCACTA
CATGCACAAAATGTACCACAACCAATTCCGACTTTAGCAGCATTTCAGAGTAATGGACTACTGCCCACGCAAA
ACTCTCATCCCAGTTTCAACCAAAACCCTAACATTCATAGCTCTATTGATTTGTCTCAACAGAGACCTACTCA
AGCTGCCTTTCTCCCTACCTTAAATTCTGCCCCCATTCAAAATCAGGCTTTTAGTGGA
CCAAATCCCATCACTATTCAAGCTGCGCCAAACTTAGCACCTCAAAATTTAAATAGTCAACAGCTTCCATTTC
AATCTTCTCTCGGCCAGTCTCAATTATACAACAGCCAATCTGCGTCCCAACTACGAGACTACTATGAACAGCA
ATCTAAGGAAAATCTAGAAAAATTAAAAGAACTACAAGAAAGGCAAAGACTCATCCAA
AAACATCAAGAA
>1002052424_2 Heli.2-DT663858.3.5
TGTGTTGTGTGTGAATATTAGAATTAACGGATAGCTATTAGTGGATTTCATAAAGAAATTGTAATTGATAAAT
CATCAACATGGGCAAGGATTACTACAAGATTTTGGGTATAGCGAAAGGCGCTACCGATGACGAAATCAAAAAG
GCGTATCGCAAATTAGCCCTGAAATACCATCCGGACAAAAATAAATCCGCTGGTGCTG
AAGAAAGATTTAAGGAAGTTGCAGAAGCTTACGAAGTATTGTCGGATAAGAAGAAACGAGAGATCTATGATGC
CCACGGCGAAGAAGGCTTGAAGGGTGGAATGGGAAGCCACAATGGTCCAGGTGCTGGCCAATCATTCTCTTAC
ACCTTCCACGGAGACCCTAGAGCTACCTTTGCTCAGTTTTTCGGTTCAGCGAGTCCTT
TCCAGGGCTTTTTCGATTTGAACGGAACCCCTGGCAACACATTCTTTGACCGCGACATGGATGTCGATATGGA
TCCTTTCAGTAACATAGGTATGGGTACTGCAAGACCTGGTGGGCCTGGTGGAGCGTTTAGGAGTCACAGCTTC
AACTTCCACGGATCACCCAACCGGAAGGAAAAGACTCAAGATCCGCCTATAGAACACG
ATTTGTATGTCTCACTCGAAGACATTGCGCGTGGTTGTGTCAAGAAAATGAAGATTTCACGAAGAGTAATGCA
GCCAGATGGCGGATCGAAGAAGGAAGATAAAGTGCTAACTATTCATGTTAAACCTGGATGGAAGGCTGGCACT
AAGATCACTT
>1002052425_2 Heli.2-EL597471.1.5
GGTTTAGGGCCAGTTCTAATGATTGTAATTTAATTGGCCGTGAACGCTCAACCGGGTGTGACTCTCGATCGGT
TTTAGTGATACGTAATAGAAGTTTTCAGGCTGGGCAGGGTCTTCAGAATGAAGCTCTCCCTGGTGTTTGAGCT
TCTGTTAATATTCGTACTGCTTGCGTTTGCAGCGTCGAAAAATATCGGTCGTCAGAAA
AACAATAGAAAAGGAATTCAAAAAGCAAGTGACGGCATTCAAATAGAGGACAACAGTATAAATAAATTTTGCA
AATGTTCAGAGGCTCTGTGCAATTGTTGCAGAGATTTTGCGGTGCCTATTATAAATCTTAATGGTCCAGGATG
CGCTTCATTAATGTATTTAAATGGCGACAAAATGTCCGTATCACTCAGCTTCGGTAAC

AAAGTCATTACAAATCGAACCCTTTCAAGCCGCAAACCAAGTCCTGTTTGTATGCCTCTCCCCGGAGGTGTTT
CAAAGTTTTGTGGACGAGTTTACAATATTGCGAGGATTGGAGAAGAATTTCGTGCTTGTTTAGGCTTAGAGCT
TCAAGCTAAAAAATCTGTAGAAGCCGCAGTTAGAGTATCCTGTTTTAAATTTGGTCCC
AGAGGTGTAATTTCTGAACCAGCTGATCCTTTGCCATTAGTGCCACAGGATGAGAAGGATACTGATGACGATG
ATGATGACGATGACGACGACGATGATGATGATGATGTCGATTTTGGACTCGATGCGGAT
>1002052426_2 Heli.2-DT663298.3.5
CATTTTCTGTCTTTTTATGACAATTTCTGGGCTAAAAACTTATTAGTAAGGTAGCCCCAAACGTCCACTAGGA
ATAGTTATAAAAATGGCGTTTTGCCGTTTAGTATTCCCTCTTTTAGTGCTAAGTGTCGTGTCGAGTTATGCCA
ATATACATGTACCAGTATTTCTTTGGGGCGATTTGAAAACATCAATAAAGTCGAATCC
CTTGTCAACCGTGCCTCAAGAAGACTTCGAAGCTATTTTGAAGAAAGAATTAAAAGCCGATCCATTCGTCGTA
ATTTTCGTCGACGAAACTTTGTCTGTTGAAGATTTCTCACTCAAAAACTCCGATGGCGACTCTTCATTCCCCT
ATTTACACGCTAACATTGGTAAAGCTGTATACTTACCTGGCGTGGAGAACGCTTTGCC
CGTCTTGAACAACTTGGCGAACCCTGAACAAGTGGACCGCGTGAAATTGACAGAAGATGGGCTTTCTGCTGAA
TTTGAACCAAGGGATGTAAAAGTACTCTTTATAACGCTTAAAGATGCTCGCGAGGGAGAATCAAGGGCTGAAC
TACTCCGTCGTCATAACGATTTCATGGCTGACATGCTCACTAAACTACAAGAACAATA
TGATAACATTGTAGCTGTATACACTGCAGAGAATCCTTCCTGGACCATTGCTGAAAACCACTCTCGAGTCCGT
CGTCAAGCTGGAGAGAGTCAAAGAGACTATGTAATGGATGGCCTGAGATTGTATGTGAAGAACATTATTCTTT
CCGTTGGTAGTGAAAAAACAAATTTGAATAACATGGCCAGTTTCTCTAGCAAATTCAA
TGAAACAGAAATGTCCACAACAATGAACTTTGGAGAGAACTCCATCAC
>1002052427_2 Heli.2-DT662779.3.5
GCACGAGGGTCTTTTGAGTTTTCTCCGCTCCTTTGCAATCATGGCGGTGAACAACATTTCCAAAAAGCGAAAA
TTCGTCGGTGACGGTGTTTTCAAAGCTGAATTGAACGAGTTCCTTACTCGGGAGCTTGCTGAGGATGGTTACT
CCGGCGTGGAGGTGCGTGTTACGCCAACCCGTTCAGAGATCATCATCATGGCGACGAG
GACACAGAGCGTGTTGGGAGAGAAGGGCCGCAGGATTCGTGAATTGACCTCTGTAGTTCAAAAGAGGTTCAAC
ATCCCTGAACAGAGTGTTGAACTTTACGCTGAGAAGGTTGCCACGCGAGGATTGTGTGCTATCGCTCAGGCTG
AATCTTTAAGATACAAGCTCATTGGAGGGCTGGCTGTGCGTCGTGCCTGCTATGGTGT
ACTCAGATTCATCATGGAATCTGGAGCCAGGGGATGTGAGGTTGTAGTGTCTGGTAAACTGAGAGGGCAGAGA
GCCAAGTCCATGAAGTTTGTTGATGGTCTCATGATCCACTCTGGAGACCCATGTAATGACTATGTTAACACTG
CTACACGCCATGTACTGCTAAGGCAGGGAGTGCTTGGTATCAAGGTGAAAATCATGTT
GCCATGGGACCAACAAGGCAAGAACGGCCCCAAGAAGCCACAACCGGACCACATCCTGGTGACGGAGCCCAAG
GACGAGCCCGTGCCCTCAGAGCCCACGTCGGACGTAAGGCCCGCGCCCGCGCCCCTTCCGCCGCCCGTCGCAG
CCACTGCTTAGACTGTACTATTATAAACATTGAAAATAAAAAAAAA
>1002052428_2 Heli.2-DT662581.3.5
AAGATTTGATTGTTGAATCCATTTTAATCCTGAGTTTGTGCTGTTATAATTTTGTGAAAAATGGCCATGTTTC
CAGGAACAGTGGCCTTCGATGAGTTTGGGCGACCATTTATTATTTTAAGGGACCAGGAAAACCAGAAACGGTT
AACCGGTATCGATGCTCTTAAGTCTCACATCCAAGCCGCAAGGCAAATAGCAGGTATC
CTTCGCACGTCCCTTGGTCCAAGAGGCTTAGATAAGATGATGGTATCTTCAGATGGTGAAGTGACTGTCACTA
ATGATGGAGCCACTATCCTTAAACTCATGGATGTGGAACACCAGATTGGTAAACTTATGGTGCAGCTTGCTCA
GAGTCAGGATGATGAAATTGGTGATGGAACTACTGGTGTAGTTGTGCTGGCTGGTGCT
CTCCTGGAACAAGCATCAAACCTATTAGACAAAGGCATACACCCCATCCGTATAGCCGATGGTTTTGAAATGG
CTGCTGCCACTGCCTTAGCCCACTTGGACAGTATTAGCGAGGCTTTTCCAGTAAATAAAGCCACCAGGGAACA
TTTGATCAAAGTGGCCATGACTACACTTGGTAGTAAAGTTGTTGTGAAATGTCATAGA
CAAATGGCAGAGATTGCTGTTGATGCTATCCTATCAGTAGCCGATTTAGAAAAGCGGGATGTAAATTTTGAAC
TAATCAAAGTCGAAGGTAAAGTAGGTGGTCGCATGGAAGACTCAATGTTGGTACGCGGTGTTGTCATCAACAA
GACCATGAGTCACCCCCAGATGCCAAAAGTTTTGAAGAACGTAAAACTAGCAATTCTG
ACCTGTCCATTTGAGCCCCCTAAGCCCAAGACCAAGCATAAGCTCCAAGTTGGCTCCGTCGAGGAGTACAGAG
ATCTTAGGAAGTATGAGCAGGAGAAGTTCCTGGAGATGGTCAAGAGG
>1002052429_2 Heli.2-DT663234.3.5
TCGTACTACGCGCTATATTATGACAGTAACGTGATGGAGGGCAAAGAATTCGGTGCGGTCCCCGGCTTGCCCA
CCGGCCTGGACTACTTCATGATGCCCCGTGTGAGCCAGCCGGCGCCGCAGTTCGACTTTAGAAAATTAGGCGC
CAGTTTCAGCGGTCGGGATGAAGATAAATGTGAAGAGCGACGAACCCCCGAGTACGTC
CCGGAGCGTCGCGACCCACCGCCCGCGCCGTGGCCTCTCGGGTTGGGGGTTCAATTCGTGAATCCCGCTACGG
GAAAGAAGAGAGTGCAGTGCAATGTTTGCCTTAAGACTTTTTGCGATAAAGGCGCATTAAAAATACATTTTTC
CGCGGTGCACTTAAGAGAGATGCACAAGTGTACAGTGGAGGGGTGTACTATGATGTTT

AGTTCGCGGCGTTCTCGGAACCGGCACAGCGCTAATCCGAATCCTAAGCTGCACTCACCGCACGTTAGACGTA
AGATCTCTGCCCACGATGGTCGATCTGCGCAGCCGTTCCCCCTGCTCCCAGCGTTGGCTCGGTTACCATTGCC
GCCGCCTGCGCTTCTTCCGCCTGAACTAGCAGCTCGTCTTCCGCCACCTCTAACTGCA
CCACCTCCTAATCATCTACCTCCTCGTGTACCTCTCGATGATCTCCGAAACTTCAGTGAAATCGAAAAAATGT
ACAGAAAAAATACCTTCTCCAGAAAATCAAACTCGTCACCCATTAGATCTTGTGAAACCTGTGCTACATGAAGA
GGAATCAATTAATTACAGTGAAAACTATGAAGACGGTTCGGATAATGATAATAAGGAC
GAAATTAAATCTCAAATTAAACCTGAAGATCAAAACCGGTTAAACTACGTCGATGAACCCGAAGACCTAAGTG
TAAATAAAAAGAAAGAAGATGATATTAAACCTAAGCAATCTAATCCTAGTGTAAGTACTGACTTGACCACCAC
AGATGATAAATCTCCTTTAATTCCAAACAAACGTAAAAGGAAAAGTGGAAACCCCACG
CGTTGCTCACAAAATAATGAATATAGTGTATCAGATGAAGAGTATAATAGTGATTTATTTAGAAATATGTCAA
CGCCCAGTTCTAGTCGAACAGACGAGGAACCTTTGTCTCTAAAAAAACAAAAGCCAGAAAAATGGGAGCCATT
TCAGTCCAATGAGGAAGCGACTGTGGACTCGGAATCAACAACGAGAGTAAAGGCAGAG
AGTGAGTCCGACGATGAATCGAGTGCGCCCAGCGTGGTTCGGGAAGGATTAAGGTTACGTTCTGATCTCTATA
CACCCAGTGACAGCGGGAGTGATTTACATACCTTAGAAGAGAGACTTGCTCGTGTTAGATCTCCTTCAGCCAG
CAGTGACAGG
>1002052430_2 Heli.2-DT662927.3.5
GCACGAGGTGACACTGCAGCGGACGGTAATAATACGCGAAAATGGACGGAGAAACACACAGCCTGGAAGCCAT
TATTAACAACAACCTCACTGGTCGGGATCTGGATGAATTTAACAGAATCTACTACGGCCGAAAGAATCATCTA
GAAATCAAAATTAAAGACTCATCTCTTGCTGCCGCTAAAGACAATGACTTCGATATAG
CAGCATATGCTTTTCCAGCAAAAAAAGAATCCACAAGACCACCCAGAATTGTGAAAGTGGGTGTTATCCAACA
TTCCATTTCCGCGCCGACCGACCGCCCGATCAATGAACAGAAAAATGCAATTTTCGCTAAAGTAAAGAAAATC
ATCGATGTCGCCGGACAAGAGGGCGTCAATATTTTATGTTTCCAAGAGTTATGGAATA
TGCCATTTGCTTTTTGTACACGTGAAAAGACGCCGTGGTGTGAGTTCGCTGAATCTGCTGAGGAAGGGCCCAC
AACACGCTTCCTTCGCGAATTGGCAATTAAATACTCAATGGTAATAGTTTCTTCTATTTTGGAACGCGACGAA
AAACATGCTGATATTCTCTGGAACACTGCGGTCGTTATCAGCGATACTGGCAACGTGA
TCGGCAAACACCGTAAAAACCATATTCCAAGGGTTGGAGATTTCAATGAATCTAATTACTACATGGAAGGTAA
CACGGGACACCCAGTGTTCGCGACGAGATATGGTAAAATAGCTGTTAACATTTGCTTCGGACGTCACCACGTT
CTTAACTGGATGATGTTTGGACAGAATGGAGCTGAAATTGTATTTTATCCATCAGCTA
CGATCGCTGCCGATGCTGGAAGCG
>1002052431_2 Heli.2-EL604454.1.5
AATTTGGATTCGGTTGGCGGGCGCGGCCATCTTGGTGTAATAGTGACCTGGCGATTCGGGTTTAGTTAGTGAA
AAATCATTTGTAATCCACCATGTCCGAAAGGGAAGATAACGTGTATAAAGCTAAGTTAGCTGAGCAGGCCGAG
CGCTATGATGAAATGGTGGAGGCAATGAAAAATGTGGCCTCACGAAATGTGTCAGATA
ATGAACTCACAGTTGAAGAGAGGAACCTCCTCTCAGTGGCATACAAGAACGTGATCGGGGCGCGCCGGGCGTC
GTGGAGGATTATCTCTTCAATTGAACAGAAAGAAGAAACAAAAGGCGCGGAGGACAAGCTCAACATGATCCGC
GCGTACCGCAGCCAGGTGGAGAAGGAGCTGCGCGACATATGCTCGGACATACTGAGCG
TTCTCGACAAATACCTCATACCCAGCTCGCAGACCGGCGAGTCCAAAGTTTTTTATTACAAAATGAAGGGTGA
CTACCACCGGTACCTGGCGGAGTTCGCGACGGGCAACGACCGCAAGGAGGCGGCGGAGAACTCGCTGGTCGCG
TACAAGGCGGCCTCCGACATCGCCATGACGGAGCTGCCACCCACCCATCCAATACGCC
TCGGCCTCGCCCTTAACTTCTCAGTATTCTACTACGAAATCCTGAACAGCCCGGACCGCGCGTGCCGGCTGGC
GAAGGCGGCGTTCGACGACGCCATCGCCGAGCTGGACACGCTGTCGGAGGAGAGCTACAAGGACTCCACGCTC
ATCATGCAGCTGCTGCGCGACAACCTCACGCTGTGGACGTCGGACATGCAGGGCGACG
GCGAGCCGGGCGAGCCCGAGCCCAAGGAGCCCGCCGCGCCCGACGCCGACGCGGACGACGTGTCGTAATGCGC
CCGCCCGCCCCGCCCACCCGCCGCCGCACAAACACACAGCCCCCTTTACTTGCGAGTATTGTGATAGAGATTT
TTTGTATATT
>1002052432_2 Heli.2-DT666661.3.5
CTCGCAGCACAATGGTGGCTTCAACGGACAAAAAGGCTGCGGCCGACAAGAAGGCTCCGGCTGAGAAGAAGGT
AGCGAAAGGCAAGGAGGACAGCAAGAAGCTGCCAGCGGTCCCCGAGTCAGTACTAAAGCACCGTAAACGCAGA
GATGCTCTAAGAGCTCGTCGCATACAGGTAACAATCAAAAGGCGTTCAGCTGCCATCA
AGAAGAGGAAAGAAATCTTCAAGAGGGCTGAACAATATGTTAAGGAATACCGCATCAAGGAGCGTGATGAGAT
CAGACTGGCTAGACAAGCACGCAACCGAGGCAACTACTATGTTCCTGGTGAAGCCAAGCTTGCCTTTGTCATC
CGTATCCGTGGTATCAACCAAGTCTCACCAAAGGTCCGCAAAGTACTAAAACTGTTCA

GACTGCGTCAAATCAACAATGGTGTGTTCATCAAGTTGAACAAGGCCACAGTGAACATGCTCCGTATCGCGGA
ACCCTACATCACATGGGGATACCCGAATTTGAAGAGTGTGCGAGAGCTCATCTACAAACGCGGTTTCGCCAAA
GTGAAGGGACAACGCGTCGGCATCACGTCCAATAAGATCATTGAGGAGAAGCTCGGCA
AGAGCGATATCATCTGCGTTGAAGACCTTATCCACGAGATATTCACTGTGGGAGACAAGTTCAAGTACGCAAG
CAATTTCCTATGGCCATTCAAGCTAAACAACCCAACCGGAGGCTGGCGTAAGAAGACCATCCATTATGTAG
>1002052433_2 Heli.2-EL597465.1.5
ACGAGGCTCTTGAGCAGTTCTAGTGAGATACCACCCAACGCCATCAATATGAAATTCTTCGTGTTCTTCTTCT
TGTGCTGTGTTGCTCTGGCATCTGCTGCTCCCCAGTTGCTCACCTGGCCTGGCGTCGTCGCCCCTGGGGTATT
GGGACCTCGGACCATCATAGCCGGACCCACCGTAGTGAACGGAGTGGACCCAGTCGGC
CGGGTCGTTCTCCCCGGGGTCGTCGGGGTCCTCTAGGCGTCAGGCGTCAGCAGCTAGGCTTAAATTTACAACG
ACACAGCTTTACACCTGTCCGGACATCACCGAAGTTATTGGCACCCAAATTATTATGTATCGTATACGAATAA
ACGCATGCTCAATTTAAAAAAA
>1002052434_2 Heli.2-DT663853.2.5
ATGGGTATAGCCGTGATCACTGGCATAAACGAAGGGCCACTGGTGGCAAACGTGCGCCCATTCGTAAGAAGAG
GAAGTATGAGTTAGGTCGCCCGGCTGCCAACACTAAGCTTGGCTCTCAACGTATTCACTTAGTGCGAGCTCGT
GGAGGTAACATTAAGTACCGTGCTTTACGTCTTGACACTGGAAACTTCGCATGGGGCT
CAGAGTGTGCTGCTCGTAAGACTCGTATTATTGATGTTGTGTACAATGCATCAAACAACGAGCTGGTTCGTAC
TAAAACCCTCGTAAAGAATGCCATTGTAGTAGTTGATGCTACACCATTCAGACAGTGGTATGAGTCTCATTAC
CTACTGCCACTTGGTAGAAAGAAGGGTGCCAAGTTGACTGAAGCTGAGGAGGCAATTA
TCAACAAGAAGCGCAGTAAGAAAACAGCAAAGAAATATTTGTCAAGGCAGCGCCTGTCCAAAGTGGAAGCTGC
TCTTGAAGAACAATTCCACACTGGTCGCTTATTAGCTTGTGTAGCCAGCAGGCCTGGTCAATGTGGCCGTGCT
GATGGCTATGTCCTTGAAGGAAAGGAATTGGAGTTCTACCTAAGAAAGATCAAATCCA
AGAGGGCAAAGTAGGGTTTTAATGTATAAATAAAAGTAAAAACAACAAAAAAAAAAAAAA
>1002052435_2 Heli.2-DT667068.3.5
TTATAGGCGGTTAACACTGCCATGCATAAAATCAAGATTACAAGATCTTGATATGCGGTGATAGTCTCCAGAA
TCAAACCAGTTCAAACAAAAATTAATACAACCTCTTTCCAGGCCAAAAAAAACGCACAAATTACTCACCGTAC
GGGCAAAACTGCACGCCAATAAAAAAATTGGCACAGGCTTATTATTCTCGGCACACCG
AGATAGGTCTTCCAATAAATCATTGTATAGAACGTTTTTTATAAACATTTATTCAAAATGTCTGAAACCAAGG
ACGGTATCGAGGATATATGCAAACGCGTATCCGAATTATCCGTTTTATCGTTCAGAAAAGTGGATGAAATAAA
AAAGTTGGCTAAGCATTTCAAATTTGTGATAAACAATCCGATCACTCAGCAAAATGCT
GTTGTTTTGCGCTACTTGAGGGATTTAGATGAAACCCATCGAAAAATCTTCCTCAAAAGACGGGAAGGTGTTG
AAGTCGAATATATATTTACGACTGAGAAGAAAAGTAACCTCCAAAGACAACGTTTCAACAGTCTTCCCGTTAG
TGAAGTGCCGGAGTTATTGAAAACTGAATTTTTGATAGCCGACACTGCCAAACAAGTC
CACATTTGTACAGACTGTAACGTAGAAATTGAGTTAAACGACGAGATTCCGCTAATGGAGTCGCTTCAAAAGC
ATTTTAACTTGGAGGTGCATTTGCCGAAGTTGAAAAGAGAGAGTATTGACGTTGCAGAACCAATAATCTTACC
TAAAATGTCC
>1002052436_2 Heli.2-DT668889.3.5
CAACATGGCTCGTGGACCCAAAAAGCATTTGAAGCGTTTAAACGCGCCCAAAGCATGGATGTTGGACAAACTA
GGAGGTGTGTACGCACCTCGGCCGTCCACTGGACCGCACAAACTACGCGAGTGCTTGCCGCTTGTTATTTTCC
TTCGTAACCGTCTGAAGTACGCCTTGACCGGTAACGAGGTACTCAAAATCGTAAAACA
GAGACTGATCAAAGTAGACGGCAAAGTACGCACCGACCCCACATATCCCGCGGGATTCATGGATGTGATCTCA
ATTGAGAAGACAAATGAGTTGTTCAGGTTAATCTATGATGTCAAGGGTCGCTTCACCATCCACCGCATCACAC
CTGAAGAAGCAAAGTACAAGCTGTGCAAGGTACGCCACGTGGGCACCGGCCCCAAGAA
CGTGCCATACCTGGTGACACACGACGGCCGCACGCTCCGCTACCCTGATCCCCTCATCAAGGTCAACGACTCC
GTTCAACTAGACATCGCCTCCGCTCGCATCATGGACTTCATCAAGTTTGAATCAGGCAACCTGTGCATGATCA
CTGGTGGGAGGAATTTGGGTCGTGTGGGTACTATCGTATCCCGTGAGCGGCACCCGGG
GTCCTTCGACATTGTGCACGTGCGTGATTCAAGTGGACACACGTTCGCCACGAGGCTGCACAACGTGTTCATC
ATCGGCAAGGGCACGAAGCCGTACATCTCGCTACCGCGCGGTAAGGGTATCCGCCTCACCATAGCGGAGGA
>1002052437_2 Heli.2-EL598992.1.5
GCACGAGGTGGCATTCAAGTTTGTGGTTCTCGCCTGTTTCGTGGCTGCTGCTAGCGCTGGTATCTTACCGGCT
GCTCCCATCGCCTACGCTGCATCTCCACTCATCCACTCAGCTCCCGTGGCTTACGCCGCCCCCGTAGCCAAAT
ACGCAGCCGTCGCACCCGTCGCTAAGGTTGCCGTCGAAGAATACGACCCACATCCCCA

GTACAGCTTTGCTTATGATGTACAAGATGGTCTCACTGGTGACTCCAAGAGCCAGCACGAATCTCGCGATGGA
GATGTAGTTCAAGGTTCCTACTCCGTTGTCGACCCTGATGGTGTTAAACGTACCGTCGAATACACCGCCGACC
CCCACAACGGTTTCAACGCTGTCGTGCACAGGGAGCCTTTGGCAGTTAAGGCAATCGC
CCCAGTCGCTAAAATCGCACCTGTAGTGCACGCTGCCCCCGTGGTACACGCTTCTCCAGCTGTATACTCCTCT
GGCCCCTTGGTGCACGCCGCACCTCTGGCCTACAGCGCCTCCCCTGTCGTCCACGCTGCCCCCATCGCGAAAC
TTGCGTACTCTTCCCCTTACTACCACTAAATAATGCCAAATATAAATTAAATCATGAA
CTGTTGTGACTACTAGTGATGTTCTAGAGATTTCTCGACATTTTTAGGATAAGTAAATTAAATCGTTTTTTAC
CGTCAAAAAAA
>1002052438_2 Heli.2-DT665501.3.5
CACGAGGCAACACTTGAAAATATGAAGGCTATAGTTTGTGTACTCCTTTTGGTCGCGGCCGTCTACGGTGCAG
AAGAGAAAAAGCAAGAGAAACGTGGCCTGCTAGGTCTCGGGTACGGTGGTGGTCTCTCTCTCGGTGGCCACGG
TCTGGGTCTCTACGGTGGCCACGGTCTGGGTCTGGCCGCTGCACCTATCGTTAGCCAT
AGCGTCGCCATCCCTGCTCCTATCATCAGCCATGGCATCGCCGCTCCCATCCTAGACCATGGTCTGATCGGTG
GTCACGGTCTAATCGGTGGTCACGGTCTGATCGGTGGTCATGGCTTGATTGGTGGTCATGGACTCATCGGGGC
ACCACTGCTAGGCCTTGGTCATGGCGGCTGGCGTTGAGGCTACACTATGTAAATAAAA
AAAAATCAAAATTAATCGTACATCTTGCAAGCACACACGTTGCGTGGGTCTTTCAGTGCCAAATTAGTTTTTT
TGGGTGTTTCTATATTATTAAGGTGAATAAAAATTAAAAACATAATAAAAAAAAAAAAA
>1002052439_2 Heli.2-DT668438.3.5
AGCAACCCTCAAGGAGCAGAGAATTATAGTTTTCTAGTGACTGGCCCACGCGAACCGGGCACAAGAACAATCA
AAATGAGCTTCCCTGCCGCCGAAACTGCTCTGGGGAGCAGCACATGGGCAGCAACCAACCTTTTCTACGTATT
GTTGGTGCCAGCGATCATCTTGTGGTACACCTACTGGCGTTTATCCCGGAGGCACTTG
TATGAGCTCGCAGAAGAAATAGCCGGGCCCAAAGGATTACCACTTATAGGAAATGCTCTAGAATTTACTGGGG
GCTCTGCCGAAATTTTCGACAAAATCATACAAAGAAGCGAGGAATTCAAACAGGAAAGTGCTGTGAAAATGTG
GATTGGACCCAGACTTATTGTGTTCTTGTACGATCCTCGCGACGTAGAGCTTATTCTA
AGCAGTCATGTACATATCGATAAAGCCGAAGATTACAGATTCTTCAAGCCTTGGCTTGGAGATGGTCTTCTTA
TCAGCACTGGTCAAAAATGGCGTTCTCATCGCAAATTGATCGCACCTACATTCCATTTGAATGTTCTTAAGAG
TTTCATAGATCTATTCAACGCTAACTCCAGAGCTGTCGTTAACAAACTGAAGAAAGAA
GCTGGAGAATTCGACTGTCACGATTATATGAGTGAATGTACCGTGGAAATCCTTCTTGAAACTGCTATGGGTG
TTAGTAAGAATACTCAAGACCAAAGTGGATTTGAATATGCTATGGCTGTGATGAAAATGTGCGACATCTTA
>1002052440_2 Heli.2-DT665413.3.5
AGTTGAGAAGATTCTCTTGATTCTTTTTGTTATTATTTAAATATATAAAACAGTATTCAAAAATGGCTTTACA
TATACCAAAAGCCCCTGGAGTACCGCAAATGTTAAAAGATGGAGCACGAATGTTTTCTGGCTTAGAAGAAGCG
GTATATCGCAATATAAATGCGTGCAAACAATTTGCCCAAAGTGTTCGTTCAGCTTATG
GTCCTAATGGGATGAACAAAATGATTATCAACCACATCGACAAGCAATTTATTACAAGCGATGCCGGTACTAT
TATTCGTGAATTAGACGTAGAACATCCAGCTGCTAGACTGATGGTACTGGCCAGCCAAATGCAAGATTCTGAA
GTCGGTGATGGAACCAACTTTGTCATAGTTTTATCTGGGGCACTCCTTGAGGCTGCAG
AAGAGTTATTGCGTCTAGGTGTCACTACAAGTGAAATTGCAGAAGGATATGAAAAAGCCCTTGATAAGTGCCT
AGAAATATTACCAGAATTAATTTGCCACGAAATAAAAGATTGCAAAGATATTGAAGCTGTGGTTCATGCTATA
AAACCAGCAATTATGTCAAAACAATATGGAAATGAAGAATTTATTGCAAGTCTTGTAG
CCAAAGCTTGTGTCGCAATCTTACCTGAAAAAACTACATTTAACGTAGATAATGTGAGGATATGCAAAATACT
TGGAGCTGGCCTGTTACAATCTGAAGTGTTATCAGGAATGGTATTCAAAAGGGAAGTTGAAGGAGACATAACA
AGTGCTAATAAAGCTAAAGTAGCAATATATTCATGTCCAATTGATATAACACAAACAG
AAACAAAAGGAA
>1002052441_2 Heli.2-DT668373.3.5
GCACGAGGACAACACAAAGATGTACGCTAAGTTGATCGTCGCACTTTGCGCTTTAGGCGTTGCCCACGGCAGT
GGCCTCCTCGCGGCCGCCCCGCTAGCGTACAGCAGCCAAGCTATTGTCGCTCCCGCGGTAGTGTCCAAGACTG
TTGTGTCCCCAGCTGTCTCGTCGGTGTCTTCCTACTCCAGCCACACCGCCCACTCCGG
CCCCGTCGCCACCTATGCCGCCGCCCCAGCCATCGCCAGCTATGCCGCCCCAGCGATCACCAGCTATGCCGCC
CCAGCGGTCGCTAGCTACGCCGCCGCTCCCATCGTGAGCAAGACCGTCGTGCAGCCAGCCAGTTCTTCCTACT
CTTCTTACTCTAGCAGCGTTTCCCACGGAGCACCAGCAGTCGCATCTTACGCCGCCGC
TCCCGCCGTAGTCGCCGCCCCCGCCGTCGCCGTGTCCCGCGCTGTCCCCGCCGTGTCCGTCGCCGCCGCCCCC
GCCGTAGCCGTCTCTCGCACTATCTCCCCCGCCGTATCTTCTTACTCTTCCTACTCCAGCCAAGTCGCTCACG
GTGTTAAAGCTATTGCTGCCCCAGCGGTCGCCTCCTACGCTGCCCCAGCGGTAGCCTC

TTATGCTGCCCCAGCAGTCGCCTCTTACGCTGCCCCAGCCATCGCCTCTTATGCCGCCCCCGCGGTCGCCTCT
TACGCCGCCCCCGCGGTCGCTTCTTACGCCCGCCCCGCCGTCGCCTCTTACTCTAAGATCTACTCTGCGCCCG
CCATCTCCTCTTACTCTAAGATCTACTCTGCGCCCGCCATCTCCTCTTACTCTAGACT
GTACTCCGCCCCCGCAGTTGCCTCTTACGCCGCCCCCGCTACTCTACTACGCCGCGGCCGCCCCGTCTACAAT
CAGCTATCTCTACTCGGCTGCCCTGCTGTATCTAC
>1002052442_2 Heli.2-DT665998.3.5
TCTCAGCCGGTTAAAGCATTTTCAACATATTTAAAAGAAATAACATCTAATTTATAATTTTAATGATACTTCC
CTATTAAGTTAGATCAAGTTTATTTCAACCTTACCGCATTTAAACTACACATCAAAAATGAAGCCGCCATACA
AAATTGCTGATGAAAAGTTAGCTGAGCTTGGACGTAAGGAGATCATGTTAGCTGAGAA
AGAAATGCCAGGATTAATGGCTTGCCGTAGAAAGTATGCTCCAGCAAAGATTCTGAAAGGTGCAAGAATAGCT
GGTAGCCTTCACATGACTGTACAAACTGCTGTACTTATTGAGACTCTTATTGAGCTTGGAGCTGAAGTCCAGT
GGTCAAGTAGTAATATTTACAGCACACAAGATGAAGCAGCAGCTGCATTAGTGGCAGT
TGGAATTCCAATCTATGCCTGGAAAGGGGAAACTGAGGAGGAATATGTGTGGTGTATAGAACAAACACTCATC
TTCCCTGATGGAAAGCCCTTAAACATGATATTAGATGATGGAGGTGACTTAACCAATTTAATTCATACCAAAC
ATGCAGAACTTCTAGAAGGTGTTCGAGGTATCTCTGAAGAAACAACTACAGGTGTCCA
CAACTTATACAAAATGTTCCGAGAAGGTCTATTAAAAGTGCCAGCTATCAATGTAAATGATTCTGTCACAAAA
AGTAAGTTTGACAATTTATATGGATGCAGGGAATCACTTCTTGATGGTATTAAGAGGGCAACTGATATAAT
>1002052443_2 Heli.2-DT668061.3.5
TCGCCAGAGGGGTCGGACCGCAATCTAGTCATTGTATTTTTAAATATATTTTTAATATCTTAATCAAAAGGTC
ATCATGAAAGTGTTCATCGTGTTGGCAGCAGCCGCCGCCCTTGCGAGCGCCCAATTCAAATGCCCCAACAAGG
ATGGCCAATACGAGGATGACAGACAGTGTGACAAATTCTACGAATGTTCTGATGGTGC
TGCTGTCACCAAATTGTGTCCCGATGGTCTTGTGTTCGACCCCACCATTAGAAAGATCAACAAATGCGACCAG
CCCTTCAATGTAGACTGCGGCAACAGAGTCGAGCTCCAACCCCCCAAGCCCAGCGCTCAATGCCCCCGTCGTA
ACGGGTTCTTCGCTCACCCTGACCCCACCGTCTGCAACATCTTCTTAAACTGCATCGA
AGGTGAAGCTATCGAAGTTAAGTGTACCGCCGGCCTCCACTTCGACGAGTACTCTGGTACCTGCGTGTGGCCC
GATACCGCTGGAAGACAAGGATGCGTCGCCCAAGAAAAGAAGACCAAGGATGGTTTCGAGTGCCCCAAGGAGC
AACAAGTTGATGCCCAAGGCTTGGCCGTCGCTCACCCCAAGTTCCCCCACCCCAATGA
CTGCCAACGTTTCTACGTTTGCTTGAACGGTGTAGAACCCCGTGACCTTGGTTGCCAAGTTGGTGAAGTATAC
AATGAAGAAAGCCAGAAATGCGACGCCCCCGAGAATGTTCGTGGATGCGAGGACTGGTACAAGGACTCCGAAG
ACGCCGCGCC
>1002052444_2 Heli.2-DT662995.3.5
TGTGTTCAACAGTTACCATCGGATATTAGACATTAAAAATGTTCTCATCCTTGCTGGAGACGGCGCGGAACTC
GCCCTTCCGCAGCCCTTTGACTACCGCGAGCTGCGAACCGGCCCTTGTTGATTCACAGGGCATGGCCGCGTCC
GCTCCAGTCCAGGGCGACTCTTGCGAGTTCGGTTCACCCAAATACTTCGCGCTTTGCG
GACTTGGAGGAATTCTTTCTTGCGGTATAACACACACAGCAGTGGTGCCCTTGGACTTAGTAAAATGCCGTCT
ACAGGTGGACCCCGACAAGTACAAGAATGTGGTCAACGGGTTCAAGGTGTCCGTACGGGAGGAGGGCATGAGG
GGCCTCGCCAAGGGCTGGGCACCCACCTTCATTGGATACTCTCTACAGGGACTCTGCA
AATTTGGCTTGTATGAAGTGTTCAAAGTAGGTTACTCTGGCCTCTTAGATGAAGAGATGGCTTACAGCTATCG
TACGTTTGTGTACTTAGCTGCGTCTGCCTCAGCTGAATTCTTTGCTGATATTGCCCTTTCTCCATTGGAAGCT
GCTAAGGTCAGAATCCAAACTATGCCTGGTTTTGCGAACACCCTAAGGGAGGCGTGGC
CCAAGATGGTACAAAATGAAGGTTATGGCACATTCTACAAGGGCTTAGTGCCTCTGTGGGGAAGACAGATACC
ATACACCATGATGAAGTTTGCCTGTTTCGAGAAGACCGTGGAATTGTTGTATAAGTATGTAGTACCCAAGC
>1002052445_2 Heli.2-DT663065.3.5
AACACGTCTTGCTGCGTAATTTCTCGAATTCTTACGGCCAGTCCGTGTTTTTGATTGATTCGTTTTGCAAAGG
CTTAAATACGATTTGCCAAGATGACCAAGTTGATACAACAGTTAAATTTTAGTAGTAGTAATTACTACGACGT
TGCTGGTGTCGTAGTTGAACCCCAAACCCCGAAAGCGAAGACCTATCGTGGTGATGGC
GCGTCCTTGAACGCGGCCGGCAGCAAGCGCACCGCTTTGTCGGCGTCCGACGCCGAGTGTTTCTCGCTGTGCC
TGGGCGCCGGGCCTCTGTGGTGGTCCTGATGTCCCTGCTCTCGACTCCGCACCGCCCGGAGGGGTGAACAGCG
GGGCCTCGAGCCCCGCAACCCCGTCCACCCCCGTACACGACGACAACAGCCGTGACTT
GTTGAGCGCCTTGCTATGGGCGAGCGCGAGCTCTCTAAGCACCAGCGCGGAGAGTCTCACCCCCGAGCCCGTG
AACACACACCCAGCACTCAACCTCGCTGAGAGACAGACGGTCATCAATAAGATCTTGGAACTCAAGGACAAGC
AACCGGTTAAGATCGACTTTAAAATCTTCCTTACCGAAAACACTGTTGTGCGTTGGGA
GGCCGTAATCAAGGGTAATACCATGTACCTACGCGTCCCCGGCGTACTGCAAGCTGGTAGCAAGGAAAAGTTT
ATGCTTTTGCT

>1002052446_2 Heli.2-DT665352.3.5
GCACGAGGTATGGCTCCACCTCAGCCCAAGGCGGTGGCCAAGGCCCCCGCAGCCCCAGCGGGCGGAGTTAAGA
AACCAGCGAAGGTTGGCAAACCAAGGAATTATGACTTAGGAAATGGAATCGTCAGATTCTCCAAGGCTAAAAT
GTTCCACAAGAAGGCTAAGTACAAGTTCATTGGCAAGAAACACCCAAAAGCACCTAAG
CCCAAGAAGCCCTCAGTTGTAGTAAAGCCTATTGGTGGTGAGAAGAATGGAGGAACCCGTAAAGTACTCTTGC
GTCGCAGGAAATCTTTCTACCCCACTCAGGACAAAATCCGCAAAATCCCCCACCACAAGACGTTCAGTAAACA
CGCGAGAAACATTCGCCCCAGCCTCACAATTGGTACAGTGTGCATCCTCCTGGCGGGC
AGACACGCGGGCAAGCGCGTCATCCTGGTGGGAGTACTGCCGAGCGGCTTGCTGCTTGTTACTGGGCCTTTTG
CTTTCAACTCATGCCCACTCCGTCGTATTCCTCAACAATATGTGATCGGTACCAGCACTAAAGTGGATCTCGG
AGACTTCAAGTTACCGGCCCACTTAGACGATGCCTACTTCAAGAAGAACAAGAAGAGC
GTCAAACGTAGTGTCAAGCGTAAAGAGGGAGAAGATATCTTCGCTTCAAAGAAAGAGAAATACGTTCCATCAG
AACAACGCAAGTCGGACCAGAAGTCAGTAGATGAGGCGGTCATCAAGGCGATCGGCAAGCGAGCTGACAAGAA
GATCCTCCGCGGATACCTCAAGTCTGTGTTCGGTCTGCGATCCAGCCAGTTCGCGCAC
CGACTTGTATTTTAGATACATAATATAATAAATAAC
>1002052447_2 Heli.2-DT665624.2.5
AAGTTTTAACCCTGTGGTGAAGTGGGGTTATCGCAATCATGGGGAAGGGAAATAATATGATTCCTAATGGCCA
TTTCCATAAGGATTGGCAAAGATTTGTAAAAACTTGGTTCAATCAGCCTGCTCGGAAACACCGCAGGAGACAA
AACAGAGTTAAGAAAGCAAAGGCTATTGCACCTCGTCCTGTCGCCGGACCCCTTCGTC
CAGTTGTTCGCTGTCCAACTGTGCGATACCATACAAAAGTTCGCGCTGGGCGCGGATTCACTCTTCGTGAAAT
TAGGGCTGCCGGCCTGAACCCTGCTTTTGCTAGGACTGTTGGAATTTCTGTAGACCCAAGAAGACGCAATAAA
TCTGTGGAATCACTACAGATTAATGTACAAAGATTAAAGGAATATCGTGCCCGTCTCA
TTCTTTTCCCTAAGGGCAAGAAGGTACTGAAGGGCGAAGCATCCGAGGAGGAACGTAAAGTAGCAACACAACT
TCGTGGACCTCTTATGCCTGTAGAGCAGCCAGCACCTAAATCTTCTGCCCGTGTTATCACTGAAGAGGAGAAG
GACTTCAAAGCATACCAATACCTCAGAGGAGCTCGTTCTCTTGCAAGGCTTGTTGGAA
TCCGGGCTAAGAGGTTGAAGGATGCAGCTGAAAACCCAGATGATGTGACTAAAGCTCCATCTGCAGTTAAGGA
AGCTAAAAAGAAGTGATTTCTCTTAAATAAACAAATAAAATCTTAAAAAAAAAAAAAAA
>1002052448_2 Heli.2-EL604074.1.5
ACATTACCAACCAACTATACTACAGTATATTCTTATACGCGGTGACTTCACGGCGAGAACCCGTGTCTGTGAT
TTAATTTATTTATTGTACAAAATAGTTTTAGTTAAAATAACTTACTTAGAAAACATGGCCAACAACAGGGCCG
CCAAATCTGGATTTGCCGCCGAAGCCCAGAGAAAGATTAACAGCAAATACAGTGAAGA
GCTGGCTGAGGAGTGCTTGGAATGGATCCGAGTGATTACCGGCGAGCCGGACAACACATCTGGCGACATGGAC
AACTTCTATGAAGTGCTGAAAGACGGAACTGTACTTTGCAAACTCGCAAATACCTTGAAGCCTGGTATTGTGA
AGAAGATCAACGAGTCTAAAATGGCGTTTAAATGTATGGAAAACATAAACGCTTTCTT
AGAAGCGGCCAAGCAGTTCGGTGTGCCCGCACAGGAAACTTTCCAAACTGTCGACTTGTGGGAGCGACAGAAC
TTGAACTCTGTTGTCATATGTCTACAGTCTTTGGGTAGAAAGGCGGGCAACTACGGCAAACCTTCGATAGGAC
CAAAAGAAGCTGAGAAGAACGTGAGAAACTTCACTGAAGAACAACTCAGAGCTGGTCA
GGGGGTCATTTCCCTTCAATATGGCTCCAATAAAGGTGCCACACAAAGTGGAATAAACTTTGGCAACACAAGA
CATATGTAAAGCTTTACTAATACTTGTCTCTTTCTAATACACTAGATTAATGGTACAGAGAAATGGACAGAAA
TTTATGCTTAAAGTTCAAAATTTATGTAACGTTT
>1002052449_2 Heli.2-DT663329.3.5
GAAACGTCTCTATTTATCGGCCAACTTTATTTAACATACGGTTTTAGGTTAATACCGGGAAGATGGGTGTTAA
ACCTGCAATTGGAAGGAAATCTAACAAGAACCCCCCAATTTTTAGCCATGAATTTGTTATCCAGAACCATGCA
GATATTGTTTCATGTGTTGTAATGGTTTTCCTGGTGGGGCTTATGGTACAGGCCACAA
GTCCCGTTGCGAGTTTATTCATCAGCTTGCACCATAATGTTACTGGAGTAGAACCGTCGCGCGATCTTCCTAA
GGGCGAACCTTTCTCGTATGAAGCTGGTTGGAAAGATGCCTGTGCTGTTTTCTTTTACTCACTAGTATGCATT
GTGATGCATGCAATTTTACAAGAGTATTTCTTAGATAAAATATCAAAGAAATTTCACC
TATCCAAGTCAAGATTAAGTGCTCTAAATGAATCAGGCCAGCTTGTAGTATTCCAACTAGTTACATTGCTTTG
GGGAGGAGATGCTATTCTTCGTGAAGGATTTATTTTCAATATTTCTCAGCTATGGGATGGATATCCAAATCAC
CCAATGAGTTTTCTATTGAAGTTATGGTGGGTTGTCCAAGCTTCTTACTGGGTTCACA
CCATTCCTGAGTTATACTTCCAAAGAATTAAGAAAGACGAATGGTCAGGACGCATAAGACATGCTGCAGCTGC
ATTTGCATTTGTAGCTCTAGCTTATGGTTTCAAAT
>1002052450_2 Heli.2-DT665411.3.5

GCACGAGGCTTTTTCTTGCTGTATATTTATTCACACATAAGATGCAGGCGTGGTTGGTGGTGGCGTGCGCAGC
GGCGCTAGCAGCGTTGGCGTCTGCCCAAAAACCCGGACGGTTCCTCTCCCTACCGATACCGCAGAAATGCGCT
AACAGACCCAAAGAATTTTCTTATCGCGGACACAATTACTTCTACACCGGCCACGTTC
CTGAGCTCGCTAATAGGAAATTCGACTGGCTCGATGGACGCAATATTTGCCGTGAATATTGCATGGATCTTGT
TTCAATGGAAACACAAGAAGAAAACAATCTGATTTTCAAACTAATTCAACAAAGTGATGTCCCGTATATCTGG
ACCTCGGGTCGTCTCTGTGACTTCAAGGGATGCGAGTCGCGTCGAGACCTCGAGCCTA
AAAATATTTTGGGTTGGTTCTGGTCCGCTAATCGCGAAAAGATATCAGCAACTAATCAGATCTCACCCGGTTG
GGGTTACAACCCGTGGTCTCAAACCGGTCACAAAAAGCAACGTCAGCCCGACAACGCCGAGTTCGATATCAAT
GGAACCACCGAATCATGTCTGAGTGTTCTGAACAACGTATACAATGATGGTATAGCAT
GGCACGACGTCGCCTGTTACCACGAAAAGCCAGTTGTATGTGAGGACTCAGAGGAGCTCCTCAATTACGTCGC
GAGCACCAATCCTGGCCTTCGCTTGTAATTGAATAACATATTTAAAT
>1002052451_2 Heli.2-DT668453.3.5
CGCTTTTGCGGCGTAGTCTGTGATTTTTTAGTGTAGCTCCAGATAATCCAAGTTAATCTACGATGGCGGATCA
GCTGACAGAAGAACAGATCGCGGAGTTCAAGGAGGCGTTTTCACTGTTCGACAAAGATGGCGACGGCACCATC
ACCACCAAAGAATTGGGCACCGTGATGCGCTCGCTAGGACAGAACCCCACAGAGGCTG
AACTACAGGACATGATCAACGAGGTTGATGCGGACGGCAACGGAACGATAGACTTCCCAGAATTCCTTACAAT
GATGGCGCGCAAGATGAAGGACACAGACAGCGAGGAAGAGATTCGCGAGGCGTTCCGCGTCTTCGACAAGGAC
GGTAACGGGTTCATTTCCGCGGCCGAGCTGCGCCACGTGATGACCAACCTGGGTGAGA
AGCTGACCGACGAGGAGGTCGACGAGATGATACGCGAGGCCGACATCGATGGTGATGGACAGGTCAATTATGA
AGAATTCGTCACCATGATGACGTCGAAGTGAGCCGCCGGCTAGTGTGTGTGTATAAAGCAGAGAATTTTAATA
TACGTTTTGTTTCATGACACACAATATTACCATCGTATTGGACCTGCATTTCTCTAGT
GTTTGCGATAAATTAATATTGTCATCGTTTCAGTTTAATGGTATATCGGCGGCGCTGCGGCGTCGCCCCCCTA
TTTAGACTTTAAAGTATTCCGTTAATTTAAATAAGTAAGGAACACGCCGGCGAACGCCGCCGCCCGACGCCGC
CGCGCCAACACATCTTAATGTAAATTATAAAGTACGTACGTGATGA
>1002052452_2 Heli.2-DT666312.3.5
CCCATCTGAACTAACACAGACCACGATGAACTCGCTGGTGGTGTTGCTTTCCGTGATGGCGCTTGCCACAGCT
AAGCCCAGCCTCTTCAACGCTGGCGTTATCAGCTATTCTGCCCCGGCTATCGCAGTGGCTCCAGCTGCAGTTT
CTCATCAATCACGCGTTGACATTAAATCATCTCCAGCCATAGTCGCAGCTGAGGTTGT
AGCTCCTGTCAGCCGTGCAATCGTGGCGGAACAAGCTGTAGTAGCTCCTGCAGCTTACTCATCACCTGCCGTG
TACGCAGCTCCAATCATTGCCCCTGCTGCAGTTTCATCTCAGTCTCGTGTTGATATTAAATCATCTCCAGCTG
TAGTTAGTAGCGTTTCTGTTGGCCCTGTAGCATACGATGCATCCATTGCTGCTCCTCT
TGCATACGGTGTATCTCTTGCTGCTCCTGTAGCGTATGGAGCATCTTTCGCTGCGCCCGTACCGTACGGTGCA
TCTATCGCTGCACCCGTAGCATATAGTGCATCAGTTGCAGCGCCTGTAGCGTACAGTGCTCCTTTGGCTGGCT
TTTCATCCATCATCTCACCTGCCATCTCTGCAGTTTCTGAGGTCGCCAAACCTGAAGT
TGCTGAATTACCAGTCGCCCCAACTGAAACACCTGAAGTTGCTGCCGCTCGTGCCGCCCATCTTGAAGCCAAA
GCACTTGAAGAGTCACATAAAATTGAGAAACGATCTGTTGCTTTTGTATCAGCTGCTCCACAGGCATCCCTAT
ACGTGGCATCCCCAGTTGCTAGCTACGCTGCACC
>1002052453_2 Heli.2-DT664379.3.5
ATACAAGATGCAACCTCAAATCCTAGTGCTAAAAGAGGGTACAGACCAGTCGCAGGGCAAGCCTCAGCTGGTC
TCCAATATCAATGCCTGTCAACTTGTTGTAGATGCTGTCCGAACAACACTTGGACCACGGGGCATGGACAAGC
TCATTGTGGATCACAATGGGAAAGCAGTCATTTCTAATGATGGAGCTACTATTATGAA
ATTGTTAGATATTGTCCACCCGGCTGCAAAGACATTGGTTGACATTGCCAAATCACAGGATGCTGAGGTTGGT
GATGGCACCACATCGGTAGTGATACTCGCAGGTGAACTCTTGAAGAGATTGAAGCCTTTTGTTGAGGAAGGTG
TTCATCCTCGTATAATAGTGAAGGCAGTAAGAACGGCAAGTAAACTAGCCGTGGACAA
AGTTAAAGAATTATCGGTTAAAATTGAGAATCGATCACCTGAGGAACAGAGAGAGTTGCTAACAAAATGCGCG
GCAACTGCAATGTCATCGAAACTAATTCACAGGCAGAAAGATCATTTCTCCAAAATGGTTGTTGATGCTGTAT
TGTCGTTGGACGCACCATTGCTACCGTTGGATATGATTGGTATTAAGAAAGTGACAGG
CGGTGGTTTAGAAGATTCCTTCCTCGTTGCCGGGGTTGCTTTCAAGAAGACATTTTCCTATGCTGGATTTGAA
ATGCAGCCTAA
>1002052454_2 Heli.2-DT667427.3.5
GTTTCTCACGAGTCTTGTCGGCAAGGAGATAAAACATGGGTTCCGTGGAACGTGCTGGTGATGCGACATCAGA
GGCAGAGTCTGGAGATGAGGAAAGAATCGTAGGTGGCGAGGAATTGGCAGCACAGTTGATGAAAAGTGGTATC
ACCCGCAATGAAATGCTAGCTGCTATCACAACGCGTATCCACGCCGAGGCGATGGCGT

CCCTACCACCCAATGTTCGTCGAAGAATCCGCGCTTTAAGATCCTTACAGAAAGAATTTGTAGACATTGAAGC
CAAGTTCTATTCAGAAGTGCATGCGTTAGAATGCAAGTACGAGAAACTGTACAAGCCCTTATTTGAAAAGCGT
GCTCTCATAGTGAATGGGGGGTATGAACCAACAGATGATGAATGTCAAAATCCATGGA
GGGATGACACAGAGGAGGAGGAGCTCGCTCGTGCCGTTCAAAGTGCGGCTATCACTGAAGGTGATAAGAAGGA
GGAGGAAAATAAACTTGTTGAACCACCCATGGACCCCAACGTCAAAGGAATTCCCGACTTTTGGTATAATATA
TTCAAGAATGTATCAATGTTATGTGAAATGGTGCAGGAATATGATGAACCTATTATTA
AATCTTTGCAAGACATCAAAGTGCAAATGCATGAAGACCCCAGCGGTTTCACATTAGAATTCCACTTCGCTGC
AAATGACTACTTTACCAACACAATCCTCACTAAGGAATATTCCATGAAGTGTAAACCTGATGAGGAGAACCCC
CTGGAGTTTGAGGGACCCGAGATTTATTCATGCAAGGGTTGTGAAATAAACTGGAAGA
AAGGCAAGAATGTAACGGTGAAGACAATAAAGAAGAAGCAAAAGCACAAGTCGCGTGGCTCCGTGCGCACCGT
CACCAAGTCTG
>1002052455_2 Heli.2-DT664293.3.5
AAAAAGTGTGACGAGCGTTTGTTTTATCAATTTCTAAAAATCAAATTAATAAAAAATGTCGACTCTTGCTGCA
CCTTTAACTGTTGCGGGGACCAGATATTCTGGAGCCCCAGTCAGAAGTCAGAATGTAATGGCAGCTGCAGCAA
TTGCCAATATTGTCAAAAGTTCTCTGGGTCCTGTGGGTCTGGACAAGATGTTAGTTGA
TGATATTGGAGATGTCACAGTAACTAATGATGGTGCTACTATTCTCAAAATGTTGGAAGTTGAACATCCCGCA
GCAAAAGTATTAGTAGAGCTTGCTCAACTGCAAGACGAGGAAGTTGGTGATGGCACCACATCTGTTGTTATTA
TTGCTGCTGAGTTATTGAAGAATGCTGATGAGCTAGTCAAAAATAAAATTCACCCAAC
CAGTATTATTTCTGGATACCGTCTAGCCTGTAAGGAAGCTGTAAAATATATCCAGGACAACTTGACAGTCTCT
GTAGAGTCTTTGGGCAGGGCTTCCGTCATCAACGCAGCTAAAACCACCATGTCATCTAAATTGATTGGAGCTG
ATGCAGATTTTTTCTCAGAAATGATTGTAGATGCCGCACAAGCTATTAAAATAACTGA
CGTCAAGGGAAATTCATTATATCCTATTAAAGCTGTCAATGTTTTAAAAGCCCATGGGAAAAGTGCAAGGGAA
AGTGTTCTCGTCAAAGGATATGCGTTGAATTGCACAGTAGCATCTCAAGCTATGCCTAAGAAGATTGTGAA
>1002052456_2 Heli.2-EL603347.1.5
GGTTCATTTTTGTAAAAGTGCAGTTGGAGTCTTATATTGAACAAAAGTTGAATGGTTTCATTCGAATAAATC
GGTTGATATTTTAGTTAGGATAGTAATATCACAGTAAATCAAAATGGGAATAAAATTCCTTGAAGTTATAAAA
CCGTTTTGCAGTATATTACCGGAGATAGCGAAACCGGAGAGAAAGATCCAATTCAGAG
AAAAAGTATTATGGACAGCAATCACATTATTTATTTTCTTAGTATGCTGCCAGATTCCACTGTTCGGTATAAT
GTCATCAGACAGTGCAGATCCTTTCTACTGGATCCGAGTGATCCTGGCTTCGAATAGAGGAACCCTCATGGAA
CTTGGTATTTCTCCGATTGTAACCTCAGGTCTTATCATGCAATTGCTTGCTGGTGCTA
AGATCATTGAAGTTGGTGACACTCCGAAGGATAGAGCATTGTTTAATGGAGCTCAAAAATTATTCGGCATGGT
GATAACAGTGGGTCAAGCTATTGTATACGTTATGACTGGAATGTACGGAGAACCAAGTGAAATTGGTGCTGGA
GTATGTCTGCTTATTATTATTCAGCTGTTTGTTGCTGGTTTGATTGTGCTGTTGCTTG
ACGAGCTCCTACAAAAAGGTTATGGTCTTGGATCTGGTATTTCTCTCTTCATCGCTACAAACATTTGTGAAAC
AATCGTCTGGAAAGCATTCTCTCCTGCTACCGTTAACACTGGTCGCGGCACAGAATTCGAAGGTGCAGTGATA
GCGCTGTTCCATCTCCTCGCGACCCGCCCTGACAAAGTACGCGCTC
>1002052457_2 Heli.2-DT668465.2.5
GGCTAGGAGCCGTAGTTTCGGTTTTTATTATTTTAATCGCCCGTAATCCTACAAAATGAGAATTTATAAGGAT
ATTATTACCGGTGACGAGATGTTCTCGGACACATACAAAATGAAACTGATTGACGAAGTTATCTACGAAGTCA
CCGGTAAATTGGAAACCAGGGTCCAGGGCGAGGTAAAAATCGATGGATTCAATCCGTC
AGCTGAAGAGGCGGACGAGGGCACCGACACTAACGCCGAGAGTGGTGTTGATATAGTCCTCAACCACAGGCTC
GTTGAGAGCTACGCCTTTGGTGACAAGAAATCCTTCACATTATACCTTAAAGACTACATGAAAAAATTAATAG
CAAAATTGGAAGAGAAGGCACCTGATCAAGTAGATGTATTCAAAACTAACATGAACAA
AGCAATGAAGGACATACTCGGAAGGTTTAAGGAGCTGCAGTTCTTCACTGGCGAGTCCATGGACTGTGACGGC
ATGGTTGCCATGTTGGAATACAGGGATATTGATGGAGTATCAACTCCCATCATGATGTTCTTCAAACATGGTC
TTGAGGAAGAGAAGTTCTGAACAGCATATGTGATACTAACTCATAATATTCCAGCCAG
CCTAATAAATTCCTTTTTTTCTATTTATTTACTTTGTTTTTACCAATCATGTCTGTCCGCTCAGCAGTACACA
GCTTCGGCAACTCCCCGTAAAAGGCTGAACTTTTGTAAAGAATTGATGTTGCATATTATTTAAAATAAATAAT
TTAATGAACT
>1002052458_2 Heli.2-DT662503.3.5
AACCTCGGCGCGCCCGGCTCCGAGCTGAGCGAGCGCAACAAGCGGACCATCGGCATCCTGCGGCAGCTGTTCC
CGACCCTCACACAGAACGCCATCGCGGATGAGAACCAGCTACTGTCTGGAGCGGAGTCCCAACAAGCTGAGGT
GCGAGTCGCGTTCGGTGACAACCAGGCGGCCGCCAGCGACGACCAGCAAACTGCCGCG

CAAACTAACTCCGCGTCCATCGACGAGATCACATTAGACGATGCTGATGACAGCGAAGAAAATAGAAATAAGA
GGTTCCTTAACTTCGGAGGATCAGCTTCAGCATCAGGAGGCGGCTCTGGCAACTTCTTATTTGACATCGTGCG
ACTCGTAGCGGGATCTTCGTCCGGAGGCTCCAGCGACGGCGGCGCGGCTGAGGGAGAG
GACAACGGCGCCGCGAAGGGCGACAACCTGACGGAGGGCGTGCCCGGCCCCATCACGCGCCTGTTCATCATCG
CCAACCGCGGCATCGCCAACCTCATCCAGGACCTGATCCTGCGCATCGCGCAAACCTCCGAGAGGATAGTCAA
CTTCAAAGCACGATTGATCACCTCTATCATTTAAATGCGTTCGCGCTTAGAATAATGA
CGAGAGGCTTCGGTCCTCGCGCCCTCGTATCCCGACTCGGACATTAAACAAATAAAATAAAGCGCTCTATTTC
AAGCAACATTTCCTCCGAGTCGTGATCCGAGCGGCGCCGAGTACCGCAGAGTGTTACCGCGGCGAGCACTCCC
GCTACTTGACTGAACACGAAAATATTGAGTCAGGTGCCTTCAGTCGTTACGGCAGTAT
CGATTGAAAGAAAAATATCCTTCTTCATTAGAGCCGGCAACAAATATTAAGGCCGCGTGG
>1002052459_2 Heli.2-DT665515.3.5
GAATTAGTAGATCTTGACAGTTTTAAAACGCACTCTGGAGATATTCCAGCACAATATAAAGAACCACCTACAA
GATATTCGTCAGAAAGCAAAACTACTACAGAAAACACAGACAATTCTTTACTTGCCAATGAACCATCTCATAC
TAAAGAAAGTCAAAGAGAAGGACATAGTTTAGCAAGTGGGATTAGATTCCAAGAAATA
CAAGATGATTCCAAATTGCCATCTGATTACAAACATACAACTACTTATGAACAAGACACTACTGAGTATACAA
CAATGACAGCGATGGAGAAAGTTGCTCTAGACTTGTACGCTTATTTAGCTGGTGAAAATCTAAATAATGAGAT
CAGTCCAGCTGGGGACCTGATGAGTTTCGACGGTTCAACTACCGTAGATGAAGACGCA
TGGACGACGGAGTTGGTCAGCACGACTGAAGAGGAAACTACACCGACAACTACGACCACGACCACCACTACCA
CCACCACCACCACGACTACAACTACGACTCCAGCGCCCACGACGACCGCTGCACCAACCCGTCCTTCAAGATT
CAAGGGCCGCCCCGGATCGAGGGCTCGCGCACCTGCCTCTACCAGCGCTGCCACAGAA
GCACCTCAAGAATCCTCTACAAGAGCTCGAGGTCGTTTTGGTAAGCCCAATGGAATCAAAAAGACAACGGCAG
CTGCTGTAGAATCTTCCACCTCCTCCGCTCCAGTCCAATCACCGCAACCAAAGCAGGCGTTTGGACGGCGCGG
CTTGTTTTCGGGTCGCATACGCCCCAGCTCCACGGCTGCTCCTGCGTCAGAGGCCGTA
AGCAGCGCTCCCAGTGAAGCAGCGGCCCCGAGAGCACGTCTTCGCCCCGGATTAAGGAGACTTCCATCATCTA
CTTCTGCCGCACCGTCTTCTGGAGAAGTCACCGACACGCCAGCGTCTGTTGCTAGTCAAAGTGCAGCATCCGG
AGAAGTCGTAGAAACTACGCCAATTCCTTCTAGAAGTATTGGACGTAAACCAGGGGGT
GTCAGACCAGTTTCACTGCGACCCGGACCTAGACTCAATATAAGACCAAGTCCTCGTCTGAGACCCGGATTGG
CGAGCGTAGCACCTACAGAAGCTGCCTCAGAAGCACCCGCTGAAACATCAGCTCCCGAAGTTCCTGCAACAGA
GGCAGAAAATGAGGGCTCCTCCCCCACGCCTGCACCTGAACAACCTCGTGGTGTGTTA
AAGCTACGTAATAGGCTTCAGGTGTCTCCGTCGCCAAAGCCACGCGTCGCCGTCACTCCGCCATCCCGTCGCC
CTAACCCATTGTTGAAGAAAAGGTTGCCGGCTACTGAGGCCACAACGGAGCCACCAAAGAAGCCGACAGAATC
AGTAGAAGAAGAAGCTAGCGCAGAGAAGAGCGAGGCCGAGGCGGAGACAGAGGCCCAG
CCAGCGGAGACCACCGCCGCGCCGCCCCTGCGCGGCCTTGACGCGCTCATCGCCCGAAGGCGCGCCGCCGGTA
TCGGCGCCCGCCCCGCCCGCCCC
>1002052460_2 Heli.2-DT665479.3.5
TGAAAACTATTTAAAATTTTTTTTGGTGATATTCAAGAAAGTGTTAATAAAAATCATGCGATTTTTTATATAT
AGTTGTATTTTGCTATCGCTAACTATAGCAAGCATATCAGCTGACTGGTCTTGGGGAGGAGATGAGAAAAAAA
CAGAAAATGCGGACGGAAAATCGACTGAACTATTACAAGGAGAAGAAATTAGTGAAGC
ACAAGGAAAGAGTTTTAATGCTAACGCAACTGTCCTAGATGATATTGTAGACGAATTAGTTAGCAGTAAGCAG
GGTAGAAGTTTAAGCGGATTTGATGACGTGTATAGTGACCCCACCATTAAAGAAGCGCTCGATTCCGGTGACG
ACTTGGAAGCACGAAATTTGATTAAAGGACGTCTTTGTACATTAGGGCTTATCCAGTG
CGATGAGGAAGATTCACAAGAAAAAAGAACTTATCTTTCACCAGATGAACTAATTTATGCTCAACCCGTTGAC
ATAAAGCCTATTGGTAAACCAGTAGCTTCAATTCCAGTGCGCGGACCACCCAGAGCTTACGGACCACCTAAAC
CAATGCTATACCCTCCCCGCCCGCAAAAACTACCCCCAAAAAGACCAGGATACGGAAA
TCCAAGACCTGGATTTTCAGACAAATATGGCGTAGCCGGAAATAATTATCAGTTTTCTCAAAGCAATGGTATA
TATAATGGGTTTGAAGCTAACTA
>1002052461_2 Heli.2-DT666484.3.5
GCACTAAGATGTTCAAATTAGTAGCTCTGTGTTGCTTCCTCGCCGTAGCCTCGGCTACACCTGGTGTTGTCGT
ACCACTGGCATATTCATCGAATGTGCTAGCATCGGCACCAGCAGTCGTGTCAAGCTACCCCAGCAGCCTGGCA
TACTCATCTCCTGTCTTCTCTTCGGCGCCATTGGCCTACCCCGGATGGGACTACCAAC
AACACCTCATCAAGAAACGTTCTTTGAGCTACATCGCGCCGTCTTCTTACATCGCACCATCTTCTTACATCGC
GCCGTCTTCCTACATCGCACCCGCTGCCTACAGTTACCCCGCTGCTCCTTTAGTCAGCTCTTACTCTGCAGTT
GCACCTCTGGCTAGTTACCCAGGGCCCGTTTACACCGGTGCTGCACATCTGATCAAGA

AGAGGAGCGCTGTACTCTTACCTAACAACTACGTCGCACATGCCGCATACTCTGCATCCGCACCTATCTTAGC
ATCAACTTATGCGGCTGCCTCTCCCATTGCACCTGTTTGGTCTGGCTCTTTGTATCCCGCTGCTCAGTATGCT
CAATTCATCAAGAAGTAAACTGCTCTTTTAGCTAAATCCACGTTCCGTCCGTTCTACC
ACTTCTCGTACCTCAAAATGCGAATTTTATCTTCTATTCCCTAATCATTGACTTTATTATCTCTTTGCGGACA
CTTGTGCCATCGTCTTGTGTACTTGCCTTTTAAGTTAATTTGATTTAAGTGTGCATGCTACACATACATTT
>1002052462_2 Heli.2-DT663969.3.5
AGTCCTGCCTTGTCTACTAGCGAGTGCGAAGCGACACCGTGAACTGTGTTTACACTCAAACGTCTCCCAGTCA
GTGTTTGTTTAGTTTCGACAGTTTCCACACGACGCACACGTGAAAATGGCTGACAGTGGTCTGAAGAGAAATA
TTCCTATCAAGCTCGGAGATTTTTCAGTTATCGACACCGAATTCTCAAGCATCAGGGA
AAGATTTGACGCCGAAATGAGAAAAATGGAGGAAGAAATGAGCAAATTCCGATCGGAGCTCATGAACAGAGAG
AGCAATAACTTTTTCAAGAGCTCTACAAGCACCACCACATCGTCACAGCACAGTGACAGCAGACAGCTGGCTG
AGCCCAGCCACTGGGACAGCTTAAACTCTCCTTTAATTCAGGATGAAGGCGATGGCAA
GTCTCTTAAACTCAGATTCGATGTCAGTCAGTACACGCCCGAGGAAATCGTCGTGAAGACAGTCGATAACAAA
TTATTGGTTCACGCCAAACACGAGGAGAAGTCAGAGACGAAGTCTGTATACAGAGAGTACAACCGCGAATTCC
TTTTGCCCAAAGGAACCAATCCTGAGGCTATCAAATCGTCGCTGTCCCGAGACGGCGT
CCTGACTGTGGAGGCGCCGTTGCCGCAGCTCGCGATCACCGACAGGAACATCCCCATTCAGAAACACTGAGGC
GGTTGTCGATTCCTCTCTCGACAGCAGCTATTCCATACTACTGTTAATAATAATTTATTATAATTTACTATAA
GGAGTATTGGATATATTCAGATATTTATTTTGAATCTTAATTATATGGAATCTCATCG
TTTTTAACATGTGTATTGTTAGAGTTTTGAATTATTTTTTTTTATTATCCAATTATACCTGCTCTTGGCAGCG
GAGCACAGTTGCGGTTAGCACTGTACGCATTAAATATTGCTTTTAAGTCAATGCTTTTTCTGTGATCGTTAAA
AATGATGGAAATTATTTCGATTTCACGAAGATCACTTTTTGATATTCAAGCGGCATGC
TGTTTTGCACCGTTTTAAATGTTTACCCACGATTTA
>1002052463_2 Heli.2-DT667140.3.5
TATTCTCGTCGGAGTCGCGCGGGAAACGCACGCGCAGTCGTTACTTTTTTAATTTTTTTATCGCTGTGGAATC
AAAATCGAGACGAGAGCAAGTGCATTCCGTAGTTAATGGCTGTATAGTGAATTGTGTTTTGTGTTTAAATATA
AGTGTTGCCAGTGTTAAATATATATTTTTAAAGATGGGTCGACGGTGGCCTATGTTCT
GTCTGCCGATTTTACTCGCGGCGTCAGTATGTGGAAGCGACATACTGATGATCACGATGGGAGGCACCAAGTC
CCACAAAATACCATTCTTAGAACTAGCGAGAGGACTTATAAGAAGGGATCACAACATTACATTAATCAGTCCC
TTCCTTCCGGATTTTCACATTGAGGGCTTGGAAGAAATTACTCCCCAAGGTCTGGCCT
CTTTCGTACGAGGATATATGTCCTTCGATCTTGTTGGCGCAAGAATGAGAGGGGAGGAGCCTTTGCCATATAT
GGATATTATAAGATATGGATATGAGGCATGCGATTCTTTTCTCAACGATATCGAGACAAGATCATTTCTACGT
TCAGGGAAAAGCTACGATCTCATAATATTAGATGGGGCATATCCAGAATGCGCTTTGG
GCCTTGTGTATAGAATGAAAGTTCCTTTCATGTACATAAACACTGTAGCCTTTTACGCTTCACCAACAAGTAC
TTCAGGAAGTCCTGCACCATATTCTATAATGGCATTCTTTGCTCGATCATTTACGGATA
>1002052464_2 Heli.2-DT662513.3.5
TTATAGTGTTATAGTGTTAATATAAGCTTATTTGTCATACAAAATACGAAACAAAATGGGGGGTATAAAGACC
CTCATTTTCCTGCAGCTGATCTACATCACCACATGCATGGAACAGCTTGACATCATCTATGAGTGGACTCAAT
TGGACTTCCAATTCCCAACTCCCGAAGCTCGACAACAGGCTATCGACAGTAGAAGTTT
TATACCAGAAAATAATATACCCATGGGCCTTGAGATATTTGGAGATAGACTCTTTGTAACAGTGCCTAGGTGG
AGAACTGGTGTACCTGCTAGTTTGAATTATGTGAATTTAAAAGATAATTCCACCAAATCACCGAAACTGATTC
CATACCCGAGTTGGGCAGCTCACACCCCGGGTCCAGACGGTAAACCAGAAATTGTATC
GCCGTTCAGAATCAGAGCAGACAAATGTGCCCGTCTTTGGGTATTGGACAATGGAAAAATTGGAAATCTAGAA
AATAATACTACCAAATTTCTACCTTCAATCATAATTTATGATTTAAAAACAGATACTCTTCTCAGAAAGTACG
TTTTCCCAGAGGATCAAGTAAAGGAAGAATCTGGTTTTGCCAACATTGCAATTGAAGA
TACAGATTGTGATAAAACATATGCGTATGCCGGTGATTTGGGAAAACCGGCTGTCGTTGTTTATTCTTGGGAG
AAAAACGAATCGTGGAGAATAACTCATCATTTCTTCCATCCCGATCCTTTAGCTTGTGATTTTAGTGTCAAAG
GGCATAATTTTAGCTGGACAGATGCTATATTCGGTATTGGCATTTCAGCACCAAACTC
TGATAACTTCAGTACTTTATATTTCCACCCAATGGCGAGTTACAATGA
>1002052465_2 Heli.2-DT663556.3.5
GGTGAAGTTAGTGGTGGAATTTTTACGTGTATAACATCAAAAAATGGCGTCTGGTGTGACAGTATCGGACGCG
TGCAAAACAACCTACGAGGAGATCAAAAAGGACAAAAAACACCGCTACGTAGTGTTCTACATTCGTGATGAGA
AGCAGATCGACGTTGAGACCGTAGGCGAACGCAACGCCGAGTACGACCAGTTCCTCGA

GGATTTGCAGAAGGGCGGCACTGGAGAGTGCAGATATGGGCTCTTCGACTTTGAATACACGCACCAGTGCCAG
GGCACGTCGGAGGCGAGCAAGAAACAGAAGTTGTTCCTAATGTCGTGGTGCCCCGACACCGCCAAGGTCAAGA
AGAAGATGTTGTACTCTAGCTCCTTCGACGCCCTGAAGAAGTCGCTGGTAGGCGTACA
AAAGTACATTCAAGCGACTGATCTCTCTGAAGCTTCGCAAGAGGCCGTCGAGGAAAAACTACGCGCCACCGAC
CGCCAATAAGCATTTACACAGAACGAATAATGATAATGTCGCAATAATGCTAATAAATAACACTTAAGTTTCT
TAAGAACTTTTTTACTGTCCCCGAGCTCAGTCATATTGACTCGTTGTTGTCACATTAT
CAAATTCACAAGCTGTACTGTTTTTTCATCAGTTTCTTTTTATACTGAAAGGGTCTACGCGATGAGCAGTGAT
AAGATAAATAAATAATCATTGGTTCTTTAATTACATCTAATAAGCTTTCCTGTCTTGTC
>1002052466_2 Heli.2-DT664658.3.5
TCTGTTCTAATTCTTATTATTTCTTAATATTAGTTATTATTATTTTTTATTTAAGTCTTTGAGATTTTATCTT
AATAGTCACTTTTTTATTATTATTGCATAAAACGACTTAAGTGGTACTTTTGCGTTTAGACGGGAGCCATCTT
GCAATCTCTAATCAAAGGTCCTTGGCTCACAGTTTCGTTGTCTAGTTGAAAGGCAGTA
TATTCAATCATAAAATAGTCAAATGGCGTTAAAACGAATTAATAGGGAATTACAAGATCTGGGTAGAGACCCG
CCGGCACAATGTTCAGCAGGACCACACGGTGAAGATCTTTTCCATTGGCAAGCCACAATAATGGGTCCAGTTG
ACAGTCCCTATCAGGGTGGTGTATTTTTCCTCACCATACATTTCCCTACAGACTATCC
ATTCAAACCACCAAAAGTTGCATTCACAACGCGCATCTATCACCCTAATATAAACAGTAATGGTTCCATTTGT
CTTGATATTCTGCGCTCACAATGGTCTCCAGCACTTACCATATCCAAAGTGTTGCTCTCAATCTGCTCACTTC
TATGCGATCCAAATCCCGATGATCCTTTGGTGCCAGAAATTGCTAGGATCTACAAAAC
TGACAGAGAAAAGTACAATGAATTAGCCCGAGAGTGGACTAGGAAGTATGCCATGTGATGAGTCAGATGTGAC
ATCAGTAAACATATACACACAGATCGCCACAGCCCGGGCTTATACCCTTCCTGTTCTCATCCACGCCCAATTC
CTTTTTTGTCTGAAAACCTTACCAATCACTAAAATTACTCAAACTTTGTTCAATCTAC
ATGGTTTTAAAATCTGATTGTTTCTTGTAATTTAAATGCCAATTATTGCGACTAAGTAAAATAATACACATGA
TATGCAAATAGTTTCTGTTCATGTAATTAAGTACCATTTTCTATAAA
>1002052467_2 Heli.2-DT662894.3.5
AATAATATATTTAATAATGGTTAATTATAATATATATATATTAATAGTATTTTTAGTATTCACAGTTTGTGAA
GGGGCTTTAGGGTTATCAATTTTAGTGTCAATAATTCGAACTCATGGTAATGATTATTTTCAAAGTTTTAATT
TAATTTAATGATAAAATATTTATTTATAATAATTTTTATAATACCTATATGTTTGAAA
CGAAATATATTTTGAATGGTTCAATTTATATTTATATTTTTAATGTTTATATTTATAAATATAAGAATTTCAA
TTATGAATTTTTGTAATTTAAGATATATGTTAGGTTGCGATATAATTTCATATGGTTTAATTTTATTAAGAAT
TTGAATTACATTTTTAATAGTTATAGCTAGAGAAAAATTAAATAATAATGATTTTTTT
AAGAATTTATTTTTATTTAATATTATTTTTTTAATAGTAATATTATATCTAACATTTAGAACTATAAATTTAT
TTTTATTTTATTTATTTTTTGAAAGAAGCTTAATTCCCACATTAATATTAATTATTGGTTGGGGGTATCAACC
TGAGCGAATTCAGGCGGGGATATACTTATTATTTTATACATTATTTGCTTCTTTACCT
TTATTAATTGGGATTTTTTATATTTATAAAGAAATAAATTTTATAATTATATATATAATA
>1002052468_2 Heli.2-DT668272.3.5
GTCGGCCGTGTACGCTTACGGAATCGTCCTCATACACTTTATGCGTGATTAATTGAAACGTTTTTCTTAAAAA
ATAACATCGAAATTAAAGTTTAATGTTGAAGAAGCACTGAAGTACAAAATGAACTCGTAAAAACAAATATATT
TAAATTTCACAAAATAAACAGGAATAAAGGAAATTTCTCGAAACAAATTACTAACAAT
GTCAAATGTAGAGGCGAATAATAATATGAATAAAAGTGAAGCTAATAATCAGAAAGTGGGAAAAGACATAGAA
CAGGGGAAATTTGGAGACAGTGCAAGTTCTGGTTTGCCCTGGAGTCGCGTCTTCGTCATGGTGTTTGCAGCGG
CAGTGCTGGTGGCAGCGTGTGCAGTGGCATGGGTGTTGTTTAGCTGGCAGATCAGTCT
TATTGTTTTTGCGGTTCTAGTTATATGCTATACACTAGCTTATTTCTTCAACTGGATATGGATTGCTGTACAA
ACTGCTCCGAGAGACTTAAAAGCTTTATACTGCTATTTCAAGATTTTAAGATTATCCAGTCAATTTACGAAGA
AGAACTGGTCTATGCCAGATATTTTTCATGAAGTTGTAAAGAAACATCCAAAAAAAGC
ATGTTTCTTATTCGAAGATGAAACGTGGACTTTCCAACAGGTTGAGGAGTTCAGTCTGCGGGTTTCTGCAGTA
CTGCAGTCGCGAGGAGTTAAACGCGGCGACACTGTAGCCCTCATGGCTAGCAATTACCCAGAAATGCCGGCTA
TCTGGCTTGGTATCACACGTGTTGGGGCCGTTGCACCACTTATAAATACCAACCAAAC
TGGCAATACTCTACTACACTCTATCAACATTGCCAAATGCGATTATGTTATTTATGGCAACGAATTCGAATCA
GCTATTCAAGACATCAGAAAGGAGATAAAAGAATCTGTTAAATTATTAAAATTTACACGCCGTCCATTAAATA
CATCTAACGATAGCGTCCAAGTAGTCAATTCTGAACATGATTTTACACATTTATTGGA
AACAACCCAACCTGCCCCTTGGTTCTTGTCCGAAGGGGAAGGATTTACTGGGAAATTATTGTACATTTATAC
>1002052469_2 Heli.2-DT665436.3.5

AGGGTCGTGTGGCATTATTTAGTTTATTTTATAAAGTGAATTAAAACAATAATTGTGCTTTATCTTAGTGATG
TCAGAACACGTACTCCCGGCTGAAGAGCCTATACGGTTCGTAGCCCCAGTGATTCAAGATAATCCTACAGGAT
GGGGCCCGTGCGAGATGCCGGAGCAATTTAGGGACATGCCCTACCAACCTTTTAGTAA
AGGTGATCGTCTTGGCAAAATAAGTGATTGGACTGTAGTACAAGATAAGAAATACCAGAATAAGTATGCTTCG
CAATTTGGCGCTGGATCGTCATATGCGTACTTCCATGATGAGGACGAAAGTACGTTCCATCTGGTGGACTCGA
CTCGCGAACAGAAACCTCCGTACCAACGAGGTCGTGGTCGTGGTAACCGAGGCAGAGG
AGCAAGAGGCGCTCGCACACCTGGTGGCATGACAACTTTGAGCAAGCAACGTGAACGTAAGCTTGGCAAGAGA
TGGGGCCAGAGAGGTGCTCCTATGAAGATACGAGATGCATCTGTAACTGTCCGACCTACTTGGGTGACAATTG
AAGACATGGACTTCCCACGTCTTGCTAAGTTATCTTTACCTGGAATTAAAGAAGGTGA
AGATATTGTATGTTGTGGTACACTGGAATACTATGAAAAGGCTTATGACCGTGTAAATGTGAAACACGAAAAG
CCCCTTCAGCGCATTGACCGCATCTTCCATACTGTCACAACCACTGATGACCCAGTCATTCGTCGTCTGAG
>1002052470_2 Heli.2-EL596932.1.5
GCACGAGGCTTAACCGCCATGGGTCGTTCGTTGCGTTCGCTATCAGTTTACACTCGTCCGTTTGCCCCCATAA
ACTGCCGTCAGGCCACCACAGGATGCCCGTTTTCCAAGCGTCAACGTTCCCAAATAGCTCCAGCTGCTTCCCT
GTCCGAGGAAATTTTTGCAAACGCGAAACCGTACTCCGAAGTTCCTGGACCTAAGCCT
ATCCCTATTTTGGGCAATACGTGGCGGATGGTCCCGGTTATAGGGCAGTTTGATATTTCCGAATTCGCTAAAG
TCACTAAATTATTCCTAGAAAGATATGGCAGGATTGTGAAGTTAGGAGGGCTTATTGGGAGGCCTGATCTGTT
ATTTGTGTATGATGCTGACGAAATCGAAAGAATGTATAGGAGAGAAGGACCCACTCCG
TTTAGGCCAGCGATGCCGTGTCTTGTGAAATATAAATCTGAAGTGCGAAAGGATTTTTTTGGGGAACTGCCAG
GTGTTGTTGGAGTTCATGGAGAGCAGTGGAGACGGTTCCGTTCTAAAGTCCAAAGGCCTATACTCCAACCTCA
AACAGTGAAAAAGTATGTAGCACCAATAGAACTCGTGACCGAGGACTTCATCAGATAC
ATGGAAAACGCAAGAGATGAAAACGGGGACTTACCTCATGAATTTGATAATGACATCCATAGATGGTCCCTT
>1002052472_2 Heli.2-DT662733.3.5
CGTATATAGGTAACTTTTCTAAGAATATTACTAACTAGACTGATATTTTTTCCTTAAAAATGAAGAAAGTAAT
ATTTTTATGCGTGTTGTCACTGTCCTTGTGTTTGAGCAAAGCAGCTGAGGACAAAGCCCCACAAGCTCCACCA
GCTTTAAATGCGACAGATGCTAAACTTGCTCCACCAGTAAATGCCACACCGCCTCATG
AAAAATCTCAAGAAGCGCCAGTAAGTAATAAAAATGAAACAACATCTGACAAACCTGCGCCTCCACCCTCACT
GCCACCACATATAAATACAACTAAGCCAGAAGTTGATAATAAGACTGCAGACAAACCAACTCCTAAGGAGGAA
ACTGCTAAGCCGAGTAACAATGAAACAAAAGTAATCCCAGATAAAGAAAGTAAGGATG
TGAAGCCCAATGAGAAGCCTAAAAATGAGACTTCGACCGAGAAGGTCACGGAAAAACCCACTGAAACACCAAA
AGTCACGGAAACACCTAAACCTATGCATATTGTTGAAAAGACTAAAGGTTTTGATGGACCAAGTTTCATTGGA
GGCATCATCCTCACCTTGGGTCTCCTTGCCATAGGATTCATGGGGCTCAAATACTATA
AGAATCAAACTGAAAGAAACTACCACACACTTTAAGTGAAATATTTATACTATTAATTATCTATGTGAGTGCA
CTCTGCTATTTACTTATAATATTATAGCTATATATTTTGGTTTTTCTCTATAAGTTACGAGATTTTTTGTTTT
TTTTTGTGCACTTTACATTTTA
>1002052473_2 Heli.2-DT666827.3.5
GCACGAGGAGAAAACATCTAACACTAAGTTATATATAGTTATATTACATACTTACACGAAAAGTGCTTGGTTT
TAGTGGTACTTGAGTTACAGATTTTGATACTCGGCTACCGGCATCTAGTGACAAGAATCATGACTACGATAGT
GTCCCTACTCTCCGCCGTGGCACTGCTGTCGCTTCAATGTGCAGCATTCGGAAATATA
AATGAAAAAGTCGAAATCGACAATAGTTTGATACCAAATGCAATACCAGCCCCACGCCATAGTCCGGTTCGAA
ATTATGTAAGAATGATGTCACCACCCCCTGAAACTGTGAGGCACATACCTGAAACTTCTCTTGTTTTAATTTG
TCAATTGAGAGGAAATCCGTCACCTGTTGTGAATTGGTTGAAGAATGGTGTACCTATT
ACAGATTTTGAAGAAGACGTGAATGAAATTCTTTCCATACCACCGTTCAGTCCCACTGAAATGACAAGTAAAC
TTGTTGTACGTTCTCCGTCTAACGCCGACATTTTTACTTGCGTAGGATCCGCTGGATTGCTCGAGATCAGTGC
TTCAACTACTGTTCTCATTGTTGGAGAAAATAGGCCTCAACCACTAACTTCAATTATT
CAATCCAAGCCTGTAATCACCACATTTTATAACAATTTATTCCAATTTATTGGTTCCAACGTAATTCTACCCT
GCCGTGTCGACAGTCCTACTAAATCCCAAGTTGTCTGGTCAGTTGAT
>1002052474_2 Heli.2-DT666934.3.5
CGTCATATTATTAAATCAGTTAACAACCTGCACTGCACCACGCATGAAACAATCATCTTAGCAAGATGTTACT
AAGGGTTGTCTGTATAGCAGTGTTTCTAGCAACAAGTGACAGTTTTAGTTCCTTTTCTCAAAGAAAAGCTATA
GAAACCTTGTACCGATGGAAACAAATTGATTTTGCTTTTCCTACACCACAACACAGAC
AACTAGCTATACGAAATGGAGAATTCAATCAGATAAACGTTATACCGCTAGGAATAGAGCGATGGCAGGGTCG
TGTTTTTGTAAGCACACCTCGATGGAAGAAGGGCGTCCCGGCTACCTTATCGTCATTGCCAGTTGCGTCCCAA
GAAGAATCTCCGTTATTGGCACCATATCCTAGTTGGGACTGGCATAATGCAGATAATT

GTACGGGATTTACATCTATATACAGAATGAGTATCGATGATTGCGGTGTCATGTGGGTCCTTGATTCGGGACA
AGTGGAAGCCTTTGAAACTGCTCGTCAGCTCTGCCCACCAACGCTTTTCGCTATTAGCCTAGCGACGGACACT
GTCGTAGGCCGTTTCCCGATTCCCAGCGAGTACGTTCTACAAAATTCTCTAATCACTA
ATCTTGTAGTCGACTCACGTGACGCTCAATGTCGAGATCTACATGTATATATTGCTGATGCATGGAGATTTGG
ATTGATTGTGTTTAGAGATTCTGACACATCCTTCTGGAGGTTTAATCATTATTCATTTTATCCAGAGCCAT
>1002052475_2 Heli.2-DT665653.3.5
TATTGTGATCGGCTGTGAATTCTGGGAGTGAATAATTTTATTATTTAAAGTGTAATTTCTTTGTCTTCGTTGT
TCGGATTTTAAGACCCAAGGGAAGACAGTGATTATGGGTGCTAAGCAGAGCAAGCGCTCGGTGGACATAAGCG
GCAAGGAGGCCGAGGGCGCGGGCGAAGTGGCCGCGGCCGGCGCGGGCGGGGAGGGCCG
CGTGGAGCAGCTCGCCGACGCCGACGCGCTCAAGCCGCAGCTCAACGGCGACGCACACATCCACGAAACCTCA
GATAAAGAGAAGAACCTCGATAGCGGTACGCCAGAGAATGAGAAAGATGCGACCACAGAGAAGGAATCTAAAG
AACAAGAGGAGGATAACAAAGAAGCTGCACCCGTAACTAATGGTGACGCCGAACAAAA
GGTAGAGAATGGCGAATCTACTCCTTCACCCGAGGACGGCAAGAAACCCAAAAAGGAGAAGGTTAAGAAGAAA
TGGTCTCTGAGATCGATAAGCTTCAGCAGAAAGGATAAACCGAAACAAGAAAAGAAACAAAAAGACGATGAAT
CTAAAACAAACGGAGAACCAGAGAAAGTACCTGAAGAGTCGGCTGAGCAGAGTCCCGA
GTCGGAGAAAAACAATATCGTACCCGAAGAGACTGTCGAAGCTGAGGCTAAAGATGAAAAAACACCAGAGACT
CCGGTGACTGAACCAATTACGAATGGCAGCATGACACCTGAGTCACCGAAAGAAGAATCCCCTCTTTCGGAAG
AACCAGTGTCTAATAATAAGGAAAACGACAGGACGGAGACTCCTAAAGCTGAGGTTGC
ACCAGAAACGTCACCTGTCGTCAATGGCCTCCCCGCAGAGGAACCTAAGAGTGAGACCCCCGAGCCGGTCGTA
ACCGAGCTCGAGACTAGCGAGCCTAAGGAGGCTGTTGTCGAAAAATCGGAGCCCGCTCCCATCATTGAAACCC
CTGTCGAGAAAGAGGAGCCCGCACCAGTCTCTGACAAGCTTGCTGAACTGATTCCTGA
AATTCCTTCAGTGCCAGAAATAAACAATGAATTGGAGGCGCCGAGCGCGGCGGTGGTGGCGGCGGGGAGCGAC
TAGGGCGGCAGCGGGCGGAGGACGGTGGGCGCTAATTTGAAGACGAGCCTTTTATTTATTCCCGAATAGCGAG
TATTCCCGGACGATATCGAACCTAGTTCGATGCCGACATCGAACGTTCACACCACCCA
CGACTAGTGTAGTTTAAGTTAGCGTGCTTTGCTTTTGTAAAATTAAGGTTTTATATATATATTATAACAAAGT
TATGTAGCAGTATACGGTGCGTTAAGTTGGTGCCATGATTACAGGGGTATTAGTTACGTCCCTCCTCGGATAC
TTCGCGAACGCTAGTTACTCCTTTATGATATCATTATTTAGAGAATTATATTATGAAT
>1002052476_2 Heli.2-EL598440.1.5
GCACGAGGCACCAACAGTCCTCCAGTGAACACAAGGAGTTAGAAAACCAAAATGGCAGCGAAAGTGTTGTGTA
TAGCTCTCGCATTATTTGGGGCGGCTTTGGCTCAACCCTCCAAACAAGGATTCTGGAATGGAACTCCTATGGA
TAGCATGGTGGAGGAGATGAGATCTGGATGCGCTGAAGGATCTGATCCCACTGCTTGC
ATCAAATACAAGGTTATGTCCTTGTTAGACAGCATCTTCAAAAAAGAGACTTTCCAGATCTCCGACGCCGTGG
AAGTGACAAAGAACGGTGCAGGTGAATCTTCAGCTCGCTCTAATGGTGATTTTATGGATTCCATTGAAAGCTA
CATTCAATCTCATGATGTTACTTTCCAATTGCCCATCGCTGACACTAAAATAACCGTC
AGCCCCAGAAACCTTGAGAATGATGAACTAAGCCTTAACATCAAGTTCAACAAAGAAGGTGCCCGCTCTGTAG
GTGAGGCTCGCAAGGCTAAGCTCAAGAAGATCATCGTCCCCATCCTCGTGTTTGTCCTCTTAAAGGCCATGAC
CCTTATCCCTCTGGCTATCGGAGTCCTTGGTCTGAAGGCCTGGAACGCCCTTCAGCTG
TCTTTCTTCTCTTTCGTCGTATCCGTCGCTCTGGCCATCTTCCAACTTTGCAAGAAGATTGCTGCGGACAACT
CGCACCCGCAGATCGCTGCCCACGGACCATGGGACCCATGGGACGCCGCCTACGCCGCC
>1002052477_2 Heli.2-DT662771.3.5
GATAATTATAGTAATTTTGCTGTAATTTAACTTGTTGAAATTAAGTGAAAGTACTTTCCATTGGAAACTGGTT
TAGTCTAAAAGTAATTCATCATGGCAGACTCGCGGAAAAACCATCTTATTATAGATGGGGGTCAACATGCAGA
CAACAGTAACCCTGCCTCTTACAGGCCTATCCCGGGACGTACTTGCTAACTCCGGGCC
TCCAAGGCTAGTCTACACAAGTCCAAGGAGAAGGTATCATCTGACGGCGCTGAAGAGAAGCTGCTTCAGAAGG
AGGACGAGGCGAAGATCACCACGCGCGTGGACATGGCAGATGCTAAATATGTGGTCGGCGACCACAGGAATGG
AGATGCTAAGATCGAGCTAGATGCTAATAAACGGCAATTCACGGGCCTCACAAAGGAG
GAACTGATGAAATACGCAGAGGATCCGTTCTGGGTGCGGCTCAGGTGGTTCATGTTCATACTGTTCTGGGCTC
TCTGGTTTTGTATGCTGGCTGGGGCCATCGCCATCATCATTCGGGCCCCGAAATGCGCGCCGCCACCGCCGCG
GACTTGGTATGAAAAGGGCCCACTAGTAGATATGGCTTCTGTAGAAAATTACAACGAG
GTAGCTTCAGAATTGGACAATCTGGCAAGTTCTCAAGTATCTGGCATGTTTGCCTTCTCTTGTAAGGAGACTT
ACGAGGTGTTGGAAGACCCCTCCTGCCTCGATCAGTTCAAGCAGTTCGTTGCGAAGACCAAGGAAGCGGGTAT
AAAAGTGATCGTAGACCTCACGGCGAATTTCGTATCGAAAACTCACAAGTGGTTCGAA

CTCAGTGAGAATCGATCATCGGTGTACAATGACTACTTCATCTGGGCGAAGGGTCAGGAGTTCGATCCTGAGA
AATCTGTGCCAAAACCACCTAATAACTGGGTATCGAACCTAAACACACCGATGTGGAGCTACAGTGACAAGCG
CAAGGAGTTCTACCTTCACCATTTCAACGAAGATCAACCTGACTTGAACTTCCACAAC
CCTGAGGTTGTTAAACAGTTTGATGCTGTACTCAAGATGTGGATGGACGCTGGAGCTGATGGGATTAGGCTGC
AAAACGCTCGCGATTTACTAGTGAACTCGTCCCTTCCCAACGAGTCCCCCCACGTGGGCGCGGGCAGTGATCC
CGGAGCCGACCACACCCAGCGAGCCTACTGGAAGCGCCAGTACACCAGCGACCAGCCG
CAGCTAGACCAACTATTGGCCCACTGGTCGCATTTAGTTGCTACCGCTAAGCCTGCTGAACAAACAGTATTCA
CGATATCAGAAGCCGGCGGGCGGCCTGAACTGTTCCTGCTACAGCGTAACTTAACCTCGCTGCGACCCGCGTC
CGCCGCGCCG
>1002052478_2 Heli.2-EL603472.1.5
GACGCACGCTGGCCGAGCTATGTCGTAGGCCCCATGGTGTACCCCGAGGCGTCCCGCTGGGGAGCGCCCGACT
CGCTAGCGCCCCTCTACGATGACGTCTACCGAGATATCTCATCCGCAGCGGACGGTGTGCCAACAGTGAACGT
GGTAGAGGCGTTGCAACGTTTCTGGCAGTCGAAAGCTTCGAGAAATGGCAGCGCCGGG
AACGGCGGCAGCAGTGCACTTGTCATTTACGAGTCAGTACCGGCGGCGCGTCCTCCCTACGTGTGCTATGTGA
CGCTGCCAGGCGGAGCTTGCTTTGGCAGCTTTCAGAATAGCCCTACAAAAGCAGAGGCACGGCGCAGCGCTGC
AAAGATCGCGCTTATGAATAGCGTGTTCAACGAGCACGAGTCGCGGCGTATATCTGAG
CACTTCATCGAGAAAGCAGTAGCAGAAGCGAGAGCGTCCTTCGCTGGAGACGCGGCGCGCCACCAGGACCCTA
ATCCCGGAATAGCTGCTTTTAGATTCATGTTAGAAGCAAACAAGGATCGCACAATGCTAGAGTTCCAGGAGCT
GATGACAGTGTTCCAGCTGTTGCATTGGAACGGGTCGTTAAGAGCGATGCGGGAGCAG
CAATGTTCGAGGCAGGAGGTGGTCGCGCACTACTCGGCTCGGACCTTGGACGATGTGATGCGTGAACAGATGG
CGCGCGAGTGGGCTTCCAGGGAAAGAGAAGCTGTGGCCAAAGGTGGTGGCGTATTACGAAGTGAATTAGCAAG
AGCAGAACTAGAACTACAGACAGCGAGGCTAGCAGCTCGAGAACTACGCTTCCCAAAG
GAGAAACGTGACATCCTCCTCCTGGCGCTACGCCTCGCTCCCCCACAGCAACAACAGTGATCTCACCGCGCGA
CTATTTGTTATTTAAGTATAGTTATAGTATAAGAATATTTTGTGTTTCATTTTCCTTTACCTTGCGTTTGTAT
GCTTTCTTTTTAAGAATCTGTAATGATCTGAATAAGAGCTTTTTTATGTCATAAAAAA
AACAAGTGAATTGAAAATAATTATATCCTTTTTCATTATATGTATAGT
>1002052479_2 Heli.2-DT663678.3.5
TGGAGTGGCGGCCATATTTAATTTATTGTTTTTTTTTAATTGTGAGGATAGGCTAAGGATGTGTTTTAGTCTG
TAATTTTTTTTGTGTTAAATAATTGTTTTATGTTATTTATTTTGGTGGCAGAAGAATTGTGAGCATTGAAAGA
TAGAAGAGAAGACAGTATACAATGTTGGTTTTCTTCATTATCTTCATTACGGTAGCAA
CCTCTCAAGAGATACCAACCCCGGCCAGCTTGATCGCGCCCAGGAATTTTAACTACCACCCGTATCAGGTTCA
ATTCTTGAAAGAAAATAATATTGAGCCTACTAAACAAGGCCCCGTCCTCTTTCCGAATGACGGCCCCCCACCA
CCTAAACCTCCTCTCGTGGTCACTTCCCGACCCCTCATCGAATCTATCGCCAGAAGTG
ACTTGAGTGACAATACGACCACAGCTGCATCGAATCCCAACAAAATAACCATATATGATCAGAATTTTGCCAA
AAATCCCGAAAATATATATGATCAAACATTATTTGGAAATGATTTTGCTAATAATGTCGAATATCAGGCGTCT
ACGTATAATGGGATCCCGTATGGAGATGTCCTGAAGAATTTAAGAGTGAATGACTTTG
GGAGAAACGATTATAATGATTATGGGGAAAGAACCGTTCTACCTCCAATTTACAAAGCATTAGAAGACCACGC
CAATCAGAATGTCTTAGACTTGCAGAAGAAGCAACTTAAACATTACAACGAACCAAAATATGTTGATTACACA
AACCAATATGAAAACAACGATGACTCACAGCAAAGCAGTGGGTCAG
>1002052480_2 Heli.2-EL598071.1.5
GCACGAGGCCGGTGCCTTGCCTCGCCAACTCGCCACGCCACACTATACTCTAAACTTTCCTCCATTCTCTACA
TCTCTATTTGCCGTCATCATGAGGGAATGTATCTCAGTTCACATTGGCCAAGCTGGTGTCCAGATCGGCAATG
CCTGCTGGGAGTTGTACTGTCTGGAGCATGGAATCCAGCCTGATGGCCAGATGCCCTC
AGACAAGACCCTGGGGGGAGGAGATGACTCGTTCAATACCTTCTTCAGTGAAACTGGTGCTGGAAAGCACGTT
CCTAGAGCCGTTTTCGTCGATTTGGAACCTACTGTAGTTGATGAAGTCCGCACGGGCACGTACCGGCAGCTGT
TTCATCCAGAACAACTTATCACTGGTAAGGAGGACGCGGCGAACAACTACGCGCGCGG
ACACTACACCATCGGCAAAGAAATCGTCGATGTCGTCCTCGACAGACTCAGGAAGCTAGCTGACCAGTGTACT
GGTCTTCAGGGTTTCCTGGTGTTCCACTCGTTCGGCGGCGGCACCGGCTCCGGTTTCACTTCCTTGTTGATGG
AGCGTCTCTCCGTCGACTACGGCAAGAAGTCCAAGCTCGAGTTCTCCATCTATCCCGC
TCCCCAGGTATCAACAGCGGTAGTAGAGCCGTACAACTCCATCCTGAACACGCACACCACGCTCGAGCACTCG
GACTGCGCGTT
>1002052481_2 Heli.2-EL597165.1.5

GTTTTGAGTTTTCTGTCAAGGTAGAAATTGGAGAAAAAAAGGCCGTCCCTTTTGGGATTCTCTGGTTTAAGAA
CTCCAATTGATCCAAAAACACCACCAAGATGAGATACTTCTTGACTTACCTGTTTGTGCTGCTGTTGTCGCTT
GCCATACCCATATTTTCGTTATACTACCTACTGACTGGAAAGGGTGAGCAAATAAGTT
TCGGATGGTTCCTCGAGAATACTTCCCCCTACATGTGGGGCACCCTGGGCATAGCGTTTGCTGTGGCTTTCTC
AGTAGTGGGCGCCGCGATGGGCATTCATACAACCGGAGTGAGCATAGTAGGTGGTGGTGTCAAAGCTCCAAGA
ATCAAGACCAAGAATTTAATCTCCGTTATTTCTGTGAAGCTGTGGCCATCTATGGAT
TAATTACGGCTATCGTATTGTCTGGTATGCTTGAACAGTACAAAGAACCATTCTTGGATATATCAATCAAGCA
ACAAAACTGGATGGCTGGCTATGTGATGTTCGGTGCTGGTCTGGCCGTCGGACTCGTGAACTTGTTTTGCGGT
ATCGCCGTAGGTATTGTGGGTTCAGGTGCCGCTCTCGCTGATGCTGCCAATGCTGCCC
TTTTCGTAAAGATCCTAATTGTAGAAATCTTTGGATCAGCTATAGGGTTGTTCGGCCTTATTGTAGGTATCTA
CATGACATCAAAAGTCAAAATGGGCAACCAGTAAC
>1002052482_2 Heli.2-DT668739.3.5
GAGGGCGAGGTCTTGATTCGCGTTAAGGCATGTGGTCTAAATTTCCAAGATTTGATCGTACGGCAAGGCGCTA
TCGATTCTCCTCCTAAAACTCCATTCATCCTCGGATTTGAATGTGCCGGTGAGATCGAGCAGGTTGGCGAGGG
TGTCACCAATTTTAAGGTTGGCGATCAAGTTGTCGCTCTGCCTGAGTACAAAGCTTGG
GCTGAACTGGTGGCTGTGCCCGCCCAGTATGTGTACGCGTTACCCGAGGGAATGTCGGCATTGGACGCGGTCG
CTATCACCACCAACTACGTCGTGGCTTACCTTCTCCTCTTCGAGATGGCTAACCTGACCCCTGGCAAGAGCCT
CCTCGTTCATTCCGCTGGAGGTGGCGTCGGACAAGCCGTAGCTCAGCTGGCCAAAACC
GTGGAGGGTGTGACTGTGTTTGGCGTTTGCTCCAAGAGCAAGCATGAAGCTTTGAAGGCAAACAACAATAACA
TCGACCATCTCCTCGAGAGGGGAAGTGACTACACCAGCGAAGTCAGAAAGATTTCTTCTGATGGCGTTGACAT
CGTGCTCGACTGTCTCTGTGGGGAGGAATGCAATCGAGGCTATTCTCTTCTCAAGCCG
ATGGGACGATACATCCTTTATGGATCATCTAACATCGTGACCGGTGAAACCAAGAGTTTCTTCAGCGCAGCGC
GTGCCTGGTGGCAAGTGGACAAGGTGTCGCCCATCAAGCTGTTCGACGAGAACAAGAGCCTGGCGGGCCTCAA
CCTGCGCCACCTGCTGTTCCAGCACGGCCGCGGCGACGCCGTGCGGCGCGCCGTCGAC
GCCGTGTTCGCGCTGTGGGCCGCCGGGAAGGTCAGGCCCATCGTGGACTCCACCTGGGCCTTGGAAGATGTGG
GTGAAGCCATGCAAAAGATGCACGATCGCAAGAACATCGGCAAGCTCGTACTAGACCCTTCATTGGAGCCAAA
GCCTAAACCAGCAACGCCTGCCAAAGGCAAATCTGGCAAAGAGAAGAAACCAGCTAAA
GAGAGCTCTGAAGAAAAGAAGGACAAGGAGCCAAAAGAGGAGGAGAAGAAAGCGGAGAATGGCGACAAGAATG
AGTCAAAGGAGAAAGAGAAGGAGAAGGAGAAAGAATCTAGCTGAATTGTTTCGCCCGAGGACAGGACCAGACA
AACCACCGCCAATTGATTATTTTAAATTTGAAGCTACTCTCGTCTCGTTCGGCTTGTG
CTTACACCGGCCTCATGTTTCCTTTTATAAGTCTGAGTACGGCACTGTCCGATCCCTTCATTTCTTATTGTTT
ACAGCGACGACCTCAGTTCGGACATTTTAATATAGTTTCTATTAATTTTAGTATAACATCACATTCTATCATT
TAGATTTAAAACAAAATATTATTATTATTATTATTCAAGTACCACATTCGAACAGCGA
ATTCGTATTAAGTTAATATGACACACATTCTATTTTGAATGTGGCCTAAGTTAGCTTCCGAACTGTCGAGTCT
TATAGTTTAATGTTTAAGATTTTATTTTTAGGATATTTTAGTCTGCAGTTATTTTGAATGGCTATTGTATATC
TAAGGTGCAGAGATGCTTAATCTTTATGTATGTTAAGCTTTAATATTTTATTAGTAAA
TGTTTAAAATTTGGCCCCTGGTGACCCACCCCCGCCCTCGCGCGCCCCCCGGAACTGCAAACGAAATCCTTGC
GTGTGATATTTTGTACTAGCATGCCTATGCTGGAGGTTCTGCGGTATTATTATGCTGCCTATACATGACGTTC
ATTTTGTTTGGGCTTAAGAAAACGATTGAGTGTTTTATAATTATATTTTTTATACTTA
GAAGTTTTGTAAATTATTTTCTAGTTAAGAGAAAAGTCAAATCAAATACATCGCATCTGCCACTGTCTGGTAT
GACACTAGCAGTGGACTTGCCAGGTATACAAAGCTCGAAGTACCGTCACTCCCTCATCGAGACTAACCAAATA
ATATTTGTAAAATGTTTAAAGTGACTCCTTGAATTTGTGGATCCCGCATTCGTTCAAC
ATTTAAGTATTAATATAAGTCGTATTTTTGTAAATCAAATGTCGACGCGAGATCTCTTTTATAAGTATAAAAA
CTTTTATGTAT
>1002052483_2 Heli.2-CV525981.2.5
GTTCTCTACGTCTGCAGTTTGGTTGTGTACCAGTTCAAAAATCATTTCAAAATTTTTGCCATAAGGAAAAAAG
TTAAGATGACGGACAAGCCGAAGCGTCCTATGTCAGCATATATGCTTTGGCTTAACAGTGCAAGAGAACAAAT
AAAGGCCGATAATCCCGGTCTTAAAGTAACTGAAATAGCCAAAAAAGGAGGTGAAATA
TGGAGAGGCATGAAAGACAAAAGTGTTTGGGAGGAAAAGGCGGCTAAAGCCAAGGAACAATATACAAAAGACT
TAGAGTCATATAACGCTAATGGAGGTGGTGGAGAAGGTGGTGGCAAAAAGGCACAAAAGAGAGGAAAGAAGGG
CAAGAAGGCGGCGCCAGCTAAGTCAAAGAAGAAGAAGGACGAGTCTGATGATGAAGAT
GGTGGTGAGGAGGAAGAGGAAGAAAGCGAATGATCTCCCAATCTATAGGCATTCTGTTCATATTGAATAATTT
ACTTGGACTTAATTTATTGCAAAGTAAAATGGGACTGACTTTCCAAAGTCTGTATTGCATTGTCTTTAATTTT
TATATGAGTATTTTAGAAAAAAATAATTCAAAGAATAATTAATTACACATTTATTATT

GTATTTATAAAGTTTTTGGTCGACACATAATTAAATTTGTTGATTATTTAATGACAGGTGTTATTTACTAATT
TACTTTGTAGTATTGAGTAGTTGATAGGTTAGAAGAGTGCAGAATTTGGAAAGTTTCACCACAGGCTAGGGTC
TCTAGGTTTC
>1002052484_2 Heli.2-EL602668.1.5
TCATACCGTATTATATCTTGCTTATATTAAATTTGTACACAATTATTGGGTTTTACAGTAGTTCGTATTTCAA
CGCCTATTTAATATCAACCAATAACTCACAATGGTTTCGCTAAACCCTATAAGGATTTTAAAGAATGAGGCTG
AAGAAGAAAAAGCCGAAGTGGCTCGTATGTCCAGTTTTATCGGGGCCATTGCAATTGG
TGATTTAGTAAAAAGTACCCTTGGTCCTAAAGGCATGGATAAAATATTGGTATCTTATGGAAGAAATGCAGGC
CAAGTTGAAGTCACAAATGATGGTGCTACCATTTTAAAATCTGTGGGAGTTGACAATCCAGCCGCTAAAATCT
TAGTTGATATGTCTAAAGTTCAAGACGAAGAAGTAGGTGATGGTACTACATCTGTAAC
AGTTCTAGCTGCCGAGTTGTTAAGAGAGGCAGAAAAGTTGGTTGAACAGAAGCTTCACCCACAAACAATTATT
GCTGGTTGGCGCATTGCTGTGGAAGCAGCCAGGCAGGCTTTAGCTGAAGCTAGTTTTGATCATGAAAAAAACA
TGAATGAAGCAGCTTTAAGGAGTGATCTGGAGAACATTGCTCGTACCACACTTAGCTC
TAAAATCCTTTCTAATCATAAAGAGCATTTTACTAAATTAGCTGTTGATGCAGTCTTAAGGTTAAAAGGTTCT
GGAAATCTTAAAGCCATACAAATCATTAAAATTTCTGGTGGTTTGCTTGAGGAATCTTT
>1002052485_2 Heli.2-DT663193.3.5
GCACGAGGCGCGACGCTTGTCTGTGAGCTCGATCCCTCAGATACGCTGCTGGTTTACGGCGTGCCGTAACACG
ACACGTTTGGAGGGACCTGCTGCTACGTGTGTGCCTGGTGCTCTGTGAACATCGGATACTGTGCTTCTAGTGC
TGTGACAATGCAAATGTGCTGATATTTCAAATTAACATAATCACCTTAAAGGATTTTT
GACGACACAGCCAGTTCCGGATCAGCTCAGCCGATAATCACCATGAAAGTTGTTAGCCTTCAATTGCCATCGG
GACCCAGTATGGAGCGGTTACCGTTACCATTCAGTCCGACGGAGCTATTATGGCGATACCCTTTGCCTTGGGC
ACCACCTCCACCTTCACCTTTAGGTGATACTAAAGCACAGCTGCCAGCTGGTCTTCCA
CCAGAACCTAGATTATGGACTCGTGAAGATGTTGCCATTTTTTTGAAATGGTGCGAAAGGGAATTTGACTTGC
CCAATTTTGATATGGATCTTTTCCAAATGAATGGTAAAGCTCTGTGTCTTTTGACGAAAACTGATTTAGGAGA
GCGTTGCCCCGGTGCTGGAGATGTTTTACATAACGTTCTTCAAATGTTAGTACGGGAT
GCTGCGATACTCGGAAGAGTGCCTTCATCTCCTGTAACACCAACCGCACGTGCTGCACCATACCCACCGTCAC
CACATTCGCATCCACCGACCCCTACTTGGGCAGTAGACGGATTTCATCATTTTCATAGTGCTGCAGCTGCAGC
AGCGGCGCAACCCAACTCTGTCACTTTAAGTCCTGCTCCTTCAGTTGACAGTTCTGGA
AGCCCTCAAAGAGGAGATACTATGACTTACGCCCCTGCTTATGCACAACCAGTTCCTGTAACTACTCAAGCTG
CTAGTTCCGGAAGTAATCATTCTGACTCGGATGAGGAAGCTCAGTTCTCAGCTCCTCGATCGCCAAAAGAAAC
ACCTTTGACCAGTCCAGCGCCACAAACGCATGTTGTACCTCAACATTCACACTATCGC
ACTCAACATCGCGAGTTCTTCCCTAATGATATGCCAGAGTCTAACACAAATGGTAGGCTTTTGTGGGACTTCC
TGCAACAACTTCTTAATGACCCTACACAAAGATACACAAACTATATAGCATGGAAAAAT
>1002052486_2 Heli.2-DT665562.3.5
GAATAGCTCGTGCGTACAGTTGATCTCAGTCGGCTAGTGTGTGATGTGCTCTGATTGGACCACTTAAATCATT
GAGGAGTTGTCCTCTCGTCCTGTGACTCTGGGACCCATTCACGTAAAATGATGCGTCTACTGGTGATACTCAT
AGCAACTACGCTTTGTGGTTTGACTGATTCTGTTCTATCGCCACAGAAATCGCTCAAG
ACATTTACTCTTGAAGAATTGGTGCCTTTGCAACCAGATTTTTTTCCATTCAGAGTATCTGTCAATTGGATAT
CAGATACGGAGTATTTAATTATAGAGCCTGGTTCAATAAATAAATATGATGTCGTTGCTGACAATTTTACTAC
AATTATAAATGAATCGGAATTTAACAATTTAAGCCGATTCTCCGTATCATCATTTTCA
AATGACCAAAAATATTTGCTTTTAACTGCAAATAAGAAGAAGGTGTATCGGTATTCTACTATAGCAGAATACT
CGGTGTATGATCTCGAAAACAAGTCTATATCGAACATTGGCAATGGTTCACTTCAAGTGGTGGTGTGGGGCAA
CGATCATGCGCTCGCGTATGTGCAAGATAATAATGTATACTACGTGCCTGATGTCTCC
AGCCCCGATATAGTTACTCCACTTACTACTGATGGAGTAATAGGCAATATATACCATGGTGTCACTGACTGGA
TCTATGAGGAAGAAGTATTTAATGCAGCAGAAGCGACGTGGTTCTCACCTGACGGAACATATTTAGCAGTAGC
TTCCTTTAACGACTCTCAGGTGGAATCTGCCGTA
>1002052487_2 Heli.2-DT665714.3.5
CGAGGTGTACCTTAATTGTGTGTTGGTTAGCGCTCGGTGGGTTTAAAAATGGCTCTATTCAGGATCACCTCTA
CAAAATTAATTGATATACAGAAAGCATGCCCAACGGCAACTATTCTGTTAAGAAATTTGAGCGCGGAGCAAAC
CAACCTGAAGAGTGTACTTCAGGAGAAAATCCCCAAAGAACAGGAGAAGATCAAGGAA
TTCCGTAAGAAGCATGGCAACGCTAAAGTCGGCGAAGTCACCGTTGACATGATGTACGGTGGTATGCGTGGTA
TCAAAGGTCTAGTATGGGAGACCTCAGTGTTAGACGCCGATGAGGGTATCAGATTCCGTGGTCTCTCCATCCC
GGAGTGCCAACAACAGTTGCCCAAAGCGAAGGGAGGGGAAGAACCTTTACCTGAGGGT

CTCTTCTGGCTTCTAGTTACTGGGGAAATACCCACCGAGGCTCAAGTGAAGGCTTTGTCTAAGGAATGGGCGC
AAAGAGCCGAGCTCCCAGCCCATGTTGTAACAATGCTAAACAACATGCCAAGCAAACTGCACCCGATGTCTCA
GTTCTCAGCAGCCGTAACAGCACTTAACAGTGAGAGCAAGTTCGTCCAGGCCTACACG
GAAGGTGTACACAAGTCTAAGTACTGGGAGTATGTCTACGAGGACTCAATGAACCTGATCGCCAAGTTGCCCG
TCATAGCCGCCACCATCTACCGCAACACCTACCGCGACGGCAAGGGTATCGGCGCCATCGACGACAACAAGGA
CTGGTCCGCCAACTACTGCAAC
>1002052488_2 Heli.2-DT664833.3.5
AGCGCACACACACACAAGAAAAATTGGAAGTTATTTAATTGTGTCAGTGCTTACCACCAGCGGCTCATCTTTACT
ATCAAGGAATCCATAAAAAATCAGGATGAAGGTCTTATTACTGTGCATGGCCTTCGCGGCTGTGAGTCTGGCT
ATGCCTGTCGCTGAAGAGAAGCAAGAAGAGCCTATTGCACTTCCATCTGCCCCTGAAA
TCAAATCTGAAGATGTTAAGCCTCAAAGCGATTCAGCGCCAAGTCCTGTAGAAGAAAAAAAACCAGAACCAGC
ACCTGAAGCAAAAAGCGTTGAAAAACCTGACGAGGTCGCTATTGAAGCCAAGAGTTCAGATGTTGCAATAGAC
GCTTCTCCGGAGGCTAAGGCGGAAGCCAGCCCAGAAGTCAATAAAGAAGAAGTACCTG
CAGCTAAGGCAGCAGACGTAGCAGATTCTGCAGTTGAACAAAAAGCAGACGTTGCTGAACCAGCCAAACCTGA
AGAAAAAGTTCCAGAAGTTAAAGCTGCAGTTGTAGAAGAATCTAAGCCAGATGAAAGTCAAGCTAAGGTTGAA
GAAGTGAGCAATGTACCTTCAGCAAAATCTGCTGTCTCTGAAGATGTAATCGACCTGG
TCAGTGCTATCAAACCTGAAGCAGCCGTTGCCGATGATGTCGTTGACCTTGCCGCGATTAACCCATCTGACTC
TCAGCCAGTAGTTTCTGCTAAAA
>1002052489_2 Heli.2-EL600143.1.5
GCACGAGGTGATTATAGACCATGACCTTTAACTGGTGCTATTGGAGTAATAACCTTAGTTACCGGAATAGTAA
AATGATTCCATAACTTTAATATAAACTTACTATTTATTGGTTATATTATTGTATTATTAACTATATTTCAATG
ATGACGAGATATTTGTCGAGAAGGAACATTCCAAGGTAAGCATACTATTATAGTATCT
AAAGGATTACGATGAGGAATAATTTTATTTATTATCTCTGAAATTTTCTTTTTTGTATCTTTTTTTTGGGCAT
TTTTTCATAGAAGTTTATCCCCCAATATTGAAATTGGATCAATTTGACCCCCTACTAGAATTACCCCATTTAA
CCCTTTTCAAATTCCTTTATTAAATACAATTATTCTTATTACATCCGGAATTACAGTA
ACATGGGCTCACCATGCTTTAATAGAAAATAACTTTACCCAAGTATCTCAAAGATTATTTTTAACTATTTTTT
TAGGATTTTACTTTACTATTCTTCAAGCATATGAATATTTAGAAGCCCCATTTACTATTGCAGATAGAATTTA
TGGTTCTACTTTTTTTATAGCAACCGGATTCCATGGATTACATGTAATTATCGGAACA
ATTTTCCTTTTAACATGTTTTATTCGACATATTAATTACCATTTCTCAAGAACCCACCACTTTGGATTTGAAG
CAGCAGCATGATATTGACATTTT
>1002052491_2 Heli.2-EL602531.1.5
GCACGAGGGTCACTTTTGCTCGATGAGAAGATTGTTGAGTTAGTTTACGCGGCGTCTAATTTAGGATACTTCA
TAAAACATTAAATCATGAATATATTCCGTTTACTTGCGGACTTGTCCCACCTGTTAGCTATTATAATATTATT
ACTTAAAATATGGAAAACCAGATCATGTGCAGGAATATCAGGCAAATCGCAGATACTA
TTTTCTATAGTATATACGACCCGGTATTTGGACTTGGTGACAACGTTCGTGTCTCCATATAACACTATAATGA
AAATAGTGTTCCTGGCTGCATCATACGCGACTGTTTATTTAATGTATGTCAAGTTTAAAGCCACCTACGATCA
CAACCACGACACATTTAGGATTGAATTCCTCTTAATTCCATCTCTTATTCTGGCTTTA
CTGATTAATCGTGAGTTCACAGTAATGGAGATATTATGGACATTCTCTATTTATTTAGAATCGGTTGCAATTC
TTCCACAACTATTTTTGGTTTCTAAGACTGGAGAAGCTGAAAGTATCACATCTCACTACTTGTTTGCATTGGG
TTCCTACAGAGGCCTATATCTTTTGAACTGGATATACCGTTACATTGTTGAAAACCAC
CATGAGCCAATTGCCATTGTTGCTGGCATAGTACAGACAGTTTTGTACTGTGATTTCTTCTACCTCTACATTA
CTAAAGTTCTC
>1002052492_2 Heli.2-DT667174.3.5
GCACGAGGTCGACTTGACATATTGAGTGTGGTATTGAGGAATTGGACCTTTGAAGTTGTTCCGAGGGTCAAGA
GATGGCGTTCAGGTCTATAGGAGTGGTGCTCTTGGCGTGCCTTGTGGTATTAGCATCGTCAGCGGTGCCTAAA
GGTGGTAAGAGGAAACCAGCTAACCCACCAGCCCGCGTCGCCGCTGATGCAGAACGAG
ATGCAGAAATAACGAATTCCTGTCCAGATGATGGCTTCTTTGCTGATGCTGAACAGTGTGACAAATATTATGA
ATGCAGAGCGGGTGAAATAATTGAAAAGCTGTGCCCTGATGGTATGGTGTTCAACGACTACAGTGCCCAAGAA
GAAAAATGTGACTTGCCATTCAATATCGACTGCTCTCAAAGGCCCAAACTACAGACAC
CTATCCCTGCCCAGCACTGTCCACGCCAAAACGGTTACTTCTCCCACGAAGATGAGAAGGAGTGTGGCAAGTT
CTACTACTGTGTAGATGGTAAATTCAACATGATCACCTGTCCTGATGGCCTCGTGTACAACGACAAGAGCGGT
ATCTGCACCTGGCCTGATGAAGCTAAGAAGAAGGGATGTGGTGCTGCTGAGGTGTTCC
AATTCGACTGTCCCGCTGTAAATGAAACTTTCGGTCTAACTCACCCACGGTACGCGGACCCTGATGACTGCCA
GTTCTTCTATGTATGCATAAACGGTATCACTCCTCGTCGCTCTGGCT

>1002052493_2 Heli.2-DT666740.3.5
GCTTATTTTTGCTGGAAAGCAGCTTGAAGATGGACGCACACTCTCCGACTACAATATACAAAAGGAATCTACC
TTACATTTAGTACTGCGTCTGAGAGGTGGGATGCAAATTTTTGTGAAAACACTGACAGGAAAAACTATTACTT
TAGAAGTAGAAGCTTCTGATACTATCGAAAATGTTAAGGCAAAGATTCAAGACAAGGA
AGGCATTCCTCCGGACCAACAAAGACTGATTTTCGCGGGAAAGCAACTGGAAGATGGGCGCACACTCTCAGAC
TATAATATCCAGAAGGAATCTACGTTGCACTTAGTGTTGCGTCTAAGGGGTGGAATGCAAATATTTGTTAAAA
CATTGACAGGCAAAACTATCACATTGGAAGTGGAAGCTTCTGATACAATCGAAAATGT
TAAGGCAAAGATTCAAGACAAGGAAGGCATTCCTCCAGACCAACAAAGACTGATTTTCGCGGGAAAGCAATTG
GAAGATGGGCGCACACTCTCAGACTATAATATCCAGAAGGAATCTACGTTGCACTTAGTGTTGCGTCTAAGGG
GTGGAATGCAAATATTTGTTAAAACATTGACAGGCAAAACTATCACATTGGAAGTGGA
AGCTTCTGATACAATCGAAAATGTTAAGGCAAAGATTCAAGACAAGGAAGGCATTCCTCCGGACCAACAAAGA
CTGATTTTCGCAGGAAAGCAACTGGAAGATGGGCGCACACTCTCAGACTATAATATCCAGAAGGAATCTACGT
TGCACTTAGTGTTGCGTCTAAGGGGTGGAATGCAAATATTTGTTAAAACATTGACAGG
CAAAACTATCACATTGGAAGTGGA
>1002052494_2 Heli.2-DT665532.3.5
GACTACGTGTAATTGACCTAGCGATCGTGCAATTTTATCGTTATACATTAACGTAGAGATAGATAATTATTAT
ACTTATCAAAATTATATCGTATTTGTGCTCAGTTCGAGACGTGTGTAATTAGAACGAAGTAAAAGGAAGTGAT
ATTTGAAATAATCTATCTATCGCAGTGGTCTACGCAGTTCGACACAGATTAATCGAGG
TAATTATAAAATACCAAAATGCCTAGAAAAGTTTACGTAGTCGGTGTAGGTATGACGAAATTCGTCAAACCAA
ATAGTGGAAAAGATTATCCAGATCTAGGCAAGGAGGCGGTCGTGGACGCGCTCGCTGATGCTCGTATTAGATA
CGACGATGTCCAACAAGCGGTCTGCGGCTATGTCTTCGGCGATTCCACTTGTGGCCAG
CGAGTACTGTACCAGGTCGGCATGACCGGTATACCCATATACAATGTCAACAACAACTGTTCCACAGGTTCAA
ACGCTCTTTTCCTCGCAAAACAACTCATTGAAGGTGGTGTCTCTGATGTAATACTCGCTGTTGGGTTCGAGAA
AATGACCCCAGGAGCTCTTGGTGGTGGTGCTTATACAGATAGAACAAACCCATTAGAT
AGGCATACTCTTAAAATGGCGGAAATCACAGAACTTACTGCGGCCCCAATGACAGCACAATATTTTGGTAATG
CAGGCATGGAACACATGAAAAAGTATGGCACAACAGAAACTCATTTAGCAAAGATCGCT
>1002052495_2 Heli.2-EL602474.1.5
GACACCAACATGAAGTACACGGTTGTATTAATAGCCTCGTTGGCGGTGGTGGCCTTTGCCAGTGAGGAGAAAG
GCACACAAAAGGTAGCTGCCGAAGAAAAGAAACAAGATAAACGAGGAATTTACGACATCGGTTCCTACGGAGG
CAGTTACGAAGATGGCGGTCACGGAGGTCATGGTGGATTCAGCTCAGGCCAAGGTAGC
TACGAGAGCTACGGTCACTCAGGTTCCGAATTCGGAGGATCTTCCGGAGACTCGTGGAAACCAATTGCCAGTG
ACCATGGCCATCATCACCACGTGAAGACCATCGAAGTCATCAAAAAAGTGCCTGTCCCGTATACCGTTGAAAA
GCACGTCCCTTACACAGTTGAAAAGAAAGTTCCCTATGAAGTTAAAGTACCTTATCCC
CAACCCTACACCGTTGAGAAGAAGGTGCCAGTCACTGTCAAGGAATACGTCAAATACCCAGTTCACGTACCAG
AACCTTACGTAGTTGAAAAGAAGGTGCCTTATGAAGTCAAGGTCCACGTTGACAAGCCCTACGAAGTGAAGAT
CAAAGTACCCACTCCTTACACTGTCGAAAAGAAAGTACCTTATGAAGTCAAGGTCCCC
GTTCCCCAACCCTACACCGTCGAAAAGAAGGTCCCATACGAAGTAAAATACGAAGTTAAGGTACCCCAACCCT
ATGAAGTCGTCAAGAAGATCCCCTATGAAGTGAAAGTACCCGTCGACAAGCCTTACCACGTATATGTAGAAAA
ACCTTACCCTGTCCCAGTTGAGAAGCCTTACCCAGTAACAGTTCATAAGCCAGTTCCT
TATGAAGTTAAGGTCCCAGTTGACAGACCCTACAAGGTTGAAGTAGAAAAGCCCTACCCAGTCCCCGTTAAAA
TACCAGTACCCAAACCCTATGATGTATACAAGAAAATCCCTTACACCGTCGAAAAGAAAGTGCCTTATGAAGT
CAAGGTACCCATCGACAAGCCCTACCCCGTATACAAAGAGGTTCAAGTACCTCTAGTC
AAAGAGGTCCCCTACCCCGTAAAGGTTCATGTACCTATATACCTGAAAAAGGAGGAGGAACACCATGGCTGGC
ATTAAATTAAA
>1002052496_2 Heli.2-DT662890.3.5
AACATGAAAGCGTTCTTGGTATTAGCAGCCGTTGCGGCGATCGGCTCGTCGGCGAGGCCCGAGGCGGGATACT
CCTACAATGCGCCCGGTGGCGGTGGTCTCGGTGGTCACGGGGGCGGCATCGGAGGCGGCATCGGAGGCGGCGG
TGGCGGCCACGGCGGGCTTGGCGGTGGCTTCTCCTCCGGTGGCCTGAGCTCCAGCAGC
TTCGGCATCGGTGGCGGATTCGGCGGAGGTATCGGTGGAGGTATCGGCGGAGGTATCGGCGGAGGTAGCATCG
GTGGAGGATTCTCCTCCGGCGGAGGTTTTGGCGCCGGAGGCGGCGGCGGCTTCGGAGGTGGCTTCGGAGGAGC
CCCCATAGTCCAGAAACACATCTACGTCCATGTCCCACCCCCAGAACCTGAAGAACAA
CGCCCACAAATCATCTCCGGCGGAGCCATCCCCCAGAAACACTACAAGATCATCTTCATCAAGGCCCCAGCAC
CTCCCGCACCTGTTGCCCCCATCATCCCCGCTCAGGCCCAAAACGAAGAGAAGACCCTCGTATACGTGCTGGT
CAAGAAACCCGACGAACAGCCCGACATCACTATTCCCACCGCCGCCCCTACTCAACCC

TCAAAGCCCGAAGTATACTTCATCAAGTACAAGACCCAGAAGGAAGGTGGTTCCATCGGTGGTGGTGCTATCG
GCGGTGGTATTGGAGGTGGTATTGGCGGTGGTATCGGCGGTGGTATCGGCGGTGGACTCGGTGGTTCCGGCAT
TGGTGGCGGTATCTCTGGTGGTAGCATCGGCGGCGGTCTCTCCGGTGGCAGCATTGGT
GGCGGTATCGGAGGCGGCATCGGAGGCGGACACGGTTCCTCGGGCTCCGTCAGCTCCTCGTACGGACCCCCCG
GACACTCCGGCGGCCCATACTAAACCCGCACACCGCGTGCTAACTTGTGATACCTGCTCAGCCGGCGACAAAC
CTAGTTTATAAGTAGAGAAAAAATTACGGTGCCTAAATGTATTTATGTAAATAGTAAT
TTTAATGTATAATTTTAGTTGTATTATAAATCTTTTTTGCATAAAAAACTCGATAAACCA
>1002052497_2 Heli.2-DT664808.3.5
AAGGAAGTGCCATTGTTGACTGTGATCCTGTGTTGTAAATAGTGTACGAAATGTATTCAAAGACATTAACTTT
AATGATATTGTCGATTGGATTTTTGGACTTGGGTGACTGCCAATCTTTACAATGTCCTAAAGTGCGCACCACC
AGTGGATGGTTAGATTTATTCCCAATACCCTGTCGAAAAAGTACAGATTGCAGTGTGA
TGGGAGCCCAGCATCTTTGCTGTAAGGGATTCTGTACGAAAGGAGCAAAAGTGGCTCAACTACGAAACAAGGA
GGCGAGTAGTGTAGTTTCATCGAGTAGTTCAAGCCCCAGTACTAGCACAAGCACAAGCACTAGCACAACCACA
ACAACCACAACAACGACAACAACAACAACTACTACAACGACTCCAAGTACTACCAGTT
CGACGACCACGACGACTACCACGACCCCCAAGCCGACCACCACCACGACGTCAACGGAACCGCCGCGCCCGTT
CCTCCAGTTGCTGCCTATACAAACGACACCTAAAGCTCCCGCAAAAATAGGTAAGAACCAAAAGCTGATATGT
CCAAAGAATATAGCAATTGTGTTGTTCCCAATTCCCTGCGAGACCAACGCTCAGTGCC
GCACGAGCAGCGGGCCCGGACAAGTTTGCTGCCAAGGCCAGTGTGTGAAAGGAGTGCCCGCCCCTCGACCTAC
CGTGTCGGAACAATCACACCAACCTATTCTCGGAGTCCCTCGCGAGTAGTGCCCAGCGGCGCCGCTGGGCGAG
CTGCTGTTCGAGGTGCAGACGTGCAAGTCGGACGCGAACTGCTGGCCGCGCGTGTGCT
>1002052498_2 Heli.2-DT664312.2.5
GCACGAGGCAACAACCAAAATGTCCGCTAAGGTAGTAATCGTCCTTGCTCTGGTCGCGGCCGCCTCAGCCCTG
CCCGTGCTGCAGTACGCAGAGCCCGAAGCACCCGCCAACTACGAATTCCAATACTCCGTACATGACAACCAAA
GCGGTGATGTGAAGCAACAACAAGAGGCCCGCGCTGGAGACGCCGTCCACGGCTCCTA
CTCGCTCGTGCAGCCTGATGGAGTCCACCGCATCGTGGACTACAGCGCTGACAAGGTGAACGGATTCAACGCC
GTCGTGCGTTACGAAGGTCAACCCATCGTTTCTGCTCCCGCTAAGATCGCCTACGCTCCCGTCGCCAAGCTCG
CATACGCCGCTCCCGTCGCCAAGGTGGCCTACTCCGCGCCCGTTGCCTATGCCGCTCC
CGTCGCCAAGCTCGCCTACGCTGCTCCCGTAGCCAAGGTTGCCTACTCCGCCCCCGTCGCCTACGCCGCCCCT
GTAGCCAAGCTCGCCTACGCCTCTCCCCTCGCCCACGTCAGCTACTCATCCCCCGTCATCTCCTACCAACACT
AAGTCAATAGCTATAGCTGATATTGTCTTTAAAGTTAATTAATTTATTTATGAAATGT
GATAACAGTTGTTGCAATTAAATATTAACAATCATGTTAAAAAAAAAA
>1002052499_2 Heli.2-DT665987.3.5
TTGTGGTGTGGTGACCAACGCGGTATTACTATTTTGCCGCCTAGCTCAAGTGTGATATATATCTTTAATTGAG
TTGAACTTTAAATTTTTAACACACATATTAGTTATAGTTTACAGAAATCTCTATCTTTATTAAACTGAACAAA
AACTACCAAGATGTTTTGGGGACTTATAATGGAACCGAACAAGCGGTATTCTCAGCTT
GTGGAGAAACCGTTTCATATATCACAAGCCGCGATGGACATCTCAACGGGTGATAATGACCCATGCCAAGTAA
TGGTTGTAGTTGACGGCAAAAATTTTCTCGTGTGCACGTTACAAAAGAACAAATGCATTCAAGTACCTTTGGA
TCTGTACTTTAAGACTGGAGATTCCATTTCATTTTTAACAAATGGTAAATGCAATGTT
CACTTAACGGGCTACTTGGATCCTGAATTTGAAGAAGAACCTTCTGATGAAGAAGAAGAAGAGGAAGAAGAGG
AGGAAGAAGCACCAGCACTTGTTGCCGCCAAGAATAAAAGGAAATTAGAAAATAGCAATGATGGTGCTGCCAA
CAAAAAGGCAAAGCAAGACAAGAAAGCCAACAAGAAGGCTGCAGCAGCTGATAGCTCT
GATTCTGATGAAGATGGTGAAGATCAATTGCAAAAGTTTTTGGATGGAGAGGACATTGACACGGATGAGAATG
ATGAGTCCTTCAAAGTGAATACCTCGGCTGAAGCTGACAGTGATGACGATGAAGATTCAGATGAAGATGAAGA
GGAGGAGGAAGGAGAAGAAGAAGAAGATGATGATGATGATGAGGAA
>1002052500_2 Heli.2-DT666925.3.5
CGTGCCTTTCGTAATCGGCAAAAATCTAAACAATCCTTTTTCTATTACATATTATATTTAGTCATATTTCGTG
CACAAACATTATATCAAATGGCAGACAGAGACACAGATGAGTTAAACATCGATAACGTAATAAAAAAATTATT
AAAAGTGAGAGGCGAGAAGCCTGGCATGAATGTACAGTTAACGGAAATCGAGATTAAA
GGGCTATGCTTGAAATCGAGAGAGATATTTCTGTCACAACCGATATTACTTGAGTTAGAAGCACCGTTGAAAA
TATGCGGTGACATTCACGGACAATACTATGATTTGTTAAGGTTGTTTGAATATGGTGGTTTTCCACCTGAATC
AAATTATTTGTTCCTCGGAGACTATGTAGACCGCGGCAAGCAGTCGCTGGAGACGATA
TGCCTGTTACTAGCATACAAGATCAAATACCCTGAAAACTTCTTTTTGTTGAGAGGGAACCATGAGTGCGCCA
GCATTAATAGGATTTATGGATTCTATGACGAGTGCAAAAGGAGGTACAACATCAAGTTATGGAAAACGTTCAC
GGACTGCTTTAACTGCTTACCTGTTGCCGCGATAGTTGACGAGAAGATCTTCTGCTGC

CACGGCGGCCTCTCCCCCGACCTGCAGGCGATGGAACAGATACGCAGGATCATGCGGCCCACCGACGTGCCCG
ACCAGGGGCTGCTCTGCGACCTGCTCTGGTCCGACCCTGACAAGGACACGGCCGGATGGGGGAGAACGATCCG
TGGCGTCAGCTTCACTTTTGGCACGGAGGTGGTGGGCAAGTTCCTCTCCAAGCACGAG
TTCGACCTGATCTGCCGCGCGCACCAGGTGGTCGAGGACGGCTACGAGTTCTTCGCGAAACGACAACTTGTCA
CCCTCTTCTCCGCGCCCAACTATTGCGGAGAGTTTGATAATGCTGGCGCCCTTATGTCCGTGGACGAGACACT
GATGTGCTCGTTCCAGATATTGAAGCCCGCGGACAAACGTAAACTGTACTCCGGTCTC
AACATGGGCAGACCCAACACACCGCCTCGTGCACAGCCCAAGAATAAGAAAAATTAGCCATCGCATCGAAGTC
GACGACTTGCATGGATCCCCGCGATACAACACATTGCCTACGTTTTGACAGCTTTTCTTATGAAAAAACAACC
CTTTTCTTACCTATATGTCCACAAATATTCACTTTTTCTCGTAAATGTTTTAAAATAA
ATAGGTTTTGAACTTTAAATTCCGTGATTTTTCGATTCCATACTTATTTATATCCATATGAATTAATTTGAA
GATGAATTTGATGCGTAACAAATTCAAATTGAAGTTTAAAACCTATATATTTTACTTATTAATTAAATTATGC
CCCGACTCCCATTCATTTTTGTATGTTTTGTATAAAATGTAAGATTAATTCTCAATAG
TTTTAAGTTCAATTCCCGCTCGCATTATCAACAATTGCTGGACAATTGTGTTCAGTGGACATGAGCTTTTGGA
CTCGAAGAACTGTCAAAATTGTGAAAAAAATATAAGCTTAGGCTGTCCTGCGTAAAGCCTTTGCATGTTCCAA
ACAAATGTCAAACGTCAACGTAAACTGTCAGAATAAGAGAAAAATTATAAAGCCTAGG
CTGTATACTTTCAAGTATTTGCTTGTTCGAAAGAAATGTCAACGTCAACGTCAAATGTCAATATCTGTCAAGC
CAGTTGGATGATTTGTAAGCTGCCAGTGCGTTTTA
>1002052501_2 Heli.2-DT664177.3.5
CACAACTCAACTTGTCTTCGATAATGACCGGTGGAAGCTAGTGGTGCCGTGTAGTGTCTGAAAAATTTTTTTG
TGTTGTGTATTTCAGTTTTCATGATGATTTCAAGAGCGCTCCTAGTAGCGCTGTGCATCGTGTACGCGACGTC
GTTGAGCCCATTAAGTGTTCCTGTAGACGACGACTGTGCACTCTACCTCACTGGTCCA
GGAAGATCGTCGGCATATGACTACAGCTTAAACCTCCTTAAAGGAAATTTTGCATACGATGGTCAACACACCT
GCATCACGTATGAGGCAATCAATCAAGCGTATCTTGACGCCAGGTCCAGAATTTTGGTGTCACAACCAAAAGG
CGATTGGAAGGGTGAAGACTTCGCTAGTGTCGGGGAGCTGATTCTGGATATTTCCATT
AGTTTGGCACGCACATACGGTTTAACATATGAAGAAATAGAAAAGGGTCTACCTCTCATAGACACTTCCCGTA
CACTTATCCGCGAGGTGTGTCCACCAGTTTTCTCCCATGTAGAGTGCCGCGCGGGCAAGTACCGCCGCCTCGA
TGGACTGTGCAATAACTTAAGGCATCCCACATGGGGCGCGACTATGGCGCCTTTCCAA
AGATTGATCGGTCCCCTGTTCGCGGACGGCATCAACGCGCCGCGCATATCGCACACCGGACGCGACCTGCCCC
TGTCCCGCGTG
>1002052502_2 Heli.2-DT668048.3.5
CACGCGGAGCTGTGGACGTTAAATTGGTACTTTAAAAAAAGTAGTTTGACTTTGACTACTACTTGTTCGTTCT
CGTGTTTTATAATTATTAATTATTTTTTTTTGAAATGGCTGGAAATAATGTCAAGCGGTTATTTAAATATGTT
TCTGATCAAACTATTACATCTACGAGGAATTATGCTTCAAAAAGAGTTGTTGCCTCTA
AGCCTGTTGTGGAAATGGATGGAGATGAAATGACAAGAATCATTTGGGAAAAAATTAAAGAAAAGCTTATATT
TCCTTATGTCAAGTTAGAAAGCTTGTACTTCGATTTGGGCCTCCCGCACCGCGACGCCACTGATGACCAGGTC
ACAATCGACGCGGCGCACGCTATTCTGAAACACAATGTTGGTATCAAGTGCGCTACTA
TTACACCTGATGAACAGCGAGTGGAAGAATTCAAGCTGAAGAAAATGTGGCTTAGTCCAAATGGAACTATAAG
AAATATTCTCGGCGGTACCGTGTTCCGTGAACCCATTTTATGTAAAAGCATTCCTCGCGTAGTTCCTGGATGG
ACGAACGGTATTGTTATCGGTCGTCACGCTCACGGTGACCAGTATAAGGCCCAAGACA
TTGTTGTTTCTAAACCTGGCAAGATGGAGTTAGTATTCACAGCGGAAGACGGTTCTGTGGAGAAGCGACTTTT
GTATGATTACAAATCACCCGGTGTGGCTATGGGCATGTACAATATTGACGAATCCATTCGTGCCTTCGCACAC
TCTAGCTTCC
>1002052503_2 Heli.2-DT664781.3.5
AACGCCGCTTGCGATGTTCAACTCCGCTTGGCGTCGTTGCCACTGGATGAGCTGACGCTGATTGATGAGGAGA
CTAGGCGACAGATGTTCGAGTCTTCTCGCAAGCCCCCATCTGACGCCAGCAAGTGGAGAGTAGGTGGTGAGGA
GTTCGAAGCCCTGATGCGTATGTTGGACAACGCTGGGTTCCGCACCGGATACATTTAT
AGACATCCATTGGTCAAGAACGACGTGCCCAAGCCCAAGAACGATGTGGAAGTGCCACCAGCAGTCTCTTCCC
TTGGATATCTTCTTGAAGAGGAAGAGTTGTATAAACAAGGATTATGGAAGAAGGGCACAGGCAAGGCCGGCGA
ACGCACCCGCTGGCTAAACTCCCCCAACGTCTACCAGAAGGTCGAGGTCCTGGAGACC
GGCACCAGCGACCCTAAGAGGATCACCAAGTGGGTGGACGCTAACAACGAAGAGTGGGTGCAAGATGGTTCTC
CGGCGCACTCTAGTACTCCAGTGAAAATTGATACGCTACAATTTGTGCCCAAAAACACAAATCCTAAGGAATT
TAAGCAGCTGCAACAACAGATAAAAGATAACCGTCGCGCTGACAAAATAAGCGCGGGC

CCGCAGTCCCACATACTAGAGGGTGTTACATGGGATGAAGCGTCCAAGCTGGTGGGTGAGGACGCCGTTAATA
CCCACACTGGGGATCATGTGGTCCTGATGGGCGCAGCGTCCAAAGGTATAATCCAGCGCGGCTACCAACACAA
CGCAGCCGTATACAGCGCGCCCTACGCCCGCAACCCGTTCGACCAAGTCACCGACAAC
GAGATAGACGAGTACCGACGCACTGTTGAGAGGAAACAGCGGCACGATTACGACACCGACATCTCCGAGTCGG
AGGCGGTGAGCGCAGCGCAAGTCACGTCACCCAACACCGCGCCTTCTGACACCGAGGATGAGTCACGTGATGA
GCAACGTGTTCTCCGGATAGAGACCAAGCAAGTGCCCGTGCGCAGTCAGCCGGAGGTC
GTGTTGAGTGATGTGGACACGACAGATTTCTTGTCAGCGGAGCGCGAGCACGCCGATAGGACCAGAGGCGAGC
ACACTGTGAACGGCGACCACTCTGATGCCCATCAAAGCACCTTCTCGCACAGCAGTAAAGAGGGTTCTCCATC
CAAGGAGGTATCAACGGAGGAG
>1002052504_2 Heli.2-DT668242.3.5
ATACGATTAGTTTTCCATTTTAATTTTCGTCGTTATCGACGAAAGGGAGTATATTATACTCCCTTATTGTGTG
TAAAGTTTTACTCTGTGTATTCACCGATTATCTAAATATTTTATTTAAGAGGGATATATTTTCTCTTTGTTTT
GTATTATAAACATGGCTTTTTAAAATGTCGGTCCTGAAGGTAAAATCTTCGTCTCCTT
GCGGAGTATCCCGCTCAATTGTTATTGTGGATGTTGGAAGATTCCAGGATGGACAAAAAATCTATAAATCGTT
CTACTATACCACAGTCGAGGGCCGTAGCCATCGACTGTAAGAAAAATACGGAATTGACACACAAACCTTGGCC
ACGAAATAACAAGAAACGTGAAGGATCTGGCAGTTTACCTAAAAACGAAACTTATCGC
AAAAATGTACCATCACAAAGGGGTAGAGGTCAAATGGATAAGCGCCCGCGCTCTCGTGGTGCCACCAATTTCG
CTGTGGGTGGAGCGCAGAACACCGGGCTAGAAGAAGAGGAAGAGCCTAAAATAGGCTCAGTGTTTATTCCTGG
AAGCAAAAAGCAAAATTTGAACCATTTGCTGAATTTCATGTACCCCTCCCGAGGCAAT
ACAGAACGGAGGGGTCACGCTCCAAGACGTCAAGTCCAACAATGGGTCTCTTATAAAAATGAACATGATCTCT
ACCTTCGAGCCTACTGTCAATTTGTGGTTAAAGAGGATGGAGATTATAAAGTAAATTTAATGGACCCTGACTT
GCCTCTAAAATGGGAGCAAATTGAAGAAATTGTTGTAAGAAGCACTGGTAGATCTGAA
TGTCCCATATGCCTCGGTCCCCCGGTGGCTGGACGAGTAGGTCACTGTGGACATGTTTATTGCTGGGCATGTA
TTTTACATTATTCTGCAGCCCATGAGAAACAACCTCCACCATGTCCAGTTTGTGCTATGTCCTTGCAAGTTGC
TGATATGAAACCTACTCGGATGGTGCAGTGGGAA
>1002052505_2 Heli.2-DT664480.2.5
GGTCACTTTGTGTCACGATGGGGCGCCGTCCAGCGAGATGCTACCGGTATTGTAAAAACAAACCGTATCCTAA
ATCCCGGTTTTGTCGTGGTGTACCAGATCCTAAGATTCGTATCTTCGACTTGGGAAAGAAGAGGGCTACAGTA
GATGACTTCCCACTATGTGTCCATCTGGTGTCTGACGAATATGAACAGCTTAGCTCTG
AGGCGCTAGAAGCTGGCCGTATTTGCTGCAACAAATATCTCGTAAAGAATTGTGGTAAAGATCAGTTCCACAT
TCGCATGAGGTTGCATCCATTCCATGTTATCCGCATCAATAAAATGTTATCGTGCGCTGGAGCTGATAGGCTC
CAGACTGGAATGCGTGGTGCTTTTGGCAAGCCACAAGGCACCGTGGCCAGAGTGCGTA
TCGGCCAACCCATCATGTCGGTGCGTTCCAGCGACAGGTGGAAGGCGCAAGTGATTGAGGCTTTACGTCGTGC
CAAGTTCAAGTTCCCTGGTCGTCAGAAGATCTACATCTCCAAAAAATGGGGTTTCACCAAGTATGAGCGTGAG
GAGTTTGAGAAGCTGCGTGAGGATGGCCGTCTCGCCAATGACGGTTGCAATGTCCGCT
ACCGTCCTGAACATGGACCTCTTGACACCTGGAGGAAAGTGCAGAATGAAATCTACAGTGTTTAACTGATATC
TAATAAACTGTGAAGTTTTTATC
>1002052506_2 Heli.2-DT662347.3.5
ATATGTCCCTGTATCGCCAACTGTACAATCCTATGTATCCTCCACTCCCGTAACTATCAATGAGAATCCCATT
CTCAAGTATACAATAAAGGCTGCTCCAGTGACTTTAGTTAGCTCTACTTATGCTCCACAGTCTTATAGTCAAC
AAACTGTTCACAAGGATAATTCTCAATTAGATGTTGTATCAGATAACCAATATGTTGC
AGGAATAAAATATGCAACGCCAAAAGCAACCTATTACGCGCAGCCATCTTTGGCTGTTCAATCCACTGTTTCT
ACTTATCAACCCCAAGCTTACAGTCAACAAACTATCCATAAAGTTGAATCAGGTAGAGTGAACGCCTATGATT
CGTCAGCTTTGGGATATGAATATTCACAACCAGCTGTCAGTTATGAAACTTCAAATAT
AGCATCATCAACTGTTTCGCCAGTAATCACCTCAACAAATGGTCCAAAATCATACAGCCAACAAACTATTCAC
AGGGTGGAAAAGCCAAGAATTTCGTCAACACCACTTCCGGCTGTTGAAGTAACTTATCAACAACCGTCTGTTT
CTATTTATGAAAATCCTATATTGAAATACACCCAAAAAGTCACACCAGTCAGCTATAT
TGAACCTACCGCGAGCGTATTTACTCAAACTTATAAACCAATTCAGCCTAAAGTAAGTTATGTTAGTAGGCCA
GTCGAATATGAAAGTTCAGGTCATCAAACGTATGAAGCTGGTTACTCTTACAACCAACCAGAAATTCAAAGAG
AAGTCGAAACACTTAATACTGCAAAATATTCTGACGCGAGTTTAGTTTCTCAGCAATA
TTTCACGTTATCACACAATAGTGCAAACACACAAAGTGAATCTGGCAAAAATGTAGTTTTTGTATCTACAACT
CCATCTTCTCTAGTTTATGAAGACCATTATACTGAGGACCAATCTGCCAAAAGAATAAAAGCATATAAAGCTC
CAGAGTACATACCACCGAAAATTGAGTATCAGCAAGATTTTGTATCTTCAACAGCTTC

AATTCCTATTCTTCAACAAAATTCATTTGTCCAGACACAACATCAATACAACTACAAACCTGCTGAGTATTCC
CAACAATACGAAGTTCAGTCATCTTCACAATACGGAAACAGTCAATCTAATACTGATCAACCACAATTTGTCG
AGTCTATACATTTTTCGTCTTCCCCAGAAGTGTCAGCCAAAACTCAGTATTTCCAAAC
CAATTACCAATCACCTGAAGTTCAAGTAGCCTACAAAGCTGAAGAATATTTACCACCAGTAGTATCAACTGCT
TTGCCTCCAGTATCTTCTACATATAGATCACAATACTCAACGACAGCGTCGAGGGAATATTTGCCCCCAACTT
CAAAATCTAAATACACTGCACCAGAATACTTACCTCCGGTAGGGAAAGCATCTGCGGA
ACATTCTACTCTATCGCCGGTTACGAAATCTTTACAATACTTACCACCATTTGAAAGTGTCGGTTCAAGGGTA
GTCAATTTTGATAGTTTTGGTTATAGCAATGAAGGGGAATCCATAGCCAAATTAAGCACTTACCAAGATTATA
GCGTACCTTCTGTTACACCAATTATAGTGTCATCAACAGCCGCCCCTGTTACAAGAAA
GTCAAATATTGTAGTCGAATTAGCTAAATCTAATTTGCTAGGTTTTGGCACGGTTGGGCCTGATGCAGGTTTA
GTATCACATTATTCTACTCCTGTTACTATTTCATCCAGTCCTGACGATTATTTACCCTCCATTTCATCTACTT
ACGCTCCCGAAGTGTCATTAGTTCCAAGAAAATCGAGACCAAAATATATAAGACCTGC
GGTGAAATCCAGTACTGAGTCTTATGTCGATTCAACTACTGCTTATAGAGCTCCTGAATATTTACCACCATCG
GAGGAGTATGTACAAGATAATGTTAAATCTTTCGAGTACCAAAGTACAGCAGCCCCCCAAATTAAATACAATC
CTTATCAAAAACAAGTACCAACGGTAGTATCAACAATTTCACCTCCTATAAGAAAACA
AAATATTGTAGTAGAAACGGCTAAATCTCACTTACTTGGCTTTGGAAGCGTGGGGCCTGACGCTGGATTAGTT
TCCGAATATAGCACCCCGGTATCTATTGAAATATCGAAATCAGGATCCGCATACTCTACAATCCCCATTAAGC
AAGAAATAGTTGAAGTGGCACAAGTTATAAGAAAATCTAAACCTAAAGTAGCCGTTGT
AACAAAATTAAATGACTTCAATCC
>1002052507_2 Heli.2-DT665452.3.5
TAATGTGGATTTATCTTTACATAGCCCTCATAATATATGTTGATTAGGTTCTTGGGCTTTCCATTTTGTTTTG
ATGCCCCTATTCCATATTTCATTAGTTTACATATTATTAACCCATAAATAGAATAATATTGTACTTAGTTTTC
CAATTATTGATTATATTTGTACTGTTTTGACTGGATGGGGCAATGTTTTTTTATAACT
TTAATAATATTAGAATATATTTTTTAGATCTGATTGAAAGGTTCAAATTCTATTAGAATATTATGTGCAGATA
TTCTTTTCATTTCACTAAAAACAGAACTATTTTATGGGGAAAAATTATTACTGTCCTTTTCACGAGTCTAAAT
TATTAGTGATTATCTTTCAGATCAATATGTATGTTTAAAATAAGTGTGTTAGGTTTAA
ATCTGGTTTGTTAACTGTTGACCATATCGGTATTTACAATGAACTAACACTACTCTCACGGCATTGGCTTTCT
AATACTTTGTCTTAAGCATTTATATGAAGCATTTACAATATGTGGTAATGTTTTCAATAATGTTATTTATAAA
GAGGTTTTTTATTTGAGAGATATGTTATCACTTATAGTTTTATATAAATTGTTATAAG
AATACATAGTAATTTACTTGAAGTAATAGACAAATTAAGTGCTGCATATAAAATATATATGGTCCTAACAGTC
CTTCAGTCTATAATTAAAAAAAATATCTCACTATAATCAATCACATATTTACAATGTGATATTGGTTTTGACG
TATTTGTTTATAAATGAATACTGTAGAATATGTTTTTATTATTACGAAAAATGATAAA
ATAGAGAAGTGTCAGTGAAATTTTTGTAAGAAACTGTAAATACGGTCA
>1002052508_2 Heli.2-DT666076.3.5
GCACGAGGGTGCGGTGCAGGAGGTGAACTTACTGCCAAGAATTCTCTGAATTCTACATATTTCATTTCAATTA
GTGACTAAATATAAAATACAGAAATGGCTCCCCCTTACTATTCTGATCTCGGAAAGAAGGCCAATGATGTCTT
CGCCAAGGGCTACCATTTTGGAGTCTTCAAACTAGACCTCAAGACCAAGAGCGAGTCT
GGAGTCGAGTTCAGCAGTGGCATCACCTCCAACCAAGAAAGTGGAAAGGTATTCGGTAGCCTTTCCTCTAAAT
ATGCTGTGAAAGACTATGGTCTGACATTCACAGAAAAATGGAACACAGACAACACACTTGCCACAGACATTAC
CATCCAAGACAAGATCGCTGCTGGTCTTAAAGTTACACTTGAAGGAACTTTTGCACCA
CAAACTGGAAGCAAATCTGGAAAGCTGAAGACCCAATTCTCCAATGAAACAGTAGCTGTCAACAGCAACTTGG
ACCTTGACTTGGCCGGACCAATAGTCGATGTAGCCGCTGTCCTCAAGTACCAGGGCTGGCTTGCTGGTGCACA
CACACAGTTTGACACACAAAAGGCCAAGTTCTCTAAGAACAACTTTGCCTTTGGCTAC
CAGACCAATGACTTTGCTTTACATACCAATGTTGACAACGGCAAGGACTTTGGTGGTTCCATCTACCAGAAGG
TTTCAGACAAGTTGGATTGTGGAGTCAACATGAAG
>1002052509_2 Heli.2-DT668658.3.5
GCACGAGGGTGTGGCCGTCTACTTCGCTACATTGTGACTTGACAAGTTAGTGTTCTTCTACGTAACCAGCCGT
ATGCGCGTAACGAACGTTGGCCGTGCAATTTCTCTCGAAAATAATTAGTATGTGAATGCGAATTTTTTATTTG
TTCTGCGTGAAAATTAGTGAAATGCAACCGTTGTTCGCGTACTGACTGACAATCTTCG
CGTCTCTACAATTTCGAACAACCTTCATGAGGAATTGGTTGTTTATATTCTTTTAAGATAATCTGTACCCGTG
GCTCCCAGTTATGATTATAAGAATACCATGGCTACTTCAAATAGTTGCAATAATTACAATAACTATGACATCA
GGTAGTCCGTTGTTTTTGCAGAAACAATCTAGCAGAAATATTAAACCAGTAATAAAAT

GGAGTCCTAAAACGGATACATGGAGTTGGGATGAAACACCTCGCAATGATGAACCTTTTTTCATGCCCTTAAA
TTTTACTCATAAGTTACCTCCATCGGCCAATGAAACAGAACTAATTGCACACATGGCTGCTCTCAAAAGAAAC
TACTCCCGCTTATTGTGGGACAGTGATAAAAGTTCAAATTTACCTCTCTTCGTAGATA
AAGCGCTAAAATTATTAAATTTAAAACAGGTCTATTATGAAGTTGAACATTCTGTTATGTCAAAAGTTTCATG
TACAGCGTGTAAGGCTGGCGCTGGTCTTTTGCAACACTATATGAGACTTGGAAAAAGTAAAGAAGAGATAAAT
AAAATGATATATCAATTTTGTGTTTCATTAAATATTCAATCTGCTAGAGTTTGCGAAG
GCATAACACGACTCTTCGGGAGTGAAGTTGTATATGTCCTGAAACGTATAACGATTGGTGCAGATGAAATATG
CAGTTTTGTAATTGGTGATGCTTGCGGCGACGTTTATAATCCATACCACGAATGGGAAGTAGCATTTCCACCT
GTACCAAAACCAACAGCGCGTGCCCTTGATGCGCCTATAGAAAAAGCTCCTACTTTTA
AAGTTTTACAAATATCTGATACACATTTTGATCCTTACTACGCAGAAGGGGCAAATGCTGAATGTAATGAACC
TCTTTGCTGTCGGGTATCTAGTGGCCCCGTTATTACACCTGGTGGTGGTGCGGGACGATGGGGAGATTACCGA
AAATGTGATACACCGAAACGTACCATCGATCATATGCTGAAACATATTGCAGATACTC
ATACCGATATTGACTATATTTTATGGACAGGAGATTTGCCACCACATGATGTTTGGAATCAGACAAAAGAGGA
AAACCTGAAAGTTCTCCAAGAAACCGTAGCCCAAATGTCAGACATGTTTCCGGGTGTGCCTATCTTCCCTGCA
CTCGGAAATCACGAATCATCGCCGGTGAACAGTTTCCCACCTCCATACATTTCATCAC
CAGAATCTAACA
>1002052510_2 Heli.2-DT665342.3.5
ATTTGAGAATTTGGTTGATTTTCTTTTCTGATAGTTCTCTGAGTTTTGTCTTTTGTATTTAATAAATTAATTT
CAGCAAAATGTACGGACAACAACCAATTATAGTACTTAGCCAAAATACAAAACGTGACTCCGGCCGGAAAGTT
CAGTTGGAGAATATTAATGCTGGAAAGACGATAGCAGACGTTATTCGAACATGCCTCG
GACCACAGGCTATGTTGAAAATGTTGATGGATCCCATGGGTGGGATCGTAATGACAAATGACGGCAATGCTAT
ACTCAGGGAAATCACTGTGCAGCACCCTGCCGCCAAATCCATGATCGAAATCGCAAGAACACAGGATGAAGAG
GTCGGAGACGGCACAACATCTGTAATTGTCTTGGCTGGAGAAATGCTTGCTGTGGCTG
AACCTTTCTTAGCCCAAAACATTCACCCTACAGTCATTATTAGAGAATATAGACAGGCTTTAGAAGACGCTGT
CAAGTTGTTACAAGAAAAGATTTCCACACCTATTGATTTGAATGACAGAGAGAAAGTGAAAGAAGTTATCCGC
TCATGTGTGGGAACTAAATACATTGGTCGCTGGGCCGATCTTGCTGTGGACATTGCTT
TAGATGCAGTGAATACTGTCACCATTAATGACAATGGAAGAGTTGAAGTTGATATTAAAAATTATGCCAAAGT
AGAGAAGATCCCTGGTGGTACAGTGGAAGACTCCAAAGTACTAAATGGTGTGATGTTCAACAAAGATGTCA
>1002052511_2 Heli.2-DT666699.1.5
GCACGAGGATTTAGTTGATTATAGACCCTGACCTTTAACTGGTGCTATTGGAGTAATAACTTTAGTTACCGGG
ATAGTAAAATGATTTCATAATTTTAATATAAATTTACTATTTATTGGATATATTATTGTATTATTAACTATAT
TCCAATGATGACGAGATATTTGTCGAGAAGGAACTTTCCAAGGTAAACATACTATTAT
AGTATCTAAGGGATTACGATGAGGAATAATTTTATTTATTATTTCTGAAATTTTCTTTTTTGTATCTTTTTTT
TGAGCATTTTTCCATAGAAGTTTATCCCCTAATATTGAAATTGGATCAATTTGACCCCCCACTAGAATTACCC
CATTTAACCCTTTTCAAATTCCTTTATTAAACACAATTATTCTTATTACATCAGGAAT
TACAGTAACATGAGCTCATCATGCCTTAATAGAAAATAACTTTACTCAAGTATCTCAAAGATTATTTTTAACT
ATTTTTTTAGGATTTTACTTTACTATTCTTCAAGCATATGAATATTTGGAAGCTCCATTTACTATTGCAGACA
GAATTTATGGCTCTACTTTTTTTATAGCAACCGGATTCCATGGATTACATGTAATTAT
TGGAACAATCTTTCTTTTAACATGTTTTATTCGACATATTAATTTCCATTTCTCAAGAACTCATCACTTTGGG
TTTGAAGCAGCAGCATGATATTGACATTTTGTTGATGTAGTTTGATTATTTCTTTATATTTCAATTTATTGAT
GAGGAAATTA
>1002052512_2 Heli.2-DT664842.2.5
AAAAGAAGTGCACGATGAGATTTCTGATTGTAGCCGCCGTCTTCGCTTATGCCTCAGCCGCACCTAGTGCGCT
GTTGGCCTCACCTTACTCCAATGGCCTCCTCGCCTATGGAGCTCTGCCTATAGCCTCCTCCATTGTGGCACCA
GTCAACTCAGGAGACCTCCAAGGCGCTGCTATCGAAGCAAACGCTAAGGCCGCCGACA
ACGTCGTCGCCGCTGTCGACGCTGTCCGTGAATACAACGACCAGGCCGCTGAAATCCAAGGCAGAGCTATCAA
CGCAGCTGAGGACAACGCTTGGCAAGCAGTTAACGCTGCCCAAGTAGCTGCTGCCAAGATCGACGGCGCTGCT
GCCAGCGTATCTCCCGATGCCGCTCGCGCTGTAGCCGGAAGCGCAGTAGTAGCCCCTA
TCGCTGCCTACTCTGCCCCAGCTGTCGTTGCCCCTGCTATCTCTGCCTACTCTTCCCCCTTGATCGCTGGCCC
CTCTTTCGCCTACCAACGTTTTGGTTCCCCCATCCTACCTTACGGCGCCCGTGGTTTCATCCAATATTAAATT
TAATTTCTAGGCAAAGCAAATCAGGCTAAACGAAGATGTGTAAATAAAATATAAAAAC
TAAAAAAAAAAA
>1002052513_2 Heli.2-DT662891.3.5

TATGTTCTCAAAAATTATTGGTTTGAGCGCTGTGCTGGCTGTGGCTGCGGCAGGTTTACTACCAGAGCCACAC
TACTCTTCAGCTGCTGCAGTGTCATCCCAAAGTATTGTGCGACATGATCAGCCCCATGCTGTTGTTGCCAGTC
CAGTTGCGTACCATGCAGCACCAGTTGCCTACCAGGCAGCCCCTGCTCATTACTCTTC
AGCTGCGGCAGTCTCTTCTCAGTCTATCCTACGTCATGACCAACGTCATGCTGCCCTCGCTGTTGCCCCCGTT
GCCCACTACTCCGCTGCTCCTGTTGCCCACTATGCTGCTGCACCTGTTGCTCACTACGCTGCTCCTTCCCATT
ACTCATCCGCCGCAGCAGTATCTTCTCAATCAATTCAACGCCATGACCAGCAACATGC
CGCTATTGCTGTAGCTCCTGTGGCTCATTACAATGCCGCACCTGTAGCTCACTATGCTGCTCCCGTAGCGCAT
TATGCTGCCCCCGTCCACGGCGTTGTTGCCAGCCATCAAGAGGAATATTCTCACCCTAAATACGATTTCTCTT
ACTCTGTAGCCGATGGTCACTCTGGTGATAATAAGTCCCAGCACGAGAGCCGCGACGG
TGACGCAGTACATGGCGAATACTCTCTGGTCGAAGCTGATGGTTCCGTCCGCCATGTTCAATACAGCGCTGAT
GCTCACAGTGGATTCAACGCTGTGGTCAGCAACTCAGCACCTTCGGTACACGCCGCCCCCGCCCCAGCTCACA
TCCTCGCTCATCATTGATTTAGCACAATTTTAGCTTTTAATTATTATTTATTATAATT
ACGAAAATTGTCATTAAAAAGATC
>1002052514_2 Heli.2-EL599500.1.5
CATGCGTCGCGCGTCTGCCCGCGCTTTTTTTTACGCGCCAATTTTGTGAAAATTTTAAATTATAATCTGTGCT
TACAAACTAATCAACAATAAATAAAATACAGTTTTATTAATATAACATGATTATAAATAATAATTATTTTCCG
TTAAACAAAACGCAGTGATATATTTTCGAACGATACTTTTGATAAAATAAAACCAAAA
TGGAATATAAATCAAATAAAAGGACTAAATGGTGTATGTATAGAAAATTAACGACTTTTAAAACAGTTTATGC
GGGATTGATATTGTTTTATGTGCCAGTTTTATGTGACAAAGATATATCCCGACCACTGACTTATGACTATATC
TACAACAGTACCAACTACGCTCAACTGCAGAATGTAAAGCAAAATCCTAACCTATATA
ATCTTAATAATAATAATCACACCGAAACCAAAGGAACAGAAGAGTTCAAATTTAGCGATAAGTATGAGATAAA
CAAACCCGACGATCTGTTGGTGGGTTTCGCGCATGACCCATTCAGACAGTCCATACCGGTAGCTGATTATATG
AAAGGTGTCAGAACTATGGATCCAAGCAAAATGAGCAAGGATCGTGGGAAGCGGGGTG
TTTTTCATCTATACAACATGTTGGCGTGCGCGACCGGGTGCGACCCCGTCAGCTATAAGGGCTACGGCTGCTA
CTGCGGCTTCC
>1002052515_2 Heli.2-DT667859.1.5
TGGTGGCCAAATTCGTAGTTTTCTTCGGAATGGTGGCAGCAGCCTCAGCCGTTGCTGTCCCCCTCCTCCCCGT
CGCTAAGGTGGCGTACGCTGAGCCCGAAGCCCCAGCGCACTACGAATTCCAATACTCCGTCCACGACGAACAA
AGTGGTGATGTGAAACAACAACAAGAATCCCGCGCTGGAGACGCCGTCCACGGCTCTT
ACTCTCTCGTGCAGCCTGATGGAGTTCACCGCATCGTGGACTACAGCTCCGACAAAATAAACGGATTTAACGC
CGTCGTCCGCTATGAGGGATCCCCTATCCCAGCCCCCGCACCTGTCGCCAAGCTCGCATATGCTGCTCCCGTT
GCCAAACTCGCCTACGCCGCTCCCGTGGCCAAGGTTGCCTACTCTTCCCCCGTCGCCT
ACGCCGCACCCGTCGCCAAGTTGGCTTACGCCGCGCCCGTCGCCAAGTTGGCTTACGCCGCTCCCGTCGCCAA
GTTGGCCTACGCCCCCGCACCCGTCGCTAAGCTAGCCTACTCTCACGCACCAGTAGCCTACCAAGAAAACCTC
GGCCACGTCTCCTTCTCCTCACCCGTCGTCTCTTACAGCCACTGAATATTAAAATAAT
TGTTAAAACACTATTTATTCTGTTAAATACAAAACT
>1002052516_2 Heli.2-DT668041.3.5
CGTACCCTCAGTCGTGCACACTGCACGCGCGTCGCACTAGCGCAGCTACCACTCCTAACAGATCACAGAGGTA
CGCACGTGCATTACTGTTCTAGTCTGCACGCGCGTCGCACTAGCGCAGCTACCACTCCTAGTGAATTTTAAAG
ATGAGCCGGCACGTATGCCTGGCGGCCGCGGCGCTTCTGGCGTTGCTGTGCGTCGCCG
ATGCCCAGCGGCGCTTGGCCCTGCCTGACCCCAGAAGTTGCGCTAACAGAGTTCGACACTCAACGTACCGTGA
CGCACGCGGTGTCCTTCATTCGTACTTCTTCAGCTGGGAACATGCTCCGACTCGCAGCCTCGAGGTAGATTGG
CTCGATGCAAGAAACATTTGCAGACGACACTGCATGGACGCCGTTTCTTTGGAGACCC
CACAGGAAAATGAATTCGTGAAGCAAAAGATTGCTCGTGGTAATATCCGCTACATTTGGACTTCTGGCCGTAA
ATGTAATTTTGCTGGCTGTGACCGAACTGACCTGCAGCCTCCCAATGTCAACGGCTGGTTCTGGTCCGGATCA
GGTGCTAAGATCGGACCCACCACGCAGCGTAACACTGGAGATTGGTCCTACACTGGCG
GGTACGGCCAGGCCCAACCCGACAACAGGGAAGCTGCCCAAGGCAACGACGAGTCCTGCCTGGCAATATTGAA
TAACTTCTACAACGACGGCGTCAAATGGCACGACGTCGCCTGTCACCACGTCAAGCCCTTCGTGTGCGAGG
>1002052518_2 Heli.2-DT665467.3.5
CCTACCACTACCACTACAACTACAACAACCACCACAGTGCGACCACCAAGACCACCGCGACGACGACCACCGA
CCAGATATGATTATTATGATGATGATTATTATTATCCAACTCGAGATGATTATGATTATGAAGATAGAGGAGG
CCGAAGAAGTAGACCAAGGAGACCAGGAAAGCGCAGACCTCATGATGATTATGATGAT

AGATATGAAACTCGGACAAGACCTCGTGATGAACCAGAAGAAGATTATGAAGATAGACGGCCATATGACAGAC
CGAGAAGTAACAAAAGACCATATCCTGATTATAGGAGACCATATGATGACGAAGAAAGAAGGCCATTCAAAGG
TAACAGAAGGCCAAATAGACCTCGAGATAATGATGAAGACAGGTACAAGCCGGATACT
GATGAAGATGATAGACCAAGGGGTGGTAAAAGAGGAGAATCGAGACCTAGAGATAAGCTGAGACCACGAGATG
AAGATGAAAGACTTAGAGAGGATATCAGGCCTAGAGATGAAATCAGGTCTAGAGATGAAATTAGACCTAGAGA
CGAAGTCAGACCTAGAGATGAAATCAAGCCCCGAGATGAAATCAGACCTAGAGATGAA
ATCAGATCTAGAGATGAAATCAGACCTAGAGATGAAATCAAACCTAGAGATGAAATCAGACCAAGAGATGAAA
TCAGGCCTAGAGATGAAATTAGGCCTCGAGATGAAATAAGGCCACGAGATGAAATTAGGCCTAGAACTGAAAC
TAGATCAAGGGACGAAATAAGGTCTAGGGATGAAGTTAAACCCATTAGAGACGATTCT
CGTTTAAGAGATGAAATAAGACCTAGAGATGATTTGAAATCTAGGGACGAGAGAAGGCCGTCTGTAGATGAAC
CTATAGAATCTGAAGGTAAGCAGAGAGAAAGGCCAAGAGATGATCGATATCAACAGAATGAGGGTAGACGTCT
TTATGATCGCCCATACAGAAACCGAGATGAAAGGCCTTATTTAAAACCTGAAAGAAAC
TATGAAGGTGAAGAAAGAATTCAAGAAGAAAAAAGACAAATTCCAACAGCTCCTGAAAGTCAAACGTTGGTTA
AACCAAATGGTCACGGTATATTCAGTCAGCCTCGAAAGCCACCAAAATTTAAACGACCGGTCCCATTAAACGA
GAAAGACAAATATGAGTACGTACCAGTATCAACTACTAAAGCACCCCCAAAACAGGAT
GATGAATATTATGACGATTATGAAGATGAAGATACTAGAAAACCATTGCCTTCTGCGAAAACAAGTACAATAC
AACAAAGACCAAGACCAGATACAATCGAAAAAGAAAAGTTCAAGCCA
>1002052519_2 Heli.2-DT665220.3.5
GCACGAGGCTCAAGCGTTTCGTTGCAGTTGTAGCTATGGCGTGTTTAAGTTCGAGTAGGCTTTGTGCTCGTGT
TCAGGATAAGTTAATGTGTCAATTCGGACCTAAAGTTAGAAGGCAAACGGTATTTGTGAAACTGAAACCTCCT
GGACCCTCTTCGATGCGGATATTTGGAAAGACCTGTCAGCGAGTAACTACATGTGGTC
CAGCCTGTGGGTCAGCCGGGCCCGGGCCCATCACTGGTGCTGCTCGACGGCTTGCGTCCAGTCTACAGAGCAC
GAGCACAGGCTCTCTCCCAGATTACGGTACACAGATTGTAGACCCATACCGCTTACTAGAGGATGACCTAAAC
GGAATATATGAGGATATAAGATCAGAATTGGAACGCAACACAAACCAGCCAGAGCTGA
ATACCATAGCTACATACTACTTCGACGGACAAGGGAAGGCGCTCAGGCCAATGGTGGCCATACTTATGGCTAA
AGCTGTTAATTACCACGTATATGGAGAAAATAGCGCAATACTGCCATCTCAGAGGCAAGTTGCGATGATCAGC
GAGATGATCCACTCGGCTTCGCTCATCCACGATGACGTCATCGACCAGAGCGACTTCC
GCCGCGGAAAGCCCTCTGTCAACGTGCTCTGGAACCATAAGAAGGTGGCCATGGCCGGCGACTTCATCCTCGC
TGTGGCGTCGATGATGATAGCTCGCCTGCGCAGCGATGAAGTCACTCTTGTACTCAGTCAGGTGGTCACAGAT
TTAGTTCAAGGAGAGTTCATGCAGCTCGGCAGCAAGGAAACAGAAAATGAGCGGTTTG
CACATTACTTAACAAAAACATACAGAAAGACAGCCTCCCTCATCGCTAATTCTGTTAAAGCGGTGGCGCTTCT
AAGTGGTGCAGATGAAACTACATCCGAGTTGGCATTCCAATATGGTCGTAATCTGGGTCTGTCCTTCCAACTG
GTGGATGATTTGTTAGATTTCGTATCATCTGCACAGGCGATGGGAAAACCCACTGCAA
CAGACCTTCGACTTGGACTCGCGACCGCTCCAGTTCTTTTTGCTTGTGAAAAGTATCCAGAACTGAATCCAAT
GATAATGAGGAGGTTCCAAGAGGCCGGCGACGTAGAGAAGGCCTTCGAACTGGTACATAAATCACGTGGCCTC
GAACAGACGAGGTTCTTAGCTAAGAAACACGGGCTCGAAGCCGCGCGCCTCGCCGCCG
AACTCGCGGACTCACCGTACCAGAAGGGACTGGTTGTGACCACCGATTTAGTTCTAAACAGAATTAAATAAAT
TACTAAGAGCTACTACGAACGTA
>1002052520_2 Heli.2-DT664382.3.5
GCACGAGGCACTTATGGCTGCGGCCGCCCGCAACCGCTCGATTTCATTGTTATTATTCAATTCAATTTAATAT
AAAAGAAAAATTGCCAAAAAAATCAATGGTAAAGTTTTTGTAAAAAATAATTGAAACATGCCTGCTGATTCTA
CAATTATAGCAGCGGGGGGAGAGCGGGAGAGTGTAATTTTGTTTCAAAGTCTCTCGGA
GAGATCGAAACATGAAGACTTTCTACGTCGGGCGGCAGATCTGCTGGTGGAAAGAGTGGTTTTCGGTAGGGCG
TCAAGGAGTTCGAAGGTTGTGGAGTGGGCGACCCCTGAGGAAATAAAGAAAACAATTGATTTGAAACCTCGAA
ATGGTCCCGTGTCCCACGAGGAGCTTTTGGCTTTTATGGCTGATATTGCCCGGTACTC
AGTCAATACTGGGCACCCATACTTTGTGAACCAGCTTTTCTCGTCTGTAGATCCTTACGGCCTTGTCGGCCAG
TGGTTGACAGACGCACTAAATCCAAGTGTGTATACCTTCGAAGTAGCTCCCGTGTTTACTTTAATGGAAGAAG
AAGTTCTTCGTGAAATGCGTACAATAGTCGGCTGGCAGGGAGGTGAAGGTGACGGTAT
ATTTTGTCCAGGAGGGTCCATAGCCAATGGATATGCTATTAGTTGTGCAAGATTCTATCTTTATCCAGAAACC
AAGACCAAAGGAGTACACGCTGTGCCAAATCTTGTGTTGTTCACATCAGAACTAGCACATTATTCAACTAGGA
AAATGGCAGCGTTCATGGGTATAGGTAGCGACAATTGTATAGCAGTAAAAACTGATGA
ATTTGGCAAAAT
>1002052521_2 Heli.2-DT662154.3.5

ATACCGCATAAGGTATAAAAGAGCACGTGTCCTTCAGGCAGAGCAATTGTTCTGTTCATCTCTAAGAAGTACG
ACCTGAAATAAAAAATAAATAAAATGGAAAGTCTTTTGAGACACGATGACCACGATCACGATCATGATCACGA
TCATGATGATACAAGCGGAGTGGTTATAGCAAAAGGGGTTGCAATGGCGACCCTCTTC
TGTGCCTCCATGATTTGTGGTATTGTACCACTGTTCCTGGCAAGGCGTTTCAGATGGATCTCCGCTGATGATG
CTGGAAATTTAAAATCGAAAAATAGAGTGGTTATGACTTTGCTCTCGTTCGGTGGAGGTGTGTTGCTGTCCAC
AACATTTCTTCATTTGCTTCCTGAAGTCGATCACAACATTGAAGATTTGCAATCATCG
GGCCTGATGGGAGAATTTGATTTCTCCTTGGCGTCTCTACTGATGATATGCGGCTTCTTCATAATGTATCTTG
TTGAAGAACTGGTTCATATATACATACACCGGAGGGAAAAGAGGAACGGCCGCAATTCACCGCTAGTAAGGAA
CCTCAGCGTTCGTAGGAGTGTATCAAGTAGCGAGAGCAAGGAAACAGGTGATAAGAGC
GTTACCAATTCCACTGCTGATCTCATTGATCCCGCATCTATTATCAAAAGCAAGGACTTGGAAGTAAATCATA
ATCACGCCAACCACGTGCACCATGGCCATAGCCACATGCCCATCAGCAATGCCGACGATGTGACCTCAGCTTT
GCGCGGCCTACTCATCGTACTAGCTCTGTCTATTCATGAACTATTT
>1002052522_2 Heli.2-DT662361.2.5
GACGTCTGCCACAGGCTTCCCAAGATGGGCGCTTACAGATATATTCAAGAGTTGTATCGTAAAAAGCTTAGCG
ATGTTATGCGCTTCCTTTTGCGTGTCAGAGTATGGCAGTACCGCCAATTAACTCGCATGCACCGGGCTCCTAG
GCCTACGAGACCCGACAAAGCTAGAAGACTAGGCTACCGCGCCAAGCAAGGTTATGTT
GTCTTCAGAATCCGTGTTCGCCGTGGTGGCCGCAAGCGCCCGGTTGCCAAGGGTGCTACCTACGGCAAGCCCA
AGAGCCACGGAGTCAACCAGCTGAAACCCACACGTAACTTACAGTCTATTGCCGAGGAACGTGTTGGCCGTCG
CTGTGGTGGTTTGAGAGTACTCAACTCCTACTGGGTTGCACAAGATTCTTCATACAAA
TACTTTGAAGTCATCCTTGTTGATCCTTCACACAAGGCCATTCGTCGCGATCCTAAGATCAACTGGATAGTAA
ACGCAGTGCACAAGCACAGAGAAATGCGTGGTCTCACTTCAGCTGGCAAGAGCTCACGAGGTCTTGGCAAGGG
TCACAGGTTCTCACAAACCAAGGGAGGCTCACGCCGCGCCGCCTGGATAAGACGCAAC
ACCCTACAACTGCGTCGCANACGATAATTTCATATATGCTATTGACTTTCTATTTTTGTATTTATTCTTAAAT
AGAAAGTTTGTAGTTTTAAATAAAAAAATATAAAA
>1002052523_2 Heli.2-EL599972.1.5
CGTGGTCCGAAAAAAAAACGATATGAGATCCCTCGTTATATTTCTGAGTGCAGTGGCTGTGGCCAGCGCCGGT
CCTGTGGCCCAAGAAGATTCAGTGATCAAATTGGTTGCCGGAAATTTAGTGAATTGCATGAATAGTGATTTGA
GTTTGTGCTTAAAGGAACATGCGCTCAAAGCGGCTGAAAGACTAGGAACTGTTCGCAA
GCTGAACATCATCGAAGGGGTCACTCTATACAACAATGTACCAAAAGAAAGCAGGAGCTACGAACCTCTTTCT
ACTGAACCAGAACTTAGAAACAAGCAGATCACTGAGAGATTGTGGGAAAGTACCGGTGATCTGCTGCAGAAGA
GCGACTTGGAGCTGAGCTTCAATGGCGGAGAAGAAGAAGAAGAAGAAGAAAGTTCTAG
AGCTATTGGAGATGTTGACGAAAGCCGGGGCAAAAAGAAGAAGCAGCTCAAGAAGAAACTGAAGCTACTCATT
CCTCTGGCTATCTTAGCAAAAGCCAAAGCTGTCGCACTTGTAGTAATTTCCCTTTTGATCATCGCCGTTTCTG
TCTTCAAAATCGCCATTTTAGCCAAAATTGCATTTATCGCTAAGGTTATAGCCATAAT
TAAGGCACTTTTGGCCAAGAAGAATGCCCAAGAAGACCACGGGTGGGTATCTCATGAAGAACATGTACATCC
>1002052524_2 Heli.2-DT663170.3.5
ATCCGCGCCACTGTCTTTTTGTTTCGGCCATTTTTGCCGAAAGGTTGGTGTTCTTTTTCGGTCACGTATCCGT
TCGTGGCCGTTAGATCACTTTATTAAAGTTCATACAAGATGGTGCAGAAGAAGCCTAAGAAGAAGGTAGGAAA
GAAGGTAGCGGCCGCTCCTTTGGTCGTGAAGAAAGTTGAACCTAAGAAGGTTGTAAAC
CCTCTCTTCGAGAAGAGGACAAAGAACTTTGCTATTGGTCAGGATATCCAGCCAACTCGTGACTTATCCCGTT
TCGTGAGATGGCCCAAGTACATTCGTATTCAGCGTCAAAAGGCAGTGCTCCAACGCCGCTTGAAGGTGCCACC
ACCAATCAACCAGTTCACCCAGACTTTAGATAAGACTACAGCCAAGGGTCTTTTCAAG
ATCCTCGAGAAGTACAGACCAGAGACTGAGGCAGTCAGGAAGGAACGCCTCAGGAAAGCTGCTGAAGCTAAGG
TTGCAAAGAAAGAGGAGCCCCCAGCGAAGAGGCCGAATACTATTAGATCAGGTACCAACACTGTGACTAAGCT
GGTGGAGAAGAAGAAGGCACAGCTGGTCGTCATCGCTCATGATGTTGATCCTATTGAG
CTGGTACTGTTCCTGCCAGCCCTTTGCCGTAAGATGGGTGTGCCCTACTGCATCGTCAAGGGCAAGTCCCGCC
TCGGTGCCCTCGTACACAGGAAGACTTGCACGTGCTTAGCGCTCACACATGTTGAGTCCGGTGACAGAGCC
>1002052525_2 Heli.2-EL599180.1.5
TCATAAGATTGGTATTTTTGTTTGGATTAAAAACTTAATTTCGTGGTGTAATTGCAATAGTTTGTGAACACAA
TGGGAAAAGATTATGGAGAACCGATACGATACGATCCAGATTTCAGTGGCCCCACACACAACCGCTCTTGCAC
GGACATCGTCTGGCTAATCATCTTCATCCTCTTTTTAGGAGGATGGGGCTACGTCGGA
TATTACAGTATGACAAAAGGCAACGTTGAAAAACTTTTAGCTCCAATTGATTCAAAAGGTCGCCGTTGTGGTT
TAGACTCCGGATTAGAAGACAAAAAATATTTGCTATTTTTTAATATAGTAAAATGTCTTTCACCTGGGACACC
TATAACAGGGTGCCCAACCACTCAAGTTTGTGTATCCCAATGTCCAAGTAAAACGATA

TTATTTGAAAAAGAATTACGCTTAAATCCAAGTTCATTTGAGCTCATTCGTAAAGACATGGTATGCAGTGACG
AAGTAGAGAACGTGCAAACAATGACTCCGGCTCAAGCTTTACAATACATTCAAGAAGAAAAATGTGCCAGTTT
TATATTACAAAGTCAACCAGTG
>1002052526_2 Heli.2-DT666650.3.5
TAAGAGCTCCGTTCTGTGCTACACACTAAGTGCTTCAAAAGTATTGCAACCATGGCTAACATTCTAGATGTTT
CAACCGATGACAACCTGCAGTACCAATTTTTCCCGGTATCCAGCGGTTCCGTCCAGTTTAAAATAAGAGCACC
TAACGATGCGCATATTGCTCTGACTATGGGTCCCCAAGAATCTGACCCCATGTATGAG
ATATTTATTGGTGGTTGGGGCAACACGAAGAGTGTTATTAGGAGAAACAGAACCAAACCAGATAAAGTAGAAA
TTGAAACACCAAATATTTTGAATGGCGGCGAATTTCGCGGCTTTTGGGTGAGATGGGAAAGCGGCATCGTGTC
AGCGGGCCGTGAAGGAGAGTCTATTCCTTTCATTTCCTGGTCTGATCCAGAACCCTTC
CCTATTGGTTTCGTTGGAGTGTGCACCGGCTGGGGTGCTTCTGGCACATGGAAAATCGAAGATGGAGCCGAGT
TCAATACGCCTGACAAGTTAGAATACAAATTCGGGCCTGCTGCATCTGGTTCTTTGGAACTTGAGTACCGCGG
CCCTCACAATTGTCACGTTTGCCTCACACCTGCACCCGCTGAAGTAGATCCGATGTAT
GAAGTCATTCTTCCCCTTTACGTGGCTAGAGCACAGCATGAAGGCGCTCTGTTACCTGGAAAGCTGGTCTCCT
CTCACGGATGCCCTGGTATAATGAATGCCAACGAGTTTAGGAAGTTCATCTTTGAATGGCGTTGCGGGAGGTT
GTCAGTGCGCGATGGTGTTTCTGGGGCAGTGTTG
>1002052527_2 Heli.2-DT663814.3.5
GGGACTAAGTTTTTAGTTTTGCGATAAGTATTAAAAGTTCATAAGTGCCCTTCATTTACAATAGTGTTTTTTT
TAATAATTAAGTTAAACAAGACTAGATTTGCACGATGTCAGACAACACACAGGATATACTAAAAGAAGCGGCG
ACGGCGGCTGCGGTCGGTGGTTCACTGTTCAGTACTTTTGACATATTCATGCTTGTCG
TTTTATTTGGTGCAGCTGTTTGGTGGCTATATAGTTCTAGAAAAGAAAGTAAAAAGGATGATTTACTTCTTAG
TAATTACGCCATACAGCCTGCAGGTTCTATTCAAGTCACAGAGAATTCTTTTATAAAGAAATTACAAACATCA
GGTAGAAGTCTTGTTGTATTTTATGGCTCACAAACGGGTACAGCAGAGGAATTTGCTG
GCCGTTTAGCAAAAGAGGGCATTCGCTACAAAATGAAAGGGATGGTAGCTGATCCTGAAGAATGTGATATGGA
AGAATTGACTAAACTCAAAGATATTGATAACTCTCTCGCTGTGTTTTGCTTAGCAACCTATGGTGAGGGAGAT
CCCACGGATAATGCCATGGATTTTGTTGAATGGCTTAAAAACGAGCCCGAATTAATTG
GACTAAATTATGCTGTATTTGGCCTGGGGAACAAAACTTATGAACACTATAATTCTGTAGCTATCTTTGTAGA
TAAAAGACTTGAAGAACTTGGAGCCACCAGAGTTCATGAGCTTGGATTGGGGGATGATGATGCAAATATTGAA
GATGACTTCATTACCTGGAAGGAAAAATTTTGGCCATCTGTGTGTGAAAAATTCAATA
TTGAAAGTGCAGGAGAGGAAGAATTAACTCGTCAATTTCATTTGATAACACATGATCCAGAGGAAATTTCTCC
AAATGAAATATTTACTGGTGAAATAGCTAGATTACATTCACTGGAGAGACAGAGGGCGCCTTTTGATGCGAAA
AATCCTTTCTTAGCACAAATTAAAGTAAACAGAGAATTGCATAAAGGTGGTGACAGGT
CATGCTTGCATATTGAATTTGACATATCCGGCTCAAATATGAGATATGAGTCTGGTGATCACTTAGCTGTAT
>1002052528_2 Heli.2-DT664476.3.5
AGACGTGAAAATGGCCAGCGGACGAATTGTCGTATACGGTGGCCGCGGTGCTTTGGGCGCGGCTTGCGTAAAT
CACTTTAAGAAGGCAAACTACTGGGTTGCGAGCGTTGATCTGAATCCTAATGAGAGCGCAGATGTAAACATTA
CAGTCCCAAAGGATGCTTCCTGGGTTCAACAAGAAGAGCATGTTGTTAATGAACTAGG
TGAAGCTTTGCAAGGTCAAAAAGTGAATGCAGTAATTTGTGTGGCCGGTGGTTGGGCTGGTGGTAACGCGGCC
AAGGATTTGAGCAAGCAAACTGATCTTATGTGGAGGCAGTCAGTGTGGAGTTCTACAATTGCTGCAACTTTGG
CATCTAAATATTTAAGTCCTGGAGGATTAGTTGCTTTGACAGGTGCTAAAGCTGCATT
GGAAGGGACCCCCGGAATGATTGGTTATGGTTTAGCGAAGGCTGCAGTACATCAGCTTACCAAATCTCTTGGT
GCTAAAGATTCAGGTCTACCAGAAAACTCTCTTGCCGTTGCTATACTACCAGTGACACTGGACACAGAAATGA
ATAGAAAGTGGATGCCTAAAGCAGACTTTGGATCTTGGACACCCCTGTCATTTGTTGC
GCAGCTATTTGATAAATGGCTAAAAGGAGATGAAAGACCTGCAAGTGGAAGCTTAGTTGCGCTTGTGACTAAA
GATAATGTAACAGATCTGATTGTCCAGTAAATCTTGCCAAAATCTCTCACCACCACTAACTTATGTATGATCA
GAGTATAACATTTTCAATAAAATATAATGCAACTATATGTTGGCATTTATAATGAACT
TAGTTCATTAGTCAAATCGCTTTT
>1002052529_2 Heli.2-EL601168.1.5
GCACGAGGCCGCGATCCTGTGAGGGTGCAACGAGAGCTAAGATCGTTTTCGTTTACGCGCGAAAAGTGACATA
AATATAATTTTCAAAATGGCGGTTACAAGTGCCAGGAGTATTGTGAATATTAAGGAGAAGGGACATTTCGAAC
ATGAAAAAAACAAGGATTGATAAATATACGTTGGCCGACGAGCTGCCAGCTCAGCTGTC
GGAGCTGGCCCTCCGGCCACGGACCATGGCCCCCTCCTACACCCTGCGCAAGTTGACGCCATATGATAAAGAG
CGCGTAATAGAATTTTTAAGAAGGTTTTTCTTCCTCGACGAACCGTTGAACCTGACGATCAACCTATTAGAGA
CTCCCGAATCCCGTTGCGTAGAGCTGGAGGAGTACGCGTCCAGCACTCTCGGTGATGG

CGCGTCACTAGCTGCAGTTGATGAGCACGGAAAATTTGTTGGAATGGTCATCAATGGAGTTGTTAAGAGAGAG
GAAGTAGACTACACAGACAAATCAGAAGACTGTCCGAATCCAAAGTTCCGGCGGATCCTAAAACTCCTGGGTC
ACTTGGACCGTGAAGCCAGGATTTGGGACAAACTGCCGCTAAGTTGCCATACAGTTGT
GGAGATCAGGATAGCATCCACCCACTCTGACTGGAGGGGGCGAGGCCTTATGAGGGTGTTATGTGAAGAGACT
GAACGAATAGC
>1002052530_2 Heli.2-DT667892.3.5
AAATAAATTATATATTGAGTGATGATGAACCATTTTTGGTTTGAAAATGTGATTCAGTCAACTGAATTTTGAA
ATTTTTTTTTCATGGATTTTTTTTATTTGTTTGATATTTGACATTTTGACAAAGTGATTCCGTGGATTTTAGT
GGAGAAGTGATTGTTTTTAAGATGGCGCACACAGTGGTAGCCTTAGTGGCATTGATTG
CCGCGTCTACAACTGCAAAAGAGCAAACGAGATGGTCCCGCCAGATTAGCTCTTACACAGCCGACATAAGCGA
CTGGGTTCCCTTAACAAGCCCAGTGGATAAAGAAGAATTGCCACCAATCAAGAGGCAAGCAGTGGCGGAACCT
AGGATATTATCTGAACCTTTTCCGGGCTTTATAAGACCAACTGGTTTTAGTCAAGACG
GATTTCCATCAAGGGCATTCCTTAACTCTCCTGTTAATCGACAATTATATTTACAATCAGTACCGTCAGCTCC
TCAAAATTATCTCTCAGATCAGGGTTTCAACCAAGGCCTAAGATTTGGCTTGACTCAACCGAATTTCCCTTTA
AGTCAAGGATTTATAACTCCTCAGTTTAACTTTGACAGTGTTCCACAATTAAAATCTC
GACCGCACAGTTCTCTTAATCCGATCAAATTTGAAAACTCTATCAAAACAACTCATCCACTGCCATCTCCAGA
ACCTTATGTCATCAGTCAAACTC
>1002052532_2 Heli.2-DT665417.3.5
ATTAGTCAACATAAGTCTTCCTCCCCCGCTCCCAATGCTACGCAAGTACCTTTATCATCAAAACCTCATGGAT
CAGAAAAAGAAGATAAGTCATCTCTAAATGAGTTTACGAAAGAAAAGTCGGAAACAAATATCCACGAAAAATT
ACCAAGGCCAAAAAGTCCTTCCAAGCTGTCTGTTAAATTACCAACTAAATCGAAAAGT
CCAATGCGTACTGCTAGTCCTGATAAAAAAAGACCTACGTCTCCAACCAAGTCATCGCCAGCAAAACCTAAAT
CGAATCGTTTCAATGAATATGCTTCTGCTTATATGAAAAAGGTGGGACTCAAGGATGAAGAAAAAACAACAGA
GATTTCAAAAGTTAAGAAGATTTCTAATGATGAGCAAAGAATTAAGAAAATGGAAAAA
CATACCTCAGAAGCTAATGCATCGTCGATTATATCCAAAAAGTCCCCTGAACGAACTGTGTCACATGACAATT
TTGAAGTAAACACAACACAAATTAATGGAAAAAGATCACCATCGCCTGAGAATACTATTAATAAAAAAATCTA
TGAATCTGATAAGAATCGCGTTTCTCAAAAGCGTACTTCTCCTGTCCGAAAAACGTAT
AGTCCCGAGAGGATAGCACATAGTCTAGAACGAAACATCCCCGTGGCACGTTCCCCAAGTCCTAAAAGACCCC
AAGAATTAAAGCAAAAATCAGACACGGATGGATCTAAAACACAAACAAAGGAAGTTCTTATAACAACAACAAT
TGATATTGATAAGAAAATTCCTCAAAAACAAAAACAAGAGGAAAAACCATCTTGGGTG
GTGAATAGAAATTTAAAAAAGACATCTGAGACTCGTACGTTTACAACAAAAAAAGTGGAACCCGAGAAACCAA
AGTACCGAGCTGTGAGTCCTTCTAAGGTGATCTCAAAGCCTATCGATGTCATCACTTCGAGCTATGGCCCTGG
ACCCCTGGATGCTGACGGCAGGCCACTCTTTGGTATCAAAGCATTAAGAAACGGAGCT
TCAAATTATCAAGTGAAAGGTACAGTCATTCGTCAAGAGTTCCACTCGCGGAACGGTGGTGAACCAGAGGGGA
CTGTGTCTGTGACCGCCTACTCCACGGAGCCTGAGGACTTGGAAAAATTGTTGAACAGTCAGGGTGAGAAACC
GTCTAGGATTCACGGTCTTGCTGCCATCACAACGACGAAGAAGTTCGGCGGCGACACC
GGCACTACGTTTAGCGAAGTTCATAACAAAGAAGAACGTGCAGTTCTGGACCAGTTCACACACAGCGATCGCC
GCGTGACAGACAGTAGGGTCACCACGAGCAATTTTGAAACATCCGACGACTTCATTCAAAATGAGAAATTAGT
TAATGTAAATAACGTTATACATGTACAAGACAAGAGCGCAGAAGCAGAAACACATGAA
AGCCGTGATCTCAAACAAATGAAATCTGACAAGGAATCTATTAAGCAAACTACCAAAATTCACAGTGAAAAAG
ACAAAAAAGTTGGAATGGAAAGAAGGGACAGAGAAAAGTTTGATAAAAGAGAAGACAAGAAGACAGTGCGACA
GAGTTCGGTGAAATCGCTTACTGAAAAATACATTAAAAGTGCAAGTGAAACATCTAAA
ACCGAACGCCACGTGTACCCCAAGGCTGGTCTCATCCTGCGCACCTCCACTATGAAGGACAACGTATCGAGCG
ATGCTGGCCTCACTCGGACAGACAGTGAACAAAGCCTGGGGTCTGATGAAGAAATAGTGACCACTACGACCAC
AGAACAAGTAAATGACGGTGTGAAGACCACGACCACTACCACCACCAGAACAGGTCAC
TCGCATGGTCAAGAGCGATCCTTCCTCGACAGTTCTACTAAGGTCACCGGGGTACAAGATATTCTTACGAGAA
TGAAGAATGCTGATATAGTAATAGAAGAAGGCGACACCAACGCTGACACAGAGGCGCGTGCTTTATTGAACAA
GTTCCTCGGAGCCACTGTCCTGATGGCGGGCATGCAGAGCTACGTCACGGAGAAACCC
TCCGGCAAAGTCGTGGTTAAACAGGAAACCGTACAGAGCAGTGGTGGCAAGGTGACCAGCAGCCGCCGGGTAG
AGGAGTTCGATATCGACCAGTGCTGGGACGAGCGTGTTTTACGCAAGCTTTTGGACGAGTGCAGTGACTACGA
ACAAAGGCGCCGCCTGCGCGCGCGCATCCGAACCCTCATGGCTGAACAAGAAGCGTGC
ACGAGTGCGGTGACGGAGGCGCTGGCGGCGGCGGGCGCGGCGGACGCGGAGCAGGCCGGCGAGCGTGAAGAGG
AAGTAACTGTAACATCGAGCGTGAGGAGAAACTCGTCCGAGAAAACCGTTAGCAGTAGCACTACCACTATAAA
AAATTCTAAAGTGATCGAGAGCATGACCCGGCCGGCGCCGAAGCCTGTGTCGCCCTTC

GCCAAGTTTCGTCAGCTCGAGAAACAAAATTCTACTAACAGTCCCAAATCCCCCCAGAGCCCGCAGAGCCCGG
GCTCTCCGTCGCAGCCCTACTTCAAGTTCACGGAC
>1002052533_2 Heli.2-DT665094.3.5
GCACGAGGCACCACTATACGACTAAAGTGAAAAGAAAATAAAATCGAAAAGATATGAAAATAAATACTTAAAC
TAATTTAATCCTGATATTGTTTGTTTATATAGTGATTGTTTATGTTTAATGTTTACCTAATAACTGTGTGGCC
TTTGATGTGAACAAAATCGATGAAATCGTGTGTTCAGTAATTCCAGACAATATGTGCA
GTGTTGATTGCGATTGACATCTGACGTAAAACAAAAGGAAAACGAAGTTGGAGAACTACTTAGAGGATTAAGG
ATGAGTTCTACTGATACAATTAATGTAACAGCACAACCAATCTCGGATATAACTCACGAGATGTTGACGTCTG
TGTTTTTGGAGACCCGCTCTGCGCAGGTCATTGCGGGCATATTTGTATGGACAGCATT
ATTTATTACTTGCCAACAGATTTACCAGCACCTCCGCTGGTACACGAACCCGTCGGAGCAGCGTTGGATAGTG
CGCATATTGTTTATCGTGCCCATCTATGGATGCTACAGTTGGATCTCGCTCTTGTTTTTCAATGGAAATTCTT
ATTATGTTTACTTCTTCACAGTAAGGGACTGCTATGAAGCCTTCGTGATATACAGCTT
TCTGTCGCTATGCTACGAATATTTGGGCGGGGAAGGGAACATAATGTCGGAGCTGCGAGGACGGCCGGTGCGC
GCCTCCTGCGTCAACGGGACTTGTTGCCTGTCAGGGGCCACGTACACTATCGGATTTTTGAGATTCTGCAAAC
AAGCGACATTGCAGTTCTGTCTCATCAAACCAGTGTGCGCCTTCATCATCATATTCTT
GCAGGCGGCGGGTCACTACCACGACGGTGACTGGAGTCCTGACGGCGGTTACATCTACATAACGATAGTGTAC
AATTTCTCCGTCAGTTTAGCGCT
>1002052534_2 Heli.2-DT664368.3.5
TCCGATCACCCGCGAAGCCACGCGGTCTACATTGTGAAAGTGCAGTTTCTCGGTAGTGAGAGTTAGCCGAGCG
CCCACAACGCTAATATAAGTGGTGTGCATTTCTTGTTAACATAACAGAGTTCAATGGAAGCCAGGTTCGTGAC
CTGCCGGAGCTTCCAGCAGATCAAGCCGCCCGAGAAGAGTTTAGCGCCGCCACCGCCG
ACGGTTAAGGGGCTGCTGTCTCGTTTCGGAAGCCAGGCTCAAGCTTTATTCGCGCCGAAGAAGCCAAGATTCG
GGTCGTCAGTTGCTATTCATAAATTACGCGAGTCGAAGTGGTTCAAACATGAAGAGCAGAACATCTTGTGCGC
TGTCCTCGAATCCGGATTCGTACTGGAAGTCCGAGCTGAGGACCCTCTGCCGCCAGAC
TCGACACTGTCCCCTGACTACCATATGTACGCCATTGCCATGTGGAAGAAGGGTAAAGAAAAAACAAAGAACC
CGGAACAGATAGAGGTGTACACGGTGCAACAGAAGGGTGTGCGCAAACGTATTATAAAGACTACTTTGGGTGC
ATTCTGGAAGAAAGACTCTGTCATACGTATCAACAACGTAGACGACAAGCAAGAGACA
CCCAACAGTGAAAAAGACATCAGGGCAAAGCTCGACATGGCTACTAAGTCAAGATTTGAAAGGAACTGGCATA
ACACTCTGCATTTCGTCCACTGGTGCCGATACGGAGAGACTCCCCAAGAGGCTCGTATG
>1002052535_2 Heli.2-EL603054.1.5
CGAGGGTTGTTTTTGCAAATGAAAGACAATACTTAAATAAATCATGTTTATGTGCTGTTTTAGAGTTTAATCT
TATATAGCTACTTTTGTTTTTATAGTTTAATTGTTATATGTACAAGTGTGATTACAAAAATGAGTGCCAAAAA
GGGTTTTGAAACAAAAGTTTTCGATGACCAGGAGAGCGATGTTGGTTTCGAGTCCGGA
CCGTTGAGTGAACAGCTGTTGTCTTCCGAGGAAGTGGATATAGAGGAAGATAGTCCAGTGGAAAGTAATGAAC
AAGTGAACAGTGACACAGGGTTAGATAGTGGTTTGTGCTTGTCAGAGCCTCTGTCTAAAGTTAACCTTAGTGA
ATCGCCCGTCGCACAACAAGGGTCATTAGTTAATATTGAAGGTGAAAGACCACAGGAT
ATCCCACCGATTTCGATACTGTTCCAGCAAGACGACGATGGTGATACGCAGCTCCACATTGCAGCTGTGCACG
GATGCCTGAAATCAGTTGGTGTCTTGATAAGAGTGTGTCCAGACAAAGCGTGGCTAGATATACCAAATGACTA
TGGTCATACACCGTTACACCTTGCTGCTATGAGTGGTAATGCAGTTGTTGCCAGAATG
TTGGTCAGAGCAGGAGCCGCCTTAGACGTGAGGGATTCCACAGGAGAAAATCCTCTACACAAAGCAGTTGGT
>1002052536_2 Heli.2-EL599998.1.5
GCACGAGGCTTTCTTCTTGTGTGTTGTGCTACTTATGTGGATTATTAGGTGATATTCTTCTTTTTATTGGATT
GTCATCCTAAACCGCAAACATGCCTAAATCGATGAACGTCCGGGTGACGACGATGGACGCGGAGCTGGAGTTC
GCGATCCAGCAGACGACGACCGGCAAACAGCTCTTTGACCAGGTCGTGAAGACCATCG
GCCTCCGCGAGGTCTGGTTCTTTGGACTCCAGTACACCGACTCCAAGGGTGATCTCACATGGATCAAGCTCTA
CAAGAAGGTGATGCAACAAGATGTGAAGAAGGAGAATCCGCTCCAGTTTAAATTCCGGGCCAAATTCTATCCT
GAGGATGTCGCTGACGAACTGATTCAAGAGATCACGCTCAAACTGTTCTACTTACAAG
TTAAAAACGCGATCCTCTCCGACGAGATCTACTGCCCGCCCGAGACGTCGGTGTTGCTCGCGTCGTACGCGGT
GCAGGCGCGCCACGGAGACCACAACCCGTCCATCCATGGACCTGGATTCCTTGCTAATGATCGTCTGTTGCCG
CAACGTGTTACTGATCAGCACAAGATGTCCCGTGAGGAATGGGAGCAGAGTATCACCA
ACTGGTGGCAGGAACACCGCGGGATGCTGCGTGAAGACGCCATGATGGAATATCTCAAAATCGCCCAAGATTT
GGAGATGTATGGTGTCAACTACTTCGAGATCCGCAACAAGAAGAACACCGAGTTGTGGCTCGGAGTCGATGCG
CTTGGACTTAATATTTATGAGAAGGATGATAAACTGACCCCCAAGATCGGGTTCCCGT

GGTCGGAGATCCGCAACATCTCGTTCAACGACCGCAAGTTCATCATCAAGCCGATCGACAAGAAGGCGCCCGA
CTTCGTGTTCTTCGCGCCGCGCGTGCGCGTCAACAAGCGCATCCTGGCGCTGTGCATGGGCAACCACGAGCTC
TACATGCGGCGCCGCAAGCCCGACACCATCGACGTGCAGCAGATGAAGGCGCAGGCGC
GCGAGGAGAAGCTGGCCAAGCAGGCGCAGAGGGAGAAACTGCAGCTCGAGATAGCGGCCCGCGAGCGCGCCGA
GAAGAAGCAGCAGGAGTACGAGGACCGCCTGCGGCAGATTTTTGAGGAGATGGAGCGCTCACAGGCTAATTTG
GTCGAAGCACAAGAGATGATCAGACGGCTGGAGGAGCAACTGCGTCAGCTGCAGGCCG
CCAAGGAGGAGCTGGAACAGCGCCAGAACGAGCTCCAAGCCATGATGCAACGCCTCGAGGAGACTAAGAACAT
GGAGGCAGCTGAACGACAGAAGCTGGAGGAGGAGATAGCGGCGAAACAAGAAGAGGTATCTCGCATCCAACAA
GAGGTGGAGATCAAGGACTCTGAGACGCGCCGCCTCCAGGACGAGGTAGAGGAGGCGC
GCCGCAAGCAGGACGAGGCCGCCGCCCTACTGGCCGCCGCTACCACCCCCACACACCACCACGTGACTGAGGC
CGGCGGTGACGCCGACGGCGACGGCGGTGACGACGGCGAAGGCAGCGGCAGCGAGGCCGGCGGTGGAGAGCTG
GCACGAGGCCCCGACGACCTGGTCGACCCGCTAGAGGAGCGCCGCACACTGGCCGAGC
GCTCGGAGCGGCTCCACAGCCAGCTGCGAGCCCTCAAGCAGGACCTGGCGCAGTCGCGCGACGAGACCAAGGA
GACGCCCATGGACAAGATACACCGCGAGAACGTGCGCCAGGGACGCGACAAGTACAAGACGCTGCGCGAGATC
CGCAAGGGCAACACCAAGCGCCGCGTCGACCAGTTCGAGAACATGTAACCGCCGCCAC
GTCCAACTTGTACACTATTTATGTTAGGGGTCGTTCCTGAACTACGCCACACGTACATGACCATGTTTGTCAA
GGAATGGGTCTTAAATTTGGTGACGTCACATTTCGAAATATAATATACTAACATCATTTTGAATGATTGAAAA
AAAAGATTTATTAAAAATATGTGACGTCACACCAAGGGTTCGAAAATCATGAAATTCG
TGTGACGTAGTTTACGTACGACACCTTAGATGTATGTACGTGTAGCCCGGAACGGTCTCTTCGACCGGTGCTC
TGGTCGGGCGTTGTCGGGACTAAGTGTAAATATTATTGATTATTATTATTACTTAATAG
>1002052537_2 Heli.2-DT663837.3.5
TCGGTTCCGTAAAGGGACTACTGAAGCTAGACTCTGTGTGCATCGACAACAATGTGTTCCGTTTACACTACAA
AGCCACCGTGATCATCCTGATCGCCTTCTCGCTGCTGGTCACCTCTCGCCAGTACATCGGGGACCCCATTGAC
TGCATCGTCGATGAAATCCCATATTCAGTGATGGACACCTACTGTTGGATTTACTCGA
CGTTTACGATCCCCAATCGCCTCGTCGGACGCGTCGGGAAGGACATGCCCGCGCCCGGCGTAGGAAGTCTCAT
TGAAGGAGAGGATGAGGTGAAATACCACAAATACTACCAGTGGGTGTGTTTTGTGCTGTTCTTCCAGGCTATT
CTGTTTTATGTGCCTCGTTATCTGTGGAAGACATGGGAAGGTGGCCGTGTAAAAATGT
TAGTACTGGATTTAAATTCCCCTGTGGTCGATGAAAATTGTAAATCGGACCGTAAGAAGTTACTTGTGGACTA
CTTTCACACAAACCTACATACGCAAAACTTTTATGCATTCCGCTTCTTTATTTGTGAAGTTTTGAATTTTATA
AATGTTGTGGGACAAATTTACTTCATGGACTTTTTCTTGGACGGAGAGTTCTCAACGT
ATGGGCGAGACGTCGTTCGTTTCACCGAGATGGAACCGGAAGAAAGAGAAGACCCTATGGCTCGAGTATTTCC
TAAAGTTACAAAATGCACCTTCCACAAATACGGTCCTTCGGGAACCGTGCAGAAGTTCGACGGGCTGTGTGTA
CTTCCATTGAATATTGTAAATGAGAAGATCTATGTATTCCTATGGTTCTGGTTCATTA
TTCTGTCAATTCTCAGCGGCATCTCCCTTATCTACCGCGCCTGTGTGGTCGCCGGACCGCGTATACGTTTATA
CCTGCTGCGCGCGCGCAGCCGCCTCGCCCCGCAGGATCAAGTCGAAGCTGTAGCCCGCAAGCTACAAATCGGC
GACTGGTTTG
>1002052538_2 Heli.2-DT668290.3.5
GTGTTGCTAAAATTTAAAATTTCGAATTTTTATTTTTTTAACCTCGACCGCGAAATGAAGGAGCTCAATGGAA
CTACTGTATGTGAAAAATATATAGGGAGGCGTCGAGCAGTCCCCGTAGTATACGTACAAGGCTCTCACTATGA
AGTCGGCTTCGATGTGGGTCGAAATTTCGCGAGCGTCATCAAAAGCTTTTTATCCTCA
TACGCTAACCTTCGGGACTTCGAAAAGGAATACAAGACGGAAGTAGGCAGGAATGCATACGAAAAAACCCTGT
CCAATATGAAAGAAAGGTTTCCTTACTATGTAAAGGAGATGCAGGGTGTCGCTGACGGCGCCGAGGTCCCATT
CCACCAGCTTTTCCTTTTGCAAATGGATGATATAATTGGAACTATCAACGACAACCAT
ATTCCACGCAACGACACTGGAGGCTGCAGCTCTCTAGCTATTAAAACTCCGAATAGTGCGGTCTTAGGTCACA
CCGAAGATGCTTTTAGTGAGACGCTAAATCACTTCTACATTATGTCGGCACATATTATACCAACCGACGAAGA
CAAGGAACGTGGGGCAATTGAAGAACGTTTTGCTTCGCTGTGCTACGCTGGTCACCTA
CCCGGATACACTATGGGCTACAATGAAAACGGTCTGGTATTTTCCATCAACACTCTCAGCCCCCTGGTACTGA
AACCTGGAAAC
>1002052539_2 Heli.2-DT665247.3.5
TCGTTCCGCGGCCCTCGCCACTACACTACAGCTACACTACAGTCGCACACCGCACGATCTGTAGAACGCTCGC
CACAGTGGGACAAAAATGAGTAACGAGTTTTAGTGAGCTAAGCAATCAAGTGATAGTGTTAGTGTTTTGGAAC
TGCCACTTTTTTATATAACCAAAGGAATTGACTTCGACGATGATGGCTCCGGAGACAG

TAGTGACTGTAGACCACAACAAACCAGCACAGCAGCAGGCGCCGCCGCAGCAGCAGCAAGGCGGTGCTCTCGA
TTGGATTAAAATTAATATAAATTATTTCAAAACACCGCCCGGCATATTAAAAGTTATCCAACTGGTCTTGGGT
ATTCTATGTATGTCACTGGGCACACCTTGGGCATCCGCGTGGTATGTCTTCGTGGCAG
TAACAGCTTTTATAACCACTTTAATGTGGAGCTTCGTTTACTTCCTTAGCATCAGAGAAGCCTTGAAGATACC
CATCAACTGGGTACTGTCTGAACTTATTAGCACTAGTTTGGAGACGCTCTTCTACCTGATTGCTTTCATCGTC
ATGTTTGCGTCTGTGTATGGCAACTATGGGCGTAATGTGGCTGCTGCAGTATTTGGTA
TGTTCAACATGTTGGCGTATGGGGCCAGCTCGTACTACTTGTATCAAGAGCACAGGAGTAGCGCAGCGAGCGC
GGCGGCGTAGATATAGTCTGCTCTTGCGCATGCGCAACATAAGAACAATGCTTCTTGTTTTTAATATTATTTC
ACAGAAAACTTTATTATAGTGCCTTGTACAGTCATCTAATGTTTAAATTATATTTTGA
CGAAGATAAATAATAAAATAATTTACATATCTTTTCTATATTATTAAAAAAATAAAAAAA
>1002052540_2 Heli.2-DT664331.3.5
GCACGAGGCCAAAATGTACGGATATAAGGCAACCTATGTACTTGTATTGTGTATAGCGGCTGTACAAGAAGTC
GCTTTAACATCAAAGGAAAGTAGAACAGGCGGTCTGAATATACCAGAAAAAATTATAGGTGGAGTTATTGAGA
TTGTTCAAAGTCACAAAAACAAACCAGTTCCGGGAGCGCAGCCAGTTCAGCCTGCACC
TTCTTATCCTCCAAATCCTCAATATAATTACCAACAGTATCCGCAGTACCAACAATGGAATTCCCAATCAAGT
TTCCAAAATCAAGGATTTCAGGGTCCACAGTATCCAGTACAGTATCCAAGCCAGGGACAATATAACTACCAAA
ATAATTACAACCAAGCAGGTAATTATCCTCAAGGAAGCTATAGCAATCAAGTCTATCC
TACTGGAACAGGACAGACGTTTACCCAGGGACAAGCTCAACAAGGACAGTATCAAGGAACTTACCAGAACCAA
AATCAATTCAACCAGCCTAATAATTTCCAGAATCAGCAATCGAATTATTATACACAAAATCAGGGTAGTAACC
AATATCAAGGAAGTACCTCTAGCTTCCAAGGACAGACCAGTCAAGCCCAAGGACCAGG
ACAAGGTCAAGGACAAGCGATTGGTGGAAATGGACAATTTACTCAAGGGTTCCTGGTGTCAGGTCAGGGTAAT
GCAGGAGGACAGGGACCGACCTGTGTATGCCAAGCTTGGACCAAACCTCAGCCAAACCAAGAAATACTAGCTG
ATACTCCGGC
>1002052541_2 Heli.2-DT665869.3.5
TAAGCTGAATACGCGTTCAACCGACTATCAGTTGCGCGCGAGCTTCTTAGTGCTCTATCCGCACATTTGTTAC
CAAATATTATTTTTATCTACAAAAAATAAATACACTGACGAGATTACGCGATTATTTTAATCATAATTTACCT
ATAAATTAAAAATAATAATTTAAAATATATAGTTACTATGTATCGAGTAACGGTCAAG
TGACGTGAGAGATCATATAGTGAAATCTGTGCCAAATTGCCTCATAAGAAAAAATTGCGGTGAAGGGTCTATC
ATAATATTGCTTCAGAAGTGCTTGAGGCTGGATTTGGTTTCTTTTCACACTAGCATCTCGTTGAAAAATATAT
TTTATTTCGTAAAGACTATATTAAATATAACACGTTAATTTATTTTTGGTCCAAATCG
ATTTGCCATAAAAATGGCATTACAATTTGAACCCGATGGCACTCCATTTGTTATGTGGGCAGAACATAAGATT
AAATTGGAAATATCACCAATAACCGAGGATTTTTACGTCGAAAAGGCAAAAACAGAGTTAAGGGAGACACCGG
AAACTATAGAAAACAGTCTTAAAGAATTAAGGCAACTCCTTAAACGAGAGACAAATCT
TGTGATACCAATAGAAGACGACAATTTTCTACTAAAATTTCTCAGACCCTCGAAATTTTACCCGGAGAGTGCA
TTTAAAAAGATTCAAGCGTATTACAAATTTAGGTTGTCACACCTTGATTACTGCAGGGATCTATTTCCTAGTA
ACATTCACTCCGCATTCGACCATTCCATCATTTCCATACTTTCGCC
>1002052542_2 Heli.2-DT666641.3.5
AAACATTTTATTTCACTAATAAATTAAATCCAAAATCATCACGATTGTGAAAATAAAAATTCTGATATTTAAA
TAACTAAAGCACTGCAGCTTATATTATAATGGCTCCTAAAGCCGGTGGTGATTCCGCTAAAGTTAATTCAGCT
GTATATAAAGATAAAAGTAAACCTACTGATATTCGCCTTAGTAACATCAATGCCGCTA
AAGCCGTTTCCGATGCTATACGTACGAGCTTGGGTCCTCGCGGGATGGATAAAATGATCCAAGCAGCAAATGG
TGAGGTGACGATTACAAATGATGGAGCTACTATCCTAAAACAAATGAGTGTAGTACACCCAGCTGCTAAAATG
TTAGTGGAGTTGTCTAAGGCCCAGGATATTGAAGCTGGTGATGGCACAACATCAGTGG
TTGTTATCGCTGGGGCTTTACTTGATGCAGCTGAAAAACTATTGCAAAAGGGTATCCATCCCACTGTTATTTC
TGATGGTTTCCAAAAAGCTCTTCAAATGGCACTGCAGGTTGTTGAAGGCATGTCTACACCTGTAGACCTAACA
AATGAGGATGCGCTTCTAAAAGCAGCTGCTACATCTTTAAACTCCAAAGTGGTATCAC
AGCATTCCACTATCCTAGCACCAATAGCAGTGCAAGCTATCCGTGCAGTGATGGAACCAATAGTGGGTGGAGT
AGGTGCGCGTGTAGACTTGAGAGATGTGAAAGTGATTGAGCGTATTGGAGGTACTGTAGAAGACACAGAACTT
GTCAATGGACTGGTCATCCCACACCGTGCCTCAAATGTTAATGGACCCCATACAGTAG
AGAAAGCCAAAGTTGGCCTCATACAATTTTGTATATCACCACCAAAAACTGATATGGATCACAATGTGATTGT
ATCTGATTATGCTGCAATGGATCGTGTCTTAAAAGAAGAGCGTCAGTATATATTGAATATTGTCAAACAAATT
AAAAAAGCTGGCTGCAATGTATTATTAGTACAAAAGTCAATTTTGAGAGATGCACTCA
GTGATTTAGCTATTCACTTCTTGGATAAGATAAAGACAATGGTAATCAAGGATATTGAGCGTGAAGACATTGA
GTTTGTATGTA

>1002052543_2 Heli.2-EL600235.1.5
TTTAGTGTTTTATTGAGCGGCCTGCCAAAAGAAAACTGAAACATGGCAAGAAATCTATCCGCTACCGAGGCGA
ATAAAGAACATGCCCTGGTTGTATCCAGGGATTTCATCTCGCAACCCCGTCTTACTTACAAGACAGTATCCGG
AGTAAATGGGCCACTGGTAATCTTAGATGAGGTGAAGTTCCCCAAGTTCTCTGAGATT
GTCCAGCTCAGACTTGCAGATGGCACCCTACGATCTGGTCAGGTACTGGAAGTCAGCGGCTCAAAAGCCGTTG
TCCAGGTGTTCGAGGGTACATCAGGTATTGACGCCAAAAACACTCTTTGCGAGTTCACCGGCGACATCTTGAG
GACTCCAGTGTCTGAAGACATGTTGGGTCGTGTATTCAACGGCTCCGGTAAGCCAATT
GACAAAGGTCCACCAATCTTGGCAGAGGACTTCTTGGACATCCAGGGTCAGCCCATCAATCCGTGGTCTCGTA
TCTACCCCGAGGAGATGATTCAAACTGGTATTTCCGCCATCGACGTGATGAACTCCATCGCCCGTGGTCAGAA
GATCCCCATCTTCTCGGCCGCCGGTCTGCCCCACAATGAGATTGCCGCTCAGATCTGT
AGACAAGCTGGTCTTGTTAAGGTCCCAGGCAAATCAGTATTAGACGACCATGAGGACAACTTCGCCATCGTAT
TCGCAGCCATG
>1002052544_2 Heli.2-EL602529.1.5
CACCGCGACTCGGCTGGGTGAGTCGACAGTTGTAGTGAATACGAATATACATTATTTATTAGTTGTAATATGT
TAGCAACTACTGTGTTTCTATTGGCACTGCAATATGCGGTTGCTGAAATTCCCCCGTATATTAAAATATGCCA
GCGTAATGATCCCAATGTTGACAAGTGCATCATAAACTCCATAGAAGAACTCCGACCA
AAGATGAAAAAGGGTATACCAGAACTCGACGTGCCTGGAATCGAACCTCTAAGCTTAGGTCAAATAGGACTCG
CGCGAGGTCCACAAGGTGCCAAGTTGACTGCAGTTGTGAACGATGTTAAAGTGCGAGGTCCCAGTGATTTTAT
TATAGAAGAGTTGAAATCAGACCTAGATAAAAACAGGTTTGACTTCAAACTATTGCTG
CCGAGGCTCGATTTCGCCGGCAAGTACAAAATGGACATCCAAGTGCTGCTATTGCGGTTGCAAGGACGGGGGA
ATATTACTGGATCATTTAAGGATTACGCCTGCAACGTCACGATGAGAGGTCACAAAGAGAAGAGAGGAGACGA
GGAGTACCTCAACTTCGAACCAATGAAAGTAAAACTTCGTGTTGGTGAATCATCCATA
TACTTGACCAACCTCTTCGACGGC
>1002052545_2 Heli.2-EL602549.1.5
AGTTTACCCGCACAGTATCTTATTTCTTATATTCAATTTAAATATTCGAATACACCCTTTAAAATGGCCCGAA
GTGTTACTGCTTTGACTCTGGCCACCCTACTGGCGGTGTTCGAGATTGGTTCGTGCCTAAAATGCTACCAATG
CAACTCCCAATCTGATCCCAACTGTAAAGACCCCTTCGCCGCTAAGGATAAATTAATT
GACTGCATAAGTCAAGATTCGATTAATTACAACCGTAACTACTTGAGAGAGATCCTCCCTCGTGAGCTCGTAG
ACGGCGTTGCTGGAGCACCTCGCTACTGTCACAAGATTGTTATGCAAAACGGCGCCACTGTCCGTACTTGCTT
GGACGCCAACCCCTCTAACCTGAGTGAATCCTGTCGTCTTCTAGAAAACCTGAAGCAA
TCTCCAGCTGACCCCAGCAAGCAGATCAAATCTTGCGCAGTCTGCGACAAGGATGGCTGTAATGGAGCCGGAG
CCATTCAAGTTTCTCTACCACTAGCCGTTATGGCTGTACTTATCTCATACTTGTACAAACAATAATATATACA
AACAAAATATAAACAACGGTCTCTTTGGCCTTGTTGACCATGCTTCATACTTTTGCAG
TATATAACTGAAATAACTAAAACATTTTTCTATGTCTATAACATCCAATCGCTAAAAGCTATTAGGGCCAGTA
TTCTATGAGTAATTAAAATAAAA
>1002052546_2 Heli.2-EL598813.1.5
TTGTTTTAATCTTCATACAATTCTATTATGCCAGTACAGCGGCGTCCAATCGTAATATACGGGAAGGAAAATT
AATAGGTGGTCATGAGTCCAGACCTTACAGCCACCCTTACCTCGTATCTTTACAGATCAGGTTCCTCTGGATC
AGGACGCACGTGTGTGGTGGCAGTATATTAAACGATAAATGGGTTTTAACTGCTGCGC
ATTGTATTAAGGAATCATTTCTATTAAGATGGCTCCCCATGGACGCAGTTGCTGGAACGCACGATGTCAATTA
CTTTAGTCCAAAGGCCCAAATTATATCTGTGGCTGAGCGTATGGCACATCCTAAATATGCTGGAGGCGTAGGT
CCTTACGATATAGCACTTTTGCGAATGAGCAAACCATTTATGTTCACTGATGAAGTTC
TGCCAATACATCTGCCTCATAGCTACGAAGTTACCAATTATTCCCTTACCTTGGCTGGTTGGGGTGCTTTACG
TACAACTTACTTTATACCAGACGTTCCGAGCAAGCTGCAAGAAGTAAATGTCACTTACATCCCATACGAGAAA
TGTCACAATGCTCTCGAACAACTATTGGAAGATGGAGAAAGTAATCCATTGGATAAGA
ATTCGAATATATGCACCGGACCGCTCTCAGGCGGAGTAGCAGCGTGTAGCGGAGACTCAGGTGGACCACTCAT
GCAGTTAGTACCTTTGAGCGTCTACAATCGCGACAAAATAACTGATGAATACGAAGAATACGACGAGAATTAT
AACGAAGAGA
>1002052547_2 Heli.2-DT666681.3.5
TTTATTTTATATCTAAAATAAGGACACATTCAGTCATATTTCAATATCAGCTTCGACGTCTTCTTTTACAATA
TATTTTTTAGTTTGCACTAAGCTATTTAGAAGTGCAAGAAGGTGGTGGGATGTCTTAACGGTTGAAGCTAATT
TTTGTTTGGTGACGTCTAAACGACAAGATGGCGTGGCTTCCTATATGTCTTCTTGCGG

CCTCACTTCTTCTATCGCCGGCATCGGCTGCCGTCTGCCCTAATAGCTGCGTGTGCAGCACCACGCGGGATGG
TCTTCACCGCGTCACTTGCAGTAGTATCGCTGACATCTACAAATATACTATCCGACAGAAGCATCATAACATT
AACATTCTAGATCTTTCCCACAACAATATCACCAAAATCACTCACGAGCTAGATAAAT
TAACAGAAGTCGTCACACTTGATCTATCCACCAATGGACTTACAGAACTAAATAAATTTTTACTTAATGCAAA
AAAATTAGTTCACTTGAACTTAGCACATAATAGAATACAAAAACTCTCATTGACGCACTTGCCCACTAGCATA
AGTTCATTGGACCTAACGGGTAATCTACTGAAAGATGTGCCTTCAGAAATATCACATC
TGCCAAATTTAGAACACTTAGAACTAGATGGAAATCCTCTAGATTGTTCATGTGACAATATAATTGCTCGTGA
CCGGCTTTTACAGGCAAATGTATACATAGATAGTGCCAAATGTCATACTCCAAATTATTTAAAGGAACATTCA
TGGTTGGAAATAAAAACAAAAGATATCTGTAAAGGCTATAAAGCAGATTTTATGGATA
TAATGATGGGTGATCAACCAATGGATGCAGTGCAAGTTGGTGAATAAACTACTGCACTAAAATCTATGCCTTT
AGTAGCTAGTAGTGATTTAGACGACGGTAAAGTAATACATGGTGCGGACGCCGAGGTTGATGATGATAGTTCG
CAATTTATAAAAGTAGGTCACT
>1002052548_2 Heli.2-DT666775.3.5
CAAAACTTTGGCCTATATGTTACCTGCGGCAGTTCACATTGTTCACCAGCAGAGGATTCAACGTGGTGATGGT
CCCATTGCTCTTGTTTTGGCTCCAACAAGAGAATTAGCTCAACAAATTCAATCTGTAGCTCAAGCATATAGTG
CTCATGGTTGCATCAGAAATACTTGTCTCTTTGGTGGATCCCCTAAAGGTCCACAAGC
TAGGGACTTAGAAAGAGGTGTAGAGATTGTTATTGCTACCCCTGGACGACTGATTGATTTCTTAGAACGTGGT
ACCACCAATTTACGACGATGCACTTATTTAGTGTTGGATGAAGCCGATAGGATGTTAGATATGGGATTTGAAC
CACAAATAAGGAAAATAATTGAGCAAATCCGCCCCGATCGTCAAGTCCTTATGTGGTC
TGCTACTTGGCCCAAAGAAATTCAAGCACTTGCAGAAGATTTCTTAACTGATTATGTTAAAGTAAATATTGGT
TCTCTAAATCTTTCGGCTAACAATAACATAAAGCAAATCATTGAAGTCTGTGAAGAACATGAGAAGGAAGTTA
AACTAACTAATCTATTGAAGGAAATTGCTTCAGAGAAAGACAACAAAGTTATTGTATT
TGTTGAAACCAAGAAAAAGGTGGACGACATAGCTCGTGCAGTGCGACGCAATGGACATAAAGCATTAGCCATC
CATGGTGACAAGTCTCAACAGGAGCGTGATGCTGTTCTCACAGAGTTTCGTAATGGAGCAACTACCATACTGA
TTGCTACCGATGTGGCTGCTCGTGGCTTGGATGTTGAAGATGTTAAATTTGTTGTTAA
CTTTGACTATCCAAATACTTCAGAAGACTATATTCATAGAATTGGTCGCACTGGTCGCTGTCAACAGTCTGGC
ACAGCCTATACCTACTTTACTAGTGGAGATGCAAGACAGGCTCGTTCACTCTTGGCTGTTCTGAGGGAGACTG
GACAGAACCCTCCAGCAAAACTGAATGATATGGCACGAAGCAACAACAACAACAACTC
TAGCCGCAATAGATGGCAACAAAGAAAAGAAAATAATAGCGGTGCAAGTTCCCCACGCCAAAAGAGTAATCAG
TGGAATAACAAAATGGCTAACATGACTAATGAAGAGAATCCTAACAATTATCAAAATAGAAACAACACACAGC
AACAAGCTCCTCGGTTCTCCAATCAGAACCAAAATGCAAACCAGGGATATAGACAACA
AAATACTTACCAAAAGACAGTACCTTTCCCCAGTCCCAATGTATTTGAGTCTATGGGAAGCTACCAAGGGGGA
TACAATAATGGATATCAAAATGCTTACAGTAACCAAAATGGCTATGGTCAGAGGACCTACAACAGTGAGTATA
ATCGCCCAAATGGCGGTCAGCAATATCACCGTAACACCAACTCTGTGGGCAACAAATC
TGCGGGATCCGGTTCGTCTTATGGCTCGCCGCCTCCTCATTTCGCTACTCCACCTCATTATCCACAGATGCAT
CCTCACCATCAGGATATGCTTGGAGGCAAGTACTACGGCGGTGGCGTCGGCGGAGGCGGTCCGTGCGGCTATC
AAGCGGCCGTAGGCGGTGGAGTGCCCTATCCGCATCTGCCACCTGGCCCGTTGTTGTA
CGCGCCAGTCCAACAGTAAATTTATAACATTTTATTCCACATTATTGTGCAACTGTTCAGTCTTTTTGCACGC
TCAATATACTGACTATAATATATATTTAAAAGCACAGAATATGTCGGTATAATATTTAGATTAATATAATATT
TTGGTTAACCGTATTGCTTAGCAGTGTTCATTTTGACAGACTGAAC
>1002052549_2 Heli.2-DT666586.3.5
GTGAAATTTTGTTCAGGTGATAGTGCTAGTGGATTTGTGTAAAAAATGCAAATTCCCAATGAATATGTCTCAA
CTGCAGAAGATGTTGCTGATCAGGTGATAAGAAAAGGCAAGAATATACTACCAACAGTAGCGCGACTGTGCTT
AATATCAACATTTTTAGAAGATGGCCTCAGAATGTGGTTCCAATGGTCTGAACAAAGA
GATTATATGGATATGTCATGGAACTGTGGCAAATTTCTTGCAACTATGTTTGTGATAATCAACTTGGTGGGTC
AGCTGGGTGGCTGCGTTATGGTCCTCGGTAGGCTAAAAGTGGACATCGCGTGCGGAATATTGTTCTTCATCGT
TGTACTACAGACATTCGCCTACAGTATACTATGGGACATGCAATTCTTGCTCCGCAAT
CTGGCCCTCATCGGAGCCCTGTTACTAGTACTAGCGGAGGCCCGAGCGGAAGGGCGAAGTCTCTTCGCCGGAG
TACCGTCCTTAGGTGAAAACAAGCCTAAGACATACTTGCAATTGGCTGGCCGTATTCTATTGGCCTTTATGTT
CATCACGTTACTGAGATTTGAAGTATCTTTCTTACAGATCGTCCAAGACCTCCTCGGC
AGTATCCTCATGGTGCTCGTAACAGTGGGCTATCGTACCAAGCTATCAGCCCTCATGCTGGTACTTGTTCTG
>1002052550_2 Heli.2-DT667554.3.5

AATGTCTGTAACAGATACTCTACCCCCTGGATCCCCACGGTCAGCTGGAGGTTCCCCTCCTCAACCGCCACCA
CAGCGCTCTGCTAGCCGGTCTAGCCAACACTCGCCCAATTCATCAGGGTCGAACGGCGGGCGGCGGTCGTCGG
GTTCGCGGCACGTGTCGTCAACGACTTCGGTGTCGTCGACGGAGGCCGGAGAGGGTGC
AGAGGCCGCAGCCCCTGCGCCTGCGCCTCTTCTAAAGTGCACCCTCTGCCAGGAGCGCCTCGAAGACACACAC
TTCGTACAGTGCCCGAGCCAGCCGCACCACAAATTCTGCTTTCCATGCTCCCGTGACTCTATCAAAAGACAGC
AAGGATCTGAGGTATACTGTCCAAGCGGTGACAAATGTCCCTTGGCTAATTCGACGGT
ACCCTGGGCTTTCATGCAGGGCGAGATAGCCACGATTTTAGGGGAAGAATTCAAGCCGAAGAAGGAACGGGAG
ACCTAGGGCAATGTGCCAAAGACCCGACTTTATCTCTTGTCCCTCTCGATGTAAATGAGCACGAGAGGAGTTT
TGCCCGTAAGGGAGCTTTCGATAGCCTTGCGCGTCGAAACTGCGGCTCCGATAGGGAT
ACGCGGCCAGGCCGTGATAGACCGATCACTTGTATATAAATTGTAAATAAATCCATATAACTTTAGTTCGCTC
ATCAAGAGTGGCGATTTGGAATTGGTTCTCCGGATGGTCGGATATAGACCGTATTCAGCGTAATGGGTTCTTT
ACGATTCAGCCGATTGTAACGAAATCGCTGGTTTTTGTTTTAATGAGCTTTGCAATAT
TCTGAATATAGTACATATCTGTTTTTCGAAGATTTTATTATTGTCTTGATTCTTGACGATCCTTTAGAAACAC
GACATCTTTTGATCTGAAGTAACTCTCGATGTACGACGGCGATGTCGCGTATATACATGTTATATGGGATTTC
TTTGGATGCCATTTTCTTAGTTACCTTTAGGTTCCGAACCATAGTTAACTTGGCATTT
ATTTATATTGTGATTCTTATACACTATACGAGTATTATGCGATATATGATCATGACATTTATGTAGTCGAGTA
CTGACTGTAAAAAAGTAGTTTTTCTGATATCGATGGCGCACCGCGCCCTGTACAGATAATTAGCTCGGTGTAC
ATTTGTATTTATTACATACATTCGCATGTGAAAT
>1002052551_2 Heli.2-EL599236.1.5
GAGTGAGAAATTATTTCTTTTAATTCAATAAGAATTGTAATATCTATTTTATTAGACTGAATATCATTATTGT
TTATAATATTTGTATCTTTAATTTCTTCTTCTGTTATTTATTATAGAAAAAGATATATAAGATCAGAGTTAAA
TTTAAATCGATTTATTTATTTAGTTTTAATATTTGTTTTTTCAATAATATTATTGATT
ATTAGACCAAATATAATTAGAATTTTATTAGGTTGAGATGGTCTAGGGTTAGTTTCTTATTGTTTAGTGATTT
ATTATCAAAATATAAAATCTTATAATGCTGGGATATTAACTGCTTTATCTAATCGGATTGGTGATGTAATAAT
TTTATTATTAATTTCATGAATAATAAATTATGGGAGTTGAAATTATATTTTTTATTTA
AATTTTATAAGAAATGATTTTTCAATAAAAATTATTGGGTTATTAGTTATTATTGCTGCTATAACTAAAAGAG
CTCAAATTCCTTTTAGATCTTGATTACCTGCAGCTATAGCAGCTCCTACTCCTGTATCTGCTTTAGTTCATTC
TTCTACATTAGTTACAGCTGGAGTTTATTTATTAATTCGATTTAATAATTTATTGATT
>1002052552_2 Heli.2-DT666929.3.5
GCACGAGGGTTGCGTCGAACTTACTGCTTTTTGTACATCATAATTCTGTACAAAAATGGCTGATAACAAACTA
TATGAAATATTAGGTGTTTCAAGAAGCTCAAGCGATTCGGAAATAAAAAGAAGTTATCACAAACTCGCAAAAG
AATTCCATCCGGACAAAAATCCAGCAGCTGGCGATAGATTTAAGGAAATAAGCTATGC
ATATGAGGTTTTATCTGATCCTAAAAAAAGACAAACTTATGATAAATATGGATTAAAAGGTTTACAAGAGGGT
GGGCAAGGTGGAGGATTTGCATCTGACGACTTGTTTGGTCATTTCTTTGGTGACATTTTCGGAATGGGAGGTG
GTGGTAGAGGCCGACGACACGCAAGAGGTGAAGATACTATACATCCTTTAAAAGTTTC
ATTAGAGGACATGTATGTTGGCAAAACAGCAAAGCTTCAATTAAGTAAAAATGTTATATGTGGTCCTTGCAAA
GGTATAGGTGGCAAGCCAGGTGCAGTGGTTTCCTGTAAAGATTGTCATGGTCAGGGTATAAAAGTTTCTTATC
AACAAATTGGACCAAACATGACTCGGCAATTTCAATCTCGCTGTCCAACCTGTCAAGG
ACAAGGTGAAACTATCAATGATAAAGAGAAATGCCCAAAATGCAAAGGAAAAAAAGTTCTCAATGAGATTAAG
ATATTGGAAGTTCATGTAGAAAAAGGAATGCGTGAAAACCAGAAAATTTTCTTTAGAGGTGAAGGAGATCAGC
AACCTGACACTCAACCTGGTGATGTCATTATAGT
>1002052553_2 Heli.2-DT665460.3.5
GCACGAGGATGGCCTTTAACTTTACCAGGATGTATTAAATTGTTTTATTAAATTTGTGAGGTGCAGAGACGAT
AGTTAAGATCAAAGATGCAGATCTTCGTAAAATCCCTCACGGGTCACATTGTCCTCGCAGTTGAACCCACTAA
CACCGTAGAGCATGTGAAGTCTAAAATTTACGACAGGGAAGGCATCTTTCATGAATAT
CAAAGATTAATATTTGAAGGCAAACAACTAAATAATGGAAGAACTCTATCTGAATATAACATTCAAAAGGAAT
CGACTCTACACCTTACTCTTTGCCTTCCTGGAGGGGGCAAAGGAGCCATGGTCATACCACCGGACCTAGTAGC
GCTAGCTCAGAAATTCAACGTACGTAAAATGATATGTCGCAAGTGCTACGCGCGCCTC
CACCCTCGCGCCACCAACTGCCGGAAGACGAAATGCGGTCACAACAACGACTTACGACCTAAGAAGGAACTAA
AGAATTAATAATAAATTAGAAATTACATTGTTTACAATATAATAAATAATGTTAATAGAAGATGTTGAGTGAT
GTTTGGTTAGAGGTTGTCGTATCGCAGCGTCTCAGTGTCTGATTGTGAACAATGGTTG
AGAGTTTGATTCTCGGGGGGATTAATTATTTATCCTGCTCTATTAATAAGACAAATCGCAATATTGATAATTG
AGCGGAAAAAGGGAATGTAGCGTTTTTAAGGTTCCGTACCTTTTGAG
>1002052554_2 Heli.2-DT667359.3.5

GCACGGGGTGTTCTTCGCTCTCGCCGCTGTGGCTTGCGGCTCAATCATCCCCTTGGCGCAGCCCGCGCACCAT
CCGGCTCTCGTGCTGGACCCTCACGGCCGCCCCCTGGACACCGCTGAGGTCATCAACGCTCGTGCCATTCACC
TCCAAGCTAAAGCCCTGGAGCCCCTCGGCCACGCCGCCATCGCTCCCCTCGTGCACTC
CGCTGTTGTAGCCGCCCCCGTCGCTCATGCCGCCATCGTCGCCCCTGCTGTGGTCGCCGCCCCCGCCGCCGTG
TCCCACCAGTCCCGTGTTGACGTACGCACCAGCCCTGCCATCATCGGTCACAGCATCGCCGCTCCTCTGCTGA
GCCACTCCGCCCACGGACTCGCCGGAATCGGACTCGCCGGACACGGACTCGGACTCGC
CGGACACGGACTCGGACTTGCCGGACACGGACTCGCTGGACTCGGACACCTCCTCAAGAAACGCTCTCTCGGC
CACTGGGCATACAGCGCGCCTCTCGCCGTTGCCCCCGCCGCCGTCTCCCACCAGTCCCGTGTGGATGTAGTGT
CCAGCCCCGCCGTGGTATCTCACGCAGTAGCCCCCGTCATCTCTCACGCCGTGGCCGC
CCCAGTGCTCGCGCACGCCATCGCGCCCGCCGCAGTGTCCCACCAGTCCCGCGTTGACGTACGCAGCAGCCCC
GCTGTCATCGCCCACAGCGTAGTCGCTGCCGCTCCCCTCGTCCACGCTGCTCCCCTGGCTCACTCCGCTGCCC
TCCTCCACGCCGCTCCCTTGGCGCATGGTGCTGGTCATTTAGTCCATGGTTGGTGAAG
AGCCCCTCTGACGAACTGTTGATA
>1002052555_2 Heli.2-DT662578.3.5
TACGATCAACTTACATGGAATACCAGTGTGTTAGCGGCTGAATACAGTAAAAAAACAATACTAAACAGTGCGT
CGTGTTTCTATTGACAGGTAGCGCGTGTTAAATACCAGAATGAACACCGAAAAGCTGAAAAAACTGCAGTCAC
AAGTCCGCATAGGCGGCAAGGGAACACCACGGCGCAAGAAGAAGGTCGTCCATGCGAC
CGCCGCCACAGATGACAAGAAATTACAGTCCTCCCTCAAAAAACTATCAGTAAATACCATCCCTGGTATTGAA
GAGGTGAACATGATCAAAGATGACGGCACTGTGATACACTTTAACAACCCTAAGGCGCAGGCATCACTCGCTG
CGAACACTTTCGCTATCACCGGCCATGGTGAGAACAAGCAGATCGCGGAAATGCTGCC
TGGCATTCTCAGCCAGCTCGGTCCTGAAGGATTAAACCAGTTAAAGAAACTCGCCTCCAGCGTGGCCCCGCCG
AAACCTATCGACGAGGACGACGAAGTACCTAACCTCGTCGGTAACTTTGACGAGGCCTCGAAACAAGAGGCTA
AAGAGGTAGCCACTGAGGAAAAAGAGAAGGAAAAGAAACCAGAAATAGAAACCAAAGC
CGCGGACAACAAAGTTGATTAAATAGACCCCCATGATATATAGTTTTTAATTTTGCTCACCTGATGAAGTGAA
TACTATAGTGGGTTTCCAAAGGCATCGAGACTGTGTCTGTGTCTCAAATAATTTTTCTTTTTTTGTATTTTTG
TGTTCCTAAGCGTTCTGACTGTTCGGTTGGTGAT
>1002052556_2 Heli.2-EL601954.1.5
GCTACAGATAGCCGTGACCACCGAGATAGGGGCCAGTACACCAGTACACTCGGCAGATACAGCCACTCTACGA
GTCGGCTGAATCCGAACAGTGAAGCTGAAGAAGATTATACACGGAGTGCTCAAACACTACCGCGCAAGCTCCA
CGAGCGTAAAGAGAAGGCGGAGAAGCCTAGCTCCAATAAATACTGGCAACGGCTCACT
TCCAACAAACGTTCTACTAGTGCCATTAATATTCCAAACAACGTATCGTCAAACGCAGAAGGAAAAATAAACG
GGCACCCGCCGGGCCCAGCGAAGCCGGCGCGGACCTATAAAGGTCTAAATAGAAGCAAAAGTTTTGCTATGGG
AGCACCCGAACAAACACATCCAAGATACGTCTCAGGAATGAATAGAAACTATTCTACA
ATGTACAAATCAAACCCACACTTGAGTCGGCTCGACGAGACCCCTGAACAACTGAAAAGCCCAGGCATTGTGT
CGATTATAAATAGGAGCCAACGCGATCTAGCCGATGCGGTTTCCCGGGAAACTGAACGCAGACGTGACGGATT
ATTCGGCGCTCGATACGGCAAGACGATAACTAATGGCCACACGAACGGTTACAATAAA
TCCTTCGATGAAACTGATAAAAAGAAAATATTCCTCAAGGGCCTTCGTGAGAGAGCTCCCGAACTGTATCAAA
CTTTACACGACGACGAATCCGACGGTAGTAGATTATCTTCACGATATGGCACCCCGTCGCCTCATTATAAGGA
ACGGATATCGGAGGAGCGCAAAATACCATTGTAC
>1002052557_2 Heli.2-DT665565.3.5
CAGTTTTCTAATTCCTGTTATTTCCCTATAAAATATCATCTATTCATAAGATAGTTGCCATCATGGATACACA
ACTGACAGGAAATGAGATTAGAAAAGCATTTATAGACTTCTTCATCAGTAAAGGTCATAAGTATGTCCATTCC
TCCTCCACAATACCACTAGATGATCCAACACTGCTGTTTGCAAATGCAGGCATGAATC
AATTTAAGCCAATATTCTTGGGATCAGTAGACCCCAACTCAGACATGGCCCAATATATAAGAGTAGTTAATAC
CCAAAAATGTATAAGGGCAGGGGGGAAACACAATGACTTAGATGATGTTGGAAAAGATGTTTATCATCATACT
TTTTTTGAAATGATGGGAAATTGGTCTTTTGGTGATTATTTTAAAAAGGAAATATGTG
CATGGGCTTGGGAACTTCTTACTCAGGTTTATAAATTGTCTGGAGATAGATTGTATGTCACATATTTCGGAGG
TGATCAGTCTTCAGGGTTAGAGCCAGATTTAGAATGTAAAAATATCTGGCTGGACTTGGGAGTTTCAGAATCA
CACATTTTACCTGGTAGCATGAAAGATAATTTTTGGGAGATGGGTGAAACTGGGCCTT
GTGGACCATGTTCCGAATTACACTATGACAGAATTGGTGGACGAAATGCTGCACATCTTGTAAATATGGATGA
TCCCGATGTCCTTGAAATTTGGAACTTGGTGTTCATACAATTTAATAGAGAATCCGATGGATCATTAAAATTG
TTACCGAAAAAACATATTGACTGTGGTTTGGGCTTAGAAAGATTAGTGTCTGTCATTC

AAAATAAAAGAGCTAATTACGACACAGATTTCTTTATGCCTATCTTCCAGGCTATTGAAAATGGTACAAAAAT
AAGACCATACAGTGGAAAAGTTGGTTCAGAAGATAAAGATGGGATCGACATGGCATACCGCGTTCTAGCTGAT
CATGCCCGAACTTTAACCATAGCTTTGTCTGATGGTGGATACCCCGATAACACAGGGA
GAGGATATGTTCTTAGAAGAATTTTAAGAAGGGCTGTTCGTTTTGCTTCAGAAAAACTCAATGCAAAACCCGG
ATTTTTTGGGTCGTTAGTTCGTA
>1002052558_2 Heli.2-DT667242.3.5
GCCGTCGCAAACAAGATGACAAAAATTTAGAAATTTTACGTGAACTTATATCTTTGAATGGCAATAAATATTG
CTTAGATTGTAATCAAAGAGGTCCTACTTATGTTAATACTACGATAGGCTCTTTTGTGTGTTCAAAATGTTCG
GGAATGTTGCGTGGCCTTACACCTCCTCATCGTGTAAAATCTATCTCGATGGCTACAT
TTACGCCAGAAGAAATAGAATTCATTAAAGTAAGAGGAAACGATTATTGCAGACGCGTGTGGTTAGGTCTTTA
CGAGGGTGAAAGTGTAAACTTTACTGATGAACAAAGCATTAAAGACTTCATGTCTGATAAGTATGAAAAGAAA
CGATACTACTTGGAGTCATCTTCTAATAATGTGACTATTACAAATGGTAGTACATTCG
CGAAAAGTAAAGTTAAAGATACAAAATCTGGAAGTAGTAATGTGGGTACTACTCCATTAATATCAATTACACC
TCAGGTATCTAAATCAGTCAGCAACAATAATAATAATAATGTGATCAACATAGCTAAAATAGTAAGCAATGAA
ACTAACCATAAATTGCCAAGGCCTGCAAATAATTTTGTTTCACCACAATTGAATAAAA
TACCAACTATTTCAACTGTTTCGAGCCCAGTCCCAGTACCTGATTTTCCTGTTGATTTTACTACAGCAAATAT
TTTCAATAGTGCCCAGTATAATAATACTGTCACCA
>1002052559_2 Heli.2-DT665208.3.5
GACGGAGGCGTGTTCGTGATAATTTCCGGTCAAGTGGGTTTCATTTAATAGTCTCTTAACCTTAATCAAAACA
TTGTATATTTATTACATTATTCATTGCGTTATACGTTCTCCATTAGGAAAGTACCCGTGAACAAGTTGTCGTT
TTCTTAAATAAAAATTAAATATTTGGAAGACATCGAAATGGCTCTATACAGTTTAAAG
AACAATCTTCAGCTGGCTACATTAAAAACTTTGTCCCTGGTCTCCATAAGCGTCATGTCACCACAGCAAAGTG
TGCAAGAGTTATTCCGGACTGCGGACTGCGTATGTTTCGATGTAGACTCTACTGTGATCCAGGATGAGGGTAT
CGATGAGCTGGCCAGATTCTGTGGAAAGGGAGATGAAGTGAAACGACTAACTGCTGAA
GCCATGGGCGGCTCTATGACCTTCCAGGAAGCCCTTAAGAAAAGATTGGACATCATCAGGCCCAGTGTAAACC
AAGTTAGGGAATTCTTAGAAAAGTTCCCCATACATCTTACTCCTGGAATAGCAGAATTAGTGAAGACCTTACA
TGACAGAGGTGTGATCGTGTATCTAGTGTCAGGTGGCTTCAGAAGTCTCATAGAACCA
GTTGCTGTCAAACTAGGCATCCCTATTAGCAATATCTACGCTAACAGACTTAAATTCTTTTTTAATGGAGAAT
ACGCTGGTTTTGATGAAAATGAACCCACATCGAGGTCTGGAGGCAAGAGTTTGGTAGTCAGGCGGCTAAAGGA
ACAGTTTGGTTACCAAAGGGTAGTAATCATTGGAGATGGCGCCACCGACGCTGAGGCG
AGTCCACCAGCTGATGCCTTCATAGGTTTCGGTGGTAACGTTGTTAGAGAAGAAGTCAAAAGGAAGGCGGCGT
GGTACGTAACAGACTTCCAAGAG
>1002052560_2 Heli.2-DT665463.3.5
TATTAGAATAATTTTGAGTATTAGACTATTATTTTAGAACTAAAACTCGCAGAGATGGACGGAATATGTAGTT
ACAAGACATTTATAGCAGCGCATAAGCCGCAACTTTTACTTTCGGGTGTACCAGAGTATTTTTGGCCTACTTT
ATGTAAAAAAATTGAAAGATCAAATATTTGATTCTGGTGCTGCATTTCAATTAGTAAAA
ATTGATTATGATGAAGAAGAAAGGAGACCATATGATCCATTATGGAGTGTTATGGCTATAACTGATATAGACA
GAACTGATTCTAGTCATATTTATCTTATAGATCACGCTTGGACTTTCAAAGCTAACAATATAAAAAATAACTT
AAGAAATGTACCAGTGCTTTTAGAGAGAATGTGTAATCTTATGCAGATCACTTCTGAA
AATATTGAAGAACAAATTAACGATGTATCTAAGTGTGTATGGAAATATGCAAACACATACGCAGTTGAAAGTG
AAGAATTATCAGTAGAAGACAGAGTGCCAGTGTGGTATGTTATGGATGAGTTAGGTTCAGGAATTACACATTC
TGATGATCCAAATTTTCGGATTGTTCCATTCATATATATCCCAGAACAAATAACATTC
ACATTGTTGTTTCCAGTTGAAAATGTTGAGGAAGGTGACATAATTACAAGAAATTTTATTGAAGGAAGTTACT
CAAACTCAAAACAAAGAGAAACTATGCTTATTCCATGGCAACATTAT
>1002052561_2 Heli.2-DT663246.3.5
GCACGAGGCGGCGGGGAACTTTCGCTCCTGCTCCGTACAGATACGCTTTAGCTGAACTTTTACGAAAATCGCG
TTCGGAAATTTAAGAACCGGCACCCATTTCCGATTCGTGACAGATTACGATAACCGTTGAAAGTGAAACCGAA
CGATTGGGACGTGATAAGAGTTGTGTAAGGATTTACTTTGGCAGGTAAATTGAGTGAC
TAAGTGGGACGATCTAGTTGAAAAGTGAAATTACACCGGACTGGTTTCGATATCGGCACAGTTTTGCAATAAA
AACTGCGTTTTGAATATTGACACTCGGTGAAGCTGTTTTATTTCCTTATATGGATAATTCACAATCTTGTTAA
TGACAAGACGTGGATTGCTTTTTAAATCTTATACATATGTCGACAGTGGTGTTTTCTT
AAGTGTTGTGAGTTATATCGAATCTTGGACTTTTGTGCAATGAAATGTGAATTTTGTGTTGATGCAGTTTAAA
TCGTTTGTCGTGTGATAACTTTGCCCTGATGATTTATCAAGTGTAACTTTTTTTACTGTTTTCTTAATTGGTT
TACTGATACTCTTTCGCTATGGAGACCGAGGAACTCACTAAACTCGTGGACGGTATAT

ACAAGAACATCTTGGACAAGTTCAACCCCGGCGCCCGGCAGATGATAACAGCTGGCAAAGCCTATTTGAAAGC
GTTACATGGTGCGGCGGCAGCGTCCAGGCTGTATGTGGATGCTGTGGGCAAGCTCGGCCGACAGGCGCAGCAA
GGTACTTGGGGAGGATGCGCCGATATAGGCACTGCCCTTATGAAGGTCGTGGAAGTGT
ACCGGGAAATCCAAGATCAACAAATGAATATCCTGAAAGCGTTCTACGTAGATTTGCTCGTCCCCCTGGAAAC
TAATCTGGAAAAAGATACAAAAGTTGTACAGTCTGAACAGAAGAGATTTTTGCAACAACATAAGCTGCGTTCA
GAAAGCTACAGCAAAGCGGCCGCTACTATTAAGAAACAGAGAAAAAAGAAAACTAATG
TAACAAAAGTTG
>1002052562_2 Heli.2-DT663074.3.5
CACTAAAACTTTCAGTTGTTGATTGCGTGAAATATTTTGTTTTGTGTAGTGATTTATTTTGAAAAAAGAATAT
TGAATATAATTTACCATGGATCCCCTAGCAGTGGACGTCGTACATTTCTACCCACGGGAGAACGCGCAGCCTC
ATAACACTGTAAAATTTGAAGCTGTGAACGATGAAAACCCAACCACTGCGATAGTACG
TCGTGGTCAACCCTTCAACGGCGTCGTACGGTTCACGAGACCGTTCGATGAGAACGAGGACATTGTACAACTC
GTGTTTACACTTGGTGATAAGCCACAGATGGACACGCAAGGTTCCATATTTTTAAGGAGAGACGCCATCCCTG
AGAAGCACTCTTGGTACGCTAAGATCGTTGATATACAAGAGGATACAGTATCTTTTGA
GGTCTCAACACCAGTCAGCCTACCAGTAGGTTGCTGGGCTCTCCGCGTCGTCACCAGACTGAAGAGTTCTCAA
GCGCGAGAAGTTTATGATTACGATCAAGATCTGTACATTTTGTTTAATCCATGGAATCCAGATGACCAAACCT
ATTGGGCAGACTTCGATCTTTTGCAAGAATACGTGATGAACGACGTCGGCAAAGTATG
GGTCGGTCCTATAAACACAACAAGAGGCAAACCCTGGTTCTATGGACAGTTCGACGCGGTTGTTCTACCAGCG
GTCATGTTCAT
>1002052563_2 Heli.2-EL596237.1.5
AACGAGGAGGTTGAAGAAGCCAAGATGTCGAAGGTATCCCGTGATACGCTTTACGAATGTGTAAATGCGGTCC
TTCAATCATCAAAGGACAAAAAACGTAACTTTTTGGAGACAGTGGAACTCCAAATCGGTCTTAAGAACTATGA
CCCACAGAAGGACAAGCGTTTCAGCGGCACCGTCAAGCTAAAGTACATTCCCAGGCCA
AAAATGCAAGTATGTGTTCTTGGTGACCAACAGCATTGTGATGAAGCTAAAAACTTGACTGTACCATGCATGG
ATGCCGAGGCTCTAAAAAAACTGAACAAGAACAAGAAGCTTGTCAAGAAACTAGCAAAGAAGTATGATGCTTT
CCTTGCCTCTGAATCACTCATCAAGCAGATCCCACGTCTGCTGGGTCCTGGCCTCAAC
AAGGCTGGTAAATTCCCTGGTCTCCTCTCTCACCAGGAGTCCATGACGCAAAAGATTGATGAAGTTAAGGCTA
CCATCAAGTTCCAAATGAAGAAAGTACTCTGCCTCTCTGTGGCTGTAGGACATGTGGACATGACACCTGATGA
GTTGGCACAGAATGTTCACCTTTCAATCAACTTCTTAGTGTCTCTATTGAAGAAGCAC
TGGCAAAATGTACGATCACTTCATATGAAGTCCACCATGGGTCCACCCCAAAGATTGTACTAAGTCCTCACAA
TAAATTTTGAC
>1002052564_2 Heli.2-EL602760.1.5
GCCGTCTGTGTCGTTAACAGTTTTGTGTTTCTCTTGTATATAGTGGTTTAGTTTCGACAACGACATCTAAGTT
TATATAAGCATACGAAATGAAATTCACGGTGATTTTCGTAACTCTGTTGAGTATATCTACATCATGGGCTATA
AAGGCAAAAAAAGAAGAGCTAAAAGATAAAAGGGAAGCTGCTTCTGGAAGTTATTTAC
CTCCACATGGCTCATCAAGTCATAGTTATCAAGGACCTGCCTTAAGTCAAGAAGATGCTAACGCTATAAGTAT
CGGAGCCGGATACAGCATTGGAGGCGCTAAACCTAGTTACAACTTCCTAACTCAAGGTTCAGGTCAACCATCG
TACCAAGTTCAATCAGAGGGTCTTCCAGCCAGTGGTCACGGAACCATTCAACTTGCAC
CTATCACTTTACAACCGAGCCAACCAGGTCTTATTTCTAATGACCTCTCTCAACTCATGAGTCAATTATCTCA
TGGAATTAATTCTGGGGCTATTGCCTTACCTTCATCTGGCCAAGGTGCCATATATCAATTCGCTGGGCAAAGC
GGTCACAGTGGCCAAGACTTGAGTGCACCTCAGTTTAGTTATGGAAGTCCTAAATTAC
AACAGTATAGTTTTGGTGAACAGTCAAGTCAAGCCGGCCAAATAGTCCCCGCTTATGCATTTGGAGCTAAAGG
CCTCAGTAGCTATGGATCTACAGGACCTGTCCTAT
>1002052565_2 Heli.2-EL602533.1.5
GCACGAGGGACAACCAAGAATTTGCCAAATAAGGAACTTTACTAGATGAAGACAACAAGATGATAGTAAAAGA
CGAAAGCTACTTCATATAATTAAATTAATATTATAAAGCTTAAAATGAAAAACAAAAGGCTGATAAAATAAAG
ACCAAAAAAATTGAGATCCAAACAATAAAACCCGAAAATGAAGCTTTAATACTAATAA
AACCCAACAAAGATGAAATAAAAACAAAGATAAAAATAAAAACGAAGACTAAAGATACAAGGAATTCAAAACT
TTAATTGATTCCAATGAATATTGATGCCGACGAAAGCCGAAAAATAAGGAGACCGGCGTAGAACAAGATCGAA
GCATACGTCGATCCAAGCAAATGAAGGCCTAAATTAATGAAGGCCGAAACAGAAGAAT
ATCGAAGTAAATGAAGACACTCAAGCAGAAAAGACAGAAGCAGTTGAAACCTTTAATTGATGACGACAAGGAA
GATTGACGACAGAAAGTAGACGAAGACCGAAGCAGATGAAGACCGAAAAAGTTGAATATCGAAACAGATGAAG
ACGAAGCAGATGAAACCCGAATCAGAACAAAATAAAGCTAGAAAAAGATGAGCAGAAT
AGACCTAAGCGTTTAATATTCTAAGCAGATAAAGACTAAACCAGATGAAGACATTAGTAGACGGCCAGCAGA

>1002052566_2 Heli.2-DT662253.3.5
AACGTAAAAGATCTGATATTTACCATTTATATATACAGTTTAATTATAAGATTGTATTGTTTGTTTTAAAATG
CTGAGTTCACGCTTATTAGGCGTTATTTTATTAGTATCGGGTACGTATGCCCTTTATGACTCGTCGTCTGATG
TAGTTCAATTGACACCAAGTAATTTTGATAGATTAGTTTCAAATTCCGACGAAGTATG
GATCGTAGAGTTCTTTGCCCCTTGGTGCGGGCATTGTAAGAACCTTGTTCCAGAATATAAGAAAGCCGCCCGC
GCTCTTAAGGGCATTGCTAAAGTAGGAGCAGTAGATGCTGACCAGTATAAAGAACTTGGTCAGAAATATGGAG
TGTCTGGTTTCCCTACCATTAAAATATTCATTGGCAGCAAACACTCTCCTTATCAAGG
CCAAAGAACAGCCGAGTCATTTGTTGATGCCGCACTTAAAGCAGCCAAAGAAAAGGCATATGAGAATCTTGGA
AAGAAATCATCTGGATCTTCTGGAAAGTCGGACGTAATCGAGCTAACGGACAGCAACTTCAAGGAATTAGTTC
TGGACAGCGAGGACCTGTGGTTGGTGGAGTTCTTCGCGCCGTGGTGCGGTCACTGCAA
GAACCTGGAGCCACACTGGGCCAAGGCCGCCACTGAGCTTAAGGGCAAATTAAAACTCGGTGCCGTAGACGCG
ACAGTGCACTCTGTGATGGCCTC
>1002052567_2 Heli.2-DT667031.3.5
GTGTTGTCGTTCTCGACAGAGGCAACAATACAACTTGTACCGTAAATCTTTTCGGTGCTACAGTGGTATCATG
GCGGGTTAATAATCAAGAACAGTTATTTGTAAGTAAGCAAGCTGTGTTCGATGGGAAGAGAGCAATTCGGGGA
GGAATACCGTTTGTATTTCCTCAATTCGGACAATGGGCGTTCGGTCCCCAGCACGGGT
TCGCGCGGGTGGCTCGCTGGCACGTCGAGAAGATGCCGGAGCGACTACCCAGTGGAGACGTGGAAGCTGTCTT
CAGCCTCATGGACGATGACTTTACGCGGTCCATGTGGCATTTCCAATTCAGACTAACATACCGCCTTATTCTA
CGGGAGAAGGAATTGCACTTCAACATAGGAGTGTACAATCCTAGTAAAGAGCTGACGT
TCAGTTGCCAGCTCCTCCTCCATACGTACTTCAAGGTTCCCGATGTGCGACGCTGCCAGATCACAGGCATGCA
TGGCTGCATGTTTATTGATAAGACGCGAGAGGGAGCCGTGTATCAAGAGACGCGCGAGGTGGTCACTATCAAC
GAGTGGACGGATCGCATCTACCAGAACACCATGCAGGAGCACATCATCACCAACGTGG
TCAGCGGACGCAAGATGAGAATACAGAAGTATAATTTCCCTGATACAGTGATCTGGAACCCGTGGTCGGAGTT
CGCGAAGGAGA
>1002052568_2 Heli.2-EL596747.1.5
AACTTGCGGTATATTTAAGTGAAATAAAAATGAAGATTTCCATAATATTGGGAATAATAGCATGCTTTTCAAT
AGCTTATGCTGATCAAGAAACTCTAGTTCTAGTGGACAATTTGAACATTAGAGAGACTCATTCACAGTTTTTT
AAATCTCTTCAAGATCGGGGCTACAATCTTGTCTTTAAGTTAGCTGACGATGCTAACC
TAGTGCTATCTAAGTATGGAGAATATTTGTATAAAAATCTCATTGTCTTTTCACCATCCGTCGTCGAGTTTGG
TGGTCAAATAGACACGGAAGCCATCACAAAGTTTATTGATGATGGTGGAAATGTTCTCATGGCTGGTAACTCT
GCGGCAGGCGATGTCTACAGGGAGATCGCTTCTGAATGTGGATTTGAGATGGATGAAG
AATCTGCAGCTGTAATCGACCATTTCAATTATGACAGTCTGGACGATGGAGACCACACTAGGATAGTAGTGTC
TCCCAAAAATTTAATCAATGCTCCTACAATTGTTGGTTCTCAAAACACACAGCCATTACTCTTTGAAGGTACA
GGTCTAATATTAGATAAGGATAACAGCCTGGTCCTGCCTATACTGACAGCAGATAGTA
CAGCTTACAGCTATAATCCTAAGAGTCAGGTTAAAGAGTACCCTCATGCAGTCGGTCGTAAAACTGTCCTCAT
AGCAGCACTTCAAGCTAGAAACAATGCTAGGATCATATTCAGTGGAT
>1002052569_2 Heli.2-DT664180.3.5
GGTGAAATATAATTTTTAGAAAAAATTTCACGTGCTTGAACCAATTATTATTGTTTACTTCATAAATTTTCAT
ACACCAAACCCATAAATCATGGCCGACAATGACGATCTTCTCGACTACGAAGATGAAGAGCAGGCGGATCAGC
AAGCGGCGGACGGAGCGACCGAAGCGGCGCCAAAAAAAGAGGTTAAGGGATCCTATGT
GTCGATTCACAGTTCAGGATTCAGAGATTTCCTTCTAAAACCTGAGATTTTGCGAGCCATCGTCGATTGTGGC
TTCGAACATCCTTCAGAAGTGCAACACGAATGTATACCGCAAGCTGTTCTCGGTATGGACATATTATGTCAAG
CTAAGTCCGGTATGGGGAAAACGGCCGTTTTTGTTTTGGCGACGCTACAACAACTGGA
GCCTTCCGAAGAGCATGTATACGTCCTCGTCATGTGTCACACGAGAGAGCTCGCCTTCCAAATCAGCAAAGAA
TACGAGCGCTTCTCCAAGTATATGGCAGGAGTTAGGGTGTCCGTGTTTTTCGGGGGAATGCCGATTCAGAAAG
ATGAAGAGATATTAAAGACTGCGTGCCCGCATATTGTGGTCGGTACGCCGGGACGTAT
CCTGGCTTTAGTCAACAGCAAGAAATTGAATCTGAAACATTTAAAACATTTCATCCTCGATGAGTGTGACAAG
ATGCTGGAGTCTCTGGACATGCGTCGTGACGTACAGGAGATCTTCAGGAGTACTCCCCATGGGAAGCAAGTTA
TGATGTTCTCGGCTACTTTAAG
>1002052570_2 Heli.2-EL600004.1.5
AACGTTTCGGTCAACATTTTAACGTTTTTAACATTTTGCTCAATATTTTAGACCTTGAACACTTATCAAACGT
TTAAAAATTTGTTTGTAAAAGGTAGTTTTAACATAAACAGTTAAAGATGGCAGTCAATGTGTATTCTACAAAT
GTGACGTCGGAAAATCTATCAAGACATGATATGCTGGCGTGGGTGAACGACTGTCTTC

AGTCGAATTTTGCTAAAATCGAAGAGCTTTGTACTGGTGCAGCATATTGCCAATTTATGGATATGTTATTTCC
TGGTAGTGTTCCTATGAAACGAATCAAATTTAAGACAAATCTAGAACATGAATATATTCAAAATTTCAAAATA
CTACAAGCAGGTTTCAAAAAAATGTCTGTAGACAAAATAGTACCCATTGACAGGCTGG
TGAAGGGTCGGTTCCAAGATAATTTTGAGTTCTTGCAATGGTTCAAGAAGTTTTTCGATGCCAACTACGGAGG
CACGGAGTACGACGCGATGGCGCAGCGTGAGGGGCTCCCCATGGGGCATGGAGCGGCCGGGGCGCCGTCCCGG
GTATCCGCTCCCGTAGCGGTTAAGAAGCCGGCAGCACCAGTTGCCAAAGTCGCCGCTA
GACCCCAAACCATTGTAAAAGCAAATCCGACAGTCAGGACTCCACCAA
>1002052572_2 Heli.2-EL598128.1.5
AGGGTCGTCCCGCCAGCCGCGTCAATAAGTAAATCCGTGTGTGACATCAATTTGTGAATTGTGTGTGAGAGGT
CTATCAAAAATGGGGTGTGGAACGAGTTTTGTGAAATATGTACTGTTTTTCTTCAATCTGATTGTCGCGCTTT
TTGGCCTAGCCGTCATTGGCATAGGTGTGGCCGTGCTCCTGAACTGGGCGGTGATCAA
GGATGAGCTCCAAGGTCACCTGACAGTGGCACCGTGGGTGTTTATCGTGATAGGCGCTATCATGTTCATCATC
GCCTTCTTCGGATGCTGTGGAGCTATACGCGAAAGTCACTGCATGACTGTCACGTACGCGATCTTCCTCCTCG
TGATAATCATAGTGCAAGTGGTACTCGCGGTACTCATGTTCACATATGCTGACAACAT
CAAAGACGCCCTGGTCACCTCCGTGAACAAGCTCTTCGACAAGCGCAGTGTGGACCCAGCGGCGGCTACCGTC
TTCAGCAATATTGAACAACAGCTCGAATGTTGTGGTAAATACAGCGCTACGGACTACTCGGTAATCCTCCCCA
AGTCCTGCTGCTCGAGACTCAGCGCTGTCGGCAAGTTCCTTGGCGACCAGTGCACTTT
GGCCGATGCTAATATAGTCGGTTGCAGTGTCAGAGTTGGCAATTTATATGAAACTTGGAATAAAACTATTGCT
GGTGTCGCCAT
>1002052573_2 Heli.2-DT665842.2.5
TCTGTTAGAATTACTATAACTGCGTTAATTTCCGATAAAAGTTGTGTTTTGTGTGTTAAATTGATCCAGCAAT
AACTTAAGAAAATGGCGCGTACCAAGCAGACTGCCCGTAAATCTACTGGAGGTAAAGCTCCTCGTAAACAATT
GGCAACAAAGGCAGCACGTAAATCGGCCCCTAGCACTGGAGGTGTGAAGAAGCCCCAT
CGTTATCGCCCTGGTACCGTGGCGCTCCGAGAAATTCGTCGTTATCAGAAATCTACTGAGTTGTTGATCCGTA
AGCTGCCCTTCCAGCGTCTCGTGAGAGAAATCGCTCAAGATTTCAAAACTGATCTTCGTTTCCAGTCTGCCGC
CATCGGCGCTCTGCAGGAAGCAAGTGAGGCTTACTTGGTAGGTCTGTTTGAAGACACA
AACTTGTGCGCCATCCACGCTAAGCGTGTAACCATCATGCCTAAAGACATCCAGTTGGCCAGACGGATTCGAG
GAGAACGCGCTTAAATTAGCTTTAATATACCTAATTTTAAATTATTCATAATTCAAATAGACATTAATTCTAT
GAAAATTTTTTATGTTGTCCCACATTTAATTTATAGTAATAACATTAAAAATCGTTTA
ATGTAAGGAGGCAGCAGTACTGATTTTTTTTATAAATTTCTTATAACATAAGTTAGTATGTTGTGTGTACACG
GAGCACTGCCACGTCTTGCAGAGATTCTCTCTTGGCCAGCGTCTTGTACAAACAGCGTAGTGTCTATATCTTA
GTGACTTGTGGTATATCTAGGTAGTACTATAGGCAATATTGTAACC
>1002052574_2 Heli.2-DT667177.3.5
GCACGAGGGCCAAGTTGATCGTCGCCCTTTGTTCTTTAGGCGTTGCTCATGGCAGTGGCCTCTTAGCCGCCGC
TCCAGTTGCGTACAGCAGTCACAGCATCGCGGCTCCCGCAGTATCTTCTGTATCATACTCAAGCCAGACTTCC
CACGTAGCACCTGTAGCTGCCTATGCTGCCCCCGCAGTGGCCAGGATTGCCACTCCAG
CCTATACTACGTACTCCGCACCCGCCATCACCAGTTATGCGGGCCCCGTGGTAGCTAAGTCCATCTCACCCGC
CAGCGTGTCCTACTCATCTTACTCGAGCAGCACATCTCATGGTGCCCCAGTAGCCTATGCGGCCCGTGCTGTT
GCTGCCCCCGCCTTAGCAGTATCTCGTAGCATCGTCGCCGCCCCCGCCCTCGCTGTTT
CTAAAACCATTGCTCCTGCAGTGTCATCTTACTCTTCCTATTCTTCACAAACTTCCCACGGTGTTAGAGCTAT
TGCCGCTCCTGTAGCAGCCTACTCTGCTGCCCCAGCTATTGCGTCATACGCTGCCCCAGCGATCGCCTCTTAT
GGTAGATCCTACGTCGCCCCAGCGATTGCTTCTTACAGATCATACGCCGCTCCCGCAG
TAACCTCTTATGCCGGCCCTGCTGTTGCCTCTTACTCTAAGGTCTACTCTGCCCCCGTCGTCTCCTCATAGTC
TAAGCTGTACTCCGCCCCCGCAGTTGCCTCTTACGCTGCCCCCGCCTACTCTACCTACGCCGCAGCCGCCCCC
GTCCTTAAATCCGTATCCTATTCGGCGGCACCTGCTGTCTCCACATCTCTTACAGCGC
TCACGGTGCTAACTACGCTTGGTA
>1002052575_2 Heli.2-EL600473.1.5
TTTCTTTTTAGTATCTCGTGCCAGTCAGTGTTCTTGAAAGAAAAGAAAAATGAAGATTGTTTTCTTGATACTT
GCTCTCGCTGTGTGCGCTCGCTCTGAAGACGCCAACAAAAAGGTCGAAGTTCAAAATAAGGACAAAAGGCAAA
CCAACGAAGGCACTCCACAACTCGTTTATAGGACGGCTAGAAAACAGGAACAAGTAGC
ACCGGTTGAAGAAAATCAAGAAGATGAAAAGGGCGACAGAGTTCAACCCCAAGAGTACCGGCCCGGCCAAGTG
TTCTCTCTCAACGCGCAAGAGCTGTTAGAGTTGCAGCCGGAAAGGAAAGCACCAATTCCAAGCGGCCAACAAC
TACAACAGCTGTATAGCAACCCTCAACAGGAACACAGGCAAAATTTACAGCAGTTTTA

TTATTTAGAACCACAATTGAGTCGACAGGTAGCACTGCAACCTTCGCATGCAGTGATTGCCCGTCCGCACTTC
ACATCGAATGGTGGTGAAGCTTCCGTTGGAGCAGCATTATCAGTCAGCGATACCGGTGCTAATTCAGCTGAAT
CCCTGGATCAAGAGTTGCTTGCTTTATTGGGTCACCAACTGGCAGGCAGGCAGGACGA
TGTACGACCTCAAACATTTCCACAACAATCACCTCAAATTCAAGCACAAAATATAGCGCCTCAGTATCAGCAG
GTGGATGTGGCGGCATATATCCAAAAACCGAATAA
>1002052576_2 Heli.2-DT664958.3.5
GCACGAGGCTCAAACATGATCGACGTTATCAAACCCCGCGATGGATGTCTCTACGAAATCAGGCAGAGGGGAC
AAAAGGAACCCTGTTACGTGGTCGTGCCTGATACAAAAGATCTGGTTAAGAGCCACTTGATGTTCGCGGTGCG
CGAGGAGGTGGAAGTACTGAAGGAGCGTATCGCAGAACTGATGGAGAGGATCAACCAG
CTTGAGGTAGAGAACAGCTACCTGCGCGCGCACGCCAGCCAGGACACACTGGCGCAGCTACCGGCCGCCGGCG
CCAAGCCGCCGCAGCCGCAGGGCCCGCAGCCCCCGGTGTCGTAGGCCCCCGCCCCCCAGGAGGGACTCGGGAG
GAGTTATGGCTGTGCTATGTTATATCGTACGTTCACGGGAGAGACGACAACGAGTGTC
CCAAAGTGTCCGCGACCCTGCACTATCGTAGTGTCACAGTGTCGCAGTGCCGTAGTGTCGTCCGCGACCGTCG
CGCAGGAGCCGTCGCGAAAAACTACTATGCTTGGATCTATAGATATATTTTTCGTTATTTAATATTTAGGGTG
CAGAATTTCGTGCTAACTGCCATGCTTATGCTACGCGTCTCGACGACGAGCCCGATAG
TGTATCCATGTATTGACTATATTTTATTCAGTTATTCCATTTAAGTATCTCGTAAATCGCGTGTTATTATAAT
ATAATACTACTACATACTGGAGTAACTCTACGCAATGAACGATGACTGTTAATTTATTTTTCATTATTTTAGT
AGATGTCGTAATTTAAGTTATTTATCGAGTTTTATAATGTTGAATACAGATGAAATGA
TATTGTTGTATTTTGTGAAGTCATTCTGCAATTGCTGGATGTTGTTGGATGTGTGGCAGT
>1002052577_2 Heli.2-DT665155.3.5
CTCTAGAGTTATTTTCGCTCTTTCATACGTTTTTTCAGATTCTATTCATCATGCATTATTTCTCGCTTCTCTT
ATAAAATTAAAATATTTATACAGAATAGAGACGAAATTGATTGCTCAAATGTTTATATATATACCTATTTTTA
GGAGAATTTCAGAAAGGAACTATTTGCTATATGTTATTACGGTACGGAGTGAATTTTT
GTATATTGTAAAACGAATTTTTAAATGTTTGTAACGTTTCTACGTACGTTGACTCGACTGCACTAAAACATAA
CTTCCTACCTCAAAACTATGCAAACTATTACATAATATATTGAATATACAATTTATTGGGAAAAGCAGTTTTA
CATGCAATAAGATATAGTGTAATAAGAGTTTTATTAAAAAAAGTATTTACGTATAAAT
GTTTATATTATAAAAAAACAAAAATATAACGCCCTCAGATATATCTATTATATATGTATTGCCTGTTTGGTGC
TTTACGGCCATTTGCATATAAATATTTACCTTGTTGAAGCAATCGATGATATTCTATGTACAGTGAATCTGTT
ATCGGCCCTAGCTGTTGTTCCTTTGTTGCGGCTGGCCTGGTCCTGGTCCTCCAGGTCC
TCCAAAAGTGGTCCTGAAGACATGAGGGCAGATGGGTTAAAGAAAGTAGTAAAAGAGCGATAACGAATGATTT
TCGCATTGAATGAATGATATGAGATAATACTAGTT
>1002052578_2 Heli.2-DT668720.3.5
TCGCGCCTACAAAATTAGAAGATTCTATGAAAGTGGTAGGACCACTTGGTAGGCGCGGTGCCGACACGACGCT
AAGACGCAGCTGGAGTGACCCTTCAGCTCGAATTCCTGAAAGAAAAATAAACAATACTGATACAGTGTCAGCG
CCAAATCTTTACTCTTTATTTCCACCACCAAGAAAAAGCAGTTCTCCAGAGCCAAGAT
CTCCTCTGGCTTTGGAAATATCCTCGGAAATTGTTACTTCTATAAATGGCCATCGTGATTTGGAAATCAAATT
ATTTTCGACCTCGCCCCCGGCCTCGACCTCGGAACGTCGCTCTTTCTTCGACGATAAAAGTGAAAGTGCCGTT
ACCGATAGATGTGCGAACACAAACACTAAGCTAAAATTAAATCGTGCAGTGAGCAGTG
CAAAAGTTGTGTCGAGTGCAAACAATGATCGTAAATGTGAAAATTTGGATTCGGGGTGCATAAAGGCACTTGT
GGCTGGCGAGAATACGCGCAACATTTGTGATTTAGCCTTAGTTTCTGCCTGAGGTCTTCGCCATGGCCATGTT
TTATACTAATTATATTTGCAAGCGTACGATTACCAGTCCAAGAGCTAGGCTCGCCATT
TGAGCAGGACTCAAATTATTATAGCGAGGCATAAGCCGGAGCGTCTCAACGCGACCCCAATGTAAATGAGATC
TCCGAATCGGTTTCCTTTAGATTCGATAGTTAATTTATTTTTATTAACCATAGCTTAGTAAACTTCAATGGAA
CTTTGTCTGAACGATGTACGTTTTGTGATTCGCCGTTATGACGCTTAAAAAGAAGTTA
AATCATGTCATTCCTTGACGAATGATCTGATATATT
>1002052579_2 Heli.2-EL602061.1.5
GTTGCACGCGGGATCTAGTGCGATTTCCCGCCATTTTTAATTAAACTGTTGACTGTGATATTGGATAACTAAA
TTAGATAAAAAAAATGGCGTCTGTGGAGACCAACGTCCAAAAAAATGGTGATATATTATCAAAGGCACCTTGG
ACCGACGAACAAGGCATCGGTATGAAGATTTATCAAGGAGTAGTGTTGGCACTCGAAA
TTATCGTTCTCGTCGTGAAAATGTATGCGACATGGATGTACTGTATTTACAAGTTCTTCGTGCCTCCCGAACC
TAAGAGCGTTAAAGGAGAAATTATTTTAATAACAGGTGCAGGACACGGTATGGGTCGAGAAATGGCACTTAGG
TTCGCGAAGCTGGGGGGCGTTATAGTATGTGTCGATATCAACCCCTCTGGTAATCAAG

AGACAGTAGAACTAATTAAGGAGGCCAAAGGAAAAGCGCATAGTTACCAGTGCGACGTAACAAGTCGGACATC
CATAAACGAAATGGCCGATAAAATACGTAAAGAGGTTGGCGATGTGTCCATTTTGATTAACAACGCCGGCATC
ATGCCTTGCAAGCCGTTACTGCAGACTGGGGAGAAGGAAATACGGACCGCATTTGAAG
TTAACTGCTTGGCGCATTTATGGACCCTACAAGCCTTCCTGCCGGCCATGATGGAAAGAAACCACGGTCACAA
GTCGCCATGTCCCTCGATGGCGGGTGTGATCGGTC
>1002052580_2 Heli.2-EL602725.1.5
GACTATTATTTTTATTTTTCGTCATAATTATATAAATTTTTGTGCATCATGTCTTCACGTAAAAAGGTTCTTC
TAAAAGTTATCATTCTTGGCGACAGTGGTGTTGGGAAAACATCTTTGATGAATCAGTTTGTCAACAAGAAATT
TTCCAACCAATATAAGGCAACAATAGGAGCTGATTTTCTCACGAAAGAGGTAATTGTC
GATGACAGGATCGTCACAATGCAGATCTGGGACACAGCAGGGCAAGAGCGATTCCAATCTTTGGGGGTAGCCT
TTTATCGCGGGGCGGATTGCTGCGTCTTAGTTTTTGACGTAACTGCCCCTAACACGTTCAAGTCCTTGGAGAG
TTGGAGAGACGAATTTTTGATACAGGCGTCACCACGCGATCCTGAAAACTTTCCATTC
GTTATATTAGGTAATAAAGTGGATTTGGATAACCGTGCTGTGTCAGCAAAGCGTGCACAGCAATGGTGTCAAA
GCAAAAACGACATTCCTTATTTTGAAACAAGTGCCAAAGAAGCCGTGAACGTCGAACTTGCTTTCCAGACCAT
AGCACGCAATGCATTGGCTCAAGAGACTGAAGCAGAGCTTTATAATGAATTCCCTGAT
CAAATTAAGCTGAATGCCAATGACAATGGCCGCAACAGGGATGGAGATAACTGTGCTTGCTAAATTCCACATC
ACTTTTAAACA
>1002052582_2 Heli.2-DT666265.3.5
GTCAAACCATTTTAATTTCACAATTCACAAATTTTAATGCGTTAGTAGCGAAAAGTCGCTCGCGTCCGACCGT
GTACTTTAAAAATAAATTAAACATAAATATATTATCGGAATAATTCAATCAAACGACGGGATAAAAGCTGCGT
TGCAGCGAATCAACCATGAAAGTGTGGGGAGCACATGGAGAGTTCTGCGCGAGACATC
AATGGGAGGTCATAGTAGCCACTTTAGCCTTGTTAGCCTGTGCAGCTAGCGTGGAAAGACATGGCACAGGTAC
CCGAGCAGAGAGATGTGCAGGATGGGCTCGAGCCTGTCCAGGGCTTGAGGCAGAATATCAAGCAGCGGATGCT
GTTATCATGACCTTTGTGAGATGTGCTGCCCTCCTCTACGCGTACTACCAAGTCTCCA
ATCTCCAGAAAATCGCGTCGAAATACCTTCTCATAATAGCCGGGTTGTTCTCAACGTTTGCCAGCTTTATATT
TACTTCAGCACTCGCTAGCTTGTTCTGGAGTGAGCTGGCCAGTATAAAAGATGCCCCTTTTCTGTTTTTGTTA
GTCGCTGATGTAGCAAGGGGCGCAAGAATGGCCAGGGCCGGTTGGAGTGCGGGCGAAG
ATCAGGGGAAGAGAGTGGGGAAAGCGCTTTCGTTACTTGGACCGACAGCGACGTTAGATACACTTCTAGCGGT
CCTACTTGTCGGTGTTGGTGGTTTGTCGGGCGTTCCTCGGTTGGAACATATGTGCACGTTCGCTTGCTTAGCG
TTATTAGTGGACTACCTCGTATTCGTTACTTTCTATCCGGCGTGCTTGTCACTTGTTG
CTGATTTCGCATCCGGAAGAAAGGAAATATCGCCAGATAGCCCATTTTCTGAGGCTGATTTAAAACCAAATCC
AGTTGTTCAAAGAGTTAAAATGATAATGGCTGCTGGCTTGCTGTGTGTTCATTTGACTAGTAGGTGGCCGTGG
ACCAGAGAGAGCGGAATAATGGAAGGATCTTTGACAAACGACTTCAAATCAGGATCAC
ATGATAATGTTTTGTTTCACTCCTATGTTAAATGGTTTTCTGTGAGCGCTGACTATATTGTTATTGCCACTTT
ATTATGCGCTTTAATAATTAAATTTATCTTTTTTGAAGAGCAAAGGAATTGGATCATAGATATGAACGATTTG
ACAGTCAAGGAAGTGGTTAAAGCAAGCGATAAACCTAAATTCTCTCTCGGCGAGGACA
TTAAATCTGAAATGTACACTCAAACTGAAGATATAGGAAATTGTGAAATGTCGGAGTGGTCCTTACTTTCGCC
GAGTTCATCAGCAGCAATGTTGAATTCTAAAAAAC
>1002052583_2 Heli.2-DT666965.3.5
GACTGGACAACAAGACGGAGCGAAGTAGGCGAAGAAGTATCGCGCGCGAACTCTATACGCTGCTGGCGATGAC
CGCATTCATATGAGTTTTTATTAAATTGTGAGAGTGCTGTCGTTTACCCCGTCCCGCTAAGTGTTCACGAAAA
TGCTGTATAGGTGAACTTCCAGAAGTAACGTGTTGTGATTGTGTTAACGTACCCTATC
TCTTCTATCTCGCTGTTTCTTGAAGGTTGTAAAAATGCCTTGCTCTGCGGTAACTTTGTCTATAGCGACCATT
ACGGCGATCGTGGCTGCTGCTTTAATGGCAATCGCATTCTCTACAGACAACTGGCTTTATATTGAAGTTAAAC
GGAACAGTATACAGACATACGTAACGGAGAATACGGACATAAACTCTCAGGCCATCCT
CGACAGCCTCAACAGCAAATACTACTTCTACACGCGGACGCGAGGGCTCTTCCGCTATTGCTACCCCAAGGAA
CGTCCACCTACAGTTGAAATATACCTGTCGCCGGTGGAAACTCACTGCAGCAACGTCGATTACTTCATACCCG
ATGAAAACAACGAGACCAGAGGTCTGTCCGAGGATGCGATGAACAGGCTACACATGGC
TCGGTCCACGGTGGCGCTGTTCATCGTGGCGTTCCTGTCTCTATTCATCGCGTTCTGGACCGGCGTGGTGGGC
TGTTGGAAGCGCAGCCCTGGAAACATAACAGCCACCGCCATACTGATGCTTGTTACTTGTTTACTGTCAGCGG
GCGCCATGGCATTATGGCATGGAGTGGAGTTCTACGAGAAGGAAAAAGTTGTTGGCGA
AGAGTATTATCAGCAATGGCCTAATGTATTAAAAGACTACTCGTCGATTTGGTACGATTGGTCGTACATCCTC
GCTTGGTTGTCCGTTGGTGTTTCCTTTGGGAGCTCCATACTTTTCTTCTCGGCGGCCATTTGTCTCAGCAAAG
AGAAGCGTCGTGAGCAACAGAATAATGTGCAGTACATAATGCCAGTGTACCCCCAAAA

GCAACAATACGCGTACGCCGGTTACCCCCCGCCCCAGGCGTACCCCCCCGGCCCCTACTACCACGGCTCCCAG
TACGGACCCTACAACTACTGAGCAAGCGACCAGATGAGGGACATGGTGGCGTACAACGCGCCCGCACCGCAGG
CACCCGCGAGAAACTCCCCACAACAAAACAACCCGTCCATATTAACATACAACTCCCA
CCGGAAAATTAAACGACCGCTTTTAAGCGATAAGAAAGTTTACAGACCGATAAGAAGGCTAACGTTGTCTAGG
ATCGATGAAATACCGCGACCGAGATCGAAGAGTATACAGCATTCCACGGATTTCTCATCAACCAGACCCATGA
TGAGATCAAGGTTCAACGTCTCAGCGGACACTAGCAGATCTGTACCAGTATGCAATCA
AAGTCAGGATATATCGAATCAAATGGAAAGCGTTAGTCCCCAATATGCGGAACCCAAATACGATTCCGGTTCA
TATGAAAGACCAGCCTCCATGATGGATTTACCAATAATGGTTCCGATGGACCCCTTAACCGGGGCGTACATTC
CGTATCCAGAATACGCGTACTATCACGACGATAACTGTAGACTCGGTTGTTATAAGAA
AAAACAATCGAAAAATAATTTGTT
>1002052584_2 Heli.2-EL599350.1.5
GCACGAGGGTCGCGTGGCGCGGCCGGCACTCCCACTCGCGGGTCGCGGTCGCGCTCGTTCACTTTAGTTGTGG
GCCGGCTGCCGCGGCGGATGCACGCCACGAGACACGCGCCTCCGATGTTAGTTTACGATTACGACATCGCGCA
CGAACTCGGACTTTTGCCGTGACATTGAATTTATCTGTGTCGAATATGGGTTAAACGA
TCCTATCGACGAAATGAGTCCGGGCGAGCGCGCCGCCCTGGGGCGTCGCGAAGCCGCTCGCCCTCACTATGGT
GCCACCGCACCGGGTTTCCCGCCACCATACTACCCGCCCTACGGTGTGCCACATCCCAATGCGTGGCTCGGTG
CTCCCCTCATATCGATGGCGGGCCGCACGCCGCAACGCGATAACCCCGTATCCAAACT
GGCGATGCACGCCCAGGGAGCTCCGCTTATTATACACAACAGACACGATAGAAGAAAATATACAACCATACCT
GAGCAGCGCAACCATCGTCCTATGGGCCAAATAGAACATCACACGAAGGATCGATACGATATGTCAGAAAATA
ATAGGCCGAGAAATCAACTTCATCATCATCAACAACAACAACAACAACAACAAAATCG
ACCATCAAGAAGTGGACGTAGTAACAACACTGACGCAGTCAGCGTCGCCAGTGACGAAAGTTCCGGCTCCACC
AATTCGGAGACAATGTTACCGAGAATAATTAAACCACGAAAGCGTCGTAAAAAAGATAGAAAACCGAATAACA
TAGTGCCACATGATTTAACATCCGTTTCACTGGATCTCAGCGATGTTGATCACTGTGC
AGTTACAAATAT
>1002052585_2 Heli.2-DT664423.3.5
GATCTTCCATGAAACAGTGGGAAATTAAAAGATCACGCACTAGGATTAAACGGTCACAAGGAAGCATTTGCTA
TGGAGAATTCGGTTGCTTTGAGGATTCTGGTCCATTTGCATACTTGGAGACGCTACCAAGTCCACCGTCAGAA
GTTGGAACGCATTTTCTCCTCTACTCTACTACGAGCAGAGGAGACCAACCACTTATAG
CTGTCCCGGCGAGCAACATGTCGGCAGCGTGGAGCTGGGCCGCGCGCGCCTTCGACACCGTCCGACCCACACG
CGTCATCGTGCACGGCTTCGGCTCAAACTGTGATAATGTATGGGTGTACGAGATGCGCTCCGCCTTAATGGCA
GTGGAAGAATGCAACGTAATTTGCGTAGACTGGGAAGGCGGTGCTACTATGCCAAATT
ACCTTCGAGCAGCAGCTAATACGAGACTTGTGGGCAAACAGTTGGCAATGCTGCTACAAGGTTTGACTCAACA
TATAGATCTGCGATTTGAGGACGTCCATTTGATTGGGTTTAGCTTGGGAGCGCATGTCGCAGGATTTGCCGGA
TCTGAATTGAGAAATATTAGTCGTATAACAGGATTGGATCCAGCGGGACCTCTATTCG
AATTTCAAGACCCGAGAGCACGGTTGGATAAAACTGATGCAAAGTTTGTAGATGTGATACATTCAAACGGTGA
GACGTTAATACTCGGCGGGCTCGGCGCCGCGCAGCCTCTCGGACACGTCGACTTCTACCCCAACGGCGGCCGA
GTGCAACATGGCTGTTCTAATTTGTTCGTCGGTGCAGTTTCCGATCTTGTTTTGCCAT
GGGCCGGCGCGTCTCCAGAAGGACGGTCATTGTGCAATCATCGTCGCGCGTACAAGTTCTTCACTGATTCTGT
TTCGCCTAAGTGTCACTTTCCAGCGTTTCCCTGCACTGATTATGATACTTTCTTGGAAGGTCGGTGTTTTCCT
TGTGATGGCGACCATCGTTGTGGCAATATGGGTTACTACGCCGACCGTTCTCTCGGAC
GTGGACAATTGTACCTTCTCACGAGAGAGGAAGAGCCTTTCTGTGCACATCAATACCACGTGGCCTTGTGGGG
AGCTAGTGAAGTAGGCCAAAAACCAAACTACGGTCGAGTGACCCTCACTCTACATGGGGATTCAGGGCTAAAT
GAATCTTTTCCTATGACCAAGCGCGAGGCCAGCGACCGCAACGTGCGCTTCGGGCGCG
TGCTGGTGCCGCACCCCGCGCTGGGCGTGCCGCTGCGCGCCTCCGTGCACTACGCCGCCTACAACGGCTGGCT
CAGCGCCGGCGCGCGCGCGCTCCTCGTGCACAAGCTACTCATTACTGACAGCTTTGGGAAGACTTCATCATTC
TGTAAGAACCTACGACTTCTATCCGACGAGTCGGCTCAACTACCATTGCAACCCGGGG
ATTGTCAAATTCAAGAAGTTGAAGAACCGACTAATAGCACTCTATTGGCGCAGGAAGTTATTCATCTAGAAAA
TGTAACTAGCGCTCCTCCAGATGACCCGCACGATAATGAACTACCCGAAGATCCGCAGCAGAGGCCTTTTGTC
GCCGAGTCTTATGACTGGGAGGAAACAGTCGACACTGGTAGAGCATTCGGTATGACCA
ATACAAAAACGGCAGCCAGTGGAG
>1002052586_2 Heli.2-EL596523.1.5
ATTCTTCGTGTTTAATAATACTAAAGGATTTTTGTTTATGTTATAACGATGCAACTACTATTAGCTGCTGCTA
CCCTAGTAGCCAGTCTCATAATAGTTGAGGCTCAAGTAAACTCGGACAGATTGGACAGTGTCAACATAGACGA
GGTGCTAGCAAACAAGAGGTTGCTTCAAGCCTATATAAAGTGCACTCTGGACAAGGGA

AGGTGTACGCCGGAAGGGAGAGAGTTAAAAACCCACATAACTGAAGCATTGCAACAGGGTTGCGACGGTTGCA
CAGACGGACAAAGGGAGAGCGTACGAAGGGTTATACGTCATTTAATAAGAAATGAACCGGACTACTGGCAGCA
ACTCGTGGACAAATACGACTCAGAAGGAGTGTTTTCTAAGAAATATGAAGACGAACTT
AATTCTCTTTAGGTAGCGACATCAAATGATTTTAGGAATATAAATTTAATTTTTGCGGCGTTCAATTCCGCCA
CGCAACACGCAGTGCTTGGAAATTCGGTAAAAGTTAAAGAAAATACAGCGTGAAACTGAAAAAAAAGTAAACA
TTTGATTTTAAATGGAAATATTTTCAACGAAATATTGCGTGTAATTTATATAATAGTA
GGTATTTACTATTAGGGTAAAAGTTAATTAATTATACTATTATAAGGT
>1002052587_2 Heli.2-EL601795.1.5
GCACGAGGGATTTCCCCTCGATAAAAAAAAAAAAAAAAAAAGGGTAGACAGAGAGAATCTGAGAAAGTAGTGTT
AAGATACTTTGTTTCCATTAACATATTTCCATTCTGAGGCATAGCGTGGCATCTAATTCCGCCACTCAACACA
CAATGCCTACAGATTTCAAAGGAAAATGGGTAACGGAAGTGTTAAGATCAGTATTAAG
ATACTAGATTTCCAGGGTCCTGAGAGAGCCTTCGGAACACAGCGTCTAATGTGTGGCCAGACCTGGATTTAGT
GAATTAATTAAAGAACACACGCTTTAACGTAGAGGAAAACAAAAAAACAAGAACAACATTGTGATACATGTAT
GTGATAATTTAATTAATATTAATATTAGGTTTTTACATATGAAATTGGTTTTTAGTAT
GGGAGGAACCAAAAGTCGAATACTTGTTTATGCAATATATTTATATGATATATTTAGTTATCTATTTTTTTAA
AATTTAGTTATCTATGAAAAAAATGGTAATCTGTTGGAAAACTGTAATGAGCGACACTGGCACTAGCCCATCA
AACGCTTATAAGTAACTCTTTTTTGGTCAATTTTCGTTTGGGGCAGGATTTGGCTGAC
TTGCTAGGGTCCAGCTTCAGATTACCGTTGAGAAACCACTTGTCATCACAGGTAATGTAAAACCTCGATGGTT
CGATGTAAAAACCTTTCATTATTTTTGCGGTTAAGTAATGTAATGAAACAATTCGCTTTCACTGAATTAAAAC
AGTTGTATCA
>1002052588_2 Heli.2-DT662608.3.5
TCAACCAAACAACCAACAGCAAAATGGTCTTCAAGTTTGTGGTCTTCTCCTGCTTGGTGGCTGTCGCCTACGG
AAGTGCATCCGCCGCCATAGCAGCAGCTCCTGTCGCATATGCAGCACCAGCCGTTGCAGCAAGATTAGAAGAA
TTCGACTCGTTCCCTCAGTATAGATTTGGATATGACGTAGCTGACTCTCTAACCGGTG
ATTACAAGAGCCAATCAGAACAGCGTGATGGTGATATTGTACAAGGTCAATACTCTCTTGTGGAACCTGATGG
TACCCGCCGTGTTGTTGACTATGCTGCTGACCCTGTTAATGGTTTCAACGCGGTTGTACGTAAAGAACCCCTC
GTGGCTGCTGCCCCAGCTGTAGTCGCAGAGCCTGCAGTCGTCCCTGCTAGAATCGCCG
CCGCTCCAGCCGTGGCAGTTGCCGAGCCAGTAGTAGCCGCCAAGGTCGCCGCCCCTATCGTGGCCCAACCTGC
TCCTGCTGTCCTTGCGGCTCGTTATGCCGTTGCTCCCGCTGCCCGTTATGCTGTAGCTGCAGCGCCCGTAAAC
TATGCAGCTGCTGCTCGGTACGTGGCCGCCCCCGCAGCTCAGTACGTTGCCGCTCCAG
CGGCTCAATATGTCGCTGCTCCAGCCGCCCAATACGTCGCCGCCCCTGCTGCTCAGTACGTCGCCGCCCCTTC
TGCTCAATACGTCGCTGCCCCTGCTGCTCAATATGTCGCTGCACCCGCTCGTTATGCCGCCGGCCCAATCGTA
GCAGCTCGTTATGCTGCTCCAGTATCAGCATATG
>1002052589_2 Heli.2-EL602821.1.5
AATAATATATTTAATAATAGTTGATTATAATATATATATATTAATAGTATTTTTAGTATTTACGGTTTGTGAG
GGGGCTTTAGGTTTATCAATTTTAGTGTCAATAATTCGAACTCATGGTAATGATTATTTTCAAAGTTTTAATT
TAATTTAATGATAAAATATTTATTTATAATAATTTTTATAATACCAATATGTTTGAAA
CGAAATATATTTTGAATGGTTCAATTTATATTTATATTTTTAATATTTATATTTATAAATATAAGAATTTCAA
TTATAAATTTTTGTAATTTAAGATATATATTAGGTTGTGATATAATTTCTTATGGTTTAATTTTATTAAGAAT
TTGAATTACATTTTTAATAATTATGGCTAGAGAAAAATTAAATAATGATGATTTTTTT
AAGAACTTATTTTTATTTAATATTATTTTTTTAATGGTGATATTGTATTTAACATTTAGAATTATAAATTTAT
TTTTATTTTATTTATTTTTTGAAAGAAGATTAATTCCTACATTAATATTAATTATTGGTTGGGGATATCAACC
TGAGCGAATTCAGGCGGGGATATATTTATTATTTTACACATTATTT
>1002052590_2 Heli.2-DT664236.3.5
GCACGAGGTGGAGTCATATTTTTGCGTAATAAAATCCGATTGTAACACTGTTGGAAATTTATTGAAATTATGT
CATTTGTGTATCTCCTCTCAAATCGTTCGTGGTCAATGCAGAAAAGCTTCGATCGTTTGACAATATCCGCCTG
AGAATTGTTATTGTTAATAAGATTGTCGATCCGGGAGACATCTTGTGTTGAAAAAGTT
ACCAAGTTGTACGACTTGTCCGATTTTGTCTTCACGTGAAAAGTTTGCAAAATTAGATCGGTGCTAGAAAGTA
GGCAAACATGGATGTTGCGTGGGACGAAGATAATTTTGAACCTAAACTTCCGACCACGTTGACATCGTCCAAC
AAATGGGAGGGCGAGGACGAGGAAGAAGCTGTCAAGGACAGCTGGGAGGACGAAGAAG
AAGAAAAGAAGGATGAAGAGAAAAAAGATGTCGCCGCTCCACCACCGAAGCCCAAAAAGAAAATACACGACAA
AATTGCAGAAAAGGAGCGCTTAGAACGTGAGAAAGCAGAACGCATAGCAGAGAAAGAAGAAGAAATGACGCCA
GAAGAAAAACTTGCGGAAAAACTGCGAAGACAGAAATTACAAGAGGAGTCTGATTTAC

GTCTGGCTATGGAAACATTTGGTATATCCGAGAGCGGTGTCGGCAAGTTGGACAGCTTCCTGCCGACGAACAA
AGCGGAGTTCACAGAGTTTGGGGATCTCCTCAGCAAAAAGATCAACTTGTTCAAGGCCAAGGAGGAGTTCCCC
GCCTTTGTTGATGATCTGGTCAAGAATATTATAGTACAAATGGCGTCCGCTGACATAA
AGAGGATAAAAATGACAGTTGACAATTTATACATTG
>1002052591_2 Heli.2-EL599616.1.5
TTTTTAATATATAAGTGAATAATTTGTTGCGCAATTCGAACGCGGGCCGAGGCAAAAGATTCACATTCCAATC
GACGGTACGACAACTGGAAAGGCGACGGTTAGCGGAGAAGCTGTCTAAAGAAGCAGACCTGAAGGAACAGCAG
AGAAGAACCGAGCTGGAGGCCATGAGACGAGTAGAAGAAGAGTTTCAACGTAAGAGAG
CGAGGGAGAAGGCCAACATCCGGCAGCAGCTGAGGCTGGTGACCAGTGGTGCTAGCAGCATGCCGCCCTCTCA
ACATAGCAAGACCAGAGACGAGCCAGACGGTTCCTGCCGGGGCTCCCCCGCGTTAGATCGAACTCGTGACCAT
CGAGTCAAACACAGCAATGCGTCTTCTGAGGGCAGTGGCAGAAGCAAAAGTTCTACAC
CAATTCGTAGTGTAGAGCTATCAGAGTGGCGCACGGAGGGCGGCGCGCGCGTGTACCGCGACTGGGCGGCGGC
CGGCGTGGCGGCGCACGCGCACCCGCCCGCCTGCGCGAGGATGCCCGCCACCGTACACGCTTATGCTGAAGGA
GAAGCCTTCAGCGGTAGTCCTCGCTCAGACAACTATCGCTTAGAGTTTGCTCGTGGTC
GCTCCCCGCGCACGCCGCGACCTCCTCGCCTCTTACACTCCCCCGCCTCGTCTACTACTGGCAGTGATTTGTC
ACTGCGACAGCCTATTAAGATTA
>1002052592_2 Heli.2-DT665593.3.5
ATCCTACAATGGCAGATCCGCGCATCCGGCAAATAAAAATTAAAACCGGCGTCGTAAAACGAATTGCCAAAGA
GAAAGTAGTTTACGAAAAGGAAGCTGAACAACAAAAGAATAGGATACAGAAGATAAAAGACGAAGGGCAGGAC
GAACACAACATAAGGAAGCAAGAGGAGGTCCTGCAGGAGTCACTGATGATGGTCCCTG
ACTGTCAGCGACGATTGGCTAAAGCATTTGCAGATTTGAAAGGCATTCTAGAAACTGAACAAGACCTCAAAGA
AAACGAGGACTACATAGCAGCAGAGCAAGTATTAAAAGACGCTGAAATCCAATTACCAGAATCTGTTTAATTA
TTTTTATTTATATTTTTATACTAAACAATCATGTCGCAAATATTTTCTAACATTTTTA
CATTCCAAAATATGCTTCCTGTGCCCCCACCACCACCGCCACCACCACTGATGACCCAAGAGCTATCAGATCA
AGAATTTGTTAAGTCATTTGAGTCTCGGGTTGAAATATCTAAGAAACGAAAATTGAAAATAAATTGTGGAAGC
ATCTCCCAAACTAAATATTGTTTGGCGAGTTTGGTTTCTGCAATAAGAGATATAAAGG
CACAAGAGAAGTTGCTTGCTGGGAACAATTTAATCAGTGATGAAGAATGGAAATCAGCCATTGAAAGCATAAA
AACGAATAGATATGTTATAGATTCAACTATTAAAAGAGTTACTGGAACATTTATAGAGATTAATAGAAAGGTG
TTGACAAGACGTGCTGCTAAAC
>1002052593_2 Heli.2-EL597979.1.5
ACGTAGTGTACTTATTAAGGCACTGTTAAGAATATATCAAGTTGTGTTGTTGTGTTTTTATTGCTTTTAATAT
GATGCTTCCGTCATCTCTTAATTTGAAGTCAAGTGCTTCCCAACGAGTGTCTTGAACACTACAACTTACAATA
ATTTTACTTATTTATTAACAATACATAAGTTTCTTAATGTTAGCACCTAGATATCAGT
TTCTTATCATTTTGCTCGAGATAAAACACACCTAAGTAGCATATCTTTCTATGTTTTATAACAGAATTGTTGT
GTTTTATTGTATTAAGTATTTATTAAGTTTATTATAAAAAGTAAAAATTGTAAAGTTTATTTGCGGCATTTTA
TCAAGATGGCAAGTGATCCAGAATTAAAGGATGGATTTACTCTTCCCGTTTGGATGAA
AAGTAAACCAACCAAGGAGTTTGAACCGTTCGCTTCGTCCCCACCAGCTCCAGAGGATTCGTTCTTCTACATT
CGCTATCCCAAACCGGACGTCCTGTTTGGCAAAACGAAGTTCAGCAATGACCTTCCTAAAGTCCTGACTGAAC
AACAGGAGTTGTCAGCGACATACAACGTGATCAAAGAGAAGGATTGGTCGAAACACAC
GAAGCCGTGCCAAGACGTCTTGCACGGCATTGCGCCTATTAATACTTCTGTTAAATCGGCCAACGTTAAACCC
AAGCCAGCGGGCACAGATGATGGATTCAAAGGGGAGGGTGCGGCGTGCGGCAGTTCCGTCGTCAAGGTGGC
>1002052594_2 Heli.2-EL598902.1.5
GCACGAGGCGATCGTGTCCACCTGACAGAGGTCAATTCCGATACCGGACGGCCTGGTTATGGACCCGATGGCG
CCCACAGCACTTGCAGTTCCAAATCAACTCATGGATCGCATTGGCAGTGGACTGACCATCCTCAGAGATCACG
GCAAAAAGTTCCAAAAGTACCTTCTGAAGAGTAGAAAATTTGAAGTGCATAGAAAATC
TTGGTCTTCAATAGTAAATATAGTGTTGATAGAAGCAAAAGATTTACCTGATGCTCCTTCGAATGGTTCCAGC
GGCCTTTATTGCAAATTCAAACTCGGCAGTGAATCTCACAAATCAAAACAAGTCCAAAAAACGAAACCGATAT
GGCGGGAACGGTTTAATCTGTATTTGTATGAAGACAGTTCCTTAGAGGTCAACGTATG
GCACAAAACGAAACAGAAAAACTTTATGGGCAGATGTGTGGTCGATCTGTCGCAATTAGAAAAAGAGCGAACA
CACGATCTGTGGCAGAAATTAGACTGTGGTTACGGTTCAGTGCACCTACTCATCACACTGAGCGGATCAGATA
GGTGCACCACCGACAACGTCCTCTCAACGACCAACGGCGTCCACAATGATGCTCCTAA
AGACGATAAGTTCCTATGGTACAGACTTAACAATTTAAATGAAGTCGGCCAGCTAACAGTCACCGTGCACGGA
GCCAGAGGTTTAACTGCATTAAATATTAGTGGCAACGTGAATGCTTTTTGCGTGTTAGAACTCGATAATTCAA
GAATACAAACACACACAGTACG

>1002052595_2 Heli.2-EL597915.1.5
GCACGAGGCGGTTGCATTATCACAGTCGGTGGTAGCAAAGTTTGTAAAAATGTGGAAATATCTTGGTTTACTT
GCCGTTTTGGCGGCGGTTCATTGTAACCCAGTGGATCGTGGTTTGGAGGAGAATTTAGTGGGTGCAGTGTCGG
AGTGCATTGATAAGGATACTTCACTATGTTTGAAGGAAAAAGCTTTAAAGTATACCGA
TAGATTGGCCTTCGCCAAAGATATCAGTATCTTTGAGGGCATGAGCCTTATCAATACTGGATCAGCCCGTTCA
TCTCGCAGTTATGAGCAATTGTCTGAGGACCCAAAGACCAGAGAATCTCAGATTGAAGAAAGGATAGCCAGCA
ATGTCGGAGACTTTTTAGACAATCATGTATTACAAATGCGCTTGTCTGAAGACTCAGA
TGAATCTAGGGCCTTAGATGATGAAGAAGGCCGTGGCAAAAAGAAGAAGAAGATCAAGAAGATCCTTCCCCTT
CTCCTTCTCTTGAAGCTCAAGCTTGCTGCTCTAATTCCTCTTTTCCTTGGAATCATCGCATTTGCAGCCATCA
AGGCGGTGTTCCTTGGCAAGATTGCCTTTGCCATTAGTGCTTTTGGCCTAATCAGGCG
ACTTCTATCTAAGAATAGTTCAGGATCCTCTGGAGCTACAATCAGTTATTCTCCCCATCACGCGGAGGAACAC
CCTGGATATTC
>1002052596_2 Heli.2-DT667202.3.5
GCGGGCCGCACGTCTACTGGTGGTTAGTGTTCGATGTTCCGTATCTGACACTGGATAGATATGTCTAAAGTCA
AGACGATGTTGAAGATAGGCGGTGGCGCGGCGCTAGTCGCGGCCATAGTAGCCGTTTTCGTAGTGGCTACTCA
AGGGAGGGACCCCGACTTAGAAGCTTTAGAACAAGAAGGTAGAGAATACATTTTGCAT
TTGGACAAAATGGCTGGCCTCAGGAAGAATAAAGCAAGTCTTGCCGAATGGGCGTACACATCGAATATAACAG
AGGAGAATGAGGAGAAACGGATACAAGTCCAGCTAGAATTATCAAAGCAAGAGAAACAAGCGTGGGAAGAAAC
GAAGATGTACAAATGGGAAGATTTTCAAGATTTTTCTTTACGGCGAATGTTTAAAAAG
TACAGTCAACTAGGAGTTTCCGCTTTACCCGATGACAAATATAAAATGTTAATGCAATGTGTTTCTGGAATGG
AATCCAACTATGCCACTTCGAAAATTTGTGATTATAAGAACGCTACCAAATGTGACTTGGCGTTGGAACCAGA
TATCACAGAAATTTTCGCCAAAAGCCAGGATCCAGAAGAGCTAAAACATACTTGGTTG
GAGTGGCACAAGGCGGCCGGCGCTAAGGCGCGAGAGAACTTTACTAAGTACGTAGAGTTGGATAACGAGGCAG
CTAAGCTTAATGGTTTCAAAGACGTAGCAGAGTGGTGGCAATCAGAA
>1002052598_2 Heli.2-DT662088.3.5
TCCGTCTGGTAAAGAAATTTTAGTGAAGGCAATTAAATTGATTATTCTGAATGAATTAGTATCAAAGTAACTT
TCAATTGCTGGTTTAATTAACCGCTAACCAGCATATGTGATCTTGCGCATTAAAATGGCTACATATGAAGAAT
TTATTCAGCAGAATGAAGATAGGGATGGAATACGGTTTACGTGGAACGTGTGGCCATC
TAGTAGAATTGAAGCTACAAGGCTTGTCGTTCCTTTAGTATCTTTATATCAACCATTAAAAGAACGCCCTGAC
TTACCACCGATTCAATATGAACCAGTATTATGTACACGTAATACATGCCGCGCTGTACTGAACCCTATGTGTC
AAGTTGATTATAGGGCTAAATTATGGGTCTGCAATTTCTGTTTTCAAAGAAATCCTTT
CCCCCCACAATATGCGGCCATATCAGAGCAACATCAGCCAGCTGAGTTAATACCTAACTTTTCTACTATAGAG
TATACTATAACAAGAGCCCAAAGTATGCCACCTATCTTTCTATTAGTTGTAGATACATGTCTTGATGAGGAAG
AATTAGGTGCTCTTAAAGATTCTCTTCAAACATCTCTTAGCTTAATGCCACAAAATGC
CCTTGTTGGTCTTATTACATTTGGGCGTATGGTCCAAATTCATGAGCTAGGTACTGAAGGACTCTACAAGTGT
TATGTTTTCAAAGGGACCAAAGA
>1002052599_2 Heli.2-DT668374.3.5
CACTAAGTTCATAATATATTGTGTCACAAATTAAATATTAGTCATGGTTGCTTAATTTAATTATTTTTTACTT
ATAGTCTTTTGTTTTCACTTTGAAGTAAATTAATAATACAATAATCAAATAATAACAAGATTCAACAATGGGC
GCCGTATTAGGTTTATGTTCAGCAGCTCAGTTGGCTTGCTGCTGTGGTAGTGCAGCAT
GTTCACTTTGCTGTTCTGCATGCCCTTCATGCGCCAATTCGACTTCTACTCGCCTCATGTACACCCTTATGCT
ACTACTAACTGTGGTAGTGGCTTGTATTACTTTAGCTCCTGGATTACATGAAGAAATGAAAAAAGTACCATTT
TGTGAAAATTCTACAGGGATAGTACCAGGAAGCTTTAAAGTAAACTGTGATCAAGCTG
TGGGATATTTGGCTGTTTACAGAATCTGCTTCATAACATGTCTATTTTTCATATTTATGGCATTAATTATGAT
AGGTGTAAAGTCATCAAAGGATCCTAGAGCCGGAATACAAAATGGATTTTGGGGTATTAAATATTTGGTTGTG
ATTGGAGGCATTATCGGTGCTTTCTTCATCCCTGAAGGCAGTTTTGGATATACCTGGA
TGATTTTCGGAATGATAGGAGGCTTCTGTTTTATTGTCATACAACTTATTTTGATTATTGATTTTGCTCATGC
TTGGGCTGAGAAATGGGTCTCAAATTATGAAGAGAGTCAGTCCCGCGGCTGGTATTCGG
>1002052601_2 Heli.2-DT664826.3.5
CGCCTAGCCACATCGTGACAAACGTCCGCAATTGTGATCAAACATTTTGTCTTACAGATTTGAAACTTTATTC
TTGATCATAGAGAAAAATCGGAGTAGAAGTCCGTCGATTTATATTTTTTTAATATATTAAGGTAAAAGAAGAT
AAGAAGTAAAGATGATGTCCAGCCCAGCCATGGAATTGGAAATCGAGGAAGCCCCATT

GGCCTTGCGCCGCTTGTGGAATCTTACTCGGGCCCGTCTCGCTGGCACTTCCACTGCTTTGCATGAGGACCCA
GCTTTAGTAGAGACTTTACTTCCTGCTCAGGAACCAGAAGTGACCTCAAAAACCTGCATACCAGTCCCTAGTG
ACTACGAAAGTGATGGTGAAGAAGCTTTTCCAGAACTAGAAGTTCATGAGAAACCTGA
GCTTCCAATTAGATCTTTGTGGAGCGAAGGTGCTTATGAAAAAGAAGTCCTGTTGGATGCCAGCACTCTGGGA
GATGTGCCCGCGGTATACCGCGTACCGGCGCCAGCGCCGCACCAAATGCCCGTCATTGGAGTTTATATCGATC
CTCGAGTCAGGACTGGCTTCAGATACAAAATTAGACCGATGCAGACTTTGGATGCTCC
TCCACTATCGGTGAAACCTCGATACTTGTTCGACGGGAAGGCTCTTGTCCTGCAATCGATTGGCCGTGGATTT
GCCAGAAGACTAACGTTTGAGCCTCATGATTCTACTTTAAATGAAAATAACAATTTCTTTTGGACCGATTCAC
GTCCTGAAGGCTTTCTATTTGAAATTGAAGCTGTTTCTGTTGGAGACAAGTTCACTAT
TTATGATGCCAACCACGAGCCGCAAGGAATATTGGAAGTAGTGCAACAACAGAAGAACCAAATCGAGATTGAT
CAAAAAATCTCAGCAGATGGACTAATTGAAAAGAAAGTGAAAATTAATACACTTTGCAAAGTGGAATGGTATG
AGAATGATGGAATCACGAAATTAGTTCCAGTCAC
>1002052602_2 Heli.2-EL600074.1.5
ACAAGCAACACGCACTTTCACCAGTGACGAGTGGCTACCCCGTGGCAGATATCAATAAGTGTTAGTGTTTTAG
TGTTTACCCTTTCAAACAAAATAAGCATGCAGCAAAAGGTGACAGGATGGGCATTTCTGGCCCTGATCTGCTC
GTCGACGATTCTTCACACGATGGCGTACCCGCAACATATGCCTCTTGTTTCACAGGTC
AAGAGCAATGTTCGGACTCAAAACCCACAGTATTATAGTAACTCATTGCCAGCACCTCACCGAGAACAAATAG
CAGAAGAACGAAAATTTGCCGAAAAATCCAATGCGCTGAAGAAAGTAGCTCTCGATGATCTAGATGATATCCA
GAACAATTCTATATCAGATGGCGGTTCCTCATGGTCAAACATGTTAGGTATGATAATG
CAAATGATTTTCAATCCTGCGGGCGCCGTAGGACCAAACAAGAGCGACAATCTAGATACAGATTCGGCTGCAC
CATCTCCTTGGACATTCTTATTCTCAATGGGTCTGAAAATCCTGACCGCAATTCTAGGAGGCGCAACCAACAA
CACTGGAATTGACAAAGTGGACAACGAGTCTTCACCAATGCAGTTTATCAACATCGTT
GTAAATCTTCTGGATGCTCTCAAAACCTCGTTCTCTCATCGGTCACTTGCTGCCCGGTCAATGGGAAAGAAGG
ACTCGGTCAGTGACGCCGCTATG
>1002052603_2 Heli.2-DT662242.1.5
TGGTGTTGCGCTATCCAAATGGGTAAACCCCGTGGAATACGCACGGCGCGTAAGCACGTGAACCACCGCCGCG
AACAGCGATGGGCGGACAAGGAGTACAAAAAAGCCCACATGGGCACAAGGTGGAAGGCTAACCCCTTCGGTGG
TGCATCTCACGCTAAGGGCATCGTCCTGGAGAAAGTTGGTGTAGAAGCCAAACAGCCC
AACTCCGCCATCCGGAAGTGCGTGCGTGTGCAACTGATCAAGAACGGCAAGAAGGTGACCGCCTTCGTGCCCC
GTGACGGTTGTCTCAACCACATCGAGGAGAACGACGAAGTGCTAGTCGCGGGTTTCGGTAGGAAGGGTCACGC
CGTCGGTGATATTCCTGGAGTCAGATTTAAGGTGGTAAAAGTGGCCAACGTATCACTC
CTTGCGCTGTACAAAGAAAAGAAGGAAAGACCTAGATCATAGATCTACTTCATTGTGATAAGTGCTGCTGAGG
GAGTCGTCGTTTTAAGTGTAACTTTTCAAAAATGAAAAAATTAAGTATGCAGTAAATTG
>1002052604_2 Heli.2-EL597351.1.5
CACCGCCTGCAAACCCTGCCCTTAAAAAGGATAAGAAGGAGAAGAAGGTTCCTAAAGACCATTCCAAAAATGT
TATGCGGAATCTGCACATCAGGAAATTGTGCCTGAACATCTGTGTCGGTGAATCTGGTGACAGGTTGACACGT
GCTGCTAAGGTGTTGGAACAGCTTACTGGTCAACAGCCTGTGTTCTCCAAGGCTCGTT
ACACCGTCAGGTCCTTTGGTATCCGTCGTAATGAAAAAATTGCTGTCCACTGCACTGTCCGTGGAGCTAAGGC
TGAGGAAATTCTTGAGAGAGGATTAAAAGTCAGGGAATACGAATTGAGACGTGACAACTTCTCAGCAACTGGA
AACTTTGGTTTTGGTATCCAGGAACACATTGATTTGGGTATCAAGTATGATCCTTCCA
TCGGTATCTATGGTCTTGACTTCTACGTCGTACTTGGAAGGCCAGGATTCAATGTAGCACACAGAAGACGCAA
GACTGGCAAGGTTGGCTTCCAACACCGTTTGACGAAGGAGGACGCCATGAAGTGGTTCCAACAGAAATACGAT
GGTATCATCCTTAACAGCAAAAAGTAAATTAAGTAATAAAAAGTTA
>1002052605_2 Heli.2-DT665861.3.5
AGCGAGCGCTTTGCTAGGCACCTGCCGGCCAATCTTCACGATAACATTCTAGTCACCGCGCAGCAGAAGCTAG
CGCAACGCGGTGTTGGACCGCGCGCGTTCCTACTAGCAGTGATATGTGCAATAGCTCTACTTACAAAATAATT
GTGATAATTTATTTAAATTAGTTATTAGTTATACGCGTTTAGACCGTGATACGGCTGT
GAGTGAAATAAATATCGGATATGTTGTGACTGCCCGACTGGAACTTGTTACCTGTGATCCAAACCAGTGTTAG
TTATTCTTGCGTTGGAGGACCTTTTGTTACCATGGTTGGGAACAACGGCACTATGATGGCGATTCGTATCGTC
CACAGCAAGCTGCGCAAGCGCGAGGAACACTCGGCTTCGGTGCATCCTGCCGAAGCTG
TGCTCCAGACTGCGCCGCAACCAACGGCTGATCCTCTTGCCTTTCGACAGCAGCCCTTGTGGCAGTTCCCACC
GCCGCTGCCTCCGCCTTACGTCTATCCTCACGATCAGGACAACTTGATGCAGCCCATTGGTAACGAGCGAGCA
AGTTTTCGCAGTTTACGTAAGAATATCGGTGGTCGTTGGAAGCGACTCGTTAAAAAGA

AGCCCGAACAGGAAGTGTACACGATCCCGCCTGAGCTCAAGCCTCAACTGAAACAAATATACGTGTACTAGCA
ACTTTCTAAGAACAGCATAGAAGCGGGATACTTCGATGCCACTTCAAGACATGCTCCGAATGGGAGTTCTGTA
GTTATAACTGATAAAGTCATAT
>1002052606_2 Heli.2-DT666452.3.5
GCACGAGGGGAAGACGCATCCACTCCGTTGCGTTTAGTGACTTGTGTTTGACATACATACATACATACATACA
TACATATATGTATATATTGCGAGTGTTTTAAGGATAATATTACTTTTAGTTGGAAATCCTCCGGTCTCAGCCA
GACGATTTGACAGTTGCATGACAGACTAGCACACGTGACCAAATAAACATGACTCTTC
CGCTTAGCTTCCGTGTGTTAACTTGCCTGTTGGTGTACGTGTCCTGCGTACACGCAATCAAGGTGACGTTTGG
CACTAAGAATGGGCCGATCGAGCCGCCGACGCCGGAGCCGCTGCCGCCGCCGCAGCCGTACCGCGTGCCCGCT
CCCGTCTGGGAAGAAAGATCTGATGACTCACCAGACCCTAACGCCCATTGGAGACCTC
AACTCTTCAAACCTCAGCCGAGATACACTCAAATTATATTCGCTGCACCAACACCGACGCAACCACCCCAAAA
CAATGCGTTGCGCTTCGTGAACGCGTACAAACCAGCTCATCAACCAATCCAGGCACAACCCATTAAGGAATAT
TTCACTCCATCCCAATTCCTGAGTTCACAAAGTCTACCAGGTATCGGCCTTCGATACT
TCGTCCCAGCCTATATTAATGAACTGCAAGCTAGGAAAGAAGAGAAGAAACAAGAGGATGCTAAGCACAATGA
CATTGAAACAAACGACGTAAACGGAGCTAACAAAGATTCCAGCTCTGATCTTTTATGGAAACATGAAAAGGAT
TCATCGAAAAGATACTTAAGAAATACTTTAGAAGTCACAGCAAGACCAATCTATGTGT
GGCCGGCGTACGTGCAACCTAGGCATTAATAGATATGAATTAGTTTAGTATTTCTCTAGGTTATAGCTTGTGT
AAGCACCACAGAAGGCAATGTGATTTAAAATGGAGAAGCGATAAGAAATGCCAGTATTAGATATTTTATATTT
CCTATATTTC
>1002052608_2 Heli.2-EL603044.1.5
CTTCGGTGCTGTTTGTTACTAGAAAACGCAAAAAATAGACGTTACAAATTTTAATAGACACATATCCTTGTAT
TTATTACTACCACGTGACTTAAAATAATCGTTGAGTGAACGTTAAAGTAGTCAGCTAATATTTTTATTTAAAG
TTTATATCACTGCTCTAGCAGTGGGTACATTAAGCGAGTGTGGTAGCGTGGGCATTAG
TCCCACGCTGTCCGAAACAAGACTGGGAGCCGTGGAGATGGTACAAGAGGGGGAGGGCGTCGGCGATGGACTG
ATCGCCCCACGCCGCTTGCCCAACCCTTGTTTAGAGAACCCCGTACGAATGGATTTGCATAGAGAACTAATGT
TCAATCAGAAAATAGGCAAAAATGTTCTCAATCAAAAGAGTGAACTACAAAAAGCACT
TTCGAAACATAAAGAGAAGCAGCTCATGAATCAAATAAAGGAGCACAAGGAAACGCCAGAACTAGAAAGGGCA
ATAGCAGAACGAGCGCGGCGCCTCGAACAGGCGGAGCAAGGCTCGGAGGAAATGGAGCCAGGAACAAATCCTA
CGTTACAAGAGGTCCGCGCCCGATTAAGACACGCGGCACCCGCGGCCTCAGCGCATTA
AGTATCGTTTAACATTTTACATTTTATCATTAAAAACCGATTTATATTTTTCCTAATATG
>1002052609_2 Heli.2-EL599553.1.5
GCGCCTCGTACTTTGTTACATTACCATTCACGTACTTAATATGTGACTGACTTCATTTTTTCACTTATCTACA
ACAAATAAAATATGATGGTGACGTTTTGTATAAATTAGATTAGTGTCGACTGTTTAATATTTCACTCTGACCC
ACTTCTCAACAGGTGTACGCCGTAATAAGGCTTTGTTTCAATCCGAAAGAAATGAAGC
GACGCGTAACATATTTTTTGATATTTATCCATTTAAAACACTTCAATTATGACAGCAAATGTGAATAAAGACT
TAGCGTTGTGAATATATTTAGATTATTAGCTTTAGAAACTTTTGAAGTTTATTCGTTTAAATGAATACATTTT
GAATTAATTGTCTTGTATATATGTTTATAATTATAAATATACACAATGGGATTGCTCA
TATCGAAAATATGGAGCCTCTTCGGAAATGAAGAACACAAATTAGTGTTAGTGGGCTTGGACAATGCTGGTAA
AACTACAATATTGTACCAGCTGCTGCTCGGCGAAGCTGTTCACACTAGGCCGACCATTGGATCCAATGTGGAG
GAAGTAGTGTGGAGAAACCTTCGCTTTGTGATGTGGGACCTCGGAGGACAACAGAGTC
TACGCTCAGCTTGGAATACTTATTACACTAACAGTGAATTTGTAATAATGGTGATAGACTCGACGGACCGCCA
GCGTCTGGCCA
>1002052610_2 Heli.2-DT667651.2.5
GGGTGTCGACATCAACCATAAACACGACAGGAAAGTTAGGCGTACCGAAGTCAAATCTCAGGATGTGTACCTG
AGATTGCTCGTCAAATTATACAGATACTTGGCCAGACGTACAAATGCCAAGTTCAACCAGATTATCCTTCGCC
GTCTCTTTATGAGCCGTATTAACCGGGCGCCTATCTCCCTGTCGCGGTTGGCTCGTCA
CATGAAGAAGCCCACTCGGGAAGGTCTGATCGCAGTAGTGGTCGGCACTATCACCAACGATGTCAGGCTGTAC
AAGGTTCCCAAACTATCTGTTGCCGCTCTTCATGTTACTGAAAAAGCACGCGCCCGTATCTTGGCTGCTGGAG
GTGAAATCCTAACTTTCGATCAGCTTGCCCTTCGCGCTCCCACTGGCCGTAAGACAGT
TCTCGTCCAAGGCCGCAGGAACGCGCGTGAAGCGGTACGCCACTTCGGGCCCGCACCTGGCGCCCCACGCTCG
CACACGAAGCCCTATGTGCGTTCTAAGGGCCATGAGCGCTCTAGACCTAGCCGTAGGTCTAATGTGTAATCTT
TAATATAAGG
>1002052611_2 Heli.2-DT666665.3.5

ACATGTCGTCCGTAGTAGCATTAAATTTATTATTATGCACAGTGCTATGCGTTCTAGTGAGTTCCAATTCACA
GACAGTTCACAGGCGTTTCGAATACAAATATTCCTTTAAGCCGCCGTATTTAGCACAAAAAGATGGTTCTGTA
CCTTTCTGGGAGTATGGAGGAAATGCGATAGCGTCAGGCGAAAGTGTAAGACTTGCTC
CATCGCTCAGAAGTCAAAAGGGTGCGATATGGACAAAGACACCCATCAACTTCGACTGGTGGGAGGTGGACAT
CATGTTCAAGGTCACAGGACGGGGGAGGATTGGTGCTGACGGTTTGGCTTTCTGGTACACCTCCCAACGCGGA
GATTACACAGGCGAAGTGTTCGGCTCCTCTGACAGGTGGAACGGTCTCGGTGTGATCT
TTGACTCATTCGACAATGACAATAAGCACAACAATCCATATATCATGGCCGTGCTCAACGATGGCACAAGAAA
CTTTGACCATAAGAGCGATGGTTCCACCCAACTCCTCTCAGGGTGTCTACGAGACTTTAGGAACAAGCCGTTC
CCGACGCGAGCTCGTGTGGAATACTACCTCAATACATTGACAGTGTACTTCCATAATG
GTTTAACAAACAACGAAGCGGACTACGAACTCTGCTTCCGAGCTGAGAACGTTGTTCTTCCTCGTGGTGGTCA
CTTTGGACTGT
>1002052612_2 Heli.2-EL596740.1.5
CAAGATGTCCAAGACCTACTACACCAGAGGTTCCGAAATGTTATCCTGGTAGCTCTGATCTACGTTGTCCGAG
ACCAACAACAACTTCAAAACCTATTTGTTATCCAGGATCCCCAGATCCAAATTGCCCTCAACCTCCAAGACCA
ACAACATTAAATCCTCCAACATATTTACCTCCTACTACTTCTGAACCAAAATGTTTCC
CAGGAAGTAGCGACCCAAGATGTCCAAAGCCCACTACACCAGCACCTCCAAATTGTTTCCCTGGTAACACAGA
TCCTCGCTGTCCGAAACCTACAACGCCCGCTCAACCTCGATGTTATCCAGGAAGTAGCGATCCAAGATGTCCC
AAACCCAGTACACCTGCGCCACCAAATTGTTTCCCCGGTAGCATTGATCCCCGTTGTC
CTAAGCCCACAACGCCCGCCCCACCTAGATGTTATCCAGGTAGTAACGACCCAAGATGTCCAAAACCCACTAC
ACCTGCGCCGCCAAATTGTTTCCCCGGTAGCACTGATTTCCGCTGTCCTAAGCCCACAACGCCCGCCCCACCT
AGGTGCTATCCAGGTAATAACGACCCAAGATGTCCAAAACCTACTACACCTGCGCCGC
CAAATTGTTTCCCCAGTAGCACTGATCCCCGCTGCCCTAAGCCTACAACACCTGCCCCACCTAGATGCTATCC
AGGAAGTAGCGATCCAAGATGTCCAAAACCCACTACACCAGAAGTTCCGAAATGTTATCCTGGTAGCTCTGAT
CTACGTTGTCCAAGACCAACAACACCTTCAAGACCAATATGTTATCCAGGATCACCAG
ATCCAAATTGTCCTCAACCTCCAAGACCAACAACATTAAATCCTCCAACATATTTACCTCCCACTACTTCTGA
ACCAAAATGTTTCCCAGGAAGCATTGATCCAAGATGTCCAAAACCTACTTCTCTAGCACCACCAAATTGTTTC
CATGGTAGCATAGATCCTCGCT
>1002052613_2 Heli.2-CV526012.2.5
GCTGTGTGAATTGTTTACGTGTTTTTAACGATAAATGTTGTGTGTGTTACAATGGCGAATCTTACACTGAGAT
ACCTGGATGAGCTCTCCTATGTCCTGAACAGACGGCACCTGGCGAGCCAAGCGAAGGCATCTTCCGTCGGCGA
TCTTCTCAACACAGAAGAGGAAAGATTTGCACCGTGGATCACGCCAGAAGAATGTGAA
GTGTACTCGAGAGAGAGGAGACCCCCGCTCGTCATACCTCGCATCACGCAGTATAGAGCGCCACGCCCGCGAC
CACAAATAAACTACCCTGAGGATTCCATGCAAGATGTGCCCGCCAAATCTTGGAACTGGTCTCCCACGCCGGA
GCGGAGCCGTCTCGATATCGAGAGGAGCTTCAGCAAAGCAAATTGTGACAGTCCACCG
CTGTGGGTCAGCAAGCGACTGGGCGGTTCAACAGAGAACCTCCTGGCCGCGTCGCGAAGGCCCAACCACCTCC
AACTACCAGCGTCTTCCTCTGGAGACAGCCTGGATGTGGAGGTGCGGGAGCGGGATCTAGGATCGAGGGTCAA
GGAGAGGAAGTCCAGATCGATGGTGGCTCCTAAGCAGAAGCCAAGACCGCCACTCACT
TCCCCCAGGCTTATGATGCATCCGCCTGTTGAGGCAACAGTAATATACGAAGGGCCAATAAAGAGAACCAAGT
TTTCGGAGGGCGGTAAGAAAAGTAAGAAGAATTGGACTGACTGCTTCATGGTGGTGACGCCCACCGCGCTGGT
GTTCTATAAGGACCAAAGGACCTACTTAGCTACGAAAATGCCAAAACCTCTGAACACA
CCACCGAGCCCCACAGCGCCGCGAGCCGAGCTCGTCCTGCCTCTGCTCAATGCGCATATTGTCCAGTGCATA
>1002052614_2 Heli.2-EL596938.1.5
GCACGAGGTGAAACGAGGGTTCAGACCTTAGGTTTTAGTTTATTGTTACTTTAAGTCTAAATATAGTAATAAT
AATAATGGCTAATAACGATAATTTTGCACAAGACGTTACTGAAAATCAAGTGAATGGTAATGCAGAAAACGGT
GGAGGCGATGGCCAAGAACATAACAGTGCCGACGCCCCGGGACGCGATGACGACAGAA
AACTTTTTGTGGGAGGTTTGAGCTGGGAAACAACAGATAAGGAATTACGTGATCACTTCAGTGCTTATGGAGA
AATTGAAAGTATCAATGTTAAGACAGATCCTAACACGGGGCGGTCGCGGGGGTTTGCATTCATTGTTTTTAAG
GCACCCGACTCCATTGACAAGGTGATGGCTGCCGGCGACCACACCATCAATAACAAAA
AAGTAGACCCAAAGAAAGCCAAGGCGAGACATGGGAAAATATTTGTTGGTGGACTCAGCAGTGAAATTTCCGA
TGATGAAATAAAAAACTTCTTCAGTAATTTTGGAGCAGTCATAGACGTGGAGATGCCCTTCGACAAGACAAAG
AACCAAAGGAAAGGTTTCTGTTTCATCACATTTGAATCTGAGCAAGTTGTCAATGAGC
TACTCAAAACTCCCAAACAGACCATCGGCGGAAAGGAGGTGGACGTGAAGCGCGCGACGCCGAAGCCCGATGG
CCCCGGGGGCATGGGAGGGCGAGGGGGCCGCGGCGGTAGAGGCGCGCGCGGGGGCCGCGGCGGCCGCGGGGGC
TACGGCGGGCAGGGCGCGTGGGGCAACCAGGGCTACGGCAGCTACGGCTACGGGCAGG

GCGGCTACGGCG
>1002052615_2 Heli.2-DT662597.3.5
CTGCTCACATTCGCTCCAAAGAAAAAAACATCGATCGAGTGCTTTCAACGACGATCGTGCCAGAAAATAAACA
TGAGGATTTTACCATTGATTGTATTGGTCGCATGTCTGGTGTACCAAGCGCGAGCGCGACCCCAAGACGAATC
AGAACCGCGTGCTATATCTAACAGGGGACTCTTAAAACGAGGCCTCGTTGGTAAAGCA
AAAACTACAACCACCACACCAGCTCCACAGGAGGCACCAGAATATGAAGATGAAGGTGATTATCCAGAAGAAG
AACCTCAAGAGCTCTCCACTGAAGCGGCACCTTCTTCAACGGAGGGAAAGAAATTAGTAGGAAGTGGAGTTAG
ACCCTTTAGAAGTAACACTGATCTCCTAGAAGCCTTAAAGAGGAGACGACAACAGGTT
GCTGAAGCAAAAGGTTCCGGTTCGCCTGTAGTACAACAACAACAATCTGAAAACCAACCGGAGGCGCCGCCCA
AAGCTAGTTACAGCAAAAAGCGTTTCAACACTGCCACGCGGGAGACTAAAGCTGAAGATGCGCCCGCGCCGGC
AGCTGCGCCCTCCAAGCCAACAAGAGGACGATTCAGTCGACCATCATCGAGGTCCGTC
CAAGAAGCAGAACCTGAAGAACAAAACGACGCTGCTCCACCCGCGAGAACTGGCAGAAATTTCTCTAGACGAG
GAAATTAAATAAATTTATCCAAAAGTGCCCTAATTTCCTTTCCTACATTCTCTCATAAT
>1002052616_2 Heli.2-DT664189.3.5
GCACGAGGCTGCGAAGCGCGGGAAAGTTTGACGTTTGCGTTCGGATATTACAAACATGATTTTCAAGGCACTG
TTACTCTGTGCAGTATGCGCGTGCGCATATTCACAGCACCAAGGACCCACCACTACTCCTGTACCGATTCTCA
AACAGATAAACAGACAAAACGACGACGGCTCATACAGCTTTGGGTATGAAGCAGCTGA
CGGATCGTTTAAAATTGAAACAAAATACCCTAATGGCGATGTAGCAGGGAAATACGGATATGTCGACGAGAGC
GGGAAAGTCAGGGAGGTTTCTTATGGTGCGAGCAGCCAGAGAGGATTCGAACCTGAAGGTCCAGGAATTATGG
TGCCACCACCAACGCTTCACGACCCATCATCAACAAATGCTTTGACCGACGGCCAAGA
AGATGATGGCCAATACAGAGAAGATCCTAAAATTTACGAAGACCCGAAATACAATGGTAGAGCACAGGCCCGA
CCTTCAGGCAGCTTCAGGCAGTTCCAGCAACCAGCTCAACAAGCGAGTTTCCAACCGCAACCAAAGTTCCAAT
CGCAACCACAGCAAACGTTCCAGTCAAGTTTCCAATCGCAGCCACAAGCTCAACCACA
ACAGTACCAACAACAATACCAGCCTCAATATCAACAACAACAACAATATCAGCCACAACGGCCTTCATTCCCT
CAACAAACCAACCTCTTCAGTCAGTCACCTCAACAATTCCCTCAAGAGTACAGGCCTCAAAGCCAAGTCTATC
ACCAACAGCCCCAATTCTCCTATCAAGCTTACACTCCACAACCCTACCAAAACTACCA
ATCTCAGAATTACCAAAATCAAAATTACAACCCCTTCCAAGGGCACCCTGCCCAAAACTTCGACCCTAACACT
GGCTCCTACTCAATCAACTTCACTGGCAAATAATTCTTGACTTAATTAGAATATTATAAAATAACTAGCTTTA
TAGTAATACAATTTTTTTAATT
>1002052617_2 Heli.2-EL597986.1.5
TAAGATTATTAAAACTGCGTCTTATACAATACAAATTGTGTGATAAAAAGTCAGTCTAGTGCTAGTAGTGATT
ATAATCAACTTTAATCCAAAGAAAATTTGTTATAATGGCATACGCTTACTTGTTTAAATATATCATCATTGGT
GACACAGGTGTGGGCAAGTCTTGCCTGTTACTTCAGTTTACAGACAAAAGGTTCCAAC
CAGTACATGACTTAACTATTGGTGTGGAGTTTGGGGCCCGCATGATAACCATTGATGGGAAGCAAATAAAACT
GCAAATTTGGGACACTGCTGGACAGGAAGCATTTAGGTCAATCACTAGATCATACTATCGGGGTGCAGCTGGT
GCGTTACTTGTGTATGACATTACTCGACGAGACACCTTCAACCACTTAACTACATGGC
TTGAAGATGCCCGCCAACATTCCAATTCGAACATGGTTATAATGCTTATTGGTAATAAGAGTGACTTAGAGTC
ACGCCGTGAAGTAAAGAAAGAGGAAGGTGAGGCGTTCGCGCGGGAACATGGACTGGTCTTCATGGAAACCTCT
GCCAAGACTGCGGCGAACGTGGAAGAAGCATTCATCAATACTGCTAAGGAGATTTATG
AAAAAATCCAGGAAGGTGTCTTCGATATCAACAATG
>1002052618_2 Heli.2-DT667905.3.5
CGGGTTAGTCGTACGTTTATCTACAATATTAGTACAAACGTCAACGATTGGTGAACAATGCATAAAAATCTTA
TATATTTAACAATATTAGTAGCCGTTGGCACAAATGCAACTAGATCGCAAACACCTTTAATAAAAAATGTATA
TCGTGGTGGAGAGCATTTCGAAGGGCCGCATTTCGCAAAAGACGAGAGTGCATTATAT
TGGGTGGACATTACAAAACAGAATGTTTTAAGGCTCGATATTGACACAGGCAATGTCACCAGTAGGCACATAG
GATATGGTCCAGTCAGTTTAGTTGTAAGGGTAAAAGACTATCCGAAGTTAATATTAGTGACTGTAAGATCGGA
AATATATCTGTTGTCGTGGGATGCACCTGAAGAAGAAAACGCCTTGAAATTATTGAGC
TCGGTAGACATGGGTTTACCTGACAATAGGTGTAATGATGGCAAAGTGGACGCTAAAGGGCGACTATGGTTTG
GAACAATGGGCAAAGAAGTAGGAGATATTATCGATAAGGATCAAGGAACTCTTTATATGGTTAATGAAAATAA
CTATAAGAATCCTGAGATAAAAGTTCGTCCCGTTTCTATATCTAACGGCATCGCTTGG
ACATCCGACAACAAGTTCATGTTCTATATCGACACTTCCACGAGAAATATAGACGTGTTCGATTTTGATTTAG
ACTGTGGCTGTATCANAAACAGAAGAACATTATTTAACTTTCAATCTAACAATGTTACTGGTTATCCTGATGG
TATGACCATAGACAGGGATGGT
>1002052619_2 Heli.2-DT668897.3.5

GAAAAAGTTTTCAAAATAATTCCATATGTAATATAGTATATAGATTTATGAAATTATTATTGTATTAACAGTA
GTGATTAAAATTGACTATATGATACTCGAGAAACTTCACCAAAGGTCACAATGAGGCTTTTTATTTACTTAGC
TTTTATAGCTGCTATACTACATTGTTCATTTGCACCCCCTGTTACGCCTGATAAACGT
AAAGAGGAAGAAAAGGATGAAAATGATTTGGCGCAATACACAGAGTACCACAGGTACCTGAAAGAGGTTGTGC
AAGCGCTTGAAAGTGATCCCCAGTTTAGAGATCGATTGGAAAGAGCTAACGAAGAAGATGTTCGGTCGGGTAA
AATAGCCGAGGATCTCGACTTTGTACACCACAATGTAAGAACCCAGTTGGATGAAATA
AAACGGAGGGAGATAGAGCGACTGCGACATCTCGCTACTAAGGAATATGAACTAACAAACGACCTCGACATGC
CCGTGCATGTACCTCCCGTCTACCCAAACTTGGATAGTCAACATTTGGACCACAAAAATCCCCATACGTTTGA
AGTTGAAGATTTAAAAAAGCTAATACAAAAAACCACCGAAGATTTGGAAGCCGTTGAT
AAAAAACGTAGAGAACAATTTAAGGAATATGAAATGCAAAAAGAATTTGAGAAACAACAAAAAATTGAACAAA
TGGACGAATCTCACAAAAAGGAGTACATGGAGACCTTGAAGAAAGAAGACGAAGCTAAGAAACATCACGAACC
GCTTCACCATCCCGGCTCTAAGCAACAGTTAGAGGAAGTATGGGAGAAACAGGACCAC
ATGGACCAACAGTTCGATCCAAAAGCCTTCTTCATGATGCACGATGTCGACGGCAACGGCGCTTGGGACCCAG
ATGAGGTTAAAGCACTTTTCATAAAGGAATTAGACAAGATGTACGGACCGGAAGGGCCCAACAAGGATCTCCA
CGAGCGCGCCGAAGAAATGGAAAGGATGAGGGAGCACTTCTTCAAGGAAAACGATCGC
CACCACGATGGTCTGATTGAATATAGCGAGTTCATG
>1002052620_2 Heli.2-DT668156.1.5
AGGGTTAAGACGTGACAATGGGTCGTGTAATTCGGGCTCAACGTAAAGGTGCCGGTTCGGTTTTCGTATCACA
TACAAAGAAAAGGAAAGGAGCACCAAAACTCCGTTCTTTAGATTATGCTGAACGTCATGGATACATCAAAGGC
GTTGTTAAGGATATCATTCACGACCCTGGTCGTGGAGCTCCTCTAGCTGTGGTCCATT
TCCGTGACCCATACAAGTTCAAAACACGCAAGGAACTCTTTATTGCACCTGAGGGTCTCTACACAGGGCAGTT
TGTATATTGTGGAAAAAAAGCTACCCTAGAAGTTGGAAATGTAATGCCAGTGGGTGCTATGCCTGAGGGTACG
ATTGTATGTAACCTAGAAGAAAAGATGGGTGACCGGGGTCGGCTGGCTCGTGCCTCTG
GTAACTTTGCTACTGTCATTGGCCACAACCCTGATGCTAAACGTACAAGAGTAAAGCTTCCGTCTGGAGCCAA
AAAAGTGTTGCCTTCTAGTAATAGAGGAATGGTTGGTATTGTTGCTGGTGGTGGACGTATTGACAAACCCATC
CTTAAGGCTGGTCGTGCATACCACAAATACAAGGTGAAGCGTAACTGCTGGCCATATG
TGCGTGGTGTGTCCATGAACCCCGTTGAGCATCCCCACGGTGGTGGTAACCATCAACACATTGGTAAGGCTTC
GACTGTCAAGAGAGGAACATCAGCCGGTCGCAAGGTCGGTATTATTGCTGCCCGCAGAACTGGAAGAATCCGC
GGTGGCAAGACTGAGACTAAGAAGGAGACGTAAACATACTTTCAAA
>1002052621_2 Heli.2-EL596274.1.5
AATATTTGGTCTTAGCGCTGTATTGGCAGTGGCTGCCGCAGGTCTGCTGCCCGAGCCACACTACTCCTCAGCG
GCTGCAGTTTCTTCTCAAAGCATTGTGCGACATGACCAGCCTCACGCCGTAGTCGCGGCCCCAGTGGCGTACC
ACGCCGCCCCTGTCGCTTACCACGCCGCTCCTGTCGCTTACCACGCCGCCCCCGTCGC
CTACTCCTCAGCCGCAGCCGTGTCTTCTCAGTCTATCCAGCGTCATGACCAGTCCCATGCCGCCATCGCTGTC
GCTCCTGTAGCTCACTACGCCGCCGCTCCCGTTGCACACTACGCCGCCCCTGTAGCCCATTACGCTGCGCCCG
TACACAGCATTGTGGCCAGTCACGATGACGTGTACTCTCACCCCAAATACGACTTCTC
TTACTCTGTAGCCGACGGTCACTCCGGTGACAACAAGTCCCAGCACGAGAGCCGCGACGGTGACGTAGTACAA
GGCGAGTACTCTCTGGTCGAAGCTGACGGCTCCATCCGCAACGTCAAGTACAGCGCCGACGCACATAACGGAT
TCAACGCTATTGTCAGCAACTCTGCACCCAAACACGCCATCATTGCTCACCATTAAAA
TAAAATGTATTTAATTTTATTTGTTTTAGTCATTTTAATTGATATTTAAATACAAACATAGTAATAATTTAT
>1002052622_2 Heli.2-DT663391.3.5
GCTAATCAAGGCACCAAAAGAAAAATGAGCAGCTCGGAGGAGGTATCATGGATTTCATGGTTCTGTGGTCTGC
GAGGGAACGAATTCTTCTGTGAAGTCGATGAAGATTACATTAACGATAAATTTAACTTAACGGGGCTTAATGA
GCAGGTGCCACACTACAGACAGGCTTTAGACATGATATTAGATTTAGAACCTGATGAT
GACCTAGATGAAAATCCAAATCAATCAGACTTAGTAGAACAAGCTGCAGAGATACTATATGGCTTAATACATG
CTCGATATATTCTAACAAACCGAGGGATTGGGCAAATGTTAGAAAAATTCCAATCTGGTGATTTTGGCCACTG
TCCACGTGTCTACTGTGAATGCCAGGCAATGCTTCCCATTGGTTTATCAGACGTGCCG
GGTGAGGCGATGGTAAAACTCTACTGTCCTCGTTGTATGGATGTGTACACGCCTAAGTCGTCACGCCACCACC
ACACCGACGGTGCTTTCTTCGGCACTGGCTTTCCTCACATGGTGTTCATGGTGCACCCGGATTATAGGGCCAA
GCGCCCCGCTTCACAGTTTGTGCCTAGACTTTACGGTTTCAAGATCCACCCGCTCGCG
TACCAGATCCAGCAGCAGGCGGCGGCCAACTTCAAAGCGCCGCTCCGGACCCTCTCGTACAACAACGGTAAAC
GTTAGGACGCCCACAAACCAACCCCTTTGATACAGATTCTACCTGTGCGTCTGAAGGCCCACTAGTGATTGAT
CGATTACGTCACGTGACAATGGTTTATATGTTGA
>1002052623_2 Heli.2-EL596919.1.5

TGAAGGCTCAAGGTATAGAAATAGGCTCTGAAAAAAAACCGCCGAGACCAGGAACCAGAATTAAGCCAACAAA
ATTAAAAACACAAATGTCACAAGATGCTCCAAGCAGCCCCGCTGAAGAGAAGAAGATTGAATTGGAAATATCA
GATAAAAAGGAAGAACCAAAGAAAGAAGAAGAAAAGAAGGAATCCGATAATGAATCCG
TTAAAGATGCTTGGGACGCTGAGTCATCTGACGAAGAAATTGAACCACCTAAAGATGTTAAGTCTGAACCTAC
CCCACCTACTAATGAATTAAAAAAAGCTGATGATAAACCAAAGGAGAAGGAAGATGAAGAGGAAAGTTCTGAA
GAAGAAGATGATAGCTCTGAAGAAGATTCAAGTTCCGATGAAGACTCGGATGACGAAC
AAATGACAGACGCACAGAAGAAACGTGAAATTGTTTTGAAGAGATTAGAGAAACGTCGCGAGGATAATGAGAA
AAATAAAACAAATAATCCACTCCGTGCCGCCGTCGTCTGTGTGCTGGGACACGTAGATACAGGCAAAACGAAG
ATCCTAGACAAACTAAGACGTACTAACGTGCAAGATGGCGAAGCGGGAGGTATTACAC
AGCAGATTGGGGCCACTAATGTTCCCATTGAGAATA
>1002052624_2 Heli.2-EL596323.1.5
CCGCATCGCTACACGCACGATTCGCGCGCGGCTTTAAGGACTTTATCAAGGTTAATTAAAAATCCACAAAATG
GCTGACTCAGGCCTCGTTAAAATAGAAGATAATCCAAAGATTCTGTCAGGGCCAGTCCTGACGAACTCTCCAA
ACGCAGTATTATCCAAGACCCTGTTGGGTCGCTACAACGACCTGCCGTTGCCCGTCGA
CCGCATCCAGGCCACTTACGTATGGATAGACGGCACTGGCGAACACTTGAGATGCAAGGACCGCACTCTGACC
TTCATTCCCAAGAAACCTAAAGATTTGCCCGAATGGAACTTCGACGGCAGCTCGACTGGCCAGGCCGAGGGGA
ACAACTCTGACACATATCTGATCCCCCGTGCAATCTACAAGGACCCCTTCCGTCGCGG
GAACCACATCCTCGTCATGTGCGATACCTTCAAACACAATCATCAGCCTACAGAGAGCAATCATCGTGTCAAA
TGCGAGGAGGCGTACGAAAATTGCAAGGAGGACGAGCCCTGGTTCGGCATTGAACAGGAGTACATTCTCTTGG
ATTCGGACCTGCGACCCTTCGGCTGGCCGCCCGGCGGCTGTCCCCCACCACAGGGCCC
CTACTACTGCGGTGTGGGCGCCAACAAAGTGTTTGCCAGAGATCTTGTTGAAGCTCACTATAAATGCTGTCTC
TACGCTGGTGTTCCGTTAGCTGG
>1002052625_2 Heli.2-DT663278.3.5
GCACGAGGGTGAACCGGTCGCTGACTGACCTTACGCGGTAAAACCAAATAGTGCACGCCGAAATGAAGGCAAT
TAGCTTTATTATCGCACTGACAGCGGTAATAAGTTGCAAGGCAGCGACCCCACAACTGCGTTTCGCGTGGAAA
GAAGTAGATTTCACATGGGACTCCCCAGAACAGCGGGAAGCTGCTATTAAAGATGGAG
TATTTGTAAAGGCCAATAACCTTCCTTTAGGCTTAGCGAGGTGGAATAACAAAATTTTCGTGACTGTTCCAAG
ATGGAAGAACGGAGTTGCTTCTTCGCTAAATTACATAGACCTAGACGGTGCTCAAGATCAATTATTGAAGCCA
TATCCGTCATTAAAAGAGAACCTTGTAGCTGACAGCGCCAAGGAACTGCCATCAAACA
GCTCAATTATTTCTGTCTTTAGAGTGTTTGTTGATCCTTGCGATAGATTGTGGGTAATGGACTCTGGGTTGGC
GGATATATTAGGATCAGCAAATCAAGTGGTTGGTCCGTCTCTTGTGATATTTGATCTCAATACCAATCAGCTG
TTACATCGATACTTCTTCAAGGTGTCAGATATGAAAGAAGATTCATTCTTCGCTAATA
TCGTAGTGGACGTAAATAAGGATACTTGCGACAACGCGTTTGCATATGTGCCAGATTTAGGTGCATACGGCGT
TGTTGTATACAGCCTTAAGCAGGACGATTCGTGGCGTGTGAGCCATCACTACTTCCACTTTGAGCCATTGGCT
GGGTCTTACAAAGTGGGTGGTGTGGAATTCCACTGGACCGACGGAGTTTTTGCTTTAG
CACTCTCAGAGCCAAGAGAGAACGGATACCGCACGATGTTTTTCCACGCGTTCTCTAGCACCAAGGAGTTCTG
CGTGTCAACGGAGCTGCTTCGCAATTACACACACATAGACAAGAACGAGGCATTCCACGACTTCAAGCTGTTG
GGTGATCGTGGTGAGCGTACACAGTCTTCGGCCAGTTTCTATGATGAAAATACTCACG
TTCTATTTTACACGCAGATAAATCGTAATGGTGTTGGTTGCTGGAATTCTAATAAGCCTTACACTCCACAAAA
CAATCCTCTTATATTCAGTGACCCCGAAATATATGAGTTCCTCAATGACTTNAAGATTGACAACGAAGGCACT
TTATGGTTCCTAAGTGACAAGATGCCACGTTTCATTTTCAAATCTC
>1002052626_2 Heli.2-DT662267.3.5
GCACGAGGCTGTACCATTGCATTTCATGGATAGTGTGATTGTGTTTTGCAAGCTTAGTTTGTTAACTAGTTGT
TTATTGTGTTATAAATTACGTCCTTATTCGTATTTTCTAATATATTATTTGGGTTTTATAAAGAGATACTTTC
ACAATTATGCGTCGTAAAAGTGAACGCACCGCCACTAAGAAAAAGTCTTCTCCAGACA
AAAAGGAAAAAGTTGAAAAAGCAAAGCGCAGAGGAACACGTCGGACTCGCAAACATTCTTCATCATCAGAAAG
TGGGTCTGCTGAAGATAATACTCCTAAAGAAACTGGCGAGGTTAGCCAAAAGGAAGCGATCCCGCCAAAATCT
GTCAAAGAGGACAGTCCCGAAGAATCTCAGGAGCAAGTTTGGCATGTTAAAGCAACAG
AAACTTCAGGTGATGTTGGTGAAATTCAAAAGTTAAAAATTTGTCTTGCTCGTCCGCCTTCAACTCCGGAAAG
GGCAGACAGGTCACCTAGAAGTAAAAGAAAGCATTCTCGTGCTACTAGTTCCAGTGACACTCAAAGTGTTGAG
GGAGAGGAAAAAAAGAAATCAAAACATCGTTCGAAACGATTGACAGAATCAAAAGAAG
CACCAGAAACGAGTAAAGAGGATCATGAAGAAGTAGAAGTTCAGCCAGCTTCCTCAGCAGTTGAACCCGAGTC
TACTAATACTACTACTGAAGTAGAGAGCACTGAGCCGAATAAAGTAGTAACAGACGAGACACCCATGTCAACT
ACTAATGAAGAAAACAAAAATGAGAAAGTAGAATCAACTGAATCGACAGAGAGTGAGG

TGAGCCATGAGAATGTACCAAAAG
>1002052627_2 Heli.2-EL600778.1.5
TTATTTCAATATTACAAACATTTTAATTCAAGTTATTATTTGACTACACACAGAAAAACTATTTAGCGCATAT
TTTTATTGCCTCTCGAAACATTATAATTTAAGGCTGCTAAAATGAGTGATTCACCAGATCAAAATGAGCCACC
AAAAGATCCAAAAGAGCTGGAAAAATTAGAGGAAGCAAAATTAAAAGCTAAGTTTCCA
AATGCAGTTCTTGGTCGAGGACCTGGAGGGCATTCAGCTTTTCTTCAGAAGAGATTGGCTAAAGGCCAAAAAT
TCTTTGATTCTGGTGACTATCAAATGGCTAAGCAAAGACCGAGCAACCTGGCTGCCCCATTCAAGGCTCCAGC
GCCCGCTAAGTTGCCTACTGGTGAAGCCATACCCACACCGGACACAGTGCCATTACGG
AAGACCTCCATCATACAGCCCAAGTTCCAGCCCACCCACGGACAGCCATCCTAGCCTTAAATATCCAAACTTT
ATAAATATGCTGTGTAAATAACATGTTGACACTTGCTTTGTTTTATTCATCCATTCCAGCTTATATTCATCAT
GTTGGTCAAGCTTGTTTTGAGTTGCAAAAGTCATCTATGCCTTGCCCTTGTTAAATTG
TACTGAAGAAATGTTATGTATGTATAAAATTTTTAGGTAAATATAATTAAATTGTGAATGAAATTTTGCATGT
GGTACATATTA
>1002052628_2 Heli.2-DT668085.3.5
ACCAAAGATTTTGGACTCCGGGGGGAGTATGGTTGCAAAGCTGAAACTTAAAGGAATTGACGGAAGGGCACCA
CCAGGAGTGGAGCCTGCGGCTTAATTTGACTCAACACGGGAAATCTCACCAGGCCCGGACACCGGAAGGATTG
ACAGATTAACAGCTCTTTCTTGATTCGGTGGGTGGTGGTGCATGGCCGTTCTTAGTTG
GTGGAGCGATTTGTCTGGTTAATTCCGGTAACGAACGAGACTCTAGCCTGCTAAATAGGCGTCGTCATTTAGG
TGTGCGCGATTTCGGTCGCGCAACTCACTGGCGACGTATTAAAATTCTTCTTAGAGGGACCGGCGTCTTCGAG
CCGCACGAGATTGAGCAATAACAGGTCTGTGATGCCCTTAGATGTCCTGGGCCGCACG
CGCGCTACACTGAAGGAATCAGCATGTTCTCCCTGGCCTAGAGGCCCGGGCAACCCGTTGAAACTCCTTCGTG
CTGGGGATTGGGGTTTGCAATTATCCCCCATAAACGAGGAATTCCTAGTAAGCGCGAGTCATAAGCTCGCGTT
GATTACGTCCCTGCCCTTTGTACACACCGCCCGTCGCTACTACCGATTGAATGATTTA
GTGAGGTCTTCGGACCGACACGCGGTGGCTTCACGGCCGTCGGCGTTGCTGGGAAGTTGACCAAACTTGATCA
TTTAGAGGAAGTAAAAGTCGTAACAAGGTTTCCGTAGGGGAACCTGCGGAAGGATCATTAACGTGTGTGTGCG
TGTTGTCGTGTTTCTCGCCCTCACCGGCGACGAAGAAGCCCTCATCCACATGACAAAG
ATATGATTTATAAAAAGAGATAAAGGAGAGGCGGGAGCGAAAGCGCGCGCGCGCGCGCATACATTTCGACGAA
GGGACGTGCAACGCGCGAGCACACGACGGTGCATC
>1002052629_2 Heli.2-DT662732.3.5
GCACGAGGGACCGCCGCGATAGCTTGACGCTTTACACCCACGAAAAAATATATAAATAAATATATATAATTGG
CATTTCACATTAGTGCTCCTTTCATTTGACCAACACTCACTTTTCCCCCCTGGAAAGTTGGAGTGGGCAAAAG
ACAGTGTATTCGGAAGTTACTAGTGTTGTTAAAATTTTGTGATTTCTAGGATTGTTGC
GTGTATTAACGGATTGATCGATTAATTATTGTCAACAAAATTGTGGAATCCGATAGATAACGTTTCCACAATC
GCAGGAATTTAATGTTGTGAAGGTACTCAACAATGACATAAGAGTTATAAACAAAATGGCGTTACGACTCAAA
AAAGATATTAAAAAGGCGTCGTACTACGTGTGGTTCCTCGGTGCGCAGGAGTCTCGTG
GCCTGAGGGGTGAGGAGTTCGCTATACCTGCAATACGACTATTAGAAGAGAGGGCGAGGGATTTGGAACCTTT
CAAAGTTACTTTGCAGGTGTCACACAAGGGACTAAAAATAATCCAGAATGTAACAGCAAAAGGCAAACAGCAA
ACGATCAAACACTTCATACCGCACGGCAGCATCACCAGCGCGGTGGTGCAAGGGGACG
TGGTCGCGTGTGTTCTGCTGCTGTACAACCCCATCACCGGCTGTCCCGTCCACGTGCACGCGTACCGATGCGA
CTCAGATCATACGGCGGAGATGCTATACACACATCTACTAGCGTTGATAGAACGTCCAGAAAACCAGAAGAAA
TTTGCTGATATCGAAAGAAAACTTCAAATGCGAGGTGCTTTACCTTCAAAGAAACCAC
CTGACTCTTCCCTCGGAAGTGAGGTTTCCAGAGAATCCGACTCCGGCAGCAACGAGGATAGGAACGTAGCTAA
TCTATACGACAGTCTTGCAGCCGAATTGAAGCAAAAGTTGTCAACAGGTAAAAAGGGTCTAGGCAAAAGTCCA
ATCCTTCTGCCCCCACGTGACTACGACACCGTGCACCGCCAGAAAGGCAACCTAAGCA
ACATAGACACTAGGCGGTGTCTCAATCAGAACATTGTAGGGATCAACGCACGCCGCAAGCTGGAGTCCTCCGG
TGGCAGTTCTGGAATAGGGAGCGACCTCGCACCGTCGCCAGAGAGGAATGAATATCTACGACATGATAACCAT
AGCACTAGCGAGGAAGATTGGGCAGAAAGCACAGCTTACTTAATGCACGAGGCATTTG
ACGCATCTCCACCTCGTCGAGCACACTCTCCACGTCGAGCTCACTCTCCACATCGCTCTTCTCCTCCTCCTCG
TCGTACCTTGTCTCCGCGCCACTTCGAGAGGGCTTACAACGATGACTATAGTGACCAATACCGTGACCAAATG
CCTTCATTCCGTGACCAAGCAACTTTTGAGCGACGCTTCCGTCATGAATCTCTTGAAA
GGAAGGACAAAACAGATAAATTCGATGACGATACGAGCTATAGGCGACGGTATGTCGCAGAAACAAAACCTTC
CAATAGAAGCATCGATAGAGTCGAAGATCGCAGATTGGGTAAGCTTCAAAGGGGTGCAAGCGAAGGAAGCCTC
AAAGTTGTTGACGCCTCCCCAAAAGAACGGTTTAACGTCGCTAAGGAAAAGTTCATGA

ACTTGGAAAGAGAGAGGTTTAATAAAGAACTTGAAGCTCACATGGCCATGAGGAGAAGCATGCTAGAACGGAA
CAGTCGCTCGA
>1002052630_2 Heli.2-DT667825.3.5
GCACGAGGCCGCCAGGGGAACCCCCGAGTTTAAGGAACTAGCGGACAGGGTATCCGCAGCGCTGGGCGCCGAA
TTGGGAAGGGTTAAAGGATACCGGGAAGTTGTTCTGGATGGATTTGTACAGAACGAAACTCAATCTGTAATAG
CTGATCTAACACTAAAGACCGTCGAGGACACAACTACGCCAACAACAACAAACACAAC
AGACGAACAGAAGTGGGAGGAGGCGGTGCGGGACATGCTCGCCGCGGGGAGTGTCGGAGCCTTCCAATTGGAC
CCCTTATTCTTTGAGTTCGAAGTCGCCGGTGCCAAGCCAACAAGCCCGGCGCCGCCGCGCGCGGCGGGCGGCG
TGCTGGGCGGCGCGCGCCTGTGGCTGGTGGCGGGCTGCGTGGCCGCGCTGCTGCTGCT
GGCGCTGCTGCAGGCGCTGTGCACGTTGGCGCACTCGAAGGCCAGGAAGGAGCAGCTAATACCACCTACACCG
TGGAAAGAATACACATCGGCCAACACTAACTACGCCTTCGAACCTTTCGAGAACGACGACAAATACCCCCACT
CCCTGCCACGTTCTCCCCCACATTCCCTTCCCCGCTCGCCCCCACAACGACCCGCCGC
TCCCCCTCCCCCACGACCTACACACGCACCTGACAGACAAGAACGGGCCAATAACAGGCATGAACTTGCCAAT
AACAAGCACGAACATGGCAACAACAGGCACGAACATGGCAATAACAGGCACGAGCTTGGCAATAACAGACAAG
AACAGACCCATAACAGGCACGA
>1002052631_2 Heli.2-DT668594.3.5
ATTTAAATTGTCCTGAAAGGCACAAATAAAATGAACTAATGTGTGCACACATGTAATGATGGCATTAAATTTA
GAGAGCTGCGACAGCTTGCCCCCAGATGAAGAGCCTAAACTGAAATATGATCGTATGGGCAATGACGTTCAAA
ATATACTTTTAAAAGACGCTGTAAGCTGTATTTGCGTACATACAAAATTTATTTGCCT
TGGAACACAATGGGGAGTGATACATTTACTAGATCATGAAGGTAACACAGTGCCTATATCTCAAGATAATAAC
CAAAAGGACTTGCAAGCTCATGCCATCGCAGTAAATAAAATCTCAGTGGATATTAATGGGGACTATATTGCCA
GTTGCTCTGATGACGGCAAAGTATTAGTGTATGGCCTCTACACAGATGATAATACCCA
TAATTTGACACTAGGTAGAGTAGTGAAATCAATTGCTTTGGACCCTTTTTACTTTAAATCTGGCTCTGGAAGA
AGGTTTCTTACAGGTGATAACAAACTTATTCTCTACGAGAAAACATTCCTGAACCGTCTGCGCAGTACGGTCC
TTTGTGAGTGTGAAGGCTATGTTCAGGCAATAGCGTGGCATGACAGATTCATAGCGTG
GGCCAGCGAAGTAGGTGTACGCGTCTACGACCTCGTAGCAAGATGTTCATTAGGCTTAATTCAATGGGAAAGA
AATCCTAATAGATCCATTGAAGATTTTCGGTGCAATCTTCTTTGGTCTGCCCCAAAGACCCTTATGATTGGTT
GGGTAGACAC
>1002052632_2 Heli.2-EL597349.1.5
AAATATTTCTGTGTGAATTTTTTTTATAACAGTTTTAGTGTGTGAAGTGCAATGGTTTACTTTTTGTTTTTTA
TTGTTAACATTGATACAAAATCGTCTGCACAATGCAAACCGTTCTAAATTTACATATTATTATTATTATTTTT
TCTACGAGAGTGTGTTATTTGGTTTTACAAACATGTTGACGGGATTTGTTGTTTTTTT
GGTGGTTTTTCTCCATTCACTACAGTTTGCACACTCCCTAAGTCTGTTATGCGGGCACACAAAGGAAGTCGAA
GTGGGCGGCAACGTGGACGCCGGAGCCGCGCTCGCCCTCACCCTGCTCCGGCCGCCCTCCTCCACGTCGACCA
GCGCCTGTCAGTTGCGTCTGACAGCTCCTGATGCTGCTGCTTTCACTGTTCGACTTAT
TGATGTTAAGGAAAATCTATCAGAATGGGAGCGCGGCGTCACCGACATGCAGGCGCCGACCGCTGGAGCCCTA
TCACGCAAGTGGAGCATGAGGAGTGCGCATGCGCAGGACACGGCCCCGGATGAGACCCCGTCGGTTACCAGTT
CAAATTCCACGGACGCTTGTAAATTGCTTGTGTACATCGGCGAGTCCAAGGCGCCGAT
ATGGAGGCTGTCTCTATGCGGCGGCAACGCGGCGGCGGTGGCAGCTCGGGCTGGAACCAAGTTGCTACCACCA
AAAATTCGTATAGTCTGGAATCCACCAACCACACCTTACCATCATACTGAGAAACTGAGATTAGTCGTCACAG
CTGTTAATAGTGGTTCAGTATGCAACAACGACTCCCAATTCACATG
>1002052633_2 Heli.2-EL597725.1.5
CGTGTGCTATCTTGGCGGTTGGCGCTGTAATATGCGCATCCCAACCGGTGGAGAAGTTACCAGTTCTAAAGGA
ACTTCCTGCGGAAGTCAACTTTAGAGTTCCAAATAAAGAAGATTTGGTCCTGAACTGTGAGACTGTTGACAAA
GATGTTGGAGTCAAATATTCCTGGCTTAAAGACGGTAAACCATTCAGCCTACGATCAG
ACGTAAGCCAAAAGGAAAACGAAGGTACACTCATCTTCAAAAAACCAGTAGATTCTGACGAAGGCAAATACCA
ATGCTTGGCTGAAACCAAATTTGGCGTTGCCAGTTCTCGTATTGTGCTCGTAAAAAAGATTTTCATAGATAAA
CCTCAAGTGTCTCTACAAAAACATAAGCCTGTGAATGGAAAGACTTATAAATTGGAAT
GCGCTATCCCCAAATCTTATCCTAAGCCAGAAATTTCTTGGATAGTGAAGACAGGAAACGAGGCAAAACCTGT
TGCTGGTGCGAAATTTACTATTTCTCCGGAAGGAACTCTATACATAGCAAGCATCTCCTCAGAAGATGTAGGC
AATAAGAAATACGTCTGCCTCGCAAACACTCCAGCTGAAGACCAGCCTATAGAACTGG
CTGAACATGTTCTAGAAGAAGTCATCCAAAATAAAGAACCTGCTGATCATGAAGTTGTAAAACAATACGTCTC
TAACGAAGTGG
>1002052634_2 Heli.2-EL603032.1.5

CGACTTTTTAGAGCACGGAGTTAAATCTCTAATTTCTTGAATAATCTTGAATCAGATAAATAAATTACAGCAA
AAATGGCAGATGATATGCCCACATTCAAATGTGTGCTGGTAGGAGACGGCGGCACTGGCAAAACAACATTCGT
CAAACGACATTTGACTGGAGAGTTCGAGAAACGATACGTCGCTACACTCGGTGTTGAA
GTACATCCACTAGTTTTCCACACAAACAGAGGCCCTATTAGGTTTAATGTTTGGGATACAGCTGGCCAAGAGA
AGTTCGGAGGACTCCGAGATGGTTATTACATCCAAGGTCAATGTGCGATCATCATGTTTGATGTAACCTCTCG
TGTCACCTACAAAAACGTCCCCAACTGGCACAGAGATTTAGTGCGCGTTTGCGAAGGA
ATCCCAATTGTACTTTGTGGTAACAAAGTGGACATCAAGGACAGAAAAGTCAAAGCTAAAACTATTGTATTCC
ACAGAAAAAAGAATCTTCAGTACTATGACATCTCTGCCAAATCAAATTACAATTTTGAGAAGCCATTCTTGTG
GCTTGCGAGAAAGCTGATTGGTGATGGCAACTTAGAATTTGTTGCTATGCCTGCTCTT
GTACCCCCTGAA
>1002052635_2 Heli.2-DT665373.3.5
GCACGAGGGGATGCGCTTGCGTGATGCCGGGTGCGTGTTATTTTAACTTTTAAAGTGGCGTCGTTTTAGTGCG
AAACTGAATTATTTATTAAAAAGGATAATATATTTTGCATAGCTATAAATTGAAAATGGTCGCCCCGAAGAAA
GGCCGTGGCGCAAAAAATCAAGCGGTGAAGGCGAAAGCTAATCAACCCGAGGAAGTTG
CCGACAACGATATCGCGGAAAATGTAGAGGAAAATAATGGCGAGGCACAGCCGGAATCGGAGCAGGCTGAGCA
GGCTGCTGGGGCGGACGAAGAACATGAAGAACAAGCTGAGGGTGCAGAAAATCAAGAAGGCGATGAAGAAGTG
AAAGGAGAAGAAACAAAAGAAGAAAAAGTCGAAACTGGTAAAATACTAGTAGAGAATC
TACCACCAAGTTACCTATTTGACTATCAAGATAAACTTAAAGAGCTGTTCTCCAAGCATGGAGAAATCATCAG
TGTGAAACGTGGTCCAATCATTGTTACTGAACAGACCACATCTCCAACATTATCGGCTATAGTCGAATTCAAA
AACAAAGATTCCCTGGAGAAGGCATTAACAGAAGATGGTACAGCCCTCGATGGTACCA
CCATATCAGTAGCAGCGGAGTCTAGAGCCGAAACTGCAGTGTTGGTGGGAGTTCCATATGAAGCTAGCACTGA
TTATGTGAAGCTGTTGTTTTCACAGTGTGGAGATGTAGCACATATACATGAGTTTAGCAAGACTAAATTTAAA
ATTTTAAGAGTAACTTTCCTCGAAAAGGAATCTGTGGAGAAAGCCCTCAAGTTAGATC
GTGAGTTACGCA
>1002052636_2 Heli.2-DT662124.3.5
CGCGCTTCTAAATAGCTTGTGAACTGTGGCTGTTGAACTTTTGTGCTTTGAGTGTGTGTTTGAGCTTATTTCT
TTTTAAGATAAATCGCCGTATTACAAACTGATTTACTGCGCTTGATAAGCTGAAGTACTATTGGACGAAACAT
ATACGCAGCCACAACTACTAAATTGTTGATAATCGAAAACACAGTGTTATAGGTATTC
CCATAGTCACGTCTTACAAAATGTTGTTCCAGCACAGTCTGTTCCAGTCGTGAAATTAAAGTGAAGAACAGTA
AACGATCACCATGGACCATCGGATATTCATCTTTTTGGCAGTATTTGCCCATTTTAGTTGCACATTGGCACAG
AGTCCATTTTATGATGGTGGACCAAAAGCAGCAAACCCAGCGCCAGTGGGTAACTTCT
TAACACAAACTGTTTACGGCTTCCTTGATTTCACCACTACAATAGGCAATACAGTAATGGTTTTCTCACCGCA
GTCCGCCCCACCACCAGAACCACCGTCAACTGAAAAATCTGTACAAGAAAACATTATAGAAACTAAACCACCA
CCAATAAACAATGTAAAACCTGAAGCAATCAAGCCCAGCAAAACTGTGGACAAGGATA
CAACAAATAAAGTGGGAATTCCTATAGGTGCCTCCAGTGCTATTTCAGTTATTAGTTCTCCTCCTAAAATTGA
TATTATAAATATACCAAAATCTGTGGCAAAAGAACAAAAGCAAATTATCACAAAAGTAGAAGTCGTCGCAGGA
CCATCCAAAATCGTAGAAAGTAATGCTGCCAAACAGTTAGCTAAAGCCAACAAACAAT
TACCACAGGTGAAAAAACAGCAGCCGAAGAATCAACCAGTGAAAAGTGTTACTAATATAGTGAGCAGCAAAGT
AGAAGTGAAACAAAACCCCGAACCATCTTCAATAGTTAATTCAAAAGTAAATAGTGTGGTCGAAATCCAATCA
AGTGAAAGCGAAGAGGAGCCGGCAATTCTAGTGTCAAATAACATTAGAGAACCAGAAT
ACGATTACTTATCACGCCAGCCCT
>1002052637_2 Heli.2-DT663055.3.5
GCACGAGGGTTCAACATGAGATATCTAAGTCAGGTGGCCGTGATTATATTTACTTTTTGGTTATGTGAAATAG
TGAAGTGCAATCCACATCGTAGATTAAAAAAAAATCAGAAGAGTGCCCACTTCGCTTTGCAACCTATACCATT
GAAACCTTTTTCGGTTTTATTAAAGAAAATAACACCGTTTGGAGAATATGGAATTAAG
CATTCTATCAAAAAAAGGCAGATTGTCCAACCTAGCGAAATACCCAATATTGAAGGTGGTACACTTGAGGATA
TTATAAAAATACAAAATATTGGAGTAGCAGACAAAGCAGAAGATATTCCACTTGAACCAAATTTAACCCCACA
AGTAATAATAACATCTATCATTAAGCCTATAGAAGATAGTGTGCCTATTTCTATACCT
TTACTTTTGCCAGAGGGTTCAGATTTGCCAATAGACCAGAAAAATATAGAGGATTTGAAGCCTCCGGACGAAC
TTATACATAAAGAATCAGAACTAAAAGTCATAGAAGTAGCATCATTATCAGAAAATGTTATTGAAATTACTCC
AAGGCCAAAGATAGAAGAAATACCGCCAGAGTACCTCGAAATTAAAAATGTAGCCATA
AACACTCCAGTGGAAAGTACAGTACCATCAATATCGAAAATTCCTCTTATAAAAAATCCAAATGGTCCCTTTT
TATTTACTGATATTACTAGACTACCTTTAAATGGTTTTTATCCTGCTCCATCTATTGGTGTTCCTGAATATAT
TGTACCTTCACCACCATTACCACTACCACCGCCTGTAGTTCAACCACATATAAGCCAG

TACTGATACTAC
>1002052638_2 Heli.2-DT667042.3.5
ATCAGGAGCTGATAAAAACAAATCTGACTTCTACAATGTCGCACACGGTCACAATTACGAGGACAACCACTAC
CACATCTGGAGGTGCATTCTTCACGAACACCGGGTACCTTAGCTCTACTCCAGGGTTCCTCAAACTTGTACAA
TTGCTTTTAGGCGCAGCTTGTGTTGGCGTGGTGGGTTACTATTTAGATCCCGGTTATA
GTCGGTACAATGGACAAAAGCCGGAATTATTTATTTACTAGTCGCGGTGACGTTCCTAATCGGTACCTTCTG
CTTGTTCATATCATGCCTGATGTCCTGGTCAACGGCTTCTGTGATATCAAAAACATTATATGAAGTTATCTAC
CATGGCGTTGCATTTGTTATGTATTTAGCAGCAGGTCTTACACTAATGATCGAAGTAA
ATCATCAAAAAAATAGTTACAGGCGAGATTTCGAGCCATATTTGGCAGCAGCGGTAATGGGACTGGTAATGGC
GGTTCTTTATTTATTTAGTACGTTCATTGCAGTCAGGTCCTATCGCGGTATTTAAGTCGCCGAGAATCTCTAT
AACACTAGCTTTATCAAAATGCATTTCTGTGTTGTTTGTATATACCAAGACGTACTTT
GCTTTCTACTTTTTATAGGCCTTTTAACTATTCCTAGACTCTATATTAACTATGGCGTCGGCATGGAGATATG
TCTACTGACGCTTTTGCAAATATGTATATAAATAACGATGCCGTTGCGACGCTAGGTTAGTTATAGTTATAAT
TGCTTCCCTGTTCAGGTCAGACAGACAACGACCA
>1002052639_2 Heli.2-DT668665.3.5
GCACGAGGATTTCCCTAACGTCACCATATCGGCTTTGCTAGTGGTAGCAAGTGCCCAGCACCATTTAAGTGGT
CATGCCACGTCATCGCAATCTATAATTCGTCATGACATCGCACAAAATCATGGATACCAAGGCCAATTAACTC
AAGCATTTCCTACTTCCTACCATCAGGCTCCCATCGTCCACCAAGTCCTTCCTATTGC
TAGCCATGGAAGTCTTCAGTCTGCTGGTATCTCACAACACGCATCTCCTATAGTTTCCCTTCAACACGTTTCT
CCTGTTGTCCAGCGCGTTTCTCCTGTTGTCCAGCGCGTTTCTCCCATCCACTATAACCAATATGAATCTGGAC
ATGGACATCAAGACTATCATGGTCACCCCAGATACGAGTTCGCGTACAATGTAGCTGA
TCACCACACCGGTGACATCAAGTCGCAGCACGAAGCTCGTGATGGTGACCACGTGTCTGGTTCATACAGTCTT
CACGAATCCGACGGTACCATCCGCACCGTTCACTACAACGTTGATGGACACAGCGGATTCAACGCCCAGGTGG
AGCGTTCTGGACACTCTCAACACGTCCAGCCCCAGCACCGTGCCCCCAACTACGTACT
AGCACATCACTAAAAATTAAACAATGTTCCTAATAGAAAGTTAGCATATGTATGGACTCGTATACCCATCAAT
CAAGCTAATGACGAGTCTAGTCTTTTAATGTATGTTCCAATTTTTGTGTATCGATTAAATATTATATAATAGT
GTAAAAAAAAAAGAAAAACCTC
>1002052640_2 Heli.2-EL604282.1.5
GATATAACGATATGTATTTTAGGATATTTATAATTTAGATTTAGGCTGAGGCTATTTACTTATTTTTATGTAG
TATTAAGCTTATATTACATATTTATAATATGTCTGGGGAATTGGAGTTTATTACGAGAAGTGGCAAGTTCCGT
TAGAATCTAAAATGGCAGAGTCATCGCCCGGTTGACGCGTAGATCCTTGATTGACCTG
AAAATCAATTCAAGAGAAATACCAAGGATAAAACATAAGAGTCGGTTAAACTCATCGAACATCGGTGGCAGGT
CAATACGTACATTACAATAGACAGACTCCAATACAATTAGCATCACGAATAATGCTTGTTGAATGGTTTGCGC
TGGTACGTTCAGGATAGCCGGTCTCCAGTAGAAACGTCCCCGTTTAGGGATCTTTCTA
GTAACAGTTATTCAGATGTTTAGTAATTAAGAATTGTTTGTTCCTATTTTGGCAGTTAGCATTTAAATGACTG
TTATTAGTTATTATCATTAGTTTAGTATGGATACTAAGCAAGTTTCCTTCGCTATTAAAGCTTTTTGGATATT
AAAGCAAGTTTCCTGCCCCTTTCTTATTACATAAACTTTTGAAATG
>1002052641_2 Heli.2-DT666443.3.5
GTTCGAATTTTTAAATACGAATATGTGTAACGTTTGCACACGCGATGTCGAGGTTACGCCGGCCCCGGCGAAA
CCTAATAAATCCTTGGAAAGACTTAAGAAGAAGCATAATATACAGACTAACCCTACTGACGAAGAGAAACAGA
GAGAAGTGGAATACCAACCAGATTTATTGGCTGTGTCGACACAATATTCCAAAGTGGC
ATACGATTTGTTCAAGCAAGCTGTTGTTAGGCTGCAGGAGACTAAGGCTTATGCTATAGCAACAAAAACGACA
CAGCCGTTCCACGTAGCGCTCGCTGTGCTAGTAATAGCGGCGTGGCTTACTAAATCTGGGTCCAGTGCCAACT
TGAGGGGCTGGAAGGCGTTGTACGTGGGGGCGGTCGCGACACACCTGGGGGCGCAGAT
ATGGATGACTTTAGTGTCAGGCATAGTGCTGTATTTCTCCCTCCCGCGCCACGAGTTCGGTCGCGTCCAGACG
GTCTTGTTCCCCGTGTATTACGCCTTCAACTCCCTGGTCAGCCTGTTGGCAGCGCTCGCATACTTCCGAACGC
AATGTCTCACCCGCTTCGAGAACACTTCCTGGGTACAACTAGCCCTGCTGTTAGTAGT
ATTCAGTATCGAAGCGTACGTCCGCCTCTGGCTCGTCCGGCCGATGCTGCGTGCGAAACACGTCAAAACACAA
ATGGAGGAAGCCGCCGGCGGTGGACAGGAAGTCGGCCGCCTAGTTCTCGGTGAGCTTGCCCACTGCCCCCGCT
ACCTCCGCGTCCTAAAGACCTTTAGGGCCTACCATTCATCTATAGCGATGGGCACCAT
GATTACTTTAGGGTGCTCCTTCTA
>1002052642_2 Heli.2-DT662784.3.5

CCGATACGAAGTGTGCGTGTGGCCTGGAAGCCTGCCTCACTCACAAGCCTGTCCTGGCTCCTCCGAAATAGCA
CCAGTACCACATTCCCGATTCCAATGTCCTGGGCATGAAGGTTACTACGCTGACCCCGAGAATTGTCGGTGGT
TCTTCGCTTGTCTTGACCATGGAAAATCTCCTTTAGAAGCTTATGAGTTTCGTTGTCC
GTTCGGCCTTGGATTCGACGCTGCGAAGCTCAAGTGTGATTGGCCCTGGCTGGTTCCTAACTGCGGAAATATA
GGAAGATATGAAGCTGAAGCCTTTGGGTTCTCTAGTGCTGCTTTATCTGGCGCTACTGGATTCCATGGGAAGA
CAGCAGATTCAGTGAACATTGCTGCCCACCAAAGCTTAGTATCCGGAGCATCTCTAGA
CAATCTTGTTGGAATACAGAACGGCTACCTATCCAAGGACGACATCCTCGACTCAAATTTCATTGCTTCTCAA
GAAGCTTCCAATCTAGGTTTCGCTGGACCACAAACTTATCAAATTGATGAAGGGTCTATTGACTATGCTTTGG
GTGGTCAAGGAGAATATAGTGGACTTTCCTACAAGTATGTATCAGCTGATAATATTAA
CAAGGGTCTTGTGCAAGCAACGAATTACAATGACGAAGACAAATATACAAGTGGATCCATAATTCTAGATGAT
TATAGATTACCGAGTAAAACGTTAGTTTCTTCCGGAAAATATATTCCTGAAATTTACTCAGGCAAATCATCAT
CTGGTATCGATTCTGGAAAATATAGGGGCAACGGGGCTTATGCCGGTGCTGCAGCAAA
TTACAATGATGGAAAATACAACAGCGGTCAGTATAGAAAAGATAAGTCTGGTGCTTATGTTCATAATCCAGCT
GGAGACAGAGCACAGCCTTACCAACATATTGACGTACCACCAGTACCTTATGAACATTTAGACTCTAAATATA
CACAAAATAC
>1002052643_2 Heli.2-DT668423.3.5
GCTGCACGTGCACCAGTCGGGCTTGCCGTGATCATGAAACTTGTAGTGCTCCTCGCCGCAGCCGTCGTCGCGG
CGGCCCAGCAGCCGCAACGTCGGGAACAGTTCAGAGTCATATATGAATGGCGCACTATAGACTTCCAGTGGGA
GTCACCTGCGGATCGGGAAACTTATGTTAACACCAGTCGATACATCCCGCAAAATGTG
CTCATATCTGGAATCAATTATTATGAAGATAAAATATTTTTGACTCTACCAAGAATGCTCGACGGCGTGCCCG
CCACGCTCGCTTATATACCGGCGCAGCAGAACGACACTACGGCGCCGAAACTGAAGCCGTTCCCGAGCTGGGG
CGACAATACCGTTGGTAACTGCGACGCCCTACAATTTGTGCAAAATATAGAAATTGAC
AGCAACGGGATGATGTGGATTTTAGACAACGGCCGCGTCGGCACTCTGACTCAAAACCCCAGTACTAAGTGTC
CTCCGTCTATTGCATTCATTGATTTAAAAACTGGAGAAAATAGAATTGATCGTATACCATTACCTCCGGACAC
GGTGAATCCTAATACCTCATACCTAAATGACCTAGTGGTCGACAGCCGTGATGGTGGT
TACGCGTACATAACCGACAACAGCGCGGTCGATCCGGGCATCGTCGTATTTCGTCTGAGCGACAAGAAATCAT
GGAAATTGCGTGACTCGAAATCGATGCTGGCCTCACCCGACGCCGCTTTATTCCGCATAAATGGCACAACAGT
GAACCTGCCAGTGAATTTAGATGGTATCGCACTCGGACCACAGTTCCTCACAGAGGAC
GGGAAGGTCGATCGTACCGTCTACTACTGCCCACTCGCAAGCTACCATCTATACGCTATCAACGCTTCTGTTT
TACGAAACGACTCAATCCATGCACAAGACGAGCTGGCCGTTCGCGCGAACGTGGTCGATCTCGGAACGAAA
>1002052644_2 Heli.2-DT667017.3.5
GACCCACAACTGAAGCAACTACTGTACAATATCAATCGTCAGAAGAAGAAGAAGATAAAGAATCTCAAGAATC
CGTATTAGAAAGAGAAATAACTTCACAATCAAATGAACCACAGTACAATTCAATTTTAAGAGCTCGCGCCACT
CTCCCCCCAGAACCAGAATTGAGTAGTCCGGAACCCACTACTGTTTTGTCAGTTCAAA
TTTCTTCCTTATTAAACGAACCAAATTCAGATAATCAAAGTCCCGAACCTGAACCCGCTATACAAACGACTGA
AGCCGAAACGACGACCACATCTACTACCGTAACGACCCTCTCATCGACTACACAAACAACATCAGCTTCATCA
ACTAGACGACCTCTAATAAGACGTCGAGGATCTACAATACAAACGACTAGTACTGAAG
CACCAACAACAAGCACACAGGTACGAGAGCGGCGTCCATTCCTTCGTCGTACCAAAGCCACTGCACCCCCTGA
TGTCACTGACTCCGACATCACCACTCCATCCCTCGAAACAACCACGAACAAGTTCACAAGACGAGGCAACAAC
AGGTTCAAGTTACGGAGAAATGAAAATGATGTCGAGAAAACGGAAAATAAGACATCTT
CAACAGTCGCGCCGCAGCGTAGTGAGAGACCGCGCAACTTCGTACGCCGGCGGCTTGGAGGGGCGAGCTCCAC
TACAGCCGCGCCAACCGTAAGTTCAACTCAAGCTGTCCTACCATCACGTCGTCCGTTCAGAATAGCGAGTCGA
CGTCGTCCCCTTTCCACTACCACTACCACCACAGCCAAACCTACTACTGCACTAGTCG
AGAGTGAAGAGGCTTTACAGGATATTGGAGATATTGATGCCATAGAAGATCCTTCTTTAACACCGAGTACACC
TCGAACGTCTCTCAACGTCCAAAGACGGCGTCCTCTCGTCCAACTCAAGATAGATGACCAGAATCCATCAGCA
ACAAATGAAGAAGAAAAGAAAAGACAGAGTAAGAAGTTTAGCGCCAGCTTCAAACAAA
ATCAATTGGACGAATTGTTAAAACTAAGGGCGAGTGCGGAAGAAATTGATATAACTACGGAAGGAAAAACTAC
TTCAGATGATATAAGTGCTGAAACTGCAGTCGCATTAGCAGCACACCAGCTCTTAGCAGCTCCCATACCAATC
ATACCAGATTACGACGAAGAGTTCTTCACGACGAAGAAAACACCTAAAACTATAGTAG
ATTACAAATTCACGAATCCAGCTTACACTGAAGAATATTTCAAAACACAAAGTTATTCAGACGAAACTCAAAC
TTATAGCACAAGGCAAAGACTAGTGGAAACTTCTACACCTAATTACAGTCCTACTGACACTAATACACCAAGG
TTAGCTGAGGGCAGTTTTACTACATCTTCATTCACCAGTCGATTCTCCAAACCAGATT
CAAATGTGAACCCAACACTTTCCGGAGTAACACTCAGTTTGGGTTCACAAAACGCGGAATCCACAGCTCGCTA
CAACCCTACAGAACCTACACCGTACACAACAGAAAGTTACGAATCCC

>1002052645_2 Heli.2-DT663239.3.5
TTATAAGCCAAGAATCATTTTATGAAGATCTACTAGATAAGCCGGCGCCGAAGGGATATTCCCAGCAAGAGAT
TTTCAGAATGACAAAGTCATTTTTCGTACTTTTTCTATATAGAGTCGTTCGTTTAACCCCAGCTTACGCTTTT
GTTATTGGACTGAATGAACTAGCACTAAGATATACGTACGATAATACTGTATTTGAAC
CGGCTATCTTCGATCATATCACTTGCGATCACTATTGGTGGAGAAATTTGCTATATATAAACAATTTGTTCCC
GCAAAAAGACATGTGTATGGTATGGTCTTGGTATATGGCCAACGATACTCAATTCTACGTCGTCGGTATAATA
CTACTATTAATATCAGTCAAACACACGAAGATTGCAATGGCATCTCTGTTTCTGTTAA
TGGTTAGTTCCTGGGGAACCACCATTTACATATCAGTGTGGCATCAATACAAGGCTCGCATACAGGAGCCGTT
CGAAATGTTCGACCCCCTATACGACAAGCCTTGGTCTCGAATTGGCCCATATTTAGTTGGAATGATTATTGGA
TGGTACTTACATAAAACAAAGTGTCAAGTTCGTATACCATATTGGTTAGTTGTGATTG
GTTGGCCCGTGTCACTGGCCATCATCGGCAGCCTGATCTTTGGAATGACTGATGGATATTTTGAGGTTTGGCC
AACTGCGTTCTATGTGAGCATCGGTCATACAGCATGGGGAGTGGCGTTAGCGTGGATAG
>1002052646_2 Heli.2-DT666296.3.5
GCACGAGGAACAACCTTGTACTCCAAGGTCGTAATCCTCATGTGCGTCGCTGGCATCGCCTCAGCCGGCAATC
TTCTCCAAGCCGCGCCAGTTGCGTCCGTTGCGTACAGCTCTCCCGTTTCCTCCGTGTCATACTCTTCGCAGAC
AACAGCTCATGGCTCGCCAATCTCCTACGCAAGCCCCGTTGTAGCCAAGACGGTTGCC
TATTCTGCAGGACCAGTGATTGCAAAGAACATTTCCCCGGCTCTATCTTACCAGACCATCTCTCATGCTCCAG
CTATCGCAAGTTACGCAGCGGGACCAGTAGTCGCTAAAGCCATCGCGCCCGCAGTGTCATACTCATCAGTATC
TCGGTCTTCGCCCATAGCGTATGCTGCCAGCCCTGCAGTGAGCTATGGAGCTCCTATC
GTAGCAAAATCTATATCACCTGCTGTATCCTACTCTTCTGTAGCTCATCCTGCATCATTAATACATGCTGCTC
CAGTAGCATATGCTTCTGGTCCAGTCGTTGCTAAAACCTTAGCTGGCCCAGCAGTGTCTTACTCATCTCTGGC
GTCTCCTTCATTATCTTATGCTGCGGCTCCTTCTTACTCCTATGCTGCAGCTCCTTCT
TACTCCTATGCTGCGGCTCCAGTCCTCAAGTCGGCTGTAGCTTACTCCGCCGCACCAGCAGTCTCCCATGTTA
GCTACTCTGGTTTGGGAATCAACTACGGATGGTGATTAAACAAAGACTTAAATATAATTAGGATATGTGCATA
TAATTAATAATTATTGAAATGTATATTAAAAACAGTCAATAAAATTTTAAAAATTAAA
AAAAAAAAAAAA
>1002052648_2 Heli.2-EL599556.1.5
AACGGCGTGTGGTAGTTCCAGTTCGACATGCGTCAACGCTCTGTGTCATGACTGTGACACAACAACGTTCGCC
ATCCACATTGCGCATCAGTCTATCGATAACTGACCCACTCGATTCAAACTGCCAAGTTTCGATTCCATCAGCG
CGCTCACTACTCGAGTAAAATACCAGACGTCTGGACGTCGCATCACGTATCCATTGCA
TACGATGCGGTAGACAGCGTACGATAGTGATGCGCTTTATGTAGATTTGAAATAATAGTAATATACTGGCCTG
CCAAATAAACTATTCTAAATTCAAATTTCTATCAGTTTTGACGGATTGCCGCTCAAGTGTCGTGTAAATAAAT
AATAACCAAAGTGTTTTTTATAGTGTTTTAAAGATTTAGTTGTGTATAAATATATAGT
GCACGATGACTTCGCGATCTAAGGAGAAAGTGGCAGCGGCTTTTCGGAAGCTGTTTCGCTCTCCAGAGAAACT
TGACAACGAGGGAGCCGGCCCGAGCGGGTCGCCAGCTAGACGCGGGCTATTTCGCCGTCACAGAGATGGTGTG
AGTCCGGCGGAAGCCCCTGCGAGTTTACCGTCAAGTCCTGCGGCTTCATCGATTGCTA
AGAAAAAAATCTGGAGGATCACACGACGTGCGGGAGAGAGCAGCAGCTGTGCGGTCACGGAGACTCATGAAGG
>1002052649_2 Heli.2-DT667705.2.5
AGCAAATAGTTGCTAACCAAAAAGTGAAAATCCCAGAGGGATTAACTGTTCATGTGAAATCACGGCTAGTGAC
AGTGAAAGGTCCTCGCGGAGTGTTGAAAAGGAACTTCAAACACTTGGCTGTAGACATTCGCATGGTGAATCCT
CGGTTATTGAAAGTGGAAAAATGGTTTGGTTCAAAGAAAGAGCTTGCTGCAGTAAGAA
CTGTATGCTCACACGTTGAAAACATGATCAAAGGTGTAACTAAGGGATTCCAGTACAAAATGCGCGCTGTATA
TGCCCATTTCCCTATCAATTGTGTCACTACTGAAGGAAACACACTTATTGAAATCCGTAACTTCCTTGGAGAG
AAATACATCAGGAGAGTAAAGATGGCACCAGGTGTGACCGTTACTAACTCTGTTAAGC
AAAAGGATGAGCTCATTATTGAAGGTAACTCCCTTGAAGATGTTTCCAGCTCAGCAGCTCTCATCCAACAATC
TACAACTGTCAAAAACAAGGATATCAGAAAGTTCTTGGATGGTCTTTACGTATCTGAGAAAACTACTGTTATA
CAGGATGAGATATAAGGACATTAAATTTATAAGT
>1002052650_2 Heli.2-DT662390.3.5
TTACATCGTCGCGAACGTAGCGTAGTCATAATAAAAGTAATTAAATATAATAAAATATGAGCGAAAAAATGAA
ACTATTCACCAGGGAAGAGTTGAAAAGTCACAATTCACGCGACGACGCCTTTATTATTATTGATAATTCAGTA
TACGATGTCAGCAATTTTTTGGATGAGCATCCTGGTGGAGAAGAAGTAATTATGGAAC
TGGCAGGTGAGGACTCCACTGAAGCTTTCGAAGATGTCAGTCACAGTTCTGATGCCAGAGCGTTAATGAAGAA
GTTTAAAATAGGCGAAGTGGTGGAGGCTGACAGGAAACAGACGAAGACCAATCTGCCGGCGCAATGGAACAAT
GACCAACAATTGGAGCAGAGCAAGTGCGTAATCTGGGTGGTGCCGCTGCTGCTGGGCA

TAGCTGCCACCGTCGTCTTCAGATACCTATTCATCTAATACCACGACGAATTACTTTAATTTCACCATAGTTT
AAGAGATTTTCTTACAACTATATAGTTATATAAAGAAATTAAGACTCAATAACGCTATAAAAACAATTTAAAA
CGATTATTTATTAAGCGCAAACTTCAGTGCAGCATTTATAAAATTGAAATCTTCATAT
ACATATATTATATATGTATATAGGAATATTAATTTATAAATAAGTATTTTTCTACATGAAACAGACAGAATTC
CTTTAGTCCATATTGTAATTTAATATCATTAAATGACATTAGTGTCTGTACCTATAGATAATTGTAAATAGCT
TTTCTACTGTATGTTGATAAGAAATAACTAACAAATGCTTGACCTGATATACTTAAGA
ATTTACACTCGGTTACACTATAAC
>1002052651_2 Heli.2-EL597981.1.5
CGCCCTAGCCGCCCCGACCGCCCTACCCGCCCTAGCCCGCCACGCCCACGCCCTAGACTAACTGCAACTGCGA
CATAGACTAAACGGTGTCAAATTGACGTCAATGAGAACAGATAATATTTACTCACTATAGGAAATATATGAAT
CTCAAGCGCTGTAGACTCAAGAGCAATAGAATATTATTGTGTAAATGTACACCTGTAA
ATGTAAAGCTTATATTTGCTGTTCAATACACATATGATTTGCTCTAAAGATCAATTAGTACAAACGTTATCTT
ATGATTGAAACTACATGACAATCTCAACTTTCCAGAAATTAAGAGATATTTGATGAATTATGCAAGTATTTTT
CCCATCGCTTTATGTTTAATCACAACACCTTATACTTCTCTGTATATTGAAGCTGTTA
TGTAAAAGCTAAAACGTTAACGCTAGTTCCAAATAATCTAGCAAAAATCGTACAAAATTTTGATCAACTAGCT
TTACTGTAATGTAGAAGTAAAATCAACGCCGAATATAGGGCTGTCCATAGATTTCGTCACACGAATTTCATGA
TTTGACTCACTCCCAAGCCTTGTCAGCTGTGACAATATCATATTTGTGAGACCCTTAC
ATAGTGTGACGTCATATATGTAGCAATTTTACATCTAGGAATTATTGA
>1002052652_2 Heli.2-DT668315.3.5
AGGATGCATGTTTTAAATGTTTACGAGAAGTTTTAATTTATAATTAACATATATCTAGTTATATTTAACGTAT
ATAAAAAGCATGTGAGATTGTGTTTTCAATTGTGATATAAGTATTTGAGGTTGCTAAGATAAGTTATAAGTGC
TTTCATTATTTACGATTGTTTGTATTGAAATACTTCGTCGGTAAGTTTTTAATAGATT
ATTATTAAAATAAAGAAGAGTGATATTAATGCCGTCAATATCCAGAGATAAATATTTTTTATGTTTTTATGAG
GTATATAAAATTACAATTATATTACGTAATGTACGCAGAATGCCTGCCGCCATCTTCGTTAGACGCGTGTATG
GGAATTGTGAAATGAAAGTTAAATAGGTTTGGTGAATTATAGTAACTTATAAATGTAA
TTTCTATATATATTAATAAATATTTAACATGTTACTATAAAAGGAAATATAAATATAATAGTATAATTTATAA
TATGATGTAAAACTATTATATATAAAAATGTATATTTACAATTTATAAATATTATATAAATATTATATAAATATC
AGTATGTTTAATGTCCTACGATAAAAGTAATCTTTCAATATGTTTTATATATTACTTT
AATTAAATATACTAGACATTAGACATTAACCAAATATTAATTATTTACGATTTATACGACTGTTAGAGTTCAT
TGACCCTTGTTTTAGTATATATTATATAAATATCATATAAATATGAGTCTGTTTAATGTCCTACAATGAAAGT
AATCTTTCAATGTTTTTTTATATATTACTTTAATTAAATACAATAGACATTAGACATT
AACCAAATATTAATTATTTACGATTTATACGACTGTTAGAGTTCACTGACCCTTGTTTTAGTATTAACTAATA
TTATAGTCAGAACCGACTGCTGCTTGGCTTCGTTGGTACTGGTGTGCTTGGCTAAGTGAATTGCCGTGGCATT
TAATTTTATATATATTATATTTAGATAGTCTCGTGGTGCCTTGTGGTGCGTCAACGTT
AACACTTGGTGTATGATGGATAGGTGTGCGATGCTATTTTATGTCCTGGAGCTCTGGTGTATGTAGTAGTGTC
CGCCCGTCGCATACGGGTGCGCGATGCTATTTTATGTCCTGGAACTCTGGTGTATGTAGTAGTGTCCGCCCGT
CGCATACGGGTGCGCGATGCTATTTTATGTCCTGGAACTCTGGTGTATGTAGTAGTGT
CCGCCTGTCGCATACGGGTGCGCGATGCTATTTTAT
>1002052653_2 Heli.2-EL602250.1.5
TGGAGGACGCGCTCACGCTCATCGTTTTAACACTGTTACAACTATGTCGAGCCGAAATATCAAACACGGAATG
GTTCGCCGCAAATTTAATAAACGACCCAAATTACACGCCAAAACCCATCACGGAAATATTTACTACTGATATT
ACATTTATACCGAGAAAAACATCAACGTCGACCCAAGCAACTTCGCGAGAAACACCTT
CTCCAACAATCTCGACCACATTTTCTGGGAGAGAAATGGTTACTCAGGATATGGGTACCGAGCGAGTACCGCA
AGTTATAAACCCTGCAATACAACAATCCCAAGGAAACATGCCACCAAATAATAACATGCCTAGTGCAGTTTTA
GTGTATGATCCAAGCCTTCAAAATAGTCAAATATATCAACAGCCTTCATCTACTCCAG
CAACAGCTGAAGCGGAGTTATCCACAACTTTTGTGACACAAATGTCACCTATGCCTCCTTCTCCACCCTCTCC
TCCGAATTTAATACCAGCTGCTCAAGGCCCTACAATTGTGGTACGGCAGCCCCTAAATCAGAACGGTAATCCT
CCACTAATACCAATTGGCTTAATACCAAATGTACAGAATGGAAATTCTGCTATCCCAA
TGTCAAATATCCCTGTGGTAAACACTTCTTCTGGCTTGATATCGCCTTCATCGGTTATGCCTGACCAACAAT
>1002052654_2 Heli.2-DT667274.3.5
CGCAACATTTTTAGACTGAATATTTTATAATTTTGCATTTCAATGAGAACATTGACATAAATATACCTTATTA
GAACCGAAACAATTGTGAACAAAAGAATAAAGTAACGTATGTGTTTTGTGTTTCAATTTCGTGACGTAACTGG
ATTTCTTGTGAAACGCGAGTAACTGTACTCAACCCGCAAACGAAGGTCGTATCTCTCG

GATGGTTATTACTTTCGTATAGTTCCGTAACGGTACATACACAAAATGTGGGAAGTTGACTCTGACACAGGGT
CCCAAAGTGCGTCGTCGGACGGGCTTCGACGTCGGCAAGGATGGGACCCAGAGGCCGAGAGTTTGGCTTCTCA
AATCGACGAACTCGACGAAGTGCTTGCGGAAGAAGAAGAAGGTTGTCCACTGCCTTCT
ACTCCAGAAGATCAACATTTACTCGACGCAGAAATGGCTGAAGTGTTGAAAGCTGGTGTGCTATCCGACGAAA
TAGACCTCGGTGCATTGGCACATAATGCAGCAGAGCAAGCGGAGGAGTTTGTACGAAAAGTATGGGAAGCGTC
GTGGAACGTGTGCCACTTCAGACACCTGCCGCGTTGGCTACAAGACAACGATTACTTA
CACAAAGGCCACAGACCTCCACTTCCATCTTTCAGCGCCTGCTTTGCTTCTATATTTCGCATCCACACTGAAA
CTGGCAACATTTGGACCCATCTGCTTGGTTGCGTGGCGTTCATCGGTGTAGCTATTTACTTCTTAACTCGCCC
ATCTATTGAAATTCAAATGCAAGAAAAAATGATCTTCGGAGTGTTCTTCGTTGGCGCT
ATTGTATGTCTTGGATTTTCATTTGCATACCATACTTTGTACTGCCATTCTGAAATGGTGGGTAAACTATTTT
CTAAGCTGGATTATTGCGGCATTGCTCTACTTATTATGGGGTCATTTGTTCCTTGGCTGTATTACAGTTTCTA
CTGCCATTATAGGCCGAAAATAATTTACTTATCTGTAGTGGTTGTGCTAGGAATATTA
TCAATAATAGTTTCACTTTGGGATAGATTCTCAGAGCCTCACCTCCGTCCATTGAGAGCTGGGGTGTTCATGG
GTTTCGGTCTGTCTGGAGTAGTACCCGCCATACATTATGGCATAACTGAAGGTTGGTTCAGTCAAGTTAGTAT
GACTTCTCTCTTCTGGTTAGTACTAATGGGTTTGCTATACATATTAGGGGCCATGTTC
TATGCTCTGAGGGTGCCAGAACGCTGGTTCCCAGGTAAATGTGACATTTGGTTCCAGTCTCATCAAATATTCC
ATGTTTTAGTTATAGTGGCAGCATTTGTCCATTATCATGGAATCAGTGAGCTAGCATCC
>1002052655_2 Heli.2-EL600974.1.5
TATTTTTTAAAGTATTTGTTGTATTGTTCTTAAACAAACCTTGCAACTTTAAAAATAGTTAACTAAAGTGACT
AATGTAATTATTTACCTCTTCAGTTGGGTTCTTTTGTTCCTAGTGTAATCGTCAAATATATCGAAAATGCCTA
TTTTATTCAGCGTTATTGCTCGTGGAACCGTAGTTTTAGCTAAATACGCTTCATGTGC
TGGAAACTTCACTGAAGTGACAGAACAAATTTTGTCCAAAATACCACCCCACGATGATAAACTTACCTATTCT
CATGGAAATTATTTATTTCATTATATCGCGGAAAACAAATTAGTTTATTTTTGCATTACTGATGATAAATTTC
AGCGTTCGAGAGCGTTTCTCTTTTTAAATGAAATTAAAAGAAGATTTATCTCAGCATT
TGGGGACACTGCCCAAACTGCTATTCCTTATGCTATGAACAGTGAATTTGCTAGAGTTTTGGCTACTGAAATG
AAGCATTACAGTGAATCCAGAGACTTGGAAACAATATCAAGAGTGCATGGAGAGTTAGATGAGTTAAAGAATA
TTATGGTCAAAAATATTGATAGTATGGCAATGCGTGGTGAGAAATTGGAGCTTCTCGT
AGACAAAGCTGATAATTTAGCTACAAGTTCAGTATCATACCGCACCTCGGCAAGGACTCTACAACGTTCGCTA
TTCTGGAAGAATATAAAAATGTATGTTATTTTGACATTAATAGTTGCGTTCGCCATATATTTAGTTGGCGCAA
TGGCATGTGGCGGTCTCGCTTGGAAGACATGCGTCGGATAAACATAATGTTCCTATCA
>1002052656_2 Heli.2-EL602985.1.5
AAGCTCGCCCATTCAGTAGATATTAAACGTTTTTAATTAAATTCAATTATAATCTTATTTTCTTACGCATTTT
TAATATAAAGTGAATTTTAAAATGTCGGCTAAAGTGGCGTTAGCCTTTGTGGCAATGCTAGCATTCGTTAACA
GTCAAAGCTATTTAGACGCGCTCGTGCAGCAGATAATGCCAGAACCCAGTGATCAACC
GAAGAGTAACTGCTTGTGCAAAGGTCCAGCCTGCATATGTTGTGTGGATTTTAATATGACATTCCTTGATCTA
GGAGGTCCAGGGTGCATACACATGAAATACATTTCGCCAGAAGAAGGATTATCGGTAAAAGTTACATATGATA
AAAAATTTCTACAAAGTTCTAAAATAAAAGGACCTCATCCAACTCCTGTTTGCGTGAA
CTTATACGGTGTACATGCCCAAGTTTGTGCCAATTTCAATGATCTTGCTCCCACTGCTGATGGACTACGTGGA
TGCTCAAAATTAGAACTTAAGTTACTTGGAAGTTCACAACTAGAATTCCCAATAGGATGCTTCAAATCGTCTG
CTGGCGTGATGGAAATGGAGGACCCACCAGTTGAATCAGAAAAGGAAGAGACCACAGA
AGAACCTACTGCAGAAGACACCAATATATTCGACCCCGAAGCATTTTTGATAAACATCTATCAGACAGCCGAA
GAAGGTGTCGCTTTTCTTGGTAGCCTTTTAAATAT
>1002052657_2 Heli.2-DT667587.3.5
GCGCGTGGCGTGACTTACGTGATGTTAATGTAACCGTGCTCATTATACTATAGTGTTCCTGTTATAGTATTGT
CGGAGTGACGCCCCGTTTAAGCATGCTGATGGTACAGACTAACGCTCACCACATGAAGACCACTAAAATAAAA
GGACCTCCACCCGTGCCACCGCGGCCTAGTCAGAGCATGGTGGCCGAAGCACTCGCTA
AGACTAGGAAAGCTGTAGCAGATTCGAAAGCCACACTGTCTAAGACCAGAACTTTTACTAAAAATGAAGTTAA
TCGTGTTTCGTCGAAGGCTAAAACATTGGACAGGAACTCTACACCGAGCAAGCCTTCCGTGTCGCCACGACAA
AACGGGCTCGCGCGCGTCAAGTCATTCATTAAAGACGTTATTAGTGATCGTAGCTCTT
CGTCTGATGAAAAGAAATCGAGTGGTCAGAACTCGAGGCGTAGCTCGAGTGATAGCAATTCGATTCACTCTCC
GACATCTAATAAATCAAATGAATCATTGAACTCTAAAGCCGTTAAGACATGTAGGCAAATATTAGTACGGTCA
CTTTCTACTTCTAACAAGTTAGATCAAGATTCTAAATGTAAAGTTTCTAAGTCTTCTA

GCTTTGCTGAAAAAACTTTACCCTTACGTAAAGCCCCACCACCTCCTTTATCGCCAAAGCCCAGATTGAAGCC
AATGAAACCTGTGCCACCTCCTGTACATAGAAGTATAGATGGCAATAAACATACAAAAGAACCTGATATTAGC
ATATACACATTGCCAGTGGATGCTAAGACTCAAATTTTTAGTGCAG
>1002052658_2 Heli.2-DT666964.3.5
GCACGAGGCGCTACTTAGCATGGCTATCCTGGCATTATGCGTATGTGCGCATGCGCAGTACGCGGAGGAGCGC
GCGCCCCGGTACATCGCCTCGGAGCCGAAAGTTACATCGACACCCGTGCCAATTCTTAAACAGATTAACAGGC
ACAACGAAGATGGCTCATATACCTATGGATATGAGGCAGCCGATGGGTCTTTCAAGAT
TGAGACAAAATCTCCTTCTGGAGAAGTCAAAGGAAAATACGGATATAGAGATGATACTGGCAAGGTACGAGTC
ATCGAATATGGAGCAAATAAATACGGTTTCCAGCCAGCTGGAGAAGGCATCACTGTCGCTCCCCCCACATTAG
TTGACGAGTCCACACGAGAACCTAACAAGCAGGGTGGCCGATCCCAATACCGTGAACA
ATCAGTCGACTACGACTATGAAGAGCCTGCACCAGCCCCGCGCCCTCGTCCTACTCCACAGCCCGCCTACCGT
GCTCCCCAGCCCCAACAACAATACAGACCTGCCCCACAGCCCCAGCAACAATATAGACCTGCCCCACAACCCC
AACAGCAGTATAGACCCGCACCTCAGCCTCAGCAGCAATATAGGCCCGCCCCTCAACA
AGCCCCAGCTCCACCTAAGCCTGCGTTTTTCGCGGGTGCCTCACCAGCGCCAGTAGAAGACAATTTCTTCAAC
CCAGAACCAGCCCAGCCCCGTCAACAGCAGTATAGGCCGAAACAGGACTTCAGGCCTGCCCCACAGCCCCAGC
ACTTCAGCCCTAAACAGACCCAAGTCGATTTTAACCAGGACTATAATGCCCCAGCTCC
CCAAAGGTTTCCACAAGCGAATCAAAGGAGTCAATCATTCTCAATGCT
>1002052659_2 Heli.2-DT664557.3.5
GCACGAGGCAGTAATTTACGTCCCTTCCTATCGTGTGCTCCCATTGCCTTAACATGGGACCGGTCTGCCCACA
CTCAGGACATCACCAAGAACATTCAGTACATTACGAACGACAAGATCGATCTGAAGACAGATCCGAAGACCAT
GAAGCTAGACAAACAACAGAATCGTCCAACTTGCGTAATGATCAATTGGCTGCTCGCT
AGGCAGAAGCATGTCATGAAGTATGCCAATCTATATTTGGAACAGGGTTTCGATGTCATATCTGTATCGTGCA
CACCATGGCAACTGATGTGGCCGTTAAAGGGATCTCAGCTGGTAGCAGCTGATCTTATAAAGTTTATGGCGGC
GAATGAAAACGACCAGCCCACTGTAATACATGGCTTCTCTGTCGGTGGCTACTTGTGG
GGAGAAGTGTGCGCGCATGTCATGGATAACAAGGAAGTGTATCAACCAGTAATAGATCGTGTGACGGCACAAG
TATGGGATTCCGCCGCTGATATCACCGAGATAACGATAGGCGTGCCGGCCGCAGTGTTTCCCAAGAACAAGAT
TATGCAGAAAACACTTAAAGCTTATATGGAGTACCACTTAAAAACGTTCCACACGGCG
GCGACGGTGCACTACATCCGCTCGTCGCAGCTGTTCCACACCAACCTGTGCCGCGCGCCCGCGCTGTTCCTGC
TGTCGGCCAGCGACCCCGTCGGCGCCGAGCGCAGCAACCGCAGCGTCTATGACAGTTGGTGTAAAATGGGCGT
CAAGTGCACCTGGCAATGTTGG
>1002052660_2 Heli.2-EL604245.1.5
GAGGCAGAGCGCCGCGTCAAGGCGCTGGAGGCCGAGGCCCTGCAGCATGCCGAGGAATTGGCGGCAGTGGACA
GGGCGAGGAGGCACGCTGAGGCCGAAAGGGATGATAGGGATGACGAGCTGACCGCGACCGGTGCTAAGGTCAC
ATTGCTAGTGGATGAGAAGAAACGTCTAGAAGCGAGAATAGCGGCCCTCGAGGAGGAT
CTCGATGAAGAACAGTCGAACAACGAAATACTTAATGATAGACTTAGAAAGGCACAGAACCAAATCGACCAGC
TCACAATGGAGCTGGGAACAGAGAAGTCCGCCACTCAGAAACTGGAGAGCGGCAAGCTGGTGCTGGAGAGACA
GAACAAGGAGCTGAAGGCTAAGCTGGCTGAACTGGAGACGGCTGGACGGGCTAAGACT
AAGGGCGTCATAACCTCGCTAGAGCTCAAAGTCTCCAACCTGGAGGAACAATTGGAGGCCGAGTCCCGCGAGC
GTCTCGCCCAACAGAAAGCTTCTAGGAAACTCGACAAGAAAATGAAGGAACTGGCCCTCCAATTAGATGAGGA
ACGTCGTCACGCCGACCAGTACAAGGAGCAGATTGAAAAGATGAACGCTCGAGTGAAA
GCTCTGAAACGCGCAGTGGACGAGGCCGAGGAGGAGGTCCAGCGTGAGAAGGTCGGCAAGCGTAAAGCTCAAC
GTGAACTGGAGGATCTGCTGGAGACCCACGAGACTCTGGCCAGAGAGTGTTCCAACTTGCGTAACAAGCTTAG
GCGGCAAGGCGGTCCCATAGGTCTCTCCGGGGCGACGTCCCGCAGTAAACGATCGTCG
CTGGCGCCCGGCGGGGCGGCGTCCGGGGACGAGTCCTTCGACGACGGCACCGACGCCGCTAACTCGCTAGAGT
GACGTCACTAGACACGCAGCCGGCCCAGTGATGTGACGTGACGTGACGTGACGTTGTACTGTCAGATGGCAAT
TGATTGTCATGACAATCATAGATATATCATAGTCGATTGATTGTTAATCGATTGTGAT
AGTCAATTATTGGTCATTGTTAATTGATTCCCAAATGATTGTCAAGTGATAATCAATTGGTAGTCAAGTAATA
GCCAATTGGTTGTCAAATGTCCGACAGACGACGTGACGCCATTGTGATTACGATTATAGCGTTGGTCTTGTAA
TCTTTTTTAATATCTTTTTTCT
>1002052661_2 Heli.2-EL596069.1.5
ACGTGTTACATTCCACGCTAGGTTATTTTCTGGTTTGTCATTGGCTTTTGCTTGTCAAATGTGACAGCCCGCC
CTTAGCTTCGAGCTTCTACGTCAGTTCTATGCACAGATTATACAGTTGCCAAAAGCTACTTTTAAAATATTTT
TAATCTGTAAAAAATTATATAAATCGCTTTAGTCTAGTAAATATAGTAGGCCTATAAT

GTTACGTGTAAATTGTTTGATAGACTCTAATAAATTATGTACACAAGTATATTAGATATTGTAAAAAAAATAT
TAGTTTCAATTTCTCATTTCTATATAAAATAATACGTAATTCAAGTAAAGATTTAAAATGTTTTTGGTTTTCT
TGTAAATTGTCCATAGCAACGTGCGTAGAAGCGTTACTACTTTATTGTTATTGTATTT
ATTACTTAAAATAGTGATTTTTTAATGTAATTTATTTGTAAAGGGTGGTTGTACGGTCTGTAACGTTTAAAGG
GAACGATTTTAGCTTTGCAGTCAATATTGATTGAAAATAACTTTGTGACCAATATTGACTATTAAGTTGATAG
TTTTTAAGACCGCGGTTTTGCGGCCAATGTTGGCCCTGAATGGAACAAACTTTAGCTG
GCCAAAGAGTTCTTATATTGAATCAGTAACTGTTATACTTGGCCAACGTTGGCCAAGGAATGGCTGGAGGACC
AGTCAAAGTTATGTTTTGGAATATTGATTATTATGTTTTTATAATCACTAACACCGGGAATGGGTCATTGTGG
GTGTCAAGGTGTTTAGGTGTAATACTTTGAAACGCTTGTCAAACAAGTGCACAAAGTT
TAGACTTCAGCTAGTGAATAAGCGGTTTTACTGTTGCATATTGACAGTTTTAATTCTGCTTGTATGTATTTAT
TTAAATATTATAACATGCCTACAATAACATAGAGGCGTTTTGTAAATAGTTTGAACGAGAATAAAGCTTAAGT
AAAGGTAAACAATTCCTATACATGGCTCGTGACACGTATTTCTTCAAGTGCTTAACGG
CACTGTGTACAAAGTTAGCGTATA
>1002052662_2 Heli.2-EL596296.1.5
TTTTTAGTATTATTTTTCTTATATATATGTGATTCACGATCATCTTATATAGATGAATATAAAAGTTATAAAG
ATTACAAATACTATGTAGTGCGAGGCGATCAAAACGACTTGGCTGAATTGAAGAGGCAGATGCTGGAGAAAGA
TAATTCAGTCATTTACTTGGAGCAAGGGAAAGAGCACCAAGTCTTAATTGCTCCGGAC
TTAAATCCATTCTTTCTTGAAATATCTTCAAAGGAAAAATTAAACGCCACGCTTCTACACGAGGATATTTCTG
AAGTAATACGGTCGGAAAAACCTATAAATTGGCGTTACAAGAATCAAAATTTTACATGGGATGCTTACTACGA
CGTCGATTACATATATAAATACCTTTCAAGTGTGAAAAAATCCTATCCCGAAATAACT
GAGGAAATAATTGGTGGCGAGAGTTACGAGGGTCGCCAGATACGAGGTCTGCGTATTAACACTCCTGCAGAAG
AGGGCGTTGAAAAACCCGTCTTCTTTATCGAATCAGGTATACACGCCCGAGAATGGATCGCACCAGCTACTAC
AACGTACTTCATCAATCAATTGCTCACAAGCTCCGATCCAAATGTGACCAGACTAAGG
GATCAGTTCGATTGGCGCATCTTCCCCACTGTTAATCCTGATGGATATCATTACAGCTATGTATTTGATAGGT
ATTGGAGAAAA
>1002052663_2 Heli.2-DT664141.3.5
GCACGAGGCAGTCCCTTTCAGACTAACGCATAACTAAGTCGTATTTCCCGTTGCATTCGCGCCAAAGTCGGTA
CGAAATCTGCAGTGACATTTGAATTTAGAATTGTGTTTGTGATTGACATTTCTATCCAGCCAGTGGACGTTTT
GGAACTTTTTGGTTTCGGATTACGGCGGAGGCGCGCGAGTAGTTTGGGCCATGTCGGC
AGGCGACAGGGAGGCTGAGGCGCAAGCCAAGAAGGGTGACGATGGATCGTCCACCGAGGACAGCAGGGCCGCC
GCCGCACTCAAGCAGAACTGGTGCATCGGTTTGAGGGTTATTGAGTTGATCCTAGCGGTGATCGCTATTGGCC
TTATAGTGGGCGCTCTGACTGCTCCACAAGTCGTGCAATCGAATGATCGTCACATCGC
ACTTATTTACTCAGCCTATTCGAGTTTCATCATCATCACCGGTGTACTGATAGTCGCAAGATTATTTGGTGAA
TCAGCAGGATGGAGGACCTCTATCGGCTTCTCCATTATTGGCTTCATTATGTTCACTGCTGCAGCTGGTATCA
TTTTCTATGACTGGCACAGATCATACTACACGAACATTCGACCTAATAAGGAGGTTTA
CAACCTTCTTATTTCATCTGGCGTTTTTGCCGTTGTCGACGCCGCTGTGTTCCTTGTTCATGCTTTTCTTACT
TTTAGGAAGGAAGCTGACTACTAAACATGAAATTTAAATAAATATGTATGTATATAATAAAATTTGTCAATAA
TTTAATTATTTTACGAATACAGATTAAAAATATAACATAGTTATTCAGTTATTGTAAA
CATATATCATTTTTATTTACTCCAAAGTATTAAACG
>1002052664_2 Heli.2-EL602717.1.5
ATTTATTGTGTAGTTAATGTAAAGTTGCAAATATTAAAGTTTTTGTAGAAAAAATACTTTTTTAACTATGGGT
CAAAACCAATCTGGAGGAGGCAGTGGTGGCGACAAAAAAGATGATAAAGATAAGAAAAAGAAATATGAACCTC
CAATCCCTACTAGAGTTGGCAAGAAGAAGCGTAAAGCAAAGGGTCCTGATGCCGCTTT
AAAGTTACCTCAAGTGACACCTCATACGCGATGTAGGTTGAAGTTGCTCAAACTGGAAAGGATTAAGGATTAT
TTGCTTATGGAAGAAGAATTTATTCGTAACCAAGAGAGACTAAAACCTCAGGAAGAAAAAATTGAAGAAGAAA
GATCTAAGGTTGATGATCTTAGAGGGACACCAATGTCAGTGGGAACCCTGGAAGAGAT
TATTGATGATAACCATGCCATAGTTTCTACATCTGTAGGCAGTGAGCATTATGTCAGCATCTTATCCTTTGTT
GATAAGGATCAATTAGAACCTGGTTGCTCAGTGTTGTTGAACCACAAGGTACATGCAGTTGTAGGTGTACTAG
GTGATGATACAGATCCTATGGTTTCTGTAATGAAGTTGGAAAAAGCTCCACAAGAAAC
CTATGCAGATATTGGTGGTCTCGATACCCAGATACAGGAAATCAAGGAGTCAGTAGAGCTGCCACTTACTCAC
CCTGAGTACTACGAAGAGATGGGAATAAAACCCCCTAAAGGTGTTATCTTGTATGGGCCCCCTGGTACAGGCA
AGACTTTGCTGGCCAAAGCTGTCGCCAATCAGACATCAGCCACATTCCTCAGGGTTGT
>1002052666_2 Heli.2-EL602715.1.5

CACGAGGCGTAGCTGACGTATTTTATATTTATATGAAATAGAAATTGTACAGATTTTTAGTATAAGTGACCTA
TCTGTGTTTATTTTATTATATATTAGTAAATATTGTTAATGACTAAGAAATAATGTCCTTCGAGTACTTGCCC
GTGGCCGAAGATGAAAATGAAGAACCCATAGAACTTCCAATCGAAGAAGATGGGACTT
TAATGTTAACAACAGTGTCTGCGCAATTCCCTGGATGCTGTGGTCTTAAGTATAGACATCCTGAGACAAAAAC
GTTCAGAGGAATCAGATTAAGAGATGGCAGGTTGTATCCACCTCCTGAAGGTTGGGGCAATTATTTATTCATT
TGCAGTTTTCCTAAGGAAAATAAACGCAAGTCAGGCGAAAATTCTGAAACATCTTCCA
TCAAGAGCAAAAGAAATGACAATTTATGCTCTGATTTAATAGTTTTGGGCTTGCCATGGAAAGCTACTGAGCA
AACCGTTCGTGAGTACTTTGAGAAGTTTGGTGAAGTTTTAATGGCACAATTAAAACGCGATCCTAAAACTGGT
ATGTCAAAGGGGTTTGCGTTTATTCGATTTTCATCTTATACATCTCAAATGAGAGTAT
TAGCGCAAAGACATATGATTGATGGTCGTTGGTGTGATGTGCGGATACCTAATTCTAAAGAAGGTTCGGTCGC
TTCTATGCCTTGCAAAGTGTTTGTTGGCCGTTGTA
>1002052667_2 Heli.2-DT662893.3.5
CCGAGTCCGATGCGATCGCGAACGCGACGTTTTGTCTCCTAATCTTTTTGTTCTAACAATTCGTTTAAATGGA
AGTATTGAGGACACACCTCATTTCCCTTTGTTGCGATTCTCTTTAACATGCTGTGAATATTCTTTGTTAGTTT
ACAAAAGCAGTGTTAGTTCTACTTTCGTAAAATTTGAATAAAACAAACCCCCCTGTGA
ATATAAATTTGAAATTCTACATCTAGACGAAAATAATTAAAAATAACTTCGAAACATTTGCAGATTCTTTCAG
ATCCAGTGTACTGTTGTGATGAACAATGTATAAACAGAAGCTATTTTTGCTGATGTTCGCGTACAGTGCGCTG
GCTGCATTAGACGGCTACAGTGCCCAGCAAGATCCTGATCAGTTCCATATACAAACAG
ACGAAGACGACGACCGTTATTTCCTTTATCAGACACACAATGGGCAGTACCGTAAGGAGCGGCGCCTCAAAGA
TGGTTCTGTTGTCGGAACCACAGGATGGGTGGGCGCCGACGGATATCTAAGGCTGCAAGATTACATAGCAGAC
GGCCAAGGATATAGAATTTACAAATCAAAAACTGTTTACGTCGGTGAAAATCGGCCTA
TTGGTGAATCTTTAAAAATAGCAAAAACTGCTCCCACAGACTCAGGTTACAATATAACGCCTGCGCCGGCGCC
TCATCCGCCACGAGGCACACCGCGTTTCACTACCACAACGCAATCTCCATTGTCTCATTCTACACCAAAATTG
CACAGCCAATATCCCTTAGATGTATCTATCACACCACCACCGCAAACCTTCTCTTATC
>1002052668_2 Heli.2-DT666102.2.5
TTCGGCACGAGGGTTCAAGCAAACGTTTATTTACGGCTTAACACGTTTCGTGTCTTTACGACTTAAAAAATGC
GTTACGTGGCCGCGTATTTGTTAGCCGTGCTCGGAGGCAAAGCTGCCCCAGCCGCTGCCGACCTCGAGAAGAT
CCTTAGCTCCGTCGGTATTGAGGCTGATGGAGAGAAACTGAAGAAGGTGATCGGTGAA
CTCAATGGCAAATCTGTTGAGGAACTGATCGCGCAAGGTCGTGAAAAGCTATCGTCCATGCCGGCGGGTGGTG
CGGCCCCTGCTGCTGCTGCTGCAGCACCAGCGGCTGCCGCAGCTGAGGAGAAGAAAGAAGAGAAAGAGGCCAA
GAAGGAGGAGTCTGAGTCCGACGATGAGGACATGGGCTTCGGTCTTTTTGACTAAACT
TTTTAATACGGTGCAGAGCACGCGGTTTTTATTCCATGTATTGGTTTAAGCGTAAATTGACGGCAAGTTTGTT
TCTGTGAAATATATCTTGCTAAATAAAAAAAAAAA
>1002052669_2 Heli.2-DT665997.2.5
TAATAAGGCCATAGTAATCGATGGCCGCGGCCACCTCTTGGGCCGTCTGGCTGCAGTGATTGCAAAAGTACTT
CTTGAGGGAAATAAAGTTGTTGTTGTAAGATGTGAACAACTCAACATCTCAGGAAACTTCTTCAGGAACAAAC
TGAAGTTTATGTCATTCTTGCGCAAACGCTGCAATGTCAACCCAGCCCGTGGTCCATT
CCACTTCAGAGCACCATCAAAAGTTTTGTGGAAAACTGTCAGAGGCATGATCCCACACAAGACTGAAAGGGGA
AAGGATGCCCTAAGAAGGCTCCGTGCCTATGATGGTTGTCCACCTCCATATGACAACCGCCGTCGTGTTGTTG
TACCTGCAGCACTCCGTGTGTTTTGTCTAAAGCCTGGACGCAAGTACTGTCATGTTGG
CCGCCTTTCCCACGAGGTTGGATGGAAGTACCGTGATGTTGTCCGCAAACTTGAGGACAAGAGGAAGTTCAAG
ACTATCCATAAAGTTTCCTATGAAAAGAAACTCAAGAAAATCACCAAGGAAGCCGGTGAGAAAGTAGCGAAGG
CAACAGCTCCCTTCAACGCTGTCATGCAATCCTATGGATACAATTAGGACTAAGGAAA
TTATATAAAAAATAAACCTTTGGTTGCCCAAAAAAA
>1002052670_2 Heli.2-EL601889.1.5
AAGCTACGTCTCGTTCGTATCCTCCGTTCCACCGTGATGGTGCGAGTGGGCGGCGGGTGGGTCGCCTTGGACG
AGTTTCTGGTTAAAAACGATCCGTGTCGAGCCAAGGGTCGCACCAACATCGAACTGCGAGAGCAATTTATTCT
GGCCGATGGAGTGTCGCAGAGCATGGCTGCATTCAGACCGCGTACGCCTCGCTCTAAT
ACAAATACACCTCCATCCACCGGACCTATTACCAAGGTAAGAGAGCGTACCGTTCGCTCAGTGCCGATGTCCG
CGGGAGGAGCCGCAGGTCGAGCATCGAGATCATCACTAAGTGCTGGAACACCTGATTCGCTTAGTGACAATGA
AGCTGCAAGTGGTCTTGGTCAAAGATATAGGAAGCCAAGTGTACCAAGATCAACATTA
ACTCCTGGTGGATCACGACCAGGTTCTCGGCCTGGGTCAAGAGCAGGTTCGAAACCACCTTCTAGACATGGAT
CCAATTTGTCTTTGGATAGTACAGATGATGTTCTCACACCATCACGTATACCAATGCGTAAAGTGACGAACAC
TCGCACTTCAATAGCTCGGGCAGCCGCCAATGCCAGTAAGTTAGGAGTGACCACTCCC

AATGGAGGCTCCCGACCAAGAACA
>1002052671_2 Heli.2-DT664019.3.5
ATCACTTCCCGAAGATATTTTCTCAGAGAACACTGAATTGGCTACTCTTCACATTCAAAGAAACCCAATTGAT
TCTGTATATGGACTTCAAATATCCGATTTACTTACTTTAAATGCAGGTCAAACAAATATTAAATTTATCGGAC
CATCCATGTTTAATGGTATGACATACATTGCCAATCTTAACCTTAGCGGAAACAACAT
TGAAAAAATTCACAACCAAGCTTTCCACAAACTGGTGGAACTTAACTACTTGGATCTTTCCTATAACGACTTG
GATTTCATTTCGAGCATTCTTATCAAGGAAAATATAGAATTGGATATTTTCAAAATATCCAACAACCCTCGAT
TAACACATTTGCCTACTGAAGGCTTCAATTGCTCCGCAGAACAATTTAACATATACTT
GTTCGATGCATCAAACTGTGGCCTCGAAGAAATTTATGATGATTCATTAAGAACATTTACTGCTCTCTCACAA
ATTAATCTGTCCGGGAACAAGATAAAAACAATAAGCAACCGAGTTTTTTCGAGAAGTCCTAAACTTATCGAAA
TCAATTTGTCATATAACTTGTTGACAACTTTAGAGGCCAAAGTATTTGAACAAAACAA
AGACCTCGGGAAATTAAACCTCCAAGGAAATCCCCTGAAAGTACTTTCAGCTGAAGTCTTCATCCACACACCA
ATTCTCACCTGGTTGGACATGAGTCATGCTAAACTTACAGCTCTTTGGAAAGTAGAGAAAAATCAATCAAATA
CACTCCTTAACAACTTGAGCTTCCTAAATGTATCACATAACCACAT
>1002052672_2 Heli.2-EL597590.1.5
CTTTTTTATGATATTCCCGTTCCTTGCACTTCTTCTAGTTTAATTTAGTTCTAATTTATATTTATGCGTCTAA
ATGCGTTTAAATAAAATGGCGAATCAATCCCAGAAAATTGTGTATAAGCTGGTACTGACTGGAGGGCCTTGCG
GCGGAAAAACTACGGGACAATCCCGACTTAGCACATTCTTCGAAAACTTGGGATGGAA
GGTGTTCCGGGTGCCGGAAACGGCTACCGTCCTATTAAGTGGTGGCATCAAGTTTGCAGATCTCAGCCCAGAT
GAAGCGATAAAGTTCCAGGAGAACTTGCTGAAGACCATGATTCAAATAGAGAACACTTTCTTTGAACTGGGGC
GAACTTGCCAACGGAACTGCCTCATTATATGCGACCGAGGAGCCATGGACGCTAGTGC
ATTTATATCCAAGGAGAAATGGGAGGCGATGCTGATCGCAAACAACTGGAACAGCGTTGAGTTGCGCGACAAT
AGATACAATCACATCGTGCATATGGTGTCCGCTGCGAATGGAGCTGAAGACTTTTACTCTACTGAAGATCACG
CGTGTCGCTCTGAAGGCGTTGAAATGGCGCGCGAGCTGGACTACAACGCGGCCGCCGC
GTGGATAGGACACCCGTACTTCGATGTGATCGACAATTCCACAGATTTCGATAAAAAGATGAACCGCCTCATA
GCTTGTGTGTGTGTCAACGCGTCGGTCTCGACACCGGGGACCGGCTCAACGTCAACTCCAAGAAAAGGAAGTTCC
TCATCAAATCCCCCCTACTCCCCGATACAGAGTTTCCGCCGTTCCAAGATTTCGACGT
CGTTCACAATTACCTGCAAAGTGATTTCCGCAAAGCGCAGGTTAGGTTGCGTAAACGCGGACAGAAGGGCCAC
TGGTCGTATATCCACACCGTACGCAAGTTCCATCCGACCAATGGTCAATCGGTGGAAGTACGCACGCAACTAA
CGCATCGGGACTATCTGAATATGCTTCCGCAACGCGACGATGCCCATTTCACGATATT
TAAGAAACGCCGTTGCTTCATACACAACAATCAGTACTACCAGCTCGATATTTACAGGCAGCCCACGCATCCC
AGGTGTCGTGGCTTAGTTTTGCTTGAGACGTACAGTGCCGCGTACGATCAAAACGCATTATTAGCTTCCTTGC
CAAAGTTCCTAGCTATTGAGAAAGAGGTGACTGGTGATCCAGCGTATTCTATGTACAA
TTTATCTTTGAAAGAAGATTGGAAGACCTCCACTAA
>1002052673_2 Heli.2-DT663062.3.5
GCACGAGGCCACGACCTACATCCACACCGCATGTGCCCGCCATCACGGACGGCCACGACCGCCACGACCGCCA
CGACCGCTCCCCGCCGCAGCCGCATAATGAATCTCCTATTTCACCTCAGATGAATGGAAGTCCAGAAAACAAA
GCAATGACACCACAAAAACCAGTCAATTTGCTACCAGAAGTTCCCAGCCAGACTTCTA
GAAAGCTCTCTGATAGAGAACAACACGACTGCGATGTTATTGAACGGTTGATCAAATCATACTTCTACATAGT
GCGTAAATCCATTAAAGATTCAGTTCCGAAGGCAGTTATGCATTTCTTAGTGAACTATGTGAAAGATAATCTG
CAATCTGAGCTTGTGACACACTTGTACAAGTCGGACCAAGCGGAGAGTCTGTTGAATG
AGTCCGAACATATTGCTCAGAGGAGGAAGGAGGCTGCTGACATGCTTAAGGCATTACAACGCGCCGGTCAGAT
TATAAGCGAGATACGTGAGACGCACATGTGGTGATGGTCGTCTGGAGGCTCTAGTCTGTGGAATAGTGAAATC
GAGCTCTGAGCTCTGTAAATAATACTTATTTAGACGAATTATGTAGGCATTTTTACTT
ATTTTAAAATGTTTGTACTTTCAAGTCTGTGTACAGTACGGACGACGAGCCGTGGCAGTCCTGAAGTTAGCTA
TAGATGGCTGCGCATATTTCAATGTTAGTATTTTACTTACTTTGATTTGTTTTTTCTCGAGTGAACAATACGC
AAAATTTTCAGAAACCCTTACTTCAGGGCGGCCATGACTCGTCGACCGTACTGTAAGT
CTCATTTGGACGTCAGCGACAAAACTGTCCTTACTTTTCGTTAAGTAT
>1002052674_2 Heli.2-EL598288.1.5
AATTATATGAAGAAGATTATTTCTTTATTGTATCGCAGTAACACAAAGCAAAATTCCATCAAAATTGTGCGCG
TTCGTGCCCGATGTCTAGTGCTGAAGTCGCCGATAATTCCGTTGACCCCCCGGCAAAAAAGCGGAAGCTAAAT
ACAGGAGAGGCGAGTTGCAAATCCTCAGTAATGGCGAACAATGGAACGCGCGTGGAAG

ACGAAATCGACGAGAGCCTGTACTCCCGGCAGCTCTACGTACTGGGACACGATGCTATGCGCCGGATGGCCAG
CTCTGACGTCCTGATCTCCGGCCTCGGCGGCTTGGGAGTGGAAATTGCTAAAAATGTGATACTCGGCGGCGTT
AAGTCCGTAACCCTTCATGACGACCGGGCATGCACTATAGTTGATTTATCTTCGCAAT
TTTATTTATCTGAGGCTGCCATTGGTAAGAATAGAGCTCTGGCGTCATGCGAGCAGCTTGCGGAGCTCAATCG
TTATGTACCAACAACGGCGCATTCCGGAGTTTTGGATGAGGAATTTCTGAAAAAGTTCCGTGTCGTGGTGCTA
ACTGGAACGTCTTTGGCGGAACAGCAACGCGTAGCTGCCATCACGCATGCCAACAACA
TAGCCTTGGTCATTGCAGACACTAGGGGTCTTTTCTCGCAGGTATTTTGCGATTTTGGGCCGGAATTTACCGT
TTTGGACGTGACTGGCGAGAACCCAGTGTCTGCAATGGTCGCTGGTATCACTCATGAATACGAAGCGGTAGTA
ACTTGCTTGGATGATACCCGCCATGGTCTTGAAGATGGAGATTATGTTACTTTTAGTG
AGATACAAGGAATGTCCGAACTAAATGGCTGTGAACCACGTAAAATTAAAGTGCTAGGCCCATACACTTTTAG
TATTGGAGATACCACCAACTTTTCTAAATACATCAGAGGTGGCATTGTCACTCAAGTTAAAATGCCTAAGAAA
CTACATTTCAAACCCTTAAGCGAATCTATTAAAAGTCCAGAGTATCTTATCACCGATT
TCGGTAAGATGGACTACCCTCAACAGCTTCACGTAGCTTTCTCAGCTCTTCATAAGTTCCAAGATGCAGAGGG
TCGTCTGCCGAAGCCCTGGAGTGATGCAGATGTGGCTAAATTCAATGATTATGTGAAGAATATTGTGGATAGT
GAAGAATTGTTTAAGAATGGTGAAATTGAAATAAATAATGAGCTTATGGAGATTTTTT
GCAAGGTATCATCTGGAGATCTAAACCCCATGAACGCCGCCATTGGTGGTGTAGTTGCTCAAGAAGTGATGAA
AGCCTGTTCTGGAAAATTCCATCCCATAGTCCAGTGGCTGTACTTAGATGCTATTGAGTGCCTTCCCAAAGAC
AGATCTGGCCTCAACGAGGACAACTGTAAGCCCACGGGCTCCAGATACGATGGACAGA
TTGCTGTGTTCGGCAAGGAGTTCCAGAAGAAACTAGGTCAATTGAAGTATTTTATTGTTGGTGCTGGCGCCAT
TGGCTGTGAACTTCTAAAGAACTTCGCGATGATAGGCGTGGGGGCTGATGGGGGACGGGTAACTGTCACCGAC
ATGGATCTGATAGAGAAGTCCAATCTGAATAGGCAATTTCTGTTCCGACCCCATGACG
TACAGAAACCCAAGTCCAGCACAGCTGCTAGGGTAATCAAACAAATGAACCCCCTAATGAATGTAACAGCACA
AGAGAACAGAGTATGCCCTGAAACAGAATCAGTGTACGATGATGCATTCTTCGAGCAGCTGGATGGAGTTGCC
AACGCCCTGGACAATGTTGATGCTAGGATCTACATGGACAGACGGTGTGTGTACTATA
GGAAGCCCCTGTTGGAAAGTGGTACGCTCGGCACTAAGGGGAATACTCAGGTGGTAGTACCATTCCTAACTGA
GTCCTACAGCTCTTCACAAGACCCACCAGAGAAGAGCATACCAATATGCACCCTGAAGAACTTCCCCAACGCC
ATCGAGCACACCCTTCAGTGGGCCCGAGATGAGTTCGAGGGTCTGTTTCGTCAGGCTG
CAGAGCACGCTGCCCAGTATCTCGGAGACCCTCACTTCTTGGAGAGGACCATGAAGCTGCCGGGCAGTCAGCC
CCTGGATGCTTTGGAAAGTGTCAGGAACGCAATCAACGAACGGCCATTAAACATCGACGACTGTGTGACATGG
GCGCGTCTACACTGGGAGGCACAGTATTCGAATCAGATCAAACAACTCCTCTACAATT
TCCCCCCAGACCAGACCACCACCAGTGGGGCCCCGTTCTGGTCTGGACCCAAGAGGTGCCCGTCGCCCTTGGA
CTTTGACCCTGAGGATGAATTGCATATGGACTATGTGGTTGCCGCGGCTAATCTGAGGGCTCAAGTGTATGGT
CTACCGCCGTGTGTGGATAGGGAGAGGATGGCGAAGGTGGCTTGCGAGATTCAGGTAC
CGAAATTCAAACCGAAGTCCGGCGTGAAGATAGCAGTAACAGATGCCCAGCTGCAGCAGAACAACGATGACAT
GGACCAGGACA
>1002052675_2 Heli.2-DT668780.3.5
GCACGAGGGCTAGCCTTTATGCGCGCGCACATAATGTCTAACACCCTCTAGCGCGATCCACCAACTTCTTGTA
CCGACCCTATATCGCTGGCGCGCTGATTGTGCTGATTGCTGTAAAAAGTATGGCAGCCTCGTCGTGAGTGACC
TTCGACCGTTAAGTGCCGATGGCAGAATGACTGAGGAAACTGAGCAGTGCGACTCTTT
CAACTCCTGGGAGCTCAACGGCCTGAACTCCTTGGACCTCTGGGATTATACTGTCGAGCTGGAATGTCTGCAA
GGAACTGAAGATTTGCAGCTCGCGGCTGAACTCGGCAAAACACTCTTGGAGCGAAATAAGGAGCTAGAAACTG
CGTTGCGCCAACACCAAAATGTTATTGAAGACCAGGCACAAGAGATCGAGTACTTAAC
AAAGCAAACGGTAGCATTGCGCGAAGTAAACGACTCAAGACTGAGAATCTACGAGCAGCTGGAAGTGAGCATA
CAAGACTTAGAGCGCGCGAACCACAGGCTCGCGGTCGACCACGCCGCCGACAAGAAACACATTAAGACTTTAT
GCAGCACCATCGAAAGCTTGGAAAACAAGTGTGAGGAGTTCCAAAAGACTGTGGACGA
TCTGAACGCGCAATTAGAAATCGTCAGACGGCGAGCGGAGAGAAAACCTGAAAGCGAAAAACCCAAAGAAAAA
GAACTAAAACCTACAGATACTGCGATACCAAACAACAAAAATGTTAACATCACACCACAAAAATCAGTGGCGC
CTCTTCCGGAACTGACAAAAGAGGACGAGGACTTGCTGAGGTTAAGCGATGAGTTGAG
AGAGAACAAGGT
>1002052676_2 Heli.2-DT662325.3.5
AACACACTGCATCAGTTCTTCAGGGATATTGCTGATGAGGAGTCTTGGATTAAGGAGAAAAAACTCCTCGTCG
CCTCAGACGACTACGGCCGTGACCTCACCGGCGTACAGAATCTCCTTAAAAAACACAAGCGTTTAGAAGCGGA
ACTGGCCAGCCACGAGCCCGCCGTACAAGCGGTGCAAGAAGCGGGCGAACAACTCAAG

GACGTCAGCAATGTAGGCGTAGCGGAAATAGAGCAGCGCTTGAAAGCGCTCGCTCAAGCGTGGGAAGCGCTCG
AAGCACTAGCGGCCGAGCGCGGCACTAAGCTGCAGCAATCATTAGCGTATCAACAATTTTTGGCTAAGCTGGA
TGAGGAGGAAGCTTGGATTAGTGAAAAACAACAACTAGTTGTCGTAAGCGAATGCGGT
GATAGCATGGCGGCTGTACAAGGTCTTCTCAAAAAGCACGAAGCGCTAGAGGCGGAATTGGCGGCGAGAGGAG
AACGTGTGAGAGATCTCTCCGCTGAAGGAGAACAGCTACTTGCAGCTGGAAATCTGCATTCTGAGGCTTTGTC
TCATAGATTGGAACAGTTGAAGGCTAAATTAGAAAAGCTAACAGCACTAGCCGCCCGT
CGCAAAGCTGCTCTCGTAGACAACTCTGCCTACCTCCAACTCCTGTGGAAGGCCGATGTAGTAGAATCCTGGA
TCGCTGATAAGGAGACTCACGTGCGGTCGGATGAGTTTGGACGTGATCTGTCCACTGTCCAGACGTTGCTGAC
TAAACAGGATACTTTTGATGCCGGTCTAGCCGCATTCGAACATGAAGGCATACAAAAC
ATTACCGCCCTCAAGGAGCAGCTAGTGGCAGCTGGCCACGAACAGAGCGCGGCCATATCCCGTCGTCACGGTG
ACGTCATCGCCCGCTGGCAACGCCTCTTGGCCGACTCGGCGGCGCGCAAGCAACGGCTACTGCAGCTTCAAGA
CCAGTTCAGGCAGATTGAGGAGCTATATCTTACGTTTGCTAAAAAGGCATCCGCATTC
AACTCTTGGTTCGAAAACGCTGAAGAAGATCTTACGGACCCTGTCCGTTGTAACTCTATTGAGGAAATTAGAG
CTCTTCGCGATGCGCATGCGCAATTCCAGGCATCACTATCATCTGCTCAAAGCGACTTTGAGGCTCTAGCAGC
ATTAGATGCTCAGATCAAGTCGTTCAACGTCGGCGCTAACCCCTACACCTGGTTCACC
ATGGAGGCCTTGGAGGAGACATGGAGGAATCTGCGCAAGATTATAGCGGAACGCGATGTTGAGCTAACCAAGG
AGGCTCAGCGTCAAGAGGAGAACGACAAGCTGCGTAAGGAGTTTGCTAAGCACGCCAACGCCTTCCACCAATG
GCTTACAGAGACACGTACGTCTATGATGGAGGGCACGGGCTCATTAGAGGCGCAATTG
GCTACGCTGCGTCAACGCGCTACCGAAGTGCGCGCTCGTCGAGCTGACTTGCGTCGTCTGGAAGAGCTTGGCG
CTGCCCTCGAGGAGCACCTGATCTTGGACAACCGCTACACGGAGCACAGCACGGTGGGCCTCGCACAGCAGTG
GGACCAACTCGATCAGCTGTCTATGCGCATGCAG
>1002052677_2 Heli.2-DT667621.3.5
GCACGAGGCCAATATTTTTCATATATTGTTTTGTTAAATTAGGCGGGAATTGAATTTGATCAATTTAGAAGTA
AAAATGAGCGTCGAAGAGGATGTAATGAAAATACAAAAGAAACTAACAAAGATGACATCAGACGATGGCACGG
GTCAAGAAGAAGCATTGGAACTTTTGAAGGTTCTACAAACTATGGCGATAAATTTGGA
CGTCTTGACTAAAACCAGAATAGGTATGACTGTTAATGCACTACGAAAATCTAGTAAGGATGAAGAAGTAATT
TCTCTTTGCAAAACTCTTATTAAAAACTGGAAAAAGTTTCTGTCTACACCAGCAACACCATCTAAAGATTCAG
GCAATTCATCTAAACCTAAAAAGGATTCAAGTAAAGATAAAGAAAAAAAAGATGACAA
AGAAAAAGATAAAAAATTGCCTGCATCATTCCCACCACAGTCAAACACTACCGATGCTGTCAGACTTAAATGT
CGAGAATTATTAACTCAGGCATTGAAGATTGATGGTGAAAATCCAAATGCTTGTGCTTCTCCTGAAGAACTTG
CCGAAGAATTAGAGGAATGCATTTATGGAGAGTTCAAAAATACAGATATGAGATATAA
AAATAGGGTTCGGTCCAGAGTGGCCAATTTAAAAGATATCAAAAATCCTACTTTAAGGACTAATTTCCTTAAT
GGAGTGATTACTGCATCACGTCTTGCCAAAATGACCCCAGAAGAAATGGCAAGTGACGAAATGAAAAAATTGC
GGGAGAAATTTATTAAAGAAGCTATTGATGATGCTCAACTTGCAACTGTTCAGGGTAC
TAAAACGGAGATGCTTAAATGTGGGAAATGCAAGAAGAAGAATTGTACATACAATCAACTTCAAACAAGAAGC
TCTGATGAACCTATGACTACTTTCGTTCTCTGCAATGAATGTGGAAA
>1002052678_2 Heli.2-DT668844.3.5
GCACGAGGCAGTTCCTTAAAACTTGATTAATTTGATTAACATTTTATTTTTCAATTCCTATTACATATTTTGG
TTCGAGCTTGACCAGAAATGGCTTTGCTTCCACCTGACCCGGTTTATACAATTCGAAATGTCGACAATTCGCC
TGTGTATTCCTTAGCGTTCAGCTTTCTACCGGGAGGACTCGAAAGGTTACTAGCCGGC
TCGAAAAATGGATATGTTTATGCATACAACCTTCAGACAAACCGGGTCCAGCAAAAAATCAAAGTTGGTCAAG
CTCCCATTCTTCATCTCATCCATACAAGCAGCCAAATGATAACCCAAGAAAAGGGTGGTAAGTTCAAGATTTT
CAACTTAACTAACAGCGGCTATCAAGAAGAACACACAATAGACATAGATTATCCAGGA
TTTTGCCGTTTCGATGCCAACACAAAACTCGAAACACTTTATGTACCTGACTCTGAATCCAAAATATACATAT
ATAACTTTTCTGGAGAAAAATTGGACTGCTTGAAGCCAGAAAACCCAAAACTTGGAGACCCAATGTGTATAAA
ACATATTGAACTGTCTAATGAACAGCCTTGTCTTCTAGTAGGCTACGAAGCGGGGTGG
CTTCTATTGTGGGATCTCAACACTAGTCAATGTATCAGTAAATTGCAAACAAAAGAATGTCCAATGTCAGTAG
ATTTTCATGTGGAACAACAGCGGGGCATGGTGGGCAACGCCTCGGATGTGATACAAATA
>1002052679_2 Heli.2-EL601199.1.5
GGACGTCTCGGCGCAGACAAAATAAATGGAACGGAACCAGTAGTGGTTGTGTTGTGTGTGTCTGTGTTTATTT
TTGTTGTGTGTTTACTCTTGAGACAAGAAATAAAAAATAGTGAACTATAAAAATGCAGGGCCGTGATAAATTG
TGTGCTAAACTGACGAGTGCATTCCTAAGGAAGTGGTGGTTCGTCCTCGCGTCAGCAG

TGGTCCTACTATTGTTAGGCATCATATGCGCTATATTCTTCGGAACGTGGATTCGAATGTTTATAGATCATGA
ACTTGTTCTCCGCCCAGGCTCGATGACATTCGAGTGGTGGGCGCGACCTCCAGTCCGGCCGTTCGTCCGAGTC
TACGTATACAACGTGACGAACGCTGACGAGTTCCTAAATAATGGGTCCAAGCCTATCC
TGGATGAGTTGGGGCCTTATGTGTATTCCGAAGAATGGGAGAAGGTGAATATAACAGACAACGACAACGGCAC
ACTCTCGTTCCACTACAAGAGGACGTACACGTTCGTGCCAGAACTTAGCGCCGGGCCCGACGACGACTCAGTG
GTCGTGCCTAATATACCTATGCTGAGTGCCACATCTCAATCCAAACACGCCGCTCGTT
TCCTCCGTCTGGCTATGGCGTCAATCATGGACATCCTCAAGATAAAGCCTTTCGTTGAAG
>1002052680_2 Heli.2-DT667354.3.5
ACTAGTCGCATGTCATTCGTATAAAAGTGTTGTTGTTAATAATACTTTTTAAGACTTATCCGCGATGTGCCTT
CGGAAGTGACACAGTTCTAGTGTAAAGTGAATTTATAAGCAATCAAAATGCTCGCAGACGGTGAAATAGTTGG
TGACGGATCATGGAACCTCACGATCTACGTGACGGACTTGAACGAGAAGCGTACCATG
GTCGTGAAGGGAGATATGCACATCGGTGGAGTGATGCTGAAGTTGACGGAAAGCTTCGGTAAAGACTTCAAAA
AGGACTGGTCAGATCATGCACTATGGTGGCCGACAAGAAACAAATGGCTATCTCGTCCAAAACACACCCTGGA
CCAATACGGCGTCCACGCAGATGCTGCCCTACATTTTACACCGATGCACAAACCTATC
AGAATTCAATTGCCTGACCTGAGATACATTGATTGTAAAATTGACTTCTCGATTGATACATTCAGTGCCGTAG
TACAACTCTGCAAAAGTCTCGGAATACGGCATCCAGAAGAACTGTCTTTGTGCTATCCTCTCGAACCTTCCCA
CCTAAAACAGAACTACCAAAATTTGAAAGAAGCGAAGAAGGTGAAATCTATTCAGGCG
CCCGATACGAATACGTTTATAGCGGCAACAAGAGGCTCGTCGAATAGCCTGGACAGGTCCTTAGCGTGTCCTG
CGACGCCGCCTCCACCGCGATCGTTGTCAGCCACGCCAGTTGCTTCACAACAGAACGGGACTCTCCGCCGCTA
TGGGGGTCACATCTACAGTACACAGAGCGACGGCTCAAGTGACGGGGGCTACTGCGGA
ACCCCGCCGCGAGCAGCTTCCATGGACGCGTTAGACTCTCTCGCGGAATTTTTTTGGCAGACTCACCTCTCG
AACCGGACAGCCAGTCTCGGGAG
>1002052681_2 Heli.2-EL600176.1.5
TGTGTTGTAATTTAATTTTACTTCTTCCATTTACTCAAAAATTCTGTTTAAAATTGCAAGAAAGAAAACTAAG
CTGATAATCAGCATGGGTCTCCTTGCAACAGCACTTGGCGTTCTTTTTATTGTTGCCACGGCTTGGTCTGCGG
ACGCTCCTAAAGGACCTAAGGTCACTCACAAGGTCAAATTTGATATAAGCATTGGCAA
TAACCCGGCAGGGACAGTAGTTATTGGTCTCTTTGGTAAAACTGTACCAAAAACAGTAGAAAACTTCTACCAG
CTTGCTCAAAAACCAGAAGGTGAAGGTTACAAGGGTAGCAAGTTCCACAGAGTTATTGAGAACTTTATGATTC
AAGGTGGAGATTTCACTAAGGGAGATGGAACTGGTGGTCGCAGCATCTATGGTGACAG
ATTTGAAGATGAGAACTTCAAACTAAGCCACTACGGTGCGGGTTGGTTATCTATGGCTAATGCTGGAAAGGAC
ACAAATGGATCTCAATTCTTCATTACTACCACTAAAACACCATGGCTTGATGGCAGACATGTTGTTTTTGGAA
AAGTACTTGAGGGCATGGATGTAATTCGTACTATTGAGAAGTCGCCCACTGGAGCCAA
TGACCGTCCAACAAAGGATGTTGTCATAACCAATACGCATGTTGAAGTTGTAGCTGAACCCTTCAGTGTTACA
AAAGACAGTGCTCAATAAACTTTAGTTTAAAATGTCAATATAACAATAATAATTTTGTTTAAAATGCATTTAT
TATTAAGTTCACTACTAATATTCATTAAGTGATCAAAGTATAAATTTCACCTTTATTA
GTTACCCTTATCCCCAGTTTATGCCAATAATCTTTTTAAATGTCACAGGGTACATAAAAATAGATATGTTTAT
AAAATCATTTTATAATAAGCTATAATTTGCAGTGA
>1002052682_2 Heli.2-DT665077.3.5
GGTGTGGTGTATTTTTGTTGATACTGTTGATACCAAATTTAGAGTTAAAATATTATTGTATGTAATTAATTAA
TTATTTTTTATTATTAACATTCCACTTATTGTTTATCGTTTCTCGATGGATTATTACTTTTATTGTATATGAC
GTTTATTTAATTGATTTTGAATGTAATATAAGATTTATTTACAAGTAAGTTGGCGTAT
GAACAGTTCGTACATTGTTTCTACGGGGCCGATGCGAACCACTGGGTATTTGTTATCGATGTAGTGTAATGAC
AGAAATTAAAACACTCTAGGATATATCTCATTCGAATAAGCTAAAAACCAAAGGTGCCCTTCATAGAAAGATT
ACGTTTTGTTGATAATATTTCAAGTTGAATGATTTTAAATGAAGTTGTATGTAATAAT
CGAATCGATTTTGTTTTAATCTCGATAATATTAGTATATTTAAATGAATTTTCACGACTATCTCAAATTATAT
TTACCTAATGCTTTTTTAGTAATTTTTATAATGTTCAATCACTCGTTAAAGCGCTGGCACACTAGCGTCGGTC
TTACGATCCTTTCTATCTGTATATGGATATTTGCAATAATTGTATAAATAAAATATAG
GGGCCGCCCTCGGTTCCGCAATACAGGAGTGCAATACGCCACTGGCTCCGGCTCGCGCTCGCACCGTGTACCG
CGCACGCGCTGGCTCCCACGGGTCTCTTGTGCCTTGCGGAACTCTTTTCCTAGAACCGCGTATATTCTTTATC
ACATCACTTTTTACGTTTCCTT
>1002052683_2 Heli.2-DT664847.3.5
AAACATAAACTAGCCACAATGAGTTGCCCATTTAACTTTAACGAAGAACAATTACTGCAGCTCAAGGCGTTTG
TAGACTTATGCAAGTCCCAACCTCAAATTCTTCAACATCCAAAGCTATCTTTCTTCAAAGAATATTTAATATC
ACTCGGGGTAACAATACCTTCAGCTACATTCGAGAATTTTGCACCCTCCGGAGACAGC

GACAGCAACTTCAGTAGTGCTTCTGCTGCTGCTCCTCCTCCTCCTGCTGAAGCCTCATCAGAAGAAGATTCCG
AACCTGAATCAGATGTTGAACTGGATATGGAAGGTGTCATATCTGACTCGTCAGAGGCAAATCAAGATATGGG
CGATGAATCCAAGGAAGTATCTGACGAAGAACGTGATCAGTCGGACGAGAAACGATCT
GAGGCTATGAGGGCGTTCTCGGAACAACAATTTGACGAAGCGATTGTATTGTACACTGATGCTATAAAACTGA
ACCCCCAAAGTGCTTTGCTTTTTGCTAAAAGAGGACAGGTATACCTCAAACAAAATAAGCTACATGCGTGCAT
TAAAGACTGCAGCCGCGCCTTGGAGTTGAATTGTGACAGTGCAGCTGCGTATAAATTT
AGAGGACGCGCTTATAGACTTTTGGGCAAATTTGAAGAGGCGTCCCATGATCTCTGCGAATCTCTCAAGATAG
ATTACGATGATCAAACAAATGAATGGCTCAGTGAGGTGAAACCTAATGCTGAAAAACTT
>1002052684_2 Heli.2-DT668444.3.5
GCACGAGGCAAAATCATATATCTAAGTCAGGTGGCCGTGATTATATTTACTTTTTGGTTATGTGAAATAGTGA
AGTGCAATCCACATCGTAGATTAAAAGAAATTCAGAAGAGTGCCCACTTCGCTTTGCAACCTATACCATTGAA
ACCTTTTATGGTTTTATTAAAGAAAATAACACCGTTTGGAGAATATGGAATTAAGCAT
TCTATCAAAAAAAGGCAGATTGTCCAACCTAACGAAATACCCAATATTGAAGGTGGTACACTTGAGGATATTA
TAAAAATACAAAATATTGGAGTAGCAGACAAAGCAGACGATATTCCACTTGAACCAAATTTAACCCCACAAGT
AATAATAACATCTATCATTAAGCCTATAGAAGATAGTGTGCCTATTTCTATACCTGTA
CCTTTGCCAGAGGGTTCAGATTTGCCAACAGACCAGAAAAATATAGAGGATTTGAAGCCTCCGGACGAACTTA
TACATCAAGAATCAGAACTAAAAATCATAGAAGTAGCATCATCATCAGAAAATGTTATTGAAATAACTCCAAG
GCCAAAGATAGAAGAAATACCGCCAGAGTACCTCGAAATTAAAAATGTAGCCATAAAC
ACTCCAGTGGAAAGTACAGTACCATCAATATCGAAAATTCCTCTTATAAAAAATCCAAATGGGCCCTTTTTAT
TCACTGATATTACTAGACTACCTCTAAATGGTTTTTATCCTGCTCCATCTATTGGTGTTCCTGAATATATTGT
ACCTTCACCACCATTACCACTACCACCGCCTGTAGTTCACCACCAT
>1002052685_2 Heli.2-EL603248.1.5
GCACGAGGGCCGGTGCGGCTGCGGCGCGGAGTCGACTTATACCGCTCAAATAATAATTCGAAGTGCGTGCTGC
CTATAGTGAAAAATTTCAATATAAAGTTTATCGTCAAAACAGTATTAAATAAATTCCACACGCAATTTTTATA
TATTGAACTGTATTTTACCGTTGTGAATGATATTTTACTTATATGTTAGATTATTGTG
TAGATAATGATACTTCATCGATAAGAAATCTGTTCAGGTCCCAGTCATCTTATTCCACGATGTGCTGCGAAAC
TGTTATCATCCGAGTGTTCTGAAGTGGATTATTTATTTAGACAATTTTTTAATATAAATAATAAAATAGTCTA
AAATGGTAAAATTGTTAAGTAAAAATGTATCAGTCTCAGATTTATGGGTATCAACTAA
TGGTAAAATAAGTGATTTTTATTTGACATCATGGCAACGAGGAGATTCGCCGTTGCCCATGCTGCTGGTGAGG
TTGTTCCTGAGTTGTATAGCTATCAGCATTTTCACATGGTCTTTGTGGAGCGGTGCCAGCCCTTACTGGCTGA
TATACCTTACGAACTGGGGCCTATTACTTGTCACATTATTAACACTCAGCGGTTTCCT
AGTGTCCTGTTGCGCTGTTTGCAAAAAACTTCCTGACGGAAGTGAGTTGCCTTGGTACGTGAGTATGTATTGG
CTGTTTTACAACATGACCATCACAGTGGCCATCATGATTACATGTCT
>1002052686_2 Heli.2-DT664638.3.5
TAACTCTACTCAACATGATGTAATAAAACGTGAAACTATACCAGAAATTCCTCAATTTGACTTGAATTCTTCA
TTGATATCCCAAGAAAAAAAACTATCAAAAACTAAAATAAAAGAAAAAGGAGTTGAAGCTCTCGATAGCACTC
TTGAAACTAAATATCCATATTACTCAAAAAAAAGTAAAATTATTAACAAAAATTCACC
ACTTAGATATGCGCAAAATTTCGCATTCATCCCTAAAAAATCCAATGGTGGCACTGAATTTTATGATTCACGT
TCTAAATATGTATGTCCAGAGGTAGAAGATGTTGTAGATCCCGTACCGGAGAAATTAAAAAAAGATGGTCATC
CAAATGATAATGAAAATGCAAATAATGATTCAGGAGATAACAGCGATAAGGAAAAATT
CGAAGAAATAGAAAACAAGCAACGATTAAAGGGACTCGGTGATAAAATTGACTGTTTCAAGGCAAAATATTTT
GATTCTAATCCATTAGACAATCCATTTTTTAAAGAAGAAGTAATTGAAGATCCTGAGCCTGTAACAAAGCCAA
ATTTATTTATTTTTGAGACCGGTAAAGCAAGAAGTTACGTTCATCCTAATCAACTAAA
TAAGAATGATGATATTTTTTCGATACCCGCAAATTC
>1002052687_2 Heli.2-DT662316.3.5
TCAATTGAAAAAGCTGATGATGATGCAGCTATCAAAGTCCTCATTGAACTTACTGAATCAGCACCTAAATTCT
TACGTCCACAAGTGGAAACCATATTCCAAGTTTGTATGAAACTAATTGGCGACACTGATCAAGAAGATAACTG
GCGTCAGCTAGCTCTGGAAGCTCTTATAACATTATGTGAGACAGCACCGGCAATGGTA
CGAAAGGTTGTACCAAATGCAATTCGTGTCCTCACTCCACTTATATTGGAGATGATGTGTGAATTAGACGAAG
AACCTGATTGGTCTGTGCAAGATAACGCTGCTGATGATGACAATGAACTGAATTACGTCGCTGCGGAATCAGC
TTTAGACAGAATGTGCTGTGGGCTTGGTGGCAAGATTATGTTGGGGCTTATTGTAGGC
CAAGTACCTGAAATGTTGAGCTCAGAAGACTGGAAACGACGCCATGCTGCATTGATGGCAGTCTCCTCTGCTG
GTGAGGGATGTCACAAACTGATGGAACAAATGCTTGACCAAGTTGTTTCAGCAGTTCTTAACTACTTAACTGA
TCCACATCCACGAGTCCGTTACGCCGCATGTAATGCAGTAGGTCAGATGTCAACTGAC

TTTGCACCAATATTCGAAAAGAAATTCCATGACAAAGTTGTACCTGGGTTATTGATGGTACTTGAAGATAATG
CGAATCCTCGAGTGCAGGCTCATGCAGCAGCTGCTCTGGTAAACTTTAGTGAAGACTGTCCAAAGCCTATCTT
AACTCAGTATTTAGGCCCATTGATGAACAAGCTTGAAGTTATTCTTACTGCTAAATTC
AAAGAGTTAGTGGAAAGTGGCACAAAGCTTGTATTGGAACAAATTGTCACCACAATTGCCTCTGTCGCTGATA
CAGTGGAGAAA
>1002052688_2 Heli.2-DT662470.3.5
TAATTATTTAGCAAATTTCATCCTTCGTGATTGTCTCAGTTGGAGAAATTTTATTTAAAAACGAGGTAAACAT
ATTTCATCAAAATGGTGTTGGAAAGTACAATGATTTGTGTAGATAACAGTGACTACATGAGAAATGGAGACTT
TCTGCCAACGAGACTGCAGGCACAGCAAGATGCTGTTAATTTGGTTTGTCATTCCAAA
ACAAGATCCAACCCGGAGAATAATGTTGGGTTATTGACTTTAGCCAATGTGGAGGTACTGGCTACATTAACCA
GTGATGTCGGTAGAATATTATCAAAACTTCATCGTGTCCAGCCCAATGGAGATATCAATATCTTGACTGGTAT
AAGAATTGCACATTTAGCACTTAAACATCGACAGGGCAAAAATCATAAGATGCGAATA
GTTGTATTCGTGGGGTCGCCAATTAATACAGACGAGAAAGAATTGGTCAAGCTAGCGAAGAGACTGAAGAAAG
AAAAGGTCAATTGTGACGTCGTTTCGTTTGGCGAGGACTCTGAGAACAACCCTCTTTTAACATCCTTTGTTAA
CACTCTTAATGGTAAAGACAATACAACTGGCGGAAGCCACCTTGTATCCGTGCCAGCT
GGTGGATGTGTGGTGCTCTCTGAAGCTTTAATAACCAGTCCTATTATTGGAGGAGACGGTGCTGGCCCATCAG
GATCTGGTTTGTCACCATTCGAATTTGGTGTTGATCCGAATGAAGATCCCGAACTTGCTCTTGCTCTTAGAGT
ATCTATGGAAGAACAAAGGCAGCGTCAAGAAGAGGAGTCTCGTCGTCAACAAGCTTCC
ACTGAGGGTGAACAAGGAAAAGCAGAAGAAGCTCAAATACTGGCATGGAAGAGCTCTAGCTATGTCTCTTGG
>1002052689_2 Heli.2-EL597566.1.5
GCACGAGGCCTCGTGCCGGTGTCGGTCTCCTTTCTATCCACATAAGTTTTTGTGTCCTACAGATATGGCTAGC
CAGACACAGGGGATCCAGCAACTTTTGGCTGCTGAAAAGCGGGCTGCCGAAAAGGTCTCAGAGGCGAGAAAGC
GAAAAGCGAAACGCCTAAAGCAAGCCAAGGAGGAGGCTCAAGATGAGGTTGAGAAGTA
CAGACAGGAGCGCGAGAGGCAATTCAAAGATTTTGAGGCCAAGCACATGGGCACAAGGGAAGGTGTCGCCGCT
AAGATTGACGCGGAGACAAAGGTGAAGATCGAAGAGATGAATAAAATGGTCAAGGCACAGCAGGAGTTGGTGA
TCACGGACATCCTCAACTTAGTGTACGACATCAAGCCCGAGCTGCACGTCAACTACCG
CTTAGCATAAGCGCATCTTCGTTACGCATAAATAAATTATAAATTTTTTCGCTATTAAATCTTTGCGAAATAA
TTATAGTTATTATTCTTGTTTATCTGTAGCCATTAGGGACGCGTGCAAATGTTGCCAATTTTCAAATGCACTA
TTTATCTTTTAAATATCTATGTAACTCGAAATAAATGTTTTTAACATCTAAATTATGA
TTACCATTCTATTAATAATATCATCTTTAATATGTTTTTGTCTTTATTAAATAGTTATTGTCGATTTATTGTG
CATTGAAAATGAGTGACAGTGACCAGCTAAAAAGGGGGAAAAGCGAAATGGTTGCCATACGCTGTATATACAC
GATTTGCTAGTATTGCCATGTTAAATAATCGTTAAATACCAATATTTAATTCAATAAA
ATCAGCTCGATA
>1002052690_2 Heli.2-DT663687.3.5
GCACGAGGATTCGTATAAAGCTGTATGCGTGTAATGATCGTTCTATTATAAATGACCCAGATGAAGTATATAG
TAGGATCTTGAACGCAAGTAATCGACTATTGGGTATCGCTTAGCTTGTCTTCTACACTCGTCTTTGCTCACAA
AATGTACCAAAGAGTTAGTAGTGTATGGGCAAATGTTTGATAGTTTTATAGTAACGCG
TAACTTAAAAGTGTTTTGGTAATAAAATGTAAACTTCACATAAGCATATCTTTTACTATAATACATATCGTAT
ATTGTTACATATGTAGATCATTACAGTATTGTACGATTCATTAGTTTGTTGGAATTAAACTTCAAACATTTGC
CTGTATTTAGCGACGTTTTAAAATAAAAAGAGTATATAAATTTATAATCTGTTGCGTA
TTTACTCTATGGCATTATTATAGGTGTTGCCAGATAATAAAAATTTTAATAAAACGGCAGCTCTCACTATAGA
CACTGCCACTCCATGCGTCGTATAGTATTATTTTGAAAAGAAAAACAAAGGCTGTTATCGAGGCCGTCGTGGC
ATAAGACGGCAAACGCGACAGTTGTGGGTTCAATTTCCTTTCGTGTGAATGAATTGTT
TTATATTTTCATTTCCTTGTTCTGGTAAGGAGGCAAAGTAGGCGTTGAGTTCCGCCAATCAACACACAATGTG
TCGAAATTTCGGCGGTGCTTGGGAAATGGAATATCTTAACACTAGGCTACCTCTGTCTGCCATGCTGTATGCG
GGATAAAGCACGAATTTTAAAGAAATGTGCCAAAAATGGACCATTCGGATCAAATTGC
TGGAATGGTAATGTCTATAACGTA
>1002052691_2 Heli.2-DT666034.2.5
CGGCACGAGGCAGAGGCGCGTACCTCGCGCTCGTTTCGCTTGCAGCGTCCGTACTCGCCGCACCTGCTCCTCA
TCCACGCGACCGTCTCGGCTTCGAAAGAGAAATAGACACTAGATACAACCAAGTGCCGAAACTCCTCGACTAT
GTTCTCAGTGAGGAATACCTCAGTTCGCAGAAGAACAGGGCGAAGCTCGTCACTAAGC
TGCGGCCGGAGGAAGCTGTGGTCGCTGACAACGTGATTTTAGAATCTAACGTTGTAAAAATTGTGAAGCCGCT
TAGGAAAATAGAAAATGGTGAACTGGTGAGACACCGAAGAGGCTACAACCTTGAAAACGTGATAGTGCCCGCT
AGGTACCCCTACCTTCCCGTGGTGCGTTATAACCGTTTCCGCCGCTGGGGCTAGTGGC

CGTAGTGGGCCCCGATGCGACGGTAAATGTCAAGGTTGCATTCAAGCTAATTTGCAGCGAGAGTCATGTGCGA
ACAGCATCTGTGAGGCGTCTTTATATAGTCTGGACTCTATGTTTACGTTGTTTATATAAGTGTTTGTATAAGT
GGCTGTGACCAAACTCTCAATTTATTGCATTATTTTGGAGATATGCATTAATTTGATT
GACACAGAGTATATGAGACTATTGTTAGTTTTAAATAGTGAAATTTTAAGTGAATTAAGATGTTTACCTATTA
AAAACATATAAATATTGTTAGTGTTTCTATATATTTTGTTCTCTACTATTATTAAATATTTATTAATTATCCT
ATCCTAAAAC
>1002052692_2 Heli.2-DT667394.3.5
TACGATTTTTTAATACCACAATAACTTAAACGATGCATCTCGACCCACCGCACCCCCAGTGACTATAGTGTAA
TACAACATTATCACTTCATAGTGGTATTTGTGATGTTTTGTGCCAAAATCTTCAGTGCCATATATATATTGGG
GAACACTTAAATTTATATCAAAAAAACTGATAACAAGCTGGGTAAGCGATGAGGGGGT
GGTTTGACGCTTTCCGCGAGGAAGGCGGGCCAACCCTATATGCGTACCACAACAGGACCGCAGTCGCTGCTGA
TGTACCGGCACTCGCGCTACTAGTCGCTGCCCTCACATTATACCTCGCGTTCTTAGCCATCTTCCCAGGCATT
AGGAAGGAGAGATTCTCCACTTTTACAATCGTCACGCTTAGTTTATTCGTAGGAACTG
TGATTTTAGTATGTAAGCATGGGTCATCGTGGCATGTCGCGGGGGCACGTGTGGCCCGCGCCGCGTACCGTGC
GTTTTCCACGGACCGCCTGGACTGTTGGCTTGCGGTCCACGTAGGCTTGGGCCACGTTAATGTTACCCTCTCT
GCGTTATCGTGGGGCAACATATCAAGAGGTGATCCTGGCGTGGACTACAATGAACAAT
TCAGGTGGGAAGAAGCGGGGGCGATCCAGGAGTGGTACCGTGCTGGCCTCTTACGAGGACTACCTTATCCCGT
CCTGTCTGTCGCTGAACATTTTGCAGCAGAGCATGAAGGATTCGAATGGGGTGCGAAGTACCGAGCCGCTGGA
TATACCACAACGACATTGCTTTGGACGGCGCTTGCTTTGTGGCTGTTGATGAATCTTC
TCTTAGTGGTTGTGCCAAGATATGGAGCATACGCGATGGCTTCCCTAGGAGTTACATTATGTGCGGCAGCGGG
AGGGTACTGGGCCTCACTGCCTCATGTACCTCTAATAGTTAGGATAGATGGAGCTATGTTATTTTTTTCATTG
GGTTGGTGTTTTTGGCTAGTGTTAATTGCAGGTGGTATTTGCTTAGTCGTTGGGTTAT
TGATAGCTGCGCTAGACCTAGTATGGCCGCATAAGTTCTCGACAGTATTAGAGGTGGACTATGATACACCATA
TGATAGACACGTGCTTATAGTGGACAGTCGGCAGCGAGCCAGACCTCAGACACAAAGTT
>1002052693_2 Heli.2-EL598533.1.5
ACGCGCCAATTTTGTGAAAATTTTAAATTATAATCTGTGCTTACAAATTAATCAATAATAAATAAAATACGAT
TTTATTAATATAAGATGTTTATAAATAATTATTATTTTCTGTTAAACAAAACGCAGTGATATATTTTCGAACG
ATACTTTTAATAAAAGAAAATCAAAATGGAATATAAATCAAATAAAAGGACTAAATGG
TGTATGTATAGAAAATTAACGACTTTTAAAACAGTTTATGCGGGTTTGATATTGCTTTATGTGCCAGTTTTAT
GTGACAAAGATACATCCCGACCACTGACTTACGACTATATCTACAACAGTACCAACTACGCTCAACTGCAGAA
TGTGAAGCAAATTGTTAATTCATATATTCTTAATAATAATAATCACACCGAAACCAAA
GGAACAGAAGAGTTCAAATTTAGCGATAAGTATGAAATAAGCAAACCTGACGATCTGTTGGTGGGATTCGCGC
ATGACCCATTCAGACAGTCCATACCGGTAGCTGATTATATGAAAGGTGTTAGAACTATGGATCCAAGCACAAT
GAACAAGGATCGTGGAAAACGTGGTGTTTTTCATCTATACAACATGCTGACGTGCGCG
ACCGGGTGCGACCCCGTCAGCTATAAGGGCTACGGCTGCTACTGCGGCTTCCTGGGCTCCGGGAGACCCACTG
ATGGGATTGACAACTGTTGCCGTTTACACGACGAATGCTACGAAAACATATACTGTCCTGGCAACACTGTTTA
CTTTCAACCATACTACTGGAAGTGCTTTAACGGAGAACCATATTGTGCCCTGGAGAAC
TATCAAAGAAGAAACAGAGTGTCCAATAGCTGTTCAGGGAGGTTGTGTGAGTGCGACAGGCGCTTCGCGATGT
GTGTCAGGAGGTACCGCTGCCCCAGGGGCAGGAGCTTTTGCACATCCGACCCTTTGAGGCTTATACAGAATAT
ATTGATGTTTAAGTAAATCGATTTTTTTTACATTTTTGGGCACATCGAATTGGTTTCG
CAATCGAATATAATTTGATTTGTATGTGATAGTAGTTTATTTTATTATTATTTTTTATGTTGATTTTTAATTT
TTTTGATTACATTTGTTTACGTATTACTGGAATGCTTTAACTGTGAAACACAATACACTTCAGAAAACTATCA
TAGTAGGAACAGAATGTTGAATTCCTGTTTAGGTAGAAGTGATAAAGGCTTTGCGGTG
TGCGTCAGAATGTCTCATTACCCTAGACCACTGGTTCCCAACCAGTGGTCCGCAGAAGCCTAAATATGGTCC
>1002052695_2 Heli.2-DT662056.3.5
CCGCCAGTGAGGAGCAAAAGGAAAGAAAGACCTCAAAACAAGAAAAAACAAGAGAAAAGGATCACATCATCTG
ATTACACTGCTTGGGAGAAGTTTGATGTGGACAAAGCATGTGAAGAGGTTGATATGGCAGACCTGGGCCCAGT
GTCCCTTGATAGCAAGAAGAGTCAACCTGCGAAACTAGAGAAACTAAGAGAAGAAGCA
CAGTATGAGAAAGAGAGGGGTAATAATTTCGTGAAGCAAGAGAAATGGGATGAAGCAATATCGTGTTACAATC
GTGCAATAGAATTGGTTAAGGATGACGCCATTTATTACGCTAACAGAGGACTTTGTTACTTGAAGAAGGATAG
TTTACACCAAGCGGAAACGGACTGTACCCAAGCGTTAAACTTAGACCCAACCTACGTA
AAAGCGTTACAACGTAGAGCCACGGCCAGAGAGAGACTTGGGTCACTAAGATCCGCTTCACACGATTTAAACG
AAGTACTGAAGCTGGAACCTCATAATGCGGCCGCTAGGAAGCAATTGGAGGCGATTAAAACTAGAATGGGTAC
TAAAGGGTCAAAATCAAAATCATCACCGGCAACCACACCGACAACCGAATCCAAACCG

ATAGTAAAACCAAAAACACAACCAAAAATAGTCGAAATAGAGGAAACTAAGAGAGAAATG
>1002052696_2 Heli.2-EL601435.1.5
GCACGAGGGTTAACCTTCGCTAAAATGATTAAATTAGTAGTTTTGTGTTGTTTCCTTGCCGCTACTTCGGCTA
CACCTGGTGCTATCATAGCCCCTTTGTCATATTCCTCGAATGTGCTAGCACCAGCACCAGCAGTCGTGTCTAG
CTACCACAGCGGCTTGGCATACTCATCTCCTATCTTCTCTTCCGCGCCATTGGCCTAC
TCCGGACCATGGGACTACCAACATCTCATTAAGAAACGTTCTTTGGCCGTAAGCAGCTACATCGCGCCGTCGT
CCTACTTCGCACCTGCTGCCTACACTTACCCCGCCACTGCTCCTTTAGTCAGCTCTTACTCTGCAATTGCGCC
TCTGGCTAGCTACCCAAGTCCTGTTTACACCGGTGCTGCACATCTGATCAAGAAGAGG
AGCGCTGTACTTTTACCTAACACATACGTGGCACCTGCCGGATACGCTGCCCCCGCACCTTTCGTAGCGTCAA
CTTACGCGGCTGCTTCTCCCATTGCACCCATTTGGTCTAACTCATTGTATCCTGCAGCTCAGTATGCTCATTT
CATCAAAAAACGATCTGCCCCTTTGGCGGTAGCTACATACACAGCCCCAAGTTCTTTC
TCTCATACATCAAGATATGATTTCGAGGCTGTATCTCCTGCTCTTAGCTACACTTCCTTCACAGGCCCTTCAC
CATTAGTATATAATACTCCAGTTCTTTCTCGCCTGGTTTAAGCCAGCGTATCTATGTAACTGCAACGGCCAAT
AATGATCGTA
>1002052698_2 Heli.2-DT662130.3.5
GCGCGGGCTACGCGGGCGGCGCGCTGTACGGCGGCGCCTACGCCTACGACGAGCAGCTCATGAGGAGTGGACT
GCCGCACCATATGGGTGGCTACTACGAAGTAGGTTATAGCGCGCGGGAAGGCACGTTCGGTCTGGGAGCGGGA
GAGAGGTTCGGCAGGACCGACGCTGCATCTCCGCAACAGGTGCCAGCAGCGCTACCCC
CCGGCTATGCATACTTCTACCAGCCACCACCCACCACATACCAATATGGCGTTTATCCTACAGCTTACGGCGG
TGGATCAAGTGTAGGCGGCGTAGGCGGTGTAGGCGGAGTGGGAGGCGTAGGTGGCGTAGGTGTGAGTGGAGTC
ACGGGCGGAGGCAAAGTGTCCGCCTACTCGCAGCAACAGCCACCTTACGATGCACAAG
ATTCCTATAAGAGCGCGGGCCCATACACGGCGAACGCTAACAAATCAGCCGGCGGTGCTTCCGCTGATCTCTC
AAACGCAATGTACGCTAAGACACATGTGGCGCTTAATAAAGTTAATAGCTACGAGAAGGCGGGCTTCCACAGC
GGCACGCCGCCCCCCTTCGGCGCGGGCTCGCACCTCTACATACCGGCGCCGCCGCACC
ACCACCCGCACCACCACGCGCCGCAGCACCAGGAAAGCGCCTCTTCGGGCAGATCGGGTGCAGCTAAGCCGGC
CGCCAGCAAGCCCACTTACTCGCAGTCGTACTGGGCGCCTAATTAACTTGCAGGGACCGAACAGACAATATGG
AGACGTGTAAATACAACGCCCGACCTCTACTCAA
>1002052699_2 Heli.2-DT667981.3.5
TTCCGCCATTTTGATGGAAGTGCTTTGTGAATTCATTTTGTGGTTATATTAATGACACTACGGACAATATCAA
ATTATAATCTAGTGTGGATTAAGTTATAATTTTCAGCCTTGATTGATTTACACATATTAATATGGAGCCGGCT
CTGGATGCGCCATTGGTGGCGCAATCCATCAACTACCATGGGCAACAATTACAAAAGG
CGTGGGAGGCAGAACGAGGAGAAGACGATTTGGGCAAAATTGGTGTAGGACCACTGGACTTTGCAGTTTATCA
GTCCAGACATAAACATCTTACATTCCAAGACCGAGGAAAGCGGTTAAATTTACATCAGTTTATTGCAAAAGAA
GCAAATGCTTTATTTGACGCTTCACTACTAGAAGAAACTCCATCATCTTCCAGTTTAG
GGGCTGATGCAACTGCACCTGAAGATAATTTGTTTGCATTAATGCCGCCATTTGAGACTTTTTTGAATGTGGA
TAAAACTGCAAGATTGCGACATTTTTTTGATAATGTGAAGACTGGTGAACTTATCATTGGGGCAGTGATCAAT
AGAACAGCCTCAGGAATGATGTTGAAGGTTCTATGCACAGCAGCTCCAACTCCAAGAT
ATGTTGCTGATATTAATGTTAAGGCATTTTTGCCAGTTGCAAATATCATACCAGCAGTGGACAAGAAAAATGT
GTCAAGAACCTACCTGATGAATGACACAGTTTGCTGTGAAGTTATAG
>1002052700_2 Heli.2-DT663061.3.5
GGGANAGGAAAAAAAANGCCAAATCCCGAGGCTGCGGAATCGGCCGAGGCGCCAGTGACGAGTCTTCTCGCAG
TTGACGTGGCTACTACGTACCGTCCCGTTCGCCCACTCCGGACGCAGCATATACGGTGACGAGGAATACGTAA
CTGGTGAACGAAGTGGGCATGGACGGAATGGTCGAGGAGAACGGCACATCTGGCGCTG
CGGGCGTCCCAGATCCCTTGGCGGGTGCAGGCTCGTCGGCCAGTACCACGCCTCACGTCGTTGTAACCAGCAT
TGTGCAACTGACGTTGCCTACACAAGCACCGTCAGCACAGGTCCAGTCAGTCATCCAGCCAAATCAACAGTCT
GTTATTCAAACAGCATCTAATATACAGTCAGTGCAACTTCCTAAAGGAAATGTTATAT
TGGTTAGCAAACCAAGCTCAGTTATACACACTACACAAGGAACATTACAAACATTACAGATTAAACCTGAACC
AAATAGTGTAGTCAGTACACAAGGACAATCTTGCAGTGATGATAGCTGTAGTGATGATGACAGTCCTAAAAGA
AAATATAGAGAAATGTTAACACGGCGTCCATCATATAGAAAAATTCTGAATGACCTTG
GAGGAGCAGAAATTGCTGTGATTCCAGCTGGAGCCCTGCAAACTGATAGTGGCTTACATACATTGGCAGTGTC
AGGTACCACAGGTGGCGGTGCTATAGTGCAATATGCGACAAGTCAAGATGCACAATTTTATGTTCCGGGTGAG
TATTGTTTATATGACTTTTGTACTATTATTAAACTATGGCAATGATAAAATATTTGTA
GAGGGAACGAACAAATCGTGCTATTATTACAGGACCTATTCTAGAGGATCAAACACGTAAAAGGGAACTAAGA
TTATTAAAAAATCGAGAAGCAGCAAGAGAATGTCG

>1002052701_2 Heli.2-EL604338.1.5
GACACTATCATACCTTATCGAAACGTTTTTATGTGGACTGTAAAGTTAAGACTGTTTCGAAAGTAATATTTAA
GATGGAATTATTAAGGCAAGTGTCGAATACCCAGTTTCTATTTCCTGTGGCAGCTGTAGCGGTTGTTTTAGTA
TGCGCTGCGCTCGTATTTATATTCGGATTTCACACAGCAGAACAACCGCAATTCGATA
AATTACCACTGGTTGCCGATGACAGGAAATCATCCAATAAGAAGAGAAAAAATAAGGAGAAGAAGTCTTCTCC
TAACCGCATTTCTTCCGATGACGCCAAAGCAAAATCAGAGAGCTCTAAGAAATCTCCTTCAAAGGAGAAAAAA
GAGGAGAAAGTAAAGGAAGTTGAAAAGCCCAAGCCTAAAGAGAGAGTTGAACCCAAAA
TTGTAAAGAAAGAAGCCCCAGTTGAGGCTAAAAAGGGTAAAAAGAACAAGGGTGTTGCAGAGGCGGAGAAACC
TGTTGACTATGATGAAGGAGTGTGGGAGGAAGTGCCTAAGAAAAGTGACAAGAAGAAGGTTGTGAAACCAGAA
GAAAAGGAAAAGAAAGAGAGCCCTGTTAAAAAGAATAAGAAGAAGGTGAAAGAAGCAG
ATGTTGAAGCTGCTCGCCCGGCCGAGGGCTCAAGTGACAAGATTAAAGTATTGAGTGCTGAGGGGCCAGGAGT
GAGTGAAGATGCAGCTAGAGCTCTACAAGCTCAAGTAGAGGAACTGCAGCGAGTTCTTAAAGAGGCAGAACAA
AGAGATCAGGGTATATCTGAAGACCTCAATGAAAACGAAATACCTGAAGTAAAGGATC
TCAGAAGCAACAAGAAGAAGGAAAATAAAGAAAAACAGAGCAAGAAGAAAGCAGTTGAGGCTCCAATACTAGC
CAACAACATCAAAGAAGACAAGGACTCTGAATCTGAGAAAAAGGAAGATAAACCAGCAGGCCCTGTATTCGAT
GAGCTTGGAGATACTTGGACAGATGCTAAAGTATCTAAAAAGAGCAAAAAGAAAGCAC
GCAAGGATCAGTGAGTGTGACATAAGGCCCGGGTCTTTGCTACCCTCTAGCTCAATGCACTTCACATCACTCA
CAAACTACAATCAATGGGGATAAAGCGAAAAGTGTCACATGTCAATAGTGGCCATCCTTAAATTACGTCACAC
GATTTTCATTATTTTATGACCCCTTTTCACAGCTGATCACATTTGGGGGTCGGTGAAG
TGTGACATACATTCATATAAGAGTAACTCGTATTTATATATACATCAATTGGTCCTGTAGTCAAATTAAAACA
ACTATTCCGAAATTATATTTGAA
>1002052702_2 Heli.2-EL600997.1.5
CGCGGAAGAGGCGCGCCGCCGCGAGCCCGGGTCGCCGGGCGGCTCGCTGGCGTCGCTGTCGTCGGGCGGCTCG
CTGGCGTCGTACAGCGACGGGTCGCGCTCTCCGCGCTCGCCGCACTCTCCGCTGACGCCGCAGTCGCCGCAGT
GCCCGCACTCTCCGCCCTCCTTCGCCTTCCGCCGCGCGCCCTCGCCCGACCCCGAGGA
GGAGCGCCTGCCCGTGTTCAACCGTCTCTCTTCCGCGTTCGGCGACATCGTGATCGCCTAGCTCCAGCGCAGT
GCCCCCTCGACGTCTTCCAGACCGCGCCCTCACCCGCTCAACGAATGATCTCCAGTCAACCTATGCTCTTATT
ACCGCCATGTAATGTTTTAAAGTTAAAGTGTTCTGGCGTCTCGTAAGTTTGCGGTGTG
AATATAAGTAGATATACTTACATGTTTTAGTTATCGGCACGCACTTGTAAATGGCACCCTATGTTCATAGATA
TTTATTGCGTAGCTCGCGTAGCGGACGCCTCCCCGACGCGAGCCTCCGCCGAGCGGTGGTCTTGTAAAGTTAG
CTCATAGTGACGTTTTATTTATTCGTATTGCCTCTCCGCGATCGGTACCGCACCGCGA
GACGGCGCTCGTGATTTATTTTTAAGGATATAAGATAATAATTTATTATTTATTTCGAGTCTCTTTAACTTTA
ATTATTATTATTTTGTAACTTTCGCGATGCCACACCGCACACGGACGAGGTACGCGTCGACGGGACGTGGCTC
GTTGTATCAACCTCCGCTCGGCCGGTCCCTTGATTGACTATATTCTGACAACTTATTG
TGGACGGAGCCGGCCGGGTCGGACCGGCAGTCGAGAGAGAGATGGGCTTAAACACCTCGAAAACATTATTTAT
ACTCTTAATTT
>1002052703_2 Heli.2-EL597152.1.5
GCACGAGGCTCGTCTCAGCGTCAGCTCCACAATTCACAATGAAGGTTTTCGTTTTAGCTGTCCTTATGTCGTC
AAGCGTCTATGCGGGTCTGTTACCTGCGCCACTTGGCTATGCCCGCCCCTACGGCTTGGCTGCTCCACTAGCT
GCTCCCCTGGCAGCACCTCTTGCAATCGCGAGGCCTGCTTTAGCTCCATACGCGGTGG
CTCGGCCAGCTCTAGCGCCCTACGCTGTAGCCAAAGTAGCAGCCCCTGTCGAAGACTACGATCCCAACCCACA
ATATTCGTATGCTTACGACATTCAAGATGCCTTAACTGGTGACTCGAAAAACCAGCAAGAAAGTCGATCTGGG
GATGTAGTCCAAGGTTCTTATTCGTTAATAGATCCAGATGGTACACGCCGTATTGTAG
AATACACAGCTGATCCTCAAAATGGTTTCAACGCAATCGTGCGCAAGGAACCTCTGGGTGGCGTTGTGAAGGC
AGTTGCCCCTATTGCTGCTATTCATTAAAATTAAGCTAATGTAAGCTGATTCAAACACCTCAAGGAAACTTAT
AAAGGAAAGTTACTATAAGGCTCTAAAAACGAAAAATATAATTTATTTTTCGAATTAT
GCCATTGGTTGATATCGTTTCTAATGAGGACTTGAATCTGCTCCAAAGCTTTTATTCACAGTGACCTGCATCT
TTGTTTTTTTATAAATTGTTGTTAAAGTATGTAAG
>1002052704_2 Heli.2-EL600258.1.5
AGTTAAAATATATTTTAAGAAAACTTTGTATATTTTGCATTTAGTGACATTTTCTCTATTGTGAATTAAAATG
GCAGAAACAGAACCTAACCAGCAAGCTGGAGATACCGGCCCACCGAAAGGCATTCCCGCATTAAAAGCTCATG
TAATAGCAAATAAAATAGATGTAGCATTGTGGGGTATTCGTGTACTCACAGTCCTTTG

TACATTTGGATATGTGTTTCCTCTATTTAATAACCCCGTCTCAGCGTTCTACAAAGCGCTCCTAGCGAACGCC
GCGACGTCCGCGCTAAGACTCCACCAGCGTATACCGGCAAGAGAGATCTCGTTATCAAGAGAGTTTCTAAACA
GATTTTTCCTAGAAGACAGTGCACATTATCTGTTCTATTCGTTGATATTTATGAACGT
GGCGCCCAATTTATTGATATTGACACCCATCTTCCTATTCGCACTTCTCCACGCGGCATCATACTCCCTAACA
ATACTAGACACGTTAGGACAGAACTCATTGTGGGTGGCGCGACTCTTGATCTCGCTGGTGGAGTTCCAGTCCC
GCAACATCCTCCGCGCGGCCGCGCTCGCTGAGATCGTGCTGTTCCCGCTCGTCGTGAT
TATGGCCCTTATTGGGTACTGCGGGCTGATGACGCCGTTCGTGTACTA
>1002052705_2 Heli.2-DT665832.1.5
TAAATATAATTTAAATAAAAATTATTGAAAATGATAAATAATTTATTTTCAATTTTTGATCCATCAACTAACT
TATTTAATTTTCCATTTAATTGAATTAGAACATTTATTGGTTTAATATTCATCCCTTTATCTTTTTGATTTTT
CCCTAATCGTCATTTCCTATTATGAAATTTTATCGCAAATAAACTTCACAATGAATTT
AAAACTTTATTAGGACCTAATAGAATAAATGGATCAACTTTTATTTTTATTTCATTATTTTTTTTTATTTTAT
TTAATAATTTTTTAGGATTATTCCCATATATTTTTACTAGAACTAGTCACTTAAATATATCTCTTTCCTTATC
ATTAACATTATGATTAAGATTTATAATTTATGGTTGATTAAATAATACCCAACACATA
TTTATTCATATAATCCCCCAAGGAACACCAACAATTTTAATGCCATTTATAGTATTAATTGAAACTATTAGTA
ATATTATTCGACCTGGAACTTTAGCAGTACGATTAACCGCTAATATAATTGCAGGTCATTTACTTCTAACCTT
ACTAAGTAACACAGGTATTAATATACCTAACTATTTAGTGATTATT
>1002052706_2 Heli.2-EL599548.1.5
GCACGAGGGTTTTTCCTTTTGCCGCACACGTGCTGAGATTTGTAATATTTTTTACTCAAAAAATTTAGTTTAG
CACGCGCTGTAAAGTTTCTTGAAACTGAGTCTAAATTAATTATATATTCAACAAGATTCTTCAAGGAACATCA
TATTAAAATAGCAGACTACCTAACTTACATGGCAATGGTGTCTGAGGCGAAGGTTAAT
GGCAGTGGAGGTGAAGCACCGAACGAGCTTCTACCAGCACTAGCAGAGAAGCCAGTAGGCCTTCCACCAGGAA
AGTATGTCTTAGTTGGGTGGGACATGGATACAACTGGAAGAAGGCTAATTGATGAAATATGTCAAGTAGCTGC
CTTCACACCAAAGCAAACATATTCCCAATACATTATGCCATATGGGGATTTAAATCCT
GGAGCCCGTAGGCGTCACAATGTACGAGTGGTTACCGTTGGAAGATTTCGTATGTTAAAGGATACTAATACTA
ATAAGATATTAAAAACAAAATCTGAGATATCTGCACTTTCTGACTTCTTAGATTGGTTAGAGAAGGAAAAAGG
AGATGGCAGTGTCATACTCATATACCATGAGCCACGTCGCTTAAGCCCCACTATGCTG
TTAGAAGCTCTAACTCGCTACAAGTTGTTGGACCGTTTTAAATCAATAGTAGCTGGTTTCACAGACAGCTATG
CCCTAGCGGCAGACAAATGTAAATCTACAGTAAAATCTGTTTCTCTACGAGTATTGGCCAGAGTTTTACTCGA
TGCCGACACACTGTCTGTGGAC
>1002052707_2 Heli.2-DT661824.3.5
GGAGTCCTCATGCAGATTTTCGTGAAAACCCTAACGGGTAAGACCATCACCCTTGAGGTCGAGCCCTCAGACA
CTATCGAGAATGTAAAGGCCAAGATCCAAGACAAAGAGGGAATTCCCCCAGACCAGCAGAGATTGATCTTTGC
CGGTAAACAATTGGAAGATGGCCGAACCCTCTCCGACTATAACATTCAAAAGGAATCC
ACACTACACTTAGTGCTCCGACTCAGAGGTGGTGCTAAGAAACGCAAGAAGAAGAATTACTCTACACCCAAGA
AGATTAAGCATAAGAAGAAGAAGGTCAAGCTAGCTGTACTTAGGTTTTACAAGGTGGACGAGAATGGCAAGAT
CCATCGTCTCCGCAGGGAATGTACTGGAGAACAGTGCGGTGCTGGAGTGTTCATGGCC
GTCATGGAGGACAGGCACTACTGCGGCAAGTGCCACTCCACCATGGTGTTCAAGGATGATGACAAATAGATTG
GGGTCACTAAGTGTTAGTGTCTAATGTTTAAGTCCCAGACTGCTGTGTATGATGGATTTTTAAATAAATCTTG
ACACAGTTGT
>1002052708_2 Heli.2-EL601180.1.5
GCACGAGGGCTATTTAGTGGTGAAATATCGACTGGTTCTTCATTAGAATCATTAACTTGGTTAGGCTTAGATA
ATAATAATATTCATTTTATATCGACTGATGATATACAAAATTTTCCTTCATTAGAATATTTAAATTTGGATTT
TAATAAATTAATCGAATTTCCTAGTAAAGTTAGTAGAACAAGTGGAACGTATAAAATT
AAGGAATTAAGATTATCATATAATTATGTAAGTAGAATAGATTCTGAGTTTTTAGAAAAAATTACGGAATTAC
AAAGCATAGACTTATCTAACAATAGAATGCACAATATCAGTGAACGAAGTTTCTGTAATTTACAGAATCTGGC
TTATTTAAATTTAGCTGGCAACGTTATAGAGTTAATAGCCGAAGAAGCTTTTGTTGAT
CTTCCTAAATTAGAAATATTGGATTTACAGGAAAATAGCTTAATTGAGTTTTCAACAAGATATTTTTGCAACA
TATCAAAGGAAGAAACGAATTTTGCAGTCAATGTTAGTTATAATAAAATTTCGTCCCTTATGGGTAGTTTAAC
AGTTTCAATCAATATCTTAGACTTGTCACATAATATGTTGGAAATAATTCCCTCAGAT
TTTTTCTTTTCTTTAGGTAAACACATTCGACAAATATTTTTATCCTACAATAGAATAACTATTATAGACAATA
CAGCATTTTGGTTATTGGCCTCA
>1002052709_2 Heli.2-EL602762.1.5

GCACGAGGTGGAGACCGGCACGGAATAAAGTATTATATAAAATATTTTATATTTTAACTTTGTAATACATTAA
TCAAATTACCATCATGGGTCGTAAATTCGTTGTTGGAGGTAATTGGAAGATGAATGGAGACAAAATCCAAATA
AATGAAATTGTTAACAACTTAAAGAAGGGTCCCCTCGATCCTAATGCTGAGATCGTAA
TTGGAGTACCCGCTATCTATCTTGCTCAAGTGAAAAGTATTGTTCCTAGCAATGTTGGAGTTGCTGCACAAAA
CTGCTGGAAGGTTCCTAAAGGAGCATTCACAGGTGAAATTTCTCCTGCAATGATTAAAGATGTTGGAGCTGAC
TGGGTCATCCTTGGTCACTCTGAAAGGAGAACCATCTTTGGTGAAAAGGATGATCTGG
TTGCTGAAAAGGTGGCCCATGCCCTTGAATGTGGTTTAAAAGTGATTGCCTGCATAGGTGAAACTTTAGAAGA
GAGGGAGGCGGGTAAAACTGAGGAAGTGGTGTTCAGACAAACTAAGGCTCTATTGCCTGCCATTGGCAACAAC
TGGGCAAATGTTGTGTTGGCTTACGAACCTGTATGGGCTATTGGCACTGGCAAAACTG
CTTCACCACAACAGGCTCAAGATGTCCACGCTGCTTTACGTAACTGGCTATCCAGCAATGCTTCTCCTGACGT
AGCTGATTCTGTTCGCATCCAATATGGTGGCTCAGTGACAGCGGCAAATGCAAAGGAGC
>1002052710_2 Heli.2-EL596856.1.5
GCACGAGGCGTGAATCTTTAAGGGGGTGTTGTAGGGCTGGCCCCGTATCGTCTTGTGATTTCACCAAATAATT
GCTGTTTCCAATATTTAGTGTTAAACAGCATTAAATTTTAAACACCGGTGATCAAGATGAGTGAATATTGGCT
AATCAGTGCCCCTGGCGACAAAACCTGCCAACAGACTTGGGACACCTTGAATAATGCC
ACTAAATCAGGCAGTCTCAGTGTTAACTATAAATTCCCTATACCTGACCTGAAGGTTGGTACATTGGATCAGT
TGGTAGGATTGTCTGATGACCTTGGCAAGCTTGATACTTTTGTCGAAGGTGTCACTAGGAAAGTAGCTCAGTA
TCTTGGTGAGGTATTAGAGGATCAACGTGACAAGCTGCACGAGAATCTAATGGCAAAC
AACAGCGATCTGCCCACGTATCTGACTCGTTTCCAATGGGACATGGCCAAATATCCCATAAAGCAGAGCCTTC
GCAACATCGCCGATATTATCAGCAAACAGGTGGGTCAGATCGACGCCGACTTGAAAGTGAAATCATCAGCTTA
CAACTCTCTCAAGGGGAACTTACAAAATTTAGAGAAGAAACAAACCGGAAGTCTTCTA
ACTCGCAACTTGGCGGACTTAGTGAAGAANGAGCATTTCATTCTGGACAGCGAATATTTGACCACTCTTCTCG
TCATTGTGCCTAAGTCGATGTTT
>1002052711_2 Heli.2-EL596673.1.5
GGCCGTATTGTGTCGGGTGAATGGCTCTAAGTCACCACTAAATAACTCATTTTATTAAAATACGTCCATAATA
TAACACAAAAATGAAATTAATCTACCTTACGTTAATAGTTCTGCCTGCGGCGTTACTCTGCTTCCAAAGCGCA
AACACAATCTTTGCTCGTGCTGAAGAAGATGAACTTGATGATGTAGTGGATATTGAGG
GCGAAGACAACCAAGTAGTGGGTGAAGATGCGCTTGACGAAGATGACTCAGTTGTAAAATCATCACAGGATGT
TGATACTACTATTTTGTTCACAAAACCCATAACAAACTATGGTGATGTGGCATTCGATTTACAAGCTGGCTTC
CCTGTCGAGTTCCTTGTTGGTTTCATTAACAAGGGTTCTGAAGATTACATTGTTGAGA
CCATGGAAGCTTCCTTCAGATATCCCATGGATTATACATATTATATTCAGAATTTCACTGCATTGCCCTATTT
TAAGGAGGTTAAGCCTAGAGAGGAAGCTACTTTTGCTTACTCCTTTATTCCCAATGAAGCTTTCGCTGGACGA
CCATTCGGATTGAACATTCAACTCAATTATAGAGATGCCAGTGGAAACTTTTACCAAG
ATGCAGTCTACAACCAAACAGTCAACATTGTTGAAGTATCTGAAGGTTTGGATGGGGAAA
>1002052712_2 Heli.2-DT662579.3.5
GCACGAGGCTTGTCCCTCGCACATCCTCGGAACGTGTACGCGAATTAAGGCACTTTTTTTATTGTTTACAGTG
AATGCATTTCTTATTAATTACGCAAAATGATATTAAAATCAAAATATAGATACGGGATTTTGTTTGTAAATGT
ACTTGTCGGCTTCAGGATTGTTTTCGCAGATGTACTTAGGACTGAACTTTCAGAAGAA
AATGAAGTGTACAGCACTGATGATGCCGTGTTTATTAACGTGAATACGGAGCGAGTAAAACGCGAATCAAACA
GCACTCGAGATGGAAAACAACTATTGTTACGATCTAGACAACTTTTAGGCTACCCGGTTGCCGCACCTCCACA
GCAAGAGCCAGACGTTCAATACCTCGGACGGCTGACACCATCATTCCCGATGCCATTA
ACCAAATTACAAATTCCTGCGGGTTTGCCACAATGGTACACCAACTTTTATCGTCAGATACCAGCACAATGGC
CGCCTGCGTTACCGCCGATACCAACACATCCACCAGACCATTCCGCGCCTACTCATCCTCCATCATTAATTGC
AGGTGTAACAAGCCCAAAACCTGTTGAAGGACCTTCAAACACGCCTCCAACTACCACA
TGGGGAACAAAGCCTCCAGTAAGCACTACTAAGCAGACTTGGCCTCCCGTTAACCCAACACAACCAACTAGGC
CCACGGCGCAACCAGGCACAGATTCCTCATGCGGCATCAAAAATGGACCACAGGTATATGGCAGCACATACGA
ATCGAAGGATGAGGAACGTATAGTGGGCGGGCACGACGCCGACCTGAACGAATGGCCA
TGGATAGTCGCG
>1002052713_2 Heli.2-EL599302.1.5
ATTTTCTCTGTCGTCCGTAGGTGCCGTTCGGATACGCTGGTGGCCGCTCGTGGTCACTTCTGACTAAGTCAAT
TTCTTTTAAATAATGCGTTTGCTAGTATTACTTTGCTTTGTGGGGATAGCCAGTTGCTATTCTGTAAACGAAA
GAGCTGGACCACCTGGTCAGGAGAAAGGCATACTGGAATGGATAAACACGTGGTTGAC

AACAACTTCGACTACGAGTAAACCGACAAACGATCCGCCAGAGGAATGCCCAACATGTCAGTGCGGCATAGCT
AGGACTCGCAGGCGAATCGTGGGAGGATATGAAACCAAGAAGCTGGAGATACCATGGATAGTGGTGTTGATGT
ACAACGGCAGGTTCTACTGTGGAGGCTCCCTTATTAACGATCTGTATGTGCTGACTGC
GGCACATTGCACTTCTGGATTCCGAAAAGAAAAAATAACGGTTCGTTTCTTAGAACACGATCGTTCTACTGAC
AACGAGACGAAGACCATTGACAGGAAGGTGGCCAGTATCATCCGTCACCAGCGGTACAACCCCGGCAACTATG
ACAACGACATAGCTATGCTAAAGTTGAATGAAAGGGTAGACCTCAGCACAGCTCTGAA
GAGAGCTCGAGGTGATGAAACAGAGGAAGACAAGGACGTCGGTCTCCGGCCTGTGTGCTTACCGTCGGCTGGA
CTATCCTATAATAACTATACCGCGTTAGTAGCTGGCTGGGGCACTAC
>1002052714_2 Heli.2-DT664214.3.5
CTGTATCCCGCACGTAGCAGGGTGGGCAGAGGGAACCTATTGTTAAGACACTCCGTTCCCCACTTGCCGCCAA
ATTCTGGAGGCATTGCGTGTAGAGTGGCGGAACTCAACACCGCGCTTTTCTTTTTGTGATTTTGTTTTTAATT
TTTAAGAAACATGGCGGCGAAAATACCCTTTCCATATGAATTTAAAAACGTAACGGAG
GAAGAGGATAAAATAGTGAAAAAATATTACAAAGATTACACACGTCCGTTCATCCGCGTGGGCCCGCACGGCT
ACATCTCCATGGCAGGGTACGGAGACCATGCGGCTGATATCTACAATTTGGAAGTTAGACCGGACGATGTATG
GGTGATTGCCTTTTCTAGATCAGGCACTACTTGGCTCCAAGAGCTAGTTTGGCTGGTT
GCGAACGATCTGGACTATAGCGCAGCCTCAGCCACGCCACTGACTAAAAGATACGCCTATATTGAATATCCAA
CGATGGCAGCAGAAACTAAGAAGCATGAACCCCCACCGCCACCACCACCACCAGGTCGACCTCACCACCGAGC
CACCTTCGACGACTTCAGAACCGTCCACACGTTGCCTTCTCCGCGTTTCATCAAAACT
CACTTACCATTATCTCTCTTACCGCCGAAACTTTTGGACACTGCAAAGATTCTTTACGTAGCACGAGATCCCC
GCGACGTGGCCGTTTCATTCCACTTCATGCACAAATTGTTCAGATACTTCGACGGAGGGGTGGAGTTTAAAGA
ATTTTGGGATCTCTTTAAGAGAGATTTAATAATGCACACACCTATATTTCCACACATC
AAAAAGTTCTGGAACGTCCGTGATCATCCCAACTTGACATTTTTGTTTTATGAAGAAATGCTAATGAATCTT
>1002052715_2 Heli.2-DT662342.3.5
TCTCCACCTCCACAAGTGCGAGATGGTAGCATCGACAGCCGCGAGGGCTCCATAAGTCCATCGCGACGTCGGA
AGAAAGCCCGTCGTACATCCACCGAGAGCACCCCCGTACCAGCGCCCGAGCCCGAGGACCTGCCACTGAAGGA
GGAAGTATCACGAGATGGCGTGGAGGATTTGACGTTAGATGAAGAAGCAGAGCCGTCT
GTTACACACAATGATGTTGTACGCAATTTTCAATGGCATATGGAAAGATCTCAAGATGAAATGATGAATTCAA
ATGATAGTGTTCGAGAGACTCAAGAGGTGCGCAAGGAGGCCCTGCCGTCGGCCTTGCTCTCGCTGCTGTCGGC
GCAGCGCGCGCAGGCGCTCGTGCCGCGCCTCCTCCCCCTCCCCGACCTCCCCGAGCCG
CTGCAGGCGCGCCTGCTGCAGCGCGCCTTCCTCGACAACAACAACGACGAGCCCGACGAGCACTCGGGGCCCG
ACTGCGACGACACGGACGACATCGTGCTGCCCGACGACGAGGCGCGCCGCGACGACGACGCCAGCCGCCAGTT
CGAGTGCCGCCACTGCGGCAAGCGCTACCGCTGGAAGTCCACGCTGCGCCGCCACGAG
AACGTGGAGTGCGGCGGCAAGGCGCCCAGCCACCAGTGCCCCTACTGCTCCTACCGCGCCAAGCAGCGCGGCA
ACCTGGGCGTGCACATCCGCAAGCACCACAACACCGAGTGGTACATATACGCCAGCAACAAGGTGCGCCGCAA
CAAGAAGACGCCCGCCTAACAC
>1002052716_2 Heli.2-DT664086.1.5
AAAACTGCAGAAGAGGCTAGCAGCCTCTGTTATGCGATGTGGTAAAAAGAAGGTGTGGTTAGATCCAAATGAA
ATCAATGAAATCGCCAACACTAACTCCCGACAGAACATCCGTAAGATGATAAAGGATGGTCTCGTCATTAAGA
AACCAGTTGCAGTGCACTCGCGCGCCCGCGTCCGCAAGAACACCGAGGCCCGTAGAAA
GGGAAGACATTGCGGTTTTGGTAAAAGAAGAGGTACTGCAAATGCCCGTATGCCACAAAAAGAACTGTGGGTA
CAAAGACAAAGGGTGCTCCGTAAATTACTTCTCAAGTACAGAACAGCCAAGAAGATTGACAGACATCTCTACC
ATGCCCTGTACATGAAGGCGAAGGGTAATGTGTTCAAGAACAAGCGTGTACTCATGGA
GTATATTCACAGGAAGAAGGCCGAGAAGGCTAGGACTAAGATGCTGAGCGATCAAGCCGAGGCCCGCCGTAAC
AGAGTGAAGGAAGCCCGCAAACGCCGTGAAGAACGTATCGCAGCTAAGAAGGAGGAACTGCTGCAAACGTTTG
CGCGTGAAGATGAGGCCGCCGTCACAGCCAAGAAGTGATTTATACATAAATATAAATA
AATAAATATAAA
>1002052717_2 Heli.2-DT662324.3.5
GTGTCGAGTTATTTCTATGATTCATACGATAAAGTAATGATTCATACGATAAAGTACGTAATATCAAGTAATT
ATTACAACATTACATTCTTCAGTACAACATTACATTCTTCAGTAATTTACTACACTGCACGCTTGAAAAGTGA
AAATGGATGCAACCGAGGTACAATACCGGCCTATACCGTTTAGTGTTCACGTCTGGAG
TGCATTGAGAACATTAATCGATGTGATCACCGCTATTTTTTTTAAATTGTATTATGGCACACAAGGAAATAGG
ATACCGCCAATAAAAGACGACATTCTGAAGCAACCGGCTGTAGAGGTTGCCAGAAGAATAAGGGCTAAACAGA
TAAGCAGTGAAGATGTAGTACAAATATGTATACAACGGATTCGGGACGTAAATCGCGA

GTTAAATTGTTTGGTAGAAGAACGGTTCGAACTGGCTCTGGCTGAAGCTAAAAAGGCTGATGATCTGGTGAGA
AGCGGTTCGAAAACGGAAAATGAATTGGCTAAAGAGAAACCTTTCCTAGGCGTTCCGTTTACGACTAAGGATT
GTATTGGTGTTGAAGGACTTCATCAAACAGCTGGAGTGGTTATGCGAAAGGATTTCAT
AGCGGAGAAGGATGCCGAGTCAATTAAATTATTAAGAGACGCGGGTGCCATTATTCTAGGTCTGACAAATGTT
CCGGAACTTTGCATGTGGTACGAAACTTACAACAAAATTCACGGTAGGACAAATAATCCTTATGATACTAA
>1002052718_2 Heli.2-DT663662.3.5
GCTCTTGTATTTTGGCGTCTGTCGAAGCAAATTTCATCTCATTACATCTGGAATTATCCAGATATTAAAGATT
AATTGTAACAAGTGCCCGTGAATATATAATCTAAGATGTCTATCGGTGTGCCTATTAAAGTATTACACGAAGC
GGAAGGCCATGTAGTGACCTGCGAAACAAATACCGGCGAAGTTTACCGCGGGAAGCTT
ATAGAGGCCGAGGATAATATGAATTGTCAGATGACCCAAGTAACAGTCACTTATAGAGATGGAAGAGTCGCGC
AATTAGAGAATGTATACATCAGAGGATCTAAAATCCGTTTCCTTATATTACCTGATATGTTGAAAAATGCGCC
CATGTTCAAACGACAGGGGAATAAACCCACGGCTGGGCGTGGGAAAAGTGCTATACTT
CGTGCACAAGCTGCTGGTCGAGGCAGAGCAGGAGGTAGAGGTGGAGGGCATCGTGGAGGCTGGCAGGGTGGCT
CAGGACCTTCGAGACGATAAACCTACAGTTAACTTAAGTTCAGTTTGTTTAATACCTATGATGTCCAAACACA
TTTGTCAGATAAGGATAATTATTTAATATTAAGATGTGTTTTGATATTGAGACATGAT
TGAACTCAGTGTGGTTATTAAAACAGCATTTATATCAAAATGTAGCTTTATTTGAATTTTATTTAGGACATGG
ACAACAAACTGTAAATATATAAGTAACTGTTCTTTATCATTTTTAACCTATGAATATTTGTCTTTGCCAGGGT
GGTTAGGAAGCTCCCCAACTGTCCCTACATTTATTAGATATATCAAAACAAAATACAA
TCTTTTACCACAACTAATTAAAATATAAATGTTTTG
>1002052719_2 Heli.2-DT667253.3.5
GCACGAGGGTTCACCCTGGCAGGAGATCAGGCGCAATTTTACCAATTACTAAATACAATATTGTCTATAGATA
ATGACATCAGATCCCAAGCAGAAAAAGTATACAATGATATACCAACAGAAACAAAGGTAGTCCATTTAGTCGG
TGCTATACAAAATGCGGATCTTGGACAAGACTGTAGGGAAACAGCTGCCGTACTCTTA
CGGCGGCTGCTTAGTGCAGAATTCTTTGAATTCTTCCCAAAACTTCCTTTTGAACAACAAGCCATGCTCAGAG
AACAACTTCTCCTTACACTGCAAATGGATGTTAACCAAAGTTTAAGACGAAAGATATGTGATGTAGTATCAGA
GTTAGCAAGAAATCATATTGATGATGATGGAGTCAATCAATGGCCTGAATTTCTTCAA
TTTATGTTTAATTGTGCTAGTGCACAAGATCCTAATATTAAAGAAGCTGGCATTAGGATGTTTACGTCAGTAC
CAGGAGTTTTTGGTAATCGACAAAATGAAAATTTAGATGTCATTAAGAGAATGTTGCTCTCAGCACTACAGCC
AACAGAATCTGAAGCATTGCGCATGCAAGCGGTCAAGGCAGTGGGGGCTTTCATCTTG
CTGCACGATAAAGAGCCAGCTATTCAGAAACATTTTAGTGACTTGCTCTTGCCTTTTATGCAGGTTGTAGTTC
AATCAATTGAAAAAGCTGATGATGATGCAGCTATCAAAGTCCTCATTGAACTTACTGAATCAGCACCTAAATT
CTTACGTCCACAAGTGGAAACCATATTCCAAGTTTGTATGAAACTAATTGGCGACACT
>1002052720_2 Heli.2-EL601015.1.5
GCACGAGGGATCAGACCTCTCTCCAGGAATGCTAAGAAAATCTAGATTTCAAAAATTTCTAGACTTCTGCGCC
ATGCAGCCAAATTCCCGAATAGCAAGAAATTGGAGTATCAAAGAAAGGACCAGATCTCTAAATTTGGAGGCAG
ACCAACTTGAGCTGCAGTCTAGGACAACATCCGGGCATGAGGGTGTACAGTGGTGCAC
TCGAGTATTACGAGACATGCAGATCGGTCGGTAGGGAAGCGAGCTGTTGAAATCTAACCAGACGTCAACAATT
CCTGGCCGGATGAGAAATTTATTAATGAAATGTAGAGTTTAATCGATGTATTCTAGTTTTAAGGGTGGATGTA
ACCAAATGAAATGCTGAACATATGGGGACCCAACACCTGTGTCTCGAGACGACGGGAG
CGGAATAGTGCAAGTCATAAAGTTACTCTGTAATATGAAGTTACTCTGTAACTAAGTCACCTTGTTACATGAA
GTCACTCTGTAAAATGAAGTTACCCTGTAACGTANAGTTACACTGTAACACGAAGTCACCTTGTTACTGGATA
TTAATCAGTAACATAAATCACTCTGTAACATGATGTTACCCTGTAACATGCACTTACC
CTGTAACATTATGATGGCACTGTCTGCTACGCTCGTCGACTCATACTTAAGCGTATTATCTCTTCATATACT
>1002052721_2 Heli.2-DT666338.2.5
GCACGAGGAACACCTTCAGAGCAACCATGATTTCTACAATCGTCATCATATCGGCTTTGCTAGTGGTAGCAAA
TGCCCAGCACCATTTAAGTGGTCATGCCACGTCATCCCAATCTATAATTCGTCACGACATCGCCCAAAATCAT
GGTTACCAAGGCCAATTAACTCAAGCTATTCCTACTTCTTACCATCAGGCTCCCATCG
TCCACCAAGTCCTTCCTATTGCTAACCATGGACGTCTTCAGTCTGCTGGTATCTCACAACACGCATCTCCTAT
AGTTTCCCTTCAACACGTTTCTCCTGTTGTCCAGCGCGTTTCTCCTGTTGTCCAGCACGTTTCTCCCATCCAC
TATAACCAATATGAATCTGGACATGGACATCAAGACTATCAAGGTCACCCCAGATATG
AGTTCGCTTACAATGTAGCTGACCATCACACCGGTGACATCAAGTCCCAGCACGAAGCTCGTGATGGTGACCA
TGTGTCTGGTTCATACAGTCTTCACGAATCCGACGGTACCATCCGCACCGTTCACTACAACGTAGATGGACAC
AGTGGATTCAACGCCCAGGTGGAGCGTTCTGGCCACTCTCAACACGTCCAGCCCCAGC

ACCATGCCCCCAACTACGTACTAGCACATCACTAAGAATTAAACATGTTCCTAATAGAAAGTTAGCATATGTA
TGTACTCGTATACCTATCAATCAAGCTAATGACGAGCCTAGTCTTTTAATGTATGTTCCAATTTTTGTGTATC
GATTAAATATTATATAATAGTGTAAAAAAAAAAA
>1002052722_2 Heli.2-EL597438.1.5
GTCACATCTGTACAGTGCTCCCGCGAATTTTCAAACAAGTGAACTATTGTGTGTCAAAATGCCGAAGAATATT
TTAGCCGAGATGGGCTCGCAAGTGGGACTGACCCCCGCCCGACAAAATAAAAAGGAAGCGGAAGAATGGGCCC
GCAGACAGAGCGGCCCTATGGTCAATCCTTCCTTCGAGCCCGACGATTTTCTGCCACA
GAGTTTATCACCCGTCAAAGATGAAAAGAAAAGGCCGGAAGACAAAGGCATCTTCCTCGTTAACATGAAAGAA
AAGAACGTGCAAGAAGTAGAAGAATACGAGCCTTACGACAACAGAGTTGTGGAACATCCTACCACAAACACAG
AAACCCTCTTGCATTTGTTAAAAGGCAGTTTAGGTACGGGTATTTTAGCAATGCCTCA
CGCCTTCTCTAAATCGGGGTACGTAGTGGGTACTATCGGAACCATTGTCATCGGGGTGCTGTGCACATATTGC
ATACATGTGCTCTTGGACTCTTGTTACGTGTTATGCAAGCGGCGGAAAGTGCCCTCCCTCACGTACACTGCTG
CGGCCGAGGCAGCGCTGTCTGAAGGCCCTGACTGGTGTAAAGCCTGTGCACCTTACGC
CGCGCACGTGGTGAACGCTTTCCTCCTTATTTATCAGATCGGCACCTGCTGCGTGTACGTAGTTTTTGTGTCC
GAAAATATACACTACGTTCTGATAGAACACTTCAAGCTGTCTGTAACCGTCACAGAAGTGATGTTATACATCC
TTTTGCCTCTGGTTCTTATCAACTGGGTTAAGGATCTCAAGTACCTGGCGCCGTTCTC
GGCTATAGCTAA
>1002052723_2 Heli.2-EL597851.1.5
GCACGAGGCAACACTCTACATCGTGGCTGCCATGGAGTTTGCCCGTAGCCGTCGGGCTGAGTGTGACGGGTGA
CGTGGTCCTAGTATCGCGCCACCTTACCGTCCAATCCACGGGTCGCTTGCCGCATTGCCTTCCACTTCACTTT
GAGCTCTAAACGCTACCCAAACCAATTGCATATATCGATACCGCGTTGAAACACGTCT
CGCAGTGCTGTCTCTTATACTATAAAACACGTGAATAATTTTATATTTTTAATTCTTGTCTCAAACGTGACAG
GCCGCCGGCGGATTATCTAAATAAACCTTGCATATGATAAGTGTGTGAATATTGTCCTATTGTTGTTTCGTTT
TTGAAATTAATCAATAGACAGTGCTGTAGCCTTAATATATTATTTGTAAATTATTGAA
TAAAAAAAATATAATAAAATGGCACCTAATTCCGGAGATGCAAATGGAGTTCTATTCGAATCAGATGCCGCAA
CACCTGATATGGCTTTACCTATAACTCCTATACAGCAAGCTGATCAATATCCACGAAAATTAGTTTGGAGGAA
TATAATTGCTTTTGCATATTTACACTTAGCTGCTTTATATGGTGGATTTTTGTTTCTG
TTTTCAGCTAAATGGCAAACAGATATTTTTGCATACATCTTGTATGTGATGTCTGGACTTGGAATTACAGCAG
GTGCCCATCGTTTATGGGCTCACAAATCTTATAAAGCAAAATGGCCACTTCGAGTAATCCTTATTATGTTCAA
CACCTTGGCATTCCAGGATTCCGCAATTGACTGGGCCCGCGATCATCGCATGCATCAC
AAGTACTCGGAAACTGATGCCGACCCACACAATGCTACTAGAGGCTTCTTCTTCTCTCAT
>1002052724_2 Heli.2-EL601087.1.5
GCGAGTGATAGTGAGGTGCACGTTATCGATATAGACACAGATACAGAAACACCGGTGATATTTAAACTAGTGG
ACATCACTATGAACGGAAACGGAACATTATCAAACTCCAAACCAAAGCCAGTTAACATACCGGAGGTTGTACA
AAAAGCCCATGACACATACAACAGCGGTTTAACGAGGCCAACAGAATGGAGGAAGAAA
CAACTAAAGAACCTTATGAGGATGTATGAAGAGAACAGGGGGGCTATGATCGACGCCCTAGTAAAGGATCTAC
GGAGAAGTAAAACTGAGGCCATATTACTTGAAGTGGACTACTTGGTTAATGATCTTAAGAATGTATTAAGTAA
ATTCGACGAATGGGTCCAGCCTGAACGGCCACCAAAAGGATTAGTGAACATTCTAGAC
GACGTGGTCATATTCAACGATCCCTATGGAGTCGTTCTCATCATTGGAGCTTGGAACTACCCATTACAACTTC
TACTACTACCAATGGCTGCAGCCCTCGCCGCTGGTAACGTGGTCATACTGAAGCCAAGTGAATTGGCAGTGGC
CTCCGCGCAGTTCATAGCTGAAACGTTGCCTAAATATTTGGATAATGATGCCTTAGTA
ATAGTGGAAGGTGGTCCGGAAGAAACAACAGAGCTGTTGAAACAAAAGTTCGACTATATCTTCTATACTGGTG
GCACAAATGTAGGCAGGATCGTGTATGAAGCGGCG
>1002052725_2 Heli.2-EL603536.1.5
GCACGAGGGTGCGTGCGAGGCGACGGGCGCGTAAATTGCGGCGTATAAGCGGCTCGACAAGAGTCCCCACGTT
GCAAGTAGCTATAGGTACTTCTTATCATTAGTTAAGAGGTCACGAATAGTTCGTAACTTCATTACCAACTACA
TTTAGGAACGACGGACCGGAGATCAATTTAGTCCGGTGCTGATGAAATCAACTATTAT
TCTCGAAGAAGTAGTGAATAAATTAGACCATCTCGCTTCACCGTTACCTGCCAGGATGCCTAACATTAAAGTT
TTCAGTGGTAGCTCCCATCCCGATCTGGCACAAAAGATCGTGGACCGACTCGGAATTGATCTCGGCAAAGTGG
TAACCAAGAAATTTAGCAACATGGAAACGTGCGTTGAAATCGGAGAATCCGTCCGCGG
CGAAGATGTTTACATCGTTCAAAGTGGTAGTGGTGAAATTAATGACAACCTAATGGAATTACTTATTATGATC
AACGCTTGTAAAATAGCCTCAGCGTCTCGTGTTACCGCTGTCATCCCGTGTTTCCCGTATGCAAGACAGGACA
AAAAAGATAAAAGCAGAGCGCCCATCACTGCTAAACTTGTTGCTAATATTTTATCTGT

TTCTGGAGCTGATCATATAATAACCATGGACCTTCACGCGTCGCAAATCCAGGGTTTCTTTGACATCCCCGTG
GATAATCTGTTTGCGGAACCAGC
>1002052726_2 Heli.2-DT665376.3.5
AGTATATACCGACTTCGACTTCGAGCTTTGTCCATCATATTTAGAATGAAGTGTTATCTGTTTCCCCTTATAA
CGTATTATTAAGCATACATAGTGTCAGTGATAGTGTGCAAGTACACTAGCCAGTGTGTTTTTGTCGTTGAACC
GTCGACGTTAACGGTTAAGTTGTGTCAATATCCTTAATTTAACTAAGTTCGTGACAGT
GTTTTTATCAGTGGGATGTGTTCGAGTATTTAGAATCTTTTGAAAAATACAGGTACAAGTCATGTAATTTCTT
CTGTTTTTAATTGAAGCGGCTTGGCGCGGCGTAGCGGGCGGGCCGCGCCATGGGTAACAAGTGCTGCAGCCGC
CGTCACGACCCCGACAGACCCTTGGTATATCCAGCGTACAAGAAGAACGAGGCGGGCT
TCACAACATGTCCCTCGCTGGAGACGCGGTACACCGGCGAGCCGAACAACCGCGCCGTGTCGCGCCGTCCCGC
GGACATCGTGCGCACACGCAACCCTGGGGCGTGTATATCAAACGGCGGGCGGCGTGTGGTAAAAGCACTATGC
GCGTACACAGCCCGCGCGGAATCCGATATCTCCTTCCGCAAAGGCGACCGCATGGAGG
TCCTCAGCGATGCGGAGACGGACTGGTGGCGCGTCCTCCACCTCACCACGCGGCGAGAGGGTCTGGTGCCAGC
CAACTTTGTGGCCGAGGAGAGTTCCGTTGAATGTGAAGATTGGTTCTTCCCGCACGTATCACGCAAAGAAGCC
GACAAATTACTTTTAGCAGAAATCAATCCCCGAGGAACTTTCCTAGTTAGACCTGCCG
AGCATAACCCGCACGGGTTCAGCCTCAGTGTTAAAGATTGGGAAGAAGGAAGGGGTTACCATGTTAAACATTA
TAAGATAAAGCCACTGGACAACGGCGGTTTTTATATCGCCACGAATCAAACATTTCCTAGTCTACCTGCGTTG
GTCATGTCGTATACAAAGAACGCGTTAGGCCTCTGCCACGTGTTAGCCAGGCCTTGTC
CAAAACCTGAGCCGCAAATGTGGGACCTGGGGCCAGAACTGCGGGACAAATGGGAGATACCCAGGAGTCAGAT
ACAGTTGATCAAGAAACTTGGACAGGGGAACTTTGGCGAAGTGTATTATGGCAAATGGTGTAATAATATTGAG
GTCGCCGTGAAAACTTTAAGAGAAGGCACGATGTCCAAACAAGCCTTCCTTCAAGAGG
CGGCCATTATGAAGAAATTCCGTCACAAACGCTTAGTCGCCCTCTACGCGGTCTGTTCCCAACAAGAGCCCGT
GTACATCGTACAAGAGTACATGTGCAAGGGATCTTTGTTAGAATTTCTAAGGAACGGCGAGGGGAAGTCCCTG
CATTTCGAAGATTTAGTATACGTTGCTGCGCAAGTAGCTTCAGGCATGGAGTATTTGG
AATCAAAACTTCTCATCCACAGAGATTTGGCGGCCCGTAACGTCTTGATAGGTGAGAACAATGTCGCCAAAAT
ATGCGATTTCGGTCTAGCAAGAGTGATAGAAGACAATGAATACTGTCCAAAGCAAGGCTCCAGATTCCCAGTG
AAATGGACAGCGCCGGAAGCGATCATTTACGGACGGTTCTCCATAAAGAGTGACGTGT
GGTCCTACGGGATATTACTCATGGAGTTGTTCACATATGGTCAAATACCTTATCCAGGATTACATGGAAAGGA
AGTGATAGAACAAGTGGAGAGAGGGTACAGAATGCCAAAACCGGTGGGACATTACCTACCGGACGACATAT
>1002052727_2 Heli.2-EL596824.1.5
TACAGGTCATCCAAAACCCCGTGTAATGTGGCTTATTAATGGAAATACAATTGTTAACGGCTCTAGATATAAG
CTAACATATGATGGAATGTATCATCTTGATATACCAAAAACTAGACAATATGATACTGGTAAAATTGAAGTAA
TTGCCCGAAGCTCAGTAGGAGAGGCATTAGCAACGACCGAACTTAAAGTTATCCCGAG
ACACGACGATTATAGAGGTGTTCTGAAAAATGCACCAAGACCTTGGTATGATGAAACAACTCAATATCAACGC
AATGAATTTTAATTAGAGAAAACATTTGAAGAAAGACAAACTATGCAACGGCAAGGAGTCATTGATCATAGAC
CTGAATTAAAATCAAAGGTAATTAAAGATTCTGAAACTGAATGGCAACAGACAGTAAA
GAAGAAGAAGAGTGAAGAATACTATAATAAGCTACAGGAGCTAGAGAATGAACAAATTGTTAAAGAAAGTAGA
TTAAGAGAATCATCACATCAATATGCCATCCCTGGAGAGAAAGTAACTAGCTCTTCTGTGGCCAGAAGTATGG
CGCAGAAATATGAAGATAATTTGGAGACAGAAGAAGTCGTTGAAAAGCAAATCCAAAG
TAAAGTACAAAAGCCTAGAATTCCTGGACCTTCAGAATCAACTGTTCATGGCAAAGAAGTGCATGTTGCAAAA
CAGAAACAAGTTCAAAAAGAAGTAGTCGGGGACACTGAAATAACACGCAAAATAACATCTACAGAAACTACTG
AAGTAGAACATAAGGCACAAACTCAAGAAAGGGTGGTCGAAGGTCCAGTTAAGCCTAG
TACACCACCTGTGTTTACCAAAAAAATGCAACCCTGTCGCGCTTTTGAACATGAACAAGCAAGATTTGAAGTA
GAATTTGACGGAGATCCATTACCAACAATAAAATGGTATCGTGAGAATTTCCCAATTAAAAACTCACCTGATT
TCCAAATTCACACGTTCAGTACCAAGTCTATATTAATTATTAGACAAGTCTTCATTGA
AGATTCTGCTGTCTTTGCTGCTGTAGCTGAAAACAGAGGAGGAACTGCTAAATGCAGCGCTAATTTAGTCGTA
GAAGAACGAAGACGACAGGGTAGAGGTGGCGTTATTCCACCTAGCTTTACGTTAACAGCTCAAAATGTTAACG
TTACTGCAGGACAACTGGTAAGATTTGACACCAAAGTTACTGGAACTAAGGCTATTGA
TGTCTATTGGCTAAAGAATGGAAAGAAAGTTCAACCAGATATTCGAAACAAGATTCTGGAAGAAGATGGTACA
TACACTCTCCTCATTTTAGAAGCCTTTACTCAAGATTCTGGTAAATACGAATGCGTTGCAATCAATAGTGCTG
GTGAAGCGCGTTGCGATGCTGAATGTGTCGTGAATGCTCCTGCTACTAAAGATAAACC
GAAACCTCAAACTAAGGCTGCTAACTCTCCGCCAGAAATTATTGAACCTTTGAAGGACAAGGTAGTAACTGAG
GGCCAGGCTATTGAATTCAGTTG
>1002052728_2 Heli.2-EL603552.1.5

GCACGAGGCCACGTCTCTCTAGCCCTGGAACTTGCTCGGTCGTCAGTGAATTTTATGTTCTTATTCATTTACC
ACGTTGTCTGAATAATTTGTATCAAAGATGAGTGCTCCAGCACCAACGCCTTCCTATAAAATTGGTATCGTGA
AGCAGGTACTATCAGGAGACACGGTGATCATTAGAAAACAGCCTCAAGGTGGACCACC
ACCAGAGAAAGTGATTGCTCTATCTGGCATCACCGCGCCGAAACTAGCTCGTCAAAGAACTGCCAACAATGAC
ACTGAGACAAAAGATGAACCTTTTGCCTGGGAGGCTCGAGAGTTTCTTAGAAAGAAGTTAATTGGTAAAGAAG
TTATATTCACTGCAGAGAAACCACCTAACTCTGCAACCAGAGAATATGGGTCTGTTTG
GGCTGGAAAAGATCCCTTAAAGGATGAAAATATGACAGAAGCCCTCTTGTCTGAAGGGCTTGTTAAAGTGAGG
GAGGGAGGTCGTAATATTCCTCAATTGAAACGTCTTGTTGAAATTGAAGAAGTCGCTCGTTCTCAGGGAAAGG
GAATCTGGGGATCTGAATTGCAGAGCCATGTGAGAGATATCAAGTGGACAGTGGAGAA
TACAAAACAATATGTGAACAAGTTCCATGGACAACCAATAAAAGCAGTTATTGAGTATGTCAGAGATGGATCT
ACAGTCAGACTGTGCTTGTTGCCTGATTACACACCTATAACAATGATGCTGTCTGGAATTCGATGCCCTGCAG
TAAAGCAAGATGGTGATTCAGAACCTTATGCGGAAGAAGCCCGTTTCTTTTTGGAATC
GAAATTGCTGCAAAGAGATGTGGAAGTGATCTTAGAATCTGTAAACAATAACAATTTTGTTGGTACAATTTTA
CACCCACAAGGCAATATTGCAGAAGCTTTGCTTAAACAAGGTTTTGCAAGATGTGTTGATTGGTCACTAGCTG
TAATGAAATCAGGTGCTTCTTCACTAAGGGCGGCAGAACGTGCAGCAAAAGATGCTAA
ACTACGAATCTGGACTAACTATGTCAGCAATGCCCCTGTTATTGCTGCTAAGGACAAAGAGTTCTCGGCAATT
GTAATGGAAGTTGTAAACGGAGATGCACTTGTTGTTAAACAAATTCCCACAAATATCCAGAAAAAAGTCTTTT
TGGCTAGTATTAGACCACCACGTGAAAAGAACAGTCCAGATGAAGAAGGCAAACAGTC
ACCTAGGCCTAAAGGCTTCAAACCCCTGTATGATGTTCCCTGGATGTATGAAGCAAGAGAATTTTTGAGGAAA
AAACTGATTGGAAAGAAAGTAAATGTAACCGTTGATTACATTCAACCAGCTAAAGACAACTTCCCTGAAAAGA
CCTGTTGCACTGTTGTCACTGGTGGAACTAATATTGCTGAAGCATTAGTAAATCTGGG
ATATGCCACTGTGGTCAGATATAGAAATGACAATGACCAGAGAAGTTCCCACTATGACAAATTGCTTGAAGCT
GAACTCAAGGCCCAAAAGTCTGCTATTGGTGTCCATGCTAAAAAAGATATTCCAACTCATCGCATCCAAGATA
CTAGTGGAGATTCAGCTAAGGCAAAGAAATTTTTCCCATTCTTGAAGAGAGCTCAAAA
GACAGAAGCTGTTGTAGAATTCGTCGCTAGTGGATCTCGTATGAGAATCTACATCCCCAAGGAATCAGTTCTC
GTCACGTTCTTGCTTGCGGGTGTCAACTGTCCGCGGGGCGCGCGCCC
>1002052729_2 Heli.2-DT666598.3.5
AACTCAACGACAAGAACGAGCAATTGGCCAAATTCATAATGACTTACGTGTGAAAACAAATCTTATTATTGAA
AATTTAGACATGGTAGAGAAGCGTTTAAAAAAACAAGAAGATGATGTGGCAACTCTTGCACAGCGACCAGTTC
CAGCTGAACTGCTTTTGGATCCCACAATAGACAGACTTGTGGAATATGCACCCAACAG
ATACAGAGTGGATGAGCCTTCAACGGACCCTATTACCACAGTTGCCTACACTACTCCTGCGACTTCAACTGCT
AATAACAGCCCGAGCATCCCGAGCGTCCCGAGCAATTCGAGTACAAGTCCAACGAGTGCTACCGTGACAGTGA
CGCCAACAAGTACGGGCAGCAGTGCGGGTCCTACCAGCCCGCGCCCCACGAGCCGCAA
GGGCGGCATCATCTTCCCCAGTGTCAAGAACAAACCCATAATCGGCAACAATACCTTTGCATCGGAGATTGTC
GCCAACTATAAAGATGTTAAAGGTTACTCTTGTGTTGATCTGTCGAACGCCGGCATGCGAGAATCTGGTGTGT
ACTATTTGCAGATACGAGGCACCACTTACTGGTTCCTGAAAGTGTACTGCGAACAAAA
CGTCGCCGACGGTGGATGGACGGTAATTCATCGTCGTGATGACTATGGGATACCAGCGGAAAATTTCAATAGA
GACTGGAGTGACTACAAAAACGGATTTGGTGACCCCAGCAAAGAATTCTGGTTGGGCAATGAGAACATTTATA
TGTTGACTAATAATGATGACTATATGTTAAGAGTTGAGCTGGAAGACTTTGATGGTAA
TAAAAGGTATGCACAGTATTCGCACTTCAAAATATATTCAGAAGCGGAATATTACAAATTGGAAATAGATGGA
TACGAAGGCAACGCAGGCGACTCTTTGAATGATCCTTGGTACGGGTCTAACAATAGTCCTTTTTCGACTTATA
ACAGAGACAACGACAGATCTTCGTTGAATTGCGCGTCTATGTTAAAAGGCGGTTGGTG
GTGGAAATCATGTGGACGCGGTTTAAACGGTCTATATCTGCACGACCCTCAAGATCTTACAGCACGACAAGGT
ATAGTGTGGTTCCGCTGGCGTGGCTGGGACTATACACTAAAAAGAGCTTCTATGATGATCAAACCTAAGGGAC
TGCAGCCAAACACATGAAAATAAAGAAACAACTTCTACAATTTAATCTTATTTGGCCT
CTGTTCAATTGTTACGTGGATTTTGGACATAAAAAACTTATGAATAATATAACAGTATTCACTTTTTTTATCA
GCAAAAAGATTTAAATAATAAGCACTGAATATTTCACAAAATAACATTCGCAGATATTTTAACAATTGGTACA
ATCGTATGCATATTACAATAAATAAAGAAAAAATAATGAAGATTCAAATCCGTTCTAC
ATAACCATTAGATCTTAACGTTTAAAATAACTTAACTTTTACACTTTAATAATTATTTACCAACGAGTTATAG
CTGTCTTAGCTTCTTGTGCTATATATTTAAAACTATAAGAAATTTGGTACTGAATCCACGTAATGACCTAG
>1002052730_2 Heli.2-DT665487.3.5
GCACGAGGATTTTACTTTATTAAATATTAGACAGTATAATCTTATGTAGACGTTTCTTACAAAGATCTATCGT
TAAAGTAATTCCAATACTATGCCTCTTTTTGGGAAATCTCAGAAGAGCCCCGCGGAGTTAGTAAGGTCTTTGA
AAGATGCTGTCACAGCGTTAGAAAGAGGTGATAAGAAAGCTGAAAAGGCCCAAGAAGA

TGTTAGCAAAAATTTGGTCTTAATCAAGAACATGCTCTATGGTACCTCTGATGCAGAACCACAAACTGATATC
ATTGTTGCCCAACTTGCACAAGAGCTTTATAATACAAATCTGCTGCTGCTATTAATCCAAAATTTAAATCGTA
TTGATTTTGAAGGCAAGAAAGATGTAGCCCAAGTTTTCAACAATGTTCTTAGACGTCA
AATTGGCACTCGCTCACCTACAGTTGAATATATTTGTACTAAACCAGAAATATTGTTCACTTTGATGTCTGGA
TATGAACACCAGGAAATTGCTTCCAATTGTGGTACTATGTTACGAGAGTGTGCTAGATATGAAGCCCTAGCCA
AAATAATGTTGTATTCAGATGACTTTTACAATTTTTTCCGTTATGTAGAAGTATCCAC
TTTTGATATTGCCTCTGATGCCTTTTCTACTTTTAAGGAATTATTAACTCGACATAAAATATTATGTGCCGAG
TTTTTAGAAGCAAATTATGATAAAGTGTTTAGTCACTACCAGCGTCTATTAAATTCCGAAAATTATGTCACTC
GACGGCAGAGTTTAAAACTACTTGGGGAACTACT
>1002052731_2 Heli.2-DT667252.3.5
GTCGGCATATTGTTTCAGATAGGGGGCGCTGGCTGGTTCACGGGGAGGTATAGCTTTCAGTGCCAGCCGGTTG
ATCACTCGAAAAGTCCACAAACAATGAGGATGGTTCATGTTTGTTGGTGGTACTACTTCTCAAAATTCACAGA
ATTCTTTGATACGATCTTCTTTATCCTAAGAAAGAAATTCGATCACGTATCAACTTTG
CACGTAATTCACCACGGAGTGATGCCGATGTCTGTTTGGTTCGGTGTTAAGTTCACTCCTGGCGGCCATTCCA
CATTTTTCGGTCTTTTGAACACTTTTGTACACATCATTATGTACTCGTACTATATGTTGGCGGCGATGGGACC
GCAAGTTAGGAGGTATCTGTGGTGGAAGAAATACCTCACTGCCCTACAAATGGCCCAA
TTCGTCGGTATAATGGTGCACGCCTTCCAGCTGTTGTTCATCGAGTGCGACTACCCGCGCGCCTTCGTGTGGT
GGATCGGAATGCACGCCGTCATGTTCTTCTTCCTATTCAAAGACTTCTACAATCAATCATACTCCAAACCCAA
GGTCCGCGCGAAATCTCCACAACCTGTAACAACGGAGATCGAAGACGACGTCAAGCTC
TACAAAAACGGCTCCGTCAAGAACGGCTACACGAACGGGCACGCGAACGGGCTGGTGCGTGCGCGCACCGTGC
TGCCCGCCGGCAACTGACCGCGGACGCGCGCTCGCCGCCCGTCGCCACACTTCGTTACCTAATGGGCACAAGT
CAAAAATTATACAGCCTGCGCACAATGTTGTGTTTCATGAAAAAAGTACTGGCCATAA
GTAAAACCTTTTATGACGAATTTATATACTCGCGCGCTCGAACTTGCAATGCAAACCTCAATATGTTGTGAGT
TAATTTCGTTTAGAAAAGTTTTGTATAAAATGAGAAATAATGTTAGACGAATGACACGTTAAAATATGAAATA
ATAAATTTTTGACTTGTTGTTT
>1002052732_2 Heli.2-DT663779.3.5
CTCCTATAAGCCCGGGCTGCAGGAATCGGCACGAGGAAAACATGCTTTCGTCGCGACTGTTGATTATTTTCGC
ATTTGCAGCACTAACGGCTGCCTATCCCGCCGATAACGGCCCGTTCCCGCGGCCTATTACAACTATCACAGAA
ACTGAATTCGATGATCCGTCACCATGGTTCAGATTCCCGGCGTTTGGTAATATTTTTG
CACCGCTAACTAGATTGTTCTCTAGTTTCGCTGATATTGGTCCCAGGATCGAATCTGATGACGAGAAATTCAG
AGTTATAGTCAACGTTAAAGATTATAGCTTGAGTGATTTGAAAGTTAAGGTGAAAGGCGACTTTATCCTCGTG
CAAGGATCCCACGAAGCCAAACAAGACGATCACGATCTGTTTGCCAGCCAATTTGTAC
ATACATATTCTTTGCCGATCAACGCTAGCGCGTCGGATGTAACAGCGAAACTAACATCTGATAAATATTTAAT
CATCACAGCTCCACTTAACGGTGCGGGTGCTGATGACGGAAAAGCAGTAGACAGAGAAGTACCTATTGTAGAA
AGTAAAGAAGCATGGAAGAGTGAAGACAAAGAAGATAAAGACAAAAAGCCAGAAGATG
TGCCAGTTCAGGCCAGCGGCGTATTGCCAGTGGAAATTGATGAAGCCAAGGGCACTACTGATCTTCCAGCGAC
GGCAGCTGAGGAACTTACTACCCCGTCCAACCGTGAAGAAGTAACGTCCGTGATGTCCTAACGAAGTGACC
>1002052733_2 Heli.2-EL602671.1.5
TTCGGCACGAGGCCAACCCCGGTCATAGCGTCCTAAACTCATAAAAAGGACGCGGGCGGAACCTCTTCCGCTG
GAGCCTGAAGGACTCGTCAGTGAAAAGTCAAAGATACGAGATGAGGTCGTTGTTTTTATTAGCGGCGTTGTGC
TGCTTAGTCTTGTCGGCTTATGCTGGCCCGGTTTTCCGATCTGATGAAAGCCAGAAGG
AAGTAGGCAGGCAATCTGCTGCACCGGCTGCGCCCGCGCCGGCTGCTTCTGATGACGACGACGACGACGATGA
TGATGATGATGATGACGATGACGTCCTCGATGACCCATTATCAGATGATGACGACGACGACGATGATGATGAT
GATGAAGCAGATGAGGAATCCGATGATGATGATGAAGATTATTTAGAAAGATTCTTTG
ATGACATCTTAGGAGGTGATGACGATGATGACGACGACGACGAATCTACAGGTGTTCAATCCGTAGTGGCTGC
TGCCGACACCGTCGCTGCCGCACCAGCACCTGTAGCTCCGGCTGAAGAAGCTGCAGCATCTTTGGAAGAAGGA
AATGCAGCTGACGCTGCTGAAGAAGTTGCTGAGGGCGATGAGGCTGAAGCTGGACAAA
AACAACCCGCTAGCGCCGAAGTAGAATCACCTGTTGCCACGAGTTCAGAAGTAGCTGCTGCTTCTGACTCAGA
TGATGAAGAAGAAGATGATGATATTGCTGATGCTCTCGACCCCGAAGACGATGATGATGAAGAGGACGATGAC
AAGGAGGATGATGACGACGATGATGATGACCTAA
>1002052734_2 Heli.2-DT663097.3.5
TCTTGCCAGAAACTTACATACTAGTTTAACCATGAATGACAAACTTTTAAATGGAAATTTATGGGACACAGAT
CCTGAATTGTTTGATATTATTAAAAAAGAAAAGCAAAGACAAGCAAGTGGATTAGAATTAATTGCTTCCGAAA
ATTTTACTTCAATACCAGTCCTACAATGCCTTAGCTCATGTCTTCATAACAAATACTC

AGAGGGTATGCCACATCAAAGGTATTACGGTGGTAATGAATTTATTGATGAAATAGAAATTTTGACTCAGCAA
AGATCCCTGGAAGCATATAGACTGAAACCTGAAGAATGGGGAGTAAATGTTCAGCCATATTCAGGATCACCAG
CAAATTTTGCAGTATATACAGGAGTTGTAGAACCACATGGTAGGATTATGGGATTAGA
TTTACCAGATGGCGGTCATCTTACTCATGGCTTCTTCACTCCCACTAAGAAAATATCAGCAACATCAATTTTC
TTTGAAAGCATGCCGTATAAGGTGGACCCCCAATCAGGTTTAATAGATTATGACAAATTAGCAGAAACAGCCA
AATTGTTTAAGCCAAGATTAATTATAGCTGGTGTTAGTTGCTATTCTCGTTGTCTCGA
CTATAAGCGCTTCAGACAGATCGCAGACGAAAACGGCGCCATTTTGATGGCGGATATGGCACATATATCTGGA
CTCGTTGCTGCAGGTGTTATTCCAAGTCCATTTGAATACTGTGACAT
>1002052735_2 Heli.2-DT664348.3.5
TTGAATTGACCTTATTTACTCAATCCGCTGTTCAAGTGAGGTCGATAACCTTTCGCTTATTAGTTACTTAGTT
TTTACATATCAGTTCGCACCGAGAGACGGCCCCGGCTCGTTTGTGTTTATGGCGATACACTGACTCCGACACC
CGTGACAATGGTCGAAGTGAAGATAACGGAAGACGTTAAGGTTGGCGGCAACAATCCC
TGCTTTATCATAGCTGAGGTTGGGCAAAATCATCAAGGTGACATTGGAATAGCAAAAAAGTTGATCCGGGCTG
CTAAGGAAGCCGGCGCGAGTTGTGTTAAATTTCAAAAGACATGCCTAAAAGAGAAATTCACAAAGAAATATTT
GGAACGGCCATATAACAATCCAAACTCTTGGGGTGAAACGTATGGCGATCACAAGAAG
CATTTAGAATTTAGTGAGAGCCAATATAGAGAACTTTTCAAATATGCACAAGAAGTTGGCATTCTTTTCACTG
CATCGGCCATGGATATGGTTTCTTTTGACTTCTTGGTGAACATAAAAGTGCCGTTTATTAAAATTGGTTCCGG
GGATTCAAACAATTTATTATTCTTGAAATATGCCGCGTCGAAGAACGTCCCTCTAATC
ATATCGACCGGGATGGTTGACAAGGAAGCAGTAAAAACTATTTATGACATAATATCGGCTCATCACAAACAAT
TTTGTTTATTGCACTGTATATCGGCGTATCCTGTGCCTTTTGAAGACTGCAATCTTACTGTATTACAAGACTA
TAAAAACACATTCGATATCCCTGTTGGGTATTCTGGGCAGGAAGTTGGTACTGCAGTG
GCTTTAGGTGCAGTTGCGTTGGGA
>1002052736_2 Heli.2-DT665875.3.5
GCACGAGGGACGCGTCTTACCTACAATATAAATAGTAGAACAAAAAGATATTTATCTTTTATTATATTTTCTT
AGTCTTTAAAAACTTCGAAAACGTTGCGTTCAAGTGAGAGTGTTACCGTCGTAGAGAGTTTGGCGAGTGATTT
GATATTCCATTACAAAGAACCAGAACCCAAGATGACACTCGAACAGCCATCAGGAGAG
ATGTGGAAGAAAATTCGCGAAGAATTAAACGAAAACGCTGACACAAAAGATCAAGACCTGGCTCATATCAAGG
AATGGCTGAAGAAAGAGCCCCATCTACCTGATGAATTTGACGATCAACGTATAATGACCTTTCTCAGAGGGTG
CAAGTTTTCCCTTGAGAAGTGCAAGCGTAAGCTGGACATGTACTTCACCATGCGTTCG
GCTGTACCAGAGTTCTTCAACGACCGAGATGTCAACCGCCCCGAGCTACAGGAGATTATCAAGATTGTCCAAA
TGCCACCAATGCCAGGTCTCACCCCTGATGGTCGCCGAGTCATCCTTATGAAAGGTATTGAAAAAGAAGTCCA
AACTCCAAATGTTGCCAACGCTTTCAAGCTGGCTCTTATGCTTGGTGACGTTAGATTG
ATGGAAGAGAAGGAAGGTGTAGCTGGAGATATTTACATCTTGGATGCTTCCGTCGCTACTCCTACTCATTTTG
CCAAATTTACACCAACTTTGGTCAAGAAGTTCTTAGTTTGTGTCCAGGAGGCGTACCCAGTAAAATTGAAGCA
AGTCCATGTCATAAATATATCA
>1002052737_2 Heli.2-EL597307.1.5
GGCGGCACTACAAAGTTTACCTTGGCTCTTTGTGATTTTTGCGGTGAATTTGGCTGTACCAACTCCAGGAAGA
ATACCGAAGGTGTACAACGCTTTGATCACGTCTAATCAAAACTTGGAACCAAGCAAGGCTTTTCCTGTATACC
AACCGGTGTTGCAGAATGCCTTTCCATTTCCGCTGCAAACGGTCGTCTACGGAGATTA
TCCATTAACCAATGGTCTGGTCCCAGTTCCTGCCGTGCCTCCAAAGGATGTGAATGCAAAACCTGACGAGACT
CCTTCATCTACTGCAGCAGTTCCATCTGAGAAGGCAGCCACAGAAAAAGCGCCTTCACCTTCTTCTGAGCCAA
GTTCTGCTCCAGAGAGTGAAGCGACACCTTCACCACCACCAGTTCCAAAAACTGAGTC
ACCAATACCTCTTAACGAATTTGGTTTACCTCCGCAAATATTACCTCTCGGTCGTATAAATCCAGGGTACCAG
TACACTCAGTTAGCCACATATCCTTACACTTATCCAGGACTGAGGTTTTACGATCCTTATGATCCCTTTGGAT
TTCATACCTATGCAAACTGGCCTCTGTACCAACCACTTACAAATTTCATAGGACAAAC
>1002052738_2 Heli.2-DT668361.3.5
GCACGAGGCGGACGTTTTATAAAGTGACGCAAGCTAATGCTTTACGGCATGCGTTTATCTAACATTTCGATAC
TAGAAATTGTTGATCAATTACTGTCGCTATCAATAAACACATGACCAGTTAGGGGTGTATTCATATATAAATA
ACAAGAAATGGCTCGTGAGTTTGATCACCTCTTCAAACTACTGATTATCGGTGACAGT
GGTGTAGGTAAAAGCTGTCTCCTACTGCGGTTTGCCGACAACACGTTTTCCGGTAGTTACATTACAACCATTG
GTGTTGATTTCAAAATAAGAACTTTAGAGATAAACGGTGAACGAGTTAAACTGCAGATATGGGATACCGCGGG
CCAAGAGAGGTTCAGGACTATAACAAGCACGTATTACAGAGGGACTCACGGCGTCATC

GTTGTGTACGATGTCACTAATGGGGAGTCCTTTGCCAATGTCAAACGGTGGTTACACGAGATTGAACAGAACT
GTGATGTCGTTAACAAAGTTCTAGTTGGTAATAAAAATGACTGTCCATCGAGAAAAGTGGTTGTAACAGAGGA
TGCACAACGGTTTGCCAGTCAAATGAACATACCATTATTTGAAACCAGTGCAAAAGAG
AATATAAATGTTGAAGAAATGTTTCTCACTATCACAAAAATGGTATTAAGATCTAAATTAGAAATGAAAGAGA
GACAAAATGTAACAGCAAATGACACAGTACACTTAAAGAAGACCAACAAAACAAAAAAGCGATGTTGCTAGTG
ACCACAGACAATGGCACTCGGCCAGGGATGTCACTTTAACAATGCTCAAATATTGAGA
GACAGTTGCACAACCCTAGCAGGACACTATGGGACCTCAGTCATATGTAATGTCAGCTAAGAATTGATAAGTT
GTAAATTCTTTAATGGACAGATA
>1002052739_2 Heli.2-EL599786.1.5
TCCACAACGCAAAAGGCACCGGTGGCAGCGGCACAACCTACCCGGCCCTCCTGCGGCCCGACGAGCAGTTCCT
GGTGCCGGACCGGCTGCTGGTGGTGCTCGACATGGACGAGGGCACGCTGGCCTTCTGCGCGGACGGCCGCTAC
CTGGGCGTGGCGGCGCGCGGCCTGCGCGGCAAGACGCTGTACCCCATCGTGTCCGCCG
TGTGGGGGCACGCGGAGATCACTATGAAGTATATCGGCGGGCTCGATCCGGAACCTCTCCCTCTAATGGAGCT
GTGCAGACGCGTGATCAGACAGCGCGTGGGGCGCGCGCGCCTGCGGGCCGCCGCCTCGCGCCTCGCGCTGCCG
CCCGCCCTCACCGCCTACCTGCTGTACCGCGCGCCCTAGCCGCCCCGCCCGCCCTAGC
CGCCCCAACCGCCCTGCCCGCCCTAGCCCGCCACCCACGCCCTAGACTAACTGCAACTGCGACATAGACTAAA
CGGTGTCAAATTGACGTCAATGAGAACAGATAATATTTACTCACTATAGGAAATATATGAATCTCAAGCGCTG
TAGACTCAAGAGCAATAGAATATTATTGTGTAAATGTACACCTGTAAATGTAAAGCTT
ATATTTGCTGTTCATTACACATATGATTTGCTCTAAAGATCAATTAGTACAAACGTTATCTTATGATTGTAAC
TACATGACAATCTCAACTTTCCAGAAATTAAGAGATATTTGATGAATTATGCAAGTATTTTTCCCATCGCCTT
TATGTTTAATCACAACACCTTATACTTCTCTGTATATTGAAGCTGTTATGTAAAAGCT
AAAACGTTAACGCTAGTTCCAGATAATCTAGCAAAAATCGTACAAAATTTTGATCAACTAGCTTTACTGTAAT
GTAGAAGTAAAATCAACGCTGAA
>1002052740_2 Heli.2-DT667054.3.5
ACGCGCCTCTCGCGCAATCGATAGGAATGCCCAGTGATACCTTTACCTAGTATGAAGTGCTTTGGAAAATAAA
AATCTTAAAAGCGAAACTATTGACCATAGATAAAAGGAGGAGCGCGGATGAGTCTGCAGCGACCGACGGGCAG
CTGAGGGCCGGCCCGGCCTGATGGCGAACGTCGCGGTGTACGCACTGCTCGTTACGGC
CGTCACGGCCACCCTTGCGGAGGCTGGTGCGGCGCGCTTCGGCGACCGCCTGGCCCCGCGAGTGCCCGCGCCG
TACGCGCCTCGCAGTGCACCCGCGTCGCGACACTACGTCGAAAATGAAGAATATGATGCTTATGAACAAGATT
ATGAAGAAGATGAAATACCAATTGAGACCAAGCCACCACCTCGAAGACCTACACGTAC
AGAAGCTCATCGTTTAGAAATGAGCACGGAATATTTTATTATTCCCGAAAGACCTTCGTCGGCACCGTCCACC
TCTTCAACATACCGAGTATTTACTGTATCGACATTAAAGCCATTAAATGTGACGCCCGTTCATATAATACCAT
CCCCAAAGTTCAATATACTTAGAACAGAATCCTGGGCAGTGCCAATTTTAGCGTTATC
TAGCGCAAGCATGATCATCATAGCGGGTTTTGAAGCATTCATAGTGTGGGGAGCAAGTAAGAAAGCTCCGAGT
CAGCGACACCTCCTTCTTGGTCAAAGTTTACTTTTTGGACTTTTTAC
>1002052741_2 Heli.2-EL601888.1.5
GCACGAGGCTCGAGTTTCATGTCTTTACTGAACAACAATAATTAATTATTTTATGTTACCAGCTATAAAACGT
GATAGTTCGTGAATATAGTGCATTATGGATTCTTCGGAGTTTCGCGAATGTGCAAAGGCCACTGTCGATTTGC
TTATCAATTATATAGATAATATAAGAGACAGGAATGTTTTACCATCAGTGGAACCTGG
ATATTTAATGAAAATGCTACCAGAAAGCGCTCCAGAGGAACCTGAAGATTGGAAAACTCTACTTAAAGATTTT
AATGAAGTAATTATGCCTGGAATGACCCACTGGCAGTCACCGTACTTCCATGCTTACTTCCCTACTGGGACAT
CATATGCCAGTATGATTGGGAATCTCCTGAGTGACGGTTTAGGAATCGTAGGATTTAC
ATGGATGGCCAGTCCAGCATGCACGGAGCTAGAGGTAGTGACTATGAACTGGTTAGGAAAACTACTTGGTTTG
CCCGAGGAGTTTCTCAATTGTTCAGCAGGTCCTGGGGGCGGTGTTATTCAGGGATCTGCAAGTGAAGCAACAC
TAGTGGCATTATTAGCAGCGAAGGATAAAAAAATTCGAAAGATGAAAGAAATAGATCC
AGAGCTAGATGAAAGCATAACAAAAACAAAATTTGTGGCATACACATCTGAACAATGTAATTCTTCTGTAGAG
AAAGCAGGAGTACTTGGTTCGATGAAAATGAGGCTTCTAAAAAGCGATCAAAATGGTAG
>1002052742_2 Heli.2-DT664192.3.5
TTTAAATTTATATATTTTTGCTCTTTTTGTGAGTTTGTTTTTAATTTTTAAGAAACATGGCGACCAAAAAACA
CTTTCCATATGAATTTAAAAATGTAACGGAGGAAGAGGATAAAATAGTGAAAAAATATTACAAAGATTACACA
CGCCCGTTCATCCGCGTGGGCCCGCACGGCTACATCTCTATGGCAGGGTACGGAGACC
ACGCAGCTGATATCTACAATTTGGAAGTTAGACCGGACGATGTGTGGGTGATTGCCTTTTCTAGATCAGGCAC
TACTTGGCTCCAAGAGTTAGTGTGGCTGGTTGCGAACGATCTGGACTATAGCGCAGCCTCAGCCACTCCGCTG
ACTAAAAGATACGCCTATATTGAATATCCAACGATGGCAGCAGAAACTAAGAAGCACG

AACCCCCACCGCCACCACCACCACCAGGTCGACCCCACCACCGAGCCACCTTCGACGACTTCAGAACCGTCCA
CACGTTGCCTTCTCCGCGTTTCATCAAAACTCATTTACCACTATCGCTTTTACCGCCGAAACTTTTGGACACT
GCAAAGATTCTTTACGTAGCACGAGATCCCCGCGACGTGGCCGTTTCATTCCACTTCA
TGCACAAATTGTTCAGATACTTCGACGGAGGGGTGGAGTTTAAAGAATTTTGGGATCTCTTTAAGAGAGATTT
AATAATGCACACACCTATATTTCCACACATCAAAGAGTTTTGGAACGTCCGTGATCATCCCAACTTAACATTT
TTGTTTTATGAAGAAATGTTAATGAACCTTCGCAGCGTTGTAGACAAAGTATCGGCCT
TCCTCGGCAAGA
>1002052743_2 Heli.2-DT665798.3.5
GCACGAGGGAACGGCTCTAGAACGCCAGCGTGGATAACGTCAAACCAAAAATCTAAATGACGTTACTGTAAAA
AAACATTCAAAGAAACTTTTTAAATTGTGTGAACATGGATTTCTTCGAAGTAAAGGGGAAAATTGGTTTAATG
AAAACGCTCCTAGTTATTTCGTTTTCGTTTACCTTTGTTGCAACAGCAAACTTAGAAT
ACAGTGTCTACAATAGTCCAGTGAGTCACAGCAGCAGAATAGCACCAGAGATTATAACATTTGAGAATAGTAG
AGTCCGTCGAGATGTCGAGTCAACCAATGCCTATGCCGTCACCTCAAACTCTTCCAACACCAATCCAGGTGCA
TCTAACAACACTGCCAACAGCACAAAAATGACAAATGAGACTGAAATATCGTCTTCCA
TCGCGTCGACAGTTGTGACTAAAGTACCACTATTAGGAGTGAACGGGTCGGGGATTTTGCACGTTAACGTCAC
TGCCAATTCACCGAAAGTCAGCGATCCAATAGTACCAGATGACGTTCCAGATATCAACGATGTGTTCAAAGAG
ACCCCAGAAGTTATCAAGGCCCAGCAAAATCTTTCAGAAATTATTACAGACCATCACA
GCTTCTACAACAGTTCGTTCATTGGGAATGTCGACTACTTTAGGGAGTATTGGGCCAATATATCTAAAGTTCC
AGCCGATGTACACGAGCTACTTAGCAACTCACATCGACGTGCTACTACTCTAACCCTTAATTCGACTTTCCGT
TCTACGGTCATTCCGTTCCAACATAACAGTGGCG
>1002052744_2 Heli.2-EL599260.1.5
ATCAGCAATCCGCAATTTTACACTATTGGCTATAAAGTAGAACTTTGGACTGCAAGAGGGGGCGGAAAATAAG
GAAGAACCGGTCCAGACATTATATTTAATCTCTTGTCCACATTTGTTCACTGGCATATATCGGAACAGGGACT
CACCAATTTACAATCACCGGTTACTTGTTATAACCAACATAGTATCACAGTACAGTAT
CAACGTAGTTATCTAAAACACATAAAATGGCTGCACCTATCAAAGTGGTTGTGACTGGAGCCGCTGGCCAAAT
TGCTTACTCCCTTTTGTATCAAATTGCATCTGGTGCAGTTTTTGGACCGGAGCAGCCAGTATACCTTCACCTT
CTCGATATAGCTCCTATGATGGGAGTATTAGAAGGTGTTGTTATGGAATTGGCAGATT
GTGCTTTACCACTGCTTGTTGGAGTTTTGCCCACCGCCAGTCCCGAGGAAGCTTTTAAAGACGTAGCTGCTGC
CTTCCTTGTGGGTGCTATGCCAAGAAGAGAAGGTATGGAAAGGAAAGACCTCCTCTCTGCTAATGTACGCATA
TTTAAAGAGCAGGGCCAAGCTCTAGACAAAGTAGCACGCAAGGATGTTAAAGTGCTAG
TTGTTGGTAATCCTGCTAACACTAATGCGTTTATTTGCTCCAAATATGCACCTTCAATTCCTAAAGAAAACTT
CTCAGCTATGACTAGGTTAGATCAAAATCGCGCACAATCTCAACTAGCGGGAAAACTCGGAGTGCCGGTACAG
GATGTCAAAAATGTTATTATCTGGGGCAACCACTTCCTCCACACAGTTCCCAGAACCA
TCTAACGCTGTAGTCAAAATAGGTGGAGTTGAAAAA
>1002052745_2 Heli.2-DT662606.3.5
TGGTGCATATTTATACGGCCATGAAGCTGTTTTGGTGCTGCTTGGCAGTAGCGTTACTGACGATTACTCAAGC
TGCTGTAGATGTTGAGAAAACAGTACAACAAGTTCAAAGTATTTTGAAAACCAATGCACTTCTACCTAGACTA
ACCAGGGAAGAAATTATACAATTATTAAATGACATAAGAGCAGAGGATGCAAAAAGTA
CCTTACCCAATAATAAAATCAACAATGTAAGAACAACTACAAAGGGTCCCACCAAAAATGTTTCAAAGAAAGT
AACTGAATCCTTAAAGTACAATAACTTACAAGATAATGAAATTGCGACTCAACTTTCACATGTAGAAGTAAGT
AGCGACAGCCTTGTTAAAGATAACGATGATACAGTATCAGCTTCTTCAAGCCCGATAG
AATCTACATCAAAAGCAAGTCCAACGTTAACTGTGGTTTTGCCTTATACGCCTAGAGACGGATCGTCCTTGCA
AGAACTGTACACAAAGCCTCCACGTCTTCAGATCGTTACTGACCAAGTCACTCCGAAACCAATCAAGCAAACG
AAATTAAAAAATGACATTAAGCAAGTACCGAAAATAAAAAATACAAAGAAGCCAGAAC
TAGACCTTCCAGCTGAATTACAGGCATTTTTAGAAGCACATGGCTTAAGAGACAAACCAGGAAAAGACAACTT
CTTGTTACCACTGGAAGGTTTCAAGCCATTACCACCACCAAGGATTGTTGATGGCACGGTACAGTTACCAGAA
AATTTATTACTTACTTATGATC
>1002052746_2 Heli.2-EL602165.1.5
GCACGAGGCCTCGTGCCGAATTCGGCACGAGGCGGACCGTGTACACCCGGTGCGATGTGAAGGCACACCTCAC
TCCTCTGTCTCCGACGGCAGCTTTTGTTGCTTGTGCCGCGTGTGTGGTGAATTTATTAGCTGTTCACGGCTGA
ATCCGTTCAAGTTTTATTCAATGTGTATGGCTGTGGAGCTGGGCAACTGTTCTGTGGC
GACATTGATGGAGTTCCGCTTGGACAGCGCGGAGTACTGCCAGGCCCAACACAAATAAGTTCACGGGCTTCCG
TGGAGACGAGGTAGCGGGCCACGACTAGACCCATGCTGCCGGGAGCGCGCGACTCACGCACCCCAAGCACTTT
GCGATATACACCCACCCATTTTACCTTAAAATCGGATCCCACGTTTAATTGGACCCTA

TAGTTTGCAGGACGCGTCGTCTTATGTTCGGTCGCATAATTTATTAGTTTCGTTTCCCAATTCGGAACCTTAG
TTTGTTAGTCGTCGAATGTGCTCGAGTGGTTGAAATCGTCATCCATGCGGGTGGTTCTAATTATATAATTGGA
CCCTTTTTAAACAGAAGCCACGGAACTGTAGTGTGTTGTGTGGAAATAGAGTGACAAT
TTGTCTTAGACTCTGACGAATCGTTACTTGACGCGTGTGAATTTGCCATCTTGGTGCGAGAGTTTAACTGTGT
ATTGTGCGTCAGTGAAGTAAAGTGGTCATCATGAAGCAGAAAGACATCAGGCCGGCGCCGTCTCTCATTGAGT
ACAAGAACATGCGCTTCCTCATCACCGACCGACCATCGGATGTCACCATCCAAGGCTA
CTTACAGGAGCTAAGGAAGCACAACGTGTGCACCGTGGTGCGCGTCTGCGAGCCGAGCTACGACACGGCGCCG
CTGAAGGCGGAGAGCATCGAGGTGCGCGACCTCGCCTACGACGACGGCACCTTCCCGCCGGCCAACGTTGTTG
ATGACTGGTTCGAGATTTTGCGCGACAGGGCCGCCAACAAGCCGGAGGCGGCGGTGGC
GGTGCACTGCGTGGGCGGGGCTGG
>1002052747_2 Heli.2-EL596112.1.5
AGTCTGTGCAGAGACGGTACAGAAGACGCTATAAACTTATATAAACATATATAAAAATATTTAATCGGCAAAA
CAAGCGGTCGGTCTGAAGGAAGGCATTGTGTTATAATGGTAATGAAATTGAATATTAATTCGGATTAAATAAT
AATTTTAATTAATTGAAGTTCATATGGATTTTATAAAGGTGTCTTCTAATTTGTGTAC
ATCGTAAATTAGTTCTGAAACCTGAAAACGTAAAAGGAAACATAAGCAAGAGGTGACAGCTTGCGGAGTGCAA
ATTGTTGCCGTGACAAGAAAGTGACTGGAAAGTAAAAATAAAGTGTTTAAGTGTATGTGTGTAAACTTAAGTG
TAGAAGTGTTAACTTGTCAAAATGTCGGGCTTCGTTAAATTCGCCATAGATGACTATT
ATTCAGACAATAACTGGACAGAAATCATCAATAAGTATTGGATGCTGGCGGAACGAGTTTCAGATCCTCGTGT
ACAAGGATGGTTTCTCTTCGATACTCCTATAGCGACACTCGCGATGGTCGTAGCGTACCTAAGCTTCGTCATG
GTAGTGGGCCCACTGTGGATGGCAAACAGAAAGCCTTTCAAAATTAAGAATATATTAG
TCGGTTACAACGCAGCGCAAGTTTTACTTTCGTCATATATGTTTTATGAGCACTTAATGTCAGGATGGTGGTA
CGACTACAGCCTCACTTGCCAACCGGTCGATTACAGTGACAGTGATAAGGCGAGACGGATGCTACACTTATGC
TGGGTTTACTATTTCTCGAAGCTTTCGGAGTTCGCGGACACTGTGTTCTTCGTTCTTA
GGAAGAAGAAAAGTCAAATCACGTGGTTACATCTCTATCATCACTCCTTGACGCCATTTGAAGCTTGGCTTTT
ACTTAAGTTCATTGCACGTGGCCACGGAACGTTTTCTATCTTTATCAACAATTTAGTACATGTTATAATGTAC
AGCTACTACATGATGGCTGCCATGGGGCCTCAGTACCAACAATATTTGTGGTGGAAGA
AACATTTGACGACTTTGCAATTGATACAATTCTTTATGGTGCTGTTCCATTCAATTAGCGCGCTTGTATACGA
CTGTGGCTATCCAAAAATCATCGCATCCGGCCTCATCCTACACTCGACGATATTCATTATCCTTTTCACCAAT
TTCTATATTC
>1002052748_2 Heli.2-DT667652.3.5
CGGTTGGATCTTCGGGATGGAATCGAAAGAAATATTAGACAGACTTCGCTTTTTATTAGAATTCTTGGATCTG
CCGAGTGAAAACAGAATGGTTAAGAATTTGAGTGGTGGTCAACAACGTCGTGTGTCATTTGCTGTGGCTCTCA
TGCATGACCCCGAGCTGCTCATCCTCGATGAACCCACAGTGGGCGTGGACCCGTTACT
CAGACAGTCCATATGGACCCATCTCGTTCGCATCACCAGTTCCGGCGATAAGACTGTTATCATAACAACACAT
TACATTGAAGAGGCGAGGCAGGCGCATTGTATCGGTTTGATGAGGAGTGGACGCCTATTAGCTGAAGAATCAC
CACAAGCCCTCCTCGCCATGTACAGCTGTATTTCGCTGGAAGACGTCTTCTTAAAGCT
GTCAAGGAGACAAGGTCAAGCCAACCAAGTAGTAGAATTAAACGTATCGGGCGGAAGCCTAGGGCTCAACAAG
ATGTCCAAACGCGAGGAGGCGCCGTACGCGGCGGAAGACAACCAAGTAGTCGGGCTGAATTTCCATCAAAGCA
AGGAAGTGTTGATCGTAGAACACGCGGCGGGTTCTAATGGGGATCTTCCAGGCAAAAT
GACTGAAGCTGTGAAGTCGGAGTGCGAAGATTGCGGGAACTTTTTCAACCTAACATCCAAAGGCAAGATTAAG
GCTCTCTTACAAAAGAACATACTCCGGATGTGGAGGAACATCGGCGTCATGTTGTTCATCTTTGCACTTCCTG
TCATGCAAGTGATACTGTTCTGTCTTGCCATCGGTAGAGATCCTAGCGGGCTGAGGTT
GGCGATAGTGAACGACGACGTCAACATCATCGACGGTAACTGCCCATACAATGCATCGTGTTCAATGAAAAAC
CTATCATGTCGTTATTTAGCACATTTCAATAATAAATCAATAGTAAAAGAGTACTACGCGACGGTGCCGGACG
CCATCGCGGCCGTGCGGAGCGGCGACGCGTGGGGCGCCGTCTACTTCAACGACAACTA
TACTGATTCACTTGTTGCTAGGTTGGCTTTGGCTGATACAGCGGACAACGAAACTATATCGTCGTCCGAGGTG
CAAGTGTGGCTCGACATGTCCAACCAACAGATCGGACTCATGTTGAACAGGGATATACAGTTCTCATATCGGG
ATTTCGCTAAGGATCTTCTTTCCACCTGCAATTACAATCCCAAAGTGGGTGACATCCC
CATAGACTTCCAGGACCCCATTTATGGTGACAGTAACCCATCGTTCACTGATTTCGTCGCACCTGGAGGTATT
CTTACGATCGTGTTCTTCCTCGC
>1002052749_2 Heli.2-DT665558.3.5
GACGAGGCAACGCCTTCTGGATGTGCCGCTAGCTGCCCCGCACACTGTGAACAGCGAGGAGACTTACCGTGTA
GTGTTATGATGCTAACTTGTGTTGATCTATGGACGGGGTGATATATTATAATAGAACTTGTGATAAACACGAA
CAAAAGGATCCAGTGCAGTGTTAATTAGCGAGTAAACATGCCGAAGATATTCCTTATA

AAGAAACGGCTGCACGAGCAGCAACTCGGGCTACAGGAGGGTCAGGAGCTTCTAGGCAAAGGTGACCCGCTGT
GTCCTGGCTCTCCTCTGGACGATGGTCCCATACCCCTTCTCTCTAAGAAGGATAGAGATTGTCTTGATCGCGA
TGTATTTGGAGAGTGCAACACCAAAACTGCACAAGAAAGACGTACTAAAGAACCTAGG
AGATTTATTTCATCAATACTCGGTGGCGAAATCCCATACGGAAGCCATAGAGGCATACATGTGCTTACTCAAG
CAGAACGAAAGCAATATTTACCGGTTGTAGCAGAAGATAAAAAAAATGATGATAAACCCCTTATCAAAGAAGA
AAAAGACTTATTAGAAACTGAGCCAGTAGTTTTACCATCTAGAACTTCACCTTCACCT
GAAACTGCTCCATCTCCTGGTATCGATAGTAATGTCACCTGCCAAAAAGTTTCCGTAATACAAAGAACACCTT
CTCAATCACAATTTCGCGATAATAAAAAAGAATTATCAGATGTAACTATTCCACCTACTTTGCCTTCTTCTGA
ACCGGAACAAGATCAACCCATTGATTACGTTATTGCTAAAAAAAGGGGTGAATCTGAA
GACGAAGAGACTGAAAAGAAAATAAGAGAACAAAGAAGGACTAGCAGCTCTAAAATTGCTAATGGCATTTTAG
CCAGACCTGTTTTAGTATTACGAAATCGTCCTGGGGGTAAAGTTCCCGGTATTATCAATGCTGCAGCTGGCCA
CGGGCGTTCAACAAGCGGAAATAGTTCAGGTGGAAATACGCAGAATAGTGGAAGTTCA
GGAAGCTTCAGTTCTAGCTCTGGGAGTGCAGCTCCATCATCAGGTGGAGGAGGAGCTTTAGGAGGAAGATCTG
GTAGTGGTGGAACGGTCGTGATGGACGTCAAATAT
>1002052750_2 Heli.2-EL599919.1.5
GTGCGCGCTCACAACCAACCGACCCCTCCGACCACAATTCAAAAGGAATTTTCAATTATAAATTAAATAACTT
GTGGTTGTATTGGTAAATATCTGTCGCAATTGTGCGGTGTTCGGTGTTAAACGGCGGAACAGTGTCAAAATGA
AGACGGATTATCCTATGGATGAGATGCATAAGAGGACGAGCTCAATCATCGGTTTGGC
CGATGTAACAGACAACAAGCTGATCTGGAGGCAGCTGACGGCGGAGCTGATCGGCACGTTCCTGCTTACCTCC
ATCGGTGTCGCTTCGTGTATTGCCATATCAGATGCAGCAGTACCACACGTCGCTAGTATTGCGTTGTGCTTCG
GTCTTCTCGTTGCGTCTATTGTTCAGGCTATAGGCCACGTGTCCGGCGGACATATAAA
TCCAGCAGTGACATGCGGACTCTTCGCGGCTGGCGACGTGAAGCTGTTGAAGGCGATATTCTACATCGTGGTA
CAATGCTTGGGAGCTATAGCTGGAGCAGCGTTCATCAGGTTGGCTGTACCTGAAGATAGAGTTATGTCTTTTG
GAATGACTCTACCTGCATCTAATGTCACCGAACCACAGGCACTGCTAATAGAGGCGCT
GATCACGTTCGTGCTGGTGATAGT
>1002052751_2 Heli.2-EL604196.1.5
GACGGACGAGTCACGCAGTGTTTGTCGTTGTTGCTAATCTATTCAGTTTACTTGTGGTTCGAATAATCGAACA
CAAGTGAGACAATTATCATATTGATTACTATTTTGTTATAATAAAGTTACAATTTATTTGAGAAGCAGCCAAT
TTACTTAACTAATTAAAAATGTCGGTTATAGGCAAATTTGTTGTGCCTAACGTTGCGG
CCCGGAATTTTACTAAAATCGGTGCCGCGCTACAAACTGTTAAATCGAACAGAACTTATTTTACTTATACACA
AGAATTATCGCAGCCATTGGATAGGAAACCTGAATTCCTCAGTGCCAAGCAAGCATTTGAAAAATGTTTAAAA
TCAGGTCAGACAGTGTTCTCGCAAGGCGCCGCGGCCACGCCCGTGCCGCTGCTGGACG
CTATGACAGAAGTGGGCAAGGCTGGCTCATTGAGAGACATCAAAGTCGTGCATATGCACACCGAAAAGGATGC
CGCGTACGTTGCACCGGAATGCAAGGATATATTTAGATCCGTATCTCTATTCATGGCGGCAAACGTAAGGAAG
TCAGTAGCGGAGGGTCGCTCGGACGCGATCCCTATCTTCTTACAAGACATTCCCAAGT
TGTTCCACAGGAAGATCATCCAGCCCGACATTGCTCTTATCCAGGTTTCTCCCCCGGACCAACATGGATATTG
CAGCTTGGGTACTTCAGTGGACT
>1002052752_2 Heli.2-DT663150.3.5
GCACGAGGGTAAAACGCACGCAAACACAAAACATGTTTTCGTCGCGACTGTTGATTATTTTCGCATTTGCAGC
ACTAACGGCTGCCTATCCCGCCGATAACGGGCCGTTCCCGCGGCCTATTACAACTATCACAGAAACTGAATTC
GATGATCCGTCACCATGGTTCAGATTCCCGGCGTTTGGTAATATTTTTGCACCGCTAA
CTAGATTGTTCTCTAGTTTCGCTGATATTGGTCCCAGGATCGAAGCTGATGACGAGAAATTCAGAGTTATAGT
CAACGTTAAAGATTATAGCTTGAGTGATTTGAAAGTTAAGGTGAAAGGCGACTTTATCCTCGTGCAAGGATCC
CACGAAGCCAAACAAGACGATCACGATCTGTTTGCCAGCCAATTTGTACATACATATT
CTTTGCCGATCAACGCTAGCGCGTCGGATGTAACAGCGAAACTAACATCAGATAAATATTTAATCATAACAGC
TCCACTTAACGGTGCGGGTGCTGATGACGGAAAGACAGTAGACAGAGAAGTACCTATTGTAGAAAGTAAAGAA
GCATGGAAGAGTGAAGACAAAGAAGATAAAGACAAGAAGCCAGAAGATGTGCCGGTTC
AGGCCAGCGGCGTATTGCCAGTGGAAATTGATGAAGCCAAAGGCACCACTGATCTTCCAGCGACAGTAGCTGA
GGAAGAGGACAGAAAAGAACTTACTACCCCGTCCAACCGTGAAGCAGTAACCGTCCGTGATGTACCTAACGAA
GTGAACGAAATCAGCAGCTCTAATGAAGTCCAGCCTTAAGTGAATATTTTAGTATGTA
TGTATATAATATATATAAAACAGTATTGTAATAAATATTTTTAATACTAAACAGTGTTTTGTTTGCGTGTGGG
TATGCGCTTAGTGCGTTCATTGTTAGCGACCTGAATACAAATCACGTATGGCTAAACAAAGATTTCAATTATG
TTTTTTATCAATTGCACGCATCTCTTGAATTTTGCATGGTTGTTAGAGAAACTAGAAC

GCACAACATTCACATACTTATATTGAATATTTTATTTGAAGAANAGGAAAACATACAACCTGGTATATTAATT
TTTTTTTAAATATATTATATATAAGTATATATGTATGTATAATTTAGCATATAAATCGATGCGTTTTGGTAAA
TACTATCCAAATGCGCCGCAGT
>1002052753_2 Heli.2-EL599315.1.5
ATAACTTCACGAAAATTAACCTGTATGGATCGCTAAAATGTGAATTGGTGAAGGTCACCGTCACGTCATACGT
CGCTGCCAAAGCCAATTGCGATTTGCGTATTTTTCCGCGTCGCGCTGAGACTCGAGCCGCGAGCCGCGAGCCC
TGCAGCAGTGAGTGAGCGTCGCGCCCCTTTACGTAACGAGCCTACGACAACTAGAACA
TAATACGTTACCACCACTTGAGATAATCTCCGCTCGCAGGACAAGAAAAACAAAATGATGTCTACAATATGTG
CGAGGGCCGCGCTCCGCGCCCGCTCCATGGAGCGCTGCGCGTCGAGCGTCGTCGAGCTGCCCAAGTTCCAGGA
CTTCGGCATCTACAACGAGCCTGTGCTGGGGTACCGCGCCGGCAGCCGTGAGCGTTCC
GAACTGATGGACGAGCTGAAGCGCACCTCCGCCGTCACCGAGGAGGTGCCTATCGTTATCGGGAGTGAGAACA
TCAAAGAAGGCCCGCCGCAGTACTAGGTGATGCCGCACGACCACGGCCGCAAGCTCGCCAAGTACTACTATGC
CAGCGAGAAGACAATACAGAAAGCGATCCAGGTGTCGAGCGAGGCACAACGCCGCTGG
GACCGCACCCCTCTAGATGAACGTATCCGCATCTGGCAGCGCGCGGCGGACCTTATGGCCGGCGAGCACCGCC
AGCGGCTCAACGCCGCTACCATGCTGGGACAGTCCAAGACCGTCGTGCAGGCTGAGATAGACGCCGCCGCTGA
ACTCATCGACTTCTTTCGCTTCAACGTCTTCTTTTTGAAGGAGAATGCTAAATATCAG
CCAATCTCCGAAAATCCCTCCGTCACGCGAAATTCGCTCAGGTTTAGAGGCCTGGATGGCTTCATAGCTGCTA
TCAGCCCATTCAACTTCACAGCCATTGGAGGTAATTTGGCATACACCCCAGCTCTGATGGGTAATGGAGTAGT
GTGGAAGCCTTCTGACACTGCTGTCCTATCAAATTGGCGTATCTTTAACATTATGAGG
GAGGCAGGTCTACCTGACGGAATTGTTAATTTTGTGCCAGCAGATGGCCCTACATTTGGTCGCACTATCACTA
AATCCCCGAACTTGTCAGGTATTAATTTCACTGGATCTGTGCCTACTTTTAACTGGCTCTGGAATGAGGTTGG
TAAAAACCTTGATATGTACAAGAACTACCCCCGGCTCATTGGCGAGTGCGGTGGCAAG
AATTACCACTTCATCCACCCATCAGCTGATGTACAATCAGTTGTAACATCTACAATTCGCTCTGCTTTTGAAT
ATTGTGGACAGAAGTGCTCCGCATGCTCAAGAATATATGTTCCTAAG
>1002052754_2 Heli.2-DT663667.3.5
CACGGCTGCCCCCGGCACAGGATACGTCCAGCAAGGCGAGCTCATCGAGTACGCGGGGGGTGCGGCAGGTGGC
CCGGCAGGCCACCACCAAGCCTACCGACGACATCCCCGTGTTCGACCGCAACAAGGTCAGCCTGGACTTTCCG
GGGAGCCTGTTCGGGCCATCCGTGTCGCTCTTCATCCGTACCACTAAAATTATTGGCG
ACGTTATTCAGAACTCGGCAGTACGCTATCAGTCATTCTTACGATTGTTCCGCCCCCTGTTCCGCGGTCCCTT
CGAAATCAAGGGCCTGGACCCTCCGACCACCACCACCACGCGCCGCACCACCACGACGAGGGTCACCACGACC
ACGACGCTCGCACCACCCGCTGACAATGAAATCAATCGTAGAAGATAAACCTAATTTT
TTGCCATTCCAATATGCAAACTTTAAAATATTGAATAAGATGAGTCATTCATTGACTTTAATACTCATATTTT
ACATTAGTTGTTAATGTTATAAAAAAACCGGCCAAGCGCGAGTCGGGCTCGTTCACCGAGGGTTCCGTACAGA
AATTAGAAGTCTCAAGCTTTTTCTGAAATTGGGGCTTACTTTAGCCTAGCCAGAGCTC
TCTGATACCTCACTAGATCTAGCGGCATTAATACCAAATTTTGTCCCCCTTTTAAGGTTATTATTTTTTAGGA
TTTAACCATAAATGTATAGTTTTCATATTTTTTTCTGAATTAGTAATCCCTGTAGCTGCTCACTGCCAAATTT
CATAACTATATAATCATATAATCAACCCGAAGTACCCTCTTAATTTTGATACCCTTTA
TAGTATCGAAATACGTGTTGTTTTGGTATATAAACTACCCTACTATAT
>1002052755_2 Heli.2-DT664848.3.5
GACGAAGGACGTTCATTCAACACAAAATTCTTCGACTAATAATATTTTTTATATATATGTGCATAAATGTGAA
TAAAATGTGGACTCTATATATTTGGGCATTATGCCTGGCGGCCATGTGTCAGGCGCAGCTGTCGGACATCGAG
AGATATTTATTGACTATCGCCGAAATGTCACCGTCGCCCCCAGTACAGAGTAACGAAA
AAGCATGTCAAGTTGATAGTTCTACAAACGGTGTTTGTGTGAAACGTACACAATGCAACTTGGACGCGCCGGC
AGTGGATCCGGCTACTACTTTCATACTGCGTGAAGGAGGATCCACGCCTAATGCATCGGATGTGGCGAACGCC
CTAGATACACAGTGCGCATGGCTTCAAATCTGTTGTCCATCTGATAAAGTCCGGGAGG
AAGAACGCCAAGTGAAATTCGAACAGCCACTTGGCTGTGGCTACAGCAACCCCGGCGCCAACGTCTTCAGGGA
ATCTGCTAATGCTTACGGCTATGCAGACTTCGGGGATTTCCCGTGGATGATTGCTTTATTACTGAAAAAAAAC
GACAAAACAAAATTCAATGATCAGTACATAGGGGGTGGAACATTAATTCATCCCTCGG
TCGTGATGACTGTGGCTCATAAAGTAGACACGCTCGAGTCACCTAATGAGTTGACGTGTCGCGCTGGAGAATG
GGATGCAGTAACAGAGGACGAACTCTTTAAACATCAAGACCGAGATGTTAAAAATATTGTCATCCATAAAAAT
TATCTATAAG
>1002052756_2 Heli.2-EL601238.1.5

GCACGAGGAACATCAACTATAATGTCAGCTAAGATTGTGATCGTCCTCGCCCTTGTTGCGGCCGCTTCCGCCC
TGCCCGTCTTACAGTACGCAGAACCCGAATCGCCCGCCCACTATGAGTTCCAATACTCCGTACACGACAACCA
AAGCGGTGATGTAAAACAGCAACAAGAGGCCCGCGCCGGAGACGCCGTCCACGGCTCC
TACTCGCTTGTGCAGCCTGATGGAGTCCACCGCATCGTGGACTACAGCGCTGACAAAGTAAACGGATTTAACG
CTATCGTGCGTTACGAAGGACAACCCATCGTATCTGCTCCCGCTAAAGTCGCCTACGCACCCGTCGCCAAGCT
CGCCTACGCCGCTCCCGTCGCCAAGGTGGCCTACTCCGCTCCCGTCGCCTACGCCGCC
CCTGTCGCCAAGCTCGCCTACGCCGCTCCCCTCGCGCACGTCAGCTACTCATCCCCCGTCATTTCCTACCAAC
ACTAAGTCAATAGCTATAGCAGACATTCTACGTTAAACTTACTATATATATAATATAAATATGTTATTGATTT
TAGGATTATAGCAATAAAAACACTTTTAGTTTCTTCTGTTAAAAGAAACGACATTAAA
TGTTTATTACTACTGTTACAAAACTTCTATATCACAAAATTTTACAATGTACACGTTTCTCATTAAACGGATA
AAATTTAAAAG
>1002052757_2 Heli.2-DT664297.3.5
GCCGCCGATACTCGATAGTTGGATTTTGTGAGGAGTAATCCGTGTATTTGTTTTTGTTTGTGACTGAAATACT
GCTTTAAAATGTCATCCAAATCTAAAAAGACTGTAACATCGTCAACCAATATAAGTGTTGTGTCATCAGTGCA
AAGCCCTCAGCAATCTAGCACTCCGGTTGGTAGCCGCCCTTCAAGTTCTGCCGGTCGA
CCTAACAGCCCATTAAGCCCAACAAGACATACACGTCTTCAAGAAAAAGATGCTTTACAAAATCTTAATGACC
GTCTCGCAGCGTATATTGATAAAGTTCGTCAGCTTGAGAGTGAAAACTCTGGACTTCGTCGTGAAATTCAAAC
AACACAGGAGGTGGTCACACGTGAAGTCTCTAACATTAAGGGAATGTATGAAAATGAA
TTACAAGATGCAAGAAAGTTACTAGATGATACATCAAGGGAAAAAGCAAAACTTGAAATTGACTTAAAGAGGT
TGTACGAAGAAAATGACGATCTTAAGAAACGCTTGGACAAGAAAACTAAGGATTGTCAGCAAGCCGAGAACTT
GGCGCGTCATTACGAAACTCGTTACACCGAGGAAAGTAACAAATACAACACTGCCCTT
GCTGACAAGAAGAAGGCTCAAGATGAGGCCAGGGAACTGGCCAAAGAGCTTGAGAAGTTGCGTAAAGTATACG
CCGATACCCGCAAGACCCTGGAAGAGGAGATGCTGTGTCGCATCGACATGGAGAATACCGTGCAAAGCCTGCG
CGAGGAGCTCTCCTTCAAGGATCAGGTGTTCCAGCAAGAGTTGCAGGAGACCCGCACT
AGACGACAGGTTGAGATATCTGAAATTGATGGTCGTCTGGCTCAGCAGTATGAAGCTAAGCTACAGCAAAGCC
TACAGGAGCTGCGTGAGCAGCAAGAGGCTAATATCAAGGCCAATAGGGATGAAATTGAAGCCCTTTATGAGAA
TAAGATGAAGAACTTGCAATCAGCGGCGACTCGCAACAGCAGCGCCGCCACCGTGGCC
GTGGAGGAACTGCGCACCATGCGCACGCGCATTGATAGTCTCAACGCTACACTTAATGATTTGGAGAACAAGA
ATGCTTCTCTTAGTAACCGGTGCCGTGAATTAGAACGTCAGTTAGAATCCGAGCGCGCCCGTCATGCCGAAGA
TCTGGCTTCTATGGAGCAGGAGTTGGCCCGCCTTCGTGATGAAATGGCGGCTCAGCTG
CGTGAATACGCCACGCTTATGGACATCAAGATATCCCTGGACCATGAGATCGCTACGTATAGGGCGCTTATTG
AAGGCGAGGAGGACAGGTTGAACCTAACATCCCAATCGCCCGGGCGCGAGTCGCGCGCGTCCGTGAGCGCGGC
CAGCGCCAGCGGCGGTCGCGCCACGCCTGGCCGCCGCGCCACGCCGCTGCGCGCCGCG
CGCAAGCGCACGCTGCTGGACGAGAGCGAGGAGCGCAGCCTGCAGGACTTCAGCGTCACGTCCAGCGCCAAG
>1002052758_2 Heli.2-EL597724.1.5
GCACGAGGCCTCGACTTACGTAGTATAGAATTACATCCGTGAGTGGAAAAACTTTTAATATTAACAAAAAAAA
ATGTGCACATAGTTTTTTTAAACATAACACAAAAATGGGTTCTAAAATAGAGTACGTTGATTCATCTCATTTA
TACGCCACAAACTATGTCCGGAATTCGAAAGCTATCGGAGTGCTTTGGGCTATATTCA
CGATTTGTTACGCGATTATAAGCGTTGTGGCGTTCGTTACACCGGAGTGGATAGGTGACTTAGAGACGGAGTA
TCCCAGGAAGTTCGGCCTCTTCCAAATATGTAGGACGGATGACTCCATCGAGGACTGTAAAGGCAGGTTGGAT
GACTTCCTGTCGATCAATGGTTTTGTGTTCAAAGTGGCCACCGTGCTGGTGGGAGCGG
CGGTAGCGCTGGCCTTGTTTACCATCTGTGCCATACTTTTGTTTTTCTTCTGCCAGTCGACCACTGTGTTCCA
TATATGTGGATGGTTGCAGCTGATTTCGGCTATGTTCATGATCGGTGGTGTGGCGGTGTACCCCGCGGCGTGG
AGCGAGACCGCCGTGCAGGAGACCTGCGGCCCGACCGCCGACCAGTACAATATTGGTC
GCTGCCACATCCGCTGGGCATATCTTCTGGCGATCATCGGATGTTTGGACGGCGTGGTGCTGGCAGCCCTCGC
CTTCATACTGGCCACGAGACATGTGAGACTACAGCCCGATACCTCGTACCCTGCCGATTTGTATAAGGGTGAA
GTAAACAACGCATACGTAACAGATGCCACCTCGGTCTCAGGTTCCCGCAAGTCCCTGG
CCCTCCAGCCGGTACTCTTGATGCATCCTCACGCTCCTCTCGACGTCGACACCTACTCGCATTACTCGGGTCG
TACTGCAAGATCTAAACATGGTATTTATGCAAATACAATGCATAATTATCAGTTGTAGAATTATTATTTTTTA
TTTTTTAATTAGATTAAATTTAAATGCGCGGCGTTGAACTCCGCCGCTTAACACACAA
TGTTTTGATTTGGTGAAGAACTCAGTAGTTAATACTGATGCACTTATATTATATTACTCCGACCGCATTACGA
GGTCTAACATGGTATTTATTTTTGTGGTAATATAATGCATAATAATCGGTCGCAATATTGAATTCACAATGTT
ATTATTATTT
>1002052760_2 Heli.2-DT666080.3.5

AGCAACAGGAAAAAGTAGAAAAATTACATGAAGAGTTTATAAATAAACAAGCAACAAAATCCATACCAACTTA
TACAACGACGGAAAGTTACGAAGAGTTCATTAAACGAACTCAAGAAAAGCGAAGACCTTTACATCCACATGAA
ACAGATTTATTTAGAAAAGCTGTTGAACTTTATGAAAAAGATCACCCGACTACAACGA
CTACGACGACGACCACCACAACTACAACAAAACGCCCGACCACTGCAAGATACAGAACTCGTTCACCAACGCG
AACCAGACCTCCTCCTCAAGATAGGAACAAACAAAAACTTTATAATGAAATAAAAAATCTTCTCGAAGAAAGT
GACACTAAAGGTTTTGATGATAACTTAAGAGCAAAGAGTGTAGCACTTTTGCAGAAAC
CAGACATATTAAAGCAACTTAAAGTAGCTTTGGCGGAAAATGCAGAAGATTTTAATGAGAAAAATTTTACATC
TCGTGAAATTTCTTTAAACGGACAGAAATTTGAAGTAATAAGAACCACAAATCCAAATCTTATTCCACAGGGA
GCGATTGCAGCAGATAGTCCTGACTTAGGTAAACTTATAGCTGCTACTCAAGAAACAC
CTAAGGAAAGTCGCATTTCTTTCGATGATTTGACAAAAGGTGTCCTCCCACCTGGTGCCAACTTCGAATTAAT
CAAACAGGCAGATAATGGAAAATTGGAAGAAGTATCTAAAATACCAAATACATTACAAAACAAAAAGAAAGTT
ACTTTCGTTTTCCTTGAAGAACAAGACGATGGTTCATTTAAAGTAAAAGGTGTGAAAG
CCAATGGGCAACAAACGGAAGAGGGTCCTGAAGTGGAAGATATTTTAAGCAAAATCAAGAAAGGTGAAATACA
ATTGCCCGGGCCAACAAAAATTTCCAACAGTGGATATACAACTAGCACTCCAAGTCCATCCACTTATACAACT
GATTATATAGCGGAATCATCACATCCTCCTTCAACATACACAAGTTTCGTGACAACCT
CTAATTACGGGGATTCTGGACACACTAACCCTATGCAAACAACCCCTGCACCGACATATCGCACGATAATACC
ATCGACAAAACCTACTCCTCTGGCTCAAACATTCCCCTCTAGCACACGAGGAACTACAAGTGAAAGATATTAC
ATAAAATCTTCTACACCAAACTATATTAAAAATAGAAATGGTTACAGTTCCCCGTCAA
GAGGCCACGTCCAAAGTAGTACAGTATTAAATACAACACCTGTTTACTCAACATCTTCATTCCGAACCACACC
TCAAGACATAGTCGTTATAGGCTCGAGCGCCGCCCCTAATTTTGAAGAGTCTACCATCAATTTAAGCTACGCC
AAGACTGGCAATCCAGATCTTATAGATATTTTGAAAGAAAACGGTCTGTTTGCAACCG
CTAAATATCTGAAACAGTCAGGTTTGGATGCGATATTAAATGAAACTGGGCCGTACACAATATTTGCACCGAC
AGACAAAGCATTTAGAACGTTGCTAGTTCAATTGGGTGGTCCCGATAGAGCAGAAGAGAAGTTCCGCGATAAC
CCAAGGCTATTGAGTGGGCTTCTCCTCCACCACGTGATCCCTGGTGCTTTCGACATCG
GCTCGTTACAAGATGAGATGACAGGGGTGTCCCTCGCGGGCACCCAGCTCAGGGTCAACC
>1002052762_2 Heli.2-EL602296.1.5
GAAGGATGCATGTTTTAAATGTTTACGAGAAGTTTTAATTTATAATTAACATAAATCTAGTTATATTTAATGT
ATATAAAAAGCATGTGAGATTGTGTTTTCAATTGTGATATAAGTATTTGAGGTTGCTAAGATAAGTTATAAGT
GCTTTCACTATTTACGATTGTTTGTATTGAAATACTTCGTCGGTAAGTTTTTAATAGA
TTATTATTAAAATAAAGAAGAGTGATATTAATGCATACAATGCCGTCAATATCCAGAGATAAATATTATTTAT
ATAGTAAATGGAAATAAATACATGAAGATGTTTTATGACGTATATAAAATTACAATTATATTAATGTACGCAG
AATGCCTGCCGCCATCTTCGTTAGAAGCGTGTATGGGAATTGTGAAATGAAAGTTAAA
TAGGTTTGGTGAATTATAGTAACTTATAAATGTAATTTCTATATATATTAATAAATATTTAACATGTTACTAT
AAAAGGAAATATACATATAATAGTATAATTTATAATATGATATAAAACTATTAAATCAAAATATATATTTACA
ATTTATAAATATTATATAAATATCAGTCTGTTTAATGTCCTACGATAAAAGTAATCTT
TCAATATGTTTTATATATTACTTTAATTAAATATACTAGACATTAGACATTAACCAAATATTAATTATTTAC
>1002052763_2 Heli.2-DT663601.3.5
GCACGAGGCTCAAACTTAAAGAGGACCAAGAAAAGATAGAAAAAGAAAAGGTAGAGAAAGAAAAAAAAGAAAA
ACTGAGAAAAGAGCGAATTGAAAGAGGAGAAGATGTTGAAGATGAAGTGGAAAGCGATACTGAGAACAAAAAA
GCTGAAGAGCTTGCTAATGATAAGAAACTGAAAGAAGAGAAAAAACAACGCCGAAAAC
GTCGAAATGAGGATGATGATGACTCTTGGGATGGTGAGTCATCCAGTGAATCTGAAGAGGATGGTCTCAGTGA
TGATGAAACTGTGGTTATTAAAGACAGAGATGATGTTATAGACGATGTCAAAGACCCAATTAATATGGTGATT
AAAGGTATTGCTGGTAAAAACGATAGCTACTTTGACTGTTCATTAGGTCAATTTTTTA
TAAACATAGGTATTAATTTAGTGCAAGAATATGTCCAAAGTGATTTGCTAAAAACACAAAACAGAAAACTTTA
TAAAGAAAAGAAATCTGGTCGTAGTACTAGAGCTACTGAAACTGCTATAGCCTCTTTGACTCAAAATCTAGCC
TACAGTAAAAAACTAAATGCTCCTTATGCTCTTGAGCAAAAACGATGTGATTTTTGTA
GTTTTAAAACTGAATCTATTTTAGTTATGGCTCATCATTTGGAGGCGCCTCATATGAAAAACAATATATATAA
ATGTAACACATGTGCATTTGAAATACGAAGTCCTCATGATATTCTGT
>1002052764_2 Heli.2-DT662629.3.5
GCACGAGGTTGGCTCCTTGCATTGCTCATTAAAACAAGTAGAGTAGAAGCGTTCCTTTCGTTTATTATTGTGA
TTCACTTTGGTTTATTCTACTTTTAGTGTAACTGTCTACATTTGTTTATAAATTAAATAAAATAAAATGCGCA
AAAGGAAGTCCCTTAAGAAAGCAGACGACGAATCCGACGCTTCGGAACCTGGAGCCGA

ACCAGAAGAATTTCAGGACTCTGGCGAAGATTGGACGCCGGATGCCGATTCGAATGAACAGCCAACCCGTGGT
GGCAGAAAAAGGACCTCAAGAACATCATTAAACAATTCGAAAAAGAAACGCAAAAATTCATCATCCGAAGAAA
GTGAAGGTGAAGCTGAGGAAGATGAAGAGGGAGAAGACGAAGAAGCTGAATTAGATGA
AGATGAAGGTTCCGATGATGAGAAAAATGGCTCTGATGGAAATAAGTCTGACTCCAGTCAGTCAAAAGACATA
CCAAAGCATTTTCATTCTGGAAATTTTGTTTTGTTAAAATCTGACGTGAAATGGGATGGGGACACAGTCATTT
CAAAGTTGGATGAATTAAATCTGTGGAAAATAGATGGAAAAGCTTTACTACAGAAATT
CATTCCTATGGAATCAAATGGAAAAATTTTACATAAATGTACATGTGTGTACTCTGGATGGAATGTTGACAAC
CGGGACAATTACTACCCTATAACTGATATATTGGACAGAAATCCACGTACAGACTCTAAAGAAATTTGCGTAG
CTTTAGATCTTAATGATCTTATTAAAGTAAGAGA
>1002052765_2 Heli.2-DT662299.3.5
GCACGAGGTGACACTGTCGAGCGGAAGGTCGTCGACTCAGCGGCTGAAACTCGTGAATTACAAACATTACTTC
AATACCGCACTATCTCGTTGTTACATCATATTGCAATCGAAATCAGTAATTAGACTCCAAGAGTCCTACATAG
GCGGTCGTGTTGTGCATTAAATAAATAAAAATGGGTTCACGTTTCCAAGTCGTCGAAC
AAGGTCCACCTATTGAAGTTTTCCAACTTAACAAACTTTGTACTGAAGATACATTTAAAAATAAAGTAAACCT
TGGTGTGGGGGCCTACAGGGATGAAAATGGCAAGCCCTGGGTTCTGCCTGTTGTCCAGAAAATGGAGAAGCAA
ATGGCAGCTGATGCGAGCCTGCTCCATGAATATCTGCCAGTACTTGGCTTAGAACAAT
TCAGCAAGGCTTCAGTGTCTATGCTTCTGGGAGAAGACAACCCTGTGATTGCCGCTGGCCAAACTTTCGGAGT
CCAAACACTGTCGGGAACAGGTGGTCTCCGAGTCGGCGCTGAGCTTCTAAGCAAACACTTGAAATACTCCACA
TTCTACTATTCAAATCCAACATGGGAAAACCACCATCTAGTATTCGTAAATTCCGGCT
TCACCCAGCCGAAGACCTACCGATACTGGGACGCCAAGAACCGCGCAATAGACTTCGAGGGTATGATCGAGGA
CCTGAAGAATGCTCCAGAGAATTCCGTGATAATACTCCACGCGTGCGCTCACAACCCCACCGGTATCGACCCC
ACTCAGGAGC
>1002052766_2 Heli.2-EL598831.1.5
GCACGAGGCTTTCTTGTGTGTCTTAAGAAGAGAAACAGTTAAAAATTAACAACTTATTTTAACTTAATTGTTA
TTGTTAATATAATAAATCTGACAAGTGTATTCTTATTCTCAATCAAATATGCCAGTTGCTGATGAAATCCAGT
CCTCATGGGCAGATGAAGTTGAAATTGATCAAGGAGCCCTACCACCTCCTTCTGAAGT
AGTTGAAAATGGTCTAAAAATTGTAACCGAATATAAATACGACAATGACAATAAAAAAGTAAAAATCGTACGG
ACATACAAGATCGAAAAAAGAGTTGTATCGAAGAGTATTGCAAAACGCAAGACATGGGCCAAGTTTGGTGATT
CTGCAAGTGACAAGCCTGGACCAAATCCAGCCACCACTAATGTATCTGAAGATGTCTA
TATGCAGTTTATTACAAGCAAGGAGGAAGCACAACGTCCAGATGAAGGTGATTTAGAGGCACTTAAGGCTAGA
CCAACTAAGGCTCTGTTCAAGTGCCGTACTTGTCATGGGGAGCATTGGACCACAAGTTGTCCATTCCAACACA
CAGAACTGGCTCAAGCTAAAGCAAGTGAAGCTGCTAAAGCTGCAGAGGCAAAAGCGGC
GACTACGAACAAATATGTCGCACCTAATATGCGAGAGGGAGCCAGCCGTGTTCCCGGTCGTGAACCTCCAGGT
GCCAAGCGAGACGATTTGGCAGCAATTCGTATTTCTAATCTCAGTAACTTTGCTGTTGAAGCTGATCTGGATG
ATTTGGTTAGAGGATTTGGGCCTGTTCACAACTT
>1002052767_2 Heli.2-EL599535.1.5
CGGCACGAGGGCAAATTTTGCCTCCACTCCTCTTAAACAACTTACAGTGTCAAACATAACTTCAGACATTTAA
TATCTGATAACAAAGTATAGTGAGATTATAATAGATAAAAAGTATTGTGTAATAGTAAATAATGCATCTATTT
ATCGCGATAGCGACAGTTGTGGCCGTTTTACTGCCGGTGGACGACGCGCTTGCAACAG
ACATGGCTAAAAATCAAAAAGTACTCGTATCGTGCATCGATTTTCCAGAAAAAGCATTGAATTTACTTGGAGA
ACATTTCACAGTACTCCAAAGTAACTACGTAAATGTCGAGAAAGGCATCGTCACGGTAAACAATGAAGATCTA
CTGAAAAAACTACCTGGAGTATCCGCGTTGATCTGGGCGTCCCATCTTCCTATAACTA
AAGATATATTGGATACAGCAGGTCCTCAATTAAAAATCGTAAGTACAGCATCAGCGGGCTACAATCACTGCAA
CTTACCAGAACTGAAAGCGCGAGGAATCAAACTGTCGAACACACCCAACGTGCTCAGTGCTGCTGTCGCTGAG
GTGGTCGTCGGTTTGCTGCTCGGTGCGGCGAGACGGTTCACTGAGAACCTGGAGAAAG
TTCGTAGTGGCGAATGGGAAATTGGATTTGGTAAAATGTTGGGGCAAGATGTTCGCGGCAGTACAGTGGGCAT
TGTTGGCTTCGGGGGCATCGGGCAAGCCGCCGCGAAGAGACTGTCCGGGTTCGAGGTCGGCAGGTTCCTGTAT
AGTGGACACAGGGAGAAGCCTGAAGCCAAAGCCCTCAACGCGGAGTTCACTTCACTTG
ACAATTTGTTAAAAGAAAGTGACTTCATAATACTGGCGGCACCCCTCACCGACGAGACCCGGCACATGATCAA
CGGGACCACGCTCGCGAAGATGAAGAAGAACGCCATACTTGTCAATGTGGGTCGTGGAGATTTGGTCGACCAA
GAGGCTTTATACAATGCTTTGAAGAACAACGACATCTACGCGGCCGGCCTCGACGTCA
CCTCGCCGGAGCCGCTGGCCCAAGACCACAAGTTGTTGACTCTGCCCA
>1002052768_2 Heli.2-DT661961.3.5

GCACGAGGGATCCCTTAGGGAAACATGCTCATGTGATTGCCGCCAGATTTCTAATTTCGAACACTTTTTTTCA
CAATCGACTGGCGTTTTGTGTTTTGTGTAATTTTATTTTATTTTTATCTAATTGAACCGTAACCCGTGATTTG
TTTGTTTGTGTGTTTTATTTACAAAGAAAATGACACGTAAAAGCCAAACACAACGCAG
CCGTTCGTACGAATCAGACGAGCACGAGAGGGGTGCTTGCGAATGGTGTGAATATGCCTTGATTATCACACTA
GCTACGATCCTCCTGATAGGAGTGTCGGCTCTCACTATATTCTGGACCTTCTTCTATAGAGAGGGTTATGGAT
GGGCCGACGATATACCCGAGAAGCAGTTCAATTTACATCCAACGCTTATGGTCGCCGG
CTTTATTACTTTTAGTGGATTCTCGGTACTCCTCTACCGTATCTGCCGGTGCTTCAGACGCATCTACGTGAAA
CTATTACACGCTATATTCCACGCGTTGGCGTTTCCTTGTATTGTCATCGGTTTCCTTGCTGTCCTCGACTTCC
ATAATAAGAAGGGCATCAACAACTTCTACTCGCTTCACAGCTGGATCGGTTTAGTGGC
TATGGGACTGTTTGGAATTCAGTATGCGGTCGGCTTCTTCAGCTTCCTTTTGCTATTGATCTGTAATAAGGGA
ACAGCCAGTTTCCGCGCATCTTTGGTGCCTATCCACGCCGCGTTTGGCATCTTGACTTTTGTTCTCGGAGCCA
GCGCCTGCCTCACCGGACTCACTGAGAAAGCTCTCTTTACACTTGGCCCGGAACGGTA
CAGCGGTCTCCCGGACGAGGGTGTGATCCTGAACGCGATCGGCGTCACCATCGTTGCCATACTGGCCGTCGTC
ATATACATCCTGTCCACGGACAAGTTCAAACGAGATGCCCGTGATATAAGAGCTTCTGTTGATCTGTAGATGT
AATAAATACATGAAAGTTATTT
>1002052769_2 Heli.2-DT667570.3.5
GCACGAGGCCTTTCAATTTCTTGAGTACGATAATTTATTACGAACCTGTAACAGTTGTTACTTATAAATAAGT
AAAAAGTGTGTATTAATTAATAATTATGGTAGTTTTGGAAGGAGATAATGGGGACGCCCAGCTATTAACTGCA
GATAATAAACAAGATGGGAAAGTAGCCAATCCTTTATCATTAGAAATATTTCGTATAA
CTAAGGATGGTCAACAGCAGCATGGTCTTCGTCATGGAGATTATCAACGTTACCGCGGCTACTGCTCGCGGCG
TATTCGCCGTTTGCGCAAAGTGCTTAAAATTCCACAGGGAGACAGACGTCACTATCGTCGGCGTGACATAACA
TCATCTCATCTTACCGGCACCAATGCTGAGAACCGTTTGCTTTGCGTACCCTTACTCC
AGGCTGAGAGGGCCTGGGCCCATGCAATGCAATTACGGCAAGAGGCCAACACGGAGCCCAGAAAAAAGTTCCA
TCTTGTATCCCGCTTGAAGAAAGCTTGCGCTCACGCTCATATGCTTTTGCAACTTTGTGAGGAAAGCGGGGCC
TGCAACGCTCGCACACAACTGGAGGCGGGCGCGTACTCGGCGTGGTTAAACGGCGTTT
TATTACTAGAACTGCAGCAATGGCGTCCGGCCGCTGAGAGCTTGCAGAGGGCTCAATTAGTGTTGGAGAAACT
GAGTGCCGCTTTGCCTGAAGAAGAAAGAGTCATTT
>1002052770_2 Heli.2-DT668826.3.5
GCACGAGGGGCCCATACTAAACGCTATGACGGCGCCAGACGACGAAATGTTCATGGACCCTAGAAGCATGAAC
GATATGGTTTCTATTGATACTGGTCTACCTCTTGTAAAAAGAAGAAAAATTGAACAAGAACAACTACAACAGG
CTCTGAGAGGCGCCGGTCACGTCATCATCAAACGAGACCCGGCACAAGGCGACCATTT
GAGTGATTATGCTTTCATGGAACCAGACCACATTGGCAAAAGGTTCAGGCGGAAAAGATCGACCGAGTCCCAT
AGCAGACAGAGGCGTGAAGCACCATATGTGATATACCCAGAAATTCTTGTGATAGTAGACTATGATGGTTACA
GATTGCACGGAGGAGATAATTTACAAATTAAAAGATACTTCGTTTCCTTCTGGAATGG
CGTCGATCTGAGATATAAACTATTGAAAGGACCCAGAATAAGGATATCAATTGCCGGTATTATTATATCTCGG
GGTCGAGACGCGACACCGTATCTAGAAAGGAATCGTGTAGGACGAGACGCGATAGACTCAGCGGCCGCGCTAA
CCGACATGGGCAAATATCTATTCAGGGAAAGAAGGTTGCCCGTTTACGATATAGCTGT
CGCTATTACTAAACTAGACATGTGCAGAAGACAGTATGCAAACGATGCGTGCAATAGAGGGACTGCAGGATTC
GCGTACGTCGGAGGTGCATGCGTGGTGAACAAACGATTGGAAAAAGTCAACTCAGTTGCTATTATTGAAGACA
CTGGTGGTTTTTCTGGTATCATTGTGGCCGCACATGAAGTTGGACATTTGTTAGGAGC
AGTACACGATGGCAGTCCCCCACCCTCATATCTAGGAGAACAAGAGCTCTCTACTCTACGTAGTTTAGTCATT
GTATAATCTGTCATTTAGATCACAAAATCATAAACCTCAAAATCTATTTCTGGAGACCTTCGACTTTATGACT
CACATGCCCAACATTACCAATTGGTACTTTCGTAAAGCATGCGATGGATTCTTTACTT
ATCAACTAAAACAAACGTAGCATTTAAAGTGTTGCTTTATATATTACC
>1002052771_2 Heli.2-DT661662.3.5
GCACGAGGTCGAACCCTGAGATCGGCGCTGCGCCGCTTAATTCGTATTGCCGTTGCCACCCACTCCGCTCGCT
TGCTTCAGCGCCTCCAGTCATTCAGTCCCGACGCGATCGCCGTTTTAGTCACACGTGGTCCTCGCTTTCTCTA
CGTGCAGTCCAGTGTTCAAGGCGAAACTGTTTCGACACGGCATTCGGTTGCGAAAGTC
TCGCGATGATATACAAGTGAGAGCTACCATCGGACGCCAGGGTAACGCTCATCGCGCACGGTGAGCCAGTTTG
TTACACGAGAGACATTCAAAACTTGCCAACCAAGGGACGGGGGGCGCACGACGCCGTCGGGTCGCACACGCGG
GAGCAACGATTCCTACGCATACTTACTGTTAGAGAGCCACGGAAACCAATTTTTAATG
TGTCAAGTCAGTATAGCTTATTGCTGCTACGTCAGATCGCAGAACGCCTCGAGTAGGAATTACAAGGTTATGC
AAATGAGTTCTGAACGCTTCTACACTAGGGGTAATCAAAAGACCATTCATAATTTTAATAAACTTTAACGTTC
GGTCCCCATACTGTGTACTGAGTGCTTATCTGTATCGTATTTCTATCCTTCATTATCG

TTCGGTGTCGCGATCGCTAATTGCTAATTACTTACCTGGAGTCGAGTGATAAGGCTCGAGATCGGTGGCCGAG
TGTACGACGTACGCAGATCAGCACCAGCACGCGGCGGCGATGGCGGCCTTGCAAGAGGAGACCCTGCTCTC
>1002052772_2 Heli.2-DT668172.3.5
TGGCGAGCAGGTCGAAAGTGAAGTGAAAGAGCAACTAGAAAATGTGTTTAAGAAAATGCAAGAGATTGTGGAT
CGCAAATCAAACAAAATCAGTAGTAGAGTTCGTTTCATGATCCAAGATGTCATTGAACTCAAGAAAAGGAAAT
GGGTGGCTAAAAATGTTGTAGATTCCCAACCTAAAATGATGGATCAGATTCAAAAAGA
AGCTGAACAACAGCAAAGACATATTGAGTTGATGAATGCGTCACCAATGGGCGGTGGAGGTTTCCGACGTGAG
GATGGTGGTCGCGGCAAGCGTGGAGGTGAGAGACGCCAAGGGTCGAACTCCTTTATGGACAACCAGTGGAAGA
CCTCGCGCAGTAATTACGTTGTTGACACTTCGAAATTAAAGGCTGTCACACAAAAGAG
TCTTAGTAACATTAAATTGGCCCCAACAAACTTTGCCTGGAATCAAGGCTCGGGTACAAAGACTCCAGCTCAA
ATAAGCAGCAACTCAATGATTAGCATTTCCAAAAATATGTATAGTGTGCTTGAAAATGTCCAAGCTGATCCTA
CATCTCTCAGAGCAAACAAGGATCTAACACCAAGTTATCAACATTCAAAGTCGATTGA
GAGATCGACATTCAATTCCAGAGGTGATTTCAATAGTGGCAGTGGAAGTCGCTCAGGGTCTATGGGACAGACG
CGCTCCAACTCAAGTAGCAGAAGTTCGAATGCTACACCGGCACCTGCCCCTGAACCAGTGCCCTCTGCACCCG
TGGCTCAAGAGCCTCTCCCTGAGGCCAAGAAGAGGATTGTTAAAATCATGATCATGGA
AATTATTGTTAATCCTAATGATGAAGAATTGGTTATAGAAGTCAAACAAACCTTCCCTGCTCAGTACCATGCA
GCAGTGGTCACTGAAGTACTTAATGTTGCCCTTGAAAAGTCAGCAAAGGAAACTATGGCAATTGCGAAAGCAC
TTCACCACCTTGTTTCAACTGGGACAATATCATCTGACAATTTATTGGCAGGATTAAA
TGAAATCTTGGAGTTTGCGCCAGATTTATATATAGATATTCCTATGTTATATGATTACTTAGGAAAGTTTATA
TCACCTCATATTGAAAAGAGGCATATCACATTTGTACAGATATTTAGGTTGTGTGAACAAATCGTCAAAGCAA
ATCATGGACATATGCTCCTAAAAGCCATTATTAAAGAATTGAAAGAAAGCATGGGACC
TTTCTTTGTTAAAACAAAGTGGCAGGAATCCAACTTGGAATTCAAACAATGGATGGATGAAGAACAGGTTCCT
AAATGGATAGAAGACAATAAATTAGAATTTATGGAGGGAGGTGACGCCATCGAAGATACGAAAAAGATTATGA
CCCCAAGTGAAACACAAAGCAAATTACTCCAACTCATGAATGCTGATGAGAGCTGTGA
CTGTATCCGAGGATGGGTGCAGGATAACCTCGGCGCATCGTCCAACGAGGACTGGTTCATGCGTGCACATATT
CAAGCGATATGTGAGCACGCGCTCTACGGCAACGACGGCCGCGATGTGCTGCACTTCAGCCACGACCGCATGA
ATAAATATTCGTCGCTGATCAACGAGTTCGGCGAGACCCGCGAGCAGCGCGAGGCCAG
CTGCCTCTTCGGCATACAGCAGCTAATACATAGACTGGAACATCCACAAGGATTAACACTAGATATATTCCAA
TACTTGCATGAACAATATATAATTAGTGTTGAAGGATTCATAGCGTGGGAAGTATCTGAAAAGGAGCCCGAGG
GAAAAGCCGTTATGCTAAAGGCGCTGACGTCGTTCTTCACGAACAT
>1002052773_2 Heli.2-DT666777.3.5
AAAACATGTAATATTTTGACTAACACTATCTAAAACTAGCATTAATAAGTATTGCTGCAATATTCTAGAAAGA
TTTGTAATTAAGTAAGATTTTGTGAGAAATATTGTGTGAAAATAAACTATATATAGGTTATAAAGTGCAATGA
ATAACAATTGGAATCGTAAACCGCGCGGGGCGTATAGAGGAACGAGAGGATTTGGTAG
AGGACGAGGTTCTGGAGGCTTCTTCCCTAATAGAGGCTTTAGAGGAGGCTTTTCCCGTTTTGATAGAGGAAGA
GGAGGATATCATTACGATGGATATGTCAATAATCAATGGACACCTAAGCCTAAACCAGAAATGCCCTCTAAAA
GGCTTTCTGAACAAGATATTTATGTTACGGAGTTCATCAGCGAACATGAAGGTTTCAA
TGGCATTATTAAATCAAGATTTTCAGATTTTCAAGTGTCAGAAATAAATTTAAAAGGTGAAGTAGCCAAGTTA
ACCGATACTTCCCCACCTGAAGTTGTTAAAGAGGAATCAGATGTTGAAGATGATGAGGACTTGCTATTAAAGA
AATATAACCTTGAGATCTTGCCAATGGAGACTTGGGATAGGATCAATAAATTGTCATT
GTCTCCTAAGTCGCAAGCATGGCAGAAAATAGAAATAGATGTAACAGGTATGTCAAAGGAAGACAGAACTAAA
ATACATGATGCTGTCAAAAAGGCATTTGGAGAAAGCATTGTTGGTAGCACAGTCAGTGTGGATGATAAGAAGT
ATGTTACATT
>1002052774_2 Heli.2-EL602648.1.5
TATTTCGAAACTTTAAAACAATTATTTATTACGTTGTGGAGTTTCTTTTTTCATTTATTTTATAATATAAATA
CTTAAGTGGGTCGTGTAAAGTGTTTTTAAGTGAAAATATTTTTACTGGTAATAGTTAGTGTAGAAATCAACGA
GTTTTGCGACGGACCGGTCTTTATAAATATACACTATGTTGCCGAGGAAAAGACACAA
TATGGGCATCATACTTATGACGGTACTAATGTTTGCCAAATACTCACATCAGTATGGATCTGGTGCTCCACCT
ATGGCATGTTTAGACCAAACACCAAGGCACAGTGAGATCCCCGCACAGTCATCAGTCCCACCATACAGTCTCA
TCCCATCATCATCGCAAGTCAGGCAGGGTGATGTCTTAAATATCACGATCGGCAGTCC
TTTTGGTGCTCCCACACCCATTGGTGGTTTTATATTACAAGCTAGAGAAATACAGGATATGGAAAAGATCGTG
GGCAAATTTACAAAAGTACCCAGTTCTGATTTGGCACAAATCAAGAGCTGTAGAGGTCAAAACGACTCGGTAA
CTCATACGAATCCGGAAGATAAACAACCTTTGACCTTCACTTGGAGAGCGCCTGACGA
TTTTTTGGGAGGCGTTGCATTTAGAGCAACGGTCGCACAAAGCTATGC

>1002052775_2 Heli.2-DT667224.3.5
AACATCTCGGNCCCGGGCTGANGAATCGGCCGAGGTGAAGAGTGCCTGCTCGTACAAAATACATTCTATATAA
TAATATAATGTTTAAAATAAAGATTACTTTGATATCAGCGCTTTTGGCCTTAGCGTCATGTGGTCGTCTGGAA
CCTCAGTACCTACCACCGAGGCCTGGCGGAGGTAACGCAGGATTGGGAGGACTGGGAT
CTGGAGGAGCTGGAGGTGGAGGAGGTAACGGAGGACTTGGCGGAGGATCTTTTGGCGGCGGAAGCGGCTTCGG
TGGCGGTAGCGGTTTTGGAGGCGGATCCGGAGGACCAAATATACCCATACTCCGATATGAAAACGAAAACAAC
GGCGACGGCACTTACAAATTCAGCTACGAAACCGGTAACGGCATATCAGCACAAGAAA
GCGGAGCGCCGCGCGCCCCCGGCCCCGAAGGACCAGCCGTCACAGCCGAAGGCGCGTTCTCCTACCGCGCTCC
CGATGGCCAACAAATCTCCCTAACCTATACAGCTGATGAAAACGGCTTCCATCCCGTTGGTTCCCATCTTCCT
ACACCTCCCCCCATCCCGGAAGCTATCTTGCAATCAATCGAATTTAATAGACGCAATC
CTTCATCTGAGGGGTCGTACAACGGTGGATCAGGCGGCAGCAATTTCGGAGGTTCCAGCAGTGGGTTTGGATC
TGGTGGAAGCGGTTTCGGGGGCTCCGGTTCTGGTGGTGGTGGCGCGGGCGGCTACCATTACTGATCTCATTTA
TAGAAGTATCTAAGATACGTCACTGAATCGAAGTTTTCATGGATGCAATGTCCTAAGC
AACAGATACTTTAAATTTCTTAGATATTTTTTATTT
>1002052776_2 Heli.2-DT664663.3.5
TATACCAACAATTCAAGCGTCTGGCATCACAATGGCGGCAGCGGGTGACTCAGTCATCTTAGAGTGCAGAGTG
GAAGCGCTGCCCAAACCTACCATCGCGTTCTGGAGAGACCCTAATGGCAGAACACCGGTTATAGATGGACCAA
ATTATACGATACAACTGCTTCCTGATCCTGAGGAACAAACCAAATACACAATGCGCCT
CGTAATAAAGAAGATCACGGAAGCTGACGAGGGGGATTACTTCTGTCATGCGGAGAACGCTTTCGGCAAGACA
CTGAGGCCGGTCTCTGTACGTTTGAGGTCGAATAGTCCTCACCACAACGTCACTGAATGCTGTACTCAGATGA
ACGTATCATCCGCGTGCATCGACGCCTGCAGCTTCCACTTGGACATGGACAACATCAT
GGATCGTCCGGAGTGTATGAACGATTTTGACAAACTCATGAAATGTGCTGCTGATGGTTCAGATCATCGAAGC
TGCTGTGCATCATGGGGAGTTCCCCGTAACTGCCTAGAACTTTGCCGCGGCGGCCCCGTCTCCAGGACTTGTG
CCCTACAGCACGCAAGACGTGCACTGGCCTGCTTTAGAGATTCTGGGGCCAGAATGCC
TGGACCACCCAGAAACTTGAGGGCTCATGCAGCACCTACACCACATGCTGTTTTATTAAGTTGGGAGCCACCG
CGCAAGAACCCCGATACTGTTTACCTATATAGAGTGTTCTGGCGAGCTTACGGTGCTAAGGTACCAGAGAAAT
TGGACACTAGCGAAACAAGCGTTGTTCTAACTGGATTACAGGATGATGTCAGATATGA
ATGTGTCGTGAAATCTGCTAATGATGTCGGCACATCTTCCCTAAGCCAGCCAATAATGTTCACGACCGCTGGT
CAAGAGACAGGTGCGGCGGCTGCTCCTGTATCGACTTCGGGAACAGCTTCAGCAGTCGGAGTAGCCGTGGCCT
GTATATTAGTGGCCGCTATTCTCTTAGCTGCCGGATTTTATTATAGGCATAGAAAGAA
TCTTAGACTTAAAGCACAAGGAGGTGTTGCTTTCGAAAACCCAAGCTACCTTCGAGAACCAAATCCTGACAAC
GCTGTTAATGGCTCAGTACCAAACGGTACTTCTGGAGAAAACGGCCTAGCCAACGGCATATCAAACAGCGCGG
CCTGGAGGCAAGAGACCCAGAACCCAACAACCACTGGGCCCAGGGAAGTTGATCCCAC
ACTGTACGAGGAGCTGAAGCTCGGTCACGATGGAGCGGGCTTCAAGAGACTGAAACCATAGCCCGAGGTCCGC
CGCTCGCCGCTCCGCTCGCCGCGACTGTCGGCGCGCGTGGCGCCGTACTGAGCGGCTTTACTCGCGTTGTCGG
CTCTTTTACTGGTGTAGGGTTGTCGAAGCGTATTATAAAATATATCGGTGTTTATAGT
CTGTGGTATATTTTCTATCTATAGTTTTATCTGTAGTGATATATATTCTATTCTTTGGCAGTAATGACAGCCA
AGCCATTACCTAAGTTTATATAATTTACTTTGGGCCATAGTTGTGGCTTGACTTTCTTTACCACCAAAGCAAC
GATATCTATATTGATATCTAAAATATTTTTATGTACGTATGTCAGTATTTGTAAGGTA
AAGTTACCATGTTATTGAAATTAG
>1002052777_2 Heli.2-DT667771.2.5
GCACGAGGATTCACTTCAATAACAATCAAAATGTCCGCTAAGGTAGTACTCCTCCTTGCCCTGGTCGCGGCTG
CGTCCGCCCTGCCCGTCTTACAGTACGCAGAGCCCGAATCGCCCGCCCACTACGAGTTCCAGTACTCCGTACA
CGACAGCCACAGCGGTGATGTGAAACAGCAACAAGAGGCCCGCGCTGGAGACGCCGTC
CACGGCTCCTACTCGCTCGTGCAGCCTGATGGAGTCCACCGCATCGTGGACTACAGCGCTGACAAGGTGAACG
GATTCAACGCTGTCGTGCGTTACGAGGGACACCCCGTCGCCGCCCCCGCCCCCGCCAAGCTCGCATACGCTCC
CGTCGCCAAGCTCGCCTACGCCGCTCCCGTCGCCAAGGTGGCCTACTCCGCCCCCATC
TCCTACGCCGCCCCTGTCGCCAAAGTTGCCTACGCATCTCCCCTCGCACATGTCAGCTACTCATCCCCCGTCA
TCTCTTACCAACACTAACTCTATAGCGGATATCCTTGTTAAAACTATTTAATTTATTTCTAAATATGTGATAC
AAATAGCTGTAAATAAATATTTACATAATAAAAAAAAAAAAAAAAA
>1002052778_2 Heli.2-EL603159.1.5
ACGAGGCCTCCTTTAAGAATAATTTTACCAAAATTGGTGATTTTCATAATATTAACACTAGAAATAAGCATAA
TCTTGGTGCACCTGTAACTCGGTTGCATCGAATCCTTAATTCTTTTATGGGACAATGTGTACGCTTTTACAAT
AGGATCCCAGAAAACGTCAGAGAATTTTCTATATCACGATTTAAAAAAATTGTGAAAA

GACGTTTGTGTGTTAAAGGTTATTACCAAATTAATGATTTTGTAAATGATAACACGCCTTGGGAATGAAATGC
TCGCTCCAGTCTGTTTCAAAGGTATCATTGTCACAAATAATTAAATATGTATTTGTTTAAAATTGATTTTAAA
AAATAGGTCCCGCTGAGTTTCTTGCGCCGGTTCTTCTCAGGTCTGAGGCATTAATTTT
AGTGAACTGGTGGTAAATTTGACGTTCAAAAAATGAAGCATGTAACTTCTTATTTTGAATAAAATTATTTGAA
TTTGAATTTGAATTTAAAACTTGTACATTTTCATTTTATTCTTTGCTATTCTTATTTTATACAAAGATATTTT
TCATATAACATTCAAGTACAACAAGCAATAATGCTATATATAGAAAACTCCACGTTAA
ATAAATTAAATATAAATAAAAAAATACAATAAAGTTTGAGAAACAGAAATTTTTTGTTTACATTTTTTGTCAT
GCCATAATGAATCGAGTATAGATACACTTGCCTCGTTGTTTTTAGGAAAAGGACACAAAGATTACTTCATAAG
TTACATAACTTAGTTGTATTAAATGTTAAGCTAACTTAATAATATAAATAATTTTTAA
AAAATGTTTGCAAAAATTATTATTATGAAATTAACTACAAATATACATTTCTGAAAGTTTTATTGTAATATAA
ATATAAAAAATTGACATTTTTCGGAGATTTTTCCCTTATTTTACTTTATGTTCTCTCACTTCTTGTCNAATTT
TATAATTGTAAGTCTACAGGAAATATTCTATAAGATTTTGCGACAAATTTTGATACTA
AATGCAGCAAAAACTATACACATAAATTACGTAAATTCTTATATCGTT
>1002052779_2 Heli.2-EL599439.1.5
GCACGAGGGTCGATACTAGTGAATGAAGACTTAGTAAAATTTTCAAATTCTCTCATAAGAGAGATACTTATTC
AAGCGTTTACGACCACGTAGGTACTACCTACAAAGAAACGAACATAAAACAGCACTATGCAGTTTAACGCCTT
CCAAAATCATCATCCAATCTATAGACCTCGACTAGACCGGAATGAACAATTCGGTAAA
ATGCCTTTCATCAATAGTCTGCATGATTTCGGTGGGCCAAAAAACTTTCAGCCAAGAAATGGGATGGGTAATA
AGAACTTTAGGCCTCGAAATGATTTCAATGGGATAAGAAATGATTTTAACAATATGAAAAATGAAAATGGAAA
TCCTAGTGATTTTGGCGGACCGAAAGAATACAGACCCAGAAATAATTATAATAATCAA
ACTCAAAAAAAGAATGATTATGATGGTGAAAAAAATTCAGGAGGCAATATGCAGTTTTATAATGCAAAAGGTG
ATTTTGGTGGACCTAAACAACAAAACTTCCAAAAGGGTTTTGCACCCAAGAATTTTAACAATCAAAATGGGGG
CAGCAATGGGCAACAGTCATTTGTTCCTAAAAAAATATTTAACAACAGTTCATCCCAG
CCATCTTCAGATTTCAATGATCGCAAATTACAAAGCCGAAAAGCAAAGTATCCTGGTGATGGATTGATAAAAC
CAGTATGGGACATGGCTAATCTG
>1002052780_2 Heli.2-EL602431.1.5
GCACGAGGGTTTACTATTTGGCTCCACAACTTTTCGTAAGTTCAAAATGTCGTTTCGCTGTATACTCGCGTTG
CTGGCTCTCGCCGGAGCCACGCTGGCCGGGCCCGAGGTCACCGAACTGAAGGTAGAGGTGGTCAGCGTGCCAG
AGGGCTGCACGGCCAAGTCCAAGCACGGCGACATGCTGACCATGCACTACACGGGCAA
GCTCGAAAACGGGCACAAATTCGATTCCAGCTTAGATCGCGACCAACCCTTCACCTTCCAAATCGGCGTTGGA
CAAGTTATCAAAGGATGGGACCAAGGTCTCTTAGACATGTGCGTTGGTGAGAAACGTAAGCTGGTGATCCCGC
CGTCCCTCGGCTACGGCGAGCGTGGAGCCGGTAACGTAATCCCCTCTAAGGCCGTCCT
CTTTTTCGACGTCGAACTTATCAACATCGGTGACTCCCCTCCATCCACCAATGTCTTCAAAGAGATCGATTCT
GACAAGGACAATATGCTGTCCAGAGAAGAAGTAAGTGAATACCTTAAGAAGCAAATGGTGCCATCCGACGGCG
CTGAGATGAGCGAGGATATCAAGCAGATGCTGGAGAGCCACGACAAACTGGTGGAGGA
GATCTTCCAGCATGAGGATAAAGGACAGAATGGCTTCATCAGCCACGAGGAAGTCTCTGG
>1002052781_2 Heli.2-DT668252.3.5
GCACGAGGAAACCAACATCCGCTACTGCGCTTAATACGTCAACAGATGATGATGATATAATAATTGATGTTCA
ACAAGCAAAATCTTCACGTGAGAACTCGCCAGATCGCATTTGTCCAACCCCAGTAGGATTTTCGGAAGATGTA
GGAACACCAAGATTTCCAGATGAAGTGAATGAACCAGACGATGAATATCAAAAAAGAG
CTCATCAAATCATACACGAAGCTGAAACTATTGTAGATGATATTGTGGAAATTGATGAAGATGACGAACTATT
CGTGAGAAAAACAGATTTCAGTGAATATGTAGAAGTAGATGAAAGCGTAAATAAAGTTTCGAAGACTACAAAT
AAATATGAAGCTACGTCACGTTTCAAAGATACTGAAAAAAAAGATCACTCTGACTTTT
TGGATGAAAATCTAAAATCAGATGATTGCTTATTATCAGTTTCTGAAAAAGTTAATAAGTTTGCCAAGGGACC
TACTAACACAGAAGTCAGAAAGAGCCCATCTCGAAATATTATTGAAGAGTTTGACAAGCATACATCATATCAA
GATGATTATACTAAACTAAGTGTGAATGATAAAGCTCACTTATTCATAGAAACAGCAG
AAAATGTAAAAGCAACAAAATCTAAGCCTAAACAAAAAGTAGAGCGCCCTAGTTTTAACGATATAGATGAAAC
ATTAAAAAGTGACGATTGCTTACTTAGTGTTTCTGACAAAGTCAACAAATTTGTAAAAACAGCTGAACAATTT
TTAAATGAAACCCATGAAGTTGAAGAGAAAGAAAAGAAAATTGTTGAGGAACATGAAA
AGATTATGAAAAAAATTGTAGGCAATATTGACGATACCACTATAAAATCACATGACGAAAATTATGCTTGCAA
TAATAATACTTCTAGTAGGAATACCGATAATGATGATAGGCGAAGCTCTTTAACAAAAGAAACAGTTTCATCT
CATGCGAAAATCAAGGACCCGGTACACCCTAGTATTAAAACTAGTGAAAAATCTGCAG

TAAGAATAACAACGTTACGAAGTAGTGATGGAGTGAAAAAAGCGAAAGCATTATTTGAAAACATTTCATCTAC
CACCCCACAAAAAGTTGCCGATATATCTCACACTAAAATCTCTAAATCAACAGGTACACCCAAAAAAATAACA
AAAAATAATCCTAAAAATATAAATTCCTTAGAAAACAATTCTCGGGCTATTTAAGAAA
CGGAAAAAAATAAAAGTTTCTACTT
>1002052782_2 Heli.2-DT665055.3.5
GCACGAGGTGAAGACGAAGTAAAAACTGAAGCTCATGTACCAAAGATGACTAACACAACAAGTGAAGTCGCTA
TCAGTGATGAAATATTGAGACAAGATTTTTCAATAGACGATGAATGCGGCTGGAATAATGTAGGAACATATAT
ATTTCCCAATAAAACTAAGGACCCGCAAATTTACGCCAATTATGGAGAATATAGATGG
ATGATTGCACTTATCAAGAAAAGAAAGAATGTAACCGTCTGGGTGTCCGACGACTATATTGGTGGAGGTTCGT
TAATACATCCATCTGTAGTAATAACTGCTGCACATAAAATACATAACATCAAACCTCGTGAGCTTAAGTGCCG
AGGCGGTGAATGGGATACACAGACGGAGTTAGAAAAATATAAATTTCAAGAAAGAGAC
GCCAGAAAAATTATGATCCATCCAGATTACTTTAGAACGTCTTTGTACTACGATGCGGCTCTAATATTCCTCA
AGAAACCTTTTGATTTACAAAGTGCCCCTCACATGGGCTTAGCTTGTCTGAACAAGATACTGCCGGAGCCCGA
TACTGACTGTTTTACAATGGGATGGGGAAGGTTATTTAATGAGAAGAATAAATATGCC
GTGGTATTGAAAAAGACAAAGCTATCTTTAGTGAGTTCACAAGCGTGCGAGACCAAGTTACAAGCAACAAGAC
TAGGCCCGTACTTCAGGATCCACTCGTCTCTGACTTGCGCTGGAGGGCAGGAGGGTGTCGACGCGTGTGACAA
AGATGGCGGGTCCTCTTTAGTGTGTCCTATAAAGAAAAATGGTGAAAACGTTAGATAC
GCGATGGTGGGAATGGTAGCGTACGGCATTGGTTGCGGGATCAAAGACCGTCCCGGGGTCTACGTGAATGTAC
CAGCTATTTACCCCTGGGTGGATAAAGTTATGACAGATGAGAAATATGGCAACGTCACTTATACTGTATAAAA
AATGTACTTCGTTATATAAATAACTATAGTTGTAGCTTTACATAAAATAAAAAGGGGC
>1002052783_2 Heli.2-DT665135.3.5
GCACGAGGCGACCCCTGCGCAGCTGGTGTGCGGTGCGGCTGCCGCTGTTGCCACTCGTATTACTTCCAACGAT
TTAAACATCACTAATTTACGTATCCCGAATAATATGACGTAAAACTTACGTGACCAGAAGCCATCGAACGCGC
GGCCGTGTTTGTGTTGTGATCCGGGGGACGTGCGCGATTTAGGAAATCTCGACGGGTG
AAAGAAATTACAGTTACTTTTGAAAAGAGGAGAAGAACTAATCAAAAATGGCGGTGTTCGGAATGGCCTCCGC
GGTGGCGGGCTTTGTCAAAGTCCGCTACCTGATCGACAAATTTGTCATCGACAATATGGTATTTAGGATGCAC
TACAGAATTACTTCTGCTATTATGTTCCTGTGCTGCATCCTCGTTACTGCTAACAACC
TAATTGGTGACCCGATCTCGTGCATCAGCGATGGAGCTGTACCTGCACATATATTGAACACTTATTGCTGGAT
CACTTACACCTTCACGCTTCCCTACAGCGGGGCTAAAGGCATCGCCCACCCAGGTCTCGGCAACGATTACGAA
GAAGAGAAACGCATTCATTCATACTATCAATGGGTGCCTTTCATGCTGTTCTTTCAGG
GCGTCCTTTTCTACGTCCCTCATTGGATCTGGAAGAATTGGGAAGAAGGCAAGGTTCGCATGATCTCCGACGG
TATGCGTGGAACTACAGCCATAATTGCCGATGACAAAACTAACCGTCAGAATCGCTTGGTTCAATATCTGCTT
GATACACTTCATATGCACAACACCTATTCTTTTGGATATTTCTTCTGCGAAGTTCTCA
ATTTTATTAATGTCGTTGGCAACATATTCTTTTTGGACACGTTCCTCGGAGGTGCATTCTTGTCCTATGGAAC
TGATGTAGTTAGGTTCTCAAACATGAATCAAGAACAACGTACTGATCCTATGATTGAGGTATTCCCTCGTATA
ACTAAGTGTACGTTCCACAAGTTCGGAGCTTCTGGTACGATCCAGAAACACGACGCCC
TGTGTGTTCTTGCATTGAATATTCTCAACGAGAAGATTTTCATCTTCCTCTGGTTCTGGTTCATCATCCTATC
TGTCGTTTCTGGCTTGGCTATCGTGTACTCGGCTGCAGTCATACTTCTGCCTAGCACTCGCGAGACTATACTT
AAAAGGCGATTCCGTTTTGGATCTCCCAATGGAGTTGAAGCTCTCGTTAGAAAAACTC
AGGTTGGGGATTTCCTTCTTTTGCACTTGCTTGGCCAAAACATCTCCCTTCGTGTGTTCGGCGAAGTATTGGA
TGAGCTAGGACGTCGACTTCACCTCGGTTCTAACCCACCGTCAGCACCGTCTACTCTAGAAATGGCACCCATT
TACCCCGACATCGACAAGTTCTCCAAGGAAACTGAGACGTAAATTGAAAAATATAAGC
AGTATACGAGTATTCTGTAATCACTTTCAAAATAGCTCAAACACATTTTTTAAGATCTCTTACACTAGTTAAA
ACTTTCATTCATGGCTGTTTCTTAAAATTTGTGTTTGACCTAATATTGTTTCAATTTAAAAAAAACGTTCCCG
CCCCTATATCTCAATATTTTCTTTATTATTACTTTAGTTATAGCGTGTTTGTGTATGT
TAATAGGTTCCAGTATGGGAATTTGAAGTTAATGACATTCCTTATTTT
>1002052784_2 Heli.2-EL601178.1.5
ATCGTATTTTAAAATATGAGAAAGTAATTGTTACAGAAGTTACTCCAGAAGGGACATTCTATGCTCAAAACAT
TGAACTGGGTAACAAATTAGAAACACTCATGGACAAAATTCACCAAGAGTTTAAAGCTAACGCCCCCCTCCCT
GGATCTTACGTGCCAAGGAAAGGTCATATTTGTGCAGCACGCTTTTCTTTAGATGATC
AATGGTACAGAGCAAAAGTTGAGAAGCTTTTGGAAGATAAACAAGCCCAGATTCTCTATATCGACTATGGGAA
CAGAGAGGTAGTGAATCAGTCTCGACTAGCACAACTGCCCTCTGGCACGGAGTCGGAGCCACCTTACGTGACC
GAATACGCGCTGGCGTGCGTCAAGTTCTCCAGCGACGCAGACGACAAGCTGGAGGCCG

TGCGCGCCCTCTCGCTAGACACGCTCAATAAGAAGCTGCTGCTCAACGTGGAGGTCTTCGGCGCCCCGCCCGC
CGTCACGCTCGTCGACCCCACCGCCAGCACCGACATTGGCAAGAACCTCATCAAGGAGGGGCTCGTGTTGCTG
GAGCACAGCCGCCAGCACCGGCTGAGCGCGCTGATGGCGGAGTACCGCGCGGCGCAGG
AGCACGCCAAGAGCTCGCGCCTCAACCTGTGGCGGCACGGCGACATCACCGACGACGACGCCGTGGAGTTCGG
CGCGCGCCGCTAGCGCGAGCTGCCCTGGTACGACGCCACCGCCTCGCCCTCGCACAAGCTGCACTGCGCCACG
TGCACCTCCCCGCACGTGGAGTCCATCATATACACGGTCATATACGACTTCCTGTGAA
GGCACTGCGGCGCGCGGCACGGCAGCGTACCCGTGAACCGATTTAGGCGTGTTGTTTGATATATCCAAGTTTC
GGATCTGCCTTCCCAAAAATCAATTAACTTCCTTTACGAGTTCGATACATTTTTAAATATTGTGAATGTTATT
TATGTAACAGAGCGGCGGCGGCCGTCCGAACGCGCTCTCGTGTGGCTATTCTTGTGTC
CAAACTCTTGTAGTGTAACGATGAGTGGCACCTTTTGCCCTTTGTACCTACTATGATACAATAATCACTTTAG
ATATTGTCTACATATTTTTATATTTCGCTAAGACAAACTGAATTATTAAATATAATAGTGAATTATACATAGA
TAATTGTAGAGACGAGGAGCTCCTCCCTACTCCTCTGATTTAATTTATGGCATCATAT
TAAATATTAATA
>1002052785_2 Heli.2-DT665299.3.5
GCACGAGGCGCCGTTTTCAGTTTCGTCCTCAATCCTCTGACTCCAGTCCAAGAAACGCGTTGCCTGCTGCAAA
ATGATACACAGGCTAACAACCCTATGGCCATACCACAGCGGTCGGTGAACACTTACTATATGGTGAAGTTACA
AGTGAGATCAGAAAATTTATGCTTGAGGCCTTGGGCATTTGAAAGAGTACCTGGCAAA
GCGCTTCGAGGGTTCGACAACAGTATAATATATACTTCTACTAAAGAGGCCTGCCTTGCTTCTTGTCTCAGCG
AGAAAAAATTCGCGTGCCGGTCCGCCGAGTACGAGTACGGGAGCATGAGGTGCTCGCTTAGTGATTCTGATAG
GAGGACCTCTCAGCATATTGCGCCGCTCGTGGATACGCCCGGCACTGATTATTTTGAG
AATCTATGCCTGAAGGGGACCCAAGCGTGCAAGGGCCAGAGAGTATTTACTGCCCCGCGTATAGGAGTCGCCG
AGGACAAAGTGGCGCAGTACGCTGGTCTACATTATTACACAGATAAGGAGCTGCAGGTGACATCAGAGTCAGC
GTGTCGACTGGCTTGCGAGATCGAATCCGAATTCCTTTGCCGTTCTTTCTTGTATATG
GGCGCGCCACACTCCGCCACGTACAACTGTCGATTGTATCACCTCGACCACCACACCCTGCCCGATGGACCAT
CAGCATATTTGAACGCTGAACGACCGTTGATCGACGATGGTGAACCAATTGGGAAATATTTTGAAAACTTCTG
CGAAAAACCACCTGCCAGTCCCAGTGGGGAACTGCCCGTGTCTATAGATCACCAACAA
GATACGAACATGTCCAGCAACTTAACGAGGAACGACGCCAACTGCGACCAGACGGGAACT
>1002052786_2 Heli.2-DT661753.3.5
GCACGAGGGAGAGTTCTGATTGTATCCGCCCTCTTCGCTTGTGTGGCAGCGGCACCCGCGCCCTCATTCTTGG
CACCACTGCCTTTCGCCGCAGTGGCCCTTCCTCGTCTTGTCGTAGTACCAACTATACCACCTGGTGACATTCA
AGCTGCTGCTATTGATGCTCAGGTAAAAATCGCAGACCAAGCTCAATTACTCGCTGAC
CAAGCTCGTGAATTGGCCGAACAAGCTGCCGAAAATCAAAATGAAGGCGTAGTGGAATCAATTGATTTGGGCA
AGGAAAAATCTGAGGAAGCCTTTTGGGCCGCAGAAGAGAAGAAATGGCAAGCAGTCAACGAAGCTCAAATCGC
TGAAGCTAGAAATGCTGCATTGTTGGCTGGAAATGCTGAGGCATTAGTCAAATCTGAT
GAAGCTATTGTCCCTGGAATTAAGTCAAACTCTGAATCAGGAGTCGCAGTAGAATCAGCCGCTGAAGCTAAGC
CAGAAGCAGCTGTAAAAAGCGAAGCTATCTCCGAAGTTAAAGAAGAAAAAGAGGCTGTTGTAAAATCGGCTGA
GGAAAAACCTGCCGAAAAGGCTGAAAGTGAAAAGAAGGAAGATTTACTTGCTAGTGCC
AAATCTGCACTCGACGATGCCCTTAAGGCACAAAATTTGGCTATCCAAGCATACAATTCAGCCATTTTCCCAT
CTGGTGTAGCTGTAGCTGGAGTGCCTGCTGGCTTGGCGGTGGTACCAGCAGCGGCCATC
>1002052787_2 Heli.2-EL600409.1.5
GCACGAGGGTGATTTTCAATCTTCTTGTTACCCACATTCCAAATCTCGTGCTTGTTTTGTACATTCCTTCACT
GTACTGAACAATTATAAGTTAACAACAACTCGATAATACCTTAAATTTCATACTAAATAATTTAAAGTATAAA
ACTATAAACATGTCTGAAGTAAATGAGCAAGACAATGGTGAAACAGAACAAAGACCTA
ACGGCGGTGGTGATGAAGGCGACGCGTGGGGAAGTCATGAATCTTCTTCGATTCGTCACTATGCTAAAATGCC
TGTGGAAAGAAACGCTCCATATTATAATATGAACCATAAAAATCGTGGTATCGCGGTTATATTTAACCATGAG
CATTTTGATATCCACAACTTGAAGTCCCGCACAGGAACTAATGTGGACTGCGACAATT
TATCAAAAGTATTAAAAACTTTGGGATTTCGTGTAACAATATTAAATAATTTGAAATTTGAAGATGTTAACAG
GTACTTGCAACAAGTGGCTGAAATGGATCATACAGAAAATGATTGTTTGCTCATGGCAGTATTGAGCCATGGA
GAAATGGGTATGCTGTATGCAAAGGATACACATTATAAGCCTGACACCCTCTGGTATT
ATTTTACTGCTGATAAATGTCCTACTCTAGCTGGAAAACCAAAGCTTTTCTTTATTCAGG
>1002052788_2 Heli.2-DT662160.3.5
GGAGTGAGTCGGCACGAGGCTTTAGCTACTGCTATATCGTTGGCTGTAGAAACATTCTATTTTAATTTCGTAC
ATAGCTGTATTTAATATTTACCAAAATGTCGGACGATGGATCAACAGCAGTTGAAAAGAAAGGACGCGGTAGA
CCTAAATCTAATGGGACACAATCAGAAGCCAAAGGTGATGGTAAGAAAAGAGGAAGGC

CAGCAGTACCAGCTGCTAAATCTAAAGAATCAAAAAATTCTTCTGATGATGAACAAGCACCAATAGCAAAACG
AGGAAGAGGCAGACCCAAAGGCTCTAAGAAAAAGGCAGCTGCACCTAAATCTAAGAGTTCTTCTGGAGAGGGA
CGATCTCGTGGCCGGCCACGCAAAGATGCACCTCCACCTAAAAAAGATGCAGGATCTA
CTGAAGAAGAACAAGAAGATGAAGAAGAAGAGGAAGGTTCTGATCAGTAAACACATATGTTTCTCTCACAACT
CACTCTTAGTGTAATTTGATTGTCATCATAACTCCTTACAGTGTAACTTTTCATAAAAAATATTTGTGAAATG
TTATATGCTATGTATAGGGGTTATCATAACAACAAGTCTCAAATGAGATTGCTCCTAA
ATTTTAATCATGTAAGGAAAGTTTTAAATTAAAAGTAATTAACACATAAATTAAGAGATCGTTTTTCATGATT
TTATTGCATTTACTTCGTCTATTTTACTGCAGATGGTCTTACATGGTTAAAATTTTTGGCAGATATAGCTCTC
CCTAGCAACGTACAAGTCTAAGATATAATTTGTAAGATCTTTTTTTTGTAGATTTATT
CTATTTCTATTATAATAAGTATTTTGTAATCTCCTGATTATATTGTAA
>1002052789_2 Heli.2-DT668631.3.5
TTCGGCACGAGGCGGTGTCATTACGCGTGGTTAAGAGGCGAAACTGGTTGTGTTTTTGTGATCGAAAAATTCT
CTGAAAGAAAGTTGTGATTATTGTGTTTATGGTAATTTAACAAAGAATACCAGAAAAGCTGTGAAATATGAAC
ATAATATGGTACAGCGTAGCAATACTGGTTTTACTGGAAAAAGGCGTCAACGGTCATG
GGAGACTCATTGAACCGCCTTCTCGAGCATCGGCGTGGCGCTACGGATTCGACACACCACATAACTACAATGA
TCACGAACTGTACTGCGGAGGATTTTCCAGACAATGGAATAAGAACAATGGGAAATGTGGTGTCTGTGGTGAC
GCCTGGGATGCACCTAAGCCTCGGCCTCACGAACTTGGAGGCAGATTTGGTCAAGGTG
TTATCGTCCGACGATACGCGCCCAAAGATGTCATAGTTATAAAGGTAGAGTTAACGGCGAGCCACAACGGATT
CTTTGAATTTAGAGTATGTGATGAACCGAAATCTACGAATCAGGACTGCATGGACAAACATGTGTTACGGTTG
GAGGGGAAGGATAACACAAAATACTACCCACGTGAAGGAAACAAAATATATGAAATGA
AGTACGAACTACCAGAGGGTTTAGAATGCGCCCATTGTGTGTTACAGTGGCGATATATAGCCGGGAACAACTG
GGGTACGTGTGCGGACGGAACAGGAGCCGTTGGCTGTGGACCCCAGGAGGAGTTCAGAGCGTGCGCTGATATA
GCTATAGGTAGCAAATTTACTACAACAACGAAAC
>1002052790_2 Heli.2-EL600392.1.5
GTCTCCCGTGTGGATGGTTCTCTTATTACTCCCAATCCTCGGGGGAGCAGAAAGATTTAACTGTCGGGGACGC
ATATTGGGTGCATATTATGCTGACGCCAAATCTGGATGCAAGGCTTTCCATGTCTGTGTAAGAGTGGCTGGCG
GTGGTATTCGAGACTTCAGGTTTTTCTGTCCACCCGGAACCTTATTCCACCAGGAAGC
TCCTCTCGCTTGCGACTGGGGCGATGACGATCCTCTCGCTTGCCCTGCCGATATCTACGATGGGTTCGACACA
AAGAAGTTGTCGTCTTCAGGGAATCGAGAAGAAGAACCTGAATTTGGTCTACAAAGAGCAGAAACTGGTGACC
GACGTCTTTCAGCAAATAATAATGGAGGAACATCAGACCTTCGAGCAGCGCACTCTTC
AGATTTCTTCACCGGCCAACGTGATAGAGGACGCGACGAAGCTGCAGTTCAAACCCAGTCACCGATATCCCAT
GCACCTGCTAGGCAATCTTTCAGGCGACCCACAACAACACGTGCTCCTTACACAACTACACAAAATTCTTCTC
CACCTTCACCAGTATCACAATCGTCAGGTCATTCTCAGTACGATCACTCAAAACGGAA
ACTTGTCAGGAAACGTCCAGTCTATACACCTACACCTCCACCCACTACACCAGCTCAAACAGTCCCTCCTCA
>1002052791_2 Heli.2-DT663887.3.5
CCCTTCCTCGATTACCGTACGTACGCTATGAAAATATTGTTCCCTAATATCGACGATCACGTCGTCCTCCAGT
GGGAACGCCCAGAGCTTATTAGAAAAGAAAAGGGTCTGAGAATGTTCGGACAGTTAATTATGAACAAAACATT
CCTTCTATTATTTATTCGTACTTTAGAAAGCAATAGGTATTTTTCAATGCGCGATCGT
GTTAATGTAGCTTCACTTATAATGGTAACTTTACAAAGTAAGATGGAGTACTGTACGGACGTTTTAAAGACGC
TCCTAGCGGAGCTTATAGAAAAGTGTATGGAGAGTAAGAGCCATCCTAAATTATTGCTTAGGCGTACTGAAAG
CGTCGCCGAGAAAATGTTGAGCGCCTGGTTTACTTTCCTCTTATACAAATTCCTTCGC
GAATGCGCCGGCGAGCCGCTTTACCTTTTGTTTAGGTCAATGAAAGGTCAAGTAGACAAAGGGCCGGTAGACG
TTATAACCTCCGAGGCGCGATACTCTCTGAGCGAAGAGAAACTAATTAGGCAGTCGATCGATTTCAAAGCAAT
GACCGTGAGCGTGTCTATATCGCAGCAAACCATCTTCGTGAGCGGCTTAGAGGCTACG
ACGGAAAACGTGCAAGTAAAAGTTCTCGACTGTGATACGATTAGCCAAGTAAAAGAAAAATGTTTAGACGCTA
TTTACCGCGCCACGCCGTACTCGCAGCGGCCGAGTCGTGACGAATTAGATTTAGAGTGGCGCACGGGCGCTTG
CCGCCGTTTGATACTGTACGACGAGGACAGCACCACTAAATGCGAAGGCGAATGGCGC
AAGCTTAACACCCTCAATCACTACCGCGTCCCCGACGGCGCTTGCTTGAGTTTAGTCGCGAAACAGAGCTCGG
TTTATAACCTTTCCAATATGGAAAAGAACGATAAATCGCACACTTAC
>1002052792_2 Heli.2-DT663031.3.5
GGAAAATATACATTTTTGTTTTATAAAAAAACAAGTTGTGCGTGCGACAGTGTTCTACCGGTTTCTCAAAGTG
CCAAATGACGTAACCTCAAAATGAAGGATCTCGGGTTGAAAGTACCTACGCGTAATGTCCTCACTGGCGCCGT
GGTCAACTGGGACGACGACGAAATGGCAGGACTTGGAGATTCTCCAGAAAACCTAACG

CTATCTTGGAAAGACCTATCAGTATATAGAAAAAAGAAAATTCAAACAAGTATATGGAGGTCACCGACTTATG
AAGAAGTTAAAGTTTTACATGGAGTGAGCGGAGTCGTTTCGTCAGGCAACTTAGTGGCTTTAATGGGTTCAAG
TGGAGCTGGAAAAACAACTCTCCTAGCTGCAATCAGTAGGCGGGACAAAAGCGCTATT
TCCGGTTATTTAATGTTGAATGGCCGCCTCGCTGGAGCCGACCTGATCGCCAGGATATCGGGATTTGTGCCTC
AAGAGGATTTGTCGATTGATGATCTGACGGTCGCTGAGCATATGGAGTTTATGGCTCGTCTAATGATGGACAA
GAGAGCAACCACAGCGGTGCGCGCGAGACGCGTGACGCAGCTGTTAGCCGATTTAGGA
GTAATGTCGTGTACTAGTACAAAACTTAAAGCTCTGTCTGGAGGAGAGAGGAAACGAGTTGCGTTGGCTGTAC
AGCTACTCAACGATCCACCTATATTGTTCTGCGATGAGCCCACAACGGGTTTGGACAGTTCAGCTGCAAGTGC
AGTTGTATCACGTTTAAGAAGGCTGGCCATAGGTGGTAAACTAGTCATCTGTTCCGTG
>1002052793_2 Heli.2-DT663126.2.5
TACGAAAATCATCAAAGCGAACGCTGTGGAGCCCGATGGCTTCGAGACATCAATCTCACAGGCGCTGGTGGAG
TTGGAGACCAACTCTGACCTGAAAGCTCAACTCAGGGAGCTCTATATTACAAAGGCTAAAGAAATTGACCTGC
ACAATAAGAAGTCAATCATCATCTATGTGCCGATGCCCAAATTGAAGGCATTCCAAAA
GATCCAGATCAGGCTGGTCCGTGAATTAGAGAAGAAGTTCAGTGGCAAGCATGTTGTGTTCGTTGGTGACCGC
AAAATCTTGCCTAAACCAAGCCACAAGACTCGTGTTGCAAACAAACAAAAGAGGCCCAGATCTAGGACCCTTA
CCTCAGTATATGATGCTATCTTGGAAGACCTGGTATTCCCAGCTGAAATTGTGGGCAA
ACGCATCAGGGTGAAACTGGACGGCTCGCAGCTCATTAAAGTGCACCTCGACAAAAACCAGCAGACCACCATT
GAACACAAGGTGGACACCTTCCAGTCAGTATACAAGAAGTTGACGGGGCGCGAAGTGACCTTCGAATTCCCTG
AACCCTACTTGTAAACTTAGACTATGACTTTACAATAAAACTAAAA
>1002052794_2 Heli.2-DT667452.3.5
GCACGAGGGCGCGTCGAGTCGCGATATGCGTCGGTGGTGAAGTGTAGTGCAGTGTCGTGTACAGTGAATTAAG
ATGAAGTCCCTCAGCGCTACCGCGCTTTTCGCACTAGCTGTTATAGCAGAATGTCTCGGAGCACAGGACCGCG
GATCACGGCAGCGCTCCGGCCCTATACGAAGCGTTCCCGTCGCGGCTGATATAAAGAG
GGAAGTTGATTTTGACTGTCCTGAAGAATTTGGATACTACCCTCATCCCACCGATTGCACATTGTATTACGTT
TGCGTATTCGGCGGCGCGTTACTCGAATCATGCACTGGTGGCCTAATGTACAGCCACGAGCTCCAAACATGTG
ATTGGCCACGTAATGTGGGCTGCGACGCCACTGGTGCTGTGGTAGCAGAAGACTTGGA
ACGATTAAATGAGCGCGAGCCTCCACCCCGTCCCCCACCACGTCGTAATCCTCCTCCACCACCGAGGGCACAG
CCGAATCCTGTTATCACTTCTCGAGGTCAACCTAAATTTAATCGACAAGAATACGAAAAGCAACAACAGTTGT
ATGCGGAAGTAGATGATTTACCACCAGTAGAAGAACTCGAGAATGACCGACAACAACG
AGTATACAGAGGTCAGCCTTCCACGATTGGGCAAGTTCAGAAGGACAGAGATGGATACGGGTCTCAATCCATA
AGTTCTGGAAGAACACTCAATTCAAATATTATACCTTCCTCGATTAACCAGAATAGTAAAATTGGTTCATTCT
CATTTGGAACTCAAGTTGACGACAGGAGAACAGCCACTGTAACACCAGCGCCTCAAAC
TTATAGCTTGTATCCGACTGAGAGCATTACTCCGTCTTCATATTCAGCTAGCCCGCGGCCGAAGATGCTGTAC
AATGGCTCTCAAGCCTATAGTCCCGACCAATACGATCCTTATTACGCCGTTTATGACGAGGATGGAGAATTAT
ACAAGGATACAGTCCAGCAGACGTACCGCGGCACACCTCCTCCAGCCCGCCGTCCCGT
CGAAACATATACACCCAGGCCTTCTCTGGATGACTACGAGGATACTCTCATTCAAGGACAGATAAGCAACCAG
AACCAATATCAAACACCAATTCGTCAGCCATCGAGGGGCGAAGGTAACGAATTGGGCTATGATCATATTCCAA
GCAGCGTGAGGACGACTGTATACGAAGCTACTTCCCTTCGCACAACTCCTCCTACTAC
TAGCACAAGCACGACCACTACCACCACTACTACTACTACCACTACCACAACTACACGACGTCCAACAACTGCA
CCTTACACCGAAGCTATGACCCCGTCTCGACAAACTTTAAGGCCATCCATTACAAGAGGACGTGGTTCAGCTC
ATTATTCCACTTCAGGTGCATCGGGGTCTTCACAACAGACTCCTAATAGAGGAACACC
TCCAACTCGCAGCCGTCCTACTTTAAAGCCCTCTACAGCAATTGTATCAAAAGTATCTGAGGTGGATATCTAC
ACAAATCCTCCTAATCGGCCAGCACCTGTCTATCCTCAACCAACTCCCGACAAGACAGC
>1002052795_2 Heli.2-DT666521.3.5
GCACGAGGTACTATTCTTTATAAGAATTAATTAATATTTTATTAGAAAAGTGCGTGAAATGTTTCGTAAGTGT
ATCATATTGGCGGTGTTAGGGTGCGCTGTCGCAGCCCCACAGTATCAAATTCAGCAACAACAAGATGAAGGCA
TTCCTCCGCACCTACTGAGACAGTACATCGGTCAACAACAAGCACAGCATATACCGCG
ACCTGCACCTCAACCAGCGCAAGCACCTGCCAGACTTCCTTACAATGTACAAGAAGAGCAATACCAGCCCAGA
GCCCAATACCAGCCCCAGCCCCAAGCGCAAGCGCAGCCCCAGTACAGGCCACAACAACGCGAACCTGAAGATT
ATGACCCTAATCCTTCCTACCAATTCGGTTTCGACGTGAACGACGATCAATACACGAA
CTACCAGAACCGTAAGGAGCAAAGAGACGGTGATGTCATCAAGGGCTCTTACTCTGTAGTCGACAGCGATGGT
TTCGTACGAACTGTTACTTACACTGCTGACCCTAAAGAAGGTTTTAGGGCTGAGGTTTCACGTAAACCCACCG
ACATCGTAGTAAAGATCCCCACACCCAAACCTCAAATTCAGCAACAACAGCCCCAACC

TCAGATTCAGCATCAACCCCAAATTCAGCAACAACTCGCTCAGAGACAACAACAGCAACAGCCTCAGCAATAC
TATCGTTATGAATAAATAATGCGAATATAATATCCACTCTTACATATTAGGACCAAAATCTAAATATTTATCC
TCCATTTGTCGATTTTTCTTACTACATCTTTATTAAAAACGAAATGTTTTAACATTTT
CTCTTCATTATCTTTGTCAACTTCGTTTAGAAAAAAAAAGATAAAAAATACGATTAATTTTCAAACATTTTA
>1002052796_2 Heli.2-DT667018.3.5
GCACGAGGATTGCCCCTAGATCAAGAAAGCAAGAAGATAACGAAAATACAGAAAAAAGTACAGAAGTTCCAAG
TACGACTGACGGATGCAGATCAAATAGTGGATATATAGAAGACAACCTTCAACCGACCCTTCAAGTAATTTCT
TCGAAAATATAGTTGTTGATGAACAACGTGCAGGAAAATTAAAAATATTTTCTTATAA
CATTTCATTAGAATTAATGGAGTGAAGAGATGTCAATTATTTTATACTATTTAAAAAAAGCCATAATACCGCC
CATCGCCATTTTTAAAAGAAAAGGAGGTTATCAATTCGACTGTATTTTTTTGTGTTTGTTACCTCAGAACTTT
TGATAGGGTGAACCGATTTCGATTATTCTTTTTTTATTTGAAAGCTAGTGATTCCCGT
GTGGTCCCGTTTTAATTCTTTCTTGTCCTTTCTTGTCAAGGGTATCCATGAGAAAATTACATAAGTTTTAAGT
TTGCATTAGGTATGTGCGCGACAAATGGACGAATAACTCATTACGCTAAATGATTGCGAAAATTCCTCTTTTG
ATTGAAAAGGATACACGTTAGTGGAAATTTGATGAAAGTTTGATTAGTTTATGGGATC
CGGGTAAAATTGAAGAAGTCGGTCTATTTTTGTTAAAAATTATAAATATGATTTCACTATGACATTACCGTCT
AAACGCTTAGTGTAGACACGCCTAGCAACATGCTA
>1002052797_2 Heli.2-CV525911.2.5
CGGCACGAGGGAAATACTCATACAATACTATTTTTATTTCCTTCTTCTATGTATATACGTACAAATTAAAGTT
AATTGATTGGTTATTACTAAACAAAGTAATAAGTGCGTGTGCTGTATTCATAAGAAGATTTATTGTTTGTTTT
TGTTTGCTTAAAATACATCAATAAAATGAGAAATCATGTTGCTGTTGTAAGATGGCCG
TTTCGTAGACTATGCGAGCGTAGTGTTGCCACTTCCGCTAGTGTGAACCAAGCAACTGAGGGATCAGATCTAG
GTCATTTATCACGGCCTCAATCGTGTCCAGTGAAGAATCATCGTGCCAGGTCTACACATGCCGTAGCATCGGT
ATTTGAAACTCTATCGCCTTCAGTAAAACCGTGGGAGGAGGTTCCAGGTCCCAAACCC
TTGCCACTACTAGGAAATACTTGGAGATTCATTCCATATATTGGTGGATATTCAGTAGAGCATATAGATAAAA
TTTGCTTATCACTGAGAAAACAATATGGCAAGTGTGTCAAAATGGCAGGTCTCCTGGGAAGACCGGATATGTT
GTTCGTGTTTGATGCTGGCGAAGTTGAGAGGGTCTTCCGAGGAGAGGACAGTGCTCCT
CATAGGCCATCTATGCCATCTTTAAATTACTATAAGCATACACTAAGGAAGGATTTTTTTGGTGCAGAAGAGA
ACTGTGCTGGTGTGATAGCAGTTCACGGCGATTCATGGGCGGCGTTTAGAACCAAAGTATCTCGAGTAGCGCT
CAGTACTGGCGCAGCAGCGCAATACACAGAACAAGTAGGGGAAGTGGCAGAGGCATTC
TTTAATAGGATACGAAAAATCAGAAATCAACAATCT
>1002052798_2 Heli.2-EL601005.1.5
GCACGAGGGAAAAGATCTGAACTCACCACCCAATACCACCCCTAAACCTACAACTACTTCTACGACTTCTACT
ACGACAACTACAACAACAACTACTACTACTACTACACCAGAACCTCCAACAACAACACCATTTATTACAACAA
CAACAACAACAGAAAAGCCAACTACCACGTTAGCTACAGAAAAACCCACAACAACAAC
AGTGCCAGAAGGAGAATACGAATATTACGATGAAGAAGATATAGAATACGAATCCAAAGATAAAAAGGAACCG
ACTACTACCGTAATAATTAAACCAGCTCAAATTAAAGAAGTACCTACTACTACAGTGTCATTCTCCGAAGAAC
TAACTACAGAAAACAAGGCAAGAGACTTAGCAACAAAAGTTTTGAGAAATTTCAATGA
TAACTACGAAGCTATTAAAGAACAGCTCGCATCTACGCTGACACCAGGGGATTATCTAAAACCATATTTAGGT
CAAACAATAAATAACAAGAAAGCGGAACCACAACCGGAAATATCAAAAGGCTATACAGCAACTGGAAAACCGT
TACAAATACCTGACATAAAACAAAATTTGGCAGATTCTTTAGAAAACGACTACGATGT
TAGACTAAATGAAGCATTAAGCCCTAAAATAAGGATACCCAGTGGTTTTATAGTTCCTGATAGAGATTATACG
TTTTCGAGATTCAGAAATAACGTGCATTCCGAACCGCAGTTTGCTGCTTCAGAAATTAATCATTACCAAGTTA
GGAAACGACCCCAAATTTCTGGAGTTTCGATCAGAACTCCTTCATCATACTACATTCA
ACCCCAAAGAGTGGTCTACGAAGACCTTGTGAGACCAGCTCGGCACTTAGTGTACAGGGAGTTTGGTAATATA
TTTTAATATAGATAAGGGTAACCTTTGAGAGATTCAAAAAACTTTGAAAGTCACCTATAGACTTTTGGAGTTT
TTAAATATATGAGATATACATATATATATATTAGACTGACATACAATATATAGAAATT
TAAATGATATATTATGTCTTAAATAATAAATTCAAGTATTTAAATATAAATTAAAACGTCAAATTATCGTCAT
TTAGTAGGGTAAAAGTAACTTACGTATTAATTCAGGATATAATCCTTTGTGGCAAATTTCATCAAAATCTGGT
CAGTAAATGTCAAAATTTGATTTTTTTATAATAA
>1002052799_2 Heli.2-DT663440.3.5
GCACGAGGGGTGATTCGATATTGGGAGTAAAATCCTGAATAGGGATAAAAAGGCTTATATTTTCCTAAATCAA
TATGTCTGGAAAAGATAAATTGCCCATTTTCCCTTCTCGGGGAGCTCAGATGTTAATGAAAGCCCGTCTGGCT
GGAGCACAGAAAGGCCATGGTCTCTTGAAAAAGAAAGCTGATGCTTTACAAGTGAGAT

TTCGTATGATCCTCAGCAAAATTATCGAGACTAAAACCCTTATGGGTGAAGTTATGAAAGAAGCTGCATTTTC
CTTGGCCGAAGCAAAGTTTACTACAGGAGACTTCAACCAGGTTGTTCTTCAGAATGTTACCAAGGCTCAAATA
AAGATCCGCTCCAAGAAAGATAATGTTGCTGGTGTAACCCTCCCAATTTTCGAGTCTT
ACCAAGATGGCTCTGATACCTATGAGCTCGCTGGTCTTGCTCGCGGTGGACAACAACTGTCCAAGCTCAAGAA
GAACTTCCAAAGTGCTGTCAAATTACTTGTCGAATTGGCCTCCTTGCAAACCTCATTTGTGACTTTAGATGAG
GTCATCAAGATTACTAACAGGCGTGTTAATGCTATTGAGCATGTGATCATCCCCCGCC
TGGAGCGTACCCTGGCTTACATCATTTCGGAGTTGGATGAGCTTGAGCGTGAAGAGTTTTACCGGCTGAAGAA
GATCCAGGATAAGAAGAAGATCATCAAGGACAAGGCGGAGGCTAAAAAGCAAGCAATGCTGGCAGCGAGCAAA
GAGAGAGATGTGCGCGACATCGCCAATCTATTGGACGAGGGTGACGAGGATTTACTTT
TCTAAATATCCCATCTGGATTAAA
>1002052800_2 Heli.2-DT664400.3.5
GCACGAGGGTGAACTTCATCAAAGATTAATGACCAATATGGAAATATACAAAAACAAACAACAATAGGTTACT
CCACGGAAACACTACCTCAAAAAGTGACGCAAATGCAAGGAGGAAAATCTGTGGAATGTTCGCAGAGTGGATT
TGTGGGTGACCCAAATGATTGTTCTAAATTTTATAGGTGTGTCGATAGCGGTAACGGG
AAATTTATGAAATATGAATTTTCTTGTAGCCAAGGCACGGCGTGGGACAGTACAGCCGAAGCTTGCAACCATG
CGTGGGCTGTAAAGGAATGTGGTGGACTGTCACCAATTAATAGTGTTTCCCCTAGCACTACTCTAACAACATC
TAGTGATAGTTTTGTTTCTAGTAGCAGTTCAACGACTTATAAGATTACTACAATTAGC
ACTACTAACGATGGTTTAATTAATGAAAATAATGGTAATGATTACCTTAATCCAATAAAGGTTCAAGAAGAAA
CAACCAAGCCCACTACTGTATCACTTATTTCTAGTACAACATCTACAACAGAGTCTGTTAAAGAATGTAAGGA
TACTAGCTTTGTTGGAGATCTAAATGATTGCCAAATATTTTACCGCTGTGTTGATAAT
GGCAACGGAGGTTATACCAAGTTTGAGTACAAATGTGGAGAAGGTACTTTATGGGATCAGGATATCGAAGCGT
GTAACCATGCGTGGGCTGTAAAATCTTGTGGGTCTAAGTCAACTACAAATATAACAAAAGTCGAAAAAGAAAC
AACTAAAGTATCAGATAAATTTACCACTAACTCATATGTCCAAGCCACGACAATGAGC
GTTATACCAAGTAATAATTATAACACAGAATATAATGCACAAGAGACACAAACTAAGAGACCTGATAAAAAGG
AAATAAACGAGGTTTCAAAAGAACCGGAAACGAATAAAAATATATGTCAAAATGTTGGTTTTATCGGTGATTC
TAATGATTGTAGGAAGTTTTATAGATGTGTAGATAATGGAAACAGA
>1002052801_2 Heli.2-EL598633.1.5
CGGCACGAGGGTCGGCCGTGTCGAGAGGTCCGCTGTGTAGATTGTATTTGAATTAGAATTTGAAACCTCCAGC
GTAAAAGACAAAATGTTCTCCCGCGCAGCAAAACCAGTAGCCACAGTCGCCCAAAATGGCATCAAAAACTTCT
CTACAACATCACAGAAAAACTTCAAAGTTGTGGTGGCGGGAGCTGCTGGAGGTATCGG
TCAGCCCTTAGCCCTATTATTAAAGCAAAATAAGCTTGTAACTAGGCTGGCTTTATACGATATCGCTCCAGTA
ACCCCTGGAGTAGCCGTGGACCTCTCGCACATGGACACCGCAGCCAAGGTATCTGGACACCAGGGCCCAGACC
AACTGGGTGATGCCATTAAAGGCTCCGACGTAGTAGTAATCCCAGCCGGCGTACCCCG
CAAGCCTGGCATGACCCGCGACGACCTGTTCAACACAAACGCCTCAATTGTCAGAGACATTGCTGCCAGTATT
GCCAAGAACTCTCCGAAGGCCATCGTGGCCATTATAACAAACCCAGTTAACTCGATGGTGCCTATTGCTTCTG
AAGTGTTGAAGAAGGCAGGAGTATATGACGCAAACAGGGTCCTCGGTGTCACCACCCT
AGATGTAGTCCGGGCTGCCGCCTTCATTGGAGAGATCAATGGGGTCGACCCCCAGTCTGTGTCCATCCCGGTG
ATTGGAGGTCACTCCGGGGTCACCATTATCCCTGTCTTGAGCCAGAGCAAGCCAGCGGTCAAGTTGTCAGAGC
AAGCCAAGATTGATGCGTTGAC
>1002052802_2 Heli.2-DT667094.3.5
GCACGAGGGCGACGACCTTCGTTTCTCTTTTATGATAAATACAACATGAAGTACGCGGCGCTTTTTGCGCTGC
TGGCGGTCGCCTGCGCCAGGGACAGCGATCCTACAGCGGTTGACAATTTACGCAATAAGTTTTACGCGCTGGA
AAAACAATTATGGTTGAACGTGACGAACCCTGAGTGGAGTCTGGGAGGCCTTGGAGGA
GACGTCGAGCTAACAAAGGCCTTTGTGGCTTTCGATGAGCAAATACAAGCGGTGCCGCCTCCGCCGCGAGTAC
CGTTCGAGACCTGGCTTTGGGCGAAGGCGACGGAGAAGTTGCGGGTCATCGAGGGGTACTACAAGAATTTCAT
TATATTCGTGAGGCGTCAAGCCCAGCCCGGCATCGTGCCCGCGCCAGTGCGCGAATGG
CTGGACCTCGCCCAGCAAATGATGGATGAGAAGTCACCTGTTGTGCAGGCCACGAAAAAGATCACCGATATGC
TAGAATTGGGCGACATGTTCCGTGGTGTTTTGCAGGAGGAAAATTCAGACCTATGTGATCTTCAGCTGTCGCC
GCATCAGTTGATCTACGATATGTACAACACCATCTCCCTGACGGAGATAAAGGGGTAC
GCCATGACGCAGTTCTCGTGGATGCTGCTCAGGATTTACGGCAAAGGAAATTACACGCAAGAAGCAAGTTTGA
CGCGTCGAAGGTATGGAGAAAGAACGACTAGGACGGCGGCCGCTGCTCGATCTGCTTTGGCTATGGCCAATAG
AGGTTTATACCGGTGCGACCCGCCTCAGCATAAACAAGGAGAGACATACGAAGAAGTA
ACACGATTGCTACAAGGCTATATAGAAAACGAAGTGGATATGAATCCT
>1002052803_2 Heli.2-DT663811.2.5

ATTCGCTCTTTTGAATCTTGCCATTTGTGAAGAGAGATCACATAAAACACACTATGTTGATCCCTTCGCGCTG
ATTGATAAGCATTTGTCACACTCCCTTACTTATCACTATCTGTGGCCTTGGGGACAATTGGTGAAAGCAGCAG
CGGCTTTGGACTTGGAAGAAAGCTTGGAAGATCCAGAAATTATATCTGATTCTGAGAA
ATATCAGATCAGCTTGAATGTGAGGCGGTTCAAGCCTGATGAGCTGAAGATTAAAGTGAAGAACCGGTACATT
ATTGTGGAGGGAAGACATAAAGTAGATAGTGACAACAAGTTCATGGCAAATCACTTCGCTCAGCGTTTCGTTC
TGCCACCGGGTACCAAACAGGAGGAAGTCAAGGCTGTATTAAATGAGAAAAGTATTTT
GATCATCTCAGCACCAAAACACGAACTTCCACCGCCGATTCCAGAACGAGAAGTGCCTATTGAAGTTATTTTG
CCCGAAAAGAAAGTTGAGGAGCCTACAGAAAAAGAGGACGGCAAGGAACAAGCATCATCAGTTGCTCCTATTG
AGCAGATCGAAACAGAAGCTACTACCCATGTAGGCAAAATAAGGAAAAAGGAATTGAA
AACGACCACTAAAACTACTAAGGATAACGAAGTTACGAAAGGTGTGGATGGAAATGGGCTGGACTACGCTTTG
ATTGAAGCTGAGGAATGATTGTAAGATATTTTATGGAAAAGACTGAATAAGCTTTAAAG
>1002052804_2 Heli.2-EL599564.1.5
GCACGAGGGAGAGACCTTGAGAGAGAGAGAGAGAGAGAGAGAGAGAGAGAGAGTGCTGACGCCTTGCTATCCA
CACAAAAATGCCAGTCGACCTATACTACGTTCCCGGCTCAGCGCCCTGCCGGGCGGTGCTGCTCACCGCGCGT
GCTCTCAACTTGAACCTCAATCTGAAACTCGTGGATCTTCATCATGGAGAACATCTTA
AGCCTGAATATCTTAAGATAAACCCACAACACACAGTTCCTACCCTCGTGGACGACGGCCACCCGATCTACGA
GTCGCGTGCTATAATAACCTATCTAGTGAACAAGTACGGCAAAGGCAGCGCGCTGTACCCAGAGGAACCCAAA
GCCAGGGCCCTCGTCGACCAACGCTTGTACTTCGATATTGGCACTTTGTATCAGAGAT
TTGGTGATTACTTTTATCCACAAATCTTCGGCGGTGCGCCCGCGGACAAGGACAAGCTAGCCAAAGTAGAGGA
TGCCCTTAAACTCCTGGATACCTTCCTCGAAGGTCAGAAGTACGTCGCCGGTCCCAACCTCACCGTGGCCGAC
CTGAGCATCGTCGCTGGCGTGTCCAGCTTTGAAGCATCGGATATTGACTTCAAGAAAT
ACGCTAATGTAAAGAGATGGTATGAAACAGTGAAATCGACAGCACCCGGCTACCAGGAGGGCCACGGAAAAGG
ATTAGACGCCTTCCATGCCGCTGGTCAACGGGCTCCTCAAGAAGTAA
>1002052805_2 Heli.2-DT661960.2.5
GCACGAGGAGTAAACATCAAACATGGCTAAGTGGAGTATTGTCGCCCTCGCTCTCATCGGCTGCGCCGTCGCC
GAGCCTCCAGTAGGCTACAGCTACTCCGCACCCTCTTCCCTCCTGGTTGTCGGTGGCGGACACGGCCACAGTT
CTCCTCTCGCCAGCGGAGGTCACTACTCCTCCGTACCGGTCGGTCATCAGTCCTCAGA
GGGCTACCATATTGACCCTCATCTATTGGAGAAGATCCGTCACATTATCCTAAAGGATGAGATCCAGAACCAG
GCCTACCAATCATCTATCGGAGGCGGACACGGTCACGGTATCTCTTCTCACTACGGTCCCCCTGCCCCCGTCT
ACGGTGTGCCCCACGACAGAATCGTAGGCGTTGAGCTCGAATCACTCCAACAAGGCAT
CCAGGTAGCTCAATACCACCAAGCTCAAGAAGACTACTCTGGCGGTTACGCTGGTGGTTACTCTGGTTACTCT
GGCGGTTCATCTGGTTACTCCGGTGGTTACTCCAGCGGTGGTCACGGATCCATCTCCTACAGCCCGTCATCCA
GCTACAGCACCATCGGCGTTCCCTCTGGCTCCTATGGTATCCCATCCCCCTCATCATC
ATACGGCGCCCCCCACCATTAAACGAAAATTATAATAGTCATCTCATTTCATTAATGTTATGAAACTCATGTG
ATCGCTAGACGTTAACTGTATTATATTTGTTGTTAATATAAGCTTTCTGTAAATAAATCTATCTTTTTTGTAA
AAAAAAAAAA
>1002052806_2 Heli.2-DT664556.3.5
GCACGAGGTTTGGCTTGGCGCCTTGCACGAGGGTTGTTCTCTTGTAGTGATAGTGTGTTACACGTTTCAAAAT
GGCTGGTGGTAAAGCGGGCAAAGATTCTGGCAAAGCCAAGGCGAAAGCTGTATCACGATCGGCTCGAGCAGGC
TTGCAGTTCCCTGTGGGTAGAATTCATAGACATCTCAAAAATAGGACTACGAGTCACG
GCCGTGTCGGCGCCACGGCTGCCGTTTATTCTGCAGCTATTCTCGAATATCTTACAGCCGAGGTTTTAGAGTT
AGCGGGCAATGCATCCAAAGATCTTAAAGTAAAACGTATCACTCCCAGACATTTACAACTAGCTATCAGAGGA
GACGAGGAGCTGGACAGCTTGATCAAAGCCACCATTGCAGGAGGTGGTGTCATCCCAC
ATATTCACAAATCACTCATCGGTAAAAAAGGAGGACCGGGGGCACCTGTTTAATAAGAGTCTTGCTTCAATTC
AACATTCTAACTGCATTTGAACCGGACACTATCAGCGTTGTAAATTCATCAGCCAGGCAGTAAACATAGTTTT
CTACAGGTAGTGATGTGGACAGTGGAGTGACTTCAGACATATTGATCCGGACTAAACT
TATAGTTGTTAAGACTAGTAATATTTTTTAGTATAAGGAGATTTAAATATAATTGAATTAGGATTAAGTTCAT
TTTCTCGTTTTGTTACGGGACATCTTTATTTTATTAGACATGTGTACAATTTATTTCAGCTACTGCTAAAATT
AGTTCATTGTCAGTTCGGCGTGGCCTGAACAACAAGGTTCAGATCGACTGCTGCAGTG
ATTTAATTTTAC
>1002052807_2 Heli.2-DT666676.3.5
CTGCTGATGGCGGCCGTGTTCCTGGTGGTGGCGGTGCTGGTGGCCGTGCTGGTGCAGCGCCTGGTGGAGCCGC
GCCCGTGACGCGCGCTGGCGGCGGCGCGGCCGCGCGCCCTGGCCCGCCTGTAGCCGCCCGAGGCGATGGAGTG
CAATTATTCGATTTATATAAACCTTCGACGAGACGCCGCCCCGCGGGAGAGAATCTGA

AATATTTATATGTATATAGTAGATAATAAAATAATCAAATTGTACGCCGCCGCCATTAAATGACGGGACGCGC
GAGGTAATCGCTGTCTTTAATCGCTTTATTTTTAATATATTTTGATTTCGGTTAATGAATATTCAGATGAACT
AGACGATGAGTTTATTATATCTCCAATATTTTAATCGACTGTACTAAACGAAAGGCCT
AGGGAACGTCGAGTTTAACGAGTTTTTTTAGATTTCACGTATATGTATTTTTAGTGAATTTACAATTATGAAT
TTGCTATTTTGATGCGAATGTATATGTAGGAAGTGTTAACTTTGCCTAATCCAATCTGTTTCGCAGATCTTCT
AAGTTCTAGTCTATGCTTTTAAAATTTCATATCTTCTGCTGAATCGTCTATTTTACGT
AAATTATATGAAACATAAAAGTTGTATTTATTGTGATATAAAATATACTTCTCGATTATAAAATCACCCTGCC
GCTTGTATTTCCCATACATTTCTATATTTTTATTAATAAAAAAGATCTGTTGCATGTTTTATCATATTTAAGT
ATAGCAAGTTAGCGACCAAGGTGCTCTAATATTAATGTTCTGTTACGAGGGACAAGGC
CCCTCGCTCTCCAACGAGTTTCCAGTGTGTGTGCTGTGATCACTTCCTTATGCGAATGAGACTGATTTTAAAA
ATATATGCTTCGTGTCTTAGGATTGTTTTGACAATGCTGTCGTTCATTTTGATGAAAACCGAAGTGTTTTATG
ATTTCCCGAGAACTTAGCAACCCGCCAGCTGAGGCGCGGCGACCGACGCGCACGTCTC
TTTTACCCTCTAATGTACCTTCTG
>1002052808_2 Heli.2-EL603369.1.5
GCGGAAGGCGCACGAGAGCGAGGTGCGCCGCGAGGTCGATAAGTTCAAGACTGAGTTCCTGGCGAGGGGGAGC
CCTGATCTCGGACAGCTCAGCTCGAGGCACCAGCAAGAAATGGAGGAGATAAAGCGTGAGATACTCTCGTTAT
CCGAGAAGTATTCAGTTAAGTGTGTGGAGTCGGCGTCGCTGGAGGAGCGCCTCGCAGC
GGCCAGTTCACAGCTTGCGCACGCGCACAATCACATCATGCAATTGGATGCCAGGAACAAGCAGCTCCGAGCT
CACATAATGTCTGAAGCTAACGACATGAAGAACTCTGAAGCGTCCACACTTGCTCAACTCATTGAAGACACGG
CACCGGGGAAATCCCCAAGGTCGAGCGGGATGGAGCGAGCACGTGCATCCGGCCTGCA
GTTCTCGAAGACGGAGTCTCCCAGAAAGCAGTTGTCTCCCGTTCAGAACTAGAGACGGAATCTAGCAGCGTGC
TCGCAGGCGTCGTCGCAGAACGGAAAAAACAATTTGAAACATAACGACGCTATTTGATTGTATTTTACGAGTA
CCCGCCATGTCTATATATAAGTATGGCGGGTCCCGCACTTACCGGGTCACAAGCTTCC
CACCACACCGGCACCTGGCCACTTAGCTTCTAGCTATAAATGGATATAAAATATTAATATAACATTAAATTAA
ATACCTGTTAGGTATAAGCTAAG
>1002052809_2 Heli.2-DT667901.3.5
GGCGCCGGGCTGAGGAATCGGCACGCGGCTCGAGTAGATTTGATTTGAATTTTAATTTTTTATAACATAGAAA
ATATTTTACTATAATCATATTATAATAATTAAGTCGTTTCCGACAATACCAAAAGACAATTACTTTAGCATTC
AGTATGAGTAGTGAACCTGTAGAGGAACGGTCCAAAATGAGTGCTTTAGATCAACTGA
AACAATTTTCTACTGTTGTAGCCGATACAGGTGATTTTGAAGCTATGAAAGCTTATAAGCCCACTGATGCAAC
AACAAATCCAAGCCTTATTTTATCTGCTGCTGGAATGGAACAGTACCAACACTTGCTGGACAAAGCCATTAAA
TATGGCAAGGATTGTGGTGGAAATGTAGATGAACAACTGTCAGAAACATTAGACATGC
TCAGTGTACTATTTGGCTGCGAAATCTTGAAGATAATTCCTGGAAGAGTATCAGTTGAGGTGGACGCCAGGTT
ATCTTTTGATAAAGATGCTAGTATGGCAAAGGCTATTAAGCTTATTACTATGTTTGCAGAGCATGGTATTAAA
AAAGAAAGGATTTTAATAAAATTGGCCTCAACTTGGGAGGGAATTCAAGCTGCAAAGG
AACTGGAGAAGAAGCATGGTATACACTGTAATTTGACGTTACTTTTCTCGTTATACCAAGCGATAGCATGTGC
GGAGGCGAACGTCACTTTGATTTCACCGTTTGTTGGTAGAATTTTAGATTGGTACATTGAGCATACTAAGAAT
GCGTATGAGGCTAAAGATGATCCAGGTGTGCTATCAGTGACTCGTGTATATAATTATT
ACAAGAAGTTTGGATACAAAACCCAGGTGATGGGTGCGTCTTTCCGTAACACTGGAGAAATAAGGGAGCTTGC
TGGTTGCGACTTACTAACAATCAGTCCAAAACTTTTACAAGAGCTTGCTGGTAGTGGAGAACCATTAAGAAGC
ATTAGACGCC
>1002052810_2 Heli.2-EL601630.1.5
GCACGAGGGGCGCTAGTGAGCTTGGCTATCCTGGCACTATGCGTATGTGCGCATGCGCAGTACGCGGAGGAGC
GCGCGCCCCGGTACATCGCCTCGGAGCCGAAAGTTACATCGACACCCGTGCCAATTCTTAAACAGATTAACAG
GCACAACGAAGATGGCTCATATACCTATGGATATGAGGCAGCCGATGGGTCTTTCAAG
ATCGAGACAAAATCTCCTGCTGGAGAAGTGAAAGGAAAATATGGATATAGAGATGATACTGGCAAGGTACGAG
TTATCGAATATGGAGCAAATAAATACGGTTTCCAGCCAGCTGGAGAAGGCATCACTGTCGCTCCCCCCACATT
AGTTGACGAGTCCACACGGGAACCTAACAAGCAGGGTGGCCGATCTCAATACCGTGAA
CAATCAGTCGACTACGACTATGAAGACCCTGCACCAGCCCCGCGCCCTCGTCCTACTCCACAGCCCGCCTACC
GCGCTCCCCAGCCCCAGCAACAATACAGACCTGCTCCACAGCCCCAGCAACAATATAGACCTGCCCCACAACC
CCAACAGCAGTATAGACCCGCACCTCAACCTCAGCAGCAATATAGGCCCGCCCCTCAA
CAAGCCCCAGCTCCACCTAAGCCTGCGTTTTTCGCGGGTGCCTCACCAGCGCCAGTAGAAGACAATTTCTTCA
ACCCAGAACCAAAACAACCCCGTCAACAGCAGTATAGGCCGAAGCAGGACTTCAGGCCTGCCCCACAGCCCCA
GCACTTCAGCCCTAAACAGACCCAAGTCGATTTTAACCAGGACTATAATGCCCCAGCT

CCCCAAAGGTTTCCACAAGCGAATCAAAGGAGTCAATCATTCTCAATGCTCGACCAACTCCTTAAAGAGTACT
CTCTGCCTCAAGGTGGATCCGCTCCACTCCACGATGTCTCATTCGGATCCTACTAGTTCGATAATTTGATTAC
ATTTTGTAAATATGTAGTGTATTATAATTCTTTTAAAGGTTTAAATTATTAAAAAAAA
>1002052811_2 Heli.2-DT668481.3.5
GTGAATATTCTTAAATTAGATCCAATTGATTCTACAAAAGATTTCATCGACAATGAAAACGTAGATTCGTCAT
TTTTATTACAAAAAATTAACAAAAATACAGACTCTGAGAAAATAGTATTAGAACCCTTTGAGGAAAAAAGTAA
AAAGGATGGTGATGAAAATACAGAGAGACATTCAGAAGAAAATGTAAAAATGATTAAA
GCGGCCAATAAAGGACTCTGCGATGAAAATAAACAGGAAAAGTTTCCGTCTAAGTCATCAGAAGAACCACTAA
ATTCACCATATAAATTTGAAGATGATAAATCATCAGATGTACTACATGTGAAGTCTACAGATATGCCAAAAAT
CATACCTATCGATGGCAAGAAACATATTACAAAAGAAATTATAGAAGAACCAGATGAT
ATACAAGAAATTGAATTAGAGCCGAAATTGACCTCTTTCGTCGGAGAGCATAAAACAGAATTAAATAGAGCAA
CACCTTTGCAAAATGTTCAATTTATTGTAAACCAAAATATAATTTCAGAAACAGAGGAACCAATAGATGAGAA
CATTTTAAGAACAAGTGTCACGAGACAGGTAGATTCACAACATTCTCCACAATCAAAA
AAAACTAATGAAAAATTATCAGAAATATCTTTTAGTCCAGTTAAGCAGTCAATGGAAGCTGAGAGTTTTGATG
ATCTACCACCATCGTTTAAAAGTATTACGGAACAATTTTTATCTCAA
>1002052812_2 Heli.2-EL603108.1.5
GCACGAGGCGCAGCGCGTGCGAGATGACGCGCCGGTGAATTTTGTATTTTAGAAATTGACGTCATTTTACTGT
GTACGCTCTATTTATAAGGTGAATTCTTTTATTATGAGATGACGCGCCGGTGAATTTTGTATTTTAGAAATTG
ACGTCATTTTACTGTGTACGCTCTATTTATAAGATTTCACAATGGCCGATCAAGTAGA
TTCAATGTCAGACGCGGAGCTCCGCACTAAACTGGCCGAGCATGGGTTCCCTGTTATGCCAATTACAGCATCT
ACAAGAAAGTTGTTAGTGAAAAAGTTAAAAATGGTATTGGACAACAAAACCAAACCCAGAAACAGTGTGGACG
CCAAAGTCGAGAGCAGACGTTCTTTAGCCAGGTATTCCAGTGGTGAAGAATCTGATTT
AGAATCTAATAATGCGAAAGAAAGACGAGGTCGGCGAGTTACAACTGGCGGGGCTATGCTACCTCCTGTGACC
AACAAAGCTAGACGAACCCCAATAAAAAAAGATTCACCTATCAAACGTGATGCTGATAAAGGCTCTGAGTCCG
AGGATGAAAAATCACCAGTCCGCACTCATGAAGAAGTTGAAACTGTTACAACAAGAAG
AATAACACACACCTATGCAACAAATGACCAAGATGACTATGAAACTGGTTCAGATAGTGATGTTGACAATGAT
AAGAAAACATCCAGTCCATTTAGAAGTAGCTCTAGGTCTAGTGATTACATTTCTAGCACTCTACCCACTAATG
ACGCGTCAACAAGCCCACCCAAAACTTCTATTTTTTCCCGCCCATCTTTATCTCAGTC
AAACTTTTCATCATTATCATCTTCAGACCAATTGAATTCAATAAGATCTAGACTCGGGCTAACTTCATCTTTG
GCTGACAGGCCATCTCTTAATAGTTACACACCAAG
>1002052815_2 Heli.2-EL595937.1.5
GCACGAGGACAAATTCTAAATTGTAGGGTATTTTGTTTTGTTAGTCTTCATAGAACCGACGACGAAAGTGGAC
AGTGGATTTTAAACGTTAACAATGACAAACGCATTTGCAGTTTGTCTTATTACAATATCATTCTTTTGTTGTA
CAAGTTTGTCAACTGCTCGACAAATTCCAAAAGAATGTTCTAAGGGACCAGAATATTG
GTGTGAAAATTTGAAGCGGGCTGCAGACTGCGGCGCAGTGCAACACTGCATCGGTACAGTATGGGAACAAGAA
CGAATCGAACTCAAATCCAATGAAGTGTCCGACAAGATCGTCCGTCTCTTCAGACAACTTAAGGATGTCAAGG
ATATGATTAATGATGAATATTTAGGAACTCGTGTGAGCTCCGCTTGCCGCGATGTGCC
GTCGCCGGCCATCACGGCCGTGTGCAAGGAGAACACAGCGGAACTACAACAGTACATGAGCCACCTCCTCCAA
TCCGACACTACTCCTGAGACCATGTGCAAGATAATCGGCATGTGTAACAACCCTAAAATTGATCGTTTGATCT
CGAAAAAGAAGACTGAAGAACCTACCAAACAAAAGCCACATCTATTGGGAGCTACGAA
ATGTACCTGGGGACCTTCTTATTGGTGCAGCAATTTCAGCACTGGTCGTGAATGCAAAATGACGCATCATTGT
GTGCAACGTGTGTGGTCCAAAATGACGTTCCCACAAGACGATGACGG
>1002052816_2 Heli.2-DT662505.3.5
GCACGAGGGACACGCATTAAAAGTAAGAAATAATCATAAGTTTGACCTTTTAGCGTTGTAACAAAAATGGCAC
CATCAACTGATTGGGATGAAATCAAACGTTTAGCAGCTGACTTCCAAAAAGCTCAACTAAGTTCAACTTCACA
ACGATTATCAGAACGAAATTGTGTTGAAATTGTATCAAAATTAATAGAATTAAAACTA
TTAGACGTCATTTTTACAAATGATGGAAAAGAATATCTAACCCCACAACAACTTATAAAGGAAATGAAAGATG
AATTATATGTAAGAGGAGGAAGGGTAAATACTGTAGATTTGGCAAAGGAGCTTAATGTGGATTTTAATCATAT
AAATGTCCATGTAGCTGAAATATTAAAAGGAAAAGATGTACAATTAGTTTCAGGATCT
TTAATTGCAAACTATTATTTGGAAAAAAATTGCTAGAGAAATTAATGAGAAACTTCAGCTACAAGGACAAATTA
CTGTTGGGGACCTGATATTGCAATATGACCTACCAGCAGATCTTCTACAACATGGTATTATGGAAAAAAATCT
TGGTAAGATTATCAATGGAAACCAAGATCCATATGACCCGAGGATATTTTATACTGCA

GAGTATATTATGAGAATGAAAGCAAAAATTAGAGGGGCTTTAATGGGTCTGCTTAAACCTACACCTGTAAGCT
TAATCATTTCTCATTGTAACATAGGTGAAAGACTATTCATGTATCTTTTTGATAAGTTAAATGCACCAGGCGT
TCTCACTGGAAGACAAGCTGGAGCTCAATATGTACCTTCATGTTACACAAATCCCAAA
ATGAGTGGGTTCTAAGCTTTTACAACAAAACATTAT
>1002052817_2 Heli.2-DT666571.3.5
GCACGAGGGTTAAACATAAGTGAAATTTAAATTATAATTCATAATAAATACAGTTAAAAACAAATAAGTCAAT
ATGATAAATGCGGTCAACGAGTTCCTAGATGACCTGCGTGGCGGCAGAATGATTGATTCACCGGTCGTGAGAG
TTGAAGAGGGTGAATTACAGGGGAAACTGGTGAACTCACCGAGTGGCAAGGCGTTCTA
TAGCTTCCAAGGCATACCCTATGCTAAACCACCTATTGGATCTCTAAGATTTAGAGCGCCACAACCACCAGAA
CCATGGGAAGGTATACGAGATGCCACATGCGAAGGCAACATTTCTGCTCAAATACCACCTTTTGAAAACCCAT
CATATTCGGGAGACGAGAATTGCTTATTTTTAAACATCTATACACCAAATCTCGACGG
TGAATTCCTCCCAGTTATGTTTTTCATCCATGGAGGAGGATATCGTTTTGGATCTGGCAGTGCCAGTCTTTAT
GGTGGCGATTATTTAGTTGAAAAAGATGTAGTCGTTGTAACCATCAACTATAGAGTTGGGCCATTGGGTTTCT
TAAGTCTCAATATTCCGGAAGTGCCAGGAAATGCTGGAATAAAAGATATGGTTCAAGC
TTTACGATGGGTTCACCAAAACATAAAGAATTTTGGTGGTAATTCGGGAAATTTGACCGTTTTCGGACACAGT
TGTGGTGGCGCGGCCGTATCTTTGCTCACAGCGAGTCCATTGTCGAAGAATTTGATAAACAAAGCTATTGTCC
AGTCCGGCTGTTCGTTAAGCTGTTGGGCAATGCAAAAAACACCGATCGAAGATGCCAG
ACGCTTAGCGGAAGAATTGGGCTGTGAATCATCAGATGTTTCTGATATTTTAGAATTCCTATCAACAACACCA
GTGATCGATATCGTCGAAGCTACCGAGAGAGTTTTCCCATTTAATGATAACATTAANAAATTCACAAATTTCT
TCTGTCCGGTCGTTGAGAAAGAGTTTCCTGGAGTCGAAGCCTTCATGACGGAGCCTTT
>1002052818_2 Heli.2-DT668368.3.5
GCACGAGGCTTGAACTTTACAAAGCTAAATTTGTCGATCTTATTTACTGTAAAACCACCCATTCACCCATTTT
ACATAGCTATTTCAAATTAAATAAAATTTAATAATCATATTCACATCTCCAATCACTAACAATGCCACGCGGA
AAATATACCAACCACAAAGGGCGTAACCGCAAATTCACTAGTCCTGAAGAGTTAGAAG
AACAAAGGAAACAGGAAGAGCTAAAACAAAAATGGAGAAAGGAACATGGTGATGTGAGTTCTAGTGAAGAAGA
CTCTGAGGGAAACAAATCTGGTTCAGATAACAGTGACGATAGCGATTCAGATGAAGATCATCCTACAAAAGCA
AAGGGAGTGTCAGGTCTCATTGAAGTAGAAAACCCAAATCGAGTAGTAAAGAAGAATA
AAAAATTATCTAATCTAAATACCCTTGGAGAAGCAGAGAAACCTCAACTCTCAAGGCGTGAACGTGAAGAAAT
AGAGCGTCAGCGAGCAGCCGCTGCGTACCAGAAGGCCCACGCAGAGGGTAAGACCGACCAGGCGCGGGCCGAC
CTCGCGCGCCTCGCCATCATCAGGCAGCAGAGGGAAGAGGCCGCCAAGCGACGCGAGG
CCGACAAGAAGGCTAAGGAGGAGACTACTAAGAAGAGGTAGATAAATACTCATCAATTTTCGTCGATGACGAG
ATGCCATTCTGAATTTCGGCGCTTCAAATCCATTGTCTCAGTTTCTTTTGTGATATTTGTTTTGAATAAATAC
AAATTTTTTT
>1002052819_2 Heli.2-DT666966.3.5
GAAATTGTTAAATACAATCCGTGTGGTGAAGATAACGATTACTATAACTACTCTTTCGATACGAAAGTGCAGG
TGCTCAATAGATTCGTTTTCAATTTGGCGGTGGCGTTCTCCAGATGCATACCTAGTGTCACGCTAGCCAACTT
CGGCTTCATCGCCGTGACATCTGGGCTGTTCGCTATTTTTAATATGTCTTACCTGGTC
GTATATCCGTTTTGCAGATTGGTATTTGGGACTCTCTACCCAGCATACGCGTCATACAAGGCTGTACGGACAA
AGAACCTCAAAGAATACGTAAAATGGATGATGTATTGGATTGTTTTCGCACTATTCACATGCACAGAGACGTT
CACTGACGTTTTTCTGTCCTGGTTCCCCTTTTACTACGAAGTGAAGATAGTACTCGTT
TTGTGGCTGCTATCGCCTGCTACGAAAGGCTCGTCCATTCTCTACAGAAAGTTTGTTCACCCGGCGTTATGCC
GGCGAGAACAGGAGATCGATGAGTATATAGCTAAAGCCAAAGACCAAGGTTACCATACAGTACTGAATTTAGG
AACTAAAGGCGTCAACTATGCAACTACTGTTATTATGCAAACAGCTATCAAAAATTTG
AATCTGCCGGGTCCCGAAGGCAATGTTCGTGCTATAGCGGACCGTGACGTCACCGATTACCATGATTACGAAG
AAATGGAGTTCCAAAGAGAAAATACACCTAGAGTAGTAGAAATTGTCGAATTAGATGAGAACGCTGGGGATAG
TAATGATCCGGATTACGAACCGACGTCGGCTAGAAGTCGTGGGGAT
>1002052820_2 Heli.2-EL596364.1.5
GTATAGAATTAATTCTCAATTATAAAATTATTGCTTTAATTGTTTACAAACGATGTTATATGTTTCACTTAGT
GGCGCGATTAGGTTATATTTTTAGTCTATTTTAAACTATAAGCATTCAAGTTGAATTAACGGTTCAATTAAGA
GGATCATTAGAAAAAAAAAAACCAAACAAAATGAAACTTTAAAGTGACGAAAATTTATT
GTTACGTCCGTGTTATTGTCCAAATTGATATATTTAGTGCTAAAAATGTTAAATTTAAATGTATTTATAAATA
ACTTTCTCGGCTGTATTCAAACGGCCCGGGACTCCCCCTGGCTGCGGTGGCTGCTTCAGCTGCTTGCTATTTC
ACATGCGATCTCGCCGGTAGGATGGCCTGCTTCTTATCCTCTGAAAGATGGAGACACA

TTCGACTTTATAGTGATAGGTGCAGGATCTGGTGGTGCGACAGTGGCGGCCAGACTGAGCGAAATTCATCACT
GGAAAGTTCTTCTTTTGGAAGCAGGGGGAGATCCTCCGCCAGCCAGTGTGATACCCAGTTTGTTTGCAGTTAT
GGCACATACTGAGAATGACTGGGATTACCACGCTAAACTGGATGAGGGAGTAGGGCAG
GCGCACCCCGGGGGCATCATATACATGACACGAGGAAAAATGTTAGGAGGGTCATCGTCTATAAATTACGAGA
TTTACGCAAGAGGCGTTCCAGAAGATTACGACGACTGGAACACTGTAGCTCCAGGGTGGGACTGGGGGACAGT
TCTTCATTACTTTAAAAAACTTGAACATATGATAGATGCAACGATATTTAAAAATCCA
TATAATGCCTATTTACATTCACAATCTGGTCCAGTAGCCATATCCAGACCAAAAGTTAATGCATACTTCAAAA
GTGTAAATGAAGAAGTTTTAAGTTCCTATGAGGAAATAGGTATAAAAAGAGTTTTAGAGAACAACGGTCCAGA
GCATTTTGGCGCTTCGACACCTCATTTTACTTTT
>1002052821_2 Heli.2-EL600440.1.5
GCACGAGGCTTTCAGGCAGTTGCGTCACCCACATGGTCTTGGTCAAGTGAAATAAAACGAAATAATTCATAAA
GTTAGTTAAAAACGCAATAAATATGCTCACAGCAACGCGGGTGATAAGCCGAAAGGCTTTGGAATGCACTGGT
ATATCAGATTTTTCCACACATAGAAATTTCTCGTCGATCCTAAAAAGTACAGCAGCCC
CCACAGTGCCTATTTATCAGCGCCATGGACTCCAGTACAGACAGAAGTCCGAAGGTGTTCGTGGCGCCGTCAT
TGGTATCGACTTGGGAACGACAAACTCCTGTGTGGCAGTTATGGAGGGAAAAACACCTAAGGTGGTGGAAAAC
AGCGAAGGTTCCAGAACGACGCCGTCCCACGTGGCTTTCTCTAAAGAAGGTGAACGTC
TCGTAGGAATGCCAGCCAAGAGACAAGCTGTCACTAACAGTGGAAATACATTCTATGCGACGAAAAGATTAAT
AGGTCGCAGGTTCGAGGACCCGGAGGTGCAGAAAGATATGAAGAACCTGTCGTATAAGGTTGTGAAAGCATCT
AATGGCGATGCTTGGGTGTCAGGTACTGATGGTAAAGTTTATTCACCAAGTCAAATAG
GTGCTTTTGTTCTTATTAAAATGAAGGAAACGGCCGAGGCCTACCTAAATACAAGTGTTAAGAATGCAGTGGT
GACAGTTCCGGCATACTTCAATGATTCACAGAGGCAGGCTACTAAGGATGCAGGTCAAATCGCGGGTTTGAAC
GTGTTACGTGTGATCAACGAGCCGACAGCTGCTGCACTGGCTTACG
>1002052822_2 Heli.2-DT663531.1.5
GCACGAGGGGCAAGATGCGTTCAGTCACACTGAAGGATGTTGAACAAGACAAGGTCGTCAAGACCGTGGCTGC
CCATTTGAAAAAGATGGGCAAAGTTAAGGTGCCAGAACACATGGATCTGGTTAAGACTGGTCGTTTCAAGGAG
CTAGCGCCTTACGACCCCGACTGGTTCTATGTACGTTGCGCTGCTGTACTCCGTCACA
TTTACATCAGATCTCCAGTTGGAGTAAAGACTGTCACCAAAATCTTCGGAGGCCGCAAACGCAATGGTGTCAC
CCCTTCACATTTCTGCAGATCATCAGGCAGCATTGCTCGTAAAGCACTTCAGGCGCTTGAAGCTCTTAAGTTA
GTAGAAAAGATCCAAGATGGTGGCCGCATCCTCACCACCCAGGGCAGACGTGACTTGG
ACAGAATCGCTGCACAGGTCCGTCTAAAGGCCAGACAAGCCGCCAAACAGAGTGTAATAGTTCTGTAATTTTA
AACTTAATAAAAATATTAAACCT
>1002052823_2 Heli.2-EL603885.1.5
ATTGAAGCAGGAAAACCGAAGCAGAAGAAGACCCATGCAGATGAAGACCGAAACATATGATGATTGAAGCAGG
TGAAGACCGAAGCAGATGAAGACCGAACGAAGCAGATGAAGACCGAACGAAGCAGATGAAGACCGAAGCAGAT
GAAGACCGAAGCAGATGAAGACCGAAGCAGATGAAGACGAAGCAGACGAAGACCGAAG
GAGATGAAGATGAAGCACATGAAGACAACAGACGAAGATTGAAGCAGGAAAACCGAAGCAGATGAAGATTCAA
GCAGTAGAAGACCCAGGAAGATGAAGACCCAGGCAGATGAAGACCCAGGCAGTTAAAGACCCAGGCAGATGAA
GACCCAGGCAGGTGAAGACCCAGGCAGATGAAGACCCAGGCAGATGAAGACCCAGGCA
GATGAAGACCCAAGCAGATGAAGACCCAGGCAGATGAAGACCGAAGCAGATGAAGACCGAAGAAGATGAAAAC
CGAAGCAGATGGAGACAGAAGCAGATGAAGGCCAAAGCAGATGAAGACCGAAGCAGATGAAGACCGAAGCAGA
TGAAGACCGAAGCAGATGAAGACTGAAGCAGATAAAGACCGAATCAGATGAAGACCGA
ATCAGATGAAGACCGAATCAGATGAAGACCGAATCAGATGAAGACCGA
>1002052824_2 Heli.2-DT665230.3.5
GCACGAGGGATTGTCCTTTTAAACATCTAGTTTGTAAATATTCGTATTGCTAAAAGAATTGATATAAATAGGA
ATATTAAATTCCGGAATTCAATAAGGAAAATCTTGTATATTGTCAATATAATTTATCTTTTTCTTTAACTATG
GAGAATAAATGTGACTTTGAGTATCCTCATACTTTTGTACTTCTCGGAGCATCAGGTG
ATCTTGCTAAGAAGAAAATTTATCCAACACTTTGGTATTTATATCGTGATAGCCTTTTGCCAAAAAATACCAC
ATTTATTGGTTATTCAAGATCGAAGTTAACTATAGAAGATATAAAAGAAAGATCAAAAAAATATATTAAAGTA
CGTCCAGGCGATGAAAAAAAACTTGAAAAGTTTTGGGAAGTTAACGATTACGTTGCAG
GTTCTTATGATAAAAGAATTGATTATGAACTTCTTAATCAACACATATCCAGACATGAAAAGGGTGCAAAGTG
TAACAGAATATTTTATTTAGCAGTTCCACCTAATGTATTTGAAGATGTAAGTGTGAATATTAGAAATGCTTGT
TTAGCAATTAAAGGATATACGCGAGTTATAATTGAGAAACCTTTTGGTAGAGATTCAG

AGAGTTCCGATAAATTAAGCAATCATTTAGCTAGCTTGTTTAAAGAAGAGCAAATTTATAGAATTGACCATTA
CCTTGGAAAAGAAATGGTACAAAATTTGCTGACTATTAGATTTGCTAATCAAATTTTTAGCCCATCATGGAAT
AGAGAAAACATTGCTTCAGTTCTAATTTCATTTAAAGAACCTTTTGGCACAGAGGGAA
GGGGTGGTTATTTTGATAATTTTGGTATTATAAGAGATGTGATGCAAAACCACCTTTTACAAATATTATCACT
TGTTGCAATGGAAAAGCCAGTAACTTTAAACCCTAATGATATTAGAGATGAAAAAGTAAAAGTCCTTAGACAT
ATAAAACCTATAGAGCTCAAAGATTTGGTTGTTGGGCAATATGTGGGTAACCCAAATG
GGCAAGGGGAAGAGAAACTTGGTTACCTTGATGACCCTACTGTGCCTCCAAATTCTATCACTCCAACATATGC
TTTAGCTGCTATGTATATTAACAATACCAGATGGCAGGGTGTGCCATTTATACTGAGATGTGGAAAAGCGCTT
AATGAAAGGAAAGCTGAAGTAAGATTTCATTTAAAGATGTACCAGGTGATATTTTTTG
GCACACAAAGAG
>1002052825_2 Heli.2-DT667630.3.5
GCACGAGGGTCGGAGCGGTTTGCTGCAGAATAGATACGTCAGAGAATTCTAGAAGATTGTACATAATGACTCT
GGGATGTCTGTATCCTTATAGAAGATTAGGTATTGGTACTCTAATGGTGGAACATGTGCTCAAGTATGTTGAA
CAGGATGGCAACTTTGACAGCATTTTTTTGCATGTGCAAGTGAATAATGAAAGTGCAA
TAGATTTTTACAAAAAATTTGGCTTCGAAATTGTTGAAACAAAAGAATTTTACTATAAGAGAATAGAACCAGC
CGATGCCCATGTATTACAGAAGACCATTAGACAGCCACAGCCAGCATTAGTTAACGGGAATGTTCCTCATGCA
AAAACCAACGGCCACGATTGAAATAACAATAATTAGAATTGTCTCAACTCTATAATAG
TGAAAAGGAGCAAGTGATAAATAACTTAAGAATAATAATCAAGTCACTTTTATATTTACATGTTTGATTGATT
AAGATGAGAGATTATGAATGATACTTAACATTATTGTAGTGGGTTGTGGTAAAGGAGATTCTATCTTCTCTAG
TGCCCCTTGTAATCTGACATGTGTAACTCTATTCATTGTATATAAATATTTTTGGCAA
GCCTTAATGGTATCTTATCCTTATATTTTTTCAGTTAAATTTGATTGTATAAATGTATGTAGCAGCCTAGAAG
GCATTCAGTTGTTCAATGTTTATTAATTGTGTTGTATTTACATGCCCAAAAGCAAAAGCATAAAACTTATAAT
TAAAAGAAAATGATGTTTTGGATCGCATAAATTGTAATGTATCCTTCAGTATAGCAAT
TAACATGGTGTTTTATATAATTTG
>1002052826_2 Heli.2-DT663879.3.5
GCACGAGGGTTTGTGAAGGCTTTTTAGACACGGACCCTTACCGTCCTTGACAATTTGTCTTTAATGAACACTA
AAGTCATAGGAGCCATTAACAACGTAGATTAAGTTTAAATTAAGGTCGATTTAGCTTAGTTACTTGCGTATTG
TACCTACGTTATGTTTTTAATTTTAAACCAATTTTGTTTTCTTTAGCGTTTTGTACAG
TCAAACGAGAGTATATTTTATTTCTGAACAATCAAAGAATAATATTTTTATTTATTATACAGATATATAAATT
ATTTACATAAATAAATATAAATAAAATACCTATTTATTTAAACTACTTTTTCGTATGAAATATCTAAAGGCAG
TGATTTCGAACAAAAACTGTTATTGTAGTTTTACATTATCGAAACTGCCACTAGAGCT
CTTTAAGTTACCGACTCTACATACAAAGATATACATTTAATTATATTTATAATACATATTTTAATTTCACTAG
TTGCACACAATTTCAAATCATTCACTATAGTTTTTGTGATAACCCTCGCTTGTAATATAACTAATCTATTAAC
AAGATGTACTCTTTGAACTTTAACTTTTTATGTTGGATAGACTCATGCATGACATGTG
ACTTCAAAACTTATTAGTTTTTCCGAATGTGATTAACATTTTTTTTTAAATTAACTATCAAAATCTCTGAATG
GCGATAAAATGAATAAATGATTCAGTGATTACAAGCAAAAGGGACCAAATACAAATTGAATTATATTACAAAC
AACACAATCGTAATATTTTAATTATTAACAAAAC
>1002052827_2 Heli.2-DT668995.3.5
GCACGAGGGCCTGTGAGTGTTTTCTTTACTAAAGTTAATTTTAAACTTTGTTTAAACTTTATTTGTGTAAAGT
GATACTTGTGTTGCTCTGTGAAACTTAGTGTCTATATATTTGTGTCAATAATTGTGCTAATAAATATATAACA
CGATGAATAAAACTTGTGCGAGGTGCGAAAAAACAGTTTATCCTACTGAGGAATTGAA
ATGTTTGGACAAGGTGTGGCACAAAGGCTGTTTCAAATGCCAGGAATGCGGAATGACACTCAACATGAGGACG
TACAAGGGCTACGGCAAGCTGCCGTACTGTGAAGCGCACGTGCCAAAAGCGAAGCACACCACAATGGCCGAAA
CGCCAGAACTGAAACGGATAGCGGAAAACACAAAACTACAGAGCAACGTGAAGTATCA
CGCCGACTTCGAGAAGAGCAAAGGCAAGTTTACACAGGTCGCAGATGATCCAGAAACACTCAGAATTAAAGCA
AACACAAAAATTATAAGCAATGTTGCCTACCACGGAGACTTGGAGAAGAAAGCTCAAATGGAGAAACAGAGAC
AAATTAACGAGAACGGAGAGATAGTGGACGTAGCGACGAACGATAACTACCATCAACA
AATAGAGAACTACGCGACGGAAATGCTACCACCAAACGTACCACCACCAAACTTGCCACCCAAAAATCATTAT
CAGACACCCGTCGTCGCCCAAAGTCATCGTCAGTATCCGGAATATCGTCAACAAGAGGAATATTATCAACAGC
CAAAGTACGAGGAATATCCTAAAAATGCGAATTTTCATTACGGAAACCAAAAGATTGG
CAGGATTCAAGATTATGATCCCCTAATCGATGCTCCGAGAGCTCCCTTAAATGTTCAAAGGGCTTCGGCTAC
>1002052828_2 Heli.2-EL596369.1.5

ATTTATTTACATATTGGTCGAGGAATTTATTATGAATCTTTTAATTTAAAATTAACTTGATTAGTGGGAGTAA
TTATTTTATTTTTATTAATAGCTACAGCTTTTATAGGTTATGTTTTACCCTGAGGGCAAATATCTTTTTGAGG
GGCTACTGTTATTACTAATTTATTATCTGCTATCCCATATCTAGGAAATATATTAGTA
AATTGAATTTGAGGTGGATTTGCTGTAGATAATGCCACACTAACTCGATTTTATACATTTCACTTTCTATTTC
CATTTATTATCTTAATATTAACAATAATTCATTTATTATTTTTACATCAAACAGGATCTAATAATCCATTAGG
AATTAATAGAAACCTAGATAAAATTCCATTTCACCCATTTTTTACTTTCAAGGATTTA
ATTGGATTTATTTTACTAATTTTT
>1002052829_2 Heli.2-DT663347.3.5
CACGAGGGTTCGAGCAGCAAGTGCGAGACTTACGACGACTTCATTTTAAAGAAGCCGGCTAGGTCGAGTCCTC
GCTACGTCTCGTTAAAATTTATAAGTTAATATAATTATATTAGATTTATAAAAATCATTTAACAGTATAGTAC
GTACTATTAAAGCGTAATTAATAATTATACAAAAAAAATCTATTATAATCTTCCTCAA
AATATTCAACAAAAGTACTCGCACCACGTGTATTTAAAAGTATTAAAGTGAAATAAGTCCTCCAAGAAACTTT
TTGACTAGATACTATTCAGAAAGCATTGTTCTAATCTAAAGAAATCGAATACTGTAATAAACTTGTGCTTAAC
AAAATAATTTTTGCACGTGTTAGTTTTTACAACAATAATCATGTCAAACGCAGAAGGT
GACTTACGGGATGGACTCTCGGCAAAAGAAATTTTTGCTAACAGTGAAGGACTAACTTACAATGATTTTCTTC
TTCTTCCTGGATACATTGACTTTACCGCGGAGGAGGTTGACTTGACATCACCTCTCACCAAGAAAATCAACCT
GAAAGCACCTCTAGTGTCTACACCTATGGATACTGTGACCGAAGCTGATATGGCTATC
GCTATGGCTTTATGCGGAGGTATTGGCATTATACATCATAATTGCACTGCTGAATACCAAGCAAATGAAGTCC
ACAAGGTAAAAAAGTACAAACACGGTTTCATTCGTGATCCTGTTTGCATGGGTCCCAAAAATACTGTAGCAGA
TGTCATAGAAGCCAAAAAGAAAAATGGATTTACAGGTTATCCTATCACTGAGAATGGC
AAACTTGGAGGACGCTTGATTGGTATTGTGACGTCTAGAGACATTGACTTTAGGGAAGGTGACCCACATCTCA
GCTTAAAAGAAGTTATGACCCCTATTGACGAAATGATCACTGCACAGTCTGGTGTTACTTTACAAGATGCTAA
CTATATCCTTGAAAAGAGTAAGAAAGGAAAATTACCTATTGTAAATGGTGCAGGTGAG
CTTGTGGCTTTAATTGCAAGAACAGATTTAAAGAAGGCTCGTAGTTATCCCAATGCCTCAAAAGATTCAAACA
AACAGTTGCTGGTAGGTGCTGCTATTGGTACAAGAGACGCAGATAAAGAGCGTCTTAAGCTTCTGGTGAGCAA
TGGTGTGGAT
>1002052830_2 Heli.2-EL603226.1.5
GCACGAGGGCAACGCTCTCGTTGTGCTCTACAGTGATTTAGGTTAAATATTATTTGTGTCTATATTTCATACG
TCATAAGCAATGCCCCAAACCAACGCGATCTCTCTCAACGGACAAAATAATGGGAGCTTGAAGAATGTCGATC
AGTCCTCCGAACACCAAAATGGATACCACACGCCGCACCGGAAGACATTTGACGACGA
AGAGTTCCATCTTAACAATACGGGAGAAGTGGACAAAAACTGTGTTAGGTATCCCGATGGCAGTGTACGTCTT
CGCAATCCCAATATCGAACTTATGGACCAAGATATTCTCTATCATTTGGCGCTGGGCAGTGGTTCTCATGACC
TTGTTGAGATGTTTGGAGACGTTAAGTTCGTATGTATGGGAGGGACACCGAAGCGTAT
GGAGCAGTTCGCATACACTATCATGGCAGAGATTGGTCACAAGTTGCCTTGCGGAACCACGTTGCAGGACATT
AGTCAATTCTCGTATAGATACTCCATGTTTAAAGTCGGACCCGTCCTGTGCATCAGTCATGGCATGGGCATAC
CTTCTGTCGGGATCCTACTTCACGAGGTGATAAAGCTGATGTACCACGCTAAAGTGCG
AGACCCAGTATTCTTTAGAATTGGCACATGTGGAGGCATCGGTTATGAGGGTGGTACCGTTGTCATATCTGAA
GAGGCGGTTGACGGCGCTCTTAAAAATGTTCTTGAACTGTGCGTCCTTGGTAAAATATACAACGACCGGCA
>1002052831_2 Heli.2-DT667800.3.5
GTTAGTGCTATTTATGTGTGCGGTTGCCACTGCTGCGGCTGGCGAGAGTGTAGCATGGCCGGGTGCGATACCA
ATCACAGCATCGCAACTTAAAACCATAACACCCGGGCAAATCAAAGGATTTGCCTACTCTACCAGTCAATACA
TAAATGCGATCCCATCTGCGCCATTTGGATATCAGCCCCAATTCAGCTACCAAATAGC
AATTCCAAGTTATCCAGCTCAGTCAATAGCAAGCGCTTCATATTTCCCTCTACCGATTGTTCCATTGGCTCCT
GCTGCCCCCATTCTTCCTGTTGCTCCGATTGCACCTATTCAACCACCGCTTCAAAACATACCATCTCAACCGG
CAGAGCAACCAGCGGGAGACGAAGACTCAGCAGTAATTGAATCTGCAGATTCTTTTAA
TCAGCAAAGCTCGCAATCATCTCAACCTTCTTTAGATACAAAGAATATGCCCGGATTTCCTCAAGTTCCTCAA
GGAGCTCCGCAAATCCCGCCATTTCCTCAAAGGCCTGAGGGTCCACACATTCCACTCTTCCCTCCATTCTCTC
AGGTACCTCAACAAAACCCACCAGTTCCTCAATTTCCTCAGGAACCTCAGCAAAATCC
ATCAAGTTCAAATTTTCCACAAGGATTTCAGCAAATTCCATCAATCCCTCAATTTCCTCAGGGATCTCAAGTA
ATCCCATTGTTTCCGCAAAATTCTCAGGGACCAGCGTTCCCTCAGTTTCCTCAAGTTCCACAACAAATCCC
>1002052832_2 Heli.2-DT662026.3.5
GCACGAGGGGATTATCTATCTTGATCTGTTAAATTTTGCCTACGAATAACAATATTTAATACAAAATGCCGTT
CCAAGTATTAACTATAGAGCCGCAAAATGAACTGAAGTTCAAAGTTGACTTTTCAGGATTGTTTGAGCAGGGC
TACACAACCTACATGAAGCTGACGAATCCCTCAGAACATACCGTCTTGTTTAAGATCA

AGACCACAGCCCCAAAGAAATATTGCGTTCGTCCCAACTCTGGTGTGCTCGATCCTAATTCTAAAGTTGATAT
AGCTATTACGCCACAGGCCGTTCACGTCGACTCCAATGAGAAACACAAACACAAATTTATGGTGCAAAGTGTT
ATTGCTCCAGAAGGAAAAACTAATATAGATCAAGTGTGGAAGGAAATTAGTCAGGATC
AGCTAATGGACTACAAGCTGAAATGTGTATTTGAAACACCTAGGGGAACAAATCTTAACGATGCCGGTGATAA
TGTCGCTCAGAATGAAATCTCCAAGAAACGAGTAGCAGTCGCCGCAGCGGAAGACTCAAAGTCACTATCAAAG
GATGCTGTAGAAGGTCTCCAGAATGATGCTAAGAGGAAGGATGATGACCCAGCACCGG
GAGCTTTAAATGCAAAAACGACCGCTTTCCCAAAATCTGAAAACCTCGAAAGCGATTTGCAAAAGGCGACGAA
TGAGGTCATTCATTTGAGGGAGGAAGAAAGCAAATTAAGACATGAAAATCTGCGATTAAAAGAGGAGTTACTT
CGCCTGAACCAGACGGCGGGCGAGGGCCGCGTGC
>1002052833_2 Heli.2-DT664225.3.5
TGGAAAAAACGATTGCAGAAGACTTGGTTGTTACCAAGTATAAATTAGCAGGGCAAATTGTTAACCGTGTTTT
AGAGCAAGTTATAGCTAAATGCGTACCGGACGCTTCAGCGAGAGAAATATGTGAATTCGGTGATAATTTGCTA
TTAGAGGAAACGTCGAAAGTTTTTAAAAAAGAAAAGGATTCCAAGAAAGGAATCGCTT
TTTCGACATGCGTTTCCGTAAATAATTGTATATGTCACTTCTCTCCAATACCTAGTGAGGCTGACTATGTATT
GAAACTAGGGGATCTTGCCAAAATTGACTTGGGTGCCCATATTGATGGTTTCATAGCAGTTGTAGCTCACACA
GTAGTTGTTGGTGGTGGGGAGGCATCCGGTAGAGCTGCAGATGTCCTTCTTGCTGCAC
ATAATGCTAGCGAGGCCGCCTTGAGATTATTAAGACCAGGCAATGAGAACTATGCAGTAACAGATGTTGTTCA
AAAAATTAGTGCTGAATATGGCTGTAAGCCCATAGAAGGCATGCTTTCTCATCAATTGAAGCAGTTCCGTATT
GATGGTGAAAAAAGCATAATTCAAAACCCATCAGAAGCCCAAAGGAAAGAGCATGAGA
AGGCCTCTTTTGAAACTTATGAAGTCTATGCCATGGATGTACTTATTTCTACTGGAGAAGGTGTTGGAAGGGA
GATGGATACAAGGTGTACAATATACAAAAAAACTGATGAAATTTACCAGCTTAAATTGAAAGCATCTAGAATG
TTCTACAGTGAGGTCCGCAACAAGCATGGATCAATGCCTTTCAACCTGCGCAGTTTCG
ACAAAGAAACTAGTGCAAGACTTGGTGTTGTTGAATGTGTAAATCATAAACTCATTGAGCCTTTCCAGGTATT
GTATGAAAGGCCGGGTGAAATTGTAGCACAGTTCAAATTCACAGTACTGTTGTTACCAAGTGGCACTCATCGT
ATCACAGGACTACCATTTGACAAGAGTCAGTGTAAAACTGAACGCATAATCAAAGATC
CTGAATTGAATGCCCTTCTGAACTCGTCCGCAAAGTCAAATAAGAAGAAGAAAAAGAAAACTGGTGCTGAAGA
ACCAATGGAAGTTGAAACGGCAGCCTAAAAAAAATTCACAAAATATCTGTGGGCACTCA
>1002052834_2 Heli.2-EL603045.1.5
GCACGAGGACGTTATCGAGAAAAATGAATCTTAAACAATTTATATTTCTGTTCTTATCACAATTATTTAATAT
TTCAACAGCTGTTTCCCCGATACCATTAAAGTGTTGGTTATGGGACTCCCCATGCACGACTGTAAAAGCTCAA
GCAATATTTTCGATCCTCGCGGCTGGAGTTCCTGATTTGGATATACAACCACTGGACA
CTATATGGATAGACTTGATGCATGTTGACCAAGATGGATTAAGAGCAGACTTTAGAAGTATGAGCATTAAAGG
AATGACTAATGCAATTATTGATAATATGAGCATGGATATGCCTTCGAAGATAGTTAATTTAGTATTCCACACG
GATTTCGTCACTAAAAGTCGATATATAAACAACGGTACTTTACTTTCAACTCCCATTT
ACGGTGAGGGCGATTACTTTATGAAACTTGAAAACGTTCGTATTACAATGAACATACCTTTTGATATCATAAA
AGACGCTCATGAAATGGATATAATGATGTTAAAGTCATTTAACTACACCTATGATGTAGCGAATGGTGCCCAA
TTTTATTTTGAAAATCTTTACAACGGTGATAAGTATTTAAGTGACAATATGCATAGTA
AAATGCATCAAAATTGGAAATATTTGACAATTGCTTATGGAAAACATTTGTTTGATCCAGTAACTAAAAAGGT
GTTTGGTTCTTTCCGAAACTATATCCAATCTGATCCACTGAGAACGGTCGCTTTGTAAACTTTTCCAACTATT
TTGAAAATTAAAGTATATTGAATTATAATATGCATAATAAAAGAAT
>1002052835_2 Heli.2-EL604060.1.5
GCAGAGGATTAAATAGGCGCTTATTATTTTTGATATTTTAGGTGTTATCGCAATTTGTAAACCAAAATGTATA
TTCCATCTCTTATATTCACTGTACTAGTCACTGTTATTAACGGTATTTATTGTAAAAGTGTAGAACTTACATT
TGAGTTACCTGATAACGCCGTAGAATGTTTTTATCACGAGATTGACCAAGATGTATCT
GCCTCGTTGGAATACCAGGTTATAACTGGAGGTCAGTATGACGTTGATGTAAAGATTGAAGGTCCAAATAATC
AAATTTTGTATCAACAGCAAAAGATGCAATATGACTCACATCAATTTACAACCCAACATAAAGGAGTTTATAA
AATATGTTTCAGTAATGAATTCAGTACATTTACACATAAATTAGTATATATGGAATTA
AATGTTGGTCCAGAAGAACCTCTCCCTGGCATTGGTGAACATGCTACAGTTTTAACACAGTTGGAAACTTCAG
CGGAAGAAATCCATTCATCTCTTAATAAGATTATTGACCATCAAACCCACCACAGGCTGAGAGAGGCTCAAAG
TAGGAAAAGGGCAGAAGATCTGAATGAAAGAGTATTCTGGTGGTCGACGGGTGAAACC
TTGGCTATTGTTTGTGTTGCAATCGCACAAGTTATGATTTTGAAGAATTTCTTTAGTGATCGACCTACATTAT
ATAAAATGTAATAGCAAATTAAATAGTGTATTTATTAATATATTTTT
>1002052836_2 Heli.2-DT665270.3.5

ATATGAAATTGTTCATAGTTGCCGCTGTACTTGGTGTCTGCTCTGCAGCCCGCCTTGACAATGCCTACCTCCC
TCCTAGAGGAGGAGCTGGTGCAGGCTATGGCGCTGGTTCCGGACCTTCGGGCTTCGGATCTGGCTTAGGAAGA
GGAGCAGTTGCAGGATTTGGAGGCAGTGGAGCTTACAGCGGGTCAGGATCAGGATCTG
GATCCTTTGGAAGTGGATCAGGTTTTGGAGGAGTTGGTGCTGGTGCAGGAGCTGGAAACTATCAGGGAAGTTT
CGGAGGCAGAAGCAACTCAGCTGACGCTAACGCTCAAATCCTCCGACTGAACAGTGACGTTACCGCAGAAGGT
TTCTCTTACGACTTCGAAACATCCAACGGAATAAGGGCTGATGCATCTGGTGTTGCTA
CCAATGGCGTTCAATCTCAAGGCAGCTTCTCTTACAAGGGTGATGATGGCCAAGACTACAGCATCCAGTACAC
AGCTGACGAAAATGGTTACCAACCTCGTGGTGCTCATTTGCCCACTCCTCCCCCAATCCCTGAAGCTATCTTG
AAATCCTTGGAACAAAATGCCCGTGATGAGGCTGCTGGCATCATTGATGATGGCACAT
ACCGCGGAGAAGGCGCAGGTGCGGGCGCTGGCGCATACTCCGGCCAAGGTGGATATTCTGGGCCAGGTGGATA
CTCTGGACCTTCTAACCAATATGGTGCTCCGGCCAGAGGATTTGCAGGTGCTGGTGCCG
>1002052837_2 Heli.2-EL596245.1.5
GCACGAGGGTACGCGTGTCAGCTTTGATTGACCGCGTGTGCCAGGTCGTTGAACGGGACGGGTCACCCCAGGA
AATTTGTCGCGCTTACCTCCGCAAAATTGACCATCTTTACTATAAATTTGACCCACGCGCCGTCAAGAAAGAC
CTTCCTGCGGGTGAAGAGACAACCATTAAGAAAATGGAACGTCTCTGCAAATATATTT
ATGCGAATGATGACAGCGATCGTCTGAGAACACGCGCCATACTGTCTCATATATATCACCACGCGTTACACGA
CAACTGGTTCCAAGCCAGAGATCTCTTGCTTATGTCTCACTTGCAAGAAAACGTTCAGCATTCGGATCCTAGC
ACTCAGATTCTGTACAATCGTACTATGGCCAATTTGGGATTGTGCGCTTTCCGCCGGG
GCAACGTGAAGGAAGCCCACGGATGCTTGGCGGAGCTGATGATGACAGGAAAGCCCAAGGAGTTGCTCGCACA
AGGTCTTTTGCCGCAACGGCAACACGAAAGGTCTAAAGAACAAGAAAAGATTGAAAAGCAACGTCAAATGCCA
TTCCATATGCATATAAACTTGGAGTTGTTGGAGTGTGTCTACTTGGTTTCCGCGATGT
TAATTGAGATCCCATACATGGCTGCCCACGAGTTCGACGCCCGCCGTCGTATGATCAGTAAGACGTTCTATCA
GAACTTGCGCGCGAGTGAGCGGCAGGCGCTGGTGGGCCCGCCGGAGTCCATGCGGGAGCACGCGGTGGCCGCC
GCGAGGGCCATGCGGCGCGGGGACTGGCGCGCCTGCCTCAACTTCATTGTGAATGAAA
AAATGAATGCTAAGGTTTGGGACCTAATGGTTGGTGCTGATAATGTGCGCGCAATGCTGGGACGTCTTATCAG
AGAGGAATCCCTCCGCACATATCTCTTCACTTACGCCCACGTGTACGCGTCTCTATCCCTACGTTCTTTGGCG
GACATGTTTGAGATGCCACGACAACGCGTACATTCCCTCGTATCTAAGATGATTATAA
ATGAAGAACTCTTGGCGTCTTTGGATGACCCGAGCGAGTGCGCCATCT
>1002052838_2 Heli.2-EL598845.1.5
GCACGAGGGCAACATGTTTATGTACGAGCCACCCACTAAGCTAAAACTTACTTCAGTGGAAGAGTTCCAAGAA
GCATTCCAGCTTTTTGACTCCCGTGGGGATGGGAAAATTCATGTGGCACAAATAGGAGATGCACTCCGTGCTT
TAGGACAAAATCCAACTGAATCTGATGTCAAAAAGTGCACCTTACACCTCAAGCCTGA
TGAGAGAATATCATTTGAAGTGTTTCTGCCTATTTACCAGGCAATTTCTAAAGCTAGAAGTGGTGATACTGCA
AATGACTTTATAGAGGGCCTTCGCCATTTTGATAAGGATGGCAATGGTTTCATTTCTTCAGCTGAACTACGTC
ATCTACTTTCCACTCTTGGAGAAAAGCTGAGTGATGATGAGGTGGAGCAGTTGTTGCA
GGGACAAGAGGACTCTCAGGGGAACATCAACTATGAGAACTTTGTACACCTCATTATGCAGGGATAGACGGAG
GCGGACACACCTCCGGCCACTGTTTTATAGTATTATGTAGTACTTTTTCTTTATCTCTTCAATTGATAATGCA
TTTATTTAAATGGCTGCAGTATGGTGGCTATACTATATAATATAACACCATGATAACT
GTGATGTATTATAATCAAAAAGAGGAGAATATTCCAAACTCTACTTCTTTATGTATTTGACTAACAATATTTT
TATTATTTCCGTTGCTATAATTTAAGAGCATATTC
>1002052839_2 Heli.2-EL599267.1.5
GCACGAGGAACAGCCATCCCTGGGGCTTTCGGTTGTGGAAAGACTGTGATTTCTCAAGCCTTGTCCAAATATT
CCAACTCAGATGTTATTATCTATGTGGGTTGCGGAGAAAGAGGTAACGAGATGTCAGAAGTACTGCGGGACTT
CCCCGAGCTGACGGTGGAAATCGAAGGTGTGACCGAGTCTATCATGAAGCGTACCGCG
CTCGTCGCCAACACCTCCAACATGCCGGTAGCTGCCCGTGAAGCTTCCATTTACACTGGTATCACACTTTCCG
AGTACTTCAGAGACATGGGCTACAACGTGTCCATGATGGCTGACTCCACATCCCGTTGGGCTGAGGCTCTTCG
TGAAATCTCCGGTCGTTTGGCTGAGATGCCTGCGGACTCCGGCTACCCTGCCTACCTC
GGCGCCAGGCTGGCCTCCTTCTACGAGAGGGCTGGACGTGTCAAGTGCTTGGGCAACCCTGACAGAGAAGGTT
CAGTATCAATCGTGGGTGCCGTATCGCCCCCTGGAGGTGACTTCTCGGACCCCGTGACCGCCGCCACTTTGGG
TATCGTCCAGGTGTTCTGGGGCTTGGACAAGAAACTGGCACAAAGGAAGCACTTCCCG
TCTATCAATTGGTTGATTTCTTACAGCAAGTACATGCGCGCTCTAGATGACTTCTATGAGAAGAACTACACTG
AATTCGTACCCCTCAGAACTAAGGTCAAGGAGATCCTCCAAGAAGAAGAAGATCTATCAGAAATCGTGCAGCT
CGTGGGTAAGGCGTCGCTGGCCGAGACCGACAAGATCACGCTGGAGGTGGCCAAGCTG

CTGAAGGACGACTTCCTGCAGCAGAACAGCTACTCGTCGTACGACCGCTTCTGCCCCTTCTACAAGACGGTGG
GCATGTTGCGCAACATCATCGCCTTCTACGACATGTCGCGCCACGCCGTGGAATCCACC
>1002052840_2 Heli.2-DT668381.3.5
GCACGAGGCTCGCCACCGCTCAACCACACGCGAGTGTTACCCTTTCTCTCTACGTGATCGTATACGTGATTAC
CGGAATTAAACTCGCCACCGCTCAACCACACGCGAGTGTTACCCTTTCTCTCTACGTGATCGTATACGTGATT
ACCGGAATTAAACACCAAAGGAACAACTTTCGGACCTGTCTCAATTTATATCAGGGGC
AAAACGAGAACACGGTATGACTGCGACGCACGGTGGCGGCTGTGAAGACCACCGGCCGGACAAGGACTCTGTG
TACACTGTGAAAACTGGGTTCAAAAACGATGGCTATCAACATGATAGAGATTCCAATGATGACATCGTCAAAC
CACCACCTCTGCCTACCGAGGATGACTCTTATCCTTCAGGCAAAGTGAAATTATCCAG
AAATGAAAAATGGAGAATATTGAAGAATGTAGCGGCCGTCAGTGCAGCCTTCATGGTGCAGTTTACAGCGTTT
CAAGGAACTGCCAATCTTCAATCTTCAATAAACGCCGCCGACGGTCTTGGAACCGTTTCCCTTAGCTCTATTT
ACGCGGCTCTCGTCGTCTCTTGTATATTCGTGCCTACTTTCCTTATAAAGAGGCTAAC
AGTGAAATGGACCTTATGCCTTTCTATGCTTTGTTACGCGCCATATATTGGCGCTCAATTTTACCCTGCATTC
TATACCCTGGTCCCTGCTGGAGTCATAGTTGGCTTAGGGGCAGCACCTATGTGGACTTCCAAAGCCACTTACT
TGACTCANGCTGGAAGTGTGTACGCTAAGCTGACAGATCAAGCAGTCGATGGTATCAT
CGTTAGATTCTTCGGCTTCTTCTTTCTAGCGTGGCAAACTGCTGAGCTATGGGGGAATCTTATTTCTAGTTTA
GTATTTTCTCG
>1002052841_2 Heli.2-EL596180.1.5
GCACGAGGTAAAATGTGTTTTCTTGTAGATACAGGATCCGATCTTTGTGTGTATCCACTGCGAGCTCTTCGTG
AAACACGCGCCGCACGAGGCTATGACCTGTTTGCTGCAAACGGAACGCAGATAGCTACCTACGGCTGGATCCA
TCTGCAACTGAACATTGGCCTGCGACGCACATATAGGTGGAGGTTTGTCGTAGCTGAT
ATAACAAAGCCAATTATAGGTGCTGATTTTCTCAGATTTTATAACCTGCTTGTAGATCTGAGGAACCACCGCT
TAGTAGACGGCCTTACTTTACTGTCTACGCCAGCTCGTCCAGCAGGAGAAAAAGCAGCAGAGGTCGCTTCAGT
CAAGGCTGTGTCGGGTGAAAGCAAGTATCACCAGCTACTTCGTGAGTATCCTGACATC
ACTCGACCAGCAGGGACCCAACGTGAGGTAAAACATAATACCACTCATCATATACGCATTACTCCTGGACCAC
CTGTATCCTCACGGCCACGTCGACTTCCACCAGATAAGTTGAAAATTGCCCAAAAAGAATTTGAGGATATGCT
GATGAATGGTACTGCACGGAGATCAGACAGTGCCTGGTCATCGCCCCTACACCTGGTT
CCCAAGAAAGAGAACGGTTGGAGACCTTGCGGGGATTACAGGGCTTTGAACTCACGGACGATACCAGATAAGT
ACCCAATCAGACACATCCAAGACTTCGCACATCAACTCGCCGGCAGT
>1002052842_2 Heli.2-EL598392.1.5
GCACGAGGGTAACTACCGTTGAATCAACTACCATGCAGTACCTTATTTTCTTGGGACTTTTCGCCCTTGCGGC
TGCTTATCCTGGTATCATTCACCAGGAAGCCCCACAACTCTCTGAGGGTTATGATCATCAAGGCCTCATTGGC
GAATCAGGACATCATCATCAAGTTCAACACGAACATGCTAAGTCACATCAATCAATCA
AATTCGAGCACTTCCATCCAGTACCTGTATACGTTAAGAAAGAACATAGCCATCTGCTGAAACATCCTCTTGA
GAAGGGCCATTCTGAACAAAACTTGAAACAAATTCACCCTGAGACCCAACACAGCCATGGTGGCGGCCTTGTC
CTGGAGGACCACAGGTTAGACACTGAACAGTTCGCCGCCAGTCTCGGCCACGGTGGTC
TTGAACACGGCCTCGAACAAGGATCACTTGAACAAGGTGGATTGGAACACGCAGGTATTCAGCAAGGTTATGA
ACATTATGCTGGGAGCTACGAAGGTGGAGAAGGATTAAAATCATACGCTGAATCTCAGCCTGAAATTCAGGGA
CACTACTACTCCGAACACGCGCAAGCCCTTTCAGGGGACAGCGGAGAGGGTTACAAAT
TCGAGCATTATTAATCGCATTTAGCTTAAGTCCGACGGGCATATCGGTGTTATAACACCGTTCTCTCATATAT
TTATAAGATATTTTTCTTGCCTGTTAAGTACTGTAAATGTACAGTATGTAAGTATCTTAACTGACGCTACGCG
GTTGATAATATATCGAAATTTTATAAAACGTACCTGATTGATATGTAAGTTAGAAACA
AATTCATATAGAAAACCCGTGAATTCTTTTAGTGGTGTAAATGTTGCGAATCTTGTGGCAATTAAATTATAAA
CATGTATTTTA
>1002052843_2 Heli.2-EL596064.1.5
GCACGAGGCCACCCTCTAACGATGAAGATGAACATCATGCAGACTATGTTACTGTTACTCCTGAACCTTTATA
CATACCAGTGCAAACAACAACTCTGAGAGCTATTATAAAGCCAGAATCAGAAATTTATCATACTGACAGTAAT
GTTAAAGTCACATTCGGTAATGATCATCACCATTATGGCAATAGACGGGCAGAAAGTC
CACAAGGAGAGCAAAGAGAAGAAACTGCGCCAAGACCCGCAATTGCTTTGCCAAATCACAATTTAAATGAACC
ACCACAATCAGAACGTTCGGTATCACCTAGACCGCAGTATAATGATCATGAAATAAAATTAAATCAACACAAT
CCCGCAATTTTTCAACAAGAAAAACATGCTCAAAGCCGTATACAATTCCAGCAACCAT
TCCTTAGCCACGAACCACAGTTACCAAGGCAAGGGCCTTCATTAATTCCTTTTAGAGCTCAACCACATCAAAA
TAATTTTGGCCCATCGCCTTCGTTAAGCCCTCGTCCTTCATTTTTCAATGTTTCTCCAAGCCCTAGTAACCAT
TTCGCTAACATTGCCCCTACGCTTCCAGGTCCGATTGGTCCAAGTTTTGCAACATTTT

CAAGACCAAATGGACCGCCACAATCTTTTAATTCTCGCCCAAGTAATACACACCCTTTTGGAACAAGGCAGCA
TTTCCCACCCAGGGCAAGTTTCCATTCTGGACCTCAGAGACCGTTTCATCAACCTCAAAATTACTTTGATGAA
GCTAAATATTTACAAGAAAATTATCACACAATAACTACCGATCAAGTTGATCCGCCAA
AGGTTCAGTTGTTACCAAATAAACAGATTCAAAATTTTAATATTGGTCCTAAGCCTGGTACAAATCCTACTTC
ACATTTTGCTGATCATCAGGGCCGTCCACAACAAATAATTCCTTTCAACCAGCAATTACCTCAGCAAAGAACA
CCACAACAATTTTTAGATTCCAATAAAAATAAGC
>1002052844_2 Heli.2-DT665079.3.5
GCACGAGGGTCGACGTAGTTCTTTCGACAGATAAAAAATAAGACGCGGTCACTCAAGGACGGTCAACAACTAT
CGAGTGGTATTTCCCGTTTAACGGGCACCAGAGTGAAACACAAATGACGCGCAAACGACACCAGTCATTAACG
CCGCTAATGTATGTGGCTTTAAGGCTGGTAGGTCGCGGGCCACACTCCGGCGAAGCAT
TCTTGCAGTATAGAGTGTGCAACGTGAGGGGCCTCTAGCGCGTTTCCCAAGTTATCGGCACTGGAAAAACACG
CTACATTTTATCACATACAGTGTGTTCGCTATCAGCTGTTGCGAGCGATGCGGTGTGCGGTCGCCAACACGTC
ATGGTACGAAAATACGAAAGAATGAGTGGTCGCCAATCTTGGAGCGAAGAAGAAATGG
CAAGGGCCGTGGCCGCCGTGGTATCTGGTAAAATGGGCTATAAGCTCGCTGCTAGAACTTTTCACATACCACG
ATCAACTCTCCAAAGACGTGCAAGCAAGGTGCGATATCAGCAACCAGATGAACCAAAGCCTTTGATGGGACAC
TATCGACGTGTGTTCACAGAAAGTCAAGAGAAAGATTTGGTAGGTTATATTAAAAGCA
TGGAAAAATATTTTATGGGAGTTTCGAGAAGAGATATAAGAGAACTTGCTTTCCAATACGCAGAGGATAATAA
CCTCAATCATCCATTCGACGTCAACACTCGGATGGCTGGTGAAGATTGGGTGAGGAATTTTTTGAAACGTAAC
CCCGAATTGCTCCACAGGTCGGATAAAGAATATGAACTGGAACCTGTTAATTTCGATC
AATTTTATCACTTTATGTGTCAATCATCATGATTGTAATTGTAAACTCCTTAGACATATTGAAGGTCGTATGC
AATGAAGCCCACCTGACGGAGCCTCAAATATTGTATAATATGGTATATACTGTATGGAACGATAGATAGTAAT
TCTACTGAAGTTGTTTGCAGTT
>1002052845_2 Heli.2-EL603900.1.5
GCACGAGGCAGATACACCCGGCCTGTAATATAGTACAAAATCGGCCAGTTTGTGATATAACTTCGCGATGGCT
CAAGCTCTTAAAAAACTATCTGCGCAAGCTCTGAAAAATAATTATGCAGAGGCTTTTACAACCGGTATTATAT
CCAGAGCAAGCAGCTCGTGGTGGAGTAATGTTCAAATGGGTCCACCTGATGTCATCTT
AGGTATAACAGAGGCCTATAAAAGAGATGCGGATCCAAAGAAAGTAAATTTAGGAGTTGGGGCCTACAGAGAT
GATCAAGGCAAACCTTTTATTTTGCCATCAGTTAGAAAGGCTGAAGAAATAATCTACAATAAAGGATTAAACC
ATGAATATGCCCCTATTGGTGGAGAAGCAGCATATACTGATGCAGTAGCGAAGCTTGC
ATTTGGAGAAGACAGCCCAGTGCTTAAAAACAAAAGTAACTGCACAGTCCAAACCTTGTCTGGTACTGGTGCC
CTTCGTCTGGGTTTGGAATTCATCACAAAGCATTATGCCAAGAATAAAGAGATCTGGCTTTCGACTCCTACTT
GGGGTAACCACCCACAAATCTGCAACATGATCAATCTCCCACACAAGAAATACAGATA
CTTTGATGCTAAGACCAACGGCTTTGACTTGAAAGGAGCTATTGAAGATATTAGTAAAATCCCGGAAGGTTCA
ATTATTTTGTTGCACGCGTGCGCACACAACCCCACAGGCGTTGACCCGAGGCCAGAGGAATGGAAACAATTAT
CACAGGTAATAAAAGACAGGAAGCTGTTCCCATTCTTTGATATGGCGTACCAAGGTTT
CGCCACTGGCAGTGTAGACAATGATGCCTTCGCTGTCCGCTTGTTCGTAAAAGAGGGACACCAGGTCATGCTC
GCTCAGAGCTTTGCTAAGAATATGGGATTGTACGGCGAGCGTGCAGGAGCTCTCACTTTCCTTTGTGGTGACG
AAGAATCTGCGGCTCGCGTGATGTCGCAAGTTAAGATTATGATCCGTACTATGTACTC
CAATCCGCCATTGTTCGGCGCACGTCTTGTCCAGGAAATACTTAATAACCCACAGCTTAAATCACAGTGGTTA
AATGACGTTAAGCTCATGGCCGACCGTATCATCTCAATGCGCCAGCAGTTGCGCGCCGG
>1002052846_2 Heli.2-DT665290.3.5
GCACGAGGCAACAGTCAACAATTAACGAGGAAACGCCTAGATATATAAAGTATTATGACCTATAAGCGCAAAA
AGTACCATCACGGTGATACTCATCTGAAGAAACGGTGGAGAGTACGGAATAGAAAGAAGGATCTCGACCAAAT
CGACGACGACATAAAAGAAGAAAATGCTGATAAATTGCTAAACCAAGAAGTAGATCTC
GACGTACCAGGCGCCGCACAACATTATTGTTTGCACTGCGCACGATATTTCATTGATAATCATGCACTTTTAG
AACATTTTAAAACTAAAGTGCATAAAAGAAGGTTAAAAGCTCTAGAATTAGAACCATACACCGTAGAAGAATC
TGAAAGGGCAGCAGGACACGGTAATTTTAAACTTCCTAATAAAAGGAAGATAGTCACA
CAAAACACAGAAAGTGCAGATATTACTGACAGAGATAGTGAAGCTTTAGAAGTGAGTCCTAATAAAAAGAAAA
AGGTTGAGGATAATGCTACGTAAAATATGTTATAATTTTTTTTGTAAAAAGATAATACTAAGGCGATATTCTG
CATTGTTTATGAATGGTCCTAAAGCTACTGAAAATTTTCTTTTTGTTACACCATACAT
TGATTTTCATGAGCAAATAAAGAACAAAGAAGTCATAAAGACCGAACTGCATAAGAGACAAATAGATTTAGAT
TTACAGAAATTGGAGAATCTTTGGGATGTATATGACACTTTAAAGAATAAAAGAAATGAACTTGATTTAAAGA
AGTCAACTATAGGTAAAGAATTAGTGGAGCTACAACAGAGTAAGGAAGAGAGTGATCA
TAAAGAAAAATTGAAAATACAATTAAATTTAGTCAAAGAAACATTCAAAAATTAAAGTACCGTTGTGGTCTA

>1002052847_2 Heli.2-EL604408.1.5
GCACGAGGCATTTCCATTAATACAAGCCACATCTTTTTGTTTCTTTCCGTTCGACAAATCCGATCAATTTTCT
ACAAATCTAGCAACAGATTAGTGTGAACGTATCAAAACTTATTGTTTCGGTTTTATTGATTATTTTGTGTGAT
CTAAATAAATTAAAGGACAACCATCTGCGTACACCGTTGCCAACTGTCGAGTCGTCGA
TATTTTGCGACCGAATAATGATCCTTGGTTTCGTGCGAGTGTAACTTTGCTAAACATTACTGTTTTTTCGAAA
ATAATATTGTAAAACCAGCAAAAATGGCATCGTTTCAGCAGTTGATAGCTTTTCGCAGAGAGCTTTTGATTAC
AGACAAATATAGAATTACAAGAAGAATCGGAGGTGGTTCTTTTGGAGATATTTACTTG
GGGATAAACATCGCTAATGGCGAGGAAGTAGCAGTGAAGGTTGAGTCTATAAAAGCGAGACATCCCCAACTTC
TCTATGAAAGCCGTGTATATAAAATGCTCCAAGGGGGCATCGGCATACCACATATACGATGGTATGGCTTCGA
GAAGGAACACAACATCCTCGTGATGGACCTCCTGGGTCCATCACTAGAGGACCTGTTC
AACTTTTGTTCGCGTCAGTTCACCATCAAGACAGTGCTCATGCTTGCAGATCAAATGCTCGGGCGTGTGGAGT
TCGTCCACTGCAAATGTTTCATCCATCGTGATATAAAACCTGACAACTTTTTAATGGGCATTGGCCGCCATTG
CAACAAATTATATATGATCGACTTCGGCCTCGCCAAGAAATTTAGAGATGTACGCACC
AGAGCTCACATCACTTATCGCGAG
>1002052848_2 Heli.2-EL598220.1.5
GTCGACCGTTTTCGTCCACATTTATTTTAACGTGAAAAATGACGAGGACTTAAAAGTAAATAACAACTAAGAT
AAATTATAATATATAAGTGAAAGTGTTCAATATTAACGTCAAGAAATCAGCTTTGGATTTCTTGCTTACGTTA
TACAAGATGGCGATATTTGGTTTCGTAATTTTGAGCATTTTACTAATTCAAGCAAGCT
CAAAAGCTGCTCCAAGTTCAGACGTAGCTAAAAGTTTAGACGAAATTAAAAACATTGAAGACGGCCCTAAACT
AGACGATGAACCATATGGTACCGCTGAAGATATAAAAACTATAAACGAAAGCGAAAATAACGGACGACAGAAG
AGATATTACGATTATGTAGAGTTTGGCCTTCCACCAATGTTTGAATCAAATTTCCCCT
TTCCTAGCCGATTTAACTACAATAAGAGAGATGAATCCCAAAATACAGGTGTATATGGCAGTGAAGATTCGTT
ACTTTCCATATTAAAGGCTCTACAAAGAATTCTTAATGATGCAAGGCGGCAACAATCAACGCCCCAGCCGCCT
CGCGTTCACATACCTACCTATATACCAATATTTTACCTACCTCAAGCAGCATGCGAGT
GCAATAATAATGTACCTCAGGTACCCATAAATAATGGTAATAATAAAGAGACCAATAATAAAGATATAAGCCA
TAATAATAATA
>1002052849_2 Heli.2-DT665906.3.5
GCACGAGGCGCATTTCTGTGCTAGATTTTTCAGTTTAGCAATTCAATTAATGATTTCATTCGTATAAGAGTGT
AATTAATGAAATTATCCGAATCACGGCGACCTTGCGAGGATTTGTCGCCCAGTAAAAAGTGTGTGACGGCGAT
ATAATAACAGGGAGGGCGATGGCAACAAACCGAAGTGGTGCGGCTCAGAGGCCCAACG
GCGCGCCCCAGACTAAAGTATGTCAGTTCAAATTGGTACTTCTGGGCGAGTCGGCGGTGGGTAAATCTTCCCT
CGTGTTGCGCTTCGTCAAGGGCCAGTTCCATGAATATCAAGAGAGCACCATAGGGGCAGCTTTCCTAACTCAA
ACGCTTTGTTTAGATGATACTACTGTTAAGTTCGAGATTTGGGACACGGCGGGGCAGG
AGAGGTATCACAGCTTAGCACCAATGTACTATAGGGGCGCGCAAGCGGCAATCGTAGTCTACGATATAACTAA
TCAGGACACATTCGGCCGTGCGAAGAACTGGGTCAAGGAGCTCCAAAGGCAGGCATCACCGTCCATCGTCATC
GCGCTCGCCGGCAATAAGAGCGACTTGGCCGCCAAACGGATGGTGGAATTTGAAGAGG
CTCAGGCCTACGCGGACGAGAACGGACTGCTCTTTATGGAGACCAGCGCCAAGACGGCCATGAACGTGAACGA
TATATTCTTAGCTATCGCCAACAAGTTACCAAAAAGCGAGAGCGTGGGCGCGGCCGGCACGCGCGTGCTCAAC
AACGCGGACGCGCCGCGCGCCGCCTCCTCCTGCTGCAAGTGATACCCGCAGACCGTGT
CGCTACAGAGTGAGGGCGCGCGCGCCGCCTAGCCGCGCCGCGGGACGCCACACACACACACACACCACAAACA
CACACATACATTCAGTGTCTGACATTCATGTACTTACATGTATCCATTCTCATACCGACATACAAACATTAAA
CAACACCCACACATACAACCCTACACCCACACCCACACATACAACCCTACACCCACAC
ACATACACCCCTACACCTACACACCCTTCTATACACAAATACATGAAAGTGCGCACCTCCATGCAACAACACT
TAGCCTTACACACACATCTACACGTCCACACAATCATAATACAATACACACATTCACGTAAAGTCTACTAC
>1002052850_2 Heli.2-EL602030.1.5
CTAAGAGATTTTAAGTTTTAAGTAAATTACTATATACTAAATTAATAACCAATTCCACATAGTGGATTCATAA
ATAAACTGGGTTTCATTGAAAAAATTATCATAAAGATGAGCAAGTTGATTCCCTCTGCAGCCAAGCTGTTTGG
TGCAACAGTGACGAAAGCAGCCGCCCCAGTAGTCACCACGAATGTGAAATATAGTACA
AAATCTGAGGCATCTTTCGAAATTAAACCTTTCAAACTCCACAGGTTGGATAAAGGTCCGGCTACAACAGCCA
CTTTAACATCTGAAGATGCAATCCAAATGTATGAAAAACTTGCATTACTTAGAAGGATTGAGACCGCAGCAGG
CAATTTGTACAAGGAAAAGATTATTCGTGGTTTCTGCCATTTATATTCAGGCCAAGAA
GCGGTAGCTGTGGGTATGCGTATGGCAATGCGCGATGCTGACTCGGTGATCACTGCCTATCGTTGTCATGGTT
GGACCCACCTCATGGGGGTCGATGTGGTGGGCGTCCTAGCTGAACTTACGGGCAAGAAAACCGGGTGCTCCAG
GGGCAAGGGAGGTTCTATGCATTTGTATGGACGTAACTTCTACGGTGGTAATGGGATT

GTAGGTGCTCAGGTACCACTCGGTGCAGGTATAGCTTTTGCTCACAAATATAATGGGGACGGTGGAGTCAATT
TTGCCTTGTATGGAGACGGCGCCGCCAATCAAGGACAGCTTTTTGAAGCGTACAATATGGCGAAACTATGGGA
TTTGCCTTGTGTGTTTGTTTGCGAGAACAATGGATATGGTATGGGCACGAGCGTGGAG
CGCGCGTCGGCCAGCACGGACTACTACTCGCGCGGCGACTACATCCCGGGCGTGTGGGTGGACGGCATGGACG
TGCTCGCCACGCGCGAGGCCACCAGGTTCGCGATCGACTACTGCACC
>1002052851_2 Heli.2-EL601878.1.5
GCACGAGGACGCGACGCATATTTGAACGTTCTTTTAATAATTGAAAAAAAAATAAAGTGCGTGTTGCCAGTCG
TAATATAAATAATATAAACCACAAAATGTCAGTCGACCGGTGTCGCTCCTCTCTCATAGTTCTTATCGCCAGT
TTAGTGGTCGCGTCTTGCGCACAAATCGAAGATCCATGCAAAACAAAATCTCGCATTG
TGGGCGACGACAAGTACTGTGACAAATACTGGGAGTGCGACGGCGGTCAGTCTGTTCAGTATGATTGTCCCAA
TGGCCTCGTGTTCGCGGGGAAGCACCGGGGAGTGACTGAGGGATGCGACTACCCCTGGAGATCGAATTACTGT
GAATACCCTAAAGTACAGATAAACCCACCAATCGGTACAGAACATTGCGACTGGCTGT
ACGGCATCTTCGGACACGAGACATCCTGCACACGTTACTGGACCTGCTGGAACGGAACAGCCACTGAACAACT
GTGTATCGGTGGACTCCTCTACAATGAAAACGCTCATTCCTGTGACTGGCCCGAAAATGTAGATGGCTGTCAG
AAACATCCTCTCTGTAACGAAGATCCCAACGGCAACGTGCCCCTCGGCAAGTCCTGCA
ACAGATACTGGCAGTGCCAAGGTGGATATCCTCGTCTCCAGCGATGCCCAGCGATGTTGGTGTTCGACAGACG
CTCGCTGAGATGCGTAGTGCCACCGACTGAAGAATGTGACGTTCCAACTACTACACCAGCCCCTGTTGATGCT
GAGGAGGAACTGAGACAGAGCCCCCACATCAATC
>1002052852_2 Heli.2-EL600832.1.5
AGATCACCTTCGCCGTCGGATTGCATGAGTCCGGATACCATTAACCCTCCGTTGTCGCCTGCCGACTCCACCT
TCTCCATGGCGTCGTCAGGCCGGTCAGGCCGGGACAGGACACGTGCTTTCTCCGACGAGGAACTGAAGCCACA
GCCCATGATCAAGAAGTCCAGGAAACAGGTAAATGAATTCGTGCCCGATGACCTGAAA
GACGACAAGTACTGGGCACGCCGACGGAAGAACAATATGGCAGCCAAGAGGTCACGTGACGCGCGCCGCATGA
AAGAAAACCAAATCGCGCTTCGAGCTGGCTACCTAGAAAAGGAGAACATGGGCCTACGACAAGAAGTGGAACT
GCTGAAGAAAGAGAACCACATTTTGCGCGAGAAGCTGTCCAAATACGCGGACGTATAA
GCGAGTTCCGCTCAGCACAAACACGCCACATCATATGTTGCGGGCATCGTCGCGCGTTGCGCCGTCTCGGGCA
CACCGCTCCTTCTATTTGTAACGCTTTTCAAATGTTCGGTTATAATGTACTGTACTATACAATACTGACTAAA
CTGTTGTTAAATGTAATGCTCTTCACCACCCACCACACTCGAACTACCGATTCGCTGT
TCCCAAAGTACTTATGTTGTAACC
>1002052853_2 Heli.2-EL603538.1.5
GCACGAGGCAACAGTCGGGTGTGAGTGAAAACTCCAAATTTTTTATAATTGAAAAAAATATATATTCGAAAAT
CGTGCTCCTAGTGTAATTTATTATTTTTAATAAATCTGAATTATTAAATCACTACGATGGGAGAACTCGGTAA
AAAGCTTGGTTTGGACGAATTGTCGGGAGGTGCAGCTGGTATCAGCAAAGCATTGCTC
GCGGAATTTGTCGGAAATCTACTGCTAAACTTGTTTGGTTGTGGAGCTGTCATCAATATTGCGCAAGGTTCTG
AAGGTGCCCAGGACATTGTACTCATTGCCCTGGCTTTTGGTCTCGCTGTCTTCGCCATTGTTTCTGCCATAGG
TCACGTATCAGGTGGTCACGTGAACCCTGCTGTAACAGCTGGTATGGCCGCCACCGGC
AAAATCAAACCGATTCGAGGTATCCTCTATGTGATAGCGCAATGTGCTGGTGCAGCTGCAGGATCTGGACTGC
TTAAGGCGTTCACACCAGAACAAGTGGCCGGTACCCTCGGTACTACGGGACTTGGCAAAAATGTCACGCAATT
ACAAGGTTTCGGGATTGAGTTCTTCCTGGGATTCGTCCTCGTGTTCGTTGTTTGCGGG
GTTTGCGACGCTAACAAACCTGACAGCAAATCAACGGCGCCCCTCGCCATCGGCCTGACGGTGACACTGGGGC
ACTTACTGGCGGTGGACTACACCGGCTCCGCCATGAACCCCGCGAGGTCCTTTGGCTCTGCCCTCGTCGCCAG
CACCTGGGATGACCACTGGGTATACTGGGCGGGACCAATAGCTGGCGGTGTGGCCGCG
GCACTACTCTATGTTCACGGCTTCGCAGCACCTCCACCGGATACCAACTCGCTCTCACCTCGGTACCGCCCCG
TCGCCTCCGATGAAAAAGAGTTGAAGCGCCTGGACGGTAAAGCCGACGACATGGCCTGAAAGCGAGTCCCGCG
GCTCCGGCCGCACTCGCCGCTCTCCGTCCCGCAAGACACGCTCCCACGCTCCTACACC
CACCACTCACAA
>1002052854_2 Heli.2-EL601619.1.5
GCACGAGGGGAGGCAGCTCTGACTGGACGACACGCTCGTCGGGAAATAATACACGATGAGGAGTTACTTCCTG
CTGCTGGCCTTATTGGTCGCAGCAATTTGCTCCACTGAAACAGTCACGAAACGGCGTCGTCACCGGCGCCTGC
GCACCACCACGACGACGGAGCCGGCCATGCTGGCGGCCGCGCGCCTCTTTCCCCCCGC
CGAGGCCGAGCACATCGAACTCGGCATGGCGGAGCGCCTCGACGAGAAACGCTTCCAGGAAGCGGAACGGGCT
CGCGTCAATGAATTGATGAACGAAGTAGACGAGGAGAGCGGCAACGAGGTGTTCATGGACGACCCGTGCCTGA
AGGTGCACTGCAGCGCGGGCCGCGTGTGCGAGATCGACGAGCACGGCGACGCGGTCTG

CAACTGCATCAAGGAGTGCCCCTACGAGACCGACTCGCGCCGCAAGGTGTGCACGCACCGCAACGAGACCTGG
TCCTCGGACTGCGAGGTGTACCGCCAGCGCTGCCTGTGCCTGGACGGCAGCGAGCTGTGCCGCGGGCCCGAGT
ACCACCACGTGCAGATCGAGTACTACGGCGCGTGCCGCGAGATGCCGGCCTGCTCCGA
CAGCGAGATGTCGGACTTCCCGCGGCGCATGCGCGACTGGCTCTTCAACATCATGCGCGACATGGCGGAGCGC
CGCGAGCTGTCGCCGCACTACCTGCGCATGGAGCGCGAGGCCGAGTCCAACCTCACGCGGCGCTGGACCAACG
CCGCCATCTGGAAGTGGTGCGACCTGGACGCGCACGACAACGACCGCTCCGTGTCGCG
CCACGAGCTGTTCCCCATCCGCGCGCCGCTCATGGCGCTGGAGCACTGCATCGCGCCCTTCCTGGACCGCTGC
GACGAGGACGACGACCACCGCGTCACGCTGGCCGAGTGGGGNCAGTGCCTGCAGCTCGACGAGTACGAGCTGG
AGGACCGCTGCGACCAGCTGTC
>1002052855_2 Heli.2-EL599856.1.5
TCGTTCCTATAATAAATATATCATTTAATTTTTAAAATCAGTTCTTTTCCAACATGAGAAGTTTAATCATCGC
GTTCGCTTTTTGCGCCTTAGTTTCAGCACAGGACTTCGATTGTCCAGAGAAGAGTGGTTTCTACGCGGACCCG
TACCAATGTGACCTCTATTACAAATGCAGCAAAGGTAAATCAGAAGCCAAACTTTGCC
CAGACGGTCTTGTATTCGCTGACGAGAACCCCCACAAGGAGCTCTGTGACATCCCCTCAAATGTAGACTGCGG
TGACAGAAAGGAGCTTCAGGAACCCAAGCCTACTAAAGGTTGCCCACGACTAAATGGATATTTCAAGCATCCC
GATCCTCAGGCATGTGACAAATTCTACTACTGCTCAGATGGCGTCCCCAATGAGTTAC
CTTGCCCTCCTGGCTTATATTTTAACGAAGAGACCTCGAACTGCGACTGGAAGGAGTCAGTACAAAGACAATG
TGACCAAATCACTAAAGATACACTAGACGATGGTTTCTCGTGCCCCGATGGTGAAGTAATGGGACCTAACGGA
CGTGCTCTTCCTCACCCAACCTTCCCACACCCTGAGGACTGCCAGAAGTTCTACATTT
GCCGTAATGGCTTCCAACCTCAAAAAGGCAGCTGTCCATCCGGCAAGGTCTATAATGAAGACACTTTCATGT
>1002052856_2 Heli.2-EL596472.1.5
GTTTTGAACGGATGTGGAGACGGCGCTGAAAGTGGCGATGGAAGAGCGGCTTCCGCGTCTCCCCGCGTAGAAG
AGCCACCTCCTGTCCCTCCAGCTGTTGCTACCGCCCCACTGCCTGTACCTCCAGACGTAAGCCCTACTGCTGA
TAGCTGGGAAGCGGAAGCAGATGACGCCCTTTTAACTCCTGAAGACAACAACGAAGCT
GAGGAAGAAGAATTAGAACAACAGGATGATGGAGAAGCTGCAAAAAAAAATACCTAAAAAGAAGCCACCTAGGG
TAGAGGACACTCGTAGCAAGAAGGAGCATGTTAATGTTGTATTTATTGGTCATGTGGATGCTGGAAAAATCGAC
TATTGGGGGTCAGATTATGTCATTAACGGGTATGGTTGATAAACGAACATTAGAAAAA
TATGAACGAGAAGCAAGAGAAAAGTCTAGAGAATCTTGGTATTTATCTTGGGCACTTGACACTAACCAAGAAG
AAAGAGATAAGGGCAAAACTGTGGAAGTTGGCAGGGCATACTTTGAGACTGATAAAAAGCATTTCACAATTCT
TGATGCACCAGGGCATAAAAGTTTTGTGCCGAACATGATTGGTGGTGCCGCACAAGCT
GACCTTGCTGTTCTTGTGATATCTGCGAGGAAAGGAGAATTCGAAACAGGGTTTGACAGAGGGGGACAAACTC
GTGAGCATGCTATGTTGGCAAAAACTGCCGGCGTTAAACATTTAGTAGCTCTTGTAAACAAAATGGATGACCC
CACAGTAAACTGGGATGAAAAAAGATACAATGAATGTCGTGACAAAATTATGCCATAT
CTCAAGAAGCTGGGCTTTAACACTGCTAAAGACTTGTCATTTTTACCAGTTTCTGGCCAAACCGGTCAAGGTT
TATTGGAAAGAGTAACAGAGGAAATCTGCCCCTGGTATCGTGGTCCATCATTCATCCAGTTGATCGATGAGCT
CCCATCCCTCAATCGCAAGATGGACGGGCCCTTCATCATGCCAGTTGTTGATAAGTAC
AAGGATATGGGCACTGTCCTAATG
>1002052857_2 Heli.2-DT666567.3.5
GCACGAGGAGATTTCTTAGGGGGTAGAAAGATTTATAAATGCGACGGTTGTGGATTCGATTCCCACTCATTGA
AATGAGCTATCTTCATTTCCCTGCTATATGAAGGATACAGCGTGAACGTAAAAGATAATATTTAAAAAAATTA
AAGAATTTTAAAAACAAATAGCTTGTGATATATTCTCTTTGAAAGTAAATAGATACTT
AATAGATTTTTAACCAACTTCTGTAAAAAAAGGAGGTTATCAATTCGACAGATTTTTTTAAATGTTTTTTACT
TCAGAATTTTGAACTGGTTGAATCGTAATATTTTTTATTTGAAAGCTGGTGCTTCTCGTGTGGTCCTATTTTA
ATTTAGTTAATCTATGACAATGTCATTCATGAGAAAATCATATAAGTCTTAAATTTGC
ATTAAGTATGTGCGCGACAAAATGGATGAATAACTCAATATTCTGTCAACCGATGATTCTTTTTTTATTAGAA
AGGATAGATGATTATGAGATCCGGTTGAAATTGAGGAGTCAGTTTTTTTTGTTATTTTAAATATATACTTTTTT
GTTTAATTTTTAAAAGATAAGACAGTAACAAAATTTTCGTTTTCCAAAACCAGGTATA
AAATAAAATATTCAAAAATAAATCAAAACACAAAGCTGTAACAGAAATTTCATAGTCAAAGATAAATTCTTGA
TACTAAATTTTGATTCGTAGAAATGAAATCTTTGAAGCTCACATTAATAGGTCACTTAGAAATTACAAGTTCA
AAGTTGGCTCTTAGCAACTCATTTATCATTCATAATATTCATTTTTCATGAATCCACT
GTATATTTATTTTGATTGAATATAAATCGTTATCATTACTACTGTGCTTATATCACTCGCTACTTTATCCCAA
TCGCTTAGTTCTTGGATTTAAATTTTTGTCTAGGTATAGTATGGTGATAACAACATAATTTACAGATTTTGAT
ATATACCTATTAATGACATATTACATTCAGGCCTTTGTGGCGTAGGGGCTCACGCGTG

TTACTGCAACGCGACAGTTTTGGTTTCGATTCCTACTGGAAGGAATGTATTATTATTTATTAATATTTTCATT
TCTTCACTCTG
>1002052858_2 Heli.2-DT664552.3.5
GCACGAGGTTGACACCCACGTCCCGAAATGTAATATTATTTAAATATACATAATAATACATCGATTTTGTGTT
GTGCTGTGAAATCTTAATAAATAAGATGAAGCCGGTCCTGCTGGTTCTATTCTTGTCGGTGGTGTACGCCGAC
GAGCTCCCACTGTCGTGCAATAGACCTGTCTATTGTAAAAGCAATTTGCTACACTATG
TTCAAATGTCTAGAATCTTCTCAGATTCTAAAACTTTCGTGGATCTTCATATGAAAAAAGATGAAAATAGTAC
CATAACAGCTTTTGATGAGTTGCTCAAAAATACCAACAATATCCCAACAAACGAACAAATTAAAGAATTTTTA
GATAATTATTTTGATTCATCAAGTGAATTAGAGGATTGGACGCCTCTTGATTACAGCC
CTAATCCACCTTTCCTATCTACTATTCGAGATGAAACATTAAGAAATTTTGGAAAGAATATAAACGACATATG
GCCTACTCTAGGAAGAAGAGTAAATCAAAAATTATTTGAAAATCCTGATCAATACAGTTTAATACCGGTAGAT
AACGGGTTCATTATTCCTGGAGGTCGATTTAAAGAATTGTACTATTGGGATACGTATT
GGATTATGGAAGGTCTCCTTGTTAGTGGTATGCGAGATACCGTCAAAGGTGTAATTGCAAATTTAATACAACT
GTTGAAAAAACTTGGTCACATTCCCAATGGCAGCAGATGGTACTATGAACAAAGAAGTCAACCACCTCTTCTA
TCAGCTATGGTATCTTTATACGTTCGAGAATCGAAAGACATAGACTTTCTTAAGCAAA
ATATAAATGCTCTTGAAGAAGAATTAGAATATTGGTTAGATACTCAATTAATTACATTCAATGTGAATGATAG
AGCGTACACGTTGCTAAGATATTATGCGCCAAGTGAAGGTCCAAGACCTGAGTCTTATTATGAAGATTATAAA
GATGCTCAAATATTCGACAACCCTGACCGTAAGCAGGAATTTTATACAGACATAAAAA
GCGCTGCTGAAAGTGGTTGGGACTTTTCGTCTCGTTGGTTTATTGGTAATGATGGTAATAATAAGGGTAATTT
ATCAACAATTCACGCTAGTAAGATTATACCAGTTGATTTAAATGCGATATTTGCAAATGCTCTTCAAAATATG
GCATATTTTT
>1002052859_2 Heli.2-DT665168.3.5
GCACGAGGCACCCCTCGAGCCGGCCCCCGCCCTCGCCCTCGCCCCCGCCCCCGCGCCCGTCGACTTCATACAA
GAACATTCCAGGCCGCGTACAACCCACGGACCACCCCAAATCCCATCACCACCGTCGGTCAGCAGCAACGGTA
GTAGCGGCAGTAGCGGTAGTGCCCCCCGAGCAGGTACCCGTGAGGTCCACAACAAATT
AGAGAAAAACCGTCGAGCTCATCTGAAAGAGTGCTTCGAGCTCCTCAAAAGACAGCTGCCAGTCACACCTGAT
GATAAGAAGACGTCGAATCTGTCGATACTTGGGTCCGCAATACGGTATATTCAGGTGCTCCGCAGAAAGGAGC
GCGAGTGCGAGCACGAAATGGAGCGTCTGGCGCGCGAGAAGATAGCGGCCCAACAGCG
CCTCGCCACTCTCAGGAGGGAGGTGACTGTGAGGGCGGCGGTGCGGCCGCGCACGATCGATGCCGTTTCTGAA
GAGAAAGATAGGGACGATAGTGTCAATGGGCAAGTGTTAGGGATACCTATAAGTATTACCTCTTCGCCGCCAC
GGTCGCAAGTGAGAATGGAATCCCCGCCCCGCACTCTAAATCTAAGCACGAAGCTGCG
CACGCTGCCCATACAGATAACGCCCACCACCTCGCAGGTCGTGCGGACGTCATATGGCACAAGGCATAACACG
TTAACGCTTACAACCATTGCTCCCGCGGAACCCACTGCACAGAACGGTCAAGAAAATGGTATCAATACATTGA
GTAATTTAGTACATCCGGCTCAAATTCACCTTCTCTTGTCACAAGTGGTGAGCAGTGG
TGGTCTCGTGGTGAGTCCCGCCGCGTTACAGCTGCTCTCGACTAGCGG
>1002052860_2 Heli.2-EL596088.1.5
GCACGAGGGCGCCTTCCAGTCTTGAGTAGTATGGCCTGGTTCCAGTTAACGTAATTATCCCCATTTTCTGCAA
GAACCGTCAAATCTGTTTTTTGCCAGATCTTTCACTCGTGTTCTGCAAGGCGCTAGTCACCGTGGTGCTCACT
TGCCAGTCTGCAGCCTGCCGGTCGCGCAGTGTATCCCGCTACCCGGTTGCAGTCGACA
CAACCAGCCCTGTGTTTTGCCAGACTTTGGGCCGGGCAGTGTGACCTGGTTCCAGTCGCCGCAACCAGCCCAG
TCGCTCCTAGTGCTTGGGACGCGAAGTGTGTCCTAGTCCAGTTAACACAGCCACGTACATCAGACACACACAG
ATACTTCACTTCTGGCCTCTGTGGTCAATTCTGCGTGGTCTTAGTGACCGTGCTGCCT
GCCCGCCTGCCTACCAGTCTTCGGGCCAGCCAGTGTGACCTGCTTCCAGTCGGCGCAAGCAGCCCTGTTTCCG
GGCCAGGCAGTGTGACCTGCTTCCAGTCGGCGCAAGCAGCCCTGTTTCCGGGCCAGGCAGTGTGACCTGCTTC
CAGTCGGCGCAAGCAGCCCTACCTCCGGGTCAGGCAGTATGACCTGCTTCCAGTCGGC
GCAAGCAGCCCTGTTTCCGGGCCAGGCAGTGTGACCTGCTTCCAGTCGGCGCAAGCAGCCCGATCTCCGGGTC
AGGCAGTGTGACCTGCTTCCAGTCGGCGCAAGCAGCCCTGTCTCCGGGTCAGGCAGTGTGACCTGCTTCCAGT
TGGCGCAAGCAGCCCCGTCTCTACAAGTCTCAGGCCGGTCTGTTCGACACAGCTCGCC
>1002052861_2 Heli.2-EL600133.1.5
CGCCGGCGAGCTGGCTGGTCGGTGTTGGTAACAGAAAACAAAATGAATTGCGCGCGAATTCTAACGAACACAT
TCGAAGTTTAAAAACGATTTGACAGTTACAATAAATTTTTTGACACATCGATTTAAACTAGATTTATTTATTT
CAAACTAATAATAAATCATTTAAGAAAAAGTGTTGTTTTTTTGGTGTTGTGTGAAGTG

TTTATTATTGAAAATGTTGTTGTGAGGCAATGCTTGTGGGGGAGCAATCGCCGGCACCGCGGGTGGTGGCCGG
CTTGCGGAAACTTAGACCTGAACTGACACCAGGACCTGTGCCTACGCCAGCTGATCCTAATCTAAGGATATCA
GGTGTTCTACGCCAATACTACAATGACGATGACCAGTCGTTCGCGTGGGTGCGGGCGG
CGGTACGAGGGGGCTGCTTGCTGGCGTGGCGTGACGGCACCCTGCCCCGGCGGTCAGCTGCCCGTCTGCCCCT
CAGACACCTGCACCTTCGGGCGGCCGCCACCCTGCCCAATGCGTTCCAGTTGTCCAGGTTGCGGGATGATGCC
GCTGTTGCCACGTTTCAGGCTTGCAATGCTACGGAATACGCCCGGTGGGTGCGAGCGC
TTTGCGTTGAGATACTCGGCCAGACACCATTGCCACAAGTCAGATTTTTGGATGTCCTACCGGCCGCGGACAC
GTCCAGCAAAGAACCAAAGAAGGTACCCATTCAGGAGACGCCTGTATGCCCTCCCAGACCACCTCCCCGAGCG
AGACGAAGGTTGCTAACCGCTCCGGAGACCCAGCTTCCTAGAAGAGACCATTCCCCTG
CCGCTACGGACGAGGGCATCGTCGTAGAAGACGACGATTATGATTCATCGTCCGATCGCAGCCTCGATCTGTC
TCTGTCACTAGACGCACTGAAGACAATTGATGTGGTCGACGCGCCAGTGCGGAAAGCTGAGGTCGTGAAGTGT
GATAACTGCAGCAAACTAAACAAAAGCCCTCAACATCACACTCTTCCACGAGCGAGGA
>1002052862_2 Heli.2-DT664844.3.5
GCACGAGGAAGCTGTCTTTGAGTGTGGTACACCATTGTTGGCCGCCACACTGGCTGGACGCCAAAGACTGGAT
TCTCGCGGATCTGTGGATGAAGAAGCTCTCGGTGTAGCTCACCTGGCCCACAATGGATTAACCGTAACGGCTG
TTCGCCCTCCCCCGCTTTATCGTAAACTCGATTCTCCTACAGATTCGGCCTCGTCCTG
CGGAAACGAAAAGCATGAAAGAATTGTCGGCCCCGGTTCAGGCTGCTCGAGTCCACGATCATCTTTAGAGGAA
CACTTGGAAGACAGGCGGCCGCCTGCTCTCGCAGACGACATGCCAGTTTTAAAGCGCGCGCTAGAGGCGCCGC
CGATAACACGCCCGCCCTTTGATGTGCCACGTCCTCGCTCTCTGATGGAGGAAGCGTA
CAACCGTCATAAAAAGTTCCGCGCCATGCGCCGCGACACGGGCGAGGCGGAGGCGCGCGCAGTGCCGCTCACA
CCGTCGCCGCAGCCCCCGCAACACCCGCACCCCGCCAGCCCCGCGCACCCGGCGCACTCGCCGCGGCCGCTGC
GCGCCTCGCTGTCGTCCACGCACTCGGTGCTGGCCAAGAGCCTGATGGAGGGCCCGCG
CATGACGCCGGAGCAGCTCAAGCGCACCGACCTCATCCAGCAGTACATGAGACGCGGCGAGGCGGCGGGCGAG
GGCTGCCCCATGCGCGCCGGCGCGCTGCTGGCGTGCTACCGCGGTGCGTCGCCGGCGCCGGAGCCCGCGCCCG
TGCTGGAGCTGCAGGTGGACGTGGCGGATGCGCCGCTCAACCTCTCCAAGAAGTCGCC
GTCGCCGCCGCGCTCCTACATGCCGCGCATGCTGGAGGCGTGAGGCCGGCCCGCGGAGCCCTGCGTCTCCGCT
ACCCACCCCGACCTACTTTAAATTGAATAAACTGTAGTTAATGATCGTTCGCAAATCGGTTTAATATATTATA
TGTGATAATTCGTTTAAGACGCGTGCGTGAGTGGCCGGTGCCGGACGAGGAGAGCGCA
ATAGACTGAAACATAATTTAAGACTATATAAGTATAATTTATAATGCAATGCAGCGCCGGTCGAGGTGTGTGT
GTGTGTGTATTGTATGTGCGTATGTGAGCGTGCTCAGTCATTTGTTTGTTAATGTAATGACAGGTTGTCGAAT
TAAAATAAAACAAACTTGTAAA
>1002052863_2 Heli.2-DT664040.3.5
GCACGAGGACACTCTTAAGTTGTCAGTGTCACTTTCAGTTGTTTCGAATTTATCTGGAGTTTGGATATCATCA
TTAATAAATAAATTACTAAGTTAGATATCACCGTTAAAACTCCGAACAAATACATTTTACTAATGTTTATTGA
TATTCTAAAAATATATCTATTTTTAAACCTCTGATACTTGTGCTTTAATTTGTAAACC
ATTAACTAAATTATAATTTATATAATTTTAAGCCGGTTAAATTTATAAATAAGTGGTACATCATGGCATGTGA
TATTATGACTCAATTTTATTCTTGCTTGAATTTACCACTACAAGAAAGAATTTTGGAATTGACTAGGCTTATT
GATCGAGCTAGTCCTAATAAAGATTTGCAAGCAATATTTCCTCAGCTTGTTAGTAATA
TTTTTGCACCCTACAATCAAAATGGATGGAACCTAAGACAAGTAACATTTGAAACAAACAGATATGAATATCA
GACTTTACTGAGTTTTTTAGAACCACTCGGACCGATGTTTCGTCTTTGCTATAAACTATTGTCTGATACACAA
CTGAAGTACAATCTTCCATTAAACACCCTTCCTATAGACCTACAGATAACCCTTGAAA
GAGGGAGATGTCCACAATTTTATGCAGACCTGCTAGCTACAGACTCCCAATCAATGAATGTAGTGGCTCTTGC
TTTAAATCCTTTTGATTATTATATATTTCACTTTGCATTGTACTTAGTGAATAATAATCAAAATAAAGCCTTG
TGGGAGAATTGGAATAGTGTATATTTTGCATTAGCTTGTGACTATCTGTTGATTTTTT
TACCTTCGGATCCAAATATTCCTGTTTTACCACATATCCCACATTACACTGGAAAGGTACCAATGGCTGCAC
>1002052864_2 Heli.2-EL603647.1.5
GGGAGGACGAGGACGCAGAATGGCCGAGGTACATCGCTTTTGTAGTTGTTTTCAGCAATATCATGGCTAATGT
GCTATTTTAATTAGTAAATTAGTAGAACGTGCCTCAAAAAAGTTAATTACACGTACAGTGCAAAGACTATTTG
ATATTTTAGACGATTATATGATTACATGCTCTCATTTGACAAATTGCGCTAGACCGCA
TAAATAAAATGGCAGCCCTACCACGTAGAATAATCAAAGAGACACAGCGGTTGATGCAAGAGCCGGTGCCGGG
AATCAGCGCGGTGCCGAGTGAGAACAATGCGCGCTACTTCCACGTAATCGTCACCGGCCCCGAGGACTCGCCG
TTCGAGGGAGGTCTATTCAAATTAGAACTATTCCTTCCAGAGGACTACCCTATGTCAG

CGCCTAAGGTTAGGTTTATTACTAAAATTTATCACCCGAATATAGATCGACTGGGTCGCATATGCCTTGATAT
TTTGAAAGACAAATGGAGTCCCGCACTGCAGATCCGCACGGTGCTCCTGTCGATCCAGGCGTTACTATCGGCC
CCCAACCCTGACGACCCGCTGGCCAACGACGTCGCAGAGCTATGGAAAGTGAACGAAA
GCGAAGCTATCCGAAACGCCAAGGAGTGGACCAGGAGATACGCCATGGACAACTGAGCGCCTCCCGTCGCAGC
CAGTGCCTCCCCCACGCGCTACACTAAGTGGACGTATCTAACATATATATCATGATTTTAAGAGTGCAAGTTA
TGTTTTACAACCTAGATCGAGTAACTTGTGAGGAGACAATTGTGGATAAATTTTTATT
ATCGGCGGTAAAAGCCGCCGCGGCGCACCGGCCTCGCGCCCGCTCCGGTCAACCTCTTGC
>1002052865_2 Heli.2-DT666813.3.5
CATTGTTTTCCAGTGCGCAGTAGTTTCTCTGTCGTGAAATCGGGATTCTGTTTTAGTATTATATCTATTCTGC
CATTTTGAAAATAGTGGCGTGTATGGATTAGACCTAGTGTTTTTTATGTGACAAATATGTCCGGGAGCAATGA
TAAAGTGTCGACTACGCAGCGTATGATCCAAAGTGTCGCATTCCCTCCTAGCCACAAA
CTTACTGTGTCTGAAGTATTTGATGAAAAAACTGGAAAACCTCTACCAGATGTACTGAAGCAACACTTTATCT
TAGAGGGTCGAATTGAAGAAAATGCAGCTTTACGAATCATCCAAGATGGTGCCACCTTATTACGATCTGAAAA
AACTATGATCGAGATTGATTCACCGGTGACCGTGTGCGGAGACGTCCATGGCCAGTTT
TACGATTTGATGAAATTATTTGAAGTGGGAGGCTCTCCTTCCTGCACCAAGTATTTATTCTTAGGTGATTATG
TCGACCGAGGTTATTTTAGTATAGAATGTGTTCTCTACCTCTGGGCATTGAAATTGTGCTATCCGAAGACATT
ATTTCTATTACGCGGCAATCATGAATGCCGACATTTGACAGAATATTTTACTTTCAAG
CAAGAATGCAAAATTAAGTATTCTGAAAAAGTGTATGATTCATGTATGGATGCTTTTGATTGCTTACCTCTTG
CTGCTCTTATGAACCAGCAGTTCCTATGTGTACATGGAGGACTTTCTCCGGAGATTAACAGCTTAGATGATAT
TCGTAAACTAGATAGATTCAAAGAACCACCTGCATTTGGGGCTATGTGTGATCTGCTG
TGGTCTGATCCTCTTGAAGATTTTGGAAATGAAAAAAATGCAGAGCATTTTTCGCACAACTCTGTTAGAGGCT
GCTCGTATTTTTACAGCTATGCTGCTTGTTGTGACTTTCTACAGAGGAACAATTTATTGTCAATCATCCGTGC
TCATGAAGCTCAAGATGCTGGTTATCGAATGTATCGTAAAAGCCAAACTACTGGGTTC
CCAAGTCTTATTACTATCTTTTCTGCTCCCAATTACTTGGATGTGTACAACAATAAAGCTGCAGTCTTAAAGT
ATGAGAACAATGTCATGAATATTCGGCAGTTCAACTGTTCTCCCCATCCATATTGGCTGCCAAACTTTATGGA
TGTTTTTACTTGGTCACTTCCATTTGTGGGGGAAAAGGTTACAGAGATGCTTGTTAAT
GTCCTAAATATTTGCTCTGATGATGAACTTATGACAGAAGGAGAGGATGCTTTGGAGGAAGCCAATCTCAGAA
AAGAAGTGATTCGTAACAAAATCCGCGCTATTGGAAAAATGGCCCATGTCTTTTCAGTACTTCGTGAGGAAAG
TGAATCAGTTTTGCAGCTTAAGGGGCTCACTCCAACTGGTGCTCTACCTTTGGGTGCA
TTATCAGGTGGTAAAACCTCTTTAAAAAATGCTCTTCAAGGATTCTCGCCCAACCACAAGATTACCTCGTTTG
CTGAAGCAAAAGGCTTAGATGCTATTAATGAACGGATGCCACCACGCAGAGATGGGCAGCGTACTCCAGAT
>1002052866_2 Heli.2-EL596844.1.5
GTCGTGCGCCACGTGCTGGCGCTGCGCGCGGCGCGCTGGGGCCTCGCCGACCAGGAGCCCGACTCCAGGGTGC
TGAGCGAGCGGCGCCGGGTCAACACGGTGACAAACCACGTGCCCCCGCCACCCGCTGAAGGGGAACTGGTGGG
GTACCTGGCAGATGGACAGACGCTCACTGTGGAGGAGTGCGACTTCTTGCAAAACAAC
CTGCCCACTAAACCTGCTGCATTGGACGAGGATATACTGGAGGAGCTGGAGAACGACGCGTGGGAGACGGACA
TGGACGCGGAGATGCAGGCGGGCTTCCTCGAGTTCCTCAAGATGTCCAACCAGATCAAGCGATAACCGCGGCT
CTACGAGCAAAAACTATACAAAAAAAATATAAAATTTTATAAAACGATAAAAAGATAT
CAGTGAACGCTTTTCGATCGCTCTATAAATATGATCATGACGTACTCCGCTACTTAAACACATTGCCTATACT
CTCCCGTTACAATAGTACACGTGTCCTAAATTAAGCAATATTTTATAGAATTATAGTAACATCAGTACCATTT
AAACGCAATAATTCATATTAAGTAAAGAAAAATATATCTAGATATTATCGACGGTCCC
TACGTATAACGGACATGTCAAAAGAGCACGTTTACGGGAGAATGGAATAAAAGTTCGTCTTGCTCTTAAAAAT
TTAAATAAAATTATAAATATACAGTATACTGTTCTAATACTATACAGTTATGTTTATAATTTATTTTATTTTT
TTAAGAGCAAAATGAACCTAAAATCGTAAACCTTCTCTTTTAGCATGTCCGATGGCAC
GTACGAACCGTCGTTCTCTTAGATACGGTTAAGTATTGAACGTTGAAGGTTGTTCGTCTAGTTGAATGATAT
>1002052867_2 Heli.2-EL598475.1.5
GCACGAGGGTTTCTGTGCAGTTCAGTTTTTAATCCAAAGTAATCTCTTTGGATTTTGTTTAATATCTAAAATA
GATATTAGATTTACTCAGAACAATGCCTCCTAAGTTCGATCCTAACGAAATTAAAATCGTTAACTTGAGATGT
GTTGGAGGAGAAGTTGGTGCTACATCATCATTGGCTCCTAAAATTGGTCCCTTGGGTC
TGTCCCCCAAGAAAGTTGGCGATGATATAGCTAAAGCTACTGGAGACTGGAAAGGACTGAAGATCACAGTGCA
GCTGATTGTTCAAAACAGACAGGCACAGATTTCTGTAGTGCCCTCTGCTGCAGCACTTATCATCAGAGCTTTG
AAAGAGCCTCCTCGTGACCGTAAGAAGCAAAAAAATATTAAACATAATGGTAACATCA

CATTGGAAGATGTTATTGGCATTGCTAAAGTTATGAGGCCACGGTCAATGGCAAGGTATCTCTCAGGAACAGT
AAAAGAGATCCTCGGAACAGCCCAATCTGTAGGTTGCACTATAGATGGAAGACCACCTCATGACCTTATTGCA
GACATCAACAGTGGTGAATTAACAATTGATGAATAAATTTCATAAGATATTATTATAA
>1002052868_2 Heli.2-EL599354.1.5
GCACGAGGAACACTCGTCACGCGCCGCGACTCGCTTACTAGTTAGTGCTCGTGTTAAGTGTCTCTTACGATGA
AAGCCATAACGGCGGTGTGCGCAACCGGAGCGTCCGTACCGGCAATCGCCAGCGGACGGGTGCAGAGGCACCG
GGACGGCGAAAACGCTGAAATTCAAATGTATCTCTCCAAATTACAAGACTTGGTGCCG
TTTATGCCAAAGAATAGGCGGATCTCAAAACTGGAAGTGATCCAGCACGTCATCGATTACATCTGCGATCTAC
AGTCAGCATTGGAAAATCATCCTGCTGTGGGGCAGTTCGAAGCGGAGGCCGCACTGGCGCCTGCATGCGCTTC
ACCACCCAGGCCACGTCGCCGACCTCTTGGACCTCGCCCAGCACCCAATACCATTCTC
CCTGCTGATAGAACTCTATCTACAACACATCACTCTAATCACATGACTCCTGAAAAGCAAGATCAACCAGATA
GGCCCACGTCGTGCTAGGTGATCTTTAGAGTTAAGGACAGTTGTATTAATGGTGAATATCAAATATACCTCGA
GATGCAAGGGCCGCGCTCCGTAGGAATGAACCTGGTACGAGAAGGTGCCTCTGCACCG
GGAGCCTCCTGCATCAGTTCCAGTGAACTTCATCTCACGATGATATCCTATGCACGATCTCGTTAAGATCAAT
TATTTAATTATTTGGTGGCATTA
>1002052869_2 Heli.2-DT664021.3.5
GCACGAGGGTAATTCTATAGCGTTCTTCTGTGTAGTAAATATCTTTTTTCTACATAAAATTAACAACCATTCT
AAAACAACGAAATAATGGCTCCTGTTGAGCAAGATGTGGAAATGAAAAATGTAGACAGTCCAACCGCTGCGAG
TGATGTAGAAACTGGTGAAGTGAAGAAAGATGCCGATGTCCTAGCTGTTCACGATTTA
AGGGAACATGTTAGACAAATTGACAAGGCTGTAACGTCCAAGGAACCGAGGTTTGCAATGCGCGTTCTTAGAT
CTATTCCAAATACACGAAGAAAACTAAATGGTAATGTTTTACGTGCTATTATTAATCAGCTTTATCCTGCAAG
CACAGACAAAGAAGCTTTGATCGCGTTTGTTGAGAACCCACTGCCAGGTGCAGTTGAA
ATAGAGGCTCCACGTTCCCGTAGTGTTCCGAAAACGCCAGCGCCAGAAGTTGATGCATATATGCACCTGTTAG
TGTTGCTTCGTTTATTAGATACTAATAAACTTGAAGAGGCAGTAGAATGTTCTCAGCAGCTGATGAATAAGGT
AACAGCTCAAAACCGTCGAACTCTAGATCTAATTGCAGCCAAATGCTATTTCTATCAT
TCACGAGTGTTTGAACTTACCAATAAGTTAGACCTCATTAGAGGGTTGCTGCATGCTCGTCTGCGAACTTCGA
CTTTACGAAATGATTACGAAGGCCAGGCAGTGTTAATAAACTGTCTTTTACGAAACTATCTACATTATGCACT
GTATGACCAGGGCAGACACATTGGGTTACCAATCAGTGTTCGCTGGAAGTGCCAGTAT
TATTGATGGGCACCCTTTTGTCGAGCGTTGGCAAATGAAGGGGTAATTCAGTAGGGATCCCCAAACTTATCCA
CTAGTGGCCCT
>1002052870_2 Heli.2-EL599054.1.5
AAGCCTGCACACGCGGTGAAAAAAAATAAACTGTCAAGTTTCCCGCGCTCACAGATTAAATCGGATTTTCGCT
TTACAATTATTTAAATCATTGATTTATTATTATAGTAAATTATTTATTATTATCAGTTCTTAAAAATAAAGAT
ATAATGTCGGCGGACGTGTTAAGTGACGATTTTAAATCGCGTCTCCACTTGACAACTA
AAACGGTAGGGCAGGAGAGGATACGGAGGGCCCGGGAAAACAATGAGGACGTCGCCATATTGTCCATGTTCCT
CCCAAATGCGACAGCTGAGCGGGAAAATAAGAAGTTCTGTTGTGCCATGAAAGACCTTTGCTTGCTCGGCGAT
CCGAGGAAAACAGTCGCAGGAACCAAGAAAATGGAGTACAAAGCAAGGCAATGGCATG
AGAAGTGTTTCTGTTGCGTTGTCTGCAAGAACCCGATCGGCACCAAGAGCTTCATCCCGCGCGAGCAGGAGAT
CTACTGCGCCGGCTGCTACGAGGACAAGTTCGCCACACGCTGTGTCAAGTGTAACAAGATCATCACGCAGGGC
GGCGTGACGTACAAGAACGAGCCGTGGCACCGCGAGTGCTTCACGTGCAGCAACTGCG
CGCTGTCGCTGGCGGGGCAGCGCTTCACGTCGCGCGACGAGCGCCCCTACTGCGCCGACTGCTTCGGGGAGCT
CTTCGCCAAGCGCTGCACCTCCTGCACCAAGCCCATCACTGGCATAGGTGGTACCCGCTTCATCTCCTTCGAG
GACCGCCACTGGCACAACGACTGCTTCATCTGCGCGCAGTGCCGCA
>1002052871_2 Heli.2-EL599620.1.5
TGAACGCGACTTATCTGACTCTCAGTCAATTACCGGTCGTATGCGAGAAGGTCAATTCGAGTGATATATGCGA
GTCCCTTACGCTCAATGGAACGAAATACTGCGAATGGGGCTACAATGGTTTGGGGATCGATTACCAAGTGCTG
GCAGGGCCGGCCTTCATGGCCGTGTTCACTGTTGTCGGCGTTATATTGGGTGTAGCTG
CTGATAGATATAACCGAGCAAAAATCCTAGGTGTATGCACCTTAGTGTTCGTGGTGGCGATGCTCTTGATGGG
CACAGTGACCGAATATTGGCACCTAGTGTTACTAAGAATGATTATGGCAGCAGGCGAGTCCGGGTGCAATCCA
CTAGCTACTGGCATCCTGACTGATCTGTTTCCGGAACATCAGAGGGCTTTGGTCCTGT
CCATCTTCAACTGGGGTATCTATGGAGGTTACGGGATTGCGTTCCCGGTGGGAAGATATATACCTGCTATAAA
TGCTTGGGGTTTGAGCTGGAGAGTGTGTTACTATGGCGCCGGTATCATTGGTATAGTCATCATTATCTTGACG
TTCCTCACTCTTCGGGAACCTGAGAGGACTACTATTGGAGAAGAAGGAAACGCGAAAG

CAGGAGATGCTGCTCTAGAGGCCGGTAAGAAAATGCCTCAAGTCGCCATCTGGCATATAATCGGACAGCCGAG
GATCCTGCTGCTGTGCTTGGCCGCCTCTATCAGACATTGTGGTGGTATGACATTCGCATATAATGCAGATTTG
TACTACCGCGATTACTTCCCCGACGTGGACCTCGGCTGGTGGCTGTTCGCGGTCACTG
TGGGCATCGGCTCCGTGGGCGTAGTGGTTGGCGGAGTTATTTCTGATAAATTTGTATCAAAAATGGGTATCAG
ATCGAGAGTACTAGTACTTGCATTATCACAATTGATAGCGACATTGCCAGCGTTCGGCTCCGTCGTGTTCGGA
CCACTGTGGGCCATGATCACGCTTGCCTTCTCTTATTTCTTCGCTGAAATGTGGTTCG
GAATAGTATTCGCTATCCTCGTAGAAATAGTGCCTGTCGCTGTCCGCTCGACCACCGTGGGTGTGTTTCTCTT
CGTAATGAACAATATTGGTGGTAACTTGCCCATTCTGGTCGACCCCGTGTCGAAGGCTATAGGATATAGGGAG
TCTATAATGATATTCTACGCTGGTTTCTACGGTATTAGTAGCATACTGTTCTTCTTAA
CAATGTTCTTAATGGACGGTCCAGTAGAAAATAAAGATAAAGAAGAGAGTGACAAACGCAGCGGCCTAGACAA
CAGAGCTTATTCACACGACGAGGTTGCAAGAGATAGAATCGATAATATTAGATTATAAATGAAATCAAAATTA
TAAGACAAATATCCATATATTT
>1002052872_2 Heli.2-EL596336.1.5
TCAAAATCATTACGTGAGAAATTGTTCAAAGACCGACTTAAATCTATACAGACAAGATGTGTGACCGCAAAGC
GGTGATCAAGAATGCTGACATGAGTGAGGAGATGCAGCAGGATGCTGTGGACTGCGCGACGCAGGCCCTTGAG
AAATTTAACATTGAAAAGGACATTGCAGCGTTCATCAAAAAAGAATTTGACAAGAAGT
ACAACCCAACCTGGCATTGCATCGTGGGCCGTAACTTCGGTTCATATGTAACACACGAGACCCGCCACTTTAT
CTACTTCTACCTGGGCCAGGTGGCAATACTGCTCTTCAAGAGCGGCTAATCGAAATCTGTCTAGCAAAACCTC
TTTTCGGACATTCCATTGATCCAACTAGGGTTGCCAACATTATTTTTGACCATAATGA
TAAAATAATACAGCAAATTTAGTATATTTTAGGTATATTACAGTGAAATTGACGGATTTTTTTAAATTTTGGG
TGCTGAAATGGCATCAAGCTAAAAACTTATAGTATTTTTGCATATTGTATATTTCTTGTACACAGACAGACAA
GCATACATACACTATTCATTACAGTACAGTTGGCAACACTAGCTCGAACCCACATTCC
TATCCCAAAGGTATACTAATGGTGTCAGATTTTAATTAAAAAAAAACCTGAAGATTACCTTATTTATTTGATT
TTTGAGTTACTTTTACATTCAAGATCTTGGTAAACAGATAGTGCAGG
>1002052873_2 Heli.2-DT667388.3.5
GCACGAGGGGCAAAATGGCTTTTAAGTTCGTCGTACTCGCATGTTTCGTGGCGGCCGCCAGCGCCGGTCTCGT
TCCGGCTCCCGTCGTCTACTCTGCCCCGGCATACCACGCCGCCCCCGTGGCGTACGCCGCCGCACCCGTCGCC
AAGCTCGCCTACGCCGCCCCGGTCGCCAAAATTGAGGAATACGACGCCCACCCTAAAT
ACAGCTTCGCTTACGACGTCCAGGACTCCCTTAGCGGAGACTCCAAGAGCCAGCACGAGTCCCGCGACGGAGA
TGTAGTACAGGGCTCCTACTCTCTTGTTGAACCTGACGGTTCCAAGCGCATCGTCGACTACTCTGCCGATCCC
CACAACGGATTTAACGCGGTCGTACGCAAAGAAGCCGGTGTAAAGGTTGTTTCTAAAG
TAGTAGCACCCGTAGCTTACGCCGCTCCCATCGCTAAGGTCGCTGCCGTCTCCTATGCTGCCCCTGTGGTCCA
CGCCGCCCCCGTGGTCCATGCTGCCCCCGTGGCGTACGCAGCTGCCCCAGTAGTGCACTCTGCCCCATTAGTC
CATGCACCACTTGCCTACTCCTCCCCCATCTACCACCATTGAGTTCATTATTAGTTTT
AAAATAAACATATTTATTTAAAAATATATGTATATTATTCATCATCCTAATTAGAAATTA
>1002052874_2 Heli.2-DT667437.3.5
AATAAACCTAAGTCGTTTACTGAAGAGTTACCGTCAGAGAATATTAATATACAAGTTGGGAGCTCTACTAAAA
TTAAGGAACCTCGAGACCGTGAAATTGTACATAAAATTAAAGAAGACCATTATAAATTTATTGATGAAGAAAC
AAAAATGTTCTCCATAACAGACATTCATGATAATAAATTAACAGACACTGTCAGAACT
CATAATAAATATCCCAAAAATCGTCCGTCCAGCCCTGACAAAACTTCAAATTTTTCAACTAAAAATAGCCCTA
ATAGGGAAATATCAAACAAAAATAAACCATCAAAATTGATTGAAAATATTCCCGAATCTCGCGTATCCCCAAC
AAGAAATATATACCCAACCAATCATGCAACAGACACAAGTATTACTTCTTCTTCAATG
ATTTCAAAAGATGTATTAAGATCACACAAAAGCCCATCACCGACAATACAAAGACCATCTTCGACTGAACCTC
GTAATGAATTTCGTAAGTCTTCATCTCCTACAAGAGAGATCTCAAAAAGAAATAAAGAAATTCTTGAAGACAC
AACCGCCTTCATTCCAAAAGAAGAAAGTAATACTATCCCTGATAATACCCCTCTTCGT
AAACGTACTCCATCACCAATAAAGAACATAAAAAATCTTAACAATAAACAAATAACTTCAGAAAATAGTGTCA
CTTTTAGTTCATCGAAAACGATAGAAAGAACTGAAAATATAGAAGAAGAATCCAGTAATATCACAATTAATAA
TGATAAACAACCAAGAAAAAATGATTTTCCTAAGAGGGAGAGTTCACCGCTTAAAACA
AAATCCATTAACAGTATAAAGGATAATATACACTTTACAGATACACTGATACATCAGTATACTGACAAAAAGG
ATACACAGATAGATAGATCCCCCAATGATTGTAGTCAACAACCTTTAAATAAATCCCCCCGAAGTAGCGTGTC
TCCAACTGAATCAATTGTTCGTGAAACTAAATTTAAACAGATGTCTGATTTCATTGAT
ACTGAAAAAAAATAATGAAGAAATTAATAAGCGCTCTTTAAAAGATCGGCCTAGACAATTAATTACACCTTCTA
CAAGTCCGACACGGAAACCTAAATACATTGAAAATACAATATCTTCAGGGCAAAGTTCTCCAACAACATCTGT
TAGTGGATTTGAATATTTTCCAGCCTGTAAAGACAAAAAAGTTGTTACTGACTTAGAT

GAAAAAGAATCATACACAAAAGATATAGAAAGCTCCAATAATGAAAGAGTTAAGCAAGTAAATAATCAAATTT
CAATCAAAAATCCTTGTAGATCGCCATCACCAGAAAAACGTCCAACAAAGGAAACTCTTCCAAGAAAAAGTTC
ATTAAAGAAAACTTCTAATAGCCAGATGTCCCCTACAGAAAAACCACCTTCTAGTTTT
CTTATTTCCCCTAATGTAGAAACAAAAGAATTTACCGATCATAAAGTCACTATCCAAGATCATCCTATAAAAG
AGACTGAAAAACCCATAAAATCAAAGCCTCCATTTGAGAGACGTGAAACTTATGAAGAAAGATGCCGCAAAAT
TCTTGGTATGATTGAGACAGACACAATGGAATCGGAAGATACAATAGATTACACTATA
AAGAGATCCAATATACCGACAAGTGAACGCAGTTCAGCCAAGTGTATCACCTTGCCGTAGTCCGTCTCCCAAT
GAAAAAGCATCTAATAATGAAATTAATAAAGAAAATACAGATAAAGAATATAAGGATGATTACAAAAATACAT
CTAACAACCGCAACGTCAGTAAAATTCCAACAAGGGAACCATCACCTACTAAGCCCCA
TGATTCGGTTGAATATAAACTGGAATCTCATACGACTGCATTGAAAGATACCTCTTTAGTTACATCAAACAT
>1002052875_2 Heli.2-DT662870.3.5
GCACGAGGCCGCACTCTGCACACGATTCGACTTATAGTTATTGAAAGAGTAACCAAAAACTTAAAACATATTC
ATTATTTGAATGTAGCGTGTATATATGAATTAAATTCTTCGCTGCACAGGTATTTATTATAAAGGAAAACATG
CCTACAATATCAGTTAAACGCGATTTACTTTTCGATGCACTTGGAAAAAGTTATAGTG
ATGAAGAATTTCAAAATTTATGTTTTGAATTTGGCTTAGAATTGGATGAAGTGACTACAGAGAAACAGATGTT
AATGAAAGAGCAAGGAGATCAAGCTGGTGCTGGTGTATCAGAAGATATACTATATCGTATTGATATTCCCGCT
AATAGATATGACCTGCTGTGCCTTGAGGGACTTGTAAATGGGCTATTGGTTTTCCAGG
GAAAAAAGGATCCTCCAATATACCACCTCAAGAAATATGAAGATTGTTATTCCCTTCATTTAACACCAGCAAC
ATCTCAAATTAGACCTTATGCAGTAGCGGCTGTACTCAAAGGAGTTTCCTTCACTAAGGATAGCTATGATAGC
TTCATTGATTTACAGGACAAACTTCATCAAAATATCTGTAGGAAAAGAACACTAGTAG
CAATTGGGACTCATGACCTAGATACAATCCAGGGACCATTTGTATATGATGCTTTACCGCCAAATGAAATAAA
GTTCAAAGCGTTAAATCAGAGTAAAGAGATGACAGCTCCTGAATTAATGGAATTGTATTCGAGTCATGCCCAA
TTGAAACAATATCTGGGTATCATTAAGGACAGTCCAGTGTATCCAGTTATTAAAGACA
AAAATGGTATAGTTTTGTCCATGC
>1002052876_2 Heli.2-EL599638.1.5
GCACGAGGCCTCGTCCTATTCGGCATGCCAAAACAAAACCGTGTGTCTATCTATGAATACCTTTTCAAAGAAG
GTGTTATGGTAGCCAAAAAAGACTACCATGCACCTAAACATCCGGACTTGGAGAAGATACCCAACCTTCAAGT
TATTAAGGCAATGCAATCTTTAAAATCAAGAGGTTATGTCAAGGAACAATTTGCATGG
AGGCATTTCTATTGGTATCTTACAAATGAAGGTATTGAGTACCTAAGGATCTTCCTCCACCTCCCACCAGAAA
TTGTGCCCGCAACACTCAAGCGCTCTGTTCGCACTGAGACGGTCAGACGTGGTGCTGTTGGCAGGCCTGATGC
CCCAGCTCGTTCTGCTGAAGACAGATCCATGTACAGGCGTGCACCCACTACACCTGGA
GCACCACACGACAAAAAGGCAGATGTCGGCCCTGGCTCTTCAGAAGTTGTATTCAGAGGAGGTTTTGGACGTG
GTAGACCAGCTCCTTAACTTAAGTTAAGCTAAATAAAAAAAATACCT
>1002052877_2 Heli.2-EL600478.1.5
TGTCATCATGAGGGAGGAAAAGCCACCCCTTTTACCCCCCGCGCCTGACTACCGCGGGGACGACGCCCCCTAC
CGCCCCCCGCCGCCCTTCGCCGCCTCCCGCACGCCGCCCCGCCCCAAAGCTACCCCCACTTATAGGAAACCAC
CACCATATGTGCCCCCTTAATTACTTTACCAGTACAATCTAATTATAACAAACTTTGT
AGTGGTAACGTCTTAGAACTGACGATTTTATGATATTATGTAAAGTATATCACATTCAGTCCTGCATTTTTAA
ACCAGGTGATAATATAATTTTACCTAATTTTGGAAGGTTATTTGAACATTTCATGTCAACGGTATTAGAAAAT
CGTCAATTCTACAATACGGCCACGACAATTATACTAATACAGATTATGTAAACGTTGG
ATAGTGAAGACGCAATTTTATATTTTAAAATTATTTTGCTTGATATATTAAGATTTTTGCTTTTACTTTTTAT
ATGGAAATGTGCACTGTTTGTGTGAAATTACGTCTTCAGTATTCTAACTATATTATATTATGGGAAATTTAAA
TAGAATTTTCTAAAATCAAGTCAAGGCGAATACTTTGAAAAAATCCAATTAATATTAT
ATATTTAATGCATGTCAAGATATCTTAATTTGGATTTCTTAAAGTATTTGTGTTCGTGTATAATAAACTCGTA
CATATATACGT
>1002052878_2 Heli.2-DT664364.3.5
AACAGAAACTTGTTATAGCGCTAGACCCCACAACATCCAAACCAATCTCTAATATCAAAAATGACATGCCAAG
TGAAAATTCAGATGTGACTTTTGACACTATCAATATGCTTTATAATCGGTCCTCTAAAGCCATAACAAACGAA
GAAAACAATAGTAATGATGTAACAGAATCGAATAATTTTAAAGATATGACTGAAACTA
CAACAGACTCTGACTGGCTTTCTGAATCAGTGACAGAAATAAATTTAGATGATGCTATGAAAAAAGATCAAGT
TGAGACTACAGAAACACCGTCTTCTAAAATAGATGAAATAATGGGTAGTGGAGTTAGTAAAGATGATTTTGAG
CCCGACTATTTAAATAATATGGAATCTAACACGAAAAAGATGGACCAAAGTGACGAAA

TGTATAGTATGTCCCATGACTATGATAATGATGAAGCTAGAGTAAAACGAGTGAACAGTCAAGTGGAAAATTC
TGACGAAAATGCAAGCAACAGCGTGCATGATGTTCCAGCATTAAATTCTAATATAAATAAGCTCGAAACAACA
ACTGTAAGTCAATATATATACAAGATCGCAGAGAAAAATGTAAATGAAATGAATGTTA
ACACTGAAAGCTCAGCACATCCAGCTCCTGTCTGGGAAGATATTGAACCAGAAAATGTAGTTCCAATAACTAA
CGAACCTAGAATGGAAGAAGAGCTAAAAGTAAATGATAACGTGATTACTTCAACGACAATGCCTCCAACAACC
GTGTCTAGTGTAGAAGAAAATGATCAAAAAAATCCATCTGAAATCAACACAAACACCC
AAAATTCGACACAAATAAATAATCTAAACGTAACGATATACGAAATATCTAGTCCTAACGACAATGAATCTTT
TATCACTTCTAAACCTGCAAGCGTTCAAGATTTTGAAGACCACGAAACAGAGATGAATCCTTTCTTACCAGAA
GTGGAAAATAATAAAAGCCTCG
>1002052879_2 Heli.2-DT662966.3.5
TCGGAATCGTGTCAAGACGGCAATTGTCACGGAAATAATGAATTTGGGCCTGATGAATCACCTACGAAGCTTA
TCGTCAATTATATTCCTGAAGTTATGACCCAGGATATGATGTTCTCGCTGTTTTCAACAATGGGTAAATTAGA
AAGTTGCAAATTGATAGCGAACAGAGGGTACGGTTTCGTCGAGTACTCTCGCCCAGAG
GATGCGGTCAAAGCACGCAAGGCCTTTAATGGGTTGCTAATGCAGAATAAGACTTTAAAAGTTTCACATGCCT
TACTTAATCCAGAAATGAAGCCACCCTCAAAACCGGAAGCAGACTGGAACCTCTACGTATGTAACCTGCCTAA
TGAACTGACCTTGCAAGATTTACATGGACTATTCGCACAATTTGGTAAAATAGTAAAT
TCTCGTATTGCCTCAGGCATAGCTTTTGTTCTATACGAACATCATTATGAAGCAGAGAGAGCCATTCACAATG
TAAATGGCACTACTCCACCTGGTTTTCTACACCCATTAACTGTTAAGTATGCTAATAAGAGTAATCCCAATAA
ACATAAAAACAATAATAATAACTTTTCAAAGAATTCTCTTGTGAAGCCTTATCAATGG
ATTAATCATGTGGGTGCTATAGGTGACCATAATTCTCCTAGTACATGGTCTATATATATTTATAATATTGCTC
CTGAAGTAGAAGAATTGACTCTATGGCAGTTATTCGGTCCATATGGTGCTATTGTATCAGTTAAGATAATTAA
AGATCATCAAACTAATAAGAGT
>1002052880_2 Heli.2-DT668551.3.5
TATATTTAATTAAATTTCAAAGTTCACTATTCGTATTGAATGTAAAAGTTGACATCGCTCTCAAGCGCTTTTG
TTAAGCTATTTTTTTATTTTCCCATAAAGAGTAACTAACGTCTTCTGTGTTACCTGCAAAAAGATACTTTTTG
TAAGGTCTACTGCTGTGACAGTTTCTTGCCAAGGAGCCTAGAAGGTTAAAATTTCCGT
AGGGACTCTCGAAGAGTTCAAAGAATTCTATTCAAAATGGTTGCTCAAAAGATCACCGTCTGTCCGCGTCGAG
GAGACGATATCGGAAATCAGTTTAAGTCGAACACCGAAAGACATGGTTTTTTCGCAAACGAATGTCAATACAC
GACAACTTTTGAAACTGGTAGAGATAAATCGCTTAAACTCCGAAAAGAATCAACAGGT
GAACGGTCCAACCGGAGTGGTTATCGGCGGCATTCTCGACTCTCCTTCTCCAACGCCCGCTCCCAGCACGAGC
TTTTCAGAGGAATTTCTCAAACCGGAGCCTCTATTTCCGGAAACAATGTTACACGATTCGGACGGCGAATTCT
TCCAAGACCTGGTGCAATGGTGCGCCGCGCCCGCCGAGGAGCCACTAGTACAGTTCAT
TGATCCATTAAAAGTAAGTGAAGAAAGGGCACACAAAACAGACACACTAAACACTCAATTCTCACCGCAAGGT
TGGGAAATATTCGACGCTAACTCACCGCCGGCGCCCGCCGCCCACGCCTGCCCGGCCAC
>1002052881_2 Heli.2-EL602755.1.5
GCACAGGGTCAATTTCGTCAACATTGTTTTCAATACTATTTACTATATTTTTCAATAATTTCAATGTAAAAAA
ACTAATTTTATTTTGTAAATTCTGAACATTAAGTCATTTACAATATTTAAATTTAGTTTTTTTGTTTGTACAC
AAGGTAACTGTGCCATTTCCGTATTTGTGGAACAAAGAAGTCAAATATAGACATTTTT
TATTTTTATTTAAAATACTTTAGATTGATTCAGTAATATAGAATGGATTGTGTTACTACGCTTAAGATATTAG
TGATCGGAGAAAGTGGAGTTGGAAAATCTAGCATTATCTTAGCGTTTACAACTGGGGATTATAATTCGTCATT
CCCAGCAACAATCGGAGTAGATTACAAATGTAAGGTTATGGATGTGAATGGTTTGAAA
GTCAAACTTGGTATATGGGACACAGCCGGTCAAGAGCGGTACAGAACATTGACAAACAGTTTTTATAGAGACG
CACACGGTGCAATTTTAGTTTACGACGTGTCCGAACCGAAAACACTGCAGAAGCTCAACGAGTGGGTTGAAGA
GTTACAAGTGTATTCAACTAAGAAGAATATAGTTTGTCTTGTAGTTGGCAACAAAATT
GACAAACCACGCGCCGTATCAAGGGAGTCGGGACAGGCGTTCGCACAAAAACACAGAATGCTCTTTATTGAAA
GCAGCGCGAAAACACAAGAAGGCATCAATTTGGCATTCGAAGAACTTGTACAGAAGATAATAGAAACACCTGG
CCTTTGGGAGTCAACCGGTTCATCGTCCAACATT
>1002052882_2 Heli.2-DT668473.3.5
ACTTCCACATCGTTCAACGTTGGTATCGGTGACGGTTCGAGACTGTCTAGCAGGGTGCTCCGCCCCCCTGGTG
GTGGTCATACGGACATCTTTGGAGGTGAGCCTGAGCCCCCGCGGGGACGTCGTCAAGCGCCTTCGGCTGCTGC
TAATCTTATACAAGGTCAATCCAACGAGCCATCACAACCAACAAATGGTGATACCCCT
ACCCAAAATGGTCAAGCAAGCGAACCTGCTCCTATCATCCAAGCAGAGCCTGCCGCTCCCCAGTCCACACCAG
AGCCAAGAAGCCCCGAGCCATCGACTCCTGACTCTGCATCCGCGTCCGCATCACCCAAGGCTGTCACCCCCAC
AGAGAGGAACGATGCTCCAAAACGTGTGAGGGTACCTCCGGGTGGTTTCTCGTCGGGT

CTGTGGTAATGCCATGTGATGGATGGATGATTGGTATGAAATATGATGTTTAGTAATGTGTATATGGAATTGT
AGTCTTTTTTTATATATGTGTAATTTCAATTGGAGTTTTCTTTTTGATAATTTGTTCAGGTCAATCGAAGTCT
CCATAGCTCTCTCCATATCTGAGTCGAATTCTTTTCGTATATGGAGGGAACATTATGA
TAAGACATTTCATTTCCCACGCACAGCACAATTATTTGGGACATCACATGTTGAATGGTGGAGCTTAAGCGCT
TTCTCTTTTGTTTAGAGCTAGCCACGCAAAAATAAAAAAATGAATTATATAAATTAGAGACCGTCTAGCGGCA
CAGCGTCTGAGTGTAAGCGTGACGATTGTGAGTTTGATTCTCATTCAAAGATAACAAG
TCAAAGCGCGGCATTAAGTTCCGCCATTCATCTCGAAATGTCTCGAAAATTGCACGAAGATACAGCCGGAAGC
TAATAATAAAAAAAGTCTTACTCCTTATTCTAATTGAATTTTTATTTCGTATGTATATTATTAAATAAATAAA
TATATTTTATTACAATACATCCTCGATCCAAGGTAGTCGCTTCTTTTTGGTACTTTTA
AATCCTCGCAAA
>1002052883_2 Heli.2-EL599892.1.5
GCACGAGGGTGAACACTTAGGAGTTTATAAACTTTAAAGTGTGCGATTCGCGTACTTTCTTTTTCAAGTTTAT
TCTGCAAGTTAGTAATTAAAAGGAGAAATGTCTCTGCTACCCTATTTGTTCGACGATTACTCTTTCAGACGAC
CGCGTCGTCTCATGGACCAGGATTTCGGCTTGGGATTGACACCAGATGATCTTCTTAC
GGTTGCAGCGGGACCTCTTCTTACAAGAGAATATTACAGACCCTGGCGTCACATGGCAGCCGCGGCACGAGAT
CTAGGCTCCAATATCAAATCTGATGCAGACAAATTCCAAATCAACTTGGACGTCCAGCATTTCTCACCTGAAG
AAATATCCGTTAAAACAGTCGACGGTTTCGTTGTAGTGGAGGGTAAGCATGAAGAGAA
ACAAGACCAACATGGCTACGTTTCCCGTCAATTTGTAAGGAGATATGCTCTACCTGAGGGTACATTACCAGAA
ACCGTCGAATCTCGTTTGTCTTCTGATGGTGTCCTAACAATAACTGCGCCCAAGAAAGTTCCAGAAGCTATAA
AAGGAGAGAGGAAGGTGCCTATAGCCCAAACCGGTCCAGTTCGTAAAGAAATCAAGGA
TCAAAACGAGGGAACCAGTGATAAGCAATAAAAAATAAACATTCCAAAGAGGCAGTGCCATTTAAGACTGATA
ACGTGTGTATA
>1002052884_2 Heli.2-DT668410.3.5
GCACGAGGGTCGCGTCGGCTGAACCTGTATTTTTGGGTACACCTTGTATTCTATTTATTTATGTTAAAAATGC
ACTTTTTATATTTATAACTGTAGTTAAAATTATAAATAAAAATGGATTTGTTGAGTGAAGCAAGTAACGGTCC
AAGATTATTAGCGGCTCGTCATATAAGCGCTGACCCACCACTCCAAGAAGTAAACAAT
GCAATCAATACAACGCTGTTGTGCCATACATATAGCCTTCAACCGGATTTCGCCACATACTTTGCAGTTCTCA
AAAGTAACGCGTGGGTAATTTGGTCATGCGGTCTCGTGGTTCTGCTGACGTTATGTGTTCTCTACATAATCAC
GCTGCGGTCGGCTCTACGATATTGGAAGGAGTCTGGTATCAGCATAGCGATAGTTCTA
GCTGTATATCCTGTGGTAGCAGCTACGGCTTTACTCACCACAGTGTTACCTCGAGTTCGAATCTTATCTGAGG
CAATTGCCCAGGAGGCTGTGATGGTAGCCATGTATCACTTCTACTTTATTATATTAGGGGAATGCGGTGGAAC
TAATCAACTTATAAGACGATCCGAGGGCTCTCAGATGGAAACAAGAGTTTTGCCATGC
TGCTGTTGGCCTTGCTGCATCCTGCCCAGACCTCAAGTTCAAAAAAAAAGCTTAACTTGGTTGCGGTACCTGG
TGCTACAGATGCCTATAATCCAAGCTATACTCTACTTGATAATTCTCATACTCTGGTCAGAAGACATGATGTT
ATATATAAACAGCTTTACTTACATCCAGCCATTCATAGTGATATCTATATTATCTGGC
GTTTGGGGTATAATAAATGTGTGT
>1002052886_2 Heli.2-DT665921.3.5
GCACGAGGCCGATTCCTATTTAGATATGTAACAAATTCCATTCGTATTCCAATGGATTGGAATAATGAAATAA
CAATAATGAGATTATCTGTGTAAATTGTGATAAATATATTTACAAACTATTTTGATAGTGTATTAATCTAATA
AACTATGCCTGAGCATTTGGAATTACAACACCTTGTGGATCTCTCAGTCGGTAGCGGC
GACGAATCTATACCAAATTTGCTGGAGAACAAAAGGCCACTTATCAAGAGATGTCCTTACCTTGGATTGATAC
TAGCGACGTTATCGTCGTTATTTTTCTCATTATGTTCAGTTATCGTCAAAAGTTTAGTGAATATTGATCCAAT
GCAACTCGCAATGTTCAGGTTCATAGGAGTACTACTACCGACAATACCTATTGTGATA
TACACAGAGCAACCGATATTCCCGCAAGGTAAACGTGTTTTACTAATATTGCGTTCTTTTGTAGGAACAGTCG
GCTTAATGCTAAGTTTCTATGCCGGCAGAAACATGCCTCTAGCCGATGCATCTGTTATTGTTTTCTCTGTACC
AGTGTTTGTTGCACTTTTCGCAAGAGTTTTTTTAAGAGAACCATGTGGTATTTGGAAT
ACGATTTCCATTATTCTTACTTTGATTGGTGTTATATTAATCACTCATCCTCCATTTATATTTGGTGATACAC
CATCTGAGACTAATCAGAATTATAATAGCTTAAGAGGTGCTATAGCCGCCTTCGTTTCTACAATCTTTGGGGC
AAATGCCTATGTTTTGCTAAGAGTGCTAAAGGGATTACATTTTTCTGTAATCATGACC
AATTTTGGAGCCATTGCAATTGTCCAAACACTTTTCTACTCTTTTATATTTGGTGTTTTGTGTATGCCAAATT
GTGGTACAGAAAGATTTTTAGTTGTGTGCCTCGCCTTATTTAGTTATTTAGGTCAGATTTTACTTACAATGTC
TTTACAGATGGAGCAGGCTGGACCTGTCGCCATTGCCAGATCAGCAGACATTGTTTTT
GCTTTCCTATGGCAAGTCATGTTCTTTGATGAGATACCTAGTAAATTTTCAATATGTGGTGCAATATTGGTTA
TGAGCTCTGTACTTCTAGTAGGTTTACGTAAATGGGCATTAGCGTTGCCCCCAGATTCA

>1002052887_2 Heli.2-EL599263.1.5
GCACGAGGATTGCACATTACGTAAATCAGCGAGCAGAGTTCTATAAAGTGCCAGGAAAAGAGAAAAATGCTGA
AAGTAGCGAGGCTTATAACACAGCACGGCAATAATGTTCGTGCCCTGGCAACAGCGGCGGCCTACAAGCAAGC
CCTAGTCAACATTCCCCCAACTCAACTTACAATTCTCGACAATGGGGTTCGCGTTGCC
TCTGAGGACTCCGGTGCTGCTACAGCCACCGTAGGTCTCTGGATAGACGCTGGCTCAAGGTACGAGAACTCCA
AAAACAATGGAGTCGCCCACTTCTTAGAACACATGGCTTTTAAGGGAACAAGCAAAAGATCACAAACTGACTT
AGAATTACTCGTTGAAAACATGGGTGCTCACTTAAACGCTTACACATCCCGGGAGCAA
ACTGTGTTCTATGCTAAATGCCTCGCCAACGACGTACCTGCCGCTGTAGAAATCCTTGCTGATATTATTCAAA
ACTCATCACTCGCAGAACCCGAAATCGAACGAGAACGTGGAGTTATTCTACGTGAAATGCAAGATGTCGAAAG
CAACTTGCAAGAGGTTGTATTCGACCACCTTCATGCCACAGCATTTCAGGGTACTCCC
TTAGGACAAACCATACTTGGACCCACTAAAAACATCAAGAAGATCTCTAAGGCTGATCTCCAACAATACATTA
AGACTCACTATCAACCAGGCCGTATTGTCCTTTCTGGAGCTGGTGGCATTGAACATGGCAAGCTTGTAGACTT
AGCCAACAAGCATTTAGGTGGA
>1002052888_2 Heli.2-EL597889.1.5
GCAGTACTCGCAGGAGTCGCAGCCGCGCGGCTCGCTGGGCCGCCGCGAGCGCGTGCTGGTGGCCTCGCGCCAC
CTGCGCAACGACTCCAAGACCATCCAGCTGGAGTGCGCCGCGCCCGCGCCCGCCGCGCGCGACCTCAGCCTGG
ACCAGCTCAAGCAGCTCGCGCTCAAGAGCATGGAGGGGCTGGAGCTGCCGGCCTGCAA
GCGCGACGACGAGACCAAGCGCCTGCTGGAGGCGGGCGGCGTGCTGCGCCACCGCCGCACGGGCTCGCGCGAC
CTCAAGCCGCCCGCCGGCCGCCACAAGCGCACCTCCTCGCACCACATCACCATGGAGCCGCACGAGCTGAGCC
TGCAGCTGCAGAAGGGCCGCAGCGTGGACCAGCTGGCCGCCCCGAGCGTGTGAGCGCA
CCCGAATCTCATTGTGCACTGGACAGTTGACACTGTGTTGGGAGGAATAGAAAGAATAATTTATGCAATTGTC
GCTTCGGACGGATGATGATAACACATTAGGCTAAACGAATTATTTCTTCTATTACGCTTAAGCGACCGTGAGT
GATTTCGAACCATATCTTAAATGTAGTTTAATTAACGCTTATCGCGATTGAGATCAAA
CGTTCTCATTGCGTCTCTTACTGTAGCTATATATTTAAATAATCAAAACGGTAAGTTTTTATTTTGTGTTCAT
AGTCAAAATGGTCGTAATTTAATAGGTTTAATACTCACATCACTTTTGCTGTCACTGTGCATATTTCGCTTAT
AATTTTGGCCTAACTATGCCTAGGTAATTTCCTTAGATTAAAAAATGCAAATTGCCAT
AAAAAAAATACTATATTTTATTCTAAACTATTTCTGGAATAAAACCAGAGCTGTACTTAAATTTTACAAATAC
AGGAAAAAAATTATGTCATTGTCATAACATAACTTTTTAACATTAATGGAAATTTTATCAAAACAAGCACTAA
AGAACTCTTAAGTAAGATATAAAGCTATAAATCTCGTAATAATACCAAATATCCTTTA
CAATAATAGCTAAATATGTGCAATTTTAAATAAATAACTACAGTCTCACATATTTAAAGATCTATTAGTATAT
TGGACTGTGCAAAGCAATATTTTTATAAAAATTAAACTTTTAATAGTAATGTAATTTATCATTAAATGAAAGA
AAATAAATGTTTAATTGCTAAAAATCATTATTTTGGAAAAAAATATTCCTTTTTTATTA
AATTTAGCATCTCACCTAAACCAATATAAAATAACTGAAAATTATGGAAGGCAGGCAGGAAGAACCATCTCAT
ATGAGAATTCAGCTGAAAAAGTGTATTCTAGTGAACAATAAAGTATA
>1002052889_2 Heli.2-DT666919.3.5
GCACGAGGGGTAGATTGCTTTAAAACAACAAAGAAAAATAATGCATAATGACGAACGCATAATAAAGTTAGTA
CTGAAATTTCCTCAAATGTTCATGTGAGTGACTAATGTAATAAACTCTGATTAGTTCTCGCGCTTCGCTAGTA
AAAATTGTCCTTGAATATATTACCTTGGACCACGTAATGTCGTCCTCGGAAGAAAATG
AAAACTTACCCTCAAATTTCGATGATAAAACCAACACAGCAGCAAGAGATGATCATCAGGAAAGAGAGCAAGG
TCAGTCGTCATGGTCAGCACCCAATACCTCGACGCTAATTCAAGTAGAAGAAAAAATGCGACGTAAATTACAA
TTTTTTTTCATGAATCCAATTGAGAAATGGCGCGCCAAGAGAAAATTTCCGTACAAGT
TTGTAGTACAAGTTATCAAGATTGTTTTGGTGACACTACAACTTTGTCTGTTTGCACATAACAGATATAATCA
TGTGAACTATACCTGGGACAACAGAATCAGTTTCTCCCATTTGTTTTTGCTTGGCTGGGATTCTACAAGGGAA
ATTAATGCTTACCCACCAGGTGCAGGACCTTTAGCAGTATATAAGGTAGATGAATTTT
ATAATGCCTTGGACTATGCCTACACAGGATATTCAAATTTAAGCAATGCCATTGGACCATATTCTTACAACAA
TGAAGATAACAATAAAACAGATCCCATGTTTTGCACATACTATTACAAAAAGGGAATAATTCATGGTTTTAAT
GAAAGCTATGAATTTAATTCAGAAATAGTATACAGCTGTGTTAATTTACAAATGAAGA
AACAAACTTTTTAAATCAAAGGAATTTATAAAAATGTCGGCATAGAAGTCATTTTGCAGC
>1002052890_2 Heli.2-DT668349.3.5
GCACGAGGCGCGCGCCCACTTAGGTACCAAACGGGACGCCGGTTGAACGCCATAGTTGCTCCAATTGCAAGAT
ATCTTTCGCTTCGCTTCGCTTGGATAGGAAAGAAAGAGTGCAGCGAGTTACAATGCGGTGATCGCTGACATTT
GTACTGTGTTTAAGTGTCAATATCAAGTGCATAGCTTTCAAAGGTGTCCCAACATGTT

TATTTTAAAGGTTGCACTTTTATTTTTCATGGTCATCCTCGTGGTGGCTGAGAAAAAAATTGAGTTGCAGGAC
ATAGAAGAAGACAATTTAAGAAGTGAAAAGGAAAAGCAATTATGAAAGTCAGAGGCCCGATCTCAAGAACCAA
ATTCAGTTAGTGCTCCCGATTTACCACTGGATTTATTAAAAAATGGATTTTTCCAATA
TTTCAAGGAACCGGTCCAGACGCCTCAACAACAACGTTTTGTACATCAATATGCTGTTACAGAACAGCCAGAG
AGACCACCTCCACTGCCGACCCCTCAATATGGACCACCAGCTCCACAGCAAGCTATGGTTGGATATTTATCAA
ACGTGCCAATGCAAATATACTTAGTGCCACAATACTATAATGAACCTTCTGAACAAGG
GATCCACCACCAATCACAAAATGGTGTACAGCAACAGACCTTGGCTCAAGTATCAGGCTATGAAAATAGACAG
CAAATACAACAGCAACCCGCATACTTTGAAGTGCCGACATACATTACACCAACAGGCAAGACACTCATTCAGC
AATATACTCCTCCAGTATCTTACGTGACTTACGCTCATCCGACCATGTCGCCAATACA
ATCCACAGTTACACCGGTGCTAGCATATCAAATGCCCGTCGTTCGCTATCCTACAGCAATCTCCGCCCCTCCA
GTCAAGGGCTATTACCAAAGTACACATTATCCAGAGACCAATGTTGTCGATGAAGATCAAGAAACTGAAGTGA
ATATACCTAAGCCATATCCTAGTCACAATGATATACATTCTTCANAGGCCTTAGGCTT
GGGTTATCCGAGATATTATAATTCCCGGACCCCAGTAACAGAAGGATATAGATCTGCACCCATTGAGCTTCCT
CATCCCAACCCTCTTCTGTTAAAAGCACCCCCATCTCACCTGGCACA
>1002052891_2 Heli.2-EL600991.1.5
CGGCACGAGGGGCACTACGGCTCTCCGGCCCTCCCTCGAACTTTCACCTCCCGCGATATCTTCATCTGTGATA
AACACCAAGTGAAATTGAATGTGCGTTCCTAGAAGAGATTTCTGCGACGGCACGCGGACGAACGGCCGCCGGC
GAAGTCGATTTCTGCAAAATAATTTCTCCGGTGCATTTCGCTTCCACGAAACAGCTTG
TGTAGAGTTTACCGAGAAGCGAAGCGATAGAGTGGATTTTATGAAATATCAGTAATTATAAGTGAACTGTGTT
TCCGTGTTCTGTTGTTAGTTACGGCGTTTACATTGGATCCCAACCAGATCGCACCCCCAATGGTGGTTAAACT
ACCAGATGCATGCCTTGAAGTGGGGGTTCCACTCAACAGTTGGTCAGCGGGAGTTGCT
TGCTGTCCAAACTGCAGGCATGAGCTCCGGGTGTCCCTGGCATGCCCCAAGAACCACCATGTTCCCGTGACAC
CACTCACTCCTACTTTCACAGTCCAGCCAACATACTTGCCACAGATCCCTTTATATAATACACCTTCATCACA
ATTTATTACATCTCCTTTAAATGATGAGTTGAAACGGTTAGCCGAGACTCTTAGAGCA
TTAAGGTTATCAGGCTGGTACTATGGAAATTTAGATTGGCAGGGTGCTCGGAATCTATTGAAGGATGCAAGCG
TTGGTGCATTTGTGATCAGAGATTCAGGAGATAGGAACTTTATATTTTCTCTGTCAGTACAAACGGAGAGGGG
ACCAACTTCTGTGAGATTGCATTATGAACAAGGCTGCTTTAGGTTAGACTGTGATAGG
CCACTAGCAAGGTACATGCCTCGGTTCCGCTGCGTT
>1002052892_2 Heli.2-DT668401.3.5
GCACGAGGGGTAAAACGTCTTTGGTAGATGTTGATTATTCAGATCAAGCTAACCACCAATCACAATCTTACAG
CTCTAATGTTATTCATGAGAGTCAAGGAGTTTCTCAGCCTGCAGTTTCTATTACTCATGTTGGAACTGACGCA
CAAAATATTGACGGATATAGTTATGTCACTACTCCTACTTCATCTGGTATTCCAACTA
CCGCAAATCCTTTGCCAGTAACATACAAAACAACATATGTACCAGAAGCGCCTAAGAGCAGTATTAAGCAAGT
ATTTGGTTACACTCAGCCCGCTGTTACCTACGTTCAACCGACATTAATTCCCGTCAAAGTAACTCCACAAATT
CAAGTTACTAATTATAATCAAGGTCTAAACTATCAGTACGAAAATAAGGACTATGCGC
TTTATAATTCAGGATCAGCTAGTAATGCTGAGTCAGGATATGACTACTCAAAGCCTGCAACAAAATTTGTAAG
CACTATTACTTACACTACTCCTACTCCAACAGCTGCTGTTGTCTCTTATAAGCCACAAGGATTTGGTCACAGT
CATCAAACTATTCATAAAGTGGAATCAGTTGGATTTGAATATTCTACTCCTACCCCTA
TTATTGAGGAACCATTTAAAAAAATAGTAGCATATACAACTGGTCCATCTGTTTCTACTTACGTACAGCCAAC
AGCTCAGTCGTTCAGCCATCAAACAATACATAAAGTTGAATCCGGGAATGTTTACACTCCATCAGAAGAAGTA
ACTGGAAGCCAATCTCTTTACGAAGTTCCACAAACTGTCGTGCAATATGCAACATCGA
AACCAATTTCAGTACAAAAGGTGGCAATATCGACAAATGGACTGCAATCAGTGAGTCAAGGTCAATCAGAAGA
CAGTTATGAATACAAGCGGCCATTGGTACAGTTTGAAGAAGCGCCAAAAATAATTCAATATTCCACACCCGCG
CCTACGGCATACAAATCTCCAAGTTATAGCTCACAATCTATAACCAGATATAATACTA
AACAATATGTCCCTATATCGCCAACTGTACAATCCTATGTATCCTCCACTCCCGTAACTATCAATGAGAATCC
CATTCTCAAGTATACAATAAAGTCTGCTCCAGTGACTTTAGTTAGCTCTACTTATGCTCCACAGTCTTATAGT
CAACAAACTGTTCACAAGGATA
>1002052893_2 Heli.2-DT664699.3.5
GCACGAGGGCGCAGCGTATAAGGTTACCCGGTTGCAGTCGACACAACCAGCCCTGTGTTTTGCCAGACTTTGG
GCCGGGCAGTGTGACCTGATTCCAGTCGACGCAACCAGCCCAGTCGCTCCTAGTGCTTGGGACGCGAAATGTG
ACCTAGTCCAGTTAACACAGCCACGTACATCAGACACACACAGATACTTCACTTCTGG
CCTCTGTGGTCAATTCTGCGTGGTCTTAGTGACCGTGCTGCCTGCCTGCCCGCCTGCCTACCAGTCTTCGGGC
CAGCCAGTGTGACCTGCTTCCAGTCGGCGCAAGCAGCCCTGTTTCTGGGCCAGGCAGTGTGACCTGCTTCCAG
TCGGCGCAAGCAGCCCTGTTTCCGGGCCAGGCAGTGTGACCTGGTTCCAGTCGACGCA

ACCAGCCCAGTCGCTCCTAGTGTTCGGGACGCGAAGTGTGACCTAGTCCAGTCAACACAGCCACGTACACCAG
ACACACACAGATACTTCACTTCTGGCCTCTGTGGCCAATTCTGCGTGGTCTTAGTGACCGTGCTGCCTGCCCG
CCTGTCTACCAGTCTTCGGGCCAGCCAGTGTGACCTGCTTCCAGTCGGCGCAAGCAGC
CCTGTTTCCGGGCCATGCAGTGTGACCTGCTTCCAGTCGGCGCAAGCAGCCCTGTCTCCGGGTCAGGCAGTAT
GACCTGCTTTCAGTCGGCGCAAGCAGTCCTGTCTCGGGGTCAGGCAGTGTGACCTGCTTCCAATTGGCGCAAG
CAGCCTCGTCTCTACAAATCTCATGCCGGGCTGTTCGACACAGCTCGCCTCGTTTCTG
CCAGTCTGCAGGCTGAACAGTGTGTGACCTGGTTCCAGTCGACGCAACCAAACCCGTATCTGGCCAACTTCAC
ATCGGTATGACGTAGTTCCTGCCTCTGTCAATCTTCGGGCCGGGCAGTGCGACCTGGTTCCAGTCAACGCCAT
CAGCTCCGTCACTGCAGTCTTCGGGTTGCGTAGTGCCACCTGGTTCCAGTTGATGCAA
ACAGCCTTTGCCTCTGTGGTGTACCCTGCATGGTCTTGGTGACCGTGTTGCCTACCAGCCAGTTCTTCAGGCC
GTGCAGTGGCACCTGGTTCCTTTCGACGCGATCCACCCTGTTTCTGCCCGTCTCTGGAGCTGGCAGTGTGACC
TGGTTCCGGCCAACGCAACCTG
>1002052894_2 Heli.2-EL598396.1.5
GCACGAGGGTCGTCCTTTAAGGGTGTTAATTGTAATCGTGAATTTGTGTATTTTGATTTAAATCGTAGGGCGA
CCCGATACCTCACCTGTCTTAAGTACTTAAACCAGACAAAATGTTACACCTTAAGAATATAGCAAAATCGGTT
GTGCCGCCCCTCAAAAATAACATCCAAAATGAGGCAGTCAATAATATGCTAAAGATCA
CGCCTGCAGCTGTAAACGTTTGTTGCCGTACCTACGCAAGTCATGAGATCCCAGACAGGCTCAAAGACATACC
GACCAGTGCTAATCCCAGATTTTTTGACATGGTGGAGTATTTTTTCCACCGGTCGTGTCAAGTAATCGAAGAC
AAATTAGTTGAGGACATGAAATCCAGAGTTTCAATTGAAGAGAAAAAGAAGAAGGTGG
CCGGTATCCTCAAATTAATGCAGCCATGTGACCACATTCTCGAAATTCAATTCCCACTGAGACGCGATTCCGG
CGATTATGAAATGATTTTAGGATATCGGGCACAGCATTCTTCGCACAGAACCCCAACAAAAGGAGGTATTCGA
TTCTCAACCGACGTGACAAGAGACGAAGTAAAAGCTTTGTCCGCCTTAATGACATTCA
AGTGTGCGTGCGTGGATGTGCCCTTCGGCGGCGCCAAGGCCGGAATCAAGATCAATCCTAAGGAGTACTCTGA
ACACGAGCTCGAGAAGATCACAAGACGATTCACTTTGGAATTGGCCAAAAAGGGATTCATTGAGCCCGGTGTG
GACGTGCCTGCCCCAGATATGGGTACGGGTGAGCGCGAGATGTCTTGGATCGCGGACA
>1002052895_2 Heli.2-DT667393.3.5
GCACGAGGCTACACACCTCCTCAGGATGGACAAGATGGCCTCGCTTCTTATTGGGAACATAGACACATACACA
ATGAATATGGTCTATGGCACATTAGAGGTACGCACGAGGGAATGCTCTCAAGGGCCAACGGCAAATATAGGCC
TTTCATACTAACGAGATCTGCTTTCTCTGGTACACAACGTTACGCAGCAGTGTGGACG
GGTGATAATGCAGCGGAATGGGGTTTCCTACAGGCGTCAGTTCCAATGTGTATTTCCTTAGCCGCCGCCGGTA
TTAGCTTCTGTGGATCAGATGTTGGTGGTTTCTTCAAGTATCCCGAAGCTGAAATGATGACCAGGTGGTATCA
GGCGGGCGCGTACCAAGCGTTCTTCCGCGCGCACTCCCACATAGAGACCAAGCGCCGC
GAGCCGTGGCTGTACGACGCGAGCACCACCGCGCGCATCCGCGACGCCGTGCGCCGCCGGTACGCGCTGCTCG
ACTTCTGGTACACGTTATTCTACGAACATACGCAAGATGGCCTCCCCGTTACCAGACCTCTATTCCAGGAGTT
CCCCAACGAGGAAGAAACTTTCACTATTGATAATAGTTATTTGCTTGGTGACAAGTTG
CTAATCCGCCCAGTGTTAGAAGCAGGTGTGTCAAATGTCAAAGTGTACCTCCCAGGAAGAGAATCTCATACCA
TCTGGTATGATGTAGACTCCTACCAGGCATACCCAGCTAATGGATATATGAATGTTGATGTTAATATTGCTAA
GATTCCAGTATACCAACGCGGCGGTACAATAGTACCGCGGTAAGAGCGAGTCCGTCGT
CCTTCAGCCTCTAATGGTCAAGGATCCTTACACACTCGTCGAGGCCTT
>1002052896_2 Heli.2-DT667141.3.5
GCACGAGGCAAGCCTCAAGTCTTCGGTGAATGTTTGACGTTTGTGAAGTATATTTTTAACTCTTTACAAGTTT
ACAAGCTTACAATACGTGCTTAATTAAAAGTATTAAATACAAAGAAGAACAAGTAAAATTATCGTAATGGAAA
TACATATAATACCTGACACATAACGTCGATATAACCATTTAATTGTTTAAATTGTGAG
GTTGTAACTATACAGCAGGTGTATTTTTGCTATTATTTAATACAAACAATTTGATAATGACGGAAAAACAAAA
AACGCTCGTTTGTGGTGATGTAAACGGAAATTTTAACGCACTATTTTCTCGTGTTGAGAGCATTACAAAAAAA
TCTGGGCCTTTTGATGTATTGCTCTGCGCGGGAAACTTCTTTGGTTCAAACAATGCTC
AATTAGACCCATATCGTTCAGGCGCAAAAAAAGTTCCAGTCACAACTTACATATTTGGTCCATCTAATAGTGA
TCATGTTCCATATTACTGTGAAGAAGGTGCTGAGATAGCTCCTAATATTATTTATATGGGTACAAAAGGTATT
TTTACAACTAGTGCTGATGTTAAGATTGCTTATTTAACTGGCCTGTCGAGACGTGAGC
TGGGCAAAGATATACCAATGTGTACATTTGAACCTAGTCATTGTAGTGCTGTGAGAGACGCATGTTTTAGAGG
ACAAAGTGAATATAGAGGTGTTGATATCTTAATTACTACCGTGTGGCCTGTTGGTATACAGCAAGACAATAAT
CAAAAGGCAGATGTGGAACCAGACTGTCTATCAGACTTAATATCTTGGTTATCAATAC
ACATTAAACCTAGGTATCATTTTGTGCCTGCAAACAATAAATATTATGAGAGGCAGCCTT
>1002052897_2 Heli.2-EL596295.1.5

GCACGAGGCGAGTTCTTTTTTTTTTTTTTTATATAGTAAATGTTAAGTTTATTTTATTTTCAAATACAGATCA
AACTGAATTGGAATGAATCACATTTTGGGGCATGAACATGCTAAGCTGTAGGATATGCATATACATATATAAT
TTATAAATAAAAAAAATTAGGACAGGGGAAATTACAACTTCTTAACCTTTAGCTAATCC
TCACTAAAATATCACAGTAAAAATTAAATTAACAACAGAGTTCACTCAGCTTCAAATAGATTGAAAATCAGAA
ATAAATACAAGTTCAAGATATTTTAATGTATCAGTGATTAACTATTTCTCTAACATCAACAAAGAGACACATA
GCTCTTCTCATTCATACACTTTAAGTATTCCCATGAGGATTTGCTTAAATCTTATAAA
TTAAATTATACTATTCAATATTCGTTAAATCTCTTGTGTCCGCCTCCGCCATGTCCATTGCCCCTGCCAAAGT
TTGATCCTCTCTTGAATGAGCCACCTCCTCCGCGGCCACCTCCATACCTGTTTCTATTCCATCCACCGCCACC
ACCACCACAACGGTCAGCCATTCCTTGTAGTTGAGGACTAATCACCTGGTTAGCTTCC
TGTAATACTGATACAAGGTCCTTAGCCTGTCGCGAATTTGAAGGGGTGAAGAAGGCATATGATGTTCCTTTTG
ATTTAGACCGTCCAGTTCTACCAATGCGGTGAATATAATCCTCAGATGAATTAGGATAGTCAAAGTTTATAAC
ATATTTAATTCCGTCCACATCTAGTCCTCTAGCT
>1002052898_2 Heli.2-DT662881.3.5
GCACGAGGCCGCGGCGTCTCCGCGCACGCCGCTTGTCCATAGAGAGTTCGCACGCCGCACACGCACCGGCCTA
TGTTAAATAACTGTTATTTCGGTCTTTAGTGGAAATGTTCCGCATTCTGAACTCAATAAACTCTCATCTCGAT
TTTATTATTACATCACCACAGATCTTAAGTGGTTAGGCGCTGTTTTTCGTTAACATTT
AGTGAAGATGTTTTTACTCATTTTTTATTGACGATGAAAGTAAATGTTTTATGAATGAGCGGATGCCGAAACA
GGTTTATTAAATGGATGTGTAGTGTTATGGATTGGTGCAGAAGATTTTAAACATGGGAACTGATCAAAAATTT
TTTACAATGCTGGACACACGGAAGCCGCCCAGACGCTCTATGTCGCTCGTCGGTCCTT
ACTTGAAAAATCGTGTAAATAGAAGTGACGCTGAGGATAAAAGTCTTTCAAATGATGAAACACCGATGCCATC
CTGTCCTCGACAACCACCCGACGGTGTTGAGACGCCTTCAGATGTTGTCGAAAAAACCCCAGAGTTGCCTAGT
AGGTTACCTCCTCGACTTTCAACAGGATGGCCTGGCACACGTTCTCGTTTTTCATATT
CTTCAAGAACTCAAGATGCAAGAAGTGAAGCGAGCGTTAAAGATAAAATTGCTATGTTTTCTGTTGATTTGGG
GAAATCCACCGATCGACTCGATAAATGTGGAACTTTACCAGTTAGGTCACATTCAACTTTAAGAAAAACTCCT
AATATATACTCAAATAATTATAGCGAAGTTTCATCTTTAGATAGGAGGGTAACGAAAT
>1002052899_2 Heli.2-DT665485.3.5
GCACGAGGCAGCCATCTTTTGTGGAAATATCATTTGTTTCGTGTCGTCAGGGTAAATTTCATAATTGTAATTA
ATGTCAGAGCCACAGTCCTCGCTTTTATTAAAAAAGCAATTAGCAGAACTAAACAAAAATCCAGTAGAAGGTT
TTTCTGCTGGGTTGATAGACGACAATGACATATACAGATGGGAGGTCCTCATCATTGG
CCCTCCAGACACATTATATGAAGGTGGTTTTTTTAAGGCACATTTACACTTCCCTAAAGAGTACCCTTTGAGA
CCGCCTAGGATGAAATTTGTTACTGAAATTTGGCATCCAAATATTGAAAAGAATGGAGATGTCTGTATATCAA
TATTACATGAGCCAGGTGATGATAAATGGGGTTATGAGAAAGCCTCTGAAAGATGGCT
GCCTGTACACACTGTTGAGACTATATTGATAAGTGTTATTTCTATGTTGGCTGATCCTAATGATGAAAGTCCT
GCTAATGTTGATGCTGCAAAAGAATGGAGAGAGAGGTATTCGGATTTCAAAAAGAAAGTTGCCAGATGTGTTA
GGAAAAGTCAAGAAGATTGTTTTTAGGATCAAAGGGAATTACACAGCAGAATGTCATG
TCGAAAAAGGGCCACGTGACACCATTGACATATATAATTTAACATTAGGTTGCACACCCACCGGCGCCAAACC
AAATTTATAGTAGTAGCTGTGAGCTCATTCTCGTTCTTTTTGCAGAAAGAATGGAGAGAATCATATTCGGAGT
TTAAAAGGAAAGTAGCGCAGTGTGTGAGAAAGAGCCAGGAGGATTGTTCATAAATAAC
AGTGCCCTTTTTCATTTATTTATTTGGATTGTGAGTGTTGGGTGACAGTTTGGAGGTCTCGCCACAGTGTGAA
GACTCGCCACAGTGTTTTTTCTAACACCGGGCCAGAGACAATATTGAAATTGATTTAATAAATAATTATTGTA
ATTAAATGGTATCGCTGCCGTATTTTATAAATAATAAATTAAATGTAGAACTCGAGGT
CTTTTTCATCTTTTTTAAGACATGGTAGTTAGCTATTGTTATAATTAAAATGGTGTACGATATTTTTTCCACA
GAAAATAAATCTGCATTTGTTCTTTTGACAGATCTCTTTTTAGTGAAACCAAATTTGTAATGCAATAAAAACA
TTATTTATTAGTCGCTAAAATGTATAGTCATGGCTGAGACAAATCATCGAAAATTGTA
TCTGGCAATTTTTGTATTTGTAAATTTTAGTTACCATAGTTAATGCAAGGAATCATTGATAGGCTTGTGTGGT
CAGATTCTTAAATTACTGTTTTGTTTGTTATGCTATCTTAGTAGTGAAATTAAAACTTATTATTTTAATGAAT
AAATGCAAATTTTTTAATATTATCTTTTTATATCAATATACCTTGCCCATACTACACA
CTAGCTACTTATAGCACCAAGTCGTCTTTCATATTGACAAAGTCAAGTTACTGTTGCAGCGTAACCATTGCTT
CTAATAATTTACTGTTAAAAACCTGCCTTTTAATGATATCTAAAAATCAATGGTTCACT
>1002052900_2 Heli.2-EL596877.1.5
AGCTGCTCTGAAGAAGGATCTGACCCCGCAGTCCATTTGTCACATCAACGGACAGTGCAACTATCTATACCAT
TCACACAATTTGTACACATTTGATGATGAAACTAAGGATCTAAAGGCTTCTGATGACATTCCATGTGAATTCT
GCGAACAAGTGATCCAACACTTACGCGACACGCTTATAGCGAATACCACGGAATTAGA

GTTTCACAAAGTACTCGTTGGTTTATGCAAGCAAACGGGCGAATTCAAATCCGAATGTCTCCATCTCGTCGAG
CAATATTCTTCAACAATCTATAACTTCTTAGTATCGGAATTAAAAGGTGCAGAATTATGCAGTTTGATTGGAA
TCTGCACTAACAAAACTGAAGTAACTATTGCACCTCTCATCCCTAAGGAGTTGGCTGT
GAAAGCTGTTAAAATGTCAACCGAACTTATTGGTCAAGATGAAGCCAATAGCTACATTACAGAGAACACCCAA
GCAAAGAAACATGAGAACGTACAAATATTGGGCACTGAAGCGAGCCAGGTCCCCGTGTTGCCCATAGAGAGGA
TGTTCGTGTCCGCGCCGCGCTCCGACGCCGTGTGCTCCTTCTGCCAGTACTTCCTGCA
CTACATACAGGTGGAGCTCAGCGACGACAACACTGAGGAGGCCATCAAGAAGGCGGTGGAGGAGGCGTGCGAC
GTGCTGCCGGCGTCCATCGACGACGAGTGCCGCCAGTTCGTGTCGCAGTACGGGCCCGCCGTCATCGCGCTGC
TCGTGCAGGAGATCGACCCGGCCAGCGTATGTCCAGCTTTGGACTTGTGTCCTAAAAC
CGAGGAAGTACGTCGTGTTGATATAAATGCAGAGAAATCGAACTGCCCCCTTTGTCTGTTTGCTGTAGAGCAA
CTTGAAACCATGCTGAAGAATAATCGTAGTGAGGAAAGTATCCGTAAAGCATTAGACGGACTGTGCAACCACC
TGTCAGCGAAACTGCGCACTGAATGCGTCGATTTCGTGGACAATTACACCAACCAATT
GGTAGAAATGCT
>1002052901_2 Heli.2-DT665609.3.5
GCACGAGGAAAGATTCTCTGTTGTCTTGTTAAGACACCGCTTCAAATTAATATATAAACAAAAAATAAAATTG
GATATATAGTGTGTCGTAACCGAATATATATGAAAATTTAAATAATTACGTATAATATAAAGCCATAAGATGA
CTGCCGCTGATAATCAATATCCTAAGGCATCTAATGCCACCTTAATAGGAGCATCAAT
AACGTACGTAGCAGGACTGTTTTTATTATTATCATTTGCTGGACCGTATTGGATCGAATCGTATCCTGAAATG
TTTTCTTCGTTCAAACACATGGGCTTGTGGGAGTATTGTTTTGATCGCTTCTGATTTCCATCGTTTCAATATG
ATAAATATTTCAATGGATGTCATTACATATTTGGTGAAGAACTTTATGTCATTCGAGA
ATGGCTTCTACCTGGTTGGTTAATGGCTGTGCAAACATTTGTGACTCTTGGTCTAATGTTGTCATTTACTGCA
CAGACCCTTCTTGCTTGTGTTATTGTTCGGTGGCCTTTAAGAACTGTGTTAAGATATGAATGGATATTTGTAT
CTACATCTTTTGTTATGGTTTCACTATCAAGTGTTTTCCTATTTCTGGCTGTAGCTAT
ATTTGGAGGAAATTGTTATCGCAGGGACTGGTTACTATACCCTTCATTTAATGTATTATCCTGGTCTTATGCA
TTTGCTGTAATTGCCTTTATTTTATTTGGTCTCGCAGCAATATTATTATTCTTGGAATCACGTAAGTTATACG
AATTGCGTTC
>1002052902_2 Heli.2-EL596488.1.5
GATACACTTAAAACACTCTTAAAACAAAATATTGACAAAACAATAAAGATGCTGATTTATAGCACTAAGACTC
AGTCCGTGAGGGAAGTTATGATAACACCTAGTGCAAATTGGGGTGGACAAGGCCTTCTAGGTGTCAGTATAAG
GTTTTGTTCTTTTGAAGGTGCAAATGAAAATGTGTGGCATGTACTTGAAGTACATCCA
TCATCACCAGCAGAATTGGCTGGATTTGTAGTATTTGTTATTTATCTCTTGTTAATAATGAGTTAACTGCACT
TATATTAACTTATTATTAATTATTAGCTTCACGCTATATCCCGCACACAGCAGTTAGGCAGAGGGAACCTAGT
GTTAAGACACTCCGTTCCCCACTTTCTGCCGAATTCTGGAGGCATTGCGTGTTGAGTG
GCGGAACTCAGCGCCTCGCTTTGCCCTCGACACCAGAGCGAAGAAATGGAAATATAAATTTAAGTAAATATTT
CATCTCCTCGAGTGGGGATCGAACCCACAACCAGTCGGTTTTACAGTCACACCTCGTGCCGCTGCGCCACGAC
TGGCCTCAAATAATATATGTAA-----------------------TAATATGCAATT
ATTACAGTTATT-----------------------CAAATCAATAATTTAACATTTAAGGCTGTTTCTGAA
>1002052903_2 Heli.2-DT665416.3.5
GCACGAGGGCCCCCTGCGCATCATCTGGCAGCCATGGGCTAAACCGACCTTACCAGATTGACACCGTTACCAA
AAGCCTCTAGCTCGAAAGTGACCTCTTTCCCACTGTTTTGAACTAAATATGTTGGACTCGAGTGTTTGATCAA
AATCCTAGCGTGACAGTGATTGTAAAAAGTGGTCAGTTTCATAAAATATTCGGAAAAG
TGCAAAGGATTTAAACACAAAATGATTGAATATTCACAAAATCACTTGTTGTTGGCGACAGTGTTTATGACTA
TTGTCCATGTCACACAAGGACAGTGTCCATGGAAAGATTTTCCTGATTTACAGGCAACTTGTGTGTGCGCCTT
AAATCTAGCAAGGCAACTATCAGTGCAATGTGATCAGGTTGATTTTCCAATCTTATTG
TCTACATTGAACATTAGTGCAAGAAAAAATACAATAGATCTCTTATATATAAATAATTCAACAATCCCTGTAT
TAAGTAATGAAATGTTCGCTAACTTAGCGATACACAATTTACAAATATCTGGATGCAAAGTGAACAAGATAGA
GGACAACGCATTTAGAGGTCAAGGACAGTATTTAAAAAATTTAAACTTGCAAGACAAT
GAGTTGACTGAAGTACCTGTTAGAGCATTGAGAGTATTGACAAGTCTATCCCTATTGGATATTTCTAAGAATA
AAATAACGTATATTGATAATTACTCTTTTACGACTTTACAAGAATTAACAACTTTAAAGTTGTCAGATAATAA
CATTACGTTAGCGCCACAAGCACTGTCTGGTTTAGAGAGCTCACTCAAAAATCTTAAT
TTAAAAGGAACTAAGCAAAAAACAGTGCCTCAATGTATCAAGGGACTCCACAGTCTTGCTTTTCTGGACCTGT
CACAAAACAGTATAAGAGAACTTCCAGGACCTGATGGCGCGCTTACCTTTGATGGATTAGATTCGTTAACTGC
ACTGAATTTAGAAAGAAATCTATTAGTTAATTTAAGAAAAGATGCATTCTTTGGGATT
>1002052904_2 Heli.2-DT663059.3.5

GCACGAGGCGCCCCCCTCTCTGCTCCTCGGACCTCGACCCGTGGACTCCAGCTGCTCCTATAGCTCGCGAAGA
GTTGTGATGTGCCTCGTGAGCTGATCATGTGCAGAGTGTATACAGTGCGGGAGTGCGCGAGCGACGAGCGCCG
GTGGCCCCCGCGGCCGTAACCTGCCATGTGGACGCTCCTGATGCCGCTGCTGGCACTC
CTCACCTCGGGTGGCGGTGACTCTGTCTCCTCCCACAGAGAACTTCTAGAATGTGCTAGCCAGATCCTGTATA
TGGGCCACCATTCATATGAACTTCCAACTAACTACAGTAGAAACATTCCACCCGGTAAGAATACGACGGTTTA
CATCGGAGTGAACGTCAACAGAGTGTCCGGAGTAGACGAGAATAGAGAGGAGATCACA
TTGGACGCTTTCCTGCAAGTTTCATGGGAAGACTTGCGTCTCAAAACGCCTCATCACATGCCCTACATTGACT
TACCCTGGGAGTTTCGACAACTGATCTGGACTCCAGATCTCTACATATGGCAGTTACAGACAATGAGGATCAT
GTCAGTCCTCCAAGAAATGGCTTCGTTACGATTATATGCTAATCGTACTGTCTCTGTC
AGCATAGGTGCTACAATAACTATAAAATGCGAAATGGATTTCGTTCTATATCCATTGGACGTACAAAATTGCG
CTATAGACTTTAGCAGTTACAAATACACAACAGAAGATGTACGTTTCGAGTGGCGGGAAGTAGCGCCGTGGTT
CGGCCTCGCCGGCGGTCAACGCCGCGAGTTCCGCCTACCGAAATACGTGGTCACATTT
GTAACGGACAAAAGTGAACATTCTGCAGCCAGATTGCGAATCAAGCTTTCCAGGGAGTTGCGGAGCTACCTCC
TTGAGAGCTACTTGCCATCATCTCTCTTCGTCATCATATCGTGGGGCAGCTTCTGCGTCATACCCGAGATAGT
GCCGGGTCGTATGGTGCTACTTGTGACCACACTGCTTTCATTGGTCACCATGTTCGAT
ACAGTCAGCACTCATTCCCCAGATGCATTAGAACTAAAATGCATCGAAGTCTGGCTGATCTCTTGTACGATAT
TCGTATTCCTGGCGTTACTGGAATACTTCGTGGTGCTGTTCGGCATCCGCTACGACAAGAGCTGGAGCAGGCG
ACGGCAGGAC
>1002052905_2 Heli.2-EL600985.1.5
GCACGAGGGAAAGGATAGTTGAGTGCGTGTTATATTTTAAAATAAATCCCCAAAAATGGCTGAGCCTGTAACT
GAATTACCTATAAATATAGAAGAAACAGTAAAAGAAGTTGTACAAAATGTTACCGACAAACCCCCGACAAGTA
TCGAAGGAGTCGCAATTGCGTACTTAAGTTTAGTGATAATGGCTATTTTACCCATATT
CTTCGGATCATTTCGATCTGTTAAATACTTAAAAGAACAAAAGGAATCAGGTGAAAGAGCTGAAACTATGTCT
AATAAGGATGCTCTGATGTTCCCGTTGATAGCATCCTGTGCCTTATTTGCTCTGTATATATTTTTCCAATTTT
TCTCTAAAGAGTACATAAATCTGCTTCTTACTGGATACTTTTTCTTCCTTGGAGTTTT
AGCACTGAGCCATCTTTTAAGCCCAATAATATCTTTAATAGTACCAGCTTCAGTCCCAAATGTACCGTACCAC
ATTTTGTTTACACGCGGAGAGCGCGACGGCCGCTCGGACATCGTGAACTATAAGTTCACCTCGTACGACGTCA
TTTGCCTGATCATATCACTTATTATGGGGAGGGTGGTATTTACTGAAGAAGCATTGGA
TCGCAAATAATTTATTCGGTATCG
>1002052906_2 Heli.2-DT663425.3.5
AAAAAAAACACACCGGGCTGAGATCGGCCGAGGGTCGACTGAAGACATTGCTGTGTTCGGTGTTGCTCGCGGT
CGCGGGCACGTTGGCTCATCATCATCATCATGTCCATGAACATCCCTCTGGAACAGAGTTACCGTCGTCCCCT
GTCACCGTGTCCCCATCAGGGGAGTTCCGCGGCGGGTACAACGTGACGCGTCTCGGGC
GCCGTTTCGAGACCTACCGCGGCATCCGATACATCGAGCCGCCCATTGGGGAATTGCGCTTCCAACCTCCCAA
ACTGATCTTGTCGTACGCGTCGCCAGTGGATGCAAGTGAGGACGGACCCGCGTGCCCACAGCCCGCACCGCCC
GGATATCCCATCGACGAGGATTGTGTCACAATCAATATTTATACGCCACTCAAGAAAG
ATAGGGCAAGTAATGCTCCTCTGCCTGTAATATTTTATATGCACTCGGGCGGGTATTATTCGATGTCAGGTCG
CAGTGACCTCGCAGGGCCGCACTACCTTCTTGACCGTGACATCGTCCTCGTTACTATCAATTACCGACTCGGT
TCTCTTGGATTTCTAAGTACGGGAGATAAGCTCGCGCCAGGTAACAATGGGTTCAAGG
ACCAAGTGGCGGCTTTGAAGTGGGTGCAACGCAACATTGCGGCATTTGGAGGAGACCCCAACAGCGTCACAAT
TGCGGGATGCAGCGCCGGTTCTTCCAGCGTTATACTGCATATGATCTCTCCTATGTCTAAAGGACTCTTCATC
CGCGGTATTTCAATAAGCGGGTCTCCTGTCACGCCTCGCCCGACGCCCGATAATATGT
ATAGTCGCGCGGTGAAGCAGGCGCAGTTGCTGGACTGTCCCACCACCAACTCTAAGGCGATCATCGATTGCTT
GAAGACTAAACCTTGGAGGCAGTTGGGAGATTCCCTTATGAGACTCTATGAATTTGGATTTGATCCAATTTTG
ATATGGGAACCTGTAGTCGAGAAGGATTTCGGCCAAGAAAGATTTTTACCTATAGATC
CAACTGAAGCAATCCGGCAGGGGAAAATGCATGCAGTTCCGTACATCATCAGTCAGACAAAAGATGAATTATT
CTGGATGGCACTCACGGTCCTTCGCAACCAGACGCTGCGCGATAGGATGAATGCGGAGTGGGAGTCCATTGCG
CCCATCTCATTTCTGCTTCCGCATGAGAACACCGTGCAGGCAACCCGTACACTGAGCC
CGCAGTATCTGC
>1002052908_2 Heli.2-EL598768.1.5
GCACGAGGCCTCGTGCCGAATCGGCACGAGGGCCGTGTTGTGTTCAAACAAAGCTACCAAGGAAAAAAATATG
CAGTCCTCAACAATCATCGTTCTGGCTTGTCTCGTAGCCGTTGTTGCAGCGAGCGTGCATTATGAGTACCCAG
AAAATACGCAAGACGTACAAAATATCCACGAGTCACTGCACGAAACACCAGTGCACGA

AAGAGGCGCAAGAACGAAAAGAGGACTGCTACTTCTGAAGAAGAAACTTATCCTTGGAGCTTTAGGTTTGAAG
GCAGTGAAAGTAGGTGCTGTGGGTGCCGGAGTAGTGGGCGCTATAGCACTCAAGAAAAAACACTGGTCCGGTT
GAGGCGCTAAAGATTTCTGTTTAAGCATCCTGTGCGTTAAGAAATCAAGAAAGCATCG
TTAAGTAATATTAGAAATAGTTAAAAGCTGAAGCCAATGGGTATTTTTTAATATGAACTAGATAATAAGAGTA
AATACAGGCTATTAAGTGAAATCAATTATAAAATTCGAAGGACAACATTGCTACATCGAAAAAGAAACGGAAT
ATACTCTGCACATTATTATTAAAAATATTACACTCATGTCAGTTACGAACCTCTGGTG
ATAACATTGAAAAACAAGTAAAAAATGTAATAAATAGTCTGTAAATTGCCATAAATACAAATCTTAATACTTA
AAAGTCAGATAAAACGCAACTGATTTTGTTTATACAATAATAAATATGTACATAACATAAATATCACTTTATA
GAACATGTAT
>1002052909_2 Heli.2-DT666757.3.5
GCAGAGGATTCACGCATGCAGACGCTGTTATAGTGTCGCTGCCATTGTAATTTTTATTTTTTTTGTTATTAAC
AAGTTATCTCTTAGATAATTTACATTAAGTAGTGATTTTTAATAACTACTGTCGATAAACAGTAGTAGGACAT
TTTTATATTGTGTAAATCTTTGTGATTGATCGGACATTTTGACTGCCATTCTTTATCC
CTAACGGGATAATAGCTTGGTTCTGAGCCTTTGTTCATAAAAAGTGTCTAAAGCAGAGTGCATGCATCTTTCC
CTGCAATACACAGGATTTTTGACCGACGACTGTGATGATACGATTGTTGAGCTATCAAAACTATCGATCGGTG
ACTCTGATCACGAGGTCACTGTTTTAGAAAAATTTCATGACACCACGAGCCGTGTAGC
GACCGCGCGGGACTACGTCTTGCGACGTTGCAATCAAACAGAAGTTGTACTGTTTGATGAATGCTATCCAGAT
ACAGCCCTGAAAAACTGCCGCAAGATCGGCGAGGGTGTCTACGGGGAAGTTTACCTCTGGCGCGCTCGAGACG
GCAGAGCCCGTGTGCTGAAGATAGTGCCCATAGCCGGCCACATCAAAGTAAATGGGGA
AGATCAGAAGGACTACCATGAAATCATATCTGAGATTGTGATTGCTATGGAATTGAGCGCACTACGCGCTCCT
ATAGCAGAAATAGAACAACATTTAGATGAAGGCAAGAGTGTTGAAACTTTGGATCTACATTCTGTAATGAATG
CTTCTGATGTCTTCAATGAGGTTCTAGCAGTCAGATGCGTACAAGG
>1002052910_2 Heli.2-EL603302.1.5
GCACGAGGCGCAAGAGTAGTAGTAATACGAAAAATACTGTCATTGTTGTGTAATTGGAAAATTGGAAATTAGC
GACATGCTTCGCTACACTCGTTTGGTTCTTTGCTTACTTTTAGTGTTTTTAAGTGTTGATGTGATATTATCAA
AACGTATTTCTGGAAGACACTCTTATCCAAAACCTCATGGCGGTGGTCTCTCTGGTGG
GGGAAGTCATTCGGGAGGCCTATCAGGCGGTGGACATGGATATCCTTCGTCGGGAGGCCATATAACCCCTTCG
AATACTGGCGGAAGCCACGCATACCCCTCGCCTGGAGGAAGCCATACATATCCATCGTCTGGAGGAAGCCACA
CATATCCATCGCCTGGAGGAAGTCACACATATCCTTCTAATAACGGAGGTAGCCATGG
ATATCCACAGGGTAAAGGATTATCAGGTAACTCACCAAGCCAGAGTCATGCCACACACACTACTACAAATATT
CATCACCACTATAATTATAATCCTCCTCAACAAATAAGATATGCACCTGTACATGGTGGGCCTCCAGTGAGCT
ACCCAGTTTATCATGGTACCCCTCCAACTTATGTCTATCAGTATAAAGATTCTGGAAG
CAAGTATGGTACCCTACTTGCTGGCTTAGCCCTTCTTAATCTTGGCACTCTTGGAGTCAGTGCCTATGCTGCA
AGTCAGAGTCACAGCCATAGTCA
>1002052911_2 Heli.2-EC091315.1.5
TGACGGTAAAGCGTGGTAAGCCCCATATACTCACTTTTGCCACGCTTACGCGGGCAAGCTCTCCGGTGGGATG
AAACAAAAACTCGGTCTGGCCTGTACCCTGGTGGGCGAACCGAAAGTGTTGCTGCTCGATGAACCCGGCGTCG
GCGTTGACCCTATCTCACGGCGCGAACTGTGGCAGATGGTGCATGAGCTGGCGGGCGA
AGGGATGTTAATCCTCTGGAGTACCTCGTATCTCGACGAAGCCGAGCAGTGCCGTGACGTGTTACTGATGAAC
GAAGGCGAGTTGCTGTATCAGGGAGAACCAAAAGCCCTGACACAAACCATGGCCGGACGCAGCTTTCTGATGA
CCAGTCCACACGAGGGCAACCGCAAACTGTTGCAACGCGCCTTGAAACTGCCGCAGGT
CAGCGACGGCATGATTCAGGGGAAATCGGTACGTCTGATCCTCAAAAAAGAGGCCACACCAGACGATATTCGC
CATGCCGACGGGATGCCGGAAATCAACATCAACGAAACTACGCCGCGTTTTGAAGATGCGTTTATTGATTTGC
TGGGCGGTGCCGGAACCTCGGAATCGCCGCTGGGCGCAATATTACATACGGTAGAAGG
CACACCCGGCGAGACGGTGATCGAAGCGAAAGAACTGACCAAGAAATTTGGGGATTTTGCCGCCACCGATCAC
GTCAACTTTGCCGTTAAACGTGGGGAGATTTTTGGTTTGCTGGGGCCAAACGGCGCGGGTAAATCGACCACCT
TTAAGATGATGTGCGGTTTGCTGGTGCCGACTTCCGGCCAGGCGCTGGTGCTGGGGAT
GGATCTGAAAGAGAGTTCCGGTAAAGCGCGCCAGCATCTCGGCTATATGGCGCAAAAATTTTCGCTCTACGGT
AACCTGACGGTCGAACAGAATTTACGCTTTTTCTCTGGTGTGTATGGCTTACGCGGTCGGGCGCAGAACGAAG
AAATCTCCCGCATGAGCGAGGCGTTCGGCCTGAA
>1002052912_2 Heli.2-EL599133.1.5
CATAGTCGAGCATCGGCCGAGGCCCTGCGCAGTTGGTGTCGCGCGGCATTAGTTTTCCAGCCACGGAAAAATT
GTGTCGCGAGTGTCGGACACTATATACCTGTGCTAGAGCAGTGTCTGACTGTGTGTCATGGATTATGTGTTTG
GCTCCTAAGTGTCGTTGTGATTTTCTTTACTGCAGCACTCCAGGCCCACCCCTTCGCT

GCTGACTTCCTTAACACTTTCAACATGGAAGCGACACGACAAGTGTCGGCCCACTGGCTTTCAGCTATAGTTC
TTCTCTGCTCATTTTGTTCTTCATATTCTTTTAATGATAAGATTCACGAGCACATGACACCAGATGAGTTACG
GAGCGTCTTCCACGTAGAGCATCACAGCAACGTACCAAAATATCACTTAGTTCATCTC
ACTCACCATCTATCCAGGCGGAATATCGCAACATCACACTCATCTAATTCCCACTCCCGTACAACCAGCAGTG
GGAAAATACCCCACGAGAAGATCTCGAAACCAGCTCCATCGCTTCTAAATGATCAAATGTACATGAAAGCAAA
GGAACATATCAAGGATGTTGATATAATAGGTGATCTAAATAATACAGTGAATTTGCAA
TATAACGTGTCGAGTTCTAGTGATAGTGTAAGTGAAGGTGATAAGGAGCACGCGGTGGAAGATCAAGAAGATG
ATGTACATAGAATTGAGTTGGAAGCTTTTGGGAAACAGCTTAAGTTGGTACTGAGAAAGCAGGAGGGATTGGT
GAAGAAAGATGGACTGAAGATGTGGCGAGCGCTGACAAATGAGTCGCAGCCTCACGGT
GTTGAATACGAGGAGATGCAAACTGATGACAGCGAAGAGATTGGAGATCTTTACCATGATGACGAAAACGGGG
CCGCCCTTCTGATTAGAAGACAT
>1002052913_2 Heli.2-EL601756.1.5
GCACGAGGATTTGTTTCCTTTCGTGCTATATATTGCGTTAAGATGGGTTTCTAGATTTTTAAACGTTTTGCAC
[truncated: 873,836 more chars]
