# Supplementary material for: 454 pyrosequencing based transcriptome analysis of Zygaena filipendulae with focus on genes involved in biosynthesis of cyanogenic glucosides
Source: BMC Genomics. 2009 Dec 2;10:574. doi: 10.1186/1471-2164-10-574 (PMC2791780; doi:10.1186/1471-2164-10-574)
Supplement: Additional file 5 — Heliconius singlets. FASTA file of all Heliconius singlets. [file 1471-2164-10-574-S5.PDF]

```
>50818206_3 CO729476.1
GACTTCTGTGATATTCGAAAATCCATGCCGTTTCTGTAATTTAATAATATCCAACAGAATAATGTCCATTACA
ACAAATTTACGCTCTTTGATAAACAGAGTTGTTTTCTCTGATATAAGATGTTCTGTGAGATTTTATTCTGATC
AATCGGATGAACCTGTTGAAGATGAACAGAAACCCATTGACCCAACTAAAGATAGAAC
AAAAATTATTCCTGTAGAGACTAGCATGAGATATTTACAAAGTAAAGCATATAAACAAACTTATGGCGAGAAT
CCCGTTTGGTTTTTATATAGACGTAACCATTTAGGTGGATTTGCACCAAAGAGAACTAGACGAACTTGTGTTC
GTAATGGAATCATATCTACAGGAAATCCTTGCCCTATATGTAGAGATGAATATCTTGT
TTTAGACCCTCTAAATACAAAATTGTTAGAACAATTTATCTCCGTATACACTGGACAGATACTTGATGCATTT
AAAACTGGACTATGTCAAAAGAAACACAAAGAATTATTGGTAGCAATTGAAAAAGCATGGGATCAAGGATTTT
TAACTTATGATGTCCCATTTAGGGAATATGATTATTCTCTTTACATAAACATGCGGTT
TAATTGAAATGTAACATATATTTAGTAGT
>50818214_3 CO729484.1
CGGTCGGAATCCCGGGTCGACCACGCGTCCGCACACGGGACACGCGTGCCGTGGTCGGTAACCCGGTCTAACC
TAACCAGTTCACCGCGTCTCTACTATCTGTACGCACCTTTACACTCGTTCTGCATCAGGACTTCTAACTCATT
TTAAGATCTAACAAGTAGTTCTTATTAAAGCAAGATGGCAGCGTTCGTGGCGAAGCAG
ATGGTGGGCAACAAGCTTAGCGCTGTGAAAGGCGCAGTGGGTGGTGATGGCGATGACAGCGACGACAAGGAGA
AGGAAGAAGAAGCCGAACGCGAACGGCAAGAGGCCATCCGCGAGGCTGAGGAGAGACGCAAGGAGAAACATCG
CAAGATGGAAGAGGAGCGGGAGAAGATGCGTCAGGAGATCAGGGATAAGTACAAAATC
CCCAAGAAAGAGGAGATAGCGGAGCAACAGCAGCAAGAGCCCGATAACCCCCTGATGAGGAAGAAGAAGACCC
CCGAGGAGCTTGCAGCTGAAGCGGAGCTTGAAGACCAGGATGAGTTCACTAAATTGAAAAACACGATCGAGAC
GCAAGTGAACGAACTGAAGACGCAGATCGAGAGTAAATGCGTGATGCAGTGAGCTGCC
GCTGTAGCTGTAAAGACACGCCATTGTTACCCCCATACAGACAAAGCCATACAAGTTAACCATT
>50818216_3 CO729486.1
GAAAGATCAAGAGCTGCAATGGTTAGTCTTTGCAACATATTTATGAACCTGACAGTATTGGCACCAAAAGTGG
TAGAGAATAGCATGTTATTTAATACTCTGCTTAAATTCATATTCAATAACTTACCTGAATTGAAAAACATCCC
AGAAAACCTTGTTCTTCATGGGCACCTAGCAGTTTTAGGACTCCTCCTTCTTAAGCAA
CAGGCGAGTAAAGTAAAGAAAAATGATTTCTCTATATGCCGTTATATACAATCAACTATAAGATTTCTTTGGG
ATGCCTACAATGTTGATGAGTCAAATGACCCTAATGCATTAGTTGTATCAATGACATATAAGGAAAACTGGAA
CGAAATTGCAGATTTATGGTTTTTGGGAATGCAAACCTTAAGTGGTGTTTTAACTACA
ATCCCTTGGATCTCAGAGTTTGCTATAGAAAGTGGTTGGGCGCAAGGAATAGCAGAAATGTTGGTTAAAGTCA
AAGTTGGTACATTAGCTCCTAATGTAAAGTCTGCATTTGAAGACTTCTTATGCAGATTAGTAGATTCAAATGA
CAGTGCCATACCGGTCCTTAAAAAAGGTGGTGCCTTAAAAATGTGTCGGAATCACAGA
ATGATGGATTTAGGCAAAAAACTATTTGGTGACTAATTATTGCAAAGTCATGCTTTTGATATTACATAAAGAT
GCTTTTTATACTGAGAAGTGTACTTAATTATTATTTTTGATTGACAACTA
>50818224_3 CO729494.1
AAAGCCGCTGCCCGCGCCTCACGCCATGGCCAACTCCAGGCACGGGAAGAACACCCAGGACGATGTCTGCTAC
GTCGTCGCCAAGTACGACTACGCGGCTCAGGGTGCCCAGGAGTTGGATCTGCGGAAGAACGAGCGTTACCTAT
TGTTGGACGACTCGAAACATTGGTGGCGCGTGCAGAACGCGCGCAGCCAGTCCGGTTA
CGTGCCCAGTAATTATGTGAAGAAGGAGAAACCCTCGTTGTTTGATAGGTAGGTTGATAGTGGATATTATATT
GAGATTGGCACCTATTAACATGTCCAATCGTTTCTTGAACCGACTTTACTCACTTCAGCCCTGACTAAAATAT
TTAAGATTTACATTTCGCCGTAAATTTGATACAGCGTGTTAAATAGCGAAGCCGCACT
TTGTGTACCATGGTGGGAAAATGAAAATAATTTAAATATTATCATTTCCCCCGAGTTATATCGAACCTACAAA
CATTGCGCTTTTAGTACAATTTTAAAATACGATAATAATAACGATTTTGCACCACGATAGACAAATATG
>50818238_3 CO729508.1
GGNTCGGAATTCCGGGTCGACCACGCGTCCGGTGACGTGTATTAAAAATAATCTAGAAATTTAAAAGATAACA
ACACATTTTAATCACTAATTTTTGCAAAATGAGAAAACTAAAAGGCGTCGTTAAAGAAACTGCCAAGCAAAAA
CGTGAGCGAAAGCAGGAATTTGCAAAAATGCGTCAACAGACTCATACTATTGTTTTGC
CCACCTTTGGTATTATATTCCTCCTTATATGTGTTTATGTCTACTTTAAAACTAGACCTTCATCTATGCAATA
TGCGTAACGACTTTCTGTAATAAATTTTCTTTTATGCTCAACAGTGTTATTGACTTAATTCAGCAAATTAAAT
GAAAATAATAATAATAATTACACTATGGTTGTGAATTCCTGGTAACTGTTTTCTACAT
ACTGTAGATAAATCACATTCAATGTCTTAATGTATGTTATATTGTGTATTATTTATCTAACATTCCATGTATT
GAACATATCATTGTATATACTAGAATAACTATTGTTAGAATTATTGGATGATTTATATTTTCAATTGGACTTT
AGTTCAGTGTAGAGGGTAATGTTATATATTAAAAATAAAACAATCTATTTATTTATAA
```

CTATGTTTATTTAACAAATATCAGTACTAAATGTATAAAATTGTTGTGGAGAAATAAATTATTTAGCTATAAT
AAT
>50818239_3 CO729509.1
GGAAAACCCTAGTGTAATTATTGTATCTGGTGTATCATAGTTTATTAAGAAATATTTATTAGTTAAAAAAATC
TCGAATTTAAAAGAGTTAATTAATTAAAAAATTTAATGTTGAATAATTATTTTAAATAATTAATTTGAAATGA
AATGTTGATCGTTTTTAAATATATCTAGTTTTTTTAGAAAAAAATTTAATTTTAAATT
TAAAATATTTTTTAATTTTCTATTTAATTAAAAAATTTTTAAAATTAAATATATTAAGGGATAAGCTTTAATT
TAAATTTTTATAATATATATATATATATATATATATATATATATTAAATTATATTGAAAGTTTTTGAAATCTATC
GTGTTTATAAAATAAAATTTATTATAAAAAATTTCAAAAAGAAAA
>50818247_3 CO729517.1
GTTAAAACTCCTAAAGAAAAGCAGCAAGTCGAAATAGAGGAAGATGCCGATATCAAAAAACTTAAAGAAGTGC
TTTCCCCCAAATTCAATGCAGAACCGGAACAATTATGTCTAATTTTTGCTGGAAAAATCATGAACGATTCCGA
CTCACTCAAGCAGCATAACATCAAAGATGGATTGACAGTTCATCTTGTGATAAAAACT
CCACCAAGGCCTGAGCCTGAAGGAGCAACTCGACGTCCTCCAGCGGATATTGGTGCAACACCATTTGGATTAA
ACTCTCTTGGTGGCCTTGCTGGACTTGAAAGCTTGGGCTTAGGTCAAAGTACATTTATGGATCTTCAGGCCCG
TATGCAACAAGAGCTTTTATCAAACCCTGAAATGCTAAGACAAGTACTTGACAATCCG
TTGGTGCAACAAATGATGAATGATCCAGAAAATATGAGATCACTAATAACTTCAAATCCACAAATGCAAGACT
TAATGGCTAGAAACCCAGAAATTAGTCATATGTTAAACAATCCTGAATTATTACGGCAAACTATGGAATTGGC
TAGGAACCCTGCCATGTTGCAAGAGTTGATGAGGTCCCATGATAGAGCTTTGTCCAAT
TTGGAAAGTAT
>50818249_3 CO729519.1
AAACATCAAAACAGCACGTGTAAAAATGCGTATTGAAACTTGTTATTTCTGCTCCTCCAGGATATATCCTGGC
CATGGCATACAATTTGTGAGAAACGATTGCAAGATTTTCAGATTTTGCCGATCAAAATGTCATGCAGCGTTCA
AAAAGAAGAAGAACCCACGTAAAACTAAATGGACCAAAGCTTACCGTAAGACTGCTGG
CAAAGAATTGGCTATTGACCCATCATTTGAATTTGAGAAACGTCGCCATGCACCAGTGAAATATAACAGAGAA
CTATGGACAAAGACCATAGAAGCTATGAAGAAAGTGGAGGAAATCAGACAGAAGAGATCTAATAATTATATTA
TGCAACGATTAAGGAAGGGACGCGAAGTAGAGTGGCAGAGAGATGTCAGAGAAGTTCA
AAGAGATATCTCTCTTATCAGATCACCTGCCGCAGGTCTCAAACAGAAACAAGCCGAAGAAATGGAAGTTGAA
CCTGAAACTATTGAAGTTGTCGATGCCAAGGGAAGGAAGCAAGTGATAGAAGCACCTGTCATGGTACCCGCTG
TGGGGGAGATGATAGAGGAGGGAGGGGATGTGGAACTGTAATTGATTAGGAAATATAA
GTTTTACGCTA
>50818250_3 CO729520.1
AGAGGAAAAGAAAAAATTCTTAAAAATTTAGCAAAAGCTGGCGTACCCTTACCAGAAGGGAAAACACCTTCAG
AAAAAGCAATTGTTAAAAAGGTAAAAGAAGCTGCAGGTTTACCGACAGTATCCCCTCCAGCATCAGAAAAACT
TCGCAAAGCTAAAGCAGCTGGTCTTCTAACACCATTAGAGGGGAAAACTCCCGCACAA
AAAGAAGATTTAATAAAGCGCCGTTTAGCAGCTGGAATTCCTCTACCTGAACCAAAAACAGCCTCAGAAAAAA
AGATAATAAAAGATGTCCTTGCAAAATCTTCAAAACTCTTACATGGAAAAGACTGAATAAGCTTTAAAGTGAG
TGGTTATTATTGTTTGTTCGAAATAAACGTATTATTTATT
>50818252_3 CO729522.1
CTTGTTGCTACGTTTTGTGTGAAGTCAACGATTAAGATTGTACCAAGGAATAAAGGTAAGCCAAGAGAAGCTA
GTGCCTTGACACTCTGTTCTCCGCTTTCCGTTCGAACTCGTGGCAGTGTTGCCAGGCGGCACATAACGCCGTG
CATTGTCATATTACTAGAGCGAGGAAATGAAAATAATTCCTTTCCCATTGCGCCAACA
TGGCCTTACGGTATGAACGAGGTACTGGTTCAAATATAGTAGTCTACATTTTATAGTAAGTAGGATGAGCATA
TTTTTCTATAATTATATATAAGTGTATTGTACATAGTATGACGTAATATGTTCTTGTGTATTTAATAGACTAA
CGAGTGTCAGTCAAATGCAGTCTACGCCAAATAAATATTTTTCAAA
>50818253_3 CO729523.1
AGCGTCGTTCACGCGACACGAAGTATTCTATAATCTAAGTATTCTAAATGTGCATAAATGTGAATAAAATGTG
GACTCAATATATTTGGGCATTATGCCTGGTGGTCATGTGTCAGGCCCAGTTGTCGGACATCGAGAGATATTTA
TTGACTATAGCCGAAATGTCACCGTCGCCCCCTCTGCAGAGTAACGAAAAGGCATGTC
AAGTTGACAGTTCTACAAACGGTGTTTGCGTGAAACGTACACAATGCAACTTGGACGCGCCGGCAGTGGATCC
CGCTACTACCTTCATACTGCGTGAAGGAGGATCAGCGTCTAACGCATCTGATGTGGAGAACGCATTGGATACA
CAGTGCGCATGGCTTCAGATCTGTTGTCCATCTGATAAAGTCCGGGAGGAAGAACGCC

AAGTTAAATTCGAGCAGCCGCTTGGTTGTGGCTACAGCAACCCCGGCGCTAACGTCTTCAGGGAATCAGCTAA
TGCTTACGGCTATGCGGACTTCGGGGATTTCCCGTGGATGATTGCTTTATTACAGACAAAAAACGATAAGACA
AAATTCAATGACCAGTATATAGGGGGTGGAACACTAATTCATCCTTCGGTCGTGCTGA
CGGTGGCGCATAAAGTAGATAAGCTCGAGTCACCTAGTGAGTTGACGTGTCGTGCTGGAGAATGGGATGCAGC
AACAGAGGACGAACTCTTTTCAACATCAGACAGAAATGTTAAAAATATTGTCATCCATAAAAATTATCTAAAA
GCACATGT
>50818254_3 CO729524.1
CAAAATGTTCAAATTTGTCGTATTCGCTTGCTTTTTGGCTTTGGCCGCAGCAAAACCCCTGGTCTCCGTGGCC
TACACTGCTCCAGTAGCCGCAGCGTACACTGCTCCCGTTGCTGCGTCTTATGTAGCAGCACCTGTCGCTTATT
CAGCATATAGCGCACCATATGCTGCATATTCAGCATACTCATACGCTTCTCCCTACTC
TGCGTACTATCTCCGTTGAGAAATTAAAATTGGACTGCATTACTGATGGAAATTACAAAAACACTTATATAAT
AATGAAATAAAATGTTAAAGCTGTT
>50818260_3 CO729530.1
GTGGCATTGACTGCTGCGCTGACTTGTGTTGTGCCATTCGCGATAGAAATGAAAGGATTGATACGAAAGGCTA
ACCTGCAATTATTTTGCAGCTAGAATTCTAAGTATAACGCTTACTTTTACGTGACTCAACCTTGAGTGCGGCG
TGGTCGTATATTAGATAAAACGTCCTATTGTTTCTCTGTCATCAATCTGTTATTATGG
CTGAAAATCAGGAGTCTTTATTGCAAAAAATTAAGGAACAAGGAGATCTTGTGCGAAAGTTAAAAGCTGCTAA
GGAACCCAATGCAAAGGTTAACACAAGAAAAAAATCATGTCGAAGTCTTGAAGGGATAAATGACCATCTCTGT
TATTACAGTTATATGTGTGGTTATGTACCCAGTGCTTTTGATATAGAATTATTTAGAA
GTATTGACTCAAAATCAGATTTGGAAAAATATCCATATGTACAAAGATGGTGGTACCATATGCGGAGTTTCAG
TGACTCTGAGATAAATCTATTTCCTGTAATTCCTCCATCAGCTTTAATTATTAACATGCTTAAAGATAATGAG
AAAACTGCTGATGAGCAGATTCAAGAAGAAGTATCTAAGTTATTGGCATTGAAGGCTC
AACTGAATACTGAAGATGCAGCTCCCCAGAAGTTTACTCTTAAGACTCCAAAAGGGACAAGGGA
>50818263_3 CO729533.1
ACATTTTTTGAATCTTTCCCACTTTTAAATAAATATTTCACTTCCTCGAGTGGGGATCGAACTCACAACCGTC
CCTCTTGTGCCGATGCGCTACGACGGCCTCATAGGTACCTACTATCGAATTTTGAGGAAAATTAGATTTTGAA
GTTAAAATGGCCAAGAGTTACTTATCACCTGTTTATAATACCTTGTATATCCAGGAAG
AAATAAAACACGAATAATG
>50818265_3 CO729535.1
CGTAAACTTCGACAAGCACCGAATTTTTAAAAGAAGAATTTTCTTACACTGTACATTCCCGTAAGCTCATTCA
AAAATCGAACATGTTTTCTACCGTTGGTAGAGCTGGTCTTTTGGCCACGAGAGCGGTAGTTAACAATACTTTG
GCTGAGAAGACACCTTTGGTTGCCGGAGCTCTTGCGAATAAACGTGATTATGCTGCTA
AAGCCGCTGCAAAAGGTCAAGGTAAGGTGGTTGCAGTCATTGGTGCTGTCGTAGACGTACAATTTGAGGATAG
TCTCCCTCCCATCCTAAATGCCCTTGAAGTCCAGAACCGGTCCCCCAGGCTCGTCCTGGAGGTGGCACAGCAC
TTGGGAGAGAACACCGTTCGCACCATCGCCATGGATGGTACTGAAGGTCTCGTGCGCG
GACAGCCCGTGCACGACTGCGGCTCGCCCATCCGCATCCCCGTGGGCGCGGAGACGCTCGGCCGCATCATCAA
CGTGATCGGTGAGCCCATCGACGAGCGCGGTCCCATCCCCACCGACAAAACCGCCGCCATCCACGCCGAAGCA
CCCGAATTCGTGGACATGTCCGTACAGCAAGAGATCCTCGTGACAGGCATCAAGGTGG
TGGACCTGCTTGCGCCCTACGCCAAGGGAGGCAAGATCGGCCTGTTTCGCGGCG
>50818266_3 CO729536.1
GGTCGGAATTCCGGGTCGACCACGCGTCCGCATATTTACTGACATTATTCCATGCTACAAAAGCATTGTTAAT
AAATTGGATTCTGACCACTCTGGTCAGCTACTAGAATTCTATAGTAAGAAAAAACAGGTGGCTAAGAAAACAA
TTGCTTTTGTACCTGTCACAGTTAGTCGTTTGGAACGTATGAGAGTTAGTTTACTAAC
TAATTTACTAGTTTTAGATAAAACTTTACGTCCCAACTCAAAGTCAAAGTCAAAATTAATTTATTCCATAAAA
ATGTTTAACATTTACTTGGACATCTCATAAAAGTAGAAAAAAATTCAAAACAAAATAACC
>50818269_3 CO729539.1
TATTGAATCTTGTGTATCATAAGTTTATTAAAAAATATTTATTAGTTAAAAAAATCTCGAATTTAAAAGAGTT
AATTAATTAAAAAATTTAATGTTGAATAATTATTTTAAATAATTAATTTGAAATGAAATGTTAATCGTTTTTA
AATATATCTAGTTTTTTTAGAAAAAAATTTAATTTTAAATTTAAAATATTTTTTAATT
TTTTATTTAATTAAAAAATTTTTAAAATTAAATATATTAAGGGATAAGCTTTAATTTAAATTTTTATAATATA
TATATATATATATATATATATATTAAATTAAGTTTAAAATTTTCAAAATTTATATCGTTTATAAAATAAAATT
TATTATAAAAAATTTTCAAAAGAAAAAATAAGATTTAAAATTATATTGAAATTTTTAT
AAAAATATAATTTATAACAGAAAA
>50818273_3 CO729543.1

CATTTGTGAGAGAGCCATCTAGTGACAAACGCGTCAAACTTGCGAGTGGCTAGTGTAGTTGTGCTGTGTTTTG
GAAATCCTTACTTATCAAAATGCCTGGACGTCCTATTTGGCAGTGTGCTGGTAAACGCGAGCCTGAAAAAGAA
CAAGAGGCACAGAGGTGGATAGAAGCTGTGATAGGCGAGCCATTCCCAGCAGACCTGC
CATACGAACTGGCTCTCAGGAATGGAATTATTCTGTGCAAGCTCATGAACAGATTGCAACCAGGCATTATCTC
AAAAGTCAATGTTTCGGGTGGAGACTACAAGTTCATGGACAATATTAGCCAATTCCAAAAAGCCTGTGCTAAA
TATGGTGTACCTGACGTGGACCTCTTCCAATCTACGGATTTGTGGGACCAAAAGAATA
TTGCGTTAGTTACGCAAACAATCTTTGCTCTAGGCAGAACGACATACAAACACCCAGAATGGCGTGGCCCCTG
CCTAGGCCCGAAACCCGCTGAAGAGAATCGCCGTGAGTTCAGTGAAGATGTTCTACGTGCAGGAGAAGCTGTC
ATCGGCCTGCAGGCTGGTACGAACAAGATGGCGTCCCAGTCCGGCCAGAACTTTGGCG
CTTCGCGTAAAATCATTCTCGGAAAGTGATGTACTAGGACATCAGCATGAGCGTCAGAATAGGGGTCACCATT
TTGTCCGCGCCCACTCCATAGTCTTTGTCCCATCTCAGGATTATTACTAACAAC
>50818278_3 CO729548.1
GCGTTATCATAACCTTAAATTTAAAAGAAATTAAATTGTTTTAGTTCAATGTAGAACTTTTCTTTTTTTATAA
TTTGATTGATATAAATTAAACATAACAAACATTAAAATGGCAGACGACGGCGATAATGTAGCTGTTATGACAG
TAAAGGAACCTCTGGACTTAATTAGACTAAGCCTTGATGAAAGAATATATGTTAAAAT
GCGCAACGAACGCGAACTTCGTGGAAAGCTTCATGCATATGATCAACACTTAAATATGGTTTTGGGAGATGCT
GAAGAGACCATCACTACAGTCGAAATAGATGAAGAGACATATGAAGAAGTATATCGGACTACAAAAAGAAATA
TTCCAATGTTATTTGTTAGAGGTGATGGAGTAATATTAGTATCTCCCCCAATACGTGT
TGGTGTGTAATTTTAAGGTTAATAAAATAATAAATGTC
>50818283_3 CO729553.1
AATTCCGGGTCGACCACGCGTCCGAGAAGAAGTAAACATGTCTTGTTTCACAGATTTCCTAAGACAGAAAAGC
TAAATATTACCAAGCACAGGCTTGGGTCAAATGTTGTTGGCCTAGGACTTATTTGGAGTCATTACAATCCCTA
CTTACTTACATTAGTTTCGGGAGACATAATTTAGATGAACAATGCCAACCTTGTCTTT
GCTCTGATTGAAAATACAAAATATTTACATATTGTTATGAATGAAAATGTACTAAAAGATTTAAAGAAGAAGT
GAACAGGATACCTTACTTTGACAGAGCAAATACAATATTGTGACATTTTTAAAGTGTTATTGGAAAGAAAAAT
ATTATTTTGGGAAACTTATAAAATATCACCTCTTGTGGTGGTTGAACAGTTTTAAAAA
AGTGCTTCTAAATAAATAGCTTATAATTTATGATGAAATTGTTTTCAAAATATTCAGAAAACAGGTGGTGGTG
GTGGTCCTTTACACCTACTGTAATGGAGAGTGAATACCAAATACTCTCAGTTTTGGATAATCAAATACACCCC
AACAACTACGACTCGTCGATCATACTGTAAATATTAGCT
>50818284_3 CO729554.1
GTAAAGTAATTATCATGATCCCATATATAAAGTTTGTTTATAAGTAAAAAAAACAATAATTACCATTGTAAAA
TATTACATATTTCATTATTAACTTGTGTGCTGATGGTATCTATATCATATTGGATTTTAATAAGTTCTTTGAT
TTTCACGATTATTACACATATTAAATGAGAAAGAAGAAGAACTATTTTAACTTTAATT
TGGATAGTTTGATAAAACTAATTTTATTTTGGATAATACATCTTTTTGGGAAAATCGGACGTTTCGAACACTT
TACAGTAGTCCGTGGTTAAACGCAATTAATCCAGTTAAAATAGTTTCATTTAATATTGGATTTTATTTAATAA
AAATGTTGTATCAGTAAAGCTATACCAATAGTAAAATTCCAAAAATCTCAATACCTTC
ATAACTATTGCTATTTACATATTAAATAAGTTAAATATAATCACTACAAAAACTTCATAAAATCCAATTTAAA
TTTAC
>50818286_3 CO729556.1
AACAAATTCTACAGTGACACAGTCACCGCGATATATCTCGAGTACTCTTTGTGAATCTTACTAGTCATAAATA
TGTCTGAAGCTTTCTTTGATGAATACGACCACTACAACTTCGAGCACGACAAGCACATCTTCTCCAGCCACAG
CGGCAAGCAGCGCACCAAGAAGGAAGTGAGCCAGCACACCAACCACTTCGATCCGTCG
GGCCATTCCAGAAAGATCGTCACCAAGCTCGCCAACACAGAAAACAACAAGAAAGGCGATAACAAACATTAAA
TAGATTTATAAACGACAGACGACTATGGCGTGTAAAAGGAATATTGAATTTTTGCCGACGGAACGGAGGACGT
TATGGCGAATACCCGAAGAAAGCACGGGTCGCTCGTCTCTGAGATCAGTGGAACAGCC
GAGAAGGTTTTTGGTAACGCAGAGTTTTTATTATGCACACCACAATGCTAGAACACGGTGGCGCAGCCGGCGC
GCCTCGTGACTCGTGCCCCCACCGGTGCATGCCGATGGATATCTATTTTGAAGAACACCGTTCCAATGACTGA
TCTCATCTCTACTGGGCTTGTTGTAAAGCTCAATGTCGCTGTGTTATTAACTTCTAGG
TAGTTATTATGTTTGTGTACGTGGTACGTTACGTTAATGAC
>50818288_3 CO729558.1
TACGGTCGGAATTCCGGGTCGACCACGCGTCCGTAGAAATCAAAATATTAAAAATATTTGGTGATATTGTGGT
ATATTAAGTATTTGGTGTGGACAAAACGGGCAAATTTAATAGTCATGGCCGCGTTAATAAAATTGCGTCACAT
GCCCATAATTCAACGCTCCGTTTGGAATAATTTTATTAAAACTAGCCGAAATATTTCC
ACTTCG

>50818293_3 CO729563.1
AAAAAAATCACCTGTGTATAATATAAAAAAGGCTTTTAGTTATTGTGTATTGTGATCTTTAATACAAATAAGG
GCCGGGCTTATTAATAACATTTGAAAATTGTAGTAATAATGAGTACTTCTACTGTAAAACCAATTGAAGAAAC
GCAGGATACAGAAGAATTAGACGGAAAACGTAAACTAAAAAAATGGCTTAATGTAAAC
TTCAGGATCGAGATGACTGATGGTCGCGTTCTTATTGGAGTTTTTCTATGCACTGACCGTGACGCAAATGTTA
TATTAGGAGCGTGTTCAGAATATCTAAAGACTGTTGATGGGGAAACAGAAGAACCCAGAGTATTGGGACTAGT
TATGGTACCTGGACGACACATTGTATCTATACAAATTGATGACACAACACCACAACAA
AAATATTACTACGATGAATAAAGTAAATTACACTTTATATAATTAGGTAATGAAATATTTAAATTTTTCTAGG
ATAAGTTTTAAATAAAAATGAATAGAGTGAT
>50818294_3 CO729564.1
CGGTCGGAATTCCGGGTCGACCACGCGTCCGCAGTGCAATGCCAGTGATGTACCCGAAAGGTGACTGTTGATA
ATAAGTAGAAGTAAATTTATCTTCATTTCGCAGGTACCTTAGCGTATGCCAGCGCCCCGCGTACGTGCCTCAA
CAAACCGCGCCGCCTAGCCGCCTGATATATTGGCGGCGCGGCGTAGCGTGAGGTGTTA
AAGCGCACCCTGGAGCCGCATCGAGGAATTTTGGTGCGGTGGGCGCGGCGCCACAATGGACGACGGAGGCAGC
AACAAGCAACGCCAAGCAACGCGGGTGTTCAAGAAGAGCTCGCCAAATGGCAAGATAACAGTGTATTTAGGGA
AAAGAGACTTTGTAGACCATATCACACATGTAGACCCGATTGACGGGGTCGTGCTGAT
CGACCCGGAGTACGTGAAGGATAGGAAGGTGTTTGGGCATGTGCTCGCCGCTTTCCGATATGGGCGCGAGGAT
TTGGACGTCCTCGGCCTGACGTTCAGAAAGGACCTGTATTTAGCTGCTGAACAGATATACCCAGCAACAGGTA
CCCCAAA
>50818295_3 CO729565.1
GCAAATTCTAACCAAAGCAAAATGTTCTCCAAAGTTCTAGCATTATTCGCTATGGCAGCAGCATGCAATGCAG
GTCTCCTGCACCATGCCCCAGCTGTCTCTCACAATGTGGTTCACCATGAAGTACCAGCCCATTACGCTGTTCA
CGCGGCTCCTTCGTATCACGCTGGTCACGCTGTGCAATCTGCACCTATTCACACCTCA
GTCATCCACTCTGTCCCATCAGTCCATGCTGCTCCTGTAGTACACGCTGCTCCCGTATCTCACGCTTCCGCTC
ACGAGGAAGATCATTACATTGGTGAATTTGCTCACCCCAAATACGGTTACTCCTACTCCGTTGAAGACCCCCA
CACTGGTGACCATAAATCCCAGCACGAAACTCGTGATGGCGATGTTGTCAAGGGAGAA
TACTCTCTGTTGCAACCTGATGGCTCTTTCCGCAAAGTGACCTACACCGCTGACCACCACAACGGATTCAACG
CCATCGTCCATAATTCACCACCGGTCATCCATCATCATTAGGGTTCGTCAAACAACTGTGATATTAGTAGTAG
GTAATTTAGTTCTACATATTTTCGATTACATATCCTTCCGAAACTTCAGCTCTTGTAT
CTGTTGCTTTGTTTTTATATAAAGGGTTTCATTATTTCATTGTTAGTTTTTACAATAATATCATCTTAACATA
TTTTATG
>50818297_3 CO729567.1
TACGGTCGGAATTCCGGGTCGACCACGCGTCCGGAAAAAGAAGGATAAGAAAAACGAAGAAAGAATCAAAACG
AAAGCGCAAGGAGCAAGAGCGTGAAGAGAAAAGAAAAGCTAAGTTGTCGCGCACCTCCAACAACTCTGAGTAC
AACCTACGTGATTTAGACAATATTGGTGATAAGAAATTACGTGATGCTAT
>50818298_3 CO729568.1
GGATTCCGGGTCGACCACGCGTCCGTAATATAAAAATCTGAATTAAGAATTTTTCCAAAAGTGGTCATCTAAC
TAGCGGAAGTTAAGTCGTGTTGTGTTTACCTTTAATTTTGAGTGATAATTAAGTGTTTAAAATTATTTAAATG
TTTATACGATTTTTATTTGTAATAAAATAATTAAACAAATGAAAATAATAATAAAAAT
CTGAATTAAGAATTTTTCAAAAAAAGCGGAAGTTAAATCGTGTTATGTTTACCTTAAATTTTGAGTGATAATT
AAAGTGTTTTAAATTATTTAAATGTTGTTTAATATTGATTTTATCATTAATCGAATTTCTAAAATCGTGTTAA
ATATTACATGTGGTTACTGAGGATTTTACTTAAATGTAAAATTGTTGTGCGCCAGTAT
TTAAGCGTACTATCAAAATAGATTCTTTTAAGATTGGAAACAAACAAATATTAAGTTTTTAATATTACTTATG
ATGAAAATATTACTGGTAATAAAATTAAAAGCACTATCCTAGTTCAGTATCATTTTGTTTAAAATTTTGAATA
AAGTTTTGTGAATGGTTCTTTTGCCATGCATTATTTCTAAATAATTTTTAGAACTTTT
TAATTGTCGCGGATTTTGTGATGATGGATTACTACGAATAAATTAAGTTGTTTTGTCACCGCACTGTGAGACC
AGTCTTAATCGTTTT
>50818302_3 CO729572.1
CCCGGGTCGACCACGCGTCCGGTCTCGGAAGAGACACCTGGAAAACCGGTTAAAATAATAATAAAAAATAACT
AATAATAATGGATTTAAACGCATTGTTGAATAGTACGGTTTTAACCAATGACATCTCTTCTAACGTGGTTAAT
TCATCTTACAATATGAAGGCGATAGAAGGAGTGGTTGTAACTAAAGCAGCCAGCTTTG
GTGCCGTAAGCGGGGTTTTACTAGCACTTTTAATCAATTTTCTCTTCAGTGTTGAGTTTGAAACATTTAAGAA
GAAAGTGAAAATTAAAGTAGGAAACGAAATTGAACTGCAAAGAATTTTTATGGTAGTTATTTTAATGTTAGCT
TTCCCGTTAATATTTGCGTTTGTAATCATAGCATACTTTTATAAAACTTTATGTTCCA

TAATTATTAAAAGAAAAGACAAACATTTCGCGGGTTTTCTCGATAGTTTCGATGTGTTTTGGAGTTTAGAAGA
CGATGCTACTAAAAGTATAATTAATGTTCTCGGAGTTGTTGGCAGTAGCTCATCAGAAGAATTAGTTGAACAT
ATAAAGGAGAAATTACAGAATATCGTACAAAATGCCGATAGTGAAAAATTATTTTACA
GAAGAAGCGAGGAATATGGCTTTTATTATTGGAGAAAGTGCTGTTACGTCGATATTAGCCAATATGTACGGAT
AATCGACGTAGCTAACACTGCCGAGCTAAGTTTATCTGAC
>50818303_3 CO729573.1
ATTATTATTAATTCTGTGTTCGTGACAATTAAAAGATTCAAAAATAATGGGCTTTTTAGAAGAATGTGATTTA
AACAACATACCAAACATACCCGAATATTATAAAGGGAAAACTCTTTTAATCACTGGTGGTTTAGGTTTTATGG
GCAAAGTCCTGATAGAGAAACTGCTATACTCATGTCCAGATCTAGACAAAATTTATAT
GTTGTTAAGAAGTAAAAAAGGGGTCAAGCCAGAAGATCGATTATCAGCTATTTATTCTTCTAGATGTTTCGAT
CGTCTAAGAAGAGAAAGGGCGGATATTTTCAAGAAAAAAGTTTTCTTCATCAGTGGTGATGTCAGTGAGATAG
GTCTAGGTATTTCAGATGAAGATAAAGCTTTAATAGTTAAAACGACTGACATTATTTT
TCACGTAGCGGCTAGCGTTAGATTTGACGATTCTCTAAAAGTAGCGGCAAACTTGAACCTACGTGGAACAAGG
GAGCTGGTAGAACTTGCTAAGAATATGAAAAATTTAGAAGTTTTAGTTCACGTTTCGACATCATACGCTAACA
CGAACAGACAATGTATAGACGAAGTAATATATCCGGCCTTTCGCGACTGGAGGGACAC
CTTGGAGATTTGTGAAAAGTTGGACAATAATACTTTGAATGTTTTAACTCCCTAATATTAGGAGAACTACCCA
ATACTTATGTATTCACGAAGCAGTTGGC
>50818304_3 CO729574.1
AGAGCAACCGCAACTGGTACATCCAGGCGACCTGCGCCACCTCCGGCGATGGCCTCTACGAGGGGCTGGACTG
GCTCTCCAACCAGCTTAAGAACGCCAACCGCTAACCTGTGCTCCCACAGACTTATCTGATTACTGAAAGAGAC
TATGGTTTAAGATATGTTCACTTTATTTGCTATAAATACAATGTATGTGCGAAATTTA
TGTACTACCGTCACCCGTCGACCGAGCTCGCATCGCATACCCAACACGCTTGCCGCGCTTAGTACTCGCGACT
CTTCCTCCTGCCGACGATTGAGATATCCATATCTATCTATTGATTTGTACAAAACGAGGGGTCGCGCCTGTGC
TGTGGTGCAAAGGTATTAGTCAATGGGATTGCGTTGTTTGTTCGATCTCTGATGACGT
CGAACAAATATAGTGTGTAAAGTTATTGCAAAATTACATTTTTTTTTGTGTTTATATTTATCTAAACGAAATA
TGAAACTGTACATTTGATTAATCGCATTGTGATGTGGATAGGAACATGTGTAGCTGACTCGTGATGAAGTGGA
TCACACCGTAACATGTTTTATATGTATAC
>50818306_3 CO729576.1
GAAATATTGAGCGGTGCTCAGAGAATACACGATCCTGAATTTCTTACTGAGAGAGCTAAACACCATGGGATTG
ATGTAAGCAAAATAGCAGCTTACATAGAATCATTCAGGCTGGGATGTCCCCCACATGCAGGCGGCGGCATAGG
TCTAGAACGTGTGGTGATGCTATATCTGGGTTTGGACAACATTCGTAAAACGTCAATG
TTCCCGAGAGACCCCAAAAGAATCACTCCTTAGGGGAATTAAGATATAAATGTCTTTTTTTATTATATTATGA
TTTATAATAAATATATGTATTGG
>50818311_3 CO729581.1
CGGTCGGAATTCCGGGTCGACCACGCGTCCGCCGCATTTTCGACTCCTCTAAAATTGGATATGTATCCACTAG
GTGGTTGCTTCTTGCTGCTTTTGTTTTGAATAAAAAAAATATTTAGTTTTTTTAGTTAGCTTTAGTTTATTC
TATTTAGTTCTATAAATATATATAAGCTAATATGTAAAAATGATTGAAGTATTATTTA
GGTAATAAAATTAGAATAAATTGTT
>50818313_3 CO729583.1
CCACNCGTCCGACAGAGCGCGGGGCGGCCCCCTTTATAGAGGTTTTTTATGATTCATTACGATAAAAGACGCC
ATATCAAGTAATTCTGTTCACACGGCCATTAATATTATTATTCGTCTGTAATTTTCTACACTGCACGCTTGTA
AAAGTGAAAATGGATGCAAACGAAGTACAATACCGGCCTGTACCATTTAGAGTGCATG
TTTTGAGTGCATTGAGAACATTAATCGATGTGATCACCGCTATTTTATTTAAATTATATTATGGCACACAAGG
AAATAGGATACCGCCGATAAAAGACGAGATTCTGAAGCAGCCGGCTGTGGAGGTTGCAAGGAGAATAAGGACT
AAACAGATAAGCAGTGAAGATGTAGTACAAATATGTATACAACGGATTCGAGACATAA
ATCGTCAGTTAAATTGCTTGGTAGAAGAACGGTTCGAACTGGCTCTGGCCGAAGCTAAAAAGGCTGATGAACT
GGTAAGAAGTGGTTCGAAGACGGAAAATGAATTGGCAAGAGAGAAACCTTTCCTGGCCG
>50818314_3 CO729584.1
CGGTCGGAATTCCGGGTCGACCACGCGTCCGTCTCATCTATCGCATCAACTAACTAACTGTTACCTGTAAGCT
AATTGCCCAGCCATCCAAAATGCCTGCCCCATGTGAACCCTGTAAAGATTCCTGCAAGGATGCCGCTGACTGC
AAGGACAACTGCAACGCTGACACCTGCGCCAGCGAGTGTACCACCAGCGGTGGTTGCT
GTTAACTCATCAGCAATATTTTCTTCGCGTGTCAAGGATGCTGCAAAATGTTAATTTTATTTAGTTAAGGTCG
CAATAAATCTTTGGTGCTCTC
>50818315_3 CO729585.1

GTCGGAATTCCGGGTCGACCACGCGTCCGAAGAAATGTCACAACCTCAACAGAAAAGCTGTTCTTAATTTATA
TAAAACGCTGTTATTTTTAGGGAAAGATTGGCCTCAAGGGTATGATTTATTTAGAAAGCGTTTACATAAAGTG
TTTGTGAGGAATAGTGAGGAAACAGACCCTAATAAGATAGAAGTAATGTTAAAACATG
GTCAATTTGTGGTCAAAGAAATTGAAGCACTTTATAAGTTGAAGAAGTACAGAGCTATGAAACGAAGATACTA
TGATGATAATTAAAATCGATACAAATGGTATGTAGGTACTTATGTTAAATATCTTAAGGAAAATTAGTAATAT
TGAAAATGAACTAAATGATCATTCTTCAATTAAAACACAATTATAAGATTAATTAGTA
TTTTACATAAAATTTATGTAAATATTATTTTAAAATGATTGGATAGTTTATATAGTACTGAAAGTCTGTGAAA
AGAATTTAACTTGTTAAGTAAATCTATTTATGATCAATGTTCATTATTAGAAAGACAATGACAGCCTAAACCC
ATGCAATGTAAGCTATTACAAGAAGATCACATAAGCAATTATTTAAAGTTTGCTAATT
AACATATAAATTTAAAATTTGACACAATGTTTATTGTGTAAAATGAAACTATAAACATCTATGTAATTTTGTA
TTATAGATACTTTTATGATTCACATAATACAT
>50818316_3 CO729586.1
GATTCCGGGTCGACCACGCGTCCGCAAATTTATACATGTAGACCTTTGGGGCCTCATCTTTTAAAATAAAGCA
GCATAATAAATAAAAGTGAAGTATTAATTCAATTCTAGGTGCAAACAAATAAACATATTGCAATGGTTCCTTA
AAATATTTAGAAATATTGATTTAAAAGCTCCCGTGTCATGAGACTCTAAGCTTATGTA
ACATATTTTTTCTCAAAATAACTGTACGATCAGTGATAACTAGTGTTTACTATCGTAAAGAGTGAAAACGAAA
AACAATGTTGCGACGCTGCTCTAAACACTTGCAGACGATATACCGGAGGCAGAGCCAAGGCCTCCGGTTTAGG
TCTACAGAGGTGCCCAAGATCTGTGGCGCTGTGGCTCCAGGGACCAAACTCCTGAAGC
CCAGAGTCCTGGCTGCCCATCATCAGGTTGCCACCATCCACTTTACCAACCCTCTTCAAGCAGAACAGGATGT
AATGACTCCGGGATTCCCCGATTCCGTTTCAGAAGGAGATGTCAAATTAGACAAAAAAGTTGGAGACGCTGTA
GCAGCTGACGAAGTGGTGTTAGAAATAGAAACAGACAAAACAGCCATACCCGTAATGG
CGCCTGATAATGGTATTATTAAAGAACTGTATGTCCAAGATGGTGACACAGTGAAGGCTGGTCAGAAGTTATT
CAGACTTGAAATAACTAGTGGTGCTCCAACTAAAAAAGAAGCGCCAGTTGAGAAGAAGGCTGATGCTCCACCA
CAGCTGCAGCTGCGCCACCT
>50818318_3 CO729588.1
GATTCCGGGTCGACCACGCGTCCGAGTTCTGATTAGAATTCTCACATAAAATCCACAATGACCACATACTTCC
AATACCCCACACCTGAGCTCCAGGAAGAGCTTAGGAAGATTGCCCAGGCAATTGTTGCTCCTGGCAAGGGTAT
TTTAGCTGCTGATGAGTCCACAGGAACCATGGGCAAGCGTCTCCAAGACATTGGTGTT
GAGAACATCGAAGAGAATCGTCGTAAGTACCGTCAGCTGCTCTTCAGTACTGATCCAGCCATCTCCGAGAACA
TCTCAGGTGTAATCCTGTTCCACGAAACCTTGTACCAGAAGAGTGATGATGGCACTCCCCTGGTGTCTCTTTT
GGAGAAACGTGGAATCATTCCCGGCATTAAGGTCGACAAAGGTGTTGTACCACTCTTT
GGATCTGAAGATGAATGCACTACTCAGGGCTTAGACGATCTCGCCCAGCGTTGCGCCCAATACAAAAAAGACG
GATGCCATTTCGCAAAATGGCGTTGTGTCCTCAAGATCGGTCGCAACACACCCTCATACCAGGCCATCTTGGA
GAACGCTAACGTTTTAGCGCGTTACGCTTCGATATGCCAGAGCCAGAGGATCGTTCCT
ATTGTGGAACCTGAAGTGTTGCCTGATGGTGAACACGATTTGGACCGTGCCCAGAAGGTGACAGAGACCGTAT
TGGCTGCCGTCTACAAGGCGTTGAATGACCACCACGTCTACCTTGAGGGTACTCTTCTGAAGCC
>50818322_3 CO729592.1
GGTCGGATTCCGGGTCGACCACGCGTCCGGTCAAAAGTTAACGTGTTTTTCGTGACTCGGTAATATTTAATAA
ATTTATAAAGTTTTATGTTTCATAAAAAACGTATTCAAGCTATATATATTTCATTTTGACCTAGCATCGTTTT
TATAAAAAAAATAAAGATATTTTGTTGTAACATCATCAATTTATCTGATCTTGTTTCA
CTATGTCTGCGGCAAAAATAAACGAACTACGTAATGATGTTGATGAACTCAAAGAATTATTAAAACAAGCCAA
ACGCAAAAGAGTTCAAGATATTCTGTCGTTAGAAATACGAAAGATTGAAACAGAATTGATTAATTTGAAAGAA
AATGCAAAAGAAACACCTGTGGAAGTGTCCCCTGCACCAACCACATCTCAGACACCTA
AAAGATATCAAATTAAATTAAATGGATATGGTTGGGATCAATCGGACAAATTCATAAAAATATTTGTGACTCT
AAAAGATGTACAGAATATACCAAAAGAACAAGTGTACTGTAAACTAACTGAAAAGTCTATGGAACTTCATGTT
GAAAACTTGGATAATAAAGATTATTTGCTTATTATTAATAAGCTTTTGTACTCTATCA
ACGTCAGTGAAAGTCATTGGAAACAAAAAACAGACATGGTAGTTATCTTTCTTGCTAAATCACAACCCAACAC
AAAGTGGTCTCACATGAC
>50818327_3 CO729597.1
GTGGCAGGATTCCCGGGTCGACCACGCGTCCGGCGCGTGCAAGATTCACGTCTCTAACGCATCATGAAGTGGT
TCTACTTTATATTTACTTTGTTTATATCAATACAAAATATCGAAAATACTTTACAAAAATTGTTACCAAAATG
TGTTATTAGAAATTTGGCTGAGATGAAGAATGTACTTGAAGAAAACATACACCCAGGA

ACGGACCTTTTATATGAAAATTTTATGAACTTTGTAAACTCTCAACCTTCAAATGATGAAGAAAGAGGTGAAG
ATCCCAGTATAGACATGACTACACCACAATTGATTGCTTCCCATGGATATGTTGCTGAATCTCATACTATTGT
GACTGATGATGGATACATACTGACGGTTCATCGGATACCGGTTTCAAGAAAATCAACC
TGTAATACTGATAGTCCTCGTAAAACGGTTTTGTTGCATCACGGACTTTTGGGCAGTTCAGCTGATTGGGTTA
TTCCAGGTCCCGGGAAGGCTCTCGTTTATATATTGTCTGATGCCGGGTATGATGTCTGGCTAGCGAATGTTCG
AGGTAACACGTACTCTAGGTCGCATATATCTTTAAGTACGAATGACTTTGCCTTTTGG
AATTTTACTTTTCACGAAGTAAGTCAGTACGATCTGCCAGCTGTCATCGATTATATTGTTCATAAAAAAGGCT
CCGATGTAAAATTGAATTATATCGGGCACTCGATGGGTACGACTATTCTATTTGCTTTGCTGTCAACGAAAAC
TCATTA
>50818331_3 CO729601.1
GGTCGGATTCCGGGTCGACCACGCGTCCGTATTATCAAAAGACAAGAGCTTCGAAATATTCAGCAATGACAGT
GCGAGTAGCTCTTTAGATTTTGAAGTTATTTCAAACCAAGACCAAGAAACTAACCGTTTAAGCCTTTCTGATA
TTGAAATAGAAATTATAGAAAAAGAACCTGAAATAAATACGGAACCCGTGATTGAACC
AGAAGCTCACATAGAGTATAAGAATCGTTTTACTAAAGCGAAAAAATATTTTCAGTCGTTGGAAGAGCTTAGA
GAGG
>50818334_3 CO729604.1
CAGGATTCCGGGTCGACCACGCGTCCGATATGATAAAAACATTAAGAATAGTGTTTTCTATCCAGTTAATAAA
AACTTAGTACTTACTGCAGCAGCAGTCTGTACTCCGACGGCTATTAAGCCCATAGCATTAAGTCTTATAGGTG
CTTGAACATTGTACAGAAAAAATAAGACGATTTTTTTATTTTTAACGTCCATGCATTC
CCATGGTAACCCATAAACTGCTTCTTTTATAACTTCGCTCTAAAATCCGAAATGGTAAAATATATTATTTAAA
TTTTATTAATGTGTATAATTTTTAGTTATCTCTACTTCCTTCGACTATTTAAACAATAATGTTTTGCTTTTCG
ACTATACT
>50818340_3 CO729610.1
CGGTCGGAATTCCGGGTCGACCACGCGTCCGAAAAAAATGTAATCAAGCAAGTTAAAAAACATCTAGTGCACG
GTTTCGTGGACTAGATGTTTTTTTAACTTGCTTGATTACATTTAGTCCACGAAACCGTGCACACCTAAATTAC
CTTCTTCCTTTTCATTCATTTTAAATCTACTAATTTGAAATTAAATATTTGTTATGAC
CTTATATAATTTAACCAACTTTACGGATAGTGTGAGTTTTACCATAGTCAATTCAACTAATAAATGAAGCAAT
AAATTAAAAATGTCCAC
>50818341_3 CO729611.1
GAATTCCGGGTCGACCACGCGTCCGACTCGCTTTAATACACGTACTTAAAAAGCTTCCCCACAAGCTTCCCGT
AAAAGCTAGACTTTACACTAGCACGTCCCTCACAAAGCTTTTAAAAGCTTTTCTCTTTCTCTTTCTCTCGGGT
CTCTTTCATTCAAAATGAGAGTTTTAATTTGTTTTTTGTTAATATGTTCGGTGTCTTT
GGTGACCGGAAGAATTGTCGACAAGAGGAAGTGCCCAGAAGTGAGAGCGGTTGCGCATTTCGATTTGGCCCAG
ATGTTGGGTGACTGGTACGTAGTTGAGTACTACGCGAGTGCTGAAGAGGCGCTCTCGTACAGCTGCATGAGGG
CCGTCTTCACTGAAGACGATCATGTGCAAGCTGAAGATGGTTCAATCGAATGGACGCC
GGGAGTAACGATGAACTTCACATACCGTTTCGCGGATGACCCACTCGGTGAGAACCTCCTGGGAAATATAACT
TGGAAAATAGATTTGAACGAGCCAGCACATTGGACACATTCGGAAATGACCTATGATGGCATTTATAATACGT
ACGTGTTAGACACAGAATATAAAACTTGGATGCTGCTACTGCATTGTGCTGAACAGAG
AGAGGGCGCCAGATACCTTAGTGCCTTCGTCTTATCTAGAAAAACTACTTTAC
>50818348_3 CO729618.1
ATTCCGGGTCGACCACGCGTCCGAGAAGAAGTAAACATGTCCCCTTTAGGAGATTTCCTAAGACAGAAAAGCT
AAATATTACCAAGCACAGGCTTGGGTCAAATGTTGTTGGCCTAGGACTTATTTGGAGTCATTACAATCCCTAC
TTACTTACATTAGTTTCGGGAGACATAATTTAGATGAACAATGCCAACCTTGTCTTTG
CTCTGATTGAAAATACAAAATATTTACATATTGTTATGAATGAAAATGTACTAAAAGATTTAAAGAAGAAGTG
AACAGGATACCTTACTTTGACAGAGCAAATACAATATTGTGACATTTTTAAAGTGTTATTGGAAAGAAAAATA
TTATTTTGGGAAACTTATAAAATATCACCTCTTGTGGTGGTTGAACAGTTTTAAAAAA
GTGCTTCTAAATAAATAGCTTATAATTTATGATGAAATTGTTTTCAAAATATTCAGAAAACAGGTGGTGGTGG
TGGTCCTTTACACCTACTGTAATGGAGAGTGAATACCAAATACTCTCAGTTTTGGATAATCAAATACACCCCA
ACAACTACGACTCGTCGATCATACT
>50818349_3 CO729619.1
GATTCCCGGGTCGACCTGGAACTGTTCTGGATCCTGGACAACATCGCCTTATCGGAAGCGCTGGTGGCGCGCA
ACGGCTCCGGCATCAACAGCACCGCCGACCTGAAGGGCAAGCGCGTCGCCACGCCGTTCGTATCGACCTCGCA
CTTCCACCTGCTGGTCGGCCTGGAGCAGGTCTGGAAGATCAACCCGCGCGACGTGCGC

ATCCTGAACATGCAGCCGCCGCAGATCGTCGCCGCCTGGCAGCGCGGCGACATCGACGCCGCCTACGTCTGGC
CGTCGTTTTACACCGAGATCGAGAAGAGCGGCAAGGTCATCGCCGATTCGGAAGCCATCGGCCACGCCAGCGT
GCCGACCTTCGACGGCCTGGTGGCGGACCGCGCCTGGGCCGCCAAGAACCCGAAGTTC
ATGGCCGCCTTCACCGGCGTGCTGGCCAAGTCGTATGCGGCCTATCGCGCCAACCCCGCCGCCTGGACCGCC
>50818351_3 CO729621.1
TTCCGGGTCGACCACGCGTCCGCAAATTACAAGACACCGCCTGCATGTGATCTTTCAATGCTACAAATTATTA
TATTTTTAAAGTAGGTACAAAGATCTCAATATTTTCTTTGCACAAATGTTTTCTCATTTATATAGTAATATGA
TATAATAATTAAGGATGTTTGTCTTCTTTATATTAGCAGTAAAAAAACATAACACACT
AATGTTTAGTAACACTATTATAATACATAAGTTGCACAGCTAGCTATGGTTCCAGAACAGTACATTTTGCACA
TTTAAAATGCAAATCATTAAATTGTCGGTTATATAGAAAAGTGGTATATTTCAATGGAGATCATTAGATTAAT
ATCATTTATATTTATGAATTGAAACTCTCTAGATGATATAAGTGTCATTTCGTTTAAT
ATGGAAAAATAAACATTTATTGCTGCAAATCTTTAACCTTTAATTCTTTCCAAAATATATATTT
>50818354_3 CO729624.1
ATTCCGGGTCGACCACGCGTCCGATCAAGGGAGTCATGACCAACTTATTACTGCTTTTCAACAGGGTCTGGCA
CCCGACAAATGGCAACCGACGAAATGGCACCCGACTATAGTGGTTGGTTACAACCAAACATCCTTGTAGTCAT
ACTAATCATCGTCATCCCGATTGTGGTGATAATGCCGCTCTGCTCCATCTTCCTGTTC
CTCTACTATCGGGGCCGACTCATCCGCGAGATCAAAGGGAAAACTAAGGAGACGCCCTGTTTGGATAAACTCG
GCGTTGCTCCCGTGACTTCACAACGAAAAGAGAGCGTTGATGCGCCTCGCCGTCCTTCCAAATTCCAGGAAAC
GCTAAAGCTGCGCTATGTGCAGTCACCCACATCGGCGGTGGACCGCTGGGAAATTGCG
AGAACTCGCATTACCCTGGAAGAGGTAATTGGAAAAGGGGCCTTTGGACAAATTTGCTTGGGGAAAGTCGACG
GGACTATTTTGGCCAAGGACTCACTGTCGGATACCATCATACAGTTCAGGAATCGCAATGTGGCTGTTAAAAT
GCTCTCAGCCAACGCGAGCGATAAGGAGCAGGAAGCCTTCCTGAAGGAAATC
>50818358_3 CO729628.1
TACTTTTCGAAATTACGTAATTTATATATCGAAATTTCTTAGTATATTTAATTTGATTTTAAAAAATGGGAAA
ACATTTTGGAGAATTGGCTAAAATCAGGGGTTTGATAACTTACAAGTTATCGCCTCATGAGCAACGTGCTTAT
GCAGGAGCTATCGCTAATGGTATTCCAAATATATTTCGCAGATTCCGTGAAAGTGTGT
TTAGAGTGGCACCGCCTTTTATAATTGGATACCTTATATTTGAAGGTGTAGAAAGGGAACACCACAGACTTGG
GCGCAAGAATCCAGCAGATTTCGAAAATGATCAATAATTGAGAATTATGAAGTAGTTGTATAGTATTTAATTT
AATAAATATTTTATTTATCTCAT
>50818395_3 CO729665.1
TCCGGGTCGACCACGCGTCCGCACAGTCACCGCGATATATCCCGAGTACTTTTTGTGAATCTTACTAGTCATA
AATATGTCTGAAGCTTTCTTTGATGAATACGACCACTACAACTTCGAGCACGACAAGCACATCTTCTCCAGCC
ACAGCGGCAAGCAGCGCACCAAGAAGGAAGTGAGCCAGCACACCAACCACTTCGATCC
GTCGGGCCATTCCAGAAAGATCGTCACCAAGCTCGCCAATACAGAAAACAACAAGAAAGGCGATAACAAACAT
TAAATAAATTTATAAACGACAGACGACTATGGCGTGTAAAAGGAATATTGAATTTTTGCCGACGGAACGGAGG
ACGTTATGGCGAATACCCGAAGAAAGCACGGGTCGCTCGTCTCTGAGATCAGCGGAAC
AGCCGAGAAGGTTTTTGGTAACGCAGAGTTTTTATTATGCACACCACAATGCTAGAACACGGTGGCGCAGCCG
ACGCGCCTCGTGACTCGTGCCCCCACCGGTGCATGCCGATGGATATCTATTTGAAGAACACCGTTCCAATGA
CTGATCTCATCTCTACTGGGCTTGTTGTAAAGCTCAATGTCGCTGTGTTATTAACTTG
TAGGTAGTTATTATGTTGTTGTACGTGGTACGTTACGTTAATGACTGTAATAATTTAAGATAA
>50818396_3 CO729666.1
CTCGNAATTCTTGGTCGACCACGCGTCCGATTTCTGTTCAAAGGTACAAAAAAGCTAAATACACCAATAGTAA
AAGTGGTAGTAATATTTTTTAAAAATTCAACGACAATATTCTACTTTTATTATATTTTCATGAATATATATTT
TAAGTGTAATATAGTCTTTTTTGTGTTAAAATATTCTATTTAGTGCCTAAGTTAATAT
TTAATGTGACTATATGTATGTTAGATGTTAACATTAATATTTGACTTTCTAAAGTAAAATTGTGCTAAATTCC
ATTATAAATAGGAAGTAAAATTAAATTATTAATAATAATCAAAATATAATTTATATGATTACACAAGTTTATT
TTGCAATAGAAGTTAATATCAAACTTAACAATGGTCATTTTAATTTATACTATATTAC
TA
>50818397_3 CO729667.1
CCGGGTCGACCACNCGTCCGCGCACTGCGAGGGGGAATACCATTGATCAGTTTTGTCTTTCGTATCAATAAGA
GAAAAACCGTTAATGTATGCTGTAGAAGATCATTTATCTAAAGGATTTAATGGGCAACAAGTTATACAAAAAT
ATGAATTCGCACTAGATGAAGTGGAAGGATTTCGCTATCGATCTAGAGACGCGTGGCG
CGCCATCACACGCATCGCTGTACGAGAGGCGCGGTTGAATGAAATCAAACAGGAACTGCTTAATTGTTAGAAA
TTACAAGGATATTTAGAATAAAATCCAACTGACTTA

>50818398_3 CO729668.1
ACCGTCGCGAGTCGCACACGCACGATACGAGACACAGCAGCAGCCATGGTGTACGAGAGCGACTTCTACACGA
CGCGCCGCCCCTACCGGCCCTCCAGCTACAGCGTATCGGCTGAACTCATCTACCGGCCGACCACTCGCACCGT
GACCCGACTCGTCACGTACCCGGAGCCGCCCCACCACATCGTGCGCGTGCGTGTCCGT
CCGTCCGTCATTTTGCGGGAACTCGACAGGATCCAGTACAAGCGCCGTCCCGCTACTGCTGTTTCTGCTGTCG
ATGATTTTTACAGATCTGAATCTAATAGGATATTCGAAGACGAGACCCGCCGCATCCGCGCAGACACGGCGTC
TCTCCTTCAACGAGCTCGCTCGGTGGTGCCTCGCGCCAAATCAGTCGCGCCACTCGAT
ACTATTTACTCCTATTCTTACGGAGAGCCGGTACCATACCGTTTCAGCAATGACGCCTACGTCGCCAAGCTCT
TATTGCCTCTGCGCAGCGTCTCTGACAACATCCAGCACATCTCTTTCTATTCAGAACCCGCTAAGAAATTCAC
AGTGAAACTCCTTCCATGGAGACCTAGCCGCAAGTTTCAGGCGCCGCAGATAATGGAA
GACCCTGCTCTGGAGCTGAAGGCGGCGAAAGCAGAGCGTGCAGAAAGATTTAGGGCTACCACTG
>50818403_3 CO729673.1
GAATTCCGGNTCGACCACGCGTCCGATCTCCCTTTTTTGAAGTCCGCTTGAAAATGAGAACATCCTCTTTTTT
GAGATTGGTTGAAAAGAAATGCAACGATCAGGAATCTCACTTCTGACATCAATTTAAAAAGTTTTTGCTTGTA
ATATTACAGTATAATTAATTTTTGACG
>50818406_3 CO729676.1
GCATTGAACGTTGTGTTCTCCTCAAGTATTATTAAATTAACATTATTATAGAAATGAAGGTGTCTCTCTTTTT
TATTTTCTGCATTTTTGCCTTGTTTGCAATCAGTCAGGCAAACCCCGTTCCTGACCCCGAGCCCTGCGACTAT
GACGACTGCTACTACCGTCGGAGACCTAGTGGCTCCAGAAACAATTATATCGGCAATG
GACGCGGAAATACCAACACAGTTTATATTAACAGACCCAGACCCAGGAACTAGACATAATTTTTCAAAGAACA
GTATTAATAAGT
>50818410_3 CO729680.1
ACCACGCGTCCGGTCACTACCGCGTGTCGTGCCGGTTTTCAGCGTTTACACTTGTAATTTTCGCAGCTAACTT
CAAAATGGCAATTCACATCAAGGACTCCGATGACCTCAAGAACAGGCTTTCTGAGGCCGGTGAGAAGCTGGTC
GTGATCGACTTCATGGCGACCTGGTGCGGCCCTTGCAAGATGATCGGTCCCAAACTAG
AGGAACTGGCCAATGAGATGGCCGACTCCATTGTTGTACTTAAGGTCGACGTAGACGAGTGCGAGGACATCGC
CACCGAGTACAACGTAAGCGCGATGCCGACGTTCGTCTTCGTGAAAAGTTCTAAGAAACTTGAAGAGTTCTCT
GGAGCCAACTACGACAAACTAAAGAACACCGTGTTGAAACTAAAGTAAAAAAATAAAC
AATGTATATGTAACAGATGAGGCAAAGGAAATAACTTGCTTTACGTGTATTTTCATATATTATTCATT
>50818413_3 CO729683.1
CGAATCCGGGGTCGACCACGCGTCCGCGGCATCATCACGCACGGCGCGCTAGTTTACGGAGGGGCCCCGGGGC
TTTGCCCTCCGAGGGCCTCTGACCTCCCACCCCTGGAGCTAGGCTTCAGGAACAGCTGGAGAGAGGATGAGCT
GCTGCCGTCTACCCCGGCGAGTCAGGCCGCGTCGACGCAAAGCGGATCTTCCGCAACA
GATAAGGGACAGAACGTGGAGTGCGTCGTTTGCGGTGATAAGTCCTCCGGGAAACACTATGGCCAGTTTACGT
GTGAGGGCTGCAAATCCTTTTTCAAGAGATCAGTGAGGCGGAACCTTACTTACTCTTGTAGGGGGAACAGGAA
CTGTCCCATCGACCAGCACCATAGAAACCAATGCCAGTACTGCCGCCTCAGGAAATGT
CTGATAATGGGCATGAGGAGGGAAGCTGTACAAAGAGGTCGAG
>50818414_3 CO729684.1
GGTATTCCGGGTCGACCACGCGTCCGGCATTGAACGTTGTGTTCTCCTCTAGTGAGATTAAATTAACATTATT
ATAGAAATGAAGGTGTCTCTCTTTTTTATTTTCTGCATTTTTGCCTTGTTTGCAATCAGTCAGGCAAACCCCG
TTCCTGACCCCGAGCCCTGCGACTATGACGACTGCTACTACCGTCGGAGACCTAGTGG
CTCCAGAAACAATTATATCGGCAATGGACGCGGAAATACCAACACAGTTTATATTAACAGACCCAGACCCAGG
AACTAGACATAATTTTTCAAAGAACAGTATTAATAAGT
>50818418_3 CO729688.1
CGGCTCGGAATTCCCGGGTCGACCACGCGTCCGTGTAATTTTTTTCTAATAATCTAAACAAATTGTAACAAGC
ATATAAGAATTTGGACGTAGCTTGATGAGAACGTATGGTCCGAACGCTTGGACGGTTTCCAAAAATCATGGCT
TCGAGTGCCGTTACAATCAAACTTTTATACGTTTCCTAAATAATATATTATTCTACCC
TCAGTATAGAATAATAATTTAAAAATATTTAAAAGAAAATTATGTTGTTACATAAATTTTAACCATGTCAGCT
TCATATATTCGAAAATCATTTTTATATTTAAAATGTAAGTAACGTATAAAATGATGTCACGAGCAACTTTACC
TTCATTAAAGAAGCTAAGAGCAAAGCTATACTATATTTATTCATATATAAATATACAT
TTATAATTAATTTCATAATTATTGATTTTTTTATATGTAATTAATTTATTTTCTAGGTTAAGGTCGTACAGGT
ACATTTAATATAGAATATTTATATTATTTGAGGTTCTTTCTTTTATTTTAGCTACACGCTGTATCCTGCACGT
AGATAGAGGAAACCTAAGTACTCAATGTTAAAACTCTTGATTTTCCACAATCCACGGA

ATTCTGGAGGCATTGAGTGTTAAATGGTGGCATTCAATGCTACGCTTTGCCTCGTTACCATAGCGAAATGCTA
ATATAAATATTTCATTTTCTCGAGTAGAATTGCACCTACAACTAATCGCGTTTACATTCGTCACGACT
>50818422_3 CO729692.1
GGTATTCCCGGGTCGACCACGCGTCCGCGGACGCGTGGGGAAAAATCTTGAAGTTAGCGGTCGTGTGGGCGAT
AGTGGCGTGCGCACACGCAGCTCTGATGCTAGCCCCTTTAGAGAAGAAACCAGACAAATTTAAAGATCAACAA
GGATGTTACATAAAGGAGCTGGACGCAGTAATACCGTTCGGAGAGTCGCGGCCCTCCA
ACAACAAATGTGTAGAATACAGATGTGCAGAAGATATGGTCACTTTTGCTTCATGCGGCGTAGTAGTCGCCAT
ACCTCCTTGCACCCTCGTCATTGACAAAAGCAAGCCGTATCCACATTGCTGCCCAAAACCATCTTGTGAATAA
TTATTCAATCTATGCGTTTTATTTTTATATAAACATAATAAATATTGTAATACTGG
>50818429_3 CO729699.1
GAATTCCGGGNTCGACCACNCGTCCGATCAATCCTAATCCATAAATCGCACCCATAGATTGAAATAGACAGAG
ATGGAGGACGGCATGCAGGTTAGTGATTGGATAGACATGTCGAACAATACAAGGTTGACGTTAACTGGAGATA
GGAGATACATTATTAACATTGGGCGTAAATCAATACAATTCTATGATCCGGACAGACA
GGTCCACCCAATTAGTTCAGATAGTTGACACTCTACTGTACCCAATTTTGGAATAAAATCTATGTGTGGTAAC
TGGAGTGTTTCAATACTTATAAAAATGATAGCAGTGGCTTTTACTTTAATAATTCCATGCGTTATTTATAATA
AATAACGTATTTACGTCTTTAAATAAATTTTAA
>50818432_3 CO729702.1
GTAATTTATACAATATACTATAGTGGTATGATTTTTTATAATTTACTAAAGTATTTATTTCCAATGTTGGATG
TTTTTAATTCCATATAAAAATTTTACTATTTGATCTGAAATGATACTGGCAGACATCTCAATATTACGACCTC
GTACGTAAGATGTGAATTAGTTTTTAGTTGTAAATTTTTGTAAATCATGTACATAATA
CGAGATTAAATCTGCATTCTTATTTTTACTTTAAAATTTATTACTAAAATATTTTTTTATAAGATCTAGATTA
TAGATAGACGCAATAAGTAATGTTACATTATTGTATAAAAAAATGTATATTTTATATGCCTTTACTTGAAGTT
TATACATACATAGTTTTGTAATTGTTACAAGGTGAACATTGATTTGCCAAATTAATTT
AATGTTTTAATTTTTTCCATGAAAAGGGATTATATTTTGTAAAGATGCCAATACCGACGATAGAATGTTGATA
TTAATAAAACGTGAAAATAAAAATAATAAATCAATT
>50818435_3 CO729705.1
GGCACTAGAAAATATTGAACACCACGATCAACAACAAAATAATGATATTGTGACACAGCCAACTTTAATGCTA
AAAGAGAATACGCCTTCACATCAGTATTATTCTTTGATGGCAGCCAAAGCTCTGCACGGAGATCAAATGTCTG
CTCCAGATGCATCGAACGATAATTTTCAATACGCAGACGCACCATCAGTGTCAACGAC
CGGCTCTCCACCCGTACCGTCTACTCCTAACTATTATAATCTACAAAGTTCAGAAAATCCAAATGTGGACTTT
TCCAAGTTCTCAGCTGACATCAAATTAAATAAAATATCAGAATCAGGAAATCGACATTCGTTGAAAGATAGAC
AGACAAGTAGGACAAGGTTATTACAGAATCAAGAATCTACGCATTCGCATGCACAAGA
TAGCTTATTAGTCAAAGGAAAACTTAAATATGGTGATAAAATTTAGACTCTTTGGATAAGTAAATTTTAAAAG
TGAATTTGTTTGTATATAGTGATGTCAGAGTAGAAGGTCCTACAAGGAATGCCTATAGTGATATGAAGACATA
CTCATCATCCGGTGGTAATTTAAATTATTTATAAATTATATTATGTTTCCATTTTAAT
GTAAATAGCGCAAAACGATATCAGGCATTTCGTGTGAATTTGTAGTTAATATTGTAAGGATTAAAATAAATAT
TAAATATAATTTTACTATTAATTACGCCAACATCGAGGAGATAAAATATTTTTATTATGGACTCCGAGTATAT
ACCTATCTGTTTGT
>50818437_3 CO729707.1
CCGGGNCGACCACGCGTCCGATTAAAAAGCAGTTAGTAGACCCTTAACAACAAGCATTCTATAGTTCCCTTAA
TATTATTGTGATAGTTCTTTTAACATACCTATGTCCTATTAAGTTTCATCGAGGCCGGAAGTAATAAAATGGT
TATTTATATAATTTTTCATTCTACTTGAAATATATAAATGTAATATTTCATTTTAAAC
ATACAAATTTTAATAATAGCAATTATAATAATATTTTCTTTTCAATTTTGCTATAACTAAAGCTACGTACTTA
CTGAGTAGTCTAGATGTATACTCATACGTTAGCAATTATAAACAGTATTAGGCGTGCAATACGTTAAATATAT
TATTAAATAATTTGGCTAATTTATGGTGCCGTTCAAAAACAATAAATGAGTATAATAA
AAGGGCAACTAATAACTTTATTTATACAGTATGTCTGTTACCAGTGCTAATCAGAACATGACTTTGTATAAGA
ACACAACATATTTTTATGTCTTCAAACTATAAATATTACAAAGAATCATTTAACGTACTTACAATGGACCTTA
CTGTTATTTAATTATTTAAAAAAAGTACTTATAGTTTTTAGAAGTTAATTTAATATGA
AAATAAGCATTATGCGGTATTTATACTTAGAATTTAATCAGTATTCTTAATTTAATAATATTTTATATTGAGA
CAGATATCGATATAAACTTAGTATTGTAATTGTAGTATTAAGCCAACTTATTAGTACACACGTTGTGAATGTG
GGACCACTTTGTCTGTATAAAAAT
>50818439_3 CO729709.1

GGATTCCGGGCCGACCACGCGTCCGCTCGGTAAGAAATATGAAATCAAAATGTATAAGCTAATACTTGCTGTG
ACTGTTGCCGCTTTTGTTGGTGCTGAAGCTCACCATGGACACAATCATGAGCCCGTAGCTCCAGTGGGACCGG
CGCCTCTTCGCGCAATCGTTCGTCTTTCAAGTCCAGATGGTCATGACGTCCACGGTAA
TGTTACTTTTACCCAGCTAGACGGCCAAGTACGGGTGGAAGGTCATATTTACGGTATGGCCCCAGGACAGTAC
GGCTTCCATGTCCATGAAAAGGGAGATATCACTGGAGGATGTTTGTCGACTGGACCACATTTCAACCCTGATA
AGAAAGACCATGGCCATCCGTCAGACGAAAACCGCCACGTTGGAGACCTTGGAAACGT
TGAATTTAACTCAGAACAGCAAAGTCACATAGACTTCACAGACAAAGTGATTTCTTTGGTGGGACCCCATAAC
ATTCTGGGCAGGGCTGTAGTCCTACATGGGATGACTGATGACTTCGGCAGAACAAACCACCCAGACTCGAAGA
AAACTGGAAACGCAAGAGGACGAGTCGCTTGAGGAGTTATTGGCATATTGTAAGAATT
TATTTTATGTATTTTTATGTTACATCTAAGATTTCCTTTATATGAATTTTGAACTTTCTTCGCTGACGTTTGT
GCCATTTCTTATTCTTAG
>50818442_3 CO729712.1
ATTCCGGGTCGACCACGCGTCCGAGATCTTACAACTGTACGCTCCTGTATTACTTTCTTTAAAATAATATTGA
AAGTGACTTTAAAAAATAAGTTAAAATAAAAGGAAAAATAACAATTTGGACAATTAAAAGTTCTTATAATTGT
TCTAATTTGATTAAGTTGTGTGGGGCTTCTTAGATATACTATGTAAATCTGATTAAAT
AGTGCTCGTGGGAAAATATTGTATTGTAATTGAGTGCTCGTTCAAATCTGGAACTGTTAGGTTTTGTTAGTGA
CCTGTCACTACATTTTGTACTGGCATAGTGACTAAGTTGTAACCATCCATGATGGGAGTGTTTGTGACGAATA
CACTCGCTATGGACATAAGTCCAACAAGCGGGTCGGGCAGCGGGCCTGGCGGCGGCGA
CACGCGCCGGGGCGAAGTCACGCCGCACGCGCCACCGCAACGACGCCTCATCAAAGTTGACTTTGATAATTTG
GAGGACGATGAAGATGAAGACGACACCGAGACTTGCGTTAGTCCGGAAACCTGTGCTCCCCAACCTTTCCCCA
GGCATATAGCCCCGCAACAGAATCCACCGAAACCATGGAAGAGAACCGCTTACGACCT
GGCGACGCAGTGCCCGTCGCGGCGAGCTTCTATGCGACGACACACCGTCACACACCCGCCTTGCCCTACTACA
GATGGTGCTCAATCTTTACCACATGCGAGGCCAGGATCTCGCGC
>50818448_3 CO729718.1
GNATTCCGGGTCGACCACGCGTCCGGCACGGTGTAAAAACACAGCACGAACGGGATGATGTTTTATATCAATT
TAAGCAAGGTCGTGCTAGTATACTTGTTGCAACAGATGTTGCAGCTAGAGGACTAGATGTGGATGGAATTAAA
TATGTTATAAACTTTGATTATCCTAATTCATCTGAGGATTATATTCACCGCATTGGTA
GAACTGGACGGTCAAAATCAAAAGGAACATCATATGCCTTTTTCACCCCTTCAAATTCACGACAGGCTAAGGA
CCTTGTATCAGTATTACAGGAAGCTAACCAGGTGATTAGTCCTCAGCTACAGGGAATGGCTGACCGTTGTGGT
GGTGGTGGCGGTGGATGGAATAGAAACAGGTACGGAGGTGGCCGTGGAGGCGGCTCAT
TCAAGAGAGGATCAAACTTTGGCAGGGGCAATGGACATGGCGGAGGCGGACACAAGAGATTTAATGAATATTG
AATAGTATAATTTAATTTAAAAGATTTAAGCAAATCCTCATGGGAATACTTAATAAGTGTATGAATGATAAGA
GCTATGTCTCTTTGTTGGTGTTAGAGAAATAGTTAATCACTGATACATTAAAATATCT
TGATCTTGTATTTATTTCTGATTTTCAATTTATTTGAAACTGAGTGGACTCTGTTATTAATTTAATTTTTACT
GTGATATTTTAGTGAGGATTAGCTAAAGGTTAAGAGTTGTACCCTGTCCTAATTTTTTTATTTAT
>50818452_3 CO729722.1
ATTGGTGGGAAATTTATTTATCCCATATTTGTTAATTTCTTTATATGTAAAATTGAATCATATAGAAGAAAAT
ACAGCAATATCCAATTTCACAATAGAATTATAGTTATAATATATTTTTTTCTTAATTTACAGTAGATAATTAT
TGCGATAACATGTAATTTTCCAGATATTGCTCTAAATATAGATTGCTTTATTTGCTTC
TATACTAAAGTACTTATACAATATTATTGCAAAAAATATAATTATATTGAAGTATTCTTGTTACGGTTGTGGG
TTGGAATTTGAATGTCAATGACTTCTATGTACCATATCTACCGATATTAAGTGTAAAACCAAATGTACAAAGT
ATTTTACCAATTAAACCACTTTCATAAAAATATAAGAAATTACACTTTCTTAATGTTC
CTATAAATGTAAGTGTATTATTTTGAAAATTGCTCAGTGATACAGCTTTAAGGTAATAAAGGATTTTTTAT
>50818460_3 CO729730.1
CACACAAGGCGCACGGCAACATGAAGATCGCTATATTGTTATTGTTTTTCGTGACGTGTTACGCGGAGATCGA
TTTTAGCGGGAGAGCGAGGAATTTTAGCATAGAACTGCTATATCATACACAATTGGAGACAGATGGCCACGTT
GTTATATCACCTTTTGGCATATGGACTCTGATGACTGGCGTCACGCTGGGAGCTAGCG
GTAACAGCCGGAAACAACTGTCACGGGCATTACTTCTACCAAGTAGTGACGACTCAATCATCAATGGATACAC
AAATTTGACGAAGACCGTCCTGGATCCTCGGACCGAGGGGGTTAAGTTGAAAAGTAAAAATTTTGTATTCCTC
GATAAAGGTTTCAATATATTTCCCGAATTCAAAAGATTGTTGACAACTGAATTTGATT
CAAAGGCGATGTCCTTAGATTTCATAGATCGCACGGCAGCTAATGTAGCAGATGCTTTAATAGAGAAATCTGG
CGCCACCGTCTCCAATGTAC
>50818468_3 CO729738.1

CGGACGCGTGGGCGGACGCGTGGGCGGACGCGTGGGTAGTAAAAAGTTGTTGTGATGGATTGGTCGTTTTGAA
ATATTTTGACGGTCGCATCTATTGTTTTACGGGTCCACTTTGTGCGTTGATTTTTTTGTGGTCCTTTTGGTTT
TATTTGCTTTTTTCATCGTTTGTTTAATTCTATGAAATGAAAACATATAATATTTGTC
TGGACTAAAATTAAGGTCACCGCGAAATGGACTGAAACAACGCTAAACTGCGGGGAATAAATATTTTAGTATT
ATAATTTAGGCCATCTATATGAATTTATATTAACGGAGCTATTTATAAAGTTGAAATGACAAATTATGGTAGA
ACTATCAAAATATTTGTTTGCAATGTATGTACATAGTATAATATATAACGGGCTATAT
AGTAACGACGGTGGTCTCGCACTTGTGTGTTCGTCGACCGTACTGTACCTGTAACGCTATTTACTGCGCTAGT
GTAGCTTAACTATCGGGCATCTTTAGGTACGACAAAGTCTTTAATACATATGTTGTTCATAATAAAATTATCT
ATAAGGGAATTTTATTTGAGTGCAATC
>50818474_3 CO729744.1
AAGAAGATTTTCAAGCGACACGGCTTCAACGCCTGGTACGGATGGAAGAACCACTGTCAGAGTGGACTGCCTG
ACATCAGTATCCTGCTAAAAACTAAAAATATAAAAATTAAGTATTACAGATAATCTTTCAAAGATCAATTTTA
TTATTAAAAAAGCGTAATTAATTGTGTTATAAAAGAGAGTATTTGGACTGTGTAGATC
TTTTAAAAATTTTGGTATTTTGTATAATATAATAATATATGAGTTAAGAATAT
>50818475_3 CO729745.1
AATTAATAAGTTTCAGATCCCACATTCAGAATGGCTGGTGGAGACTCCGTCGATGAATCCCAACTTAAGGGAC
TCGCTAAATACTTTAACAGTCAAACAAATAAAGGGCGAGCTAATACTGCTAAGGCAACATATGCATTCTTTGG
AGTTGTAATACTGTATTTTGCTTTGAAACCAAAATCAAAGTAGGAAAAATTTTCAATT
CTATTTCAATCTAATTTAAGCAATTTAAATGTCTTTAATTCCCACATCAGCAATTATTTAGTTAAAGTGTAAA
ATCATGTATAATATTGTTTTTATTTATGAGATAGGAGAGTGAAAATAAATAATCTGTTATGTCAT
>50818484_3 CO729754.1
GGTCGGATTCCGGGTCGACCACGCGTCCGCTTTCCATGAAAGATCTGTGAGTGAATGAGTGTATGTATGTATG
AAAGTAACACTCACCATACAAAATCGACTAAATTAGTAAACTATAGATTATCTATAGAATACCATTCCATCGT
TTTATATGAGTGAGCCCATATTTAGGATCTATTGATGAGATTTTATTGGTTGTACTTT
TTTTATGAATAATTTTATATGTCACTACACTTAATACCCAATAAGTTTCATCGATATAGCCTTTATTATGTAT
CGAAATACAGTAATTTTAATGCATTTCTTAATTATTCTCAGTGGAAATAAAAATAAATTAGGAAATATTTAT
>50818490_3 CO729760.1
AACGCGACTAAAGCAAGATCTGAACTAATAACAAAATGCCTCTGGCACCTTACTTTTTCGAGTATGGTCTACC
GCGATGGCCGCGACGTCTTCTTAACCAAAACTTCGCTCTCACATTATCACCTGAGGACTTGTTTAGCGCAACA
TCTGCCCCAATAAGCCCGATTATCCCAAGATATCCAGTATGGTGGACTAAGGATTCGG
GTTCATCAATCAAATTGGAAAAGGACAAGTGGCAAATCAACGTGGATGTTCAACACTTCGCCCCGGACGAGAT
AACTGTTAAAACGTCAAATGGATATATTGTTGTGGAGGGCAAGCATGAAGAGAAGCAAGACGAACATGGATAT
ATTTCTAGGCAGTTTGTGAGACGGTTTAAGATCCCAGAAGACACTAATGATGAAGGTA
TCGAATCAAGATTGTCTTCGGATGGTGTTCTAACAGTTTTAGCTTCAAGAAAGGATACTCTTAAAGGAGAGAG
GAGTGTCCCCATCACACACACGGGACCAGTGCGAAAGGAAGTCAAAGATGACTCAGCAGACCAAAAATCTGAC
AAGTGATTTGATAAATTTCATAGATATTTTTATTATTTATTTTTTTCCAAATGACTGA
TTTATGAGACT
>50818495_3 CO729765.1
AGAAACTAAAGTTAACTTCGACAAGTGTTTGAAAGAGACGAAAAGGGCAATAATGTCGTTGATACCGCTTATT
TGTGAGCATCAGCCTTTAGCCGTGAAATCCAACCAGCGTTTCAACCAGGTGGTGCCACGGGACTTCTTCAAAC
CATGGGAAAATATGTTACGGCAGATCGAAGAAATCACCGGCGGATTCAACCAGATAGG
CTTAAAAAACATTTCTATATCTAACAATGAGAAATTCCAAATCAGCATCGACGTTCAACACTTCGCACCAGAT
GAAATAAATGTAAAAGTTATTAATAAGTTTATAGTAGTCGAGGCCAAACACGAAGAACGGCAAGACGAACACG
GCTTCATTTCTAGAAGATTCTTACGCAGGTACCCATTACCACAAGGCTGCTTACCTGA
TACTGTCAAGTCAAGCTTATCATCAGACGGCGTTTTAACTGTGGTAGCTCCCAAAATTTTGCCTATGCCATCT
ACAGGTGAACGTATCGTGCCAATAATTCAAACAGGGCCC
>50818498_3 CO729768.1
ATTAACCAATTTGTTGTTGTAAAAAGAAATTTTTATAATATACTATTTATCAAGATTAAAACACGTCACTATG
ATTACAAACTTTGATCAACTATGCAGTGAATTGGAAAAACAAGAACTCGAGGCCACAAATGGTGTCGCTTCAG
CATCAACCTATGCACAATTATTAGCAATATATCTTTACCAAAATGATTTATGTAATGC
AAAATTTTTATGGAAAAGAATACCTCAAAATATTACAAGCAGTAATCCAGAAATAGTTGCTATTTGGGCAGTT
GGACAAAAACTATGGAAAAAAGATTTAGCCGGCACTTATCAAGCTTTAGCTGCCTACACATGGACTGAACCAG
TTTCTAATATAATTAGAGCTTTAGAAGAAAAAGTTCGTGAAAGAGCTTTCAACCTTAT

TGGAAGATCATATAGTTCAATTTCTTTAGATACTATAGTACTAATGTCTGGTTTGAGTCGTGAAGCCGTGCTC
CAAATATGTAGAGAA
>50818501_3 CO729771.1
GTCGTAAACGGAACATTGCGAGAATTTAACACAAGGCGCTATAAGCTATAATTGTCGTTGCGTTTAAAAGTGA
TATTTGAAGAATTTAGCAATGGCTCTTCTACCATTCTTATTGGATTACGAATTCGAACGTCCTCGCCGTCTGA
TCGACCAGCACTTCGGCCTGGGTATAACTCCAGATGATTTGCTCAGCGTCGTCACCGG
TTCCGCCAACCCTTTGGTAAGCCGGGAGTATTACCGACCATGGCGTCACGTGGCAGCAGCGGCACGGGACTTC
GGTTCCAGCATCAAAAATGATAAGGACAAGTTCCAAGTGAACCTCGATGTCCAACACTTCAACCCTGAGGAGA
TTTCGGTTAAAACTGCCGATGGGTATATCGTCGTCGAGGGAAAGCATGAAGAAAGAAA
AGACCAACATGGTTTCATTTCCCGTCAGTTTACCCGCCGCTATGCTTTGCCCGAGGGTTGTACTCCCGATACT
GTTGAATCTCGTTTGTCTTCTGATGGGGTATTGACTGTTACTGCCCCACGCAAGGTTCCACTAGCAGTGCAGG
GAGAGCGCGCTGTACCAATAACCCAGACGGGACCAGT
>50818503_3 CO729773.1
GAACAGCGCACGCGACAGTCATGTACACTAAGTTATTTATTTTGTGCTGCGCGTGCGCGTTGGCGACGGCTGC
GCCTGGTCTCCTGGATGGTCTGGGCTACGGAGGGGGGCTGTCGCACTCGTACGCGGCGCCCATTGCCGTCGCT
GCACCAGTTATTAAGACCATCGCAGCCCCTATTGTAAAGACAATAGCCGCTCCAGTGC
TCAAAGCTGAACCTATCGACCCGAACCCCGCTTACAGCTACGCATATGGAGTAGCCGACCCTCACACTGGTGA
CCACAAAGACGCTCAGGAGACCCTCCAGAACGGTGTCGTCCACGGCTCCTACAGCCTGGTGGAACCTGACGGC
CACATCAGGAAGGTGACTTACACAGCTGACAAAATAAACGGTTTCAACGCAGTCGTTG
AAAGGAGCGGTGCTAGTCACGCTGCGCCTGTAGCACTAGCCCACGCACCCATCGCCGTGGCCCACGCCCCGGT
CGCCATCGCCGCCCCCATCGCCAAATACTCCCTACCAGCTTTAGGACACCCCTGGCGCTAGTCACACCACCCA
CCCCTTGTCGCATCGCTGACCAAC
>50818505_3 CO729775.1
CCGAGCGCGTACCGTCCCGGCCTCGCGATAGTGTTCGTAGCGGTGTCCGCGCAGTGCCAAGTGTCCTCAGGTC
CGTTTCAAGTGGGCACAGAAACAATTTTCAAACAGTGATGACAAACACATAGCACTATGGGGGTGACTGACGA
TTTTGCCCCGAGCTTCACGCAGAAGCCGCAGTTGCGTCAAGAGGATGATGGCAACAAG
CTGGTCTTTGAGTGCCAACTGCTGGCATCTCCCAAGCCCGAGATCAGCTGGTACCGCAGCGATGAACTGCTCA
AAGAAGATAATAGGACACAGTTTAAGATCCAGAGCATCGCCAACAATAAGTTTCTGGTGGTGCTGGAGCTGGA
CGACGTGATCGAGACCGACGCGGGGCTGTACAAAGTGAAGGCTAAGAATAAGATGGGC
GAGGTGGCCGCCTCCATCAATCTTAACTTCAGCCCCGCGGACGAAGAAAAGCAAAAGCAAGTAGACGGTAAAG
CGCCAACTTTCGCGCGAAAACCAGCCATTCGTCAAGAGGACGATGGCAAGCGACTAATATTCGAGTGCCGTAT
CGAGGCTGAGCCGGTGCCCAGCGTCGCCTGGTTCCACAACACTATGGAAGTCAAACAT
AGTCCGAGACATAAGTTAACAGTAAGCAAGGACGGTAAATCGTACTATGCGTCGTTAGAGATCAACAACGTGA
CAGTGGAAGACGCTGGCAAGTACCGAGTT
>50818507_3 CO729777.1
AGCCGGTATATGGGAGACAGCCTGCAGTGGTGATTCTGGTGGACCTCTCATGAGAAAAACTCCAGCCGGTATA
TGGGAGACAAACTCTAAGCGGAAAGGGAAGCCCCGTAAAATTAAAGTTAGGAATGCCAATATTCAGCAAGTCT
GAATGCTTTTCCAAATATAAAGGTCTTGGCGCCGAGCTGACTAACAAACAGCTATGTG
CTGGTGGAGTATTCGCTGAAGATGCATGCAGAGGTGATTCTGGTGGACCTCTCATGAGAAAAACTCCAGCCGG
TATATGGGAGACAGTTGCAGTGGTTTCTTTCGGTAATGGATGCGGAAGGGATGGATGGCCAGGAGTATACACC
TCAGTCGCTAGTTACTTGAACTGGATACAGGACACGATGGCCTCGACTAATGTTTAGT
TACTATTCATACTCTAAGATTAATTGTTCATTCTAATGCCATATTCTTTGATGCATTTGAAATCTGAATAACT
TGGTACCAGTGTTCACTATTTTTTCATATATATTAAGAAAATATGTCAAGTACAAAAACCCTTTGTAACGTTT
GATAAAAATATTAGTAATAAAAAATATCA
>50818513_3 CO729783.1
ATCTGAAAATTTCAGGGCATACGAGGTTACTGGAACGAGGCGAGGCTGCGATGTCACCTGGAAGGGAGTCATT
TGGCCTCTCCTCTGAACACGGGCTTGCGTTCAGCTATGATAAAATTTATGGAGAGGCATGGTGGGCTTAAATG
TGGAGTGTTTACTGGCATACACTCTACGTTTTCTAGAGGTGACTTCCATTCCGTTGAA
GGTATTCCTTTATCCAAGATACCTCAGAAATGGGCTGCAGATGAACCGGACAACTACTAAGATGAAGAACAAT
GTATAATGATGCAGGCGAGTGGAGACATGGCGTATGTAAAATGTGGCGAGCCTTTGCCATATTTCTGTTAAAA
AAAGGCAAGAACCTACGCTTGAATGGATGTGGT
>50818522_3 CO729792.1

CACCAGATTAAGATTTTAATCATTAAATGATTATTGTCATCGTCAATTTAACGATTTCGATCATCAATTTATG
ATTATAATCACAGGTGATGAGCTTGTTAGTAGATGGTGATGATTTTTTTTTGTTCATATTTATTCACGTCCAC
ATGTTTTGTTCGTGTCGCGTTGTATTATTTATCATGTTATTAAGAAAATAATAATATA
ATTAAAGTACATTTCCTCTATG
>50818523_3 CO729793.1
GCAAGTAGTTATATTGGCGGCTTGAAATTAAGTGAAATTTGCAAAACATTTAAACACTATTAGAGTTTTGTGA
AGATGAATTCTCTTAATAGAGTTCTAGCTTTAAATTCAGCAATACCTAAAAATTCGAGGTTAATATCGACAAC
AAGTACAAGATTGAATGCTGATGATGGCTGGCCGTCGTTT
>50818534_3 CO729804.1
CCCTCATAAGCGTTAATTTCATCCTTTTAAGTGAAGTGTAATTAAATTGATAGAAAATATGTGCGCTTCATTT
CGGATAGCTGTTGTACTTCTCTGTGCGGCTGTTGTCTGCGGACATGTTCACGGTCATAGAAGGTTACGTCGCA
TGGACACAGAGGACTATTCCGAGAATGAAGTTGAAAGCAATAGAGCAGACGAGAGCTC
CTGGTGGCCATCGGCGGAGTTCGCTATTTTACAAAAATTGTATGAAGATTGTAGTGCGCAAAAACATTTAACA
ATGTGTCTTAAAGGCAAAGCTCTCGTAGCTTTAACGAGAGCAGTGGAACAGGAAAGTGTACAGCTCGCTGATG
GGCTTACATTGGTTAAACAAGTCGACACGCCAACTTCTGTAGCAGAGCCTCGCTTCCT
CCCGGGAATGACGATGGAGGACAAGCTCGATACGATGCTACGTACTCGATTCGAGCAATTATTAAGCACTCAT
ACTGTTTCTATGGATTTAGCTTCAGAGGGCAGAGGTAGAGGATAGAAAGCTCTACCTTATTTACTGCTTGGAA
TTTTCACGA
>50818539_3 CO729809.1
AAGAAAATGCCCAAGTCCCAAATAGTGGAAAACTAATTGATATGACTGATATTACTAATGATCCTTTACTCAA
ATTTTATAACAAATATTTTGGCTACTTTAAATTATTTTTCTGCTACATATTACCAGTCAGCATAAACGTATAT
TTGTTTGGGGAGTCATGGCAGTGCGCGGTCGCGTGGCAGTGGTTCATACGGTTCCTAA
GTATGTTTCATAGTGAACTCACAGTAAACAGCCTGGCCCATGCTTATGGCTACAAGCCTTATAACAAGAACTT
AATACCAACAGAAAATCGCTTCGTAGCGACATGTACTCTCGGGGAAGGCTGGCATAATTATCATCACGCGTTT
CCTTTTGACTACAAGGCTGCGGAACACTTCGATATGTTCAACTGGTGCACCAGATTTA
TTCAAGGTTTTGAAAAACTCGGTTGGGCTTACGACCTTCGTGAGGCAACTCCTGAAATGATCAATTCTATAGC
ACAAAGATTAGGGGATGGAACTCCAGTACATTTTCCATAAAGTCTCAA
>50818549_3 CO729819.1
TACGGTCGGTATTCCGGGTCGACCACGCGTCCGCTTCTGAAGAAGCAGCTCCCATAAAAACAAAAACAAACAA
ACTCTTCCGCTTTATTATATTAGTATAGATTAAACATATTATGGCCATGGGCATAGAACAATGTCCACTTCAG
AATCGTTTGTGGCCCACCCCAAAATTGTGTCCAATAAAATTTTAAAATTAAGCCTAAA
AATT
>50818552_3 CO729822.1
GGNTCGGTATTCCCGGGTCGACCACGCGTCCGCGGACGCGTGGGCGATTCCATGCAGCGTTGAGGAGGCCCAG
GAATAACCATGGACAAGTTAAAATGGATGACAGAGATCGGATAAACATGTTAATAGAAGAGCTAGGCTTAGAA
CAAGTTAGAGATACAAATGTGGGTCGACTAACTCGTTCTGAAATCCGTAGACTTAACG
TAGCCTGTCAACTGTTGCTGGACATGGCGGTGTTAATCCTAGATCAGCCCACAAAGGAAATGGATATATTTGA
TACGTTCTTCTTAGTGGAATATTTAAGGAACTGGGCCAGTACTGGACGTGTAGTTATAATGTCGCTACACCCG
CCTACTTATGAAATATTCGCTATGCTTACTAAGGTAGTTTTAATTTCCGCTGGTCGCA
CTATGTTCAGCGGGTACAGAAGAGACATGTTGCCATATTTTGCCTCAATAGATTATCCCTGTCCTGCTTTTAA
GAACCCTTCTGATTACTACTTGGACCTAGTGACCCTGGATGATCTGTCTCCCGCCGCCATGTTGGAGTCGTCC
AGCCGCATTGAGGCGCTGGCTGGCGTGTTCGGTGGCGCGCACTCCGCGCCCGACCCGC
CACCCCCCGTCACGCT
>50818554_3 CO729824.1
CGACTCCATCGTCCGTTGCCGCGCCGCCGGTATTCGCGTCATCGTCATCACTGGTGACAACAAGGCCACCGCT
GAAGCTATTTGCAGACGTATCGGCGTGTTCACGGAGGACGAAGACACAACCGGCAAGTCATACTCCGGCCGCG
AGTTCGACGACCTGCCCGTGGCCGAGCAGCGCTCCGCCTGCGCCAAGGCACGCCTCTT
CTCCCGCGTGGAGCCCGCACACAAGTCCAAGATCGTTGAATTCTTGCAGAGCATGAATGAAATTTCCGCTATG
ACTGGTGACGGTGTAAACGACGCCCCTGCCCTCAAGAAAGCTGAAATCGGTATCGCTATGGGTTCTGGTACTG
CCGTCGCCAAGTCTGCTGCTGAGATGGTGCTCGCTGATGACAACTTCTCATCTATTGT
AGCTGCTGTT
>50818557_3 CO729827.1

CGACCACGCGTCCGCGCTATCCACACAAAAATGCCCTTAAGAGTATACTACGTTCCCGGCTCAGCGCCCTGCC
GCGCGGTGCTGCTCACCGCGCGTGCTCTCAACTTGAACCTCAATCTGAAACTCGTGGATCTTCATCATGGAGA
ACATCTTAAGCCTGAATATCTTAAGATAAATCCACAACACACAGTTCCTACCCTCGTG
GACGACGGCCACCCGATCTACGAGTCGCGTGCTATAATAACTTATCTAGTGAACAAGTATGGCAAAGGCAGCG
CGCTGTACCCAGAGGAACCCAAAGCCAGGGCCCTCGTCGACCAACGCCTGTACTTCGATATCGGAACTTTGTA
TCAGAGATTTGGTGATTACTTTTATCCACAAATCTTCGGCGGTGCGCCCGCGGACAAG
GACAAGCTAGCCAAAGTAGAGGATGCCCTTAAACTCCTGGATACCTTCCTCGAAGGTCAGAAGTACGTCGCCG
GCCCCAACCTCACTGTGGCCGACCTGAGCATCGTCGCTGGCGTG
>50818558_3 CO729828.1
CTTGATAACCGAAATTGTGCAATGTATTTTGTAAAAGTATAAACTATATATTTTTTATATTGTATTATTATTA
GTTTGTACACATTTTTTGCTAACAACAGAAACATTACTTGTGCACAAGTTTTGATACAATATTTATATTAGCT
TTTAATGTGGCTGATGTTCATTTCTATACTGCTTTATTGTACATATCATAAGAATTGT
AAAAATATTAATTTTTATAATTACTTATCTAGGATACTTATGAAGGATTTTTACATATATTTTCTACATAAAA
TACAAGTAAAATGGTAGACTATATATATGTATTTGTGTACTTGGAATTTCCTTTTTTTATCTGTAGTTTTTTG
GATTATCTAGACTTGTCTATACTCTGTGCAATTTGATATAGTGGCACATATACTA
>50818559_3 CO729829.1
GGTCCGGATTCCCGGGTCGACCACGCGTCCGCACACAAGGCGCACGGCAACATGAAGATCGCTATATTGTTAT
TGTTTTTCGTGACGTGTTACGCGGAGATCGATTTTAGCGGGAGAGCGAGGAATTTTAGCATAGAACTGCTATA
TCATACACAATTGGAGACAGATGGCCACGTTGTTATATCACCTTTTGGCATATGGACT
CTGATGACTGGCGTCACGCTGGGAGCTAGCGGTAACAGCCGGAAACAACTGTCACGGGCATTACTTCTACCAA
GTAGTGACGACTCAATCATCAATGGATACACAAATTTGACGAAGACCGTCCTGGATCCTCGGACCGAGGGGGT
TAAGTTGAAAAGTAAAAATTTTGTATTCCTCGATAAAGGTTTCAATATATTTCCCGAA
TTCAAAAGATTGTTGACAACTGAATTTGATTCAAAGGTGATGTCCTTAGATTTCAAAGATCCAACGGCAGCTA
ATGTAGCAAATGCTTTAATAGAGAATTCTGGCGCCACCGTCTCCAATGTACTACGGTCAGACGACTTTCGCCG
TTCAGAATGATTCTGACAAACGTTATATCATTCAAGGGTCTCTGGGCG
>50818562_3 CO729832.1
ATAAACTTCTATGTCTATTCTCTTGTAACTTTTTAGTTAAAAAATGATGATCATTCTTTTTAATTATATAAAA
TAAATGTTATTGTTAATAGTCTGTAAAACTTTAGTCAATTACTGTCAATAGACATTATGTCTTTGGGCGACGA
AAATAAATATTTACCATTACGAAATTTAAGAAATGTATGGAAAGCATACAGTAAAACA
AAATCACTTAAATACGGCTTACCATTTGTAATATTTGTAATAGGAGGGTCATTTGGATTAAGAGAATGGACAC
AAATCAGGTATCAGTTTTCTAAAGTCAAAGGTGTAAGTAAAGAAGAAGCTGAAAAAATGGGCCTTCATCGTGA
CAAAGAAGTCACCCTAGAAGGCGCTTATGAAGAAATTAAGACTCTTGATATAGATAAT
TGGGAAAATAAAAGAGGTCCTAGACCATGGGAAAGACATGCAGAAATTAAAAAATA
>50818563_3 CO729833.1
ATTCTTTTAAATATTATTCAAAGATAATTTAATATTATTATGTTTATGAGTGACATAACATTCTAGAAATCAT
TGTGATTTTTTTATAACATTTAGGTATTTGTAAGGGACCAATATTGTTAATATGACTGATTTTTTGGTGTACA
AAATATATTTAAAAAATGAAATATATTTTCATATATAAAGTATTCTTATAATATAGTA
TTAAATTGCTGAATATTTATGT
>50818571_3 CO729841.1
ATTCCGGGTCGACCACGCGTCCGAGTGGCTATTGTAATGCTCACATATAAACAGTTTCGTTAGTGAGTGTAGA
GACAAGTGTTGTGAAAATGTCGCCCAAATGCCCTGTGGCTCCTTTACACGAAGTGACATTGTCATTAGTAGCT
AGACAATTAATCCACGCTCTGACCCAAGCCACGTCTGAGGAAGATGTGGCTCTACCGT
TCACTATGATATCGTCTTACTACGAAAGTATGGGTGCTACGAGTGATATATTCCAAGATCTCCTAAATTTAAT
ATTAAGTTCAGAATATTTAGATCCATCGATTAGATATTATACAATGAGATTGCTCTTGAAGGAGAATGTTAAG
AGCCTGGCTACGGGTATCTTCCCTTGTCACTATTACAGGAAAGTCCTTGAACTGATTG
CAGAACAAGGCCGACATCTGACAACTCTGAATTTAAAGGCGTGTGGGTTAAAGATGAACAGCTGTCCCTTTTG
TATGATATTATTAAGAATTTGTCAAATTTAACTGCCCTAAATATA
>50818576_3 CO729846.1
AATTGTTAGGAAATGTCCACTTTTTAAATTCAAGTACTTTGCAATTGCATATCATACAGTTTTATATCACTAT
TTAAAATTCAAATCATGCTCTATATACAAAAGTAAAGGGCTAAAATCAATTGTTATTTTTCATATTGGCACAT
ATACCAATACCCATTTTTTTGTTAATTTATTTTTATCTAAAATTGTTATATTGACATT
TAGATAATGCCTAAAGAAGAAAAAATTTATGTATACAAAACCAACTGTTTGGAAATCATGTGTATTAAGTTAAA
TTTATGTAAATTGTAAAAATATTCACTAGACGAAACATGTGTGCTGGGAAATATGAAAAAATAATTCCTATAT
ATCAATTTGTTTTTCCTCTTTAAACAAAATATATTCTTGTTGAAAATGGGCCA

>50818582_3 CO729852.1
CGGTCGGAATTCCCGGGTCGACCACGCGTCCGCGCGTTCAGGACACAAAATCGGAAATTCTAAAAGTAAAAAG
ATGAAGATAAGTCAAGCGGATGTACCACCATCGGAAGATAATAGGGAGAGAATAAACACTATCAAAACTGATT
CTCTGGATGTTAATGTCAAGAATAGTGGTCAAATAGAAGTAGCCACCGAGCAAATGGA
GGAGAAAAACAACGATAACGCGAGTAGTGCATCGACTGTGGAAGTTTTAGATGATAAGTGATACGTGCCATAT
TATTTGAATAAACTTTTTTTTGCTATCCTTACCCAGTGCAATCTCCTGTTAGAATATTTTATGTTGTTTTTTT
TTTATATTATTTTTGTGATTTTATCATGGTTTTAAAATATACTCATTCTTAATTAAAA
TATCTA
>50818586_3 CO729856.1
ATTCCGGGTCGACCACGCGTCCGATCTTGCGAAATTGAACGTTGTTCAAATTTGATGCTTTTAGTTTATAAAT
TCATATATAATTAGAATAAGTGTAGTCGTGACTGAGGAAACCCTGTCGGGAAACAGTGGAGAACAAGCTGATG
GCAAAGATGTCAGCATCAGCGATTTTCATAATGAAGATGCACCAACAGAAGTTTGTGA
ATCAGTGAAAGGCCTCGACGCCAATCGCTATGCCACGAAGAAAACCGTTGCTCAGGGAATGCTCGATATCGCT
TTACTTACTTCTAACGCATCCCAATTAAAGTATGTTCTTCAAGTTGGACCAAAACACGAGTTTTACATGTTAC
TTGTGGTGCTCATATCAATCTCTATCGTGTTACAGGTTCTTTCAGCGATTTCCTGCGC
CATTCTTGGTTTCATTTTTAATATTAATCACCTACCGCATCAACGTACTGCTGAAATTTTAAATAATGTTCAT
TTATGTTCAAATTTATAG
>50818591_3 CO729861.1
GAAAAAGAAAAAAAATCGCCATGACTCTGATAAATCAGACGTAAATGAATCTTGTACTTTCGCGTTTCAAAAG
CTATTAGAATCAACAAATATTGAAAATGAAGAAGAAAATACATTACAAAATCCTGTTGTAGTACAAGTTCAAG
GAGATAACAGCAAAATTGTCGACTTTTTGTCATCTAATGAAAATGTAACAGATATAGT
AGAAAATAAAGAACATACAAAGGGTAAAAGAAAACAAAAGAAAAATAAAAATGCATCTATCGAAGCAGAAACA
AATGTAAAAATTAAAAACAAACAGGACAATATTATATTGCCTCCAGACAAAATAAAATCAGAAAATATTACAG
AAAGTGAGACTACAGCA
>50818597_3 CO729867.1
TCGGNTCGGTATTCCCGGGTCGACCACGCGTCCGGATCACTTGAATTTAATCATTTTGAATATTCGGGCATTG
AGCATTACCTATAAATAAATCATCATGTCTCTAATGAAGACTAATATGCTGGTTCGCTCATTGAGCACTTCAT
CAGCTGCAGCTCAACTTGTAAAACCTCCTGTACAAGTATTTGGTTTGGAGGGCAGATA
CGCATCAGCTTTATTTTCAGCTGCTTCTAAAAGCAAAGCTTTAGACGCTGTTGAAAAAGAGCTGTCTCAATTC
CAAAAGTCTATTAAATCTGATGCTAAACTCAAAGATTTCATCATAAACCCGACTTTGAAAAGAAATTTGAAAG
TTGATGCTTTAAAGCATGTCGCCTCTAAAACCAGCATGTCACCAACCACAAGCAACTT
GTTAGGTCTTATGGCTGAAAATGGCCGTTTGGATAAACTTGAAGCTGTTATTAATGCCTTCAAAATCATGATG
GCCGCACACCG
>50818600_3 CO729870.1
TACGGTCGGTATTCCGGGTCGACCACGCGTCCGGAACTTGTGAAACTAAAACATTGTGTGCCTTCGACATGTT
GAGGAGTATTTTACGTACCAGTCGCTGCCTGGTACGAAGACAAATCAATAAATATACTCTAGCTCCATCATTA
TGCTTATATCCATTACAAAAAACCTGCAAAACGTGGAGTTTAACATCAGCAACACGAG
AATTTTCTTCGAGTTCTCAAGAAATTACTAGTTCTGAAAGTGTAGACCCAGTAGAATATGACAAAATATGTAA
CGAAACTCTAGAATCTCTATATGAATACTTCGATGAATTAGTAGACAAGTCAATAAATTTAAAGGGTGCTGAT
GTAACATTTAGTGATGGTGTATTAACAGTTTTTCTCGGACCTAGTTATGGTACATATG
TAATAAACAGACAGACACCAAATAAGCAAATTTGGTTGAGTTCTCCAACATCAGGCCCAAAACGCTATGACCT
TATATTAAAAAATGGTGGCTATTGGATATATAAACATGATGGTGTAACGTTACATAGTTTATAACAACACGAA
ATATCTAAAATTGTTAGCG
>50818601_3 CO729871.1
CGGGTCGACCACGCGTCCGCCAGACTCGTGCAAGATGCTCTGACTGGTCGCCTCTATTGCTTTGATTACGTAC
TTGGGCGTCCGTTGGTACAAAAATGCCTGCAAGCGGCCCGGGAAGAACTTTCCACCAGGACCGCCAAGTCTAC
CAATATATGGTGCTTACTGGATCATCCTATGGCGACAGTTAAACGACTTAGGAGGATC
TTTGAGAAAATTAGCCCAAGATTATAAAACTGACGTTTTGGGAATGTATCTCGGGAGCTTTCCTACAATCGTC
ATAGATGATCCCAAGTTAATCAAGGAAGGCCTGAACCATGCGGACTTTGACGGTCGCGTGGATATGATAGTAT
CTCGATTGCGTTCATTTTGGAAAAAACTCGGTATTTTCTTCACTGA
>50818603_3 CO729873.1
CGGTCGGTATTCCCGGGTCGACCACGCGTCCGTGAAGATGAGCAAATCAACTCTTTAATGCGCAAATTCAAGT
TTGATTTGTCCAAGCAATTACAGACAATAAGAAAAATAATTATTAATTTATTGAGTTTAATTCGCTGTGGATT
GTAAACCTACGGCATTTAAAATGGATCTTTTAAACTTTTGGCAGGCTATTGATGTTTT

AATCCATAAGCCTCATGTTATTAATAAAAACCTGTGGGGATCAATAGTGTTAAAAAGATATTACATTAAAACA
GAAATAAATAATTGGAATGAGTATCTTGCTGACTATAGACTAAATACAATGGTATTTTTAAATATAGATCAAT
ACTTAGCGGAATTATCACAAATGTTCCACTTTCAAATTGTTAATGAAGAAACGCAATA
TGAAAGTATTTTCATAGAACTC
>50818604_3 CO729874.1
GTCGGAATTCCGGGTCGACCACGCGTCCGAGAAAATGAACAGCTACATCACTTTTGCCTGCCTATTAGCGGTC
GCCGCTAGCGCGTACGGTTCTGGCCTCGGCCTCGGCTATGGCGGACTCGGTTACGGTGGTCACGGCGGCTACG
GCTACGGGGGCTACGGGGGCTACGGATCAATCATAAAGGAACCCATTATCGTCAAGCC
TGCTCCTATTATAAGTCCACTGCCGATTATTAAACCTCTCCCCATTATTAACCCGCTGCCTATTATTAAGCCT
CTCCCCATAATCAAACCTCTTCCCATCATAAAGAATGTGGAAATTGCTCCCATACTGTCTGAGCCTATTTTAA
AATCTGTGCCCGTATACGGTGGGTATGGACTTAACAAGGAACTTGGTTATGGACTCGG
TCTTGGTTATGGG
>50818607_3 CO729877.1
AGCTTAAGGAACCACCGCCCTATACTAATAATAACTTAAGAAAATGTTCGCAAAGGCAATCGTCGCATGCGCG
CTCCTGGTTGTAGCGCAGGGAGGACTCATCGCGAGCCCTCATGGAGCTATCTCATCTCAGAGTATAGTTCTTG
GACATCCTGCACCTGCCATCGCCGCTCATGCTGTCGCCCCTGCATTGGCTGCTCCTGG
ATATGGATTGGGACTAGGTCACGGTGCCATCGTCTCACACGCGCCCGTGCTGCACGCGCCTGTGGCCCACGCC
GTTGCCCCTGTTGAGGTTTATGCCCACCCCCGCTACCAATTCAACTACGGTGTCACTGACGGCCACACCGGCG
ATCAAAAGAGCCAATGGGAAGCTCGCGATGGCGATGTTGTAAAGGGACAATACTCTCT
CGTCGAACCCGACGGTACCATCCGTACTGTAAACTACTCTGCTGACGATCATAATGGTTTCAACGCTGTAGTG
AGCAGACAAGGCCACGCTGCTCATCCTGCCGTTGCAGCCGTTGCCCATGCTCCCGTCATCGCCGCTGCTCCTC
TCGGNCACGGCTCCCTCCTCCATGGCTCCCTCCTCCATGGCTAAACACTTGAAAACGA
CATATTTATTACCTTTATTTAC
>50818608_3 CO729878.1
CGGGTCGACCACGCGTCCGCAACCAACGCGTTGAGGACTTTTGAAGCAATTGAAGACCCTCACCTCCAAGTTG
AAGGAAGCCGAGGCCCGCGCCGAGTACGCCGAGAAGACCGTCAAGAAACTCCAGAAGGAAGTCGACAGGCTCG
AAGACCAATTGTTCAACGAGAAGGAGAAATACAAGGCGATTTGCGATGACCTGGACGG
CACATTCGCCGAGCTCACCGGATACTAACCTAACCCTAGCACATAGTTTAAGACGATACCGTCCCAACATCAT
CCGTTCGATCCAAATCATCCTCATATGCAAAACTACGAAAATTTGATCTGTTCAATTCATGCATCTGCGACTG
GTGAACGAATAGGTTCAAGCGGTCGAACCCTCTTATTCTAAAATTTGTCTAGGACGCT
TTAAATTTCT
>50818611_3 CO729881.1
GTGTATCAGAGTTTATACCCTTATAAGATTAGTTAAAAAAATCTCGAATTTAAAAGACTCTTTTAATTAAAAA
ATTTAATGTTGAATAATTATTTTAAATAATTAATTTGAAATGAAATGTTAATCAGTTTTTCCCTATATCTAGT
TTTTTTAGAAAAAAATTTAATTTTAAATTTAAAATATTTTTTAATTTTTTATTTAATT
AAAAAATTTTTAAAATTAAATATATTAAGGGATAAGCTTTAATTTAAATTTTTATAATATATATATATATATA
TATATATATATATTAAATTAAATTTAAAATTTTTAAAATTTATATTGTTTATAAAATAAAATTTATTATAAAA
AATTTTATTTTTATAAAATAAAATTTAAAATTATATTTAAATTTTTATAAAAATATAT
TTTATAACACAAAAAAATTACAAATAATGATAAA
>50818614_3 CO729884.1
AATATCATTAGAAGAGGACCATTCGTTATATCATATGGCGCGATTAGAAATTATAGGGGTAGAAAATTCTGAC
AAAGGAGAATACAGGGCGTGTGGCTAAAAATAAACACGGTGAAGGCGTGGCAAAAATAAATTTAAATTTCCAG
GGTGGGGATAAACCAAAAATACCCGATGGCAAAGCACCTAGATTTCCCAAAAAAACCT
ACGATTAGGCAAGAGGGAGATATTTTAATAATGGAATGTATCTTAGAAGCATATCCCGTGCCCGACATTACCT
GGTTTCACGGTGACAAAGAGATTAGGGACGGTGCTAAAATGAAAATGTCTAGGAAAGCTATAGGAAAAGATAC
TTTTAACCTTACTCTAGAAATTTTAAGTCCTACACAGGAAGATGGCGGGAATTATAGA
TGTAACGCTTTCAATGTATTTGGTGAAAGCAAT
>50818615_3 CO729885.1
CGGTGTTCAGAGTTTATTACATATAAAGAGTAGCTAAAAAAATCTCGAATTTAAAAGACCCTTTTAATTAAAA
AATTTAATGTTGAATAATTATTTTAAATAATTAATTTGAAATGAAATGTTAATCGATTTTTACCTATATCTAT
GTTTTTTTAGAAAAAAATTTAATTTTAAATTTAAAATATTTTTTAATTTTTTATTTAA
TTAAAAAATTTTTAAAATTAAATATATTAAGGGATAAGCTATAATTTAAATTTTTATAATATATATATATATA
TATATATATATATATGAAAT
>50818621_3 CO729891.1

CGGGTCGACCACGCGTCCGGCATCCCAAAGAGAAAATCTCTTAAGAAGCCGTGTGGTTCCTATCAAACATAAC
AGCGCCCTTCAAACAACAAGTCCAAGCAGTCATCGACGCTGGACTCTTACCCAAAATAGTGGAGAACCTCAGC
AAAGGGGAGTTCCAGACGCAGAAGGAGGCGGCCTGGGCGGTCTCCAATCTGAGCATCA
GCGGCACTCGCGAGCAGGTGGCGGCGCTCATACAATGCGGTGTAATACCGCCGTTTTGTAATTTGTTAGCTTG
CAAGGATACGCAGGTTATTAATGTGGTGCTCGACGGGCTGAGCAACATGCTGAAGATGGCGGGCGACAACGCA
GAGCAGGTCGCCAATATGATCG
>50818623_3 CO729893.1
CGGTATTCCGGGTCGACCACGCGTCCGGTTCTGTGCATTAACGTGCTTTTAGAGAAGTGAATGATTAGTAGTG
CTGAATTGGCACGCTCGGAACGGCATAAATAAAGAACAACAACAACAACACGCGTAACATATGATACAATATT
TTATAAACAGTATTTCTGTTAAAAGTAAAAACAATGTCTGTATCTCTGCACACTGACG
TTGGAGATATAAAAATTGAATTGTTTTGTGAACAATGTCCAAAGGCTTGTGAAAATTTTTTAGCTTTATGTGC
GAGTGATTATTACAATGGATGTTTATTTCATAGAAATATTAAGGGATTCATAGTACAAACAGGTGACCCAACA
GGCTCCGGGAAAGGTGGAACTTCTATTTGGGGCAAATAATTTGATGATGAATTTAAAG
AAGAGTTAAAGCATAATGCAAGAGGTATGGTGAGCATGGCTAACAATGGACCTAATACTAATGGTAGCCAGTT
TTTCTTTACATATGGAGAACAGCAACACTTGGATTTAAAATATACATTGTTTGGAAGAATAATTGATGGTTTT
GAAGCACTTGACGATTTAGAAA
>50818629_3 CO729899.1
CCGGGTCGACCACGCGTCCGTTTTTATATTCTAAATAACATAATGTTATTTAATAATTAATACGAATCAAAAT
GCTACATCCGGAATAGCTTCATACGCCCAGGCGTCTCGGGCGCCATGCTGCCATGTATGTCAGCGAGGGAGGG
CAGGTGCGCCAGCTTGCCGCGCGCCGCCACCGCCGGCGGCTTGCTGAGCATGCGACGC
GCTACCCGGATTATATCTTCTGCTTGGATTTTTTCAATTTCATTAATAAAGTAGGATGGTGGTTTTCTCTTGC
CCGTCGCTAATACTTGTCGGCCGACATCTTCGAACACTACAGGCCTCGCTTCGAGATTCATTAACAGCATAGA
CTGTAGCTGCGTCTTAGCTCTCCTCAGCTCGTTAGCGGACACGGAGCCGGCCATGGCG
GCGAGCTCGCGCGCTACCACGAGCGCGGTATCGTGCAGGCGCGCGGGCGGC
>50818630_3 CO729900.1
ATTTTCCCTATTAATCTACAAAAATGTCTCTCCTACCATTCGTGCTTGGCTACGACCACCCGCGATATCGCCC
CAGCAGGTTTCTAGACCAGGATTTCGGCTTGTCGCTTACCCCTGACGACATGTTGACCGTTGCCGTCGCTCCA
TTGATGTCGAGAGACTATTTTAGACCCTGGCGTCAATTAGCCAATGCTTCCCGGGACG
TGGGATCCAGTATTAAAGCGGATAAGGATAAATTCCAAGTAAACCTCGATGTACAACACTTCTCTCCTGAAGA
AATCACTGTAAAAACTGCCGATGGTTTTATAATTGTT
>50818634_3 CO729904.1
TCCCGGGTCGACCACGCGTCCGGGAAATAGAGCAGTGGCTCTGTTCGCGAACAGCGTTTCGCAATAAAAATTC
GATTAAATTATATATAAATCATGTTGTCCAGCCTTAGGTCTGGAGTACAGGGCCTGTGGAGAGTATCATCGCG
GGCTCTGCAAACCACTGCTAATTTACAACATGATAGCTTATTTGTACACCGAGACACC
CCCGAAGACAATCCAAATATACCATTTGAATTCTCACCGGAAAATAAACAGCGTGCTGATGCACTCTTAGCCA
TATACCCTGATGGTCATAAAAGAGGAGCTATGATTCCATTATTAGATCTAGCACAGCGTCAAAATGGAGGCTG
GTTACCAATATCTGCTATGCATAAAGTTGCTGAACTATTAAATTTGCCCCGCATGAGG
GTTTATGAAGTGGCCACATTCTATACTATGTTCATTAGGAGACCTATAGGCAAATATCACGTTCAAGTATGCA
CAACAACACCATGTTGGTTGAGAGGCTCCGATGCT
>50818640_3 CO729910.1
GTCGGAATTCCGGGTCGACCACGCGTCCGCGGACGCGTGGGCGGACGCGTGTGTGAAGTCGTGTGTACTCCTC
GCGTTGTTCCTCGTGCTGGCTATGGCCGCTACAGCATATTGCTACGAACCTGAAGAAACTCCTAGTTTCCACA
ATCTAATGAAACGGCAAATTAGATCCGGATGCACTCTTTGGGAGTGCGCTTCCAACTG
CAGAAGACGAGGCTTCAGTGGTGGCTACTGTGCTTTCACAGGATGTTCCTGCTATTAACATATAGTTATACTC
CAGCTAATATTTATAAATATAAGAGAAAAAATTGATATATTGTAATTATTTCTGTTTCCTATTTAGTTACGAA
AATTTAATAAATATAATATATTGTAAAA
>50818644_3 CO729914.1
GGTCGGATTCCGGGTCGACCACGCGTCCGATCGCGCTTTGCTCTTACTATTCTTATTAACTGCTGTGGAGAGC
TGTAGGCATTCACGTTTTTGCCGTCATAACAGAAGATTTGGAAGTAAATTTTACCATCGCTTCCACCGTCATG
ACGTCGATCGAAGTATCAAAGACATCGCCTATAAACTGCAAAGACTAGATGCTGCTTT
CAATAACATCTGTCAGCAAAATGAGAATCGCACCACTGAAACATATGGTATGGATTATGTACTCAAATATAGT
CTCCCAGAATACGTGAACCCCAATATAACTGTCAAAACTAATTATCGTGTAATCTACACCACCGTAGAAATGG
AAGGAAAGACGTTTAACGATATACGAATTTTACCAAATATTCTTAATCTAAATGAAGC

TGGATGGGTGTTTGGAGACGGCGAATTGAAAGTGGTGTTTCCTTATGCTATCAAAATAGGCGAAGAAAACGCA
GTTAAATGTGATTCAGTTGATACAGCTATACCAGAATGGCGTGATATTAAGTACAAAATACAAGTAAGATCTG
CCAGCGGGATTGATGTTCGAAGTTTGCAATAGATCCTATGCAACTTCCAGAAAAGTTT
TATATGA
>50818648_3 CO729918.1
GAATATTCAAGAATCCCTTAAGGTGCCCATGCTGCAAAGGCCATGGGAATCTTCACAAGAACAACAAATGACT
CCTCCGCGTGAGATGCGCATTCACGTTCAACGTATCTTCGGCATCCCCGCGCCTCACAACATGCTTCCTCCTC
TGGAGAATATGGGACAGTTTGGACCACCACCACCACCACCACTGCTTGAACGTAACGA
GCAGCGTGAACAAGAAGAAATGATGGCGCCAAGACAGATGCGAGTGATTGCAATTCCTGCTCCACACAACATG
CCACCCCCACCCGAAAACATGGGACCCTTCCAGCCCCCACCGCCTCCTCAGGCTCAAGCGCAAAATGAGCAAC
CCGCCGTGCTTCATCAGTTTATACCTCAGTCTGCCCCCACACAAGAAGTAGAACAACC
TGAGGTACAAAAGATGCAAATGGAAGGTAGCC
>50818649_3 CO729919.1
TCGACCACGCGTCCGGGGGATCCTCCAACGGCTTTCTAGGCCGCGGGTCCTTCCAGAACAGGCAGAACAACGA
TGGCTACCACCCTAGGCACAACAACGACTACCAGAACAGGTCGAAGGAAGGTTACCAGGGCCGGCAGTATGAC
GGCTACCAGAACAGGCACAAGGACGGCTACCAGGCGCGGGGCGGCGACGCGTACTACG
GCAACGGCGACGCCGGCGACGCACACCACGAGAACGGTGGACACAACTACCAAGGGTACAAGGGCCGCGGCCG
GGGAGCGCGCGGAGGGCCTCGGGGCGCTCC
>50818650_3 CO729920.1
GTACGGTCGGTATTCCCGGGTCGACCACGCGTCCGGATCGTTTTATGTAATAATGACGTTGAAGTAAAAAATG
AAGAAGATTTAGGCGTAAAGTATGCCAATCGTTGTGAAGTTTGCAAAATATTAGCTACTGAGCTACAAGGGAG
ACTAGAAGAAACAGGCAAGATTCATGATGTTATTGAAATAGGATACTCTTTAGACGAC
GTTAAACCTAAAAAGAGAACAAAATATCAGAAATCCGAATTGCGGCTCATAGAATCTTTAGAAGGGGTATGTG
AAAAAATTTTAGAGTACAATATTCATAAAGAGCGTCAAGACAGTACAAGATTTGCAAAAGGTATGAGTCAAAC
ATTTAAGACTCTTCATGGCCTTGTTGATAAAGGTGTAAAAGTAGACCTAGGTATCCCT
CTGGAACTTTGGGACAAACCATCAGCAGAAATAACAAATATGAAAACTCAATGTGAAAGTCTTTTAGAGGAAA
ATGAAATGGCTATTGAAGACTGGTATTGGAAATTTCAAGGAGAAATAGATTTAAAACACCATTTGTGTACAAA
AAATGCTTTAAGAATAATGA
>50818652_3 CO729922.1
GTCGGAATTCCGGGTCGACCACGCGTCCGGATCGAAGTGAACAGTGCACAATGCTTCTCAAGTTAGCAATATT
GGGTGCTATAATATTTTTTTGTGTTAAACTTATGAATAGACGAAGATGCTTCTATTGTAATTAATAGGAGGCG
TTAGCTTTAATAAGCGAGTATTCCGAAGCGGGTATAACAGTAAGAGGTACATACGTAC
CACCAGGAAAAGCACCACCGGAAGGCGAAAGAAAATTGTATTTGGCAATAGAAAGTTCACAAGAACTGGCCGT
GGCCAAAGCCAAATCGGAGATAACTCGACTTATTAAGGAAGAACTACTCAAACTACAGACGTCAGCTCACCAC
GTGATCAACAAAGCTAGATATAAAGTTATTTAATGTATGCAAA
>50818656_3 CO729926.1
ACTAAGGCTCAGGGGATGGTGGTTCACGAGACAGTCCTTTAGTACACCGGAATAATGGATCTCCTAGTGTAAT
TCGATTAGAACGATCAGTACTGCAGAGTGTCGGCCAGCCTGAGCCAGTGTCGCGAGCAGCCATGCTGAGGTTT
GTGAAGCAGATCAGGGACGACGCCAGCAACGTGGCCTGTGTGACTGCTAAGTACGAAG
TGAATGGCACAACAAAAGTGAAGAGAGGTTGTTCCTTCTGCGCGGCGGAGGAGAGAGCGAGGCGCCGCCAGCT
GACGGGGGTCGCCCACTCCGACTGCCGCGAGTGCCTCTCCAGCCTGTGCAACTCCGGACACAAGCTGGCCATC
ACACTCATACCATTCATCATTAGCATTCTTATTCAT
>50818667_3 CO729937.1
TATGGATAGTAACTTTACTCGGATGTATTAAATTGTTTTATTGAAATTATAAGGTGCAAGGACGATAGTTAAG
ATCAAAAATGCAGATTTTCGTAAAAACCCTCACGGGCCACGTTGTCCTCGCAGTTGAACCCACTAACACCGTA
GAACATGTGAAGTCTAAAATTTACGAAAGGGAAGGCATCTCTCATGAATATCAAAGAT
TAATATTTGAAGGCAAACTACTAAATGATGGAAGAACTCTATCTGAATATAACATTCATAATGAATCGACTCT
ACATCTTACTCTCAGTCTTTCTGGAGGTGGCAAAGGTGCAATGGTCATACCACCCGACCTAGTAGCGCTAGCC
CAGAAATTCAACGTACGTAAAATGATATGTCGCACGTGCTACGCGCGCC
>50818669_3 CO729939.1
AGTTTTTTCAACATAAGTCACTTGTTTGTAACATTTTTTAAAGCGTTATTTTACACTAAGGATAGAAAAATAT
TTTTAAAATACGTATAAAAAAACTTAACGTCTACAGGAATCGAACCCCTGCCTCTTTAAAGAGGCCACAGATG
CATCCTAAAACCATTTCCAAGCTTTGCAGGTTCTAGTTCCGCTACGTTTAATTTTATA

TACGTCTTCTAGAAATATAAATAAATTGATTGCAACTTTTTTGTACTTTTGACTGACGAATAATTTTGTTATA
ATAATTACTATTTATATATTGTATACCTTTTGCCCATACATTTTTTCTATTTTAAATGTAAATTGAACATTGA
AATAAATGTTTTCTG
>50818671_3 CO729941.1
GGTCGGATTCCGGGTCGACCACGCGTCCGAACTCGAACTCGAAACGCCCTATTTCGAGAAATATATACTAAAA
CTTAATAGGTATATGGATCTCAAAATTTAAGTATTTCGAAAATAAAATCATATAAAAAATGAAGGAATCAAAG
CAAGCTCAAACGCCTATAGAAGATTTAGCTGAATTGCGTAGCTACTTTGCAAGCCATA
ACGTTGTACGCGAATACATTGCTCATAGTTCTAAAGTTCATTCTGTGGGGTGGTCCTGTGATGGAAGGAAACT
AGCTTCAGGGTCTTTTGATAAGAGCGTAGCTGTGTCTAATTTAGAAAGAAATAGACTGGCTCAGGATTTCATT
AACAAAGGTCACACAGGATCATTGG
>50818675_3 CO729945.1
CCGGGTCGACCACGCGTCCGGCAATGTGGATGTTAGTATTTATGATATTATAATATTTTAATTTATAATATTA
GTGTTTTACAACAAAATATATTAAGAATTGTAATTTTGTTGTAAAATTTAACTTAAAAAATATGATAATGGAT
GTTGAAACTTAAGAATTAATATTTTAGTTACACAAATGTCTTAAAATTTTAATTATGG
TTACCTATTTTTGTGATATTACAATTTAATAGCAATTATAAGATTTTAAAAAACACTATATACTTTTCATTAT
TATGATTATGTTTGTTGGAAACTAAATTGCTGTTTTAAAATTAGAATTATTTAACAATTG
>50818688_3 CO729958.1
CAAACTCAAAGAAGTAAGTTGGCGAACAGAGAAAAGGAGGCCTAAAAAAGAACAATCAATCGAAGATATAATA
CTTAAGCCAGCTCCTTCACAAAAACAACCAGAAGAAGAAAAAGCCGAAGAAGTACGTTACAAACCAGTGCAGA
GAGAGAAACCAAAGGAGACAGAAGAATCCAAGGATGAAAAGTGGCCCACTGGTAAAGG
AAAATCCAAAGAAGAACAACTAGTTGAAGAAGTTGTGCTTAAACCTGTTTCAAAGATTAAGAAACCAGAAGAC
GAAAAACAGGAACTTTTACGTCTCAAGCCAAATGTAAGTGATGTAACTAAAGATGAAAAAGACAAGCCTAAAG
AGGTAAGTTGGCGAAAAAGCAAGAGCAAGCCAGAGGAAGAACAATCTGTTGAAAAAGT
TATTCTTAAATCAGTATCTCAACAAAAAGAACAAGAGATAGAAAAGATTGAAGATGTATATCTTAAACCTGTT
AGTAAGGAAAAATCTAACGAGACA
>50818690_3 CO729960.1
AAGCGATAGAATTCTTAACTAAAATTATACAATTGACTTCCATTGCGGTGGAGTTTTATATCCACCGTGCAAA
AACAATAAGTTTTCCAAAATGTATTCTGTGTTGACAAGAGTAAATGCAATATTAACTTATACTCTAAGTGTTT
TAGCATGTTTAACGTTTTTGTGTTTTCTTTCCACGTTAACTGTGGACTATAGAACTAC
CGCTCAAATGAACACTGTAAAAGTAGTAGTCAAGAATGTTCCTGACTATGGAGCTTCAAGGGAAAGAAATGAT
CTTGGGTACTTAACTTTTGATCTCAAGACTGATTTGTCTAACCTCTTCAACTGGAATGTAAAACAGCTGTTCT
TGTACCTCACAGCAGAATATGTCACCCCAAATAATGAACTAAATCAAGTTGTATTATG
GGACAAGATAATATTAAGAGGCGAGAATGCTTTATTAGATTTCAAAAACATGAACACTAAATATTACTTCTGG
GATGATGGAAATGGTTTGAA
>50818691_3 CO729961.1
GGATCCAAAGAGCTTTAGAATACATCGCTGGCAACCCTCAACCACAAGATCAGCAACAGCAACAACCTTTCAG
GGGAAGACGCTAAGATATTTTAATCACAACCAAGGAACTCTTGCCACTGCCAGTTTAATTGCCTCTAGCGATG
TATAGCTTAGCTTTTATTATATAACCTTCTCTGTAATACATTAGTAATCCCTAACCTT
GTAGAAGCGGGTACGAATTGTTAAAAATGTATCATAAAAATGTAGATAATAGATAAATATAGATTTTAAGTTT
ATACTAGAAATGTACACTGAAATTAAATGTTAATAATCAGTTATTTTTATGTTTCATTGCCGAAATTTATTTA
CGATAATGTACCCGCTTTACAATC
>50818694_3 CO729964.1
GGGCGATGCGCAGTTTGCTGTGATGGCTCTTTATAATACTTACGTATATTTCACTTTTTTATGATATTCCCGT
TCCTTGCACTTCTTCTAGTTTAATTTAGTTCTAATTTATATTTATGCGTCTAAATGCGTTTAAATAAAATGGC
GAATCAATCCCAGAAAATTGTGTATAAGCTGGTACTGACTGGAGGGCCTTGCGGCGGA
AAAACTACGGGACAATCCCGACTTAGCACATTCTTCGAAAACTTGGGATGGAAGGTGTTCCGGGTGCCGGAAA
CGGCTACCGTCCTCTTAAGTGGTGGCATCAAGTTTGCAGATCTCAGCCCAGATGAAGCGATTAAGTTCCAGGA
GAACTTACTGAAGACCATGATTCAGATAGAGAACACTTTCTTTGAACTGGGGCGAACT
TGCCAACGGAATTGCCTCATTATATGCGACCGAGGAGCCATGGACGCTAGTGCATTTATATCCAAGGAGAAAT
GGGAGGCGATGCTGCTCGCAAACAACTGGAACAGCGTTGAGTTGCGCGACAATAGATACAATCACATCGTGCA
TATGGTGTCTGCTGCAAATGGAGCTGAGGACTTTTACTCTACT
>50818698_3 CO729968.1

ATTCCGGGTCGACCACGCGTCCGAAGCTCGACTCCTTATTTTCATATGTTGATTTTAGTCCTGCTATCCTGTG
CAAGCGTAGCGTTGGCGGTGCCAATAGGTTACGGTGCGGTGTACTCGCACGTGCCACAACTGATACACGCACC
AGTCGCTGTGGAGCATATTGCTTATCCAAAGTACGCTTTTAATTATGGAGTCAAGGAT
CCCCACACTGGCGATGTCAAGTCACAACAAGAAGAGAGGGACGGGGACGTGGTTAAAGGTAGTTATTCTTTAG
TCGAGCCTGATGGTACGACGAGAGTTGTGAATTATTCTGCTGACGATCATTCAGGTTTTAACGCAATTGTACA
CAGATCTGGACATGCTGTACACCCTGTTGCAATAAAGCCTGTAGCCCATTACGCCGCA
GCGCCAGCGCTTGTACTATCTTCTTATGGACATCACTAATTAAAAGCGATACGACGATGTATTACTACTTATT
TAGACACTAGCAAATATTTCTAAAGTTAAGTTTTGTTATTTAGATGTTAGATGTAGAATAGGCTAAGGTAATA
AAT
>50818701_3 CO729971.1
AATTCCGGGTCGACCACGCGTCCGTGAAGATGAGCAAATCAAATCGATAATCCGCAAATTCAAGTTTGATTTG
TCCAAGCAATTACAGACAATAAGAAAAATAATTATTAATTTATTGAGTTTAATTCGCTGTGGATTGTAAACCT
ACGGCATTTAAAATGGATCTTTTAAACTTTTGGCAGGCTATTGATGTTTTAATCCATA
AGCCTCATGTTATTAATAAAAACCTGTGGGGATCAATAGTGTTAAAAAGATATTACATTAAAACAGAAATAAA
TAATTGGAATGAGTATCTTGCTGACTATAGACTAAATACAATGGTATTTTTAAATATAGATCAATACTTAGCG
GAATTATCACAAATGTTCCACTTTCAAATTGTTAATGAAGAAACGCAATATGAAGTAA
TTTTCATAGAACTCCTTCCTAAATTTATATTGTACAGCGTGCGA
>50818704_3 CO729974.1
TCCGGATTCCGGGTCGACCACGCGTCCGCAGCCTAATATAAACCAAGACCATTTACCACATAGATCAAAGAAA
TCAAAAAAATCTAAAACAACTTCTACTCCAAATGTCAAAGAATTTAAAATGGATAAAGATGTAAATGAAAATG
TTAAATCTATTATGAAGTTATCAGTTGTCGCTGATAAGTCTTTAGATTTATCAATAAT
TGATCAATCCAGCAGTAAGCCAAAGAAAAGAAATAAGTCTGTCAGTTTTATACTTGAAGAAAATGAAGAAGTA
GTAGTAAAGAAGTCTAAATCAGAACAATCTAGTTCAAATGAAAAAAATATT
>50818706_3 CO729976.1
ATATAACGTGAATCGGCACAAGTGTGTGACTGTAAACGCGACTGGTTGTGGGTTCGACCCTCACTTGAGAAAA
TAAAATATTCATTTCAATTTATATAATAAAAATTAAATAATGTTGATTTCATAACAAATTAAATTGCTAAAAT
GATTTAAATGTAAGATAACATAAAAATTTTAAACCCTCTCTGCTTACCCTGGAAAGAA
TGAAAATGATTCATTCTCCCGAATGCGAATCAACCCACAATCGTCACATTTACATCGGCATCGGA
>50818713_3 CO729983.1
CGGTCGGATTCCGGGTCGACCACGCGTCCGGCAACAGTTGAGACACAAGGCTCTCAAGAAGGGTCTCGACCCC
GAAGCACTTACAGGCAAGCACCCCCCCAAAATCCAAGTTGCGTCCAAATATGAGCGACGTGTCGACACACGCT
CCTATGACGACAAGAAGAAATTATTTGAGGGTGACCTAGAAAAACTGAACAAGGACTT
CCTTGAGAAGGTGTGGCAAGAGAGGGCCGACCAGTTCGGTGGCAGGCAAAAGGCGCGACTACCTAAGTGGTTC
GGTGAGAGGCCCGGCAAGAAGAAGGGCGAGCTAGAATCCCCTGAAGGCGAGGAGGACGTGAAGGCCGAGCCCG
AGGAAGACGAAGAGCCCGTATTCGAGCCTGAGCCCGAAGAGGAGGAAGAAGTCGAGGA
AGTCGAGGAGGAAGAGGAGGAGGAGGAGGAAGAAGAGGAAGAGGAAGAAGAGGAGGAGGAAGAGGAATAAATA
TAAGAAACATTNCACACTCAAGGGCTAGAACGCCGACGGAGCTTCGTCGGTTACCATTTTGTAACCAATCCAT
TACTTATCGCTACT
>50818715_3 CO729985.1
CAGTGCAGTCCGTCCCTATGGCTGTGCAGTCATTATGGGAACTTGGTCAGAGCATGACGGGCCTCAAATGTAT
ATGCTGGAACCTAGTGGTGTTTCTTTTTCATACTTTGGATGTGCAGTGGGAAAAGCAAAACAGGCGGCCAAAA
CAGAAATTGAAAAGCTAAAGCTTGCTGACATGACTGTTAAAGAATTAGTTAAAGAAGC
TGCCAGAATTATCTACGTCGTCCACGATGAGCTAAAAGACAAACAATTTGAGCTAGAACTCTCCTGGGTGTCA
AAGGACACTAATGGCCAACACCAACTTGTACCCAAGGAAGTAGCTGCTGAAGCAGAGAACCAAGCGAAGCAAG
CGCTCGCTGATATTGAAGACTCCGATGAGGGTGAAATGTGAATAGATAATTAATAAAA
TTTTATTTACTTT
>51896052_3 CV133737.1
CGTCCGCACCAACCGTCCTTCAGTGAAACAAGGAGTCAGAAAAGCAAAATGGCAGCGAAAGTGTTGTGTATAG
CTCTCGCATTATTTGGGGCAGCTTTGGCTCAACCCTCCAAACAAGGATTCTGGAATGGAACTCCTATGGATAG
CATGGTGGAGGAGATGAGATCTGGATGCGCGGAAGGATCTGATCCCACTGCGTGCATC
AAATACAAGGTTATGTCCTTGTTAGACAGCATCTTCAAAAAAGATACTTTCCAGATCTCCGACGCCGTGGAAG
TGACAAAGAACGGTGCAGGTGATTCTTCAGCTCGCTCTAATGGTGATTTTATGGATTCCATTGAAAGCTACAT
TCAATCTCATGATGTAACTTTCCAATTGCCCATCG
>51896063_3 CV133748.1

GTCGGATTCCGGGTCGACCAACTGGTTCAGGTAATCGGGCAACAACTTGCCTTTTTCATTCTCGGTGAGAAAG
GTCCCCAGGTCGCGGCGGTGCTCGTTGATCAACTGCATGGCCTTGCCCAGCCCCAGCGCCTTGCTGGTGCGCA
GCTTGCGCGTCACCAGCTCGGCGGAGATATTCACGCTGTTGAGTACGTTGCCGACGTT
GTGCAGCACGTTGGTGGCAATTTCCGCCATGCCGGCCTGGCGGGCGCTGTCTAGGAGTTCACCCTGGGCATCC
TTGAGTTCCCGGGTACGCTCCTCGACGCGTTGCTCAAGGTTTTCGTTGACCACATGCAAAGCCCGGTTGACCC
GGTTGATCTCGGCGAAACTGCGCAACAGGCGAATCGCCAGGTACAGCAGCAGCGCCAC
CAACAAGGTTGAAAACACCAGCAGGTAGCGATGGTATTTCTGGTCGAGCAG
>51896066_3 CV133751.1
ACGGTCGGAATTCCGGGTCGACGCACGCGTCCGCGATCAGTGAGCATCATCAGGATCGAAATATAGGCGAAGC
ATGAAGACCGTAATTCTAGTTGCATTGATGTGTTTGGAGGCAGCAGCATGGGGGAAACCAGCCTCCACTTATG
CAGACAAATGGGATAATATCAACATACATGAAATTCTGGAATCCAACCGGCTTTTGAA
GGGTTACGTCGACTGTTTACTTGACAGAGGTCGGTGTACGTCAGATGCTAAAACCCTAAAGGAAACATTACCT
GATGCTCTGGAACATGATTGCAATAAATGCACTGCGAAGCAAAAGTCTGGTTCTGATAAGGTCATAAGTCATC
TGGTTAACAAGCGTCCAGAATTATGGAAAGAATTATCTGTTAAGTATGACCCCAACAA
CATCTATCAAGAGAAATACAAGGACAAGCTTCAAACTATCAAAGCCTAAATTTCTTTTTTCCTTTTAGGTTTC
TTCTTTCAAAATTTTTATTGTTCTGGATCGATGTAATTTGTACAATAAAATTCTTTATTTTAT
>51896070_3 CV133755.1
CGTCCGAACGGTTGTACTAGTCAAAGAAAAGCAGCAGCTATGGTGGACGCCGCAACCATTGAGAAATTGGAGG
CTGGATTCAGCAAGCTCCAGGCCTCTGACTCCAAGTCGCTGCTTAAGAAGTACCTAACCAAGGAAGTCTTCGA
TGCTCTAAAGAACAAGAAGACCTCCTTCGACTCCACCCTCTTGGATTGCATCCAGTCT
GGTGTTGAGAACTTGGATTCTGGTGTTGGAATCTATGCCCCTGATGCTGAAGCTTACTCAGTATTTTCTGATC
TCTTCGACCCCATCATTGAGGACTACCACAATGGCTTTAAGAAATCCGACAAGCACCCAGCTAAGAACTGGGG
TGATGTTGAGACCCTTGGCAACCTGGACCCTGGCAGCGAATTCATTGTCTCCACCCGT
GTCCGCTGTGGCCGATCCTTGGAGGGTTACCCATTCAACCCCTGCCTAACTGAAGCCCAATATAAGGAGA
>51896075_3 CV133760.1
CGTCCGGACTTTTAAGACAAGGAGGAGGTTCTCAATTAAACATTTTCGATTCGATATTCGTTTATTTATTTTA
GATTCGATTTCGATATTGAATTACTCGAGTCGATTTTTGTAATATGCAGTCATTACAGTTATTTTACAAAGAA
TATTATAAATTCTTACTTAAAATTAAATTATTTATTGACATCAACACATTTGACCATC
AATTCTGTTTACTAAATGAATAATCATGTAGTTCAAGCGGGCGAAGCCGCGGGGGGGTCGCTATTTCTTTAAT
ATGAACTGTATTGAAATAAAAGTTATTTTTAAAATAGGCAAGGATTCCAACTGGACTATCTTAACAACAGAGG
TTCTGGAAGAATAAGGATAGTATTAAGGATTCCACAATATGTAAACTGTTATTTATCA
TTATCGGCAAACATCAAGCAACAATGATGACTGTTATGATATGCAAAAATATGTTTTATTAAAAGACTGGTCA
AATGCAAGTTCGACTCGCAAAATTACCAAGTACAAATAACAGTACCAAGTTCAACATACAATGTATGCAATTA
TGTTGATGTATGAATTTATCTACTATTATAAGTTATAATGATTTCTTTCTATGAAACA
TCTACGAAATTTCACTTTTTGCATATG
>51896077_3 CV133762.1
CGTCCGCCGAACTGGTGTCGATCACACGTCCGCCAGCCAGCCCGACGTATATAATGATGGAAAACTTCCGTTG
GTGGTGGCTCGTTGGTGCAATGGTTCTGGTGACGCTCGCTTCTTCTGAGCCTCTCGAGCATGATGACCACGAG
CTCCTCGTGCGCAACGAGCGTGGCGCCAAGAATCGAGAAACTTGTCGCTACGTCCGCG
GCGCTTGGTCGGAGTGCGACCCGAAGACCAACATTCGGTCCCGCACACTCACTTTGAAGAAAGGAGACCCCGG
CAGTTGCGAACGCGCCAAGACCATCCAGAAGAAATGCAAGAAAGCATGCCGCTACGAGAAGTCATCTTGGAGT
GAATGCAGCCCCAATGGTGAAATGTCCAGGACGGACATGTTGAAGGCGAATAGTGATC
CCACCTGTGACCAGAGCAGGCGCATCACTAAGAAATGCAACAAGAACAAACAAGTCAAGTCCATTAAAG
>51896079_3 CV133764.1
CGTCCGAAGAATGAAAGTATCAACTCACTGAAAAATGAAACAACGGAAAAAGAACTGTTTACAGACACTTTGG
AAGGTTCCAATGATATTTTAAAAACATTTAAAAAAGAAGATTGTAAAGATTCATTGAACGTGGACACAGGATA
TATCAGTGAAAACGACAAATCCAAAGATTCCACAGTTAAAGTTAGTAACAAAGTTGAA
GAAAATAAATCTGTTACAGCTGAATAGTCACATTATACTTACTTACCTAGAAATAAAATTTCATCAATGTATT
TCATGTTCTAAATTTACATAATATTTACGAAATCTTACACTACTGTGTATAAAAAAATCTCATTAGGCAAAGC
CCAAACAAGATGGTGGTAACCCATTATTATTTTGATAATCAAGGACCTGTGATCATTG
AAATTTGTATGGTTCTGTGACTCTTGATGTTTTGTTATTACTTAATGATAATAATTTTAGGTTCTCACGTTTT
TAATTAAGAACTTCTTGTTTCCATTTGTTAATTTTTTGTCTTATATTTTCATTTTATCTTCCTCTTTAAATAT
ATTGTTGTTATAGTTTGTAATACTCATTTTTTATAGTGATTGTTTTGTCAGTATCCAT

TCCTAGATAGGATCTTTACTTGAAAAGTATAATATTGTTTTTTTTTATGTTTAAGTTATTATTACCGTAAAAC
TTCATTATTATAGTCTCTAGAGCATAACATAAAGTAGCATTTAAAATATTTTGA
>51896082_3 CV133767.1
CGTCCGCGGACGCGTGGGTGTAGTTGCACGCATCGCGACAGTGCCAACAAATTTTAATTCATGTATCAATACC
GGAACATATTTTAAGATGTATTCTAGTGACGATTACAATGAGGGGGGTTATGAGTCTTATGGAGACTATGAAC
CCCATACAGGCGACCCTCAATATGACTTGGAGTATGATAGGTCATACTATCAAATGCC
CGATTTGGTCAAGAAATTTCTTGTATACTTTCGTAACATGATTACTGAGGGAATGACTTTTGAAATCCAAAAT
CTTTATGAAAATACATTCCCCAAGCTCACAGAACAATACTTTGAAAATACTCCATGGCCTGATGAAAAAGAAG
TTGCACCAATTGTAGACAATGATCATGTATTCATGATCCTGTACAAGGAGCTCTATTT
CCGTGATATTTACGCTCGGGTGCCTGGTGGTCCAAAACCAGAGCAAAGATTCCTGTCGTTCTACAATTATTGT
GACTT
>51896087_3 CV133772.1
CGTCCGGTGAACGCTTACTTCATTTCACGAGTTTTAAACTTTCAAGTGTGCGTTTTCGTACTTTCTTTCTGAA
GTTTATTCTCCAAGTTATTGATTAGAAGAAGAAATGTCTCTGCTACCCTATTTGTTCGACGATTACTCTTTTA
GACGACCTCGTCGTCTCATTGACCAGGATTTCGGTTTGGGATTGACACCTGATGATCT
TCTTACGGTTGCGGCAGGACCTCTACTTTCAAGAGAATATTATAGACCCTGGCGTCACATGGCAGCCTCAGCA
AGAGACCTAGGCTCCAATATCAAATCTGATGCAGACAAATTCCAAATCAACCTAGACGTTCAGCATTTCTCTC
CTGAAGAAATATCCGTTAAGACAGCTGACGGTTTCGTTGTAGTTGAGGGTAAGCATGA
AGAGAAACAAGACCAACATGGCTACGTTTCGCGCCAATTTGTAAGGAGATATGCTCTGCCTGAGGGTACACTA
CCAGAAACCGTTGAATCTCGTTTGTCCTCTGA
>51896088_3 CV133773.1
CGTCCGCAATTTCTGTTGAAATTATTATTGATGCGTTGAATTAATTCTGTGGATACATATGTTTTGCACATAA
ATAATAAAAATGTCTCTAGCTGACGAGTTACTTGCTGACTTGGAGGAAAATGATGATGGGGAACTGGAAGCTA
TGATTGTAGACTCTACCAATAACGAAGCCAGTGACTTTGCAGTACCTTTTCCAGTAAT
ACCAAAGGAAGAAGAAATTAAAAATGTCTCTATTCGTGAATTAGCCAAACTTAGACATTCAGAACGCTTAAAG
CGGGTTATAACTGAAATTGAACAAAATGCTGGTAATGATAGAAAAAAAAATTGAAGTAACTGGTTTAATGGAAT
CAGACCCAGAGTATCAGCTAATTGTAGAGGCCAATAATGTAGCAGTAGAAATTGATGG
AGAAATAACTACTATTCACAGATTTGTTCGGGATAAATATCAGAAGAGGTTCCCTGAACTGGAGTCATTAATA
ATTACTCCATTGGAATATATAAGGAGTGTTAAGGAACTTGGCAATGACTTGGATAGAGCTAAGAATATGAGAT
TCTTCAAAGTTTCTAACTCAAGCAA
>51896089_3 CV133774.1
CGTCCGGCACAAGCAACACGCACTTTCACCGGTGACGAGTGGCTAGCCCGTGGCAGATATCAAATAAGTGTTA
GTGATTTAGTGTTTACCCTTTCAAACAAAATAAGCATGCAGCAAAAGGTGACAGGATGGGCATTCCTGGCCCT
AATCTGTTCGTCGACGATTCTTCACACGTTGGCGTACCCGCAACATATGCCTCTTGTT
TCACAGGTCACAAGCAGTGTTCGGAATCAAAACCCACAGTATTATAGTAACTCATTGCCAGTACCACACCGGC
AACAAATCGCAGAAGAACGAAAATTCGCTGAAAAATCTAATGCATTGAAGAAAGTAGCTCTCGATGATCTGGA
TGATATCCAGAACAATTCTCTATCAGATGGCGGTTCCTCATGGTCAAACATGTTAGGT
ATGATAATGCAAATGATTTTCAATCCTGCGGGCACTGTAGGACCAAATAAGAGCGACAATTTAGACACAGATT
CAGCTGCACCATCTCCGTGGACATTTTTATTCTCAATGGGTCTGAAAATCCTGACCGCAATCCTAGGAGGCGC
TAACAACAACACTGGAATTGACAAAGTGGACAACGAGTCTTCACCAATGCAGTTCATC
AACATCGTTGTAAATCTTCTGGATGCTCTCAAAACCTCGTTCTCACATCGGTCACTCGCTGCCCGAT
>51896095_3 CV133780.1
CGTCCGCAAGCATTCGTTTTATGCCAGTGCAAATATTTACAGCATCGTAAGTAGATGCTTGATGTTTATTAAA
TAATAATTTTATTTAATTTAATATCTTTTTTATTTACTTTAGTATAAAATGCATAAATTAATATTGATACTAG
TGATTGCACTAACAGTGACTAGTGTCATATATGCAAAACCGTGCTCACAAAGAAAACG
ATGTAATTCAAATGACAGCGACGAGAATAATTGCAAGGATAAGAAAAGATGCAATGAAGTATCATCATCATCA
TCATCGTCGTCGTCATCGTCCTCATCGTCTTCATCATCATCTTCATCCGAGGAAGACGATGATAATTGCAAAA
AAAACGACGTAGATAGTGATAGCTCGTCCTCATCGTCATCTTCATCTTCATCATCTTC
AGAGGAGGAGGGAGATAAGAAAAAATGTAGTAACTCTATAGAAAAATCTAAACATCATCACAAGAAGTCCCAT
CATTTTAAGAAGGACAAAGGTGTTTGAATTGGTGCTACTGGACTAGGTTTATCTTAGTATCCGCTTAAACTAG
ACTAGACTACT
>51896103_3 CV133788.1

CGTCCGAACCGAAAGTTACGTGGCTATTGTAATGCTCAAATTTTAACAGTTTCGTTAGTGAGTGTAGAGACAA
GTGTTGTGAAAATGTCGCCCAAATGCCCAGTGGCTCCTTTACACGAAGTGACTCTATCATTAATAGCTAGACA
ATTAATCCACGCTCTGACCCAAGCCACGTCTGAGGAAGATGTGGCTCTACCGTTCACA
ATGATATCGTCTTACTACGAAAGTATGGGTGCTACGAGTGATATATTTCAAGATCTCCTAAATTTAATATTAA
GTTCAGAATATTTAGATCCATCGATTAGATATTATACAATGAGATTGCTGTTGAAAGAGAATGTTAAGAGCCT
GGCCACGGGTATCTTCCCTTGTCACTATTACAGGAAAGTCCTTGAACTGATT
>51896104_3 CV133789.1
CGTCCGCTACTTGGATATTATTAAGCAATCCTGTCAACAAAGATAACGATTCTGTTTCAACGGAACCAACAAA
AAAAAAAAAAAAAAAAAAAAAAAAAAAAAAAAAAAGGGGGGGGCCCCCTTTGGGGGAACCCATTTTTTGCGGGGG
GGGGGGGGGCCTTTTTTTTTTTTTTTTGGGGCCCCAAAATTTTTTTTGGGGGGGGGTT
TTTTTAAACGGGGGGGGGGGGGAAACCCGGGGGGGANTNNNNTTCCNGCGTGCTATTGTGGGAGC
>51896117_3 CV133802.1
CGTCCGGAAGAATGATCGGTATGGATTGTACTGAAAACTCCAAAGAGGTCAGATGCTGTCGGTACCCGCTGTC
CGTGAATTTCGAGGAGTTTGGATGGGACTGGATCATAGCGCCGAAACAATACGACGCCAACTACTGCAGCGGG
GAGTGTCCGTATAGTTTCATGCAGAAGTATCCTCACACTCATTTAGTCCATTTGGCGG
CTCCTCAAGGAAGTGGTGGGCCTTGCTGCGCTCCTAGACGTATGAGCAGCATCTCCATGCTATACTTCGATCA
CGATCTAAATATCATATACGGAACTATCCCTGGCATGGTAGTAGATAGCTGCGGCTGTTCATAGCAACCGCTC
CAAGAATTATTTGGAAAATTAATTTACACTTTTGGTTCAAAAATGGTCTAACCTATCA
AATTTTGGACTTAAAATTATGTAAAATGAAGATCTGAGATTCCTTTCTTCTCTTGNGGCTTTAAGTGTAGTGT
TCTTATTGTGATACATTCGGTTGAAGACTGCGGTGCGGACCCTTGTGGTGTGTGTGTGTGTG
>51896121_3 CV133806.1
CGTCCGCGCGCAACACAAAATGTACAAATACGCACTGATACTAGCTCTCTTGAATCTTGCCGTTTGTGAAGAG
AGATCACATAAAACACACTATGTCGATCCCTTCGCGCTGATTGATAAGCATTTGTCACATTCCCTCACTTATC
ATTATCTATGGCCTTGGGGACAATTGGTCAAAGCAGCGGCGGCTTTGGACTTGGAAGA
AAGCTTGGAAGATCCAGAAATTATATCTGATTCGGAGAAATATCAGATCAGCTTAAATGTGAGACGATTCAAG
CCTGATGAGCTGAAGATTAAAGTGAAGAACCGGTACATTATTGTGGAGGGAAGACACAAAGTGGATAGTGACA
ACAAGTTCATGGCAAATCACTTCGCTCAGCGTTTCGTTCTGCCACCGGGTACCAAACA
AGAAGAAGTCAAGGCTGTATTAAATGAGAAAAGTATTTTGATCATCTCAGCGCCAAAACACGAACTGCCACCG
CCGATTCCAGAACGAGAAGTGCCTATTGAAGTTATTTACCCGAAAAGAAAGTTGAGGAGCTTA
>51896126_3 CV133811.1
CGTCCGGCCGCTGATGCCACTCTGGATTCAACGCCATCGTACGCAAAGACCCGGCTCTCATTGCTCACGCCGC
TCACGCCGTTGTAGCCGCACCTCTTGCCCACGCCGCCCCTGCACTTGTAGCCGCTCCTCTGGCTCATGCCGCT
CCCCTCGCATACGCTGCCCGTGTAGCTCCCATCGCTCACGCTGCTCCCGCTGTCATCG
CTGGACCCGCCGTGTCTTACTCTGCATCTTCAAGCTCAGTAGCTCATGGAGGTTATGCCGCGCCATTGGCCCA
TGGTGCCATCGCCGCTCCCTTAGCTCACGGTGCCCTTGCTGCTCCCTGGGGCCATGGTGCACTCGCCGCTCCC
TTAGCCCACGGTGCCCTCGCCGCTCCCCTTGGTTATGCCGCAGCTGCCCCCTTGGCTC
ATGCTGCTCTGGCTGGCCACCTCGGTCACGGAGTAGC
>53910664_2 CV526285.1
GGCCTGCAGAATCGGCCGAGGCATGGTGTAGCATTCATTGTTAAAACCAGTGAATTTTAAGTAATTAGATACA
TATTTTGCACATCGTGGGTTCTCCGTCCATGATTAACCTTTTTTTCCACTCGACGCAACCTATACGACATAGA
GCCACTAGTGTTGTACCATATAAATTGTTTGTTTGTTATCGGTAAATATTTTTAGTAC
ATTTTTTTGTACTTTTTTAATATGAAAACTCAATTAGCCTTCAGTAAAAACTGTGAATTGTTTGTTGCTATTC
TGGTGACTTACTTGGTGACCTAATAATGAACTAGCTTCTACCACTGACTGTAGCGAAACATCGACGTTTTATT
GCAGATTCCTATTTTCTATATTAGATTTAAGAATAATATGATTAGCACTCGGGAATGT
TAAAATTGGTTATAGTAAAAACTTTAGATTTACTCGCTTGGTAAAATTATAATTTATTATTGAAATTATATTG
TAATAATTCAATGTCATGATCATCGATAACTTTATAAGTTAAAACATTAGGTGTATTTCAGTAGT
>57963547_3 CX700430.1
CAGGTACGGTCCGGAATCCGGGTCGACCACNCGTCCGGCAAAATCTCATTCAGAGACACGTACTACTAAAGTG
ATGGGAACTCATGATAAAGTAGTTGAAAAGACTCATATAGTTTCCCGTACTAAAGGTCACGATGGTCCATCAA
ATGTAAACCGTAATAAATTGCAAGACACTTCTATTACATCATCATCAAAAGATGTAAG
TCAGTCATTATCACGCGATACTACCGACCGGAATGCAACGGATCGTTCACATAAAAGACAATCTACGGAAAGA
AATGTTATAAATAAGAGTGATACTACAAATATTCAACATTCATCTTCTACTAATAAAATAATAAAACAAATAG
CAGAGAAGAAGTTAATTGCAGGGAAATGGGTAAATGTAACTATAAATGTGGAA
>57963557_3 CX700438.1

ACCAGAGCGCAAAGGAGGCCTGTGTGTAATGTTGAATTTGATAAAATGGCAGCCACTTGTATTACACCGGGAT
TTGACGAACGCCAAATATGCGTAGGCTTCGGCAGAGGTCACATCCACCAGGTCGATCTTCGGCGAGGAAAACC
AGACAAGGGCTATAAGGGTTGCGTGGGTGCCGTCACTAGCATCGACTTGGTGAAAGGC
CAGGATGAGGGTCAGGGTTTGGTGGTCAGCAGCAGCTTGGACAGGTGGTTAAGGGTGCATAATTTAGAGACCA
AGGAGTTGGTTTATAAGCAATACTTAACATCGAAGCTAACATCGGTGTTAGTCCAAACGAAAACATCTACTCC
CTTACAGAGGTTGGTAAAGAAGGAAGATCAAGAAGATAAAGAACTGAAGGAAGAGGAC
TTGGATAATATATTTGATGGCATGGAGACTGTCGGCGAAAAACCACGACAGAAGCGTACCGAAGCACCCGAAA
GCGAAACAATAGAAAAAAGCGAAGGAAATGAAATCGAAGCGAAGCGATCGAAACCGTCTCTAGAAGGCAGTAC
TATAGTAATAGATGAGGACGAAGCGATCATGAAACTTTTGAGAAGCACCGAGAAGCA
>57963559_3 CX700440.1
TACGAAATTATGTTTGGACCTGATATTTGCGGACCTGGCACCAAAAAGGTGCACGTCATCTTCAGCTACAAGG
GCAAGAATCATTTGATCAAGAAAGAAATTCGTTGCAAGGATGATGTCTACACTCACGTCTACACTTTGGTCGT
CAAACCTGACAACAAATATGAAGTCCTCATTGATAACGAAGTTGTTGAGTCTGGAGAA
CTTGAAGCTGACTGGGACTTCCTTGCACCTAAGAAAATCAAGGACCCAGAAGCCAAGAAACCAGAAGACTGGG
ATGACCGTGCCCAGATCCCTGACCCTGATGACAGTAAACCTGAAGACTGGGACAAGCCTGAGCACATCCCTGA
CCCTGATGCCGCCAAGCCTGACGACTGGGATGATGAGATGGACGGTGAATGGGAACCT
CCCATGATTGATAACCCTGAGTACAAGGGTGTTTGGGCTCCTAAGCAGATCGACAACCCTGCATACAAAGGTC
CATGGGTCCACCCCGAGATCGACAACCCTGAATATGTTGAAGACAAAAACCTTTACAAACGCGATGAACTTTG
CGCAGTCGGTCTCGACCTTTGGCAAGTCAAATCTGGTACTATCTTTGATAACGTCCTT
ATCACTGATGACATTGAACTGGCCAAGGAACGAGTGGAGGCTGTCAAGAAGACCATTGAGGGGAAAAAAAA
>57963560_3 CX700441.1
AACTTTAATTAAAATGGCTCGCGAAAGCAGCGCAGGATTTGATAGACATATTACTATTTTCTCTCCTGAAGGC
AGATTATATCAAGTTGAATATGCATTAAAAGCTATTAACCAAGGAGGCCTCACGTCGGTAGCTCTAAGAGGCG
CCGACACGGCAGTAGTGGCGGCTCAGCGCAAGGTCCCCGACCGCCTCTTGGATCCAGC
TTCTGTCACACATCTGTTCCGACTGACTGACCACATAGGTTGCGTGATGACTGGCATGACTGCGGACAGCAGG
TCTCAGGTGCAAAGGGCCCGCTACGAGGCGGCCAACTGGACATACAAGTACGGCTCAGAGATCCCTGTGCACA
TCCTGTGCCGTCGCATCGCGGACATTTCGCAAGTGTACACTCAGAACGCTGAGATGCG
CCCACTTGGATGCAGCATGATGGTAATAGCATACGACAGTGAATCAGGCCCTAGCGTGTACAAGACGGACCCG
TCCGGCTACTACTGCTCGTATAAGGCGGTGGCGGCGGGCTCCAAGGCCACCGACGCCAACGCCTACCTCGAGA
AGAAGCTGAAGAAGAGAGCCGACCTCAACCATGACGACGTGGTGCAGCTCGCCGTCGC
CTGCCTCTCGCAGGTGCTTTCTGTGGACTTCAAGGCGT
>57963561_3 CX700442.1
CAAATACAGGTGCTGTAAAAATAATGGAGAAACTTAATGCCCTGACATTTCAAAAAATTGGTAGACTTGTGTC
TTCAGTGGATTCGCAGCCTATGTTTGATGGTGGTGTTTTAATTAATGTTCTCGGCAGGTTGCAGTGCGACGAT
GACCCACCACACCCATACATGCAAACATTTGTGTTGAAGCCTCTCGGAGAATCTTTCT
TTGTACAGCACGACATATTCCGACTCGGCATCCACGACGTCGCCTAAACTCACTCTATCACCATTGGATGCTA
ACATAATTGCTAATTTCCATCTTGTTTTGGTTATCTAGGTATATAAGCTAGTTGGTCACTGCCCACTAAATTG
AAAATTGTAAATTAAATCACACTATTTGGCATCATTCGGCTTAGATTAATTATTCGAG
ATGGCTTTAGTCAATGTAAAGACGGACTTTCAGTTACATGTATACATTTTATAATAAATACATTATTTTCT
>57963577_3 CX700456.1
CAGTACGGTCCGGAATCCGGGTCGACTACGCGTCCGCGGCGCCATCTTGACTTATCTTCGAGCATATGATCGT
TGTTGTTAGTTCTCTGTTAGAATTAGTAAAACTGCGTTAATTTTCGATAAAAGTTGTGTTTTGTGTGTTAATT
TGATCCAGCAATAACTTGAAATGGCGCGTACCAAGCAGACTGCCCGTAAATCTACTGG
AGGTAAAGCACCTCGTAAACAATTGGCTACAAAGGCAGCCCGTAAATCGGCTCCTAGCACGGGAGGTGTGAAG
AAGCCCCATCGTTATCGCCCTGGTACCGTGGCGCTCCGAAAAATTCGTCGTTATCAGAAATCTACCGAGTTGT
TGATCCGTAAGCTGCCCTTCCAGCGTCTCGTGAGAGAAATCGCTCAAGATTTCAAAAC
TGATCTTCGTTTCCAGTCTGCCGCCATCGGCGCTCTGCAGGAAGCAAGCGAGGCTTACTTGGTAGGTCTCTTT
GAAGACACAAACTTGTGCGCCATCCACGCTAAGCGTGTAACCATCATGCCTAAAGACATTCAGTTGGCCAGAC
GGATTCGAG
>57963578_3 CX700457.1
GCAGGTACGGTCGGAATTCGCGGTTCGATTTGGACATCCCCCGTGATGCCCTGGTGGTGTTCACCGGCGTGTC
AGGCTCCGGCAAGTCCTCGCTGGCATTCTCCACCGTCTACGCCGAAGCCCAGCGCCGCTACTTCGAATCCGTC
GCGCCCTATGCCCGTCGCCTGATCGACCAGGTTGGTGTGCCCGACGTCGACTCCATCG

AAGGCCTGCCACCTGCCGTGGCCCTGCAACAGCAGCGCGGCACACCCAGCACACGGTCCTCAGTGGGCAGCGT
CACCACCTTGTCCAGCCTGATCCGCATGCTCTACTCCCGCGCCGGCAGCTACCCGGCGGGGCAACCGATGCTG
TACGCCGAGGACTTTTCCCCCAACCTGCCCCAAGGCGCGTGCCCGCAATGCCACGGCC
TGGGCCGGGTGTACGAAGTGACCGAAGCCCTGATGGTGCCCGATCCTTCCCTCACCATCCGCCAGCGCGCCGT
AACGTCCTGGCCGCTGGCGTGGCAAGGCCAGAACCAGCGCGACATCCTTGTCACCTTGGGCTACGACGTCGAC
TTCCCATGGAGAGATCTGCCGAAAAAGCAGCGCGACTGGATCCTCTTCACCGAAGAAA
CCCCCACCGTCCCGGTATATGCCGGCTTC
>57963582_3 CX700461.1
CACGCGTCCGACGGACTCTTCACCGTCACTCTCTTCAAGAAGGTAGTGGACGAGTTCAAGCTACACGCGCGCG
AACGTAAATTCATAGTTCGCGAGTTTTCATACAACGAGGCCGACCTCGCCGCCGGCAAGAACGAGATAACCAA
GCTCGTCACTGATAAGAAGAAACAGTTTGGTCCCTTAGTGCGGTGGTTGAAGGTGAAC
TTCTCAGAATGTTTCTGCGCCTGGATACATGTTAAGGCGTTAAGAGTTTTTGTTGAATCCGTTTTGAGGTACG
GGCTGCCGGTGAACTTCCAGGCGGCAGTGCTGGTGCCGGCGCGCAAGGGCGCCAAGCGCCTGCGCGACGTGCT
GCAGCAGCTCTACGCGCACCTCGACCATTCCGCGCACCACCACGGGCACTCCGCGCAG
GACACGGCGGAGTTGGCGGGTCTCGGCTTCGGGCAGTCGGAGTACTTCCCGTACGTGTTCTACAAGATCAATA
TCGAGATGGTCGACATCAAAGCTTAGATGTTCTTCAGTTTAGTGATGGTATTTATAGGTATTGTTACCGGTCA
TGCTGTCGTGTTGTCAAGCTACCAATAGTTACAAACTACAACC
>57963585_3 CX700464.1
CCAAAACAACGTCAATTTTTGTAAACCATAATAATTATAACCTATTTGTAGTAACTACAAAATATCTTTTATG
TGCATATAAAGAAATATTTACAATAACAAGTATACTTTTATAGTAGTACTTGTTAATAATTGTGAATGATTAA
CAGATATCGTTATTTATATGTGATTTTTTTTATTATAAAAAGATTTAAATCACCTGCA
GCAATATGAATGAAGAAACAGAAAATGAAATGAGTAAGATATGTATTCCTGGAATGAGACTGAGTCCATCAGA
CAAAGAACATATATCTGGTCCAGGGACATACGAATTAAAAGGCTACATATATGCATCTGAGGCGGGAGTTCTA
CAAAGGGAAGAGGATCATGATAGAAAGGCAACTAGATTATGTGTGGACAGCCCTAGAA
CACCCAGTGTATTACCTATGAATGGTGATATAG
>57963589_3 CX700468.1
GCGGTACGGTCGGAATCCGGGTCGACCACGCGTCCGATACAAATATAATATATTATATACAATCATCTGTGTC
ATGAAAATGGACTCGACGGAATGGAGCTCATGATTTCAGTGTTTTATATCGCTGCGATAAAAGTGATAAGTGA
TAGTGAACTATTGTGTGATACACATCTGATACGGACAATTGAGATACGAGCATGAGGA
GTTGGAGCGGTGATGCGTAAATCTTATTAAGTATATCAAACAATGTTGAGACTGGACTCGGTCTTGGCGTGAG
TTCAACTTGCAATTGACTTGAAATGTGGATAGGAGTGAGGACTTAAAGTGCGTGTGTAAAAAACGATGCATGG
TTTGTGAGGTCAGCAGCACGGCCATGGACATGAGCGAGGGCGCGGGGCANCAGCGCTG
GTACGACAGCCGCGTGAACGGCAGCCCCGCCGCCGGCGACGTGGAGGCCAATGGGGACTTCTTCCACTCGCCG
CTGGAGGGCGGGCACCACAGCCGCGCCCGGTACTACCACCAGCAGATGCACGCTCCTTATTCTGCCGCGGTCG
GATCTCATGGTCGGACGTTAANCAGCGGCAGCGGGCAGGTGTGCAGGCCGCATTTCCA
CACGCCGTTGCATCCTTGGCTCGATTCGAAAGCTTTGGGTAGCGGCGCCTGGGGGAGCGGCTTCCAACAAGAC
GCTGC
>57963593_3 CX700472.1
CTAGCGTCCGCACTCGCTTAGTTTAAACCATATTAAAACCAACCACCAAAATGTTCAAATTGTTCTTCCTCAT
CTGCTTCCTGGCCATCGCCGCAGCCAAGCCCCAGGTGCTAGTATCAGCACCAGTCGCGGCGGCGTACTCTGCA
CCAATTGCTGCAGCATACTCCGCACCTGTAGCATATTCTGCGTACTCCGCCTACAGCG
CTCCTCTGGCTGCCTATTCTGCCTACCCTTATGCGTCCGCTTACCCCGCGTACTATCTTCGTCGTTGAACAAA
TCCAAAGAAACATCCTTGTCTAGTCCAAATATTAAAATGAATATAAATTATATTTATTTGCCATAACCGAATA
AATAAATACAAAGT
>57963600_3 CX700477.1
GCAGTACGGTCCGGAATTCCGGGTCGACCTAGCGTCCGCAGAATATGAGTCAACGCCAGAAGGAAGAAAAATA
ACGAAATTGGATCAAATATTACTCAATGGAAATAATATAGCAATGTTGGTGCCAGGAGGCGAAATGCCGGGTG
ACTCGTACGACGGATAATAAGTTTTAGGTTAAATAAATGTATTTTAAGTTTC
>57963605_3 CX700482.1
TGCAGTACGGTCGGAATTCCGGGTCGACCACGCGTCCGCACAAAGATCAACATCAGAACTCTTATAAAAATTA
AAAATTTTAAATTATTTACTTAGCTATGAATAAAAAAATGTCATTGTATTTACTATTGTTCCTAAATAATTTA
GTCGAAATATTAGGCATTCCATATCAGGAGAGCTTATTCGACGAATACGAACATTTGG
AAAATTT
>57963606_3 CX700483.1

GCGGTACGGTTCGGAATACCCGGTCGACGATGTCGTCCGGCGACTTGTACTCCAAAGCTATGTTGGGTTTTGA
ATTACTTCACGAAAAAGCTGGTATATTTGACAATCATATCCGGTATATCTCCCAATGTATAATGAAGAAGAAC
GGTATTACTGAGTTAGCTTCAGTCAGACATAAAACACAGACAGAGGTGTACGTTGCTG
GACGAATAGAATGTGATGCAGACGCAAGACTTAATTCTAAGAGTGTTATTTTACAAGGGACATGGGAGGAGTC
TCTCAGTCAGGCTGTGCCAGTAGACTTAGATAAGGGGGGGGAGTATTCATTGTTTCCCGGGCAAGTGGTGGTG
ATGCGCGGAATCACCCCGCGAGGTAACTCGTTCATAGCCCAGCGAGTGTATACGGACG
CGGCTCGCCCCATACCAGATGTTGGGGCT
>57963607_3 CX700484.1
GCAGTACGGTCGGAATCCGGGTCGACCACGCGTCCGGTGTAGTGGGCGGTGTAGCAGCATTTAACTTCCCAGT
AATGATTCCACTATGGATGTTCCCACCGGCCATTGTAACTGGTAACACTTGTTTGATCAAACCCTCGGAACAG
GATCCCGGTGCCACTCTCATGATGATGGAACTCTTGCAGGAGGCTGGAGCTCCACCTG
GTATTGTTAATGTTGTCCATGGAACCCACGACACTGTGAACTTCTTTTGCGATCATCCTGATATTAAAGCAGT
GTCCTTTGTTGGTGGAGATGCAGCAGGAAAACATATTTATACTAGAGCCTCCGCTGCTGGCAAAAGAGTCCAA
AGCAACATGGGCGCCAAAAACCACGGAGTAATATTACCAGATGCAAATAAAGAGCATA
CGCTCAATCAGCTAGCTGGAGCAGCCTTTGGAGCAGCTGGACAACGGTGCATGGCCCTGAGCACTGCAGTTTT
TGTTGGTGAAGCAAAGGAATGGATCCCAGATTTAGTGAAACGGGCTGAAGCACTCAAAGTGAATGCAGGCCAC
GTTCCCGGAACAGATGTTGGTCCAGTCATATCTGTTAGAGCTAAAGAACGCATTCATA
GGTTGATTGAATCAGGTTCCAAGGACGGCGCAAAAGTAGTTCTCGACGGCCGAGGCATCAAAGTGCAAGGAT
>57963609_3 CX700486.1
CAGTACGGTCGGAATCCGGGTCGACCACGCGTCCGGTGATTTGTGAGGCGGATTGCGTTTGATCAATTTTTTC
ACGTGGTTGAACGTTTTGTGATAGTTAATTTACACGTTTTAAAAGATGTCTGGTGAAGTTCCTGTACCGCCTG
CAGTTTTATGGGCACAGCGCAAGGAAAAAATTTTGCTCACATTTAGCGTGGAATCCAA
AGATCCGTCTATTAAAATAGAAAAGAATTCCGTATATTTCAAAGGCGTCAGCGCACCAAAAAATAAAGTCCAT
GAGGTAACTATTCAATTATATGACGCTGTAGTTCCCGAAACTAGCGGTTTTGTTAATAAGGGTCGTTGCATAG
AAATGGTACTTCGGAAAGAAAAAAAGGACGCGCCGTATTGGCCTTCATTAACGAAAGA
CAAAAAGAAACCACATTACCTTAAAATAGATTTTAATAAATGGAAAGATGAAGACGATGAACAAGATGATGAA
GAAGGTGGTGGTCCTACTGGTGATCAATATGATATTGAAGAAATGCTTCGGTCGATGGGTGGTGGTGCCTCTG
ATAAGGGTGATAAGCCTTCATTTGATGACCTGGAGAGTGACTCTG
>57963616_3 CX700493.1
TGCAGTACGGTCGGAATCCGGGTCGACCACGCGTCCGAATATATTTAAAAGTTTATTTTATATTTACAAGACC
TTGCTCATACTTTTATCTGGAGAGTTGTATAAGACATTGACACAAGTGCCCGAGATAACACACTGCAGGTGCA
ACAGATTAAATGGAAATAATTTGTCTGATGTACATAATACAGCTTCTAACTTTCCTCA
ACAAAAAGAGTATCTAACAATTTTTCAATTCAAACCACTACTTCTTGAGAATAGTGCATGCAACCAAAGAAAC
TTCACTTTTATAATATTAGTATTTTATTAAAATCTGGTTATAAAATTATCCTTATTGAGACAGATTTTCATGT
GTCGCTTGAGTAACCATGACTACAACGAGATAACATGAGTTTAGAGTTGACTGCACCA
GGTGTTCGAGGACTTTGCGTAAGAAAGGAAGAATTGAAATTGGACGGTAGGTACTCACTATCACAAGTTTAAC
TATTCTTTTTGGGTAATTAGAGCCCAGTCGTGATACAGGGGCACAAAATGTGACTAAAACCAACCGAGTCGAT
TTCGATCCCCACTCGAGGAGATGAAATATTTATTTAAATGTATATTTCTTTCTTCTTC
GCTCTGGTGTCGAGGGAGAGGGCAGCGTTGAGTTCTGTCACCTCAAGACGCAATGCCTCCAGAAACGG
>57963619_3 CX700494.1
TGCAGGTACGGTCCGGACATCCGGGTCGACCTAGCGTCCGACTGTCTATACAAGTTATATACCTCTTAACCAA
ACTTTAAAATTAATTTATATCGCAAACTTTCTGATGGCTTTTACGGGATCAGCAGACAAGCCCAGCATGCAGC
TGGCAGCTGCTGATGCGGGATCTCATCTCCAGCACATGTGCTCCTTAGATATTGATTC
TAAGGCTTCATATGTGCGTCTTTCTGGAATTATCTGTACCATTGGACCCGCATCTCGCGAAGTGCCAATGCT
>57963621_3 CX700496.1
GCTCGGAATTCCGGGTCGACCACGCGTCCGATTTATTTTATTTCTTTAACATATTTAGTTTAAGTTAATCTAT
CATATAAAATGTCTAAAAATAACCATAGAAGATGTGCTAAGTGTGGCCAAACACGTGCTGATGATATGACTAT
TTCCTATTTTAGGTTTCCTAAGCCTGGAATATATAATATCACCAAAGCACAGGCTTGG
GCCAAATATTGTTGGCCTGCTAAAGATTGGACTACTTTGGAAGCATTACTCTCTGTATATCATTCCAATAAAG
TCCTATGTGCGAGACATTTTACCACATCAAAGTTTTCTGATCTTGAAAGACAAAAACTGCACTCATATTCTGT
GCCAGATGTTGTTGAGGAACAAGATTTACCTGTAAATCAAAAAAATGATCAGATAGAA
AATAACACAGAAGATTTAATCAAATGTAGAACAGTCAGCGGAGAATTAAAATTAAAACCGGACACAGGTCCTC
ATATAGTAGACTGTCAAGTGCTGTCGGAAAGTGAAGAATATGAGACGGAAACAGGAGAATACCTGACACTACC
ACTCTCCGAGGACATTGTAATTATAGAGGTTACTACCTCTGC

>57963623_3 CX700498.1
TGCGGTACGGTCGGAATTCCGGGTCGACCTCGCGTCCGAAATAATCAAAATACAGGTGATTTGTACGAGTCGG
ATGTTGTAGATTCCAATAAAGAGAGACGGGAAGTCGCAACTTTTATTGAATCGGAACGAATGGATACCCATCT
GCGTCATATGACTGAATCCTTGAACGTAGCAGAAAAAAGAAAACCAGAGGCATATGAT
CAATCAATTGATAATTGTTCAAAATTATTGACATCGCATGTCATAGAAACAAATACTGAATTGAATCAACCAT
CACAAACTATTGATATTTCAATGTCTCTAAAAAAATTGGATGCCAATATGAAAATTGAACCCAACCTTGAAAC
AGATATTAAAATACAAGATTTTGAAATAGGCAAACCGGCTTTAATTAAGAAAGATATA
CGAGGTTACCTACCCGCTGATATAAATATTACTGAAGAGTATATTAATATACAAGACAGTAATAAAAAACAGG
ATGATGCTAGCCAATTTTTGCCAGTTTCGGAAGAGTTGACATCTGACCCAAAAAATTATGAAATTTTAGAAGA
GAGTGAAGTAGAGCCCCATGATATATCCTTAGAAACAGAATACATAGCAGAGACAGAT
AGAAGCATGGGAGTCACTGAAAGTTCATCTT
>57963635_3 CX700510.1
ATGTCCTGCAGGTACGGTCGGAATCCGGGTCGACCACGCGTCCGGAGTTGACGTGTCTGTCTGAGAAGTCGTT
GTGCTTTCCTTAGTTTCCTTTGCTGTCAAATAATTATAGTTGAAATATTTAAGCTTTGTTAATAATATGGATT
TATTTAGTTCCATTCCTACTCACATTGATTATGTGGGTCAAGCAAAGGCAGAAAAATT
ATATAGAGCAATAATAACACTATTTAGTATTGTTGGTTTTATATGGGGTTACATCGTTCAGCAGTTTTCTCAG
TCGGTCTACATTTTGGGAGCAGGATTCATCCTTGCTGCAATTTTAACCCTGCCACCCTGGCCCATGTACCGCA
AAAATCCACTTAATTGGCAAAATCCTAGAAATGCCGAAGAGAAGTCTACTGGCAAAAA
AGGGAAAAAATAACATGAACATTTACATGACGTGTCGCTTTATTATTGATCTTATATATCTTGGTTTATAATT
AATAAATAGATTTTAGGGT
>57963636_3 CX700511.1
GCAGTACGGTCGGAATCCGGNGTGACCACGCGTCCGGGTGTAGTGTTGTTTTAATTTTTCTTGTATTTTAATC
ATTATTCTGACGATTAAACGTGCTTGAATAGTCTAATTGAACAAATAAATTAACAAAATGGCAAAGTTTAAAT
TGAAAAAGCCATCAAAAAGGCAACCCGCACGCCTTCGATATAAAATAGAGAAAAAGGT
TAGAGACCATAATAGAAAACTCAAAAAAGAAGCAAAAAAGAATCCAAAATCTAAAAGAACTAAGCCAATTCAA
ATACCCAATATTTGTCCTTTTAAAGAAGATATATTAAAGGAAGTTGAAACTGTTAAAAAACACAAGGAAGAAG
AGAGACAAAAGAAAAGAGAACAAGCAAAATTAGATAAGC
>57963638_3 CX700513.1
ATGTCCTGCAGTACGGTCCGGAATTCCGGGTCGACCACGCGTCCGCACAAAGGTGCGATAAGATGTGTAGAAT
TTGCGAACGAAGTCAATGCTGTATTGACTGGCAGTTGGGATGGTACTGTGAAAATGTGGGACAGCAGAGTGCC
TAATTGTGTTGGTACTTATAATCAAGGAAATGAAAGGGTTTATACAATGAGCATAGTA
GGCGAAAAGTTTGTAGTAGGCACATCAGGACGTAAAATATTTGTATGGGATGTACGTAATATGGGCCATGTCA
ATCAAAGAAGAGAATCATCACTCAAGTATCAAACAAGATGTATCAGAGTGTTTCCTAACAAGCAAGGTTATGT
CCTAAGTTCAATAGAGGGTAGAGTGGCCGTAGAATATTTGGATAGTAACCCAGAGGTG
CAGAAAAAGAAATATGCTTTCAAATGTCATAGGATCAAAGATGGTGGCCTAGAAAAAATTTACCCAGTGAATG
CAATAAGTTTCCACTCAATGTACAACACTTTTGCCACGGGAGGATCTGACGGTTATGTCAATATATGGGATGG
TTTCAATAAGAAGCGGCTTTGTCAGTTCCATAGGTACAATACAGCGGTATCATCGCTA
AGTTTCTCCCATGATGGTTCAGCCC
>57963643_3 CX700518.1
CGCACGTACTGTACTTCCGTCACCTCGTGCCGCGCAAATACACCGCCACCGGGCAGGCCTTGCCAGTTATTGC
GCACTTTTCTATTGGGCGGTGTATCGGCGCCTTGGTGAGCGGGCTGGTGTCGCTGCAGTCGGCGCTGCACGCA
CAACGCGACGCGTACCGCGCGCTGGGCGTGGCGGCGCTGCTGGCGGCCGCCGTCTACC
TCGCGCTCTACCACCTGCTGCTGGCGCCGCGCTGGACAGCGCCCGCGCAGCCGCCGCCCCACCACCTGCTGGA
AGGATTAAACGCGAATGGCGGATCGAACGGCAACTACTCCC
>57963644_3 CX700519.1
TACGGTCGGAATCCGGGTCGACAGCCTGCCGACTTCTGCGAACAGGAAGACACGTATCCATGGCGCCGGCTGC
GGGCAAACGCCTGTGGGAAATGGCCGAAAACACCCGCGGTGTATTGGCGGTGGAATGGTTGGCGGCGTGTCAG
GGCCTGGACCTGCGCAATGGCTTGAAAACCTCACCTGCCCTTGAAAAGGCGCGCGGGA
TCCTGCGCAGCAAAGTGGCGTTTTATGAGAAGGACCGGTTCTTTGCGCCGGACATCAATGCCGCCAGTGAATT
GTTGGCGACGCGTTGCCTGAATGAGCTGGTGCCGGCGAAGTTGCTGCCGAGCTTGTAAGAGCGACACCGATCC
AGTGTGGGAGCTGTCGAGCCCCGGTGAGGCTCCCACATTTGATCTTCTACAGATTCGA
ATTTTCTCAGGATAAAAATAATTAAGGACGAGAAATGCACCAGCAAGAAAAGACGCTAAAACGCGGGCTCTCC
GCCCGACATATTCGCTTCATGGCGCTCGGTTCCGCCATCGGCACCGGGCTGTTCTACGGCTCTGCCTCCGCCA
TCCAGATGGCCGGCCCTGCCGTACTCCTGGCCTACCTGATCGGTGGCGCCGCCGTCTT

CATGGTCATGCGCGCCCTCGGTGAAATGGCCGTGCACAACCCGGTGTCCGGCTCCTTCGG
>57963646_3 CX700521.1
TTCATCCCCAGCTCTTTGATCTTGCGAGTCAGGGTATTGCGGCCCCAGCCCAGCAGCACGGCAGCGTCGCGAC
GACGACCGGCGGTATGCTTGAGGGCGGTTTCGATCATGATCCGCTCGAAAGCCGGCACAGCGCTGTCCAGCAG
GTTCGACTGGCCACGTGCCAAGGCCTGATCGGCCCACTGGCGCAATGCCTGCTCCCAG
TTGGTCACCGGCGCCGAATCCTGCGGCAGGCTCAACAGCTCAGGCGGCAGGTCGCTGATATGCACTTCGCGAC
CCGAAGCCATCACCGTGATCCAGCGGCAGGTGTTCTCCAGCTGACGCACGTTGCCCGGCCATGGCAGGTTCTT
CAGGTATTCCTCGGTCTCGCTTTTCAGCAGCTTCGGCTCGACAGCCAACTCCTGGGCG
GCGCGGCTGAGGAAGTGGCGGGCCAGGGTCGGGATGTCTTCGCGGCGGTCCGACATCCGTGGGATGTGGATAC
GGATCACGTTCAGGCGGTGGAACAAGTCTTCCCGGAATTTCCCGGCATGCACCAGGGTTTCCAGGTTCTGGTG
GGTCGCTGCGATAATGCGTACATCGACCTTGACCGGCGTGTGCCCGCCTACGCGGTAA
AACTCGCCGTC
>57963649_3 CX700522.1
ATGTACTGCAGTACGGTCCGGAATTCCGGGTCGACCTACGCGTCCGGACTGACAGGCAGGTCAGTCACACAAC
TAATTTATGAGATTTTCGTAGTCCTAATTGTATATGTTTGTACTCAAGATTATATAAATTATGCCGAAAAATA
AAGGAAAAGGAGGTAAAAATAGAAGGAGGGGAAAGAATGAAAATGAAACAGAAAAACG
TGAGTTAGTGTTCAAGGAAGACGGACAGGAGTACGCCCAAGTCACAAAGATGCTCGGTAATGGCCGCTTAGAG
GCCATGTGCTTTGATGGCATAAAACGTCTTTGCCACATCAGAGGAAAATTAAGAAAAAAGGTCTGGATAAATC
AAGGAGATATTATACTCATTGGTTTAAGAGATTATCAAGATGCCAAAGCTGACGTCAT
CTTAAAATATACTCCTGACGAAGCAAGGAATCTGAAGACCTACGGAGAATTCCCTGAAACTGTACGCATTAAT
GAGACTGTTGTATATGCGGTTGATGGCTTGGATGAAGACATTGAATTTGGTGACGAAGTTAGTTCAGAAGATG
AAGCAGATGCTGTTGATGCTATATAAATATTTGTACATATTTTGACCATCTACAGTAA
GTTAAATGTAAT
>57963651_3 CX700524.1
GCAGGTACGGTCGGAATTCCGGGTTCGACTGGACTACCACGTCAAGCTGCTGCCCCAAAGTGATCAGGCAGAG
GTGCGCCTGAGCCTGTCCCAGGGCTCGGCGGTGCGCAGCCTCAACTTCGACCTGGGCCGCGACGGCGACTACA
GCGATTTCAAGGCCGATGGCCAGTGGCAAGTGATGCCGGGCAATACGCCGGGCGCCAG
CCGTGGTCTGTGGCAACCCAGTGCCGACAAGGCCAGCCTGACCTACCGCGTTCGCCTGACCCACGCCCGCAAG
AAGGGCGTTTACGAAGCACGCATGACCCCTGGCTGGGCGCTGTTTCGTGGCGAAGACCTGGTGCCCACCGCGC
GCCTGGATCAGCAGGACGGCATTGAACTGGTGTCGCGCCTGGTGTTTGAACTGCCCGC
CGGCTGGAAAAGCGTCGAGACGTCCTGGCCGCGCATCGGCAAGAACAAATTCCGCATCGATAATGTTTCCCGC
CTGTTTGACCGGCCCACCGGCTGGATGCTCGCGGGTACGTTGGGCAGCCGGAGGATGCGCCTGGGGGAAACCG
AAATCACCGTGGCG
>57963653_3 CX700526.1
TGCGGTACGGTCCGGAATTCCGGGTCGACCACGCGTCCGCGGGAAACTTGTTTTTTAGTTCTTATTATTGAAA
GTGACATATAAAAGCTATAGAAAAAGAATGAAGCTCTTTTGTATTTGGGCTTTAGCCCTAACAACGGTGTACG
CGAGGACGGCGACCAAGCCACAGAGTATATCACCAGTGATAACGTCTTTATCTGATAA
GGATGCCAAGCTGAAAGCAAAAGTTCATCCAGCATTTGCAAATGCAGGACAAAAAGCGGGCCTTGAAATTTGG
CGTATAAAGGACTTTGAGCCAGAAGCTGTACCGAAGGCTGACATCGGGAAATTCTACAAAGGAGATTCTTACA
TCGTTCTAAAGACTACATCAGATAAGAAAAACAATCTATCCTGGGACATCCACTACTG
GATAGGAGCTGAGACCAGCCAGGATGAGGCCGGAGCAGCCGCCATACTGACTGTTGGTTTAGATGACAAGTTT
GACGGAAAAGCTGTGCAGCACAGAGAAAGCATGGGCTATGAGAGCTCTCAATTTTTAGGTTATTTCCCTGGAA
AAGCTGTACGCTATGTAGATGGTGGTAATGCATCTGGCTTCAATCACGTTCAAACAAA
TCC
>57963656_3 CX700529.1
TGCAGTACGGTCGGAATCCGGGTCGACCACGCGTCCGATTACATCTTACATTAAGCAATTTGGAAACAATTAG
TTTGTTAGATACATAGCTATAATAGTGATCTTTACTACTTTTTACTTAGTAGAATAATTAATACTACAAAGGT
ACAATTTTAATTGGATCTGAAATAGCTACTTAACTTCATGTCATCGGTCATAGTAATA
TCACCAAAGATACTAAATTGTAATACTCGCAATAAATAAAAGAATCACTTTCG
>57963659_3 CX700532.1
GCAGTACGGTCCGGAATCCCGGGTCGACCACGCGTCCGGCGAAGAGCTGAAGAAGTGAAGAGTTTATTATAAT
ATTTTATACATCTAAGGGGCGTTGGTGATTTTTTGCAGAATAACTAATATAATTACATAAAAATGGGTGATCC
ATGGATGATTCAACCCCATGAACATATAAAATTTGCAGAACATTTCCGCAATCTCGGC

CCTGTCAATGGTACTTTGACTGGGGAACAAGCAAAGAGATTTATGTTACAGTCACAATTGCCACCACCAATTT
TAGGGCAGATTTGGTCTCTCGCCGATACAAATGCTGACGGCAAATTGGATTTGAAGGAGTTTTCTATCGCTTG
CAAGATAATAAATTTAAAACTACATGGTGTGGAAATACCGAAGGCTCTACCGCCTTCT
CTCCTTGCCTCACTAAGTCCAACAGGTGCAAAACAACAATTTCCTCCCCCTAAGCCACCTATCCCACCTATGC
CTACTATGACCCAACCCCTAATAGCCGGGATACCCCCTCAACCCGCTCAATCCAATCAATCTCTTCTAGGTGA
TTTCAATCAATCACTCCCCCCAAAACCAGCAGTCCCAGATTTAATTTCCGGTGTTAAA
CCACCTGTTCCACAACCGTTGATCGGGAATGGACAA
>57963662_3 CX700535.1
GCAGTACGGTCCGGAATTCCCGGGTGACGGACAGTGCCCCAGGCGACTGGCGGCCAGGGCGTGGCACCAGTGA
GCGCCATCCAGGCCGTCGAGCAATTGCGATGGCAACAGCATGACGCCCGTTTGCGCATCACCTTCGGGCGGCA
GCGACAGGGCGAAATCCGGGGCCTTGAGGGTTTCGAACAGGTCCAGGCACACATGACC
GTGGCCCAGTTGGTGGCTGGTCAG
>57963663_3 CX700536.1
TGCGGTACGGTCCGGAATCCGGGTCGACCACGCGTCCGATCAGGCCAATAGGGATGAAATTGAAGCCCTTTAT
GAGAATAAGATGAAGAACTTGCAATCAGCGGCGACTCGCAACAGCAGCGCGGCCACGGTTGCCGTGGAAGAAC
TGCGCACCATGCGCACGCGCATCGACAGCCTCAACGCTACGCTTAATGATCTGGAGAA
CAAGAACGCCTCTCTTAGTAACCGGTGCCGTGAGTTAGAACGTCAGCTAGAATCCGAGCGTGCCCGTCATGCC
GAAGACTTGGCGTCAATGGAGCAGGAGTTGGCCCGACTTCGTGATGAAATGGCGGCTCAGCTACGTGAATACG
CCACGCTTATGGATATCAAGATATCCCTGGACCATGAGATTGCTACGTATAGAGCGCT
TATTGAAGGCGAGGAGGACAGGTTGAACCTAACATCCCAATCGCCGGGTCGGGAATCGCGCGCCTCTATGAGT
GCGGCCAGCGCCAGCGGCGGTCGCGCCACGCCCGGCCGTCGCGCCACACCTCTCCGGGCTGCGCGCAAGCGCA
CTCTACTCGACGAGAGCGAAGAGCGCAGCCTGCAAGACTTCAGCGTCACGTCCAGCGC
CAAGGGGGACCTGGAGGTGGC
>57963664_3 CX700537.1
TGCGGTACGGTCCGGAATTCCGGGTCGACCTCGCGTCCGAACGGTAGAAAAAGGAAGAATAAAAGAAAGAGGA
CAGCGCAGCGGTGGCGGTCGCGGTGGGAGGCGCGGTGGCATGAGGGGCCCCATGGACCGCAGAGGCGGCGGCC
CGATGCGTGGTGGTCCCGGCGGTCCTCGAGATGCACCCTACCCTCATATGCGCGACTC
CCGGCCCATGGGCCCCATGCGCGGCGGTGACATGCGTGGGCCCCCCATGGGCGGCGGTGGCATGCCGATGAGG
AATGGTATGGGCGGCGGAGCACCCTACGACCGCACGCTTGAAAGGCCCCCAATAGGAGGAGGATATGACGATC
GCTACAACGGCTACGGCGGTGAAGACCGTCGCGGATTCGCTCTTATGGACGGCCGCGG
CGCTCGTGACCAGTACGGAGCTCCCCCGCCGATGTACGCAGATGACAGACGAGGATACGGCGCCCCAGTTGAC
>57963665_3 CX700538.1
TGCAGTACGGTCCCGGAATTCCGGGTGACATCATCGCCGACAGCCCGGACTGGAAATGAAAGAACCCGCACAC
CAAACCCACGCCGTCATTCGCCTGGCTGTCCAGTGGCACCATTGGCTTGCGCGGGCACTCCTGGGCACCGGCG
GCGGTGCCATCGCTGGACAAGCGGTACGCGAGATCCCGCAACAGGAACACCGCATCGC
CGTTGTTCAGTGGGTAAGGGGTCGGGTCGCCATCGATGTGCATCCAGCACTGCCCTTGCACCACCAGATGAAA
ACTCGCGCTCGCCAGGCCGTGGGTGCTGGCGTGCCAGTCGCCGCAGTAACGGCCGACGTGAAACAGACTGGTG
TTGAGTTCGAGGCTATCTAATAACCAATCGACGAGCGAACTGGACGAATTCATCTAAC
GGAAAGACTCTAGAGCAAGTAATCGCTACTTTAGACTATTGAGCGGATTTTTTATAACCAACAGACTTGGTAC
ACGATTCCATGAGGAGACCACTCCATGTCCCGCGTACCGATGCTAACCCTGGAAACCGCCCCTGAAGCGGCCA
AGCCCTTTCTGGAAAATGCCCTGAAAGGTTCCGGGTTCATCCCCAATCTGTTGGCAGT
GCTGGCCAATGCCCCGGCAGCATTGGAAACCTACATCACCGTGTCCGGCCTGAACGCCAA
>57963669_3 CX700542.1
ATGTCCTGCGGTACGGTCCGGAATCCGGGTCGACTACGCGTCCGGAGCTCCTTTTGACACTATAGCTAGTGCA
CTCTGTTTATTCTCTTCGGAGTTTGAAACCGACTGTTGGTTCGAGCTTTGCTGTTCCGTCACGTTGTTATAGT
TATTAGGAGATTTTTCCGTCGATTGGGGTTTTCAAGCCTACCTTTAGTTTATAAGCCT
ACGTTGCTGAGACAAGAAGTTCAGCGTAATTTCAAATAAACTGCACGAAGATCCGATAGGCAGTATGAGAGGT
AGGGGCCGACGAGGACGCCGAGGTAACAATGTGGCCATTCCCGTCTACAGCACCATCAACCTTGTGCAACCTG
TATGCACGGTACCGTATAATTTTGTAATAAACGGTTCTGGTGATCAGAGTGTTGATTT
ACCGTCGTTTATGAATGGCGTTGCATGGAAGGTTACTTCCATCAATGCTACCTTCTGTTCGTCGGGTGTTGCA
TCATGCATCATCCGACTGTATGGCGCCGTCTCTGGGTCTAATCCCCCAGTTGGCGCCACGGATATAACGGCGC
GCAGCCGTACATTTTTGATTACGAATACGCCAACGCAGATTCGTTTTAAAAATGGACG
GCATGTTCAACACGGTCAGACGGGTAGTGGCGTTGTGATAGCCCAGTTTGGGTTGTCGCAGGTCACGT
>57963676_3 CX700547.1

AGTCCTGCAGTACGGTCCGGAATCCGGGTCGACCACGCGTCCGGTTCTAATGGATTTAAAAGCAATTTTTGTA
TTTTAAAGTAATTTGGTCAATTTATAATATAATAGTTGTAAGTGTCTTGTTGTTAATACTCAGATGATTGCAA
TATAAAAAAAGTTGAGCAG
>57963679_3 CX700550.1
AGTACGNNGTCGGAATCCGGGTCGACTCAGCGTCCGAAAATGACCAAAGATCTAGTTATCTGCATTCATGGAA
TGGCCAACACTAAAGAAGGAATGTACTTGAACACTGAAGACTTTCTTGAAGCCATTGCTGAGCAACTTGAACG
TAAACTTAACAAATAAAATATAAAAATAATTAAATAAGCAGTAAATAAAATAAATTAC
TTAGATTTGTTATTTGTTCCTTAATTTGGGGACCATTTTTGTATTTTGGTAACAGCCTTGTTAGAAGGTTCAG
GTGCTATTCTTAGTAAACAAATGATTTTTAATAAAATAAATCCAGCATTTATATATTGTTCTTTTTCTAGTAA
ATAAAATTGTGAATATTTATATTTATCACAAATATGTATTACATTTTAATAATAAATT
ATAATAGTATTG
>57963680_3 CX700551.1
TGCGGTACGGTCGGAATCCCGGGTCGACCACGCGTCCGAAACTATTTAGTATGGACTTATGCGATGATAGCGG
TGAAATAAGAGCGACCGCATTTAAAAATGAATGCGATAAGTTCTATGATATGATTCAGATAGACAAAGTTTAC
TACATAAGTCGCTGTCAACTGAAGACAGCAAACAAGCAGTTCACCACATTAAAGAATG
ACTATGAAATGACCTTCACGGCTAACACCATAGTGGCGGAATGTATGGACGACGCGTCCTCTATGCCCACCGT
GAAGTACGACTTCGTGCCCATTACTGATATAGCTGATAAACCTGCTAACACTTTGCTAGATATTATTGGAGTG
TGCAAAATGGCATCTGATGTGCAAGAATTAACTGCAAAAAGTACAGGAAAGTTGCTTA
AGAAGAGGGAAATTACACTTGTTGATTCATCTGGTGCAGGTGTAACTCTGACTCTGTGGGGCAGTGATGCTGA
GAACTTCGATGGCAGCAGTACACCTGTTGTTGCTGTTAAGGGTGTTCGCGTGTCGGAGTTCAACGGCAGCAAG
TCTCTGTCGCTGCTGGCGGGGTCGTTACTCCGCGTGCAGCCCGACCTGCCCGAGGCGC
ACCACCTGCGCGGGTGGTACGACCGCGCGGGCGCCACGGCCAGCGTCACAATATATCTGCACGCTCGGGTGAT
GGAGGCATGGG
>57963683_3 CX700554.1
ATGTCCTGCGGTACGGTCGGAATCCGGGTCGACCACGCGTCCGGGTGTGAGCTGAAATACTCCGAGCTATATA
TCCAGACAATGGGTATTCTGCTGAAACGGGAGGAATCATGGAGAATTTGCGAGTTTCTACAAGTAACACTAAA
GTTCGTGATTTTCATCGGAAATTTTACAGAGCTGAAAATTTAACAATCATCTTAACAG
GCCAGATTGAAGCTGAGGATGTATTTAAAGCACTTGCCACTGTAGAGGATGACATCATTGCAAAGAAAGAGAA
GGAGCCACAGGAAGAGTGGGTTAAGCCATGGCAAACAATCCCTCCTCCGCCAATTTATGGAGAACTTAAAGAA
CAGTGGCCAGCTGACACTGAAGACTGTGGACAGGTATTATTCGGTTGGCGCGGTCCGC
TGCTCACGGGCTCGGGCTCGCAGCGCGAGTTGACGGCGTGTGCGCTGCTCCTGCGCTACATGTGCGACA
>57963685_3 CX700556.1
GCAGTACGGTCCGGAATTCCGGGTCGACCACGCGTCCGGATAAGGACAGATTCTTATACCTGGTCCAAGAGTT
AAGAAGAGCGTTTATCAGAGCTGGCAAGGGTTGGGAACTCACTGCTGCTGTACCCTTGGCCAATTTCCGACTG
ATGGAGGGATACCATGTGCCTGATCTTTGCCAGGACTTAGATGCTATTCACGTAATGT
CCTATGACCTGCGAGGGAATTGGGCGGGTTTCGCTGACGTGCACTCTCCTTTATACAAGCGTCCCCATGACCA
GTGGGCATATGAAAAACTTAATGTGAACGATGGTCTCAATCTATGGGAAGAAAAGGGTTGTCCGTCAAACAAA
CTCGTCGTGGGTGTTCCTTTCTACGGAAGATCCTTCACGCTGTCAGCTGGAAACACTA
ACTACGATCTTGGAACGTATATAAATAAGGAAGCTGGTGGTGGCAACCCTGCACCTTACACAAATGCTACAGG
ATTTTGGTCTTACTATGAGATTTGCATGGATGTTGACAAGCCAAAATCTGGCTGGACCAAGAAATGGGATGAC
CATGGCAAGTGTCCTTACGCGTACAAAGGAACCCAATGGGTTGGAT
>57963686_3 CX700557.1
GCGGTACGGTCCGGAATCCGGGTCGACTCAGCGTCCGCTCGCCGTCCAAATGGATACGTTAAATATAAAACCG
TTAAGTCAGGACTTGGAGTGGTCTCCCGAGGCGCCCGTCGGCGTAGGAGCCACTCGGGACGTGGTGGACCACG
CTCCCACTGACATGGCGTCGACATCGAGCGAGGAGAGGGCCATACGCCGCGTCACTGG
CCGCTGGGGTGCCGAGTGGTTGAGCTGACTCGGCACAGGCTATATCAAACCTCTCGGGCCCCAGTGGCCCGCA
GACCACCCCCTACCGACGCCACGGTGGGGGGCCCCGCGTCCAGCTTTACACTTGTCTCTCTCCAAATACGAGG
ACTTGGTCGCTAAAGCTGAGGCGATATTGAGCCGACTCGTCGTCGCGGAAAACTATGA
CTCCGTTAACAACTTCCTTACTCTATATGATACCTATATGGCGTCTACCGATGAAACTTTGAAGGAGTTCTTC
CCTAAATACAATCCGCCGATTCGCGCTCACAAGCACACCTGTGTGGGCTTAGGTATGGAGGTCATGAAGCGTT
TGAAGGTTCTAGAGAACGACTTCCCTGGTATCACCAAGTCCATGATGCTAGTATCATG
TGATGAAAACATCGAAGATCTTTTAGACTATACCACATCATTCCCTGGACCGCAAGGCTTCTTGATTGAGACG
>57963688_3 CX700559.1

GCGGTACGGTCGGAATCCGGGTCGACCACGCGTCCGGGCGACCCACGGACCTGAGACCTCCGGAGGGGAATTG
TAATCTATCCCCTGCCTCTGGCTTGCCTTTATTGGCCGGCAGAGTGGAGCGCAAGAGCTATATGCTCGAAAGG
GTGGAAGTGCTCAATCTGCGTAATAGCGGAGAAACCCGCTCACGAGCTCCTCGTGATG
GTGAAACCGGTGCACACACCTCTCCACACCTTTAGCCGACCACGCCGATGTTGCGCCCGAGGCACTACGCCGC
TTTGTGGGGGCCCGTCTAGTGGGAGGCGAGTCACCGTCCGCCAACCAATTTGAGACTATTGTAAAGGCGGACG
CCCCTTAGTCTCATTCATTAACCCAGTAGCCAGTCCATCCAAATTCCATTCCATAGCC
CTAGTTTAGATTTCCACATTTTCCGTAGTTCCTACGCATTAACTGGCGACCATCTCTGGGCAATTACCTGTGG
TTACCAACAGATGGATCCCGGCTGATGCGTCACAGTAAAACATTTGTATATTCTCGTAGGGGCCTCTACGTGT
TGGACCTGTGTCCCCACGCAAGCTTTCCAAAGTGAAGTGTATCTTTACAGAGAAACAT
AAATAATAATAATAGACGCCGTCTTCCAAACTATACTTAAATATAATATCCACAAATTTAAACATAATCACTT
TATTTT
>57963693_3 CX700564.1
GCAGTACGGTCCGGAATCCGGGTCGACTCACGCGTCCGGACTTTTCTTGAATTTTCAATTTTGTAACTTGTAA
AAGTTTTCGAAGAATTAAACATTGCGGAATTTATGTTCTAATAAAATAACATTTATTTATATCTAAAAGGTGT
ACCTTTTTAGATTTTTAAAATGGCCGCTCCACAAATGAGCGAAGCTGACCAGCTAAAA
ACGTTTAAGGATTTTCTGGTTCAATATAACAAACTCTCAGAACTCTGCTTCAATGACTGTATCCATGATTTTA
CGTCAAGAAATCTCAGGCCTGCAGAAGATAAATGTACATCCAATTGTATGGAAAAGTACTTAAGGACAAATCA
GCGAGTGTCACAAAGATTCCACGAATTTCAGATGATCGCTAATGAGAATATGTTAGCA
TTAGCCCAGAAGTCTGGGAATCCTAGTTAAGCTATTGAATTATTGTATATTTAATATATTGTTTTAGTGTTTT
GGTTTGACTGTTGCAAAATAAATATTGTTGATGAACTT
>57963695_3 CX700566.1
CAGTACCGGTCGGAATCCGGGTCGACCACGCGTCCGCGCTTCACTTAAGTAAGCCCCTAGTCTACGACTTTTC
GGTACTCATAAAAACTTCGATATAAACAAATATAAAATGCTTATTAAATTATTTTAAATTTTGTTTTCGTCGT
TATTTTAATACGGTCCTAGTATTGTGAATAAACGTTTAGTTATATATAGAAAATGTGA
TGTGCTAGTGAAAGTTGGATTTATATAAATGCAATTTGAGATGAGGACCTTTGTGGAAACATAGGAAACCTAC
ATGAATATAAGTATTAAGGTGATGCGAGGAGAGATAGCAAAGTTATGCTTAGTGATAGCTATCCTGGCGCTGG
CTGGCGGGGCTGTATCGGGAGACGGCGCTTGCAAGCGGGGGCACACTTGGTGGCACCG
GGACAGCGGTGCCTGAATGCCATGCACCCGCTGCGACCCGCGCCGCCTCGCAGTCAAATACCCCTGCGAACTA
CACCGTGACACCATATGCCAACCTCTGTACGAGGTCCGCATATGGGCCTTCAACACGGAGAAAGACAATGATG
TCAGTGAATCCTCGGGTGATTGCGAGTATTACGAAT
>57963701_3 CX700570.1
ATGGTCCTGCGGTACGGTCCGGAATCCGGGTCGACCACGCGTCCGCGCAGTGTCATCTTGCCGCGCTCCAACC
GAGTGTATAATTCATACTGTTAAGTAAGCTGTGATAGTGTAGACGTGAACGTATTAGCGGTTTCGTTTCTCCT
AGTGCTAGGCTAAGTAGTGGATCGTGAATGTAAATACAATTCTGTTCGTTTCGATCGT
TTTCCATTTTGATGATGTATTTTTAAGGGTTACTAGAGAAGGTTATGCATTTTGTATTTGTTCGAATTAAGTA
GTTTAAAGTGAGGTGTAAGGTTGTTACTATCATTGTTGTGTCGTACATACAGACATACAGTCGCTCCATCTCC
CGAACTTCAGAGTGAATTCTGTACTATTTATACTGAACAGTTATTTTATGACCGCATC
GAAGTCTTGCAGAGTGATATGCCAAATACAACATGCAACAATGATTATATGGTATAACGTTTCTTAATATAAA
ATATACAATTATTGTTGGAAATTTTCTAAATATGATAAATAAGCAAATCATGAAGAATAAATTATTT
>57963706_3 CX700575.1
GCAGTACGGTCGGAATTCCGGGTCGACCACGCGTCCGAATCATTTAATTTGAGTCAAAGTTTTAAATAACTTA
CCCATCCACTAGTGTATGTTCCCCACATACATACGATTCATTTCTAACATCTACTGTTGTATTTTGATTCTGT
AGGGATTACACTAAGCATAGTTAAGTGTGATGGTAAAAAAACTTTACCCTTGATGTTC
CTACAAATGTCATTAGGTAGGATTGGTGTAATAAACTGAAATTATGTTACTGATAAGTATAAAATATAAATGA
TCATCAGTCAAATCAATTGTGTGGATATAATGTTTTCATAGTATACTATTATATAATTTTAGTATATTGTATA
GTATAGAAATAAATAAATAGAATAAGTTATATGTATTTACATTTACTAAAATAATAAA
ACAATGGTATATG
>57963712_3 CX700581.1
GTCGGAATACCGGGNTCGACTACGCGTCCGATTAAAAATAATCTAGAAATTCAAAAGATAACACATTTTAATC
ACCAATTTTTGCAAAATGAGAAAACTAAAAGGCGTCGTTAAAGAAACTACCAAGCAAAAACGTGAGCGAAAGC
AAGAATTTGCAAAAATGCGTCAACAAACTCATACTATTGTTTTGCCCACCTTTGGTAT
TATATTCCTTCTTATATGTGTTTATGTCTACTTTAAAACTAGACCTTCATCTATGCAATATGCGTAACGACTT
TCTGTAATAAATTTTCTTTTATGCTCACCAATGTTATTGACTTAATTCAGCAAAATAAATGAAAATAATAACA
ATAATTACACTATGGTTGTGAATTCCTGGTAACTGTTTTCTACATACTGTAGATAGAT

CACATTCAATGTCTTAATGTATGTTATACTCTGTATTATTTATCTAACATTCCATGTATTGAACATATCATTG
TATATACTAGAATAACTATTGTTAGAATTATTAGATGATTTATATTTTCAATTGGACTTTAGTTCAGCGTAGA
GGGTAATGTTATATATGAAAAAT
>57963713_3 CX700582.1
GCAGTACGGTCCGGAATCCGGGTCGACCACGCGTCCGTTTGGCTTGGCGCCTTTCCTGTTGGTTGTTCTCTTG
TAGTGATAGTGTGTTACACGTTTCAAAATGGCTGGTGGTAAAGCGGGCAAAGATTCTGGCAAAGCCAAGGCGA
AAGCTGTCTCACGATCGGCTCGGGCGGGCTTGCAGTTCCCTGTGGGTAGAATTCATAG
ACATCTCAAAAATAGGACTACGAGTCACGGCCGTGTCGGCGCAACGGCTGCCGTTTATTCTGCGGCTATTCTT
GAATATCTCACAGCCGAGGTTTTAGAGTTAGCGGGCAATGCATCCAAAGATCTTAAAGTAAAACGTATCACTC
CCAGACATTTACAACTAGCTATCAGAGGAGACGAGGAGCTGGACAGCTTGATCAAAGC
CACCATTGCGGGAGGTGGTGTCATCCCACATATTCACAAATCACTCATCGGTAAAAAAGGAGGACCAGGGGCA
CCTGTTTAATAAGAGTCTTGCTTCAATTCAACATTCTAACTGCATTTGAACCGGAGACTATCAGCATTGTAAG
TTCATCAGCCAGGCAGTAAACATAGTTTTCTACAGGTAGTGATGTGGACAGTGGGAGT
GGGACTGACTTCAGACATATTGATCCGGAC
>57963716_3 CX700585.1
CAGTACGGTCCGGAATCCGGGTCGACTACGCGTCCGGACCGACTAGGAATAGAGCTTTCAGCTTTGGGACCTT
CTCCATGATGCATATTCGTACTCCAGAAACTACTGATACTAGTGATTAGTTGACTAATTGTTATACCTCCTAT
GTATAGTACGGTCGAAAACTTGTGGCCGCTCTGTTGGGTAATTGGTTTTCTGGTTACT
TAATTTATGTGCATTGTTCACTCGGAGAAGAAAATAGCAAAAAGTAAGAATAATCCTAAAATCAAATCTGTAA
AGTCATCACAGCTAACTTCAGGGCAGCCACAATTCATTGATCATACTGTATTTACCATAATAGTGCAGCCTAC
AAAACAAGTGAATAAATGACACAGGTTTAGATATCAGTCCAAAAAACCGTCAAAGATA
CCATCAATGTAATCTGCTTAAATACATAATTAAAACACC
>57963719_3 CX700588.1
GCAGTACGGTCCGGAATCCGGGTCGACTACGCGTCCGCGACGAAATCTTAAAATTGAAGAACATTAAGATTTC
TGCTATCGCTAACTTTTAAATTTGCTGCTTATTTATATTTTAATACAATGGCCGCTGCAGCTAAATTACCGTC
GTTGGTTAGCGCGGCACTCGCCAATGCTAGGCCGAAATTCAACATTTTCATGAAATAT
GCCAAAGTTGAACTACCTCCTCCTAAACTAAGTGAAATTCCCCAAATAAAAGCTGGTCTTGGCAAATTGGTCA
GCAGCGCTAAGAGTGGAGCATGGAAACAGCAAACTGTTAAGCAAGCTACACTTAACATGTTAGTTGGTGCTGA
AGTACTCTTCTGGTTCTATGTCGGAAAATGCATTGGGAAGCGTCACATTGTAGGTTAC
AAAGTTTAAGCTGTAGTATTTTGTTTGTAGATGGAATGTTTGGCAAAGTAAATTATTAAATC
>57963722_3 CX700591.1
GCGGTACGGTCGGAATCCGGGTCGACCACGCGTCCGTGGGGAACTTACATTAGTGGATGGGGAAATGAATACT
ATTTCGATAACTTGTTTGTACAGGCTCGTAACATTAGATATGTACACATACCAGAAGCTATATCAATCTTAGG
AAGTATTAAAAACGAGTTAAATAAGCTAATTCATAAAGATGTTAACAAAGATTTAATG
GCTACAAAAAAATCTAGAAAAGCAACTAAGGCATTACAGCAGCACATGAAAACAGTGGCACAATTAGAACTCA
ACAAATAAAATAAACAACAAGTAT
>57963729_3 CX700598.1
CTGCGGTACGGTCCGGAATCCGGGTCGACCACGCGTCCGGATAAATGTGGTCTTAGTGTTCGCAAAAACATTA
TTTAGTTATGCGGAAATAAAGAATTTTGTGATTAAAATATAATAATTATAGTACTAAAAATGGAAGACGAATA
TATGGATACACAGGACTCTAATATAAATGATATAATAAACCGCGCGAATGACAATAAC
GATAGTACAAGTGATCCTAGTAAAGATTTTGCCGATGTTTCCAATTATGATGATGTTTTCGATGATGATGATG
CTGGGAACGGATTCCGAGGTGGTCGTGGTGGTCGGGGTAACTTTAGGGGCAGAGGCCGTGGCCCACCACCAGG
CTGGATGAATGGGCCCCCACCACCTATGAGGTTCCGTGGTCGAGGCTATGGACCTGGA
GGTCCACCAATGAGAGGCAGAGGTTTTTTCAGAGGGAGAGGTGGTCCACGCTTTGGACCCAACAATGGACCTA
ACTTTGATAACAACTGGGGTCCAATGGGACCACCCCCACCTGGTATGATGGGAGGGCCCCCTCCGTTTGGACC
GCCACCTGGTATGATGCCGCCAGGTGGTCC
>57963731_3 CX700600.1
ATGTCCTGCAGTACGGTCCGGAATCCGGGTCGACCACGCGTCCGCCCACGCGTCCGCCCACGCGTCCGGCGCC
AGCGGCTTTTAGCATTTTTCTGATATTGTATCTCATTATATATTAAATATAAATATTATCTCAGTGTATTGTT
TATAAGCTAAATTATAATAATGTCTATCACAACATTGTATTATTTACAAATGAAAAAA
GAAAATGCTCACGAAGATGCCATATGGTGTTGTGGATGGAGCCAAATTACACACGAAAAGACTAAAGAAGCAA
GTGAAAATGCTGAAAATATAGAAAACTCACAAGATTCAAATCATTCGCAAGAACCTCCAGAAAACTATATTAT
TACAGGTGGATTAGATGATATAATAAAGGTGTGGCAATTTGATAATGGTAAGTTAGAA

GTAAAACATCAGTTAGAAGGCCATTCTCTTGGAGTAATATCAGTTGCTGTGAGCCCTGATGGAAAAACACTAG
CAAGTAGTTCTCAAGATTCCTCATTAAATCTATGGGACATTACAACTGGGGAAAAGTTAAAAACAATGGAGAC
AGGCACCACAGATGTATGGACATTAGATTTTCACCTGATGGTAAACATGTGATCTCAG
GCAGTAATGCAGGAAAAATATTAATATTCAGTGTT
>57963732_3 CX700601.1
AGTACGGTCGGAATCCGGGTCGACCACGCGTCCGGTCACGTACAAAATATATTTTAGTAGATATATTTTATAT
GTGATTCGGCACAGCAACGTGAAGATGCACGCTGGTGCCATATTATTAACTTAACGTTTATTGTTGTTTGTAA
ATATCTTGTTGATTATAAGGTGATCAAGTTATTTTAATAAATACGCATATGT
>57963733_3 CX700602.1
TGCAGGTACGGTCGGAATTCCGGGTCGACCTACGCGTCCGGTTCTAGATCGCGAGCGGCCGCCCTTTTTTTTT
TTTTTTTTTTTTTTTTTTTTTTTTTTTTTTTTTTTTTTTTTTTTTTTTTTTTTTTTTTTTTTTTTTTTTTTTT
TTTTTTTTTTTTTTTTTTTTTTTTTTTAT
>57963740_3 CX700607.1
AGCACTTTGAGAGCAAAACTTGAGGCAGCCATTTATTAATTAATTATATCCTGAACTATCACATAGTTTTGCA
TTCAAAGTTAGGTTTTATATTAGTGTGCTAAAGAATATTTGAATAAGTTTTTTTTTTTAAGTAAAATAATGAC
CCACAACTTTTGTATAATTTTGATACAGTAAAAAGTGTCAGTTTGTCATTATCAAATA
AAATAAAGTCAATATTCCAT
>57963744_3 CX700611.1
GCAGTACGGTCGGAATCCGGGTCGACCACGCGTCCGGGACTAAGCTCTTGCTGCTGTGTTTCAGTCAATGGGT
ACACTAGAGGAACTCGCGATGCCCCAAAATGGGATATACCACGTGGGTGTATCATCTCTTTCTGAAGCCTTTA
AGCACAATCCTCGGCTGAGTCGGTTAAATTTAAACGACAATACTGTTGGACCTCGTGG
TGCTGTTGCCATAGCTGCGGCTTTACCTAAATTAAAACATTTGAAAGCTGTCAATTTTGGTGATTGTCTCCTT
AAGAGTTCAGGAGCAAAGGCTTTGGCGAAAGGATTC
>57963751_3 CX700618.1
CCTGCGGTACGGTCCGGAATCCGGGTCGACCACGCGTCCGATTTGCAGTTATTTTTCATCGTTTTCTATTGTT
TAAACAAAATCAATCAAAATGTTTCAGTTTGGTTTCAACATGTTTCACGAAATTACAAGACCTTTTATAATGA
CTTATCGATGTTACTCCGTTTCTATGCTACCTGGAAATGAAAGACAAGATGTGGAAAG
AGGAGGAAAAATTATAATGCCACCTTCAGCTTTAGAGCAGCTGACTAGACTGAACATTGAATATCCTATGATA
TTTAAATTGACAAATAAAAAAACTAAAAGGATTACACACTGTGGAGTATTGGAGTTTGTTGCAGATGAAGGAA
GAGTATTTTTACCACATTGGATGATGGCAAATCTTGTATTAGAAGAAGGAGCCTTACT
TCAGATAGAGAGTGCATCTTTACCAGTAGCTACATTCTCTAAGTTGCAACCATTGTCAGAAGATTTCCTTGAT
ATTACTAATCCAAAAGCAGTGCTCGAAAACTGTCTGAGAAATTTTTCATGTTTAACAAATGGTGATGTTATAG
CAATTAAATACAATTCTAAAGTCTACGACCTATGTGTACTGGAAACTAAGCCTGGGAA
TGCAGTTATTATTATTGAGTGTGATATGAA
>57963753_3 CX700620.1
TGCGGTACGGTCCCGGAATTCCGGGTCGACCACGCGTCCGCCCACGCGTCCGCAATGATGATGCCGTTCAAAA
GATTTAATGATGAAATAACTACTGAACTTATGTTAAATTTGGGAGATTTCGAATATACGTTGGAAAATAATAT
ACAAATGATCGCATTGCCTTATAATGACAGTGAAACCATTCTATATGCTATCAAACCG
TTGAGGCCTGCGGAGCTAACATTAGCAGATATGATGAACTCTTTAGATTACAAAAAAATCGACACTTTAATAG
ATCAATTGAGAGTTCAAAAGTGTGTAATACGATTCCCAAAAATGGATATAAAATTTAATGCCAATTTAGAAGG
TCCTTTAAAAGCTTTGGGTATTGAGAGTATGTTTAATCCCAGTCAAGCTAATTTCGCT
CTTATGTTGGATGATGAAAACGCCGATAGCAAAAACGAAACTAAAATTTTAACGAGGTTCGCAAATGGTGATG
AAGTGGAAAAAAGTAATTTAAGACATATAATTGACAGCCTACCCAATCCTCGTATACATATAGACTCTGTTAT
ACATGATGTGCGCATAAAAATAGATGAATACGGCACAGAAGCAGTGGCAGCCACGAGC
GGTATTTTAGCAAGATCGGCAAACGCTTTCTACGCCAATTCACCATTTTATACATT
>57963756_3 CX700623.1
GTCCTGCGGTACGGTCCGGAATCCGGGTCGACTACGCGTCCGCCACCAGGCCTGTCTTTCCCTACGGTCAACT
CTACGCGTTTCTCTCTACAGTCACCAGGACCGTGCTGAATAATCAAAGTAATTAGTTATGTTGGTGAGAATTT
AATAGCAATGATTTAGACGAATAATTTTCAGTTGTTTTCAAGGATTACTTAAATTACA
TATGTATAAAAACATGGTAATGATGATAATATAAATTAATCTTAAACTTATTAAAATTTTCCAACAAGCCTGA
TACGCATATTCCACTTAAAAAATACTTGATTTAACAATGGTAAGTTCTGTATTTTATTCGTTTAGGCTCCTAG
ATTCGAGGTTAAGATTTTCAGGGGCAAATAATTGAACGAATGAATGAATTGAAGCTAG

CTAGCCATTGAGGCAACAAATACAACTGAGATTGGACCAAAATTTAAAAAGAATAAAAAATGAACGACGAACG
ATAGAAATAAATATATGTTTTACAAATTGTCATAATGTGAAAGGAAAAAGTTGTCTGCTGGTTAGTAATACTC
TTGCTCTTCCAAATAATGTAAGATAAAGAGATTTTTTATCGAATAAGGATAATGGCAT
TTATTACAGGTTCGTAAAAGTAGGGTAGTTAAGAAAATTAACTA
>57963757_3 CX700624.1
CGGTACCCGGTCGGAATCCGGGTCGACCACGCGTCCGGTTTGGCATTGTTATGCCTTATTATTATCACATTAG
TAGTAACATTGATACTAAACCAGCTCAGAAATGTATACAGTCTACTCTCTCAGTCTCCATTAATGATGAATTG
TAAAAGTTTCTGCATTTGACGTCATGTATAGTTTACCATTTATTTATATTGTACAAAC
GTACTAATGATTTCGCCTTTCGATATGCAGATAAAAATTACTAACAT
>57963761_3 CX700628.1
CGGTACGGTCCGGAATCCGGGTCGACTACGCGTCCGGAGAAACTTACCTTCCGGTCTTTTTACTCCTCCGTGG
CATGGCGGATGGTCTTTACCGCCGCATTTAGTGTTTATTTGGAGGAATAACTTCGCTTATATAGCGAAATGAG
TCTATCAGTAGCCCGACCCTTAGTGTCGGTATACTCTGAGAAGAGTGAGAAGGTGGCA
GATGCCTCGGTCCCTTTGCCATTCGTTTTCAAGGCCCCCATCAGGCCTGACCTGGTCAACAATGTCCATGTTT
CCATGTCCAAGAATGCAAGACAACCTTACTCTGTTAGTAAGGAGGCAGGCCATCAAACCAGTGCCGAGTCATG
GGGTACAGGACGTGCTGTAGCACGTATCCCCCGTGTCCGTGGTGGTGGTACCCATAGA
TCAGGCCAGGGTGCGTTTGGCAACATGTGCCGCGGTGGTCGCATGTTCGCACCAACCAAGCCGTGGCGACGTT
GGCACCGCAGAGTCAACCTACGCCAGCGCAGGGCTGCAG
>57963769_3 CX700636.1
CGGTCGGAATTCCGGGTCGACCACGCGTCCGCCGAGTGGACCTCTTTGTAGCCAAATCAATGATGAAAATAAA
CCAAAAGATAAAGATAACGCCACCATAGAAGAAAAAGATAAAGCTATAGATAAGAAAGAAAAAGAAAATAAAA
AGAAAGAAACAGACGACGATAATGATAAAGAAGAGGATAGTAAAGATGAAAACGATAT
TGCAGATGTAACAAAAGAAAATGATGAAAAAC
>57963779_3 CX700644.1
TGCAGTACGGTCCGGAATCCGGGTCGACCACGCGTCCGGTGTTTGCAGATTTGATCGGTTTTAGTAAATAACA
AGATTATATTATAAGTGTACATTTTAATTTATAAAGAGCTTAAATTCTATTTCAAAATGTCGAAAGCACATCC
TCCAGAATTAAAAAAATTCATGGACAAGAAGCTATCAATAAAATTAAATGCCGGACGA
GCGGTTACCGGCGTACTCAGAGGTTTTGATCCTTTCATGAACTTAGTTTTGGATGAGTCTGTCGAAGAAAGCA
AGGATGGGCAACGGAACAATATTGGCATGGTGGTGATCCGAGGAAACAGTATCATCATGTTGGAATCACTAGA
TAGAATATAGTATTTAGTTAAATGTCTTTATTGTAAGTTTATATTAATTAATATTCAT
AATTCATAAAAGAAGTCCACTGAAGTGCAACTGTGCAGATTATTGGAAAATGGTGAATGTATCCAAATACATG
TTTTACAATGATGCCTAATAAAAAATATGTGA
>57963782_3 CX700647.1
GCGGTACGGTCCGGAATTCCGGGTCGACCACGCGTCCGGCGACGCACCGGCCAGTCTTTGCCGAGCGCCATGG
CTCGATACGCGTACCTGTGTCTAGGGGTATTACTTCTCGCACACTTTGTGATTTGCGAGACACGAGTGAAGCG
GCAAGATGAGGATAACGAAGATGAAGTGAATCCGGAAAAGCTTTGCGACGGTCGTCCA
GCTGACGAGTACTTTAGGTTATCTACTGAAGGTGACTGCCGAGATGTCGTCAGGTGTGACAAGGGAGGTGAAA
ATGGTGTGACTAGGCTAGCTTCAGTGCGCTGCCCCGGCGGACTGGCTTTTGATATCGACCGCCAAACATGTGA
TTGGAAAACACATGTTAAGAATTGCGATAAATTAGAAAAACCAAGAAAAATATTACCC
ATCTTGAAAACGGATGAACCAATATGCCCTGAAGGCAAATTANCTTGCGGAAGCGGTGATTGTATCGAAAAAG
AGTTGTTCTGTAATGGAAAACCTGATTGTAAGGACGAGTCTGACGAAAACGCCTGCTCTGTTGACCTAGACCC
TAATAGAGCACCAGATTGTGACACCAACCAGTGCAGACTTCCCGATTGTTTCTGTTCC
GCTGATGGCACACGG
>57963785_3 CX700650.1
TGCAGTACGGTCCGGAATCCGGGTCGACTACGCGTCCGGCGCCGGTCACGCACGTAAAAATAGTAATGGCGTC
GCGAAAATTAAATCATTTCGTTGTTTTATCTAGAAATTACAGTTCAAAAGCTGACAAAAATGTCGCCTTCCTC
GGCCTTGGTAATATGGGAGGGTATATGGCAGCGAATTTAGTTAAAAAGGGTTTCAACG
TGCGAGGGTATGATCCGTCCAAGGATGCCATTTCTGCGGCTGCCAAGAACGGTATCTCCGGTGCCAGCTCCAT
AGCCGCGGCGGTAGACGGTGCTGACGTGGTGGTGTCAATCCTGCCAAGCAACAAAGTTGTCCTTGAAGCGTAT
CTCGGCAAGGATGGTGTTGTTAAACATGCGCCAAAAGGTTCTCTCCTCATCGACTCCA
GTACAGTGGACCCCAACGTGCCCAAAGAGATCTTCCCGGTGGCCATCGAGAGTGGAGTCGGGTTCATCGATGC
TCCTGTTTCTGGAGGTGTAATGGGCGCCCAAAATGCCACACTAGCTTTCATGGCAGGAGGTCGTAAACAAGAC
TTTGACACATCCTTACCACACC
>57963788_3 CX700653.1

CAGGTACGGTCGGAATTCCGGGTCGACCACGCGTCCGGATAAGACATGTACCTAAGTTTATTATCTAACCAAC
ACAACTGTCACTAACAACACCCCCGAGTGCACCAGCATGTGCATGTTGCATGTGCATGTTGCCTACATGGTTA
TTGCACGTCCACGTTCACGCAGCATGTGACTCTCCCCTAGAGTGGTCTATATCAAAAG
AGTGTCCAGGCGTGCGAGCTGCCGGCGGTGCAGAGCGAGCACAGCCACTACGTGTGCGACGACCAGGGCGAGC
CCAAGTGCCTGCCGGGCTGGCAGGGGGACCTGTGCGACGTTCCCATATGCAAAAGGGGGTGCGATCCTATGCA
AGGTTTGTTATTTCTCTTGTTGTTTTATTTCATTTAAAGGTGATCCAGGAAATAAAAT
ATTTCATATTTTCATTTCCTAGCTTTGGTAACGAAGTAAAGCGCGACGTTGAGTTCCGCCTCCTGAACTCGTG
TAGACTTCTTGTAGTAGGTTAGTGATCTAAGATCCCTTTGCCTGTATTGTGGGGGCCACAGCGTGAAGCTAAA
AAGTAACTTACTTAGATTTGGGGAGAAAATTAGGAAAATTATGTAGGTAGCAG
>57963789_3 CX700654.1
GACTAAGGCCTGGAGTAGGGGTGAGTCCCACCTGCCTGTGGATACTTGTTCCGTCATGGAAATCACCTTGCTG
CAAAAGAGGTTGGGGGAGGGCGTCCTTGCTGAGGCCTCCTACACATCGGGCAACAAATTATGCTTAATGATAA
TGATTGTCAAATGAGAAGACGAACTAATCTTATGCTTTATCGAGAGGTGTGAACGGCG
TCTCGCTGACTAATTATTTCCAGGGGCCATCCGTTCTCATGGGTGACAGGCCAGTTAACAAACCGGCCAAGGA
TCAGCGCCTGGAGGAGGACAAAATGGGTTTTTATCGTGTGAGCGGGAGTGGTCTGAAATGAACACGCCGGCTC
GATTACGCCTGTTTTGCCTGCCGTATTCCGGCGCCAGCGCCATGGTCTACAGCCGTTG
GCGGCGGGCGTTGCCCGAGTGGTTGCAGGT
>57963791_3 CX700656.1
CTGCGGTACCGGTCGGAATTCCGGGTCGACCACGCGTCCGCCGATGGTCGCCCTGTTGGTGGTCAATTGAAGA
AGTTTGTGTTCCGCGGGAAATCTACAAACAACGGCGATAATAGAGGAGCGGAGCGCGGACTGGTCCATTGAAG
CTCTTCTGAAGAAGTGGGGGTTGAAAGGTGCTCTTCTGGACCTGGACTCGCTGAGTGA
GAGCTCGGGTGTGGACTACAGGGCCCTCATAGGCGAGAGATCTTTGCATTTACTTGAAATAATATCTGTATAG
GGTATGGCCAGCGCAGCTAGAGCAGCACGCCTCTATGTTGGCAACTTGGCATGGACAGTCGGCCATCGTCAGC
TTAGAGAATATTTCGCACAATTTGGTCCAGTTCAAAGTGCTAGAGTAATTTTTGATAG
ATCAACTGGCCTTAGTGAAGGCTATGGTTTTGTAGAATTTGCCAGTCCTTCTGGTGCCGCTGATGCGACTAAC
AAGCAGTTACACACCCTCGAGGGATTAAATTTGAATGTTCAAATACAAAACAATTAAATTATCTGGTAGTTTG
TAGTTTTTATAGTGGCAGAGGCATTACCAGAGAATACAAGTTCAGATTTTTCTGATGA
TGAGC
>57963793_3 CX700658.1
TGCGGTACGGTCCGGAATTCCGGGTCGACCACGCGTCCGAAAATCTTGAAATATTTGAGAATCATTTTGTTTC
ACTTATAAATTAAATCCAAAATCATCACGATTTTGAAAATTAAAATACTGATATTTAAATTACTAAAGCACTG
CAGCTTACATTATAATGGCTCCGAAAGCCGGTGGTGATTCCGCTAAAGTCAATTCAGC
TGTATATAAAGATAAAAGTAAACCTACTGATATTCGCCTTAGTAACATCAATGCCGCTAAAGCCGTTTCTGAT
GCTATACGTACGAGCTTGGGCCCTCGCGGGATGGATAAAATGATCCAAGCAGCAAACGGTGAGGTGACAATTA
CAAATGATGGAGCTACTATCCTAAAACAAATGAGTGTAGTGCATCCAGCTGCAAAAAT
GTTAGTGGAGTTGTCTAAGGCCCAAGATATAGAAGCTGGTGATGGCACAACATCAGTTGTTGTTATTGCTGGA
GCTTTACTTGATGCAGCTGAAAAACTATTGCAAAAGGGTATCCATCCCACTGTTATTTCTGATGGTTTCCAAA
AAGCTCTTCAAATGGCACTGCAGGTTGTTGAAGGCATGTCTACTCCTGTAGACCTAAC
AAATGAAGATGCCCTATTAAAAGCTGCCGCTACATCACTAAACTCCAAAGTAGTATCTCAGCATTCCACTATC
CTGGCACCAATAGCAGTGCAAGCCATCCGTGCAGTTATGGAACCAATAGTGGGTGGACTAGGTGCTC
>57963799_3 CX700664.1
GCAGTACGGTCCGGAATCCGGGTCGACCACGCGTCCGACATTTGACAGTTAATGGCGTCTATGTGAAATAATA
TTTTCCCGCTAACGAAATACCAAAAATTAATCTGTAATTTCGTTTATAATCATTTCGTAAAGATGACTATCGG
AAAGAATAATAAAATGCAACAGCATAAATTACCGTGTAAGGGTAATTTTACAAGATTC
ACGTACGTTTATTGGAACTTTTAAGGCTTTTGACAAACATATGAATTTAATTTTGGGTGACTGTGAAGAATTT
AGGAAAATCAAATCAAAAAACAGCAAAACTGCGGATCGTG
>57963800_3 CX700665.1
GCGGTACGGTCGGAATCCGGGTCGACTACGCGTCCGGTTTCCTGGTTTTAAAGATGACCATTCGTTGGGGGAT
CGTTTCTGCTGGGAAAATTCTCATGATTTCGTTAATGCTTTTAACACATATCCCGATGTTGGAGATCAGGTGA
TAGCAGCTGTAGCAGCTCGGGACAGGTCCAGGGCAGAGGAGTTTGCCAAAGTACATAA
TATTGAAAAGGTTTTCGACACTTACGCCGATCTAGCGAAGAGTAATGATATCGATGTGGTGTACATAGGAGCT
CTTAATCCAGATCACTATGAGATAAGCAAATTATTTCTTCAGTCGGGCAAACATGTACTTTGCGAAAAACCGT
TATGTTTAAACTTGAAAGAAACAGAAAGTTTGCTCAAAATCGCGAAAAGCAGTAATCT

TTTCTTTATGGAGGCTGTGTGGTCCCGTTTTGCACCATCATACATAGCTTTGGAGAAAGAGATTCAATCGGGT
AAACTTGGGGAAATACAATATGTTGAAGTTAACTTTGGTCTTCCAATTATAAAGATTGATAGATTATGCAAAA
AGGAATTAGGTGGCAGTGCAATATTGGATATAGGAATTTACGCTCTACAGTTTGCACA
ATAT
>57963806_3 CX700671.1
GGTACGGTCCGGAATCCGGGTCGACCACGCGTCCGCTCGCGCCCGCTCTGACAGCACGCGCCGCCCGCCCCGC
GCCGCTCGATAATCACAACTTTAAGAAGAAGAACAAAGTTTGACCGCCCGCCCCATACCAAATCAAAATGAGA
AGTGCAGTGTACCTACTGGCCGTCCTGGCGCTGACGAGAGCGGCGCCCGAGCCCGCCG
CGCAGCCCGACCCCGCCGCGCAACCCGACCCCGCCGACCAACCCGAGCCCGACA
>57963809_3 CX700672.1
GGTCCTGCGGTACGGTCCGGAATCCGGGTCGACTCACGCGTCCGCGTCGCCGCCCAAGGAAAAGCAAAGTGAA
GAAATAATAATAAAATAATGAATTTATTATTTTATTTCTTCACAATAATGTAGTAGCAATACGACTGTATAAC
AATTTGAGAATTGCTTTACGAATATTTTTCGTAAATTTAAATGTATTATTTATGTGTT
GAATCTCGACATGATTGGAATT
>57963814_3 CX700677.1
CTGCGGTACGGTCCGGAATTCCGGGTCGACCACGCGTCCGCTCTTTTATGATACATACAACATGAAGTACGCG
GCGCTTTTTGCGCTGCTGGCGGTCGCCTGCGCCAAAGACAGCGACCCTACAGCGGTTGACAATTTACGCAATA
AGTTTTACGCGTTGGAAAAACAATTATGGTTGAACGTGACGAATCCCGCTTGGAGCCT
GGAAGGCCTTGGAGGAGACGTCGAGTTAACAAAGGCCTTTGTGGATTTCGATGAGCAAATACAAGCGGTGCCG
CCTCCACCGCGAGTACCGTTCGAGACTTGGCTTTGGGCGAAGGCGACGGAAAAGTTGCGGGTCATCGAGGGCT
ACTACAAGAATTTCATTACATTCGTAAGGCGCCAAGCCCAGCCCGGCGTCGTGCCCGC
GCCTGTACGCGAATGGCTGGACCTTGCCCAGCAAATGATGGATGAAAAGTCACCCGTTGTACTGGCCACGAAA
AAGATCACCGATATGCTAGAATTGGGCGACATGTTCCGTGGTGTCTTGCAGGAGGAAAATTCAGATCTGTGTG
ATCTTCAGCTGTCGCCGCATCAGTTGATCTACGACATGTACAACACCTTCTCCCTGAC
GGAGATCAAGGGGTACGCAATGACGCAGTTCTCGTGGATGCT
>57963825_3 CX700688.1
ATGTTCCTGCGGTACCGGTCGGAATCCGGGTCGACCACGCGTCCGCCTGGCCTTGGCTCAGACGCATTTTGCA
AACCATTTGATAGCAGGTCAAAATTTCTCAATCTTTCGTAAAAAAATCTACTATAATAAACTTTTATTACTAT
TAAGCACAATGCAAAATCGTACAATGTTATATATTTTTCTATTATTAAATTTCCTTGT
GGTTATAAGTGGTTATAAATTGGAACAAGTGGTTATTCTGAGCCGTCACAACATAAGGACTCCTTTGACTAAG
AATTTGGAGCAGTTCTCTTCCGATCCCTGGCCTGAGTGGAAAGAAGCTGGAGTTTTAACTGACAAGGGAGCTA
AATTAGAAGAAGACTTAGGTGAATATATTTCACGGTGGCTTCAAAAGGAAACCTTGTT
CTCTGAAGATTGCCAGAACGACTCTATTTTCGTTTACTCCAATACTAGGCAAAGGACCAAAGAATCAGCAAAG
GCATTCGTAAAAGGTGCGTTTCCAAAGTGCAATGTAACAGTTTATTCGATAAATTCTGACAAAATGGATCCTA
TGTTCAATCCTATTATACGTAACGATTCACTCATATTAAAAGATATTGTAATAAATGA
AATGGAGGCAGCTTTAGATAAATTAGAACCTTAAGAGTCTTATGATTTATTGGAGAAAATATTAGACCTGGAA
AATTCTC
>57963832_3 CX700695.1
GCGGTACGGTCCGGAATTCCGGGTCGACCACGCGTCCGCGGTATGCAAATGCCACATGTGAGTCTTTGGTCGC
TGACTGACGTACGAGTTATGTTGAACTGTATACAGAATTCCACACCCATGCGTCACAAGCGCACACATTTTGT
GAACATACCGCACTATGGCGTCAAGTTCTTCGAGTTCGCTGTGTCATTGCTCAGCGAT
AAACTTAAGGATCGTGTTATGTTCCATCGTACATCGGAAGACTTGACAAAATATGTAGACCCCGCCATATTGC
CTAAGGAATATGGCGGTACAGTTCCACTAAAGGACATGATAGATGAACTTAAACGTAAGCTACTGAAACATAG
AGAAGATTTACTCGCCCTGGATAATATGTGTATTGATTTGTACGCTCTTGAGAAAAAT
GATCTTACTCAAGATATTCATTCTACTGCGGGGTCGTTCAGGAAATTGGAATTAGACTAGAATAGTAGACATA
AGTTAGATTTTTTAAAGTTATAATTCTAATACTCAGGCTAAGATTTTGTTTTATAAAAAGT
>57963835_3 CX700698.1
CATTGGGCTTTGTGCACAAGAAGGAGTTGCTCAAGGAATACCTGGCCGGTAACGAGCCGAACCTGGAGCACTT
GGCGCGCCGAGCGGTCAACCTGCTGGAGAGTTTTTCGATTCTCAATGCCCTGGAGCAGATGCGTCAGGAATCT
ACTCACATTGCGTTCGTGATCAACGAATTTGGTGACTTTATCGGGGTGTTGAGCATGA
CCGACATCCTCGAGTCCATCGCCGGTGAGTTGCCGGATGCGAGTGAAGTCGAAGGGCCAGACATCGTTGAGGA
AGAGGGTGGCTTTCGGGTTAATGGTGCCTTGAACCTTAACCTGATTCGCCAGCGCACCGGCTTCAAGGCGGTG
GCGACGGAGGATTACCACACCCTGGCCGGGCTGGTGATGAGCCTGCTGGATCGCCTCC

CCGCCGTCGGCGATAGCCTGGAACATGAAGGGTGGCGCCTGACCGTGGCAGCGGTGGAGGAGCGGCGAGTTAC
TCAGGTGTTGCTGGCTCCTCTCTAACGGGTGAATTTCATTCGACGCTGCGCTGGCCTTTGGATAGCATCGGCG
CTCATTGAACCGTTAAGGAAGGGTTACCCGAATGACTTTGGACGTGCTTAAGATTTCT
GCATTTTGTGACGGTAACCAGGGGCGGTACCCGGCAGGCGTATGGATCGGTGACAACCTGCCGGAAGCCG
>57963837_3 CX700700.1
TCAGGTTGAGCGTCCGAACAGGCACACCACCACGATCAGGTAGGCCACGATCACCGGGCGGTCCAGCGCGTTC
AGGCTGTCCAGGATCAGTTTGCCGGCGCCGGGCCAGGCGAAGATGCTTTCGGTGATGACGGCGAAGGCGATGG
TCGAGCCCAGCTCCAGGCCCAGCACCGTCACCAGCGGGATCATGGTGTTGCGCAGCAC
GTGCACACAGACCACCCGGAACGGCGACAGGCCCTTGGCGCGGGCGAACTTGACGAAGTCCAGCGGCATCACG
TCGCGCACGCCGGCGCGGGTCAGGCGGATCACCAGCGAGATCTTGAACAGCGACAGGTTCAGCGCCGGCAGGA
TCAGGTGGCGCCAGCCGTCGGCCGTCAGCCACGACCACTGGAAGCCCAGGAATTCCAC
CGTCTGGCC
>57963841_3 CX700704.1
GCGGTACGGTCCGGAATCCGGGTCGACCACGCGTCCGCCACTACACATCTGGAGGTCATTCTTCACGAACACC
GGGTACCTTAGCTCTACTCCAGGGTTCCTCAAACTCGTACAATTGCTTTTAGGCGCAGCGTTTGTTGGCGTCG
TGGGTTACTATCTAGACCCCGGCTATANNNNNNNNNNNNNNNNNNNNNNNNNANCTATT
TTATCTACTANTTGCTGTGACGTTCTTAATCGGTACCTTCTGCTTGTACGTATCATGCCTCATGTCCTGGTCG
ACGGCTTCTGTGATATCAAAAACACTATATGAAGTTATCTATCACGGCGTCTCATTTGTTATGTACTTAGCAG
CCGGTCTTACCCTAGTGATTGAAGTAAATCATCAAAAAAGTAGTTACAGGCGAGACTT
CGAGCCATATTTGGCAGCAGCGGTAATGGGACTGGTGATGGCAGTTCTATATTTATTTAGTACATTCATAGCA
GTGAGGTCCTATCGCGGTATTTAAGTTGCCGAGAATCTCTATAACACTAACTTTATTAAAATGCATTTCTGTG
TGGTGTGTATATTCACACCAACAAGTACTTT
>57963845_3 CX700708.1
ATGTTCCTGCAGTACGGTCCGGAATTCCGGGTCGACCACGCGTCCGGAATGCCTCAACACAAAAATCCTTTTG
AAAAAGGTAACTTGTATATTAAATTTGATGTTACTTTCCCTGAAAATAATTTTGCTACTGAAGATCAACTTAA
AAAAATTGAAAGCATTTTACCCCCAAGACCAGCATTTGTAATGCCTACTGGTGAAGAT
GTAGAAGAAGTCAACTTGATGGAGTATAATGCTAGTGAAAGAAATCGTGGCCGTGAAGAGGCCTATGCTAGTG
ATGATGATGAACATATGCACACAGGTCCTGGTGTACAATGTGCCCACCAGTAGATTAATAAATTCGATATATT
TTCCTAAATTGGGATTTGTACTCAAACCTCACCATGTAATTTTATGTTATCTGTTAGG
TAGTTAATATTTACATAAATATGTGACACTTAAATTTACAAAAATATTCACAATTTTATTGACAGTTTTAGTT
TTATTAATTAAAAATTAAAACAATTGAGTTGCATGGTTCACTGTATACATTGCCAGCCACACCTATATTAGTA
ATTATTAGGTTAAAATTTATTCAATTGTATAGAATGAATGAGGTCACATCAGAGCCTA
ATGATTCATTATAATAATCCTTCTGTATTAACAGTATTTAAGATATTAATAATAAATTCTTAATGGAATGAGT
T
>57963851_3 CX700712.1
TGCAGTACGGTCGGAATTCCGGGTCGACCACGCGTCCGGGGGTAGGGGTGGCTTCGACCGGGGCGGCCGCGGT
GGTGACAGGGGAGGTCGCGGTGGTGACAGGGGAGGTCGCGGCTTCGACAGGGGTGGCAGGGGCAACAGAGGCG
GCAGGGGCGACCGCCACTCGTGGGGCGGTGACCGAGGAGGATTTAACAAAGGGGGATT
TAATGACAGGAAAAGCTTTGAAGGAGGTGACAATTCTCAGAACAAAAAAATTGTATTTGATTAAAATAATAGG
GTGGTGCTCGCGGGTCATGGGGCGAAAAAGCCAACCTGGACTTGAAACATACACGCGGCAAATCATTCAAACA
TGAGAAAACTAAGAAAAAACGAGGTGCATACCGCGGCGGTGCCATTGACACGTCTGTT
CACTCCATCAAATTTGAGGATTAACATCTAATGGATGATAGTCGATTTGATTTATTTTCCTTAGCTATCCTTG
TAATTTTGTTTTACTTGGATATTATCATTTATATTTAG
>57963854_3 CX700715.1
GCAGTACGGTCCGGAATCCGGGTCGACCACGCGTCCGCATTATTAAATAAAATAAATTTTCGTTGCATCTTGG
GAATTTTGTAATTTGATCAAAACAGTTGGTAATTTTGTATATATGAATAAATAAATAATATAGCACATACAAA
ACGAAATGGCGCTAGCACTGCAAAGAAGGGCAAATGTAAGACTACCGCCAGAGGTCAA
TAGAATATTATATATCAGAAATTTACCTTACAAGATTTCAGCTGAAGAAATGTATGATATTTTTGGTAAATAT
GGAGCTATACGTCAAATAAGAGTTGGAAATACACCTGAAACACGAGGAACAGCGTTTGTTGTTTATGAAGACA
TATTTGATGCTAAAAATGCCTGTGACCATTTATCTGGATTTAATGTTTGCAATAGATA
TTTAGTAGTATTATATTATAGATCAAACAAAGCATTTAAGGGCATAGATATAGAAAAGAAACAAGAAGAATTA
GACTCTCTTAAGAAAAAATATGGTATTTCAACTGATGAACTTCACAAATAAATGTTTTCTATTTT
>57963860_3 CX700721.1

GCAGTACGGTCCGGAATTCCGGGTCGACCACGCGTCCGCTCGCGGGTCGATGAAAAACGCAGTTAACTGCGCG
TCATAGTGTGAACTGCAGGACACATTTGAACATCGACATTTCGAACGCACATTGCGGTCCGTGGGGTTACATC
CAGGACCACTCCTGTCTGAGGGCCGGCTGCAT
>57963861_3 CX700722.1
GCAGTACGGTCCGGAATCCGGGTCGACCACGCGTCCGAAACAACCATAAATAATTTGAAGAAATGCATATTTT
TCTTAGTTTGTCAAGTGTAAAATATTTTAAGAATTTATTTCTTAGAGACTTAAGATGAAGTACTACTGTTTTA
TTGTAAGGCTTTGCATTGTTTGTTTCATATAACAATTACACCATTTTATATACTGTAT
GTCTACAAAATGGCTTGTCTACATATTCATTTGAAAGTAGATCCCTTTAAGCCATATATTCCACTTTGCGTGA
TTTTGTTGTTACAAACCAAAATGCTAAGTCAAATTCTTTCATAGCATTAATATTTTTACCAATGTATAACTAG
CAAAAGGTAAATAAATCAAATATTACTGTC
>57963870_3 CX700731.1
TACGGTCGGAATCCGGGTTCATTCGTGGGTCACCAGTCGAAGCCGGTGATTTCCGCCTTGGCGATTCACAGGC
TTCGGTCAGGGTCGCGACCAGGCGACGCTCGATGCGTGCGCTGTCGCGTTTGGTTGAAGACATGGGGATTCCT
TGAGGAGCGAGGAGGGTGATGATACGCGAGAGATCAACTGAGGGAG
>57963873_3 CX700734.1
GCAGTACGGTCCGGAATCCGGGTCGACCACGCGTCCGAATATTTATTCTACAATATCTTTTTGTCATCTAGTT
CTATTGTGGTAAGATAGTATAATGATACCTCTAAGAGTATGTCTATTGTAGATTAAATAATAATAAAATAATG
CTACTTTTATTTAATGCTCGAACACATTTTTATTATTATGAACTATCGTTATTGTATT
AATTATTATTTAAATTTAAATGCATACATTTTGTAAAGCTTTTTTGGATGTTTAATAAAAATCGAATTAATAA
AGCTTTATGTTCTCC
>57963880_3 CX700739.1
GCGGTACGGTCGGAATTCCGGGTCGACCACGCGTCCGATCGGAGAAAACGGGACTAACCGTGATAATAGCAAA
CGTCGAGGGTCCTTTAGTAAACGGATTTTTTTGTCTTGCAACTGAAGCCCACGATGATGATGGCTTACCGCAC
ACATTAGAACATTTAATATTTTTGGGTTCAGAGCGGTATCCTTATAAGGGAATTTTGG
ACTTGTTGGCTAACCGTTGTATGGCACATGGAACTAACGCCTGGACAGATACAGACCACACTTGTTACACTAT
CTATACAGCGGGTGATGCGGGTATGCTGACATTATTGCCCATCTACCTCGATCATATTTTAAGACCTACACTC
ACTGATCAAGGTTTCATAACTGAAGTCCATCACATTGATGGTGATGGGGAGGATGCAG
GTGTTGTTTATTGTGAAATGCAGGGAAGAGAGAATACAGCAGATAGCAGGTCATATTATTTATATCTAAATAT
TTGCAGTTTGATTTTCCAATTGGAAATTGTGCTGTTATATTTTAAATACTCTTTATAATTAACAAACGGCATA
AATTATAAAATATAACTGAATCTATTAGGTGTGAGCTGAAATTACTCCGAGCGATATA
TCCAGACAATGGGTATTCTGCTGAAACTGGAGGAATCATGGA
>57963883_3 CX700742.1
GCAGTACGGTCCGGAATCCGGGTCGACTACGCGTCCGTGCTTATGAAAATAATTATGTTAGTTTAATACAAAC
ATCTTTTACAATGAGTAAACTATACGTGCTCTTCGAGCACAGCGCGGGCTTCGCCCTTTTCCGCGTGGCGGAG
TTCGAGGAGGTCGCAGCATTTCTACCTCAAGTGGAGGAATCAGTCACAGACTTGCAAA
GATTCAATTCAGTGGTCACACTCATTGCGTTCCAGCCTTTCAAATCTGCCGTGTTAGCTTTGGAGAACATTAA
TGCTATATCTGAAGGTATTCTACCAGAAGATCTAAATCTCTTCCTTGAAGGAGCATTGCCAAAACGCAAGAAA
CGCAGCAAATGTTCCCTCGGAGTATCTGATCCCAAACTCGGTGCAGCTATCAGCGAAG
CATTAGAGATACCATGCTCTCACACTGGAGCTGTCCCGGAGCTCCTCAGAGGTATAAGACACCATTTCCACGC
TCTCATCAAAGGTCTAACACTCAAAGCATGTAGTGTAGCCCAACTGGGGCTCGGCCATTCATATTCGAGGGCT
CGAGTCAAGTTCAATGTACACAGAGTGGATAATATGATAATACAGTCAATAGCACTGT
TGGATCAGTTGGATAAGGAT
>57963890_3 CX700749.1
GCGGTACGGTCGGAATCCCGGGTCGACCCGGTTTCGCCCTCGGTGCCCTGGAACAAGCAAAAATCCATGCACG
CCAGGCCCGGCCCTGGATCGACAGCCAGCAGGATCACGGCTGGCAAGACCCGTTCACCATCGCCGCCATCGGC
GACCTGGAATGGCGGGTCCACGGCACCGAAGCGATCCTCGCCAAGGCCGGCGTTGCCG
TAGACCAGGCCCTGGCCAACCCCAACGAAGACACCGTGGCCAATGCCTCGCTGGTGGTCGCCCAGGCCAAGGT
GCTGTCAGCCGAAACCGCGTTGCTCGCCAGCAGCAAACTCTTCGAACTGGCCGGCACCCGCTCGGTGTCCGGC
AAGCACAACCTCGATCGTTTCTGGCGCAACGCGCGCACCCATACCCTGCATGACCCGG
CCCGCTGGAAATACCACTTGATCGGCAACTTTGTGCTCAACGGCGTCAAGCCCGCACGCCACGCCTGGAACTG
AGGAGCTTCATCCATGACCGCACTGAATCAGGCGCGCCAACTGCTGGAAAGTACCCGGCGCTTTGTCGAGAAA
AGTGATGACCCCTACGTGATCAGCCGCTTCGGCGATTTGCAGATTCGCGTGGAGGTC
>57963891_3 CX700750.1

AGTACGGTCGGAATTCCGGGCCGACCACGCGTCCGGGAGTCATGTTGTTAGCGAACGTGAGTGTAGCGGAACA
ATTAGCGAAGGAGTTTCCGCAGTGCGCCCTATTACGTCTGCACCCGGCACCACCGCCGGCCAACTTCCATACC
TTCCTGAAGGCGGCCCGACAACAGGGTTTCGAACTAGATGTGTCAACAAACAAGTCTT
TCTCCAAATCTCTCAACGAGGCAGTGATACCCGACCGGCCATTTTTCAACACCCTATTACGCATAATGGCGAC
TCGTTGTATGCAACAAGCTGTGTATTTTTCAAGTGGAACTAAAACACAAGATGAATTTTACCACTATGGACTC
GCGTGCCCTATTTATACGCATTTTACGTCGCCTATTAGAAGATACGCCGACGTAATAG
TACATCGCCTACTAGCTGCGTGCATAGGAGCGGACGTGACTCACGCGTCTCTGTTAGACACTAAGGAGGCCGA
TTCCTTATGCGACAACTTGAACTACAGACACCGGCAGGCGCAGTACGCGGGGAGGGCGTCCGTGGCCCTCAAT
ACTCATATTTTATTTAAAAATCGTGAAGAGATTCAGTCAGCTGTAGTATTGGCCGTCA
AACGTAATGTGTTGCAAGTGCTCATCCCGAAGTACGGACTCGAAGGACCGTTG
>57963892_3 CX700751.1
CAGGTACGGTCGGAATTCCGGGTCGACCACGCGTCCGGACTATTCTAGATCGCGAGGGCCGCCCTTTTTTTTT
TTTTTTTTTTTTTTTTTTTTTTTTTTTTTTTTTTTTTTTTTTTTTTTTTTTTTTTTTTTTTTTTTTTTTTTTT
TTTTTTTTTTTTTGGG
>57963894_3 CX700753.1
GCGGTACGGTCGGAATCCGGGTCGACCACGCGTCCGGACATTTGCCGTTTGTTTGCGCGATTTTATTTTAAGT
ATTCTTTACATAATATATATGTTATTAGATATCTTAATTCGCAAACCAGCTAAATGAATTTTACAAGAGACTC
CGAAAATGTTATGTTACTTTGTGCAGACCATGTATTT
>57963898_3 CX700757.1
TGCGGTACGGTCGGAATTCCGGGTCGACCACGCGTCCGCTAGTTCTAGATCGCGAGCGGCCGCCCTTTTTTTT
TTTTTTTTTTTTTTTTTTTTTTTTTTTTTTTTTTTTTTAAAAGATTTTAATTTTTTTCTAAAATGAATGGTAT
TATATTGGTGGGCCCCTTCGG
>57963900_3 CX700759.1
GCGGTACGGTCGGAATTCCGGGTCGACCACGCGTCCGCTTTGATACTGCCCTCTGACAGACGGACAGGGGAGT
CTTAGTAATACGTTTTACCCTTTGAGTACGGAACCCTAATACAAATGATTGAACATAGGACAATGTATATTGA
TTTCTAGACAATATCTTATTTTACTGTTCCTTTCGTTGAGAGTTAACCTATTGTATAT
TTAGTGTAATGGTTGGCATGCCAACTACTAGTACTGCGTAATTATAATATGTATAGTAAGAAGTATTGCAATA
TTTTTTTGTATGAAGATATAAGCTAGTCTCATCGGCTTAGTGAAAGCTATAAGAATAATTATTTTTTCAAAGT
TATATAACGGTGAAATTGTATGTGCATTCATTAAACTTATTAGTCTGAGGTCGTCGTT
GCGCAGTGTGACAGTGTCTGACTGCAAACGCGATGGTTGTGAGTTTGATTTCCGCTCGGGGGATACAAGTATT
TCCTTTCCTTGCTCTGGTAAGATGACAAAGTGCGAAGTGTAGTGGGGAATAACTTGCAGGATAATTAGTACAT
TTCTATATATACCTAAAATTG
>57963901_3 CX700760.1
GCGGTACGGTCGGAATTCCCGGGTGACGTGGGCGGCACCAACAAACGCAGGCCCAGGGTGCCCACGGTGCTGG
CGGTGGCGCCATCGGCGTCGAAGTCACCCACGATCAGTATCCGCTGGCGCTGTTCCAGAGCCGTCACCAGCAG
GTCCACGGCGGCATCGATGCCCTTGAGCTGCTGATACGGAATCAGCCGGGCCAGGCTT
TTATCCAGCTCGGCTTCCGATTGCACCCCGCGTGCGGCGTAGAGACGGGTCAACAACGGTGGCAATTCACCGA
GGAATGGCAGGACGGCGGGCAACGGGCGAGGATCGATACGCATGGGGTGACGGAAGCTTCTCTTTAATGTTGA
AAATTAGCGGTGATCCCTTGTAGGAGC
>57963905_3 CX700764.1
CAGTACGGTCGGAATCCCGGGTCGACTCAGCGTCCGAGCCGAGACGCCAAGTTAGGGCTTGTGGAGACTGGGA
GGGGCCTTATTCCCGGTGCAGGAGGCACTCAGAGGCTACCGAGAGTAATTCATCCAAACATTGCTAAAGAACT
GATATTCACTTCAAGGATTGTTAGCGGAGAGGAAGCTAAACAACTTGGTGTAGTGAAC
CATGTGGTATCACAGAACAGCAATAACAATGCAGCTTTCGAGAAAGCCATGAGCATAGCTCGGGAGATTATAT
CCAATGCACCTATAGCCCTACGATGTGCTAAGCAGGCTATAAATGAAGGAGTCCAGCTCAGTATCAAAGATGG
ATATGAAGTGGAACAGAAATGCTATGAGAAGAATATTCCAACTAAAGATAGGCAAGAG
GGCATGATCTCATTTATAGAGAAGAGGAAACCTCAGTACAAGGGACATTGATGCATTTATTTACCATACTGTG
TATGAAACAAAGATGTTTACTCATAATATGAATATAAAGGAACTACTATACTGTAAAATTAATTGATGAAAGC
CTTTATTAGGTATAAGTAAATTTTAAGTTCACTAATTTTATATTCAAATGAATTGATA
AACTATCAGCCTGTGAAACTTTAGTCAACTGTGAAAATTATCTGTGGTTTATAGTTATGACAATATTGTGATG
TGTAAGACTAACTA
>57963906_3 CX700765.1

GCAGGTACGGTCGGAATTCCGGGTCGACCACGCGTCCGCGCTACTCGTGCTGACGCCTCGCTATCCACACAAA
AATGCCAGTCGACCTTTACTACGTTCCCGGCTCAGCGCCCTGCCGGGCGGTGCTGCTCACCGCGCGTGCTCTC
AACTTGAACCTCAACCTGAAACTCGTGGATCTTCATCATGGAGAACATCTTAAGCCTG
AATATCTTAAGATAAATCCACAACACACAGTTCCTACCCTCGTGGACGACGGCCACCCGATCTACGAGTCGCG
TGCTATAATAACTTATCTAGTGAACAAGTATGGCAAAGGCAGCGCGCTGTACCCAGAGGAACCCAAAGCCAGG
GCCCTCGTCGACCAACGCCTGTACTTCGATATCGGAACTTTGTATCAGAGATTTGGTG
ATTACTTTTATCCACAAATCTTCGGCGGAGCGCCCGCGGACAAAGACAAGCTAGCCAAAGTAGAGGATGCCCT
TAAACTCCTGGATACCTTCCTTGAAGGTCAGAAGTACGTCGCCGGCCCCAACCTCACTGTGGCCGACCTGAGC
ATTGTCGCTGGTGTGTCCAGCTTCGAAGCATCGGATATTGATTTCAAGAAATACGCTA
ATGTTAAGAGATGGTATGAAACAGTGAAATCCACAGCACCCGGCT
>57963908_3 CX700767.1
GCGGTACGGTCGGAATCCGGGTCGACTCAGCGTCCGACGCTCTGCACTTCTGCGTTTCGCGTCAAGTGGACAA
CCAACACGTCAATTTCACGAGTTAAAAAAGAACGAACGACTTGGTGTAACCAAGTTACGTGAAGTTCTAGAAG
ACAGATTACAAGACATAAAGCGAGCTAAGACGTGGAAGCACGAGAGGGTCATCACGTC
GCCTCAGGACACAAATGTCAGGGTGCAAGGCGAAGAAGGAAATTTCTTAAATTTTTGCGCAAATAATTACTTA
GGATTAGCCAATCACCCAGAAGTCGTAGAGGCCGCTAGAGAAGGGCTGAGTGAGTATGGAGCGGGACTTAGTT
CTGTCCGTTTTATTTGTGGAACTCAAATCATACACAAGGAGTTAGAAAATCGTTTGAC
AAAGTTCCACGGTCGTGAAGATACAATACTATACGGATCATGTTTTGATGCTAACGCTGGTTTATTTGAAGCT
TTACTGTTGCCCGAAGACGCTGTCTTCTCGGACGCCCTTAATCACGCCTCCATCATTGACGGCATCAGGTTAT
GCAAAGCTCAGAAGTACAGATATCCTCATAGAGACCTTAATGAACTTGAACATTTGTT
GGCTCACAGTGAAGCGCGACTAAAACTAATAGTAACAGATGGTGTGTTCTCGATGGATGGCAACGTTGCTCCT
>57963916_3 CX700775.1
GCAGTACGGTCCGGAATCCGGGTCGACCACGCGTCCGCTTTAGAGTTAAATTTTTCAAAATCCTTTCTTAGTG
GAAGTCCAGATTACGAAAGTAAACGTCTGTCCAAACTTTACGTTTCTACGCACAGCGGTTACGGCTGTGCGCG
CGTCTTACCTCAGTCAGTGAGGACAAGTCCTTTTATGTATATAAATAATAAATGGATT
AACATCCTTAATTATTATTTATTATAATTTAAACATATGTTTTAGTACTTATTATACTAAGAGTATTTGCATA
GTCGATTAACGTATTTGATATTAAATTATTATTACAATATTTAAGTACTAAGTCCTATATAAGATCCTATAAG
GGTTCTGTTTTATTGCTTTCGAGGTACAGAACCCTAAAACAG
>57963919_3 CX700778.1
GCAGTACGGTCGGAATCCGGGCCGACCACGCGTCCGTAAATTTCCTCACTCTGGTAATTAAACAAAGTGCGTC
GTTGAGTTCTGCACTAAAAGTAAAGTCGCGTGTAAGAAGTGTATGCGCGGCACGTGTGTCTGTTACCTTTCCA
GTGCAGTATGTTAGAGTAAATCATGGTTAATACAATTTATTTATTACGATATACAATA
TGATGTATGCGTTCAGTCAAAGTGGGTCAAACGTCTGCCAGTAAACGATACATACTCGTAC
>57963921_3 CX700780.1
CACGCGTCCGACTATAGAACGCTCGCCACAGTGGGACAAAAATGAGTAACGAGTTTTAGTGAGCTAAGCTTTC
AAGTGATAGTGTTAGTGTTTTGGAACTGCCACTTTTTTATATAGCCAAAGGAATTGACTTCGACGATGATGGC
TCCCGAGACAGTCGTGACTGTAAACCACAACAAACCAGCACAGCAGCAGGCGCCGCCA
CAGCAGCAGCAAGGCGGTGCTCTTGATTGGATTAAAATTAATATAAATTATTTCAAAACACCGCCCGGCATAT
TAAAAGTTATCCAATTGGTATTGGGTATACTATGTATGTCACTGGGCACTCCCTGGGCCTCGGCGTGGTATGT
GTTCGTGGCAGTGACAGCCTTTATAACGACCCTAATGTGGAGCTTCGTTTACTTCCTC
AGCATCAGAGAAGCTTTGAAGATTCCCATCAATTGGGTACTGTCTGAACTTATTAGCACCAGTTTGGAGACGC
TCTTCTACCTGATTGCTTTCATCGTCATGTTCGCGTCTGTGTATGGCAACTATGGGCGTAATGTGGCTGCTGC
AGTATTTGGTATGTNCAACACGTTGGCGTATGCGGCCAGCTCGTACTACTTGTACCAA
GAGCATAGGAGTAGCGCAGCGAGCGCGGCGGCGTAAATCTACTCTGCT
>57963922_3 CX700781.1
GCAGTACGGTCCGGAATTCCGGGTCGACCACGCGTCCGCTAGTTCTAGATCGCGAGCGGCCGCCCTTTTTTTT
TAAAAATAAACATAAATAATTGAATAAATGATCCATAAATTATGATTATAAGAAAAAATTACCTTAGGGATAA
CAGCGTAATTTTTTTTTTTAGTACAAAT
>57963923_3 CX700782.1
GCAGGTACGGTCGGAATCCCGGGTCGACCACGCGTCCGGTATCAATGAGGACTATGACTCAGACTCTGATACT
GAAAAACCCAAGAAGCCTAAGAAAAAGAAAAAACAACAGCCTGTTTCAGAAGAATCAGGTGGCAAAAAAGGTA
AAAAATCTTTAAGACAAATTAAAAGAGAAAAATTTGCTCAAAGACAAGCTGAAGCACA

GGCAGCGGCTAAAGATCAACTTAAATCACAATGCCTTAACTACTTATCACAATGGAAACATGATAGGCAAAAT
TGGAAATTTATGAAGGCAAAACAAGTATGGCTGTACAAAAATAAATTCTCAACTCAGTTAATACCAGACTCAA
CATGGCCAGTATTTTTGGAATATTTTGAATCAGCGACAGGAAACATAAAAAATATGTT
ATTAAATGATGCCAATAAGATTATAAAACAAATGGATGATTGGACAGAATCACAAAACAAAGAAGGGTCTAAT
GAAAATGAAGAGGAAGGCACAACAGAGGACCTAGGGAAACCTAATGACACAGTTTATAATAGAGCTAGAAGTT
TAATACAATGTTTACAAGAGT
>57963924_3 CX700783.1
GCAGTACCGGTCGGAATTCCGGGCTGACCACGCGTCCGATTTACTTTAATCTTATCAGATTTAACCTCCTCAT
TTACTTTTTAATTCAGTTATAATTCTTATTAGTTTACAATAATGTCTTCTCCCGAGGAAAGCTCTCAACTCAA
AGCCGGCCATCCCCCCGCGGTCAAGGCTGGCGGCATGAGAATTACCCAACACAAAGTT
CCACATGCAAAAGATGCAAAGGAGGTACCTAATGAAGATCTCACTGGTCTTGCTGGACCGTCTCCTGTTCCGT
CGAACCCGGTATCAATTTCAGGACCCCCCAATAGAGGTAATGCGGATTTCACGCCTCAGGCTGCCCAAATTGC
TCATAGCCCTAAGCCGCCTGCACATATTAATGTTAAACCTAGTCCAAATATCCAACAG
CCCAGAAAGTAGACTGAAATAATAGGTTGTGATAGGCATATCAACATCTTGTATGTTTTGCTAGGTGTTTTAT
GACATTTAATTGATATTAGAATATTTTTATTATTCACAAGCATAATTTAAACAAATATATTGATTATTTCTAA
TTCTTAACTACATTACAAAAAACTGAACAGAAATAATTATATGTTGAAAACTTTGAAA
CCAGAGGTCTGTAATCTATTGATACAACAAAATAGAATAAACAATAAGCAACAATCATTTTAATTATTATT
>57963925_3 CX700784.1
GCAGTACGGTCGGAATCCGGGTCGACCACGCGTCCGATTTGATTATAATTTCTGTAGGTCGGTGGAGTTCGAA
TTTTGCTATCTATGACGACGTTAAAATTTATAAAAACACAATTAAAATTGTAAATAAAATTTGTATAAATATT
TTGACAATACTTCATGGATTCGATGCTAAGCTTACTTATAACATAACATATTTTGTAT
AAATAATCGTAAATTCGATTTCGTTTTACTATTACTATTTGTTGTACTAAATAACGCTACAAATCAAATTAAT
TTTTTACAAAATCGAGTATAGATTAACATATATTAGAATTGATTAAATTCAACAATTACTCTTAATATCTTTG
GAATCAATATTTTTACCATAATTTTTAAAATTGAAAATGAACTGTTTTTAAATATATT
TTCTAATAATCATTATTTCGTCATTTTTCATTTTACAATGTAATGCATGCGTTTATAATCATTTAATAATAGA
AATGAAAATAATATTGTGCTTGGAATTTCATATGGAAGAAGGTATTCTTCCCTACTCAACTGGTGTCGCTGTA
AAGGCAAGGCAAGTAGGATAAACAGCATTAACCTAAAGTCCTCAACCTCGAAAAGAAA
ACAAAACATTTGAAACATCATAACACGTTGCGCATAAAATATTGGCATACATACTAATTTTGTCTTTTATCAA
TAAAATT
>57963928_3 CX700787.1
CAGTACGGTCCGGAATCCCGGGTCGACCACGCGTCCGGGCAAAGATTCTGGCAAAGCCAAGGCGAAAGCTGTC
TCACGATCGGCTCGGGCGGGCTTGCAGTTCCCTGTGGGTAGAATTCATAGACATCTCAAAAATAGGACTACGA
GTCACGGCCGTGTCGGCGCAACGGCTGCCGTTTATTCTGCGGCTATTCTTGAATATCT
CACAGCCGAGGTTTTAGAGTTAGCGGGCAATGCATCCAAAGATCTTAAAGTAAAACGTATCACTCCCAGACAT
TTACAACTAGCTATCAGAGGAGACGAGGAGCTGGACAGCTTGATCAAAGCCACCATTGCCGGAGGTGGTGTCA
TCCCACATATTCACAAATCACTCATCGGTAAAAAAGGAGGACCGGGGGCACCTGTTTA
ATAAGAGTCTTGCTTCAATTCAACATTCTAACTGCATTTGAACCGGAGACTATCAGCATTGTAAGTTCATCAG
CCAGGCAGTAAACATAGTTTTCTACAGGTAGTGATGTGGACAGTGGGAGTGACTTCAGACATATTGATCCGGA
CTAAACTTATAGTTAAGACTAGTAATATTTTTAGTTTAAGGAGATTTAAATATAATTG
AATTAGAATTAAGTTAATTTTGTCGTTTTGTTATGGGACATGTTTATTTTATGAGGCATGTGTACAATTTATG
TCAGCTACTACTAAAATTAGTTCACTG
>57963934_3 CX700791.1
ATGTCCTGCGGTACGGTCGGAATCCGGGTCGACCACGCGTCCGGTAGTTCCTGGATGGACGAACGGTATTGTT
ATCGGTCGTCACGCTCACGGTGACCAGTATAAGGCCCAAGACATTGTTATTTCCAAACCTGGCAAGATGGAGT
TGGTATTCACAGCAGAAGACGGTTCTGTGGAGAAGCGACTTTTGTACGATTACAAGTC
ACCCGGTGTTGCTATGGGCATGTACAATATTGACGAATCCATTCGTGCCTTCGCACACTCCAGCTTCCAGGTA
GCATTACAAAAGAAGTGGCCACTTTATTTGTCTACAAAAAATACTATTTTGAAAAAATACGACGGCCGCTTTA
AGGATATTTTCCAAGAAATTTATGACAGCAACTATAAGAAGCAATTTGAAGAAGCTAA
GATTTGGTATGAACACCGGTTAATTGATGACATGGTAGCACAAGCACTTAAGGGCTCAGGTGGTTTTGTTTGG
GCCTGCAAGAACTATGATGGTGATGTGCAATCTGATGTTGTAGCACAGGGCTACGGTTCATTAGGCATGATGA
CCTCAGTCCTCATGTGTCCAGATGGACGTACAGTCGAGTCAGAGGCAGCTCACGGCAC
AGTGACGCGTCACTACCGCATGCACCAACAGGGCAAGCCTACTTCCACTAACCCTGTCGCATCTATATATGCG
TGGACTCGAGGCC
>57963936_3 CX700793.1

CTGCAGGTACGGTCGGAATCCCGGGTCGACCACGCGTCCGCGTTCACGCGACACAAAGTATTCTTCTAAATGT
GCATAAATGTAAATAAAATGTGGACTCAATATATTTGGGCGTTATGCCTGGCGGTCATGTGTCAGGCCCAGCT
GTCGGACATCGAGAGATATTTATTGACTATAGCCGAAATGTCACCGTCGCCCCCTCTG
CAGAGTAACGAAAAGGCATGTCAAGTTGACAGTTCTACAAACGGTGTTTGCGTGAAACGTACACAATGCAACT
TGGACGCGCCGGCAGTGGATCCCGCAACTACCTTCATACTGCGTGAAGGAGGATCCGCGTCTACCGCATCTGA
TGTGGAGAACGCATTGGATACACAGTGCGCCTGGCTTCAGATCTGTTGTCCTTCTGAT
AAAGTCCGGGAGGAAGAACGCCAAGTTAAATTCGAGCAGCCACTTGGTTGTGGCTACAGCAACCCCGGCGCTA
ACGTCTTCAGGGAATCAGCTAATGCTTACGGCTATGCGGACTTCGGGGATTTCCCGTGGATGATTGCTTTATT
ACAGACAAAAAACGATAAGACAAAATTCAATGACCAGTATATAGGGGGTGGAACACTA
ATTCATCCTTCGGTCGTGCTGACGGTGGCGCATAAAGTAGAAAAGCTCGAGTCACCTAGTGAGTTGACGTGTC
GTGCTGGAGAATGGGATG
>57963940_3 CX700797.1
AAATTCATATTATAAAGAATTCTATGACGTCTAATAGTAAGATTAGTTTTAAAACTTAAAACAGTTTCTTTCA
TGCTTATTTATTTACTTAAAAGACTGAATGTAAATAATAGGGCCCGTATATAAATACATATATATCAAATCTG
AGATCGCACATTTATTTTTTAATTTTTTTGTAGATAGACAGACTCTAGT
>57963943_3 CX700800.1
ACTAAAATTTAAAAAAGATAACTTATTGAATACTATGGTATAATCTGTTTAATTGTCAGTGTAACTTTACTAA
TGTATATATTATATCATTTTTTTATCATGTCTTAAATAATACATATATTTATATGATAACTATACACAATAAT
TATCTATTGAATTTCTCATGCATCCTGCCCTCAACACATTGTGTGCTAATGACATAAG
TCATTTGTAACTTTTTTTATAACTTTTCTAAATATTTTTAGTAATAAGTTTAATTTTAATTGTATATTCCTAG
GCTATTTTGTTATAGTTTTATATTTTTTATGTGTCCACTGCTTAAAGATTCTATTTAATGAAATTAGTTAAAG
ATTAAAGAAAACTAGATTTTTTAACATTTTAATGTGTGAATATAACTTTGTGCTGCTT
TGTCATGTAAGCCATCATTTTATTTATGAGTTTGTGAGATTTCATAAAAACCATTGTTGGTGGTGAGAGGTCA
AGCTACAAGTTATTCATATGTGACTAATAATATTTTAGAAACCATATAAAAATTTCAACTTGCAGATACCATC
TAGGGCCGTCTCCAGCCTTAATTTTTTTATACATTTTTAGTTCCTTAGTGTAGACAAC
AC
>57963944_3 CX700801.1
AAATATTTTACTATCCTTAGTGTAAACTAAGAATGTAAAAAATAAAACTAAAATCCCAAATTATTTTTTATCT
CTTCATAAATATTATTTGCAAGATAATAGAATTTAAAAATTGAAAAACTACAGATACTACACACAAATTTCAA
TAAAGTTAGTTTTTAGTTCATTAATAGTTTAACCCTACAAGGCACTTCGACTCTAATA
GTTCCTACAACTACACAACTACACAACTACACAACTACACAACTACACAACTACACGACTACAAGTAGCGGCA
TCTGTTGACATCTTGATCTTCTATCAACAGCAATGTTATAGCTAATTAACGGCTCTTTACCGAAAAATGTTAA
TCGTGTCATT
>57963948_3 CX700805.1
GGTACGGTCGGAATTCCGGGTCGACCACGCGTCCGAGTAGCTTTACTGGAAAAATAAATATCTTTAAAAATAA
TTGAATTATCTGATTTTCATTTTAAAACTATATTTTAAATAACGGATAAGTTGTGTTTCGCAAACAATTAAGA
TCTAAGTAATAATGTACGACGATAGACGTGGTCGCGGTGGACGAGGTGGCTCGAGAGG
CCGGGGTCGCGGTGGAATGAGTTCCAGAGGTCGTTCTAGTGATAGATCTTTAGACAGAACCCCTAGGTTTGGT
GGAGGCGGCAGAGGTGGTAGAGGCGGAGGTCCCCGTGGTGGTGATTCCCGTGACGATTTCCGTGGAAAATTCA
AAAACGATCAACCCGGTGGTGCCTTACGTAAAATCCGTTGGGCTGATGTCCAACTTAC
ACCATTCCAAAAGAACTTTTACGTTCCCCACCAAAATGTCCAAAGGCGATCCATGGCCGACGTCGAGGCGTAT
AGGAGTGAGCATCAAATAACTGTTAAAGGCCGAGATGTGCCAGCGCCTAGCATATACTTCGAGGAAGGAGGTT
TCCCTGACTATGCTATGAAAGAGATATTGAAGCAGGTCTTCCCCAATCCTACACCTAT
TCAAGCTCAGGGTTGGCCCATAGCTTTGTCTGGCCGAGATATGGTGGGCATTGCTCAGACTGGTTCCGGAAAG
>57963950_3 CX700807.1
CGGTACGGTCGGAATCCGGGTCGACCACGCGTCCGGTTTATTTAATTGTGGATATCCGCGGTAATATGTAAAT
ATTTGTCGTAATTTATTATCGAAATCATTAGGTGTAATGTGACTTGTATATTTGGGCCGTCAACTTAACGCCG
GCCTCGTAAGAATGGATTTGTTATTCGCCACCCGCGTGTTCACGAAGCCCATTCCGGT
TGGTAACTAGGCAATTTGTAGCTAAATTAATAATTATTTATTTTATTTTAATTATTTGCCAACGGAATGCGAC
TATTGTACAGATATTAAAAAAACAGTAGACAAATATAGAAAGAATTGTTTTCGCCCTTGTCACATGTGTCGAT
GAGTGAGCGAGTTTAAAGAACTGTAAAATTGTTAGTTTGCAAGTCAGTGTAGATAAAG
GGGAAACATTCTTTGTATAAGAAGGATAGCTTCTTAATTTAGGAAAGGATGTACGCGGACTAATGCGATGTCA
GCTCGTCCTGCTCCACCTCATCGATGTGAAGTCGTTGAGTTAGTGCTTTCAATACACGTGCAGTACACGCACG
CTGCACTATAATCCTAACTTAGTTAAAGTATACGGATAATATCGCCGAAGGTGATAGC

GACAATGTTGAAATGATCGGGCTATCGTCCCGACATTTCTTAACAACTGATTTCC
>57963954_3 CX700811.1
CGGTCGGAATTCCGGGTCGACCACGCGTCCGGCGGTGAACAACATTTCCAAAAAGCGAAAATTCGTCGGTGAC
GGAGTTTTCAAAGCCGAATTGAACGAGTTCCTTACTCGGGAGCTTGCTGAGGATGGTTACTCCGCGCCCGCGC
CCCTCCCGCCGCCCGTCGCAGCCACTGCTTAGACTTTACTATTATAAACATTGAAAAT
>57963956_3 CX700813.1
CTGCGGTACGGTCGGAATTCCGGGTCGACCACGCGTCCGCTAGTTCATCGCGAGCGGCCGCCCTTTTTTTTTT
TTTTTTTTTTTTTTTTTTTTTTTTTTTTTTTTTTTTAAAATTTTAAAATTTTTTAAAAATTAATTTTTTTAA
ATTTTGGGCCCCTTCGG
>57963957_3 CX700814.1
GCGGTNACGGTCGGAATCCGGGTCGACCACGCGTCCGAAAGAAATGATGTGTAGAGTTGTTACTTGCAGTCAA
TCCGTGAAAAAAAATATTTATATAGCAAGTTTTATTTATTTTACACTATTGCTTTTTTAAACTTAGTTAAATA
ATCATTAGCCTGTTTATAGCAGTGTTTACAAAGCTAACGTTATTAATAATAATAATAT
TTTATTTGAAGCGCTATCTTACAAAAATATTTAAATATGGCTTTAGCGTATTTACCCCTTTTTTTAAAAGGTC
AAGTTGTAAAAGGATTTGGAAGAGGTTCGAAGGACTTAGGTTGCCCTACAGCTAACTACCCTCGGGAAGTAGC
ACAGTCACTACCAAAAGACTTAAAGCCGGGGGTTTATTATGGATGGGCACAAGTAGAC
TCGGGGCCAGTTCATAAGATGGTAGTGAACATAGGATGGTGTCCCTTCTACCATAATAAGGAGATGTCTGTTG
AAACACATGTAATGCACAATTTCAAAGGAGATTTTTATGGTTCCATATTAAAAATTTGTGTAATTGGATATAT
GAGATCAGAAAAGAACTTTAATTCTTTAGATGAATTGGTGGAAGCCATTAAGCAAGAT
A
>57963958_3 CX700815.1
CACGCGTCCGGTTTTACGTAACAATACGACGAAAAGGAAGATTCTTAAATTTTCGAATTAAATATTTTTTAAG
TATAAAATTTTTAAAATGATTGGTAAATTGGGCAGACGCCACGCTGAAATTGCGGCCGGTTTCTTACCAACGT
TGGCTGGATTCGGAGGAGGAGCGTTCCTGGGCCTCCTGTACTTCACCGACTGGAGGGT
GTTCGTCGATAAAATCCCGTTTTACAATGGCAAATTCAAGGGTCTTGAGCCATCCGAATAAGGAAAAGAAATA
AGTCATCTACTTTTGTTATTTAAACTAGTATGTAATTATAAGTTTTAGTATATG
>57963961_3 CX700818.1
GCAGTACGGTCGGAATCCCGGGTCGACCACGCGTCCGCTAGTTCTAGATCGCGAGCGGCCGCCCGCGATCTAA
AACTAGGTTTACAGTCGCACACATGTGCTTCTGCGCCACGACGGCCTCAAAATAATTTCTTTTGTATAATTTA
ACGAATATCTACTTAATGTATTTTATGCGAAAATGTCTCAATTCTAAAATTGGTTTTT
TGTTAGAACTTAGAGCTATTAAAGTTAGTATTCTGTAGAAATAAGAATTTTATAATTTATTTAAAAAGTGTAA
TATTGTTGAGACTGTTAATAAATATTTTAAATT
>57963964_3 CX700821.1
CAGTACGGTCGGAATCCGGGTCGACTACGCGTCCGGGACACTTCAACAAAGTCTTGAATTTGATGAAAGAAAA
GTGCGGCATAAATAGATTATCCGTGTGAAAAAAAGTGGAACTTTACTTTAATGATAACCTAACCATTTTGTGA
AAGCCAATAATTTTACGGCTTTTTGTTAAGAAATTAGAAAACATGTTGAATTACAAAT
TTCTAGCATGTTTAGGGATATTGTTCTGTCACAGTTTTTACAATCCAACCGATAGTGGAGCTGTTCAAATTAC
ACAGAGTAATTTAGATATGGTTCTTGCTTCCAATGAAGTTGTGTTCATTAACTTTTACGCTGAATGGTGTAGA
TTCAGCAATATGCTGATGCCTATATTTGATGATGCGGCTGACGAATTATCAAAAGCCG
GCTATGACACTGGCAAAGTAGTAATGGGTAAAGTAGATTGTGATAAAGAGGGTGCCATAGCAACAAGATTCCA
TATATCTAAGTATCCTACTTTAAAATTATTTCGAAATGGTCTACCAGCTAAAAAAGAGTATAGAGGGCAACGC
TCAGTAGAAGCATTTTCTGAATTTATTAAAAAACAGTTGATTGACCCTATCGTCAATT
TTGGTTCCCTAAAAGAATTACATGATTTGAGT
>57963965_3 CX700822.1
GCGGTACGGTCGGAATCCCGGGTCGACCACGCGTCCGAGCTGCTTCTGTTAGCGGCCGCCCTCGCCGTGGCCT
CGGCCTCTATTCTCAAAGACGACCATCTTGTAATGATTGGCAAGGAGCGCTTAGTAAACATAGATATCAAGAG
CAAGGAGGTCCTTTGCATGAAATTGTTGAACTACATCTTGCAACCTACCGTTTACGAT
GATATCAGAGAAGTCGCCAGGGATTGGATCATTGAAGAGAACATGGATAAATACCTGAAGGTTGATGTAGTGA
AGCGCTTCATTGAAGTCTGGAAGATGGGCTTCTTACCTCGCGGCGAGATCTTCGTACACAGCAACGACAAGCA
TATGGATCAAGCTATCTTAGTTTTCCGCATGTTATACTTCGCCAAAGACTTCGACACT
TTCATGAGAACAGCTTGCTTCTTCCGCGAACGTATCAACGGTGGTATGTTTGTCTATGCCTTGACTGTCGCCG
TATTCCACAGAGAAGATTGCCGTGGAGTAGTTTTACCAGCTCCATATGAAATCTATCCATACTTCTTCGTCGA
CAGTCACGTCATTAATAAAGCTTTCATGCTTAAGACGAGCAAGGCTGCTAGTGACCCC
GTCTTATACGACTATTACGGTATCAAGGTGACTGACAAGAACCTCGTCTTCATTGACTGGCGTAA

>57963968_3 CX700825.1
TGCGGTACGGTCGGAATTCCGGGTCGACCACGCGTCCGACTAGTTCTAGATCGCGAGCGGCCGCCCTTTTTTT
TTTTTTTTTTTTTTTTTTTTTTTTTTTTTTTTTTTTTTTTTTTTTTTAAAAAATTTAAAATTTTTTTAAAAAAA
AATTTTTTTTTTTTTTTGCCCCCTTCC
>57963969_3 CX700826.1
GCAGTACGGTCCGGAATTCCGGGTCGACCACGCGTCCGACTAATTCGTTTTTCGTTATTATAATATATATGTA
GAAAGTAAATGAAACTTTTTTAGTATTTCACATATATTTAAAAATGTTTAATTTACAACTCTTAAGTCAGTTT
TCACGCTTGGCGATAAATGCGACATCTAAAGGAACGATAAATACAAGAAATATTGCTA
CATCTGCTATTGTACAAATTAAGCTTACTAACCAATTATGGGCAGAACCTATGAAGAAGAAGAAGAAGATAGA
TCCAGCTATTATAAAAGCTCGTGAAGATAGGCGAAGGAAGAAAATAGAAAAACAAATCAGAAGATTGCAAAAA
AATGCAAGGCAACTTAAACCTATTGAAGAAATGGAACCACCTTTACATATTTTAGATC
AGCTCCGTAAACGCAAAAGGCCTGCAGTTGTTTTATCAGAAGAAGAAAATGAAAGGCGAGCCTTACTCTTTAA
AGAGTGGTCGAAGTATAAAAAGCAAGAGCATATGGCTAACATAGCACAAATTGATAGAATCATGGCGGCTCAA
AGGAGAGCTTTAGATATGCTTTATGAAGAATCTGAGGAATTGTATAATGAAGCAATTA
TGCCTGACTTAAAATTAATACCTTATACTATTAATGGCACTTATTCAACACCACCTATCAAGAACTATGACTC
TCCAGATGGTGAATATGTCGATAT
>57963986_3 CX700841.1
AGAAAAAAATTTAATTTTAAATTTAAAATATTTTTTAATTTTTTATTTAATTAAAAAATTTTTAAAATTAAAT
ATATTAAGGGATAAGCTTTAATTTAAATTTTTATAATATATATATATATATATATATATATATATATAAAATA
AAATTTAAAATTTTTAAAATTTATATTGTTTATAAAATAAAATTTATTATAAAAAATT
TT
>57963987_3 CX700842.1
CCTGCAGGTCCNGGTCGGAATCCCGGGTCGACCACGCGTCCGCCCACGCGTCCGAACTAAGCTTAGCGCAAGT
TTATGAAACCGAATATTTAAAACAAAAACAAGCTCAGTCAGGTGATGTAGAAGAGGAGAAAGAACCGGAAAGT
CATACTGAAATACGTAATGCTATGACAAAATTGTTTGCACAATTAGACGCTTTGTGCC
ATTACCACTATACACCTAAGCCACCACAGGCTGATGTTAAAATTGTTAGTAATACTCCAGCCATTTCTATGGA
AGAAGTTGCTCCTGTAGCAACAAGTGATGCAGCCTTACTTGCACCTGAGGAGGTTAAGAGAAAGCGTAAAGGA
GATCTGATAAGTAAAGAGGAAAGGACACAAACAGATAAAAATAGAGAAAGAAGGAAAA
AGAAGATGTTGCAACAGAAAAAAGGTCATGTGGCAGATGTAACGAAACACAGAAATACAGTGAAAGGACATGA
AAGTAATGATAAATCATTAAAAACATCAAAAGCATTCTTCCAACAACTCAATGATGATTCTAGTAGTTTAATT
AAAACAAAAAAGAAATATGTTAAAAATAAATAATAAATTGTTTTTTTTGTGTGTC
>57963995_3 CX700850.1
GTACGGTCGGAATCCCGGGTCGACCACGCGTCCGGTAAGAAATGAAAATTAATTATTTAAAAATTAACAAAAC
TAAAGACTGTAAATAAGCCTTTTTTTATATTTATCTTAAGTTAGTAGACTAAAACGACGTAAGACTTCTTATT
TTGATCACTTTTCAAGTATTTGTTTCAACATTTACTCTTTTATTAAATAAGAACTTAA
TTGATAACCAATAAATAAACCTTGTATCGGTGCTAATTTGTATACAATACCTATATTTATATTAGAATGCTTA
ATTCATATACATACTAATACATATGTTATTAAATAATACTAAGAAAATATTTCAAAATTACACGAGAGTATAC
TGTTTTATGAAATCTTATGCAACTAGTTTTTAATAATACCGCCTTAAC
>57963998_3 CX700853.1
GCAGTACGGTCGGAATCCGGGTCGACCACGCGTCCGGTCATAGTGGTCAATCTCAATAGTGATTAAAGCTGTT
CTTGGCTTCGAATGTTTTCTTTTTCTATTTGAAATTTTGAAGAATTGAACTTTAAACATATTTAAAGAAATAT
CAAAATTAAGAAAATGGTTTACGTAAAAGGAGATGGCACAGTTGTGGAGAGCAACTCT
TTTAGTATTTCCGGGTGGTTTTGGGGCGCAGTAAACTTTATTTATTTACTATTTCAGACCCTCATCAATCCAA
ATTACAATAAGCATGGGGATAAGTATGTGAGAGATTTCCGTCCACCTGGCAGTGGTCCTCCTAAACCACCATC
TAGGAAGTTTGGTGGATTTGGGCCTTCCAGCTCCGGACCCTCACCTCCACCAATGGGA
GGTGGTGGATGTGGCTGCGGCTAAACCATTATGTGCAGTAAATGTAAACATGTTATCTTCTTCTTATTATTAA
TTTATATAGTAAAATATTCATGCTATTATATAAAGAACTGAATAAAGCAATATTAATCTGAATTAATGCAGAT
CATAAAATTTAAACAAATGTTATTGTAATCACTTCTCTAACTTTTGATTTAATATTAT
TAAACTATACCCAATATATAGTATATATCCA
>57963999_3 CX700854.1
TGTCCTGCAGTACGGTCGGAATCCCGGGTCGACCACGCGTCCGATTACTACGAGAAAATCTATAATTTATAAG
TGTATTCCTCGCATATTTAAGTATATTTATAAAAAGTCTCAGCAACTTAAACTCATCACCTATACAATGTCA
TTTTTAGTCAAGCAATGTATCCGCAGGGTACTGGCCCCAGCTGTAACAGTAGCAAAAA

AATCATGTTTTTCAACTACCAGTATAGTATTAGCTCCAAAAATACAAAAACCAGCTCCTGACTTTGCAGCAAC
TGCTGTTGTAAATGGAGAATTCAATCAATTAAAACTGGCTGATTTTAATGGAAAATATGTTGTCCTTGTTTTC
TACCCTTTGGACTTCACTTTTGTCTGCCCAACTGAACTAATCGCTTACAGTGATAAAG
CTAAAGAATTTGCAAGCATTGACTGTCAAGTGATTGGAGTATCTACTGATTCTGAATTCAGTCATCTTGCATG
GATCAATACACCAAGAAAGGATGGTGGTTTAGGGAAATTAAATATTCCATTGCTGGCAGATTATAAGAAAAAG
ATATCACAGGATTATGATGTTCTATTGGATGATGGTTTTGCTTTACGTGGCCTTTTCA
TAATTGACAGAAATGGTATTTTGAGACACCTGTCAGTAAATGACTTGCCGGTAGGACGGTCAG
>57964000_3 CX700855.1
CGGTACGGTCGGAATCCGGGTCGACCACGCGTCCGGTGTCCCAAAAATCTCAATAAATTAGTGAAATCACTAT
GTCAGACGAATATTTTTACGGTGTCACCTTATCATCTTCACACAAGTCCGAGACATGGGACCCGGAAGCGAAA
GCGGAATATCCACGCAGTAATAAACTGCTCATACGCCAGGCACTGCTGGGACCAGACG
CTAAACCTGACGAACTAAATGTTGTTCAAGTGGAGACCATGTGCCTCCAAGAGTCTATCAAAATCCCTGTGGC
CATCCTCAAAGTTGGTGAAACGAGACAAGCACGTCTTGATATTGAGTTTCCTGATGCACCAGTCACTTTTACA
CTCATACAGGGCTCAGGACCAGTGCATTTAATTGGGCAACATTTACTTGGTGCACTGG
TTGAAGAGTTTGAGGACATGGAGGAGATGGAGGAAGAGATGCTGGACGAGGAGGAAGGAGAAGATTGCCAACT
GAAGGAAGATGAGAATAAAAGGAAAGGACCATCAGGCAAACGCAAGACGAATGAGGATGAGGATGACGAGGAG
GGCGAGCCGAAGGGCAAGAAGTCGAAAATTTCAAACAATGCTAAAGGGAAGGCGCCGT
CGCCCAAGAAGAACGCTAAAAAGTGAACCTTCAACACAGGCGG
>57964006_3 CX700859.1
CCTGCGGTACGGTCGGAATTCCGGGTCGACCACGCGTCCGGAGGAAGTCGATCAGTGCCTGTTAGTGCGCTTT
AACAATTTTATAAACCATCTTAAAAGTGGTTCACCTGAGTTGAACATTGAACAGTCAGATTCAATAGTAATAG
ATGAGTTGTCGATCGCACTGGGCGGCGGCCCCGACGGCTACAGAGCTACCTTCAAAGA
CATCAACGCGAGCGGCGTCGACGACGTCACCATTACCAACGTTAGGTCAGACCTGGACACGTACCAGTTCCAG
CTGACGCTGTCCATCCCACACATCAGCGCCACCGCGCGCTACCGCTCCTCCGGCGTGCTGCTGCTAGTGCGCG
CGTCCGGGGGCGGGGACTACTGGGGGGAGTACGAGAACATAAAAGCCAAGGTGTATTT
CCGCGGTGAGCCCTACAAGAGCAAGGGCAAGACGTATCTCAAGCTGAAACAACTGAAACTGGACTTCTCCGTC
AAGAACATCAAGATGGGAGTGGAGAACCTGGAAAATAGCAACAGTGTGCTGCAAGCCGCATTAAATCTCTTCA
TCAACACGAACTCGCAAGAATTAC
>57964007_3 CX700860.1
CTGCGGTACGGTCGGAATTCCGGGTCGACTCACGCGTCCGCGGCTTTGAATTCATGAAAAATCATAGCCACGA
GTTCGACGTAATCATAACTGATAGCAGCGACCCTATTGGACCTGCTGTCAACTTGTTTCAAGAAAACTATTTT
GCCCTGATGAAAAGTGCCTTAAAACCTAATGGAATAGTTTGTTCTCAGGCCGGAACAT
TTTGGGATAGTATGGATTTAGTTAAAAATACACTGCGTTTTTGTCAGAATCAATTCCCCGTAGCCTCTTACGC
TACTACTTCAGTTCCCACGTACCCTTGCGGTCAAATTGGCTTCGTGCTCGGCTCTTTAGATTCAAATGTCAAA
TTAAATGAACCTGTTACCATCTTTTCTAGTAAGGAAATCGTCGATATGGGCCTGAAAT
ACTATAATAGTGATGTGCATAAAGCCGCGTTTTGTCTTCCTTCTTTCGTGAAACAAGAAATAAAATCGTAAGG
CGATATCGTACTTAGAAATGCATTTATTTTTCTAATACCTATTTCCACTTTTTAAATGAAACTCTTGAATCAA
ATTCGTACACACACTTCCTAGAACGCTTATTATACTAAAAATATTTGATACTAACATA
ATTAATACTAAAAAATATAAATACTACCAAAATTAGATCTAGGGGGCTCATTTAAAATGTGTAAA
>57964012_3 CX700865.1
CGGTACGGTCGGAATTCCGGGTCGACCACGCGTCCGGCGTACTTGGCTGCCAGCCGGTGAAGCCCTGCTTCAG
ATGATTGCCATCCACTTGCCTTCCCCCGTGGTCGCGCAAAAGTACCGTATGGAGATGTTGTACGAAGGACCCC
ACGACGACGAAGCCGCTATTGGTATCAAGAGTTGTGACCCTGAAGCTCCTCTTATGAT
GTACGTGAGCAAAATGGTACCGACCTCTGACAAGGGTCGTTTCTACGCCTTCGGACGTGTGTTCTCCGGAAAG
GTCATCACTGGACAGAAGGCCCGTATCATGGGACCTAACTTCCAGCCTGGCAAGAAAGAGGATCTGTATGAGA
AGACGATCCAGCGTACCATTCTCATGATGGGTCGTTATGTGGAGGCCATTGAGGATGT
ACCCTGCGGAAACATCTGTGGTCTGGTCGGTGTCGACCAGTTCTTGGTCAAGACTGGTACCATCACCACCTTC
AAGAACGCACACAACATGAAGGTCATGAAATTCAGTGTATCACCCGTCGTACGTGTAGCTGTGGAGCCCAAGA
ATCCTGCTGACTTGCCCAAGCTGGTGGAAGGTCTTAAACGTCTGGCCCAGTCTGATCC
TATGGTGCAGTGCATCAACGAGGAGTCTGGAGAACACATTGTTGCTGGTGCTGGAGAACTCCATCTTGAAATT
TGTTTGAAGG
>57964015_3 CX700868.1

CGGTACGGTCGGAATCCGGGTCGACCAGGCACCGCCAATGGCGCCCCAGCACCCGCGGGATCGCCGAGGCCGC
CACCGCCAGGCCTACGGCGCTGGGTAGAAAGGAGCTGTCGCGGAACAGATCGGTCACCAGCCAGACGATGATC
GTGCTCTCCAGCACGAACTCGCCGAAGGACGAGAGCGCCTGGCCCAGCCATAAAAGTG
AAAAATTGCGGCTGATGAAAAAACCGGCAAACCGGCTTTGCCCTTGATTCGTCCTGAACACCATCCCTTCCCT
GTGCGAAACGCTGTGAAATTTTCCGGCCGCTGCGAATTGTTGTGAAGCCGGCGATAAATGGCCAATTGCCCAT
GCTAACGTCGGCTTCGATGTCATCCAATCCATTGAGGGAGCAGCGCCGATGTCTACTA
CCGTAACACCCGCCGGTGGCGGACCGACCACTCAAAAAGCCTCGACGTCGGTATTCGACGACAAGTTGAATAT
TTCCAAGTCGAGCAAGGTGATCGCCGATTACATGCGCCAGTCGGGCAAATCGGCGATTACCAAGCAAGAGCTT
TCGCAACTGGCCAGCAATGCCTNCGGGCAGGTGCCCGCCGATGTGAGCGCTGCTGCCC
AGTACATGGAACGCCATCCCGACGTGTTCACCGCCATTGAAACCCATGACGTGGCGGGTGCCGATAACCTGTC
CGGGGTGTGGAACTTCG
>57964016_3 CX700869.1
GCGGTACGGTCGGAATCCGGGTTCACAGCTGGCGGCCAAGCTGCTGGGTGAACGCCCGCTGACCCGCAAGACC
TTCGAGCGCTACACCACGCTGATGGGGCGTATCCCGGGTGTGACGTTGCAGGCACAAGTGCCGCCACCCGGCA
CCACCGATGGCGGCACGCACATGCAGATCCAGGCCAGCCGCAAACCGTTCACCACCAG
CATGAGCCTGGACGAAGACAACCGTAGCGGCACGCAGGCGCTGCTGGCCGCCACCAGTAACTCCCAGACCTCG
ATGGGCGAGCAATTGAGCCTCAGTGGCCTGTTCCCGCCGGGCAAGGATGATGAGCATTACTACCGCCTGGGCT
ACAGCCAGTTCATCAATGCCGAAGGCAGCCAGTTGGTACTCAACGCTGAACGCTACCG
CGCCGACCCCAACAGCAACGTACAACTGGACGGTGGCTTTGAGCTCAAGCCGCACCAAGCGATTGACCGCTAC
TCCATCGGCCTGTCTCACCCCTTGATTGCCTCCCCCACGGAGTCATTGACCCTTGGCACGCGCCTTTACGCGG
TGGACCAAACTACCCGCTATCAACTCGTGGGTTACCCGCTGCGCTTCGACGTGGAAAC
CGACCTGCGAGCCCTCGC
>57964017_3 CX700870.1
GCAGTACGGTCGGAATCCGGGTCGACCACGCGTCCGGATCGGAGGCCGCATTAAAATCTGGGAACACAAAGAT
GCTTGGTACCCAAGTTGGTTCTCTTCAGATATGATTGTTGATTCTGTCAAAGTATATGCACTGTAATGAAATA
AATATTTTATATTTTTATAAG
>57964018_3 CX700871.1
CAGTACGGTCGGAATCCGGGTCGACTACGCGTCCGGCTTTTATCACTTGGGAATTATTTATTCTACACTATCA
TACCTTATCGAAACGTTTTTATGTGGACTGTAAACTTAAGACTGTTACGAAAGTAATTTTTAAGATGGAATTA
TTAAGGCAAGTGTCGAATACCCAGTTTCTATTTCCTGTGGCAGCTGTAGCGGTTGTTT
TAGTATGTGCTGCGCTCGTATTTATATTCGGATTTCACACAGCAGAACAACCGCAATTCGATAAATTACCGCT
GGTTGCCGATGACAGGAAATCATCCAATAAGAAGAGAAAAATTAAGGAGAAGAAATCTTCTCCTAACCGCGTT
TCTTCGGATGACGCCAAAGCAAAATCTGAGAGCTCCAAGAAATCTCCTTCAAAGGAGA
AAAAAGAGGAGAAAGTAAAGGAAGTTGAGAAACCCAAGCCTAAAGAGAGAGTTGAACCCAAAATCGTAAAGAA
AGAAGCCCCAATTGAGGCTAAAAAGGGTAAGAAGAACAAGGGTGCTGTAGAGGCAGAGAAACCTGTTGACTTT
GATGAAGGAGTGTGGGAGGAAGTGCCCAAAAAAAGTGACAAGAAAAAAGTTGTGAAAC
CAGAA
>57964021_3 CX700874.1
GCGGTACGGTCGGAATCCGGGTCGACCACGCGTCCGAAATAACTCAAAGAAAAACAAAATGAGCGTATTTATT
AGGAATATTAGGTTAAATGCCGTCTATAACGCCCAGATATTGTGGCGTTTTAATTCAACAACTGCTGAAACGT
TGAAACAAGGTGACGATGAAGAAGAATTTCGCGTCCTCGATATTTTACAGAAAAAAGA
TAGACAGAAACGTAAAGTAGCACGCAGGGCCGATATACAGCCAGATAGATCGGAAAAAATGCGTACAGACCAG
AATTGGGGCAATGTCTGGCCAGGTCCAAGAACCTTTCATCCTTCATCAGTACCACTACCTTTAAGACAAGGTT
ATGTACCAAAAGGGCAAGCACCACCTGGCAAAAAAGCAAACGCCGAGCTAATGAAAAT
ACCAAACTTTCTTCACCTCTCACCTCCTGTTATAAAGACACAATGCGAAGCAATCAAGCAATTTTGTACTGAA
TGGCCAAAACTACTAAGTTCAGAAGAAGCTATAGAGAAACACTATCCTGTAGAAGTAATTACATCTGACTATT
GTCATGCCAGTCCAACCATAAGGAATCCTTTAGCCCGTATAGTCACTTTGAGAGTTAA
ATTGGCAGATTTGAACCTAAACACGAGGGCCAAAG
>57964023_3 CX700876.1
GCAGTACGGTCGGAATCCGGGTCGACCACGCGTCCGGGTAGATGTGCAGAAATCATTCTTCGTGGTACAGTCA
TCGGAAAAATTGGCGTTATCCATCCGAATGTCTTAACTGCCTTTGAATTAACTAACCCATGTTCTGCAGTAGA
GTTCAATATTGAGCCATTGTTATAAAGTCAAGTTATCTTTGATTTTCGAACCAGGCCA
CTGTGTTAATAGTAACCTACTTATTTATAAAAAATGTCATTAAACAGCATTTTTACAAATT
>57964024_3 CX700877.1

CAGTACGGTCGGAATCCCGGGTGACTACCAGGTCACTCGATTCATTCACAGGGCATCGATCGCCTGGCGCCAG
GTTTTCAGGTCGAGGCCACCGCGCCGGACGGCCTGATCGAAGGCTTTTCGGTTCCCGCGGGCAAGGCTTTTGC
TTTAGGAGTGCAATGGCACCCCGAATGGCAGGTAAGCTCTAACCCGCACTACCTCGCA
ATCTTCCAGGCATTTGGCAATGCCTGTCGAAAGCGGGTGATGCAACGCGACGCGGATGCTGCGTCAACTTACG
CCTGACCTTTAATGGATGTCAGGAAACACAGCCCAGAGGCATTTATGAGTAACAACCTCGACCAGCTCACCGA
TTGGTTGAAAGACCACAAGATCACAGAAGTCGAATGCATGATTGGCGACCTCACCGGC
ATCACCCGGGGCAAGATCTCGCCGACCAACAAGTTCATCGCCGAAAAAGGCATGCGCCTGCCCGAAAGCGTTC
TGTTGCAGACCGTGACCGGCGACTATGTCGAAGACGACATCTATTACGAATTGCTCGACCCGGCCGACATCGA
CATGATTTGCCGCCCGGACGAGAACGCGGTGTACCTCGTGCCCTGGGCCATAGAGCCT
ACGGCCCAGGTGATCCACGACACCTACGACAA
>57964025_3 CX700878.1
GCAGTACGGTCGGAATCCGGGTCGACCACGCGTCCGGACTAGTTCTAGATCGCGAGCGGCCGCTCGCGAAAAA
ATCAAATATATAAAATATATCTACATTTTACTTAAAATATCTAAGCTTCACTACAAAAATGTATGTCTGTCTT
TTCATAAAAAAAGTAAAAGGCTAAAAGGTAATTCGATCTCGACGTAACAGAAGTGAGG
GGCCACAACTAATTTAATATAGGAAGCGAAAATGTGAATATTTTAAGTGTAGCACCAAAAAGCATTTATATTT
TAGTTAATTAATGTTTTTATATAGGATAAGATTTGAATAATGGTTTATATTAATGTTATTATGCGATATTAAA
AGCTGGTTTTCTCGATT
>57964031_3 CX700882.1
CAGTACGGTCGGAATCCCGGGTCGACCACGCGTCCGGTACAGGGTTCCCGCTTTTGAGGTTCAAGTTGGACTA
CATGAGTTGCTTGGACACGGCAGCGGAAAGTTGTTGCGACAGAACGCTGATGGCTCTTTCAACTTTGACAAGG
AGAAAGTTAAAAATCCTTTGACTGGCAAACCAATAGAATCCTGGTACACCGAGGGTGA
AACATACGACAGCAAGTTCACCACCCTCGGCTCTGCGTTTGAAGAGTGTCGCGCTGAAGCGGTCGGATTGTAC
CTATCTTTGCAGCCTGAGATATTGAAAATATTCGGTTACGATGGTGAAGAAGCTCAAAATGTGATTTACGTGA
ACTGGCTCAGTCTATTGTGGAATGGTGCTGCTAAGGCCACAGAGATGTACCAACCTGC
TAACAAGACTTGGTTACAGGCACATGCTCGAGCACGTTTTGTACTGATGCGTTTACTAGAGCTAGAAGGTTCT
GGGTTGCTCACAGTCACAGAAGTGGAGCCTGGGACAGATTTGCTACTTACCCTTGACAAATCCCGCCTCGCCA
CTGACGGGAAGAGGATCATTGGCGACTTCCTAGTAAAATTACAAACGCTAAAAGCAAC
GGGCGACGTATCAGCCGCAGAACAACTGTTCGACCGCTACAGCCGCCT
>57964034_3 CX700885.1
GCGGTACGGTCCGGAATTCCGGGTCGACCACGCGTCCGGTATTATGGCTCTCTAAAGATATATGTTAAGTCTT
ATTATTTTTAAATATACTAAGTACGGGCACTGATATTAAATCAATTGTAAATTTATTCATGTATGCTGCTAAA
ATACTTAATATTGATGTGTACTAAGATGTATTTGTAAAGAGAAATAGTCTAAGAAAAA
TGTTGTGATATTTGTATTTGGAATATTTATAACATTTGCCTATGTAACAAATTCAAGTTGTAAGCTAGTAAAT
CTACAGTTTTTCTAAATTAATCTCAATTATTATTGATTGAAAATAATATAAGACTCAAATATTTTTACGAAAG
AAAAAAATTCTATCAATTGCTGCCTATAATTCATAGTAATAAGTATAAATTGTAATAA
AAATGTCCAAGGAGTTGTTTTACATGTAAATTCTAAATATAATAGTACTAGTAATGATCTCTTTTTTTTGGCT
CAAAGTTTTATAAAAT
>57964035_3 CX700886.1
GCGGTACGGTCGGAATTCCGGGTCGACCACGCGTCCGCTCGAAAGAAAATGTCTGCCTTAGTCCACGAAAGAG
TTTGGGTTGACAAAAATGTCTATAATGATGCCGAACGAGCTTATTACGAATCTCTATCAAAAAAACAGGCAAC
GGGTATAGCTAGCTCATCATTAGCTAGTGAAGTTGCAAAAGCTAGACAACACATTAAA
AACTCACTGGAATGTATGGATAGTGTTTCGGCATTAGCTGGTGTTCCAAATACTGAAATTAACAACAAGTTAA
ACAGCCTAGAAAAAGAAAATTTTGAATTAAAAAAAGTTGTTGATGATTTGCGGAAAATGGTTATAAGTCTACA
AGCCAGAGTTGAGTCAATTGAATCAGCTAATTCTGGAAATAAAGCTGAAGCCCCTAAG
GCACCAGTGCCTCAGGCTCAAGAAGATTCAGATGATGGAGTTGATTTATTCGGTTCAGATGATGAAGAAGAAA
GTGCAGAGGCAGCTAAGATAAGAGAAGAACGCCTTGCTGCATATAACGCCAAAAAATCCAAAAAGCCAGCACT
TATTGCAAAATCCAATATTATATTGGATGTGAAACCTTGGGATGATGAAACTGACATG
AAAGCAGTTGAAGATGCAGTAAGGAGTATCAGTAATGATGGTCTTTTATGGGGTGCCGCCAAACTGGTACC
>57964045_3 CX700894.1
CTGCANGTACGGTCGGAATCCCGGGTCGACCACGCGTCCGCCAGTGCCGAGTCATGGGGTACAGGACGTGCTG
TAGCACGTATCCCCCGTGTCCGTGGTGGTGGTACCCATAAATCAGGCCAGGGTGCGTTTGGCAACATGTGCCG
CGGTGGTCGCATGTTCGCACCAACCAAGCCGTGGCGACGTTGGCACCGCAGAGTCAAC
CTACGCCAGCGCAGGGCTGCAG
>57964047_3 CX700896.1

GCGGTACGGTCGGAATTCCGGGTCGACTCACGCGTCCGATTCTAAATAAAAACTATTTCATTTAGAAACATTG
ACGATTGAAGTTTATATTAGTACAAAGTTTTGTGATATCGGAAGCTATTTTAAGTCAATATTATCAAGATGAG
GTCATCTATAGTATTCTTAGCGATACTATACTATATTGTCGGGATACAGATGGTCAGT
TCAAGATCAGTTCACGAGAATGATGGTGAAAAGCATCGCCATCGTCGAAGATCATTTTATGATTACGACAGAT
ATTATGAAAGAATCGATTATGGCCGTCAACATGAGGAGTTGTTGAGTGAAATAACGCGCTTATTACAAGAACT
ATCGTCATATGTAAGACGACCACCGAGCCCTCCACCACCGCCTCAAATTGTTTATGTG
CCATATCCTGTTAACTTTCCAGTGCGCGTAGCGTGTTCGGGAAATAGCACAAATTTAGAAAACCGGTTTAAAG
TGGAAGACGAACGCCAGATTTGGAATTTAGTACCTGGTGATAATGGACTTAATATGGATAATGGCAACGATGG
TGCGAGACCTATATCTTTCGATATCTTTCAACGGCCGGAGTCAGCTCCGGAGATGCCA
CCAGTCGAGCACGGGAGCAGTCAAGCTGGGGTACTATAT
>57964048_3 CX700897.1
GCAGGTACGGTCGGAATTCCGGGTCGACTCAGCGTCCGATTTATTACAGTATATACACGAAATATTTTCAAAT
TTAAAAGAAGAAGCCGAAATTATCAAATCATTTCTTATTTTTAACAAATTAGTACCGTTTTTCTATGATTTAG
TCCATTTAAATAAAGTTTCAACTAACAAATATATAGTGGAATTGTTAAAAGAAAAACA
TGATAAATTCAAACGTAATCCAAAAAAAGTGCCTGATTTGGATACGCTTGTATTTTTTAAATTAATATCAATT
TTGTATCCCACTTCGGATTTCCGACATCCGGTGACGACGCCGTCTTTGTGTTTTATGGCGGAAATACTAACTT
TGTCGCGATTTAGAGACGCTCAGTCGATTTCTAGAGGATTCTTTATAGTTGCATTAGT
CTTAGAATATACGTTTTTATCAAAAAGATACGTTCCGTCCGCTTTAAACTTTCTGAGAGGCATTTTCTATTTA
TGCGCAAACACTTCTATTTTAAATCCAATTCAGGTAGTACCGCCATTCCGTTTACATAAAGACGTCAAAATTT
TGAATTTAGAAAGCGACTGTTCGAACACTGACGTCTATTTAAAAATGGTGGCAAAAGA
TTTAGTTTTGACAGACGTTAATGATG
>57964050_3 CX700899.1
CAGTACGGTCGGAATTCCGGGTCGACCACGCGTCCGATAATCCAAGAATTTGACGTCACAGAATCTAGTACAG
ATAATACAGAACACACATCCAATGTTGCAATATAACAATAGTTTCATAATGCCAGTCATCATCCGATGTTGA
ACAACCGGATGTTCAATCAAACGGTTAATCATCCTATGTTTAGGAATGGGAACTACGG
GAATGTTAATAGACACACAAACAGTGAAGACAGTGCGAGTGATACAGACGAATATGCAGGTTTGATGTCGCAA
AGGGAAAAACAATGGCTGATAAACATACAAATGCTGCAGCTCAACACTGGAACACCCTATATACATGATTTTT
ATTATACTGTGTTCTTAGAAAGGCAAGC
>57964051_3 CX700900.1
GCAGTACGGTCGGAATCCGGGTCGACCACGCGTCCGGTTGGATATACTAAAAAAGCCTTTAAATTATTTAATG
ATATGAAAAGAAGGGCTTTAAAACCTACTGGGGATACATACACATGTTTATTTGAGGCATGTATGAATAGTCC
ATGGCCTGCTTATGGATTAAAAATGGCTACACATTTAAGAAATTCAATGATTGAGAAA
GGTATAGAACCAAATATTACCAATTATAATGTAATGATAAAAACCTTTGGTAGATGTTTAGATTTAAATACAG
CATTTAAGATTGTGGATGAAATGGCATC
>57964052_3 CX700901.1
GTACGGTCGGAATCCGGGTCGACCACGCGTCCGCACTGTTAAATAATAAGATGTTTTCTAGTTGTGTTTTTAT
TGCTTTTAATATGATGCTTCCGTCATCTCTTAATTTGAAGTCAAGTGCTTCTCAACAAGTGTCTTGAACACCA
CAACTTCCAATAGTTTTACTTATTTATTAACAATATATAAGTTTATTAATGTTAGCAC
CCATTTATCAGTTTCTTATCATTTTGCTCGAGATAAAACACACCTAAGTAGCATATCTTTTTATGTTTTGTAA
CAGAATTGTTGTGTTTTACGGTATTAATATTAAGTATTTATTAAGTTTATTATAAAAGCTTAAAATTTCAAAA
TTTGTTTGAGACATTTTATCAAGATGGCATCAAGTGATCCAGAATTAAAGGATGGATT
TACTCTTCCTGTTTGGATGAAAAGTAAACCAACCAAGGAGTTTGAACCTTTCGCTTCATCACCACCAGCTCCA
GAGGATTCGTTCTTCTACATCCGCTATCCCAAACCGGACGTCCTATTCGGCAAAACAAAGTTCAACAACGATC
TTCCTAAAGTCCTGACTGAACAACAGGAGTTGTCAGCGACATACAACGTGATCAAAGA
GAAGGATTGGTCAAAACACACCAAGCCCTGCCAAGAAGTTTTGCACGGCA
>57964054_3 CX700903.1
GCAGGTACGGTCGGAATTCCGGGTCGACCACGCGTCCGCAGGAAACGACTCTGAGATGCAAACTGCTCAACAG
AGTTTCCCGAATGCAGTACCAGTGCCATATAATTGTGCTCAATGCCAAGCAAGCAGAGATATCTATCGATACT
TTGGCAGAGTAAACAGTGGCCGTAGTGTAGATCTTAAGGATTTGAAGGAAATTAAATC
CTTAACGGAACTAGAAAGTTTATTGAAAGATCAAATAAAGCCTCCAAGAAAAAGCTTAAGAAGAATAGCGGTT
CACCCAAGGGTCCTCGACGATGTAACTAATGATAAAAAGACGAAACAACAAACGAAAGCGTAAAATATGTCAT
AATTTTAAGTATTTAGTGGAAGTGTTAAAGGGAAAATAATTTTGAAAACGTCTGACGT

TATCTTAGACGATTTCAAAATTTTTATAGTTGATGCTTAATATTTATTTTCTGATTAAATCAGAAATTTATAT
ATTTATTATATACGTCTATCGAATCATCAACTCCCGCCACAAGTATGTAATATTTACATTTGTAACTTGTATT
CATATTTTTAGTCATATTCTTTGTATACATTATATTAAATCTTGTATACGTATTTATA
TTTAATACAATGAAATAAATATTTAA
>57964057_3 CX700904.1
GCAGGTACGGTCGGAATCCCGGGTCGACCACGCGTCCGACTAGTTCTAGATCGCGAGCCGCCGCCTTTTTTTT
TTTTTTTATTAATTAAATAATATTTTAAACCCTAAAAAAAATAATAAATATTTTAAAAAAATGGGTAAATTTC
TTTTCCAACATAAATATTTTATTTTATCATACCAATAACGAGGTAAATTCCCCCAACC
TC
>57964059_3 CX700906.1
ATGTTCCTGCAGGTACGGTCGGAATTCCGGGTCGACCACGCGTCCGATGGGCTCATTTACAAGCAAATTTGTA
CGTTCATCTAACATGGCGAGCTCCTCAGAAATTCAACAATTTATTAAAGAAGCTGTTTCTCAAGAAAAAGTAG
TTGTGTTTTCAAAATCTTATTGTCCTTATTGTAAATTAGCGAAAGAAGTTTTCCAGCA
GGTCAAACAACCCATAAAGGTTTACGAGCTTGACGAGCGCAATGATGGAACTGATATTCAGAACAACCTAGCG
GAAATTACCGGTTTTAGGACAGTACCTCAAGTGTTCATCAATGGCAACTGTGTGGGAGGAGGATCCGATGTTA
AGAGCTTGTATGAGTCTGGAAAATTGCAGCCAATGCTTATCGGATAACTTTTATGTCA
AATAATGAAATAAAGCACTCACAATAAATATATTTTTGTTCTATATTTATATATAAATTACTATAGAAAAGTT
TATTAAATTATTGTTGCCAAAATTCATCTGTGTTGTATTTTATAAGTAGATTGGTTTTAATTTTTTTTTGCAT
TTAACTTTATTCTAAAATGTTTACAGCTTTATTATGTTACAATTTTATTATAAAGATT
TATTAAAAATTACTTAAC
>57964060_3 CX700907.1
CTGCAGTACGGTCGGAATCCCGGGTCGATAGGAGCTGTTCCTGGGCCACGTAGACCGCCCGGGCATCCATCCG
GCGCCGTACCTGGCACGGGCGCAGTGGATCATCATGGAAACGCCCTACCCGCTGGGCGCTGAGGAACTGCGTG
GCCTGTTGCAGCGTTCCCACCAGTTGGTGGTGAGCAAGCTGCCCAAGCGCACGCAGGT
GGGGCTGAGGCTGGATGACTGATACACAGCCATCAGGCCAAAAAAGCCACCCTATTGAGTGGCTTTTTTGTAT
CTGAGGAGGAATCAGCAACCCGTTTCCGCAGCGGGTTGTTACCGTAGTAGCGGCTAAATAAGCACGCCCCCGC
ATCGACCGTTACTCCAGGGGCAAACCTTGCTCCACCAGTGAGGCCAATAAGCCTGCGC
CGAGGGGGATGATGCTGTCGTTGAAGTCATAGTGATCATTGTGCAACTGACAGGAACCGGCGCCCTGGCTTGG
GCTGCCTCCCTGCCCCAGATAAATATAGGCGCCCGGTCGTTGTTCCAGCATGTATGAAAAGTCTTCGGCCCCC
ATGGTCAAGGGTTCGTTGCACTTCACGTTTTCCTCGCCTATCAAGCTACGGGCAACCT
GGGCGGCGAACAGCGCTTGCTCAGTGTGATTGACGGTCGGCGGATAGCCTCGCTGGTAATCACACGTGGCCGT
GG
>59637383_3 DN048386.1
GTTTGAATGCACTCCTCGTTGCATGTCCCGCGCGTCGTTATGAGAACCCCGTACTGGCGATCACGTTGACGTC
GCGCGGTGCCGCCATATCTGTCTCGGCCACCAACTAGCAAAATTAATAGGCTCAAAAAAAAGTTTCACATCAC
TAAAAGAATGCGATGACCCACGCATTCGCTTTTTGAAATTACGCCTTACCCTGATGGA
ATGAGGAGTGCACTATCCTATTGTTTCACAGTTTCTTATGTTTGAACATCATTTGTTTTTTTGTTTTTCGGAC
AATTAGCGAGTTGTCATTTTCTTCCAAAATTACAAAAAATTGTAAAATAAGAATGGCGGAGGGTAATGGAAAA
ATATCCCTAAAAGATGTCTCCGGGAAGGACTCAGATGTTGGCCGGACACCAGATTACC
CTAAACTTATAGAATATGGCTTGGATCCAAAGGTAGCAACCAAGCTCGATGATATTTATAAGACTGGAAAGCT
AGCGCACGCTGAGTTGGACGACCGTGCGTAAGATGCCTTAAAAGAATTTCCATCCGACGGTGCTTTAAGTGTT
CTAGGACAATTTTTAGATTCAAATCTCGAGCATGTATCTAATAAAAGTGCTTATTTAT
G
>60203152_3 DN172636.1
TGATCCCCGAACAGCTAGATCGGGATCGCATCAGCTCGATTATGTAGAAGCGTCAGCTCGTAGCGTGGTACGG
TCAACAGCTCCGTTAGGCTGAATGATTGCCAAGCTCGTATGCGCGTAGCATCTGTCCATAGCTCTAGCCGAGA
TTCGTACTGAGAGTCAAGCTT
>60203154_3 DN172638.1
CGTCCGGGAAATCAGTACAAATTACTAAAAATGTATTTTTTAACGCGGTTAGGACAATGTATTATAAAACAAT
CGATACTTTCTTCTTCTGTACGTTCAATCTCAACAACTAAACAATTAAATCTCAAAGAACTTCGCGAACATAA
AGAAGGCACAACGCTGGTGGTAGAAGGAGTAGATGTTCCTTCTCCGCGTTCACAATAT
TTAGTACGCGCCGAAAACTTTATAGACTTGGCTCATGATGGAGAAAACTGTCAAACCTGCTATATGTGCGCTT
TAAAGCTAAATGTAAAACATACTGATGTTGTGATCCTTAGCCAGTTTGTACGATCGGATGGGTGCATGTTGCC
TCGCCGTATAACAAAGCTGTGTCGTCGACAGCAGAAAAAAATTGGTAAAATGGTCACA

ATGGCTCAGAAAGCAGGTTTGATGATAAATTTGACTCCATCTTACTGTAAAAAGGATCCTAAGCAGAGATTTG
GCCCTAAGAAATTTAATACATACTTTGATGAGAAGACAATATTTATGAAATATATACCAAAACCTACAGATAG
ATTTAAGAGATAAAATTTAATAATAATAAAAATATGAATAAGTTGGAATAAGATAATT
AAACAATAAATCTCATAA
>60203157_3 DN172641.1
AGGTACGGTCGGAATCCGCGGTCGACTCAGCGTCCGCCCACGCGTCCGAAAAGTGTGACGAGCGTTTGTTTTA
TCAATTTCTAAAAACCAAATTATTTAAAAAATGTCGACGCTTGCTGCACCCTTAACTGTTGCTGGAACCAGAT
ATTCTGGAGCCCCAGTTAGAAGTCAGAACGTAATGGCAGCTGCAGCCATTGCAAATAT
TGTCAAAAGTTCTCTGGGTCCAGTGGGTTTAGACAAGATGTTAGTTGATGACATTGGAGATGTCACAGCAACT
AATGATGGTGCCACTATTCTCAAAATGTTGGAAGTTGAACATCCCGCAGCGAAAGTATTAGTAGAGCTGGCTC
AACTACTAGATGAGGAAGTTGGTGATGGCACCACGTCTGTTGTCATTATTGCTGCTGA
CTAATTGAAGAACGCTGATGAGCTACTCAAAATTAAATTTCACCCAACCAGTATTTTTTCTGGATACCGTCTA
GCCTGTTCGGAAG
>60203158_3 DN172642.1
GCAGTACGGTCCGGAATCCGCGGTCGACCTAGCGTCCGGCCCTCATCAAAGTCAAAGCCAAACGGCAAGGCCA
CAACTGAAGTCAGGGCACCAATAGCGACATCAGAGTCTACAGCCGAATCACCAGTAAAAATGCAAGCATCTCA
AGCAGCAGCCGCTCCAGTTCCTACACCGACATCGACGCATTCGTCAAGTCAATCTTAC
AGACACACAAAGCGCACTGCTAAAGATTATATATTTGGAAAGCTGATCGGTGACGGCTGCTATAGCACAGTAT
TTTTGGCGAAAGATATTCACACTGGCAAAGAATATGCAATTAAAGTATGTGAAAAGAGTCATATCGTACGCCA
TCAAAAAGTACAGTATATTAAACGTGAAAAAGATGCTCTTAACATGCTCGCTAATGTC
CCGCATGGGTTTGTGAAACTTTACTGCACATCTCAAGATGAAGAGCGACTGTATTTCGTGTTGTCTTATGCTA
AGAATGGAGAATCATGACATTATATAAACGTAGTAGGCTCCTGTGAACTAAATGTAGCGAAATATTATGCTGC
TGAGTTATTATGAGCGTTAGAAAAAAATGCACGC
>60203165_3 DN172649.1
TCCTGCAGTACGGTCGGAATCCGGGTCGACTACGCGTCCGATAACATTCGTTGTTTAGTGATTTTATACAATA
AACTGTTAATAATGGAAGAAATTGGAATTATTCTACCTGAAAAGGATGATCAAGCCCCGGACACCAAGGGACT
TGCATTTGTAGGTCCACAAACCTTTGATGAATTAGAATCAGAAGATCTTTACACAAAA
TATAAGAAATTACAAAGAATGCTTGAATTCCTTGAAGTACAAGAAGAATATATTAAAGATGAACAAAGAAATT
TGAAAAAAGAATACCTCCATGCTCAAGAAGAGGTTAAAAGGATTCAATCAGTACCACTTGTTATTGGACAATT
CCTTGAAGCTGTTGACCAGAACACAGGAATAGTCGGCAGTACAACAGGTTCTAATTAC
TATGTGCGCATCTTGTCCACCATATACTGGGAGTTGCTGAAGCCGTCAGCATCTGTCGCTTTACACAAACATT
CTAATGCATTGGTAGATGTGTTGCCCCCAGAGGCTGACAGCTCTATATCTATGTTACAAGCAGATGAGAAACC
TGATGTCCAATACTCAAATATTGGAGGTATGGACACACAGAAGCAAGAGATTATGGAA
GCTGTAGAACTGCCGCTCACACATGTTGAGTTGTATCGTCAGATCTGTATTGAGCCT
>60203171_3 DN172655.1
CGTCCGTGTCGCTGTTACTAAGCCCTATTATTATATCAACGAGTGTTTCTAGAAATTTTGAACAATTGAATTT
ATTTTTATTGTAATTCTTACATATTTTTATTTTTGAAATTTTGTATATGTTCTAGAAACACTGTGTTTTGTTT
ATACATAGATAGGATCTTAGTGCCAACTTTAACTAAATATTTTTTATGGTAGAATCTA
AAATGTGTTAAATATTATTATAGTAAAAAAATATCATATGTTTAATTTCTACACAGTGCCATTTTTTTTGTCC
CTTAAATTTGTGCATTATATTTATAATTCGATTGCATTTATATGTATTACTTATTAGATCCTATCTAATATCT
ATTGTAAATTTTGGATACCACTAGAGTAAATATAAAATTATTTTGAGATGTTGAATTA
ATTAAAATACTTTTATATTATGTATCAATTCTTAATCAAATTTATGTATTGTAAATCTTTTTTTTATTATTTT
TCAGTATCGGCCAATGTCGATCATAATTATTTTGTACAAATGTATATATTATTCCTAATGGTTAGTGAGGAAG
ATTTTATCTGTATGTACTATACTGATATAATTGGTGCCGCCAGTACCATTTAAGAACT
TTTGTACAATATTACAATAA
>60203173_3 DN172657.1
TACCGGTCGGAATCCGGGNTCGACTACGCGTCCGCTGCAGCCCGACACGCTATACCGGACCATGAAGTTGTTG
GCATTATATGGCAGTGCGTTATGTCCCGCGGGGAGTGGAACAAAAAGGAGGAACTGTTGGCGGAGCAGGCGGC
CAAGCATTTGAGACATTACACGCCGTTGTTGGCCGCCTTCGCGCAGTCTGCCAAAGCG
GAGATCGCGCTTTTGACTAAGGTGCAAGAATACTGCTACGAGAACATGAGCTTCATGCGCGCGTTCAGTAAGC
TGGTGCTGATGTTGTACAAGACGAACGTGCTCTCCGAGGAGGTGATCCTCAAGTGGTACCGGGAGCCCAACTC
CAGCAAGGGGAAGGTCATGTTCCTCGACCAGATGAAGAAGTTTGTGGAGTGGCTGCAG

AGTGCCGAGGAAGAATCGGAGAGCGGCGAAGAAGAAGATTAGAAATAAAGGCAGCGTGAGACTGAACACATAG
CGCCGTCCCACACACACACACACACACCCAACAGTAAACCAGGAATCATATACCAACCCACAACCAACCACAA
ACCACAACC
>60203185_3 DN172669.1
CGGTCCGGAATCCGCGGTCGACCTACGCGTCCGAACGCGAATATATGTATAGATTATATCAATTAATTGAAAA
TATATAGTTAAATATAATTTATAGTGAGAGCTATGGGGAGATACGCGAGTGTGTTTCTGGCGGCGTCGCTGCT
GGTGCAAGGATTTTGTATACCACCTAACGTTGTGCGACCTAGGAGGCAGTCAGAAATT
AGTGATAATTCAATAGCGGAATCAAGTAACAGTGTATCAAGTAGCACAGTCAATTACGACCTGCCTCCACCAA
TACCTTTCAACCACGATGATGATGAAGATGATGATGATTTAGACA
>60203196_3 DN172680.1
CGTCCGGCGGGATACACGTGACGCTAAAGCTAAGCTAAATGAGACAAAGATGAAACATTAATATTCAATTGGA
GCTAAATGATTCCGGCCCTCTATCTTAGAAAACGTGTAACTACGAAACAGATTTTAATGCGATTTTCACTAGA
GAGGGTAATTAGAGGATAAGGTTAATAACGACGTAAAAACACTTATTTTCAAAACAAT
GTGAAATACGTCAAAGATAGCCGAGAATACGCTGTTTCACCAAATGATACAAATTATATATTAGTGTATTTCA
ATATGACAAAACTACGAGTGGAACTACCTTATTTATGTAACTGTATGATTTCCTTAAAAGAATGTAATAAATT
ATTGTATTAAATTATGTGAATAGTACATATCGAGGGCGGGAATTAATTAACCCAAAGA
AAAAAATGG
>60203199_3 DN172683.1
TCCTGCAGTACGGTCGGAATCCGCGGTGACATCATCACGCTGCCAGTGATAAACCCGTTCTCCATCGCCAGTA
CGTACCCGGCAGCCACTTCTTCGGCCTGCCCCACTCGCCCCACCGGCAATGCCCCGCCCGCCTTGGCAAACAT
TGCCAGTCGCTGCTCCTCGGATAAGCCCGAATACGCCTCGGTATCGATCACCCCAGGA
CTCACCACATTGACCCTGCGTGGCGCCAGTTCCTTGGCCAGTTGCTTGCCCAGCGCTTCAGTGGCCGCATTGA
TCCCGGTCTTGATGAATTGCCCTGCAACCGTTTTGCGCCCCAACTGTCCCGACGTCAGGGTGATGCTGCCGTG
CTCGGCGAGAAAGGGCAGCGCCGACTGGATCGCCCGCAGCGAACCCCAGAGTTTTACG
TTGAAGTTGTCTTGCGCCAGGTTCAGGTCCGTCTCGATCAATGCCTTCGCCTGTACCGCAGGGCCTGCGGTAT
AGACCAGATGATCAAACGGCCCCACCGTTTCGAACAACCGCTGCAACGAGGCCGCGTCGGTCACGTCTACCTG
CTCGCTGCGCACACCGTTCTCCACGGTTGAAGACAAGCGTCGCCCGGCCAGCACCACG
TGGGCGCCGCGTGCCACGGCCTGCTTGGCCACGGCGGCACCAATGCCGCTGCTACCGCCGATGACGATGATGG
>60203200_3 DN172684.1
CGTCCGCAGCGGTTTGCTGTTATTAATTCACATTTCTGTTTTATTTTTACATCTTCAACAAAATGCCCCTCCA
ACTAACTAAACCGGCGCCCCAGTTCAAAACCACCGCGGTGGTCAACGGCGAGTTCAAAGACGTAGCTCTCTCA
GACTACAAGGGCAAATATGTTGTGCTGTTCTTCTATCCCTTGGACTTCACATTCGTAT
GCCCAACCGAAATAATCGCGTTCTCAGAACGCGCAGATGAGTTCCGCAAGATCAGTTGCGAGGTTATCGCGGC
GTCCACCGACTCACACTTCACTCATCTCGCTTGGATCAACACTCCGCGTAAACAGGGTGGTCTCGGCCCAATG
AACATCCCAATAATTAGCGACAAGTCCCACCGCATCTCGCGCGACTACGGCGTCCTTG
ACGAGGAGTCGGGCATTCCTTTCCGCGGGCTGTTCATCATCGATGACAAGCAAAACTTGAGGCAGATCACTGT
TAACGATCTGCCTGTGGGACGATCTGTAGAAGAAACCCTAAGGCTGGTGCAAGCGTTCCAGTTCACCGACCAA
CATGGTGAGGTCTGCCCCGCCAGCTGGCAGCCCGGTGCCAAGACAATCAAACCCGACA
CTAGGGCCGCCAAG
>60203201_3 DN172685.1
CGTCCGGGTGAACAACATTTCCAAAAAGCGAAAATTCGTCGGTGACGGAGTCTTCAAAGCCGAATTGAACGAG
TTCCTTACTCGGGAGCTTGCTGAGGATGGTTACTCCCGCGCCAGCGCCCCTCCCGCCGCCCGTCGCAGCCACT
GCTTAGACTTTACTAATATAAACATTGAAAAT
>60203210_3 DN172694.1
CGCGTCCGCTCACGAGGAATACTAAGCATCATGCTCAAATACGTTGCCGTCGTTTGTGTGCTCGCGGCATTGT
GCACTGGAGCTCCTCAAAATCCACAGGATGTACAGATCCTACGGTTTGACAGTGATGTGCAACCTGTATGGCT
ACAGCTTCGCATATGAGACCAGCGATGGTACGTCAAGACAAGAACAAGGCAAGCTTGA
TAATCCACAATCAGAAAACGCTGCTCTTACGGTAACCGGTCAGTATTCATACGTAGCTCCAGATGGCAAGCAC
TACACTGTCCAGTTCACCGCCGGCCCCGAAGCCTTCCAGCCTATAACTTGTTTGGGTCAGAAGTAATTTAATA
TATAATTCTGGTGTACCTTTGCCCATGTAAATAAATATTTGGTATATACCAGTGAAAT
CACTATTATAAGAACAAAATACTCGATCTCAACAATTGCTTATCGCACAGCTACATACGAGAATCACTGTATT
TAGTGCCATAGGATATCTTTTGTATTACTACTTTTGTAAT
>60203222_3 DN172706.1

TGCAGTACGGTCGGAATCCCGGTCGATTCGACGTCCGCTCCTTTCGCTATTTTTCGTCTCCGATTTATATTAT
TGTTATAATATACGTTTTACAAATTTTTCAATACAATGGCTGAGGAGGTACAAAAGAAACTTCAAATAGAATT
CGATGTTTTCAACAATTTGAAAAAAGAATACCATAAGGCCGTTGCTCAGAAACAACAA
CTAGACAGTAAATTAAATGAAAACAAAGCAGTAAAAGAAGAACTTATGCTACTAAAAAAAGATGCCGAAGTCT
ACAAACTTCTAGGTCCAGTGCTAGTGAAGCAGGAATTGGAGGAAGCAAGACAAAATGTGTCTAAGCGGTTAGA
GTATATTAGCAAGGAAATCAAGAGATCAGATGGTCAAATTTCCGCAATAGAAAATAAA
CAAGAACTAGTTCAGGAAAATGTTAATAAATTGAAAAATGATCTTGCTAATTTAAAAGTAGCTTAAATAAGAG
TAAAAATATATTAATATTTAACTTTTGTATACCAGTTTGTTGGAGTAATATTATAAATGTTCATTTATCCAAT
ATTATAAACTAACATCAGCTGCTATGAACAAAAGGCATTTGTGTGTAATCTATTTATA
CAGAATCTAATCATTACAGTTG
>60203224_3 DN172708.1
CGGTCGGAATTCCGGGTGACCACGCGTCCGGTTTTAATTTCATTTTTGACGGATATTCTAGTTTTTGTATTTT
CGATATACGATTGTGTAATATGATTCTAATGTTACCAAGAACGATTTTAAGGGGAAATATCCTCAAAATTTCT
CCGAGATTTCATAGCGAAATCTCTGGCCAAAATCCAAGTGCTACTTCATCGTCGGCAC
CACTACCACATGCTAAAGTTAACGCCAATGTTCCTGGTCTTAGCAGTGCAGTCATCCATCAGACCTCTGAACC
GTTAGGCCCAGGTGTAGATCCCAATAAAAGTGGCCCATATAAAGTACCGGAATATTTTTGTTACGACAATATG
AGTTATTTTGAAGCAGAAATAGAAATGGCGAAATTTAGATTACCACAACCTTCTGCTG
TGAAGAAGTAGAATAATAGAAAATATACTTATATTATTTGTGTTAGGAAACTCATAAATATATGTTAATAAAT
TATAATTTCAAACCT
>60203245_3 DN172729.1
CGTCCGGATGAAGGCGTGGTTGGTGGTGGCGTGCGCACCGGCGCTAGCAGCGTTGGCGTCTGCTCAAAAACCT
GGAAGGTTCCTTTCCCTACCGATACCGCAGAAATGCGCTAACAGACCCAAAGAATTTGCTTATCGAGGACATA
ATTACTTCTACACCGGCCACGTTCCTGAGCTCGCTAATAGGAAATGTTACTGGCTCGA
TGGACGTAATATTTGTCGTGAATATTGCATGGATCTTGTTTCAGTGGAAACACAAGAAGAAAACAATTTGATT
TTCAAACTAATACAACAGAGTGATGTCCCGTACATCTGGACCTCGGGTCGTCTCTGTGACTTCAAGGGATGGG
AGTCTCGTATAGATCTCGAGCCTAAAAATATATTGGG
>60203246_3 DN172730.1
GTCGGAATTCCGGGTCGACGCCGGGCAGCATCAGGTCGAGGATCAACGCATCCCAGCCGCCTTGTTCCAGCAG
TTGCACGGCCTCGTTACCGTCGGCGCTGTGCACCACCTCGAACTGTTCGTCCCGCAGGTGCAGGCAGATCAAA
TCGGCAATGTGCGCATCGTCCTCGACAACCAGGACACGTTTGGGCTGATCCATTGGCA
AAACCTTTTCTCGTGATTCGCGCATTGTGCGGGTTTTGCAACAGTGTAGTTATCACGAATTGTTTAACTCTGC
GTGAGGATTCCGCGACCAGACCGGGCCTAACCTTGACTCATCGCAATGGCATAAGTACGCAACCCCATTGTGG
CGGGGGGATGCACGGGCTGACAATCGTCAGATACGCCGCCCAAATACGCGATAGCCAA
AAGCCACATGGGGTATTCCCTTATAAGAAGACATCGAATTAAGCCGCGGTGAACGTCATGGCTGCGCCGTTCA
TGCAATAGCGCAGGCCGGTAGGTGCCGGGCCGTCATCGAACACATGCCCCTGATGACCGCCGCAGCGGCGGCA
ATGGATTTCCTTGCGTTCCACACCGAAGGAAGTGTCAACGTGGCTGGCCACGGCGTTT
TC
>60203247_3 DN172731.1
CGTCCGATTATATATCATATTCTTTAATGTTCATTATTAAATATCTATTATAAAGCTTTTACATTATTTTTAT
TATATTATAATATTAATATTGGTAATAAATTATGGTTCCATTTCACGTAACTCGCGTAACTTGCTAGTATTCT
CACATTCACAAACTTAGCTTAAGTTCTTTAAGTTATGAAAATTAATAAAAGTATTAAC
GCT
>60203249_3 DN172733.1
GCAGTACGGTCGGAATTCCGGGTCGACGCCCATTATCAAAAAACATTGAAAGAGACTGCTCTTTTAGCGGGTT
TTATCTTCGAGAGAAATAGCCAATCATCTGTCCATCCCGTTCATCACGTGTCTGGAGTCGAATATGTCCGCTG
CCTTTAACGTCATCGCCACGCTGATCGCCAAACCCGGCCAACAGGCCACCCTGGAAAC
CCTGCTGCGCGAGCTGCTGGAACCGACACGCATCGAAGCCGGCTGCCAGCAATACGACCTGCACCAGGACCTG
CAACACCCCGAGACCTTCTACATGCTCGAACGCTGGAGCGACGACACCGCGCTGGCCGACCACGACCAGAGCG
CTCATATCCAGAATTTCCGCACCAAGGCTGCCGACGTGCTCGAGCATTTCGAACTCAA
GCGCCTGAAGTATCTTGCCTGATCAACCGCCCCCTTTATGGAAACCCTCATGAAAGCCAGCGCCTACTACGCT
GCATAGCCCATCAACGATCCAAAATCCCTGCAAGACATCGAACTGCCACAACCGGT
>60203252_3 DN172736.1

CTAGCGTCCGCAACGACCAGGCCAAGCTCTATCACCACAACCGGCTAATCCCAGCCCTGTACCACAAAATCCT
GTAAGTCAAAGCGCACCTCCGAAGAACCCTGAAAGTGTACCTCAACCGCAAGCAGATGCGGTTCCCGTGCCTG
CATCTAACCCAGTTGCTGCATCAGTCCCAACCTCACCTGTAGTAGCACAACCAGTGCA
GTCCGCTGCCGCTTCTCAACCTGTTGCCGTTCAACAAGTGGCTGCCCAAGAACAACCTGCTCAAGGCGGACCA
ATACAACAACTAGTTCAAAATAATCCCATTATCAAAGGCATTCAATCCGCAGTACAGAGAATACAAGGAAACC
CGAGCTCAGCAGATACGCCACGATATGAAGTTGTTGTAGACGAG
>60203258_3 DN172742.1
CGTCCGTATATTTATATCATGAAATATCAATTATCAATGTTTATTCTTATATTTGCAATAGTTGGGAATTATT
GATGGTTGTAAAATTTTAATTTGACGGCAGCATCCTATACTATGTTCACTTAAATCTATAAGATTTGACTGAC
CGCAGCATAGTGTCAAATGAAAATTTTGCAATCTGGCAACACATTCCTATCTACATGT
TTAACTTGTTTTGGTCCTGGCAATATTGTTTGTAGCTTGCTTTCGATACAGTACTGAGTGTAATCACTCTTAA
AATTTCGTATCTATGTATGTTTCCCATAATGGCACTGTTAAAATTATTATGTAAATATATAGATGTGTTAGGT
AACCAAAAAAAAG
>60203261_3 DN172745.1
GTCCTGCAGTACGGTCGGAATCCGGGTCGACCTAGCGTCCGTAATTAACACCGTAAAAAACATTAAATATTGC
TACAATGAACTTTGTTAGCCGACCATATTTTTTATAGTTACAATTTTAATTCGAACGGAGCCGTCGGTGGTGT
TGTGAGTGTTCAAATAAAGACATTAACAAGTATGCCGTATATAATGGTTATGGGCAGC
CTCAGCGCACCGCATCTGTCCAAGGATGAAGGTGCTACGGTCTACGGCCTGAAAAGTGAAGAACGGGCAGCGC
TAGTTAGGGAATTAAACTCATCATTTGGCATGGTGATAGCTACATCATCGGACTTGGAGAGAACAGTTCGCGT
TACACAAAAAGGTCTAACCCTGGTGGTGGTCAACCGGATCCACGGTTTGCTGGGCTAC
GACGTGGTCTCCCACGCCATGGCCATGACCAACGCCAATCGGGAGCTCATATCTTTCACCATGTACAAGAAAA
CCAGTAGTAATTCCCATCAATCCCACAGTCACGGTCACAGCAGTTCGCACAAAAGCCATTCACACAGCAGTGT
GGTCACCCTTTGATAATGGTCCATTGTTAGTCGCGACTCGTGACATACTCATGTNTTG
TCATACGTCTGTATTGCAACTGCAGGCCGATAGTCGATTTCAATGTTTTAAGTTAGCGTCGCGATCGAGCGAT
GATAATTTATTGTGATCAAACGGTTTCTATTGTATGTATATATG
>60203264_3 DN172748.1
TCCTGCGGTACGGTCCGGAATTCCGGGTCGACCTACGCGTCCGAGCTTTGTGATTCAGTTTTAAAATTGAAAC
ATACCAATAATATTATTGTACACTTTTATGGACTTCAAAGAATGTGATAATGCTAAAAAGAAGTTAAACTATG
TATCTGAAAACTTTTATAAGATTTTATGTGTTGAGAACTGTGCCACAATCTGCAATAA
TTTATATGAAACATTATCATATTCTTTAAACCTCAAAAAACTTGCAATCTGCAAATTGGAAAAAGATGTATAT
TTTAGTTTTCTTGCCCAATGTGAGGAACATCTTAAAGAAAATGTTGATTTTATATGTAATAGTGTCTTCCACA
TAACAGTAAAAAGATTTTGTTTAGTTAAAAATAAACAATTTGCAGATTATCAGAAATT
GTTAAAAAGGAAAATAGATATATTAAAAATAAATAATTGTATATTAATGTTAGATTTAATTTGACCAACACTG
TATTAAGTTCATTATACCTACTTTATTAATTTTATCTTTGTCTGTAATTTTTTTTAAAAAATAAATTCAATAA
CACAACAATCAAGTACCTGCCACAATCTTATAAGTAACTGCTAAGTAACTGCCCACAA
TCTTATAGGTTAATAAATATATAAATTATATATTTTTTGCAGATTATGTTCTATTTAACTTATAAAA
>60203267_3 DN172751.1
CGTCCGAGGAGATGGAGGAAGAGATGCTGGACGAGGAGGAAGGAGAAGATTGCCAACTGAAGGATGAGGATGA
CGAGGAGGGCGAGCCGAAGGGCAAGAAGTCGAAAATTTCAAACAATGCTAAAGGGAAGGCGCCGTCGCCCAAG
AAGAACGCTAAAAAGTGAACCTTCAACACAGCCGGCTCCGCACCCTGCCACGCGCCTG
TCCTCGCTCGCCTCAACATTGCACCAGAAAGACTGATACGCTCTGGTCCACAGCGGCAGGGCGCCGGAGGCGG
GGCGCGGAGCCCTAGTTTACTCATAGTGTGGTAATTCTCTAGGTTAGCGTTGTATCTCGTGTGTGCGAGCGTG
GGTGTGTGCGTGTACCGCTGGGTGCAGTCGTATATCGATCACGATTGAGTGTGATTTA
TAAACGTTTTTTCTTG
>60203273_3 DN172757.1
CGTCCGATGATACTTTGCCACATATCTATTGCACATGATACTTTGCCAAAACTAAGTTTGTGCAATATAAAAA
TATATATATAAAGTTATAATATTTTTAACAAACTTATAATTTCACATAGCTTAATAGCCATATGGCTTAGTGG
TTAGAGAGCTTGACGGTTTCCCGGAGGAGCTAGTTCCGATCACAATATGTTCAATATT
TTATGTGTGCTAAGTCATAGTCCTTGAATTACTACAAAGCATAGTATTGTAAAAATGTATAATAAAAAAAATG
CATAAACATGTGTTAATTTACAAATGAGAAGACTACGTTTGTACTATACATATATTGATTGAAATTATATATG
TATATGACGATTACTTTGTAAAAAAATCTAAAATTAGTGTGAATGTATTCATTAGCTT
GAAAATTATGTGATAAAATTATAGCGAGATAAGAGAATCTCGGCCAAATGCATTAATTTTCTGAATCAAAAT
>60203275_3 DN172759.1

CGCAGTACGGNTCGGAATTCCGGGTCGACTCACGCGTCCGGTTGTATCTTGAGTGGTGTAATTTTTATGTTGT
GTGAGCATGTTCCTAATTTTCTAATTTTGTAGTCGTTAATAATTTACGTTACTTCTTTAAATTTATAAATATA
TTAATAATGTCGATATGTTTTCTAGTTAAAAAAAGTAAAGTAATTATATAAATTTAAA
TTCAATGTGTCGATTTACGTGTGGTGATAACGTTGTCAAGCTCAGCTGGAACGATGAGCGGTAAAAATTTCGC
GAGTTTTTCTTAAGTCACGGAAAGCATTTTTTGTGCTCTATTTCTTCAGCCAAGGATACTGAATGATTAGAGT
TTCTCCATATTAAATATGGGTTTGGTAAAGTAAACACAAATAAAATATAATAAAAAGT
GAATAAAGTGCATCAAAATGGATGACAAGATTGAAACAAAGCAAGAATCAGATCCTCCAGCGCCATCCTCAAC
GTCATCAGAAGCAGCATCAACAATAAGTAATGCTCCTTCAAGTACACCAAAGTCTGAAATTTCACGACCATCA
ACATTTGCAAAGCCATCTGGCCTGAAACCACCTACAAAAATTGGACGACTATGCTCGA
ACAGCGCGCCGAAACCAGCAGTTCCTATATCTCCAAGAAGTGACGGCAGCTCTACGGAC
>60203281_3 DN172765.1
GTACGGTCGGAATTCCGGGTCGACTCAGCGTCCGAACAACGACTGAACAAATAAAATTACAGGTTTTGGTGGT
GAATAACATTTCTCAATAACTTTAAGGTTCTATTTATTGCATTAAATGATGGCCTCGCCTTTAGAACAATTTG
TTAATAATGTGAGAACAACTTCTTCAGCAGGCAATTTTCGTGAACTTTGCGATATTAT
CGGAAAATCAGATGAAGTGTTACAACGGAACAGTGCCCATCTTAATACGGTTTTGGAGACATTGGACATTCAA
CAACATTCTCTGGGTGTATTAGCAGTTCTTGTTGCTAAATTTTCTCTCCCACAAGGAAATATAGATTTTGATA
GATCTACTATGTTCCAAGAAATTCATGAGTTCATAACCAATTGTAATGGAGAACAAGT
ACGATTCTGTCCTGAGTTGTATGCTGACTTATGTCATTTATTAACAGAACATCTCGTTGAACTCAAGCAACCA
ATTCGAGGAATTGAAATTTTAAAAAAAGCTATTAGAAAAATTCAACTTTTTGATTCTCAACTAACATCAATTC
ATGCAGATTTGTGTCAATTATGTCTTTTGTCAAAGTGTATGAAGCCAGTGCTAGAATT
TTTGGAAACAGATGTTACAAGTATTGGCTCAGAGCTTGGTGGTGTAAATGACTC
>74326481_2 DT662302.1
GCACGAGGGCTTACCCTCTTTCGAAACTATAGATATTATTTGACAAATAGTAATAGATCTATTGAAATCGGAC
TAAGTAAATTTGTAAAAAAGCATATTAGAAGGCTTTCTAACTAAACTGTAATACTTTTTATGAAAAAATGACG
AATGAAACACCAGAGGTAGTTAAAATCAACAAATGGGATGGTGCAGCTGCTAAAAACG
CAATTGATGATGCTATTAGAGAGGTTTTTACTGGAGAATTGAAATGTAAAGAAAGCTTTGCACTGATTGATGG
ACGCTTATTCCTTTGTGCTTTGGCTGTTGGAGTTGCGCTTTATGCCTTACTTTGGGACTATTTATATCCGTTC
CCACAATCAAAATTGGTCCTTATAATTTGTGTTTCTTCATATTTTATCCTCATGAGTA
TTTTAACACTTTATACAACATTCAAGGAGAAGGGCATTTTTGTGGTCGCAAAGGAAAAAGTTGGAAATAATAA
CAGAGTTTGGGAAGCAAGTTCTTATGTTAAAAAGCATGATGACAAATACAAGTTAGTATTGGTGATGAAAAAT
TCTAATGGAAAGTCCCGAGAAGCCTCTATCAATAAATCTTTTGCTAATTTCATTGACA
CCAATGGCACAGTGGTCCAAAATCTTATTATAAATGAAATTACTAAACTTTACAACTCCCCTTCTGCTGAGAA
GAAGGAAAAGTGATTGTCTCACATGCACAAATACATATTATATAATTTAATCTNCATAAATTATGTGTAATGC
T
>74326719_2 DT662540.1
GCACGAGGATTTAACCCTAAGAATTCTTATTATAAATATATTTTGATTATTATAAGTGGTGTAATATCAGTGT
TATTGTGGATTGATTATTTAATTTACAAAGAAATTTAAACTACCTAATATAAAAAGTGTACTTCAATATTGAA
ACAAGTTAAATATGTCTCGACTTGATAACATTGACGCATTAGAAAAACAATTAGCTGA
CTTGCAAATGGCTAAAGATTTACATTCATCTGGAAAAATAAAAAAAATACCACCAGCTGTACCACCCAAAAAG
AAAGCCCAACCTCAAGTTCCTCACAGTGCTGTCGTTACCGCGGTGCGCGAGCCTTCCTATTCTGCGAAGATAA
CTCCGTTACAAAATCTACATAATAATGTTGCACCTCCGCCTCCACCGCCTCCGCCAAC
CTATAGTAATGTCGAACGACAGCCGAACAATATGCATAAAGATTACGCCAATGTAACACAACTGTCACTTACA
AACCATTCCTTTCGCCATAATGCTCGGCTGTCG
>74326780_2 DT662601.1
GCACGAGGGAATTGTCTTGAATTAAAATACAAAAATGGTATTAGTTGTTATTTTACGTGATAGTTAGATAGTA
TAGTGATTTTTGTGAATAACAATGTTTACATTTTAAAAACTACTTATAGTTAAAGTTTTTACTTGTTTGTAGG
TATAAATCATCATGAGCGTCATCATTAGACTGCAGAATCTGCCGTGGTCGGCTAATGC
TCTAGACATTAGGAATTTCTTTCGTGGTCTTTCAATACCTGAAGGAGGTGTACACATCGTAGGCGGCGAGCTT
GGAGATGCGTTCATTGCGTTCAGGTAGCTATATGAAAACAGATTATAAGATACTTTTAATATTTTTAAAGTTT
ATGCCACACTTAGGAAAGAAATTTACTTTTCTAGAATACATAAAAAAATTATGTAGCA
GGATTTGAAATTATGTACAACATAAGTTATAGATGAACAATTTTATAACAATTTAGACATATATGAAAATGTG
TAAATTGTTTAGTGAGTAATTTTCAAGAGTTACGGAATCAAACCCCGCTATGTTAAAAAAAATATGTTTACTA
AAAAAGTATAAACTATGAGATATTTAAAATGAACATTAATTATTACATTTACATAGAA
CTTCGATATTTATCATCTAAGCTAGATTAACATTCTTGGAATAAATAAAACAGTTATT

>74326799_2 DT662620.1
GCACGAGGCTCATACATACGATTATATTTTTGTAGTTAGACAAAGTGTACAAGAGTTTTTACTTAACACAAGA
GTCGTTGTAAGAGATTTTTTCCCCCAAACGATGTTAATACAAGGTTTGTTAATGATGTCAAGTAAAGAGTTTC
TGAAAATGGAACAGTGTTGTTAGAATCGCGTGAAAATCACGATATAATGATGTCGGCA
CAAGTACATGACTTGATCTGAAGGATTAAATTAAATTATTTTTACACTAAAAATAATTTAGTATGCTGTGCTG
GAAACCAGTCGTGGCGCAGAGGCTTAAGCGTACTACTGTAAGCGAGACAGGTCGTGGGTTCCATTCCCACGGC
GTTAAAGCGCGGCGTTGAATTCCGCCACCAGATACGCGGCGTAAAGCTATAAAAAAAA
TAATATGTAGCACGCAGTGTTTGAAAACTGAAGGACTTACAGGTACTGTCTATATAAAAAAGGGACGAACATA
ATTACATTAATAACAATAAAAAAAAACATCAAAATAAATATCTAAGCTTATTCAAAATCCTCTCCAACTTAAA
AATCTTATAAGCAGGTTTTGAATTTGTTGCATATTTTAT
>74328143_2 DT663964.1
GCACGAGGATATATCTTTATTGTAGAAGGAAAACCATCTGAATTTTGAATATGGTAAAACACATCAATAAAAA
TATACACCTCAAACGACGTATCTCTTCGTTAATGTTAAGTTTTAAAGATATTTTCCCTTTTTTATTGTTATTA
GACGTAAATAGTAACCTTTTGTGACTTGAAAACATTACGAATTTTGTGGAAATAAAAT
ATTCATTATGGCTGAAGATATGAAGAAGAAAGCGTTATTTGTGTGTCTTGGTAATAGCTGTCGGTCACCAATA
TCCGAAGGTGTGTTTCAACATACAGTTAATAAAATGGGCAAAGGACATGAATGGGAAGTTGATAGTGCAGCAA
TAGCTGATTGGAATGTTGGTTATCCACCTTCACAAAGAGCAATAGATACCATGAAAAA
GTATGGAATAAGATATCATGGGATAGGAAGACAGATAACAACTGAAGATTTCAATCATTATGATTATATATTT
GGCATGGATGGAAACAATATTATGAATCTCAATAGCTTAGCACCTCCAGGTCATAAAGCGAAAATACTTCTGC
TAGGAGATTTTGATCCAGAAGGCGACAGCATTATAAGAGATCCTTATTTTGACAAAGA
TGCCAAAGGCTTCGACAAGTGCTATCATCAAGCAGCAAGAAGTATAAAAGGATTTTTGGAGCAGTTAGACAAT
GGCAATATATAATGAACACAAAGTGTTATTAAAAAATCATAATAAGTAATATTTTTAATATATAACCCTTTTT
ATCATGCTATAATAAATTATAATAATAAATATTATTTTTGTAAT
>74328144_2 DT663965.1
GCACGAGGCCTCGCGTTGAATTCGGCACGAGGCTGTAAACAATTTCACTTGCATAAATATAATGTTAAGTGTT
ATTCGAAATACCCTAAAGAAATCTATTAATAATGTGGTGCGCACCTTACAAACCTCTTCACCTGTAGCCACCG
AAAAGTTTGTTCATAAAAGGATTCCTTTAGATGATGAAGGTGTTCAAGGAGAGAAAAT
CGTTGATTTAGATGCTGTCTTAAAGTCAAAACAAGGTTTGTTCCCTACTGCTGAATCTGATAATCTGTTATTT
GGTGGCACCCCGTACAAAAACCTTGCGATTTGCAATGTGAGAGTTAGCCATAATAATACAATATTTACTCTTA
CTGATGCTGAAGGGACAGTTAAAATGATAAGATCTTGTGGTATGGATGGTTTTAAAAA
CACTAAGAAAGGAACTAATATAGCTGCTCAAACTACTGCTATTAATCTAGCATTAAAAGCTATTGATAGAGGC
TATAAGGTTATAAGAGTAAAAGTTAGGGGTTTGGGTCCTGGAAGACTGTCAGCTATAAAGGGCTTGCAAATGG
GTGGTTTGGAAGTAGTATCTATCACTGACAACACCAGAGTGTCATGGAATCCACCAAG
ACCAAGAAAAGCTAGAAGATTGTAATTTATAAATAATATTAAAATTAATTAGTTTTATAGTGGTAAATAAAAA
TATGCTAATT
>74328196_2 DT664017.1
GCACGAGGTAGCACCTCTATCACCACACACCACTTCCACTTTAGTTCAATGTTAGTCCACTGGTTTGTTATAA
ATGTGCCAGTATGTCGATTTCGGAATTGGGTTGGTTTATATGGGTGTCGTTGAGCGCTGGGGAACCTGACACG
GTGACTATGACGGGCTGGGCCTCAGTGTGGACTATTGTCACAAGGTGCTCATCCGGGT
CCTTCTCCTGTTCTGTGGGCTTGGGTAAGGCTTCTATGGTGTCGTCAGCATATTTGAGGTCTAGTGGCGTGAG
TGTGGGCAGTGTGTCTGAGACCAGCACGGGGGGCTCCGCCCGAGGCGCGCGATATGAAGGGGGTCCGCTGTAT
TCGGACCCACACCACGCGGCTCCCGTTGTTCCTCTGAGTCTATACTCTTGCATTGAAG
CTTGGTAAGTGGGGGGCGGAGGCCTATAATGGAACTCCGGGTACAGCATGGCCGCGTACGGGTGGTGTGGACG
AGCATAGCTCGGGGGCAGCCGGGGGACGAGCCGACGACAGGACGCTCGTCGGAAACCTAATAATGTACAAGGG
GCAT
>74328614_2 DT664435.1
GCACGAGGGAAAACGTCAAAATGGTGAACGTACCAAAACAGCGCAGGACCTACTGCAAAAAATGCAAAGTCCA
CAAGGTACACAAAGTATCACAGTATAAGAAATCCAAGGAAAGGCACGCTGCCCAAGGTAGAAGACGTTATGAC
CGCAAACAGCAGGGTTATGGTGGTCAGTCCAAGCCTATCTTCAAAAAGAAGGCCAAGA
CAACCAAGAAAATTGTGCTCCGTCTTGAATGTGCTGACTGTAAAGTGAGATCCCAAGTTGCACTTAAGCGGTG
CAAGCACTTTGAATTGGGTGGTGACAAGAAGAGGAAGGGACAGATGATCCAATTCTAAGCTGATTAATAAATA
AGTTTAAAATCT
>74330073_2 DT665894.1

CGAATTTAAAATCAAATTTGCGATTACACATAAGAAGTCATACAAAAGTTATGTGTTTTGAATGTCAATTTGA
AGATTGTATTTACCGAAGTAGTTCAAAAGCATCTTTAAGAAATCACATGTTAAAACATTTTCCAGAAAAAAAT
ATGTTGTTTTGTGACAAATGTAATTACAAAACTGTTTATAAGCATTCATTGAAAAAGC
ATAGTGATAGTCATGAAAGGAATTGTACAAGAATAAAGTAAAGGTTTGATAAT
>74330082_2 DT665903.1
CGGACGAGGTGTAAGCTCCCTACTTGCACCTGATATTTAAACAAAATGAAATATATCTTTCTACTAGTTGTGA
TAGTTGCTGCAACAGCTGCAGACATTAGCAGCTTGCAGCCGTTCTTATATCATCAGCAACCACAATTACAACA
GCAACAACAGCAGGTGTCCACGACGGAACCCATTCCCATCATAAGACAAGAACAAATA
CTCAACCCTGATGGATCTTATAAATGGAATTATGAAACCGGAAATGGTATAAGTGCTGAAGAAACTGGCTTCA
TAAAAAATTTGGGTATTCCTGAACAAGAATCAGCGGTTGCACAAGGACAGTACAAATATACAGCACCCGATGG
ACAAGTTATTCAGCTACAATATGTAGCTGATGAAAATGGCTTCCAGCCGCAAGGTGCT
CATCTTCCTACACCACCACCAATCCCTGTTGAAATTCAAAAAGCTTTAGACCATCTCGCCACACTTCCCCCAC
AAGCGCCTGAGCAAAATCTATATAAATTATTATAAATCAATGTTACAAAGAAACATAATTTATTATGACCATA
AGAGGCTACAATACAAACACAGGCATTAAAGTATAGTAAATTATATATAAGTGTGTGA
CTTAGTTTTAAGTACAATAATAATTTATTTTATATAGTGGAGCTATTTAAAATTACATCGAATAAACAATTTG
ATGTT
>74330714_2 DT666535.1
GCACGAGGCGTATCACTGTCACACCTTTGAAAGACAATAATAAAAATTTATATCTTAATACGAAAAGTGCTAT
GTAGCGTTTTTAAATTTTAAATGTTGCTTGAATGTGTTCAAATTATATTTAACTAAATGTTACTATATTATTA
CTGTTATAATTAATAACATGTTGAGTTTTCAAATTCTATGTATGTACTTAATTAAAAT
CTTGTTTTCTGT
>74331427_2 DT667248.1
GCACGAGGAAGAGCGTACGTGGTGGTTCCGCGGGAGCGAAATTCCGCATCTCACTGGGTCTCCCGGTGGGAGC
CGTAATAAACTGCGCAGACAACACAGGGGCAAAGAACCTGTATGTGATTGCCGTGCAAGGTATCAAGGGTCGC
CTGAACAGACTGCCAGCCGCTGGTTCAGGGGACATGATTGTCGCCACTGTAAAGAAGG
GTAAACCAGAACTAAGGAAAAAGGTAATGCCTGCAGTGGTGATTCGGCAGCGGAAACCGTTTAGAAGGCGTGA
CGGGGTGTTCATATACTTTGAGGATAACGCAGGCGTGATAGTAAACAACAAAGGTGAAATGAAAGGCTCAGCT
ATCACAGGACCAGTGGCTAAGGAGTGTGCGGATCTGTGGCCCCGTATTGCATCCAATG
CTAGTTCTATAGCTTGAAGTTAATATATGAATAAGG
>74331581_2 DT667402.1
GCACGAGGTGCCGCTTCAGAAAATAGTAGATCAAATACAAAAATGTTGCGTTCAGCTTCCCGTTCGTTAGTAA
ATGCATTTAAAACTACCGTCGCCCCTACATCCAGGGTTGCCGTCGTCGCCATCAGGAAGTCTCATGATGGCCC
CGTGGAGTCCGATGAAGACTTCGATAACAGATATGAAGCCTACTTCAACAGGAAGGAC
ATTGACGGTTGGGAAATTCGTAAGGGAATGAACGACCTCTGCGGTATGGACTTAGTTCCCGACCCTAAGATCA
TCAAGGCAGCCATGCATGCCTGCAGGAGAGTGAATGATTATGCCCTCGCTGTGAGGTTCTTAGAAGCTTGCAA
AGACAAGTGTGGGCCCAAAGTTGGAGTAATCTACCCATACATTATTCAAGAAATCAAA
CCTACTCTTAATGAACTGGGAATTGAAACACCAGAGGAACTCGGCTACGACAAGCCTGAATTAGCATTCAAGA
ATGTCTATGAAATGTAGTGTGATAATAAGCTGTAGTGGAACTTAAACAATAGTTATGTTATTAATTTTATTTA
ATTGAATGCTGTTCAGCTTAATAATTCCATTGCCACCATCTTAAATGTTGAACACTTG
ACTGTGAAGAAGGTTATTTAAAATGATTTGTTATTTCATTCAGTCTTACTAAAACATGTAGAGATTTTAATAA
AAAGTATTCAATAC
>74331856_2 DT667677.1
GCACGAGGCCTATCTGCTATAGCCGCATTTTATATCACAAAGAAAAAGTAATCTTTGAATAAAGAAATAAATA
CATAGTATATTAATATATATTATTAAACAAAATGAGCGAGGATAAAAAACCGCCAGTGGAGCCTGCAAAACCT
AAAATTAAACATGACTGGTATCAGACAGATGCACTAGTAGTAGTCACAATTTTGCTCA
AAAATGCTCCTAGTGACAAAGTGAAAGTTAATTATGGAGATAAGAGTCTTTCAGTATCAAGTGTAATACCAAA
CTCAGATTCAGAATATAGCTTAGAATTAGAATTGTCCCATGAGATTATACCATCAATGTGTACACATGTAGTG
TCTCCATCTAAAATAGAAGTTAAGTTGCGAAAGAAGGAAGGTATAAGATGGAATCTTT
TAGAAGGAGAGAGTCAGAAGGAAGAAATTAAAGCTATACCACAAGCTGTTGTAGATGCCTCTGGACCTCAGAA
ACCACCAAAATTATTCAAGAAAGACTGGGATAAGATAGAAAAAGATATTATAAAGATGGAAGAAGAAGAAAAA
CCACAAGGAGATCAAGCTTTAAATGCGCTCTTCCAAAAAATATATGGGGAAGGTTCAG
ATGAAGTGAGAAAAGCAATGAACAAGAGTTTTGTTGAATCAGGAGGTACCGTATTAAGCACTAATTGGGATCA
AGTGGCCAAAGAAAAAGTTGAAATGAAACCACCAGAAGGCATCGAGTACAAGAAATGGGATTAATATTTACAA
AAGAGATAATTAATTAAATATATACTTTAATATTATT

>74332120_2 DT667941.1
TTTTTTAGTTAATACATTTTATATTAGGTACTTTATAACAATACTTTTAACAGTCTTAAAACGAACGACTTAG
TGACAATCGGCGGTCAACGCCAGTCTATATATACAATATGTACAAGAAGTCAAGCTTGCCAACATAAAGGAAA
ACAAGGAGGTCACTTTTGGGAAGCTTTAAAGTTCATTTTTGGCATTCTGGGAGTAGAG
TCAAAATATGGTCAATAAGAAATGTCATTAGCAACTAAACAACTGTGAGGGATGTGAAAATTCTCGACGCGGG
GTTAGGGGGTGCACTGTATCTCTGCAAGCCTGAACACTGTCCTGCGCCACGAGCCACCGCTTCAAAGAACTGT
CGTAAGTCGCTCACCGACCTCGTCGGAGGTGCTTGGAGGTCATGCAACGAGGCCGCCC
TCCTCGGAGTATCGTTCACAATCACAGGTCGAGCTAACTGCTCACTACTTCTTGCCAAACATCCACCGCTTTT
TGAAAGCGACTCAAACAGAGCCCGCCTCTCACGCACTGGAACGTACTTTGACACAACTTCCCGAGAATTTGGG
GAGCACGATCTTGCGCGATTCTCTAGTGATGCTGCCTTTCTTCTTGCCGCCACCATCG
CAAACCTGCCCCGTTCAGCACCTCCTGTAGTGCCCTGCCAACGTTTCAAATACCTATGCCTCCCTATATCGGT
GTCGCTAGTCCTCGCTCGTGGA
>74332456_2 DT668277.1
TTCGGCACGAGGAGGCAACCCTCAACGTCCGAAAGTCAAGAACAAACACCAAAAGAAGTTGAAGAAGTCAATA
AACTCGAACTCGAGAGAAAAGAACGAGAAAAACAAAAGGCCATCAAAAAGAAGGCCAGAAAACTTAAGCAAAA
GCTACTTGCTACTGGCAATTCAAATCCAATATTTGATGATACAATCACAACTGCCGAT
ACATTCCTAAATCCTGTTACAAGTAAAGTGTACAGAGTTATCAGTTCAATAAGACATATTATAGCGCCCTTAT
TGCGGGACCTTAACGTTACTGTTCACGTAAATAACGTAGAAAATGGCGAGGATAAAATCGGAAAAAAGAAAAA
GAAAGCTAAATCAGCCAAAATAGATGACACCGCTAACGCTAAAGAAGTTAAAACTGAT
GATAATAGCAATAATATAACTAGTGATAAAATTAATGATCAAACTAGTGCCGTTGTTAATGGTAACATTAATG
ATAGAGATGATGATAAATTGAATAGTAATGGAGCTATCAAAAAAATTAAAGAAAATAAACTAAATACAAACAA
TAATAGTAAAGATACTAAAATCACTAATACTAACGAAATGAATGTAAGTGATAATAGT
AAAAATAAAAACAATGATACATACAAAAATATGGAACATAACGAAGCANATACAAGCAATAGTTCAGAGAAAG
CTCCAGAAGTCGAGAGT
>74332988_2 DT668809.1
GCACGAGGTTTTAACTATTATCTCTCAAAATGAAGGCTAAAGGAGAGTTGAAGGAATATGAAGTCATCGGTCG
CAAGCTGCCGTCGGACAGTGAGCCGAAACCTCCTCTATATAAAATGAGAATCTTCTCTCCGGACCCCATTGTT
GCTAAATCACGTTTCTGGTACTTCTTAAGGCAACTGAAGAAGTTCAAGAAGACTACTG
GCGAAATTGTCTCTATCAAGGAAATCCCAGAAAAGAGTCCAGTCAAGATCAAGAACTTTGGAATCTGGCTGCG
TTATGAGTCTAGGTCGGGTGTTCACAACATGTACCGGGAGTACCGTGACCTGAGCGTGGGCGGTGCCGTCACA
CAGTGCTACCGTGACATGGGAGCCCGTCATAGAGCTAGGGCTCATTCCATTCAGATCA
TCAAAGTAGAAGTAATCAAGGCGTCCGCCTGCCGCCGGCCACAAGTGAAGCAGTTCCACAACAGTTCCATCCG
CTTCCCACTGCCCAAGCGCGTCCACCATTACAAGCGACTCAATACATTCGCTTACAAGAGACCCAGCACTTAC
TTCTTGTAATTTAATGATATAAACGTATAAGAATCGCC
>78073031_2 DV501006.1
GCACGAGGATCCCCTTAAGACTTTCTGACTATATTCTAATACCTTTGACAATCACTCTCAATTGCACCGATTG
ACGGTTATTTTTTCCCTTTTAATTGTTATTTAAATAAGCCTTAATCTATTCGTGATAACCTGTTTCCAACATT
TATGCGTATTCCCATACCTACATAACTAATAACATTAATAACGACAATGTCCTTGGAA
TTAACCCTGTAGTTTCAGATACATCTACAACAACATCTCACGAACTAGTACTTCATTACAATAACACAGACTG
ATTTGCTCCGCCATCAATGTCTCTACAATAAAGTACATTTATTATTACTATCTCCGTTTAATATAAGCGATGG
CGAAGCGCTTTATCGAAATACCTTATAAAATTGTTGTTAAGTTCGTACAGGACTGATA
TGTCATTACGGTGATTTAATTTTTCTTTCCAGAGTTCTAGAACTATCCCGCCCGCCGCTAGAGAGTCTTTACA
AGATCTCCTCTCTCTATACCCTACACGTGACCCACAATCACGCACATGTTGACTTCATAGAGAGCTGCCCTGA
GAGTCCTATTCAAGTAAGTGAAAAAGACTTCGTACTTCTCAACCAAACCACTAAATCG
CCCCAAATGGGTCTAAATCATGTGCACTCAGTGTCCGGAGCCTTAATTAATCTGCACTTATGACAATTACACG
ACTTCGCCCACCTACGAATCTAACATGTTGCATCATACGAATCCCAAAAAAATAGTTCTCCGTTAAAAAAAAA
CAATAGCATTTCAATCTTTTTTTTCTAACCTTTTAAAAAGAACGCATTACTTTCCCCT
GAAGATTATTACGAAATTCGTCCATAATACCCAAAAAACTCGTTAATGCATATATTCTTTCAAACACAGAACA
ACATCCCCACGGACCC
>78073158_2 DV501133.1
TGGGGGGGGGGCCCAAATCTCCCCGCGTGAAAAAATTTTACCCCCNNNNTCACNNAAAAAGGGNGGNANNNAC
AAAAGNNCNTGGGGAGNCNNTCCACNCGNNCGNGNTGGGNCGNGNCCGNCNTCNTAGNAACCTCCAATGTGAA
TCCGNCCCGGNNNTACCCAATTTCGNNNNNNNNNNNNNNNNNNNNNNNNNNNNNNNNN

NNNNNNNNNNNNNNNNNNNNNNNNNNNNNNNNNNNNNNNNNNNNNNNNNNNNNNNNNNNNNNNNNNNNNNNNN
NNNNNNNNNNNNNNNNNNNNNNNNNNNNNNNNNNNNNNNNNNNNNNNNNNNNNNNNNNNNNNNNNNNNNNNNN
NNNNNNNNNNNNNNNNNNNNNNNNNNNNNNNNNNNNNNNNNNNNNNNNNNNNNNNNNN
NNNNNNNNNNNNNNNNNNNNNNNNNNNNNNNNNNNNNNNNNNNNNNNNNNNNNNNNNNNNNNNNNNNNNNNNN
NNNNNNNNNNNNNNNNNNNNNNNNNNNNNNNNNNNNNNNNNNATCCACGTAAAGGCGAAAACCGCTACAGGCG
ATGGCCTACTGACCTCACCTATCAAGTTTTGGGCGAGGGCGTAACCTAATCGACCTAA
CGACCCCGATAACTGACGGAAGCGGCACTGCAGAAGAGGAGAAGCAGGACGGGCAGCGCTGAGGACGCAGTGC
GACACAACGCCGTATGCCGCCNGNTACGGGCTTCGATGGCGACCTTGTATTCATCTCATGACGTCTGATACTG
AAGCTAATGAAGAAAGTCATCTGCTATCTTGGTCTCGTTGCACTGATAAGAATTGGCA
GACAGTACGACATCCAATGCGCTATGTGG
>78073278_2 DV501253.1
TATCATTAAAGGTCCGGGCTTCNNGNNNCNNNNCNNNCCTTCTTGAAAGGGACCTCATTGGGGGCCCGGNNCC
CNNGCGCCCNGGGGGGGNCGAAGNGCNGGCGCGGGCCTCCAAAAGANNNNNNNNNNNNNNNNNNNNNNNNNNN
NNNNNNNNNNNNNNNNNNNNNNNNNNNNNNNNNNNNNNNNNNNNNNNNNNNNNNNNNN
NNNNNNNNNNNNNNNNNNNNNNNNNNNNNNNNNNNNNNNNNNNNNNNNNNNNNNNNNNNNNNNNNNNNNNNNN
NNNNNNNNNNNNNNNNNNNNNNNNNNNNNNNNNNNNNNNNNNNNNNNNNNNNNNNNNNNNNNNNNNNNNNNNN
NNNNNNNNNNNNNNNNNNNNNNNNNNNNNNNNNNNNNNNNNNNNNNNNNNNNNNNNNN
NNNNNNNNNNNNNNNNNNNNNNNNNNNNNNNNNNNNNNNNNNNNNNNNNNNNNNNNNNNNNNNNNNNNNNNNN
NNNNNNNNNNNNNNNNNNNNNNNNNNNNNNNNNNNNNNNNNNNNNNNNNNNNNNNNNNNNNNNNNNNNNNNNN
NNNNNNNNNNNNNNNNNNNNNNNNNNNNNNNNNNNNNNNNNNNNNNNNNNNNNNNNNN
NNNNNNNNNNNNNNNNNNNNNNNNNNNNNNNNNNNNNNNNNNNNNNNNNNNNNNNNNNNNNNNNNNNNNNNNN
NNNNNNNNNNNNNNNNNNNNNNNNNNNNNNNNNNNNNNNNNNNNNNNNNNNNNNNNNNNNNNNNNNNNNNNNN
NNNNNNTAAAAATTTCTAAATGTTACCTTTTAGAGATAATAACCTGACGATGTTTCAT
ACATTTGAAAAGGGAAAAGACAAGGATTGATTCTTCTGGGGCTCCGTTTTCTTTTTTGGGGAATTTTGCCTTC
AGTTTATGTCACTAAAAAATTGTTGAAAAAAAAATTCTAAAAT
>78230504_2 CV525691.2
GCGAGCAAGACACTTATCATCCTTCCCTGTGGCAGTAGCCTTCGCGCACCGCAGGTACCTTCGGAGCCGATCC
CAATTTTGCGTCAAGATAGCAACATCAACCCTGACGGTTCCTTCCAATATTCTTTTGAAACTGGCAATGGAAT
TAACGCGGCGGCTAATGGAGCTCTAAGAAACATTGGCGCTGAGGAGCCAGCTCTGCAA
ATCCAAGGGGAGTTTAGCTACCCTAGTGAAGATGGTGGCAGCATTCAATTATCCTATGTGGCTGATGAAAATG
GATACCAACCCCGAGGTAGCGCTCTACCTACTCCTCCTCCAATCCCAGCTGACATCCAACGCGCTTTGGACTT
CCTCGCCACTGCTCCCCCTCAGGCCCAACGCTAATGTGTTAACTAAAAGGAGTGGTTA
AATATCCACGAATACAAAACCTAGAAGGCTATTAGTTATAATTATATCAATAAGGGACTATATTTCCTAAAGG
CGACTACGCATCAGTTAAAATCGTTTTGAGGCTATGGAATAAAAACATTATTATAAACAAAGTATTACTAATT
ATTTATAAAAGTAACATTCAATTGATTGCAATTGTAAATCTTAAGAAAATGTTAACTG
ATGTGTAAACGCTTGAACAGCAACATAAAGATGTGAGGTCGATATCATGTTTGTATTTTTGTACATACCATTT
TATATTTTATTTAAATAGTCTTATATGAAACCGACGAAAAAAACCCCGGGGGGGGGCCGGGTCCCAATCGCCT
AAGGGGGCGTTACCTCCGGCCGGTTAACGCGA
>78230507_2 CV525694.2
GGCCGCGGCTAGTTATATTACAAAATAACGAGCCTGCTCCACTGAAGCAACATTAGAGCGAAGTAATTCTAAC
TGTAGTGGTAACTACGTCTATATCAAACAATCAGACTCAATAAATGAAAATAATTCGTACACTGCACATTTGT
AACACATACATTCATAACTTTGTAACCTTATTATGAGTTCATACACTGTAAATGCGAA
AAGTTTATTAGGAATTTACATCATGCCTCCCATACCCCCCATGCCTCCCATACCGCCCATACCACCCATGCCC
GCCATAGGGTTGGACTCTTATTCTTGTGGGATGTCACAGATTACAGCCTCAGCTGTGGTCAGGAGTGATGCTA
CACCGCTGGCATCGGTTAAAGCTGTTCTTACTACCTTAGTGGGGTCAATGATACCCTT
CTCAATCATGTTAACATATTCATTGTTTAATGCATCATATCCAAATTCAGTGCCAAGTTCCTCTACTTTAGCA
ACTACAACAGATCCATCAATACCAGCATTTCTAGCAATGGTCATGCATGGCATTCTTAAAGCTTGTTTGATGA
TTTCAATACCAGTAGCCTGGTCTGAGTTGGAGGACTTAAGTTCATTTAACATTGGGAT
GCATCTGAGCAGAGCTGAGCCACCTCCAGGTACAATACCTTCTTCAACTGCAGCTCTGGTAGCATTCAACGCA
TCATTTACACGGTCCTTCTTCTCATTGACCTCAACTTCACTGGATTCTCCCACCTGTAAGACAGAT
>78230508_2 CV525695.2
CGGGTCGGGACTGGCCGAGGCCGTTGCGTTGGTCTGTAAACTATTATAATTTAAACGGATATTTTAAGTTAAA
AAATTTAATAGACTACTTCAAAGATGTTCTTAACTCGTTCCGAATATGACCGAGGAGTTAATACCTTCAGCCC
CGAAGGAAGGCTTTTCCAGGTTGAATACGCTATAGAGGCCATTAAACTGGGTTCCACA

GCTATTGGCATCGCTACTTCGGAAGGTGTTGTGTTGGCCGTGGAGAAGAGAATCACTTCGCCTCTGATGGAAC
CAACCACTATAGAAAAGATTGTTGAAGTAGACAGACATATTGCTTGTGCTGTATCTGGTTTGATGGCTGATTC
TAGAACTTTGATAGAAAGAGCTCGTGTAGAGTGCCAGAACCACTGGTTTGTATACAAT
GAGCGCATGACAGTGGAGTCTTGTGCTCAGGCTGTTTCTAACTTGGCTATACAATTTGGGGACAGTGATGATG
ACAGTGGCACTGCTATGTCAAGACCATTTGGTGTGGCTGTTATGTTTGCAGGTATTGATGAGAAAGGTCCTCA
ATTGTTCCACATGGACCCCAGCGGTACATTTGTACAGTATGATGCAAAAGCTATGGGT
TCAGGAAGTGAAGGAGCTCAGCAAAGCTTGAAGGAAGTCTACCACAAATCTATGACACTCAAAGAAGCGATCA
AGTCCGCTCTAACAATTTTGAAACAAGTAATGGAAGAGAAATGTCCGAG
>78230510_2 CV525697.2
AGGCGAGCGGATACAGGGATCAGCTCTGAGGAACACTGTGTATCCACATCAGGACAAGGGCATATACATCCTC
TGAATAGTGTTTTTCATGTATTCATACATTAAAAGCTTGTTATAGAGCTATTTTTTTTTCATTTCTTAGCAAA
TACCCTCTTCAAAGTAGCTATTGCAAAGGACTATGGGATACCTTTTGGGCATGTGTCT
TTTTGCAATTGACAAAAAAAGATGTAAACCAATTTTTTCTGTATCACTATCCTTGGTAAATGCTACTTGGAAA
AGAAGGGTCTATTGAGAGTGGAATGTGTGTGCCAGTGTCACTACAAAAATGTGTGCTTGGTCAGCTATAGAGC
CAACTTTGTTCATGTTTGTGACTTAGTTGGTACTGCATGTTGGTACTGCAAGTTAATA
CATTCCCATACGTTGCCAGTTGGGCTTTTTCTGTAGCAAAAACTTCAAATTTATGTGACTAGATGGTTACATA
CATCACCACCCTGCATACATCACCACCCTGGACAGCATTTTAAAAGCTGTAACTTAAGTATCAGACAATGCAA
GTTCTAGCTTGAGGTAGAATGCTTGAGTGGTAAAATATATCAGTATTTTGTAAATAAT
CACAAGAAAAAAAATACAAAAAGAAGAAACCCCGGGGGGGGGGCCGGGACCCCAATGCCCCATAGAGGAAGGC
GGAATCACAAAACGGGGCGGCGTTTGAAAGGTTCCGAGGAACCCGGGGGGGAAACAAATGGGGGGCACACAAA
CAA
>78230514_2 CV525701.2
CCGGGCTGAGGATCGGCACGAGGAATCAGCTTCATGCTGTATCCCATACAGCAGGATAGCCAGTGGTAACCTA
GTGTTAAAACATTCCTTTCCCCACTTTATGCTGAATTTTTCAAGGCGTAGTGTGTTGAGTGGTGGAACTTAAC
ACCGGGTTTTTGCCTTGGTGCTAGGGCCATAGAAATTAAAATATTAATAAATAATGAT
TTATTCCCTCGAGTGGGGCTTGAACCCACAACCTTACATTCACACTCTTGTGCCACCTCTCCTCTTCTAAATT
TTTTATCTCTCTCTCTATCTATTTTTCTGGGCTAATCTCTGAATTTTGACTGAAACATAGAGGATGTTGTGCA
ACAAATGGGCTACTAATTATCCCAGAATTCCAGCAGTTTTGGGAAAAACACAGGGACA
GCCTTAAAATTTGGACAAAGTTGCAGATGGGTTGGTGGTATTAAATATATAAAAAAAAAAATCATACTCTAGG
TACAAGAAGCCATAAAACGCCGCGGCCACAAACATGAGTCGGAGGGCAAAGCTTTATCCAAACCTTATTTAAT
GGAATTAGAACGTCAATACAAAGAACAATTATTTGCGAGACATATCTACGCACGCAGA
AGCTTCTGGTTATGATTGGACTGGTGGTGGTGAAGTTGCAGTCGTAGTCAGAAGATATAGAGCGTTAAACTTT
GGACCAGTATACGGGACACATAAGACCCCAACATGAATGGACTGGTGCCTTGCAGGAAAGCTGGCAATGGGGT
TGAACAGTCAGTGCTATAACATATTACACGAATACCTGCAGAATTCTATCAATATACA
AAATTTTGATGTGCCGAA
>78230515_2 CV525702.2
GGGCTGGGATCGGCAGAGGCTGAAAGTACAATTTACAATTATTTTTTAGGTTATGTTAATATTTATACATATA
CAAGGGCTTCTACATGGTTTGATAAAATTGGAAAGCCACGATTTATAGTAGCTCCCATGTGTCGATGCCAGTG
AGTTGGCTTGGAGGCTTCTGTGCCGACGACATGGTGCTACATTATGTTACACTCCCAT
GCTTCACAGTACTGTATTTACTAAAGATCCTAAATATAGAAAAGAAAATTTTACAACATGTAGTGAAGATCGG
CCGTTGATTGTTCAATTTTGTGGTAATAATCCAGTGACAATGGCAGCCGCTGCTAAATTTGTGGAGAATGACT
GTGATGCTATTGATATAAATCTTGGATGTCCCCAATCTATAGCAAAACGAGGACGATA
TGGATCATTTCTTCAAGATGATTGGCAATTACTACAACAGATAGGCTAGTAATGTTATTATTATCATTGCTAT
TATTAGAATAATATAGATGGCATTGTAGTGCAGTGGCACAAACATGCAACTGTAAACACAATGATTGTGGGTT
CAGTTCACACTAGTGGAAAATGATATATATTTTTCTTTCATTTGATTATGGTGGTAAT
GAGGCAAGAAACACGACGTTAAGTTTCTCCACTGATCACAAAACTCTTAATTCAGATTCAAATTCAATGAAAC
ATGAAGGATCTAAAGTATCTTAATAGACACAAGAGTGTGTTTACAATAGTTCTCAGGTCCCTATGCTACTC
>78230530_2 CV525717.2
CGGGCGAGGACTGGCCGAGGGTATACATCACGCCATGTAGAAGAGGCTTTACAAGAATGCCAAGCACGTGGCA
GTAGCAAATCTCTTTTGCAAGACTTGGTGACTGCTGCAGGTTCTAGAGTGGCTGCTATAGCGGCTCTGGATAT
GTTCCTCGTGGGAATTGATACAACATCAAATGCAGTAGCATCTACATTATATCAGCTA
TCACAAAGGCCGGAAGTTCAAGAACGATGGTATGATGAAATATCTAATGTTTTAAAGGGGCAATCCCTAAAAC
CTGGAGATATTAACCAAATGCCATACTTAAAAGCATGTGTCAAAGAAGTTCTCAGAATGTATCCAGTCGTAAT
TGGTAACGGCCGCCAGCTCTCCAAAGACACTGTTATATGTGGCTATAATATTCCTAAA

GGGACGCAAGTAATATTCCAACATTACGTAATGGGTAATAGTGAGGAATATTTCACGAACGCATCGGAGTTCC
GCCCTGAACGTTGGCTGGACCGATCGATGTACAAACACCATCCGTTCGCCTCTCTACCTTTCGGTGTTGGTAC
ACGAATGTGCCTTGGACGTAGATTTGCTGAACTTGAAATTCATGTTATTATTTGCAAG
ATGGTACAAGCTTTCAAGATGGAGTATCACCACGAACCACTGGAATATCACATTCATCCGATGTACACACCAC
AAGGCCCCATACGTATTAGATTGATTGATCGATGAAATTGTATCATTTGCATTAAAAACACCAAAACTGGTGA
TTTCAATGTACTTGTAATACT
>78230547_2 CV525734.2
GAGGCCGAACGCGTGCCAGGAATCGGCGATAGCACGTAATTTGTATTCACGTATCAACTAAAAACGTATTTTG
AAAGTTTAGCGAGTAACCATAATTCCATTTCCTTCCCAATTTGCTATAGAGTCGTAGTGGTTATGTGATTCTG
CTTATTGTTGTAAAATTTGAATTAAACCTATTTGCTACACTTTAAATCAAGTTGGAGT
GTGTAAAACATACGTTCTAATTAATAAAAGATATAACGCGTAACTGAATATTTTCTAAACCTTGTGTAATAGT
AAGGCGCTATCAAAATGGCTCTATATCCATCATTAGAAGATATGAAAGTGGATAATATGATTCGAGCACAAAT
GGCTCAACCAGACATGGCACAGTCTTCTTATCATCAATCCTACAAGACACCACCAGTA
TCAGCTNNT
>78230550_2 CV525737.2
AAAAGGAAAAANCTCCGACCGGGCCGGATGCCGAGCTTTCACTATGATGACTCAATAGTTCTATAAAGCGATT
GAACCCGCTCTTTCATTCCAATGTTCACAAGTAGCAACTAAATTATAGTGTTTTGGAATTAAACCTTCATATT
TAAGAAATTTTAACTCTCAATGAAATATAAAGTTATAATTGCATACTTACATATACTA
AAATATATAACTTCTTTTCTAAGTTAGTATCATTTTAGTTGTTATTTTGCTTTAACATTTTTTTTGTGTCTTT
TGTTTTAAAAATGACGAATAGATTTTTGACGTATGGATTTGTATTAAAAATATTCAAAAATCTTCAAAAAAAT
TGAAGGTCTTCATAGCGCAGCGGCACAGCGTTTATATTTAAACGGTTGTGAGTTCGAT
TCCCAGTAGAGGAATAAATTATTTTCCTTCACTCGCTCTGAACTTGTGCGCGGCGCTGAATTCGGCTACTTAG
CGCGAAATGTCTCGAAATCACTTAGCGTGATGCTATTATTCAAAATAAAATAAAAACTTCTGAAATCCAACAT
TTTGAAATAACACAAATATCTTTAGAATACCATTAACTAATTGGATAAAATTATATTA
GAAACTTGAGTCATGTTGACTTTTACNNNNCC
>78230561_2 CV525748.2
GCGGGCATAAAACGAGACATCAAGGAGGCATATAACCTTCAGATAGCGGAGCCGATCACAAAAACAGAGAGCC
CCCTGAAGATCAACTACTTATACGGATCCGAATATGCTGTAGATGTACATCCGGCCATATGATATAATTCAAC
TATGTGTGAATAATAACATAACGAAAATAAACACATAAAACGGCCAAAGTGCGAGATA
TAGTAACGCACGATGGATTCCGTACCATTATCTATCATCACATAAGAGGCCTGTGATAAAGCCCTATGACAGA
CAGACAAACTAACGGACAGTGGAGTCTTAGTAACACGTTTTACTCTTTGAATGCGGAACCCTAATAAAAATGG
TTGAATATTATATAATTTCTCTTGATTGATTATAGTGCTGCGACTTGATTGCGGTTTG
ATTTCTAGAAAATATTTATTTTTACTGTTCCTTTCGTCGAGAGTTATCTTATTGACTTATTGACGTATATTTA
GTATAATGGTTGGCATGTCAACTACTAGTACTGTGTAGTTATAATATGTATAGTAAGCATATATATTGTATTT
GTATTGCAATAATTTTTTTTATGAAGATTGAAGATATAG
>78230562_2 CV525749.2
GAAGGAAAGAAGCAGAGGACGAGAATGGCCGAGCCATGTTATATCATTGAAAACAAACAAAAAAAAATATGAA
GAATCAGCCTAACCCGAAAAATAGACCACAGATAAAACAGAACGTACAAATCCGTATCGACGAGCAGAGAACT
GTCAACTGTCAAGTTCCTCCAGATTGACGTCTGCTTTCTATCCTCAACGTATTCTANT
CTTTATCGCGACATAAAATTAATTATGTTTGCAACTAATTGTAATGTAATATTATCGTAATTCGTATAAGTAT
ACAGTTAAGGTCATATATTTAAAGTAAGTTTGGCTGGGAGTGTAATATAAAGGTTAGTTCACCTCCTAGTTAT
ATTAATACCAAGTCCCGGTGCATTAAACGATCACAAAAATTGAAAAAAAAAATCCCAA
CTGAGAAAGCCTATTTGTAAAATTGTGACGCATTCCAAACAGTATTTAGTTTACTTACGCTTTTTATATGAAT
TCAGTTTTTTTTTAAAGTTCGTATTAATATGGGTATTATAGCATAATTAAGCCTTGTAATATAAATATACCAT
TATGTAAATCACGACGTGGCTTCTAATTAATATATGGTATGGTCCATAGTTTTATTTG
GTCGCAGTGTATTAAGTTAACATATATATCCTCNNNG
>78230568_2 CV525755.2
GGAGCGGAAGGATAAACAGAGTTAGAATTACAACGCGTGCACCGGATTCAAAAAGACAAAAATGTAACGGCAA
CTAATAATGAAATTAAATTGACAGACTTTGTACAACAGAAATAATATTAATAGTACAAATAGAAAACTGTGTG
TATCCTCGGAAATTCAAAGACAAAAGTGGTGTAGCGCTGATACTTTTGGAAAAAAATG
CTTTCAAAGAAGAAGATTTAGACAACAATGGTTAATTTTCTGCTGAAAGTGAATTAAAAACGTTTTTCCAAAA
CGATATTTACGGAAATTTCGAGTGCTACCCCAAAATTAGTATTAACGGTGTAAAAACTACAATAATATATCCA
GCCACAGACAAACATATAGCCAAGTACAGTCAACAAGAAGTACATATTATATTAGAAA

CACCAGAGTGTTATAATAAGGTGACATTGCCACAGATACAAAAAGATCAATTTAATTTACAGTGGGTATACAA
CATTTTGGATGGTAAAAGCGAACAAGATAGAATTGTTTACAACAATAAATGTGAGAAGGAAGGTTTTGTACTA
GTTCCTGATTTAAAGTGGGACGGACTTACAAAAGAAACATTGTACCTNNNA
>78230571_2 CV526398.2
GGGAANNNNNNNTTGATATAGACACGCCAGTTATCCAGACAGAATCCTGGGTGACTCTCGTACTAGTACTGTA
GTCTTGTGGAGAATTATATNAATGAGGCCCCCACATTGGTTTGTGCCCAGGCCCCCCACAAAGGTAAATCCGG
CCCTGTGTTTCCCTGTTACAACCAAAGTTGATTTAGAGTTTGAATGAATGAGGTCATC
AATTTCACCCAACATCAATCCATTCAAAATGATATTTCAAGCAAACCTTTTTTGTAAATAACAAAATATTACA
TTTTTTATCCCCATTTACAGATATACTATGTGTTCGTTGAGTGACAGGGGTTTCTGTTTTGTAAGTTATCGCT
TGTTGAAAGTAGACTAAGATTTCGTTTATCACGTGATATAATGGAATTTTATATTTGT
CTATAAAATATTTGAAATTTCTTCATGCCCATGGGAGCACTAAGTGGGGATGGGGGTAGGCATGAAGTCCCTT
TCAGGCAGAATATTTGAAATTTTAGGCAAAATTCATACTATTTCAAGCTAAGATGTGACATTTTTGCATTACA
ATTTCTGGTACTCTTTATTCCCCTCCATTTTCCCTTCCCATTCCCCCTCCCCGTGGTT
TCGTACGAATATGGCTCANNNG
>78230572_2 CV526411.2
ACAAAGCGCCGCANANNTNTCGCTATTAGACATCGCGAGTTACTTGATGAACTTAGAGCCTACCTTTGACACA
CAAAAATATTTCGAAAATCAATCCAACAAGTCTGAACAACAATCGTTAGCTCCTAAGACTTTACAATCTTTGG
CAATTTACTACGCAACAGCTTTAGATAATTTGTCCACCATTCCTCCTCCTGAAGACAG
CGAAACTACTACCAGTGTTAACAATGAATATGGATCTGTAGATCAAAACTTGCCAGCTTTATTTAGTCCTCAA
ACCATATCAAAATATGGAAGTCTGTTTGGACACGGTAATAATAACGACGACTTACTGGAAGATATTAAATTAG
ACCCTAATGGTACCTATAACGAATTGGCAGAAGATTTAACAGCACAAAATGAGTCAAG
GCCCGTTAGCAACATCCCCACAAATAAGAGAGCTTGCTCAAGTATTTACCCATGCTCTCTCTGCTTATCTTCA
AGATCCAGTACAGTTTAGAAAAGTGGGATCTGATATTAGGGCAAACACATTCCATCGGTTTCGGGATTGGA
>78230575_2 CV526414.2
GGGGGAAGANGGGANNNNNNTCGCATTGAGCACGGCGAGGTTATCATGTTCTAGAATACAGGGCNGNTTCCAT
CGTAACGGAGAAGAGATCGCAAAACACGACTGCGGTGAGAACAGTATGAAAATAGTGACAGAAAAGAAAATAG
AAGATGACGCTTCAGAGTCCTCTGGCGGAGAAAACGTTGATATAGAAGATAAATCTCT
ACCAATAGTAGAGCCACCCTTAGAGACACCCGCAGAAGCTCATTCAGCACAATGTTCCTGTCCAAATTGCGAT
GAAGTACTGTCTAGGTTTTCAGAATGCAGTATTTGCTTGGAACCTTTACAGTGCTCCGGCCCATGCGTGTGTC
CATGGTGCGGCGGGGTGTGGTGTTCAAGGTGCTCCCGACGTATGTCCCGCTGCGCGTG
GTGTAGAGGCACCTTACGGGCGCCGGCTTCACCGTGTTTAGCTCTGCAGAGACTTATCAATGATCTGATGCTG
CCTTGTAGAAACTATAGACGTGGCTGCACCGAGTTATTATCAGCAAACATGAGAGCGAAACACGAAGAAGAAT
GCAAGCACGACACAATGACATGCCCCATCACGTCCACC
>78230578_2 CV526417.2
GGAGAGGGCGANNNTTGCAGTGACATCCCAGCTAGTTAGTATACGGATCTCGCATGGTTACCTTTTTTAATCG
CAGATATCCTGCGATAACACAATTTCTTTTATGAAAATAAATTGATATCTTGTGTGTCATAAGTTATTAACTT
AGTAACAAGTACAGTAAGAACCTAACCATGCCGCCGAAAAAAGCTGGCAATGCAGCCA
GCAAGAAGACAGAACAGAAGAAGAAGGAGAAAGTTATAGAAGATAAAACTTTTGGATTAAAAAACAAAAAAGG
TGCAAAACAACAGAAGTTTATACAACAAGTAGAAAAGCAAGTGAAAAGTGGGGGGATTCATCCAGCAAAACCT
ATGGAAGATAAAAAGAAGGACAAGGAACAAAAAATGAAAGATCAAAAGGAATTAGCTT
TGCTTTTTAAACCAGTCCAGACAC
>78230580_2 CV526419.2
ANAAAGGAGGAGGAAGNNNNNNNNNTGCTAGGTCTGAGATCCACCGAGGTTAACTGAAGCTGTTTTGGTCGAA
CAACTAANTCTATGCATCGAGTACTCTTCATATTCCTTGNCTGTAAATTGAAGCTACTACCAGGAGGACAACT
TGGCTTTCAATGCAATGACCCAGCATTATCACACCCTTACACCGGTGATACCGTCAAT
TGGAAATGGCTTATAGGAATCACTATGTTCTTGCCATTTGTTGTGATGATGATTGTAGAAAAACAGCAAGATA
AATCGAAACATGCCAAAGAAAATGCTCTATGGTGGTACAAGGAGTATTTGTATGGATTCCTCATAAACTTGAC
GGTAGTGCAATGTATCAAGCTCATAGTGGGTTCACCGAGACCCCATTTCTTTGACACG
TGCAGCCCCAAAGAGGCGCTGACATGTGAAACGNT
>78230584_2 CV526423.2
GAAGGGAGNNNAGAAAAAAAAAAAGNNNNTGCTGTTGAAGGTGCCCGAGGTAACGGCGCAACATTATTATTGG
ACGCTCATGAGTCGTCAATACAAAGGGGTTAAGTTGTGCTCATCCGTTTTGGCTTACCATACCAGAAAAACGT
TCTTAGCGAGTTGCTGATGAGGNTTGGTGGTGTATTCTTTTGAATCACGTGATAGCAT
TCTGGTACAAAAACCCGTGTGAAACGAATTTATTGCCCTCCCAGAGCATCTCTTATTTCAGTNA

>78230585_2 CV526424.2
AAAGAGGGGCCNAGNNNNGNNNNNCAGTAGAGAGTGGCGAGTACAACATGGGTGTATCTCGGGAACGTGATAA
TAGCGAAGTCATGCAGGCACGCGGTTCAATCTTCGCGTCACTTCAAGAACACATACGAAACAGCTATGGCTAT
TAAGAAATTGCCTCTGCGTCGCGCCGNNTACGTTACCTTAAAAACGTAGGNTTAGAGC
AGAGAGAGTGTATACCATTCCGTCGTTTCAACGGTGGTGTTGGTAGATGTGCTCAAGCCAAGCAGTTTGGTAC
CACACAAGGTCGCTGGCCGAAGAAATCCGCGGAATTCCTTTTACAATTGTTAAGGAATGCCGAATCTAACGCT
GACTACAAAGGCTTGGATGTTGACCGGCTTGTCATTGATCACCTTCAGGTTAAACGCG
CCCCCTGTCTCCGCAGACGTTCATTCCGGGCTCACGGTCGTATCAAACCCTACATGTTGTCCCCCTGTTACCT
TGAAGTGTGTCTCACTGAGAGGGAAGACGCGGTAGCACGCGCCGGCCCCGNNNNA
>78230587_2 CV526426.2
CTTAGTTGAGCGTGCAGAGGTATATGAATCAATATTACTTAGTTATAATTAATAATAGGTTTTAATTACAAAT
TGACCTCGTGGTATTCAGCAAAACAATTTTTCTTTTCATTTACACACAAAAACACGTTGCAATGAGAACATTT
ACTATGAGGTTTCGACTGAATCTTTTTCAAACTACAAACCTCACACCTTCTCCTATTT
TCTACGAAAGTAGGCCAATGGATGCCTCTATTACCCAAGCGAACGTCTTTTATAGTAGAGTACTCAGATTTGC
GTCTTTTGGAAGGGCCAGCTTTCGCAGTATTATCAGTAGAAGTCTTTCTTTTCTGACTAACATCTTTCATTGT
CATCAAGCCTTGAGTAACAGAGCGTCGAAAATCTTTTACAGTGGTTTGTTTCATTATG
AGTCCATGAATGACATATGAATTGACAAATGCGATGTCCAGAAGACCCCAAAACAACCTGTGCCACCATTTCG
GAGATTTGCGATCTATACAG
>78230588_2 CV526427.2
GGAGAGGCGCTGANNNGNNNNTGCAGTTAGGTGGCCAGTAAGACAATGGAATGGAGTGGCTAGTGGATATGAA
ACCGGAATCCATCAATACACCGTTACCGGATGACGCACCTCCACCGGCCCCACCCCCACCTCCACCACTACCC
GAAGATAAACTTACAAACACAAGCACTCCTAACAAAGCAAAAGTTAATAAATCACCTA
AGAAGAAAACAAAAAATAAAAACTATGATGCTAATGAAGCTAATATAAAAGACAATGAAAATAATACAGAAGC
TACAAAAGTAAATAAATATAATGAAAGTGAAAATAATGACAGAGCTAAGACAAATAAAAATAATGAAAATGAT
AAGAATGATAATGATGATTTGGAAGTATTAGAAGTGTTGCACGTCCCCGACATTCCCG
TGCCTGGTGATGATTTTATTGTTATTGATGAAGACTATAGTGTTGATAATATTTCAGTAGATAGTCCTACTTT
AGCTGACTTAGAGGAAAAGAAAAGACAACTCCTAAGTGCGCTTAAAGAACC
>78230590_2 CV526429.2
CGTAGTTAGAGATCGCCCGAGTAACAGATGTGTATAGGCATTGCAGAGTTNGTTATAATTTAGAACCAAAAGC
AAGCTAATTTAACCTCCGGGGGCCATTTATATAAGACATTAAACGGAAAACCTACCTACTTATCTTACTACAG
ATGGCTTTGCAACTTTATAAGCTCGACGTCAGTCCACCAGCGTGTGCTACTTTGATGT
TGATTGATCATCTTAAAGTTTCTCCAATTGAATTTGTGGATGTCAGTATTCTGAACAAAGATCATTTGACACC
AGAGTATTTAGAGAAAAATCCTATGCACTCTATACCATTGTTGCAAGATGGAGATCTATTTATCCATGACAGT
CACGTCATCATGACTTACTTAACTGAAAAATACAGCAAAGAAGACTCTCTATACCCTA
AAGATCTGAAACAAAGAACCTTGGTTGACCAAAAATTATATTTCGACGCACAATTTTTTACTCGTTTCAAGAA
CATTAGTTATGCAGCAATCTATGAAGGAGTAAGGAAACCATCTGAAAAGGCTATCAATGACCTACTCGAGACA
TATGATTTCCTAGAAGCTTTTCTCTCCAAAACAAAGTTTGTTGCCGGTGACAAGATGA
CAATTGTTGACTTT
>78230591_2 CV526430.2
GGGGGGGGAAGGGGCGCGGNNNNTNTCAATGAGACGCCGAGATCAATGTGTATAGCTGCAGAGTGTAATAATT
TAAACCAAAGCAGCTATTTAACCTCCGGGGCCATTTATATAAGACATTAAACGGAAAACCTACCTACTTATCT
TACTACAGATGGCTTTGCAACTTTATAAGCTCGACGTCAGTCCACCAGCGTGTGCTAC
TTTGATGTTGATTGATCATCTTAAAGTTTCTCCAATTGAATTTGTGGATGTCAGTATTCTGAACAAAGATCAT
TTGACACCAGAGTATTTAGAGAAAAATCCTATGCACTCTATACCATTGTTGCAAGATGGAGATCTATTTATCC
ATGACAGTCACGTCATCATGACTTACTTAACTGAAAAATACAGCAAAGAAGACTCTCT
ATACCCTAAAGATCTGAAACAAAGAACCTTGGTTGACCAAAAATTATATTTCGACGCACAATTTTTTACTCGT
TTCCAGAACATTAGTTATGCCGCAATCTATTAAAGAGTAAGGAAACCCTCTGAAAGGGCTTCATGACCTACTC
GAGACTATGATTTCTTGAAAGCTNTCTCTCCAAAACAAGTGTGTTGCCGGTACAGATG
ACATTGCTGGCTTCGTTATGTCTCTATGCTGCGCCGTTCGTGTCTCTGGTGAGAGTTCCTATCTGCGGTTTTT
TGGAAGCCGTTTTATTTGGGCCGGGCCGGGGGACCCCGAAGTTTTTTTTTTTTNTGC
>78230592_2 CV526431.2
GGAGGAAGCGGCGGANNNNNTNTCAGTCTGAGGACGCACAGGTTAGTAACTCGCGTGGCCGCGCATCAAANTT
CGATATTATAAACATCGACGTTTAAAAACAAAATGTCATAAGTGACCGTATTGCTTTTAACTATAGACTGAAT
TAAAAAAAAATAAATTTAAACATGGAAGTAAAGAAGATGGATACCACTAGTGATGTGA

TCAGGAAGCCAGATTACATGCTCATGTCCTTGCAGAGGATACTTGTGTTGTTTGTAATAATGGCCAGTGTGAT
TGTTGTTGTTTACTATACCCCGATGCCAGAGGAATACTTTGAAAACTGTGATCGTCAGTGTCATGAATTAGAT
TGGCCCATGATTTGCCGAGTGAAGCTGGTCATCGAGGTGTATAAAACCTATAGCAAAT
CCTGCAACAACTGTGCAGAAGATGGCAGCGATTGCCCGCCCATGTGTATAACAGCAGATGGTCGTGAACGAGG
AGTTTTATCGGCTAATAGGCAATTACCGGCCCCGGCCTTCCATGTCTGCCAAAATGATATTCTTGTTGTCGAC
GTCGTCCACCGTGCGCCTGCGCATGCCCTTTCAATACATTGGCGCGGTCAACCGCAAA
AAGAGACTCCTTTNNNT
>78230593_2 CV526432.2
GACGACGAGGANNNNNAGAGGGCAGTANGCGCTACCTGTAACGAGNCNNGNCTACNNCATCTGTGTCGAATCA
TATAGCTGCATCGCTTTGATAATGAAGATCCGTTATAGTATTGGTGTGTATTGGTATTGTGTCATCGAAAATT
TACCGGACGATTTCCCCAGTGCAAGCGGAATGACCCGGAACTTGACAAATGTGTTATC
AAGGCAGTGGACGTAGTGAAACCAAGACTTCTTAATGGAATACCAGAAGTGAATGTGCCAGCTTTAGATCCAT
TCTTAGTACCAGACATCGACATTAGACAGAACAGCAAATAACTTAAGGTTGAAGGCTACTATTAAGAATTTTC
AGGGGGTATGGGGGTTCCAACTTCCAAAATAGAGAAGATCCAGGTTCAACCTCTACAA
ACCAGTACGGTTGGAAAAATCCGACTGATCATTCCCCAATTATTTGGAACTGGTGGATTCGACGTTCGTGGTT
CCAAGAACCTTACTCTGGACCTTCAGTGGAAACGGAAAGCTCCGGCATAATTTTACTAAATAATGCGGGAATG
GCTAATGGAAGTTCCAGCCCACCTAAAGGAGGTGTCAATTACTTCAGTGAAAAATTTT
CCATTGAAATTGTCCTGACAATCCCATGATATTGCAAGACAATGGTGCCATAAACACACTCTCATGCAGTCTG
GTCGCTGAATTAACCTGATAAAAAGGTCTATCAACTTCACAAATCGAGANNTG
>78230595_2 CV526434.2
GGGACGGAAAANNNNNNTNNNGNGNNCNTCCGATAAGGCNTCACTATATGTGTAGCGAGTTCAAAAACAACCA
ANTATGCCTAATATTATTGTGACTACTGTGACACTTATTTAACACATGACTCTCCCAGTGTACGAAAAACCCA
TTGCACTGGGCGGAAACATAAAGATAACGTAAAATTTTACTATCAGAAATGGATGGAG
GAACAAGCTCAACACCTTATCGACGCTACAACGGCGGCATTTAAAGCTGGTAAAATTGCACAGAATCCATTTG
GAAACAAAGGAGCAGCAATACCTCCTCCTTGGGATCCATCTGTTATGGGTCCTGGTGGACCCAGACCACCGGT
ACCTGCTCCTGGTGGTCCACCTGGCATGATACCCCCTCCAACCATGGGCCCTGGAGGA
CCAATGGCGCCACCAATGATGATGGGTCCACATGGGCCAATGCCTCCTATGATGGGAATGAGGCCACCTATGA
TGGGCCCTATAATGGGTCCAATGGGACCTATGGGCCCTATGGGGCAAATGAGACCACCTTTGATGAATGGACC
TCCAATGAACAAATAGTGTTAAATATTTACTTTGTGAAGTTNNC
>78230599_2 CV526438.2
GGGGGNNNNNNNNAAAGGGAGGANNNNNNTTCGTAAGATGCCGAGTAACTTATATTATTATTTGTGCTGTATG
AAAATGAAGTTGAGATTAGTATTGANATACGTATGACTGTAGGTGTTGGTTGTGGGTTCTTTCATGTACACTC
AACGAAAATGAATATTTTTACTTCTTCGGTCTCATAAGATATAGCGGGGCATCGAGCT
CCGCCACTCAACACGCAGTATCTTGAAAAAGTGTCTTGACACTAACTTGCCCCTGCTTAAACAGGGTGAAGTA
AAAAAAAGTATTCTACATAAAACTTAAGCCATATTTGAAAATTTCAAATTTTTTTATTATCAGATATTACGGA
TTTAAAATAACGTATAGAAAAGACATTGTGTAATTAATTTATCATTTGTTAGCATATC
ATAAATTGTTTCAATAAATAAATGTTATAATGTAAGTCAATTCAATAATCTGTGTTCCTTTTAATCAACTCAC
ATTGAAATATTGTGGCATTTAGCGAATTTAAAATATGTTTTTAATTTACAATTTATCTAAATTTTATATACAG
TATAAACTCGATTGTTCAACCCTCAGTTTATATTAATTTANNT
>78230600_2 CV526439.2
TTTAGTAAGGTGCAGAGGTAACTCGTGGCGGAGAACTTGGACAATAATTAAAATGGCTCGTCTACCTCAAATA
ACGACGGTGCTAGCTGTAGTAGCGTTTTCCTCAAGCAGCATTGTCAAATCGCCAGAAACCCAAGAAAATAACT
TCGAATTGCTGATATTGCACAACAACGACATGCACGCGAGGTTTGAACAGACATCACA
GCTGAGTGGGGCTTGCACCGTCGCTGATAGGGAAGCTGGGAAATGCTACGGGGGATTCCCTAGGGTTGCTTAC
GTCGTGAAGGAGGCGAGAAGAGCAGCGGCAGCTGGTGAAGGTCCACCTGTCCTATACCTGAATGCTGGAGACA
CGTACACTGGGACCGCATGGTTTACCATATACAAATGGAAAATTGCCGCCGAATTTCT
TAATGCTTTACAGCCCGACGCCGTTTCACTTGGAAATCACGAATTCGACCACGGCGTCAGCGGGTTGACCCCA
TTCATAAATAATCTCACTTGCCCTGTGTTGGCTGCCAATCTAATAT
>78230603_2 CV526442.2
GGAGGGAANNGGGCGCAGGANGCAGANNGGCTTNCTCATAAGCGTGCNCNAGTTAAATAAGTTATCCTTTATA
CTTCATTATAANTATTTCTTTTTCTTAATCTTTTTGCNTTTTAGTGGCCATATATAAATTACGTCACACTTCA
AGGGGTCCTTCTTCCACCTACCTTGCATTACGCGAGATACGTGATGAGCGTGACACAG

CGTGTTAAGAAGTTTTTGTGGGGTGGAAAAACTTGATATTGTATTGCGAGAGGTGGGAAAGAGGTCTAAATTT
CGTCGCATCATCACATTTCAAAATATAATATACAAACGTTATTTGTCATAACTATTGTCTTACATAATAATT
GCGCATATNTAATATTATACATTTTTGCAAGCCCGCCGTTTTGTACACTTTGCAGTAG
TCCGTGGTCACGGGTAGACTGACAAAGCGCAAAATATACTATATTAATTTCAAATTGATGTTTTAATTAAATA
ATTGTTTATATGTCCTCGTGACCCAGGGCACAAGCGTTCAATTTTTGTTGGTTCAATTCTCGTTCTTAGGAAG
AAAATATTTAATATTTTCTGTGGCGAACGAAATTGTCTTAATGGGAACAGAGTGTACA
CTAGGTTGCACTCGTCATGTTTACAGTATAAAGCTAAAAATAAAAGTAAAAAGGCCAAAATTGTGACGTTCCC
TAGATGAAGATCTCCCATGTGGCCCTTTACCGGGCGGGGGGGGGCGCCCCTCAAAAACAGCGGGTTTANNNNA
C
>78230605_2 CV526444.2
CGAGGGAAACCCGCGACGCGCAANGCTCTCTCTCTACGTTCTAGGTTAAGACGTCTCGCCGGAGCTCGCGTCG
CTGCGGNATCGTGCGGTGCGTTTATGTTTTGTTTAGTGTAAGGATTTACTATATTACTGTATAAAAATGCGGC
TTCAGCGACGAGCGTCGACTTTGACGGCGGCACTGTGGGTGTTTTTTACGGTTGTATT
ACCAATTGAAACACAAATGGCGAATCCTATTTCATCAGTAATGCAATTTTTACAAGAAGGTACAAATCAACTA
GATAATGAACCACAAAGATCAACAGAATCTCCTTTCCCGAATATGATTTCATAATAGTGGGCGCCGGCACCGG
CTGGATGTGTACTAACAAATTCGATTTACCAGAATTACCCGAATTGAAAAGTCCTTTC
GTGAAAAAGCCTGGTTCCAATTGAAAACCTTGGTTATGGAATATTCCCCAATTTAACCCAAACATAATTTACC
AATTTAACAAATTGCCTAATTCGCAACTTTAAAACAAAAACCTTTCAGAATAGGTTTTGTGCCAGGAATTCCA
AATTACGCACTGTACGTGGCCACTTGGTAAAGTCTCTCCGCGGGATCCAGGTTTTTAA
ACTACATGATTAATCCTAGAGGTTCCCATATGATACGACAATTGGCAGCCTTGGGAACCAAGACGGGGATGGC
ATGACGTCCTTCTATTCAAAAAATGAAACTTATATCCTTCTTGACGAGTTAATTCCGGCAGAGGAATTTAAGT
AAATCACGACCGAATACAGAAGCGTTAAGCGCACAAAGTCAAAGAGNNNTA
>78230607_2 CV526446.2
GGAGGGGGGGGNNNNNNNTGCGGTGAGATGCCGGTTTGATTGACTGAATCTATAGGATTTTTATTATCATAGT
ATATTTAACATGTTGCCAAAAATATTTAGAATTACACTTTAATTTAAAGCGTATCTATCGAAAAAACCACCAA
ATTGTTTTGAAACCAGGCCAGTTATTGTTAAACTAAAATCCATTTCCAATTTATATTT
GTTGTGGCTTTAAACACCATATCAATGTTACCGTTCACTTTCGAAACCCACAGTCAATATCTACTACTAGCTG
TACCGCGGCTTCGTCTCCACTATTAAATGGACATATATGAGAATGCCATGAATGGCAAAAAT
>78230610_2 CV526449.2
AAAGAGAGAGCAGNNNNGACGGAAGCATCCAGGTACCAGTCCACAGCAACGGCAANATATATATATAGGTATT
ATAATAGGCGTATCGTATCCGTTTATATCACTCAAGCTTCGCACGTGCTGTGGTAGTTATCGTATTATGGTAA
CATGTTTATTGTCTTGTGAACTCGACCTTTTTTAATCGGAGTGAATGTTTTTGCCTAA
TTAGTTTATTTCCTCGATAAAAATGCATCTCCAGACTTTATTGATTGTTGTGGCTCTCTCTTTTGCATATGGA
CAAGATAGGAAAATTACTTTCAAAACTTAATCCCGGGATGCCAGAAAGCTGTACAAAAGAAAACTACCTTTAG
TTTATGTTTAAATGTGATGGGGGAACTCCGAATGGTTATTCCACCCAAATTAGGGGAC
TTTTACCCAAGGCTTACACCCAACTTGTTCATTATTTGGCTATAGGAAAGCTTTAAATTCCACGTTAAATTGT
GACTTGGGACAGGGATGTTACCAAAGACAATATCTGTATCAGAAAAACCCATGTACAGCTTTGCAACTGCAAT
TGACAAACTTTATGAATATGACGATTTAGATGACAGTGGTCATATAGAACCAAAGTCC
CCCAAAGGGAAGTCAGACTTAACAGTATGGTTTGGCATCTAAATGAAAGCAATTGATTGACAAGAAGCATGGT
ACCGTCTATGGGTATTGATAAAATGAACCGGAAACCGGATGTATATATTGGGCCCACGNNNA
>78230614_2 CV526453.2
CGACAAACGACACCCAACAGCGCAACAGAACAGCCAGCAACAACCACAGACAAAACCAACAGGACCCCATACA
ACACAACACACGACGAGAACAAACAAAGCAAAATACAACACAACACCACAAGCACGACCAACGACCAAAGAAT
GANGGANGGGGGNCNGNCGACCGGTGCACANCCCCCCTNCCCATATACTGGCCCGGCT
TATTCGGCGGCTCGAGAAGGCTGATTCANGATATCACGGTGATAAGATGTAAAATGGAGTCTCATGAAGAAGT
GGTACGTCTTATACACGCTGGGGGGGATCCCTTACATTGCGTCTTGCAACGCCCATGGATCGGGCTTGTGTTA
AGTCACCAGAAACCCACCAGACCAATTCAAATCAGGGTTCAGTATCAGCAGCTTCCTC
CTCCAGCGGCTCCAGCGCCACACGACGGCGTGCACCATGGAACCCCTTCAAACGACATAGCACGCGGGATGAT
AGACGTGAACATACTAATGTTATTTTTAGATAAACTACATGTTTGAGTAGTGATGGGTCACTACAGTTTAATA
GGTCCCCCTTGAAATAACATAACACTCGAAACGTTCAACGGGAATCCCTGATTGGGGA
TTACGCCCCTTGGATATTGGGCCCTTGGGTCCCTACGGCAAAAAGTATGAATCTGTGGGAAATGATCGAAAAA
ATGTCGAATAATAAACATAACGGAATAATTATTTCAATTATATAAATAATATCAAATATCTTTATGATGATTG
GAAATTATTAAATTTAATGTAACATGTAGAAGTGAATCCGATATTTGATCAGCGTATA
TAGCTTAAATCTTAACGAAGCAGAGATAGGAATTNTT

>78230615_2 CV526454.2
GGAGAGAGANGAAANNGNCTTCAGTTAGCGTCAGAGGGTAACGCCTATAATATAGTCCGTCGAGATTGAACGC
GACGATGGTCATTATTTATAGCAGATATCCACAGAGAGGATAGTGGCACGTATTTATGCTTCGCGACTAATCC
TTCGGCCAACGTCACGGCGGAGGTCGTTCTTAACATAGATCCTATGACAATGTTTGAG
ATTAAAATGTACAGTTTGTTATGCGGAGCTATATGCGCAGCGAGTTTTCTAGGCCTCACTTTACTTGTTCAAG
CGATGCGATACATATTCTACAGGTTTCGCCTACTGGAAACTTGCTGCAGTTGTTGCACTTGCGTCAGTCGGGA
TGCGCCTCGCACTCGACAGATATACAGCATGTTGGACAACATTGAACAGTATAAACGA
CTGCAGTTGGAGAAACTTAGAGAAAATTACGCCGTTCAAGTTCATCGGTATTAAAGAGAACTGCACTCAGCAA
ATGGAGTGGATTCAAAGTAGCTACTCGACTCAAGCGGCTCATCTACGCAATATCAGAGACATCGGCACTAACC
ATTTGACCGCTATGAAAGATCAATATTATGATCAGGTGAAGCGCGTCCGCGAATACTC
CACGTCACAATTAAATTGGGTGCGGGAAAACTACGTTTTTCAACGAAACAAAATAAGAAAATTCAGCGCGCAT
CAGATAATTAGAACCCGGGAGGTTTTAAAATACCGGCACGAACTTAACAAGTTTGGAGAACTGCGNNNNNNG
>78230618_2 CV526457.2
GGAAGACGAAGANCCNCTGGGGCGAGATCCGCAGTTAGTCGTGCTAATTATAAACTAAACCATTATAAGAATA
TATAATTGAGAATCATCATGGAAAATAAATCTGAAGATTCTGCTGCACCCGAAAAAACAATATTTAGTGCCAT
AGCTCAAGGTGACTTGGCTGAATTTAAGAATATTCTCGCTCAACATAAAGGCAGTGTC
GATTTCTTTGACGAAAATGGTATGACGGCGCTCCAACATTGCCGCTTATAANGGGCAGTAAGGACATGGTGCA
ATTACTGCTCGATAGGGGTGCAGACGTTAACTCTGGGAAACATG
>78230623_2 CV526401.2
AAGCACACACAGACCACAACGAAAAGATCCGAGGACGAGGAATCGCTCGAGGTGTCTACAGTCGTGCTAGGTT
AAAGGTATTAGGCACTACGCGCTCGGGGGAAGTATTATTTCGCGATGATCGCTCCGGGACTCGACACATTCCA
TCGCCGGCGTCAATACCAGCGTGCCCGGAGTAAGATATTAGCGTTATTTTTTATTTTT
GAATTGTAATATTGCATATATTTTTTTTGTTAATTTATATCCACAAAAAATACTGATGCAATTAGCCTTATTG
TGATGCAATATTAATTTATGTCCTACCACCATGGAAATTTATTGTAAATGACAGTGTAAATCTAATAATAAAA
TTCAAAAGTCCAAGGTGTAGTTTTATTCTCCCGCACTCATTCGGTGTCTCTCTCTTGT
TTATATATGTTGTGTCGGACATGTCATTTCATCTTATTAAATACTTAAATTTAAGTAATCAATTATTCACACC
TAGAACTTTAAGGGAAAATATCTTCATAAATTATATGCTTATTGTGTTTGATAACATTAGAAAATATTAATTA
AACAAACAAATATTTAAGTGTATTTTACAATCTTTCAGGAATTAAAGAAGGTAATTGG
AAATATTGTCGTCCAGATGAATGTTATGTACCTTATATGTATCTGTCCCTTGTGAGAACATCATAGAACTTCA
ATCGAAATGTTCCTTAGTGCA
>78230624_2 CV526402.2
AAAAAAAGAACGANCNCACCGGGCGGGATCGGCCGAGGGCGCGTGTGTTGTAGCTCTCGTAGCAGTCGATAAG
TTCTTATAACAATGCGTTAGTAAATATAAATAATAATAATAAGTTATCAATGATAGAAAGAAAACTGTGACAC
GTTTTTTTTTAAATAAAAAAGTGTAAAAATATATTTTTTTTCAAGTTTTCAAAGGATT
TTAGCAACTTTTTAAAGTAATATCCTGAACAATATTATCTTAATTTGACTACGTATGACAAAACATGGGGAGC
GTAGGCAACAATACCACATTATATGACGTACAAATTCTTAATCCCAGCGAGCTTACTCTTCAAAGGGAATTCA
CCGGTATCCTAACAAATGTGAATACGTGGCCTATAGAAAAATGTTTAGAAGCACATTT
AATAAACAGTACATTGGGTGACGAGTTAACTGGCCATGTTTTTATTGTAAATGGTACTCAGGTTCTTTGCCTG
GAACATGCGCCTGTATTGACCAAAAATACTTTAATTAGAGCAAGTGTCCTAACCGCAATGGCAATTTTATCTT
TTGTTGGAAATCTTGCAACAATGGTAAGCGTGCAGAAAGGAAAACGCTGTCGAAGACG
TGCAAGGCCCTCGTGGACAGCAATATATAGCCTAATATTTCAACTCAGTATAGCAGATTATTGGTCAACCAAT
CTGTATAAGAGAAAGCGGTTGGCATCCCAGTCACTCGTTGCCGGAATTTGCTGCATATTCATCTCCNATGCGC
TCTTTCTCGTCTCTTGCTACTGGTCAATGCAGGGAACACAATG
>78230625_2 CV526403.2
TGAGAAGAGAAGAAGAACGACCGTGCGCCGACGAAGCAGGCACGGCACGAGGGTTACTTCAAGCTCGTTAGAC
ATGTGGCTTAATAGTCCACTCTCTTTACCATCTATAAGATGATGACGGGATGTCATACGAGAAGCCACTTTCC
AAGGACATCATACCCTAATAGTAATTAAAGGCCTACGGTACGGTATGATCCTGTCTAT
AACATCAGAAGATTGTTTCTTCTTCGCCATTTATATGACGCCTTCTTCCACAGAACGCTTAGCCCCAAGAGTG
GAAATTGGTGTCACCTGGCCTCCTGCTGGAATAAACCCCTTAAAACCCTTTCTAGTACCACTACTAAAAACAG
CAGTACTTCTTTCCTCAGGAGTTACAATAACCTGAGCACATCACAGCATAATACGAAA
AAAACCGAAAGGAAGCTATTCAAGCCTTAGGTCTAACAGTAACCCTCGGCCTATACTTTACCGCTCTTCAAGC
ATGAGAATATTAGGATGCCCCATTCACTATAGCAGACAGAGTCTACGGGTCCACATTCTTTGTAGCTACAGGA
TTCCATGGTCTACACGTCATTATAGGATCCACCTTCCTAGCAGTGTGTTTCTTCCGTC

TCCTTAACCATCACTTCTCCAAAAACCACCATTTTGGATTTGAAGCAGCCGCATGATATNGACACTACGTAGA
TGTCGTTGAATATTCGTTATGTATGTATTACTGATGACGATCATCGAGAAGAGACGATTTACACTCATCAAAT
GATCAAGTAAAGCATAGCATCGGCATTCTCTCATTTATAAACTACAACAAGAAATCAA
CAAAAAGCGCNNNNT
>78230626_2 CV526404.2
AGACCCGGACGAGGAATCGGCCGAGGATTCATTTATATCACCTAGGTTTTAGGCGAAAATTGACAACCAGATT
TAAATAACAATGTCGGGATATCCATATGGAGGGAATCCTCAGTACCCACCTCCTCAAAATCAGGTGTATCCAC
AAATATATAACCCATCAAACATGCCACCTGGACAACCTGCACCTCCAGGATACCCTTC
AGGTCAAAATCCTCAATTACCTTATCAACCAGAATTTGCAGTGTATCCCACTCAGTGCAGGTCAACTGCCACC
CGCTGCTATGTCTTACCCGGGACGGCTGCCAACTGCACCGTCATATGGATATGGGTTTCCTCAAGTTACACCA
CCGCCGCGAGGTGAACCAATAGTAGAATGGGTACCAATACTCCCAAATAGACGCATAT
AAGCTATCCAACCGTGCACTCATAGCGGGCTACGAAAGACACGACGGCAGTCCACTGTGGGTTATACGAGCAA
GGTTCGAATGGGACCTCATACCTGGAACATTGGCCGAAAACACAAAAAGACTATGTGCCTAGGGGAGGACGTG
AGAATAATGTGCAGAATATTGAGGTATGTTGTGCTCGTCCGGAGACAATTCCTTGGAT
CGATAGCCGCGATGGCCGTCTGCCACTATATAGCATAGTCGCTGGCACCCCTTCATATGGTGACCCATTATCT
GTGGGCAGAATGACGGAGCAGGCTCTCTCACTCGGGCACTGTCTTCTCGCCACGGCTTGTCTATCTACGCGCC
ATC
>78230655_2 CV526394.2
AAACCTATGCCTAAATATGCCGTCCTTGTTGCGTACTCTGGTGCCATAATGTAGGATAGCTTGTGCCACATGC
AGGCACATACCATGCAACAGCTACCATCATATATGCCTTCTCCATCATCTGAGTGACCAGTTGTTATCAGGAG
GTAGGCTATATGGACCAAAAATCATGGTGAAAGTGTGATTTTTGTGAGCCATTTAGTT
CCTATTCATTACCATTTGAAGGTGCTTAAATGCATAACGTTAATGTTCATATTCTTATAATTAATTAACCGGA
GTTTGCATATCTTCTTTCACAAGTACCCAACTGTTGCATCACTACTGGAGTAAATGGACCCCACCATTCCCCT
AAGGAAGGTCCCTGGACCACAAGTTCCCACTACACTGATTGTTTATTTTGGTTTTGGG
GTAGTGCACCTTTATTGAAAATCATTCAATTAGGCTTCTCTTTTTCAAATAGTTGGACAACTTTGTGTTAAGG
TAACTGCAGAAAGGACCTTTCTTTGTTTTGCAGAATGAAGCTGCACATAACATCTAGGTTGAATGTTGAGAAA
TTTTTCTGGTGTATTCATGTAGTGATAGCTACATTTGAAAGCAAATATGTTAAACTAA
CTTCTGCTCTTCTTTCAGTTGAACAGATTAAACCGTGAAATTTATGTATTATCACTGCTA
>78230656_2 CV526395.2
GGAGAANNGTTGCGAGAGGATCGGCAGCGGGTCATTTCTCACTAAGATGCTAGAGGCGGCGAAACAGCTAGGT
TACGTACAGGAAGTGCGTCAAGCTGTGAGATATGCTGCGGCCGACTTAAAAGGCGAACCTCTGCCTGGATTTT
GTTTACCTAAGGGTATAGCACCTATCCTACCGCTGTCCGAGAGGCGATACTTAATGGT
TTACAGAAGAGAAAGAAAACGCAGCCCTTATGTTAGGAGAAGTAATCAAATTAACAACAGCACAAGCCATCCA
GCCCTCTGTGGTGCACATCACCGGACCTCTCATCCGTATCCTGGGTGATAGGTTCAATTCTAGCGTCAAGGCT
GCCGTTCTTGAAACACTGGCTCTATTATTATCTAAGGTTGGAGTAATGCTGAAACAGT
TCCTGCCTCAACTACAAACAACGTTCGTAAAAGGTCTGTCAGACGCCAACCGTCCAGTGCGCATCAAGGCGGG
GCTGGCCCTCTCCCAGCTGGTGCTGATCCACACGCGTGCTGACCCCCTGTTCTTGGAAGTGCACAACGGCGTC
AAGAACGCTGAAGACGTGGCTATCAAGGAGACCATGCTGCAAGCGCTCCGCAGTGTCA
TCACCAATGGTGGTTCCAAGATGTCCGAAACGACAGCCCTCGCAGTGCTCGCTATGCTGACGAGCCCCGGCAT
GCTCGCGCATGCAGAAGACGCGCCCGCGNNG
>78230660_2 CV526309.2
GNNTGAGACAGGTCAGAGGATCGGCCGAGGGTTGCGGCCGGACGGCACCATGTCATATCGACCTGGAAACATG
GAAGAAAGTCCTACGAAACCGCACAGTAGTGGATTGCCACGTATCCAGGTGGTGTGGATGATTTTAACTTGTG
AGGTACTGTGTGGTTAGAAAATTCGGCGGCAGGTAACAGGTCAAACAGTTCTTCAGAA
CACTCGACATGATAAATGCGGTATGAAACGCACAGCGAGGAGACGTACCTACGCATAGCCAAAGCCTTTAACA
GATCATTGGGTCTCCAATGATTCGAATGGATCTATGTAGTATGCGATCAAATGTTTCAAGCTAATACTTTGAT
GCACCGGCCCAGAGATGAGAACAGTACTCCATATGAGGTCGAACCTGGGCCCTATATA
GGGCCAAACGGTGTCTTGGCGTGAAGTACCGTCTCGCTCTGTTGATGACCCCGAGTTTCTTCGAGGCCAACTT
TGCCTTGCTTCCCAGATGATCGCAATACTGGCAGTCACTCGAAACATTTATGCCCAATATACTGATACTGGGC
GCGGCTGTAAGAGGAGTGTTTCGAAAGACTGGCGATCCGATGAATGGGACTTTGTGAA
TAGTTAACGCGCAGACTTGTGTCCTCCAGGGGTTAAACTGAACTAAGTTTCATTCACCCCATGAGGAGATATT
GTCGAGGGATGCCTCAAGCAGAAGACCCAGTTTATTTCCGGCCTGTTCAGCGTTTATCCGAGA
>78230662_2 CV526311.2

AGGGGNCNCGGCGNNAGGGNNCTACATAGGTTAGAGCGATCGGCCGAGTGATTCAAATGTCAACTTTCATTGT
TTCTTAACTCTGTGGAAACTGATTCCTTCTTACATCGTGCAATCAGCTGCAATGTACTCGTGCCATAGGCTTT
GCTGATGTAGTTGTCACCCCCATGCTTGATGTGCTTCANTTTAAAAGTAATTTGAAGT
GAGTGATATGTAAAGAGGTATTAAGACATTATAGAGATTAAGATGTTAGCAATCAGTCAGATTGGTCAAAGCC
TTTCAGTACTCATCCATGCCAGAAATCAGTAGAGGCCACTCAGTTTGCTATCCTTAACAAACTTTACTATCTT
ATCGTATATTCTTTAATAAACGCCTTGGGAGTTGCATCTTTGAAAAGGGGGGAGGCAT
TTATTTGAGGATATATTTTATTAAAAAAATAACTTCTGCTACAGCCTACACTAGTGTAACCTACATATGCTAC
ACGATTACATGTGTGTGTATGGCGGGCAATACGTTGGTCAGCGTGATGGCTTCAACTGTGCATGCAGCTATTA
GCATGGCATTGCTGGACTTGGGCCTTATAGGTGCACGGATGATATACTGTACCGCGAA
TGCATGAAGATGAACTTTCATTCACAAAATACTTTGTGAAAAATTATAGTGAGGCANNT
>78230667_2 CV526316.2
ANATAGCATAGGTTGAGCATCGGCCGAGGAGAATGTTGGACATGGGTTTTGAGCCGCAGATCAGGAAAATTAT
TGAACAAATCAGGCCAGATAGACAAGTCCTCATGTGGTCAGCAACATGGCCGAAAGAAGTCCAGAACTTAGCA
GAAGAGTTCCTTCATGATTACATACAAATTAACATTGGCTCTCTTTCCCTGTCGGCCA
ATCATAATATTCTACAAATAGTCGACGTCTGTGAAGAGTGGGAAAAGAATGATAAACTGTTGACTTTGTTAAC
GGAAATATCGTCCGAGGAGGAAACTAAGACTATTATATTTGCTGAGACTAAACGGAAGGTGGACGACATAACA
AAAACAATAAACCGCGCCGGGTGGCGGGCCCTGGCTATACACGGCGACTAGAACCAGC
AGGACCGCGACTACGTGCTGCAGCAGTTCAGGCACGCGCGCTCCTCCATACTGGTCGCCACCGATGTCGCTGC
TAGGGGGCTTGATGTCGAGGACGTGAAATTTGTGATAAATTACGATTATCCAAACAATTCTGAAGACTACGTG
CATAGGATCGGAAGAACTGGCCGATCTCAAAACACTGGGACCGCTTATACGCTGTTCA
CGCCTAATAATTCTGCTAAGGCTAAAGATCTATTGTCAGTTCTACGAGAAGCGAATCACGTAGTGACTCCCAA
GTTGTTAGAATCGGCACAATGTGGAATGGGCTGTCAAGGAA
>78230668_2 CV526317.2
GGGGGNNNNGGNCNNAAGCGAGCCATAGGTCTAGAGGAATCGGACGAGGACGTCGGCCGTAGAGACGTAGAGA
CGCAGAGATGTAAACACAGCACAGTGTGTCTGCACGTTACACTTCAGCCTAGGAACCGGTTGCTCGATCAACA
GATACAACGCTACGATACTTGTGAGTTGTGACATCGATGTGTTCAGTGACCTTTTTAA
CTGTGTTCAATTTGAGAAAAGTGTGAATAACGAGTGTCAGTGGGAACTTTAATGGTGCGATGATCTGTGCTTT
TACTGGTTGTAAATTGGATTTATTTTGGTTGGATTTATACGCAGGGGGTGGCATTAAAAATGGCGACTGGAAA
AATGTATAGACTCTAAACAACATGGCCGTCGCTGGTGCGGTCTTGGTGTTGCTAGCGT
CCGCCGCACACGCGTGGATGCCTGATGCTAATGCAAAGATCCATGTCAAGTTTGGTGATGATACTACAGAGCA
ATTTGCGGGGAACACAACGGGACCAAACCATGTTCGTATTTCAGATAAAGATGACTTCTCAATTGTGGTCGGC
GGCAGGAGTACAGTCTACGAGTCTACGTCCTTGACGACATTATCTGAAACTGTCGAAC
AGCGTTCAAAGTGGCAATCGACAGACGCCCACGGAGAACTATGCCAAGTGACACGCAAATCAGCTGTTGATTG
GCCGAACCAGTTCGTTCTGCAACAGGGTCCCCGTNAG
>78230671_2 CV526320.2
AAAAAAAANANGAAGGAAGAGGAANAAAAAGACCCTAGTGCTCCATAAGTCAAGCGATCGCCGAGGCGCGCGC
GCCCGCGCTTGTGCTGTGACAGTGACAGGTGACAGTTTATTTGAAATATACAGCCAGTAAGAAGAATGAGGAT
CGCCTTTTGGGTCACAGCGTTTGCTGTGCTCAGCATAGCATTTGCAAAGAACTCGACA
GCGAAAACGAAACCGAAACGACAGTTAGAGAGTATAAGAACTCCACAGAATTTACAATATTCATTAGTTCTTC
CCCACTCACGAGTAGCACATTTTCGATCATTGACAAGCTCGAGGAGAATCTCAAATGTAGCTCCACGTGGAGG
TAAACTGCCGCCTTATGCCGTACAAATGGAACCAGTTGTGCAATATTCACAAAAAGAT
CCACTATACGGCTCCAATCACGAACACATAGAAGAAACTGTGGCAAATAAAAATGAAATTTATGCTCAACCAA
AAATCCTTAACGTTCATGATGGGCCCGAAAGTTCGTTTGGACCTCCCTATGGCAGCCTACCAACAGCTCCGCT
TTACAGCGAGGAAGCTCACCCTCAATATTATGATGCACCAGAACCTATTATTGAGATT
ATTATTAAAGAATCTAATGAAACACTGCCTGCTCCCCCACCACCTCCAGTACA
>78230675_2 CV526324.2
GGGGANNNNCACAANNANTTCTACATAGTTAACGATCGGCCGAGGCGAAGCAGGGAAACCTGATGGAGGACCG
TAGCGATTCTGACGTGCAAATCGATCGTCGGAACTGGGTATAGGGGCGAAAGACTAATCGAACCATCTAGTAG
CTGGTTCCGTCCGAAGTTTCCCTCAGGATAGCTGGCGTCGATTATTAACAGTCCCATC
CGGTAAAGCGAATGATTAGAGGCATTGGGGCCGAAACGACCTCAACCTATTCTCAAACTTTAAATGGGTGAGA
ACTCCGGCTTACTCGAACGATGAAGCCGGAGATCTGATGACGGTGCCAAGTGGGCCAATTTTGGTAAGCAGAA
CTGGCGCTGTGGGATGAACCAAACGTAGTGTTAAGGCGCCTAAAAAACGCTCATGGGA

CACCATGAAAGGCGTTGGTCGCTCATGACAGCAGGACGGTGGCCATGGAAGTCGGAATCCGCTAAGGAGTGTG
CAACGACTCACCTGCCGAAGCAACCAGCCCTGAAAATGGATGGCGCTGAAGCGTTTTGCCTATACACTACCGT
TACGGGCACGTGCGGCGTGCGCGTTTACGCGTGCGTCTGCGTCATTATGCCGTAACGA
GTACGACGTGCGCGGTGGAGAGCGCCCAAGGGTCTGGGCGTTGAGCCCGGTTGTAGCCTCCGGTTGTGCCGAT
CTTGGTGGTGTAGCAATACCCCGGGAGGCCTTGATNCG
>78230677_2 CV526326.2
AGAGAAGAAACGAAAGATTGTCTAGTCTGAGCGATCGGCCGAGGGAAAGTCTATAGAAATAACATGAGATGGA
TCAAGACATGGCTTTGGAGGCGCCATGGTGTGGACGGTGGACATGGATGACTTCTCCGGCGACGTTTGTGGTG
GAAACGTGAAATATCCACTTATTGGTGCTATGAGGGAAGAACTACGTGGAATATCTCG
TGGCAAAGACGCTAAAGATGTGGACTGGGTGTCAGTCGCCGGCACGGTGGTCATAGAAGTGACTGAAAAACCA
CAACCAATCAAAATCAGTTTATCCGATGTGCTGTCTAAAGTGAAGAAACCAACGAAGACTTTAGATTATAAAG
AATAATAATGCCTTGGCTGTGGATAATACAAAACGCGATCCCCATATCCTCTGCTATC
TAACTTCGTGGTCTTCAAAGAGCCCGAGCTCAGGCCGCTTCATGCCCGAGAACGTCGATCCCAGCTTGTGCAC
CCACGTAATATACGCCTTCGGTACTCTAAAAGACCAANA
>78230679_2 CV526328.2
GAGGNANGGCANNNNAAGGTGCACAGGCTGAGGAGCTGCGACGAGGATTAATCTAAACTAACGTTTATTATTA
TTCTAGAATTGAAAAGTAAACTGTTCTGTGAAAATGTTTGAGGCACGCCTTTTACGCAGCTCCATCTTGAAGA
AAGTCCTTGAGGCCATCAAAGATCTTCTTACACAAGCAACGTTTGATTGTGATGATAA
TGGAATACAATTACAAGCTATGGATAACTCGCATGTCTCCCTTGTATCTCTAACACTGCGGGCAGATGGTTTT
GATAAGTATAGATGTGATAGAAATATATCCATGGGAATGAACTTAGGAAGTATGTCAAAGATATTGAAGTGTG
CTGGTGACAAGGACACAGTAACAATGAAGGCTCAAGACAATGCAGATACCGTCACATT
TGTATTTGAGAGTCCCAACCAAGAGAAAGTGTCGGACTATGAAATGAAACTCATGAACTTGGATTTAGAACAT
TTAGGTATACCTGAAACTGAATATAGCTGTACTATCCGACTTCCAAGTGCTGAGTTTGCAAGAATCTGTCGTG
ATTTATCTCAATTCGGAGAATCTATGGTTATTTCATGTACCGGAGAAGGTGTTCCCTT
CTCCGCGACCGGCGAGCTATGTTCCGGCCATGTGGGGTTAGGGCAAACAGGGTTCCTTGTACCGGGCGGATAA
ACCCTTTTCCTCTCCCTGGGTAACCGTTGCCCTCCNNTG
>78230695_2 CV526344.2
AGNAACCTGCTGAGGACNTGCCGAGCTGTGCGAGTTTTATTGGACAAATACAGGCGAGATGATGTAAATGCTA
TACTATATTTTAAATTGGAGAAAAATTAAGATAAATATTATTAATTCTGTAAAGAGTAAAATATTTCTGTAAT
GACTTTTATAGTTACTCCTGCTCTTAGGATTAGAAAAAGACTACAATTTTCTTTAAAA
ATTACTATATAACAATTTTTATTAAATTTTCTTTATTTTTCTAAGAGCACGCTTAATATATTTTCCTTACAGA
ATTAATTTAATTTGTACTTAATTTGAACCTTAAGTCAGTGCTAGAGAAAAATATTTTTATGTAACTAGAAAGA
TAAATATAATGTTAATGTTTTCTACCGAATACTTATTAGTATAATGTAACATTTGAGA
CATTTTAAACCAGGTTTTTGCTTTACAAGCTAACCACGTATTTGCGTATAAAATGTTCATTTATATAAAATAT
ATGCACATTTAAAAAAAATACAAATCGCATCATAAGAATGAAAGACTCAAATCAACATTTACGATATTGAGAT
ATACTCTATATCTTATAATTGTAATTTACTATAGAATACATTTTTATAGACGTTTTTA
GTAAATTCTTTCATTCTCGCTGTGATAATATAGCGCGTGGGGTGTTTGCAAGCATTAGACTTTGGGATTAGTT
TTAGACATGTTCCTTGTGCCCTCTGTTTNNT
>78230697_2 CV526346.2
AGAAAAAAGACACCATTGCGTTTCTGTGGCTTATGAGTCTGGNCGAGGGAACCGAGAAAGAGAAGGAGAAGCC
ACCTCAAGTCCGGTGAAATACCGACGATAATAAAGATACAGAACGTGACACAGAGAGATCGGCCTCGCCCCTT
TTCGGTGCCAAACTGAACGAAAACGAAACGTGGACGAGTCGAGAGTCAACGCGATGGC
GCTGCTGCGCGCGCAGCGCGACGCGCGCGCCACCCGACGTCGAGGTCAAAGCAGGAGGAATGCGCCCTGGAAG
CAGGGCCAAATGAATGAATGACTGAGCGCCAGACAGCAGGAAACTTAATACGGTGAGTACAATGTAAACCCAG
CAGTGTCAAAGATTAAAATGCAGTCTGAGTATCTATACCGGACGACTACAAGGAATCA
GACTTCAAGAAGGATACATAAACTCAACAAGGAGTCGGTACGAACGATTCAAC
>78230700_2 CV526349.2
GGGGGNNNNNCCCNCANATTCTACATAGGTTGAGCGATCGGCCGAGGCAGAATCAATGAAATATATATTCTTA
TCTTATAATATTTAAATAGAATACAAGCAATTTACAATATATATTGTATCATAAATTTTAACTGTCAATCTCA
TAATTCTTCTGTATCGAATCTTTTTAGACTTTATTTATGCCGAAACTTGGACTAAATT
CTTATAATAAAAAAATACGTCAAAATTTTACAGTTCCTTATTATTTTTTTATTGTTAATCGTTTTGACCACAA
AATACAACGACACAAGTTAATATAACCATTATTGTACATATTTAAGAACAGATTGCAACCACATTTGAAATTA
TTGATATGAAATCTGTAATTTAAATAAGATGTATGCGTGTTGTAAAATGTGATCTTTA

TTAATAATAATTAGAATTATATTTCAGTTGCTAAAATATCTAAATATAGCTAAAATTAAATTAACTATGCTAC
TAATAAATTTCACTGTCATTAAAGAGTAGTTAGAAGGCATAGTAGAAGGCGTAGGCTTTATGTAATACAATTT
TATACATAAGTAAGTGTGTAATTTCTAAAAAGTAAACGTATGTCAAAAATACATATCT
ATTTAGATTACAATACATATTATATTACATTAATTTTAAAATAACAAAATAGGTCACTTCGGTATCTCATCAT
CACATTAATTAATAAGCAATATTAGTTTAGA
>78230703_2 CV526352.2
GGAAANNNNNGNNGGAGNGNAAGACTAGGTCGAGCGAATCGGCCGAGGGTATGGCGACGTAGAAGTAATGAAT
GTGTGAAGCTGTACGCCAACGGCTGTCTCCCCAGGATTACGTACCTGATCTACCAGAGCGCGGGCTTACTGGG
CGCTGGGGCCATGACCATTGCGTTCATTCAGGTAATCGGCATCGTATTCTCATTCTCG
CTGGCCAGTTCGATTCGCAAGGCGAAGTCTGAGCGCGAGCGCAGGCGTTGGGAGATACAGGAGCGTATGATCA
ACGCGTACACCTCCCTTAACCCGGACAAGGAGAAGAACCAACCCATTGTATACGTGCCATTCCAGGGACAGAC
AGCTTAAGCTTAATATTGTCGATATTTGTATTCAAATTTATGGTAATTTGTCCAAATT
TTATTTACTTATTTGACTTTCTTATAGTGAAATTAGATTATATTTGTTTTGACGCACTTTAATATTAAAGCGA
TACAGTTAATATTAATTAAATTATGAATAGTTTGTTTTATAGGAGATTTCGTACTGCTCTATTAAGATTTCTT
CATGCAATGCTATCTATAATAACAAATAAAATGTATTGCACACAATAACTTAAATTTC
ATTTTTGAATATACTTTGAGAATAAATATAGTTAAACTATCGTCATAGACTCTAATTATGCAAATTTATATAT
GCGAAATGTCATGGTTATTTCTTAAGTGATGAAATGTTGATTACGTAATTTAATAAATTAGTTATNNCC
>78230705_2 CV526354.2
GANNNNNNAACAAAAAGGCTTGCAGAGCTTGAGGATTGGCCGAGGGGCAGCGGAAACGCGCCGATGTTGTACA
GAACGCTAAGCGAGCGGTGCTCGCAACTAAGAGCAAGCGACCGCCGCCGCCGGTGCACTCCACTGCAAGCAAC
AGGAATATAAGACTGAAAAAGGGTATGAAGACGGCGAAACAGCGCCTCGGAAAAAATCC
TCAAAATACACAAAATGATATATTAGTGTAATATTAGTTGTAATTTAACATACGAATGTGTAGAGTTATATTT
TATATACGTATATATATGTATGTAGATCGGACCAATATGTGTTTTTAGATCACGTCTTGATTTCTATTGATTT
AGTTTGATTATATTACGGCTGGATACCGTTATAATGTGTTTATTATATAATTTTACTA
ATGTCCTTTTCTTGCTTATTTATTTACTTGAACTTGATTGTCGATTCGATTCCCGATTGAAGTCTTTTTTGTT
GTTTATATATAAAAGTTAGATATACAAATTTGTCTTGTAGCATTATGTATTATTCAACACTTTAAACAAAAAT
TGTACAATCTAAATACAGATAATCAATTTAAATTATGAACTAAACAGATTAAAACTGC
CTAAAAGATCGTATGTCAATATGATGCAATATACAACAGTTGAAACTGGTGTGATAGTCAATCAANNNA
>78230709_2 CV526358.2
GAGGGGNNNNNNNNAGCCACAAGGTGTTCTGCTGGTCGGAGCGATCGGCCGAGGAGAAGCACTGTCGCCACCT
AAAAAACGTTTCCGTATGAGTGATGCCTCGTTCCTGCGTCGGGATTCCTTCATAGACAGCATGTTCGGTGGCG
TGTTACTGACGCGAGTCGAATGCACCGTCTGTCACGCGTCTTCACTATCACGCGACGT
GTTCCGAGACCTACAGCTTGCCTTCCCGGAGAAGCCTGAGGGATGGCAGCATAGTGTTCAGAGCCTGCTAGAG
TACTACTGCTCCAAGGAGAGCCTCTCCGGCGATAACAAATACGAGTGCCGGGACTGCGGCGGTCTCCAGGATG
CTGAGAGGAGCGTCCTCATTGAAACCACCCCCAAGTATCTTATACTTGTATTGAAGAA
CTTCAAGTTCGATTCCAAGCTGCAAGTGCAAACAAAGCTGATGCACACAGTATACCACAACCACACGGTGACG
CTGCCCACGGTGCGGGCGCAGGCCGTGCACGCCGTCTACAACCTGTTCGCGGCCGTCATACACGCCGGCACCA
CGCTCGACTCCGGCCACTACTACACGCTGGCCAAGGACAACGACACCTGGCACAAGTA
CAACGACGACGAGGTCACCTTCGCGGACGAAAACCATCTGCACACGCTGTCCCGGTCCAGCACACCGTACGTG
CTGTTCTACAGGCGCACAGACATCGAGGAGAGCACCNNA
>78230713_2 CV526362.2
GGCNNNNTAGCCTGNGNTCCTAGAGGTCAGAGCAATCGGCACGAGGAGTAAGGCAGGCGATCTCTTCCCAGCC
ACGTTCCCGGATAAGGGGCCAGAACTGTCTCTTAGATGCGCCAGGTACATAACTGGAGGATTACATGCTGCAC
AGGTGCTGGATATATACGACAACGGCGTGGGCATGTTGCGCATCGGGCCGTTCAACTA
CGAGCCGCTGCGCGGCGTGGACCTGTGGCTGGAGCAGTCCGACGACTTCATCCTGCAGCACCTCAGCACCACG
CCCGCCGTCGAGCCGCCGCACTTTGTGCACAATATACGTTCAACACTCAAGTTCATACAGCAACATCCGTTCC
CCGCCGTCACCGTGTTCCCGGACAACCGGCCGCACTACTACCGGCGCGACGAGCAGAC
CGGCTGCTGGGTACATGTTCGATTCTAGTTCAAACTCATACTTTTAATTGAAATATACTAATATTTTCATTGA
AATATATGTGATGTTATGGAATGCTTTGATCGTGACGATGAACTAAAAAAGTATTTGTAACCGTAACCGAATA
CGATATTGAAGTGAAATGTAACATTATGGGACTTATCGTTAAATTCCTCGTAAATCTG
ATTCGGTGTCCAAAATTTCTGGATGATCTGCAAAACTCTCATATCCGCAATCGAAACCGAGGCCTTGTCGTTC
ACTAGAGTGACCAGTGTTGCTNGG
>78230717_2 CV526366.2

AGGGGGNNNNNCACNANNNCCGCCGGGTTGAGGATGGCCGAGAGGAAATAAGATGTGTTAGAAATTCATGTTA
CATTCGAAGATGACCGTTAGCTGTTCCGGAAATTAAACTGCTTCTTGCTGCCCATAAAATTGCTACATTCTAA
TTCTCTTTGAAGGATCGATACCGAGAAAAGTTGCGAATATTTGCGAAAGGAATAATTT
GTTGGAAATTCAAAGAAAACTATTTAGTTTGTTAACTAAACCAAAAGACTACTTGGTTTGCTCTGAAGGCCTA
ACAAATTCTTCTCACTTTTGGGTTTGTTATAAATCGTAAATAACATCTTTTGAAAGTTTCAAACCCTTCGTGG
GCTAGGGAATCAAATAGTGAACAGCTAGTTGACTACGGTTTTATAGTAGCCTTCTCTT
GGAAGACTGGGTTTGGGTCTTTTTAAAACTGACGTAAAATACTTCCCGTGATCAACTAGTTATTACGTTGAAC
TCTTGCCTGTAAACTTGATCCAGGCTTAAATGACACTTAATGATCGATCATGAAGGTTAATCTGTCTGGAAAT
GTTATTATGTACACAATACACTGTGAGCCGATATTGCGTGCATATATATGTGGGTACA
AAATATAACAGAGTGTACATGTATGTATGCAACGATTACCGGCGAATTAGAACAACGTGGGGAA
>78230720_2 CV526369.2
GANANAACCAGGGNNACGTGCAATGCTTACGAGCTGGNCGAGGGATCTTAGGATCACTCGAGCTGTCCAGTAA
GATTATGAGGAAGAAAAAATTATAAAAGGGGAGCCACAGTTCTTGCCTAAACTAAAAGTGCAACGTTTGTTCC
AGTGATAGAAGTACATAAGGACACTAAACCATACAATAAATTAAATAGTTTAGCGAGA
TTATATTATAAGCTTGTAACAAAGTTTCAATTAATCGATATGCGAATATCGAGTTTTTCCAAAAATAGATTTT
CTTAAGCAGTTAATCGAATACGTGTTCATATAAAAATAATGATACAGTAAACATTATATAATTACAGATAAAA
AAAAAATACAAATAAAAAAGTATTGGATTTTCGAATATCGATTTTTCTGAGAAATAAT
CGTTGCAAGTCTAGATTTCTGTACTTAATTCGTTTATTGTTCATATAACTCGGAAGATAATCGTAAATTTAAG
TATGTTCAAGAGATCTATAGATATATGGTTAACTTTTTATAAATAAGTGATACATTTAAGTGCAATTTACATT
TTAAATATATAGATTAAATAATTAAGAAAGTATAAGTGAAGTGTGACATTGTGTGGGG
GATTGAGGTTGAGGAAGTGTGACATTGTGGGGGATGGATCCTAGATCCTGTGATACCACGAAAAATATTAGCT
CNNC
>78230729_2 CV526265.2
CGGGTGAGATCGGCACGAGGAGAATGTGTCCAATGTCCCCGGGAACTTTTCTACTACGTTCTCCGACTCCGAG
CTGGGTGGGATCACTATTGCGTGGGTTGGTGAGGGTAACGAAGTGTCAGCCTCCAGCCGTTCACCTCGCGTGA
TCTAATGCTTAGATCGCTCGCGGATCGTATCTTGGACCTCGCGCAGTTGCAGTTCCTG
TACCCAAATGTCGCCAAGGACGATGTGTTCTCTAAGTACTACACTAAACCTGAAAACGAAATGTTAAAGAACG
GTTACGTGAAACCAGTGTTAGTGACGACGCTACCGCCGTACATGTCGTCGTCCCCCTACGCGCACTCGCCCGA
CTCGCATCGCAACACCCCCAGCGTGCACAGCAGGTACATGATTGAGTTTTGACCGATA
CGTCCGATTTTTTATATACTTTATTATTATTTTTTTGGTGTTATTTAATAGTCACATCCTTTTTTATTAAGTA
TTATTTAATGTGAGCTTCCAAAAAATCCATTTATATAATCT
>78230730_2 CV526266.2
TAGGGCTGCGGAATCGGCACGAGGTTATTGCAATAATCATATTCCCATCAAACAAACAAAGAATTTAACAAAA
AAATCAAAGAGGCGAAGAATTCTAAAAATAATGTAATACAAATATAGCTAACAAGTGCACTACATTAAAACAT
AAGTAATTCTTATTTAGTAACACGCCAATTGGTTGCTATTTAAAATATGTCTACGAAA
AATATTGTCTTTCTTTTGCAGTCACATAAGACTCGGTTTCAATTTTCGTTTACAATTTACATATTACTGATAT
GATTTGTATGAATTTTAATGTTAAAAAAAATTAAAACCAAAACAGCAGCCATATCATATATAAAAAAGAAGTG
CAAGATTAGAAAAGCCCAGGTTCACTTGAAATAATTTTCATTATTATCTAATACTAAC
ATTATGTTTGCCAATCTCTTAAAATAGTTTATTTACGAGTTCAGAGCTCAGATATTTATTTACAAATATTATT
TTCCATTTTCAATCTCATAAAATATTTATTTATATTTATGAACACTTACAACAATTTAGAATATAATTTTTAT
TATGTCTAATACATACCACAATACAATAGTTGGTTTCATACCATACAAGTTGTCACTT
TACATTAATATAATTTNNGT
>78230734_2 CV526270.2
GGGCACTGCAGAATCGGCCGAGGATGGACACCTGGCTTGCGGTAAAACTCTGTTAGCCAATGCCATTGCTAAC
GAGTGTCAGGCCAACTTGATATCAGTAAAAGGTCCTGAACTGCTCACCATGTGGTTTGGTGAATCTGAAGCTA
ATGTGCGAGATATCTTTGATAAGGCCCGTTCTGCATCCCCGTGCGTGCTGTTCTTCGA
CGAGCTGGACTCCATCGCCAAGTTCGCGCGGGGGCTCCGTGTCGGACGCGGGCGGCGCCGCGGACCGCGTCTT
CAACCAGATACTCACCGAGATGGACGGCATGGGCGCCAAGAAGAACGTCTTCATTATTGGTGCGACTAACAGA
CCTGACATCATCGACCCTGGCCATCCTCCGACCTGGTCGTCTCGACCAGCTGATCTAC
ATACCACTGGCTGATGAGAAATCCCGTGAGGCCATCCTCCGGGCTATTCTACGCGAGTCACCCATCGCTGAAG
ATGTCGATCTGTCTCATATTGCTAAGGTGACCCAAGGGTTCAGCGGTGCCGACCTGACGGAGATCTGCCAGCG
CGCCTGCTCGCTGGGCATCCGGCAGGCCATCGAGGCGGAGATTCACCGCGAGCGCGCG
CGCCAGGCACATGCCGCCGCCGCCGAATATGGATACTGGACGAGGAGGACCGGTGCGGGAGATTCGCGCGCGC
ATTCGAGGAGGCCTGAAGTNNG

>78230736_2 CV526272.2
GAGGAAGCCCGGGCGGGATCGGCACGAGGAAGTATATAAAGTCTAAACAGATACACTTATTATAAGTATTGCT
ATTTTAGTTTTAATTTATTTGCTTGTAAGTTGCCGAAATATTAACTAGTGTTTTAATTTAAAAATAAATTCAA
AATGAAGTTTTGCGCCAACCTGTCCTTCATGTTTACCGAAACCTCGAATTTGTTAGAA
CGATATATTTTTGCGAGAGATGCAGGTTTCAAAGCCGTAGAGAGCGGATTCCCTTTCGGTTATACATTGGGGG
AGGTCAAGCAAGCAAAAGAATCCAGTGGTGTTGAACAAGTTTTGATCAATCTCAAAACAGGTGATGTAACTAA
AGGTGAATTAGGAGTCACAGCAGTGCCGGGAAAAGAAAATGAATTTAAAGACAATTTG
AAAACGACCATTGATTACGCTAAAGCT
>78230740_2 CV526276.2
CGGGGCTGCGGAATCGGCACGAGGAAATACTAAATAATATATACACACGTACCTTTTTGGTTTAGAAATTAGC
ACAACATACGCGAGAACCAATAATGTCTTCACCACGCCCTAAATCACCTCGTACTCCCGTTTTACCAAAGAAA
GAAGACAAAGAAGAATCATTTTGGGATAAAATTGGAACGATTGGTCGCAAAAAGCGTA
TAAAGGAAGTGCAGGAAGTACAGGCGGAAGGAAAATACGCGATCGACTCACCTGGTAGTCCAACAGCTCCAGA
AATTCCACCAGAGGAATATAGCTTGCATGACAATGAAGAAAGAGCAATAATTGAACCTAGGTGCTTGGAAGAT
CCTAGAGTAAAAGACTTGATCCAAGTATTGATTGATTGGATAAATGATGAACTGGCAG
CACAAAGAATCATTGTCAAAGATATTAGTGAAGATCTTTATGATGGTCAAGTGCTACAGAAACTTCTGGAAAA
GTTAACAGAGACTAAATTGGATGTACCTGAAGTGACACAGTCAGAAGAGGGACAACGGCAGAAGCTGGCTGTA
GTGCTGAGAGCTGTAAATCGGGTTCTCTATGGGACACCAAAACCGGTACAGAAATGGA
GTGTCGATTCTGTGCATTCAAGGAATGTAGTGTCTATTAAACATAAAGTTAGTGGCTCTCGCCCACCATATCA
GAGCTCCTAANNC
>78230742_2 CV526278.2
AAGGACCGGGCTGAGGATCGGCCGAGGATTCAGAATCTTTTAATATAAAAAATAAAGTCACTGCTGAACTTGC
TTCAGCTGGTATGCCTTTACGCAAATGGAAATCCAATCATGCTTCACTTGTAACTTCCTCTACAGTTAGTTCG
TCTTTGAATTTAAATATAGGAGCTATAGAAAACGCAAAAACTTTAGGACTAGGATGGC
AAACAGAATCTGATGAAATTTGTTTTTATATTAATCATTCAACTCTGAAAGAGCTTACAAAACGAATTATATT
ATCAATAATTGCACAAATTTTTGATCCTATGGGGTTAGTTAGCCCATGTGTTATAACAATGAAAATTTTATTG
CAAAAATTATGGCTTATTAAATTAGCATGGGATGAACCATTGCCTACGGAGACTTTAA
GAAATTGGTACAATGTCTCTAAAAATCTTCCTTTATTAAGTAAATTACGCGTACCTCGTCTCGTGGTCTGTGA
TTCATACTCTTAGTAGGAATTTCATATCTTTTCTGACGCATCAGAAAATGCCTATGGTGCCTGTATTTATGTC
AAAAGTGTAAATAATCGTGGTGAAACATTCGGTACAGTGAT
>78230743_2 CV526279.2
GGCCCTGCAGGAATCGGCACGAGGTTTAATTCAATGATCTCCTTTTCGTATAATTGCTTTAGTCCTATCAAGT
TCATATTTTGATTAAATTCAAAGTATATTACTTGTTCTATGAATTTCTAATTTGCTATATACACTGTATATTT
ATTTTTACATAACGTCGGCTTAGAGTGTAAATCTGCTAAATCAGTTTTTAGCCATTTT
TATATCATTAGAAAGATTAAAAACTGGCCTTTATTTTATTTCATGTTTTAGAGAATATAAATCTTTTTAAAAT
ATTCTAAAACATTTTATCTCCTGAGAATTTGCTTAATAGACTTACCAGGTTAACCCTCTAAGCTGATGATATG
AATCATACTAAGTATACCTTATTAAAAGGGAACAGAATGTCTTAACACTAGGTTCCCT
CTCGCCAACCTGCTGTGTGCGGGATAAGGCGAGAAACTGACTCATTTAATTTTTTACTTTATTAATTATGATT
TTAATGTAGGTAATCTTATTTTTGTACTTAATTATATTGCCAGATCGAAATAAAATTTATCATCGTGTCTCAA
GCTTACGATTGATTTCTTTAGGAATGATCACAGTTTAGCCGAGACGAGAGTCATTTTA
AAATATTTGAATACCATATCCATCTATATAATATTCTAATTGCCCTTTTGCATGAGCTATACAGATATCTGTT
AACGTATATTTTTGAAAAACTGGGAAAAGNNG
>78230747_2 CV526283.2
CCCGGGCTGCGGAATCGGCACGAGGATTTTTTATACATAAATGCATTACANTAGCGCAATTTAATTAATAAAT
TTTAAAAATAATCGCAAAATAACGTTTTAGGCATGATTTATATTAACAAGCAGCGTCTAAGCTTTGGGGTGCA
CCCATCCCACGGGTATCGGTGAAGCTTCGTGACTCGTATCTATGAGCATATCCCATTC
TGACCTAACATTTATAAACTTTGCCACATCCCCATTGAAATCCACCTGCGGATTACTTTTAATACCAAATAAT
AATTTCTGCCATTTTGATAGATTTTTTATTGTTTGTTTTTCAATTTCCGTGGTGTTGTCTTTATCTTTGTTTT
CTTCTAATTTTTTGCGTAGTTGTTCTTCTTCTATTTCGTACGAAGCTAACTTAAATGT
CCAGAGTGATTTGAGTCTTTTAAGATTATTAGCAAAGGCCACTGTCTCATGTTCTTGTACCTGTTGCCCCTTA
CCCTTGGCAGTGTTCTGTCGAGCCAGCCTTAATTTAAATAGAGCATCTAATTTCTCCATGCTTTTCTTAGCAT
CATGTTTTCTACGGAGCACTTCTTTTAATACCATATCTGCTTTATGCTTAGATTCTTG
TTCCATTGTAGCCCTCTCCAGATTCTCCTTAGTCTTTACCAGATTCTCAGTGATTTTTCGAGAAGTCTCCTCT
TTTCCTTCAAAGCCGCTTCTTCTCCTCCTTATANNNG

>78230751_2 CV526288.2
CCACGGGTCGCAGAATCGGCGAGGATGTGCTTATAAACTATAAATATAATATTCAGACAATTATACATAAATA
TAATTTTCAGCAATATTGTAGTTTTCCATTTATTACACTACTAATTACGTGAGTTGTCTAAATTGGCTGCGTT
TTTAATCGCGGTTAGTGTGGGTAAAATGGTGATAGAAAAAGTCCATGGCTTGCAAGCT
ACTAGTATATCTCTAAATGATCGGTTTACAATACTGGCGTCAACTGCTCCAATAGGTCGTGTGATAAAACAAC
GGCGACGATCTTCTGGACTGTTCGTACATGACCGAAATATAAATAATCGAGCTTTGATTGAACAAATAGCGCA
ACATTTTGGTCAGAATGCAAAAAAAGCAAGCAGTTAGACAACGCCTGGGCATGCCTGG
CCTGAGACGGTTCGGCAGCGAGGGTAGTTTGGCAGGACTTAGGCGTTCAAATAGCTTCAACAATTTGAACCAA
AGGAATGTTATGAAAAGAATCTCCTGGAGGCAATCTAATGGAAACCTAAGTCGATCAGCGTCTTTCAACAACT
TATCGCTAGGCATGTGGCGTGGACGTGGGTTCAGACGAAGGGGAGGACGTATGGGACT
TGTAAGGGGAAGGATGAGATCCGCTGCGCCAACCGTCAACACTTAGTGAACAGAATGCGTAACCAGCAACGGG
TGAACCGGCGCCTATTGGGCGAACGGGGGGAACCCGGGGCAACGTGTCTACGCGNNT
>78230757_2 CV526294.2
GGAAAGAGGAGTCGGCACGAGGCCATGGGTGTAGCATTCATTGTTAAAACCAGTGAATTTTTAAGTAATTAGA
TACATATTTTGCACATCGTGGGTTCTACGTCCATGATTAACCTTTTTTTCCACTCGACGCAACCTATACGACA
TAGAGCCACTAGTGTTGTACCATATAAATTGTTTGTTTGTTATCGGTAAATATTTTTA
GTACATTTTTTTGTACTTTTTTAATATGAAAACTCAATTAGCCTTCAGTAAAAACTGTGAATTGTTTGTTGCT
ATTCTGGTGACTTACTTGGTGACCTAATTATGAACTAGCTTCTACCACTGACTGTAGCGAAACATCGACGTTT
TATTGCAGATTCCTATTTTCTATATCAGATTTAAGAATTATATGAATATGCCCTCCGG
AATGTTACAATTGGATATAGTATAACTTTAGAATTAACGCTTGGGTAAATTATAATAATTCATGAACGTTTAT
GTCATAATCACTGTCCTGATCTCGATCACTTATAGATTAAACACTTAGTGTGTTTCCCTACTTTAAACACAAA
AGTTATTTTCCATAATAAAAAATTTTTAAAGGGGGTATATCCGCGGTGATATATATCC
CAAATTTTTTTACCGCGCCTATAAAATAGATCTACAGTACGAACGAAATACCAATTTGGTTAAATTAATAGTT
CATACGTCCAGAGGTCTATGGGAAAAAACCTGGCTAAGTATTTGAGAGACTTATTTACTAAATTTCGTAAAAC
CACACCAAATTTTGGTGGAGCATCTTACATTGTGATCTCTGAATCAACTTTCAATGTC
TGATGCGAGCTATTAACACTCTCGGAGGAATGGATATAAATGTATAACAGTTTGGNGNTG
>78230760_2 CV526297.2
GGAGAGCCCGGGCTGAGAATCGGCCGACGATTACTATAGGCTCAGTTTGAAAGTCATTCAAGTGACATATGCT
TTAGATATTCTGTTATTAATATACTTGTAGTTGTAGGTCTGTCGGTATTACATCGTTTAGGTTTAAGTACACG
TTAAGGTCCTACGTTAGATACTAAAAATAGTACTATATAAGCATACTATTGATATTTT
AATGGCAACGATCGCACTTGTAAATGTTCGTTTATGATGAAAAAGTTTATTTACATTATTTTTTTTTTGTTTA
TTTACATTTTTTAATTTATTAATGGTGTGCCACAGAGTTACTCTGCCAAAATATATCATTACCGTAATGTGTT
AGCGGATAGCTATTTGTAAACTAAGAGAGCTGATGCCGCCGTCTAAGACGGACCCACC
ACGCTTTGCGATCCACCGGTCGCCCCCACAAAGCGATTGACTCTATGGGGCCATTTGTCAATTGTGTCACGCG
ATTACGCTGTCTTTCATTTTCGCGATGTATATTGAAGCCCGTATTTATAAATCGTTCTCAAGTCTAATATCGA
TCTTGAAATGTCGTATTAATTCATAGATATGTAATTATTAATGCAAACGGCAAGTCAG
GATCGATCTCAGATTTGAGAGCGGTTAATAAATACGGGTCCTATAGTATTGTAACCATTTTGGAAACTCGACG
CGAA
>78230761_2 CV526298.2
CCCGGGGCGCGGAATCGGCCGAGGGACAACAAAAGGAAGGTATAATAAATTCTCCGACAAAAATGGACGAGTA
CTGTCGGGAGAAAGGCTTCGCTGGATGGTTCGAGACGTCGGCAAAGGAGAACATAAATATAGAAGAGGCGGCA
CGTTCGTTAGTACATAAGATTCTCCTCAACGACAAATTACTGCAGAGCAACGACAAAG
ACGGTGAGAAGTTCGCCCTCGACCACAAGATCGCCAATGGGGAGAACAACCGCGACCAAAGCTCCGGCAGATC
CTGCGCCTGCTGACCGACCACACCCGACCAAGCACTAATACTCTGCAAAATTATTACTTTTTTTTCTATATTT
CCAGACTTTTAATAGGCATTTTTTGCTGTGTAAAGCAAAATTGGATAAATATTGAAAT
AACTTTTTATGTAGAGTTTTTTTACTTAAAACATGGAATTTTATTTTATACAGCAAAATTAAGTCGTAACTTG
GGGTTTGTATTTAAAATGTCGTTCCTTTACGTATAGTTTCATAATAGTTCCCTATTATTGACCGTTTGGTTAG
TAAAATAATTTAATGGTACATTTTGCAGTGTATTTCCTATAGTAACTCTACTATCGAA
GTCCCTGTACGTCATCGGTTGTAGTGTAAATCATACTTACATACATACTTATACTATGTCCTTGCATGACATT
CGTAAAATATGGCGAAACACGCAGATTTATACTAAACAACGCCAATTAACGGGAAAGAATAAATCGCTNTNTA
>78230764_2 CV526301.2
CCCGGGGCTGCGGAATCGGCCGCGGGTGATAACANTATGATATGCAATAAATAAATAATAAATTGGAATTAAT
ATTTTCTTATTAAAATACATTATGTACGACACTCTGAAAAAATCAGAATCGAGTTTTAATACATAGTTTTAGT
TTATTTAATATACATGTAAGTATATCAAACATGTGTAGACCAAATCAACTATATTGTT

CTATAATTTTCTAGGCGCTTTTTTTTTGGATTTTAACAGTGTTAAAGAGGTTGTGCAACGTGACTTAAATATT
CCTTTTTGGTTTACTGAAATATTTGTTTATGATTTTATAGTGGTCCGGAAAGTCGATTTCCATAGTCATTGAA
CACGTATATATTCCGAACTTGACTGTAAACGTAAGCCGCGATGAATCATTATATTTTT
AGAGAGCTTAATACCAAGGGTTATACGTTGAAAAAGATGATTTGAATCTAGGCAGTAGTGTTTTGTTGGTGAT
ATTTTAAATATGAAGAACAATAACCAGCACTGGGACTTACTCTCATATTCCATAACTACTAACAAGGGTCAAA
GGCTAACCAAGCCAGATAGCCATTATTCAATGGTGTAGGTGGTTGTGTGCCATGTCGC
CAGTGTAGACTGGATGGCTTGGTCGCAGGCATCTGGATGTCATATCCTTGTGGACGCGGTGAGGGCAAAGACC
GGTCCGTTGTGCCCGGGCGGCG
>78230780_2 CV526210.2
GGGACGCGGAATCGGCCGAGGGAAAACTTTGATCTCATATTACATCTCGAAAAAAAGTAATATTGTATTTTAA
CAATTGTAATATGACTAAAATATATCAAAATGAGTTTTGGACTACCTTCATTAAAAGTAAAACCAGAGCATGC
CGGAAATATCCCAGTACAAGAAGGCCCTTTCGGGATACCTAGCCCAATGGTTGCAGGA
CTTGCGGCCACAAAAAACAAATTAGGCATGTCTCATCCTCTTCAGGCTTCTGAACAGAATTACCATCTTAATG
AAGAAAAAATGAATATGGCTATGCTTCGAAACATCCAAGGTCTCCATGCGCCTTTAAAGCTTTCAATGGAGCG
AAAGTTTGCTAGCAAGGTTGGCCATCTTCCTTTCCTCACCAGCTCAAATTTGCAACAT
GATGTTTTTACCGGCAGATATCTTGATATTGGTTTCGAAGATATTCTCAACACCCCCGAATTCCTTGAAGTCA
GCGGGCAACCTCACGCAGTTGTTGAAAAATCTTTGGGCGTACTTGAATGCACTTATAAATTAAATGTATACTT
ATTTTGTAATAAATATCAATAAAAGAATTAAACATTTTAAAAACAAAAAACACA
>78230799_2 CV526229.2
TGAGGCTGAGAATCCGGCCGACGGTGTATATGTGGTTGTCCGCGTGATGTCTAAGAGAAACATTATATGTGAA
ACTGAAGATGTTAGTTTTGTTTTCGCTTTTTTTTTCTTCAAAATTAGAATCACTGAAACTTAAAAGTATATAT
TAATACACCAAAATTCTGCTTGGAAAAGGAAAAAAGACGCGTGGTTATGCTCGAAGCA
TGCCGTCTACGTCGCGGTCGCGAGGCCTCGGGGCGCGGGCCTGACGGCGCGCCACGGAATCAAGTCATAGCAC
CTATGTCGGAGCGGCAGCAATTGGCTCTGGTGATTAAAATTACATCTCAGGATGCCCCCCCAGTGCCGCTCCG
ACACCAGCTGAAGAACGCATAAGGCAACAGACAGCAACTGAAATGAACGCGGCGAAAT
ACCTTTGCACGTAGTCTCGCCATTAGAGGATACCATGACCAGGTCAAAAGACTGCTTGACCAAGGTCAAGATC
CTGATGTCCCAGAATAAGCTGGCTGGACAGCATTACCCGAAGCATGTAATTAGGGGTGGCTAGAAGTGCGCGT
GGTCCTCGTTAATGGTGGGGGTCCCGTCAATGCCGAAGGTCCAGATGTCGATTTCCCT
TTCCCATGATCCTACTACTTTCAAGTCCTTGCGCTATGGGCAATTTCCTCTGGGNT
>78230802_2 CV526232.2
GGTCTGCAGGAATCGGCACACGGCTCGTTTGACCACTTGTAAAACTAAATATATAGTGTTTTCTTATTTGTTT
ATTTATAATAATTTATTTATATGAAAGTTTAAGCTATGATTTCAATGAATTTAATCCCGCTTGTTTGCTTACT
GGGCGCCGCTTTAGCCCAAAGACTGACCGACGTGAGTGACCCAAGCTACATCAGACGA
GGCGGGGGCTATGGACTGCTGAGACCAGGATATAACAACTATGGTAGCGGTTACGGAGGATATGGGAGTGGAT
ATGAGGGCTATGGGACAGGATACGGTGGCTATCCTGGACAATATCCTAATTACGGAGGATACGGTGGTTATCA
GGGAATCGGATCAGGATACCAAGGAAATGGGGGCTACCGCCCTGGATACAGGCCCGGA
TACGACTACCGCCCGGAGATATGATTACCAGGAATCAAATTATCCCGGATACAGACCGGGATATCAATCCAGA
CCTGGATACGACCGCTATCCGGGATACAGTAGTAATTATGGATATGACGGTTATGATAACTTTAGATCATTGA
CGCGTAGAGCTGGCGAGGTGAAGAAGGATACCGTCTAAGAACTAATTTAGAATTAGCT
TATCAGTATGTATCTCATTTATTTCATATTTACAAGTGTCGGCCCGGATCCGGCGTANNA
>78230804_2 CV526234.2
AGCGCTGGCGTCTGAGGAATCGGCACGACGTGAATGAACCCTCACAGGCCATCAGTCCCTCACGTTTGGCATG
AAGCTCCATTCCAATATACTGGTCTCTGGCAACGCTGATTCTACAGTTAAAGTTTGGGACATCACTACTGGAC
ACTGTCTTCATACTTTATCTGGTCCCAACAAACACCAATCGGCGGTGACGTGCCTGCA
GTCGAGCAACCGCTTCGTGATCACGTCGTCAGACGACGGCACCGTCAAGCTGTGGGACGTGCGCACCGGCGAG
TTCATCCGCAACCTGGTGGAGCTCGGGTCGGGCGGGTCGGGCGGCGTCGTGTGGCGCATCCGGGCCTCCGCCA
CCAAGCTCATATGCGCCGTCGGCTCCCGCAACGGCACCGAGGAGACCAAGCTGCTGGT
GCTCGACTTCGACGTGCCCGGCGCCTGTCGCAAGTGCGACGAATAGCATCGACTCAACCGGCGCCTGCTGCGT
CGATACGCCAACCTGTACGCGCCGTGGAACTGTTAGGCGAACGGTGGACTATATGACACAGTGCACTACCATT
CATACATTCTAGTTCTAACTAGTGTAATTTGTTCAGTGACGCGTTCGGGGGTGCCGGC
TCGTCGGTGATGGCGCACGTCACCACCGCTGGAACTGTTGTGGCGGCGCTTTCCATCGTCCATTGCCAGTTCT
GTTGTTATTGCGTCGTTGTCCCAAGAGTATGAGNNT
>78230815_2 CV526245.2

CCCGGGCTGAGGAATNGGACGAGGCCGGCGACGCCTCAGAGGCGGCGCGCCTGCGCAGCTTCAGCGACTGGCC
CCGCAGCATGAAGCAGAAGCCCGAGGAACTCGCCGAGGCGGGATTCTTCTACACAGGCCAGGCCGACAAAACT
AAGTGCTTTTACTGTGACGGCGGGCTCAAGGACTGGGAGAACGACGACGTGCCGTGGG
AGCAGCACGCGCGCTGGTTCGACCGGTGCGCCTACGTCCAGCTGGTCAAGGGCCGAGAGTACGTCCAGAAGGT
GATCACGGAGGCGTGTGCCCTCCCCGCCCCCAAGGAGCAGCCCGAGAAGCCCGCCAAACCTACGAGTAATAAC
GCTGACAATAACATAGAAGACAATAAATTATGTAAAATCTGTTACAACGAGGAGAGAA
ACGTGGGTTTCGTGCCGTGCGGCCACGTGGTCGCGTGTGCCAAATGCGCGCTCTCGACGGACAAATGCCCCAT
GTGTCGACGGACGTTCACGAACGTAGTGCGGTTATACTTCTCCTGAACTGGTCCGCGCGGCGCTGGTTGTAGT
GAATACGCACGCGGCGTCTGAAACACGCGCGTGGTCGCATTGCCGCTCCTCAGTACCT
CGCGGCGGACTCGATGACTCCGACTCAATCATAGTGGAACTTGACATGTGTCTAGATCGTACATATAATTATT
GTTCAAAATTCGCATTGAAGAATTGTTATCCACATAACANNNG
>78230832_2 CV526153.2
AAAACAAAAAACACACAAAGAACAACAACGCTAGTCGCTCCGGGCGCGGAATGGCCGAGGCTTGATAACTTAC
TCATATATTACATTACAAAGTTGTAAAATTGAAAATTAAATTTTGAGATTTCTTGGATGAGTTAATTGGATTT
GGATATTTATTGAAAATAATATATTTTCCTTAAACTTTTTATTTTTTTTCCGAAAGTT
GTTAGTTCATACGATTTTAGGCAAGCCACATAAATATGTGAAATACTAAAAATATATGTATTTAACCACCAAT
AATAATAAAATCACACAAAGTCAATAGAATATCTCAAAACTACTTGCGGGAAATAATTATTATTTTAATTAAA
ATAACTACTTACATAATATAGAGTAATATAATCTTACATTTTGATTTGTATCAACATT
CAAATTATTAAAAATATCGAATGGTAAATAAATATCCACTTTCGTAAATTTTATGTTAATACTCACAATTCTT
TTAGACACTGATACTAATATAACAGTAGGGGAGCCCATGATGCAAAATCGAGATTTATCGGAGCGTCATAGAG
ACATATTTGCGCATCTTAGATAATAAAAAGACAATATGTCATTTACTATATATCTTTT
AATCGGAGAGGTGAGCGGCTATAGATTTGAAAATTACAAAACAACTATGNGG
>78230833_2 CV526154.2
GAAAANNNNNCNNCNNANNGAAAGCCGGGTCTGCGGAATCGGCCGAGGTTACCAGCAAGTCAGCAAACACGA
AGTGGTAGATATGCCTCAAAATACTCGCTCATCGACCTCAGCAAACTTAGCCAAGAGACGGTTACCGCCCGAA
GTGGAAGTGACCGTAGTGAACCCGAATAAGAAATTACGATCTGACGATTCCATAGACT
TGGATCATCAAAACAATTTTTCTGGAGGACCAGAAGATAGGTACTGTAATATTTGTGACAAGGAGTTCACCAA
GCGAGCCGCATACCAGCGGCACATGGACGAGGTGCACCAACCGAATTCTGTATTCTGTCCAGTTTGCGACAAG
TCGTTTACACGAAAGTCCACCTTAATTGTACATATGAAGAAACATTACGAGGGCGGAG
AGGGCAGCTCCGGCACGCAAATAGACGAGGACGTCCACGCGTGTGAAGTGTGCGGCGCGCAGTTCGACACCGT
GAAGGATTTGAACACCCACCACGCGATGGAGCATGGGGACGAAGTGGACGACACGGGCGAATCTGACGACGAC
GGAGGAGCTGTGGCGCCACCGGGGGAGTTCCTTGCGGCCAGTGCGGGGACGGTGTGGC
GACTCCTCGCGACCTGATAGCTCACCGGACATGCAGTCTATTCCAATAATTCCTCTGCACATATGCAAGGTCA
CTTCGCGAGAGCCTTGAACTTTCTTCCACAGGCGGCGAAGCATCCGACAACGAGNAC
>78230837_2 CV526158.2
AGAAGGANGGGGAGGAGAGGGAAACGAGGACGAAGAATCGGCCGAGGGTTGGATTATACTAGTTATATAGTGG
ATGTTCCAAGTGATGTTTAAAATCTTTTATTAGTAAATATATTTAAATTCGATATAAGAATTTGAAATTTTAA
ATAATCCACTAAAATCTTAATCTCTAACTTACATAATTCATATGTTTTCATTACTATT
TTTCAACAAAATAACTTTATGAGGTTGTAACTTTATATAATCGGTTGTTGAATTCGTTATATTTGACCCGTGA
GTCATATCTAAGTACATGGGTTGTCAACTTTACTCAGGTTACTATAATTTTATAAAAATACTCTCATTGTAAT
TTATAATTTAAAATATTATAGAACTTTTTAATGGCAACACTCTATGTCAAAGCACAGA
TTAAAGGCCTGTATTTATACACCGTTATCTGAGATCGATCTTAAGATGCCGTTTTCATTCATAAATTACGTAT
CTCAAGATCTATCTCTGACTTATTTTAATCTGACTTAAGAATACGGGCCTAATACTCACTAACCGAATGTGCA
AAAGTAGAGCTTTTTTAAAAATTGGCTGTCGTATTTATAACTTTAAAATATAATTCAT
GGTATATATATTACTATCAATTTGCTCTATTTAGTACTTTAATAAAACCCTCAAAATCACTGCCAATTGTAT
GTATACTTTTTCGTATCTTATTCCNG
>78230838_2 CV526159.2
AGAGAACAAAAAAACAACGAAANTCGGCCGGGTTGCGGAATGGCCGAGGGGTCATATCATATTCCCCCATTCA
TTATTCCAATATTGCTGTAAATTATTCACAACGACAATTAATTATTAAATTGAACCTTCAAAATCTAAAATGG
CCGATCTAGACGATTTCTTCGCAAAGAAAGACCGTAAAAAGTCAAAAGTCAAAAAGTT
CGCGACGGCTGGTGAACTAGCAAAAAAACTGGAAGATACCAAGCAAAGATCTGACGTGAGGCCTAAGAAGGAG
AGACCAGCGCAGGAAGGTGAAGAAACTGGTCGGGCTGGGGAAGAGGAGGAGTGGAAAGATTATGAAGAACCAG
TGAAAGATTACACGGGCCTCAAGATACAAGTGCTTCAAGGTGGGGGCCGGTGCTCCTG

AATCTGGTAGGGATACAACAATCGAGGATAGTGCGCCTGAGAACACTAAGAATAAAGGGCCTTGGAATAAGCC
TGCGGAACCTGAGCAAGCAACGCCGGTGCCCCCTCCTCCGCGTGAAGAGAGAGCGCAAGCAGCAGCCTACGTG
CCACCGCAGTACAGGAACAAGGCAGCCGAACCTGTGAGGCGGCATCAAGCTAAGAAAG
GCACCGAGATCCACAANG
>78230839_2 CV526160.2
GGGAAGAAAAAAGAGNGCAGAAACCGGTGCGCGGAATCGGCCGAGGTAAACTTACTAACGTTATTGTATTTTA
AATAAAAAAATACATATTTTTGTGGCGACCAGTGATGGCTGGGAAACTGAACAAGTTCGAAACACCCAGTTCG
GCTAGCTCGGATAGTGACACAGAACTTGCGCTAGAAATAAACGAACCAGTGTTCATAG
ACGACGATGACTACTCGCGCGCGCCGGCCTCGCCCACCATCGTGCCCAATGTCAGGCCATACCGACCTCCTTC
CACGGGTTCGAACATTCTCAAATCGCCTCGAGCTCGTCGCGTCCGTACAGCGTCCATGTCTCAATCAAACAAG
AGGACGACAGCAGAAAGCATCTTACATGCGGACAGACGGACCATATACACGGCGGGAA
GACCACCGTGGTACAATTGCACCGGCGGGCAGGAAGTTGAACCTGTTCTCATTGGCGTCGGTGGCGCGAGTGC
TTCGGGAAAGACCACAGTAGCTACCAAAATTATAGAATCTCTGAACATACCGTGGGTTACCATCGTCAGTATG
GACTCGTTTTATAAGGTACTTAATGAAAACAGCACCAAGCGGCGATGCGTAACGAGTA
CAACTTCGATCATCCGGACGCGTACGATATCGAGCTCCTAGTAACTGTGCTGCAGCGTGCTCAAAGAGGGCAA
GAGAGTGGACGTGCCCATTGATAATCCTGTGACACTCCAATGGAACTCGTACG
>78230842_2 CV526163.2
GAGGNNGGGGCAAGGAAAAACCGGGCTGGGATCGCCCGAGGATGGTAGCGCCCGTCGGGACTACGGCTGTTGT
ATATTATAGAGATACGCTATTATATTGCATATATATATATATTAGCACTCCGAGTCGTCGATACGAATTTTTT
GAGGGTTAAAAATATTTATTGGTGCAATATATTTTTTATCGGAATACAGAGGTTATCT
GTGGCGGTAGCTCGGTTAGTCGATTTATTAGAGGTGTTGCAAGGAACTTAGGTTTATTTATGTATTATTTATA
TATATTGTATACATATGTATGTATGTTGGTTTGCGGTAATCCACTAAACATAGCACGGGGGAGGTTCTGACGA
GCGCGCTGCAACACCCTAGAGTGATAGCAAGTATGTTTTAAGTTGTCGTTTAACAGAT
CAGTATTTTAATAATTGAGTGACATTCCAGATTCAATTTGGATCGAGACGATTAGCTCCCATTAGCTCTCTCT
CTCTCTTTACAAGCGGTAAATGCAAATGGTGCATATTTGTGTTAAGACTCTTGATATATTAACTTAATTTATT
ACTGTCAGTTCGAAGTGTCAAAAGTTGTAATTCACTAGACTGGCTGTTATATTGTGCA
CGTGATGTGTACTAAGAGTTCCTTATGTGGGAAGGTTCCAGGGGTCCACTAGTGTTTGATTTTCCATCGGGCT
TAAATAAACCAATGTAATATCTTCCAAGAATACTCCCGTGGAGGTATCTTAAATACAGGGGGGCGTGAAATTT
CAGGCCGGGAGNT
>78230844_2 CV526165.2
GGAAAAAAAAAAAACACACGGTCGGTCCGGTGCTGGGAATCGGCCGAGGAGCAAAATCGTATGGCAATAGACA
TGCCAGAAGAAACCCTAACCCAACTTTCAATCAATTTCACTAATTTGGAAAAATTAAATATATCTGTATGTGA
CTATGAATGGTCCGATCTCCTTGCTCTTTCTCACCTTTGGCCAAATATTAACGAACTT
ATAGCAGCCTATAATAGAATAAAAAAAATAACTCCGCCAAAGGTCACTTTACATACCCTGACTGTTATTAGAC
TGGATGGAAACCCTATAGATTCTTGGCAGGAAATATTAAATCTTGGCATGTTGAAGAACCTTAAAGTTCTGAG
TTTGAATGATTGTATCATCGAGGAGATTAGGTTTAATGATGACCCAAATAATGATAAA
GTGGACGTGTCTGACAATTTGGAACTTTTATACTTNNT
>78230846_2 CV526167.2
GAGGCGCGGATGGCCGAGGGGGTATCACCTGAGCGAATCAGGCGGGGATATATTTATTATTTTACACATTATT
TGCTTCTTTACCTTTGTTAATTGGAATTTTTTATATTCATAAAGAAATAAATTTTATAATTATATATATAATA
AAATTTTATGATTATAATTTAATTTTATTATATTTAAGTTTAATTTTTGCATTTTTAG
TTAAAATGCCCATATATTTTGTTCATTTGTGATTACCTAAGGCCCATGTAGAAGCTCCAATTTCTGGGTCAAT
AATTTTAGCCGCAATTATATTAAAATTAGGGGGTTATGGTTTATTACGAATATTAATAATTATACAAAAAAAT
AAATGTAAGATTGGGGTATTTTTGAGTAGTAGTGAGATTAATTGGGGGGTTTTTTATT
AGTTTAAAGTGTTTTTGTCAGGTTGATATTAAATCTTTAATGGCTTATTCTTCCGTTGCTCACATAAGATTAG
TAATTGGGGGGGTAATAACAATAAATTATTGAGGATTTTTAGGTTCTTATATTTTGATAATTGGTCATGG
>78230849_2 CV526170.2
AAGAAGAAGAAAGAAGCNGAAACCGGGCTGCGGATCGGCCGAGGTAAGGTACTTTAAAAAGAGTCAAAATATT
GGATAATAAGCTTTCTAAATTAAATATTACTTGAATACGTAATGGCAGATCCCAAATATGAAAATCTTCCCGG
CATACTTATGACCAACCTGATGTTACGAAACCGGTGATTACCAGAAGCTGATCAGCCA
GAACCCTATGAAGAAGAAGAAAATGAATCAATTGAACAGCTACATCTTTCGGTTAAAGATTCATTTAATAAAT
TTAAAGGCAAATTTCTTACTGGAAACGTTGATTTTGCGGATAGACTGAGCAGAAAGTCTCGAATCGGAGCTGG
AGAATGGGAATTGGCTGCTGAAGGTGATCCTGAGACTACACTGGAACGCTACAATAGA

CTCCGATGTGAATTTTCTGAATTATTAGAACAGGTTGCTGAACTATAAAATCGAAGCTACTGATAGTGACAAG
GATGAGCACTCCAAGTTGGCAGCTCATATTAATACAATCAAAGAAACTTTTGGAAAAACTCAAACTTGAGGAG
GGAGAAGAAATCGATCCTAAAGCAGAAAAGCTGAAGGAGTACCTCAGTATCAATGGGA
CATAGAAACAATGTGACCTTGTTTCAGGACACTTGAAGCTTTAACTAAAATTACCTACACAATTACACGATAG
CACATGAGAGCCCCACTTCGCAATTGGAGCAGNNA
>78230852_2 CV526173.2
GGACGCAGCCAACACAAAGACCACCCCCGGGCTGAGGAACTGGCCGAGGATGCGTTGAAAAGTTGATGCGTAT
GCGCATCTTCCAAGTAAAAGAACGCATCTCGATACATCGTATCGTTATCGATAACTTTGTGATAGTGGGTGAA
GTGATGATGGTAAAGCTGGTGTAGAGTATATTGAAACGGAACCTTTGTTACCATATTG
ACACGATGAATCAACAATGTAAAGTATGCGGAGAGCCTGCTGCTGGATTTCACTTCGGAGCTTTTACCTGTGA
AGGATGCAAGTCGTTCTTCGGCCGGACGTACAACAACCTGTCCTCAATATCTGAATGCAAGAACAATGGCGAA
TGTGTCATCAACAAGAAAAACAGAACAGCATGTAAAGCATGTAGACTACGAAAGTGCT
TATTAGTTGGTATGTCCAAATCGGGATCTAGATATGGAAGAAGATCGAACTGGTTCGGGATACATTGCCTATT
ACAAGATCCCGCAGCAACAGCACATGCCACAGATGCAGACGAGTAAATCATCTCCAAACTATAACTCTACTCT
TATCCCAGCTTTCCTACCTACTAATCTATTGCCTGCTGCCGCTTTACCAGAATCTTTC
GAAATNAG
>78230853_2 CV526174.2
GAGAAGAAAGAAAAAAGGCGACCCGGGCTGCGGAATGGCCGAGGTGTTACCTTTTCGCGGTATATTACTAAAT
AATAAACACAGATCTTATACAATTATAGCTGTTAAGTTAAGACCAAACTACACTAGTATATTTAAACAAATTC
TTTTTCCTAAAACTTTCAATAAAAGCTTTAATATTAGAAACAGTTACACAGCTATGGG
TGCCCTAAGTCCGTTAGACCCGTCTTCTTTTTCAAGACCAGAATTGGCTGTGATAAAACATGTGTCCCTATCA
TATAAAGTGGACTTTGAAGAGAAAATACTGTATGGATCAGCAATTTTAAATATTGAAGTAATTGATTATATCG
AAAATGTGATATTAGATACGAAATGACTTAACAATAGAATCAATTGAATTCCCAGATG
GTACAGCATTGAAATATAAATTGGACGAAGCGGTGCCAAGATTACGGATCAAGATTGACTATATAGTAGCCTC
AATCTGCAGCACCAGAACAAAAGCTTGAGATTAAGATTAATTATGGGATCTATCATCATCTGCGACGGCGCTG
CAATGGTTGGAACCAAATCGGACCTCAGGGAAAAAACATGCGTATATGTTCTGCCAAT
GTCGGGCAATCCAGGCTCGCGCTATCTGACCATGTCGAGACAGACCCTTTGTGAAGTTCACATATGAAGCTGA
ATTAATTGCCCCAGAGGAGTTTACAGAATCGNNNA
>78230854_2 CV526175.2
AGAAACCCGAGGCTGCAGAATCGGCCGAGGCGCGACGAAGTCGGTTCACTCGTTTTAAAGTGAGTGTTCAGTG
ATTAAATTAAAAGGAATAAAGATAACATGAGTGCCGTACCCGTGGGTCCCAAGCCCACCACTTTGGTGTGTCC
TTCCTGCAGATCCACTATCGTCAGCAGAGTGGACTTCAACTCTACCACAAGAACACAC
ATTATAGCCTGTCTTTTGTGTTTATTTTTATGTTGGCCTTGCATTTGCGTGCCGTATTGTATGGACTCGTGCA
AAAACGCCGACCACTATTGTCCGAGCTGTAACGCGTATTTAGGCACATACCAAGGTTGACTTACCTTCCGTAC
CAGCACTTCCGTATATTATTATATAGTATATTGATCTCCTATTTATCTATTTCAATTA
GATCATGAACTGAGATTAACTATTATTGTTATTTTACTATGGCACCTAAGTTATTGTTAATGCCGTTTAAATA
TTTTGTAATTTTTTTACCTTCTAGTGTTACTCAGAATTTTATGTATATCTATGGTATATCTATGGTATATCTA
TGGTATATCTATGGTACTTATGTTAAAATCGTAACGGGTAGGTGTCTGTTCATGGAAC
ATGTATAAGAAACAAAAAACA
>78230856_2 CV526177.2
GAGAGAAAAACAGAAAAAGACGACCGGGNCGCAGAATCGGCCGAGGGTTATGGTGGACTTATATTTTTGGCTT
CTTTATTTTATACAAATTTGGCTATATATTCTGAACATTTTGCATTTGTGATATATAGTGATTGAAATAATTT
AAAAATATGGGTATAGATGGAGATTCTATGTTATATTTATGTAATTTTGATTGTCAAT
TAAGTGACACAGATTCAGATATTATAAAAAAATTGTGGTCAGGACAATGGGAAACATTTGAAAAAGATTTATG
TAAAGATATCTTGAATAGTGATTTGGAAAATGAGGAACTTGATAACAAAATAAAGAGTCATTGTCATATTGAT
GTCAGTGAACCGAAAATTCTACAGACTGCCATTAATGTGTATTGGCTATACTGTGAAC
AAAATTGGAGTGCACATCCCAAGGAAATTGTGTTAGAAGAACTTTGGGGGGTAGAATGGACAAAATCGATTGA
TGTTCTAGTGTACCTTACTGAGAGATTCATAACCTGGGTATTCTATTTTATTATCTCCTGAGGGGTTACATGG
CCCATCGCTATAATTCAATGCGCTTTTTTCTGCCAACCCTCAGTAGCTGGGGTTTCTG
GTGGTGTTTCCGACGAAATGGCGGTCGTCACCTGGCCTTTGAGCCCGGCGGTGTATCTGGCTACTGAATTTTG
CGCTATTCCCGCGAGGTTTTACGGGATCCACGATCTCCCNGG
>78230860_2 CV526181.2

GCAGAAGAGAAAGAAAAGAAAAAGAACCGAGGTCTGCGGAATCGCGCCGAGGGAAGAGAATGAAGATGAGACA
GAGAGCCTCCAGGAAATAAAGCGTAAATGTAGACCAGACGAGGTCTTCGAGGTGGACTGTAACATGTGTCGAT
GCTCCGTAGACGGAATGTCATTCTCTTGTACTCGTAGGGCTTGCGTACCTGAAGACGA
TGGGAAGGATGCATCACTACTTCGAAAAGCTCGAGCAACATCGCAGGGGACGCGGAAGGCCTGCCAGCCGGGA
CAGGAGTTCACGATGGACTGCAACAAGTGTCTGTGCAACAACGAAGGCCAGGATTACTCTTGTACGCGCATCA
ACTGCGCCGAGTTGAACAGTAACGGCAATAACGGTGCCAGAGCTAAGCGAGAAGTGGC
AACCCAAGTGAAGGCAGACTGCGTGCCCGGCAGCGTGTTCAAGCAGGGCTGCAACACTTGCCAATGCACGGAA
GACGGGAACCGCGCGACGTGCACCATAAAACGATGCACAGAGGACAATCCTGATGAGAATGAGGTTAACTTAC
CAGAATCCGATCCAAGTGTCCGCTGTAACCCCGGAGAACAATACAAGAGGGGTTGCAA
CGACTGCGCTAGTTCAGCCGACGGATCAAGCGTGTTCTGCGCATTACGTCTATACGACCGGGACCTTTCACTT
ACCGTCCTAATTGGATGCTATTGGGCACGAAGTAGAAAACGCGTATATAGGCCCTT
>78230864_2 CV526185.2
CGTCCGGTAATCCTACTCCATCACCGCCACGACGTGAGTCCTCCCAAACATCTGGTAATTGGTTCAAATCCAT
GGACAGATTGGGCAAGAAACGATGAGAAGAATAGTAAGGTCGAGAAAGAAAGCATTATCACAACAGAAGACGA
ATCAAGCCGTCGAGTGTGGTCAACACCATCAAAGCCTCTTAACTCTCCGGCGAAAAAC
TTAAGATTTTTCGGTGACACTGACCACGAGTCAGATGGAAACAAACCCACACTTAAACCAAAGTCCAAAAAAC
CATGGCACTTTACGTAAAACTATTTCGAATTCCAGCGCAGGCTTAGACGAAGTCGACCACAGCGAACTATCAA
ACAAAAGTTATTCTCTTTCAAATTTACATAGAGATGATAGAAGGGAAAGAGAAGGCAA
GAGTGAATCTCCTAAAACTAAGGAGTTTAATCAGACAGAATTCGAAACATCTTTCCGAAGTTCAAGTAATTCG
GAAAATGATGGTCACTTTGATAGGAAAATGACTTGGAAGCCACGATAGTCCTTATGTAGATCCAGAGTACAGA
GTCCAGACTTGAAAGGCAACGAGTGATTGAGAGGANA
>78230867_2 CV526188.2
AGGGGNGAAAGAAGGNAGAACCGGGCTGGGATCGGCCGAGGCTCTTTAATAACATCAATCAAAATCAACTATA
TAAATCTTTCTTTTTTTCATATAAAAAATTGATTTCTACTACGTGTAGATTGTTTCGTAGTACTATTTGTACT
GTAAGATAAAGAAACAGTTAAGAAAATATTGTAACGGGTCCCCGTTACATTTTCTCCA
AAAACATTATTATGCCTCATGATTTGAAAGGACAGGGTGTAAGGAAAGTGATAATGGCTCCCTTATCTCCCAC
TCCATGTGCAGAAATCAGTGATGATGAACTGGTTTCAATTTCTGTCAGAGATCTAAATCGACAGCTTAAAATG
AGAGGTCTTACGAGAGATCAAATAGTTAGAATGAAGCAGAGAAGAAGGACATTAAAGA
ACAGAGGCTATGCTGCCTCGTGTCGAATCAGACGAATAGAGCAGAAAGATGAATTGGAAACTGAGAAAAGTCA
GGAATGGCAAGACATGGAAGCGATGCAAGAAGAATACACAAGAATACGAGATGAACTTGAAGCTCTGAGAAGT
AGATATGATGCGTTAAAACGTTTTGCCCACATGAAAAGTATTCCTTTGCCAGCCGAAT
TGGAAATGTTGCCATAATCATAGAAAA
>78230870_2 CV526191.2
AGGGAAAGAAAAAAAANAACAACCCGGGCTGCAGAATCGCCCGAGGAGTTTTATTTATTAAATTGTAAAATAA
AGAAATGTGTTTTTAATAATTTTTTGGGCTGAACTTGGCAACATTTCTTATATTGAATGACAGTTACACGCTC
ACACACAGACAGACAAACAGAGTTAGTCACATTAAGTATAGAACCATGTGCGAGTTAT
ACAGTGTTGCCGTATATGAAAATGAAAATAAAATAAGTAAACATTTCGTCCTTTTTATTTATTAAATTGTTAA
AAGAAACAAATTATGTTTTAGCTTCACGCTGTATCCCGTATAACAGGGTAGGCAAAGGGAACCTGGTGTTACG
CTCCGTTCCCACGCACTGCTCGATTTATGAGATACTACGTAACTCAACGACCTTTGTC
TTGTTAAGAAGGAGGAAGGCAAGTAGATGAAAATAGCTCATTCCCCTGAGTAGGTATCGTACACACAACTATC
GTGTAAATTAAATCGTAAATTACAATCATGGTTAAATATATTAAAATAATTATGTATTACATGCTGGTCTAGC
TGAAAAGAGATAGCTGTGGATTCCCCTTTGCAGGAGTGGTAGTCTTTAAATTCTTTTC
ATTTACTCGATCTGGCAGCGGCACTATGTTTCCCCGCTCAACACGCAATGTGAAAAGAAGAGAGGAGTGTTTA
AACAGTAGGTTCCCTCTGNCTAACTTGCTCTATGCGAGATATACNGTGAAGCANNT
>78230873_2 CV526194.2
GGGAGACCCGGGACGCGGAATGGCAGAGGGTACTGTTANCATAGTACCGTATTTAATATTTATTTTATAAAAA
GTAGGTAAATGTTTTATATGTACATGTACATATTTCACTAAATCAAATTAATATAAAAGGTAGGCTTATATTA
TAAGATTAATATGGAATTACAAGACACTTATTATAACAAATCAGAATATGTTGAAACA
GCTTCAGGAAATAAAGTCAGCAGACAAACAGTGCTATGTGGCTCTCAAAACATTGTATTGCATGGTAAAGTTA
TAGTGCAAAGTGATGCTATCATCAGAGGTGATTTGGCAAATGTTAAGACTGGTAGATTCTGTATTATAAGTAA
AGGCTCAGTTATACGGCCACCATTCAAAAAGTTTAGCAAAGGTGTTGCATTTTTTCCC
CTACAAATGGGTGATCATGTGTNTGTAGGGGAAAATACAGTAGTAAATGCTGCAGTTGTTGGTTCATATGTTT
ACATTGGCCAAAATGTTGTAATTGGCAGAAGATGTGTATTAAAAGACTGCTGTATGATTGAGGATCATTCAGT
ATTACCAGCGGAGACCGTAGTTCCATCATTTGCGAGATACTCTGGGAATCCGGCGAAG

CTGATAACAACTTTACCTGAAGCTACTCCAGACTTCGTGACCGAATTTACTTATGAGCTACTATCAGGCTTTC
TTAACTACAACTGTTAATAAGCTTTTCTATTTNNA
>78230880_2 CV526109.2
GGAGGNGAAAAAAAAANNCAGCCCGGGCTGCGGATCGCCCGAGGTTTTATTTATTAAATTGTAAAATAAAGAA
ATTGTGTTTTAATATTTTTTGGCTGAACTTGGCAACATTTCTTATATTGAATGACAGTTACACGCTCACACAC
AGACAGACAAACAGAGTTAGTCACATTAAGTATAGAACCATGTGCGAGTTATACAGTG
TTGCCGTATATGAAAATGAAAATAAAATAAGTAAACATTTCGTCCTTTTTATTTATTAAATTGTTAAAAGAAA
CAAATTATGTTTTAGCTTCACGCTGTATCCCGTATAACAGGGTAGGCAAAGGGAACCTGGTGTTACGCTCCGT
TCCCACGCACTGCTCGATTTATGAGATACTACGTAACTCAACGACCTTTGTCTTGTTA
AGAAGGAGGAAGGCAAGTAGATGAAAATAGCTCATTCCCCTGAGTAGGTATCGTACACACAACTATCGTGTAA
ATTAAATCGTAAATTACAATCATGGTTAAATATATTAAAATAATTATGTATTACATGCTGGTCTAGCTGAAAA
GAGATAGCTGTGGATTCCCCTTTGCAGGAGTGGTAGTCTTTAAATTCTTTTCATTTAC
TCGATCTGGCAGCGGCACTATGTTTCCCCGCTCAACACGCAATGTGAAAAGAAGAGAGGAGTGTTTAAACAGT
AGGTCCCTCTGCCTACCTGCTCTATGCGAGATATT
>78230881_2 CV526110.2
GAGANNNNNAAAACAAACCCGGCCCGGGCTGGGATCGGCCGAGGTGTTTAAAAAGGCAAAATGAAACTCTTAT
GTGCAGGGCATGAACGTGCCATAACGCAGATTAAATACAACCGCGAAGGAGACCTACTGTTCTCTGCGGCTAA
GGATTCCAAGCCTAACGTGTGGTGGTCTTTGAACGGTGAGCGTCTAGGTACCTTCAAT
GGACACGGTGGTGTTGTTTGGTGCCTTGACGTTGACTGGCAGTCCATCAATCTTATAACCGGAGGTGGTGACA
GTTCATGCAGGCTGTGGGACCTGGAAACGGGTAAAAATATAGCAACAGTAAAGACTAACTCTTCGGTAAGAAC
ATGTAACTTCAGCTACAGTGCAAACCAAGCAGCCTACACTACTGATAAAGCTATGGGC
CATCCTTGTGAAGTGTTCATAATTGATACTAGAACAATTGATGAGTCCTTATCAGGACAAGGACCTATATTGA
AATGGGAGATTACGGACTCAAAAGTTACCTCAATGGTCTGGGGTTGCCTCGATGAAACCATCATCACTGGTCA
TGAGGCTGGAGATCTCATCCAATGGGATCTAAGGACTGGGAAGAAGGTCCACTCGGTC
AAAGAACACACTCACCAAGTAAATGATATGCAACTATCAAGAGATGGCACCATGTTCATTACAGCATCTAAAG
ATCAAACTGGCAAACTTTTCGACACCAGCTCCCTTGAACTTCTCAAGGAGTACNNT
>78230883_2 CV526112.2
GGCAACCCCAAAGCCACCGTCGGCCCCGGGGCAGTGACTTCGTACTAGGGTCATACGTGACCTGGCCAGTCAG
TGTACAACGTCAGTATAGTATAACATTCTTTTAAATAAACTAGGTAGGTACTTTAGGGTAAAATTGTAACTAC
TTTACTAAACATAAATATACATACCACTTCCTAGTGGACCTCTAAAAGAGCACAATTA
ACGAGCACTGTGACCCTAAACGTGAACTCTACCATATAAATTGCCACGCTCATTGGGCAGAATGGGTAGTAGG
TACCTACTGTTGTAGTCGTGTACCTACGAGAGGTGTAATTTAGGGTAGGGCTCAGTTTTTAAGAGCTTTGCCA
TAAGTGGATTTATTTGAGATTTTTTAAAGCGTTATTTATTCCACACTGAGAAACCGCC
ATGTTTAAAAAAATTATGCAGGTTTTCTAAAATATGTATTTTTTATGTGGTGTATTTAAATATTGTCGCTTTG
GTGGTGTAGGGAGTCGACCTAAATATTTGAAGTTTAAGAGTATTAAAGTATTGCTTATGTTCACGTTATGGAA
CGGTCTAACTACGGTCGAGGGTAGTCGTGGCGAAGGGGCACAAGGTGTGACTGTAAAA
TCTAGTTTCGGTTGTAGGTTCGATCCTAATTAATAACATATCTATTTGAATGAAATATTCATCTAAATTTATA
TATTCATTTCTTCGCTCTGGTGTTGTGGCAACNNT
>78230887_2 CV526116.2
GGGAGNCCAAGAGAACAAANAAGGACCGGGGCTGGGAATCGGCCGAGGATCACTCAGACTTTAAAGCAATGTA
CAGAAATTAGGATAACACACACAATCGCCACAATCGCCTCAACTTTGTATTTAAAAGTTAAATTTTCTTGTCT
TGTAAGAAATAAAATAACACATTCCTACCTGATAATTTTGTGCTATATACACTCCAGT
ATAGACTCCAGTACCAAAAATAAGCTAAAAAATATAAGAAATTAGTGTCAATGAACATAATAGAGACTTTATG
ATTCTGCATATACTTACACCAGTTTTCAGAAGTGACATAAAACATCCCATATTGATGGGGTTTTTGAAACCAA
AATGTTAGTAAGGCTGTTTAAGCATATTTATGTATAGTATAGTAGTCCCTTACTTTGT
ATAATATACATGTCTAAAATTTCATTATTGTAAATTTGGAAATTGTATAGACATAAAATTAATATAATTTGAC
ATCTGACAAAATGATTTTCCATATTCAAATTTTTTGTACAAATTTTAGATGGCAATATTTATTGCACATTGTT
GGCATTTTATAGTAGAAAAATATTTTTTTTTATGAATTGTCTTAATTAATATGTATGT
TTAACAAAGTTTGAGACTTTCAGAAATAGAAAATATTAAAACATTCTATACAATAGTGAATAAATTGTATTTT
TCATAAAAATATGAAGCAGCTCAGATTATAGAAATAATAGGTAATAAGAANT
>78230896_2 CV526125.2
GGANGGAAAAGAAAANGAGAAGCCGGGCTGAGAATGGCCGAGGCTAAACATATTTATTTAAAAATTCTATATA
AATCACTTAACAATTCTAATTCTACATAAATTTATATTTAAATTTAAAACTATATTGAAAGTAGATTACAAGA
TTTTTTAAATTTACTTTGAAGCATTTATTACATAATGTAATAAAATGAAATGATAAAC

GTATTAAATATATTTAATATACACTTAATAGAGTGCTACTTTAAGAAGATACAAATAAATCTTATTGGCAAGA
TTTACTTTTTTATTATTGAAACATTCTAAATTATATTTTATATTCTACATCACTATACTAAATATATTATTTA
ATAACTATCTTACATATAAACATTATTACAGTTCCTAAGAATTTTAATCAATTATAAA
CAGCTTATTGACTTTCAAACTATTTGTCAATAACTTAAATATAGGTTTGGCAGTTAAATATTAGATACTATAT
TGAAATTAGTTGTTTTCTATGTTACTCTTTTATATGTCATAATGTATATCATATGTTTATTCGCGTTTATCTT
AGCTCCGCACGCACTGCTCAATCATAGAGATTGTCCGTGTTTGAATGGCGGACCTCAT
CGCTTCGCTGTGTCTTCTTAGGAAACCGAGAAAATTTATAAAAAA
>78230898_2 CV526127.2
AGGAGAAAAAGAAAAGACAAAGGCACGGGCTGAGGTTTGACAGTCATGTTAATGCATGTGAGTGACTGTTATT
GTAATTGGCGAAGTGATATGTGCTCAACTGGCACGCTCGAAACGCCATAACTAAAAAACCAACCAACTGTTAT
TGTAATTAACCAAGAAACATATACTTTATAAAATATTTGTTATTAACTGAACGTAATG
ATTGTTGATTTGAACTACATTAGGGCTCTACTTTATTACATATAAATATAAGTCATAATAAAGAAGGATAATG
AATATTTCTAATCATTGTCTCTTTCTTACCTACCAATAAAGTTTTTACTTATTTTCCAATATTTTCATATAGT
GATTTCTTTTCGGATACAAGTGATAACCCCTTTCTAATTATATTTCCTTAAACATATA
CGATCTAGTCTCTTACAGTAGACTCATGTCTAAATGGTCAATTTATAAATATTTTGTTTGTAAAAAATAATTG
GCATGTTCGAATCGCCATAATTAAAGAAGTGAACTAAACTGAACTGTAAGAAAAAATGTCTTTAACCACGGAT
GTTCCACCTCATATACTCGAGCTTCAAAAGCAATTTTCTAATATTGAAGATATAAATG
ATCCCACATTAATGGATAACATATTATCATGTCTAGAAAAGATACATTATGAAGATGAGGAATTTCTTGAGGC
TCTCTTGGACANT
>78230910_2 CV526139.2
GGANGCAAAAAACCCAAATTTGAGGCCGTGGCTGAGGATCGGCCGAGGATAGGAAGAGCAGATTGTCCTAGTG
ATAAGGAAGAGGTAGTTCCGGCAAAGAAAGGAAAAAAGAAGAATGTTGAAGCTGTTACAAATAATATAAATGG
TGATGGTATTTCAGAAGAAGAAGTATCTGCATTAGACAATGAAAATGATGATGAATCT
CCACCAAATGGCAATTCAGATGATGTTGAAATACCAGTAAAAGGTCGTAAAAAGGCTACAAAAAAAGATGAAA
AGCCAAAAGAAAGCAAACCAAGAGGTAAAAAAAATGTAAAAGCAGAAGTAGATACAAATAACAAAGATGTTAA
TGAAGATAAGCCCAAAGGCAGAGGTAGAGGTCGGAAGGCTCAACCAAAAGCTGAACCC
GAAGACGATGAGAATGCTGCTGAAGAAGAAGAAAAGCCAGTTGCAAAAGGTAGGAAAAAAGCACAACCTAAGA
GTCAAGAAAAATCAACCGATCAGAAAGTGGAAGACATAGAAATGGAAGTTGAAAATGGAGATGAAAAGGAAGT
GCAGGAGAACATTGACAATGAAGAAAGATTGGTAGAGCCAAAAAAGAAAGAACCTAAA
AGGACCGGAACAATGCCATAAAGGCTCCACTTCAGGAGGAACACAAGACATGATGAAGAGTCTAGAAAAGCTC
TACCAGCAAAAAACGGAATGTACTAAAAGGACGATCAGGANTG
>78230919_2 CV526148.2
GAAAGAAAGAGACAAAANACTTTCCGGGCTGAGGATCGGCCGAGGGTGAGCTCGCGTGTTCCCCGCCATCCGA
CAGTCCAGTCAGCAAGCGACCCAGTCCTGAACCACATACAGTAAAGTCACAACAAAACGGCACATCTGAATCA
TCCAGTCCCACACTTCAACAGCAGTCGCAAACCAAACACAAAGACAACCACAATCAAA
ACCAAAACCACACAGAAACCAAAACCAGCAGAAGTAAAGACAACCACACAAATGGGCCCAGCAAAAAGAAAGA
CAAATCGAAACAGAAGAACTCACATAGTACTAGCGAAATTGAACCAGAAATTAATGAAACCAAGGAACAAAAT
AACACACAAAACTCATCAGTATTCGAAAATGACACACACTATCTATCAAATGAATTAG
ACGAATTAGAATCGGATAGACACAGTTTATTGGAAAATCATGATTTATTAGATAACAGTGATCACAGTTTGTT
AGACGATGACACACACAATCTCCTAAATGATGCCAACAACGAAGTCATGATGATGCAAAGGAATAAGGAAATG
TTGTTGGGACTCATTGATAATCATTTAATTCCTCAAATGAAACCGTTAAACGGGTATG
GGTTGTCAGATAGGGATATGTATCTTAATACTGATAGAGCGTTAACTCACACGGNCCAAGGTCAAATGATGGA
TCAGAAAGAGTTAATGACAAGCAGGAACTTGCTGNNTT
>78230926_2 CV526059.2
CCGGGTCGCAGAATCGGCACGAGGTGAGAATAATCTTAAATATATTTTTTTATCAACTAGTTCGCAGAATTT
TTGCTAATTAAAAAAAGTTTAACGTATCACATTTAATTTCTCTTGTTCTTTATAGCTGAACTCTTAATATTTA
TATGATTTACCAATATATGATATTCTTCATATTAGAAATCTGAAAGTTATATGTAACA
TTATGTAAACTATTATTTTCAACTACCACAAAAATGATACAGTTACATGAATACAGAGCAATCTAAGACCTCT
TAAGAAAGTGAAAGTGTTAACGCGTGATTTTGTGACTAGTTGTCTTTACTGGTTAAATTATAATCTTTTAGGT
TTAACTTTCAAAAGTTATTCTATTATAGTTATATATCATAATAGTTGTAGCAACTGTT
TGATCGTATAGTTTAAAAAGCCAATGATCTGAACGTCAAAAAATCGTGTAAGCGTATATTCGTAATAACCTTT
ATTGTCCTCAGTTCCTGAACGTTTACCATGGAAGAACCATAGTCCACTGGAAACATTCAGTTCTTCAGAATCA
CTAATTTGGACCCCACAGTGGGTCCCGCAACTTCATCCCATGGAGTTCCTTCGGACCT

CTTGTTTATTGAGACCACGTCTTGTAACCTCACTTTGATGGGGTACTTTCCCATTCACTTCCTATACTTGTTG
GCGGGCCAACGCCCCGGATTGCCTTGAAGCCTTACATTCCTGAAGGGCTTGTGGAGNG
>78230930_2 CV526063.2
CGGGGTGCACGATCGGCACGACGGTGATGTGACCGCTAGGCCAGTAGCTGGCGTATTTAGTTCAACCGTGACC
GTAAAAACACAAAGCCGCCAACATGGACGCGATTAAGAAGAAGATGCAGGCGATGAAGCTGGAGAAGGACAAT
GCCATGGACAAGGCAGATACCTGCGAACAGCAGGCCAGAGATGCCAACCTCCGTGCTG
AGAAGGTAAATGAGGAAGTCCGTGAGCTCCAGAAAAAACTTGCCCAAGTTGAGGAAGACCTCGTCCTAAACAA
GAACAAGCTGGAACAGGCCAACAAAGACTTAGAAGAGAAGGAAAAGCAGTTGGCCGCTACCGAGGCGGAGGTG
GCCGCTCTCAACAGGAAAGTACAGCAGATTGAGGAGGATCTCGAGAAGTCCGAGGAAA
GGTCTGGCACTGCCCAGCAGAAATTGCTTGAGGCTCAACAGGCTGCTGATGAGAACAATCGTATGTGCAAAGT
ATTGGAGAACAGAGCACAACAGGATGAGGAACGTATGGACCAACTTACCAACCAACTTAAAGAAGCTCGTCTG
CTCGCTGAAGACGCTGACGGCAAATCTGATGAGGTATCACGCAAGCTGGCCTTCGTTG
AAGACGAGCTCGAAGTCGCTGAGGACCGTGTAAAGTCTGGTGACGCCGAGAATCTCAGAACTTGAGGAGGAAT
GGAAGGTCGTAGGTAACTCCCTTTAATCTTTGGAAGTATCTGAGAGAAAGGTCGACCAGCGCGTTGAGGAATT
CAGGAAGCCAGTTTAAGACGCTCCACTCCAAATTCGAAGAGAGGCGAGAGCCGGTTCC
AGATACACCGGAGAAAACGGTCTGAAACACANNNNCG
>78230932_2 CV526065.2
ACCGCGGCTGAGGAATTGCACGACGCCAGTGGTATATCTTTTGTCACCCAATCATTGAAAAACCACTGACGAA
TATCCAATTGATTAAAAATGGAACATGCCCAGATCCAAACATCAGACGGATCTCAAGTGATGACCATCAATGG
ACAACAACTGCAAGTGCTGCAAATGAACAATAACCCCCACATGCTACAAGGGCCTAAT
GGCCAACATATTGTTATACATTCTATACCATCTTCGGCTCCAGCTATACAAGTGGCAGCTCCAAGTGGTCAAC
AGTTGCAACAGTTACAAGTTCTGCCCTTATCCAGTCTTCAAGGCACAAGTAACAACATTCAGCCTATGCAATT
AGTGCAGACACCAGATGGTCAAACATTTATATATCAACCCACACCGAGCACGTCTCAG
CCACAGATTGATCAACATCAGATTATACATCAACCTGGTACCCTCATAAATCTTAATGGTAATCTGGTGCAAG
TGGCCGGTGTGGGCAATACTGTGCAACCACAGCAAATTGTAAATCAGCCTAATATTGTTATGATGGTAAATGG
CAATACCGGTGCCACAGCCTCTTCATCAGAAGGTGCCTCCCAAGCTGGTTCAGACGAG
GAGCCTCTGTTATATGTAAATGCAAGACAGTACAAGCGTATACTGAGACGTCGAGCTGCACGAGCCAAGCTAC
ATGAACAGGCGAAGATACCTAAGGAGAGGC
>78230940_2 CV526073.2
CGCCGGGCTGCAGAATCGGCCGCGGCTGATGTATAAGCCATACGTTCTAAATAAGCTACATAAATTGGTATAC
ACTTATAACGTAGCTTGATAAGAACTTACGGCTCAGTGCTCAGAGGTCTTAGACGGTAAACATAAGGCACGGG
TTCCAGTTATTTAAGTTCCAGCTACGTTTAAACTTTTGTATGTTTTCTATTATAAAAT
AATATTTTGAATGTATAATATTTTTTTTGCAAATAACACTTTGTTCTATGAAAATTGAATTATATCAGAATTA
AGGCCTTAAAAGACAAAACTATATAATGAAACACCAAAATTATTTTTAATGACGATAGAAATAAAGAGAAAAT
GTGTAAATGTCATTAGTAAATTTTTTTTCGTTTTTGAATTCAACTTTTTATTTAGTTA
AATTTTTGTATGACGATATACGTTTTTTTCTTTGTTTCTTGATCAAATGAATTACCTTGTATAGAAATGTGTA
TGAAGTAAATTGTTTAATTGTCCATATTGTTTATAGAAACAGTACTGTAAGTGGTTTAGTTGAAATTGTATTT
AGGTTTCTTATCATTAGAACTTTTAAAAAGTACTTCCTTTTTAAATAAGAAAAAATAT
TTTTAATTCATGATACAATATTACGAATAAAACAGTTTGCTTTAGCGAAACA
>78230943_2 CV526076.2
CTGGTGAGGATCGGCACACTGCAGGTAACNCCGAGGCGGGCGCCGCCCCGCGGTGGTACCGCCGTGTCGCCCA
GTATAACTGCCAACAGATAAATGATAATTATGTTTGTAAGATAAAAAACGTGATAAATTGATTTATTTAGTTT
AGATCTGTAAAAAGAAAAGTGTTAGGGAAGAGCGATAATGTCACTGATATTATGACGC
GACTAAGCATTTTAAATCCGTGGTTTTAAAATCTAAAAGAACCTGTCGGTGTATTAGTTAACAATTAATATAG
ACATGTATTTATTTTATAAAGAAAAGTTCTAACATTTTATTTATATATTATCCTTGACATCTTGTGTCATATT
TCCATCAAATAATATTTTTCCCTTCCCACAAATTTCTATGAATCTTATGAATAGGCTA
AGGTTATGTTGTACAAAATATTATACACTTATTTATAGTTATCGTAATGTTTTTAATATATTCAGTAATATAG
ATCGAGCATGAAAATATTTGTCTTGTAACATTATAAAATTGTTAACTTAATCCAATAATTACCATGAATATTA
TACACATACGTTATGTAATTTATGATTTTGATTTTATTCAAAACAAATATACGTAAAA
ATTTAAACATATCTAATACATTAGAATTATTATCGAAATCAACTACTTATTGTNT
>78230947_2 CV526080.2
CGTGGGTGGATCGGCCAGCTTAGCTTAATGTAATTGTGAGGGCTGCCTCGGGTGTAAATTTGTTGTGGCCTTC
CCAATGTGGCGCCCTCTGTTGGGCACCAGTCGTCTCTAAACAAATTTACGATAATACAAGATAAAGCTTGTTT
ATTTTAAATTTGATTAGAGGCATCGGCTTGCCGACTGCTGTGATAGTGCGTGCTGTGC

TCTCCTCATTCCTTTATTGATGATTTTAATATGGTATCATTAATTTAGTAATATATAATATATATATGTATGT
TAAGGGCCTATTTCACGACGTAATATTATTCACAAATATTTGGTCTAGATACTAGATAGGACAGCTTTGTACT
GGTGAAATGTTTGTCTTTGGCCCGTTATGCAATAAGATTATTCAAAATTGGTCGCAGC
TTGATCTGTCTCGTTATAAAATGTTTGCTTATTTTATAGCTTATAGTGTAGACAGTGGTCCGGGTAAGGTCAG
TTTTAACAAAATTCAAAATGGATATCTATCTTGGGTACCCAAGAAAATTATTTTGAATATAACATTGTTATTG
TAAATTGATGATTGTCGATGTATCACCTATAGTGATCTGTCTATGGTGACCGTGTAAA
GTTAATTGTGTGCAAAGTCCTTGCAGTGGCATATCATACTAATGGTGATTCGTAACCTTGGTTAAATTGCTTC
AGTTGCCCTCGTTATTCCACATGTGNNT
>78230957_2 CV526090.2
CCCGGGCTGCGGAATCGGCCGACGAAGAATGTAAATTACTATTTATTTAGGAAATGTATAAAAATTTAACGTA
GCGGGATTCGAACCCCGCGCCTCGTGAAATGAAACTAGTAGTAACAATACAGACCTGGTCGGTATTGTCATTA
CTGGTACTATTTTCATTTCTTCACTTCACGGTGTTGAGGCAAATCGCGGCGTTAAATT
TCGCCACGACACACGCAATGCTTCCAGAATTCGGCGGACAGTGGGTAGTGGAATATCTTAATACTAGGCTCCC
TCTGCCCAGACTGCTACATGCGAGATACAGCGTGAAGCTGTACATGTTAAAAACTGTATATTTTATCTCTATA
TATAATAAGACAGTAAAAATATATTGTCTGTACATTTAGTAATTTTGACTGAAATAGA
TAGAGGGACACTTAAAAAAAATACTAGAAATGCAAAAAGTATTGCTTTAAATTTCTTGTCTGTCTGTCTGTCT
GTCTGTACGACATATATATGTACACATTTTGCTCTGAAAGTACCGAACCGATTTACTTGAAAATTTCACCAAT
TGCGTAGTGTTACCTCGACTTCAATACTAAAGTTAGTCCATCAAAATCGGATCAGTTA
CACCAAGGTATCGTCAGATGAACGAAAGTGTGATGAATTCAACCTCAGAGATTAGGAACATCTTAGGGAACTA
TCGGTGACTCGAAATTTTAATGGACGACATTATATAAGTACTATTACGNT
>78230964_2 CV526097.2
CTGGGGGTGCGGATTCGGCAGACGGAAGATGCATCTGTGATATGTCAATAGCGCAATGTAGATCGCAAGAAGA
ATATTATCCTACCGACTTATCTGTAAAAGGAAATTATGATACATTACAATCATATGAAAAGCGCAGAATCCAA
GAAAACTGCTCAGAAATTAATGTCGTTCAAGATTTATCACATTCTATGATGCAAGTGC
AACATATGTGTCATACAGGGATACCAAGTCATGGACTTGTAAGTGAAGAACCAACAGATTTATCAAGTAGAAG
AATTGAAACTCATTCGAATACTTTATCATTATGTATGCCAGAACAACAACCGTTGAATTTAAATATATGCACA
AATAGTACTGAATCTAACAGAGACGAGGTTCCATCTGATTTATCATCCAGAAGTAACG
AAAGAAGGCATCGGATTTATGTAGACTCACAAGGAACTGAATATTATGAAAAAATCCTTGTCGATAGTGAAAG
AGGTCCTGTACACCATGTTGAACCGCAACTATCTTATTAACCGTATGGATACGGAATCAATGATTAATGTCCG
TTATGCCTCACCACCAATGTCGAATAGTACCTTATCTATTATGACTTATTAACCTCAA
AGTACTGTTGACCGCGGGCAAGAGTTTACTACTTCCAATCGTATAGGACGAAAAACGCTAAGCTACCTGTGAT
TAAAGACTTGAATTCAGTGGTATCTGGGNA
>78230969_2 CV526102.2
ACTCACGGGCGCAGGAATCGGCAGCAGGGCGTAAAGCCGCNCGGCCGGCGACGGCGACGAGGATCTCGACTTT
GATCCCTTCAAAGAAACACAGAAAGCGTTAGCTGAAATGATGGAAACGGAGGTCATGCTGAATAATATATCTA
GTGGTGACAATATGGACCGTGTCCGCCGGTCTCGACTCCCACCACCCGGCTTCTCTCA
CATGAACGCGTTCGGTGTCGGTGTGCCTCGCCACCAGGGACACCATCAAGGTTACAATACGAACGCGATGTTC
TCAGATTGGACTCAAATGGACCCCGCCATCATGTCGACCTCCGTGAATTTTGGCAAGACGCCCCAGACCCTTA
GTCAACAGCAACAAGATCTATTTGCCAGGTTCAACCACCTCCAGGTTCAGCCCAACGG
TGTCAAGCTGCCCTGGCCCCAACAGAAGCCCTGGCCCCACCAACCGGCGCTCTACAACGCCATCCCCGTACCA
CCGGGCTTCGTTCCACGACAAAAAGAACCAAGACCAAGCCTAGTGTATAGACCCAAAGTCACTCCAACGCGAT
GACAAAACTTCCTGATTGCAATTACACAGTAGATGTGAANC
>78230974_2 CV526107.2
TGGTCGCAGAATCGGCACGCGCAGATGTAAACATGTGAATAAGATCGGTTTAATGATTTCGGTGTGTAAGTAA
TTACTACTACGCCTTTTGACATCTTTTGCGTGGTTAGTAATAGGGAGGAAAAATGGATAAAAGAAAATTATCA
ACTGCTGGCGATAGTCTATATCAAATACTGCAGGTACCTAAAACTGCTACTGCTGATG
ATGTCAAGAAAAGTTATCGTAAACTAGCGTTGAAATATCACCCGGACAAAAATCCCAACAATCCAGATGCAGC
AGAAAAATTCAAAGAAGTGAATAGAGCCCACACGATATTAAGTGACGCTACGAAGAGAAATATCTATGACAAC
TATGGATCCTTGGGATTGTATATAGCTGAGCAATTTGGAGAAGAAAACGTCAACGCTT
ACTTTGTTGTCACTAGTACGTGGTGCAAGGCCCTATTTGTCGTATGTGGTATCCTGACGGGATGTTACTTCTG
TTGCTGTTTATGCTGTTGTTGTAATTGCTGCTGCGGAAACTGTAAGCCGCGCCCTCCAGAAGAATCTGGTGAC
TATCACACGTCGGAACGAGATGATTCAGATGGAGTTCAGCCGGTGACGATGCAGCCAG
GAGGGGAAGGGCGTTCTACAGGTGAGCAAGCGCCACCTATCGCCATGCCGGGCCCAGCTCCGGGTGTCTCCGA
AAACCCAGGCTCAACNNNA

>78230979_2 CV526011.2
CCGGGGCTGAGGACTGGCCGACGGGAAGTATATCTATGACGAAAATTTAAGAAAATATGTAAACAAAACAATT
TCGACTAATTGATATCTATCCTTTTACTCTAGAAGCGATTTTTTCTGTAAAATGGATAAACTTACAGTTATAT
CTGGAACGCTTTTCATGGCTGCAGATGTTTTCGCTATTGTGAGTCTTGCAATGCCAGA
CTGGATAATTACGGATGTTGGAGGTGACACGCGCTTAGGATTGATGTGGTCATGCATAACTCTATACAACCGT
CCACAAGTTTGCTTTACTCCTGATCTACAACCAGAGTGGCTATTGGCCCTGATTTGCATCTTTATGGGATGCA
TCTGTATAACTACAACTGTAATTCTTTTAGCATCAAGCCATTTTGATCGCAATGTGAT
ACCTTATGCGCGTTGGGTTGGATTTGCTGCAATGGTTGTATTCTGTTTAGCTGCAGTTATATTTCCTATGGGA
TTCCATGTTGATGAAATTGGGGGACAGCCTTACCAACTTCCAAACAGCCACCAAGTTGGAATATCATATATAT
TATTTGTTCTTTCACTATGGATAACTGTAATATCAGAGTTATTTGCAGGCAAAGTATG
CTTACCACATTTCTAATACATGTAATAAATGTATTAACAATGAAATTTTATACTATCCAATTATTACTAAGTC
CTTANNNT
>78230982_2 CV526014.2
GGCCGGGCGGGAATCGGCCGACGTGTATGCTCGCCATCATGGTGAACAACAGAGTACCGTCGGTCTTTTCGAA
GACTTATGTCACACCGCGTCGTCCTTTCGAGAAGGCGCGTCTAGACCAGGAGTTAAAGATCATCGGTGAATAT
GGTCTAAGGAACAAGCGTGAAGTGTGGAGAGTCAAGTACACCCTCGCCAGGATCCGGA
AAGCCGCTCGTGAGCTGCTCACTTTGGAAGAGAAAGACCCTAAACGTCTTTTCGAAGGTAATGCACTCTTACG
TCGTCTCGTCAGGATCGGAGTCTTGGATGAGAAGCAAATGAAGCTTGATTATGTGCTTGGCCTCAAAATTGAG
GATTTCTTGGAGCGCCGTCTCCAGACCCAAGTGTTCAAGGCTGGTCTAGCTAAGTCCA
TCCATCATGCTCGCATTTTGATCAGACAGAGACATATTCGTGTCCGCAAGCAAGTTGTAAACATCCCCTCCTT
CATTGTCCGCTTGGACTCTGGCAAGCACATTGACTTCTCCCTGAAGTCACCATTCGGAGGCGGCAGGCCTGGA
CGTGTCAAGAGGAAGAACCTCCGCAAGGGACAGGGCGGTGGAGCCACCAATGATGAGG
AGGAAGATTAGATATTACCTGATTAATGTCTAAGCCAAAAGAAAAAAAAACCCCCGGGGGGGGCCGGTACACC
AATGGCCAAAAAGGGG
>78230985_2 CV526017.2
CCGGGCTGCAGAATCGGCACAGGCAGGTGTGTCCCTCNCGGGCACCCAGCTCAGGTTCACCAGTACGATATGC
ACGACGTCGACGTGGAACGAAGTACGAGTGACCACCATCAACGGTGCCAGAGTGCTGGATGATAAGAAGGATA
TACATATACCTCAGGCTATAGCGCACGCGGTGGACCGCGTCATGTTCCCACTGCCGGT
GGGCGACCTGGTGCAGACGCTGCAGGCGGACAGAGACCGTCGCTTCACCATATTCCTCAAGGCGATACTTGCT
AGCGGCTTCGCTGACACCCTGGCTGAAAGTAAAACCTACACAGTATTCGCGCCGACCGACACCGCATTCTCTC
GCCTGACAGCCTCCGAGCTATCTCGTTACTCGGACAAGGCAGCCGCTAAGGCATTAGT
CGCGCGGCACGTCCTACCCGGTACATTATACAGTGCGGGCATGAGGTACTACCAGCTCAGGAACTCTATGGAA
GACGCTAAGCCGCTCACTTTGCAAAAGAGTAGTGGTCGCATCAAAGTGAACAACGCGCAAGTGATAACCCACA
ACATCCCCGCCACGAATGGTGTCATACACGCCGTCGACACGCTCTTGTAACCCGCCAA
ATTATTATTAGACTATATCTATTTCATTATATAAATCCCTTTTTTAACTTCTGAAATCGCCTTCAAAATTTCT
ATTTATTTAGAAAATTTATAGAGACATGATCATTGAACAACAAAANGT
>78230990_2 CV526022.2
CCGGGCTGGGATCGAATACGAGTTGATCCAGCGACACGATAGGGGGGCCCGGTCGCGATGCTGCCCGTATAAT
GAGTGTTTCACACATCTCAGTCCGTGCTTAAATACGTTGTAGCTTGTGTTATTTTGTGAAGGCATGACAGATA
GAAAAGCTAGATCAAGATCACCCATTAGAAGGGATAATGGCCGTAGAAGGGATAGTAA
GAATATTGAACAATTAAAAAATCTGGACAATATGCAAAATGTAATAAATTCAATGATGATGCCAAATATTTAT
CCTCAAGGAAACATGATGATGCCAGGAATGTTTAATATGATGATGCCAAATCATATAATGGGAGGAGGTATTA
TGCAACCAGCTGGAATGGATATGATGCCGGGCCCAGCAATGGAAATGATGCCTCAGCC
ACCCATGAATATGGCATCTATGGGTCAACCAATGGCACCTGCAGGCCCACCCATGGACATGGGAATGATGGGA
GGTGTTATGGAGCCCATGATGGGAATCTTTCAAAATATGGGAAGTGAATCACAACAAGACAAAAAGGAACTTA
TACTGAAACACTGTAAACTTTTCCCTCCACTACCAGGAACTCCTTCACCACCATGCAG
AGCAAGACCTCCAGGTTGCCGAACTATTTTTGTGGGAGGCTTCCAGGATAGATAAGAGAAAGTACGGTAGAGA
AACTTTTGAGCGATATGGCAGAANNG
>78230993_2 CV526025.2
CCCGGGCTGGGAATCGGCACGAGGGTTAGTTAGAGGTGATTATTGAAGTAACTAAACAGTGCATGTAACTCAA
CAAGCTTACTAATGGGTGGTATCATATACTAGTTTTCATCATGGCTAATGAAAAGAATAAATTTAACTCGCAC
AAGTCTTCATCTAGAACTAAAAGAAGTAGTAAAGTTGATAAACTGCAAACACTATTAA

TAGGTTTGACAATGACAAGATTACAGAGTTGGTTATGAGAGATCAAACTTCTCCGAATTCAGATGACATTGAA
GAACCTGCAGAAAAGGAAATTGTGAACGGCACTCAACTTGATACAGTGATTTTAAAGGATTCAATAATAAAAA
GAGAAGTACATGTTCCAAAAGACAGGTTTCTGACGAAACAAGGATATCTTAAACGTAA
AAACAAAGATAATTTGCAACTTTGTAATGACATACTGAAAAATGAATTAATGATTGTGGACAACAGAGAAAAA
CAAATTAAAGCAGTGCATAATAACATCAATAACAATGATTTAATTACAAGTGATCAAAATAAGAAAACATCTG
ACAAAGAAATTGAAAAAAATAATAAAACAAAAAGGAGATCACCGAGGAATAGTGAAAA
AAGTGATCAAAGTAGTAGTTCATCAGAAAATACATACGACAAAGCCCCGGGGGGGGCC
>78230997_2 CV526029.2
CCACCGGGCTGCGGAATCGAGCCGAGGCCCGCTCGCCGCCCGCCACGGCCGCGCCTGTCCGTGTCGTAGTGCC
ACAGTGCCACAGTGCAGCGAGATGCAAGTGCTAGCCGCGCTGCTCATAGCGGGGTTCATCGCCGGCGCGAGGG
CCGCCATAAGGACCCCCTCGAGGCCTTCCGCCCGTCCGGAGAGACGCACGTGCGGAGA
CCGGAGAAACTGCCCCTGTTGCCGCACAAACGCACCGCCCAGCCCAAGAGCGGTCTCAAGATTCTCTATCAAA
CTGGAGTATCGGAGGAGGATCTCCCTGACTGCAGAGCGCGGCAGGTGTGCAGCAAAGTGGACCTTTACGATTC
AACACAACCCTGGATTGAGAGGAAGTGTCGGTGTCTCGGGCATAGACCGTGTTCTAGT
GAGCTATCTGATGAAGACAACCACACGTTAGCAGACAAAACTACATTATACAAGACATGTGAGCCAGTGAAGA
GGTTACCAAAGTGTCGATACTTCAAAGACGCAGCATGGATCATATATTCGTTCCCCGACAGCAACGCCACGCA
ACAAATTGTCAATTGCCACTGCCCGAAGCAATCCGTTACGTATCTATTGAAGAAACTA
CCGTATACGACACCTAGCGGAGAGCCGGGGATCAGTCCCAGTTTGCGTGGTTCGCCGCAAAGTAGATTACGGT
GTCCTNC
>78231000_2 CV526032.2
ACGTATTCATCTTATGTTCTTGGTTAGATCCAACGCTAAAGCCATGTGCGACGCCTCCGTTCTACTTGAAAAG
GCGAATGTTTATTAATTTATATATACAATTAGAAATATAGAATATAAGAATCGAAAAGATGGATACCTAAAAC
ATATTTCTTCATATATTATTGTTTTTGTCTGCAAATTCTAAAGAACATAGATTTTTCG
ATATATATTCGATATTATATTCATATTTGATGTTATTTCATGTTTTTGTATGTCCCTTTCTAAATAGAGATAT
CGAAACTATTCTAAAACAAAAGTCAGAGAAATGGTTTTGTTTTGTACATTATTTGTCCAATGAAAATTATGAT
TTTTATACTCGAATTATGTCTAAGTTGTGTTTGAATAAATAATAAAATTATATGTATA
CGTAATTAAATATTTTATATACATTTATTATATACGTATTTAAAACTAAATATATTATGATATAATGCAAACT
AACTTTGAACGAGTCATTCGCACGAAATTATTTTAAAAAGAAAATGTGTATTTAGATACAGGGCTTATTAGCT
TAGGCCCTTAAGATAGACGAGTTTTCTAGGGNAC
>78231004_2 CV526036.2
AGACAGTAAACAACCAGTGGCAATTTTTAAGCACCTACAAACTTACAATATTGCGGTAATTGTCTCACGATAT
TGATTTATGAATTTGATGAAGATAATTCATAAAAAGATGATTGGTTTTTTAGGAATATGTGGAAAAAAATATC
CCGTGCAAAAAGGTCCAAACAAAATCGGCAGGGACCCTAATACTTGTAACATAGTTTT
GGATTTAAACTCGATATCTAGACATCATGCTGTTATCAATGTATTAAATAAGCATGAATTTATGCTTATGGAC
TTGGATTCCGCTAATAAAACTAAACTTAATGGTAAAGCATTGGAACCATATATTCCAAGTGCACTGAAAAATG
GAGATATGGTGCAATTCGGTCAAGTGTTTGGTGTATTTAGATTGTTTGAAGAAGAGAA
TGATTTACCTATGACACAAGCTATAGATGTTCCAGAAACTCCAGTACAACATACATGTATATCAAAAATAAAT
AACCAATGTACTACTATTCCTGAATCACCAGATGTCAGTGATAGGGATGATTCTCTAATTGGTGGGTCTCAGC
AAAAAACAAGTAATACATCTAGAAGTCCAAAAGATATTTTTCCGAAAATGTGTGCAAA
AGCAAAATCACTTGAGCCACTCGGTCCAAACAAATCACCATGTGTCTGCATTCTCACAATNNT
>78231005_2 CV526037.2
CCGGGCTGAGATCGGCCGACGCTGTGCGATTCGGCACGAGGCGATCTCAAAATGCAAGGAATATCGTATCACC
GCTCTTCTGGCTACCGCCATCGCTGCTCCACTTGGCTTGGAGTACAACGCGTATGTGCCGCTCGCCAAGTCAA
CATTCTCATCGAGCAGTCAGGTCGTCAACCATGGAAGTGCCCATATCGTCCATGCCCC
CGTAGTGGCTCATGAAGTCGAATACCACGTGCCTTTANCCAAGTCCACTGTGACCAAGAGTAGTCAAGTAGTA
GACCATGGATCCACTATCGTTGCCCACGCTCCAGTAGCTCACGCTGTGGTCGCGCCAGTCTCCCACGTTGTAG
CTCACGCTCCTATTGCCCACGCTCCAGTATACTCTGTCAGCTCCTTCTACTCTTACCA
TTGATTGTTTGTTAGTTGTTAAATAAATTATTTCGTTTTCATAAC
>78231012_2 CV526044.2
CCCGGGCTGAGGAATCGGCCGACGGTACTGCACTACCTCCTGCGTTTTGCGTCCAGTGGACAAGGAACACGTC
AATTTCACGAGTTAAAAAAGAATGAACGGCTTGGTGTGTCCAAGTTACGTGAAGTTTTAGAAGATAGACTACA
AGACATAAAGCGAGCTAAAACGTGGAAGCACGAGAGGGTCATCACGTCGCCTCAGGAC

ACAAATGTCAAGGTGCAAGGTGAAGAGGGAAATTTCTTAAATTTTTGCGCAAATAATTACTTAGGACTGGCCA
ATCACCCAGAAGTCGTAGAGGCCGCTAGAGAAGGGCTAAGCAAGTATGGAGCGGGACTTAGTTCCGTTCGTTT
CATTTGTGGGACTCAAGTTATACACAAGGAGTTAGAAAATCGATTGACAAAGTTCCAC
GATCGTGAAGATACGATACTTTATGGGTCATGTGTGGATGCTAACGCTGGCCTGTGCGAAGCTTGATTGTCGC
CGGAAGACGCTGTATTCTCGGACGCTCTCAACCACGCCTCTATCATTGACGGCATTAGATTGTGCAAAGCTCA
GAAGTACAGATATCCTCATAGAGACCTTAATGAACTAGAACATTTGTTGGCTCACAGT
GAAGCGCGACTAAAACTGATAGTCACAGATGGTGTGTACTCGATGGACGGCAACGTTGCTCCGATAAAAGGGT
GCGGGAATGGCTGATACGTATAGNNT
>78231017_2 CV526049.2
CCGGGGCTGCGGAATCGGCACGACGGGCAGTGCCAATCCTGGTNTCGAATTTATATAATTTAATTGTTATTTT
TATAATGAAGATCCTTATAGTATTGGTGTGTATTGGTATTGTGTCAGCGAAAATTTACCGGACGATTTCCCCA
GTGCAAGCGGAATGACCCGGAACTTGACAAATGTGTTATCAAGGCAGTGGACGTAGTG
AAACCAAGACTTCTTAATGGAATACCAGAAGTGAATGTGCCAGCTTTAGATCCATTCTTAGTACCGACATTGA
AATTAGACAGAACAGCAAATAACTTAAGGTTGAAGGCTACTATAAAGAATATGAAGGCGTATGGTGGTTCAAA
CTTCAAAATAGAGAAGATCAAGTTCAACCTCAACAACAAGTACGTGGGAGAAATCAGA
CTGATCATCCCCAAATTATTTGTAACTGCTGACTACGACGTCCGTGGTTCCAAGATCCTTACTCTGGACATCA
GTGGAAAGGGAAAGCTCAGCAGTAATTTTACTAATATTGCGGTAGTGGCTAAGGGAAGTGCCAAGCCAATCAT
GAAGGACGGTGTCGAATACTTGCAAGCTGAGAAAATTATTACAAAATTGAAAATCGGC
CATGGACAAGTCGCCATTGATGATAGCGAAAGACCAGTGGCTGCCACTTCAGCCGGGCACTTCTATCTTGCAA
GTCCTGGTGTCTGCTGGACATTCTCACCCCT
>78231023_2 CV525959.2
CCCCCCGGCGCTGCGGAATCGGCCGAGGCTCGTGCCGAAAAAGAACTAGAAACTATACATTATCTTCAACAAC
GTGGCTTGATCGATCCTCACGGCCGTCCCGTGCCCGGATCAGAACATCAAATTCACCAGGCCATCCAGGAGTT
CCAACACATGCAACAACAGGCTTACGGACAATACCAGCACCATCCGCCAGGGCAAGTG
CCCCCACCGGGAGAATACCATGGACAAATACCGCAAGGACAGTACCAGGCTCAGGTACCACATGGACAGTATC
AGGGACAGATACCGCCAGGATCGTATCAAGGACAGTACCAAGGACAGTACCCAGGACAATATCAATGACAGGT
TCCGCAATGTCCGGCACAACATGGACAATACCAGGGTCAAGTACCACAAGTGACAAGT
ACCACAGGGACAAGTTCCACAAGGACAAGTACCACAGGGACAAGTACCACAGGGACAAGTACCTCAGGGTCAA
GTTCCTCGAGGGCCATGTACCTCAGGGTCAAGTTCCGCAGGACCAAGTTCCTCAGGGTCAAGTCCCTCAGGGT
CCAGTTCCTCAATGTCTAGTCTCCCAGAGCCCTGTTCCTCAGGGCCATGTCCCTCTAA
GTCTAATCCCACGAGCTCCAAGCCCCCAGGCTCACGTTCCACACGTTCTCGTACCACNAGCGCAATATCAGTC
GCCAGTATCTCCCGGAGCAGCGTGCTCAGGGCAGTGCCCCCTGTTCATGTGTGGGCCCCAACCGTTTCCCTTT
CTCTAAAGTCCTCGGTTTTGCCCCGAGTTTGCCTCGATTTTCGTTCGCTCGCCCCTTT
TTACCTACCGGGTAGCCTCTTGGGCTTTAATGCGAACTCTGCTTTATCGCGCCCCTGTTTCTGAATNC
>78231027_2 CV525963.2
CCCCCGGGCTGAGGAATCGGCACGAGGTAAGATCTCATCCCAGACTTCTTAGGAGGACCTGNCAAGAGTTTCG
TCCACGAAGGTGGATTGGTGCCGAAGAGCTTGTACGTAAGCGGTGCCTTTACGGAGCGCGATGGGGATCCTCT
TAGCGAAGACAGTATATACAGATCAGTTAGCTTGGGAAAAGGACAGGTCCACGAGGTA
ATAGTACAGAACCGAGACCCGCAAAGTGTTCTAACATGGGACTTCGACGTGATCCGCCACGACATCTCGTTCA
CGGTGTACCGCAGCGACCACGAGCTCGACCATCCTGACCATGCCGCTGCTATGTGTGTAGGCGGTGTAATCGC
AGAGGAGTCCCGCTCGGTGTTGGACAGCGGGCGCGAGGGCGAGCACTACCACCGTGTG
GAACCCACCCTTGTGTGCCACGATGGGGAGAGTATACAGGGCTCACACGTGATGGCCGTATGTGGTAGCTACG
TGCTGCAGTGGCGGTGTGAGGCGGCGGAACGAGCGCAGCTCATGTATTTCCATGAGACGCTCGCCAGCCATCA
TTACCGCGGCTCGATGTCCAGTCTCCAATCAGCTACAAGTGGATTTTCTTGTCTAAGC
AGCAAATCTGCGACAAGCTCGTGCCCCTCTCGGTGATGCCACCCATTAAACCCTCCACAT
>78231036_2 CV525973.2
CCGGGGGCTGCAGAATCGGCACGAGGGTGCTTTGTGAGGTTTTCTTTTAATTTTTGTTGTATTTTAATATATA
TTATTCTGATTAAACGTACTTGAAAAGTCTGACTGAACTAATAAAAAAATAACAAAATGGCAAAGTTTAAATT
GAAAAAGCCATCAAAAAGGCAACCCGCACGCCTGCGATATAAAATAGAAAAAAAGGTT
AGAGACCATAATAGAAAACTCAAAAAGGAAGCAAAAAAGAATCCAAAGTCTAAAAAAACTAAGCCAATTCAAA
TACCCAATATTTGTCCTTTTAAAGAAGATATATTAAAAGAAGTTGAAACTGTTAAAAAACACAAGGAAGAAGA
AAGACAAAAGAAAAGAGAACAGGCAAAATTAGATAAGCAAAAGAAATTAGAAGAGAAA

AGTAATACTACAGTCAGTATGGATACCTTGGTTAAAAATGCACAAGCAAGGGGTAAAGTCCATGAGGCATTTA
AGAGTAATGAAAATGAAAGTGATGATGTACAATTTGGTAAAGACAAACAACAAGAGAACTCACTTGGAGCTTA
TTACAAAGAATTTAAGAAAGTTATCTCAGAAGCTGAAGTTATTTCGGAAGTTGTAGAT
GCCAGAGATCCATTGGGTACAAGATGCATCCAAGTAGAAGAGGCAGTCAGAGAATCCGGCAAGAAGCTTGTTC
TTGTCTTGAACAAAGCTGATTTGGTC
>78231046_2 CV525983.2
AGAAAAACAGAGAAGAAAACACACAAAAAAATATCACCTGGGCCGCGGAATCGGACGACGGTCTAGATCACCA
TTCAANGGTTGTATAGCAGATGCACCATTAATGGAAGGGTTTTGAACCTTTTAGAACCATTTAGTAACCATAG
TGTTACGTTTGGAAGGTGTGGCAAGACGATTTCTACTGGTGGTGTTATCCAAGATCTA
GGATCATGGCCGGCACCACAGCGAGGAGACATCCTACCAACACCGAGTATTATAGAGCCTATAGAAGAAGTCA
CCAGTAAAAACGTAAACGTAGAATACATACCAGAAGTGCAAACAACAACGACACCATTCCCAACAACAATCCC
ACCAACAACTAAGGCGCCCGTCAAAAAGACAACACTAGCGCCTATAACTACTACCCGA
CTACCGCCGCCACTACCGGAGCCGGGGTGCGCGTTGGCGTACGACCAGCAATATACTACCGGTGATCCAGAAG
AGGGCTACAGGTTTGGTTCAAGAAACGACAGTCGTATAGAATACGGTAAGCTGCCGAGCCGACAGCTAGAAGG
CTTCGACCTCACTGTGGGCTTCCGTACGTTTGACAAACATGGCGGGTTGATATTTTAT
GCGGCAGCTAAACGAGCCCCAAGCCAGTTTCTCGCACTGTATATGAAAGATGCTAAAGTGCACTTCACATTCC
ACTGTGGGTGGTGAAAACGGTTTCGTGGTATCGAACCAGACTTACAACGACCCGGAATGGCACCCTGTANNA
>78231053_2 CV525990.2
CTCCGGGCTGAGGAATCGGCACGAGGGGTCACGTTCGGACGCGCAAAAAGCGCCGTGATCCATGACAGCGTTA
TTAGATATCCTTATGAATAAACAGTGTTATTGTGTTTTGTGTTGACAATGAGTGGCACGGGCGTGACCTCCCG
GGGACCTGAGACGGTGTCCATGCCGGCCGATCTGCCTGTCCGGCTGTACATGCGCGCA
CTGCGCCAGTACCAGAAGTTGATCGACTTGAAGCGATTCACCCAGGAAAAAGTTGCTTCTGGCAAAGATGAGG
AACGGTGGCGCAGTAAGAACGAGGGGGCTCTGTTCAGACGGTGGAAACGCAATACTGCCTCAGTAGAGTCCAG
CGCTCCTGTTCCTACACCGAATACTCCATCTCCGCAACCCTCAGATCTACTTAAATTC
TTAGCAATTAATGCATTAGAACTCTCAGCACCAGCATCTGATGCTTTACTCAAGTCACGATCTTCAGAATGGT
TCCAGTTGTCCGGACACCCAGGATCATTGGCACCTGCTGGCCCTGGCACTGTTTGGAAAAGACGTGCCGCCGG
CGATCATCCTATGCACAATCCAGAGCGAGACACATACGAGGCCTTAGCTGCCTGCCCT
CATATGCGCTCTGCCATACCACGCTATTACAGAGAATTAGAGTACGGTGGTGAACANTCATCGAACTAAGGAT
CTTGACCGGATTNT
>78231060_2 CV525997.2
CCGGGGCTGCGGAATCGGCCGACGATGGTGAGCGCCCGTTCGGGACTACGGCTGTTGTATATTATAGAGATAC
GCTATTATATTGCATATATATATATATTAGCACTCCGAGTCGTCGATACGAATTTTTTGAGGGTTAAAAATAT
TTATTGGTGCAATATATTTTTTATCGGAATACAGAGGTTATCTGTGGCGGTAGCTCGG
TTAGTCGATTTATTAGAGGTGTTGCAAGGAACTTAGGTTTATTTATGTATTATTTATATATATTGTATACATA
TGTATGTATGTTGGTTTGCGGTAATCCACTAAACATAGCACGGGGGAGGTTCTGACGAGCGCGCTGCAACACC
CTAGAGTGATAGCAAGTATGTTTTAAGTTGTCGTTTAACAGATCAGTATTTTAATAAT
TGAGTGACATTCCAGATTCAATTTGC
>78231070_2 CV526007.2
AGAAAGCACCACCTCCGTTCTTCCGGGCTGAGACTGCCGAGGGGTATCTGGTACAAGTGGGGCGAGCTACATG
GACTATTTAGTTACTGACGCAGTGACGTCACCCGTAGAACTGGCAAGCCAATATAGTGAGAAACTAGCCTACA
TGCCCCATACATACTTTGTAGGCGATCATAAGCAAATGTTCCCGCATTTACAAGAAAG
GCTAATTGTTAGTGATAAAATCAAATCTCACAATAACATGGGCAGTGTGGCAGACAACGTGGCTGTTATTAAC
GCGACTGATTTGTCGCCACTAGTTGAAAATACTGATATTAAGGAAATTAAAGAAATTGTAAGAGCCGCTCGTC
CTGTAGAGATTTCATTGAAGGTAGCCGAATTGCCTACCACTACCCCCATTGAAACTAT
GATAGCGTCGGGGCAAGTGCAGACATCAGTAAACGGAGTAATATTACAAAATGGCTTAGCGACAACACAAACG
AATAATAAAGCGGCGACCGGCGAAGAAGTGCCCCAGTCGATAGTGATAACAACTCGACAGCAATATGGCTTAC
CCGACGATGCAGTGGTTTATTGTAACTTCAATCAGTTGTATAAAATAGACCCATTAAC
TCTACATATGTGGGTATATATTTTAAAACATGTGCCCAATAGCGTGTTATGGCTGCTAAGGTTCCCTGCTGTT
GGTGAACCCAATTTGCAAGCGACTGCTCAACAGTTAAGATTACCGGCCGGTCGTATAATATTCTCAAACGTGG
GTGCAAAAGAGGACATGTTCGACGAGTCAATGACAACGTTGTCTGATACCTCTGTGTA
CGAAACCCACAGTTTGACTTTGTGACNNNNT
>78231071_2 CV525901.2

GGGGNNNNAACAAANNNNGGGCCGGGCTGGGATCGGCCGAGGTATATTTGTGAATAGTTTATGGTTTTTTTA
TTATAAGAATTGAATTATTTTGGTACCCATTAACATAGACAATTTTTTTATTAAAAAATTATTCTATCTACCT
TGCACAATACAACGTGAAGTTGATTTTTTTTTAATTACACGTGTTTCAGTTTATTTTG
ATATCATTCATCTGTATATGTGATTGTTGAACTAAAACTGACACGGGGCGCAAAATTGGAAAAAGCTCGGATA
TACGCAGTGGAATCATTTTAATTAGAAATGGGTTTTCGATAAGCATCAAAAGAAATAAAAGTACCGTAAATTA
AAAAATTCCACCTTAACTCCAGTCATTTTTACTTTCGTCATCACATAGACGATCTCTA
TCCATCCTAACAAAATTGTCGCAGAAAAATTATTGATTTAATTTTAGGAGGACGAGAGAGATAAATAAGAACA
TGAATTTGAAATTTAAATTCAAAATATTCTGTATTCAAATAGACTATTATAAGCACTTTTGAATCGTCGAATG
AACATTTCCGACCGGTTCGGAAGGCAGTTCTGCTGAGAAGAGCCGGCAAGAAATTCAA
CAGTTGCTCTTTTCAAAGTCAATGATATTTTAAATTGTTGCAGTGGCAAACTTAGAATACGTTACGGAATTTT
ATTATATACCTACATGTATACACAGACCCTTAATAAGATTTATTAACTTTTTGGAGGCGAAAACTGGCGAAAT
AAGCTTATTTATTTCTCGCTCTTATTGTTGCCTATCATTATTTATTGTGACTCGTCTG
TGTCGACACCAGTTTACTTCATTGGGACCTGNNNNA
>78231082_2 CV525912.2
GAAAAAAAAAAAAACCATNTCTTGCGGGCCCGTGGCTGAGGAATCGGCACGAGGAATATGACTACAAACGGCG
GAAGTCATTAATAGCACAGAAACTATTGAACAGTACGATGATATTACTACTGAATCTGGCAAAGAGATGTGGA
AGAGTACTGACACGTCCACTTTACCGCCCACGCTGTTGTTTTCAGAACACGAGAAAGT
GAAAGTGGATCGAGTTGATAACAAAGATAATGATAAAAAGGAAGAGTTGCACCCGAAACCTGTGCAAGAGACG
GTGCGACCTTCGGTCATAAAACTCCGGGGAGCGAACGCGTGTGAGACTAAAGAAGAGATGGCGGCTCCGTTCT
GGGCGAACAGCACCCGCGGAGAGATGCTAGCACTCCTCAACATGTACCCCTTCGAGCA
ATACATACATTTAGAGACGTGTGTGCATAAACACAAGCAACAATACTGCAGGGAGGGGTGCAGATGCGAACAG
CAATACCGTCTACATCGTCTCCTCGCCTACGACCCGCGCAACGAATGCCGCGGTATCTTCGCCGATTGGTTCA
AGTTCCCCGACTGCTGCGTCTGCAGATGCTACGATGTACCTCTGGAATTCCGCGCGAG
GTCACCTCGTATCCTCCATCCACAGTATGATGAGGACGTCAAGAGACTTATTTATGAAGATGTGGCCAGAGAC
TGGTATACCGTGTCGTATGGGGAGGACGATGATGATCTGTATTAGACAATCAAAATGGTTTATATATCGTCGA
TTTATAAATAAATTATATAACATTCGTCGCTCGTCGACATGTGACTCTGAAACGAGAT
CTCAAAATTATTGCGCCTCGAATATAAAACTTATAAACAACGTTTCAATCCTGGCCGCATCTACATCTATTTG
GGCGGCAACTCGTTCTGTTTTAACAACAANNNNGG
>78231084_2 CV525914.2
AGAAGAAGAACAAAAAGAGAAAAGAAAAAAAAAAAAAACAAATTCTGCCCGTGGCCTGAGGCGAATACAATAT
AATATGTACCTAAATGTATGTATAATTTCGAAAATTCGAAAATTGTGCAATATATTGGATTATCTTTATTATA
TATATTATACCCTTTGGTATTTACCGCGTATTTCTTTTTGTTGAATGTGTTGAGAATA
AAAAGAAAACATGTGTATTTATTACATAATTATAGAATTTCCTAGAAAACTATAAAGATCTTTATTGTAATTG
TTGTGATAACTTGAAGTCGTTCGAATTAGCAGATCTTTTGATGAGCATTGTCTATTCTTTTAGAGAAAAAAAA
AACTGACAGATTGTGTATTCGAAATTGTAGTGATTTTAACATTGATTCGACAAAATTA
GAAATTGTAAACATTCAACATATTAAAAGCTAAATATTCTTCTAAGTAGCAAAAAAATATTTCATAGAAAAAA
TTGTAGACGAAAATTATTGGTGAACTACGAATTTGTAAAAAAAATATATCTATTCCTTGATTTGAAAATTATA
AAAAAAAAATAATTACTCGAGTGTAATACGCTGTAATAATCGTATTGTTGTAACAAAA
TTGTACAACTGTAAATCTTTTCAAAACATTATTAATGTTTAGAGAAACGGTACCTACAGAGAGCCTTCGTAGT
GGATGCCTATACATGGTGCATTTGAAACAACTATATTAAGTATATTCACAATATCCCAATCTCATATCAAATT
ACTTTTTTATTCAATATAGTATATTAGTCATACGCGCGAAGATCAATTCAAGGGAACC
TCTTAAATTTCATTGTACATACAAAATAACACCATGAAAGGTGCGCTGTAAATCAGACCTCAAGTAA
>78231089_2 CV525919.2
GCCNNAAAGAACCANTAGCCGTCCGGGCTGAGATCGGCCGAGGTCACGTTGTGAAAACCGCCCGAAAGCTGGC
TCACCGCTGTTCAATTTCTGTCGAAACTCAGATTTTGATTACGTCGGTCAGCATACGAAAATAATGGCAACAT
ACAAGGAACCAGGCAAAGGGGGACCATCCGAAGAGGTCCAAGCTCACAGAATCCGCAT
CACCCTCACCAGCAGAAATGTCAAAAGCTTAGAAAAAGTATGTGCTGATCTTAAGAACGGAGCTCTTCAGAAG
AATCTGAAGGTGAAGGGACCCGTCAGGATGCCCACCAAGACTCTTCGAATCACCACGAGGAAGACGCCATGTG
GTGAAGGGTCCAAGACATGGGACAGGTACCAGATGCGTATCCATAAGAGGATCATCGA
CCTTCAGAGTCCCTCCGAGATTGTCAAGCAGATTACGTCAATCAGCATTGAGCCGGGTGTTGAAGTGGAAGTC
ACCATCGCCGATGCCTAAACTGTAAGGCTGCCATTTAATTTGCAGAGATGTACATTCCAGGAAGTAGGAAGAG
TTTCATTTCCTGCAGATAATAAATCAAAAGTTGTAAACTG
>78231098_2 CV525928.2

TACACAAAAAAAAAAATACAAAGAGGATCACATCAGCACATATACGGCCCGGGCTGAGGAATCGCGAGAGGTTA
AACATCATTCCAGATCGTACGAGCTAATATCGATATTATCATCCATTCATTCATTCTGAACGATTATTTGTGA
CCGAATGTTATTATGTTGCATTCGATTTTGATACGCTTATATTGTGACACGTGTGTTT
GTCGAGATAATCGATTTAATACTTTAAATATATAAATATTCATATATAACAATTTATACATAATAGTTTATAC
ATTTTGACATTTTATACGTACCTAACAAGAAAAATTATAATTTCATTTCATGTTTCCATTTTGTAAAAATTGT
TATTATTTGTAAAAATAGTTTAACATATCTAAAATATATAAAATATACGGACACGACG
ATATTTTAAGTAGGATAGGATATATTTAACTTATGCTACAAATTATAGCAATCTATACAAAAAGGGAAATATA
CGCTTGAAATTGTGATTTAAGTTGTGTTATCTATGTATACAATATCAAAAAGCCTCTATATGTATAATAAAAT
ATTATTTTTATATATGACTGTGTATATTTACTATAACTGGTTAGCCACAAACGAATTC
ACGTCAAAAACAATCTATCGGATCTAAGAACAAATAGTACACGATGAGCGGCCGTAGTAGTAATGTTCCCGCG
TAGTCCATTATTATAGTTAATACTGTATTAATGCTATGGCGTAACGTTAGTAACGTAAGTTAATATTACCTAT
TATTGATATCGTGAATGTCGTTGTGTTGTAGATGGGCACTGCCTAAAGCGGCGCAGAT
TATCATTTCAGTACTAACTTATTAAGCTAAGAATATGGGNNG
>78231104_2 CV525934.2
GAGCAGCCCAGCGAGAAAAAGACGCGGCCGGGGCTGAGGAATGCGAGAGGGTATTAATATCCTCACTGATACC
GAACAGGTAGTAATCAAAATTATCAAGAAAATGTTGATATGATCACTCCCATTACTACTCTAAAACAAAGTGA
CAATTCTATAGCTACTAATCAATCAGAGCAAAATATTACAGTTACTACTCAACCAGAG
CGAAATATTACAGTTACTAGTCAACTAGAGCGAAATATTTTGGCCCATGCCCCATCTCAGACCACTAGTATAT
TACAAGCTGGCCATACAGAATCTGCTTTTAAACCTGTAAGAAAACAAGTTTTACCACCACCTCAACCTCAGGA
ATTCTCATTGGTAACTGAAAATATTCAAAACCAAAAGGAATCCAAATTATTTGAAAAT
ACAACGACAGTCTCTTCTCTTAGCTTCTCACCGGCTGTTCAAAGTTCCGAAATTTGTTCAAAAACCGAATCAA
TAAATACAGGATTACATAGACCCGAAACTATTCCACCTTATCAACAAAATTAGGAATATTTGGCATCACATAG
GGGTCAAACGCCTGATTTGTACAGTGCTCCGTCTGATCCAAACAACATTGCGAAAACT
GTAGAGCGGCAAATGGCATGTCCATCTGATCCATAAGTATCAGATCAACATGAATCTCTAAGATTGAACTCTA
TACAGGCATCGGCTATTAGAATCAAAAGCCGAGATTCATCTATACCAATTCAACCAGTATTAGATGACCCTAT
GGCGACTAACATCACGGCATTCAAGACGTACAATAACTCTTAGAGTGTTACATAAACG
GGGGCGATAATACTCGACACAATGGACTATTACCGAAAAATACGTCCNAAGNNNT
>78231106_2 CV525936.2
GGGGAANGGTTCACNNTTGCGTCCGGGTCTGATGATCGCCCGAGGTACAAGGCAACGAGCGTTCGGGTCACCT
GATGGTAAGTGATTACCGCCGCCCACGGCCCCGTGCAACACCAGAGGAGCTACAGGTGCGTTGCCGGCCTTTA
AGGTGGAGATAGACGCGCTTTTTGAAGGTACCCATGTCATATCGACCTGGAAACACTG
CCGCGGGTAGGCCATTCCACAGTTCCGTGGTGCGTGGAAGGAAGTTCCTACAAAACCGCACAGTAGTGGATTG
CCACGTATCCAGATGGTGTGGATCGGCGGCAGGTAACAGGTCAAACAGTTCTTCAGAACACTCCCCATGATAA
ATGCGATAGAGAACGCACAGCGAGGAAACGTCTCTACGCAAAGCCAAAGTATCAAGCC
TTTCACAGATTATTGGGTCCCCAATGATTCGAATGGCTCTGCGTTGTATGCGATCAAATGGTTCAAGCTGATA
CTGGGGTGCACCAGCCCAGAGATGAGAACAGTACTCCATATGAGGCCGAACCTGGGCCCTATATAGGGCCAAA
CGGTGTTTAGGCGTGAAGTACCGTCTCGCTCTGTTGATGACCCCCAGATTCTTCGAGG
CCAACTTGGCCTTGCTTTCCAAATGATCGCGATACTGGCAGTCACTCGAAACATTGAGGCCCAATATACTGAT
ACTGGGACCAGAAGATCACCGGTTGAGCGACCATGGCGGAAGCCATATCGTCGGTCATAATCAATCGGGGATC
CTCTAGATAACCGAGAGCGACATTGTTATTGCCTCCTGACTTTGAAACAGGCAGGGAT
TAATAGGCGTCTTCATTGTCGACCCTCCCTTCTGGATCGGGAAANNGC
>78231107_2 CV525937.2
AGAGAAAAAGACGATAATTCAGGCCGAGGCTGAGGAATCGCCAGAGGTATGAAATGGCGGAAAGTGGGGGACG
GAGTGTCTACACTCTAACACTAGGTTCCCTCTGCCTACCCTGTTGTATGCAGGATACAGCGTGACGCTAATTG
AATGTATCAAGATGGATGAGGTGTCTGTCATTTTACTTCATTAGGTAATTAACATATT
CAAGGCAAATATCGATAGCAAGTTTAGCAATGAGATTATTGTTTCTCAATAAAATTCCAACTATTGTAATTGA
TATCTTGTAATAACGACGTCCATTGTTAATGGATTCCAGTCGAAACATCAATTTGCTAAATGACTCGATGAAA
ATGAATGTATCGCAGCCGTATCTGGTACTCTCTTACAATCAATATCGAAAAACTTAAA
ATAGAAGTGGTTTAACTCAAAATTTTGCTATAAACAGACCATGTAACGTTTTATTTTAAGTATTGGTGTTTTT
TTCCATATAATTCAGTTTTTTAGAGTTAGATCACTTTGATCATAGTGCGATGTAGATAAAACAGAATCTAAAT
ATATTTAAGATTGTTTCCAAACGTTATTTGTATTTCATAAATTGTAACACGATTCTTC
CTATACAGTGCTAAATAACCGAAAATTTATTGTAAGTCGACGTAAGACAAGCAATAGTGCCATGCAAGTTAAG
TGTTTACTGGCTAATGTATAGTTAACGAGGTTTAACGGATGTCAAATAGTAGCCAAACCATCACTATATCATT
CGTAAAAAATTATTCTGATACTATAGTAGAGGGGGGGTCAACCCGCTATAGTGTAACT

GATTCCTGAGATATTGTCTATCCATCNNNAG
>78231109_2 CV525939.2
GAAGCNCCAGCGAAATACGATCCGGGCTGAGACTGCCGAGGGTTGTATGTTCGTTACGAAATAACAGCAAAAT
TATGGATACTTAGCTAAAAAACTGCTGCAATTTTTATACAATTACATCCAATATGTCACAACAATTAATTAAC
GAAAATAGCAAATATATATTAGACTTATTTTCTGAGCAAACAATAGATTCACAAAGCT
TGGAATCGATATGTGCCCAAGTGCAAAAGAGGTTTCCTAAATCAGACCACTTTAACTTATGCCTTCTCCTTTC
TTCACTAATAACTGGTGGTGATTTATCACTGCCTGGGCAGAGAGTGGTTGCAATAGCTCTAATATTTGACATT
TATAAAACAGATAATCCATTTAGTTCCCTTTTCCTACATTTGTTAGAAGGAAAACCAG
GGTTACTACCATTAACTCCACAGGAAAGGATATTCATTGGACAATTACATGGATTCATTTCAATTAACATAAG
AGATGTACTAAAAAAAACAGCAAAACAAGTCATCCTAACAGAAGTATTAGCTAAGGACTTGGAGTTCGATTAC
TCTTCACTCCAAGGGTTGACAGCAGAAAGATCAGCTGACATCAGTTCTGTTGCTAAAG
CCACGGACCCCGCCTTAGTGCCACTTAGTGATGGAAGCCCCCCGAATCGTATAGCAATGAAGGAACTAATAGA
AGCCCTTATGTCCAACGACTATATGCCCTTACACNNNA
>78231111_2 CV525941.2
GGAGNANAAAAAACAACATGCTCCCGGGCTGGGATCGCCACAGGTCGAGCGCGTCGGGCTCCCGCACCCCGCG
CCTGCTCGGCCACGCGATACCAACTCCCGATTAAAACCAAAAGTCAACCGCGAGTGCATTATGACACTGGTGA
GCTAGCGGGCCGTTGTGACGGTGTATGTGTGTCTGTGTTCTCGTGCTTGTGTGTGGTG
TTAGCCGATAAAAACGTTCGTGGTGGCCAGCGGAGCCCTGCACCCGGGCTCGGGCCGAGCGCTCATGGCTCAG
CCTAGGTACGACGAGAGCCTCCACGGCGGGGGCTACATGGAGGGCGGTGCGATGTACCACGAGCACCGGCTCG
CGCACCCCGACATCCCCCCAGTGCACTACCCCCCCACGGCGCCTGCGCACGCGCTGCC
CGGCGAGCCGTTGGTACACAAGCGGGACAAGGACGCGATATATGGGCATCCTCTCTTCCCCCTGCTGGCGCTG
ATATTCGACAAGTGCGAGCTGGCCACATGTACCCCCCGGGACCCAGGGGTGGCCGGCGGCGACGTTTGCTCGT
CCGAATCATTCCACGAAGCATATAGCCGGTCTTCAGTAGACGGATACGCCAAGAGAAA
CCCTAATTACATAGCAGACCCAGAGGTCGACTCACTTATTGGTGCAAGCGATACACGGTCCTACCGCTTCACC
TAATAAACTAGACAAAGTGGCATCTATGCACAACTNNNA
>78231129_2 CV525841.2
GGCCGCGGAATCGCCCGAGGTCATTTACTAATATGTTTTATTTTAAAAGGTACAAAAACAATTACGCTATCAG
TCATACGCTCTATAAAACTAAGAACACAGCATACAAAGCCGGTGATTTCACAAAATATAATTATGACGAACAT
GCAACATTAAATCTAGGTGAACAACAAGAAAGTCAAATAAAAAAAAATTGAGCACGAT
ATGCCGAAACAAAATAAAGAAAGGAACTAAAGCTCATGTTTTGGTCTAATTACAATACACTCATTATATTAAA
CTGATAATAATATAAAAAAACAGAAAAATCTCGAATATTATAGTTTACAGAGAAATAAAAACAGAAAAAAAAA
ACCGGTAGGAACTTTTGAAATGGACAATGATAATGATAGTCAGGGTCTAAAGTATATA
TCCAACGTTGGGAAAGAGTTCACATGTAACCTGATATCATTGTCTCAAAAAGGCACCAATGTTGGTACCAATA
TCGGTGTAGAGTCATCCCATTATAGCATTAGTCGACCAGTACTGCTTTGTCCAATGCGGCTTGGACTCCTTAG
TGCGATGGTCCCCTTCGATCTACCTCGCTAGGTTCATACTCTTTATCCTTTACCATTC
TTCCTTTGCCGTAACAACTTCCCAAGGATTCTAAGCGACCTCGATACGAATCGGGCCAATATATCCCGGGGTC
TATCTATCCCCGTTCCATATCTGGTTTTCCCTTGGACTCCTTCTCCATCTGCCCGGAACTCCGGCGGTTTCCA
ATTCTTCGCCGAATAACTTATCTTCGTAACATCGGTACTTACCATCCGGGGCCGCCGG
TGTAACATCCTTCGGATCGGATTGGAGGCCTGGGACCCATTCTTGGACAGCTACGTGGGCTCTGNCNGG
>78231130_2 CV525842.2
CTTGGGCTGAGGATCCGGACGATGCTACATGAAGGAGAGTTAACAACGGAATGAACGGAAAGATGAATAGCTT
AGCGAGATCTAATGCCAGTATATACGGAACTAAGGAGGACTTGTATGACAGGTTGAAGCGTCACGCGTACCCG
GGTAAGAAAGATAAGAGCGACAGCGACAGCGAAGGTCAATAAATCGTGGAGAAACTAC
GTTTTTCGTATTCTGGTCAAACTTGCATAATACTATCGTATTCATTAAGATTATATACATTTCTAATTAAAAA
AATATATTTTCGTACAAAAAAAAACTGTTAAAATTGCGCTACTTATATGTCATTTAAATATCAAAATTAAAAA
CAATCTTTACAATGTTAAACGAAATCGTGACTTAGCAGTAAAATATAAAACATCACAT
AACAATAATTTTAATGATTTAATATTTTAAAAAAATATATTACAATTTTATTATTCAAGATGACATTATATTA
CATATTTAATTTGACATTTACAAACTGACTACTTTTCAAATTGTTCTATAGTTCTGTATCTAACACGAATATA
TGCGGAATACGTATAGTATGTCATCTAAGAGCGTAAGACTCAACACTCGGGTATCACA
AAGGGACCATGCCTTATGCATATGCGTGCTTATCATCACATTTCGTCATATGCCGAATACTGAATNT
>78231138_2 CV525850.2
CTGAGGCTGAGGATCCGGACGATTCACAGATCATGAAGTAAATATTCGACAAAGAAAAACTGATTATAAAAAC
ACAAGATTAAAACGTAATTCAAGTACATCTAATAAAAATAAGAGTAGTGAAAGCAACCAAGAAATAAGAAAAC
CTGAAGATATATGTAACTATAGCAAAATCGAATCAACAAAAGAAACTACTTCTCACTC

TAATTCTTTGGGAAATGGTGATGTTCTCAAAAATGTAACTCCTAAGAAAAAAAATGATATTATTATGCATAAG
ATGATGGAAATTACTGGTGAAAAAATTCAAGAAAAATGTAATGAAGTACTTTTGATAGATAATAAAAAAGAAC
TAATGAAAAACACAATCATAAATCAAACTTCAACTGAAAACTCCCAGCCTAAAAGTGA
AATGAGAATATTAAATAAATCATTAAATACGATTTCGGAAACAGATCAAGTATCAAAAGTTTCAACAGATATT
TATCGTCTCAAAAAAAAGATTGGATCAGGTGTGGGACTGATACTTGCTGGCGGTATTGATCGTGAAGCTAAAG
ATGTTACAGTACATAAGAGTCATGGACGATGGCATCGCTGGCAAAGCAGGGATCAACA
CGGGATCAACAGACCATAGTCTAAACGGCAATACATGGTCAGGTTGAAACCAGCCCAAACTGTAAGCTCTTAA
GGAACAGGTCCAATNTA
>78231140_2 CV525852.2
GGGAGAGGGCCGGGCTGGGATCCGCGAGGACACTCCTGCGTTTTGCGTCCAGTGGACAAGAACACGTCAATTT
CACCAGTTAAAAAGAACGAACGGCTTGGTGTATCCAAGTTGCGTGAAGTTTTAGAAGATAGACTACAAGACA
TAAAGCGAGCTAAAACGTGGAAGCACGAGAGGGTCATCACGTCGCCTCAGGACACAAA
TGTCAGGGTGCAAGGCGAAGAGGGAAATTTCTTAAATTTTTGCGCAAATAATTACTTAGGACTGGCCAATCAC
CCAGAAGTCGTAGAGGCCGCTAGAGAAGGGCTAAGCAAGTATGGAGCGGGACTTAGTTCTGTTCGTTTCATTT
GTGGGACTCAAGTTATACACAAGGAGTTAGAAAATCGATTGACAAAATTTCACGATCG
TGAAGATACGATACTTTATGGGTCATGNTTTGATGCTAACGCTGGTCTATTCGAAGCTTTACTGTCGCCGGAA
GACGCTGTTTTCTCGGACGCTCTCAACCACGCCTCTATCATTGACGGCATTTGATTGTGCAAAGCTCAGAAGT
ACAGATATCCTCATAGAAGACTTAATTGAACTATGAACCTTTGTTGCGCCACAGGTGA
AGCGCGACTAAAACTAATAGTCCCGAATGTGTTGTCTCGATGGGACGGCACGTAGTCCCGAATACAGGGGTCA
CGGAATTGGCGAAATAGTTTAAGCTTGGTACGCATCTGATGAATTGCAGCAAACAGGATCTTGGAAAACTGCA
NGNCT
>78231143_2 CV525855.2
CGGGGCGCGGATCGCCCGAGGTCTCAGCATACAACATGAATTTGATTGTGACTCTACTTGCAATTTTGGCGAC
ACCGCCTTAGGAGAAGAAACAAAATCTGACTCAAAGGCTGATCCATCTACATTAAAAGCGATTTTAAATAAAG
AATATTATCTAGGCTCGGGTGCAGATCGCTCGAAAAATTCACATGATCACCGAGATAA
GGAGCTCCGTTTAACTGAGGAAGAAAACAACATCGTGCGAGATGTCTACAGTCCTGATTACTACGAACCCAAT
GTTATTCTGGATTTTAGATACATGAAATCAGGAGGCAGAGGATACGCTAGAAGTGATGGGGGACAGATCTTGA
TACTGCCTAAACGTAATCAAAATCCTTCTCTGTACAACAACTATGACACTCTCATCGA
ATACTTACGCCTTAAACACTTAGAACCAGCAAATAAACATTTGTTATTCGAGTCCAATCGACAGAAATATCAC
GGATGGCAAAAGACCTACCATGAATATGAAGATGAACAATCAGACGCAGAGTGAGAATGGGCAAAATACAAAA
ACTAAATACAATTATCCATCGATTTTTTTCAAATTTGAACTCTTTTAGGATAACTATT
CGCTTATTTGAGAGAAAGAGGCGCAGGAACCCGAGGGGGCCCGACCCATTCGCCTTAGGGACCTTTAAATCCT
GGCCGGTTAAAGGTGAAAACAGAGGGAAGGGCGAGACAAAGCGAGAACAGTAGAGGTGNCT
>78231147_2 CV525859.2
CAGGAATCGCCCGAGGTTTAAACATGTTTAAAATACTTAGTTCATTTATTTCATATATAATAAAAAAAATGCA
ATTTCATTTTTTATATATTATTTTAGTTTATTCTAAGTTGTAGATCCGCATGTATTACATTACACAAGGATTG
TTGATTAAAATCATTTTGTCTTATTTTTTTCTTTGCTATATTATTTGTCATTATAATG
TGGTTTAAGATTTATAAAAAATATATTAACAATTTATATTTCGAATTCGTCAATGAAGACAAAATTGGAATTT
ATATCTATTTTTATGAGTAGGTATTGTAATTTTCAAAATGCCTTATTGACATGTAACATTTAATAAATAAAAA
TGTTGTAATTCTTTTTAAAACAACAATTGGTAAATAAAATGTTTTTATTACAAATGTA
AATGCGATAATGTGTCATACAAAAGCCATGTAATTGTTATGCAAAAGACATCTTTTTATTTTAAATATTTTAT
TACTAAATAGGTTTGTGCCATATATGGCCCAAATAAATCATAGATTTCGTTGCCAATTAATTAATCTGGTTTT
GGGCCAGTTTGATCAAGTACGGTATGGTTACTCAAATAGCGGTTAAGTACAAATCCAA
TCTACATCTGTAAAAGATTTCCAGTTTGTCCAATTGGTCTTCGATAATTATTAGATACCGATGTTGGTTGTTT
ACGGCTTTCACCAGTTTCTTGGGCACAGATCATATCGAAAGATTTTGCCCTGTGTTAATTGTGGATTAATGGT
CCTGGTCTTAAAAGTCCAAATTCAAAGTTACGTGGATTTTAAACCCTTTACCTGTTGG
GGAAANATCCTAGAAGCGATTATAACCCGATTAAGTTGGTGG
>78231152_2 CV525864.2
GCCCGAGGGCGCGGCTTACCGATTGGTTATAGATTTGAGTTTTAGTGATCATAAACAGTTCCGACATAAACAC
ATTCAATGAAAATATCGTGCAGAAACTTTGTTTAAGCGCTCTTAAGAATATCAAAGTTGGCAATCAAGACGCC
GACATAGAAAATATACAGCGAATAGCATTGGATATATTAAAAGAAATGTATACTAAAA
AAGATAAAATAAAAAATGGTATAACAAGTGAGTTGTTGCATTCAAAAAGAGATCTTGACACTCCAACTGAAAT
TTTATCAGTACACGATTATGAGAATATATGTGCAGTCAATATTGCTAGAGAAGCTTGGGGCTTACGATCGTGG
AATGGATATTGTGACATCGAAAATTGGTTACATGATGGCAGCGTTGTTAGAGATAGAA

GTCGTGACACCCTTACTTCGGGCAGTTCCGAAATAAGCAGCTGTTTATCTCAAGAATTGAAAAATGCCATATT
ATCAGCTCCAGCGTTTCCCAAAAAGCCGCACACCTATTCTAATGTCTTTGTCAAATTCGACAGAAGTCAGGAG
GACTACGTCAACACGAGTGATGTGCCACTCCAATGATGACACATTCTTGGGCTAAACC
ATGTATGATAGACACGTTAAAGGACCCAAATCACTTGGAACCGATTTAAAAGAATCCATGATCGGGGGACATC
GTATGTCTGTAGAGT
>78231153_2 CV525865.2
CAGAGGCTGCAGATCCGGCCGAGGCTTTGTGCAAAATGTTCCCCTTCGTGTGACGTGTTTCGTGCTAATATTT
TCTTTTGCCATTTGCTCTGCAAACGTACATCCATTCTCCGAAGAGTTCATAGATATTATTAACTCTAAACAAA
CAACATGGAAAGCTGGCCGAAACTTCGCGATGGAAATGCCGTTGAAAGCAATTAAGAA
TTTATTGGGAGCTCTTAAGGATGACTATTATGAGAGGTTGCCGACTGTTACACACGACGTTGAATTAATAGAT
AGTTTGCCGGAAAACTTCGATCCTAGAGATAAATGGCCTAATTGTCCAACGCTAAATGAGATTCGAGATCAGG
GATCTTGCGGGAGCTGCTGGGCGTTCGGCGCGGTCGAAGCAATGACCGATCGGTACTG
TATCTATTCGAACGGAACGAAGCATTTTCACTTCTCCGCAGAAGATCTACTTAGTTGCTGTCCAATTTGCGGC
CTCGGTTGCAATGGAGGAATTCCTACATTGGCGTGGGAGTACTGGAAACACTTTGGATTGGTCTCTGGAGGAA
ATTACAATTCCTCTCAAGGGTGTCGTCCTTATGAGATTGCTCCCTGCAAGCATCACGT
GCCCGGCGACAGAATGCCATGTAGCGGTGATTCGACGACTCCTAGGTGTTACAGAACTTGCCGAGAANT
>78231178_2 CV525890.2
GGGCCGAGATCGCACGAGGTATACTCTACTTAACGCCCTCTCCTTGGCAACATTTATTTTCAATTAATCAATT
CAACTTCTTAATAATTGACTTTATTTAAAATTGTATCATTTTTTTGTCTTTATATTTTAAGTGACTTTTCCAT
ATACTTTGCAAATTTCTTTTAATCCGACAATGTCTAGTGACAACGGTTTCAACAATGA
CGATTCTCCCAATAAAACTTTAACCCATCTTCAATTACCGAGCCCTATTATAACAAGAAGAAATCGAACAGCT
TCAACTTCTGAAAGAGCTCTACAAAATCCCATTGAATATGGCAAAATTAAAAAGTTTTGTCGGGAAAAAGGCC
ACGGTTTTATAATTCCTCTAAAAGGCGGCGAGGATATATTTGTCCACATTTCCGATAT
TGAAGGAGAATATGTGCCACTGCCAGGGGATGAAGTAACTTATAGACTATGCCCAATTCCACCCAAATTTGAA
AAGTACCAAGCTGTTCATGTCAAAATTATACAACTCGTACATGAAAGGCCTTGAAATGGCATGAACCCCAAAT
GTTAATTGCTCTAAAAGTACTTATTTATAACCGTCTGTTGGCACTTATAATATTACCT
TGTATTGTCTTTATAACCCTTTAAGACCAATCCTCTACTATGATCTGTGTCAACTGCTTAATTTCCACGTATT
ACTCGTTATGACTACCGAAAGCTTTTTTCCACCGGTATTCTATACTCCTAAGGTAGCATTCGACATCTGTGCC
ACATCTTGAGGGATTCCCCTCGGTAGTTTAGATGCCCTTTAGTTGTAGCAACCTGGCG
CGGCTTGACCGACATTCCTTATTTCTAATTACTCTCAACAACCGCCTTGGGCTATTAGATGGACCCCCGGTCT
TTCCACATTTGCCGGTCCCCTTGGGCTGCGCGCATTCGTCTGATCGCCNGNNG
>78231180_2 CV525892.2
CCGAGGGCTGAGATCGCCACAGGTTCATGCATACTGAAGGTCTCGACTGATTGTGCCTGGTGTCGTGTGCGTG
TATGTGTGGACAGCGTACTGCAGATATTGGTCACGTTAAGGCAGCGAATCGCTAAACTGCTTGGACGTCTCGC
CGTAAAACATCATAAGCGTGGGATTTTATCAGCTGTTCCACCGTTTAAGTTAGGACAT
GACATACCCCATGTAACTCATTCAGTATTGAAGCCATTGGTTGTGACATACCCACCTACCGCGGCTGTAGCAG
CCGTGAAGATACCGGTACCTAATCCTCACGTACCAAGGTTCCCAGTACCAGTCGGACATAGAGTACCTGGCTT
GCCGCATCCACACTATGGCTTGAAGTTTCCACATACAAAGTTTGTATCAAATTCTGAC
CACCATTTCCATCACCATCATCATCATTTGGCACCAAGGCCGATTGTACCGGTTGTTGCCGCTGCTCCTGTAC
CTGCAACTCCCGTCACGGCATATTTAGCTCCAGCACTTCCTGGACCTCAAGCAAGTATTGCTGTAACGATTCT
TGCCCTGTATCTTGGCTTCCAGTATTCTTACCAGAACGGGTCTAACTAGGTCATAATT
AACGGTTTGCCCCAGTTCCAGTACTTATTGTGCCAAATTATTTACATTTAAAGCAATATTGGCTTGAGCAGAT
TCCAGTGGACTCAGACCTGCGCATTGATACCCGGGCCAATATTCTCAAAGAAGGCTACCTAATGTAACGCCCG
ACGTCGCAAACCCGGTGTGACTCGGCAATTCGGGAAAGCATGGCCTGTCACTGGGTTA
CAAGTGGAAACCCAACGATCATCCTCGGATGTGGGTACGCCTATAACGCCCAACCGCGCGTTTCGCGTTAGAG
G
>78231184_2 CV525896.2
CCGGGGGCTGCAGATCCGCACGATGAAATGGTCTCTATAAAACAAGTGTACACATCTGTAGCTTGCAACCGTT
GCCCTGAAATAGCAGACTGGAATAAACAGGGATTAATATGCTATGGCGCTTCAAATGCTGTAGTTATATATGA
TACGAATATAAAAAACTCCGATCCACTTACGGTGCTAAGCCATCACAAATCAAAAATA
AACTCAGTAAAATGGCTACACAGACCTGATAATAGTTGTATAGAACTACTTTCTTGTTCAGCTGATAAGACTG
CTGCAATATGGACTTTAGAAAATGATATTTGGAATGTTACAAGTGTTTTATCAGGACATGGAGATGGAGTGAC
TTGTATCAGTGGATTATATATTGAAGATGATTTAGTAGTATATACTGGATCTATTGAT

ACTACTATGAAAATGCGGGAACGTAAAAATGGTACAAAAACCCTTAAACAAACTATAGATCTACATTCCGGGC
TCTGCCTAACTCTTAAAGCCCATATTTTACCTATTGCCAAAAGACCCATACTTCGTTGTGCATTAGATGATCA
TAAAATACATCTTATCACGGAAGAAAATGAATATCATAGAGTCCATGGCTCAGTGGGT
CATGAAGATCGGGTTCAACGACTACATGTAATAAGTTTAGATACAAACAATATGCTACATCAGTTCACAGACA
CTA
>78231197_2 CV525808.2
AGGGGCCGGGTTGAGGATCGCGACGAGGGCTGCCGGGCGGTTGTGGCGCAGCGAAAGCGTTTGTGAACTTTAC
TTATTTAAAGTTACGTATAAACATTGTGTTATTTAAGAACAGTCTAAGAGTAGCTTAAGGAGTACTACTTTAC
TATAATTGTGGTGTTATCGAGTGATTTTGTGCTAATAAAATGGATTTGTCTTGTGGAG
AGAATCTACAAAATTCTGGACACAACAGCAACAGCAGTGGGAGAACAGTAGATATGTGCATCGCTGGACCTGA
CCGGGCTCTGGACCGCGATCCAAGACTTTTACTCAACCTTTTGACTTTGGAAAGGGTCCACGCACTACATACA
GATTATTTTCAACATGTCCAAATAGATATACAACCTTTTATGCGAAAAGTTGTTACAA
CTTGGATGCTGGAGGTTTGCGAGGAACAACAATGCGAAGAACAGGTGTTCCCACTCGCTGTAAGCTATATGGA
TAGATTCTTGGCTCAGCGCGCTATCTCAAGACAGCAGCTACAACTGCTGGCCGTCACTGCCTTGCTGCTGGCC
TCCAAGTTCCGACAGTGTCATCCATTATCAGTAGATCTCCTTTGCGCGTACACTGACA
ACTCTGTTTTTCCACATGAAGTAAGGCAATGGGAAGTATGCTGCTAAACGACTTAATTGGAGCTATCCATAGC
AACAGCCTTCACTTCGTCGAGCTCTTCTCGCGCGGGTAACGGGGGCGTCCAACTCCCTAGTGAAAATCAGCTC
TTACTTACTCATTTGTACACAAGACGAATCCGATAAGCCCCGATTAATTCGGACGGTA
ACGGGGACGCCATGTGGTCGCCCNNC
>78231214_2 CV525825.2
CGGGGCTGGGCTACTGTGTTATATGATTCATAATTCATTTCACGTAATAATAGTATATCATCGCCCTATCCTT
TGTGCCACTTCTGGAACGAGGGCGTAGTTTTCCTTTGAACACAATGGGAGATAACAGGAAAAAGAAGAAGTTT
ACTTTCGTAAACGTAGAAATAAATACTTTTTGGAACCCGGCTTTAGAGGTTTCTTTTG
CACATGTAACTTCAGGGAAAAGGATTGTGTCAAGGAAATGTATAATTTGTTAAACGAGTACGCAGGCAAACTA
TACCCAAACGAAGAAGATGTCCCGCAGGAACGGCCAGCTTCTGTTGAATCCGATGAGCGATCTGACAGTGAAT
CTGAAAATGAGACTGATATCGGTGATATGCTCAAGCGTGAGGTAGAGTCTATGAAAAA
GGATTCTCAGAAGTCTCTGAGGCATAAGCGCTTCCAGGTTGTTGAAACAGGAGCTTCAAACTGTATCTTTATC
AAGACTAATTTACCTTCCCCTGAACAGTTAACAGATGCTATTATGAAAGACTTATCTGCTACAAAAGTGCAAA
AGACGAGGCATGTCCTGCGTCTATTACCAATAATGGCTACATGTAAAGCAAACTTGCC
AGATATAATGGAGTGTGCAGGGAAACTCTTTGACGAGTATTTGTTGAAAGAACCATCTACTTTTGCAGTCATT
TTTAATAAAAGATTTAATAANNG
>78231225_2 CV525836.2
GCAAGACAACACAAAAAACAAGCAAACCCGGGCCTCGGAATCCAATCAGGTTGTCTATGACCGATAATACTCC
AGAGGGGGCCCGTGTACCGAATGCTATCCTAGCATGTGTCTGTACTACTATTCATAGACCGTCGTGTTGCACG
TCTACGATTAGCTGGCTCAAGAAAGAAGGTGAAAAAACAGAAAGTATTGAGAATGTCG
TGGAGGGCCTCAAAAGAATTTATAGGACGAAACTGTTGCCACTTGAGTTGCACTATCAGTTTCACGATTTCCA
CTCCCCACAACTGGAAGACCCTGACTTTGATGCTAAACCTATGATACTGCTGGTCGGTCAATATTCAACGGGC
AAAACTACGTTCATCAAATATTTGCTAGAAAGAGATTTTCCAGGAATTCGTATTGGAC
CTGAGCCCACCACAGACAGGTTCATCGCGGTCATGTTCGATGAAAAGGAAGGCATGATCCCCGGAAACGCACT
TGTCGTAGACCCAAAGAAACAGTTCCGCCCGCTGAGCAAATTCGGAAACGCCTTCCTTAACAGATTCCAATGC
TCTACAGTAAACTCTCCGGTGCTTCGTGGGATTTCCATAGTTGATACGCCCGGTATAT
TATCGGGCGACAAACAACGCGTGGACAGAGGATATGACTTTACCGGCGTTCTCGAATGGTTCGCGGAGCGTGT
CGATCGTATCATCCTGCTGTTTGACGCCCACAAATGACATATGCNNT
>78231253_2 CV525784.2
CCCGGGGTTGCGGAATCGGCAGAGGGTCAACGTCTCCCGNTCGCTCGCGTGAAGTAATACAAGTTTATAGTCG
ACATGACCGGTCGCGGTAAAGGTGGAAAGGGTCTGGGGAAAGGTGGAGCGAAGCGTCACAGGAAAGTGCTGCG
CGATAACATCCAGGGCATCACGAAGCCGGCGATCCGTCGTCTCGCCCGCAGGGGCGGC
GTCAAACGTATATCCGGCCTGATATACGAGGAGACTCGCGGAGTGGCTGAAGGTGTTCCTCGAGAACGTGATC
CGCGACGCCGTCACTTACACGGAGCACGCCAAGAGGAAGACCGTCACCGCCATGGACGTCGTGTACGCCCTGA
AGCGGCAGGGGCGCACTCTGTACGGCTTCGGCGGTTAATAATAAAACTGAAGACCGCG
AAGACCAGTACGCCGCCACTCTAGTGAAACCGACGAGAGCGAGAAGAATTGTGAAGTGTGCGGGGGTGGGTGG
GGGTTGAGGGGTTGGTGTCTCTGTCTCCGTGCGTGCGTGTGTGTGTGTGCGCTGCGTGTGTGCACGGACGCGG
TCAGTGTAATCGTTTACATCTAGATTATGACACCCTCGAACCCGCCCGCCCGTAATCT
CTCCGTCTCTCCGTCGGCTTACTATTGTGGCGACAGAAGAAAAAAAAAAAGAAAAAA

>78231255_2 CV525786.2
GGAGNNACACAACAATTCAGTTCCGGGCTGAGGATCGCGACGAGGTGAGACATGGAGATTTAAAAGATTGGTG
CTACGAAGGAGTTTCAATATACTGGCAACACCCATTAAACAATGCAAGCAACTTGGCTACCGTCTTGACTCTA
AAATATTAGATATAGAAAATATTGAACTTGTTATGGGAAAAATGATGGATCAAGGCCC
CGTATTAGTGGTCACCTTTCAAAGTCAACAAATAATGTGCGTCAGAGACGCCAAGAACAATGTTATAGAGGGT
GATCCCAATAAAGTAATGAGAGTTAATTATGTATGGGTGTTATGTAGAGATCCTCAGGAATTGAACCCCAAAG
CTGCGTGGAGGTTGCTCGAGCTGTCAGCCAATAGTGTGGAACAATTGATTTAGTGAAA
CTTTAGCGTTATAGTGTAGTGATTGTTATGTATTTTGTTGAAATACTGGGTCTGTATGTTTGTATGTAGTGGC
ATTTGAATTGAGTTTTGAGTCTTACATTATTAAATAATAATTAAAAGATATTTTCTTATTAAAGCAAGCAAAT
GTGTTATATTCAAATTATCGTTCAATTATTACTAAGGAGAATGTCGTGTTGCAGAGGC
ACACCAGCTGTAAATGCGAAGCTCGTCTCACTCAAACTACAATTATGCACATTTATCCACAGTGAATGACGAC
TCTTAGCGTAGAGTCCCGGCACTTAACACGCACTG
>78231257_2 CV525788.2
GGCGGAGGGGGCCGGTGGCGAGGAATCGCGAGAGGTCAGCCCTACAACCTCAGTCGGTTCAATTTTCGGCGCC
CGCTCCCCTCATGAGCCTCAAGCCACATAGCGCTCCACTGCGCCCGCAAAAGAAATTCCAGCTTCCTCCCGTG
CGCAAAAATTTCAACCCGACCAAAGTCACCAAACCCAAGACGCAGCCAATCCAAGCGC
CCAACAAGCTCGCCTCGTATCCCACCGAGCAAACTAAGCAATTAGTTAACAACGCTATGAAAGCGATCGACAA
AAAGCCGTCGCCCCATGAGATATTGCGGGCCGACCGGCCGCCAAGCGCCAAGATGAAGGGACGGCTGGAGCTG
GCTCTGGGTATCATCCTCAAGAAGATGAAGACCCTGGAGCCCGAAGTGGCGGAACTGG
AGCTCTTCTTGCAACGGCTGCTGAAGCACACAATTCGCGCTCGCATCCGCGCCCTCATGCTCAACAAGTACGT
GGGCGCTGCCGAAGACATAGTGACGGCCTACCGCCGGGTCTACCCCAACGACACCGACCGGGAACTGCTCACC
CTGGCCAAAAACGCCCACACCCTGCAGTTGGTTGATGCAGATGATCCTGAAGAATCTT
CAAGCAAAACATGACAAATATATCGAATACCAAATTGGAAGATATGTGTGTAAAACTGGAAAAACTATGTAGA
AAAGAGGCAACTAATCTAGATGACTACATTGATAAA
>78231261_2 CV525792.2
GACCGGGCTGGGATCGGCACGAGGCTTTATAATTTTAAAACAAATCTGCTGATGTGACTTTATAATGTTAAGA
TACTATGGACTTTATAAGTCACATTAGCGGTTAGCCCGCACTCTATATATGTGTATATAAGGCGTGCTTCTAG
AATGATAATGTGTAATTATCTATAACGTTCGCCAAATTGCTTAATAATGGGTAACCAA
AATAGTTGCTGTTGTTACCGAAGTCCTTCGCCAATCAGGAAGGATATTGTTAAGCTTGAAGACTACCTTCCTG
AAGGACAAGTAAGCTCCAATAATATTCAACATATTAGTGAACGAGAACCGGACGATGGTGATATTGATCCATC
TCAAGATCCAATTGCAGGAACAATTTTTATGGAAAGGTCCAAAGCGTCAATTGAAAAT
GGAATCACAAGAAAACGTAGTCAACATCAAATTGCAGATAATAAATTAAAGAAAAGCAGTTCCTGTGCTACAA
TATACTTAGATGATAGTACTGTGTCTCAGCCAAATTTAAAGAATACAGAGAAATGTGTCGCACTTGCAATATA
TTATCATATAAAAAAATAGAACATCAGAGAGACGGGTAGATATATTTGATGAAAAACT
TCATCCTTAAAGTAAGGAAGGAGTAAGTGACGATCATGATAAATATCATCCTGAAGCTAAACCACTATATAAA
TTAGTAACAACTTGGTTAATTGCATCCAAGTAACAACCAATGTGCTATATACTTAAGTATACTGCAAGGTACT
ATATGTCCGAGTCATATTTCCCTCTATAGACAAATTTTTGGTCCCAAACCTGAAGCAC
ATGGCTACACGCCTGATTGTCGCAATAAGTAAAAGCGGACAAAGAAAACTTNNNG
>78231264_2 CV525795.2
AAAGACCCGAGGCGAGATCCGAGAGGTTAGTTGAGTCTATAATATTACTGGTAAATTAAACAGCTTGTTCTCG
ACTCATTTTAATTATTTTTAAAGTAATTTAAGTGTGTGAAACTATAAGCTTAGCTTTTTATTCTGATTTATAT
ATAGTTTTTACGAATAAACGATCTGAAAAAATATAAAAATGGTTGTAAGACAGTACGA
CGAAGAGTTAAAGTATTTAGAAAAAATAAATCCGCATTGCTGGAGAATTAAAAAGGGCTTTCAACCAAATATG
AATGTTGAAGGAATATTTTATGTTAATAATACTCTTGAAAAGCTAATGTTGGATGAATTGAGAAATTCTTGCC
GGCCCGGCATGACAGGAGGGTTTTTACCGGGTGTCAAACAAATAGCAAATGTAGCAGC
GTTGCCAGGAATTGTAGGCCGCTCTGTCGGACTACCAGATATTCATTCAGGATACGGATTTGCTATCGGTAAT
ATGGCCGCATTTGATATGTCTAATCCAAAATCTATTGTGTCTCCTGGAGGTGTGGGTTTTGATATCAACTGTG
GAGTGCGGCTTTTAAGAACAAATTTACTTGACAAGGATGTGCAACCAATAAAGGAGCA
ACTAGCCCAAAGTCTTGTTGATCATATCCCTGTTGGAGTGGGCTCCACTGGAATTATACCTATGATTGCCAGG
CATATGGAAGAAGCNNTT
>78231267_2 CV525798.2
ATCAAACCGAGGCGCGGAATCCCCGAGGTATGTGTGAACAAACTATCAACATTTGCGAATAAAGTAAAATTTC
TTACAAAATCATGACAAGTAGAGCACATTACAAAAATACTCGAATGAATTACAAATTTCAAGATGACTTACCA
GATGCAAAAGGTACTAAACCCATCAGGGAGGCGTCGTCAATATTAGAAATTCTTATAC

TTATGATAGTGCACATTTGTAAGAAAATATTGTTCTTTGACACTAGTTTAAAAATAGCTGTTTATTTAGGTGC
TCTCTTTCTACTGTCTCTAATTGCAGATGTATTGACTTTCCCAAAAAGTTATTTTTCTAGAAGTGACAACATA
TCTAATCAATACTTTGTAAAAATTGGATGGTTTTGGACATTATTTTTGTCAGTGCCTT
ATGTTCTTCTGACTTCTTATACGACATGTTGTGGCAAAAGAAAACTTATCTCAACTGCTCATTTACTACGGCT
ATTGATTGCTACTGTGTTTCGGTACACATGGACAACTGTGTCTCATATAATAGAAATGAAATATGGAAAGTGT
AATTCAGGACTCTTACATGACACAGTTGACTGTCCTCAAAATGGACATTTCTGGAATG
GGTTAGACCTATCTGGACATAGTGCCATTCCGATTACTCAAGTCTAGTATACTTGAGGAACCAAACCATTNG
>78231269_2 DV570503.1
CCTGACTGAGATCCGCGGCGGATATTAGGCAAAGCTTCAATGGTTTCCAAGACTATTAATCATATCCTGCTAT
GTTAGATCTTATACACATTTTAAATATATCAATGAAATAAAAGTAAAATATATTTTAGTATTATTAAATTGAT
TTATAAATCGCCATCGCTATTCAACTATCGGTAGTAATTAGCCGTCTAGGCCTGAAGG
CCCTTATATAGTGGGTCAAACCTGCAATACATCCCTTGTGATAACTTTTAAGTAGTTATAGTGGAAATTATAC
TTTTTTCCATATTACTTTATCTATCACTCGTTAATGTGTCAAACTCTATTCTCTCCTTTTAATCTGATCACTC
AAAGAGAGTATTGTCCCAATTAGGTACCTGAAGTTTGTCGAAAATTTACATGTATATC
CCTTTCTCTCGTACAGAAATCATCGCAACGTATTATTTTTATTTGAATAAGCTACTTTTTTAATGATTTCATT
TTTATAAAGTTATTATTGATTCACCTTAGTATATTAATTACATCATGGAACAGTGTATCACAAATACCAGTAC
TCATATATTTTAAAAATCTATGAGTCTAAAGTCTACGAATAAGTCCAGTAGGACCAGT
AACCACCTAACCACCACACCAAGCGAAACTAACGCAATACGTAGTCGCCAACCATGGAACAAGATACAAGAAA
TGGAACAAACACCAACACTCAGCACCGAAAATACCCGAACAAAAAAGGAATAACTGCCCCCAGACCAGAACAC
TACACAACCGCTGC
>78231270_2 DV570504.1
TGACCGAGGCGAGGAACTGGACGAGGGCTATGCCGTCCACACACCCGTACGCGGCATGCTGTACCCGGAGTAC
CATTACAGGCCTCCGCACCCCCACTTACCAAGCTTCAATGCAAGAGTATAGACTCAGGTTGCTTCTCCTAGAC
AGAAGTGCGCCCGCGGTATCGCCACCTCCCACTTACAGATCAAACTCAGCTACTCTAG
TCAGAGGAACAACGGGAGCCGCGTGGTGTGGGTCCGAATACAGCGGACCCCCTTCATATCGCGCGCCTCGGGC
GGAGCCCCCCGTGCTGGTCTCAGACACACTGCCCACACTCACGCCACTAGACCTAAAATATGCAGACGACACC
ATAGAAGCCTTACCCAAGCCCACAGAACAGGAGAAGGACCCGGATGAGCACCTCGTGA
CAAGAGTCCACACGGAGGCCCAGCCCGTCATAGTCACCGTGTCAGGTTCCCCGGCGCTCAACGATACCCATAT
AAACCAACCCAATACCGAAATCGACATACTGGCACATTTATAACAAACCAGTGGACTAACTTTGAACTAAAGT
GGAAGTGGTGTGTGGTGATCGAACACGTGCTATGTGATTACAGGTGCCCATAACGACT
AGAAAACACTAGAATACATACACACTGAGTACGTATATCATATCTCAACACCAACAGATCACTGACATCAGAC
ATAGTAAGGCGACACAGTAGCACTACGGCACACACAACAACCGACGCACACTAGTACATCAC
>78231276_2 DV570510.1
GGGAGAGNGNGNNNNNNNCCGGGCGGATCCGAGTACTTCACTGTCTCCCCTCCCACCCTTCTATCTCCTTGTG
TGAAGAAGCACAAGTAACATAGAAAGACGAGAAGACCCTGTCGAGCTTAGCCTATAAGTGAGCAGCAACCCAA
CATATCACCCAAAGGCTTTGGTTGGGGCAACCACGGAGAAAAAAAATCCTCCAGAACA
AATAGAAGAAACCCCTTCACTATTGATATAACCTAGAACCAGGAACTGGTATTCGGAAAAAGTTACCGCAGGG
ATAACAGCGTTATCTTCTCTAAGAGTCCTTATTGACGAGAAGGTTTGCGACCTCGATGTTGGATTAGGGCCCC
CTCAGGGTGCAGAAGCTCTGAACGGTTAGACTGTTCGTCTATTAAAGCCCTACATGAT
CTGAGTTCAGACCGACGTAAGTCAGGTCAGCTTCTATCTTCTTTCAGATCTTCCTAGTACGAAAGGACCGGAA
AATCCCTTTTAATAGGCAACCTCAAAAAGGAAATTGAAACCTAAATATTTTCAAGATTTT
>78231278_2 DV570512.1
GGCCAGCTGCGGATCTCCCGCAGTTAAATGTACTAATGTGATTCGCATGATGATACATATCCAAAAGAGATCT
ATAGTAAACAACTGTAGAGAGTACTATGACCTTCGAAAAAGAGTGGCGTAGATTACGGGCCAATGCTGAACGG
TTCGACCGATAACGAAACGAGTAGTTCGAGCGTAGCGTCAGAAACTAGAGACCACATG
ACGCGCTGGCACTGCGTCGTTGGAGCGCGAGATGTCCAGCACCAGACGACTACTCTGGGGCGACCACGTGAAG
GAAGACGTGTATCGCCGATGGGCATACAAGGCTTCCACATTTAATTCTTGATCGAGCCCAGGGCACTACATAC
AGCAGGAAGGCGGGCCGTGTGCTGCCATCGCTCCAAGTGCAGGGATTCCTTTTGAAGA
TCCTCCTATCCGAGACACCTGGACATAGTCTAACACGACTTGACCTCGGAAAAGTGCAATTCCTTAATATGTT
AGGGCTGTGTGTACTATAATAAGTACACTTGTCTAACTTCTACATACAATAGTTGCTAGGCACCGCAAAGAAT
GATCCAGCAGGCGAAGAACCAGCACCAGCCTCTATGTAAGTGCCGAGGACCCCTCTTG
GGATAGTATTATCCACTCACTAGACGGATAGAAGTCATTCTTTACAAACTTGTCCCACNNCT
>78231279_2 DV570513.1

AACCCACACCAATTTACGCCGGCGCGATCGGCCAGATCAATATATAACTTTTGACCTATAATTGCTTCTCCCC
ATTTAAATGACTTTTCAATATAATGGTCTATTAGTCATTAAGCAAAAATGCGTGTTAAGTTTGTTTATCTGGT
TTAGTGTTGGTTTAGTAGTAAAATATGTGGAATCTGCGGCGTTGATTGATGAAAACAT
TGAAATGGCCTCCGTACATGATGCAAGTGTGGATATAAGGAAAAGACTCTTTTACACCGCCTTAGAAGAAATA
GGGGATGTGCTCACTCGAGGCCTACCAGCTACGTATAGAACACGTAGAAGTAGCTGTCGAGGACAGTCCAGAC
TTAACGCAGCCACCGTTCTACCTCACGGCTCCTGGCCTCTTAGGAGATGCTAAACTCA
TAGAGTTGGGAGGTCCTCCTTACTTAGTGCCGCTTGTACAAAGGGACACTGTGTACGATTTGGAGGCGTCGTC
GAAGCACTTGGGCAGACAGCCCGCGTTACTGGCATGCGCTGGAGCTGGACCATGGCCATATATCGGCGTCAAT
TGTGAGGGAATAATAAACCTGTCCCTGGACAAAGGTGTGATATCACAAGGCACGCGTA
TTAAGTCGGTGTCGCCGGTGGGCGCACCGACAGGCTCAGCGGGTACTGCAGAGACCTACCATTATCGACAGAC
CGTTCCCGAACACGCTGCGAGGAACGCAGGCCAAGTGAAAAAACGATACTACCGCAAACTNNNNG
>78231282_2 DV570516.1
ACAAAAAAAAAAACACGCCCGGCGAGATAGCGAGGTTGTCATCATATCATAAGTAATAAGATATCAAATAACG
TATATATAAATAGTCTTACATTAGTTATCATAATCATGGATAGTGTAAAGAAACTTCGTGACTCTATCAAGCA
TTGGTACATCAGGTATTTAGTTTCTACGGAGTCGTATATGGTGGAACCTTGGGAGAAG
GTGGTCATCCATATTATGTTAGCAATATTCTTCACTTCGTTCTGGTACTTCAACTACTCTATCATCGTCAGTG
CAATATCTCACTATCGGGAGTCATCGGGAACCGTCGCAGAGTTATACTGTTATTTAAACTATAATATTGTTAC
CATTAATATTTACATTTAATACATTATTTGTTACATAAACCAC
>78231283_2 DV570517.1
AGAGNNNNNNNNGNNNNNNNNGAAAGACCGAGGCTATAGGAACTGCACGAAGCGCGCGCGCCCGCGACTAGCT
GCTAGTGACAGCTGACAGGTGCACAGATAAATTATGAGATTATTACTGACCACATAAAGAACGAACTGAAAGA
CTCAGCCCTCCTGTGCGAGTCCACGATGCCTTATTAGCTTGGTAGCCTCTAGGCGATC
ACCCAATATTTGCCAACAAGCAGATCTCCGGAGCTAGCCGCATACGATCGGAGATATCCTGCAAAAACCGCAC
CGAGGATAATTGCAAGAACGTTATTAAATGAAGGGGTGCGCTATCTAGAAAAGATTTGACCAAGAATGAATCT
GCCACTATTAAGNTATGCATATGCCCACGCCAACTTCCTACCTGAAGGTTAANGCCGA
GCAAACTTTTTTGCCGTAATTCAAATGTAGAAGCAATATGTCGTGCAGAAGGAGGACGTAGAATTCGTGCTAG
AAAATAGATTAGGTCCTACACTAACTGATAGTGAACGAGCTAATACAGCGTAGCCCCTGACACTTGTGATTCG
CCACGGTTGAGCTATATATTCGGGGACACCACGAGGATTTAGTTGGGCCTATTACATA
TGCAACCCTANGAATAAGGAATTCCCAACTNANCTAGCAGGGACTTCCCCTATTCACCTGAAAACANCAGTGA
GTAACAGAAGAACATGATGAGCAGAGATAACAGAAATTCAAGAGTTTCCAATTGCCTTGCAACCACAGGGAAT
AGTCATTCTCAAACTGCTTACANTGGAAATGAGGTCCCACTANTAATGCTGTCAGGTA
ACTGAGAACTCTTACACNCGTAGATGGGTCCAGGGGCCCGTCACTGCAAGGACACGNCTTTCACGAACGTGGG
CATCAAAGCCGAGNACGCGGAGAATGNCTTGCTAACACGCCCTTCTAGATTTACTAGTAATCGGAAATAGGCT
ATCCCAACCGAGAACACATGACTGTAACTTTAGTATGCACTGGTACTTTAATTGTACG
GAAGGATAATCCTTAGACTTGGAGATAGCAAAGCGTGGTACTTTGTCTTTCACCCGCGCAAAAACGAACTGCG
TACCCAATGTTTAAGCCGGGTTTAGTCACCGTAAGGATTATTTCCAAGGAACCCACACAGTGGGAGAGGGGGG
GAGCCCCTGGTTGTGTGTTTCCATTTTTTCTGGGCCCAAAAAATNNTTA
>78231284_2 DV570518.1
GAGAAAAAACAGCAAGGTCCTTTCCGGGCTATAGGATTNGCACGAGGTGAAATATGCGCTCGAACATAGCGCA
CGCACGCTACGACAAGCCGACGCCGGTGCAGAAGTACGCGATCCCCATAGTGCTGGGGCAGCGCGACGTGATG
GCGTGCGCGCAGACCGGCTCCGGCAAGACCGCCGCCTTCCTCGTGCCCATACTCAACC
AGATATACGAGGCGGGGCCTATTAAACACGTGGGACAACCAAAACGCAGTAAACAGTATCCCTAAGGGCTGGT
GCTCGCGCCGACGCGTGAGCTCGCCACACAGATATTCGACGAGGCCAGGAAAGTAAGCAGTAGCGAGACGCGT
GATGCGGTCCATGTGTCTATATAACGGTTGGGATGCCCCGAATCAGTCGAACNC
>78231285_2 DV570519.1
AAGAGAAAAACCACCAGGGCCTTACAATGGCTTAGCGATCTGGACGAGGGGAGCTCCCTCCACGGGACGGACG
TATCGTTCAGCTGTGTTGGACGAGAGAGCCTGGATGTGACAGGGCTGCAGCGTCGAGCCCAGGAAGGATTGAC
ATCACCAAACAGCTGAACATGAGCGACTTTGAATCCAACACCATTGTCTATTTTGGCT
TTTTTAACGGGCAGGATCCAAATCCAGCTCAGGAGCCTGGATATATCATCATGTCGTCTTTTCTCATCTTCTA
CAAGTGATTTTACTTAATGAAAAGAAAATCTTCTCAATTGTGATTAATATTTCTCAGACTGTGCAATCAAAGA
ATACATTTTTATTTTTTTCC
>78231289_2 DV570523.1

TGTAATGGCCTTAATTCCCTTGTCTCTTCAACATTCACTAAACGGTTCTTTCTAGAGGGCCAAGAACTAGAGA
CCATATGAAACATAATCCCCGCCTTAATACTAGTATTTATAGCCTTCCCCTCCTTGCAGCTACTCTATCTAAT
GGATGAAGTCAAAAACCCGTTCTTAAGCGTAAAGGCAATAGGACACCAATGATACTGA
AGCTATGAATACACAGACTATCATGAAATCGAATAAGACTCCTACATGATACCAACCAGAGACCTATACGAGG
GCCAACCACGACCAACTAGAAGTAGACAACCAGGCTTATACTTACCTATCAGAAACCAATACGTCTACTAATC
TCCTCAGCTGACGTGCTACACTCATTGGGCCGTTCCTTCACTAAGGAGTGAAAATGGA
CTCTGTCCCTGGACGCCTCAGCCAGAAGTTCTTCTTTCTAGACCCGACCTGAATATTAACCGTGCCACTGTCA
ACGATAAATTGGTGCCAACACAGAATTACTGCAAACATATATGACTCCGATACTGACTAATCAATTCGAATCT
TGAATAAACAAAACTAAA
>78231291_2 DV570525.1
CCCTGGCGTACATGAGTCCGTATAGAGACTACGTGTGAAATGCACTGGTCCAGTCCGATTTGACACCGCTCAT
ACTAAAAGACATTGTTAACATGGCCCGTATATAGTAACGAAAACGAAGGTGGTAATATAGACCCAGCTTATGT
TGCCTCTAATGTAAAGAGCTATATAAAACAGGATTTGATCAAAGAATTACTCAATGGA
TCATTTCAAGATAACAAAACTAAATTGGGGAATGATGCTTTGACTCTCCTAGTAGAAGTAGCGAAATGTCTCG
TTACAGAAACATGTCTGAGAGCATCAAACGAAGCCCTCCGAGAAGGTTCTGATGAAGTTGACTTTAAACATGT
TGAAAAATGCTTACCGCAACTGATGCTGGACCTCCCCTAGTGCTTAAAATATTTAATA
TGTTACATTTCTATCATATGTATTTTTTTTATAAAAAAGAATGTTTTACAGCAATTGGCATGTTTAGTAGATT
GTAAAAGAAAGTGTAATTATGGATTTAATTTCTAGATATTAACTTTATAAAAAATATTTTAAAAAATACACAA
TAGTAGTAATTTGTTTAAAAAAATAATAATCATAATAAGTAAATCTACAATAAATATA
TTAGAATATCAATGGTCTCTACAAATATATAGACATGTCATAAGTGATGTTTAACAGATGAATTGAGGTTCAT
CTTGTCTTTAAAAACTTGAAAAACANNTT
>78231294_2 DV570528.1
CCCGGGCGGACGTGACTTGATGGCTCCGTTTCAGCCAATTCCTCACCGCACTGTTAATGACTGCGCCTCTTGC
GCCGGAAGATACGGTGATTCGTATTAAAGGCGATCTGGTTTCTAAACCTTATATCGACATCACACTCAATCTG
ATGAAGACGTTTGGTGCTTGNAAATGAAAATCAGCACTATCAACAATTTGTCGTAAAA
GGCGGGCAGTCTTATCAGTCTCCGGGTACTTATTTGGTCGAAGGCGATGCATCTT
>78231296_2 DV570530.1
GGAAACCCACGCCCAGACCAGTCCGGGGTTACCACGTCGCGCAGTCTGCTCTGCGGTCAGACCGCCGCGGCGT
TAATCGCATCCATGATGGTCAGAGGAATATTGGTAATCGGCTGCTGACCCGATTTGCTGACTTCGCCGGTGAC
GTACGCTTTTTGCGATCGGAACGCCGCGACGCTGACATCGACTTGCGGGCTTTCAATG
ACTGAATCGAGACGCGCTGTAATCTCGTTGCGTACCTGGGTAAGTGTTTTCCCGGCCACTTTCAGGCGACCAA
TATAGGGGTAGAAAATCGCACCATCGGCGTTAACCCAGTTGCCGGTATCACTGGCGCTACGGTATTGCCCTGC
GGGGGTGGTTAGTTCCGGGTGATCCCAGACCGTAACCATCAACACATCACCAATCCCG
ATACGATATTCATAGTTTGCGATTTGCTGCTCCAGCTCCGGGTTAGCCTGCGACATAATCGGTTTGGCGCGCA
ACTGATCAATCAATCCTGGCGTGACGGGATAGATATTGACCATTTTGTCCAGGTCATACTGATTGTCAGGTAG
TTCAATGACATCCTTGTTACTGGTCG
>78231298_2 DV570532.1
GAGTAACAACAGAACAACAGGGGCCAGGCTGAGGATCAAGATGAACTACAATAACGACCCTTCCGTGTTCCAC
TGGCCGCGAATTCTACTTCGCGAGTGAGGTAAAGCCGTATAATGAGTTCCAGTGAATTTAAACTCGAGAGAAT
GTGGAGTTATCGGAGGAGACGAAAGCGATTGCTGAGAAAGAATTGAGGGAGACTCCGG
AGAGAGTGCGCGAAGCACTTGAGAGGCTTAGAGAACTTCTTAAGGAAGCCAAAGACATACACTTTGGCGACGA
TGACGAGTTGCTCAAATTATAGTATGCGTCCTTGCCAAAGTGGATATCCGGAGAGCGCCTAGGACACTAATGC
GTCCGGATCGCCAGACGTTTAACAACGTTGATAATTGCGAAGCCTCCTTGCGATGGAT
AACTTTCCAGAATCAGGGAAAAACGACTGTTTTCACTGGAAACAATAACATTTTGTACAACGTCAATGAACCG
GAACATGGATTGACTACGGGAAACGTTTCATGGCCCGCTGGAAAACTTGTCAGGCGCGGACCGTGGGAAATCC
TTCACAAATCTGTTACCATACATAATCCAGAATTTACCACGCGCCAGTCATACCTCTA
AACTCAATNNNC
>78231299_2 DV570533.1
GGGCCCCGGGCTGTGGCAAACTTCTGATATCGTCACGCGTTTTGCGCACTCTTCTCGTAGGTACTCAGTCCGG
CTTCTATCAGCATCACCGCTTCCTGAACTTCTTTCAGACCATCGATGGCCATACGACCGGAGCCTATCCAGTC
GCAGTTCCCCCAGGCACTGCGGGCTTCCTGAAAACTGAAGCGCGCTTTTGAAGGTAAC
GTCACCACGCGGCGAACGATGGCCTCTTCCAGCCAGCACAGAAACATCTGGCTCGCCTGACGGGATGCGACGA
ATTTTCGCCGCCCCATAAAGTACGCCCACGACTCGTTCGCACTGGCCCGTGCCGTGGAGTAGCTCATCTGGGC
GTAATTCCGGGAAAGCTGCTCATACGAGACACCCAGCCCGGCAGCGATATACCGCAGC

AGTGACTGCTCAAACACGGAGTAGCCGTTATCCGTATCCTGAGCCGTCTGCAGGTTCAGTGAGTCACCCGGCA
TCAGGTGCGGTACTTTTGCGCCTCCCAGCCGGACCGGCGCTGCGGCGTTTATACGCGGGCATTCCCCAATTCC
AGCCGGTCAGCTTTTCCCGGTGCTCCTGATTGTTCGCGCCCAGAATAGATCCCTTCGT
TGACGGCTTTTCCGCGTCCATCCCCAATGGTGCGCGGCTTACTTCGGCTTCCCCAAGGGCGCCTCGGCGCTAG
GATGTCTGGGGGGTGTAGAGCATCTCATCTGGTCCACCGGCGTTGACCTTTTGGCCCGATTTGGCCCGCCCCG
GGTTAAACCGGTGAACAGGCCCCGCGGNCG
>103774661_2 DV501510.2
GCACGAGGTTACTGCTCTGCAGGAGCTATGCGCGTGGGTGGGAGCGGCGGAGGAGGGCGCGCTGTTTGCTTTA
CACCGTCTCGCTAAATCGGCTAGAGCGAGATCTGCTGATCGAGATAATCACACATGGAAGCTCCCAACGTTAG
AAGGCACTCAGCGCTACACGGCGAAGCCGCTAGACTATATATCCGGGGA
>103774683_2 DT668985.3
GCACGAGGGGACACCCTGACTGAGATTACGGCGGAAGGTGATAGTGGTGTTGACACCGCCCATAATTATTCTG
ATGATGAAACTTGTAAACCTGAGAAATGGACAGTATTAGATCTCCAATTTGGCATTCCATTGTTTGATGAAGA
TTTATGTGAGAAAGTATGTAAGAGCATCGTAGAGAGGATAGCAAAGCCTGAATTGTTA
GAAAAAGTAAAAGAGGATAATGAGTTCGTAAGATCAGATGTGCTTAAATTTGTTTCACAGTGTCAGTATTATC
CTGGTGAAGATATGGGTATAGTGAAACGCGGTACACTAGTTCCTCTGCCGAGAAAGAACTTGGTGTTTGAAAA
TGGCAAGATTAGTGAATGGAATGGAAAATGAAAAAAAAAAACGTTATTAATTTCAATAG
ATTTTTTTTGTATATAAGAGTTTTTGGATGTAGTTTTTTTTTATATATATCATATCTAATATTGACTTGCTTG
TTTCAAAAATCTATGCTCGTTTTAACATTATCCATATTTTATATTATATTTTGGATGAAATCAGATGAATGAA
ATTACTTTTTTCTTTTTCTTTGGCTATTACAAAAATTGAGAAGTGATAAGGTTTATGC
TAATAATTAAATCAGTATTTGAATTATTTTTTGAGATATTCCATTTTTTTATTATTTTATGACATTAAATGTC
AATTTCCATTGTGTCATTATGGTAAGTTTAGTGTTNAATATATAAAATAGAAATGATTTATGAAATTAATTAA
TATATACATATGTAGTGTTATATAAAAATATAACAAAGTTTTATTTAGACGGTATTTT
TACTTACCTTAATTTTATTAATATAATATCTATTAAAAA
>103774697_2 DV501513.2
GTTGAGAGTGGGAGCTGACGGATAGGAACACCAGCTGATGTTCATCTGGGCTATGACTATTGTGCATAAAATC
AATGAGAAGTCACCACTTTATAATTTGTCAGCATCAGATATGTTGCGGGAGAGATTTGAAATTGTAGGCATGT
TACAGGGTGTCATAAAATCAACGGGTATGACAACACATGCTATGAGCAGTTACTTACC
ATCAGATATCCAGTGGGGGCACCGTTTTGAAACTATGGTATCATTTCGCAAGGACACCGGAGAGTATGAGGTG
GATTATACAAGATTCAGCAACACATACGAGGTGGACACGCCGTTGTGTTCCGCAAAGCAACTATACGAGTTAC
GCGCTACAGTCTCAACTTCACAGACATTACATCGTATGCTACGAACCATTCCGAATAC
ATTCTCCAATGACACGTTAGACATGAGCTCTGTAGATTCGATGTCA
>103774719_2 DT669011.3
CGGCACGAGGAAAAAACTTAAAGATAATGATTATTATTTTCGCTATTTATACATGATCGATGATTTTTATAAT
TAATCGAGGGTGTCCCAGTAGTGCGACTATTGCCGTTTTCCTCTTTTAATTTCGAATCCGTTTTAGATGTTGT
TTCCTTTAACAGTTATGATGTTGTTGAGTTTTAATTCTAATCTTCGTACAGGTATCTA
ACAGTGGTCGTTGTTTTGTGTAAACAATGATGGATTTAGTAAAGAGCTGCTAATAAAATTTTACTTTATATTT
ATGGATTAATTTATTAATGATTTAAATTTAAATTAAATAATAAAGACTTTTAATAAATCTGTTGCCCGATCAC
TGTGGGATACTGCATGGAGTTTTTTTTTCGTTTTCGCTGTTTTTGTGTACCGTTAACC
TAAAATAATCACAGGCAACACAAAGAGAAGAATCGTTGGAGGATCAGGTGCATCTCCTCGACGCTCAACTCAA
AGAGGTAACATGAAGGAAACCAAGGAACCTCGTAGATAGGAATGGTTGCGGTTTTAGTCTCGATTCATGCTCA
TGTGTGTTTGGTGAAGGAGATCCTCTTTACTGCCACAATATAAGAAAATAGTGATGTA
ACATAAATTACAATTTTATAATAATATTTTCGTAAACGGGAGTTTTTGCTTCACGTATTAATATATATGTAGA
TGTATAAAATTGTAATTCTTTTTGTATATATATTGTGGATTGTTATATAAGGGCATGAGGATCTTTTTCCCTG
GTGCAGGTTCGTTTAGTCCAGTTGGCTGAGGTTGGTGCTTCATCTATGTATCTTTAAA
GCG
>103774722_2 DT668973.2
GCACGAGGAAAAGCATAAGTAGATTTCCTTAGTCAATCTGAACTGTCATTCTGGAAAGACCTTATTGATAAAT
ATCTATATCCTATAGATGAAAATAAGGAAGAGAAGGTAAACTTAAATGGAAATATAAATATTATCATCTTATA
TTTTTATGTACAATTGTGTTGTTGAATATGTTATTTTCAAAAAAAAAAAATAATCAAT
GAATATTGAATAAGTGAGTGAATAATTTAAAAACTGTTTATTTTTTATTCCAACTCAAATTTAATGCAAATAT
GGCCATCATGCAGATGGTTAATATTTGTACAGATTTTTATTAAAATTATTCATGGTACTACGTTTTAGTATTA
AAAATATTTAAATTTATATGATGTTTAATTATAACTCTTTTTTTCAGGATTTTTGTTT

TTATTAATATATAATTGTATTATAAGAATAACACTTTTACCCCGACTTTTAGGCACGTATATCCAGAGATCTT
AAAGAATTGAGAGATTCGTCAGTATTTTCCTTTATAATTATTAATGCTCTTTTTGTGCTCATTGTTTTCTTAC
TGCAACTCAACAAGGACAATCTTCATATAAAATGGCCATTGGGAGTTAAAACTAATAT
TACTTATGATGAGGTTTCGCAAGAGGTAACGTGAGAATAAATTGTGTTCAACGATCGGCCTTCAATTGATGAC
TCTGAACGACATGCGAAACCTCAAACAACACAATGTTATATAAGTCTTTCTGAAAAGTCGCATTCTACGTCAC
CATTGAAAGCTGATATATTTAATG
>103774724_2 DT669012.3
GCACGAGGATGATTCTTTGTACACAACTTGGCTGGCAATAACAATGTCGTTTTTTAAGAATGTTACGTCCAGT
TTTGGAATTCGTAGCCGCGACACAATTCTCAAGGAATCTCTTATAAATAACTTATCTGAGTGCGTAAAAGATA
TTCAATACAATAGTCAATTTGAAGAAAATTTTCACAGCGTTCTTGGTTCTACAGATTC
TACAAATACATTATGTATGGCTTTAGAGGCAGTGTTTCTCCATGGTCTCAAGGATACCTTTTTAAGAAAAGCG
AAAAATGTTATTTCTGGTGATCCTGACTACCGTCCACAGTCTAGTTTTTGGCCTCTTATACTCGTGCTTTCAC
ATAGACAAAATATTGACCAGATATCATCATTACCTCAAATTAATACAGAGATAGGCCA
ATGTAGAGCTTGGCTCAGAATAGCACTCAATGAATGTCCCTTGTCTTCATATATGTCAACATTACTTAAAAAT
ATTTCAGCAGTAAAACCATTTTATGCCAAATCTGCTTTTGTCTGTGATTCAGAAATATTAGAAGTTGCTCAAC
AATTAATTCAGGGCCTGGAAACATGTGTTCAATTTAACTTGCCCATAAACTCCAGTTT
GTTGAATAACTGGCCTGAACAAGTTTTAATGCAAGCTGGCATCTGGGTACCAACTATGAGAGTAGCTCCCATA
TCTGCAGGATTAGATGTAGCAAGTACATTGTCAGATGATATAAAACCTAAACTAGCCACAACCCCAACACATA
ATACAAATATAACAGG
>103774735_2 DT669013.3
GCACGAGGAGGTTCTTATAATCTAAATTTACTTTCATTGCCTTGCTCATTTTTGGATAAGATGGAACGTTGCA
CAGATTTTAATTCAGATATGTCAACTCGATTGTAAATCGATTGTTCTTAATGACATATCGGAATTGATTTTCG
TATCTGCAAATCTGAATAACAGCAAGCGCACCGTTTAAATGTTTTCATTGAATAAAAA
TCGCTAGAGTTTTAGAATTTCGATTAATTAATTGTGTAAACTACATCGGGTGTTCACTTAGATCATAATTTTG
ACTCTACTTATAAGGCTCGGTCCACACTATCGACTTCCGTAAGGTCTTATCTGTCTTGTTGATAATTATATAG
GTTTGTACATTTTATGATATCACTTATTTAATGAAAAACAATTGTTTTATATAAAAAA
AACAGCATTTAATTATTTGAAAAAAGAAACTTAATTTTATCTATTAGTAGTGTTTTTAGGAAAAACAGCATTG
GAAATCTCTGAATACAGGTCGGGTCGTTAAGTTACTACTGTATAGGTGATATGCCCGATATAACCGGATATAA
CAGGTGACCGACACAGCGATCTGCCGACGACATATACACAACATTGTCATTTCATTGA
ATAAGTGATATTATATATAGGTTACTCAGTTAAGTCCTAATTAGCCTCTTATATGANAATCGTGTAGGTACAG
ACCGAATGGATAGACTATTGTGGACAGCGTCATATTAGTTTTGTTCACAAGCAATAGATAGGAGGATAAACGC
CGA
>103774742_2 DT669003.3
GTATGTCTGGTGAAGAAGAAAATATACCACTATTAGATCGTATTTCATCTAAGAAGAAGAGAGCTGCCCGTGA
AAGCGGAGCAGAAGAAGACATACCTTTACTTCAACGCCAAAAATCAAAAAAGAAAATTAAAAAAGAATCATAT
GATGATTATGATGAAGAAGAAGCACCTAAGAAGAAGAAAAAGAAAGCTGAAAAAACTA
GATCCAATAAAAGTATGAAATCTGAAACTGAAAATGGCCCTAGTCCTACAAAACGCAAGAAAAAAGAAGAGGA
AGAACAAGAAGTTTGGAAGTGGTGGGAAGAGTCTAAAAAAGAAGATGGTACTAAATGGTATTTCCTTGAACAT
AAAGGCCCACTGTTTGCACCTCCTTATGAACCATTGCCTGAAAATGTTAAATTCCGCT
ATGACGGTAAAGTCCCTAAATTGTCTCAGGACACTGAAGAAGTTGCAGGGTTCTACGTGCGGATGTTAGATCA
TGATTACACTACCAAATCAACATTTAACAATAATTTCTTTAGTGATTGGCGCAAAGTAATGACACATGAAGAA
ACAAAACTTATAAAAGATCTTTCAAAATGTGACTTTAAGGAAATGCAAGCATATTTCC
TAAGTGTATCAGAAAAAAATAAAAATCGTACAAAAGAAGAGAAGGCAGCACTCATAGCAAAAAATGAGGAATT
TCAAAGGAGTATGGCTTTTG
>103774748_2 DT668982.3
GCACGAGGGACAAACTTTATAACGTAACTAAAAGTAATAATTAAAAGTAACAAATAAAATATTAAAATGTATA
TAATTTTAACAACATGTATCTTAATAGTGTTCTTAGTGAGATTTTATCAAAAGAAAAATAATGCAATGTGTAA
ATCAAATAAAAAATTGCTCGGAAAAACAGTGATTGTAACTGGTGGTACTACGGGCATT
GGTTTACGTATAGCCATAGATATGGCTTCTAGAGGGGCCAAAGTAATCATAGCATGTCCCGTCGAGAAGGAAG
GGAATAACGCTAAAAGGATTATAATACGGGAAACGATGAACCGTATTGTTGTATACAAAACCCTAGACCTAGG
TAATTTGAAATCTGTTAGGAATTTTGCCGAATATATCATTCAAAATGAAGAAAGGTTG
GATATTTTAATGAACAACGCGGGCGTCGGCGTCGTCGGTGACTATCCCACTGAGGACGGAATGAATGTTATAA
TGCAAATAAATTATTATGGGCATTTCCTTTTGACACTCCTTCTATTACCACTTTTAAAGAAAACTAGTACAGA
TAAAGAGAATAGTAGAATAGTGAACACTACGTCGATGATGCGATCTTTAGGTTACTTG

GACGTAGAAAATTATAACAAAATAGGTTATTGGGGAAAAATTTCTATTTATTCCAACAGCAAGTTATGTACTG
TTTTGTTCTCCAATGCTTTGACAAAACGGCTGAAAAATCTAAATATTGTAAATCATGATGCCGATCCAGGACT
CGCAGCTACTAGAATATATCAAAGTTATAATTACATAATTG
>103774756_2 DT668968.3
GCACGAGGAAAAAACGTTTCTTAAATTAATTATTATATTATGTGTAGTGTATCAAGTAACGTGTTTAAATTTA
AATAGTGATTGTGAAGGCTGCAGAAATGACTATAGGAGGATAAAACGAGCAAAATACTATCAAAGTCCACCTT
TCAAGTTCTATAATCGACCACCTTTCAACCCCCCTATTAGGGGACCATTTTCCTATAC
CCCACCTCCAGGATACACTGCCAACGATGTTGGATCACCAAATTCACCTCCAAACCTCAGGCCATATAAGGTT
AGTAAACAACCAGTAAATGAAGGTTTGGGAGATGAAGATATCAATAATTTAGTTAAATATCTCTCTAAGAAAG
ATTTAGATAAAATCATTGAGTTTGCTAATGTAAAACAAAAACATGAAAGTCAACGGGA
GCCGATTATTAATAATTATGAATATTCTGTGCCAGATGATATGACCAGTAGCAACTTTTTTAAAAATCAAGAT
TTTGGGAAGGACAAGATTGTGAACATTCAAAGTTCAAAATCTCACGAGATTAAATATACGCCTGACGATAATC
CAACGAGATATTCATTCAATAGTCAATTATATGGTCCAGTTGAAAGTCTACAATCACA
AATACCTTTTGTTAATGCATCAGAACAAAATGAACAATTTTCTTTCTTAGATACATTTATTCAGAAAGAGATT
AAAGATATGACTATCAACAAAAGTTATATTTACACAGATAGCAGTATAATGCAAGAGGAATCATTACCAAGCC
CTGTGAATCTA
>103774762_2 DT668971.3
GCACGAGGTTAGATCTTGCGTTTGTTAATCTTAAATATTTCACATATTGTATTAAAAATGGCAAATGTTGAAG
AATTACAAAAGAAAGTGATTGAATTGGAAGCTAAGTTAGCAGCGTTGCAAGGCAATGCTGGGCCAGTGCGGCA
GAAAATCGATGTTATGTCCTCCGAAGTGGTAGACTCTAATCCGTACAGTCGCCTCATG
GCCCTCAAAAGGATGGGCATAGTTGATAATTATGAAAAAATCAGAGAACTGTCTGTAGCAGTTGTAGGTGTTG
GTGGAGTTGGTAGTGTTACTGCTGAAATGCTCACAAGATGTTTTATTGGAAAGTTGATCCTATTTGACTATGA
CAAAGTGGAACTGGCAAATATGAACAGGCTTTTCTTTCAACCTCATCAAGCGGGGTTA
AGTAAAGTTGATGCGGCGGCGGCAACACTACAAGCAATCAATCTACATGTGACTATAGATACTTATAATTATA
ATATTACTACAGTGGATAATTTTCAGCATTTCTGTGACACTATAAGTAAAGGGAGTCTATCCAAAGGCCCAGT
GGACCTTGTGCTAAGCTGTGTGGATAACTTCGAAGCTCGTATGGCCATTAATACAGCA
TGCAATGAATTAAACCAGCGCTGGTTTGAATCCGGTGTGAGTGAAAATGCAGTGTCTGGACA
>103774768_2 DT668991.3
TATACGTATCTTCCTTAATATACAAATAATTTTATAGGATATGTTGTTATCAGGTGGTGACTGTTGAACGACG
GTAGCACGTGACCTTTTGCAAAGTAACAGGCTTCAATGATGCTGAAACACATGTTTTAAACAGTGTCTGTGAA
ATTGGATCATATTTTTATGCTCAACCAAAACCCAAAATCAAATTATTTTTTTTAACAT
TTTTTTTAAAAAATAGAGTAAATAGAGAACGGAGAGATATTTCACAAAAATAATCGAAATATTCTTTATCTAA
ATAGGCCATTACAAGTAGTTTTGAGTCAAATTACATAGATATGTTATTAGTGTCAGTCATAACCGTAGGTAGT
CGTCACCAGTTTGGAAAGCAGTTCTCCTGAGAAGAGCCGAAAAAGAACTCAACAGTTG
CTTTTTTCAAACATCCCTTACATTACATACTTAGCACATAGATAAAAAAAAGTCTGCCTCACGCTGTATCCTG
CAAACTACGAGACGAGTATCTTAACACCGTTAACTGTTCTGCTATAAGCGGGTCAAAGCGTAAAGGTAAATAA
AAAAAATATTCATTTCTTCACTATATCGTCTGGTCAAAGGCCAGTCATGGCGTAAAGC
TACAAGCGTGTGCAGTTTGCGCGGCAGTTTGTGGGTTCGATTCACACTCGAATAAATGAAATATTTAATATTT
TCATTTCTGATAACGAGGCAAAGCGATATAATCCACAATATCTACCGAAA
>103774770_2 DT668987.2
GCACGAGGGTAATACTTAATAATAGTAATAGTTACAAAATGAAACTATTAATAATTGGCGTTGGAATATTTGC
TCTTATTGAAGCTCATACTATCCATCTCTCACACGGACAAAAGGAAAAGGTAAAAGAGTACGCAGCAGAATGT
ATGAAAGAGAGTGGCGCAAAACCTGAAGTTTTAGCTGACGCTAAGAAAGGCCATTTAA
TAGACGATGAAGGACTTAAGAAATTCATATTGTGTTTCTTCCAAAAGACTGGGGTACTGTCTTCTGATGCCAA
GTTGAACACAGATGTCGCATTATCGAAACTACCTGCTGGTATAGACAAAGTCACTGCTGCCAAAGTACTAAAT
GATTGTAAAAACAAAAAAGGAGCTACTCACGCTGATACAGCATTTGAAATATTTAAAT
GTTACTACACTCATACTAAACAACACATTCTGTTTGAAAAACCCTGAATGTAGCTTTATAATGTGATTATTAT
TTATTTATTTATGAAAGCTTACAGTGAAGCGTGTGGCTAAATATATATTTAGTCATATTTAATTTATTTTGTT
ATTAAATATTTTTTAATTTTAAATTATTATAAATACACAATATTGTGCAATTCCTTCG
AGGCAAAGAGGATTGAAATTTGTGAATAAAAATTGCAGTTTTTAAAAAAAAAAAAAAAAAA
>103774772_2 DT668989.3
GCGCTGACGCGGCTCTGCAACGGCACGCCGCCGCCCGTGCTGCTCACCGCGCTGCGCCGCCTGCGCGCCGCCG
CGCCGCTCACGCACGGCGTGTTCAGGAGAAGTGCGAACGCGAAAGCTCTTAGATTGCTAAGAGATAAGTTGGA
CGCATTAGGTCCATGGGCGGAAGGATGTGTCGAGTTGGACCGCGCGCCGGTGCTGCTC

GTCGCCGCCTTAGTGAAAGAGTTCTTCCGTGCTTTGCCACAGCCACTGCTTGCCTCTGATCTTTACCCATCTT
GGCTACAAGTGCTGAATTTTTTCCAAGCTGAAAAAGTGACTGCCGTACGCTCCCTGTTATTAAAACTGCCTAA
AGCGCACTACAACATGTTAACCTACTTCGTATGTCTCTTGCAAGCAATCGCTAAAAAA
TCTAATATAAATCTGATGTGTCCGATGAACTTGGGTGTATGTGTTGGGCCAAGTCTGCTATGGCCTAACGCGT
CCTGTGACAAAGCTCCCAAAGCAGTGCCAATCATTGTAGAATTACTTATAGTAAAAGCAGAAGCGTTATTTGG
ACCACAAATATCTGGACTTCTAGGAAGCGGCAGCCCTGAATCTTCGAACGCTTTACTC
GATTCGGGTGCAGAAGAAAGCGATAGCCTTCATTCTGTCCGGAATATCTCTAGATTCTATGGAACTATCGACA
CCTAGAAAGGAAAAAATATTCTTTGA
>103774780_2 DT668988.3
GCACGAGGCGTCACAGTGCTACATTACATATTTACAATTAATTTTCATATAAATTCGAAGACATACTATTAAT
CATTTATCTAGAATTTGTGACATAATTAATTAAAATATACCCACCTGGTCATGGCTATGATATCCTCGTGGCA
CCCAGACAAAAAACACACAGATGTGGAGAGATGGATCTGCATATATCCAGCATACTTA
AATAGCAAGAAAACACTTGCACAAGGACGCAGACTTCCAAAGAATAATTGTGTTGAAAATCCTACTCATCAAG
AAATAAGAGATGTTCTATTTGGCACTGGATTGCGTATAGGTGTTGAGAATAAGCTCTACCCTCGTGAACGTAG
CAAAGAAATGTTGCATAGAGGCCGCATTCGTGTAGAGATAAAGAATGATGATGGAACT
CCAATTAAACCAGAATATGCAACTAGAGAATCTGTAATGAAATTCATTGCGGAGTCAATACCAAAACTGAAAA
CTCGACAAAATAGACCTGCAGATCAACAGCCTCAAGCTGTGCAACAAGGCAGCAATAAGAGTAAGGGGAAGAA
AGGAAGGCGATAAATTCTTTATTTTTAATTTTATACTTATAGTATTTACAAAATAAAA
ATATTCATTTGTTAAATATTTATTATTATGTCTACATTTATTTCACTGTACATATTTATAAGTTTATGAGTGA
ATGTGAATGGCCTTTTCAGATAGTTTATTTTCTCCATTAACATCTCTCGCCACACTATATCCATCAAATATCA
TGACTAATTTTCCAGGGAACGTGGAATTTGACAAATTTGTTAGATTTACTGTTTTATC
TGGTCTACTAATTTCTTGATTT
>103774788_2 DT664445.3
GCACGAGGGGTCCCCTTAAAGCTGCAGAATTTGAGCATCTAATAGAGCGCTCATGGTATGCAATAACAGAGAC
ATGTCTAGCATTTACTGTATTTAGGGATGATTTTAATCCAAAGTTTATAGCTCTGTTTACTCTCTTATTATTT
TTGAAGGCATTCCATTGGCTAGCTGAAGATAGGGTTGATTTTCTGGAAAGGAGCCCTG
TTATTGGCTGGCTGTTCCATGTTAGGATTCTTAGTTTACTTATGCTTCTAGCACATGTGGACTTATATTTTAT
TCACCATGCTTATATATTTACTGCCACGAAAGGCCTTTTTTTTCAATTGGTCTTCGGATTTGAATACAGTATA
TTGATAATAATGATTGCAAACATATTGATTAAGTATGTTCTTCATGCAATTGACTCTC
ACTGGTAAGCACCAT
>103774807_2 DT664860.3
GGCATTACGAAACTAATGTAACAACAATTGTATAACATAAAATAATTAATCTCGCAATAATGGCGTTGAACGC
CGTCGCGGAAGAATATTTTCGTTCAATGAACCCTTTGGCTTCAAAAATACATGAAGATATACAAGAAACTAAG
AATTCTTATGAAAATATATGGGATACTCTCACGGAAAAGGAAAAGGCTGAAATAATAA
ATGAATCAATAATAAAACCAGAAATAGTATTAAAATATGCTTTATTAGACACATTAGAATTTGATATAAACAA
TCCACCTGTCCGTAAAGATTATTTTTTGTCTTTTTTTGGACATGAGCATGGTTTGAGAATGATACAAGATGAA
AATACATCATATCGAGATGAGCATTCAGCTCCTTTCTTATATCAAACCAAAAGTCAGT
TAAACCTCTGTGTTTTAACTGAACATAGGGTGTCTAAAGACGCTAAATGTACATCACCTCCACCGATTTTGAC
AGAATTAACATTATCCCACT
>103774815_2 DT664124.3
GCACGAGGCACGACGTTCAATCGCACGTCCACAAGGCAGACCGACGTTTATATATATAAAATGATGGAAAACT
TCCGTTGGTGGTGGCTTGTTGGCGCAATGGTACTGGTGACCCTCGCTGCTTCTGAGCCTCTCGAGCATGATGA
CCACGAGCTTCTCGTGCGTAATGAGCGTGGCGCCAAGAATCGAGAAACTTGCCGCTAT
GTCCGCGGCGCTTGGTCGGAATGCGACCCGAAGACCAACATTCGTTCCCGTACACTAACTTTGAAGAAAGGGG
ACCCCGGTAGCTGCGAACGCGCCAAGACCATCCAGAAGAAATGCAAGAAAGCATGCCGCTACGAGAAGTCATC
TTGGAGTGAATGCAGCCCCAATGGTGAAATGTCCAGGACAGACATGTTGAAGGCGAAT
AGTGATCCCACCTGCGACCAGAGCAGGCGCATCACTAAGAAATGCAACAAGAACAAACAAGTCAAGTCCATTA
AAGACAAGGGTCGCCGAAATCGTCAGTAAATTATACACAAAAAGTGTAATTTCATTTTAGCGAAATAAAAAAA
TTTCATTGTGAAGTTGTTTCTTTCCCGCTCTTGAAGGCAGTCTCTCATAGATAGCCTT
ATTAGGTATTTAAATAGTGCCAAAATAGTATGGAAACATTATTTCTATTATACTTCTATTCTACAGTTCTAGT
TACAATTATTACATTAACAATGCATTGTCTTTAAATGTTAGATAATAATTTGTTTATTATTATTATTAATAAG
TCAAGTCTTTGATATTA
>103774820_2 DT664222.3

GCACGAGGGGAACCATAAAAAAGTTTATAACATTTGTAGCGTGTAGAGTATTAGCTAGCTTTCTTATTGTTTC
CAAGGATACAGTCTATTAAAAATGGTTACAACAAATAACAGAAAATATATATTTGTTTGATAGGATAAATAGG
ATCACAGTACAAAAAATTAACTTCAGCTTAGACATAATTAAGAGGATAGTTGAATGGA
AAGATGTTGAGCAGCATCCATTAGAGGAGAGTAGCCTTGTAAATACATCTGGTAGTGTCCAAGTGGCAGAGGA
TAATCCTTTTAGAAGTGGTCCAAGTTGTGGTAGCAGTAGTATTTTTGCACAAATCCCGTCTAGTTCTCGTAGT
GATAATTTACATAGTGCTTTCTCTAATTTACAAACACATAGTAGTTCACCTTCGACAT
CCTCAAGCGATAATGGTGATAGTAAATCTGTACAGTTTAAACTTAGCGAGCCAGTTTTAAAGTTTCCCCTTGA
TACATCAAATACTAGTATGGACCATTCTGAGTCACCTGAACCTCCCCATGCAAGAATAGAGAGAGTCAATGAA
CATGGTTGTGTTACAGTTATACTGACTGACTCTGAATCCTTTGATACATCCCATATTA
AATGTTTAACGATAAAAACGTTAGAGCATCACTGGCCAATATTGGTTAAAAATATGAAGTTGCTTCTTCTAAG
GTACCTGAATTTATCTAATGCTGAAAATTGTATATTAACATTTTTTTCCCTATGGGAGAACATTATTAGTGTA
AAG
>103774824_2 DV501128.2
GCACGAGGTGTCACCTCTTATTGTAATTTAAAAATAACTATTTAGTAGTTTAGGTGATGAATGAACACGATGT
GTACAAATTATTTCAGTTGCCATAGAAGAAATATGTTTATTGTTTTATTTTTGTGAGAAAGGTGATTTTTTTT
ATTATTATTTTCCTTGTTAACGTTTTCTGAATGGGAAAATTATATAGTGACACATTAG
AAAGTCGAGTATTGATATGCTGTCCAAGTTAAATATTTAATGTAAAGTTTTCAGCCTATATTATATTATATTA
TATTCGTATTATCATATCAATGTATTCGTCCATAGTTCTCGATGCTTGCTCGGTTGTAGTGACCGGTTTCAAT
GCGAAAGTGTTGCGTTCCTCATAGTTTTAACATTCCCCCGTTGCAAATGTTAAAGTTT
ATTTGTTTTTTTCTAGTTTTTTTTTTTATCTGTTAAATTGTTTTTTATTTTTCTATTTTCAATGTTGCAATAG
GACTTTCATCTCAAGTGCCTATCCGGAGCCATCAGATGAACTGTAACTCGTTAACTAGGGGCCGTCCGTGGAT
TATGTCACACGGAGTTTATAACTTTTTTGACCCTTCCCTTGTCCTTTGCCCGCAACTT
TACCCCTATGAGTTATTAAAAGTTTGTGAGTTATTTTTGGTCTCAGGAATTTCGTGTTTTGATACTTATTANA
TGAAAGTGATGTCCTTTAATGTTGGACCCCTACTCCTCTCACAGTGTTACACTTGGTCGAATCCCCGGCCTCC
CCCTCCCCCC
>103774826_2 DT664046.3
GCACGAGGGTTTTTCTTTTATTTTTAGCTATTCTGATAGTGCTTTTATAGATATTTTTTTATGAATTGCTTTG
TTAATATATTCAATAATGTAAACATTATAAGCGTGCTTGAATAACATAGGAAAAATATATTTCTTTTCGCATT
AGTGACAAAACGAAAAGCATAATAAGGATATTTCTATTTTACAAAGATGTAAGTGACT
ATCAAATTAAGAATACACATTCACATTAAATAAAGTGTGCAAATGTATACATACATATATATTTCTATTTCTG
ACATTTTATTTCAAACATTTACCATGCTTATATCAAAGAATGGTATTTGTTATATTAAAATTATATATTATTT
TAATTTTTATTTCTTAATTTCATCGCGAGAACCTGTTTGGCACCCATTAATTATAACA
TATTATGCATAAGAGAAATGTAATACAACTGTACAGTATGCTAATGGAAGATATAACTTAAAACATATGTTAA
GTCAGCAGTTAGAAAATAATACATAAAATAATACGAAATATATGATGGTGGTTGAATTTTACGTAATCTATAC
TTTTTTTGCAGCTTTACAAATCTACTTGGGTAAATATTTTGTCAAAAGATCCATTACA
ATGACTGGATGATGAATGAATGATGACTGCACAAATTTGTCAACCACTATACATAATATAGAAATTTAAATTC
TTATATCAATATATATAAAAACGTAATGAGATACTGCTGCATAATTTTTTTA
>103774830_2 DT665018.3
TCTGAATCCGACCGGACCTTATAGTTGTTTACTGTGTACAGATTGGTTTATAAATAGATCTAAAATGATTTTA
CATTATGCATTAAATCATAAGAAAGATTTTTGTGGTATTTGCAGATATTTTGTACCAGACAGGAGTGCATGGT
TTGAACATGAGAAGTTTCATTCACCGTGGCCCTGTTCACAATGTGTGGAAACCTTCTC
TACTGACGTTCAGTTATTTTTTCTTCTCAGCTCTATTCATAACTTAGTGCACTGTCGCCTTTGCCACTTTAGG
GTGACAGCTGATGATGATTACAGTTCTCATTTGTTCCAAAAACACAATGTTACCAATGCTGCTTGCCTAAATG
ATGAACAGTTTTGGCAAGTAGATTATGATGGAGATGATAAGTTTATGTGCTCACTATG
TTCAAAGCCAGATTTACTGTGCTCAACTTTTTTTGCACATTACATGGGCTTCCATCATTTCACATTGAAGTGT
T
>103774835_2 DT663580.3
CGAGGCTCACTCCTTTAAAGGGAACATTCTTCACTGTTAATCGTATGGATTATTTTAGGGATTCCAAATTGTC
ATGGCGTTTAGAGCAAGCCTTACTCTCTCAAAACTGAGTATTAATCATAATGCAGTGGAATAGATGACGGTCA
GTCTACTCTGATGAAGTTTATAACGTTCGTTTCTAACCAAGATTGGGTGGATCGAGTT
TTATGTCCTGTCTCCGAGTCTTGCTGAAAGGACCATTCTTGGTTATTAAACATAGTGTTGTATAGGCTTGACT
ATATTCTCAAGAATCGTATCTTGATACACTTGTGTCGATTTTTTTACACCTTTACAAAAATAAGGCTCAGTCA
CTTCCTTATTTATACTTAACATGTGGGATGTTATGATAAACTATGACTGAAGTCTATA

TGGGATAATGTCCACTTTGTACTCTGTTGACTAATTGGGAAGCTTATTTAGAGCTTTCGTTTAAATTGCTGAT
TTGTAAAAAGAATCTGTTCTGTTTTGTTCTTTATAATTATCAGTTAAGAAATAAAAATTGCATATTTTATAAG
CTGCAAGTCCTAAGTCATCTTTTAAAATACACGACATAGTTCTAGGTGCTATCTTCAT
TTCCCAAGATAAAATCTTTTGCTTTCGGGCAGTATTTCTTTTGAATACTTTCCTTCACTACTTTAATCATTTT
CTTTGTATAAACACTATGTCAAGGGTAAGAACTTTTTCTGTCACGA
>103774856_2 DV501168.2
GCACGAGGAGTTCCCTTGAAAGGGAGTTCGAGCGCAGTCCAATTCGCCGTTGGAATTCAACGCTATTTTCATT
CAAGGCAGTGATAAGCCCGTAAACAAACCTCGTGTTTTGTTAGTGCACGTACGTACGTTGCTGTACCGATAAC
ATTTAATTCGCGTTCTTTAATCTATCGCCTATTTTGAAACTGAATATCGACGGATGAC
TGTCCTGTTGCCTTTTTTCTGGGACCGAGAAAGAGGCGCAATGGAGAGTTGTTACGAGTTGTACACATGGATC
AACCACACGCTGCTGATGCTGTTGGTGCAGATGTTCTGGAATGTCAGTCAAATATCGGACTATTTCAAGCACA
GGTCTGTAGAAGAAAAGAGAACACACAGCCCTTTCATGTTTTCTCCGCTCACAAAAGA
ATCACAATATAGAAGGCACATCCGATCTATGTCATATGGACAGAGTAGTACTCCATACCGCCTACCATTTAGA
TCAAGAAAAGGATTCCATCCCACTCTCACATATGATTCTGACACAAGAGCGAGAAGGATAGCGTCCTACAACA
GAAAAAGGCTGCAAGATTCTTCAGACTCTAGTGACTACTTTGAAGCCCCAAAACATTT
AAGCATAGGACGCAACATCATTAAGCATTCTGATTCTATGAGGATACTCTGAGAAGAGATGATCTGAAAAAAA
TGACTTACTAGTAGAATACTAATAATAAATCTTTGACCATGTTGCTTTGAGTAATATGTATAATAATAATATA
TATTATGGACTGCGATTATACTAGTTACACGATCCCATCGCGATCT
>103774872_2 DT664622.3
GCACGAGGAAATACATTTAAGCTTATTCCAAACTGGGTGACACAAAGATGGGTTGCTATTCTCAGTGCCGAAT
ATCCCACTGTTGCTTTTCATGCATCAATGACTCATCCATTTGGGAAAGGATCTCTTATAAACTTATTGCGACA
ATTTGCAAAATTGCATATAGATAAGAAACAAATAAGTGTGGGGCTAATTGGCTATCCA
AATGTTGGCAAATCATCTGTTATAAACACACTTAGAGCTAAGAAAGTGTGCAAAGTTGCACCTATTGCAGGTG
AAACCAAAGTTTGGCAATATATTACATTAATGAGAAGAATTTTCCTAATCGATTGTCCAGGTATTGTGTATCC
TTCTGCAGAAACTGATACTGAGAAAGTTTTAAAAGGAGTTGTTAGAGTTGAATTAGTG
CAAAATCCTGAAGATTACATTGAAGAGGTGATAAAAAGAGTTCGAAAAGAATATTTAATAAAAACATATAAAG
TAGAAGGATGGAATAATGCTACAGAATTTTTAGAAAAAATGGCAGCAAGAACTGGTAAATTATTAAAAAAGGG
GGAACCTGATATAAGTCAAGTAGCTAAAATGGTGCTTAATGATTGGCAGAGAGGTAAA
CTACCTTTTTATGTGGCCCCAGAGGGTTTTGAAGTGCCCTTGACTAAGCAACAAGAAAGTACAGAAAATAAAG
AAGTAGACACAAAAAATGTAAATGATGAAAAAGAAGAGCAAAGTCT
>103774888_2 DT664323.3
GCACGAGGGATTTCTATAAGTTAATAGAACTGTAGAACACAATTACTTTTGCTTTCTCTTTCGATATGAAGTT
ATATAAAAGACAAAATTAAATTATGAAATTGCTTTGGATAGTTTTCTTGTTCCTCCCGTTATGTACTACAGAA
AGTGACCCATATTTCAAAGAACCATATCGTCCTCTTGGCCCATTGGTGAAATGCAGTT
TTCTACCCCTCGAGTTCCTAGACTGCGACGAGCCAGTCGATCACAAAGGCAACGAAACCGCTAAAAAAGCAGT
AAAACATGGTTGCGTTAAATTTGGAGGTGTTAGATACGAGAATGTAGAACGAACACAAGTTCAGTGTAAGGCT
TTAGATGGTATTGAGTGTTACGGGAACCGTAGCTTCTTGAAAGACGGATTTCCTTGTG
TTAGATACTCAGGACATTATTTCACTACTACTTTGATTTACAGCATTCTTCTTGGTTTCCTTGGAATGGATAG
ATTTTGTCTCGGTCAAACTGGTACAGCTGTTGGAAAGCTTTTAACATTAGGTGGAGTGGGTATTTGGTGGATT
GTTGATGTTGTATTATTAATAACAAATAATCTACATCCTGAAGATGGAAGTAATTGGA
ATCCATATGTTTAAATTAATTCTGTGGACATTCTATTGATAAAAATGTATATAAGTTAATGTTTCACGTTGTC
TTTGAAAGATACTGTATAATTAAAGTGTAAAGGTTTTAAATTATACAATTGTGTTATCAATTTCTAAT
>103774900_2 DT664151.3
GCAGAGGGCATGACCGTCACGGGCGCGGGCGCGGCGGAGCGCCTGCGCGGCGTGCAGATGGCGGTGTGCCACG
ACTGCAAGGCCATGGTGCTGCAGATCATCAAGTCCTCGCGCGCCGCGCGCGCCGCCTCGCGCGACCGCGCGCT
GCGCCACCTCACGCTGGACCTGGCGCCCGTCTACACCGCCGACTGCTGAATACGTTGA
GTGTGGGCTGCTCATGTTGAGTGCTTGCATTTTTTTTGTATATATATTTGTAGATTATTTCAGTTACTGCTGT
GTATAAAGACATGTTATATTTATAATTGTACATTTTTATTTTTAATTGTCAATTATTATTGTTTTGAACTTTA
TGACTTGTATTTAAAAATTATTTTGAAATGAATGAATGTATTACTTTGTTAAATATCA
TGGAAGGTCACTATGAATGTTTTATATGTATTTTTTAAATATTTTACATTATTAACATGGCAGCCTTAACTCA
ACTAAATATGTGACGTAAAGCGACTTAACAACACGACTCGGCTCCGTTTCCGTACAATTTACTAGGCACAATC
TATTCCTTACAATAATTTATGAAACACCGCCATTGTCATGAGATTGAGGAACCTAAAT

AAATAGAAATTATTGAAAAAAAATATTAGAAAAAAATATTTTTTGTAGTCACGAGAATAAAGAGCTTCCGAGG
TTGTGTAGAATTATGTTGTGTTTGTAGGTGGACTGTGATCCAACACTCGACTAGAATGACTTTTTGACATACT
ATATGAC
>103774913_2 DT664226.3
ATAGGTCACGTTAACGAAGTTTCAATGGGCCACTTGTGTTATGTTTCCTAAACATTTACATAAATTGATTACA
CGTTCAATTACCCATGTCAGTTACGTTTGAAATGCTCTACAGGCCTTAGTATATTTTTAATTTAATATTAACT
TTTTACATTTTATTATTTTGAGTCTTTGATTCAAAGAAACCAAAAATATAAGTGTCAC
ATTCAAGAGCAATTTTAGTGATTTGTCTCTATTTGTATAGTCATATATTTATACAAATATTTCATTAAAAAAC
GTATATATTTTTTAGGCGGAGAGTGTACATGTGGGTGCGGCGCTTGGGAGAGGCGGAGATACGCAGAATCACC
GAGCAGTGTCCACCGGTACGTCAGCGCTGTTACTGATAGATGGAGTGGAAGAGAATGC
GCGTGCAGAAGGCGTAGATGGCGCCGGCCCGCGTGCGTGTGCACGGCGTACAGGCGGGTCAGTGACGCGTGCC
ACGATGATAGACTGGCAGCGGTCCTGACACTGGGCGCTAGTGATTTAAGGAGGGAATTAGACG
>103774916_2 DT664642.3
GCACGAGGGTCATCTTTAAAGTTGCCACTAGGCCAGCAGAGAACCCGATGAACGTGGTGGCGCGCCGCCTGCA
GCTGGGCGGCGCCATGCGCGACGCGCCCAAGCTGTCGCTGCCCGCCAAGCTCGTGTTCCCGTCGATGCACCAC
CAGGGGCACTTCTCTATGCTTGGTTTGGGCGACATAGTGATGCCGGGATTGCTCCTTT
GCTTTGTACTGCGTTACGACGCCTACAAGAAGGCAACACTCGTCTGTCAGATGGGACAAGTGCCGGGCCCCCG
GTCTATGGGTTCCCGCCTAACCTACTTCCACTGCTTTTTTTTCGGCTACTTCCTGGGTCTGCTGACGGCGACG
GTGTCGGCGGAGGTGTTTAAGGCGGCGCAACCGGCTTTACTGTACCTCGTGCCTTTCA
CGCTCCTGCCTTTACTTACTATGGCTTATGTGAAGGGTGATCTCAGAAGAATGTGGAGCGAGCCGTTCATAGC
GCCCCCTGCGGGCAAGGGGGGTGCGGACTTCGACGTGTGAACACCTCCCCCCCGCAACCCTGCCACCCCGCGC
ACCCCCGCACGAGGCCACACTGTTAGC
>103774920_2 DT664654.3
AATAATCAATTGCCAAATAATTTAACGCAACTTCAAAATTTAATAAAACGTGATCCTGACTCGTATAAAGATG
AGTTTCGTCAACAACTCGCACATTTCGAGACGACTCTGGAAATTTTCAAACTAAACCCAACACAATATAACAA
GAAATTAGATGAGCAGGCAATGTTTTTGGCCCAAGTGACGCAGTGCTATCAAAGTGAA
ATGAAGACTTTCCCCCAAAAAATTGTTGATATTTTTAAAACCCACAATACAACTTTACATAATGAAATGAGAT
TATCATTATGCAAATGTTTAATATTGCTTAGAAATAAAAATTTCATCACAGCTTTTGATTTACTTGAATTATT
TTTTTCATTAATCAAATGTCAAGACAAACATCTAAGGGAATATCTAAAGACCCATATC
ATAACAGATATAAAAAATATGAATATGAAACATAAAGATATGAAATTAAATTCAACTCTACAAAATTTTATTT
TCTCAATGTTACGTGACTCANATGTGAAAACAGCTAAACTGGCTATTGATATTTTGATTGAAATGTATCACAA
AAATATATGGAATGACCATAAAACTGTTAACATTATTGCTGACATTGGATGCTTTGCT
AAAGTTACAAAAGTCATGGTGGCATCTTTAAAATTTTTCTTG
>103774922_2 DT664324.3
GCACGAGGCACAATTATTGTGGTGAAATCAAATCGTTTTATAAATTTTATTTCTTCTGTTGACGCATGTGTAA
AGTAGGTAAAGAAAACATAAATGTTAATTGCTTCTATGTGAAGAATATGACATAAACATACAATGCAAAACGT
TTGTAGCAAAGTTTTCTATTGTGAACATAATAACTTTAAAAAAGATGTCATGCAAAAG
AAATATTCCATTTATTTAATATTACAGTGAATCATAGGAACATGTTTTTAACATAGTAAAATAGCTAAACTCA
AAAGTTATAAAAATCATGGTCATAAATTGCTCGGCAATGATTTCCCCGGAAGAAACTGACAATATTCCTGCTG
CTAGTAGTTTCTGTAGAGATTTTATTAGAGGCTGCTGCCCTAGAGAGAATTGTCGGTT
TATACATGAAATACCACCGAAGGTTTGCTTAAAAGAACTTTTTCGTTTCTGTCATGATTTTCAAAACAAAGGC
TGTTTTCGACCCAACTGTAAATTTATGCATGTTAGCCTGGAAGATGAAGAACATTTTTATAATACTGGAATAT
TTCCTCGAGATCCACGTAATATACCAGCTATATGTCCAGCTTTTCTTCATGGTATATG
TTCCAATCAAGACTGTAAATTTATTCATGTCAGAGAAATTGCTAACAATGAATTGAACGCCAAACGTAATGTA
CCTAAGATAATTTACAATCGTAATCACATCAGAGACGAAGATTCTCCTCCTGAAGCTAAAATAAGAAGGTTTG
CATTTGATGAATCTGGAATTGATACACACCAAGTATGTGGTAACTG
>103774928_2 DV501233.2
CCTGATCCACCATCAGTTGGGCCTCCAATCCAGCGACCTGGACTTGCTCCGGCCACAGCTGCACGACAACAGG
AGCTCCTTAAAGCAGCAGCCCCGGCTGAACCTCCACCACCCAGGGATCCTCCACCAGACTTTGAATTCATTGC
TGACCCACCATCCATCTCTGCACTTGAGCTTGATATTGTTAAACTTACTGCCCAATTT
GTAGCCAGAAATGGTAGGCAGTTTTTGACAGATTTAATGAAGAAAGAAGAACGCAACCATCAATTTGATTTCT
TGCGTCCGCAGCACTCCCTCTTTCAATATTTTACAAGGCTTTTAGAACAATATACTAAAGTTTTGTTGCCTCC
AAAAGAATTAGTAACGAAATTGAGTG
>103774934_2 DT664512.3

GCACGAGGGTTTCCTGGAAATGTATACACAGACAGACAGGCAAAGATTATGCTGTTAAAATAATATTATCACA
GAAGAGAGATGTGAAACAAGAAATAGAATTGCTCAAAGCGTGTCAAGGGTGTCCCTATATTATTACTTTGCAT
GAAGTTTTCTATGATTCTGCCTTCACCTACATAGTGACAGAATTAGCGACCGGTGGAG
AACTATCGGCGGCGTTAAACTCCGCGGGTGTCGGGGGCGGAGAGAGGATGGCACAAAGGTTTCTAGTACAACT
GGCACTGGCAGTACGACATATGCACGCCAGGAGAGTTGTACATAGAGATCTAAAATTGGAGAATATTCTCCTA
TCAAGTACTCGTTTAAACGAGGCTAAAGTGAAAGTGGTAGATTTTGGTTTCGCCCGGC
GCATGCCCGAGTGTCACGACCGGGAGAGGATGATGACACCGTGCTTTAGTCTGCCGTACGCGGCGCCGGAGGT
GGTGTCGCGCGCGCGCACGGGCGCGGCGCCGGGCTACGGCGCGGCGTGCGACTTGTGGAGCTTAGGAGTCATC
TTTTACTGCTTATTGAGTGGAAAAGCTCCATTCCAACCGACGTCCAAAAAAGAACCTG
TTACAGCATTCATGGATAGAATAAGAAGTGGGAGTTTTACTATGGAAGGTCCAACATGGGATAATATTTCTGA
AGATAGCAAANAGATAGTATCCGGTCTTCTAGCGGTAGAACCAACAGACAGACTGACCATAACCGA
>103774937_2 DT663542.3
GCACGAGGGTCATGCCTTGTTAGTGAGAAACGAATGCATGCTACGAACCGACAAATATTTACAGTAATGGCCA
TTATGTATATCTGTATTCTATATATATATGTATTCTTTTTATTGCAGATTAGTCATCAGTATTTGATTACAAA
AAGTACTATTTTTTCTGATGTAAAATAAATTACATATTATTAATTAATTTTAATGCTT
ATGATCAGATCTAAATAAAAAATATAAATAATGTTTTAATAACTTCGTTACGATTGGTTGATTTTGAGTTTTG
ATGATACAAAATCGCAAAATATACCTGGGCATAATACATATTATTTTAATTATACAATTTTTCTATTATTTTA
ATTTACATTATTTCTCTATTCGGTTACTTAAAAAAAATTCTAATTATGTTATAAATTT
TACGAGGCAAAGTAAGGTTTGTCACAAAATAAAGAAAATAAAATTACACATTTTACTAATCTATTTTTTCTTA
TCTATGTTTTAATCTTACGATTGTACCTGACTCATATTAATAACTTTTTTGTCATTTTTAAAGAATTTCTTGT
GAATCGGTTGCAGCTTAAAGCTGATTTATAAAAATTGCCAAGATTTTAAGTATTGATG
ACCAACGGATAACGTGTTGATAATATATCTACTGTTTAGTAAAAATCTCTTAAGGAATACTTGCTTCATTAAA
ATCTTAATGTTGAGAACCTTGTTAATAATAGATATCATCTTAAATCTAGTTATGAGTCATGGATGCGT
>103774943_2 DV501167.2
GCACGAGGAACACCTTTTGTTAGAAGAAATAGTTCTTGTGGAAAATGAATGGGACAGTAACAATGTGGAATCA
AAATAAATCGATAAACTAATTGGTGAAATGATCGCTCTTCAAACTTTACCTTTTAACTTTGTGGAAGGCGTTG
GCTTTCCTCCATTGATCACAACATTGGCACCTGCGTATAATTTTCGCGGGCAGAGATT
TTTTACTGATTTTGTATGCGAAGAACTTTACGGACGACTTGCATCGAAAGTGAAACCATTAATACAAAAATTT
GATTACCTGTCCTTCTCCTGTGACATTCGATCCCACCCAAGCTCAAATTATACTTTACTTATCTTCACTTGTT
ATGGTTTAACAGAAAATTTCAAAAGATCGTCAGCATCATTGAAATGCTAAATAATTTC
CGAAGACCGCCATATTGATGATACAATTACTGAAAAAAATTGACGCGACACTTTCTGAATGGAATATTAAAGT
AGAACAAGTCCATGGTATAACTTGGAAGAAAGGCTCCAATATTAAAAATGAAAAAGGAGCTATTAATGATATA
GATTGCATACTTCATAAAATACAACTGTGTATTCGCAGTTGCTTACATTCACAGGATA
ATATAAAATGTGTTAAACAAAAATGTAAGGAAATATCAGCACACTTCAATGACTCCACTATCGCTCACAAACA
ACTTGAAATAATTCATGACAAAATGAATCAACCACACCACAATGTGTACCTAGATGGTATAACGCGATGGAGC
AGTGCATATTACATGTTTGAGCGGTTTTAAAAGTAAGGATGCTGTGAGTTTTTATT
>103774955_2 DT664385.3
GCACGAGGTGTCCCCTAGTATTCCAGTTATTGTGAAATTACTAGCATCGTCCGTATCTATTGCTGGCCGAGCG
GGGAAAATTGTACGAGATGTTATGAGCAAAGGAGAACTCGGTATCGTTGAAAAGGGCAAAGATGATTATCAAA
CAGAAGCTGATAGATCAGCACAACGATGTATTATTGCATCACTTGCAGCACAATACCC
AAAACTTAGTATCATAGGCGAAGAGGACAATACGGATACGGAGGGTGAAGTTCCGAGTGACTGGCTGGTAATA
GACTCAGACAAGGAAGTCTTGTCACTACAATGCCCTTCTAAATTCCAAGGTGTAAAAGAAGAAGATATTGTGG
TGTGGGTTGATCCTCTTGATGGCACTTCAGAATACACTCAAGGTTTCCTGGAACATGT
GACAGTTTTAATCGGTATATCTGTCAATGAGAAACCAGTGGCTGGTGTGATACATCAACCTTATTATAAAACT
TTAGTAGGTGGGGAGAAAAA
>103774970_2 DT664166.2
GCACGAGGGAACATCTTAAGGATAAAGTACGCGGCGCGCGACGAGGCCGCCCGGGAGCGGCCCGCAGCGCTCA
TTTAATATAATATAGTATGGATTTATAGATTAACTCGAGTATTATTGCTGATTAGATTCAATTTGACATTATA
GTATCGAATTCTTACCTTTGAACTGATTCTTATAGTAATAATTATACAGAGCTTAAAC
ATAAAGTGTAGAAACAGCGGCGTCCCGCAGCGTGTGCGCGTGTGTGTGTGCGCGAGTGTGTGCGCGTGTGAGC
GTGTGTGCGAGTCAGCGGGCCACTCCACTCCGCGATGAAGAGAAGGATCTCATTGCAAACAATCGTTGTAGAT
TTGTATTCATTTGGAGGCGGAACAAGAAACAAATCAAAATCATGTATTTTATACAGAG

CGGCGTCCCTCCGCCGCCCGCCGCCCTCCTCCCTCCGGGGAGCGAGGTGGGGCGGGGCGGGGCGGCGCACGGT
AGTGTAATAAGGATCACATGAACAAACTGCTGATATACAACTGTATGTCCAAATAAAACTGTAAATAAAATGT
TGACTTTTTGTGTAACTACATGGGTGTTTGTTTGTGGGGCGTCCACCACCACCATCAC
TACCACCACCACCACCACTACAACCACTACAACCGCCAGCGCTCCACTGTCGTCTTGTCACTTAATGTGTAAT
GATTGTTGTTGTATTTGTGTTGAACGATGATTTGGATACAAACTAAGCCTAAGTACTTCTAACTTAAGTAAAG
ATGGGTCAAATAAATATGGGTACAATATAAAAAAAAAAAAAAAA
>103774979_2 DT664905.3
GCACGAGGGTTTTTCCTTTAAGGGTTTCATAAAAAAAACAATTTATTTCATTCTGGGTATCTAAAATACTATT
TAGTGCGTGTAAATATACAGGTAGTTCAAAAATGTGTTCCTTAACTCTACAATGATATTTACATTTATAATTG
TACAAAATCTTTTTTCATACAAATTTTGATCGAAACATCACAATAACATCTTTCTTTG
CATTTTTTTAAAAAATAAGTTTTTACATCTAGAGGCATTTTAAACAAATAATTTATGGCGTTAACACGTTGAG
AAACGATTATAAAGTATTTTTACACCTCCTTACTTATTATAATTTTTTAAGTATAACTGGCTGTACGTTTAAA
ATTAAGGCCTGTATACAGCGCTCTCAAGTCTAAAATCGATCTTGAGATAATAAAAAAA
ATATAAATGAAAACATAATCTCAAGATAGATCCCAAAATCGACAATTACAAAAACAGGCCTTAAATTCACAAA
AAAATAATTTAAAACTGAAAATATAAATTAAATCATCTTTTTTAATGATACAGCGTGCTAGCCCAAATTATTA
ATTTCTTGGTTCAAAACCACATCTTACATCTACATCTGGAGTTTGCCCAGAAATATCA
CATTCAATAACATTATATTTGTGTATAACTAATTCTATTCCACTTCTATAGAAGTGTAGTCTATATGACTAGT
TGGAATGCTTTTTAAATGCATTAA
>103774983_2 DT664407.3
GCACGAGGCTAAACCTTATTTAAAATTTTAATTTTAATACTTACTCAAAATCATCGCGTACAGTATATCTTTT
GTTTCAATATAAGATATTCAAATAGGACAAACCCAATTAACCGGACTAATGTTGTTTTTAATCATTCATATAA
ACCGAAAATCCAGATAATCGAACGTATTTAATAGAAGTTTCTAAAATCAGAGCTTAAA
TGTTTGGAACAAAATTAATAAATAATTATTTTTGTGTCAACATTTAAAAATATTAAAAGTATTCCAATCAAAT
GTTACGCGTATATATACACATATACATGGCCGTATAATCGAGTCTGCACTGTTACTAAATTTTTTTTTATTGC
TTCATGCTGTCTCGCACATAGCAGGGTAAGCAGAGAGAGCCTAGTGTTAAGACACTCC
GTTCCCATTAAGACACTCCCTTGTTCTAGAGCGAGGAAAATGGAATTATTAAATATTTAATTTCCTCCAGTGG
GAAATGAACAACCTGTTTTGTCGCGTTTATAGTGGCAAGTGCCCCTGCGTCACGACCGCCTGACCTGGCCTCA
TTGTTCTATATAAGTATTTTTTTTTTCTTTAATGCATTAAGTATTAAGAATATTTATT
TTTACGGGTTACACCACTTTTATATATAGCAGCAAATAGTCGTTGCAATATATGCGTGTTTGCTTGTTTGCGT
TCCAACATGGCGGTGCGTTTGCTTGTAATAAAAAATAAGGTAGCTTTTTATAGACGCAATATTTATTTTTCTT
CTTTCTTTTGTCACTTTGGTAGTGTAATAAAAATCATTTCTTTTCACGGCTTTGTTTT
TTTGTTTTGAAAAAGACATTATCTTT
>103774989_2 DT664560.3
GCACGAGGCCTGCCTTAAAGGGGCCACTTCTGCCGCGCCACCTTCTGCCTGCATACCCTGTGACCGACGACAA
CATTTCTGACCCCAACTCTCTCAACACAAATTTCACAATCATATCCCAACTATAGCGAACGGAGTGTGCGATT
TCGAATCCGATGGACGCTGTTAATTTTAGTGTTTCGAAAGACAATGGACAAACTGTAT
GAGCTTCGTTTAGTATTTCGATATATTCATATTGTGTAATCCAAATGTGTATGTGTATGGAACGATTAGGAAT
AGTGAACGGTGAACATGAACTTTGTCATTTTGGATTTTTTGATGGGTCTCTCTCATAGATTACGCTTCTAGCG
GTCGCTCTGATTTTAACCTTTAAACTTTACCACTTTACTGTTATTTCTAAATGGATGT
GCCTTGAAATGCTTTCCTCGATTCGACTTGTAGTATAGAGCGTTTTTGAACCCTTTAGAAGGAATTTCTCTTC
TCTGTTTGTGTGGAAGTTCCCGTATTATAAGTGCAAAACACATCATTACCGTGCCTTTACCGTTCTATAGACT
TCGTAAGGGACGTAATTAACAATAGGTTTTAAAAAAAAACGCTTTCCCGATGGAAAGA
CAAACCAACCCAAATATTGTTTGTTCTTTGAATTTGTGTAATTGCCAGCTTCTTTGTTTTATGTTACTAACAA
TTTTGTGATCTAATGATATTAAAGAGGAAAGTGAGCTAGGACCAAAGTCTTTCTCAAATGCGCGTCAATTT
>103774995_2 DT663770.3
ATTAAGTCAGGAAGTGGCCGGTACCCGTCGGGATATCGACAGAATGTCCAACAAGAACACTTCTTCTAAGCAT
CATCCAGTGAACTCCTCGACTCTACCTCATCACGACACGCTTTCTCGTCAGCTGGCACAACAAAGCCACATTC
ATAGTCCATACCCGGACTGTGATGGCCAAATAGCTCCCGGATCCCCTAGACACGTGAT
CAAGGATGAGGAAAGCCCACTCCATCATCAAGGATACCAAACGACCAGAAGCTACAACCACGACTTCAGAAAT
CCCCACGTTTTCATGTCATTTTTTTCGCCCGGAAGTCAGGAGATGATTTTCCGGAAACACTACAAAGACGACA
ACCGACGAAAACATCATCACCACCACGGCAGCCAAAATATGAATCACACGTACTCAGA
AATAGCTTCTCAACAAGGAAGGATATACAGACGGGGTTCAGAAGAATTTCGAGTAATACAAGACGACCCAGTG
TATGAAGAGATCGAAAGAAACGAGACGCTTATGTCGGATATGTCCGACGATAATTCCGGACCGGATAACTGCC
GGCAGAACCCTGGACACCACGGGTCGTCCGGCTCCAAGTTCTTCGGAGACCATCGGCC

ACTAATCTCCTATAGCCCTGGAGACAGGCACCATCACGAGGAACATTATGGGAAGTACGG
>103774997_2 DT664091.3
GCACGAGGCAGAACGTTGACTGTTGAATTCATGCATGCAGTAATTGCAGCTAAAGCCCTTCGTAAGGTATTTG
TGTCAGTAAAAGGTTATTATTATCAAGCAGAACTTGAAGGGCAAACAATAACATGGATTGTGCCACATCATTT
CTCATTCCAACCTCAAAACAAAGATGAAGTGGATTTCAAAATTATGTCTACATTTGTT
GAATTTTACATTATGATGTTGGGATTTGTTAATTTCAAACTGTATCATTCATTAAATTTAGTATATCCGCCTA
AACTAACTAGTGCATTCAATTCTGAAACTGAGAAAGATCTTGTTGATGAAAATGCCTATGTTGCCGAACGGAT
TGCTGCTATGAATTTATCTGTAGCAAAAATCACTGGTGCAAATGAAGTAGAAGAACTA
CCAGAAATAGACATATTTAACACAGAAGATGCGGACCCTCAAAAATTAGAAGAAGCTAAGCTTGAGGCGGAAA
AAGTAAAAAAACTAAAAGCAATGTTCAAAGGCCTCAAATTTTTCATTAACAGAGAAGTCCCTAGGGAACCCTT
AGTATTCATAATTCGTTGTTTCGGAGGTGAAGTGTCTTGGGATAAAGATTATTTTGTT
GGGGCTACTTTTGATGAATCAGATGAATCTATTGCTTATCACATAGTTGACAGGCCTAGTATGGATAAGCAAT
ATCTATCTAGATACTATATTCAGCCACCATGGGTATTTGAT
>103775002_2 DT664914.3
GCACGAGGGCCGCGCCCCTCCTCCTCGCGCCTTCGCCCCGCATTCTCGGCAGTACCCGGCGGACTGGCGGCAC
GTCATGCTGCCGCAACAGGCCGCCAGGCCGCAGTTCAATCATCATCATCAAGGATTCAATATGGGGAATATGG
GCGGTATGGGTAACGTGAGTGGCGTCAACGTTGGTGGTAGTATGGCGCAGCAGCAACA
GCAACAGCAGCTACAGATGCAGCAAGCGCGGTTACAGCAACAACAACAACAGCTTATGCAGCATCAACAACAA
CGGGCGTCGAATCAGCAGCCGTCAATGTCACATCTAATTATGCAACAAAATCAAATGATGAGCGTGCAAGGAC
AGATGCCAAACATCCATATGTCGCAGTCGCAAAGTATGAGTATGCAACAAGGCGGGCT
CTCACACGGTCTTGGAATGCAACCACCCACCTCACACTCTCTACAAAATAATCTAATGCCACAAAATGGCAAC
TTCCTTCACTCGTTTGGCCCTACTGCGGACTTCAACTTGGATTTCCTAGACAACATGCCCTCTAATGACGCTG
GAGATATGACGGCGCAAGAATTACTAAACTCTTTGGACAATTCGTTCCTCAATGATAT
TCTATAGATGATGTTTACTCTGGTCGTCTTCCTATACTAAAGTCTTTAGGCTCGAGATCTCCTAATTTCTTCC
AACCTCATGCCACAGAGTGATGTTGCAATGACAGCGCAGTAACTGATAAACTGTTTGGACAATTCGTTCCTTA
TAGGTATCATGTGAAATGTTAACTTGATTGTCTTCCTACACT
>103775010_2 DT664463.3
GCACGAGGTATTACTACAGCTGCTACGACTTTAGCTTCAACTGCTAGTAATATTGCAAAATCGACCGGAGAAC
AACTTTTGAATGTTATAACTTCGACGTCTACCGTTCCTCCGACAACAGCGCCTACGATTAACAACGCTACAGA
AATATTTGAATCTTTTGATGGTCTCAATCCTGCGGCATTAATTGCAAATATCGATAAC
ACTACTGTTTGCGGTAGGAACCCAATTATGGGTACTATAGTGTCGGATTCTGCTCCATACCTGTATCCTTTCA
TCATAGAATACTCATTGATCGGAGCTGCTGTCATTTATGTTATGTGGAAGCATATTGGACGATACCCGAGGTA
AATATAGTTTTACACTTTTTTTCGACACTTTCTTCTTATCATACTTAACAATAACTAA
CGTTATGTTTTGTGGTGATATAAAAACTCAAACGACATAACTCCTATAAAGATATTTTTATAACAACTAAGTC
ATACGTTCTTAACAAGCGAGTGTTGCTACATATAAATTTATATTCCTCTTTCTTAGTTTGTAATTACTTTACG
AATATCTCGTTGTATGGATAAAATACGAAAGAGTTTGTACAGTAGAAAAGTAGAAAAG
AGAATGATACTCAATAGGACAGTGCATGTTAAAACGAGACCCTGTTAAAATACTGCCTTTACCTATGTATTAA
CATGTACCTTAAGATGCACGATTAATAGCACGAACAATAGAAATTAGGCCGTCGTGGCGCGTGGATCGTGTGA
CTAAAGACGATATTTTCATTTCTTTACTCCGGTA
>103775017_2 DT664870.3
ATAAAGAATTATAAATTTCCGAATATTGTTTATAGAGTATTGCATATAGGGCCAGGCCATTCAATAAAATGGA
GTCCAATATGTCTTTCTTCAGTTTACCTTTTGTTGACTCGGAGACTTCAGATCTAGTATTTAATAAGAAAGTT
GATGAATGTGATTATGACTTTCAAAGTGAATTACCTTCGCAAAGTGCTTTACTTGGTA
CGGATGACGAACTATCCCAATTACTGACCGCTGATGGACCTTTAGAAGAACCAGATTTAAATGGACCGGCCAC
TTTACATTGCGATATTTTTTTTTAAAATTTGATAATGCAAAGAAATATTGTGGACATTTGAGAGTACATTCTA
AAGATAATCTTTGGATTTGTGAGAAATGTCCAAATCAAAAATTTCTACAAAGCAACA
ATTAATGAAGCATAGTCTAACTCACAAGCCACTGGCAAGGGTATGGAAATGTCCTCAATGTTCCATGGCTTTT
GAGTCTCTGTGGCGTCTTCAGCAACATGTCTTTACTAAACATTTTGATTATATACCTCATAGGTGTGAACCAA
TGTGAAAAATCAT
>103775019_2 DT664730.3
GCACGAGGCCTCCCGTCAATTCGGCACGAGGGGCGTGCGGTCACGATGTGCTTGTGTGGTGTTCAAGTGAAAG
TTCGTTTACGTTAGAGAAATGCGTCTCGATATCGCCTTTCTGTTTGTGGTGACATGGAGTACAGTGCGGTGCT
ACGACTACGCGTCAGCATGCGCCCGCCCCTACCGCACCAAGCGGGCGAAGGCCATCCC

CGATGTGCCCTGCGATCTGAGCAAGCAGAGCTACTGCACGACACCGGGGAACAGCTACCCCTGGCACGCGATA
CGGAGGTTCATCAGGGAGAACCAGGGTCTCATGAGGATTTTTTACGGTGATGAGCGACATATAGCTGTACTTA
AAGCAGAATTGGAAAATTTTATAGACGACGACGATGATGATGACAAAAGCGAAGACTT
CGCTGAAGACCTAATTAAGACCAAAATGTTATATACAAAGAAAGGCCACGGACGCGCTATGAAGGATAAACCA
CATTTCAGGCCGATACAATCAAATAGCGACAAACAGAAAAAAATAGACAGTGAAATATTAAAAGTAAAACCGC
TTGATAACACAATCAATAGTACAAATATAAAGGAAAATGAGAATAAAACAGATGTCAG
TAATAACGAAACGTTAAACGACGATAAAGGTATATCGTACAAAACAAAATTAGAAAGTATAGCTAATATAGAA
ATAAACAACTTAGATCCAGATGTGATAACG
>103775036_2 DT664212.3
GCACGAGGGTTATATTATAGATAAATATGTTTTAATAATATTTAGTAATATTCGTCAGTTCCGAGTATACGTA
TTAAGAGAAGTAAAAGAAAAATCACTAAAGTTATCTATAATTCAGATTACATAGAAATACATAGAAATATCTA
GTATGTAATATTATATGTAATTTTAGATAACAAACTTCAATGGTTGCCACAGAGACCT
GTATACAATTACCATCACTGCGAGTTCAGGTTTTGGAGGAATCCCAATTTGAAAGCGATACCTTATTCGCTAT
GGTACCACATGTTCAGAGGACAGCAGACGAGGAGTCTTAATATTGGGTGCCTTAATTACAAACCTTCACATCA
AAACACCTACAATGAAAGCTGCATTTTACAACTAAACCAAAACATAGATATAAATTTT
CCTAAAACATATAAGCATATTTTAGATAAGAACGTTGTAACAGTACCTAGTCTTTTTGAACTAAGCAAAAGAA
AAATATACGAAATAATTAGCAATACAGCGAAAAGGCTAACCAATGTTCCACCAAGTATATATTCTGATATAAG
TTTCTTTGTTAAAAACAACAATAAAGAAATTGATTTTCAAAACAACAATACATGTGAA
AATGGAGATATTAAGAATAGAATATTAATATTAGTAAATAATATAAAAGATAGATATTGTGTTCCGGCGACCA
TAATTGAAGAATACTTTTATTATTTGCCCAAAGTAATGAAACATGATCTTTGGTACGGACCAATATCGAGATG
CGAAAATGTTGTTTGTAAAAAGCCATATTCGATTACGTTTATTTGGAATTTTGTATAG
AAAAAATATTCTCAT
>103775040_2 DT664737.3
CGGCACGAGGGCGACTTTTATTAAACAATTTTATAAAATTGTGTAAGATTAAAAGTCGTTAAAAATTAAGAGC
GCTCAGTATGGTTTTTTTCTAACTTTTATATTATAAGGCTTAAATAACTTATACAGTTATTAAATATTAAACT
ACTCACAAAATGTCTTATAACATATAAACAATCAGATAACTGAGTATTGATTTTAATA
CATTCGTCTAGGCTAGTGCAATAAATATTTTCCGTAAAAATGTCAGATCCGACTATACAAACATTTTGCTGCC
ATCACACCGGTAATAAAGAAGATTTAGCGATCAAAATAATGTTTGAATTTAATACAGAATCATATAATACTGT
TTGTCTTGTGTCTTCAACGATTGGGATCTTGGGTTCTCTTTACCAGATAATACCAAGA
AATACATCAGATTCTTCCTATAGAGCGCAAAATTTACCTACTACTCGAGGTCGTCATATCATCATCTGGCTAG
CTGTTGCAGATTTATTGGCCTCTCTAGGTGTTTTAGTTAGATCATCTTTATGGTTACAATATAAAAATATTAT
GCCTATGCCCAATGATGATGTCAGTGTTTTATTTTGTAGTATTATATCTGCTTGGACC
CAATATTTTTATACAGCTACATGGTTTTGGACATTATTTTACGCAATAGATACTTGGAACTCAATTAGAGGCA
ATATACACATCTAACACTCTATCATTCCTTAGCTTGGGGGCTACCTGCTGCTACTACAAGTATTGGCTTATCT
ATACTATACATTCCAAATGCTACATGTCACAATATAACATCAATCTCAACAGCC
>103775047_2 DT664227.3
GCACAGGACGGCAACATACTTTTAGCCCAATGGCTTCGACAGCCATTAAGAGACATTAACCTTATAAATGAAA
GGTTGGACATAGTCGAACTATTGGTGGAAAATTCACAACTAAGATTGCAATTACACGAGGAACATTTGAAAAG
GATACCGGACTTGCAAGCATTAGCGAGACGCCTCACACGAAAAAAGGCAAATTTACAA
GATTGTTATAGAATTTATCAGGCAATAAATAGACTACCACATTTATTACAATGCTTAGGGGAAGCGAACAGTC
CAACTGTACACTCATCTCTAACTGAACCTATATCTGAGTTAAACTTATGATTTAGACAAATATCAAGAAATGA
TAGAGGCCACTGTTGATATGGATGCTATTGATAGAGGAGATTTTCTGGTAAAACCATC
ATTCGATGAACAATTGCAAAGCTTAGCCGAGGAATTGGAGTCATTTCAAAATTTAGCAGAAAAGGAACTTAGT
AAAGCTGCTAGGGACCTCGGTATGGATGCTGGAAAATCCATCAAGTTAGAAAACAACAATCAAAACGGATTTT
GCCTAAGAGTAACCTTAAAAGAAGAGCAAAATCTCCGTGGAAATAAAAAGTATACAAT
AATAGATGCACTCAGAAGCGGAGTCAGATTTAAAACTAGTGGTTTAGATAAGATTACTGATGATTATAACCAA
ACTAAAAATGCTTATGAAAAAGAACAAGACAAAGTGGTGGCAGAAATTATTGGAATTGCTGCTGGGTACTC
>103775061_2 DV501134.2
GCACGAGGGTTGACTGTGGCATTCTGAGAGGCTACTTTGGACCACAGCTGCGTTTATCGGTGGTACAAAATGT
TCTCAGAAGGCCGAGAAGATGTGAACGACGAAGAGCGTACTGGACGTCCGAGCACTTCAGCAACAAAGGAAAA
AATTGATGAAGTGAAGAAAATGGTATTGGCCAATTGTCGAATCACCGTTAGAGAAGTT
GCTGAGAACCTAGAGATATCGATCGGCTCGTGTCATTCGATTTTTAGCAATGTTTTGGGCATGAGACGAGTCG
CCGCGAAATTCGTACCAGAATTGCTCAATTTCGACCAAAATCAGCATCGCATGAACATTGCTAATGAGCTGTT
GGACTCTGTGCGCGACGACCCAAATTTGTTCCAGAGGGTTATAACTGGTGACGACTCG

TGGGTTTATGGTTACGAATATGACGTGGACACCACAGCTCAATCATCTCGGCTGAATCTGCCGTACGAAGCAG
GACCGAACAAAGCGCGCCTAGTTCGGTCGTATGTGAAAGTTTTGCTTACAGTTTTCTTCGATTGCAGGGGCGT
GGTGCATCATGAGTTCTTGCCACACGGTAGAACG
>103775063_2 DT664133.3
GCACGAGGGAAAAGCATTACACTGAGCGTTTGGAACGCGCGCGTGCGGCGCGCTCGTCGCCCCGGCCGGCGTT
CGCCTTCGGTTCATCCACACCCCGGTTACTAGAGCCGGTGGATTCAGCTGGATTCTTTTGGGCTGCGCGCAGG
GCGGCATCAACAACAAATGTGATGTTCGCGTCTGCGCCGCTCACGAGACGCGCGTCCG
CACTGCATCTGGATACGTCCGACACTGACACTAAAGACGAACCCGACCGCTCCCCGCAGCTGTGGTCGTCGGT
GGCGCGCCGCCGCACGGACCTGGTGCCCACGCTGCCCGCGCGACCAGGTAGGGCTCACTCCATGACGCGCCTC
GACCGCCTGCGCCCCGCCGCCTCCACGCAGCACCTCTCCATACAAACTCGATCAGGCG
AGACGACCCCAGGTTCGAGGCCGTGCAGTGCAATGTCCAATACGAACGTGGCCAACGCGGGCGCGGTGGTGCG
GCGCGCGTCGTCGGCGCCGCGCAAGCCGCGCCCCGCCTCTATTGCCGGCTCGGAGGCCACGACCCCGTCGGTG
TCGCGCGACGCGAGCACGGTGGTGGCGCGCGCCGCGTCCACGCCGCGCCGCCCGCGCC
CGCTGTCGCTGCACGGCGCGCACGCCAAGTCCGCGCACTCTACGCCCGTGGGTCCGGGAGAAGGCAAGCCCCC
GCTACATAGAAAGCCCAAAGCATCCAAAGATCATGATAAGAACGCGGGCAAGTCGTTATCAGCGTCGCCGGGG
CGCGCGCTGTCC
>103775082_2 DT664788.3
GCACGAGGGTTTGGCGTAATTAGATTGTAAATCATATTAATTAAGTAGTGTTATTGATTCAATATAACTAACA
GTTACAGACATAAAAAGAATCATGGCCATTATATATATAATATTTTAAATTGATGACTGTAAACTGTTGGGGA
TTGTGGGTTCCCTATCCTGGAATATCAATTATTTTCATTTCCTTGCTTTGGTAACTCA
GTATGGAGTTATATTAACTCTAATACAGTAACATTGAAGTGATAAGCAGAGAAAGAAGTGTCTTGACACCAGT
TGTCTCTGTCTACCCCGTTATATACAGTATATAGCATTAAGCTGAAGAAAAAAAATAATGTGCCAATCATAAT
GACAAATGTATGATGATTATGAGAATTCAGTTCAACTATATAAGAAAAAAAGTATAAT
ATATATTTCTAACAATGTTGTAGCAATAACACTACTTAATTAATTAATGGCAATGATTTGCTTGTGACGTATA
AATTTTCGTATTTTAGCCTTGTTTTAAAAGAAAATGTTTAGTCTACTGATTTTGATTGAACACAGGCTGTGAA
CAACATTTATTGTGAACATCTACCTCAAATGCAAAAATATAATATTTTAAATATTCTT
ATGAACAGCTTTCTATATAATGATATAGGAATACAGAAATAAGTATCGGCCAAATTATTTTTTTTAATAAAAA
AACAAATTGCTCATTTATCAAAATGGCTGTTACAAATTCTGCAATGTGCTGTAGATTTTAGTCAAATAAATAA
AAAATAGATACTAATTTAGCCATAATCTGTGCACAATGATATCTACAATATGACAAAT
GTCAAAACAATAGTGTGATAATTATGTATGTAATCAATTTAAACAGGGTTC
>103775090_2 DT664383.3
GCACGAGGACTTACTTTTTTTATTGGAAAAAAAATTCATCCTATATTGAAAGGCAACGTGGTATTGTATATTT
TTAAGAAATGACGACTTTATAAAGTACCTAAATACTGTAGTTAATGGACAGGTTATATATAGTGTATAGGAGC
CGGATGCGACGGATGGCACTCATTGTAATGTCATACAAATACAAATCAAAATATATCT
CGGGAACAAGTACACGCCACCGCACTGACACATATGGAATGCCTTTATTACACTTAAAAAATAACATTATACA
CATCCTATAAGCATACAATTAAATATTATACCTTTTTTTTTATAATTTTGTTATGAAAAAATTTGTAAACTGA
TTTAGGCCGGAACTTAAACTATGATATATGAATCAATGAACGGGTTCGACCGACTCTG
AACTCTTATTAAAGAATTGAGAGTAACAAATAACGGTTTCGAAGCTAACACCTAATCTACAATATATAATTAT
AAATGATTATAAAAATAAATAAGGGCCGTCGCCCCCTCCCTCTACCGCGCGGACTCCTTGAGCAACTAGTTGT
AAACATTTTAGAAAATATGGTGTTTGACACTTGACTTGTAACAAATCGCTTACAATAA
TTACTTATAATTGAACAAGTTTGCCTCGATCATATCTGGTAAGGCGGTTGCCCGCGGATTTGCTCGCGTTTCG
CCTGCCTTGTTTGTAGCGACTCATGTTTCTTAGAGTTGAGTCGCTTCATTTATAATGTTAAGTAGTATAGAGG
CACTCAAACAATTTAATGCAAAT
>103775092_2 DT664070.3
GCACGAGGCTAGTCCTAAACACACTCAAATATTTACTTGGTTTTGGTTCTGTGAGCTACTATTTGGGCTATAG
TGCGTTGACAGATTTCTTCCCTACAATGACATTAAGACCAAACCCTCAATGTGAAGATTCCTATTGTCGCCAA
AGGCAAGCAGAGTACCGCGCCAAGCCGGTGGTGGAGCTTGCGACCGAGGTCAAAGAGG
ACGACGCCCCGATACACGCGGAAAATGAATGGGGAATTAGTTTGGTGGATGAAAATTCTCCCGAAGAAGAAAC
CCCTAATTTGAAACTTGTTGATGGCGTACAGGTGGCCTACACACTACCAGCAGATATAAGTACACCAGAGTCG
AGTACGGGCACCGCCATTGCTGCTTCGGAACTGTCTCTTGAAGATTTAATGCAACAAA
TGAAGAATATGTAAATGAAAAGAGGATTTGAACCGGCTGCCCTACCACTAGCTTGTTAGTGTTAATATATATA
GGTTTTGTTTTTTCAATTAATTTCTGTCTTGTAAAATATGGCCTTTTGAAAAAATGAAAGTAAACATGAATTC
GATATGTAAAAAATAAATTTGAGGCGATCGTGTTGCGGCGTGGGTTCGATTCCCACTC

GCGGAATAAATTGTCATGTGGGGAGCGGAGTGTCTTANAAGGAGGTTCCCTCTACCCTGCTATGTGCTGGATA
CAGTGTGAAGCTAANAACAAAATGAATTTGAAGATGAAAAGCTAAAACGTAAGTATGTAATCCATCAAATTAA
AAAAAATAGTAATATTTATATTATATATAGCAGCAGTCTGGATATGCACTGCATCCAT
GTAAAGAAAAACTGAAAAATGGGTAAATTCATGAA
>103775097_2 DT663623.3
ATTGACGACGCCTTCGAGGAGGAGACTGATGAAGCCATAAAAGTGTACGGCGCAACGGGCGATAGGTCCCCTT
TACAGAATAAATTTAAGAATGGAAGCGATTATATGGCGAGCAAAACCTACAAATGTTCAGAGGACACGACGTC
ACGTGACTCCGACTCCCTCATCCACGAATACGTAGAACCTACACAATCTCTCTACTGC
TGGAACCATCCGAAAGTTATTTTTTTTTTGAAAACAGTATGTGCCGCGGTCATACTGTTGCTGGTCGGCGTTG
GTCTCTTGGGTATGGGAGCCTTCGCTGTGGCGGAGCCCGACAATGGACTACAAGGTGCAGTATTCTTCGTCGC
CGGAATGATTTGCTTCGTTCCCGGCGCGTACCATGTAGTCTATATCTGGTTGGCCGCC
CGAGGCCAGAGAGGCTACGATTTCTACCACCTCCCACTTTTTACTTGATTTCCTTATGTAAATCCTAAGTTGT
TTAAGTAAAAACGAAATTGATACGCTCTCTATTATCTGTTGTCGCCTGAAACACCTTGGTAGGGTAGCTATCG
AAATTATTTTATCTGGTATCCACGAATCTATAGTGATATATTGAAT
>103775103_2 DT664541.3
GCATCGTAAATAAGTACCGTAATTTTAAATTGTTTATTAAGAAAATGTTAAGATGAGTGTTAAACTTGTTATA
CTGTATAGTGCATCGAACAGTGTGTGAAGATGAAGAACGTTATTGTGCGCCATTGTTTATTAACTCGTCCATC
CCGACGCCAACTATCTACGTCATCGGTCAGAAGAACCTCTAGATGAGGTTAAAGGCGC
AATGTGGCTGCACCAGCTACAGTTACCCATTATAAGAAATTC
>103775105_2 DT663558.3
GTCTTCCGCGCATATCATCCAGACGCTGCTCCACATCATCCAGTCGACCGCCTCCTACATCCTCATGCTGGTG
TTCATGACTTACAACGTGTGGCTCTGCATGGCGCTGGTGCTGGGCATGGCGCTGGGGTACTTCTTCTTCGGCT
GGAGGAAGTCCTCCGTGGTGGATGCTACAGAGCATTGTCAATAATAAGATTTTTTATT
TTAAACCGGATTAATCGAGTATGTTTAATATTATAATTTTCGGAAACAAGTCACGACTTTTCGAATAGTTTCC
GAGTAGTCCGTGGTCATTTTTTTAGATTAGTCCGGTTTAAGACTTTAATTTAAAACATTGTCAATAATGTTAG
ACATCATGGGCGTGGGAGTGAGGGGAGCCCACCAGCAAAAGGTTTACTGGCAAAAAAA
TTAAGTCAATTAAAAAATAAAACCTTCAGTCAAAAAGATCAGTTCAAAGCATGACTTTCAAAGCCGCTGTAAA
CCATCTCTAGGTACGCCCATGTTAGACATACTTGTGTAAGACGCCTTGGAGATATATTCGTTTTTTTTTTATT
TTAACTATTTGTCTATTTGGTTTA
>103775111_2 DT664971.3
GCACGAGGTTTAGCTTTACGTAAAGATAAACAATTTGTAAATGAGGTAACAAGTGGTCAAGAATGTGGCATTA
TCCTAGATAAAACTAATTTCTATGCTGAGCAAGGCGGTCAAATATTTGATGAAGGATACATGGTAAAAAGCGA
CGATGACAGTGTAGAATTTACTGTTAAAAATGTGCAAGTTAAAGGTGGTTATGTTCTT
CATATAGGTAAAATCGAAGGAACGCTGAAAATTGGTGATTCTCTTGTTTTGCATATTGATACAGAAAGACGTA
GACTAGTTATGAATAATCATACAGGAACTCATATTTTAAATAGTGTTTTAAGAAAAGTTCTCGGAAATGACTC
TGATCAGCGAGGATCACTTGTAATGCCCGATCGTCTTAGATTTGATTTCACTAATAAA
GGCCCTATGACAGTTAAACAGATCAAGGATGCTGAAGATGAAATTAAATCGATTATTAATAATAATAAACGTG
TCTACGCGAAACATACTAGTTTAAGTGAAGCTAAAAAAATTAGAGGCCTTCGTGCTATGTTTGATGAGCAATA
TCCTGATCCAGTTCGCGTAGTTTCTGTAGGTATACCAGTAGAAGAATTAGAGCAAAAT
CCAGATGGGTCATCTGGAATGGATACTTCAGTAGAGTTTTGTGGTGGTTCGCATTTACACCAAACTGGGCATA
TTGGAGAATACGTCATAGTTAGTGAAGAAGGTATCGCAAAAGGTATTCGAAGAATTGTAGCATTAACTGGTCC
TGAAGCATAAAAGGCATAAATAAAATGAGTATTTT
>103775121_2 DT665023.3
GATTATAAGGGCCACCAGGAACACCGGAATGGTGATATCGTCACAGGGTCATACAGCGTTGTAGACCCTGACG
GTACCAGAAGGATCGTGGACTACTCCGCTGACCCTCTTAACGGTTTCAACGCTGTTGTCCGACGTGAACTTTT
TGTATTAGCTGCTCCTGCTAAGGTCATCGCTCCGGCTCCCGTCGTAGCCCCTGTGGCT
CCAGTCGTTGCCAGGGCACCCATCTTTGCCCCAGCACCTGCACCCATCTTAGCGCCAAGATTACCATACTACG
TCTAGATTAATTACTACAATTTTGTTGTGTTGCTGTATATACCTATTAAAAATAAGGCTTTTTATAATTGTTA
TTTATGTTATTAAATGAATGATTTCAAATAGAAAAAAAAAAAAAAAAAA
>103775126_2 DT663714.3
TTTTTTAAGTTGTGTAACTTTGTGAAGAAATGCGTTTATATTTTTATATGAATAAAAAAATATAGAATTGTTG
ATGTATTTATTCATGTCTATATTATTTTAGAAATCATTAAAAAGAAAATACTATAATGAAAAATGTAAAACCT
GTTTATTGGAACAACTTTCGTCTCTTAACGCTGATTAATGTCTTTTTATTTTTGTCAT

TGGGGTTTTTTTTAATTCTCATTTTTCTGTAGTAGTGTATTCCTTTAAATATGTTTTTAAAATAATATTTCAA
ATAATTATACATCTCTTTTTTTTTCTCTATTTGTAAGATGCTTTATTTTGACTTCAAACCATTTTTATAATAT
AGCTGGACTATTTATAAAAAGGTTTTATAAAGTAAAATGTACGTGTATTTCTTTACCA
AGTAGCAAAAAATAAATGTCAAATAAAAAAAGAAATGTACAGCGAAGTGGACGCTACAGTGAAGTGAAGTGCA
TTGTGTATGTAATATAATTTATATAATAATTTAA
>103775138_2 DT663614.3
GCACGAGGGTAAAACGTATGTCAATGAAGTTAAAGAAAAAGACGCTGCTAAAGAAATTGAACCGCGCCTCCTT
GGGTTGACTGGATACGGCGGCAATGGCGGTAATAACAACAAACAAGTGCAGCCCACGTTTGGTGTCTCTTTTG
GGTTGCCCCAGCCTTCCTCTGGTTATCCTATAAATCCCTATAATTCCAATCCTTTACA
CAATCCTTACGGCCCTGCTTTAAACGGAGGTGGTCTTAACTTAGGTCTTGTTTCTGTAAATCCATTACTAGCC
GTCCAAGTCACCAAAAACGACTATGGTGAAAAAATTGTCAAGCCATTTGTTAATTTACATGTGACCCCTAACG
AACATGTTGTCAATAAATTAGGTCATCTATTTCATGAAAAAAAACAATACCTTTTAAA
CAAACATGAGCACCACCATCACTACAATCCATATTCATATAACAATTATGCGCATCAACCATATCCTTCCCAT
CCATATCGTCCCATCGTTCCTCCTGTGAGTCATCCTCCTCCACCTTATCCCGGCATTGGTCATTTACCTATTA
GTCATGCTCCTGTAGATTATCATGAGGGATACGGAATACATAGGCCACCCACATATTC
ACCACAATACCCGTATAACCATCATAATCAATACAATGCACTAGGAGCAACTATAGGTAACGAAGACTATTAT
GACGAAGATAACAATAATTACGATGATACTCTTNCATACAATTCATACAATAATTTTGGTTTCGAAAGATCTC
TAAATAATAGTGTAAATAATAAGGGCCTCACCTATGCCACTCGATACGCGTATTCACG
ATCCCTTAACGTTCCTTCTAATTCTGGGGTAAACGGGGAAATCA
>103775140_2 DT663809.3
GCACGAGGCTTTTGTCCTTAAAAGATAAAATACGCTTATAAATTATCTAACTCTCCAAATAGTTGATTCAATG
GGCAATTCTGTTCTGAAACAACATTATGAAACTGCATCTAAGACTGGTGTTTTATCAATATCGAATCACAAGT
TAAAGGAAATACCTGAAGATGTCTTTGGATTATCAGAACATTTACGAAATTTGGACTT
ATCTAAAAATAAAATATCTCAAATACCAGAAAGTATAAGTTCATTGAAATTATTAAAACAACTCAATTTAGGA
ACAAATAATATACATTCTTTACCAGGATCGTTAACCAATCTTAAGAAATTAGAGCTTCTTAATGTATCTTTCA
ACTCTTTATCATCATTGACAGACTCATTTTCGAATTTAAGTAGCTTAAAACAAATTTA
CTTAAATGGAAATAAATTTAAAACATTCCCTAAACAGTTACTTGGTCTTCAAAATCTGGAAGTCTTAGATCTC
TCTAATAACAAAATGATAGAGATACCAAATGGCATGTCTAACCTTTATGTGACAGAATTAAATGTTAGTCAAA
ACGAGATTTCCATTTTAAGTGAGGATTTAAATCAAGCCCCAAGATTAAAAATATTAAG
ATTAGAAGAAAATTGCTTAAGTTTAGATGCTATATTACCTAGTTTATTAAGAGATTCTAAAATACATACTATC
AATGTAGATGGTAATCTCTTTGAACCAAAACAATTAGCATCAGTAGAAGGATATAATGAATATAC
>103775144_2 DV501145.2
CGTTAGACCCGCCTTCTTTTTCCAGACCAGAATTGGCTGTAATAAGACATGTTTCCCTATCATATAATGTGGA
CTTTGAAGATAAAATACTGTATGGATCACCAATTTTAAATATTGAACTAATTGATTATATCTAAAATGTGATA
TTAGATACGCAATGACTTAACAATAGAATCAATTGAATTCGGAGATGGTACGGCATTG
AAATATAAATTGGATGAACCGATTCCGAATTACTGATCAAGATTGACTATATTGTTGCCTCAATCTGCACCAC
CATAACAAAAGCTTGACATTAAGATTAATTATAAATCTTCACCATCTGCGACGGCACTGCAAAGGTTGGAACC
AAACCAAACCTCAAGCAAAAAGCATCCGTATATGTTCAGCCAATGTCAGCCAATCCAC
GCTCGCTCTATCTTACCATGTCAAGACACACCCTTTGTAAAGTTCACATATGAAGCTGAGATAATTGCCCCAG
AGGAGCTTACAGTACTGATGAGTGCTATCCGCGGGGAAAGAAAGGTGAATAAAACTGTCTTCAAACAGCCTGT
ACC
>103775148_2 DT664892.3
GCACGAGGTAATTAACTTGCACAGCTTAGTCTTTTTTTTTCTATTACAAAAATATATTATAATTGGCACGCCTA
TTGACATCAAAAAAATAAAACGGTAAATCGAAGTATTTTCTTGTTAATGTTTTCGATATGATTAATATGAGCG
AAGAACGAAGATATTTTAATGATATAAAAGTGATAATTTCTTTGATTGTGTTCTTAAA
ATAAACTTTAATAATTAATTACGATTAGCTATTAAATTACTTTATTTTTAATCTAAGTTAAGTATCATGTACA
TTATGCTATAAACACCAATGTGTAATTAATTTTATATAATTATTTTATATGTGTACATAAGTGAAGTACCTAT
GTTTTATATTTTGATTTACACAGTTTTAGTGCAACGGCTGTAAGCACAACGAGCGTGG
GTTCGATTTCAAATTGTAGAATTTAATTATTCTCATTTTATGCTCAATTTTCGAGACATTACGTGTTCAATGT
TAATTATCGTCACTGTCTCAAATCCCGCTATTTGCTTGTACTGCTGCGTGAAGCAATTTTTTTTTGTTTCTTA
ACGCTGCTATATTATGGCGTAATTAAAATTAAGTGAAAATATAAATATATTCAATGGA
AACATTACCTTATATCTTGAAGTAAAATTCGAATCCACAATCCAATTACCGAGTGCTAGAATATTTAAAATTT
AATATAATTAAAAATGTATTACAGCCTTATATTTATAAAATAAAAAAAGTATTGGACGAATTAAATAT
>103775155_2 DT664702.3

TTTAAGGTTATGTTGTCTTATCAGACATTTTTGACAAAACGATAATAAATGTGACCGGTTATAGTTCTGTTTG
TGATTGAAGAAAGTGCTTGACGCAATTTAGTTTGTATGAATTTATATAGTGGAAAGTTCTATATCTATTGTTT
TCAGTTAATAAATACCTAGATAACAAATTAACATGGCTGTAAATTTGACGAAATTCTC
TAAATTTTGGAGTGGACTTAGTAATAACTGTAACAATATTAGAACGTTATCTACATCGGGCTTGAACAACGCA
AAAGTGAGTTTTAACTGGGAGGATCCTTTGAATTTAGATCGACAATTACGTGACGATGAAAAGGCTGTTCGGG
ACTCGTTGAAAGCTTATTGTCGAGAGAAACTAATGCCTAGAGTGGTTGAGGCAAATAG
AAACGAAGTGTTCGATCGTGCGATTTACAAAGAAATGGGCGAATTAGGGATCCTTGGCTGTACCATTAAAGGC
TATGATTGTGCTGGAGTGTCATCTATAACTTATGGACTTATTACTAGAGAATTGGATGGCGTAGATTCATCGT
ACAGATCAGCTATGAGTGTACAAAGTAGCTTGGCCATGGGTGCTATTTATATGTACGG
GTCGGAAGAGCAAAAGCAAAAGTATTTACCGCGTATGGCTAAAGGTGAATTAGTTGGGTGTTTCGGCCTAACA
GAACCTAACCACGGAAGCGACGCCGGTGCATTATTTACGAAAGCGAAATA
>103775157_2 DT664495.3
TCGGTTTTCATATTATTATTTGTTATTATTTCTATCTTATTTATTTTGTAATCTAATTTTAATGATTATGTTT
AAGCATTAAAGTAGATTTACTGTTATTTATGCATTTGAAAAATGTTTATTTTACCATTTTTACTATTTTCTCT
ATCCTTGACGTATGGTGAAAAATCAGTGCAATATGCTGCTGTTATATATAGACATGGA
GACCGGACTCCAGTGGATCCATACCCTACAGATCCTTGGCGCAATGAATCTCTCTGGCCAGTAAAATTTGGAG
AGCTAACAAATATTGGCAAAAGACAACACTATGCTTTAGGAAAATGGCTCAGAAAACGTTACTTGCATTTAAT
TTCAGAAGAATTTGACCCTACTGAGATATACATAAGGTCAACAGATGTTGATCGTACA
TTGATGTCAGCTATGGCTAACTTGGCAGGCATGTATCCTCCAAATGGTACTTCTGTTTGGAACACAAATTTGA
TGTGGCAACCTATTCCTGTCCATACTAAACCAGAAAAAGATGATGAAGTATTAGCTATGAAAAAAAAAATGTAT
TCCTTATATAAAAGAAAAAAAATAAGTACATGCATTCTGCTTCTTATAAAGAGAGACTG
AGTAAATATCAAGGCCTCATGGATTATTTAACTGCTTATACTGGAAAGAAAATTAAAGATTACAATGATATAA
ATGAAATATATAATATATTGTTTATTGGAAGTTTATACAACTTCACTTTACCAAACTGGACCCAATCTGTTTA
TCCCAACAAGTTAAAAGAACCAG
>103775170_2 DT664883.3
GATCGTTTGGGGTGCCTACTATAGATTACAACGATCCTGAACAATTAGGTTTTGGATATGTCCAGACAATTAC
AAATAAAGGTCATCGGCTCAGTGCTGCTAAAGCTTTCTTACATCCACACAAACGTCGTAAGAATCTACATATT
ATCACAGAAGCTAGGGCTACCAAAGTTGTTATAGAACCCCAATCTAAAAGGGCATATG
CAGTAGAATACATCAAAAACAACATCAAACGAACTGTACGTTGCAGAAGAGAAATTATTCTATCTGCTGGACC
TGTAGCATCACCTCAATTATTTTTGTTATCTGGCATAGGTCCCAAAGAGAATTTGCAAACACTCGGAATACCT
GTTTTTAATGATTTAAAAGTTGGAAGGAGTCTTTATGATCATATTAGTTTCCCTGGAG
TTATTTTCAAGTTGAACTCAACAAACGCAAGTCTTATGGAACCTAAAGTTGCAACACTTTCAAATTTTATGCA
GTGGTTGCAATTCGGTGATGGCCTTTTAACGACACCTGGTGGAGTTGAAGCTATAGGATATTTAAAAACAGCT
TATTCTGATTCTAATCTACCAGATATTGAATTGGCCAGTATCGGTGGATCCATCACAC
TTGACGCTGG
>103775174_2 DV501189.2
ATTTCTTATTAAGTCCAAAAGAACATGCTTTTAAAAAATAACGATTTTGAGCAAATTGTAGGTGATGGCAACA
CAGTACTGAAATAATAATATGCATATGATAGAACATTGTAAAAAACATTATTTATTATATAAAAGAGTGTTGC
CAGAATATACAAAAATCAAGAACGATTGTTTCCAATTTTCTTAATTCACATTAGTCAA
AAGCACCCAAAATTAAAATAATAAAATTGTCTATCAATTTATATATTTTTTTTTTAATATTTTTTTTTGTCAT
AAAAACATTCTAAGAAAAGAATAACAGTATGTTTATCGACTTAAAATTACTATGAATATCAATTATTAAAAAA
GAAATCAACTTTAATTATGTCTTTTTGGGTAGAATCTCATATAATCATAGTCAAAACG
TTTAAAAAAAAATACGCGCCCATAATAATTATAAGACGTTGTATATATTAAAATTCAATATTTATTGTATTTC
ATCAAAAAATTTCAAAATTATAATAAACTAT
>103775197_2 DV501254.2
GGCAAATATCATGAAGTATATAAAAAATAAATTTGGTGCGGTGCGCGACCGAAGCCGTGCGAAGGACGGAATG
TGTCGGCAGGCAGATTTCGGGAACCACATGTCATTTCCTTTGGAGCCCTATGCGGGGTAGGGGCGATCGGGAC
GATTTCTATACCCCCCGAACACTAAACGAAGGGGG
>103775202_2 DT664384.3
GCACGAGGTGAAGCATTTCGGCTCGGCTAAATCCATCAGCTCGGCGCAGTTCTTCGGCGAGTCCGGCGGCGGC
GGCGGCGACCGGTGGGGCGCCGGCGGCGACCTCTCGCGCTTCGAGGGCAGCAGCAGCATATCGTCGGCGGAGC
TGTTCGGCGGCGGGGCGCGCGGGGGCGGCGGGGCGCGCGGGGGCGGCGCGGGCTTCGC

GGCGGCGCCGGACCTGGACGAGGTGCGCGAGTCGGTGCGCGCGGGCGTGACGCGCGTGGCGGGCCGCCTGTCC
TCGCTGGCCAACGGGGTCGTGTCCTCCATACAGGAGCGTTTTTTGTACTGAACGGACGATACCCGTTTGAATG
TACAACACAGAAAGATACAAACACTTTCCGTAATATACTTAATGACTTATTTCCTTTA
AACTGTTTTTCAGGGAGAGAATTAAAAATAGTTAAATTTGTAAGAAAATTTTAATATTACTTTTGTATCATTT
TATATATTATATGGAGGTTACGTTACTTGCGTACTGTCAATTGATATATAAATTTATTAAAAAGCATTCAATT
TTAATTACATGTTTGGATAAGTGGAAATTTGCATATTTACATATATTTTTCTATTTCT
TTATTCTTGCGTATTTTACAAACTTGCTATGACTGTGTTATATTTGTATTTTTAGCCATTAAACCAGGATCAC
CGATTTGTAATATTGTAATATAACAGTAGTTATTGAAGTAGTGTTAGTGTCACTGCACTGCCG
>103775216_2 DT663538.3
GCACGAGGATTCTTATAAGGGTCAAAGTAGTAGTACCTTAATTGATGAAAGTCTTTCACGTTCCTTAATTAGA
ATTATTATTTTGATTGATCAAAATATAATAATATTAAAAAATGTTTTAAATTAAGTCCATAAAAAATAGAAGC
TTTTTATCTAACATTTTTTTTGCGTTCGTTTATTTTACTGTACTCTGTGTTGGGTGAC
TAATGTAATAGTTATTTTTTTCTTTTAATATATTTGCTCAAAATGTAGTCACTTTTGAACTATAACCTGGTCA
TGATTAGATGGCGTTACGAGGAACCTCTTTTTTTTAAGACTCTACTCATACTTCACCATCTCGTATTCGGTCT
AGATACCCCGATATCATGATTTCATGACGTTTTAAGAGTTTAGCATAACTGATGTTTC
TAAGTTGTGATGCTACGTCATTTGGTGGTTGCAGAGCCGATTTTTGTTTTTAGAAGAGTTTTTAATTTCTTTC
TTTACATTTTTCTATTTAAAAAAAATAATGAAATTACTAAATATATATATTAATCTTATTGACATTTTAACCT
GATCTTATCGTTAAACCTAAAATA
>103775238_2 DT663629.3
GCACGAGGGTTGACTGGAGACGGACCACAAGAGTAACTTAGAAATTATATCGCGTGAAGCAACAAAAAACGAC
AAGAAATATACGTAAATCGTTTTCAGCTTTAAATAGTTAAAGTCGTGACTGTTAATTATACTTTTTATAACAC
TAGATATTTCGAGCCATGGCATCTTACTCTACTAAGGTTTCTTTGAATGAAGTAGTCA
TTGTATCAGCAGTAAGAACACCAATTGGATCTTTTAGAGGAAGTCTAGCAAGCTTATCAGCAACAGAGTTAGG
CGGCGTCGCAGTAAAAGCAGCAGTTGAAAGAGCCGGCATTCCAAAGGAAGAAATTAAAGAGGTATATATGGGA
AATGTATGTTCAGGATTTCTTGGACAAGCTCCTGCAAGGCAATCTGTAATTTTTGCTG
GTCTCCCAAAGAGCACAATATGCACTACAATCAACAAAGTCTGTGCATCAGGAATGAAATCTGTGATTTTAGC
TGCACAAGGACTTCAAACTGGCACACATGATGTGATTCTTGCATGTGGAATGGAATCTATGTCAAATGTACCT
TTCTATTTAAAGAGAGGTGAGACATCCTATGGTGGTATGCAATTAGTAGATGGTAT
>103775241_2 DT663703.3
GCACGAGGGCGCGCCCCTAAAAGGCATATATCCTGGTATAAAGACGGGAAGCCAGTAGCTGATATAGCGCTAC
GGAGATTCCGGGTGCAGAATTATAGGAGGCGCTCGGTACTAGTAATAAGGCATGCTCGCCGCGACGACTCCGC
GTCGTACGAGTGCAGGGCGCAGGGCGCTGTGGGGCCGCCGGCTATCGCTACGGCGAAT
GTTAGCGTTCTACCGCAGATCACTGCTACGCCGGATACACCACCCGGTGCGCCGTGTCCCATGCCCGACCCTG
CCTCCTACTGTCTAAACGGAGGCACTTGTTTATTCTTTGAAGTATTTTTTGAACAGGCTTGCAAATGCCCAGA
AGGTTTCAACGGGCAACGCTGTGAGAACAAGGACGTGTCGAACAGGAGTAGTATGTAT
CATTCCTACACATGCAAACTGGGCCTCTCCACGTCCTACTACTGTTAGCTGTTACCCACCAAAATTGTTGCTG
CATCTTTTTATAGAATTCCAATTTTATTTGTCTCAAATATGTAAAAAAATAGCAATTTTGACATAAATTATGG
TATTATTACTAAAAATATGAAAAAAACCTATTTTGTTATAAAAATAATTTATTGAAGA
ATTTTTTTTTGTTATATTAAGTAAAATTGGAATTTTGAAAAAAACTGTACGCAAATAATATAATTGGACACTC
GTATCATAGACATATAGCATGTGATT
>103775245_2 DT663756.3
GCACGAGGCGCTTACCTTTTAGCTTAAATAGAACATATCAAAAAAAAAAACTAAGACATTTTACATAAATCAG
CATCAAGAGTTGTTTCTGGCCGCTTGAAACAATAACTGTATACTTCAAACCGTTACAAGGGATCAGGTTAAAG
CAATTTTTTGACAAAAATTTATAATAATCAAGCGGGCACTCTAAATAAATTATATGAG
GTATCCGTTTTGTAATTATTTGTTTGGACTCGAAGTCAATGATGTTTTAACCTGATTTACTAATGTTTATTTT
TTTTTATAAGCGGTAAAGTCACAGTATTATTAGAAGAAAATATCTTGAATCATCAATGGGTTGTACAGTGACA
AGGAAAGATATATTTTATACTTCGTTTTTATTTGCAAAAGATATTTTTCAAAATAATA
CTATTTGCACCCCTGTCGACCCTTTGTGCTACAAGAGAGATGGTAAAAAAAAATGAAAGTTGTATAACTGTGA
CTATTCAACAAAGTGGAGGAATCAATTCATCATGTTACGGTTTGCATAATGAACATTGCTCTGCTTATTGGGA
AAAGAAAACGAGAGCATTTGTGTTCAATTAATATATCGTGCTCTCGGGGGGGGCTTCA
AAATTCGGGATGTAACGTGAAGAGTCACTAATTTGGTCACATGCTTTAATTAAACACTTTTTGAAGACTAATG
ATTGCCTGCCGATACTGAGAATATTTATTTAGTTTATTTTTTAGTTGGTGCACACTTTAATTTTAAAAATTAA
ATTGAAAAACCTTGAAAGCTTCCTTCAGTTTACT
>103775247_2 DT664664.3

TCGGCAGCAGGAGCAGATGGCAAAAATCAAGGAATATGAGGCAGCGATTGAACAGTCCAAAGTTGATCAGAAG
CGTGTTGATTATGAAGAGAGGCGTAAAACTTTACAGGAGGAAACTAAACAACATCAAATAAGGGCGCAGTATC
AAGACCAACTGGCGAAGACTCGCTACGAAGAACAATTGCTCCAACATCAGAAGAACCA
GGAGGAAGTGTTGAGAAAACAAGAAGAATTTGTAGCAAAACAAGAAGCGATGCGTCGCGCGACAGTTGAACAC
GAAATGGAGTTGCGCGAGAAGAACAAATTGAAGGCAATCGAGGCTGAGGCCAGAGCTCGGGCCAAAGCCGACA
GAGAGAATAGAGATATAACACTAGAGCAGATTAGGTTGAAGGCGGCGGAGAACAGAAC
CACTATATTGGAAAGTATACAGACGGCGGGCAGCGTGATAGGGACAGGCCTAAACGCGCTAGTGACAGACTGG
GACAAGACCCTGGCGGCGGCCGGCGGGCTGTCCCTCTTGGCCCTCGGCGTGTACACCGCCAGGGGTGCCACGT
CTGTCACCGCCAGATTCATTGAAGCCAGGATTGGTAAACCAACACTAGTAAACGAAAC
ATCAAGATTCTCGCT
>103775253_2 DT664804.3
GCACGAGGAAAATCCATAGTTATCCTTTATACTTATATAATATTTTCACTTTTTCTTAATCTTTTTGCTTTTA
GTGGCCATATATAAATTACGTCACACTTCAAGGGGTCCTTCTTCCACCTACCTTGCATTACGCGAGATACGTG
ATGAGCGTGACACAGCGTGTTAAGAAGTTTTTGTGGGGTGGAAAAACTTGATATTGTA
TTGCGAGAGGTGGGAAAGAGGTCTAAATTTCGTCGCATCATCACATTTCAAAATATAATATACAAACGTTATT
TTGTCATAACTA
>103775260_2 DT663684.3
GCACGAGGGTCAAGTCTTAAATAGAATAAATCTAAATCATTTTTCTCATAGAATGTTTCTTATAGTTTTTCGT
TTAAACTTGTGTTTTTAATATATTAATGTTTAAAATATTAGTGGATACAAAATGAGTAGTGACGATTCAGATG
ATGATAATATTATCCGTTACGGAACTCCCCTGGAACCTTTTGAAGAAGATGAGGTTCC
GTCAAAAAGAAAATTTCAGCAACAACAAGATCAATATGCTGTTGATGCACATGGAAGGCGAAGATTTCATGGT
GCTTTTACGGGGGGTTTCTCAGCAGGTTTCGGTAATACAGTTGGAATATCAGAAGGATGGACACCCAGTACCT
TTAAGAGCTCTCGTGCAGATAAAGCACAAATATCTAATCAGAAGCCAGAAGATTTTAT
GGATGATGAAGACCGTTCAGAATTTGGTATTGCCCCCCGTCAATTGCAAACTAAAACTGATTTCTCTGGACAA
AAGCGCCCACATCAGAAAAGCTTTCATGATGGACCTATACCTGGTGATCCAGTATTAGGACAATTAATACGAG
CGGTCCACGAAACAGCCGCGGTGAAGATGCTGCGTAAAATGGGCTGGAGACCAGGACA
GGGAGTCGGGGAGAGAGTGTCACAACGCCAGAAGAAGAAAGCCAAAGATCAACATAAAGTGTATGGATGTTAT
ATACCAGATGAGATGCGAAAGGAAGCACACAATCAGACAGCTCAAAATTCAGAATTTGAATATGACACATTAT
TCGCGCCAAA
>103775267_2 DT664738.3
CAATGGGCATGCAAGTAGCTCAACCAAAATTGACCAACTATAGTGGGAAAATGATGGATCTACAGAGAATATT
ATTGATGGCAAACGATAAGAACCTTAATATATTATTTGTCATTGTTCCTGCACGACTGAAGGATGCTTATGAC
AGAGTAAAATAATGTTTTATTATTTTTTTATGTTATTTGTATTACATTCTAAGCCTGT
GTGAGAGTTTTATATGTAAATAACATTAGACAGTACTTATTGCAAAGGTTTATTATATAAGATGTATTAAGAA
TTACAGTGAAATTAGATTTTTAATGACTTAATTAATCACATATAATTTGCTATGATGATAGTATAATGATTTT
TTGTATGTACAATTTTTGACATAAATAGACTGGACTAGTTTAATATAAGCCAATTACA
AAAATCGAAGAACTTATTGTTTTTTTATATGGTTTCAATTGGTTAAACTAATATAAACATTATTCTTTTATAT
CGAAGAGGCCATCGTGGTGCAGAAGCACAATCGTGCGTGCTCTAAAAGCGGCTGGGGATTCGATTCCTACTCG
GGGGAATGAAATATTTAATATTTACATACATTTCCTCGCTCTGGTAACGAGGAAAAGA
ACGTCAAGTCCTGTCACTCAACAACGCAATAGTAATGTGGGGAAAAGAAGTGTTTTAATGGGAAGGGAGTGTC
TTAACACCAGGTTCCCTCTGC
>103775285_2 DT664415.3
GCACGAGGTAACCCCTTATAGGTCTACACTCTTGTTTCAGGGAGTAAAAATACCATTTACGAAATAATCCAAG
TATAAAGATGTATAAAATCTTTAGTAATTCTTACCTACCGGTAAAATGAGCTTTTCAGTCTTAAATGGAAGCT
CCTTTCGCTCTAATATCGGATATCAGATAATATATTTCATATTGGTGCTTACGAATTT
CTACTATATTTTTCTGGTTAAAATTTATATTTATCTAAATAATATGAGTTCTAGTATTCACAGTATTAGTAGA
AGGTGTATCAGTAACACTGGTGTTCAATAGTTTGATTGGGTTGTGCAGAGACATGCTAGCAACAACAACATAG
TGGTGGGCGAGAGCGGGTCGCTGGCGCGGAGCTACGTGCGGCCGTCGCGCGAGTCGCG
CGACTACAACGCCAACGTGGCGCGCACACACACGCACGCGCGCCTCGTGCACGCACACACACACGCGCACGCA
CACATGCGCCACGACAACACTCTCGACATTATACTTAAGCCTCTCATAGAAAGCACTCAGTAAGTAATACAGG
AGGTGAGCATATGTGTCACACCGCCTAGAGGAATGAAATAGCTGCACGTTGGGTTGTC
CCCTCTCCTCTCGGTTGCTCGTGAAGGTATTTATTTGTGTCTTGTATTT
>103775295_2 DT664035.3

GCACGAGGAGCAGCCCTTGACGGTGGACCATCAGCATCATTGGCCATCCTGGGGCGGTTCGCTGTGAACATTT
CATACAACATCGGTCTGCAGTATGCGGCGGAGTTGCTACCGACTGTAGTTCGAGCGCAAGGAGTCGCTTTAAT
ACATATAATGGGATACGTTGCGTCAATCGTAGCACCGTTTGTGGTTTACTTGGCCAAC
ATATCACAAGACCTGCCATTATTAATCTTGGGAGCGTTAGGCATAGTGGGAGGCGTGTTATGCCTCTTACTAC
CAGAGACGATGGACACAGAAATGCCTCAGACCCTCGCTGATGGCGAGAACTTCGGCAAGGACCAAAGATTTTG
GGATATCCCTTGCTTCCCCAGGAAAAAGAAAGAAACCAGCACAGAAATACCTACCCAG
CCACAAGAGGATCATTTCATCAGATCGCTCCCAGCAACTGGGTCCAGAGCGTCCGTACGGGCCTCAATAAGAC
TATCGACGAGATCCACAAAGAGAAATCAAGATGAAAACAAAGCGTAAACTAAATATATTAATTATTAATAGTA
ATTTTATTTATTATTTTGTAATTTTTATTAAAAGAGATCTCTGTATAGTAATTTATGA
TTGGTTGTAAATTTCGTCAAAGAAAATGATTAAAATTTGAAAATTAAGTTATTTTAAATTATCGAGCAAAATA
GCGTTGATTTCGTCATCTGATGACTCTTGAGCGGAAATGGAAGGGATG
>103775303_2 DV501183.2
GGGAAAAGGATGTTGGAGAAATTGAGGAAGTCAATGCAACTAGTAGTTTTCATCAGGATCCATCTGAACAGAG
AACGTCTTGTGAAACCCCTGCCATGTCGGAAGATAACTTCGACGGGGATAGTGTTCCGTCGCCGTCAACGCAA
GATGTCAACACAAGCACGGAAGCGATTTTGGATATGATAGATGAGATCGTCGACGGGC
CTGGAGCGCCGAAGCGATTACCTTTAGCTTTAGAAAATG
>103775305_2 DT664620.3
ACGAGGGTAAGATTAAGGAGGTCGGGAAGCAATATATGGGCAACTTTTTATCCTGGTATTTCCGCGGAGTCGG
GATTCACACGAGGAAAGCCACGGTCACCGCAAACGTGTATCTATCGCCGAGATACATTTAAAATAACCCAACA
TTTTTCTATTAAATAAGACAATGAGGTCTCATGACGTAAGGGCACAAACGTGTGACCG
TAAATGTTACGGTTGTGGGTTCGATTCTAATTGGTGTAATATTTAATATTTTCATTTCAACAAAGGTAACAAA
ACAGCGCGGCTTGGATTTCCGTCACTAGACACACTTTTTTTTGAGAATTCGGCGGAAAATGCAACGGAGTGTC
TCAACACTAGGTTTCCATGTTCTTCCGGCTTTCCTACCCAGCTATATGCGGAACATAT
AGCAGAGTAGGTAATGGTACGCAGCGTGAAGCTGCAAATTTATAATTCTTTCCTTTCCTCTCTTGCACCATCA
CTTTCACTCATTTTAGGTATCTTTAATCGAACTTATGACCTAACAAATATATCTCCACAGCACCACAAATCGA
CCTATACAACCAGACATCAGTGAAGCTTTTCTCCCCATCGTCTAAAACGGGCAGCGTC
ACTCCGCGCGACGTGTTCCTAGTGTTCCCGGGGGCCACGCTCGACTTTGACATGCTATCAGTCCTACCGCCGG
TATCAATGGACTCGTCTTATGTTGGGAT
>103775307_2 DT664478.3
GCACGAGGATACCCTCCTATGGGACGGTAAGAATCAACTTATAAGTACAGCAACCAGAGAGAATGTTTACTCC
ACTTTTTACTAGGAATATGTAATGTTTATAAACACACTAAATTAGTCATTACTTCAATAGTGTTTTAAAATAT
TCTTTCTAAAAACTTTCTATAGAACTTTGAAAATTTAATTTAATAGATGTATTTTTAT
TCTAAAATTTAGATTTAAAACTTATTATAAAAGCTTCTTAATTAGTGATTCTTTGATAAAACAATGTCTGCTT
CACCATTATTATAGCAATAATTCACGTTTATGTTTTGCATGAAGTTACATATTCCTAGATTTAGCTACAATAC
CATAATATGATGTATGTAAACTAGAAGATGTTACACATTATTAGCATTTCCGGCACCT
GTACTGAGTTTGTATGTGAACAATAAGTTGATATATAACAGATAAATAGTAAATATTATAGAATATGTTAACA
CTAGACACAGCTCTGTGCTAGGAACTTATTAATTAATTAGATAATATAATGGTGGCGATTGTTTGAATGGGGT
GGTATATGGAATATGGAATAGTCTCTGGTTGCTCGGGTCGTTCGGTAGTGGCGACGCT
GACGTGGACTTGAGGCGGAGTAGATAACAGGCGTGTTGTTGTGTAGCGGCGGCGGAGGCGCGGCGCGCGGCGC
TCGGGACGTCCCGCGGCGCTACTAGCGGCGAGCGGCGTTAAACGCGGGCGCTATTGCGGCGT
>103775309_2 DT664698.3
CAACCAGATACTCACCGAGATGGACGGCATGGGCGCCAAGAAGAACGTCTTCATTATTGGTGCGACTAACAGA
CCTGACATCATCGACCCGGCCATCCTCCGACCTGGTCGTCTCGACCAGCTGATCTACATACCACTGCCTGATG
AGAAATCCCGTGAGGCCATCCTCCGGGCTAATCTACGCAAATCACCCATCGCTAAGGA
TGTTGATCTGTCTTATATTGCTAAGGTGACCCAAGGGTTCAGCGGTGCCGACCTGACGGAGATCTGCCAGCGC
GCCTGCAAGCTGGCCTTTTTGCAGGCCATCGAGGCGGAGATACACCGCGAGCGCGCGCGCCAGCAACAGGCCG
CCGCCGCCGTTATGGATATGGACGAGGAGGACCCGGTGCCGGAGATCTCGCGCGCGCA
CTTCGAGGAGGCCATGAAGTTCGCGCGGCGCTCCGTGTCCGACAACGACATACGCAAGTACGAGATGTTCGCG
CAGACGCTGCAGCAGAGCCGCGGGTTCGGCACTAACTTCAGGTTCCCAACGTCAGGCGCGGCGTCGGGCGGCA
CGGGCACGTCGGGCGGGGACCAGCCCACCTTCCAGGAGGAAGGCGGCGACGATGACCT
GTACAGCTAGACGCGTACATACTGACATTACTTACACAACTTACATTACTT
>103775315_2 DV501149.2

ACTCTGTCCTCTCGGGATCTTTCAGACAAATCTTCCGCCCCGATTTTTTCATGTTCTGACATTCTGGTGCTGG
TTACAACTGTTCCAAAGTACATTTCTTTGAGGATACCCATTTCTTTAACTCTTTTTTATACATCTTACTTTAT
GTATCTAAA
>103775319_2 DV501220.2
GGTCGGTGAAGATCGCATACAAATGAAACATTTGAAATTATTTACGAGAGTCGAAGTACATTAGATGTAGATG
CGTTACAATCGACCTCGGACTCGCTGACGTAGCTTAGTGTTAGATTAGCGACAGAGTGAGAGGGTTAAAGACA
ATATTTGTAAATAACATAATCGTAATTACCTGGAAA
>103775353_2 DT663609.3
GCACGAGGAAGATCCTTCAAAGAGAACCATGAATCAAGGCGAAAAAAGAGTATGCCAAGAGACAGGCCCGAGA
CTTCCAGGGAATATAGAGATCGTTCTCCATCTTACAAATCTAAGAAAGACTCTAGAAAAGAACCAGAGAGATA
TTACAAGAGTTTCAGACCGGAACAATCGTCAGAATCTGAAAGTGAAACGCCAAGAAAA
CGTAAAGATAGTAAATCACCTAGTAAGAAATCGAGATACCACAAAGACCACGAATCTCCAGTTAAAGAAAAAA
GGAGGCGAAGCGTTTCAAAACCTCGAGAGAGTCCAAAGGAATCTTCTAGAAACAAATCACCCGAACGAAGGCC
AGTTGAGAAGAAAGAAATTAGCAAATCCAATGCCAAACCTGTAGATAACAAGAGGCAT
GAAAGTAAATCGAAACGGAGATCACCTACGCCCGAAGATAAACAAAGACGCAAAAAATTAAGAAGCAGGTCAA
AATCTTCGTCAATAGAGCGTAAGAGCTCTAAGAAAGAGAAACAAAAGAGTAACACTCCCAGTATTAAATCAAA
GAAGAGTTCGGAACGACGAACTCTATCGCCACGACCGGAACCAGCTAGACCGGAGAGA
AATGAGAAAGAAAAGAATGATTTAGAAAAAAGTAAGGAGTCTCGAAAGGATCATACTTCCCGTTCTAAAGAAC
GCAGTCGTAGTACCTCGAGTCTTAGCTATTCCCCAGCTCGT
>103775361_2 DT663653.3
GCACGAGGACTTGCCTTAAAGAAGACAAAGGAAATCATGAAACTAAACACGACGTAAAAACCAAAAAGACGCA
GTTTATTGATAAGATTATATTAATTCGTAGTAAAAAATAAAAAAATCGTATTCGGGGTGATGTTTCAGTAATT
TCTTAGTTTTTAATTAGAAATTACGTGTTTAAGCCATGGCCGCGGCCCTCGCGAATGA
TTCTCCCAGATATAAGAACAGTTTAATCTGCTGCAGCCCTAGAAGTGCTGACCTTGTTTCTAGATCTTCTTCA
TCCACGTCAGGATGTGTCTCCGCTGACGAGAGCCTCGACTCTTTTTACATCCCAAAATCGATAGCCAATAAAA
AATTAAAAATTCACAGCTCAGCCACACGAGAAGAAATAGAAAAAGCAATTAATCAATG
CAAGGAGCTAGTTTTAAGCAGCCCCCAGTGTTCAGACGAGCGTAAGTGGCTTGTACGTTATTTAGTGGAACTA
CGACTTAGGTTAGAAGATCTAAAGGAGAACGATGGACAAATAAAATCAAAAGTCGCGATAAAAGGACACCATT
TCGAACAACAAGTTAATACAGGTAATAGAAAACAGTATTGTGACCATTGCAGTGGAGT
AATATGGAGTATTGTTCAAAGTTCTTACATTTGCGCAGACTGTAGCTATGTCTGTCATTATAAATGTGTAGAA
GATATATGCAGGGTATGCGCTCATGTAATAATGACTGAAAGAGGTTCGTTTGAAATGAATA
>103775367_2 DT664420.2
GCACGAGGCAATTCTTTAAGAATGCCGAATCTAACGCTGACTACAAAGGCTTGGATGTTGACAGGCTTGTCAT
TGATCACATTCAGGTTTTCCGCGCCCCCTGTCTCCGCAGACGTACATACCGTGCTCACGGTCGTATCAACCCT
TACATGTCGTCCCCCTGTCACATTGAGGTGTGTCTCAGTGAGAGGGAAGACGCGGTAG
CACGCGCCGCCCCCGCTGACGACGCGCCCGCCAAGAAGAAACTCTCCAAGAAGAAGCTGGCCCGCCAGAAGGA
GAAGATGATGAGGGAATAGACTATTAATAAATGACTTTAATTATTAAAAAAAAAAAAAAAAAAA
>103775370_2 DT663622.3
GCACGAGGCAAATCCTTTAAAAGCTATTCTATCAAGTAATGTAAATAACTCTTTCTTAATTTCTATCTAATTT
CTCAATTAATTATCATTATGATTTGTACGCGACTCCTTATAGAAATTTGTTATTTAGTGTATCGATATGTATA
ATACGAATAGTAGTACACTATCGCTAGATGTAAACTTATTTTTTATTTATGCTCATAT
TCACGATTTCTCGTTTCAAATCCTGTATTTGAACCTATTACCCATCTATTTAACGGTTTTAACATCAGGATTT
TTAAAGTGAAATTGTGGCACATTCAACTCGAAATGTATTTTTTTAAGTATAAATGTCAGATGTCCTTTCGATAT
GTCAAAAGGTCATAGACCTCTGGAAGCCATTAGTCAGGTCACGTGATATTCATTTTAG
CCAATTAATGTTTTGATTAACAAATCAGCCGTCCCGTTCATATCGTGTATCAAATAGCATGTTGTTTTATCAT
TTTGTGTACATATTGTGGATTGCAACTACATTCTACACTTGGGCGGGTTATAATAAATATTTCATATTGGAAT
GGACTTTTTTTTTCTATTAAAGCAAATAGAACTTAGTGACGATAATTGTAAAAAAATT
ATATATTTATGTGAAAATTGTACTGTTATTTAAAGGCAAAATTGGGCAAAGCAGATAGATTGATTAGTGATGG
AATTAGATTGATTTTTAAAATCAAATTAATTGATATTTTATTTTAAATTAAAATTTGTCATTTCATTTATTTA
TTTGGTCTAA
>103775372_2 DT663582.3
ATTTGCACCCTCAAGAACAGTTTGGCAGAGAATATAAACAAAATGATGTGTTATTAGTAAATGTCTTCACCTC
AAATCCCAAAAATCTTGCCTATCTTATTGACTTTTATACATACAGCACTCGCGCTTCTGTTGAAGACCCTCCT
TGTCATATAGGATATACTTATGTTTTACCAAATATGTTTAAGCCATCAGAAGGTGCAT

TAGAATTACCGGTAACTTGCAATGTTAAACATCGCCCACTTGGCACAATTAATTTTGAATATCTTATTATTCA
TCCAATGGAAGAAAACTTGTTTTTTTTGGGTGTTTCTTACGCAAAATATTGGGATCCATCATGGACAGGATTA
GAAGTTGGTCACAGAGGACTAGGTGCTAGTTTTAAAACAAAAGAGTTTACCATTTAGT
AGAGGGTAGGAATCATGAAATCCTATTTTTCGATGAAGATTTAGAAGAGCATCAGCCATTTCCTACTTTAGAA
GAGGCTTTCAAGAAGTTAGATGAACATGTAGGATTTAATGTAGAGTTAAAATGGACTATGGAATTGAATGATG
GAACCTTTGAACTTAACAACCCTTTTGATATGAACACATATGTTGATAAGGTGTTAGA
TGTAGTTTTGAAGCACGCGGGAGAGCGACGCGTGGTCCTGTCCTGTTTCAACCCAGATATTTGCACGATGGTT
CGATATTAACAGAACAAGTACCCCGTCATGTTTTTACAGTGGGAATTTCC
>103775377_2 DT664785.3
GCACGAGGATGCGACCTTTTATTCTCAAAGTTCTCGAAAATAACACATGAAGGATATATAATATATGTGCAGT
TTATTGAAATAATGAAACCACATCAACAAAAGTAATAATCAAAATTTCATTATCAACAATTTACTTTAAAAAA
TACGTAAACACTATGTCATGTTAAAGTGACTTTGTTTTCGTATAGAAAATAAATAACA
ATGGATCAACCGCTTTCGTTGAAATTGTCGTCCATGAGTTTGATTTCTCAGCACAAGTTGAAATGTATATTAA
CAAAATGTGGAGATAAAGCAGATTTAATAATAGATCCAAGACTAATAAAACCTTTGGAAAGAATCTGTGGAGT
CAGTTGGCTCAGGCAACATGGAGTTGATAAAATATATAAAATGGATCCTCAATTGGGC
GCAACAACAAACGCCAACCGCATCTATTTCATTTCTGCTTGCACATTAAAATATAAGTGTGTTTTGGACCAAA
TATCTTCAATATTGAGTCAAAATTCATCTTTAAGTGAGACAAAATGTTTCAATATCATAATAGTGCCTAAAGT
GATAGCAACTTATGATTCCATGCTAGAAAACAGAGGTCTATATGATATCGTGCAATTA
CATTCATTTTCATGGGAATTTGTTTATTTAGATGATCAGTTACTAAGCTTAGAACTACCTTTTTTGTATAAAC
AATTATTTGTAGAAAAGAATCATTCTCTTCTATCTAGTATTTCCATGTCATTATGGAGTTTATTTCATGTGAC
AGGAAAACCAAATGTGTTTTATCTTTTGGCAACTGTCT
>103775394_2 DT664050.3
GCACGAGGGTCGCCCTTCGCGTTCGTGCAGATGGTGCTGCAGCTGGCGTACTTCCGCGACGCGGGCCGCTTCT
GCCTCACGTACGAGGCCAGCATGACGCGGCTGTACCGCGAGGGCCGCACGGAGACGGTGCGCTCGTGCACGCT
CGAGAGCTCGGCGTGGGTGCGCGCCATGCAGGACGCGGACGCGCCGCTCGCCCGCCGC
ATGGAGTTGTTCCTGGCCGCCGCCGCGCGGCACCAGCTCGGCTACCAGGACGCCATGGCAGGCCGCGGCATCG
ACCGCCACCTGTTCTGCCTGTACGTGGTGTCCAAGTACCTGGAGCTAGACTCGCCCTTCCTGCAGGAGGTGTT
CAACGAGCCGTGGCGCCTATCTACCAGCCAGACGCCGCACGGGCAGACCAGCCTGCTG
GACCTCAAGAAGTACCCGCGCTGCGTGAGCGCCGGCGGCGGGTTTGGCCCCGTGGCGGACGACGGCTACGGCG
TCTCATACATCATCGTGGGCGAGGACACTATCTTCTTCCACGTCTCCAGCAAGATCAGCTGCCCCATCACGAG
CTCCTCGAACTTCGTGAGGCAGATCGAGCGCTCGCTGCAGGACGTGCACGACATGTTC
GTGGAATACAAGTCGAAGTCTAAGAGCAAATAGAAGAGTCGTGTGACCGCCGGTCAGTGCGCTACGCAGCTGC
CCCTCTCGTCTTGGTACATATACTCGAATAGGCGCTGCGCCCGTGCCCGTGCGGGGGTACCCGGACGCGGTAG
ACGCGGTCGACGCGTGCGCGTCAAATGTTAAAGATATTTATTTACACTTGTTATAAAT
ATTTAATT
>103775396_2 DT664617.3
GCACGAGGCACCTCTTAAAAAATTGAGTCCATTGCAGGAGCGCAAATCGCCGCCAGAAAATAGTGATTACATG
GTTATTAAATTATCTTCACCTGTACAAGGGAATGGGGAACCGTTTGATAAGTGCGAATCCCCTGAGATATTTA
CTGAAGAAAAATCTCCGCCACGTGAACAATCACCTACAAGACAAGAAACTACTACAGT
CAATACAAGTCCAAGTTCTTCGTTATCTTTAAAGAAAAATAGGCCACAAGTTCGTTCAAATGGACGGGCCGCT
CAAATGTTAGGTTTATGCGTCCCAGATATACGAATGGACTATTATTAGAAAAGTCCATCGGTTAATACATCAG
CTCGTAGAAATTTAAGGATTCTATACAACGCTAGTGGAGAACAAAGCGATAGTGTTGA
AGAAAGTGAAGAGAGCGATAATTTTCTTAAATTTAAAAAGTTATTGCCGTCTAACGATTGTAGTCCATCTGGA
CCTATATTGAAGAGGAAGTTGGCGGATATTACTGACGAGGCCACCGCGTCGCCAGCTAGTAAGAGAAAACGCG
TGAGCTTCCACGATCCGCCTGTATCCACGACTATATGCGTTAAAAAATATATAGAGCC
ATGTGGGTTGAGATCTCCTCAAAACTCCGCTTATAAAAACGACAAGAAAGACTTCTAAGATCACAACT
>103775403_2 DT664363.3
GCACGAGGACAAACCATAAAAACATTTTTATAATGTCCTTCACTACAATACGTAGGAATTAAACTTACGAGAG
TGCATTTATTTTAACAATCTTGAAAATAGTTGACATTTTTATACATCCTTACCATCAAAGTAACGTAACATAA
CACATTTTTACCCACAATTTTTAATGTGGTGGGAATTAAACCCACATCCTGTCGTGTT
GTGTCTGTCTAATCAGACAGAGACAGTACAGGGGAAGAAAAAGGAACAAGTGTCAAGATACTATTTTGCTGAC
GAAAAGTTTTTTTCGTCTTGCATTTAACTGGTCTTTTGTTAAAGTTTTGTGAGACATTGCGTATTGAGTGGCG
TAACTCAACGCCGTGTAACTCACCTAACAAGATCAGCCAACGTGTGTCGCGGCATCAA

ACACACTCTCTCCATAGCGTCTTCACAGTACGCGAGATAATAAAGACATATCGACACACCGGTGGCCGCTACC
GATGGACGTGGGACTTCTAAAAATTTCTGTAATATATGATATTAAATGAAAATTATATTAAAATAAAGATATA
TTAGTGATCCTAAACAAGAAAGCATAAAGGCATGAAAAATAATAAATATAATACATAT
ATATAAACTTAATGCGGTCTTTTTCCACTACATTTCTTTTCATAATCATACTTAATAGCTATAATAGGTTATA
ATGAAGTCTTGTTCTTACATATTTTTATGTAAATTCTTTACATATTATATTTTTTTTTACATTTTTAATGTTA
TTTCTCACTAACAAAAAATCAAGAGT
>103775437_2 DT664135.3
CACGAGGCTCGAGTCCCTGAAGCTGTGGGAACTATGTGCGAATAACGTAACACAAAACAGGGACTCAGCATGG
GGGCCTTTTTAAACAATCCTGAAACTAAGAAGTATAACGAGAATGGAGAAGGTAACGGTCTTCGCTACGGCGT
GGCGTCGATGCAGGGCTGGCGCGTGGAAATGGAAGACGCTCACCACGCACAGCTCACT
CTGAACGGTACACTATCGGACTGGTCGTATTTTGCCGTATTCGATGGCCACGCGGGTGCCCGGGTGTCAGCGC
ACTGTGCGGACAATCTTCTCGAGTGTATCTTACAAACGGATGATTTTAGACGAGAAGACATAGCGGAGGCGAT
ACGCGCCGGCTTTCTAGACCTGGACAAGAAAATGAGGGAACTCCCCGAGCTCTCGAAC
GGCGAAGAGAAATCGGGCTCCACAGCAGTGTGCGCTTTCGTATCGCCAAAGCAGATATACATAGCAAATTGTG
GCGATTCTCGTGCAGTCCTTGCTCGTAATGGAGCACCGATATTTGCAACGACAGATCACAAACCGGAAATGCC
TTCAGAAAAGTCTCGTATAGTACAAGCTGGTGGCTCTGTTATGATACAGCGTGTAAAT
GGTAGTTTGGCTGTGTCTCGGGCGTTAGGCGACTACGAGTATAAGAAGGTGCTGGACCGTGNGCCGTGTGAGC
AATTGGTATCACCAGAGCCCGAAGTGTCTGTGCATGAGAGGCTAGATGCAGAGGATGAGTTTCTGGTGCTGGC
CTGTGACGGTGTTTGGGATGTAATGAGCAA
>103775439_2 DT664475.2
CGGCACGGGATTATAATCTGCGCCCTTGTGGCTTGCGTGGCCGCTGATAATGAAAAACGTGAAAAGCGTGGTT
TCCTCAGTGGTGGCCACTCAGTTGGACTTGGAGGTTACGTCGGGTTTGGCGCACATGGATACAGTGGGGGCTA
TGGCGGGTATGGTGGGCATGGTGGTCATGGAGGATATGGTGGATACGGAGGCGGATAT
AGCTATTCAGCACCTGCCAAGATTATTTCAGTAAACAAAGTAGTGAATGCTCACAAAATTGTTGAAGTTCCCC
AAATTGTGAAAGTACGCAAAGTTGTGTCTGTGCCACAAGTGGTTTCTGTGAAGAAGATCGTGTCTGGACCTAG
CTATGGTGGTGGATATGGCTATGGCGGAGGATTTGGATATGGCGCTGGTTACAGTTCA
GACTATGGTTATGGCTCAGGCCATGGCTCGAGCTACGACTTAGGCCATGGTTCAAGCTACGGCTCAGGATACT
CTAGTGGTTGGTGGTAAACGTGTTTATCTAGTCTTTTTAAATTAGTGTGTTCTGGTTTCATATTAGTGAATTT
ATCTTAAGTCGATTTTAATATGTATATATGTATTATATATATATATATAGAAAATGTA
TATAATGCCAAAATAAATATATGTATATCGTAAAAAAAAAAAAAAAAAA
>103775443_2 DT664709.3
GCACGAGGCTCACCTTAAAGGAAAATTCGAAATTTAAACTTTTTAAGTTTTATTTTATGTTTAATTAAACGAA
GCAATGGATAACATATTGCAGCCAGTTAGAGAAAATTTTCCGAAATTAACAATCGATAAACAAAAAAGTCCAA
GTTATGTGGAAATTTATACAAATAAAGATATCGAAATTGTTTTGGCTTATATAAAGGC
TAAACAAACAATTAATTCCTCGGAAAATAATAAAAACAACAGAAATGAGACTGAAGAGCTCGAGTTGACAGGT
TCTCCTCTCAAATTGGATCTAAGCTTGCATGCTATATTGTATGAAACAGTACTCCCTGATGAACCTCAGCTTT
GGAAGAAAGAAGAAATATTGCATGCACCTGTAAATATTGATACAGCAAGAGACATAGC
TAATCTATACAACAAAACTTTAGCTAAATTAGCTAAAGAAGAAGCAGTACCTATGTGGATATTATGTAATCCT
AGTAACATGGGGCAACCATTACTATTAACTATTCAATCAAATGAAGATTGTTTTGGCCGAGGCATAGTAAATT
ATGAAGGTTGTATGGTGTTAGAAGAGGTTGATGTAGAGAAGTTTATTAGTGATTTTGC
TTCTTATGAAAGTGTTAGTGATGAAACGATAAAAGTAACAGTGGATT
>103775455_2 DT664876.3
TCCCCATCGGAGGGATTGAAGAGAGGATGCCGTATCCAGTTATTATTGCTTTCGGTATATTGAAGAAGGCGGC
CGCTAAAGTCAACATTGAATTTGGTCTGGATAAAAAACTAGCCGATGCGATAATGCAAGCCTGTGACGACGTG
ATCTCTTTTTTTTTGTACAGGGAGGGGCACTTCCCTCTGGTCATCTGGCAGACGGGCT
CCGGCACACAGTCCAATATGAATACCAATGAGGTAATTTCAAATCGTGCCATCCAAATACTGGGTGGCAAACT
AGGCTCCAAGGACCCCGTGCACCCCAACGACCACGTCAACAAGTCTCAGAGTTCCAACGACACGTACCCCACA
GCGATGCACATCGCCGTGGCGATGGAACTGAGAGACCGCCTGATGCCCGGTTTAGTCG
CCTTGAGGGACACCTTAGAGTGCCAATCGAAGGATTTTG
>103775457_2 DT663525.3
AATAAATCTCTGAAACTGAACAAAGTACATCCCTACAAGGAGATTACAACTTGACATGAGATAGCACAAGCGT
CGGAACAAACCCCACGTCGGTGATTAATTGCTTTCCAAAAGTCCGTTCAACTTTTATGATTTAAAAGCATTAT
CTTGCCCGCACTATTATTTAGATACGTAATGTAAATAGATAAACGTTGTTGAATATTA

CCGGAAACGACTGTTGATACAACGTGGGTTTTTTTTAGTAATAACTAAATAGTGTAAATGATAGTGCAGGTGA
ATGTGCTGCCATAGGTAGGACTGGTCAGCATGGCCGGCCAGGGGCAATGGATCGCGGGGTTGGTGTGG
>103775459_2 DT663801.3
GCACGAGGCGCAAACCCCTAAAGATGGGAAACAATTCAGTAATAATTTGCGAAAACCTAATTTAAATGCTATG
GCACCCGCCCCGCAAGTAACTGACAAACAAAATGGCAAAGATGACTGGATTCGTGTTAATAAAAAGAACAACA
GATTTAGAGGATTGCCAGGTACAGCCAGTGTACACTCGGGAACAAAGTTAAAAGCTGC
GGATATACAAATTCCTTTGTATATTTATAATATTTCTAAGGAAAATACCGAACGTGATGTGGCAGAATATGTT
TATGCGAAGACACAGATAAATATAGTACCGGAGAAAATTAATATGAAATTTTCTAGGGATTACAATTCGTTTA
AATTTTATGTACTTCGAAGTAAGTTGTCGTTATTTATGGACAATAGCTTGTGGCCAGA
AGGAATCTATTTTAGAAAATATTTTTTGTTTGGGAATAGAAATAAGACTTCAGTAGATCAACATGAACAATTA
AATGTGTATGAGCCAAAACAAAAAACACACGAATAATTCGTTAAAACTTGTTAGCTTCAATTGCAAATCTGTG
AAGCGTTCTGTGGAGAATGTGAGGAGTTTGTGCATAACTGCGGATGTGGTGGCTCTTC
AAGAGACATGGCTGCTGCCTCACGATCTGACCTATCTCAGCACGGTGCATGACGATTTCGAGTGGACAGGTAC
TTCA
>103775467_2 DT663769.3
GCACGAGGCAAAAACACATCAAAGGAAGAATCCAAACGTAAGAAACGTCTCAAAGTGGACACTTTTGAGATAA
TAGATGAGAAAGAGGACAATACTATCGATATTCGTGATATAGAGTTCCGTATGACCAACGACGCGAGTCGCAT
ACGAGCTCTGATTAACGGATCTAGCACCAATAGTGGAAGGGATCTTATTATGTTGAAG
TTAGTGCTATGCCGAGCCCTATATCCACAAGTAGCTGTAGCAGATGAATTCAACCACTGCAAGTCAGTTTCCG
ACCAATTATATCATACTTGGAGTAAACCGTTCGTTTTTCTTCATCTTTTTTCCTATTTCGGTAGAAATTCCAA
ATTATTACAATTACATGAAGATGATATACAGACAGACTTGCCTATAGGATATAAGAGC
AAATTGCCGTTGTCGTCGAAACATCAAATTTTGTGTTATTTGTCGCTTCTGGAAACAACGAAACCGTATATAG
TGAATTCAATGCGAATGCCGGCAGCTCAAACTTTGTTACTTCTAGCACATTCTATAGACACTAATATTGGTTT
TAGTAGACTGGTTTGTGATTCGTGGCTGTTATTGGAATTCCCTTACCCTTGAAGTGGA
CTGAACCTACTAATGAAGGCGACTAAACTACGAC
>103775475_2 DT664187.3
GCACGAGGATTAGCCTTTGAATTTCTCAGCTTGTCCCAAATATTGTTAAATTATATATTTTAATACATTAAGT
TATAATTATAAGAAAATCATAAAAATCTAAATGTATTCTAACCTAGTAATAACACCAAGGCAGTGGTCAAGAT
TAGCATGCTTATGTCGATCAGTATCTTCTATAGAAAGTCAAAAACTTCCACAAACTAG
TAAATCCAGTGATGGTATTTCTCCACCGCCCCGAATATTAAGAGGACCTACGGATATTTTACAAGCTTTAGCG
GCTACTGTCGGCGCCGATCCCACTGCGCCTCATTACAAATATCATGATGACCCATTTCTTATTCCACTGTCAA
ATTACCGAAAACGAGCATACGCGTTGTCTGCTGAGGCTGGTAGAAAGGCAGCAGCTTG
GATAAGAGATGAACACTCAAACTTGTTTACTACAAGAGAATGGGATCCTCCAATGAAAATGAAAACTCCATAC
TTCAATGCAGATCCAAAAATTGATATATTTGCCCCTAAACCTATTTATAATGATGATAGTAAGGTCACAGAAG
ATGACTTAAAATACACAATTCAAAATGCACTGATAGAAGATTCAATAAAAGTATTTTG
AGCTCCTGGGTGGAGTTGAGAATTTGAATGATGATTTAAAGTTATCTTTATTACAACTTTTATGTTTCTATAA
TGAAAAAGAACCAATTGCAATGGAATGGCTTGAGGAAAGATGGTTTGCTGGATCAGCCAAAGAGAGACATGGT
GCTACATGGAAACTTGGTGGCT
>103775481_2 DT664138.3
CGGCACGAGGGCCATACCCTTTTGGGTACCACGTGGTGCTGGTCGACACTGCTGGGAGGATGCAGGATAACGC
CCCGCTGATGAAGGCACTCGTTAAGCTGGTGACGCTCAACGAACCGGACTTAGTTCTGTTCGTGGGGGAGGCG
CTCGTGGGGAACGAGGCCGTGGACCAGCTGCTGAAGTTCAACAGGGCGCTGGCCGACC
ACAGCGACTCCAGCCAGGGGGAGGCGCGGCTGGTGGACGGCATCGTGCTCACCAAGTTCGACACCATCGACGA
CAAGGTGGGCGCGGCCGTGTCCATGACGTACGTGACGGGGCAGCCCTTGGTGTTCGTGGGCACGGGGCAGACC
TACGCCGACCTCAAGGCGTTGAACGCCGGCGCTGTGGTGCACGCACTCATGAAGTGAC
CCGACACATACCTAAAAATATTCTTCTATTTTTTGTCCGTCATCAATAAATTTTATTTTTATCAAGAATCCTA
TGCCCAAACAATTTACTCTATTTATTTATTTCATCAATCAGTTTTACTTGTAAACAATACAAAACGTTTTAAA
TATATGTATATTTTTCATAAAATTTATTTTGGCATTAAATGAGTCACAAATATGAATT
AAAAATTGTTTTAAAAATTATACTAACTTAAATGGTTAGA
>103775485_2 DT664792.3
GCACGAGGGAGAAACGACCCTCCATTTAGTTGGAGTCAGGCGAACCAGCACGCGCTCTGCGCCTGCGCAATGA
GCTGCAACGCGACGTGCGCCGCGAGGTCTTGGAACGAACGAATCGACACGAGCAGGAAGTAGGGTTTGTACTT
ATAAGAATGTTGAGGTGTAATTTCATAAGAAATTGACTGAGAAATATGACTGAGTAAT

GTAGGTAGTGCCGGTCTCGGAGTAGGCTATGATAGGGTAGGTGCGGCGGGCGGCGCGGCGACGGGACTCTGTT
GCGGGACGAACGTGACTATTAGTAGTTAGCTATTAAACGTGTATCTATAATTATAATAATTTGTAAATTATTT
ATTTTATTTTCCATAGAGAAAGTCGTATTTTGTTAACGTTTTAGTTGATTTTAATCTC
TTGTCCCCTAGTTCCATCCGATGTAGGTGTGTAGGATGTCATACGCTTGTATTTTTAGTCAGCCTTATGTGTA
TTATTTAACTGTTCAGTGGTCAGTGGTCAGTGGTGGGCGGTGTGTGCGATGTCTACAGTGTCTCTCACTTGCT
TCTCTCGCAGTCTCGATATATAATTAACATTTATAAGTAACACACACACTATGACGCT
GTGACATTTATATTCCTGCGACACCGCGACACCGACACTACGACACCGCGTCAGCGAACTTCCCCATACTACC
TAACGTCGCTTATTCTCTTACTCGCTAAGTATTTTGAATATTTTAATAACAAGAAAGCTTGTACGCTCATTGT
AAGTTGAAGCAACATTTACAACGTATAAATGGACAATATTTACTTGTGTTTGTGTCAG
CTTACACGACGCGTCCCTT
>103775495_2 DT664634.3
TAGACGTTCAAATTCAAACGCTAGAGAACAAAGAAGTTTAATTATGGCAAAGTGTACTCATTGCTCTAAATTG
ATAGCGAAAAAGAATCCTGGACTTGAATGTAACAAGTGTAGTGTTATAGTGCATGCTACAGCGATATGCTCAG
GACTAACAGCAAAACAACTAAATTCTCTGAGATCAGCAGAAAACTTAGAGTGGACGTG
CCGTGACTGCTCCCAAAAATCACCTCGAAAAAGCTCATTTATTACACTACAAGAAGAAGCAGAGGACGACGAA
GAAGTACAAAATCAAAGTTATTATCCATTCGACATGAAAAAACTACTTAACGATTTAACAACAGAAATAAACA
AAATTATTAAAAAGGAACTAAGGGAAACAAATACAGCTCTCCAATATTGCAGCGATAA
AGTAGACGAATTTCTCGAGTGTATGGAAGCTTTCAAGGACAAAGTAAAACAATTAGAAAAGAAAACCATGGAT
TTAGAAAATAAATATAAATCAACTGAAAATAAAATTGGTGCTTTAGAACAACGTTTAAATGAATACGAGCAAA
GAGAATTAAATAAAAATATTGAAATAATTGGAATACCTTATCTAGAGAACGAAAATTT
AGAAGAAATATCAAAAGCGATAGCGAAAAAATTGCACAAAGACGTTGCAGATATAAAAAAATCAAGCGGGTAT
CCACACCACCTGGAAGATTAGGTAAAATAAAATT
>103775506_2 DT663646.3
GCACGAGGGTTTTACATAAGAGGTCTCCTTATTTATGTACAACAATTGTCTTAAATTTAAGCATGTATTTATA
TCGAGGCCTAGTATTATTATATTTAAGCTTTATTATTACGTTTCGTTGCACGCATTTGCTTAATAGTTGTCGC
AGCGGAAAAGGCGCATCAGAACCTTCTAGACGGCGTTGAGCACTTTGACAAGACCCAA
ATGAAGCACACGACGACGGAAGAGAAAAATTCTTTGCCTGCCATTGAAGGTTGTTACCGCTTTATTTACTGCT
TAGCACGTTTATGTTTAAAGTATTTATATTTTTTATTTTATTTTTGAATTTATATTATTATTTTTGAGTGTAT
GGCAATTTATTTTTCGCATTATATGTATATGTATGTGCCAACACGAGGAAACTAATAT
TCTAATGCAATACTTGTGCACAGCAATATGCATTAAAAAGACCGTTTTAAATATATCTGGCTAGTATCGAAAC
TTATTGAATATTTTGACGACAATTATTGAATAAGTAGAATTGCCTTAATAAATTTATAACAATGATCAATTGT
TTTCAATGTTATTATTACACATAAACGTTACAAAATAAATATTGTTAAATCTGTATTT
TACATATAATTTCGGACAAACTAGATAATATCAATCAGATGTGCTTTGTACTTCCAAAGCCATTGTAACTCGA
CATTTACGTACATAATTATTTATTTTGATAAAAATTATTAACT
>103775550_2 DT663627.3
GCACGAGGGTTTTCCTAAAAATATTTTTCTTTCCTAAGTGAGGAATAACACTTTAAAAATGTTCTAGAACAAA
TTAAATATTTATAAAGAAATTGTTGGAAACATTGAATTCACAAGTGAGCTCTAAATTTGCTATTGTTAATAGC
ACAGCTGTATCACAAAACTGAATTTAAATATTTTATTTATGATTTACTTATAAGTAAC
GTCAATTATAAGTCACCAATTTGAACTTTTAGTATAATTTTCTTCTATAATTGTAACAACTTGACTTCCTGAA
ATCAACGCCGCTCCTTTTCTTTTTTGCGTTTGCGAGGAAACTAAAGTGATTCTACTGAGTGGAAATTGAACCC
ACAACTGTCACGTGGACAGCCAGTCGCTGTCGCCGATTCACGACGGCCTCAATTATTA
TTTAAAATATTATTTTAAGGTTTTTTCATGTATATACTTATTGATATAAGGCTATACTTTAAATATATGTATG
TTGATTTGAAAATAAATATATCGTGACATAACGTCATATTGTAAATAGTAGACAAGTTGAAATTTGTGACTCA
ACCAGTGTTGACGACTATTAGAAAATAAATGAATAATTTTGTATGTATCTTAAGATCT
GCATACTATATGTATATTATATTTTATATAAAATGCAATTTTAATATTACATTGTAATCGCTCACTAACAAAG
GTGGGGGTTTAGTTAGCAAATAATATAATTTGTGATAATATCATTGAATGTATGTCATTATTATTACAAACAT
CGACATCGATGTAACGAAC
>103775553_2 DT664741.3
GCACGAGGGCCGCCATTAGCTCGCGGCCGTTTGATACAGTGCGGTGCGTTTGTTTTGTTTTAGTGTAAGGATT
TACTATATTACTGTATAAAAATGCGGCTTCAGCGACGAGCGTCGACTTTGACGGCGGCACTGTGGGTGTTTTT
TACGGTTGTATTACCAATTGAAACACAAATGGCGAATCCTATTTCATCAGTAATGCAA
TTTTTACAAGAAGGTACAAATCAACTAGATAATGAGCCACAAGATCAACAGAATCTCCTTTCCGAATATGATT
TCATAATAGTGGGCGCAGGCACGGCTGGATGTGTACTATTTTATCGATTAACAGAAATACCCGAATGGAAAGT
CCTTTTGTTAGAAGCAGGTTTCAATGAAAACTTTGTTATGGATATACCAATTTTAGCA

AATTATTTACAATTTACAAATGCTAATTGGAAATATAAAACAAAACCTTCAGATAAGTATTGTGCAGGATTCG
AAAATAAGCAGTGTAACTGGCCACGTGGTAAAGTCATCGGTGGATCTAGTGTCTTAAACTACATGATTTATAC
TAGAGGGTCACAATATGATTACGACAATTGGGAAGCAATGGGCAACCAAGGCTGGGGA
TGGGATGACGTCCTTCCATATTTCAAAAAAATTGAAAACTTTTATATACCTTCATTTGACGATGATAAATTTC
ACGGCCATGATGGATATTTAAACGTAGAGTACGCTCCATTC
>103775557_2 DT664864.3
CGAGGCTTATCCTTTAAAGGAGTGATAATAAGAAAATTCTATTCTATTGTAAAGAAAGACACAAGTGCCCTGA
TTTATAAAAATAATAAAGTGGTGATGAGTGCAAATATTAAAGAAAACAATTTATATGAGTTGAATATAAAAAC
AGTGGTTCCAGATACATGTAACTTGGTTCAAAATAATCAAAGTGATATGAAAATGTGG
CATGAAAGGTTAGGACATATTAATTTAAAACAAATTAAAAACATGAGTGCAAATAGTATTGTGAACGGATTGA
AAATAATTGACAATGGACAAGATAATTTTGTATGTGAATTTTTTTTATATGGGAAACAATGCAAGTTCCCATT
TCAAAAATCTGAGCGAGGAGAATTACAGCCTGGTGACCTGGTTTACAGCGATGTTTGT
GGACCTATGAGTATTTCATCTATTCAAGGAATGAGATATTTCTTACTTTTTAAGGATGCTGCAACAAGCTACA
GGCATGTATATTTTATTAAGCATAAAAGTGATGTTTTAGAAATCTTCAAAAAAGTACAATGCAATGATTAAGA
ACATGTTTTTGCATAATGTCAGAACACTACATACTGACAATGGAAC
>103775561_2 DT664163.3
GCACGAGGAACCCCCTTTTAAAGTTTATATGGATTCCAATATGGAGTTCCCCTCTGTACAGCTGAATGTGCAA
GGCCAAGATTTGTCACTTTCTAAAAAAAATACATCTGAACCTGATATAGTAAATAGTACTTCTGAATCTCTTG
ATTCTGATGTAGTACAATCTAATTTAGAAATAAGGGCAGATGATACAAAATCGAAAAG
TAAACATCAGAGAAAGCAAGTGTTGACTCCCGTTGAAGAACCCGAATTAGAATTAATTGAGCCTAGTGATTTA
GGAATTGAAACGTCACCTAAAAAGAAACGTAAATTAGAATATTTTTTTGAGAAAATAACTAAGAGTCTCGTAC
CAAAGAAAAACTATTTACTGAATCGCAACGTTATTGAAGATAAAAGTACAAATTCTCA
AGAAACATTGAAAATCCCTGTCAAGGAACCTGAAATTGTCAGCGAAACGTATACAAATCATACGGTCGCTATA
TCTGCGTTGGATAATTTAGTTAAAGCGGCAGAATTGATTGAAAATCAATCGGAACATAATAACTCTTTATCAA
GTGTTCGTTCAACGGACAGTCCCGTTACTACTCCTGTTAAAAGAGGTACAGGTCGTCC
AAGAAAATACCCGTTGCCTGAGTCAGGACCTGAAAGAAACAAAGTTCCTAGTCCTCAAAAAAAAACCGCGCTTA
ATAGACGCTAAACCTCCAAAGAAAGAACTAGATAATTACACAGATTCAGATGAAAGCAGTAACGGGGAAATTG
TGAAAGAAAATTGGACAATGGGCAAAATTAATGAA
>103775577_2 DT665047.3
TAGGAGATTTTGTATAGTTACTTGCTATGGGGGCTAGGTTTAATTGGTTATTTTCATTTATATGAGACAAAAA
ACGGCTAGAGAACTGTTCATCAAATTCTGTAAAGTTATTTGCAGACCTATGTCGAAAACAAGTGATATCGCTT
GTACTATTAGTGCTAATCATGAGAACGAATCATACAAAGGTGAAAAGTTAATAAGTGC
TGCAGCATCTTTTTTTTTAGACCCTATACTACAAGTTTTAAAAGGAGGGGGAATAACAGACTCTGAGCAGCTT
GAAGAGGAAAGCCCTTTATTTTTGGAAATATCATCAGGAACAGGTCAACATGTAGCATATTTTGCACCTCACT
TTCCTGGTGTAAAATTTCAGCCATCAGAGTTTGACAAAAATGTTTTTGGAAGTATTTC
TTTTTATGCTTCTAACTGTCCTACGAAGAATATCCTTAAACCTATGTTTATTGATATTCAAAAAAAACTTTCA
CAATATGGTTTTGATGAAGAATGTGTTGATTATATGTTTAATGGCTAACATG
>103775583_2 DV501197.2
AAAATGCCGACTTGGCCACGGAGCTGAAGGCGGCCAGCGCCGGCCGCGCCGACGGCGAGCGGCGCCGCAAGCA
GGCCGAGGCTCAGCTGCAGGACGTCGCGGCCAAGCTGCAGGACTTTTTTAGGGCCAGATCGGAGCTGGCAGAC
CAATGCGTTCGCCTGCTA
>103775591_2 DT664779.3
GCACGAGGCACCGATATCACGCCCGAATTCGATCTCAACGGGTTTAGCGACGAAAAAACATAATGTTTCTCAA
ACTTTACAATTTTAACCGCCATTACAAGTGGATGGTGGTTTTTTTTCCAATATTCTTAAGTGATAAGTGATTT
TAAAGTGTAAGTGATTGGACAATAAGTGTTGCACTAAATTCCATATCCAGAGGTTAAC
AAGTAAGCTCAATCAATCATCACCTGTTCAATACACGTTGCTTTTCCACTTTCTAATTACCTCTAAAAAAATG
TGTGAACAAACGGACAGACTCGTAGTGTTGTGAAACTTAATAACAACGGACGTTGCGATGCGGGCGACGAAAA
TGACGTCGTCACACTGCCGCAGCGTGACCCCGCCATTGTCACGGGCGGGCTCCAGCGA
CATAGTGCCGTGCTTGTCACCGGACCTGTCACGCCGTGGAATATCTCCTGACGTGACATTCCCACGTCACAGG
GCGTTATCACCAGATATACTAGATAGGATAGGATCACCGGACCTCCCGCGCAGGTCCCGATCCCCCGCGCGGC
CTCGATCCCTAATGGAGACACTATTAGTGGCTAAAATGGAAGCGTTGAGCACCGGGAG
GTTAGTTCGCACGGACTCTTTGGACAGCTGTAGCAGTTTCGGCTCCATGTCGTCCCTGCCCAGTGATGTGTGT
AGATGTGATGATTGTTTGCTGGGAATCGTGGATTTTTATCTGCCTGCGCCTGATTC
>103775632_2 DT664380.3

GCACGAGGAACAACTGTTTTTGTTAAAAAAAAAACATAAAAAAAATGTGGAAACTGACTATTTTGTTAGTTTT
TGTGCTAAACAGTGCGTCTAATGGATGTGTTGAAGAAGAAAGTATAGATATCTCTGATGGAGATCGACTTTCT
AACGGTGATATAAAATATGAAGGCATTAGATACAGACGAAATGAATACGTTACTAATA
TAAATGGTACACGAGGATGTATTTGCCAGAAAAAGACTTGCATCCAGAAATGTTGCCCTTTCGGCTACGCTTA
CAGCCTTCCGGATAAAAAGTGTATAACATTTGAAAAGCGATTCAATTTGACAGTTGTTAATAAATATTTAAAT
GTTCTAAATATTAATCCGATGGAATATTTCCATTTCAAACACAAAAAGCCAATTTGTA
ACGAAGAGAACGAAGAGGTTCGTGTGCGCTTACGACAAGTGTACAAACAAAAGTCGTTTGTTCGAAATGACGG
AAGTTTATACATAGAAGTGCCTAACAGAATACCGTCATGGATAATACGAGATCCCAACAAATATTGTGTGGAT
ACATTTATTTTCGATCAAGAAAATGGCACCAAGACCTCCAATTTCGATGCCTTGGTTT
GCTTCGAGGAAGATGAAGACAGCGCGAATTATCTCCTTAGCTCCACTTGCATGATAATATCCTGCGTGTTCAT
ACTAGCGACGGTGACCGTGTACGCGTGGCTGCCGGAGCTGAGAAACTTACATGGGCGAGTCCTTATGGCGTAT
CTAATAAGCGTTTTCGTTGCCTTCTCATACTGGGAACATGCAGATACTCTATTTCTAA
ATACATCATGCTGACCGTCTGTGTGACTATG
>103775650_2 DT663718.3
ACAAAAAATATCGATGTTAACTACAATGAAATCATTAAATCTTTTGAAAAGAACCTTGACAAAATAATAAATG
AACCACATATAATAAAATATGGAGCCCATTACTCTGTTTTAACTATTTTAATTATAATTACTATTATTTGTTA
TATTATATTTAGATTATATTCTAAGTTGTACAAATCTAACAGCAGCCTTTCCCATTCA
AACTCTTTCAAATCCCCAGAGTCAACCTCTTCCAAGTCGCCTACAGTTCTTTTAGAAGTAAATGAAGCTGAAA
GACAGACTGACAAGACTTTTTTTTTAGTTTCACCTGCTAAACTTAGAATCAGGGTTTAAAATCTAAAAAATAT
CATAATAATTTAAATTTAAATCTCTTATTGTACATAAACTTCTAAAAGTAGAAGTTTA
TTTCTTTAAAGGGGGAGGTGTTATACCAGGACCCT
>103775652_2 DT663624.3
GCACGAGGCCTCCCCCTAGCGCACGCAGATGATTAACGCACTCGAGCTCGACGGGTGTCCGATCGAGGAGTTG
GGTCTGGACTTCATTTTGCCGGGCGACGGCTGCACGGAGCTGCGCAGGGGCGGCCGCGACCTGCCCGTCACTG
CGCACAACCTCCACGAGTATATAGCTCTTGTCACACATTGGTTATTATACGAAGGCGT
ATCTAAGCAATTGGATGCATTTAAAGAAGGTTTTGAATCTGTATTCCCTTTGAATAATCTGAAAATATTTTAC
CCCGAAGAATTGGAACAAGTGTTCTGTGGAAGTCCGTCAGGTGGATGCGATCAAAGATGGGATCCACGTATGC
TTGGTGAATGTATACGCCCTGACCACGGCTACAACGCAGAATCACGCGCTATAAGGAT
GCTCATTGATATTCTAGCCTCATATAACCGAGAGGAGCAACGTCTCTTCTTGCAGTTCGTTACCGGAAGCCCA
CGCTTGCCCACTGGAGGTTTCAAGGCACTAAATCCACCTCTGACAGTTGTGCGCAAGTCGCTGGAGTCGTCGC
TCGACCCCGACGAGTACCTGCCGTCGGTGATGACATGCGTGAACTATCTGAAGCTACC
GGACTACAGTAGCGCGGAAGTGATGCGCGCCAAGTTGCGCCTCGCCGCCTCGGAGGGCCAGCACTCGTTCCAC
CTGTCTTGAACACTCCCTCTCACCATGCTCCCGTAGTGCTACCTGTCTTGTATAATCTGTGTGAATGCGGTTA
GTGATATAATTAATCAGTTTCTCGTAATAGCGGCGCGACAGCTCCGGACTCCTT
>103775662_2 DT663690.3
ATACATCTAAAATGGCGAAAGTGGAAGTTAAATACACTAAATTGTTCATCAACAATGAATGGGTTGACGCTGT
CAGCAAGAAGACATTCCCAACTATCAACCCACAAGATGAGTCTGTAATCGCGCAAGTTGCGGAAGGGGACAAG
GCTGATGTGGATATTGCCGTAGACGCTGCAGTAAAGGCCTTCCATCGTTACTCAGAAT
GGAGACTATTGGATGCTTCTCAAAGAGGCTTATTACTCCTCAAATTGGCAGACCTGATTGATAGGGATGTCAA
ATATTTAGGAGAACTTGATTTTTTTGACAATGGAAAACCAGTGGCGCAAGCAACTGGTGAAGTCGCGTGGTGT
GCATCAATTATAAGATACTACGCCGGAAAAGCTGACAAAATTCTTGGAAATACTATTC
CTGCAGATGGTGAAGTGCTTTCTATGACACTAAAGGAACCAGTTGGCGTCTGCGGCCAAATTTTACCATGGAA
CTACCCAGTGCCTATGTTCGTATGGAAGATTGCTCCTGCATTAGCCGCAGGTTGCACCCTTGTTCTAAAGCCA
GCTGATCAAACACCTCTTACCGCGCTTGCTTTGGCTGCTTTAATTAAAGAAGCTGGAT
TCCCTC
>103775678_2 DV501227.2
GGATTGGTCGGACCGAGCGGCACTCTCGCCTAACGATATACAGCAAACTAACAAAGGTGCAACTGAAGCATTT
TACATTTAGCTGTAAAATCATCTTTTTGAAGATATTCAATGATTATACAGTTTTTTTTATTATTCTGTCAAAA
TTCTTCGAAGACAGTAAATGTTATATGATATATTATTGTACAACTTTAAAAGTTTGAA
TCGATTTTCGATACACAATAAGGTAACAACGGTGCCACTAATACGTTATAGGTATATATAGTATAACATCGTC
AGTCAGATTTTCAGTTTTTTTTTTTTTTTTAAGTATAACTTAACTTATGCACAGTTTAGGGTTTGAATCACCTT
ACAATATATAAGTACCTAATACAGACGCACAAACTTTCTGCTGATGCTTTATATGTAT
ATGTAATCTGTAACTCCACATCTATACTACAGATATGGAGAGATTTAAAGCAGCCTTTTTTTTATTATTCATA
GTTTTTAAATT

>103775682_2 DT663477.3
GCACGAGGCGTCTTTGTTTTGCTTACACGTTACTGTTAATAATTAATTTGTTAAAAACTACAATTCGGAATAA
AAATGTCAAACAATCCCGAGGACCAAATGGAAGAAAGTATGGGTGAGAGTGATGATGAAGACATGGAAGAAGA
AGGAAGTGATGATGAAGACGCCGGCCCCAAAACATATCTTCCAGATCAACCATTGAAA
GAAGATGAGCATTTGGTATGCGATCAATCAGCGTATGTTATGCTACATCAGGCACAAACTGGAGCCCCATGTC
TTAGTTTTGATATTATACCGGACAACTTATGATTTGATCGGTGTCAATTTCCAATGACAGCCTACTTAGTAGC
TGGTACTCAAGCATCGAGCGCACAACTTAATAATGTTTTAGTGGTCAAAATGTCAAAT
TTAAATCCAACATCAAAGGCTGTTGATGAAGAAGCGTCAGAAAATGAAGACGAGGATGATGATGATGATGAAG
ACGAAGATGAAGAAATGAAACCACAAATGACATTTGCATTTATAAAACATCAAGGATGTGTTAATAGGATAAG
AGCAACAAATTTCAAGAACTCTGTATT
>103775687_2 DT663658.3
GCACGAGGGCTCGTCTTTAATCGTCGCATCGCGGCGATAATATCGCCGTGTGGAGACTGTTCCATATAGAGAA
TGTACAGAAATACGTGGCACGACGATAATATCGCCGCGATTCTCTCGCTCGTGGAGTCAGCGCCAAACAAAGG
CAGTATCCTGTATACCATAATGATAAAGTTTTAATAACAGAGTTTCATGGTCTACACA
ATCAAAGGCTTTGGATAAATCACAAAGCACTCCATTTGCATTTTGTGACTTCTCCCATGCGTCATAAATGTGC
TTGAGGAGTGCTGCTCCCGCATCTGTAGTGGACTTGCCTTTGGTGAAACCATATTGCTCGCTATGGAAACAAT
TATTAATATTTAAGAAAATTGTGAACTGATTAAATATAATTTTTAACTATTTTACATA
AAGACGGCAAAATAGAAATCGGACTGTAATTTCCGGGGTCAGATCTATCTCCACATTTATAAAGCGGCACTAT
TTTGCAATATTTCAGTAAGTCAGGAAAAATACCGGATTCTACACAATTGTTCCCAATTAAAGCAAGATAAGGC
GCAATATTAGGTATAATTGTATTAAGAACTTTAACAGACATGCCCCATAAGTCACCAG
GGGCCTTACTCTTGAAAGTAATTTCAATCCCATTTTGACGTCCAATATTGTCCAATGCAATTCTGCGGCGACG
TTATTTTGGTACTCTTGAAACCAATATTCTTAAAGTTGGATTACTATTTTATTCACCAATCCTAACGCCGTT
>103775697_2 DT664275.3
GCACGAGGCAACTCTCTGCTCAGACTATATTAGAAGTATTGTCACCACCACGTATCACTTTACGTCCGCAAAC
GCAAACAGTGCGACCGGGACAGTCGCCAACGGTTGAATGTCTTGTCGAAGGCGATGATATTATAAGTGTTTCA
TGGAGACCAATCAATACATTCTCCAGTCGTGTCGAAAGTCGAGGGCCGATACTAGTAT
TCCGTCAAATAGAAGTCGAGGACGCAGGAAAATATGAGTGTTTTGCAATGAATAGAGTGGCAAATGCAACTGC
TGAAGCCGAAGTTATAGTAAATGAAGATTCGGATCGAGTCTCGGAGTCTAACGATAACCAGCAATGGGCCCAC
GCCGGTTCGGAGGTCCATCTCAGCTGTAACGCCTCCCTGCCGAGACAAAAAGTTACCT
GGACCAAGAATGGAAGGCCACTTCCGAGATACGTGACACAGAAGCAAGATGGCTCTCTGTTCATACGCCTTGC
GCAGAAAACAGACAGCGGCCGCTACGTCTGCATCATTGCCGACTATTACGGACGGCAGATTAGCAATTACATC
GATCTCCACATAGAGGGTGCCCGCGAATCTTCAGCGTTGGTAGCAATTGATCAACCAC
GCAGAGCGTATAGAGTTGGAGAGAAAGTAGAGATCTTGTGTAGAGCTATGTCCAAAGATATCAGGGTTTCTTG
GGAGCGATATGGAACGAATCAATATGTAGCATATAGAGTGTATGGCGACGGTGCACTACTGCAAATAACGGAA
GTGCAATCTTCTGACGCCGACGTTTACAGGTGCACCGGTTATGATGGATTTGGACGCA
GCACCTATGACGACTT
>103775719_2 DT663854.3
TTTAAGTTTTACGGAGTTAAAAAAAATAATTCTTCATAATGGAGAAAAGGAAAGAGTCTTTAAACATAGTGAA
AATGTGTCGCTTTTGTCTGACACAAAATGAAACATTGGAGAGCTTGTATGATAGAAACCGTTCAGGAAAGAAT
TCACCAAATCTTCATTTGAAAATTTTATCGTGTGTTTCTATTGAGATATTTCCCTCTG
ACAAAATGCCCTCATTTATTTGTCATCGCTGTAAATTCTTCATGAATCTTTTCTATGAATATAAGAAAATTGT
TCGCCAAGCTGATGAAACAATCTTACTTTATATTCAGCATAACTCTCCAATGGAAATAATAACATGGCCCTCA
ATCTTTTCTAAAGTATATAATAAAGTGAATGAAACTCATATAGTCAAAACTCTAGAAG
GTGCTACCGTGCAAGTTTCTTCTCATGATAACTCGGATAGTGATGAAGAACATGGAAATGTTTATAATATAAA
GATTGACGATGGTCCAGATGATGATACAAAAACTGCTTGTGTTAAAATTATTACCAGTAAAGAGAGCAAAGAA
ATGGAAGATCCATTGGGTCAAGGATTTGTCTCGGTTTCCAAACTGGCGAGGCACGTCA
TGAGACACCCTGGTGAACGTCCTTATCCTTGCAAATTTTGTGA
>103775723_2 DT664075.3
GCACAGGGACGAGCCCTCCTCAGATTTGCCACTAAACCTGTCGAAGCACTGAAGTGACCGAGGTCTCCGTCGC
CACCGAGGATCGCGGACGCGAGCTCGTCCCAAATATATGCAAAGCTAATCAAAGACTTTTAGATTAATTAAAT
CGCAAATGAAACTGCAATGTAAATTTTCGTATGTTTTATATTTTCTAAAAGGAGTCGA
GCTAAACATTCCAATGTCGGCTATCAGTCTCACCCACCGGTCCACGCCAGCGCGCAGCTCGCCGCCCCCGCGG
GGCGCGTCGCGGGGGCGAACTATATTTTTATACGAGAAGAGGACTTCCGGCGGACCCTACCGCCTGGAGGTCC
ATTTATATATTTACAGAGTTATGTATAGTGTACTGAAATAGCGTTATTTTACTATAAA

TGTATGAATGCAGAGTCACGAGTGTATTTTCGTATGAATTATTCGAACTACGACTAAATATTTTATTATTGGC
TATTACTTTAGTATAGGACGCTAAGATCTAAACGGAACAAAACACAGTTGTCGATTGTTAACGATCAGTGCCT
TGTAAGAGATGAAGATATTATATCATATTTATGAATGGGAAGTATTTGTGCTCCCGAT
GTACAGTTATCGCTATCGCGGCCGTGAGTCCATTTTTGATACGATGATTTACAAATTAGGTACAAAAAAGAAA
TATTTCGGTCCATATAGGGCGATGCCCGCAAGAATGAATACGTTCGATCTCATAATTTAATTTAGAAAATAAA
CGAGTAAAGACTAC
>103775731_2 DV501213.2
TATATCGATATATTTTCAATGTTAGCATTGTTGCGGTTGATTTGAGTCCATTCGGTACATATGATTATAATAC
TTTAGTAGCACACATTACTGATAACATTTTGTATCTTATAAGATGAAGTAAATTTTCCAATTTTGAATATCAA
TTTATGTATCTATGTAATAATAAAAGATGTATTTAATTGTAATTGTTACCGT
>103775743_2 DV501187.2
GCACGAGGAGACCTTTCACACCATCGATTTCCAATACGATTTTTTAGCTTTTCTCAATGAAAAGTTTGCGTTG
AGTGCATTAATATATAAACCAGTGGAAAAGTGCAGTGCTCTTATAATATCAAGAGGAAATATATCGGTATTTT
ATAGCTTATTGAAAAAGTGTCACGTTTCAGGAGCTGAATGTTTTGTAAACAAGCGTGA
ATCGTCGTATCTAGCGTGAGGTGTCGTTTAACTTAAAGAGAGCTCCAGTAATGTGCTGACATGCCAAGTACAG
CGATCTACCAGGAAACCAAGGCGGTGACGCCTTTTTCTCCCACTACATATGAAGAAGCTTTGGCTACTGCTGA
AGCGGGGGAGACCATAGCCACAACCTAAGAAGAGGAATGGGTAACTCGTAAAACGAGT
GAAGTGACGACAACACGTCAGATCGCTACCCGCGTGAAGCGCGAGCTGGTGCTGGAGGACGGGCGCATCCTCA
GCGACTCCGGGCCAAAGATATCTACCTCAGTCAACGAAGACACCCACACAACTAATTACCAACAAACTGAGCA
TAGAGTACCAGAGAACCAAGGTGGTGAAGAATATGCTGAGCTGACATGTGAAGATGGT
GCCA
>103775745_2 DT663877.3
GCACGAGGCTGGTGCGTCGCGCCCGACGGCAAGCCGCTGCCGGATACCTCGGTCAGGAACGGCAGGCCGGACT
GCACCAGGACTGGAAAGTCACAAACTAAACGGAGATCATCGGTTCGAGGTCAACGCAACAAAAGAAGTTGCAC
TAGAGTAGATCGCGCTCAGTTCAATGGGAACTTAATAAAAATATTCAGCGGAGAATAC
GACCGGGCGCGGTCCGACCAGGGAGGGACCACTGTCGATCCCCGAACGGTCGCCGACTGGAAGTTTAGGGAAC
TCGACCGGGATAGAAGTGGCACTCTCCAAAAATCGGAGTATAGAGGGCTACGACGCCTAATTAAAAAGGTAGT
GAAACCAAAGAGATGTGCGCGTGCGTGGACGCGCGGTTGTGACAGCGATGGTGACGGG
GAGATCGCGCGCGGCGAGTGGGCCTCCTGCCTGCTGGCCAATCCGGAGCCCCCGCACCGGACTACGACGACA
ACGGTCCAGAGCCGGAGCCCGACTACAACGAGGAGGAGCTCACGCCAGATCCTAGCTCAGTTTTACCAGGTAT
CATGAGGAATTCCTTCGCGCCCGACGGTTCCGTGGCGAGAGAGGACGAAGCAAACGAC
TGTCTCACAGACAGACAGGCAGTGCTCGATGAACAGAAAGCGGGTACTGCAGTACTCTACGTGCCAGAATGTA
CTGGCGACGGGAGGTATGCTCGGGCCCAGTGCTACCGCTCCACCGGGTACTGCTGGTGCGTGCACCAGGATAC
CGGGGAAACAATCCCGGGATCCTCCGTCAAGGACAGAGGCCCGACTGCGATGCAGCCC
CGCACACGCCAGCCCCA
>103775761_2 DT664171.3
GCACGAGGGTACACCCTTAGATACTGTTATTCTGTGCCTGGGTGGTATATTTTGCGAACAAGAAAAAATATAA
GAAAATATGTGCGGATCTGATAAGAAATTGGGTGAAATACGTGCCCTTATCCTAGTCGGCGGATATGGGACTC
GTCTTCGCCCTTTGACTTTGAGTAGACCTAAGCCGTTAGTTGAATTTGCTAACAAACC
AATTTTAATGCATCAAATTGAGGCATTAGTTGATGCAGGAGTAACACAGGTTATTTTAGCTGTTTCATATAGG
GCTGAAGATATGGAGAAAGAATTAACTGAACAAGTTTCCATATTAGGTGTATCTTTAACATTTTCACATGAGA
CTGAACCACTTGGTACTGCTGGGCCTCTAGCTTTAGCTAGGGAACTACTTAGTGCAAG
TCCTGAACCCTTTTTTGTTCTGAATTCTGATGTTATATGTGACTTCCCATTCAAAGAGCTGGCCAAATATCAC
AGAAGTCATGGCAAGGAAGGTACAATTGTAGTAACAAAAGTAGAAGAGCCCTCAAAATATGGTGTTGTAGTAT
ACAAAGATAATGGGGAAATTGAAAGCTTTGTGGAAAAACCACAGGAATTTATATCAAA
CAAAATAAATGCAGGAATGTACATACTCAATCCATCTGTTCTTGACAGAATTGAGTTACGTCCCACATCAATA
GAAAAGGAAGTATTTCCTTTTATGGCAACAGATGGTGAATTATATGCCATGGAATTGCAAGGTTTTTGGATGG
ATGTTGGGCAGCCTAAAGACTTTTCTACTGGAATGTGTTTATATTTAACTAGTCTACG
TCAAAAGAATTCAAATATGCTA
>103775780_2 DV501243.2
GGGTGGAGAAGAGAATTTAAAGTTATATTGGCTGAATTTGTATCTACAATGCTGTTGCTTTTTCTTGGCTGTA
TGACGACTGTTCCTCTTTCGGGTTTTGACAACAGCCCCCAATGTACTCGCCTATTGCCTTTGGTATGATAATT
TTATTTTGATATCAC
>103775800_2 DT665014.3

GCGCCTACTATTTATGGACTTTGTCGCTTTCTGACAAAAGACCCGAAGGATGGCCGCTCGTAGATTCTCCAAC
ACCGACATTTATCTACACTTTAATATACCTCTTCATAGTATGGATAGGGCCTAAGCTCATGAAGAACAGACGC
CCGTTTAAACTGAGCTGGCTTCTAGTGCCCTATTTTTTGGCTATGGCAGCGTTAAACG
CATATATAGGGGGGGGGCGACCAACAGCGTCGTTTAGACTTCGAGCTAGTTATATTTGCGAACCGT
>103775804_2 DT664952.3
GCACGAGGTAATTGTCATATTAAGAATAAAACAGTTCTGACTAAATAAAAATATTTATTTATTAAACAAATAA
TTAATGATAGTTAATATCATTAATTAATTGATCAATAATTTATCAATAATTAATTGTAGTTCAGCAAAAAAAT
CAAGGTCACAATTGTAAGAAATTAAGTTTTATTGTAAAATAGGATGCATAATATCATA
TGATACATTTCAGAATATTATTGATCTAATTCCAAAAATGGAAGTTGATTAAAATCTTGGACATCAATAGCAA
TATTTTCAGATTTAGCATATGTATCATCACTTTTGTTGAAATCCTGTGGTATATGTATACTACCGTCCATGTA
AGGTTCTTTGTATAAAATAGCCCATCTTGCGGCAATATCTTTATGTAAAGATTTTCTA
AATGCCATACAATGCATATGTCGATATTTTTCATATTTAATTCTAGAAAACCCCATCAAAGCTAATGAATACC
TCCAGTTTTTCATTAATTTACTATTTGCCCCAACATGTTTTGAGTCAGGTGTATTTATTATTAAAACACCACC
TACTTTTAATAATTTATAAGCATTATTGCAGGCAAGTATTCTTAATTCTGAGGAAGGT
ATATATTCCAAGAAAAAACAAAATGTTACTATATCAAAATAATTTTCTTGAAGTTTTATTACTTTGTTTCCAT
CTATTANAGTATCTGAGGTGATAGGAACATTCAGAAAATCACATTCTTTTACAGAGTTGTTGGCAGGGCAGAG
ATCAATAGCCAGGACATCTAGAAAATCATATATTTTAAAAGGATT
>103775815_2 DT664186.3
GCACGAGGAATATCCATTCAGACCAAGGTCTTATTCCAAGCAGAAGTGTATCCTCATGGGATACAGGCGTCAA
ACGAGTGCTAAAGCAAGATGAGGAGCCAATTGCGAAGCGTGTAAAACGCAGTCCATCGCCGTCTTTGAACACA
CCACCAGCTCCAGTTAACACTCCATCCTCAAGCGTCTTAATGAACCTCCTGGACATCC
ATCCAGGTGGCAAACAGATGCAGCCACAAGCTCGTCCCAACTACCAGCATGTCATGAATACACCACGAGCGCA
GTCGCTGAGCCCCGTCAACGGGAACATCCCGATACCCGTCATAAACATCATTCACTCTGCACCAAATAAACAG
ATGATGCGTGCCAATGTGCCGATTGTGCCAAACCAAGGGCATCCGGTCGCTGGCCCAA
TGTGTCCTCTCTCTTTAAATGTGGGAAGCCCGATGTACTCACCATGTATGAGCCCAAATACCACATACAGTCC
AGCCATCAGCCCAGCTCCAAAAGACCGCGTACTAAGCCCTTACTCCACCCCTCAATCTTTGTCACCTGCTGGC
AGTTACCAAATGTACAGCCCCAATAGACTACTATCTCCAACCGGAGTAATGCAAGGTT
TCGATCCATACCTGACGAACAAAATGCAGACGAGCCCTGGATTCGCACTGCAGTCCTCAGATATACTCCTCGA
TTCTAACATACCACTGACTTCGACAGACTTCTGGCCGGACTCCGAAATATTGCAAGGCACCAGCGACCTGCTC
ACCGCCTTCGAC
>103775826_2 DT664961.3
GCACGAGGACAAATTCTATATTTCAGATGAATTCAGACGAAGTATCGAAGCGCTGAGAGGTCACGATGATAAA
ATTCGTATTGTGTTAAACAAGGCGGACATGATCGACCATCAGCAACTGATGCGTGTTTACGGCGCTCTCATGT
GGTCACTAGGTAAGGTGCTGCAGACACCAGAGGTGGCTCGTGTGTACATAGGTTCATT
TTGGGACCAGCCCCTTCGTTATGACGTCAACAGACGGCTATTTGAAGATGAAGAGCAAGATTTATTTAGAGAT
ATGCAATCACTTCCTAGAAATGCTGCCTTACGTAAATTAAATGATTTAATCAAAAGAGCGCGACTTGCTAAAG
TTCATGCATACATAGTTAGTGAATTAAGAAAAGAAATGCCCTCAATGTTCGGAAAAGA
TGGTAAAAAGAAGGACCTCATAAAAAATTTAGGTCAAGTGTATGATAGAATCCAAAGAGAACAGCAAATTTCA
CCGGGAGACTTCCCTGATATCAAAAAAATGCAAGAAACTCTGGCTAATCATGACTTCACCAAGTTCCATCCAC
TGAAGCCCAAGTTGCTTGAGGTGGTAGACCATATGCTGGCGACTGACATTGCTCGGCT
AATGGACATGATCCCGCAGGAAGATGTCAATGTTGTAATGGAGCCTCTTATCAAGGGCGGGGCGTTCGAGGGC
GTAGAAGACCAAATATCGCCGTTCGGTTACGGGCGAGGCGAGGGCGTCGACGCCGGGCACGGGGACCCCGAGT
GGATCTGCAGC
>103775828_2 DT664404.3
GCACGAGGCAACCCCTCACGTCTCTGGCTATGCCCTTGACTGACAATATGAATGCTATTGGGAAACGACAAGC
CTGTTTTGTGGAATATCAGCTAGGTGTAAATGAAGGAGGTGAGATACAATATGCAGACGTGTCTTATTACTGC
GATTGTGGCATCTCGTACAGTGACACGTCTGCTGGTGACATTGTCAGTACAATATTAC
TATACTATGACGCTGCCCGGTGGAGTGTCACTGGGTACAGTGTGCTCACTGACAAGGCCAGCAATACATGGTG
TAGAGCGCCTGCTACAACAGAAGCTACAGCAATCGCTGAACTCATAATCAGACGTATATCACATATAACACAA
AAAGATCCACACGACATACGCAAACTAAATATGGACAAAAGCCACGCTACTTTAATGG
AAATGGTGGAAACGTTCAAAATGGATACAGAATACGAAGAGAGGCTAAAGCAGATAAAAGAATTTAATAGAAA
CAACGCTTGGAAGAAAAAAGCTTTAGAATTAACAGTAATGTGTTTCCCCATGTCGTACTTTGGGAACTTCCCA
GTTACAGTGTCAATTTACCATGGTGACGGTACAGTAATCATTTCCCATGGTGGTATAG

AAATGGGGCAGGGTATTAATACTAAAGTAGCCCAAGTTTGTGCGTATATCCTGAAAATCCCATTGGATATGGT
TAAAGTTAAAGGGGCGGACACTTTTGTGTCCCCAATTCTATGGTGTCTTCTAGTAGTATCACAAGCGATTGCG
TTGCTTTTGCTACTGTTAAAGCTTG
>103775840_2 DT663896.3
GCACGAGGATGCACCATTTTTTAATCCAGCAATAAATGTTCTTTGGGTTCTTTTAATGATTGTTGGACTTAGT
TCAGCTTATTTTCATGCAACATTGAGCTTAGTTGGTCAACTTCTTGATGAGCTTGCTATTCTGTGGGTTTTCA
TGGCTGCATTTGCAATGTTTTTACCTAAGAGATACTTTCCAAAATTTTTAGGAGGTAA
TAGGAGAGTGCTAGCGTTCTACTCCAGTGTCTTCTCAATGCTTTCCACTGGCTTTTTAGTTATGCATCCGAAA
GCAAATGCGTTTGCACTCATGACTCTAGCCTTGCCGGCCATCGGTTTCTTGTGCAAAGAATTAAATAGGGTAA
AATGCGCTAGAGTTTATCGACTGGGCTTACGATGTGTTGCAGTTTGTTTCTTGGCAAT
ATTCTCATGGATTATAGACCGTATGTTTTGTGATACTTGGCTCTCAATAAATTTCCCTTATATGCATGGTATT
TGGCATGTACTCATTTTCATAGCTAGTTATACAGCGTTAGTGCTTTTCGCATACTTCAATGTATCAGAAGAGC
GACCTGAACAAAAACCGCAATTAAAGTACTGGCCAAGGAACGATTTCGAACTCGGCGT
GCCCTACATTACCATTAAACATCCTTGCTAAAAAGACAATCATTTAGCTATATAAGACGGGACGATTGCAAAA
ATGCGAAAAAATATTTAAAAAATTTAGATATTCTAAAATAAACGTGTGATTTGTAATGTCGACGTATTGTTAA
TAACGAAATGAGTAACGTTATTCTAAATATCATAGTAACTGCTAACTACAAAATGAAG
AAACCAGCCGTCACTAGTGCACTTGGACTTGGCGCACTTTGACCACTTTTT
>103775847_2 DT663620.3
GCACGAGGATCGTCCCTTAAAGCCCGCCTCTAGTTTTAGGTATAGCAAGCTTCGAGTGCCACATTAGGTATTT
ATTTTTTAAACTCAGAAAGTTAATCGATCGCGATCCGATGACTCGATGACAGGGAATAGTGCTGAGTCTTAGA
TGAATATCGATATTTACGTGTGTTGTTTGAAATCTCGTAATATATTTAAAATGCCGAC
TGGCGTATAATTAAAGTCTTGATTTGTGCGTGTTTTTATTAAAATATGCAATTAGGTTAAAAATTATATTGTT
ACCCAAAGCGTGTCGTCGCTTTACTTTTCATAATAATTAACTTGAGTTCTGTTGATCACACTACATGTTATTG
GTCTTATAATTTTATATCGGTTGTTGTGATATGACGAGAAAGTATAATGAAACTATTT
CGTTGTGCGCTTATTATAGCAAAAACTCTACGTGTTGTTATGTACTTGCATCGATCTATCGATTTTTTATCGG
TTATCGTATCGTTTCTACAAACAATCTTTTTAAAAATATTTTTATTATATATGAAATTTAGTTTTACTTTGAT
TTTTGTTTTGTAAATTTCTGCTATAATAATCGGATAGTGTGGAGATGTTTCCGCAGCG
GCTTTTGTTCCGGCCCGCGACAGTAGCATAATAATGTAAGGCTCGCCTTACCTGTTATATTGAATTCCATGAA
TTTGCATTTATAGCGAAATGTGTATCGTCCTAGAGATTTATCATATTTAAGATTTAGATATACGCATCTAAAG
GACCTAATTGAACCGTGTAAAATGATTTTTTTTCGGTTGCGTCTAGTCTTTGCT
>103775866_2 DT663857.3
GTGGCCTGGGGAACCCATTGAACCTTACTGGCGAGAACAGCTCGATATCGCTGAAGAAGAACTCCGCCCGGGA
GCCTTGGCGTCGTGTGAAAAACGAGTGTCGGCCCCCAACCTGGCGGACAGTGGCCGCGCTAAATGGATACTGG
CCCGAGCGATAGATGCAAGTGCTGAAGCAAAAAGAGATAATAAACTTCTATCAAAAGC
TATTTCTGCATACTTAAATCTTCTCAAAATTAACGAGCGTCTCCCAGATAGGAAATTGGTTGAAGTTGCTCAG
CGGACTATCGACAGAATTTATTTTAGAGGTACGTACCTGAACGCTGAGCCAATTTATCGATTGTTAATTCGAA
GATTCCCACAAGATATCAACTACCGTAATAACTTGACAGTGTCCTTTTTAATGGCTAA
CAGAGCTGATTTAGCCGATGAAGTTCTAAGAGAAACACTCAAACTGTGGCCGAACGACCGGGTGGCGCTCGCC
CATCACGGCTTTGTTTTAAAAAATCAATATAATAAATTGGAAGAAGCGGTTGTCGCTTTACAGAAAGCATTAG
AAGGTGACGCCGGACCAGCTCACCAACCTCGGTTCTACTACCATTTAGGCGACGCGCT
TTTACTTCTCGGTCGGTCAGAGGAAGCTCATCAAGTACATGTACGTGGAGCATAACTGGGGCACTTCCTATCG
CCTT
>103775870_2 DT664329.3
GCACGAGGCTATTCCTACGATTATTAATAAATATACAACCATAATTAAATTACAAAAATGGTTGAAGCACAGA
AATATAGAATTGAGCAAGAGATGACAAATCTCGTAAATGAATTAGATAGAAGTTATTTAAGAAAAATGCAAGG
TGACATGCATAGATGTGCGGCAAAATGTTGTGATGATCCGGATTCTTCCTTAGAAAGA
GTTCATGGATGCATAGAAAGATGTACAGCACCTTTGAATCAAGCTAATAATTATGTGCAAAATGAAATAAATC
ATCTACAAAACAGATTGCAAAGGTGTGTTATGGACTGCAATGATTCAGCCCGAGACAGACTAGGTCCAGATCC
TAGTCAAGAGACTATTGACAAATGTACAATACAATTTGAAAAATGTGCAGTCACTTGT
GTTGATAAACATATAGGATTGATTCCTGGGATGATGAAATCTATGAAGAAAATTTTAGCCAGTGGAAAACCAC
CCCCGCAAAGCAATCAATAAAAAAACTTAAATGTTGATTATTGTATATGTTTGCACTATGGTCAATAAGTTGT
GGCTACCCTGAAGTTAGGCTTTCTGATCTTTTACACAATGTTAACTCAGTATGAAAAT

AATCAAAAGTAAAAAAAATCTTAGCAGCCAACTTCCAGGCTGAAACAACTGTGAGACTATACTGTCTATGGTT
ACTTGCATTTTTTATATAACTGCTTTTTTAGTGATAGGAGGGTAGTTTTATAATTTTTTCTTTAGTGGAAACA
GTGTGTCATTGACCATCTACT
>103775879_2 DT664795.3
CGGCACGAGGGGAACTTCTATTACCTCGAATGATGGATCCATTTTTTCCTACATGGTTAAATCATGTCATGCA
CTCTAATATTGTAATTTGTATTATTATAGAATTAGTTACCTCATTTAGGATGTATTCTACCCGAAAGATGGGG
CTGTCCATATTAAGCATTTTTATGTTATGCTATGTAGTGTGGATTCATTTTATATACT
TTAAAACAGGAAGCTGGGTATATCCAATTCTCTCTGTTATTAACTGGCCTTTAAGAATATTTTTCTATTTATT
CAGTCTTGGATTTGTTTTTGGTCTGTACATACTAGGTGACTCTTTGAACAGAGCAGTATGGTCTAAGGAAGTT
GAATCAACTGTGAAATCAGGGAAGAAAAAGGCTAAATAATAAGTCTAAAAATATTTAA
AGTCCAGTATTTGAAAAAGAAATAAATTAAATTGGATGTGTGATTACATAAGATATTATTGTGTTCTGGCTAT
TAAAAATTAGGTATAAGATAAGATAAATTTGATGTAGCTTGATATTAAATTCTTGTATAGGAAAAATTATAAT
ATATTCCATAGTCAGATTACGTAAATTAATATATTTTTAAATTTTTATATTATTTATT
TACAAATGTTTTATTTTGATATTATGATCACTTCATTTTAATTAAGTTTTGTATGTATTTATTTGTTGGTTAA
AAATTGTACAACAAATGAACATATGCAAAACTGTGCAGTATTACAGTCAATAGGTTGTGGGTTCGATACTCCC
CTTGAGGATAATAAATTGTTTTCATGTCCTTACTCTGGCAACA
>103775891_2 DT664144.3
CGGCACGAGGATTTTAACTATCTCACTTCCTGTCGGACTATATCACGAACCACTTACATTGTAAAAATGTAAA
ATGCTTACATTTTAATATGAACCAATAAAAACGATTCTTTTCTTTTAAAGCGAAATGAAGATAAATGCTTGTG
AGTTCGAAGCGGTTTTTTTTTGTTGTTGTAGATTTGCTACGCCTTTGCCGTGTGTTAT
ATTTTTGTATATAGTTACTATTGTACATACTATGTATGCTGTGTGTAGAACTGTAGATTGTTGCAAGTTGTTA
GGATATATAAGTCGAATTTATGAGTAATATAATTAATTATTACTTATCATATAGTTTGTTACAGCTGATATGT
TTTTAACGTATTTGTAGGGCGCATTCTAATTTGGACAGTACATTAATGTGGTTTTTTT
TTGGTGCAATTTTTTTTTATTTATTTACACATTCAGTCATAATATTATATATAAATTATATACATTTTTTTTC
ATGATAAGTTTTTTTTTCTTCGCTCCACACTTTTTTTTGTTATAAATGAAAATTTGTAAGCAATAATAAATTT
ATTCTGTAATGAAATTTGAGACTTATAAATTCATCATTGATTGTAGTGTACATTTGAA
AAAAATTTAAAAACAAATTCTTTCTATATATTGAAATATTATGCAAACAATCCTGGGCATTAATGTTTAGTTC
AGACAAAGTAATAGAAATAATAGAGTTGTTCTATTAATATATATATATTTGAATAGGGCTTGTTTGATGTATA
TGAAACATGTATAATATGTATAGTTTTTTATATGTTTGACACACTTTCACACTG
>103775893_2 DT664815.3
CGGCACGAGGAGCAGAACTTGAAATTAAAATTATTATTACTGTTTTATAAAAAATGAAAGACACTAAAATGAG
CACCCATCTATCTGGTGGTCGGTTAAACGTGGCTATTCTACAGGAAACTATGAGGAAGGAATTATTAAATTTG
CTTCAGCAATGCGAAGGACCTAAAGTCACAATTTGGGATGAATGGCTAGCAGGACCTG
TTGGGCTTGTAGCTCAGTATTCACTACTTATGGAACATGAAGTTGCTGATATGTTTCCTCTAAGACCTGGAAG
CTTGCCTACAATTTCTGTTAAGCATATTATCTTCATTGCCAGACCAAAACTAGTCTTGATGGATCTTGTTGCG
GATTATATCCTGTCGTTAAGATCTAAGCAAAGTACTCCAGTGGAATTTCACTTATTTT
TTGTGCCTAGAAAAAGTGAACTATGTGAAAAACATTTAAAAAACAGAGGTGTGTTTTCAAATCTTACTCTACA
AGAATTTAAATGTGATATATTCCCATTTGACAGTGATGTTATGTCATTAGAGCTACAAAATGATTTTCGCGAA
AATTATTTGGAAGGGGATCCAACTTGCCTATATAATTCAGCACAAGCTTTACGCACTA
TACAACAACTATATGGAATAATACCTAGAGTATATGGAAAGGGTCAAGCGGCTAAACAGGTATGGGATCTGTT
CTGTCGATTGAATAAGGAGGAACAAGGCTTGGGGCCGAGAGGAGCTTCCACATCTTGTATAGACCAATTGTTG
TTGCTTGATCGCGCTGTAGATTTTACTAGTACTATGTGCACTCAGCTCACTTACGAAG
GGCTCATAGATGAATTATA
>103775907_2 DT664625.3
CGGGGGAATGAAGTAATATTTTCATTTCATCGCTCTGGCAACGACCCAAATCGCGGCGTTGAGTTCCGTCTCT
CAGCAAGCAATGCCTTGAGAATTCGGCTAAAAGTGGACAACGGAAATGTCTTAATGAGAACGCAGTGTCTAAA
CACTAGGTGCCCAGATTTCCTCCGGATACTCTGCATATATATGTGGGATACAGAGTGC
GGGAACCTAAAAAAATATTGAAATAAAACACTTCATACTTAATTATCCGTTTATTTAACAAAATACTTCTGTA
AAATTAATTCCTTATTTTTTGACTTAACCTATGATTATTTTTAATTATAAATTAAATACAAATCGCTTTTAAT
TATGTAATTTGACACAACTTTATAACCAATACTTCAGGTGTTCAGACTATAATATATA
TAAAAAAAGTATATTTCATAGATTTTTTTTATTTCTTAAATTTATTGTATGTCACATATTACAAAGTTATAGG
TTCCAGTATATATTTAAATCCTTTAAAACCATTTGATATTTATAAAGCAATCGACCTAAACGTCGCAGATCCA
AAGCAATCTCCATCAGAACATTTAAAAATAAGAAAACTTAATGGAAAACAAAAGAAAA
GCACTATCTTAACCCAGATAAGCCTACTGTCCTATATTTAGGACAGTAG

>103775940_2 DT664494.3
GCACGAGGCAATCTTTTCGAAACTGGATATTAATGTATTCTTGCTGTTTGAATTAAAATTATTTTTCGTACCG
GTTTCCCTCAAATAGGTTACGCAAAGGAATAAAATAATGTCTGAACGGGAGCCTATTAGTGGATCCAGCCGTG
ATTACGGAGCTACGGCTGCACCTCCTACAGTAGCTTTTGCTGACTTCAGTCCAACCGA
ACTTTACAACTTGAGCGAAGGAATTGCGGATAATATCAATACAATAAATAATGGATTACGAGCATTGCAAAAG
ACAATGAAACAGATTGGTGGACCTAATGATAGTGTGCAGCTAAGAGATAAAATACATGATACTCAACAAAACG
TTAATAGTTCTGTATCAGCGACTGCCCGTGATATACAAAGGCTTAGCGTGGTAGTGCG
TAGGGGGGATAAACCGCAAAAATTACAAGTGGAACGTTTGACTCAAGCATTCAGAGATGCATTAGCTAAATAT
TCTTTAACACAAAAGGAATTATCAGAGAAAATGGCTGCCCACATGCCAACACAGGGTCGGTCTAGGAATGATC
CGGAAGCGTTGGAACGGCAAGCCATGGCTGATGACGAAGAAGCTTCCCTTTTAGCTAA
TCAACAGGCTCAGGCTCGTCTCGTCCAATTCGAAACGAGCATGCTATTAGAGAAAGAAGCCTATATGAATAAA
ATAGAAGCGGATGTATTAGATGTGAATCAAATTATGCAAGAACTAGCTGATATGGTCAGTGCTCAAGCGCAGA
ATGTTGATACGGTGGAAAGTCACATAGAAGCTGCCGGTGCTAGTGT
>103775942_2 DT664963.3
GCAGAGGCCTCGTGCCTTGAGTCGTCTCTTATTGTAACTTATAAAAATAGAATAATATTGAGTAATATTATCA
TGATTCTCTGATTATCTTGTGGTTATCTTGGAATTAAAAACAACACTTACTGTCAATATCTACCGAGAGGCAA
TTTTTGTCGGATTTTGTAGCTGGAGCGGAGAGCACAGCAGGTGGTCGACTAGCACGTT
GGCTGGCTCCAGGACCCCGTGTTGAGCCTTCGAGGTGTGAGCTCAAGCCACCGCTCGTACCCGCACGACCCGC
TGCAAGGCTTAACTTGCCAATAATTGTCAGAGATCAGTATGGGTATATAGTCTGCTCTCCAGCCATTAAAATA
GAGGTGGTTGTTCAACGTATTGAAGACAGCATTTTACGAGCAGGTCACTCATTGGCAC
CCGAACGTCGCGGCAGCCTGTCGGACGTTCCATACCAACCAACTGTCCGAGATACCATGTGGTTTCACGCAAT
AACGATGATGAAGCCGTATCAAAATTATTCATTTGAAGAGTTACGGTTTGCGAGTGGCGCATGGGGTGGCGCT
GGTGCCATGCTGGAAGGACTTGGAGGCGGCGGCCGCATACCCGCCGAGCGGCTGATCG
TTGATGGACAGCGAGATGGATCCTATATGGCTGTATGGGTGCCAAGGTCTCCCGGGACTTATGTGTTTAGATG
CACTATGGATGATTATCCTACGGCACAGGATTTAAAGATAGATGTTGCTGAAAGTGCAAACGATGAGTTTGCG
GCGGAGCGATGGTTGGGTTCCG
>103775950_2 DT663758.3
GCACGAGGGCTTTGACTATTTAGATTACCTCATTAAGTATTATATATTTGAAGATATTTTCGTTGCTGTAATG
GTGATCGTCTCTTATTTAAATTTTGAAGCTGCTCTTTCTTAGTATCTTATGGAAACATATGCTACATTAATGA
ATAAAAGAAGATTTTTAAGAATAAAATGTGTTCAAGATCGATAGCAACATTTTTTAGC
CAGTGGAGACTATGGATTCGTCCTATCTTCATCGCTACCTACGTTATATTAGTGCTGGTATTGGTACCATTGC
TGATAATTCACTCAATTAACAATGGATTTACTAAGTCTGATCTTTGTACTTTAGTTGGGGGTGGCTTTGTACT
ACTGGCTGTACCAATATCTATTTGGCAAATAACTCAGCATATTGTACATTACACCAAA
CCATCTTTGCAGAAACATATTATAAGGATATTGTGGATGGTACCAATTTATGCACTAAATGCTTGGATTGGTT
TAGAATTCCCAGAACAGTCTATTTATATGGACTCTTTAAGAGAATGCTATGAGGCATATGTAATCTACAATTT
TATGAAATATCTTTTTAATTATTTAAATGAAGATCATGATCTAGAGGCAGTGCTAGAG
ACAAAACCACAAGTGTATCATATATTTCCGCTATGTTGCTTTAACACCATGGGAAATGGGAAGCGAGTTTTGT
ACACAACTGGAAGCATGGAATTCTTCAATATACATTAGTAAG
>103775960_2 DT663523.3
CAGATCTGTCATTAAAGACATCATCAGCGCCATTACACGCGACATCCACCTCATCCATCTCAGGGTCTGGATC
CAAGCTGTCACACAAGCTATCTGGTGCAGAAGTTGGAGCAGTTCGACAGCTAATTACAGGATATCGTGAATCT
GCTGCTTTCCTTCTACGGTCAGCAGATGAGCTAGAGGCTCTACTGTTAAACCAACCCT
AGGACACACCTTTTTTTTTCTCAGGTCTATTCGGATTTAAATTCTGATACTTCTGAATGAACCCCGTGCTTGT
GTTTCGTTTGGAATTGTGGTTTACTAACGTTTGGACACGAAAAAAGAACTTTTTAAGAAAATAACGGTTTATA
CCAATTTGGATTGTAGTTTGAATGTAAATTTATAATACGAAGTAGGGATTTTGTTTTT
ATTTTAGTAGCCACAAGCTATATATTGTGAGATGAAA
>103775967_2 DT663562.3
GCACGAGGCGATTGCTTCTACATTGCTCCGCTACGTTGCATGTGATACTAGTGCTCTGTGTACCATGGTGACA
GTGTTTTTCTTTTCGAGTAAATAAAATTTGTGAAAATTTTCAAGGGGAGCGAAGACCGATCTCTCGCACACCG
CAACACGTTCGGCGGCGAGATGTCGGTGATGCAGAAGCGCCAGCACTGCTACTTGTGC
GACTTGCCCCGCATGCCGTGGGCGATGTTGCACGAATTTTCAGAGGCGGTATGTCGCGGATGTGTTAATTATG
AGGGAGCGGATAGGATCGAGATTGTTTTGGAAACGGCAAGACATCTGAAGCGCGCACATGGTTTTCAAGAAGG
AAGGGGTCCTACCCATGCTAAAGGAGCCCACCGAGCACCGATGGAAGCACATCAGAAT

GGAGGAGAAAGTACTGCTAGGCCAGCTCCACCGGCTCATCATGCTCCATCTGCAGGATTTACTTTACATCATT
CAAGACCCACGACAAACGCTGGTCTTTTGGCAGAGTATGGAGGACGCAGAGAGGAGCACGACGGTAGGCGGTT
AACTCACTTACCGCTAGCTCATCTGCCAACTCATGCAGGTGGTCGCCTGGCTCCGGGA
TTAACGCTAAAACGACCTTTAGATGACGATGACCACCAGGCACATCATGAACCAACAAAGAGGCTTTTAGAGG
AGCACAGTCGGCCACCACTAACTCGTGGTGATTCCTTACCCGCGGTGTCCTTACCCGCTCCTTTTGCCCCTGA
TCAAGTCTTCAGCAAG
>103775970_2 DV501212.2
TTAATACTATTTGTGTTACGAAATTAAAACCGGATTTAAATAGCCTTTCGCTATATTAAGGGAGGAAGGTCAA
TTGGAGGGAGGTATATTAAAAATATTTTCATAATAACCAGAAGAGGATTTTTTTCAAATTTAAAAATGATTTT
ATTTTGAGAATTTTTAAATTTAATTTAATTATATTTTTTACAGATTTGTTTAATATTT
ATTAAGTGAATACTTGAACTAATC
>103775982_2 DT664754.3
TCAACTTTGTAAATTGTGTTGACGATTGTTAATTTTTGTTCTCTTTAGTATTTCGCTTGTGAAATACTTACTT
TTTGATTCCATTTTGGTATCTAATTTTAGCAAAATTGCTTCCTTTGAAACCATGAATCGTAGACGAGCTCATG
AAGAAGAAGATGGATACGAGCGCCTTCATAGAAAACGACGCCGTGTATCGGAAAATCA
AGAAATTGAAGACAGACTAGAATCTCTTATTTTACGAGTAGGAGAGAAAAGTAGTTCTAGTTTAGAAAGTAAT
TTGGAAGGTTTGGCTTTTGTATTGGAAGCTGATTTGAGTACATTTCGTGTAAAAATTTTACGTATTTTAACAG
AATGTGCTATACGTATGCCAGAAAAATGCACTATATACGCCACACTGGTTGGATTGTT
AAACGCAAAAAATTACAATTTTGGTGGTGAATTTGTTGATTATATTGTTAAAACTTTTAAAGAAAACTTGAAA
ACTGGCAAGTGGAATGCAGCTCGTTATTGTCTGAGATTTATATCTGATTTAGTCAATTGCCATGTATTAGCAG
CTTCATCTTTATTGACATTACTGGAAACCTTAGTAGATTGTGCTAATGAAGATGGTGT
TCCTCAAGTCAGAAGGGATTGGTTTGTGTTTGCAGTACTATCAACTTTACCATGGGTTGGGAGAGAGTTATAT
GAAAAGAA
>103775985_2 DT663989.3
GCACGAGGGTTTGGCATACTGTGTCGATAATAACGACTTTATGCATTTAGATATAACATCAGGTGGTTCAGAG
CTAGGCAGTGCTAGTGACCTGTTAAAAAGTTCACCGATAACCGTAGCTGGCGCGGACACCAGCTCCGCTAACC
AGATAGATTCTCAAAAACTAGATACAATCAGTACAAATTCAGTTGAATCTCAAGAAGA
CGTTAAGAATGTAGTTATTGAATCTAGAAGAAAAAGAGGTCGGCCTCGTAAAGTGAGAAAAGATGAATACGAC
AGTAAAGGTGCTGAATCTCAAAAACAGTCGAAGTCTCGAGTCAATGTAATTAATGATTTTCACAATAACAACG
ATCCGCCTAACGTTTCACCTGACTCTGGTATATTGTCTAATCACAATTCACCAACACA
TTCTCCATTGCGGCGGCATGATATAGAAGATAGTCATAACAGGCTTAGCCGGAAATCCATTCATAAAGAAAAT
ACCATCGGTGAAAGGAAAAGTGCCCGTTCAAAATCTAAAACAAGAGCCAGAGCACACAATATATCTGACTCAA
GTGACTGTGACTTCCAAAAAAAGCGAATAGAGAATGAAATTAAGCAAATTAAAACGGA
AGCGCCCTCACCCATTCCACTTAAACAAGAACAAAATAAGTTTGAAAAGAACAAGAAAAATGATCCTAAATTA
GATATTGCTGCCTTAGATAGGATGCTTTACGCAACTGATAGAGTTTTATATCCGCCAAGGAAAAAAGTTGGCC
GAAAGCCTCAAAATAAAGCAAAACAACGAAGTCGACATCTAAAACGCTG
>103776003_2 DT664196.3
TTAAATCAATATAAATTTTACTTTTTGGGTACACGGTATGTTTTAACGTTGCCCTCGAAGGTGCAATCCGTCC
GTGACGGGTACACACTCTTTAGATTTCCTTCCGTATCTACTACTTTGATCTGTTGCACAGTCAAAGTATGGAA
CCCGCTATCCTCTCCATCGCCAACACCAAGTAAAAGACACTCATGTAACAATTTGTCC
ATAACAGTTTTGCACGCTCCAAGCGAGGAGTGAGACGTCACTTCCACTAACATTGCTTTGCTTTCTACGGACA
TCTTAGTATTATCAGAATTAGTGATAGGAGGGAAGCTTATCACTCTGCCTGAAGAATCTTCTACACATGGGTA
CTTGGGCTTGCCTTCTAGAAGATAAAGATATTTATGAATTCCAGAATATACATTCCTC
TTCTTCTCCTTCCTTAAGGCATCAGCTTCTGATGACAACTGCTGGAAGAGCTGTTCTCCAGTGAAAGCTTTGT
TTCTTGATAGTGGTACAAGTGACAATTTAGACGGTACTTTAGCTGTATATACTATATCACCTGGAGGGATTTT
GCTTAGATCGTGAGTAGCTATAGTCGCTACATTTCTCTTATCGCACACAGTATCATGC
AACTTGGTTTGCATTTGAATAAATTTCTTAAATAAGACATCATTAAAATTTAATTTAGTTACGATACAGCATA
GGATATAAGGCCTAATGTTCCATACTTCAGGTTCAACTATTTTAACTTTTATTGTATCATCTTCAACATGTAA
TATTTTTAGTGAATGGCATAAATCATTAGTTTCATC
>103776020_2 DT663921.3
GATTTTATATTTAAATTGAATTAAATTGCGTTTTATTTTTGTTCGCAATAATTTAAACAAACCCATGTTTCGT
AAATTTATTTAAATAAACATTTATATTAACACATCTACATTGATATACAGAGGTCCCGCAACCATGGCTCACT
GTATTAGAGTTTTGCATGGCCAAGGTAGCAGGACGAGGACAGCACTCGTGGAAATTCA

AAAAAGATGTTTCAGCGTGTCCCCCCTTACCGCCGCTGCGGCTCAGGTGGCTATGTCGAAATTCGACAAAGCT
CCCTTGCCCTACGAAAACTTGACTCAAAAAATGGAGATTGTGAAGAAGAGGTTAGGCCGTGACATGACCTTGT
CTGAAAAGGTGTTATACTCTCACTTGGATGATCCTAAGGGCCAGGAAATCGAAAGGGG
CACAAGTTACCTACGTCTCAGACCAGACAGGGTGGCTATGCAAGACGCTACAGCCCAAATGGCCATGTTGCAA
TTCATTTCCTCTGGACTCCCAAGGGTGGCTGTGCCATCTACGATTCATTGTGATCATTTGATTGAGGCTCAAA
TTGGTGGTGATAAGGACTTGGCTAGAGCTAAGGATATCAACAAAGAGGTATACAAATT
CTTGGAAACAGCTGGAGCTAAATATGGTGTTGGTTTTTGGAAACCTGGTTCCGGTATCATCCATCAGATCATC
TTGGAGAATTATGCTTTCCCTGGTCTTCTCATGAT
>103776042_2 DT663731.3
GCACGAGGCTGTCAACTTAATTGCCAATAGCACTTCTACATTTTCACATCATTTCACAAAAGATGATTCATTC
ATTCCTAGTGACGATATATGTCTTGCTAAATCATCTTCATGTGATAAATCCAAAAGTTCATCGCCATCCAACC
AATCACCACAAGCATCAAAACGGTCATCAAAAGATGAATTTCAACAGGTCCTTAGTTT
GCTAGCACAAGAAGCTATGGATACTAGTAATAAGCTAGGAGTTGCTTTAGCCGAACTTGACAAGAATCGAAAT
AAAAACGAGACATCTGATATTCAAAATAGAATATCTGATAGTAGAACGAATTTTTTAAATGGTTTAACCGAAA
AAAACTTTGACACTGCTCAGGCTGAATTGAATCATGACGAAGATTTAAAACATACTGA
TTCTCCTGTACGTGAGAAATGTAAAGCTAGTCATGATGAAAATTCTAAAGCTAAAAAAACAGAAGATAGTTTG
TTGGTAATTTCCGATCAGCTTCATAGAGTTGAGGATCAATTAAAACATAATTTTGAGAAAGAACTGAATGTAA
CTGAAAATACCTTAACACCAAGTATTATTAATATAGAAAATTCTGCAATTTCATATGA
AAAGCGACATACACCTGTTGTCGATAGTTGCATAGCTAATGGTGTACCCAGTAATATACCGCAATTACTTAAT
GAGAGTAATGAATACGAAACTGCAAATAATGTCCAATTAGATAAGCCAGCTC
>103776046_2 DT663902.3
GCACGAGGGTCACACGTCAAATATCCAATCAAAATCATAATTTAAATAAATATATATTATTTTAAATAAAAAT
TACAATGTTCTCTATAGACGCACCCGTAGAAATTCAAAATAGTGAATATTGCAACTATCGTTCGCCGTTAAGT
ACAAGATATGCGTCGAAGGAAATGCAATATAATTTCAGTGACCAGAAGAAATTTTCTA
CATGGCGAAAACTGTGGATCTATTTGGCAAAGGCGGAACAGGAGTTGGGTCTTGACATAAGTAACGAACAAAT
TGCTGAACTGGAATCAGCCATCCACGACATAGATTTCGCATCTGCAGCGGAACATGAAAAGCGTGTGCGTCAC
GACGTTATGGCTCACGTCCATACTTTGGCTGAAAGATGCCCTACTGCTGCGCCCATCA
TTCATCTTGGGGCTACATCATGCTATGTTGGTGATAACACCGACCTAATAGTCCTCAAACATGGTCTGGACCT
GCTCTTGCCGAGGCTGGCTGCCGTAATCGCCCGTTTGTCCAAATTTGCTGATGAATATAAATCTTTGCCAATT
TTAGGCTTCACTCACTTGCAACCAGCTCAGTTAACGACAGTTGGAAAGAGAGCGTCCC
TTTGGCTCAGCGACTTATTAATGGACGAGCGTGCTTTATCACGTGCGCGAGATGATTTACGTTTTCGCGGTGT
CAAGGGCACTACTGGGACCCAAGCTTCCTTTTTGCAACTTTTTAAGGGCGATAGTGCCCAAGTAAAAGCTCTA
GATAAGAGAGTGGCAGAGCTCGCTGGATTTGATAAACGATACATCGTAACTGGGCAAA
CTTATT
>103776072_2 DT663535.3
CGCACGAGGACTTCACAATTTCGGTGATAAAAACTAATAAAATGCTTATGCAATGACCGATGGTCGCCCTGTT
AGTGGTCAATTGAAGAAGTTTGTTTTCCGTGGGAAAACTACAAACAACGGCGATACTAGAGGTGAATACTTAG
AGATCGTTATACCAGAACTACTGTCGGCGGGGTACTCGTTCTACACGTGGCCCTCCGC
ACCGCTCTTAGCTTGGTATTTGTGGACGCAGAGAAGGAATTTACGTGGTCTCCGCGTACTGGAGTTAGGTTGT
GGTACAGGTCTGCCAGGTATCTTGGCGGCCAAGTGTTTTTTTGCGAGTCACATTAACTGATAGTGTCGCACTT
CCACGGTCCTTAACACATTTGTCAGCGTGTTGTGAGGCTAACGGCCTGGTCCCAGGTC
GTGACGTCCAAGTACAAGGGCTCTCTTGGGGATTATTCTTAGCCGACGTACATAATTTACGTCCTGTAGATTT
ATTACTTGCGTCCGACTGCTTTTACGAACCGTCCCAGTTTGAAGAAGTAATTTCCACTGTAGCTTATTTTTTG
GACGGTACTGACGCACGATTTTTGTGTGCTTACCAGGAGCGGAGTGC
>103776080_2 DT664123.3
GCACGAGGAGGACCCTTAAATAAAGTAAATAAAAATATAGAACTGATTACTGAATCTACTACTGAACAAACTA
GCACTACTACAACAACCAGTACTACTCCACAACCTGTAAAGCTTTTTACACTAAAAAGTACACAATTCTCGGT
TGCAACTGAGAGGTCATCTCCAGATCCATTGCCAGTACAAATCAAAAAATTTACTCCA
CGAGTTATGACTTCAACTTCTCCTAAGCCAAAGAAAAAACTAGTAACTGTAATGGATGATCTATCAGGATTAC
TACCGGCAGGATACAAATCAAGACAGTCTTACAAAGATAAACGTACTAGTTCTACTACTACTACTACTACTAC
TACAACTACAACAACTACAACTTCTACCACTACTACCCAAGCCCCACCATCAACTATC
AAAGATAATATAATAAATATCACACAAGGCCGGTCTTCTGGTATAAGTTCTAAAAACAAAGTCATTATTTTAG
ATGACAAGTCATTACTTCCAAAAGAATATAGGCCGAAAGAAAAAAATCAAGAAGATATATTTAAAAAAGTTCA
AAAGGATGATATTATCAAATTTTTACCTCCTGGGTACAAGCCCGACGTAGCTAACGAG

ACAGAGGACAGTAATAAGCCAAATATTTCCAAAAATATAGAAAAGTTAGATATATCAGCATTTCTGCCTAAAG
GATTCAAGCTTAATGCTTCAGGTTCATCATCAACAACAAGTACAACCACCACAGAAAAGACTAANACACTTCC
TAAACTACCTATAAATGCAGTACCTGTGGACATTTCCGCCTTGCTTTCCGCAGGTTTA
>103776087_2 DT664416.3
GCACGAGGCAGTCCCTTAAAAGAAATTACAAATATTTAGCAAAATATAACGAAAGTGAATTTTACGTTTTATA
TTGTTATACAGAACAATTATTGGACACTGTCCTCTAAATTTTAATATTCTTTCAGCTTAAAATTCTAGAAACT
TCTTAATATGTCTTACGCTAATCCAAATGATTATTCTTGGCAACCTCAAACAAATAAC
CAAAACTATTCTTTTAACACTTCAAATACATTCGGAGATTCACAAACGTTAGACTTTCAGAACTTCACTGCAG
ATCAGCAGAATTATTCTTTTGACCAAGCTCCAACACCGGCATCTCCCAACAATAATCAATATTATAATCCAAA
CTTATTCACACCTGCCCCCATACCTGGGGAACAAGAAGCCGGTGTAACATCAGAATTT
GATGAGCCACCACTGCTAGATGAACTAGAAATATATCCTGATAGAATATTAGAAAAAACACTAGCTGTACTTA
ATCCTTTTCATGGTCAGTCTAAAGCAGATGATGCTAATTTTTTACTGAGAGATACAGACATTGCTGGCCCTAT
TGCATTTTGTTTAGCATTAGCTGTTTGTTTATTTCTGTCGGGTAACAAAGCACACTTT
GGCTATGTGTATGGTTTATCAGTAATGTCAGTTATTTTAATGTACTTTTTACTTTCACTTATGAGTCGAACAG
AGGGTGTTTTCACTGTACTTAGTGTTGCTAGTGTACTAGGTTACTGTATTGTACCTATGGTAGTTTAG
>103776093_2 DV501255.2
TATAGTGGACGATTACTGATCGTTGGAGGAGAAATCAGTGTTTTTTATCGACAAAGCTTGGGAGTACTGATGA
ATGTCGTGGATTGATATACTTTTTTTTTAAACTGAGTTGCGGAGTGAAACGTCGCCTAGAGTTGTGTTTGTTG
CTCAAATTGCATATCTTGACGTTGAAGGGTTTGACTAAGGGTGTGGCTGCGGTGCGCT
GGTTTCCCTCCATTGTCCACCTTATGTTGTCTGCGGTTATGGTTTGTTAGGTTAAGATTTTGGAAGTGT
>103776097_2 DT664916.3
GCACGAGGTCACAGTCTTTACAACAAGTGTTTACTGTTTACTTTGTGAATATTTTTTATAAACAAAAAGTTTA
CTTACTTATCTACTCCAACTTAATTAAATAATCGCTGTTGCGTAACATTCTAATAATTATAAGTTTGACCGTG
AAATCGAGAATATTGTGAATTTCATTGTTAAGGAAAAAATTACGTCATATTTTACTCT
ATAATTATTGCTTCATTAGGTTTTTCCTAAGTGCAAAGCGTCAGTAAGCAACTGAAGAAGTATACAGGACATC
GATACATGTTCCTGGATCGATATATTAAATAAATTTTGATGATCTTAGAAAACTCCTGGGACATGCCTCCTGT
TAAACGCGGGAGACAAGGAGCTAGTGACGTAGATGACGATGACGATGACTATAGGCGA
CGAAGAGATCGAAATAACGAGGCTGTAAAGAAAAGCAGGTACAAGTCGAAGCAGCGAACGCAGGAGACATTTA
CACGAGTAACAAAATTAAAAGCAGAGAATCAAGAATTAGAGGAGAAAGTTAAATCATTGACAAAAGACCTAAA
ATTTCTTAAAGAGCTTTTTGTGGAATTTGCTTCAAATGCACAGAATCCTAAATTTCAA
ACAATGGATCTTGATGCTCTTACAAGGGATTCAGAAAATGACAAAAAAGATGAAAGCACCAGTAAAGGATAAT
AATAATTAAATAAAACAAAATACATGATTATTTCATATTTACATAATTTTTCTCACTGTTGCCAATTGGGTTA
TTCTGT
>103776121_2 DT663628.3
CTCGCTCTAAACGTCAATGTGAACGGGGATCTGTTCTCTAAAGCCACGAAGAGCAGAGCAGATGAGAAAAATT
TACAAAACAGCGTGTTCGACTTCATAATAGTGGGCGGCGGTACTGCTGGGTGTGTGCTCGCTAATAGGTTATC
AGCCGTGGCTGACTGGAAGATCCTAGTTCTTGAAGCTGGAGATGAAGAAAACATAGAT
TACACCATCCCTGGAGTACCTTTTTTTGAATTTCGCCCAATTTTATGGAATTTCAGAACAGAAAGAAATGGCT
TTTCATGTTTATCAAGACCTGGGGGAAGTTGCGAAGTAAAAACGGGGAAAGTGCTAGGAGGATCTAGTATTAC
CAACGATATGAAATACACAAGAGGGTCAAAGAAAGATTACGACGCTTGGCATAGTAAT
GGAGAAATGGGCTCTTTGGACTGGAAATTTGAAAATTTGATTGAACATTTCAAAACTTCTGAAGATAACGGTG
ACTACGATGTTCTCATGAATTCTTTTTACCATTCTCGCGGCGGAGAAATGCATGTACAAAGATTTAAATACAT
TGATCGTTACATGGAACTATTTCTGGGTGCGTTTGCAGAATTAGGATTTCGATCTGTG
GATATTAATACGAATGTACCAGAAGCTGCTGTTAATAATCAATTTGTTATGTATAAAAA
>103776124_2 DV501245.2
TTCGGAAATTGGCAAAGTACAGTACTTGATCCTATTACATTGAACGACGGCCTTAGCCCCACAGGACCAGAAC
AGGGTGTGTCAGAAATTGCTCAACAAACCAACACCTGGGCACTCGACACTGCAACAACACAATCATCTTACTT
AACAACCAATGAATTTTTTGTCTCATCAACTGTGAATGTAGAATTTTGTGGGGGTGAC
AAGCAAAACGAAAGATCTGGTAAGCTTCTCAGGGACCCCACATCCACGGAACCCGCATTGGATATATCAACAG
TAATTGATACAATTGTCGAGAAAGCTAAAGAAATAATGGGAGGTATGAATGCCACC
>103776139_2 DV501234.2
GCACGAGGGAATATTCTTTAAGATCTATAAGTGTATAATAATTTGTTAGGTTTAGTATTTTTTTTTTATATTT
TTTTTGTTCGCATTACCTAACACGCTTTGTGAAATATTTATAAGTTTTATGGTCCTTAATATAATAGTCGGTT
AGTTCTTGTGCCATATATAAGTGACGGAAACACCATTTAAAATAAAATATGTTACCAC

ATCAAGGCATGTCTAATGTAAACAATTAAGCCAATCCTTCCTAGTAACTTGTAATATAACGCTATTTTTTTTT
GCTTTCTAATTATTATTATTATTAATAATGTCGTTATTTTTTGTTATAAGTAAATATATATTATTATATTTAA
AGAAAAATCTATTTTGAAATTATTGTATTTCTTATTTTCTTTTTGTGTTACACTTTTA
TTGATTGTAAGTAGGGAATTATAATGTACGTTTCTATGTTAATACGTGAGTACGGGGGTCGCTCATTTTTTTT
ATATTCTGTATTGAAATGATATATTAGTGAATAATATATTGTGTAGGTATTATTTATTTTTGTGTTGGTAGCC
CTGTCATATTTGACAAAACGCCATTTTTTTTTTTAAATTTATATTCGTAGTTAATGCA
ATTTTTTGAAGCTCTTGAGATTTGAAGTTAGTCGAACTTATAGTTTGAACGGGCTTTACATCTTTGTATGGTA
AGATTTCTAATATATATATTATATATTTATAATTGTACGTGCAATAAGAGCGGCCCCGCTCTGTTTAGTATCA
TTGTGAAAATACATGGCGTCTTATATACTTATTGTAACTTAAAAAA
>103776143_2 DV501147.2
GCACGAGGATTACCCTTACAACATATTGATTAACTTATTAAAGTATAATTTATATATAAATTTCGTAGAAATT
ATTTAATTTAAGTTTTCTTAGGAACGTTAAAGTTTTGTGGCTGGAATTTCAAAAAGTATATAATAGTAAAATA
GTTGTTAAGTAAGGTAGCTAGTCTCTTTAAGTATTAAGTGAAAAAAAGATTTGAAAAA
GTAGCAGTATAGACAGAGAGAGTAGAGTCAAGACATTACGCACACTTTCCAAATTCGAGAGTCAAGACATTAT
GTGTTGAATGGCAGAACTCAACGCTGCACTTGCTCTTATTATATATAGTATAGTATTATTTCTACAGCCAATC
ATAAAATAAAGTTCAAAATTTCCAGTAAAAGCATTAAAGTTTCTTCATAACGAACAAA
GACTCAATCAAAGGCCAGCATTAGAAATGGCAACAGGATGTACCACTGCCACTACGTGACTCACATTACAAGG
GGTTAAATGATACACATCTAAAATTACCCCATGCACGTAATAATAATTATAAAAGTGTTTTTTTTTTTTAATA
ATACTATAGCGTGAATGTTTTTTTTTTTTATTTTGGGTAAATTAACTCTACAAGACTTA
TAAATGCCATGTGTAAATGATATGACTTTAAACAATTGGTTGTAGAGTTGATTTGATAGAGTAAGAAGTAACA
ATAGTTTTGTTCTTTAAAACGGTATTATTTTATACGTTTTACTTTATAGACGTATATTTTTGCTCTATTACTG
GGCGAATTTTCTTTAATAAATTCATCAAAAATTTCACGCAAAAATGAAATTTAT
>103776147_2 DT664580.3
GCACGAGGCAACCCTTTAGGAGGTTGCTAACGATTGTCTAGACTACGAAATCTGCTTGTTCGATATGGACGCC
TGCTATGAAACCTTTCATAAAGATGGTGACGGGCATTTGAATCTCGACGAGTTCCGCCTCATCTGCAAAGCTC
TGTTCCGCAACGACAAAGGCCACATTTACCCGCTGTCTGAGGAGAGGGTGCGTCACAT
CTTCCAGGTGTTCGACAAAAATGATGATGGCTTCATAGATAAGGAAGAATTCTCCTTTTGCTGGAACCATTGG
ATTAAAGTTATCGTGCGTCCAGTCAGCGCGTTTTTTTTCGTCGACGTACAGAATGACTTCATATCTGGAACTC
TAAACATCAGCCAATGTAACGCACAGCAAGATGGAGGCGAGGTGGTGGACCCTATCAA
TAGGCTGCTGGAGTCAGTGTCCTTCGACTGCGTCTTCTACTCCCTGGACTGGCATCCTCCAGATCACGTTAGC
TTCATCGACAACGTGCATATGAGGGAACTGCATCCTTCTAGCCCTGTGTCGGCGGACAAGGCGCAGGCGTACG
ACACGGTGATGTTCGCGGGCCCGCCGCCCATGAAGCAGCGGCTGTGGCCGCGGCACTG
CGTGCAGGACACTTGG
>103776153_2 DT663956.3
GCACGAGGGTCAACCCTAATGGTTGCCCAATTTCGGCGACATTCTATGTTTCTCATGAGTGGACCGACTACAG
TCAAGTCCAAAATCTTTATTCAGCTGGACATGAGATGGCATCACATACAGTGTCTCATAGTTTTGGGGAGCAA
TTTTCTCAAAAGAAGTGGAACAGAGAAGTGGGTGGGCAAAGGGAAATTTTATCAGCAT
ATGGTGGTGTAAAACTTGAAGATGTTAGAGGCATGAGAGCCCCGTTCTTGTCTGTTGGTGGTAACAAAATGTT
TAAAATGTTGTACGATTCTAATTTCACTTATGATTCATCGCTTCCCGTTTATGAAAACAAACCGCCGAGTTGG
CCGTACACTTTGGACTACAAATTATTCCATGACTGCATGATCCCACCATGCCCAACTA
AATCATATCCAGGAGTTTGGGAAGTACCTATGGTAATGTGGCAAGATTTGAACGGCGGTCGTTGCTCTATGGG
AGATGCATGTGCCAACCCACCCGATGCGGATGGAGTCTACAAAATGATTTTAAAAAATTTCGACAGGCATTAT
ACTAGCAACAGAGCACCATTCGGTCTATTCTACCACGCCGCGTGGTTCACTCAACCTC
ATCACAAAGAAGGGTTCATTAAGTTCTTAGACTTTATTAATCAGATGAATGACGTATGGATTATTACGAACTG
GCAAGCTATTCAATGGGTGAGAGATCCAACACCTATATCACGGTTGAACAACTTCCAACCATT
>103776157_2 DT664362.3
GCACGAGGGTAAACCTCTACCGTTGAAGATGTTATTGTTACCAGTAGTAAAGTAAGCATCGATTGTTTTATAT
AATTACTGTCGGTTAATATCGTATGTTATTAATCGTTTTTTTTTTCTCGAGAGGAGGTTAATATCTTGTGTTA
ATAGAAACTGTTAATATCGGAGGACATTGTAAAAATGCATTCCATTATTCTGAGTTTT
TGCTTAGCACTGTTCATTGATGCACTCCGCGTGTATCTTATAATCTATAATTTCTAAATGAATTATACACACA
TCATGTTTGTCATCTAAAAATCGATTTTATTTACCAAATTTTTCTATACGAATACGAGGTAAAGCCACTTAAT
ATTTTAATATTCACAATCATTGTCTCCATTTTAGTATAATTTGTTTGTGTTAAATGTT

AAATCTTATATATATTATATCTGTTTATATTATAGTGCATCAATTATGTTACAGTGTACAGACAACTCTAAAG
TGAACTTAATATATTGTAAATATCTTCTATAGCTCATCATCGATAATCCTGTCACAGAGGGCGCTGTAAAACG
TCACCCGTTGCCCGATTGTTACCCTATAAGTTATCAGATGGCCCGACAGATTTGTATT
TTGTCATATTTATACAGATATATCTCGGATGAAATATCATAGTCCATTGTACAGTATAGGCGATACCTAGTGT
AGTGAGGTCACTTACGTTTTCTTAGATCCTAGTTAGTCATATATTAGTAATACTAATTCCCCAATGTATATTA
GCGGCGGGCGCGTGACGTGAT
>103776169_2 DV501218.2
ATTAGAGGTTTTATATATTTTTTTTATTATTATATGAATTTATTTAAGAATCCATGGATGTATATGTTGTTTC
TGAGGTATCGCTTACCATTAGTAGATCCATTAAATTTTTACATTTTAGCGACGGCGTTGAAGAGGAATTACAA
GAAGAGAAGATAAGTGAACTTCAAAGTGCGCCTCATGATAGTGAAAATGTATACACTA
CATCTATGGCGTGGGGTCCATGGTTATCATAGTATGCTTGGAAATCTAGTATTAAAGTTCTAAGTGCAATAGA
TACTGCAGGTGATTTTTTTTTTTGTTTGTTTGGAATAACAACTCCAAAATATCGCATTGAAATAAATGAGTAT
GAAAGAATGCTCGAAGAGAAGAAGAAACTGGAACAAGAGGCCACTGGATGGGTGCCTA
AAAATACTGGAGGGGACGTGCCTTTAGTATTGAACGAACCATTAAAAGATATTGATAGAGCACAGATTGCCTG
AAATATTTTATTTGTATTCGTTTTTATGGTAGTATGG
>103776179_2 DT664067.3
GCACGAGGGGCAGTCTTATTAATATGTGCTGTTCAGATAACATGCATCGCAGCCGCTCTGAGACAATTTCCTG
ATGGATTTAAATTCGGAGTATCCACTTCTGCGTATCAAATAGAAGGTGCTTGGAATGTTGATGGCAAATCTAC
AAGTATTTGGGATAACTTCACACATACGAATCCAAATAGAATTAGAGATGGTTCAAAC
GGAGATGTTGCTACAAACTCTTATTATTTATATAAGAGAGATGTAGAAATGTTAAGAGAGCTTGGTGTTGACT
TTTATCGTTTCTCTCTATCCTGGACGAGAATTTTACCAACTGGATTTCCTGATAAAATTAACCCTGCAGGAGT
TGAATACTATAACAACCTTATTAATGAACTGTTAATGTATAATATAGAACCAGTGGTA
ACTATATATCATTGGGACTTGCCACAGAACTTGCAAGATATGGGTGGGTGGGAAAATCCCAATATTCTACAAT
GGTATGGAGATTTCGCTCGCACTGTGTTTGAATTATTTGGAGATAGAGTAAAATATTGGATAACAATAAACGA
ACCAAGTGAAATTTGTTTATTTAGTTACGAAATAGGTGTGCTAGCTCCTGGTATCAGT
AGTAGTCCCGGTATATCTAATTATCTGTGTGCTAAAAACCTTCTTTTGGCTCACGCTACGGC
>103776181_2 DT664875.3
GCACGAGGCTCAGGCTTTCAAGACGCGTCACCGCTCCCCCACTCGCTGTTCACGCACGAGCCTCTGTACCAGT
TCTACAACGCGGCTAAAGCTGAGTCAGCGTGTAGAGAAGGCGATTCGGATTCGGACGCGTACGAAACAGGGGG
CTCCACGACCTCCTCGCGGCCGTCCGCCATGGCGCTAGTGGCGCCCCGCGGCCCCGCT
CGCACTCTGTGGTGCGAAGTGCCTGAAGTAGTTAATTCTACTGTGCTTAGTTCTCTTGCACCAGCCCAAAAGA
AACTTCAAGAGGCGAAATTCGAACTCCTAACATCTGAGGCGTCTTATTTCAACTCGCTAAATGTACTGGAGGC
CCACTTCATTTCACATCCCGCCTTCAGAGACCCACACATACTGCCCAGGGAGGATTGG
GAGACTTTGTTCGCTGCTATTTTACCTGTACGAAGATGTTCACAAATGCTAATGACGGATCTCGAACGATGTT
GGCAAGAGAATATTTTGCTCCAAGACATTTGTGACATCGTCCAACGGCACGCTGAAGCGCGGTTTAACGCTTA
CGTCAAATACTGTGAGAACCAACCGCTTATGGTAAAAGCACTGCAACGCTTGAGGGAA
CGACCGGCCTTTGCTGCAGCGCTAAAAAGACTGGAAAGCCACCCGGCGTGCCAGAGCCTCTCGCTGCACTCGT
TCCTGCTGCTGCCCATGCAGCGCGTGACGCGCCTGCCGCTGCTGCTGGACGCCGTGCTG
>103776183_2 DT663995.3
GCACGAGGTTTTCCCTTACCTTTTCGCGGTATATTACTAAATAATAAACAAAGATCTTATTCAATTATAGCTG
TTAAGTTAAGACCAAACTACACTAGTATATTTAAACAAATTCTTTTTCCTAAAACTTTCAATAAAAGCTTTAA
TATTAGAAACAGTTACACAGCTATGGGTGCCCTAAGTCCGTTAGACCCGTCTTCTTTT
TCAAGACCAGAATTGGCTGTAATAAAACATGTTTCCCTATCATATAAAGTGGACTTTGAAGAGAAAATACTGT
ATGGATCAGCAATTTTAAATATTGAAGTAATTGACTATATCGAAAATGTGATATTAGATACAAATGACTTAAC
AATAGAATCAATTGAATTCAAAGATGGTACAGCATTGAAATATAAATTGGATGAAGCG
GTTCCAAATTACGGATCAAGATTGACTATATTGTTGCCTCAATCTGCAGCACCAGAACAAAAGCTTGAGATTA
AAATTAATTATAAATCTTCACCATCTGCAACGGCACTGCAATGGTTGGATCCAAATCAAACCTCAGGCAAAAA
GCATCCATATATGTTCAGCCAATGTCAGCCAATCCACGCTCGCTCTATCTTACCATGT
CAAGACACACCCTTTGTAAAGTTCACATATGAAGCTGAAATAATTGCCCCAGAGGAGTTTACAGTACTGATGA
GTGCTATCCGCGGGGAAAGTAAGGGGAATAAAACTGTCTTCAAACAGCCTGTACCGATTCCATCCTATTTGTT
GGGCATTGCAGTGGGTGTCTTGGAATCGAGGTCTTTGGGACTAGATCTCATGTATG
>103776201_2 DT664927.3

GCACGAGGCTAGAAACGGTGCCAAAGCCATGGCAGGTAGTGCTTTAGTGGGTGGTATCCTCTTAGCTCTAATA
GAAGGTGTAGGTATAATGTTCACTAGATTATCCTCAGAGCAGTTTAAACCCCAACAGCCTATATTCGAAGATC
CTTCAGTTTTAGGACAAAGTCAGGGACAAGGACAGAATTTTCAGTAAATGATTGTTGA
TATGTAGAATAAAATAAAATTGTGCAGTTATTTAAACATATGTGCGAGATCTGATGCAATAATTGCAACTAGT
GTGAAAATTCTTCATTTATATACATATGTATATTATAAATTGTGCTTTTTGGATTTGTATAATCAATAATTTC
AATTTTCTTACTAAATTTATCTATTTGAGAATAAATCATTTCTAATATTGAGTTAGTA
GCTGCTTATTAAAGTTATAGATGAATAGTTCATGACATAGGTTATCATCCTAGGGTCGTACTCATTATGAAAC
TACTCATAATTATAATTGACAAATTAGAAATTGAAGCCATTGTGGTATCAAAATTCAACTGTAAGTGCAATGG
TCGTAAGTTTGACTCCCACTTGGGGCAATAAATTCCTCAAAAAATAGTAAAACTGCCT
ATCCAGCTATATGCAAAAAAGTATGAAAGTAAAACAAGAAAAAATAATAATACAAGTTGATATGATTTTGATA
GTATATAAACAAATTCATATTACATAAATCAAAAATTGTCTTTTTATTATTAAAACTACAATTTTTAACGGTG
GTTCAAATAGTATTTTACTCATATATACCAAGAAATTATCACAGCTGTTATTATTTAA
GTATGTATAACATTTTGGCAGCATCATTATTTTTTCATTTTTATATAACTGT
>103776207_2 DT664773.3
GCACGAGGGTCAACTACTGAAGAGTCGTGCCGGTTTTTAGCATTTACACTTGTAATTTTTGCAGCTAACTTCA
AAATGGCAATCCACATCAATGACTCCGATGACCTCAAGAACAGACTTTCTGAGGCCGGTGAGAAGCTCGTCGT
GATCGACTTCATGGCGACCTGGTGCGGCCCTTGCAAGATGATCGGGCCCAAGCTAGAG
GAACTGGCCAATGAAATGGCGGACTCCATTGTTGTACTTAAGGTCGATGTAGACGAGTGCGAGGATATCGCCA
CGGAGTACAACGTAAGCGCGATGCCAACCTTCGTCTTCGTGATTTTTTCTAAGAAACTTGAGGAATTCTCAGG
AGCCGACTACGACAAACTAAAGAACACTGTGTTGAAACTAAAGTAAAAATATATAAAT
ATATATATGTAACAGATGGGGCAAAGGGAATAACTTGCTTTACGTGTATTTATTACATATATTATTCGCAAAA
AAAATTATAGTATATAATGCTTATATGAAAGGAGCGTAAATGATTATTGAAGTCTCGTTTTCTACTCTAATTT
CACCT
>103776209_2 DT664257.3
GCACGAGGAGTCAGTGTCGACATGGTTCACAGAATTTTATTGTTTTTGTGCGCGCTAGTGTACTTTTATATAG
CTGCTGCTCAGACCGGCAATCGCGTAAGGAGTCCAGCGACGAGATTCACGTGTCAAGGCCGCGCGTCCGGCTA
CTACGCAGATGTCGAGACCGGCTGTCAGGTGTACCACATGTGCGACGGCCTCGGCAGG
CAGTTCAGCTACTCCTGTCCGAACACAACGCTGTTCCAGCAAAGAATGCTGATATGTGATCATTGGTACATGG
TCAATTGTACTAATTCCGAAAGCGATTATGATGCTAATTTGTTAATAGGTCAACGAGACAAGCCTTTCGTATC
GGAATCTGACATGCGCCTGCGCACGCCTCGTCCAGACATACTCTCAGTACCACCGAAC
AATAAATATTATGATGGATTAAAAGAGGCTGAGTCAAAGTTCACCTTTCATCCTGACAACAGTATAGTTGGGA
TATCTGATACAATCAATCCAAATGACAACGAACTGGACAGCGACAAACGAAACTACAGACCGCCCTCTTCCTG
GTCTACCAGTTCAAAGAAAAATCGACCTACGATAACTAAAACTGACATCGATGATTCA
AATATCGGTCAAAATAAAGCCAGAACGAATGTCATAAAAACTAATTTCAAAGATACTTCTTTAAATAACAAAT
TTTCTTTAAACACTAATAATAATATAAATAGGCCATCTCAACATTTAGTTCCTCCATTCTTGCCATCTACTAC
AGAAAACGATTTAGGATCAAATGTTAGAGTGAAATCAACACCAGATCC
>103776211_2 DT664447.3
GCACGAGGATGGCATGCCGAAGCAGACGATTTAAATGGTCACGATGGATTGACGCGGTTAGTCGCCGTTAAAA
CTATAAAGGAATCAGCTTCACAGAAAGAAAAACAAGAGCTCTTACACGAAATATATATAATGCAGAAGATTGG
CACACATCCCAATGTTGTTACTCTTCTTGCTTGTTGCACTGAACAAGAACCATATCTT
CTCATAATGGAGTATGTGATGTGTGGAAAGCTCTTGACTTACCTTCGTGAGAGGAGATCCAGACCAGATCGGT
TCAGTGGCAGTGGAGCTTTAACATCTCGTGATTTAACAGTGTTTGCATATTGTGTAGCTAGAGGAATGGATTA
TATTGCGTCCAAAGGAATCGTGCACCGCGACCTGGCCGCGCGCAACGTGCTGGTGGAC
CACAACAAGCTGTGCAAGATAGCGGACTTCGGCATGTCGCGCGCGGCGGGCGGCGGCGCGCGCGGCGGCCGCG
GCGCGCTGCCCGTGCGCTGGATGGCGCCCGAGGCGCTGCTCTACAACGTGCACGGACACCACACCGACGTGTG
GGCCTTTGGCATCCTGTTGTGGGAGATTGTTACTTTAGGTTCAACACCCTACGCGGCC
ATGTCAGGAAGAGAAGTATTAACAGCCGTAACGGAAGGCTACCGTTTAGAACGACCACCTCATTGCAAACCCC
AGTTATATCGAGCAATGCACGCTTGTTGGCATTCAGATCCTTCCCAGAGACCTTCATTTGCTACTCTTAAATT
CTCATTTAGC
>103776222_2 DT664309.3
GGCACGAGGAATGACTTATAGCAAAATCAACAAACAACAGTGAAATGAAATTTTGTATGACATATTATAAACT
GATCTATTTTTACTAAAAAAAAAACTTTTTTTTTTTTATTTTGTTAAGGAATCTAATATTTGTACGTACCTAC
TATTTAGAAGAATATTTTCTAATTAACAATTGATGAGCTATATGTATGTGTAAGTAAA

ACTAATTATATTGCAAGGTATAAATTTAATTGATATCAACTATTTAATTAATTTTATCAAACACCAGTCATAT
ATTTGTAGTAAAGTGTGAAATATTATGTTTTTCAGAAATATTACTTAAGTGATTTTATACCTAATCTAAGAAA
CAATGTGTTACGTAGATATCTTATAAATAGGTACAATTTAGTATTGCTAAATGGTAAC
AATTGAACAAAAATACTTTTTTTTTAATATTTTTTTCAGAATGATAAAGATTTTTTGCATTTACTTATATATT
AATGTATTCTATAACGAGGCACTATAAGTTTTCAACGTGTACCATGTAAACTGAATAAGGCAACTATAATAAC
TTCTAAATATCAAACGGTGCCTTATATTACTAGTATTACTATAAGTAATGAATTTTGT
TTTACAAACAACGATCTGCCTAAATAAGAAAGCTTATTGAAATTAAATGTCACTCAGTCGAATATAATAGAGT
AAACCGTTAGTGAGTGTTTTAACAACTATTTTATAGAATTCTGTATGTAAATAAAAGAAATGAAATTTTTAAT
ATCTAGTACCTAAATATCGCTATAGGAGAAATATACTTTTATACGGACAGTATACATT
AGATTCTATGTAAAA
>103776228_2 DT663530.3
TGTGCTCAAGATAGTCATTACAGAATAATGTTTATTTAAAATCATTATTATGTTTTATTTGTTTATCAAAACC
CTTTTGATTCAAAATTGAATAACATTTTGAAATATGAGCGCAATCGACAGCTTAGATGAACCAGTCAGTAAGA
GGCGTAAAATAGGTCCCACATGCACGGATATTAAATGTGAATCGGAAAATAAACCAAA
ACCAAGGAAAAAACATATTCCAAACTTTCGTTTAAAAGCCACTGGCGAAACTGCATCTTTAAATTTACCAGAT
GATGAAAGAATTTTTTTTATTACTTACAGATGTACAACATTTACTTCTACATTCTTTATTGGGGAATCTTAAT
TTAACACAGCCACCTCGTTGGTATGTTTTAGATAAATGTAGTCACATAAGTCAAACAA
CATGTCTAATAATTGAAGGTGTATCTATAGAACATTGGAAAAAATATCAAAATGTTATGACTAATACTACAAA
GATATTTGATAATTTTGT
>103776236_2 DT663666.3
GCACGAGGTGCGCCCTTACAATATGCGTTGATATGTGTACGTTTCCACAATTTTCTTGGTGTTTGTGCAATAC
CTGAACGCGCAGGTGCCACAATGTCTCGTGAGGACCTCAGAAATAACTTCATAGACGAGTCTTCCTATAAAAA
CGTCTCTGATGATGACAAAGTTGAATTAAGAAAGGACAAAACTAGTGTTAAAGATATG
ATACTTCGGTTTGAAAAAACTGGTATCAGTCTAAATGGTTCTAGTGAATCTCTTGAACGTACTCGGGACCCCG
CATTAGGTAGTTTAAATAACATTACAAACTGTTTACAACTTTTTTTTCACCATCTTTCGCCAGTGTCTAAATC
ACAAGACAGGAATTCGAATGTGTCAGTGAATTCTATTTTATCTAATGTATCGGAAATA
ACTTGTGAATCGAGCTCCTCGGGTTTTGTTTCTGATAGAAAGTCATCTTCCTTAAAGCCCCATAGACGAGCAC
CTCCCCCACCCGAGCCGTTCAAGGATAATACGTCTGTATGTAGTAAATCCCCCGGCTCCAGAGGAGCCTGTAG
CGCTTGAGAGGCGGTACTCTTTCACCACTCCATCTTCATCACCTGTTGTCCTTCGTCG
CTCAAGGCAACCTCCTGTGATACGTAGAAAATCGTCAATCGGGGGACAAGTACGAGCACGAGAAACTCATTGG
TCTTAC
>103776246_2 DT663878.3
CAAGGACTGGCCGCTTACACCCTTTAAAAGGATAGAAACATGACATAAAATCAAATTATAATATAAATTCATG
ACAAAAAGTATTTGTTATTTTCAATATTTTGTTTCATTTAGTGTCGTGCAGTGTTTTGTTTGTTGTAAATAAG
TGAAATTTGTTTCTGGGATTACAGAAGTAGAGCGGAAGAATGGCGGGAATGAACCTAC
GACTGGCGTTCGCCATCGTATCCTCAGCATGCTGGTCAGCTTTCCAACACGGCTATAATACTGGAGTCATAAA
TGCACCACAAGCTGTTATATCAGAATGGCTACACAAACAAGCTTTATCCGATACAAACGTAACTCTGCCGGCC
CTAGACGACCCCAAAGTAACAACGATATGGTCTGTCGCTGTCTCCATATACTGCGTGG
GCGGGATGATAGGCGGCGTTATAACGGGGGTCGTGGCTGACAGATTTGGTAGAAAAGGTGGTCTTTTACTCAA
CAATATATTGGTTTTCATAGCCGCAGCGTTACAAGGGAGTGCTAAAATAGCAAAGTCAGCTGAAATGCTGATT
GTTGGGAGGCTAATAATCGGTATAAACAATGGTTTAAACGCCGGCTTAGCTCCTATGT
ACTTGGCTGAAATATCACCCACATCGTTACGTGGATCGATCGGTACAGTTTACCAGTTAGTGATAACCATAAC
CATTCTTCTCTCACAAGTGTTGGGCTTGGAGTCGGTCTTAGGTACAACCAATGGTTGGCAGTGGTTGTTCGTT
ATAACCGTGATACCTGCGGTTATACAATGTTGTACTTTACCCTTATGTCCTGA
>103776265_2 DT664455.3
TTTTTTTTTTGGTCAGATCCTTAAAAATTTATATTACATTGTATATTTACATGCTTATAAATTGGTTTTTAAA
TTTTAACTGGCTTTGGATGTTATATAACCAAAACTGCCCCGTTCCAACTTCCTAATCCTGTCCATGGGGTATT
CCATATAAAAATACATATTTAAGGAAAAAGTTATATTTTTCTGAATTAGTTTTTAAAT
ATTACACATGTACACGTTGACTGCAATTCCTTCAGACTTTTCATCACAAACCAATTTTATTTTAAATACTATA
GCAGTCTTATGACTTACATTTAATGAAATTATTACAAACAAATGTATGACTTTAAAATGTGTGTTACTAGACA
TCCGAATAAAGAACTAGTTACCTACATAAAAATAAGTAATTAACAAGATTTATTTGTA
CTATATGAATATATAAAACAAATAACTAATCTCTGCTATTAAAAATTAAAGAAAGTTTCCAAAATTTGCAATC
TCTTTGGTTGCTTGAATACATTTAAACCCACAAAATTAAATGACGATGGTTATATTGTAAATAACCTATATTT
TACACATTTTACTGTCATTGCAAAACTTATGAAGATATATTAAATGATAACTTAAACC

GTCACGGTTGTGGATTCGCTTCCTCCACTCAGTGGAATGAAATATTCAATATTTTCATTTCCTCAATTTAGTA
GACAAAGAGCGGCGTTAAGTTCCACCATTCAATGGACAGTGCCTCAAGAATTCGACAGATAGTAGAGACTAGA
AGTCTCTTAATGGAGTATAGTTTCTAAACTTTAGGTTCCCAGGTGCCCTCTGCCTACC
CTGCCATCT
>103776271_2 DT664704.3
CGGCACGAGGGTCGGACTTTCAGTAGATTCTATGGAACTATCGACACCTAGAAAGGAAAAACTATCTTTGAGC
CGGGATTCAGGGCTGACACTTTCTGAAGACGACTCAAATAGTAATGGAGGAAATAGCCCAGCTCCCAGGAATA
TTTTACATAGTCTTTCAGCGCCAACTTGCAATCTACAAGATCCTAAACAAAATCGATA
CATTACTGAAAATCACAATGTAAACGGTCTTCCACAAATATCGCAACGACAGCAGAATATATACTCTAACAAA
GCTGAATTATATAGTACGGTAAATGAAGACCTTTATACGATGCTTTATATGGCAGAACGTTACGTACACAACT
CAGTTAACGTTGGAATAGCAAATAGTATCGATGACAGAAATTACATTTCTAAAACGGC
AGTGCAGCGGTCAACTGCTGATATCTATGGACAGAGCCCTGCGAAGTACATAACAGGTTTGGAACCGCAGAAA
CCACCGAGAAGCAATGTTTCTAAAGCAGCTAGGACCTCAAAAGTATACAGTATGTACTCAGAATCTCAGGAAA
CTGACAAGAATCTTACTAATAAGAACTTTAACAGAGCGAAAAGTTCTGACAATTTGTT
AGAGTCAAAGCAAGAAACTGATAATAAGCGTGACATGGTTTCCAGTCAAGAGAATATAGTGTACAGCAACATC
CACGACCTTGCCATGTTTACCGAAAATATTCAGAGGCAGAACCAGTACCAGTCGCATGGCGAAATTATGTCTA
ATATG
>103776273_2 DT663631.3
AAAAGACATAGAGAGAGACTCAACGCTGAGCTGGACACGCTGGCGTCACTTCTGCCCTTCGAGCAGAATATTC
TGAGCAAGTTAGATCGTCTCTCCATTTTGCGGTTGTCTGTCAGTTATTTACGAACGAAAAGCTATTTTCAAGT
GGTGATGCATAAAGAAAAAGAGGATCTTCACTCGCATAGGCGGGACCACACGCACTAC
GATCATATCATGCCTGATGGAGAAATGTTTCTACAGGCACTCAACGGCTTCTTGATGATATTAACATGTTTTG
GAGAAGTATTCTTCGCGACACATAGTATCGAGAGCTACTTAGGCTTCCATCAGTCAGATATAGTCCATCAATC
AGTGTACGAATTAGTCCATTCGGAAGATCGGGAAGAACTTCAAAGGCAGCTATTATGG
AATTCCTTCCTACCTCCAGATGTTAACAATTTAACTCTCCAAGAGGTGCTCAGATCTGAGAGGGCTCATCTCT
CCCAAAGAAGTTTCACAGTCCGGTTTAGATGTTTATTAGATAACACTTCAGGGTTTTTGAGGTTAGACATCAG
AGGTAGAATCAAAGTCCTCCATGGTCAAAATAAGAAGACGGAGGAGCCTCCTCTCGCG
TTATTCGCTATCTGCGCACCGTTTGGACCTCCGAGCCTTT
>103776279_2 DT664502.3
GCACGAGGGTTCCATCCCCATATATGATACCAACGCCGGTATACGCGCCAATTAAACACGCGACAACGATACA
AGTAAACGGCTATCCGATGCCCCCATACAGATATCCAGCAGTACCAACGGCACAGCTGATAGAATTAGAAGCG
CCATCTTTGTACGAAAATGGAAAATTCGATAGACATGAAGAAAAGAGTCACAGAAAGA
ACGTCCAAAAACGACATTCGAGATCAGGGTTCAGTGATGCATCAGTGCCAAGTTTGCCCAGATCAGATACACA
ACCTACACTAAGCAAAGCCAAGGAGGACGGTATGGGTACATACGAAAGTTGGGACTACGTATTTCGGAATTTG
TCGAGTAAAGAGCAAGATGGTGACAGCAGAAGTGGTTTCTCTCAATCACTGGACCGAG
ATTCAAGAACATTAGACAGATTGGATAGAGAAGAAAGGAGATCGAAATATCAGCCGACAACCCTAGATTTAGA
AGATGGTCTCCAAGCGTTGAATCTTGACAGAACTTACGACGAACATATGTTCAGAACGGCGAAAGTCAATGAG
AGTTTAATGAGGCTAAAATTAGAGCAGGAACAGAAGAAAGCGAAACAATTGGCAAAAA
AGTATTCAGATGAGAAACGACCTAAAAAACCTTTAGAACCCATAGGCAACCCTAAAGCGGATGGTCTAGTCAC
ACAAAAGGTAGCTCCGGATAAAGTTAAATTGTTGACTA
>103776306_2 DT664204.3
GCACGAGGGGTTGCTGGGCGCTAAGTCTAAGTGGTTGGCTGGTGATGTCCCAGCAGCCAGAGGTATTCTTTCA
CTAGCGTTTCAAGCTAATCCCAATTCAGAAGAGATTTGGTTAGCGGCTGTTAAACTTGAGAGTGAGAACAAAG
AATATGATAGAGCGAGACGGTTGCTGTCTAAAGCGAGAGCATCCGCTCCCACACCACG
GGTGATGATAAAATCAGCAAAACTAGAATGGGCGTTAAACAACATGGACGTCGCATTAAATTTATTAGAGGAA
GCGATAAAAGTGTTTGGTGATTATGCTAAACTCCACATGATGAAGGGGGAGATCGAGGAACAGATGGGGAAAG
ACGATGACGCGTACAATACTTATTCTCAAGGCCTAAAAAAATGTGCGACCTGCGTGCC
GATGTGGATTCTACTCTCAAGGCTGGAAGAGAAACTGAAACATGTCACCAAAGCGAGATCTGTATTAGAAAAG
GCGAGGCTTAGAAACCCGAAGAACCCCGAACTGTGGCTGGAGAGCGTGCGGCTGGAGGCGCGCGCGGGCTGCG
CGGAAGGCGGCGGGCGCGCTGCTGGCCAAGGCGCTGCAGGAGTGCCCCGCGGCCGGCC
GCCTGTGGGCCGCCGCCGTCGCCATGGAGGCGCGCCCGCAGAGGAAGACCAAGAGCGTGGATGCATTGAAGAA
ATGTGAGCACGACGCCCATGTTCTACTCGCTGTGTCACAACTTTTCTGGACGGAGCGCAAATTGAATAAATGT
AGAGAATGGTTTAATAGAACGGTAAAAATCGATCCGGATCTAGGCGATGCATGGGCTT
ACTTCTACAA

>103776308_2 DT664031.3
AACAATTCAGTTAAATATTTCATTTAACGTTTTATAAAAATGCCTAATAAAAAAGCTTACGCTCCATACACTT
AAGCCAAATTGAATTTAAAAAAAAACTAACATATAAATAATTTTCTGGATCAATTTCATTCTTGGTGTTTATT
TAGATCGTAAACTCAATATCTTTAAAATGCATGAAGCATATGAGGTATCACATTTGCT
TAATGCAACAGTGCAAATAGAAGCTATTGCAGCTTATGATGGAAATCTATTTTTGGGAACAAGGCAAGGGCAT
CTATTGATGTATTCTTTATTTTCAACTAATGGTAATCAAAAGTATGAACTACAATTACTTAGATACTGCAAAT
TTTTTAGTAAAAAACCTATCCAGCAAATTGAAGTAATACCTGAAGATAACTTACTCCT
CTGTTTAACTGATAATATTCTTTCATCCTATGATATAAATGGAGTTAATTTCCCTTTAATAAAGACTTTCTCG
GAAACAAAGGGAGCTTCACTTTTTGCATTAGACAATAAGTCTGCAACATCAATGACAGGTGAATCTAATTCTG
TAGTACACTTGTGTGTAGCTGTAAGAAGAAAATTGCAACTTTATTATGGAAAAAATGG
TGAATTTAAAAAACATCTGTTTGACTTTAC
>103776333_2 DT664706.3
GCACGAGGAATGACGTAGGAGATTCACCTGATCAAATTTTTAACGCCGATGAGACGGCATTATATTGGAAAAA
ATTACCATCTAGGACCTTCACATCGAAAAATCAAAGGCGTGCTCATGGATTTAAACCTTCAAAAGAACGTGTC
ACCCTTCACGTGTGCAGTAATGTATCAGGGAGTCTGATGATAAAACCAATGATAATAA
ATCGCTCTTCAACTCCCCGTGCAATGAAAAACATTGATAAAACCCAGTTGCCTGTATTCTGGCGATCTAATAA
GAAAGCTTGGATGACTCAAGATTTATTTAAGGATTGGTTTTATTATTGTTTTATCCCGGCAGTGGAAACCCAT
ATGATAGAAAAAAATTTGTATTTCAAAGTTTTATTGATTCTTGATAACGCTGGTTGCC
ACAACATAGAACTGGATCATCCAAATGTGAAAATTGTCTTCCTTCCTCCTAACTGCACGTCATTAATACAACC
TTTGGACCAAGGAGTCATTCATACTTTAAAAATGTACTACACACGCCAAGTTTTCAAAACCATTTTCGACAGA
TTAGAACGTAGTGAAAATAAAACTCTTACTCAAGTGTGGATGGAATTTTCAATTTTAG
ATTGTATTAGAACCGTGTCATTGGCTTGCGCGGAAATTAAACCATCAACGCTTAATGCGTGTTGGAAACCTCT
TTTACCTCAAATGGTTGAAGCAATACAAGATGATATTACAATTTCACTCC
>103776344_2 DT663792.3
GCAGAGGGGAAAAAACTTCAAAGCGAAGCAAAATGGGCTCGAAAGAACGTGATATTAAAAAGAGTATAGATTT
AAAAAGCCAAAACACATACAGGATAGATAAGAATTCAAATGAAAAGAAGTTATTGCCGAAGTCAATAGATAAC
AGAATGAATGGTCATAGCAAGCCAGTGCCAAAACTAAACAAGGAAAGTTCGACTCAGT
TAGTGAAGAAACCCATAGAGACCAAATTAAATGGACAATTGAAGGATAAACGTTTGGAGAATAGTAAATCACA
ACAGAAAAGGCCAGAGAGCAAACCAGGCGCGGGTCCTAGTAAACCTGTTATAGGAAATTCATTTGATTTCGAC
AAACATGTAAATTCCTTAAGCAAAAATGGCGTCCGACAACCAGGCGACCCTAAAAGAA
AACAACAAGATGATAGAAGAATGCAGAAACGTCGTGTCGTAGAATCGGATTCAGAGTACGATTCAGAAATGGA
TGACTTCATTGATGATGGCGACGAAAACATGGACTATTCGAAACACATTAAAGAAATATTTGGATATGATAAA
TCTAAGTATCGGGACATGGACGACGATGACGACCCTACAATGGAGTCGAGCTTTGCGC
GACAACAACGAGAAGAATATTTGAGCAAGAAAATAGGTATTATGGAAGACTTGGAAGACATGAGGATGGAGGC
GATGGAAAAGAGTNAAAATAAAAAAAGGAGACGAATAAGCGACGACTGACCTGAGCTAATATGAAAATTATTC
AATTGTGTTTGTGTGCTTAAATTGTATTATATTTTTGAT
>103776372_2 DT664711.3
GTTATCTTCTCGTTCCTAAATCTTTATGAACGATTTTATAAAAATGCTTTAATATTTAATTTTATAGAATTTA
GTTAACTATTTAAATTTTTGATTTTGTGAATGTAAGAAGTGTTTTGTGAAGGAACTTCAGTGCAGCACCGATT
GGCGCTTCAGGATGTCTCGCCACGTGCTCTTGTCACGTCTTGCTGAGCATTTTCAAGC
AGCATCGCCGCCGGGACAACGGTTCCCATCTCCTGAAAGATTTTTTTCTCCTCGTTCACCGACCTCTGCCAGC
TCGCCCCCGCACTCTCCCGATTCTGTGACGCAACAGCGCCTCGATATGATACAACTTGGAGATATTGAGATCG
CCGGAAGAGCACTTAAGCAGTACGCCAGAGCTACTGAATTGGGAGCATTGATGTACTG
CAGTGGACTAGGAGCGCTCTACAGCGGTGGCGTGTGGCCTCTGCTGCTTCCACATACACCTCCTTTCGCCTCT
GGTATGGATCATGCCAGACATACGCCACCAAGAGATGATGATGAAGAAGAGCTACCCTTAAATTTATCAACAA
AAAATCGTCAAATTTGGTCACCGGGCAGTGCCTGTGAACGTGAACACGTCGAGATGGA
GTCAGACCCACAGTCTCAATG
>103776391_2 DT664874.3
GCACGAGGCCGATGTCTTGAATGGCAACATGTAGACTGTATGGGTATAGACAGAAATAATATACCAGACGCGT
ATATGTGTGAGTTGTGTCAGCCGCGACACGTAGACCGGCGACACGCGCGTTCCATACAACTCAGAAAGAGAGA
GGAGCTTAGTGCTTTAGGGGCATCTGACTCAGACTCTTCTGAATGCGATCGATCTGGA
CAAAGAAGGAAAAGGCTACTCACTGTCACAACTTATACAAACACTAGCGGTTCATGTGTTACCACGTATAATT
CAAACTTGCCAGTGCTGCCGCCCTTACCGCAGCCGACTTTAATTTTTTTCAAGCGGGGACCAAAGCGTCCTAA
GAAAGTAGAAGTCATAAGAAAAGGCACGAAACGAAAACTTTCTGAGAAGAGAGTGAAG

CGGAAAAAAGAATTAATGTTAAACAGAAGTAAATATAATAATTCCAATAGCTTATCGTCAACTCAATCTCATT
GGAGAGATTTGTATGAACTAGCCATGACGAATCACTACAGCCCAGAGTTAAGAGCGAAAATCATGAAGTACAG
CAGCAAATCAGGCAATTCTCCGAACATGGCGTCAGCTATAACGGCGCACTTGTGCACG
ACAGTGCCACACGCCGGCGGCAAAATTCTTATAGCAACGAAGGACCTTAAAGAAAACACTCCAGTCATAGAAT
TGAGAGGAAAATACATGTTGTCCAACCAGCACAGACCGCAATTGCAAAACTCAGCTC
>103776393_2 DT663613.3
TTTTTTTAAATAAAAGACAATTTTTATTTCCAAAATTTACTTACACTCATGTACATTACGAAGTATTTTACCA
TGCAATACATAATACAAGTAACACTATCTACTTTGACTTTAATAATAAATAAATTTAACAAATATTTTAAAAA
GAATAAAGTTTGTATGACAGGAAGAGAAAAAATATATCTTTATTAAAGTTAGTATTAT
TAAAATATATGTAAATATTTTAACAAAAATATAATTTATTTACCATATAAACTTTATTCGATGCAAATTTATT
ATCTATGTATTTTATTTGTGTTACTAATTTCGAATCATAAGAGCAAGACATAGTTAGATTTAGTAATAAAACA
TTAAAACTTTTGTAACGTTAATAAAAAATAAATAAATTTCCAATTTTATTAATTGTTA
TTATTTCTCATGCATTGACTCTTAGATGATTAATAACATTTAATTTATATACAATTATATAGCTACTGTTTAA
TAACTAAGTATACAAAACCCACGATGTTCTTACACTCTACTGTGGATCTAGAAAAATATGGCAATCATGCGTG
TGTGACTTTACAATACAAGCCGTTCTACTATTTTGTACACATTTTATCTTATTCATAA
TAATACATTGTAATAATTATCCGAAATAATGACAACTTAAATATACGTTTTATTACATTTTTGAGCATGTGAT
GGACAGTTAGATTGTACAAAAAAAAAATTCG
>103776406_2 DT664399.3
GCACGAGGATCGCCCCTTTAGAGATCGATGTTGTGAACGCAGTGGAACTGTACACTGTTGTGAAGCGCTGAGC
GAATCTGGTCATATTGTGCAATTCAAGCTAATGTTTTTAATTTAAACTGGAATATTTTATCGATTTTTCTAAA
GTGCACGTTGGTGTGTGGTTTCCGATGTTGGCGAACGCCAATATTTGTGAATACTTCT
CATAAGTTCGGAGCGTTCAAAAGTACGTGTGTCTTTCTATCAAGGGGCGCGGCCGCAGTGTCTGTCCCCGCCA
CCTGTACAGTCTGACTCACACATCCTATACGCCGATTTGCTCTCAGCTGCTGCTAACGTCGATATGGCAGACC
ACACCATGTACAACCGGCAGATCAGCACTGAAAGTGGATGGGACAATCCGTTCCGTCC
GGATGGCGACCTAAGCCGTGAAGCCGATGAGATTGTCTCTCTAATTAAGGGAGGTAAACCAATTACACCAACT
CCACCTGTTGTGAGCCAGTCACCGATTAGCGATGAAAAAGAGGAAAAAGTTCACGCAAACAATGTCAATGCTA
GTCCTCCCGCACAGACGAAGTCGAGCCCAGTGCAGCAGACCACCCCGAAGAGTAACGG
CACAAAGAACGGTGTTGCCGTGCCAGAAGTGCGTTCGGGGCAAGTGGAAGTTCAGCGCTCGCCCCCACAGGAG
CCTCTGCAGCCCGAACATGTTACTATCAAGAAGAAGCAAAAGTGCAAATGCTGTGT
>103776410_2 DT663945.3
GCACGAGGTGCAGACTTTTTAAAGAGTCGTATTTAAGTACGATTTTTTAATATATTATTTACATAGACTCTTG
ATCTTTTTATATTTTTACTATTTGCTTCTTTTGTTTGTTGGGTTATTGTTACTATCGATGTTATATTATAAGA
CGTGAACACTTCACGAAAATAACGAATTAGTGTTAAGTTTTTCAATATTAGACTTAGG
ATTGCTAGAAATTACAAACGTTGCTTAATAAATTTAAATTTTAAAAATGTTTAAAATTAATACTGGTTATAGG
GCTGCCACTTTTATCTTAACAATAGTAAGTGATGAACCAAAAATGTTCTTTATGCATTATATGTTGAAATAAA
ACTAATCACTTTTATGTTTAAAATATTGGAACAAATATAATACAAGGTCTCGAAAATT
GCATGAACAGATTTTGCTTACTTTGCTATATATAAGATAAAATATATAATTTACCATATATAATAATAACACT
AGGTCTAATGTAAATGCCCTCTGATAAAAATATATATTTAAATAATATTATTATTAAATTCAGTGGCAACCCT
AATTAAAAGTGATCTCTAAAAACATTTTGAGGCTTTAATAAAATTGTTTGATAAATAT
TTTGATCTGCCAAAATCCTAATACAAAGACTTACATACCATATCTTCCTATTTCATAAAAAAATTACAACATC
ATATGAGGAGCTTTTTATATATTAAACCAAAGATTTGTTTATATATGAGCTAGGACAAAGAAATACACCACAT
TAAATGATATTTTTACAAA
>103776434_2 DT664054.3
CACCCGCCAAGGCAGGATTACACGTCAATTTTACCCACAAGGGCGAAGTGAAGGAGAGAAGAGAAAAATTCCT
AACAGCTAAATACGGGAGCCACCAGATGGCACTGATCAGAAAACGCCTCGCCGTTGAGATGTGGCTCTACGAC
GAGCTCCAGAAACTATACGAAACTCCGAGCGACGCGGCCGCCGACTCGCAGGAGGTGG
AGGTGGACGTGGACGAGCTGCTCGACATGGACAGCGACGAGATACGGCGGCGACATCTCGCGACCTTACTAGC
AGACGCCAAGAAACCACAAAAAGATGTACATAAATTCATAAATGATTTGTTAGAAAAGGCTAAAACTCTATAA
ATTACTGTAAGTGGGAGCAAATCTCAGAAGGCTCGTGCGGGGAAGTCCCGCGTTGGTA
TATATAAGAGTAGGTATGTATAAAAGGGAAGGCGGCCGAGCTGCTGCCTGCTGGAGACATATCACGCGTCACT
TCCCGCTCCAAACCAAAAGTTACATTAAGTAGGTTTTCTTAGAAATAATATGTATATATGAGCGACCCTGTTG
CAGGCTTGCGGGTTGGCCTATACATATAAATAGAATAAAAATTTTTTGCATCAGTGCA
TTTATGCCGGTAGCGAGATATTGTTTTATATACATACATTAAAGTAT
>103776437_2 DT663799.3

GCACGAGGGTTTGACCCTTAAACGTAAGGCTGACTGAATTTATAAGACCCAAAGCTAGTCCAGATAAAAAAAA
TATAATAATAATAAAAAAACGTGAAATCATATTTTTGTGAACGTAATTATTAATCGTTAATTATTGTCTCATA
AGTAATTTGTACGGTTAGTGTTTAAATGTCTGTCATAATATAAATAAAAAAAAAATTG
TAACAGTGTGCGTCAACTGTTCTCGACCGAATGTACATATGAAGGTTATGTATACATATAAGCTATGTAAATA
GTATAGTGTTGCCGTCGCGGCGCTTCGTTGGCTGCCCGGTACTCGTGTTTGGATCGGTGCTGCCAGACGTTCC
GATTTTTTTAGCCCAAAATTAAATGGTTCCGTGTTTTTTTTTTAATTATTTTAAATTG
GTTATTAATAATTTCATAGAGAGAGATATTTAGATTCTGAAGACTCGATTGGTGATTTTGATGTATTTATTAA
AATCGATTCATCAGTAATTCAATTTTAAATTTAACCTCAGTATTTTTTCAGTTTCATTTCATGTATTTCGTTT
ATGATACAGACAATGTAATTTCTTTTTGTTAAAGTAACAAACGCTTGTAATTAACTTA
ACCATACATCTGGTTCACTTTGTAGTTAACTGTTCTAAATATAGTACAGTATCTTTTAGTAATTATTGATTAT
TAATATTCTAACGCTTGTATACGTCACAAGATCTCGTAATTTATTAAATTTACGATGTAAAAAAAAATGATCA
AAACTATATAAATGAATACTTTTGTAACGCATTTTTAAAC
>103776440_2 DT663974.3
TAGGTTTGAAATATTTTACCCTATATGCTGTTTCCGTAAATGGAGCGCTGCCTCTTCCACCCTGTATATATTT
TTTTATCAAATTATATATTTATCAATTTAATTATTAGTGCATATAATTGGTTGGTATGTTTTTTTTATAAATA
AAAATATTGTATTAATTTCTTTCTCACAAAATAATGCTCAGTCACCTTTATGACGTGA
AAGGATATATTTAGTGGTTTTGCCATATAAATGAATAAAAAGATTAAAATTTACCGCGGCGGCACAAATAGTA
TCTAAAAATTTAAAAAAATTGCAAGATTCTTTGTATTTACATTATCTGAGAATAAAGAGAGTTGACTGCCATT
AACAACACAAATTTGCGATGCACACAATACTAATGAGATTAGCGAGAAATAATATTAA
AATTAAAAGGAGCAAAACGCACATATATTTTGCTCTTTAGTCATTTACGAAAACAATTAAAATAAGTAATTAA
AATATATAATTTGCTTACACTTCCCAGTACTTAGGTACAAGTT
>103776452_2 DT664679.3
GCACGAGGACTAACCTCAAAGGAATATTTGGATAAAAAAACACTTACACAACTTACTTAAGCGTAACGTACTT
ACCTACTACCAAGTTACTTTCATCGAGATTAGAACTTGCTACCCATATTTCAACCAGAAGGTTCCAATTAAAA
ATAAATATTTTTTCACATTTCCGAATGAGAATCGATTGTAACACGAGCGGGACATCTG
TCGCCGTTGCCTGCGAATTGGCAACAGCGCCTCTTCTTACAGAACACCATATTAATAAATAATTATGAAACTA
TTTCTTAAGATAAAAAAAATAATCTGTGAACGCGAAGGTTGTGGGTTCGATTCCCATGAGGGGTAATGAAACA
CGTAAAGTCTTCGAAATTGGACGGTGCGAGGGGGACGGAGTGTCTTAATACTAGTTTC
CCTATGCCTGGCCTGCTACTTGCAGGATTCAGCGTGAACCTAAAACAATATTAATATTTACAACTGCTGGATC
TCAACGCCATACTTTGTTCGATTTAATATTTAAAAACAAAATGAATTTACTATTAGCATATTAAATTTAAAAT
GGGTTGGACTTTAGCTTTGAACAGATGTTCGTGTAAAATAAAACGTGTAAGTCTGAGT
TCTTGAACTCAACGCCGCGGTTTATCTTCCGTGAGTTGAAGGGAAATGAAAATAATTGTAACACAATAATTAC
ATCTCACGGGAATCAAAGCCACAATCCTTACGTTTCCACTTAGACG
>103776460_2 DT663890.3
GCACGAGGGCGAGGCGTACGCGTCGCTACACAACTTACTTTAGTTTCACCTGTATTTTTTTCAAATCCGAGTG
TTTCTAATATTGCGATATCTGTAGAAAGGATTTCTGTGAACGTTCTATTAACAAATTCTTTTAGAAATTAAAA
TAAAAAAAGTGTTAAGTTAGTGCATTCCAAGCCGGTGTCGTTCTTTAGTGGACGAAGT
GTACTGTGTGTGTTGGCCAACTGGTTTAATGCTCTGTAACGTGAACTAGATAAATAAACAAGAATTATTATAG
TAGAAATATGACACTTAAGTGAATGCAGAATGTAAATTTTGCATTTGGCAGCTAATTTTGTGGTAAAAATGGC
TCGAGGCATGTTGAGTGAAAAAGAACGCCATTGCTTGGACGCGGGTGCAAAAAGTCAG
CCAGATCGTGTGCAAGATGCAGAAAAAACATTAAGCGATTGCAAATCTAACGGACTGAGAGTTCGCTTCCTCG
ATGAAGAGGGGAAGACGGAAAGGACGGTCAGCACGGACACGGTCGACTGCGCAGTATCACGAGTCAAGAAGAT
TCATATACTAAAAAACAGAAGTTCCAGCGAAACCAGCATACTCTTGAGAAATCCATCT
CTGTTTAAGAGACGCGTGAGCTCAGAGCGTCGGTACAGCGATGGCCAAACAGACACCCTCAACAATAATTCCA
TGAGTGNAAACTTGAATGGGAAGTCACAAACCGATTTAAACCAGACTGGAGACCAAACCAAAGAGAGCCAGAA
CTTCAGAAAGAACTGTAACGTTTACAGTGTTAACCAAGCGAAGACTTTG
>103776466_2 DT664255.3
GCACGAGGGTCCGCCTTAGATGGTTAGCGTTATTTGAGGGTGTTTTCAATTTCTAACCAGCTGAAATTTAGAC
TACTCGCATAAATTTCCAAAATGATGTCCTCGTGAGAGTATATATACTTAATTCTGCATTATCAAGGCGTTCT
CTGCCGATTTAATACCATTTGGTTTACAAAGTTTTGCCGTTAAAGTTACGAAAGAAGC
GGGGCAATGGCATCGTTTAGAATTCACGAAGATCAAGAAAACTCAGCATTAGGATTGAGAAAAGATGGTGACG
TATTTGCAGCTGCCTCCCAGCGTCGTGCCCTCGGAGATCTCTTTCATTTTGCTTGCAATCAGAACAGAAATGT
TAAACATGCTGGATTGACGAATGGACCTTGTAAAGTTCAAGATGAAAATAGAACAGTA

CGCCAAATCAAAAATGAAAAAAATATTGTGCCCCCTGTGGCCCAGTTTCGCGCCTTCAGTGTATACGAAGATA
AACCTACAGAGGTCGAGGTTAAAAAAAGAGAACCGACATTCAAACCTTTCGTTGCTAAAGAGATAAGAAAGGA
TAACTTCTTCCTTAATGCTGCAGAGAACGTCAGAGCCCTATGTGTCCAAGCAGAGAAA
CAGAAGCAGCAAGAACAGAGCANAGAAGTTCCGCCAGTCAGTCAGCCACTAGAGGAGAAAAAAGATAAAAATG
TTGTGGAATCACCTATGTCTGTTGTGGACTCCAGTATACTCTCTAT
>103776468_2 DT664305.3
GCACGAGGTAATACTCATACATCACAAGTTTCCGACACAACCTCATACTTTAAACTAAATACAATAAATATAT
CTATTTTGTTTTACATAAGTAGTTTATTATATTTTTATTTCTTTAACTGGCAGCATTTTCACTTAAATCGTTA
ATCAGAAGAATTTGTTTGATTGAACGCGCCAATCTTAGGATAGGTTCAAATTTAAATA
TTGTTTTTGTATTGCATTTTAACAGATTCATCAAGAACACAAGATGGTGAAACATCGAACCTTATTAATATGT
AGAAAAAAGAATATATATAAAAAATAAATTTATAAATACTATATAGATACTATCTAGTAGATAGTACCTACAT
ATAAATGTCTTTTATAATAAGATTGGAAGCCCTTAGAAAATCCACGATAATAACAAGT
AATAATAGCAAGAATTGAGCAGAAAAATCTAAACGCACATCAAAATTTAACAATATAATATAATAAATAAATA
GAGAGAAAAAGACAGTTAAAAAAATTGTGACATCAAATTTATATACTTTTTTAAAGTAAAGGCCATTTCCATT
AATTGGAAAAAAATAATATTAACTATATTATATAGTATAAAAAACACATTCTATAACA
ACCATAATATATCTGTTTACAACTACTAACACAATAGGTTGTCGGTTCAAATCCATACAACCTTCCATACATA
ATTCTACAGTTCCTCTTTAACGGCTGGTTCCTGTTCCTCAGCCTCCAATTCCTCGATATCCTCATCGTAACCC
TCGGAGTCTTCGATATCCGGCATCATGGACTGGTGGGGCATGGCACCTCCGAAGCCGT
AGGGATCTGGGCCGTGGATTTTAACGTA
>103776477_2 DT665026.3
GGGCTTCAAATGGTGTAGTTATATATGATACGAATATAAAACACTCCGATCCACTTACGGTGTTAAGCCATCA
CAAATCAAAAATAAACTCAGTAAAATTTCTGCACAGACCTGATAATAGTTGTATAGAACTACTTTCTTGTTCA
GCTGATAAGACTGCTGCAATATGGACTTTAGAATTTTTTTTTTGGAATGTTACAAGTG
TTTTAGCAGGGGGGGGAGATGGAGTAACTTGTATCAGTGGATTATATATTGAAGATGATTTAGTAGTATATAC
TGGATGTATTGATACTACTATGAAAATTTGGGAACGTAAAAATGGTACAACAACACTTAAACAAACTATTGAC
CTACATTCCGGCCTCTGCCTAACTCTTAAAGCCCATATTTTAACTATTGCCAAAAAAC
CCATACTATTTTGTGCATTAGATGATCATAAGATACATATTTTCACAGAAGAAAATGAATAT
>103776479_2 DV501180.2
GCACGAGGCTTTCCTTTAACCGAATATATACTGTATACAGTGTGAAGTTAAAGAGAATCTACTTACATTATGG
AGTTACAAAAACGTAAATACATTTGCGTATTTTATTTTTGATTCCTTTTCATTAGGAATTGAACCCACAACCT
ATCGAAATTATAGGCGCACGCTTGCAGCCCTTCACCATACGAACATATAAAAAGAACA
TAAACGCTTATTTACGACGTTTTCTTAGGCACTCATGTTAATTCTATATGGTAACTGGGGCGTTAAAAATTGT
GAAGCGCATCCGTTAACTTTCAAATACTTTCTTTTCATGGTATACATAAAGTTAAATGTGGAACACGATACGA
CGCAACGGCTGAATATATATATATATATATATATATATATATATATATATATAAGAGAAG
AGGAGAGTAGAAAGCAAATTGTACTGCATAAAATAAAATGATTGTATCTTAGAAT
>103776485_2 DT663977.3
GCACGAGGTAGATACTATTTCCCGGTGTGTAGTTTGTGAAGTGCCAGCTAACGTTATAGCTGTCCATAGTCAA
ACTCTTGATATACCAAATTGCCCTGCAGGCTGGAATTCTCTATGGATTGGATACAGTTTTGTTATGCATACTG
GCGCTGGTGGACAAGGTGGTGGGCAGGCGTTGGCTAGCCCAGGCTCATGCTTGGAAGA
CTTCCGAGCTACACCATTTATTGAATGTAACGGAGAAGGCGGAACATGTCATCATTTTGTTAACAAGCTCAGT
TTCTGGCTTACAACTATTGATGACAATAAACAATTCTCCATGCCCGAGAGAGAAACTCTTAAATCTGGACGAC
TTCTGCAACGGGTATCTCGATGTGCAGTTTGTATTAAAAATACAACATAGCAACATAT
TGTATGGACATAATACCATTATATTAAATTAGCTGATAATTTATTTTTGTTTTCCTTTACAAATTTTATCAAT
AAGGATCGAAATATCAGGTAATGCTATAAAATAACATTAAAAATTGAACATATTTTATATTATAATGTACACC
AAGGCAATGTAACCTTAAGAATTAAGGTATTGTATAGTATATTTAATTTCATATGTAA
TAACAATAAGTGATAAGATATTTTATCTGATTCTTAAAATACAAAACTATTACAATTATTGCTTAGCAATTAA
AATCTGTATTACAATATGGATTAGTACCTATGTATTGCTACTAGTCACAAAAAATCTTTAGAAAAACCTTTTG
ATATTTATGAACATCTTACTGAGAAGGAACAATTTTGACAGAGCTGGCTTTTGAATTT
ATATAAGAAACGTTGTATATATGTATACTTGTAAGTGTATTTAAATTTTTT
>103776493_2 DT664902.3
GATGAAATGCAATGAATTTGAAAAAAATAAACCTCGCCATCATATGAACCTGGACAATTTGGACAAGGCATTG
CACGACTTGGGCATGTTGAGCGCAATAACCGAAGCGAGGCGTGAATTTTTAAAGGAATCTAAATCTAATTTGG
GCGTAATGCGACTGAACACGCGGTACGTGTCGAATGTCACTGTGGGGTATTGCATGAA

AAGAAGCACAAACAAAAACAGACTTTGCCCGTACGTTTTACCCGTGAGGCATAGAACTAAGAGCGAAAACTCC
GATGCCATTGGTTGTGACGTGATTTACAGCAGCTGTGATTTTAAATATAACTTGCTGGAACCATCTACTAGTA
CCTATATGAGAGAGAATCTCGATATAAAGAGCCCCGCGAAGCGATGCCAGTCACTGGA
GAATTTAAATCTCAATGTAGAGACACAGCCGAATGCGGAAGCTTCTCCTGATATGGATTGTGTGTCCACAAGA
ATTCAGAAGTTGCAAGTTGATGAATGACAGTCAAATTATACCTTCAACTCTGTGTAGTGCATTATGCGTGTAC
ATTACAGTCGGTAGCTAGAAGAGTTGAGTGATCAAAGGACTGCAAATTTATTTTTTGT
GTATGAATTTGTATGATAGTACGAAAGTTAGTAAATGATTATGAATGTTCCTAGATCCTAATTTAATTAATTT
TGCATGTTGTCTTACAAATGATGGGTGATTA
>103776503_2 DT664351.3
GCACGAGGCAAAAACTTAATTGGTCTGTGTTGTGTTATAAATGAAGAAAACTTTTATCCAACAAAAGAGAACA
TTCAACTTGGAAACAAGGTTAATCTATCTGATCGTGTAGGTTCCAGCCTTGATGTGATTGCATTAGAAAAAAC
AATGAAAGCACTAAATTTTGTAGTTATAAGTAAACATAACTTGAGTCGAAATGAAGTT
TTTCAATTTTTAAAGGATGTGATACGGGATAAAATTCTACCAGAAGATAGTATATTTATGTTATGCATACTGT
CTCACGGAGTCATAGGTCATGTCTATGCGGCAGATTCTATCAAAATTAAAGTGCAGGATATACAAAATTTATT
AGACTCTGATGAATCTCATAACCTTTATGACATACCAAAGGTGTTAATAATTCAAGCT
TGTCAGAGTGAAGAAGAATCAGGTGCAAATTTGAATATTGTGTCCGATGGGCCAAGTTCACGTGATAAACATT
TAAAAAAAACTAATTTTTTAGTTTTTTCGGCTACTGTACCTGGATATGAAGCCTTCAGGGATATAGATGTTGG
TTCAATATTTATACAATGTTTGTGTGATATAATTAGAAAAAAGGCAAATCATGAAGAT
TTATTTAATATTTTTACAGAAGTAACTGATTGTGTTTTATCAATATGCACAGAATTGAAGCGAGACCAAGTGC
CATCATTTGAGTCCACTTTGAGAAAAAAGATTTTTTTACGTCGGAATAATATACTATTGAGCTATTCCCATTC
GTGGCTCTGACTCGATGCTACTCAAAATGCAATGTC
>103776505_2 DT664354.3
GCACGAGGATTATTTTTTAAGCATAATTAATTGGAATGATTTGCGTAAATTGAAATATACAAGATAATATCAT
GTAAAGCACCACGGCGCACCGTCCTGAGCACAGAGGCAGAAGACGCAAACGCTTTACATGTCGCACGTGATGT
AGAAGACAGTTCTAGATACTGAAGAAATACTTTAGAAATTGACACTTAAACTTTAAAT
AATATAAAAAGAGAAGAATGAGATTCTTGATCTCCTAATTTAAACTAAATAAATAAAAACAGAGCCAACTAGT
GTGGTGACATATGCAAATTACGGAATACCTAGTTAATTTTTTTTAACATTATTGCATATTGCAGTGAGCATGG
ATTTGTTATGTTTTCATTTTAAAATAATATTTAAAATATAGAAATTAAGTTACATCAA
AGCATTTTTCACTTTTCGAATAAATTATGCCTAATTATTTATTATATCTTTGTTATCATGTGATAATAGATAC
AATATTATGATAAGCAATGTTTAATATATATAGCCATATCAGATTACTAAACTAGAATTGTGTATTTTTTGCA
ATATCCACAAAAAAAATTTTTTAAATATAGAAAAAGTATAAGTGCAAATACTCACAAT
TTATTGGAGGTACTGTCGTATATTTTTGTAATTTTGTGAAAAATATTTACATCGTATAATATATTTTGATTGT
ATATTATTAAATGTAAAAATAGTTTTGTTATTCTCGCTCGCTTAATAAAATACAAAAGAATTTAAAAAAAAAC
ATTTTTTATATTTTTAT
>103776511_2 DT663990.3
GCACGAGGCTAGACCGTTCCATTTCACGGACTTGGGCGGTCAGTTCGAAGCGAACGAGGTGCTGTCGCCGTTG
AGACACGAGCAGCTGGTGACACCCTTCCCTTTTGAACAAAAGGAGCTGTATTCGGCTTCGCAACCCGCGTTAT
TCCTGCTAGACGATGGTAAGCGCGTGTGGGTGTGGCAGGGGTGGTGGCCGCGCGACGC
GAACGACCTTGACCCCGCTGAGAGGAACGGTGTAGGAGCATTCGCAGCACGCTGGCAAGCGATGCGAGCTGCC
GCATTACGTACAGCCGAAGCCTACTATCGCATCTCCAGAGGTCCCGGGGATGTTGGCGGTGTTGGGGGTGAAC
CCGACGTCCGAGTAGTTGCAGCCGGCTTGGAACCCCTGGCCTTTACGCACCTGTTTGA
TGTATGGCTAGAACACGATGAGGCCGCTGATGCTAATATTGCTCACGGGTACAAGGCCGGCGAGGCGCTGCTC
GGCACGGTGGAGCTGTCGCGGCTGACCAGCTGCGACGCCGTGCTGCCGCTGGCCGCGCTGCAGCGCCGCCCGC
TGCCCGACCACGTCGACCCCCACCACCTGGAGAGACACCTCACCGCTGCTGATTTCCT
GGAGGCGTTCGGCATTGACCAAAGAAGAGTTCGATGTTCTACCCGCGTGGAAGCAAACCAACCTGAAGAACCA
TGTCGGCCTATTTTGAC
>103776515_2 DV501174.2
GCACGAGGACCCCCTTTACCGGTATCTTATCCCGACCACGTCACACGCTTTCATTACTGTAGCTGCTGTGAGT
GCTATTCAGGTCCCCTCTATCCACATCTCCTCATGGTTGCTACAACTCTTTGGACGAGAAACAGCGGTTATGA
CATTTTTCACTGTTGGCGGCCCATGCGTGGTAACACTAGGTCTAGCTCCCGATAATGT
CTGGATAAACTTAACTTTAAGCTGCGTTGGAGTCAGCTGTGTCTCGATAGTATCTACATGTGATTACATCTAC
ATTTCGGAATTATTCCCTACAGTTGTAAGGAATATGGGCATGCGTGCTTGTTCTATGACAATGAGAATGGGTT
CGATGGTGGCTCCCTTCATTGCTTAATTTTGCTGAGACGAGACCGTGGCTGGCTACTA

TAGGGTTTGGAGTCGCTCCTATAAGTGCTGCGTTGGTGTGTATTTTATTACCTGACACCAAAGCTACTGCTCT
GCCTGA
>103776545_2 DT663722.3
GCACGAGGGAATAACCCTGCAATTACTACATCAACTATATCAAACTCAGCAATCTGTTCGACTGAACCTGTTG
AGCCCAAGGAAAATTTAAATCATAATAAGGTTTCTACAGATGGAATTTTTGAAACATTAGGTAACTCAAAAGA
GGAACTTAAACAAAATGATGGCCCCGTAAAGGATGAAAGCAAATCAGAAAATGTACAA
ATTGATACTAATAAACAAAACAGTATTAGTAGCAATGCCAATGAATCTATTTTAAGTCAAAGTTCTCTTTTGT
CAAATGCACCTAATAAAGTAGATACTGTAAGTGAACATACTTCAAATAATACTGCACAAGTTAATTTAAATTT
GGGCCATACATCACCAGCAGTAAATACAATAAACAATAAATATGAAAATACGGCAAAC
ATCAACGTTTCAAGAAATCAAACATATCAAAGTGGTACCTTTCAAAATATGCAAAGTATTATTCCTCAAGGTG
TATCATCTAGTATTGTGCAAAGTATTCCAAATAGTATGCCAAGTAATCAAAGTCACAATAACTCTACTACAAA
TTTTCAAACAGCAGTTCCACAGGGTTCATCCCCAACATTGCAAAACTTACAAAGTAAT
GTTCCAGTTCCTCATTCAATACAACATGCAAATATAACTAATACTGTATCTTCTAGTATACCTGTTCACAATT
CACAAAATAATATGCCATGCTTACCTCCTAATGTTCCGAATATATTAGGAAATGTTCCACATGCTGTTCATAA
CTCACCAAGTAATATTATTGGTGCTCAAGTCAATGCTCCT
>103776550_2 DT664755.3
AGCTTTAAAACATTAAATAAATTATTATCCAAGTTTAATTAATATGGCTCGTGAAAGCAGCGCAGGATTTGAT
AGACATATTACTATATTCTCTCCTGAAGGCCGATTATATCAAGTTGAATATGCATTAAAAGCGATTAACCAAG
GAGGCCTTACGTCGGTGGCTCTAAGAGGCGCCGACACGGCAGTCGTGGCGGCTCAGCG
TAAGGTTCCCGACCGCCTCTTGGATCCAGCTTCTGTCACACATCTGTTCCGTCTGACTGACCACATAGGATGC
GTGATGACTGGCATGACTGTTTACAGCAGGTCTCAGGTGCAGAGGGCTCGCTACGAGGCGGCCAACTGGACGT
ACAAGTACGGCACAGAGATCCCTGTGCACATCCTGTGCCGCCGCATCGCTGACATCTC
ACAAGTGTACACACAGAATGCTGAGATGCGCCCACTTGGATGCAGCATGATGGTAATAGCATACGACAGTGAG
TCTGGCCCCAGCGTGTACAAAACGGACCCATCTGGCTACTACTGCTCGTACAAGGCGGTGGCAGCGGGCTCCA
AGGCCACCGACGCCAACGCCTACCTCGAGAAAAAGCTGAAGAAGAGAGCTGACCTCAA
CCACGACGACGTCGTGCAGCTCGCCGTGGCCTGCCTCTCGCAGGTGCTCTCTGTGG
>103776568_2 DT664690.3
GAGGAGAACCTTTATTACTTCAGTAGCTTTAATAAATAATGCCAAAAATCTTGCTGATGGTAATTGGCACAAG
CTAACAGCTACAAGAAACGGCCGAGTAATATCACTCAGTATGGCGTCTTGTACTAACCATGGAGATGTTTGTA
GTGAATGCGAACCAGGTGACGACGTATGCTACGGAGACGATACGGGACAAGCAGGAAC
TTTAAACTTCAACAACGAGCCACTGTTAATTGGTGGGCTTCACAAATCTGATCCTGTATTAGAACGTCCAGGG
CAAATCCACTCTGATGATTTCGTTGGATGTATGTTTATTATTACAATAAACGGTCGAATCTTAAATTTAACTA
ATCCTCTGGACGCT
>103776582_2 DT664533.3
GCACGAGGAAACTTTAAAAAAAAAAATACATTTCGTAATATATAAAAGCATCTAGACGATTTTATTGCAGGGA
GGTATATCTTAAAATATTTTATTCATAGTTAAATTACAGTTTAAGATGTAACTTGCATGTCTACTTTTCCATT
CGCGACTTTTTATGTTACTTATCGATATTTAGAGGATCGGTTCCGTGGCATAAAAGGA
TCGCATCCGACGTGTTTCGTACTCTTTTCGGTAGTAAGCTTTTACATTTAAGGGTTAAGCCTGTCAGTAGGCG
CAGAGTTCCGGGCCGAGATCGTATCGCAATATTTTGAGCTCGTCGGCAGCTGTATAGGTTGGTAGGGGCTGGT
CCGGCCGACCAGACCGGAGACAATAACGCGGTTTGCGACCGCGCGTTCGCCGCGCGCT
ATCTCGCTCGTCCACCAACACATATTTTATTCTATCTATTTTTAATATTAATGTCAAATATTAACATAATTAC
TATACTAATACATATTGCGCATATTTTACCTATGAATGGAATTATTTTATGCCTAATTCGAATTGTATAAAAT
CACCTCGGCTATTCTGTTTCTGTAGAAAAATTCAGGATTACTTCCCTATAAGACTATG
TTAGTTACGCGTATACATAACTTTC
>103776594_2 DT663821.3
GCACGAGGCCGTCACACTATCCCCGTACCATCTGGTGGTAGATATGTGTCACCACCCGGCCGACATGTTGTAC
CATCGGGTCGACATGTTTCCCCAACTGGCCGGCGTGTGTCTCCATCTAGACAGATGTCTTCTAACAGAGAAAT
GCTAACTGAAAGGAAGATGTCTCCTTCAAGAAGAGACTCTCCGCAATTAAGAAAAGAG
GAATATGTTAGAGGAAGGAGAATGTCACCTCCATCACGTTCACCGATTGACAGACGTCCATTACATCAATCAT
CGGACAGAGACGCCAGAGACATACCTTCCCACGTCCGTCACGAGTCACCAAGACGGCAGCCAAGATTTACTTC
GCCACATCGTATTGATCAAAAGCAAGCGTCATATCAAGATGATCATCACGCTGCCATG
AAGAACATAAGACCTGCCTACGAACCACATGTACAGGCGCCAAATGAAGCTATATACTCAGGTGGTTATCGTC
CCAACATACGTGATGAAGCAATTAAATATCAATCGCGGTCGGAACACTGGAAGGAAAGAGAAAATTTTTCCTC
CAAAGGTTTGATCGAAGATAGAAGAGACGGTCCAAGAATACAGCAATTTGAGGTTTCT

AGAGCATCAGAAAGACCTACTGAGCAAGATGCGAATCGTTCCCGGTCGCCGAAGCGTGATCGATCTCCAATAC
GCGACCGCTTCAAGCGCCATTCTCCTTCTCCTCGATCCCCAAGGCGTTCTTGGGCTTTAGAAAAACGTAGAAG
TCCAGAAATTCGTGAACCGCCGCCGCCTCCGGT
>103776608_2 DT664967.3
GCACGAGGACTGTAACGTGTATTTTATAATGTGTAGTGATTAAGGATTGGACGGTAGTTTTAAAGTTATTAAA
CTTAAATGTAAATTTGATATGTCTTGATGAATTAGGTACAAATTAAATTAAGAAATAATATATGTATGTAATA
GTAGTTTAAAAAAATATTGAAAAAGTATACAATTATTAAACGAGCAAGATGTCAAAAA
GAAATTCACTCGTTTTGCCGCCAAGAACTGAAATACGTATAAAATTAGTGCCGCCCGATGGAGGATGGGGTTG
GATGGTTCTATTGGGAACCGCTCTTTCGAATATTTTCAATCAGTCTATGTTGTCTCTCTTCAGCTTGCTCTAT
GGGGACGTTATATACTCGATGGGTCATGAAACCCAGGGTGCAGCCATTGTTTTAAGTA
CCATGTTATTCGTCACCAATTTCGGAGGTCCAATTGCCGGAGCCGTCATAAAACTAACGAGCCCCAGATTCGT
CGCTGTCTCTGGCGCCTTTTTCTGTACTATTGGCATTTTATTCAGTGGATTTTCAACGAATTTATATCATTTG
ATCCTTACTTATGGCGTGTTGCTAGGTTTGGGTCTAGGCTTAATACAGAACGCATCGT
TTGTATCTATCAATAGTTACTTTAAAGTGCGGAAAAGTTTAGCAGTCAGTCTTGCCATGGCTGGCACTGGTGT
TGGACAAACACTCATGCCTCACGTAGTCCGATATCTTTTAGAACACTATGGATTCAAAGGAGCCTGTTTGCTA
TTGGCATC
>103776610_2 DT664111.3
GCAGAGGGTGAGACCTCTTCAGAAGGAAATAAGGATGACGGAAATGGCTTAAAGAAATGGAAAGGGGTTGCGT
TGTCACCTGAAAACGACCGAAAAGCAAAACCTTTCCCAACTGATATTCCACCGAAGCCAAAAGAAGTTGACAC
CGCGACCGTCCAAAAAGAAAACGTCGAACCTGAAACCCCGGTGGAAGCGACTACCACA
GAACCTACGATAGTGACAGTGGAGCTGAACCGCGGCTGGAACAGTCGGCTCGGGTTCAGCGTCCAGAGCCACC
CGGAGTCGGGGCAGAGCTACATCTCGGCGGTGTACAGCGACAGCGTGGCCGCGCGCGACGGCCGGCTCCGGCG
CGGCGACGTCATTTTACAGGTGAATGACGAAAACGTAACGTCAATGAAAACACCAGAA
GTAATCGATTTATTGAGAATATTGCGTGGATCGATTTGCATCACAGTTCTACGACCGCCAAACGTTCAATGAA
ATGTAAAATTTTATTAGAAAATTTGATCAATACTATTTAATAAACGCACAAGACCTAAAGCATGATTGCTGCG
TAAAGATCTTAAATTACTTATTTTATTTAGCTCAATCAAATAAAAGTACACAAGTGAA
TGCTCACTATAACATAAATGTTCTAGATCTGTCATATTATATACTAGGAATATCATATAGTCTTTCCTAATGA
CGATTAGTATGTAGATATGCCTAANAAATACACACACAGCTTATGAAGTTTATGATATGACATATAGTAAATA
TGTATGTATATATGCATAAATAAAATTTTTATGTGGATGGATATTCCTAATAAGTAGT
CATTTTTTTATATATGAACTGTCTGTTTGGGTGTAATGTTTGA
>103776618_2 DV501236.2
GTCTCGTCGTCCACACTTCAGAAAATCAAGAAAGTCCTGTAGATTTTACTGTATTATTTGGAGATTGCGGATT
GGTTCATGTGAACGGCCTGGCGAGCTTCGTCCTCGTGATGCAAAAACACCCGAAATTAGTGACATCGAATGCC
AAAGCTTTCCATATTAAATGTATATACAAAACTGGAGAGCAGAATGTGACACTTGCGT
TTAACGTGTCAATGTTGACAACGGCCGGTACGATCGCCAACACTGGGCCACCGCCCACTTGCTCTATGCGCAT
CGTCTCTCGCACTGGCGACCAGGTCAACTCCGCAGAAATCG
>103776622_2 DV501229.2
CTACATATTATCACCTAAACTAGGGCTACCATTGTTGTTATATAACCCCAATCTTAAAGGGCATATGCACTAG
AATACCTCCCATACAACATCACACGAACTGTACGTTGCAGAAGAGAACTTATTCTATCTGCTGGACCTGTAGC
ATCACCTCAATTTTTTTTTTCTATCTGGCATAGGTCCCAAATAGAATTTGCGAACACT
CAGAATACCTGTTTTTAATGATTTAAAAGTTGGAATGAGTCTTTATGATCATATTAGTTTCCCTGGAGTTATT
TT
>103776626_2 DT663797.3
GCACGAGGCAGGAAACTTTTAGAAAAGAAATATTATCTTGTGAATTCTCTCGCATCTAAATCCTTCATAATGT
GAATTCTGTGTTGTAACTAGCATTTTAAGGTTTATTTTAGTTAATTATTAGTTAGTAATCTATATGTGTAAAT
CGTCTAAGATGGTTTCTAGCGCGACGAGGGTGATACAAGTCACCAACATTGCTCCTCA
AGCGACGAAGGATCAAATGCAAACTTTATTTGGGTATTTAGGAAAGATAGACGACATAAGGTTATACCCAACT
ATCAGAGACGTATCCTGTCCAGTACAGTCTCGGATATGCTATGTTTTATACTATGATTCGACCACGGTTAATG
TAGCTCAACATATGACAAATACTGTGTTTATCGACAGAGCATTAATTGTCATTCCCAT
GCAATCGGGTGAGATTCCCGATGAACACAAAGCTTTGGAAATGTCCAGTAATGGGACTCTAGTTCCAGGCCTC
AATACAGTGGAACCACGTTTACCAGCCCACGTTATCAACACTTTAGAAGGTGTGCCACCAAATCAAGTTATAC
AAACTTATGATCCTAAAATCGCAGCTGCGGGACTACCACCTTACCCACCTCTTCCTGC

TGCTTATGATTCCAGAAAAATAGAAGAAATTCGAAGAACTCTTTTAATCATTGATCTTGGTGCGCTCACACAT
CAGCATATCATTGACCACTTTTGTGAAGCAGGAGAAGTTAACTACTTACGTTTTTGTGAAAGAGACATTGATA
AACTCAAGTATGCCTTATTTGA
>103776647_2 DT663763.3
CCGGGCTGCAGGTTTCATTGAAGGTACAGATTACATAAAAAAGATATTTAAATAAAAGAAATATATATATAAA
AGTTAATTAAAACAATTGAAGAATGTCAATCTTAGCATACAACGGTGGTGCTGTGGTGGCTATGAAAGGCAAG
GATTGTGTAGCCATTGCTACAGATAAACGATATGGTATTCAAGCTCAGACAGTTTCAA
CAAACTTCCCAAAAGTCTACCAAATGGGCCCAACTCTTTTTGTGGGTCTCCCTGGATTAGCAACTGACACACA
GACCGTACATCAAAGATTGAAATTCAGAATGAACCTATATGAATTAAAAGAGAACCGTATAATTTTGTCTAAG
ACATTCTCAGCAATGCTCTCAAATTTGCTGTATGAAAGGCGCTTCGGACCTTACTTCA
TTGAACCTGTTATTGCTGGATTGGATCCCGTTGATAGTAAACCTTATGTCTGCAATATGGATCTGATTGGCTG
CCCCAATGAGCCTGAAGATTTTGTAGTGTCTGGTACATGCACAGACCAGCTGTACGGTATGTGTGAAGCTCTG
TGGGAACCTGATCTAAAACCTGATGAATTATTTGAAACTATTTCACAAGCGTTGGTGA
ACGCAGCCGACCGCGACGCAATCTCTGGTTGGGGCGCCGTCGTGTACATC
>103776659_2 DT664326.3
GCACGAGGCCTCGTGCCGATTTCCACGGGTTGAAACGCGTTAAGGAATTTGTAAAATATAATGTATTTGTCAT
AACATTAGCTGATAGTTTAATTTATTATAGTTTGTTTTCAATAAAAACATCGCGTGCACCGTTCTGCGCCGCC
AGCGACGTGAATTTGTTACAATTTAATTCGCTTTTAGGTTATGTTAGTAACGAGTTCG
TATTTATTGTATAAAACATTTAAACGATGACCGAGGGAGAGTTTGAGAACAATAGCCGGGTCGTAGCGATACC
ATGCTCCTTTGCGAAACGAAAAGTGGAATGTGCTGGGGGTGCCTTTCTGAGATATTACAATATAAAATGTCAC
GGCGAAGATACATTACTGCTCACTTCGAATTCAAAAGATAAGGCTGAAGAAGTAATAT
TTGAAGATGACGTGGAGTACCTGCGGAGCTTGGACCCTAAAGAGTGGAAGAAGCAAGACCACTACGCCGTGCT
AGGTATAAAAAAATTACGTTACGAGGCAACAGACGATGACATCAAACGTGCTTACAGACAGAAAGTACTTAAA
CATCATCCAGACAAGCGCAAGGCACTAGGCGAGGAGGTGAGAAGCGATGATGACTACT
TCACGTGCATCACAAAGGCTTACGAGACCCTCGGAACTCCTAAAATTAGACGATCATACGATTCTGTTGACCC
TGAGTTTGACGACTCCATTCCGACGACCAGTGAAATAAAAAAAGACGGGTTCA
>103776665_2 DT664084.3
GCCGAGGACGGTCCTTTTTTTTATTTAACCACAAATATTAATATAATATATATATATGAAAAATATAACTTAA
AATAATACAAAAAAAAATAGTCGGTAGATTCAAATTAGTTGAATTAGAGTGGTCTTGTCAAGGATTTTAGCAG
ATATTTATTTATTATGATCTCAACGTTTGTACAGAGTTGTTGTGTGATAAACTGAGTG
ATTCATATTTAATTGTATATAGTGTTGTGCTGTGTTGTGTTGTTTAGTAAACGCGCGCTTGTTGTTGATGTTG
TTGTGGGCAACACTCTTTGTTGGTGATAGCGAATGTCTTCGTAATTTCTTTTGTGTAAAAATATTATAATGTA
CGAGTGCTTCCTCGAGATAGCGTTCGAGGCTGAGCTGAGTACGAAGGAGGACCCCGAA
CTACTAGCTATTGCACCAGAACAATGCAACGAAAACGCATCCACCAGGGACACGGCCATAGCGGAGTTGCGAA
AAATGATATATGACAGGGGAGAATGCACGCCACACCGAACTGACGACGCGTACCTCTTGAGATATCTCCGTGC
GAGAGATTTTATAGTACCACGTGCACATAAGCTGCTAGTCCGATATTGTTCGTTCCGC
GAGGAGTACCCTCACCTGTACAAAGGAGTGGACCTCTGGGGCCTCATGAAGGTGAACGACGCGTACGAGGGCT
CGATGCTGGACAGGCCCGATGTTGGCAGACTATCTGTTTTTAGATTTGGAACATGGGATCCTAGTGAATTT
>103776671_2 DT663782.3
GCACGAGGGCTTGACCCTCACGGCCATGGTTGTGGATCTATTAATAGGGTTACAATATTATGTAATAAGTTTG
ATGGACATCCAAAAGGATTTGCCTACATAGAATTTGGAGACAAAGATAGTGTACAGACAGCAATGGCCATGGA
TGAGTCTTTGTTTAGAGGGCGTCAAATAAAAGTGATGCCAAAACGTACGAACAAGCCG
GGCCTCTCGACAACGAACCGGCCGCCGCGCGGCATGCGGGGCCGCGCGCGCCCCTCGCGCGGCTACACCCCCT
ACTACTCCGGCTACCGGCCCCCGCGGCGCGGCAGGTACCACCCTTATTTTTTGTACAAGCCGCGAGGTCCAAA
CTGAGCTTTGCGAGTGTGTGAGTGTAGCTCTAACGTCACGACCTCTTGTACTGACAGC
AGCGTCACATAATTCATTCTTAATATTTTATTTTTATGTTCGATTTATTAAAAGTTAATGATAAAGTTTGCGC
TTATGGAAAACTGTATGTGACAAAGAAGTAAAAAAAAAGATTATAGAGACATTACTGTTCTTGATCAGATTAA
CTCGAATGACAAATCAGTTGAGAGTAAAAATTATAAAGATGCAATGTCAGAAGGGCAA
ATTATAAAGATAAAAAAAAAATAATGTGGGATTTTTAAAAATTGTTTTCTATTAATAAACTTAATTT
>103776673_2 DV501135.2
GTACCAAACGCTACGAGTTCTGCATATATCATACTTCTGACCTTTCCGTCGTCCCATAAGACGCGCCCACCAT
GGGGGAGACATCATGCTTTTTACGTGACCTTTCTCCAGCATCATCGACATGTTTGTCCTCCTCTGCCTCTCTC
AGTTCAATTAATTATAGATATGTTACCCATCCTTATTTTGCAAACATCTATTACTAAT
TGCAATTTACAAGTAATGTTTTCT

>103776675_2 DT663569.3
GCACGAGGGTTTTCCCTTCAAGACATTGTCTTCACGTACAAATATTTTTAATTTCGTAAACTTTAGAATTTTC
CAAAATGGAACTCTCCGCCGAAGGAAAAACCCCAGAATATATGGCGTTAGCTGGAATCAAGTTCAAGTTATCA
TTACCTCAATTTAAAGATAATCCTCAACTTAAAGAACAATTGTTACAAGGAATTAAGT
CTGGTCATATGGCCCCATATTACAAAGAAGTATGTTCGGACCTCGGTTGGAACTTCGATCAAAAATTATTTGA
TGAAATGGCTAAAGAGAATCAGGAGCGACTTGCTAAATTTGAAGATTATGATTCTGAAACGCCAATATGGCAA
GATCGATTGGACTATTTATGTTCCATTGGAGACAAAGAAGCTGCAACAGCTCTGGCGA
CATCTAAGTATGAGGACTCTACTTTAACTACAAATCGTAGATTAGATGCTATATTTGCTATGTTTAGGATTGC
TTACTTCCATGGATGTAATGTGAAAGAAATGGGCAAAGCAATCAACAAGGCTCATGAATTAGTAGACAAAGGT
GGTGACTGGCGTTCCAGAGACAAACTTAAAGCATATGAAGCAATATATTGCTTAGCTG
TGAGGGATTACAATCATGCAGCTGAGCTTTTTATTGATTGTGTATCTACATTTGAGTCATATGAATTAGTAAA
TTTTGGCACAATCATCCAATACTGTGTG
>103776689_2 DT664394.3
GCACGAGGGCCACATTTATATTATGCTAGTTTCTAAAAAACTACAAATTTACTTTAAAAATATCACGTTTATA
ATGAAGTAATTACTGTCTAATTTTATTTTAATATTCTATAGAAGTAATCTATTAATAATTTAAAAAACTGCGA
AATGTGCGATCTTTGCTTTTGAAGCATAAAACAAAAAGTATCTGTCAGTCAAGTATTA
GTCAAAATAAAAGTCAAAGTCATAACTAATATTCCAAATCCAATACTCAATAGCATATAGTTAATATTTATAG
AAGTTAAAAATTGAAAATTAAAAAAACAAATTGCATGATTAATTTTAAAAAGCTAAACTTTTACATAATTTAT
TGAATACTGATTAAATAACTTATCCACATATATTGTTGACCTGTTTTTTTTAAATTAC
TACAATAATGTTTAACCTAGGATTTATTGGTGGTGGAAATATGTCCACAGCAATATTCAAAGGAATTTTAAAA
AATGATGATCACCCTGCATCTAATATTTGGGTATCTGGGCCTCATTTAGATAACCTCCAACATTGGAAAAATC
TTGGTGCTAATGTAACTAATAAAAATGGAGACTTGCTTAATGTATGTGACTTGGTGTT
CCTCGGAGTGAAGCCTGTAATGTTGCAAAATGCCATAGCAGATTGCCTTAATACTTTTTGTGATAAATCTGTT
ACAAACAAACACATTGTGTTTATTTCAATGTTAGTT
>103776714_2 DT664288.3
GCACGAGGCATTTGTTGGCACACAGTGAAGCGCGACTAAAGCTAATAGTCACAGATGGTGTGTTCTCGATGGA
CGGCAACGTAGCTCCGATAAAAGGGTTACGGGAATTGGCTGATAAGTATAGAGCTTTGTTAGCAATCGATGAT
AGCCACGCTACTGGGTTCTTTGGAGAGACTGGCAGAGGTACAGAAGAATATTGCGGTG
TAAGCGGCGCTGCTGACATAATATGCTCGACATTGGGCAAGGCGGTGAGCGGAGCCGCCGGCGGGTACACTAC
GGGACCCAAGGAACTGATCGCTCTCTTACGAAATGTATCGCGCCCCTATTTGTTCTCCAATGCACCCCCACCG
CCTGTTGTGACTGCTTGCTTGAAGTCTCTGGAACTACTAGAAAATAGCAACGATCTGC
GCCAACGTTTGCGGGACAACACTCGTATATTCCGTGATGGTATGAAAGCCATTGGCTTTACCGTGGCGGGTGA
AAATCATCCGATATGCCCTGTAATGGTGGGGGAGGCTCCCATGGCGATGGAGCTGGCGACTGGAATGTTGGAA
AAAGGAGTGTACGTGGTAGCGTTCAGTTACCCAGTAGTGCCTAAGGGTGGAGCGCGAG
TGCGCGTACAGTTGAGCGCTGCGCACACGCAAGACGACGTACGACGCGCAGTGACTGCCTTTGAGGTTGTCGG
AAAACGTATTGGATTACTTAAATGATTACAGTGAAAAAATATGATAATAAATCGATGGTCCCTAAGTAAAGTA
TAAAAATTGAAAAATTGTATTTCTTTTTATTGCTGTTTTAA
>103776730_2 DT663515.3
AATTATCCTTTTATGATAAGACGATACAACGCGCTATCAGCTTGTGATAAGTTAATTGTGATACTAGATAAAA
AACGAAGTGGTCGACGCCTGTATTTCTGTGTGGACGCGTATTATGTAAAATTCTGAAGAGTGGGGGTTCCTTA
CGGGGGGTGCAATGATGACGACGATGGCGGGGCCTCGGAAGACTATCTCCATTCATGA
TTATCCTGAACATCTTAATCCGTTTTTTTTTTATGATAATCATAACAAAATCCGGTTTTGGACTATTGGTCGG
ACACTAAGCAGGTCCAATAGCATATCGTTTTCTGGGATAAAAGACCTCAAAAATTCGTGGTCTCTGCGGTCTT
TTTATGAGGAAAGGGAAAAAAGGAGATCAATCTTCCA
>103776739_2 DT663541.3
GCACGAGGCTTTTCCCTTAAAAGCCTATTTAATTAAGTGTAAAACTTTGAATTTTTTTCTTAACATGGCTTTC
TCTATGTTCTTACGCACACCAGCTGTCGCAAGTCGACTTCTATCTCAGCAGATGACCAAATTGGGAACACAAC
CTGCCTTAAAGCCAATTATAGCTCAACGTACTTTAAGTAATGTTAATGCACTTACACA
AACAATATGTAAAACTGAGGCTACACCAATATTAAATTCAGTTCGCTCATTCCGCACTTCAGCTGTAAGGTTA
GCTGGTGAGAAGGTCCATGACCACTCTAAATTATGGGTGGTGGATAATATAACCAGTGCACTCTTAGTGCCTA
TCATACCTTTAGCGTTAATTGCACCCAACAAGGTGTTTGACTCTGCCTTGGCTATTTT
AGTAGTTGCCCATAGTTTTTGGGGTCTAGAAGCTATTGCAGTAGATTATGTTCGCGCCGGCGTTTTTGGGCCA
ATCATACCTAAAATGGCCATAGGCCTTGTCTACCTCATCTCTATTGCCACCCTTGGAGGACTTTTCTACATCA
TCACTCACGATATTGGTATTGGCAACTCTATCAGGCAATTCTGGGCTATTAAATCTGG

ACAAAAGGCATAATTTATTTTTGTTTATGGCAACACATTGTGATCATCAGTTGTTTACCGTACAAATTGTTAT
TTATCTGTATT
>103776741_2 DT663688.3
GCACGAGGATTCAACCCTTAAAAATTTGTTGTTAACTTTTTGTTATTAACAAATAACTACAAACAAATTTATG
TATATGTTTTCTCAATAAATATTTAAAGTAATAAAAAAAAAAATAAAAATAAATATCATCAACAATACATATG
ATATAAAACATCTGAAAATGTCTTGTGACGCATTAAATATATCTCGTCGGAAATTAAA
CGATGTTTTAGGAGATAAATCCACGAAATATTTCAACCACATGAAACAATGGTTTCGAATGAAACTTACCAAA
GAAGAGTTTGATACAGAAGCTAGAGCCTTATTAAACCCTGATCATTTTTATTACCATAATGAATTTTTACTAG
CTCTGTTAAATAAAGTTGAAGGATTGGCTGAAACCTCAATAACTATTGCTCAAGAAAA
AGCTAATTCACACAATAGAAATAGTCGAAGACATAAAAGAAATTCTCGAACATCTGAAAAATCTAACTTTGAG
CCAGCTGATTTGATAGAATACTTACCACCTCATTCACCTCCAGGTGCTGGAAGTGATGGAGTTAAATATGCAA
CACAGGAAGTATTCCTCCCAGACCATGCATTGGTTGTAGGAAGGTTCATGCTAGCAGC
CTGGGAACTTGGTTTGGAAGGTGCTGAAGATGATGCAGCAGATATAATTGTGGTTGCTGTACAAAACTTCCTT
AAAAATATTATT
>103776746_2 DT663574.3
CAATGACTTATTCGTCCATGTCAGTGGTAATTCGCCCAGTGTTCACCCTCTAGAAACATTGTGGATCGAAACT
ATTTCCAACACAGACTCTGTCCAGAATGCCATACACCTTACAACTCCTGAGGAATTAATATGTGTTACAACAA
TATCAGAAGAAGATAAAACTGAATGGATACATTGCTTGAATTCTTCAATACGAATAGT
TTTGAACAAAGAATCTACTTACAAACCACCAGCAGTACGAACCGCTAGTTACACGTTTTCTAATAAAAACTCG
TTCTACAAAGAAGCTAAATATTTTTGAAGGTGGTTAGATGGCAAAATACATGGGAACGGCAAAGTAGAGTGGC
CCGATGGGAAGTATTATGTGGGGCAGTTTCAACTGAACACATTATGTGGTCATGGTAA
AATGGAAATTCCTGGAGTAGGATTATACGAAGGCCAGTGGAAAGATAACTTACAAAATGGTTACGGCGTCATG
AAGTACACTTCTGGTGACATTTACGAAGGCTACTTCAAAGATGGTCATCCCCATGGACATGGTATAAAGAAAC
AAGGGGATTTTACGTCGTCAACTGCTACCATATACACAGGAGAATGGGTAGCGGGCGT
CAAACAAGGTTACGGAGTAATGGACGATATAGGAAAAGGAGAAAAATATCTTGGGCACTGGGCC
>103776774_2 DT664688.3
CGGCACGAGGGTTGTCCTTAAAATGCCACTGTAAACGTTTCGTATTTCCACTCGAGGAAATGTGAATCGATTT
GGAAAATGGCGTATTTACAATCAATGCCTAAATCAGAAACAATTAAACTGCGAGAAAAACACATTGGCGCCGC
CTGTCAGTTGTTTTTCCGTTCGTCTCCATTGAAAATAGTGCGAGGTGTCGCCCAGTTT
ATGTATGATGAGACGGGCGAGAGGTACCTCGACTGCATCAACAACGTAGCCCACGTTGGACATTGCCATCCCC
ATGTAGTAGAGGCTGGTCGCAACCAGATGTCATTGATCTTTTTTTATAACCGTTATCTCCACGACGAGCTCGT
CATCCTCGCTGAGAGGCTCGTGAATACTATGCCTGAATCACTCTCCGTCTGTTTCTTT
GTTAATTCGGGATCTGAAGCCAATGACCTGGCACTTAGATTGGCTCAAGTTCACACCAAGAAGAAGGATGTCA
TTACACTCGACCACGCATATCATGGGCACCTTACGTCTATGATTGATGTGTCTCCATACAAACTAAACTTACC
AGGAGGGCCAGAGAAGCCAGAATGGGTTCACGTGGCACCAGTCCCTGATGTATATCGG
GGTAAATACACGTGTCCAAAGGATTCTACGTCAGAAGAAGAGTTGGGAAAATTGTATGCCAAGGAAGTGGGCA
AGATATGTGAGGAGGTGAAGACACAAAA
>103776801_2 DT664216.3
GCACGAGGCGTGACCTTTAAATTTCCTTTATAAACTAAAGGTTTCTTGTTGAAAAAGTTCTTTACCCGCCAGC
AAAATAAAAACTAAACTCTCCGGGCGTCGTGACATGCAAAGGAGAGCACTCTCGCATAATAAAGATAACGCGA
GAGCTGCTCACACAGGTCGAACAAGGGATGACAGCAGTAAGAAAGGCAATGCAAGTGC
AAACAGCAATTCTAGCAAACGAAGCAACATGGAGGGCCTAGCGCAGGGCGAAAATTTCGAGGGCAAGGAGGCC
TCGCAAGAGAAAGGCAATAATGTTGAGAATAGAGATGAGGGAGTTCTACTTACTAGTGGAGTGGTATCATCAA
CACAAGATGAAGTGGGCACCACTTCAAAAAGATCATATGTAGAGGATAATCAGGAGGC
AGAAGCCACAGAGGATGATACATCAAAACGAAAAAAGAGAAAAGACACTGAGGAAAAAGAACCCATGGCAAAA
CCTGCCCCTGTGGCAAGAGGAAGCACTGGCCGAATCCTAACTGGTAGCAAACCTCCGGGACCCGCAAGTAAGA
CTGGTGCAACACCATTGGGAAAAGGTTCTCAATCAGGAGGCAAGGGAGGCGGATCCTC
GGGGAAAGGTGGACTAGCGCAGGGAAAGGGTGGCGGTGGAGCACAGGCTAATGCTCATAATACGCATCAGGGG
AAGTCTGGAGCCGGTAAAAACTCATCAGGATCACAATCTGGTAAGCAAGATCGAAAGAGCCCTGT
>103776803_2 DT663897.3
GCACGAGGAAAAGCCTTCAACAAAAATAACATAACTTTGTGAATAAATGTTTACGTTTCTACTGTATTTATTT
TTATTGTGTTTATGAAACTCGAAAATTAATAATTCTAATTGGAATACATACGTACTGTTGAAAGTGTACAATA
CTTATTAGCATAATGATTGACCCCAGTAACTAAAGCTACAAAAATAATTTGGTGCATA

AGCAATTATTAACCTCGAGGCATATAACAATAACTATTTTTAATTGTCTATCCTTTATTTAAATGGATTGGTT
ATTACTTAACGTTATTTATGAAGTAAAGAATCAATTGACATTTATTAATATAAAACACTAATATATAGCAGCA
GATTATATGTTCATTTAATAATAACTAAAAATATTTCAACATAAATTTATTAACATGT
CTCACTCTACTGACTACACTATAAACCAATCAATGAGTTTAAACGACAATATAAAAATCAAAAAAGAGGAAAG
CAATGAGTTAATATTAGATATAAAGCCTGAAAAAGAAGATATTAGCGAAGATAATCCATTTGAAAATATTCAA
GTAGAAGTAAAACATGAATTTATAACACCAAGCTGTGAGGATGATAAAATACATTATA
TAGAAACAGACAATGATAGTACCCANATAGATCATGATATTAAAATGGAAACACCATATGAGTATCCAAGTAT
AGATGATGGAAGCATGGACAGCGAGGAAGCTTCAGCTTTGAATAATGTAGAAGCATGCCTACAAGAAGAAATT
GATGTTAAAAATGAGATATTT
>103776805_2 DT664437.3
GCACGAGGCAAGACGCTACTGTCAGAGTATGGAACATAACACAAGGCGTATGCATATCGATATTTGGTAATCA
TTCGCAGTCCAGTTTAGGCCTGTCATGGAGTGCATTCCCACATTTAAGTACTGTTGTTTACTCTGGCGGAGCT
GATTGCTGCTTGAGAGCGTGGAACTATGAGAATTATCCTCCTGAGAAGTTTACTGAAA
TCAAACACGAGGTTATGCCAAAGAAAGAAAAAAAGAAAAATAAAGATAAGAGAGAAGAAAATAAAACCGAGAC
TGAGACTATTGAAGTCAAAAACGATTCAAAACTTGCTACAACTCTCGATATAAATGTTAAAAAGAAGAATTAC
AAAAAATTTCTTCTACCAAGTCTGGTTAGAAACGCAATTACTATAGAAAGCATAAGTA
AAATGTCGGAAAAATATTTAAAAGGAACAACTAATGGTGATACACAAGAATCAAATAATTTTACAAGAAAAAT
GGATGATGATGATAATGATATAAAAAACTATATGAATGAAATCAATGGTGATAAAGAAAGTATTGACATTGAT
GATGATGATGATTTGAAAGATGATTCTAATGATGTCAAAGATGATTGTTTTGAAATGG
ACTTTATAAAGCTCTTCGGTACTACCAATGAGTTTAATGAGTTGTTGGATCTGGAAGTACAAAAACACCAACA
AATGCGTAGATGTGAATCTTACACGATGCTGTGTATATTCCGCGGCCACATCGACGCGATGGTGCAGTACGCG
GCGCAGAGGGACGCGCTGTGCCCCTTCATTGTCAGTGTCACGCCTTGTGTGTCTTTTA
AAT
>103776810_2 DT663571.3
TTACCTCTCTGCGCTGGCCATAGTAAGTGCGGCGCCTCAAGTTACACGGTCTGGAGACTTGGATGCTATCATA
TCTGAAATATTCGGCCCTCCGCCCACAACTGCAGCTCCATCTATTAAACCATTTCAACCCTCTACTATTGTGC
CACCTGTTAATTCTACAGCTTCAGAAAAATCAGTACAATGTGTAACAGATGAAGGAAA
AAAGGGATTTTGCGTCCACTATTACTTGTGTAGTACAAATAATTCCATCATTACCGATGGTATAGGAGTTATT
GATATAAGGGCACTTTTTTTTAAATGCCCATCATATTTGGACGTCTGCTGCGCTTTGGGTGACACTAGAGATC
CCCAAGATCCCATCACACCAGCGCCAGATAAACTGCCTCAGCGTGAAGGTTGCGGTTG
GCGGAACCCAGATGGTGTAGGTTTCAAAATCACAGGAGCTAAGGATGGTGAAGCGGGCTTTGCTGAATTCCCA
TGGATGGTGGCCGTATTAAAGATCGAGCCAGTAGACGAAAACGATGTGAACTCCCAGCGGTTGAACGTGTATG
TCGGTGGTGGTTCCCTGATTCACCCGAACATCGTACTCACAGCCGCCCACTATGTAGC
TACCACTAAGTCACTCCGGGTTAGAGCAGGAGAATGGGATACACAGACCACGAAAGAGATATAC
>103776815_2 DT664827.3
GCACGAGGTATAGCTCTCTAAAGGTTTTTGCAAAATTAAAATTACCATAAGTAACGCAGTATTCTCTAAAATA
TTATGGTAATAGAACTTTACATCGTGTTTCTTGAACTCATAATTACAAATAGATCTTACTAACAATGTTAAAG
GATAAAGGGCAGTAACATTTGAGGACAATGGCCATCCTGCGTNTATAGTTTTAAAATT
GCTTAACAAGAACAGTAACCCAAACTGAATGGCAAGATCTTTTTGTAGCTGTACTTCAGTCTGCTTATGGGAT
GACGAGGCCCACATA
>103776816_2 DT663733.3
AGTTATTGATGATGAAGAACTCTTACCGTTAGCTGCTAATCCTATGGAGAAGTGTCTAGTTGATAGTAAATCG
GCCACTTGCTTCAATAGTGGTGATGTCCGTGCCAACATGCATCCATGGTTAACTAGCCTCCATTCTTTATGGG
TTAGAGAACACAACCGCATAGCCAGAAAGTTAGCTTCCTTGAACCCAAGCTGGAATTC
GGATAAATTATACCATGAAGCAAGAAGAATTGTAGTTGCTGAATTACAGCATATTACATTTAGACATTGGCTG
CCTGCTTTAACCGGTAAAGTTTTTTACGAATTGTACGAGAGCTACGACATTGGTTACAATCAGGATATCGATC
CTACTATATCTAACTCGTTCGCGACTGCCGCTTTCCATTTCGTCAATAGTTTGTTAGA
TCAAGATATTGAAATGGTCGATGATAATAATAACGTGACGTCACATCGTTTGGGACAAAATTTCTTTAAACCT
GAATTATTAACTGAGAAGAAAGGCCTAGAAAATATATTACGAGGGATGGTCAATCAGAAAAGTCAAGGATTGG
ACTTCAATTATGATGACGATCTCCGTCACCATTATTTCGGTGGTCTAGACGTGGTAGC
GTTAGATATACAGCGTGGTCGTGACCATGGTTTACCCGGATACGCGCATTACAGAACGATATGTGGATTACCT
TTACCAACGACTTTTGGGACAGTGAACGATG
>103776824_2 DT663765.3

GCATAGACACTGGCACATTGGCAACTTGAATATTGCTATGGTTATTTCTTTCGTTTTTTGTCATAATTTGGAC
TAAATACGACTATCGATTTCCTTAAAACTATCCTAATATTAAATAGCACGACATTAAACTAGAACGAATTTTA
TTAAAGCAATAAAATAGCGGCAGTAGTAGTAATAACCAGAATAAATTACAGTAAGAAA
CTTAGAATAGACTAAATTACCAATAAGAAAATTTAATAAAATATCGTGCCAAGAAACTCCGCACTTCGCTGCG
TTCTTCGCAAAAGATATTTTTAAAGTTATATCATATCCAACAGTTTCATATTTCCCTGAATGTTAGAAACGTC
GTCCTTTAAGGATTTATTATAGTTTCCAATCTCGGGAACATCTTGCAGAGACAATGCA
CGTCTGTGAGTAGATTTTCCCATTGCATTCGGTAACGTAAATGAAAACAATAAAACGGTTATTTGAAAGGTTT
TGACAAGTTGAACTATTGTCCGATAAGTTCCTGGTAAGGTAATGCCGTCTCTTTCTGTTTATTATTATAAGAA
AGAGAAAGAGAGACTATGAATATCAAATGCTGGAACAGATCTATATAGGACTATACTT
CTTAGTATACTATTTAATACCAACTAATAAAAAAGAATCTTTACTCAGCATTTTTTGAACCATAGTCCTAAAA
GTAA
>103776828_2 DT664188.3
GCGCGAGAGGAGATCCAGACCAGATCGGTTCAGCGGTAGTGGAGCTTTAACATCTCGTGATTTAACCGTGTTT
GCATATTGTGTAGCTAGAGGAATGGATTATATTGCGTCCAAAGGAATCGTGCACCGCGATCTAGCCGCGCGCA
ACGTGCTCGTGGACCACAACAAGCTGTGCAAGATAGCGGACTTCGGCATGTCGCGTCC
GGCGGGCGGCGGCGCGCGCGGCGGCCGCGGCGCGCTGCCCGTGCGCTGGATGGCGCCCGAGGCGCTGCTCTAC
AACGTGCACGGACACCACACCGACGTGTGGGCGTTTGGGATTCTGTTGTGGGAAATTGTTACTTTAGGTCAAC
ACCCTACGCGGCCATGTCAGGAAGGGAAGTTTAACAGCTGAACGGAAGGCTACCGTTA
GAACACCACCTCATTGCAACCCCAGTATATCGAGCATGCACGCTTGTTGGCTTCCGATCCTTCCAGAGACCTC
TTTGCTACTCTAATCTCATTA
>103776845_2 DT664167.3
GCCGAGGAGTGGGACCTTCTCTATTTACAAATGAAACTTTGCAATATGCTTTGCAAGTTTTGAATGCTGAAAT
GGTCGTGACTGAAGACTTACCGGATTATCCCGTTGACTATAGAAGACAGCTTGCCATTAGCTTATTTTATAAG
GGGCTTCTTTCACTTTACCCTGTAAATAGGTTGCATCCTCGTTACCGTTCTGGAGCTG
TGAAAATACACGATACGCGTCCGGTTTCTGATGGCAGGCAAATATTCACAACTAATCCCACACTCTGGCCCTT
AAACAAACCTATACCTAAACTAGATGGATTGATCCAATGTGCCGGTGAATCTCAATATACAGAAGACCAACCG
TCCCTCCCCCGCGAGGTTTTCGCTGCATTTGTCCTCACGACGGTACCACTGGGCACTA
TTGATAATATTGATCCTAGTCAAGCTTTGAAAGAACCTGGAGTCGTGGCATTTTATAAAGCATCTGATATTCC
CGGACCAAATAGTTTCATTAACCCAGTTAACATATTTTTCCCACTTAACGAAGAAATCTTATGTGATAAAGAA
GTTAAATATTACAATCAACCCCTAGGCATAATCGTGGCGGAAAGTCAAAAAATTGCAG
AAAAAGGTGCTAGTTTGGTTAAAGTTACTTACAGTAACATGAAAAGACCAGTCGTTGATCTAAAAGTTAACAA
AAACGATCCCAGCAAAGTGACTGTTCTTCAAGCAATACCCGCCACTAGAACTGGTGCTGACGTAGAAAAGGTG
ATAAAAGGAGAGAATACTATAT
>103776854_2 DT663502.3
GCACGAGGCCGCCCTCTAGAAGGGCTACGGCATGTAGGGAACCGCCGCCCCTGCCTGGGGGTCCTTTGCGCAC
TTCTGAGCGACGTGCCGACACGCCGACACGACTCGCCGACACCGACACCGACATGCCGACACCGACACGACAC
GCCGACATACATACTTATTAACTATTAATACTAACGCCATTCATTAAGGATAGCATCG
ACGTCGCCCTATAGTGTACATATTACTATGCTTATTATTTGTGAATCGTATTATAAATTAGATGTTGTTTAGA
GTATACAAGTAGACCCAGTGAAAATTGTAATATATTTTTACGTCGGAGTGTCATTTCGGTTGTTTCTTTAGAA
GCGGAACTCTCTGCATTTTCGAAATCAACTGTCATTTTAGTTTATGAGGTCAAACTTG
TGAAATAGTCTAGATTTTATACACAACGGGTTTACGTTAGTAAGCGGCATGGCCTAATTGAGGGTTTGTTTTT
TGGTCATTGTAAGTATTAATCGAAAATGCACTGAGATCTTATAAAATAAATAAAGGGAAAATATCGTATTATA
TTAAAATTCTATAGCTCTCG
>103776860_2 DT663852.3
CCCGGGCTGCAGGAATTCGGCACGAGGGGCGTCCTTTAAAGACCCAGTCCAGTGAGACAATCACAGCATTATT
GCTTCTATTCTTTGATTTTAACCTGTTCACAGGGTCTCCTCATCACCTATAGGATGGAACGTATTCTAAAAGC
CAGGTGTAATAATATCGGTATTAACATATTATTATATTTTCTTTTGCGTACAAAAAAA
ATATCGATTAATAATTCGAAATATTATATATATTTTTGCTGTCTTTTCGTATCTACGTATTTCGCGTCAAATT
ACAGTTGAACGATCACGTGCAGGTTCCGGTGCTGACCTAAGATGTAAATCTCTGTCCAGT
>103776862_2 DT665028.3
GTTACCACACGCGCTCGTCTGGGGAGGAGTTGGTTACCCTTCTGCTCCTTTACTTTTACCACCTGCTCCTCAT
CCCTCTCATCACCTGCAATTGCTACCAGGCGACCTTCTAGCGTCCACTGCTACGTTGCAACATACACACACGC
ATTCAACACGACTTGTAACATTAGCTCCCGCGCCACCGCAGCCTCCTCCACAGCATAC

AATGGACAAATTTTTTTTTATCATGCAACCTATGATGCCACCTATGGGTGGGCCACATACTCTCATAAAAATA
GAACCTGGACAGCAAGAAAAACCGCCTACGTTTGCCCCGGAGCCAGTGCCGCAGCCGATTTTAACAACACATT
TGTATTATCAATCAGATTTTGCTGAACAAGCGTGTCGAAGTCAAGCCACGTCACCTGC
TACACCTCCGACACCACCCCCTGAGGCACCTGAAGTTCCAGCTCATAGGGATGCGTCAAATTCAACTGATCAT
ATTGACGGATGATGATGATTCTC
>103776863_2 DT664403.3
GCACGAGGTTTACCTTTGTCGTTTGACAATTTTGTGATGGCCACGCTTGACGCGAACCTGTGTTTTTCAGATT
AGAATTAATTGTGATCTCGCCAGAAGAGGTCATACTTCTAAATAACTCTCCAAGGAGCTATTGGGACGGTCAC
CGTTACAAAAAATAATTTTCAAGTTTACTGAGCTTCAACCAAAATGACTCTGGGTACC
TACAACAGACATCAGGCTGAGCGAAAAAAGCAGGCGGCATTGGCAGCTGCATTTCCTCAAGGCATACGCTGTC
AAAAAATGCCTAGAATATGGCCACTGGAGCTATGAATGCATTTGAAAACGCAAAATGCTGGTGCGTCCATCGC
GCTCACAAATATTGAAGAAAAACTTAAAGGCAAAGCAAGATAACGATTGCAGCAATGG
TAGATGCAAGGTGCCAGCTAAAAAGAAACGAGCTGAGAGTAGCTCCGAAAGCTCCGGAAGCAGTTCATCAGAA
AGCTCATCTGATTCATCTTCAGGAAGCTCAAGCTCCTCAAGCGACAGCAGCAGCGACTCTGAGAGTGACAGCA
GCAGTGGGTCCAGCAGTGGATCCGAATCAAATAGTGACTGTTAAACATTATTTAGATT
TACATAATTTAATTTATTTTAGATTATTTTAATTATATTGAATATTTTACCATTTAAATAGTTTAGTACTGAT
GCGGCTTGAAGTCGTGAATGTAACTTTTAAATGTATTTGTGGGTTTTATGATTTTTTATGTTTGTTTTGAAAT
GTGCATTTTTTTTTATTTATGTGTCTAATCTAATAACATTTC
>103776875_2 DT664929.3
CCCTTATAAGCCCCGGCTGCAGGAATTCGGCACGAGGATCGCCCCCTTAGAGAGCTCTAGGTTCTGTTTGCGT
GAGACATTACGAGTTCATGTTGCGGCCAGAGCTGAAGGAGTTCTACCATCAGCTGCTGACGTCAGAGTTAGCG
CCTGTTGAGATGAAGGCAGATGTGCTTAGAAATATTGAGATGTACTTACAGGAAGAGG
AACAAAGAATGATTAGACAAGATAAGGAATGGTCAAAGAGGTCCAAACATGAAAATCTAAAAGAGATGGGTGA
CGTGTCCTCTGGCATGGCGTCCACCGTCATCCAGTTGTATCTGAAGGAAATCCTTGGTGCATTTTTGCATGTC
TTCTCTGTAGTACGTTCCAGTGCTATGAAGGTAGTACAACTTGTGCTGGCCCAAGGTC
TGGTGCACCCAGTACAGATTGTCCCATACTTAATATCTATGAGCACTGATATAGAAGTAACAGTATCTCATAC
GGCTGACAAACATCTCCAAGAAATTGATAAGAAATATCCAGGGTTTATCCATATGAAGGCGCAACTTGGCATA
AAGTTATCGTACCAGTTACAAAAAATATTACAACGTAGTAGAAAGGGCGTTATAAGAG
GATTTAGGAAAAAAGATCAAGATGACTTGCCATCCGCGTTAAATGGTTTTTTATATTCACTTCTGCGTAACAC
GCGGCCTCAACGACGAGCGTTAGTTCTATCACTACTGAAGCAATTTGATGATGTTTCTACCGCTCCATTGGAT
CAAATGTTGTACCTGGCTGATAATTTAAGCTATTTTCCTATCAAGTTTCAGATGAACC
ACTTTT
>103776876_2 DT663706.3
CTCTTATAAGCGCGGGCTGCAGGAATTCGGCACGAGGTTTTTTACATAAAAGATTTATTATCGTAAAATACAC
AATTATGTATATCTTTTGGCCTTAAAATAATATAAGAGACTAAATTCCACTAATGAGACACGATACAACAGTT
ACAGCTAATATACACAAAAAACATTTACTTATAATTATCAGTTATAGTTATCGAATAA
AATATCTTGGAATGTTGTAATAATAAAGCAGCAATGTCATTCATTTTATTTTTTTTATAAAATAACGTAATTT
TCAAAAATATTGTAAAATATAGTTTTTGTTTTATAAAAAATATGTTTAGTGTAATAGGCTATAATAACGTAAT
TTTTCTATTTATTATTCATTAACTGTTTAAAAAATACTAAACAGTTAGAATTTTTATT
TTTTAAGAATATAAATTCATTTTTCAATAAAAGTAGTATATTTAATTAAAATTATATTAAAATATAATATTAT
CGATACATTTCCATTAATTACGTAAACAATTTGAAGAACCTCAATTTTAAAAGAGTTATCACAGTACAATGAG
GTGATCTCCAAATTTTTTAAAAAAGTTTTATCATTATTTGTATATTTATATAAAAAAT
ATAATTTTGAATAAATTTATTTAAGGCACTGTTTGGGAATGACATCGAGCAAATATTTTTCAAAAGTTTTTGT
TTCAATAAGTATAAAGATAGTGTTATAATGTTCGTCACATTACTGGTTCAAGAGATTCATATAATTTATA
>103776905_2 DT663700.3
CTCCAACAAGGCCGGGCTGCAGGAATTCGGCACGAGGTGATTCCCATAAAGAGGACGCTGTAATAGAAATAAA
CGAGGATACTGATGAAAATAAAAACAATAAGGAATTAAATAAAAATATTGATAAAAACGCAATAATAGTGAAT
GGCGAGGCTAATGATGAGACATTGACAGAGTCTGTGATTGAAAATGATAAATTGCTAG
TGTCTGATATTGAAAATTTTCTTCTTGGTTCAAATATTTTGCCCAGCATTAAACTGCTACAAGATTGGGTTTT
GACCGAGAAAGACCTTATTGTGTCATGTGGAGATAGTGGCGAGTCACTGTTTCAATGTGTTGTGGACTTGCTG
ATTTTATTTCTAAATTACTTTAATCCGAAAAGTACTGAAACAAAAAATGGTGAATGTC
AAATATTGATTTATGCTAAAAAGTTAGCTAAGAAACTCAAACTTGAATACAAATCTATACCTTTGCCTGAAGA
CATTAGCCTACGAGGTACTGACATCTGTAAATTTGATAAAGACGCTGCTGAGTGGCAGATAATGGAAAAGTTC
AAGCCGACACCTATTGAAGAAAATGTTATAAGAATTTTAAATTTCATAGATTTTGGAT

ATCAAACTGCCAAAATAATACCAAAAATACGATATAATAGATCACTGAAAATATTCTATTTCAAGAAAGTACT
TCCTCCGAAAGTTAACACGAAAATTAACCACAAGAAGAGCCGGGAGTGGCACAATTCTAAAAAGCAGGCAAAA
CAAGGCGAGACTGGCTTACTGCGTCGTCTAAGGCGCCTCTGGCTCGCCTCGCAAGTGC
GGG
>103776913_2 DT663719.3
CGCTGATATGGTTCGGAGTTTGCGTGCGAATGGGATCATAAAATCAGATGTAGTTGCAAATGCAATGGTTGCC
GTTGATCGAAAGAATTATTGTCCACATGCACCTTACCATGATTCACCACAGTCCATTGTATATTCTGCTACCA
TTAGTGCTCCTCATATGCATGCACATGCCTTAGAAATATTAAAAAAGCAATTGATTCT
TTTTTTT
>103776925_2 DT664055.2
CTTTCCGTGCGGGCTCCAACGTGGAGTTCGGTACGGGGCTCAGCACGCCCAGCACGGGGCCGGCCGGTGCCGA
TGTATATGCGCTGGATGAACCAGCGAGAAAAGGTCGTGACTTTAGCAACCCTATGTATGAAGCTGTTACGAGA
GCTACAGCAGCTGGTTTTTTTTAACCGCCTTTGCCCGGTATCTACGAGGTGCCGGAAG
AGATAAAGGACAAGGTGGTGTCAGCGGTGATATCGCCTTCGTCGACGGAGACGCGCGAGGGGCGCGGCAACAA
GCGCACGCGCGCGCTCGACCCCGCCGCCGACACCGGCGTCGACACGCAGCATCTCGTGCGCGAGGACCACGAC
TGCTAGCGCCGCCCTACCGGACTTACTATAACGTTTTCTACTTATATTGTTTAGTGAC
GACTTTTAATGATAACAATTATTTGTTAAACGCTATTGATGTATCCCTCTACC
>103776949_2 DT664170.3
TTCGGCACGAGGCCTCGTGCCTAATCGGCACGAGGGATTGACACGCTTCCTTTTTATATTAAGTACACGTATA
ATATAAGCTGCGATTATTTTATTTATTATTATAACGTATTAAAATATCAGTAAACATATATAAAATCTGCAAT
CATGAGTACTATACTGGAAAAAAATTGCGGCTATAGAGTCCGAGATGGCTCGCACGCAA
AAAAATAAAGCTACAGCTCTTCATTTGGGCTTACTTAAAGCGCGATTAGCTAAGTTAAGACGTGAACTAATCA
CCCCTAAAGGCGGTGGCGGTGCCACTGGTGAAGGTTTCGATGTTGCCAAAACTGGAGATGCAAGAATAGGATT
TGTTGGTTTTCCATCAGTGGGGAAGTCGACATTGCTTTCTAATTTAGCAGGCGTGTAT
TCTGAAGTGGCAGCATATGAGTTCACAACACTCACAACAGTGCCTGGTTGTATAAAATATAAGGGAGCTAAGA
TTCAGTTACTTGATTTACCTGGTATCATAGAAGGAGCTAAAGACGGCAAGGGTCGAGGTCGTCAAGTAATAGC
CGTAGCTCGAACTTGCAGTCTAGTATTCATAGTCTTGGATGTCTTAAAACCTTTGCAA
CATAAGAAGCTATTAGAACATGAATTGGAAGGTTTTGGCCTAAGATTAAATAAACAACCGCCCAACATATATT
TTAGAAAGAAAGATAAAGGTGGCATCAATTTGAATACTACTTGTCCACAGTCTGAGCTAGACCTAGAAACAGT
GAAGACTATTCTCTCTGAGTACAAATACACAATGCTGAATATACACTACGTTACGACG
CCAACAGTGACGACTTGATCGATGTCATTGA
>103776955_2 DT664749.3
TTGAAGTTGGTGCCCAAGGCTTAGATACTTTAAAACAGATATTAGAACTCGACAGAGCTGACAACGAAACAAT
AAACTATGCACTCGACACACTCAACAACATTGTTTCACCGTTACAATTCGAAGAAGAAGAGGACAAACCACAC
TTACCCATCAACATTGGAGATCAGTTCACAGAGATGTTCATTAAAGATCCCCGGAATA
TACAACTAGTTTTGGATCTTCTGGACGAATATGATTTTTTAGTAAGATTGTCTACAGTACAATTCCTTACTTC
TCTCCTAACGAATAGGACTAAAGATATTCAAGAAATTATACTTGATAAACCCATGGGTGTATCTAAAATGATG
GATTTATTATCCGACAATAGAGAAGTTATTAGAAATGAGGCGTTATTACTGCTTATAA
GATTGACCAAAGGCAACGCTAACATACAAAAAATCGTAGCTTTTGAAAACGCCTTTGATAGATTATTTGAAAT
TGTCAATACTGAAGGTTATTCGGACGGCGGCATCATAGTAGAAGATTGTCTTCTACTAATGCTAAATTTGCTA
AAAAACAACAGTAGCAATATAAATTTTTTCAAGGAAGGCAGTTATATACAGAAAATGC
TGCCCATGTTCAACATGCCGGAGGAAGCCGAGGATGTCGGTTGGTCT
>103776960_2 DT664337.3
GCACGAGGAGTCTCTTCTGTTCTTAAGGAACTCAGCAAGGCTTTCATGCTTAAAAAATGGTATCACGGGATCA
TGTCCGCAAAGGAAGCTGAACACTTGATGATGGAGAAAGGACGTAATGGATCATTTCTTGTTCGCGAATCCCA
GGCGCACCCTGGCGAGTATGTACTTTCGGTGCGCGTGCGTGGTAGAGTTAGCCATATT
ATGATCAAACGTCAGGACAATAAGTACAATATTGGCAGTGGCGAACAGTTCGATGATTTGGTAGGCCTCATAG
AACATTTACGTTTATACCCCATGATTGAGAACTCTGGTGACGTCCTAAGACTTCTCCAACCGGTCAGTGGGAC
GTGCTTACGAGCCAAGGACATACAACAAAGAGTACAGGAAATGGACGAAGTGCGAGTT
ACAGAAAATTCTGGTTTCGATGGCGAATTTCAAGTAATCAAAATGACAGAAGACATCAACGTTTACACAAACT
GTGAGGGGATGAAAGCGCACAACTTAAATAAAAACCGATACAGTAACATATTACCATATGATCAAACTCGTGT
TATATTACGTTATCTAAACAAAAGAAATGAAACAGATTATATAAATGCCAACTACATT

CGTTGCAGTCGGTTACGGGACTCTTCTAGCTCTGTGCAATCATCAAATGANAGTCTCAACAGTGTTAGTTCTT
TAATTCTAAGAAGCGAGCCCCAGCGGCCGGTACCACTAGTCACTAAATCTTTATCAGATGATGCTCTTAGAGA
TGCCAAAAATTTATATCTGCTGATAAAGCAAATGAAAATGAAAATA
>103776999_2 DT663875.3
CTTTAAAGGGAAATAATTTTTATTTTAGTTAAAAAAAGAAATGTATATGTAATATAATATATTTTAATGAATA
GAATTAAAATGATTTCTTATAAAAAAATAAGTTTGGTTAATTGGGAGAAAAAGTTANAAATATCCCAACGCAG
CCAAAATATGGCAAAACAAATCCAGGAATGTAATGGCCCAATTTTACTCACGACCTTA
TCATATTAACACATAATATAATGCAATGATAAATGATGAATGTGATGAATTAAAAATATTACATATAAATATG
CTATACCAAGTACAGTATGCAAACATATTCTTTTAAATTAAATAATAAAAAAATACAGATATATATATATATA
TAAGTATAAAAACATAAATTGGACCATCCATCGACAAATTGCTCTTAAAGATAAAATA
TTGACATTAGAAAAAAACGTTTAAAAATCATGGTATTAGAAATCATAGTGGAGCTACTTTCTTATGGCTGATC
ATCAAAATTAGTATAAAATATATCACAGCAATTTTTAGCCCAAAAGATGAAAAGTTTATGAGATCGAAAAAAA
TAAAACACAGAAAAGGAGGGGGTAATATACAATTACAATTGCTATAATTATTATATTT
TATACCAGTCACTTCATGCACTCGGTCCAATTTTATCTAGACAAACACACAATGTGTGTACATATACAAACTT
TTTTTTAAATATTTTTACCAT
>103777001_2 DT664290.3
GCACGAGGCCAGCCGCATCGCGGCAATCGGTTACTTATTCTTTTAAAGGTGGATAAGGCCGATCAAATATGCA
TCTGTATAACCTGACACTGCAAGGCTCTACAGCCATTACCCACGCTGTGCATGGCAATTTTTCTGGCACAAAA
CAGCAAGAAATTGTGATATCTCGTGGGAAAACTGTGGAACTTCTCAGACCTGACCCCA
ATACCGGCAAAGTTCACACGCTGATGAAAGTTGAAATATTTGGAGTCGTCCGTTCAATGATGTCATTTCGCCT
TACCGGCGGAACTAAAGATTACATAGTAGTTGGTTCGGACTCTGGCCGTATTGTGATATTAGAATATATACCT
GCCAAAAATATACTTGAAAAAGTTCATCAAGAAACATTTGGAAAGTCAGGATGTAGAA
GAATCGTCCCAGGACAATACCTGGCTATTGATCCAAAGGGCAGAGCTGTAATGATTGGTGCAATTGAAAAACA
AAAATTAGTATACATTTTAAACAGAGATGCTGAAGCCAGATTAACAATCTCATCACCTCTAGAAGCACACAAA
TCAAACACACTGGTCTACCACATGGTGGGTGTTGATGTTGGTTTTGAAAATCCAATGT
TTGCCTGTTTAGAAATTGATTATGAAGAGGCAGATGCTGATCCAACTGGTAAAGCTGCTCAAAACACACAACA
AACGCTTACATTTTATGAATTAGATTTGGGCTTAAATCATGTTGTACGAAAGTACTCTGAGCCTTTAGAAGAA
CACG
>103777009_2 DT663775.3
GCACGAGGCTTTTACCTTAAGAGTTTTGCTGGGATCAGACCAGACTTCTAGAGCTTGGTTTGCAACATTCTTG
CGTAACTCACACAAACGGCGAAAAGGGGATGGACATATAACTTTAATAAAATTACGTCAAGAACTTTTAAACA
GATTAAAAGAATCAATTGCAGGAGTTGAAGCCTCAGCTTTACTTAGATTATACTGTGC
TTTAAGAGGCATAGCTGGAATTAAATTCCAGGATGATGAAGTATCTGGATTACTAAGATTAGTAACTCAAAAA
CCTCCACCAACACCAGCAGGAGTTCGATTTGTTTCCTTAAGTCTTTGTATGATTTTAGCATGTCCGTCTCTTA
TGGCTGCACCAGAGCATGAAAAGAAGGCAATTGAATGGGTACAATGGCTTGTAAAAGA
GGAAGCTTATTTTGAAAGTAACTCTGGAATAACAGCATCTTTTGGAGAAATGTTGTTACTAATAGCCATTCAT
TTTCATTCTGGACAATTGGCGGCCGTGGGTGAATTGGTGTGTGCCACTCTTGGCATGCGTGTGCCAGTTCGAC
CTAATGGATTGGCACGCATAAAACAGGCATTTACACAAGAAATATTTACCGAACAGGT
TGTTACAGCGCATGCTGTGAAAGTACCAGTGACTGCAAACCTAAACAGCAACATAACCGGATATCTACCTGTT
CACTGTATTCATCAGTTACCTCAATCACGAGCATTTTCTAAG
>103777015_2 DT663859.3
GCACGAGGATTTTCTTGAAACTGAAAAGATCGTACAAAAAGCTTCAGCCATGGACTCGATGAGAGAAAAGGCA
TTTCAAGACTATCGCAAAAAACTAATGGAACATAAAGAAGTAGAATCAAGATTAAAAGATATGCGAGAACAGC
TAAGAGATCTCACAAAGCAGTATGATAAAAGTGAAAATGATTTAAAAGCCCTTCAAAG
TGTTGGCCAAATTGTAGGTGAAGTCCTAAAGCAGTTAACAGAGGAGAAATTTATTGTAAAAGCAACAAATGGA
CCGAGATATGTAGTGGGTTGCCGTCGTCAATTAGACAAAAATAAGTTAAAAGGAGGCACCAGAGTTGCCCTTG
ACATGACAACACTGACTATAATGAGACATCTTCCAAGAGAGGTGGACCCTCTAGTATA
CAACATGAGTCATGAAGATCCCGGTGATGTTACTTACTCGGCTATAGGAGGACTGCAGGAACAAATCAGGCAG
CTGAGAGAGGTAATCGAACTGCCACTCCTCAATCCTGAATTATTTGTCCGTGTTGGCATCACCCCGCCCAAAG
GGTGTCTTTTGTATGGACCACCGGGCACTGGAAAGACTTTACTAGCTAGAGCAGTCGC
CTCACAATTGGACGCTAACTTCTTGACGGCGGTGTCCGCGGCCATAGTGGACAAATATATAGGTGAATCAGCT
CGTCTCATCCGTGAGATGTTCAACTACGCGAGAGACCACCAACCTTGTATTATATTCATGGACGAGATTGATG
CTATAGGTGGCAGACGTTTCTCCGAAGGAAACAGCGCCGATAGAGAAATACACGGACA
CTGATG

>103777019_2 DT663986.3
GCACGAGGACCTCCCTTGCCTATATCACCAGCGAGAACACCCGATGATGCAACTCCAACTCCTCGGAAGTCTG
ATAATCTTCTAAGACCACCACCAAAGATCCACATCGACACTTCCAGTCATAGATCCAGAAGTCATCACAAGAA
ACGTGACGAAGTACCCCAAAAGAAACCCAGCTACTTAAATTTAGCTTGTTCAGTCAAC
GGTTACTCAAATCTAACAACATACGACTCAAAATTACGCCAAGATATCAACAAAAGTCGGGAAGCGTCGCCTA
TCAGACCTATCACTCATACATATCAATATCGGAGCGAAAATAACTCCCTTCTTGTTCCTATTCCGGTCAGCGC
TAACAAACTGTTGGTACCAACCTTCGGACCTCATGATACTCGCAATAATTTGACAGCA
AAAGCGCCTTCCAAGGCGCACACAGATCCTCAGATAGCTTCCCCGATACATGCCTATATGGCTGCTGAAAATA
AGCAGTTGAAAAACGATTTTTTAGGCCCCAGCATGACGACTACCAGTCGTCCTTACATATCTAACGAGAGCAA
GAACTTCGCTGCGTCAATGCTCCATCAGAAAGATGAATTGGATAACGCGAAGGAATTG
ACATTTAAGTCGAGTTATTCGGAAACAAATTTTCGACAATCAGTTACGACGAATGGCAAGGAAACAAGATTTT
CCTCGGAATCCTATACGATATCTTCAAATGGCGTATCAAAACGGGTAGAAATTACAAAGGAAAATGGTGAAAA
GCTGACCAGTCCTATGAAGAGT
>103777021_2 DT664882.3
CTTCTTCATGTCCAACACCACCGACGTCGAGCTGGCGGCCATCGACTCTGACAAAGCAATAGGCGTGGAAATA
AAACACGATGACAAACTGACAGCAGAAGAAGGTGTATACATACAAGCGGCCCTCCTATACACGCACGTGAGTG
GTCAGCGACGTTTGCGAGTGTTGAACTTGGCACTGGCACTCGCACACCAACTGGCAGA
TGTTTATAGATCCGCTGAACTGGATACTGCTGTTAATTTCTTGACCAAACAAGCGGTGTGGGCGCTGCGCGAC
GCGACGCCGCGGCAGGTGCTTTTTTTGCTGACGGGGCGCTGCGCGCGCTCGCTGGCCGCCTACCGCCGCCACT
GCGCCTCGCCCTCCTCCGCGGGCCAGCTCGTGCTGCCCGAGTCCATGAAGCTGCTGCC
GCTCTACACCAGCTGCGTGCTGCGCTCCGACGCCGTGGCGGGCGGCCCCGACATCACCTGCGACGACCGCTCC
TGCGCCATGTACCGCGCGCTCACAGCTGATGTCAGTCTCTCCTTAGCGTATACGTACCCGCGACTACTCGCCT
TGCACACGCTGCCTGCGACGCAACCGACGCGCTTGCGGGCATCGTTGGATAAGATGAC
CGAACAGGGTGTTTATTTGTTAGAAAACGGTGTCCACATGTTGATCTGGGT
>103777050_2 DT664879.3
GCACGAGGGAGAGCCCTTTAAAGGAGGCCTACAAGGAAAAGACATAATTGGTTTGGCAGAGACTGGATCAGGG
AAGACTGGAGCTTTTGCTTTACCAATCTTACAGGCTTTACTAGAAAGTCCTCAGAGATATTTTGCCCTCATAT
TGACACCAACAAGAGAATTGGCATTCCAAATTTCTGAACAGTTTGAGGCACTGGGAGC
AAGTATAGGTGTAAAATGTGCAGTAATTGTAGGTGGGATGGATATGGTGACCCAGTCGCTCACTCTGTCTAAG
AAACCACACATTATTATAGCAACACCGGGCAGATTGGTCGATTTTTTTTTAAACACAAAGGGATTCAATTTGA
AAGCTTTGAAGTTTTTGGTAATGGACGAAGCCGATAGAATATTAAACATGGATTTTGA
AGTGGAGGTTGATAAAATCCTGCGCGTAATTCCCCGGGAGCGAAGAACTTATCTTTTCTCAGCGACTATGACT
AAAAAAGTGCAGAAATTGCAGAGAGCATCCCTCCAGGACCCCGTCAAAGTTGAAGTGTCCACTAAATACCAAA
CTGTGGAGAAATTGCAACAATATTATATATTTATACCTGTTAAATATAAGGACGTATA
TTTGGTGCACATCCTCAACGAGCT
>103777072_2 DT664861.3
GCACGAGGCTTCTGCCTTAAAGGCGGTGCTTGCATGTGGTCTATAACAGAAACACGTGTTTTATAAAAGTATA
ACACTCTTTCTCATATTTATATAATTTAAATCATAATTAATAAGTATAATTTGCCGTATAATCTGAATAGATA
CAGTGTAGAACTTTGAAACCATTATTTAGTTAAGTGCGCCTTAATTTTTGCACGCGTG
CATCTACACATCTCGATTAAGTACAATTTGCTAAAAATGACGGAAGAAAAAGAAGTAGTAGTAGCAGCAGAAC
AAAGCAATGGGCAGGAAAACGATAAAGAAAATAATGTTGAATATTTTTCTGATCTGGATAGCGCTATCATACG
ACAGTTGGAATATTACTTCAGTGATGTCAATTTGCCAAGAGACAAGTTCCTCAGACAG
CAAGTGACGCTGGATGACGGCTGGGTGCCACTTGATGTACTTACAAGGTTCAACAGACTTGCCAAATTATCTA
CAGATACTGATGTTATTGCTAAAGCAATTAATAAATCATTGTCGGGATTACTAGAGGTATCAGAAGACAATAA
GAAAGTAAGACGAAATCCAGAACAGCCAATACCAGAAATGAATGAAGAAAGGCGTAGA
GAGCTATCTTCTAAAACAATCTATGCCAAGGGCTTCCCAAAAGACTCTGTTTTAGATGACCTACTAAAGTTCT
T
>103777086_2 DT664120.3
GNACGAGGATTTGCATTAAACTTTCGAAAAACTCGAGAATGAGCTGCGTGAAGTGAATCAAAACGCCGAGGCG
TTAAAGAGAAATTACTTGGAGCTAACTGAGCTCAAGCATATCCTAAGGAAGACGCAAGTATTCTTCGACGAGC
ACGAGGGTGGTGCCAATACCACTGAGTCGATGACCCGCGCGCTGATATCCGACGATCC
GAACAAACATATGGGACAGGTCCAACTAGGTTTCGTTGCGGGTGTAATTCTGCGTGAGCGTATCCCGGCCTTT
GAGAGGATGTTGTGGCGCGCCTGCAGGGGAAACGTGTTCCTTAGGCAAGCTGAAATTGACACGCCGTTGGAGG
ATCCGGCGTCGTCTGACCAAGTATACAAGTCGGTGTTCATCATATTTTTCCAAGGAGA

TCAGTTGAAGACTCGAGTGAAGAAGATATGCGAAGGCTTCCGCGCGACCCTCTACCCGTGTCCCGAAGCGCCA
GCTGACAGACGCGAGATGGCCATGGGCGTTATGACCAGAATTGAAGATTTGAATACGGTCCTAGGACAAACCC
AAGACCACCGTCACCGTGTCCTAGTGGCAGCAGCAAAGAATATAAAGAACTGGTTTGT
AAAAGTTCGCAAGATAAAGGCGATCTATCATACATTGAATTTGTTCAACTTGGATGTCACACAGAAGTGTTTG
ATCGCTGAATGCTGGGTGCCCGCGTTGGACTTGGAGACTATACAGTTGGCTCTTAGGAGAGGA
>103777096_2 DT664745.3
AAAAGTTTTTTAATAAAAGTATTTTATAGACTTTTTAACTTAAAACAACAATGGTTTTAGATTTAGATTTATT
TCGCGCCGACAAAGACGGCAATCCAGATAAAATTCGTGAAAATCAACGTAAACGGTTTAAAGATGTAGCACTA
GTGGATACTGTTGTGGAACAGGATACCTTATGGAGAAAATTACGACACGATGCCGATA
ATTTAAATAAACTTAAAAATGTATGCAGCAAAGAGATTGGACAGAAGATGAAAAATAAAGAACCAGTTGGTCC
AGAAGATGAAGCAGTTCCTGATTATTTGTCTAATAACCTAATCGATCTTACTGGCGAACAACTTCGGCCTTTA
ACAGTCAACCAAATTAAAAAGGTGCGAGTACTCATAGATGAAGCAATTGCAAAAAATG
AACAAGCTTTATTAGCAGCAGAAAAAGCACGTTCAGTAGCACTCAGAGAAGTTGGTAACCACTTACATGAGTC
AGTACCTGTAGATGATGATGAAGATCACAATGCTGTTGAAAGAACTTTTGGTGACTGTTCTGTCAGGCAAAAG
TATTCTCATGTGGACCTTATTTGTATGATAGATGGTATGGATGGTGAAAGGGGAGCTG
CAGTATCTGGTGGGCGAGGCTACTATCTTAAGGGTCCTGCAGTTTTCCTTGGAACAGCCTTGGGTCAACTTTC
TCTTA
>103777102_2 DT664178.3
GCACGAGGCTTAAACTTTTTCTTTTCATAATTTAACAAAGTTAGAATTACGTGTGCTGGAAATTTATTTGTTT
AATATTTTCGTTAAAGCTATAAAGCTCAAATTGAAAGTGCATATTTTAATATTCCTCACCTATGCCCTTATAC
ATTTACCGCAGCCTGTAACTTTCCCGTTACCTGCATTAAAATCAATATTTTATTTGTG
CCGTGCCACGTTCTTGCGGCAGTTTATTTTTATATTCTTAAAGGGATGTAATTTATTTTTCGAGACATTTTTC
ATTTAAAGAAGCAGTCATGTCTAGCATGTCAGATCGTAAAGCTGAACTGGAGCGTAAGAAGGCCAAGCTCCAG
GCTCTAAGAGAAGAAAAGGATCGCCGTCGAAGGGAAAAAGAACAGAAAGATGCAGAGG
AAGCATTGCAAAGAGCAAGCACAGCCTCAAGTCTTGACAGCCGCAGGGACTTAGATGAGATGCTGTCATCTCT
AGGAGTGGCTCCAGTTAAAGATGTTCTGTCGTCTTTATCTTCAATGACATCTTTGACACCACCGCAAACAGCA
TCACCTGATGCAAGCCTGCCGCATACTGATAAATCTAGCTTACCTCCACATGGTGGTG
TAAAGAAGCCTCCACAGTTGCAAGTAGTATCCGTACAGTCCACTGACATACCTCCAAAGGAGAACGTCACATA
CGCAAAACAAACTCAAACAACAACATCTGGCGTAACTGAATTGCGTGATGGATACATGGAGGACTGGTGGCGG
CCGCGGAAAGCGCACGCAACTGACTACTACGTGCTTACATTCGACGGGGATGGCGCAC
GGCAAGGCG
>103777108_2 DV501194.2
GCAGAGGGATAAACTTTTGATTGATTATTTGGCAGATATACATCTGCCAAATATCATCTCTTTCTTATTATAT
TTGTAAGAAAAAGATAATATTAAAAATAACTGTCTGATAAACTAACTGGAGGTTTTAAATTTGCTTAAAGATA
ATTTCTTTTGTATATTTTCACTGTGATTTATCTAGTTACACAGCTCTAGGTCAATTTT
AGTTAGCGCTAAATTTAAGTTCTAAATTTTAAAATTATTGTATATGAAATTAATTTTAAAATTATTGTATATT
AAATAAGAGAAAAATCGTAGTGTTTATATATGAGTGTTTTTTTTTTTTTAACTAGAAACTCGTGATAGTGTCA
TATTGTATTATTTGACTATTGTTTTCGTTTAGAAGAAAATAAAGAAAACAGATAAAAA
AATCTAATAAATATATTTATTTTTAACATTTAAAAGTTGCTGTTTTTTTTAGCTTCAACTGATTTTTAGTTTA
TGTATGTATATGCTGTAAATATTGCTAAATAGGAATAAGTTATTGTTTGATGTATTCAAATTTATATATTCAT
TCACTAATTTGCACATTAATATTGGTAAGATAAAAGTCAAAAAATGTTCGAATCGCATT
GTTTAAATTATATTATCATAGACTTCATGTTTTAAAAAAATATATCTTGACTAGTCTAGTCTAACTATCATCA
GTGTATTTCTAGAATTTACTTCAAAGGCAGCACTGGCAGAATCTTTTTTGATTTGGTTATGTGTTTGGACCGT
GGAAG
>103777110_2 DT664836.3
GCACGAGGCTCAAGCTTAAGAGCGTCTTAAATTCCACTCGAGCCTTTCCAATAATTCTACTAAAGAATATCAT
AGCTTAGCTGTTGCCAGTCATGAAGGAATTAACAGAATGGTAATGCAGTCAGATTTGCGAGATGTATATCATG
GAGTGCATATAAGTGGATTCTATCCTATTGAAATAAACCCACCTCGAGGTGAACCATA
TACGGGTGTCATTAATGACTTTTATGTACAGCTGTCCGACAATGCACATGAAAGTAGACTTAAAGAAGTTATA
GAGAAATATTTGCGAAACAATAATTACAGTCTTGGAGGAACGGAAGTACACGCAGCAGAAGAATTAATGGATA
GACTAAATGTCAGCGATTTCGACGAATGTACTAGCAGACAATTCCATGACTGTTCAGA
ACACGCGATGTGTTTCAATCTTCGCGGCACTTATACTTGTAGTTGCTTAGAAGGCTTTGCTGATCTTAGCGTT
AATACTTTGTATCCCGGCAGAATTTGTTCATCTGACACAGTAGGCTGCGGGGGCTGTAACTATCACGGCACTT
GCTTCGAAAGAGAAAGTGCTATGATCTGTGAATGTTTCAAGTGGTACGCCGGTCGGAC

GTGCCAGGTCAATCTGAAAGCGGTGCTTATAACAGTGACAGTAGTTGGGGCTGTTATAATATTAGCAGTTACT
ATTTGGGCTTCGAAGAGATGCTGCAGTCCGAAAAATCCTACGACACAAACCTTCGTAATTGGTTGTA
>103777112_2 DT663683.3
GCACGAGGGTCAAAATCATAAAGGTTACGACAAGGGCTGAAGAAGAAATTCCAAGGGAAAAGCTAGTGGAGGA
GCTAAAATCAAAGATTCAGCTTTTAGATTTAAAGGAAATGAACGGACCAATGCCTAAGTAAATTCAGTATTTG
ATATTTTATTTGTACAAATTTTTTTGGAGTAATAAATTATGTAAACTAACCCTTTTTG
TCTTTTTGGTTAACATTTTTTTTTTATTTTAAGCTAAAGCTCTTGATAAAAATGAGTGTGATGATGTAATATC
AAAATATTTTTGTTTGTTGTATATAGAAATTGTATTCCTAAAGTTCTTTAGTAATTAATTTTATAGATTTTAT
CTATTATTATTTTTTCATTTATTTTGTCTACCTATAGACTAAAGATTGACTTGTTATT
ATGAAGATTTTCTTCAATATAGCAAATTTGTCTATAAAAACGTAAAAGATATCTTTTATTCTATTAAATTTAT
TTTCCGTTTGTAAGAAAATATCAAAAATTCTGATTCATTCCTTACATTATACTCAAAAAGACTCAGCATTTAA
ATACCTATAAACTCAATGTAACGAAGCATTTTTTAAAAGTATAATGTGATAGCATTTT
TATTTAAGATGTTCTATTGTAATTTATCTAATTTTATTTAAAAAAATTTGTTCTTATTATTTAATGTTTTTTT
TATATAATTGGGGAATATATCTAAATACTTTTCCTAGCCTTAATCT
>103777116_2 DT664791.3
GCACGAGGCAACAGCTGACACTGTAATCATCTATGATTCAGATTGGAATCCTCATAATGATATCCAGGCCTTC
TCTCGAGCCCATCGTATCGGTCAGGCCAATAAAGTTATGATATACCGATTCGTAACTCGTAACAGTGTAGAAG
AGAGAGTCACTCAAGTAGCCAAGAGAAAAATGATGTTGACTCACTTAGTAGTACGGCC
CGGTATGGGCGGCAAGGGCGCTAATTTCACCAAGCAAGAATTAGATGATATCCTTAGGTTTGGTACAGAGGAG
CTCTTCAAGGAAGAGGAAGGCAAGGAAGAAGCTATTCACTACTATGATAAAGCCGTCTCAGGGCTACTCGATC
GTTCTAAAGAGGGTATTGAACAAAAAGAATCTTGGGCTAATGAGTATCTAAGTTCGTT
TAAAGTGGCTAACTATTCTACCAAAGAAGGCGACGGCGAGGAGGAAGTCGACACTGAGATTATTAAACAGGAA
GCTGAAAATACTGACCCTGCTTATTGGATCAAACTGCTCAGGCATCATTATGAACAGCATCAAGAAGATCAAG
CGCGAACTTTGGGTAAGGGTAAGAGAGTACGCAAGCAAGTCAACTACAACGACGGTAG
TGTTGCCCAGACGGANAACAGAGAGGACTCAACGTGGCAAGAAAATGGCTCAGATTATAATACTGATTTCTCT
CAAGGAAGCGAAGATGACAAAGAGGATGACGATTTTGATGAGAAGAATGACAATGGCGATTTACTCAGTCGCC
GCAGTAGGAGACGCCTGG
>103777118_2 DT664096.3
GCACGAGGCCTCGCGCTGAAAAAGAAACTAGAAACTATACATTATCTTCAACAACGTGGCTTGATCGATCCTC
ACGGCCGTCCCGTGCCCGGATCAGAACATCAAATTCACCAGGCCATCCAGGAGTTCCAACACATGCAACAACA
GGCTTACGGACAATACCAGCACCATCCGCCAGGGCAAGTGCCCCCACCGGGAGAATAC
CATGGACAAATACCGCAAGGACAGTACCAGGGTCAGGTACCACATGGACAGTATCAGGGACAGATACCGCCAG
GATCGTATCAAGGACAGTACCAAGGACAGTACCCAGGACAATATCAAGGACAGGTTCCGCAAGGTCCGGCACA
ACATGGACAATACCAGGGTCAAGTACCACAAGGACAAGTACCACAGGGACAAGTTCCA
CAAGGACAAGTACCACAGGGACAAGTACCACAGGGACAAGTACCTCAGGGTCAAGTTCCTCAGGGCCATGTAC
CTCAGGGTCAAGTTCCGCAGGACCAAGTTCCTCAGGGTCAAGTCCCTCAGGGTCAAGTTCCTCAAGGTCAAGT
CCCCCAGAGCCATGTCCCTCAGGGCCAAGTCCCTCAAGGTCAAATCCCACAAGCTCAA
AGCCCACAGGCTCAAGTCCAACAAGTTCAGGTACCACAAGGACAATATCAGTCACAAGTACCTCAGGGACAGC
CGTCACAAGGACAAGTGCCCCCAGTTCAGCATGGACAGGCCCAAAACCAAATCCCAGCTAATCATTAAAAGTG
CATACTAAAATATGTACATCAATAGATTATTAAATTACAAAAATATATTCATATTATG
CATAGGC
>103777120_2 DV501119.2
GCACGAGGGAAAATCGTCTGCCTTAGTCCACGAAAAAGTTTGGGTTGATAAAAATGTCTATAATGATGCCGAA
CGAGCTTATTATGAATCTCTATCNAAAAAACAGGCAACAGGCATAGCTAGCTCATCACTAGCTAGTGAAGTTG
CAAAAGCT
>103777126_2 DT664253.3
AATATAGTGCTCAAATTTTGCTGCTGTACCTAGTGCTTTCGTGATTTGTGAGTGCACAGTAATTGGAGCGCGT
GGCTGGTTACTCTTTGTTGTTGGATTCGGCGTATACCAGGTTCAAGTTGGAAGACAGCAATCAGTTAAGACAA
TAAAGTGAGGAGTGAGCTCATTCGGCTCGCAACAGACGGTGTGTGACAGTGTAAAGTG
CGGTGCGGAGTGTGCGCGTGCGC
>103777128_2 DT663774.3
CGGGCTGCAGGAATTCGGCACGAGGCACGAACCTTAAAGGGAATGATAAAGTAGGGAATTAGACTTTCTAAAA
TGACAATAAAAATTCAACAGTTTTTATTTGTAGTTTTGTTTTGAGACCGTCGTGGCGCGGCGGCACGGCGTGT
GACTGTAAGCGCGTTGGTTGTAGGTTCGATTTTCACTCTTGTGATTGAATTATTTTCA

TTTCTTCATTCTGGTGAAAAGACAAGCCTCTTTCAGTTTTGTCACTCAATACGCAATGTCTAGAAAATTGGGC
GGAAAGAGAGGACGGAGTATCTTAATCTTAACACTAGGTTCCATCAACTTGTCCACGTTTTTTTTGTGGAATA
CAGTGTGAAATTGAAAAAAATACGTTCAATGCTTACTAAAACGTGAATAGAATATATC
TTTAAAGAAACCACTGTCATAAATAGATTTCTATTAAAGCCGATACTTCAATTGCTTTGAACTTAACCCCAAA
ATATTTATATAATGATCGACCCCAGAACACTTTCACGAGGTCGAACAACAAAATGTATGCGAAGAAATTTANA
ACCTAGTAACAATTGTTAAAACGGAATGAAAATATAAAAAGACTAGCAAGTTTCTACC
TGAGCCCTTTGTACGAAGTGTCACTTTAAACAAATTGCGCAGGCGCAGCATTTATAGGTCTATAGAGTTACTT
>103777132_2 DV501151.2
GCACGAGGACTCCCCTTTAATAATCACTCTAAATCATAATAATGGTGTTCCACTAACAAAAGACCGTATATAT
ATAGTTGTGCAAAGAATTTCTTAAAACAAAAAGACCACATTCGCTTATAAAAGCTTTGGGTGAAGCTTTCACA
CAGCCACCAATATTACCTATATGTTTTCAAAGGAAAATGAACCAGATCGATAATGGTA
ATGATGGAATCTAAAAAATATATCAACAATCGAAAGCAATGTGATCCTCAAGTGATCCATATAAACATGTAGA
CCGTGTGACTGGACCATTTACTAAATGTGGTCTCGAGTCCGATATTTTTTCAGTATCTTGCCTAATCGCCGGC
ATATTATTA
>103777146_2 DT664003.3
GCACGAGGCCTCGTCCTGATACGGCACGAGGCCACAGCTGTGATTCGTATCTACTACCATCACACCCCATTAG
CGTGGTGGCGCACATATATCGATACTTAACTCTCATCGTAGATCGTAGGCCTAAATTTTTGATCTCATATTCT
AATTTGCTTAGTGATAACTTTCGGAATCGTTAGCGGTCGCGACTCGAAATTCTAAATT
GTTTGTTTTTTTTTTATTATCTGTCACTCGCTATCGTCCCGTCTGTCGGTCGTCCGATCGATCGATCATTAGT
CGTAGTGGAATGTAACGATATCTTATTGCGATGCTTTAACTTAGATGTCGGTGTAAATGGAGTTCAATAATTT
TATAGAAGTGTTTCCGAGACGTGAAGCCTCCCCCGGTCCGTGGGATGCATGACGCAGT
CCTCAGAGGCAATAAAATGAGTCTTCACAAGATAAATTTAATATGATGTTTATTGGAGAAATCCAGAGAAGTG
CCTGTTGCAACAAAAAATTGGCAATATTTTTTAATCGTATTTCGTTCATAATAGATATTATGTTCGGTGTTCA
TGATGTGATCGCCACTAGTCGACCGGTGGCATGTATGGCAATAAGTGAACCGGAGGCG
GCGGGTCGTACAAGCTTCTTACTAAAACTTTGTGTGCCTTCTAGCGGGTAGCGTTAGGCGTACACAACAAACA
AACACCATCGACGACGCTTCATTTTCAGTTTCATTCGTCGACACTTTTTTCGCGA
>103777160_2 DV501209.2
CAGTACTTCCAGAATACTTTTTCTCTATGTGATAGATTATGTGTGTTAAACCTTTCACATCATACATTAAGTC
AACTACCACGTGTGAGACGAGCGTCACCGCTTTGCTTGTAACGTCTGTACTTAACTGCTAACTGTTTATCTGA
TGAAGTTATAGATGTTATTAT
>103777162_2 DV501259.2
GGAATAATGTTGTTTTGTGTTGTGATGGTTGTTGATATGAAATGATAGTATTGGTTTGTTTTTTTTAATTATT
ATCCCTTGATTTGTGAATTGATTAACTTTGTTGATTAATTAATTGTTAGTGACAGAGACTTTAGATTTCAGTT
GTTTTCCTTTGTATTGTAAATATCGTTTTGTAGTATTTTTTTTTTTTTGTAATTTGTGT
TCTATTTTTTTTTTTTTTACAATTTTCAAAAGTTACGTTTTTTGGGGGGGGGAAAATCTACTTACCTAAATGTA
GATCCGTTTTTTGTTAATTCTGTCGCTTTAAAGTTTTGTTTAAAAATTCTAATATGTTGATTCTCTACATATT
AGATATTGTAACAAACAAACAGACATCCAATATATCAATCAACATTTGTACTAGTCAG
CCTGCTGATAAATAATTTGTTATTTTAAGAATTTAAGTTCATTCTGCCCTAACGATTGTTA
>103777164_2 DV501161.2
AGGTTCATTCAAAGTATAAAAAGTATGCTGGAACAAGATATGAAAAATGATAAAGCGATTGGTAAGTTTGGAT
GTGAAGCAATACTAACGGGCTTAGAGAGTGAAAGCTATGTGAGAGTATTAACAAATTGTATTACTGGATCGCT
ACCAACAACTGGGTATGGCACTGCATTGGGAGTTATTAGGGCTTTGCATGCTG
>103777182_2 DT664881.3
GCACGAGGATCAAGACTTAAAGGGAGTTGTCTCATTAAATAATAGTGATAAAAGTGATAAGGGATCTACTACT
ATCGAATTAAATAATAGTGACATAACAATTCACAGTTCTCCATTAAATGAAACGGCAACAGATACATCAGTTT
TGGATGAAAATGAGCGGAAAGCGGCTTCTCTTGGTGATTTGTCAAGATTTGAACTTAG
AGCTAACACAAGTAAACCATCTACAGGGACGTTAGAAAGAGCCCAAAGCTTAGACATATCAGCTGATGATTCT
GAAATTCAAGAAACACTTTCTCCTAAAAAACGTAAAGCAATGTTTTTTTTTGAAACAACATTTTTCGACTCGG
GCAACGAAGATATAATTCCGGAAGTCATGGATTCTGAAAAAGGAGCTCTGATGAAACA
AAAAGAACCAAGACTAAGTTTAAACATAGCGAAAACATCCGCAATGGAAGGATTAAACACGTTCCAAAGGAAT
CGTTTGAAGAAAGCATCATAATTTGGCAATTTAGAAGACGCAATTGTAAAAGGATCCCATTCTTCTATAGATT
CTGGAGAGCATAATTCACAA
>103777207_2 DV501146.2

GCACGAGGCCGACTCCTACATACAGCTCCTGTGCTGATGTCAGGAAGATCCCGAACTACCACAAAGAGGGAGG
TACGCGCTGCTACCTTAGCTCGTGGACTGCATTGTTGATTCGTTGTACGATAGAAGATCTTCTAGTGGACCCT
AATCCCGAACAATAACTCATAGCTCTTTCTATTGGGGATCCCACCACTTTTGGTAATT
GAAATCCTCCTGAACAGGAATTGCAAGCGGTTCAAGATAGTGTTCAGTTACCTGAAAATCGTGGGATATGCGC
CGGCTAAAGGGCACTATGAAGCTAGAGCCGCTGGGGACAATTATGATGCCCATCCAGGAGATGTTACTCCTCA
CGACGTTATCCTATCCCGTGGCTGTTCGCATGGCATTGAATTAGTGATAACACCTATT
GCATAATGTGGACTATATATTCTTGTCCCGCGCCCTGTATTTTCAATATATCACACATAGGCAGCTGGCTTAT
GCATTGTCATAGGATATTACAGTCTATTGCCAAATGAACCATGGAAAGTCGATCTGAATGATTTAGAAAGTAC
TGTTGATGATGATACTGTATCCGTAAAACTTATAAATCCATCCAATCAATGTGGGTCA
GACT
>103777215_2 DV501152.2
ATCTGGAAAAAGCCGGCCCTAAAAGCGGCGTAAATGTTTACGTTGAAAAACAAGGCGCTAACGGCATTGAAGG
GCGTTTAACGGCGGATCAACTCAATAGTGCAACCGCCTGTATTTTTGCGGCTGAAGTCGCCATCAAGGAGAGT
GAGCGTTTTAATGGCATTCCCGCGCTTTCAGTGCCTGTTGCCGAGCCGATTCGCCATG
CAGAAGCGTTGATCCAACAAGCGCTTACCCTCAAGCGTAGCGATGAGACGCGTACCGTACAGCAAGATACGCA
ACCGGTGAAAAGTGTCAAAACGGAGCTGAAACAGGCACTGTTGAGCGGAATCTCTTTTGCCGTACCGTTGATT
GTCGC
>103777231_2 DT664543.3
GCACGAGGCCCCTTTAAAGCGTGATCGCCACTGCCATGTGATATAGTCTGTGATAGTGACGCAACCAATATGT
CACTGTGTAACACCTTCGGGTTGTTCGTATTAAGCTTGATCGTTGTGCTATCTTGTATTGACACACCAGCCGC
TTTTACGCTACATCACAGAACTAGAGATCAAGATCTCGTTCATGAAGAATCCTCTCTT
GCAGATGTTGTTGTTACAAATGTATCAAATAGCAATATAAAAGAGAATCTATCAGAAAACTATCTTAGTAAAA
GTTATAATGTAATTAAATATGGAGATGATATTTTTTATGTAAATTCTCTACAAAGTCCTCTAGATAATATCTC
GACCGAGGGGCTTATTACAACAACCACACAAGGTATAGGAGATCCAGGATTTGATTCG
ATAGCTATGAGTACAGAGAATTTCTCAGATAAATCTGCTTTTGGTACTGCGTCTATCACAGGGTTCTTATTAA
CGAATCCTACGTCAGAAAGAACTATAAATGTAAATGGTCA
>103777248_2 DV501225.2
GCACGAGGATTTCCCATTGATACATTCACCCCAACAAGCAGATATTCAACTGATCCTATAACGATTCCATTTT
TACTTTACAGAACTCTTACTACAAGAAAACAATTCAAGAACCACAATAAATCAAAATCGGAGATGAAAAAGTC
TCACTATTACCATAAATATGACTTGGAAATGGAAAGAACAAGTAGTCCAATTACCTCG
ATATACAATTGATGGCTTGGCGGATATGTGATATGGGATCCGCTCTTTTTTACACACTGATCAATAACTTGCT
CCTTCATGCAGAAGAATGGCCGCACACAGGCTTTGGGAATGATAAATTGAATTATTTCACAATATTACTGAAG
ACATGCGATGTTATCGAATTAAAATTTTAATTTATATGGACAAAAATTGTATTGTAAT
TGAATATTTCATTCGACTGACAATGCGATGCTGCCAAAAATTGCTTTTTATATATATATATAGTCAAACACAA
TATCTAATACAATTCTTGTTGTAATATAAAACACACCTAAACTTATCACAAGAAAACCGACGAATACGCCTCC
TTCTGAAATCAAGT
>103777307_2 DT664760.3
GAGGATTTCCCTTCAAACTGTACTGTTATGCTAGCTTACAATAAAACAAAAACTTATATACGTTGCCGCTTTT
GTTATGAACTAAACAATAAAATTTTTAGTTATGGATAGTCTTAGCTCATTGAAAGAAATAGACAATATTGAAA
TAGCAAACTCAGTTACACTAGACATACAGAACCAAAGTAGAAATCAAAAGTATAAGTT
AATAAATCAACTTACAGTCATAACGCAAAACATTCGTAGTATATATAAAAACTTTGATGACCTACAGGTCACT
TTGTCACGGTTAACATATGACTTTGATGTATTAATTCTCACGGAATGTAGATTGCATATCACAAAACCAGTAC
CTTATTTAACAAATTATACCTCATACCAAACGCAAAATCTTTTAAATCAGAACGACGG
CGTTGTAATTTACGTTCATAATAAACATAAAATATTAACATCTGAACTTCGACTTTCTAATGCTTCTGGCCTA
CAATTAATTATGAAAAAATTTGCTATTTTAGGAATTTACCGTTCCCCGTCTCACTTGGACGCTAGTAATTTTA
CGGACTCCCTAGATGAATATCTAAACAGTATGTCGAATTACAAAAATATCTACATTCT
TGGAGATATAAATATTGACATTATTACCACACCAAAAGAGACATCTCAAGAGCGCAGAAACAGATTACATTAC
CTAAATGTCCTATCAACTCACGGTTTACTTCCCGGCCATACATTGTCCACTAGAGATGACAGCTGCCTGGACC
ATGTATTCTTGA
>103777314_2 DT664405.3
GCAGAGGAACATACCCTGCAATTTCTGAGGAAACTGGAAAAAAGAGTGTTTTGACACTAAGTCATATTTGTGG
GAGCCAGAAATAAATTGTACCATGAAAAAAAAAAAATTATGCATATTGTAAATGAATCTTCTCTTCACTTTTG
TTTTTGTAAAAGAGGTGCTAGGCGTTAAAAATACTTCGTTTCATCCCCGGGGCCCGAC

AAAATCCCTAACAAAGTTTTAAAGTCCCTTCCGATTCACTTCGTATCATTACTAGTCATGATCTTCAATGCTC
TTTTAGCAGGTTGCTCCTTTCCTACCGAATGGAAGGAAGCAATATTTATAGGCATTCGCAAGCCCAAAAAACC
CGCCAACCTTCCCTCCAGCTACAGACCCATCAGCCTTATTAGCAGCTTAGGGAAAATC
TACGAGAGAATCGTTCTCAGTAGATTAAAGGAAATAGTCGAGGCAAAACAACTGTTCATCAACGAACAGTTCG
GTTTTAGATCCCACCACTCGTCCATACACCAAGTTCACCGGCTTACCGAACACATCGCGAAATATAACGTCCG
TAGATCGATTCGTAGCGACACGGCGGCCATTTTCTTAGATATCGCCAAAGCCTTCGAC
AAGGTCTGGCACAACGGCCTGCTCTTCAAGCTCCGTCGTATGGCTCTGCCAGACTCTCTCGTTCACATCATTC
GGGAATACCTGTCGAATCGCACGTTCCGATATAGATTAGAAGGGACCCTATCGTCCCCTAGACCCGTTCGCGC
CGGAATCCCGCAAGGCTCCACTCTTTCACCGTTGCTATACCCGCTT
>103777316_2 DT663927.3
GAGGATTGACCTTTAAATTTTGAAGGTTTTGTATATTTTAAATATGTCTCTTATAACCAGAAACGTTTTTCGT
ACTTTAATTCAGGCTTCACAGCACGTTGGCAAAGGTTTGCACACTAGTGCGGTAAACTCCGATAAAAACCAAA
CAACTAAGGAGGGTCGCATTAAGTGCACTCTGATCCCCGGAGATGGTGTTGGTCCAGA
AATGGTTTACTCTGTTCAAGAAGTTTTTAAGGCAGCAAGCATCCCTGTGGACTTCGAATCGTTTTTCTTCTCC
GAGGTGAACCCAACTTTGAGTGCACCACTTGAAGATTTTTTCGGATCCATTGCCAGGAATAAGATATGTATTA
AGGGCATCTTAGCGACGCCAGATTTCTCCCACACTGGTGAATTACAAACTTTAAACAT
GAAATTGCGTAACGCGCTCGACTTGTATGCTAATGTGGTGCATGTGAAGTCATTACCAAATGTAAAATGCCGC
CATCATGATGTCGACTGTATTATTATAAGAGAACAAACCGAGGGCGAATATTCCGCTTTGGAACATGAATCTG
TGCCTGGAGTAGTAGAATGTCTTAAGATCATAACGGCTGCTAAATCCGAGCGTATAGC
AAAGTTCGCCTTCGACTACCCGGTCAAAATGCGCCGCCGTAAAGTTACGGCTGTACACAAAGCTAATATTAT
>103777317_2 DT663655.3
GCACGAGGCGTACCCTTAAAGGCTTTAAATTTAAAATATTTACTTCATCGGAATTGAATTCTATATCTTGGAA
CATCGTTATACAAATACGAATCGTATACTTATCAAATCAAATCAAAATGCAAAGTTTCCCCAATATAAAACCT
GCCCTCATAGTGTTTTTTGTAAACTTCGAAATGTAAGTGTACTGTGGTCAGTGATATA
GTGGTCATTCTTGACTTTTATAAGTGAGTGTTTTTTTTTTATTATTATATAATTATATATAACATTGAATTTT
ATAAAGAAATCGATGGATGGAGGTAATATAATTATGCTATTGTTAGCGAGCCTTATGAGGCTTCGCCCGCTTA
AACTTATTTTTTATTATAGACAAGATATACAAAATTTTTATATAAGTTGTAGCCTATG
TGTCATTCTGATGTATAAGCTGTAAAGTTTGATGTTGAATTATTTCCTATTTTGGTGGGTAGGTGTAGAGTGG
CAAGAGTTAAGTAGCGGAACCCAACGCCGCACTTTGCCTGAGCGGACCCCTGAAATTGCATAAAATAAATTAA
TATACCCATCAAACACGCGCCATCACGGCAAATCCAACGATGTCATCATTTTATTAAA
ATCGGTTTACTGGTTTAACAGTTACAAAGTGCATAAGAATGAACATATATACCATATGTACCACTAAAAAATG
TCCCTTTTGGGC
>103777323_2 DT664483.3
GCACGAGGAGACCCTTCTAGATAATACTATAGATGTAATGGTGATATGTGAAATTTATTCAGTGAACTTAGGG
ACGTTTTTGATTACGGATCTTACAAGAGTAACATTGAGTGGGGAACGACAAACGATTGTACGTAAAATTTAGG
CTTGTATTTAGAATCGGTATAGAAATTTCAAAATTCATTTGAAAACGTTATTCATTGG
CGGAGTTTGTTGTTTACGACGGAAACATTTGTTTTGTGAACGAATTAAAACCCATTCGTGACTTTAATTTGTG
TTTTTGCATTGTTTCACTTTGGTTATTGTGCAGGTATAATGTAACAAAGCGTTTGTTCCGGCCCGCGCCAAGT
TTAATGACCATAGAGATTGTGCTAACGTGAATGAATACCAACTTGGCGCTTTAGTCGG
ATGCCGTTTCCCATTCTCTTGGCTTAGTTAACCCTAATTCGGCTCCCAAACACCAAATTAGTGAGTAAATTGT
ATAGAAACGCAATACATTTCAGTGTATATGGTTTGTTGAGTTTTTCTATGTAATAAGTGGAGGTTCACCCGTT
GAGTGAGGCACCGGCATCTTGCGATGTGATGTAACATAAGTATATATTGTTTGATATT
GAGCATTTAACTGAATGGTCCCTTTATCTTATACTTGTTGCCTAACGAATTAATATCAGTTTAATTTTCCAAT
TTTTTTATTAATTTTACCCCCCTTTTTACGAGCTTTTTTATATTATTTTATCATTAAGTAACAAGTATAAAAT
ATCG
>103777329_2 DT663676.3
GCACGAGGGCCCTACATACCAGGACCCGATCACAGGACAGACTGTGTGCTCCTGCCAATATGATTTTCTGAAC
TATCAACGGCTAGCTTCAGGGGTGCCGCTGTCCATGTACAGCGCGCCGTACCCCGATGCAGCTACAGCGGCCG
GTATGGCTGCGTACTTCCCAGCCCTAGCCGCTGATCAACCACCCTTTTACGCTAATAC
AGCTGCCGGGATCGAGCTCAAGGAGAATCTGGCTGCCAGTGCGGCTAGCTGGCCGTATCCCACGGTGTACCAC
CCGTACGACGCCGCCTTCGCTGGGTATCCGTTCAATGGATACTGAATGGACCTAAATGGCGCTCGAAGAAAAA
ATGCCACACGAGAAACTACCAGCACCTTAAAAGCCTGGCTTAATGAACACAAGAAGAA

TCCATATCCAACAAAAGGCGAGAAAATAATGCTGGCAATAATTACAAAAATGACACTAACGCAGGTGTCGACG
TGGTTCGCAAATGCCCGACGACGTTTGAAGAAAGAAAATAAGATGACATGGGAGCCGAGGAACAGGGTTGATG
ACGATGACAACAACAATGACGATGACGATCATAAGAGCAACGACGGCAAAGACGCTTT
AGACGGAAAAGACTCTGGGACGGGATCGAGTGAAGACGGCGAAAGACCTCAACAAAGGCTAGATCTACTCGGA
CAACGAACTGAATCAGAATGGTCAGAGTCACGTGCGGACAGCGGA
>103777331_2 DT663583.3
AGGATAATATGTTTGTTTTCAAAGAGTTATGAATTGTAACTTATTTATTGTTGGATAATATAAAATCTATAAT
AATAGAAACATGGAGGTTCCATATCTCGTCTTATTCTTGTGTATTATCTGGCATGGAGCCGACGCTATTATGT
GGAGTTTGGCTCCTAACACGCAAAAATGCTTGAAGGAGGAACTACACGCGAACGTTCT
CGTTGCCGGTGAATATGAAATAACAGAAGCAGATGGCCAGAGAATTGACTATATTGTACGTGATTCTAAAGGA
CACATTCTAGCCCTTTTTTTTTTTATCACCAAAGGAAAGTTTACATTTGTAACAGAAACATATGACACATTTG
AAGTTTGTTTCATATCAAAAGTGCCTCAAGAGCGCCGTGGTGTAGCACAGGATGTGAC
ACTAGATATTAAAGTTGGCATTGAAGCTAAGAATTATGAAGGGATCGGTGAAGCAGCAAAACTGAAGCCCATG
GAACTTGAATTGAAACGGTTAGAAGATTTGTCTGAAGCAATTGTCCAAGACTTTACTCTAATGAGAAAGAGAG
AAGAAGAAATGAGAGATACTAATGAATCTACAAACAACCGAGTGTTGTTTTTCAGTGT
ATTCTCTATGGTGTGCCTATTGGGACTGGCCACGTGGCAGGTGTTATACCTACGCAGATATTTCAAAGCCAAA
AAACTTATTGA
>103777335_2 DT664924.3
GCACGAGGGGAACACCTTACAAGCGTGAACACCTGTAAATAATGGTACTAACATAAACAAACATTGTTTAATT
CTGATTTGTGTTTTAGTATAAAAAAGTGATAATGGCTAATAGCTATAATATGAAGGAATTCAGCTCGACGGCA
GTCATTACAGAAAATGGAGTTTTTCCGTCCAGTATATCTATCAGCACGATCAATACAA
AATGTAAAGAAAACGAGATAGAAAATCCTTATGATCCCTTCCAAAATAGGAAGTTGGAGCATCCTAATTCAGA
TGTCAGATCGTTCGCAAATCTTTTAAAATCATCACTGGGCTCTGGTATCCTCGCTATGCCAGCTGCGTTCAAA
AACGCAGGAACTATTGTTGGTATATTTGGCACGATCATCTTAGGGTATATTTGTACAC
ATTGTGTGTATTTATTGGTTAAAACGTCTCAAGACGTGAGCAGAGTATCAAAAGTGCCATCACTGGGATATGC
TGAGACCGTAGAAGCAGTATTTGCTACTGGACCAAAGCCATTACGGAGATTTTCAAGATTCTCTAGGATATTT
ATAGATTGGGCGATGGCCTTCACAATACTTGGCGCCTGCGCCGTGTATGTCATCCTAC
TAGTGGAATCTGTACAGCAGATCGTCGACCATTTTGTGACAGACAACGGCCTCAACACAACTATGTATTGCCT
TATATTTTTGGCTCCAATACTTGTATTCACTCAAATTAAAAATTTAAAATACATTGCACCGTTTTCTGGATTT
GCCAACATTCTATTGGTACTTACATTTCTTATATGTCTCTACT
>103777352_2 DT664868.3
TTGATACCAGGTTCCATAGCAGACAGAGAACGTAAGAAGTGGCTTAATTACGTTGATATGCCCAACAACCCAT
ATTCTCCTGAAGCAATAAAGAAGAGACTCTCAACTAAAAGCACATCTTCACTTTTTGATTCTATAACGTCCAA
AAAAGAAGCAGATGAAAAAAGTATATCAAATAATAATAACAATAATGTCAAAAGCCTT
AAAAAGCAAAATTCTTTAAATGAATCTAAATTATATTCTAATGTCAACACTGAAAATATAAAAGTAAATGACA
GAAGGTCAATGAGTCCATCTTTTTTAGTTTTAAAAGATGTGCTCTCAGAGGAGATTCCACAGTACAAACGATA
TGATCGAGATTACTATATCAAAGAAGCGAGATCATCTTCTGGAGAGCGAAAAAAAAAC
AGCTTCAGTGGCGCGAGTTCTCTCTCAAGTTCCATAAACAAATCGACCAGCTTAGAGAATTTCGATAGGGAGT
TCGCACCTGGACTATTGATACAACAGTTCAATACGATCGCTACTAAT
>103777354_2 DT665044.3
CTTTTTAATAAATAGTGACTATATCAAAATATTGTGATGTAACAGAACCAATTAGAATTGAAATAGTTTTGTA
CCTTTTGTAATCGATAAAAGAATGTAAACATAAATTCAATAGAATCGAGAGATAAAAAATGAATGTTGTGTAT
AGTATAAAGTGCATATAACGACGCCAAAGGAACTCCGCCGCCCTGAAGTTAGCTGTAG
ATGGCTGCGCAAATTTAATTTTTATGATTATTCTTATTTTTATTCTTTTCTTCCCGAGTGTACAATGCGCGGA
AGTTCAGAAGCCTAATTTCAGGGCGGCCACGATTTGTCGACCGTACTGTATCATTGTATAAAAAACAGACTAA
ACCAACCAAACTTAAATGATTTCGACAGTTTATGGAGTTTTTTTGGTAAATAGAATTA
TGTTATTTGGACTTTATACTGTTGTACCGATTGACTTTAAAATAGTTAGTAGTTAGACATTGATTTTTTTTTA
AACACATCTATAATTTGGTTTGTATGTAATTTCCACCTCGCTTTATTTAAGGATATAAAATATGCATATATAA
AACAAAATGGCCGATTTAAATTCTCATCAATAATTGTAG
>103777364_2 DT663686.3
CTCTTTAAGGCCGGGCTGCAGGAATTCGGCACGAGGAGGTTATATTTTAAAAAAAAAATATAATCAAATATGG
TATTAACAATATCAATATTTTCTGTTTGCAGGACGCCGGAGCCGGGCCCGCTATAATGTAAGCGGCGCGAGAC
GACGTGCGTTTTCCCTCAAGGTGTTAGCGATGTGGTATCCGTGGATCGCGTTACTGGT

GTCGTGTGCACTAGGGGCGGAGACTGAAGGCCCAGTAGCCACAACCACCCGAGCTCCCCCGCGCACCCTAGAC
CGACAGGCCAGCGAGCTCGCGTGGGGGTCCTGGCTGCAGAGCTCAGAGAATGGAAACCAGAACAACCCGCCTT
TTTTTATCACGACCAAGTCATTATTCATAACTCCACTAGTCTGTCCAAAAGGACATCG
CCTCGATGGAACTGCTTGTGTTCAGGTGGTAACAGTAAGCAAAGACGAGCACGAACGCATTTTGTTGGACCAA
CTTAACGCTGTATTTACTGCAGCTCCTAACAATGGCGAAGTATATGATTACGGCGACGAAGATCCGGGTCCCC
TACAACTATCCATTCCAATTGGTATCGATCCATCTGTACAGGATCAGCAACAAAACTT
TATCAAAAAAGGAGATAACTCTGATGCAAGTATAGAGGCTGAGCTGGAACTACTTAAATTACAACAAGCTCAC
GCCAAACTAAACGGCAGTATTCCCCAGAGCTCTAACATTTT
>103777380_2 DT663831.3
TTGCGCAAAGAAAATTAAAAAAGACGAACAAAATGAAAACGGCGCTATAGAATCGAAATCCTAAAAGAAAGAC
TTTGTTCCAACAAAAAAATAAAGAAGAGACAAATAGAAAAACTGTGTGATCCTCGGAAATTCAAAGAAAAAGT
GGTGAGCGCT
>103777384_2 DT663660.3
GCACGAGGGTTTTTCTTTTAAGTTTTTTTATATTTAAATAGTAAATTTATTATGCGAGTAAGCTCTGCACTGC
CATGTTCAGGGCCCCCTCGTAGGAGTTGTTCCCTCCTATGAAGCCGGTGCTGTGACAAAACACGCAGCCTTCA
ATCCCCGACACCTTACTTAGCTGTTCATCACGGAGACCACACCACTCATTTTTTAGAG
GAGTCCGTAATATAAAACTGGTAGGGCTAACAGGGACAGCTTGAACCCTCCATGATTTATCTTCAAACAATAT
GTACTTAATTTCATTTTGTATACCGAGAACTTCCTCTATTTTTTACAAATGCTCCTTCCAAGGCAGTCCTCCA
CATTCAAACTTCAATATTTTACCAGATTCGTGAATAGAAAATCTTTTTTCTACGGCCG
ATTGCACATGGTTCCGGGCTGGCAACCAGACTGATATAAAATAGTCTATAGTATGAAGAAATTCTTCGCTAAC
TACTAACATTGCTTTTTGAAAAAGTTCATCAAGGTGTATGTTCTTGTCAGAGTTCCAAGTGGGATTTAACCTC
TGTACTCTAGAACTGAGGTGTGTGCGGATCTTGTATCTTGGTTCTGCATCAGTCATGG
GCACTCCATTGTCAATAGCATCTACCTCTTCAACTAAGTGTTCATAAACTTTTTTGTAAATTATGTTTAAGTG
TAGAGAAGACAATTGTTTGTCTTTAGCTAATAGTTTAATAATATCTTCC
>103777386_2 DT663903.3
CAATGCCAATAACTTTTATTTTAGTTAAACAAAGAAATGTATATGTAATATCATATATTTTAATGAATAAAAT
TAAAATGATTTCTTATAAACAGATAAGTCTCGCTCATTGCGAGAAACAGCTACCAATATCCCAACGCAGCCAA
AATATGGCAAAACAAATCCAGGAATGTAATGGCCCAATTTTACTCACGACCTTATCAT
ATTAACACATAATATAATGCAATGATAAATGATGAATGTGATGAATTAAAAATATTACATATAAATATGCTAT
ACCAAGTACAGTATGCAAACATATGCATTTAAATTAAATAATAAAAAAATACAGATATATATATATATATAAG
TATAAAAACATAAATTGGACCATCCATCGACAAATTGCTTTTAAAGATAAAATATTGA
CATTAGAAAAAAACGTTTAGAAATCATGGTATTAGAAATCATAGTGGAGCTACTTTCTTATGGCTGATCATCA
AAATTAGTATAAAATATATCACAGCAATTTTTAGCCCAAAAGATGAAAAGTTTATGAGATCGAAAAAAATAAA
ACACAGAAAAGGAGGGGGTAATATACAATTACAATTGCTATAATTATTATATTTTATA
CCAGTCATTTTATGCACTAGGTCCAATTTTATCTAGACAAACACACAATGTGTGTAAATATACAAACTTTTTT
TTAAATATTTTTACCATAGAGTTAATTACTGAACAGTAAATATTATTTATAGTTTTATCATATTTTAGGCAGT
CAAAGATACGTCAATCACAGG
>103777390_2 DT664740.3
TTTAAAGTTGCTTATCGCAGCTAAATTAGGTAGCAAGAAAATCGAGCTATACAAAGCAACGTTTGAAGACTTG
TCTTCAGGGCCGAAGGTGTTACCGCTGCTGGAGGTAGACGATCAGCTCAGTTTCTTCAGTAGTAATGCTGCTG
CACAGTACCTGTTCCCAGTGTTGGATCTATCAGAAAATGGACAGTGTCAACAAATGCA
AGAGTGGGAGGCGACCCGCCTTTTACTTTTTTTGACGTCAGTATTAACAAGTAAAAACGTACCATCTGATCTC
AAGCAGACATTAAACTCTGCACTGCTGTTGATTGACGGTGTTCTATCAAAACATAAATATGTTATTGGGGATA
AAATATCAGCGGCAGATATATCAATATGGAGTACATTGTATCCACTTAACTATAATAA
CGAATTGAAAACCGAGTATTTATCGCAATATAACAATATTGTGAGATGGGCTGAGGAAATTAATGCTGCGAAA
GCGGTACAGGAAGCCATCAAGCAATGGGGCGGTTCAGCCAAGGGTCCGTTCGCGCCCATCTCCGCTTTGGGAA
TACCGCAATTAAGTAATTTAGTTTCTCCAATAGGATCTCCAGAGGAATCTCCCGAAGT
GATTGTAACGGCGGAGGAAATAGAGTTAGCCAAAGAGAATTGGGAAAATGGCGTCAAAAACTTACA
>103777411_2 DT664635.3
GCACGAGGCGACCCTTAAAACAGTGTGGTGCGATGTTATTATATTTTTATTAAATATATATTAAATAAGTATT
AAGATTCTATATTTTTAAAATGCCAAAAGTAAAGGTTGATAAAAAAACCAGAGTCCATAATGAAATTGCTAAA
CAAGGAATCCAATTTAATAAGGACTTTGGTCAACACATTTTAAAAAATCCATTAATTA

TTACGTCAATGCTAGATAAAGCAGGATTGAGGCCTACTGATGTAGCTCTTGAAATTGGTCCTGGAACTGGTAA
TATGACGATAAAAATGCTAGACAGAGTAAAAAAAGTTGTTTTCTGTGAAATTGACACAAGGTTAGTTGCAGAA
CTTCAAAAGCGTGTCCAAGGCACCCCCTATCAAGCAAAACTACAGATATTAGTGGGTG
ATGTTTTAAAAACTGAATTACCCTTCTTTGATATATGTGTGGCAAATATCCCATATCAAATAAGTTCACCTTT
AGTGTTCAAATTACTTCTCCATAGACCATTCTTTAGGTGCGCCGTCTTGATGTTCCAGCAGGAATTTGCACAG
CGATTAGTTGCATTGCCTGGTGATAAGTTGTACTGCAGGTTATCAATAAATACACAGC
TCTTAGCAAGAGTTGATATGCTAATGAAGGTGGGAAAGAATAATTTTCGACCTCCACCCAAAGTAGAATCAAG
TGTAGTGAGAATAGAACCAAGAAATCCACCACCTC
>103777423_2 DT664279.3
GCACGAGGGTGTAAATGTAAACACATAATTTATTGTAACAATATACACATTAAGTAATATTATTAGTGCCTGC
TTTACATTTTTTTGGACAAAAGACATATTTTGAGTAAAACTTCTAAAAATATTAGGAGTAGTGTTCGGAACAC
TCGTTGCCTATGAGGTATCGCTACTTTAACGTATGAGGCTTCCTAAAGTAAATTTTAA
TAATATACGTTAAGATTATGTTGTTATTATTAAATGCATAAACGCTTAGCCATTAAATTTGTTTAGTTATTTG
ATAAGCTTTATTACGATACTTTTAAACAATTGGAAGACTAGTGATTTGGTGCTAAATTGGAACGATGTTCGTT
AGTGAAATTAGGAAAAATGGGATTATCCAAAAAGGATCTAAATTGTTTTTAGAAAAAA
AAAACAAAAATTCGCAACATTCCGTGCACATGAGAGGGAAAAAAAATGTTAAGTTTTATTTGCAAAAACAAAC
TATTGCAAGGTTTTTGAACGTAAGACGTAGATTTGTTGTTTTTTGTGTAGTAATTTAGTGATAGTTTTTATTG
TTAATTTGGGTTTGGGAACTAAGTTAGTTGACTATTTAGATATTAAGGTTTATCTGAA
TTCGTAGCGGTAATAGCATCTACATGTTTAATTTACGATTACAGGGATGTGCTGCTAAGTGTGGCAGTCAGTA
ATAATTTGGCTATCGATGAACAATCGAAAGATCTTGTGAATTTTATTTAGAAAAAAAATTATTCAAAAAAAGT
TATGCCAAAATTATAAGCGCTATAGACTGATTAGACTAAGTACGCATTTTATTGTACA
CTATTAGGCGTATTTCTAGATAAAATGTACAATTATTACT
>103777447_2 DT664567.3
GCACGAGGATGCCTTTGAAAGTTCGCCAGATCAAGCTTTAATTGTGATATTTGTTGTTAAAAATTAAACTTGT
GATAGCGTTATAACACCAACATGTCTGACGTGCCAGATCTAAGTTCGGATGAAGAGCATTTCGAGGAAGATGA
GTGGCAAGAAATGGAAACTAGCAATGAACCTGTAACGTGTTTATTTTGTATAAATATA
TTTTCAAATATTGAGGAAACGATATCTCATTGTGAAACTGTTCATAAGTTTAATTTGAATACATTAAAAACTA
AATTTGGCATGGATTGTTATTCATATATAAAACTTTTCAATTACGTGAAAACCCACAAGTCAAGTTCGGTTGA
AATCATGAATTCGGAAAAAGCAATTTGGGATGACGATAAGTACTTAAAACCAGTTGAC
AACGACGAGTGGTTAATGTACGACTTCGATTCTATAATTGACAAGCCAAATTCTCCTAAAACTTACCACGCTA
ATGTTGAGAATGGTTTGGTCACATTATCTCAAGCTCATTTTACAGAGCTTCAAAGAACTATACAAACGCTGAC
AGAACAGTTAAAAGAAAGTCAAACTCATCTTCAAATGGTAAAAGAAGATGTGGGCAAA
ATGCAAGAATCCATGAAAATAATAGTAGATGGAGGTTCTGTTAATAACGGCACTTCTGAAAGCACAA
>103777449_2 DT664720.3
AAGAGTATCTGGTGTGTTCGACGAGAAACTAACGGAAGCGATAGCTAACTCTAAAATACTCGTTGTCGGTGCC
GGTGGTATAGGTTGTGAAATATTGAAGAACCTAGTTCTAACCGGCTTTCCTCAAATTGAAATAATCGACCTTG
ATACAATCGACGTAAGCAATTTGAATCGTCAGTTTTTGTTTCACAAGGAGCATGTAGG
GAAATCGAAAGCGCAGGTGGCAAAAGATTTTTTTCTAAGTTTTAATCCAAACGTTAATATTATTGCTCATCAT
GACAGCGTTATAAGCAATGATTATGGAGTGAGTTACTTCAAGCAATTTAATATTGTAATGAATGCATTGGATA
ATAGAGTTGCTCGCAACCATGTTAACAGAATGTGCTTGGCTGCTAATGTGCCACTTAT
AGAGACAGGAACTGCTGGTTACGCTGGTCAGGTAGAACTAATAAAAAAAGGGCTCACTCAATGCTATGAATGT
CAACCTAAGGCACCGCAGAAGACATTCCCCGGCTGCACAATAAGGAATACACCTTTCTGACCAATCCACTGTA
TTGTATGGGCCAAACACCTTTTCAACCAGCTCTTTGGTG
>103777463_2 DT664644.3
GCACGAGGAAGACCTGATATATTAAACTTTTCTCCGTCTTGATTAAGTGAGAAACGTAGTTAATTTAATAGTG
AATATTTTTAGTTTTAATATAAGTAGCTTTCCAGAAACCACTACCACCATGCCTAAAGGAAAGAAAAAGGGAA
AATACGAGCACAGAAGAACTGAAGTTCCTTACTCATCAGATGAAGATGCTGGCATAGA
TATGACTAATGACAACTATTCAGAAACTTCCGGACAATCAGATCTTAGAAGTAACCATGATGATGCAGAAAAC
GAAATCCAAGAAAAAAATTGAAGAGAAAGTTCTCGAAACTATTGATGCTTTAAGTGCAAGAGCTAATGCAGCAA
GAGCAGCGGCTTTAGTGTCTCTTAGGAATTTATTACAAAGACGCTATTTAGCACATCT
TTTGAGCAGCCAACGAGTGACCCTCGCTGATCACATTACTAGGGCTTTGAGAAGAGGCAAAGATGGCGAGAGA
AAAGCTGCAGCTGCTGTTGCACCTATTTTAGCTTTACAGATTGGTGAAGAAGGCACAGAGGAATTTATGGCTG
AAGTCCGACCTGCCCTTGCTGCAGCCGCCGTAGACAGAACAGCTTCACTGGAAACACG

CACTGAGTGTTGTTCATCACTAGCAGTACTTTGCTACCTTCTAGAGGAAGATCTCACAGAAATACTAGAGATC
ATGAAGATGTATGAAACAATATTCAGCGGCAGTTATCTTAAGGGTGACGGTACAGTCAAAGTGTCAGGTTCGG
CAGTAGAAGAAGCACATGGCATGCAGCTGCCTTAGACGCTTGGGCACTACTAC
>103777467_2 DV501116.2
ATATAAATCTAAAACAGAGCCAATTTTAGAATTAACCGTCTGCATACGAACTAACATAACTACGTATGTATAA
GTTGCAAAATCAATATTCTTGACACGACAAATGACGCGAAACAAAGATATAAAACAGCTTCACGCTTATTTTC
CAAACTTTCTATTTATCTGTACATTTTATCTCCTTGTAGTAAAAATGAATAAAATAGA
GATATTGTCGTTTTATATTATGATCACAGTAGTTGTTTTTTTTTGGCGCCACGCATTGCCTCGTTACCAGAGC
GAAGGAATCAATATAATTAATATTTCATTA
>103777469_2 DT664851.3
GCACGAGGCGACCCCCTGAAACCACTGGCAACTACGAAAAAAAACAAAAAAGAACAAAGAAAATAGTGAATTT
TAGTGCTGTATTTATCAATTATTTAACAATCGAGTTGAGTTTTTAATAATAAATCGAGATGAATCAACGTATT
TTCAAAATTCTCTTCGTTATCTTCTTCACGATCGTTTGCCATAATGGGGAGGCTGCGG
AGAACGATCCGGCTATACCGGAATACTTGCTGCCGTGCAGTCGAAGCGACCCAGAGCTGGACAACTGCATAAA
GAGATCCTTCAACCACCTACGGCCGTACCTGGCGAGGGGTCTTCCTGATCTGGACGTTCCCCCTGTGGAACCT
TTGTTCATAGATAAGCTGGTTATGGCAAACGACGCTGGGCCTGTGCGAGTGAGAGCTA
CCTTCAGTAATATCACCGTAGTGGGACCTAGTAACTATACTGTGACTAAAATAAGATCAGATTTGAAGAAACT
ACGAATTGATATGGGTCTGGTGCTTCCTCGTATCGAAGTGACCGGTCACTACGAGGTCTCCGGACAAGTGCTG
CTGTTCCCCGTCCGCTCGCAGGGAGACTTTTGGGCTTCATTTAACGACGTCGTGGCTA
TCGTAAAGATATTCGGCAAGGAAATCGAACGCGAAGCCGTCAAATACATGTCAGCTGATCGCATGCAGGTCGA
CTTTAAGCTGAAAGGTTCCCGCTTCAAGGTCAGAGACACGGTGAATCATGGAAGTGTAATCGGAGAAGCAATG
AACCAGTTTCTAAACA
>103777481_2 DT663645.3
GCACGAGGAAAAACCTTAAAAGGGTCGTTGTTGAGGATGACGATCAAGGGCGTGAACAAGAACATACATTCGT
ATTTCGCTTACACAATGAGAAAGCCTGTAAACACCTTTGGAAATGTGCTGTGGAACATCACACATTCTTCCGC
TTAAGAGCTCCGGTCAAAGGCCCGTCTGCAAGACAAAACTTCTTTAGAATGGGTTCAA
GATTCCAATACTCAGGTAAAACAGAGTATCAAACAACTCAACAAAACCGCGCGCGTCGGACAGTTCAGTTCGA
AAGACGTCCAAGCCAACGTTTCGCTCGAAGGCAAAGTCACGTCTTGAGGGAAAGGGAAAAACAAACCACAGTT
GTAAAACCAGAATCCCCACAACCAGAACCAAGTACATCTGCTGAGGCAATAGCATCAG
CCATTGAAAGTGGTTTCGACACGTTATCCA
>103777497_2 DT664886.3
GCACGAGGCTCAAACCTTTAAAGGGCCTAGAATATATTATTTATTTATTTGAACAGAGCACTCGTATAGGGAC
CAAATATATACGAAATTATAATATTATGTTTTATTTATTTATTTATTTATCTAACGTCCATTGCGCTTTACGG
GCGATGCATACTAAAAAGACAAACCATCTTTCCATATAAAACGCTCGTATCGTGTTTA
TAGTTCTAGCGTTCGTGTAGTGATAGTGTACAATACAGAAGGGCCTTTAATTTGATAGTATTTAAAAAGTTGC
TTGTTATCTTCGTTTATATATATATTTTTGTACGTTGACGTCCTTTTTTTTGGATGGAGGTGGGCGATTATGAAT
GATTTTACATTTTATTATTGTGAATATTTTAATCTTACAATTTACACTTTTTTAAAAG
AAAAAATTGTTTTAAATCGAACTTTTATAAAAAAAAAATCTTCGACGAATTTGTTTTTTTTTTATAACACTGA
CGACGACCACAACGGTGAAGTTTTTTTTTTAGATACATTTTAAACATGTCGAATTTGATTTGGTCATTCGTGA
TCGTCCCCCGTCTAGGTAC
>103777499_2 DT664011.3
CGAAATATCGTTCCCATCTGCTTTCGACCCAGTAATTCACAACGGTAATAACGAACGTTCGACCGTGAAGCGA
TTACTGGAGAAACTTATTCTCGACGACGAACAATTCGATGTGCGAAACATCCTACCGAACACGGTGCCGGACC
CCGCCAGCTTGAAACTCGAATCCGACTACGCTTGTCCTATGGGACAGGTTGTTGTGGC
ACCTGATTGTGTATCGTGTGCGGTGGGCACGTACCTGGACGCGGCCAGCGACTCGTGCAAGCCGTGCCCGGCC
GGCACGTACCAGTCCGAGGCGGGGCAGCTGCAGTGCACGCCGTGCCCCGCCATCGCGGGCCAGCCCGGCGTCA
CGCAGGCCGCCGGCGCCAGGAGTGCGGCCGATTGTAAAGAGCGCTGCGCCGCCGGCAA
GTACTACGACGCGGAGGCGGAGCTGTGCCGGCCGTGCGGGCACGGCTCGTACCAGCCGCGCGAGGGCGCCTTC
GCGTGCCGCGCCTGTCCGCGCGCGCACACCACGCGCGCCACCGAGGCCGTGTCCGCCGCCGAGTGCCGCGAGG
ACTGCCCCTCGGGCGAGCAACTGAGCAGCGAGGGCGGCTGCGAGGCCTGCCCGCGCGG
CACGTGGCGCGA
>103777501_2 DT664735.3

GCACGAGGGTTCCCCTTATATTTATTAATACACTCGCTATGTTTCTGTTAAGTGATAACCTACGGTCAATTTA
CTGACTATCAGTCATACGTGTATCGTCTCAGTTTACGGTTCTAATAGTGTCGATATATTTAAAGAAATGTCTA
ATAAAATGCGTTATGTGTTCGGTCTATTGATTCTGCTGCTCTTGTCAACAGTTATTGC
GAACGATGATTGCAATTCAGATGCACAATGTGTGAACTTGAAGGATTGTAAAGAGCTATATAGACAGAAAGTA
GAAGGGAAAAGAATAGATGTTCTACTTAAAGGGCTTTTATTTGGTTTCGACGTGGACGATCCTAATATTTGCT
GTCCGCCATACTTCCAAAATGTGCGGCAAGAGTTTGATCCTGTTAGTTTACTGCCAGA
CACAAATGTATGCGGTGTACGCAACAGCGACAGAATTGTGGGTGGTACACAGGCAGATATCGACGAACATCCT
TGGATAATTTTAATCCGGTATGAC
>103777505_2 DT664142.3
GCACGAGGCTGGCCCTAACAGCAAGGCGAGATCGCCGGCCCTCCAGACACTCCGTACGAGGGTGGCACCTTCC
TTTTGGAGATAAAAGTCCCCGAGACCTACCCGTTCAATCCACCTAAGGTACGCTTCATGACCAAGATCTGGCA
TCCGAATGTGTCTTCCGTGACGGGAGCTATTTGTTTGGACATTTTAAAAGATCAGTGG
GCGGCTGCCCTCACTTTGCGCACTGTTCTCCTCTCCATACAGGCTTTGCTTTCTGCTGCCGAACCAAACGACC
CTCAGGACGCGGTCGTTGCTAAACAATATAGGGAAAACCCCGCCTTATTCGCCATGACTGCGCGACACTGGAC
AAATGTGTACGCCGGGGGGCCCTCCCCCGTATGGGAGTTTAATGAAAAAGTCCAGCGT
TTACGAGACATGGGCATCGATGAGCACGAGTCCAGGGTAGCTTTGTCCACGTACGATTGGGAAATCGAAATGG
CCACTGAGCAGTTGTTTAGTTAAACTTTGTCCATTCTTCTGTAATGTGTAGTGTCCTAAAGATTTCTCAACGT
CAACAGCATTTCAGCTTTTTGTTTTTCTTTCCCCAATTTCTGTGATTTTATCATTGTG
ATTATCACGTCATATGATAAATATTAGGTGTAAGAGCTGAAGCGGGAATATATGATAGCTTTGAGGGAATATT
TTTTAAACGGTGGAAAGTATTTTTGAATGGTTACGACGGGTATAGTTGATGGTCGATTGTCGATTGTTTACCT
CTGATTTTTGCTACTTATCTAACAAAATATGTATTTTAACTCCGCTTGAGATAAATTT
TAGCTTGCTAAAACTAAAATAACTAAATTTGTGGAAGAAAAGCC
>103777521_2 DT664900.3
GCACGAGGCTGCGTCGTTTTTATCGAATCCACTAAAATGTCGTGCTGTAAACTTGGGCCCGGGTACGCGTCCC
CACTTGACGCATTCAGGAACGGCCCCAGGGAGGGGTTACTTTACGTGGTGTGCGTTCAACCAGACCAGAGCAA
GCAAGACTACCTATCTACGGTCGATGTTGATCCAAACTCACCTACGTACAGCCAGGTT
ATTCATAGAACCTACACGGGCAGCGTGGGGGACGAGCTCCACCACAGCGGGTGGAATGTGTGTTCAAGTTGCC
ATGACAATCCTGAGCTCAAAAGAAACCTACTCATATTGCCCAGTTTATACTCCTGCAATGTTTTCGTTGTTGA
TGTCGGCACAAATCCTCGAAAACCAGAATTGTACAAGGTAATCGATGGTTCTGAGATG
AGATCGTTCAACTGCACTTTCCCCCACACGACCCACTGCCTCGCCAGTGGTGAAATCATGATTTCAACTATGG
GTGACGAAAATGAAAACGGAAAAGGGGATTTTGTGCTCATCGATGGGGAAACTTTGAAAGTGACAGGTACATG
GACTAAGGGAAAGCCGGCGAATTTCGGTTATGACTTTTGGTACCAACCCTACCATGAT
GTGATGATTGCGTCCGAATGGGGTGCACCAAAGTTCTATAAGTCAGGCTTCCATGCAGCTGATATTCCAAATG
CTGAACGATATGGCACTTCTCTCAACGTCTACAAATGGTCCACACGTGAATTACAACAAGTC
>103777527_2 DT664390.3
GCACGAGGAAATGCTTTACTTTGTGAACTAAGTGGCAGAATTCACAGTGAGACATATACATATGTATGTACAT
ACAGGTATTGTAAAAATATTGCTTGACCGAATAGGAAACCCATTTAAATTACGGCAGGAAGACATGGGCGTAT
ATTTTCTTGGACTGGGCTTTGTACAAATATGTTCGTGTAAATGTAATTTTTTAAATGT
TCCGGGTCAAATTTATTCTTGGTAAAATTTCTGAGTACCAGACTATTCTGGGGTGGGCGCGGACCACCCGGCC
CCCGTCTACCTACGCCCATACACGACAACGACGATGACTATTTAAAAAAAATAGACAGATTTAAATAGACCCT
AGGGTATAGCCCCAGTTACCATTACTGGTGGCTATTAAAATTAAATATGACCATTTAA
TATATTTCAACTAATAAGCACACCAAATGTAAGTTAGTATTTATTGTATGCACCACATACATACATTTTACAT
CCGGGAAATCTAAACTTAATTTGCACGAAGAGTAATTTAAAAATAACTATAGAATACACAAATGATTGTTCAA
CACGCAACTAAATACTTATTTATTCGTGCAATATACATTAACTTAGGTATATAAAATT
ATAATATATCGTTTGAATTATCAGTGTATTAATATTTTACACTTTGTAAATATACATAAGTATTTGTAAATTT
GTTTGTATTTGTAGAATGTTTTATGGTTTTTGGAGAAATGTCGCAATACAATCGTTTTTAACTATAGGTTATA
ATTAATTATAAGTATCTATATATATAATACGTATAATTATGAT
>103777535_2 DT663776.3
GCACGAGGATTTTTTCTTTAAGGGCATAAAATATTTTGGAGATGTATTTTATGAGCAAATATCAAAATAATTA
CATAGTGTGACTATTATATCATATCATATGGGATGAAAAATATGTAATAATGGCAATTATTCGAAGAATTTTC
TTAAGATATGCGAAGTGTTTAGCTCTTATAGTTGTGACAACATTTTTTGCTCAAATTT
TAATATCTGTTAGTCTTTTTCCTTCTGTTCATGATAATTTACTTAAGAGAAATGGATATACATCAGCAGCTAG
AAATAATATAGGAGATATTTCTGCGAGAAAATTAGGAAATATTGTTGGGGACGAAGAAGATGCGTTAGCCGGA
AAAAACCAAAATAAGATTATTCCTCACTTAAGATTAGAAGAACTAGATTTTATACCTG

CTTGTGACATCAAAAGTAGAGAAGCTATTTCAGCTATACATCGTGCCAAGACCCAAATTTGTAAAGAGCAAAT
TGTAAACAAGACTTGTCATATACAAAATGGTGATTTTTACCCAAAACGCTTACCTAATTACTGCACGTCGGAA
ACAAAAACTTACGGTCGCCACCTCGGGTGCTATTTAGACGAAAAAAAATTAAGATTAC
TATCTAGTTATTATAGTAACTTTGCTATGTACAATTCTCCTACATTTTGTCTGGATATTTGTGTACAAGCAGG
ATTTCCTTATGGCGGGAGTTCAGTA
>103777543_2 DT663507.3
CGAGAATAAAATAAGGAGAAAACGAGATAACGATATCGTTAAAGCCGAAGCTTACACTGATAAAGATGGACAA
AGAAAGCACGTTGTAAATATGAATTGCCAAAAGAAAACTGCTAAGTGTATACAGTTCAACTGCGTAATCTACG
TACTGGGAAGGATGCAAGCCTCCACCATCACGGTGAAAGCGCGCCTGTGGAATTCTAC
ACTTGTGGAAGACTACCCTCGTGTTAGCCACGTTAATATCGCTTCCACTGCCTATTTTTATATTCCGGAACAC
TACAACATCCATCAAAGCAAACATCATGATGATACTGCTACAGTGGAAACGGTCGCCTATCCGGACTTGAAGT
TTAGTGAGCCTTCAGAAGTGCCATTGTGGGTGATCATAGTGTCAGTGATACTTGGACT
CTTAGTGCTAGTAATTTTAATATTTGTGTTGTGGAAACTTGGCTTCTTCAAAAGGAGTCGACCAGATCCGACC
TTGTCAGGAAACCTGGAGAAGAATCATGAATCTAGCCCGTTCATAGTGCGCGACAGAAATAGTATACGATGAG
AACTATAGAAATCGAATTTTGTACATAAAATCAACTGTTTCGGTACTTTAGGACGAAT
TAGTTT
>103777551_2 DT664053.3
GCACGAGGGTGACCCTTCATTTCGTTAATATGAAACTCGTTCAGTGCGTTGTATTTATAGTTATAACGTTATC
GTGTCTCGTCTTTGGACAAGAAACATGCATTACACCAACAGGTAAAGATGGTGTCTGTATATTGATAAAGGAC
TGTAAGGCTCTCAGAGATAAAATAAAGAAAACAGCAGAAGAAAGGGAGACAATAAGGC
AATGGGAATGTGGTTTCGAAGGGGAGGTGTCTAAGGTATGCTGTCAATCTGCCACACCTCTCATAAGCATTCT
TTCGACCACCACAAGTACCACCACAATTACCACTACTACTGCTACAACCACCATAAAACCTCCAAAACCTGCG
GAGGAGACACGTTGTGTTACCAGATTTGGAACAATTGGTACTTGCATTCGAATTCAAG
AATGCCCAAAGCTTCTCATGATGATGGCAAACACCACTAGGCAAAATTTAGATTTTATACAGCGATCAAGGTG
TCCTGGTCGGGGTCTTAGTGTGTGCTGCGATGAAGTTATTCCACTGAACAGAATTCAGAATTCCCAAAACTGT
CCTAATTCGGTAACGCCTCCTGCACCTGGCAAAAACTGCTGTGGCGTAGAGAGTCCTT
CAGGGAACAAAATTATAGGTGGTACACAAACATCTATAGACCAATATCCGTGGCCAGTTCTAATAGATTACAA
TAGGCCCTACAGTTGCCGAGGAACACTTATAAGCAGTCTGTACGTCCTGACCGCCGCACACTTGTCTAGT
>103777553_2 DT665013.3
CAGGATGACGATGCGTATTTCACGCTTGGGGTGTTTCATCCAACCCAACAACAGCTCCTAACAGCGACTTCAT
CAGGAGATATCAGGATATTTAATTTATTCACCGGCGTATAATAGAGTTCTTATCAAGTCCACGAGTCATATAT
ATATCATATGCAGGCGAGCAGAGACGGGTTGCTATTGCTAGCATCATCGACCACTTCA
TGGCGGACGACGTCAGCTCTATGGAATATGAAAGAATTTGAACAATTGT
>103777557_2 DT663618.3
GCACGAGGCAAAGACCTTAAACGTAAAATTTGAATTTCAGCTTTGTAAGCCATATTTACCATTTAATATTTAT
TCATTTGAGGATATTAATAATAAATTTTTTGATGACGAAATTATTAAAAAATGAATTTAGGTGACCCTGAATT
GGAGTTGGTAGATCCAACACCTAATGTTCACTCTTTGTTTTTACACTTCGATAAATTA
TTTTTTTGGGCCAGACTATCAAGCAGAGCTGTAGTACGATGGAGCAAAAGGATGTATTCATGCGCTGGGATAT
GTTCATATGACAGAGGTGGCTTATGTGACATAGCATTAAGTGAACCACTTCTCAAATTAAGATCACGTAAAGA
TCTAGTGGAAACTCTTTTACATGAGATGATCCATGCATTCCTCTTTATAACAAACCGG
GACAGAGATAGGGATGGACATGGACCAAACTTCAAAGCCCATATGCATAGAATAAATCAAGCTGCTGGCTTAA
ATATCAGTATTTATCATGATTTTCATGATGAGGTCAAGCTATACCAAACACATTGGTGGAGGTGTAATGGGCC
GTGTCAAAATAGAAGACCACATTTTGGCATTGTGCGTAGATCTATGAATAGAGCACCG
GGCCCATCTGATTGTTGGTGGGATGACCATAAAAGGAAATGTGGAGGCACTTTTCAAAAAATCAAAGAACCAG
AAAAACAACAAAACAAAAAACAAGACAAAAAACGCGTTTCTCC
>103777571_2 DV501158.2
GCACGAGGAGGTACACTGAGGTGGCATTCGTCTGTCAGTGACATAATTATTATATCAAACTACCGCCAACGAT
TTCTTAATTGCCTCGCGTACTGCCCTGGATTCTTGCAAAATGAGCTGATAGTATCTTGCCCTCTGGTTAACCC
ACCATGGGTTTGAGACTTCAATGATTTAATGAGTGGGTCCCAGGTGGTAGAAAGTTTC
CAGTCATCCTCTCTATTGAAATTAGGATGTTTTTGTATTTCAATAGCCTCGCGGATCATTCTAGGAATGTATC
TGTTTTCCTTACCGAGGATTTGGGGTTTATCACACCTAACGAAATGACTGGGTTTATCCACAGCATGCTCACA
CACAGCTGACTTCGATACACGCCCATTCTTGATATCGGAGATGTGTTCCTTCACCCTA

ACACCGATGTTTCTCTTTGTTTGACCGATGTATAATAAGCCACAGTCACAGTCTAGTTTGTATACGCCTGCTC
TCTGAGTATTACTTTTTACTGGTCTCAAGAATTGGCTCACTTTCTTGTTAGGCTTGAATATGGTTTTTATGGA
AGCTCTTCTTAAGATAGTTGCCAATCCTGTCTGTAACCCCCTTCACATATGGTAGAAG
GGCCGTTGCCGGCTGTCTCTCTACTGTGAACTGGAATTAAATGAATTTTGGCATACAAATAGTTCAACGCCTA
ATGCACCTTGTAGGATGCTTTTATGTTTTGGAGTAT
>103777583_2 DT664251.3
GCACGAGGGTCGTGCTGTACACTTCGCTTCTTGTCGATAGATTTATTGGAATGTTTATTTTTGAATCCCGAAT
GGTTTATCTGTCGTTAGTACTAATATCTAATTTTACTGGAGGTAGTAGTATGCTTTAGACGTAGCGTTTAAGT
AAAAGAGTACTCCGCGGAGTTCTTCTTACATATCACTGAGATTTCTCCTCGTAATTAT
GTTTTAAATATTTTTTCTACTGTATCCAATTTACAGTGCTATCTGATATACATTCATTTTACTGAACAAATAT
GCGTACGGGTCCAAACCCTTAGAAATCGTAATTTTAGATCTAACCTTTGCATTAGGATTGTAAAATATTGTTA
ACATCTATAAATTCGCTCAACGTTTATACCAAAGCACAAATACCCATCGATTCATAAT
TTAGCGGAGCTGTCGCGCGTCATTCATCTTCTCATTAGATTTAATAGTCTTTCAGTTAGAAAAACTTAGAAAA
TCTACTAGTTATACGTATTAACACCACATAAGATTTTAGATTATTTAGCTTTTCTACATCGTTTTCTTTAACC
TCCTACGTAATCGAGTACATCGCAGATCTGACGCACTCAGTTAAGTCGCGAAGGGAGT
CCACGTGTGCCACGTGTCCGCGCGTGTACCGTGTCCGTTGTCAATTGATAAGATGCATGGTGCCGTCTGCATT
AGCTTTAATTACCCTCACAATAACGTTAGAGACGAAGGAAGCGTCTCCGAGACACCTCTGATGTATATTTCGA
CTCTATAGAAGTTTCTAGTCTTTTCGCGCACAATGGTGTAGGTTCAAAGGTGCTCGCA
TTGCCCCCCACTGGTTATTATTATATTATGT
>103777585_2 DT664209.3
GCACGAGGATTTATCTTTACTTAGTACCTACGTAAATCTATTAATATAATGCAATAGCATTAATAAATCTTTA
AACATTGAATTTCTTTTTAAGATGTAAATAGATTATAATCAAAAATTCCAAAAGTATTATAGTTCTTAAGATT
TTAATTAAAACTTCATCCCGCTAGATACTTAATCCAGCAAAATGATGCAATTCATGCT
ATTATTCAGTCGTCAAGGCAAGTTAAGGCTTCAGAAATGGTATGTAGCACACCCAGACAAGCTGAAGAAGAAA
ATAACCAGAGAACTGATTACCACTGTGCTTGCTCGAAAGCCAAAAATGTGCTCATTTTTGGAATGGAAAGATG
TTAAAGTGGTTTATAAAAGATATGCATCATTATATTTTTGCTGTGCAATGGAGCAGGA
AGATAATGAGTTACTAACACTAGAACTCATACATAGATATGTGGAGCTTCTAGATAAATACTTTGGCAGTGTA
TGCGAATTGGACATAATCTTCAACTTTGAGAAGGCTTACTTCATTTTGGACGAGCTGGTGCTCGGAGGAGAGC
TGCAGGAGACCAGCAAGAAGAATGTCCTCAAGGCGATAGCGGCACAGGATCTGCTACA
AGAGGAGGAGACCCCGCAGGGCTTCTTCGAGGACCACGGACTTGGGTAAAATGATCTACTGCAAGAGGAGTTT
TAGAAGAGGTAAAGAATACATATTCACAATTGTAATCATTAAATAATTAATTAATTATATAGATTTACTAATA
ACTACTGATATAATTATTTACATATG
>103777599_2 DV501258.2
ATAGTATATTTTTCTTCCAAAGAAAATATAGTGACATTTGGAGGAGGGGGAGAATTACTACCATTATTATGTG
TAATAGTAGAATGTTCTACTGAATATACAAAAGAAGTAGTAAAATATGAGGGTTGTATTCCAAGAGATTGTAA
GAGATTTGTCACTGATCAAGTAGTTTCAGAAAGAGAAGCAGAAGAGTTGTTGAAACTA
GTACAAAAAGGCTTAAAGCATGGAGGTTCTTATGGGGGAGCTTCCATATTAG
>103777603_2 DT664095.3
GCACGAGGCCTCGCGTTCTTATACGGAGAGCTTTCGCAATATTTTAAATAATATGGCTTCCTGGGATATCTAG
ACCCAATTTGTTTCGTAGAATGAACGGTGACCAAAGTCCTGGTAGTCCTTTAGACATAAATGATCGTGAACAA
GCTGTACTAATGGATCCTATTGATCATGCTCGAGCCAGATATCCTTATTGCATTGTGT
GGACACCTATTCCTGTTCTCACGTGGATATTCCCTTTCTTAGGGCACATGGGTATTTGTATGTCAAATGGTGT
GATTAGAGATTTTGCTGGCCCATATTTTGTATCTGAAGATTTAATGGCATTTGGAAATCCTACAAAATACTGG
CAATTATCTCCTCAAAAATCTAATAATGGACAAGCTGGTTGGGACGCAGCTGTTGCAG
AGTCCTCAGAGATTTATAAGAAAAGAATGCACATAATATTTTATGATAACTGTCACTCTCATGTGGCCACAGC
ACTTAACATCATGAGCTATGATGGATGTAAAAATTGGAATATGGTTAAGCTTGCTCTCTACATGATACCATAT
TCAAGATATGTCAGCTTTGGAGCTTTTATGAAGACATGGCTGCCTTTTTTCATTATAG
TTGCATTTATATGTGGCTTAGCCGCTACTATATAAGCTGCATTTAGTATGCAGCTGACTCTACAATCCTCTTA
AAAGTTTACAAATTTTTGTAACAATCTAAAGACTTTATGCTAGGGTTATTTTTTTTACTCTTATTAATATCAG
TGTTTTGTATGAATCAATTATCACAGTGATAGTTTTTCCATCTCAAATTTA
>103777607_2 DV501124.2
AATCGCTATAACCAAAATGACGGTAAATATGCTTGAGCGCTTGGAAGAAAATTTGGAAATTTCTGGAATAAAA
CCAAAACCAGGCTCTGCGGCGCAAGGCCTAGCGATATATGGATACCCATCAGATGATGCGATGAAATATGAGA
AGTCATCGATGACGATTACTAAAGCCTCGCAAAATCTAGTCTGGTCACGATGCACAAA

ATACGGGCTGGCGACGGAC
>103777616_2 DV501162.2
GCACGAGGATTTTCCTTTTTGATTTTTAACGTTTATTTAAACAACATTTAGTGTAATAAATATTTAACAAAAT
GATTGCTAATCTAAAATGCTTTTTTTTTATTTAGCTGTGAAATGTGACTAGTATTCTCTGGAGTCGTCTTCGT
TTTGTTCCCTTTGAGCGTATTGTTGAGGTGCGTGCGGTTGACCTCCTGGCTTCTTGTG
CACTGTCGCCTGAAAACCATTTTTGGGATCAGCCGTGTATTTTACTAGCCGAATTGAACCGTCAGGCTCGACA
AGAGAGTACTCGCCGTGTACGACGTCACCATCGCGGTACTCTTTGTGATCTTGTTGGTTGCCGCTGTGGGGAT
CTTTAACGCCGTACGCATAGTGGTAATCTGGTTTAGCCATGTAATCAACTGTTTTCAA
GCAATCATATTTAACTGTCTCCGGATCAATTTTTCCCGCTTGGTGGTCGTAGCCGTAGTCTGCGTGCTGTTGC
GCCGGGACGCCTCGTGCCGCAAGAACCTGGACTTCAATTATTTTTCCACTCACTGGACCACTGAAACGGTTGT
AGGAGTACCCGCCTGGAGCCTGGTTGCCCATCGCTACTGATCCTACCACTAATAAAGT
AAATGTCTTGAACACCAAAACCATTTTTATAAGTAACTATTAAAACCTGCACACCGTTAAATATTTATTTGCA
CCACCCGACAAATCGGGCATGATCTCAACGCGCTAACGAACAT
>103777660_2 DT663864.3
GTTTTCACATCTGTTGTCAACACGAAATAACGTGGATTATGCAAGGTGGAATGAGATGTGTACAAGTGTTAGT
CAATAAGACTATGAGGGTTGTAAAATAATGCAGTGGCTGCACAAAATATTGACGCTGGCAATAATTTGCGCAG
CTGTGGAGACGAAGGAGTGCCCTTTTACTTGCACATGCACACCAACGAGGCTGGCGTG
CTCCGGCGCCGCCATACCCGGGAACACATTCACCAGAGAAGCCATATACCACTATGGGCCCGAGCTCCAAGAG
ATCATCTGGACTTTCTCTAACATACCCGTCATACAAATCGACGTCTTCGACGGTCTACATAACTTAGAATACA
TCGATCTGAGCAGAAACGAAATCCAACGAACCCCACACGGACTATTCGCGAGACTGAG
TCGTTTAAAACATTTAACTTTATCCAGAAACCAAATAGACTACATACGTACGTTCACTTTTGTAGATCTAGAA
AATTTACAATTACTAGATGTGTCCAATAATAGACTACAATCGGTACCATTCCAGGTCTTTGGGCCAATGATAA
GATTGCAATATTTGGATATT
>103777666_2 DT664398.2
GCACGAGGCCCAACATGAGTAAGGAGGAAGCGAAACAATTTGTTACTAACACTTTAACTTTGGCCATGCTGAG
AGATGGATCTTCTGGTGGAGTCGTCAGACTTGGTGTTATCACTGAAGCTGGTGTGGAACGGTCAGTCATCTTG
GGTGATCAGCTACCCAAGTTTTATGAAGGTTGATGATTTTTAAAATATTAGTATGGTA
ATAATAAATAATTTTGCACTAAAAAAAAAAAAAAAAAA
>103777675_2 DT663936.3
CGGAGTTACGAGAATATGCCCGTCTCCAGTGCGGTGAAGATCCTAACACACGGCTTCAGGCGATATATGAGTT
GAGAGACATGATATACGAGAGAGGCGAATGTAGTCCTCACAGAATGGATGATGACTTCTTGCTACGATTTTTG
AGGGCCAGGAACTTTATACCTCAGAGAGCTCATAGGCTGATGGTGAATTACTATCAAT
TTATTTATTAAAATCCGGAACTATTTGAAAATGTTTACCCTTTGGATTTAAGAAGAATTGGAGATGCAAATGT
GATTGCCGTACCTCCGTACCGTGATCAGAACGGGCGCAGGCTACTTCTATATAGAATAGGTTGCTGGGATCCG
AAATCAGTTGCTGTAGAAGATATCTTTAAGGCGACGATTTTTGCATTAGAATTGGGTA
TGCTGGAGCAACGCACTCAAATCTTGGGTGGTGTAGCATTGTTCGACTTGGAGGACTTGAGCACTAACCACGC
GTGGCAAGTGACGCCATCTGTTGCGAGCAAAGTTGTTAAACTACTTGGTGTCTAGTTTCCCCTGCAACAACCC
C
>103777681_2 DT664258.3
GCACGAGGCCTGTGCTAATAAAACTACATTTTTAACTAAAAATTTAAAATAAAAAATTACTTAATTTTAGTTA
TTTTGCAAGTATTATAAACAGTGTGGACTACTTCTAGATATATTCTATAGTAATCATGGCGGACCCTACGTAC
GGTAGAGGTGTTCAGGTGCTGACGCCCAATGAGGACCACACGTTTTCTTTGAACGAGG
AAGAAATGGAAGAACTGTTCCTCAGTGAAGCTGTCAAGGACAAGCCAGCGGTGGTCATCACCATCGCGGGGGC
TTTTAGGAAAGGCAAATCGTTTATACTGAACTTCTTTTTGCGTTTCATGCATCAACGGTATAATTTAAGAAAT
CCTAACTCCGACGAGTGGCTCGGGAAGAAAGACGAGCCTTTGAAAGGGTTCAGCTGGC
GCGGCGGCTCCGAGCGCAACACCACTGGACTGCTGTTTTGGTCGCAGCCTTTTATTGCGACTCTGGACAGCGG
GGAGGAGGTAGTCATATATCTGATGGACACGCAAGGAACATTCGACAGCAACTCCACTGTAAAGGACAATGCG
ACGGTGTTCGCGATGTCCACGATGCTCTCTTCGGTGCAAATTTACAACTTGTCATCCA
ACATTGAAGAAGATGATCTACAACACCTCCAGTTATTTACAGATTACGGAAGGCTGGCATTAGAGGGCAGCGC
GGGCAAACCGTTCCAGAGACTGCAGTTCTTGGTGCGTGATTGGAACTACCCTTACGATCACCCTTACGGCGCG
GTGGGGGGGAAACAATTACTCGACAAACGACTTGGTACAAAGGAAGATCAGCATCC
>103777695_2 DV501256.2

GCCGAGCCGCTTTTTTTTTTGCCTATACACTTCGAACTGGTAGTCCCCGACGGCGTCGAACAGCGTGTCGTCG
TCGGCCAGGTCGTTGTAGAGCGCCGCGTCACGCAGCGACACGCGCGACAGCGAGTAGTCGATGATGGTGGCCG
CCACGCCGCGCCGCCGCACCGCGTACGTGCGCCCGCGCAGCACGAA
>103777703_2 DT664450.2
GCACGAGGGAAGCATGCAAGCGGGGCGCTATCTGCAGTAGCGGCGCTATTACCCGAGTGTTGGCACCACAACC
CTCCTGTGCGGCTCACTGCTCTCCGCATAAAGAAAACACTAGCTAAATGTCGCGCGGAAAGCACCATCAAGCT
AGTTTGAACATTTTCTTTGACTTTGGACCGACCGACGCACACAGTTCCTCCTATTTAT
TACCCCCCCAAAAGTTCCACCGCTCCCCCAATCTCAGT
>103777717_2 DT663555.3
GTTCTTTCAATCTATCGAAGTGTCAAACGGTTGGGGATACCTGATAGCCAGATTATACTTATGATATCTGATG
ACATGGCTTGTAACCCTTTTTTTTTTCGTCCTGCAACTATATTTAATAATGCTCATGAACAAATCAATGTTTA
TGGAGATGATGTTGAAGTTGATTACAGGGGATATGAAGTATCAGTAGAAAACTTTGTT
CGTCTTCTCACTGGCCGAGTGCCACCTGACACTCCGAGGTCAAAGCGGCTACTCACAGATGAAGGGAGTAATA
TTCTTATTTACTTGACTGGCCATGGTGGAGATGGATTTTTGAAATTTCAGGACTCTGAAGAAATTACTAGTCA
AGAATTAGCAGATGCCTTATAACAAATGTGGCAGAAAAGAAGATACAATGAGATTTTT
TTTATAATAGATACATGTCAAGCATCATCTATGTATGAAAAATTTTACTCTCCTAACATCCTTGCTAC
>103777721_2 DV501224.2
GCACGAGGATTGACCCTTAGATATTGTTTCTTTGTCCGCTGTTTCATGTTTGATATTTAATAATTTTAGAAGT
AAGGTTGAAAATATACATCAAGTAGTCAAAACTATGTTCAAACATGGATGCCCTGTTTGATATTTGTTATCTT
ACGCCTGATGCTAATAACACGGACCCCGACCATATTCCTAAAACAAAATAAATGCGTT
TGGACATTGCGAAAAAAGTACAGTGCAATTCATGATTTACATCGTATAACACGAGATATAAGTTCCATAATTT
GGTGTACATATACAGAAGGATTTGCACCTATAGGAGATGAACGGTTAACATCAGATAATGGATGGGGTTGTAT
GTTACGATGCGGCCGAATGGCATTACGTGTGGCATTAATGATGATCCCCTTATCTTCG
GACTGGATATGGACACCCCATCCGAGAGATCCTGGATATTTAAAAATAGTTCATAGATTTGACGACAGGATCC
ACCCCCCATATTCAATACATCAACTACCACTTATGGGTGCCAGTGAAGGAGATCATGTGGGTCAGTGGCTTGG
ACCAAATACGGTTGCTCACGTTCTG
>103777727_2 DT663895.3
GGCACGAGGGCGCCCTAAAGTCACTGTCAAAATGACGCGTCTAGTTCTGACGTTTTTCGTTTTTCTGAACTTC
ATGAGTAAAATGGCGAATGCTAGAAATCATTCTGCCCAATTTTTTATTCAGCGTTTGGAGAGAAATTTAGAAG
TTAAATTCTTTGATTATGCGAACAGTATTGCTGTATGGTTGGAAGGTATCAATGATAA
ACACTTAAAGGATAGTGATTTGGGAAGATTTTTAAGATATGAAGAAATGAAACCTAAATTGAATAGAATGTAT
GAAGCGCGAGGCTCAAAAAAGCTCTCCTTAGCCCTCCTCCCTATCATATTCCACGTGGGAGCGACGACCTCGT
GGTTGGCTGTGACCACTATCCTGTCAGCCAAGTCAGTCGCCATTGGTCTGTTGCTGCT
GATCTTCAAAATAGCTGTCAGTTCCGCTAAGATATCATCATTCTTCACCGCGTGGAAGTTGAAAAAGCATCAT
GATGAAATGTCCTGGATTCCTCATCATGACCACCACGCATCCCACATGGACTACCACTACCAACACCCGCACA
AAAACGACTGGGGCGAATACAAAACGTTACCGAACGACCCCGAATTGGAATACGGAGA
AAGCAAAAAGGTCCAAAAAGGCGCCGTTTATTCTTAAGATTAAGTTAAAATATTTGAAATTTACACCTTACTT
CCTCAGTAGTTAAATACCTAAATGTACTTATTGATTGTGGGTACAAATTTGAATTGGTAGAATGTCTATTACT
ATTATTATTATTTT
>103777743_2 DT664443.3
GCACGAGGCTGGACTATAAATCGTCGTAGTTGTCCAGGACGTGCAAGACATGCCTCCTGTGTTCACATCGGCG
CCACCCATCACACACTTGCCTCGTCAAGTGGCGCCAGGTGATATGATAGTGAAAGTTCGTGCTGAAGATGGCG
ATAAAGGTGCGCCTCGGCAAATCAGATACGGCCTTGTATCTGAAGGGAACCCGTTCAC
ACCGTTTTTCAACATCAACGAGACATCTGGTGAAGTGACACTTGAAAGGCCAATTGAAGAAATTGCAGCAATA
TCACATGCTGGAGCTCCAATACTTCTTACTGTAGTTGCTGAAGAGGTGCGTCTCTCTAGAGAGGAGCCTGAAG
CGATGTCGTCAACAGTACAACTTGCATTTATACTTCCTGAACGTGACAACTCGCCACC
ATACTTTGAGAATGACTCATACATAAGCTATTTAGACGAAAATGCTCCTCAAGGTACAGCGCTTGTATTTAAC
GATCCTTATATTCCTCAAGTGAATGATAATGATGCAGGAAAAAATGGCGTGTTCTCTCTCACACTAGTTGGTA
ACAATGGCACCTTTGAAATATCTCCAACAGTCGCTGAAAGGCATGCACAATTTATTAT
CAAAGTTCGAGACAACACCATGCTGGATTTCGAAGCCAGAAAATCAGTTATCTTTCAGATTTTGGCACAGGAA
CTTGGTCCAGCAACGAACTTATCAGCAGTTACAAACGTAACTGTTTATTTGAACGATGTCAACGACAACCCTC
CTGTATTTTTAGCTCTCTCGTACGACGT
>103777751_2 DT663961.2

GCACGAGGCCGCGCCTAAGTCGGTTTCAATCGTTTTAAAGTGGTGTTCAGTGATTAAATTAAAAGGAATAAAG
ATAACATGAGTGCCGTACCCGTGGGTCCCAAGCCCACCACTTTGGTGTGTCCTTCCTGCAGATCCACTATCGT
CAGCAGAGTGGACTTCAACTCTACCACAAGAACACACATTATAGCCTGTCTTTTGTTT
TTATTTTTATGTTGGCCTTGCATTTGCGTGCCTTATTGTATGGACTCGTGCAAAAACGCCGACCACTATTGTC
CGAGCTGTAACGCGTATTTAGGCACATACCAAGGATGACTTACCTTCCGTACCAGCACTTCCGTATATTATTA
TATTATATTGATCTCTTATTTATCTATTTCAATTAGATAATGAACTGAGATTAACTAT
TATTGGTATATTACTATGGCGCCTAAGTTATTGTTAATGCCGTTTAAATATTTTGTAAATTTTGTACCTTCAA
GTGTTATTCAGAATTTTATCTATATATCTATGGTACTTATGTTAAAATCGTAACGGGTTGGTGTCTGTTCATG
GAACATGTATAAGAAAAAAAAAAAATTTGTGGCATTTAAAAACGCAAAGAACCAAAACA
ATGAATTTCAATATTTCTGTCTGTTTGTCTGTGCGCTTGTTTACGCTTATCTCAGAAACTCCTTAACCGATCT
AATTGCAGTTTTCCCTATAGTATTTTGGCAAGCTTTTTTAACATTATGATGTTTGTTTTAATTTTAATCAGAT
TATAAATAACAAAGTAATAGCATTTTTAAAAAGTTGTTCGACGAATTGCTGAA
>103777755_2 DT663593.3
GCACGAGGCTTTATTCCTAAAAAGTTAAATAGCACTTATTTTACAATACAATGTACTGACCACTTTCTTAAAT
ACTGAAATAAATATGAATGAGGTAAAACTTAATCTAGGGTTTGTAATATAATTGTAATATTTAAAAAAAATAC
TTCACACACTTTACTGAACTGACTTCAGAGATTAAGATTATTTTAGAGTAAGTATATA
GAGATACTTTTGTCGATAGCATAGATTTTAAGATTTTCTTTATTTACTTAATTGTGAATTTTGATTGTTATTT
TGTTATACTCTCAAGTTAGGGTTCGTATATCTAACGTATTTTTTTTTATCATCGTTTTCTTCGCACACGAAGG
GCTTGCGGTGGTGACAGGCCACGTCATGCCAGTGAACACCGTCGTTGTAGAAATTGTT
CAAGATCGCCACGCAGTGTTCCGCAGCTCCGCCCTGGATCAATTCCCGATTGTCTGGTTGAGGTTTTCCGATG
CCACCACCCTCGGACCAGTCGTTTTGTTGACGGTTAGTGCTAGGAGCCAGCTTTTGCAATTCGGCGGTCCAGA
ACCAGCCATTGATTTCGTTTGGTAGCAAGTCTGGGCGATTACATCCTTTGAAATCGCA
CAAGCGCCCGGACGTCCAAATGTATTTTACTTTATCCTGTACAATGCGGGCTTTCACCCATTCGTTTTCATCA
CTTGTCTC
>103777761_2 DT663807.3
GCACGAGGGTTTAAACCTTAAAGGGCCCAAACGACGCAGATGAAATAAACGATGTAGTTAGACTTAAAATACC
AAATAAACCGGAAATTACTGACCATCTTTTCGAAAAATACGCTGACCTTAGGAGTAACACAATGAGAAGCGAG
TACGTTCGTAAGCGGCGACCAGGCATCAATATAGCTTTCGGTGGGAGCGTCAGGCTAA
AAAGTTTTCATGGAGAACTTTGGTGATTCTACCCTTTAGTTCCAATTAGTACACCTAACTCTAATTTTTCTGA
CAATGTAGATATCCCAACCGACAGGACGAATCTCCATTTACTATGTTTATTACCAACATAGTCTTTATTTATA
AATTAAGATATTATATTCATGCGATGAGATGGTGAAAGGATATTATTATATCATATTA
ATTTAAAACTATCTTCGTTTATACTAAATCATCTGTGTGAAAGTTTTAGCATGATTTTAAATATTTCGACTGA
CTAACAGCACTATGCACTAGTATATCGACTAGATCAGCACTCCATAGACTTGTGCCCTTTAAAGTTTTCAGTA
TAACAACAGTAATCCTCCAAGAAGTTAGTGGGACTATTGATACAACAGTTCAATACGA
TCGCTACTAATCAACAGTACACAAAATTCAGTGGTAAAGATAAAGAAAACATATCAATTACAACAATGCCTTC
CGAATATAAAAATGAAATTATAATAGAATATGAAGACGCCAACGAGCATACGTTATTTCCAAATAAA
>103777763_2 DT664012.3
GCACGAGGCCCGCCCTTAGATGCACAGATTAAAAAAACAGTTCTAAATTCCGCGAAAAATATAATATTGAGTC
ACACATAATGCGGTTAGTGTTTTTGGGCGTTCTCACGCTCTTCTCTCTGGTGTATGGCCAGGAAACGTGCCAG
TCCGCTGATGAAGAACCATACCTCCTTTTTGGAACAAAAACTGCATACACCTTCGCCA
GTGTAGGAATACCATTGAATAAAGTTTATGATGTTCCTGGATGTCAACCCTTAGCCATATGGTTGATGAACCG
CCACGGCTCACACAACCCAGAAGCGGATGAAATAGCCAACCTACAAAAATTGACGGACTTCAAAAATAACATC
ATAACAAACTATAGAAATGCCAATTTTCGGGAAACAAACATGCGTATGTGCACATCTG
ACGTGAACCTTCTAGAACGCTGGCAATGGAATCCCCGACAGAACCTAACCTTCGCTGGCGACTTAACGAGCGA
TGGCTATATCTCCACTCAGCAGTTGGCACAAGCTTGGAAGCAGAAGTTCCCTGGACTGTTGACTGATAATCTT
CATGATTATATGTTTAAATATGTAAATGATCAACGTTCAAGCACTACATTCCGAGCGT
TCACCGAAGGCCTGTTCCGGGCTCAGGAAGATCCTGCAATTACAACCCAGGAAAGCGATGAGAAATTACTCCG
ACCTTATAAATTCTGCCCTTCTTGGACACG
>103777773_2 DT663551.3
AGAAATAAAGCCTATGAACAAATGGGTCGGAATTATAGAGAATATCGGCGAGAATCTAGTACTAATCATTTCT
ATAACAGAGATTATGAGTCAGATCGATGTAATTCTCGAAATCGATGCACTAGCTCAAGAAATTATGAAAGCAG
ATATGAGAATGAATATAGAAAATATGATGATGTGGAAAGACGTTCTTATCAACGTGAA

AAAAGTGTCGGAAGAACTAGAGAAGTTCGTGACCGATTTCCAAACGCAGGATCATTGCGAGATCGTAGTGTTG
GACGTTCAATCTCTAAAGAATTTTATTTACACGAGAATCGCACATTTGCTATTGATACAAGTATTAATAGTAC
ATTTCAATCACATAGAAATGATAAAAACACAACTTTTGATAAAAGAAGACAACGTGCT
TCTAGTGTCGGAAGATCGTTAATTAGAGATTCATTTTCTAGCAGAGATTTAAATGAAACTCGTTTTACAAATG
AAAGTAAAGAAAATGATAACAAACATTTAAAGTATAAACGAGCATGCAGTGTAGGAAGAGAAATTAAGGATAA
TAAAACACTTAACTTTAAAGACATTAAGGAAGATTTAAGGTCTTTCAAATTTCAAAGT
AATTTAAGTAGTGTTAAATCACAAACAGAAAGGGATGAATTAAAAAAAAATAGTGATTGCAAAAAGAATTGTT
ACAGTCGCAGCCGGAAAGAATATATGAAATCCAACAAAATAGCAAAT
>103777775_2 DT663996.3
GCACGAGGAGCAATCTTAACCATAAGCATCCAATGGATATTATTGACCAAAGTCCTGAAGGAGTTATGTTAAC
TGATATTTCTAGTCCTGAATTAGATATTTTGACACCTGACAATGGTTTGTCTACTCCAGAACATTCTAAGAGT
CCTTTACGAAAAAGTAGGGCATCCTCTGCCACACCAGATCTATTATTTAAAAAATGTC
CACAAAAGTCTGAACCATTATATATAGATGATGATGTTGATGCTGAATCAACTCATTCATCTACAGGCACTTA
TACGATAGAAGGTGATAATTACACAGAAGAACAAAAAGCTAGGATGAGTATAGATAGAAACTTTGGTACCGAC
TCACATAATGATTTGGAAGATACATTAAAGAGACACAGTGTTGAAAAGGACCCAGAAA
TAATATTTGATTATCCCAATCAAAGGGTCATCACTAAGGAGCCGAGAAGAGATGTATGCTTAAGAACTCCAGT
TACTATAAGTAAATGTGATCCCCAAAAAGTGTCAAAAGACAAGAATGTACTAGAAATATCATGTTGCTATGAA
TCTCCAAATGAAAATGATTTAGCGCTACAAACAACTAAACAAATTAAGTCAACCAGAA
GTTATTTAGAAAAAATAAAAAACAGAGTGAGGACTATAACAGAGAAAACATTTGCTAAGTCGCCTCCAAAGTA
TGAAGACAACTTAGACCTTGGTACATTCACTTCTGTTANCACTTCTGGTGTCTTAAGCTCAAAATTTCCCATA
AAAATCGAGAAACCAAGTCGTAGACGTTGTAGCTTACAAAATCAGAGATTTGATCAAC
AGATTATGTCCATAGATTATCTCGAGAGTCCTCTGTACCAGTTTCTTTG
>103777778_2 DT663789.3
GCACGAGGGTCGTCTCATTAAGGAGTGCGGAGGCGCGGGACCGGCGCCGGCGCACCGGCCGCTCTCACCTCAG
TACGATGGCTGTGTTAGCAACGTGCGCCGTGATTCTCTTGGCGGTGGGCGCCCTCGCCAACGATTTGCAAGTA
CTGGATGCCGGTACAAATGGCACCTTACCTGCGGAGCAATATTTCATTGACAAGATAT
TTGACAAGTATGGGGACAAAGGTGTTATCACGTTTGAAGGTTTCGAGCATCTGCTGGACAGTCTCGGTTTAGG
TGGACGGGTCTTTAATTCACCACACGACATTGCCCTTCACAATATTTTTGGCTCCTTCCAACAACTGCATGAT
ACGGCCCACCGGCACAAAAGATCGCCAACATCTAAATCTCCCATATTGAACAAATCAT
GCCTATCTCCAAAAGAAATTTTGGAAGTTTATGGAATGGAGAACGAACCAGGCATTATAACAATCAAACCGAA
AACCTTTTTAGAAATGTGCCCTGCCCTTGTATACCAGTTAGATCAGAGATCATGTTACAAGACAGAAGCACCG
TCACCTAAACTTAGTCGGCTATGGACGTGGATATATGCGACGTTAAGTGTACTGGTAA
TAAGTGCAACCCGGATTATTAGGGGTGGCTATAGTACCGATGTTAAAATT
>103777787_2 DT664802.3
GCACGAGGGCCATCCCTGTCGGCATGGCCCTCCTCGGGATCATTATCGGCAAGTTCCTCATGTAGACCCCCCT
ACGCCCGACACCACCCCACGCCGCTGTTACACCACCCACTAAAAGGCCTCCCGTTAATTACGTCACTCTTCCC
CCGTCATTATCAAAATTGGTCACATTTGTGAGACGCCCTACTTAGTGTGACGTCACGT
GTTTTACAATTTTTCACCTAGATGTGTGACGTAATTTATGGATGGGCCTACGGGCAGTTTAACTATTACGGAA
CGCAATTTTCTGGAGGATTTCTAACGTTCTCATGCAACACGCTGTGACGTTTTCCTATACCTCTTTATCACTT
TGTTACCAAACGTTACGTAACGCTCAAGCGTTCGTTTTAACTCTGTAACAATTTATAT
TAGTAGGTGAAGGAAGGGCAAAATGATATATTTAAAGGAAACCACTCGTCATCAACAAATTACATCTTTATTT
TATTGATAATGGATCCGTTACTAGTCTTATGCTCCCAGTACGAACGACGACTGCAAGCCGAGTTTTGAGTCCC
ACAGAGGACGCCCTTGTCGCCGTCGACATCTAGCAGCCTTGTAGTGTTTGCAAATTTT
TCATGGACAGAATACCCACCCGGTGTCACTGTAAAAAAAAATAATGCTGCCTAATGAAGTGACATAATGGGTT
CGTTTTGCCATTTCAATATTTTTGATTCTAGGTGATATTCTT
>103777793_2 DV501215.2
TAATGTAATTTTTTTTTAATATTAAGAAAAAAAATAATTTGTCTTGAAAAAGNGTCGCCCTCATTAATAATAT
ATGTAAAAAATAGGTACTGTGATTTATTAATGTAAAAAACAAAAAAAAAATATTCCATATTTTTTCTAGATTT
TAAGCAAAAGTTGAGTGAATGAGTGTATAAGAAAAAAAAATGTAAAAAAAAAATCCAC
CGATATAATTTTTTTCTCTAACTTAGTATTATAATATTGTAAATGTATTTTTTTTAAACTGTATACTGTTGAC
TTTTTTTTAAATTTAAACTTGTTGATTTAAATTAACTTTAAATAAAACAAGTCTATAGTATGCAGTTTTTATG
TATAGTCTCGAGACTAATATAATTTTTTCGTTTTATTTCACT
>103777799_2 DT663905.3

GCACGAGGTTCACCTTTCGCAAGCTCTGAAACTAGCTATGGGGATCGACGGTAACAATATACTAGCCGAAAGC
TTAAAAAAGCAGGGTGTTGAATATGCATTTGGAATTGTTGGGATTCCTGTTATTGAAACATCACTTGCATTTC
AAGCCGCTGGTATAAAATATATAGGAATGAGAAATGAGCAAGCAGCATGCTATGCTGC
ACAAGCTATTGGATACTTAACAGGTAAGCCAGGTATATGCTTAGTTGTATCTGGTCCGGGTCTTTTACATTGT
ATCGGTGGTATGGCAAATGCTCAAGTCAATTGTTGGCCACTTTTGGTGATTGCGGGTTCTTGTCCAGAGGATC
ACGAGGGGATTGGGGGTTTTCAAGAATGGCTCCAGGTGGAGTCCAGCCGCCTGTACTG
CAAGTACGCGGCGCGGCCGCCGTCGCCGCGCCTCATCCCGCTGCACGTGGAGAAGGCGGTGCGCTACGCGGCG
GCGGGGCGGCCCGGCGTGTCCTACCTGGACATGCCCGCCACGCTGCTCACGGCTGAAGCAGATGAAGATAAAG
TTCCCTTAGACTACTACTCGGCTGGACCACTGAGGCTGGCGCACCCCGACCCCGCCCT
CGTGGAACAGACAGCAGACGTCATATCGAAGGCTGAAAGACCTTTAATTATTGTTGGCAAAGGAGCTGCTTTC
GCAAAGCTGAAGATTCTATTGGGAAACTTGTGGAAAACACCAAAGTTTCATTTTTACAACGCCTATGGGTAAG
GGCGTGGTGCC
>103777816_2 DT663494.3
GCACGAGGGTCCCTTTCACGTCCACAAGGCAGACCGACGTATATATATATAAAATGATGGAAAACTTCCGTTG
GTGGTGGCTCGTTGGCGCAATGGTTCTGGTGACCCTCGCTGCTTCTGAGCCTCTCGAGCATGATGACCACGAG
CTCCTCGTGCGTAACGAGCGTGGCGCCAAGAATCGAGAAACTTGCCGCTACGTCCGCG
GCGCTTGGTCGGAATGCGATCCGAAGACCAACATTCGTTCCCGTACACTAACTTTGAAGAAAGGGGACCCCGG
TAGCTGCGAACGCGCCAAGACCATCCAGAATATTTTTTAGAAAGCATGCCGCTACGAGAAGTCATCTTGGAGT
GAATGCAGCCCCAACGGTGAAATGTCCAGGACAGACATGTTGAAGGCGAATAGTGATC
CCACCTGCGACCAGAGCAGGCGCATCACTAAGAAATGCAACAAGAACAAACAAGTCAAGTCCATTAAAGACAA
GGGTCGCCGAAATCGTCAGTAAATTATACACAAAAAGTGTAATTTCATTTTAGCGAAATAAAAAAATTTTCAT
TGTGAAATTGTTTCTTTCCCGCTCTTGAAGGCAGTCTCTCATAAATAGCCTTATTAGG
TATTTAAATAGTGCCAAAATAAGTATGGAAACCTTATTTTCTATTATACTTCTATTCTACAGTTCTAG
>103777820_2 DT663554.3
GCACGAGGGTCCATTCTTTTTAGTTTTTTTTGACACAAATATATTTAATGAAATAGTTACATAATAATAACAG
TATAATATATATAAAGAAGTTATTTACCAGTAATCTATACAATTGTAATTTACGTTATAGTATATAAATTTAT
AAGCTTTGGACTTCAACAATCATTATTTAATTTTAGCTTGCACAATTACAAAATATAA
AATCACAGTTATATATTTTTTGATGTCAGGTGTAGATAAAAAAATTATAAAAAAAAAGATAATACAAACTTAA
ATAAAAATAAATCTAAAAGCAATTATAATACGAACAAGCAAAATTTTCTATCTGAAGTAATAATACTTAAAAC
TAAAATTGGTAAATATATTTAAATACTAAATTAGCCTATACTACACATGGTGACTAAT
TTGTTCTTCAGATAAGTTCACTTAAAAAAATAATGTAAAATATTATAACTTAAAACAACTAATTAATAATTTT
CATACTGACTCCTGTACTAATTCTTTGAAAATGAGATAAATTAAAAAAAAGGTTTAATTTTATGATACCTAAA
CTTAAATCCATGATTTTCACATAATAACTAAACATTTATAATAACATTTTTAAGTTTA
ATCCTCCAGTGTGACATTTTTGAAGAGATATGACATGGACTCTTTGTTGTGTATGCGACGGATTGCTTCACTT
AAAAGCACTGATATGTCGATTGTTTTTATTTTATTGCATTGCATCTTCTGTA
>103777827_2 DT663561.3
GCACGAGGCTTGGCCCTTAAAAGAAATAGTTGAGGTTAGTTTGCCGGTGTGCGGAAAAACTAAAAATATATTG
TTACTTAAACTTATACAAATCTCCCTTTTAATTTAAAAAAGAATAAAGATGGGTGACCAAGGATTTTTAAAGC
AAAAGCCAGGGTATGCTACTACAGCAATTCATGCAGGTCAAGACCCAGACCAATGGGA
TTCTAGAGCTGTTGTTGTACCCATTGTAACAGCAACAACATTTAAGCAACCAGCTCCTGCAGAACCAAAACAA
TTTGAATATGGTAGATCTGGTAACCCAACAAGAAATACACTTGTTTTTTGTTTAGCTGCTCTGGATGGTGGTA
AATATGGTTTTGCCTTTGCTTCAGGCTTAGGTGCCACAACCACCATTATGTCTCTACT
TAGCCATGGTGATCAAATTATATCCTGTGATGATGTATATGGTGGAACTAATAGATTATTTAGACAAATAGCG
GAAAGATTTGGTATAGAAGCAGAATTCGTTGACTTCACTAAACCCGAACTCCTAAATGCGGCGATACAAAAAA
ATACAAAGATGATCTGGATAGAAACACCAACAAATCCCCTACTAAAAGTATTAGACAT
AGCAGCAATTTCCCAAATAGCTAAAAGCCACAACGAAGATATCTTAGTAGTGGTGGACAATACTTTCGTCACG
AGTTACCTGCAAAGACCCCTTGATTTTGGCGCTTG
>103777837_2 DT663509.3
GCACGAGGCTCGCTTCTTGGCCCCAAAATTCTTGATTTTATGATGACTTTAGTGCAGTGAGTGTTTTAGTGGT
AGTGCCCCGTGAATTTTCTATAGAACAGACATGAAATGCAAAGAGTGACAGCTATGGCGACCAAAGACCAAAC
AATGGGGCAGTACACTGAAAATGTATTAGATAAAAAATACTGTACCGATTGTGATATT
AATATTAATAATAGTATAGATTCTAATATGAACAAGCAGAAGTTTACGGAGGTTCAGGATTTTTATAATGGAA
AGAATATATTGATTACAGGAGCTACAGGGTTTCTCTGGAAAATATTAATGGAAAAACTGCTGCGCTCGTGTCC
GGGTGTGGAAAATTTATATTTATTAGTCAGACAGAAACGTGGGAAAGATATCTACACA

AGGATTGAAGAAGTCTTCGAAGACCCGGTTTTTGACCGTCTCAAGGAGGAAGTACCAAAGTTCAGGCACAAGA
TTGTGGCCATACCAGCGGATTGTGAGGCGGCGGGGCTGGGACTAACTATAAGTGATAGGCAAACTCTTATAGA
AAAGGTACACGTAATATTTCATTCCGCAGCGACAGTCAAATTCGATGAGCATTTACGC
GCGGCTTTAGTCATCAATGTGAAGGCGCCCTTATATCTTTTACAGCTTGCTAAGGATATGAAAGGGTTGAAAG
TATTCATGCATATATCAACAGCATATTCAAACTGTCACTT
>103777845_2 DT663673.3
GCACGAGGGAGAACTTTAACTTATTGTAGTCTTAATACAAATGTATATTCTTTTGCGCAAAGTTACTTAATAT
TTTATTATAACAGCTTGTTTGTTATTGTAATTTGAGTAAAAGGCAAAATAAAAGCAGCGAAATGGTTGTTTCA
CCGCAAAGCCCTGCCAGTGAAACCCAAAGCGTACAAGTAAAAAAAGTCAATATGGCGT
CCAAACAAACTTCGGTCGCTGATACAAGAGCTGAACTTGCCGAATTAGTTAAAAGAAAAGCGGAGGTTGCTGA
AACCTTGGCTAACTTAGAACGCCAAATATATGCATTTGAAGGATCATATTTAGAAGACACACAACTTTATGGA
AATATTATTAGAGGCTGGGACAGATATCTTACAACTAACAAAAGTACTAATTCAAAAG
CAGACAAACGCAACAGAAAATTTAAAGAAGCTGAAAGATTGTTCTCCAAGTCTTCTATAACTTCTATGGCAGC
TGTCAGTGGATTAGTAGACCCTGCTGATGCGAAAAATGAACGAAATAGTGAAACTGAATCACAGACAAATGAA
GATTCATCTGATACTCAAATAAGTAGTGGTATGATTGGAACTTTGAACCATACAACTA
TTCATGGTTCTACTGAAAGTATTGCATCAAAACCCTCTGGCAAACAACATACTAATGGTAGTATTGAAAAAAC
GATAACTAGTGCTTTAACAAAGCAAAGTGCAACACAGAAGATTGCCTCATCATCTAATGTACTGCAAAAAGCA
TTTGGAAGTGATAATAAGATGCTAACAGCTGTCGGCTT
>103777847_2 DT663836.3
GCACGAGGGAAATTCCTTCACAATTTAAAGATGCCATAGATAAAGCGTTGAGAGAAAAATTTGGCGTCATTAG
TAACCAAACCAGTGAAACCTACAGTCAAGATTCAGAGGATGTTATATTTATAACAGAAATACAAGCAACTAAT
GAAGAAAAAGAAAAAGCAAAAGAACTTATGCTTCCGGAAAATTTCTTTACCTATAAAA
ATAAAGAAACATGTAAAGGATGCAGGGGCTGCGACAAGGACGAGGATGCTAACAATGAATCTGATCAAGTGTC
TCGTATTCAACACTTAACGTCGTTGACAAAACAAAGTTCATTAACCACGCCGCTTAAAACATCAACATTTAAT
TTCCAAAGTCCAAATAATTCTTTATATGGTACACCTAATAATTTCAACAACACTATTG
ACACTACAATATTCCGTACTCCTTTGAGTACTAATGGTTCTAATACTAAAAGTTCTTCAAATACTACAGGAAA
CTTAAGCAATACTGAAGATGATTCAACAGATAAGGAAAATACCTTAACAGACAGCTCACCTAATGTTATCAAA
CCAGTATCTTTTGTGAGTGCTGGCCAGTTGTCTAGCACATCAACTGTTCTAGCCCCAC
CCAAATTTACTGGCATAAATGTTATATCAAATAGTAATGGGTCGCCAGCTCCTGAATCTGTATTTGGACAAAC
GGAAGCAAAATCTATATTTGGATNCACAGAGACTAAGTCCATTTTTGGTCAAAGCAACCCAGTTTTTGGTGAA
AGTAAATCTATATTTGGAGAATCAAAACTTTTGTTTGGTTTGTC
>103777855_2 DT664267.3
GCACGAGGCTGACCCTGGAGCCGGCGGGCGCGGGCGAGGCGGCGCCGGCGGCGGGCGAGGCGCCGGCGGGCGA
GGCGCCGCTGGCCGCCATGTCGCAGGCGGTGTCGCAGGCCGTGACGCAGGCCGTGTCGCAGGCGGTGTCGCAG
GCCGTGTCGCAGGCCGTGTCGCAGGAGATGACGGCGCCCGTGCAGGGCCTCACGGACA
TGAGCGACCAGGACCTGCTCTCCTACATCAACCCGAGCACCTTCGATCAGGTGTGAGGGGTGACACTGATGCA
TTTGTATCGAGTTCGGGCTCAAAAGCAGTTTTTATGTGAATTATTAGCGTAGTATAAGCTATTATAATCTCCA
TGGATGAAAGTATGCTTGGTCAATTAATACGTGAGTCAGTATTACAGGGCTAATGATT
AATGTATTGTAAAGTTTGACGAAACTGATCTATTTTGTTTATTATAAAAAATATATTTACTCAAAAAGTGTTA
GATAAATATAAAGTATATAGTATTCATAATTTTCAATCATTTATTTAAGACTGCTGATTGAAGTCTCGATTAA
GATTTTGAGGTTTCTTTTTTTCACGTTTCCTATTTCTGCAATTTAAAATAAACGTTTC
GTTGAACTATTAACGCGTCCTAAAAGATAATTGTATGTATCCAGGTGAATGTACGTGAACGATTGTGTTAGGT
TATATTTTCGAATTTTATTATGTAAATAGTGATAGCTTTGTATTTATGTCCTTGTTGTTGACGTGTGGCGGCC
ATCTTGGAAATGCGTACATTTTTCTAACATTCCACATTATGTTCAACGCACAAAATGA
CAAAGTGAA
>103777859_2 DT664491.3
GCACGAGGGGCCCGTTACCATTTGGGTATACCTACTACTAGAGCTGGGGGGCTGGTGGTAAGCTATGACCACA
AAGTATTCAGAGATAAGACCTACAGTGGCAGAGCGAGACAAGAACCCACAGGCATCGTGCTTAGACTGGCGCC
CGCGTGGTACAGACTGGGCTCTTTTGAAATATTGTATAAGAGGAATGAGCCAGAACTG
GAAAAGAAGTTGGCGGATTTTATTATTAAGCATTACTTCCCTAATATTAATTTAGAAGATGATGACAAATACG
TGAAATGGTTTACAGAAGTTGCTCATAGAAACCTCGACATGGTGGCGACTTGGCAAGGTCTAGGCTTCACCCA
CGGTGTCCTAAACACGGACAACATCAGTATTCTAGGTCTTACCATAGATTACGGTCCG

TACGGTTTCGTGGAGCACTATTACGAACACTATGTTCCCAACACGTCAGACGACATGGGAAGATATGCGTTTA
ATAAACAACCTGATATTATTCTATGGAATTTGGAGAAGTTTATAGAAGCATTGCAACCTATTTTGTCTGAATC
AAACAAGCAGAAGATAAATGATGTAATGGGTACATTAAGTGAATATGTGAAAAACAAA
ATTCTAAAAACATACCTATTAAAGCTGGGTTGTGAACATATCCAAGACGGAGACGATAAACTCGTGAAGGAAT
TCTTAGAACTAATGCAACAAACTATGGCCGATTTCACATGCACATTTAGACAGTTAGCTGAGGTAAACCCGGA
AGATTTGAACACAAAGATATATTAGAAACTAAATGGTCGTTAAATAAAGTTAGCCAAG
>103777861_2 DT663802.3
GCACGAGGCTTCAATCTTGATAGCTAAAGCTCTAAAGTCAGTGGCAAATAGTGGACGTGTTGTGGCGTGTGTG
ATACACCAGCCGTCATCACAGCTGTTCGCCTCTGCTGATGACGCTATTCTACTTGCCAACGGAAGAACTCTAT
ACTCCGGAGCGATAGAAAATATTCCTGAATTATTAAGAAAATCAGGCTTCCTGTGTCC
TCAGTATTACAATATGGCAGATTACATTTTAGAAATTGCGAGTGGGGAGCACACTGGTAATTTATCAATTTTA
GAAGATGAAGCAAAAACGTATGCTAATGAAATGAGAAAACTAGCATAAAAAGAAATCAATCAACAAAACGGCA
AAGACAATTCAGCAGAAAGCGAAGCGCTATTAAATGAAAACATAAGTATTGTAGATGG
ATATAAAGCTAATTTCGGACAACAAATGAATGCACTTCTTCGCCGCGGTTACATTGGAGCACTTCGGGATGTT
CACTTAACACAGATACGACTCGTGACTCATTTAATAGTCGGTTTGTTATTAGGCGCTTTATACAACGGGGCTG
GAGCCGAAGCTAATAGAATGATATCAAATACTGGCTGCTTATTTTTTTTCTTGTTATT
CCTATATTTCTCCAATGCAATGCCAACTATACACACATTCCCTGTGGAATCTACTGTGGTACTTCAAGAACAT
CTTAACAAATGGTACTACTTATCAACGTACTGCGCGTCTAAATAATTATAGATTTACCGATACAGTTATTGTG
>103777873_2 DT664361.3
GCACGAGGATTTACCTGTTGTTTTCAATGAGATCAGAGTTGCAAAGTGAATGATACACATTAATCTGGTATTT
GATAAAGTTTGTTAGATATTAGATGTAGGATTTTGAATTCGGGCTGTACAAATAATCTACTTAGTTATTTGTA
GAGAATTTTACATTGTGAAAGCATTGTTATAGTCATTAGTTTAAATATCCTGGGATGG
TTACTTAACCAAATATGCTTGTTTCACGCTTGCAATCAAAAAAATTGAGTAAATTTAAATTAGATATGTGAAT
TTGGATAATATGAATATGATTGTTTCATATGTGTGAAATTATTGAGTTAGTGTTGCCAGTTTAATGTACACAG
AATGTTGTAGTTTATGACAAACTATGGAAGAGCCATATTGTTACAATATTTATTATGT
AACACATTTTATGTGCTTTATTTATTATTAAAATGTTGCTTAAAAGCATATATGTAATGAAGCTTAAAATCAA
ATATGGCTACTTATGAAATACTAATGTAATTTTATTATTATATCTGGTAACACTGGCT
>103777877_2 DT663607.3
CGGCACGAGGATGTTCTCTTCAAAGAGTTCCGTAAATTGTTTGTACCGATAAAATTTGTAGTTACAACGGTAT
TTGTGAGCAATACAAATAACTTTTTCGCATTGTGTATTTTATATTTTAAGTTAATAATTAAATTTAAGTGTAA
TTTAATTGTTTTTTCATTATAAAGTAATTGTAAAAAAGAGACAATGGCAGTGCTAGCG
GCAGCACAGCTCTTAGATGAGTTAATGGGAAGGCATCGTAATACAAATCCAAACGAAAAAATAAAAAAACCGA
ATTGGGAAGACCCAGAATATTGTAAATATTACATGGTTAAATTTTGTCCTCATGATTTGTTTGTGAACACAAG
AGCTGATCTTGGGGCATGCCCCAAAGTACACGAC
>103777883_2 DT664938.3
TACCTTTAAGGCCGGGCTGCAGGAATTCGGCACGAGGCGGCGGCCGTAAGACGGCGTCGGCGGCGCTGCCCCC
GCGCGTGCTGCTCGTCAAGCTGCTCAACGTGGCCATCACGCTGCTGCAGCTGGCCCTGCTGCTGGTGGCCACC
GTCGCCGGCGTCGCCATGCCCTTCTTGAGGACTAGAGTTCGAGTACTAACAACAAGTT
TAGCAGTAATGGTCGGCGTCATGGTACTCAAGCAGTGGCCCGAAGTGACTCAACTCTCCGAACATCTGCTACG
GCGACTCAAGGAGTACCTACTCGACAAGCACCACGACAGGTATGAATGCAAGTCGTTGTGTGGTTGATATCTG
GCTTTTCGGAGATTTTGGTAATATTGCAATTTTGTCCTGACTTTTGATTTAAGTCGAT
GGCCATTATTTTTTTTAAACTGTTATTGGTGGCAGATGGTAAGATAAATAAGTATAATTTACTTATTGTTTTC
TTCTTTTTTTTTGATTTTATGTATATTGACAAGATTTCAACTTTATTTATTGTATATCCGATTGAATAAAAAAA
ATCTTTAATTAAATACTAATTAATGATACTGTATTCTGTGGTTTTATTTTGCTATTTT
AGTAAATTTTGTATGGTGATCAAATAAATTCATTTGGAAATGGTTATGTTGATATACTTTTATGATGTTTATC
TTACCACTTAACGCCGTATAATAAATATTGGTGATTATTATTATATTAAAAGGTAGAAAAAATGAAACCGTTT
AATTATTTTAAGTTTAGATATGTTTGAAATATTGCACATAAATATTTCGTGGGGCAAG
AATATAAAAACTTATTGTGTAATTTG
>103777891_2 DT663608.3
GCACGAGGGTTTCGCCCTTCAGGTGACATTTAATTTTAATGAGAACGTTGTCGTTCGCCTCTCGCAATTCGGA
GGACTTGCATGAATGAGTCGGATGCAGCTCGACGTGTCGTCGGATCATCGGAGCATTTGTCACAACTCACTCT
TTACTATAATGTAACAAAACGACAACACTGATTGGCCCTCGTATCGTATATGATATAC

GCTCGTATTATTACATATTTGTATGTACTAATTACTTATAAATTTTTAGTATGTTTAAGATTTAACGCGCTCG
GCGCACGTTTTCAAATCACATAGTGGAGAGCTTAGTTGTCGTCTTTGCGGCTATTCATTTGCTGGCACATTCT
TTATAATTCGTGTAAATATATTATAATTATTATCTTTATGGTATGACTCTGATAAACT
TAATCGGTAGAGAGCGGTGGCTCGCTCGCTGCGCTGCCCGTTCGCTGGGCGCAGTGAGTATTACTATAGTTTA
GATACGCATATTTTTTATTAAGTCTTATATCTATTATGAATTTTTTGTAATCGACGTTGCTTCTAAAGACCTC
TTCTGTGTAAAAAGTTAAGACATACTAGTGACCTGAACGTTTGAAGCGATGCTAACGT
AATGTCTTCGTGCCACTGAAATTAAACACTTTCACGATTCCCGTGCACGTTCACTAAGACGAGAATCATCCCG
TAAGACACATTACTTCGAAATGGGGTTGACCTATATAAATTATTTAGAAAGAGTTTTCTTATTGCTAGTACTG
TAAAATTA
>103777893_2 DT664496.3
GCACGAGGTAGACCCTTAATAATTGACAATATATAATCACATTTTGTAATCCAAGTGTCGTAATTTAAAATTT
CTTTAATTTCTAATATTCCTATAGAAAAATAATAAAAAATCTGTACTAAGACTCGATGGTTATTTTCCACAGT
ATATTGAATTAAGTCACTTTTATTTTAGATGTGTGATAACGTTATAAAATAATATGTC
GAAAGCTAGTAAATAATTGTTTAATAGATATCTGTAATTAAATAACATATTCATATGGAATCCTACAATAATA
ATCGTCGAGGTCCCCCTCGTAAGCAACCACATACAGTAAGATCGACGAGTCGTGGCCACTCTTAATTTAGCTG
GTAGCTGCGCAGATTTAATTTTAAGACTATTCTTACTTTTAATTATTTTCTTCCCGAG
TGAACAATGCACAAAACTTTAGAAAACCTAATTTTAGAGCGGCCACGACACGTCGACTGTACTGTAGTGATTG
CGGTTTGTCGTGGACAGACAATCTCCGTAACCGCCAGTAGGGCTAAAAGTACTAAACTTACGTTCGAAAATAA
AACATTCATAAATTAAAAGAAAATAAAGTTAATTGTGCCTTGGAATAGGTATTTCTTT
TTTTACCACTATTATCTGCAATGGATCTATGTAAATTATTTAATTGACTTANATATTTTAGTGTTTTAAAAAT
CCTAAGTGACGAATTCAGACATTTTTTTTAGGAATCAAATACAATATTAACATTAAATTGATTTTTATTTTTT
CAGCTTTACATTGTCTCCCGCATATAGTAGGGTAGGCAGAGGGAACCCATAGACACTC
CGTCCCTCCGTT
>103777905_2 DT664633.3
AACAACCACCCGCCGGGCAAGCACCACGACAAGGACGACCAGCACCAGAACGCGAGCCAGCGGCCGGCGAGCT
CGGAGCCGGACATCTCCTCCAACCCGATGTTCAACCCGTTCCAGCAGGGCTTCTACCCGGAGTTCCCGCCCGG
GTTCCCCATGCCGCTCAACATGCTGCACCTGCTGCCGCCCAGCGAGAAGGCGCGGGAG
CTGTACCAGTCGATGCCGATGGCGCTGGACGCGCTGATTTTTTGTGCCCAAGGAGGAGGACTGCAAGAGCGAC
CGCTCCAAGGAGAACTCCGCGGACGAGGAGGGCGACGCGTACCGCGCGCCGCACGCGCCGCTGCCGCCCGACG
CGCGCGCGCTGCTGCACCACAACGGGCAGGACTAACTGCATCGGAACAGCGCGCCCGC
ACCGCCCGCCTCCGAGAGGGACTCGGAACGGGGTCTTTATCATTTATGGACGTTTGGTCGTTGTGAGCAATGA
AGCGACGGTGAAACTGGAGTTTTGTTAGTGTATGCCCGAACTCGGTCTTTATTGTGATTTATACTTATTGTGT
ATTTACTTGGATCTTATCTCGTGCAATAATCTTATCGAAGATGAATTTTATTAATGTT
T
>103777933_2 DT664997.3
GCACGAGGCACCTTTTTAATATATTGTAAAGATATTAAGATAATTTCTAGTTAAAAAACGTGTCTAATGTGCT
GTTTCAAGTGAAATCAATATGAAATTTCTAATTGCAATCATTGCGTTGTGCATTTTAGAAGTAAAATCACAGC
GTTTCAACAATGGTCGACAACGAGTGTTCTCCGTAAGCCCAGTACAAAACAATCAAAA
TATTGGTGAAAATATTTACCTTAGTTCAAGAACCTCAAATGAAGTTAAAGATTCTTACGATAGATATTTGCAA
GACATAGTTAATCAAAGAAATACTGCAGCGCATTCTAATCCCAACAAAGTTCAAGATTCTTATGATATATATC
TGCAAGAAGTAATTAAACGAAAAAATTCTGATGCTCAAGTGAATCTCTATAATGACAA
TGCTAATCAATTATCATTGACCAATAATCGTGATAATATAAATTCCATCAATCGTGTTACAACCAATAGACCC
ATCGACTTAGACGAATCTTCGATTGCTTCAAATCCAGTTAGATTTATTGGTGTTCCAAAAACTGATGTAGAAC
AACTCAACAATATAACATACGCAATAACAACATTCGGAATAAATTTAATGAAAAGTGT
GAATAGTTTACAAAATGGCAACATAATAGTGTCTCCCACTTCCATTGCAACGGTTTTAGCTCTGTTACAACAA
GGTACAGTAGATGAAGCTCAAAA
>103777941_2 DT664598.3
CGGCACGAGGGGGCCCCTTGAAGGCGCCCAGCGAGCAGGGCGTGCCCGACGGCGCGCTCGTGCTGGAGGCGGC
GGCGCGCGCGCACGCGGGCCTGTACCGCTGCGCCGCGCCCGGGGCCGCCCCCGCCGACACCGTGCTGCGCGTC
AAGGACATGTACGCGGCGCTGTGGCCCTTCCTGGGCATCTGCGCCGAGGTGTTCGTGC
TGTGCGCCATCATCCTGCTCTACGAGAAGCGCCGCACCAAGCCCGAGCTGGACGACTCCGACACCGACAACCA
CGACCAGAAGAAGTCCTAGGTCACTTGTTATTTAGTTTATTTTTTTTTACGAAATTATAGAGAATTTTTATTT
TATCCGAACTCTTCTATGCTCTATACAAACTTAATATAATGATCACTTAGCTTAACCT

GAATTTATTTGTATTGTTTACTTGTGTTGTTTTCGCACATATCGTTGTATGCTTTTATTATATTTCGCGCACT
CGCCATTTTCATCTCGTTTTAGCGTACTTAAGAGTAATTGTTAACAAAGTAGTGTAATGATCACCCCGACTGG
AGGCCCCCGGCCTCCTTCCCCGTACTGCCACTGATACCACAACTATCACTACTACTAC
TTCACTTATCAATATATTAACGACCAGTTAGTTTCTATTTAAACAACTGAATTCCGGTGGTGTAAGCAGGTGG
AATT
>103777945_2 DV501264.2
GAGGTAGAGGCCGGAAGGCGAAATCTGAGGGAGAGGAGGAGACCGACAAGTCGGAGAGTGACAGCGAAGCGGA
GTCTGGCAGCGAGGGGGAGGAGTCCGATGAACCGAAGAATAAGCGCGGGAGGCCGGCAAGTGCTGTGGGCAAA
GGACGCAAGGGCGCCGTGGCTAAGGCCAGCGCCAAAGCGACGCCTGCTAAACGGAAAG
CGCCTACGCCGCTTTTTTTTTAGAAGGCCGGTGCCAAGCCAGTCGGGTGGGGGGGGGAGAAAGGCAAGAAGGC
ATCTTCCGAGGACCCCGGGGAAGGAAGTGAGGAGGAAGAAGAGGGAGGAGGGC
>103777947_2 DT663576.3
TCTAATATTGGGAACTGTGAATTCTGAAAATTCAACATTATCTGACATATCAGAAGACTCCTGCGAAACTCTA
GATATAGGGCCATGGAGCTTAGCGCTGGAGGACTGGGCCGTCGCCACTACGTCCGGCAGACACGGTCGAATCA
TGGCTTTACCGCCATCAAAAGGCTATTACGTAACAGAGCGTATAGACACGCCTTATTT
GACACCTCCCCCACCTCTTCCTATACCCCACTTCACCCCCATGCATTACAGCCCAAACCAAAACAACAAAACC
TACACTTTAAATCCAGACCAAAAAATATCAAAGCTAGGTCTTGCACCAAATCAAAATCATAAACCTAATAAGC
CAATATCCCAAAACGAGAAGCTGTCCTTGAATTCCCAGGAAGTAAATACTGTGAACCA
AGGCCGTATCTCGCAGTACCCGGGACAAGTGTACCAAGGATATGGAATCTCACCTCACAGCTACAAGGAGTGG
GAGACACCACAGTACACTCCACCAGGTGTGAGCAAGATCGTGAATAGACCTCCCAAGCCTCACCAAGACAAGT
ACAAGCCCAGCTATCCAAGTCCACCACGTCCAGCATCAGACCGTGTGGACGAGACCTC
ATCTCCACAGAACCAGTATC
>103777951_2 DT664051.3
GCACGAGGAATTTCCTTAAATATATTTAGCGTACGGTAGGTTAAAATAATAACAGTTCGTAAATAAGTCGTCG
ACGGACCTTCGCTGATATAGACACATGCTTAGGATAATAACGCGTTCGGTCTCTCGCAAGGCGATTGTGAGTT
CCATTCCGGGCTTTTACAGTTTACAGCGTTTAGGCTCAAAACACACGGACGACGCTCA
AATACAATAATACGTTTTTACCCTTAGTTGAGAGTTGGACCGCTTTGTGTACAGCTTCGAATTAGTTCGACCC
ATTTTAGTTTCGGTTTGAGCGCGGGATAGTGCAGGACAGCGCGTGACAGTGCGGACAAGCGCGTGACAGTGCG
GAATAGCGCGTGACAGCGCGGAAAAGCGTGTGACAGTGCGGAAAAGCGCGTAAAAATG
TGTGACAGCGCGGACAAACGCGGAATACCGGGGACAGGCGGAACACGACCGCAGTGTGTGTGATCCGGAGGAA
ATTTAGGTTCGGCAGGGATTTTATTATTAGACGTGCGTTACGCACCATTTGTAACGAATTATGTTCGGAGACG
CAACGGAGTAGGTATCCTCACCTTCGTGTACCATACCGTGTGTCGCGTAGTCTAACGA
TATTGGTGATAGATGCCAAATAATGCTATAAAGAACTGATTTTATATAAATA
>103777953_2 DT663893.3
GCACGAGGGTGCGCACTGCGCAACCCGTCTGTTTAAACTTTGGAGGTTCAAACGAAGGTGTAATACATTCAAA
AGGTTATTCAAATGGCGCCTTTAAATTGTGTTTCTTTTGTGTTTTTTGTTCTGGCACTTCACCAATGCTCGCC
ACGAGGTCTACCAGAAGAAGACTCAGTTGTGAGTGTATATACGACACAGTCCACTACA
GATGGAGTGACGCACGTCGACGTCTACCACCAGATCGCTAACAATATTGCTGAGAGGATCACTTCGCCAATAT
ATAAATTCCTAGGCCTCAATAAAAATAAAACGGAAACAACAACAAAACGTTCATGGGACAAAATCGAACTGTT
AGACGAACCTGAAGAATCCACCAAAGTCATAGCACCAATCAATAATGATATATCTAAT
AAAGACGTAGAAGAACTATCTGCTGAGGCTCTAAAAAAGATAGATAAGAAACCAGAAAAGATAACACTATACT
CAAGCTATTTACCCTCAGAAAAGATAGAAGTGAACGAGACTATTAACGAAGTTGATAATGAAACGTTTGGCTT
AGATGATGTTGATGATACTGATGATAATAATGATGATATAGATAATAGTAAACCTGAA
AGTAAAATAGGTCCTCTAGTAATAGTACTGGAGGCGATCGGTAGTTTCATACAATTAATCTACGGTGGATAAG
TAGGGATTTTTTCATCTTATAAATCTAGTCCAAATTAAGCTTAACTAAAAGAAAAAAAAGAAATTTGTATTAT
TTTATAATAAATGAAATATATATTAAAGATTCCGGAATAATGAAATGTTTGCTATATA
TTTTTAACT
>103777971_2 DV501249.2
ATTGTATGGAAAGGATTTAGTATCAGACCTAAAAAGTGAAACCAGTGTTTTATTCGAAGATCTCCTAGTGGCT
CTCATGACGCCGCTACCCCAGTTCTATGCGAAGGAACTCCATGACGCCACAGCCGGTATTGGAACGGATGAAG
ATGTGTCCATCGAGGTCATGTGCACCATGTCCAACCACGAGATTAATGTTATCAAGCA
AGCGTATACTGCTATGTACGGAACTCTTCTTGAGGATGACTTGCACGGTGACACCTCAGGCAACTTTAAGAGA
CTGATGACATCACTGTGTATGGGTAACCGGGTCTGAAGAATTCCACGTGGACCAGGAGAGGGCTAGAGA
>103777985_2 DT664300.3

CAAAAAACTGGCATACCTAACGATGTAGTAGACTACATAGTGTATGGCACTGTTATTCAAGAGGTGAAGACAT
CAAATATTGGGCGTGAGGCTGCTCTAGCAGCAGGCTTCAGTGATAAGACACCTGCTCATACTGTGACCATGGC
CTGTATATCTTCAAATCAGGCTATTACTACAGGTGTGGGGATGATCGCCGCGGGTGCG
TACGACGTGATCGTGGCTGGCGGTGCTGAGTTCATGTCCGATGTCCCGATACGTCATTCTAGGAAAATGAGAT
CTCTATTGCTGAGACTCAACCGTGCTAAGACGCCGGCGCAGAGAATGTCCTTGTTGGCCTCTATTCGACCTGA
TTATTTTGCACCTGAGCTCCCAGCTGTAGCAGAGTTCTCATCTGGTGAAACAATGGGT
CACAGTGCGGACCGCTTAGCTGCGGCGTTCGGGGCGTCCCGCGCGGAGCAGGACGAGTACGCGCTGAGGTCGC
ACAAGCTGGCCGCGGAGGCGCAGAGCAAGGGCTACTTCACCGACCTAATACCTGTCAAGGTGGAAGGCAAAGA
TGGCTTGGTGGAGAAGGACAACGGCATCCGAGTGTCCGCCCCGGAGCAGCTCGCTAAA
CTGAAACCAGCTTTCATTAAACCCCATGGCACTGTTACTGCAGCTAATGCTTCGTTCTTGACAGATGGTGCAT
CAGCCTGTCTCATCATGTCGGAGGCGAAGGCCAAAGAGCTCGGTCTCAAGCCCAAAGCTTATCTACGAGACTT
CACTTATGTTGCTCAGGACCCCGTGGACCAGCTGCTTCTC
>103777987_2 DT664866.3
GCACGAGGAAGCAATCATAGAGGAGAAATTGAGATCAGCTCTATTAGAATTAGAAAAGGATTCTCTGGATGTT
AGAAGTGAAAATGTGTCCTTGCGAGGACAATTAGCATCAGCTGAACGTCGAGTCAGGGCTTTGGAAGACAGAC
TGACATCTATGGAAAATATTTCTTTATCAAACCAAAGGAAGCTTAGTGAAATGACAGA
ATACACATCGTCATTAAAACGCGAGCTACAAGAGAAAGAACTGCTTCTAAGCCAGAACAAGCTCGACAAAGAA
AAACAAAAGAAAATTTTAGAACGTAAATACAACCATAAAATTGCTTTTTAACACGAAAAAGCTAAAGAAATAA
ACACCGAGGAGGAACGACTTCAAAATGCGATAGAGGAAAAGGAAAATCGATTGCGCAT
TATAGCGGAGGCACTGAGCGGCGATCGGGGTGACTTGGATTTACCAAAGAGTACAGGAGATATGGCAGCTCAG
ACAACCATACGAGATTTTACACCTGGATCTACACCTAGGGCTTTACCAGCTCATCTTCTAACTCCTTT
>103777989_2 DV501238.2
GCACGAGGATTTCCCCCTTACGAAGCTAATTGTAACGTTTGAATGTAAGAATTGTATATATATGCATTAAACC
GGACATTAACACATCCTTCTTTAAATATAAGATGAAGAATAATATGATGCATCGTTTCTTTATACCAATCGTG
GGGCACGGGCACTTAGTGTTATGGGTTCCATCCATTTTCTGGGAAATTAAATATTTAT
TTAAATCTGTATTTTCATTTCTTCGCTCTGATGTTAAATCAAGGCAAATCTCGTCGATGAGTTCCGCCACTCA
ACACGCAATGCCTTCAGAATTCCTCTACATATCAAGTTCCCTCTGTCCAGCCTTCTGTGTGCCGAATACAACG
TGAAGCTGATTGAGTGATTTCATTAATAATATATATTACTAAAGACAAACTATATTTT
GTCACCTTAAGTCACTCACAGCACATACCACTTCTCGCTTTGTTTACTTACCAATTACCAACAAATTAAGTTG
ATTGCAAATTTGTAATAGTCATTTAACTAATTTTTAGTAAAAATTTTGTATGTACTGTCCCGATACCAATACT
GCACTTTACCAGAAGACTCTTTCGTTTGGAGGTCAATGAAAAATAGTATAAATAATGT
TTATTATCAAACTGTATGTAGTACTATATTTTCGATTTTTTTGTAAGATTTATCAATTATTATAGACATATG
>103777995_2 DT664228.3
TTTTACCAACATTTTAATATATATTTTTATTCAAATGAATTCAATCATACATTAATTTTTACGGAATTCTTGA
ATTTAATGTACCATCTTGTAAATTATAAATAATTACAAAATAATAAACCTTACTGTGTTCTTACATACATATA
TAAAAAGTTAATGCTCTCCTTGCACTCTCGACGCCGTATAATTTCTATCCATTTAATG
TTTTGAGCAAACCTCTTTTATTTTTTCATCGAACAAAATCAATATTGTGCTCTTTGCCGCTATCATAGAAGCT
ATATGGATCTTTAAACTTTTTTATGCACCATCCAATGTTCACTTCGTCAATAGTAACAGTGATTTTTATTTAT
AGTTTGCTAAAACGTTTCGGCCTGTCTAGAACTATTCATCAATTCTTTTCTAAAACAT
ACTTGGACACTAGTAAAATAATTTCAAATTTGTTTAGATCAAGGAGAGGAGCTCCAGCAATATAGGTGGACAT
TACTGCATATTTAAGGGAACTTTTAACCAATATTTAGGTCGTAAGCCCCAGGCAATTCTTTAAGCTCCTATAT
CTAAAATTTGATAGGCATTCGTATGGTAAAACCAAGGAATGCAAAATAGTATCTTTTG
CACCATTCAAAGTATACATTGTTAATGATGGGTACTAATC
>103778001_2 DT663829.3
ACATTTATCTCCACTTAAGGTTCACGTTTCGTTCTCACTTGGTGGTGCAACACAGTTGCCGACATTTGTAGGC
ACAGTGCTACAGAGTATCGGTGTTACATTGACTGACATGAATGACGTCGTTTTTAAGTTGTCGTATTATGAAA
GAAACTACGAGTTCCTATCGCAAAAGGAATTGATAAGTCAAGTACAAAGCCACTACAC
AGGACAAGCACTCAAACAGTTGTATGTATTAGTTTTGGGACTTGACGTCATTGGAAACCCGTATGGACTCGTC
ATTGGCCTCAAAAAAGGAGTGGAGGATCTGTTCCATGAACCTTTCCAGGGCGCAATCCAAGGGCCGGGCGAGT
TCGCGGAGGGGCTGATGCTGGGCGTGCGGTCGCTGGTGGGCCACACGGTGGGCGGCGC
GGCCGGCGCCGTGTCGCGCATCACCGGCGCCATGGTCCACGGCCTGGCCGCCCTCTCCTTGGATAAGGATTAT
CAGAGACGTCGT
>103778019_2 DT664291.3

GCACGAGGCTTAACACAATAGTGACTGGAAACATTTTTGTTTATAGTTTAGAGCAATATAATCCTTTGGTTGT
TAGGTAACACACTAAATTAATTAATACTTAATACAATAAAAAATCTTAATAATAAAGTAGAAAAATACATTTT
TTACATAAAATTTTATTTCTTATTATTATTAATTGTTATAAAAAATTGTCATATAAAT
AACTTTTGATAATAATCTTAGTTTTTATTATTATATATGCATTTTTTTTTATTTAGCGGAGGAAATTTATTGGT
ATTTCATTTTAAATTAAATTCTTCAATAATGTATAATAACATTGTATTTTAAAGCACAAAGAATGACTTTCAA
TAGGCCAGAAAAACAAATTTATGCTATAAATTATAAATGTTCTACCAATTTCATTGAA
ATGTTTACTAATATATTTATAAAGTAGTCAAATCGAGATTTTTATTTATAATATAACTCCAATATTATTCAAT
TATATTTGAATTGTAATATAAGTACACTCACTGGAAAATACTAAAATTAGTAAAAAAATCTAAGATATGTAGT
AGAGTCTTGGTTTATAATTGTTACAATTTATTTATAAAGAAGTCTTGTTTTTGTGCCT
CTAACAACAGTATATTCTATACACTAGGGTCTTAGCCGCCCCGAAGCTAGGTTTTCGTAACTTTTGTCCATTG
TTTACTTAGAGAGATAATAATAAATAAAAAAGAAGACAGAACCTTTAGAAACAAATCTGCGCAGCCACCTAAA
ACTATTTCTAGAGCGGCCACCAATTGTCGATCGTAATGTACGACGTTGATTATCAAAC
GTCCGATAATTAAATACAACTGCCATTT
>103778021_2 DT664173.3
GACGAGGATGAATCTTTAGTATATCAGACGATTTATTTAAGTTTATACTGGAGCATGAATCCAAGCATCATCA
TCAAGCAACTAATACTGTCACGGACGTCTGTCTATGCAACTCATTTTTTATCCTACTACGCGATTCAAGGGGA
GTACTATTAATAGGTGTTAAAATATAATTGTTCGGTTCCTTTCATAAATATTTTTTTC
AAATCCCCAAGATATTAACTCCCTAAAGGAAATACTAAATTTTCCGTCTTAGACTTACCTAATAACGAAGCTA
ATACGTTCTAACGAAAAAGTATAAATAAAAAAAAAAACCTCAACTACCCACTCAAAACTAAAAAGTAAAAACAT
TATCTATTTTTTACTAGAATTAACAATCGGGACCTATTCTTTAATCAATGAAATCTTC
ACAAGAAGTCCTGTGAAATTTAAATAGTCCCGACTGTTAATCCTAATTAACTATTACACTTGTTGCTTACTTT
TTAGCTTTATGCAGTACTTTTTTTTATAGTAACTTTTTTAATATCTGAAATTTATATATTTTTTACATCATGA
ATGTGTCTTTTCGTCACTTATTTTGAAATTCTGTCATTAAATTGTTTAATTGGTCGTG
TTTTATTCATTTTCTATTAGTTATAAAAATTATACTTGATTCTATTTGAGTATTCTAAATTTTTTGTTTATTT
CAGAGTATCAGTTGTAACATTGACCACAGACCGTATTGCGGCGATGAATAGATTGCCCCACATTCTATTATGT
AAACACTTACTCTCGCTTACGCTAATAGTATTGATATTGAAATCAGGAACTTGCTGCA
GCGTGGCTTATTTTTTGTATTTTTTGTA
>103778030_2 DT664269.3
GCACGAGGATTCACCATAGCACCAGATAAATTTTCTTTTAGCAGAATAAAGTTTTTTAAGTATGTTGCCACGG
CAATATCTAACTGAGCAAGATATAAGAAGACGGCAGATCGACACATTAAAAATTTTCAACGACAATGTCACAG
AAATAAAAGAGAACACAGAATACAGAGTCGACTTCTGTACTGAAGGTCGAAATTTGAG
CTTAAACGTGGTATTACCACCAGACTTTCCTAATGAAAAGCCTAACATATTTATTAATCCTGTTTTCCCACAC
CCATGGCTTGCTGAAAATTCTAACCAAGTTATCGGAGCTCCGGGCTTAGTGAATTACACTCCTCATGCGGATT
TAGGTCGTATAGTACAGGCTATTATACGTGAATTTCAGAGAGCTGTGCCTAATTTATT
GTGTCAAGAAGATAAATCGACAGATACAAGCCCTCAGTCTCAATATAGTGCTCAATCTTTAATGTTTCCTGAG
TTAGGTGAATTAACTATTGAAGAATTACAGGAAATAATGGAAAATCCAGATTTGCAGGACAAACTCTTAGAAA
CAAACCCACAACTAATAGAACTTGACCTAGAAACGGAAGAACTAATGACAAACATAGA
AAAAATAGCCCAAGACAACATATTAAAACAGCAAATGCTGGACAATCTAAAAACAGACGTCTTAGACAAAATA
TCTACTATAGTACAAATGAAAATGGCGTACGAAGAATTGAATAGAAAACACCAGAAGTTGTCGGAGATGTACG
ACCCGCACAGGATCAAAGACTGTTTGAGA
>103778035_2 DT663950.3
GCACGAGGCGCACCCTCACGCCGCGAGATGACTCGTTCAATATTCGCTCTCCTAGCGCTCGCCGCCCTCGCAG
TCGCCACTGCCGATCCGCCGCGACCTCGGCTCATAGACTTCAATCCTTGGTCCTGGCTACCCAACAATATTAA
TAAATCCCCATCTGTGATGGATTACCTGTTTCCTCCGTCTAGAACCCCTAAGCAGTCC
ATCCTAGATCCACCCGCAGATAGACGTCCCCCGCCTACCGTTCAGACCACGATACAAGACATCCCCACAATGA
CAACCCTTCAAGAGCGCGCTACGACGCTAGGCAGAAAATCTCATAACAAAGTCAAAACAACTGCTTCGTCAAT
TCGTCCGACTGCGTCCACACCAACAAAAACGAAAAAGAATTCCCATAAAAACGTCGCT
ACTGATACGACGGTCGACACCAAAACGACGCAGACATTGAAAACCGAGACGGTGCCGACTACGAACTTTGTAA
CTGCTACGGTGAAGCCGCCCAAACGCTATACCATACGACCGGTGCGGACAACGGCGACCCCCGCGGCCCGTGA
GACAGAATCTACTATTAACACGAATAGTGATGTTACAGAAACTGTATCAGTTAGCGAC
GTATCAGGGACTACGGCTGATGTGGCTAAGGACACAGCCTCAACACAGAGTACTGTCGTTGAGACGCACTCCA
CCGACGGCACGACCAGCACCGGCGTGGAGTCGACGACGCTAACAGAGAGCACTGTAACGG
>103778051_2 DT664838.3

GCACGAGGAATCCGCTAGTAAAATATGTGCAATTGTAATTAAATAAATAATATCTTTTACGTGTCTTAAGACT
TCAGCAAGTTCATTATTATGTTTTGTAGAAAGTTCATCAAATATTAGTTACATAAGTCTTAAATATTTATTGA
TTTATTATTATGGTGTTGTAAATAGTGAAATTTAATAAGACTTAAAAAATTATAAAAA
TGAAATATTTGTGCTTGCTTTTATTTAGTCTCATAGGAGTGTTAGGTATAGAACATGACTTCAAAGGATTTGA
CGATTCTATATTATTTGGTATTGAGTGGCCAGGACCTTTAGATAATTTACAAATATCGGACGAGGACTGGATT
TCCAAGAACAAAGATCTAATAAGGGTGACTACTTCTGATAAAGAGAAGTATGAATGTC
AGATACCAGAATTACATGTAAAAGAAATGCAAAACAGCGACAAATATGAGGGGCCAACACCAATAAACCTATT
AAAGCCATTGTTCTCACAGAAAATATGTTCGTACAGACTTGAGAGTTATTGGAGCTATGAAGTCTGTCATGGG
AGATACGTTAGACAATATCATGAAGAAAGAGAAGGGAAACAAGTGAAAACACAAGAGT
TTTTCTTGGGACATTGGAATGCAGAAAAGCAATTAAAGCTTGAGGCAGATTTGAAGGCAGCATCAGAAGCTTT
GGTCAAACCTAAGATGACTAAAGTGGAGGGAATGTCATTGCCATATATAGAAATGGCTATGAATGATGGTACA
ATATGTGATTTAAGTGGAAAACCTCGTCTCACAAGAGTGGTGTATGTGT
>103778055_2 DT664718.3
GCACGAGGGTTAACTTAAAATTTTTTTAACATTAAATATAAAAATTGCATATGATCATGTGATAGCAATGATG
ACTTTTTGAATTAAACATATATTTTTTGATGTTTGAAGCCAGTATTTTAACTAGATTTTTTTTTTATTATGCA
TTCTTCTATTGTGTAACTACTAAGTCATTGTACACTAGCTCAACTGCACCTCCTTTTG
ATATGCTTTTTCCTACTTTTTATTGGCCATTAACTACGAACTGAACTGAATTATTTTTTTTAATATATAAACT
TAATTGTCTATATTTTTGTCCTTAGAATTATATCTTTTTTATTCCAATGACATCTGTATAGATAATACACAAA
TATATAGATACAAGTTCATTGAGAACGGTGAAATTGTTTTCTAAAATATGAAACTAAT
ATATGTAGGTAGTTTTTATAGTTTTTTTGAACATTACAATATAAACGTTTAGTAATATTTTAGGTCTGTGGCA
TATTTATTATCTTTTAAATTTTTGTCTTCTATCAATTTGACAATGACAATAGGTGTAGTCATTAAGTTGAAGC
TTGCACTTTAAAATGTATTTTTTTTAACAAAAGCTCAAAAAACGTTTGCCGTTCCTTA
TTTAAAATTAATTTGGCAGTAAATTATAAATAAAACATTTACCAATACGCTACAATGACTTACGGTGATGTTC
ATAGGTTCTTTTTGCGAACTATAGCAAATCGTGTAGTGCTAAC
>103778073_2 DT664513.3
GCACGAGGATTCCTTCTTCAGTTAAAATTTTAATTTGTAAAGGAATATATATATATATATATATATATATATA
TATATATATAAACGTTTATTCTTTAATATAATAATTGTTAAAATAAATAAGAATGAAGGAAAAGCGTGAAAAA
TTAATTTGATTATAAATTAATCTATTTGTAATGTGCAAGCTAAGTGTACTCTCGATGT
GAGGAAACAATAATTTAGTGATTTATATATAATATTGCTTTTGGCACTTTACGTACAGTAACATTTACAAATA
TGGCAAATAGAAAACCAATGTGGAACAATTATAGTGGATATCAAACATTTTACCCTGATAGGCTGTCTTATTA
TGGTGAAGATGCTTATTACGAGTATTACTATCAAGACCAACCCACGGTGTCTTATAAT
AATTATTATCCTCCCAATGGATATTTTCAACAAAATACTAATAACAAAGTACCAAAAGGGCCAATGCAAAACA
AACCAAAAGAAAGTGTTGTAGATAAAAGTAGCCTATATAGGAAACCTGCTGTCACAACTCCAGTCCATAATAC
TTGCAATTTTAAGAAACCTATACACCAAAATCATCTTAAATGGACGGACCAATATTGG
CAAAAGCAAGATGAAGATCTTGAATCCACAACTAAACAAGGAAATATTCAGCANAATGTTTCAAAGCCAGCAA
ATAAAATAGAAAAAACATTAAATGAGATGCTTGCGCAGCCTGTACATATGAGTAAAGGTAAAAGTATGTTAGA
AAAATGGGATGGACAGGCGGCGGCCTCGGTAAAGATGGCGATGGTATAATCGAACCTA
>103778085_2 DT663480.3
GCACGAGGTTTTCTTTTAAATTTTGTGTTATTACGTTTTATTATTGAAATCAGAATAATTTACATTGTAAAAA
ATACCCATACTCATCCCACTAGGGTTAACAAGTTTGGATCTATGTAACAATTTTATTTTATACTTTTTATTCA
GAGAAATAATTTACAAATTTCAAATGAAGACCTTCTTTGTTATTTCAGCCTTATCAAT
ACTGACATTTACTTTTAGGTCGCAATCACAAATACGATTTTTTAAAGTAGAGCAAATATTTTTTTCTTATTTA
ATTGAAACACATTGATTGATGATTATAATAATTTTTATTAAAAAACATTCTTTATATTATATAATATAAAAAT
TATTAAGCTTTCATGCTATTTTTTAATTTGAAATTGATGGCAACTTACTTAAAATATTT
GGGATGGACTTTTTGCAGTGATATCAAAATTAAGCTTAGTTCTTTGGCATAAATAAGCATATTAACTCATATA
CAGTGATTTTTCTGATACACAATAGGTAAATTAAAAAAGATACGTACTTAGAGAATCACTTATATTAAATATA
TCCCTATAACATTTAAATTATATTATAATTAATGTACTGAAATTGAAAGCAAAATGCT
ATCTCACACTTTTACTGTAAATCTTACTCTACATACAGACTTAACTATGTTTTTACACTTAGGAGTATTTAAA
AATAAAAGCTGCA
>103778109_2 DT663815.3
GCACGAGGAGAAATGCATGTCGCAAACGGAGTGTCGTTGGTTCTATTGAAGAAATTCTGAAAGCAGCTAGAGA
AAATAAAGAAAATGAGATTTTTATAAGCAATGAAATTGACGATGGATTAATCGGGCCAATGTCACCATCGCCT
ATAGAAATGGTGGCATTAAAAAATGACAACACAATAACACCCGAAACGAAGGATGTTT

TTACTGTCTACGCTGTATCTGTAAATCCCACTAAGGAAGAAAAAACAGATGTATCGAGTACACTAAAAAATGA
AGTTATTGAAAGTATTTCAACTCACCCAATAGATGATAAGAAACAAAACACAGAAACTGACATGTCCAACGAA
TTAGATGTAACCACTAAATCTTTTAAAGTTTACGAAGTGTTCCCAACAACTAAATTGG
AGGAAGAATTTAGTCCTTTGACTGTTGATAACAATTCCAATGATTTCACAGTAAAAAATGTGAAAACCGAAAC
TACAACTGAAGCTAAATCTGAAACAACGCCATCTGTTCCATTTGTACCGAATCGACCGAAGCCAACGTATGCA
CCAGAAGTTATTACATACCCTCCATTAACGAATGGCGGTATATTCGACTTTATTAAAA
GACTGGTTGAATTCAAATACAGATTGGGCTTGAGCATCTTGCAAAGTACATCGGAATCTTTAAGTAGATATTT
ACGTAATTTAGATGATAAATTGGCGAACTAATTATAGAAAATTTGATACTCACATTTTAATATTCGTAATTAT
TAAGGAAATATTTAGCTAAGGTTTGATTTTTTTTTCCAAATAGAGAAAAATATAAATA
TC
>103778111_2 DV501222.2
GCACGAGGGTTTGTTTTAAAGGCGGTGAGAGATGCTGTGTTGCCGAAGGGGAAGGGCGCAATCCTCCATTCAT
TCATGATGGCGACAGATTCTCAAGGAGCAGCTCAGGACAATGTCTCCGTGATACAAGCGCTGGAACTAGCGAC
GGAGAGAGTGGCCAGAGAGAAGAGATTCAAAGGGGTATTTACTACTAATACCAGTCCT
TTGACGCAGCAACTAGGCACAGATGTTCTCGGATTCCAAACGTTACTCGACTATCAAATAGACCAATATGTCG
ATCCTAATGGCGAAAGGACTTTC
>103778115_2 DT664231.3
GCACGAGGCGTAAACTTTAATTTTTATTTTATCTAATTATCCATACAATATTTAAAATACACTCAAAATGTGG
CATGAGGCGAGGAGACTCGAAAAAATGATTCGTGGAATGATAGTGGATTATCGTAGAAGAGCTGAACGTCGTA
AGGACTTCTATGAAAAGATTAAAGCTGACCCGACCCAGTTTCTGCAGCTTCACGGTAG
AGTGTGTAAAATACACCTGGATCCAGCTGTAGCAGCTGCCGGCGAAGGTCCAGCTATAATGATGCCGTGGCAG
GGTGACACCAACAACATGATAGACAGGTTCGATGTTCGTGCTCTTCTGGATATAATTCCTGATATAAAAACAC
CCGAAATACCACCAGAAGAATTATCTCAGGAAGAGAGACAATGCAATTATGAAAGATA
CAGAATTTTGGCTCAAAATGCTTTTCTAGGAATAAGTGAAGACAAATTTCTTCAACAACTTGCTATCGAAGAA
CAATTTGGAGTTACTGTTGAAGAAAAAGAAACACAAAGGGAGAAATTGAGTGAAAAAAGAGGGACAGGGGCTG
CCATAGGATATAATTATAATGAACCTAATCAACTGTCTACTTCTAATGCTGCTCCAGA
AGTAAAGAAACCTGAAGCTAAGGATTCTGATGATGACTCTGATGTTGACATGATTGATGTGGATTTAAGCATT
GACGTTAATAAGATGGAGGCTTCACAGACACATGAGTTAAATGCAGTTGGCCCTCAATTTG
>103778139_2 DT664181.3
GCACGAGGCTTGTACTAAGTACACCGACAGCCATACGACGGACTAAATATGGCCAAAAACAGTGAGATCCTGT
TGCGCGCCCGCGCCCTTATATCGACTAGGTTGTGTAGGTATTTTGAAAATGTAATGTAAAATAAGTGTTTGTA
GTCGTTTTTGGTGAGATGTATATTAAAATCATAATCAAATTTTTGCCCTCAAATTATA
TCTCCATCCACATTCGAACAATAAGCGGTGATGTGTACATTTTCCGTGACGACGCGTCCAGGCGTAGTGTAGC
TCACCTAATGTATATTCCATCCTCTTAGAGTAATCGTAAGTACATATATAGATAGCACTGTGAGAACACTCAG
CGTCGTAGCCCGCCACTTGCCTCCATTCCTGTACACTATGTTGTATATTAATATTAAT
AATGTAAGGTTATATTAAAATATTAAATTTATCAAATATTCAGCCGTTTTTGACTATGCACTCAGTGTCAGAT
CCAGCCCATAGCAAGCTTGCTAGACTGTCGTACTTAAACATTCCATTGCATACATTTATCGTTCTACTGAACC
TAATTAAGTTTTAAGTTACATTTTTTATAGACAGGATATGTTTTTCTCAAATTTTTGC
ATTTTTTCTTAAATGAATTTGATGCGTAGGTCAGTGTGTATGTGAGAGGAGTTGTCGCTACAATCGATATTAA
CTTCGAAAGTCGATAAATGAATTTATCGATTTTAAAATTACAAAAAAAATAATTGTAACTTACATATTATATT
AATTTGACTATTGGATGATAACGCATACAAATTAAATCGAT
>103778141_2 DT663579.3
CCCCTAAAAGGCCGGGCTGCAGGAATTCGGCACGAGGTTTAATTCTTTCAAAATATAATTTTGTTTGCTTTTC
AAACATAAATTGCTATGTAATTATTACTTGATGTAAGACTAACCTATTACAACCTTGCTCCTTGAACTTGAAA
AATTCGTCGTAAATAATGTCTGATAAACCAGTGTTAGCGAGTGGCAATCAGCCCATTG
TCAAGATCGGACACTATACACTAGGAGCTACTTTAGGTGTTGGTACATTTGGTAAAGTAAAGATCGGAGAACA
TCAACTCACAAGACACAAGGTCGCAGTGAAGATCCTTAACCGTCAAAAGATCAAATCGTTAGATGTAGTCTGT
TTTTTTAGACGTGAAATTCAGAACCTTAAGCTTTTTAGGCACCCTCATATTATTAAAT
TATATCAGGTGATATCAACACCAACAGATATATTTATGATTATGGAATATGTCTCTGGTGGGGAGCTCTTTGA
TTATATAGTGAAACGTGGCAAACTACAAGAACATGAAGCCAGGCGCTTCTTCCAACAAATAATATCAGGAGTA
GATTACTGTCATAGACACATGATAGTCCATAGAGACCTGAAACCAGAAAATTTATTGC
TAGATCATAATATGCATGTTAAAATAGCTGACTTTGGTTTATCCAATATGATGATGGATGGTGAATTTCTGAG
GACTTCTTGTGGATCACCTAACTATGCAGCTCCAGAGGTCATATCTGGTAAACTATATGCTGGGCCAGAGGTT
GATGTGTGGTCTTGCGGTGTCATTCTATACGCACTACTTTGTGGCACATTACCATTTG

ACA
>103778165_2 DT663635.3
TTTTTCAATATAAAATTCTCCATTTTTTTAGCTGAAGAAAAGCTCTTAAATATTGTAAATATATATATCACAA
GAGTGTCTATAATTGTTAAATATACTTTTTCTTTTTTTTTGCACAGAAATTAAGTTATCAGTTTGAACTTGTA
TCAAACGATAAAATATTAAATATAATAAAGTATCCTTAATACTACAGTATATAATACC
TACAGTTATCTCCATTCCATCTCTTTTTTTTGACAAAAAAGGAGATAATAGTTTAATTCGTATTAGCAATGAA
TGCCATTTGTATAGCTTGAGTTAATTAAGCTATAAGTCCACATTCACTACGACCATACTTTATAAAGTAACCA
TATTTTTATATATTTTTATAAATTTTTCATATTTTATATAATTTTCTCGCATATA
>103778169_2 DT663578.3
GCCCTGCAACACCAACCCGCTGTGCATATGTCGCAACGATCACTTCGCATGCGATTCTGTGTCCTTTCATCGG
TTTCCCGATACCGAGGAGAGCGTATTGCACGTGGCAGTGTCGGCGGCGCGGCTGGGCGCGCTGGGCGAGGCGG
CGCTCGACGCGCGGCCCTTGCGGACGCTCGTGCTGGTCGCCTCGCATCTACACTATAT
CGAGCCTACTTCCTTTCTTCTTGCCTCTTTGGACTTAGGTTATAATGAGTTTACAACGATACCTATAGAAGCA
CTGCGACCGTTGAAAGTACTTTTTTGGCTAAATTTACAAAAATATAGTTATTGCGACGCGTCACTTGGAGGCA
CTAATAGTTCTCATACGTAAAGAGTCACTAGACACACAATTTTCATCTCTACGCGCAT
CTAAACTCCGTTCCTTAGAAGTCCGTGGCGTAGCGATGCGGTATGTGGCCCCTCACGCCTTCGCTGCGTTAGG
AAGACAACGTTCACTGTCACTGCGACTGACGAACTCTGGAATTGTGGAGTTACCCGATGGTCTCGTGTTGCCA
TTGTTTCGAGTCCCACATCTCGCCCTCGATCTCACAGCTAATAAAATTGTGAATTTCG
GGCCGACAGTTTTATATCCAAATTCAACAGGATGGCACCGATTGGCCACAAAGCTATTGCCAGGAGGTCTTGT
CCTTGCCGGAACCCACTTCG
>103778171_2 DT663504.3
GCACGAGGTATTCCTTTTATTTATTTATTAGCTCCACTCTACCATGCATATAGCTGGGTAAGGAGAAGGAAGC
TGGGAGTCTAATGTTAAGGCACTTTGTTCCCATTAAGAGAAATAATATAGAAAGAAATTCTGAAGGCATTGAG
TGGCGGAACTCAACGTCGCACTTTGTCCACACCAGATCAAAAAAAAATAAAATATTAAA
TATATTTGTTTATATTTATTTATTTCGCACAGCTTAGTATATGATATGGACAACTAATTTCACTTTCACGTCC
ATTCGGTCAGTGTGTCCATCCGTTTATTCGTCTTATATAGACAGTGTAATAACTCATAAATGATCCTATTTCA
ACACTGCTAAAGCTCAAATTCAGCATGACCAGTTTTGAGGACGCCCTCGCTTCAGCCA
CAGTGAGCTGAAAGGCTGTGGCTGGCTGATTTGTGTAATCGAGTAAGGAGATACGCTCGAGTAGAGTAACGAG
TTGGTTGGGTTTTTTTGTTGCCATTTTTCTACTATAATTTTTTTTTAAACTTCAGAAGATGTTTTAAGCGTAG
AATCCAATATTGTTTCCGTGGAGTATAAATACGTAAATTACAGCTTTTTCGGAAAATC
ACTTATATATTACCCAGATGTGTTTTGTCAGTCAGTCACTTTATAACAATACATAATAGTATTTTTTTTCAAG
AATCAAGTCAGCTTCACGCTGGATCCCGCACACAGCAGGGTAGGC
>103778212_2 DT664713.3
TAAATTAAAATATATATTATAAAATCTCCATTGATATGTAATAATATTTGATTTTTATTATATAAAAGAATGA
AATGAAACTAAAACATGGTTATTACGACATTTTCGTTCATACCTGTGCAAAATATTCTTCCCAAATTGCGGTT
CGACATTATAACGATGGAACGTACAAAAACTACTCTTATTCTGAGCTATACGGGGTGT
GTGAGTACATATCACAAAATTTACAACAGCTAACATGTAAAAAAGGTATTTTTTGATTGGTTTCCAACCGGAA
TATCTTAATTCCTTGTGTTATTGCTGCAGTACATAAATGTAGCCCAACATTCATGTTTCTAGATCCAACTCAA
CACATAGAAAAAATAGCTAATGACGTCAAATTCACTGCGCTAATTGCAATCAGAGAGT
CAGAAAACAATACTTCGTTAGAATTTTTTGATAAAAAACCTGATAAATCTGTGACAATTTTCAATTTAATAAT
TGATTTGTATGCCTTAAACACAAATTACAATCATAACAATTATGTGCCTGATCATTCTTACATAGCAATGACA
TCTGGTAGCACTGGAGAGCCAAAACATATTAAAGTCCCTGTACAATGCATGCAACCAA
ATATTAGAAGATTTGACTAAAATATTTGGAATTACACCAAATG
>103778214_2 DT663960.3
GCACGAGGGTTGACCTCATAAAGATGGCGAAAGAAGATTTATTCAAGGGAGTGCTTTACGTAGTGAATGTTAT
TTACGCGGTGTTTGGTCTCGTCACCGCCGCCACTGGAATATGGTTCTTCGTGCAATTATCCGAGTTTGTTTCG
CTTAGAAACAGCAACCATTATTTGTTGGACTACAGAGTGTACTGGCCGCAAGTGGTAC
CATGGCTCTTTATATTATTGGGATTATTCGTTATGATTGTCGCCTTCTGTGGATGGTGCGGCACGAACAAGGA
AAGCAGAGGATTATTGGGAATGTACGGGATTTTCATGATTTTGATTGTCTTGGGACAAGCGATAGCGGCTACA
TTGATCTTTGTTTTCGTTGATGGTGAACATACTGATAAGTTTATCAAAGATACAGTTT
ATGATGGATATTACAATTCACAGTCTAACCCTGACGTCTTCAAGGCATTTGGAAGGATTGAAAGGAAATTGAG
ATGTTGTGGTGCCAATGATGCTCGTGATTATAGAAGCTGGAGAAATGATTTGCCTTTGACTTGTTGTTTGGAC
AGCTATTACCGAGCTACTTGTGAATTCACCGATAAGGAGGCCAATGAACGTTTGGGAT

GTGCTAAAGTAGCAGCTGTTTACACTAAGATTATAAGCTCATCTGTTGCTGGTGCATCGCTACTTATATCTAT
TGTAGAGATTACTGGAGTCATACTTGCTTGGAAACTCTTNCATTCATTACAAGAAGTGGAACATTACATCACA
GAGAGAAAGGAGGGAGAGACCGAGTGCTAATTTGAACTAAAGGTAACGTGAACTAGTG
TTGCCTGATTT
>103778216_2 DV501230.2
GTTTTATAAGTTTCTACAATTTCCTTTAACATTGTCGTTGACACTATATGTCCCATGAGCTTCCATGTACAGA
CACCGATTATTTACAATGTTATTATAACTTTAAAGGTAAAGTCTATATTCGTGTTGGCCTGCTTGTAGCTACG
ACAGATATGGGGGCACAAGTTATTTTGGGACACGTCAATAATAAACGTATTTTGATCG
TTCTGACGTCATCTGTTTTTTATTTCGAAATGTAGTTACTGCCTCTGCTATTTTATAATTATTTTTTTTATTG
ATTTTATTTTTTAATTTTTTTTTTTACAGTAATATTTGTTTGAAATGAAATATTTCAATTGTTCTGTTTATTT
GTATCTTAGCGTTTACTTTAGTAATTTTAGTATTAATATAAGAAATTAATATATTTGT
ATTAAGTATTAAGGTGGTAATCATTTCACATTTCTTGGAATAATATACCTTTGCAAATAACTAGGCTTCTCGT
CGCCCAAAGAATAAACGTAAAAATCTTCTGTCTTTTTATCTTCCTTTATACGTTGTATGGGTTTTCGGAATTC
CCTCAT
>103778239_2 DT663518.3
TTTTTTTTTTTTTGGATTTTATATCAGCTGTTTTGGGTATTTGATTTGTCTTGATCTCAGCTTCTCTAGAGTT
AGCCTTTGGTGTATCTTTGGGGGCAGCATTTTGTTTTACTGGTGCTTGATTTTTTATTTTTTCAGGTTGTATT
TCCACGTGAGCTTGTCTTTTTGCTTCTTGAGGCTTTTGTTTAGGCTGATCCTTCTTTT
CCACAGGCTTATCTGCTTTGGGTACATCTTTTTTCTCTTCTATTTTCAGTTTATTTTGGTTAGGCACGTTTGC
CTTTGGTTTTTTTTTTACTGGCGATTGTTTCACAGAAAATGCTTTCTTTAATAATGACCGCGCTGGTACAGAT
CCAATAAAATTTTGCTTGTGTGGTGCTGCCCAATATAATTTATCATTAAATACTATAG
CATCGCTAATACCATAGAGCTTACTA
>103778254_2 DV501164.2
GCACGAGGAAATTCCTTTATCATCTAAATATGCCCAAACTTTTCCATCTAATTCATAAAATAACATATCCATC
AACCTCATGTTGAGATTGTGACGCATTAATCAGGCCAAATGGCATCACCTTGAAGTGATATAAGCCCCTACCT
GGTACCGCAACATGCAATTTTCACTTATGCTAGCTTCTTCCAGTCCAATTTGCCAAAA
AGCTGACTTTAAATCAATTGTGCTGACAAACTTAGCATTTCGTAAATTGTCCAATATAGAACCTACTCTTGGT
AAAGGATATGTATCTCGTTTAGTAACTTTATTAAGTTGTCTACTATCCAAGCACAGTCTATTTGTGCCATCTT
GCTTTTTTACCAGCACAACATGAGAACACCAGGGACTATGCGAAAGTTCCACTACATC
TTTACATAACATGTCGTCAAGTTCACGTTCTATTTCTTTTCTGATTATTGGCGAAAAATTATATTGTTTTTGA
TAAATGGGCTTACTGTCCCCAGTATCAATTGTGTGTTTTATTATTTTCATCCTACCCAGTCCATTACCTATAT
TGCAACGTATTTCATTTATAAGCTCCATAAGTCTGTGTTGTTGATCCTCATTCAAATT
TTCCTTACTTACAAGGGCAGACTGTTGCTCCAAAATTGCATGTATTGCAAGTTTATTACATATAATTTCCGAT
TTATACTTAACATAAATTCCGAATAGATCAAAAAAATCTTTACCCAAAATTAAAGGCTGATTG
>103778260_2 DT664913.3
TAAAAAAGAAGATTTGAAGCCTCCTATAGTACCATTGACTGGCAATGTTCAAACTTCCCAATCCGTTATCGTA
GTTGATCTGAAAAAACCGGCTGCTGAAGCAAAAATAGAAAAAGTGCCATCCTCAATATTCAGCGGTACTCCAG
CTGCGCCTCCGCCCACAGTTCCCAAGCCACAACAAGGATTCTCAGCTATATCAACACC
GAGTGATACATCAACAATACCAGATAGTTCTACAAAATCTGAACCTACACCTAAAAGTACGACATTCGATCAA
ACGAACGCCACAAGTGTAACTGTGACATTGCCTAAACCAAAAGAATCGGATTCTATAAAAGCAGATGCCCAAC
AGGAGAATTTAAGTGTGAAACCTATTGCAGCATCCACGCCTGCGACTACTAGCGTTGA
ATCGAAACCAGTAGAAAGTCCTGCATCAGGTGCTACTTCAAATTCTTCAGTGTTTTCATCCTCTACAAATCAA
ACTACTCAAGGATTGAATTGGTTTGGAACACAAACATCAAGTTCTCAAAGTATCTTTGCTTCAAGCACTCAAG
CGTCTACTACATCTGTCTTTGGTACTTCGGCAGCTCAGAGTCTATTTAGTGTGGCCGC
GTCTAAATCTTTATTCGGAACTTCTATTACCACGACCCCGAGTACTGTGTTGTCCCAAAATTCTGTTTTCGGT
ACATCGCAGTCTACACCTAGTCAAGTTTTCGGAACGTCCACACAAGGTTCTATATTCGCAACCACTACG
>103778266_2 DT664762.2
GCACGAGGAAGGCTATTTTATTCGCGTGTGCAGTAGCTGCTGTCCAAGCAGGAGGCTTAATCGCTCCTGCTAG
CTACTATGGCTCGTGGAATCCGTATAGCTCCTATCCCGCGCAACCCGCCATTGCAACACAACAATCAAATATT
TTACGTTCGCCATTTAATTTAGGACAGGTATCGACCTATTCGAAGGCTATCGACACTC
CTTTCTCGAGCGTACGTAAAGCAGACGTACGAATTAGTAACCCAGGGTATGCTGTCGCACCTGCATATGGTAG
TTATGCACGTTTGGTGTCACCCGTTGCTGCTCCGTTATCTGCACCTAAGGTTGCAGCTGGTCTCTTAGGTGTG
GCGTATTCTGCTGCCCCTGCCGTATCTCACATGACCTACACTAATGGTCTCGGCTTGG

CGTATGGGTGGTAGACTTCCATAACTATTTGTTATTTGTTATACTCTATTCTTTTAAATGTGTTAAAGATTCC
TCTGTTACCTGATAGTTTTAACATAAGTACATATTTATTTGAATGTTTATATAATAAATTTGTTTAAAATAAA
AAAAAAAAAAAAAAA
>103778276_2 DT663912.3
GCACGAGGGTTTACGGTGATCGTGCTTTTGTGTGGTATTCATAATTCTTTATTATTAATATAATGTCTTTTAT
TCTCAAGACAGCAGTGTGTCTTTCAGTAGCACCTGATAATAAAGAGCACCACCAATTCCCTCGCAGCATGTCA
AATCCAGGCTTAACATCAGAGGCGCAGATGCGATACATTGTTCAATGGTTTGGTGAAT
TCAGTGAATTGCAAAGAGAGGACTTCCTTCCCATATTAGTTGCAGCTCATAGCAGACAATCAGATGAATTAGC
TTCTACACTTGCATCTCTATCATGTGAAGATAAACCAGTCAGTTTGTTTCAATGTCGTATAAAGCTATTCAAT
GAATGGTTTCCAACATGGTCTGAAGAGCAAAAACAAAGACTTGTAAAAGCAGTATCAG
ATATGGATTTGGATTTTGGTTCCAAACTGCAAGATGCACTTATAAATGGTGTTAAAACAAATGGCTTAGATGA
AGAACATTTAAAATTTGACTCTGCTATCATTCCACCAGAGGTAAATGACACAGAGGTAGTCAATAATGCTATT
GAATCATTATCCATCTCACAAGATAATTTATCTGTGGAAATTGCAGCTGCATCCTAAG
TATAGAAATTTTATAAAATAATTTTATATAATTGGATTGGATGAAGAACATTTAAAATAATATGACTGCTATC
TTTCCACCAGAGGTCAATGACACTGATTGTGTCAATAATTATATTGGATCATTGTCGGTCTCACAAGATAATT
TATCTGTGGGAATTGCAGCTGCATCTCAAACATAGATATTTTA
>103778297_2 DT664615.2
GCACGAGGCGCGCGTTTTACTAGTGTACAAAATGCAGAACGACGCCGGTGAATTTGTTGACTTGTACTGCCCG
AGAAAATGCTCGGCCAGTAACCGCCTCATCCACGCTAAGGACCATGCTTCCGTGCAGCTGGTAATAGCAGATG
TGGACCCCGCCACTGGCCGTGCTGCTGACTCCAGCAAAATGTATGTGGTCTGCGGAGC
CATTAGGCGTATGGGAGAGTCCGACGACTGCATTGTTAGACTCACTAAGAAAGATGGAATATTGACTAAGAAC
TACTGATGTAGTGGTGTAAGAAGCTTACTTTATAAGAAATAAATAAAAAAAATTAAAAACTGAAAAAAAAAAA
AAAAAAAA
>103778307_2 DT663545.3
GCACGAGGCCGCTGCCCTCGAGCACTCCTCGGTGGAACTTTGAGAGATTCACAAAACTATAATATATCGTTTT
TAATTATTGTCTATTCTGACTGACTGACCTACTAAGAAAATCTAAATTTAAATTATTAATAAAAAACAATACT
AAATATATCGTAAACGAGTTAGGACGTTTATTTCGAAGTTGTAGACTCCATTTTTCCT
TTTTTTTCAATTTCAATTAAAAAACCTACCATTTACGCAAATTGAAAAATAAAATTGAAAATGTACAGTAAAT
TACAAATGAGTATACTAACAGTATTATTACTGTTAACAGTATTTTTTTTCGTAAATAGTTTCCCCGACGGGGC
GCCGGTGGACGCGTGTGTAAAGGATCGCGCGAACCAGCCCAACCACGGCCAGCACAGG
ACGCAGCCGGCGTCGACCTTGCCATATAGGATACTGGCGTCGTCTTCGTACTATGGACCTAGCTCTATGATAA
CAGTACACGTCGACGGCTTGGAACCCTTCAAAGGTTTCTTCATCCAGGCACGTTCCATAGAGACCAACCAATG
GATAGGTTCGTGGGAGGTGACCCCTAATACCACACTGATACCGGAATGCTCCGCCATC
ACTCACGCTGATCCCAAAGAGAAAATAAGAGGTACATTCGTGTGGAAAGCACCCGAATAGTGAACAAGGACGG
GT
>103778311_2 DT664263.3
GCACGAGGATTAACCATAACTTTAATTGGTAATAACAATTAAATGGCGTCATACAAAGCATGCTTACATGCTA
ACTGCAATTATTTAATAAAATGCGGTCATTATACTTTCGACAAAGTCGTGGACGTTATATACAAAAGGTAGAA
AAATATTTTAAAGTTGTAGCGAGATATTTACAATAATTATATCTCTTTAGGCAAATAA
ACGTTAAGAAAATAGGTACTACAGATTGAAAGGGTTTCGCCGGATAAGCATGGGAAAGCTGCAAAGGCTTCTA
ATGAATTATTTTTACCTCTATTTTTTTTAAACATTTGCACTAATTTTATTTTTTATTTTATTTTGAGACATGT
CCCGCTACTTCGATTTTTTTCTAATTTATAGCTAACTAATATATACATAATATAATTT
TCACTCAAAAAATAAGTCCCAATATAATAATGAGCTTATTAATAGCTACATTGGACTTTAAATTTATAATCTC
CAATAAATTGCGAGAAAATAAACTTTATCGTTAAGGATTGTTAACACTATAACTCCCTTACTGTATGGTGTGT
ACCTTATGTACAGGGTATGTAGAGAAAAATCGTGAGTCGCTATTTCGGATAAAAAGCA
ATACAATCATAGTAAAAATAATCAGCCAGAAGCCACTGCCCCTTTTCCATGCTTATCCGGCTAGATTTTTTAA
TGAGCACCTTTATTTAACAACTAAAAAAAAAATGT
>103778331_2 DT666677.3
GCACGAGGAGAACATGAAGACCCTGGAGCCCGAGGTGGCGGAACTGGAGCTCTTCTTGCAACGGCTGCTGAAG
CACACAATTCGCGCTCGCATCCGCGCCCTCATGCTCAACAAGTACGTGGGCGCCGCCGAAGACATAGTGACGG
CCTACCGCCGGGTCTACCCCAACGACACCGACCGGGAACTGCTCACCCTGGCCAAAAA
CGCCCACACCCTGCAGTTGGTTGATGCAGATGATCCTGAAGAATTTTTCAAGCAAAACATGACAAATATATTG
AATACCAAATTGGAAGATATGTTTGTAAAACTGGAAAAACTATGTAGAAAAGAGGCAACTAATCTAGATGACT
ACATTGATAAACTGCCTGAGCCAAATAAAAACAATGAAAAGAAGACCGAACTGACAGA

AGCACCCAAACCATTAACCATCGAAGAGATGTGTGGAGAAGACTTGAGTTTTTCCATAATAAAAGAGAAAATG
GCCAAAACGAACCACTACACTAAATTCATGGAGGTATTAATCGAAGATCAACTACCCAAAGTCCTGCCCAAGT
TTAAAAATCTTCTACTGTCAATAGTTTTTTGCGACAAAGAATTCATAGGCACCAAAGC
CATGATAACTGCTCAGCTGAAGAAAAAAATAATCAAGACCATAGAAAAAGATGACTCCTATTTGGTTCGCAAT
AAGAAAGTTAATACAAGTAGTGATTCTGAAGCTGCAAAATTGTCTATAGAAAAAAATGATTCAAACAATGAAT
CTATTACGAATAATGCTGAAGTTGAATCTAAAACATCTGAAATGAATGATTCAAACGA
AAACATTGAACTCAATCATGAAGTTATTACT
>103778353_2 DT666539.3
GCACGAGGCTCAACCTTAAATTCTTGATCGTAGTCGCCCTCGTTGCCGCAGCGGTATGCGCTGAAGAGACTAA
GAAGACCGAGAAGCGTGGTCTATCCGGACTGGGCTATGGTGGACTTGGTTACAGTGGAGTGGGTGGTCTTGGA
CTTGGCTATGGTGGTTATGGAGGCTATAGTGGCTACGGAGGATACGGAGGATATGCCG
CCCCAGTAGCTGTCAGCAAGGTGTCATACACCACTGCCCATGGAGGTTACGGCGGATATGGCGGACTTGGCGG
ATACGGCGGCTACGGCGGATACTCCGGCCTTGGTGGTTATGGAGGTTACGGTCATGGTGGTTACGTAAGCGCA
CCATTAGTCAGCGGCCATGGTGGTTACGCCGGATACGGTGGATACGGAGGATACAGCG
GATACGGACTTGGCGGTTATGGCGGATATGGACATGGCGGATACGGCGGACTCGGATACTATGGACATCACTA
AGCTACATCT
>103778363_2 DT665425.3
GCACGAGGCACGACTTCGAGCTATAAGACCTGACGAAGTTCAGACTACAACAAATTTCAAGAAACCAGACGAC
GGCAGACGCAACAACTTTCTCGAATACAAAGCCGGGCAAATCATCACCGTGCTCGGTAGAGAAAATTATACTA
AATCAGCTATGTGGTATGGAGCTCTTCCAGGTGGTGCCTGTGGCATGTTCGATCCGAA
CCAAACGAAACCATATTTAAAGCATGAGAGAAGCTCATTCGCGCCTTCACTGTCACCGCACCCAACTCGCAAC
TCTTCCCGCTCCATACGTACTTCGCTATTGCGCTCTGACGTCAAACGGCACACGTATACGGGGAAACGTACGA
TACAACGGAGCATGATATCTAGTCCACAGGGTGACGTCAAGCATACAGGCCACGTGGG
CCTCGATGGTGCCTATTTTGGGGATTTGTCATTTTTGGATTCAGCTGGTTGTCCACCTCGTCAAATAGTGACT
CCTTACAAGCCATCAGAGGACTTGGAACAAGTGCCACTGATCAATCCGAATTCACCATCACATTTGACCACTA
CCAATACTTCGCCATATCCACTACTCACCTCTTCTAGTTCCAAACCCAAGGAGTGTGA
TGAAGTTGATTTATCGTATGCTGACAAGTCTAAGGCAAGCAAAAAAGGCCACAAATCGATCTTAGAGCCAACA
ACCAGTCTTCTAAATAAGGTGAAAAGTGCAACACTCGGTCGCACCCACAAAAATGAAGAGACCCCAAAACACG
ACGAGGTTCATGAATATCATGAAATATCTGATACTGATACGGAAA
>103778366_2 DT666159.3
GCACGAGGATTTTCCTTGACTTCAGCCTTATTAGTTATTAAAAAAGACAACATTTATCATTTAAGATATAAAT
GATGAGAATTATAAATATCCTAATAAATTACTGTGAATACATTCTGCTCTAAACTTAAAACAGCTTTAGTAAT
TCTACAATGTTATTCTGATAATAAAATACCTAGTAATTATCAAAATATTAAAAAGCTT
TGACTATTTTTTAATTTTAGTTATTAATCCAGAATTCAATGCTCCCTCGCCGTCGAAACGTTGGTGTGTCGGA
GAAGACACCCTTGCTATCACAAGACGAACAAATTCCTTATAACAAATTAAACAAAAACGGATCTACATTCTAT
CAACCCGTGTTGCCTGAACCTGAAGAAAGATATCAGAAATATTTAGTAGAGAGTGATA
TTAAATTTGATATTTTGGAAGAATTTATATTTGAACCAGTTATGGCTTCAAGAGATAGAACTCAAGAATTTGT
ATCTACTGTGCGCAGCTTACAAGGAAGGACTTTGGCAAGACCTATTGTAAGAGATGAAAGAAAAGCAGCAGTT
CTAGCAACTTATTCACAGTTCATGAGTATGGCAAAAGTTATAAGTAAAAATATTACTA
GCACTTTTGCTAAACTAGAAAAACTGGCTATGTTGGCAAAAAAAAAGTCTTTATTTGATGATAGACCTATGGA
AATTCACGAACTAACCTATATAATAAAAGGTGATTTAAATTCACTTAATCAGCAGATTGCTAGATTAGGTGAA
ATGGCCAGGGGGGCGCAAAAGTATGCATAG
>103778369_2 DV501492.2
AGATGTCCCGGCTCGGAATAGCCATTTAAAAAGAATTGTTTGTGTGATGTGTTGAGAAACGTTACTGTGATAT
CAATATAGTGTTAATTTGTAAACATTTAAGATGAAGCATTTTACTTGTAGAATAGGTTTTTTGGCGGTATGTA
GAAGCAC
>103778373_2 DT665183.3
GCCAGCTTAAGAGAACGCATCGTAAATAAGTACCGTAATTTTAAATTGTTTATTAAGAAAATGTTCCTTTGAG
TGTTAAACTTGTTATACTGTATAGTGCATCGAACAGTGTGTGAAGATGAAGAACGTTATTGTGCGCCATTGTT
TATTAACTCGTCCATCCCGACGCCAACTATCTACGTCATCAGTCAGAAGAACCTCTAC
ATCACCTCAAAGGCGCAATGTGGCTGCACCAGCTACAGTTACCCATTATAAGAAATTCAGCGATATACCGGGA
CCACTGGCCTTGCCAATTTTGAGACACCACGCTCATGTTCTTCCGAGGATTGGAAGCTTCCACCACACCGTGG
GACTAGGCCTACTAGAAGGGTTACAAGACCGCTATGGAGACTTGGTACGGCTGGCCAA

AGCAACTAGATCGAGACCTGTGCTTTACGTGTTTGACCCTGAGCTTATGAGACAGGCATATCAAAGCAATGCC
ACCGAGCCTCCCCAATGGGCCAAGTCGCCATTGTCTACACACAGGAAAACTGCGCACACTCAATGTCCTATGC
ATAGTGACGAAACAAAAGCAATATGGGCTGGTATGAGGACATTGTTGCAAGATGGCAC
GCTTCTTCGAAACTATGAAAAAGCATTCGACGGTATAGCCGCT
>103778376_2 DT666255.3
GCACGAGGATTTGTCCTGTGGTGTATGTTGGGCGGCGGTCCCCAACGCCGCACTCGTTGCCACTTGATACACC
AGCGGGGATGCACTGGTGCGCTCCCCCTTAGGCAAGCGTCCTACTAGTGCACTTTTGCGAGTTGTACGTGGCG
GGTGGAACGTTTGCAAGGGTTGGCAGGGGTCCTTGCGGGTAATCCTTCCATCGTGGAT
GAAGTAAAATAAAACAATAATAAGTATTATAAGCGGCGATAATGTCGGCAGGTGGACCGCGAGATGCGCACGC
GGCGCGTGCCCAAGGCGGGCGGCGCGGCGCGCGCGCGCGGACGCGACTTCTAGCGGCTGGTGCGCGCGCTGAA
GGCGGCCGCCGCCGCCCTGCGCGCCCTGCTCACGCACCGACCACGAATTTAAACTGAC
GCGTTTATAATTATTTATACGTTTTATAAGAAGCGCGCCCTCGTGTGTTAATGATGTTGCGATCTCTGCGTTA
TCGCCGAATGGATATTTTGATATTCTATTGCTCCCCTATATTTTGCACGTTCCGATGAAATTTGATGTATTT
TTGTAATAGCGTTGGTGTATTTTATTAATACGCGAATGTATTTACCCCCCGCGCCGCG
TCCCCCCGCGTCCCCCCCCGGGGCCCCGCGGGGCGCGCGCCCGCCGCGTCCTTTTGTATTATATACATGGTAA
TATATATTGTACCGTCGAACACGGAGACTCGTGTGTATGTATGTATGTAAATATAGAATCCTTTACTCGATA
>103778378_2 DT666313.3
GCACGAGGCGAACCCCTTAAGTCTTTGTAAACAGAGCGTTGAAACCCGGCTGTAGTCACTAGTGCAATTAATA
CGATTTAACGAGAAATAATTTGACAAGCGAATCGAAAATAAATAATCAAAAATGGTGTTGCTTTTAAGAAGAC
TCACAAAAAAATGCGCCCAAATTGCACTCGGATGCAACAGGGCAGCGCATCAAGCAGC
GCTCCGTGGCGCTACCAAGCCTAACCCTCAACTTGCTGCATTGACACCGAAGGTTCGTGCCTTCATAGAGCGC
AGCGCAGCGCTCTGCGAACCCGACCACGTGCACGTGTGCGACGGTTCCGACGCGGAGGCGGGCGCCTTGTTGC
AGCTCATGCAGCAGCAGGGCACCATCAAACCACTACCCAAATACGATAATTGCTGGCT
CACCCGCACCGACCCGGCGGACGTCGCTCGCGTCGAATCCCGCACCTTCATCTGCTCTGAACACGAGAGAGAC
ATCGTGCCCGCAGCCCGCGCCGGGCAGAAGTCCGCTTTGGGGAATTACATATCTCCCGCGGATTACGATAAAG
CGGTCGCGGAGAGGTTCCCTGGCTGTATGCGAGGTCGCACCATGTACGTGATCCCGTT
CTCGATGGGTCCCGTGGGTTCCCCGCTGTCCAAAATAGGTGTAGAAATAACTGACTCGCCGTACGTCGTGTAC
TCTATGCGCGTTATGACTAGAATGGGTAGCAAGGTGCTAGAGGCGCTCCGCAAAGACGAGAGCTTCGTGCGCT
GCCTGCACG
>103778388_2 DT666696.3
GCACGAGGCAACCATATAGATATCTTGCGCTTTGAGCACAAATTACTCGAGGATTACTTTGCACCAAGCTCAA
ATCATATCATTTCTCATTCTCCAATACCAGCTTATTCCCGTTGAGTAGGCCGACAATGCTTTTAAATTAATAT
ACAAATTCTTCCCCGGAACTGTCATGATTTTTGAGTGTCTGATAACTGATAATGGCTT
TTTATACGTCCCTGGGGCATGTGCTTCATCTTAACGTTCACTGCCCTCACCGGCTCCAGGGGTACATACTACG
GGTTATTGTACACTCCACCCTTACTGCAACCTATTCAAAGAGATACATTCCCACGTGAAGTGGGAATTTATTT
AATTTCACTTCACTTTTAATAATAATCGTTTCGAGTGCAGACTGATACATCTAATCCA
GCCGTACTAGAACTCCCAACTAAATTATCTAATGACAGTAACGAGATTAATATGCGTTTGCCAAAGTCAAAAC
CACTATCATCGAAGTAATTTCCAAGACAAATTAAATTGTTCACTAAATTCCCAAAAAAGCTACATGGTAAGAT
TTTACTTACGAGACAAATAAATGTCTTAAGAAACTCCTGGATTGTCACAAGCTCTACC
AGATTCAATTGGACCTGACAACCCGCCACATACATTTCATAAACACGCCGCAATCATTTCAATAATTGTTAAT
TTACACAATGCCAATGACAATGTATATAAGCGAATAAGAATTAAAGTAAATGTATTTAATTAATATTATTAAT
TATATTTATAATTAATTGAATTTGTAT
>103778394_2 DT665965.3
AAGGGAACATGTCACACTAGTACATTTTAATACCCTTCAAGATACATTACACAATTATTTAAGGAAACACAGA
TTTTGTGCAGATTGTAAAACTAAGGTATTAAGAGCTTATCAGTTATTAGTAGAAGAGAAGGAGCCACAGAAAG
AGAAAGGTTATGTTGGTGCCTTATATGGAGGTATCAAGAGATGTTTGTTAGACAAACA
TTTACACTTGCAGGCCAAAACTGATTACATTGCGCATCTCATAGCCAGAGCTGAACTTTATTTGTTTGGTAAT
CACAGGGAAAGGCATGCCGTCACATTAGAAATTGCACAGGAGGAGGTCTTAATATGTCTCGGGATATGTATAT
ACGAACGTCTACAACGCATCTCATTAAGGCTCCGCGAGGAAGAAGGCACATGTCAAGC
CTTAGCGGCCATTGGAGTTGAAGCTTTATATAGGAAATTTGAAACCGCCGTTGAAATGAAGAGCGGTGTCTCC
AAACTTCAAATTGTTATATGATGAAATTACACAGGAGGAACTCTCGAGACAGCAAAGGAAAGAACAGAAAAAA
CTTAAGAAACGCAAGAAGAAGGAGCGACA
>103778398_2 DT666324.3

GCACGAGGATTTTATTTTACAGAAATAGCAATTAAACTTATCGTAAACCGGCACTTGTTTAAAAGAAATCATG
GCATCACAAATCGCAAAAAAAATTATTAAAGCAACTCGCCCTACTTCTACAGTGAAATTTGACAGCACCAGAC
GCGCTTTAAGTGTGGGAGCAGCATTGCAGCAAAAAAAAAGTCTTCCAGATAAGACTGG
TAAAAATGTTGTGCTGGTGGATGGAGTCCGGACACCTTTTCTTGTATCATTCACTGACTATGCCAAGATGATG
CCACATGAGCTTGCCAGACATTCATTATTGGGCCTTCTCCAAAAAACTGGCATACCTAAGGATGTAGTAGACT
ACATAGTGTATGGCACTGTTATTCAAGAGGTGAAGACATCAAATATTGGGCGTGAGGC
TGCTCTAGCAGCAGGCTTCAGTGATAAGACACCTGCTCATACTGTGACCATGGCCTGTATATCTTCAAATCAG
GCTATTACTACAGGTGTGGGGATGATCGCCGCGGGTGCGTACGACGTGATCGTGGCTGGCGGTGCCGAGTTCA
TGTCTGATGTCCCGATACGTCATTCTAGGAAAATGAGATCTCTATTGCTGAGACTCAA
CCGAGCTAAGACGCCGGCGCAGAGATTGTCCTTGTTGGCCTCTATTCGACCTGATTATTTTGCACCTGAGCTC
CCAGCTGTAGCGGAGTTCTCATCTGGTGAAACAATGGGTCACAGTGCGGACCGCTTAGCTGCGGCGTTCGGGG
CGTCCCGCGCGGAGCAGGACGAGTACGCGCTGAGGTCGCACAAGCTGGCCG
>103778417_2 DT666087.3
GCACGAGGGGCAAACCTTAGGGAAAGTGTTAAAAAACTAAGTAATACTAGTAATAATAAACCTGTAGCTAAAG
AAATTCCAGTCAGTGGTAACAGTGAAAAAAATAATAAAGAAGAACCTACTCAGAAAGAAAATTCTGTTCAAGT
AAAAGCAAAGAAGAAACTTAAACCTATTGCTACCTCAGACTGGGCAGTAGAGTCAATT
GACAGTTCAATATTGAACAAAAAATTTATGACTAAATCTGTTGATGTAGCATTTGATCATTTTGAAAGTAAAG
TTGCTGGTAAAGTGGAAAAGAAAATAAAAAAGTTGAAGAAACAAATATCTAAATTAGAAAATGAACCAAATGA
GAGAAGGCTTAAAAAGGAAAAGAATGACAAAGATACAGACAATTTAGAATATTTAAAA
TTAAAAAATAATAAAACTAAGCCTATTATTGATGAAGAATTAATAGAGACACACAAAGGAACAGTAGTTAATA
ATAAAGTATCAACATCTAATATAAACTTGGACATGGATGAAATTCCACCTGAAGACAATAATAATATGAATAT
TGATCCAAGCAGATTTATTGAAGTTAAGCCAAAATATTTAAACACTGCTGTATCAAGA
GTAGAAAATGGTATAGACGATTTGGATGATGAAGAGCAAGTTGTTCCAAAAGTTGACATTGAAGAAGTGTTTG
AGGAAGATGATGTTGTTGCAAGTTTTAGACAAGAAAAAGAAGATGAAAATCACAAAGATATTCCACAGGATAT
AGATTTGAC
>103778419_2 DT666245.3
GCACGAGGATAAAACCTAGAACATACGGTTACTTCTATAAAAAAACAAGAGAAATTAAGAAAATTAAGGCAAA
ATGATTACCGACATTCAACTGGCAGTATTCTCCAACATTTTAGGAGTTTCTATTTTCTTATTGGTCATCCTCT
ATCACTACATCAATGCGAATAGCTCCAAATAGACCCGGTATTGCTTGTTTATATATTT
TAAATACAGTGGTGACACGATAACCTGGCCCTCAGTCGCATTCTAGATTATCCATATGCATATACCTACACAT
ATAGCAGGGTATGCAAAGTAGACTTGAGAGCCTAGTGTTAAGAAACTCCATTTCCATTAACACTTCCATACTT
CCCTTTCTGCTAAATTCTGGAGGCATTGCTTGTTGAGAGGCAGAACTCAACACCTTGC
TTTTTCTCATTACCTGATAAAGGAAATAATAATATAAAATATTTCATTTCCTTGTGGGTGTGGAAATCAAACC
AATATTGAAATTTTTCTTTCTAATATTCTCTATCTGGATTTATTCTTCGGGCTAATAGACTTTTAACCTTATA
TTATAATTAATTGATCAATGAAGTTTTTTATGATATAGACAATTTATAATATGTTTAT
TATCATTGTTTATATGCTTATAATATAGTCTTAGTAAGTACACTTCATGATTCCTAATTTAGCACTTAAGTAA
ACATATATTGTCCAAACAATTGTGCCAATTATTTATTATAAGAAAAGGATTTACATTTAAAAAAAAATAACCT
TCAAAAATATTTATTGTAAGGAATTTGTCACATCTTAATGTAGTAGTCAATAGTATAT
TACCATACAAGTATAAAATAAAATGTATATTCATTTTAG
>103778442_2 DT666482.3
GCACGAGGGGTAGACCTTTTAACCTTATTTAGAGGCGAAAATAATTTAGACTTATTTGAACTTAATTAATCTT
ATAATTAAAATGTCGGGGCTCTATGAACACAATCGCCAAAGGAGATATTCTGGGAAATCACAATCTGATGATG
ATATTCTTACATCAAAACACCCATAACAGAAGTCAAGATCTTAATTTTGTAAACAATC
TATTTGACATAAACATAATAAAATCTTTTTAACATTTTTAAAGTGCCATTCCACTCTTAAGAAAGAAAAATAT
TTATTTTAGCAAAGTATACAAATTTGAAAGGACGGGCGGGAGTACGAGTACACCCAGTCGAAGGAGAGCCTGG
TGCTGCAGCTGTTCTACCGGCAGATGCGCTCCAAGGTGCGCTGCACGGTGTG
>103778454_2 DT666223.3
GCACGAGGGCGGTACATTGATAAGGAATCGATTTATTTACAATTCATAATGAGGCTGTTTATTACAACAGTCG
CACTATGGGCTTTGGCATCTGCCAAGCCTGAAATGTATAAAGAGAAGGAAGACTTTCAATTTTCGAGAAGTTC
GTCTGACGATGGTTCAAAATCTGGTTACTACGGAGCTCAGAGAGGAAATATGGGAGGC
AATTACGAAAGAGCACATAACATGGATTCCTTAGCACAACATCAAATGAGTACTCTCGTAAGACATGTAGACG
GAGAATTAGGTGAAGGTTCAAATACAAGAACCGGAAGTGTTTTCACTTCAGGTAATTCCCGTGGCATTTATGG
CAGCGGAAATTACGATCTAAGTAATTTGAAAGGAAGAAATTTTGAAGAAAGCGGTGCA

TCATACAATGATCTGTATTCGTCATCTTCATTATCTCAAAATTCCAGGAACCATGCAAGTTTATATTCAGGAA
CGAATAATGCGAAATATAGTGGCAATCAACTAGAAGCATCTGGGCTTAGTGCCGGATACCAGCATGGAAGTCT
AGCATCTCAATATATTCAAGCTCAGGATAAGCAGCAGGCTGAAAATCAATATCATGGA
TCCTCTAGCAATTTCCAATACGGTGGTCAAGGCGCAAGTCAATATAAATATTACTCTCAAGGAAAAAATGACT
TTGACCAGAGCGCCAATTTGCAAAATAATAATAATTATAATGTCAACACTA
>103778460_2 DT666942.3
GCACGAGGCCTCGCGTCAATTCGGCACGAGGAAGAAACCAGACCCTGCATTTGATGAATGTGAAGTAGAAGTG
AAAATAGCTGATCTAGGTAATGCTTGTTGGGTACATCGTCATTTTACTGAAGATATTCAAACAAGGCAGTATA
GGTCATTAGAAGTATTACTCAGTGCTGGCTATGGCACTTCCGCTGATATTTGGAGTAC
TGCATGCATGGCTTTTGAATTAGCCACTGGTGATTATCTCTTCGAACCACACTCTGGGGATGGTTATAGCCGA
GATGAAGATCACTTAGCTCATATTATTGAGTTGCTTGGTGATATTCCTAAACGTATTGCTGCATCTGGAAAGT
ACTCTAAAATATTTTTTAATAAAAAAGGTGAACTTAGAAATATAACTGGGTTAAAACC
ATGGGGCTTAGTTTCCGTTTTAACAGAAAAATATGAATGGAGTCAAATGGATGCAGAAGAATTTGCTGATTTT
TTGAAACCCATGTTGGATTTTGATCCCAACCGGCGAGCTACTGCTTACGAGTGCCTTCAGCATCCATGGTTGA
AGATTACAAAGCCAGAACAAAATGAGTCTGAGGAATAATTGCTCTCTTACATTGGATG
TTTACACATTCATTAAATCAGTAGTGACAACATATTAAGTATTTCAATGTTTTATGTTAATAATCAAAGTTTA
TTTATGGATTTGTTTGTGAATAGTTTAATTTGTATCTAATTTTTTGTTCACAATCTTTGATTCCAG
>103778463_2 DT666831.3
GCACGAGGCATTTACATTGTTGATGACAAGGTAAACAAATTTATTACCTATATTGCTAGTCAAGGAAAATATT
ACCTCAGTAATAACGTGATTATTACCATGGGAGGCGATTTCACATATCAAGACGCTACGATGTGGTACAAAAA
TCTTGATAAATTAATTGAATACGCAAACATGAAAGCTGCTAAAGATGGTCTCAATATA
AAATTATTTTATTCAACTCCCGACTGTTATTTGAAAGCGGTTAAAGATGCGAATCCGACTTTACCGACAAAAC
AAGATGATTTCTTTCCCTACGCGAGTGATCCTACCGCGTATTGGACGGGCTATTTCACTTCGCGACCAACTAC
TAAGTATTTTGAAAGGGAGGCTAATAGATATTTACAGATGGCGAAAAATCTTCAAATA
CTAGCGAACCTTCCAGAACATAATAAATTTGTTTTGGACGAACTTAGAAGCGCTATGGGTGTAATGCAACACC
ACGACGCTATTACCGGTACAGAAAAACAGCACGTTGCAAGAGACTACGTACGATTGTTGGATAATGCGTTGCA
AGACTCCTTGCTTATTGCAAGACAGGCTTTCAACAAACTACACGAAAATGGCCAAAAT
TTATTTTCTTATGATAGATGTCATTTTAATGAATCAAGTTGTGCTACAAGTGAAGATGGCGACCAATTTGTCG
TGACAATATACAATCCCGTGGGATGGAATGTGGTAGAACCAATACGAATTCCAGTTAAAAAAGGCACTTTTGA
AGTTTACGCTCCAAATGGTGAAAAGTACCATCACAATTGGTATCTATACC
>103778467_2 DT666098.3
GCACGAGGGCAAGATGTATTACAGATGACTCCACATCTTAACCAATATTGTGTTAATTATCTGATGCGGTGGC
GCTGCTAACCAACAGTGATGCAGGGCGGAGGCGCGCCCGCCACCGACGCCGACCACGCGCACTTCCAGGCAGA
CCAACGAACTGTTTACCATCATCCTCTCTTCCCACTCCTGGCTCTACTCCTACAAAGA
TGTGAGCAAGCCACTGCTGGGGCGGAGCCTCCAGCTGCTGACGCCTTCGGTGCTGACCTCCAGGCCTTTGTGC
AACATCAGCGCACGGATAGACGGCCTTTTCTGGTTGATGATCCTGATATTGATGGACTAATGAATAACAGTAT
ACAAGTCCTCAGGATCCACCTCCTGGAACTGGAGAAAGTTCAAGAGTTATGTAGAGAC
TTCTGTGGAAGGTACGTCGCGTGTTTGAAAACCAAGATGCAGAGTGAGAATCTTTTAAGGACTGATTATAACG
CTGGTGGTGTTGAGAGTAATAACAATTTATCTGGAACGATGGACGATCATTCAAGTACGTCTAATTCGCCACA
GTATCAGTCCCCAGCCGCCCCGCTACCTGCCCCCGCG
>103778471_2 DV501499.2
GCACGAGGGTTCCTTTGCGGGTGAATATTATAATTCTGCGATAGCTATTAATGGATTTCGTGTTTAGATTGTA
ATTGTGATCTCATCATCATGGGCAAGGATGACTACTACATTTTGGACAGCCCGAAAGGCTCTACCAATGACAA
CTCCTCAAAGGCGTATCGCAAATTAGGCCTGACGTACC
>103778473_2 DT666654.3
TGGTGATGATCTAGGTAATGGATCTCCTGTCGATTCGTTAATTGGAGACTCTTTACTATCTTCTCCCGAACCT
CAGGAAACTGAATCATCTTGCGAACAGTCTTCCCTCTTGACAGAGCTGTCATTAGATGCATTTAGTAATAGGT
CAGATAATGACATTGATGATGGAAATTCGCCATTTATCCCCACTACTGATGAGCTTCC
GCTCCTGGAGCCGGCAGTTATGTGGGGAGCACTCCCAGATAATGTAAGGCAAGCGAGGCCACAACCCTCGGAA
CTTCAGAGTCCGGTTTTTTTTCTGCAGCGCTTGCTGGCTGCCCCACCGACTGGTCCGCCGCCACAAGATCTCA
TTACTAATATTTATTCAGACCAAGGTCTTATTCCAAGCAGAAGTGTATCCTCATGGGA
TACAGGCGTAAAACGAGTGCTAAAGCAAGATGAGGAGCCAATTGCGAAACGTGTAAAACGCAGTCCATCGCCG
TCTTTGAATACACCACCAGCTCCAGTTAACACTCCATCCTCAAGCGTCTTAATGAACCTCCTGGACATCCATC
CAGATGGCAAGCAGATGCAGCCACAAGCTCGTCCCAACTACCAACTCGTCTTGAATAC

ACCACGAGCGCAGTCGCTGAGCCCCGTCAACGGGAACATTCCGATACCCGTCATAAACATCATTCACTCTGCA
CCAAACAAACAGATGATGCGTGGCAATGTGCCAAATGTGCCGACCCAAGGGCGT
>103778475_2 DT665785.3
GCACGAGGAAATAACCTCCTTTTTACAAAAGTCGTAATAATAACTTCTATCATTTGAAGTAACTGTGAACTTT
AAACAATTTACAGTTATAGTGACGAACATGGTGTATACTGAAGAGTATTTAAAAGAGAAACTAACGAAGGAAC
TGGAAGCCGTTCATGTCGTGGTAGATGAGAGCGACGGATGTGGTGGGAAGTTTTCTGC
ACTAATCGTCTCTGATAAGTTTCAAGGGAAGCCATTGCTCGCTAGACACAGGCTGGTAAATTCTGTACTTCAA
GAAGAATTGAAGACCATCCATGCCTTCACACAGAAGACTTTGACTGTTGATCAATGGAACAAAAAATAATGTT
CTAATTATATTTAATGGTCTAGTTTATCCGTCTTTTATTGCTCTATCCTATAATATTG
TGTTGTCAAAATGTCGCTTTTTGATGTTAAAGTGTCTGCACCCGGAAAAGTGATTTTACATGGGGAACATTCG
GTTATGTATGGGAAGTTTGCCATTGCTGCCAGCCTTGGCCTTAGGACATCTATTGTTATTAAGGAAACCGATT
CGAATGACTTATTCATCCACTTAAAACTACGACCTGTTGGCCTAGAAGAAAAATTACC
TCTAAATCTCATAAAAGACAACTTACTAAAATTATCCAGCAGTACATGGGACCAACCTAAAGCGCTACAGCAT
GAAAAACATTTAGAAAAAGTCAATACATGCCTTCAAAAGACTATACCAAATTTTGATGAAATTCACAGCGCAA
AAAAGAATTCTCTGCGTGGATTTCT
>103778481_2 DT665341.3
TAAAATTAAGCCTTACAACAAATCTCACCGTCTAAAAATATATATTCCATGATAAATCAAAAAATATGTTCCT
AATATTATAACTGGTGTTTGAAAAATGGCGAAAGCAAAGCGAATTTCCGAATTGACAGGGTCCTTTATAATAA
TGCAACAATTTTTGGGTATTTTTGTAATTGTTTAGACTTAGTAATCTTTAAGCTAAGG
ATAAGAAATGTAGTTGTATATTAAAAATAATCCTTTATACACTTTTTATATGTAACTTTTAAATTTTTGTGTA
TAACCTGTAGGCATAAGTCACAATGTAAAAAACAAATCTGATCTCAATCGCTTAAATAGTTGTTAAAAAGTCA
CCCCAAAAAGACTGATGTTGATAAAATTTTGATGATTTTTTCAATATAAATGTGTGTG
TAGGGCAAAAAATCATTATAATTCAGAAAGACGGCTATTTTAATGTTTTTTT
>103778489_2 DT665200.3
GCACGAGGCCAGCCCTGGCCGAATCATAAGTCTCTTGGACACGTTAGCGCGCGAACGTATTAAAAAAAAGAAG
TGTTTCCCGCGTTTTTTTATTATCTGTTTAGTGTTTGCGGATTTTAATTCAAAATGGAGGGCTGCGGGATGAA
TTGTATAAAATATTTGCTTGTTGTTTTCAACGCTTTGTTTGTGATTTTCGGCATCATC
ATCATATCAGCAGCATGCGTGGACATGGGAATGATCAAAGGGTTCGCTGGTATGGAGACAACTGGCCATGAAA
CTGATATAGTACTTATTGTTGTGGGAGTCCTCATCATCATTGTAGCGGCTTTCGGGTGCTTCGGAGCTTGGAA
GGAGAGCCCCAAGCTTTTGTATATCTTCGCCGGCTTCTTGATTCTGATCATCCTGCTA
GAACTTTCTGTTGGCATTGCGGCTGCTGCTCTCCGACCTCAAATTGAGGGTACAATGAAGTCTCAACTGACAG
CTTCTTTCATTAAGAACAAGTCTACTCGGGAGGAGGACTCCACTTATAGGGAATTCTGGGACCGCATTCAGAG
TAGCCTGGAATGCTGCGGCGTGACGGGCCCCGACAACTACGTGAGCGGCGAGCCGCGC
CTCAACCCGTCGTTCAGCTGCTGCCCGCGCGACGACAGCGAGCGCGCCGACGAGATCAAGCGCGCCGACTGCA
TCTCGCTCACCAAGTACTACGGCGCGGGCTGCGAGGACAAGGTGCTGAGCGCCGTGCACAGCGCCGGCATGAC
CGTCATCGTCTGCGGCATTCTGTTCTGCTTTTTGGAGGTGGCCGG
>103778502_2 DT665949.2
CAAGTGAGTGTCACCGATTAGTAAAATGGCGTGCCCTTTCTTGGGATCGATGAATCAAACTTTCGTGCCCCCT
TACGGAAAAGTGTTGCTCAAGCAGTATGGGAACTTCTGCCCCATACTTTCTCGGGGCTTTTGCTCTCTCGGCG
TCGACGAAACGAAATGCCCCTTCATACAGTCTAACTCCATCGTCTCCGAGGCAAAAAA
GGTTTTTTTTTAGGATATCGTGGACAACTCGAGTTATTGGTATGATAAATAATTCACCGAGCAGATCAATGCC
AAGAAGAAGGATTACTCGTATCGTATATTCAGGAAGGTGTCACGCCTGGTTTTTTACGGTATGTACCCGCAGG
CCTTCTAGGGCCACGACAACCGTAGTGTGAAGGTGTGGT
>103778506_2 DT666106.3
GCACGAGGACTACCCCATGAAGTTCCAGACGATCTTCGACAACAATTACTTTCCTCAGGAATTTTAGACAATG
CCGACATTAGTGTTTTGGACTACGATAAAGTAGGTGAGACGCCTTTAGAATCCTTGCCACCAGACCAATTAGC
AAACTTTTTCAGTGCAGGAGGAGGTCAGCAAATATCAGCTAGTGAACACAGGCCTATT
GTAGTCAAACCTAATGGTGGCAGCATTGAATCTA
>103778514_2 DT665115.3
GCACGAGGCGATACCTTAAGTCACTACGCCGGCTCCCCGACGACGTTACGTACGGACGATCGCGACTCTCACT
TAACCAATAAATCACTTAAACTGAACAAAGTACATCCCTACAAGGAGATTACAACTTGACATGAGATAGCACA
AGCGTCGGAACAAACCCCACGTCGGTGATTAATTGCTTTCCAAAAGTCCGTTCAACTT

TTATGATTTAAAAGCATTATCTTGCCCGCACTAGATTTAGATACGTAATGTAAATAGATAAACGTTGTTGAAT
ATTACCGGAAACGACTGTTGATACAACGTGGGAGAGGGCGAGTAATAACTAAATAGTGTAAATGATAGTGCAG
GTGAATGTGCTGCGATAGGTAGGACTGGTCAGCATGGCCGGCCAGGGGCAATGGATCG
CGGGGTTGGTGTGGGCTGTGTTAGCATCTGCTTTGGCGGACTCCTGGACAGCTTACCAAGAACAGCCCTGTTG
TCGTCCTGTGACGCATCATCGAATTCGTCACCACCGAGATCATATGCGGGAGTTTTCATGCGGCAATCTCTTT
TACCGGACTTTATATATTAACGAAGAAAAGAATACGCTTTACGTTGGTGCTATGTAAG
TTGTTTATTTTTTTTGTATAATATATATAAAAGTAAATAATACCAATATATTAATATAATATTTTATATATTT
AATATAACATTTTATATACGTTTTCTACAAAACACATGATTTTGTTATACACTAGCGAGTCTAGCGACCCGTT
TGCGGCTTCGCCTGCTTGT
>103778518_2 DT666099.3
GCACGAGGTTTTTAGATTAAAAGAAAATATACATAATCATTGCAAATATATGATATTAATATTTTAAACATCA
TATGAAAAGTGTTTACTCTTCAAGAAGCAATTACTATTTGTAACAAGCTTATTTTTGGGTAGGTAGTGAGCGC
CGGTGAAATTGTGATCATGGCGAATCCGTCATCAGGCGTTGTGGTTCCTAGAAATTTT
CGACTTCTAGAAGAACTTGAGCAAGGTCAGAAGGGAGTGGGCGATGGAACAATTTCTTGGGGACTCGAAAGCG
ATGATGATATGACCCTGACTCACTGGACTGGCATGATAATTTTTTTTCCTAGGACTCCTTATGAAAATAGG
>103778531_2 DT665715.3
CGGACGAGGGCGCTACGCCGTGCTCATGGAGCGCCTGCACCACCACCAGGCCGAGCGCGGCGACCACACCGCG
CACGTGCGCCTCAGGTACCTGCTCCAACTGATGACGGAGGCCAGTTGCGTGGAGTGGGCGCTGGTGGTGGCGG
TGGCGCTGCGCGACGCGCTGGCCGTGCTGCGCTGCGTCAACGCGGCGCGCGGCCCCGA
CGTGGCGCTGAGCGCCGCGCGCCGTCTGCGCGCCGCCGCGCTCGACCTGCGGGTGTGGACCGAGGCCGAGTGT
CTCGGCTATAAGCCCTTCATGTTGGCCATTAGTAACCAAATACCATTCTTCTCTTCAATAATCAACGCGCGAG
AGCGACGCATTTCCCTAACACAAACAAGAGCAAGAACTCCAAGCACCAGTTCCTTAAC
AGAACAAAAACAAGCCCCAGTAGAAACAAAATTACCGCCGCCCCAGAAAGCCCAGGAAGTCTCTAAACAATCT
GTAAGGAAAGATTCACCGAAGTCTATGCCTGAACCGGAAATAACTGAAGTGTTACCACCAAGACCTTTGGTAC
CACCACGGGAAGAAACTGCATCTAATTGTGCCATAATGTAAAAAGGTTTAGGATTTTA
TCATAAATATACATGGCGGCTTGTGTTAAATGATTCATTTTAAATTCTAAAGATTATTGGTGATATTTTTTTA
TAAAACCATTTACGATTTTAAATACTTTATACTCCATTTTGGGGAGTGTAAAAAACGGCTTTA
>103778542_2 DT666902.3
AAAAATAATTTTGTTAAGTTTAATCATTGAATGTTAGTTAGAACTTCAATATAGTGTAATTTTTATTATTTAC
TCTTCACCCTGTCCAGCCGAGTCCAGGATCACTTCGAAGGAGAATGGCTGGAATAGATATCCTTCACCCGAGT
AGAATTTGCTTTGGAATTCTACCCAATGCAAACGTAAAGTATGCAGGAAAGCGGACAG
CCCCTCCATGAGGACCAGGATGGAGACAGAAATGGCCGCCCATCCGGCGAACACTATATATAAGTATATGCCT
CCTTGGTAGCCGGCTGACTGCAAACCTTTACGCAACAGCATGTTCCAGGCGACCTCGGCGAGCTGCGCGTGCG
CCAGCGACAGCGCCCACAGCCGCAGGTAGGACGCCGTGTGCGACACGCTGCCCAGCAC
GTACTCGATCGTGTGAATTGCCTGATGTATAAAAACTTCAGTGATATCATCATCATGATTGTGTGCAGCGGGG
ACGGGAGCTCCACCGGCTGTGCCGTTTTCAGCCGCCGCTTCGATGCTTTGGTGACCTTGGCTAGCGCGTTGCT
TCTGCTCCTTCATGATGAAGTACGGCTTTCCGAACAGCATGACTGGGACGCAAGCGAG
GGCCATCAGCACGAAAAACTTTTGCAATCCATTCTGTCCGGCGTACATGGTGT
>103778555_2 DT665736.3
GCACGAGGTGGAAATCATCGACAGACATAAATAATTGAACTGTGTTGTGTAATTATCTCGAAAACGACTTTTA
CGGGAGTCAATTATAATAAGAAATAAGACATGGTAGTTCGCTGGGCGAACCACGCATAGTTGGCAAGTAACAA
TAGAACAATGGGTCAACAAACATCAAGGAAAAGCGGAGAGTGCACATGCGGGTGCGGC
GCTTGGGAGAGGCGGAGATACGCAGAGTCACCGAGTAGTGTCCACCGGTACGTCAGCGCTGTTACTGATAGAT
GGAGTGGAAGAGAATGCGCGTGCAGAAGGCGTAGATGGCGCCGGCCCGCGTGCGTGTGCACGGCGTACAGGCG
GGTCAGCGACGCGTGCCACGATGATAGACTGGCAGCAGTCCTGACACTGGGCGCCAGT
GATTTAAGGAGGGAATTAGACGCCATTGTCATCAACACTGAAAGTGACAATAACAGAGAGAACGGTGAGCCCA
CGGCGGAGGTTTATGTGCTGTCAGTCGCTACTAGGAATGACGGCGAACCTTCTCAAGAAGGCCGGAGGACAGA
ATTGGCTCAAGCCAACGACCCCTCTATAAGACGATTTCAGGTGGTCTGCGGCGGTGAG
CTCCGCGCGTTGTTGTTCAGAACACCGCCGCTCGCACATCAGTCGATAACTCCACCCACCACTGTACATACGC
AGGTGGACTACAAACATTGTCTAGTGCCAGATCTGCAAGAGATCACGGCGTGCTC
>103778571_2 DT666917.3
GCACGAGGCCTATCTTTCGTAAGCTTTTCTTTTCTTTTTCTACACGAAAAGTTTTTAAATAAAGATTTGTGGG
TGATTTAGAATGCCAATAAATGTGATTCGGATGATGATAACATATTTTTAAAGAGATATATTGTTAACAAATG
TTGAGAGTTACTATGACCTTCGCATTTTTAGTGGCGTAGTTTACGGGCCAATGCTGAA

CGGTTCGAACGATAACGAAACGAGTAGTTCGAGCGTAGCGTTAGAAAATAGAGACAACATGAGCGCTGGCACT
GCGTCGTTGGAGCGCGAGCTGTCCAGCACCAGACGACTACTCTGGGGCGACCACGTGAAGGAAGACGTGTTTC
GCCGATGGGCTCAAGGCTTCCAATTTAATTCTGATGAGCCCAGCGCACTCATACAGCA
GGAAGGCGGGCCGTGTGCTGCCATCGCTCCAGTGCAGGGATTCCTTTTGAAGATCCTCCTATCCGAGACACCT
GGACATAGTCTTCAAGACTTGACATCGGAAAAGTGCAATTCCTTACTTGTTAGGGCTGTGTGTACTATTTTAA
GTCAGTGTCTAGCTTCTAAGTACAATGTTGCTGTTTACCGCAAGAATGATACAGAGGC
AGAGACCAGCACCAGCCTCAATGTTAGTGCCGAGGAACAATCTTGTGATGCTATTAATCACTTTCATAGACGG
ATTGAAGTACATTCATTTCAAACATTGTCAGAAGTTGAAGCATTCTATACAAGAAACATTCGCATTTTGAAGG
ACAAGTATGGTGTTCTGCTTTTTGTATATAGTGTCATTCTAAGCAAAAGAGTAAGTAC
TG
>103778592_2 DT666492.3
GCACGAGGTGGACCCCTTAGACAGATTTGATCACTTCCTCCCTAACTCTTTCAATAACGATAAAATATTTAAG
CAGATGATCGCATCCGACTTTGAATATTTCTTAAATCTTAACAATAAGTCACCAGAATTCTTGTCGCTGTTTA
TTGATGGAAAACTCAAGAAAGGAGAGAAAGGCATGAGCGAGCAGGAAATAGAAGCGGT
ATTAGACAAGACAATGGTGCTGTTCCGTTTTCTGCAAGAGAAGGATGTGTTCGAGCGCTACTACAAGCAGCAC
CTCGCCAAACGTTTGTTGCTCAACAAGTCCGTCTCGGACGATTTTGAGAAGAACATGATCTCAAAACTTAAGA
CTGAATGCGGTTGCCAGTTCACATCAAAATTAGAAGGCATGTTTAAGGATATGACAGT
TTCTAACACCATCATGGAGGAGTTCAAAGAACATGTGCTCTCATCTGGTACCAACTTACACGGCGTGGATCTG
TCGGTGCGTGCGCTGACGACGGGCTTCCG
>103778595_2 DT666946.3
GCACGAGGCCTGCTTCCTCCAGAGGAGCTTCGAGCCCTTTTCCCGAATTTGCCGGGAGTTAGAGAGAGACATA
CTAAATTGTACTCGGAGTTAAGACAAGTGAGAAATGAAGCGGTCAACCACGTGGTTGAGATACGACAAGTAGC
TGATGCTATGTTGAACACGCTTGGAGAATCTGGTTACGCGCCATGTCTATCGAAGTTC
TGCAGTGGCCAGCGTATGGCGTTAGACTCTTTAAGAGAAAGGCGTAGAAAAAACAAGGAATTACATCAGTTTT
TGGCTGGTAGAGAACAACTGCCCAGATGTGGAAGATTACAGCTGAGAGATTTACTGGCTTGTGTATGGCAGAG
GTTAACAAAATACCAGCTTCTTCTAGAAAGTATTCTCAAAACGACAAGCGAAGATGGC
GAAGATTCCGATAGTACAGATGATATTTGTCAACTAAAGAAGGCGCTATCAGTAGCGAAGGATGTATTACACA
GTGTTGATACAGCGATACGAACCCAGGAGAATTATCATAGATTATGTACAATTCAAGACAAAATAGAAGTGCG
TACGTTACCAGAGAAGTGTGGGACAGACTTCGAAGAACTACGCAAATTGGATTTGACA
ACACACACATTGAGATTAGAGGGAGAATTAGCGTTACGGACTGATACTAAGAAAATACGTGTTTTGGCGCTTA
TGTTAGAAGACAGTCTCGTTTTACTACAACGTGAGGGAGAAAAGTTTATACTACGACCGTTGCCGCAAACAGG
ACATAATAGTTATATCACACCTATTA
>103778601_2 DT666158.3
GCACGAGGCGCACGCTTAAGCGACGCGCGTCTCAGTGCCCGCCATTTTGTTTATACAGCTGTCAAAGTTAGTT
TTTTTTTTATTTTCTCTGTAATCTGTGCTGTGCTATACGTTATTGTGCCGAGAAGAAGGATAAAGTCCTAGTT
AAATTCCTATTGGATTAACAATTCCAGTGTCGTGAAAAAAAACAACATGGCGTTTTTA
AAACTAGTATTCTCTTTAGGCTTTATACACATGGTAGCTGCTAAAGGTTCATAAGCTCAAGTAGAATGTACGC
CAGAGTACATGCGTGTAATGGTGCCGATGGATGGTGATAGAATTATATCTTATCTGGACCAATTGAAAGACTA
TTCACCTTGCCAGCCAACGATAGAGGGACCCATGCACACGTACGTGCTCGACCTTGTC
GACTATCACAAGTGCGCAGTTACCCGCGTCCTTAACACTTACACCGGCAAACGGACATACTATCACAAAATCG
TGATAGAGGACTCATCAGGAGGCAGAGAGACTGTCAGAGCGCGGTGTGAGGTGACCGGAAAGACGCGCGCCGT
CGCAAAACGCGCTGTCGAAGCTTTCCCACTGGATTTCAATGAACCTGATGTATTGAAC
ATCACAAGGTACATGGAGGGTCACGCTGGTGATCCTATTTTAGGAGCAGCTGTAAAGCACAATGGAAAACAAG
TGTCTGGTGAAATATCTGTGAGTCCCGGTACTCCGCTGTCCATGGAGATATTCCTCGATGAGTCATCCAGCTC
TGTTTACGGCATACTTGTTAATTATATGCACGTCACCGATACT
>103778606_2 DT665742.3
CGGCACGAGGCTCTCTTCCTTTTTTTTATTTCGAAATATATATTACACAAAAATTATATCCAGATTATATAAT
AAACCTAATATGACTTATATTATTTCGTTTATGTGCTAATTTCACAAATTGTCGTTAACAATAAATGTGATTT
TGTTTGTATTAATATTACTTATTATCTTATAATTATGTCGGCTGTTTATTTCATTTAA
TTAATTTATACTGCCTTGTGCTACACCAATTGCATAATATAAGTAATGTGCACAGCCATCAGTAAATAATGCT
AAATAGCTATCTAGTTAAGTCTATTTGATCACTTACTTAATAAAACTACTGAGTTATTCATGAGACCGGATTG
GACATATACGTCACACGTATTTTATTATTTTGACCCCTCTTATTAGATTAAATTAATA

CAGCGGTTCCGAAATTGTTTGTATATTATTTTTTGAAATATGATGTCACGAAACTTAGAACCCTTCCTCACCC
CTTGTGTCACACTTCGACCCCTAACGTGACACGGAATATATGGATGGACCTTAAGTGCCTCTAGACGTCGTTG
GCGTCAGTGCAAGACCTCATAATTGCAATTTAAAAAAAAACAAGTCTTTCAATTTTTT
TTCGATTAAACAGTACAATCTCCAATATCTTTATAGTATTGACGTCACTTTTAAGGTACTTTTTACTGTCTAC
TGTAAAAACAGTATTTTTGAATTTCCTGTACAGAAGCTGACGTTATGCATATTCAATTTAGCTAATCCATCTT
TATTACCATTAAAATCCAATTATATTTAAATTTTTAAGCTATTT
>103778615_2 DT665708.3
GCACGAGGTCGAGTTCGTCCTTTTATCTTACTCGTTTCTCGTTTCTTATTTTGTATTTGTACGTTGTAATCCT
AATTTGTAATAAAATTTGGAACATCAAAGAAGAAACAATCGCTTTTATTTTTAAAACTATTTCGATTGCCTTA
AGGTATTCTAGGAAGTAGTGATTACTTATTTTCCTCTTTCTTTTGTTCAATGCTTGCT
AGATTTGTCAATTCACTGCCATTTGGGCTATCACAGCGAATAACTAAGCTGTATTTTCAAGCTTTAGTTTGGA
ATCAGCGTCATTCACATAGTGTACAAGAAGTCCACGAATACAAAGCAATGGATATAAGAATATTGCCAGCTTT
ACAGGACAATTATATGTACCTAATAATTGACAAGACTACTAAAGAAGCTGCTATTGTA
GACCCAGTGGAACCACAGACGGTCTTGAAAGCTATAAATGGTCAAGGAATCAAATTAACTACTGTATTGACTA
CTCATCACCATTGGGATCATGCAGGAGGAAATGAAGATTTGGTTAAATTACAACCCGGCTTAAAAGTATATGG
AGGGGATGACAGAATTGGTGCCCTAACAAACAAAGTTCAACATGAAGACCAATTTAAT
ATTGGCGAACTCAGTGTGCAATGTCTTTACACACCTTGCCATACTTCAGGGCATATTTGTTATTTTGTTTCAC
CCAAAGACGGCAGTGAATCAGCAGTTTTTACTGGTGACACTTTA
>103778625_2 DT666422.3
GCACGAGGTGTTACACTAATGACACGTTAAAATATGAAATAATAAATTTTTGACTTGGTGTTTGTTAGAAATT
AAGCGACGTTATGGACCACGGAACTTTACGTTAACCATTATGGACCACGTTATTAGCAAAAGACAGGGGCATA
TGAAATGTTCCATTAACTGTGATCATAGTTCAATATAGTCATTTTATAAACTGACTCT
CTCACTTAGAACATAATACAAACCCTATGTCGGTAATAGTAAATACTCCATCAGTTTGATGATTTTGAACAAA
TACTTTGCATTTCATTATACTTTTTCTTTCTAAGTTATTTTTTTTTTATTCTGCTAATGGCAACATTGTTTTT
ATGTGGGGTGAAGTATTTTAAGAACTGAATTTTTACATTTTTAATGTGTTATTCCTCA
TTAAGATAATGAAAATATTTTAGAAAATATATAAAGTAACAGTAGTCGAAACCGCACCGGATAGTTGTCAAGA
GGCGCGGGTTCGAGTTCCGCTACATTTAAAATTTGAACGTTTTCTAACAATATTTTATTTCGTAAACATTAAA
TACAATATGGAGTTGTTTATAAAATGGCCGTATCAGGATTAGTATAGACAAACAAATT
AGACTATTGATGTAATGTGGACCTTCCGGGCAGTGGTGTTGGACTGTTAGTGTTAAATGTGATTTATCTAGTG
TAATGTATACAAATCGATACATTATTCGCAAACCAGCTAACTCGCATCTTCGTGAACTTAACAAGAAACGCCA
AAAACGTTGTTA
>103778635_2 DT666407.3
GCACGAGGATCCAGCTTTAAATTTGGAGAGCAACGAAAAACATTTTGATAAAATGGTGAATCTCGCTTCGAGT
TCCATCTCAACTAGAATGGATAAAAGCAAAACCGACAACGTTCTTTCTTCAGTAATTCCACCAGAAGCAGCCG
CTCGCTATCCTAAATATGTTCCCGAAAAACATCGTCTTACTTGCGGAGCTCAGGGCTG
CTCTTATATATCTGTTGATGAATCGATGTTAAAGTGTCACTGGGAGACCCTACATTCCGGTACGAGTGTATTC
CATTGCGTGCATTGTCCACCATATCAACATTTGGATACTACTATTCCTCTAACGGCAGCTAGAATTATCGCTC
ATTTAAAAATGCACGATATGAATTTATATGCGTGTTCGTTATGTTCATACTATCATTA
TAGAAGGCAAATATTAGAGCATCATATATCCGATGTTCACAAAGGTGGGAATGTGTTGATAGTTAGAGAGGAA
GGAGCTCCAGTACCGGCAAGTCCAACTGCATCTCAAGCGGCTGCTCCAACCATGGATCTGAAGCCTTGGCAAT
GTGGACTCTGCAAATTTAAAACCTTATTACGACTGAACGTTGTCGAACACTGTGCCAA
AGAACATAATTCCAAAATGCAATATAAATGTGCGTATTGCGCATTTAGAACTTCAAATTCTGAGAATATACTG
AAACATCAAAATAAATCTCATGACGACAAACCAGAAGAGATATTCTATTATTATTATCAAGAAGGTACCGTAT
CTGATTGCCCTGATGGTACACCTCGATGGCAAAAGCAAAGCGAA
>103778651_2 DT665235.3
GCACGAGGCAACGCCTTTAGAGGTTGCTAACGATTGTCTAGACTACGAAATCTGCTTGTTCGATATGGACGCC
TGCTATGAAACCTTTGATAAAGATGGTGACGGGCATTTGAATCTCGACGAGTTCCGCCTCATCTGCAAAGCTC
TGTTCCGCAACGACAAAGGCCACATTTACCCGCTGTCTGAGGAGAGGGTGCGTCACAT
CTTCCAGGTGTTCGACAAAAATGATGATGGCTTCATAGATAAGGAAGAATTCTCCTTTTGCTGGAACCATTGG
ATTAAAGTTATCGTGCGTCCAGTCAGCGCGTTCCTCATCGTCGACGTACAGAATGACTTCATATCTGGAACTC
TAAACATCAGCAAATGTAACGCACAGCAAGATGGAGGCGAGGTGGTGGACCCTATCAA
TAGGCTGCTGGAGTCAGTGTCCTTCGACTGCGTCTTCTACTCCCTGGACTGGCATCCTCCAGATCACGTTAGC
TTCATCGACAACGTGCATATGAGGGAACTGCATCCTTCTAGCCCTGTGTCGGCGGACAAGGCGCAGGCGTACG
ACACGGTGATGTTCGCGGGCCCGCCGCCCATGAAGCAGCGGCTGTGGCCGCGGCACTG

CGTGCAGGACACTTGGGGCGCGGAGCTGCATAAGGATCTGAAGATCGTAGACCACGCGGTAAAAAGTGTACAA
GGGCACCAACCCCGAAGTGGATTCGTACTCCGTATTCTGGGACAACAAGAAACTGACCGACACCAGTCTTTTG
CACCAGCTGC
>103778686_2 DT666153.3
GCACGAGGCAAAAAACTATGACGTGTGATCTGTGAGTAATTTAACACAAAGAATTCTTATTATAAATATATTT
TGATTATTATAAGTGGTGCAATATCAGTGTTATTGTGGATTGATTATTTAATTTACAAAAAAATTTAAACTAC
CTAATATAAAAAGTGTACGCCAATATTGAAACCAGTTCAATATGTCTCGACTTGATAA
CATTGACGCATTGGAAAAACAATTAGCTGACTTGCAAATGGCTAAAGATTTACATTCATCTGGAAAAATAAAA
AAAATACCACCAGCTGTACCACCCAAAAAGAAAGCCCAACCTCTAGTTCCTCATAGTGCTGTCGTTACCGCGG
TGCGCGAGCCTTCCTA
>103778696_2 DT666825.3
GCACGAGGTGACACCTGGACTGAAGCGGACTGGGCAACAGAATGCGTGTCTCGCGCTGAAATCCTCCGGCTCA
TATACCAAGGGCGCTTCCTGCACAGCTCGGTGACGCTCGGCGCGCTCGGCCTGCCCCTGGGGCGCACCACTGT
GATGCATCTAGTACCGCGTGAACACTTGCCCGAGCCTAACTCACATGATCAACGACAA
AAGAGTAAAGGCGGTTCAAGCAGTTGTTGCTCCGCGTCGTGCTGTATATTGTAATGCGCTTATATTAGATCGA
TCGAGCGATCGCGGACAATGTGTTCACACTCAGTGATGTAGGCACCAACCCCTTCGCCCAAACCTAGTGTTCA
CTTGTGATGTTTTTAAAAGACTCGTCTATACCTACTTTACTTACGTTCATTTCGGATA
TTTCAGATCGGCACTTTACTTCCTTTCGTAAATTGTAATTTTAAACGCACATTTTTTGGGGTACTATTAAAAA
TATCGCAGGTATCGAAATCAGCTTTACTAAGCGTTTATACTCGTATCTCACCGCGTATTTATAGTTCATAGTA
ATAACCGCCGTCGACTGCTAGTAAAAAAGTTGTTGTGATGATTTGGTCGTTTTCAAAT
ATTTTGACGGTCGTATCTATTGTTTTACGGGTCCATTTTGTGCGTTGTTTCTTTTTGTTGCCTTGGTTTAATT
TGCTTTGTTCATCAATTTTCTATCTCTAAATCTAATGAATGAAAATATATAANATTTGCCTGGACTAAAATTA
TCCCAGAAAACTAAGGTCACCGCGAAATGGACTGAACAACGCTAAACTGCGGGCAATA
CTATTTTAGTATTATAATTTAGACATCTATATGAATTATATTAACGG
>103778707_2 DT666747.3
GCACGAGGCAATCGTCTACTGGTAGTCCTAATTGGTACTTGAGACCAGAAGATCAAGGATATAATATGCAACC
ATCTTATGAAGACACAGATGTATTAATGGGTAATAATACAGATTTTTCAATGGAAGAGCAAAACAATATGGAA
CCAGATGCTCTAAAATATGTAGAAATTGTAATAGAAGATAGTCCAGTTAAACATCACA
ATGAGACAGGCAAAGAATTAGCTATTACAATTGAAGATAGTCCAATAAAACCTATTACAAGCAAGGCAAAGGA
TATTGATAGTAATAGTGAAAAATCTGATAAAACACCATTAAGTAAAAAAAAGCTGCAATATCCAAAAGAAACA
AAATTAGACAAAGAAGTTGTGGAAATTGAAATTGATATGTGTGAATCCAATTTTAAAT
GCAATGATTCTAAGGAGCCTAGTTGTCAAAATTTAAAAGGACAAGAGTGTGAAAAAAGTGAAAAAGACAATGA
TGAATCATCAATTAAAAATATAAGCCATAACCTTACAGCTGAAAAAGAAAGTAATGAAGAGCAAAAAAAAATGT
GATGAATTTCATCCAGAATACCAAAAATTTATAGATTTATGTTTTGAACTTGAAAACT
CATCTGACATGACCAAAATAGTTGAAAAGAAAATTAAAGCTTATTACAAACAATGCCCAAAAGAATATATAGA
ATCTGAAGAATTTATTGAAATGGTTTCTAGTANAGTGATATCAATTAAGGCTAGCCCTGAAAAAATGTATTTA
TATATAAAAGATATAGTAGATGAGCTAAATTTACAAAGAAAGATGAATAAAGTTGTTG
TTGCCAAAAAGAACCTCGGC
>103778719_2 DT666138.3
GCACGAGGCCTCGTGCTGGTTTGTGTAAAAATTTTGTTTTAGAGTGAAATATAACAAATATAATTATTTAAAT
CATGTCACAAAGTAATGATAATTCTGATGAAGAGTCCTTGAAATCTATATTTGATCAAGCTATGAAATTGTTT
GATAAAATAGAAAATGATAGTGAACCTACTAATAGCGAACCGGTGCAGTTAAGCATAA
AATCAGCAATTTCAAAATTTGAAAAATCTACTAATCTAGTATCTTTAACTGGAATGTTCAGTAGAAATGAAAG
CTTAGAAGAGCTGCCCACAGAAACTTTACAGTATCTTCTACTGCCGGCTTTGCTCGGCACACTCAGTCTAAAG
CTGTGCAATCAACCAAGGAAGGACATTATTAATGTTGCTGAAATATATTTTAGAGACT
TCTTACAAAGGTGCAAAGATTATGGTGTTACGGATGTAGAGGTGCCACAGACTAGCAACAACGAGGTTTCTAC
TAGAACACAGACTGAACAGGCTAAAATTGCAGCCATGGTCCTATCCCGTGAAGCTAAAATTAAAAGGTACAAG
GAAATGAAGGAATTGAAAGAAAAGTTAACAACTTTGTCCAAAGCAATGTCTGCACCCA
ACATAGATGATGAGACCAAACGACAGTATTTCACAACTTTACTATTAAACTATGTGAATCAAGCATTAGATGA
ATTAGCTAGTATGGAACAGGAAAAGCCCATATTGGAATATATGGCGAACCGCGCT
>103778731_2 DT666131.3
GCACGAGGGAAATTTCCTAAAAGTTTATTTGGATGCTGTTTTCAAGCCAAATTTGTTGAGATTAGATTTCTTA
CAAGAAGGTTGGCGGCTGGAACATTCTAAGCTAGATGATAAAACATCAGATTTAGTGTTTAAAGGAGTAGTTT
ATAATGAAATGAAAGGTGCTTTCTCTGAAACTAGTTCCCTCTTTGGTCAAAAATTCAT

TAATACAATTTTACCTAAAGGTACATATGGTTATGTCTCTGGTGGTGACCCCCTATGCATTCCAGAACTGACA
CATGAGCATTTACAGAATTTCCACTCCACCTATTATCATCCATGTTATGCAAGGATATATTCCTATGGCAATT
TTCCTCTTGAACAAAACCTTAAATTTGTAAATGACACCTATTTGAGTAAATATCAATT
TTTGGATCCAAAACATACAATAGTGGCTCCACAGGAGAGATGGAAGATAGTAAAACGGGCAAATACATCATGT
AGAGTTGACCAATATGGAGGTCCAATAGAAAAACAAAATCAAATAGCATTAGGTTACGCAATGTCTGACATAA
CAGACATTTATGACACCTTCATGCTTACTGCATTAGCTGAATTAATGATAATTGGTCC
AAATTCAGCTTTCTACAAAAGTTTAATTGAGAAAAACATTTCAGGGGGTTATAATTCTTTAACAGGATATGAA
AATCAGATTAGAGACACATTGTTTGTTGTTGGCTTACGAGATGTGGAAAA
>103778741_2 DT665170.3
GCACGAGGAAACTCCTTAAAAAAAAATACATTTCGTAATATATAAAAGCATCTAGACGATTTTATTGCAGGGA
GGTATATCTTAAAATATTTTATTCATAGTTAAATTACAGTTTAAGATGTAACTTGCATGTCTACTTTTCCATT
CGCGACTTTTTATGTTACTTATCGATATTTAGAGGATCGGTTCCGTGGCATAAAAGGA
TCGCATCCGACGTGTTTCGTACTCTTTTCGGTAGTAAGCTTTTACATTTAAGGGTTAAGCCTGTCAGTAGGCG
CAGAGTTCCGGGCCGAGATCGTATCGCAATAAGTCGAGCTCGTCGGCAGCTGTATAGGTTGGTAGGGGCTGGT
CCGGCCGACCAGACCGGAGACAATAACGCGGTTTGCGACCGCGCGTTCGCCGCGCGCT
ATCTCGCTCGTCCACCAACACATATTTTATTCTATCTATTTTTAATATTAATGTCAAATATTAACATAATTAC
TATACTAATACATATTGCGCATATTTTACCTATGAATGTAATTATTTTATGCCTAATTCGAATTGTATAAAAT
CACCTCGGCTATTCTGTTTCTGTAGAAAAATTCAGGATTACTTCCCTATAAGACTATG
TTAGTTACGCGTATACATACTTTCTTATAATAATAATATGTATTATCATATAAAATTTTATCCTAAGGATAAC
ATGATCATATTTACGATTCATAAGAACATTTGACAAGCTAAGTCGGAGACATTTCAAAATAATTATCTCTCAT
CGCATT
>103778745_2 DT665471.3
CGGCACGAGGCGCGCCTTGAGGCACAAGTCGCGCGACTGAAGGCCAGGCAGGACCACCAAGACGAGCACGAGG
AGATGCTGAAACAGGAGAGGCGGAAGCTGCAGAGGGAGGCGAGAGAAGCACTAAACCGAGTAGAAGAACTAGA
AACAGAAAACAGTCATCTAACAAAACGTCTTGACAAACTGAAGAACGCCAAGTCAGCT
CTATTAAAGGACCTGTGACAAGATTGAATACAATTTGGTGAAAATACCATGAAAATGCCGATAACGAAGGCGG
GCGTAGATAGTATATTAAGTGGATTTGGATGATAACCGAATGTGCTAAACCTTTGTTTTTAGGCTACGCCATA
TTTTATATTTAGGATTCTATGGATATGCCCTTTTAGTATTGATTAGGTTAGTATAAAG
CTGCGAAGAAAAATTATAAAATTACTATTTTTTGTTTAAAACTAAATCAGTGAGGTCGTTGTGGCGTAGTGAG
TCTGCGACGGTAAACGCGACGGGAGTTTTGGGTTTGAACCCAACCCGGGGGAATAATTAACTATAGTTAAATT
ATTTTCATTTCTCCGCACTGGTAGGTAAAGCACTGTATTGAGTTCCGCCGGTTACGGC
GTTCTCAACACTTATGCAACAAGCATCTATGAAATTTTGCCGTGCGTCGGGAACAGGGTGTCCTAACACTAGG
TTCTAGTAATATATGTGGGACACAGTGTTATGCAAAAAAAAATTAAAACTGAGCTTCATTGTTATAGATGGCT
ATATGAAGCATTTAAGCGGATGTTTGGCGTATGTCTCAATTTT
>103778763_2 DT665488.3
GCACGAGGAAATGATAACATACAATTCTCAAGCAACGAAAAAATTGTAAATATAAGTAAATATTCGTCACATA
TCAAATCAATTATATTTGTAATAGTTTGAATTTGGTCAGTCACTACTTTAAAAAGTTAGATTCAACATCATAT
CAAAGTAAAAGTGATAGTAAATCCATCTCAAGATAAAGAGCACCACCAATTCCCTCGC
AGCATGTCAAATCCAGGCTTAACATCAGAGGCGCAGATGCGATACATTGTTCAATGGTTTGGTGAATTCAGTG
AATTGCAAAGAGAGGACTTCCTTCCCATATTAGTTGCAGCTCATAGCAGACAATCAGATGAATTAGCTTCTAC
ACTTGCATCTCTATCATGTGAAGATAAACCAGTCAGTTTGTTTCAATGTCGTATAAAG
CTATTCAATGAATGGTTTCCAACATGGTCTGAAGAGCAAAAGGAAAGACTTGTAAAAGCAGTATCAGATATGG
ATTTGGATTTTGGTTCCAAACTGCAAGATGCACTTATAAATGGTGTTAAAACAAATGGCTTAGATGAAGAACA
TTTAAAATTTGACTCTGCTATCATTCCACTAGAGGTAAATGACACTGAGGTAGTCAAT
AATGATATTGAATCATTATCCATCTCACAAGATAATTTATCTGTGGAAATTGCAGCTGCATCCTAAGTATAGA
AATTTTATAAAATAATTTTATATAATTGGATTGGATGAAGAACATTTAAAATAATATGACTGCTATCTTTCCA
CCAGAGGTCAATGACACTGATTGTGTCAGTAATGATATTGAATCATTGTCAGTCTCAC
AAGATACTTTATCTGTGGAAATTGCAGCTGCATCTCA
>103778766_2 DT666123.3
GCGGGCTGCAGGAATTCGGCACGAGGCGCGCGCCTTAAGGGGGAGCGCGACGCACGCCAGCGCGTCAAGCGCG
AGCAGGACGAGGCCTACCAGCAGAGCTTGGAGGCGGACTGAGGAGAAGAAGAAATAAAGAAACAACAAGAGCT
AGAGAAAAAATTTAGAATTGGAGAGAGCAGAATCGGAAAGACTTATGGAAGAGGCAAAG
AAAGAGGCGCTGCGCGCGGGCGCGGCGGCGCGCGTGCCCGCGGAGCCGGCGGCGGGC
>103778796_2 DT666172.3

GCCTGTTATACGCAAGATACAGAATTATTCTCTGAAATATATGAACATATTGAAGTTCTCTACTTTTCTCTTG
AATTTGGGCCATTGTATGTCATGGAACTCAGCACCACTCTTGCCAGAGTGAGGAAACTAATTCATTCCTTGAA
TGAAAACGGTGTTGTGTCTCTGTGTCTTACTAAGTACTAATGAACAATTTACTGTAAA
GCCACTAATATTATGGGAAAAGTATACATTATTTTATAATAATTTATCTGTTTAATTATTTTTGTACAACTAT
ATGTAAAAATGACATGATAGTAACAATAGGTGACTTGAATGTAAATTGCATTCTGTACACCAAATGATACAGT
GCTCATTGCATCATCAGAACGAGTTGCAGGCATTAGTCACCACTTTGAAAGGAGGATG
TGAAAATAAAGGTCCAAGCCTGAATGCAAGTAAGGTAAAAGAAATGAAGACAAAATGGGATGCCTTGTCAGTG
TAAATGGTAAGATATTAGAACAAGAGAATGAAGTGCTATATTTGGGAAGTATGTTCAGTAGAGATGAAAGATA
CAAGATGGATGTGGAAAGGCGTATTGCTGTCGGTAACAGGGCTAACAGCGCTTTAGCT
GCATTAATGAGACAGCGAAATGTCGGTACAGCTGCACATTTGGCTGTACACAATGCAGTATTTGTACCAATGC
TTATATGGCAGTGAAATTGTAGGTAT
>103778798_2 DV501488.2
GCCCAAGGCGCGACCTATCGATACCGACATCGGATTTCGAACGACGGGGAGCGAGACCGAGACAGGGTTTCTG
CTGACAGGAAGGAGAACGAGAAAGAACTGGAGAACGACAAGGATCATAAGGAAAAAAGGGCTTCGGGACAGAA
GGATGGGGATGATGAG
>103778815_2 DT666236.3
GCTTTCGGCAATTTTGATACTTTAAAACTACATTTTATACTTAATATTAATTTAGCAGTATGATTGTAATTTA
TAAAATAATGTTATTTATGTTAGTTATCTTACCTTTCGCATTGTGCAGCGATGGTGATAGATCGCCATTCTTC
CAGAAGTGTTTAGTTAACTGTAGAAAGGCAAATTGTACTATAGATGGAAAATTTCAAC
GACATGCTGCAAAATTGCAAGATAAATGGTGTCTAGTACTCTTTTGGTCTTGTAAAGAAGAATGCATGTATAG
CTGCATGTGGAGGACTGTCAGTGGCTTTGAGGAGAGAAACTATGAATATCCTAAATTTTATGGAAAATGGCCG
TTTGTACGACATTTTGGTATACAAGAGCCAGCATCTGCATTGGCGTCAGTTTTGAACC
TATTGATGCATATATATATGTACCAATATGCATTCTTAGATCTACCTTTTAAAAGGAAACCATTGTTAATAGG
TTTCTGGCATATATTTGCAATTGTATGCATGAATGCTTGGGTTTGGTCAACAATATTCCACCATGCGTGACCG
CTACTTTACCGAGTTCATGGACTATGCATGTGCTTTGTCTGTCACTATTATGTTATTC
TTATCAGCTATTGTAAGATTAATGTATAAAAGGAAA
>103778826_2 DV501437.2
GCACGAGGTTTGCACCTTAAAAATCAAACGCTGTCTAATCTCACATGACAAAATGAAAACAGAATACGAGTTC
ATGACAGCCACCCCATTGTCCTGTATAACCAATTGTGCGTACGGTGAACCGAGTGTCTAAAACAAAATGTCTT
GTCCTGCCATAGAACTTCTGATGATATATTTCAAACGCTTACAAGAGGATCCACCTAC
TGGTGTATCTGGAGCACCGACTGACTACAATATACATGATTTGGAATGCTGTGATATTTGGACCTCATGATAC
TCCATTTGAAGATGGTACCTTCATATTAACAATTGAATTTACGGATTATTACCCCAACAAACCACCAACTTGT
CAGATTTGTTTCTAAAAGGTTTCATCCTAGTGTATATGCAGATGGTGGTATTTGTTTA
GACATTTTACAAAACACATGGATCACTACGTATGATGTTTCAGCAATTTTAACTTCTATAGAGTCATTATTAA
GTGACCCTAAACCATATTCACCAGCATATTGAATGGCTGCACAGCTTTATAATGAGAATCGCCAATAGTATGA
TAAAAGAGTCAGAGCATGTGTATAACAGTCATTTATTGATT
>103778836_2 DT665878.3
GCACGAGGGTGAGCTCTTGGAAGTGATGTGAAAATTTTTTTACAAACAATATTCCGGCGTTGAGTTACGCCAC
TCAAGTTGCACTACAGAACACACAGAGAACAGAAGTGCCTTATCAGAATGTAACAACTAGTTTCCAAAGTTCT
GCCTACCCTGCGATGTGCGCCATAAAGTGAAGTTAAAAAAAATTTTTTCCACATGAGC
GCGGCGTTTTCTTCCACTACTCAATG
>103778853_2 DT666097.3
GCACGAGGCTTTCTCTTTTCATGTGGTCGTTAACGTTGGTTTGTTTGGTGTTCGCGCCTTTGGTAGTGTCAGA
AATAATATTATCAGAAAGGGTTCCCGTAAATGACGAGGATAATGTTCTCGAAACAGTCGATGTTGGCGACAGG
ATAGTGGGTGGGGAGGAAGCCAACATAGAAGAGCATCCGTACCAAGTATCATTTATAG
TAAACAGCTCATACTTCTGTGGAGGTTTTATTATCAGCGAAAATTATATCTTAACAGCGGGACACTGTGCTCA
GAATGTCGATCCATCAACAGTAGTTCTCCGAGCAGGTAGCTCCTTCCGTAGGAACGGCACAATCATTCCCATA
GCTGAGGTGAAGCCTCATCCGGAATACGACAATCCACCCTTCGACAAGGACGTCGCGT
ACATGAAAACTGCTGAGCCTATACAGTTTAGTGATACCATACGGCCTATAGCTTTACCGCCCAGGGGTAGGCC
TTTGAGTGGTGAAATTATGGTTAGCGGTTGGGGTAGAACTAAGCAAGGAGCATCCACAATCCCCGATCGTCTC
ATGCACGTGGAGCTGCCAGTCGTACATTTCTTCAGATGTTTCCTCGTCTACCCTACAG
TCTTGACGAGAAACATGTGGTGTGGAGGAAAGTTCTTTTTAGGAGGGAAAAGCACCTGTCAGGGTGATTCCGG
AGGAGCAGCCATACAAGATGGGATGGCAGTCGGTATAGTTTCCTTCGGCAGAGGTTGCGGACAGGCCCTCTCA
CCGAGTGTCTTCGCTGACATCGC

>103778875_2 DT666579.3
GCACGAGGGCAAACCCTAAAATTATAAATATTAAGAATTTTAGAAAAGTATCGAAAGCTTTTACTTAAAATGT
CTTTTTCTGAGTTAAAACATGCTTTAAGAAAGCATGAATTTCCAGCCTGTGCAAAGGAAGGTCTTTTAAAAAT
AGGTACTTGTTTCCATTAACATTACTAACCCTGAAAACGTTACAAAATGAACTATTTA
CACTTTTCTTATTGTTTTTTTTTTCAGAACAATTGCTTGTGGGAAGATCAGCCCCTACTAATAAACAGCTGGA
TAATGCAATGGAGCTTATATCAGAATTTGTATTTTGTGAGACCGACCGCCGCGGAAATCGACGTGGATTGAAT
CCTCTGCAGGAATTACAGCTCATAGATATTATTTGTGATTATATATCAGCTTGTACAA
ATGAGACTACAAAAAACACAATATTTTTATCTTTATTTGGGGGAATGGAAAGTCAGAGAAAGCTGAAAATTTT
AAGCATTCTTGCTAGCATGGCTGTTTCTGCCTCAAGTACTCCTGTGTTGTTAGCAGTAGGTGTGTGGCTTCAA
CAAATGGGTTGCTCCTCTCAACAATCATTACAATTAGTTGAAAATGTAATCAGAGACC
ATTTCTATTTGAATACTTCCAACCAGAGTGCTTTAAAGACCCTAGCAACAACTGCTCCACAGTTTGTATCCAA
TTTTATAACTGCTGTAACTGAATTGTACATGCATGATGGACAAAAAGCTAAGAAAATGCCCCCCAAAAATTTA
>103778889_2 DT665791.3
GCACGAGGACACAATCATTTCTCAAATTTTATTTACACGTGAAATAATCAGTGCTTGATAGCGAAAGTGACGA
ATAATATTAAAATAAAACGCATCATGAAATCGATATTAGTCATAGCTACTGCATTCCTTTCTAATGCACTAGC
TTATCACGATCCTGACCTCAACTACCATCTGAGTAAATTAAATAATGCACAAGATTGT
GGCCATTCAGGATATTCTTATCCTGCCCCTAAAGTGCAGTTGTCTTTAGCGAGAGTTAAAGCCACAGCAGCGC
CGGGATTACTACAATCTCTTTCTTTAAGTTCATCGAGCGGGCAAAAGAGTTCAAGTGGTTACTCGCAGTCTGT
ATTTTTCAAATCCGCGGCACCCCAAATTGCCTATCAAGCACAGCCAATTTCTTACCAA
GCTCCTATCGCCATTCAAGCTGCTGCTGCTTTAGATGCAGAATATTCAGCGCAAAAGGAAGAACATGGTTATG
CCACTTCTGCAGGACTATCGGCAGCTACTAGGACAGTTATTCCACAAGCAACTTATGCTCAAGCACCAATAAT
ATCAAAAATAACTGCTGCCCCATTGCAGGCTAAGTTTTCTTTAGCTCCAACAAAAACA
TACGTTTCCCAAAACTTAATATCGCAACAATCTGCTTATTCCAGTGGTTCTGCAGCAAAAGCTTCTCTTCACT
CTTTCACACAATCAGTAGGACCAGTTGTCTCACAAGTTTATGCTGCTCCTTCTGCTGGATATTCAACTTCACC
AGCATTAAAGGGCCAATCAGCTCAATATGCTGCCCCTTCTTATTCATTTGTCAACCAA
TATAATCAAGCTTCATTAGCTAATGTTGCATC
>103778903_2 DT666201.3
GCACGAGGTGTTTCTGAGCCCAAACTTAGAACAGTGCCTGATGTTCCAAGAGCAAATGAACCAATAAGCTGTT
GGTGTGTTATAAGCTCTGGTTTAAGATCGTCAGCAGTTATAGGGTTCCTAGTTTGCAGGGATAAAGAAATCTA
TAGATGTCAACTTGGTGAATCTAGGCCAATATTATTGCGCCCAGAAATAACCAATCCG
TACACACAAGTTCTCACAATCGTGCCTTCACAAAATGGCCGCCATGTTGCCCTGTTCACTGATTCGGGAATAC
TGTGGATCGGCTCGTCTGATCTTAAGAATAAGTACTGTGAGATCGACACTGGATATATTAAACAGCCGAAGGA
GTTAGTTTGGTGCGGTTCCCAAGCGATAATAGGTCACTGGGACGACACAATGTGTATC
TACGGCTTCAATAGCACCACTATAGTATATCCCTACGACGGCCCGTTCCATATAATACCAGAAATGGACTGTG
TTAGAATTATATCGGAAATGTCACACGAGTTACTGCAAAAAGTCCCCGTGGAGGTGGAAAAGATTTTTAGAAT
TAATAGTACTGCTCCTGGATCGTATTTGGTTGAAGCGTCTAAGCAATTTCAGAAACGT
AGTCACAGAGCTGATGAATACATACGCCTAGTANAACCAGATTTACCGACCGCAGTACAAAATTGTATAGATG
CTGCCGCCTTCGAATTCAATACCGATGTCCAGAAAATGTTAATACGCGCAGCTCAATT
>103778907_2 DT666781.3
CGGCACGAGGTAAGTAACTTCTACTACGCCTTTTGACATCTTTTGCGTGGTTAGTAATAGGGAGGAAAAATGG
ATAAAAGAAAATTATCAACTGCTGGCGATAGTCTATATCAAATACTGCAGGTACCTAAAACTGCTACTGCTGA
TGATGTCAAGAAAAGTTATCGTAAACTAGCGTTGAAATATCACCCGGACAAAAATCCC
AACAATCCAGATGCAGCAGAAAAATTCAAAGAAGTGAATAGAGCCCACACGATATTAAGTGACGCTACGAAGA
GAAATATCTATGACAACTATGGCTCCTTGGGATTGTATATAGCTGAACAATTTGGAGAAGAAAACGTCAACGC
TTACTTTGTTGTCACTAGTACGTGGTGCAAGGCCCTATTTGTCGTTTGTGGTATCCTG
ACGGGATGTTACTTCTGTTGCTGTTTATGCTGTTGTTGTAATTGCTGCTGCGGAAAATGTAAGCCGCGCCCTC
CAGAAGAATCTGGTGACTATCACACGTTGGAACGAGATGATTCAGATGGAGTTCAGCCGGTGACGATGCAGCC
AGGAGGGGAAGGGCGTCCTACAGGGGAGCAAGCGCCACCTATCGCCATGCCGGCGCCA
GCTCCGGGTGTCTCCGAAAACACAGGCCTCAACACAGGGAGCAGTATTCCAAAATACACATGAAGTGTTTACA
TCTAGTCTAGAGTTATTTGTTAAGAGAAATGAGGAACTGACTACTTATTACCACCAAGCAGCTTCTTACTGAT
GTAAGCCTACAGTTATTATAATATGTTTCCTTTAATATATTTTTTTCTTTTTTAGATA
TTTTTTTTGTATTTAATTTATTTATATTTTTGAAGTATTGTATATATAAATGATTTTTTTTTACGTAGG
>103778911_2 DT666522.3

GCACGAGGCCTCGTCCCCGACAGTACACGCGGTAACATTCATTATTTTTATATATATATATTTCTAATATTTT
CTAGACCAAAAAGTTTTTTTCTTTTTATATATAAAGAGAAATACTGTTTAGTTTTAGTGACAATGGAGTTAAA
AAGTATCCTGCTTGTGTTTTTTTTTATTTGTTTTTGTTTATGGCCAACAAACAAATTTT
GAACTGCCAGTGCAATTAGTAGGTTTTCCTTTTATAGTTGGAAGTGTTAGATTAACTAATTTTTTGAAGAAAT
TATCGTATTCCCTTAGTCCTGCAACATACCGTTCCCGTAATCGTCGTGAGCTAACTTTCGCAAGTGAAGTTCC
CGAGTCGTTCGACGCGCAAGAGGTTGAAAAGTATATCATAGGAGAATTCGGCGCACGC
GCATGCATTTTCGAAAGAGTATGCGCCCATTATGCAACTCGAGCTCGGGAACAGCCAAAGCCACAGATGGACT
GGCCACAAGTTTTCAGCCAATACAAAAATTCACCGGACCAGGCAAAGGAGCTATACCTACTCAGTGTCTTTCT
CGGTGACATAGTTGGATCTCCACAGCTCTGTCACCAGCTGGGGAAACGGAGGGGATGC
GACGAGAAAGTCCTTGGTGATATTCAAATTTAAACATAGATATATGTTTATAAGATATTAGATATTAATTTAA
ATTATAGCATTCGGTACAAGTGTAAGTAGTAAAAATATTTTTTTATATATATATTGTTATTTTGTAATGATTT
ATTTGGATGTTGTATATTAAAAAAAA
>103778938_2 DV501435.2
GCACGAGGGAGCCCCCTTCTATGCTCCCGCCTACAGCCTGATGCCTGTTCCCGATCACTATATTGTTATTTTA
ATGTTTATGTTTGTTCTTATTGTGGATCCTGGTAGGATTGTTGTTAAAATAATTGTATTTACTGTATTGGTGG
TGTCTAATTACCTATCTCACCGTACTGTCAACGAGTCATTGGCCGCTTTGAAGTTAGG
TTTTCAGAGGTTTTACCCCTTCTTCACTCTGGGAAGAAATTGGCTTGACGTTCGTTGTCGGCTTCTCTAACGT
TATCTATCTAGATATATGTATCTATATAACTCTGTGTATACCTTTTGCTCCTTCAGATTTTGATTGACCAAGA
ATCTATTTTTTACCAATAAACATAATAGGTTTACCTACCTGTTTTATTTTGTTAAAGT
CATGGCATCTCTTTAGCATAAATGATTGTAAATAATTATACACCGCCGATTTGCGCAAAGCATTAACGGCAGC
TATATACGATATTTTACATATATTTGTCACTATAATTACTAAAATAAATCAAAACACTTAAAACTTAGATTTT
TATACGAACTGAAATACCTTAAATCACACTAATGTACAAACAAAAAAAATGTAATTCG
GAAATCCGACATCTATAATCTATAACCAAAACTTCGGTATATTCAGTTTCATTTAGTTTATAATAAAAAAAAA
TTCTTGTAATTGCGCTGTACATATTCTTC
>103778942_2 DT666303.3
GCACGAGGTACGAACGGGACTATTCGCGGCTATTTTATCTTGATTTTTGTTTATTCTAAACGTTTTACTTAAT
GAAAATACATTGCTTTTTTTAAATAATGTGTACTGAAGATGCCAGTTAATTCTCTGTCGGAAATAAAACAAGG
TGTCGAGAAAACTAAATTTTCACTTTCACTTAATAGAAATAATGCAACTAATTTAAAC
AGAGAAAATAAAGTTGCAGACATAAAAGAGAATAATACTCTGAAAGAAACTAACAACGATAAAGAGAATAAAC
TCGGAAAAAGACCCTTGAAACGCACATATTACAACACATTGAATGCAGCGAAAAAATTAAAACCTTTGACAGA
AGTAGCTAAACCTAAAGAAAAGGTGTCAGAGTCAAAGTTAATAATTTTGGAACCTTCA
GGAGGAATTTTGTCTCTAAGTAACACTGCCATGTTGAATGGCCATCACCCTGACAACCAGTATGGAGGGAGCA
CTCAATGGAAAACACCCATTGCTACTCTCTCCAACCTTGGAAACACATGTTTTCTGAATAGTGTTCTATATAC
TTTGAGGTATGCCCCACAATTTGTTCATAATCTCCATCACCTGGTGTCAGATCTCAGT
CGTGTAGAACAAAAATTGGGTAGCATCCGGTTGAAGAGTTCCTCGCTAGGTAGAAGTGCAGCTGGTCTCGCTT
CATCCGGCACCAGATCATGGAGCAGTAAGGATTTACTCTCGTTGGGACAATCTGACAATAGCTCGGCTAAGAG
TAAATACAAATAGCAACAGAAAACTTCACGAGAATACTTAAGTCTCCGTGCGGC
>103778949_2 DT666656.3
GCACGAGGGAGACTTTTACGTTATCGCTGCGTACTACAATATAATCTTGTATTTAAAACATAACTCGGATACT
TTTGTCCCAAACCTTTATTTTGTATTGATAAGTTGCCTTCTGCACCAGTGAGTGTAAAGGCACCTTTCAATAG
GACGTTACTGTGCTATCGTTTAATTATTTATCGTAATTCAGATTTTTCTATACCCTCG
TCAAAAATATCTGTGATTTTATTATAGAAAAATGAAGACGATTCTGCCTTTCCTCGTGATTTTGGCTGTCTGC
TCTTCAGCATTCGCCACACAATGCAACGTGAACCCGGTGAACATCCCCAAGCACTGGATCACGATGTCGCACG
GCTGCCGCAGCAGCGTGCGCCAGCAGATACAGATGGAGATAGCTGCGTCGCTGCAGTA
TCTGGCCATGGGGGCTCACTTCTCTAAGGATTCGGTGAACCGTCCAGGCTTCGCCAAACTGTTCTTCGAGGCC
AGCTCTGAGGAACGCGAACATGCCATGAAGCTGATAGAATACCTACTGATGAGAGGAGAGCTGACCACCAACG
TGTCCTCCTTGATCACTATTAGGCCCCCTGACCACAAACACTGGAACTCCGGCCAGGA
GGCCCTCGAGGAGGCTCTCAAGATGGAAGCTGAAGTGACAAAGGCCATCAAGAACGTTATCATCAAATGTGAA
CACGACAGGGACAGCCAGGGACACGACAATAATGATTATCATCTTGTCGATTATCTAACCGGT
>103778953_2 DT665542.3
GNACGAGGGACTGGCCCTTTATTCAATATATTCGCCTTTTAATTTCGTATAAAATTTCTTTGAATAAACATAG
TTCGTTTGTGAAACATTCACAGTGTTGTGTAAATATGAACGAAGATAATACTGTCAGTGACGACCTGAAGAGA
CAAGCTGCTTTGTTTTACAATTCAACAGAAAGGGAAACAGCCAATGTAGCACCAAGTG

GTAAACAGGAGAAATCATGGCAGTTTTGGAAGAAGAGACGGCTAATTGTAGCCTTATTAGCTTTCTTCGGATT
TTTCAACGTATACGCTTTAAGAGTCAATCTTTCTGTGGCCGTATTCGCTATGACGGAACCTGTGGAAGTGGAA
CTTGAGAATGGAACAGTCGTTTATGTTCCAGAATTCGACTGGTCGTCTCAAACAAAAG
GATTAGTGCTAAGCTCCTTCTTTTATGGTTACATCATTACTCAACTACCTGGTGGTTGGCTTGCAGCTAAGAT
AGGAGGTAACAGGATGTTCGCGATAGGGGTTGGTACTACTTCCCTATTGACGCTCTTCACGCCGCCCTTAGCG
CATACAAGTACTGCCTTGCTTATTACAGTGCGTGTGATTGAAGGATTATTTGAGGGTG
TAACCTACCCCTGTATCCACGCGGTGTGGTCCCGCTGGGCGCCCAGTGCGGAGCGCGCGCGGCTCGCCACGTT
CGCATTCAGCGGCAGCTATGTGGGCACAGTCGTCTCCATGCCTATC
>103778970_2 DT666448.3
GCACGAGGTTTTTTCTTTTTAATTAATGTCATCTCATTTGGCATTTAGTACGAAACCTAAACATAACCAATAA
AACTTTATTAAATAATGGTTTTATAGCACTTAAATATAAATCGTTTTAAGTTATCAGTGTTTATTGTGGTGCT
CTGTTGGTTATTCTAGGTAACTTTATTTTGTCACGTGATGGATTCAGACATGAAAGAG
AAAATAAATCAACTTCAATTGAAAATAAAAAGTTTTCCCGATTTTCCTAAAGAAGGAATATTATTTTGGGATA
TATTTTCAGCTTTACAAGATGGTGCAACTTGTAAATTATTACAAGATATACTTGTAAATATTATTAGGACGAA
ATATCCAGATGTTGAAGCAATAGTTGGTATAGAATCCAGAGGATTTCTATTTGCATTC
TCTATAGCTGCCGAATTGGGTATAGGATGTCTAGTTGTTAGAAAAAAAGGAAAACTGCCAGGAGATGTATTCT
CCTATAAGTATGATCTGGAATATGGATCTGACATTTTAGAAGTTCAAAAGAGTGCGGTGAAACCAAATTTAAA
ATGTTTAGTCATTGATGATTTAATAGCAACTGGAGGTACAATTACTGTTGCAAGTAAA
TTACTACAAAATTGTAAAGCAAATGTCCTGGGCTGTCTTGTTATCATTGAACTTGAAGAGCTAAATGGTAAAA
AGAATATTCCAGAANGCATTTCTCTACATTCCTTGATAAAATACTAATGTAGTGTTAATTCCATGTAATATTA
TTGAATCATGGGACAGTGCATTTTATGACATTATTGAGAGTTCCCTTTACTAAAAACC
TCTGTCTTCTCTATTTTAACGGAATACAGT
>103778987_2 DT666839.3
GCACGAGGCAAAGACCTTAAACAATTATAGCTGTTAAGTTAAGACCAAACTACACTAGTATATTTAAACAAAT
TCTTTTTCCTAAAACTTTCAATAAAAGCTTTAATATTAGAAACAGTTACACAGCTATGGGTGCCCTAAGTCCG
TTAGACCCGTCTTCTTTTTCAAGACCAGAATTGGCTGTAATAAAACATGTTTCCCTAT
CATATAAAGTGGACTTTGAAGAGAAAATACTGTATGGATCAGCAATTTTAAATATTGAAGTAATTGATTATAT
CGAAAATGTGATATTAGATACAAATGACTTAACAATAGAATCAATTGAATTCAAAGATGGTACGGCATTGAAA
TATAAATTGGATGAAGCGGTGCCGAATTACGGATCAAGATTGACTATATTGTTGCCTC
AATCTGCAGCACCAGAACAAAAGCTTGAGATTAAAATTAACTATAAATCTTCACCATCTGCGACGGCACTGCA
ATGGTTGGATCCAAACCAAACCTCAGGGAAAAAACATCCATATATGTTCAGCCAATGTCAGCCAATCCACGCC
CGTTCTATCTTACCATGTCAAGACACACCCTTTGTAAAGTTTACATATGAAGCTGAAA
TAATTGCCCCAGAGGAGTTTACAGTATTGATGAGTGCTATCCGTGGGGAAAGTAAGGGGAATAAAACTGTCTT
CAAACAACCTGTACCGATTCCATCCTATTTGTTGGCCATTGCAGTGGGTGTTTTGGAATCGAGGTCTTTGGGA
CCTAGGTCTCATGGTTGGTCAGAGAAAGAAGAGATAGAACAAAGTGCATGGGAGTTTG
C
>103778991_2 DT666457.2
AGAAAATGTCTGCCTTACTCTACGAAAAAGTTTGGGTTGATAAAAAATGTCTATAATGATGCCGAACGAGCTTA
TTATGAATCTCTATCAAAAAAACAGGCAACAGGCATAGCTAGCTCATCACTAGCTAGTGAAGTTGCAAAAGCT
AGACAACACATTAAAAACTCACTGGAATGTATGGACAGCGTTGCGGCATTAGCTGGTG
TTCCAAATACTGAAATTAACAACAAGTTAAACAGTCTCGAAAAAGAAAATTTTGAATTAAAAAAAGTTGTTGA
TGATTTGCGGAAAATTGTTATAAGTCTACAAGCCAGAGTTGAGTCAATTGAATCAGCTAATTCTGGGAATAAA
GCTGAAGCCCCTAAGGCACCAGTGCCTCAAGCTCAAGACGATTCAGATGATGGAGTTG
ATTTATTTGGATCAGATGATGAAGAAGAAAGTGCAGAGGCAGCTAAGATAAGAGAAGAGCGCCTTGCTGCATA
TAACGCCAAAAAATCCAAAAAGCCAGCACTCATTGCAAAATCCAATATTATATTGGATGTGAAACCATGGGAC
GATGAAACTGACATGAAAGCAGTTGAAGATGCAGTTCGGAGTATCAGTAATGATGGTC
TTTTATGGGGTGCCGCCAAATTAGTACCTCTAGCTTTTGGTATAAAAAAACTACAAATATCCAGTGTTGTAGA
AGATGAAAAAATATCAGTTGATTGGCTGATAGAAGAGATTGAAAAATTTGAAGACCTTGTACAGAGTGTGGAT
ATTGCTGCATTTAATAAAGTGTAACAGTGGGCCTACGTGAATAAAAAAAA
>103779011_2 DT666332.3
GCACGAGGCAGGAACATAGATATGTCAGGGCACAGCACATTGGGACGGAGTAGCGGCAAGCCCAGCTACATGC
CGGCCCCGAGCCCGATGCCGCCGCTGGACGGCTCCTACTTCAACAACACCGAGTCCTACCTCTCTTACCCACC
GCTCATTGGAGAATATTTGCAACAGCAAGCAGGACGTACCCCGACGCCGCCTCAACAG

TATTCCTCAATCCTGCCCAAACCTCATCTAATGAACGGAGTACCACCGTCCCCCTCGCACATGGCAAGCCCGC
CACTGAACATGGGGTTGGATGGTCTGGACCGGCAGTCCGCGGGGGGCACGCTGCGGCGGCAGAGGTTCGACAA
GATGGCGGTCCCGCCGCCCGACGTCACCGTGCTGCACCAGGGCGGCTGCAACCAGCAC
CCACCAATGAACCAAACGCCAGCTATCAAACAGCCACAATCAATCCTCAAAGATCCCTCTAGACACAAGTATC
AGTATGGGAGCCCGATATCTTCAAGCACCCCTCAGAACTCCAGTCAAATACTCACAGTTCAGAATTTAACTTC
AGACGTACCCCAATATGGTACCATTAAAAAGGAGAACAAAAGGCAAAATGTTACTATA
GATGAATCGTACAACAAACGCAGCGAAACGCATGTCGTTTAAATTTTACAAGAAAACAAAACTATTCAAATAA
TAATAATAACTATAAAGTGTTTAAGTGTATGTGACTTTAAGTGAAAGACAGAAGTGAAGTGCAAAACAAGT
>103779023_2 DT665847.3
TTCGGCACGAGGCACAACTGCTTCTAAGAGATATCTAAAGTAAATAACTATTCGACACCGTAAACGAATCTTA
TATAAGTTCCGGATAATTTCTGATCTGGTCTAATGGTACTACATGAGACTGGTTTCACTGGTTATTTTCTAGC
TTTCAGTACGAACTAGCGATACGTATAAAGAGCTTTTGTTAATAAAAAAGACTATAAA
AACAGCTGTCAATAACAAACTCTTTAAAAAAAATCGTGATTTTTCGTCTTTAAACTTTCTCAAAAATCTAATA
ACGATATCCTTAACCGAGATTATCGGAAAATAACTGTTGATCCGAAATAATGTTATATCCGTTACCGAAACTA
AAATGATATCCGTATCCAGATCCGTAACGTTCCGTAACATTCCTGAACTTTATATTGC
AATAATCTTGAATATGTCTAGAAGACTAGTTAAGATTTCCCGTCGTTGTTTAACATTTAGTTCGATTTAGTTG
GTTGCCGTGTTAGTGTTGATTTTTGTGCTATAGATGTGCTATAGATGCTTAATGTTCGGTTATTTTAACAGTT
TATTGTGTTCATAATGAACTCTTAGGTACTGTTTAATCGGTTGTGTTAATATTTTTGT
TATTTAATACGATTTATCAAAATCAAAATTGATTATTTGTTTAATTTTGATTAGAAATAGTTATGGAAAATGG
TTTATAATAATTTTTATTTCGAACAGAGTAAATTTTAATATATAAAAAAAAAAGGTTTACAAAACATTTTTAA
AATTTATATTTCTAGTGACTAACTGTGGAAT
>103779031_2 DT666374.3
GCACGAGGGCCACACCTTTGAGGCCTAAAGGGACTATGCAACACTTAGGAAACCTAAAGGGACTATGCCACAC
TTAGGAACCCAGACTCATAGCTCAACACAACTTGGCCTTAAAGAGCATTTCACTCACTTAAGCGTACCAAGTT
GTTAAAGTCGTCTTGCTCTTAAAGAGTATTTTTCTCACTTAAGAGTACCAAGATCAAC
TTTGAGCCCACATGTCAATATTCAAATGATATTATAAAATTATTGAGCAATATTCCAGATTATACTCTTAGTG
TATATTTTATCACGGCTCGCTTAAATTCCAGTGGTTTCTTTCTGTTTGGTCAACCTGATCACACCAGTCTGGA
TCTGGATTGAATGAAGCAATATCACTCAGTCTTGGTCTTTGTGTTGTTGTAGTTGAAT
TACTAAATAAACCTGCAAGGAACTCATGATTAACTTGCATACTGGCCTGTTGTTGCTTAGACAGCGTCTCCAC
TCTCCAGAATATTAGCCAATGCCAAGAGTGTCTCCGAATTCCTTGGTATGTTTCTTCCTTCAGCGTGAGAGAA
TGAACCTGCTTGGTCCATCCCACTTCTGATGTTAACGGTGGGCGTAAATAAAAAACGA
GTAGAGCCTCGGCCATGTCCTGGCCTTTGGTTCTAATCATATGTGAACACATGACACATGTACTAACACTCAG
AAGCTCGGAGCAGTGCCTGGGACTTGCGACCACAGGTGTAGGCTTCA
>103779033_2 DT665834.3
CGGCACGAGGAAAAAGCGCGAAGCTTTTTTTTATATGTTAGGTGTAGCTGTTCTGAAACCTAAAATTCTCCCT
TATATTTTTTGGAGATATATCAATGTGATGTTTTCGAAAGTAATACTTTGAACTATTTTTATATGATACCGTA
GTAGTTTCTTTTTTTATTTGACATCTACTAATTTTTTTTAATTAATAGTATGCTAATT
TTACAAAATTCCTTACATGTTTTATTTATGTAAGAGAACTAAAACTACCTAAATTCTAAATTTACCTCAACAA
AACTTTGGACTCTCATTTGTATATGTTTCCGCAGATTTTACTATTTTCCGTCCTTTCACACCACTAATTTGAT
TTCAGCTTGTTTAATGGTTTGCGTGTAATCTGGACACTTGTTAATTTATTGGTAACTA
TTCCATCCCTATCCTCTCCTATCCTATTTTTAAATTTGTCATCCTTGAGGTCTACCACAAACTTTATCTCTAA
CTCGATGCTGATGCGAGTCCAAAGTTTTCTGGGTGAAGTTTGTGGAAGATCTTGTAGTTCGTGTAGGCTCATA
GATGTTTTAGTAGAATATCAATACATGTAGATGTCTGTAGCTGTGTTGTTTTCAGTGA
TAAGTAGGTCGTGTTCAATGTCTGGTGTGGTGCACAGGTAAACGTAAGCACGACGGGGGTCAGCAGAGCGCTG
GGGGGGAGAGCAAGCGGCGGCTGGGCGCGGCGGCGGGGCGGGCCGGTGGGGGCGCGGCCAGCTAGCGCGCGGG
CGACCCCTGTCCCGGAAACACTACAAGCTGC
>103779036_2 DT666591.3
GCACGAGGCCTCGCCTCCCTCGCCCTCCGCCCCACCACCGCCCCCCAGGACTGACAGCCTGAAGCTTTCAACA
GAGGATATGGTACTAGTGGCCAACGCCGGCCTCCGGACTCCATCAGGTACGCTAGCGCACTACCTGACGCCCT
ACACGCCGGAAATATTCACACGCAGCGATTATTTACGACCACAACTGAGCACGTACAG
ACCACAATCGTTCACCAAAAGCGATTATCTCCGGCCGCCATCTTCTCCTGTCCAACCGCGGTCGTCGCTTTCC
CTTAGAACGGATAGTATAGAGGTACACGGTATTATACACACGTCGAGCGTTCACTTGATAAGGCAGGCGCGCG
TGCGCTCACCGCCGTCGCTGGAGTGCGACTGTCTGCCCGTGGACCCAATACCGCCTCC

GGAGCTGTTCGATTTTTGAATTTCGTTCGAATTTTAATTTTGTATTTTACGATTGCGATTGATTGATCTTTTA
TTCAACCATCACCCCAATACCACCTTCGGAGCTGTTCGATTTTTGAATTTTATATATTTTCTTTTTATTCGAA
TTTTGAACTTATAGTTTTTTAGTTCTTTTGTGTCTACGTTTACGGTTGATCGATTTTT
GAAATAAAAAAATGGAATTTTAATTTTATGTCTTACGATTGAGATTGATTAGTTTTTATATTTTATAATTCCT
GTAATTATTAGCTTTGTATGTAGGTATTTTTTTAATCTATAATATGATTATCCCTAGTAATATAATTCCTAAN
ATAGTAATTTGGATTGCCTACATAACAAATTTAATATTATTTAAATAACTGGGTTAAT
TGTCAT
>103779038_2 DT666566.3
AAAGCTAAAAAGAAGTTTATAGACCGAAAGAAAGCAGTCACGTTCAATCTAGTACACAGATCGCAAAGAGATC
CCTTAGCTGCTGACGAAAGTGCCCCGCAACGTGTTCTTGTCCCTGTAAACGCTAACATACCCTCTAGAAAAGA
AAAAGATCCTGAGTTAACCCTAGAGCAGAGAAGAGAGGAACAACATAAATATGGAATC
TATTTTGATGATGACTACAATTATTTACAACATCTTAAGGACACTCAAGAAGTTACTATGGTTTTACAACCTA
AATCAACCCACAAAAGGAAAGAGAAACCTACAAATCAAAGCCAAGATCAAGACATTGATACAGATAAAGAGAT
AACGGACAACATAAAGCTACCGAGCTCAGTGTTTGCTTCTGAAGTTGAAGAAGATGTA
GGATTATTGAATAAGGCAGCTTCACAAGGCTTATGCTTAAATTTGGATCCAGAAGTAGTTGCAGCATTAGATG
AGGACTTTGACTTTGAAGATCCTGACAATCAATTGGAAGATAACTTTATAGAATTGGCTATGGGGGATGGAGG
TGATGAAGAAGAATATGAAGGAGAAGAAGATGATGATGATAGTATGTATGATGATGAT
GACTCGGAGAAGGCATTTGCATCTGATTTGGACTCTGATGATGATAGTGGAAA
>103779053_2 DV501441.2
GCACGAGGGTTGACCTGATCTCCGGTTCAGCGCTGAGCACTTTTTAATTGGACAGTTGATTATCTCGACCGGT
AAATCTCATAGGCCTGGCATCCGCCGTCGTGTCACGGGAGGACATCTAGAAGCCCCAGGCTGAGGTCTCACCC
ATCCACCGTATCCTTATCACATTGACATCCCGCAATGTACGTTCTTTACAATAAGTCT
GTGCTGATTTGATCAATGGTGCCAAGGAAACAAGAATTGCTGTGTAAAGGGGCCTGTCCGCATGCCTACCGAT
ATTCTCCGCATCACCACCCGCAAGACACCTTGTGGTGAGGGTTCCTAGACCTGGGACAGGTTCCAGATGAGGA
TCCAACCGAGATTGATCTAACTTCTTTCTCCTTCCGAGATTGTGAACCAAATCACCTC
AATCAGTATTGAACCATGTGTAGAAGTTGAAGTTACTATTGCGGACACGTACGCACAACTGCCTTAATAAATA
TTTTATAATTG
>103779057_2 DT666920.3
ACCAGGTACTTCTTTGGGAAATGGTAATTGTAGTGACTTGTTGATAGACAATCCTCCTGGTCTAGGTTCTGGA
GGCTGAACTCTAGACTGGGGTGCTGGGCTTGCTTCCAATGAGTCATCATCAATATTAATAACTTCATGTGATC
TTATATCTATTTCTCTTGACAAATCTACTGGACTATTATTATTGTTCATTAAGGATTC
TTCACTTTTTACTCTAATATTTTCACTTTCATGTGAATTTTGCAATCGTGAGATCAAAGTTTCAGACTGTTTT
TTTAATTTTTCAGAAACAACCTCTGGAGCATTCTTATCTTCTTTAGATTTGTTCGATATTCTTTTAAATATAT
TAATTTTTGATCTGTCTGGCTGTGAAGCCAATTTTTCATCTTCATCTATGACTTCCTT
TTTAATTTCAACTTCTGGAATTATAGAATTTGTATGATGTTTATTTACAGGTATAGAATTTGAAACAAGTGGA
GATTTTGAAATAGGTATTTTTCTTGGTACTGGAATTGCCTCTTCCCCTGGTATTAATGAACTTCTAGGAGTTA
CAGGTGGATCAATTGGTTCTTGTTTAATAACAGGTTTTACAGGTTCCAAAGCTACTGG
ACTTGGAACAGGTATTTGTGGGGGTGGCATAGCTTTCGGAGGAGGCGCAGGCCTTGGCCCCGGCATAGTTACA
TGATTATGGTGTGTAGGGGGTACATCTTTACTTTTCAT
>103779083_2 DT665993.3
GCACGAGGGTCGGTGGAGGCGGAGAGCAGCGGCTCGGGCTCGCTGAAGCTGTCGGTGCAGTACGCGGGCGGCG
TGCTGTCCGTGCTCATCCTGCACGCGCAGTCGCTGGCCGGCGCGCCGCAGGGCCTGCCGCCCAACCCCTACGT
CAAGGTGTACTTGGTCCCCGATCCAAGCAAAGAAACGAAAAGAAAAACTCGCGTACTA
AAGCGCAACTCCCACCCATCCTTCATGGAGATGCTCGAATATCGCTTACCATTGGACGTGGTTAAGAACCGCA
GCTTGCAAGCCACAGTGTGGCATTATGATTCACTACAAGAGAATCAGTTCCTCGGCGGTGTTGTAATTCCACT
GGGTTCTTTGAACTTGAGGCAGGAAACTGTTGCCTGGTATCCTTTAGAGTATATTCCC
AGGTAACTGTTGCTTTCCCGCTCTGCTATATGGAGCACGGATATATGACAATATGTCAAATGTATAGGGCCAT
CGGGAAATTACGTCACATATTAATTCAAAGTGTAAACATTATTGTGAGGCAAGGACTGTGGAGGGTCTTAGAT
TTCGCGACATCACATTTCAAAATATGATAAGCAAACAATAATTTCGCACTGTTCTCTT
AATTTTGTAATTTGCGTGATACTTACTTAAAATTAGGTGTGACATAACAAAATATGTGACGTCACACTAGCTA
CCTAGTCACACAAATTGACCAGCTATACGAGCACAGGCGTC
>103779085_2 DT666446.3

GCACGAGGCTGAACCATACATTTAATGTTTATACAGAACGGTCAACGAGTCATGGCCGTCCTGAAGTTAAGCT
TTCCGAATGTTTGCCCAATTTTTCACTGGAAAATAATATTATTAAAAGTAAGAAATAATCGTAATTCAATTCT
GCACAATCATCGACAGCTAGGTTCAGGGCGGCCACGACTCATCGACCGCACTATACCT
AAAATAGTCGCCTTATTGTATCATTCACTCGTTCTTCATTCACTTTCAGCACTTAAGCATTCCTGTATAAAAG
AAAACTACCTAGTAGTGAATTTTACTGTTTATCTCACTTTCAATATTGTTAATTTAAGTATATTAGCTATAAG
GAATAAATAAAATAAAGTTTTCTAAATTTTTGTTATTTGAAAATATTTTACATGCAGG
TATTCTGCCTCTTTTGCTCAATACCTTCGTTTCTTATGAACAAAGTGTTTAAATTAAGCGTTGTTATAGCATA
TTTCTTAATTTACACCTAATATATTTTAAAATACCTGCATATAAAATTCATATTTATATTCTATTGGACAATC
TCAAGAGCTCTTTGTTTTATTTTTTCTTTTTTATGTATTAACAAATCGTTCTTAATTT
AAATGTCGCTATTAGCGTTCTTAAGAAGTACCTTCAGCTGATTTATTAACGTATATGTTATAATAATGTTTTC
TGAAACTTTAAAGAAATTCGTAATGTATTAAGTAACCATATAAGTATAAATGTAGTTAAATATAACATATACA
ATATTGAATTTATTAATAAGTGTGTGACGTAATGGCGTGCGAGTAGCACTATTTCGTT
TGACATTACAT
>103779087_2 DT665236.3
AGTGATATTTCGACAGGTAATATTCAAAATAAGTCATATGCCACTGATTCCTATTCAAGTGAGACTCCATCCC
AGCCGTCTGTCAATACAGAGGCATTAAAAAAACTTGCAGAGGAAGAGAGACTGTTTGACATTCAATTTAAAAA
ATGGGAGGAAGAAATCCAAAAGTGGAAAAGAGAAAACGTGAACCATCCTGATAGGCAA
GCTTACAATGAGTATGAACAGAAATTTGAAGCTTGTAGAGCTCAACTGTTGGAACGTAGAGATCAAATGAAAC
AAAAAAGAGCCCGCCTAATGGGTAATGGTTCCCAAGTGCTAAGCTCTACAAACACAGGTAATAAATCAACTGA
AATTACTCCCATGCAAAATACTAACTATCAACAAAATATACAACCTAATACATTACAA
AAAAATAACCAAAATAATTATATAAATACATCAAGTATGCAGTACTCATCTCAACCATACAATGAAAAACATC
AAGCTGTACAAGCTTTTAATCAAAATAATAACAAAAGTCAAGGTTATGAAGACAAATATGAATCATTCAACAA
GAGCATTGAAGATTACTCCCAAAAGCCACCACCTTTAAATTCTAATTTTTTAACTAAT
CAATCAAAAGGTATACCAGGATTAGACTTAGTACCTGAAGGTGAAAGGCCTGTTACTAATAAGCAATATGAAA
TTGTTGATGTAACAAAAAAAAATCAAGCTTACATCATCAACAAACACCATTA
>103779091_2 DT665086.3
AAGTTTGGATCTATGTAAAAATTTTATTTTATACTTTTTATTCAGAGAAATAATTTACAAATTTCAAATGAAG
ACCTTCTTTGTTATTTCAGCCTTATCAATACTGACATTTACTTTTAGGTCGCAATCACAAATACGATTTTTTA
AAGTAGAGCAAATATTTTTTTCTTATTTAATTGAAACACATTGATTGATGATTATAAT
AATTGTGATTAATTACCATTCTTTATATTATATAATATAAAAATTATTAAGCTTTCATGCTATTTTTAATTTG
AAATTGATGGCAACTTACTTAAAATATTTGGGATGGACTTTTTGCATTGATATTAAAATTAATTCTTATTTCT
TTGGCATAAATAAGCATATTAACTCATATACAGTGATTTTTCTGATACACAATAGGTA
AATTAAAAAAGATACGTACTTAGAGAATCACTTATATTAAATATATCCCTATAACATTTAAATTATATTATAA
TTAATGTACTGAAATTGAAAGCAAAATGCTATCTCACACTTTTACTGTAAATCTTACTCTACATACAGACTTA
ACTATGTTTTTACACTTAGGAGTATTAA
>103779095_2 DT666907.3
GCACGAGGCTAACCCCTTGATCGCTGTCTCGCTACGACGACTCGACACGTCATCAACTTTCCCAAGCGAAATA
AGTTTCACATTTAAACTCGATAACCTCAAATATTTAAACTGATTAATCAAAACTTTAGTTAAATTCAACTTTT
GTGTTATTTTTTTGGGGGGAAAACTGGATTTCGGCAACGTGTGCAGTGTGTGTTGTGT
GTTGATGTGTTTGATAGTGGCGTGAAGATGCAGGGCGCCGAAGTGGCGTTCTTGCTGACGCTGATCCTGGCGG
CCGTCGTAGCTCAGGACACCAGGAGATTATCAGGGGACTTTTTATGTCCGACCGGCATGTTCCGGTGCCCGGA
AGGGAAGTGCATCCCGTCGCTGTGGGTGTGCAACTACCAACGTGACTGTGAGAAGGGT
GAAGACGAGTTTCAGTCTTGCCCTCCCCCAGAATGCGACGCCGGCCAGCTAACGTGCGGACAGTATGTATGGA
ACAAGACGTACTGCATACCGCCACACTACCGCTGCGACATGCACGTGGACTGCGTTGATGGCACCGACGAGGC
TGACTGCACATACCGCAAATGCCAAGTAGATGACTTCCAGTGCGGCATGGAGGGCGCG
AGCGCCGGCAAGGTGTCCTCTCAGGGGCCTTGTATACGAAAAGAGAAGAAATGTGACGGTTACC
>103779097_2 DT665934.3
TAAACCCATTTAGCCTTATGGTAAAGACGGTACATTATATAATTTTGACATTTTTTAATGTTATTAAATTAGC
TGTAGATTCTTTAATATTTCTCATAATAAACAATTGAAAGATGTTCCAAACTTCTTAAAATAATTTTTTCTAT
TTAGTTTTTGGGTTTTATTTTTAATTTTTATACTATATAGTAATTAAATGGAATAGTT
TTTTTTTATATTTTTTATTTTTAATCGACGAACAATATTTTGTTTAATATTTAATTTTATATTATATTAAAAT
CAAACAAAATAAATCACAATGTTCATAATTTTAGTGGTTTTAAAATATTTTTGCTTAAAAGAACCTATATGGA
AATCTCATACATAATTTATAATGATGACATAAGATATATATAGATTAATCAATTATGC

TTAAACCTTTATATCTTACTTCATATTTTTTGCAATTGCTACTGTTTTCTTATATAAAGGGCTAAAAATAATA
TACATTTTAATATAATAATTCTTCAAAAACATGTTAAGTGATACATCTAAATG
>103779099_2 DT665907.3
GCACGAGGATTTTGACTTTCATAGAAATTGGCTTGCTGTTACACATAATCTTACAATAGATAAATGGTATTAT
GATTCGACTTCTGAGTGGACGTTAGATTATCCTCCTTTTTTTGCTTGGCTGGAATACGGTCTTTCTTATGTAG
CTAAGGCTTTTGATCCAGAGATTGTGCAATTAGAAAACTTAAATTATGATTCTGACAT
GACTGTCTTATTTCAAAGATTATCAGTGATCTTTCTAGATTTTGTGTATATCTATGCAGCAAATAGATGTGCA
AATTTAATAGCTGTTGGGAATCTCTTAGTATACATACTACTTGTTTCCAATCCCGGATTACTAATGATAGATC
ACATACATTTTCAGTATAATGGTTTTTTGTTTGGAATCTTATTGCTGTCTATATCAAA
TATGATACTAAGTAATTTTGTTTTGGCAGCATTTTGGTTTGCAGTGTTGTTAAATTTTAAACATATATTCCTA
TATATAGCACCTGCATATGTTGTGTATTTGTTCAGAGCATACTGTTTTACAGTATCATCTAATGATGGAGTTC
ATACACCTTGGTATTCTTTTTCAATAACAAATTTAATTAAATTAGCATTAACAGTAAC
AGCTGTATTTAGCATCTCTTTTGGACCCTTTATTGATCATTTACCACAGGTGCTATCAAGGCTTTTTCCATTC
AAAAGAGGGTTATGTCACGCATACTGGGCTCCTAATTTTTGG
>103779122_2 DT665818.3
GCACGAGGGTCACGTCTTCCGATCAATTTACCTCGTTGTGTAACTGTATAACTTGTTATTCTTACATTATACT
AAGGTCAAATAACCTAAAGGAAAAATGTATATTGTATCAAGTGTTGTAAAAGTGGCAATTCCTAATTATTTGG
CAAGTTTACCTATTCCGGATTCCATCGGTGGATGGTTTCGTCTTGGAGTTCGTGATTG
GCTAGCATTAATACCTCCAACTCTTGCCATTGGTGGATTATCATATTATTCCTATCAAACTATAAAAAAAGCA
AATCAAGCCGGAAGTGGACAAATCAATCCGTGTATTAGAAAGGACATAAATAAAGTTGTAGATTTTATTGATA
TTGAAGATATCACAGAAAAGGCATCTCTGTGTAGATGTTGGAGAAGTAAAAATTGGCC
CTATTGTGATGGTGCCCATGGAGCTCATAACAAGGCCACAGGAGATAATATTGGACCAGTGGTCGTAAGACAT
AAGGAAAACAAGTAAAATTGTTTGCTAAAAAATATATTTGCACAGGTGATAAATTGTGATCAAAATTATTAGA
CATTTTAAATGTAAATCATCATCTTCAATTATGTGAATTACTAATTTCATTTTTTTTG
TTTTTTTCTTTATATTTATATATTGCATTTGTTTCTCAGTACAGTAAGTAGCTTAAAACTTTTATCATAATAT
AATTGCTTATATTATGTAACATAGCAATTAATTCACATCAAGTGTAAGATGAGAGATACTCAATCTACAGTTT
TTTAATTGATATTTTTTAT
>103779126_2 DT665543.3
GCACGAGGCTTTTCAACATTGAAATAGGAGACTAATCAAATTGTGATACGAATAAAAAAAACAATATTAAACC
TATTTTTTCGCAGTTGCATTAAAAAATAATAATAAGTAAGATTTTTTTTATAGTAATTTTTATATACTGATAT
AAATTAAAATATCTACCTAAAATATAAGATGCATAAACGCTTAGAAATGCGAGAATAT
CTAAAGAATGTGTTAGATAACTATATAACCTTCATTTATTAATTTTTTTATATGTCGTATAATATATTAACAT
TTTATATGATTAGAAATATATTTAGAATACTTATTCTTTGTTGAAAAAAAAATGCCTTTTCTACAAAAAGAAAT
CATTTTTTTTAAGCATTTAAGTAAGTTTATTTTGACTTTGTTGAAGTATTTTGTGTTT
TTTGTTGTACTTAATATTTGTCATATATGTTTTATACTATAAATATAATATATGTAAAAATTCAAAACTATTA
AGAAAAATAGGTATTTTTCATGTATGTTATCTATTTAAAATAAGTAAAAAAGATTTGCATAGACTTTGTAATT
TTTAAAAAAATAAAATTAAATCACTTTTTTATATTTATACTTATATAAACACATATAA
AAACGCCTTAGCATTAAACTTAAAAATTTCCTTGACTGTATATTTTATTAAAATGTAATCATAAAAAGCAGCT
TTTTAAAAAACGTAGTATATCTTTGACTCTTTGTCCTTTCTAGTCTATCATAAGAGTCAACAGTGATTTCTAA
CATTTTTTTCTTTGATTAGTTTACCTTTGTTTGTGGCTACGACATATACATATGAGTT
ATTTATTAATAACGCCGTTGCCTAT
>103779128_2 DT665758.3
CTTTCAAAAACTTATATTTAAAAATCATTATTACAATACGAGGAGGAGCGCGGCGTTAAACTAAGCACCTGAA
ACATTATTGTGCTATAAACCTCCTCTAAAAGAAATAAATATAAAAAAATTATCAAACAAATTCCTTTACATTA
CAAACATACAGACAGTATTTAATGGTCAAAATCGAGTCACTAGTAATTTTCTATCTTG
TAAATACAGTTTATAAATACAAGCTTTCAAATTTTACTTACTTCGTCAAGTAAGAACCAAGCATGATAAATTA
TTATATAAAAATAACCACACTCAGTACAAAAAAAATGTTATACTATCAAAAGCAAATGTGACTTCGGATGTTG
ACCATTAAAGAAACGATCGCTCCGAACGGCCAGCGCCTCTCACCGTCTCGCCGTTACG
CAATATAATTAAAATCACAACAACTTAAAATTATTACAAATAAAAAAAAACTTAACCGAAATACATGACATGG
CGCGACTGACTTAAATAATATATTACGTACTACTTATATACTATGTAAAAGTGAAAATGGACGGTTGATCCGT
GTGTATCCGTGTGGGCATGCCGTTGCCGAAACCGGCGGCTGCGGTACTGACAGCAGAA
CACGTTAGCATATTATGTAAATGTAATAAGCATCCATATAAATCGTCAATCCTATTTACGCAACGTGCTCCAC
CGTACCGTATGTCGAGTCTACAAGTTTAGTTTCAGATGTCGATTTTGCTTTTTGAGTTTTGTGCTCACAACTC
AGACATGAAATCTCTG

>103779130_2 DV501403.2
GNACGAGGGTCACCCTTAAAAGTCTTACTTGAATTGTAATTAAGATTTTGAAACTTTTATGTAAAAAACCCTC
TGTATATTTAGTGAACCAAGCTTTACAAAACCTAATTAAGCATTTATAGAAATGGATGCACAACTTTTCGACC
GCATTGGTCAGCTAGGCTTGAGTGTCGCCCTTGTGGGGGGAGTCATAAATTCTGCTTT
ATACTACGTTGATGGTGGTCACAGAGCAGTTATATTCGATAGATTTGGTGGTGTGAAAAATTTAGTTGTGGGT
GAAGGAACGCACTTCTTCATTCCATGGGTACAAAGACCTATTTTATTTGACATAAGATCAAGACCAAGAAAGG
TTCCTACAATTACTGGGAGTAAAGATCTTCAAAAGGTAAACATTACACTTCGTATCCT
GGTCATACCATTCCCTGACCAACTTCAGAGAATCTATACCATTCTTGGTGTAGATTAAGATGAGAGAGTATTA
CCATCCGTCACATCGGAAGTGCTAAAAGCGGCAGTTGCTCATGTTGATGCTGGCGAGCTCATCACTCAAAGAG
AACTTGTGTCACAACAAAGTAAATGAAAGTCTAACATAAAGAGCTGGACAGTTTGGTT
TAATATTAGATGATATCTCAATCACACATTTAACAT
>103779131_2 DT665808.3
GCACGAGGGACCAACCCTTAATACTTTACTACAAGGGCAACTATTCTATGTTCAAAAAAATGTACGCGCAAAA
ACGTAAGGAAATGATAAAGGAGTTTGAGAAACAAGAGAAGCGAATAAAGGAAATGAAGGCACATGGACAGTCT
AAGAAGGCGGCTGAGAAAAAACAAAAAGAAGTTCTCACACGTAAGCAAGAGAAGAACC
GCAGCAAGTCGCAGCGCGAGGAGGCGGAGGACAGCGCGGCGCCCGTGACGCTGCTGCAGCGCCCCAAGGAGTA
CCTGGTCAAGTTCTCGTTCCCCGACCCGCCGCCGCTGCAGCCTCCTATATTGGGATTGCACAATGTGGACTTC
AACTTTCCCACTCAAAAGCCTTTGTTCAAGCAAGTGGACTTCGGTATCGATCTCAATT
CCCGTATCGCGATTGTGGGTCCAAATGGTGTTGGCAAATCTACTTTCCTTAAACTTCTAGTGGGTGAACTGAG
CCCCATTGAGGGAGAGCTGATCAGGAATCACAGGCTGAGGATCGGTCGCTTCGACCAGCACTCCGGAGAGCAT
CTAACAGCTG
>103779135_2 DT666487.3
GCACGAGGACAGTACCTCAAGTGGACGTGGCGCCAACTATCGCGTGGCTGTTAAACGCCCCCATCCCCGGGGA
GAGTACGGGCAGGATTCTACCTTCGTTGCTGCCTTTGGACATGCAACAGCAGCTGTATTTACTTCATGTACTC
GCTGAACATAATAAAATGTATTTGCCTAAACATGCTGAGCTGTACAAGCAGACGGAGC
GCGCCCGCATCCAGTACGCGCACTACCTCGCCACGGGGCAGCTCAGCGCCGCCAAGATATCCAAGGAGTTCTA
CGAGGAGGCTTTGGACGAGATGGCCAAGTTCTTGGCGGACACCAGCACGGATTTTGATCTGTTCGCCATGGGC
GTTGGCATTGTTATGCTTTATTTTATAGTCGGTGCCCTCACATGCGTAACTCTCTTCT
CTCTCCAACTTGAATCGCATAGAAAAATAAGTATAACAAAACACAAGCCGAAAGCTGGAACGCTGTTAGCTTT
CACCATAATGTACGCCATTTTATCAAGCATATTAGTGACGGCGTGCTTTATAACAGAAACTAAAAGCGAATTT
TGCTCATTTAAACCGGAATGGTGCTTAATATTCCTAACCACAGCTTTCACTTTAAATT
TGATGTATTTCATAATTAAATCTGGTATAGAAAAAACGAGAAATCTATCGCAGTTACAAGAATTGAACGCGAT
AGATTATTTATTAATAATAGGAACCTTGTTCCACTCTTGGTCGTTCTTCGGAACCAGTTTCATA
>103779137_2 DT665490.2
GCACGAGGTTTCCCTACCTTTGAAAAAATCGAAAAAGTGATAAGTGATAGGCAAAGTAGTGATATAGCGGAAT
TGCATTTACGCGACGATGATACAGTGTTCTCCGGTTCAAAGGTTGGACCGGTACAAAAAAGGCAGAAAACATA
TTCTATTAATGAGGATTATGTTTTATATGAAAAGTGTGACTACGGTACGATTGATAAG
TATGAGGTGGAGTCGCCTAAAGAGGATCAGAATGCCTTCCATTATCATGATGCTGTCAA
>103779141_2 DT666835.3
CGGCACGAGGCCCGATACTTTTTTGTGACATAATATCACTGCGATTGAAAGATATTTATCATAATATTAGTTG
TAAATAAAATAAACTCCATAACAGCATGTGGAAGGGACTTAGATTAACACAGTCTCAGTTAGCTAAACTGCAT
TTCAATGGCCATAAATTGCAACCTATTGGAGTTCCTGCTTTAGATTCTGTATTGAATC
AGTGGTATGAATTTTGTCAGAAACCACCTAAAGGGTTCGAAAAATATTTTCAACCTGGTGGTAGTCCGAAACA
GGCGAAGAAAGCAGAATCAAATGAATCGCCAGCTCCCTCAAAGAGCTCGCCGCCTCCACCAAAGCCGAGTACG
CCGCAAGATAAATGGAATATGAACATGTTCTCTGGTGGCAGTGGCGGTAGCAGAGGCC
CTGGTCGTGGTGGCTACGAGGGTCCAGATAAAGAAAAATGGATGATGTTTGGTGCTTTCGGAGTAGTCACTTT
ACTTGCTTCCATAGCTTACTTTGAATTAAGATATAAGGAAATAAGTTGGCGAGATTTTGTCAACTTATATCTA
AATAAAGGTCTTGTTGAAAAACTTGAAGTAATAAATAAGAAATGGGTCAGAGTGAGAC
TGCAGGGCTCTGCTTTGGAAGGAAAGATAATATGGTTTGCCATTGGCAGTGTAGATTCCTTTGAAAGGAATTT
AGAANATGCACAAATTGAAATTAATGTTGACCCACCTAATTTCGTTCCTGTAATCTACAAGACTGAGGTAGAA
GCTGCAAGCTTAACAGGAATGTTGCCCACTTTG
>103779143_2 DV501431.2

GCACGAGGCGCCGGACCCTGCTTGGGGAGGCAGGGCAGTGACTGTGCTGACCACGTGTGGTCGACTAACGTCA
TTGCTAGAGACAGTGGCGGTCTACTCCGCATAATGTCTTCACCATCGGGTCTGGTTTACGACTCAAACTTCAT
ATCCGGTACAAGGGAAGAGGTAGTCGCCTCGTACAGAGCCACCTGTTGCTCGCCTCGC
GTCATCATAAACGCTGTTGACGCGTTCGGAAACACCAATAGTTACACTATTGATATATCTAATTACATCAACG
AAGCTGGCATAGCGGCCATAGCGCTCGGCGTTATACTTATAGTACTTATTATCGTCCTAATCATTGTCTTGAT
ATATTGGTGCGTTAAACGCAGAAAGGAATCCAGAGAGTTGCCATCTTACGCGTCTAGG
AATATAAGTTAAGGCCTTAATATCTTCTTGTCTTTAATTTTTTTTTTTGGTACAAAATGAGATAGAAGTTAAT
TCACAAAGTCAAAAAATTTGAAATGGGACAGCTTCTGACTGCACGCGCTATGGTTGTAGGTTCGATATTGAAT
TATAATACTTCTACAATATTATTATATTTAACAATAAAATGGTTTTTTTTTGTAAAA
ACATTTCGCANATTGGTTTGATTGTGAATATTAACTGTTGCAAAATTTACATTCATCAGTATAAATATAGCAT
GTGTTTCTTTTAAATCAAAATATAAAAAATTAAAAGTCTACAAAACTAACATCAAAAATAGTGATGAATTTGA
ATGTCTTCATGTTATGTTAATTACCGT
>103779166_2 DT665935.3
AGGCAAGAAGCTAGAGGACTATATAAAATCAGTTCCGGTGCCGACCCACCTCTACCGCACCGAGAATCGTTCG
GGCCTCATCCGCGCCAGACTCCTGGGCGCTAGACACGTGAAGGGCGACGTTATCACGTTTTTGGACGCTCACT
GCGAATGCACTGAGGGATGGTTAGAACCGTTATTAGCGAGGATAGTAGAAGACAGGAG
TACGGTGGTGTGTCCGATTATCGATGTCATATCGGACACCACGTTCGAGTACATACAAGCTTCTGACATGACG
TGGGGTGGCTTCAATTGGAAATTGAATTTTAGATGGTATCGGGTACCTGAACGCGAGATGGCCCGTCGCGGCG
GAGACCGCACGGCTCCCTTGCGCACGCCAACTATGGCCGGCGGGCTGTTCGCTATTGA
CAAGAATTATTTCTATAAGATCGGCTCGTACGATCAAGGAATGGACATATGGGGTGGAGAAAACTTGGAGATG
AGCTTCCGGGTGTGGCAGTGCGGCGGGCGGCTGGAGATAGTGCCGTGCTCGCACGTGGGGCAACGTGT
>103779170_2 DT665164.3
AGATTTCACACCTGCCATGTATAGTTGCTGTCATGCTGCTGTATGATCGATAAGAGCGTTAGTTATGTTATTT
CTATGTAAATGTGTCTCTGTTAGCCATTTTGTTAAGTTATCGTACATACATAAAAATACATTAGAAGTGATAA
TGATTATAAAAATTATGTTCCATTGTGACTAACTGATGTATGTATGTATGTATGTATG
TATGTATATACACGATAAATGTCCTTTCAAATATGTTAACATAAAAGAGTTCTATAAGTTTATTTATCATCGC
CATTGCGTCATGTTGTGTATTAATAAAAATATATTTTGTATATTCGCAGTTATTATAAAGTCACGTTTGTATT
GGATGTTGTTTTCAATACTATGAGCTCTATCATTTCAAAGGCAATTTACACGTAGAAA
ATTATGTATTTGATTACAAAATTATACCATAATTATTGTTCAAGTTTTATTTACATCAACGTTCCTTGTCATG
GTAATATCAATCTATGATGGTGGGTGTTTCGCTCTGCTATGCTATAAATAGACTATATATAAGTATATTTATT
AGGTTAACCGAGGGTTCCGCATATAATTTACATTGGGAATATGTAACTGCAATATATA
TTATTG
>103779184_2 DT665214.3
GCACGAGGCCTGCCCCTAACGCGCCACTTCTGCCGCGCCACCTCTGCCTGCATACCCTGTGACCGACGACAAC
ATTTCTGACCCCAACTCTCTCAACACAAATTTCACAATCATATCCCAACTATAGCGAACGGAGTGTGCGATTT
CGAATCCGATGGACGCTGTTAATTTTAGTGTTTCGAAAGACAATGGACAAACTGTATG
AGCTTCGTTTAGTATTTCGATATATTCATATTGTGTAATCCAAATGTGTATGTGTATGGAACGATTAGGAATA
GTGAACGGTGAACATGAACTTTGTCATTTTGGATCTGACGATGGGTCTCTCTCATAGATTACGCTTCTAGCGG
TCGCTCTGATTTTAACCTTTAAACTTTACCACTTTACTGTTATTTCTAAATGGATGTG
CCTTGAAATGCTTTCCTCGATTCGACTTGTAGTATAGAGCGTTTTTGAACCCTTTAGAAGGAATTTCTCTTCT
CTGTTTGTGTGGAAGTTCCCGTATTATAAGTGCAAAACACATCATTACCGTGCCTTTACCGTTCTATAGACTT
CGTAAGGGACGTAATTAACAATAGGTTTTAAAAAAAAACGCTTTCCCGATGGAAAGAC
AAACCAACCCAAATATTGTTTGTTCTTTGAATTTGTGTAATTGCCAGCTTCTTTGTTTTATGTTACTAACAAT
TTGTGATCTAATGATATTAAAGAGGAAAGTGAGCTAGGACCAAAGTCTTTCTCANATGCGCGTCAATTGGTTA
ATATTAAAAAATAATAAATCCATGGTAATAGGATAGTATAACAATGTGA
>103779198_2 DT665904.3
GCACGAGGTACGAATCTTTTAAAATGTAAAGCTTTTGAAATATGACCAATCAGTTATTAATATTGGCTGCTGG
CTGAAGTAAAATTAAACTAGATTCCATTTCCACAGACATTCTAAGTAGACCGGTGATAACTTCGCGTATTTCG
CTCTTGCCACGACGACGTTAACTTAGTTGGAACATTTTTAACGAAACATGTATTAAAT
AATTGTGACATTTCAGTGATAACTCAATAAACTAATAAAAATGGGTATGGAAAAACATCATGGTATCGAGGAT
GGTTATTCAGAATATTTATATTGTCCAGAAATTCTTTTAAAGTTGGACTTTAGTAGGAGTCCAGCTAAGTTTA
ACCCTCCCATAAGTGCTGCACAGCCAGGTGAAGACTGGATGGTTGTAAGACCTTTACA

AAGGGCTGATTATGATAAAGGGTTTCTGCAGCTACTTAGCCAACTAACAAGTACGGGAAATATTACGAGAAAA
CAATTTGATGAGAGATTTACACAAATGAAGAGTGCTGGGGGTTACTACGTAACTGTGATCGAAGACAAACGTA
TATCAAAAATTATTGGTGCTGCAACACTTACTATTGAACAGAAATTCATCCACAACTG
CTCTGTCAGAGGTCGTCTTGAAGATGTAGTGGTAAACGACACATATAGAGGAAAACAGCTGGGGAAATTAATC
GTCGTGACAGTCTCGTTATTGGCTCAAGAGCTCGGCTGCTANCAAATGTCGCTCGACTGCAAAGACAAGCTTA
TTAAGTTCTATGAAACTCTGGGATACAAACTCGAGCCAGGCACTCTAACGCTAT
>103779206_2 DT666453.3
GCACGAGGATTTTTTCTTGCAGAATATATTATATTCTATTTCTTATTTATTTATATATTCTATTTCTATTTCA
TAAATATTAGATTATTGGTGCATTGAAAAAAATTTTCAATATGGATTCGAATTCAAACGTTCACCTCGGTCAT
TTGATGTTTGACAGCATGAAGAAACATAAAAATGTGATATGTCAAATAGACGCAGCAA
CTGGGGAGGAGGAGACTTATACTTCCGTACTGTCAAGATGTATACGTATGGCCAAAGCGTTGAGAGCGTTTGG
TCTAAAGCCAGGCGACGTTCTCGCTTTCGGTGGCAAAAATCACATCGACATTCACATTCCGTTTTACGCGGCC
CTCTTTAACGGTCTACCCATTGTTGGGGTCGATCCGTTTTTCAAATATGACGAAGTGA
AGTCCCTATTGAGTCTAACAAAACCTAAAATAGCGTTCTGCCAAGGCGATTCATACGACATATACAAGCGAGC
ATCAGAGAACCTTGGTCTTAATATGAAGATTGTCACTTTTGATAAAGGATATCCGTGTACATTCACAGAGTTT
ATGGATACATACGCAACAAATGAAAGGGAAGATGATTTTAAGGTACAAGAAATCGATA
TAGAAAAAATATATGCATTTCTGGTATGCACGAGTGGTACTACTGGTAAAGTTAAGATCGCAGCTTTCAACCA
CAAGCCGTTCATGTTCAAAATAACGAAACTATTAGAACAATACAACTATTATAAGCAACCAGGAAAACGAATA
CTGATTCTATCTCCAGTAAATTGGATATCAAACTACTTTTTGTCTGGTGGTGGTCCGC
TG
>103779208_2 DT665727.3
GCACGAGGCACAGTTTGTGCGATAAGCTACTTGACAGATGGTGGCAACGAACAAATACAAATGGTCATAGACT
CTGGTATTGTGCCTAAATTGATACCACTACTGTCACACAAATAGGTGAAAACGCAAACGGCGGCGTTGCGCGC
CGTGGGCAACATAGTGACGGGCACGGACGAGCAGACACAGGTGGTGCTCAACTGCGAC
GCGCTCTCACACTTCCCCTCGCTGCTGTCGCATCCCAAAGAGAAAATCTGCAAAGAAGCCGTGTGGTTCTTAT
CGAACATAACAGCGGGCAACAAGCAACAAGTCCAAGCGGTTATCGACTCTGGACTTTTACCCAAAATAGTGGA
GAACCTCAGCAAAGGGGAGTTCCAGACGCATAAGGAGGCGGCATGGGCGGTCTCCAAC
CTGAGCATCAGCGGCACTCGCGAACAGGTGGCGGCGTTGATACAGTGCGGTGTTATACCACCGTTCTGTAACC
TGCTCGCTTGCGAGGACACGCAGGTTATTAATGTGGTGCTCGACGGGCTGAGCAACATGCTGAAGATGGCGGG
CGAGAACGCATAGCATGTCTCCAACATGATCGAGGAGTGCGGCGGTATCGACAAGATC
GAGGAGCTGCATGGACACGAGAAGGTCGAGATATACAAGATGGCCTACGACATCATCGAGCAGTACTTCGCCG
CCGAGGAGGATGACGTCACGCTCGTGCCCGCCGCCGCCGACCACGGCTTCACCTTCTAGCCCGCGCCCGCGCA
TCACGATGCCTTCCGGTTCTG
>103779223_2 DV501459.2
AATTTGTTTGTATTTGTTAATAGTTGAGTTGTAAAAATTAATTTTAATTGTATATGAATATAATTGAAGGTTG
AGAATAGATTTGATATTTTGTTCTTTTTTTTAAAGTGTAAAAAAAAATTATGAATTAAAGTGTAAGTTTTCAT
ATTATGTAATTGTTTGCAATTTATATATGTATTTAGAATATGAAAAAAAAAAAATGTA
TATGATTATAATAAATAAATTGATATA
>103779231_2 DT665112.3
CGGCCTACCAGAGTGCTCTGTTATCAGCCATGGCGGCGCACACGCATACGTTCCCGAATCTCACTGCTCCCAC
AGATCTGTCATTAAAGACATCATCAGCGCCATTACACGCGACATCCACCTCATCCATCTCAGGGTCTGGATCC
AAGCTGTCACACAAGCTATCTGGTGCAGAAGTTGGAGCAGTTCGACAGCTAATTACAG
GATATCGTGAATCTGCTGCTTTCCTTCTACGGTCAGCAGATGAGCTAGAGGCTCTACTGTTAAACCAACCCTA
GGACACACCTAAAAGTTTCTCAGGTCTATTCGGATTTAAATTCGGATACTTCTGAATGAACCAAGTGCTTGTG
TTTCGTTTGGAATTGTGGTTTACTAACGTTTGGACACGAAAAAAGAACTTTTAAAGAA
AATAACGGTTTATACCAATTTGGATTGTAGTTTGAATGTAAATTTATAATACGAAGTAGGGATTTTGTTTTTA
TTTTAGTAGCCACAAGCTATATATTGTGAGATGAAATATGAAAAATTATTCATTCTGTAGCTTGTGGTGCTAG
TTAAGGTCGGAATTGGGCCTTGTCTTTTGGAAAATATAATAGTTTCTCGTCTTTACTT
GCCAATTCAATGATTACATCTTCAGGAGATGGAATTGGGAAGTAAAATCCCTAAAAAGGTGTTTACTCTGAGA
ACTTTTGTAACTTATTTAAAATAAAATTGT
>103779239_2 DT665419.3
GCACGAGGCTCGCCTTATAAATGTTCAGTGTTTGTTAATGTTTGTGTTTAGTCCTTTATGTAAATTTTTATGT
AAATATATTAAACATAACAAAATTAAACTCTAAATTAACACCCATGTAAAATATTATTTATTACTAACCTAGT
ATGCTGTGTATATACTAAAAATATGTATTTGTTTTGTTTTAGGTGAAGGCTGTACTTT

AACTGGATCTCTTTTTTTTACATGTATTCTATAATTATGTATAGTTATCTTTTTTTTTACCGGTCAATTTCCT
TCGTGTGTTTTCCATACAACGTGACATTGGCGAAATGAATTGTGCTCAACTGGCACGCTCGAAACGCCATTAA
TTAAGAAATCAATCAATCAAATAGATGTTTGTTAGAATTTAGAAGTATCTTTTTATCC
AGATGGATAACTATATGCATCAAATATGTTCAGTTTGAGAAAATACTTGTTTGTATTCATATAAGCATGTTTT
TATATATATTTATTAAAATATTTAGGTCTGTATTTATCAACCCCTCTAAAGTATGAGATTGATCTTAAGATGT
TGTTCTCATACATATCCATAAATGAAAACTGCATCTCAAGATAGATTTCATACTTGAG
AGTGGCTTATAAATCCCGGCTTAAGCAATATTAGTAGTATATCGGATTTTTTTTCTAAATTAGTTTTTCAATC
TAATGTCCACTCTACATACCACATGTACCTGCTTAGTAGTCTGAATAATGTCGAAAATTTATATAAAGAAAAA
CCTATTTTAAAAGAT
>103779253_2 DV501480.2
GCACGAGGCTTGCTCGTAACAGGTATTTGTAATAATTATGCATCATTTTTATACTACATATAATTTAAACGAT
ATCCATAGAATCAGTACAATTGTGTGTGTACAACACTCAATATTTACATTTTATTTAGTTATAATTATCAATG
ATATACTATTAATATAACATGAAATATTTGTATATTATTTGTATAGGCCATCGTGGCC
TAGTGATACCTACCCGGGGAAATGAAATATTTAATATTTTCATTTCCTTGCTATGATAACCAATCAAAACACG
GACTTGAGGTCCGCCACTCCATACACAATGCCTTAAGAATTCTTCGGAAAGTGAGGAACCAAATGTCTTCTTA
GAAGTGAAGCACTT
>103779255_2 DT666664.3
GCACGAGGCCTCCAGCAACCTGCTCGGGGCCTACGATAGGACCTAGCCGCCTCGTTTAAATCATGTTGTCCAA
ATTGTTTATGGTGTTTGTGTTGTATTCAGCGACAGCTGCTGAATATTACAAAACAAAACCTAGTTTCGTCCAG
ACCTGTCTTAAAAGTGATCCAGACTTCGATGATTGTTCTACAGAAGCCGTACAAAAGT
TGTTCATAGCTTTGGGCCCAGGTCTTCCAGAAATAGAATTGCCTCCTATAGATCCTCTAAAAATTCCAAAATT
AAGAATACTCCAGGGTGAAGGACCAGTTAATGTCAACTTCTGCGCTTGATAACGTGTTGGTGACAGGTTTTGG
GAAAACTGAAGTATTATTGAGCCAGGTTCACAGTAAGTCATACGATTTTTATACGAGA
GTTCGGGTTCCAAAAATAAGAATAGAAGGCACATATGATTTAAGAGGAAAAATTTTGTTAATTCCTTTAGTTG
GTAGCGGTTTGTGTTGGTTTGAACCAAGTAATATGACCATAGACATCGTAAGCGACGTGAAACTTTACGAAAA
AGACGGTCTACCATTCTTCAATGTTACTCAAGTTCACGTAAAATATAATATAGGAGGG
CTAAAATTACATATGAGTAATTTGTTCGACGGTATATCGTCGCTCGAGGATAGCACGAACGCTTATCTGAATG
CAAATTGGCGACCAGTATCCGAGTCTTTGCGGCCGATCTTATCAAAGACTATAGAAGATATTTTACTGGGCTT
CATTCACCAATTTTCCACAATTTACC
>103779269_2 DT666853.3
GCACGAGGCTGAACCCTTAAGTTGCAGAACTTAAAAACGTTGATCTTGTTGAAGTTACATCTTCAGTTAAGGG
ACTAGAGAGCAACGCCTCAAATCTTTTGCGATCGGCTGAATTTGCAGCCACAGACAGCGATAAGCAAATATTA
AGAGCCGCTGAGTTGACTAATGCAGCAGATGAGGTACTGGCCTCAGTGAGAGAAAACA
GTCAAAAGGCCAAAGAGGTCGTCAACCACGTGGGCGACCTGGCGACCGGACTTGAACTAAGTCAACAACCTAA
AGTGGATAGCGCTTTAACTGAGGCACGACAAATTAAAAGTGATATTGCAGAAAAAGATCTGACACCCAAACAG
ACGCAAGCATTCAACGCTCACACAAACGCATCAAAACAGATAGAAAGAATGAACCTTT
TCGTCCAACCAGTAAATGAACAGATAAAGAAATTCGAATCAGTCATCAATGCAACTCGTGAATTGAAATCTAA
ATTAGATGAAATGCTCGAATACACAGACCTTGCACAGCACAGAACTACGAAAGCAGAAAAACTGAACGAAAGC
AATAGGAAATCTAAATTCGGAAATAAAGTATTATCAGTGACAAAATTATATGGCGCTG
CTATGAAAGATCTCATAGACGCAGCTATAGATATCAACAACGCGACGTATTTCACAGCTAAAGTTGGGGAAAT
GACCAAGAACGCAACGAAGGTTCTAAATGACACCATCAATATTAATAGCGAAACGGAAAACAGTCTACAGAAC
TTAACTGATATACTTCCTGAACTGACTAAC
>103779284_2 DT666704.3
GCACGAGGGTTCCAAATAACACAATTAACTGTGTCCAATATTAAAATATGGATTACAAATATGAGCAAACAAG
TGAAGTAGAAGCCCTAGATTCCATATACTATGGTGATATGCAAATTTTACAGGGAGAACCAGTACATAAATTT
GGCATAGGTATAAAATCTGAAGAATATGACGAAGGCGAAGGATTAGCTTGTCAGTTAT
TATTTACATATACTCCAAAATATCCTGAAGAATTACCAATCCTGGAAATAGAGACTGACGAAAGTTTTGATGA
AATAGTTGACAAAGATGAACTCTATAAGCATCTTATGGAACAAGGTCAGGAAAATTTAGGCATGGTAATGGTA
TTTACATTAGTGTCAGCAGGTCAAGAGTGGCTCAATGAAAAGTGGGACAGTATAAAGA
AAGAAAAGGAAGAAAAGATATTAGCCAAAAAGAAGGCAGAAGAGGAGGCAGAATTGAAAAGGTTCGAAGGGAC
AAGAGTTACAGTGGAATCATTTCTAGCATGGAGAAAACAATTTGAGATTGATATGGGTATTCCAGCAAAGAGG
GAAAAGGAGTCCAAGGACAAAACTAAATTAACAGGAAAAGAATTGTTCTTAAGGGATA
TTACTCTAAATGAGTCTGGATCTCAAATTCCTT
>103779296_2 DT665776.3

GCACGAGGGGAACACGCGTACTGACACTCGCGGATAGAGGGAACCCACAAATATACTATATATAAACAAAATA
AAAGGAATTTACAATAGAATACGTATTAAAAGCAACTCTGAGGATTTATTTTTATAACATTATTAATCAATAA
CGTGGGATATTTGATTATATTAAGATGCCGGTCGAAACGAAAATTAGCTTTTCTGACG
GCAGCTCTACGGAAACATTGAGAAAGTTGAGCGA
>103779306_2 DT665201.3
GCACGAGGTTTTTCTTTTTTTATAATATATCTTACACCTTTATTCATTTATTTACATTTCTTATTAAGTCCAC
AAGAACATGCGTTAAATAACTAACGATTTTGAGCAAATTGTAGGTGATGGCAACACAGTACTGAAATAATAAT
ATGCATATGATAGAACATTGTAAAAAACATTATTTATTATATAAAAGAGTGTTGCCAG
AATATACAAAAATCAAGAACGATTGTTTCCAATTTTCTTAATTCACATTAGTCAAAAGCACCCAAAATTAAAA
TAATAAAATTGTCTATCAATTTATATATTTATAACAAAATATTTTTTTTTGTCATAAAAACAGTCTAAGAAAA
GAATAAGAGTATGTTTATCGACTTAAAATTACTATGAATATCAATTATTAAAAAAGAA
ATCAACTTTAATTATGTCTTTTTGGGTAGAATCTCATATAATCATAGTCAAAACGTTTAAAAAAAATACGCGC
CCATAATATTTATAAGACGTTGTATATATTAATATTCAATATTTATTGTATTCATCAAAAAATTTCAAAATTA
TAATAACTATCACAAAGAAAAAAAAAAGAAAATATGTAAAAAAAAATCGCTGTGATGC
GTAAAACAGGTAACAGAAATTACGAATAATTAGAAAAAATAAACATTAAATATGACTAACTTAGCTAAAGACC
ATATCTCCGTACATTTAAAACTTAAAAAAAATAGTGATGGTAAAAAAAGTTAAGTACCTAAACCTAAGGACGG
AAATAGACTAGTCTATCGGTAAATATTTTTATTTTTATTTACAAATCACATCAAAATG
GGTCAAGCATTGA
>103779310_2 DT666136.3
GCACGAGGGTTGTACTTAGGATCTGAAATAATGAGAGAAGTGGGATGGGTTGTGTTTGATGAAATTCATTACA
TGAGAGACAAAGAAAGAGGTGTTGTGTGGGAAGAAACATTAATCCTATTGCCTGACAATGTGCACTATGTGTT
CCTATCGGCCACTATACCCAACGCGCGTCAGTTCGCTGAGTGGGTATGCCGTTTACAC
TCCCAGCCATGCCACGTGGTCTATACAGAGTACCGGCCGACGCCATTACAGCATTATATATTCCCAGCTGGTG
GAGATGGCATACATTTGGTTGTTGATGAAAAGGGGCAATTCTTATAAGACAATTTTAACACTGCAATGACTGT
GCTAAGCAATGCAGGGGACGCAGGAAAAGGGGATCAGAGAGGACGGAAAGGGGGTCCT
CGTGGAAACAATAATACAAATATATTTAACATAGTAAAGATGATCATGGAGAGGAACTTTGCTCCGGTCATTA
TATTCAGTTTTAGTAAGAAAGACTGTGAACTGTATGCCATGCAAATGGCCAAATTAGATTTTAATACAATTGA
AGAGAAGAAGTTGGTGGACGAAGTGTTTAACAATGCAATGGACGTGCTTTCTGAAGAG
GATAGGAAATTACCGCAAGTGGAAAACGTAATACCATTGTTACGACGCGGCATAGGAATCCATCATGGCGGCC
TTTTGCCCATATTAAAAGAAACTATTGAGATATTGTTCGGCTTGGGACTTATTAAGGCTCTTTTCGCTACTGA
GACATTCGCTATGGGTCTTAATATGCCGGCCAGGACTGTCGTTTTTAC
>103779314_2 DT665800.3
GCACGAGGGTCAACCCCTACCACGACTCCGCCAAGCTGCAAGATCTGCTGGACGGATTTATCCGCAAATTCGT
CCTCTGTCCCGAGTGCGACTATCCCGAGACGGAGCTGATAGTGTCCACCAAGAGGAACACCATCTCGCAAGGA
TGCAAGGCGTGTGGATACCACGGCCCCTTGGGAGTTCATCACAAACTAAATACCTTCA
TACTGAAGAACCCGCCCGCTGC
>103779325_2 DT666811.3
CGAAAGCGGTGGTTTCGTACCCTTCTCGCTGTTTGATACGCCATGGCCGGAACAAAGATCATTAATTTTTATA
CTGTACCATTTAATTATACTACCAACCTACCTACCGTCCGATGCAACGAGGCTTTGGTTTTTTTTATGCACAA
TTAAGGTTAAAAATAAAATTTGTTTCTTAAATAGAAAAAAGTTTTTTTTGTTGCCATT
AAAAAAAAAATATTGCAAATGATATTCTTAGAGACTATAATTTTTATTATTTGCCTGTTATTAATAAACATTTT
ATATTATCTGTATTTAAAAATTAATAAAAAATATTTTTAATTACAATTAGGGATGTGTATTTAGACAATTAGT
ATTACAACCAATCATAAATAAAATAAAGTAAAATTTAATTTAATTTCAAAGGCCTAAA
TAAACCTCAAAACAGCTATTTGCCATATATTATTTATGGTGTACTTACTCTGGTACCTGTCTGTACAATCGTA
AGCAATAATACCGTATAAGTAGAACTAGAATTCGTATTTATATTATATGAAATAAAAAATAAAAATTATATAT
ATATGTCGTGTCAAAACTGTATGGTCGAATAATTTTATTTAGTTTTGTTTTTTCTTTT
CTTTATTATTTTACCTTTTCACCTTTTGTTTTTTTATATAAAAAAGATGGTTTTTTTTGTTTAATATTTACAT
ACTTACTCTGCTATAAATACAAGTAACTTTAACTAATGGAATTGATATATTGATATTTAACATTGAATATTAT
AATATGTATATAAATGAATATATAATTAGAAAAAATTATATATATATTTAGTGTACCT
ATTTTTATTAAAAAAGTAAAAATATGATTTATCTGCCCCGCG
>103779327_2 DT665531.3
GCACGAGGGTCATTTCCTAAGTGGTCTTGACCGTAGTCAGATCTGGAATGATTCGGGCTGCGGAATATTGAGA
CTTCTTCTTGATAGGACTTACGGTGAAAAGGTCCTTAAGTGTAAGCTAGGATATCTCCATTTTTTGTAAGTTG
GTATTAGATGCTTTATAAAATTTATATTACAACGGTTTTCTTTCTTAATTTTTGGGAT

AATTCTGTTTTTATCTCATAAAATTTTTAGAAAGATTTTTTTCCTTAAATAAATCAGCAATATCTCCTGTGTT
TCAAAACGTTTTTTTATTCCATGAATATGAGTTATAAATGATTATAAAATTTTGTAAAGCATCTCTACTCAAA
AATTATTTTTACAATGTAAGCAGTGTATGTCAGTGTGAATGATTAATTATTCATGTTA
TAGAACTCTTATTTCATTTCTAGCGTAGGTATACTGGTTATCTACATTAATTTTAATATTGGGTGTGGTCGTG
ATATACACAACGTACATGTATTTTACTATGATCTCTTGTTTACTTACAATTTTTCTGGAATATGGCTCGATGT
GAAAGAAAGATAAAAAGAAGGATAGGATAGGAAACTCGTATGAACGCCAATACTTTAA
GCTTAAATGTAACAGTTAAGTGCTTTTGATATTTATAAGAACCATTTTAAAATATAGATATACTTCGTTTGAT
ATAATTTATAATATAAATTAATGATACATTACAATGTATATTTTTATAAATTTGCATTAATTGCATAAGGAAT
TGGCACAATTGAACCATAAATTAAGCGCTTAGATTATTCTGTTTACTATAGACAAA
>103779344_2 DT665519.3
GCACGAGGGAAGACGCCTCGTCTTTTCTAGATATAATTATTTAATTTTCACACTAACTTCAAAACTGAGTGAC
TGTGATAATGCCTAACATTATAAATGAAGTTAAACTAGATTTTAAAGACGTATTATTAAGGCCGAAACGAAGC
ACACTAAAAAGCAGGAACGATGTGGACCTGTACCGCGAGATTACGTTTCGCAATTCAG
GCCAGACGTATCGGGGAGTCCCCGTGATGGCGAGCAACATGGACACCGTTGGCACGTTTGAAATGGCCAAGGA
ATTGTCAAAGCACGGCCTCTTCACCTGCATACACAAATATTATTCATTGGACGACTGGAAAAAATTTGCTGAA
GAAAATCCGGGATGTCTAGAACAAATGGCCGCAAGTTCGGGAACAGCAGAATCGGACT
TCGATCGTCTCTCAGAAATTCTAAATAACGTGAAAGACCTGAAATTCGTTTGTTTGGACGTTGCAAATGGATA
TTCACAGCATTTTGTGGAATATGTGAGGAAAGTTCGAGCTGCGTTCCCTAGTCATACTATTATTGCGGGGAAT
GTCGTGACCGGAGAAATGGTAGAAGAGCTAATACTGTCTGGGGCAGATATTATAAAGG
TGGGAATTGGCCCTGGTTCAGTGTGCACGACGCGTATGAAGACCGGCGTAGGGTACCCACAGCTATCCGCTGT
TATAGAATGCGCGGATGCTGCTCATGGATTAAAAGGACATATTATATCGGACGGAGGCTGCACGTGTCCCGGC
GACGTGGCCAAGGCGTTCGGCGCGGGCGCGGACTTCGTGATGGCGGGCGGCATGTTCG
CGGGGCACGA
>103779352_2 DT666515.3
GCACGAGGCTGGTCCATACTACTGGCACATAGCGGTCAGAGTCTTGGTTTTTGGACACTGTTGACTGAAATGC
CCTCGTACATGGATAAAGTACTGGGTGTCGATATAAAGAGCAGCGGTTTCCTGTCAGCGCTACCATACTTCGC
CATGTACGTGCTCAGTTTCATCTTCAGTTGGACTGCCGAGTTCCTGGTCAACAGAAAT
ATTTTGTCTCTTGCCGCGTCGAGAAAAATTTTCAACACTATTGCGTTCTGGGGTCCGGCGGCTACACTACTCG
CCTTGTCATACATCCCGGCGGGGCACTTGCCACTCGCTGTCATAATGTTAACACTCACTGTGGGACTGAACGG
CGCACATTACGTTGGATTTTTGATATCACACATCGATTTATCACCGAATTTTGCCAGC
ACCCTAATGGGCATCACAAACGGCTGCGGCAACATCTTCTCTATAATGGCACCTTTGAGCGTCTCAGTGGTTG
TAAAAGACGAAACAAGTGCCGCCGACTGGAGAAAGGTGTTCTTCATATCGATCGCATTCTACTTCCTTAGCAA
CCTGTTCTTTATTCTGTTCATGTCGGGCAAAGTACAGGATTGGAACGAGCCGAAGATC
AAAACGTCGGTTGAGGATGGAGTGAAGAAATCTAAAGATAAAAAGAAATCAAAAATCGAAACAACAGGCGAGC
ACAAATTCTGAAACTTTATTTTATTTATATACATAATATTTACTAGTCAATATATTATTAAAATAA
>103779397_2 DT666667.3
TTTAAAAATGAAATAAATACGGTAAAAAAAATACCAATCTCTTAATAAATTTATAAAGAAAGTTACATACTAT
TATCCTAATACAGACACAAAGATTGGGCGTTTACTTATTTTTTTGCAGAGAAATGTTCTAAAAGCATTTATTT
TGGTTCTTCCCTTCTGTTTTCGTCATCTCGATATCAGTTATAGGCAAAAATGTTATTG
GTTAACTTCAAAGCTCATTTTAAAGTTGGAATTATTTGCTTTAGTAGTATTTTAAATTGATTGCATATTTTTT
AATTACTTTGAATACTAAGTAAGATTTTACGACAATAACAAAACGAAAACTGCCTGTAATTACTAGAACTAAT
GATTCATAAAAAAAACTATATGTTATGATCAATTACCCATTTTAACTTTGTCTAAATG
CAATAAAATTATACAATATATGAATTAATATTAGCCAATGTATATATTAGTAGATATATATAAATATGTTAGA
AGAACCCACCAGGCTCATCCCAAGACCATCACAAATAGGTTATTTGTATAGTGGAACAATTATTATAAATCAC
GCATTATTATATGAATGTATGTTGTTTTAGAGAAATTTTAGTATGTATTAAAAATATA
TTTTATATTTGTAACGCGATCTGAACTAAGTATAAATCTAAATAATCCAAATATGATAGATTTTAGACGAAAT
ATTAGTAGGAAATATACATCAAATTATACACAATGACAGTCAAAGTACCTATATAAAGTTGAATAACGTAGCT
GCCATTCATTTGTTGGCCTTCAAGATCTATAAAAAATCATAATTTTTATGTAATTTGT
ATT
>103779403_2 DT666546.3
GCACGAGGCAGTGTCGTGGTAGTGCCCCGGGACTCCGACCCTTGTAACAGAACTGTTTTCTTTTTTACGTGTG
ACTATACTTGTAATTTATATTTATAAATTAAGTATTTAAGTTTTTATAATATTTTTATAGTCGCATGGTCGTG
TAGTTCGCTTGTTTCCATGCTCAAATTTATTTCATAATGACTAGTCATAATTAAATTT

TATTTGTAGAGAATGACGAGATGAGTCACACGGTTTACATAGAGTGATGTTAGTCTAAATTATTGTTTAATAT
TTTAATGATTATACAGTTTAAGGGTGCTGGTTTACATTCTCACGGATGGGCCACTCGTCTCTGCGCGTTAGTG
ATCTATCGCGTAGAGTAAACAATGCTTTTATGTTCATCATGTAATATAAGTTAATCGT
TGTACTTGTAAAAAAATTAAGACAAAAGTAGGGACTGTGACTTGCCTTCTAATCGACTTGCCTTTTTGTGTAC
TTGAACATAGCGAGGACCTGCGTTTACATAAGAGTTTACATTACGTCTATAAGTTTCTCAACCGATAGATTTC
AAGTACGCAATCACGAAGCTAGTTACAATCACATATCAATTGAAGTGTAATACCATTT
GAGATATTTGTAAAAATCGCCCCAAATATTTCGGGGCAATCTACAGGTCCGATAGATTGGAAATATGTTAACA
TCGACCTTGCGATCGTGAGCTCGTTCGACCATTTATGATAGTAATATATTTTTTTAATTCGTATAGAGCTCGA
TTAACGTAATTAACTGTGTTATGACTGCAAGTTATATCATGCTGTCAATGTTATGAAT
AATTATCTAT
>103779413_2 DT665375.3
GCACGAGGCAGACCTTTATTACGAAAGACAACCAGCCTCTGCATTAGTTGAACCAGTTGTAGAAAGTGGATTT
AAACCTATTTTTAAAAATCCCCCGAAGTCACAAAAAGCCCCATCTATACTTCGTCTGTTCAACAAGACGAAT
TGTCGTATCATAACTATTATATTGAGAATCAAAAGAATCCAGTCGATGATTCAACTTT
AGATGTGCCAAGAGATAATTCGTATAAGCAACAATATATTATTGAACAACCTACCGTACCTGTATCTACACAG
AAACAGGAATCTTCAAATTCTGTTACATTGGCAGAACTTTTAAATTCTTTACAAATTAATAAATCTATACCAA
AACCAATCACAAGAGAAAATGTAGGCGATTCAATAAGAACATTACTTAATGTATTGAG
TACATTAAAAGCTGCTCCATTACAAAATGAAGGAGTGGCAGTTGTCAGTACGCCAAAACCGTTTGAACCGCCT
GAAATTTCACATGTCACACCTCTCAATTCGGTATCATCGACAACTCCTAAGGCCGAGGAACCAGATGAGGAAT
CGTATCTTGCACCAATACAAACACCTTCGCAATATCTAGACGATTATCCAACTAGCAG
TGGAAGTACGCAAAGATTTCCTCTTCCCGTGACCTCGGACGAGGAAGGTGGGACTCCTGGCCGCCCTGGCGTT
GATTACCCTATATTGACTGTCATCCCACAACAAAATTCAACTGCAAACACAACGTTATAAAGGTTTCTTTGCA
GATACTGGAACAAGATGTCAAGTCTG
>103779419_2 DT665602.3
GCACGAGGCGTCTGGCTATTAAATGTGTTTGTAAATTGTACTAATTGGTAATGGCGGCCCCAACACGCAAAAC
AGTGTTTTCATGTATAACTATCCCGGTGGCGTCCTACAACGACCGCGCGTTCGCTCTGTGAAATAACCTGTGA
CTGGTGACAGTTAATACTATCGCACGCGAGTCTATCGCTAGGACGGATGCATTATGCA
TATGTCGAGTGTATGTGTGCGTGGAGTGTTCACAAATAGTCAGTGCGAAAGGGGTTCGGAATGATCGCTTATT
GTACCAACGCTACTTCTGCAGAATTATGGTACATGTAAAAAATTAAATATTTTTATTTACTATGGATCCAATA
TTAGAAAATGAAGCTGCATCGTCTGACTCATCAAGCAAACATGGTAAATCTAAGCATA
GAAATAAGAAAAAGAAAAACCAGTCGGAAGGACCAAGTGAGAGTCAGACAGTTGTAGGTCAAAGGAAAAAATT
CGTAGTTACAACATTGACATCTTCGGATGACCGAAATATTCCATCTGAACAATTACCGCAAAGTAATGTACAT
ACAATTCACTTCGGAACCCGCGGTGTTGAATCACAAAGACCTCATGTTAGATCGATTT
TTAAAGATAGATCTGCAGAAACATCTAGGCCGTCGAGTGCTGAGAGGGAGAGATCCAATAAAAAAGAATATGA
GACATTTCACGGAACGTCATCTAGCCCTCGAAGCTCTATTTTTGATATATTTCGCATAAGACGGAAAAGTGAC
TCTAAGAAACATCAATCACTTCTGGAAAATGTCAGAGGTGCTCTGGGTGGTGC
>103779429_2 DT666829.3
GCACGAGGAACTTCCTATTTTTAAACAAAATAGAATTTTAATTGGATTTTAATTAAAAATGAAAGAAAAACGT
CATAAAAGAAAACATAAAAAAGCTCATAATTTGGAAAGTAAGCGTCAAGAAAAGAGACACAGTAAAAGAAGAA
ATTCTTCATCTTCAGAGAGTTCTGATTCTGATGAGTGGGTGGAGAAGAAAACAGAATC
TAAAGAAAGAAGAGAAAATACGTCTCGTGATGACTGGATGTCTATGACAGGATTGATGAAAACTTATACAAAA
GATGATATTCGACCAAAAAAAGAAATAAAAGAGAAAGGTCATATAGATTCGTATAACCCTTCAACAAGTAGTA
GAGAACTCAATCCTTATTGGAAAGATGGTGGCTCTGGTGTGCCACAAACATCAGAGAG
CTACCAGAAATCAAGACAATTTTTAAAACCTTCAAATGATGACGACTATTATTCATCTTCCAGAAAAGACCAC
AAACATGAAACATCAAAGCATAAAGAATCAAGTGTAAACAAATCATTCAATTGGCGTAAAATTACAAAAGACA
ATAAAGAACGTATTCAAGAGGACATATCACAGAATTTGTATGAAGTTTCTCAGAGTAC
TAACAAAGCATTTGAAAACCTAAATGAAATAGAAAGTGAAAAACCTAAAACTAGAGACAAAAACAGTTTATAT
TTGTCAGATGAGAAGATGAATAAACTGGGTGGCAAAATTGTAAAGGCTGAAATACTTGGAGATATGAGGTTAG
TTGAAGAATTAAAGCTAAGTTAGAAGCAGCACGAGAATATA
>103779437_2 DT665982.3
GCACGAGGAAAGCAACGATGTGAAAATAACTAGTCCCATATGCAAAATAAACATAGATGACATTTTGTCATCT
ATCAAAGAAACCACAACTAAAGTAATAGAATCGAGCACATTTAATTTGATTGATATGGAAAAAGTGCCTGCAA
GAAAAGAGAGAGGATTTAACGTTGTAGAAAGCGACAAAATAACGTCTCAAGAAGTAAA

ATTAGAAGATGAGAGTAAGTTAGAAAAAGATGAACTGGAGAAGTCGTCTGACGACGATAACGCATCCCCCGTC
CTGTCCACTGACAGCGATAAGGATGATAAGAAGGGCGGCGATGCCTCCAGTGTTAACACGCCTTTGTCAAAGC
AATCTAAATCTAAAAAGTCTCGTAGGAAGAAGAAGTAAGATACGTGTTCAATTTGAGC
TTCATTATAGGTTAATGCATATTTAGTGGGACTTTCGCAGCTATCGTTGTAACTTAGTTAAGCAATTTTATGG
CGTGTGTGCATATAAAATACAAAAAAAAAATCTTTACGTGATATGCATAGTATGGTTTACATCGCTGGTAGTT
CTTGTCGACTTCGATATTGATAAAAACTATAAAATCGACGTATGTAACCGTCATATAT
CAATAAAAAAAACAAATAAAAAATTTAAAAAACGTAGGAAAAATTATAAAAAATCCTTTTTATCGAGATTCTT
GTTGAGACTCGCGTCAACGAATAAAGCAAATGTAGTTTGAATAGTTTTCAGTTTTCAATGTATAATATTATTT
TGATAAAATTGAATATTTGAATATTAAACATTTTAACTATTATTTATACAAATTGTAT
CATATATATAAAAAATACAAC
>103779458_2 DT666241.3
GCACGAGGCACAATCTTTAATTAATACAATTAATAATCGATTAATAAGTTATTCATCTTTACAAAGAGAAAGT
TACATAAAGAATAACTGACTAATATATTATTTTGTATATGTGGAGAGTCCGCATGCATGATGAAATACTCAAC
TTCACCTACTCCATCCACTTCTTCGAATTACCCGAGGAGTACTTCTCCACTGCCAGCC
CCAGCCAACTATCAGCCCACCACAGCTAGTGCTGCAGTCAAACGATACAAATATCTTAGAAGGCTATTTAAAT
TTAATCAAATGGACTTTGAATTTGCAGCTTGGCAAATGGTCTATCTTTTTGTTTCACCACAAAAAGTGTTTAG
AAATTTTAACTATAGGAAACATACTAAATCGCAATTTGCTCGAGATGATCCTGCTTTC
TTAGTTCTCTTAAGTGTATGGCTTTTCTTATCTTCGGTATGCTTTGCTTTAGCTATGGCATTAAGCTGGACAG
AACTAACATTATTTGTACTTTTTGTTGTATTTGTGGATTTTATTGGAGCTGGAATTGTAGTCTCCACAATATT
TTGGTATGTAAGTAACAAATACTTAGCGGTAGACGGTGCTGGGGGAGATGTGGAGTGG
GGGTACGCCTTTGATGTACACATCAATGCATTCTTCCCACCGCTGTCTTTGTTACACTTTCTTTCAGATTATT
ATATTTAAAAATCTAAATTTCTTATCATGCTTCGTGGGCAATACATTTTGGTTGGCGTCCATCATGTA
>103779460_2 DT665950.3
TATTAATTTATTATATTTACGTTTTGTGTATAAAAAATATAAAAAAATCATTCAAAAAAAAAAGAACATTGGA
AATACGGACATCAAATGCTGAAAAAGAAGCAAAAATAATAGAATTGGAGCTGGATAAAATAATAAACGATGAT
CTTAGTTCAGTTGGTGATCCGAGGCTGGATAAATTGCTATCCGATAATGAGAAACTCA
AACATCGTCTAACTATTCTTGAAAATGCTATAAAATCAGAACAGGGTAGTTCACCGGATTCGGTGAAAAGAAA
TTTTTTTAGTTTAATAGTCGACATAAAGTCTGTCGAAGGTACTATTTGCATAATGGATGAATTAAAAAATGTT
TTCGAAGTCGCTATAACTTCGGCATATCCTGATTTGGTGGATCCACCAATTGTTATAT
CATTATCTGGGAATAATCCAAAGTTTGGTGATTATCAGTGTAATTCTGCTATGCCTATATCGCAACTCCTTAA
AGGCCAAAAATATAAAAACGAATCCTAGAGAAGTAGAAAAAATATTTTAAATAAGACTCCTTCTTCAACAATT
ATAAAAAAAATGGAATTTGCTGGTGCTGGATTTTTGAATATCTAATTAAAATAAAAGC
TAATGGCTGAACA
>103779470_2 DT665885.3
GCACGAGGGCCTTGCGTGTCGCACTGGAAGAACGCTGAAGGTTACACAATCCCCCTGGACAAACGTCTAGCGG
CAGACGGCCGTGGCCTACAACAGGTACACATAAATGAGAACTTTTCAAAATTGGCCGAGGCTCTATACATAGC
TGATAGGAAGGCAAGGGAGGCGGTGGAAGCGAGAGCACAGTTAGAGAAGAGAGTGGCG
CAGAGAGAGAAAGAGAAGAAGGAGGAGCACTTGAGAATGTTGGCGCAGAGGGCTAGGGATCATAGAGCAGGTA
TCCGTAACCCGGACGAGGAACCTGAGGAGGATCCTCTTGCGGCTCCAGGTCCCGCTGAAGGAGACCTGTCCGC
CGCCGAGAGAGACAAGCTACGGGCGGACAGACACCGGGAAAGGCAGCGGGATAGGAAC
ATAGCGCGTGCAGCGCCTGATAAACGATCCAAGCTAGTGAAGGACCGTGAACGTGACATTTCAGAACAAATAG
CTCTAGGCCTACCAGCTAAAACTAATCAAGGAGGGGAGGCCATGTTCGATCAGAGATTGTTTAATAACAGCAA
GGGAATGGATAGTGGTTACGGTGATGATGAAGCATATAATGTATATGACAAACCGTGG
CGCAACCAAGACTCTATAGGCTCCCATATATACAGACCTTCTAGAAACGCGGAGAAGGACAACTATGGTGATT
TGGATAGCTTGGCCAATAATAAACGTTTCGTAGCAGACAAAGGATTCGCAG
>103779497_2 DT666166.3
ATCATTCGATAGTCTCAGATTCTCTTATCGGCGGCCATTCCAAATGTACAGCCCTAACGTCCAAAGCTAACAA
ATCAACTTTACAAGACAAAGCAGCACCCAACGACGGCTGGGTTCTCCCAAACTCCCCGTGGACCCATTCCTTC
ACGTAAGTCCCCGCAGTCGTTTTGACGAAAATACGAAATAATTGTGGGTAGTTTGGTA
CCGTGTCTCCAGTGAGTTCCAATATTTCCCTGGCGCGGGTGAGCAGCGGCCGCCGGTGCAGGACGCGGATGGG
CGTGTTTCTGTTGGATGAGCCCGTGCTCTTTCGCGAACTTGTTTATTAGTTCTATATCTTTGGCCGTCACTTC
GATTTGACTCGAGTCATCATCATCCTTAGAATGCGTTAGTTTAATGCATAGAGCTTCA

TACGTTTTACTTTTTGTTTCTTCACCCTTCTTCAATTGTGCTAGATCTTCTCGTTTAATAACAACTAATTGCT
TCACGATGACGTCACCACTCTTGCTTATTTCTTCACACGCTTGAGCTAACTCCTCCTTTGATAACTGTCTAAT
AGGATCCGTTATCTCTATTGCAAATGGTCTTCCATCACCCAAACATCTTACATCGACA
TCTTCTCTACCGGCTGCTATAATTTTAAATCTACGATCAGCTTCTTCTTCGTCGAATTTATATAACCCGGCGA
GGGGCGCGTATATAATTTCTTGTACAGACGACGGCAAACATCTTTTGCCGTCTAAAAT
>103779514_2 DT665507.3
GCACGAGGCAACAAATCATAGTCACTTGAGTAATTACATGAACATATCTAAAAATCTTTATAATTAATGCGGT
GCCTACTTGGTTTTTTAATTATAATTACATTATCACATAGTGCACCAATACAAAATGAGAAAGAAGCGGAAAT
TCTTCAATACGAATATAATACTGATGGAAAAGGCAATTATAATTTCAGATCGATGCAG
TCTGACACAGTCCTGGTAGAAGTAAAGAGTCAAAATCCCTCGACTAACACCGAGACTGCTGCAGGCTGCTTGA
GATTAAACTCAGTCAACAATCACTACGATGGCAAACAAGTTCTCATCGCTCCTAGAGGTGAACGTGTCATCAA
TAAGGAGTGAATGGCTAGGTTAAGGGGAGATTGCTGATAAGAAGGAATCACCAACATT
TTATAAATTTAAAACTGTGGAACGAAATTTAAGACGAAGAGTTAAAAGTTTACATTCTTTCAAAGTTAAAAGT
AGCTTATGCTTTAAAACTTCTAACGGTATAACAAGATCAGAAGTAGGAACTGTAATGGATGAGGGTCTTTCCA
GTCAGTATATATTGGTAGAAGGTAGAGTTGTATATCAAAATCCTGACGGAAATGAGGA
AGCATGGACTTATACGGCAGATAAAGACGGATTTCACCAAATGCAAATTTCAGCAAATCTTCCAGAAGTAGGC
ATTCCGCAGGCTGCACTACCGTCAGCAGTTGTGGCATCTTTACTCGGCAAATAAATCGCGTTCTCTATTACTG
CAAGAGATACCTTTAATGCGGATATTATTATTTCCTCTGGGCCCTCACAAGTGCCTTT
GA
>103779516_2 DT665969.3
AGATGATGATAATATTATGAGTTATGGAATTTCTCTGGAACCTTTTGAAGAAGATGAGGTTCCGTCAAAAAGA
AAATTTCAGCAACAACAAGATCAATATGCTGTTGATGCACATGGAAGGAGAAGATTTCATGGTGCTTTTACGG
GGGGTTTCTCAGCAGGTTTCGGTAATTTTTTTGGAACACCAGAAGGATGGACACCCAG
TACCTTTAAGAGCTCTCGTGCAGATAAAGCACAAATATCTAATCAGAAGCCAGAAGATTTTATGGATGATGAA
GACCGTTCAGAATTTGGTATTGCCCCCCGTCAATTGCAAACTAAAACTGATTTCTCTGGACAAAAGCGCCCAC
ATCAGAAAAGCTTTCATGATGGACCTATACCTGGTGATCCAGTATTAGGACAATTAAT
ACGAGCGGTCCACGAAACAGCCGCGGTGAAAATGCTGCGTAAAATGGGCTGGAGACCAGGACAGGGAGTCGGG
GAGAGAGTGTCACAAC
>103779524_2 DT665955.3
GAAATGTTATAAGGGAATTACCCCCAGCTGCGTTCGAGCACTTTAAGAACTTACGACATTTAGATTTGTCAGG
AAACCTTTTAAATTCTATATCGGCGGATGTCTTCAGTGGGTTGGAGACTAGTCTAGAATTTTTATCACTTAAT
CAAAACAGAATTTTAGGATTTACAGGAGAAGAATTAAAATTTGTTAGTCTTTGGTTTT
TGGATATATCAGGAAATCAAATATCAGAAATTCCAGTCACTGCTTTCCATTCTATCCCCAGCTTAACACATTT
AAATATGAGTCATAATTTACATATCAGTGTCTTACCGCAAAATGTTTTTCATCAAAATGATGCATTATTATCT
ATAGATATTAGCAATGTAGGGCTGAAAGCTCTTCCTGTTAATTTATTTTCAAAATCTC
ATAATCTACAACGAATTTATTTATCTCATAACTACTTACAAGAAATATCTGAGGGTACGTTTAAAAATCTTAA
AAATTTAACTCACCTTGATCTTTCATATAATAATATTGTAACCATAAGGACTCCTGCTTTTGTTAACGTTATG
GCGATACAATATCTGTCTTTAAAGGGAAATCAGTTAAATGCCTTTAAAGGAGAATTCT
TTAATACAGGTACAAGCTTAGAAGTGATTGATGTTTCAGACAACCAATTAAGTTATTTATTCCCATCTTCATT
CAAAATTCATCCCCGTCTCCGGGAAA
>103779544_2 DV501410.2
CAAGTATTATCTAAAATTAACACTTTAATATGTTCAGTTAATTAGAGAACGACTTAGTCGTGACAAAAATATG
TTCTTCTTGATAGCTCAATCAGCTAAGAAATAAAAAAAACGTATTCAAATAACTTAAATAAAAGAAAAATATG
TGGTTAAAACCCAGCACCACACTGTCTATCCATGATGGAAAAATATTTAAAAGTTAAG
AAGTTGCTATATTATTTCAAATGGGACTTAAACCTAAAATCATCGCATTTTAAGTAAAATTGTAAAAATTTTG
TTTAATAAAAAGATATTAAACATATGTTAAGTCATTAAAAACTTTTT
>103779548_2 DV501428.2
GCACGAGGGCGACACCATACACTATTCTCCTCTTAACTCTCATAACCTCCCGGTCCACCCTGACCTCTATTGT
CTCTAGCCCCATTCTTCCCTATGCGTTTCCACAATTGGTCTAAAAATTGTAATTTAAATTTAAAAAAGAATGT
CAAAAGTGTAAAGTCGCGTCAAAAGTAAGAAAAAAAAATGTTACTTTATATATGTATA
GGTATTGTTTATATGACGTCATAATTATAGTAGAAAAAGTGAAAAAAGTGTTTTTTTTTATTTTGAAGCGACT
TTGCTCTTGTGTATGTGTAGAAATAAAAAAAATGTATGACATAGTATTACTTTATAAAAGTCCATCAACTGTT
TATAATCTGTGCTTTTTTATATAATATTTATGTGAAAAAACATTAAAAGGCAAAGGTC

GTACAGTATTTTTCCTAATATTTTTTTCGCTATTAATAGCGTGTAATAATAAAAATGCTTATTAAATAAGTGT
GCATTGTTTTAAATATGTAAATAGTACATATTGTTTTTTTTTTTGTTGTTAATAATTCCAAACTCCGACTCCC
ATATTTAACTTATAAGTGTTCAGCCTTTGCCTTTTTAATTTTAATCGAATTTAATAAT
ATATATGATAAAAATAAGTCAACGAACAAAAAATAACTATAAATTATTTATTATATTATTATAATAATAATAA
AATAATATTATAAACATTTGGTGTGACTTCAACCATACATCGATATCGATATCTTCTAAAATAAC
>103779555_2 DT665901.3
CGTGCGCGCGCGTCGACAGCACCTGTTGTACTTTTACCTTATATGATCACAATCATGCTGCGACAAATATTGC
TTTTTACAGTTCTTCTTCAAATTACTAGAAATGTGGTCCAATCAGCGCAACAGGTCCGGCTATCAGACTCATA
TCTACCAGTTGCAATGCAAATAATACACCACCTGACCGATCAGATGGCCTTCGAGGTC
AAGGATGATCCTCCTCCAAAATCCACAACACCCAAACCCTTAGACTTGACTACACCGTTCCATAAACCTGGAC
TGTTCGCTCCACCGTCTCCACCAAAACCATCAGACAGAGTGATCCCAAAATCTGCGAATTACGTCCTCTTACC
TGTTGAAAACAACTCACAGCATACCGTTATAGAACCTGAAGGAGTAAGAACAGAGGAA
CCTCTTCTTGCTGCTCACAACGAAGAAGTTAAACCACTATTGGAACCGATACCTGACGTTCCACACCAGCTTC
TGCCTTATGATAAAGGTTTAAAGATATCCAGTCGCTCAGATAACTACGAAGATGAGATAAATGAAGAATCGTT
GCAACATTTGTCTCCAAATGTCAGAGATTTAATCAAAATGGCCCATGATCCTAATGAA
GAAAGAGTGGTAAAAGTTTGGGAGAGCTTGAGAGCGAACCCTGGGGAACCATCGACGAAAGGGAAGCTCTCAT
CCTCTAACTTGAGACTTCTCTTATTGTATGATCTTCTAAGTCGAGATGCTAAAAAGCAAAAATTATCTGATTA
CAGTGGTTTCTCACCGAGTGTAATGAAAAC
>103779559_2 DT665545.3
GAGGGAATTATTCTTGAAGAGAACAATTTTACAAGATCAATTAAATAATGCAGATAAGAAACTAAAAGATAGT
AACGACAACCTTAACGCTATTACATCTAAAATAAATTTAGGCAATGTGACCCTAGATGGATTGAGAACGAGTA
TTGGTCACTTAAAGTCGAAAACTCTTGAATTAGAAAACAATGCCACAAAACTCCAGGA
AGCGAATTTAGAAGGAGCCCTTAACCTAACGCGTGAAGCAAAAGAAAGAGCTTTAAAAGCAGCAGACGAAGCT
GAAAGTGTGCAGATGGTAATTGCTAATACTGATAGACAAATTAAAAATACAGATCGCTTAATAGAAATGCAAT
ATGTGAATTTCAATAATACACAAAATGAAAATGACAAGAAGTTAGATGAATTGCAGCA
ACAATTATCAGATTTAAAATCGCAATTGCCAAAAATAAATGAAAATATGTGTGGCCAGGAAAGTGATAGCTGT
GACATATGTGGAGGTGCTGGATGTGGAAAATGTGGCGGGATATCTTGTGATCAAGGAGCTATCACGAAAGCAG
AGCAAGCATTGGATTTTGCAAATAAAACAGAACACAGAATCAAAGAACATGAACTTAC
TGCTGAAGACTTATTTAGATCAGTTTCTCAAGTCAAACAAGATACAGTTGCAGTACGATCTAGAGCCAAAGAT
TTGTTTAATAGAGCTAACGATTTCAAATCAGCAGCAGAAAGAGCAACTAATGAGAGCCAAGAACTTACATCTG
AGTTAAAAGAATTTTTAT
>103779563_2 DT666073.3
GCACGAGGGTTTCTCATTTTAATTATTATATTATGTGTAGTGTATCAAGTAACGTGTTTAAATTTAAATAGTG
ATTGTGAAGGCTGCAGAAATGACTATAGGAGGATTAAACGAGCAAAGTACTATCAAAGTCCACCTTTCAAGTT
TTATAATCGACCGCCTTTCAACCCCCCTATTAGGGGACCATTTTCCTATACCCCACCT
CCAGGATACACTGCCAACGATGTTGGATCACCAAATTTACCTCCAAACCTCAGGCCATATAAGGTTAGTAAAC
AACCAGTAAATGAAGGTTTGGGAGATGAAGATATCAATAATTTATTTAAATATCTCTCTAAGAAAGATTTAGA
TAAAATCATTGAGTTTGCTAATGTAAAACAAAAACATGAAAGTCAACGGGAGCCGATT
GTTAATAATTATGAATATTCTGTGCCAGATGATATGAGCAGTAGTAACTTTTTTAAAAATCAAGATTTTGGGA
AGGATAAGATTGTGAACATTCAAAGTTCAAAATCTCACGAGATTAAATATACGCCTGACGATAATCCAACGAG
ATATTCATTCAATAGTCAATTATATGGTCCAGTTGAAAGTGGACAATCACAAATACCT
TTTATAAATGCATCAGAACAAAATGAACAATTTTCTTTCTTAGATACATATATTCAGAAAGAAATTAAAGATA
TGACTATCAACAAAAGTTATATTTACACAGATAGCAGTATAATGCAAGAGGAATCATTACCCAGGCCTGTGAA
TCTAAGGGATACCGAATACGAGATATCTTTTACGAATAATGTTCCGTCTGTAGCTAAG
CCTGAATCA
>103779573_2 DT665817.3
GCACGAGGGCCAGGGGTGCCACGTCTGTCACCGCCAGATTCATTGAAGCCAGGATTGGCAAACCAACACTAGT
AAACGAAACATCAAGATTCTCACTGCTGGAGGCCGTAAAACATCCGATATTAACAATTCAAAGGGCAGTGGCT
AGTTTTAAAAAGCCTTCTGACGCACTAGCCGGTGTCGTACTATCGCCTACTTTGGAGA
ACCGTCTCAGAGACATCGCAATTGCGACCAAAAATACTAAAATGAATAAGGGTTTCTATAGAAATTTACTAAT
GTACGGCCCTCCTGGTACGGGTAAAACGTTATTTTCTAAGAAACTAGCAAAGCACTCGGGCATGGATTACGCC
ATAATGACGGGTGGCGACGTAGCCCCCATGGGCAAGCATGCGGTGGCAGCCATACACA

AAATGTTCGATTGGGCCAATACCAGCAGAAAAGGAGTTCTATTATTCATCGACGAGGCCGACGCGTTCCTCCA
CAAGCGTTCATCGACACAGCTGAGCGAGGACCTCCGGGCGGCCCTCAACGCTTTCCTCTACCGCACCTCAGAC
CAAAGTAGTCGTATCATGCTGGTATTAGCTTCAAATACGCCGCAACAGTTCGACTCTG
CCATAAATGACAGGTTGGACAAGATGATCGAGTTCGGTCTACCAGGGTTTGAGGAGAGGGAACGTCTTATTAG
ATTGTATTTTGATAAATTCGTGCTGCAGCCAGCTTC
>103779577_2 DT665570.3
GCACGAGGGATGGTTCATAATGTGAGCAAATATTTACTAATATAATTGGGTGTGTGGTAATGTGTACGTATTC
GACCTATAATTCACGAGATAATTTATCAAATTGCAAAATACTCTTATTCTAAAAATTGTGGACTATAGAAGAA
GAGAATCTTAGACGGTACTCGACAGTCCGAATTATTTAGTCATCCCTACAAAATGTTT
TGCGGATGGCAGCAGGTCCAGTGTTATCTTTTTTATTCTTATTTTGGTAGAAAAGTACTGTTGGTAAATTGTA
GTATTTAAGTTAGATTAGAATATTCCATAAAATATTTTTTTTTACTAAAAAAGGAATTAAATGGTTTAAAAAG
TAGAGTATACTAAAATACATACACACAATGACTAGTGGAAAATTTACATAACTATATC
ACTTCTATTTATTTTACAATTTAAAATTATGTAATGCATAGTTCTGAGATACTTTTATATTCCTTTATAAAAT
GAACCGAAATTGAAAGGCTGAAATGTGTGATGATTGTTATTCAATAAGATATGTTGGCGTTCTTTGATGAACT
AACTAATGGATACATTCCTATTTTGGAAGGAACTTTCTAGATTGTCCGATCCAATTGT
TCCTATTAGGAGAAACTTTATTTATTAATGCTTTATCAGTTCATAATGTTGTTATCTTAGTTTAGAAACGATT
TACAATTTCATGAAAT
>103779583_2 DT666739.3
GCACGAGGCTTAATCTTAACAACAATTTATGGTACAAAGTGTAATTTTAGTTCCATAGACGTTTAAGATTTAC
AGAGTTTATATTTAGAGAAGATTAATTAAATAAATAAAATGATTATTATATACGATACGGCGGAGCGCCCGCC
CCCTCTCAGTCAATAAAGTTAATATAATGATATATTATGTACGACGAGTGTGGCGTAA
AGTTAATATAAATAATATAATATAATAGTGTCTACGTGTCTGCGTGTGGTCGTTACGGAGTCGAGTAGTGTAC
GCGAGAGATACCGTCGGCAACGAGCAGTAGTACGTGAGGTGTGGCTCTAACGCAAAAGGGTTTTGATTTTCGG
AATTTACGTATTTTGTGTCTGGCCGGCGGCACCGTACCCTGTCCCCTTTCCCCAGGCT
TACCTGTAGCTGTCGCGTTCGGACGCGTTGCTTCTACATAGTCTTTAGATAGATTATAAGACCTATTTGATTT
CGCTATTTTGGAAAATTTCATAATCGTCACTTCCTATTTTTTGTTTTATTTAATCGTTTTAAGTATTTACTAA
TACATACATTAATCTGTCCACAAATACTTTACATAAAGCCCTCCATCCCCCATAAAGC
TCGATTAGCTATCGTCGTGTGTGTAGTACGGTGTCCGCCGGCGGGCGGCGAGTGTTCGAAGCTGCCTCCTGTG
ATACTCGCGCCGCGCCGCATCCCCCGCCGCGCCACCGCCTATTATTTACGAATTGGTTTAGCAGATCTAATTA
AATATTCTTACCAATTTTCGTGGATTTGTTCTTATTTTTTCCTCCTT
>103779591_2 DT666884.3
GCACGAGGAAAAACCTTAGGGGGATGGCCACTAAATCATATAGTAAGCATAAAGAGTACAAAGAGTATTCCAG
CAGCAGAGGCACTGTCCCTTAGACGGACGAACAATTCGATATGATAAAATCGGAGTTAATACCGAAAGGCAGC
AAGTTGGACTCATTAGGACGTGGCGGGGGCACCAGGGAGTACCACTATGAGTACAAAG
AAGAGACGCACCCGTCTGGGAAGTATGATAGAAAAGACTTGCATACTGTGGAGGAATTCACTATCCCTCCAAA
GACTGTACCAAAAGCGGCCGAGGCCTCGTATTACTATGAGAGGGAGGAGAGGGAGCTGCCCGCCATCACAACG
GGCACGAGGACGTATAACTACGAGAGCTCGCCCACACCTGGATATTCAAAAGTGAAAA
GCTCAAAAGTGACGAAGGAGATGACACAGAACGTGGACGAGCTGGACTCTCTGCTCGACGACTTGCAGAAGGA
TCAAGAGCGACAGCTTTACAAGAGTGGATACACTTCTGATACAGCTGAAAGCTCATCATTTGATTACAGGAGA
GGTACGCCGGCAAAAGCTCCTTCAAGCGGCTATTCCACTCTAAAGCACGAGGAGAGAA
TAGAGAGCTCGTCGTGGGGCTCCAGCCCTCCGCCCGTCGTCAGCAGGGCGGCGGAACTGCGCCAGGAGGTGTA
TAGGGAGGAGAAGACTGAGTCTCGCGTAGTACCAGCTCCAGTACCACCCATGCTTGCCCCAGCTGTGTGGTGC
CCACTGCCACCACCCACCAGGCACTGGTACTCTGTCCCGCTG
>103779593_2 DT665963.3
AGGAGGAAGGAAAATTAATGTATGGAATGCTTTTTTCCATAAAATCATTTGTTGCAAAAATCTCACCTCTAGA
TCCAAAAGATGGTTTCTTGAACTACAAAACATCTAAATATACTCTTCATTGTTTGGAAACTCCATCAGGGCTT
AAATTTGTTATGAACACTGATAACCAAGTTTTTGGTGTAAGAGAATTATTAAAGAAAA
TCTATGGAGAAATATATGTTAAGTATGTTGTAAGGAATCCACTGTGCGGTATTGGAGAGCCTATTATTAGTGA
ATTATTTAAAAACAAATTAGATTCTTTTATAAAACAGGCACCATTGTTCGCCGTTAGAGCTTCTTGATGATAA
AATGTATTATTATATTGAAATTTGAAATTGCTTACTTGAACATTTATCACACTTTTTG
TCAATATTCAAAATGAGCATACTTCATTTATAAATAGGTTAAAACTCATTACATTTATTTTCTCATACCATAT
GACTGTATATGCAGTGGTTGTTGGTTTGGTCCCTACTCCAGTGAAATTTAAATGTGTTTTTTTTAA
>103779602_2 DT665146.3

GGTCCAAAAGTGTTTATAAATTGTTCAGGTTTCATCAAAATCGATACAAGTAGTTTGGGTGATGGTACTGAAG
CGTATATAGAAGTGTTAGATGGATCTCGAGTTCACCCTGAAACTTACGAGTGGGCGAGGAAAATGGCGGTGGA
CGCCTTGGAGTATGAGGATGAAGATGCAAATCCTGCGGGAGCGTTAGAAGAGATTTTA
GAAGCACCAAAAAGATTGTTTTATCTTGATTTAGATGCGTTTGCTGAATAATTAGAGAGACAAGGCTTTGGTA
ATAAAAGTATAACTTTATATGACATTATAGCGGAATTGAACTCTAGGTACAAGGACCTTACGGTACCTTACCG
ATCAGCAACCGCTGAGGAGCTATTTGATATGCTAACAAAAGAATCGCCCGAACCGTTT
TTCGTATGATAAATGGTTATGGCTAGTGTGAT
>103779611_2 DT666112.3
TGACTTTGGAGGTGACCATTCAGGAGGAGGATATGGCGGTGGACACGATATAGGAGGCGGCTATGGCGGAGGT
CACGACAGTGGTCACGGAGGTGATGGCGGCCATGGTATTGATTTAGGAGGCGGTCACGGAGGTCATGACATAG
ATGGTGGCCACGGAGGTGATTTTGGAGGTTATGACCTTGGCGGAGGTCATGGAGGAAT
TGGCTTCGGAGGAGGAAGTGGTGGAGGACACGGAGGTGGACATGGAGGTGGCTTTGACGAAATTAGCGGAGGA
GGCGGCTATGGCGGTGTTCTTGTTGGTAGCATCAGCAGTGACCACGGCAGTCATGGAGGAGGTTTTGGTGGTG
GTCATGGAGGTGGTATTGGCGGCGGTCATGTAGGTGGTTTCGGCGGTGGCCAT
>103779657_2 DV501412.2
GGACGAGGGATACCCTTTAGAGAAGAAGTTGTTGCCTGTGAAGGATGCCTTCAGCTGCGAATGCGAAGTCTGA
AAAACCAGCTTCTTCGGAAGCTGTTGATAATTCACCAATACGTCAGAATATAACTGGTATTGAACACAAAATT
CGTAACTTGGAGAAAAGGAAGAACAAGCTGAAATCATATCGCGACTTACAAAAAGCAG
GGAAAGAACTTAACAGTGATCAGAAAGTTGCTGTAGCTAAATACGAAGAAATGGCTCAAACTTTAAAATTTGC
CCGAAATCTTTCGAAACAAGTGACTATAATAGCAACTGCTGCTGAACGTGAAGCCAAGAAAACAGCTAAAAAA
GAGGCATGGGTGCGTTATTCAGTGGAAACTAATAAAATACGCGAGGTTCTTCTTATTC
TGGATTGTTTAGTGCAAATGGGTAATAATGAGGCCAGAGAGGATTTTTTAAATGGTTCCCATGGTGCAGCCAA
GTTGACTGAAGAAGACTTGAAGAATTTGGATAATCTATACCCAGAAGTTACCCCAAAACATGAGGTTAATGAA
GAAGGACAGGCTGGGTTTCATACACACACAATCAAAGCTGCCGAAC
>103779661_2 DT666843.2
GCACGAGGGTGTTGCGTAATCACACAGAAACAATTTTAAAAACATATAAAACACTTCAGCAATGTTAAAAATA
ATAAGTACTACATCAAAGCTGTCTCGAACAGCTCTATCAAGATCCTTGCCTGCATTTTCAACATGTTCAATAC
GACTAGCTGCTGAAGACCCTATCAGTCGTAAAGAGGCACCTATAATTGATAATGCTGC
CGTGATAGCTCTGCCCGAAGAAGTGAAAGAAAGACGTGTTCAAGATACTACTATTTCTGTTCCAAAGAAGGTT
GATTTGTCACCAATCACTGGAATACCAGAGGAACATGTTGGAAGTCGTAGAGTTCGCATATATCAACCTCCAA
AGAATTCCATGCAAAGTGGAACTAGCAACATACATCATTGGGAGATGGAGTTTGACAC
TCGTCAACGTTGGGAAAACCCATTAATGGGTTGGACTTCAACTGGAGATCCATTGTCCAATATGAAAATTCAA
TTTTCATCACCAGACGAAGCTATAGAACATTGTGAGAAAAATGGCTGGAGCTGGTATTTAGATTGTCCTAAAC
TAGACAAAGAATTTAAACCTAAAAGCTATGGAGAAAACTTCTCATGGAATCGCCGTAC
CAGAGTATCTACCAAGTAGATCATGGAGTTCAATAATAGTTTTTTTTTATTTATAGACAGTGATATAGCTAAT
TTTATTAACTGTCTTTGTCTAGTTAAAATTATATGTGCGATATCAACTTTTGTTAAAAAACATTAGTTACAAA
ATAAAAAAATTATATGAGTTGAGTCAAAAAAAAAAAAAAAAA
>103779671_2 DV501469.2
GCACAAGGATTTTTCTCTTGTATTATGATCACGTCATGCTTTTTACATAATGGTGAAGAAAATCTCACACAAT
CGTGCCGACCGCTGCGTCTGCCGCATCGGAACTCAATTCGGGAACATCCTCTCTGACGACCACATACTCCACG
CCCCTGACGCCTCCCCCCCGGACTCCGATTTCCACCTCGAGCACCTCCATGTATACTA
CGATGAGGC
>103779693_2 DT665495.3
GCACGAGGGAAACCGGCTTTCTTTTATTTGAGTGCATGTTTCGTTAAAATTTAAGATTATACTCAAGTGAATA
TATAAATGCTATAAATTTGTGTTTGAATTGTAATTTGATAAGACCTAAACAAACAGTATGGGTATTATAGGTT
TATCAAAGTTAATAGCTGATATAGCACCGCAAGCAGTAAAAGAAATGGAAATCAAAAA
CTATTTCGGAAGAAAGGTGGCTATTGATGCATCTATGAGCTTGTATCAGTTTCTAATAGCCGTAAGAAGTGAA
GGTGCTCAGTTAACATCAGTTGATGGCGAAACTACATCACATTTAATGGGAACTTTCTATAGAACAATTCGTT
TGGTAGACAATGGTATCAAACCAGTTTACGTATTCGATGGTAAACCACCAGACATGAA
ATCTCATCAATTAAACAAAAGGGCTGAAAGGAGGGAGGAAGCTGAAAAGGAACTTCAAAAGGCTACTGAAGCT
GGTGATAAAGCTTCAATAGAGAAGTTTAACCGACGTCTAGTTAAAGTGACAAAGCAACATGGTGAGGAAGCAA
GGCAATTATTAAAATTAATGGGAGTTCCTGTAGTTGAAGCTCCATGTGAAGCAGAGGC

ACAGTGTGCAGCTTTAGTGAAAGCTGGTAAAGTTTATGCAGTGGCTACAGAAGACATGGACGCCTTGACATTC
GGTTCAAATGTTCTTTTAAGACATTTGACATTTTCTGAAGCAAGAAAAATGCCTGTGCAAGAGTTCCACTTGG
ATAATGTGCTGAAAGG
>103779697_2 DT666249.3
GCACGAGGGCAAGACCTTAAATATAACCTTAAAAGTTGAATAAACAAAACTAAGAGAGCTTCAAAAATGGTAG
CCGTAAAACCTTTAGCGAAACAAGTAAAGAAAAAGAACCTTAAAAATAATAAAAAGAAGAATACGGTAAAGAT
CACAATACCAAAACCAGTTGTAGAAAAGACTTTGGATAAAGAAACAACTAATGTAGTT
AAATACAAATTACCATCCAAAATAATAAATGAAAATCTTGTTACCCAATGTTTAAATGCACTGGAAAAATTAA
ATACACTTCAAGATAAGAAAAATGCCATTTTTGGCGACGAAAGCCAAATATTTATGGAGATTCGTAGTATCAA
ACTATTAAACGCTATAGGAAATTTAAAATTTGTTTTACCTCATAGCACAGTAGCATCT
ACTAAAGAAATTTGTTTGATTATACCAGATTTAAAAAAAGGAAGAAAGGTTGATCATGAGCCTACCATAGATC
ATTGGGAACAATTAATAAGAGAAGCTGGTGTTACAAGTGTTAAAACTGTTTTGCCAATGCGTCAGTTAAGAGT
TGAATATGATCAATTTGAGTTAAAGCGCAGACTGTTAACTCAACATGACCTCATAAAT
GGTTGATACAAGAATATTAAATCATGTATCACATCTCCTGGGGAAAATGTTTTTCAAAAAGCACAATATGCTA
ATACCAGTGAAAATAAATGAAAAGAAGGATTTAAAAAAAATATTGATGTTGGTGAAAGAACTGTCATGTTAAG
ATTA
>103779703_2 DT666916.3
GCACGAGGGCAAGCCTTAAGCGTCTAGAGAAAGAATATTACTTGAAGAATCGATCTATCAACGGAAACGACCC
TATTGCTTTGGCGGAGCTTAAAGCACTTGATGATCAGGTGAACGAATGCAAAGAAGCGGCATGGTCGCTGGGC
GAGGAGCTGGACGCGATGGTGCGCGCGTGGCGTGAGACGCGCGTAGCGGCCGACGCGC
CCGCGCCGCGCGCGCTGCACGCGCCGCCGCACCGCTACAACGAGATCTGCTTCAAGTCGGCGCGCTGGCGCCT
CACCGACGCCGACGGACAGCTCGGCATCGCCGACCTGCTGCTCACCAACTTCTTATACACAAAGACATCGCGG
TCCGATGATTCAGTGGAACATCAATTAGAAGTTGGCTATGTCCGGGTAACGAACCTGC
TACCCAATGAACCTTTCCCAGAAGTTTTAGCTCCCCAAGCTGCCTCAGGCCGAGCGCCAGTGGCTCGCCGCGC
GGCGTTGCGTGTGTTTTGCCGCGATCGTCCTCCAGTGGGTGGTATTTCTGTGAAAGAACATTTTGAAGTTAAT
ATCGTGCCTATACGGATAGGACTTACAAAGAAGTTCTTCAACACAATGCTCAAGTTCT
GTTTTCCTGAACGAGACCCTGATGCAATAGAAGAAGGTGAGGAAGAAGGTGAAAAACCTGGTTCGAAGAAACT
AAAGAAGAAACCCAAAGACTCTAATTCTAACTTCTACGTGAAACGAGATAAGAAAGATAAAGATGATGTCGAG
AAAATGAGGGAGCGAGCA
>103779706_2 DT666552.3
GCACGAGGATTCTCCCTACCTAGGTCCTGTTAACCACTGGGGGAATGAAAAGCAAAAGCAGGAATTTGTAACA
CCATTTTGTACAGGAGACATAATTGGTTGCTTTGCCCTGTCTGAACCTGGTAATGGATCAGATGCGGGTGCCG
CATCTACTACAGCTAAAGATGCTGGTGACAAGTGGATTCTTAATGGCACCAAATGCTG
GATAACTAATGGTTATGAGAGTAAAGCATCAGTTGTTTTTGCTACTACAGACAAAAGTCTGAAACATAAAGGC
ATATCTGCATTTATTGTACCTAAACCAATAAATGGATTAGAGTTAGGTAAAAAGGAAGACAAGCTCGGAATCA
GAGGATCCTCAACTTGTTCACTCATATTCGAAGACTGTGCAATTCCTAAGGAAAATAT
TCTTGGTGAGCCAGGATCTGGCTTCAAAATTGCCATGATGACATTAGATGCAGGAAGGATAGGTATAGCTTCT
CAGGCATTAGGTATAGCACAGGCATCATTAGACGTAGCTGTGGATTATGCATCAAAAAGAACAGCATTTGGCA
AACCAATCACGAAGTTACAAGCTATACAGAACAAACTTGCGGATATGGCTCTCCAATT
AGAATCAGCCAGGCTGCTCACTTGGAGGGCTGCTTGGTTGAAAGACAATAAGAAACCTTACACAAAAGAGGCG
GCCATGGCGAAGTTGGCGGCATCCGAGGCTGCTACGTTTACTTCTCATCAATGTATACAGATTCTAGGTGGTA
TGGGTTATGTGTCCGACATGCCA
>103779712_2 DT666142.3
GCACGAGGCTCAGACTCTTCAATTTAATTCATTTATGGTACCCCAAATTAACTGTTTCTTAGATGGTTGTGAT
ACTTCCGTGGTTGTTTTTGGACAGGCGAAATCAGGGAAAACTTACACTTTGTTTGGACCAGGTTTTGAATGCA
TACACAGTGAATGTGACCAAGGAGTAGTTCCACGTTTCCTAACGGACATATTCCATAG
GCTCAGTCAAATGCCTGAGAGGGAATATATTCTACACATAACATGGATGCAAGTTGTAAATAATAATATATTT
GATGTGTTAGGAGCCGGTTTAGTAAGATGTTCTAATGTGGATTAAGCTTTTCAATATTTACAATTGGGCTGGA
GACAAAGGTGTTTAGGAAATAATCATACTGTTTTTACTGTAAGCATGGAGCAAAAATG
GGTTGGGACTAATGGTGTTCTTAACCACCGAATGTCTACTGCTAGTTTCTGTGATTTGGCTGCTTACCCTGAA
CCAGGGTTATATGCTTTAGAAAATATAATTAAGGAACTAGCAGCTGGAAATCCCCCCAAACAAATAAACTATG
ATAGATGTATATTGACAGCCATGCTACGAGACTCATTTGGTGGTAGGGCATTTACCTG

GGTAATAGGATGTATAAGCACAGAACCTGATGATTACCAAGACACTTTGAAAATTCTCAATTTATCCTTATGT
TGTAAAGGTATTAAAAATTTTGTTACTGTAATTTTATTTGCTGATAATATTACTCCTGTATATTCTGAAAAAA
CTACAATAACAACTGACAATGCTTTCCATTTTACATTTGCAACAACTC
>103779726_2 DT666418.2
GCACGAGGAAACGTCTTTAAACATGGCCGATGTCGATGTTGAAATTCCCAACAACCCCGTCTTGAGCGGTGGT
GCTATGGACGTCAATACTGCACTTCAAGAAGTATTGAAAACCGCCTTAATCCACGGTGGACTCGTCCATGGTC
TTCATGAAGCCGCCAAAGCTCTGGACAAGAGACAAGCTGTACTCTGTGTGTTGGCAGA
AAACTGTGATGAAGCTGCTTACAAGAAACTTGTACAAGCCCTCTGCAATGAGCACCAAATCCCACTTGTAAAG
GTGGACAACAACAAGAAACTCGGTGAATGGGCTGGTTTATGTTTTATCGACAAAGACGGCAAGGCCAGGAAGA
TTGTTGGTTGCTCTTGTGTAGTAATCAAAGATTTCGGTGAGGAGACACCAGCGCTGGA
TGTGCTTAAAGACTACTTGAAGTCGTCAAGCTAATCTTTAGGTGTAATGTCTTTAAATAATAAAAGCCAAAAA
AATTAAAAAAAAAAAA
>103779734_2 DT665509.3
GCACGAGGGACAACTGCTGCTTGCAATTTTATTTTTTGATAGTGCCCATTGTGATTATAAAGTATACGCTCAG
CGACAATAATATTAGAAAAGATGTACGAGGAGAGGGTTCTCAGCAGCCGGCCCGATAGAGAAATAGCCAAACT
ATTGTAGGACCCGGTGACTTTACTATAGTCCCTTATGAGGACGCTCGCTGCAATCAGA
CTAAAATGTCCATGAGGGATAAATGGGCATGCCATAACATTCCCGAGAGTCG
>103779736_2 DT666538.3
AGCTCAATTTTACCTCACAACAATATACAAAATTTAAATAACATATTCGATAATGGTATTGATGTAGTTGACT
GTGCCATGCTACCGCCACCGCAGCAAATGTTTGAATTACCTCCTTATCCGAGTCCAATGAATTCTGTGAATCA
CTCAAGACAACCCAGTGAAGAATTTCCTCCTCCACCACCTCCGATAGATTTAACGCCG
TTACATGACGAACTGATAAAACTATTTCAGCATTTAGCAAGTACTTGTGAACATACGACACATGACAAAAACG
AAGTACCAAAAGATTCTTTATTGGCCCAATTACAAGAAAAACGAAGTCAAATTTTAAGGAGTGACTCAGCTAA
ATTAAGTCAATTAGATAACATTGGCACTGCTGGTGATAATTGGCTGAGAGAGCTACAG
GCTAAACAAGCAGCTATAAAATTGAAACGGTCAGGATCATTAGACTGTCAATTGACAACGGCCAATGACAACA
TATCTCAAGAGAACATAAATAATGTAAGAAACATTGCATCAAAATTTGAAAACTCTCATCATTTATTTAATGA
TGGAGACAGACCTCACGGAGGATATACAAGTTCTACTAATAGCAGCCGTGATGTAATT
AACTGTTCTAATCAATCTAACAATAGAATATATAACAGGC
>103779743_2 DT665678.3
GCACGAGGTTTGCCCCTTGGAGCAGGCGCGTTCGACGTCGCCGGCCACACGGAGAGGGCGAGAGAGGCCTAAA
CAACAATTGTCAAAGAGACGAATGCAAGTCCGGCAGCCATGTGGACCAAGTGAATGCAGTGTTCAGTGACATA
ACAAGTGCATCTTTTACAAACGTTCGTGCTGATGATACAAAAATCATGGATGTCAAAA
CTTCGATTTCAAGACAAGAATGGCATATTCCGCGACCGTCGTTGGCGAGCAATAATGGCAGCAACAACGACGC
GCGCTCCCGTGGAGGCTCCGCCTCTAGCCAAGGCTCGTCGAGTAATCACAGCACACACAGCGTATCTTCAGGA
TCGCTGTTGTTAAGCGCAGCGCATTTGGCTAGAATGAACGGCAGAAATTCCCTGCCAG
AAGAGTGCGAGTGCTATCAAACTCAATCACGGTGTATGAACATCGAAGGAAAATCAGCACCGGCCAATGCTAT
AGTATCGATACCTGAAGAATCACAAGAAAAAACGGACACATTTTGGTCTGATGTGAAACGCGGATCTAACGCG
GGGCCTGACGCGGGTTCCGTGGCCAGCTCAACCCATTTCACTGTCGTAAATGGCTTTA
CGAAGCATCGCCCCAGCAAAGAGCCAAAATCATGTTGTTGCGATCATTCACACCAAATTACGGTGCTAGTTAT
TTCGATGACGATTTTGTTCTCAGCCTGCATACTGGCCGCTATATGCTTCGTTGAAATGCGGATGCGTAGGGAG
ACTGGTATGTA
>103779747_2 DT666359.3
CGGCACGAGGACTTTGCACTAAACAGTCGTTTATAATGTGGAAGGTTATAGCATTCGTCGGTTTAGTTACGGC
CGCTCCGAACAATTTAGTTAACATAAACTTTAATCCAATCAACGAGTTTTCTTTAAAGTTACTCCAAAATGCG
TATACGTTTCAAGAAAATTTCGGTGATAAGAACTTGGCTCTATCACCGTTATCAGTTT
GGAGTATATTCTCGTTGTTAGCTGAAGGCTCGGCGGGAGACACTTTTACGGAATTAGTGAGGGAACTTCGACT
GGCGGACAATTTGAAGGAAACACAATTATTACATTCGGCAGTCTCTTCCTTGTTGAGGATAAAGGACGAAGAC
GTCACAATCAACAGCAAATCAGCCATGTTTTCCGACAATACACTGAAAATTTATCAGG
AATTCTGCGAATCTGCTCTATATTACGACACTGACGTGTTCTCTGTGGACACAAACAATACAACAAAACTTGC
AAGTGATATTAATTATTATGTATGTGTAGCGACTGAGGGGAAAATACGTTCAGCGGTTCGACCGGAGTTACTT
GATAATTTAAGACTAATATTAGTCGATGCACTTTATTTCAAGGCCAGCTGGACCAATC
CCTTCGACCCAACTCANACTAAGGAGGAAGCTTTCTACAATTCACAGGGTAAAACGATAGGTACTGTTAATAT
GATGTACCACAAGGCCCCCCACAATTTCGGAGATTCGAACCTAATTGGGGCACAGATTTTGGAAATGACTTAT
GGAAAAAGTGAACAGTACTCCA

>103779755_2 DT666793.3
GCACGAGGGGAAAACTGCCAAGTTTGCCAACGCACGATCGTTGTGTAGTTTAGTTATTAAAATGTCGTTTTCA
GATTTGTATGCAAAATATAATTGTACATATTGCCAGGAAGAAATCAATGGCGTTCGTGTAAGATGCTCAGAAT
GTAAAGACTTTGATATTTGTTTGCAGTGTTTTTCTCTTGGTGCTGAAATTGGATCTCA
TAAAAATGATCATTCCTATCAATTTATGGACTCTGGTGCGTTTGGAATATTTATAAGTCGAACAAGTTGGTCT
GCTAATGAAGAAGTTAGATTATTAGATGCTATAGAACAATTTGGTTTTGGCAATTGGGAAGATATTGCTAAAC
ATATAGAAACTAGAACCCCTGAAGAGGCAAAAGATGAATACATAACAAGATATCTAGA
AGGAAGTATAGGCAGGGCGACATGGGGTAATGTGGAAAGTACCAGTCGTCCTTCTTTACATTGCGCAGATAAA
GATGAAGGGCCCCTTAGTCCAAGCGCTGTGTCCCGTTTACCCCCCTTAGCTATAAGTTCTGATGAAGCCTCTC
AACTTGGTTATATGTCTAACAGAGATGATTTTGAAAGGGAACACGATCATGAAGCTGA
ACAATTAATATCCACATTATCACTCAATCCTGAAGATGATGAATTAGATGTTGCCTTANAGCTCTCTCAAGTA
GACATATACACTAGAAGGCTCCGTGAGAGAACAAGGAGGAAACGTCTTGTTCGTGATT
>103779758_2 DT666111.3
GCACGAGGGACACCCCACATAATTGGTGGCGTACGAGCGCTCCTGCCCATGCGCACACGCTGCACTCAGGTGA
CGATGCAACGCCGCCGGTAGGCCATATAACGTGATTCATCTCGAACGGACGTTCAATTTTTTTTTTTTTAATTG
TGAAGTGAATTATTATTCTGTTTAAACATGGGTTTAGACTTTTTAGAACATGGAGCGG
ACTTCGAAGCCGCGATTTCGGCGACCGGTTTTGGCCGTTTCCACTTCTGTCTACTGGCGATCACGGGGCTGAT
CTACGCCAACACCGCCATTGGCATCACAATCGTCTCGTTTGTGTTGCCCGCTGCGACATGTGACTTCCGTATG
ACATCTTCCGATAAGGGTTGGCTGACTGCCGCCCCTATGTTAGGCATGGTTCTCGGTT
CATACTTCTGGGGTTGCTTAGCCGACACTAAAGGACGTAAAGTTGTATTGGTTTCGACTCTGCTCCTCGACGG
GTTTGTGGGTATCATCTCCTCCTTTGTCCAAATTTTACCCATATTCATGGCTTGCAGATTTATCAACGGTTTC
GCTGTCGCAGGCGCAATGGGTATCTGTTTCCCATACTTGGGAGAATTCCAACAAACCA
AATACAGAGAAAAAATCCTGTGCTGGATGGAAATGTTCTGGACCCTCGGTGTCATTTTGCTGCCATTGATTGC
GTGGGGCATCATCCC
>103779766_2 DT666899.3
ATATGTCTTGATTAGGTACAAATTAAATTAAGAAATAATATATGTATGTAATAGTTTAAAAAAATATTGACAA
AGTACACAATTATTGAACGAGCAAGATGTCAAAAAGAAATTCACTCGTTTTGCCGCCAAGAACTGAAATTCTT
ATAAAATTAGTGCCGCCCGATGGAGGATGGGGTTGGATGGTTCTATTGGGAACCGCTC
TTTCGAATCTTGCTTTTTTTTTATGTTATATACTCGATGGGTCATGAAACCCAGGGTGCAGCCATTGTTTTAA
GTACCATGTTATTCGTCACCAATTTCGGAGGTCCTATTGCCGGAGCTGTCATAAAACTAACGAGCCCCAGATT
CGTCGCTGTCTCTGGCGCCTTTTTCTGTACTATTGGCATTTTATTCAGTGGATTTTCA
ACGAATTTATATCATTTGATCCTTACTTATGGCGTGTTGCTAGGTTTGGGTCTAGGCTTAATACATAACGCAT
CGTTTGTATCTATCATTAGTTACTTTAAAGTGCGGAAAAGTTTTAGCATTCGGTCTTGCCATGGGTGGCACTG
GTGTTGGACAAACACTCATTGCCTCAC
>103779772_2 DT665114.3
TTTATTTTTATCTTAAAATTTTATTACATATGTTTATTTATTATTTGACCGTTTTGAATTCATATACATAATT
ATATAAATTATTTATACATACACAATTATATAGACTTCTATTGTTGTATATATTATTGTATTTTGAAATTCTT
TCTTCGGTGCACGACGTATCAAGGTAGACGGAGGGAACCTGAGAACCTAGTGTTAAGA
TACACCATATTATTTTTTAAGAGATTTTTTTCAATAAGATTCTAACCCACAACCTGTTGCGTTTACTGTCGCA
TGCTTGTATTCCTGCTCCACGAGTGTTGATGTCATTAATTTTCCTGTTAATAAATGGTTAATTATTTATATTT
TTTTATGTAAACTAAAAAAAACTATATAAATTTTACTTAATTACAATGACTTAATGAA
TATATAATTACTGCTATTATTTTTTTATCTGTTTTTAAAGATTCTTTACAAATTAAGATTGGTATTAAATATC
TTTTGTATTGTGTTTTTTTTTA
>103779774_2 DT665945.3
AACACTTATTTTACACCAACTTTGTACGTAATGGCACTGGTTAAAAATCAGTCGTAAGTTTTAAGCCCTGAAC
TTAGGCTAAATAGTTAAGTACTCTGTACGTATATAATAGCTCCCTTCTCTCCGTGTTAGGTTAGACGATCGAA
GATTAGTATGTGAAATATGTCCATAGCTTTAAATGTGTCACACCCGTTGTATCAACGG
GTCCTTCACGGTTTTTTGAGCTATAAAAATGTAACGTTAGAAGTAGGATATCATTACCGTTAAGATCATGTAA
GTGCCTCGGATTTAAGGGGTGGTTTGTTTTATTTGCGGTTCGGCTAGATTCGGTTCGATTCGGTTCAGTTCGA
CTCCGCTCGGTTCGGTTCGGTTCGGCTCGGCTCGAGGACAACACGCGTCGCTTGCACT
CCTTGCGCTCCTTTTCGTCACTCGCATACTCGCTCGTATTCTTGACGATTATTAATTTTTGTTTTTTTTCTTA
TGAAATAGTGACTGCAATATTAATTTTGTCAATAAAAC
>103779778_2 DV501406.2

TGTATCCCCAAGAAATGAATGCTGCGCTGCCAAAACATATCTACAGCTTTATAGATAAAATCTCCATTATAGA
CAAGCATATACATAAAGTACCAACTGTTTGAAATTGTTATTATGTATCGGGTGGTCTACTATTGGTATCTCCG
ACGAGCATAATCACACCCTATCATGATGTCAAAGTAATTCGGGATGCTGCGAGGAAAG
GAATGATCA
>103779801_2 DT666675.3
GCACGAGGGAACAAGTCTATTATCGAAACAGGCTCGCCGTCCAAATGGATACGTTAAATATAAAACCGTTAAG
TCAGGACTTGGAGTGGTCTCCGGAGGCGCCCGTCGGCGTAGGAGCCACTCGGGACGTGGTGGACCACGCTCCC
ACAGACATGGCGTCGACATCGAGCGAGGAGAGGGCCATACGCCGCGTCACTGGCCGCT
GGGGTGCCGAGTGGTTGAGCTGACTCGGCTCAGGCTATATCAAACCCCTCGGGCCCCAGTGGCCTGCAGACCA
CCCCCTACCAGCGCCACGGTGGGGGGCCCCGCGCCCAGCTTTACACTTGTCTCTCTCCAAATACGAGGACTTG
GTCGCTAAAGCTGAGGCGATATTGAGCCGACTCGTCGTCGCGGAAAACTATGACTCCG
TTAACAACTTCCTCACTCTCTATGAAACCTATATGGCATCACCTGATGATACTTTGAAGGAGTTCTTCCCTAA
GTACAATCCGCCGATTCGCGCTCACAAGCACACCTGTGTGGGCTTGGGTATGGAGGTCATGAAGCGTTTGAAG
GTCCTGGAGAACGATTTCCCTGGTATCACCAAGTCCATGATGCTAGTATCATGTGATG
AAAACATCGAAGATCTTTTAGACTACACAACATCATTTCCTGGACCGCAAGGCTTCTTGATAGAGACGGAGAA
AGATCACGTCATGATGGCCATCCACGTCAGGGTCGAGGGAAGACCTGGGATATTTTTATCAGATTTAGGTTAT
CACATTTCTCGTGTCGTCACCGTTATGGCTGAACGTTGCTACCACATACCGGCTGGTC
ATTCATCTG
>103779821_2 DT665551.3
GCACGAGGGCTACTTTCTTTCCGAAATATCGTGAACAATATTTAAAAGAATGTTGGCCTCTAGTTCAAAAAGT
TTTAAAAGAACATCAAATCGTAGCTGAACTTGATCTAATAGAAGGAAGTTTGACGGTCAAAACAACAAGGAAA
ACGTGGGATCCCTATATAATTGTTAAGGCTCGAGATTTCATGAAACTTTTATCAAGAA
GTGTTCCATTTGAACAAGCTGTTAGAGTATTAGAGGACGAAATAGGATGTGATATTATCAAAATAAATTCATT
TGTAAGCAAAAAAGAAACATTTTTAAAACGGCGGCAACGATTAATAGGTCCAAATGGGGTTACGTTAAAGTCT
ATTGAATTATTAACAGAGTGCTATGTACTTGTGCAAGGTAATACAGTCTCAGCTGTGG
GACCATATAAGGGGTTGGTTCAAGTTAGGCGGATAATTGAAGATACGATGAAAAATATTCATCCAATGTACAA
TATTAAAAGTCTCATGATAAAGAGAGAGTTAATGAAAGATCCTAAACTTAAAAATGAAAGCTGGGATCGTTTT
CTACCTAAATTTAAAAGTAAAAATGTTCCAAGAAAACAACCTAAAAATAAAACTAAAA
AGAAACCTTACACACCTTTCCCTCCACCACAACCAGAAAGCAAAATTGACAAAGAACTGGCTACCGGTGAATA
TTTCCTTAAAGATGAACAAAAGAAAGCTAAACGCCGACATGAAAAAGAAGAACAACAGGTGCAAAGCCAAAAA
GCTAAACAAGAACAACGTAAAAAGGACTTTGTTCCACACAAGAAAATCTGATAAGAGC
CAATGTATC
>103779823_2 DT666260.3
GCACGAGGGCGGATCCTTCTGGGAATCGGCTGTTTGGACGCCATCTTGTTATGCAAAATGGTGGCAGTGTTTA
AAATGGCGGCGGTTGGAAGCGACCCCGGGTACAGTACGGCCGATGTGTGGCCGCCTTGAAGTTAGTTGTAAAC
TGTTTGCACACTCGGGAGGAAAATAATCAAGTGAAGAAAAAAAATCCCAAAATAAAAT
CAGCGCAGCCATTTACATCTAACTTCAGGGCAGAGTTGGGTAAATTTTAAATGCAATTGACAGCAAAATACTA
ACAATTGATCAATTGTAACCATCAATAATTCCGCACAAATAAATCAATTGCCATCTAAAGCCCCCCAACTGTG
TCACCGACAGGTCCGGCTGTCAGTCCTAGTACTCCCAGTGCGTGGCGGCGGAGAGGTC
CTCGCCGGCGAGGCGGAAGTTCCCGTTCTTGGCGGCGGCGAGGTAGGAGGGCGGCGCGGCGGCGCGGATGGCG
AGGCGCGTCGGCTCGCGCAGCTCCAGGTGGAAGGGCTGCGGGCTATCCGAGTCGCACGTCACCGCCGCGCTGT
CCGCGTGCCAGTACTTGCCATTTTGTCCTTTGAAGTAAACAGCTCCCTTGGGTCCCCT
CACCACCTGTATGGTCTCGTAGTTGGCCTTGTTGCACTCCAGCTTAGTGCCCTTCGGCCCCA
>103779845_2 DT665142.3
TAATTTAGTGTTGGGTGAGACCCGCCCAAGCTCCTTCGGTCCAGCAACTTATCAAGATTTCAAAGATATAAGG
TCAAGGTGCTTAGCTGAGGGGCGACTCTTTGAAGATCCTGAGTTCCCTGCCATTGATCGCAGTTTGTATTACA
AAGAGCGATTAGATAGACCTCTGACATGGCTACGACCTGGGGAAATATGCGAGAATCC
TCAACTATTCGTGGAAGGGTACAGTCGTTTCGACGTGCAGCAAGGGGAGCTCGGCGACTGCTGGCTTTTGGCC
GCTGTTGCCAATCTCACTTTACATAGAAAACTATTTTTCCAAGTGGTGCCTGATGATCAAAGCTTTGATGAAG
AATATGCTGGTGTCTTCCATTTCCGTTTCTGGCAATATGGCCGCTGGGTGGACGTCGT
CATCGATGACCGTTTGCCCACATATAGGGGGAAATTGGTCTTCCTTCATTCTTCTGACAGAGGTGAATTTTGG
AGTGCTTTACTTGAAAAAGCTTATGCTAAATTACATGGTTCATACGAAGCTTTAAAAGGCGGTTCAACATGTG
AAGCGATGGAGGATTTCACCGGCGGTGTAACAGAAATGTATGAAGTAAATGAACTACC

GCCCAACTTCTATACTATACTGCTGAAGGCGTATGAGAGAAACTCCCTTATGGGCTGCAGTATTGAGCCGGAC
CCCAACGTTCTAGAAGCTGAAACCCAGCGGGACTTATCCGGGGGACTGCATATTCAGTGACCGGAGTGAAATA
CG
>103779849_2 DV501452.2
TTTCCCGGACACAGTAAATATCATAGGTGGGGGGTGCACGGCCTGAGTGGATATTCACCGCATTACGCTATG
TTGGTGATCTCAGCGACGGCGGGTATGACAAAAATAACAGAGGAGTACATCGGGCTCCTGCTTGCTTTGGACA
TGCCGTTCTTCACAGTTATCAATAAATGTGAATTAAATAATAACATAACTAATATTGT
TACACGTTTGGAACAACTTCTGGAACCAGTTAAGAAGAAACCTTTATTAATAACTGATGAAAAATTGGCTTCG
AATTTGTGTTGTACCTC
>103779855_2 DT666220.3
CCGGCGTCTCCCCGCACATATCCAAACTAATCAAGATATGTATGAACGAGGACCCCGGGAAACGACCCTCCTT
CGAAATGATCTTACCCATCCTGGAGAAAATGAAGCGCTAAAACATACTACACTGTACAGTACGGTAGACGAGT
CGTGGCTGCCATGGAGATAGGCTTTCTGGACTTTTGTGCATTGTATTCCCGGGAAGAA
AATAATCAAAAATCTGCGCAACCATCTAGCGCTAACTTCAGGACGACCACAGCCACGATCGTACTGTATAATA
TATATGTATGACATATACTCGTGTATGAATATTTATTTGTTTTGTAACTTTGATATATTAAAATTTAACATGT
TGATAAACATCATGAAGTTAGAATAGATAACAAAAATGATAATGATTAATGACTAAAC
GTAATATTTATGAGAGATGGTAAAACGATGACGATAATAACGATTTTTAATCACATTGAACATTTAATATTTT
TGCTTTCTGCAAGTAATGTAATTTTCAAAATTGATGAAATTGAAAACTTTAACTGGTTGTAGGTTCGATTCCC
ACTCGAAAAAAATTAAATATTTTCATTTTCTCCCTCTGGTAACCAGACAAAGCGCTGCG
TTGAGTTCTACCATTCAGTGGCGAAACACAAGAGACGGGAAACGGAAGTGTTTTAACATACTGCATGCTCTGC
CTAGAAGCTTAAAGGATAAGAGATTAATGCAAAGTTATGATATACCTTTATTTAACACTCTACAAACTGATAT
CGATAGTTTTATAAAAAAATAA
>103779856_2 DT666435.3
CACTGAAATCTTATAACATAACCCACCAAGTGCATTAAAAATAGTTTATAAGCGTAATTAAATTGTGTTATTG
TGAAAATGGCGTTAAAATCATCACCGGGTCTATTTGGTCTCCTCTCCCGTATATCTCGAAGGTCTTTTTCTAC
TTCTAAAGCGTTGGCTTCAAAGCCTGTGACAGTGAGAGATGCTTTAAATCAAGCTATT
GATGAAGAAATGGAGAGAAATGAGAAAGTATTTGTGTTGGGTGAAGAAGTAGCTCAATATGACGGTGCTTATA
AAATTACCAGGGGCCTTTTTTTGAAATACGGAGACAGACGAGTAATTGACACGCCCATCACAGAAATAGGTTT
TGCTGGTATAGCTGTGGGCGCTGCATTCGCCGGACTGAGGCCTATATGTGAATTCATG
ACTTTTAACTTTTCTATGCAGGCTATTGATCAGATAATAAATTCAGCAGCAAACACATTCTACATGTCAGCGG
GTACGGTGCCCGTGCCCATAGTGTTCCGCGGCCCCAACGGCGCGGCGGCGGGCGTCGCGGCGCAGCACTCGCA
GTGCTTCGGGGCCTGGTACAGCCATTGTCCCGGGTTAAAGGTCATAATGCCGTATTCA
GCTGAAGACGCTAAGGGTCTCCTTAAAGCGGCCATTCGTGATGATGATCCAGTAGTGTTCTTGG
>103779861_2 DT665677.3
GCACGAGGGCGAGGCATATCAACATCGCTGTTGTTACTCAGATATTCTATTATGAACTTAACGCTTTGTTACT
GGGTGAAGAAAACCTCTTGAGAGAAGGTATATTATCTCCTACACCTACACATAGAGATTTGTACAATGAGAAT
GTACCGGCGGTGTCTATGGAATACGGTATGATGTTACGATGGATGCATACCATTCAAG
AAGGCATAGCTAAAATGTATGACACAGAAGGCAACTATCTCAAGAGTGTCCCAATCGTCAACCTAACATTGAG
GACTGGCTACCTAGCGGATCAGAACCACATAGATTACATTACACAAGGAGCTTTTAGACAGCCGGCTGCCAAA
ATAGATGATATTGTGGACCCTGATATAGCAGAAATAGGGCTTGGACCTCTACAACGCG
TTTCGGACTTACCAACAAGTGACCTAGCAAAGAATCGCCTCTTCAGTCTCGCACCTTATACCTGGTATAGAAA
AGCCTGCTTCGGAGAAACTATACGAACATTTGACGATCTTATAGTTGCTATGAACCCTGAGAAAATCGAGCTC
TTCAAGGAGCTATACAGCTCTGTGGAGGACATCGAGCTCCTGGCGGGCGTGTGGACGG
AGCGCGCGGTGCGCGGCGCGCGCGTGCCCGCCACCTTCTACTGCCTCGTGGTGGAGCAGCTGCGCCGCTCCGC
CGCCAGCGACCGCCACTGGTACGAGGCCAAGGACCGCCCCAATGCCTTTACCATGCATCAGCTACTGGAGATT
AGGAAAGCGACGATAGCGCGGTTGATGTGCGACTTCGGCGATGGTGTTAGGACCATAC
AACCTAGAGCGTTCATTATACGGGGACGGGAAACGAAATCGGCAGCTGCAGCA
>103779877_2 DV501434.2
GCACGAGGCCAAAATCGTCACAGAATATATTTTAACTTTATTCGCTCTCGCGATTAGCGGCTCCTTTGCACAA
AGTTGTCAAAATCCTCAAGTAGAAGCTGCGTCTTTCACGAGTTTAGATGCTACCGTCGTCACACAAATAGCTT
ATATTACAGAATTTACATTGAACTGTGATAATCCTGTGGGAGAGAACTATGCCCT
>103779887_2 DT665392.3

CGGCACGAGGCTAACCCATTTGGAAATATACCGGTGAGAGCTGTGAGTCCTAGAGTGAGATGGGTTGAAGAGA
AACCTTCTAGTGACCAATATGGCTCGCAACAGATTACGACATCACAGCAAAACTTAAATATCATAGGGGCTGA
AGAATCTTGGACTACTATTAGATCACCATCAAGAGTTCAGCAACCTATCAGGGCTGTA
AGTCCTAGAGTCCGGAGAAACACTGAAAATGATGACTTGCAAAAGGAAGTTCAAAGTAACGAAATCGGGGAAA
TACCAACCGCTGGAGAACTTTTGGGGAGGAGTCACGAAGAACTTGTTCTACTTTTGATACAATTACGACGTCG
ACACGCCATCACGCACCGTTCTATTGAAAGCTGCTGTGCCCAAATAAATAGTATTGAG
GATTGTCTCAGTATACTAAAGGCCAAGGAGCGCGAGGAAAGCTTACAACGTTTAGAACAATTAAAAATGCATC
TGATGGAACTAGAAAGCCAGTACGAGAAAAGTAAGCCTTTAGTACAGCTCGTCGATAACATGGTGAAATTAGG
TTCCCTATATAATAGACCTGGCTCTACTATTGAAAGATTGGAAAGAAACCAACGGCTA
AGACAGAAAGTTCTAACGGAGCATGCTATAGAGAAACAAAGATGGATAGAAAGTGCAGCAGCTGGTAAGCCAC
TAGAAACCGAAGCGGCGAGGGTCCGCGTCGCGGAGCTGTGGGCTCTGGAGCAGGAGCTGGCGGACGAGGC
>103779895_2 DT666726.3
GCACGAGGCGAAACCTGTAGAAAAAATTGAAATCCTTCGGTTATTTTTACAAAAATCAAGTATGGACTGGTAT
TCTTCTATGATGATAAGACTGAGCTTGCAATCAGAGTGGTCAGAATGGAAATCACATTTTTTACAAACCTATG
GCAATAGGGGTTGGTCTGCTAGCAAATATGCCCTATCTTTCAAGTATCAAATTGGTTC
TTTATTAGAGTATGCTATTAAAAAAGAAAAACTTATATTACAAGTAAGAAAAACAATTGACGAAGGGACTCTA
ATAGATATAATTGCTGCGGGCTTACCAGATTTTGTAACAGATAGAATCAATAAGGAAGAAATTGTATACACAA
AGAATTTATTTAATGAACTGGGCAAATTAGAGCATTTACTTACTAAAAAAAAAATATAT
TAAAAAAAAAGAATATTCTAATGACAGAAAAATGTTTAAAGAAAAATGTAGTATTTGTGAAAAGTTAAACAAG
GGAGAACGTTACCATCCAGAAAGTTCATGTTGGTTCGCTTCAAAAATAAATAGTGAAAAAGAAAAAAAACAAA
TAAAGGTTATTAATAATTCAGAATTAGAATGTGAACTTAAATATGAAGACCAAAAAAA
CTAATATTGCCACCATTAATTAAAGTAAAATTATTATTGAATAAAAAAATTGAAATATTGGGAATCTATGATT
CTGGATCTAATGTATCATTGATAAATTCAAAGTTACTAAAGATAAAAAATAAAGATTTGAATTATTCTAACAA
TACTAGTTTGAAAACAATTAATGGTGTTAAAAAGGCAATGGTATAATAAAACTAAATA
TAACTATCTTTAATATTGAAAAAGAAATGGATGTTTTTGTAT
>103779901_2 DT666589.3
GCACGAGGGCGCTTCTCCCTCACAAAGGCTGAAGAGAAATCTCTTAAGAAAATTAGGAGGAAAATAAAAAATA
AGATATCAGCACAAGAAAGCAGGCGTAAGAAAAAGGAATACATGGACCAATTAGAGAGGAAGGTAGAAATCTT
AGTATCTGAGAACTCAGATTATAGGAAAAGGGTGGAGACCCTGGAACAGTCCAACGCG
AATCTTCTGAGCCAGCTGGCGGCCCTGCAGGCGCTGGTCACGCGAGGCGCCCGCAAGTGACGTCACCAACATG
ACGATGACGTCGGCGACGTCACCACGCTGGCCATGACGTCACTGACGTCGGCGCCCACAGAACTGCACAGGGA
CTTAACTTTCTGAACATATTAAAATCAATTGATCACGTTAACTTTAACTCAGGCCTAA
ACTTAAGCTTTTGAACTTATATACATTTGTTTTTCAAATTTCATACCATATATACTATTTCTTGACAATTCTG
TGTTTTATATAGAAGTTGTACCAGTATTAGCCGATTATAGTGATCAGGATAAAAGACTGTGAGAAGTGATAGC
TCTCATGTTGTTTGCGAAGTCCGTTATACAACGACGTCCCGTAGCGAAGTGTATTTGT
ATATTTTGGATGTAATTTTACACAAGTCCAAAGGAAAGAACTGTTGATTAGAATGGTTGCTCATTGTATTCGT
TAGTAAATAGTTATTGTTGTGGTGCTGGATATGATATACCTGCATTTTGGTATGAGCTCGGCGATTATGAAAG
ATAAATCGATATCATTTTACGTACTGTGTGTACTAATATATTGAAATATTTAATTAAA
TCGATTACGATCGTTCGCAATCGCCCGCCTCTAGTTTTAGGTATAGCAAGCTTCGA
>103779918_2 DT665459.3
TCCTTCAAGACGCAGCACAAGGATTAAATCTAACACAAGCATTGTATCAGAGACTGCAGTAAATATTGACTCT
CCAAGAGCCAAAAGGGCTGCAAGAAGAACTTCTGCAGTTGGAAGTGACTCTGAAACTCCGTTAACACCTGTAA
GACAAACAAGAAGAAATCGAAAAGATTCTGTCTCCAGTGTTGATAAAGCTGAAATACC
TGCTTCTAAACCAGCCATTCAAATAACAGAAATAATAGTGGAGGAACCTGAAAACAATGAAAGAAAATCAATA
AGCAATGCTGACAAAGACAATAGTATTTCTCAGAATAGTGTGCGTAAAAGCCCAAGGCTAGTAGAAAAAAGTA
AAAAGAATAGTCCAGTGAAGAAAGAAAATCAAAATGAAACAACACCTGAGAAACAAGA
TGAAAATATGCCATCTAGGAAATCCATTGATGCTTCTCTACCGAATTCTCAAAAAACAAACAATGATAGTCAA
ATAGATCCACCCACAAATAATAATGAAACAAAAGGCAGATTATCAATATCAAGTGCAAATTTAATCAAAGATT
TGGAAGTAAAAGTTTCTA
>103779930_2 DT666818.3
GCACGAGGGACGATTCCATCGAATTTATTTATAGATATTATGAATTAACGATGTTGACGCGCGCCGCCCTACG
CCCTGCGTCGCCTGCGAGTCGGGGCGGTAGGCGGCGCGGCGCGGACTAATGTAAAACCGTTGCTACTATTGTA
AATTTATGATTAGTGATGAGACGAAAGCGAAGCTCGAATCGGGTCAGACCTTCGCCCA

GTGAGGAATACGAAAGAGTCAATAGGTGCAATTTTTGTTTTAAGTGCTCGATAAAGCCCGCGAGGCGGCGCGC
CCGCTGCTGCCCGGCATCAGTGCGCCCACTGGGGCGGCCAGTGGGGCGCCAGTGCTCGCGCTGATCCGGGCGG
CGGTGCCGTCGCGCTGCGCCGCGAGGCTGCTCACGTAACTTATTGAAAATGAAGCAGA
TACGATGCCAAAATGGTTTTATTCCTGTTTTAATTGTTTTGTTGTTTTTTCGTTTGTATGTAATTGTGGCCAA
TTTCGGTACTTAATTTTTATATAAATAGGTATTTTTTTTATATAAAGTAATGTTATTTCCACGTATATGATGA
TATATATAAAAATGTTCTTTATAGAAGTGAGATATATAGAGTCCAGTCTCTCCGGGCC
GGCACTCTGCCGGTCCAGTGCTCGTCCGTCAAGGGCACGCCACACGCTGCGCACGACACTGCGCCGTACTGTT
TGTTTGTCCGGTTGTTTGTTTGACTTTGTTACGGACGCGAGTGCCGTAGTGTATGCTAGTGCAGTGCGCGAGC
CCGATACGAACCAACCCAATGTTGTAAATGTAATTA
>103779952_2 DT666783.3
GCACGAGGGTTCATTGTGAACGTTGAGCCGATAGAAGTTATCAAAATGGCTCTCGTAATCCCCGACAAATTTC
AGCATATTCTTCGTATTATGAATACTAACATTGATGGCAAACTAAAAGTTATGTTTGCCATGACTGGTATTAA
AGGTGTTGGTCGCACATATTCCAATATCGTATTGAAAAAAGCTGACATTGATTTAGAC
CAACGTGCTGGAAAATGTACTGAAGAAGAGGTATAGAAAATTATTACCATCGTGTCAAACCCCGGGCAATATA
AAATTCCGGACTGGTTCCTTATCAGACAAAAGGATATTGTTGATGGTAAATACGGCCGGCTAACGTCTTCTGA
CTTGGACTCTAGACTCCGTGAAGATTTAGAAAGGTTGAAGAAGATCCGTGCTCATGTA
GGTATGCGTCACTACTGGGGTCTTAGGGTGCGTGGTCAACACACCGAGACTACTGGCCGCCGTGGAAGAGCTG
TTGGTGTGTCTAAGAAGAAGTGAACTGTACAATAAATAATATAATTTCTAATACAAAACAAAAAGAGTATAGT
GACCGCCGGACGGTTAATTACAACCAAAGACAGCGGGGTATTCGTTAAAATTGTCACT
TAGTATAAAACAGTTAATTAGTTTCCTTCATTTGCTGTCAACACCAATTTATTCAAATGTACGACCGTTTAAT
TATTTATTATTATACAGTATATTTATGTATTGTATGTATGGTATGTATGGA
>103779961_2 DT666803.3
TTCGGCACGAGGATGTCGACTTGATGTTACTTGTGTTTGTCTAGTAATTATTGATTCAATATACACATTCAAA
ATGTTTTTAAAGTAAATAATTAACGTAATGTGTTTTTATGTATCTTTTGTCTATAAATAATTAGTTCGTTACG
CGCTTTATGCCCCAGTGTTCACTGTCAATTATATTGGTGACGCTGGGCATGCTTTGCA
AAAATGGTTACTTCTAATAAACAATCGGATAAAAAAGTTGTTAAAATGGCAGAGCAGAATACTACTGTTGTGG
TGCCTCAAAGAACATTACTTGGTGAAGTTAATGAGCACATTACGTGTCCGTTATGTCGAGGTTACTACATAGA
CGCAACCACCATTGTTGAGTGTTTACATTCTTTTTGTCGAAGTTGTATTATAAAGCAC
TTACAAATTAAGAGTTACTGTCCAGTTTGCGAAATGATGATCAACTCGGCTAAACCTAACATTAAGTTAGATA
AAGCATTGCAAGATATAGTTTACAAACTTGTGCCTGGCCTTTTTCAAAAAGAAATGGAGCGACGTCAACAATT
TTATGCTTCAAGACCAGGTCCAGCTGCGTCTGCCACTCCAGAACAAAGAGGTGAAGAT
ACAGAACGAATTATATTTAGTCCTGAGGATGTCATTTCATTCTCTCTGGAATATGCTGATGCAACTGATACCG
ACAGTATATCTAGTAAATCTTCCGATAGCAATGAGCCCCAACCGCTCTCTGCTACTACAAGAAGATATTTACA
ATGTCCTGCTGTTGTTAATATCAACCACTAAAAAAATTTTTAAGTATGAAATTTGATA
TTGATAGTACACAATTTGCGATTGATATTTTATAT
>103779965_2 DT665731.3
GCACGAGGTGTCATCTTAAAAAGAGGCATATAATAAGCAATATGTTAAAGCAATTGTAAAGCAATTCTATGGC
GTTCCTTAATATTGTTATTTTGTTATTTAACTAAGACTTACACCGTAATGTTATATGTACTTCTAAAGAATGC
AGTTTTCATACTAAACAATAAGAAAGTAAATCGGACCAGTAGATCCAGAGATCTTAGC
GTTTAAACACACAAACGGACTCTTCTGTTTTATTATATTAGTATTGGCACCGTACCGCACTAGTCAATTGAAA
CGTTATTTTACTGAAAATGTTGGGTGGAGCTGCTAAATCACACCTTATCAAATTAGAAGTAGTCCAATGTGCT
ATACTTAAGGTGGCAACTTTTCGCCCATATCTATTCTCGTCAGAAGACTTGTACAGGT
CATGCGGATTATATATCCATTAAATGAATATCGCTGTAAAAAGGCAGTATATGACTGGTTGTCTAAGCTTAAT
TATGAGAAAACTGAAAGACTGCTCCATACAGTTATGTGAACAACACTTTGTCATAAATGCATAGATCGCATAT
ATCCTTACGCATGCACACACATGCACACACGCACTCACACACATGCACACACACACTC
ACACACATACACACACACACTCACACACATGCACACACACACTCACACACATGCACACACACGCACTCACACA
CATGCACACACGCACTCACACACACACACATGCACACACGCACTCACACACACACACACACACATGCACTC
ACTCACACACACCGCCTATTCAGCTTCATACATAATAATGAAATGTTCTAACTTAATG
TTAGTT
>103779994_2 DT666086.3
GCACGAGGGCCAAGCGGTGGGAATATATTAAAATTTTATTTTCGTGTTTATTACCTTTATTTCTACATTACTA
GAGTCTATCTAAAGGTATCAATATTAACTATTACTGTGACTTTGATTAAATTACTTATCGTTTTAATGTGTGC
GGCGGAAAACATACAATGTCAGAAAGTAAAAACATCAATATTAATCCTGATCAGGATA

AAAAGATCGAAGTCCCTGATCTCAATATAGCAGCGCCTAGCGATGTGTCCAGCGTTGCAGGAAGTGTTAAACA
TACAAAGCATGCAACATCTTCTGAAAGACAAATAAAATTTGAAAGAGCTCAGAAATGCCTGGAAAATGCAGCT
CTTGTGTCAAAGAGAATTAAGGAACATCGAAAAGCGACTGCTGAGCTACTTGGTCGTC
CTTTCGAGGAAGATGTTGGAGACACAGCGTCTGAGGTGGCAACCACCATAAGTGAGAGGACTGGCTACTCTGC
TGCAACAGATACATCTGCAACATTATCTGTTCAAGATGCTTTGAATATTCCTGGAATTAGTGAGAGTTTGGCG
AATACATTGAAACAAAAAGAGCTTTTGATGGAAAGAATTAAACAATATAAAGAAATAA
GTAAAAGACCTATGAAAAAACCAGTACAAACCACAAGAAAGGATTCTGTCAGTGAAACATTTGACTTGAGAAA
GAGCTCAGAAAATCCTGATATATCTAAATTA
>103779996_2 DT666132.3
CGGCACGAGGGCAAAGACCGTATACGGAACGTGTAGTTTAGTGCATATTTGATTTTGAACTTATGCAAAATAT
TTGTGTTTTGTCTCAAATTTCTGATAAATAAAACCAAAGATGGATCCCTTAGCCCAGATCGAATTTAACGCTA
CTTCGTACACAATGAAAGACGAGTATCCTGGTTTCGGATCTCCAGCTTTAGACCGTGA
CAAAAAGGCATTATTTTGGATGAGTGGTGTGATGGAATGTTTAACAACTGTTTGGTCTCTTTTCAAAAGTCCA
GACTACGAACAATCGCAAGCAGAAAATGACTGGGAAGTTCCTTTTGATAGCCTGACCGATTTAGTTTACCTGG
GTTCTGGGGCTCAAGGAATTGTCTTTGGTGGAAATCTTAAGGGTGAAATGGTTGCAGT
GAAAAAGCTAAGGGACAAGAGTGAAGCGAACATCAAACACTTGAGAAAACTTAACCATGAAAACATAGTTCGT
TTCCGAGGAGTATGCACAGTTACACCGTTTCATTGCATAATAATGGAATATTGTCAATACGGTCCTCTATTTG
AGTTTCTTCATAGTGGGGTATCTTTCACACCAAAGCAAATTGTACGCTGGTCCCGTGA
TATTGCTTTGGGCATGAGCTATCTTCATTCACACAAGATTATACATCGAGACCTTAAAAGCCCCAAGTAAATA
TGTTTCTTTATTATAAATACAGTGTAGAATTTATATAATGACATAGAACAGATAAGTGTATTATATGGAGTTA
TATTAAAAGGATAAACATAACTATGAC
>103780008_2 DT665753.3
GCACGAGGCGCGCGCCGTCGCACTCGCGCGCCGCGCGCCTCTACGTGGGCGGCGCGCCGCCGCACGCGCTCGA
CGCGCATATACCCAACTCGATAAAACGCGCGGGCGGCTTCATAGGTTGCGTCCGACGAGTGACTCTCAACGGG
CGGGCGGAGGACCTGGTGCGGGACTCTCGCGCTCACTCAGCTGTGGGGCAGTGCTTCC
CTAGAGTGGAGCGCGGCGCTTATTTTGCTGGCGACGCGTACGCGGAGTGGACAGACGTGTGGCACGGTGATAG
TGAGGGTGAGGAGTCAGTGGAGGTGCGACTTCAATTCCGGGCGGTGGGAGGAGATGGGGTGCTGGCTGCAGCT
GCCGGCGCGTTGTTGGAGCTTAAAGATGGATTTGTAGTTATATCAATGGGAACACGTC
GAGTAGAAGCACGAGCGCGGGTCTGCGATGGACAGTGGCACGCCGTCAGAGCGAAGCTGTCCCCGCGGTCCGC
TGTGCTCGCGCTGGACGCCGCTCCGGAGGAGAGAGCTCCTCCGGCTCTGGCCACTCTGCCCTCTGTGGTGGCT
GTGCGACCACCGTCTATATATGTGGGGGGATTGCCTGAAGGAGCCACAGAATTAGCAG
AGGGCAGAGAAAACTTCAAAGGTTGCATTCGAGACGTGGTGGTAGCGGGTTCACAGCGCGACTG
>103780045_2 DT665594.3
GCACGAGGAGATTACCTTATGTATTTCGTGAGAGAGGGCGGTTACTTCCATCTACATGCTACAAAACCTGATA
CGATCGGTGCCGCTCTGACGGACACCCCCGCTGGCATGGCAGCGTATGCGCTCGAGAAGATTGGCATTTGCTG
CCATAGAAACCAATTGGACACTTTGCACGGAGGCTTAGACAATTTGGATATTGACGAT
CTGTTGGATACTATGACGATAATGTGGGCGAATGAGAAAATAACTTATTCTATTAGAATATACGCTGAAGCGT
TCGCGTGGCCTGAAGTGTTTGTTCTACATAACATACCAACAAAAGTACCAACGGCTGCAATCAACTTCGCACA
CGAAGTGATATATCAACCTGATTGGATACTTAGAGACAAATTTCCAAACTTAGTCCAT
TCGACCACTTTAGACTTCGGCGGACACTTCGCCGCTTTGCACACGCCCATAGCGCTAGTCGACGACTTTTTTG
AATCCGTCGCCGAATTCAATAAGTTTAATGCAAAGTACAAACGTACTTAATATTATTATTATAGTTAATTTTT
CAATTTTCGACTGATATCTATAAGTTGAACTTAAATTTGGGTGATTTGTGTGTGTTTT
TTTTCTCAGCCATTCATAATTATTATTTTTACTGGCGGATATCGATATATATAACGTTTATACGTCATATAAT
CAGGGCTTAATTAATTTATTACTTTTACGATATATTACTTTAAAACTATATAAATCGATATCGACCACGTTAG
AAACTATTTTTCTAT
>103780047_2 DT665643.3
GCACGAGGCTCCGTGTGCGCTCGATACAGTTTAACTAACAATAAGATATATATATATATACTTTGTGCAAAAT
GTTTCCCCTTCGTGTGACGTGTTTTGTGCTAATATTTTCGTTTGCCATTTGCTCTGCAAACGTACATCCATTC
TCCGAAGAGTTCATAGATATTATTAACTCTAAACAAACAACATGGAAAGCTGGCCGAA
ATTTCGCGATGGAAATGCCGTTGAAAGCAATTAAGAATTTATTGGGAGCTCTTAAGGATGACTATTATGAGAG
GTTGCCGACTGTTACACACGACGTTGAATTAATAGAAAGTTTGCCAGAAAACTTCGATCCTAGAGATAAATGG
CCTAATTGTCCAACGCTAAATGAGATTCGAGATCAGGGATCTTGCGGAAGCTGCTGGG

CGTTCGGCGCGGTCGAAGCAATGACCGATCGGTACTGTATCTATTCAAACGGAACCAAACATTTTCACTTCTC
CGCAGAAGATTTACTTAGTTGCTGTCCAATTTGCGGCCTCGGTTGCAACGGAGGGATTCCTACATTGGCGTGG
GAGTACTGGAAACACTTTGGACTGGTCTCTGGAGGAAATTACAATTCCTCTCAAGGGT
GTCGTCCTTATGAGATTGCCCCCTGCGAGCATCACGTGCCCGGCGACAGAATGCCATGCAGCGGTGATACGAA
GACTCCTAAGTGTTACAGAACTTGCCGAGACGGTTACAGCACACCTTACAAGCAAGACAAACATTACGCAAAG
CATGTCTTCTCTATACGAGGAGGTGAAGACCACATTAAAGC
>103780049_2 DT665423.2
GCACGAGGATTTCTTTAATAAAATGGCAGCTAAGTTTGTAGTATTCCTGTGCGCGGTAGCGACCGCCCGCGCC
GGAGGTCTATACGGCGCGTCGTACGCACCCGTTTCCTCCTACGCTGCAGCACCCGTAGCTTCATACGCCGCTC
CCGTTGCATCGTACGCTTCGTACGCCGCTCCAGTTGCCTCATACGCTTCATACGCTCC
CGTTGTAAAGAGCTACGCTGCACCCGCCATCTCATCCTACGCTGCACCCGCCATCTCATCCTACGCTGCTCCT
GCTGTGGCATACTCTGCGCCTATCGTAAAAGCCGTGGTTCCTGCTGTGTCATCAGTCTCCTCCTACTCTACCC
ACACATCCCACGGCGCCCCATTGGTGGCTAAAGCCATCGCCCCCGTCGCGTACGCCTC
GGCACCCGTAATCAAGAGTTATGCCCCTGCCATCTCGGCGTACGCTGCTGCCCCAGTCATCAAGAGTTATGCC
CCAGCCCTTTCTTACGCAGCCCCAGCTATCTCATCCTACGCGGCTGCCCCAGTCCTGAAGAGTTACGCTGCCC
CCTTGGCCTACTCCGCACCCGTTAGCTACGCGTCACCTGCTGTAGTCAAGTCCATCTC
TTACTCTGCCGCACCCGCCCTCGCCTATGGCGGTCTTGCCGGTCACGGCTGGTGATCACTTTACCTTTACGTA
CAAAATCTAGTTAATATATAATAATGTCTATATTTATTTTTATACAACGCGAATAAAAATATAAGAAATTCTG
AAAAAAAAAAAAAAAA
>103780057_2 DT665188.3
GCACGAGGCCGCCCTCTCGAGTGATCGCCACTGCCATGTGATATAGTCTGTGATAGTGACGCAACCAATATGT
CACTGTGTAACACATTCCGGTTGTTCGTATTAAGCTTGATCGTTGTGCTATCTTGTATTGACACACCAGCCGC
TTTTACGCTACATCACAGAACTAGAGATCAAGATCTCGTTCATGAAGAATCCTCTCTT
GCAGATGTTGTTGTTACAAATGTATCAAATAGCAATATAAAAGAGAATCTATCAGAAAACTATCTTAGTAAAA
GTTATAATGTAATTAAATATGGAGATGATAAATCAAATGTAAATTCTCTACAAAGTCCTCTAGATAATATCTC
GACCGAGGGGCTTATTACAACAACCACACAAGGTATAGGAGATCCAGGATTTGATTCG
ATAGCTATGAGTACAGAGAATTTCTCAGATAAATCTGCTTTTGGTACTGCGTCTATCACAGGGTTCTTATTAA
CGAATCCTACGTCAGAAAGAACTATAGATGTAAATGGTCAGCCGACATCCGAGAATTATGCAAAAATTATATA
TAAAACAGCGGAGCCTGAAGAGATTACTTCAGAGTTACCTCAATGGAAAAAATACACG
ACAATCGACCGAAAACGATCATCAACTATATTCGACGACGTTACTGTAAGTAATAAAATTGAGGAGATGCTTA
AAGCAGAAACTAAAGCGTTTAGCGTTTCTTTAAACGATGTTGTTACGTCAGATATATCTTCACTTGAAAAATT
AAAAATGACTTGTT
>103780067_2 DT665977.3
AAAAAAACTTCACTGACTCTCGCTACATGGAGACTTGCACTGATTGTCTTGGGGGGGGTGGGTGGTTGGGCGA
TTTTTTTGGAACAGGTATACCCGGTAAAGATAACTATATTCCAGATAACCTGAAAAGAAAATAATAGGCATAG
AACCTATGCTTTACTTTATTTGTGTTCACGTTGACGGCAGATACTATATTTTT
>103780081_2 DT666070.3
GCACGAGGCGCGCGCCTTTTCGCTGAGTGGGTATGCCGCTTACACTCCCAGCCCTGCCACGTGGTCTATACAG
AGTACCGGCCGACGCCATTACAGCATTATATATTCCCAGCTGGTGGAGATGGCATACATTTGGTTGTTGATGA
AAAAGGGCAATTCAAAGAAGACAATTTTAACACTGCAATGACTGTGCTAAGCAATGCA
GGGGATGCAGGAAAAGGGGATCAGAGAGGACGGAAAGGGGGTCCTCGTGGAAATAATAATACAAATATATTTA
ACATAGTAAAGATGATCATGGAGAGGAACTTTGCTCCGGTCATTATATTCAGTTTTAGTAAGAAAGACTGTGA
ACTGTATGCCATGCAAATGGCCAAATTAGATTTTAATACAATTGAAGAGAAGAAGTTG
GTGGACGAAGTGTTCAACAATGCAATGGACGTGCTTTCTGAAGAGGATAGGAAATTACCGCAAGTGGAGAACG
TAATACCATTGTTACGACGCGGCATAGGAATCCATCATGGCGGCCTTTTGCCCATATTAAAAGAAACTATTGA
GATATTGTTTGGCTTGGGACTTATTAAGGCTCTCTTCGCTACTGAGACATTCGCTATG
GGTCTTAATATGCCGGCCAGGACTGTCGTTTTTACGAATTGCCAAAAGTTTGATGGCAAAGATTTCAGATTTA
TAACTTCCGGAGAATATATCCAAATGTCAGGTCGTGCGGGTCGTCGTGGCTTGGACGACAAGGGTATCGTCAT
ACTAATGATTGACCAGAAAGTGACCCCTGCTGTTGT
>103780087_2 DT666331.3
GCACGAGGAAACGACCTATTGTATTAATATATCGTATTTATTTCTATTCTGATGCAAATATGATATTGTAATA
ATATAATAAAATTAAATAATAAATAATATTTTTAAATTGTTAAAATTATAAAGTTACATTTATTATTGACTAA
ATAATAAAAAATACAATTTGAATTATAATGATGACTTCTATAAATAAATAATAAAAAT

TATAACATGTAACTATAGTAAAATTCAAATTTATGTGTACTTATGAATTATATTTTTATTCTGTGAATCTCAT
ATCTTTATTATAAAATAGAATAGTGTTAAATATATGTAATAGCATATTTTTTCTTCAGTATTACACTAATTTT
ATTATTATATGACAATGACGAGTTTTAGCAGATATTGATCTAAATAATTTTATATTGA
CATTTTTGGTAATTGTACTTAATTTTTTTATATAAATACAATATGTAAGTACAACTTCATTGCAATTGATAAA
AATATGTGTTATTAGAGTATATAGATGAATGATGTGTCGGGACAGTGTCTACCTGACAGCATAATGGTTGTGG
GTTCGATTCCCCCCAGAAGTATGAATTATTTTCCTGGCTTTGGTAAGTTTCTACAGGT
TTTTAAATTATTTATGTATAAAAAAATCATATAGGTATTATTAATGTATCATTTCGACGTAATCTCTTTTACA
GTATTATTTAGTTTATTTCTTTTTGTTGTTTTTATTGTATTTAAATGTTAATAAAT
>103780117_2 DT666333.3
GCACGAGGCACTTGCGTCTTGGCTCAAGCACTGCCTCAGTCACCTGCAGATATCGTTTTACAATTGAAATAAT
ACATACTGTGCACAATTATGTCGCCGCAAGAAAAGGATAATGTTGATCAATCTTATGAATATAGTTATAAAAA
AAACTTTCGTACTGCCTTGCCACAGTTTTTGGCAGTCAGTGTGAAGAACCTTCTACTA
TTAAGTTATGGAATGACTCTGGGTTTCACAACAATCTTAATACCGGCTGTGAAAGAACCAAAGGAGGGAGAAG
TTCTACATTTAGGAAATGCGGAAATATCTTGGATAAGTTCTATCAACTTAATTGTGGTACCCCTAGGCTGTGC
CTTGTCCGGCGTAGTCACGTCTCCGTTGGGTCGCAGACGCGCCATGCAGATAGTGAAC
TTGCCCTTTTTTATTGCATGGTTGATCTTTCATTTTTCAACTCGGACTGCTCACCTTTATGGCGCTCTTGTCC
TCACTGGATTAGCAGGAGGATTGCTTGAAGCACCAGTTCTTACGTACGTTGCTGAGATCACACAGCCTCACCT
TCGTGGAGCACTAACGGCCACCAGCTCCATGTGTATTATCATAGGCGTGTTCACGCAA
TTTCTCTTCGGCTTGCTGATGTACTGGCGGACTGTAGCCCTCGTTAATATCATATTTACGGTATTTGCAGTGG
TTGCACTTTGTTTCGTTCCGGAGTCGCCTCATTGGCTCGTATCGAGGATACGACACGACGAAGCAAGGAAGAG
TCTGCA
>103780125_2 DT665556.3
GCACGAGGTCTCGAGCCTTTTTTTTTTTTTTTTTTCACGTTTAATAATTATTTATTAATTTGGAGTTACGTGCA
TTTATGACCTTATTAAAAATGGAGACAAACCAACCGTAGTCGACCGATTTCGACTTGCATCGCTTTAGAGGAA
TACTGAGATCCATTACATTATCACAAGGTAAAAAAAATAACATCGGATTTAATATTCC
AGTCATTACGTCGGTGTAACAGTAATTATAACAATAACAGGTTTATTTTCAGAATTTGTCTCCAAACACACAC
TCAAAATCAAGATCTATTCATTTACACATTAAATTTATCTGTTGCTAGCGTTCGGCGCCATCTTGTGGCCGTT
CGCAAAACGTGTTCTTAAAAAAAATAACAATTAAAATGAAGATCGTCCTGTATTTACAA
TGTTTTTTTTTCTGTACTTAAAATATACGATACATATATATGTACCAAACATCTCCTCTAACGCTCCGTCTAA
CCTAGTCTATATACAATTTTAGTAAAGTATGGATTTTGCGTGAAAAAAAGGGGAAAAACCGTCTAAATATTAA
ACATGACATTATGAAAATGGATACAACGAAGCGTCCGAGGAGATTAGACTCAGTTGTT
GTTGTTCTAACTTATTAATCTAATATGACATTCTATAATGATTTAGTAAATCAATAATTTTAGAAGATACTAT
ATATAGCCTACAATAGCATTAGACCGTCGAAAATAATAAGCTGTTAGACGATGAGCGTATTAATAAAAAAAA
>103780127_2 DT666551.3
GCACGAGGGCCTGACGTCCGAGTTAGTCGCCTTGAGGGACACCTTAGAGTGCAAATCGAAGGATTTTGATAAG
ATTATTAAGATTGGCAGGACGCACCTGATGGACGCGGTGCCGCTCACTCTCGGACAAGAGTTCAGCGGCTACG
CGACGCAGCTCACGTACGCGATCGAGCGCGTGTGCGCGACGCTGCCGCGACTGCACTA
CCTGGCGCTGGGCGGC
>103780131_2 DT666514.3
GCACGAGGTTTTATCTACCCTATATGCTGCTCCCATAATAGGAGCGCACACGCTTTCACCCTGTATATGCCAA
TGCTAACAGATAAACATTTCATCATCATGTGACATAGAGAAGAACTAAACCATTCGAGTTGCGAGTACAATAT
AATAATTAAGTAGTGAAAGTATATATGTAAACATAAAAAACACAATAACTAAGTATCT
TCTCCTTATAAAGAAATTTAAATTACTCATTAGACTGCTTAACCTGACAACTTTGCTAGATTTAAGATACAGT
GTAAAGTTGAAAAAAAAAACTAAAAATCTCTTACCTCCAATCTTTTAAACAATTCATCAATCAGTCGTACTGC
TTGCAATTTCTTCTGACTTTTCAATAGTTTCCTTTGTTCTTCAGCTACCTTTCTGTTT
AGATCCACATCCTCCCGTTCCATTAACTTTGCAAGTTGTTTTTTCTTACGTCTCTCTTCTCTTTCTCGCATTT
TCGCTAGCTTTTCTTTCTCATGATGCTCAACTTTTTGTCTTAAAGATTTAAAAATGAATCGTGAGACTATTTC
TTTTTGTCTGCAATGCAATGAGCCTAATCATATTTAATAAATAAGTTTATAAAATCAT
AATAAAACTATACATTGAAATATAAAAAATAATAAGACTAAGACTGACTATCACCCAATATTAATAACCTATC
TCCAACATCAATCTTAGATTGTTTCATATATCTATCCAATCTAGAGATTGCTTGATTGATTTGCTCCAGAANG
TGGGGGTTCGAGTCTCGCTATGTTAAAATTTTATACTTATCTAAATAAGTATTTTCT
>103780137_2 DT666459.3

GCACGAGGAATCGGCGTTATATTCCTTCAAACTCATCTTGGTTGCTTTAAGTCGAGTTTTAAACTCATAATTC
ATTTGCTCAATTGAAAAGCAATTTTTTTTATAAAATATTTTTTTTATTATTAATCATATTTTATTCATGCACT
TAAAGTTAAGACTCGCAAAGCTACCACTAAGTACAATAACTAGTTATAGAATAGGATA
CTGCGTTACCGGTGTCAATTATATAAAAATATATTTAATTATTAGTTTTAATTTGTTATCACTAAATATGACT
ACAAATTCATATAATATATACGGAACACTTTATGTAAACCCCTAAAATATACTTATAATAAAGTATACAGTAG
GGTCAACAAGCCGTGGTCACCCTGAAGTTAGTTGTAAATGGTTGCGCAGATTTGCTTA
TTTCTTCATAACTTTCTTATCGAGTGAAAAATGCGCAAGTGTCGGAAACTTAACAACAGGGCTGGCACGACTC
GTTGACCGTACTATAGTACATTGTCGTTTTTAAAGAGATTGCTTCCTTAAATCGAGTGCATAATTTTTTATAT
ATATACCAATATCTATATGTATAATATAGTATGTATATAATTGTAAATATTATGACAG
TAGTACTAAAACTTGCCAAACTATCTACGTATGTATGTAGTAACTTCTATCTATTTATAAACTTTATGAACTT
CGGGAACAAATTAAACGAACTTGATATATTTATAATCTGCCTCTACAACTCGACTGTACGTCTGTCTGTGGCA
TTTGACTCTCTTAGATAAATATATTATTATAATACATTCCAATGTTCTATTAAAGGTC
ACACTCAGTCCTGTTTTCTTG
>103780139_2 DT666939.3
GCACGAGGAATACCCTTGGAGACTGACTTTAGAAGACTGTCTGAAAGACGTGGTTCCGGCGGGCGGGGCGGTT
GTTGCGGATCAACGCGTTATTTTAATGCAATTTTTTATTACCAAAATTTAGTTATTATTTTAACATTATTTTG
AATAACGACTTTATTGGCATTTATATAGTGGCATGACTATTTTCGACGTAGCAATTAT
CACATTTGTTCAAACGTGCGTATTGCGTTTCTTATAGTTTTTAGTACGTCGCTAGAGAAATGACTGTTTCATT
AAATCGATTTTAATTAACAACTAATTTAGCGTTAAAACTGTTCTTGATATTCCTTGACGTAGTGCTTTAGATG
GTTGATAACTGATAGCTACTAGTCTTAAGATCCTCCACCACGACATCTTCTACTTCTA
CTTATACTTCTACGTTAAGTATAATGTAAGTCGCTATAACGCACTTGTTTAAATTAGATAATTTATAGATATT
CATCTACAAATTGATTATTTTTTCAATTAGTATGTAAATGTGTGATTGTAATTTGTAAAATCAAAATTTGTGT
TAGTCCAAATATTTTAGCTAAATTGTTTCATCAAAATTATAAATCGACTTTTAGTCTA
TATTTAGTACGATCGGCGTTAAGTCGGTCGTATTAGATTATATATAATACACTGTGTTCAATCAATATGTGTT
CACTATTTAAATATAGTTTAGAGTAACGTATTCGGATCGAGCGGTTCGAATGGCCTC
>103780140_2 DT665505.3
TTTTTTTTTTACATTTTATAATTCTATTTCATTTTTTACATTACATTTCACTTTCTCACTAATCCATATTAAA
ATAAATAGTGTTAAACCTAGTACTAGATTTAAAATTTCTTGTAAGTTGATACACATTTATATTTTATACATAT
ATCACTGGTTGTATTAGATATAATAATCTCGCAACACTTGATCCTCTGCTAGTATGTT
TTACTTAATACTTCTTAGGAATATCGCTTCAGGGTTTCGTGTTTATTCAACTTGGATAATACGAATAGTATTA
GTGTATCCGGATCCCTGTTTCCATCCAGTGACGACCACCACATTGTCCCCAGTGTTGAGGAATCCACGAGCGC
GGCCAAACTTCAGACCATATTGGACACGGTTGTCTACATCTTTCAGCCAATCGCTGGC
GGGGCTTTCTTGATAAACAATGGGCAATAGGCCCCTAAAGAGATGTGCCTGACGAGCAGTTTGCGGCTGACGG
GTAACAGCTATGATGGGACACCTAGGGCGGTACTGGCTTAGCAGATGTGCCGAACGACCAGATGTAGTTATCA
CTACAATGGCGGATGCCATACATTTGGTTGACGCTTCTACAGCTGCTATGGCCACAGA
TTGCGCTGGTTCGAGAGATTTAACCTGAGCGCTAAGATCAATAAACAGCTGTCTATGCCAGATAGCAGCCTCA
GCCTCCTTACAGATATTGGCCATAGTGAGTACGCACTCCAACGGGTAATCCCCCTTAGCGGTCTCTCCTGACA
ACATGACACAATCGGCTCCATCCAATATGGCGTTTGCTACATCAGATGTCTCAGCGCG
GGGTGGGTCGGGGC
>103780144_2 DV501477.2
GCACGAGGTTTTTCCTTTATAACGATTTAATATTTCATAGAAAACAACAAAGAAAGTAAAATGGGGCTCAAAA
TCATATATACGCAGATGTAAATACATAAATAATAAACGATATTTCAAATATATTCCACTTATTTCTAAATATT
CTATTCGGACGAGAATAACCTCCAACAGGAGTTGAACTTTATTATTCAAGTTAATTTC
ATTTTGAAAAAGGCAAAATTCTTACAATATTCAAAGTATTTATATTTTTGTAAGATTATATATTTTTTTTACT
ATAATAATAACAGAAAATAAAATGTTTTTTAAGAATATATGTTTTTTTATAACGATTATGCTCGCGCGAACTT
TGCAAAAGGTCACTAAAAATACAACTATACATTACAATAGTTCATTTTTTTTATACAA
ACATTGGAATGAAGGTGGCACTAGCATGGAGGGCACTCATTTCTTTAATTATTTTTTTTAATAAACACTACGT
TTTTAATATATTTGAAGCTCAGAATTATTGAAATCGTTGTTCTAAAAATCACACTTTCAAGGCCGATTTAAGG
AAAGCGTTTCTAAGATCTAAGAATATAACATTACATCATTTTTTCTCGAATTTAAAAA
AAGTGTTAATTATTTCAATTTCGACAAAAAACGCCGTATCTATGACATCTACATCACGATTCACACACTTAAA
TACATTTAATTATTTATTTCATTATATGGTATATGAACAGGCACATAGTCGGTTATATGATAAAAATCT
>103780153_2 DT666485.3

GCACAGGCAGGCCTCCCCGCAGGTGCGTCACATCTCCGGGCAGCGCATCAAGGAGTTCAAAGACTTTCTCTTG
AAGCATCCCGACACCTTCCAGATCATCGAGGATAACGTTGTTCTAGTCAGTGAGAATCATAGACGTGATAATG
CACATGGCAACTCGGAGCATTTTCATAATTTGCCTGTTGCTAATATTAACACAGACAC
TGCTCAGCAATTGTTAGATTATGTTGCACAGTGTATCGAAGCCAAGGGTCCTGTTATGGTAGACCAATTATTC
CACTTAATAGTGTCACGCTTCCCACAGGAATACTGGTATCAAATGTTTAAAACACCAGCAGATTTAAGTGCGT
TTTTGAAAATATTCTCAGACAGCTTCCATGTACAATCAAACCTTGTCACATTAATAAG
TAAACCAAAAATACCTGTAATGTATCTAAATGCAAAGACAAAAGTAAAGACTGAGCCACCAAAGCAAGTAATA
GAGGAGAAGCCATTATCCCCTGTGCCCCCACCAGTCCCAGCAAGCCCTACCCCATCAGATGGCATGAAGAGCC
CGGCAAACAGAATACTTAGCCCAATAGAGTCTCCCCGAGGCCCTCAAGCCAATATAGC
TAATCAAACTCTTAAACAAAGAATTAACTCAATTGTTATAAAAAACTTAGCAGAAAATATGGTAAGAGACAGA
GTAAATAATCACTTAAATGCTCGCTCTGCCGAAGAACAAATGGAATGGCTAGCTTAAGAACAATATGA
>103780203_2 DT666187.3
AGACCTTGGTTAAAATCTAAAGAAATAAATGTAAATTGTAAGACGGAGGATAATAAAAAAGAAAAAGAAAACG
AAGGTGCAGAAAGAGTAAAGAGATATGTGGTACAATCTGAGGATGATTACATAGATGAAGATACAAAATACTA
TTATGCAGACCGTGCTGTACAATATATCAACGAAAAATCTGATACCAACAATTTGCAA
AAGCTTATAACTGTTCATGCATTTCAGAATAATATAAACATGGGTGTAAATATGGTGAAAATGTATATCGAAA
CAGCTTACACTTACTGTTTGCGATTTCAAGATGAGGCAGATATACCAAATTGTGAAGAGTTATCGGGAATGTA
CCATAGACTCTGTTACGTAAGATTATGGCCCTCTCCTGACGATGATTTGGTCGTAGAC
AGTCTTGTTATCGTGTGCGATGACGAAAAGGACTTTAAGAGCATAACTGGGTTATCAATGAAAGAATTATTAA
AAAAATCCATAGATGAATTAGAAGCATCACCAAAACTAAAGAATAAACTTATCGCTTTAGGGGAAAAATCTAT
GATTCCTAGTTTTGATTCTAAGACGCCGATTAAATTGAACTTTATAGTTAGCACGACA
AATTGTTCTAAGCAGATAGACCTACACCAATCTCCGTCGTTGTGTGTCGTTGATACTGCTAAACTTTCTAAGT
CATGTACATCCTACTTTTGGATGACCCAAATAATAAGAAATTTAAAA
>103780205_2 DT666824.3
GCACGAGGGAGCTCTTCTTTGCCTATATCAGTTAATCAAAAGTTATGAGTACCATGCTGCTGAAAAACGAGTT
CCCCTTATTGAAGCGATGAACTTGTTGTTACCTATGATGTACAACCTCATTGTAAATCTCCAAGCTGATCAGT
CTGTTGAATCTGTCCTTATACAGAAACAAATACTCAAATGTTTTTATGGCTTAATAAA
GTACTTTTTACCCCTGGACTTGATCACTAAAGATATATTTACAAAATGGATGGAGGTACTGAGATTGGTTATG
GAGCAACCTGTACCAGATCATACATTAGAAGTGGATGAAGATGAGCGCATGGAACTACCTTGGTGGAAATGCA
AAAAATGGGCAGTGCACACCCTATACCGCTTATTTGAAAGGTATGGAAGCCCAGTAAA
TGCAAGAGATGAATATGTTCAATTTGCCGAGTGGTACTTATCCACATTCACTGGAGGTATCTTGGAAGTACTG
TTGAGAGTGCTGGATCAATATAGAAACAAAGTATATGTATCACCTAGAGTGTTGCAACAAACCATTAGCTACA
TCGACCAGTGTGTCAGCCATGCACACTCTTGGAAGTTGCTGAAACCACATATGTTTGC
AATTGTACAAGATGTCCTTTTCCCACTTATGTCTTATTCTGAGGCCGATGAAGAATTATGGTTTACTGATCCC
CATGAATATATTCGTATTAAATTTGATATATTTGAAGACTTTGTGTCACCTGTAACAGCAGCACAGACCTTAC
TTGTCTCCCTGTGTAAAAACGAAAAGATATGCTAGAGCGTACATGCACCTTTGCATGC
AAATTTAACAAGCCAAAGTGGGGAGTAT
>103780210_2 DV501501.2
GCACGAGGCTCCTACCTTTAATAAACTTATTACTTTGTTCTCAAATGCTCTCACCACGAGCTCTCTCCTGATT
GTATCACAAGGAGGACCTTTAGCGAATTATAATTAAAAATGAACATTATTATATCGTAAGCTGTAACAAGTAA
GCTCAGAGACTATTATCGAAATATTAGTATATTCTATACATTAGTTAGACCAAATTGT
AATTCGCTTTGAAGAAATATTTATTTTATATTATTTTTTATTTTAAATAACCATATGATAAGTAGCACCGGTA
AGCGGGAGGGAATCCGGGAACCTAATGATAAAAAATGCCGTCCCTATTAACACACTTCCGTCCCTCAATTTCC
GAGAAATTCTCGAGTTATTGCGTGTTGAGTGGTGGAACTCGACGCCGCGCTTTGCCTT
GTTACCGAATCTAAGTAAAGAAAATACTTTTTTAGAAAATGTAAAGAATAACCTTGAATTTAACATATTTAAG
CCCGCACTTCTT
>103780216_2 DT665947.3
CGAATTGAGCGGCGGCCGCGGTCAAGAGGTAGAGTCGAGGTTCACTCTTAAAAGGAAGATACAAATACAAACA
GCAACCTGGTTAACGAATCTAACGGAAGTCTGGCTCCGGCGCAGGAGTTGGAATCTTTTCTACCTCAAGATGA
GAAGAAAGGACAAATCGTAGAAAGTAAAAAAATACAACTTAACCAAAGAAAAAGATGC
GGAGTCCGGCGACTTTTATCCTTTCTCAGAAAGACAGGCGGAGAATCCTACTTCGAACATGGACACACTGACC
CACTTGCTGAAGGCGTCGCTCGGCACCGGTATCCTCGCCATGCCTAAGGCCTTCAAAAGCGCGGGTCTCATAT
CCGGGATCTTCTTCGCCATTATAGTAGCTGTTGTCTGCACACACTGCGCCTATATCCT

GATAAAATGCGCTCACATCTTATATAAGAAGACGCGAAAAACTGCTATGAGCTTTCCTGAAGTAGCCGAATCT
GCGTTGGACAATGGACCACAAGGACTTAGAAAATGGGCTTATAATTTCAGAGTTTTTATCCTCGTGAGTCTAT
TCATGACGTACTTCGGAACCTGCTCCGTCTACACTGTTATTATCGCTAAAGAATA
>103780229_2 DT666064.3
GCACGAGGCAGAAATCATCAAAAAAGAGATATTTCACTTAATTTTTTACACCACTACAAAATATGAATGTATA
AAAATAAAATATAACTAACAAATATCATATTTATAAAAGGACATTTAGAGTAGACAAAAAAATCTTATATAAA
ACAATATTAAATAGCTGAAATCTTTTGTTTATAAATATAGCAATACTGTAATGTGCAT
TAATTCGCATTGATACAGCTGACTAGACCCAAATGGAAACTACTGACGTCTGGTCTGCATAATTAATTTAAGA
ATACATTTATTAATGAATTAACACTTTAATGGCATAGTTGGGACGTATTTAACTACAAATAATGTTTATGGAT
TGACTGGTTTCAATAATATATATTTTATTGTTTTGTAATTTCGTGCTATATATTGAAT
AGGTGTAAGGTGGGCAGAGGAAACATTAGAACCTAACTTAGAGGGAACGAATAGGTTCCCATTAAAACGCTTT
CTTTCCCCAATTTCCGCCGAATTCTGAATTCTGAAAACTGCATGTTGAATGTGTGGATATCAACACCGCGTTT
TGCCTTGGTGAGGAAATTTAAAATATTAAATATTTCTTCAATATCGAACCCACAGATG
TCGTGTTGACAGTCGCACGCTTGTGCCTCAGAGCAACGACTGGCCTCAACATTCATTGATATGGAACGTGTCA
AATTACAACATAATGCATAAATAATTTATATAATTTCCCAATTCAAAATATATGTAAGTATCCCCCTAAACCA
AACATGGGCCCTATACATTCGACCAT
>103780231_2 DT666370.3
GCACGAGGGTAAACCCTTAAAAGCATGGATTTTAGTATATACATAATAGGGATTGTCATCACGTTTCACGGTT
TGCACAGATTGCACTTGCATGCCTCTAATGATTGAAGGTTTGTTTAGTTTTGCAAGCTTGTAAATAAATTGTT
TTAAAGCGTCTCGTGATAGTTTATCGTGTTGTTCATGTGGATACACAGAATGCGTAGA
TCAACTTAACAACGCTTACTTCATATAAGTTAAAACAATAATTTATTTCAGACAAGATATCTGTGAATATAGA
TTATTTTGTATATGTGCGATACATGTGGTGGCTAACCGTCTTTTTCATCGGCAATTGATAACATTTTTTTAAA
CAAGCATGAAGCTAGATGGGGTTATCCGGTTTTAGACATGGTCACGAGTTTGGTAAAA
TATTGCTACAAAGTGTGAAAAGGCTTGCATTTTTATCCAGCATATATTGTCTGCTGACTATACTAAATGCTTT
TTATGCCTTTTAAAGTTATTAGGATCATTTGACCATCGGCAAACGCATTACGGTACGTAAAATATGTAACAAT
ATTACATTTATGTCTT
>103780233_2 DT666784.3
GCACGAGGGTATATCTTAATTATCAGTACAATGGAGCTCAATAACTTTTATTATTCTCAAGTAAACCTATTTA
ACTGTTCTGAAGTAACAAGCCTTAGTTGTGGTGATACAGTGTGTGGTACAAACAAATTCGTTTACCAAGACTC
GGGACTCAGAGGAGACCCTCAAATAAAAAACGAGGACTTCGATTGTTTTAGAATAAAG
CACAAGCCCTTCCCTTATTACAATAGATTGTGTATTTTTAACATGTTCGCGTGAACACAAACGCCTTAGCATC
CTCTCAGAACCTGTAAAATATAGTGTACGTATTGTTAATAATATAAAATTTATATAACAAATAGTGATAATGA
ATACGATAGGTAATAATATGCGCAATAAGCGCAAGCTTCTTGATATAAACAGTATGTC
ACTCCAATTTCCTATGTCTAACTATTCTACGGACGTCCAAAAGCAAGAAATGGAGCAAATTTACGGAGACAAC
CTAATATTGAACAAACTACAACTACTACCAGACCCTTTGAAAAAAAACCTGGCAGATTCTGTCATTACTGGCG
AAAAAACAATGAACCATAAGGTAGAAAGAGTAGCAACAAAAGAATATCCATCAGATAA
GCCATCTAATAATCAAGAAACTTATAATTCATCTAGAGCTACAATAACTTATATTATGTCTGGAATTTATAAT
ATTGTAGGTGCATTATTCTCTTCACGCTCACAATACCAAGATTGTCAGAGTAAATCTGAGGAGATGGCGCCAC
AGTCTTGGCAGCCTT
>103780243_2 DT665483.3
GACCACCTCACGGCCACCGTCGGACACCTGCTCATATACAAAGTGCCCGAGGATACATTCTTCGACCCTGAAG
ACGGTAGTACCCGCAACCTGCAGCTGTCCCTCAAGTACAGTGATCGTTCAGAAATACCGTCGAACCACTGGTT
GCAGTTTGATGCCAGAAATCAAGAATTCTACGGACTGCCCACTTCAAGTGATGAAGGA
AGCGTGCATTATCATTTTTTTTTTGAGGACTCTACAAGGAAGAGCGCCTACGACAGCTTGATAGTAGAAGTGG
CTAAGGCACCCACCATCCGTCCTACAGTTGAGTTCCAAATGACAATGGACCCTTCACCCAATTTAGTGGACAA
TGCGAATAATAAACGAAAAGTAGTCGAGAAACTGGCAGCTCTGTTCGGACAGAGAGAA
ACTGATAATATAAGAATACATAGTATTACGGATAATCCCACTACTATTATATGGTACAACACAAGTTTACCAA
TGGATAGATGTCCGCAACGAGAAATCGAGGAGCTACGAAAAATGATAATAGTAAACGAAAAAGGAGCCATCGG
CGGGAATCTCAAAGAACACGTCGATCAAATATTCTACAAAGACTTAAAAGTTATGTTC
CTTTCGTCTCATACC
>103780251_2 DT666493.3
GCACGAGGACAACCTGGGGAAAATCGCTATCATTATTGTCGCAACAATGACGGGGCTCGCAATGGGATGGGAG
TTCACGTTTCCCGATTATAAACGGACTACACTGGGTGACTCAGTTGATCTAGCTTCTATGAAACTCTTGAAGG
AAGTATATGAAGCATCAAGCGACAAGAACGTAGTGGCATCTCCTCTTGGCGTATTGAC

TCTACTTTCACTATACGCTGCTGGCTCTGAAGGAGAAAACAGAGAAGAAATCATGAAGCTACTTGGTTCTGCT
GACTATAAACAACTTTCAGAGTCTTACAGTGAACTGAGCGATAGATTCGCAAGAATGGACCGAAGTTACCTGA
ATTTGGCGAACAAAGTGTTCGTTTCGGACAAATATTCTCTAAACGATGACTTTTCTAA
AACAGCGCGGTCGTTCAAGAGCGAGGTGGATAGTATTAACTTTGCTAATACTCAAGATGCAGCTAAAATAATG
AATGAATGGTCCTCCAAGAACACTCAAGGAAAGATAAACCACCCCGTGTCCGACTCAGACATAGACCCGGCGG
CGGCTGTCGCTCTCCTCAACGTTATTTTCTTCCAGGGACATTGGCACGTTCCGTTCAA
CGCGAGTGAGACCAAAGAACAGGACTTCCATACATCGAGAGGTAAATCTGTGAAGAAACCGATGATGCATCTG
TTGCAATCATTGTACTATCACGAAGATGCAAATATTGGGGCGAGAATGATAGAACTACCCTACAAAGAAGATA
AGTTCCGCATGATCATATTACTTCCAG
>103780255_2 DT665435.3
GCACGAGGAAAGTCATTAAATTGACGCCTGAAATGGAAGACGCATTCAATAAATGCAAATCTAGTCTGGCCCA
TGCTACTTTATTAGCTCATCCAATTGTAGATGCAGAGTTAGCAGTTTACACAGATGCTTCTGACACTTCGATT
GGTGCGGCACTGCAACAACGTACTGGCGGCACGTGGAAGCCGTTAGCATTTTTCTCGC
GGCGTTTGTCAAGTGCCCAGAAAAAATACAGCCCTTACGATCGGGAGCTGTTGGCTATTTATGATGCCATTCG
CCACTTTAGACATATGGTAGAGGCTCGTACATTTACAGTATATACTGATCATAGACCATTGACATATGTTTTC
CAGCATAACCGTGAGAAATGTTCGCCACGTCAGTTTCGTTACTTAGATTATATATCAC
AGTTCACTACAGATATACGCTACGTGGCGGGCAAGGAAAACGTAGTCGCGGATGCTTTATCGAGAATAGAAGC
CGTATCAGTGCCAATAGACTATGAGAATCTTGCACAATCTCAAGACAAGGATCCAGAACTGCAAGAACTTCTT
AAAGACCAGACTACAGCACTAAAACTTAAGAAAATAAAAACCACTGATTGGAACAGTA
GCGTCTATTGTGACGTTTCGCACCAGCCACGACCGTACTTGACAGCCGAGTTCAGAAAGCCAATTTTTGACAC
TTTACATCGCTTAAGCCATCCAGGGATTAAGGCAACTGTCAAACTCATAACACAACGGTTCGTTTTGGCTGGA
ATAAAAAAAGACTGCCGAGATTGGGCAAAGAAATGCACAGATT
>103780266_2 DT665569.3
TTAAGAAAACGTATAAAAATTTCATCGTAGCGCCTGGTCGGTTTCCAGGAGGCGCGGGTTCAAATCCCGCTAC
GTTCAAAATTTTATACGTTTTCTAAATAAGTATGTTTAATCTACTTCTATAAGAGCAGGAGATTAGGTATGAG
TAGTCACAAACTTATAAAAAATTCAATAAAATGTTCTAGATAATAAATAGATTGTAAT
ATTAGTTACACTTCAGGGTAAAAGGTAACAAATGAGATTTAACCTGATCATTCCCACTAGGGATTGGACTTGA
CCCGAGTTCTCTTTTAATATAAACTGGCCACAAACTTTCATTACAGCTAAGCCTATAAAGGAAAGAATAGGTA
TTTCTCGGATAAAAAAATTTTACAGAACAATCTAGATGGATAACATAGTTAATACAAT
AAATAAATGTTATTTTTAGCTAAACTTCATATAAAAAATATACTAAGAATATTTAAAAAAAATTGTTTTAAGT
TGTATCACCCACACGGCATTGTAAGCAGAGGGAATCTAGTGTTGAAACACTCCGTTCACCATTTTCCGCCAAA
TTCCGGAGGCATTCTGTGTTTTGTAGTGTGTTGTGTTAAGTAGCGGAACTCAACAACT
CGACATCAGAGCGAAGAAATGAAAAAATAAATTGAATTATATATTTTATTTCGTCGACTGGGAAACTCTCAAC
CAGTCGCGTTCACTGTCACACACTTGGACCCCTGCGCCACTTGCCTCGTAAACATTTGAACTCCATATTAAAC
AATTTATTAACATTTATC
>103780284_2 DT666222.3
CAATTATTATAAAGAAAAGTATAAAAATAATAGTTTTAAATATAGTGTATATTTTTTTAATATGTGTTTCGAA
GAGCAAAAATATTGAAGTTGAAATGAATTAAATGAATGTAAGTTATTATTGTCATAATAAGTATTAAATTTAA
TGTGCTTACGTGGATATGATCTGGTTCCTATAAACATTTGTTGTACAATATTTTGAAT
AATAAAACTATAAAGAATGCATACTGATGTAAATTATTAATAAATAAATCTAGGAATTGAAAATAAATTATTA
TTACGTTTGTTGCGATGTTTAGTTCCGCCCTTTAATAAGCAATGCCTTCAGAAATAGGCGTAAACTGGGGAAC
GAAAGTGTTTTAATGAAAATGGAGTGTATTAAGACTAGATTCCCTCTGCCTACCCTAC
TATGTGTGTCTAATGCGGAATACAGCGTGAACCTAAAGAAAACTTATTTTATCACTTATTAAAAAAGAAATAA
CAAAGAGTTGTATGTATTTCAAGCTCACAGAAGCTCTCGTTTCTGTTACATTTTGCACCTTCATTACGCTTCT
ATTGGCAGCGTGATGTAATATAGCTTATAACCACCTTCAACCAGAGTATACAACCCGA
TAATATTTTTTTAATTCCCATCAGTAGTTCCGGAGTTTA
>103780294_2 DT665900.3
GCACGAGGAACGACCCCTCGCGCCGCTCTGATTTGGTACTCAGCTAACACGTGCTTGGACTTGGGGTGCGCAC
GCGCCGGTCGGCTTGGGATCATTTTTCGCAAAAATATCTAACTGCAATGGGAATACGGATATTGGTACTGTTG
ACGCAACTTGCGTTAGCGACGGCGTCTCTGGAGGTCATCAGCCAATGGAGCTTGCTGC
AATTCGACTTTCCTCCATACCCAGTGTTGCTAGAAAAGTTTCAACCCGAGAATACGGTACCGACTGGACTATA
AATTGGATGGGATCGCATATACTTGGGAATTCCCCGACTGCATGCTGGTGTACCTGCCACTCTAGCGTGGATC
CCACGGTCTCTACCACCCGGCGTCAGTCCAGATCTGCAGGCGTATCCGGATTGGTCAT

GGCACACGGCCGGGCGCGGTGACATCAACTGCACAGGCCTGATCTCTGTGTACCGAGTGCGCGCGGACCGTTG
CAGCCGCCTATGGGTGCTCGACGCTGGAGTCATCACCAGCCTAGATGACTTCCGCCGCGTCTGTCCCCCAAAA
ATTCTAATCTTCGATATGGCAACTGATCGTTTGGTGAGAAGCGTTTACTTCCCGCGTG
AGCTGCTGCGGCCGAGATCCCTCCTCACTAACATCGTATTAGATGACAGCAGATCATCAGCTT
>103780298_2 DT666894.3
GAGGATTCCCCTTTAAATCGAGGATGGAAGGGAAATGGTTGAGGAGTATAATGTTGATACACATGTGTTATTG
AGAAGGGCTTGTCCGATGAAAGGTAAACTAGGAGGAGAAGGCAAATGGTCTGTAGAAGTTGGCGATCCTATAC
CTGATGCAACACCGAACACTGATGTTCTTGATATTATTGAAAGTAAAGATCAACCCAT
TGTATTAAAAAGAAATACAAGAGTCAATATAGAATGGAGAATAAGAAATTTGCCTTATCCTATAGAAGTTTAT
TCTATTAAGGCAAACAATGATGAGAAATATATAATCTTTTTTACTTCAAATAAAAAGTACTATAAGAAACTTG
AAGTATTAGAATTAGCGAGATTAGGATTACCCATAGAGCAAACAAATATTGATGCAAG
CCACAAATACAATACACTCATAATAACATACAAGAAACCACCGCAATTGCTTGATATGGACAAAGATTGGTAT
GAAGAGTTAAGTAAAGTGAAACCTCTAAAAGATGTACCAAGTGAATGTAAAACTCAATAAAAAGTATAATATT
AAAATACCTAATACATAACTTACACTTTGCCTTTTTTTAATGTATTGTAAACACTGAA
GAATAATGTCTTATTTTGTTCACTGCTTCTAATCGCAAATTATACTTGATGAAGAAAATATGTACAATAGAAA
TAAGTTAGTATAGTTTAAAAATTAAGACTAAGATAATAAAATAGTGTATAAAACTAATAGATAGTTAACAAAT
TTCTATGTTAACTATGTATTAATTAAAATTATT
>103780306_2 DT665286.3
GCACGAGGGTTTTCTTTTTAACAGAAAAGATTAGTTTCTCTTGGTGAATATATTAAAAGAATGGCTAGGAAAA
ACAGAAATGTTAATAAGTTTGCTCAACGAAAAAGAGATCGAAAAGAACAGGCTAATAACCCAGAAGAGAAACC
GGCAGGTGATAACCGAAAGCATTATGAAGATATCGTGCGCGAAAATGCTACATTCGAA
GAGTACTATAAGATACAAAAAGTTTGCCCTGAAGATCAATGGAAAGATTTTATGGATGCACTTAAAATGAACT
TGCCCACTGCATTCAGAATAACTGGCTCTAAATGTGAAGCTAATGCTTTAATGAACATTGTCAAAAGTGAATA
TTTTTCTGAAATTCTTAATGCCAAAATTAAAGTGGAAGGAAAAGAAGATGAAGAGGAA
ATCAAACCCTTTAATTTGCCTTGGTATCCAGGGGGCCTAGCATGGCAATTACGTATCTCACGTGTAGACATAC
GCAGAAGTGAACCTTTGTACAAATTGCACAACTTTTTAGTAGCAGAAACAGCCGCTGGTGGTGTATCAAGACA
GGAGACTGTGTCTATGATTCCTCCTGTTGTATTACAAGTTGAACCACACCATAAGGTA
TTAGACATGTGTGCTGCCCCTGGATCTAAAACTGCACAACTTATTGAATTTTTACATGCAGATGAAAGTATTA
CACCTACAGGTTTTGTAATGGCCAACGAAGTGGACAACAGCCGTTGTTACATGTTAGTACATCAAGCTAAAAG
GGTGAACTCACCAAGTATAGTTATAACTAACCAT
>103780309_2 DT665119.3
ATTTTGAAATATGAGGGGAATCGACAGCTTAGATGAACCAGTCAGTAAGAGGCGTAAAATAGGTCCCACATGC
ACGGATATTAAATGTGAATCGGAAAATAAACCAAAACCAAGGAAAAAACATATTCCAAACTTTCGTTTAAAAG
CCACTGGCGAAACTGCATCTTTAAATTTACCAGATGATGAAAGAATACCATTATTATT
TACAGATGTACAACATTTACTTCTATATTCTTTATTGGGGAATCTTAATTTAATATAGCCACCTCGTTGGTAT
GTTTTAGATAAATGTAGTCACATAAGTCAAACAACATGTCTAATAATTGAAGGTGTATCTATAGAACATTGGA
AAAAATATCAAAATGTTATGACTAATACTATAGAGATATTTGATAATTTTGTGGAGAT
TCTTACACCTTCAATATATGATGGTTCCTTATTTAAAGAGTTAGCCTTAGTGCCTCTTTCAGAAACT
>103780317_2 DT666708.3
GCACGAGGTTTCAATTTTACAATAGAAAGCAATATAAATAAAATGAATTTTAAAATAATATCACTATTGTTGA
CTTGGAATTTTTTATTAATTAGTGCAAAGAGGGATTCCTTGGAAGATGATGACTCTCGAGCTAGAGAAATAAG
GAATAAAGAGAATAATCGAAATCGGACTACTTTTAATGGGACATTGACAGATTTACCT
GAAAGAAGGTATTTGAGGCACCGAAATAGCTCTCGTTTTCGCGGCGATAGACGCAGATTTTGGGAGCGTGAAC
ATGAAATCCGACGTGATAGAAGAAGAGGATCAGATGCTCACTCTTCTGAAGAATACATCAACAATCGGACTGA
TCCGTACGCCAAAAAAGATGAGAAAAACCGTAAAACGGAAAAACACTGGATGAAGAAT
ACTTCAGAAAAAATAAATGAAAGACGGAAACAGGGGAATCAAAGTAAGCCAGGTCGTCCGCTGCCTGTAAGAG
AAAATACAACTAGATTCAAGCCAAAGTTGACGAATAAATGGCAAAGTGGTGGATATATTCCATATCCACAACC
ACGAGAAAAGAAACCCAATATAATACTGATTCTAACAGATGATCAGGATGTTGAACTT
GGAAGTTTGAATTTTATGCCGAGAACGATGAAAGCTATAAGGAGTGCCGGAGCAGAGTTCCGCCACGCGTACA
CGACGACGCCTATGTGTTGTCCGTCTAGGAGTTCGCTGTTAACTGGTGTCTACGTCCACAACCACAACGTGTA
TACCAACAACGATAACTGCTCTT
>103780343_2 DT665143.3

GCACGAGGTTTTTCTCTAACAACTTTTAGCCCGTCAGGGAAATTGGTGCAAATTGAATATGCCTTAGCTGCAG
TAGCCGCTGGGGGCACCTCCGTGGGAATTAAAGCGTCTAATGGTGTTGTAATTGCAACTGAAAATAAGCACAA
GAGTATTCTTTATGATGAACACAGTGTTAATAAAGTGGAGATGATCACTGGCCATATC
GGCATGGTATACTCTGGCATGGGACCCGACTACCGTCTTCTTGTAACCCAGGCTAGGAAAATGGCACAACAGT
ACTACTTGATGTACAAAGAGCCGATACCTACAGCTCAATTAGTGCAGAGAGTGGCCACAGTAATGCAGGAGTA
CACTCAGTCTGGTGGTGTCCGTCCATTTGGTGTTTCGTTGCTCATCTGTGGCTGGGAC
GGAGACCGGCCCTATCTATTCCAGTGCGACCCCTCTGGAGCCTACTTCGCGTGGAAGGCCACTGCCATGGGCA
AGAACTTTAATAATGGCAAGACTTTCTTGGAGAAGAGATACACAGAAGAACTAGAACTCGACGACGCAGTGCA
CACAGCTATACTAACACTTAAGGAGGGCTTCGAAGGTCAAATGACAGCGGACAATATA
GAGGTGGGAATCTGCGACCCAAGCGGCTTCCGAAGACTCGACCCCGCGCATGTGAAGGACTACCTCGCTAATA
TACCTTAATTGTGATTTTGTATTGTATATGTAATATGGATGGATATAAAGGAAATTCTGTTTTTAAAAAAAAA
>103780345_2 DT666040.3
GCACAGGCACGGTCTCTTGACGAAGATCCCGTTCAACGTGACCCCCGGATCGGAACAAGTGCGCGCCACCATC
GAACGCGACGGCATTGCCCTAAGACGACGTGAATTTGGTGGAACTGTATTAGCAAACGCCTGTGGCCCCTGCA
TCGGTCAGTGGGACCGCAAGGATGTCAAGAAAGGTGAAAAGAACACCATCGTGACGTC
ATACAACAGGAACTTCACCGGACGCAACGACGCCAACCCGGCCACCCACTGCTTCGTTACCAGCCCCGAACTT
GTTACCGCCTTGTCTTTAGCTGGGCGCCGACACTTCAACCCGGTGACTGACGAACTAACAGGTTCGGATGGCA
AGAGGGTCAAGCTGTCACATCCCTTCGCATATGAGCTCCCGACCAAGGGCTTCGATCC
CGGCCAGGACAGGGGGAGCATCCTCCCTCTGATGGTTCCAAAGGCAAGGGTGACGAAACACCAACATCCGACC
GT
>103780355_2 DT666475.3
GCACGAGGCACCATCATAATTGTGCAATTAAAAAAAAATTAAAATAATAATCGACATCAAAAATTGCCTTCCA
AAAAACAGTGGCTTAAGTATTACAAATGTAGTGTTCTTGTAAAAGTTTTCGATTAATAACATTTTTGGGACTT
AATTTTGAATCTGTGGATTTTTGTAATTTCACATTTTAAAGATGGTGAGCAATTTGCA
GGAGTTAGCAGTGCAGTTGTTCGATGTGGGCGCGGTGCGCCTCGGTGATATCGAGGCGAAGGTGGGACGTCGT
ACGCCGATATACTTTGACTTGAGGGTCGTCGTGTCACATCCTAAAATAATGATGGCAATCTCCAAGGAGTTAC
AAGTGTTAGCATCAGAAATAGAGTACGACATCCTGTGCGGGGTGCCCTACGCGGCGCT
CCCCTTCGCCGCCGTCATGTCCGTCAACACAAACACCCCGATGATCATGAAGAGAAAGGAAACAAAGCTATAC
GCGACTAAGAAAATTCTCGAAGGCGTCTACAAACCTGACCAAAAATGTTTAGTTGTTGAAGACGTAGTGACAT
CTGGCGGCAGTTTACTCGAAACTATTAATACTCTGCGCAACGAGTTATTATCTGTGAC
GCACGCTGTCATAGTTCTTGACAGGGAACAAGGTGGCGCTAGCGTGCTCAAAGCAAATGGCGTCCATGTGAAA
TCGATTTTTACCATGACAGAATTAGTAAAAATATTAAAAGATGCGGGCAGAATTACGGATGAAACTGTCGAAA
TCGTATCCGATCATA
>103780363_2 DT665361.3
GCACGAGGAGGGTCTTTGTGTTTTCTTTTAAATTAGTTATATTATTCGCATATTTTTTAAACATTGAGTATAA
TATTAGTCTTAAGATTTATTTCCATTCTAACTCTGTAACTGGATTACTTTTGCATCTCGCCCGTCTCGGGTAC
TACAAACTGGCGGCGGCGTCGGTAGTGGAAGTGGAGACAGAGCAGCGTACGCGCAACG
GAGTACACCATTCACAGCCGAAGGAAATCAGAACTAAAATAATACCAATGGATCCAGACAGTCTCGTGCTTTG
GCGGAAGCCTTTTACGACTCTAGAATACTTTTTTCGGGAACTTCTAATATTAAGTTCCACAGGACTTAAGAGG
TTAGTAGCATACAAGGCACTCGCGTTAGCGCTGCTCTCCGTCATCTCCGCTACGGGTG
TTTTATATTTTATTAATGGTCCACACCAACCATATGTACAGGATGTAGTATTGACCCTGGCGTGGTGGGGCTG
GTGGGTCATGCTGGGTGTCTGCAGTTCGGTGGGACTCGGCACTGGTCTACATACATTCCTATTGTACTTAGGG
CCGCATATAGCGCGCGTTACGTTAGCCGCTGACGAGTGCGGTGGTGTGAACTTTCCAT
CTCCTCCTTATCCTACTGATATAATCTGTCCAACAGAAGTTGATCCAAAATATGCAGTGTCGATATGGAACAT
ACTGGCAAAAGTGCGTATAGAATCCATGATGTGGGGTATGGGAACCGCCTTGGGGGAACTGCCGGCATACTTC
ATGGGGAGG
>103780379_2 DT666282.3
GCACGAGGGAGGACGCTGTCGAGACCATCAGAGACCAGCGTCGCGGCGCCATCAACGCGAAGCAGCTCTCGTA
CTTGGAGAAGTACAGGCCGAAGTCGCGCCTCAAGAAGAACGGCCACAAGAACTCGTGTTGCGTGCAGTAGGCC
CCGTAGGCGAATCGAGCTCTGCTTTCACACACACACTCACACATACACACGACTAACT
AGTTGTAACTTCCCTCAAAATTCCCCTCCCTTACTTTACTCTCCCCCCCACTGTTTGCTAAAGATCATGACCA
AAAGTTATTACTATATTACATTATATATTGGTTTTGTTTATCAATGCAATGTTTATTCCCTATTTGTACTCTT
AGATAACTTTCTGAACTTGCTTTAGTATTGCATTGCAACAGTCTAACGTCCATTTAAT

GCAAAAGCTGATTTATTTACAGAACGTCCACTCGGTTATGATCTTTAATGAAAATGTTTTTGAATCGTTCGGT
GTTTTCTATTGTACAAGTATCTATCTATGTAATTTGAGAAGTAATGTAATTTCAAGGTATATACCGTCGTGCG
GTGGGACAGGCGTAGACGCGAGCCACTATTTGTGTTCATAGTCGACAGTTACTTGGTT
TTTATAAAAAACATCATAATAAGTTAATGTCCATAATAAACTGACAACTTACCGCTTATTTTTAATTGTGTGT
TGAAATCATTATATATTTTCTTTGTAGACACAGTATTGTAATTGAGACATAATAGAAGAAATGGTTTTTGTGT
TTATGGTGCGACTTAGTGTCGACCAATTTGTATAATTTTAATTCTTATTTTATCGATG
>103780403_2 DT666468.3
GCCGAGGAATACCCCTTACACAGGTTGTTTAAATCAATGTACATATTGCAAAACTAAGCATGCTAGAGGTGAA
CTTGGAAGTTATCCTCCTGAGGAAATTGTTGAGAGGGCTCGTCAATCTTTCAAAGAGGGTGTCTGTGAAATAT
GGCTGACCAGCGAGGATACTGGTACCTATGGGCGAGATATAGGGACGTCTCTACCAGA
ATTGTTATGGAAATTAGTTAAAGTTATACCGGAAGGCTGTAGACTAAGGCTCGGTATGACAAACCCACCGTAC
ATTTTGGAACATTTAGAGGAAGTCGCTAAGATCATGCATCACCCTAGAGTTTATAAATTCCTCCATGTTCCAG
TACAATCAGGTAGTAATATGGTGCTATCAGACATGAAGAGAGAGTACACTAGAGCTGA
TTTTGAGCAAGTTGTAGATTTTTTAAGAGAAAAAGTACCAGGCATGACAATAGCAACGGATATAATCTGTGGC
TTTCCAACTGAAACTGAAGAAGATTTTCAACAAACAATGAGTCTTTGTGAAAAATACAAGTTCCCATCTTTAT
TTATAAATCAATTTTTCCCAAGACCCGGAACTCCGGCTGCTAAAATGCCAAAAGTCCC
CGGACAGGAAGTGAAGAAGCGTACGAAAAAACTATCAGAATTCTTCAGATCGTACGAACCGTACGGGCACAAG
GTAAATCAAATACAAGAAGTTTTAGTGACAGACATATCTCATGATAAAAATTATTTCGTATCACACAACG
>103780406_2 DT666246.3
GCACGAGGATCATACCTAAGAATTTATCATAATTTATATTAATAAAAAGTGAAGTGATAATTAAATTTAAATA
ATAAAATGATTTTTATATGATTAAGGAATGTGAAATATAAACAATAATTCAACATGTCAGAAGGAAAGAAAAG
CAGTCATATATTTACCGGGGCGCGAGTTTTAGGTTATGTCAGTACACATGTTCCTTTT
GTGGCACGATTTATTAAAAGAAGACGACAAACATTGCTTTGTACTAGTGTAGGAAAGTGGTTTCATACCTATG
GATGTGATAAATTCCGTTTACTAAGTGTAAGTGGCGAGCATCCCGCTCCTATAACATGTATGAGTGGAGACAG
TTTTCATGTATATACGGCAAGTGAAAACGACATTTACGCGTGGCGGCGAGGCTGTGAA
CTCAAACATGTTTACAAAGGGCATCAGGCTGCTATACATAAAATTTTACCATTTGGTATTCATCTTATATCTA
TAGATGAAGATAATATCTTAAAAATATTTGACATTAAAGAGGAAACAGAGTTTTTAGATCTTCCTTTTGATGA
ACAACACTTTAAAATTACAACAATATGTCATCCACCTACTTATTTAAATAAGATTTTA
CTTGGTAGTGAACAGGGTCAATTGCAGCTATGGAATATTAGGACATCAAAACTTGTATATACTTTCAAAGGAT
GGCAATCTCCAGTTGTAGTGACACAACCGGCACCCGCAGT
>103780408_2 DT665959.3
TCTACCTCTAGTCAAGTTTCACAAAGTGAAACAACTACAAGTTCTGATAAAACTCCCCAAAAAACTGAAAATC
GTAATAATAGCTCATCTAAAGAAAAGGGGAAGCAACAATCCTCTGACATAAGTAGTAGTGAAGGGACCTCCAA
AAATCTGAGCTTGGATTTATCTTTTGAACCTATCGAAAATTTTGAAGATGCGCTTACT
TCATGCGCTGAAGACGTCAATAAAACTTTTGATATTATTGCAAATGAAGCCACCCCAAAAACAGAACACTTAC
ATTTTTTTTTTACCCCACAAATAAATGTGATTGCTTTTTCTGAAGATGGAAAAGACTATAAAGGAAAAGGTTC
TCAAACCAAAACTCCTATTTCTCAGCCAAAAAACTTGCTAGGCCATCCCAAATATTCC
TGTATCATCGAATAATAATGATTTTAAA
>103780421_2 DT666653.3
CCAATATTGGCAGTGTGCCTTCTTATCTTCGTATGCCATCTTAAGCACAATTCCCCTATAATATAATTTTATA
TTTGTTAAGCAAAGGACAATGGGCAAAAATGTATGAAACCTGGGACCACCCACCGATAATAATAAACTGTGTC
ATTCGATAGGTTATTTTCGTAGGAATAACTTTTGTGAAGACAGCTTTGTTGTCCCACT
TGTCCATTCAGAAATGTAATAGTTATAACTTTCACGTTATAACTACGCAACTTAGGGATCATCTACTATAACG
TCATTTAAAAATATTACTGGTGCATTTAACTCTTGACCCATCCCGTGCCTTACGGCCGTACGAAGGCGCGCTT
GGGCATGCGTCAAGACAAGATAATGTGCGAAATAAACTATTACTACTAAATAAAAGTA
GTAAGCACAATAAAAAACGTTATATTACCTGGGCGTTCTATAATGCTGGCAGAATCTTACGTATTGTATGAAG
GAAGCAGCGACAGGAGTATGTGGTAAAGGGAAAAGAGATTTTAGCAAAAAACGTGACGGTGGGATGATGAAAG
GCAAGATGCTGTAGAAAGAAAGAAGAAAGTATGATAGGATTATAAAGCCTCGGGTAAA
ATTAAAGTATGAGAGAAATAAAAAAGAAAGAGTATAAGCTGCAAAAAAACCAAACGAAAGTACTGATAAAGAT
GTAGCGTGGAAAAGCAGAGAATGAAAAAGATGAAAGTTAACGAACAACTTCAGGGAAAACACGAAGCTTTTCT
GGCGAGAAGTAAGTGTATCTCACGGTTGCTCGCTAACCTGT
>103780429_2 DT665943.3

CATATACGGTCCTGATAAACCCACACAATTAAATTATCGGCCTGATTGTAGAAACACAACTCCCGACCTAGTT
TTACCTTTGAATATTCAATTGCAAGATGATGTACAAACTCTTTTTAAACTGTCATCAAATCACTTACCTGTTA
CTTTTAGTGTTAATGGAGCTGTACCTCGACTTGATCCTACTAAGATCTTCAAATATCA
CCAGGCTAATTGTTTTTGTTATAGACATTCACTAGACACAAAACTTAGGTATAACTTCAAGTACATACTCCTC
AACTTCCCAAATTGATGCTGCTGTGGAGCACCTCCATACGGCTATCATTGAAGCTCGTAATACGAACGTTCCA
ACAGCATCACAAATACCTCTCTTCATTAAGCTACCTTATCGAATAAGAAGCCTCATAC
GTTATAAAAATTATATTCGTCGCATAGAGGCTCTATGTAACAACTTGGCACTCAAAAAACGACTT
>103780441_2 DT666881.3
GCACGAGGAAGATCCTTTAAGAACGCGCTATTCCGCACTTGCGCGATATCTTCGGCCTCAATCAACAGTACGA
TGGCAAGGCCTACGATTTCAACGAAGACAAATCTAAAAAATCTGGGAAAAAGAAGAAAGAAGACTATCGCACG
TACGCTAATGGCGACGTCAAAACTCTTGAAAGACATCTTAGTATGAAAAAAACTATAC
GCAAGAAAATAATGCGCGACCTTCAACAGGCATTTGTAGAAGACCCTAACGAGTTTAAGGTCGAAAATACAAG
TCCGGAGAAACTAAAAGCGGAACTCAGCGCAGAAGCTGTAAAATTTTAAAAGAATGTTCGAAAAAATGATCCA
ACTTTCTTAGATATGCTCCGAGGCGAGACAAGGGGAGAGGAGGCTAGGGAGGAACAGC
CACCGGCGGAAAAGACCAGTTTCTGGCGACGTCTTACCATGAAGAATAAAAATAAAAGATAATCTGCTTAAAT
TCGCCGTGAAACACGTTACTCCGTTTATTATCCGCTTGTTCTCAACGATTCAGATAAGACTCTGTTATTTAAA
CGTTATCAACAAACAGGTCACGTCCCTTTGTCCCGAGCTCGCTAACCGATTCTGTTTT
TGTGATTCCAAGTTAATGAAATGTACACAAAAACTGAAACAAGATTAGATGTCAGTTATTTTAAGTAATGATG
ACGGTGCTGAGTATAGTTTGCAATGTCAGCCAATATTTATTAAAAGTTTCTAAAAGTTATTTTAATTATTAAA
ATTCCGATTCACATATTTGAAATTTCAGCTCTGAGAGTCAAATGTGCCCAATTTAAAT
AATTTAAATATAAAGAAAAATAATAATGA
>103780443_2 DT666279.3
GCACGAGGAAAGACCTTTCCTAATGAACCGCAAAAACCTGTATCAGCATATGCATTATTTTTTCGGGACACAC
AAGCTGCAATCAAGGGTCAAAATCCAAATGCAAGTTTTGGAGAAGTATCTAAAATAGTAGCATCTATGTGGGA
TGGCTTGGATTCAGAACATAAAAGTGTTTATAAACAAAAAACTGAAGTAGCAAAGAAA
GAATATCTGAAAGCCTTGGCTGCATATCGAGCCAGCTTAGTATCTAAGGGAGGTGAACAAGAAAACCAAGTAA
TGTACAATCACAACAATTCAAATGCAAATTATGGGAATTATTATCAAGGCCAAGCGTATGGAAATGGACACAC
TCCTCAAAGCTATGCGCCAAATTCCACACCCCAAGGTTATACACCTCAAAACTTTTCT
GGAGGTCAACCACCATCTACATTCGCTGGACCTTCCCCTCAAAGCTACCCACCCAACCCCCAACAGTCGCCAC
AAGGCTATCAAGGGAATATGGGACATAATCCTCAAACTTACCAAGGGGTAACAGGTCAGTCACCTCAAGGCTA
CCAAGTTAATTCAACAACTTCTCCTCAAACGTGTCAATCTAATATGGTGCAGTCTCCG
AGAAGTTATCAAGCAGCACAGTCTCCTAACTATGCTGGTAATTCTGGATTATCACCCCCAGGCTATAGACAAG
TTCAATCACAATCTCCTCCAGTCCAATCTGTTCATGCTGCAATGCAGTATCATCATTCTCAACAGATGCAACA
GGCTCACCAAGTGCAACAATATCAACAGCAGCATCAACA
>103780477_2 DT666873.3
GCACGAGGGAAAACCATGACATTCAGGTTACGTGTGAAAAAGAAAATGCGGATTCGAGAGTAAAAAGACATAT
CCCTGGTGGTTTTGTAGATCATAATCCGAATGAACCTAAATATCAGCTTATGGCACAAGCATCATTAAAAAAA
TATTTGCAAAGCAAAAAATCTACAAGACATCATGAAATACTTCAGGTACAAAGTGCGT
CAACACAGGTTGTGGCTGGATTGAATACTCTAATAAGATTTATTATTACACCAACAACTTGTTTACTGGACAG
CAATATGGAACCAACATCATCAAATTGTGTAATATTGGATAATAATAACTTGATTTATTGTTATGCACAAATT
TGGACACAGCCTTGGGCTAACAAAGAAGAAACTACAGTTAAATGTAACCACAATGAAC
ATTTAAATTCTACTGCCTTTAAAGATTTTCAAGAGGATTATAACCCAGAAGAAGCCAAATATACCATAGTGAA
ACCAGATGATTCTTCTGATAATTTGCAGAAACATAATAGACAACGAAGGTCATTGAAGGGTTCCCCAAAAAAA
GAAAATCCAAATAATGTAGAATTTAAAAATTGGGCTGAAAAATCATTCCGGAAATATT
TAAAACGTGCGAATTTAAAATTTAGCCACTCCATTAAAGTCCATCGTGTAACGAGCCAACTTGTTTCAGGGAA
AATTTATAAAATAGATTATGTAGCAATTCCAAAATCGTGCTCCTCGAAAACTTCACCTGATTGTAATGAAGGT
CTAAACATTATTGT
>103780485_2 DT666017.3
GCACGAGGCCAAAGCCCTCCAAGATGATAGATCGTAGCGATCCATATTCAGAAATTCCTGCAAAGCATTTAAA
TAATTTAATGCAAAGATATGGTTCACCAATTATGATACTCAATTTGGTAAAGAAAAGAGAAAAGAAAAAACAT
GAATCTCTCTTAACAGATGTAATAAGCAATGCTGTAAAATATCTTAATCAGTTCCTGC
CACCAGAGAATGCTATTCAATACTATCATCTGGATATGGCCAGGATGAACAAAGGAGCCGATGCTAAAGTACT
TGACAAGCTATCAGCTATAGCCCGAACAGTTGTAAGAAGAACTGTTTTTTTTTTAAGTGGAAAAAATTATGGT
GAGGAAATGGGTGTGGCAGGACATCAAGGGGGGAGGCTTCAGACGGGGCTGGTGAGGG

TCAATTGTGTAGATTGTCTCGACAGGACTAATACAGCACAGTTTGCTATTGGGAAATGTGTTTTAGCTCATCA
GCTGTATGCACTAGGTCTTATTGAGGAACCTGTGATTGAATTTGACTCGGACTGTGTGAGGCTCTTGGAGGGG
TTGTATGAAGACCACGGCGACACATTAGCATTGCAATATGGAGGATCACAATTGGTAC
ACAGGATTAAGACATACAGAAAAACAGCGCCATGGTCTTCTCACGGCAATGATATTATGCCAACTTTTGAGTC
GATACTACTCCAACACGTTTTCTGACGCCGAGAAACAAACACAATGAATCTATTTCTTGG
>103780488_2 DV501404.2
GCACGAGGTGCCCTCCTTACAAGATATACGTAATATCACTGCCGATCACGGTCGATCTGCTCCTCCGTTCTGC
CTGCTCTAACCCCTGGCTCTCCTACCTGTGCCTACGCCCGCACTCCGGCGACCTGAACTCCCCCCTCGTCTTC
CCCCATGTC
>103780496_2 DT666054.3
GCACGAGGCACAGACCGTCTTAAAGGTGAACTAGGAGATCAAGGAATCATTGGGTTTTCTGGAATTAAAGGAG
CTAAAGGCCAAAGTGGTGACTACGGTCACCAAGGATTAGTTGGGCAAATTGGAGACAAGGGGGTCCCTGGTCA
ACCTGGACGCCGTGGCATTCCTGGGCGCTATGGGATTAAAGGTATTTTAGGACCAATT
GGAATTCCTGGAAAACCCGGACTTGTTGGTGACGTTGGCGTTTACGGTGAATCTGGTAAAATGGGTGCGCCAG
GTTTGCCTGGTGATTCTGGAATACCTGGTTTACAAGGCATTATTTGTGATAAAGGTTCAAAAGGAAAACAAGG
TGATATTGGCCCTAGTTCTTATTTACCTGCAACTAAAGGTGACAGTGGTGATATAGGT
ATGGAGGGTTTACCCGGCCTACCAGGTGACTACGGTGATATTGGAATTAATGG
>103780500_2 DT666640.3
GCACGAGGCACACTTTAGGCAAGACGTTCGTGTTATTAATAAATTATTATTTATTTATTATATTTTAAAGAAG
AATTTGTAAAACATTTACAATATGGAGGTAATGTCGCCATTTGGAAATGGTGTCTCGTCGGAATTTCCAAAAG
ATAAGCAAAAGCCGGTGCACGGCGGCAACTTCGCAGCGGAACCTCGCGATACGAAAAA
ATCTACTTGGCTGCTCTTCTCCCCTGTAACACCTGACCAGGCGGGTTCCTTGGCTAAGTTCTTAAGCATATTC
TCGACTCATGGGGTGAATCTAAGCCACATTGAATCCCGTTCCTCCGCAAGAAGACCCGGGTACGAATTCATGG
TGGAATGCGAGTGTGGCTCTGGAGATTTTGGCGCAGTACTAGCAGAACTCAATGAAAA
CACCGGCTACCTAAACATAATCTCAACCAACTACAAGGACAATCGATCTGCTGTGCCGTGGTTCCCACGTCGT
ATCCGTGATTTGGATAGATTTGCAAATCAAATACTCTCTTACGGTGCTGAGCTTGATTCCGACCACCCTGGCT
TCACTGACCCTGAGTACAGAGCTAGGAGGAAGTACTTTGCGGATATTGCCTATAATTA
TAAACACGGCCAACCCCTACCACATGTCGATTACAGTCAGGAAGAGACTAAGACCTGGGGTATAGTGTTCAGA
AAACTGACTGAGCTGTACCCAACTCATGCTTGTAAGGAACATAATCACGTTTTCCCACTTTTAATTGAAAATT
GTGGATACAGGGAGGAATATATACCACAATTAGAAGATGTATCTAATTTCCTTTAAGA
CTGTACTGGATTCACTCTT
>103780505_2 DT665781.3
GCACGAGGATTTAACCTATAAATATCAGTTTCTTTTACAAGAGTGGTTCTGTAGTTAATAACAACCAGCTACT
GAAACATGTGTCCTTATCGGGACCATATAAATAAATATATAGTATATAATTATCCTTCTAAATTATATACAAA
TGAATCAATTAATTTAGCATATCCCAATTATTATTATTATTAACAATATTATATATAT
TATATTATATATGATGGCATTTGATGCGAAATACGGTCTATACGTAATATATATATAGTGTGATTTATATGTT
GTTATATTTTTTACATTGTTTCAAATAGTTTTAAAGGCGGTCGTGTGAGTTGGGACCAAACCGGTGCACCGAT
ATTGGGAATGGGGTATTTAAATATATTTACCAGAATGTCTTACTAAATAATATTTAAA
GTTTCATTGGACCTTTAAAGTGGGCTGTTTTAATATCTACCGTATGGCGCACAACTTTTACAATAAATTTTTT
ATCTGTGTATTTTTGACAGTGTATAAATGTTACGGTCTTTGCACCAACTGTTTTAAAGTTTCTTGCCTAATAT
TTAGCAAAAATATATGCGTTGTAATTATGAGAAATAACTGATTAGATCTTGCCTTTGT
TACTCGTTCTGCTGGAAAGTTGAATAATTTTTTTTTT
>103780509_2 DT666258.3
GNACGAGGGATTAGCTTTAACAGATAAAATCCACAATGACCACATATTTTCAATACCCCACACCTGAGCTCCA
GGAGGAGCTTAAGAAGATTGCTCAGGCAATTGTTGCTCCTGGCAAGGGTATTTTAGCCGCTGATGAGTCCACA
GGAACCATGGGCAAGCGTCTCCAAGACATTGGTGTTGAGAACAACGAAGAGAATCGCC
GCAAGTACCGTCAGCTGCTATTCAGTACTGACCCAGCCATCTCCGAGAACATCTCTGGTGTAATCCTTTTCCA
CGAGACCTTGTACCAGAAGACCGATGATGGCACTCCCCTAGTGTCTCTATTGGAGAAACGTGGAATCATTCCC
GGCATCAAGGTCGACAAAGGTGTTGTACCACTTTTTGGATCTGAAGATGAATGCACTA
CTCAGGGCTTGGACGATCTCGCCCAGCGTTGCGCCCAATACAAAAAGGATGGGTGCCATTTCGCAAAATGGCG
TTGTGTGCTCAAGATCGGTCGCAACACACCCTCTTACCAGTCCATCTTGGAAAACGCTAACGTTTTAGCCCGT
TACGCTTCCATATGCCAGAGCCAGAGAATCGTACCCATTGTGGAGCCTGAAGTGTTGC
CTGATGGTGAACACGACTTGGATCGTGCCCAGAAGGTGACCGAGACTGTATTGGCTGCCGTCTACAAGGCGTT
GAATGACCACCACGTCTTCCTTGAGGGTACTCTTCTGAAGCCTAACATGGTGACTGCGG

>103780515_2 DT665850.3
GCCGGGCTGCAGGAATCGGCACGAGGGAAAATACATTAAAAGGTGCTAATGGCTCTAGCTCTAAACACAACAG
TTTTAACAGCAAGGCAAACAAAAATACAATAATTTATCAAACAATAGCAGATTTTTCTGAAGTACATTTGTAA
GTAAATAAAAAAGGAGAGAAAATTTAGCTATTTAAATAAAACGACTTCTTAATAAAAG
AAATGTATACCTATATTAGTATATACCTATATACTAATTAAAAAAAACTCTTACATTTTTTAATATAATAAAA
TATAAGTAATGTTTGTACCATGAAATTTAATTTACACAACATTTAACTGTTTTTCAAATTTATGTAAATGCAA
AAAATGTAATGGTAACGAATTGTGCAATTCTAAATTTTACGCTATCAATCGGTGTACA
ATTTTGACGATATTTAGGGTTCCGTACCTTTTAAGGAAAAAAGAAACTCTTACAAGTTAACTTTGTTGTCTGC
CTGTCTGGCAAGAGGCTTTATCTCAGGAACGTTTTGAGATATAGAGCTGAAATTTTATACCCAGATCTACGGT
CCCATGATTCCTTGAAAAGAAAAAATATATTTCTCTATTTACTTAATAAAAAGATATG
GTCGCTTATATAACAATAAACCCGTATTTCGACACTATAAAGAGATCAAAATTAAAAGAATCATCCAACTTCC
GGTTGACCTAGAATTATAAAATTTGGCAGCTAGCATCTACATCTAGTACTTG
>103780526_2 DT666061.3
GCACGAGGCCTGGAGCATACACGTGGGTGTGGCTTACGAGTTATTCGCGAGTGCCATATAAAGTCGTACAAGG
ATTTTGTAAGGCAACACAAGATTATTGTCCTCCTGGAGTGCAAGGGCCGAAAGGACCGACAGGTTCACCTGGC
CCAAAAGGTGACAGGGGAGATCCGGGCGCACCCGGAACAGCAGGAAGTCCAGGGTCAA
GAGGACCTCTTGGGCCACCTGGACCTAAGGGTGAAAGAGGTTTCCCCGGAAATCCTGGCCTAGATGGGAGAGA
TGGAGTGCCTGGTGAACCAGGTCTGGACGGCCTATCAGGCAGATTTTGTGCGGATGGAGCACCAGGGAAAGAC
GGCCGTGATGGAATACCTGGAAAAGATGGAAGCCCCGGCAAGAACGGAAAAGATGGCA
AAGATGGTAAGGCAGGAGCTCAAGGCCCTGCTGGTGTTAGAGGGCCAAAAGGAGAGCGCGGTCCTATGGGTCC
TAAGGGACTACGTGGCAACGATGGGAAGGATGGATTGCCTGGAAAACCTGGATTATCCATTTATAATTATACA
AAAGAAAATCAATTATTCATTCCTCCAAGCTTTGCTTTGGACAATCCAAGGTTGATAG
TTCGAGAAAGTGACACAATGCGCATGGATTGCAATCCTAAGGGTTTCCCTGAACCAACTATAGAGTGGCGTAG
GTCTGACGGTACACCAATTATTCAGGGTTCCTGGCGCGACGCGTCAGTTAGCGGCCACGTGCT
>103780568_2 DT665968.3
TGGAAAAAATACAACTGAAAACAGAAATTTAAATTCTAATTCAATATTACAAGTTGAGTTCTCCAAAGAGGAT
CCAAAAAATTAAAATTCCCTCCACTAATTGAGATTAAGGTTATATTAAATGATACATTAAAAACTAAGGGACT
ATATGACCCTGGCTCTAGAATAACATTAATAAATTCAAAGTTAGTTCATGTTACCAAT
ATAAAACAAAATAAATTTCATGGGACAATAAAAACAATAAGTGGAGGGGGCAAAACCGAAGGATTGATTACAT
TACATGCAAAATTATTGAATAAAGCAAATAAAATAAATGCATTTATCTTTGAAAATGAAAATTTTGACTATGA
TCTAATCTTAGGGCTAGATACAATCAATAAATTTGGGTTGACTCATGATGAGAATTTA
AATATCCAAATACAAAAAAAAGAATATTTAAAAATAAATAGTAAAAATATAGAGGAAAATGAGTATGGAGTAA
ACTTTAATGAACGTATAGATACAAACAAATTTAATATAGATACAGAACACTTAAATAAGAATCAAAGAGAACA
AATAAATAATTTATTGGACAGTTACAGAGATATTTTTG
>103780576_2 DT665511.3
GCACGAGGCTTTTTTTCAGTGTTTCGAAGTTAGAACTCAGAGACTTCTCTGACTTTTCTCCTCTTTTATAGTG
GTAACTGAAAACAATATACAATTAATAATGCTGTAGTAATACAATGCTGTCAAAGACTAGTGTAGTATAATAT
TATAAAAAGATCTATTCTAATAAGGCGATTGGTTACAGAGCAGACTGTATTTGTACTG
GACCTCTTTTTAATTGTGTAACTTTATCTCTTCACTTTTACTTTTTAATCTCCCTTGTGTGTTGTTCATATAA
TGTGCCTTTGACAAAGTAAATTGTACTCATCAACTGAAGAACTAAGAAGGAACTGACCTCTAACTCTCGTCTA
TATATCACTTTACAGCAATTACATTTATCAAAGTTTCTAGTAAATGAATAGAGTTCTT
ATAAATTGGAAAAAATAGGTCACATTAACATAACATATCTTTTTGTAAGTAAAAAAATGTCTAATGTGTCAAAA
AATGCACAAAACTCTGCTGCTGGTGATAATTCAGATGATTACATAAATCCACGTGCAAATTTAAAAAGGCATG
CATCCGATACAGATAATGATATACCTGCTAAAAAACAAGTTTCACATGCAAATATTGT
TGCTGAGCACTATAATCGTATAACAGAAAAAGACTTGAGAGAAAGGTTTAATTCACCTATTTTTTATCTTAGA
AATTTTAATAATTGGGTAANAAGTGTACTCATTCAAGAGTATACCGACAAAATTAGACAGAAAGATTATGGAA
GACGTTTGAAATATTAGATATATGTTGTGGGCAAGGAGGTGATTTATGTAAATGGCAA
AAAGCTCATGTAGATCATCTTATTTGTGCTGATATT
>103780591_2 DT665475.3
GCACGAGGCCTCCCGTCGAGACAGTTGACATAATTATTATATCAAACTACCGCCAACGATTTCCTCCTAAATT
CTCTGCAAGAAATATCTAGAATCTCATTCTCAATATAAGCGATTAATCCGGCTATTAATAGTTTCATTTAAAA
TAAAAAATATTGTTTTTTATTTTCGATATCACATATTTATTCATGTATGTTTTCAATT

ATAACATTTTCATACAAACATTCCGCATATAGCACCGTGGGGAGAGAGAACTTGGTGTTAAAACCCTCCGCTC
TCCACTTTCCGTTCATTACGTCTGATTACAAATTAGATGTTCGTGGGTTCAATTCTAACTCGAGTGAATAAAT
AATTTACATTTCTTTGTTCTGGCATGGCAAAACACGGCGTAAAGTTCCGCCACTCAAT
GTCTCAATAATCGGAAAACGAAGTGTCTTAACACTAGGTGCCCCCTGCCTACACTGCTGCATGCGGGATACAG
TGTGAAGTTTAAAAATGAAATGAAAATAGTAATATCATGCCACCATAGGTTGATAAGTCGACGTGCATGTATA
TTATGTTTTTAATGATATAAGATATTTGCCTCCGATGTTGTATTGTACTCATCTATCC
GCCCTATAAATATAATAAATCAGTAATAATCTTTATCATAAATAATTAAAAATTTGTATATGCTACTCGACTT
TTCTTAACTTAATCATGATATATACATACATTCAATAGCAAAAGAGATTTTATCTGTTATTTTACTCTAGTTC
GAAGTATGTAATCGTTTATTAAAAATAAACGATTTGGTAATATGAAAGAAAAATTTTT
TTTTTCTTTTTTTT
>103780625_2 DT665692.3
GCACGAGGGGGAACCCGTCAGAAAGACAGATTAGCGCATTACTACGTCTCTCTTAAGAGAGAAGACGCAATAC
AACCGTGGAAACACGAAAATCATATAGATTTATATTATATGTTGATCTGTGCTATATCCGAAATACCAAATTA
CGATAGAGGACTTAATAGCTTTTAAATTATACTTAACTCTCTAAACTTAAATATTGAT
AAAAATAATATATTGTATTAATCTTTATATAGACTAAAGTATTATCGGTATAACCGGCCCTCGCCTTGACCTC
ACCCACCTACCTACAGGGAGACATTGATCTACACCACTGCATAGCACTGCTACGCTCTAAGGGCTGTTACGTA
TTAGGTGCCGTAGAAAAGTAAAAATATATTTTTTATATATATATTTAGAACATTTACA
CAAAAAATAATTTCATACGATAATATCCGCATTAGTTAATTAATTTGATGTGGAGGGTGACGTTTTACATTAG
ATCTGGTGTCAGATTCCGTTGCGGCAACGGCAAAGCGTTTGGCGGTGACCAAATGGTGGTTCGATTTCTACTA
AGGAGAATATTAGTTATTTTTATTTACTTCTTTGACTAGTAAGGCAAAGCACACATTG
TCTTCAAAATTAACTGGTGCGTAGAGAACGGAGTATTTTAACACGAGGTATCCTCCTATAAAAGGATACATTT
CCTTACCTTTCCACGGTATGTTTTACATATGAACATTGCGTGATGATAAAAAATAAATACTTGATTGCTCACC
AACGCCCCTCGCCATTGAAAAGAGATAATAAAACAAATTTAATAAATGTATAATATTT
T
>103780629_2 DT666202.3
TCCACGGAGCCAAACGATCTATTACTAAAACAACAGGTGGCATCTACGAAGCTCGCTGGTACCCCCCATCAAC
AGAAGTGAGATCACGTGAACCCTGCCTGCAATGTGTATGCGCACAAGGCGCGTTGTCGTGCAGACGGCGGGCC
TGCGCTCCTCTGCCTGAGCCCCCTCCACGGTGCCATGTCGTACATGAGAAGGGAGAGT
GTTGTGCTGAAATTAAATTTTTTTATGACCTGAAGCATTTACAGATCACATCAGCCGCTCAAGTTGACGATAC
AGAAATAGAAACACCGTCCTCAATTGGTCATGCGTGCGTGGAAGGCGGAGTAGTCTACGCTGCGGGATCTGCG
ATGAGTGGAGGTTCAGCTTGCGAGCAATGTTTTTGCCTGGGGGGAGCCCGTCGGTGTG
TCAGACCGATTTGCCTCCCACCACCACCCGGATGTAAAGCACGAGCTGCATCCGGTGCCTGTTGCCCTCAGCG
ATACTACTGTGAACCCAGCCCGTCTACTGATGTAGCTG
>103780633_2 DT665952.3
AATAAGGAATTGTGTAGATTGTTGGATCACGATAATGATTGGAAAGAGCTAGCTGGCAATTATATGAAATATT
CCGCCTTCGACGTCAATGAAATAGAACAAATGGCAAAACGACAAATGTCATCTCCGACAGATCAACTTTTTAG
AACATGGGGTCAATTAAACCATAGAGTAGAGGAATTATTTGTTTTATTGCATCGTATG
AAGCATGTACCATTTTTTTTGTGTTTAGTTCCGATAATGGATCATTTGTTTGATAGTTTATTACAGAAACATG
ATAAAAATTTAAAAGATAAACATCCACATACATCAAATGATATCAATCAACAGAAACAAAATATACAAAAAGT
GGAAGCCAGTGATGATAATTTACCAGTTCCTGTGCTACGTATTAAAAAGGGACTCCCG
ACTTCGCCTCCACAACCTGTGATTATTAGATCATCAACAGATAAATAGTAATAGTGATAGTTCTAAGAATACA
TTATCAACACGAGGCGTACACGATGAAATATT
>103780638_2 DV501416.2
GCACGAGGCTCCCCTTTAAAAATGTACGTTTCAACAGAGCTAACTAGCCTGTTATCAAACACATATAAAGAAT
ATTATTGAATATATGGATGAGGATAGCAAATCTAGGCTTTCAGTATCATATGGTGAAGTTATGAAATAAATTA
ATTCCTAAATTAATTAAACCAATCACTGTACAAGATGACATAGAATCTGTTCCTAATA
CGACTCCTTACAGTTTGGAGGATGTTAAAGATGCTGCGCATGTTTATATAAATAACCTAGATACTTCTAATAT
TTTGACTGATACTATCCTCCAAAGTGTTATAGACACAGACATTGTATGTAGCCCCAAAAAGAGTATTGAGACT
GATGTTACTACCTCTATTCCAACACATGCTGTATATAGCACTGTTGCACTAAATAAAT
TATATAATACTGCTACATCTGCACTTGGAGCTCTGTCTATATCACTTACTTCAAACAATGACGAATTGAATAT
TGCACAATCTACTGAACCAAGTAGTATTAATTTAAACACTCCTCAAGATATTATTTCTACCATGAGTTTGCCC
GAAATTGATATAGCATTAAGTTCTCCAGGGGCAAATGCGGTAGTTGTACCCAGCGATT
CAAGTTTGAATTCGGATAATGTCACTAACAGTCCTCCTCTAGTCCCTTTCCTAACTAATTTCGAATTAAATGA
TTTTAATGACTC

>103780640_2 DV501377.2
TTGAAGACTTCGGCAATGGAATGACATAAGCGTTCTTCCACTGTGAAGGAAAGACATTACGTGACAGTGAAAA
ATTGATAATGTGGGTGACCACTGGTGTAATGTCCTCCAACACAGGAAGAATCATCTCAAGACTGAGATTGTCA
CTTCCGATGGCTTTAGTGGAAATGGAGTGGACACACTTCTTGACTTCCTCTTCAGAAA
CAGTCTTAAATGAAAAAGGAGTTGAGTCTGGTAGGACGGTACAAGATAAGTAGCAAGTGTTTGTGATTTTATT
TGGGGATTTATATTTTAAGTTGGGGTTTGTAAAGTGAGAATTTAAACATTAAGATC
>103780651_2 DT666342.3
GCACGAGGGGGGTACCTGCGCGGGGCGACATGGCATGTGCCTGGACGCGCTCGAGAGCAAAGAGCCACAGGTT
GAACAGGTTCGGAAAACTCGAGCGAAAATAGGCCTAGGGGTGACATTGTCACTCGAATCAGACGGGGTTTGGC
TTTACAATCGAAGCCAGGAACCCGTTTTCGTCAGCTCCCCCGCTTTAGACGCGGCGGC
GGCCAAAGCCCTACTCGTGTGGCGAGTGGCGCCAGGTCACTGTCTATGTATCTTTGACCCTTCAACCCCTCCA
CCAGCTATAAATCTCCCCCATGTGGGTCCAGTTGACCCCCGATCAGTAAGAATATCGTTCGCCAAGGGTTGGG
GCCCAAAGTACTCGAGACGTGATGTCACAGCGTGCCCTTGCTGGCTGGAGGTACTTCT
GGCACCTCCAAGCTGACGCTAAGACAAAGTGGACCAAAGTGATTAAACTATCTATGAACTATTGAATAGTCTG
TGGCATTTGTCATTGTCAGTGTCAGTGTCAAAAGGACAATGAACTTTAGTGGCGGTTATTTTTGTCGTTGTCG
ACAAAATGGCGGTCAATTCCGTTTCAGTGACAGAATTTTCTTTTTTTATTTAAAACTT
TCTGTCACTGAAACGGAAGGTGTTTTTGACGTTTTCAATACTACCGCGTGTTGTAGACTATGGGTTTTTCGCA
CTTTTTTTTGTTTCTTCCCATAGCTTCGACATTTTGGTGGAGGCGATTGACCCAATCTGTATACAGACAAAAT
ATTTATTTA
>103780655_2 DT666189.3
AATAATGTTTTTTTTTTCATATTTGGGAAAAAAAATACAAAAAATATTTTTCTTTTTCATGTGATACCAACGA
AATGGTTAAGGATAATTAGAATAATATTGTGATAAAGCACGACCGTCAGTAAAACAACACCTCTTGACGATTA
ACATTTATTATAATAAAATTTTGTATTATATTTCAGTAGTAAAGGATTGTTTGGAAAT
GTTATATAAAAATTACGTTGAATTTAGTAAATCGTCATTATTATTACCATAATTATTGTAATAGTCATTTACT
ATGTGGTCTTAAGGGAAGTGGGATATAATAAGTAAAAAGATGTTAATATAAAGATTTTTTTAAAGTTACGACT
TTTAACATAAGGCCAGTCGCGGCGTATGGGCACAAACGTGACTGTAAACGCGGCAGGT
TGTGGGTTCGATTCCTACTCGAGAAACTAAAATATTTAATATATTTATTTCTTTATTTCATAAATTTAAATAA
ATATTTCATCTCCTCGAGTGGGGATCGAACCCACCACTAGTCGGTTTTACAGTTACACTTTGTGCCCCTGTGC
CATGACTGGCCTCATGTAAATATAATATAATCAGTGATACTATCCTTATTAATGCAAA
AATTAACGTTAATTGTTCGTAATTAAATTTTTAAACCGAATTATAATTGAATCGTTGTTTGTTTAAATTAAAT
GTTTGGAGCAAATTGGAAAGTATGTCAGTGTCAATTTCTTTCGTAACTCGAGTACGCCAATATG
>103780657_2 DT666953.3
GCACGAGGGGACACCTAGCAAGTGACCTTCCACCCCCTTTATAGTTCAGGAACAGCTGCAGCTAAAGGGGACA
CACCACTTTTACACAATTACCATTATTATTGGGAAGTTAAGATACTGACTGAAACATATGGCACTGATATTAT
GGTAGGCCTAGGTTCTGACAAAGTGAATGTAGTGGATTCCCAATTTATATTCACATCA
CTGTTAGGCAAGGACGACGAATCCTACGGCCTATCGTACACCGGTGCTATTAGACATAATGGGGTTATTGCTA
AAGACTCCGTGGGCTTCTGCCGTGGCAGCATTGTTGGTGTCAGAGTTGATATGTGGCGAGGGACACTGGAGTT
TTATGTTAATAGAAAGCCTCAAGGCATAGCATTCTATAATCTCCGCCGCCACGAAGCC
CTGTACCCAATGATCTGCTCGACTGCGGCCCAGTCGTCCATGAGGCTCATATACGCCGCCTCCTGGCGCGCCT
CGCTCCTAGTAGACGCTGCGAAGATATTAGCGAGTACACTGAATAGCGATAGGCGGTCAGAAATACCGCCTGG
ACTTCAATATACGCTTAGGTCGCAGTTTTGGTTGACTTTGCCTTCTGAGGGTGACGAT
ATAATTGAAGACAGTGATAGCGAAGACATAACTGAGACTGAAGTGAAATTTAACAATAGTGTGACCCTGTCAT
CCTGCATGAGGAGGCGCCATTGGCAGAGGGCGCGCCCATATTTCGTTTTATCTAACCAATCGAAT
>103780673_2 DT666226.3
GCACGAGGGTTAGTCCTGCAAGTCAGGTGGGGAGGCTAACAAGTGCAGCTCGAAGGCTTTATTCACATGCGTA
TCCGAAAATAACTACACCTTACACAATTCATCCCCGTGATAAAGATCCAAGATGGAAAGATGTAAATATGGAA
AGGGTGGCAGAAAAGACAGACATTCTTATTATTGGAGGTGGTCCAGCAGGTATGGCAG
CAGCTATCAGAGCTCGACAAATTGCAGAAGAAAAGGGAGCTGAAGTCAGAGTAACTTTATTAGAAAAGGCAGC
AGAAGCTGGTGGTCACATTCTATCAGGGGCCTGTGTAGATCCTATAGCATTAAATGAATTAATACCAGATTGG
AAGGAAAAGGGAGCACCAATAAATACACCAGTTACATCAGATAAGTTTGGCCTATTGA
CTAAGAGTGGGAGGATACCTTTGCCTGCTTTTAAAGGATTACCTAACTACAATCATGGCAACTATGTAGTTAG
GTTAGGTCATTTAGTTAAGTGGTTATCTGAACAAGCTGAAGCTGTAGGTGCAGAGGTATGGCCAGGTTGTGCA
GGAGCAGATTTAATATTCACAGATGATGGTTCCCTCACAGGAGTGGCTACGGGTGACG

TGGGTATAGCATAAGACAGTAGCCCTAAAGACATGTTTGAGAGGGGTATGGAGTTCCATTCCAGAGTTAGCAT
ATTTGCTGAAAGATGCCACGGACATTTAACAAAGATGGTCTCGAAAAAGTTTAATTTGAGAGAAAATAGTGAA
CCCCAGTCTTATG
>103780677_2 DT666134.2
GCACGAGGATTTAGTCTTACATCTTAAAAATGCTTTTAGTGTAAGTAGCTGTATGTACAAGCTACTCATAAAT
CACGTACACATCTAGAAGAAGCAGTTCAATATTATTATCTATAAGTAAATGTAATAATTAAACTAAGGAGAAA
GTTATATATATCATAAAAATGATTTCCCTTAAAGAATTGATCTACCTCCCTAAGCTAG
CAAGGTGCAACTCATCAGCGTCTTCTGTATTTATAAATAGAAATCCTAGAAACTTGGAACGAATGCGGATTGC
TCGTAAACCTGATGGATACCATTTAGAAAAACCCGGACGCCAGTATTGGCATAAACTAGCACTTACGGTAACT
AACAGATTAGTTAAAGCAGAAGTAATTCACTTTATAAATGGACCTGTGATTGAAGCAA
AAACCTCAGAAAAAGCTCTACGCAACCAACTCTATAGTATAACTGATACATGTGCTTATAAAAATCTTGGTAA
AGTTTTTGCCCAAAGATGTTTAGAATGTGGCATTTCTGAGTTGTATTGCAATATGGAAGTGCCTAAGGGAGGC
AAAGTTGAAAAATTTTTGGAAGAAGTTTCTAAAGGAGGGGTTAGGCTGGAAGAATCGA
ATGTGTATAAAAAACCTCAACCATGGGATATGCATAGACCTGAAAAGCCATGGGAAGTTACAGAAGAATGAAT
ATTAGATGTATAAAATGTAGATATGCTTAGAATTAAATATATGTTATAAATAATAAAAAAAAAAAAAAAAAA
>103780679_2 DT665083.3
GCACGAGGCGTCCATTTTTTGCTTACACGTTACTGTTAATAATTAATTTGTTAAAAACTACAATTCGGAATAA
AAATGTCAAACAATCCTCAGGACCAAATGGAAGAAAGTATGGGTGAGAGTGATGATGAAGACATGGAAGAAGA
AGGAAGTGATGATGAAGACGCCGGCCCCAAAACATATCTTCCAGATCAACCATTGAAA
GAAGATGAGCATTTGGTATGCGATCAATCAGCGTATGTTATGCTACATCAGGCACAAACTGGAGCCCCATGTC
TTAGTTTTGATATTATACCGGACAACTTAGGAAATGATCGGTGTCAATTTCCAATGACAGCCTACTTAGTAGC
TGGTACTCAAGCATCGAGCGCACAACTTAATAATGTTTTAGTGGTCAAAATGTCAAAT
TTAAATCCAACATCAAAGGCTGTTGATGAAGAAGCGTCAGAAAATGAAGACGAGGATGATGATGATGATGAAG
ACGAAGATGAAGAAATGAAACCACAAATGACATTTGCATTTATAAAACATCAAGGATGTGTTAATAGGATAAG
AGCAACAAATTTCAAGAACTCTGTATTAGCAGCCAGTTGGTCAGAATTGGGTAGAGTA
GATATTTGGAATATTACACAACAATTACAAGCTGTGGATGAACCTACACTTCTTGAGAGATATAATCTTGACA
CAGTAAATAATCCTGTTAAGCCATTGTACTCCTTTAATGGACACCAACAAGAAGGATTTGGTATGGATTG
>103780681_2 DT665547.3
GCACGAGGATTTGTACCATATTACCCCATGGCTGTCAACCCTAATGTACAGACAGATCACGCTTTACATTACG
TCAATGTATATAAACCCCCTCCCCCTTATCCGTCAAATGGTCTAGCTTCCAATTCGACCCCAGATCTAGCAGT
GGCAAGCCAAGCCTTAAATTATCATAGAGGTTATATCAATTCGCACGTATCAGGTTCA
AGTCCAGATTTGGTATCGACTAGAACTGCCTTAAATAGACAGTTACTGGGTTATGTTAATCCTGCCCATAATG
TAGTTAATTATACTCGACCTAACATTTTACCCGCCACGCACGGAACTTATAATAATTTGACGTCAGTTTTAGA
ACCTAATCCTCATATTATAATCGATCCTCATCATATATCCGATAATATCCAAAAAGTT
TACGACGAAAGAGGGAATATAATGTATTCTATGCCAATGAGACGGGTTTCATATCAAAGACACCATATTTTGC
CCCAACCGCATACAGAAACTCAGGAACCTATTTACGAAAATGTACCGTTACCATGGCAGAGTGATGGCAAATT
AATACGAGAGAGAGCTCAAAGTTTGACCACGACAGACGAAATTAATCGACTTAACGAA
AAGAATAGCAGTCACCCAGCGATAAATAACCACGGATTAAAACAAAATATCAACCTGTCCTATTCAAAAATCG
ACAATGACAGCCATTACGTAAACGCTCAAATAATAAAAGGAGTAAGAGAATCCAGTATACCAAAAGATATCAT
AAATTCCAATAAATCGCTGAATACCGAGCAAATAACACAAAATTTAGACCAAATATCA
TTGAAACTGA
>103780686_2 DT665695.3
GCACGAGGAAAACACTTTTTTATTGTTGTTATTCTGTCAATATTGAAATGTGAAAGACAAAAATATATTTGAG
GAATTCATGGATCAGTGACGTATCATATTAATTATTTATTTATTTATTATGCCTACAGGCCGCGCTGTTTTAC
TATTACGACGCTTGTCGACAGGTGACAATAGATTTAAGAATATAAATTTGGGTCAAAA
TAGTCCAAAAGATCCTTTACTAACACACCCGGTGTGCACATATCGGGATTTCTACTTTAGTAAGAGAATTTCT
TCAATTGGTCACAATCACAACAATTTAATTGTGCCTCCGCTAACAAGAAGTTTCAGTGATAATATAATTAAAA
TGGCTACAGGCAGCACAGAAACAAGTAATGCAAAACAAGAAAAATTTACAAATAGATT
AACCACTGAAAAATCACCATACTTATTACAACATGCTCATAACCCTGTAGACTGGTACCCTTGGTGTCAAGAA
GCAATTGATAAAGCTAAAAGAGAAAACAAGCTTATATTTTTATCTGTGGGTTACTCAACATGTCATTGGTGCC
ATGTCATGGAGAAAGAATCTTTTGAAAATGAAGCTGTTGCAAAAGTAATGAATGAACA
TTTTGTTAATATAAAAGTAGATCGTGAGGAGCGACCTGACATAGATCGTGTTTATATGCTTTTTGTTATGGCA
ACATCTGGATCTGGAGGTTGGCCTATGTCAGTTTTTTTAACTCCAGATTTACACCCAGTAACTG
>103780712_2 DT666719.3

GCACGAGGGCACGGCCTCGCTCGGTTTTTTTATCTTTTTAACATAATATTTTTTTTAAATAAATTGTGTAAAG
ATGAAGTATCTGATTGTACTCGCCGTCCTATCTCTTGCCTTCGCGGCGGAAGAGGCAAAGGAGGAGTCGAAGA
CTTTCAGCCGGCTGATCCCCGCTGATGTTTTAAGAGATTTCCCTGGCCTCTGCTTCGC
GTCAACCCGTTGCGCCACAGTAGAAGTTGGTAACACCTGGGACCTCGCCCCATTCTGTGGCCGCAGCACCTGC
GTCCTCAGCCAGAATGAGCCTAAGAGGCTTCTTGAGCTGGTCGAAGATTGCGGACCCTTGCCCGTGCCTAATC
CTAAATGCAAACTCGACTCTGAAAAGACCAACAAGACCGCTCCTTTCCCTGGATGCTG
CCCAATCTTTACCTGCGAGGACGGCGTTGTCTTAGAATACCCAAAACTCCCAGTAGTCCCCGAAGAGGAAAAC
AAAGAAGAAGTTAAAGCCTAAATTTCCAATCAACAATTGAAAACCGACTAAAATTCTAAACCAAATATTTCAG
ACATTCGGACTATTCCAAAATTTGGATATATTTTTATATTACGTTATATATGATTATG
TATGTCTATTTTAATAGTATTGATAGGATATTTAAATTATTTCTGTCGTCGGTTACAAACTTTTTTTTAGATA
TCTATTCGACATTACTTTCTAAGTATTTGTTAACAATTACCTATTGTTTGCCTCCATTTCCGTTTTACACAAA
GCCGTAACAGAATAGACCTTTTGTCTATTTAATAGGTTTGTTGG
>103780720_2 DT666216.3
GTAATGATTTTCTTCTTTTTAAATTGAATAATGATAAGTATTCATAATGGGAGGACAAAAATTTGAATATGAT
GAGAGTGGAGCTACTTATTTTTATTTTGTATTATCGTTTCTTGCTCTTGTATTAGTACCGGCGACATTTTATT
ATTGGCCAAGAAAACGGAAAGAAGATCCAGTGAAACAAGCAGAACTATGTCAATGTCC
AAATTGCAAACAAAAACAAATTATTATAGAACAATCAGAACCATACAAAAAAGTAAAAAACTTTTTTGTTATG
CTAGCAATAATATCAGGATGGCTATTATTTGCATTCCTTGCTGTTAAAGTATCTCAATTTGATTATGAAATGT
CAAATTTCGATCCTTATGATATATTAGGCTTGCCACCTGGTGCAACTCAAGCAGAAAT
TAAAAAGTCTTATCGTAAACAATCTTTAATCTTGCATCCTGACAAGGAGACAGGAGATGAGAAAGCCTTTATG
AAACTCACAAAGGCATATCAAGCTCTCACTGATGATGAAGCTAGAAGAAATTGGGAGAAATATGGAAACCCTG
ATGGTCCAGGTGCTATGAGTTTTGGTATTGCTCTTCCTAGTTGGATTGTTGAAAAAGA
GAATAGTGTTTGGGTGCTTGGATTATATGCTTTAGTATTTATGGTTG
>103780722_2 DT665349.3
GCACGAGGCGTGGCCTGGATCAAAACATTGAGTACGATCGTATGTTGATAGACTACACTAAAACAATAAATAA
TAACAGACAAACGTTCATATGTGAATTAAAAATATTAAAAACAAATATTAAGTGAAAGGCCGAAGTGAAATAC
GAGACATTTAAAATAAATTGACTATAGTGTTACATTTTAATTATTGAAGAAAATATGA
CAGCTTCAACGATGATTGAGCTGGAATCCTGGATTATCTACCAGTGTGTTCTTAATGAGTGCCATGTCTCCAG
CTCGTGAGCTGAATGTGTCGCGTATTATTTATTGGCACGAGTTTTTTTAAGTTATCTTCATGGTAGCCACTTC
GATGGACGGCGGCGCGTGGTGCGCCCGCGACATCTCCCTGCGGGCGCAGAAGAAGTTC
CTGTCGCGAGTGGGCGGCGCGGCCAGCGCTCGGGCCCTCGTGCTGGATGAACACGCCGCGAAGCTGCTTGATC
AGTTCTTATGTGTACTACGCGAGCGCGTAGAGAAGAGAGAAGCGGAGAAGCTGGTCAAGCACGTGATCAAAGC
GGCGGTCAAGATAGGAGTGTTGCGGCGGCACGGACAGCTCTCGCCCGCTGACGAACGC
GCCCTGGCCGCCTTCCGTTCTAAATTTCATACGGTATTAATGGCGATAGTATCGTTCTGCGAAGTGGACTTCT
CTTATGATAGAGGGTTTCTACAGGATGCTTTACGCGAATCACATCAATCACTCAAGTCAGTAGTGGAGCGACA
CTTGTC
>103780730_2 DT665611.3
GCACGAGGCTAATATCTATCACTTTTATGGTTTTTATGGAATAAATAAAATTGATAGAGAGAACTTAATATCA
AGTTACTCCATATATTTATAAATATACTCTGCAGGTTTGACGGAAACCAATGCCGTAAATTAAATATGTTTTA
ATATTCTGCCATACGATAATTGCTTATTATTGATACCATTTAATTTCTATTTACAAAC
GTTTAATTTTGCTTAGAAAATATTATAGCATCAATTTATATGATTATTATTATTAAAGGTTTATCCAATGAGA
TACAGGCTCGTCGTCCATTATAGTTCGTCGCGCCGCACGGACGCAAATATTAGAGCGTTCGTCACACGTCTGC
ACTGTTATTGTTTTATTATTCAAATGGATGTGTCACACCAGCCGACACGTATAGTAAT
CATGGCGTTATACGTACATCGTGATCTATTCTATTCAAATTACTGATAATATTAAGAGATTCTAGCCGAATAG
CGAATCAATTTATGCAGGAAAGATTTATTTAAGGAAAGTCTGAAATTTTGAAAATAAAAGCAAAAAATATATC
CTCAATTTAGGGTATTAAGATACGATGGGCCACCTTCAATTTTATATATTGTCTAACC
AAAATGGTTCAATATAAAAAAATCATGAAATGGATACCAACAAGTTTCGCAATGTAATCAAACATGATATAAA
TGATTAACTAAAGTAAATATGTATCTATATCAATCTGGATTTAATATGATACACTCAAAAAGTTTATTTTGAA
AAAAGGCTATCT
>103780734_2 DT665860.3
GCACGAGGATATAATCTTTTATATAAGTTTATTCATAAATTACATAATTACAGGTAATGGTCAACATTTTAAT
GTCATATTTATGTAAGTTAACATTAAAATAAAGCAATATTAAGATCTTTTTCAATTTACTTCGTTCGTCGTTT
GTATCTATGGTTCACGCTCCTGGCAGAACATCAATAGCATAAGCTTTTTAATTTTTTA

AAGCTTGCGTCGCACTCTAAGTTTGTGATACGAAGGCCTTGGGACATACAGCCTTTCGTTGCTGTTGGCAGAT
TGTTCTAAGCTTTCTTCTAAAGGCTCAGGTCGTGGTGTGAATATCTTTCGCCTACTCCGAGTTATGCTTTTAG
CAGGACAACTCTGTATACCGAGTACTGGCGGTAGATTCTCATTGTCATTCTTCTCAGG
CGATGGTATATCTTTGAACTCTTTGGCGGGAGCCGGTTTATCTAATGTCACATCAGAAAACTCCTTCTGTAAA
TTTATTATATCTTTTAATTTCGCTTTTTCTATGTCTTTTACATTTGTACCTGCATCTATATCTTTAGATTCAT
CTTTAGATCTATCTTTAGATCCATCTTCAGAATTATCTTCAGATGCGTTTTGACGTCG
TCGTAGTCTTAAATTCCTTTTTTTTGGTTTAGCTGTTTCACTTTTTGGCGGAGAACATTCCAATACTTTTTTT
CCTCTTCTCGTTTGATTTTTAGGG
>103780738_2 DT666615.3
GCACGAGGACGCAACACAGCACGTTACTTCCAAAATTTGCTTAGCTGAAGCGTATTAAAGTAATATTAATCTA
AAATTAATTACATTTTTAAACTATATATTATAATTTGAAGAAGTCCGATAGTGTACAATTGCACTAAGGATAA
TTTGGAAAAGTAAACAAATTATTTATTTGCAGTAGCTGTAACTTTGTTTTCTATAATA
ATTTCAAAAGACAAAAAATAATAACATTTTTGTAATGGCTGAACAAAATGCAGAACCCGTAACAACTGATGTT
GAAAGTAATTATAGTGCACCATCTAATGATCCAAAGAACATGCAAGAAGTTACACAATATGTACAATCTTTAC
>103780760_2 DT665627.2
GACGAGGGTCTCGACCTTTTATTTTTTTTTTTTAATTCTTAACAATAGAAACTTAACTATAAACGTGTACTAT
ATTTTTTATTGATATGAGTTTTCGATGATATGATACTATTTTATTCTAAATCACTTGTTTTAGTTTAATTGAG
AATTTAAATATGGATCCCCAAAATTCTTCTTGTGAAGGAAATATAGAAGCCAGTCAAG
CATCTACATCACAATTATCAGATTTACTGCAATCAGCAACTTTAAATATCTCTTCAAATGAAAAAATAAATGA
AACAAAAAAACAAATCGTATCAGCATCAACTAGCAATTCTTGTAGTAATGAGGAGAGTTCTACTAATTGCCAC
AATGCCAACTTACCAGAGTCAAGTAAGAACATAACTATCAATAATGATTCTTCTCAAA
ATTCTCCTTCAAAGACGTCTAAAAAAAAAAATGCTAAAAAGAAAGAAAGGTTAAAAAAATTACAAATAGAAAT
GGCCAACGATAATAAGCAACCTGAAACCATCCAAACTGCTCCAGCAGCTCCTACACAGAATGGTATGACATCA
GTACACGATTTTTTTTTAAAACATATTACACCATTATATGCAAGTGCTAATTCGAGGT
ATCTAGAAAATCTAACTCATCACCCACAGTATTTTCTTATGCAGAATGTACATTTTGGAGAAATGTATCACAA
CCATCCAACAGCTGCAAGTCTACCAAATTCTCCATTTCTACAACAATCTTATCCATCGAATTCTCAAAATGCA
CTCTTTCATAATACACCAATAC
>103780767_2 DT666290.3
GCACGAGGCACAGTCCTCTTTAGACAGACGTTATTTCATTTTTTCCAAAAAAATAATCTTAAAGCTACATATA
GGGTACATTATATATAGGTATGTAGTTAAAACACCCCACGCACTCAACATACGACTTATCAACTTCTTACTTC
CATTGTTACAACGAATTTTGATAGTAACAATAGGTTTTAAGATCGAATGTCCCATTAT
ATTTTTAATATAGGGGGTGGTTGAGACCTTTATGATGCTGTGGGTCAGTGTTTGCCTATAAAAGCAATGGTTG
TGGGTTCGATTTTCATTAGAATGAATTCATTATTTTACTCGAGTAATCAAACGCGGCGTTCAGTTCCGTTACT
TTAAAAGCGATATATTGAGGGAACACTCGTTGCCTTTGCTAACCCTGTTATGGGAAAA
AATGCTTGAAGCTATATATAAATTTATATTTAAATTATTTTCTCTTACCCACTTTAGTAAATAAAACGTGGCC
TTAAGTTTCGCCACACTCGAACACCTAATTTCTCGAAAGTTGAACGATGCATACTGAACGCAGTGTCTTAATA
CTGAGAATCTCTCCGCCTAACCCGCTATATGCGAGATACAGCGTGCAACTGTATATAT
AGGATGGTTAAAAAGGGATAGGGGGCGAAGATTCTGATTCCGCAACGCGTTTGATAGCGTTAGTTTTTACGGT
AAGGTTTTATATATTCCGTAATTCTGTAACTGAAATTTATTGTACTAATAAAATGTGAATGTTTTTTAGCTTT
TACAATGTTGTTTTATGTT
>103780784_2 DT665743.3
GCACGAGGATTCTGTCTTCTTACAAATGCGACATTAGAGATAACATCATAATTTCTGTAAACTACATATATAA
CAATTATAAACGTTACCTGATGTTTAATAATGCTTAAATTGTATAAAAGTTTAGAGATCAACATACAGAAATG
GAAATATAGTTATAAAATATTTTATTTTAATTTAATTTTAGTTCTAATTTTAAGTATA
TATTTATTAATATGTAAGTTTTATTAAATAGTTATTTCCACTAACAGTCTTAAGTTATTAGTACAATATAAAT
CTATTGTAACACGTCTTGTGTAACACGATTATATATCTAAGTTAAGTTCAAATCATGAGGCAACAAATATTCG
TAATGTGATATCATAAAATCCTAGTTTATTTAACCAGTAGCCGCCAAAACGGAAAATA
ATCTAATAAATAGCCAACATATTGTTGTTGATTAGAGAAAACTGTAGCGGACTGTTTTTGTAAGTACATTTAG
TCTATCGGGCAAGCAAATAAAAAGAAATAAATATTTAATATTCTCAAAGGTTACTTAATATTCTTAAAGATTA
TTTAGTATTAGTTAGTTTATTATCAGTTATAAAGAGGAAACTTTTAGATCTCAAAAAA
TATATACGAACGTATTTTGTTTATTACAGTATAGTACGTTTTATTACAGTATAGTTTCATGTTTAATGAGAAT
CTAGAACTTCAAAAGAATACATGTCTAGCTATACAATGTCTGCAGTAAAGACTTGCTCTAATA
>103780790_2 DT666750.3

GCACGAGGGACAGCTCTGTCGTTTTACTAAAAGTACAAATAGTGTGCATGTTTTATTTATTATATTTTTGTTA
TTATCAGTAAATTGAGACACAATTATATGTTAATAACAAATTGAACGTTTAAATGTAATGTGTATCAAGATGA
TATACGTGTAAAATGTGGACAGATTGATTTTAGTAAATCTGTTTTCTGTGAGAAAATC
ATAAATTAAAAAATACTACATAATAAATTGAAGAGAGATGAGTGCAGGAGCAAATGCTGCAAACAACCTGCCA
CCTGGAGTGAATCCAGAGAATAATTTATTGCCAGGTGGTCCTATTATAAATCAAGGAGCAAACAGAAATAGAA
ATAACAATCAGAATCCACTATTCAGTGTTAGAGAAAGACTGTTTCATGCTTTATTCTT
CAAGTTTGCCTTAGCATATGCTAGAACATTCCCGAGGCCTATTAGAAGATTTTTTGAATTTCTTGTGTTGCTG
AAGGCACTTGTGGCATTCTTTGTACTTGCATACATACATATTGCATTCTCAAGAACTCCAACTACTTGTTTGA
ATCATATCAAGGATTCATGGCCAAGAGATGGTATACTCAGAGTAGAAATCTTAAGAAA
TCCTTCTCAAGATTACACAGTTGAACAAAGTTATGCTAAGGAAAGGAAGTTAANAAGAGATAAAGATGAGCTA
AATTCTATGCTGGGAATGCTCACGACTGAAGGGTTTTACAACCTAGAGTCGTCCACAAGTGAGGACATTGAAG
AAGATGAGTATTTGCGAGACGGCACAGACTATGGCAACATCACCCGTATTTAT
>103780798_2 DT666479.3
AATTATCAACAAAATGTTCCTTAAGGTACTGTGTTTCCTCGCATTTGTGACAATTGCAGTATGCAACCACGTC
TACAGGTATGAGCCTGCCTACTCCTCGCAGGCTATCTCACGTTACGACGGACCTGCAATCGGACATGGATACG
CGAGCCGATATGGCTATGGATATGGTCCTAGATATGGTCTCGGATATGGTCCAGGACA
CGGTTACAGACACGGACTCGGATACGGGCATGGATACGGGTACGGACACGGACTCGGATACGGTCATGGATAC
GGTCTTGGACACGGATACGGACTAGGATACGGACTTGGACACAGATACGGATACGGACACGGATATGGACACG
GACACGGACACGGATACGTTGATTATTATGCCTATCCTAGGTACAACTTTGGGTACAA
AGTAGTGGACCATCACACTGGTGATATAAAGGAACAACAGGAACACCGCGACGGTGACGTAGTTAAGGGATAC
TACGCACTACATCAGCCCGATGGCGTTCAGAGGATAGTACGTTATTATGCGGACGACCACGTTGGAGAAATCA
ACACCCTTATACCACGAGAGTATAAAAAATCCATACCTATATTATAAATTCGAAAGTA
ACTCTGTCTGTCTGTCTGTCTGTCTGTCTGTCTGTCTGTCTGTCTGTTACGCAATCAGGGCCAAACCGCTAATTCAA
TTGTGATAAAATACTCATAAAGAGATACTTAAGGATCTTCGGATGAACATAGGTTACTTTTTTTTCTCATATT
TGTAATATTGTCGATATATACTAATATATTAAAAAACTTTTTAGTTTGACAGGC
>103780800_2 DT665761.3
GCACGAGGTAAATGTCTTATGTTAGGTTTGTGATTTTTTTTTTCATGTTGTAAGGCTGTATGTTTTATGCTCT
TGGTGTTTTTTTTTTCTATGTTAAAAAGATTAGTTGCTGTAGCAGTGTCTGTCTGTTAACTACTCTGTGCTCC
GTATTCCATATTTTCAAATATCTTTATAAATTTTTAATATGGAAACATATGTTGCTCT
TCTATTGTTTTCTAGCAGAGTATAGAAATTATTTGTGATGACCAGAGTATAAAATATACAATCTTATTTTAAA
ATTAAGTGGAAATTGTAAGCATTTTCTTATAATTAAAATTTGTTACTCGCTGGACCTTCTAAGATCTACACAA
GTTTTCATAATATTCACATATAATAATGTAGTTTTCTAATAAATGTTTCATAAGTCAA
TTTTATATATTACTAGGTGACATGATGACAATTGTCTACGTAAAAATAATGACACTTATAAACTTTTTGCACA
ATAATTGTTCATGTTTATTTTTTTTATAAATTATTTATAGCGTCTTCTTGTCTATGATCATTGTTTTCTTTTA
CCTTCGGTTGTTATAAGTACAGTTATCAAAAATTCATATAACACTAAATGTATTGTAG
TTGCATTCCGAATTTACTGTAATAAATATGTAAATATATTCGTACTTATTGACTGATGATTTGTATATTAAAA
AAAAATATAATTACTGTAAAAAAATAATTTTTAAATACAGTTACGACGAGTCTATAGACGCAATGTTACGATA
TATTAGCACTATTCTTCCTTGTTACAATTATTTCTACAAAAATCTATATGAAAGACAA
AAAAATATTT
>103780831_2 DT666124.3
GCACGAGGGCAACTTCATGCAGGCGCAAGAGTAGCCTCGAGGAAATTGGCATCTCACACTTTAATTCCTTACT
GCAACACCAGCAGCAGCAGCAGCAACAGGATGCGAATGCTATTAAGATAGCTCTGAACGGCAGCATCGGGCTA
GAGGTGACGCCTCCAGACGAGGGCCCTACAGATCGGCTAGGCATGGTCGGCACCTATC
AAGATATAGCATCTGCGCTGCCATCTACATCGACCGGCTCCGGGTTGCCGCCAGCCTCGCCGGATCCGCAGCA
TCTACAGGGAACTATCGTATGATACAATCTTATGTGGGGACGGTTACTCGGTTCCGTCTTGAGGAATAGGAGG
AAGTGAGCCGGGCCTCTCCGGCTTTCCTTGCTCTCCTCGGAACCACTTCTTGTTTCGT
AGCGTCTCTACTGTGCACGTATACGTAGTGTCTCAACTAAATGTCTCCAAAGAAACTACTACTTGAGTGTGTG
GATCAGGACAATACGCATTGGTGAAATCAGTGTATTGTATGCGGGATCAACTAGTTAATATGTAAAGGGAATC
GTGTGG
>103780836_2 DT665990.2
GTTGTTTACATTTTATTTAAATTAAATTGAAAGTTATTAAACATAATATAATATTTGAAAATGTGTTTTTAAA
ATGTATGGATTTTGATAGCAACGTCTTAATAGCTGCAAACACAAAATGGTACACATAACTATCACACAATCAT
CCCAAATCAAGAAAGAGGACGGCTTAGCCAATTTAATCGGACATTTCGGGAAGTGGCA

ATGCATTTTACTCCTATCAATATCCCTAGTTAAGCTATCCTCAGGATGGGTGCAAATGACGATCATATTTTTG
ACCCCAGAATTGACTTTTTTTTTTTAAACCTTCAAAAATTCCACTACGATTGGGGAAAATATGAAATGTTATG
GGGACTGTGTGGAGTAT
>103780844_2 DT666901.3
ATGATGAGGAGACTCCGAAGAGTTAATGTTGATCATTTTTTTTTTGGATGGTATCAAAGTGCGGATGTTGGCA
ACTTTTTGAGTCAATCTCTATTAGAGTCACAATATCATTACCAGACATCAATTGAAGAGAGTGTTGTAGTTAT
ATACGACACTAAGAAATCTGCAAGGGGATTCCTGACTTTGAAGGCTTACCGTCTGACT
CCTCAGGCAATAGCTATGTACAAAGAGAGAGATTACACACCAGAAGCTCTGCGTAACCTTAAGATTGGTTACG
AGAGCCTCTTCATAGAAGTGCCGATAGTGATACGTAACTCACCACTCACTAATATCATGATGTCTGAGCTGTC
CGAGATGATACCTGAGGAGGAGGGTTCTTAGTTTCTGGACCTTGGAACTGCTTCTGTG
CTTGAAGGTCAACTACG
>103780846_2 DT665453.3
GCACGAGGACTACCCTTTGAAATTTTATATTTTAATTTAGTGCGTATTTTTTAAAATACCAAGTGTATTGTCA
TTTTATATTTCTTATCATAATCATTAGTGTAAGGAGACAGTGAATCGAAACACCTGAACATATTTAATATATG
TTTGTGATTTTTGTGATATAAATTGTGCGACAATGGCAGACAATTCAAAATTGACCGA
CGAAGAGCTATCAGAAATCCGGGAGCAATTCTCACAGCTGGACACGAGCGGGAATGGATACATTGACTTGAAG
GAGTTGAAAGACGCCCTCGACAGCGTCGGCTACAAAATACCACAATGGAAGGTACGATGCATGATCGAAGAAT
ACTACGACAACGGTGTCAAGAGCGGGCGCGGCGTCAACGGCAGCATGAACGGCGACGC
AAGGAAGAACGGCATCTCACTATCAGAGTTTGAGGAACTCTGCGCCAACCTTAAAGCACAACAGGTGTCGAGC
ACATTCAAGCAGGCCGTCAGTAAAAAGGAGAACCTTGAGCATTTGGGCGGCATGAGCGAGGCTTCAAGTGACG
GCACAACACATTCAGTGCGTCTGGAAGAACAAATGGCGTTCTCGGGGTGGATCAATAG
TAATCTAGAGCACGATCCCGATCTCAAACACCTGCTGCCCATCGACCCCGAGGGCAAACAGTTGTATGAGAAA
CTCAAGGATGGCTTAATTTTGTGTAAAGTAATAAACCATTCGTGTCCGGACACAATCGACGAGCGCGCCATCA
ACAAGAAGAATCTAACGCTATACA
>103780860_2 DT665477.3
GCACGAGGTGTGCCTATCTCTCTTCTAGTATTAGGGATAAGACTGAGTTTACTAACTTAACTGCAGTTAAGTA
TATCTTTGTTTAAGGTTTAGTAATTTTACTTTTACTTCGAAGCTGTAGTGCTGTAGGTGGATGAGTTCTAGGT
TTGATACTCACTTGGGGGGGCATGCTTGTATTTAAATGAATATTATGTTTCGAATAGG
CCAAACTTAGCGTTGAGTTCCACCACTCAGTACCCATTTACAAGACATTGTCTTGTAAATGGGGAGAACGCGG
AGAACGGAATGCTATAACATTAGGTTCCTGTGCCTACCCTACTATGCGGGGAAAAGCGTGAAACTACTTTATT
GTCACGTCAAACTCTTGATACTTTAGCATATTTCAATACTGTGGTAAATAAAGGTTAT
GATTAGTACCTAAAAACACTTTTTTTATATGTTATAAAGTTATTTATAATTAAATAAGATGTAAAGACAAATG
TTTATGCTCACATTAAAAAAAGTGATGTTAGAATAAAAATTTGTAATGTCCATTATAGTTTATATGAACAATG
TTGTAATTTAGTCTTGTAAATGTAAACTCAGCCTTATATTTATATATATAAATATATA
GAAAGCTGCACTGCTCTCATTTCAGTCGGCTTGAGTCTATGTTAGCATTTAAAATCACGTAACTACCTGTTTT
TATATAATGTCTATATAAGGGTAATAAAAAAACTGGCTTAAAGTTAAGGGAAAAATATGAGAGCTATATGTA
>103780866_2 DT665788.3
GCACGAGGGTCGCACCCTCAACTTTGTCAAATTACAAGAGATCCATTTCAATAATTCGGTTGTGTTAGCAAAT
GGAACAAAACATACCTACTATAACCAATGTGTTGTGTGATAGAAGCGAGTGCATTACTACATTCTCTTTTGTT
TTATCGCTGACGCTCCGCTGAATGGAAGCGTTACGACGGCTCTCATTTAACGATCAAT
CTACCATTAAAATGATGGATGATGTCGCCTTTTGCAAAAACAGTGTAAAGTGTTAATATTAATATATAATGTG
AAGGGTTTATTTCGTGTTTATATACGTTTGTGCTTGTGATGAAAACTGCGTCGTAATTTGTCGTACACATTGC
ATAAATGAGTGGAATGGCGTTGCGGAGCGCGCGGGCCGGAGCATGCGCGCCAGTTTCA
TCGCGACCGCTGGGAACGCACTGAGTATCCTTCGTTTATGTGCATATCAAAATGGGAAGGTCTAGGTTCCCCG
GGAAACCGCTAAAATTCCAGAATAGAAAGCGTATTAGTGTCCTATCTGGTGCTGTCTATCATGAAACTGCACC
AGAGAATCACTCGGCCAGCCGAGGTTCGGCTGCCAACGATTTTGCTGGAGAGAAAGAG
GAAGAAGAGAAGAAGGAAAACAATAGTGAGAATGAGGCAAATACAGCAAATGAGAATANAGTAACCAATAGTA
TTGTTAAAAATAAACAGGATAACATAGATAATACTACTACTGATTCCTCTAAATCTCCAGTGCGGACAAGATC
ACAAAATAAAATGGATATGCCTAAAAATGATAAACCTAAA
>103780870_2 DT665274.3
GCACGAGGGAAACCTTTAAAAGTGTAATAGCAGGTATTAATAAAGAAAAGCAACAACTAACTGATGAATTACA
GTGTATGACCACAAACATTGATTCTATCAATGAAAATTTAATTGTATTGAAATCGCAACTGAATGCAAACGAG
GAAGAGATTACCCTATTAAAAAGACAAAAAGAAAAAATGCACGAGATCAGTTCTACTG

TGACTTTAGATATTGTAAATAAAGAGAAGGACCTAAGAAATTTCGTAATAAACAGCTATAAAAATATGTTAGA
TGACAAAGTTATTACTCAGAACATAGATACATTGCTTCCCATCCTCGAAATTCTTACTATACTTCTTGATTCT
ATTTCTACACAAACCAAGTACAATTTTGAACAAGTAGAATCAGACAAACGTAATATTG
TACTTGAAAACGAAGAATTAAAGACAAATCTTAAATTGAAAGAAAAAGAACAAGCTGAACTCTTTGAGGACAA
TAAGAAATTGACTGCCAATCTAATAGTCAAGGAACAGCAATATCTGTCACAAATGAATATATACGAAGATTTA
AATAAAACATATCAGACAAAAGTAGAGGAAAACAATGTGAATATATCAAATATAAAGA
AACTAACGGACGAACTTAATATCTACAAGGAAACTATTACTAGCAAAGATACACTCATTGCCAGTTTAGAAAG
TAAAGTTGAAACTCTTTATGAGAAATCAAATGATACTAAAGTATCTGAATTAGTGCAATCTGCGACTAGTTTA
CAAAAAGAAATTTGACATTTGAAATCTATTAACGAAGTTATATCAAAAGAAAA
>103780872_2 DT666409.3
CCGCGCGAGTGTTAAATTGAAAGAAAACAGTGTGAATTATTATTGAGAAAGAAGAAATTTGTGCTTTATTGTT
TAGTTTATATAATGCAATGAACTCTTGTGGCGTATATAAATTTTATTTGAAGGATTTGATTACGAACTTTTGA
TATAATTTTATAGAATGTCGAATTTTCAACGAACAAGAAGCAAGGTCTGGTGGGGTGA
CGCATCGCCAAATATTGGCAGTCCTGTGGCACAAGCCGAGTGGAACCAAAACAATCGACGCGAGGATCAAGAG
TGGGAACCCCAGCTGCTTTTTTGGATAGCGAATCAGCCCGAACAAAGGAACAATAGCCACGATAATTTAGGAA
GGGTAAACAATGTACAGAACCAATGGCCTCTACGCCACGGTAGCCCTGGCAGTCACAA
AAGTCAAGATTCTGGCTTTTCTGATTCGGACAGTTCCCCACCGACATCCCAGTATTATTCATCCCCAGATAAT
AGCGATGATAAAAACAAATCGACAAATAGCAGTGACTCAAATAATAATGAGAATGTTACAGTTAAACAGTCTA
ACGATTCACAAAACCATTTAAAAAGTAATGATGTTGTTGATAGTTCAATAACTTCAGA
TAAAGTTAGCACCGGTGTACCTACACCAAAACCTCGTTTAAGGAGCAGTAGCGTAATTCAAACTCCTTATGAT
TTACAAAAGTATTGTGAAATTCACCATGATGAAGAACATTTAGAATTATTAAAGGAC
>103780874_2 DT666384.3
GCACGAGGAGGAACCTTACAATGACAGAACAATTAGAAATAACTCATAATACTGATGTTCTAATTGGTATGCA
TGGAGCTGGACTGACTCATTTGCTATTTTTACCTGATTGGGCAGCTGTTTTTGAGGTATATAATTGTGAAGAT
CCTAATTGTTATTCTGACTTAGCTAGGCTTCGCGGCTTGAAATATATCACATGGGAGG
ATAAAACAAAACTTGTGCAACAAGATCAAGGTAACGGACCTGGTGGTGTGTCACACGCAAAGTTTACAAATTA
TTCATTTGATGTAAATGAATTTCTGAGGTTAGTAAAGATCTGTGCAACTTATGTTCGTCAGAGACAGGATTTC
AAGAATTTCATCGAAGCCTCAATGATGAAAAAGTCACATGAAGAGCTTTAATATTACG
TTCTCAAAACTATACATAAATATGTGATATTTTTGTTTTTTTATTTGTGTTAATTAATTTAATTTTTAAGGTC
TAACAATTATGTCACGTAGAAACATTATTTAATATAAACGCATAACAATTGCACGTCATAATTTANAAAAAAA
TAACAAATTATGTATCAAGACTTAAAAGGTAATTTGCTTTAGTCTGTAGCGCTATTAA
ATTCTGAAATCAGAGCTCAGCTCGAATGACACAGTTAAAGTGAGATTAGATCATGACGTGTTTCTATCTCTAA
TTATATTTAATATTTCTAATTTTAAAATAACGTTGCGAACTAACCTCCTTTCACTTGTTTTTTTTTAATTTTT
CATGACGCT
>103780889_2 DT666743.3
GCACGAGGGCCGATTCTCGTAGTTTTCACAACCTTCGCGATGAAATTCCACCTAACCGCTAACGTTGGTACTC
CCACCAGCGTCACGTAGAAGCAATTTTACGTAAACGTTACTGCGTTTTCTTCTGTGTTCGGGTTTTTTTTTAT
TCTCTTCTTAATTTTTTTTTTTTTTATTAATTCCATTGAAATAAATAAATAACAATATG
GTATCGAATCGTGGAGCGCACGTGCTGGTATGGGATCAGCCTGGAATGGAAGACCAGAGACCCAAAAGTTACA
GTGGTGGTCTCGGTCACCGTCACTGGGTGACGTTCATGCTGTTCCTGGGCATGGCGAACGCGTACATCATGAG
GACGAACATGTCGGTGGCGATCGTAGCTATGGTCAACCACACGGCGTTGCCTGTCGAC
AAGGAAGCTGTAGACACTGAATGTGGGAATGTTAATCCAAATGCAACTGATGCGTCGGCCGGTCAAGACGGGA
CCTTCGTGTGGAGTTCGACGCTACAAGGTTACATCCTATCCTCTTTTTTCTATGGTTACGTCATCACGCAAAT
TCCTTTCGGCATCCTGTCTAAGAGATATGGTGCCCGTCTTTTCCTTGGAATTGGCATG
TTGATCAACTCGGCATTCGGCCTACTAGTGCCCGTTGCTGCAGAAGCTGGCTATGAATGGCTGATCCTAGTGA
GATTTATACAGGGTCTGGGCGAAGGACCCATCGTGCCCTGCTCTCACGCTATGTTGGCCCAATGGATCCCACC
CAATGAAAGAAGTCGGATGGGTGGCGCCGTTTATGCGGGTGCTCAATTTGGTACGGGT
ATCTCTATGCCGCTCTCCGGTCTACTCTCT
>103780899_2 DT665578.3
GCACGAGGGTCGTTCCGTATTGGGTTAGTCTAGTCAGTGTGCGATCGTCACTCGTGGTGTTCTCACGTCAGTT
ACAAGCAAAATAAATAAAAATGCCCGAATACAAGATTTTCTACTTCCCCTTCAGAGCTCTTGCTGAAGGAGCT
CGTTTGTTATTGGCCTACGGTCAAGAAGGATTCGAAGATGTCAGAATTACTAACGAAG

AGTGGCCCTCATACAAACCAACAATGCCGTTCGGAACAGTCCCTGTCCTAGAGGTCGATGGTGTTAAGTACTC
ACAAAGTATCGCCATCGTCAGATACCTCGGTCGCAAATATGACCTGGCTGGGAAGACATTGGAAGAGGACCTC
GAGATCGACCAAAACATGTACTTCCTGAATGACATTCGTCTAGCCGCTGCTTCAGTTG
GTTACGCAACAGACGAAACTCTTAAGGCTAAACTGAAGGAGGAGCTTGAGGCCAACTTCTACCCTAATGCGTT
AAAGAAACTGAATGAAATTATCACCAAGAACAACGGACACTTAGCTTGTGGAAGGCTCACATGGGCAGACTTC
ATGTTTGCAGGCGTGTACGACCGCGTCAGGTTCTTACTTGGCATCCCCAACTTAGACG
AACAGTACCCAATCTTCAAACAATTACAGGCTAAAGTGCTAGCCATCCCACAGGTTAAGGCTTACAACGACAA
AGCACCTAAATCTTTGTGGTAAACTGTAACTATTGTACTTTAAGTTTAGGTTAAATGTATTAAATTATTAATT
T
>103780903_2 DV501424.2
GCACGAGGCGACTGTCATAACAGTGTGGTGCGATGTTATTATATTTTTATTAAATATATATTAAATAAGTATT
AAGATTCTATATTTTTAAAATGCCAAAAGTAAAGGTTGATAAAAAAACCAGAGTCCATAATGAAATTGCTAAA
CAAGGAATCCAATTTAATAAGGACTTTGGTCAACACATTTTAAAAAATCCATTAATTA
TTTACGTCATGCT
>103780907_2 DV501372.2
TCCCAGAGAGGATGATGACAAATCACAACCTATCCATGACACTGTCATCCAAACATGGGGTGTATATTTTAGT
GGTCTTAGATACATAGGGGCTAATTTAGCTTTAAATTTTACCTCGGATTGCTTCAAAAGCAATACTCTGGTGT
TCCAAATGCACGGTGGCTACTTTTGCTCGTATAAAAGTTTAAAACTAGACATAATTCC
GCCTGAAAATGAATTGG
>103780909_2 DT666580.3
GCACGAGGGTTTTCCAGGGATCTTTTAAACTGGCCGGTCATCCCATGTACTACGCGAGTGGCACTCACATACA
CAGCTTCCCCGAACACGGCGTGCTGGTCACCGCTCAGCTATCGGATCAGGGGCAACAAATTATGTCAGGCGAC
AAGCCGAATGGAGGTGGGGGTGCGTCGAACGGTAAAACGGTCGACGTGCCCATATTAG
GCCTGCTGCAGACGGACGGTGAAACTAGAGATTATACTAATGACACCAACGATAAGTTACCGAAGGCAGGTCG
GCTAGTCGTGTATGGAGACTCGTCCTGCCTTGAAGGTGGTGCAGCGAGACCTTGCCACTGGCTCCTATTAGCT
GCCCTACAATATGCCCTTGTAGGCCATATGCCCTCATCACTTAAAGAGGCGGCCGCTA
CTGCACACCATCGTGATGTTCATATTATACCATCTGATCTACCAAAACGCGCTGAAGGTGGTCGTCTCCACGC
GTACTCCCGAGTCCTGTCGCCAGACGGGAGTGGACCCCGCCCCATACCGGAGTGCGTGAAGACTCCAACCCTG
GAGCCGCGCCCTGTCCATGCCCCGCCATCAGCGCGCACGCTCGCACCAAGACATCAAC
CCACAGATCCTAAGAGTATAGGTGCACCAGACTTAGAAGGCACCGAAGCAGCGCCAAGAGCATGGCGTGGAGC
AGGCGCAGCGCCATCACGCTCCCATCCTAACACCGAACCTGCTACCTCAACGTTCTACAACCGACTGTTAACA
TTCACTGGCATAATCGCTATTATATATTGCGTAACAGCATTTT
>103780926_2 DT666496.3
GCACGAGGCCAACCCCTTAAGGGTATCCTGTCAAGGCGAGACCCCAGCGGACAAAGAGAACATAGGGCCCATA
AGATATCTACCCTACCCAGGATTTCCGGGATATTTCTATCCTTATAATAATGCCGAGGGATACTTAAGCCCAC
TAGTTGCTGTACATTTGTTAAGGCCTAGAACTGGTATAGTTATCAACATAGAATGTCG
AGCTTGGGCAAAAAATATTAAATACAATAGACGAGACGGTCTTGGCGTCGTCCATTTTGAGTTAATGCTTGAG
TAAAAAAGAAGAAGAAATGACCAACTTAAAATATACGGTGTCATTTAGAAGACCAGTAATAAGCAATATGAAT
TTTATAATCTACTGTAAAAGACTATTTTAAATTGAATCTCTTAAATTTTATTATATGT
ATTTTTATATTTGAAAGACGTAAGAAACATATTCTTGATATTTTCTTTATAAAATTATGATTAAAATATATTT
GTTACTGTACTGTCTTAGTATCCATAGGTTTTACCTTTTGCTAGTAAATCTTTGGATTAAGTCCAGAAGATTC
TCCTAGAATCTAATCTATCGTAATAGTCACTCATTTTGGTAAATCCTAAGACTTACTA
GTAAAATTTATCTTTTATATTTACCGATATGCAGGCTTTTACTGTTAGATGATTAAATTAAAACTGACAAGGT
CTTATAAGAAGACTAAATTAAGTAAATAATAGATTTTGAGCCATTATAGTTATTATTTTG
>103780932_2 DT665157.3
GCACGAGGGTTGACTGTATAGTTTTCAAGAACCTGTGCGTTTTTAGACGACACGTAAACGTCGTGAATATGAA
AATAAAGTACATGTGTAGGTACAATATTCTGTACCATGGATCCGTGAACATAATTTTTTAGCGTTATGGTGAA
TTAGAATGGTGCCTGTGGTTTGTCAAGCTCGCACGTCGACAGGTTACCCGTTTGAAAC
GTAAATATCATCTAGCGGGCGTTTTATATTTCAGATATGACAGCGTCGCAAAACAGTAGATAATGGAGAGCTC
AGAAGACGATGCGGGCGCCGCGACGAACGCTTCCCGCCGCGTATCGGCGCGGGAAAAGGACGTTGGACAAATT
GAGGACGTCAATGCAACTAGTAGTTTTCATCAGGATCCATCTGAACAGAGAACGTCTT
GTGAAACCCCTGCCATGTCGGAAGATAACTTCGACGGGGATAGTGTTCCGTCGCCGTCAACGCAAGATGTCAA
CACAAGCACGGAAGCGATTTTGGATATGATAGATGAGATCGTCGACGGGCCTGGAGCGCCGAAGCGATTACCT
TTAGCTTTAGAAAATGACACAGACAGTAGATTTGCTGGTGGGGGAGATTCCAATAAAA

TTGTAGATATTAAAAATGAAGATAAAGAGGAGTCGTTAAATATTTCCCATTCGGACCCTACCACAAGTGCCAA
AATTAATGAAGAACAAAGTTTACAAAAAAGTTTAACAAATATACTCAATAT
>103780947_2 DT666948.3
GCACGAGGTGGTGGCTGGTGTATTTTAGTCTCGTATTTTATTTGACCTTTTGAACTGAGATATCAAGAATATT
GTTACGAACACTCCTGCCAGATATCTCGTACAAATCACTGGAATATTCTTTCATTCATAAAAAACATGTCGAA
TGTGGTTGTAGTTAATACAAACCCCTACGAGGGCCAGAAGCCGGGTACGAGTGGTTTA
CGTAAAAAAGTTAAAGTTTTTCTCCAAGATAACTACACAGAAAACTTTATTCAAAGTATTTTGGACGCTAATA
AAGATGCTATTGTTGGCTCGACTCTCGTCGTCGGAGGTGATGGCAGATACTTGGTGAAAGAAGTTGTGGACAA
AATAATAAAAATATCAGCTGGAAACGGGGTGGCAAAACTTTTAGTGGGTCAGAATGGT
ATTCTTTCTACACCTGCAGTGTCACATATTATCAGAAAATATCAGACTCTAGGTGGGATCATACTCACAGCGT
CCCATAATCCAGGAGGCATCAACAATGATTTTGGTATGAAGTTTAACTGTAGTAATGGCGGCCCCGCACCCGA
TGCCATTACTAATGAAATCTACACAATGACTACTGTTATCAAACAGTACAAGATTGTG
CCGGACATCAAGTGTGATATAGACAAGATTGGCATTTTTAAATTTAAGGTAGAAGATCATGATTTTATGGTGG
AGGTGATAGATTCGGTGAAAGATTATGTAGATTATATGAAGGACATATTTGACTTTCCCAAGATCAAGTCTCT
TTTACAAGGATCTGAACAAAACAAGCCTTTCAAAGTTCTCATTGATTCAATGAATGGA
GTAACGGGACCATATGTGAAGCGTATCTT
>103780959_2 DV501380.2
GGTCGCCTATCCGGACTTGAAGTTTAGTGAGCCTTCAGAAGTGTTATTGTGGGTGATCCTAGTGTTAGTGATA
CTTGGACTCTTATTGCTAGTAATTTTAATATTTGTGTTGTGGAAACTTGGCTTCTTCAAAAGGAGTCGACCAG
ATCCGACCTTGTCAGGAAACCTGGAGAAGAATCATGAATCTAGCCCGTTCATAGTGCG
CGACAGAAATAGGATACGATGAGAACTATAGAAATCGAATTTTGTACATAAAATCAACTGTTTCGGTACTTTA
GGACGAATTAGTTTAAAATAT
>103780984_2 DT665422.3
GCACGAGGCAAAACTTAAAGAGTTAAATCTCTTGAAAAAAATCTTTGCAGGAAGAAAACATTAAAAATATTTT
ATATCATAAATTTACTTAAAATAAATATAAATATTTTTATATATATCATACCAAATCATCAAAGATGTTAGAA
CTGAAAGGAAACAGCTTGTCTAAAAGGCAAAAAGAATTAAACGTAAAAGTTAATGAAA
CAGTATTACAGAAAATTGATATAAATAAAATCAAGATTTTGGAGGAAAAGTCTGATGTAGATAGATTATTTAA
AATTGATGTGGCTTCAGAATATAAGGAAGTTGATTATATTATGGACACATTTAAAAGTGGTGATTCACTTTAC
ATTTCAAGAGCTTTAAAGTCAGTGTGGCTCTTCAATGATGAATATGCACATATAATTA
ACCCTGTACACTTACATGAAAATATTTTCCCATTAATGTCTGTAAAAATGACTAAAAAAACTGCTAACAACTT
TATCCATTCATTTAAGAAATGAACAGAGGGCACTCGACTTCTACAATTACTGTATTAAGAATAAATTTACTAA
TATTGCATTTAAATTTCTGCTGTTTACATCTGAGCAAAATAAACTGAATTATTTGAAA
AGCCAGAAAGACATTGATAATTCTATAGATATTGCCCATTTTATTGGAAACTCATTCCAGTTACTTGAAATAT
ATTTGCACCAGTTACATAATAAGAAATACTATGTCTTAACAGGTGTTCTACATAACAAGCTTCGTCATCTTTA
CTCAGTATCGAGTGAGAGATATTTAGATTTATTTGAAAACTACTGTAATATATCATGT
ACGGATGAATTTGGA
>103781020_2 DT666875.3
GCACGAGGTTTACCTTTTATGGACACCAGCGAGATAACATGTAATTTAATTTGAATGATTATTTTTATGAACC
ATTATCTACCTGTTTTTAAGATTAGTTTTTAATATTTTACTGAAGTAATTTTCAAACTATGAAGACATGCTCA
ATTTTTTATGGTATTTATGTTAAATGTGGGTTAAGCCCACGTGTTCCTCTGAAAATAA
CTGGGAGTTGTTTGGTAAATCACACATTCTAAGTGCCAGAGGTAACTGTGACAGCAGGTACACTGGGCTACAA
CGAAAAGTTTAAAGTGATGTGATATAGTCTGTAGCCTTCCTCTATAAATGGGCTATCCAAATCAAACAAAATC
TTCGGTTTTATAATTACAGACATATCTATAGGATGGAAGTGTCAGCGCTCCTTTTACG
AGAATAGCATATAGGGTAAAATATTTTAAACCCATACCTAAAAAAAATAATCAAGATACCTCAACTATTATAT
ATTTTGTTTACATACATAATTTTATTATTTATTTTGTTTAAAAAAATCACTTTTTCATTTTTGACAGAAAAAA
AAAATGTATAAGGCTTTTATAGAGTTAAAACTGTGGGTTTCGTTTAAAGTGGTGCTGA
CACTTCCAGCCTGTATATTAGCTTANAAGAGAAAAACGGAGGATTGTCCAAAATCTCTCTGTTATAACTGTAA
AGTGAATTACTTAGGCTTAGATTTACTAGTAATAGAATATATATTATATGCAATTATTAATGATAATATATGT
ATTTAGTTAATTAGTTTTTTTTATAAAATGTTTTTTTCTATGTTCAATTAAACATTTG
AACATAAAAAAAAACACATTCTGTATATATGAAATATGCTC
>103781028_2 DT665975.3
GAGAGACAATTTAGTCCTTACTTATCTATTGAGGATTTATAAAAGGGATTACAAAATGATACATTATTGGAGG
GTGTTTTGCGAATCAATCCTAGGCAATTTCAACATTCTTACGTTTCTAGTGCTGATAAAAGTGAACAAGATGT
TCTCATTGATGGGGTAAAAAATAGAAATCGTGCCTTGGAAGGAGATATTGTTATTATA

CAATTCATTGAGGATGATTCTGAAGAAAATAAAGCAAATGGTGAAGATAAACAAAAGAAAGGCAAAGTAGTTT
TTATAAAAGAGAAAGTTCATACTCGTTTATGTATTGGGAATTTAAAGTTAATGCCTGACAAAAGTAGAACAAG
AGCCCTGTTTGTGCCCAGGGATTACA
>103781042_2 DT666217.3
GAAGTATTGATTCGCCAAAACTTTTAATGCTATCGGGAGTGGGGCCTCAAGAAATTTTAGACAGACATCACAT
AAACGTGTTAGCTGATCTTCCCGTAAGCCAGAATCTACAGGATCACAGCGTTGTTCTATTAAATTTTATGGGG
CAAAAAGGCTTCCAAACAGCTATTCAAAATTTATTAGCGCCAACTGAATTGAACGCGT
ATCCTACACCAATACAATCTGGTTTGATAAGAGTAAATACGTCTTGTGACGTTTGTTACGGATCAAAACCACA
TATACAAATTACTAACATACTTATTGGCGCTACCGCGTCTCCTGCTATACTATATGGCTGTCAAAGTATTACC
AACTTTGATAGAGATTTATGCTTTTCATTTAGCAAAGCGAATATATATCGTGAAATTG
ATGCTACAAGCCTTGTTCTTCTTCATCCTTTATCAAGGGGACAGGTAACATTAAAAAACACCAATCCTTTCCA
CGATCCTATCATAGAATTGGGATATTTTCGAAACAAGCAGGATATTACCGTCCTTACTGAAGGGATTAAATAT
ATGCTAAATTTACTTTGGACGACACATTATAGAAATGTGGGTGCTGATGTAGTTAGGC
TAAATATAAAAGGTTGTGAAGGTTTGATCTTTGGTTCGGATGAATATTGGCGCTGTTACGTCAAGAACACGGT
GACGTCGCTTTTACATCCCGTGGGTACGTGTGCTATGGGTCCTAATGGTGTTGTTGATGAAAGATTACGTGTA
CATAACGTTAAAGGTCTAA
>103781046_2 DT666745.3
CGGCACGAGGGCACCGCGTCGTCCAGCGCCATCGCGCGCGCCGTCAGCGACGCCGCCGCCGGCGAGTACGCGT
CCGCCATCGAGACGCTCGTCACCGCCATCTCGCTCATCAAGCAGAGCAAGGTGGCTCATGACGACCGTTGCAA
GATCCTCATATCATCCCTTCAAGACACGCTTCACGGTGTAGAGACTAAGTCCTACGGT
GGAGAACGCCGACGTTCGCGTTCCCGGGAACGCGACGCGCGCGCACATCACCGCGCGCCGCGGCGGCGGGACC
GGTCTGCGTCCCGGTATAGAGATAGATCGCGTGACCGGGATGACTTGTTTAGGTATTATAGAGATTATCGTGA
GCGTGAACGTGATCGCTCCAGGTCAAGGGATCGGGAACGTACTGACCACTATAATAGA
GGACATAGTCGAGAAGAAAGGCCGCGCAAGTCGCCGGCGGAGGCGGCGCCCGAGGCGGGCGCGGGCGACGCGG
CGGGC
>103781070_2 DT665134.2
CAAAGTCTCTTTGAAAGACTAGTAGTGCTCGGTATTCGACCCTTCAGGCTGGAGGTTCTATATCGTATGCACC
CTGAATTGTCACGGTTTCCATCTGACTTCTTCTATGAGGGGTCATTACAAAATGGAGTCAGTGCGGAAGAACG
TCGCCTCCATAAAATTGATTTTCCGTGGCCGAGACCTGATCGTCCTATGTTCTTTTAT
GTTACTCAAGGACAAGAAGAAATTGCAGGGTCTGGAACGTCATATTTAAATAGAACAGAAGCAGCTAATGTTG
AAAAACTTATTTCTCGCTTCCTGAAGGCTGGAGTACGTCCTGAGCAGATAGGCATAATAACACCTTATGAGGG
ACAAAGGTCCTACTTAGTGCAGCACATGCAGTACCAGGGGAGCTTGCATGCTAAACTC
TATCACGAAATCGAAGTGGCGAGCGTCGATGCCTTTCACGGCCGAGAGAAAGATATTATAATAATGTCCTGCG
TGCGCTCCAACGAACACCAAGGCATCGGTTTCTTAAGCGACCCGCGGCGCCTCAACGTAGCGTTGACTCGCGC
CA
>103781085_2 DT665855.3
GCACGAGGGTCTAACGCTCTTCGCGTCTCGATCACCTCACGTGTTATCTTGATAAAAAACTGAAAATAAGCTC
ATATATTAAGTTTAATTTATTTTTACTTATTAATGTAGCAACTGCTAAACATCGAAACACCTTATTGACTTTT
GAATAAATAAATAATGCAGACAATTGTGACCGTTTGATTGTGTAATACAAACATATAA
ACCTACAAAAATATACATATTTAGTGATATCCACTGTGGCTCGTGTTTTGTAGCCAACTCGGAATCAAGACAA
GTGAAAGTTCCTTATAATATACTCTGTGGGACATTCGAAAAAAGTGAAAAGGTCTAAGACAATGGACCATCTT
TTTCGGCAACGTAGGATTAGCGTGCCTAAACGTACAAGTGAGCTGGTGGCTCTAGTGG
CCGTTTCATGCACGTTGTTCCTGTTTCTTCACACGAGGGACCTCAGCACAAAATTACAGCGGATGCAGCGCCT
TAACGATGACGTTACTGCCTTCAACCATCTTACCGACAAAATAGACACAGACAGAACAGAACACGCTTACGCG
TCGGCACAAGAATATAAAGAGGCTCTCGAGGCGCTACGAAGGAACACCGGCGTTAAAC
GCAGCAAACCCCAACGTACACTTGAAGCGGATGACAAATCCAATGATATATCAGAAGAGATATCAGAAGAAAT
TATTCCAGATCCATGGGAACTGAACAACACGGCGAGAGCTGGCACCGAGATTTTATTTTTCATCGCGTGCCAA
AAATCGGCAGCCAGACGTTCATGGA
>103781104_2 DT665686.3
GCACGAGGACTTATTCTTACACAAGAATGGCCTGCAACTGCTTGTAAAGAATGGAAAAAGAAAGACCACTCTC
ACAAATGTTCTATGCCAACAACTGTGGATAGTTGGACCATACATGGCATTTGGCCTACAAAACTAGGAACAAT
GGGACCTTCCTTCTGTAATAGAACATGGATGTTTGATCCAGAGAAAATTAGGCCAATT

GAAAATCAATTATCACAAGTATGGGCCAATATTTTTGGAGGTACACCAATTTATCAGCTATGGTCACACGAAT
GGTCCAAACATGGTACATGTGCTGCTCAACTCAAGCAATTCAATTCTGAATTTAATTATTTCTCAATGGGTAT
CAAGTGGTTGACAAAGTACTCAATGCTTAATATACTTAAATCTAGAAACATAATACCT
TCAAATGATATACAATACTCCATAACTGATATAAATGATGCAATAAAAGCTGAGCTTGGTGTAAATCCAGCCA
TTGAATGTAGGAAAGAAGATGGTGAAAATAACATATCAGAAATACGAATATGTTTTACAAAAGATTTGGAACT
AACAAATTGTGATGGTGTCCGTGCTAGACAAAGCTTTGGGGAAAGCTCCATCTTGACT
AACTGTAATCCGTCCAAAAACATTGTTTATCCACATTATAAAATTAATAGTATGTATGTACAATTATATAAAT
TAGTATCATGGTTACAATGGTTCACTTTATAAATTGACTTTATTTTTAAAACATATAAATATGAATTTCAAAA
CTTTTTGGTGCATAT
>103781117_2 DT665224.3
TTTTTACCAAACAATATATTATGTAGAGACGAAATGTGAACATAGTCGGTCTAGCACCTTTTTCAGACATGAT
AAGGCAATCATTAAAATTTCGAAAAACTCTTACCATCGTTTGTGTTTTATCTCTACGCAAGATAAAATAAATA
ATACTGATAATAGAAAACAAAACCAAAGAGGAAACGATGAAATTCAAGATATAACATA
TAGAGATTTTAACCAGATGCCATTTATAGATTATTACAACGTCATACAAAACAATTTAAACGATATGGATACA
AGTAAAATTGATGACAGTACAAAACAAGAACTTCAGGAAGCTAAAGCACGCATAGAACAATTGGAAAAGAAAA
TTGCCTTTTTAGAAGGAAGAATACCACAAAAATATCCTGATGTAAAATATCTCGGATA
TAAGGAACGAAAAAGAATATTGGTAACAGGAGGTGCTGGATTTGTAGGATCTCATCTTGTAGATGTTTTAATG
ATACAAGGCCATGAAGTTATTGTTGTTGATAACTTTTTTACTGGAAGAAAAAGAAATGTGGAACATTGGTTTG
GACATAGAAATTTTTGAAATGATTCACCATGAC
>103781121_2 DT666415.3
CTGCTGTTTTCTTTCTCATTTGCTGAACTTATTTTGATGTGATTCTCTATAATGTTGTGCGAAACAATGAGGC
ATAAGGTGTGCGAGAAGATGGTGAACTGCAAAGGGCTGGCGTTGATGCGGTCTTATCTCGATATATTGAATTC
GAGCGGCAGCGAGCCTCGGTCGCGTAGTGCGACCCCTGATTCATTGGACTCGTTGCCG
AGCGGCGGCTCCGGCAGCCCCCCCACCGAGTGGGCAGATAGCGCTACGCCACCAGATTTTGTTGACACGTACC
CGCCACACACGCACGCTTTTATTTCTCGCACGCCGTCCGCCCCGGCGCGGCGCGTGCGCCTCTCGCCGCCCGC
CCACTCGCCCCCGCGGGTGCAGGAGCTGTTCAAGCCGAACGACTTACATTGGGCAGAT
ATTGCTTCTAATACTGAGGGTATTGAGTTGCTAGGCTACCAAAGGTACGGGACAGTGCATCGGCCGCGAATAG
GCAAGGAACGTATCAATGGATCCATACTGAAAGACAACGACCCTTTTATGAAACATGCGCCTGTATCGCGAAC
AAAGAGCGCGGCGTCAGCGCAACAGCCGCTCAGCTTCGAACATAAGTTTAACTT
>103781123_2 DT666741.3
GCACGAGGGATTTGCATAGATTGGAAGTGAAGCAATAATTACATATACATATGTCTTTGTTATTTAGTCATGT
AAAGATAACAAAATCGTTCTAATTTTTGATTACAAGTATGGTGATAAGATTTTAATCGTATTAAAATTAAAAA
AAAACGAGATCATTAAAAAATAATATTTTAAAATTAACACAAATAATTCTAATTCTTA
ACATTTTGTTATAGATAGAGCTAAAGAATTGAATTTTATTCTATAACAATTAAAAATAGTCCATACATATATC
ATACATACAAAAGGATGTGTTGTCATGTTTGTGGATGAACAATGACTAGTTTTCATATTTTTATTTTATATTC
ATTATTTTATACTTGATTTTGCAAAGTTTACGGAAACGGAAATTTTGTAAATAGATCG
AAGTTGTTAAACGAACGAACACCACTTCCGTTCAAGAATTTTTACATACAGTCTGTTTGTTGTAATATGAAAT
ACCTCGTTTTAATAATATCTTCACTCACACGTTGCTTCCATTATTAACTATATATATTTTTAAAATTTTTGGT
AATTATAAATGAAATAATAATATTTTTTAATGCTAGCAACAAAATAAGTTGTAACAAA
CATATAAGAATTTCGTAGCTCGATGAGTGCGTGTGTATTAGTTGGTCAGAGTGCTTGGACGGTTTCCAAGAGG
CGCGGGTTCAAACCCCGCTGCCTTCAAATTTTTATGTTTTCTATATAGTATTTTTCTTTCTTGTGTGTGAAAT
AACACTCTAAAAATGTAAAAAATGTTTTAATTTTAAATTATATTAATAATAAAGTAAT
G
>103781137_2 DT665702.3
GCACGAGGGTTTTCTCTTTAAAGATCCACAAGCCACAAGTGTTATTACCACAAGCTGTATTTTGTCATTTTTT
AAATATACATTTTATAACCTCAGCGATTGTTGAGACATATAACGACTGTACTACGTACAATATCACTATTAAA
TAAAATTGTGGTTTTAGATAAAATCTTTTTAACGCTTCATCCTCATCTTAACCTATAA
GCAGAGAGTGATACGTTTTTACTTAAATTATATTTACGTTTCTTATTTTCATTGTCTATTTCAGAAATAAATC
TGACAGGTATATATAGATATTTATTGCTATATAAAACAAAGCTAATATCATTTGGCTTTACTTTTACTTTGAT
TTTAACTTTAACACATATATCTGGAACAAAGTGTTTATTGTTGTTGTTTCTTATGTTA
AGGCTTTTAGGCGTGCCAATTGAAGTATTTATTGTGATATAAACTTAAAATCCGACGTCTTTTACAGTCAATC
AATAGTGAATGGCAATCAATAAGGACATATAATACATCAGAGACGTTCCGGCATATTTTGCCATTCATTTATA
GTAGTCGTCCATGCTTTTACCAAATATGGACAAAAGCGGTTCTTGACAAAACAGCCCT

TATTTAGCCTAGAAACGTTGAGTCAGTCGCGTTCGTTCCAAACAGTCACTTCACGTGAACTATTGGTTGGTAA
TAATAAAAATAATGATTCATTGCTAGAGAATTTTTCATACAGATGTAGCTCATAGGTGTCGTATGATGTAAAT
TAGTTCACTTGAAGAATCGTTGGTCATTCTAATTATATTGTTTCCTGGTTCTTACATT
TA
>103781145_2 DT665614.3
GCACGAGGGCGCCCTCCTCGCACCTCGTCATCGAGTAGCCGTCACGTCACACCCCGACGTGACGTGCCGCGTG
CGTGACGCCCGCGTGACGCCCGCGTGACGCCCACGTGACGCTGAACTGATGTGTACAACTAATGTTATTCATA
TGTTTTTGTATAGTTCAATACTATGCTTACAATAATTATTGATTTGTGAACGTTACAG
ATAAATAATAATTTCTTAACCAAGTCATTGATGACAAAACTATGGAAACCAAAACCAATGATTATTGTAAAGT
CCCACCACTAGACACCGATTTATGACGATATATACGATTTAACACTATATAACGAATATTCGAATTTTGCCTC
ACCGATAAATAAATAGAACTTAAATACATATACATAGTAGAAGGTTCAAATATAGGGA
CGCTTTGATACACACGTAAAAGTGATTTTATAGCTTACTTAATAATGGAATTTTATTCATACAAATGAAAACT
AATATTTAGGCGATAAAATGCTAACAGTATTCAAAGTTTATTCGGCAATAATAAATAATTAATATTCAGCTCA
ATATTTCGATACATATATACATGGTGTTCGTGAGGCTATAAACATTTTTAACATTTAT
TAAATATATAGTAATTGACGTATGGATAATGTGATTTTTTGTATGTTTTCTAACGCCCGACTAATAACCCGCC
AAGTTTACACAGAGCAATTTCTTATGTGAAAATATATGCATTCCAGATATAATATCTCTTTTTTTTTAAATCT
TGGCTCTTTTATACGGATTACACGGATGTATAAAAAACAAAATGGCGCGACGTAATT
>103781149_2 DT665187.3
GCACGAGGTGAAGTTTAATTTTTGACTATAAAACAAATATTTTCATTACACTTCATATTAACGTTGATTTAAG
CTTAACTTAAAACAACATAATAAAAAAATATCTTATATAATAACAAAGTTTAGGTAGACCATGGAGGATTTAA
AGAAAGTTTACATTTCACCTCTCCCTGATTCTCCTTACGTGAAACCATTTAGATTTAA
TTACACACAAAATGGTAAAGAGAAGACTTGGGATTTACTTGAAGTTCACGATAGCGTTGCTATCGTAGTGTTC
AATGTTAGTAGAAAGGTTATGATATTCGTGAAGCAGTTTAGACCCGCAATTTATTACAATTGTATTCATCCTG
ATGACCGTCAAGAAGACATAATAGATACAAACAAATATCCAGCATCTCTAGGCATTGC
ACTTGAAATGTGTGCTGGTATTATTGATAAAAACAAGCCAGTGGAAGAAATAGCAAAAGAGGAGGTGTTAGAG
GAATGTGGATATGATGTGCCGCTTGAAAAGTTACATAAAATTACTTCTTATAGATCGGGTGTTGGTGTTCAGG
GAGCGCTACAAACATTCTTCTATTGTGAAGTTACAGATGATATGAAGACTGAACAAGG
CGGCGGCGTCGACGACGAAATAATAGAAGTGGTGGAGAAGACGATACCAGAAATAGAGGAAATGGTGAACTCC
CCCGGCCCCATACAGAGTCCTCCGAGCTGCTTGGTCGCACTGATGTGGTTCCTACATTACAAGGCCGATAAGT
ATAGAAAGTAAGTTATACTTAACATT
>103781170_2 DT666247.3
ATGAAGAAAAATCTTATTCTTTATTTCATTGGTTCCGCTATATCATCTAAAAACTTACAACTAAATATAAACT
TAACAATACACACAAATCAACATAACAAAGTATTCAGTGGGTAAAGAATCTCGTAAATATCGAGAATTTTTTG
ATACTTTATATATTTTAAAAAAATATTTATAAAACGAATGGTAAAAATCGATTGATTT
AATAATAAGTATGGCAGTCTTAATATTAATAACTATACTTGATCAATAAATTTAATAGACAACCACCTATTAT
TAATAGTATATGAGATGGAAACAAGTTGGAGACTTATAGTAACGGTAACTCGTCGATAATATTTCACGAAGCG
AGAGAATTTGCAATCACGAACGATCTAATTTATATACCCTCGTTATAGCTTCATTTAA
TAACAACATTTTCGATCCAATGGAGAATGAAATCTGATATTCTGGATTGTGACTGGCAAACGAGACGGATCGA
CTGTAAATTGGGATTCTGTAATTGCAGTTCACTTGTAAAGTTTGATAGTAAAAATTCGAAATTTGTTGATGCA
CTCGATACTTATGTGCTTAAACAACAACCTATTAAAGCTTTCCACTAGAACAAGGTTT
AGTGAAAATTAAAATTTTATTCAATTTTAATTTTCACTATAACAAATATCGTAACTAATTGTACCATAGAACA
CAGTCGCATCGCACGCACATTTCTTCGCAATGCTCTATATAT
>103781188_2 DV501438.2
TATGATGATGAAGACGTTCCCACGAACGCCTCATCGTACCAGATCGATGTTGAAGATGTTATTAGCATGATAT
ATAATTATTTCTAAGAAGATGCCTCTAAAGATCCCGAACTTCCTAACCTTCACAAAGTTTCTCATGCCCGTTT
TTTAGAAGAATGTCTAAGAGATCTGCCTGACAGTTATAGTTGCTTACAAGCAAGCCGA
CCATGGTTAGTATATTGGATTTTACATGGTCTTTGGACTTTAAAAGATATGCCTGATTC
>103781191_2 DT666499.3
GCACGAGATGCGATTAAAATAATAAAGAAAATGGTATCTATAAAACAAGTGTACACATCTGTAGCTTGCAACC
GTTGCCCTGAAATAGCAGACTGGAATAAACAGGGATTAATATGCTATGGCGCTTCAAATGCTGTAGTTATATA
TGATACGAATATAAAAAACTCCGATCCACTTACGGTGCTAAGCCATCACAAATCAAAA
ATAAACTCAGTAAAATGGCTACACAGACCTGATAATAGTTGTATAGAACTACTTTCTTGTTCAGCTGATAAGA
CTGCTGCAATATGGACTTTAGAAAATGATATTTGGAATGTTACAAGTGTTTTATCAGGACATGGAGATGGAGT
GACTTGTATCAGTGGATTATATATTGAAGATGATTTAGTAGTATATACTGGATCTATT

GATACTACTATGAAAATTTGGGAACGTAAAAATGGTACAACAACCCTTAAACAAACTATAGATCTACATTCCG
GCCTCTGCCTAACTCTTAAAGCCCATATTTTACCTATTGCCAAAAAACCCATACTTTTTTGTGCATTAGATGA
TCATAAAATACATCTTTTCACGGAAGAAAATGAATATCATAGAGTCCATACATTAGTT
GGTCATGAAGATTGGGTTCGAGGACTAGATGTTATAAGTATTGATAACACAACAATAATGCTAGCATCAGCAT
CTCAAGACACCTACATCAGGTTATGGAGGATCCAGCGTCATGAAGATGAGGTGGTCTCTGGGATCAAGGTGGA
GGAGAAAGTGTTTCAAGCTCACAGAGTAATTTCGTCGGTAAAACTTGATGCTGTTCTC
GCTGGACATGAAAG
>103781227_2 DT666721.3
GCACGAGGTCTTTTCCTTTATTAGTAAATATAACCAGCCAATTCGTACTTTATAATTTGTGAAGATTTTAAAT
AAATAAGCTATAAAATACATATCACAATTTAAAGTAGCAAAATCATCAGTTATTCACAATGAATTCAGCGACT
CCAGGTGTGCCTCAGGTGCCGCTGCTCGACGACGACACCATCGCGTTTGGTGAAGAGG
ATAATGCCAATAAACAATTTGTACATCCCTACATAGTGTTCTTCCACTTAATATTTAGAAGTGCAGCCATAGT
AGTCTACATACTGTGTGGATGGTTCTCAGACTCATTCATTGCAAGCTTTGTATTGGTCATTTTACTGTTATCT
GCGGATTTCTGGACTGTTAAGAATATTAGTGGGAGGCTACTAGTTGGTTTGAGGTGGT
GGAACTATGTGGATGATGATGGCAAGTCACACTGGGTGTTTGAATCTAAACAGAATCGAATCAACAGCAATGA
AAGCAGATTGTTCTGGATGGGTTTGATCCTGTGTCCTCTCATCTGGTCTGGCTTCTTCATTATATGTTTGTTT
GGACTTAAATTTAAGTGGATGCTATTAGTGTTAATAGCGTTAACACTGACAGGAGCGA
ACTTATATGGTTACGTTAAATGCAAATTTGGCGCCAAAGAGAACCTCCAATCAGCGACCACAGACTTCGTANA
GAAACAAATATTCCAAAATGCCTCATCGTTTATGTTCTCTCAGCCAGCCCCGCCCACAACTGGCAATACTGGC
GTTGTATAGACTAATTATTAATAATAAATTGTAATTGTAATATTAA
>103781262_2 DT666954.3
GCACGAGGGTCGACCTTGCGAACGTTGGGAACGGGCTACTTCAACAAGTGGGCTTCAACCGCGCATCGGGTAT
CCGGAAAATTGTGAAAATTATATGTGATTAGTGCGAACATAATGTAAAAGCATGTATAGATATAGATATAGAT
GTGCATTAACTGCTTGCAATTCCTTCATACATTATATAAGAAAAGAAATATGTTAACA
TACAAAAGATTTTATTGTAGTGAAAATCAAGACAAATAATTTTATAAATGAATAGAACTCTGTGAGAATGTGA
ACTTAGTGGAAACAAAGCCGACACATGCAGGAGCCAAGTTTAAGTGAACAATGACTTCAGAAAGTTTAAGTTA
GATGATAAGTGAAGTTGGAAAGGCAAGATGATGGGCAGGTACGACGGTCGGGTCCTCT
CCATTATGTTCAGCACTTTGCTACTGGGACGAGCTATTGCTGCTCGCGGCCACACAGAAGTCAGAGTGGAATT
ACAAATGGAGAGATGGGCGGCTCGCGGCGGATCTGTCAGGCTTCGCTGTGTTCATGATGTACCACCTCACTTA
CTCGATAAAGTTGTCTTCCTACGCCACGGCACCAAGATCTTCCAATATATAAGAGACA
GAAAACCACCATATAGAAATTTCACTACACCTGGCGCTGTACTTAATATTGCACTAGCAACAGAGAACTCTAT
AATCCTTCAAAACTTGGACTTTGCAGCCAGCGGGAGCTACTCGTGCGAAGTATCGCTTGACACACCAATTTAC
ACAA
>103781266_2 DT666408.3
CGCACGAGGGAAGGCCCCGTTGGAGGGGAACGAGGAGCTTAAGAAGGAACAGGAACGTATAACTGAAGAATAT
TCTAAAAAACTAGAGGAACTGGAACAAGAGAAGCACCTTCTACGACGACGGCTGGACACCGAGCAGGGGGAGT
ACGAGGCGAGGTTACTGGAGCTGCAGAACGACATCAAAGAGCTGACCACTAAGATAGG
CTCCAAGGATACATCTGTGAAACAACGTGATGAGGAAAAAGCCAGCCTTATCGCTGAACTGACAGCCCAGAAC
TCTCGTCTCACAGCCCAGCTGAAGGAGTCGTCGGCGACAGAGGCACTCCTCCTGGAACAGCTGGAAGGGCTGA
AAGATCAATGTTCTATGAGAAAAACCGATCTACAGGACCATGTCCAGAGCCTAAAATC
GCTTAAAGCAGAGCTAGCCTTGATGAGTGATAAGCGATCTGAACTAGAGCGGAGACTCTCCACCTCTCTCCGT
GATAAGGACAGCCTCCACCAACAGATAGAAGAGGCGAATGATAGGATCACTGCTCTGGAGAGACAGTTGAAGG
AACAGGAACATCTGTACCAGAACACACTAAAGGAGTTGGAACGCCTGCAAAGATCCCA
CGACACGCTAGCAGAACGAGTGGGCGTAGATACAGTGGAGACAACAGAAATAGCCCGTTCCCTGCACGCTGAG
ATGGAGTCGGAGCCAGAGGAAGACGAGAACTGGCTGAGGAATGAGGCTGTACAGGTCTTCAAGCAGTTGAGGA
CGCTGGCGTTGCAATTGAGTACCGGCCATG
>103781291_2 DT666530.3
GCACGAGGTTTAGACTCGTATTGTGAAAAATGATATTACATTCAAAATATTCAATATAATATACTAGTTCACT
TGCTTACATAAATAACTTTTAATTATACATTTTTATACAATAATGTTTCGAAACTTTATCTCGTTTACCATTT
TGCTATTTTTATCAAGTTATGTGGCACCAGAAGAAAGTGCCGTGTACGAATATGGACC
AGATAATTTTAAACAACAAATAGAAAATATGGACGGCAATTTTATCATGTTTTACGCGCCATGGTGCAGTCAT
TGTACAAAATTTCACCCTATTTGGTCGGAATTGGCAGAACTAGTAAATACGAAAGAGTCAAAATTTGCAATAG
CACAAGTTGATTGTACAATATATGCTAAATTGTGTCATGAAAATGACATAACAGGGTA

TCCAACACTTCTTTACTTTCATAAGAACTCATTTTCCCCTGTGGAATATAAGGGTGCCAGAGACCTACCATCA
TTGACACTTTTCCTGAGTGAAGTTTTTACTATGAAATCTGAGGGTCTGGAAGTGGAAGAAACTGAAGATGTGA
AAATTTACAGTGGTATGGCATATTTACATGATCAAAATATTGAAAAGTTTTTATCAAA
AGGACAACATTTTGTTATGTTTTATGCTCCATGGTGCAAAGCTTCTCAGAGATTAGCTCCTACATGGGCAGAC
TTGGCAGTACATTATGCTCACAATGAATATATTCAAATTGGTAAAGTCAATTGTTTGAACAGTGAAAACAGCT
GTAAAATTTTGATATAAGCAATTTCATATTTGCTTTGGGTGGTA
>103781307_2 DT666888.3
GCACGAGGCCGTCCCTCCATCTATGATCACTGTCACTGAGGAGGGGGAAACAACACCAATCCTAGAATGCCGG
GTCCGCTACCTGTCCTTCCTGGGCATCGGCCGCGACGTGCGGCGTTGCGCCTTCATAGTGCACACTCCGCACG
ACGCGTTCGTGGCGCACGCCTTCCACGCGGAGCCCAGCTCCGGGGCGCTCTGCAAGAC
TGTTGAGGCTGCTTGCAAGCTACGTTATCAAAAATGCCTGGACGCTCACGGCGGCTCAGTGGTAGCTGGTTCA
CTTGGCGGGTCAGTCCCAGGGTCAATGCTGGGTGCGGGCGCGGATGGACGCGCCTCTATAGGACAAGCTCTTA
AGTCCTTTGTGGGCTCGTTGACTGGACGACGCGCTTCTTGAGCGGCTTCAATGCTAGC
TCCCAGCGGTTGACATCGAGAATAACCGCAACACACCGAGCCGTTACCGTGACTGTGACAAAACCGTGACGTG
ACGTGAGCGCCACGTCACTGTCATAGTGACTGCAATCATGGTCACAATGTCACTTTGACGTCACCGCTTCATT
GTAACTGTGACGTGACAGCACCGTCACTGTGACAGTGACATGACGTCGCTGTGACGTG
ACATAATCGTTACTGTAAGCGAGCTTTTTGCCGCCTTTACTCGCGCACGCTTCGAGTATCGTTATTATATGAA
AAATGTATTGATGTTAATATTATTATAATATAAAGATTAGAGTGTACAGTCAGTAAGTCAAAATGTTCAGAAT
TGTGTTTACTGGTCAGTCATGACATTTCTTGGATGTCAGT
>103781323_2 DT666302.3
CGGACGAGGAGAATGTTTAATGGACCTGGACCTCAAAAGCTACTCAGTCATTATGTTAGATGAGGCCCATGAA
CGCACTATTCACACTGACGTCCTCTTTGGTCTTCTCAAACAAGCAGTTCAAAAAAGACCAGAACTTAAACTAA
TAGTCACCTCAGCTACCTTAGACGCTGTAAAATTCTCTCAATATTTCTTTGAAGCCCC
CATTTTCACTATCCCAGGAAGAACGTTTCCTGTAGAAGTCTTATACACTAAAGAACCAGAAACTGATTATTTA
GACGCGTCTCTGATAACAGTAATGCAAATCCATTTGAGAGAACCTCCAGGAGATATACTCCTCTTTCTAACTG
GTCAAGAGGAAATTGATACAGCATGTGAAATTTTATATGAAAGAATGAAGTCTTTGGG
GCCTGATGTACCAGAATTGATAATTTTACCTGTTTATTCTGCCTTGCCATCTGAAATGCAGACGCGGATATTT
GAACCTGCACCACCCGGCTCAAGGAAGGTCGTTATAGCCACTAATATAGCAGAGACGTCGTTGACTATTGATG
GTATATACTATGTAGTGGATCCCGGTTTTGTCAAGCAAAAGGTGTATAATTCAAAAAC
TGGTATGGACTCGTTGGTTGTTACGCCTATTTCACAAGCAGCCGCTAAACAACGCGCTGGTCGAGCTGGACGC
ACAGGACCGGGTAAATGCTATCGGCTGTACACAGAGCGGGCTTACCGGGACGAGATGCTGCCTACACCCGTGC
CGGAGATACAAAGGACTAATCTGGCTACTACAGTCCTCCAATTAAA
>103781337_2 DT666100.3
GCACGAGGGTGATAAAATGAAAGTAGATAGTAAGAACGTTGTGAAATGGTCATGTGTTTATTTATTTGGCCAG
CAGTGTTAAAGTGTGCCAATCCACATATTTCTCACTCCAAAGTTTGTCGGAAAATTAGACATAACTGAATATA
ACTTATAGTTATAACAATATAACACCTTAAAGTATCAAAAATTATAAATATATTGAAA
TTCCTTCTCTTGTGTCTACTTCATATACAATCAAATAAGGCAAAGATTTTATATGAGGATTTCAATATATTTA
TAATAGAATATTTTCTTTGTATACATTTTTTTATATTGATCGCGCCATGTAGTAACTTCTACGTAGTTAATAG
AACCAGTGCCATGGTAATAAGTATAATGTATAGTATTTTATAGTTGTACCTAGAATAA
ATTAAAATTAAATATATTATATTTCAAATGAATTTGTGCCATGTAAATAAGAATATAGAATAGTGCCTAAATA
TCAATACGGCAGTACAAAGAGCGTTGCCTTCGCTATACATACAGACATACATACATATAGAATTATATTAAAA
CATAAAATAATTATATATACATATATATTTCTATTATAAATAAATAAAGAATATTGAA
GTGTTGAATGAGTTTCGACATGCCATCTTGACTCATGTTGAGTCCAATTGTTGTAATTTAGAATAGATTAAAT
TTTTTGTATTTTTATATTGTTAAATGTAATATTGTTTATGAGACGATGTATTTATTAATTTTAAGTTGCTGTT
CAATTTTTTTG
>103781341_2 DT666770.3
GCACGAGGATCGCACGTGATGCTACAAATATTTGGTGCCCGATAATTTGTTGCATGTTACGACATACAACTCA
ACAGACTAGATGTTTCTACTATGCGTTGTTTGCTTCTACGCCTGGGTTCCGCGCTTGCGAAACTTTTTTGCAT
TGAGTATCGTTCCAGGGTATTTCTATAGTCAGATTTATCTCTCGTCCGTAATCCCTTT
TAAATCAGTTTTTTTTCTATGGGATACTTTCCCAAAATGCGTATTTAACGATATCTTCTTTACCAACCATAGA
TATAGCATGTAAATTAAATTTAAAATTGATATTGTGATTTAATAATAACCTATCTAAACGAGAACGTACAAAT
AATAATAGGGCGTATCCGTAATGTTATAAATAACGAAGTAAAACCCTATAAGAAATAT

ATTAATATTTTTATACCCTAAACGTCATAATCGAAGTTAAACTACTGACCAATTTCGTTAACGAATCATTGAA
ATAAACGTTTGGTATTGTTCATAATAGTGAGATGAAGCTTTACAATAACCAAAACACGTAGCGATATAACATT
AAAGTTCATTCCATTTAGTTTGTTACGAGACGGGCCCTCGGCGGCGTCCGGAACACCT
CTCCGTAAGTACAAGTGATGGCCTTGGTAGCTACACGACAGCGTTCAAACACATAGTTGACAGTTAGTGAAGT
CTACGTAACTTATTGAANATATTGGAAGCATATTTATTGGATTGTTAGAAGAGCATTAAATGCCGTCACATAG
TTTTTGACGTCTCGATGTGGACGAAATGTACCCAAAGAAGGCTATATTTTTTACAGTT
TATATAG
>103781347_2 DT666597.3
GCACGAGGATCACAGCCTCCGTGATGGAGATCTTCCACTTCCCAGTGTTCGTGGCGCGCAACAACCTGCCTGC
TATCTGCATACTTATACTGCTATACGGGTTCGCCTGCGGTTCTCTAATCCACGTCGCAGAGAAGGCCTTCAAT
GAGGCGTCCCTCGCCAACATGCTGCTGTTCTGCGGCAACGCCTTCGCGGGGCTCGCCG
GCATCACGCTGCTGCTCGTGCTGGACGTCATATCGGACTCTGATGAGACCGACTACGCGCGCTGGGTCCTCCA
CAAGATCCTGCTCCTCATCCCGCAGTTCGCGCTCGGGGATGGACTATTGGAAATTGCTAAGAACACTATACAG
GCCGAGGTGCTCAGTCAATTCGGCATGGACACGTACCGCGACCCGCTATCAGAGAAAG
TGGTGGGCTATCACTACATAGCGCTGCTCGCGGTCGGCATCTTGCTCTTCGTCGTTAATCTCGCGCTGGAGTA
TGATTGCTTTGATTTTGTGATCACTAAACTTGGCCATAAAGAAACAATGAAAGCCGTCGCGGCGGTGGCGGAG
GACGTGCGCGCGGAGGAGCGGCGCGTGCTGGCGGCCGTGCAGCCCGTGCGGCTGTGCA
CCATCGGGAATATCAACGCAGGCTTTATAGACACAGAAGACATCATAAAGAACGGTTCGCTAAAACGTTCAAG
TTTCAACAAGACAGACAGTCTTCCTTTTAGAGGAGACGTATGTGC
>103781364_2 DT665730.3
GCACGAGGGAACAACCCTATAAGGAGAAACTTTCCAGCTCAACAAGTTTCAAGCATTTTTAGAAGCATAACAT
TTTTGCGCCGCATGATATAACCTCAAAAATCCTCATGGCGAAGTTACCTGTGGAAGAATCTATCAAACCTGTT
ATTACAAAAGCAAAAAACAAAGGGAAAGCAAAAAATAAATTAAATGATGTAAAAGTCG
AAGCTATAAAATCGGCTGTTTTGCCTGAGAAAAATGGAGCTAAAGAAAAGAAAACTGCTTCACCAGTTATAAA
AGTAGAAGTACAAGATATAAAGATAAAAAAGGAACCGAAATCGCCTAAAGAGGAACCAAGTGCACCTTCAAAG
GTGACTAAGAAAAAGAATAAAAAACCATTCAAAGTTTCGGCAAGTTCAGCAGTTAAGG
CAGAAGCAGAAAGTGATAAAATGAAAAAAGAAAATTCATCACCAGTGAAAAAAGGAAATAGGGCCCCACCAAG
TGTGACATCAGAATTTGCAGAAAAATATCCAATATTTAAAAATGAAGATTTTGTGGCGAAATTTCTCAGTGTC
TCATTGACTAATAATCAAAAAGGTAGGATTCGACAAACATTTAGAGATAATTTTAAAG
GCACTTCCGAAGACGGTCTACCAGAACTCATTCACGATAAAATAAAATCTCTAGCGAACGACAAGGAAAACTT
ACAAGATGGCAGTTTAAGAAAAATAAGAATATTGTATAACATGTTGAGGCAGACAATGGAAAAGGCAACCAAC
GATTTAACTGAAGTTACAGAGAAAAAAAATAAGAAC
>103781370_2 DT665088.3
AAACGATATATTTAAACCTAGTGACTTAAAACATTAATATATTATACTATAAATTTAAATGTGCCAAAAGATC
AGTGAAAATATGGTTGGTGATATGTGAAATGTTGCGAATTATTCCCTCAAGTTTATTCCAAGTGGTTTAAGTG
CTGAGTGCTTGGATCCAAAGCTTTCAGAAGAGCATCAAAGGCGAGGTCACAATGCTGA
TATTGCTGTTATTCTTGCCTTTAGTCTTGCCGCAGCAGTCGTGGGTGCCATGTTCAGAATTAAACGATGACTT
AAGATATCCGTGCAAATGTCGTGTTCAAGTAGACCGAGCACTGCAGCTTCGCATCCTCATGAACTGCGATAGG
GTGGTCTTCCCTGGGGACTTCCCTCCACTGCCGTATGGAGCTCCCATAGTGTCATTTA
GCCAGCAATGGGCTGGTCAGCAGTCTCTTCCTACACAGATCTTTTCTTCATACGGCCTTCCATTAAAAGAACT
AGATTTTTCTCACAATAGTCTTCGTCGGTTGCCCGATAGACTCTTAGCAGGAATTAGGGGCAATATCACGAAA
CTAGTCCTAGCAGATAACTTATTGGGGGATAACCTGAATCCAATAAGTTCAACTGCGG
AAATTCCCAACTTACCAGCTTTAGAGGATTTAGATTTGAGTGGAAACAATATTCGAGGTTTGGAAAAAGGAC
>103781374_2 DT666592.3
GCACGAGGCTACTGTCTTTTCGAAGCTTCGAGTTTCCAAAAATGTATAAGACCAAATATATGCGATTAATTCA
GTTAAAGAATTTAATTTAAAATGTATTTGTTCAGTTCGTGAGCGAGTTATGGGCATCGTATTTTGTGTGGTCG
GAGGTGAAAAAGGGGGTAAAGGGTGTATAGAAGGAGACAAAGTATCCTTCTAAATATT
ATTAGTTTTGTTTAGTTCCGACTCGGTATCACTCCCCGTGATTTACTTGTATTTGCTACGCTCGTGCGAGCTC
TTAATTTTTTTTGTACACATCTTTTAAATTAATATAAAAAAATATACAAAAATACAGCAAAAAAATGTAGGCT
GTTAAGTGTTTTACGGTAAGTAGGTACGTAGTTATCTACGACTCGGAGTTAGTAATAC
GCCCGTTTAATAGTTTATGTTGTTCGACCCTCGCACCGAACGATGTTTGTAGGTGATTGTATAATCTATACTA
ATATTATAAAACTAAAGAAATGGTTTGCTTGAACGCGCTAACCTCAAGAACTATTGGTTCAATTTGAACAAAT
ATTTTTGTGTTGGATAGTTTATTTATTGAGGAAGACTATATAATAGGCTATTTCATTA

AGTTAATCGTTTCACTGTACCCCATAAGATTCTGCGTCGTCCTGTCCCTGTATGAATTTTGGGAGATCCTCGT
TTACTCTTCACCATAATCTCAAGGAACCAATATGGCAAATTTT
>103781377_2 DV501411.2
TGGCTCCCGTTTTAAGTGAAATATATTTTCCAGGATAATATTTAAAAATGGAATGTTTCTATGGAAGTCCTAA
CGATCACCATAACAAAAAAAAATGGTTACCGGCTCTACAGTTACCTACTAATCCCCCTATCCGGTTTAATTAT
TATATCTATGGACTGGACCTCCTAAAATACCACTATCATAATCCCCACCAGTGCTTGG
GAACTAA
>103781399_2 DT666876.3
GCACGAGGATTTCCCCTTTCCAAAATACAATGCACGCGTGGTTACCAATATAAAAACAATATTGAATTTAAAT
CACCGGTGGATTGTTATTGACCGTGATGGAACTGTGATAGAGTGATAGTGAAACTGTTGTGTTATTTTAGTGG
CGAATAGATTTTAATTTACGATGGCCGTGGTTAATTTTAGCGTATGTTTAGTTCTCGT
CCTGTACCAGCTCTCAACTGTTTTCAGTGATGAAGCGTCCTCGGAATCGTCAGATCTTGGATTTATTGATCCT
AAACAAGAAGGGGATGGAGAAGGTGTCAGGTGCTACTGCATTTCATCACATTGTATAAGCACAGGGTACATGT
GCAGGGCGCGTAAAGGAGGCGGCTGCTATAGCGAACTTCCCCAAAGAAGACACCACAC
GAGACACGGTTGCTTACATCATTTGTCTGAAATTGAAAAAGAGGCCTTGTCTCACTGCCAGAACGCGCCGAGC
CAAGGCGGACCGCCTCCCCGTGAACACACTCTACTACTGTGCTGCTTTAAAGACTTGTGTAACCACGAGGACA
GCCCGCTGGCGAAGGCGAGGCTCAATCTAACAAATGATTTAGAAGGCAGTGTAGCAAG
TGAACGTGGCGCATCATATAACAGTGAAGTGTGGTTCAAAGCTGCCACCATAGCGGTGCCGGTATGTGGAGCT
CTCATACTGTTCCTTCTGGTAGCTGTGGCTGTACGGCTATTACGAGCTGATGCCTTGCTA
>103781404_2 DT665546.3
GCACGAGGCCTCGTGCCGTGGCGAATAGCCCGCATTATTCCTATACCAAAATCCAAAGATCCAAAATCCTTTT
CCCAATACCGTCCAATCTCAATACTTCCCTTCCTGTCTAAGGTCTTGGAACGTATTGTCTCGGAACAATTAAA
TCAATACCTTTTAGAAAACCGTATTTTGTCTCAGTTCCAATCTGGTTTTCGAAGTGGA
CATAGCACAGTTACTGCTTTAATCAAAGTATGCGATGATATACGGTTTAATATGGAGAAGCAAAATTTAACTA
TTATTGCATTGCTTGATTTCAGTAACGCTTTTAATACGGTTGACACTGATTTATTGATTGCGGTCTTACAATC
AATAAACATCTCCTCTGAAGCTACTGAATGGTTCCGAAACTATCTATCTAACCGGCGA
CAGTACGTACAAGCCAATGACAAATCATCTTCCTCATGCTCACTGTCCGCGGGTGTTCCCCAAGGCGGTGTCC
TTTCCCCACTGCTGTTCTCTATTTTTATTAATACTGTTTCCCAAACCCTATCCTCTTCCTTCCATCTATATGC
TGACGATTTACAAATTTACTTAGCGGCGCTGTCCAGTGATATGGATAAAACCATTTGT
AAATTTAACGAGGATTTACATAATATTTCTGTGTGGAGTAAATCTTATGGACTTACTATTAACCCTAGCAAGT
CCCAGGTAAGCATTATAGGCAGCAGTAAGCAATTAAAAAAACTTGATTTGAAAAGACTTCCTTCAATACAGCT
TAGTGGTGTCGATTTAAGTCTTAGTGCGTCTAT
>103781406_2 DT666763.3
GCACGAGGATTTTACGCGCTAATAATAAACTGTATTTATCTTAAAAAGTCCTTATTTTTACACACAGTTAAGC
CAATAATTAATCAGAGTTCTTTATAAAATCTTAAAACAATATTAAAGATGATTGCAGATAGCATTATTGGTGA
TGGAGCTAAACAAGAGAAAAAAGTTCCTGCACTTCCTGTGAATCATCGCTTTCTTGAG
CCACAGAAAGCTGTAAAAACAATAACTGATATGGCTATTTGGGAAAAGTCTGAGGCTTATATGGAATATACAG
GTTTTATTGCTACTTTAAATGAATCAATTAAAGGCAAACCCCTTTCAGTTGATTGCAAGATCTCGAGTAATGT
GAACAAAATAGTTGATCTACTTCATAAAATGGAGAAAATAATTGATGAATTTCCACCT
GTAGAACAACCACAAAGATTTGGGAATTCTGCATTTCAAGACTGGTTAGCTAAAATAAAACAGAATTCTACAC
AGTTTCTCCAAGAAGCATTAGACATTCAATTGCATTTAGCTATTGCAGAAATAAAAGTGTATTTAGTAGAGAG
TTTTGGTAATGCAACAAGGATTGATTATGGCACAGGTCATGAAATGTCTTTTATTATG
TTTCTTTGCTGTCTTTATAAAATTGGATATTTGAATACTGAAGATAATGTAGCTACTGTATTTTTAATATTTA
ATAAATATCTTCAAATTGCAAGAAAGCTACAACAGACTTATAGAATGGAGCCAGCCGGAAGCCATGGTGTTTG
GGAGTTGGATGACTATCAATTTGTTCCATTTATTTGGGGTAGTTCTCAACTTATAGAT
CAACCAGAATATATCCTCCTGGT
>103781418_2 DT665698.3
GCACGAGGATGATACCCTAGAAAGAGATGCGAGCGAAGAGGCGTGAGGAGCGCCGTCGCGCCCACTCAGGTTC
TCGCAGCCTGTCTCGCTCGCGCACGCCTCGTTCGCCTCGCTCGCCGCGCTCGCCTCGCTCTCAGCGTTCGCCT
CGCTCGCAGCGTTCGCCGCGCTCGCAGCGCTCACCTCGCTCGCCGCGTTCGCCGCGAG
CGCGCCCGCGCAGTCGCGGCTCGAGCAGAATTAAATATTCTAGATCGCGGTCAGCGTCGCGCGGTCGATTGCG
GCGCTCCCCGTGGTCTCCTCGGCGCCGACGTACTCGCGAAAGGTTTTTGTCGTTGACACGGTCCCCGTCCCCG
CGGCGCCGGCACCGCTCGCCCCTGCGCTATCGCTCGCCGCTTCGCTCGCCGCCGCGCT
CGCCTCGCTCGCCGCCTCGCTCGCCGGCGCGCTCGTCGCCGCGCCCGCTCATG

>103781432_2 DT666574.3
GCACGAGGATTTGCCTTGCCTTCTTGAAAATAAGAATGGTATGAATGTCGTCGAGGAAGTCGAAGGAGGTTCT
TAATTCGATGTCTATTTTTTTATGATTTGGCCCAGTTCTAACAATTCATACATGATAAAATCAACATGTATCT
TGAATTTGCATTAAGTATCTGCGCGACACAGTGTAACTGATATCCCAAACTCCTTTGA
AAATATTATTTAATGAATAATGCCATGGCTCGTGATGTGTCTAGTGGTTAGAACCGTTTATAGTTGTGATCGT
GAAGAACCCAAAATCTTTTAGAGATTCAGAGGTAACAGTGTTAAGAATTTTATATTATGCAATTAGAAGGTAT
CGCTTCAATAAGATATTTGCCATCAGAAGTTGTTCTTGTGTCAGTTATTTAGATAACT
GGAAACTCGACAATATGACATTGTCATAACTCGAAGCTCGTCCGAGGAAAAGCCAAGAGCGCGTCCTATATTC
CTCTCCACTATAACTGCGAACTAGTTATAATAAAAGGAAGAAAGAGTACGGGCACCGCCCCGCCCCTACACCG
TGAGTTCCTACCCGACTGCGGCTGCAACAGTCTTATTGAACTCTCCGGGACGTTTGTG
TGTGATCTTTCTGAAGGTACTTGTTACGCTTGTTTATATTACGATTGTATAAGAATAGTGATTTCTGATGTTG
ATTGATTTGTGAGATGAATATAAGTCGTTGGAACGAACGCACTACCGCTAGACGGCCGATATGTTAATAAACG
CACGGCACACACCCCACAGTCGTGTCTCGAACCGTAGGCGGACACTACTACATA
>103781434_2 DT666401.3
GCACGAGGACGTGGCTTTCGTTATTTTAACATACGAAATACGTTTCGGGCGTGTTTTTGCCGGGTATTCTAAT
GAAAAATTGATGAATTTGCGGTCCTAACGATCCATAGGTTGGTGTAGACTGTGTTCTAATTAAACATTGTGAC
AGGATGTACGCTACATGAGCGATTAAAAATATCAGTATGAAAGCCGGATAGTGAAGTG
CAAAACTTATATCGATAAAATGGCGAGGCTTATATTAATGTTGCTGCCGCTGGCCGCGGCGGCACTTTCTTCT
GACACAGACACCTGTAACCAGGACAAAATGACCGTATACATTTTGGTTTTGCATACCTATTGGACCAGGGAGA
AATTTCCTAAACATTACCCGGATTGGAGGCCGCCGGCGCAGTGGTCGAGGATATATGG
TGTATCACACAGTCGCTCCTACGTTTTGTTCCGGTTGGGTCGTCGAGTATCAGCGCCGGTTAGACAATTTGCA
GAATCTGGTAGATCGGACTCTTTAGGCTCCAGTTCAGGTACAATGGACGTGTTCGGAGCACCAGCTATTGGAC
AAGGTGCCGGGCGGACGGAGGCTGAATTCTTTGTAGACGGAAATCATTCTAGAGTATC
TGTGATGGCACGAATGATTCCTTCTCCAGACTGGTTCATCGGAGTGGACAGCTTTGATCTCTGCGTCGACGGC
AACTGG
>103781441_2 DT666822.3
GCACGAGGCTTTTTCATTGCAATATCATGAATGCTTGAAGAATAAAGGCCACATAAACTACCTCCAACAGTCC
GGGCCTATGACCAACGAGCGTCGTGAACTGGAAGCGTGCCTGCGAGTGCAAATAGATGAAATGGAGAGGAAAT
TGAATGTTTTGGTTGCCCAAATAAATCAATCTCAAATGGAACTAGTGGACCACATGAA
GGAGAACATCGCCAATCTGCGCCAGCTGCAAAGCCAAGTGTTAGACGAAGAACTCATCAAGTGGAAGCGCGAA
CAGCAACTGAGTGGAAACGGAGTAATAATGCACTCCAACCTCAATACGATACAGGAGTGGTGTGAACTGCTCG
CTGATCTAATATGGAGTACGAGACAGCAGGTGGGCAATGTTGCCCGCATCAACGGGAA
GACGATAGCTGAGCTCCGGCAGCCTCACTTGACTGAGATGCTGGATGATATGGGGAAACAGGTGACAAGTCTC
CTGTCAACGTTGGTGACGTCAACGTTTGTGATAGAGAAGCAGCCGCCTCAAGTTATGAAGACCAATACACGTT
TCACGGCTACGGTCCGCCTCCTTGTGGGTGGTCAACTTAATGTGCATATGACGCCGCC
ACGGGTCACTGTGGTTATAATATCTGAGCAACAAGCTCAACTCCTGTTAAAAAGTGACACACAAGGTCGGGGC
AAGCAGCCGGTGGAGTGTGGCGACATATTGAACAACAGCGGGTGCATGGAGTACCAGCCGACCAGCAGGCAGC
TCAGTGTTAGCTTCCGTAACATGCAATTACGTAAAATCA
>103781443_2 DT666500.3
GCACGAGGATTTTGCTAAATATTTTAGTTCATTAAAAATCTTTTAATACTAGTATAATTTCAGCAGTAATCTT
TGCAAAATGGATTGGAATTTGGAATACGGTGAACTAGCCAACGATCAGGATTTTGTAAACAAATGTGAGGATT
TAAAGGAAAATCTGATGGAGCTACAAAAGGTTTTGGACCGGCTGGTGCCGCTGCGGGC
GCACTACGACAAGATGTCCCTGCCGGCACAGATCGAATTAGATATATTTCTAGCTTTCACACTGAATTCATTA
TACTGGGTCCAGTTGCGAATTAAAGGCGTGGATCCGACAAATCATCCACTTAAAAACGAACTCTTGCGAATTA
AGGCGGTTATGGTCAAGTGGCAGGAAGTTAAGGATCGCGACTTGAGGCCCACTATGAA
CATGGAAGCGGTGAAGAGATTTGTTCGAAGCGGTCTCTACGATCCATACAAAAACATGGGGCAGCCGAAGGCC
AAGAAAACCAAATTTAAGGATGACAGTGAATAAACTTTCTTAATTTTTATCTTAAGTACTAAAGACTGAATTT
TATGAGTAATCTGTTGTTTTGTTTATTTGAATGTAATCTACATGTTGTTGTGTTTGTT
CTTTACGGGTGCAAAATCATCATAAATGAAATATTTAATTATAGTTTCCAAATTAAAATAGGATATTGCATGT
TATATTGTAATAAGATATATTTAAGTTAACATATATTCATTCATCTTTATTTTAAGTACTAGATTTGTCTTTT
A
>103781455_2 DT665857.3

GCACGAGGATTTGACCTTTGTGTAATACAGCTTTATAGTGTAAGACTCAGGACCATATGCGTAACTAGTGTTG
CACCGATCACGTCTTGTGCAAGCATTTATTCGTGCATTACATTTAAGCACTAGCTTGTAGCGGCTTTAATTAT
CTGTGGCTTGTCATTTTATTGAATTGGCGATATTTGTTTTTTTTTGTTATTTTCAAAA
ATGTTGCCAGTGTATTGTTTTATTATTTTGTAATGCTAGCAAAGATTGAATTAAATTTTTAATATGTTATAAG
TTTAATATATTTTTTTTCATTATTGATTTTATTTTTTGCTGTGAAAGTATCCAGCTTTTGGAACATATCATAA
CATCTTTGCGCAATGCTTGCACTTTATAATGGAATAGTCAATAGATAAGGTTTTAATG
AATTTATTGCCATAACTATAATTTGAAGAAAATCGGAAATGAGAACCAGTTACAAGTATTTTATCATACAATG
TACAGAATAAATTATTTTTGTTTGTGTGTTTATTTTGTTAACGCATATCTATATTGATTAGTTTTAGTGTTAG
AAAATAATACGTAGTTTTGTAAATACAATATCACTATATATTACTTTAGGTGCTAATT
TCCTGTTACCTACTTACTGTCCTATTTAAATTTCTGCCTTCGTCTTTAGTTTTTATTCACAAATAATCTAATT
ATATAACATTATAAAGCCTTAATATAATATAAATA
>103781459_2 DT665369.3
GCACGAGGGCCCTCCTTTAAGCACGTGTATCGCATCCTACTATAAACACAATACGTGAAGCAACTCATTTGTA
ATGGCAATTAATGATTGTCAAAGCTCTCGGTAGATAACAAAAACGTTATAAGCAATCTTCCAAATGATGTGAA
TCCTACTCTATGTGATGGACATTTGATCCTATTTTAACATCTAATTGTTGGCACCACA
AGATACCAGAATTTTCTTTGGATGGCAACAGCAGCTTAAAAGCAAACGGAAGTGCCAAAGATCTGGCCGCCGT
AATGGACAACCTAAAGAAAAACAGCCTTCTTGGCCGGCTTATCTATACCCTTAACACTCAAGTCACAAGCTCG
GTAAAACAACAGATGGTGTCTAGCACAACATCCAGCATTGCCGTCACGAGCCAGAGGG
TCCAAAGCTCATCACAGCGCGCTAGCTCTATGAAGTCTGACCTTACAGAACTTAAAAGTTCTATTTCCGAAAT
GAAAACACTCAGCAATTCTGCCGCCTTGAATTTTGAACAAAGATTAAAAAGCTCGATGGAGAATATAGTATAC
CAAGATGACATATAAGAGCGTCACATTCCTGATATCAGGGATCTCAGTGACATGGGGG
ATATCGGTGACATGAGTGATATGGGTGATCTGTCTGGAGATGGACCTTTAGTGACTTTTCCCGAATCAGATAC
CCCGCCGCCTGACAAAATTGCATACTCGCTAGTAACA
>103781469_2 DT666221.3
TCGTCGCGCTCGAGCCCGCGCCCGCCGCCGCCTACGTGCTGCGGCTGCTCAGCAACGAGGGGTGCCATGAGAG
GGAGGACTGCGAGATATTGCCGGAATTAGTCCCAGCACTAAACAAACACGAGTGGGTGCTGAAGGCGACGCGC
GTGCTGTGCGCGCGCAGCATGCGTCTGCGTGCGTCCGAACGCGCCGTCGTGCATAATG
CGCGTCTCATTGGACCCTTCCACCAGGGCGAGGCTTTCTCTTTGGAAGATTTTGTGTTGCTTGATAGATACAG
CAATCAAGTATATGGAGATAAACTTTCAGAGTTGCTCAATAAGAAAAAGACTGTTACTAATGATATTTTAGAT
GATGATGATGATGAAGCCGAGTTATCTACTGAAAAGTATCTTAAAGTGATATCAGTAC
TAGCAGCACGTAGTCCTCGCGCCCGCACTCCTTTGCCCTCGGGCCTGCGTACCGAACACTCTGTTGTAGACCT
ACCCCCGATATATAAAGATGAAGCTGCTGTTGAGATAGTAGCGGTAGTGGACCCGGCGTCGACGGCGGCGCAG
CGTTTACCGCCGTTACTGTTAGTGTTGCGACGCGTCGTCAACTGTCGGCTGCGGCTCT
TCCTCAACCCACA
>103781473_2 DT665980.3
GCCGAGGATTTGCAAAATATATTTTTCAAAATAATACTATTTGCACCCCTGTCGACCCTTTGTGCTACAAGAG
AGATGGTAAAAAAAAATGAAAGTTTTATAACTGTGACTATTTAACAAAGTGGAGGAATCAATTCATCATGTTA
CGGTTTGCATAATGAACATTGCTCTGCTTATTGGGAAAAGAAAACGAGAGCATTTGTG
TTCAATTAATCTATCGTGCTCTCGGGGGGGCTTCAAAATTCGGGATGTAACGTGAAGAGTCACTAATTTGGTC
ACATGCTTTAATTAAACACTTTTCGAAGACTAATGATTGCCTGCCGATACTGAGAATATTTATTTAGTTTATT
TTTTAGTTGGTGCACACTTTGATTTTAAAAATTAAATAAAAAAACCTTGAAAGCTTCC
TTCAGTTTACTGTTTGATTATGTAAATTCTTCACTATATTCTAATTCGCTTTAATATTATTTTATTCTGTAAA
ATTGATTGTCAAAAACCTGTATATCTGTGATATTAGTTTCATCAAACATGATATATGTTGTGATTATAACATT
CTAAACAAGATGCGTGAATTTCGTATAAGGAAGAATCTTATAAACATTGCAGTTATGA
TATACAATAATTAAAATGCATTTTTGAATTTTAAAAAGTTATAGACAAGAATAACGACAGGTCTAGATTGAGA
TGTAAACATTTCCTTGATATTAAATAAAAGTAAGCTAAAATGTGCTTTATAAATAAATACATGTGTGGTAGTG
GATGTGGAA
>103781475_2 DT665984.3
GCACGAGGGTTTTACCTTTAAAAAATAAGTTTTAAGTGTAATAAATGTATATAAAGCGAATCTTTGAAAATTA
TGGGTATCGCGTTATATTGCTGCATCATCTATCTACTAGTGGTAATGGTCTTGTTGGTAGAGGCATGTCAAGC
CTGTAGACCAATCATCGACGTAGATGATAATAAAGTTTGTCCTATTTCTGAGAACTTG
AAGTCCATGAATTATGTTCTAAGCCAAGGTGTAGTTTCTGACAATAACACAACTTTAGGGCTCACTTTACGAA
AATGTGAAATTGCAACCGTTGAATTGCAAGCCTTTCAAAATTTATCCTCACTTAAGTACGTTGATCTATCACA
GAATTCCATTTCTTATCTAAAAGTAGGTGTTCTTGATAGTGCCAGGCATATAACATAC

TTAAACCTATCTAATAACGCGCTGACTGACGTTGCGCTTGGATTGTTTGATCAGACAACGATCTTGGAAGTAT
TAGATTTGAAAAAAAACAAAATACAGCAACTAAAGTTAGGATTATTTGATTCTCTAAAAAAATTAAGAAACTT
AGATCTTTCAAGTAATTTATTAGTAGGTAAGGATTTAAATCCATATATTTTTGACCAT
AATCGATATATCACTAATTTAGATTTATCTAAAAATGATATGTCAGACACACCAAGTAATTTTCTTCACGCCT
TTGAAGATTTACAATTATTAAATTTAGATAAATGTTTGCTTACTGATGTACCGACCTTTGTAGTTGCTCAAAA
TTTAAAAACTTTAAAGTGTTTGATACTTTCTAGAAACAAAATAATAATTTAGAGATGT
ATCCAATTTATCCATTTTGAAGTTTGGAAATTTAATCTAGC
>103781523_2 DV501507.2
GTAGAGACGCGCCTTCGAGGCAGCTCTATGACAACGCCCGACAGCCTGCCTACATGACTGGTTATACTGGATA
CGTGCCTGGGATGCATTTTACCTACGGAAAATCCTACGGCAGAACGGCCGACGACTGCATGGCGGATTTTACA
GCGAACCAAAGAAATTTACGACGCAAAGCCGACTTAAACCGTAGTTACGTGAGATCAC
GGAGCGCGCCCAAAATGGAAACTGTACACTCGAGAGACGAGATAAGGAGAGATTTGAGCAGGTTTAGGGAGAT
AAATAAGTATAAAGAAAACACAATATCGCCAGAGTTCCCACCAATAGCTGGCTATACGGGGCATATACCTAGA
ATAAAAGGTTCAGAAGCATCACTAAGCCAGCGATATCACTGCGCTGCGAAACGAGGGC
TGGAACTTATCAAAATGGAAAGGGACAAAAGGAAGGAGCTACAAAACGCCGACGTA
>103781531_2 DT665862.3
GCACGAGGAACCCACCATACAAGACCACCATACCATCCATACCATCTCACCATATATAATATATATGGTGAGA
TGGTTATAATCTGTACTAAAATTTTGAAAAATCTGTTTATTCAAATGTGCTTATTTTGTATGTAGCGCAGGCG
CATATTCAAAAAAAATTTAAATAATTTTAGTAATATTTACTTTCAATTACATTTTTGT
GACCCAAATTATTGACACACTTATTTACAAATGTCAAAAAAGTATCTACAATTTTAAATGCAGCATAAATTTT
ATAAAGTCACTTGGGTATATTTTTGAGATAAAATTTTGATTTATTTCTTATTTTTATATGCAAATTCAATTTG
AAAATAACAATATTTAATATAAGGAAAATTGTTCAATTGACAGTACTATTGAAGTAAC
ATGTATAATTTTTTTAAGGAAAATATGAATTTATTCAAAATCATTACCAATGACTCTGATGATTATCTATGAA
TGTATGCTTCTTATTTACAGTGATTCCAGTATTTTACAGTGATTTCAGTAATTTTGAATATAACTTCTGTTAT
TTTCTT
>103781541_2 DT665500.3
GCACGAGGTGAGGACACAATTGGAAGAACTGAAGCAAAGAAGGCAGAGAACTCAAAATGAAATTGCTGGAATG
GAAAATCAAGCACTGCGTCAACGTTTCCAAGATATCCTGCACACTTTGAATCAAGATGTCATGTACAAGGAGA
TGGAATACCAGGGACTTATAACATTGCAGAATTCGGAGGACATATGAGGAACGTATTT
TGTTAACTACTAAACCATAGGAGCTAAATTATTTGGACTCCAGTGAAAGTGACACTTAGTTAATAATGTGTTC
TTTAAGTTTCATATTACACACTTTATTTTTGTTGTTTAATTCTAAGATTTAAATGTTTAGTTACATTGCTAAC
CTACTATACATATATTGCATTGTATATCTTATTTACAAAGTTAATTGTTATTTTTGTT
TTCTTGTTATATTTGAAAATTTTGACTAAAGAAAATTTATAAATAATATTTTCAAACTTATATTTCTCAACTC
GCATAAATCATTGGTTGGAATCATGGACATCTCTATATATATTTACAAAGTGGGACATGGCCTCCACTGGTCA
CTTAAAGTATTGATCATCTTATTGACCTTGAACTAATGTTTAATTTATGAAGTAAAAA
CCTTTTTATTCATCTTCACACTGTGACCCACATATAGCAGGCAAGATAAGCAGAGGGAGCCTAGTCTTAAGAC
ACTGTTCCACAATTTCTGTCGAATTTTCGAGGCATGCATGTTAAGTGGAGGAACACGACACTCTGCTTTTGCT
TTGGTACAGA
>103781560_2 DT666921.3
TTTTTTTTTAAATATATTATAATTTTATTAGGTATATTTTAAATTTTTAGAATTTCTTCGGATTTTCCTAAAT
ATTGGGTTAAATACATATTATGTTATTTGTACATCAATACTCATATAAACTTGAAAAGATAATCAAAATGTTT
GAATTTCCATAATTGTTTTATATTTAAGTGATAATATCATTTATATGGAAGGAAACCA
AGCAGCCATACATTTTCTATATAAAGATAACAATTAGTTTAAGATAGTGCATATAAAATTTAATTTATTGAAA
TATTGTATTATAATACTTTTTTTGCACGACGATTCTTTTTGCACAATTTGAATTAAATGTTACTTTTATTAGT
AAGTAAGTTTGAAAAGTTTATATTCGTGTTAAAACTTGTCAGTGTTGCCGAAGAACAG
GAAAATATTTTATAATCAATTTTACTCAAATACAATACAAAAATGCCTATTTATTTTGCCGTTACAAATTTTA
TAACATAACATGAGATGTTGAAGTTATAAACATTTTATGTATGCACAAAAGCAACAGTTAAACAACTGCATAT
TATTATTTAATATCTGACAGCTCTTTTCCCTTAGTTACACAGCCTCATATTTTAATCA
TGTATGAAAAAAATATATTAAACTTT
>103781562_2 DT666337.3
GCACGAGGCGTGTCTCCCGGGTACCTGCCCCCGCCGTTGTATGTGCCCACTTATCATTACCATACCGGTGGGG
TGAGCGGGGTGAGCGGCGTGAGCGGAGTGGCGGGCCCCCGCCCGCGCACCACGGCAGCACGCGCCTGCCCTA
CCTGCAGCCCTACTCGCCCACCTACCTCGTGCCGCAGCACCCGCAGCAGCACCTGTGG

TACACCGACTGAGACCACCGCGCCGTGCTGTATATGGATCATTGAAGCTAAAGCCCTCGCCCACTAGTGAAGC
TATAAATGGACCATTAAACTTAAGCTATGTGAAGATATACCGAGATATTGTATGTGGGATTATTGTAACAAAA
CGACGATACGAAATAACTGTAATAATTTCAACGAAATCGATTATGAATAGTGATACCG
ATTGTGTATCGACACAATCGATTATATAATATCGATATATATTGGACGCTAAGAGTTTTAATGTTTATTATTA
TTATAACGTAACGCACAACTCGCGGCCCTTATTGACGAATCCATGTATATGGTTGGATATGCGATAGTTCGAC
AATAAGGGCCAACGAGTAATGAACCCCGAACCGTCTTCACACTGCAATTTTATATACA
AAACTGTGACTAGTGTGTTTTGAACGCGTGTTTTAATGTTCTCGTGTGTCGTCCTGCAGACCCGGTTTACATG
ACAGTTGTACGATTTTTAATATAACTATTATTATTAAAATGTATGGAATTGTCGTGCACGTGTCGTGTAAACG
GCTTTCTATTATTGTATTTGATTATA
>103781566_2 DT665130.3
GCACGAGGGCGGGCCTTTAATTCTGTTTCACTAAACTAACTGATGCTAATATATTTATCATTTTGTATGGCGT
GCTTAGTATTTATTTTGCGGGTGTTATGGTGCGTCTTATGTTAGTACTAGCGCCGGTCATGTGTATAGTATCG
GGGGTCGCGGCTTCAAGTCTGTTAAGTCTACACGTTAAAGATATTGAGCCTAAAGTTG
AGAAACATGATAAGAAGAAGAAACATGAAAATAATTTAGTTTTTAGATCTGAGGTGGGCGCTCTATTCGTCTG
CATTCTTAGTTGTCTTCTAATCTCCTATGTGTTCCACTGTACGTGGGTCACGTCGGAGGCTTATTCGTCGCCC
TCCATTGTACTCTCAGCTCGAGCTCATGATGGAGCCAGGATTATATTTGATGACTTCC
GTGAAGCCTACACTTGATTGAAGATGAATACTAAAGAGGACGCAAAAGTGATGTCGTGGTGGGACTACGGCTA
CCACATAACGGCGATGGCGAACCGCACGGTGATAGTGGACAACAACACGTGGAACAACACGCACATCTCGCGC
GTGGGGCAGGCCATGGCGTCCAGCGAGGAGCGCGCCTACGAAATCATGACGGAGCTGG
ACGTGGACTACGTGCTCGTCATATTCGGGGGGCTCGTGAGGTACTCCTCTGATGATATAAAC
>103781568_2 DT666404.3
GGTGTCATATCCTACGCGACGTTAAACGCCGCAGTCGTTGGATGGGACTTGAGGGCTCCGGGCAACGCGTGGA
AACTCCAAGGCGATCTAAAACAAGGCGTGTACACGTGCCTGTACGCCAGCAGCAGCGGGTACCTGGCGGTGGG
CACCTCCAGCGGCGCCATCTGCGTGTGGGATCTAAGATTTGGATTGCCCATCACGTCT
GTGAAACATCCTAATTCGGAGCGCGTGCGGCGCGTGGCGGGCGCGGGGCGCGGCTCGCGCGTGTGGGCGGCGG
GCGCGCGGGACGCGCTTTTTTTTTTCCTGGAGTCCACGCAGCGCACGCACGCGCGGTGGCCCGCCGGCGCCGG
CGCCACGCCGCTGCACTACGCGCCGCCGGCGTCTCACTACATAAGCGCATTGTACTGC
GGCACCTGTGATGGTAACAAGTTCCTCATAACGGGAGGATTCGGATCAAAGGC
>103781574_2 DT665728.3
GCACGAGGGCAATATGCATGTGGTGAATGAGCCCGGTCGCAGATACAACAGTCAGTTGATGAACGCGGTGGTG
CTATACGTGGGCACACAGGCCATAGCCCACATCAGATCAAAGGGACAAACACCTAACATGTCTACAATAGCAC
ATTCTGCTCATATGGATATATTCCAAAACTTCACTGTTGATTTTGACTATGAGGGACG
GTATCTGTTCCTCAATGCGATTGCTAACCAGCTTCGGTATCCGAACAGCCACACTCACTATTTCAGCTGCTGC
CTCCTGTACTTGTTCGCTGAAGCCAATTCTGAAGCTGTACAGGAACAAATTACAAGGATGCTGTTGGAAAGGT
TGATCGTAAACCGTCCGCATCCGTGGGGTCTGCTTATCACGTTCATCGAACTTATCAA
GAATCCCATTTACAAGTTTTGGACCCACGAATTTGTACATTGCGCTCCGGAAATTGAAAAGTTGTTCGCATCA
GTGGCGCGCTCTTGCATCACTGACAAGGCGGGGGGGTCCGACCGTGAACTGACCGAGTAGCGTCGCGCCCTAT
GGGAGTCTGTCCGGCCGAAGCAGATGTTTAACTGCTTCGCCGAGAGAGGCTTCCAGGA
GGCGCAACAGGGGGAAGCGCCACAAACACAGCCATCAACGTCGCAGCCCGTCAAACAGGTTCTGCAGTGGAAT
AAGCTGTTCAAAAAGTAAACTGTTTATTGTCTAATAGCCGCACGATAGGGACGTTACGGTGATTAGTGTAAGT
GATAGTGATGTGACATCGACAGCGACCAGCGACAGTGACATACGGGGAATATGCTCAA
>103781577_2 DT666608.3
CGGACGAGGCAACGCGTCCCTTGCGGTGTTACGTCCCCGCACGTCCCCGCCGCCAGCCCGAGTGACCAGAGTT
GCACCAGCACGCACTCGCAGCTCCCAGCAGCTGACCCTCAACATGGACACCAGGATAGCGCTATGTTTCCTGC
TTTTTGCTTTCCAAGTTATTTACGGTGAAGTGGACATTATAACAGAGCCACGTCCAGG
AGAAGAATATGTATTAGTGAGCTCTGGACAACAAACTCCTTTAAGAAATTTTGAAACCTCACATAAAATTCCT
GAAAAACATCCCAATCATCGATTAACAAAACAAGATGACGGCGAAAAAATATTTAAAAAATTACACAGTGGAA
AAATTAAACATCCTTCTGAGGGTATTATTTCTATAGAGGAGGATAAAAAGGCAATATT
AAAAAAGGCCCAAAAATCTAAAGTCACTGAATCTATAGAAGTAGATATTCCTGTTTCTAAATTAGAAGATATT
AAAATAAAAGAGAATGTTAAGGCTGTAGATAACAATCCTAAGCTTTCTGTTGCTCTAGAAACTATGTCAGAAT
CAGGAAAGCTAATTGATAACGATCAAACAACTACTCAGAAGCCAGTTTTAACTACGTC
AGCACCATCTAAATCACAAACCTACGATTATATTCCTATTAATTTCGACACTATAGATGATATTAACAGCGGG
TTACTTTCATCGAATATTAAGTTGGAAACAGCTGGTTCCGAACAAAACATTTCAGATTTCAGTTAAAAAAGGT
CC

>103781581_2 DT666627.3
CATTTAGGGGCCGGGCTGCAGGTTTAATCGGTATTTTATATATCTTGCCTGAGCAGCTAGTGCAATGTTTTAC
AGTGATTTATAGCATCTACCGTTTATATAGAAATTATTTGAGTATATTTGAAATTTAGATATCTATATATGTA
TAAGAATATTTTCGAAGATATAAATGGCACATAGAAAATAAATAATTATAGAAAAATA
TAGCCCGTAATCCAAATTTGCCGCGTGCCATTATTATAATAATTATCATTAATAACATGTTATGTGTACGATG
TTTTTGTCAGAAGGACCAATATTAATTACCAATTTAATCTGTGGGTGCCTTTGTGTGTTTAGCAAGTAATCTA
ATTATAATTTTCTTTCACATATAAATTATATTTAACAGTTAAACTTCGTTACATTCCA
AAGAATGATTCTATTTGCTGTCTCTTGTAAGCATTCCTCTTTATATCATCAGCCACAATTCTGTGAATGACAC
AATTT
>103781583_2 DT666419.3
GCACGAGGGTTGTTCTTAAAACGTCTTTCCCGTAGAAAATAATATAAATATCGTGATTTAACAATATTTGTGG
GTGATTGTTACGTGTTTGACTTTAATTTGTGCGTTTCTATCGGCTTATGAGTGATTAACGTCTACGCTTTATA
AGGTCGTAGAACAAGATTATATAATCACGCTGCCGTCATGACGCTGCACTGGTGGATG
CACTGGTTTTGGGTAACGATGCTCGTCATCATTATAGCGAGCACCGGGACAAACCTGGCCGCACCCCAGTCAG
AAGAAGTGCAACATTCTGCCCCAGATTTTGACGTAGACAATTTATTTGCAGACCGACAAGCGAGAGCTATGTA
TTTCACCAAAACACCTGTTACGACTGCAAGACCGCTTTTCGCGAATGATACTAATATT
CAACCACCAAAAACAGCAAATATCACCCAAAATATTACAATATTGACAAATTCTACAAAAAATCAACAAATAC
AGTGGACGCCGGTGAACACTACGGAGAATGTATCCATAATCGCCCTTCCAGACTCTAATATAACTCAATACGC
CAACGTGACTGAAAATCACAACTCAACGTTTACATCTAAACGACGTAATTTGACAAGA
CCTGAGTTTATTAAAGAGGATCTTAGCTTGGAAGGCATTTCAGAGAGCCGTATTGTGACAGAAAAACCGCTGT
CATTAGAGACGACGTTTCTACAAACTAGAATTCCACCCAACGTGGAATCTTCTAGAAGGATAATAGACGACGG
TAGTATGGACACCGGCGTCATAGCGGGTATCTCTTTTGCGGCGTTAGTCCTCGCCGCT
TTGGTCGGTATTACTGCATTTGGTTTGTACCGCAAGCGGTACTTAAACAAG
>103781603_2 DT666596.3
GCACGAGGTTGAGATCTTTCTGAATTAATAATATAAATTACAGTTAAAAAAACGCAATTCTATTTTAAAAATA
TTGTGCAAAATTTTATTTTAACCTTTAATTCAAATTTTCATAAATTAAAAAATGCCACCTAAGAAAGTGGAAG
AACCAGAAAAAAAACCACTTATTGGAAGAGTGGGCACCAATTTAAAAGTGGGTATTGT
TGGAGTGCCAAATGTTGGTAAATCTACTTTTTTCAATGTTCTCACAAAAAGCCAAGCAGCTGCTGAAAACTTC
CCATTTTGCACCATTGACCCGAATGAGAGTAGGGTACCGGTTCCCGACGAGAGGTACGATTTTCTCTGCGAGT
ACCATAAGCCAGCGAGTAAAGTACCAGCATTCTTGAATGTAGTTGACATTGCGGGTCT
GGTGCGCGGCGCAGCGGAAGGTCAAGGTCTCGGCAACGCTTTTTTGTCGCATATAAAAGCTTGTGATGCTATT
TTCAACTTATGCAGAGCTTTTGATGATGAAAGTGTAATACATGTGGATGGCGAAGTTGATCCCATCCGTGACT
TGGAAACCATTGGAGAAGAATTACGTCTGAAGGACGAGGAACAGCTGGTGCAAAACAT
CGAAAAATTAGACAGGACCGTCAATCGGGGTGGGGATAAGAAACTTAAACCTGAATATGAAGCTTTAGCTAAG
ATCAAAACTATTCTAGTAGAAGAGAAAAAGCATATTAGATTTGGTGATTGGAGTGCTGCAGATATAGAAGTGC
TCAACAAATATCTGTTCTTAACATCGAAGCCAGCGCTGTATCTCGTCAACCTCT
>103781607_2 DT666826.3
GCACCTGGAGGGCTACCAGGCGACGGGCCAGAGCCAGGCGCTGGTGCGCGACGGCGTGCTGCTGCCCACGCGC
GACGCGCCCGACCTCGCCTACATCCGCGACAGCTCGCCGCAGCAGTTCGTGCCCGACGTCTACTACAAGGAGC
GCGACGCTTACGGCAACGAGGTGAACGTGTCGGCGAAGCGGCTGCCCGTGGCCTACTT
GCTGGTGGACGTGCCGTGCGGCGTGGCGCCCGACAGCGTGCTGCCCACCTTCGAGCCCAGCGCCACCTTCCCG
CCCGCGCACCGCGTTTTTTTCTCAACACGCGCAATCGCTGCGCGCGCTGCGCCGCCACGTGCAGCGCCACGCG
CGCTTCTTGGACGCCGCCTCCGACTTCCACGTGCTGCTGTACCTGGCC
>103781611_2 DT665790.3
GCACGAGGCGAGTTTCTTTTTTTTTTTTTGTTTTCTAATGTTTTATTTCATATTATTTAAATGCGTAAATATA
ACTACAATGCTAATACTTAAATTACATAACAACCCTTTACTTCTTGGCCATTGCCTTTTCTCTTGCCTTCTTT
CCTTCCAGGAGCTTGAGCCTGTCATCTTCAGAAAGGTTTTTGTTGAGTTTAGTTATAA
CACCAGTTCCTAATGTCATGTCACCAAGCCGGAGAGTGAATCTTTGTCCAGGCTCACAAACCATAGGTTTCAG
AAGTCTCAATTGTAAACTGCAATAAAAATATTTTTTTACATAACATTCAAAACAACTTAATAATTGATCTTAA
TATTATATTAATTGTGAAAGTAGAAATTTTGTCTCTTTATCATGGTGCAGTGACATTG
TCTGACGGTAAATACAAAAGTTGTGGGCTTAGGTTATTTTTAATTTTATTATTCTATTAATTATTTTCGTTTC
CTTGTTCAGGTGAGAAAACAAAGAACTGCGTTAAGTGCCACTATCTAATACAGAAGGTTGAGGAAAATGGAGG
AAAAACTTAAGTGCAGTGTAAGTTCCCTCTGGCTATATGCAGAAAAGTGAAGTTAAAA

AAAAATTAAAACAATTGCATACTTACGTAGCATCCTCCCCAGGCATAACCATCTCCTTATCTGGAATAGTGAC
TTGAGTGGCGCAATCCCATGT
>103781619_2 DT666275.2
GCACGAGGAGATTACATATATACCACAGATTATTTTCTTAATTTTCATAATAATATTTAAATATTTGACATCT
AAAAACACCCAACCGTCAACTTGTTCCCAAACTTGAGACGCATTTTAATACTATAAAAATGAACTACTGTAAT
AAATAATAGAGCACAAGTACTGTGGACATTTTACATTAAAATGTTATGTTTTGTAATC
GGTCAGATTATATCCGTCATTTTTAGTATCGGTCAAACTGTCTTATGAACAGTTTTGTTGAGATTCCGTTGTT
GGTGAGAGAACACCAAACTCTTTCTCTGTTTCTCTTTCTCTCTTTGGTGTTCTTTTGGTGCTTTTTGTGTTAA
GTTTCTTGTTTAAGTAAGTTTAAAATTGTTGTTTTTTTTTTGTTTCTGTTATATTGTT
TTTTTTTTATATTTTGAATCACGAAGATCTAATTAAAGAGAAATTAAAAAGTTACATAGTTAGAAAGTTAAGC
TTAGTTAGAAACAGGATTTCTTTGTTTACATTTTATCTCATGCCGAAATGAATAGAGTATAGTGTAAATTGAA
ATTAAGCACAAAATGTTGTACTTAGACTTTATTGTAAAATAAAATATCTTTATATAAT
ATAAGAATTAAATTCCGTCCATCTTAAATGTCTAATTACAGTTAATAATTTAGAATTTAATTACAATAATATA
TACCTAAAGGTTTTATTTTTAATAAATTATTATTTTTATATAAAAAAAAAAAAAAAAA
>103781633_2 DT665849.3
GCACGAGGATTTTAGCGTGAAAGAAAATCGATTTTGATTCGATAGTAACATCGAAGATTATGTATTTCATAAG
ATAATATTTCGTTTGTGTCAATCATCTACTAAAATTCAATTATAAGACTTGGACATTACATGCAAAAAATGTG
TCAAAATCCATCCAGATGTTTTTTTAATCAGTCATATATTTTACACAACATGTATGAT
ATTTCATTATGTAATATGTAAATATTCAAGTTGAAACACTTCATAGTAGTATTCAAACAAATTAAATTTAAAC
TTATGGCGTTATATTATACCTGTCTTAACTAAAATATCAAAACACTTATGGTTTTCTTTTAATTTATTTAAAT
ATTACTATAATTATAATGTTGAGCTTTAGTTGCGGACGTGACATTTTAAGGTTGTGTT
AGAATGTCCTTGACTAATCGATAGCAAGCAAACGATTGTTGAATCGAGTTGGGGAAGCGAGTGCGGGACTCGC
GCTAGGTAAGATAGAGTAAGCGAGTACTGAACGTGACGGATTGATGACGATAATGACGACTGAATCGATTAAT
CGTGTGGTTGTAATGATTCATATACAATCATCATTGCGATTTTATATTATAATAATCG
AATGAAGGAATTTTTGCAATAATTGCAAGCTTAAATGATAAAATTAGCGCAATATTGCTACAATCAATATT
>103781657_2 DT665923.3
GCACGAGGATTGGTCATGAGTTATAAGAAATATATCGAATAAAATATATAAAATAATATATATATATATAAAA
TAAAATAAATATATTCAAAACATGGATCGTTGGATAGGCAAAGTGGCTGTGGTCACTGGAGCTAGTTCTGGCA
TTGGGGCCGAAATTGCTCTTCGTCTTGCCAACATGGGGCTCAAAGTGGTCGGGTTGGC
CAGAAGAAAGCATTTGATTGACCAATTAGCACAAAAAGTGACTGGCAAAGGAGAGATTTATCCAAAAAAGGTA
GATCTTTCAAAAGAATCGGAAATAAAAGAAGTTTTCCAATGGATTGACTCGGAACTGAAAGGCCCCTTCATTC
TTGTAAATAACGCTGCCTATGTGTCTCCTGGATATATTACTGATTATGGAGATCGAAC
AGTAAATGACGATGATATTGCAATGACAGTGGATGTAAACGTAAAGGGACTTATACTGTGTACTCGATATGCA
GTATCTAGTATGAGGAAACGGAATGTTGAAGGTCACATTGTGAACATGAATAGTGTGACTGGTCACTACATAC
CAATCTTCCAAACTACGAATGTGTACCCACCGACGAAACATGGACTAACTGGTTTCAG
TATTGCCCTGATCAGAGAGCTGGCTAAATTCAAAAGCAAAATCAAAGTCACGAATCTCTCCCCCGGCGTCGTT
GGTACTGCTATGCTTCCCCCTGAACTAGCAGATCAACCCCAATTGGAAGTAAAAGATGTAGTGGATGCTCTAA
TCTACGTGTTGTCTACACCACCTTTCATTAATATCTACGAACTCGGATCAAATCTATA
GGTGAAGGACTGT
>103781678_2 DT666368.3
GCACGAGGCGACCACTATGCGGGCATATAATAAGCTAGTGCAGAAATTTGGCGCGAAAATATTTATTTAACTA
GTGTTTTTTTTAAATATATGTGATGACTGAAACATTAAAATATATACTTCGATTGATTGTTGTGACTACATTG
TACTACGTTTTGATAAGTGTGGTAAAAGAATTTAAACGATATTGGACTCAGGTGGCGA
CGTAAACATTGGATTATTTGATTTCATTTAGAAATTGATATTCGAAATGGCTGATTCAGATATCTTTAACTAC
ACAGAGGATATAAATTCCAATGTTCAACAAAGTGAACTTTCCAAATTTTCACCAACACTACTAACATTCGCTG
CTGTGGTTACTGGAATTATTATGGTAGTGGGTCTTTTCGGTAATCTTTTGACCGTAGT
TGCTCTCTTGAAGTGCCCTAAAGTGAGAAACGTTGCGGCAGCGTTTATAATCAGTTTGTGCATAGCTGATTTC
TTATTCTGTGCTGTGGTACTACCATTCGCCATATCTGGCTTCTGGACGAGGACCTGGTCTCACGGGGGTGCGC
TGTGCAAGTTGGTACCGTTTCTACGATATGGCAACGTTGGCGTCTCCCTGCTGAGTAT
AGCGCTCAATACTCTAAACAGATACATAATGATAGCTCACCACAGCTGGTATTCGAGGGTATACCGCAAGCAC
AACATAACCTTGATGATCGTTTTCTCGTGGATGTTCTCGTACGGGATG
>103781684_2 DT665872.3

GCACGAGGCTTAAACTTACATAATTCATTTTTCAGTAAAATAATAATAAAGGAAAAATAGAGTTTTACTATTC
ACTTTCATTTGTATTTAAGGATAAAAATCTACAAAAAATTGGCGGTATTTTGCGCGGGAAGTCGAATTGGAAT
GAGAAACTTCGTTGATCGTTAGCTTTTGTTACTTACAAACAATCTTACTTGAATAATG
GCAGACGCTTTAGAACATTTGGTTGTCGATGGAAACGAAGTTTTAGAAATAAAATTAGTCAGATCAGCTGATG
ATATTGAAAATGATGACACAACTTTTGGCCCCGAAATGTGTCACCAAGTTTTTGGAGAAAATGAAAATATTTT
TGGTTATACAGATTTAAGAATTCAACTTTACTATACTGCAGGAAGTTTGAAGACTTAT
CTTGGAATATCATACACAGACAAGATTGAACCAAATAATTCTGAAAAAATTAAAGCTGATGATATTGAAGGGG
CTTTAAAAAAAGTCTTGGCTCCTGGCTATGTTACAAATCTAGATCTCTTTGTATCAATGATAGAAAAGGATAA
ATTATTCACACCACATGGAAATCTTGTACATTCGTTTACAACCCAACCTTATGATGGT
GGAGAAAGCAGAACATTTGAAGTTTATCACTCTGAGGTATCTACACCAGGTTTTCTAGATTACCATGAGCGTT
TACAAACATTTTTGCTATGGTATGTAGATGCCGCCTCCTTTATAGATTGTGATGATGATCAATGGACTTTTTT
TACAGTTTTCGAGAAGTTCCGCTCCAGTGAGGGCACCGCGCGGTACGCGACGGC
>103781689_2 DT665768.3
GCACGAGGCCGCGGTCGCGTTCTACTTGATTTACGTCAACACACTTTTAAAGTACTACCCAGATTGAAATGTG
CTTCAGACTTGGAACAAGTAATTGTTTCCAATGAGGAAGAGCTGATCACGCAACTTGCGGAGAAACTTATCAG
CCGTGGCGCTAAAAATATAAACATAATCAGCAAAAATATATCTAACTCACTTGTATTT
AAACAGAGATCTTGGAATGCAGAAGGAATTAAAGTTGAGTTAGAGCAAAGAAACGTGGATGTTGGTAATTTAG
TGAAGTTTATTAAAAGTAACGAGAAGGGGGCTGTAGAAGGAATTTATTACGCACTTTCTTCTAGCGGAGAAAT
TGATGATGTAGAAGAACTAACATCAAATCTAAAGAAATTGATTCCTGCACTTAAATAT
TTTGCTGTAATTAATACTGGATTCAGTGGTGAACAAGAAATAAGTATCAATAGAAGTCGAGTACCTCTGGGCT
ACAGGATATTGTTGCCAGATTTATATGGGGCTGAAAAATTAAATAGAACCTCTAAAGATTCCATATCATCATT
AAATGCAGTTGATGCTATTGAAGGAGCATTATGCTCCAAAGAACCAATAGTGATAGCG
TCAAGAAGAGATCCTCAACGCGTTCCTTTAATGGAAGAAATAGCTAGGATTGCTGATATTAAATTGGAACAAA
GTTCGGAGGTAACATTAAAGGATCTAGGAATAAATGTCCCTAAATTGGAAGCGGTGAGAGCCTATCTGTGGAA
CGAGCACAGCGTATCTGTACCCAGTAAAGACATACCGTCG
>103781699_2 DT665944.3
AAGTATGGTCAGCGGGCCAATGGGCAATATGCCCAACATGGGTAACATGCCCAACATGGGCAACATGCCGAAC
GGCGCTGGCGGCGCGTGGGGGGGATGGTGTTTTTTTCCCATGGTGGCCCAACCCCCTGGTATAGCAGCCGCAA
CGGCTGCCGTCCCAGATGCAGTAGGTGCGGCAACGCAGATGCCACAGATGGGTGCTCC
GATGACGGCCAGCGAAGCCTCCTGCGGACCCACCCGCCGCGTCAAGTAAGAAGGAGGAGACGGTAATTTCGCC
GGAATTGCAAGCGCGAGCAGCCGAATGGACTACCCATATAGCTCCCGATGGTAGACCGT
>103781701_2 DT666078.3
GCACGAGGCCGCCGCCGTGCTCGCGCGACCGCCCGCGCCAGCGCCGCGCGCGTCGCGTATCGCAGTCGAGCCC
ATCGCGCGTGTTGCCGCCCTCGGAGCACCTGCAGTACTCGCTGGCAGACCTTGACTCCATCTTTACGGAGCCG
CCTGCCGACCTGGACATGCTGGCGCCCGACGACTTCCTGAAGGACGTGATGCTCGAGC
CGGGCCTGGACCCCGACCCCGACTTGCTACAGGTGCACGACATGTTCGACCCCGACGACGCGCCGCGCGGTGA
CTTCATAGACCCTGACGAGTTCCTCATTCACAATTACTTACGCATTCCCAACGCCGCTGATGACGAGCAACAA
GAAAATACACACTAAATAACAAAACGCATGATTAAAACATGAAAATATTATATGTTGT
TGTACCTTATCGATAAATATATGGGTAAAATAATAAATATCAACTAATTATATGTTATGTTATATTGGAAACA
TAGTTGTTATTTTTAAAATTGTGCATTATTATTTACTCTAAACGATAGTTTTGTCAACTATATGAATGTCTTT
TCTTAATTAGAAACATTGTTAAACTTACCTAAATTGACTGTAAATATGGCATACACTA
ATAAATTACTGATCATAAAATTATACTTTAATATTTGAAAGTGATTTCATCAGATTTGACGATTTTTATAATA
TTTGGTGCACTTTACATATTTTTTTCTAATACAGTTGTTCATGTGGGATGTTCTAAAAACGTGTAATATTTTT
TTTTTATCCAATTACATTTTTTATACTACTTAATATAAGAAAATGCTATAG
>103781713_2 DT665189.3
GCACGAGGAGATCCCTCTCACCATCGATTTCCAATACGATTTTTTAGCTTTTCTCAATGAAAAGTTTGCGTTG
AGTGCATTAATATATAAACCAGTGGAAAAGTGCAGTGCTCTTATAATATCAAGAGGAAATATATCGGTATTTT
ATAGCTTATTGAAAAAGTGTCACGTTTCAGGAGCTGAATGTTTTGTAAACAAGCGTGA
ATCGTCGTATCTAGCGTGAGGTGTCGTTTAACTTAAAGAGAGCTCCAGTAATGTGCTGACATGCCAAGTACAG
CGATCTACCAAGAAACCAAGGCGGTGACGCCCGAAGCTCCCACTACATATGAAGAAGCTTTGGCTACTGCTGA
ACCGGGGGAGACCATAACCACAACCAAAGAAGAGGAATGGGTAACTCGTAAAACGAGT
GAAGTGACGACAACACGTCAGATCGCTACCCGCGTGAAGCGCGAGCTGGTGCTGGAGGACGGGCGCATCCTCA
GCGACTCCGGGCCAAAGATATCTACCTCAGTCAACGAAGACACCCACACAACTAAATACCAACAAACTGAGCA
TAGAGTACCAGAGAACCAAGGTGGTGAAGAAAATGCTGAGCTGACAGGTGAAGATGGT

GCCAGGACACCGGTTGAAGCATTGGAAAACGCACCAGAAAGTACAATAGCACCAGCCGGTGCTAAAGTACTTG
TTTCGTCGTGCGTGGTCGCGAACCCAGATGGCAAGGTGCGTGAAAGCCGCGACCGCCGCGTCCTCACTAGGAA
CATTACGGA
>103781732_2 DT666561.3
GCACGAGGAGACACCCCGTTGACCACTTTGCTGACCGCCGAGCTTTAGAGGCATTGCATGTTGATTGGCGGTG
CTCGATTGACGCTGCACTTTGCCTCGACACCAGAACGAATAAATGAAAAATATAAAGACGGACGATAATATTT
ACTAACCTGACGTCCACACGGCGACGACGACGAGACGAAGCGCACGCGGCCAAGCCAC
AGCGGCGGTCCCCGCACACTGAGGCGCTGCTAGCCGCCAGAGGTCGTCCTACCGTCCATCGATCCACCCTTTC
GTCACCCCATCCCCGAGCCACTGTACTCCTATCTCAAGTGAGTAAGATCTATTTTCGCAGCATCCATCTCAAT
TCCCCAATGGTCCTCTACTGACGTAACGGCAACTTGGTGACGCTGATCACCGAGCTTT
GTCATTACGAACTGCATTAAGCCCTATTATGTCTCAAGTTAAATCCATTTTGCTCGAAGGGTATTTTAGGATG
ACAAAAAAACACATAAACAAACAACGTAATTTTATTTCGTAAAATAATTGATAAATCACAACATCACTACATT
CGTTCATTATTCGCAATACAATAATGTTAATATTAAATATATAAGTTAATTGACTTAC
GTGTCAATTCGCGCAACTTGCTTGTACATATTAGGTACGGTTTATAGCTCACTATTTATTATAATACAAAAAT
GTAATTACTAATTATTAGTATATTTAGAGAAAAATGTTGGAGCGTGTAAAATACTACTGTTTATGTATTTTTT
ATTTCTCAGATAAAAGTATTTTTAATACGTCAATATTAAAATGTATTATTGATATTTA
TGAAGTTGTCGAGGTTCTATGCTCG
>103781734_2 DV501455.2
AAATATGTTGTCTGAAGAAATTTAATATAGGAATAAACTACAAATAAAAGGTTGTAATAATAGGTGTTTTCTG
ATATATCCATAATTTGTTACGGTGCATACGACATCAGACCTCAATATTTCATTTGAATTTCATTCAATTTACA
ACGAGAGTCGTGACTGACGGACACCTGAATGTTCCTAGGGGTATATAGATACAAGATT
TCCGTTTTTTTTAATTTTGAGACCCACAACATATTTTTTTTGTTATATAGTTCTTATATTATGTCGGTTATGT
GGTAATTATCGAAACACGGTCGCAAAACTCAGGTACGTACGTACGTAATTTATTTTTATCACGCTTACATATG
TTAAATGCGCAGACATATTTTTTGGTTAGTTTAACAATATGTTGCTGTTTTTACCTAT
TTTAGAGAAGAAAGGGTGTGCTCGCACCAAGTGTGGGTGTTAAAACTACTGACAGTATTTAGATTAAGA
>103781738_2 DT666523.3
GCACGAGGATATTGCCTAATAATGCCGGCGAAAGCAGTTTGCGTTTTAAACGGAGATATCAGCGGGACAGTGT
TCTTTGATCAGAAGGATACGAAATCCCCAGTTTTGGTATCAGGAGAGGTCAAAGGTCTTTCAAAGGGTAAACA
TGGCTTCCACGTCCACGAGTTTGGTGACAACACAAATGGCTGCACTTCAGCTGGCCCT
CACTTCAATCCCCTAAAAAGGACCATGGAGGTCCCGACTCCTCAGTCAGACATGTTGGTGACTTGGGGAACA
TTGAAGCAAGCTGTGATGGTGGCGTTACCCAGATAAACATCCAAGACTCACAAATCTCACTATCAGGTGAGAA
CAGTATCATTGGACGTACTTTAGTAGTGCATGCTGACCCTGATGACCTTGGTATTGGT
GGACATGAGCTCAGCAAGACCACAGGTAATGCTGGAGCCAGGATCGCTTGCGGTGTCATAGGACTGGCCAAGA
TTTAAGGGGTTTTATGGTCCTTTTGCTACTTTTTGAGGAGATGTAGGTGGATGAAGGCGAACATATAAAACAT
AGTTTTTTTGTTAAGTAGATATGTGAAAGTATATGTGTTAATAGTGCTATTTGTTCAA
AGCTCTACAAAGATTTAATAAAAATTGATTTTAAGGTTAAGCCTTTGTGTTCATTTGAAAATCTGAAATTTAT
ATGTCACCTTCATCTATGCACATGTTGAAATTCATATATACCTATATTATCAAAATTATCATTCAGCCTGTTA
TATTATAAATATAAAATAATATTCTATATTGGCAATACTGTTTCAAAAGTTAGTAAAA
AAAGC
>103781759_2 DT665967.3
CGTAAAACTAAAAAAATCTAAAAATTAAAACCCCAGTTCTCAAATTATGTTTGCTTATTACATTACATTTTAA
GATGTTATGTCTAAACTTAGGACCATGTCACACAGTATCATAGTTCGTGAATCCCCTCCATCTCCCACAGCTT
GTGATGAAATTTATGGATGGCCTCATGTTTATTATTAGTTTGCAAATTATTATATGCA
ATGTGTATATTATATATTCAAAACAAAAAATACAATGTTCTACATACTGTAAGTGAAATACCAGAAATAATAA
TAAAAATATTGCATTTTTTAAATTATAGTGATGGTGGATAAAAATGTGATAATATTTGTAAGTGATGATGAAA
CCTTTGGTAATTGTCCTTTATCTATATCAATAATTAATCTATTGCTGCTTATATTGAT
AACTTCAGCTAAAACCATAACAATTTTTATTTTTTGTTAAGCGTCTAAATAAAAAAATGCATTTTGTTTCTTG
TTTAAATTATTATTAAAAATATGCTGTTATTTTAAAGAATATAAGGAATTGGCGTATGTTAAATGTGTATTAA
AACTGAAGCACGAATATTAATTATATTTGGTTAAGCACATTCTGTAATACTAGTAGTA
TATATTCACAAGTCAACAATACTATGGTTTCCTTTCCATGTAAATTCCACTTAATTTATTATATTGGTCATCT
CATGTCAAT
>103781777_2 DT665095.3

TTTTTAACTGTGCTTTTTCTGTTGTATGGCGCCTCTTTATGTTTTAGCAATTTCTTTCTTCGTGTATTCCCCT
TATTTCATGTGCGGCCTGTTTTATTATTGGTTTTGTATTGTGTCAACTACTTTATTTATATAATATATCAATT
TAATTTTACTGTGATATTTTTTTATATATTTTTATCCCATTAATATTGTCTAACAGAT
ATGTTTAGCTGAAAATGTCCGGTAAAAAGAAAAATATTCTATTATGGTAATTAAACCACTTTTTACTATATTT
ATGAAGTAACAATAGCTTTAAATTTATATTCATTTTGCATTTTGCACTATTTTAATAATATTATTTTAATATG
ATTTACTTC
>103781787_2 DT666445.3
GCACGAGGCGTATCCTTCCTATTTCTTTTCAGACTTTCGTCGATTCTTTCTTTAATACTGACTTTTTTATTAT
TTTTAGTGATTATAGGACACCTCGGACATTGTGTAGTGTAACTAATGTGATTTATTTAGACTTTATCGTGCGT
AAGGACTTTGATAAGTGTTCAATACGTTTAAATCGCGGCCAATGTCAGCAGGCGTGTC
AGATCAGCGGATGAAAGGGCAAGGGAACCAAGTGACAAATGTACCTAAAGAGCAACAGTTAGAAAGTGGCGGA
GATGAGTTGGCCGAAGTGTCATGGCGTCACCAGCAGCAACCATCTTATGCTCCACCAATTTCATCTGCAGCAG
ATCCCTACAGCGCAGCAGGTGTATTCGGGCCATCTACCACACCATTTTCAACAGCTGC
ATTTGGGCAGCCATCAACATTTAACTATTTCCCAGGAAATGGTGATTATTCTACTTGGGGTCAGTTAGGACGT
GCAAAGCAATATGACGATTACTACAGAACAGATGGACTTTACGTCCCTGATGCTATCAAGGGCATGGAGTCAG
GTGTACAAGCTCTCTCTCTTAGCGACCATAGTAAGATCGACAAGGATCGTACACCTGA
ACTTAAAGACGTATCAAGTGGATCGCAACCCAAAAAGATGACATGGGCTTCGATTGCGAGCCCACCGGCGAAA
CCAGCGCCCTCTACCCAGAGTGGAGGACTAAAGAAGAAAGGGCCCGGCATGCCACCGCCGCCGATAGTGCCAG
GGAAACATAATATGGACATTAGTACGTGGGATGCGGGAAAAAGTGCTTCAGTAGTAAC
AGCTCCTCCACCCCCTCCCCTTCACCGCCG
>103781791_2 DT666626.3
CCAGCGCTTGCGAGATACGCGGCTGAACCACATACACCGAGTGATACTTCCACTTATGAAGATCTTTTGGCTA
ATGCTATTCTAAATAAGGTTATCGAACGATATCAAACAGAAAGCGGATCGGGTGGTCGCAGCAGTGGCATACA
CTCCGCCAGTGCCAGTCCTTCACCCTCGGATAGATCTTCTCCAAGATCTATTAACTCT
AGGAACACTAGAGATATCACTCCTCCACTCCGTGCAGATGATGATGCGTCTGACTGGGAGGAAGGTGAGGCAA
CAGATGAGGCAGTCGAGGTGCCCCGGCGTGTCCCCTTCCCGGAGTATGGAGGTGACATAGTGCATGCTTCAGA
CAATGAATACCGAGACTTTGATGAAGTTTCGGGCAGCGATATACAAGCAGTAGACGGG
ACTTGGGAGGAAAACTGGCTTTTCCAAAAGAAAAAGATAAAGAATATTCAATCAGCACCTGTACCTATGCTCG
TTCCAAATTCTAACACTCAATATAGAGCACTTATTGGCGATAGAGATGCTGACGATACCACAGACCTTTCCGA
CAATGCTAGTGATAACGAAGAACCAGACGTTAAATATAAAAGTGATATTAAAAAAGTT
CTGGATTCAAAACATGTGATTGGTGGCAAACCCAAAATAGATGAAACATTAGATTTTGAACCAGATAGTCTGA
TGATGATTGAAGATAAAGATGTTACACAAATTAAAAATGAGCGTACAGAATCTGAAGATACAGTAGATTCTGG
GTTAATTCAAAAGAATATGAGTAATGGTAATATTATATATAACGA
>103781815_2 DT665772.3
CGGCACGAGGGGTAGTGTCTTTGTAGTTATAGTATTGTTTTTTTGTATTGGAAAATGCTTTTATTAATTATTA
AGATTTTTTTAATACCTTTTTTACTATACAAATAGTTATGTACTCGTGTTACTGGTTTGAGATCTGAATAAAC
AACATTTTCTAACAGATAAGCTATTTTATATTGTAAGACATTCAGAGATCTATTTTGC
AACTAAAAGGAAATCGATTACGATTCGATATAAACAGGAAAAATCGATTTTTGTTAAATAGATCTCACACCGT
TCAATCACCACGTTTGCCTTAACAACATTCCTTTGCATATTCCTCATACGCTACGACAATAATATTTCGAATA
CTTAAATTATATTGATCTCTGTAATCGATTTGAATCAATTCCGTGAATCACACTGCCT
TACAGTATTTTATATGACATATAATCTCATTTTTATGCTTATATTTAGCTAGTACGCGATTTTGTATTAAGAA
TAAAAAATATTTTGTAAATGTAACTTTTTCATTTATTAACATTTATAAATAAAAACAAAATCTTTGAACGAAT
TCAGATAACATTAACATTAACCATTATTTAAAGAAAAAACTATCTGTATGTAAAGTAA
CGTAGAATCTTGTTAAATATAATGACCACTCGAATTTAAATGAAAGCTAAAAGCAAATATATAAAATTTGAGT
GTGCTTGGTATGTTTTCTGGTAAA
>103781817_2 DT665770.3
GCACGAGGCAGGAATCTGGGAAAATAACAAAAGTATTGCTGATAACTTATCTCCTGTAAATAATATAGCACAG
CCTTGAGTATTCTGAATGAGTGTAAATTGCATTAGATAAATGACTGTTAATATTTCTCGTATTAGTGGTTAAT
TGCAGCACGCGATGTGTTTAAATAATTTGTAGCGTCCAGTGGACAAGGAACGCGTCAA
TTTCACCAGTTAAAAAAGAACGAACGGCTTGGTGTATCCAAGTTGCGTGAGGTTTTAGAAGATAGACTACAAG
ACATAAAGCGAGCTAAAACGTGGAAGCACGAGAGGGTCATCACGTCGCCTCAGGACACAAATGTCAGGGTGCA
AGGTGAAGAGGGAAATTTCTTAAATTTTTGCGCAAATAATTACTTAGGACTGGCCAAT

CATCCAGAAGTCGTAGAGGCCGCTAGAGAAGGGCTGAGCAAGTATGGAGCGGGACTTAGTTCTGTTCGTTTCA
TTTGTGGGACTCAAGTTATACACAAGGAGTTAGAAAATCGATTGACAAAGTTCCACGATCGAGAAGATACGAT
ACTTTACGGGTCATGTTTTGATGCTAACGCTGGTCTATTCGAAGCTTTATTGTCGCCG
GAAGACGCTGTATTCTCGGACGCTCTCAACCACGCCTCTATCATTGACGGCATTAGATTGTGCAAAGCTCAGA
AGTACAGATATCCTCATAGAGACCTTAATGAACTTGAACATTTGTTGGCACACAGTGAAGCGCGACTAAAGCT
AATAGTCACAGATGGTGTGTTCTCGATGGACGGC
>103781829_2 DT666211.3
GCACGAGGCAAAACCCTTTAAAGACAGAAACCAAAACCAGCAGAAGTAAAGACAACCACACAAATGGGCCCAG
CAAAAAGAAAGACAAATCGCCACAGAAGAACTCACATAGTACTAGCGACATTGAACCAGAAATTAATGAAACC
AAGGAACAAAATAACACACAAAACTCATCAGTATTCGAAAATGACACACACTATCTAT
CAAATGAATTAGACGAATTAGAATCGGATAGACACAGTTTATTGGAAAATCATGATTTATTAGACAACAGTGA
TCACAGTTTGTTAGACGATGACACACACAATCTCCTAAATGATGCCTACAATGAAGTCATGATGATGCAAAGG
AATAAAGAAATGTTGTTGGGACTCATTGATAACCATTTAATTCCTCAAATGAAACCGT
TAAACGGGTATGGGTTGTTAGATAGGGATATGTATCTTAATACTGATAGAGCGTTAAATCACACGGCCCAAGG
TCAAATGATGGATCAGAAGGAGTTGATGAGAAGCAGGACCTTGCTGATGAGGCACAACGAAATGCTCCGGCAA
CACAGTGTTATGGTGGAACCTAAACATTATATGCCAACACCCAGTATAGGACTTGAGT
CTGCTAGTGAATTATTAGGTGCTAATTATCATCCAAATCACAATATGCTTCCACCGTTCGGCATGAGAGTAGA
CAACTCAATGGGTACAAGTTCTTTAGGGAATACAA
>103781841_2 DV501467.2
CTTAAAGGCCTTCCGGCGTCAGTGTGAAGCGGTTATAAATCTCCTTAAAGGCGGTATCCGTACCGATTTCGGT
AAGGTAAACCCCGTTTTTGTTTCGCTTACGTGGCATGCTGGCCACCGGCTTTCCGTAGACGGATGCCCCTTTA
ATGGGGATCACCCGGAACAGCCCATGTTTTT
>103781843_2 DV501486.2
ACGAAGACCAGCAGCAGTTTTTTTCAGCACCCAAGTTACCTGATGTCAGAGCATGTTGTATAACGGCGGGAAT
GAATCACTACGGTTGTGTGGACAAACTGTGTGACCCAACCAAAACATTTGAAATTGGGTTGCAGGTCACAGAC
CTGATGATATGCGCACCGTGGGCTGGAACCACCTTTGGGTGTCTGGCTAATGGTCTCG
ACCACACACCGTGCTGTAGATCTAGGGGACTTCCCGAAGAATGCTTGCCGTTATGCAGTGGAAATATAACTTC
GTTGGATTTTCATCATTTTAAGTGTCTCCGTTACATGACGGCGTATACCAACTGCTTATTCCAAGGATACGGC
GTTCTCCCTGGACCGCCATCACGTCTTCACCTCACTAACATCGACACCGACTA
>103781852_2 DV501464.2
GCACGAGGAACATACCCCTGGGTGATGTGGACTCCGTCCTGCCTAATGAATTCGAACTCGAATACCTGCTACT
AGAGGGTCACGCGTGGGACGCGTCCCTGGGCACGCCGCCGCGCGGCCTCCGGCTGGTGCTGGGCACGCGCGCG
GCTCCCGAAACCGTACACCCCTTAGTGATGGCCAATCTCGGCTACTTCCAGCTAATGG
CCAACCCCGGCGCCTGGACCCTGCGGCTCATGCCTGGACGCTCGGACGAGATCTATGAGATTGTTGGACATGA
CTTGACAGACACGCCGTCGGGATCTCATGATATACAAGTGCTCATGATCTCGTTCCGCGTTCTCGTTATCATG
CTGCGGGTCAGCTTGAAGCCGGAGAATCTTCGTCGAGATCTACTCGTGGATAATGATG
AGTAGATGTCGGGTGGCATCTGGAATTCTATTGCCAGTTCATTCGGCGGCGGCGACGAACTGGAAGCACACGA
TGAGACGATCAACGTGTTCTCCGTACCATCTGGCCATCTTTACGAGAGGTTCCTGCGGATTATGATGTTGTCG
GTGCTTACATACACTATTTCGCCAGTTAAGTTCTGGTTCTTGATCACTTTGTTGAGTC
CATCCTTAAAGGACATCTGTACCATACATGGCCGAAGAATGCGGTTTCTCGTATGAGCTAGTACAGTACCAGT
GGCCGCGCTGGCTGCTTCGGCAGCTCGACCGACAGC
>103781863_2 DT666717.3
GCACGAGGAGAGTTCTTTGAAATTGATTAATTGTCTATAAATATATTTTACTTCAAAACTTAATATGTAGTTA
AGAATATTTTTCTTATCATAAACATAGATTGAATGAAATATTTACTCAAGTCAACTTTGCAAATAAAAGGCAA
TTTCTGCCGAAGTTACAAAACAATGACTAACAAAGTTTTTGTATACGGCACATTAAAA
CGAAATGAGCCAAATCACCATTGGCTTACGAATCCAGAACATGGAGTTGGAAAATTCCTAAGTGAAGGCAGAA
CTAAAACTAAATACCCCTTAATCATTGGAACCAAATATAATATACCATTCTTGTTGCACAGCCCCGGCACTGG
GCATAATGTTAAAGGCGAAATCTATGAAGTAGACGAGACCATGCTTAGCAAACTAGAC
ATATTAGAGGACCATCCTAATTATTACATTAGGGAAATTGATGAAATACTTATTAGCAAAGGGTCGACAGAAC
AAGAAACAGTACAATGCTGGGTATATTTTCTGAAGAATTTCAGACCTGAATTACTATCGAAGCCTTTACTTGA
AAACTATTCATCATACGGCGATCATGGGATGCGTTATTTAGAAAGGTCAAAACGAGAT
CCCAACGATAATTACTATCCAGAAGTTAAACGATAAAATAATTCTGAGAGTTTGTTAAATTGGTAAGAAAAGT
ACTTTTGTGTGTGTAGTGATTGATGTTGACCGGTGACATTATTTGTTACAGTACGGTCTTCGAGTCATGGTCA
TTCTGAAGGGATTGGTTTTAGAATTTTTTTTACGTTAGATTATTTT

>103781871_2 DT665746.3
GCACGAGGGCAAAATCGTGTAAACAAGAGAAGGTTGGACCTCAGGCGCACCCGTACAACCCTTATTTAGGGGG
AGGAGGTCCCCCACCCTCCGTGGTAGCTTCAATGCCCAATCCTTTCTCACAATTAGGATTTGGTTTTAGAAAG
CCCTTTGATGCAAACGCCCTCGCTTCTTTTAGATACGGCACTTCACCAGTTCTGGGCG
CTCAATACCTCGGAGCACCACTGTCTCGACCACCTCTGTTCTCCGCTCCAATATATTCTACTAACACACCTCC
ATTCCACACATTGCTCGCTGGGCTGGCCGCGCCGCCTCGACAATCTCCCGACCCGCCACCAGTGTCACCACCT
ATATCCCCCGGAAGTGAATCCCCCCCAAGCCAGCAAGGACCGGAAGTTGAAAGAAGAA
GTTCGAGCATAGCTGCTTTAAGAATGGCCGCAAGGGAACACGAACTTAGGCTGGAAATGTTAAGGCAAAGACA
TCACACAGACCTAATTAGTTGAACTAGATAAAGCTAAATAATGGTGATTGAATTGTATGAAAGGTCATATCAT
TGACTCTACTTAAATTATTATTTAGCTGTGATGTATTTATCACATTATAAGTTGAATA
AACTATAAAGGTATCTCAATTTTGTTAGACAATTTTTTTTATTCCCCAAAAATTATATTGTATTTCAATACTA
GAAGAAATATATTATTTTTTATCCAATCCTAAAATAACTTTATC
>103781892_2 DT666196.3
GNACGAGGTTATAGCTTTGACAGTTATTTCATATTTTACGTGTAAACAGTAAATTTATAAATTTGCAAAACAA
GAATTAAGATTGGATCTATCTATTTAAGAAATGTGTTAATGTCATGTGAATTTATCGTAAAATCTCAACTATT
AATAAATGCAAGGTTCAAATATAAATAATGTTACAATTTGCTGTTAATGACAGTAAAT
AGATTAATATTTTTCGAAGCGGTATCTAAATATAATGAATAAAATATGTAGAATTTGTTTAGAAGAAGGAATA
TTGTCATCAATTTTTACAAAGAATTACAACATATCATTATGTGACATGATAGAGTATATTACTAACGTAAAAA
TTAATAAAAATGATGGTTTACCCGATCAATTATGCAGTAATTGCGCTTACAAATTAGG
CATTGCGTACCATTTTAAGCAAACTTGTGAAAGTTCAGACTCACGTTGGAAGCAATATTTGGGGCTAGAACTA
ATAAGCAAAACAAAGGATGCTGAAATGATGACTGACCCTATTGTTATGACACAACACACTATAATTAAGAAAT
GCAAATGTATCAAAGAACAAAGGAAAACATCTTCAAATTACAAAAGAAAACTTGAATG
TCAAAAGTTAAAAAGAGGGCCAAAACCTAAACCTAAAAAGGAACACGCCTGCTATCAATGTAATAAGCAATTT
AGATGCGAGGCTCAGCTGGGAATGCATA
>103781898_2 DT665158.3
GCACGAGGCTAACCCATAATACAGAAAATATAATTGTATAGGATATTTATATAAAAAACAGTGTTTTTTATTG
TAATGTAACTAAAAATGTCTAAAATTACAAAAAAGATGTAATAAAATTGACAAATTTAAACTGTTAAAGTAAA
TTTAAAATCAAATATTAAGCTATTAAACAAGTAAAAATTTGATTTTTTCTGTGATTGC
GTAACATGCCCGGTTTATTCCCGGTCTAAACATGAATCATTCTATTCTGAATGTAATAATTAACGCATGTTCA
AAAGTAGATAATAGCTTCAAACAAAAATTGTTTAATAAATGTTGTAAGAGTTACGTATCAGATGACCGCGCAT
TTCTTAACTGTATTTCCTCGGGTTCCCGATGTTTCCACTCTTCGTACACTTCATCAGA
AGCTCAAAAAGATGGTGTTGAAGTACTTTTATCACCACAAGAAGTCACTGCCATACTTCGTAAGAATGAATTT
AACAAGGACTTTGTTTATGGCTCAGTCAAGTCCTATGATTCTAACCAATTACCATCCAACAGCCCAATTGAAG
ATACTCGATGTGAAGCACAATGCAAGATGACACCAGGATTTCTTATGGGAGTGTTTGA
TGGTCATGGAGGCCCTGCTTGTGCACAAGTGATATCTAAAAGACTTTTCAAGTACATAGCTGCTGCACTTTTA
CCATTGAATAAATTAGAAAAATATATGAAACAAGAACCTCGTGAATTACTAATAGAGACTTATAATGATAGAT
CTGAAATTGTTCAAGATTTGAAACATATCTACGATGAAAGTTTAATTACTATTTAAAA
AATCTTTTTGAA
>103781904_2 DT665563.3
GCACGAGGAGCAGGATCTTTAAAAAGAACCTTATGTAAAGACACTCCGCTCTCCAGTTTCCGCCAGAATTCTA
AGCAATACGTATTGAGTGGTAAAATTGGAAATTGTACTATGTCTTGCCAGAACGATTGAACCCGCTACAATCG
CATTAGCCCACAACCAAACCAATGATATAATAAAGTTTGCGTAAGCTTTCACAATAAA
TCAAGGTAAATCTCATATTTTCTCCTTTTCCGTATTAATTTCGAAACATCCACTCTTCAACCTGTAAAATTTA
AACTTACTAAACCCAGAAGTTCCATCTGTGCGTCTGTCTGTCTGTCTATCATTCAGTAACGTTACAGTTTCAT
TTATACACAAAAATCCCCAAAATCAAGCAAAACTAATGATTAAACGACAAAAATCCCA
CAGCCCATTAGACCTGAAGACTTCGTACATATTTCATACGATAGAATACGCACGCACCGACCAATACGCGATG
CTAGAACAGTAGTATTTGATAAACTACTATCATTTGCGCTGTAAAGAGTAGATTCCTCATGCAGCCTTCACCA
ACTTTTCATCCTGCCCTTATCGGGAGGAGCGTGTTGCTCGCGTTGAGCTACGTAAGCG
CGAATAAATCTAAGTGACAGGTCCCGGTGAGTTGTTGAATACGTACTTCTGAATTTAAATTAGGTTTGTACTT
TGACGTGATGTTGTAAGGGTTTGGATTTAAGATGGTAGTGGGTTCAATGCCGATTGCGGCAATTCTTTAGTTG
TAGCATCTCTTGTTCATAGTAGAATTGAGAATTTGGTGCTAAGATATACCATTT
>103781914_2 DT666405.3

GCACGAGGGTTATCCTTTAAGGCCGCGCAGAAGTATCTCTGTGATAATAGAGTATACATTTAGCTTATTTAGT
GTTTATTAGAGTGATTTATTCAGTACAAAATAGGTAAGTATAAATAAGTAGCATATTCCCGAACAAACACGTC
GGTACTCCTTACTAAATCCTTGCAAAGAACGCTGCTGACTCATTTCGTGAAATTGTGT
ATGCGTTTCAGAAACTAAAATGCCGGGCGCCGGTACTTTCAAAATCTTCGTCGGGAATCTATCCGATAAAACG
ACGGACGCCGATCTGAGACCGCTGTTCGAAAAGTTCGGTATTGTTGTAGAATGCGATATCGTCAGAAATTACG
GTTTCGTGCACATGGAGAATGAGATAGTCGGCCGCGAAGCGATACAGAACCTGAACGG
CGAGCTGGTTCACGGCCAGGCGATCAAAATAGAGGCGGCCAAGAGCCGGAAGGCGCCGTCGACGCCGACTACT
AAAATCTTCGCCGGTAACCTCACGGACAAGACGCGCGCGCCCGAGGTCCGCGAGCTGTTCCAGAAGTTCGGCA
CCGTGGTCGAGTGCGATATCGTGCGTAACTACGGCTTCGTGCACCTGGACGCGGCGGG
CGACGTGAACGAGGCCATCAAGGAGCTCAACGGGCTCTCGGTGGACGGC
>103781926_2 DT666749.3
GCACGAGGTAAACACCTAACTTCTATTTTAACATTTATTGTACATAAAGGTATTATTTAGAATAAAAACAGAA
TAACTAATACTAAAAATCAGTTGTAGTTTTATATTACAAGTTACAAACAGAACTTTAAAAATGGCACGAGGAA
AGGACAAGAAAAAACGTAAGTTAGAGAAGAAGCAAGAAGGTGATGATGTCCCTAAAAA
AATACCTCACACTTTGGAGTCCCTTAGAGAGAAAGATGAGACTATGCTTGTTAATGCTGAAGCGGAAGAACAG
GAAGAGGCACAAAAGGATATAGAATTAGATGAAATGTCATCATATTTTGAAAATTCTTATGAGCCCAAAGTAT
TAATAACTTATTCTGACAATCCTCACTCAAAAACTAGAATATTTGGCAAGGAGCTCTC
ACGAATTATACCAAATGCCTTATCTAGGTATAGACAACGATCATCAGTTAAAAGAATAGTGCAATCGGCCATC
AGAGAGAAAATGACAGATGTTATAATAGTAAATGAAAATCAGAAACAGCCAAATGGTTTCCTTTTAATACATT
TACCAAATGGACCAACAGCCCACTTTAGATTATCTAGCTGCAAAATAACTAAAGAACT
TAAAAAAGATCATAGAGAAATCACTGCTCATAGACCTGAGGTAATTCTAAACAACTTCAGTACTCGTCTGGGT
ATGACCGTGGGTCGCATGCTCGGCGCTCTGTTCCACTACGAGCCGCAGTTCCGCGGCCGGCGAGCTGTCACCT
TCCACAATCAGAGAGACTATATATTCTTTAGGCATCATAGGTATGAAT
>103781928_2 DV501405.2
GATCCCACGTAACACCGTCATCCCCACAAAGAAATCTCAGATTTTCTCTACCGCCAGCGACAACCAACACACC
GTCACCATCCAAGTTTATTTTTGTGAACGTCCAATGACTAACGACAATTTTTTACTCGGCAAGTTCGATTTGA
CTGGCATTCCCCCGGCGCCGAGAGGCATCCCTCAAATTGAAGTTACTTTCTAAATTGA
TGC
>103781956_2 DT666391.3
GCACGAGGCGGCTATCGACGCGTCGAAACGCGCCCGTCCGCGGTCCTGTTCGCTGATTTATTTACGCCGCACG
CGTTCTCTATTTACTAAGTTACTGCGTGTTTTCAACTAACGTTGAAAGCTACAACTGTTAACGGCTAATGGCA
CTGAAAGTGAATAAAATTTAAGTGACTTTTTTTTAAATAACTAAAAATGATTATGTGG
TGGGTGTTTTGGTTAACTACGTTCACTCTTCTTGCAAACGTCAAGACATCCATCTATGACGATGACTACGACA
CGTACACTAGGACCTACGATTTTTCTACATCAGATATTCTCACCTGCACGTCGTCGGAGTTTAGATGCAAAAC
GGGACGATGTGTGCCGATGTCGTGGAGATGTGATAATGAAAAGGATTGCTCCGACGGA
TCTGATGAAGAGCCTGGCACTTGCAAAGTGGAAGCGTGCGGCCCTGAAGAGTTCACCTGCCGAGGGAAGAAAG
GGGAGTGCGTTCCCCTCACGTGGATGTGCGACGACAATTTCGACTGTTCTGACGGGTCTGATGAGAAAGCATG
CAATGAGACATGTCGATCTGATGAATTCACATGTGGAAACGGCAAGTGTATACAGCAC
CGGTGGGTTCGTGATGGCGATGATGACTGTGGGGGACGGTAGTGATG
>103781968_2 DT666210.3
TTAAAATATTTATTCTTGGTTTTGCCCATTAGCGTGTATTACGTCAACGTGTTATCTATTTTCTTAAAAGTCA
AACACGTTTAACTTTGACTTAATTATTGTTCAACCTTAGATAAGAGCTAACTTAATATTTGATAAAATATTTT
GATATGTGAATCCACCATATGTGAACGTCACAATTTCGTTACGCTTCTATAGAAACAA
TATATTTTTTTGCCTAACTTTCTACGTACACTTTTTACGTTTAGATATCTTTCTAATGAACATATTGCTAAGT
GCTAACATGCTTATTTACACATATTTTGGACATCTTAAGGTGTAAAAACTACAATAATCTATCCAGTCACAGA
CAAACATATAGCCAAGTACAGTCAACAAGAAGTACATATTATATTAGAAACACCAGAG
TGTTATAATAAGGTGACATTGCCACAGATACAAAAAGATCAATTTAATTTACAGTGGGTATACAATATTTTGG
ATGGTAAAAGTGAACAAGATAGAATTGTTTACAACAATAAATGTGAGAAGGAAGGTTTTGTACTAGTTCCTGA
TTTAAAGTGGGACGGACTTACAAAAGAAACATTGTACCTGTTAGCAATAGTTAGACAG
AAAGGTATTAAGTCTCTAAGAGATTTGAATGGCAGTCACCTGCATTTGTT
>103781970_2 DT665962.3
AAGAGCTGCATCGCTGAGATCTTCAACGCCGACGGCACTGCTGGGGGCAGCCCCGTCAAGAACGGCTCCAGCC
GCCCTCGTGTCGTTAGAGACACCCCAACCCGCCCGCGCGACACTTTTTTTTGTCTTTTCGGACAGTCTGGAAG
CAACTCTCAATCATCGCCAATGATAACGGACACCATCCGGAGCCACATCCAGTTTGGT

GATAGCGAGATGAACGGCAGTCCAGCTCACTCTCCCAAGATCAACGGCAGCGCGCACTCAACACCCAGCCGAG
GTGAGATGGACCTCGCCGACCACTAAGGCAACCCAATCACTGGCGACGGCTACAAATCTCTGAACGGTCAAAT
TAACATGGTGACGTCGATCAACGGCAACAGCAGCATCCTCCACAATCGCGTGCCGCCC
GGTGGGTTCTCATCTGGGCTCTGGTAGAACTCTCCCGAATATGGCCAAAAGTATCGAATACACTGCAATCTCA
ACAGATCTTTGATTTACCTCTAAAAATATTTAGATAAGAATTTTT
>103781972_2 DT665659.3
GCACGAGGGAATGTGCATTGAGTGAAGATCAAAAATGTATCAGTCCAAGTGTACTAATAATAAAACAGATTAA
TGAGGATGTCTTTGCTAGAGTTCCTGATGATAAATCAAAAGATCTAATCTATCTTATAGCATTTGCTGGGACC
TTCAGTAATAATCCAACAATATCAAGTGCAGCGTCAAAAGCTATTAAGAAAATACACA
TAAAATTTGAACATTTCAAACCTATTCTTCATAGCATGCTCAATGCAGCAGACCCCACAGTTAATATGTCGAA
GAAGAAGAAGTCTACAGTATCAATGCTTTCTTATCAATTAGCTGAAACAGATGAGTGGCGACTTGGTGTGACA
CTACTGGAATATGTTCAGAGCAAAAAGAAAATGTCTGTTGATAACGCTTTTGTAACCA
TTTTATTCCAATTGCTAAATAAATGTTTGAGGTTCGAAGAGCAATCTTACGTCGAATATACAAAACAGTTGAT
TTTGTCTTCGCTGTGGTATTTCTGTAAGAAATTTGTGAATGATAAGGACAAAGATCAATTGAGGTTAATCAAA
AGTATATTTGATGTAGAAACTATAGTACAGTGTATAAGAGGAACACAAAATCCACAAA
CGCAGCACCACGCTTTGATACTTCTATCTCATGCTGCTTATATGCTACCAGAGCAAGTTCTCCATCATACCAT
GGAAATATTTACCTTCATGGGGTCATCTGTCCTTAGACATGACGATGCCTACAGCTTCCAGATAATAACGA
>103781979_2 DT666533.3
TTTAACCTTTCAAAAACTTTCTGTAAATAGTGTTTAAGTTATTAAGGTGATTTAAAATGTTTTCTCCATATAC
TAGGCAAATATTCTTTTCAACACAGCTGCGACGATTACAGAGCACCTTAATTGTAGCAGAACATAATAATGAA
GCTTTAGCTCCAGTACACAAAATGCTTTGAGCGCTGCCAAGAAAATCGGTGGAGAAAT
ATCAGTTCTTGTAGCTGGACTAAGTGTAGTCCAGCAGCAGAGGTATATCT
>103781985_2 DT665574.3
GCACGAGGAGCAGCTCCTGCGCTCTACACCGCGACAGCATGGACCACTCCCACACTCCATCTAACTCCCACTC
TCACGCCTATTCCCACTCCCATTCGAACGAAGATAGTCCAATGCCACCTGTTATTAAACTTAGGAAGGTCCAG
AACTCTTCCCACGCGTCACCGAGCCCTCTTCCCTCAACACCAGGCGGGGAGAAGAGGT
CACTACAAAAACAACTAAGCGCTGCCGGTGGAGAGAGACCTCCCTCTCCGCCGCGAGTGACTCTACACTCAAT
CATAACGGAGTATTTGTACAATCAACACGCGCTGTGCAAAAACCCTGTTGTCACTTGCCCGCAGTTTAATTTG
TTTGAACCACACAAGTGCCCCTCGCCGTCCCCCCCGGGGTTAACTTACGCCCCTCGTG
GTTCTCCCCCGAGTATAATATCTCGCCTAGTGCGCCGCGAGCTCGGCGCACATACGACACGTCGTGCGCACGC
CCGCCTCGCGCACTCGCACTTCGCGCACGTGCGCACGTTGAGGTTGCAGGATGACGATGCGTATTTCACGCAC
ACTGTGTTTCATCCAACCCAACAACAGCTCCTAACAGCGACTTCATCAGGAGATATCA
TGATATTTAATTTGTTCACCGGCGTAGAAGAGAGTTCTTACCAAGTCCACGAGTCTATATTTATCATATGCAG
GCGAGC
>103781987_2 DT665641.3
GCACGAGGTTAGATCCTGAATACTACAACTTTATATCATATCAACGGCATATTGAAGAATCACCTCACCCAGA
TACAGCAACATAAAACATTGTCTGATGTTTCGCATATTATCAAGCAATTTGAGAAAGTATACCCACAAACTAT
TATTGACGTTGAAAATCGTGCTCAAAATGTTAACTTGAGCGAATTAATTCCAAATATA
CCATCATCAAAATATCCACTAAACTTAATCGGACAAGAATTTATAAATTCACGTATTAAACTTGAAATATCTG
AGGAAGTGTGGTCATTCTAAAAAAAAAATATGACTTCATGTTATCAAGATTTGCAAGATTCAGAACGAAGATA
TGGCATTCTAGTGACATCCAAAAATCTTAATGAATCTGCCGAATTGTCAAGTGCTAAA
TGCATAAACACTTTAACAGACGATACAGAAAGTAAGATAAGTCAAGACAACTTAAATAAATTTGCTTATGAAA
ATGTATATTTTAAAAAAGATATTCCACCAAGTATGAATGTAAAAACTTTGGCTCAAACTACAAAGAGACATGA
AAGCCTATGTGAACAGATAAATTATAATTATCAAAATATCGCTGATGCTGAAAATATT
TTAGGTACCATTAATACAAACAAATTGAGTATTACAAGTGAATCTGTACAAGTCTCGATACAGAATTCATATG
AAGATGTTAACCATCACATGAATCACAAAGATAATACATTCGTTAGTGAACAATTATTAACATCCCAGAGTGG
TAGTTCTTCATCAACTACCGATGAC
>103781996_2 DT666314.3
GCACGAGGTATAAACATACACAAATATAGATAAATGCAAATAAACACACAAATGCATATAAATCAATGGTTAT
TTATATACTAACACCACTACAGCTACAAATTTGACAGTTTTTAATTGTTAAAGTTACGTCATTTCATACAAAA
CCTAACGTAACCCAGCCTGCAGTTTAAAACTGCTAAATCTGTAGCTGCGGTGGCGATA
GTATATAATTACCTGCATGTAAACATACATAAATGCATATAAATACTAATAAAAAAAATTAAATAAAAACATA
CGCAAATCTCTCGCATTTTGCAATCTAACTATTTAAAACTTACGTAACTTACTTTTAAATTACTTATGATAAT
AAATAAAATTATTTTAAATTGATAAAATTTTGTCATGTTAAGCTTGTGCTTCAAATCT

GTTAACGCTTTGTTTGAGTGCGACTTAACTAATGTGTTGCCAGATTATGAGATTATGTAATTAATAGAGGCAA
AATAACATATTGTTTATTAAGTAAGATCTTTTACTAAGAATACTCGGAGTTTTAGGCTTTCTTTTAATGGAAA
GTAAAAAATCGTTATAAAGAACTAAGTTTTCTCTGAATCAGAAAACAACTTAGTTTCA
TCAAGTTACTTAGTTATACAGCGTAAAAGTATTTTAAACCTGGCAACACTAAAAGTGACTAAATCTTATTTTG
TACTGTGCAGCGATAAAACGATCCTTCCGTTAGTCGCAAAAGCGAGAGACTGGTGCAATTTACGTCGAATATC
CCACGACCAAACCG
>103781998_2 DT665716.3
GCACGAGGCACCATCCCTTCAAAGATGTTCCGCAGAGTGCTCACCATAGTGCACTCGCACGCAAAGCTTGGTC
TGACAGCGACCCTCCTCCGCGAGGACGACAAGATCGCGGACCTCAACTTCCTGATCGGGCCCAAGCTGTACGA
GGCCAACTGGCTGGAGCTGCAGGCGCACGGCTACATCGCGCGCGTGCAGTGCGCCGAG
GTGTGGTGCCCCATGACGCCCGAGTTCTACAGGGAGTACCTCGTCGAGAAAATCAACAAGAAAATGCTCCTGT
ACGTAATGAATCCATCAAAGTTCCGCGCGTGCCGGTTCCTAGTTCGATACCACGAGCGTCGCGGCGACAAAAC
TATAGTGTTTTCTGATAACGTATACGCCTTACGACATTACGCTGTTAAAATGAACAAG
CCGTACATTTACGGGCCAACGTCGCAGAACGAAAGGATACAAATATTGCAAAATTTTAAATTTAATCCCAAAG
TTAATACAATATTCGTCAGTAAAGTCGCCGATACGAGTTTCGATTTACCTGAAGCTAATGTATTGATTCAGAT
ATCTTCGCACGGCGGGTCGAGGCGACAAGAGGCTCAACGACTGGGTCGAATTTTAAGA
GCGAAAAAAGGTGCATTGGCAGAGGAGTACAGCGCTTTCTTTTATACACTCGTTTCTCAAGACACGTTGGAAA
TGGCCTACAGCCGGAAGAGACAACGGTTTCTTGTTACCCAAGGTTATAGTTA
>103782006_2 DT665760.3
GCACGAGGCTGCTGCTTTTTTTTCTCATTTGCTGAACTTATTTTGATGTGATTCTCTATAATGCTGTGCGAAA
CAATGAGGCATAAGGTGTGCGAGAAGATGGTGAACTGCAAAGGGCTGGCGTTGATGCGGTCTTATCTCGATAT
ATTGAATTCGAGCGGCAGCGAGCCTCGGTCGCGTAGTGCGACCCCTGATTCATTGGAC
TCGTTGCCGAGCGGCGGCTCCGGCAGCCCCCCCACCGAGTGGGCAGACAGCGCTACGCCACCAGACTTTGTTG
ACACTTACCCGCCACACACGCACGCGCCCATAGCTCGCACGCCTTCCGCCCCGGCGCGGCGCGTGCGCCTCTC
GCCGCCCGCCCACTCGCCCCCGCGGGTGCAGGAGCTGTTCAAGCCGAACGACTTACAT
TGGGCAGATATTGCTTCTAATACTGAGGGTATTGAGTTGCTAGGCTACCAAAGGTACGGGACAGTGCAAGGGC
CGCGAATAGGCAAGGAACGTATCAATGGATCCATACTGAAAGATAACGACCCTTTTATGAAACATGCGCCTGT
ATCGCGAACAAAGAGCGCGGCGTCAGCGCAACAGCCGCTCAGCTTCGAACATAAGTTT
AACTTGGACCGACAGATGATGCATCACGAGGAGGAATGGCCGGAACAAGACAGCGTCGTAGATATCGAGAGTG
TGGGAGAACCTCAGTTAAAGAAATTACAGCCTTTACTATGGTTGGAGCTGACTACGCTCTTCGATAGATACTC
TTTACCTT
>103782019_2 DT666613.3
GCACGAGGGAAAGGTCGTTCATTTTCACTAGTCATAATAAAATTTAAGAAGCTTATTAAGCAGGAGTGTTTGA
TACAATTTGTAAACACCAAACCAATAAGCTTCATACAAAATTAAGAATGACGTCTGAAGAAATCCAGATATCC
GAAGAACTGAAGGGTTGTTACGAGAATCTAATTGTAATCGACATTGGCGCAAATTTAA
CTAACAAAAAATACGGCCGTGATCTCGATTCGGTGGTGCAGAGAGCCAAAGATGCAGGTGTCCAGAAAATAAT
GGTAACAGGTACCTCAGTCCGTAGTAGTAAGGAAGCCCTAAGATTGACCCGACTCTATCCTAGCACAATTTAT
TCAACAGCAGGTGTACATCCTCACGATGCAAAATCGATGACGGAAGAGGATTTATGGG
CGGAGCTACAAGCGACCGCGTCGGCGCCGGAGTGCGTCGCTATAGGGGAGTGCGGGTTGAACTACACTAAGGA
CTTCTCCGAGCCGAGAGTGCAGAGGGAAGTGTTTAAACGGCAGGTGGAGATGGCTTGCGAACTAAGAAAGCCG
ATCTTCGTCCACGAAAAGGAAGCTCAAGATGATTTAATAACAATATTAGAAGAATTCG
GCAACCGACTACCGCCCATCGTCATACATTCCTTCACCGGTTCCGTGGAACAAGGAATGAAATATATAGA
>103782026_2 DT666125.3
GCACGAGGCTTCTCTCTAACTCTATCTTTCTTTGTTAGAGACTAGAGTTACGTACTAGTTATTTCACAACGTT
TCTTCAAAAATCTATCAATTATTTTCATAAGTTAAGAATTTAAATTATCTTGCAAAAATGGGAAAGGGTTTTA
ATAATTACATGTGTAAGAAATTTTTCCATCCAGCATCCAGGGATAATTTAAAAAGGGT
ATGGATGGCAGAGCAAAAAACTGATGCATATAAAAAAAAGCAAGAAGAGCTAAGAGTACAGTACGAGAAGGAG
CAGGAATTACATGAAAATAAAGCATTATTAAGCAAAGACAGTAAGGATAAGTTAAGCTTAAACTTTATGTACG
AACCTCCACCGGGCGTCAAAAAAGAAAGAGAACGCGAGGACAACGAACCAGAGTATAA
ATTTGAATGGCAACGTAAATATAATGCTCCAAGAGAAAGTTACTGTAAAGGTGACAATGAAATCAGAGATCAA
CCATTTGGAATATTGGTTAGAAATGTCAGATGCATCAAGTGCCATAAATGGGGACACATCAACACAGATAAAG
AATGTTTGATGTATTCACTTTCCATGAGTGAGGCAAGAGCTTTACAAGCTTCAGCAGC

TGCACCTGAGGAAGAAAATGAAAAACCAGATGTACCAACATTAATGCAACAAATGAGAGATACAGGTCTTGTA
ATGAAGCCAGGCGCTATGCCTCTTACAGCCAATACAGTAGTGGATAATGACACAACACTCACTGAAACCGATC
TCATCAGTCAATTAACTGATAAACAAAAGAAAAACTTTTAAAAAATTGGAGAAATTGG
GAAAGAAAAAAGAAAAGAACACAAACACAATCTAAGCACTAACATAATAAAAATTGTAC
>103782037_2 DT666854.2
GCACGAGGGATAGCCATGTGACATCTTGAGTAGAATTATTGTATTAATTTAGCAATAGCCATACGAAGCTCTT
TCTTCATTTTCTTTAAATAAAATTTATCACTAAATGAAATTGAAGAATGTCAAATTGATTACACACACATATA
TATGGTGCTACCAAAGTGTAATCGAGTTCTTAAACTATTAGTCACTTATTCAGCAGTC
CAAGATTTTTTTTTAAGTCTTATTTTTTTTTATATTATCCCATTTAATAATTATTTAATAGTAGCGCTCAGTA
GTGTGAATTAGCTTAGAACTGATATTAAACTGGAACAAATGAATTTATAATATTTTTATTTAATGTCAGTTAG
TCATTTGATTGATATCTTCTGATTTAATGAGGCAGTCGTGGCGCACCGGCACAGCGTT
ATGCTAAAAAAAAAAAAATATTTGTATGCATTCTATAAAATCGCATTGCGTTTCATAAAATCTCTTAAGGCTT
AAGTCCTAACATTTTTATATTTCCTTAAAAAAGCACACTTATAAAACTTCAATTTAATCAAAACGTGTAACTC
TTATGAACAAAATTGTTGTAGAAAATATGTACGATATTATTATTTCTTTTAATTATGA
GTGGTTAAAAGTTAAATATTAATTTAAAAATACGTTTTAAAAAGTATATTTAAAACAATTATATTGTTTTTTT
TTTTTCATTTTAACAAAACTTGTAGTACCTGATGAAACCTGATTTTTTTTTTCAACTTTTAATCTTTTTGGTC
AGTCTAATATATATATATTTATTATCTATTTATTA
>103782051_2 DT666454.3
GCACGAGGTTGCAATAATATTGTAATATTTATTTTTCTTGTGATTTATATGTTTCATGTTTTAAAATGCAGCA
AGCTCATCGTTCTGGTACCTTAAAACAGAGTAATAAAGTTCATAAATCCCGTCATCGATCAAAACGCGGTATT
TCGGCTGCTGTTAAAGGCAAAGTGAATATAAAAGAAGTGGTTCGTCGTAATCGCCACA
TACTAAAGAAAGATGAACGGCGTCACCAAGCTTTGCAAATACGTAAAAACAAACGAGACGAAGTTTTGTCTAA
GAAAAGAGCTATTGGACGGAATAGAAATCCTCCGTTCCTAGTTTGCGTTGTACCCCTGAATGCCCAACTTGAT
GTTCAGTCAGCATTAGTTATTCTAAAAACATGTTCAGAAGGGGCCACTGTGAGTCACT
CAACAAATGGTATTTTACATCTAGCCTTATCAAACTTCAAGCAACGCTTTTCATTTGTATGCCCAGAAATAAA
TGATGAATTTAGTTTACTTGATACCCTGAAAATTGCTGATACAGTGTTGTTTATCAGCTCAGCTCTAGATGAA
CCAGTGGATGAGTGGGGAGAAAAAGCTTTGGCCCTGTCTATGGCACAGGGCATGCCAA
CTCCCATTGTAGTAGCTATGGACATAGAAGGAGTTCATCCTAAAAAAAGAACAACTGAAAAACAAAATGTGCA
GAAACTCATTTCTAAATGGTTACCAGAGGAAAAAGTAATGCAACTAGACAAAAGTTCCGATGGTTTAAATGTT
TTACGTCGTATTGGTAACCAGAAAAGAATATTTTACACCATAGAGAAAAGAGACTTAC
ATGCTTGCTGAAAATATTGAGTATATGCCAATGCTATG
>103782057_2 DT666316.3
GCACGAGGGTATGGCTGTGTTTTTAGCACACGCATCACCGCGAATTATACAAAATGATCCCATATAAGTTCAT
ATTTGTTTTGTTTACAGTGCAATATATTTTTCAAGTAACAAATTGCCAACGTGACTTGAAGTTTTTTCGAAAG
GACTACAAATACATAGAGGATACGCGGGCGTTCTATAAAATCCACACGATCCACAGAA
CATGGGAAGACAGCAAAGATACGTGTGCCATGGAAGGGGCGACGCTTTTCTATCCAGATGATGATGATGAGGC
GAACACTGTCATAAACTATTGGAATGAAACACAACCGTTTAGTTGGGTTTTTATTGGGATTTCAACACCAAAT
GTCAAGCAAGTGTTCGAAACCGTAGACGGTGTTCCAATAACAAGTGTATACGACAGAT
GGGGCCCGGGTGAGCCTAATGACGCCGGTGGCGAGGAAGGCTGTGTTATATTACGTAGAGATGGAACGTTGAA
TGATGATAATTGTGAACGGAAGTACCCGTTCATTTGCAAGAAGACTTTGAGCTCTCTCGAGTGGAACAACTAT
TGTGATATACCAGATCTTGGTTACGAATACGTTGAGAAACTTGGCAGATGTTACAAAT
TCCACTTAACACCCATGAACTGGACAGATGCTTACACAGTATGTAACACGGAACAGTCGTATTTAGCGATTAT
TAATTCCAAAGACGAGGCGAACTATTTAAAGGAGTTGACTGCCAAAGCCGACAAGGACAAGGTGCAGGGGGAC
TTCCTACGCGGGGCCGTACTTTGGGTTATCGGTTAAGGAAGGGCAGTTGGCAAACAAT
TA
>103782059_2 DT666254.3
GCACGAGGACTCCCCTGATCCATTTTGTTTTTTTTTTCTAGAATTTAGTGTTTTGCGAATCTAATAAACAGAA
ATAACAATTATGGCTTCTCATTTAAGTACAAAAGATCGCTTGCTATCGATAATTGATGATATGGAACTAATAG
CAAAAGAAATAATTGAAATGTCTATAGCTCCGAAATCACAAAAACTATCAGCAACTGA
CCACGCACAGTTAACAGATTTGTTATTAGCCAAAGATGCTGAGTTGAAGAAAACTTTGGAATTAGCCGATGAA
CAGGCTAAAATACAACAAAAGATGAATGAATTAAAAGCTGAAGTTGATAACAAGGATCAAGATATTCACCACC
TGCAAAGGCAGTTGAAAGATGCTGAGCAAATCTTAGCAACGTCTTTATATCAAGCAAG

ACAAAAGCTTGCTTCTATAGTTAAGTCAAGAAAGAGACCAGTTCCTTCACAAGAGCTTATTAAATTTGCACAT
AGAATAAGTGCATCAAATGCAGTCAGCGCACCACTATCTTGGCAGCCTGGAGATCCGAGACGTCCATATCCTA
CTGACTTGGAAATGAGACTTGGAATGCTTGGAAGATTATGTGATTTACCTCTAAATGG
CCACACTCCCTCCACATTGAATGACCTACATCGAATAACCACGGGTGGAGTTCCAGCATCTACTCCAAATCAG
TTCACATGGCATCCATCAGGTGAGCTACACATGTCAGTTGGCGGAGGTAACTCTGTTCAAATAGATGGCCGTG
GCAAGGATGCACCCACA
>103782062_2 DT665856.3
GCACGAGGCCGATTTGCTATGTTTCTATAGTGAGAAAAAATTCGAATATACATGGAATATTTCTAGAAAGTAA
TTGAAAAAGCTATAGTAAACATGGCTATTTGTTTACGCACGCTTTCGCATAAAATATTGCCCATAAACGGAAT
CGTTACTACGAAAAATCATTCTAATCGGCTGTCCCGGAAAATCCCCTTGCGGACGCAA
TTTCTTGAAGATTTTCTTAGCAGCAGGCGCATATCTAGTTGCCAAGCGGCTCCAAAGTATGTTGCAGAAAATA
CCATCGATGATGTTTACATTCACGAGGTTGCCATTGCCAATAGTTACCTAACCCAAAAGTCGAACGAAAAGCC
GAAACTAACCTACATAGACAAAACAGATTCAAAAGAAACCAACGACATTATAGAGAAT
TACTTTAATTATTGTGTTGATGGAATGAAAACATTAAACTTAACTAGTGATGACTATGTAGAACAGCCAGATT
CAAGGAACTTACACAAAATGCCATCTATTAGTATGAATGATGTTGATTTTTCTAATTGCAATGAGGATATAGT
ACCAACATGTTTATCTGACTTCGAGCGAGATGCAATTGCATACTGTAGTGGACCTACA
GTATCCGCACAAACAACTACCAGTGAACCTGATAATGAAGGAAAACCCAGTGAAGAACAACTTATAAAAATAT
TTACAACTCTCTCTAATAATATGCCTAATTTGTTCGTCAAACCATTAGATTATTCAATCTATCATCCCAATTT
AGTGTTTGTAAATAACATTAGGGGCACAACAACTGTA
>103782082_2 DV501414.2
AGAAGAGCCAAGCCAGAGTTGGAACTTATTGACGAAAATACTCCAGAAAACACTACTAAAACAACCAAGAAAA
CAATTACAAAAGAAATAAAAGATGCTGTTGAAGAGAAACCAGTGACTCCTTCAAGACGCAGCACAAGGATTAG
ATCTAACACAAGCATTGTATCAGAGACTGCAGT
>103782084_2 DT666039.2
GGTGAAAATAAGGATGTTGAAAATGTTGAAAAGATAGTTTTGGAGAGTGTTGTTGTAGGTATTGAAAGAATAT
ATGTTAAAATGTGGGGATGTGATTATAATAATTTGGATTTTGAATATATGGTGGGTTTGTTAGGAGTTGATGG
TTATAAATTGAGAGAAGATAAATG
>103782088_2 DT665356.3
GTAGTGACAGTTTCAGTGATAATATTGTAGTGCTTAAGTTTTTTTTTATGTGACAGCTGTTTTTAAAGAAGA
TTGAGGCAAAATTAGACAATAGAAAGGCACGCACAGCTATTTAAGTCAAAATATGAAATACTGTCGTTACTGA
ACAAGCTGGACAATAGAAAATTGAAAATTAAAAAGAAAAATAGCAAATAATAATGCGA
AGCCAAAATTAAAAATGGTATTTTTTGAACGTAACGTGAGTTTAAGATAATAAAATGGCTCACAAAGTGAAAA
ATAATCACTACGTAGCGACGCATCGCTCGGGAATCGACTCGCCTTCGTCCGGAGGGTATCTGCCTAGAGCGTC
GTCGAGGGACGAGAATGACAGTCACCGGGCGCCTTCTGAAAGAACTCTTTCAGAATAC
ACTGCGATAGACGAACGGACACGTTCTCCTTCAGGTGGGGAACATAGAAATGGACGGAGACACAATGGTTCCC
CGCGGGACTCCGGTTCAGAAGTGTACGTCACCAGCGCGGCGTACCGACCGCCATCTGAAATAAGCCGGCAGTC
TCGCGCGCCTCGCAGCGTCTACTCCTACAGAAGTGCTGTGGCACCCTCTGTAGCATCC
ACACACCGGCCCAAAGTGTCTAGAAAGGCTGGTGTTAAAAT
>103782094_2 DT666351.3
GCACGAGGGTCGTTCGTTAGCTTAGGGGAGGCTGTACAGATGGTGTATAAGAAATTAGTGGCAGAAGGCAAAA
TCATCGAAAAGGAAGAAGTAGAGCCGCCCAAAGTGCCCATGGACTACGACTGGGCTAGAAAACTAGGCATAAT
TCGTAAACCAGCTGCGTTTATAAGCACTATTTGTGATGAAAGAGGTCAGGAACTGAGC
TACTGTGGTGTTCCCATAAGTGCAGTGCTAGAAAGACAGTTGGGTGTCGGCGGTACTATTAGTTTATTGTGGT
TCCAGCGCGAGCTCCCCGACTGGGCGTGCAAGTTCTTCGAGCTGGTGGTGATAGTGACGGCGGACCACGGGCC
CGCGGTCTCCGGAGCGCACAACACCATGGTCACCGCCAGGGCCGGCAAGGACCTCATC
TCCTCCATCGTCAGCGGCCTGTTGACTATTGGAGATCGTTTCGGCGGTGCGTTAGATAAAGCAGCTCAAGATT
TCTGCGCTGCATATGACAAAGGGCAGCATCCGCAAGAGTTTGTCAACGAAAAGAGAGCTAGAGGAGAACTCAT
CATGGGGATTGGGCATAGGGTAAAATCAATAAATAACCCTGACTCCCGAGTGCGGGAG
CTGAAGGCGTACGTGACGGCGCGCTGGCCCGCCTGGCCGGTCACGCGGTACGCGCTCGACGTGGAGGCCATCA
CCACCAGGAAGAAGCCCAACCTGATCCTGAACGTGGACGGCATAGTGGCCGCCGCCATGGT
>103782110_2 DT665468.3
GGCACGAGGGGAAAGGACCTTCACCATGCTCTTCTACCGGTTCTTTATTTTGATGTTTCTATACGTGAGTATA
GCGATAGCAGCGCCACAAGACTCTAGCGGTAGCTTTGTAAACACAGAAGTCTTCGACACAATTCCTACAACTA
ACCGAACGTCTCGATACGAAAACTTTCCAATTACAATAAGCAGCTTTAACAATTTAGC

AAAAAACAATTCTAATACAGAATACGAAACCAAAAACGAAAATATTATATCAAATAAGGAAAATGGAGATAAA
GTAGAAGTATATAACGCATTATACTAACGCATCGTTTGAAAATAACGAATTCATACGCCATGTTGGAACCGAT
GACGTCACTAGTGTCACTGAAACCGACCAATCAGAAATAACCACAGACCAAGACCTAG
CAACCGAAGATGACTTTACAACCACAGTTACGCCTAAAACTACAACCACTTTGAAACCAACCCCTAAATTTCT
ATCAAAATTAACTAATATAAAGAAATCAAAGAAGAACAATGTTTCCACGAATTCAATAAAACCTTACGTCGCT
AAAGATAAGTCTTATAAAAGAAAATTCAAAAGCAGGTGCAGATGTGAAAAAATAGAAA
ACTGCCCGAAATTGCAGATCACCGTACCGAGATGTCCCGATGAATATTTCTTGTGCTGTTTCTAATCTGAACT
TAGATAAATATTTTTATTTCTTTGTAAATATTTGATATAATTACTAACCCAGGTTAACACTAAAATAAATATT
TATATCAATAGATAAACGTAGGTTATTTCGTTTTAAG
>103782122_2 DT666609.3
GCACGAGGGAACTTACATTAATTTAGATTTATTTTAAATATCATATATATATTTAGCAAAAAGTAGCTTAATT
TCGTTTCGTTATAAACTAACGTATACGTGTATGATGAGTTTCATATTTGCTTACGCGGATTCCTTTTTTTTGT
AGTACTTTGGTATGTTTTTAAATTGTCAATATATTGTATAGTATTTTATACAATTTTG
TCCCATGTCTGGGACTCGCGGTATTGCCGCCGCGTGAATGGTGGCAAGTGAAATTCAAAATTTACCATAGTCC
ATTTTATATCAATTGGCTAAGACGTTTCAAGTGACCTAAAATAGTCGTTAGACTACAGATTTATTAGACAGAT
TGTAGATGACAACCGTCGATTCACTATTTTTGATATAATCTTGTCGAAATTTGCCGTT
TCATGGATGTTTAATTAGTAAGGAGTTGCGGAAATACACGTGACGTTCGTTGGATTAGCTAGAGTAACTTTAT
TTACAACATTTTTATATTATAATGTGGATGGATAGAACATATTCGTATCTGAACGAGAAAATACTTTTTTTTA
AATCGGAATGCCTTATATATGTTTGTATTTCACATTTCTATTAAAACCTATATGGTTC
ATTATTTTTTAAAAAATCTTCCTTATCTTATTAATCTTCAGTATCTTATTAATTATGACGGCCAAAAATCTTT
TCGAATTCAGATACGACTATGTTCTCAAAGTTATAATGTGTATTATTATATTATCGTAATGGCAAGTAGCAAC
TTGAGAGCCTTTAAA
>103782137_2 DT666725.3
GCACGAGGCAGCTGCTTAATACATTCATTTCTAAGTTCTGACAACCACAGAAGTAAATAAAAATATTTACGCC
GGTTTCCGTTATTATTTTCAATTTTTATCTTATAGCGATTTCAAATTATTCCTTATCAACAGAGAACTACTAA
GCGATTATTATTTATGAACTGTGAACGGTTCATTATATTGAAATTCTATACGCCGTTA
TCACGCTTACCCTCCGTTGCTTGTGAAATTTATCTTTGTAAATTATCGCCTTTTTTTATATCAAATCAAAGTT
ATCTGGCCTGCAACAAGCTAGGTCCGTTTCCTTGACCGTTTATCTATTTATACGATAATAAACGAAGTAATTA
GTGTGTAAAATTTTAAGTAAAATGGCTGTTATAGCATATTTAAATCCGTTTAAAGTTT
TTCGAGATTTTATTCGGCAACCTATAGAGTCATTTGTTAATGACCAATATACGGGATATAAGAAGAAAACAAA
TGAAAGTATCACAAGTGTAAAAGAACTCTTATATAGAGCTGGTGTAGTAGCTGTGTTATTGTCAGCAATATTA
TGGATTTCAATATTTTTCTACGTTATATTTTATTACACGTACATGCCTAATGTAACAC
ATGTGCGGCCTGTACATTTGCAGTTCAAGTCATGTGATGAGAATGTGGGAGTTTGTTCATTTCCATCTGCCCA
TGTACAGTTGACTAGACGCAGCCAAGTGTTGATGTCTCAACAACCTTATAGGATTAAGCTTATATTGGAAGTT
CCTGAGTCTCAAGTAACAAAGAACTTGGTATGTTCATGGTATGTGCTCAATTACGATC
CACTGG
>103782144_2 DT666366.3
GCACGAGGCAAAATCTTATTTTTATTTGATTTTAATTAAAAATGAAAGAAAAACGTCATAAAAGGAAACATAA
AAAAGCTCATAATTTGGAAAGTAAGCGTCAAGAAAAGAGACACAGTAAAAGAAGAAATTCTTCATCTTCAGAG
AGTTCTGATTCTGATGAGTGGGTGGAGAAGAAAATAGAATCTAAAGAAAGAAGAGAAA
ATACGTCTCGTGATGACTGGATGTCTATGACAGGATTGATGAAAACTTATACAAAAGATGATATTAGACCAAA
AAAAGAAATAAAAGAGAAAGGTCATATAGATTCGTATAACCCTTCAACAAGTAGTAGAGAACTCAATCCTTAT
TGGAAAGATGGTGGCTCTGGTGTACCACAAACATCAGAGAGCTACCAGAAATCAAGAC
AATTTTTAAAACCTTCAAATGATGACGACTATTATTCATCTTCCAGAAAAGACCACAAACATGAAACATCAAA
GCATAAAGAATCAAGTGTAAACAAATCATTCAATTGGCGTAAAATTACAAAAGACAATAAAGAACATATTCAA
GAGGACATATCACAGAATTTGTATGAAGTTTCTCAGAGTACTAACAAAGCATTTCAAA
ACCTAAATGAAATAGAAAGTGAAAAACCTAAAACTAGAGACAAAAACAGTTTATATTTGTCAGATGAGAAGAT
GAATAAACTGGGTGCAAAAATTGTAAAGGCTGAAATACTTGGAGATATGAAGTTAGTTGAAGAA
>103782164_2 DT666806.3
GCACGAGGTGTGTACATAATATATATATATAACTTATATGTTACAAGCGCGATAGTAACTATAAAGGCCGCCG
GCACATTAATAAATATTTATTTTCTTTAATGAATATAAGCTCCTTGCAATTCGCTCTGTTGAAGACAAGTACA
TTTTTCAGAGCTAGATTACTGTCCGCAAGTATGTCTTCAAAACCTTTAAGTATCGAAA

AATTAGAAAAACTAAGGAATAATTTCTATGCTGATCCTAAGAATGAACTTGCTCAGAATGTATGTGCAAGGTT
TGATCCATTCGAAGTAGCCATAAGTAAGAGGAAGACTGAAAGACAACTACATGTTTTTAATGTTAAGATTGAG
TCAGAAGGTAAGCCTGTTACAAATCAAGAGTACACGGGTAGATGTTGGCTGTTCGCAA
TTTTGAATGTCATGAGATTGCCTTTTATGAAAAAATATGGAATTGAAGAATTTGAATTTTCACAAAGCTATCT
ATTTTTCTGGGACAAAATTGAGAGATCTCACTATTGGTTAAACAATATTGTCCAGACAGCGAGACAAGGTGAA
GAGTTGGATGGACGCTTAGTTAATTTCTTACTTAAGGACCCAATCAATGATGGTGGAC
AGTGGGATCTTATAGTGAACCTAGTGAATAAACATGGATTGATGCCGAAAAAATGTTTCCCAGAAACCTTCAG
CTCAAGGCGTAGCTTGCATATGAATGCTTTAATCAAAACTAAGTTGCGCGAATACGCTAAAGAATTAAGATCA
TTGGTTGCCAGCGGTGCGTCT
>103782166_2 DT665822.3
GCACGAGGCAAACATCGTAGTCATATTATAGCTAGATATAGGGATAGTTTGGAACAGGTCAAATTTCTATTTG
TCTTAAGGTTAAAATGTATAGATTTTAGAATATTGTTTTAAAATATAAGTATAGCTTCACTAAGCTTAAAAGT
TACAAAATATTATTTCTATAATGCGTTGATTTATTGTTTATAAGTTAATTTTATCAAC
TGACGTCAATCTATTTGGTTTTATAAATCAATATAATGCTATATTTTTATTAAAACTTGTCACTGTTTATATA
ATAAGAGTGATGGACTAAAGAAATTTATTATATTTACATATATTTATTAAATAACATAATAAATTATATACTA
TTGACATATTATGTACAGTCCGGTTGATGAGTTATACTCGTGGCCACTTTGAAGTTTA
AGTAATTTTGCGTATTGTACAATCGAGAAAAAAATTATCAAAAGTAAGAAAAAATCCTAACATCAAATATGCG
CAGCCATCTACAGCTAACTTCAGTGCGGTTACGAATTATCGGCCGTACAGCGCACTGTATAGCCCGACAAATG
CTTAATGATTTTTTTAAGTAAACATGCGTTTAATTTGCGCATATACACTTGTACAGTC
GGGGACATAATTTTTGAAATGGAGGCAATATTGCACAGTAAGTGACACTTAGCATCGCACTATACAGTTGCGG
TGCGGTGCAGTATGAGCGGGGATATCTCTATCTACGTATCAAAAACATGCATTCTGCCCAAGTGTACGCGCGT
TCCTGTGAATCGA
>103782174_2 DT666319.3
GCACGAGGTTAATACATCGTGAAATTAACCTATAAGCAATTTGAGTTCCATATGAATGTATTATTTTATTTTA
TATGTCGTGTCGGGAATTTAATCAAGGTTATATAAAGAAAAACAAATTTATATAACATATTAATAAAAATATT
ATTATCTATGGAAATAATTTTTTTATTAATATGTTTTATAAATTTTTCTTTGTTCAAT
CTGCAATGTGTAGGGTTATCATGAAAAAAAAATAAGAAATCGTCAAATAGTTTTTAATCGTGGATTTAATTTT
TTCTGGCGCGACAAATGTTTTGTACATCTACAACGGAATCATTAGCAAGTTCGATATTTTTAATCTCATCAAA
CTAAAATAATTCATTTCATTTTGTATCGTGTAATTTTCATCGCAGCCTTCATTTTGTT
CCGTCATAGTTCATATTATATTGTACTTATTCCATTAACGATATTCGCTAAAGAATACTTGGAAAAATCAGAA
AATTAAAAAAAAAAGAAAAAATATTATAATAATTAGTCAAGTTTGCATATAATATTATGTTTGATTAGATATT
AATCTAATATAATGTGCCTATGCACTTGATTTGTGTCCGCGACACCAGGACGAGTGAC
GACTACGTCGACACTACGGGAGCTTGCGATCGCCTCGCGGGGACGAGGAGATCGGCTCTTTGNCAGCTCCCAC
GCCAGCGAAATTACTGTTAAAACCTATCATTTCTCAGTGATTTCACTGGCGGCACTTATCGCCTCTAG
>103782189_2 DT665932.3
GGGTGATACCGGCCAAGGAAGTGGTCATGTGCACCGACAAGAGATTGCTGTACTTGGAAAAGAATAATGTTTT
CGGCGGCTGGCAGATCGTATGGACCTACCTATGGACAGAAATCCCTGACGTACCGACAGCGGTAGCTAAAGGC
GTGTACATACCAACGGCCAAAAAGAAAGTACTAGGAATGTTCCCTTCATCTGGTTCAG
GAAAGGTGATATTCCTGTACGACTAGCAACAAAAGAAATATCTCCTGGCGCAGTGTCAAAGACTCATGGCGTC
TCGTTGACATGAACTTGTAGAAAGAACGAGCCCGACCGAGTGGTTGAGTGAATTCTTTCTACTTATATTTGAT
TATCGCACCGCATTGCTTTCTATCGCCTATCACTTTAATCTACACCATTTGAGCCCTA
AAGGCTGCCCTTAGTTGTTTTGGAGTTGCTTTCTAATGAATCTTATTATTTTTATTGTTTTTAACAATTTTTA
AGTTTTGTTCCACACTAAGGATAGAAAACTATTTTTAGAAGACGTATAGAAAATTTAAGGGCTCTCTTGGAAA
CCCAGATAAAAATGTGAGTGGTCAGAACGCTTGGAC
>103782191_2 DT666367.3
GCACGAGGCTGATCCTGAGGAACATAAACATCGTGGAAACTAATTTCCATGAAGGCGAAGTAACACAAAAGCC
AGCTCCAGCGAAGACCGGTCCACATGGCGTCCAAGTGGTGAACACTGGGCCTGATCACTCTTTCGTGCTGGAT
GAAGAGTCTCTCGAGGAGTTGCTGCTCCAAGATGACATCAAGGACAGATCAGCGGTTG
TCATATCGGTCGCGGGGGCGTTTAGGAAAGGGAAATCTTTCCTTTTGGACTTCTTCCTCCGGTATATGCATCA
AAAGTACGGCGCGGGCGGCGGAAGCGGCGAGTGGCTGGACAGTGAGGAAGACCCGCTGGTCGGGTTCAGCTGG
CGCGGCGGGTCTGAACGGGACACCACCGGCATACTCATGTGGTCTGAGATCTTTAAAG
CCACACTCGAGGATGGTGAAAAGGTAGCAATAATCCTGCTGGACACGCAAGGCGCGTTCGACAGCGAGTCCGC
AGTGCGCGACTGCGCCACGGTGTTCGCGCTCTCCACGATGCTCTCATCTGTTCAAATATACAATCTGTCGCAG
AACATACAGGAAGATGATTTGCAGCATTTACAGTTGTTCACAGAGTACGGGCGTCTAG

CGCTAGAGGACGGGGGGCGGACGCCGTTCCAGAGACTACAGTTCGTTGTACGGGACTGGAGTTTTCCTTATGA
GGCGCCGTACGGAGC
>103782206_2 DT666207.3
GCACGAGGGCTAGTCTTTAAAGGACTAAAATAATTGAAACTTCTAAATAAGCTATGCTTATATATGAAATTGA
AACTTCATGTGAACTTTATTTTAACTGAGTTTGTTATATAGAACTACAAATATATTTATGATAGTTAGACTAG
AAAACAATTAACTTATTAGAATATTGTTATTCGTGAAGTTCAATTTTGTTGTAAATAT
GTATTAAAAATATCATTTGAAATTGGGAACTTCATGTAATTGAATTTGAAGTGTACCTAGTGTAAATATAAAT
ATCTAAATTGAAGTTCAAAATCTTAATTTAATAATCTAGAATTATATTAATTTACACCAAACTATTAAATAAG
TTTCAAAATGGACGCCATTTTCTATTTTAAGTTTTTTTTATTTATTAATTAATTTTAA
TTAGGCTGTGATTTAATTTTGTGCTCCTTATTAATAAGTGATTATTTTTAAGAAATTTAAAATTTCACCAAAA
GAATTACTTGTATTTGAAAAATAGTTTTTTTGTTCGCGACTCGGGGGCGCAAGTGAGCGGCTGTAAACGCTAC
AGTTGTGTTCTATTCCCACTCCAGGAATAAAATGTTCAATATTTTCATTTCCTCGTTC
TAGTAACAAGGTAAAGTGTAGCGTGTAGCGGCCACTAGCACTGCCTCGAGAATTCGGCGAAATTGAGGAACGG
AATTGTTTTGACGCTAGGTTCTCTCTACCTACCCTACGATGTGCGGCACACCGTCTTATTTAATAACGCAACG
GTTGTGG
>103782218_2 DT666218.3
GCACGAGGCTGAGCCTCTCAACAGATCACAGTGTTGTATTAACTGAGGATACAGACAGTTTTATAATTGGTGA
ACGTGTATGGGTGGGTGCCACAAAACCTGGCCAAATAGCGTACATCGGTGAAACACAATTTGCTCCAGGTGAT
TGGGCCGGCATTGTGCTCGACGATCCTATAGGTAAAAATGACGGGTCAGTAGCTGGAG
TTCGCTACTTCCAGTGCCCGGAAAAGCGTGGAGTATTTTCACGTCTCACGAGACTCACTCGAGAGGCACTCAT
AATTCATGCACCGCATGATGCTTCACCTATTTCTGACGCCTGATGTGTGTTCGAACGCCCGCCTTCTGGCTCC
GCAAGGCCCAGGCGTGCCCTCTCTCCAAATGGTAGCGTACGAAGCGTTGTCAGCAGTA
AGATGAATGCTTCCATTTCTACCACCACAAACGGAGATCTTCGCTTAGGTGACCGAGTAATCGTGTCAAGTAG
CCGAGGCAGTAAAGCCGGTACCCTACGATATGTGGGAGTTACAGAGTTCGCCCCGGGAGTCTGGGCGGGTGTA
GAGCTCGACGACCCTCTCGGGAAAAACGATGGGTCCGTCGATGGGAAAAGATATTTCG
AATGCGCGCCGCGCTTCGGATTATTTGCGCCGATTTCAAAAGTGTCCCGATCGCCGTCGAACCGCAAGCCGGG
CGCCTGCGCCATCCACAGCAACGGGCGCGCCACGCCGCTGCGGCGCTCGCACTCGCGCGAGTCGCTCACGT
>103782234_2 DT665203.3
GCACGAGGAATCCCTTTAGGATTTGATTGAATTTGACTTATTAGTATTTATTTTGACATTTTGAATGTATCTT
TTTATATTGTCATTTCCATTATCAAAAAATATATATTAATATTAATGATAAATACTATTAACAATTATTGATT
ATTATTAAAGACTTTTCCGCAGTCGAGTCGGAATAGGGCCTAAATTAATATTAAACTA
GGGAGATCTCAAAATGTGGGGAATAAAATATGCGAGAAATCGAACACCATTTTTATTATAATACTACATAAAG
AGGCAAAGGCATTACATCCATTCCGTTTAGTGAATTACTGTAAAAGTAGTAGAGTAAATAAGTATTGAAAGCT
GAAATTGAAAAAAAAAATGCCAAACTGTAGTTTAATTAAACTCAGATGTATCGGAAAC
CAAATTAAATTGGGTTTAGTCAAAATTTTTGTGTAAGTAGATGGACGACGCACATAGCATCAATTGGATATAT
TACATATGTAAATGTAGCTATATATACATTATTGCAAAATATGAGATTCACACCAAGATAGCGTTGGAGTTAA
AAGCCGTGTCTGCAGATCCAACATGGCGGCCAACACACCAGCGCCATCTTTGTATGAA
ACGCTTGTAAACTACTGTAACTTGTGGATAATTTGATCTGTAACCGTAGTAGAAAATGGCGGAAACGATAAAA
AACAAATATTGTTACGCTAAATGGTAAATAAAAACTTTCGAGAGTGCAGCTTTAATAACTTTTCATATTTTTT
TATATATTTAAATCGAATATGTCATGTAAAAGGTTATTTAATGTATTTATAAAAAACA
TAATTTCAACTAATTTATACATTTGGCACGTTT
>103782238_2 DT665971.3
AAAAAATAAACAATAAACTGTAAGATTAAAATACAATAAATTAATTTGGTCCCAAAATAAATATTAAAATAAT
TTAAGGGAGAAGTAAATAAAAAAGAATCAACAAATATAAAAAAAAAGTACTCATCAATTTTGGCAATAGTTAA
TGGAGTCCTACTTAATTTAATGTTAAACCTTAATAGAAATAAATAGACTAAACATACG
ATACATATTCAATCATACAAATTTTTCACACTATTTAGTCTCCATTCAGTCCTCTTCGAGACTATATTTACAT
GGTAAATGTAGCTACGGAACCAAACACTCGATGAATACACAGACTGATTAACAATTAGATAAATCCTATTAAT
GTCGCCTAAGAAAACTATAAAGCTCGTCTAAATCATTTAACACACACAGAACTAGGGA
TGTTCGATCAGAATCCATTACGACAGTGCAGGTCGATAAAAAGTAATACTATACACCTGATTTTCATATCGAT
ATTATGGTTTTTGATTCGTTAGTAGATCGACACAAGTATAATGCAAATCGGGTTGAAAAGTGGGACCAATTTA
TTAGTATA
>103782250_2 DT665657.3

GCACGAGGTGGCTGCCTTTATAAAAAAAACACGTGGCTTTGTGAATCGTTCATGTCATAACCTCTACACGGAC
GAATTGAGCGGCCGCGGTCAAGAGGTAGAGTCGAGGTTCACTTTTAAAAGGAAGATACAAATACAAACAGCAG
AATGGTTAACGAATCTAACGGAGGTCTGGCTCCGGCGCAGGAGTTGGAATCTTTTCTA
CCTCAAGATGAGAAGAAAGGACAAATCGTAAAAAGTAAAAAATACAACTTAGCCAAAGAAAAAGATGCGGAGT
CCGGGGACTTCGACCCTTTCTCAGAAAGACAGGCGGAGAATCCTACTTCGAACATGGACACACTGACCCACTT
GCTGAAGGCGTCGCTCGGCACCGGTATCCTCGCCATGCCTAAGGCCTTCAAAAGCGCG
GGTCTCATATCCGGGATCTTCTTCGCCATCATAGTAGCTGTTGTCTGCACACACTGCGCCTATATCCTGATAA
AGTGCGCTCACATCTTATATAAGAAGACGCGAAAAACTGCTATGAGCTTTCCTGAAGTAGCTGAATCTGCGTT
GGACAATGGACCACAAGGACTTAGAAAATGGGCTTATACTTTCAGAGTTTTCATCCTC
GTGAGTCTATTCATGACGTACTTCGGAACCTGCTCCGTCTACACTGTTATTATCGCTAAGAATATCATGCAAG
TCGTATAGTTCTACATGGTCGACATGAAGAACCTAGATATACGGATATTTATCATCTTCCTGCTGATACCCCT
CATTTTTATGGTTTGGATCAGAGACTTGAAATACTTAGCGC
>103782252_2 DT666191.3
GCACGAGGTTTTTGCATGAAGGGAATTCCCCAAGTGCAATTTCATAAATCCTAGTTTTCCTTAACAGGAGGCA
ACAGAATTTTTGCATCAGCTAGAGTTCAAAATGTATTTTTTTTAAATTATCGCTGTTAAAGATTGTGCGTTTA
AGAAATAAAAGTTTTAAAATATTTCATCATGAACAAAAGGGACGAACTTCGCAAAATT
CGCGCTGACGCACTTTGTAAAGTTATTAATATATTAGAAATTAACGATGATTGGAAACGAGTAATGTCTATTA
TACCAAAAAATTTACTAGATGAAAATTCTGGACACAAATATAATAATGAACACATAAGATTAATAGAAGAACA
TGCAAAAGCGAACAACCAAAAATGTGCAGAGATACTCATTGATGAATGGGGAACATCT
GGAAGAGTTCGACCAAATTTACATCATTTAAAAAATATTTTGATAAAAGCACAAATATTTAGAGCAGCTGATG
AAATAGCAAATATGTTGCAAGAACCATTGCCTGAGCGCCCATCATATGGTCCGGCTGCATTTGTTACCACTGA
TCTTACTAAAATGTTAAATGAAGAGATTTCACTTCAACAAATCAATGAAATACCAACA
CATAAAATGACGTCTTTAAACAAGCTCTATGATGGTACAACAAGACAAATGGAGTCAGTTAGTGATATGATAG
AATTTTCTA
>103782260_2 DT665421.3
GCACGAGGAGCAACGTCTATAGCACCATTGATAGATGGTTATCCAGTGCCTGATGCAACAGCTCCTATGCCTG
GTTACACAACGGGTACTGTGCAGGGCCCTACCTTCCAACACGGGTACTGGACGGCGCCCCAAGCGGAGCAAAA
CGATAGGAGCGCCAGCCGCAGTCGCGACAGACGCTCCAAAAGCAAGAATAGAGACAGC
ATAGTAACTAGGAGTAGATCGCAAAGAGATAAACCGCCATTGATCCCACCGGTAATCGAACCTATAGTAATGC
CGACGCCTATGGTCACTTCCACGATAGATGCTGCTA
>103782266_2 DV501451.2
AAACCAGTGGCGGTGCCGGTCGACAGGCCTGTGCCAGTGCCCGTGCCTCATGCTGTTCCTGTACCAATAGTGA
AACAGGTCGGTGTACCTGTACCCGCACCGTACCCAGTACCAGTAGCCAGACCAGTAGCAGTACCAGTAGCACA
ACCCGTTGGCGTACCGGTCATCAAATAGATAATTGGTGGTGGATTAGCCTCACGAATT
GGAGGAGGTATTGTATCCGGATACTATGGAGGTGGACTCCACTATGGCTACCATGGACTTGGACACATTCACT
CCTGGATCTGGAAAAAATATGTTCAGAAGACGTTCCGTTTTTAAAAAAATCTTTAATAAGCATATATCCTTGC
AAATGAAATATGGTGAAAATGTACCATCGTTTGACTTGCAAGACTATAATAGAGGAAG
GTCCTTACACATTTCCCCAAAGAAATCTGAAAAGAAGATTTTCAAGTAACCGAATGTCGGACAAGTAACGCTC
TATTTAATCACGGTTGGTATTATG
>103782270_2 DT666840.3
GAAATACATTCAGGTATCACCGTCAAGGTAATGCTGGTACTCATTTCTGGCACGCGCACACTGGTCTCCAAAA
GTTGGATGGTATTTACGGAAGTATTGTAGTTCGTCAACCTCCCTCCAAAGATCCCAATAGCCACTTGTACGAT
TATGATCTGACAACCCACGTAATGTTAATCAGTGACTGGCTTCACGAGGATGCCGCGG
AAAGATACCCAGGGCGTCTAGCTGTAAATACTGGACAAGATCCTGAAAACGTACTCATCAACGGCAAAGGACA
GTTTAGAGATCCCAACACTGGATTTATGACCAATACTCCTCTTGAAGTATTTACAATTACACCTGGTAGAAGA
TACAGGTTCAGAATGATTAACGCCTTCGCATCAGTCTGTCCAGCACAAGTTACATTTG
AGGGTCACAACTTAACAGTAATTGCAACTGACGGTGAACCCGTACAGCCTGTCCAAGTGAATACCATCATTTC
TTTCTCTGGTGAACGTTATGATTTTGTCATCGAAGCGAACAATGTCCCTGGTGCGTACTGGATCCAAGTACGT
GGCCTCGGAGAGTGTGGTATTAAAAGAGCACAACAGCTCGGTATCCTTAGGTACGCTA
GAGGACCCTACCAACCATCATCTACTCCTCCATCCTACGACGTCGGAATCCCACAGGGAGTCGTAATGAATCC
TTTGGACGCAAGATGTAACACTCCTAGAAACGATGCTATTTGCGTCAGCCAATTGAAAAATGCACGAAATATC
GACCCCGCGATTTTACAAGAGAGACCTGATGTCAAAATCTTCTTGCCTTTCCGTTTCT
TTGTCTATAGACCGGAAATGTTGTTC
>103782282_2 DT665239.3

TCGGCAAGCGGTTCCGCAAGCTGGACCTGGACAACTCGGGCGCGCTGTCCATCGACGAGTTCATGTCGCTGCC
GGAGCTGCAGCAGAACCCGCTCGTGCAGCGCGTCATCGATATCTTCGACGCGGACGGCAATGGCGAGTTGGAC
TTCAAGGAGTTCATCCAGGGCGTGTCTCAGTTCAGCGTGAAGGGCGACAAGCTGTCCA
AGTTGCGTTTCGCGTTCCGCATATACGACATGGACAACGACGGGTTCATATCCAACGGTGAATTGTTTCAGGT
ATTAAAAATGATGGTGGGCAACAATCTAAAGGACACGCAGCTCCAACAGATCGTCGATAAGACCATATTATTC
GCTGACAAGGACGAGGATGGCAAAATATCATTTGAGGAGTTCTGCAATGTTGTCGGAA
ATACCGACATTCACAAAAAAAATGGTTGTCGATGTCTAGATACTAGTGTACACACTCTACGACACCGGCCGAC
ACTCG
>103782294_2 DT665454.3
GCACGAGGAGAGCCTCGGGTACACCACCGACGACCCCGCACTGGAGAACGTATCCATGTTAAACGAAGCCGGT
CTCACTGACGCAGAAGCCGCGCTCAGTGACGTCAACTCCTGCTACTTAGACCGTGACGACCTCTCGTCACGCG
CCTCTTCCCGCCTCTTGGACTCAGAGGCGCTGGTGTCACTAGAGTCACTCAGTGCAAT
ATACGACTCGGATGAACCCGCACACCGCTACCGAGCCAATATACGTACTGTTAGCGAGTGCATCACGAGGAAC
TTTGGTCAACCCGCGCACCTTACTGACGCTGAAAGCGATGTCTAGCCCCCGCCTACCCCTAACTATTCATACT
CCGCTCTCTAGGGCTTCCAGTATTACGTAACACCATTTAAGAACATTCTCCTTCCCCA
CCCCC
>103782302_2 DT665735.3
GCACGAGGTATGCTTCTATGATAGGACCATATTAATCCAAAGATAAAAAGCTTTGATTGCAAACAAATTCAAA
CTTTATAATTTTTTTATACATTCACATAAGCAAAATGAATTAGTCTTAAAGCAATACTGTGTTACCTTAAAAA
CTAGAATGTAATCTCATTATCTAGATAAATCTTAGCGTAGAACATATTTTACTTGCTT
TTATTATCAACTCACACCAGCCGCGATATATGGTGGCTTTGGTGCACAAAAATCATTGAGCTTGATGAAATAA
CAGTAATATTATAAATAATTACTTATAAAATACTTCCAAATGAGTAGTTTAAGATTGTAAACGAAATGTTTTG
TATCTAAAATATGTAAGCACAATTTTTTCTTAGTGTTAGTGTTTCAGAAGAAGTTCTC
ATAATTGCTAAAAAAAAATAAGATTTAATTATGACAGCATAAATTAAATATTGAGATATATTAATTCTTTGAT
ATTTATTATATAAATCAAAATTGAAAATCAAATTTAATCAGCTTCACGCTATATCCCGCCAAAAGGTAGAGGG
GACCTAAGTAAATAAATATTTCATTTCCTCGAGTGGGGGATCCAATCCACGACCACTT
GATTTTACAGTCACACTTCGTGCCTCTGCGCCAAGACTATTATATAAATAAGGTTAAATAATAATCTAAGGGC
ATACATATACATATAGTTGAAACAGATCAGAAATAACAACCTGCCAATGTTTATTTTATTGTTTATTTTGCGT
CATTAATTAATAGCAAATAATATGAATTAAATATATAATCATACAATTCTTCTTGTGG
TCTTTTAAGTTGTAGTCTTTTAAAATTAATGTTTTTGTCTTACC
>103782304_2 DT666941.2
GCACGAGGATTTACCTAAATGAAATATATCGTTCTACTAGTTGTGATTGTTGGTGCAACAGCTGCAGACATTA
GCAGCTTGCAGCCGTTCTTATATCATCAGCAACCACAATTACAACAGCAACAACAGCAGGTGTACACGACGGA
ACCCATTCCCATCATAAGACAAGAACAAATACTCAACCCTGATGGATCTTATAAATGG
AATTATGAAACCGGGAATGGTATAAGTGCTGAAGAAGCTGGCTTCATTAAAAATTTGGGTATTCCTGAACAAG
AATCAGCGGTTGCACAAGGACAATACAAATATACAGCACCCGATGGACAAGTTATTCAGCTACAATACGTAGC
TGATGAAAATGGCTTCCAGCCGCAAGGTGCTCATCTTCCTACACCACCACCAATCCCT
GTTGAAATTCAAAAAGCTTTAGACCATCTCGCCACACTTCCCCCACAGGCGCCTGAGCAAAATCTATATAAAT
TATTATAAATCAATATCACAAAGAAACATAATTTATTATGACCATAAGAGGCTACAATACAAACACAGGCATT
AAAGTGTAGTAAAATATATATAAATATGTGACTTAGTTTTAAGTACAATAATAATTTA
TTTTATATAGTGGAGCTATTTAAAATTACATCGAATAAACAATTTGATGTTAAAAAAAAAAAGATCCATCTAT
TTAGACATTAATTAATAAAGCATTTCTGTAATAATATTACAAAGTACCCGTCGTTTTATTGGTTAACCCTATT
GTCATTTTTATACGAATAGAACGTCAGCTGACTAGTGTAATAAAGTTTT
>103782328_2 DT666467.3
GCACGAGGCAAGTTCCTAAGGTATTTTTTCAGTTAAGTTTTTGGATTTTTTTGAATTGAAAGGATAAATCGTA
AGCGTAAAATAAAATAGTTTTTTTTATTGAAACATTCAATCGCAAACGTAAACCAAAATTGTTCTTGTTTTTA
TTGAAACGATCAATCGTAAACAATAAATGAAATCGATTTCTTAAATTGATATTTATAA
ACGTGTTCGTGGTTTTGCTCCTGAAGTCAATAGCTTAAGTGTTGGTTTTATTTATAAGGCCAGTGTTCTCTGT
CAATATTTTTTTTTCATATTAATATTACCGTTACTACATTTTTCGTGTGTGTCTGTTTACCTTTGTTCTGATA
CGATAAAATTTATACACTATAAACTAAAATGTTTTTTTTGTGAACCTAGTTTCAAATG
TATGTGCTAGTTTGGTTTATCGTGTATATATTATATTATGTACTTAGATCGTTCGATTTGAATATTTACAAAT
CGATTATTTCTCTTTCTATTCGAATATACTGTGTCGTAACTGAGTTATAGATAATGCAATTTACTTGTAACAT
TATTAACAAGTATTTTGTTTGCAATAGACTTCGGTGGTCTAAATTGTAAGATGCGTCG

TTATATAATGAAATTATTTTCCCCTTCATCTTCTCTATAATAATAATTATCTCGTATTTTAATAATCATTTGT
AGAATAATTGATTTATCACAAATTATGTAAATATCAAACACTGTAGTGTTGCCAGATAAAATGAAATGTGTAT
TATAACTAACTGCCTTTTATAATTTTCACAATGGCAACAATAAATGTCTAAATATAAA
CTCGAGACTTTTTAAATTTTG
>103782332_2 DT665124.3
CGCACGAGGACTTCAGCATTTCGGTGATAAAAACTAATAAAATGCTTATGCAATGACCGATGGTCGCCCTGTT
AGTGGTCAATTGAAGAAGTTTGTTTTCCGTGGGAAAACTACAAACAACGGCGATACTAGAGGTGAATACTTAG
AGATCGTTATACCAGAACTACTGTCGGCGGGGTACTCGTTCTACACGTGGCCCTCCGC
ACCGCTCTTAGCTTGGTATTTGTGGACGCAGAGAAGGAATTTACGTGGTCTCCGCGTACTGGAGTTAGGTTGT
GGTACAGGTCTGCCAGGTATCTTGGCGGCCAAGTGCGGCGCGCGAGTCACATTAACTGATAGTGTCGCACTTC
CACGGTCCTTAAGACATTTGTCAGCGTGTTGTGAGGCTAACGGCCTGGTCCCAGGTCG
TGACGTCCAAGTACAAGGGCTCTCTTGGGGATTATTCTTAGCCGACGTACATAATTTACGTCCTGTAGATTTA
TTACTTGCGTCCGACTGCTTTTACGAACCGTCCCAGTTTGAAGAAGTAATTTCCACTGTAGCTTATTTTTTGG
ACGGTACTGACGCACGATTTTTGTGTGCTTACCAGGAGCGGAGTGCAGACTGGTCCAT
TGAAGCTCTTCTGAAGAAGTGGGGGTTGAAAGGTGCTCTTCTGGACCTGGACTCGCTGAGTGAGAGCTCAGGT
GTGGACTACAGGGCCCTTATAGGCGAGAGATCTTTGCATTTACTTGAAATAATTTCTGTATAGGGTATGGCCA
GCGCAGCTAGAGCGGCACGCCTCTATGTTGGCAACTTGGCATGGACAGTCGGCCATCG
TCAGC
>103782352_2 DT665449.3
GCACGAGGAATACCATCGATTTGTGTTTTATTTACTTCGATAACAATTAATAAAAGACCGCCACGTATGAAGA
TAATATAGATTGTACAACCATAGTTACTGTTATTCTGTGCCTGGGTGGTATATTTTGCGAACAAGAAAAAATA
TAAGAAAATATGTGCGGATCTGATAAGAAATTGGGTGAAATACGTGCCCTTATCCTAG
TCGGCGGATATGGGACTCGTCTTCGCCCTTTGACTTTGAGTAGACCTAAGCCGTTAGTTGAATTTGCTAACAA
ACCAATTTTAATGCATCAAATTGAGGCATTAGTTGACGCATGAGTAACACAGGTTATTTTAGCTGTTTCATAT
AGGGCTGAAGATATGGAGAAAGAATTAACTGAACAAGTTTCCAAATTAGGTGTATCTC
TAACATTTTCACATGAGACTGAACCACTTGGTACTGCCGGGCCTCTAGCTTTAGCTAGGGAACTACTTAGTGC
AAGTCCTGAACCCTTTTTTGTTCTGAATTCTGATGTTATATGTGACTTCCCATTCAAAGAGCTGGCCAAATAT
CACAGAAGTCATGGCAAGGAAGGTACAATTGTAGTAACAAAAGTAGAAGAGCCCTCAA
AATATGGTGTTGTAGTATACAAAGACAATGGGGAAATTGAAAGCTTTGTGGAAAAACCACAGGAATTTATATC
AAACAAAATAAATGCAGGAATGTACATACTCAATCCATCTGTTCTTGACAGAATTGAGTTACGTCCCACATCA
ATAGAAAAGGAAGTATTTCCTTTTA
>103782362_2 DT666610.3
GCACGAGGCATTTTTCCTCATAGCGTTAAGCATTTTAGTTGATAATGTAGCGTAATTTTGAATTAATAGGTAG
GCGGTAGTAATTTTTTAATATTTTTGTTAATTTTTTTTTCGTATCTGTAATAAATTCCTTAAGAGTTAGGTTA
ATCTTGAGCGGTCCTCGACTGGTCTAGGAACAAAAAGACAATGAAGGGATGTTGCATT
TTTTTTTATTACTAAAAATCATAAATAATCTATAAGATTTCAGCAAAATAACGTGCAATCTAACATTAAACGC
GTTTCTAATGAAATCGTCGCTCAACAGTTTATATCCGTAGCTACTTGTGCGAAGATCTCGTCGCCTATAATGT
GGACGCGCGGTTTGTCCAAAGAAAACAATAGAGATACATATTTAGATAGCTTCATCGA
TATTTTGGTAATGGAGTCGACGGGCCGCGGCGTCCACACTATATCTTTATGATCGTTATACTTTAAAAGACAT
TGCCAGATCTTATTAGTAAGCATTCGTAAACACATGTTGTTTGTTTGTGTGACTGTATGTCTGTACACAGCCG
CGCTGGCCCCGGGCGGGCGGGGGCGGGCGGGCGCACCGCCGCGTCGCCCGCGACCGCC
AGACCGGCCA
>103782372_2 DT666817.3
GCACGAGGCTTAATCTGTGAATTATTTAAACAATACTTACACATAAATATTAATAACAGAATATTAAAAACAC
AGCAAGATGACCACAAACGGTGATATAGGAAGTTTGGATGCGAGCGGCAAAATTGTTAAAATGGAGGTGGACT
ACAGCGCTACTTGTGACGAGAAGATCCCATTGTGGAAATCATGGGCGGCTAGTGGTAA
AGTTCAAGAAGCGATCGACCAATTGTTAGCTTTAGAGAAGCAAACTAGAACTGGTGCAGATATGGCTTCAACA
GCTAGGATCCTTGTAACTATTGTTCAAATATGCTATGAGGCAAAAAATTGGACAGCCCTCAATGACCATATTG
TTCTCCTTTCTAAGAGAAGATCACAACTTAAGCAAGCTGTAGTAAAAATGGTGCAAGA
ATGCTGTACTTATATTGATAAAACTCCTGATAAAGAAACTAAAATAAAACTAATTGAAACATTAAGATCTATA
ACTGAAGGAAAGATTTATGTTGAAGTGGAAAGAGCAAGACTCACTCACATTCTTGCCAAAATACGTGAACAAG
AAGATAATGTATCAGAAGCTGCAAAGATTATCCAAGAATTGCAAGTTGAAACCTATGG

ATCAATGGATAAAAGGGAAAAGGTTGAACTTATTTTAGAACAAATGAGACTTTGTTTAGCTATTAAAGATTAT
ATCAGAACTCANATCATATCTAAGAAAATAAATACAAAATTTTTTGATGAGGATGATACACAAGAATTAAAAG
AAAAGTTTTATAGATTAATGATAGCAGTAGACAACACAATGGACAGTACCTATCTGTT
TGTCGT
>103782376_2 DT666177.3
GCACGAGGCAAAAACATTCCTACAGGATGAACGCATTTATTACAGCAATAAAAAATAACCAAATTGACTTGCG
AAACGCATGTGGACATTTTAACAAATTAAACGAGATGCTAAGAAGAGCATGGGCGGTGCCTACGTATGGACAC
GAACTGGGTTACTCCTTGTGCAACACGCTGCGGACTTCTGGTGGTCTCGATATATTAA
TGGATAATTGTTTAGAATCAAATAATCCAGATTTACAGTTCTCATCGGCGAGATTACTCGAACAGTGTCTAAC
AACTGAGAATAGAGCACACGTTGTAGAAAACGGACTAGAAAAAGTTGTAAATGTTGCATGTGTATGTACAAAA
CACGCGAATTCTGTAGACCACGCTAGGATTGGTACTGGAATTTTAGAACACTTGTTCA
AACACAGCGAAGGCACATGTAGCGACGTTATAAAGCTGGGAGGTTTAGATGCTATTTTATTCGAATGCAGGAA
AAATGACGTTGAAACTCTTCGACATTGCGCTACAGCACTTGCCAACCTGTCACTTTACGGTGGTGCTGAGAAC
CAGGAAGCTATGGTAAAAAGATCTGTACAAATGTGGTTGTTCCCACTCGCGTTTCACA
ATGATGACAATATCAAATACTACGCTTGCTTAGCCATCGCAGTTTTAGTTGCTAACAAGGAAATAGAAGCTGC
AGTGTTGAAATCTGGTACACTAGACTTAGTAGAACCGTTTGTAACTTCACATAACCCGTCCGAGTTCGCTCGT
TCCAATTTGGCGCATGCTCATG
>103782380_2 DT666149.3
GCACGAGGGAACATTCTTCTACGGCATCGAGTCTCCTAATGGGAGACAGGCGTGTGTGATATCGAAACAGTGC
TGTGTGACAAAAGATCATAAAACAAAAGTTACAATGGAACTGCAAAATTCCAAAGAGGAAAATGGTGTTAAAT
CAGAAAAAGCACCACAGCAAGATCTCATTCAGAAGGTGACGGGATCTTTTGGCAAATA
TCAATTGTACTTATGTATATTGATATTTTTAACGAAATTTCCTGTTGCTTTCCATCAAATGGCCATTATATTC
TTATCACCTAAAGTAGAGTACACATGTATAGATTCTAGCAATAATGAAACATGCCCGTGTCCAAACCCAGAAT
ACGATACATCGATATTTACAAATAGTATTATAATGGAATGGGATTTAATATGTGACAG
AAAATGGTTAACTAGTTTCACACAAACTTTGTTTCAATTGGGAACTCTATTAGGGAGCGTATTTTTTGGAATG
GCATCTGACAGATTTGGGCGGAAAAAGCCTTTGTTGGTAGCCGTTGTCATCCAAGTGTCGATGGGAGTTGTTG
CAGCCTATGTGCCAGATTTTTGGACTTTTACATTCGTAAGGTTTAATTGGCATGTCCG
TTGGTGGAACAATGGTCACCGGCTTCGTGATGGTCATGGAGTTTGTTGGGACAAAATATCGAGATGTTATATC
AGCGTTATATCAAGTTCCGTTCAATTTAGGTCACATGCTGTTGCCCATATTTGGATATTACTTTAGAGATTAT
GAGAAATTTCAACTTGCCATTTCGATACCTGCTGTAATACTGCTGGCTTATTTCTTT
>103782413_2 DT668718.3
GCACGAGGGTCACCTTGAGAATTATTATAATAATTCAGCTGAAGTGTTTGTAAACTTAGATAAAATGGCAAAT
TCCGTTAACATATCAATAGAAGCACCTATCGAAAATCAAATACAAGAAATTACTTACGATGGACAGGAAATAT
ATCAAGCACCACTATCCATGTCAGAGAATCTCACACGATTAGCGCAGAAAATTGACTT
TGCTAAAAGTGATGATGAATTCTGTAACATAAAGAAAGAAGGTGAAAGTAGTTCTGATGTGAAATCAGAGGAA
GATAAAGAACCAGGATATCAGTCAAGTTTATGGCCTTGGGACTCTGTAAGGAATAAACTTAGAAATGCTTTAA
CTGAAGTATGCGTACTGGCCGATGTGTTAAGTATTGCTAAGGAAAAAAGATATATGGT
TCTCGATCCCATCCAAGCTGATCAAGTTGAAAGCCGTCCTATGGTCCAAGTTTACGGCAGAAAGAAAGCTCTG
TCTGCAGCAGGTGCAGTACTGCTCAATGGTGTGGAAAGACTAAGAGCCAGTGAAACTATGAGAATGACTCGCA
ATATGCCAGATTTTCACATAGATCTGCTAAGACTTAGACAGAACTGGAGGCTAAAGAA
AGTTTCTAACACAATTGTTGGTGATTTGAGTTATAGAACTGCTGGGTCTAAGTTTAGTCAGACTGGTGTTTTT
GAGGTAACAAAAGCGCCAGAAGAGCAAATAGCTCAAGCTATGAGCGTGAACGCGGCCGGCCCCACCCCCTCCG
CGTTGCGAGTGACCGTGCCTCCGGAGCTGCAGGGCGTTGCGTTTATATCG
>103782421_2 DT667293.3
GCACGAGGCTCCTCCTCCTACGCCACCTACACTTGCTCCACAGGGGTCACTGGAAACTGAAACCAAACCGGAT
TTCAAGATAAACCAACCAGTAACCACCGGAACCACACACTTCACAGATCACACAACAAAAGAACACAATAGTC
TACCTATTACGTCAAATCGCGTCTCCACTCTCATACAAAATTACAATTTATACGGAAA
CGTTCGAGAACAACACATATACGGAAACGAACCCCCGCCCTTCCAACACTCGCAGTCGTGTGATGACTCCAAA
ATCGATATCCCAATCGATTCTGTCAAAAGTAGAGTTCAAGCGATTGCGAAAATCGATTCGCCTCCGATTTATT
CGATTAGCAAAAAAATGGTCGATCATTCGGATCAAGGATAGAAAGAGATTTTTTTGCT
GTAGGTGTGCTTTATTATGGCTATGATAAATTGTTTGGTGGGCGATTGTAGATTATTAATTTGGTTATAATGA
GTTAAATTTATATATACGTTATTCTTAATTTTAGCATTTTTTTCTTTATTCATGAATTTCTTGAAATATATGC
TCTACACTAAAATATACACTACTGTTGTTAAGCTTGTGTTATTTTATATTAAATTATA

TTCGATTTTTTTTTATAATTATTAACAATATTGTTCATCCAATACTAATAAAATATTTGTAAAACTATTTCAA
AAAATGTTTTAGAAAACGTATAGGTGAGGTTGCCGTAACGCATGTTTCAAATTTTCATACATTTTCTAAATTT
TTCGTCTTTAGTGTGGAATAACATTTTAAAATATTG
>103782423_2 DT668304.3
GCACGAGGCGAGTCTTTTAAATTTTTTTTTTCCCAACAACAATTTTATTATTTATTGTATAATTGTTTAAGAC
TTAATATATTATGCGTTTTAAAAAAATAATTATTCTAAAGAAATAAATAATAATTAATCATTACATTTTAATA
AAATTTTAGTTTTCTGATACGAATCGTTTCTTCGTCGTCTAAGCACTGGCCGTACCTC
ACTTCCATACATTTGCTACCCTTAGCAAGATAAGCCTTCATATAGAAATTACCAAAGAGAACAATAAAGGAGA
TCATGTATATGATGAGTACGTAGTGCATCCACAGCGGGAACTCGCAACCAGTTCTGATGCCGTTAACGCCGAG
TATCAGGGCACATGTGAATTGTATCAGTTGAAGGATAGTCAAGTATTTCTTCCACCAC
AGATATTGGCTCACTCTAGGTCCGAATACGGAAAGTCCATAGTACGTATACATGAGAACGTGGATGCCACTGT
TCACCATTGCGGGAAGGAACGTCGATCCACTTGGCACCCATTTAATGCCGATCCACCAAAACCCAAACATCGT
CGAGTGATGGTAGACGTGTAAAAATGTTAATTGTTCCTCTTTCTTCCTTAGTATGAAA
AAAAATGTATCGCAAAACTCGAGAAGCTTCCAAAAGTAGTACCACCACACAGCATTCGCTATTTGCATTTCAT
CCGGATCATACTTCTGTCGACCCGGTTCACAAAT
>103782438_2 DT668918.3
GCACGAGGAACAAACTTTCAAAATTCCAATGAATTATATATTTTCAATAATATAAAATTTCAAATAATATTTT
GTTTTCTTCAACTTGCTCTTATACAAAAAGGTTGTGTGTAAATTTAAATAAATAGATTTTTTGAAAAGCCTTT
TTCTGACTCGCATAGTCAGTGGGATCGTGGCGTTTGGCTATAATATAACTCTTAAAGC
CATTGAACGGTTGCAATCGTTATTACCACGAACTATAAAGAAGAATCGAAGAAAACGAGACCAGCGGAGCACC
CTGTGCATCGAAGGTTCTGCTAAGCAGTTGAGTCCCGGCTGGATATGCAGAGGCTTCTTTGGTAAGTACTCTC
GTTATCCAAGTTCCTTTGTTATAGTTCCTTCCATACTATTTTAAAGTTCCGATCTAAT
TAATTTTCCGAATTAATTTTTGAGAAATAAATAGTAAGTAAATAAAAATATGAACATCAGAACAATTAACTCT
GATAACTTAATGTCTTAATAACCATTACCAATCAGATCGAAAATAAATTTACACTAACTAATCCCACAGAAAT
TGAAGCACTTGCGCTCCTTTTACGAGAGCAGCATATAGAGTAAAATATTTCAAACCCA
TACCTAAAAAAAAATATCAAAATTCTTCAACAGTTTAGACGTAAGATCGTTATTTTCATTTTATGAATTTTATC
TATACAGACTGATATTAACATCTACAACTACAAAAATACTACTAAACATTAGATGATCTGCCCATTTTATTCT
CAAAAA
>103782464_2 DT668026.3
AGAATCAACAGAAGATTCAATATGTGAGATCCCGGAACAAATCGAATTTACCGTATTTTTTAATAAAAGTAGA
CATGACATCACATTTGCTTATGATGCTACTGTATTAGAACTCAAAGCTCACCTAGAAAGGATATGCGGCGTAC
CACAATCAGCTCAAAAATTAATAATTAAAGGTATGGCCCGGGATAATGTTACATTAAG
AAATGCTGGAATAGTAAAAGGTGGCAAAGTAATGCTTGTTGGATCTAAAATGCATGATATATTGGCTGT
>103782470_2 DT666957.3
GCACGAGGAGACACCCTATAAGACCATGAAAGGACTACTAGTATTGTCGGTACTCGCTGTTGCGCACGCGCAG
TTTCCCAACGGACGTATCCTGGAGCCGCCAGTACCTGGACAATGTGCACAGCGCATAATACACGAGAGGTTTG
CCGATAATAAAGGTTACTTCTTTTCGTGGAGAGATCCAGCGTTACGTGGAGTAGAAGA
AGACTGGTTGAGCGCCAGAAACTACTGCCGCCAACGCTGTATGGACCTCGTGTCGTTGGAGACAAGTGATGAA
AACGAATGGGTGAAAGCCCGCATTGTACAGGATAAAGTAAAATACATTTGGACGTCCGGGCGTTTGTGCGATT
TCAAAGGATGTAATCGCCCAGACTTGCTACCAAACGAAATCAATGGCTGGTTCTGGAC
CGCCGAATTGCAAAAGCTGGCTCCTAGCACTAACCGCCAACAAAACGACTGGTCCGAGGGTGGTGGCATCGGA
AAACCTCAACCAGACAATCGGGAATTGATCCAGGGCGGAGCTGCGGAACACTGTGTGGCGATCTTGAACAATT
TCTACAACGACGGTGTTCACTGGCATGACGTCGCCTGTGACCACCGCAAGCCCTTCGT
GTGCGAAGAAAACGATGCTCTTCTCAAATACGTTAGATATACGAACCCTAACTTGAGAGTATAACAAAATAAC
AATCAAAATTCACAATTAAGTAAATAAAGAAAATCTTAAAATCTATGCTATCGACAAAAGTATCTCTATATAC
TT
>103782480_2 DT667381.3
GCACGAGGGCTTGCTAGAGGATTTCATGAGGTGGTGGACCCGCGGCTGGTGGCGGCGTTCGACGCGCGCGAGC
TGGAGCTGGTGATCGCGGGCGCGCCCGAGCTGGACGTGGCGGACTGGCGCGCCAACACCGAGTACCGCGGCGG
CTACCACGACCACCACCCCGTCATCCTCAGCTTCTGGCACGCCATCGACAGATTTACA
AATGAGCAACGTCTGCGTTTAGTCCAGTTCGTGACTGGCACATCTTCAATACCGTACGAGGGTTTCTCCGCTC
TCCGTGGCTCGACGGGGCCGCGCCGGTTCTGCATAGAGCGGTGGGGGAGAGTGGAGTCACTACCTCGCGCCCA
CACGTGTTTCAACCGGTTGGACCTCCCGCCGTACCCGACGCTGCAAGTTCTACATGAG

AAACTGTTGCTGGCCGTTGAAGAAACTAATACATTTGGAATTGAATAGGGCTTTTTTTATTTATATTGGCAGT
GCAAGTATTATGTGTAATTTCGTCAGCTTCTTGTGTTGTATACACTTGTGGTTGATATTTAAAGTTATGAGTA
TACATATATTATTTGATCATTTAATGTAATAATAATTATCTTTTTAATATTTTTGCTG
ATGATGATTTCAATTAAGCGCATTTATTACATTTACAAATTATTCATAAATTTATGACAATTTTCTTTTAAAA
TCCAAGATTTTCCATTTTATTTTATACAAACTTGCATTGTCATACTATGTTCATATTTCCAAAAAGAATGTTA
GTTTAAGATTTATTTTGTACTTTAACACATTCCACATTACTTCGACC
>103782482_2 DT667809.3
GCACGAGGCATTTGTTCTTTTAAATTCGCCAATAAGTTGCAAAATCGGGTAGGTGGTTGTGTTATGCCCTCCC
GTAGTACTCGCCGCGTCACTGTGTAGTGTTGCCATGTCAAACAGAAAATTATGGTTGTGGTAGTTAATTCAGA
TGATATATTTTCCTTTAGGGGAGTGACGTGTTGTTGAAAATTATCTAATACGAATAGA
ACGACCGCATACCCGACTTTGCGTACTTATCGATGATCGACGAATACATCGATCGGAGTGTTTAATTAATTTC
GATCTTATTTGTTTAGCTTAATTTAAAAAGTGAGACGCGTATGTACTAGTGCTGGCTCTGGGGTGGACTCATC
GATATTTCGATTTAAATTAAGCTCAACATGTAATAATGTGATATTTTGATTTTTTTTT
ACTCATTTTATTTTATGTGTAACTACATTAACGTTATTTATATTTGCTTACTTTTAAGGCGATCAGTTCTAAG
TGACACACGACCTCGAGGTCGGTACGATGCTTCCTTTTAATGTTAACAGTTTCGTGATGCTTTTTACCATTTA
TGTTATATAACCACGTCATTATTTGTATCGTGTATTGTTTTTGATGTTTTCATTGCGT
TTACTTTACTTGTTAGATAAGTATTAGATTTAAGGCAACTTATTGATTAATCAACTTTTTGAATAAATATGTA
ATCTGTAGCTAGTTGTGTAAGGCCGTACGTTATTCAGATAACACTAAGTAACCCTACTTCGTATAAACAATAG
TGTAATCA
>103782489_2 DT668491.3
GCACGAGGCATGGCTCTGCTGTGTTGTCGCATGAGCTGTTTTTATTTCAAATGGTTCATACTCTACACAATTT
TGAAAAATAGAATTAAATATAACTTCTGGTGAAATAAGGGATCTAGAAGCACCTGTGTCAATTAAAAACTTAC
CTTTAAATTCGGGTAATTCTATGTAGGGTAAATGATCTCTATCTTGATTATTTAATTT
TATTACCTCTTTACGTCTGGGTCTCGGCCTAACGTAAAATCCTGTTGGTTACTTATTTCCTCGTTGACAGGTT
CATAAGTATCCTGCTCGTCTGGTACATTATCGTAATACTCACCTGGAGTGTAATATTGAGAATATTCATTGAG
CTGCTGCTCCTGTGGCTCGTCTGTATAGAAAAGCTCTTCCACTGCGAACTGTGGCTTC
TCGGGCTTATTAGTTGACCTCATAGTCACATCCGATACCTTTTGGGGTGGATTTAATTGTTGTTGAGGAATGT
TACCTCTATTATTCATAAACGGTCTATAATTACCTGAGGGTTGTCGATTTTTCCACGGTAGCATGGACTGAAT
CCACTGTTGACGTTGTTCTGGCGTCCAATTATTTTGCTTAGGGGTAAAGTTTGAATTT
ACGTGCATAGGGGGATTGTTAAACTTAAATTGCGAATTTGGAAATGGAATAAAATTTTTATGATTCTCT
>103782491_2 DT667056.3
GCACGAGGGCTTATCGTTCCTGGGGGCGCGGGCCGGGGCTCCGGTAGCTGTCCTCGCGCTATTGTGTGTCATA
TCCACGGAGACGGTACTGTCGAACAAGGCGCGGAATGTCCTCATACTTCTGGCGGATGATGGAGGATTCGAGC
TGGGTGCCTATAGAAACAAAATCTGCCAAACTCCTAACATAGATGCTTTGGCACAGCG
CAGTGTTATTTTTAATAATGCTTTCTCTTCTGTCAGCAGCTGTTCTCCCAGCCGAGCAGCGTTACTCACTGGT
ACACCGAGCCATCAAAATGGCATGTATGGGCTGCATCATGGTGTTCACCACTTCAATTCCTTTGATAATGTTA
CTAGTTTGCCAAACTTGCTAAGAGAAAATGGAATATATACAGGTATTATAGGAAAGAA
GCATGTCGGTCCAAGTAATGTATATAAGTTTGATTATGAACAAACAGAAGAAAATAATCACATAAACCAAGTT
GGACGCAATATTACACACATGAAACTATTAGCTAGAGAGTTTCTTAAGGAAGCTAACAAAGAAAACAAGCCTT
TCTTCTTATATGTGGGCTTCCATGATCCTCACCGCTGTGGGCACAGTGAGCCTCAGTT
TGGTTCTTTCTGTGAGCGTTTTGGTTCTGGAGAGCCAGGCATGGGCCATATACCTGATTGGACACCATGGTAT
TATCAATGGGATGAAGTCCAGCTGCCTTATCATGTACAGGATACAGAACCAGCCCGAAGAGATATTGCTGCAC
AGTACACCACTAT
>103782557_2 DT667004.3
GCACGAGGTCTGTCCTTAAAAGAATACATTGTCTTCACGTAGAAATATTTTTAATTTCGTAAACTTTAGAATT
TTCCAAAATGGAACTCTCCGCCGAAGGAAAAACCCCAGAATATATGGCGTTAGCTGGAATCAAGTTCAAGTTA
TCATTACCTCAATTTAAAGATAATCCTCAACTTAAAGAACAATTGTTACAAGGAATTA
AGTCTGGTCATATGGCCCCATATTACAAAGAAGTATGTTCGGACCTCGGTTGGAGCTTCGATCAAAAATTATT
TGATGAAATGGCTAAAGAGAATCAGGAGCGACTTGCTAAATTTGAAGACGATGATTCTGAAACACCAGTATGG
CAAGATCGATTGGACTATTTATGTTCCATTGGAGACAAAGAAGCTGCAACAGCTCTGG
CGACATCTAAATATGAGGACTCTACTTTAACTACAAATCGTAGATTAGATGCTATATTTGCTATGTTTAGGAT
TGCTTACTTCCATGGATGTAATGTGAAAGAAATGGGCAAAGCAATCAACAAGGCTCATGAATTAGTAGACAAA
GGTGGTGACTGGCGTTCCAGAAACAAACTTAAAGCATATGAAGCAATATATTGCTTAG

CTGTGAGGGATTACAATCATGCAGCTGAACTTTTATTGATTGTGTATCTACATTTGAATCATATGAATTAGT
AGATTTTGGCACAATCATCCAATACTGTGTGCTAGCATGTGCACTGGCCCTGGAACGTCACGCCCTACAAGCA
GCTTTACGGCGACAAGGCGCTGCTGTGCAGGCCTTTGCGTCACGCTTTC
>103782559_2 DT668931.3
GCACGAGGTGGAGCCCTTGGGTGTAATTGCGTTTGCCCTTGTGGGCTTAGTGACCGGAACTCCTTATGGATAT
GGTGTTAAAAGTGGAGCATTAGCTAAAGCTGAGGCTAATGCTGGATCTGGTGCTTTTGGTGTGGCACAACCAA
TTTCCTTAAGTATTCCAAGTGGCGCAGGATATTCCGGAAGTTTCTCCAAGTCCTCCTC
ATCAAGCTTTGCGTCATCAAGTTCTAGCTCAAGTTCTAGTTCGAGTTCATTTACTTTTAGTGGTGCGGGTTCT
AATGGAATACCTGGCATCGGATGTCAATCTGGTGGATGTAAAAATAATGGAATAATAGCGCCCTCTTCATTAC
ATGGTTCAAATGATGGAGTGCAAAATGGAGCTTTTGCTTCAGGAGTGGCTGGAGCAGT
TGCGGGATCATCTTCGTTGTCACAAAACAATGAAAAATGTTTATCGGGAAAATGCAACTTAAGTGGCAACTTA
CAACCCCAGTCTCAATCATTGTTACCTTCAAACAATAATGACTGTGCGTCCGGTCAATGCATTCCATCTTTCT
CAGGAAGTATACCGAGTAAAGATGGATCTGAACAAGAGATTTTCAATCATGGTGACTC
TAATAGTANAAATTCCGAAAATAATGAAAAATCTGCTTACGAAGGCTCTGATTGTGCTTATGGCAAATGTGAT
GCTAATGTTAATCCTAACACAGCATCATCAGGAAACGACCACGATATTTTCGTTCCACTTTCACCATCTTCAA
ATTCTGCTGTACCAAAACCTCAAAAACTTCAGTATTTTT
>103782577_2 DT667973.3
GCACGAGGGTGTCCCCTTTAAAGTAAGAATTGATCACTTTTTATTCGTACGTCTTCTAAAAGAACTCTCAACA
TCAGCCTGTGCGTGAGGTGGACGGTGCACCACGTCAGTGTAGATTTTCAGCAGAAGTGTAGTGTTGGATTTGT
GAGAGTGCCAGTGACTACTTTGCGACGTCGTATCGTGGTCGTCGTGGATCCTTCGAAT
CTCTCGAGTTGACGGTTGTGTTTCTCAGTAACCGTATAAGGTACTTGGAACTGGGTGTGAGCCCAGATACGGT
GTGTGTAGTTTTATGTAACCATATTTATACTCTTATTGAATTTTTGTTAGTGTATATTTAAATTAACTTGACG
GTGGTAGTGTATCGCACGGGATTGAAGTGAGTGCCGTTTGTACAAAAGTGAAGTGTCC
GTACGACGACCACTATCCTGTTATTGTGCATCGCAAATAAGTGATTTGTGTCATTAGTTAATGGATTGTTAAC
CATTTGGATTTTAAGCAGCTCGATGGCAATTATAACACCCAATACAAAGGAGGAGACATGAACGCGACTGCGG
GTTGCCACTCTCATCAAGGAGGATAGCTGTCATCACAAGGTGGTGGTACTGGAGCGGG
AGTGCATAGTATGGATGACAGTTTCAACAATGTGCTGTTGGAAGGGACGCGTCAGTACTTTATGGGATTAC
>103782581_2 DV501278.2
ACGATCTGTTTCTGGTGATGAGCTTCCAAACGCTAGGACTGTTAGCAACTTTTTGTTCCCCGAAAAGAACTTA
ATTGACCCTATTTGGAACCTAAACGCCCAGCAGTGGGGTCAAATTATAACGCACGACATGTCTCTTACCGCTG
GTGTAGCACAAAGTCATAAAGATCCATTAACTTGTTGTGATGACAACGGTCAGCTATC
TGCGGATGCGCATACGAATCCCATGTGTGC
>103782589_2 DT668932.3
GCACGAGGCGGACGTTTGTTTATTTTTATATAAAATGGCCAACAGGATATGGTTGTGCCTATTTTTATTTTAT
TTAATTTTGAATTATTCTGACTGTGGAAGGTTTTCCAGTGGAGGTTCAAGATCTAGATCTAGTGGAGGTTCAA
GATCTAGTTCTAGTGGAGGTTCAAGATCTAGTTCTAGTGGAGGTTCTAGATCTAGTTC
AAGTGGGAGCAGAGGATGGGGTTCAAGCAGTTCATCGTCAAGTTATAAACACAGCTATCCCTCATCTTCCGGC
TATTCTGGATCATCGAACCAAGGCTCACATATTTACCCCTCGTCCTCATCGGGCTTATCGGGGTCAAGGTCTA
ATACAAATTATTGGAGTCCATGGAAAAGTCCTAATACTGGATTGGGAAGTTTAAGTTC
CTATCCATCGTCAACAGGGTTATCTGGCGCTGGTTCCAGTTCTGGCAGCAAATATCCGGCATCAACAGGATTG
TCAGGATCTGGAAGCAAATATCCTTCATCTACGGGTTTGTCAGGAACAAGTAGTAGTGGCTCACAGTCCTCGT
CAGGATCAGCGTCCGGTAGTAAATATCCAGCATCAACCGGGCTATCTGGCTCAGGTTC
CGTTAGTAAGTATCCAGTATCTACAGGCCTATCAGGGACAGGAAGTGGTAATTCATGGGCAACGGGATCATCA
GGATCTTTATCGGGAACCAAATATTCGCCCCCAACAAGTTTTTCAAATACAGGAAGTGGTAAGCACATTTATC
CAACTCT
>103782595_2 DT667869.3
GCACGAGGATTTGGCCTAAATTAGTTATTTAGACCCTTTTATATGATTACTTCCAAACAAATAAAACTTTCAG
GGCTGATTAGATGTTTATAGAAGGGCAACTTTATTGTACAACTTTGATTGAATTGTTACTAAGGTTTTGGTAG
AAAATGGACTACGCTACAAACTGTCAAACTGAACACGCGATGGTCGCCGGTTCAATTC
CAAACTTGCGGGATTATATTGTATTTTTTTATTATTTAAATCTGATAAAAAAAATTCAAAGTCGTCTAACACG
TAATATCTCTAAATTAGGTGGTCACAGAGTGTCTTAGTACTGGATAAATATAGCAGTGTAGTAACTCGCTTCT
TCTTAATTAAAAAAAGATATGTATAAATAATATCTTTCGTCGTCTAATAGCTTTCGTA

GCTCAGGGGCAAAAGGATGTGACTGTAATGCGACAGTTGAGGGTTCGATCGATCCCCACTCTGGGAAATAAAT
CATTATTTATTAATATTATCATTTCTTCTCTCTGGTACCAAGGCAAAAATCCGGCATTGAGTTCCACTGAAAG
TGGAGAACGGAGTGTCTCAACACTCGGTTTCCTCTGTCATCCCTGCTATATGCGGTAT
ACAGCGTGAAGCANAAAAAAAATATAATGTTTTATATAAATGTGTTTTTTATGCATCTATAAAACGTGATTTC
TGAATTATCCTCGCATTGTCATCACAATAACGCCGTTTTAAAAGTCGTTCTTCGACATTTACTCCTTTAAAGT
ATAA
>103782597_2 DT668244.3
GCACGAGGGACTGCTGATACATTTCCTAAATTTAAAATTTAAATTTTTAAATATTTTTATAAATTTATTATTT
TTATATGTAATAATTTGTAATTTATTTTTATTTTATTTTCCAATTGTTGTTCTCAATGTTATTCGAATTAAAT
AAAAAATATATATATTTAATTAAATTTTTTGTTAAATAAAAATAATTTTATAGATATA
TTTTAGAACTATTATTTATTAATAAAGTGTTATAAATTAAAAATGAAGTGTGAGACTATTTGTTTGTTGTTGG
TATTATTCACCGGTTTTACGAATGGGAGCTTTATGGAAAACGAAACAATATCGTTTGCCGATCTACCACCACT
CTACCACTTAGACGAGTGGTCAAAATGCCAGCGGCCAAAAGATGTGTATTGTCTCGTA
GACGCAGCGTTGATATCGCCAGCTCCATCTCCGCTATTCGATTTTCTAAAGGTATATTCATCACAAACGCAGA
AGCATTACAATCGAACTTTGGTCCATCGAGGAGTGTGTGTGACGAGGTGTGGAGCTCAAGGGCCTGATACGTA
CCATGAAGCAGCAGAGAAGTGTATTAATGACAGCTTGGTGGAATACGGGTTAGAAGCT
GACGTCCTATCAGTCCGTGTCTGCAGCTCACCGGAAGTAGAAAAACCAAGTATTTCAGCTAAAATCTTCTTAA
TAGTTTACATGAGTTTAATTGCGTTTACTATTGTGGCCACTGTGCTGCAGGTAGTGGGACCTCGTTGTG
>103782599_2 DT667023.3
GCACGAGGCTAAACCACCACAAGACCTATCACCCGAGGAGGAGGAAAGGAGAAAGGTCCGTCGAGAACGAAAC
AAAATGGCCGCTGCCCGCTGCCGCAAAAGGAGACTGGACCACACTAACGAGCTCCAGGAGGAAACTGACAAAC
TCGAAGAGAAAAAGCAATCCCTCCAAGATGAAATACGCAAGCTCAACGCGGATAGGGA
ACAGTTGCAGGCGATCTTACAGAGCCACATGCCAATGTGCAGACTCAACAAGCGGTCGACGAGTCCTCCAGAC
GTCAAACCCTTCCAGGACCCTTATGAATATCCAGAGTTGCTTTATGACGGCGTGAGGGTCAAGGTGGAGGTCA
TGGA
>103782603_2 DT667976.3
GCACGAGGCGCGGCCTATCAGTTTCAAATTAAATTTTAATATTAACGTTTATTTAATATATATATTTTATACT
ATTATTGTGTATTTAAAATTGTAAAGAAAGTAAAGCATCATGACTGACAAGAAGCATCTGTTACTGTTCTTCG
ACAGACCGACAGAACCATGTTTCATGCCAAAAGATGGCTCCGTCTTCCAATTACCTGA
CAATTATTTCCCGGAAAAATACAAGGAATCCAGCAATATCCTCTCAAGTCGTTTCGGGGAGGGTGTTAGCCCC
ATACCAGTGAGGAACATTGCGTTGCCAGATCTGTCTCTGCCCATGGAAATGCCCTATAATGACCAGTTCTCAT
TATTTATACCTAAACATAGGAGAATGGCTGGGAATCTTATTGATGTGTTTCTTGGTAT
GCGTGATGTCACCGACCTCGTATCCATCTGTTCGTTCTGTCAACTGAGAATCAATCCTTACATGTTTAACTAC
TGCCTTTCTGTGGCTTTACTGCACAGAGACGACACGAAAGGTCTAGACATCCCGACCTTCGCTGAGACCTTCC
CCGATAAGTTCTTGGACCCAAAGGTGTTCAGGAAGGCGCGTGAAATATCTAGCGTTGA
ACAACCGGAAGAAAGGATACCAATCCCAATTCCCCAAAACTACACAGCATCAGATCTAGAACCGGAACAACGC
GTGGCTTACTTCCGTGAGGATATCGGCGTTAATCTCCACCACTGGCACTGGCATCTCGTGTACCCGTTCGACT
CGGCGAACCGCGCCATTGTGGACAAGGACAGGCGCGGCGAGCTGTTTTATATAT
>103782609_2 DT668159.3
GCACGAGGAGAAACCTTTTTGAAACTTAGTTCGAAATCGACTTCTGTGTGCTCCGGTCATGGGGAAGGTGTAT
ATATTTAGTTTGTGTGTGTGTTTGTGTCTAAATTTAGTTTTTGGCCAATATGGATTCTTCCCAGAACGAACAG
ATCGCCGAATTTCATCACCATTTGGTTTCGCTCAAAATCTTTTTGCTGGCCCAAACGG
ACTAAGAAATACTTTGACAAACATCTTGCATCTTCGACCTGAAACACCAGCCAGTAATCCTGCATACATCTAT
CCATTTCCAAATCGTCCAGTTCAAAACAATCAATTTGATTTTTATTTATTAATTCATAACAATGAATTTATTC
AGAGTCGACCAATCCATAACAACCAATTTGACCAAAACCGGCCAAATAACAACAATCC
AAATCAACCTGTCAATAATCAATACGATGAACACTATAACAACAATCAGTTTGATCAAAATGGGCAACTTAAC
AACAATCAGTTTGATCAAAATGGGCAACTTAACAACTATCAATTTCTAGAGTATCCACCATATCATGAAATTA
GTCCACCTATAAAACCTCTCCGATTTCCAATGACCGTCT
>103782631_2 DT668267.3
GCACGAGGGAGATACGTAAAGGAGGTATCAGTCTCTCCCATCTAGGCGCTTGCAACAACATCAGTGCTCACAG
AGAGAACTGCCCCGTACACTGTTCCCAGGCTCCTTTAGACGGACCCATCTGTGGCTCTGACGGCAACGTTTAT
AAGAGTACTTGTCAGATGAAGCTGTTGACTTGCGGACAAGGTGTAGTAAGAACGAGCA

AGAAACACTGTCAAACTACCCGCCACTGTCGCGAGTCTTGCTGGAGGGCGTCAAGACCCACTTGCGGGTCTGA
CGGCAAGTTGTACGCTAACGCTTGCAGGATGAAGGCTACTATTTGTGGTAAACACGTATTCGAAGTTCCCATG
GCGTTTTGTGTGTCTCAAGAAAGAACATCTGGAGGTGACCCCTGCCCCACCGACTGTT
CAGGCCAAAAAGAAAAACTTGTCTGCGGCTCCGATGAGAATCTATATAGAAATGAATGTGAAATGAAGATGCT
TAATTGTGGAGTAAGCAACAGAAAGATGGTCAAGAAAATGGATATGGAGAAATGCAAATCTAAAATGAGCAAG
TGCCTCAAAGTGAAGTGTCCGAACGATGTCGATCCTGTATGTGGAACTGATGCCAATG
ATTACACCAATCCTTGTTTTTTGAAAGTCGCTACTTGTTTAAAAGGCGTTTCACTAGCCCACTTCGGTAACTG
CACTCTTTTACCTAGAATGGAGACTGATTGTCC
>103782634_2 DV501309.2
GCACGAGGACCGCCCTTAAAGGGGATGTCATCGTTATATAATTCGTATAGGGCAGGTGATAAAATGCTGCCCT
GGGGAATTCCGGCCCCTATAGAATGGCTGCTACACTCTCCACTCTCACGAAAAATGTCCTATCATCGGTAATA
ACATGCTCGACGTGATGGTGCCGGCCCTGAGCACTTTATGTGCTACGTCGAGCGAACG
AACTATTACACTCTCCTTTCGATTAATCGCTGCTGCACCGTAGTGACCCGCTCTGGCCACCTGCATAGTGGTT
GAGTGTCCGCTCGCCAAACTATTTGCTTCGTGGTGCTTTTTTTGTGGGAGGAATAATTTGTCTAATATCTTCG
CCGTCGCGCTGATGAGCCTTATGTCACATGATCAGGCCCTCCTTCTATGCCCGGCGGA
AAGGAGCCGGGCGGCCGTGGCGGATGCACTTTTATAACCCCACCACGCGTGGCGGGCGTCTAGCCATATGGCG
CACTAGACATTTGAGTAATGTAATTTTAAATCTGTGTTACAGCTTCAAAAGTACTTATTAAAATTACTAACAC
TAAATGACAGTTTTTATGTACATTATGTGGTCAATATTAATTATGCTGTGTGTAAG
>103782637_2 DT668126.3
ACGAGGAAATTCCCTTTAAACTGAATGAACCAAGTGCTTGTGTTTCGTTTGGAATTGTGGTTTACTAACGTTT
GGACACGAAAAAAGAACTTATAAAGAAAATAACGGTTTATACCAATTTGGATTGTAGTTTGAATGTAAATTTA
TAATACGTAGTAGAGATTTTGTTTTTATTTTAGTAGCCACAAGCTATATATTGTGAGA
TGAAATAAGAAAAATGATTCATTCTGTAGCTTGTGGTGCTAGTTAAGGTCGGAATTGGGCCTTGTTTTTTGGA
AAATATAATAATTTCTCGGCTTTACTTGCCAATTCAATGGTTACATCTTCAGGAGATGGAATTGGGAAGTAAA
ATCCCTAAAAAGGTGTTTACTCTGAGAACTTTTGTAACTTATTTAAAATTAAATTGTA
ATTAAAATTTTTCAAATCAATTAGATTCACATTCATTTAAATTAACGTCTTTATAATTCTATATTAAATGTTT
TATCGTCTTTATAGATAACAAAGCAATTATTGTTTATTTAATCATTATCTGTTATAATGTTATATTTCATGAC
AAAGATGGCGGTTTTTAACTTTATGTGCCAACACTTTAATCTAATATTTAAATTCCAT
CACGTTAACAAGTTAAATTAAATTAATTTCTATATGTGAAGATATATTTTGATCGCCGTCTATCTGTCAGTA
GAAAAATTTTCATATTATTCAACATTTTTATAAATGCTGCTATATAATATTATAAGGTATTCTAAT
>103782640_2 DT667223.3
GCACGAGGGCTATGCTCGTGTTGCTGCTGCTTGCCGCCGTCGCCGTCTACCTCGGCCATATGTATTTATTTGG
CGACCCACTAAACCGACAAACGTTTCGAGGATCATTTGTAGCATCCTCGTGGGGGACACAGGAAATATCGGCA
GAGGCGAAGGACAATAGCACACAAGAACAAGAGCTCCAGGAAGCTATATTTAAATCCT
ATAGAAATTCAGAATTACGGTCATGTTTTGTGGACGCTGAGGTTCTTGCTTTAGATAACTCAGAAAAAGGAAC
ACGTGTACACTTTGAAGTTTCTTTCGAGCCCATTTTCACAGCTGTGAGTACGGCTGAAGTGAGCGCTGTTATG
ACAAGGGATATCTTAACACCGTTTTTCCAAAATATTGGTGCAATACCAGCTACGTTGC
ATATCGAGGAAAGTTCCATGATTTCTTCACAGCCTCTAAAAGAAAGTGATGATCCTGTAACTGACGTACCCAC
AACCGAAGGAGTAACTTTTGAAGAAGAAGACCTGCGAGAGTGTTCTCCATTGACATTATCTTTATGTTCACAT
CTGCCTTATAATATAACAGCCTATCCAAATTTAGTGGGACATACATCTAAAGAAGTTT
TGTTTCGCGATCTAGTCGCCTTCAGAGAGCTGCTAGACGCCGAATGCTCTCTTTTAGCGCAAGATTTCGTCTG
CCAAATGCTCAAACCACGATGTGCCGATGAGAGGCTTGTTCGGCCATGCCGTGCTTACTGCCGCGCCTTTCAT
GC
>103782669_2 DT668246.3
GCACGAGGCTGCTCCATTATGAACTTATTATATATATTTTAAATATTTTTATTGTAATCATATTAGTAAATAC
ATGAATAGGACACTTGCACTTTTGGGTGTTAAATCTACATCAAATAGCGTTTTTTAGACCGGAAAGTCTTAAC
ACTACGTTTTCTCTATCTTCTCTTCTATATGCGGAATAAAGCGTGAAAGCTAAACAAA
CCCAATAAGCCCTATAATCATCACAATGTAAACATATACGTACAAAACGACAAATAAAATTACTATAGCGTAG
GCTTCTAGCCTTGACATTCATTGATGGCATACCTTTAATTTTAAAACATAATAACTTCATTTATAAGTGCAAT
CCAAAATATTCCATAATTTGTTGAAAACAAAGTCGACTTTTCTTCTAATTTTATCTTA
AAGTTTTTACTATGTAATTTAGTAGAGTAATACCGACCATTTTTCTGTTGGAATAGGATTTAATTTTGTTTAA
CATTGCATTGTTTTCGTATCCGGGTTGAAAACAAAGCCAAGTGACATTTAAGTCCACGTCCAGTCGGACATGT
GTATATAGCTAATGTATAAATTCATATAAACAAGACGTTTTTTCTTTACAATTTCTTT

AAGTTTACTATTTTGTTATCTTATACAAGGTATTCTCCCTTTTCAGCCCTATTTTCAGGACATTGCATGTTGA
CTGGTACACTCAACGCTGGGCTTTTTTTTGGCGCTGAGTTCCGCCACTTA
>103782677_2 DT668697.3
GCACGAGGGAAAAACTTATCAGAAGAAATGGCTGAAAACATACAAATTGACCTTTCTGGAGAGATAAACGCTA
ACACTGAAAAGAAAGGATTACAAAAGTATGTCAATGAAATGGAATTATTGCTGCACCATGTTCGGAGTGGTGC
TGGACCCGCCCTAGTCATGGGGGTACTGTCCTCGCGTAGACCATGGACACAATTTGTC
GCCACTGACAGTTTTAAGGCTCCTGCATCCATACCAAGATTATCCAGACGATTTTATCGAAATGTTGAATATT
TTCAAGCAAATTATTTGATTGTATTTTTGGGCTTATTTGCTTATTGTCTAATCACTTCACCATTGCTTCTAAT
TGCAATGGTTGCAAGCTTTTTTGGTTATAAAAAACTAACAGCAGGACCAAATACTTGG
AAGATCGGTGGATGGGAATTGACTAAGACTCAACAGTACGCGGTGGCGGCGGCAGGCTCCATGGCCTTGTGTT
GGTTGGCCGGCGCCGGAGCTGTCCTGTTTTGGGTTTTAGGTGCAACGGTAACTGTGGTGGCTTTACACGCTAG
TTTCTTCGACGCTGAAGCGTTGCCGCCGTCCGAAGATCCCGAACAATTTCCGATGGTA
GAACAAGTGTGACACGGACAGTTGCCTTTATTTTTACCAATGGCCACGAGACTAGCCCACCTTCACTTTGACC
AATGTGATGTAAATAAATGAAAATACTTCTAAA
>103782695_2 DT667933.3
CGAGTCCCCACCGTCGGCACCCTCGTAAGCAGCCACGTAGTGCTGGCACTTTCTCAGGGACAGAGTCCTCTTC
CCTACCGAATCGGTGTCGCTGTTAATGCCATTAGGGCAAAGTATTAAGGTGGAAACTGTCGTGACGGTAAGTG
TGCACATTTTCGTGTCGTGGCGTGCTATGATATTTTTTTTAACAGTACTTAACGAACC
TTTGCAGCATTAAAACAATAATAATGTTTTCTAGTAATTTTGATGAAGATCTTCAATTTATCTTAGCAATCAA
AAGAAAAGAAAAGAAGATACTGAACAGGAGATCAAATGATCCTTGTACTCTATACACTTTGAGTTATATATAG
CTTTTCATTGATGTCTAAGTTCAATAAGATCCTAAGTAATTTTTCTATAATAAATTAA
AAATGTCCGCTAGGTGCTCCCGCCACAAGCCACGCCATAACCCCTACCACCGCTCTGGCGCAATCGGTGTCAA
GCATTCGGCATCACGACAAAACGCGTTGCGACACGACACGACATGCAAATATGTCCCTAACCATACTAAATGT
ATGAAACCGATTAACTTCTAGTAGTGTGTCGCGACACGCCACGACACGTAAATATTTT
TCTGTCTTTATCCTCAAAGTCTAAAAATAAAAACAGAAATAATATTAAATAAACCTACTTAATTAAACATAAT
ATTGTAACAGCTATCTTTTTCTAACATTTCTCTTTTAAACGTTATAAAAACCAAATAGTGATCAAATATACTT
TACTGCAATCAACCCCAGTGTATG
>103782720_2 DT667106.3
GCACGAGGGTTTACCCGCAATTTACTTAAGATATCCTTGTGGACATTTTAATTGCCAAAATTGTGTTACAACT
GCTGAGGAATGTTTGTTGTGCCTAACACCACCATCTGTAATAACCAATTTAGTAGATAAACCTTTGTCTCGGC
GTACAGAGCATATTTCAAATTTACTTAATACATTTCAAGATTTATTTAACTTAAATGT
TTTTAGGCGACAAAGAATTTCAGATCAATTAAAAGCAGAAAAGCAAATTTTCCCAGAATGTATCCAGGCTCCA
TCAAAATATTGTAACAAAAGAAAAAGCACCCTTGATATTTTAGTAAAAGACAAAGAAAATATAATATCAATTT
ATCCAGGTGAAAATATTTCATCTTCCAAAGCTATAGAAATGGAAAATAAAAATGTCTA
TGTTAAAAAATGGCTGGATACAAACTTGCCATCTCAAAACAAACAACGTAAAGCTTTCACTGATTTAGGAGTC
AATAGAAATTTAACTAATGATAGTTTATTAGGATCAGCTAAACATAATATTCATAGCAGTTTTAATTGTAAAA
GAAAAATGAATAAAAGTTTACATGATGGTAGTGAAAACAAGAAAAAAATTACCAATAAA
GAAGAAGAAACTTAAAAATAAAATAAACCCTCAAAATAAGTTACAGGTAGTACCAAAAAACAATAAAATTGAA
AATGATGATAGTGGAATTATTGATGAAGAAAATATTGTAATAGATGAAAGTGAAAATGACTTTATTTGATAAG
ATATGCTTGCAT
>103782721_2 DV501270.2
GCACGAGGATTTGTCTTTTATCAATCTACATTACCCGCGAGATGAATACTTTAATATATTAATTAGGAAGGGT
GTATATCCATACCAAATAATGACTTCATTCGAAAGTTTGAATATATCATGCATTGCCAACTCAAATTCTTTTT
TTTAATTCGTTGGTTGATACTCATGTATCTAATGATGATTATAGACATGCCCTAGATG
TTTGGAATCATTTCTGATGTGGAACCATGGATGATTACACTATTTTATATTTGATTATGTTTATATACGATAG
ATTATTATGAGTAAGTTTTCGACGTTTTGTATAGTATAGCGTACCTATTTATCTATTACCTCTAGATTCCTCT
TTATGTTTGAGACCAAAAACGTTTATACTTCTTTGATTGAATTCTTCATACTTGTGAA
TATCTTTCAGTTTTACAGGATTACATATGTTTACGCAGTTAAATGTATTTTGGTTATGATGATATGAACTCCC
TCTATCTGAATTTCTTGTATTCTGGATATATTACAA
>103782729_2 DT667194.3
GCACGAGGCTCACCTTTTTTTTTTTTTTTTTTAAAATCAAAGTATTCTATAATCAATTTTTAAAACTAAAATAT
TTAAAGGTAACCAATGCCATATTAATATTAAATATTCTCGTGAAGTTCCTCTATAAAATCTATAAATTCTTAT
ATTATATTTTCCATGTTGAGTAAAAGAATATAAATATAATCTATACCCAGCTCTAAAA

AAAGAAATTATTATTAATATAATTATTGATCACCATGATCATCTAATTAATCTATTAATTAAACTAATTTCCC
CTATTAAATTTAACGAAGGAGGGGCGGCCATATTTGAAGATATTAATAAAAATCACCATAATCTTATTGAAGG
TATAAAATTTATTATCCCCTTATTAATAAATATTCTTCGTCTATTCAACCGCTCATAA
TTAATATTTGACAAACAAAATATTCCAGAAGAACATAATCCATGACCAATTATCAAAATATAAGAACCTAAAA
ATCCTCAATAATTTATTGTTATTACCCCCCCAATTACTAATCTTATGTGAGCAACGGAAGAATAAGCAATTAA
AGATTTAATATCAACCTGACAAAAACACTTTAAACTAATAAAAAACCCCCCAATTAAT
CTCACTACTACTCAAAAATACCCCAATCTTAAATTTATTTTTTGTATAATTATTAATATTCGTAATAAACCAT
AACCCCCTAATTTTAATATAATTGCGGCTAAAATTATTGA
>103782734_2 DT668774.3
GCACGAGGATGAGGCTGTATCGTCTCATATAGATAGTTTGAGGTTGAGCAGAGAAGAATTAAATGAAGCTACT
AGTTTAAACTTAAGTACAAATGATCAGCAAGAAGAATTTAGAACGATAGAAGATGTTTTAACAGAATTAAGAA
GGCATAGGGAATCTGATAAGAAAATAACTTCGTTAGTAGAAAATAAGTCTGAACCTAA
TAGTAGTGAAACGAGGCGTAAAAGAAAAAGTGCTAAAAGTAAGAAAAAAAAGAAAATGTGGTCGCCTGGCAAT
GTTATATTGGATAATCGGTCCATACAGAGCCAGAGTCCTTTGAGATTGAATTTGGACCATTTTGTTGATGAAA
GCGGCACGTCGTGCGGCACGTCAGAGTGCGCGGACGACGAGCAGATAGCGCTAGCGCT
GCAGGCGCAGGAGCTCGCCGCCAGGCAACAGGCGAGAGGGAAGTTTAAATCAAGTGAAGATCTCATACACCGT
CTATTTGTATGTATAGCGGGTGTCGCAGACCAACTACAAACGAATTTCGCGGCTGACTTAAGAAATATATTAA
AGGCCGTATTTCTACTAAATCAGACGAGTGAAGATGAACCTGAATCTGATGAAATCAA
AGAGGAAACCACTATTGAATATCAACCGACTGAGGATCAAGTTATACAGAACGGAAGTTCATTCGACAGCGTC
TATTCAGCAGAAGAGGTGTACGCGGACAGCAACTCCGATTCGACAGCGTCTGAATCCCACGCGACTAAT
>103782753_2 DT667948.3
GCACGAGGCATTTACTTACTGGTGATGAACTACCTTCTTATAATGGAAAATTGCGTTTATTTGCAATGAGATT
TTGTCCTTATGCCGAAAGAAGTGTACTTGCTTTAAATGCGAAAAATATTCAATATGATTTAGTATTTATTAAT
TTGGATCACAAGCCGGAATGGATTTTTAACTTCAGCCCCAAAGGAACTGTACCAGCCT
TAGAATATGAAGAAGGAAAAGGGATCTTTGATAGCAATATCATAAACTATTATCTGGATGAAAAGTACCCTGA
AATTCCACTGCAATCATCAGATCCATTAAAAAGGGCTCAAGATAAAATGCTAGTTGAGCAATTTGCTGGTGCG
CAGTCAGCATATTATACTGCAGCTTTTAATGCTGCAGCCCTCCAGCCTAATATGGTTG
AAAATTACCACAAAGGGCTCGAACTCTTACAAAAGGAATTACAATCTCGTGGGTCGAAATATTTGAATGGTGA
GGAGCCAGGATTAGTTGATTACACCATTTGGCCGTTTTTGGAACGATTTGAAGCTCTTCCTTTATTAGGTCAG
GCTGATTTTGCTATAGACAAGGATAAATATGAAGTGCTGGTAAGCTACATAGAAGCAA
TGCGCAATGTACCAGCCGTTAAATCTTATGCCTTGGCCCCTGATACTCATGCTAAGTTTACAGAATCTCGTGT
AAAGGGTGATGCTAACTATAACATGTTGGATACAAGTGCTGTCTGTTGTATGCGCCCCAGAAAGAAGAAAGAA
TAAAAAGTGATATTTCATTATTG
>103782773_2 DT667944.3
GCACGAGGATTTGACATGCTTATGACTCCTGAGGCAAAAGAGAAAATTACACTACTCGTTACCTTTAATGACT
ATTACAAGAAACTTATTGATCAAGCATCATTCAACATAGCTTGCTTAGCAACCATAGTAGACAGGAATTTTGA
CTACTTCGCACAAGATGATTTTAGAGTTCGTAATCCAGATATTAAAATAGCTTTTGAT
GGAAAACCAACTTCGAAACAAGAGTTCCTAGTAACTGTGACGTTGGAAAATCCTCTACCGATACCGTTGAAAA
ACGGCAAATTTTACATACAAGGTCCAGGTCTCGATAAACAGCTTAAAATTGAACTAAATGAGAACGTGGCTCC
AGGGTCTCAAGCGACAGCGCAGTTCAAGTTGACACCGCCATGGGCGGGCCGACACCAA
ATATCTGCGAAGTTCTCATCCAAAGAAATGCACGATGTGGATGGTTTCCGAACAATAATGGTCTCACCACCTG
AAAATGGAATACCATTAATAGATGCAAATGAAACGCACGACATTTGAAATGTTTTCTACTTTCTAGTACAATT
TTATCTCATTACCTTTTGCTTAAGATGATTTGTTTTCGCATTTGACTACAAAAACTGC
TTGAATATACTTTTAAATCACCAAACAAATTGTCGAAGTACTACTATGTACATTGTAATATCACAAGCTATGT
GTTCAAATACATTCTCATAAATAAGAATTAATGTGAGTTAAATC
>103782775_2 DT667440.3
GCACGAGGAACCGCTTTTATGATCGACTTGTAAATTTGAAATTTCGCATAGTTAGCAAGGACTAATGACGATG
CAATAGTATAAAAATACCAAGAGACAAGTCGTGACGCAGAGGCACAAAGTGTGGTTGTAAAATCGACTTGTTG
TGGGTTCGATCCTCACTCGAGGAAGTGAAATATTTATTTACATTTATATCCAGAACTC
GGCGGAAAATGGGGAACGGAGTGTCTTAACACTAAGTTCCCTCTGCCTACCCTGCTGTGTGCGGGATGCAGCG
TGAAGCTGATTTAATTTAATTTTTTAGTACAAAAAATACCGGTTAAGTGGGAGTCGAATTAGCAAACCGAGGG
CTCCGTACCAATTACAATTTATAATAAATATTAGTCAAAAAAAATTAAAAACCACGCT

TTTGTGAGGAAATTAAGTTTAATGTTACTTACAAACTTACACTTTATCCTAATGTATTTATAATATATTATAT
TTTTGTGCAAGTAATTTGTCCAATTACGATAAAAATACAGTGTAGGGCGATGCTTGCATATAAATATTTCATA
TTTTAAGACACAAAAACTTTCTTTATTCTATATTTTGTATGACGCAACCTTTTAATAG
AAGTCAACTATCACCTACATCAAAGCTCATGAAACATCTGACTTCTATTTTTATTATGCCTAAGTATAGGCAA
AAAGAATTTTATGAATTTATGAATTGAACGATATATCTCTAAACGACTACGCGAAGCAAACAAGTGTAATGTG
TTTGTCAGTCTATGTACGTATGTATTGAATATGTACAAATTATCAGCAGTAACAATCA
GAACCCAATCTTAAATCAATTGACTCGCATTAAGAC
>103782837_2 DT668013.3
GCACGAGGTAATCGTTTACGGATTTGTCATAGAACTATGGCCTCAACTCAATTTCGTCAATCATATCGTTGTC
TATTTCATCTTCATGAAAACACACGTTTTGCCTTGGTGATGAACTTGACGCCATTCTTGAAATTAAAATTCGA
AGACGACTAGTATGGAAACCGCAAGGTGGAGGTGACATTTAGTTTCTAATGCGCGCAC
GGACGCACAAATGCGCGCACCTTCATTTAGGGTGATGTACACGGTGACAGTGACGCAACAGTCACGGTGACAA
AAGTCACCAATGCGCGCTATACCTAACAGAACAGAATATAGAGTGGAACTTGACCTTTGATAATAATAATACG
TGTACTTAATGATCTGTACAATTAAAAAAGCTTGAATTTATCCGTAGACGCAAAGAAA
TATCTTAATATGCGCGTTTTAATAACATATTTTAAATAATAAAATATATTATATCAGTTAACGAAACGCGATT
TGCACTTAATTAAAGTGCTTTGTAAGGCTTGATAAATGTAAGCGTGCCAAAGGTATAAATTTAAATGTGAAAA
AAACTAAAACCAATAACAAAAAAATATCTAAAATTGTTATTTAATACCGTTGTATTGT
AAACTTAACAATCTTACATACGAGTATGTACTTAATATTAGTAAATTTACTATTAAATCTAATAACAATTCTT
CAATGTAAGAAAACTAANAAGAAAATTAATCATTTAAAAAAACTTCCGAAATAACTAATATTACTTACAACAT
TAAAAAAAATATAAGGCTTGACAAACAAAATGATACATAATTATATTTCAATGCTAAA
AAAA
>103782839_2 DT668591.3
ACGAGGCACACCCTTTAAATATACTCACACACACACGAATACGCACACAAACACACACCGCCTATTCAGCTTC
ATACATAATAATGATCTGATCTAACTTAATGTTAGTTATAAATAATTCTAGTCAGTTATATTAGTTAGTCTAT
AAATAATACTAACAATGTTCTTAATGTTCATGATGTTTGTTAGGGGAAGAACCTGGCG
GCCTGAAACACAGGGAATCCTAGTTCAGGCGTCAGTTCTTTTTGTTAACTGTACAGATGATTTTTGCTAAATA
AAATTATTATTATTATTTTAAGTCAAGATGCTTTAATTGTTTGTAGACAGGATATAAGCTACATAATGCACAA
ATAAACCTAAAAGCGATTCAGTCTCTCAGCAAATTATAAATAGTAACAGTATAACTTG
CCGTCTGTAAGAAAAATAGGTCCAGTCCTCGAAATGCCTATAGTGTTTTTATGTAACAATATGTGTGTGTATT
TATGTAACGAACGGTCTACTCTTGCATCTAGGGATAATGTTTAACTACAACTGTGATACGAGGTATTCCTATG
GATGTATTAAGTGATAGTGGTGATTTGAATGTTTCTTTAATATCATTGAACATTCTTA
TATATTTATCTTAGAAGGAGTAAGAAATACCATTATTAAGTTTAGAGACTTTTGTTACCTTAACTATTGA
>103782846_2 DT668690.3
GCACGAGGTTTACCCCTCCAAATTCAATAAATATATAAGATTTGAACTCTACATTACGAACAGTATTTATAAA
GATTTTATTACATAGTAATACATAATGTTTGCTTCCATAAAATCGTTTTTTAAATGGAATTTATCTAAAAGTA
ATGGAGACAAAAAAAATACATCATCTTTGTTATTTGTAATATTTGTAAATATACTTGA
AGATGTTCTATTCTGGAAAAAAATGTGGCTGTCTTTGTTATTTTTTATATTATTTAATGTTTTATTTTTCTTC
TGTGTTTATCAAGAGTTGAATTTGCTACAATTTATTTTCGGATTTTATATCATAATACTTATTGCTGATGCAT
TTGAAACATGGCTTAAGTATAAACATCGAACATCATGTCTTAAAAGATTAATTAATCA
TGATAACATTAAAATTAAGTCAGTAATTTGTAAAATACAGACATGGGTTAACAAATTGCAAATGGGCTTTATT
TATTTAAGAGATAGGAACCACACCAAAGCATTTTTGTTACTTCAAATTATATTAAGTGTCATGCTATTTATTG
GGAAGTATATGAATGGATACACTTTAATATATATTTTATGTACAGTATTATTTTTCAT
TGATAAACTCATACCTCCTTTAATTAAAATTTTAAGAAAAGTTCAACAAAGTGCAGAGTCTGAATATGAACTT
GAAGGTCTAGTCCCAGAAGAATCTGAAGTTAACTTACAGTTATTATCTATAGAACCAGAGCAAAAACCTGTGA
TTGAT
>103782863_2 DT668070.3
GAGGGAAATGCTTGCTTAGAAGGACAAGATAAAGGAAGTGCGGATGAGAATCGACACGGTATACCCAACGCCC
CTCCTCCGGGATTCCCCCAGACGACACTCCCGGCTCTCACTCCGGTACCGCTGTCAGCGTCTCGGAACAACTT
CAACTTCGGGTCCTTCGGAATTTTCAAAGAGACATGTACCACAATAGATGGTGGGGTG
GGAAGTTGCCTCAGCATCACCACTTGCGGGCCATATGTAAAGATATTGCAACAAGCCAGGACCAACCCCGAAG
CTGTCAAGATTTTGAGGAAAGCCCACTGCGGTTTCTATTGTTATAACCCTAAGGTGTGTTGTCCTCGTCAGAC
TATCCCTGATGCTCCACCCAGTGTCACTACTTCAGCACCGCAAGCAAGTACACTAGCT
CCAGTCACACCTGACCCTGGATCTGATGCAGCATCTGGCAAATCTTTACCGTCTTCGGACTACTTGGAAAGAT
TCCCCGTTCCCCCTGAATGTGGTGTCAGCAATGCTTCATTCACTCGCGTCGT

>103782884_2 DT667654.3
GCACGAGGATCACCCTTAAAGGAACGTGCCAGCACCAACACCTGCTAAAAGGGTAGAAACAAGTGAAACAGAA
TCTTCTGAGCCACAGCTGGTTGAATGGGGTACAGATGTAGCAATTGCTGAATGCAGGGAGAAATTTATTAAAT
TTATACAAAGATTTGTGGAACCCACTGCTCTAACTACTGAGCCATTATATGAATTAAA
ATTGGAAGAGATTCACACATTAGAAGAGCCATTCCTGGATGTGGACTGCGATCATGTGAAAATATTTGATTCT
AAACTACATAGGCAGCTTATCAGTTATCCCCAGGAGGTCTTATCAGCATTTGATGCTGCAGTGAATGAATTAT
TTTTTGAAAAATATCCAGCTGCAGTGTTAGAACATCAAATACAAGTGAGACCATTCAA
TGCTCCACAAAGACACATGAGAGATTTAAATCCTGAAGATATTGACCAACTAGTAACAATATCAGGCATGGTT
ATAAGAACATCTGGTATAGTACCAGAGATGCGTGAAGCATATTTCAAATGTGCAGTCTGCGGTGCTGCAGCTG
CGGGAGAGTTGGAACGGGGCAGGGTTCCGGAACCAGCACATTGTTCCCA
>103782886_2 DT668413.3
GCACGAGGCTGATGCTTGTCATCCGACAATGGTTGGAATGGCGAATCTGATGAGAGAATGCTGGCATCAAAAT
GCTTCAGTACGACTACCAGCTCTAAGAATAAAGAAAACACTGTTGAAACTAGCTTCAAACGATAATGCCATCA
GATTGAATGACGACAACGAAGTTTATGTGTAAAAGTGATGGTTTAAAGTGAATTTGTG
TGAAAAAAAATTACAAACTTGTACATTTTCTATGGATATGTTGATTTATAAGTGACATTGTCGCCAAGTTTTA
AATTTATTATTTTATATATAATAATTGAGGTCATTTGGTGTGCAGTAGGATACCTTCTGACTGTAAAGAATAT
AGTTGTGTGGGGTTCGATCCCCATATGGGCGAATGAGTTCATTTCCTCACTCTGATAA
GAAGACAAGGCAATATCTCGAAAATTCGACAGTGTTTGGGGACCGGAGTGTCTTAATATTACTTTTCCTCTGT
CTATACTGCTATGTGGGAGGACAGCGATAAGCTAAAAAAATTACATAATTTTTTTATGTATGGACTTTTTAAT
TCATAAGTGACAATATAGCCAAGTATCAAGATTATTTGTTATAAATAATAATTAATAG
TGTTATTGAGTTAGTTTTAAATGTGTATGAAAGGTTTATATAGAAATGATTGATACTGATTTATAATGACATA
TTATTTTTTTTGTAAGTGACAGATTTGAAATGAATTATTGTATGTTAACATCTAAATAATCTTATTAAATAAG
TATAAATTAATGTATGATTTAGCGGCTATTTTATATAGTTTAATATTAAAGGAATCTT
TATTTTTTCT
>103782913_2 DT668587.3
GCACGAGGGTCTCCTTTAAAAGACTTCGCTATTTCAATATTGTCTATTTCTTTCAAGGAGCTAAAACTTTCCA
TTATTAAAAATTATATTATTTAGTTCATTACAAAAGCGGCAACGTATATTGATTGTTCTATTATAAGCTAGCA
TAACAATACAGAACGAAAGTAAATAATAAGAAAATATTATATAACAATTAGATGCTAC
ACTATAGTGTGTAATAAAATTATAGCAATACATAAGGTAGTATTTTGCACACATTTTCATTGCTTGTGAGACG
TTGTAAATATATAAGCTCGTATGTATCAGTATATTATAAATATAGGATTTTAATTATGTATTTATTAGAAGGT
AGATTAGTTGTATAGCCATTATATAAGTAAGTTATTATTTTTGTATATAGTAAATGTT
TTGTAATTTTGAAAACGTAGATAATTAAATGAGGAGAAGTATAATTATGTGTTTTAAAGTTGTGGTGTATGTA
TTCTAGTATGTATGTAGGGTTTGAAATTATATAGTAGTAACTATTATGTTTGAGTATATGCTGTATTTTATCG
CACCATTTTACACAGACATTTATTATAGGAGTTTTGAAATAAAAATATGATAAAAATA
AATACTGGACTAGTCACAGCTCCTGCATGAAGTGTTGCACCTGAGCTTCGTTTTGAATAAAGATGATTCTAGA
TTGGTCATCTTTTCTCACGTATACCTTCTCCAACG
>103782943_2 DT668902.3
GCACGAGGCCTGGCCCTAAAAAGACAGCCTAATCAGGCTAAAATCGAGTTTGGCGGAAAACTAGGGTATTCAG
AGAGGCTCGCACGAACTTCTTTACAAAGGCTGGGCCCGGATCCACCTCGAAACGAATTGTTGGCGGAGTTGAT
CAAGTTAGCAGCGAAGAGACCGCCCCGGGCCATCTCTCCGCCCTTGCCCTGCGAGCCT
GAACCACTGCCCGAGCGAGCTCCGTTACGACACATCGTCATTGATGGTAGCAATGTGGCTATGAGCCATGGAA
ATAAAGAAGTATTTTCGTGCCGAGGAATTGAAATATGTGTTTTTTTGGTCCGAGCGCGGGGTCACAAGGAGAT
TACAGTTTTCGTGCCAAACTGGCGTAATGAAGCCTCTCGACCAGACAATCC
>103782976_2 DT667577.3
GCACGAGGCGTCAACTTTTATAGATGTAGACGCGTACTCTCGCACAATTTATTCCAAGTCTCGAGGGCCCCAG
CGTGGCTGGGCCTGTTATTGTCTCATTTGTAAATCTAGACGTAGTCATGACACAAATTCTAGTCAGCTTTCAT
TGATCTCTTAATCCGGTAATCTTAAAGGTAATCGCAGTTATATACTTCTGTTACTAGT
GTATACCTACTGTAAGTGAAGTTCTCGTGTTCAAATACGGCGGCGGGGGCCTCCTGACGTTCTCGATGGATCG
GACTCTTTTTCTTTTACTGTTCAGATTGCAAATACCTTCATATTATTGGTCGATTTTCGAACATTATCGATAA
ATATCCTTATTTTAGTAATATATCTTCCTCTGTGTTGCCTATTATAAATTTACCCTTG
ACAGGTATAAAAACAAAATGGCCCCGTCGAGACGTCGACGGCTCCCGCGCGCTGTTTCATCGTGTTTTAATAA
AAAAATTGATAATATCGTAATACATAATAGATATTTTCATAATCAATAGTATGCATATTGTTACTGGATTGCT
AATTTTGTATACCTAAGTACACAATTAGTATAGTTTATATACTTATGTGTCAATTCTT

ATAGCGTTTTATTACCTTAACAGAAAATAGATAGTAAGTGTTAAAAGTAATCTAAATATCAGTGTTTATCGAG
CTGTTTCATTCTTAGTTGTTGTTTATTTTTGATTTATTCGATATTTTAATAAATATGCTTCTATTGTTCTAAA
CTTTTGAGCTGTCTCTGTGTT
>103782978_2 DT667958.3
GCACGAGGGTTGATCCTGTCGAAAATTATTTACTTGATTTTAGTTAAATACTAAAAATATGAATCTTATTTTA
TGGAAAAAAGTTTTTTGAAGATAAAATGTCGTTTGTTAACGAGCCAGTGTTGAACATAATAGTTGTAGGTTTC
CACCATAAGAAAGGTTGTCAGGTGGAACACTGTTATCCTGAACTGGTACCGGGTCGTC
CATCTGAATTACCAGCAGCATGGCGCTATCTTCCAGCTTTGGCATTACCTGATGGTTCCCATAACTATCTTTC
AGACACAATATTCTTTAATTTACCTAGTTTGACTGACCCTCCTCACACAGTTTACGGTATATCATGTTTTCGC
CAAATCCCTGTCGAGCAAGTGATTCAAAAAACCGAAGATATGACTCGTAGTTCTGTGC
AAAAAAGTGTCTGCATAATATGTCGTGTCCCACTTTTTGGAAGGCTTGCTGTAAAAATGGAATTAGTTGTGAG
GGCATGGTTTCTGCAAGGTGATTTTTCACAGACAAAACTATTAGAGGATGCCTTCAAACATCTAAATAGTTGT
CCTGTTCAGATAGATCAAGCTCTTGAAGGCTTGTCAATAACAAAATTAGTAGAAAGTT
GGAAACACAAGGCTTTATTACTATTTAAACTTTTGTTATTAAGAAGAAAAGTTCTAATTTATGGATCACCAGT
TGGTGCCCTCTCAGGGGCCCTTTTAACTCTTGTATCACTCTTACCACGCTGCTTAGAGTATGGATTGTCACAG
TCTGCTAATGTAGTTCTTTCTAGGCCTCTATCACCTATACCTG
>103782990_2 DT667789.3
GCACGAGGCAATATTCCGTTTTCTGTAAATAATTGATAATTTACTTTTAATAAAAGAGATACAATGGAGAAAA
TAATAAAAATTGGTAAAACACTACGTAATAATTGGAAAAAGTCGGTGTTTGGAGCAATCGTCGTTTATTACGG
TGCGGCAAAACTGAAAGAAAAATATGAAATAAGCATTTTAATGCAAGCAGCGTGTAAG
GAAGCCTCAAAATATGGTGACTCATTAATACCCATGGATAGGAACCCAACGCTTGTTACTGTTATCCTAAATC
CTGTTGCAAATAAGCGTAAAGCTAAAAAAGAATATGAGAAATACTGTGAACCAATATTGCACTTGGCAGGATT
ACAAGTAGATGTGATACAGACTACGTCAGAGGGACATGCCAAAGAAATTGTTGAAACA
TTAAAGGGTACAGAAGCTATAATAGTTGCAGGTGGAGATGGTACCGTGTCTGAAACAGTTACAGGCTTACTAA
GGAGAAATGACAAGGCCAACAGATTTCCATTAGGTGTCTTACCTCTCGGTCGTACCAATAGTCTCGGCAACGA
TCTATTTCCTGGAGGCGATGGTGTAGAAAAAGTAAAACAATTAATTCAAGCTTGCATG
GCTATCATAGAAAATAATACAACTTGGAAAGATGTCATGAAGATTGAACCTTTGGCTGATGACGATGAAAATC
CTAAACCGAAACCTATTTACGCCATGAATTCTATTGAATGGGGAGCCTTCAGAGATACACTTGCAAAGAATGA
TAAATATTGGATTTATGGACCT
>103783007_2 DT667412.3
GCACGAGGCTAGCCTTTTGAACGGCAGTGATGAGGTCTTCAGTGCGGGCCAGGCCAAGATAGTGAAGAGTGGT
GCGGCTGATAAAGTGCTGTTGATCGGCTCCGGGGTGACCTTGCATGAGGCGCTCAAGGCGGCTGAAATACTGC
AGAAGGAAGGAGTGGGCGCGCGCGTGCTGGACCCCTTCACCATCAAGCCGCTGGACGA
GGAGGCGGTGCGGGAGCACGCTGCCGCGGTGGGCGGCCGCGTCGTGGTGGTGGAGGACCACTACCAAGCTGGT
GGTATCGGCGAGGCGGTGCTGTCGGCGCTGGCGCTGCATTTTGACGTGGTGGTGCGCCACCTCTTCGTGCGGG
AGGTGCCGCGCTCCGGACAGCCCGACGAGCTGCTGCACAAGTACGGCCTCTCCGCGCC
GCACGTCGTCGACGCCGCGCGCGACCTGCTCAAGCTGTAGGGCATATGGAGTTAGAGTTAGAGTTGAACTCAT
TTATTGTTGCAATTGAGATCTAAATATTAACAATTGCTCATTTTTGGGCGAAAATCATGTTAATTGTTGCACA
ATCAATAATTATTTACTTGTTAATTGCAAATAAAATTTGCTCAACTCTTTCTATAATA
GCTCTGAATATACAATGGACTTGCGGTAATCCGACACGCCTACCATTATTGATGGTTCATAATGTGAGCAAAT
ATTTACTAATATAATTGGGTGTGTGGTAATGTGTACGTATTCGACCTATAATTCACGAGATAATTTATCAAAT
TGCAAAATACTCTTATT
>103783009_2 DT668811.3
GCACGAGGCAAATCCCTCGGACGGACACGCGTACAAAAAGCGCGTAACTACGAATTAAATTCTAAGCGATATT
TTTTTATTTTACGAAATTTATATATCGTTATACATGTGAAATTCGAAAAATGTAACGAAATAAAACGAATATA
GGTTTTTTTTGTATTTTTTTAATTTAATGTAATTCGGAAATTAGCAAATGTGTAAACT
TTGACGTGTATTGCATTTCATCGATACGATAAGGTTTATTTTTCAATTTACGACTACGATAAACAAGCACTCG
ATAAATTTCCATGCATTTCGTAAAGATAAATGTGACTATACATGCCGGAGGCCGGACTACCAGGAGTTTTCCC
ACTTAGAATTTCCCCAAAAAACGTGGAAAGTGAAACGGTTTTCCACTTGTTAGACGCA
GAAAGATACAAAAATGCAGTTTGTACGCGCCTTCTTCAAGCGGATCACAGACGATCTCAAACAGAAAAAACTA
ATACCTCTAAAAATATTGTTTTTCGTACATGCCTCCACTCTGTTTGTTTTGTACCCGTACCTGACGATACACA
TGAGAGAGCTGGGCATAAACGTAGAAGAGACTGCTATAATGTCAGCCGTAACGCCAGT
CATCTCGATCGTGATGCCGCCCCCTCGCCGG
>103783022_2 DT667811.3

GCACGAGGCGCCGTGCCTACCATCAGGGGCCGTTCCTTGCTAGCGGATCACGTGATAAATCTGTTAAGATCTG
GGACGTGAGCACGGGGCAGTGCCTGGCGACGCTGGTGGGGCACGACAACTGGGTGCGCGGCGCCGTGTGGCAC
CCGGGCGGCCGCTACCTGCTCACCGCCTCCGACGACAAGACGCTGCGCGTGTGGGACG
TCGCGCACACGCGCTGCCTCAAGACGCTCTACGCGCACCAGCACTTCGCCACCAGCCTCGATTTCCACAAGAG
CTTGCCATACGTGATATCCGGCAGCGTCGATCAGACCGTCAAAGTTTGGGAGTGTCGCTAAGAGCATTGCCAT
TTTAGTAATTCAAACTCACCGATGATATTTTTATCATTATTTTTCACACATAATGGAG
CTGCAATGAGACAATAGTTTCAACTTGGATGGTGATCATATAATGAAACAAATAATACTTTTGATAATTTATT
GATATTTTCCTTTCTTCTGTTCATCGAGATCTTATCTGGTGTTGAAGACAATGCTATGTCCACATACCAACTT
TGCACACAACGCCCATTTTATTTTATCGATTAGTGTAACACATTCCATTAAAAATTAA
CGGCTCCTAGTTAATTTCTGTATATGAATAGTTTAATTTTGTTACGTCTGATTTTAGTTCAATTTCATTGAGT
GACGTAGCATTCGTCGCGCACCCGATCGAATCGAAGCGAAGCGAGGAGTAAAAAGCTGTGTTATAATTTAACA
TTTCTTTATTCTATT
>103783058_2 DT667870.3
GCACGAGGGCGGCGCCTTTGGCGGCCGACGTGGCGGGCTGCTCGCGCCGCGACCTGCTGGCGCTGCTGGTGGC
GCCCGTGCGCGCGCCCGCCGACCACGCCGCCACCGCGCACCACATCAAGCAGGCCTACCACTCGCTGGCCAAG
TGCGTGGCGGCCGTGGTGGTGGCGGGCGGCACGGACGCGCTGGACATCACGCGCGGCT
TCCTGCGCGACGCCGCGCAGCCGCCCACTGACACGCAGCACATGTTCGCGCTGCTGGCGCTGGCCGAGATCGG
CAGGCATCTAGACCTCAGTTCAATTTCCAATTTAAAAGAAGTGCTTTCTGAATTCCTTCGCGCCATCCTCCGA
AGAAGTGAAGTCGGCAGCCAGTTACGCACTTGGTTCAGTGGCAGTGGGTAATTTACCG
GAGTTTTTGCCTTTTATACTCAATGAAATTGAAGCTCAGCCTAAACGACAATATTTGCTGCTACATTCTCTTA
GAGAGATTATCGTCTGCGAATCTTGTACTCCAGAAGGAGTTGAAGCGCTGAGGCCTTTCATACCTCACATATG
GGTACAGCTCTCCAAGCACTGCCAATGTACGGAGGAAGGATCTCGCAATGTTGTCGCT
GAATGTTTAGGTAAATTGTGTCTACTAGAACCTCAAGATCTACTGCCTCATCTGAAAGAATTCCTCAAGTCAC
CTGACCCGGCTCACTAGGACCACTGCTGTTACTGCTGT
>103783068_2 DT668042.3
GCACGAGGCTGATCCTTAGTCAGATGATACTGGCAGTTCTATTTCGACTGTCGTTGATAGATTGGGATCAATG
GCTGGTTCTGTTGACAACAGCATACCTTCGACACCTATCATGCAACCTAAGTCAATAGATATTCAAAAAGTAT
TGAATAGCATTGAAAACATTGATACAGACATATTAAAAGATATGACTAGTGCAAAATT
TCTAGAGAAACCTAAAGAAAATATTGTTGGCCAAAAAATTGAAAACAATATTTATGCTAATGAAATGAATACT
TTTGATAATGCTGCACCGTCTTTAATACTATCCATTAGTAAACAATGCGAGGAAAATGATAAGAATAATAACT
TAACTCAGAAGGTGAATTCAAAAATTGAAGAGCCTTTAATGAAAGTTGAAAGAACTAA
TAAATCAGAAGCTATTGAAAAGCCTAAGCCAATGCCCAGAAATACTAAAGTAGAACGATCATCCAGTGATAAA
AAACGACGCCCAATACCAATCCCAGAAATGATTTCACCACCAATAACTGAACCACTGCTAACACCAACCAAGA
AAACTTGTATAGAAACATGGCTTCAAAGGTCAGAAGCAGAAATGAAAAATAAAGAAGA
GAAATTAGAAGAAAATCCTCCTCCTGAAGTATTACCTCGTCTAATTGACTTTGTTCCTAAAATGCAATTTAAG
GAAAATAATGTTGAACAAACGACCTGTATACGTCCTACAACAGCACCGCCACCACCGCCGGGTGAGCCCCGAG
AAGATGGATCCGGCGCATCACTAGACACCATAGGGGGAACTGGATGAATCTTGTGATG
TTG
>103783072_2 DT668163.3
GCACGAGGCAAGACCGGTGTTACCATTGAACTGAAGGATAATGGACAGGTTGCTTTGAACGGTGCCTCACACG
GCTACCCAGTCATAGAAAAAGACGTATTCGCGTTCAGGGAACCCAACGGACGTATCGGACTTGGATCGTTATA
CGGACTTATGGCCTTCTGTACCAGCAAACTTGAGGTGTGCTACATAGAAGTCAACGGC
TTCTATCTCGGAAAAGTTCGTGGTCTTCTAGGAGATGGAAACAACGAACCTTTCGATGACTTCAGGCTACCCA
ACGGAAAGATCGCCAACTCTGAAAGTGACTTTGGCAACGCGTACCGCCTCGCCGGCAGCTGTCCCCAGGTGAA
GGCGCCCGAGCACTCTCACCACCAGATGCACTCCCCTCTCCCTGCTGCTTGTGAAACT
GTGTTTGGTGGAGCGTCCCCGCTTAGACCTTTGTCTTTGTTGCTCGATGTTAAGCCTTTCAGACAAGCCTGCA
TCCACGCCTGCTCAGGCGACTCTGAAGACGCCCTCCACCAGGCCTGTGACCTCGCGCGAGGCTACGCAGCTCT
TGCAGCCTCCGGCCTCTTGCCCGCCGTCCTCCCCTCCGCCTGCGTCAGGTGTACTGAC
GCAGACAAGCCCAGACAAGTCGGTGACGTGTTTGAGGTGAAGATGAGCGGGAAACAGGCTGATATTATTGTGG
CTGTTGAGCTTACTATCGCCAACGAGAAGAACTACAAAGACATCGTAGTACCGCTAGTGTCACAGATCATAGA
TCAACTGAAGAGGCAGCGTGTTACTGATGTCAAGGTGTACTTAGTCGGTATTA
>103783111_2 DT668822.3

TGTTATTCTTACCATAACTGTGGCTGTAGCAGTAGCGTTCCCGAATTTAGAACTCGTTATAAGCTTGGTGGGA
GCCATTTTCTTCTCAACACTAGGTCTTCTCATCCCAGCTGTAGTAGACACTGTGTACCATTGGGACAAAGATT
TGGGAAGATTCAACTATGTATTAATAAAAAATACTATAATTAGTATAATTTCCTTAAT
TGCACTTGTATCAGGATCGTATGTGTCCATTTTAGGTATGATGGAAGATTTTTCCCCTAAATCGGATATGCCA
GTACTTAATTCTACCATTACATAAAAAAAAATATATATATATATACTAAAACATACTTGCTTGGTCTAAATTA
CCAAACTTTTTTAGTTAGTACAAATTTATTTAACCTACTTTAGTAACAAAATTTTGGT
AGCTTGTATTTGGTAGAGTTCGTATGCTTACAGCAATGAGGCCACTCGTGGCGCAGGGGTACAAGCGGACTAC
TGCGACAGGTTGTGGGTTCGATTCCCACTCGAGGAAATTAAATATTTAATATTTTCATTTTCCGAGCTCTGGT
AAAGAGTCAATTCTCAACACGCAATGCTTCCAGAATTCGGTGAAGTGGGG
>103783123_2 DT666970.3
GTGATGGCCACACTGTTTTGGTGTAAAGTATTCAAAGCTCCCGATCTACCCGGAAAGCATCATATAATACGTT
ACGAACCACTAATAGACAGTAGACCAATAAAAGACGGGAAGCCGGTACAAGAGAAGAACAGCCTGTCACCAGT
ACATCATATGGTTTTGTATGAGTGTGCAGAAGACCACGACAAAAGACAGTGGAATGAA
TGGGCGCAGAACAGCAATGATGGTTTCTTCGGGCCAAATAGACCAGCAGAGTTGGCGACTTGTGTCACGCCTA
TTGCTGCGTGGGCTATGTTTTTTAAGGGTGAATTTCTGCCAGAAAATGTCGGCATACCGCTAGGTGAGAGGGG
AGGTGTGTCGTACTACATGCTCGAGGTTCATTACGATAATCAGGCGCTACACGAAGTA
TTATATAATTCCGGAATTCGAATCCACTATACTCCATCTCTGCGCGCCCACGACGCTGGTCTACTAGGCGCTG
GTATCGGCGTGTCTGCGTTACCACGTTATACCTC
>103783135_2 DT668762.3
AATAACTAAAATATATTAATCATTTTAAGTTTCTTTTGAGATATTTGATCTTCTGAAATTGTAAAATCCTGTT
ATTAATTTTTAAAAATATTAGAGATTACAAAAAATGACTGAGGACGACAGCAATAATGTTGAATCAGACAGTA
ATACGAAATACAAAGACGGTGTTAATCGTAATGCCAGACTGATTGTTCGCAACGTATC
ATTTAAAGCTACAGAAGATAATCTTAAAGAACATTTTTCTGAATACGGCAATGTAGAAGAGGTTAATCTACTA
AAGAAACCCGATGGAAGGCTCGTTGGATGCGCATTTATTCATTACACACAAGTTCCAATGGCAAATAAAGCTT
TGGCTGCGACGAATAAGAAACCTTTTTTAGGTCGACCTATATATGTAGCTTGGGCAGT
TCCACAAAATAAATACAATGAAAATGCACAAAACAACAGACCCAAGAAGAAATTTGATAGTCCTAACGGAGAT
ACAGAAATGGAAATTAAAGATGAAGACACAGAAGATCAAAAGCCAACTAAAGAGGATAAAGACAAAACTAGAA
TAAACAGGAAAGCTCGTCTAATTGTCCGTAACATATCTTTTAAAGCGACAGAGGAATC
TCTCAAAGAACATTTTTGAGCCATATG
>103783145_2 DT668358.2
GCACGAGGACTTAAGTAAGCATAAGTAAACCTTATAATAATCAATAATGGTTCTGAGATTATATTACGATTTG
ATGTCACAACCATCCAGGACACTATATATTTTACTAAACACGGCAAAATGTAATTTTGAATCCAAACCTGTTG
ACTTAAGAAAAGGAGAACATTATTCAGAGGAATTCACAACACTTAATAGGCTTCAAAA
AGTGCCTGTTATTGAGCATGATGGCTTTATTTTGAGTGAAAGTGTAGCCATTGTGAAGTACCTAGCCAGAGAA
AATATAATACCTGGAAGTTTGTATCCTAAGGATACTAAGCAACAAGCTCGAGTAGAAGAGTTCTTGGAGTGGC
AGCATATAGGCTTGCGACTACATTGTGCTATGTATTTTAGGGCCACGTACTTGGACCC
TATTTTCACTGGTAAACAACCAGATGCAAAATCTATACAAGGATATGAACAGAGGATGGTGTCAGCATTAAAT
GTATTTGAAACTAAATGGTTAAACAATGGTACAGAATTCATAGCCGGCAATACAATTACTGTGGCCGATCTGT
TTGCTGCCTGTGAATTGGAACAGCCAAGAATGGCTGGATATGATCCAGCAGACCATTT
CAGTGGTATTTCGGTTTGGTGGAAGAAAGTGAGAAATCATTTTAACCCAATTTACGACGAAGCACATGTTACC
CTCAACAAAATAGTTAATAAGAACAAAAAGGCGAANAAAAAAAAAAAAAAA
>103783153_2 DT668380.3
GCACGAGGCGGCGCCTTAAGATGAGACCCTGCTGCTGGGCGACGCCGACATGTCGCTGAAGCAGGGCAGCGGC
ACCGGCAAGGTCCATTTCGGTGGTTTGGACGATGTTTCCCTGTACGGCACGCCTGTGGAGCCTGCGCCCCCGG
CCCCCGACGCCAAGCAGGGGTTTCTTCGCAACCAGTTACAAGCACTCTTCCAGCCTAC
CGACAACAAACTAGCCATGAAGCTGTTCGGCTCAAAAAAAGCTCTCATGAAAGAGCGCATCAGACAGAAGGCG
GCGGGCCATTGGGTCATACATCCATGCAGTAGTTTCAGGTTCTACTGGGACCTGTGTATGCTTCTACTACTGG
TGGCTAACCTAATCATCCTGCCAGTCGCCATTTCTTTCTTCAACGACGACCTCAGCAC
GCGGTGGATAGCCTTCAACTGTCTATCTGATACTATATTTCTTATAGATATCGTCGTTAATTTTAGAACAGGA
ATAATGCAACAAGACAATGCAGAACAAGTGATATTGGACCCGAAGTTAATAGCAAAGCATTATTTGAGGACAT
GGTTCTTCCTCGACCTCATCTCTAGTATACCACTAGATTATATATTCTTAATCTTCAA
TCAGGTAAACGACTTTAGCGAAAGTTTTCAAATTCTTCACGCCGGTCGAGCCCTAAGGATACTACGGTTAGCA
AAGCTTTTATCGCTGGTTCGACTCCTCANGCTTTCCAGACTCGTCCGATATGTCTCACAATGGGAGGAAGTAT
ATTTCCTCAATATGGCCTCGGTGTTCATGCGGATATTCAACCTCATCTGTATGATGTT

ATTGATCGGCCACTGGTTCTGGATGTCTGCAGTTTCTCGTCCCTATGCTCCAAGGATTCCCACCAAATTCCTG
G
>103783155_2 DT667707.3
GCACGAGGGCTTCCTGTGAGCGGTGTGCGGCGCGGCGCTGGGTGACGGCCGCGCCGGCGCCGACGTGCGCGTA
CGTGCGCGCCGCCTGCACTGCCACCACTGCTACAGCGCCGACGACGGCAGCAAGTACTCGTGCGTGTAGCACA
CCGCACCACCACGTGCGCGGTGTGCGGCGACGTGCGCGTACGTGCGCGCCGCCTGCAC
TGCCACCACTGCTACAGCGCCGACGACGGCAGCAAGTACTCGTGCGTGTAGCACACCGCACCACCACGTGCGC
GGTGTGCGGCGACGTTCGCGCCGCCTGCACTGCCACCACTGCTACAGCGCCGACGACGGCAGCAAGTACTCGT
GCGTGTAGCACACCGCACCACCACGTGCGCGGTGTGCGGCGACGTGCGCGTACGTGCG
CGCCGCCTGCACTGCCACCACTGCTACAGCGCCGACGACGGCAGCAAGTACTCGTGCGTGTAGCACACCGCAC
CACCACGTGCGCGGTGTGCGGCGACGTGCGCGCCGGCGCCGACGTGCGCGTACGTGCGCGCCGCCTGCACTGC
CACCACTGCTACAGCGCCGACGACGGCAGCAAGTACTCGTGCGTGTAGCACACCGCAC
CACCACGTGCGCGGTGTGCGGCGACGTGCGCGCCGGCGCCGACGTGCGCGTACGTGCGCGCCGCCTGCACTGC
CACCACTGCTACAGCGCCGACGACCGCAGCAAGTACTCGTGCGTGTAGCACACCGCA
>103783161_2 DT667302.3
GCACGAGGAGGAACCTTTATATCTTTTTTTTAATATAAAAAAAAAGTAAGTGTCAGTCAGTATGTTGCGTGTA
AATATTGTGAAAGGTGTAGGAAGAAGGTTTTACTCGGCGGTTGGCGGCGATTCTCTTCCGCCTGGTACTATAT
TTTTTGGACAAGAGGGTCTCAACGTGGACAAACGAACTATTGTAAGTGCAGAACGAAA
ACATCAAACATTCACTGACAGATCTCAACTGAAGTATGCAAAAAGACTAGTCGTTAAACTTGGCAGCGCAGTT
ATCACTAGGGAAGATGGCAATGGATTAGCTTTGGGACGATTGTTTTCTATAATTGAACAGGTAGCAGAATGCC
ATCACGAAGGACGAGAATGTATTATGGTTACCAGTGGAGCCGTGGCATTTGGACGACA
AAAATTGACACAAGAATTGCTTATGTCGATGTCAATGAGAGAAACATTGTCACCAAGTGATCATACTAGAGAA
GACGCCGGCAGCATTTTGGACCCGCGGGCGGCGGCTGCGGTCGGACAATCTGAACTTATGTCGATGTACGACG
CCATGTTTTCGCAATACAATGTCAAAATAGCACAGGTATTAGTAACAAAACCGGATTT
CTACAATGAAGAAACAAGAAAAAATCTATTCGCAACGCTCTCGGAGCTTATCTCGCTTCAACATCGTCCCCAT
A
>103783174_2 DT667436.3
GCACGAGGCTCGACTGTATCTTACAAACGGATGAGTTTAGACGAGAAGACATAGCGGAGGCGATACGCGCCGG
CTTTCTAGACCTGGACAAGAAAATGAGGGAACTCCCCGAGCTCTCGAACGGCGAAGAGAAATCGGGCTCCACA
GCAGTGTGCGCTTTCGTATCGCCAAAGCAGATATACATAGCAAATTGTGGCGATTCTC
GTGCAGTCCTTGCTCGTAATGGAGCACCGATATTTGCAACGACAGATCACAAACCGGAAATGCCTTCAGAAAA
GTCTCGCATAGTACAAGCTGGTGGCTCTGTTATGATACAGCGTGTAAATGGTAGTTTGGCTGTGTCTCGGGCG
TTAGGCGACTATGAGTATAAGAAGGTGCTGGACCGTGGGCCGTGTGAGCAATTGGTAT
CACCAGAGCCTGAAGTGTCTGTGCATGAGAGGCTAGATGCAGAGGATGAGTTTCTGGTGCTGGCCTGTGACGG
TGTTTGGGATGTAATGAGCAATGAGGATCTGTGTGCATATGTACACTCTCTACTGCAACTGACAGATGACCTT
GTAGCTATCACCAACCAGGTCATCGACACCTGCCTGTATAAGGGCAGTAAAGACAACA
TGAGCATCGTGCTAGTGGTGTTCCCGGCGGCCCCGAAGCCCAACCCCGAGGCCCAGCGCGCCGACCGCGAACT
GGACGACACCCTGC
>103783176_2 DT668943.2
GCACGAGGCACGCTCTTTAAGAACTTAAACAAAAAAAAAAAACTTTTTTCGTGACTCGTGTTTTCGTTGTTTA
GAATAAATTTACGTACCTAAAAAGTAATTATAATAATTATTATGATAGTTGTAAACGTAAGAAAATAATAATT
GCAAAAAAAAAAACAAAAAAAAATATATATAAAAAAATGAAAAAAAAAAAGAAAAGCAA
ATAATAAAAAACCAATGATTGTGCCTCAATAAACTGTGGTACGTTACCGTAACCTTCGGGCAGGTTGGGTTCC
CTTCGGGGGCAATGAAGTGGGTCCCGCGCCCGCGCCCGCGCCGTCGCCGTGCGTAATCATTGATGAGCTTAGC
TATGTTTTAATTTTATTGTAGGTAAGTGTTATGACATCAATCGACATTTTATATATTT
ATTATACTTGCGTGTAAATATTTTTATCTATTTATTTTCGTTTTCATGAAGTTTAACCCGAATTAACGTGTGT
ATTTGCTCGATGTTTTTTTTTTTTAATATTTATTTCGGGTCAGAGCCGATTCCATAACACATGTATGCCAAAG
TCGATGTCCTATTTGCCATTAAATACCGCGACATTCGATCCGTGGATCTCCGACATCA
CAACAAAACTATACTATTTTAATGAACGTACAAATGGGCGCCATTTTGTTTTTA
>103783177_2 DT668360.3
GCACGAGGACGTGCCCTTGAAAATCTGTAAAATAACTACTAAATTCTCGATTCGTGTTCTTTAAATTTTCTAT
TAATCTAACTATATAATATAATTAAATGAAAACGTGAGCAAGTGATTACTATACGCAATATAAAAAAATATAT
GAGCGTGGGTTAAAATCTAATGACTTAATCAAATTTCAATGTGAAGCAACAACGAAGC

AGTATTAGTAAGTCGGTCGTGCTCGCCAGTGCTTATCATATTATATATCACCTGACTGCGCTCGTCCTTGCTA
TTTGATATGGTAATTCAATAATCAAGAAGTGTGCCCGCAAGCTTCGACAAATTACAAATGAAAGATTGCCAGC
TCCAGTTGTTCCAAGCTCCCGTAGAGTACACCACGTCTCACATGTTGTAATGCGTCGG
AAGGAGTCGTTACCTCCCAGCGGTGGTAGAGGCAGAAGCTTGATTGCAACCAGAACGAGTCAACAGCAAAGCA
GAATGCACGGCATAAAGAGCCAACTACCGAGAAGCTATTCTTCACCCGGTTGCCAAAATGATGGTAACTCCCA
AGAAGAGGCCAAGGGCTATGTCATGCGGAGTTTTTCTTCACCGGAACCCAAACAGACC
AGCAGAAAGAATTCAGAAATATTTTATACACCAGAATCGCATGGATCAAAAAGTGAATTGGATTCAGATTTGG
AGAATTTTGAACAGCTTGAGGAAAGTTTGCTGGACATCGATGCAGATAGCGAAGATATTTATGGGCAATATCC
C
>103783185_2 DT668557.3
TCTTATGTTAGAATATGTAATATAATATTGTGCCCATTTTATTGTATAGTTTTTTTATAAGGCCTATAAGCG
AAATATAGCGAAGAAAATCGAAATATGTCAGTCGCTTTTGCACCTAGAGGCAATCGACCTCAATTGACACCAG
CCCAAATTCAGAAAATGCTAGACGAGAATGCTCATCTCATACAGACTATTCAAGAATA
TCAAGCTAAAGGTCAGCTCATGGAATGTCACCAATACCAGCAAGTGTTACACAGGAATTTAGTATATTTAGCA
ACGGTGGCCGATATTTTTCAGAACCTTAACATACTCTTGCCGCCACCTCACCAATTAGCTGCTGGAAATGTAC
AACAAGGTCCTTTGAATCCACCAACTAGCGGAGCAGAAGTGCCAGGCTCACAACAGCC
CTACAGACCACCATCAGGTGTTTCTTCTACACCAACAAGACCAACACAGTCATATGGACAAAGGCCTTATCCA
CAGAACCAATATCAAGGACAATACCAAGGTGCTCCTGGCTATCCTCCTCAGGGTGGATATGGACCACCAGGAC
AAGGTTATGGACCCCCTAACCCACCTCAACAACCTCAGGGCTACCCTCCTAACTCTAC
ATTATGGACCACCTATTACTACTG
>103783198_2 DT667690.3
GCACGAGGCACAACATGCACTATGATAAAAAATAGACACAAAAATTAGTCATGCTTCTTGCAGCATGAAATGA
ACCTGGGCTTCGTTGGTGATATGGATAATACGTGAATGGTCATCCTTCCGAACAAGAACTTTGCCAAAAGATG
TCCAGCAGTATTTATATTTGGTTGATTTTACCAGTTCACGAGCTAGAAAAAATAATCG
TGCAGCCTTAGGTGTTAGTTGCTCGGAGACGAAAACAGGAGTATCTTCAGATGCGTGCCCAAGGTGTTTCGCT
CGAAGTTTATTTTTGTTCTTCACATTGAACAATTTAACCTTTTTTAAAAGATCCGTTTTCAGAACAGTAGAAC
CGAGTTCCACAATAATTGGAGTGCTACTGCGCTTGTTCTCGGGTTTGGCCTGTAGTCT
GAAGATATCTTTTATATCACGTGTTTCCATTTCAAGGTTAATATTCTTGGCGAAGCAGAGTACCATTTTGGCC
AAATCTTCTGTACTTTCTCGATCCGATCTCGGAACATTTTTTATTTCTATACACGTTTTCCGAGACTGTCTCT
GCATATCCTCAAATTTATTCTCAAGAACTACTATATATTCTCGATCTTTCTTACACTG
TAATTCAAGCGTTTCAATTTTTTTCTGTAATTCCTCGTTTTGACTAGTTAATAAAGTGATAGAACTT
>103783205_2 DT667001.3
TTAGAAAGTGATCGCAAGTTAAGAAACAGTATAACTAACTAACGAAATATAAATAACAATATACAATAGAAAT
TAGGATTTATGTCTTAAAATAAAGAAAATGAAGTTGTTACTATTAGTTTTCCTCTATATAGTAATCGAAATCA
ATAGCGACGATGACTCCGGATGGTCCATAGTAGATAATCCTTTGGTTGTTGTACACCC
ATGTAACGGTTCGACAGACATACAGCTTATGTATGAACCTTATAGATCTCCCGAAGAAGAAAATAAATATAAT
CTTTGGATTCATAAGAAATTTCCCAAAAATACTGTTATTAAAATAAAATTTGATAAAGGTGTCACCGTTCTTT
TGTCAAATCAGAGTGCTGCAAGAATAGATCTTGACAAAAGTGATGAACACGCATTCAA
AATAAGATTTTTCAAATCACATGAAGGTATTTGTTTGCACGTACAAGGATGTACGCTTGGAGTCGTGCCTTAC
ATTAAAAGTGTCAACATAAATACAAAAGAGTACTGTGAAGATCATGTTATGGGTTTTTTGGATTCTTACATCG
AAGGTTATAAGCTACAAGCTCAAAGTTCTTGTGCTAAACAAAAAGTACAACAAAAAGG
CAATTGCGGAAAACGGCAAAATCTTGTAATTCCATTAAGTGTGCAAGGGGAGTCTCAGCGTAATGACTGGCCG
TGGCATGTTGCTGTGTTTCATAAAA
>103783206_2 DT667243.3
TTTTTTTTTTTTTTAGTAACATAAAATATATTTAATATATAAAAATATCTAATCAACAATAATAATTATATAG
TGCAATTTACATAATGGAATGATTTGTTTACGAAATCAAAAAATGTAAAAAAAAAATGTATAAAAAAAAAAGT
TAAAACTTATTGCACTTTAAAATAACATAGACACTTAAAACTGTATTAATTGTGACGT
AATATAAATGCCCAGCCCATCCATATCCCATAGACAATGTATACATAAATATATATTTTTTTTTATTCAAAAT
TTATAATTTCGAGCGTATAGAGTCGATTTTGCCGGCGGCGGATTTCTGTAAGTCTGTCAGTTTCATGGCGAGG
CCCATGTTGCCTTGTATTTTAATCTTGCCCTGGAAGAAGGCCTTTTGGGGATTTAGTT
TTCCAGAGATCAAATCAGCTACGTCCTCATCGTTTATCGTGAACGTAACGTCACATTTATCTTTGCCATTGTA
CTGGACGTTGCCCTTTCCTTCCTTCGCGTTGATCACCCAATAGCCTTCCTCCCCATTGGGTCCATTCCTGACT
TTGAACCCGTATATGCCTCTGACCTTCTCAATAAGTTTATCGTCGTCGTTCTTCATCG
CCTCCTCGAGTATTTTCATGTATTTGTACACTTTGAAGTTGT

>103783214_2 DT668663.3
GCACGAGGATCATTCGTCAAATAATAAGTGTATATTTTTTTCAGTAAAAAATACACAAGCAGTTGCTATGTAA
ACTGTTTGTCAGTGATAGTGTTAAGATGTTGTGAGCCTTTATTGATATTCTTGTGGAGTGGTAATAAATACCA
AAACAATGGTTCCTGTACCGAGTTGTCCCCACACATGGGATTACTGGACGGCCACGCC
ATCTGATTATGTGGAGCTGACATGTTTAATGCCAAATTCTATATATATTCCTCTAACAGTCAGTTGGGATGCT
AATCTTCAAGATGTTAAGGAGGAGCTATGGGATATGGCAGCAAAGCAGCCTTTATTTGGGATGCTGCATGAGA
TGTCAGGTTATGTGTTCCAATTCATCAATTCCCTGGCAGTTCCGGAGGAGGTGGAAGA
TGAGAACAAAAGACTTCGGGACATTAGGCCTGTGTTTGGAGTTCTCATGCTCATTGAACGTTCCATTGAGAGG
CCTGGGGAACATTTATTAAATACACAAATTAGTCATTTGATTGGCAAAGGTTTAAATGAATTTGATAGTTTAA
GAAGTTCAGAAGTTAATGACTTTCGTATGCGAATGAGATACATAGCAGAGGAGAGTCT
CATAAAGAGAGCCCAGAGTACAAGGCTAGAGAGACTAAGATACCACTGTCCACCAAGATTAGCTGATGTGCCG
TCTGTTCCCACTACACTCATAAATCATTTAAATAATAATTGTTTTATTCTGGTCACTAAAGTAGGTAACACTG
AGACGTCCTTCACATCGAACG
>103783233_2 DT668502.3
GCACGAGGATACAATCTTCAAGTCAGAGGATTTAAAACTGAGAGAGGTATTCATGCAGATTTGAAGCGGAACC
CAAATCTTATAAATAGACTGCGCCTAAATGTACAAGAAAGTACCGAAAAACCCAACCTAGGACCCGATGTTGC
TCCAAGACTCGAGAAACTTCTCAATGAAGACACACTGCTAACACATCAGCAAAAGGAT
AAAATTAAAATTGCATTCGCAGAAGGTTATTTAGCAGGTTCACATCCGGAAAACGTACGCGGTACTAAAGCGT
CGAAGTATCTCAAATTGGTACAACAGTTGTTGACTATTGTATTGTTTCTGGCCATCTTTGTCAGTCTGATGGC
GTCTGTCAGCGGAACTGTTTTCCGGATCCAGCTCGGCAACCAGGTGGAGGTGGATCCC
GAGGATATATCTGTGACATTTGACGATGTGAAGGGCGCGGACGAAGCTAAGCAGGAGCTGAAGGATGTGGTGG
AGTTCCTCAAGTCTCCGGAGAAGTTCTCTTCGCTAGGCGGTAAACTGCCCAAGGGGGTGCTCCTGGTGGGACC
GCCTGGGACAGGCAAAACCCTTCTAGCTCGTGCCGTGGCCGGTGAAGCCAGAGTGCCG
TTCTTCCATGCCGCAGGTCCTGAGTTCGATGAGATATTGGTGGGGNCAGGGGCAAGGAGAGTGCGAGATTTGT
TTAAAGCGGCAAAAGAAAGAGCACCCTGTGTAATCTTCATAGATGAAATTGATTCAGTCGGAGCCAAAAGGAC
TAACAGTGTTCTACATCCTTAT
>103783235_2 DT667672.3
ATACGATCGCTACTAATCAACAGTACACAAAATTCAGTGGTAAAGATAAAGAAAACATATCAATTACAAAAAG
GCCTTCCGAATATAAAAATGAAATTATAATAGAATACGAAGACGCCAACGAGCATACGTTATTTCCAAATAAA
TCTGAATATGCAGATGATGAAACAGATAAGAAATTCGAAGAAATATTGCAGTCTGCTT
ACAATTCTGACAAAATTATACCCACAATAGATAACAGTGAACATGAAAAATCGCCTATAAGCATCGAGAACTC
AATAACATCAAGTA
>103783258_2 DT668191.3
GCACGAGGCAAAGACTTTAGATAAGAGAAGATTTCAATCCCAATATAAGACCCAATTTTGTTAGACAAATGTA
CTTTCCAAGACCCTACAATCCCAGATTTCCCCCAAATGAAGTTTTTCCGCAAGTTATAAGGCAGCCTCTGGTA
TTACCCTTACCTAAAAGGGAGCCAGAGGTAAGAATGTTACCTGTGCTTACCCCTAATT
TACCAAGTTTACCCAATTTATCTAATCTACCTAGTGGCCTGGCGGGAAAGAAAGTGTTGATTAATCCACATTT
TAAGGGGAACTTTCAACCGCCTGTAGATGGTCCAATTAAAGATTTTGACGACGCGGCGGAGAGGTTTATAGCG
GAACAAAGGAATGCGTTGGCTCGAGCTGCCACTAGGAAAATACCAAGACGGTCTCCAC
AAAGATACATAGAAAACACAACGATAGAAATTGAAAACGACTTAGTTCGGCCTCCGGTGAGGCGTAAGAGTGA
TGACGACGACTTATTAAGGAGGCAAGAGGAGTTTATTAATGCTAACAGAGCTGGATTGAGGCGACGTATGAGA
TCGCCATCACCAGTCCGTAGATCACCTTCACCTCGACGAAGTCCCGAACGAAGACAGA
GGCCTTTAGATGAGGAAAGTGAATACAGACGGCGAGTTCGCGAACAAGAAAATCTAAGGGAACG
>103783271_2 DT668482.3
GCACGAGGGTTTTCGCATATGTCACCACGTCTCAAAATATCAAAATATGTTATTGAGCGTTTGTCTAAAATTG
ATACAAATGAAACTACAGGCTGCCTTTATGGTCTCATGTACGACAGTACATTGCTGGTCGTGGGGATGAGCTT
AGAATTTTTCGAAAATGAAAAGAAAACTTATAGACAACTACTTCTACATTTGCCCGCC
GAAATAGAACTATGTGGTGTTATAAAATTTAATGAGACTTTATCGATAGATTCAAAACCTAAAGAAATTTTCC
AGGATGTAGATATAACTGACAATCCACTTTTTCTAATAATTAACAAGGAAAAGGAGATTAAAGCTCATTTTTT
GGTACATGATAAGTTTGAAGAAACAAGTTATGATATACTACAAACTGATGAACTATGG
AAACAATTTATTCATGTTCGTCTCAATACTATTTTGCCTCTTGCTTGTGAAGCTACTATACCTGGGGTAAAAA
ATTTACTCCAGAACAAAAGAAAAAAGATAGCATCTGGGCAGGTGTCATTTAACATAGATGGAACATCAGTATA
TTTATTTGGTGTGGCATCAGATGTTGGTGTAACAGGCACCAGTATGGATTCCAATGTA
GGAGAATTTATAGACTCTGTGGGCCCAGAACAGTCAAAGAAGAAGAAATATATTGCCAATA

>103783286_2 DT667797.3
GCACGAGGAAACAGCTCGTTTCGGCGATGAGGACATTATTTGATATAATGGACGATAAGCAAACTGGGTACGT
AAAATTAACCGACATTGAAAACAGGTGGCGGGATGATCGAACTAAGGGTCTTCCCCGTGGTGTTATAGAAAGC
CTTCAAAAGGTAGCTTCACATGATGGATTATTAACATTTGAAAGTTTCTGCACGGGTC
TTAAAATATGTTTATTGAGAAATCAAGTCCAAAATGCTTCTAACAAAATAAAACCTATGAGTGAGGATATGGA
AAAACCTGGAAATCCGCAAATTCCCATTCATCGTCCTCCATTAGCTCCATTAATAGATATAGAAACCAATCAC
AATAGAGAAAGCACTTGGAATATTATGAGAAACACAGGAAGCTCTACACAAAGAACTA
TAAGTATGCCTCAGTTAATCGAAGATAAGGATACATTAGCTGAATTACTACATAATGGAAAAGTAAATTCCGT
ATCTAAACATGAAGTTACACAAAGTCATAGACCATTTGGCCCTCCTAAGCCACCTCGATTAGAAAAAAATACA
GTAGACAGGAAAGAAAATTCGAGAAATTTATACAAACCTGAAGAGGCGGTGGAAAGTG
ATGACATAGTTCAGACAAAATTGGAATTTGATATGACAAGGCCAATAGAGTGTCAAGATCGAGGACTTGGAGA
TGGCCGCTCAGCGAATGTAACACAAACAAATTTGCGAAAAACTACAAGACGACGGGAGCCCCGTCGCCATACA
CTCCATAATGGTGT
>103783290_2 DT667124.3
CGACGTTGGCCGCGTACGGATATGGTGCTGGGTACGATTTAGCAGCCAGGCGGAAAAACGCCACGACCGAGTC
CACGGCCACGCTGAAGGCCTGGCTGAACGAGCACAAGAAGAATCCTTATCCTACCAAGGGAGAGAAAATAATG
CTGGCTATCATCACAAAAATGACACTCACACAAGTGTCCACGTGGTTCGCGAACGCCC
GCCGACGCCTCAAGAAAGAGAATAAGATGACGTGGGAGCCCAAGAACAAGACGGACGACGACGACAACACCAT
GCTCTCCGACGAGGAGAATGTTCAAGATGATAAAATAAAACCTAGCAAAGATGATGAAAGGAAGGGCGATGAA
CTGCTGCAAGGGATGCATAGTCAGCTGCTCGGTTATGGCATAAAGGAAGAATCAAAGC
GCGGCACGTCAGACTGCGGCGTGCCTATTCCAGCGTCAAAGCCGAAGATATGGTCCTTAGCTGATACAGCGGC
ATGCAAGACACCACCTCCAGCTGCACAACCATGGCCACAACACGGCTACGGTCACGGCCACGAGAGAGCATCA
GCCGGAGACAGTAATTCAAATGGATTTGCATTACCTGCCACTGCGGCAGCAAGTCCAG
CCAGTGGTTCTTACGGACGATATGGCGGCTTCCCAGGAGGACAGTACGGTGGGCAGCATCCATGTGTACATCC
TGCCGCGTTTCCCGATGTGCAAACAGACACACCACCACAGACGCC
>103783303_2 DT667276.3
GCACGAGGCGGTTCTAAATGCCGGGGGTGGTATTAATCCGGTCGAGTCTGCCCATGCGTGTTGCCATTGCTGG
CGACGTGACTCTACCACACGTACTTTAGTCGCCTCTTACGACACCACCAGTACCAGGGTGATCCTATTCTAAG
CCGGGATCACACGGCTATTCTATTTTCATTGTTTGAGAAAATCATAAGTAAACATATT
ATTAGTAATTATTTATAATGCTGACATTACATTAGAGGTAGCAGGTTCAACCTACGTGTGAACCATTTAGAGC
GTAACAATAATATATTGGATTTATCTTGAAAAGATAATAATTTCAAGGTAGTTTAGCGTGGGCACAGTGACGT
TTTTACTTTAAATGCCATTTCTTAGATAAGACATACCTACCTAAAAAAAAACTTGAAA
ATTTAAATATATCAATGTAACTGTTGTTTTATATTTTTTTATAAATTATTTATATTTTATATTTTTGATAAA
TTATTTTTTATGTTTTATCTTTTTAATAATCATTTATAACAAGTATTAATCAACCAGAATTTTTTTTTATTAA
TATATTGATCAAAACCTATAATTAATATTGGGCTCATGTCAATAAGGCCAAGGTCGTG
GAATTATAAAATATATATAAAAAAACTAATGATATAATATTTATTTACATTAATATATAAAAGTTACTTGGAA
TTAGTGTATATTTAATTATAAAGGATTTAACTGTCTTTTTGCTAACGGAGTTTTCCTATGTTTTTTTATATCA
AAAAAGCAAATTGATTTACCGCATCTACAGATTATAAATTATT
>103783343_2 DT668144.3
GCACGAGGAGGACCCCTAGGTCAACCTGGATACCCAGGACAACCAGGCCAGCCAGGCCAACCAGGATACCCAG
GACAACCAGGCAAGCCAGGCCAACCAGGACAGTCAGGACAGCCAGGGCAACCCGGACAACCAGGCAAACCAGG
TCAACAAGGTCAACCTGGACAGCCAGGATACCCAGGACAACCAGGTCAACCCGGACAT
CCAGGCAAACCAGGACAACAAGGTCAACCTGGACAGCCAGGATACCCAGGACAGCCAGGACAACCCGGATACC
CTGGACAACCAGGCCAGCCAGGACAACCAGGTCAACCCGGATACCTTGGGCAGCCAGGCCTACCTGGATACCC
AGGACAACTAGGTAAGCCAGGCCAAACCGGATACCCAGGACAGCCAGGTCAACCCGGA
CAACCAGGCAAGCCAGGCCAGCCCGGACAACCAGGACAGCCAGGATATCCAGGACAGCCAGGACAGCCAGGAC
AACAAGGTGGTCCTGGTGAACAAGGTCAACCCGGATACCCAGGACAGGCAGGCCAACCTGGATACCCAGGACA
GCCAGGCCAACCTGGATACCCAGGACAACCAGGCAAGCCAGGCCAAACCGGATACCCA
GGACAGCCAGGTCAACCCGGACAACCAGGCAAGCCAGGCCAGCCAGGACAATCAGGACAGCTAGGATACCCAG
GACAGCCAGGTCAACCCGGACAACCAGGCAAGCCAGGACAAACAGGACAGCCAGTACAACCAGGTGGTCCTGG
TGAACAAGGCCAACCAGGATAC
>103783348_2 DT668804.3

GCACGAGGCAAAACCTCTCCATATATATTTTTTTGTGTCACTGACGAGGGACTCGTGAGTCAAAGATCATCGG
ATGCACACGGGGAATTACAATATTCTGAAGTCACGATGCCTAATATATACCTACTGTATACACGTGGCTTTGA
AACTACGTATTAGAAAATATTCCAATACCATGTTTATACTAGGCGCACTGTTTTAACT
TAAAGTTATTAATTTCACTGAAAAATCAACTATAAATCGTTTTTTACATTTATATACATACCTAATAAATGAC
TAGTGTTTCATTTTTTATAAATAATTCATGTTTCAATAATACAATGGCTTGTACACTTTCCATTCAATTCCAT
TCAAAGATATTTGTAAGTTAGATATGTTTCGGTTTCATTGCCCAACAGAACGGATGGA
GGGCTACGTCTGAAGCAAGGCCGTCTGGCCTACTTGCGCGCGCGCACCTTTATACGCTGAGCGAAAACCGTCT
CTAACGACACAGTAATTACAAACAATTGACTTTAATTCTCTATGCTGTAGTTGTCGACACAATACCGACTGCT
CGCGATTCACACGGTTAAGAGACAATCATTAAAAAAAAAATACCACAATACTAAAAAAA
AATTATGAATTTAAATATAATATTTAAATATTATTTAGTTGTAATTAAATGGAATA
>103783350_2 DT668877.3
AGGAGGCGCCTAATATGAAAGCTTTCCTTAGAGCGATATCTACCCGACCGCAAGAACAACAAATTCCTTCGCA
ACAACAACAACAAGACAGCAAACGTAACTCCTCCACGTCGCAGGGCTCCACCGCCGGCTCAGAAGGTTCTAGA
AGTGAAATCATAGAGGCCGATCAGGAGTGGGCGCCTCACGTCGACTTGGGGAAGCAGC
TGGCAACGTTGCACGCGTTGTTGGCGGACAGCCTACCTAAGTTACCTGCGGGGAAGATACAGGAGTTGGATCC
ATTATCTGAAATATTATATGATCTTAGTAAGAGGTTGATGAGCAACGACAACGGGCCACCGGCGCAGATGGGT
GATAATATTTTCAGATTTAATGATCCCACATGCAACCCAACGACGAAACCAAACGAAC
CAGTGACAAACGGTCCGAACAGCAACTTCGCTCACAGCTCGCCCATCATGAACAAAAATGGCGTGCAATTCAA
CATAACACCATCTAAATCTAACGTAGAGGATTCTAAATATAAGGGATCCTTTGTCTCAGTGTCTAAATCGCCC
AGCTTTAATTTACGCTCAGCGACGCTCCCAAGAAATGGATATGGATCGAGTCCAGTCA
ATCAAAACGTGAACCCTGACCGATACAGCTCTCAGTACAACAATCAAGAACATTGCGTTAATCTGAAGGTCGT
ACAGATTGGTTTCGGCTCGGACCA
>103783357_2 DT667940.3
GCACGAGGTACACACCCTTTAAATGGGTGCTTAGTTGCACTTTTAGTTATCTTTTCATTAATGTTATAAACCT
TTAATTATGGGTTCTAAATCCAAAACAGGATTATTTGCAATGGGATTCTTTATTTTCGCTTCTTTATTTATAA
CGGTAGCTTTTTTGAGTCCCTATTGGTTAGTAACTGATGGAAAACTAAAAGATCCAAA
GTTTTTACGAATAGGTTTATGGGAGGTATGTTTTAATGGCTTTGAAGATGTTCATCATTGGTACGATACTATA
TTTACTGGTTGTTGGTGGATATTTGAAGAAGAATATTATATTATACATGATATTTTACTCCCAGGATTTTTCA
TTGCAACTCAGTTTTTTTTCACAATTTCCATGTGTTGTGTTTTAATATCAATACTCTT
AACATATTTGTATTTAAATAAAGATGAAGATGACGATAATTATGTGACATTACTAATCACATTTGGCACAGTT
CTTGTAATAGGAGGCTTTTCAGGAATTATATCTGTGGTGACATTTGGAGCTAGAGGTGATGGACGTGACTGGA
TGCGAAATTGGGAACACAATGATCTTGGGTGGTCTTATGCTCTTGGCGTCATTGGGGT
TATATTCCTCTTTCCAGCAGGCATATTGTTTCTTATTGAAGCTAGAGTTCGGAAATACAAAAGGTTACATGAA
ATGCAGAGTAGGGAACCATCTTCTTATAGCATGCGTGAAAGAAAAGCAGCTTATCCTGAACATACAGATATAT
AATATGTGTATAGTTGTATACCGTATGAAAGTGT
>103783360_2 DT668669.3
GCACGAGGGCGACCTCTATGTGGACCCAAATTGTGGATTTTTAAATTACGATGATTAAAATGGATTGGAAAAT
TAAAAAAAAAAACATCCAAATGGACAAAAATCGCTACTAAGTTACTTTTTAGTAACTAAACATAAAAATAAAG
TATAATAATAAAACAAATAATACATATTGACCGACTTATAGGATGTGTAGTCGTTGTA
GTTGGAATTAAAAGCATTTTTTGTATGAGAATGTGCACGTGTGGTGCAAAAGTATACATGTCTGAATGCAATG
TGGAAGCATGGGTGACTGTGAAAAGATAATTGTTTTGTGGTTTTGCTATAACACAAACAGCCCATCGAATAAG
CGTAGAGGTAAGCGATTGCGATCAATGATTATTATTATCGAATGATAAATATGAATTA
GAGATCATTTTTATATATCGATTGTAATATTTAAATTATTTTTTAATCGTATATTATATTGACGTTTGACGAA
GTACCTAAAGTACGGGCAACAAGTCATGGCCGCTCTGAAGTTAGGTTTACTGAACAACATAATAAGTAAGAAA
AATTCTAAAATGAAATCTGCGCAGTCATCTAACTACAACTGGCTTTAGGGCCACGACT
TGATGACCTTACTGTATAGCGGCCAATTCTTTGGTGGGAATCACTCGGTTCTTCATAATCGCTCGCCCGGTTC
TAGTACTCGAATACCGAATAGGAAACTGTGGAGACGTACGAATCTCGTTGGATAAGTACATACAACAATGCTC
TACAATTACCACATACTGC
>103783367_2 DT668437.3
GCACGAGGGTGAGACATAAATTAAACAGACCAGAAAAAAGGTTACTTTACAGGTCATGATGATTGATGGATTG
ATATAGTTAGTAAATAACTACGAAGTGATACGCTGATAGCACTGTAAATAATACTATTAAATAATATTTATAG
AACATTTGAAAGCCTTTGAGACTTGATAGAAATTGAAAATTATAAATACATTAAATTA

TTGTTTTTTTTTAAATTATTTTTATTAAATAATTTACACATGTAAAAGCACAGAAAAGTCACACACTTACAGG
TGATATTCAAACCTCAATCTCATTTTAATACGGAGTACAGAGGCTGCAAACATTTTTTTATAAAATGTGACCT
TATATCATCGGTCGCTTTATATGCGCTGAGAGAATTGTTCCCATATAATTTCATTTAA
TTCTCGTTATTCCGCTTCTACTTTTGTATTTTTTTTAAGTTTCTTATTTTATTAAGCTGATTGTGTATTTGTT
TTTGTTACATTGATCGAAAAAATCTTCAATATGAACAAGGTGGCTATTGTATTGTTTTCTAACACCTAAAAAA
ACGGTGCTGGAGGATTCGTATCCGTAGAAATGTGATATTACCAATAAAGGTTTGAAGC
TTTGTTTTGTTTGTTTATGTTTTATCTCAAGTTTTTTATACCTAACTAAATAGAAGTAGTAATAATAATTAAA
ATTTCTTCTAAACTATTTACTTTTATTATAATGTAAACATGTATGTATTTTTGTTAATTAAATTAAACGTAAT
TCATATTTTAGCAAACGGCGTCATACACAACAGTTAGTA
>103783371_2 DT667090.3
GCCGAGGCAAAACCCCATTTTTTTTTACTTTAGGATGGGCTTATAATAGGCTTAAATTTCTTTTATATTTCTG
TTTTAATAGTGGGCTCGAACCTCTCCTTCCCCTTATTTCTACGCCCATGAGTATATTATATGCTTCATGGATA
GAAATAGTTATTTTAGTCTATAAAGTGGTAGTAATATGTTATGTTAAAAACTCAATGT
CTTAGAGTTCAGTATGGTGACGGCAAAAAAAATGTTATTAAAGCTTTAAGACGCCTCCGTTTTCTTCTTTTAA
TTTGTTATTTGTAGTTGTATGTTGTGAAACTTTAATTTTATTTAGTTTTCGGTGGAATTTTAAAATTATAATT
GAAAAGCAATTAGTGAAATACATTTTTTTCTAAACTAGCATTTTATTAGACTTTTTTT
ATAACATTTATTTAAAGATAAACTCTTTAAAATAAGAAGCGTCACTAAATCATACACAATAGCGAAAAAAATC
GGATGTTTTAAAGATGTGTTTATTAATATAAAATAATTATATAAATCCTTTGGTCACTCTTGGTTCATGTTTC
GTGTACTTCATCATTACATCTAGTTTTCTCGAGCAAGCGTTCATTTTCTTCCTTGAGG
GTCACTATTATTTCTTCCTTTTCCTGAAATATAAAAATATATTTCAACATTTCGAAATAGAATTATACAATTA
TAATTAGCCCTC
>103783377_2 DT668477.2
GCAGAGGGAAAAACAAAAAAGAAGAAAAAGTAAAAATCGCAGTATACTACGAATCCCTATGCCCAGATAGTAA
GAAATTTATAACAGAACAGCTTGCCCCTGTGTGGAGGGACTTTAGAGGACTTGTCAAAGTCAAACTGGTGCCT
TACGGAAAGAGTACGCATGACCAGGTGAATGGTAAGTGGGTGTTCAACTGTCACCATG
GACCCGATGAATGCTATGGAAACAAGGTTCAATCTTGCATCTTAAAGGACCGTAGCCTCCAAGACACAGATAA
AATGGAAATGATCATTTGCTTGATGAAACAAGCCAGCCCCGACAAATCTCTGGATACGTGCCTCCAAAGTGTA
AAGTCCCAGGGTAAATCAGATAAGCTATCGTCGTGCGCCAGCGGAGAACAAGGCGACA
GTCTCCTCGCGTCGTACGGCGACAAGAGCGACGCGGTGCAGCGGCCGCTGTCGTTTGTGCCCACTGTTGTTAT
CAATGAGAAATTCGACCAAGCTGTCCAAGACGAGGCCATTTCTAACCTTAAAGCTGTTGTATGTCGCGTCTTA
CCTAATAAACCTTCGATTTGTTAATATTATTAATAAATACTATTATAATAATACATAT
TAAATAAGTACATTTTATAATACATTTTATGTAAAAAAAAAAAAAAAAAAA
>103783388_2 DT668558.3
GCACGAGGCTGCCTTACTACGCACGCACGGACACTACGAGACACTACTACAGACACTATGTGACACGGTGCGG
GAAAATGTTCAGTGACTACAGTAACTGAGTGTGCATTGTATATAAGATAATTGTGAATATATTTAGATTTTAA
ATGGGGCGAAGAGTACGAAGGGAATATCAATGGGGCTCGTGCGTGGGGTCGTGCGCGC
ACGGCGGGCGCTGCGTGGCGGGCGCGTGCGCGTGCGCGGGCGCGTGGGGCGGGCCGCGCTGCCGCCACTACGT
GGGCCACGACCACGCCTGCCTCGCGCGCGCTTTTTTTTTCGCCCGCCGTCTGCGTGTGGAGCCCGTCCAATAA
CCCGACAGACCCCCGGCACTCCATACTGCGCGTGCGGGGAGGGCCTG
>103783407_2 DT668259.3
TTTTTCTTTGTGTCACATTGAAATTGCTAAAAGACATAAAGTTATTCTATATTTTTATGCAGTAACTGAACAG
TTTTAACTGATAACAAATTTCAGCTGCTGGCATTGTTAATTATTGTTCAGTGAAATAAAATATATAATACTAA
TCACCTATCAACATGTCGGCCCTATTAACTGCAGATTGGTTCCAACTTGACTCTTATT
ATCGTAAATTTGATTTGTATAATATGATTTGGTCCATGGATGAAGGTTTATTTTATATGATAGTAAGCGGAGC
ACCTTATGGTGGACCAGTAGCAGTTGTTAGGGATAGGAAACAATTTGTAAGAGTAATGGCAACATCTAAACCA
GTGATCACAATATACAACTGTGCAGGAAATGTTATATCTAAAATATTGTGGAACAGTG
GTGTGCTTATTCATATGGGATGGTCTGATGGGGAACAATTGTTGTGTGTCCAAGAAAGTGGTGATGTACTAAT
ATATGACATGTTCGGAGCTTATCAA
>103783416_2 DT667073.3
GCACGAGGGCCGCGCTCTCCACATTCCAACGTCGTTGTGCTGGTCGAGGACTGACTAAGTGACCGCTTCTACC
CGTGCGACAATAACAAATCATCAACATGTCTGACGAAGAGGAATATTCTGGCTCCGAAGAGGAGGAAGTTGAA
GAAGAAGTCGT
>103783419_2 DV501357.2

CAGCACTACCGACGCCGACTGTCGTCGCGGACGCTCAGCAAGTCGTGCGCGGAGCTGCGCGCGCAGCCGGTGC
GCGACGCCGCGCTGTGGCGCTGGCACGGGCCGCTCGTGGTCGCCGCCGACGCCGCGCTCACGGCGCTCGGCTA
CCTGCCACCGCACCGTGGACCGGGGACCACCCCACACCCCTCCCCCAGAGCATAC
>103783437_2 DT668907.3
GCACGAGGACCCGCCTCTCAACGGACAGATCAGGTGTTCGCGCGCAGGTCGCGGTCGGCGCGGCGCAGGCCCC
CCTCGCCGCCCCCCCTCCCGGACGGGGCCCTCGCGCCCGGGGCCCCCGCGCCCGCCCTCGCATGAGGCACCCT
CACAATGGAGTCTATCCTCGTTTTTTAAATGCGTCGCCGACAAACGGATATACGCGCA
CAGGCCCCTCCCCGCGATGCGCACCGCGAACAGATATTAAAACAATCCCCGAACATTTGCTCGATAATCGAAA
ATCGATTTTAATTTCATAATATCGATTGATGTTCCTGTTTTTTTTGTTGTGTCGGAATATCATCGCATCAGTC
GATGTTCCTATCAATATTGTTTATTCTAATGTCGATTTCTAGACAATGTTGATCGATC
GGTGGAAAAAAAGTCGATTTTTTTGACATAAATGTGA
>103783442_2 DT668093.3
GCACAGGCGAAGACCTTAAAGGCGTACGCTCACTCGCTCCGTCCGATACTCACGCGGACAACTGGGTCACGGG
GCTCGTACGCGTTTGCTCTTGCAAAAGAAAACATATTTGTTTAGTGTTGTGGATGATCACGGTGATAGTGGTT
TGCTGTTGTTTACGAAACAAGAAACTAGCGGCGCTTTTGAAGTGTACGTGATGCGTGC
GTGAGCGCAGCGCATGACGTGCTACGAAACGTTGAGAGCTTCGGCACCCGCCGCGGACGCCCCCATGGCGCAC
CACTTCGTCAAGTGGTGGCGCAACAAGTTTAGAAATGGCTACTTTAAATTCAGCATTGGCACAGAGAGTGAAA
GGACTGACACTGAGGAACTGGTTGGTCGTGCCGCATCGCAATGCTCAGCACCGCCCGA
CCTCTTGCCCAGTGAAGCAAGAGCTTTGTTACAACCAACGCCACCACCAGTTCCAGCCAGGAATTCTGAACAT
GTCTATAAAAAGATGACTTCCTCTGAAAAGCTCACAGAAAAAGTTGAGGACAAACAACCTAGAATGAGATTTG
AGGAATTAACGTGTGACGTTGAATTAAAACATCCCGGAATCGTCTAAGCAACAACCGT
TGCAGTTTTCATTTACCCTTCTATGACCTCGACGGGCATGGTCGAATGACGA
>103783443_2 DT668139.3
GCACGAGGAGACGGCCTGCTCCTGCCAGTACGACGGGGCAAGACTGGCCCTGACGTCGTACCCGAGGCTATCG
AGTGCAGCTGTAGGCGTCTACGGCGCCCCCTACCCCTCCACGGACCAGAACCCGTACCCCAGCATCGGAGTAG
ACAGCTCAGCATTTTATTCACCATTGAGTAACCCCTACGCATTGAAGGAGGGTAGCGG
AGAGATGTCGGCTTGGACATCAGCTGGGCTACAGCCATCGGGGGGGTACTACCCATACGACCCGACGTTGGCC
GCGTACGGATATGGTGCTGGGTACGATTTAGCAGCCAGGCGGATTAACGCCACGAGAGAGTCCACGGCCACAC
TCAAGGCCTGGCTGAACGAGCACAAGAAGAATCCTTATCCTACCAAGGGAGAGAAAAT
AATGCTGGCTATCATCACAAAAATGACACTCACACAAGTGTCCACGTGGTTCGCGAACGCCCGCCGACGCCTC
AAGAAAGAGAATAAGATGACGTGGGAGCCCAAGAACAAGACGGACGACGACGACGACACCATGCTCTCCGACG
AGGAGAAGGAGCAAGATGATAAAATAAAACCTAGCAAAGATGATGAAAGGAAGGGCGA
TGAACTGCTGCAAGGGATGCATAGTCAGCTGCTCGGTTATGGCATAAAGGAAGAATCAAAGCGCGGCACGTCA
GACTGCGGCGTGCCTATTCCAGCGTCAAAGCCGAAGATATGGTCCTTAGCTGATACAG
>103783447_2 DT668038.3
AGGTTTTAACGTATTCACAATACCCCTAAAAGGTATATTTATCTGAAACAAAAATGCGTAATGTAGAAATAAA
AGCTAAAGTACACGACGTGGAAGCCATTTGCCAAATAGCAGAAACTTTAAGCGGTACGAAACCAACAATTATT
CAACAAGATGATACATTTTACAAAGTAAATACCGGTCGATTGAAATTAAGAGTATATG
CGGATGAATCAGCTACCCTTGTAAGATACGATCGAGATGACAAACAAGGACCGAAACTGTGTGATTATGACCT
TTTACAATTTTCTGTAGATGAGAAAGAAAAAGCTAAGTTATTGAACAACATGCTTATTAAATGTATGGGTATT
CGTGGAAGAGTTGTAAAAGAACGGTTAGTAGCTTCAATATTCTAACTAAAAGAAAGCT
ATACATGGTGGGTCAAACTCGTGTACACATTGACATTGTACAAGATATTGGCAGCTTTATGGAGTTAGAAGTT
GTATTACAACCTAAACAATCAACAGAGGAGGGTCAGGTGATTGCTACGGATTTACAAGACAAACTTGGTGTTA
AAAATGAAGATCTGATACAGTGTGCCTATGCTGATTTATTAGATAAGAAATAGAAAGA
AGACAAATTATATAAAATCTCCTTTTAAGTATTTAATTGCATTTTTCCCATTTTGTTCTGTAAACGATATCTA
AA
>103783448_2 DT668881.3
GCACGAGGCGCATTCTCCATCGAGACATCAAGCCGGGCAATCTGTTGGTGAACAGTAATTGCGTGCTCAAGAT
ATGCGACTTCGGTCTAGCCAGGGTCGAGGAACCAGACGCCACGAAAAACATGACGCAGGAGGTCGTTACACAG
TATTACAGGGCACCGGAAATCCTGATGGGCGCTAACCACTACACCGCAGCGGTCGATG
TGTGGTCGGTTGGTTGTATATTCGGCGAGCTGCTCGGTCGTCGTATTCTGTTCCAAGCACAGAGTCCTGTACA
GCAGCTGGAACTAATAACTGAGCTCCTCGGCACCCCGTCCCTAGAGGACATGAGATACGCGTGCGCCGGCGCC
CGCGCCCACATGTTGCGACGCGCGCCCCGCCCGCCGGCACTCGCCGCGCTCTACACGC

TGTCTGTGCAGGCTACACATGAAGCTGTGCATTTGTTGGCACAGATGCTAGTGTTTGATCCGGCCAAGCGTAT
TTCCGTAGTCGATGCGCTAGCACATCCCTATCTCGAGGAGGGCCGACTACGTTACCATTCCTGTATGTGCAAG
TGTTGCTACAGCGCCCCGCCCGCAGCACGTCACTACTCACCCGACTTGGAACCCGTTG
CCCCCCACGCCTTCCACGACGGATGGGAACGGAAGTTGACTTCTGTGCATCAGGTTAAAGAGGAAATCCACAA
GTTCATAGCGGAACAGCTGCAGACGTCGCGCGTGCCGCTCTGC
>103783449_2 DV501294.2
GCACGAGGAAACCCTTATTGTATGTATAATACAATGTGTACAATTGTACAGTAACTTATTTTGTATGAGAGTG
AATGGAACGCGTCAGTTTGAAGTAAATGATGTATCTTATAAATAAAAATAATAAACTTATTTAGTACCTTTTT
TTTAAACATCTAATGTGGTAATGATTTACATGTGATTATTTATGACGTATAATTTACA
TCGAAACAACTTGTTTGTTTAAATATTATGTTTTAATAAAAAATATATATTTGCTTATTTTTTTTGTTAAACA
TCTAATGTTATCTCAAACTTAATGACAGTTTTGAATATATCCGCAAAAAATCTGAATAATTATAATATTTTCG
GAAAGACATCTTTTTGCTTAAAATATTGCTTTAATAAAAATATATTCTTTTTTACTGA
AGAAAACTTAAAATCTGTAAATATAATACGTAATCAAAATTTTTAACGAAACTGTTACATAAAATTTATTATA
AAATAAGTTTTTTTTTTTCTATTTTCCTTTATATTAGGGAAGTACAATAGTCTAATAGTCTTCTCGTAGTTAT
TGCAAAGACGGCGAAATCTATTTCTATTTCATTTTTTTAAAATTTGTAACTTAGTTTT
TGTTCGAGTTGTTTTTTCATTCAATACTTTAAAAGTTAGTTTTGGATTTTAAGAAATAAAAAAGGCAATAAAG
TTTGGATTGTATTAACAAAAAAGTAAAAATATAAGTTTTTTTCTAAGAATATTGAAGATCTATTCCATCCAAA
A
>103783450_2 DT668537.3
GCACGAGGTTCTCGTATTATATTATATTATATATATGAGGCCGCGACTGGAAACGCGACGTTTGTGGGTTTGT
GAGAAATGAAATGTTTAATATTTTCATTTCCTTGCTCTGCGTCTATACCCCCAGAATTCAACGGAAAGTGGGG
AATGGAAGTAGTTTAATGGGTACGAAGTGTCTTAACACTAGGAAAAAAGAGGGATTCC
CTCTGCCTATTCTGATATATGCGAGATATACTTATCGTGAAGCTAAAAAATATATATATATAATATAAACAAA
ATATTTCTTTACAGCGTTCCTTCCCGCAATGGTTTTTAGTGGCATCCACGTTCTTATGTACACATACTATGGA
CTTTCCGTATTCGGACCTAGAGTGAGCCAATATCTGTGGTGGAAGAAATACTTGACTA
TCCTTCAACTGGTGAGGATAATTTTGGTTATTTATTTTTTTAATAATGGAGCTCTCATTCTCCTTTTACATAC
AATGTTTATAATGTTGGTCTTATAAGTAAGACGATAAAATAACTTTTGGTTGATACTTCGATTGATTTTAAGA
ATAATACTTGAGTAGTAAATTAATATAATAGATTTAAGCTCTTAATTAAAAGTGTGTG
TACAATTTTTCTTACTAAAGGTATATCTTTTAGTTATTTAGAGTTAGATAGTTAGTTAGAATTTTATGAATAT
CGAA
>103783452_2 DT668082.3
GCACGAGGCCTCGTCCTGCTTGTTGTAGGTCGTACATTTGTACTGTGCTAATTTATAAAATAAACCATTATAT
CAGAATATATAATTGAGAATCATCATGGAAAATAAATCTGAAGATTCTGCTGCACCCGAAAAAACAATATTTA
GTGCCATAGCTCAAGGTGACTTAGCTGAATTTAAGAATATTCTCGCTCAACATAAAGG
CAGTGTCGATTTCTTTGACGAAAATGGTATGACGGCGCTCCAACATGCCGCTTATAAGGGCAGTAAGGACATG
GTGCAATTACTGCTCGATAGGGGTGCAGACGTTAACTCTGGGAAACATGAATACAATTACACAGCGTTACATT
TTGGAGCGTTATCGGGAAATTCGGAAGTTTGTAAGCTACTTTTAGATGCAGGTGCAAA
ACCAACTGCGACAAACTCTGTAGGACGTACTGCATCTCAAATGGCTGCGTTTGTTGGTAATCACCATACTGTT
GCAACAATAAACAACTATGTGCCGTCTAGTGAGATATCCTTCTATTCTGCGCCTCAGGGACAGCAAACTGAAC
CTCTATTACCACCTATTTTGGTGGATCCTCTACACAAATTCCTTTTAGGAGTCAACAT
ACATCCTGTCCGATTAGCATTAAATTTGCAGCATATGCCTACACTGTTAGATAATGCAGACAAAGTTAGCAAA
GTCCTAGAAATGCTGTGTAAAAAGGAAATGACAAGAGGTAGCGATACTAATGAAGTAATGGCCTTTAATATCC
CTACCTTGCTTATATTTTAAGAAAAAATTAATAATATTAGAGACAAGCAGAAAC
>103783453_2 DT667237.3
GCACGAGGGTTAACCTAGGACGCTATTCTGTTGTTCTATAAACTATAGCTATTTACTATTTTTTATAAGAAAA
ATGAGTGAAAAACTAAAAGAGCTACGTGAAAGGTCACAAAAAAGAAAAAAGTTACTGGCGCAAACGCTGGGAG
TCTCGAGTGTGAGTGAACTCCGTCATGCATTAGGAACTTCTCTCGACGTGCTTCCAAA
AAAACAAGCGGTAACTGAATATAGTGAAGATGGAAATGAAAAACCAGTGCATAAAGAATCAGACAGCCTTGTT
TATACTGATTCATCTACATTTTTAAAGGGCACACAATCATCCAACCCACACAATGACTACTGCCAGCATTTTG
TAGACACGGGCCAAAGGCCGCAAAACTTTATCCGTGATGTAGGATTAGCGGACAGATT
TGAAGAGTACCCTAAACTCAGAGAATTGATCAAATTAAAAGATGAACTTATCGCTAGAACTGCCACGCCTCCT
ATGTATTTAAAATGTGATTTAAAAACATTTGACCTGAAATCAATGGGCAGTAAGTTCGACGTGGTGTTAGTGG
AGCCGCCATTGGGAGCGGGGTGGCGGTGGCGGGACGTCTTAGCCCTGGAACTACATCA

CCTGGCACAACCCAGGGCTTTTGTGTTTTTTATGGTGTGGCAGTTCAGAAGGTTTGGACATGGGAAGAGAATG
TCTCANAAAATGGGGCTTTAGACGTTGCGAAGACATTTGCTGGATCAAAACTAACATAAAAAATCCAGGGCAT
TCAAAAAATCT
>103783459_2 DT668455.3
GCACGAGGCCGGACCTTCACGTGGGTGCACCATTTTATTTTTCTCGCGACGCTGGTGGCGCAGTTTACTTGTA
TCTGAATATGAAACGTACTTTACCCCAACAATACAATGTAAAACTTACCGGGAAACCGGAATCACAATTCGGT
ATTGCTATTGCAAATACAGGAGATTTAAATAAAGACGGCTGTGAGGATATCGCTATAG
GAAGCCCGTATGAAGGCAACGGAGTAGTTTATATTCATATGGGTGACAGAAATAATGGCTTAAAAAGTAAACC
AGATCAAATTATAAAAGCGGAATCTCTGCCAAATGTGATGCATACGTTCGGATATTCTCTGTCTGGTGGAATA
GATCTCGATTCTAATGGATATCCAGATTTATTAGTTGGTGCATATGAAAATAGTAGCG
TAGCACTTATTCGAACAAAACCAATTATCGACATCAAAACGTCAATCCGACCATCGAATACGATTATTAACAT
TGATCCTACGATACAAGGTTGCACAAAGGACCCTAATTCGAATTACACGTGCTTCCATTTCCAAGCATGCTGT
ATCATTAAATCACTTGTACGGTCAGCACAAAGCAACAGCCACGAATTAAATTATATTA
TTGAAGCTGAAACGTTTCCTGGAGGAAGAAAGTACTCTAGAGTGTTCTTCGATACGGACAAGTCCAATATCGT
TAATAAAACTATAAATTTAGATAAAGATGTAGAAGATTGTAGAGAACATGTTGTTTATTTGAAGAATAATACC
AGAGACATACAGACGCCTATCAAGTTCCAAGTGACATACAAAATAGTTCAAGTGCAAC
CGCAATATTCAAC
>103783485_2 DT667845.3
GCACGAGGTTGCAACCTGGCTGGCTAACGTCAGTGACAATGACTTAGAATCAACAACGGAGCAAGTAATAAAG
GAAGTCAACGCAGATATGGCGCTCAAAGCCAGTGTTTTCATTGGCATGGACACAAGATATACAAGCCCCCGCC
TCGCAGCGGCAGCCGTCCACGGGGTGATGGCCTTGAAGGGCACCGCCAAGGAGTTTGG
TATTGTCACCACTCCCATATTACATTACTGTGTTAAATGTCGCAACGATAACACTTATGGAGTACCCACTGAG
GAAGGTTACTACGATAAAATCGTGGGTGCTTTCAAGAATATTCGTCAAAAATTACCAGTACATGGTAAATACA
ATACAACGTTATACGTGGACGGCGCGAATGGCGTTGGTGGGAAAAAATTGAATATTAT
CAAAAAAACATTAGATGGGGAACTCGATTTAGTTCTATACAACTTGGGTGGTAATGGTGGAAAGTTGAATTTG
AATTGTGGAGCAGATTTCGTGAAGGTCTCGCAAAAACCACCCGTGGGTGTGGAACAAGTGCCTTTCCAAAGAG
TGGCGTCGCTAGATGGCGATGGCGATCGTATCGTGTATTACTATTTGGATGACAACAA
AAAAATGAACCTACTAGACGGTGATCGTATAGCAACTCTACTAGCTAGTTACATAACGCGAGAGCTGCGCGCA
ACCGGCGCTGAGTTACGCGTCGGTCTAGTGCAGACCGCGTATGCCAATGGCGCTTCTACTAATATATAACCGA
TCATTTGAAAATCCCCGTAAGCT
>103783491_2 DT668371.3
GCACGAGGGTGGCCTTGAGCTATAACCTCTGTATTTGTAAAGTATTAGATAAATAAAGTAAAATGGAGTTAAA
TGAAAGGATTTTAAAGTATTTAGAGGGTAGAGACAAAGTCGATACCCTTAAATTAGCTAGTGAATTCAACGAA
GATCACCAAAAGATAGTTGGAGCTGTTAAAAGTTTAGAAGCACTTGAAATGGTCGTCT
CAGAGCCAGTAAAAAGCACGAAATGGGAATTAACGGAGGAAGGAAATCTAGTAGCTGAAAAGGGCAGTCATGA
AGCCGTTCTATATAGAAGTAT
>103783493_2 DT668878.3
GNACGAGGTAAACCTTTAAAAATTTGTGCCATGAAAACAGCATCAGCGCTATATTATAGATTCTGTGAAATAA
GTGTATGGATTTAGATCGAGGAATGCTAGACGAATACTTGATATAGATATTATATTTCATGTAAATTACTAAA
TATAAAAAAATGACTCTCGCCGAGTTTTTTAGTTGTTCATTATTGGCCTTCGGCCCCC
CTATTGCAATGTTTGCATTAACAATCGCAAATGATCCGATCCGCATAATTATTATGATAGCAGCTGCATTTGG
ATGGCTTCTATCATTTTTAGTTTCGTCGTTAATTTGGTATGCAGTGGTTCCGCTTCGAACCTACTTAGCTTTT
GGGATGGTTTTTGCTATTATCATTCAAGAGGCTTTTCGTTATGGAATGTATGTATTAT
TAAAAAGGACAGAGGCAGGCTTAAAGGTGATATCGGAAAATCACAATGTAGGCACTAATAAGTTAGAGATGGC
TTATGTCTCTGGACTAGGATTTGGGACCATGAGTGGAGCATTTGCACTTATTAATGTGCTGGCAGATTCAGTG
GGTCCAGGCACTCTTGGTCTACACAGTGGTACGGAATACTTTTTTGTAACGAGTGCAG
CAATGACCCTGTGCATGACACTTCTTAACACATTCTGGAGTGTTATTTTTTTCAGTGGTTTAGACAATAAGAA
TTATTTAAACATTGCCTGGGTATTAGGCTCTCATTTCTTTGTATCTGGACTCAGTTTACTTAATAAAAAAGAA
TTATATATAGTAACAATTTTGCCATCATA
>103783497_2 DV501301.2
GCACAAGGACATCCCTTAATTATATATTTTATTTCCTGTATTTTACTACATAATTTACTTTTATCTATTAGAA
TGTCTAAAAACAATAATATAAAATGTGCTAACTGTGGTAAAACACCTACTCAACATATGGCCATTTCCTTTCA
CACATTTCCTAAACCCACAAAGGATAATATTACCAAATTAATAATCCCATAAATAAAA

TAATGCAAACAAACCTTCAACTTCTGGAATTACATTCACACCAATTGACAAATGTTAATGTGCAACTTACACC
AAATTATTTTCTTCAACTACCCCCATAAATTGAAGTCACACCTAAACTAACAACAAAAGAATATAATGCACAT
TATGTTATGATAAATCCAAAGTCTGAATAAACTTGTTTACTGATCCACTCTTCTAAAA
AAAGAAAAGGAATGCTCACTTATTTAAATGTGAAAAAAATATGAGCCAAAAGATAATAAAACAGGTAATGAAA
TTGAGGAAAATAAGAAAATTACAAATGTTATTGAAGCATTAACAGAGGAAGAAAATGTGCATAATTTGTGCAA
TTTAAATATGATCAGTACCTTTGCTATACTATTGCAAAGCCAATTAAAAAATTGCCCA
AACAAACTGAGAGGCACGAGACATTCTCTAAACCAAAAAAATATGGCTTTGGCTATTTATAAACAATCACCAG
CATGTTATAGACTTTTA
>103783503_2 DT667972.3
GCACGAGGGTTCACTCTATTTCTTTTTTTAACCTTTGAATTTGGGGCATTTTGTAAGCAAACAATTGTGAGCA
CGTAAGTAATAAGTTTATTATTAGAAATTCTTAAATGTTATAGCTGGTACAGAATAGGCTTGTCATCATGAAA
TAACATTTAATTTAACAAATAAAAAATAATGTTGTAGTTAAACAATAATTCCAATTTA
TGTATTTGGAAATTAATTACTTAGTTAACCTACATTTTAATTTAATAACTAAAAGAAAAATATATAATGATTA
AAAGTTCTCAAATATCGATTGTAAAATAAAAATAACCACATCCTGATGAAATCATTGTTTTATATACAATAAC
ATAAAGTTAAAACTTTATATGTTACAAATTTGTGTTTATGTCTTTTAACTCATGACTT
TGTCTCCTTAAAAATTTAAAAATAATTATAAAACACTAATATTTGCATATTTCAGACATCTACTTACTTAATA
ACGTTTATTTTCAGATAAATACCTATTGTAAGTACCTACTTAGCTATTGGTTAGTCTTTCTCCTTAATCCGTG
TCCACGTGTTTCCATAAATTTAAATAACATAACGAACAAATTGAATTAACTTTAATAA
ATGAGGAATAATTGTGGCTTGTACTTTTTTATTACTTAATACTTATGTAGTTTTTTTCAGAATCGAATAAATG
CCAAATATCTCTAGGTTAAGTATAATTATTTGAACAGATAACCTGAAGCCAATCGTGGCACAGGGGCACAAAG
TGTGCCTGTAAACGCGACTGGTTGTACGGTCGATCCCCACT
>103783509_2 DT668169.3
GCACGAGGCTCCGCCTCCGTGGACTGGAGCGTCAAGCTGTGGAGCACCAAGGAGAGCAAGGCGCTGTACTCGT
TCGAGGACAGCGGCGACTACGTGGCCGACGTGCGCTGGTCGCCCGCGCACCCCGCGCTCTTCGCCGCCGCCGA
CGCCGCCGGCCGCCTCGACCTCTGGAACCTCAACAGGGACACCGAGGTGCCGATAGCG
TCGGTGCAGTCGGAAGGCGGCGTAGCGTTTAACCGCGTGTCGTGGAGCCCGAGCGGTGGTCACGTGACCGCCG
GCGACGACAACGGCAAGATATGGGTGTATGAATTAGCTGAGATCGTGGCGCAGCCGCGGCACGACGAGTGGAG
CAAGTTCGTGTACACGCTGCAGGAGCTGCGCAACAACCAGGCCGACGACGACGCGGAC
CGCCTCAGCCTCGCCTCCGGCCCGCCCTCGCTGTCCTCGCTCACCTCGCTCGCCTCCAACCCGCTCAGATAAC
TGATGCACTGGAATGTTCGCTGCGGGTGGAAGGTGTAGGTTAGAAAGAAAGGGACTGAAGGAGACTGAAGGGA
ATTGTGGTGCACATGATTGCAGAAGTGATGTATAGTTGAAACTGAAATATATCATTAT
AATGAAAAATTCAAGTACACATCACTTTTCATTATAATATGGTATCATTATTTATTGTTTTTTAATATGTATA
TACACCATTACTTATGTAATTCACTGGTA
>103783511_2 DT668573.3
GCACGAGGCAGACATCAAGGAATGCCAAGTTATAACAATTATAGGGCGCTTTGCAATCTAAAGAGAGCTGCCA
CCTTTGAGGATTTGGCTAGAGAAATACCTGATGAAGTTATTGCGAGATTCAAAAGAATATACGCGACCGTGGA
CGACATCGACCTGTTCCCGGGCGGCATGAGCGAGCGGCCGCTGCAGGGCGGGCTCGTG
GGACCCACCTTCGCCTGCATCATCGCCATACAGTTTAGACAGCTTAAGAAGTGCGATAGATTCTGGTATGAAA
ACGACAACAGAGCGGCGCGCTTCACGGAACATTTTTTAGCTGAAATACGAAAAACGACTCTATCCAAGATCTT
GTGTGAGAACTTCGACGTCAACTCTGATATACAACGCGCTGCTTTTGACCTTCCCAGC
AATTTCCTCAACCCCCGAGTGCCGTGTGCGTCTCTGCCCAAGATAGATCTGTCCGCGTGGAGAGAGAGCTCCA
CGCAAGGCTGTTTGATCGCAGCGCGTAATGTTCCTTTGGGAGACTCCGCTTTCCCATCGCCTTGTACTTCTTG
TATTTGTACTGCTGAAGGGGCGCATTGCGC
>103783534_2 DT668526.3
GCACGAGGCTGTCTTATAAGGTAGCACCAGACTACCTGGAAGGTCTTCTCCTGGTCCTCCCCGGCGGCGGAGC
GCCCGGAGCACCTATACTACGAGACGCGGCCCTCGTCGCGGCCCTACTCCACCGAGCTGCAGGCCTCCCAGAT
GCCCCCCATCCAAGGGATTTAAAATTCCTCTTACCAAAACCTTTGTTGGCCTTGAGGG
AACCGAAACCAAATAAATGGGCGTCGTGGGTGTCCGCGGAGTGGCCTACAGTGCGTACTTTAACGCCGGCCGC
TGCTAAATCCAAAGTGTTGCAGGTGTTATCCTTTTGGCCATTATTCGGTTCATCATTCTTCGCGGTACGTCGA
GTGGGCGGCGGCGGAGAGTGGAGAGAGTATGTGCTGGCGCTGAACAGGAGAGGCGTGC
ATCTGCTGCACCCCGCCACACACGACACCGACGCGCATTGGCCGTACGCCGACCTCATATCTACTAGGAAGGT
CCGTTCAGAAGACGGGACTCTCTTCCTAGACGTGAAGTGCGGCTCGCTGCTCCAGCAGCGCGTGACGCGGCTG
CAGGCGGAGCAGGCGCACGAGGTGGCGCGCCTCATACGGCAGTACATAGCGCTGCAGC
GGGAGCGGGACCCCGAGGGAGCTCGAACACGACATCAATGATCGTGATTCCGTTTTCAACAGCCAGT

>103783541_2 DT668613.3
GCACGAGGTGATGCTTTATTGTTCCTCACTGATATTCCTAAGGAAGCCGAAGCAGCGCGCTCAGCGGGCATGC
GGGCGGCGCTGGTCAGCCGCGAGGGCAACGCGCCGCTGCCCGCCGACGCGACCGACGCCTTCGCGGTCATACA
CTCGTTCGCGCAGCTCGCCGCCTCCAATAAGCGGAAGATGCCGCAGGATGAACAACCA
GCTAAAGTTCCTAAGACAGATTCGAGTAATGATATAAAAGAAGCAGTCGAATCAAAAGCAAGCGCTGAAAAAA
GTGCTGATAAGTCTGAAAATACCAATGGCGAAAAGGCGAAAGAAGAAACTGAGAAAATGGATGTAGATGAAGA
AAGTGCACCAACGAAGAAGGAGGACAAAGAAGAAAAACAAGAACCAGCAATAGTTGTG
GAAGAAATTACAGGTTCCAACGAAACAGTTCCTATTTGTGATGCTGAACCAATTGTTACAGAAGTTATTGATA
AGCCGGAGGAGGTCGAAAAAATGGAAACAGACACTGCTGATAAGTCAGAACCTGCTAAAGAACAAAAAGGAGC
AACTGATTCAGTACCAAATGATAAAAAAACTGAAAAACTCGAAGTGCCAAAAGAAAGT
GTTGATAAGCCAGTAGAAGAAAAAGCACCGATTGCTGAAGAAACTCCTCCTGCTGTTATTACTGAAATCGAAG
AAATAACTCCTGATAAGGAGAATCTTGCAGAGATGGCTGAAATTGTAGATGATATTGTACCAGTTGTTGAGG
>103783544_2 DT668035.3
CGGCGGGCCCGCGCGCCGCGCCCGCGCGCGTGTGCTCCGTGTGCCTCACGCTGCTGCAGCCGCACCCCCCGCC
CTACTTCAGCACCAGGCCGCCTAACACGCCCGACTAGACCTGACACCTTTTAAAAAATATATTCATTCATCAT
TTGATTGCGCGCGGTGTTGTAAATTAATTCGTTTTTAATTGTTACACAACAATTACCA
TGTCATTGAGATTTCCACAAACAGATAATCAATTATACGTGTATAATTGTTGATGGCAATTATAATTTGACGA
ATACTGTTTAAAACACCTTTGTTACTGTCACATGTAGAAAATGGTACTTCAGTACCTGGACACCTTATACAGT
ACTGTATTCGAATGTGCTACTCTATAGTGTGTTATCAGATCTAGGGAAATGTTAGAAT
TCTGGAACAGTTTTTTTTGTTTGCTTCACGCTGTATCCCCAGTGGTAGGAACATAGTGAAAAAAACACTTGTT
TCCTACAAACTGCTT
>103783558_2 DT668104.3
GCACGAGGTTTTAACCTTAAATACAATAAATAATCGAAAATAAACATTTGCTTGTAAATTTAACGTACTCTAG
ATTAAATTTTATATTATTACAGTGTCGGATTGGTGATACCGTTGGAGATATTTCTCGATTACTTGTGCTGTAC
TCGTACAGATGTCAACATAACCTTACAAAGTTTTAATTAAAAGTGGTGATTTTAAGGA
ATGGCAGACGCTGCGCCATCGACAGCGCCCAAGGCGAAGGCGCCTAAAAGCAAGGCACCTAAGGAAAAGGCCG
CGAAGGCGGCCCCAGCGGCACCCGCGGAGCCCCCGAAGGCTTTTTTCAAAGTTTCATCTAAGCCTCGTCATGG
AAGATTGTACGCTAAGGCCGTATTCACAGGATACAGACGTGGTTTACGCAATCAGCAT
GAAAACACTGCTCTTTTGAAGATTGAAGGAGCTAAAGGTCGCGACGACGCCATCTTCTACTCTGGCAAGAAAT
GTGTATATGTATACAGGGCTAAAAAGAGGACTCCCATTCCCGGAGGCCCCCGTGGCAAGAAGACTAAGTTGAG
GGCCATCTGGGGCAGGGTCACCCGCCCCCACGGTAACTATGGCAGTGTGCGCGCCAGG
TTCAAGTCTAACTTGCCCGCTCAAGCAATGGGACATATGATACGAGTGATGTTGTATCCATC
>103783564_2 DT668609.3
AGCTTCATCTCATCATATAAATAGATTGCTTCCAAAATGGCAAATCAATGGGGTCAAAAGGCTGAGTTTTTGA
AAATTTCTAATAAAGTTGCTGGTTTAGGTTTAACAAAAAAGCCTGCTTCATGTCAGCAGTTAGATTCATCAGA
AGAAAGCGCTGACGCCGCGAATCCTGCTGACACTTCTTTGTTGATGAAAATAATTCGG
CAAGGATTAGTTGAATCTAAACAAGACATTGAAATTCAAAGGAAAGATCCGAACTCACCCTTATACTCAGTTA
AAACATTTGAAGCTTTACATTTAAAACGAAATCTACTTAAGGGTGTTTATGAAATGGGATTTAATGCTCCTTC
TAAAATTCAAGAAACTGCCTTACCAACACTATTGGCAGATCCTCCTCAAAACATGATT
GCCCAGTCACAGTCAGGAACAGGAAAAACTGCTGCATTTGTGTTGGCTATGTTGAGTCGTGTTGATACAACTA
AGAACTTTCCACAAGTGTTATGTTTAAGTCCCACATATGAATTAGCTATTCAAACAGGGGAAGTTGCTGCAAA
AATGGCCAAGTTCTGTCCAGAAATAAAACTGAAGTATGCTGTAAGAGGAGAAGAAGTT
CCTAGAGGAACCAAAATTACAGATCATATACTGATTGGAACCCCTGGTAAGATGTTAGATTGGGGTGAAAAAT
TCAAACTCTTTGATTTAAAAAGATAAAGTGTTTGTTTTGGATGAAGCCGATGTGATGA
>103783585_2 DT668561.2
GCACGAAGCCAACATGGGTAAAATAATGAAACCGGGGAAGGTGGTGCTGGTCCTCAGCGGGCGGTACGCAGGG
CGCAAGGCCATAGTAGTGAAGAACTACGACGAGGGCACGTCAGACAAGCCTTACGGCCACGCTTTCGTCGCCG
GCATCGACAGGTATCCACGTAAAGTACACAAGCGAATGGGCAAAAACAAAATCCACAA
GCGGTCCAAAGTGAAGCCTTTTGTTAAGGTTGTAAACTACAATCACCTTATGCCAACTCGCTACTCAGTTGAC
TTCAGCTTTGAGAAATTCAGTGCAAAGGACCTCATAGATCCCGCGAAACGCAAGAAGCTCCGTTTCAACACCA
GAGTGCGTTTTGAAGAGAGATACAAGAGTGGAAAGAACAAGTGGTTCTTCCAGAAACT
TAGGTTCTAAATACTTTTAAAAATAAACACCTAAAAAAAAAAAAAAAAAA
>103783629_2 DT668250.2

GCACGAGGGTTTTCCTTTTATTTTTTTTTAAATGTGTGATTTATTGGATGTAAATGATAGAATACAGTATAGT
TGAGGCTATATTCTAACGCATTCCACTCACTTGTTATATAAATATTGGCGTTTCGTAGGTTGAGGCAGTGTTT
TATTACTATCTAATAATATCTATTATAACTATATAAAATTGCAAAATTTGTATTTAAT
AAAACATTTTACTTTTGTGACATTTCAAATAGTTTTTTTTTATTTTGTGAAAAATTGAAAAAGCTACGTAACT
GAGTGTAAAATTTGGTTTACTCATGCTTCTAGTTACTTTTTTTTTGTAATGTTGCTGTAT
>103783637_2 DT668747.3
CTGGGTTCGGTCATTGGCGTGGTCAGGAGCTGTTCTGACCAAGACGTCACTTTGTGTTTGAAGGAAAAAGCAC
TCAAATACGCAGAAAATCTGGCATCAGCGCGCGAAATCAATCTTACGGACGGAATAACCCTTGTTGGTATGGA
TCCTCTCGCGGTGCCAGGCCTTCGAGCCTCTATCCGAAGAGCCCAGGGCAAGGGAAAA
TGAGGTCGAACGAGGCTCGTGGACCGGCACTGATTTCTTAAAACCATGTGATCACTGCTATGCCC
>103783640_2 DT668624.2
GCACGAGGGAGTACCTAGATCACCGACGGCGGCCGCGACCTTCGCTGTGGCGACCGCTAATGCTATAATAATA
CTTTGTTGAATGGGTTTAGTACTAGTATCAGCCTGTTTGTTCTCTATAGTAACTGCGTTATACACGTCGAGGC
ACTTCCTTCCTAAATATTTCTCAGTTCTTTCAAGTGCAATATGATTACACTGACATCG
ACTCTGTAGTATGTAACATTTCAGATCTGGTACGGATATAAAATTATTTCGGATTATCTTTGCTTTGTATAGT
TTCGGTAATTGACTTTAGAGACATTTACTTTTTAAAGAATCTGTTAGTGACAGCTGTTTTATGTATTGTAATA
TTTGTTGCCCTTCAAATAAAAAAAAAAATCAAAAGTTGTTGAGGCAAAATTGAAATCC
TGTCAATAAGCTTCTCTTAAATTCTGGTCCGATCCGAAATTATAACAAGCTTATATCGGATACGGATGCGACG
CTCCATGTAGTAACGCAGTAGTAACGCGGGGAGCATTATATAGTAAAGAGTAAGATAGAAATATATGTACTTT
GATTTTATGAAGAGAACTCATTGAAGCTTAGTTGCATCTTTAATTGTGATCACTTAAG
ATAATTAACAGTTGATTTAAATCGAAATCGATAGAAATATATGTAGAAAGTTATTTGTTTATTAGTTATTTTT
AACTAGTATAAGCTAATAATATGCGTTTTTGAATTANGACTGACTCATCTGTTGGTAAAAAAGCCGCGGATAT
GAAAGTGCCTTTGAGTATATATAATCATATTGTATATACACTACTATATA
>103783655_2 DT668487.3
GCACGAGGTAATAATCATGTAAAAGATTGGAGCGTAGACAAGTGTAGAGCGTTGCGTGGCTGTGCGTCGCCCG
TGCTGCCCGTTATTATGGTGGACAGCGCCGTACCAGAATAATTAATAGCAACCAGTAAATATAACCCTTAAAA
CAAACATAGTTATTTTCTTTAAATATATTGAACCATAAATTCATAGATACGTATAACA
TTATGTGAAATACATACTTATGAATGTTTAAGCGTGGCGGTCGGCAGCGCACAGCTCCGTGCGCGCGACGCGG
CCGCAGGCACTCGACACTGGGTTCCGCTAAAGCGACGCACAATCGACAGACACACATAAACCATAGCACACGG
ACGCTTTTTTTTTAATTTTAAACTTATGTAGACTAACAAGTAACAACCGACGTCGGAC
TTCGTACGTACGGTCTCGGTTTCCTATGTAGAGGTGGATCAGTAGCGCTTCGAACCATTTCTTTGTGTAAATA
TTGTATTATTATTAAAAGATTCGCCTTTAGCGTGATTCATTTTATATAGAAGTGATAAGGGGGCGCTCCCTCG
AGCGGTCCAGTCAATTATCGCATGCGCGACGCTAGACGTCACGTTTTTTGATGCACTA
TAACACTTCGTACCTTGTTTTAATTGTAGGTGAAAATAATTTATTATCGATAGTGATCATTTAGTAGGAGTAA
GTAGGCCGGAGCGATAACAAATTCGGATGAATTAAGTTTTGTGATAGAGCAGCAGTGGGCTGCGACCGCCGCC
TCGCCGCCGGCTCGACACACCTCTACACACCGACGGCGTAGATGTAGCCACATGCATG
AAAA
>103783657_2 DT667837.3
GCACGAGGCGACCCCCTTCAAGCCGCGGGTCCGCCCATCGAAATGAAGTGGCTGTGTATGTTTGTGCTTTTTC
TGTGTTTGAGGTGTGAAGCCGCGAACAAACCTAGGAGAGGACGAGGAAGTATGTGGTGGGGCATTGCGAAAGC
TGGCGAGCCCAACAATCTGTCACCGGTTTCCCCTGGAGTTTTGTACATGGACCCAGCA
GTTCACGCGACGCTGAGGAGGAAGCAGCGACGGTTAGCTCGAGAAAATCCTGGAGTCTTAGCAGCAGTGGCCA
AAGGAGCCAGTATGGCGGTAGCAGAATGCCAACACCAGTTCAAATACAGAAGATGGAATTGTTCTACAAGAAA
CTTCCTCAGAGGAAAGAACTTATTTGGAAAAATTGTTGACAGAGGTTGCCGTGAAACA
GCGTTCATTTATGCAATAACCAGCGCCGGCGTGACACACGCGGTGTCCCGCGCGTGCGCCGAGGGCTCCATCG
AGTCGTGCACCTGCGACTACTCCCACGTGGAGCGCGCGCCGCACCGCTCGCGCGCCGCCGCCGCCGCCAACGT
GCGCGTCTGGAAGTGGGGCGGCTGCAGCGACAACATCGGCTTCGGGTT
>103783662_2 DT668198.3
GCACGAGGTAATAACGTTAAAGCTGTGGATGTCTAAGTTAGAGTGGGACGATGAAGTTCCTACTGATATTAAA
CAAGCATGGTTGTCATTCTCTAGCACATTAAAGTATCTAAATAGTATTCAAATACCCAGATGGGTACTGTATA
ACAATTACATAAAAGCTGAACTGCATACCTTTTCAGACGCTTCTGAACGAGCCTACGG
CGCATGCGTTTACGCTCGCGTTGTCTGTGACGATGGCTCTGTTCACACGTCACTAATCGCATCAAAAAACAAG
GTAGCACCATTGAAACCTACTACTATGCCGCGCTTAGAATTATGTGCTGCTCTTATTGCTGCGAGACTGTGTA
CTAAAGTGTTAACTTCACTTACGTTGCGGGTTGATTGCAGATATTGGTGCGATTCCAC

AATCGTACTAAGCTGGCTAACCACGGCCTCAAGTCGGCTCAAACCGTTTGTTCGCAATCGAGTAAACGAAATC
AATGAAGCTACTGCCGGTAAAGCTTGGGGTTACGTGCCGTCTCTGGAAAATCCCGCTGATCTCGTTTCTCGCG
GGGTGAAGGCTGATGTCATCAGCTCATCCTCTATGTGGTGGTCGGGGCCATCTTTTTT
ACAAAACAGTGAGGAATCATGGCCTAAAATGCCGAATTCCAGCGAGAATCGTGATCTGCCAGAAATTGTTTGC
CACATAGCTCAAAACA
>103783691_2 DT668435.3
GGGCGCTAGTGTGGCGCGCTAGCTCCTCTAAAACGGAACCTAACCGCGGGCCTAGGCTATATGGCCGGGACGC
CATGTATGCACACAGACCAAGTACTCCGCAATGCCTTTCCACTAAGTCTTTTGATCTGCAACTCTTCATGAGG
GATTTAATGGTTATCTCCTCATCTGGTAGGGCGTTGCAGTGCATCAGCCCTGTTAGTA
ATTTGGCTGCTGCTTTTCGTACCTCAACCCTCGAATCCCTCATAAGTTTAAACGCGAACCGTTCAGCACGAAC
CGCCCAATCAGCTCTGTCACACAACATAGGCAACCCATAAAACAGGAACGGCTGAGCAAAGTCCAAGCAGGCC
AGACGAGCCCACCAAGACTTGGTTAGTCCCAGGGTCTCTAACGCTGCGAGGGCAGCAT
CAAAATCCTCATGAGAATGGTGCACTAGGGAAAGACTGGCGAGGAAGCCACCAGCTGCTCGTGGAAGTTCCTC
GTGAGGCTGTGGTGGGCCTCTTGATGCGAGTGCACAAGCTGTGCTTAGCAAAGCGTATTGTACTCCAGTTTTG
CTGTTCACGCCGCGGACTATCACGCCCATGCAGCCGCGGAGCACGGTGGTAAGTAAGT
TGACAGCGCGCGCGTGTTGGTGGTGCGCGGGGGCGTCGCCGTCCAACCGCAAAGCATTCTCCAACTGCTCCAC
CAGCTGCTTGTCTACGCTGGACTCGCAATCTTGTACCACCGTAGAGTTACTTGAAGCCTGATGACAATGGCCC
GGCCGTTGATCCAAATCATCAACTGGTGTCAACGTTGCCA
>103783695_2 DT668418.3
GCACGAGGCGACGACTGGCCTCTTAAAATTTGCTCTGATTAAAAATTATTTTTTATCTTTTTAAAAGTAATTC
AGTTTACTTATAGAACAATGAAACTGGCGTTATAAATTATACAGGAAATGAGCGGAAACATGGAATTCAACTC
CGTTATTCTCGATAAAGTGAGATAACACAAAATGCCATTACATATCTATAGTAAACTA
AAATCTATTTTAATTTTTTTACAGCTAGATTAACTATCAAAATGTGTCAAGTCCTCACATATATACGGCTATT
CATGGACAATGTGATCGATTTCATCTTCGGGCTTTTCTGGGGTTGCAGTAAAGTGGCCATTCCAGATGTTGAG
AAAAAACATTCCTTCCTAGCCGAAAGCGCGTCAAGTATGGCGAGAAAAATTAAACAAA
AGGAATTGAAATCTGAAGAGCTAGTACAAGCTATGATTGATAGAATTAAGCAGGTCAACCCGTATTTAAATGC
GATTACTGCAGATCGGTTTGAAGAGGCTTTAAAGGAAGCGAAGGAAATCGACAAACAGATAGCATTAGGAGCC
TCCGATGAACTTTTGAAGAAGCCATTTTTGGGAGTGCCTTTTACAACCAAAGAGTGTC
AGGCAATGAAGGGAATGCCGTTGACGATGGGTATCTGGTGTCGCCGCAACGAGAGGGCAACAGAAGACAGCGA
CGGAGTAGTGTTGCTGAGGAACGCTGGAGCCATTGCCATCGCCTGCACCAACCTTCCCGAGTTACTTATTTGG
CAAGAGACCCG
>103783707_2 DT667005.3
GNACGAGGATAATCCGTTAGAATATTTACATAATATGGTTGGTATTTAATTGTTATTTTTAGTGTAATTAAGA
GGTTTTTCAAAAAAGACTCTTAGGTACAATAATGTTCTATTTAAGCTCACGTAAGAATATTAGTTCTGAGTTT
AAACTGTTTTCTTAAAATAAAACTCCTTAGTGATACATAAATACATGATTTTCAAATA
ATTTCAAAACTCAAAATAATAACATTTTATTTTTTTTATTTAAGCTTTAAATGCAATTAGCTTATACGTCGCT
CTCTATTTACATGAGTAGAAAAAAAAAAAGAGTAGTTTAATTTTTTTTTATAATTTGATGATATAAAATACAAAT
AGTGCAAATAAGGTAAGTACAAATAAAACACAACAATATAGACTATTTATTTAAAAAA
ATAATACATAGAGATACATTTCATTCTTAAGTTTAAATAGTTATACAATAACTCAGGTAGATTACAATATCTG
TGTGTAAATATTATAGTTACCTATGTATTAAATTTATTTGCCTTCACGCGTATTTATCTAAAATAAAATGGCT
TCACGCTGTATCACGCATATATTAGGTTAGATAGAGGGAACTTAATGTTAGGACACCC
CGTTCCCCATTTTATTGGGTTAAATGAGGAGAAATGAACGTTGCGCTTTGTTTGGTTAACAAATCTGGTTATC
GAATACACAACCATCGCGTTTACTTTGAGATGATG
>103783709_2 DT668171.3
GCACGAGGGCCGCCTGACAGGACTTACTGACGAGATGAGAGCCAACTTCCAACTGATGCGGTCATTATCAAAC
CACACTAAGATCGGCCCTGATATGCGTATGCAAAAATTATTAACGTTCAACCGCAGGTTCACGCAAACCCAAC
AAGTTGTTGAGGAATTAGGAACATGGTCCATGAGATTATCTAAAGATTTAGTTCGCTT
CAAAGGCCGTCAGTTACCAGCTGAAAACATTCTTCAAGGAGGAAATTCTAAATACCCCGCCGGTAACACCTTA
GATGGCTGGACTAGGGACATGCGGTCAAAGAACTTGCTCAGTTTAGCTCAAATGCCTTCATGGGTAGTAATAA
CGCCTGATCGTCTCTACAAAGATACTGAAAGTTTTATTGGTAATATCATGAAAACTGG
TGCTGGAGTTGGCTTTAGAATGCCTAGGCCTGAGATTGTGACTATACAACGTGATAGCCAAATGGAATATGCG
AACATGTGTGAATCTGTAATTGCCAAGAAGAATCCCGCCATGATAATGTGTGTGTTGGCGCGTAACTTCCCTG
ACAGATATGAGGCCATAAAAAAAAAGTGTACAGTAGATAGAGCAGTGCCTACCCAAGT

TGTTTGCGCAAGAAATTTGACTAGCAAGTCGGCCATGTCGATCGCTACAAAAGTAGCTATCCAAATAAACTGC
AAGCTTGGCGGCGCCCCCTGGGCAGTAGAGATCCCGATGCCCACGCTGATGGTGGTGGGCTACGACGTGTGCC
ACGA
>103783727_2 DT667462.3
TAAAATACATTAATAAATATTGATATGTAACCAATCTTATTTCGACACTTTTTTATCTTTATAATATTATTGA
ATTATTGAATACAGTAATAATAACCTTCACTTACTAACATTGTTTGTTCTAAAGTTCTATAACGCTACTTTTT
TATTGTGCATAGAATTTATGGTCTATTTATATTTAAAATGTTTTTAGTTTGTTGTTAA
CATTAATAATGAAAGTATTGTTATAAATCCGCATAAAAATATTAGCAGAATAGAACTGGCAACACTTGGCTTT
CCGTCCAAACGTTCTAGCCAATTATTTTATACGTAATATTAGACATAAAACCTTCAATCTTGGGATTTGATGA
TCAAAGCATATTTTGCGTCGATATATTTGTCCATAATTGTCATGCTTGTTCTTTAATT
TTTTTTAATTCTATATTTTTCCAAGCCCAAGGCTTCTGTAGCTGTATTTTTTACTAATTCTGAATGAACAACT
TGTATGAAACCTATTGTAGTACGTATATATTATTATATAAAAGCTTAAAAATTGTAACGCGTGGCCTCTGTAA
TCGAAAAATGCTTAGATTTCTAGTAAAATATTTGAAACGTAAGTGTACGAAAGTGTTA
CAGCATCTATTAATATATTAAAAGAATTAAATTTTGTTTTATAACTATCAGACAAACGGATTACAGGATGTTG
TATCGATATGATGAAATAAATATTATTTTTGGACCACTGTGATTGTTGCTTCAATCCCCTCTGAGAGGAATAT
TGTTTTCTTTTCGTTAACTTT
>103783732_2 DT667739.3
GCACGAGGGTTTATCATAGCGAGTTTGGTGATATCTTTTTAAATTAAACATATATTTAAACGAAAGTCTAGAA
AAACAGATATCATATTTACTAGATTTTACTAGAATTATAAAATGGGTCGCAAAATTCCTGCTAAAAAACATCG
AGGAGTAAAAGATCCCGCTGTACAACAAGCAAGGCGGTTGCAAAGTTTAAAAGGCAAA
ATTAATGCGCCACCAAAGGATCCAGATGAGCAACCAGTACCACGTTCTCTCACAGAACTCTTTGCATTTGCTA
ACAAAGACGGAAATGTCAAAAAGAAAAAGAAAAATAGGAATATATCTTTAAAAGATGACAAAAATCAAACTGT
AACTAAACACAATCCAATAGCCTCCTTAAAAAAACTACCTGGAGAGTCTGGCCGTGGA
TTCTCTTTGCGAATCAATAGTGCTATAAGAACTTTGCATGACCCCATGGAACAGCTGGACTACCCTCAAGACA
TGGATGAAGAAGATATAAAGGGTGAGCGTATAGCGGCTCAGAGGGAGCGCCGACTGCGGAAACATAAGAAAGC
AAACAAAAATGCTCCACCAGAGGAACCAAAAATCAGTAGAGCAAAGAAACTCGCTCTT
AAGAAACAGGAGAAGAAAAACAAAGCTGCAGAGGAGGCGTCCCGGCCGGAGGTGGTGTACGAGCGCGTGGGGT
TCGGCGACGTGACGCACGCGCCGCCCGCGCTGCCCGTGCGGCCGCGGCACGCGCGTGGTGGCGACGGGGCGCC
CAGG
>103783736_2 DT668182.3
GNACGAGGGATGTCCTTTAAAATAACGACTCTACCGTATTCACTTATCGAAACAAGTTTTATCATATCGAATG
TTCAAAATTTCGTGGTCTCTTAAAATATCATTATGAACTCTAGTGTCGACCTAAAGAATCAGACTTTTAAATG
AATTTTAAAAAGTTTGAAAACTTCTAAGTGCACTTTGTGAATTACTTTATCTGTAACA
TCGCACGTACGTACGTAGGTGTTGTAACTCAGTGTATTGTTTATTGTTTTCATATAAAGGACAGAATACCGTT
GAAGACGCGGAGTTTGAATCTTATGTTGTTTTCTTCTTGTTTATAACAACGCTTTCGAGTCGCGTGTTGAAAA
GAGACAAGATGAAGTCCCACCGAGCGGGAGCGACAGGTGTGGCGGCGCTGGCGGTCTT
GTTGGCGGTTATAACAGGATGTTCATCACAAACGCTGCGGATATTTCCCATGCAAAAGCAACAAACAGTTGCT
GTGGGCAAGTCGGTGGTTTTGACCTGCCAAGCCGACGCCCCGGACCCGGCGCTCGTCACACAGCCGCAATGGA
GGGACCCAAGAGGACTCATTATAAATGAAGCTGCCGCCGGTACCAGACCAGAAATCTA
TACAGAGCCGATGCCGGCGAAA
>103783783_2 DT667500.3
GCACGAGGTATCCCCTTTAATTTTTATTAATTTTATTTTATTATTATTATTATTTTATTATTATATTTATTTA
TTTTAGGTATTTATATATTACTATAATGAGCCATATACTGAATATAAGTAGCAGTTCCTTTAATGCCGCTAGT
ATTTATGATTCTGCATCAGATTCAAATGATAGTTTTCACAGTACATCTATGCCTTTAG
ATACGCTTTTGGAATCCAAATTTTCTACTGCTCCTAAAAACTTCAATGTCATCCATATAAATTCCCAAAGTAT
CCCTGCACACTATCCTGATCTCCTTTCTTCCCTTAATGTAAAAAATATCAATGCTATACTTGTATCCGAAACC
TTTCTAAAACCTTGCCTTCCTTCAACTTCCTACTCCATACCAGGATTCCATCTAATAC
GTAATGATCGTATAGACAAAGGTGGTGGGGGTGTGGCTATTTACCTTCGTAGTCATATACCTTTCACCATACT
TGATAAATCTAACACTCAAAACTCAGAAGCTGCTGAACATATTTTTGTAGAAATCCAGCTTGGTACTTTAAAA
ATTTTGCTGGGAGTTTTTTATAGTCCATCTTTACATGTTAATTATTTTCAATCGTTTG
AAACATTACTAGAGAAGTATGTACATACAGTAGATCATACTATTTTAATGGGAGATTTCAATACTTGTATAAT
CAAAAACGACAAGCGTGCTACAAGCCTTAAAAACATCATCGAAAGTTCAAACCTGCATCTGCTTCCATCTAGC
GCTACACACTTTATCCCCCATTGCTCTCCTTCTCTCCTTGATCTGATTATTGTATCGT
CCCTAAATCACGTAAATCT

>103783798_2 DT667196.3
GCACGAGGGAGAGCTTATGTGTAATATTTATGTTTTACGGAGTTAAAAAAAATAATTCTTCATAATGGAGAAA
AGGAAAGAGTCTTTAAACATAGTGAAAATGTGTCGCTTTTGTCTAACACAAAATGAAACATTGGAGAGCTTGT
ATGATAGAAACCGTTCAGGAAAGAATTCACCAAATCTTCATTTGAAAATTTTATCGTG
TGTTTCTATTGAGATATTTCCCTCTGACAAAATGCCCTCATTTATTTGTCATCGCTGTAAATTCTTCATGAAT
CTTTTCTATGAATATAAGAAAATTGTTCGCCAAGCTGATGAAACAATCTTACAGTATATTCAACATAACTCTC
CAATGGAAATAATAACATGGCCCTCAATCTTTTCTAAAGTATATAATAAAGTGAATGA
AATTGATATAGTCAAAACTCTAGAAGGTGCTACCGTGCAAGTTTCTTCTCATGATAACTCGGATAGTGATGAA
GAAGATGGAAATGTTTATAATATAAAGATTGAAGATGGTCCAGATGATGAAACAAAAACTGCTTGTGTTAAAA
TTATTACCAGTAAAGAGAGCAAAGAAATGGAAGATCCATTGGGTAAAGAACAACATGA
GATAGATGCGGTGGAGAAAGGCTTAGAAGATGGCTGTTGGGCTTGTGACGAGTGTGATTGTACATATCCTCTG
NCACAGTTGTTGGAACTCCATAAGGCACAGAAACACAGGGAGCGCACAGTGGAGTGTGATCAGTGTGATGCAA
ATTCTTCACGAAATATGATCTATCAACTCATCTACTGCGTCATACTGATGAATTACAT
>103783800_2 DT667162.3
GCACGAGGGGCACACTTGTTGACATGTTTCTGTTTTAATATATATACGGAAATTGTGATTTTTTGGCGTTTTT
AGTGTAATTTTAGTGTTTTTATGTGTGCAGTTTCGTAAAATGAGAAAATGGTTCGAGCCATCCACGCAGTCCT
ACTTCTCGCCGTAGTTGCGGACGTGAGGTGTCAGTTCTTTGATTTCCTCTCAGCTTTT
CAACCAGTACATTCAACAACAAGATCTTATGATATAACTACACGGAGACCAAAGACCCAAACAAAAAGTACCA
CGAAGAGACCAGCATTTTATTACGAAGAAGATGAAGATAACAATAAACAAAGGAAAATTGGAGCCATCGTTTT
TGACTTATACTCTACTACTAAAAGACCTGAAAGGAAAACCACAAGAGGTTCAATAAAT
AACAATTCTAATTCTAAAAGTCATAATAATAAGTCTAACAGAGATAACAATAAAAATAGAACTAATCATAGAG
ACGTAGACGTCACTGGTATAGAGCTACCCAAAGTAAGACAACCAGAAAGAAATCAATATCAAAATATAAACAA
CGATAGATATGATACTGAAAAGAATATTATAGTTAATTATGAAACATCGAGACCTAAT
TTCAATGATAACAGGAGGTATTATAGTACCCAGATACCTTATACGAGTAGGCCTGGGGTCAGGCCAATAGCGG
CAAATGGACCTCCGATCACAAATCGACCCGCAAGGAAACAAACTGTTGGGATATCTCGTCATGATGATAGTGT
CACTCCTG
>103783802_2 DT668685.3
GCTGCAGGAATTCGCACGAAGGGACCGTCCCTGGAGGGGAAGATATTATTGGTAGCGTCTTTGGCGCTTACAG
CGTTTTGTTTTGGCGTTGAGGGTCAGCGGTACTACAACCCGAGGGGCTACTACGGCCGCATCTATACGAGCAC
GGGCGGTCCTATCCGACGAGGTGGTCAGATGTGGACCAAGCCCTGCGGAGTGGGGCTC
CTGCCTCCGGCGCGGAGACCAGCTGCGCCTGTCTCCCGGG
>103783804_2 DT667505.3
GCACGAGGGTTAGCATCTCTAGAAACAGTCAAAGATAATGAAAAAATTAGTACGGTCCTGAAAAATCAAACGG
TAATGGAACCTCTGAATGAGGTGTTAGCAAGGATACAACCAATTTTTCGGGGTAAAGACGCAAGACTATCCCA
CGAGCGCAGGATAATGGCACAGGCGGCGTTGAAAGAAGGAGATCTACAGAAAAGTCTA
GCTTTAGTTAGCCAAGCTGTATTACGAGCACCGATGACAGGAGCAGAAGAAGTTATAGACGACGGAGTATCAC
TGGCGCTCGCGTTGTGGCTTCGTTCTGAGATACTTCTGCAATTGAAACGGCCAGCAGCGGCTTTGGAAGACTT
GAAATTAGCACTCAAGGAGAGATTACCTGCTAAGATGAGAGCGGAATACTATTGGAGG
ATGGGACATTCCTATATAGGAACTGGAGAACCAACTCGAGCTAAAGTATCTTATGAGCTAGCGGGCAGACTTT
TGGGGAAAAATGAACAAGCCAAGCTTCAATTGGCCAAGGACATAGACGCTATAGACTACACGGTGAAACCTAA
ATTCCCCCCGGATAAAAAAGATGTGACGTTATCAGGTGGCGCGAAACAGAATTTGCCG
GCGTTGTCTAAATTGGTGCGAATTGTGGAGGAGAAAAATAAAGGTCGTTTCGCGGTAGCTGACGCTAGTATCA
GGACTGGGGACGTCCTGCTAGTTGACAGTCCGTACGCAGCGTGCCTACTGTCGGACTTCTACGGCAGTCACTG
TCTTCA
>103783806_2 DT667328.3
GCACGAGGCGTTACTCTTTAGTGTGTCCCCTATTGGCGTAGAGAGAGATATATCTGGATTACAGGACACTCAT
TCTGAATTAGAAGCAGCCTGTAATGAACTCAGTGTTCGGTTGCGGAGCAAGTTACAATTGTGGCGGCGATTTG
AAAGGCAACTCGAATTGGTACAAGGCGCAGTGCGTGAAGCTGATTACATGGTGGAGTT
ACTCACAGTACAGGGA
>103783817_2 DT668714.3
GCACGAGGGGCCCCTTTGACGCGCAATATAACAGAATCTTATGAAAATAAATCTAAGGAACAACTAAGTTTCA
TTTATTTATTTATTACATATTTTAATGTGATTAACATTTGGTACAGTTGAAAAGTGTAGTGAGTTTTGAAATA
AAATGACTTCCAATGAAGGCCGAGCCCAATCAAACTTGCATCAGCAAGATTTATCTAA

GCTAGATGTAACAAAGTTGTCTGCTCTATCACCAGAAGTAATATCTAGACAAGCGACAATTAATATTGGTACA
ATCGGCCATGTAGCCCATGGGAAATCAACTGTAGTGAAAGCCATTTCAGGTGTACAAACTGTCAGATTTAAGA
ATGAATTGGAAAGAAATATTACAATAAAATTAGAGCGCCTGGCCGAGTCAGTGATAAA
ATTGTACCGTGAGAGAACAGACGAAAGAAATGAAATTATTTTTGCTGAAAAACTGAGAAAAGGAAAAAAAAGG
CCGTTACATATTGATTCAGATGTGACACCAGAAAAAACACCAAGCAAAAAAAGAAAAAAACAAAAGCAAGAAC
CAGAAGAATTCATTATAGAAAAAATTTTAGATTTCACATTTGAAGCAGGCAAAGTTAA
GTTTTATATAAAATGGAAAGGATGGAATGATAGTGAAAACACATGGGAACCAATTGAAAATCTGGACAATTGT
CCTGAAATATTAAGAGAATTTTTAATTGACAAAGAGCTTAAACATTGTCAAGAAATAGAAGAGCTTAAAGAAA
AGTTGTCCTTTGATAATTTACTTGGT
>103783829_2 DT667029.3
GCACGAGGATAGGACTTTAAAGGGGATTTGAACTTCATGCTAAATATTTATAGTAGAATATGCAGTATAATGG
CGATTTTAAAAAGTTTTATTAATGTGTTAGACATGTAAAGCTATTCAACTGATTGCCTTCAGTGATGGCTATG
GCTTATACAAAATATATATAGTTAAAAATATAGTTGTATGTAGTTTAGCATGGTTTAT
ATAAAAGATAAGGCATAGTGATTAAACTCATAGGAAATAAAGAGATAAATTGCGGCGTTAGGTTTCGCCACTC
AACATGTAATGTCGATTCTCTTTACTAATAGAGAAAATGCGTGAAGCTACAAAACATTATGGATAATGCTTGT
TGGACAATTTGACTGTATAAATTAAAACTTAAGTTTTTTATTTATTTGAAATCTTTTT
GTATATATGTGATTAATTAGATTAGTTTCACACTGGGTCCCACATATTGTAGGAAAAGCGGAAAAAACTTACT
TTTAAAATATCGTAGCCCACATGCCGCTTAATGTTTGAGACATCTCGTTGGCGGAGCTCAACGCCGCGTTTTG
TGTGCATACTACAGCGAGGAAATGAATATAATTTATTCGCCCGAATGGGAATCGAACT
CATAACCATCGCGATTACACTCAGATGCGGATTATGTCATAGATCTTCTATTTAAATGTAACTAATTTGTAAC
TGATAAATATAAATTGTTTATGTTTCATTTATAATATATTCATTACAATGTAGCATAAGATATTTTAAGTGTG
ATCTTAATAAAAGTGGACTT
>103783835_2 DT668283.3
GCACGAGGCATGGCCTGGCAACGACGATGGCAAGGTGGTGAACAACCAGCCACAACGAGTGATGGACGAGCGT
AACGGCATCGGCCCACACGCCGGTTATATTGGAAGGATAACAAACGACGCCCGCGAGGACGAGATGGAAGAGA
ACATGGGCCAAGTGAACACCATGATTGGCAACTTGCGCAACATGGCCATCGACATGGG
CTCCGAGTTGGAGAATCAGAACCGCCAGATCGACCGTATAAACCGCAAGGGAGAGAGTAACGAGACACGTATA
GCTGTTGCGAACCAGCGGGCGAATAAATTACTCAAATCTTAAATCAAATTTATAGCGTCGCAAGCACTCGCAA
TATGGTGTTGCCATGCTCTATCCGATCCCTACAACGAACTCCGTGACACTAATTTTTC
TCCTAGTCATATTATATGAGTAAATAGTATAGAATAAAAAAAGAAAGCAAATAAATTTTAATTGATATTAGCA
TGGCAACATTGTTTTGTGCCGCACTGTCACCGTGATTAGATGTTAACGTGTTATTGTACCAATCGTACATTCG
ATATACAAAAAAAATGTGCAATAATTATATTGAAAATTATAAAAAAATCGTTTTCACT
CAATACAATTTGAAAATTTTACGCTTTATGGCTATTTATGANAAAACACTATATTTAATTTGCACGTAAATTT
TACTTTAGGTAATGTGCCAAAGTTTGTTTCCTTTTACATAAACATGGTGGTATTTAAAAGATTAAG
>103783858_2 DT667997.2
GCACGAGGAAAACGCCCTCACATTTTTGTTTGATTTCCTTAATATGGTAATAGGACAGAAACGCGGCGCTGAG
TTCCGCCACCTAACACGACATTTTTCGAAAATTCCGCGGTACGTGGGGAACAAAGTATCTCAACACAATGCTC
CCCCTAGCTACAATGTGGGATACAGAGTAAAACTAAAAAATATATTTTCAGATTGTTA
AAAAGGAACCTCCATTTGAATACCAACTAGACATAGACACGGATCCAGAGCACGATGACGATGAAGAGGACAC
TTTTCAAGAGTTACCTGCTTTTACTAAAAATAGCCTAGAGCTCTCCGACCCGCTCCGTACGGTGTTGAATGGT
GATTCGGGAATAGATTCAATAAGTCATTTTGGATCATCCACTCAAGAGAATAAAGAAG
TTGTAAAATTAAGAATGGAATTATTGAAATATAAATTGGAAACAGCAAAATTAGAAAGACAAAGAATACAAGA
ATTAATGGAAGCGGAGAGCGCGGAGAGAAAATCGAAAGCAATAGAGACATCCCTCCGTCTACGGGCCGCGCGG
ATAAACGCTGCCTCTGCGGAGATGAACCTCCCACCGGCGCACCCAGCACTACACTCAC
AAAAAAAAAAAAAAAAAA
>103783861_2 DT668025.3
AGATATTATTGTACTTATATCTGGTACGGTACATTGATAACGGAGTACGTGAGGCGAAGATATTTCAGTGAAA
GCAGGAGTAGCTGATTCAGTACTTCCTGTAACTACCAAGGACTTTAAGGATACGTCAACGTCTGCGCCGCAGT
TTGCTTGGTACTACTTGTCGGTGGTCTATCGCCTCAATCCAACGCCGCGCCACACAAA
CGGACCATGGAGAGGCAACGCCGCGAAGCCCAGAAATATTTTATCTGGGATAACCCATGTGATTATGACTCAA
ATTCAAAGCCAGCTCAATATTCAGCTAAGCTCGCAAAGGATGTTGCGTCGCAAGCGAGAAACGCCTTCGAGTC
AACGGCAAAATACAAGGACAAATTCGCGTCTATGTTACATAGTTTTGATACATTCGAG

GAGTTATTGAAGATCTGGAGCGGTAACGACTGGCTCACAATCTTCTCGTGGTTTCCTGAAGAAGGCTTCCCTA
AAGAAAAAGACCTATACCATAAAATGCCCGATGAATATATGGAGAAGTTAATGACTAGAACGGACGAAATACT
ACCCGCCATGTACAAAG
>103783868_2 DT667891.2
CAATTATATAAAATATTTATTTATCAATTTCTGTATCCACAACAAATACAAGTGGGTTTTGGCAAATAAAAAA
TATGTACATGTACAACAAACACGTCACATTAAGGTAAAAAAATAAATAATCCCAATCTAAACCACCAATATTA
ATTTAAACAAAATAATAAAACCATAAAAAAATATAATCAACTGGTACAACAACCGTTT
TAAAAATATATATCTTGGGATGAAATACATAAAAATTACGTAATATGTTTAAAAAAAATGTAAAAAAAATAAC
ATCTAGCGACAACTATACAATTGTTCAAATTCAACGGGAATGTGAACTAAACGCCATTATGATACAAAAATTA
TAAATACACTTACACATACACATTCTAGTTATGCTGCTGTTACGTAATAAATTATGCT
CTTTCCCATATTAATAAATTAATTTCCATTAAAATTTGAAATTCATAAAACAGCCAACAAAAGGAGTATTTCT
GGAAATATCAAAGCTTAATGTTAAAAAAAAAAACTGAAACTTCTGTTAAATTACAATCGAACAAATTAAAAAT
ATTACACATTTTGAATTCAATACAACCTTAACAAAAATATAAAAAAAAAACAGTCAAG
CGAGAAATGCACTGATTATAAAGGTGTCTCACACTGTAAATAAAGTGAGGTGTCAATGTCTCGTATTTTGTCT
G
>103783876_2 DT667495.3
GNACGAGGGTGACCTTTAAACAGGCCATCAAAGGATGAAATTATTTTTTGGCTTTTTGTTTAAAAAAGGTTTA
TGTTTACACATTTACTACAGTATTAATTAATATTCGGTGCATAAAGCTTAGTTTTACTTTAATATTAGACATC
TCGATTCATGTGCATTAGTTTACTTGTGAATACGCCCTTGAAAACCGTGATACAAAAA
GTCTAAAGTCAGCATAGCAACAACACTCTAGTTTATTTTATTACGATTTTGAAAAACACTGCGGGCTACAAAT
TGCAGTGGTGCCTTTTTGAGATGAGTACTGAAGACATATTGAGATTTAAACAAGAGAAAGTAAATATTTTAAT
TTGATAAATACATTTAAGTATAGGTGTGGTGCTTTTGTTAGTGTAAGATGATAACTCG
GAGGTGGTTCCATCCATCATTAAATGGAATAGATGCAGAGAAATTACTTTTAGAGAGAGGCAGAGATGGATAC
TTTCTTGCTCGACCAAGTATGAGTAATAAGGGAGATTTCACATTATCAGTTAGAAGGGGGTTAGAGGTCACAC
ATATAAAGATTCAGAATAATGGAGAGTTTTTAGATCTCTATGGTGGCGAAAAATTTGC
AACTCTTTCTGAGTTAGTACAATATTATATGGACAACCAGGGTCAACTTCGTGAGAAGAATGGGAATATAATT
CGATTAAAAACCCCATTGAATTGTGCTGATCCGACAACGGAAAGATGGTACCACGGACAGCTAAC
>103783905_2 DT668529.3
GCACGAGGCACTCGTCCAAGAAGCAATGGCAGCTAAGTTGGTTGTAGTACTCGCCGCCGTGGTCGCTGTGGCC
CACTGCTCAGTGGTGCCAGTGGTGCGAGCAGACGCCGACTACTCAAGCTTCGCATACGACGTTGCTGATCCCT
ACAGCGGCGACATCAAGAGCCAAGTGGAGAGCCGCAGCGGTGGCAACGTACAAGGACA
GTACTCTCTGATCGAACCTGATGGCTCCAGACGTGTAGTTGACTACGCTGCTGATGATGTGAACGGCTTCAAT
GCTGTTGTACGCAAAGAACCCGGTGTGGTCGCCTTCGTCGCCGCCGTGTCTCCCGCCGTGGTCGCCGCCCGCG
TCGCTTCCCCCGCTGTTGTTGCCGCCGCTCCCTCTGTAGTCGCCGCCGCCCCCGCCGC
AGTCGCCGCCGCCCCAGCAGTTATCGCCGCCCGCGCCTACTCCCCTGCCGTAATTGCCTCATCTCCCGCCGTA
GTTGCCTCATCTCCTGCCGTAGTTGCCGCGTCTGCTCCCGCAGTCGTCGCCGCCCGCTCTTACGCTCCCGCAG
TATACGCTGCATCTGCCCCAGCTCTCGTCGCTGCCCGCTCGTACGCTTCACCCGCAAT
ATACGCCTCTGCCCCAGCTGTTGTCGCTGCCCACTCTTATGGCCCCTCCGTATACGCCGCAGGTCCCTTAGTG
TCCTCCCGGTCTTACCTCGCTCCTTCCCTGTACTCGTCCGCGGCCCCATTAGTCGCTGCCCGTTACGCCGTCA
ACCGCCCCCTACTACTCCGCCTACAACTACGCTAACCCCATCGCCAACTAC
>103783907_2 DT668574.3
GCACGAGGGATACTTGTTAATAACCTAATTTATAAATAAAACATTATGGATAAACAAAATAAATTAGAAGAAC
TGGCCCTTAAATTATACGAAATAGATGCAGTCAAGTTCGGCGAGTTTATGACTAAAAGCAAAATAAAAACACC
TGTATATTTTGACTTGCGTGTTATTGTTAGCTATCCCGATGTTATGGAATTAATTTCG
AATTTGCTATACGATTTTGCCCTTAAAGAAAATAACTGTGATCACGTGTGTGGCGTGCCATATACTGCGTTAC
CTATTGCTACTTTACTTAGTGTTAAAGTGAAGATATCCATGTTGATGAGACGGAAGGAGGCAAAGTCATATGG
TACAAAGAAAATGATAGAAGGACATTATAAACAAGGAGATTCATGCCTTATTATTGAA
GATGTAGTTACATCAGGTTCTAGTGTATTAGAAACCATAAATGATTTAAGAAAAGAAGGTCTTAAGGCAAACG
AAGCAATAATTATTTTAGATAGAGAACAAGGAGGGAGACAAAATTTGGAATCTAATGATGTACAAATGAAATC
TTTATTTACAATGACTGAGTTGATTAATATTCTTGTTAAAAACAAAAAAAATAACTGA
AGAAGTTGGCAAAAATGTTATTAATTATCTGCAAAATATCCAAGCACCTGTTTCTGCTCCCGTTATTAATAAA
AATTTGCTCTCATAACGAAAAAAAGAGCTGAACAAGCTGCAAATCCTATAGCAAAACAACTGTTCAATA
>103783919_2 DT668073.3

GCACGAGGGCTTTTCATAATGTTAATTAAAACTCGTAATAACGTCGATAAATAGTGGTAATTAATAGTGGCAA
GCAAAATTGCGTTATATAAATATCAGAAAATGGCTCATTTTGAATTTGCTATTCCTTTACAAAAGGATGAACT
CTTAGAATCTCATGCTGGCCAGTATCACGTAGAAGATGTTGTTCAACCAAGACTATTG
CTGTCTAAATTTCAAGACGCCGCTCGAGCATACAATTCTGATGGAGTTGAATATATACTGGAACATTTTGATA
CATATTTTTCAATAATTGTTCATGGCACAAAACTAGAATGGAATATTATAAACAAAGGCTTTGATCATTTAGT
AAGAACTGTAAAAAATCTTTGTGGGCATTTAGAAGCATTACTTCAGTCACAAATAGAA
ATGGATATTCGTGTAAAAAATTTAAACATTGTTAAAATGACATTGTATTTATTTACACAAATAATGAAAATAA
AAGATGCCAAACTAGCTGCAGATAATTCCACTAAATTGACATTAGGAAAGAAGGGAAAAAAGGTAACTGATGA
GGATGAATTCTGTGGTTGGACTGAAGCAGACAAACAAGCTGCACTTGTGTGTTTGCAT
TTATTACTACAACATCCATTATCTCATTTATGGGATCCACCTTTGGCTGAAGATAATTTTGTATCGATGGTGG
CAGAGCCTTGCTATAAAGCTTTAGAAGACAAATAATAAAGACTAAAATAGTGAGAGAAATTGTTTTTCAAACA
TTAGGTGTACTTATAAAGAAGTATAACCATGGGACATCATGTGTCATAAAAATGGTTC
>103783925_2 DT668385.3
GCACGAGGCTCAACCGTGTTGGCAGTATTATAACAGCGGAAGATTTGAAAAATTATGATGCAAAGATCACAGA
ACCTATCTCAGTGCCTCTAAGCAACGGGGACGTTCTGTACACCCCACCACCGCCCAGTAGCGGAGCTATTCTG
GCCAACATTCTGAACATTCTTAGTGGTTACAACTTTACCTCGGATAGTATTAATGGGA
CAGACAACAAGATACTTACATACCACAGGATTATTGAAGCTTTCAAATATGCGTTTGCAGCTCGAACCAAGTT
GGGCGATTTGGACTATTTAGATTTAAATGGATTTTTGAGTAACATCACATCTCCTGAGTATGGAATGGAGATT
AGATTAAAGATCAATGATTCAACAACCAGCAACGATACCGCTTACTACGGAGCTACCG
AGTACCAGAAGTCAGATTCGGGCACCGCACATATATCAGTTATAGCGAGCAATGGAGATGCCGTTTCTATGAC
GAGCTCTGTTAATTACTATTTCGGCGCGGGCTTCACAACACTGAACACTGGTATCCTTATGAATAATGTCATG
GACGATTTCTCTTCCCCTGGCATCACCAACTATTTCGATTTGAAACCAACTCCAGCGA
ACTTCATTGCACCGGGAAAGAGGCCTCTCTCCTCAATGAGTCCCAGCATCATTGTCGATAGAAATGGCAATGC
AAAACTCGTGATTGGTGCTTCAGGAGGAACGAAGATAATTACAGCTGCTGCTTTAGAACAATACGCAAGTTAT
GGTTCGATCAACTATCAAGGAAGCGGTAGACGAATCTCGCATCCACCACAAATATCGC
CAATGTATATTCATTATGAATATGGACTTATCGAG
>103783934_2 DT668180.2
AAAAAACCAGGAAATATTTTTTCCATAAAATTTTACTAAATAAGATCTCAATTTAACATACACATTGACTGGC
TTCCAAATAACGGACGTCCTCGGAATCACGTAATCTTTATCAAGTTGCCAAATGTTTCAGAGTTCTAATGTAT
GTTTGTAAAAATTATAAGTACATAAAATTAATAGTATATCGAGGCGTACAATTACGAT
GTAAAATTAGATTACCATACAATATTGAATAGATTAAAGAATTTTTAGTTCAAAAATTATTTCGTGTAAGTTG
TAGTTGTATAAATACTCACGCCTTTTTTTTCATTAGATATTTTACGATATATTATATTTATTATCCTTGTAAT
TAAATTTTCATTCGTACTTACTCTTATCTATTTTGATATTTGTATTGCATTTATTATA
TTTATTATTATTTTCTGTAATGTTCTTCATTTACTAGCAGCTTTTTTATCTCG
>103783936_2 DT668445.3
GCACGAGGCGTTTATTGACATAAATTCCACTTTGCTTGTTAACTTAGAAATATATTTGATCGTTAGTATTAAG
TTAAATAATGATACAAATATAGGATTAATGCTCAATTGTGATTCAGGTGACAATATTATGTATTAATGTTTAT
GTTACACGTCGTTTTGGGACGTGATCACATTTTTTATATTAGCAATTTGTTTCATATA
GTTTTTTTACACTTACATTTAACTTTTATATATATATTAATATGATGATACTGTCCTTCATCTGTATAAATAT
AGAAGCTTTTTTTTGGGTGATGATGACCTATAATAGAGGATTTCTTATTTAAAATATTGTCGATATTACAGTC
ACCATTTATTTATCAGAAAATCTCTCAGAAAAGCTCGATTTCCAATCGAGCAAATGAA
ATATTTAATATTTATATTACCTCGCTCTGGCAACCAGGCAAAGCACGGCGTTGAGTCTCGTCACTTAACACGC
AATGCCTCCAGAATTCGGCGGAGAGTGGGAAACGAAAGTTGGAATGGAATGTCGTAACACAAGGTTCCCAGCT
TCCATCCGCCTACTCTGCTATACGCGGGATACGGCGTGTGAAGCTAAAAGACATTCTG
TGAGGGGCACCAGACCAATCTGGAACGGTGGGCACGGCCGACCCGGTCTCCCATTCCTACGCCCATGATGTTA
ATGTTAAAAGAATAATGTCGACTGTAACATAACTTGTAAAGCGATGAGTTGTATTTCATATCAAAGTTATTGN
AAATGATATCTCGACATTCGAATCATTCCATGATGTGTTGTATTTTATTATATTATAT
TATATAGCTTT
>103783968_2 DV501283.2
CGCACGAGGCTATTTCTGATTAAATTATTTTATTGTTACCTTTCGTAAACTTAAAAATCGAATTCCAAAATGA
GTTGTTTCGATAATATTACTATGCCAAGCTGCGTTTGGTTCGAAAGTGGGGAGAAGCGGAACATTCTAGCATC
AATATTTGCTGGTCTACTGGTTTT
>103783974_2 DT667493.3

GCACGAGGTTTGTCTTGAGATCTAAACGATTAATTTTTTTTTTGTAAATAAAGAAACATGTCAGAAGCAAAGC
TGTGTGGCGTATCTATGGGGACCATAGGACTCATTGCGGTACTTCTATATATAATAGATGTATCGACAGCTGC
ATTAGACGGTTATATACCGGGGGAAGACTATCCTGTGTACGAGGAAGTGCCAAAAGGC
TTATCGTTTACTTGTGATGATAAGATACCAGGATACTATGCGGATCCCGAGACTCAGTGTCAGGTATGGCACT
GGTGCGTGCCCGGCATCGGAGGGAACATGATGTATTCATTCCTATGTGCCCCTGGAACTGTCTTTAATCAGCA
GACGAGAGTATGCGATTGGTTCTTCAAGGTGGACTGTCCAAATGCCCCTGCATATTAT
GGAGTGAATGAGGATCTTTACAAAGATGAAGCGGGCAACTACATAGCTGGGAAAAAGTAGATCATAAATTATT
ATAAATAAGAAAAACTTAGCATTAATTAGTACAAATTATAAGTTACCAATTGAAGGACTGACTTAACTGACAC
AACTGACATTTAGACTTAAAAAATATTTATTTGACTTTTTCTT
>103783987_2 DT667965.3
GCACGAGGATTCACATCTCCACGCTGTCATACCCCACATTGGTGACCCCGACGTGATGTACCCCAACGGGGGT
ACCCCGTAGCCATCTGCCGACGAGATGTACTCCGACAGGGGTACTCTGCCAGCTTCAGCCCGAAGTTATCGTC
GAGACTAATACAATGATCCAATCAACTCCCGTGGTGCTATACTGGGAACCACGTGAAC
CGGACTTCAACCCAACATCTCCTCAATACAAATCTTCATAAGTTTGGTGAGTGCACATTCTAATCGGCTTCAC
ACAAGCGTGTCCCACCGGTTAGGATTTCAAGAGGCAACCCCATACTTTGAGATATCGGATCAAGATCGATCGC
AAAATTAAGTGAAAGCTGATGAAATAATGAAAAATACAACATTTTAAGAAGGCATCGA
TAAAAAAGGCTAATTTATAGTCGATACGCGCAATAACAGATTAGTCGATAGCATTACAGGATTATTAAATCCG
AAAAGGATAATTAATTCTGGCTCACTATATTTACGATTGTCTATGTTAAGCGCCATTGAATAAACACAAATAT
GGTAGAACGAAGAAACTAGGGAAACTTTTCGGTCAGTAGAGAAGCATTGCGCTGACTG
CACCCTCGCACCCTCTCGCACCCGCACGCACCGCCATCGATAATTACCGACGCATCCCAAGCATGTGTCGGAG
ACGTTCCACAGCGACAATCAAACAATGATTGCAAAACTTTCCAAGGCTCAACCCGAAGCCCAACAAGTGCAAG
TCCACCGTATATACTGCGCATA
>103783989_2 DT667868.3
GCACGAGGGCGACCCTAACGGCAAGTGGTGGAACATGAAATATTGGAGCTTCTGGGCTTAGGTGAAAGACCGA
GACGTTCACAAGCACCGCCGTTGGATCGTTCGGCGCCTAGTTTCTTGTTGGATGTCTACAAGCAGCTGGCGGA
GGAACATGAGCAAGCTCGCCCAACTCGTAGTTCCGAAATGGCCCTTAGCGGTGAAGAG
CAGCACGCAATAGATGAAAGTGATTTAATAATGACATTCCAGAGTAAAAAACACCACTTGGGTGCATTACGAC
ATGGACATGGCCGTCACGTGTGGTTTGATGTCGCTAGCGCCCCAGGGGACGCCTCCTCCCTTCTCACAGCAGA
ACTGCGCGTACATCAGGCAGCTTCTCACACGGCTGACCCCACAGCGTTATACACTGTA
GTGGCACATCGAGTGATAAGCGTGGATAACTTGGGGGGTATAGAGATGGAACGTATTGCTGCAGTCAATACTA
GCGCCGGAGCTGAAGGTTGGCTGGAACTAAATGTGACAGCAGCACTAGCGACTTGGCTGAATGCACCAGTTGA
GAATCGCGGCTTCTTCATTACAATGCACCCGCATACACAACCAGAACGCCATGTAAAA
CCAGAAGAGATTGGACTGGACGAGACTCACGGTGGTAATTTGGAGGGAAAGCAACCTTTTATGGTAGCATTTT
TCAAGAATGGCCCTAAGCTTGGTAGCACCGACGCAGGAGCGCGTAAAAAACGTGAAGCACG
>103783993_2 DT667680.3
GCACGAGGCTTGCCCTAAGAGGCCATCTTGTTACGACTGATTCTTCAAATTTGGCATTAATCTTAGAAATTAT
ATAAAAAATACATTATTTATATGAATATTAGCACAAATTTATTATAATGAACTATATATTGATATATATATTT
AAAACAAACAAAAAAAATATGAAGAATCAGCCTAACCCCAAAAATAGACCACAGATAA
AACAGAACGTACAAATCCGTATCGACGAGCAGAGAACTGTCAACTGTCAAGTTCCTCCAGATTGACGTCTGCT
TTCTATCCTCAACGTATTCTAGTCTTTATCGCGACATATTTTTAATTATGTTTGCAACTAATTGTAATGTAAT
ATTATCGTAATTCGTATAAGTATACAGTTAAGGTCATATATTTAAAGTAAGTTTGGCT
GGGAGTGTAATATAAAGGTTAGTTCACCTCCTAGTTATGTTAATACCAAGTCCCGGTGCATTAAACGATCAAA
AAAATTGAAAAAAAATCCCAACTGAGAAAGCCTATTTGTAAAATTGTGACGCATTCCAAACAGTATTTAGTTT
ACTTACGCTTTTTATATGAATTCAGTTTTTTTTTAAATTTCGTATTAATATTGGTATT
ATAGCATAATT
>103783998_2 DT668600.3
GAATATGAACGAAAATGAAGATGAAATTACAACATTTTGTGATTCAACAGCATCGTTGTTGGACCGATGTCGT
TTACCAGTTCGAATGCAGGGTGGTGATGACTGGCCCCTCGCAAAAATTATAAGCATTAAGGAAATTCATGGGC
AAAGAGGATATTATGTACATTACGTACATTTTAACAAACGTCTTGATGAGTGGGTTCA
TGAATCATGGCTGGATACAAGAAAAGTACAATTTCCAAGACGAGATGGTGCAACGACCGGGGGTACTACAACC
CCAAAGAAAACTCTTTTTGTATCTGGAGGTGGCGTCATGCCTAATTTTCTTAATGATAGCGTCTGCAGTGTTA
GCGAGATTCAAAAGTCACTTAGCATTCCACCTAGAGAAAATAATGTGAACCATCATCA

AATTATACCTAAATTACCTGAGAAAATTACTCATATTGCACTCAATGGTTCCGCACAAAAATCTGTTCCTGTT
ATACCAGTAGCACCAGGGGAACCTAAGCAGTTGGTATCACGTCCAGCAAGTCCGACGCTTGTTAATGATTCGT
CGTCCCTCGTCAATGGCGGGGCAGTACTAGCAGCTGCACCTACAAAAAAAATGAATAG
GAAAAGAACAGCTAATTCTTTGGAAAATAGTGAATCTTTAACACCCCAACCGGACCCCCCAGA
>103784004_2 DV501268.2
AAAAAATAAATATTTTTATTGATAAAAATTAATTTTAACCATAAATTATACATACATATAAAAAACAAATACT
ATGCCACTTAAACATCATTCATATTATGAAGAACTCAAATGTAAAATAAATAGCAGTGTATAATATAAAAATA
GTAATTCCTGCATCGTTTAATATTAATAAATAATTAATTACTGGATATTTTTGATTGG
GCAAAATTCTGGGCAATTGTAATTGCCTCATGCAAGGCACGGGGATTATTACCTGATGCTTGTTTGGATTCAA
ATTTTCCCCCTCCCTTTCCTCCTATGGCGGGTACAATGGATTGAACCCATTCTGATGCTAACAATCCTCTGTC
AACTAATGGTTTGGGTACTGCCCCCAAACAAAATATTTTATTAAAATCTTCATCGACA
AAAAAAAACATAGCAGCTGAATCTGGTTTCAATTGTTTAACTTGTTTTAAAGCACCATCTAGGGCTTTTGTAT
TATTCTAAGCTTTTAACTCTGAAATAATTATTTCTGGACATTTTTCTTCCAAACACATTTCTTTTGCTTTTTC
AATTACTAAATTTATAGTCATTGCTTTAGCGGCTCTTTCTTTATCATCTATTTGTTTT
TTGAAAAATTTCACCATATTA
>103784010_2 DT668302.3
GCACGAGGTCCATGCTCCAGTGGATATAACAGAAATAATTTGTTATCCGATTACGTAAAGAGCAGACAGCACG
TAAGCATTTATTTATTGTTATGTATTTGTCGAACATAGTAATGCTAGGAAAAATTTAATGATCGATGAATGGT
GAAGAGCAACTGTTTTAAATATATTAAAATCTTTGATTTGAAGAGTCCTATATTATTA
TTTTATTGATATTACAACACGGTAAAGATGGATGAAACGATAAAAGATATATAGCCATAAGGTAACACAGTTT
ATTTATACTAACTTCTTGTTCCTATTTTGAAATATTTCATTAAAAAGCTTTGTGTGGTGTATTGTTAATTTCA
ATGTGTATATAATTTTGATTATATAACTAACAAAAAAATTACGCAAGTTTTTACATTT
TCAGCTACAAACGGAATAACATTTTAAAAATGTTAAAAATATTTAATTAATATTTATTAAAATAATTTTCTCT
CCCCTACAGAAGAACAACATAAATTATGATAACGTTAAGAATTTCGAAGTAGCTTTATTTAAACGTATGGCTT
TGTTGTCAAAGCGCTTGGGCGGTTCGCAAGAGCCGCCTATTTGTCTCACTACGTTCAA
ATTCTTATACGTTCCCTTAAATGTAGACTTTAATTTATTTATATTTAATTATCGCCGAAATGTAGTTTATAGA
TTTTTAAATATAATCAGATATATTGAGTCTCACTTTGCAACTTTATGGACAAGCATATTATATGGTAAAATTA
ACAAAATATACACCCCGTATGTAGACGTGGCTGGCCACATCCATACGTA
>103784040_2 DT668468.3
GCACGAGGCTAACCCACTGAGTGCACGTGCGCAAATAACAAATAAATGTAACATAAAAAATGACACAAATACA
GATACAAGAAGTTTATCCCAAAGACGACATATGTCCTATGATTGTAAACTTTCAAAATGGTTACATTACTGAG
AAATTTTCACAACAGGATTGTTTCCTTTTCGACAATTCTTCAAATAAAACCAAAACTA
TAGCTACTGAGCTAGACGGAGTGCTATATGCTGGAGATGAAGAAACAAATGAAATCGGTAAAACTTATATTTT
AGCAAGAAATAGAGTAACTGGTAAAGTACGTTTAATTGAAAGTGCAGTAGTTAATTGTAAACCTGTACTTAAT
ATGGAAATAAGTACAGTGCCATTGCTAGAAACTAGTACTTTAGAATTAAGTAGAAAAT
TTGGTTCTAAAAAGCAAAAACAACAGATGGAACAGAGGGAAAAATTAAAAGTCAATGTGGAAACTGTTACGGA
GCAAATGCAGAATGCCACTCAAGAAATATCAGAAGACCATTTAGATTTATCTATATATAATAAAACTAATTCT
GATGACTTCTACATACCACCTATAGACAGGACAGCAGATAATGTAGATGGTGTATATG
ATATAAACAAAATTTTAACAGAAGAACAGTATGATAAAATATATTCAGAGTTAGAAAACAAAGATTATGAATC
AGAATTGTTGCCTGTACTGAAAAATATAATATCAAAAAATAAATTATCACCAAAATTAACAGTTTTAGCTATG
TATGCAAATTCTTTGATCCATTATATAATACAATGGTAAAAGAATAGTCAAAAAAAGT
TTTACATATGTTTAC
>103784046_2 DT667267.3
GCACGAGGGTTTTCCTATAAATTGTTAAAAATTTAAATTCATATTTTTCCTTTTTTCACACTCACTCTATATT
ATTATTTGGATAAATAAGTATATTAAGAAACACCTAACAAATATTAAAATCTCCTATGTCATTTCTATTAAAG
ATTATAATAATAAATGTATCAGCGAAAAACTAACAATATCGATTGTCGCTGACTTACA
ATACAACAATTAAGAATATTAAAGTTAAATTAACATTTTAACATATACATTATACATAGATGGTGGCGGTTAT
ATGAGAAACACTCATTGTAAATCATCTTTTGCTTTGCTAACCGGTGATGCTGTCATCTCTCAACTTAGGGAAG
AGGTAAATTATTATTTATTAGCATGATATTGTTGTGTATGTTTCTTATATATGTATTG
TATACTCGGACTCATTGAGCACCTGTCATTGTTGAAAAATTTTGAGGTTCTTTATCAAAGTTGATATTAGTAG
TGACAATATATAGAAGTGTTGTGTTATTCTATTAATAAAAGGAGGCGACGCGCCTGCAGGATTCGTCTCTCAA
AAACATTTTATGTTCCATGGACACTCTGTGTAAGTTATATATGTATACTAGAAGTTGA

TTCCCTTAACTGACTCCCATCCAAAAAACCAGCAATAATCAATGTCATTTTTAAATGCATATACAAAGGTACT
TTGTATATAACAAAGGTGCATTAAAGGTTGATAAAGAATGTTTCAAAAATTTCAAGATCATTTTCTTCTTTCC
ATACTTCCATACACACAAACTTCATTCAATATTCAAAAATGATTCCGGGTATACAACA
TATTAT
>103784055_2 DT668603.3
TTAGAAATATTATAATTATTAGGTGTCACATGTAATTGCGAATCCTAACCTAGATTAGCGCGACCATGGTTAA
AAAGAAGAGACAAAGCGATCCAAGTGAGAATGGAGATGAATCTACGGAGTCGTGCGACGAAACTATCAAATCC
GCATGTCCTCATGTAGCTAAAGCTGTGGACCTTGCAAAGCTGAAAAAATCATTAAAAA
CTGGGGGTTTCGAGAAAGAATGTGCTGAGTGCAAAAAGATTTCTAAGAATGAAATCGACGACCCTAACTATGA
AGAAGACCTTACATTATGGATGTGCCTACGATGCGGAAGCCAACTATGTGGTCGGGCACGGAACAAACACGCC
CTGAACCACTTTAATACGCCACACTCTGACTGTCATGCTCTAACTGCTAATTCTACTA
CTTGGGAAATCTATTGTTACAATTGCAACAATGAAGTCACAGCTTCTAGCTCCAAAAAACTCCATGAGTGCAT
CGAATATTTGAAGAAGCAAGCCTCAAGCAATCCTAAATTACCTCCTATAGCTTTACCACTTGAAAGTCAAGGA
CCAATACTAGAACTAACAATGCCAGTAGAATCAATGTCACGAGATAAAGGATAAGATA
AGGCTATAGCTCTAAACTTGCCCAGAGTTCGTGGATTGACCAATTTAGGGAACACATGTTTCTTTATTCTGTG
>103784058_2 DT668744.3
GCACGAGGCTTATACCCTTATGATTCTTCTCTCTCCTTATGTTAACAACAAGCAACGTGTTCGATCTCATCAA
TTACTTTTCCCAAACGCTGTGGCTGTCGGTGGGGGCTTCAGTAGTAGGGATGCTGTGGTTGCGGCGAACCAAA
CCGAACATGGCGAGGCCGATCAAAGTCAACTTAATTATTCCCTACGTATTTCTCATAG
CTATCGGTTGTTTGGTTATCATACCCGCCATAACCAATCCCAAAGACACCGGCATTGGATTGGCCATACTCTT
CTCTGGAGTTCCTATTTACTATTTATGTGTTAAATGGCAGACGAAGCCCGACTGCTATAACGCTTTCTCCGGC
TGTTTACTGAGATTCATGCAAAAATTGTGTTCTTGTATTTATGTTGAATCATCTGAAA
AGATGTCGAATTGATTTTACGATTTCATTTATATTTATAAAGTTATGAGCTTTGATGTGTATAATGGACCAAT
AATATGTGTATCGTAAATGTGTCAGTTTATTAAAAAAACATGTTATTGTTGTTGTAAAGTTGATAATATAAGC
TTGTTTAGACAATATTATAAAAATATTTAATCTTAGCAAAATGTTTTATATGTAATAG
TAACAATATATATTAGTAACGTGCAAAGTTTTAATACAGTATGGATATATCATATTTTGATATAATATATTAA
CTAATACTATAACGAAATAAAAAATATGACCAATGACCATAGTAAAAATGGACTGAT
>103784060_2 DT668740.3
GCACGAGGCGGTAACGTAGATTTTGAGGTTACGTAGTCAGTGCAGTGTATTCGGTCGTTTTAAACGTGTGAAA
TCCTCACAAAGTGGAGATATATTCAAGAAAAAGGATCAAAATAGCTCTGAAAATAATAAATTTCAAGATCTTT
TACAACAATATAAAGATAAAATTACTTTTAGTTCATATTTAGAACATGAAAAATTAGA
ACTTGGTCACTTCAAATATTATATTAAAACAATCGCTAAAGCCAAACGGGAGAAAGATAAAGAGTTAGAACAA
AAAAGTTTACCTCCTTTACCCCTATCTTTAAAGTATTATGT
>103784066_2 DT667057.3
GCACGAGGGCTGCACCTTCTAGACGCGTTCCTGACAATGAAGCTGCAATTACATATGTGCAGTGCGATGGATT
GGCGGTGATGAAAATAGTAAAACACTGCCATGAAGAATCTTACAGCAACATGGAAGTTGCCCAGGGTGCGCTC
CTTGGGCTAGTTGTGGAGAACCGTCTAGAGATAACAAACTGCTTTCCATTCCCTAAGC
ATGATGACACAATGGATGAACAAGAGTACCAGCTGGACATGATGAGGACACTCCGAACAGTTAATGTTGATCA
TTTCCATGTTGGATGGTATCAAAGTGCGGATGTTGGCAACTTTTTGAGTCAATCTCTATTAGAGTCACAATAT
CGTTACCAGACATCAATTGAAAAGAGTGTTGTAGTTATATACGACACTAAGAAATCTG
CAAGGGGATTCCTGACTTTGAAGGCTTACCGTCTGACTCCTCAGGCAATAGCAATGTACAAAGAGAGAGATTA
CACACCAGAAGCTCTGCGTAACCTTAAGATTGGTTACGAGAGCCTCTTCATAGAAGTGCCGATAGTGATACGT
AACTCACCACTCACTAATATCATGATGTCTGAGCTGTCCGAGATGATACCTGAGGAGG
AGGGTTCGAAGTTTCTGGACCTCGGAACTGCTTCTGTGCTTGAAGGTCAACTACGCAGTCTGATGGAGCGTGT
AGACGAGCTCAACCAGGAGGCCATTGAATTCAACCGCTACCAACAGTTGGTTGTACGTCAGCAACAAGACAAG
TACCGATGGCTTATGACACGAGCTCAAGAAATACTATAAGAGCTGCTAAAGATG
>103784105_2 DT667337.3
GCACGAGGTGCTACTATACATGGAAAACTGAACGAAGCATTCACCCGCATAGAGCAGCTCCACGGTAAGCGCG
TGTTCGGTTCCTACTTCCGCGTGTCGTTCTACGGCGCGCGCTTCGGGGACCTCGACGGCGAGGAGTTCATTTA
CAAGGAACACGCTTTGACTAAATTACCTGAGATATTTAGTAGATTGGAGAACTTCTAC
GGCCAACGTTTTGGTACAGAAAACGTAGTGATAATCAAAGACTCTAACGTCGTAGACGTGAGTTCCCTAGATC
CGGATAAAGCATACATACAGATAACATACGTGGAGCCTTACTTCGAGCCACACGAGTTAAGAACCCGAATTAC
ACACTATGAACGGAATTATAATATTAAGCGTTTCATGTACGCGACGCCGTTCACGCTG

GGCGGGCGCGCACGGCGAGCTGGGCGAGCAGTGCAAGCGCAAGACGCTGCTCACCACCGCACACCACTTCC
CCTACGTCAAGACGCGCATACAGGTTGTACAAAGGACGCAGATAATTTTGACACCCATCGAAGTGGCCATAGA
GGACATACAGAAAAAGATTAATGAGCTAGCAGCAGCAACTAGTCAAGAGCCACCAGAT
CCCAAGATCCTGCAAATGGTACTCCAAGGATGCATCGGCACTACTGTTAATCAAGGACCTTTAGAATTAGCTC
AGGTGTTCCTGGCGCCGGTGGGTGGAGGGGACTCAGCCGCCTANCAGACTTACGAATAAACTAAGACTTGCAT
TCAAAGACTTTTCTAAAAAATGTCACGACGCACTCAAAAGAAACAGAAACCTGATAGC
AGCGAGCAGCGCGAGTACCAGCGCGAGCTGGAGC
>103784134_2 DT667148.3
GCACGAGGAAACGCCTTCAGAGTGTACGAAGTCTCCTTAACAACTATTATTAAAGTGGACTGGGATGATGGCG
TTAGGAAAGAGGAATACGTTTTATCGCTACCAAAAAATTTAAAAGTCGGTAATCAAATGCTCGGCGAGGACGG
AACCCCCGCGACCGTCGAACAAACCAAGGACACTAAACTAGTTTATTATGAAATGACT
AATAATGCTAACATTAATACACAGATGTACAGCGTGATTTTAATTATTAGTTCCGTATTATTTTTTCTTTAAA
TAATCTATTTTAGTAAGATTAACCAGCTTTTTTTATTTGTGTGTTTAATTTTTTTTTAAACTAAATTTTGATA
CTCAATAGTTGCGTTTTATATCATTTCGATTTTTTTATTCGTATGTATGCGATTTCGT
AATTTAATTTCAGGTGATGTATTAATAAATAAAAATAATTAGTTTAAGTGAAAGTGATGAATAATCTATTTTT
AAGTGCCTTCTTAACTTAGAAACTGCCAAAAAAGTTAAATAATAATGGCGATAAATAACTTTCTCTTTGTTAT
CTATCATGTTTATTTCCATGTACTTTCAACTATTTTCTTGCTTACTGTACTGTACTGG
AATTTACTAAGCTGGTGGATATAAATCCAGTTAAATACTATAAAAGACAAAAAAAATAAAACGATGAATATCA
CCAAATGGCGGTAACAGTCGTTGTATCTTGTTAATGCACTTGTCTGTAGTTCACAGACAGTTCCTAAATATTT
TACATTAATTTCAGTACAAACTAAGTTTTTGGAGAAA
>103784136_2 DT668696.3
GCACGAGGGCAATCCCAAGTGTGAGCAGTCTAATGATGTTGTCGTTGAAAAGAAGGAGTCTCGAGCTCTGCAT
AAAACAACTTCTATTTTCCTGAGGAACTTAGCACCCACTATAACTAAAGCTGAAGTTGAAGCTATGTGCAAAC
GCTACGGTGGATTTTTGCGCGTGGCGTTGGCAGACCCGCTGCCGGAGCGGCGCTGGTT
CCGCCGCGGCTGGGTCACTTTCCGCCGCGAGGTCAACATCAAGGATATATGCTGGAACCTTAACAATATACGG
TTGCGCGAGTGCGAGCTAGGTGCGATCGTCAACCGCGACCTGTAGCGGCGCATCCGCGCGGTGTCAGGCGTCA
CGCTGGAGCGCGCCGTGCTGCGTGCCGACGCGCGCCTCGCCGCCCGCCTCGCGCACCA
CCTCGACGCGCGCTCCCGCCTCTGGAGCGACGCGCACCACGCCCACGCGCACTCCGCCGACAACTTCAGCTTG
AATTCAAAAAATCCAGTGCTACACAAGATTACAGAACATCTCATAGAAGAGGCATCAACTGAAGAGGAGGAGT
TGTTAGGACTGGAGGCATCCTCAGAGTCTGTGAACCAAGATCAACCGGACCCGGAGTT
AGTGCGTGTTCTCGACCGCCTTGTGCTGTACTTGCGTATAGTTCACTCGGTAGATTACTACAATCACTGTGAA
TATCCCTATGAGGATGAAATGCCTAACCGCTGTGGAATTATGCATGCACGAGCTGGGGCACCTGCTAATAAGC
CCACTCCACAAGAATCCAAGATTATATCAAGACATTCCAAGGCAAAATGTCTGCTTTT
CTCCAAGATGTTA
>103784150_2 DT668837.3
ACTGCAACATTAATTTTTTTATGTCACTCACTTGGACAGGTCATAGTTTATTATGCTTCAGTTCCTTGAAGAG
TTCTTCCTAATGTCCGTTTAGAATATGAGATTTTTTTTACTATAATACAGGGTTTTTTGGATTCTTACATCGA
AGGTTATAAGCTACAAGCTCAAAGTTCTTGTGCTAAACAAAAAGTACAACAAAAAGGC
AATTGCGGAAAACGGCAAAATCTTGTAATTCCATTAAGTGTGCAAGGGGAGTCTCAGCGTAATGACTGGCCGT
GGCATGTTGCTTTTTTTTTATAAAACTGAGTCCGTTCCGAACCTAAGCCGTTATATGTGCGGTGGTACACTTAT
TTCCAGCTATTATGTTTTGACAGCTGCCCACTGTGTCTTTCGACGAAGATCAGCTATT
GCTAAACTAACAGTGGCTCTCGGCCAATATTATTTAACACATAGTATGGAACTAAGAGCAGACGAAAAAGAAG
TAAAAATATTATTTTAGCTCCACGCTGTATTCCAAACATAGCAGGGCACAGGGCAGAGGGAACCTGGGAAGCA
AGTGTTAAGAAACTTTCGTTCCTTACTTTTCGCCGGATCTTGAGCATTGAGAATTGCG
GAACTTAACACCGTACATTGCATGCACCAATGCTAGGAAATGAACATATTAAATATTTATTTTTCGCCAAGGG
GAAATCC
>103784171_2 DT667207.3
GCACGAGGATCTTCATTTGCAGCAATACAATTCTCTACAGTATTAGCTGGTGTAATGTCATGTTGTGTGCAAT
CAGTATTTAAAAAGAGTGTTATATCACTAGCGGGTGCTACTCAAGCATTGGGAGGTCTTTTCGGAGTCCTAGG
GCTTCTGCTCTACCCTTGGGGATGGGGTGCTCCAAGGGTCAAAAGAATGTGCGGTGAA
TATTCTGAACCTTATATACCTGGGGAATGTTCTATAGGCTGGGCTCTCTACGTAACAGCCACAGGTGTAGCAC
AAGTGTTTATAGCAAGTGCATTATCAAAGCCAGCTGATAAGGCGGCCAACTCAGATAAGGTCCAGTTCCAGAT
GGATGAAGGGAAACAACTTATATGTTTAGTTTAGTTAACACTATAAGAAAAAAGCTGA

TAATTTTGAAACACTTTGCACATATTATTCATCCGTTTAGGAGCTACGATGCCATAGATAGACGTTTATGGCG
AAATAGCTCATAAAGATACATAGATACGTCAAAATTATAACACCCTTTCTTCCGCGGTAGCTTAAAAATAAGA
ATTATGAGGATTTGGTATATTTTTTATTTAAAATTTATCTTCTATTTATCTATATTGA
GGCTATCATTAGTTTTGTTGGGGTTGGTTTTATTTGGAACGGGAGGCCTAATGCCTCAGTCATCATGAATAAA
AGTTGAGCATGAATAAAAGTTGATGCCTCTTTTATTATTTTAATAAAACTTAGTGTTAAAACACTATGTTCTT
GACATCCTGTCTGTACATTATGCACTTTGAATGATTTCTGCA
>103784174_2 DT668323.3
GTGCAATTTACATAATGGAATGATTTGTTTACGAAATCAAAAAATGTAAAAAAAAAATGTATAAAAAAAAAGT
TAAAACTTATTGCACTTTAAAATAACATAGACACTTAAAACTGTATTAATTGTGACGTAATATAAATGCCCAG
CCCATCCATATCCCATAGACAATGTATACATAAATATATATTTTTTTTTATTCAAATT
TATAATTTCGAGCGTATAGAGTAATTTTGCCGGCGGCGGTTTCTGTAATCTGTCAGTTTCATGGCGAGGCCCA
TGTTGCCTTGTATTTTATCTTGCCCTGAAAAGGCTTTTGGGATTTAGTTTCCAGAATCAATCACTACTCCTCA
TCGTTA
>103784177_2 DT667333.3
GCACGAGGCTACCCCTTAAAGATATTAAAATCCGTAATAAAAATATAAAGACACTTGAGAAATCATGAATAAT
ATATGAATTATATGTCTATATTAACTGTCTAGCTGTTAAAAATAATACTTTTTGTTACAATTTGGTGTTTTTC
ATTGAATTAAAAATGGATGGTACTTGGGTCCTGGTTTTACTTGCTTTAGTTATGCTCA
TCGGCTCCTACGTAGCTGGAAGTATTCCTTTAAATGTGAATATGTCGGAGGATAAACTCCAAAAAGTGACGGT
ATTCGGTGCTGGCCTTTTAGTCGGTACAGCGTTAGCATTTATAATACCTGAAGGAGTGAGAGCTCTTTTCTCA
GAAAGAACTGTTATGGTGACAAAGGATTATACTCCTGAACCGAGTGGACATGATGACC
TTCATTCAGTCATTGGAATTTCTTTAGTCTTAGGTTTTGTATTCATGTTATTGGTAGATCAAATGTCATCAAG
ATTCAACGACGCGAGTAATCCAACGGAGAAAAATATTACTGCAACCGTCGGTCTTGTAGTACACGCTGCTGCC
GATGGTATAGCCCTAGGCGCCGCAGCTACAACTTCTCATGCTGATGTAGAAATTATAG
TATTTCTAGCTATAATGCTTCATAAAGCGCCTGCAGCTTTCGGTCTTGTCACGTTTTTGATGCCCGCCGGTCT
TGAGAGGAATCGTATCCGAAAACACCTTTT
>103784180_2 DT668472.3
GCACGAGGAAAACCCGATGCTTTGTTATAATTACGACAGTTTACATAGTTAAATATGTCTGTTATTGGGCCGG
AATTTTACAACTTTATTAAAAAGTTAGAAGACAATGGGGATAAACTTATTCCTGCCACCCCAGTCGAACAGCC
CATTGAATTTGAATTTAATAATACTAGTTGCTATGTTTGCAATGGGTATTACGGTCCA
AGTTTTGGGGAACCAGTCTGCGTAACTTGCCATACGTTTTTGTTTCCGGATTTTCCATCGTATCTACCAAGTT
CATATTTTTGTAGTGAAAAAACTGATGATGGCGACTCTGGAAATGATGAACCTTCAGATTTAAATTATAGTTC
AGAGCGTAGACTAAATAAAGCCTGCCCATTACCAGCTTGGTGGAATTATAGGAATTCC
TTAGACAGTGAGACAAGCCCTGAGGATGAAACAAGCCGCACAAGTAACTCAGGTCCTGGTGCAAGTGGTAGTG
GCAGTGGTGGCAGTGTTTCTGGTCCTTCCTCGGCACCACGAGAAGGAATCGAAGACCTTGTTGGCCAAGGCCC
ACCACCTCAGCCTCCTAGTCTAGCAAGATCTCTTCAAGCACTTTCAACTCCAAGGCCA
CCTGACAATTTAGCACCTGGTCTGGTTGAACATTTGCCTTCCGAAGTTCTGCTGTCCATCTTCCGGTACCTGG
ACGACCTGTCGCTGTGCGCGTGCGCGCGCGTGTGCGAGCGCTGGCGGCGCCTGGTGGTGGCGCGCGT
>103784188_2 DT666961.3
TTAAAGTTCAAAATTTTGACTTAAAATTACAAATTAGTGGGACAAAGTATAAATTTCATACATTTATTTATAG
TTCAATAAGAACTTGAGGTGTTCAGTGAAACATGGTGTGAATAAAAATAATATATAACTAAAACAACAGAATG
GTTGTAAATTTAATAATAAACGGCAGCTTTTTGTTATTTTTTTGCGGAACTAATTATT
AATAATAGGTTAAAAGTTATTTTAACCTATTATTAATAATTCGGCTATTATTAATAATCGGCTTTGAATTTTA
ATATGGCAACATTAGGCGTGAAATATTAAAATCTAATATAATATGATAATGTTTTAGGAAATTGAATAATTTT
TTTCTTAAATTGATATTTGAATTCAAAGCCATTATAACAAAAGTACTGAGTTCCAATA
CAGCGTGATCGTAGTATAGAATCACTTATAGAGATTAAAATTAACATTCCACATTAGATAGATTTATAAAGAC
TTGGTCTTACATTGCTATATATTTTCAAAAAAAAAAGAGCTTTTTTCTACTTCTGCTCATAAGTAACTGTTAA
TTTATTTGAAAGATATTTGTTAATTATGAAATGTTGTTTAATAGCTATCTGTCAAGTA
ATTTAATTTAACAAGCAGTTTTTAGATGGGTCCTAATTTTTTAAATTGAATAATGCTTTAAATTAATTATTTT
TTTCAATTAAATTTTATAATTTCGCTTTTAAAAAGTAACTATTCTGTGTTTCAGTATTAGAAAAATCTCATTG
AAACATTTT
>103784194_2 DT667396.3
GCACGAGGACTTGCGTTCGAAAGTTTTTTATTGGAATTTAAAAACATTTGAAGCAGCCATGATAGTCGCAGAG
GCGTGGAAAGCCAACTATCTCTTCTGGATATTACCAGCGGTCTTCGCCCTCGGCCTACTCGCTGTTTTGGGGC
TGATCGGTCTTTTCTTCGTGGTACGTTTCTACGCCAAGTTCACGTGTGGCAGGTTTAG

AGAGAAGACCAGGATGGATGGGAAGACCGTGATTGTGACTGGATGTACTAGTGGAATCGGAAAGGAGACGGCG
AAGGAACTGGCCAAGAGAGGCGCTAGGGTTATCATGGCGTGTAGGAGTATGGAGAAAGCTGAGAAAGTGCAAG
ATGAAATTGTGCAGTCCACAAAGAACGAGAACGTACTAGTGAAGAAGTTGGACTTAAG
CTCCTTTTCGTCAATCCGGACCTTCGCCGAAGACATAATAAAGACTGAAAAGAAACTTGACGTATTAATCCAC
AACGCTGGTTACGCTGAAACCTTCAGAAAGAACATTTCGGAAGACGGCTTGGAACTTACTATGGCTACAAATC
ATTACGGCCCATTTCTTCTGACCCATCTTTTGATAGATTTACTTAAGAAAAGTGCACC
TTCACGTATAGTTATCGTGGCCTCTTCATTGTATCGTTTGGGATCAGTCAATTTGGACAATCCCAACCCAGTT
AACACAATGCCTGGATACTTGTACTACGTTTCAAAGGAAGCTAATATTCTCTTCACTAAGGAATTAGCGAGGC
GTTTGGAAGGCACTGGAGTGACTGCTAACTGCCTTCA
>103784200_2 DT667262.3
GCACGAGGCCACCCTTAAGGGAGACTTGACTATTACACCGGACTTCCAGTGGGATGAGAAGGTTCACGGCCAA
TCAGAAGCTTTCTGGATACTAGTAGAGGACGTGGACTCCGAAGTGATACTCCATCATGAATATTTCCTTCTAA
AACAGAAGTACTGTCGCGACGAGCATCATGTCAAGTTGTTCGTGCCTGTATTTGAACC
CCTGCCACCGCAGTACTTTTTACGGGTGGTTTCGGATAGATGGATAGCAGCTGAAACTCAACTCCCAGTGTCC
TTCAGACATTTAATCCTGCCAGAAAAAAATCTACCACCTATTGAGTTGCTAGATCTTCAACCTTTACCAATAT
CGGCGTTACGAAACGCAAAATATGAAGAACTATATGCTGATACATTCCCGCAGTTTAA
CCCTGTACAGACACAGGTTTTCAACGCAGTTTACAATTCGGATGACAATGTGTTCGTGGGAGCACCCTCAGGT
TCAGGGAAGTCGGTGATAGCTGAGTTATCTCTACTTCGCCTCTTATCAACTAATGCGGGGGCTCGAGCTGTCT
ACCTGTTGCCGAAGGATGCCCTCGCTGATATTGTATTTGCTGATTGGTACCATAAGTT
TGGTACTAGGTTCAATTTGAAGGTAGTACAATTGACCGGTGAGACGGCAACAGATCACAAG
>103784206_2 DT667793.3
GCACGAGGATTTTACTGAGATAATTTATTCAGCGGTCGTGATTGAGCGGCAAATAAACCTTGAATTAAAATTA
ATTTATATAAATTTCATCAAACAACGCTTAAGTTTTAATCTAAGGTAGTGTACTTTAAATGTTTGCGTAGAAC
GCCGGTCTCGGTACTCGATAGGCACTGATAAAATTAATCTAATCTTTTTCAAGTGTAT
TAGATCGTATTTCAAATTGATTTGTGTTGCAAAGTGTAATTTTTAACTTCAGTTTAAATTTAAACAGATATGA
AGAAAGTCGTGGTATTCGGTTCCACCGGTAATACCGGTCTTTGCGTTGTGGAAGCCGCTGTTAGTAAAGGTAT
AGCAGTGCGTGCCTTCCTCCGTGACCCAGCCAAGCTGCCCGAAGAGCTGAAATCCAAA
GTCGAAGTAATTAAAGGAGATGTTCTGGAGCCCGACTCCGTATCCAATGCCATTGAGGGCACAGACGGTGTAA
TCATAGCTTTAGGCACGAGGGAAAGCCTGGAACCCACATCAGATCTTTCTGAAGGCACCAAGAATATCATCGA
GGCTATGAGAGCGAAGAATGTCAATAAAGTTTCTGCTTGCCTATCTGCCTTTCTATTC
TACGATGCGGATAAAGTGCCACCAAGGTTCATTCCACTCACAAACGATCATAGGAGGATGTTTGAGGAATTGA
AGTCCAGCGGCTTGAATTGGGTGGCTGTGTTCCCACCGCATATATCTAATGATCCAAGTCGGGA
>103784217_2 DT667314.3
GCACGAGGGCTGACGGTTGCCACGGACATTTAACAAAGATGGTCTCGAAAAAGTTTAATTTGAGAGAAAATAG
TGAACCCCAGTCTTACGGCATAGGATTAAAAGAGCTATGGGAAGTTAAACCAGAAAATCACAAACCAGGTTTG
GTAGAACACACAATAGGGTGGCCATTAGACAAGAACACTTATGGCGGATCTTTCATAT
ATCATTTAAATGTTGAAAATGGCGAAGCTCCTCTAGTAGCGGTGGGCTTCGTCGTCGGCTTGGACTATGTTAA
CCCTTATTTGAGCCCATTTAGAGAATTCCAGAAGTTTAAAACTCATCCATATGTAAAATCTATGTTCGAAGGT
GGTACTCGAATAGCGTACGGAGCTCGAGCACTAGTAGAAGGCGGATGGCAGTGTCTTC
CTAACCCTGTGTTTCCGGGAGGTTGTCTAATAGGAGATTCAGCGGGATTCCTTAATGTACCTAGGATTAAAGG
CACCCATAATGCTATGAAGAGTGGTATGCTAGCAGCTGAGGCAGCCGTAGATCTAGTAATATCTGGTGAAGCA
ACCCACGACAATGGAGTCATACCAACATTATATGAAGATAAGCTAA
>103784226_2 DT668646.3
GCACGAGGAATTACTTTTAATATCATTTGTGCCTTTTAAGCTATATAACATATGTTTTTACTTTTTTCATATC
AAAGACTGTAACGTACCTTGATAATTTTGAGTATTTTTTTCTCTTTTATATGACTTAAAAGGCGCAAATAATG
GCTTGGCCTTAAATAAACATTTTTCTAGGCATATAGCTCTTGTAAAATTGATAGATTC
ATGTTTATTTTTTGGCCTAACCTGTAGATTATAAATAGTTGTTAGTTTATACGTGTGACTGATACGTCTACTT
TAGCGTTGCAAACGCGATCCTTTGCCAAATGTAGAAAATTATCATAAAAAATGTAAAATATACTACAGTAAAT
CCAAAAAAAAGGTCACAATTTTTAATGGAATGGATGAAATATTTATTATGAACTGAATA
TTTTATTGCAAACTTTGTATGGCTTTTGATTGTTAACTAACTATAATTACAAAAAAAAACAACACGGCAAAGG
ACTTTGTAAATATTAGTTTTGGAGTTAAGACGCGTGTGACGCACATATTAGTATTTTATCTGTATTTCTAAGC
AAGTGCCATTATAGAATAGAAGTTAATTTTAAGTATAATTTTCTTCTTATAATGGTTT

TGCTAAAGTAATCTCATTATATGAGACATGTGTAAAACTAGTTGTAGGTTATTTTTCAGTTGTTATTTTTTCC
TTTTTACAATCTATGGTAGATATTAAAAGCATGATATTATAAGATCTCTTTATGTTTTATATTTTTTATTGCC
TTTTATTAAATTTTCACATCATTTATTTAGGCGTATGTCATAA
>103784245_2 DT668327.3
GCACGAGGGTTTGCGTATTATTAAAATATTATTATTGAAATTTTATCAAATAAAACGAGATTGTTAAGTTTAA
TTAATTCATTATAAAGTTCGGTCATCACTTACGTAATACTCTATTTCTCACGACATTGAGAAATTTTTAATTG
TAAATACAATACGTCAGAGTATATAAGCTGATAAATATGAAAGTGCAGTATTGGATTT
AAAATACAAATTTCTATGAATTCCATTGACAATTTTAAAACACAACATACATAGCATAATAAATATAAGAAAT
GGAATATGACAACGATGATACTTGGCGAAGATCGAATTCGTTTAAGGAAATTTGCCATCCTATACCAAAGAAG
TCAACAACAGCAGCAGTGATCGTATTAGGTTTGGCAGTTTTAAGCTGTCTCCTTGGTT
ACTGTGTGTTAAGCGAAACTTTACCTTATGAATCTAAAACTTTACGTTTAGTTATTATTTTTTTTCGACACGG
TGCGCGAACTCCTGTATCTTCATATAAAAGTGATCCTTTTAAGAACTATCATTGGCCTGACGGGCTGGGCAGC
TTGACAAATTTAGGTAAACTCCAATTGTATGAATTAGGAAAGAAATATCGTAGCTATT
ATGCAAATTTTATCCCTGAGGATTACTATGAAAAAAATGTGTTTATA
>103784266_2 DT667007.3
GCACGAGGTGGGACCTTTGGGTGCACAAACTAATTAATTGTGAGGAAATCATATTTTTTATATAAAAGAATTT
ATGTAATTTTGTAGAGACTTAAATGTAGTTTAGAAACAAATATATTTGGCCACTGCTCTTATGTAAGAACTAG
TAAACTAGTAACGACACACTTTGGCAACTGTTACACAATGTCATAAATTGTTCATTCA
AAAACATAAATTTCAAATTTAATAACTTAATAAGAATTAGTGGAAAAATATGCCATTTCAGTATGAAATTTAA
TGAGAATATTTATCTATTCAAGACTGTGAGAAATATAACAACCATATTATCCAATATAGTTATCATATTTCTC
ACAGTACTCAGTATCACAAAAGTAATTTTTGTAATGTTTGAAACAAATTGGAAATACA
CTGTAGTATAAGATTATAGTTTAAATATAAACATTTTAAATTAAAATATTGATTCTGTAAACTAGATGTACCA
CTTACCTCATACTTGGGCAGGTATCGTGAAGACCCTTTAACGTGTCGCTTTCTTACAAAAAACAGTAAATCAT
CGCAGTCTATGGACTGCTCCTCTTGGAAAAGAAAGTGTCGGACGAACAGATCTGTCCA
GTAGGTCGTACCTTGCAGCACTACGAACCCCGAGTCTGTAACGACGGCACTCTGTCATTATGACATCTTCGTT
GTACTTATGGTATGTCATTTGTGTAGCGTGATGCTTTACTATTTCTATGTATATTGTATACATACATAANGGT
CTAAGAATCTAGGAAGTTTATATGTAAAGTGCCTTCTAAACTATTAATAAATCTTTAA
CTAATGTAATAAATGTTTAGATGT
>103784292_2 DT668143.3
GCACGAGGTTTTTTCTTTTTTTTTTCAAAATAATTCATTCCCTTTGTTAAGCAATAAATTAGTATCATATATG
TCATAAAGTTGTTACCTTTATGATACAAAATAAAAACAAATCTAATTTACTAAATAATATTTCAATAAAATAA
TATTTGAGGCTATCCTGCCGAAAAGAAGCGGGCACAAACATGCCGGACGCGACACATG
GTCTTGTGTTCAATTCCTACTCGAGAAAGGAAGAACGAAGTGTGTTAATACTAGGTTCCCTCTGCTTACACTG
ATATATGCGGTACACTTTATACTCTTTCTGATTTAGCCCAAATATTATGTTAAATATTTATGTAAATTACCTT
CAAAAGATGCCTACGATGTCGGACACTCAAAGGTTCAAGCGGATATAAAAAGCAAAGC
CTGCACATTAATTTTAAGGGCCTACGACGCTAACCGACTTCAACAAGGAATATTTCTGCATCAGGAGTAAAAG
TTGAGTGCACTACATGCAGCCTCGAGGATATCAACTGCATTTTTTTACAACTTATACAAATGGAAAGCATTAC
AAGTTCTGGTCGTATTCAAAAACATTTATTTATTTGCGCATATATTATGTAAATTTTA
AGATTTTATGTTATGATTTTTTCGTGTTTTGTTGGTGTAACGAGATTCAAGATAACCTACAGTGATAATTTTG
CAATATGTTATTCCCTTTTTTACATTTTTAGTCCTATTCTAAAGTTTCGAAAGAAAAATACTTTTTTTTACAA
AGTCTCATCATTCTATCATTTCA
>103784295_2 DT668476.3
GCACGAGGATGGTTCCTGAATGTATAACTACCGTATCAAATATCCGCGGATGTATTAAAATTGTTATATATAT
ATATATATATATATATATATACATACATATATATATATATATATATATATATATATATACATACATATATATA
TATATATATATATATATATACACATGTGTGCGTGGCTGCGTCCAGCACTATAAAGGTT
AGCGCAAATTATAAAAAAGCAGTGCATTGTCAACCGGCTATCGTTTCACTTGTCCTTCTCTTATTGCATACGT
AACAGTGGCGACGACGGGTTACCCACATATTCTCATTTTGGAAAAGAAAAGATGTCTTCCGTAAAATTTGGAA
TTTTAAATATCTTTGACCATAATGTACATACACGGAAAACATACACGGGTCGAATTTC
GCAATATTTTATTGCAAACGAATTGAATGAGCAGACAGATGCAACAGGAAGGAAGAGGAAAGCGATCCTTCTC
AGCGCACTCGCAGAGGGTACATATAAGCTGGCAGCAGACCTAGCGTTGCCTAAAGATATTGTGGATGTACCAC
TGGAACAGATTTTACGGCTACTGGATGATCACTTCACTCCCAAACGTGTGGCAACCAG
GCGAATCTCATGCGCAATGGGCGGCAAGACTACGCGGATTAACGACACACTGCGGGTTTCTAAACGTTGAAGA
CACTCTACGTGATAAATTTATAATGGGCATGC
>103784298_2 DT667842.3

GCACGAGGTTAAAGACTTTTTAGTAGCAATTATTTTCGGTTCCGTCGGATAGTGGGTTCGATACCCACTAGGG
GAATGATTTTTTTTTATTTTGCGCCGTTGAGTTCCGCCGCGGAACAAGCATTGCTTCTAGAATTTAGCGAAAA
TTCGGGAACGGAAGTAATTTAATGGGAACGAAGTGTTTTAACATTAGGGTCTCAGGTT
TCCTCTCGTATACAACGCGAAGCTAAAAAAAAGAATTTGTATACAGTTTTTTTTATTTGTTAAAATCGTCTTT
GTTCTAAGATAACGAATTATTAGGTCGTATGAAATCAAAGTTATCAAAAAATATAAAGAAATTATGGAAATTA
TTTAGAGGCGATCTCATTTTAAACACGATGGTTGGTTAATTAAATAATGATTTATTTT
TATCTTCTCGCTCTGCCAATAAGACAAAGAAAGGCGTTGAGTTCCACTACTCAACGCTCGATGTCTGGAAAAT
TGCGCGGTGCGTTGAGAACAGAGTGTCTTAACACTAGGTTCGCTTTGCCTACCCCGCTATATACGGGATACAG
CGTGAAGCTAAAGAAATACATAGTCTAACATCAAAATTAAAAAATTGTAAACAAACCA
AAATAATGGATTTTTGTTATTTGAATATTTTTTAACTTATTTTATTGCATTAATCTATGTATGTACTTTATCG
ATTTTGATATGACTTTGATTTATACGACTTTAATATTATGCCTAAATATATATATTTTTAAATACAATATAGA
AATAATTAAAAGTTTTCGTTGATTTAGTTAAAACCATTTTATTATATTTTTTCCAGAT
TTTGGTTTTGGTTTTCT
>103784301_2 DT668644.3
GCACGAGGGGCACCATGGACAAATTTGATGTGGCACAGGACAATTTCATTTTTCAATATGTTTTGGAATTGTC
GTGTTTAAATTAGTGACAAATAAATTAGGCTGTGTCCGATTAATTAACTTTTGTAATGAATAATGGTTACCAG
AAAATGACGGAAGAAGAATCTGTGGATATAGAAAAGGAGCTCGATGAGCACCTAACTA
AAGTTCAACCAAATCTTCAAGATGTTATCAAAAGAAGCTTTACCAATGTTGCTCTGCAGCAAACAAAAAATGG
AGAAAAACCAAAACCAGATACTTTAGAAGATACATCATACTTTGCCAAAAATACACAAGTGAACTTGTCAAGA
TTAGAATTAGTTAGGCCTCCAACTTTTCACATGCAATCAGTATCTTTGAACCTAAAAT
CAATGTTCCTATCATTACGCTGCTCACTTGGGGAAGTGAGTGTAAAAGGACTGTACAGTGCATTTAATGAAAA
CCTTTACAACCTCATACCTGTCATGGCTGAAGGACATGTTCTTATAACATTGTCAAATATGACAGCAGATGTG
AAAGTTGGCTTAGTTCTAGAGGATGATGCTTATTCCTTTATTAATCTTGGCATAGAAT
TTATTCATGATGAGGTACTTGTTAAGTTATCGTGGCCATCGCCCCAAAGAGGTGGGGGCTACGACTTTCTAAC
TACAGAGCAACTTGCCAAACATATAGATGACCTGCCACTTACAGCGGCGATATCTCTTCCCCTATTTGCATTG
CTTCAACAGAAATTGGAAAGGCATTTGAGT
>103784303_2 DV501279.2
AATTACTTAAACATTTTATTGGAAAAAAATTTCCTCCTATATTGAAAGGCAACGGGGTATTGTATATTTTTAA
AAAATGACAACCTTATAAAGTACCTAAATACTGTATTTAATGGACAGGTTATATATAGTGTATAGGAACCGGA
TGCCACGGATGGCACTCATTGTAATGTCATACAAATAAAAATCAAAATATATCCCGGG
AACAAGTACCCCCCCCCCCCCTGACCCATATGGAATGCCTTTTTTACCCTTAAAAAATAACATTATACCCATC
CTAAAACCATACAATTAAATATTATACCTTTTTTTTAAAATTTTACTATGAAAAAATTTGTAAACTGATTTAG
GCCAAAACTTAAACTATGATATATGAATCAATGAACGGGTTCCACCCACTCTGAACTC
TTATTAAAAAATTGAAAGTAACAAATAACGGTTTCCAACCTAACCCCTAATCTACAATATATAATTATAAATG
ATTATAAAAATAAATAAGGGCCGTCCCCCCCTCCCTCTACCGCGCGGACCCCTTGAACAACTAGTTGTAAACT
TTTTAGAAAATATGGGGTTTGACCCTTGAC
>103784324_2 DT667301.3
GCACGAGGTACGACTTTTAAAACAAAAACAAATTTTCTCTCGATTGTCAATAGTGGTTAAAGTTATTCTTGGC
TCGTAACTTTTTCAACTTTTTTCTATCTGATCTTCCTGAAGACTTGAACTTTAAACATATTTAAACAAATAGC
AAAAATAAGAAAATGGTTTACGTAAAAGGAGATGGCACCGTTGTGGAAAGCAACCCTT
TCAGTATTTTTGGGTGGTTTTGGAGCGCAGTCAACTTTGTTTATTTACTATTTCAGACCCTCATCAATCCAAA
TTACAGTAAGCATGGGGACAAATATGTGAGAGACTTCCTCCCCCCTGGTAGTGGTCCTCCAAAACCACCATCT
AGGAAGTTTGGTGGGTTCGGGCCTTCCAGCTCTGGACCGTCTCCTCCACCAATGGGAG
GTGGTGGATGTGGCTGCGGCTAAACTTATGTGCAGTAAATGTTAACATGCTATCTTCTTATTAATCTATTTAA
TATATTTTTATGTATTTGTGCTATTATATCTCAAATCGAATAAAGCAATATTTTTGGAGATCAGGCAACTTTG
TACACATATTATTTGACTATAACCAATATATTATCTTTTAAATAAACTGCTAGTATTA
GACAACTAAAAATAAATAAAATAAAGAACCCCATTTATCATTATTATGCTATAAACATTCCATAATCATTATT
TGATTATTCATAATTTTTGGGGTTCCATAACTCAAAAGCCA
>103784330_2 DT667484.3
GCACGAGGGTCTACTTTTGCATGGTATACAGCAGAACTTAACCCCGACAACTTTAAATTCATCACTAAATTTA
CCTCCAAATATTTCCATAATAAACAGCATGAACCAAAATATACAAACTAGTATCGCAAACACTAGGAAGGATG
TTGATGTACCACACAACCTAGTGGTGCACAGGATGCCCACAAACGGCATCGACATGGA

CATATACAGGACGCCGTTGCGCAGAAACGGCTTGGAACAGTACAGAACAGAAGTGGCATCACCGGTGAAAAAT
GAAAATTTTGTCAATAGAAGGGTCTCGGAGTACGATGATTATGAGCATACAGTGCCTCAGGATACACGCGGGT
TGGGGATGGAACCCAGGGGTTTAGAGCTCTCCGAGGCTCGCTTAGATGACAAGAGGAT
AGTGAAGCCGGCCCCCATTCAAGCGAGGCTCGTGTCGGTCGTGGACAACGACGTGAAGGAGGCGGTGCCGCGG
TTTTACGTTATACCGCAGAAGTCTCTGGAGAAGAAGACTGTTGTGGTGATAAAAAACGAGCCGACTGATGCTG
TTCAGATGGACCACAAACAGCATCAGTTACAACAAATAAATAACAACAACGAGCCAGA
GATCGATCATCGCAATATGGACAATGGTGATCATGTGAACAATGTCAACAGTGCAATGATAGTCACGCCGAAT
AAACTACTG
>103784362_2 DT667292.3
GCAGAGGTTACTTCCTTAAAGAGTCAAAATATTGGATAATACGCTTTCTAAATTAAATATTACTTGAATACGT
AATGGCAGATCCCAAATCTGAAAATCTTCCCGGCATAGCTTATGACCAACCTGATGTTTACGAAACCGGTGAT
TTACCAGAAGCTGATCAGCCAGAACCCTATGAAGAAGAAGAAAATGAATCAATTGAAC
AGCTACATCTTTCGGTTAAAGATTCATTTAATAAATTTAAAGGCAAATTTCTTACTGGAAACGTTGATTTTTC
GGATAGACTGAGCAGAAAGTCTCGAATCGGGTACAGAGC
>103784374_2 DT667425.3
GCACGAGGCGTAACTTGTAATAATCCATTCTATTTAGCTTCAACTGTAAAACATTTATAGCTGAATTTGCAC
TAAACACAACATACTGCGCTGCACATTTGACAATATCGGAAATGAGTAATAGTTATCAAAAGAAAATATAAAA
GGCAAAGGTCGATTTTGAATAATTAGTTTTGTTAAATATTGTCATTATTATTATATTT
AGTTGGCTTAGCTCAAATACTTATATTGTACCTTGGTGTCAAGCGTAAAAAAAATTCTATACTTTAATATAAA
TTTGACGCAGTTCGAACAAGTGTAGAAAATCGATTGTTTCCACATTCTTAGACAAAAAGACCTTATCAGTTAA
TTACAAAGAGTTAGGTAGTGTTATCAGAGCGTCAAAAGCAAAATGGGGAATAAATCGT
CTTTGTTGTTAAGAGAAGAAGAAATTGCACAAATTCAAGAAGAAACTGGCTTTACTCCAAACCAAATTGAACG
CTTATACTCACGTTTCACATCATTGGACAAAAATGACTGTGGAACACTATCAAGGGAAGATTTTTTGAGGATA
CCAGAATTAGCAATTAACCCTCTGAGTGAAAGGATTGTGCACTCATTTTTTGCTGAGA
GTCATGATGACAGAGTAAACTTTTTACAATTTATGAGAGTACTATCTCATTTTAGACCAATTAGAAAAAATAG
GGAAAACAGGCTTAACAGTCGAGAAGAAAAGTTAAGGTTTGCATTTTCCATGTATGATCTGGACAATGATGGC
AAATATCCAGAGATGAATTACTAGCAATTCTACACATGATGGTGGGG
>103784446_2 DT667200.3
GCACGAGGCGACCCCTTCAAGCAGCCTGCTCGGGGCCTACGATAGGACCTAGCCGCCTCGTTTAAATCATGCT
GTCCAAATTGTTTATGGTGTTTGTATTGTATTCAGCGACAGCTGCTGAATATTACAAAACGAAACCCAGTTTC
GTCCAGACCTGTCTTAAAAGTGATCCAGACTTCGATGATTGTTCTACAGAAGCCGTAC
AAAAGTTGTTCATAGCTTTGGGGCCAGGTCTTCCAGAAATAGATTTGCCTCCTATAGATCCTCTAAAAATTCC
AAAATTAAGAATACTCCAGGGCGAAGGGCCTGTTAATGTCAACGCTGCGCTTGATAACGTGTTGGTAACAGGT
TTTGGGAAAACTGAAGTATTATTGAGCCAGGTTCACAGTAAGTCATACGATTTTTATA
CGAGAGTTCGAGTTCCAAAAATAAGAATAGAAGGCACATATGACTTAAGAGGAAAAATCTTGTTAATTCCTTT
AGTTGGTAGCGGTTTATGTTGGTTTGAACCAAGTAATATGACCATAGACATCGTAAGCGACGTGAAACTTTAC
GAAAAAGACGGTCTACCATTCTTCAATGTTACTCAAGTTCACGTAAAATATAATATAG
GCGGGCTAAAATTACATATGAGTAATTTGTTCGACGGTATATCGTCGCTCGAGGATAGCACGAACGCTTATCT
GAATGCAAATTGGCGACCAGTATCCGAGTCTTTGCGGCCGATCTTATCAAGACTATAGAAGATATTTTACTGG
GCTTCATTCACAAATATTCC
>103784464_2 DT668912.3
TTTCCGCTTTAGGTTGTACTCTTTAAAGATTATTATGATTAGAAGTATTTACTTTTAATAAATAAACAATATT
TATTTTAGTGGAAGTGAGAATTATCAAATGAATTGGTTTTTAGCTACGTGAAATGTGAAAATCGCTATTTCAT
AGAATTGTCGTTTTTATATCTTAATTACAATTTTGCAACATTAGGTTTTCAACATATT
AATCAAGAAAGGAAATTAATATTACATACTGTGAGATTTATTATTTGCTACTAAGTGCTAATAATAGAGACAA
GATGAATCAACTGCCTGCTCAACCTACTGGTATACAGAGTACAGCCGTGAGCTGTCCCTGTTCGAAATGAAAA
AGGTGAAATTTCTATGCAAAAAGTCAAAGTGCAAAGGTATATATCGGGAAAGAAACCT
GAATATGCTCAAGGTGTTTCGTCTTCTGAAGAATCAGACACTGAGGACTTCATAGAACAACAGAGACCTGAAC
GTAAACATCAAATAGCACAAATAATCAGCCGTAAAGATGATCATCAAAGTGAATCTGAGACTGAAATCGACGA
TCCACGTTTACGACGTTTACGTGTGGCTGCTCACTCTCCGCCACGGCGAGCGGAACAC
AAACCTGGAAATATAGATGCAGAGCCTGAACAAGAATCGGAATCTAGCGAAAATGATGCGCGGCACAGTTCTG
AGAGTGA
>103784506_2 DT667519.3

GCACGAGGCCCAGCTTTACCAGGACAGCCAGGTCAACCCGGACAACCAGGACAGCCAGGACAACAAGGTGGTC
CTGGTGAACAAGGCCTACCAGGATATCCAGGACAGCCAGGTCAACCCGGATACCCAGGACAGCCAGGTCAACC
CGGACAACCAGGCAAGCCAGGCCAACCAGGACAGTCAGGACAGCCAGGGCAACCCGGA
CAACCAGGCAAACCAGGTCAACAAGGTCAACCTGGACAGCCAGGATACCCAGGACAGCCAGGCCAGCCAGGGC
AACAAGGACAGCCAGGTCAACCCGGACATCCAGGCAAATCTTTTTTACAAGGTCAACCTGGACAGCCAGGATA
CCCAGGACAACCAGGACAGCCAGGTCAACCCGGATACCCAGGACAGCCAGGTCAACCC
GGATACCCAGGACAGCAAGGCCAGCCAAGACGACCTGGACAGCCAGGATACCCAGGACCACCAGGACAGCCAG
GTCGACCCGGACAACCAGGACAG
>103784512_2 DT667860.3
GCACGAGGACCAAATCGTCGATCCTAAATTGTATAGGTGTAAAATTTGCTCAATGTCATTCGACCACAAAGGT
GTTTATTTGACACACATAAAAAATCACGACCTTATAAAGCCGTTTATTTGTGACACTTGCGGGAAAGGGTTTT
GTACACAATACACGCTGAACCGCCATATTGACTCGCACAATGGTGTAAAGAAATTTTT
GTGTAAGCTGTGCAATATGTCACTGTCAACTAAGAGGTTGTTGAATCGTCATTTAGAAAAAATACACGATCCT
AACAGACCAATCAAAAAGAAAATAACATGCGAAATATGCAAAATGGCGGTCACGGATATGGAGAAACATAAAA
AAAGTCATACAAATAAACGTTTTCAATGCTCATTTTGTACTAGATCATATTCAGAAAG
GAATACATTGCATCGTCATATAAAACATAATCATACCGGTATTACGCATTCTTGTACTTTGTGTGAAAAATTC
TACGTACAGAAGGGTAGTTTGAGAAATCATATGAAAAAGGATCATAAAGATAGTGTTAAAATGAAAGAAGAAT
AATCTAGGATTAAAAGCAACTTCACGCTCTTTCCCGCAAGGCTAGATAGAGGAATTTT
TGTGTTTAGACACCCTGTTCCCCACGCGCCGCCCAAAGTTCTAGAAATTGTGTGTTGAGTGGCGGAATTAAAC
GCCGCGCTTTATCTTTCGGAGCTTGTGACTG
>103784536_2 DT667428.3
GCACGAGGTATTGACTTATCTCTACTGCATCGAAATGTGAAGTTCTTTCTAGAATAAAATGTTGCTTTTCTTT
ATTTAATCCAAATTCTTCGTTAACATACTCGAATTACGTTGAATAAACATCTAGCCTTTTCAGTTCAATAGTC
ATGACTAATGTATTCGGCTTGATAGTGTCGGGGCGACTTGTGCAGACAGACTTTACGC
CTGTTTCTGAGACTAGTCTGATCACGACGATACTAGACGTGGACTCTATTAACCACGCGGTGGTGTTTTTAAC
GGGCACGACGCCGCTGCCGGTGGGCACGGTGGCGGTGGTCTACTGGAGCTGGCCCGACCCCAACGCGCCGCCG
AACTGGCAACCCTTAGGACATATATCAAACGCTAAGCCCTCTGCCATATTTAAGATCT
CTAATTTAAAGAAATTACATGAATTATCTATAGGTGAAAATAAATTTATGAGTGCCTTTGGCCAACAACAAAT
TTGTAACAATGCTCAAATTGGAATTTCTATAGAACCAGAGGCAAATGTTCAACTTCTACCTAATTCTGAAGCA
CAACAAGTAAATTCATATGTGACATTTGCCCAAAAAATGTTAGAGAATTTAGTTAATT
TTGTAGCATCATTCTCTGTAACACAGGATCAGATGACTCCTACTCCAGGAGTGTCATACATACCTCTGAACAC
TCTCCACACATGGTACCAGAACTTTGAGAGACGGTTGCAACAAAACCCCAATTTCTGG
>103784539_2 DT667408.3
GCACGAGGCGAAGCCTGTGAAGGTGTCGCGTGAAGTAAAAGTTGGCAGTTTTTACGAGAATACTTACGAGAGA
GCTGTGTTTGTACTACTGCGGTAGTGAAGATGGCGCTATTCATAATAGGAATAGCTATAATATTGGGGACCGT
GAATTCTGAAAATTCAACATTATCTGACATATCAGAAGACATCTGCGAACCTCTAGAT
ATCGGGCCATGGAGCTTAGCGCTGGAGGACTGGGCCGTCGCCACCACATCCGGCAGACACGGTCGAATCATGG
CTTTACCGCCATCAAAAGGTTATTACGTAACAGAGCGTATTTACACGCCTTACTTGACACCTCCCCCACCTCC
TCCTATACCCCACTTCACCCCCATGCATTACAGCCCAAACCAAAACAACAAAACCTAC
ACTCTAAATCCAGACCAAAAAATATCAAAACTAGGTCTTGCACCAAATCAAAATCTGAAACCTAATAAGCCAA
TGTCCCAAAACGAGAAGCAGTCCTTGAACTCCCAGGAAGTAAATACTGTGAACCAAGGCCGTATCTCGCAGTA
CCCGGGGCAAGTGTACCAAGGATATGGGAACTCACCTCACAGCTACAAGGAGTGGGAG
ACACCACAGTACACTCCACCAGGTGTGAGCAAGATCGTGAATAGACCTCCCAAGCCTCATCAAGACAAGTACA
AGCCTAGCTATCCAAGTCCACCACGCCCAGCATCAGACCGTGTAAACGAGACCTCATCTCCTCAGAAGAAGTA
TCAGAAACTGATCTGTACTT
>103784552_2 DT668901.3
GCACGAGGGTCGACTCTGTATACTATCGATACTTATTCAAACATTTATTTTGTTTAATTAAAAATTGGTTAAT
TATTAAAAAAAGCTCTTTTCATTGTACGTGCGTTGCAACTATATTGTTTATATATCCTCGTATGGGCCAATTT
TGTTCAGCTTAACGGGCTTTTCCAAGTGTACTGAAAGTGAAGTGTGTATTTGAATGTC
ATAACTGAAGTGACACGCCAAAGTGATCTGTGAGGTGCGCACGGGCCGCGTGGCGCGAGGGTGTGATGCGAGG
GGCGCGCGGCGGCGAGTAGCCGCGACAATGCCCGGCCCGCGCACCGCCCACGCCTGTCTCCTGCTCTCCCTGC
TGGCCATGGTCTCCGCGGTTGTGGCTGATGGTTCCGTACAAGGCGTTATCAAAGGTCA

ACGAGCACCCTGTTCGAACGAAGGTGAAACTGTGAGACTTCAGGACGAAAGACTAGAACAAGATTGGAGTAGT
TGCTTTCGTTGTATCTGTAAGAATGGCTTTGTAGAATGTCGGAAAGGTGCAGAAGAATGTAGTTTGATGGATG
ACTGCGCGGTGATCATGCCTCGTGAGGGTTGCTGTGCACGCTGTAAAGGATGCTGGTA
CAATGGTACTGAATACCTCTCCCACACTGAATGGAACGAAGATGGGCGACTGCTGCGCTGCGAGGCCGGCGTC
GTCACTATATCACGACCAGAATGCTACGCGCCGTGCGACAGGCCCAACATCAAAGGCATACGCACTACAATTG
TCCAGTCTGTGAAGAGTGCGTCATCA
>103784589_2 DT667232.3
GCACGAGGACCTACCCTAGTTCGCAGTAAAATTGTTTTCGTATAATACCTACTCATCATAGAGCCGTTTCATA
TTTGTATCGGTTGGTTATCACCTACTTATTTTGTTGTAATCATCTCCGACTCAGGGTTAAATATTTAATACAA
TCAGCGCTGATTTGAAAGGCTACGTAAGCTGATGTACAAAACTTGTTGATACATGTCT
CACCGGAAGACGAATCGTACCTGCCTTTGAAAGAGGAAAGATTTATATCCCTTAAGTATTTCTTTTGCAATTA
TCTCTGAGTCAGTGTCCGTTTCAATATTACATTCATGTCCCTATAAGTTTCATAGGGTGGAGGGGTTCTTTCA
TACCGTATATAAGCTGCACTGGAAGCTGTTCTGATATGAAATAATTGTTGTTCGTGAT
ACGTGAAAGTGCTCTTCCGATACGAAACAATGGCGGTGAGTATTATTTTTAGGTCATTTACTAATTTATTTAT
GTTGTTGCCCAGTAAATATATTGTGTACTTTTTCATTATTGCAGCTGTCAAGTAACAACTCGTTTGATTGTAC
ATTCAAAAACTATTACTATTCTAATCTAAGCGGAGATGAGGCTGAAGAGGACGCCCCT
ATGAAAATACAGAAACCCAAACAATAGAGAGCCTGCAATGATATTAGTGGTTTCTTTGAGAAGCCAAGAAGCA
AAAGAAAATAATATTATCATCATGTGTTGCTCAATCGAATCCTGAGGGAACATCGTATATTTTCTACAACTCG
GACGATGGTTCGCAGGCAGGCATTTCATAAAAATCG
>103784595_2 DT668436.3
GCACGAGGGGCGCACTTTCGCATGCGCCAACATTACCCGCCAACGGCCGTGAGTTCGTACTACACAGATAAAA
GTAAAAAAAAAATCAAAATTAATTTAATTTTAAAAAATATATTCTAAAGTGTTAGAAATAACTTTCTCTAAAT
TCAGAAAAAATATTTATACAGCAAAAAATATATTTCATAAATTTGGCAAACGATAATA
ATTTCTTATTATTAAAGTAAAGTATATTAAAAAAAAATATTAATATTTTGAAATTATTGGATATGTTTTAATT
AAGTGGTAGTGTCGTGATGTGGTCATCATGTGGGTGGTGGGACTGCTGTTGTTGGTGAGCGAGGTCCTCGCGT
ACAATGTCAGCTTTGTAGCTTTTAATAATGTGTGGACCGTACATTACCCACAACGCAA
ATCCAACGTGCGCCGTCAGCGCACCATATCACCCGCCACGCCTTTCCCCTGCCAGGATTCAGTTCCCTGGGGC
CGCAGCCGACAGGTTCCCACGTCTGTGCACAAGCTGAGACCAGGGGATATAGATGTGGTTGCTGCCATAGGAG
ACTCACTGGTGGCAGGTAGCGGCGCCTTAGAAGAGTTCGCGCTTGGAGCCATCGTTGA
GTACCGAGGCGTCTCTTGGTGTGCAGGCGGTGACAGTTCATGGCGGGAGTTCTTAACTTTACCGAATATTCTG
AAGGAATACAATCCGAATTTACGAGGCTACTCCACCGGCACCGGCGAGTGGCTTTCGAAAAACTCAAGGCTGA
ATGTCGCGTTCCCTGTGGCTTCAGATCAAGATGCTTATAACAAGCG
>103784639_2 DT667829.3
GCACGAGGGCCGACCATGTGAGGGTGCCATTTGTTAAATAACGTTGTGATTTTAGCAATTGTTTTTGCCATCT
ACATAAATAAGTCACTGGAAAAAGAATCGAACGTAGAACTATCGCAATATCTTAATTTGTTATATTTTGTTAT
AAATAGTATAAACTTATATCTATTTATATCATTTAATGTGTTTTTTGTACATATAATT
ATTATATGTATTTATTCTTCTAGAAAATAAAATTTTAGTTTAGAACTTTAAACAATAGAAGCAATGTTATCTA
CAGTTTGCTATTTGAATATTGTAAAAGCTTATACATATTAGATTAGTTGCTTATGCTTCATTCAAAATAAGTA
AAGGGATTGCTTCATAAAATATGTTGTACTAATATATGCACCGTTAATTATAATGCAA
TTATTAACATTTACAAAAAATATGCATACAAATAGTAATATTTACAACATATAGTGACTAACTAACGCACCAA
CATATTGCGCCTTTATTTCCAGGACGCAGTACCGATTGATCCTGAAACACCAACGGTGCGTTTTATATATATA
GTTATTTCATAACAATGTAATGATTAACAATACTATATGTTAGATCATATAATATTAT
TAATTGTTATATTATTTTACTAAAATGTAATAGCACTTATCGCAAATAACCTATAGAATTCTCTAAAATGCTT
TTTTTAGTTTAATTTATATCTTCATGGATATTCACTTTTGAGTTGTGAATTTTTTATCACAGGACATATCGTC
GTTGCACGTCTATTGATTCTCTTCACCTATTGGCTTATACCAGATCTCACAAAATCGG
AATTATTCAATTTAAAACATTTTTTTTTGCACCAAACAATACCCGCGGGCAGTA
>103784651_2 DT668691.3
GCACGAGGTTTTTCCATATATTGATAAAAATTTACATCTCAATGAAAATGTTTGATTTGTTGTAAATTTCAGA
TTGGTCATTTAAGAATGTCACATCCGGAATTTCTATATAAAGTGAGCGGAAGATGCAAACTATGTTTTGTTAT
ATTTTATTACATCCTCTCAACATTGACCAATACTTTGTAATAACTCTAAAACAACGAC
ATGATAGACTAATTATATCAAACATTCGATAACTTTATACATTATGTTATTTATATTTATTATTAATATAAGA
ATTCCATGACAAAGCATTTATTTTATAAGTGGGGCCTAATTTTATGTGACTTTCGAAAATTTTTGACATGATT
TTGTATTTTTTTTCTTAAATTATTAAAAATCTTGTATTTTGGTGTCTCTCAAATTTCT

TCAAAACGCAATAAAAAAATAATAATAAAAGGATGTAGCGTTTATTAAATAGTGAACTTTAATATTTAGGATT
AACATTTTGTAAAATACTTTGTGATATTTTGAATATCGTGATACCGGGACTAGACGTGAACGCGAATCACGAA
ATGGCTAAAAAGCACAATTTTATTCGATTTACTCCAGCTTGTGACTCATATCGTCCAC
AAAGTTGCCAAAGTAGCCTGTAATTACAATTTGCTTAAATTTTCTTAAGAAATGAAGTGGGAAACGCTCAAAA
TCTGTGTGAAGTGCACGCACGTGTGCTTGTAGACATCGGGCTATATACATTACTGTATATTATGTATACGGCT
TTTATGGCAATATAAAAATC
>103784661_2 DT667681.3
GCACGAGGGTCAAACTGATGAAGGTGAAGATGATCTCAAGAAAGGTACTCAACTCTTAGAAATATATGCTCTA
GAGATACAAATGTACACAGCACAAAAAAATAATAAGAAACTTAAGGCGTTATATGAACAATCATTGCACATAA
AATCTGCAATTCCACATCCACTTATAATGGGTGTTATAAGAGAGTGTGGCGGCAAGAT
GCACTTGCGTGAAGGCGAATTTGAGAAAGCTCATACAGATTTCTTCGAAGCGTTTAAAAATTATGACGAATCT
GGGAGTCCCCGACGCACCACTTGCCTCAAATATCTTGTGTTGGCCAATATGTTAATGAAATCGGGCATAAATC
CATTCGATTCTCAAGAGGCAAAACCCTATAAGAACGATCCAGAAATCCTTGCTATGAC
GAACCTAGTAATGGCGTATCAAAACAACGATATCAACGATTTTGAAGCTATATTGAAACACAATCGTAATAAC
ATAATGGACGATCCATTTATAAGAGAGCACATCGAGGATTTACTAAGAAATATTCGTACCCAAGTCCTCATAA
AGTTAATAGGTCCTTATACAAGGATACACATACCATTCATTTCCAAAGAGTTGAATAT
TGATGAAAAGGAAGTGGAAAATTTGCTTGTTACTTGTATTCTTGATAATACAATCAGCGGTCGCATCGACCAA
GTGAACTCTGTATTAGAACTGGCCAGTGGAGCGCGGGGTGCTGCAAGGTACCTAGCGTTGGAAAAATGGACTG
CACAGCTATCTTCCTTACATCT
>103784719_2 DT667060.3
GCACGAGGTTACCCCTCAGTTGACACATCTAAAGGTGCTGGAGCAGTTGCCGGGCACTTTAGCACGATTAGAA
GCGGATGTAGCGGCTTTAAGAAAGGCTGTGGAAGGTGATAGGAGATTGATGGATCAGGTGTCAAGAGGTTGGC
GATGGCCGTGGCAGGAACTCAGCCCACCGACACTAGCACTTATACTAATATGGCCGTT
CATAGCGTTTCGTATCGCTAGTAGATTACAGAGGAAGAAAACATAGTGGTTTTAGCAGCGCTAGGAGTTATCA
ACAATGAAATGTTATCTGTTTTACACTTACGTAGGTTTTATATGTAATTTTGTTGTGGCAACATTGATCGTTG
TAAACGTCAAAATAAAATGTAGATTTCAAATATATTATGTGTACGTACACTTTTATTG
TAACTACTTACTTATTATTTATTATGTATTGAAGGCCATAAAACATGACATTTTATTTATTTATACAACATTT
GCTTTTTCCTAAATGCAATATGTAATTTTGTTATAAATACCTTTGACAGTTGTCATTTAAATAAATTTAAGTG
TTGCTATATACAATAATATACAAAATATAGAATAATATACAAAATATAGAATAATATT
CAAAATAATGAAACGTTTTTGTTTTTCATTTTTATGTGCAATTATTTAGTTTTTTGGGTTATCGGTTATTTAC
ATAATGAAATGATTGGCTCAATTTTTTTATTTTTATAT
>103784732_2 DT667723.3
GCACGAGGAGGACCTTGAGGTATTGAAGGAGAAAAAGGAGATAGAGGGCTCCCAGGGCCTTCTATTAATATTA
AAGGTGACAAAGGAGACATTGGACAACCGGGTATTGCTGGTATTAAAGGACGGAAAGGAGAAAGAGGTAGAGA
TGGATTCCAGGGTATAACAGGAGAAAAAGGAGACAGAGGTTTTACTGGCCAAAAAGGG
GACATGGGTAGAGTGGGATTGACTGGAGCAAAAGGTGACAGTGGTCCTGCAGGTCCAGTAGGCATATCTGGTA
TGACAGTAGTAGGGGAAAAGGGCTTGCCTGGGTTACCAGGATTGCACGGTAGACCAGGTATATATGGTGCTCC
TGGAGAAAAGGGAGAGCAAGGGCTACCTGGACTTCCAGGGCAAATAGGTACACCAGGA
ATACCGGGTCCTCCAGGACCTAGAGGAGAAAAAGGTGAACAAGGGCATGAAGGTATCAATGGTCCACCTGGTT
TTGACGGTACACCAGGAGCAGCCGGACCTCCTGGAATATCTGGGAAAACAGGCCCTAAGGGAGACAAAGGTGC
AATTGGGTTCGGATTACCTGGAATTAAAGGTGAGCAAGGACCTCCAGGAATTCCTGGA
TTACAAGGTCAAAAAGGCAATAAAGGGGAACCTGGTCGAAATGGTGCATTTGGGGAACCAGGGCAAAAGGGAT
ATGCTGGTGAAAAAGGTGATAGAGGATACAAGGTAGAACCGGTCAAACAGGAATTATGGGCCCAAAAGGTGAT
AAG
>103784761_2 DT667722.3
GCACGAGGCAGCTCTTCTACCCAATTTATATTCCAGAAAACCGCATTCCTGCCTTCTATACCTAGCAGCAGTA
ATGGCGGACGCACTATCACCGCCGGACACGGTTCAACCGCAAAATATCCAAATACGCAGGGAATTGAAGGAGT
TGTTACACGCGTTGTTGCCGAGAGCGCTGCAACTGTTGATGATGCAAGATGGTCTAAA
GGATAATCCGGATACGGTCGATGATCTGTTCCGATTGTGTGTTCGCTACCTGCAACGGAGTCCCCAAGCCCTT
TTGAGCGCGGAGGCGGAGGGCGCCAGCGGAGCCCTGCATCATGTGCTTCAGGTGGCGAGCGCCGCGTGCACGC
TGGACCACCGTGACGCCAACTGCTCCCTCATGAAGTTCCTCTACGACTTCACCAGACT
CGCCAACTCTAGTAAGACCGACGATCAGCTGCTTAAAGGTTTGTGTTTTTTTTCTATTGTATTACTGCAAACG
CGACGGTTTCTCGCTTTGGTTAGTCTTCTAATGTGTATAGTGGTTTTGAAGACAAAACAATGAGTTACGCTAC
TTAACACGTTTAAATATAAAATAATAGATTTACATTATTAACTTATTTTAATCTAATA

ACATAGCTGTGGTATGTGGATATCCCACTTCTGTTGATAAAAGGTATAGGTGTTGTCTTTTGAT
>103784800_2 DT667873.3
GCACGAGGCTGGACGCCTTACAAAAGAGAAAACATACACTGAAAAAATTGATAGAAGAAAAGACCAAGAAGAA
TGAGGAAAAGAAAACTAACTCACAAGACATAGAAGATACTCAAAGTATAGATGAAACGAAAGTAACAGATACC
AAAATCGCTGAATCAAATATTAATAAATCAAATAACGATGAATCAGAAAATATGGAAG
TTAACAAATCAGTTGAAGAGAAGCATGAAGAAAAAGAAACTGATCCTGACAAGGATGATATGAGAGACAGTGA
TGATGGCGCCAACGCTTCCGGAGTAGACGAACTGAATTTACTAGAAAAATTATATTCTGAAAACGAAATTAAT
TCTTCACAAGAGTCCTCTGACTCTGATGCATCTATTATAACATTAGACACTATTGAGT
CAGACAGTGACACTGAAAACAGTCAGTCACAGCCTATTGACGTCATCAGCATTGAAAATTCAAGCTACTCTGA
GAATGAATCTTCTATGGATTGTCATGACCAGGACTCAATTAAAAATAGCACAGAAAAAACGAATAAAGAAATA
GTCGAAAATATACTTCTGGCGTCATCTGATGACGAAGAAACGTCATGCGCTGTAGAAA
ATATTGTTGACTGTATAAATTTAAAGGACGATAATATATCCATAACAGACACTACACTAGATGGTGAGAGTGT
TGAGAAACAAAATGTCGATAGTGTCATAGAGAATAATGTTACACAGATAAGTGAA
>103784829_2 DT668496.3
GNACGAGGCTTTCCCCTTAAAGGTGAATAAACTGCCTTCACTTTAAAATTTAAACACAAAAATGTAGGTGAAT
GTTCGCTTATTTTTTTTTTTCTTTGCATTTTTTTATGTTTATAAAGTGACAATGTTCGGCGGAGTGTTTGTGT
GCTGATTGTTTTGTTTAAATTAATGGATTTTGTATATCAAGTTCTTCATGTATTTCAT
ATAGGAAGATGAAGCCATTTATAAAACAGGCGTTCGTGGTATCTGGCGCGGCGCTAAACATCGTAGGCCATGG
TTGCGCTCATGGTTTCCCTGCTGTTCTATTCGCCCAGATCAAAAGCGACGGTGGACCGGTCACACTCACTGAT
CATGACACTTCTTGGATTGCATCAGCAGTTGGTGTGATGGGCATCGTCGGCAACTTCA
TATCGCCAGTCCTCATGACACGCTTTGGCCGACAAAAGGCGCACCTTATGTGCACGATACCCGCACTACTGGG
ATGGATCGTCTTCGTTTTAGGAAACACCGTCCCACTGTTCCTCTTAGCCCGACTTCTACATGGACTTGCTCTA
GGTTTGCGTACGCCGTTAGCCGCTATCCTCGTTGCGGAATATACTGAGCCAAAGTATA
GGGGGGCATTTCTGGGTACATTTGCCATTTCACTGG
>103784836_2 DT668518.2
ATGCATCTCTTGAGACACTTTTGCACTTAATCCTCGTATCACAAATATTCATAATATAATAATATTCATAATA
TAATACTAATAGTATTAACTACAAACCAATTAGTTTACTTCTGTCATTCTATCCTCAACCTACTATAATATAA
AACATAATTATGATACTTTACAATAATTGTGACAGACAAATATAACTATTTGATTTTT
TTTGTTTTTTTTTACATTGGCGAAGATTGATATAATATGTTGAGGTGGCACATTCGGCCCGCCATATTTAAAG
AACCACATCATAAAATTTTTTGTTCAAAATATATTTGCATTTCTAAATTTCTTATTTAATTCTGTTATACTTA
ATTTAAATTATTGATTTGTTCAAGATAATTAAGATATTTTTACGGGAACCTCAAAACA
AATGATATCTTTTTCTTTATGCACC
>103784838_2 DT667435.3
GCACGAGGGTCGTCCTCTCCGCGAGCGCAGCCACATACATACATCTCAGGCGTAACTTTTCTTTGTTTGAGAC
GAACATGTGAACAAGAACAATGGCTCTCTCGACGAATTGCTGGAAATATCTCGTGTTGACCTTCAACGTTTTG
TTTGCGATGTCGGCCTTAGTGTTTTTCGGCGTTAGTGGGTTTCTCCTCTACCGGCTGT
TCGTCTACCGCCACTTCTTGGGCGTCAGTGTGGAGTACCCAGCTATACTGCTACTGATGATGGCCATCATCAC
ATGCTCTATAGCCTGGATAGGATGGAGAACCGCGAAGTCGATGCATCAAATACATGTTATTATGGCTGGTCTA
CTTATAGTATTAGTAGTAGTTATCGAATTCTCATGCTTCGTGTGGGCGATGGTGGTGT
GGGACAATATAGAGATAGACATCAAGACGACCATGACATCCTACTTTATGCTTACTGATGAAAACAAAGAGAC
TAACGCAGATTCCGTCCGCTGGGATCGATTGCATGTTAAATTCGAATGCTGTGGAGTGAGCGGTCCAAACGAC
TACAGTACAACGGGCCACGTTCCCTTTTCCTGCTGCGGGCAGGGGCCCCTGGACTCTA
TACAGAAGCCCTACACATCTGACTGTTCCACGCTGTACCAGAGAGGCTGCGGGAACTTGTTACATGATTACGC
TAAGCAACAGCTTCTGTTGGTTGCAATGGCTGCGTTGACAGCGTCTATATTGCAGAGGTCCGGCATCTTCTGC
ACGTTCTTCCT
>103784846_2 DT668716.3
GCACGAGGGCCAATCTTACTTCTACATTTTCACATCATTTCACAAAAGATGATTCATTCATTCCTAGTGATGA
TATATGTCTTGCTAAATCATCTTCATGTGATAAATCCAAAAGTTCATCGCCATCCAACCAATCACCACAAGCA
TCAAAACGGTCATCAAAAGATGAATTTCAACAGGTCCTTAGTTTGCTAGCACAAGAAG
CTATGGATACTAGTAATAAGCTAGGAGTTGCTTTAGCCGAACTTGACAAGAATCGAAATAAAAACGAGACATC
TGATATTCAAAATAGAATATCTGATAGTAGAACGATTTTTTTAAATGGTTTAACCGAAAAAAACTTTGACAGT
GCTCAGGCTGAATTGAATCATGACGAAGATTTAAAACATACTGATTCTCCTGTACGTG

AGAAATGTAAAGCTAGTCATGATGAAAATTCTAAAGCTAAAAAAACAGAAGATAGTTTGTTGGTAATTTCCGA
TCAGCTTCATAGAGTTGAGGATCAATTAAAACATAATTTTGAAAAAGAACTGAATGTAACTGAAAATACCGTT
GCACCAAGTATTATTAATATAGAAAATTCTGCAATTTCATATGAAAAGCGACATACAC
CTGTTGTCGATAGTTGCATAGCTAATGGTGTACCCAGTAATATACCACAATTACTTAATGAGAGTAATAAATA
CGAAACTGCAAATGATGTTCAATTAGATAAGCCAGTTCCAAGATTCCATTCTCTTAATCCATTTTTAAGTACT
GATAATAAAGTAAAAGAAATTAATGAGATAAAAGCATTCAAAGAAATAAAAGACGGTA
TATCTGATTTAATCGCTGGT
>103784865_2 DT668071.3
GCACGAGTTTTTTTCTTTTTTTTTTTTTAATTTCGTTTATTCAATAAGTATTACAGTATTAAGACTCAAACGAT
ACCTTATTACAATGATGCGATAAAATACTTTAAAATACAAAAGACGTTTCGCCTAAAGCTAAAATAACTAAAT
ATTTGGAATATAAAATCATTTTTTAAAAATCGTTATACAATGTTAACATTTAATTTTA
TATAAATCTATTTATATATAACGAATATATGAGGCCATAGTAGTCTACTCAATTTTAATTATTTTCATTTCCT
CACTGTGCTGGCAAAAAGACAAAGCGCGGCGTTGAGTTCCGCCTTTCCACAAGAAATATTTCGAAAATTTAAG
CGGGAGGTGAGCCTGCCTTAGGCTAGCTGGGAGCATGTAAAGTATGTGAAATATAACG
TGTTAAAAAAGTCAAGTTTTTAAAATATTGCATGCTTCAATAATGAAAATTACCAACAAGAGATTTATCTCGT
ATATTCCAAACATTTTTTTCGTGAATACGTTTCATACAGTAAGATTAGCAACTAGCCATAGGCTTTAAGAACA
TAGGCATTGGTAAGAACAAAATTCTTCTTCACTTCAAAACATGCACACAACAGTTTTA
ACATTAAATTTGCATTCCGAAAAATATTTATTGAAGTAAAATTGGTTCTCTCATCTTTCAAAACTTTTGTTTT
ACATCGTTAAGATCCGT
>103784867_2 DV501358.2
CGGCACGAGGCCGCGCCGCATAGATACTGTGGATACGTGGCCTTACGCGCCTCCAGACACATCTGCGCGTGGT
GCGCGCGTTCAAGTCCACGCTGGAAGACCTGGAGGAGCGCCTGTCCGCGCACTCGGCGGCGGGCCTGCGCTCG
CACCTGCCCGCCGAGCCGCCCGACATCGCCTACATCCACACGGACGAACACGCGACCC
CCGCGAACCCCGCGACGCCCGCCA
>103784901_2 DT666995.3
GCACGAGGATTTGCCTTCAAGTTTATATAAAGAAAAAACTAAACTAAAATAAGTACTGCACTGAGCTGAACAA
ACATACAAAGAAAAAAAAACCAAATTACATTTAACAAAGTACACGCTAAAACAAAACGGACTTTGATTAAACA
ATTGTCTATTAATAATAACCTATAGTGAAGTGACAATACAAAAATACTTAAAAATTAT
AAATAAAGGAGATAAAATAATCGCTAGAGAGACATGCAACACTAACTAATGTGAATATATAATAAACATGAAG
CTACTTATTTCTTTATAATTCTTTTGGTTCTTGGATTCACAATTTTAGTTGTTTTGGCATCCCTTGCCTCAGT
GGACTTGGTGGAAATTACTGAAGGAGCAGAAACAGAGTTAGGAATCATATCCAGTTCA
GCTGTAGTAACAACCCGGTGTTTTGAACCATCAGAACCTAGCACTATTATAACACCATCCCGAGTCCAACACT
TAGACACACCAAAACGTTGACGCGCTGCTAAAAATGTTTTGTGGCGGCATTTTGTAAGAAATTCTGACATTGT
CACTCCAGTACCTTTCAAGTCTGTCTTAGCCAACCAAACTTTATTTCGTAAAGAGAAG
TCCTTGAATTTGGTTGCAAGTGCACGAGGTCTTTCAGAACCAGTACGACCAAGCCGGTGACACCGACTAAATG
ACTCTGTGCCAATCTCGGGG
>103784913_2 DV501267.2
GCACGAGGATCGTTCTTAAAGAGACTATATCAATTTGGTTAGAAGAGGTTCAATATAAAATTCAGAGACAAAC
CGTAGCAGGTGAACCACTATCTGATCAGAAAGAACGTGTTATATTGTTGGAAAATTATATTCAAAATCTAAAA
CAAATAGTTTATGTGACT
>103784919_2 DT667755.3
GCACGAGGGTTTTTCTTTTTTTTTTTTATTTTTCAGTTTTTTAATCTTTCTTTTCTAACCTTAACTTTTTTCGT
TCTTTATATTCTTGAACATTGTCATATTTTCTTTTAAATAACACCTTAATTGTGTTATTGTCTATATTCATCT
CTGTACTGATGGTTTGATTCTCAAATACTGGTATTTTCTTAATAAAATCAATAGAAAA
AAAATCATGTTTATACAACATAGTATATTCTGACACCTCAAAACTTATCTTGTCATAAACAAATAATGATAAT
TTATGGAATTGTATACCAGCTGGGAAGTTTTCCAAAGAAAGTTCTCCAATAATCTTGTGACTAAATTGTTCAT
TTATATTTTTCAATCTGTAAATTAATATTTTTTTGTAAGAATATACTGATATACCTAG
AAAACTGGAGTTTTCATCAAATTGAGTAATAGTACAATCAACAACTGGTAAGCTGTTGATTTCTACATTATCA
GCATCCATAAATGTACTGAATTCTTGCTTTAATTCTTCATTATTCTTTATATCAGTATAAGTATCAATAGTAT
ATAAAAGTTTACCGTTGGTATAATTCCAAATTTTCACAGTTCCATCTCCAGAGGTACT
CAATAAAAAATCATTAGCATGTGGGACCTGTGCAATATGGTTTACAAATTCCTTGTGTCCCATGCAGTATGTT
TGTATATTGTATGTATTTGGATAACATGATACTCTGATTTTCTCGTCTCTATCACATGTTATGATATATTTTG
CATCATGAGTTTGCC
>103784933_2 DT667342.3

CGGCACGAGGCAGGACCCTTGCTGCAGCAACGACTCGGAGGTGGCGGGCGGGCGCGTGCAGGCGCGGGCGCGC
GGCGAGTCGGAGGCGCTGTCCAAGGCGCGCGCCAAGCTCTGCGAGAAGGTGGCGGAGGCGCGCTCCCAGCAGA
AGCGCATCGACGTGTACAGCACGGCGCTCTCCGCCGAGATCAACCTCGACTCCTTCTC
CTACTTGGACGAGGGCATGATGGCGGGCGACGAGGAGCCCCACCCAGTAAGTGACACCATGCCTCTACAAAAG
AATGAGCAATAAAATTTATTAGTTTTAACGAGAGCAGGTCGATATTTCTTTAAAATGTAAAGTCGGAGCGGGG
AGTGTCCGTAATGTATTGCGTATATTTTTGTGACGCCTATTATAACTGTTACGAATGC
CGAGCGACAGAGGCTAAACTTTATGACTATGATTATTTGGCCCGAACCAAAGTGAGGTTGTTATTACAATAAA
ATGCATAATATTTTATTATTAATCAATCACAACTACTGTCTTAATTTTGTTTACAGCATGTAATGTTAATAAA
ATAGCACAATTGTAAATAAATATACATAACCTGTTTTAATTATATGTATGTATCACCT
TGTAGTTCTCATATGCTATATGCTTTACACTAAATCTTGCCATTATAATGTTTATTTTGAAACCTTTTAGAAG
AGCAAATTTCGAGTGCAATTTATGCATTGTCAAGAGATGAGTAGAAAAGCATACTTA
>103784951_2 DT667671.3
GCAGAGGGTCGTTCGTTAGCTTAGGGGAGGCTGTGCAGATGGTGTATAAGAAATTAGTGGCAGAAGGCAAAAT
CATCGAAAAGGAAGAAGTAGAGCCGCCCAAAGTGCCCATGGACTACGACTGGGCTAGAAAACTAGGCATAATT
CGTAAACCAGCTGCGTTTATAAGCACTATTTGTGATGAAAGAGGTCAGGAACTGAGCT
ACTGTGGTGTTCCCATAAGTGCAGTCCTAGAAAGACAGTTGGGAGTCGGCGGTACCATTAGTTTATTGTGGTT
CCAGCGCGAGCTCCCCGACTGGGCGTGCAAGTTCTTCGAGCTGGTGATGATAGTGACTGCGGACCACGGGCCC
GCCGTCTCCGGCGCGCACAACACCATGGTCACCGCCAGGGCCGGCAAGGACCTCATCT
CCTCCATCGTCAGCGGCCTGTTAACTATTGGAGATCGTTTCGGCGGTGCCTTAGATAAAGCAGCTCAAGATTT
CTGCGCTGCATATGACAAAGGGCAGCATCCGCAAGAGTTTGTCAACGAAAAGAGAGCTAGAGGAGAACTCATC
ATGGGGATTGGGCATAGGGTAAAGTCAATAAACAACCCTGACTCCCGAGTCCGGGAGC
TGA
>103784957_2 DV501370.2
GCACGAGGCTTCTCCTTTAAAGAATATTTCTCTGAGGCGGATTACCGATTCATTCTTGGGTAATCGAGCCATC
GGAAATTCTGATTGGTGTTTTTCGAAGATGAAATTCGGCATGAACTCCAATGACTGGATGAAAGCACGTCTTC
TAAGAGATTATATTGATAAAGATTGAGCGACTGACAATTGGGGGATGTGCCGGTGACG
CCACGACGAACTGACTTTGGAGGTGACCTGATCGTATAGTGTCTGAGGCCTGGATGGGAAGATGAAGTTCTCA
GGCGGGCTCGAGCTCCAGCGATCTCGACAGCGACTCGCTGTTTAGAAAGGCTACTCTATGGATTTATACTGAA
GTAGATGCTCCAATGCCACCGCCTTGGAAGAGGATGGAGAGTATCTCAGAAGCGAT
>103784987_2 DT668818.3
GCACGAGGATTTACCTTACAGCGTTTGTAGACATATAAAAATGACTCCACAACATCACAAAACATATATTCTA
AATGAACTAGACGATGGTAACTTATGTTCAATCAATGCCGAAAGTAATGCTTTGCAATCACAAGCTTTAATAG
AGCATGAGTAATAAATAACTATGAATTAAAATTAAAACAATCAAACAAATAAATAAGC
CTAGTAAAATTAAAAGAGAGAGGACTCAACTTATTATATACGTTCGAAATTTGTAAGCTCGTAGGACATTCGT
GACGCTTGTCACAATTTTGATGGCACGTTGAGATTCGCAAGCTTTCAGTTATAATACATGTTAAAGAGCATTT
GTTAGGAGAGTGTCGCCTTGACAAGCTGGTCCTTGGCTGGGCCAATTACAAGCACCTA
GATTAGGGTCGAAACTGAGTTGAACTGGGCAGTTAAATACATGAAGGGTGAACCTTTGTTTTGTGCAGTCCCA
TCTACAAGCGTAGAATTTGTTACACTGTTTAGGATGTCTGAATAAACCTGCTCTTTTACAATCAATTGAACCT
TGTAATGTTTCATCTCTTCCTCTCCAACCACCAGGGCCGTAACGATTGAACGATTTCG
AATCAATCTCAGATACCGATGCTTCCGATTCTGAGAGAGCCGCAGACTGTTCTTCGTTGAGTAGAATTGTCTT
ATACGTTGGTGCTACAGTTACAGTCTTGATTCGTGGCTTG
>103784992_2 DT668494.3
GCACGAGGCGCCGTGCGTGTTTGTGTTTTGTGTTACGTATTTTTATTGTTGTGCCACTAATATTAATTCAATT
TGCAATATTTAATTTGAAGTTATAAATATTTTATAAATAAAATGGCTTGTCCTATGAGGTCAGGGATAGACGA
GTTGTCAGGACAAGATGGCAGCTGCTTGGGCAATGAGGCGGGCATGCTCTACTCTGAC
TACCTGATGCTGGACAAACTGCTGGCTGCACAACGAATGCTCAGCGCTGAGTCGTCGAAACCAGTACACGATG
AACACCTTTTCATTATCACACATCAAGCGTACGAGCTTTGGTTCAAGCAGATTATTTTCGAAGTGGACTCGGT
GAGGTCATTACTCGATGTCGAGGGCCTGGACGAGAGTCATACTATGGAAATCCTAAAA
CGATTAAACAGAGTGGTCTTAATACTAAAGCTATTAGTAGATCAAGTTATGATCCTGGAAACAATGACACCGC
TGGACTTCATGGATTTCCGAACCTACCTGCGACCCGCGTCTGGCTTCCAAAGCTTACAATTTAGACTTTTAGA
GAATAAGTTGGGACTAAAACAAGCTTTACGGGTGAAATACAATCAAAACTACCAAACT
GTGTTCGGCGACGATCCGGAAGCGATGGAGGCGCTGAGAANATCTGAAGAAGAACCTGCGCTTTTAGCCCTTA
TAGAACGTTGGTTAGAACGTACTCCGGGACTCAACACTCACGGGT
>103785004_2 DT668475.3

GCACGAGGATGAAGTGCGCTGTGGCTATAGTACTAGCTCTTATCAGCTTATCGCTCGCTCATCCTGCTCCATC
GGGACTGGGTGGACTAGCCCTTCTAGCACCCGTCGAAGCCAGGTCCACCTTCATCAACCACGGCATCTCCATA
CCGAGAATACCCATCGTCGTACCCATAAAGCCCATTATTGAACCCATCATCAAATATC
CCGTAATTCCCATTGTTAAACCATATTTGATTTAAACTATTTATTAACTTTGGTTATTGCTATTGACGGCCAT
GTTGCTCATTCGGTAATAAACTGTGTTGTGCTGAATTGTTTTATTTTGGAACCCTATAATTCGTTTCTTATCA
AATTATCTAATTTATTTTATTTATTTTAAGCCCTTTTTTACGAAACCATTTAAGAAAG
GAATTAGAAAACGTATAAAAATTTGAACTAAATAAGTATTTTTCTTTCTTAAGTATGGAATGACACTTTAAAA
AAATGTTTAAAAAACCATTCAAGAAGTAATGATTTATTTTTATTCGGATGTACGTAATATTACATAGATGTAA
ATCATAATGCATCTAAAGCTACATAAGTGTATAAAATTTTTTTAACGGTGTAAGAACT
GAGTGTCTTAACACTGTCTCCTCTGCTACGCTACTCTGTTATGTGGAAGCGCGAATCTGCTGCTGCTGAATAT
CGAGGTATATTTATAGCACATAATTATTATTTAATTTAGGAAATAGAATTGATAATTAAC
>103785012_2 DT667298.3
GCACGAGGACTTTCGTTTGTCCATTGAATCCAGCGCCAGAGTTGCAGTCCGAGAAAGAATTCTCTCAAAGATT
CGCCCACGTTATCCGTGGCGTGATCGACTTCGCCGGCCTCATACCTGGATTCCAGTTACTGACTCAGGATGAC
AAGTTCACATTATTAAAAAGTGGTCTCTTCGACGCACTATTCGTGAGGCTCATTTGCA
TGTTTGACGCCCCACTCAACAGTATAATATGCCTAAACGGGCAGCTTATGAAACGTGACTCTATCCAAAGTGG
AGCCAATGCACGTTTTTTGGTGGACTCTACATTTAAGTTTGCCGAACGTATGAATTCCATGAATTTAACAGAT
GCCGAAATAGGCCTCTTCTGCGCCATAGTCCTAATTACGCCGGATCGTCCTGGTCTTC
GCAATATAGAGCTAGTTGAAAGAATGCATGCAAGACTGAAGGGTTGTCTACAGACCGTCGTTACGCAAAACAG
ACCAGACAGACCCGGTTTTCTTAGGGAGTTAATGGATACGCTACCTGACCTACGTACCCTGAGTACACTCCAT
ACGGAGAAATTGGTAGTTTTTCGCACGGAACATAAGGAATTATTACGGCAACAAATGT
GGGGAGACGAAGAAGGATGTTCTTGGGCAGATTCGGGCGCTGATGAATCCGCTCGCAACCCCATAGGATCAGT
GTCGAACAGCGAATCCGGTGA
>103785036_2 DT668845.3
GCACGAGGGAGCGCGTTAAAAAGCAACGTGCCTACGTATTGCATCGCCCGCTCTAATAACGTAGCTACTTAGC
TCAGCTGCGTCTGTCTCCTAGGCGCGCAAAGAGTTCACTGGCAGTCTCTGGCCGCACGACGGGGTCATTGCCG
TACGACTGAGGCTTGCGACATGAAGCATACACCATGGAGGAGTCTCATCTGTTCAATT
CAACTTTACTTCATTTTACTACATGGGTCTCAAGCCATTAGCAGCCTTGATTTATCACCAGAAGTAATCCAAC
TGAAAGATAGTGTAAGCCAAAATTATCAACTATCAGATATTTTTTCCCAAATTGATCGCAATATTAGCATAAT
AGAAAATGATTTTGTTTTGGATGGCAAAACTATTCGGATAATGTCTGGGTCCCTGCAC
TACTTCCGCTTACCGTCAGAATATTGGCGAGATAGATTAAGAAAGCTGAGAGCAGCTGGTCTCAATACAGTAT
CTACATATGTAGAGTGGAGTAGTCACGAACCAGAAGAAGGTTTGAATTTATTTGATGGTGACAATGACATCAA
ACGCTTTATTCAAATCGCTGCAGAAGAAAATCTCCATGTACTGTTAAGACCGGGACCG
TATATATGCGCTGAGAGGGACTTGGGAGGTTTGCCATATTGGCTACTCAGCAAATATCCAAAAATAAAAGTTG
CGGTCCAATGACAAAGATTTTATTGCCGAAACTAGAGAATGGATGGCA
>103785091_2 DT667957.3
GACGAGGGAATGTTCGTACTGTTGATATGTTTTGATACGTATTTTGCTTTGCCGTTAAATAGATTCTATCGCT
ACCATGAAACGTTACAAGAACGCACTATGCGTGTAAACCCCGCCTTAGCAGCGACGAGCCAAAATGCGGGCGT
ACAAAAAGTACCATACGGACTCATATTTAAACAATCCATAGTACAATTGTACAATATA
TTTATTGTTTTCTGTGTAACTCTCACTGTGTTCCCGGTGGTGCATTCAGATATCAGACCGGTAACGCCAGATT
TTCTAGGTGTTAATTTTGTTCGAATCACGTGTTTCTTGACGTTCAACTTGACAGCCATGATAGGTAACATAAC
AGCAAGCTTGTGGCAATTTCCAAATCCAAGATGGCTGGCACTGTTCACGTCACTCCGT
ATTCTCTTCATTCCCCTATTCATGCTGTGCAACTATCAACCTAAAGGCCGACTGAGGACTTTACCCGTTGTTA
TAAACAACGATTGGGTGTACTGGGTTATGTCGGCATTACTCGGGTGGAGTTCCGGTCATGGCAGCTCTTTAGG
CATGATGTATGTTAGCGGCACCGTAGCACCAGAACACGCTTCAACAGCAGGAATGATT
GGTGGAGCCACTCTCGTTACAGGAATTGTGGCTGGCATCACTTTGTCCAAATTCTGCCCGATTCTTGTTTCGT
TATCCGCTTGGCAGAATATGTAGCGATTACTTACGTATGTAGGTACAGTGTGGAACGTACTGATACGTGGGAA
GTGATTGTAAAATCGATTATTCTAGAAGTGACCA
>103785111_2 DT667299.3
GCACGAGGCGCGTCTTCCGATTACCAAGTAATATTTTTGAAGACACCACGATGGCATCAGAAGGGTACTCGCC
CAGTGAGCTGGAGTCGATGCCTGACCGTATAGCTGAAACGTTCACCGGCTTGCACTTGTTAGTGACTGGTGGT
ACTGGGTTCATGGGCAAAGTGCTGGTTGAAAAGCTGTTGAGGAAATGTCCAGATATCG

GTAAAATAATGTTGCTCGTTCGACCGAAAAAGGGTAAAACTCCTAAGCAGCGTCTCGAAGAAATGCTCAATGA
TGAGTTATTTGGAAAACTCAGAGCTCTAAGAGGTGGTGTAGAACCATTACTGGAGAAATTGGAGATCGTAAAC
GGAGACGCGTCCGCACCAGATCTCGCTATCAGTGAAGCAGACAGACAGGCGATCGTTG
ATAACGTCAATATTATAATACACGCTGCTGCTACTATCAGATTCGACGAAGAACTTAAAAAGGCAGTGTTACT
CAACGTACGTGGAACGAAACTCGTTTTTGAGTTAGGGAAGAAGTGTAAAAACCTTCAGTTATTCATCCACATA
TCCACGGCCTACTGTCACTTGCACGAAAAGCTGTTAGAAGAACGTCCATACCCCCCGC
CGGCGGACCCACATAAGATAATCGACGCTATG
>103785143_2 DT668111.3
GCACGAGGAATTACCGTTTTGATTAGGGAGTAATGACTAAGAACTACTGATATTTTCGTTTTCTTTTTTGATA
ACGTAAATAAATTTAAAATTGTTTACCAAAACACAACTTAGTAATGGCGACGTCAAGTGATACTTCTGACCAA
AACGTAAAATCAAGAAGGCCTGCTGAATCCGCTTTTAAGCAGCAACGGCTACCTGCAT
GGCAACCAATCTTAACAGCAGGGACTGTTCTTCCAACATTTTTTGTAATAGGTATAGCATTTATACCAGTTGG
AATAGGATTACTTTACTTTTCTGATGAGGTAAAAGAACATGTTATTGATTATACATATTGTATGAAAGAAGAT
GAAAACATCACATGTGCAGATTACATTAAAATAAACAATTTGAGTGCCTGCACCTGCA
AAATACCATTTAACCTTACTGAAGATTTCAAAGGAGATGTATATTTCTATTATGGTCTCAGCAATTACTACCA
AAATCATCGTAGATATGTAAAATCAAGAGATGACAGCCAACTTCTGGGACGGTTATCACTAACACCTTCTAGT
GATTGTGCGCCTTTCGCTTATGCTGATGTGGAAGGCAAATCGGAAAAGATGCCCATAG
CCCCATGTGGTGCCATTGCTAATTCACTTTTTAATGATACCCTAACAGTAGAGTCGCAAGATTTAAACAAAGA
TGTGCCAGTACTAAGAACTGGTATTGCGTGGACTTCTGACAAAGAAATTAAATTCAGAAATCAACCCGGGGAT
CTGGAGACTGCATTTA
>103785180_2 DT668411.3
CTAGTCTCAGTTTTTTTTTTTTTTTTTTGTTAAAGTTAGTTTTTAGCATTTTCATTACCGTTGACACTTGTTT
TTCATGAGGATTTTTTATAACAGCAACTCTGTTGGAAACTTTTTCTTTTAGATTTATTTTCGATAGCGACAAT
GATTTCGACTTTGGTTTTAGTTTATAAGGTAAGCCCTTTTGCAATTCTTTGGGTATTG
ATAAAGGTTTAAAAACTTTTGGTCCTCTCACAATTTCAGTGTACATTGAATCTGTATTTGCGTCGTTTTTAAT
ATTTCTTTCTCTTTTTAGTTGTCCTTTGGTCTTCATTCCCTGCCAGGCGTTCTTACTATGGGCAGGTAGCAAC
AGATTCACGACAGGAGCGTAAAACTTAGTAACGTCCACTTTGAACCATGTTCTGCAGA
AAATTACATCGCTCATAAGGATTTTATCTTCAAACGTCGCACGGAATGCTCCTTCCGGTTTATTTAAAGCCTT
TTTAATCTGTCCTCGGATACCGGAAACAGTTTTAATCCTGGCCCCCTCAAACTTAGCCACCTCTAAAGTAGTA
GTGAACATGTCCTTTATGAAAGCTGTCTTTTTGTAAATCTTTAATGGTGTCCCAACTA
GTTTCAGCTTCTTCATAATTTGTGTAGATTTATTGATTTCGTTCACACTCCCTGTGGCCGCTATTCTAAAACC
TAATTGTTTTATTTCATTTGTTTCATTTTTAACAGTCTGTAATGCTAGAAAGCCTGTGTTTTGAGGTGTTATT
GGACCATAAAAGTGCATGTTACAAGTAACGTGCTCAGGAGTATACTTTAAATAACGAC
ACTTTAAGTCATCCTCAATCTT
>103785183_2 DT667896.3
GCACGAGGGAAATATACATAAATCATAATCTTTCCAGTGAATTTAATTATAGAAAATATATATAATTTAAAAG
ATTTTAATAATTTGATGAAACATTTGTTTTTATGTGTCTATCTTAATATGTTATAAATATACAGAATACGGTG
AAATATGTTACGCCAATGTTTTTTGCGCTGTTTATTGGATACGCATGCGGCTATAAAA
TTACCTCATGATATCGAAATATTTGTCGGTCGAAGTAAAATAACTAAAATTAAGGATCAATCATGCTCGAGAC
AACAAATTAGTTTAAAAGCTGATTGTGAACAATGCCAAGTTGAGGTCAAACCTTTGGGAATAAATCCCTCTGG
ATTAGATGGTTTTGCGTTAAAAAGAAATGGGATATATCAAGTTAAACATGGAAGTAGA
CTTGAAATCTTATTGAACAACTATATTCACGTTATTGAATTTGATCCACCGCCGAATGATACGGATAATCCTA
AACAAAGAAAAAGGAAACTAGAAGAAAACGTAGAAACATCACCCAAGAAAAAAATCTAAAATTCAATCTAAATT
AGAACAATCGGTAAATGAAGCTATGGAGAATATGTGGGAAGAGATTGATAAAGGTGAA
GTATACGTATTCACAGCTAAAGGTGTAAAATCCAGTAACAAAATAGCTGCATTTGACATGGATGGCACATTAA
TTAAGACTAAATCNTGGAAAGTATATCCTGTGGATACAAATGACTGGCAAATAGCATTTACTCAGGTAGCACA
CAAATTAGC
>103785196_2 DT667778.3
GACGAGGCCGGCGCTCAGGACCCGCACTCCACGTCTACTACCGCTTCCTTCCTGGCTAGAGCCTTTGGCATAG
TAATACGACAAATAGCCGATTTACTGTGGGAGTATGAGCGGTTAACGCCCCCGTTACAGCGTATGTTGCCGCT
TGCTTACCGTGAAGCGTTGCGCTTACAATGCTATTTGGAAAGGCAACTTAAACAAACC
TGGGATTGGTTGGTCACTGTTATGGACGCTACTGAAGCGCAATTAAGATTTGGTGCATCTCTCACATCGAATA
ATGCGAACACTTCAAACAGTGAAAGCCGCTCTTCTGCACCAGCAGCTCGGCGCACCACGTCTTACACCTCGCC
CGCTACACGTATAATTGGATTTTCAGAAGGACCACGTGCTAGAGATCGTGACCAAGGC

GTGGAAGCAGGCAGCGCCCGGAGAGAATTCTTGGCGTACTGCTTGTCTTTGCTGCGTGCCCACAGCGCCGAGC
ACGCGGAGCAGCTGCCGGTGCTCGACGTGGCGGCCCTCAAGCATGTGGCATACGTGCTGGATGCGCTCGTGTA
CTACATGCGAGCTGCACAACCGCAGCCGCAACACCATCAGCATTTGTGGACTCCGGAT
GAAAATGAAAACGA
>103785198_2 DV501310.2
GCACGAGGAAACCCTTTGCAGCTACTAAAGATCGAATATCAAAGTAATTTCTATATAATAATTATTATTTATT
TTAATTTCTGTTCATTGATGAATAAGGTTATGTTTTACTATATAAACAAGTGGTTGTAAGATTATTATATGTG
CTTAGTGTGTGGGTTCACCAAATTGTAGCACCATGAATAACTTTACGTATGGAAGAAT
CTATCAAAATGGCAACTACATCGGAGAATAAGAATAATGAGTACAAATAGTACTCGAGCACCGGAGGCGCTGT
CCCTTACACTGAGCAACGATTCGATATGAGAAAATCGGATTTAATAACGAAAGGCAGAAAGTTGGACTCATTA
GGACGTGTCGGGGGCACCAGGGAGTACCAGTATGAGTACAAAGAAGAGACGCACCCGT
CTGGGAAGTATGATAGAAAAGACTTGCATACTGTGGAGGAATTCATGATCCCTCCAAAGACGGTACCAAAAGA
CGGCCGAGTCCTCGTATTACTAT
>103785225_2 DT667423.3
GCACGAGGAAATTCTCTTAGAAATCTTTTACTTTGTAGTTTTCTTAATTTTTTTTTTTTTTAGTTTATTTCCG
TCTAGTAATTTTAAGTTCAATAATTTACCTATTACATATACGTACAAAATTTAGTTTATGATATGAAAATTAT
AAGAAAGTTTTTATTATAGATAGTGTTATAATGATAACACAAAGTTGCAATTATTACG
TCAATTGATTAACAGTTGCAAATAAAAATCTTTTAGATTAAAGTAATTAATGACAATATAGTGGCGAAAGACT
GATCTATAATTCTGAAGAAAAACAATACATACTTAAAAGATGGTTTCACGAAAAAGAGTATTTGACTTATTTC
GAACCATTAATATCGCGTCCTGCCAGTGTCCAGCACATAGTCAAAGGGGAAACTTTCA
AGTTACTCATGCTACTCCATCGAAAGATTATGCCTTTGAGATAAAATGTTCAACAGTGAGATATGGTTTAGGT
GTCACAAGAGAAGTTGGACAGGATTTAGTGAATTTGGGGGCAAAAAATGTCTGTGTTATGACAGACCCTAATG
TTGTGTCTCTAAATCCAATGAAAGCCGTTCTTAATTCTCTTACAAGAAATGGAGTCAA
TTATGAAGTATACGACCAGGTTAGAGTTGAACCAACTGATTCAAGCTTTAAAGACGCGATAAATTTCGTCAAA
CAAGGTAGTTTCGATAGCTTTGTGGCTATCGGCGGCGGCTCGGTAATGGATACGTGTAAAGCTGCCATTTATA
CTACTGCGACCCGAACGCGGAATTCCTCGGATATGTA
>103785229_2 DT668387.3
GCACGAGGCAGAACTCAACGCTGCGCTTTGCCTCGACATCAGAGCGAAGAAATGGAAATATAAATTTATAATT
ATACAAAATTCTTTTACAAAAAATTATATGAATTTCCAAAAGAAGAAAAGATAACTTCCATATTCCATTTATC
GTGTCAATCTCTTGTAGGAGCACTATAATAACATTATTTGACGCCAACATAGCGTAAT
GTGAACGTGCCTGGCTGCACGCGATGCTTTGTGGGCCGAAATTCCACTCGAAGGAATGTTTATATCCCGGATA
CGATCCTTTTGCAGACCAAATGTGGTGTTTTGTTCCGCTAACCAAACTGTATATAATATAGGGTGGCGTAGCT
TAACACTGCATTCGTTACTGACGATTGTTTATTCGATTCCCACTCGGACAAATTAATT
ACTTACTTTCATGGGTAACAGAGTGATTTAACACTGGGTTCCTTCTGTCTAACATTGTATGTGCAGAGTACAT
GGGAAAATTAAAAAGTATTTTATCGTAAAATTCAAAGAAATTATGCTATTTTACATATTACATATTAAGCAAA
GCGCGGCGTTGAGCCACTCAACACTCAACGTCTCGAAAATTCGACGGAATGTAGAGAA
AGGAGAGTATTAATACTAGGTTTCCTCTGCCTTCCCTGCTATATGCGAGATACAGCGTGAATCTGAAAAATAA
TTAGATATTTGATATCACGCAACACCCAAATCATCACACATCAAGGGCATGACTCCAAATCATCTAAAATGAC
TTACCGTTTTAGTCATCATCTAAACACAATAGATCATACGGGCATTTATTATAGACAT
GAGTCCAGTTG
>103785236_2 DT667490.3
GCACGAGGACACTCTTCTGAACTGTCTGGAATATGTTAATAGATAAATATATGTAATAGTTTCGTAGCTATTG
CTTTTTATGTGTCGGCTAAAACAGCGCGGCTTTGAATTCCGCCACTCAACACTTATTGCTTCCAGACTTAACG
GAAAGTTGTCTTAACGGAGTTAATTTATTCTTAACACTACCTTCCCTCTGCCTAACCT
GCTATGTGCGGGATACAGCGTGAAGCTAAAAAAATATACATTGGACGTGGCGCAGCGGCACAGCTTGTACTTC
CTGCACCAAAGGAATAACAACGCTTGTTCATAAATTTCTCTCTCCACATTTTTTGAAATATGGAGTTAATAAT
GATTTTTAAATCGTCATTTTCAAATTCATTAGTTAATTTCTTCAAAAGGCCTTAGTTT
CATAGTTTAACAGCTACGTAAATATACGTATTCTATAGCATATTGTTTAGTAAAATATTTTGAAGAACAAATA
AATTTAAATAATTAAACAGATCTTTATATGTCTGTTTATATAAAAATAAATTGTCGTATGAATCAATTTCTAC
AGTAAGTTGCAAAAAAATGTGAAAAAGAAATGTTTTTGCTGACACCTTTAAGCAATCGC
TTAAAATAATGATTATTTAGTGACCTTAATACTAAATATAATGATATAAATTGATTTCGACGAAGAGATTTAA
TACTCTCTTTCACAATTTTTAAGAANAATAAACGTAAATACATAATTATAAAACCTAGATTTAGTAAAAACTG
AAAACTTTCAAATTGATAAAACGTGACTGCAATTATGGAGTTGAGACGTAACACATAA
TCATATGTGATAGTCGTGAGTTCAATGCTGGTTGGATTT

>103785261_2 DT668384.3
CGGCACGAGGGTTTTTCTTCTTTGTAGGCGTTGGAATCAAAATTAGCGTGTCGTGGTACTGTTAGACCTAAGG
AACACTCGCCGAACCCGAGCGACGTGAACAAGGACTACAGGTGTGTACTCAAGAACTGAACACCAAAACTCTT
ATAGTACGACGCCAATATTACAACAATTCGCTTCACACATATACTCACCTACGGTTAC
TCCATCTATGTTCTGTGATCGGAATCACACTCCTAATATTACTAATATCTGTGATGATAACAAAATAGTATAG
AAATTTATGTAAAGGTGTAATTATTTTGGCCTACCCACCGTGATGGAGGCGAAGACACAGCTAGGGTTGCCAA
CTGTATTTCTTTATGTATAGTATTGTACTATTAGTAATGTATACTATGTATAGAGATT
AATTTTAAAACAGTTTTTTAAACAGGCTGAAAGATGCTAGCAAATTTATGTTAAATAATTTAAATCTTGTAAA
AAGATTCGAACTTGAATATGATAAAACACGTTAGTGCATTACCAATTCAGTTAAAATATATAGCTTTCATAAA
AATCAATAAGTTACAAATATATTTATACCAATTTATTATATTATACTTAAAGTTTGCT
ATATTTGTTTGCAGTGTACTGTATTTATTGTTGCAATAAATAAATAAAAAAAATACTATAAATGTTGGCAACC
CTAAACACAGCGGCCATGCCCCGCCTCTTGCACTGCGTTTCTTCTAGATTACATCTCGAATTTATATTACATT
TGGCTTCTTAAGTCAATGTTAAATGTCCTCCGGT
>103785287_2 DT668920.3
GCACGAGGCGCGCCCCTACACCCGCACGCCGCCATGGCCCACGCCACGCTCGGCCACGGGGCCATGGCCCACG
CCACGCTAGGCCACGGAGCCATGGCCCATGCCTCCATGGCGCACGGGCACGCGGCGCACGGCCACGCCACGCT
GGGCCACGGAGCTATGGCGCACGGCCACGCCACGCTGGGCCACGGTGCCATGGCGCAC
GGCCACGCAACGTTGGGCCACGGCCATGTCACGCTCAGCCAGCTGCAGGCCAGCCTGCCGCTGTGCCACCGCC
ACAACACCCTGTATCGCGCGCCATAGGCCGCCGCGCCACAGGTCACGCGCCACGCACCCCGCGCCACACGCCA
CACCACACACCAGTCGCGACTCACCACAATCATGTTTGTACTATCTTGTTAAGAGTGT
ATTCACAGTAGCTTCGTTTCATTGTTAATGTATGTATAAGTATTACTAAATTCGTGTATACGTTTAATGTAAT
CAGTAGTTGTTGTCATCGGAAACGAAGTATTCCCATTTAGGGGAACATTTAGTGTGTAATGTCAGAAAGTTGT
AGTCGCGGCCACGTGTCGACTCATCCCTCGCAATCTCATCTATCAAACCATTTTATTT
TACTGAAACTTTATTTTTAAGACATTGGTCCCTCTAATATGGGGCACTGTCTCTTTTTCACCTCTTTC
>103785313_2 DT668556.2
TCATTAAATATTGTAAGTTCGTATATGAATAAATTTAGACATTATGAATATTAAATCTTCCAAATATTTCTAA
ATTAAATGCATATTAGTTAGTATATAATGAAAGCAACAGATGGTTTATCAGAACAATCGAACCGAAAACGTTA
TTTTTTCTAATATGCGAACTCACAAAAGCTGTCTAACATAAAAAACATGAGAGGTGCG
TGAAAATAAAAATCTTATAAAAAAAAATGATACGCAAACAAGAGTTCATTTTTTATATATATATTATTGACTT
TTTTAAGTGCTAAAAATATAGCAACGCTTGATTGCAACACCGCTTTGTCTAGTATAGGTGGTTTGTGGTAGAC
ATTGGGTATAATATTTTGTCAATTGTAGTACATTTTATACATTCATATCCCTTACTTA
ATATTATAAAGAAATTGAAACAATATGAACGCCCCCAAGTAAAGTCGATATTGATTGTTATGTATATTGTGGT
GTTATACCTAATTGTACAAAGTGTTTATATGTTATTAGTGAACCTACAGCTATATAGACTAATTATTGTATTA
AATAAATGTAGCAAGTGTTATTAGAGAACAATATATTTTTTTTCAATTGATTAGAATC
TTTCATATTTATATAAAAAACGTCGACATCTTTCTCTTGCTTATACAAATATAAAACGTATGATAAGCTTAAA
TGTTGCACCCCCACTTGATATTGTAAAGCTAAAAATCTATTAAGTACTATTTTCTCTGCTACTCTCTCTC
>103785334_2 DT667612.3
GCACGAGGCACCCTTTTATTACGAAAGACAACCGGCCTCTGCATTAGTTGAACCAGATGTAAAAAGTGGATTT
AAACCTATTTTTAAAAATCCCCCCGAAGTCACAAAAAGCCCCATCTATACTTCGTCTGTTCAACAAGACGAAT
TGTCGTATCATAACTATTATATTGAGAATCAAAAGAATCCAGTCGATGATTCAACTTT
AGATGTGCCAAGAGATAATTCGTATAAGCAACAATATATTATTGAACAACCTACCGTACCTGTATCTACGCAG
AAACAGGAATCTTCAAATTCTGTTACATTGGTTGAACTTTTAAATTCTTTACAAATTAATAAATCTATACCAA
AACCAATCACAAGAGAAAATGTAGGCGATTCAATAAGAACATTACTTAATGTATTGAG
TACATTAAAAGCTGCTCCATTACAAAATGAAGGAGTGGCAGTTGTCAGTACGCCAAAACCGTTTGAACCGCCT
GAAATTTCACATGTCACACCTCTCAATTCGGTATCATCGACAACTCCTAAGGCCGAGGAACCAGATGAGGAAT
CGTATCTTGCACCAATACAAACACCTTCGCAATATCTAGACGATTATCCAACTAGCAG
TGGAAGTACGCAAAGATTTCCTCTTCCCGTGACCTCGGACGAGGAAGGTGGGACTCCTGGCCGCCCTGGCGTT
GATTACCCTATATTGACTGCCATCCCAACAACAAAAATTC
>103785442_2 DV501328.2
GAAATCCAACTACTCATCAAAATAATCGACATCGACGACCACTTACCTAAATTTGACAGTTCCTTCAAGAAAG
TTGGCGTTCGTCTGAACATTCCGATTGACACTGTCATCGCAACTGTCAAAGCCACAGATAAAGACCCAAACGC
TTTGGCCATAGAGTATAAAATTGTAAATATGACGTTCGAATCGCCGATCAAGTCAAAA

TCAATTAACAATTTGACAGATGTTATTGTGTTGAATAATACGAATGGGGAATTGAAAATTATGAAAAATTTAA
TACATTACGCCGATTTTTTTTTTTGGTTGACCATTAGAGCTAATAATTCGAATGAAACGGATCGATACAACGA
TATCTTAGTAGAAGTAGTAGTAGTACGAGAACGCGACTTGCTCCGTCTCACCGGCGTA
GGCACCACGTCCCGCCGCACTGCAGCGCTGAGTGAAGCGCTTCAAGAATTGTTAGCGCCGCGCGGTCTAAAAC
TACAGCTGCACGATGTTGTAACAATGCCGGCGCATCTAGCGCTTTATGGTGGCGGAGGACCATGTTTTCAACT
ACGGAAAACCAACACGGGCGAAGCGTTAACACCCAAAGCAACACTAACAACATTCCGC
TCAATAGGCGTTCCCCTACAACGAGTGCTACAACAACACCAAGTACACAACATAACGACATGCGGGCGCAACA
ACAATGT
>103785450_2 DT668325.3
GCACGAGGAGTTGACATCACTTTATGTTCATCAACATCATACGTATTAACACTTTTTATTATTATAACGAGTC
GTGCTCCTGTCTATTAAACCATAAAACTTTATACTCTTTGGTCAAAAACGTAAACATTAACACTAACAGTAAA
CATTAGTATCGCTCATGGTTTATGATGAAGAATAATTATAAATAGTTAATATTAGGTA
GTTTCCTTATTACAAAATGTCTTCTAGTGGCAAAGATATGGAGAAAAATGAAGAAGCACCAATGGATCAAATT
TCTTCCCAAGATACAGAAGAGAATGAGAAAGCTTTGCTCTCGGATTATGTATCTAACCTAGAAATGAGAATAA
AACAAAAATCAGAACTCCGAAACCAAAATTTAAACTGTGTGAGACCACCAGATAACTA
TTTTTCCAAATTAGATTCTGGCCTAAAAAAAATACAACTTTTGTTAAGAAATTGAAATCTTTTAGCGCTACTC
AAGTAGAAGCACTTTTAAAAGATTTAGGAGCTCTTAATTTGACAAAATACATTTCTGAAGTAGCGGCGGCAAT
AGCAGAAGCGAAGCTTAAAATGTCGGATATTTCAGCAGCAATAAATCTTTGTTCCGTC
TTACATAAAACGTACTCGGAATTTGCAACAAACTTTTTTGAAAATTGGCAAAAAATTTTGACATTTAAGGCAA
CAGACAAAATAACAAACTCTTCGAAGCTTAGAGTAGACTTAAGGTTTTATGCAGAACTTATAGCCGT
>103785457_2 DV501356.2
GCACGAGGAATTCACTTATATCGTATAGTGTTTCTTATACAAAGGTAATAAATTGTAAAATCCTCTTTCACGA
TCCAATAAATAAATACTTAGTATAACTCCTTCAGTCCTATCACATGCCCTTTGGGCAATATTAGGAGTATAAT
TTACAATACATCGAATACTTTCGATCTAGCCTTCGTAGGATTGATACAATAGTCCCCA
ACCATGGGCTCTACTCGATAACGAGACGCAGGATAGAAATATATGAGTGAGAACGCCATGCGTTTCATTCCTT
TAATAATCTTAAAATATTGCGATTCAATCGCAGCGAGTGCTCATGGTAAGGGCCGGGGCCATACTTTTTTTAA
CAAATGTACAAAAATATTATTTAAAGGTAGCATTAAGGTAGGTATGTATTGTCAAAGG
CACTGGTACTCTTATCCTGATCAATACTCTGCACGATAGGATTTAAATTCTTAAAAATAGTTTGCGATTTCTT
GACAGCTAGAGGATCTATCATACAATTTTTTTTTTTGGTTTTGGAACATAACAGGCATTCCGATCGAGAAAGA
CTATACATTAACATAAATAAACATCTAAGTTGGACTTAAAATTACGACTTAGAACTAA
CTTATAAGATAAATAGTCTTTAGCCATCGATTTTCACTATCGAGTCTTCGAATAGATGAGACAGATCTACTGC
GCTTATATTACACATTATACATAGCTCAACCACTAAAAATGCATTTTTTTAAATCTATCACTGAGTAGATAAA
TTTTATCTGCTCGAAAAGTCAGCATACCGACATTAGTCTCATATAGGGTCCACGCGCG
CGTCGACCGATGCGGCTATC
>103785467_2 DT668466.3
GCACGAGGGGCGTACCGTACACGGCGTTAAAGATACAAACGACTGACATATATCCCACACTCGCATCTCCTGC
TATTGAGGTGCCGACGAGATCATGTTTATCTGAAATAGTCGAAAACGAGCCAAGCTGGCAGCCGCATATTATA
GCAACACTACCGAACGGAATTCCGGAAGGATATCAAATTGTAATTTATGGCAGACTGC
GCCCCCTGCTGCACTCGTTCGCGCTGGACCTGATGGACGAGGCGCGCGAGTGGCCGCGGCCCAACGTGCACGT
GCACGTGAACGTGCGCGCGCACGTGGAGTCGCAGCTGGCGCGGCAGCTGGTCGTGCTCAACGCCTGGCTCGGC
GCCTGGGGGCAGGAGCGGAGACAGCGCACCGCCAAGCTAATACCCGGCACTAAGACAA
TGTTCCGAATAATGCGGGGAACTACCGAATGGTCAGTGTACGCAAACGACATACTTATAGGGGAGATGGAGTA
CCGCGCCGCTGCCGCCGGAGTTCGCGCCATACGCGTGCGAGGAGACTTGTACCCCGAACAAATATATATATGC
CCGTATAATACTTCCCCTCAAACAGAAATATCTTGCTCACCATGAATTATGTAACTTG
ATCGAAGGAAACTTAGTAAAACATGGGCGTTGGGGTGGAAGTGAGGGGGTG
>103785478_2 DT667343.3
GCACGAGGATAACCTTACTAATTTCCGTGACATTGTTATTTCTTTTTTTTCCTTTTATTAAGGTATTCATAAG
AATACCTTAGAATAACTTTAAAAATAATGCATAATAAGAAATTTGGGCGCAAGGTCTTTATCGATTTTTTATT
TAAAGAATCGATTTTGAAGAGATCTTTCTGATAGCGTTTGTGATCGATTCCATGTTTA
GTTAATTCAGCGTATTCTTTAAAAAATTTTTATATGTTTTATTTCTAAATTCCCATTCAGCTTTCTATAGTAA
TATAGTACCTTTCGATACCAAGATACGTTCCATAATATCCGATTTATTTTAATAGTTCCCGGATCTAGGTCTC
GCTCGAACCAATTCTGTTATTTCGTTGCGGCTCACGGGATCCTCCTTTCACTTCATAG

GTTACTATTGTTTTTAAGCTTTTATGAATTTCACGGCTTAACACTGCCACTTACGAATTTTCTACACGAGCAA
ACTTCTTCGATCGCATTTATTTCATCATATTATTTACTGGAATTTCGTGTGCACAATAGAAACTCTCAGTTGA
TACAAAGCAATCACCGTTATCAATATTAGAGACCATCTTGGCAGTTACGTATCTGGAT
GATGGCAGCTTAATTTGTAGCAAGGTCTCTTTACGCTGAGAGTGTTAGACCTGAGCTGTGCATTTGAGACATA
GTGAGTGTCATCGCGTGGAGGAGTCCCGTGAACCGTCTCATGTGATAGTCAATTATTATAATATTCATTAGCA
CTCGCGCCAGCGTCGACACACCGCGCTACAATACATC
>103785510_2 DT667415.3
GCACGAGGAAACCCCTAGCGAGCGACATCAAAGAGGATATTATAGTCAAATCAGAAAACCTAAAGAACGATCT
GATTAACAAAAAGGAAGAATTAAAATCTAACATTGAAAATAACGTCCAAAAAGCTAATAATGCAGTGAATAAT
AAATTAAATGAATTATCTGAATCCGTAGATAAAACGAAAGAGGATACTAAAGCTAAAA
TCGTTGATGTACACACAAAAGCTTCTTCAGACTTTGATAATATTATGTCTTCTGCTAATGGCAAAGTAAGCAA
CGTGATTGAAGTGGTTGATGATGCACAAGAAAAAGCAAAGAACAAGTTCCATGAGCTTGATTTTGCAGCAATG
GAGCGAGCTGAAGAGATTAGACTTAGAGGTCAAGATAAATTATTCCAATTAGGAAAAA
CAATCGAAGAAACATCTGAAGACGTCACAGACGCAGCACAAGACAAAACAAATTTAATTTTGAACCAAGTAAA
TGATCTTAAGAATGATATTAATAAAACTACTCAAGACGTAAAGACAGATGTACAAAATCATAACGAAAATATA
AAAGACAAAGCGAATAACGTTAAAGAAAAGGCTAAGAATACAATAGATGAAGCCAAAG
TAGCAGCTAGCGACAAACTCAATCAACTAGGCGAATCTGTTGATTCAATAAAAGAAAAAGGCCATAAATCTAA
GAATAAAAATA
>103785512_2 DT667715.3
CGGCACGAGGCTCGAACGTCATATTTTTCAGTCAACAAGGTAAAAAATTCTGTGTAAGACAAAACAAGAAAAA
TGCCTTTGCTTCCACGAGCGCGAACTATGATATGCGCCGGTAGATGTCTTAATGTAAGTTTCCTTACGACTAC
ACGAAGGATTATGTCGAGAACATTATATTACTGCGAATTTGGTGATCCACTTCAAGTG
GTCAAGATAAGAGATTTACAGACGATGCCTCTTATGTCTCATGAAGTTCTAGTCAGGATGTTAGCAGCACCAG
TGAACCCAGCGGATATTAACACCATTCAAGGAAAATATCCCGTTAATATACAACTTCCAAGTATACCTGGGAA
TGAAGGTGTAGGAATTGTGGAAGATATTGGAGAAGATGTTGATGGAATATGTCCGGGT
GATAAAGTCATTGTTACTAAACCTGGGAAAGGCACATGGAGAAATTTAGGCTCATTTGACAAAAAAGTTTTAA
AAGTTGTTCCAAATGAGCTGGGATTAATAGAAGCAGCAACCCTTACTGTCAATCCTTGCACAGCTTATAGAAT
GCTATCAGATTTTAAACCCGTAAAAGATGGCCTTGTTGTCATACAAAATGGTGCCAAC
AGCGCTTGTGGACAAAATGTTATTCAAATATGTAAAGCCTGGGGTGTTAAAACTGTAAATATTGTTAGAAACA
GGCCAAATATTAATGAATTAAAGCAATATCTAGAAGGCTTAGGAGCTACTTATGTACTTACAGAA
>103785521_2 DT668565.3
GCACGAGGATAACCTTTTGAGTTTAATATTTTATACAAATAAAATAAATATAAATAAAGTTTTATACTGTGAT
TTGTGTGTGCTGTGATTATCTATAGTAAAAATTTTAGAGGCAATGTATGAAATTAAATAACAGTACTTGAAAA
CGTCTACTGAAAATCATCATGGAGAAGGAAAATGTCCCACCGCCTATACGAAATGGAG
TGGACACATATAGCTCAAAAATCGAGCTGGCGTATAATGGGTCCAGAGAAGACCTCGATCCTTACGTACCTGC
TGAGCACAGGTCGTTGGCTTCTAATACATCGAGTTTTTTAGCATTGGCACATTTGTTAAAAGCATCGCTTGGA
TCTGGAGTACTCGCTATGCCTCTTGCCTTCAAAAACGCTGGTCTTGCTGTGGGAGTCA
TAGGCACTATGGTGATCGGCTTTATTTGTGCCCACGTCATTCATGTTTTGGTAAAAACATCACAGCAGTTATG
TGTAGAAATTAAGAGACCGTGTTTAGGTTACGCGGATACTTGTGACTTAGTCTTTCAGAATGGACCGAAGCCT
GTTAGAAAATATGCTCCATTTGCCAGATAGCTGGCAGACTGGGCGCTTGCTGTCACAC
ATTTGGGTGC
>103785541_2 DT667053.3
GCACGAGGAGGATACCTAGGACAAATAGGACAGCCAGGACAACAAGGTGGTCCTGGTGAACAAGGTCAACCCG
GATACCCAGGGCAGCCAGGCCAGCCCGGACAACCAGGACAGCCAGGATACCCAGGACAAACAGGACAGCCAGA
ACAACAAGGTGGTCCTGGTGAACAAGGTCAACCCGGATACCCAGGGCAGCCAGGCCAA
CCTGGATACCCAGGACAGCCAGGCCAGCCAGGACAACCAGGACAGCCAGGTCAACCAGGACAAGCAGGCATAC
CAGGCCAAACCGGATACCCAGGACAACCAGGACAGCCAGGCCAACCAGGACAACCAGGCATACCAGGTCAACA
AGTTCAACCTGGACAGCCAGGATACCCAGGACAACCAGGCCAACCCGGATACCCAGGA
CAGCCAGGTCAACCCGGACAACCAGGCAAGCCAGGCCAGCCCGGACAACCAGGACAGCCAGGATACCCAGGAC
AGCCAGGTCAACCCGGACATCCAGGCAAACCAGGACAACAAGGTCAACCTGGACAGCCAGGATACCCAGGACA
ACAAGGACAGCCAGGTCAAGACGGATACCCAGGACAGCCAGGCCAGCCAGGACAACAA
GGTCAACCTGGACAGCCAGGATACCCAGGACAACAAGGACAGCCAGGATACCCAGGACAACCAGGACAGCCAG
GTCAACCCGGATACCCAGGACAGCCAGGACAACAAGGTGGTCCTGGTGACAAGGCCAACCAGGATATCCAGGA
CAGCCAGGTCAACCCGGACAACCAGGCAAGCCAGGCCAGCCCGGACAACCAGGACAGC

C
>103785544_2 DT667417.3
GCACGAGGCACGCCCTAAAACAGTTGTGTTCTTATTTGATCACAACTCTTTTGTCGTGCGGACATGTTGAAAT
GCCTCAGTGTGCTTTATAAAACCAATATTAAACAATTCATATATTTTATCGTTTGGCCAATATTTGCGACTAG
GAATATTGGTTGGTCTATACTAAAACATACATGAAGTGGTTATTTTAACTGTTTATTT
TTTTTTGTTTTATATCATTTCTACTGATCTCTTTCTCTGTGCACAATGGATATGCATTGTCGCCCAAGAAATT
AATAATTAGATTATTAAAGCTTACTCGTAATATATATAATAGTGGCAGAGAGAACCTAGGAGCGTCGTGTTAA
GACACTGTTCCCTATTAAGACATTTCCGTTCCCCATTTTCCGCCAAATTCTTGAGATA
TTGCGTATTGGGTGCCAGAACTTAACGCCGCGTTTTTTCGTTGGTAGTTCGCGCATTGCGAAAGCGAGGAAGT
AAAATATTAAATCTTTCATCTCCCCGAGTGGAAATTGAACCCACAACTGTTGCGTTTACAGTCGCACGCTTGT
TCCCCTACGCCATGACAGCCTCCTCGCACCCCATTGATATTAAACTTTATTTTTTATT
T
>103785556_2 DT668090.3
GCACGAGGTGAAACTCGGGTAGTTCCAAAAATAAAAAATAATATATTTTTTTATTAGAACTAATATTTACATA
CCAAACGATTCAGTATTTCAGATAAAAATCACAACATTAAAATTAATAGTAAAAATTTGTTGTTAACTGTTAC
ACTACAAGTAGTAGGAGTTAAATGGGATTTTCGCACAAAAGAAAGAATAAATATTGAT
ATTTAAATGTTAATTGTTACTTAAATTTGCTCAACTCTGTTTTCTGGTAGTTTAGGGTTTTTTTAAGACACTT
TGAGGACCTAAACCACATTATTGTTGTCGAAGCAAGAAATACTGTATTTGGTAATTACGTATTATTAGAAATA
CCAGATTTTCGAAGTTACCCGAATGCACTATATTATTTCTACTATCATGTTTTACATT
ATAAAACAGTAAAAAAAAACTGAATATATTCCATAATTGAGAATTTGCAAAAAAGTAAATTTTCATTGGACAAA
GAGTAAAATAAAAACTAATCTAAATATCAATAAGTATTAGGTTTACATGTTAAGTTTGTTGTTTTACTGGTAA
ATTTACAGATCGAAATTCCCTATAATCTACTTACTGTATAGAAAACTTCCAGAACATA
TAGATATGGGTAAAATGGTTCTTTGTTACCGTAGAAAAAAGTATTGTATCAATTTCTTTTATTTATTTAAAAT
ATGTT
>103785568_2 DT668296.3
CGAGGCCGTCGAGCAGCTCACGCAAGCACACCACACACTTTCACGAGACACTCAAAGCACTCCAACTGTTCTT
CTAAACTGGCTTCGCGGGAAGAGCACACCAAAAGTAGTGCATATGTGAAATGTAAATGAAATAGGAAAGGTGT
AGATTATTATTAGTGCTTACGTTCCAGACTGCATTTGGTTCTGTAAAGATTGTATAAT
GTACCTATATTTAGTACTAAAATGAAACGTCACTATATACATTTTTTTTTATAAAATCTTTATTTTCTTTTGG
TAGCTCCTCGCTGTATCTTTTTTATAGCAAATAGAGGGAACTTATTGTGACGGCGGTTCAAGATGTTGAGTGT
TGAGTGGTGGAGTTAAACGCTGCCTTTTGTTTTATTAATAAAGCAATAAAAAAAATCA
TTCCTCCCGAGTGGGAATCGAACTCACAATCGTCGCGT
>103785571_2 DT668961.3
GCACGAGGAGAATGCTTTAATTTATATTCATCTGAAATTATTAAAATTGTTTAATCCACATTGTATCTATAAT
ATTTTTATTTAACTCCTTTCCCAACAGCTGCATGTACATCACTGTAGCCATCCTTAATAAGAAGCTGTGCTAA
TTCTGTTTTAATCTTTGTCACAACAGGAGGCCCATGGTAAATAAGAGCTGTATATATT
TGAACTACACTTGCTCCAGCCAAAATTTTTTCATAAGCATCTTGGCCTGTAAATACTCCACCAACACCTATTA
TTGGTATGGTACCTTTTGTCAATTTATAAACTGTTTTTATCATTTCTGTAGATGTTTTAGTAAGTGGTTTACC
ACTTAGTCCTCCTATTTCCTTACAATATTGTTGATTTTTTAAAGAAGATCGTTCAATT
GTTGTATTTGAGATTATAAGTCCATCTACTTTACTATCTTTTCTAGTGATCACAGCA
>103785577_2 DV501297.2
CCGCCCCAAGGGAACCCCTTCTAATAACCAATAATAACTTATATAAAAGGTTCTTACATGCTGTCATCACTTG
CTTACTACAGCATTGTATGTCTAGGAAAAAACTTTACACTTATTGCACTTTATAATACCATTGACTCTTAAAA
CTGAATTAATTGAGACCTAGTATAAGTGCCCACCCCATCCGTATCCCCTACACCATAC
ATAGCTATATATTTTTTTTATTTAACATTTATAATTTCTAGCGTATAGATTCGATTTTGCCCGCAGCCGATTT
CTGTAACTCTGACTGTTTCATGGCTAGGCCCATGTTGCCTTGTATTTTAATCTTGCCCTGCAAGAACGCCTTT
TGGGGATTTATGTTTCCAGAGATCATATCCGCTACGTCCTCTATCGTTTATCGTGAAC
GTGACTTCACATTTGTCTTTGCCATTGTACTGCACGTTACCCTTTCCTTCCTTCGCATTGATCACCCAATAGA
CCTTCCTCCCCATTGGGTCCATTCCTGACTTTGAACCCGTATATGCCTCTAACCTTCTCACTAAGTTTATCAT
CATCATTCTTCATCGCCTCCTCGAGTATTTTCATGTATTTGTACACTT
>103785598_2 DT667186.3
GCACGAGGTGTTTTCGTTATATTTTGCAATATAAACAATATGAATGTAAATCAACTTATCAACAACATCTTCA
GCAATTTCCAGAAAAGTTTGGACGAGGAGCAGGAATTACGTGAAGTTATACGCAACATTTGTAAAGAAGTGGA
TCAAATATCTCGTCGGATTACAACGGTTCTTCAAGTAATACACCATAATGAGGCCGAC

ATTGGCCCTGCGTGCCTTCAGGCTCGAGAATTGTTAGAAACATCTCGGGAAGGTTATGGCAGACTCAAACAAG
CCGTGCCCGCAACTGATTATTATAAATATCAAGACCACTGGCGTTATATGACTCAACGGTATTGTTACTTAAT
ATCTCTCATAATTTGGTTAGAAAAAGGAATTTTGGCACAACATGAAACTGTAGCTGAC
ATTTTAGGCATAAGCGCTGTGGAGGAGAAAGAAGGCTTTCATCTCGACATCGAGGATTATCTTGTCGGTCTTT
TGAATCTCTGCTCAGAGTTGTCCCGTCTTGCAGTGAACTCGGTGACGCGCGGTGATTATGAAAAGCCTCTGCA
AATTTCCAAATTTGTGATGGAACTCAATGCCGGATTTAGATTATTAAATCTCAAAAAC
GACCATTTACGCAAAAGATTCGATGCTTTGAAGTATGACGTGAAGAAGATAGAGGAAGTGGTGTACGACCTTA
GCATTAGAGGCCTTCTGCCCAAGATAGAGGCTCATGATATTAATGAATAAATTTCTATTATACT
>103785616_2 DT667119.3
GCACGAGGAAACGACCTAGATCCTGAACTCGACGATTTATTGGATAGTGCCTTACAAGACATGACTAAAAAGG
ACCAAGATTCAAAACCAGAAGACAGTGGACAACAAAATATGTGGAGTGAAGAGTTTATTAAGGAGGCTGCCGC
ACAGTTTGAAAGTAATATGGCAGCCATATTGGGTGGATTTAGCGGTGTTCCAGAGGAT
CAAATCAGTCAAGAGCAAATAGCACAAACATTTTCAAAAATGGCAGAGGCAGCAGCCCAAGTGTTGAAGCCTT
CAGATAATGTCAGTGAACCTGCACCTACCGATCCCGCTTCACAGGAAAAAATAGACGAGGTTTCCGCAGCCAT
ATCACAAACGCTACAAAATTTGAACACAAATTCGGAAAATCTGCAGACGCCATTCTCT
GAAGCGGACTTAGCGAACATGTTCAACAATTTCAATCTCGGTGAAGGGAATCAGGAAGGCAACATGTTTTTGC
CATTCATGCAAGGTATGATGCAATCGCTTCTATCAAAGGAAGTTTTATATCCATCTTTAAAAGAATTAGTAGA
TAAATATCCAAAATGGCTGGAAGATAATAAAGGGAAAATAGAACAGAGTGAATATGAA
AGGTTTGAAAAACAACAAGACTTAATGAAGAAAGTGTGTGCAGAACTGGAGCCCGAACAAGAAAGTGATCCAG
AAGACATGAAAAGGAAAAGATTCGAATCTGTATTGAAAATGATGCAACAGA
>103785640_2 DT668772.3
GCACGAGGGGGGCCCCTTAGCGTTGAACGTGTATTCCGTACCGCGTCGAGCAGTAGTCACGATCGTTACCGGC
ATATAATTTTCCCGTATACACTTGATCTAATTAAATAAAAATTCCATTATTTTTTTTATTTAGATTCTTCTAT
AAATATCACATGGTGATCTTTTATTCATATATTAAATGAAAATTTTAACCTCTCTAAA
AGGATATTTATTCGTAAATAATAATATAGTGTACTACTTTACGTTGTGTGTTATCAGCATCTGTTCTGTGGTA
AACCAGCGGCATTCTATTATCAGGACAAGGCTGGCCGATGGTGTTTTAGTTCGCACGTGGTGTTTCCCGCGGT
GGCTTAGCGCAGTGTGATGCAATGCTTGCGTGACGGAGGATCACGGTTTCCGCAACAG
GCCCCGATTAAACCATGTGTTCTGTTTAAATCATGGTGAACATTTTGATTAAAAAGTGAAGTTTTTTGCTTGT
TATTGTTAAAGATTTGACTGAACTCTTAAATATGGCTAAAGTATCGGCTACAGAGGGTCTCACACCCAAGTCG
ATGGATGTAGGTGAAACTGGCACATCCGCAGAAGTGGAATCAAATGAAGGGATACGAC
TGAAAAAAGAATTGAGCCTAATGAATGGCGTGGCGATCATTGTGGGAGTTATAGTGGGATCCGGTATCTTTGT
ATCCCCCAGTCTGGCCTTGAAGCACGCGGGCTCGAAGGGCATGGCCCTAATAGTATGGGTACTTTCTGGGTTC
ATGTCCATGATTGGAGCTCTATGCTATGC
>103785849_2 DT668174.3
GCACGAGGGTGACCCTTACAGCTGTACATCTATAACCGCGTACGAAACGCTAAAATTATGACTTATGGATATT
CACACGATAAAAAGTTTATCATTATAGAGAGAAAACTCAGTATTGTAGCGAGGTATAAAGAGAACGGAAACAC
AGCTGGGAGGCTGATTTTTAGTGTTTTAGTTCAGTGACTGTGTTTTGTGTAAAAGGGT
TATATATTTTTTTTGTTTTTTTTATTTTTTGGTTTTGGATTTACAATATGGTACCCCAGCAAATTTCGGATGT
TCGCTCTATGACGCCCAATTCTAATGTGCCGGTCTTTGGATCGCAATTGGTTGATAAGAATTCGTCTACGCCG
TACACTGATGCTACGCAGACGAAAAAGAACAACCCAAACCACATAAAGAGGCCTATGA
ACGCGTTCATGGTGTGGTCCCAGATTGAACGCAGAAAAATTTGCGAGCAAACGCCAGACATGCACAACGCGGA
GATATCTAAAAATTTGGGCCGCGTTTGGAAAACTTTGAACGACGAAGAGAGACAACCCTTCATCGATGAAGCG
GAGAGGCTCAGGCAGTTGCACATGCGGGAGTACCCCGACTACAAGTACCGCCCACGCA
AGAAGACAGCCAAGCCGGCGCAGAGGAGCGGTGCAATAACGAAGCAGAAGCGCAAGCAGCGGGCAGACAGCAA
CAACAACAGAGGAGTGTCCCGGCGGCGGCCG
>103785944_2 DT667048.3
GCACGAGGCGAGTTCTTTTTTTTTTTGAAACATGTGTGTTCAGCGGAATTGAATGCCGCGCTTTGTGTGTTAC
CAGAGCGGGGAAATAAAAATAATTCATTCCCTAGAGTGGGAACTGAACTCACAACCGTGGAGCTTGTAGTCAA
ACACTGTGCCGCTACATAGAAATTTAAAATTTTGTCACTTGCCATGACTTTTAAGTAT
CCCGGAGATTTAATGTTTGTTGCGGCTTGTACGACGTCGGTGTCGGGCGGCATCCAATCAGGTACTACACTAT
TGGTAAGCCCCGCTACGCGACCAGCGACTGGGTCTGATGGTAAGGTACATCAGAATGGCGTCAAGAGACAATA
TCCCCGATGAAGACATGGGAGGAGGGTCTATTTTAGCCAAGAGCCTGTTTGCAAAAAG

CTGGATGATTCATTGCCGTTTCTCACTTGATTCCGCTGCGCTCTTATCTTCTATCTGTCATGTTAGTGATACT
AAGCTCGATTGGCGTAAATCATGCTTGGACGTATTTATACCTTGTAGGACTGGGTATGTACTCGCAGATCCTC
TCTGTTTTTAATCACATTGTCAATCCACTATAATGGAATCGAATTCTCGCTGGTTGGA
TGGAGTCCAGGTATTGACATTCAAAAGCCAATCGGAGGTCAGGTCCACTGTACGTTGAAGCTTGAAGACTATT
TGGGGTTAAGTGATTTCGACATTGGATGTCATACGCAGAAACACAAAGCGTGGTATTACTAATGTATTATAAA
AGGCAGTAACTGAAGAATAACTCGTTAAATCCTGATTCTCTTCTG
>103785946_2 DT667579.3
GCACGAGGGTGCCCCCGCCGCAGTTCAGTGACCGCGTGCGCGTCGTGGCCGCCCTGCCCAAGCGCGCGCAGCT
GCACTAGCGCGCCGCCGCCGCCCGAGGAGGAGGCGGGGGCGGGCGCCGCGCCCGTGCTGGCGCCACCGCCGCA
GTTCAGCGACCGCGTGCGCGTCGTGGCCGCGCTGCCCAAGCGCGCGCAGCTGCACTAG
CGCGCGCGCGACCACCTCGAGCCAAAGATTTATACTATTTATTGTACTACCCTCTCAGTCTATTTATTGCGCC
CCCTCTGCGACGCAAACGAGTGCCATCGTGACTATTGTTTTGTAATATTTTTTATATTCATATGTATGTGTAA
ATATTTGACGCGCGCGGGGAGAGCGGACGAGGCGCCTGTATATACAAATATATATCCG
GCCAACGAGATCGAGATGAGATACGATATAATCGCAATATTTATACATATACTTTGTTATTATTGAAATCTAT
TCGTGTTTTAAAAGTAAATAATTATTTCATTCGTCCAATGTGAGTACACTTGCGGTCCGCTCTCCCGACGCGC
TCTTGGTTCTGTGCTTTAGTGCGTCGTCCATTGTAATGTACATTTTATATTAGCTCTG
CTTGTGCCGTGATTTCAATTAAAAAGTAACGCTAAATATTATATTAAAAAAAGAAATTACAAATAATAATATT
ATTTATAACTTTTCAAATTCTATG
>103785952_2 DT667779.3
GCACGAGGATAAGTCCTTACATAGTGGCATGAGAGTGAGTGTATGAGTGCAGCGTGCTGGTTCCGCTTGAAAG
ATAAGAATACTGAGCACGATGACAGCATCTACCTTTCTACGTACAAACGCCACTTACACGAAAGCAGATTGCA
ATACACCGTCTATAATTACACAAGACAAGAGGAGAGACTACGAATGGCAAATAGTGTG
GAGAAACGTGTTAGCATTTGTTTATCTCCACGTAGCCGCAGTTTATGCTTTTTACCTGATATTTAGCGGGAGA
ATAAGGATGTCGACTGTCGTATTTGCATTATTGTTCGCAGCGATGTCCGCATTAGGTATAACAGCGGGCGCGC
ATAGACTATGGGCGCATCGAGCTTATAAAGCGCGATGGCCCTTAAGACTATTCCTGGC
TCTAATGCAAACGATGGCTTTTCAAAATCATATATACGAATGGGTGCGTGACCATAGGGTACATCATAAATAC
ACGGAGACAGATGCAGATCCTCACAATGCTCGTCGTGGGTTTTTCTTTTCTCATATGGGGTGGCTGATGGTCA
GAAAACATAAAGAAGTGTTCGAAAAAGGTGGCTTAGTCGATATGTCAGATCTTGAGAA
AGATCCCATTGTAATGTTTCAGAAAAGAACTTATTTGTTTGTGATGCCGTTACTCTGTTTTATTTTACCAACT
TGGATTCCCGTAGCATTATGGAATGAGAGTCCGTGGAACTCGTGGTATGTGGCTGCCATCATGAGGTACACGG
TGTCGCTTCACTTCACCTGGCTAGTCAACTCTGCCGCCCATATATGGGGCAATCGGCC
ATATGATAAGTACATAG
>103785958_2 DT668807.3
GACGAGGCGGCAGCCGTGACCCATCGGCCACGGCTACCTCTCAGTGCACGCGCTGCCTTGCCGCCGCCAGCCT
GCGTGGTCCCACCACGATACGATACGTACGACGACACTGTCTATCTGACGATACTATCGATACTTATTCAAAC
ATTTATTTTGTTTAATTAAAAATTGGTTAATTATTATAGAAAGCTCTTTTTATTGTAC
GTGCGTTGCGACTATATTGTTTATATATCCTCGTATGGGCCAATTTT
>103785960_2 DT667557.3
GCACGAGGCACATCCTTAAATAATACTACAATGAGTATATTTGCTGTTTTGTTTACCCTATTATATGTTGCGT
TCTTTGGAGTGGGTCCCAGTTCTATTCCTTGGATGATCTTGTCTGAACTATTCGGTCAAGGTGCAAGAAGTGC
AGCTGTTAGTGTTGGAGCTTTAGTTAACTGGTTCGCAAACTTTATTGTGGGACTAACT
TTCATCCATTTATCAAAATTGCTTGGCAATTTTGTATTTATACCGTACACGATACTCTTAATCATATTTTTCG
CATACACTAAGTTCAAGTTACCAGAGACGAAGAACAGGACGATCGAAGAAGTCACAGCCTTATTTAAGAAATA
ATATCCTTGGAACGGTTAGACTTTAGCAAACATTCCTTAGGTAATATATCTATCTATA
CATTTTATGTAATAAGTGAACACTAAAATTGTGATAGTTACTATAATAACTTGTTCGTTCAAATATATTCCTT
TGAGAGGTATTTTAATGAACCTTGTATTTATTCTGTGAAATCGCCATTCATCGATTTCCGATTATTAGGAAGA
CTGACCCTACGTACGTTAAAATGTTATAATAAACTAATGATGTACTTATTACTGTAAA
CGCAACTATTAAAATAGGGTTGTATTGTTATATTTTATATTTTTAAAATACAAGGCTATTAATGGCAAAGATG
CTTGATTATTTTTTAAACTTTCTGAAAAAAAAATAACTTAAGGGTTAGCTACAGTTTTTAAATTATAAGCTTA
GCTTAATTCAAAATATATTTAATTCATTTTTATCAAATAGTTTTTTCCTAATATTTTC
TAATTCTGAATGTAT
>103785965_2 DT668963.3

GCACGAGGGGGAACCATATATGAAAAGGGAAAAAGGAGAATTGGAAAAGCCATGGGAAAGAAAAGCTGCAATG
CGTGAATGGGATGTTGGTAAAAATGACAAAACCAAGGAACCAATACGCCAAGATAAGTTGTTAAGGGAGGAGC
GGCCACCATTAGAGAAACGACGACATCGCACACCTGAAAGAAGTCCAGAACCGGCAAG
GAAATTCAAGAAGAAAGAGGATGAAGCACCTGCAAAGCTGTTGGATGACTTATTTAAAAAAACAAAATCAACT
CCTTGTATATACTGGCTACCATTATCAGCTGAAACTATAGCAGTAAAAGAGGAGCAACGACGTCAACACATGG
CCGAGCATGAGCGGCGGCTCCAGGAGCTGCGTCGTACCCACCGCAGACACTAGCATGG
TCCGTCTATCGCCCATACACACATTATACACACACATACACATCATACGTAGACATGCATAAACACGCGTTCA
CACACACAATATCCAGTCACGCGTAAAACACACACATTTAGAATCACATTCACACATCTACATTCTATATACC
CCACACAATTTACATAACATTATAATCCCACATATATTAATGCTTTGTCGCAATGTCG
ACATTCCTTTTCAGGTTTCATTGGCAATTTTAATTAAAAATTATAAGTAAAATATTGCTTAATAGATTACTCT
AGTTTGTATAGTAAC
>103785967_2 DT668287.3
GCACGAGGGTTAATCTATCGCACGCTTAATCAAATAAAGAAATAACTCAAAAATCGCTGTTTCTGTGATTTAA
TCATAAAACGCAATATTACGGTGCTAAGAATAGGTACACTCTAACTGCCGTTAGATTTGAACGAATATGAGCG
AAATAAAAGATCGTCCCTCTCTTTCTTATTAACTCAAGTCAGCAAGTTGCAACCAATA
AAACGATACATTCAGTGTTGTGCCAGTGCTGACTCAAGGAAGACGATTCGAAGAACGAGTTAAAATATATTAT
ATCTTTTTATTATTAACAGGTGCGTGATATATTTTTTTCTAAAATGGTTATTCTTAGTTTTATTACGCTATAG
AATCAAAATATTATCGCTTATTAATGAAACATTTAGACTTAGTGCATTATTCTTTTAA
ATTTATGCCATAATCGATATGTATTTTTTGCAGTTAATTCAGTTTACTTGTTATTTTATTTTAGATTTATAAC
ACAGATCGACTTACTTCGCTTTATTATAAATTCAAACGTAAAATATATCAAACAATTATATTTGTTTCTGTTT
AACATTAAAAGTGATTTTATGTCCATTGCCGTATTAGAATATAATTCCTTTTTTGTTT
AAATAAATTAAAACTTTACTCTACTGTGCATGGTCATAAGATAATAATTAATAGTAATAGTGATTGCTTTAGG
GATCCCACCGTTCCATCAAACCTCTATT
>103785969_2 DT668906.3
GCACGAGGGTATCCCCGGCCCGCGGCCTGCATTTAGCTTACGGATACCGAGCTACCTCACTAGGTAAGATTTG
GTCTAAGCTGTAAAATATGTTCTGAATTAGTGCAGTGGACGTTTCAGACTGTGAGTGCGTTCTTCTCAGGTAT
ACCAAAATGTTTCCGAATTCTACATGAATATGTTATCTAGTCGTGTATGTACTTTCGG
AATTGTAAATTGTTTATTTATAGCGTTTATATATGGCCAACTCTGTGGAGCGCGAGCGCGGGGCTGGTGCGCA
GGTGTGTGGCTCTCGAAGGCAGTAGGCTGTATGATATGTTTTAGAGAGGCGTGCTGCTTTCAACGCGAGCAAG
TACAACAAAGAGCCTACACCTTGTATGGAAATATAGTTTTAAATTGTGAGTGGCGTCA
TTATCGAACCTACTTTCGGTTATAATCAGAATTAGCGTTATGCCATCGAGACTGAGTATATCGGATTCATAAT
ATTTTACAGTTTAGTTAGACGAGCCTCGCGGCACAGCAACACTTCATACTTACCTCAGAAGCACGCGTGCTTC
CTTAGAATATAAGACAATAACGATTGGGTCTGGCCGAGATCGGGCGTACACGCCGAGC
TTCAAATTATTTTCATAAAATGCGATGTGCCAAAATTTCTATATTCTGGACTATCCTACGAAATATTCTTAGG
TAAAAAAAGCAATAATTCTTTTGGTTGAGCGAACAAAAGAAACTTATTTTGATGTCGTCTATTTTGAATTTAA
ACAAA
>103786016_2 DT667968.3
GCACGAGGCCGCAGCCGTATCAGGGGGGGCGGATGGTTACCAAAGGAGGGCTCGCGCTGGGCGGCGGGGCGCA
GGCGCGAGGAGGGAACAAGAGGTTAAAGAAGCGAGAGCAAGGCTACAACTTGAAGTCCGCGCTAGATTAGCTG
CTGAGGCACAAGCTCAGGAGCTAGCAGAATTAGCACAAAAAGATAATAAAAAATTAGA
AGAATTAAAAAATGCACAGGCAGAGTTAGAGAAACTACTGCAAGAAGAAACACAAGCAAAGCGAGACGAAGAA
ATAGTCAGAGCCTTACAAGCCAGGGTATTAGCAGAAGAATGGGAACGACGAGAGGAACTCGAAAGGTTGCAAG
AGGAACAAAATAAAATGCTAGAAGAGGAAAGACTGAAACGCAAACAATTCGAAATGCT
CCAAGCTGAAAAAGAAGCACAATATAGAGAAGCTGAAAAACGTTTAAAAGAACTGGAGCAAGAGAAACAGCAG
CTGGACGCAGAACTGAGGGCGGCACAAGAAAAGATCTCTCAAACGGAACACAACGCTCAAGCACTTCACGTTC
AACTTAGGGATTTAAATCCAATTCTTCGCAATGGAGAACGCGCGCGCCGAGCTATATC
CTTTGTGCCTACAACGAAAAGTTCATCCGACACTTTAATCGACCTTCGAGCATTGAGGCATTTGAAAACTTTA
AACGACGATGAGAAAATATAAAAGAATCTGTTGGTTGTAGTAAGAAAATTATGGGAACGTTACCAAAAGAGAA
AAGAAGGCGGAGAGGGATTGATGGATATCTAAACAAGTTTATTTCTTGAGAAACAAAA
GATATTCTTGACTTAATGTATGAATTTTTATATTGCTTTTGATTT
>103786019_2 DT666983.3
TCACATTATAGTTTACGAAGCCCTAATGGAAGACTCCATCGATCAAATATTCGGCTTCAGCCACGTGGGCGAC
GGCAGCGGCTCCACCACCGCCCACGTGACCACGTGGAACCCGATTTTTTTCTCAAGACTCATGAAGTGGGGAG
AGTAATCAGTGCCGATGCGTCACAAGGAGTTCCAACTGGTCAACGTGCCGGGCGCTCT

TAAATATATAATTGACTTTGCAAAGAGCAAAGTGTCATCCAAAATTGGCGAAAGACTTCACATCCACACAAAT
ATGAAGAACATGCAAAGCCACGTGGACATATCGTGCTTGCCCACTTCCTTTGGTGGTCATATACCATTACAGG
ATATGATCAAATTCACAAGAGAACTACTTATGGACAAAAGGCAGATCATAC
>103786022_2 DT667929.3
GCACGAGGATATTCAAAATGAAAGTTACTCTTGTTCTTAAATAAAGCGACATATTTAAGATAAATAATAAAGA
AGTAATAAATGTAAATAGAAATTTGAACTCAATTTTGTGTAAAAAGTGGAATAAAAGATTGTTTCTTTTTAAA
TAAATAAGTCACGTTCAGAGACATGTAAAAATCGAATGTAACAACTATTAAATATTTT
ATCTGGGAAGTAGCAATATCGTAAATAATAATAGTTAAAAGTCTGTGCTATTCATAAACATATATATAATCTG
TATGAATATTTAGTGTTACCAGTGGTAATAATATAAAAATCAATTTTAAAAGTAGACTGTGTAGTATTAAGTC
TTAGGCAATATGGTGAAGAAGTCTTAGGAATTAAATAAATATAAAAATGTGTTTTCTG
GGGTATAATTTTCGATTTTTTTTTAACAATTTATTGCTATTTTTATTTATTTTTTATATTTTGTTTCTAAATT
AAAGTTTAAATATAGTTAACCTTGGCTACATGTATGAATCCTAAATCTTGCATTTACTTTTTTTATGATAAAG
TATAAAAGCCAAGTAAGTTGTGTTTAATAACTT
>103786030_2 DT667970.3
GCACGAGGTTTTTTCCTTTTTAGATTTCTTTCATATTTATTATAAATAAAAAAACCAGATATATAATATCAAT
TCCATACCGCAACACTATTCTTCATATCAGCAACAACGCTGCTAAACTCCGGGAAGTGCAGCTCGGGGGCGAA
GGGCAGCGGGTGCGCGAGGTCACCGGTTCGACGTCCAGCTGTGATACATTGGTTCTCC
AGTTTACGGTTGTTTGAGACAATGGCGGCATGGTATTTGCCTGTTGCTATTCCCTGTGCCCCAGTTAAAGTAG
CGACGTCCACAATGAGATTAGCTTTCAGATCTCTCTGCGCGTATACGACGCCGCCGCTGAGCACCAAACGACC
TTCTGCGTCGGTATTGTTGATTTCGACAGTGCGTCCAGAATACAGCTGATGGATATCA
TCAGGTCTAGTAGCATTAGGCCCGACACTGTTTTCAGCTAAACAGAGCACGGCATGCACATTGATGGTGGGTT
TGGACTTGACGAAGGCTAGGAAGGCACCAAGCACTGCTGCAGCACCACCGCAGTGACCTTTCACGCCTGCCAT
TGACGTCCTGGCTTTAATGCTGAGGCCACCAGTGTCGTAGACGATGCCCTTGCCGACC
CAGGCCACGCTGGTGTCGGCGCCGGGCGCGCGGTGCGA
>103786049_2 DT668682.3
GCACGAGGGTCGACCTGGTAACTTGCTTTTATTAATATTTTGTGTTTAGTGAAAGAAATGGTAAAATGGATGG
TTTTATAAATACTAAAATAGAAGCGCAAACGATATACGAAATATATCGAGCATGTCGTCTCTGCGGCGCTGGT
GCTGGGTACAAAATGCCTATAATACAGAACGTTGTCGACTTGGATGTAAGAGAAGTGG
AACTCAAACAGAAAATACGAGATTGTGTTCAAATTGAAGTTCACCAAGATGACAAAATGCCCCCGCTGATTTG
TGAATTGTGTGTGGACAAAGTGAATGATTTCTACGAATTCCTTGAGATGTGCAGACAGACAAACAAAAGGACT
AGGTTAAGATTGGGCTTGCCTCCACAAACAATGCCTAGGGGTGCGCCAGATGCAGGTG
ATTGCATATTGGGTATTACTGAGCCAGTCTTTATAAATGATGATGATAATGAGCCGTTAGTAAAAACAAGACA
ATCTAAAAACTTTAAAGGGAATTTGAAAAACATCAAAAATAAAAATCTGGATAGTAAAGTTAAGATCCAATTA
CCAGACAGAAAAGAAACAAAAAAAATTAAAATTGAACCCCGGTCACTACGGAGTAAAC
AGCCAAGCCCGCCTCGCTTAAGAGGAACTAACAGGATGCATGAAGATGACGTTGCTTTGGGTAGTTTACAGAA
ATCAAAACGGTCAGTGCCAATGCCCAAGTCAATATTAAAAAAAAACTGAGAACGAAGATAACTCTTATCTAACA
CCCAGATTAA
>103786059_2 DT668300.3
GCACGAGGAAACGGCTAACGGCGGCTCGGGCTACAGGCCGCGCTGCTCGGCGGACGGGGCGTTCTCGCCGCGG
CAGTGCGGGCGCGCGCGCTGCTGGTGCGTGGACGCGGCCGGCCACGCGCGCCCCGCCTCCACCATGCACGGCG
ACTGTGAGGTGACCCACGTCGAATCGGCTCTTCTCGAGCTGGAACTGATCGGTGTCGA
CCAAACCATCGGAGACAAAGCTCAGGCGCTACTGACTTCACGTCTTCTCGCTCTCGGTGCAAATGTACCTGTC
ACTGTTAGCAAGGAGAAAGAGGTTGTTAAGCTACGGGCTGTGCTGTCTGGTCCGAGGGCTGCTGATTTGGTGT
ATCAGCTTGAAACTCAGGTAAAAAAAGAAAAGCTATCCGGCGCCAACAAATCTGAGGA
GGGCGTGCTCGGCGCTGACGTCATCCGCAGCGAGTACCGCCTCGTGGCGCCCGCGCCCGCCGTGCAGAGGGAA
ATTGTCAGTGAATCTACAGTATCTGCAGCCACTTCTTATCACACCGCTTTAATAGTCCTCGCGGCCACATCAG
CCTTCATTATCAGCGTGCTCTGCGTATTAGTTATGTTGTACAGAGCACGGCTTCAACG
GGAACCACATAAAGCTGAAAGGTTCCTGCCTGCTGCTCCACCAGTCTACGTGTTATCCGCTGACGAGAAGGCA
GAACTGGCCAGGGTCCTACACGCTCCTCCTCCACCCGTGCCGCCGAATG
>103786096_2 DT667002.3
GCACGAGGCGAGACCTTTAAGAGTTGTGCCCACCACACCCAAGCCCCTGCTAGAAGATAGGCGTAAATTGTAA
TGTTTGTAATAATATTATTCTTTTATTTTGTAATGTATTACAGGTGTTAAAATGTAGAGCCATTTCGATGTTT
ACAAATATAATTTGAATTTTACATGTTAGTAAAATATTGAAATTATTGAATTTATTAT

CATAACAAAGTAACACCACATATTTTATTTGTCAATTAAAAAATTATTTGATTTCAAACACTGTTACATACTA
TATAGAATTAATTAGATGATAATAATCGGCATTATATTATTATTTTATACTAACGTCAGCCAGTAAAAATGTA
AAATAGAATAAAAATAAATGACATCACAGTTTGTCAATAAATTAATAATTTTGCTTAC
TTATTATAATAAAATTTCTGTACATCTTAGTAGAAAAGGCCAACTTAGCCACAAATACAGTTAGCCACTTAGA
AAATATTATAAATAGAATATTATTATCCAATTCTTCTCTAATATGTAACAGTACTACATAATATTTTATTCTA
ATACACCTGTAGTCTAAATCAAACCCGTAATATTTATTGGTCTTTTCGATCAAATCAA
AGGGTTCGTGTGATATAATGTAAATCTCTCTTTATTCTCTATTATTGTAGTTATTTAGATATTTTATTAGATC
CTTGTGAGTAAGTTGATGAATCAAAAGTTGTAATATGTATTAACTATACAATATTTTACATTGATGATTTTAA
ATAAATAAATGCAATAAATAGTTAGTTTACAATATTGATTTCTA
>103786099_2 DT667211.3
GCACGAGGAATTTCTATTAATAAAAAGTGAAGTGATAATTAAATTTAAATAATAAAATGATTTTTATATGAGT
AAGGAATGTGAAATATAAACAATAATTCAACATGTCAGAAGGAAAGAAAAGCAGTCATATATATACCGGGGCG
CGAGTTTTAGGTTATGTCAGTACACATGTTCCTTTTGTGGCACGATTTATTAAAAGAA
GAGGAGAAACATTGCTTTGTACTAGTGTAGGAAAGTGGTTTCATACCTATGGATGTGATAAATTCCGTTTACT
AAGTGTAAGTGGCGAGCATCCCGCTCCTATAACATGTATGAGTGGAGACAGTTTTCATGTATATACGGCAAGT
GAAAACGACATTTACGCGTGGCGGCGAGGCTGTGAACTCAAACATGTTTACAAAGGGC
ATCAGGCTGCTATACATAAAATTTTACCATTTGGTATTCATCTTATATCTATAGATGAAGATAATGTCTTAAA
AATATTTGACATTAAAGAGGAAACAGAGTTTTTAGATCTTCCTTTTGATGACGAACATTTTAAAATAACAACA
ATATGTCATCCACCTACTTATTTAAATAAAATTTTACTTGGTAGTGAACAGGGTCAAT
TGCAGTTATGGAATATTAGGACATCAAAACTTGTATATACTTTCAAAGGATGGCAATCTCCAGTTGTAGTGAC
ACAACCGGCACCCGCAGTGGATGTGGTTGCTATTGCTTTAAAGAATGGAAAAATTGTCCTCCATAACATTCGT
TTTGATGAAATTGTTATGGAATTAATCATGA
>103786104_2 DT667009.2
GCACGAGGGCTTTCCATATTTTTTTTACGTATTTTAAAAATATGCAATCAATGCTTTAGCAAGTTGACACGAA
CTTTGAAACAATAAATGTAGAGTTAATCGAAATTGGAACTAACATAAATATATTGATATGATTAAAAATATGT
CAAACTTGCTATAGTATTCGGCATTAATTAAACTAAAATTATATAATAAAATAAGTTC
CTTTCTATATTCAAAAACGTTTTAAATATAACAGAACTTAACGCGAACACTCGCGTCGTGCAGTATTATTGTA
TTTAGGGTTTTTCTTCGAATTTATGATAAGATAGCACGCAGTTTGTCATTATAATTTATAATTAATCTTTTCA
TTTGAACGTCCACCTCAATTCACTTTTATTACATTTGATTTTGAAATAATTGCCACTT
CATTGTAATATTTTTTTCTTATTTATTTTTACTAATAAAAGTATTTTTTTATATGACAGACTGTGTGCACTCA
TGTAGACTGTTGTGTACTTATCGATCGTTTAAGTATTTATTTTTAGTACAAAATAGTTCAAGCATCTAGTATG
TAGTAATTGTCTTGTGTTTATTTTAGAGGTATGTCTGGAACCGCTTTCAAATTATTAT
TTTCAATAGAATTCACGTAAACGATTGCTATAAGGGTCTTACGTTTTTTGTTTGTATCGGTTGGTATCATGGA
ATAAATTTTTTATAATAAAAAAAAAAAAAAAAAA
>103786107_2 DT668639.3
GCACGAGGCAATACTTTAGGTTTGGAAGAAGGACTCCTGATTGGGTGTGACATTTTAAAGATATTACGCCTCG
ACCGTAACAATATGAATTTCGTTCCGTCTTCTTCACTCAATGGGCCGCAGTCATTAAAAGTTCTTTCGCTCAG
GGAAAACAGAATAGGTACAATAAGGCAAGCGACGTTTGTGTCACAGAAGACGTTAGAG
GAAATTGATTTGCACGGCAACATGATATCAACTATCGAAGGAGGAGCTTTTATCAGCTTGAGTGAATTACAAA
GCCTCGATCTCGGTCGAAACAGGCTTTCAAAGTTCAACAGCGATGTCTTTCAAGGAATCGAAAACTTGGAGAA
ATTAGATCTTTCCGAGAACTTTATAGCGGATTTCCCAACTGTGGCTCTTAAATCATTT
TCAGGCATCAAGCACTTAAATATATCTAGCAACATGATTTCGAATATGGATCCTAGCCATTTGAACTCGTTGA
CGTCACTGCAAGTTTTGGACTTGAGCCGGAATAATTTAGTCAAGTTGTCGCCAGGCACCTTCGTAGGCCTTAC
TGATTTACGTTATTTGGATGTAGGAGTCAATTCTCTAAGAACAGTGGAAGACGATGCT
TTTGATGGTTTGACTAGTCTTCAAACTCTTCTATTAAGGGATAATAACATACTGCTGATTCCTGCTGCGGCTT
TGTCTCGATTACCAAGCCTTACATCTATTCACTTAGGATTTAATCGTGTTACAGCACTTTCAAGTGATATTTT
GCG
>103786110_2 DT667805.3
GCACGAGGGCTCATCGTAGATTACATTCAGATAAAATGTTGGTTAAGAAACGTAAACGAAATTGGTTGGCGTT
GCAATTGAGGACTGAGAAACGGAAAATTGAATTACAAGATAACTTAAGTACTGAGGAACAGAAACGAATTCAA
CGTAAACTGATCAAATATAGGAAAAAGTATTCCCTACAGAAAGAAAACAGGAACCCGC
CGTGGGTACCGACACAGAAAATGGCGCGAATGGTATACAGCAGTGCGTCAGCAATGAGAAAGTTCCAGAAGAA
AGCTGAATGTATGGGTGTGGATACACATGCGTTGGAGTTTCAAGATGCACAGATGGCAGCATTAAAGGATGTG
TCCCGCCGTTGGCACGTACCGCCCGCGTTCTCCGTTGAATTCGTCGGTTACTACCTGG

AGAGGAACACCCGTGTTACCTCCTTAAGAAAGTCTTTAGAACGACTCACGAAGGAAAATGAGAAACTTGTCGT
GGAAGATGAAAAATTGAGGATGGAGTATGATGATGCGTCTAAAGAAAACACAGAGGCGCTATCAGAACTGACT
TGCACTAAAGAGGGTTTGCAAAAGATATACGATGCAATAATAGCTTTATCGCCTAAAA
AAGCTATTCCCACCATAATGGAGGAGAAGCCTTTAGTACCGCCGGACGTGCCGCGTTCAACTCCGAAAGTTAC
GCCCCAACAGTTGCACAAACGGTCCGTGCCCACAGCGGCTGCACTTAAGATGGGCGTTGGTTTTCCTCTTAGC
GACAACCCGGACGCACGCCATGGGAAAGTCTTGTCTACTTCGTTAGAAGCGAGCGCGC
TGGCGGGCTGCGGCGCGCTGGCGG
>103786119_2 DT668654.3
GCACGAGGAGAGTCATGAAAAAATTGAGTCAGAACAATTTGCCAATGATAAAATGCATGTATTCAGAGAAGAT
GCTGGGCCTAAGAAAACTAAATGGTTCAAGAATCTTAAATTATTAAAAAGTACAGAAAAATTAGATGGTAAAA
TTACGGCTATTGAAAATAGTGAAAAGTATAAGGACGTTAAAGATCCGTTTGATCCTTT
AGGGGAAAAACATAGTTCAATAGAACGCTATCAAGATATGGCTAAGTTAATTGAAGACAGATTTGGCGTTTTA
AGAAAAAATGATGGAACAGCAGAGAAAAGTCATTCTTATAAAAGTTTAACGACTTCATCTTCTGATATGGGTA
CGAGTCAATGTAGTTCTAATCACAAGAAACCAGTTCTTACTAAATCAGTATCAGTGCA
AACAGGTATGCCTGACAGCTCATACACAGAAGAAGAAGACAGTGGCATAAATATGAAACATGGCAGAAAAAAT
CTTAGTGTTGATCAAATTAACTATTGTGGCTCAATGGAAAGTGACTCAAGTTCTGGTAGAAAGTTAAAATCTC
TAGACGAAAATATGCTTAAAAAATCATCAGCTAGTAAATCTTCATCTAATCTGGAGCG
AAGGCAAAGAATACAACCAGATTTAATACCAGAAAAAGCTATGGTAAAATCAGAATCATACAATCAAATTCAA
AGTTGCGAGGATATTAATATGTTTGGAGCTGGATCTTTATACAATGACAGCGATTTTGAAATCAATTATAAGC
AGCACTCTTTCATTACCGAAAAATATGTTCA
>103786157_2 DT667190.3
GCACGAGGGTAAGCTTGCGGATGACACTAAAATGATTAATAACATCACTAACCAATTTAATGACGATGATAAT
TCTGATGTAGACGCTGACGATGATGATAACCAAGAAGAAGGTGAGGACGAGGAAGATGAAGATGAATCCGGCG
AAGAATATGACGAAGACGATGAAAATCTCGATCTCAAAGTTAAGCTTATCGAGAAATC
ATCAACTCCGTCACCAAAACCGATTGATAAAGCGCAAAGCGATAGTGTAAAGATAGATATCAAACTTTTAGAA
AACGATAACATGTATGACGACCTCGAACCTGAAGTATACATTCAAACTTCTGTGAAGGACACTGAGCACGGCC
GCTACGAGTACTTGTGGCCAATCTTGATTGCCATTCTGGGTGCGCTACTTCTTCTGAT
CGTCGTCGCCAAGCTTGTGGTGATGGTATTTGTGAGACGGAACAAGCAGATACGTTACATCAACTCGGTGTTT
GCTCACATGGCGCCGCCCGGGCCGCAGCGCCTGCGGCGGGACTGCGGGCAGGTGTACCAGCCGCTGACGGAGG
AGCTGACGGGCCCCACCACGCCGCGCCTCAACCGCTACGCGCCGCTCCGCAACGTGAG
CGTGAGCGCTGCCAACATGTCGTACGAGAGCAGTCCGTTCCACCACAAGAACATCGTCCCCGAGGCGGTGTAA
GTCACGGTACAAATAACATTTGTAAACACATTAACTGCGTTAACATTTTATTTANGCAGTAAGTGATAGACGT
AAGCGCTAGTCAAAACTTTCTACATATCGAAAATTGAGCATTAAGTAAGTGCCTTCAG
ATTTTTTCCCGAATGAC
>103786159_2 DT668620.3
TATTTATTAAATAACGCCGTTGCACTATAAAAGAAAAATATATAAATAATAAAAATAATATATTACATACCTA
TATAAATTTAAAAAGACCTATAGAACAATAAAAAATGTCAAAACTTATATTAACAATTTTTTTCTTTTAAAAC
ATTTTTTAGAGTGATATAAAAGGTATCATATTTTCTCCTCGTCACATATCAGTATTTA
CTTTTATTTAAATATTTATACAGTGTTATTAGTAAGAAATTATATTGGTTTAAAATTTTCTTTTTCCACTTAA
AGATTTTCCCCTTACTTTCGATATAATAAAAACATGAAAGAATAAAAGAAAAATATGAATTTTATATGAAAAA
AAAATTGGTGTTCAAAATCTGTATGAAAGTTAATAAATAAGTTTATAAATAATTATTT
GACATTAATATAGTGCTATCTTCTTTTTGATCATTGCAAAAGAAAAGGACGGAAACAAAAGAAAAGAAATGAT
CCAAAAAGACTATTTATTTTATTAAAAAATAAAAAAAATTACACACGATTTTCGTGTCGCCGTTATTATTAAA
ATTTTAATTTAAGTAAATAGTCTTTTGGATCTTTCATTGTTTATATTTTGAACTCCGG
ATCCTACAAACATATATATGTATCTTAGTAATAAACTATAGAGAACTTTAGAGAGTATTTCTGTAGATCTTTA
AGTGAATATGTTTTGGTGTATTAATAAATTATAAGTGATTGATGAGGGCGAATTTAGAAATGTTTATAATATA
ATTTG
>103786203_2 DT667987.3
GCACGAGGTAAGAACCCTAGCTTGCAAGCCACGGTGTGGCATTATGATTCACTACAAGAGAATCAGTTCCTCG
GAGGTGTTGTAATTCCACTGGGTTCTTTGAACTTGAGGCAGGAAACTGTTGCCTGGTATCCTTTAGAGTATAT
TCCCAGGTAACTGTTGCTTTCCCGCTCTGCTATATGGAGCACGGATATATGACAATAT
GTCAAATGTATAGGGCCATCGGAAAATTACGTCACATATTAATTCAAAATGTAAACATTATTGTGAGGCATGG
ACTGTGGAGGGTCTTAGATTTCGCGACATCACATTTCAAAATATGATAAGCAAACAATATCCCGCACTGTTCT
CTTAATTTTGTAATTTGCGTGATACTTACTTAAAATTAGGTGTGAAATAACAAAATAT

GTGACGTCACACTAGCTACCTAGTCACACAAACTGACCAGCTGTAACGAGCACATGAGTCAAAACAATATAAA
ATTCGTGTGACGTAATTTATGGATGGTTCCCAACGCCCAAACTTAGACTGATATTGATGTCTA
>103786247_2 DT668730.3
GCACGAGGGGAAGTTCATATTATGGAGCACTTAGCGAGTACTATTCAATGCGTGTTTGTCCTACTTCATTGTC
TGTCAAATTTCCCTTTTATTTTGCTTATTTGGGGAGGGGTTACTATATTTTTTGTTTTCTTTTCTTTGCAGTT
AATGCTCGCTGCACCCGTGTAGTGTATGGCTGGAAGGGTGGTGGCTACAGATGACCTT
TTAGTGGTACCCCTCTGTGTTTAGTGGTTGTGCTGGTGGCCGTGGTGGTTTTAACGTCTAGTGTAGTGTCATG
TAGTGTTGCCAGTGCCAGTGATGTAACAGTCTATGGAAGTGTTAGGGTTTGCTCGGAATGTTTACGAAGCACG
GCAAGGCTCCTGAGCGGTGCCATCACCCCTCGCATAGGAGCTGCAAGAAAAAAAAAGCA
ACCCCCACACACGCCGTGGAAGTACTTGATGAATTGCCCACTTGTTAGGAAATGTTTTGGTATAGTATTGTTT
TTTCTGATTTTATCATTTATTTTTTTTGTCTTATGATGTACCAAAGATTCTAATATATTATTATTTCGGTGAA
TTGTTTGCCCCCCTGCCCCCAATTTTATTATTTCGGTAATCAAATCAGGAAAAGAATA
GAAAAGATACAAAAAAATATTATCAAATTCATATAACAGAAACTCTAAACTTAGATCTGAATCTTATGTAAAT
AATATTATATATTTACAGATGCAATCTTAGGCTACGACTAACGTA
>103786311_2 DV501293.2
GCACGAGGGTGCACTCTAAATGAATTACTAACAACTTATACTGATAGACAGATTTTTTTAACATGGATTGTGG
CAAAGTATAAGATATGTTCATGACTTTCTATTTTAAAAATACCCAAAACTGCTAAGACAGCACTACTAAGGCA
CAATGACATATTCTTATTTTTTC
>103786334_2 DT668382.3
GCAGAGGCCATGGCCATTGGCATTATAAATTAAATAATTCTTTTACAAAAAATACCGCTATTATGTAGATTAC
CTTTTAAATGTTAACTGCGGTTAGTATTAAACGATAAACTAAAGATGTCCACGGTTCTTCTTGCTGTTATTGC
TGTGATTCTATGTGTTCTTTTCCGAATACTTAACGTTAACAGCCAACCTCATAAGCCT
GTTATATATGGGAGAGATAGAAATTTCATTGAAAATATTTTATTTATTGCACCATTTCTGAATGAACCTTACA
CCCCTACACGTCTTTGGGGTTTTAGTGGACATGTCCAGACCATCCTCCATAGCCTTATTGGCAGAGTTCGATG
TCCATGGCCTATTGGAGGTCGCATTTCCTTGACATTACCAGACAAATCTACCCTTACA
TATGATCTTTATGAACCTTTAGGAGCTGAACATGAAGATGATGTGACTGTGGCTATATGCCCTGGTATTGGGA
ACACTTCAGAGAGTGTCTATATACGCACCTATGTGCACTACTCTCAACGTCATGGCTATAGATGTGCTGTATT
GAACCACATTGGTGCTTTAAACAGTGTTCCTGTCACGGGCACCAGAATATTTTCCTAT
GGTCACACCGATGACTTCAATTTCATGATAGAGAATTTGCTCGAGCGATACCCTAATACAAAACTGATTTTAG
TCGGTTTTAGCCTCGGCGGTAACCTAATCACGAAATATTTAGGCGAAGACAGGAAAAGACCTAACAATATTAT
AGGCGGAAT
>103786349_2 DT668848.3
GCACGAGGGGCACCCTTGGATACGGCAGCTACGGCTACGGGCAGGGCGGCTACGGCGGCGGTTACGACGGCTA
CGGCTATGGCGGATACCGATACGACAGCTACGGGGGCTACGGCGGATACGATTACTCGGGCTACCCGAATTAC
GGTAAACGCGGCAAGCAGCACTAGCGTGGGGCCCGCTCCGACACACTACCTCGACTGA
CGTTACCTGAGTAGAGTAGACGTAGAGTAAGGCGGCGGGCTGCGGTCCGCCGCGCCGCTAGGTTAGCGTAGTG
GGCCGCCTCCGTCCTTGAGCGTCGTTAGGTGTATGTCTTTTTGTGTAGAAAATATTTATTTTTCTATTATAAC
TGCGTATTTACATTGATATTCTATCGAGATAATGTGTAGTGAATGTCGTCGGGGCGCC
GTGTCCGTCGATCCCAAGCGGACTGTAATATTTCCGAGAGATCATTTTATTTGCATTATTTAGTTTATTCCAC
TGATTTGCATCACTGTTGCGCGAGTGCCCACGCAGCTAGATCACTCTTTATCATTGTGTCACTAGGTACTATG
TTTGTACATATATGTTACCGCCTTACGTTTTATATAAGTGTTCATTTGTAATACCACT
TTTGACATATGTAGCAATGTCGCCCGAGTTTTGTTTGTTTTTGGTATCTTTTATTTTACCTATTAACGCTCTC
GCCACATGCTACATTTGGGGAATTGGGGTAAAATATGGAGAGACATTGTGAAAGTGATAAAGATA
>103786355_2 DT668448.3
GCACGAGGAAAGTGCTTGCTATTAAGAATTTTCCCGTTCCTACAAATGTTAAGCAGCTTCGTAGATTTCTAGG
CATGGTAAATTTTTACAGACGTTTTCTTTCACAATCTGCTAAAATTCAAGCACCACTAAATTCACTTTTATCA
GGTTCTGTTAAAGCTTCGCATCCTATAACTTTAAAAGATGATTCTTTAAAAGCATTTC
AGGACTGTAAAGACAGCCTTTGTAACGCTGCTTTACTTGCTCACCCTGATTCTACAGCTAAGCTTGGTTTAGT
AACAGATGCTTCTGATTTAGCTATAGGTTCGGTTTTGCAGCAATTTAAAGATGGTTCCTGGCAGCCACTTGCA
TTTTTCTCACGTAAGTTGAGTCCATCACAGACAAAATATTCTCCTTACGACCGAGAGC
TGTTAGCAATCTATGAGAGTGTAAAATATTTTCGACACATGCTCGAAGCTACCCATTTTACCATCTACACCGA
CCATAAGCCACTATGTTTTGCCTTTCATTGTCGTAAAAACAATTGCACACCACGGCAATATAGGCACTTAGAC
TTCATTTCCCAATTTACATCGGATATACAGCATATCTCCGGTCGGGATAATGTTGTCG

CAGACACTCTCTCCCGCATCGGAGAAGTGCAATCCCCTGTAGATTGGGAAGTGATAGCTAAACTACAAGCAGC
AGATCCTGAAATCCAAAATTATCTTAAAGGTGAATCATCATTACGCCTAGAGAAGAGGTATATACCTGGAAGT
CGAACTATCTTGTTCTGCGACGTCAGCACATCGACTCCCAGGCCATTCATTCCTCATT
CCTA
>103786364_2 DT668563.3
GCACGAGGCATCCATATCATCACGATTCACGTGATATAATATAAACTTATCTAAAAATGAAAAATCCTACATA
AAAATTATTATTAATATTAGTTGGGTAAAACTGTATACTAAAGTATAATAAAATAATGTTTTGGGTTCCTATC
TCATGTCTCAATAGCTACATTAATGCCTCGAGCATTATTGACATATTTCTGGATAGAT
GACACATAGACTATAGAGTAATCAATCAATCTTATGATCTATGCAATAAACATTTACAATTACAAATAAATAA
TAACAAATGAAACAAAAATTTAATTACGACTAATATCTTTTACTGTTTAACTACTACTATATATCACTGCAAA
AATATAGTCCTTTCTGCAAACAAGGCACGAGTTATATCTTTTTAGAAATAGCAATGGC
CGCTTTACACCCAGTCTTGATAACTTCAGTTAGAGCTTTATAGAAAGGGGCCTGTTCTGCGCCGTGGTCTATC
CCCGTGTCGTGGTGTTCTTGTTCCTCGTCCCTAAATTTAGTTATGGTTTCTAAAATGTCTTTGTCGATATTGG
GATCTTCTACTAAGGTGCGTAATTGGTCGTTATAGTGGTCTACGATGACTGTTTCTAC
GGCCACTGTGCACGCCATCGCTGCTTCCTTGCCTAGAAGGGCTGTACCTTGAAATTTTGTTAAAAAAATCTTG
GTAATATCATAAATGTGAAAC
>103786397_2 DT668253.3
GCACGAGGCTCACACTTTGCCTGCCAATATCTTAATTACTTCACTAAAGCTACTTCTTTGAGTCCACAGGTCC
AATTGTCAATGTCAGCCGATGTGCCGCTGGTCGTCGAATATCGTATACCGGACATCGGTCATATTCGTTACTA
CCTCGCTCCTAAAATTGAAGAAGAAGACAGTTGAATTAATTATATGCTTAAACATTTC
CTTTCAATTTTAATTATGTTGGTAAAATATGTACAATATGTATTACATACATTATCGTTGGAAATTACACTTT
TGAAAATAATTTAACAATAAAATTAATAAACACTATTTTGTTAAATATAAGAAATCTCCTTAATTGCCTAAAC
AAAAAATTAGGCCGTCGTGGCGCAGCGGCACTGTGTTTGACTGTAAACTCGACAGTTG
TGGGTTCGATTCCCATTCGGAGGATTGAGATATTTAATATTTTCATACCCTCGCTTCTCAACACACAATTCTT
CAAATATTGCGTGAAAAATTCGTAAAGTGTCTGTGAGTGTAACACACAAACACGGTAAATTCCCTCTGTCTAT
CTGCTATGTGCAAAATAATGCGTGAAACTAAAAATAATATTAGATCTATAAACAGGTG
TAATTTAACTTACAACAACAGAACTTACTAAGTTACAAAAAAATGTGATAAACCGGGAACAACATTTTTTATA
TTTAATTAAAAACAATGATGATATCCAAATGTTTTATTATGACACTATCATATAAAATGTTACACTATTCCAC
ATCCATGTTTCACATGAGTATTGAAACAAAATAAATTAATTACTAATTAAAATATAAA
TATTTTC
>103786403_2 DT667879.3
GCACGAGGGCACAGCCCCCTTACCGCCATGAGTGCTTGAATTGCCGCAGAAATCATACAGCCTAATGAATCTT
AGCCTGCCTCCTCTGCCTACAAAACACAAGTGAGTGTTGTTTGTTACCAAATGGATGACGCTATTTCGTTACC
ATCGACGCGCAATGACCTCGCTGGTATGTTACAGATGTCAGCCGTCACCGCACGAGGA
ACGGCGATTTTATCACATCTTGGAAAATGACTGAAGGTCTCCCCCCGTATAGAAATGAGAAGGCGATGTGTAT
ACGCATGTGGCTGGAGGTGGGCCATGCTTGCGAGCCGCGGCAGTCGGTGACGGGGCGCGCGCTGGCGCTCGAC
TGGAGGGTGTGGGTGCGCGGCGCTCGAGGGGACATCAGCTCGTTTGTACAGAAGGTGG
TCTTCAAACTGCATCCACCGTCCGCTTTTGTTTATCCTAAACGAGTGCGCCAAGAGCCGCCTTATGAAATCCA
AGAGTCGGGCTGCGCGTCCATTAACATACCTATA
>103786412_2 DT667153.3
GCACGAGGGTTGTCATCAGATTTAGCAAAACATTTTAAACTACCTCCAAAATAGAAACGAAATAATCATGTAA
TCAGTGGCGGAAACACCTATTTTCTTTTTTTATATATATTTATATTATTTTTGGTTTCCAGATTATTGTTATT
ATACAATTCAACAATTTCCTTGAAATTGTATGTTCTGAATTTTAGTTTTTTCTACTCC
TAAGTAACGTGGGGAATGTGAATGTTTGCTGGACCGTTGAGTGTACAAATATCAACAAAGAAGGAAACGTCAT
GGGAAAAAAATATCTTAGACTTGAATCTGTAAAATATCGGAAATCTAGTAAACTTTTATGAGGTAGCGAAAAG
AGTCGATCCCGTTATTACTTAAAAAATGTGCAGCCCAACGCTTAAACCTACACAGCTT
CCGCCACTGAAACATATTTATCTAATTATTAAATTAAAATAAATCAATGTAATATTTTATTTACTGTAAAGAT
TTAATACGACAAAGATTGTGGTAAAAAAATCCTTTTAAGCACATAATGCGAGAAACGCATCTCCAATACAAGA
TGCAAAATGGCGATCTAATCCGTTACGGGGTTATCTATAGGATACGTCACACGTGAAG
TGAAGTGTGACATTTTGTGACAAGGAGTGGGGGACAGGATCTAAATTTCGCGACATCACATTTGAAAGTATAA
TATATTAGTTTTATTAGCTTTTTTTTTGGTCAAATGGAAATAACTTCTCGTTATTTATGATACTTTACAAGTGA
TGCATTGACGCATTCCAAGCAAACTATAACTGTGACGTAATAAAGACTTTCGCC
>103786418_2 DT667492.3

GACGAGGGTTCATCTTTCTAGGAGCATGTATAGCTGTGAAATTGGGGGAATACCCTACGTGGATGTTTTTCCA
GTGTTTCTGCGCAATGACACTTTTCTACTGCGCTCATTGGCAGGCGTACGTCACGGGTACGCTCAAAATGGGT
CGCATTGACGTGACTGAAGCGCAGTACGCCATCATCGGGATACACTTAGTATCTGCGA
TACTTGGCCCCGAGGCTTGGACCACTCAGGGCACGTTGGAAGTGCGTTACTCGCTGGGCGTGGTCGCAGTCGC
GGGTGTAATACTAACGCTGGCGTCGCTCGCCGCCGCCATCGCGGCTGGCGGCGTTGGCAAGAACGGCTCCACT
GTCGCTGGAACAAGCATATTATCTCCGGTGATCCCGTTCTCGCTGGTCGTGGTTCCCG
CCTTCATTATTTTCCAAAAGAGCGAGTCTCATGTATACGAGAACCATCCGGCACTCTACATACTGGCGTTTGG
TATGGTCGCTGCAAAAGTCACGAATAGGCTTGTGGTAGCTCACATGACAAAAAGTGAAATGGAGTATTACGAC
TGGTCGCTCTTAGGACCGGCGATGCTCTTCCTCAACCAGTACTTCAACAATGCAATAC
CTGAATATTATGTGTTGTGGCTGTGTACTATCTGGGTATGCGCGGACCTGTCGCGCTACTGCGGACAGATATG
TCTCGAGATATGTGATCATCTCAAGATCCATCTGTTTCGCATCTCGAGCGCGCCGCCCTCGCCCAAGCACGAG
AGAAACGGTACGAGGCGACCCTCT
>103786431_2 DT667166.3
GCACGAGGATGATCGCCATCCACGGCTGGCAGGATAATGCTGGAACTTGGGACAATCTTATTCCACTTCTTCC
CACAAACACATCAGTCCTGTGCATCGACTTACCGGGTCACGGCCTTTCCTCGCATTATCCTAAAGGCCTACTA
TACTACATATTTTGGGATGGTATATCACTTTTGCGACGAATTGTTAAACACTTTAAAT
GGGAGAAAATAAGCTTAATAGGACATTCGTTGGGTGGCGCTTTAAGTTTTATGTACGCAGCAACTTATCCTGA
TGAAGTAGACCGAATTATTTGTATAGACATAGCGAGTCCTGCGGTGCGTGCCCCTGAACATATGGTCAAAAAT
ACTGCGGGCATGATCGATAAGAATTTGGAGTATGAAGATTTGCCAGAAGAAAAAATAC
CATGTTACAGTTATGACGAGATGATAGATATTGTATGTGACGCGTATAAAGGATCAGTCTCTAGAGAGAACTG
CAAGGTCTTGATGAAGAGGGGTATGGCTCCTACTCCTGCACACATAAAGAAGAAAGGTTATTACTTCAAAAGG
GACCCGAGGCTAAAGGTAGCCGGCCTAGCCATGATGTCTATAGAAACTGCGTTAGAAT
ACGCTGGCAAAGTGAAATGCAAAGTTTTGAATATCAGAGCCTTACCAGGGCAGAAGTGGGAACGATTGGATTA
TTACTTAGACGTCATAGAGAAACTAAAGCAATCAGCGGACGTTAGTTACGTGGAAGTGGAAGGAACGCATCAC
GTACATTTAAATTCGCCACAAATATAATTAGCATTATTG
>103786445_2 DT667311.3
CGGCACGAGGGCGGCTCGTCGCATATCCGGCGGCAAGTCAGTGGATTACAGTCGCCAAGCAGTGGATACGTCG
GAGGTGCGAGCGGGTGTGCGGGAGGAGCGGTGGGTCCATTAGTAGATAAGGACAACGGAGGCTGCGCCGGTGC
TGACGACGCGTCCAGCGGGTACGGCAGCCCACCTGATCCCGACGTGTGTGATGCTGCA
CGATGACCGACAATGCAACGCCGACTACTTGCCTCGGTGAAGCCTCACTGAGTCCCGGTCGGTTCGGTTAGTC
GCCATGAAGTATTCGCGCGTAGAGTCGCCCAACAGAACATTGTCTCTCAATTTCACGTTCACCTAGATAATTG
ACTTTCTCTTTTCCATTAATAGATACACAGAGTTCACCAAATTACGACCACAACGCTT
TATTTTACTTTTAACTTTATAGTGTTTATAAAAAAAGTGCAATTTTAATTTAACTAACTTAAAAAAAACGTGC
AGTCAATGCAAGTACAATTTTTAGAAACTGTTATAACATTGAGCGAGTGTAAGATGAATTTATGCAAAACCAG
CAAAGTGCATTAACTACATCGAAGTATTTCAAACATAGTTTAAAAGCAGAAGTATATT
AGGATAGACGGGCCATGGATGAGGCCACTTTACAAAATGTCCGGGATGAGACGCGATAACGTTGTCAGAACAC
GATATTTCCATTATGTATACGATTTGAGTCTACATGTTTA
>103786466_2 DT667112.3
GCACGAGGAATACCTATAAAGTACAATTGAAGTGTAATATTTTGTGCCATAATTGTTTCAGTTATTATTTTAA
AATCTCATAAAAAAGGAAATATAACAACTTTAAAAAATATTTGACTGAATTCTGACTTTGACCTTCAAAAATG
AATATACAACGATGGTAGACCTGTCGATTCGAGAAATTAGTTCTTTTTTTACATAATG
GTAATAATAACATTGACATAATGACTAGATATATATATTTAAACTAGACATATCCTGATATCGACGTTTTAGTAT
GGATAAGTATTTGGCGCTCATCTAAATATAGTCATATCCATGCTTAGTTATCGTTTGTCAATTTCAAAATGAA
GCGATCAAAATGAATTATTGTCATTTTCATCGTTCTAATAACAATACAAAGAAGGATA
TTGAGTTCTGCGTGTCAACAAATGTCTAAAAAGTAACGACGGAGTGAAATGGGCAACGAAATATCTTAACACT
TCATTTCGTCTGCCTGTTAAATTCAGGATAGCTAAAAAAAAAGAATGAACTGGGTATGTCAGTTTAAATGTTGT
TTTAAGAATTTATTTAATTAGAATAATTTCGTCAAAAGACATTAAAATTTGTTACTTA
ATTGAAAGTATTAATTTAGTGTTTTGTGATAATATTTGAGTGTTATTACTTTGTTGTTTATCGTGTTATTGGT
TTTTTTTTTACTAACTAAATATACGAAGTCGAATTTGGTACAATGACGCTCAAAACATTTTGTGCCAATTTTT
GACTTGACGACATCTTTATTGGGACTTTTTAGAATTGTGTACATAATATTATTATAAT
ATACATTAAATATTTTAATATGGAGG
>103786469_2 DV501302.2

GCACGAGGGTCCTTTGGGATCTCAATGTTGAGGACGATGGTATGTAGAGACACTACTCTATGACAATAAAGAA
TAACAGACTAACTTTCATATGTGAATTAAAAAAATTAAAAACACAATACTATGTGAAAGGCCGAACTGAAATA
CAATACATTTGAGATAAATTGACTGTAGTGTTACATTTTAATTATTGAAGAAAATATG
ACGGCTTCAACGATGATTGAGCTGGAATCCTGGATTATCTACAAGGGTGTACTTAATGAGTGCCATGTCTCCG
ACTCGTGAGCTGAATGTGTCGCGTATTATTTATTGTTTCGAGTTTTTTTAAGTTATCTTCATGGTAGCCACTT
CGATGGACGGCGGCGCGTGGTGCGCCCGCGACATCTCCCTGCTGGCGCAGAGGAAGTT
CGTGTCGCGAGTGGGCCGCGCGGCCGGCTCTCGGGCCCTCGTGCTGGATGAA
>103786478_2 DT667988.3
GCACGAGGGGCAAGACTATTAAAATGAATAGTAAACTTGTTTCAAGAAATTCTTTATATTGAACAAACATTGC
GAATAATTTATTTAAACAAAATCTATATACATAACCAATGTTATATTTAAGGTGAACCTTTAGGTGCATTCGT
TTTATATTGGGATTGGACATCATATGTCCGGACAATGTCCAATTCACCAGTAAATATA
AAATTCAAATGGGGTCTGAGCGCATCAGACCCTGAACAGAAGCCAGTATTGTTTGTGGGACAGGCCGCCCACC
TCGCTGCTTTATCCTGGCAGGATGTGAGGTGCAAACTTGAACCTAAAGTCTCTGAAGAGGCATGGTGCAGAGG
CATTAGTGTGGCTTCACCAGGTGAACTATGTGACATATGGCCGAGGGCAGCTGCATTA
GGAGTCTTGCCACTACGTCGGTCACGGCACGCAGCACCATCTAGATGCCATGCTTTGTCAAAGCTTGTTAGGA
ATGCTGTGAAATCTGTGGAGGAAGAGTTTGTGGTGCTGGTCTGCCGCAAACGTGATGTCCTACCCAGCGCAGC
GGCCCTAGCTCGTGCCGCACCTCTATACAGTGCTCGTTCCGGACTGCTGCCCAATGCC
CCAACCGCACCACCCACGTCAAGCGGAAAGAAAAAATTGATTGTGGAAATTTGCATTGCTAATGATGAAGGTG
ACTCTGATGATGAAGACCAATCAGCCCAAAGCCCCGACCTTCTAGACAAGACTCTCTCCAGCGAGGAACTAAC
CACCATCCAGGATATAGCTGATGCTACCCGACTAGC
>103786484_2 DT668430.3
GCACGAGGGTTCACCTTAAAGAATCTTTTGCAATAAATAGTATCATTGATTTAGTTAAAAATAAAGATGTTAT
TGAATCGGTTGAAATTACTCGATCTTATAGTTCACAAACTCCAACTCCATTACTAGACGATGACTTAGTTTCT
AGCATCAAGCCTGACTCTGTTAAAACTTACTACACTACATATACATTTTTTACTACAA
TTTATGTAGGTTCTGATGCAAATGTTTGTAGCAGGAGTGAAGTGTATACTAATTATGTTGGTCCTGATGAGTT
AAAGCCTACAAAAACTAACCAACTACCAAATGAAAATACAAGGGTACAATTTGATTTTAGAAATTGTATGCAA
ATCAAAGGAATGCCACAAGATAGTACTAATGAAAATTCATCATTTGAAAGTAGCGTCA
CAATGGAAGAAAAACAAGTATCAGAAAATAAGGACATTGAGAATCCACAGCCGTCTCCTATTGAAGTAGATAT
GTTGTTTAGCGTAGAATCCAACCCACTTATATCTGAAAACTCATATGTTACGGATGTGAAGTCTTCTTCTTCC
AAAGGTGAAACAAAATTTTTAGATAACAATAATGTACTTGATGACCAAATAAGCTCTG
AGAGTAATATTGATGAAATTCTTCCATCACCTACACTACTACTACAAACTAGTTACACAACATTTACTTACTT
TACTACGATGTACATAGGAAAAGATTCTAGTAAGGTAAAAAGTCGCCTCGAAACAATAACAAATGTCGTTACA
GAAACAATTACTCCGA
>103786496_2 DT667168.3
TTCGGCACGAGGGGGAGTCCCTCCGGGCCGGGGAGCCGCCCGGCTCCCAGCTGAAGGTGGTGGGGCGGCTCGCC
GACCAGTGCCAGGACGCGCTCGTGCAGCTGGGCACGTTCCTGGCCTCCTCGCACGCGCCCGACGAGTACGCCG
CGAGGCTGCCTCCCCTGCAGGAATTGTTGCGCGATTATCACGTTGACGCTGATGTAGC
ATTTTTCCTACATCGTCCAGTTTTAGCTCAAAAAATTGCAGCTAAGGTAGAAGCTTTGAAAAAAAGTTCTGAA
AGCAAAAGCGATTCGTTAGAGAAAAGTATTGAAAGGTATAATATAGCGTCTAAAGAGGCACTAGAGCCAGTTG
TGACGTCAATAACACCTCTGCTACCTGCACGAGTGTGGGAAGATATTTCACCAGAATT
TTATGTTACATTTTGGTCACTATCAATGTACGATCTTCGAGTGCCAGTCGAAAGTTACGAAAGAGAAATCGAT
CGTTTGAAGACCGCTGCCGCCAACGCCATCAAGGACTCGTCGCAAGGCACGAAGGGCAAGAAGGAGCAGGAGC
GGTTCAACACGCTGATCGACAAGCTGGCGGAGGAGCGGCGGCGCCAGGCGGAGCACGT
GGCGCGGGTGCGCGCGCGCCTGCAGCGCGAGAGCGGCGGCTGGTTCCCGGCGCGCGCCGCCAAGTCGGCCAAG
AACGAGACCGTGACGCGCCTCATGCAGCTGTGCCTG
>103786505_2 DT668008.3
CGGCAGAGGCCACGACCCCTTTAACTTAGCTGGAACATATTTAACGAAACATGTATTAAGTAATTGTGTCATT
TCAGTGATAACTCAATAAACTAATAAAAATGGGTATGGAAAAACATCATGGTATCGAGGATGGTTATTCAGAA
TATTTATATTGTCCAGAAATTCTTTTAAAGTTGGACTTTAGTAGGAGTCCAGCAAAGT
TTAACCCTCCCATAAGTGCTGCACAGCCAGGTGAAGACTGGATGGTTGTAAGACCTTTACAAAGGGCTGATTA
TGATAAAGGGTTTCTGCAGCTACTTAGCCAACTAACAAGTACAGGTAATATTACAAGACCAACAATTTGATGA
GAGATTTACGCAAATGAAGAGTGCTGGGGGTTACTATGTAACTGTGATCGAAGACAAA

CGTATATCGAAAATTATTGGTGCTGCAACACTTACTATTGAACAGAAATTCATCCACAACTGCTCTGTCAGAG
GTCGTCTTGAAGATGTAGTGGTAAACGACACGTATACAGGAAAACAACTGGGGAAATTACCGGTCGTGACCCT
CTGGTTATTGGCTCCCGAGCTCGGCTGCTACAAAATGTCGCTCGACTGCAAAGACAAG
CCCATTAAGTTCTATGAAACTCTGGGATACAAACTCGAGCCGGGCAACCGTAACGCTATGAACATGAGGTATT
CAGATCATAAACGTTGTTTTCAATTTTT
>103786508_2 DT667867.3
GCACGAGGTTGATTCCTGAAAATATTGCTTAAGAAGTTCTTTGTTAGGTTTCCCATTTAAAAATGATGTAATT
TAATTAATATAATCAGATACGCATTACAAAGTACAGTTAAAAATTTATCTAAATAATAAACAGTATTTTATTT
ATTGATTTGTGAACGACATAGTGAAAATTATTGCGTGCTTAGTACAAAAAACCATGAG
TGATTCGAAATCTTTTGTTAACTTTTCTTGCCAGAGATGTTTGCAACCTTTGAAATTAGATGAATCTTTAAAT
AATCTTGGAGAACACACCATAGCTGATCTGACACTGCAGATTCGTAGAAATAATGAAGCAGATTTGGACTTAC
AATCGTCTAGTTTGGAGCATTACGTTCCACCATTCCGTATGTCAGAGTCAGGAAATGG
GGCGAATGGTTTTATGGTTATCTCAGATGGATGGGAGACCACATCGTTAGGTCATCAATTGCATGTCAAAGCT
ACATTATTTGACCTTTTATCAAATAATTCCGATGTTGATCATCCATTATGCGATGAATGTACAGACACTCTCT
TAGAATTAATGGACAACCAGCTAAGACTTACTGAAGCCGAATGGAAAGATTATAATGA
TTATTTAAAAAAATTAGAAGATGATAAAGAGGATTTAAATTTAGAAGGGCTGGAAAAAGAATTGGAAGATTGG
AAACAGGAACAAAGTAGACTCTTGCAAGAGTTATCAGCTTTACAAAAAGAAGAAAAGGCTATGAAAGATGAAA
TTGAAATACAAGAAAAGGGAAAAGAAAGATTGGGAATAGAACAGGATATTTA
>103786517_2 DT668864.3
TTGCCTGCGATACACTCGACACTTGGATTGACACCACTGCTATGGGTCGCACAAATCTACCACCAGACAAGGT
GCCATGGGAAGCGCTAGTGACCCTCCTGTCGCAGTGCATCTACGGCGGTAAAATAGACAACCTGTTCGACCAG
CGACTGTTGCACTCGTTCCTCTGTAAACTGTTTACACCGAGGTCGTTTGAAGCTGATT
TTGCACTTGTAGCTAATGTGGATGGGGCATCTGGCAACCAAAGACACATCACAATGCCCGACGGCAACCGCCG
CGATCACTTCCTCAAGTGTTTCGAGGAACTGTCCGACAGACAGACTCCATCTTGGCTCGGATTGCCTAATAAT
GCTGAAAAGGTGCTTTTAACAACTCAAGGAAGTGACCTTGTCTCCAAGCTTTTGAAAA
TGCAACAACTAGAAGATGAAGAAGAACTTGCATACAATGCAGCTACTCAAGATCACGACCCACATGCGGCGGT
AGTAGAGGGCGATGGACGTCCTGCATGGATGAAGTCCTTACAAGAAACTTCTACAAGCTGGTTACAACTGCTG
CCTAAAGAGTTACCGACACTTCGCCGAACAGTGGAAAATATTAAAGATCCTCTATACC
GTTTCTTCGAGCGCGAGGTGGCGGCCGGCGCGTCTCTACTGCAACAAGTGTTGCATGATTTGCACAATGTCAC
TGCTATATGTCAAGGCTCGAT
>103786531_2 DT667067.3
GCACGAGGGTTAATCATAAACTTCGTTCCCATTAAGACAACTTTCCGGTGAATTCGCCGCCCATTGCCTCGTT
GAAAAAATTGCCAGCGAGGAAATGAAAATATTACATTTTTCATAGCCCCGAGTGGGAATCGAACTCACAACCG
TCGCATTTACGGTCACACGCTTGTGCCCCTGCACCACTTCTTTTTTTTTCAATTTGTA
TTGTAAGTTCTCACTTCGTAGGCGAATCACATACAAAATATAATAAAAACGATTCATAAATGCTCGTATCTTT
TAATTGTGTTGTCTTCTTGATATTGTTCATTACTTCAAGACTGTAGACCTGAATATTTGATATTAAATTCAGT
TTGATATCTCCACGCGATTTTGAGATAAATGATCTTGACAGACGAATACTCACAGCAA
TGTAATTCTATAAGGGTTCCGTTTTTTTCTTATAAGATACAGAACTTGTTAAATCAAAGAAATCATTAGGAGG
CAGGGCAGTTTTAATATAACCATATTAAAACCCGCTATATTTTTATGTGTCTAATGGGCGGTCTTAATATATT
GTATATTTGTATACAATAGATTACTTAATCAGATTGTTAAAAGTAGATAAGTTATTGA
GACCGGCCGTAAGTCGGTATGTACTACGTTCTGATTTAAATTATTATGCATTCTATTTTTACAGAAATACTGC
GCATAAAGTCAGCATGTTCATCGGCCGTTGGTTGAAGATGATTACATAAATTCTTTTATTTAATTTTCATTGT
TTTTTTTATT
>103786540_2 DT668196.3
GCACGAGGGAACAACTTTAATATCGAAACAGGCTCGCCGTCCAAATGGATACGTTAAATATAAAACCGTTAAG
TCAGGACTTGGAGTGGTCTCCGGAGGCGCCCGTCGGCGTAGGAGCCACTCGGGACGTGGTGGACCACGCTCCC
ACAGACATGGCATCGACATCGAGCGAGGAGAGGGCCATACGCCGCGTCACTGGCCGCT
GGGGTGCCGAGTGGTTGAGCTGACTCGGCTCAGGCTATATCAAACCCCTCGGGCCCCAGTGGCCTGCAGACCA
CCCCCTACCAGCGCCACGGTGGGGGGCCCCGCGCCCAGCTTTTTTCTTGTCTCTCTCCAAATACGAGGACTTG
GTCGCTAAAGCTGAGGCGATATTGAGCCGACTCGTCGTCGCGGAAAACTATGACTCCG
TTAACAACTTCCTCACTCTCTATGAAACCTATATGGCATCACCTGATGAAACTTTGAAGGAGTTCTTCCCTAA
GTACAATCCGCCGATTCGCGCTCACAAGCACACCTGTGTGGGCTTGGGTATGGAGGTCATGAAGCGTTTGAAG
GTCCTGGAGAATGATTTCCCTGGTATCACCAAGCCCATGATGC
>103786552_2 DT667550.3

GCACGAGGGAGCACCGTGCTTTTATATTTTAAAACTAATATTGTATTAATTAACTTGCATTTTAATGAAAATC
ATAAATAAATTATCGATTTAGGCAATCAATATAGACCACTAAAATGCCACAATTAGATAAAAAACCCAAAGTA
TTTCTAGAGGAATTTCGTGTAAAGAATGCTCCAGAAGACTATGGAATGGCTGATGAAA
AATGGAATTTTAAAAAAATTGCTAAAAACTTTCGTATAGTGATAGTACGTATGGACAATTCTGAAATGGAATT
TGATTTAATTGGAATACAACCTGCGTTTGCAAATGCATTCCGCAGATTGATGCTAAGCGAGGTCCCAAGCATG
GCTATAGAAAAAGTTATGATCAAAAACAACACATCAATCATTCAAGATGAAGTATTAG
CACATAGATTAGGCTTAATTCCTCTAAAAGCAGACCCTAAATTATTTGAATATCGTCCAGAAGATGCAACAGA
AGGAACAGAGTTTGATACATTAGAATTTACTTTAAAAATTAAATGTACATCTAATAAGTTGCAAAATAAAGAA
TCTTTCAGAGCTGAAGATATGTATGAAAATCACAGTGTTTACTCATCCGCAATTAAAT
GGAGTCCAATTGGTAATCAAGTATCTGTGTATAAAGAAATAGATATTGGACCAGTACATAGTGATATACTAAT
AGCTAANATGAGGCCTGGGCATGAACTAGATCTTAATTTAGTTGCAGTGAAAGGAATTGGCAAGGACCATGCT
AAATTTTCACCAGTTGCTACAGCATCATACCGCT
>103786558_2 DT667284.3
GCACGAGGCACTACTTAAAATGACTGATATTATAATTTTTTTAATTCGTTTTCAAATATCTTTTGTTTATAGC
ATGATAGGTACCTAGTGTTATATCACTCTACTTCTAGTGCACTAGGTTAAGTTTTTTTAAGACATTTTAGTTA
CTTATCGAGACCAATTTTCCTAGAAATCAAATCCACAGCTGTTGCGTTTAGAGTAAAA
CGTGATTCTGAGGGTCAAAAATATAGATAGAGTAATATCAATGTTAGTTATGCTGTAAAAAAAAGTTTAATTT
AGCACTATGATTTTACTGTGGACAGTGTAGATATTTTTAAATATAGTTTTATAATTTTAACAATTATATTATC
AATGAAATTTATGAACACTTATAGTCCAAGATCCCGGGGTCGCTAGACCTAAGTCATC
TTAGCAAAGCTGAAGTTTGGGGACTATTATGCCGGCCAAATAAGCAGTAGTATCGAAAATTAATTCTTTCCAA
GGACACCGTACGGTATATGCAATTTCATACATATGACATCTTTTTTTTGTTTTATAAGAGTATTATATTAATT
TTTAATTCATTCATTAATTAGTTTTTAAAATCTTCCTTACCTGTGTGTACCTTAAGTT
GACGCTACCTCTGAAACACACTTTCAAGTTAGTAAATACGCATACAGTATTAACTAAATATTAGACTATTAAT
CTTAAAAATTTTAACTTTGCTAATGTGACGTTGTCTAAGCGCACTCGGTTCTTAGTGTTTTAGTGGAATATTG
TTATTATTGTTTGAA
>103786561_2 DT667128.3
GCACGAGGCTCAACTATAGTGGTACTGAATTCTACAGCTCATATAGGTACACAAGAAAACAAACAATGGGTAA
TAAAACGACGCAACCTCATCGAGTTTGAGCTCGTTTGATTCATCTTGACGAGTACTTTGATACTAAATGGTAA
CCAGGACCTGATGATGGAGCTGGAAGGTGGCCATAGGAGCTCCGTAACTCCGGTGCCA
TGACAATCGCGTTTGCACTCTTCGCCATGGTGTTCATCATTGGCAACACTGAGGCTGCCACAGGCAGCTGTCC
GTTGCCATCAAAAATCTACAGCTGCAATCCAAAATGCCTTCAAAATTATGACTGTAGCAACGGCAAGGTTTGC
TGCCCGAACTCGTGCAACGCCAAATCCTGTGTAGACCTTGCTGCAGTTGGTGGTGGAA
ATAAGGACAAGTATTCGCAATCGGGTGGTGCCGGTGTATATTGTAACAACCAGAAGTGCAATTCGTTCGAAGT
CTGCAAACTTGATCCTTCCACCAAGAAGATGAAGTGTATGCGAGCTTAGATTAATATTAAAAAATAAACTGTT
AGTTCCTTAGTATTTATAGGTTATTTTAACAAAAAAATCATACATATACATACATACC
TATTGATCATAATAGTATTTACTATTAGTAATTATAAAGATTTACTACCTACATACACCTATAAAGTATTGTT
TTAGTCTTAGGTAAGAATCCTTCTTATTCACTTCTACCAGAAGCTTTTTGGCTCTGTTATGGAAATATTATAA
GGATTTTACAAAATAATAACATTCTTTAGACATTAACTCCGCTATACTACTTATTAAA
ATTATGCTTCAAAAATGTTATACTCTTTTC
>103786581_2 DT667530.3
GCACGAGGAATTGCTTTTTATTAAAAAAACATAAAACATTGTTCATTTCACTTTTAATATAGTAAATGCTTAA
GTACATAGGCAACTAACATAATCTTAAACCATATCTTAACATACTATTTACTTATATGCCTATTTTACTAATG
TATTAATTAATAACTAGTAAGGACGTTTCTTAAAATTACCTCCCGAAAAGAATGGTGG
TGGGTTCGATTCCTAGTAAGTTTAATTGTGTAATTTAACATTTTGCTTTATCAAGATATAATGTATTTTATTT
ATAATTTAAATATAAAAAGTATTAGTCAATAATTTTATTATTAACAGGGGTATGCGTCACTGGCTCTAATCGG
TCTGACCACTAAGACATAGGATTTCGTCATGCTACGCCGACACTCTTATACGTACACG
TAAACTTCTTTATGTTATTACTTTTTGTATACTTTCATTGAAGAAAACTATAGAAAGTATACATTTAAACTTA
TTTCTTAAAATTGAAGTCATCGATAGCTTTAAAAAAAACTATTTTGTAAGTATATATGTCG
>103786588_2 DT667281.3
CGGCACGAGGCGTGGACTGTGAGGAGGACAATGACGCTTCATTGCTCATTAGAGAGCGGTCGCTGGCGTGCGA
CGTGCCTCACCCGCAGATCCTGAGCACGCAGCTGGCGGGCGGCGCGCGCGTGTTCTGCACGCTGTGGCGCGCG
CGCGGCGTGCCGGGCCGGCGCCGCCCCACCGTGCTGCACGTGTACGGCGGGCCCGAGG

TGCAGACCGTCACCAACAGCTACCGGGGTATCAGACAGTTACGCATGCATATGCTGGCTGCCCGTGGCTTCAA
CGTGGTAACCGTAGACTCACGCGGATCTAAACACCGCGGGCGGGCTTGGGAAGCCGCTATCAAGGGAAAGATG
GGACAAGTGGAGCTTGATGATCAGGTGGAAGTTTTACAATGGCTGGCGAAAGAGACAC
GCTGTATTGATATGGATAGAGTCGCGATACACGGGTGGAGTTATGGGGGCTACCTGGCGCTGCT
>103786605_2 DT667226.3
GCACGAGGCCTCGCGCCGCCAATGCGGCGCTACGCTGATATCTGCTGAATGGCTCCTCTCTGCTAGCCACTGC
TTCTACCAAGCAACTGAAGCTCATTGGGTCGCCAGACTGGGGGCCTTACGTCGCGGCGCGTGGCCCCGAGGCC
CCTGGGAGCGAGTGGCGCGCGTCCGGCAAGTGGTGCTGCATCCCCGCTATGCGCCGCG
CGGCTTCAGAAACGACATAGCGCTGTTGCGTATAGATCCGCTCGCTTTGCACGCCCGCCTCAGGCCGGCGTGC
CTGCCCCCGTCGCGCGCGCAGCCCCCGGCCGGACACCACTGTACTGTAGTTGGTTGGGGACAATTATATGAGC
ATGAAAGAGTTTTTCCCGACACGTTGCAAGAGGTGGAGCTACCAGTAATTTCAACAGC
GGAGTGCCGAAGACGCACTAGATTATTGCCGTTGTATCGAGTCACCGACGACATGTTCTGTGCGGGCTACGAG
CGCGGCGGCCGCGACGCCTGTCTCGGGGACTCGGGAGGGCCACTGATGTGTCAGGAATCTGATAAGTGGTATA
TCTACGGTGTCACAAGTAATGGATACGGGTGCGCGCGAGCGGACAGACCGGGTGTCTA
CACTAAAGTTAGCCACTACATCGACTGGATAGACAACATAATGGCTACCTTCACACCTGTAAAAAACGAAACA
TTCTCTTCAAGCGACGATAGTTACAATGAAGATTTCTACTCGGATTTAGAAGCCGCTGAAAACAAAAGAGTTC
TTCATAAGACACACGACACATGTAGAGGCTTCCGTTGTCCACTGGG
>103786614_2 DT668067.3
GCACGAGGCTCGCCCCTACGCGATGTTTTTCGAGAGTTTCTACATCAACTTTTCTATAATAAGCGCTTTGATC
GTCGCACTAATATTCGACTATGTGACCAAATTCTTCAGTTATTGGTACATAAGACATGTGCCATACAAAATAT
GCTTTCCGTTTTTCGGCAGTGATTACCACCGAGTTTTGGGTATCAGGAATACATCGGA
AGAAGTGAATACGCTATGTTCCATATACAAGGACAAATATATCGGTTGCGTCAAAAGTAGAATACCCGATTTG
ATTTTAAAAGACGTAGATTCTATACAGAGGATTCTATCGACTGATTTTGCAAATTTTCATTGCCGAGGCACTG
GCTTGGACCGATCCCAGGATGTTTGTATACGAAACAATTTGTTTTACGCGGAAGGTGA
AAAATGGACTCTATTACGTGAGAAATTGGAGTTACTATTGAATAACATGAACTATGAAATTCAAGACAGTTTA
CATGACTGTTTGTCAGGAACCAATGGAGATACGAAAGTTCAACGGTTACTATCGGGTATATCTGGACGCTGTG
TTTAAGAATTTGCTTATCGATAATAAAATAGACAGCTCAGCGATGAGGGGCGTGAGGG
AGCCTATGCTGAAGCGGACCCTGTTTGGTAAACTTAAGAGCTATTTAAAAGATATATTTCCCTCAATTTATAT
ACTCCTTGGATTAAGCGGCATATCTGGACAATTC
>103786617_2 DT668786.3
TTTTTTTTTTTTTTTTTTATTCTCTACCACATGATTATTTTATGTAATCTTGGGAATAAATTACAATAACATT
TTGATGATATCTATTTTTCACTTTCTGTACCACACTTGGATTGTTTTCATTCTCTTAACCTTCTTTTGTAATT
GCAAGACTCCATAAAGTAAAGCTTCTGCTGTTGGTGGGCATCCAGGCACATATATGTC
AACGGGCACTATACGATCACAGCCTCTTACAACAGAGTAAGAATAGTGATAATATCCACCACCATTGGCACAG
CTGCCCATTGATATGACCCATCTTGGATCAGGCATTTGGTCATAGACTTTCCTTAGAGCTGGAGCCATTTTAT
TTGTTAAAGTCCCAGCAACAATCATTACATCAGATTGACGTGGGGATGCTCTGAATAC
TACACCATATCTGTCCATATCATAGCGTGGGGCAGCAATGTGCATCATCTCTACAGCACAACAAGCTAAACCA
AATGTCATTGGCCATATGGAGCCTTTTCTTCCCCAATTCAACAAGTCATCAAGTCGAGCGACAGCATATTCTG
CGGCATTACTAGTATCTTGGAAAGGAGAATAAGGTCTTTTTTCTTTTGGATTAGGAGC
AGGTAAATGAGTTTGACGAACTTGTTCCACTAGAGGTGCGAAGGCTCCTTTTTTCACTACAGAAAGAACTGTA
GAAGGAGCGGTCGCTCCTAGAGCTTTTAAAGCTTGCATTTTATAATTCTAGCGCATGAGCTATTATATCGACT
GTAAAACTT
>103786636_2 DT666978.3
ACGAGGATCCCCCGTTAAAATTGATTGTTTCTATCATATAGTATCTTATAGTTATACTATCTACCCATTAACT
AAAGTACATACCTTTACATAAGCTTATAATATGTATCAGGATCAGCTGTCACTCAATTCTAATTCAGTTGGTT
AGTGCTAATAGGGAGTCGGTGCCATCTGATCTTTAAAGCTCTTTATTAGAGCTATGAC
CTAGCTCTTAGTTTAGAGTAGTTTGTTAGATTACCATCCATGCTTAAATCTATCGAGGCTAATCGCTTAACTT
GCCAAAGCACATCCATCTTACAATTAACCCATCGAATATCTGTATTAAAAATGATTTTTTTTATTGTTTCACT
TTTCATAGTAGGCAGAGGGAAGCTAGTGCTAAGAGACTCTGCTTATTGCTCTGTTGAG
TGGCTGAACTCATCGCCGCACTTTGTCTTGTTACCTCAGCCATGAAATGTAAACAAGAATGATATTACCAAGT
GGGAATCGAGCCTACAACCGTCACACTGCACCACGACGACCTCTACATAGCCTAAACAAATATCACTTTTATT
TTCATTTATTTATACATTTGAATTTCAATATCTACTGAATATGTATCACGTATGTGGC

TCACTGATATATGTATCGCTTATCTCCATTGAAGGTACGCACGATTGCGTTTTCAATACCCTAGGCTTAATAA
TAATACATTAAAGTGTCATTTTATCAAATGGGTCAATAAGTGTACACACGCCGAGGTTAAACTGTTTTGACTA
TCGATTTTGATTTATACTTTAGTTATAATAAAATGTATAATTTTTTTTCGTTGCTTCT
TTTACT
>103786662_2 DT668815.3
GCACGAGGCGGAATCCTCTGGAGTCCCAGGATGTCGAGGTCTGAAGGGTCGTGTTTATGAAACTTTTATTGTT
CTGGCTCTTTTGGGAATACCTATGTTGGGACTTGCTTATGTTATCTCAGCATGGTTGGAAGGTGATAGATCAA
GTCTAGATGCATTGTTAAATTTATGGACGTACCTTCCGTTTTTATACTCCTGCGTTTC
ATTCGTCGGCGTCCTGATGTTATTAGTTTTTACGCCACTGGGGTTCGTTAGATTGTTCGGCGTGGTCGGCGGC
GTCCTTGTGAAGCCCCAGTTTCTAAGGGACCTTAATGATTTTTATTACGTCTATTCATTTGAGGAGAACACCA
TCCGGCGGCGTATAAATAATTCCCTCGTAACGGGTGTTGGATACATATCACCTGAACC
AATGTATCCAGATAGAGGCGACATATCAGCTCCTGTTGATCCTGATTTGTCCAGTCTGAAGGAGAGGAAATCG
TCACCCAAAGGCAAAGAAACCCCGTTACTGAGATTAAAAAACGGTGCGTTGCAAGCTGGATTAAATGAGAGGC
TCCAAAGGATTGTCGCTTTGAGAAAGCAAGTGGAGAATCAAAGGAAAACATCATCATT
ACAACGCAATGTCGTTTATCCGCTTGCTATGCTGCTGCTTCTTGCGTTGACCACCATAACTGTGCTGATGGTG
C
>103786668_2 DT667143.3
CGGCACGAGGCAAGTTCCTTTTTAGATTTGTTATACTTATTAATACTAATTATTATTAACAGCATGTCAGGTT
TATATGAATAGTAAGATTTTAATTTCTATATTAATATAAATTATATATATGGTTTCCTTCCGAATACATGATT
ATGTAGAAAAATCTATGGAAAAACACAACACAACACAGCATTATAATTAGCGAGGATC
ACTAATATTTATAAATTATGTATTGGAAAATGTTATTATTCTGTATAACTTGGCAATTGATCTTATACGTCGC
TTACACGCTTGGCAAGCTTCTGCAAGTGTTCACCAACACGGACGCTTTGACAAGCGTGTAAACTATGTATAAT
GTATTCTAGATATTAAAAAAATAAGCAACTTTCAAAGATTATAAATATTTAATGTTTC
TTATAGGAAGGCAAATGTAAAGTAGATAAATGATGTGTATTGATGTGTGTAGTACATATGCGTTGTTTTTTTT
ATGTCTAACTATGTCTATATACTATAGCATTGATGTTAGATTAGATTAAAGTAAACTACTTGTTGCAATTATA
ACTAAGGTGACCGAGCTCACATATGTAATTTGTGATTTTACTAAATTCTTTAAAAAAA
AGATCAAGTATTTTCTTATTAGGTCACTTTTAAAGCATCATATATAATGGTAACCTTCTCTTATGCAATTTTT
TGTGATTATTTTGCATCGAAATGTTTAAGCTACGACTTTTATGTATATCAGATGTTATTATTATTAGTATGAG
TAAT
>103786680_2 DT667721.3
GCACGAGGGTTCCCTTAAGATAAGTTAGGTTTAATATCAGTCGATGTCGTTTATTTAGTCTGTGTATAGTATT
CTAACGGTTTAAGTTACTGTGAGGACGTGAATTTGAACGGATCGACGGGCCCGAGCCCGAGCCGGAGCCCGAG
CCCTAGCCGGAGCCCGGCGCCCGGTGCCCGGTGTTAGTTATGTAAATCTTTAAGTACC
TAAGCGATTATTTCTGTTGCACCTAACTTTATTTCGAGAGCCGTGTTAGTTGTATTTTATTAAGTAATTGTAC
ATATAGATAGGGCGAGGTTAACAGTTTTCATTCATCTCTATTGGTTATTGTTGAATCTAATAATTTGGGGCTG
GTAAAGTATCAATGCAACGTCGCATTTGCTGTCGAATAGGCTCGTCTGCAACGAGGAC
GAGCGAGCAAACGGAACGCGTCCCTCGCATGTCGGTCCAGTGACCTCTTATAATTATTATACGTTTATAGTAC
ATACCTACATACATAAGGACCCCACATGTTTCTGCTCCTCTATTAACTGAACGCAGATGGACGCAGGCGGACT
CCTACCTGACGCATCACTTGAAACATCACTTTGTTACAGACGTATGTGAATCGTTTAT
AATGTAAATAACTGTACAATTTTTTCTTAATTTTATATGTTTGTTTTTGCACGTAGGCTAAAGTGCATAGAAT
AAGAACCGAGTACTTACTTGTGACGGTGGTTCAGTTAATACCGGAGCGAATGTTGAATCTTAACGTTGTACCG
TGTCATTACCT
>103786721_2 DT668116.3
TGCGAGCGCCAGAATTCTATCCACATAGAAAACAGGAGAGATGCTGTCGTCAGTTTGGCATTTATCATCCTGG
ACATCCTTATATTAAGATGATCGAAGAATCCGGTGATTGGCTTGTGGGTGGTAACTTGGAAGTGTTTGAACGT
ATTCGTTGGAACGACGGCTTGGACTCGTATCGACTAACGCCTAACGAGCTCAGGCAAA
AGTTTAAAGAACTGAACGCAGATGCTGTTTTCGCTTTCCAGCTTCGTAATCCCATCCACAATGGGCACGCGCT
GCTAATGCAAGACACTCAAAGGCAACTCGTTGAACGAGGATACAAAAAACCTGTTCTCTTACTGCATCCTCTT
GGTGGGTGGACAAAAGACGATGATGTGCCTCTAGAAACACGCATCAATCAACACAAAG
CAGTTTTGAATGAAGGTGTATTGGACTCCCAATCTACGGTTCTTGCTATTTTCCCCTCACCTATGATGTATGC
TGGACCCACTGAGGTGCAGTGGCACGCCAAGTGCCGCATGAACGCGGGCGCCAACCACTACATCGTGGGGCGC
GACCCGGCCGGCCTGCCGCACCCCGCCGCGCCCCCCCGCCGAACTCTACGACCCGCGC
CACGGCGCGCTCGTGCTCACCGCCGCGCCCGGCCTCGCCGACCTCGAG
>103786724_2 DT668554.3

GCACGAGGACACCTCTGTCATACCACTCAGTTTGGTCAGTTAAGCTTCTCTATTAATTCACTAAAACCCTCTA
GTCAATCTCAATTTGGAAGTCAGCAAGACTTTTCATCATTTGGACAAGGAAGTTTTAATAGTAAAGAATCTAC
GCCTTCGCAATTTGATCAATCCACTCAACAATTTGGAACAGATTTAAAAAATAAGCTG
TCAACACAAATGCCGAGTGCAACTCAAGTACAATACAATGACTATAACGAAGGAACGACTTCTAGTTTTGATC
AGTTTGTTTCAACAAATCAACCTTCAAAACTTAGTATTGTGTTTACCGGACAACTTGGAGAAGATAATGATTC
GAAAACCACGACAGGTATTCAATATTATAATAAGCAGCAAACTATGACTTCCAATGAG
CCGTCTACTTTAATCTCAAGCACATTTGAGATTCAAACCCCCACTCAAAGTTCTGAATTTGGTCTGAATGACT
ATAGGAAACCCGTTCAAGGGCTTCTAACCCCATCTCAAAAACCAGATTCGCCACAATTCCAAGCCAATATACA
ATTAGGCCAGCAAGGCTTGTTAAATATCCAAAAAGGAAAACCTGCACAATTTACACAG
CCATTATTTAACACTCCGAGCTCACAATTTAGCACTGTAAATGAAGACAGAGTAGAATCTGTTACACAACAAA
ATAGTTTAGGAACCTTTCCAAGTACACAAGACGGAGCATTTTCAACGTCTCAAAATTCTTATCAAAG
>103786734_2 DT668262.3
GCACGAGGGTCGACCTCTAAAGCGGCATCCAGCGCACATAAGTAAATCAAAAAAATATCTCAAAATGGATAAT
GCTGAACTATTTCATAAATCTAATAGTTTATCAAAAAGAGATGCATTGCAATGTTTAGAAGAATATGCGCAGA
AAATTAAATGGAAAAACGGTGCGGGCACAGTTTTAGATATTGGTAGCGGAGATGGAAG
TGTTACAACAAATATACTAACAAAATTTTTACCCAGTAATTGTATTAGAGTCGTGGGAAGTGACAAAAGTGAC
AAAATGGTACAATTCGCAAACAAACAATATGCGACCAACCTTATTAACTTTACCACCCTCGACATAGACGGTG
ACTTACCTGACGAGTTGCGAGGAAAATTTGATCTCGTTTTTTCTAATTACACCCTACA
TCGGACAACACAGCAAGAGATAGCATTCACAAATATATACAATTTAATGACGAGAGGTGGTACTTGTCTACTT
TCGTTCTTAGGATACTTTCCGATGTATATTATTTATAGAAATTTAGCTCGCCAAAAAAAATGGAATAATTGGG
TGAGGGACGTTGAACATGTCGTATCACCATACCATGATTGTCAGGACCCTGAAAAAGA
AATTAAGAAAATGATGGCTTCTATCGGATTCAGCAATATTGAAATACGTTGTACTGAAAAATCGTATACATAC
ATCGGCTTGGAGGCTATTAAAATA
>103786737_2 DV501346.2
GACGTACACAAACATGCCTCTCCACGCCAGCGTCGTCCTCGACACATTTGTGGCTATTACATGGACAAACGCA
AATGAAAACCTGTACGCTAGGAAGGGAAAAGATTTCAAAGTTATGTGTGAAGTTACAGCTGAGCCTGCACCAT
CTGTAGATTGGTTTAAGGAAGGAACATCCATATTGACAGGAGATCGATATGTTATACA
CGCAAATGGTCTCTTAATTAAAAAACGT
>103786768_2 DT667268.3
GCACGAGGGCGGCCCTTGAGGTGCACGCCTCGCTTCATTGTATTGTTTTGGCTGTGTACACCATTGTTGTATT
CCATCCAACTGCAATCTATAAGATATTTCAGTGAAACACTGCGGCCTGAGTTGTTTAATACGATAGTGATACG
ACAATAAAGCTCTTATCTATATGGATACTCTGCTTATTGATAAGTGAAACTCGATCGC
ATTATATAGGACGTTTTGTGTTTTGTTGTTGAACCATAAATAAATGTGCAGTGATCTCTGATATAAAGAATAG
CATGCAAATCAATAATCAAGTGGATGAAGTGGACCCAGCATCGTAAAGCGAAGAGCTCTTCCCGTGCGGTGAA
TAACTCGGTTGCATATTGATTTGTCGGACAGGGATCTCTGGAAACTGTCGTCCTGTTG
CGATTTTTACGTGGCGGGCTTACGGAGATCAACGCACAGTGTGTTTTTTTTTAAGTTTATACGTCTGAACTTC
ACCATGCTGCGACCAGACGAGAGTGTCAGATAGTCGGCTGTCGTAACGCAGTATGGGTGCAAATATGGGCAAG
AACTCGAGCCTGCTGGACGCGCTGCCTTCGACGCAGGGCACGCTCCACGTCGTCATGC
TTGGTCTCGACTCCGCCGGCAAAACTACAGCGCTCTACAGGCTCAAGTTCGACCAGTACCTTAACACCGTCCC
CACTATAGGTTTTAACTGTGAAAAGGTCAAAGGCACAAT
>103786780_2 DT668118.3
ACTTGCTATGGCATATCCCTATAAGGAAGACGAAGAGAGATATTATCGGTAAATATAACGAGTTACCACATCC
GTTCAAACAGTTTTTGTGGTTTTATTTTATACTTAGATATATTATATTATAAAATGTAATGTCACATTTAAAA
TTTTTCCTCCACCCATTGTCATCTCCTTAACATGTGACGTAATTTCTAGATGGCCTCC
TACTTACATTGATATTGTGCTGGAATTCCGGAAAGAATTGATAGGCAAAGAAATATGTTTAGAAGATATACTA
CTACTATATATAAAACTATATAAAGAAAAGGAAAGAAGTTTTCTATTAAAAGAGTTTAAGAGTATGCTGGTCT
TTTTTTAAACAAGGAGATTATGAAATAAAAAGATGTACATAGTACAAGTATTTGAATA
TAGGATGTGTTGATGTTATAAAAGTATGCACGAGTCAATATATAATGTAAATTAATAATTTAAGAGTAAAATA
GTATAATAATAAAGAAATGTGGAAGTACAAAAAAACATTCAAGAATAATATTTTAATTTTTTTTTCTTAGCTT
CTAAATCAGAAACTGTCGGCATCGGTCAACTTGTATAATTTCCGACAAACTGTATTGA
GAAAATAAAGCCACAACAAATAGGGTTTTGCCACGTA
>103786789_2 DT668467.3

GCACGAGGAAGCACCCTTCGAGCGCGAGCGCAAGTCTAAACAGGGGCTGGAGAAGCTCTCCATGGCGATGAAG
CAGACGCCCACCTTCGGCAGCGACGACTCGCAGCAGAACGTCGCCGACAAACTTTATCATATGAAGTCGATGC
TGACGTACCTGGAGGCGGTGCGCTACAAGACGGCGGCCAGCCTGGGCGCGCTGGACGC
CCGCCCGCCCGCGCCGCACCCGCTCGCCGCGCACATCCGCGTGCTGCGCGACAAGCAGGGCCTGCAGCAGAGC
ATACTCAAGGTGCCACCGTGGCTTCAAACGGATTATCAGAATGAATTGAACAAGAACCTACAGCCGAACTACA
CAGCGCTGTCCAAGAGCGTGAACGGCGAGGAGCGCGAGAGGAGGGACTCCATCATGAG
CCGCGCGTCCAGCAAGCTGCGCATCGAGCACCTGTCCTTCCGGCGCCACGCGGAGCACAAGAGCGCGCCGCCC
ACGCCGTGCGCCGCGCCGCCCGCCCCCGCCGCGCCGCCATCCGCGCCGCCCTCCGCGCCGCCCTC
>103786795_2 DT668353.3
CGGCAGAGGCATTGTGCATATTTAAGTGCTTCGTACACTATTGATACTGTAGCAAAGTTATTAAAAGATATAG
CACACGAGTATGATATAGAAGATAAAATTACATTAGTAGTCTCGCAGAACGCTCATTACTTAAAAGAAGCTGT
TGAACAAAATGAATGGCCGTACTTTGTTTGTTTTATACAAAAAATAAATTTGGTAATT
GAAATGTCAATTGGTGTCATTGACCCAATAGTTGCAAAAGTAAAGAGAATAGTAGTATATTTTAAAAATAACG
TAGAGGTTACACAAGCTCTCTTAAAATATCAAACGGATATCGAAAATAAGACACTTCCCCTTCGCTTAATACT
ATCAGTTTCGAGTAGATGGAATTCTGTATTTTATATGTTACAACGATTTTTAAATCTG
CAAAATGCTGTTACATCAGTTCTTGAAGATCTAACGGTGGATTTGCCAGATATCTCCGCAGAAATGTGGGGTA
CAATAAAGCAAGTGTGTATGATTCTACAACCATTAGAAGAGGCAACTAGAGCTATGTGTGGCGCGCCATATCT
AACAGCCAGCTTGGCTATTGTCATAGTTGATGGTTTAAAAAATGTGATTGAATTGATG
AAAAATAAACCATTAACCGGCGTAGCAAAGAATTTCTTTGTAAACGTAGAATCATGCTTAAAAATCTATTTGC
TAAATGCAGAAATTGAAGATAATCTGCTTTTAGGAGTGTGTACTTTTTTGGACCCACGATTCAAAATACACGC
CTTTTTGGATCATGATTC
>103786818_2 DT668733.3
GCACGAGGATTAATTTTAAACACATTATAAATCTAATTACATTTCAAACCGATGCTACAAGTTAAGAAAGTAT
TCTTAACTTGTAGCATTTATTCATTTTAGGTGCACTATACGTTTCTATAGTGACTTGTATTTTATAAAGATTG
ATTGAAGATATTAAAAATTTACAAACTATCTCGTAGTCCATGTTAAATCTAAAGAGCC
TCACACAAATATCTATAGCCTTTATATGTGAAAATATTTATATCTAGTTTTAATAAACAATAAATAAAAATAT
AGGTACCACACAGCTGACTTCAATATAGAACATATTACATTGAGGTCAGTCGTGCTGCAGGGGCACAAACGTA
CGACTGTATACCTCCAGAATTCGGCCCCAGAATTCGACTATTATAACATTACACAAAA
ACATTACATTTTTCATTGATATTTGTCTATGGCCCCTTTTAAATATTTTTCTCGTACCAGTGTTATCAAGGAT
ATACTCGTAGTTTATCATTTTTCATAGAATATCGTTTTAAGATAAAAATATGTTGCAACAAGTAAAGCAAGCA
ATCATAATAATAAGCGTAGCCAAAAAAACAGCAAAAAAAATAAAACGTTATTTTCATA
ATTTATGAGTACCTAAGGGCCATTGTGACGCAGCGTCGTAATGTAAACGCGCGCGGGTTAAATTATCTCTCGA
TGAAAGAATGAATTCTTTTCTTGGCCCCGCACGTCTTGTGCGCAAAGAGACAATGCGCTTTGAGCTAACCCAA
TCACACGTAATATTAAAAAAAAAAGATTTGGTCAGTGGTCG
>103786824_2 DT668388.3
TTTTCGTAAACAGTATTTTACGAATGGAAAATGCCGAAGAAATATTTCAATTCCTTGAAGAGTTTTCGAAACG
TAAACCAAAAACTATACCACAAGAGTTAAACGATTATTTAGCATATGTAGCGCGTACCGGCGACCCCGTCTAT
CAGTGGGCTCTTGTTAAGAGTTTGTTTAAAGAGAAACTTCTGAATGTTTTAACAGACT
TCTACGAAACTAGTCCAGGTATAGATATCCCACCATATCCAAATGTTGATCCGTTTAACTACGATATTATGAA
AAATAGTTTAATGGAACGACTAGATTCTTTTACCTCTGCTCCCTTTACTGTACAAAGAATTTGTGAACTATTG
ACATACCCACGTAAACAATACAACCGTGTGGACAAATTTATGAGAGCAATCGAAAAAA
ATATTTTAGTCGTAAGCACACGAGAACCTGGTTATCAACGTCATCCAGAACCAGAAAATGGTGAACCTATAGA
GCCAGTTGTAAATGGCTCAGACAATAATTCTGAATATAATGTTGATGTAGAAATGGAAGATATGACCTGGAAG
GAAAGTAGTGGTTCTCGGCAGAACTCTGAACCTCAACCAGGGTCTTCAGATGCTCATA
TTTCAGTGGATGATTTAGAAGCTCGATTACAAGCTAAAGAAGAAACTTCAATGGAAATACAAGATAAACCAGT
TACACAATATGAGTCAGTTACTCCTCCAGAGTTAAATGTTACAGAAACAAATGATGAGTCTA
>103786857_2 DT668233.3
GCACGAGGTTCACCTTTGTAGCATTCGAATTTTTATACGTCTTTTAAAATATTTATTTATTCTGAGTATTTAT
ACATTTCCTAACAATAGAAGAACTAAGAAAATATTTATATGAGATTTTAATAAGTAACCACGATAAACGAATT
GGGATGACCTACAGATCCAATTATTACGATAGCACAGACAGTAAATTATTATAAAGTG
TTATTACCAAAAAAAATACAAACATAACTTCTTTTTCCTAATTGAAAAATGTTAAATATCTTTTTAAAACTCA
TTTTAATAAGCTTAATCAAAAAACAATTAAGATTTGCAATTTTTTATATTTACTTTAAAATCATCTACTTCGG
TTATCCGAAAATGCATCATATTACCTTTATCCATTTATGTCTTCAATTTACACTATAG

TTCACGCTAGTAATTTAATATATTTTTTTAGCGTGAAATATAAATATGTTTGTTTATGTGATTATACAAGGAT
ATAATTTAAAAAATACAATATTAATGAGCAGCAGCTAAACATCTAACATATAATTTTCATAAACTAATATAAA
TTCATAGTAATAAATATTTTTTTTTATAAATATTCAAAAAAGGCACTTAGATTTTTTT
TATGTTTTTTTTATAGAAAAATAAAAAAAAACATATGTTTATGCCATTACATTATGGAAGTAATTAAATAATT
TGTACTCACTTGGATCACAGGCGTAAGGAGTTAAATCTTTGACGCCATTTTCTTTTAGAACGTCTTCATCAAT
TGCAAAGTTGCCAGTATATTGTTTTGGGTCTCT
>103786873_2 DT667250.3
GTCGCTGGAGCGCGACTACGACCGCTCCTACGCGCCGCCGCCGCCGCCCGACGACGACTACTACTTCCGCGAG
CCGCCGCTCTTCCTGGGCGAGCTGCAGTCACAGAACTCCGACTTGCAGCGCGAGCTGGGCAACCTCAAGAAGG
AGCTGGAACTGACCAACCAGAAGCTCGGCTCCAGCATGCATAGCATAAAGACCTTCTG
GAGTCCCGAGCTGAAGAAGGAGCGGGCGCTCAGGAAGGAGGAGAGCGCCAAGTACAGCCTAATAAACGACCAA
CTCAAACTGCTCAATTTTTTTTACCAGAAACAAGCAATGCTAGTAAGGCAGTTAGAAGAAGAACTGCGTCTGC
GCATGCGCCAGCCCGCTCCTGAACTACAACAACAACTGGAGGCGATATACGCGGAGAA
TGAACATCTGCAGAGAGAGATTGCAATATTAAGGGATACGATCAAGGAACTGGAGCTACGTATTGAGACCCAG
AAACAAACGTTACAAGCCCGTGATGAGAGCATCAAGAAGCTATTGGAAATGTTACAAAATAAAGGCATGGGTA
AAGAAGAAGAAAGGCAAATGTTCCAGCAAATGCAAGCAATGGCA
>103786876_2 DT668351.3
GCACGAGGCGTGGTCTTTTTGAGGCATTTTTTATTTGCTCGTATTGAGGGCCACGACGCACCTCGGAAGGGTT
TAGGTCGGTGTGCTTTCCTCCTATTCCGTCTCTTGCGGGATACCCAGACACGCGGGTGCCTCTAGTTAATTAG
CCATCTCTAAATTATTATTTACTGGTAAACTGTGGTGTTATTACATAGAATTCCGGAA
CTTTCAATCTTATCACGGGTTTTAGATGTGTTAGATTACATCTGACGCATTATCACTTTATCATTGCCAATAT
TGGAACGCTAAACACGAGTACGTCTATATCGATAGTAGCTTTTTTTTTAGTACTTTAAACTAAGAATGTATTA
CGGTCTAAGGCCCGTATTTATAAATCGATCTCAAATCTGATATGGAGATCATAGAATA
TTAAAACGGCATCTCAAGATAGCTCTCTTGATCACTCATAGAGTTGAGAGCAGTTTATAAATACGGATTTATG
TATCCGAGGTATCTCTAACCGCAAGTACAGTGGGAAGATTCAGTTAAAGTAAGTTGTGATGCGTTACGATTGT
GGACTTGATTCCAACTCTTATTGTGAATAAAAAACAATTGCTTTTTCCAACGTGAGCG
GG
>103786910_2 DT668701.3
GCACGAGGCGTTACCTTAGTTAAACGCATTCCACTGGCACATAACGGACAGTCAAAGCTTTCCTCTAGAATCT
TCTAGGCAACCTGAACTGAGTAAATATGGTGCTTATAGTCCTAGTAAAGTGTACAGCGCGCAGACGATCCGCG
AGGTGGTGGAGTACGGGCTGCAGCGCGGCGTGCGCGTGGTGCCGGAGTTGGACGCGCC
CGCGCACGTGGGCGAGGGCTGGCAGGACACCGGCCTCACGGTCTGCTTTAAGGCTGAGCCGTGGGCCTCGTAC
TGTGTGGAGCCGCCGTGTGGTCAACTCAATCCTACTAAGCCCGAGCTGTACGATGTACTTCAAGATATATATA
CCGATATGGCTGAACTGTTCACAACAACGGACATCTTCCACATGGGCGGGGACGAGGT
CAGCGAGCGTTGTTGGAACTCATCACAACAAATTCGGCAATATATGGCAGGAAACCTATGGGGACTGGATCGC
GCCTCGTACCTGAAACTGTGGAACTACTTCCAGAGAGAAGCGCAGGCGCGCGCTTACAAGGCTTTCGGCAAAC
GGGTGCCTCTTATATTGTGGACGAGTACGCTCACTGAATTTAATCACGTTGATCAATA
CCTTAATAAGGATGATTATATTATACAAGTATGGACAACAGGGGCAGATCCCCAAATAAAGGGTTTATTGGAA
AAGGGCTACCGTCTAATCATGTCTAACTATGATGCTCTCTACTTTGATTGCGGGTTCGGTGCCTGGGTTGGGT
CAGGGAACAATTGTTGTTCACCATACATCGGCTGGCAAAAGT
>103786916_2 DT668789.3
GCACGAGGCTTTATTCATAACTGCTCTATCTGATAAATAACAAGGAAAACCAAAAAATATATTAAATCACAAA
TAATATTTTGAGATATTAGAACAGGATGTTTTATTTATTGATAACGTAAGTTAGTGCACAAAAATTAGAAGTT
TTTAATATTTTAATATGCCTCATTTATAATGTTTTGCGGACAATATATATATTCAGTA
CATACTACTCTTGTTCCTGTATAAGCAAACTTTTTTGTTTCTTTATTTTTTTATATATAATATTGCATTGCCA
CTTTAAATTTGTAAAGAGTTTGTGCAGAATTATGAATGATATTAACAAGGCTCTTTACATTCCTTTCATGGGT
TAATATAGTGAACTATATTATCATTCTAAGATGTTCAATTTTATTATTAAGGCGTTTT
TTAAGTTTACAACTTAGCTGGTGTAATTTAAACATTTGTATTGTTTTATATAGTTTTAAATGGACGAAGTCTG
CTAAATATAATTTTGGTACATGTAATAAAGTCTTTTTTATATGAGAATAAGCATAATATTCTATATTGTTAAT
GCTTAGCGACTAATACTTTGTTTATTTATTATTTTAGTTAGTATAATAATAATCGTGT
AATCATGTGCGTATATCGCACCTCTGGTGCAACACTGTCACTTATGCAAAATTGTATTTTCACAGGAGAAGCA
TTTGAAATTTCNAAGAGCTTAATTAATGCAAGATGAATATACAATATCCATAATAATACTTTCAGCTTTAGAA
TGAACAAAACTATCCCGTATATTATTCTAATACACTTAACTATTGTTTTGTGTATGAG
GCCAGTCG

>103786922_2 DT668134.3
GCCGAGGCAAATGTCCTCTTAAGTCCGGTGACTGTGTGGTCCGACATGCTGGAGTTTTCACTACTGGTCTAGG
AATGGTGGGTGCAATATGTGACTTCTGTGAAGCATGGGTGTGTCACGGGCGCAAGTGCCTCACATCGCACGCT
TGCACTTGTCCCTTGAGTGATGCAATTTGTCTGGAGTGTGAAAGAGGAGTTTGGGAAC
ATGGTGGGAGAGTGTTCCGTTGCTGTTTCTGTCAAGGATTTCTGTGTGAAGATGACCAATTTGAACACCAAGC
CTCTTGCCAGGTGCTGGAGTCTGAAACATACAAATGCCAGTCCTGCAACCGGCTGGGGCAGTACTCGTGCCTG
CGCTGCAAGACGTGCTTCTGCGAGGAGCACGTGCGGCGGAAAGGCGCGCCGCAGCGGG
GCGCCGCGCGCGGCCCGCCGCCCTGTCCGCGCTGCGGGTACGCCGTGCACGTCACCGCCGACCTCAGCATGTC
CACGCGCTCGCACCGCTACGGGCGGCAGGGCGCGGCGCTCGACGACGACGACGACGACCGCTACGGGCAATCC
GGATACGGAGGATATGGTGGAGTCGGTGACGATGGCGGAGATGGAGGCTTGGAAGGTG
ATGACTATTCGTACTCCGAATCAGATGACGACGACGAAGATGATGACGAGGAGGACGACGAGGATGAGGACGA
GTCGGCTTGACGAACACGAGGCACGACCTTCTGCTGTTGCTACTGC
>103786931_2 DT667607.3
GCACGTGTATCGCATCCTACGATAAACACAATACGTGAAGCAACTCATTTGTAATGGCAATTAATGTTTGACA
GTGCTCTCGGTAGATAACAAAAACGTTATAAGCAATCTTCCAAATGATGTGAATCCTACTCTATGTGATGGAC
ATTTGATCCTATTTTAACATCTAATTGTTGGCACCACAAGATACCAGAATTTTCTTTG
GATGGCAACATCAGCTTAAAAGCCAACGGCAGAGCCAAAGATCTGGCCTCCGTAATGGAAAACCTAAAGAAAA
ACAGCCTTCTTTTTTTGTTAAACCAGACCCTTAACACTCAAGTCACAAGCTCGTTAAAACAACCGATGGTGTC
TAGCACAACATCCATGCAGTGCCGTCACGAGCC
>103786936_2 DV501323.2
TTTATTATTTGCAGCAACATTACCTCTATCGTCACTTGCTTTATACAGTTAAATTACAATAATAAACCATTTC
AGTATTTCTTAATTGTAAAATATACAAATTATTAAATTTATATATAAACAGAGCATTAAAGGCCATTTTATTC
TAACAAAAAATATTCGAATACTTACAATGACAATTCATTCATTTAATTATTATATATA
TCATTCTAATGTTTTTTTCGTATTAATTATACAATAATCATGGCAACACAGGCATTATAAATGGAGCTGGGAA
GCATTCAAGTGTTAATTATTCATTCTTGATACCGACTTAATACTAAACAAGGCAAGTGAAGCAAAATAAATAG
TGGACTTAAAAGTAAACATTATATGAAATAATATTATAAGTCATGCCTGAAACAATGC
ATTAGGCCAGGTTCTTAGTATACATCATTACATGTAACATATTAAATACCTCATTAAAAATAAAACTTCATTT
TTACACCAATGCTAAGTTCAGATAGGAAAAATGCCGATTTTCAACGCTCAAAAAAAAACATACCTTATTCCAT
AACTGCCAATAAAATTATTTAAATAAACAATGAACCTGACAAATGATGTGTTTACTAC
AAGTTCATATTACTTTGTTTATTTGGGATGATAATTTTACGTTTTTCTGCAATTATATAT
>103786942_2 DT668223.3
GTTGTTGTTCCAGTGTTCAAGCGCCGCACTGTTCACGCCGTGGAACATGACCTTCGAGTATCCAGAAGCTAGG
AGAGACGAGACTGTGGTCGATGATTACCATGGAACTAAGATAGCAGATCCTTACCGATGGCTAGAAGATCCCG
ATTCAGAAGAAACAAAAGCCTTTATAGAGGCCGAAAACAAAATAACCCGTCCATTCCT
CGACTCCTGTCCAGTGAAGACTGATATCAATAACCGCCTTACAGAATTATGGTATTACCCCAAATATTCAAGC
CCCTTCAGAAGAGGCGACCGTTACTTCTTTTTTAAGAACACCGGACTACAAAATCAGAATGTACTCTACATGC
AAGAGGGTCTAGACAAAGAACCTACTGTGTTCCTCGACCCCAATACCCTCTCGGAGGA
TGGTACAGTGGCACTCTCAAGCTACAGATTCACAGAGGACGGCTTGACTTTCGCATATGGACTATCTGCCAGT
GGATCTGATTGGATTTCTATACATTTAAAAGATGTTCTAACTGGCAAAGACTATCCAGAGGTGTTAGAGAAGG
TCAAGTTCGCGTCAATGGCATGGTCGAAGGATAATAAAGGACTATTTT
>103786945_2 DT667294.3
GCACGAGGCGAAACCTACTATAATCAACCAGAAAGTGGTTACAACAACCAATATAATCAAAACAATCAAAATT
CTTGGAGCAATCCTCAACAAAGCCAACCATCAAACACTTGGCCACAAAACAACGGATGGAACAATCAGCAGAA
GCCTCAAAACAATGGATGGAACAATCAGCAGAAGCCTCAAAACAATGGATGGAACAAT
CAGCAGAAGCCTCAAAACAATGGATGGAACAATCAGCAAAACGCTTGGAACCAACAGAACTCATGGAACCAAC
AGGGCAGTTCGCAAAATGCTGGTGCACAAAACAATTGGAATAAACCTCAAAACGGTGGCTCACAGGACTCCTG
GAACAAACCCCAAAACTCAAGGTCTCAAAACAACTGGAACGCAAGAAACAACCAAAAT
AACTGGAAT
>103786951_2 DT667024.3
TTCGGCACGAGGCCCGAATGTTGACACTATATTTAATTCCGCTGATGGCGCAACTTTCGTTTTCAAAGGTGAG
CATTACTGGAGGCTAACAGACAGCGGCGTGGCGGCCGGCTACCCACGCCTCATATCACGGGCGTGGCCTGGGC
TTCCCGCAAACATCGATGCAGCTTTCACATATAAGAACGGCAAGACATATTTCTTCAA

AGGATCCAAATATTGGAGGTACAATGGACAAAAAATGGACGGAGACTACCCTAAGGATATCAGTGAAGGTTTC
ACCGGAATTCCCGATAATATAGACGCAGCCTTAGTTTGGTCTGGTAACGGCAAAATTTACTTCTATAAAGGGT
CCAAGTTCTGGAGGTTCGACCCCGCGCAGCGTCCTCCAGTTAAGGCCACATATCCCAA
ACCTCTGTCTAACTGGGAGGGCATCCCTGATAATATAAACGCTGCTATACAATACACTAATGGATATACCTAT
TTCTTTAAAGGAGGATCGTATTGGCGTTTTAATGATAGAACTTTCAGCGTTGATACAGACAACCCCTCTTTCC
CACGTTCGACTGCTTACTGGTGGCTCGGGTGTAGCAGCGCGCCGCGAGGCACCATTGG
AGGTGTAAAATCATCCGCTCCCAGATCATTCTTCTGGTTCAGGAAATAACTTCGGTGGCGCGTTCCTGAATAT
TTGTACATAGAGAGTAACGTAAAGAACAAAGACTAAATTAGTAAAATAGTGGATTATATGAATACCAGTGAGA
TTATAG
>103786958_2 DT668882.3
GCACGAGGGGGACCTTTAAAACCAACTGTTGTACATGATGCATGTTTATTGATTACAGCTAATGACCGAATTA
AGTTAAATATTCGAAGACTAAAAGTGAACGGCAAAATGATCAACGCCCAGAACTTCTTCAAGTCAACAGAAAA
CAAAATATCACTCCGACTATCAGCTGAACAGAAACAGTTCATAGAAAATGTTCGTGAT
ATTTGGAAAGCAATACTAAGAATATATATAGATAATGATACAGACTTCTTTGCATCATGAGCTGGTTCCATGG
ATGTAGAGAGATTGGTGGAGGAAGTTAAAGACCTAACCCATTTATAATTAAAATATGAACATGTTTATATGAC
TACCACAGTCAAATAGTTTTATACTATGGCCATATTGAAAACCCG
>103786963_2 DT667222.3
GCACGAGGCGGCACGAGGCGCAGCCCATCGACGTGCTGCGCTCCAATCACAACCGAAATGCCACGGACATTGG
CTCCCTATCGCCACCCACTGTGCCATTCACAATGAGGACCCCTCCTTCGGCTCGACGACGAAGTGCTTCTGGG
AGTTCCCCACCACCCTCACTGTGGCAGGTCTCCCCCACGAATAACAGATCAGGCACGT
CGCCGCCGCTGTCCATGGCGCTGAAGGTGAGCGGCGAGGCGTCGCCCGCGTCGCTGCTGCGCCTCAAGGTGCA
CCACGACCCGCCCGTCTACATACCCAACTTGCAGGAGGAGACTATACTGGCTGAGGAGCATAGCAAGGTGTTC
TCGCAGTTGAACTTCGTGTTGATGCTCGCGGAGCTGTTGTCGGACCTCGCCATGTCGT
GCGGTGCGCCGCTCGCCGCGCTCATGGACACATCGGATGAGCGCTGCAACGAGAGCGTGCGCTTCGGGCTGCT
GGTGCAGGCGATGCAGGCGCTGGCGGCCGGCCTGCGCCTCGCCGCCGCGCACTACCGCTCGCGCACGCTGCAG
CCCACGCCGCAGGTGCGCAACGTTGTTTCACTAATGAACGGAAAATATAAATGGATTT
TGAACGAATCAAAAAGACTGCATGAAGCACGTGTGACACCTGCAGTCTGTGATAAAATTCTCTATGAACATGC
TATCGAACTGTGCCCAATGGCCGCCATCGAGGAGCTATTCGGCGACGTGAAGGAGTGCGAGCGTCGCTACATG
TCCGCGCAAGTGTTGCT
>103786972_2 DT668562.3
CGGCACGAGGAAGGCCCTATAGAATATTGCAGGAGCATAGGAAACTTGTTTTTGGTAAAGAAGACAAAAACCC
ACTGCCCCAACCATCAACCTCAGCACAAAATGTCCAAGATGAGACTATAAACTTCATACAGTCGGAAGCATCA
CAGTCTACTTCAGCCCAGAAAGTGGGGAAGCCAGATGAAACGGAGAAACTAACGCAGG
CTAAAGAAAGCCCTCCGTTGGAACCAAAAGTCACTGCAACTAAGAATGTTCATGCTGATGAACCAAAATCATT
GGCCCCAGCTGCTAAAATAGCTAAAACGGAAAAGACTTTTATTATAGAATACGAGGTGGTATGCGATGGTGAC
GTTTGTGTTAGACGGCCCAAAGATCAGGGTGGGCCTAGAAGCGTTGAGGACTAGCCAC
ATGAATCATCTACAGGAAACGGAAAGTGCGTCCAACGTTCAAGATAAAATGGAGAGAGCTGAGGAATTGATTG
AACCAAGGAGAAAGGAGAAAGC
>103786975_2 DT668869.2
GCACGAGGGTGACCCTTAGTTAAGTTAAGTTTAAAGGACTATAGACGTGACGGTTGTGGGTTCGATTGTCACT
CGTGGAGTGAATTATTATATTTTTTTCGCTCTGATAACTGGACAACAGTGGCGTTTAGTTCCGCCACTTAATA
CGCAATGCCCGCAGAATTATGACAGAAGTATCTTAATGGGAACGAAGTGTCTTAACAC
TAGGTTCCCGGGTTCTATCCACCTACCCTGCTTTATGCGGAATAAAAATTGCAGACGTATTTAAACAAAAAAA
CCGTTTTCTATAATATTTTTCTATCTATACTATGGAATAATACTTTAAAATAGTAAAAAAACACTTTATATTA
TTATAAAAGTTATTTTTTACATGTATTGCCAAAATAGAATAAATGCTGGATTTTTTTT
ATATACTTAGTCTTGCCATAAATATTGTTACAAAAAAAAAAACATTTTTTATTGTACGTAAAGTTTATAACTA
TTACAGTGTGTTAGTTTAATACATTAATATAAAACCAATAAATATCTATAAAGCTTATTTGAAGTGTTCTCCT
TTAGCTGCTCACACTCTCTCTATGGGAACATTCTTCGCTGCTAATCGTAGGAATTGTT
TTAGGTACTTTAAATTATCATCAGATTTTGAACATGTCTTACTCTTTAAAATTAATAAACTAAAATCACAATC
CAGTGGATTAAGATAAGAATAGATGCTAGATGACGGTCAGTCTTCAGCTCTGGTGAAGTCCACAACGTTCGTT
TCTAACCAAGATTGGGTGGATCGAACTTCATGTCATTCTTTATTAT
>103786978_2 DT668801.3

GCACGAGGAGCAGACCATCGAGCTGCTGAAATATGGATGATCACTCTCGCCTGGCTCTCACTCCTGTCAACCT
CATTTGCACTATTAACATTCTGTGCGGAACCTTCTCGATACCGCTACCCGGAGAGGCCGGTGGTCTGGATGGC
GACCTGCCACGCCATAGTGGCACTAGCTCACGTGACGCGAGGTTGGCTCGGGGCCAAG
GCCGTGTCGTGTTCGGGCATCACGCTCGCAGTGGACGGGCTGGCGTCCCCAACTTGCGTGGCCTTCTTCTCTC
TGACCTACTACTTCACGCTGGCCGCAGACGCTTGGTTCACCAACGCCTGCGTGTCCTGGTACCTGACGGCGGC
GAGCGAGTGGTCCACGGAGGCGCTGGAGCGCGCCGCGGCGTACCTGCACGCGGTGGCG
TGGGGCTGGGCGGGCGCGTGGACCGCCGCCGCGCTCGCGCTGCGCAAGGTCACGGCGGACGAGCTCACCGGCA
CGTGCGGGGTGGCTGACACCGCCGTGTCCGCGCTGGTCGGAGTTCCGCGAGGCGCACTGCTCATCACTTCAGT
AGCACTGGCCATAGGGGCGTGTCCTGGCATAATGAGAGTACGTCGGGCGCTAGATTCT
AGAGGAGCTAAAAGAGTTGGACGCTTACCAGCACGGGCCGCGGCCGGAGGCCTCCTCTACCTACTGCTGGCTG
CCTTTCAAACGGGAGCGCGGGTTATGGA
>103786990_2 DT668540.3
GCACGAGGCCCTCCTAAAAAGTACGTAATAAAAAAAAAAAACGTTAAAATAACTATCCAATCGGCCCCTTCAG
GGTCACGTTGTGCAACAATTTCCACATCCCCGCGATCCGTTTTTCATAATTTATTAGACGCGTGATCTAAAAT
AGTCGATTAGTACCACCACCGTTTGGTTGCAGGCGTTTACCAGTAGAGTCAGTTGGAG
CTCGGACGCAGTCGGTCAGTGCGCGCCTCACTTGTGATACTGTGACAGTGACAAGTAACCAACATGACCAAAT
ACGTCACAGTCGCGCTGATAGTATGCGCGTGCTTTTTGTTGACGGCGGCGCAATACAACCACCTCGGAGTCGG
TTTCTCAGATGGCTACCTCGCAACCAACTTCGGTCCTCCAAGCTTAAATGTTTTATCG
GCAGCCGCTTCCAGGGACCCTAGAGCAAATACAGGTCCAGTAGTTTTTCCTCCATCTCCTCCCGGCGACCCGT
CTCAGACCAGTGGAGTCGTAATAGGCGCTTCAGGCTTTGGTTTCCAGCCTCCCGGCTCGCAAGGTAAACCTTA
CGGCCGATAAAAATAGTCTAATGTAGCTAGAATTAATGCACAGCCAAGTAAAAGCAAT
AAAAAATTATTATATATATTACTT
>103786993_2 DT668941.3
GCACGAGGGTGGTTCTTTTGAATATATTTTCTGTGTGTGTGCTATTCTGTGGTCAGGACTCAGGTTATACCTG
GGAAGTCAAGAGCTGCCTATGGTGAATATAATAGCGTTAATACGGATGGATCTTTTGATTTTGGATATGCCAA
TAAAGACAGAGGCGGTAGTTATCACTTAGCGCACGGTAACCCCAACGGTGTGGTCGGC
GGCCGCTTCGGTGCGAGGGAAGCTGGTACTGATGAAGTGAAAGAGACTATTTACACTGCTGGACCTAGGGGGT
TCCGTGCAAAGGGTCCAAATGTTCACCGTAAAATCGATCTGTATCAACGACCACGAGGTAACATAGGTAATAA
AGACGACCCCTACTTTGACCCCAACGAAGACCCCAGTTACTCATTTAAACAAGATACT
AGAACTTACTCCAAAAACGAAAACGCTGATAGTAGAGGCGATGTAACAGGACATTACACATTCGTGGACGATG
TCGGAGAGCGACATGATGTATCCTACATTGCTGGTAGGGATACTGGGTTCCATGTGTCGTCAGCCAATCCTGA
TGCCCCAAATCTCATTGGAACTCCCTTCCACCGAGTACCACTTGTTAGGGGTGAGAGT
AAATCGAGGGGAAAACTGCTGTACAACGGGGACTCGACGGTTCATACAGATTTATTTCTGCGGGGCCTGATCA
GCGT
>103787011_2 DT668130.3
GCACGAGGATTTGACCTATGTGAAATGATAAAATGTTTAATTATTGTATTTATTAATGAAAATATATAAAAAA
TATAAAACTTCATTGTCATTACTCATAATCTAGTATTTGATGGCAGTTCCTATAATTGACCATAATGACTACG
TTACGGGAACGCCAACTCAATGCAATCAAGCAAATGCTTAACTTAAATCAGCCACTGA
CAAAAGCAGTAGCAAATGAGCCATCATGGAAAGTACTAGTTTACGATAGAATTGGTCAAGACATAATATCACC
CCTAATATCAGTTAAAGAATTAAGGGAATTGGGTGTCACTCTACATGTACAGTTACACTCAGATCGTGATTCT
ATACCTGAGGTACCAGCAGTATACTTCTGTGCTCCGTCTGAAGAAAACTTGGGTAGAA
TATGTCAAGACCTGGATAATGGCATTTATGACCAATATCACCTTAATTTTATATCACCAATAACTAGACAAAA
GTTAGAAGATCTAGCAGCATCAGCAATTCAGTCAAATTCTGTTATGAGCATTCACAAAATATTTGACCAGTAT
TTGAATTTTATATGCTTAGAAGATGATTTATTTATTATGAAGCACCAGCAATCTGACT
CATTGTCATACTATGCAATAAATAAAGGTGATACTAAGGACACAGAAATGGAGGCTATAATGGATGAAATAGT
TGAAAGTCTCTTTTCAGTTTTTGTTACCCTTGGTAATGTCCCAATAATA
>103787037_2 DT667282.3
GCACGAGGGTTGACCTCATCATGAAGTGGTTCTTCTTTATATTTACTTTGTTTATACCAATACAATATATCGA
TAATACTTTACAAAAATTGTTACCAAAATGTGTTGTCAGAAATTTGGTTGAAATGAAAAATGTACTTGAAGCA
AAGGCACATCCAGGGAGAGATATTTTATATGAAAATTTAATAAATTATATAAACTCTC
AATCTTCTAATGACGAAGAAAGAGGTGAAGATCCCAGTATAGACATGACTACACCACAATTGATCGCTTCCCA
TGGATATGTTTCTGAATCGCACACCATTGTGACTGACGACGGATACATACTGACGGTCCATCGAATACCGGTT
TCAAGAAAATCAACCTGTAATACTGACAGTCCTCGTAAAACGGTTTTGTTACACCACG

GACTTCTAGGCAGTTCAGCGGATTGGGTTATTCCAGGTCCTGGGAAAGCTCTCGCTTATATATTGTCTGATGC
CGGGTACGATGTCTGGCTAGCGAATGTTCGGGGAAACACGTACTCCAGATCACATATATCTTTAAATACGAAT
GACTTTGCCTTTTGGAATTTTACTTTTCACGAAGTAAGTCAGAACGATCTACCAGCTG
TTATTGATTATATTGTGCATAAAAAAGGTTCCGATGNTAAATTGAATTATATAGGCCACTCGATGGGTACGAC
TATTCTATTTGCTTTGCTGTCAACGAAAACTCATTATAACAATGTTT
>103787040_2 DT667887.2
CGGCACAGGGCATGTGTCCTGTGGCGCGGGGACTCCCCGCTACTAGTGGCTTAATTTTAACAACAATTTATGG
TACAAAGTGTAATTTTAGTTCCATAGACGTTTAAGATTTACAGAGTTTATATTTAGAGAAGATTAATTAAATA
AATAAAATGATTATTATATACGATACGGCGGAGCGCCCGCCCCCTCTCAGTCAATAAA
GTTAATATAATGATATATTATGTACGACGAGTGTGGCGTAAAGTTAATATAAATAATATAATATAATAGTGTC
TACGTGTCTGCGTGTGGTCGTTACGGAGTCGAGTAGTGTACGCGTGAGATACCGTCGGCAACGAGCAGTAGTA
CGTGAGGTGTGGCTCTAACGCAAAAGGGTTTTGATTTTCGGAATTTACGTATTTTGTG
TCTGGCCGGCGGCACCGTACCCTGTCCCCTTCCCGAGGCTTACCTGTAGCTGTCGCGCTTCGGACGCGTTGCT
TCTACATAGTCTTTAGATTATAAGATCTATTTGATTTCGCTATTTTGGAAAATTTCATAATCGTCCCTTCATA
TTTTTTGTTTTATTTAATCGTTTAAGTATTTACTAATACTTACATTAATCTGTCCACA
AATACTTTACAAAAAACCTCCCATCCCCCATAAAGCTCGATTAGCTATCGTCGTGTGTGTAGTACGGTGTCCG
CCGGCGGGCGGCGAGTGTTCGAAGCTGCCTCCTGTGATACTCGCGCCGCGCCGCATCCCCCGCCGCGCCACCG
CCTATTATTTA
>103787044_2 DT667403.3
GGACGAGGGATTACTTTTGAAAGCGATTAAGGAGTTTATTATCTAGATCTAGTCCTAACTGTAGCGGAAGATT
ATGCTTAGCACAAAAATTCATTCTTAGAAACTGAACCAATTTGTAATATTCCAATACTTATCCTAGAGTATGA
ATTCATTTAGAAAAAAATAAATGGAAATATTAAATAAATAAATAAGTATATTATGCGT
AATAAATTAATTTACACAAAAAATATAGACAAATCTGCGTTGGGTTAGTTAACGATATAAACAATATCAGCCT
CCTGCGCGCATAACATGGTTAATATGTAACTTTAACGTTTCTTTTAACAGCTAATAATAATTTACATAATTCA
TTCATAATAACACTTACATTTGAATCGATTTACGGTCGAGCAATAAACAAATTATAAT
AAACATATAAGTAATTTTTTTTTTCAGTATTCACGCAAAATTTGTAACTCTATTGCTAGTAACGAAATTTTAA
ACATTAATAATAATACAAAAATTTCTAAAATGGTCACAGCATTAATAATAATCGGTAAGAATTTAATATTATT
TAATTCAATTATAATTTCTAAGACGAGTCCAGTCACAGAGTCAGAGGCACAAGCATAC
GACCGTAAACGCGATAGGTTGTAGGTTCGATATCCTACTCGAGGAAATGAAATGTTTTTATATCTCCTCGCTC
TTGTAAAAGCACAGTAGTAGAAGCGCCTGCGTAAACTTCCACTACTCAACACACTATACCTCC
>103787053_2 DT667602.2
GCACGAGGCATCTTTAATCCAGAAGAAGTTGTTGCCTGTGAAGGATGCCTTCAGCTGCGAATGCGAAGTCTGA
AAAACCAGCTTCTTCGGAAGCTGTTGATAATTCACCAATACGTCAGATTATAACTGTTATTGAACACAAAATT
CGTAACTTGGAGAAAAGGAAGAGCAAGCTGACATCATATCGCGACTTACAAAAAGCAG
GGAAAGAGCTTAACAGTGATCAGAAAGTTGCTGTAGCTAAGTACGATGAAGTGGCTCAAACTTTAGAATTCGC
CAGAGATCTTTCGAAACAAGTGACTATAATATTTTCTGCTGCTGAACGTGAAGCCAAGAAACAAGCTAAAAAG
GAGGCATGGGTGCGTTATTCAGTGGAAACTAATAAAATACGCGAGGTTCTTCTTATTC
TGGATTGTTTAGTGCAAATGGGTAATAATGAGGCCAGAGAGGATTTTTTAAATGGTTCCAATGGTGCAGCCAA
GTTGACTGAGGAAGACTTGAAGATTTTGGATAATCT
>103787060_2 DV501313.2
GCACGAGGATTATTTATTATCTCTGAAGTACTTAAATCGCGATAATAACGGATATGTTGATTACATGCCTTGT
AGCGTTATTTCTGACCACGATCCGCGCACACGTGGTTCTGTTGATGGCCATATGTGGAGATGCCACTGCACAT
ATGTACCGCAATTGAATCAATGGTTAATAAAAAAGGCTATAAAGTCCCATCACTTGCT
GTGCTACGCCGCTCATGGTGTATGATTGACCAGAACTATAGCGGCCGTTGCTATG
>103787069_2 DT667660.3
GCACGAGGCGGACACATTTTTAGAGAAAATTATTTTGACCGCTGCAAATGGTGGCACTCATTTTGCTGTTAGG
TCTTTAGAGTTAGCTCTGGTGTGTGTTGGACACCATTATTATGATGTAGCTGAAATCACATTTAATTTATGGT
ATCGCTTATCAGAAGAAATATACAATCGGGACTACCAGCCCTTAACAGATACCTTCAA
GCCTCATATAGAAAGGCTTATAGAAGCCCTAGCTCGGCATTGTCAATGTGAACCCGATCACACTCGTCTACTT
GATGAGGGAGATGAATTCTATGAGTTCAGATCGAAAGTGATGCTACTTGTAAAAGACGTAGTGTTCATAGTAG
GTTCAAGTTCAGTTTTCAAGAAAATGTTTGCTGTCCTTCAGGCTGACTTGCCATGGGA
ACAGACCGAAGCTGCACTGTTCATGATGAAGGCTGTCGCTAAGAATATATTGCCTGATGAATACGAATATGTA
CCAAAAGTGGTAGAAGCAATACTAGCGATGCCCGAAAATGCACACCCGGCTGTGAGAAAAACTTGTATTTTAC
TACTCGGGGAGCTCTGTGAGTGGATTGAGAGACACCCTGAGTGCTTGGAGCCATGCTT

GCAAGTACTGTTGCGTGCTTTACAAGATCCAAAATTGGCCAGTGCGGCTGCTAGTGCTTTACAGAGCATCTGC
GCGGCGTGCCGGCGGCAGGGGGGCGCGCACGCGGCG
>103787081_2 DT668319.3
GCACGAGGATAATTCTAATTACAATTTTTTTGTTTGTTTATTTTTAGATAGTATCTAAAACGCTAGAATGTTT
TTTAAATAATTTCGGTCTAAGTTTGTAATGTGGCCAACATTAATAATTAAAGATTAATAACCAGATAATTAAC
CAGGCCTACCAACTGTTGGGCCAATGCACACATTAATTTGTTGCCAACTTTTAAAAAG
ATCATAATACAATAATATTTATTGCTGATTAGAGCATTGTCCCAACTTATGTTATGAAGTATTAAGGAAATAT
ACGCTACTCCGCAATATTTTAACGAAATTTTCAGATTTAATTTTATATTTTTTGTGGTCACATTTCCACCAGT
AGGACGGCACTCGCAACGTGACAAGAGAACGTATTTTTTTTATAATTAATTAATTATT
TGAATTGCAAGAATGTGATGTAAAATAAACAAAACATAGGCTTAATATCGTTATTTTTATACAATACACGTTT
TGTATGAATTGGAAGTACATACGTAGTTAATCGTTGTTGTACGCATGTAGCGTTAGCAAAAGAATGGAGACTG
TTTTTTTACATTTCTCATATAAATAGTGCAATTACAGTGTTTTTTATTGAACGTCTAA
CCGTAAACGCGACGTTTGTGGGTTCGATTCCCATTCGGGGAAATGAATTCTTTTCATCTCCTTGCTCTGGTAC
AAGACAAAAGCGCGGTGTTAAATTCCGCCACAATAATAGCGTTTTTCAATTGTATTGAGCAAACTCTTGC
>103787089_2 DT668021.3
AAAATAGTGTGCAAGCGCGTAACTCGTGCCTCACGACGTACACAAACAAAATAATAAACCTCTAAAGGTTTTT
TTATGTATAAAATGCATTCACTCAGTAAGTTAGACATAGCCTATTTTGTTTACATTAATCTTTATCGTATCGT
TGAGTTTTACAGCAATTATCGCTGTGCATTGATCCCCGACTCAAGTGGCGGCTCGACG
TTGAGGACGAATATTTTTAAATCAACAGAAAAATTGTTTATTCAATGTCTACGAAACTGTTATATTTATCAAC
ATAATGTATTTTTTTTCACCATTTTTCTATTCGTACGACAGAAAGGGGACTGGTGTTCTAGTAACGACCGGTC
TGGTGTCGGTTGAGGAGCGACAATAACCTGATACATAACGCAATCAACAGGGTCCCGC
TAATGCGCATTAAATCAGAATTTTTCTGTTGATAAGACACTATAATAACTCCGCGTCTACGTCGAGCCTATCT
AAGAGAAAAATAACGCAAGACTCGATTTATCATGCCTTCCCACGCGTGTGCCAATAAGCTAACACAGCACAAA
AAAAAATGTCAATATACTAAACAGCCCATAGTGTGTAAAACTCACCGATGGCGGCGCA
ACGTGGTACCACCGCGAATAACTTTAGTTATATTCACATTATCTACACCAT
>103787092_2 DT668321.3
GCACGAGGGGAAACCATTAAGAAGTAGACGTGCACGATGTCTGTTGCTTATAAACTTGGTGATTTAGTTTGGG
CAAAAATGAAGGGGTTCAGCCCTTGGCCCGGCCGGGTGGCCATTCCAACCCCTGAGCTGAAACCTCCCAAGAA
GGCTATGAATGTTCAGTGCATTTATTTTTTCGGAACAAATAACTATGCGTGGATCGAA
GAGCACAACATAAAGCCCTATCAAGAGCATAAAGAGCAGCTAATTAAATCTTGCAAGACCGCGGCGTTCAAAG
AGGCCGTGGCTCAGATCGAGGATTATATTGTAAACCCTGAGAAATTCGGTGATTTCGACGAACACGCACGTAA
TCCTGAAGAAGAGTTTGACAAACTGAAAGATGTTCTAAAAGAGGACAGCTCCGATGAG
AAGCCCGAAGAGGAAGAAGCTTCCCCCGAAGTGAAGAAAAAGAGCTCGACGCCTAAGGTGAAGCCTCTAAAAC
GGTCTTCGTCAGCGGGCAAGCCCACGCCCCGCAAGAAGGCCAAGAGCTCGCGCTCCTTCACAGACAGCGACAT
GGCGAATGAGAAACCGTCTATGCTGAACCATTCGTTCTCTAAGAAATCCAGCCTATTG
CATCGCCCATCCAATATTTTGAGACCTGAAACTCCACCATTAGACTTGGAGAACGTATCGGAGACGCTCCTGG
AGAAGAATATTAAAGCTAGCCAAATGAAATTCGGTTTCCTTGGCTT
>103787105_2 DT667757.3
GCACGAGGCGCTCTTCTTCAATTGAAAGCATTGGAAGGGAACGTAGCGAAGAAAGACCTCGAGCTGTTTCACC
ATCTCCAGTAGAACGAACTCATTGTCATCACAATGAACCAAGAGACAGAGCAAGTCCATATACAATGCGTCCA
CAACAGCATGAAAAACATAGAATGCCATCTTCAGATTTATATGAAAAACGAAGATACA
CTACAGATGAAATCATGGAAAAAGAAATGCGTTACTCGGATATGCCTGAACAACATCAAATAAGACATCCGCA
ACTGCAGATACGTCGTGATTTACACCAAGTTGTTCCACCAGATCCTGCAAAAATTAACCAAATGTCTAGTCCA
CCTATAGACCCAGGTCCCACGGGAATGTACCTCAGTACGTTTAGAAAAAGAAGTCATC
CGCCGGATATGGGAGATAATGTTAGGTCATGTGCAGCTGTGTATAGAAGTCGATCACCTGAACCTACTAATTT
GACTATGCCAAGACCGCCTATGGGCGCTAACGAAGTGATGGTCGGAATCCCACCAACAATGATGGGCATGCCA
TCATATCATAAATTTGGCTTGCCCTTGCCACCTCCGCATCCCCAAATTTCTTATGGTC
CATTGCCACATTCCGCACTAGGCCCACAGCAAACTGTTGCCCAAGACTTAATAGTAAAACATGCTCCACCTAA
AGACACAGCACCCAGTACTTCTACAATTGT
>103787138_2 DT668077.3
TTATAGGTTAATAGCATATTTATAATAAATTATGCATAATTGAAATAAAAAACCAAAATAAACAATATCTAAA
GAGCTTAAATTTATAAATTGAGTATTATATTCTGTGACTAGTATGCTTAAAGTAAAGTTATTTGTGTTTATAA
AAATAATAAGTTAAAAGTAATTATAACTAAATATTTTGGTATGTTTTATAAAATATAC

GTTAGTATTAAAATATTTTAATAAGGAATAGAAAACAAAATATTAAAAATTAAATAAAAAAATAAAAAATGTA
TATAACATTATAGGAATATCAAGATTAAGGACAATCTAAGATTTATAAGAAACTAGTGAAATGCTTACCAAAT
ACAACCCATATAAGAATAAAATTTAAGTGATAACAATTAACAATTATTTTTTTATGAT
TCTTTTAGGTCTAGTAATATTGTTTTTAGATGATTTCAACATTGTAGAAGAATTTGAAGGTAGCACAGAATTC
TCACAAACACTTTTTTGAATGGAGTTTCGCTCAGTCATTGTAGTGCCATGATGCTTTAAACCATCATCACTCC
GCACTATAATGATACCATTTTTTGTCCAGCATTTAGTCACTCCCCAGCGTTGCCTATC
TGCCGAGAAAGTCTTGTGCCGCGCCTGGGTCAGGAATTCAGACAAAG
>103787150_2 DT667744.3
GCACGAGGCAATGCATGCAAATTTGATTTAGTTTATTGTTTTGTCATAATAAGTAACCACACGTATATATTAC
TTATATTTCTAACAATGTAACACTATACCATATACTAACTCATGTAATAGTTGCAGACTTAAAACTCAAGCAA
TGAAATGTCATATACCGGGACCGAACGTTAAAGTTCTCGGTAGGACAGTTCACGCTTT
AGCAAGATTCGGAGATGAACTGTATTTGGAGTCGTTGGCAGACTGTATTCTTTTAAGAACTTTAAATTCAGCT
GAAAGCGCATATGCCATGGTAAAATTCAATAAGAGTTTCTTTTCATATTTCAATTATAACTTTTATGCTTCTG
AAGACAATGAAGGACTAAAATGTAAAATATCAATGAAATCTGCCTTAAATGCATTCAA
ATCTCCTACACATATGGATAAACAAGTTGAAAACCTTGAAATAAAACTTGATCCAGAGTCTTGTAAATTAATA
TTTCAATTAAAATGCAAGCAAGGTATTGTTAAAACACATTTTGTTTGTATACTAGACTGCAAAGCTATGCAAG
CAGTGTACACCAAGGACTTAGTACCTAACAGGATAACATCATCTCAACGAGTATTGTC
CGAAGCTCTTGGAAATTTTCAAAACTCAGATGACCAAATAACGTTAGAAGTTTCATCTCAATCTCTTTTAATG
CGGAACTATGTGGATGCTGCTATGGATTTATCAAAAATCATAAGAACACAGATATGTATGAAACCATCAGAGT
TTGCGAGCTACACTATAGGCAGCGATACAACAATAACTTTCACACTTAAAGAATTTAG
AGCACTACTTGCTTT
>103787162_2 DT668433.3
GCACGAGGGTCATCCTATAAATTGATTATATTTATCATCATTTATGTTAAAATATAGTAGAACAATATTTCTT
TTGTGCTACGTAATCAGTATAAAAATATAAAGTGGCGTAATTAAAAAGAAAATATGTCGAAATTCGAGGATAA
TCCCTTTAGTGATCCAGCCATAGACAATCCCTTTGCGGACCCAGCAGTACAACAAGTA
GCTAGAAGTACAAATAATGCGACACGAGGTCTCGATGATTACAATCCTTTTGATGGACAGCAAAATGCAAATC
AGGCTACGCCTCAGCAACCTACACAACCTGCTATAATGCAGGCAGTATCGCCACCACCTACAGGTGGCGGCCA
GTACACACGACCAAACCAACAACAAAGCCAAGGTCCACCTCAAAACTTTACCACAGCT
GATTTTCAGAGAAGACAAGAGGAATTAGAAAGGAAAGCTGAAGAGCTAGCGCGTCGCGAAGCCGAACTAAGGG
CTGGTTCTGCCGGGAGACGTAACAATTGGCCACCATTGCCAGGCTTCTGTCCAGTACAGCCGTGCTTCTATCA
GGATATTAATGTGGATATACCGTTGGAGTTTCAGAGGATCGTACGACATTTGTATCAC
CTTTGGATGTTTCATTCCCTAGTACTAGCACTAAATATAGTAGGGGGACTCTCATTACTATTTACTGGTGAAA
ATTACACCATTTTCGGTCTAGCAATACTATATTTCATATTATTAACACCGTTCAGCTTCATATGCTGGTACAG
ACCAATATACAAGGCCTTCAGGAGTGATTCCTCATTCAACTTCATGGTGTTCTTCTTC
ATATTTTTGTTTCAAATTATCATAACT
>103787172_2 DT667014.3
GGTTTATTCTTAGATTTCCAAAAATATTGTAGTGTTTTTTTTGTGTGTTTAACGTTTTTAATCATTTTTAACG
ATGTATTCTATGGATTTTGACTACTAGCGATAAGTATCAGTGTGTATCAGTGCCCTTTCTAGGACAGCTAGTG
AACAGACGCGCCATTGTTGTGTTCAAGCCAATTAATAGCTTTAAATATTTTGTTATCA
ACAACTGCAATTTACAAAGCAAACCTATTCTTCATATTTGTGATGTACCCGCATCCCATCAGCGCGGAAGAAG
AACAGAAGACGAGTTTTTTTCCGAGTGTGAACCTTCTTCATTCAAAAAGGAAAGGAATATTCATACACCCCTC
TGCTATGTACAGAAGTAGTGCCCCGTGTAAATATACAAGTTTGATATTGATAACGAGA
AGTTTTTGTGGGTAACTCATCGGTAAAATATAAAAGAGATTAAAAATGAAACTCTTAGAGAGCGGACGCCTTG
AGGCTATTAGTAGTGCACTGTCGATCCTGAATGGCGATAGTGCAGTTCAAGGTCGAGTGGAGAGCTACAGCTG
CAAAATGGCAGGTGCTGAAAAATCTTTTTATAAGCGATTCACAGCAGATGGAGAAACA
ACTCACGACCTTCAGGCTTTATCTCCACCTGAAGGTATCCTGTACAGCCGTAACTTTTCCGGTGATGAA
>103787196_2 DT667157.3
GCACGAGGGCCTTTATAACGCTAGTTGATGAATATTTACACCAGTACATTTTACTTGGTATGTACCAAGTAGT
CTTTGAAGTATTTTCGTTATATAATTTTAATACTCTTTAAAGTTATATAACAAAAATATCTAAGCTTACTATT
TTAAAATATGAACAGACTTTTAATTTAAATTTGCTTAATCATTTTATAAATTTATCAT
ATATTTTTTAAATTATTATTATCATACTCTTTTTCTTTATTATTTCAAAATATAAGGCAGGTGCGCCTAAAAA
GGACCGGTTCCAATAAGCCTGCGATTATTGAATAGTTTCTTAAAAATATCTTTCTTAAAGCTACGCGTATATA
AATAAACGTCTATATAATTAGTGTAATATGTAGTTTTTTGCTCATTTAGCATAAATTA

TGTTGATTGTATATCTTAAATATCTTTATATTTTAGATATTTATTTTTAGTAAATTTAATATTGTTATTGAAT
CAGTAGAATTCGTTTGGCACCTTATGATACAAAGCTAGAATTGTGATTTTTTATGTGAATCAAGTTACATATT
ACCAATAATAAATTTGTTCAAAATTAAAATAAAAAATATGTCTATTATTCTATGTATT
GCATAAAACTTTCATATATATTTTTTATTTATTAAGTTTAATTGCATAACTATATATGTTACCTTATATTTTT
TTTACTTATTACCACTTTTTTTTATTTAATAACAAATGAAAAATGAAAATTGGAAGTAATATTAATCTTTTTA
TGAATATATATAAGTTAAAATTAAGCAATAATAGCGCTATTTTTTGTGTAATAATTAT
TTACTAT
>103787199_2 DT667751.3
GCACGAGGGAAAACCTGGAAAGCTTGCTCCATAATACGACTCAACCTGTCTCATTGCTTTCTTATAAAGATTG
CACACTCTTTGTGCGTGCGTCTTCATCTGCGACGGATAGCACATATTTTAGATATCATTAATATTTTTATAAT
TATATGAATATTTAAATATCTGTATTTTAATTTTTTATAAATTTGACATTTATTTTTT
TATTACTAATTGTTTTTACAAATGATAAGAAAAAATCTTTAAATAAATGAAATAATGAATTAAATTATTATTT
AATTAATTATTTGTTCAAAAATTGTAGTTGAGTTAAATAATTAATTCATTCATTATTGAATGAAATAATGTAT
CAAATAATTATTTAATTCATTATTTCAAATAGTATTTTGAATTATTAGTCAAAAATAA
GATTATTATTGTTATATCCGTATATAACAATAAAAATTTTATTACTTAGATATACGTTATCAAATCAAAAAAA
TATCATTTAGTTATTTTACGTATATGATTTTGCAACTAGTAAAAATTTGTTTATCTGTCATCTGTCGGGTACT
TAAGAAGCTTTAAGTTGATGTTATTCGTGTCATCGTGTGCACAGAACACATATACCCA
AAGTGAAGAATGTTCACTTCATACAAAAAAAATGGGGGACCAAACGCCACTGCTGCCATCTCTTGTGTGCCCAC
CAGAAACAATATTTAAAATTTACCGCCTCAGATATGGGCGGGAAATTCAAAATTTGGCTAATATTTGGGTAAT
AAATCGATTTATATTCAAAGCCAGAATGATTCACTCTTTCAAAATCATTTGGAAGTTT
ACTACTTATTTTTAAAGAAAATACTTCCAAATGATTTTGAA
>103787210_2 DV501303.2
TTTTAATTTTATTAAATATGGCTTGGAGGAGTCACGGTATAAATAACGCTGATATGGTTCGAAATTTGCGTGC
GAATGGGATCATAAAATCAGATGTAGTTGCAAATGCAATGGTTGCCGTTGATCGAAAGAATTATTGTCCACAT
GCATCTTACCATGATTCACCACAGTCCATTGGATATTCTGCTACCA
>103787231_2 DT668186.3
GNACGAGGTATAATCCTTTATAGGTATTTTATAGTCCTTTTATTATCCATACCCACAGCCTTATTACGACATA
ATCATTTTGGTATAATAGACTATAATAATTGAGCAATAATTTTTCCATAAAAACATAAAAAAATCCTTAGAAT
AGTAAAGTGATAATAACTATATATTTATTATTATGATTACCATTGTGTATTTTTATGT
TTGAATAGGTACTCTCGTCTCTTAAATTACTAAAAACTTTTCTTTATAAATTTTCATCAGTATGCAAATTTTT
TCTCCCTAAAATTATTGGGCCGTTTCCTTATTAATTGCTTACTGTTACTTATCAGTGCGTTGTATACCGTAGT
CGTTCGCAGTTGAACCTCATACTTCTGGTCATGCTCAATTAGCGTAGGATACAGGTAC
TTTAGCTCCGCATGGTCTAACGTTGGAAAGAGATGGTGGAGTGCGTGACCGCCAAACCGTGTGAGGGATTTGA
AGTGGTCTCCCCCATACTCGACCCTTTCAACTACGGTATCAAGTTGATGGATGCCCCAATCTATAAACTCCTC
CCTAGGTACGTCTCCTTCAAAGAAGTTCGTG
>103787247_2 DT667946.3
GCACGAGGCAAGTTTCTACAAGGTTGTCTCGATCACAAGTTATTTCACTATTAAATGATAAACAGTTATACTG
CTTTAAACTCATACTATGGTTGTGTGTAATATTTTTTTTCTATTTAATTATTCCTACTAAAGAACTACAATAA
GCGGTAAATAACGTTTATAAATAAAATATGGAAAGGCGTTATTGTTTTATATTTCTTA
TTTTAGTGAGTTATCATGTACAATTAGGTATCTGTAAAGACCAGCGATACAATCCAAGTAGCGATTTTTTGCT
ACATGAAGGTGGTTTTACTCCAAATAATAAAAATACATATGCTGCAAATTTTCCAAGTTTGCCAAACGCAAAA
CCTAATGTAGTATCGACAGAAGGTACTAATATAAATTCTATAAATACAGCAAAGCAAC
AATCGTCTGGTAAACGCGACTATGTTGCGCCTCAGCCATCAACATCAAAGCCAACAACAGCAACCCAATCTAC
ACCTAAAGCAAATCCTACCACCAAAGCAAGTAGCACAACACAAACACCAAAAAGAGATTATGTAGCTCCTCAA
TTTCCAACTTTGAAGCCCATTGGAATATCACGTCAGCAACCTGGTGCAATATCGACGG
CCATGCCCTCTGGTATACCGAAAAGAGATTATGTAAATCCCAAATATGAAGGCACAGGAGAAGTTAAAGATCT
TATTAATTTCTACGATAGTAAGTCGACTGTGAGTGGTTTTACAACTCCGCGTCCGCCAAGTTATAGTTCAATA
TTGT
>103787257_2 DT667010.3
GCACGAGGGGGACAGTTAAGTATTCTGAGTGCTAACACTAGATTTCCTTTGCCTACCCAGCTATGCGGGATGC
AACGTTAACCTAAAAATATGCAGATGACATTTTTAATATCTGTTTTATTTGTCAAACGGTTGTGTCAATAGAG
AAAACATTTTTTTAATACTTAAAATATGAAAACTACGTAATTGAGAAGAAAATATTAT

TATTGTTCATTTATTTATACACAAAAAAATAATACTAGAAGATCAACTTTAATCTTTAATTAATTATTAATGG
GATACTACTAAAGATCTCTAAAAAAGTTGACAAACTGTATATAACTGTATTTTTTTGAAAAGTTGCCAAGACC
TTTCATTTTGTCTTCCAAAATTTATTTTTTACATTTACCTCTATAGGACTTATAGCAC
AAATATATTTCAGAAAATGTCTTGCCAAGTAATAAAATAATGGCGGCCAAATATTGTACTTGGTTGAAGCGTA
TTTTATTTATATACTTAATATATTTTTAGTTTATAATATTTTTTTATAGTTTTGGCCCTTCGAGCCAGACTCT
TGAAAACGTCAACCATTTATTTTATGGCCTTATTTACCTAGATTTTTTTAGGACGTGG
TAACCCTTTGAGATAGCGCTTGACTGTAGACGCGATGGTTGTGAGTTCGACTCCCACTCGGTAAAATGATTTT
ATTCAAATTATTTTCATTTCTCTTCTCTGATGCACAAAGCGTGGCCTTCACGTAATTTGGGAAAAA
>103787287_2 DT667878.3
GCACGAGGGTTATTTCATCCAATAACTTACAATAAAGTATTTTTTTATATTTATTCGATAATAATAATAAAAT
AACATATTACGAATACGTTATTAGACAATGTGTAAATAGACAAGTGTATAATCTTTATTTTGTGTGAAAAAGC
GTATAATTTTGAGAGTATTACACAAACGAATTGTGTGACAATGAGTGATATCGAATCT
GGAGTTAGTGTCAATCGATTTAAGAACCAATTTACAATAAATCGATTTTTAAAGACCATTTTTCATGGATTTT
CTTTTGTGTTATTTGTGTTTGTGTTATTATTTGGATGTAAAATATGGTTGATGATGGATGAATCGTTGATGGT
ACATCCATTATTGAAGGATTTGAAGATGCTAACACCGTTCTTCTTTACTAATTGGAAT
TTTGTGTTCCAAACAATCTTCCTCGGTCTCTCTCTAACACACGACGTATTAGAATGGTTAGACAAGGAAGAAA
ACAATGTAGCCCAGAAACTTAAATATTGGCGGGATGTGTGCTTCTGTGGAATGGTGGTGCCTTGCACTTTGTT
CGTCACGACTATGTTCTGGTGTGTATACGCAATAGACCGGGAGCTGGTGTTCCCTCAA
GTATATGATGAAGTGGTACCTTGGTGGTTCAACCACTGCGTCCATACAAATATAACCATCGTACTACTCATTG
AGACTTTGCTTCAGTCGAGAAGAAACCTACTGACGTGAAGATGGAGTTGATCTTGTATTGGATTGTGGCTGTC
GCTTACGCTATAGTGTACTACTCAATTTACTTCGCTACAATCGCTGGG
>103787296_2 DV501296.2
ACAACCTCTCTGCCTTGCTCCCTCAACCTATCTACGTTCATGTTTTTCTCATGGATTAAGACGGACTTTAGAC
TAATGATTGTGAATGGAACTAAGAAACAATGCTGCAATTATATGCATTTCCTTGAACGAGGGCGTGTCCTTAT
ATTTAAAACCCTTCGCCGAGCGATTACAATATATTTGCTCTTTGATCTATAGAACAAA
CATGTAAATACAAGAAAAAACTTTTTTTTTCATTTCTCTATAAAAAGTTTAATTGAAGAGTAAATTTGCAACA
TATATGGCAGTTTATTACCAGATGTTATACCTATATAATAAAAATATTAAATACATATTTTTTGTCTTCTATG
TGACCTTAAAGCCGCACACTAGTTTGGCATACTA
>103787305_2 DT668034.3
AAAGCCACTCATTTGTGTGCGTTATCACTTCTGTACGTAAATAAATTTTGTTCTCTGACTCATAAACACAGGA
TATATTTTTGTAAATGGCCAAAATGTCGAGAAACATTGTATTTGAAGGCTGGTTAACCAAATCGCCTCCATCG
AAACGGATTTGGAGGACGAAATGGCGGAGAAGGTGGTTTGCCTTGCGACAATCTGGTG
AACTCCCTGGGCAGTATTTCCTAGATTATTATGCAGACCGTAACTGTAGACGGCTCAAGGGAACAATCAACTT
AGATTTTTGCGAACAGGTGTATTCTGGATTACACATGGAAAGAGGTAGCAATGGTTCGATGGATCCAAAGATG
AGAGGTTCAGTGTTCACAATACAAACACAAACTAGAATTTATCATCTTGAAGCAGATT
GTGAATCCGAAATGGAGAAATGGGTAGATGCAATATGCAGAGTTTGTGGTTTAAGAGCAATTGATGATGC
>103787319_2 DT667724.3
GCACGAGGGTTTCCATAGGGTGAGCCCAAATAATAATATACATTTAATTTCGTAATAAATTTAAGTTACCCCT
TTTTTTCTTATTTTTACTAGGCAATTATACAATTGTAGTCAAACCATCTTATCTTATTATTGAATAATAATTT
TAAGAAAATCTTATGAAATGGTGCATAGATTTTATTTGCCATTTTTTTTAGTAACATG
CGACATCACATTCCATTTATGTACAAAATCTGTTTGATTAGCTTCAGTTTTGTGCTGTCCGTACATTTTGGGC
TAACTTAATATGACCAATGCGTACTCAGGTTGGCCCAATTTATTAGTAATTGTCGCATACTGTAGATTTTTAA
CTGGTTTATTATAATTCTATATTGTTGTAAACCACAGTAAAACGGTAGTTAAACCACT
GTTTGTCTTTTCGGTATGCGGCGTTTTTTTTAAACAGCTCATTCCTACCTTTTGTCCATAGTCAGCATTTATG
ATTTGGAATTGAAAATAATTTAATAAATATTTGTTTTCATAATAAATTAATAAAATAAATGTAGTTGTCATAT
AATCATATTATATTATTATTATTATTGTAAAAAAATATATACTCTTATGAACGTCGTA
AACTTATATTGAATGCTTAGAATTTGTTTTAATTATGTATAGAATAAAAATGCCATTAAAAATATATTAATAT
ATATTTAATTTGCTATAAAACTAAATTTATTATGTGTACCCATCTTAAAATGAAATTATAATTTTTTAAACCA
>103787328_2 DT668066.3
TAAAACAGGAGCACTCGGGACGTTCTAAATATAGTTTTTATAAAATTTTCGAGAGGTTAAACCTAAACAAATG
TTTAACTTACAAGCGTTTGTGTTTCTACTTGTGCTTTATGCGGTGAATGCGGCGATGGTTGTTAGTGTTTATG
ATGATGGATTCAGTGAAAAGCGGAATATAAAAGATTGCAACTATGGAGAATGCGATCA

AATATGTCGTCGTATAGGTTTTCCCGGCGGAGCATGCGTTGGTGACCGCTGCAAGTGTGACGTAATGCAAGTG
ACGGCAAAATCTGATGTGTTTTAATGCTACTTCCCAGACTGCAACAACGATTGTATGCATCGCGGTTTCGATG
GCGGTTATTGCTGGTTAGACCGCTGTAAATGCGTTACCTATAATAAAAGTAATGAAAA
TGTATTGTTTCAAACGAGTATGAAAGAAAAAAACGCTTGTTATAACGGACAGTGTGATGAAATTTGTCGTCGT
CTAGGATTTATAAGCGGTG
>103787341_2 DT668459.3
GCACGAGGGTCGTCCTTGATTGGCGGCCCAGTTCGATGCCGCCTAAAGCATATCCCACCTATCAAGCATTCCA
AGGAAGCAAACAGTACGAGCTATGCATGTATACATTTAAAAAAACAAAGTAAACATGAAGAAATTAAAAAAAA
AAAGCAAAAACAGTGAACACGAAGCCGCAACGAATTTTGTCATCCAGACTAATGAAAG
TGTCCTAATAATTGTGATGTCTATAACAATAATGTTTTAGATGGCGTATATTTTTGTGTGTTTTATTAAATTT
AAGTCTCAGGCGTTCGATAAATGTTCAGTGCACATATTTGTGTATGTGTGTGTGTCTATATGTGTGTGTGTTT
GGGGAATTATTTAATTTTTTTTATAATACATTGTTTATGTGCTTTACGCTTATGGGGT
TGCCTGAAATTTATGATAATGTTTTCCTAAATTTTAAACAACCTCTGGTTTGTTTTTTTTAATTTGAGAATCT
GTCTCGCTAGTTTCGTATGTGTGTCAACAGGCTTTATATTTTTATCTGTTTTACACACACGGTTATAAATGGA
ATAATTTTTTTTTTAAATTGAAATTCGCGAGACAGGCTCTTAAATGCGGTAATTTATT
TGTTTTGTATACAATAATATTGATTTTGAAATCGTCAAGACTCGCATTACATATCATATTAAGATGGCGTCTG
GTGAGAATATTTAATTTTACGCACTTGTAACATTTTTCGAACGCTGCCGCTCTAATATAATAATAATAATTAA
AATGTTAATTTTTTTTTAGAATTTTATCCACTGATATTTGATC
>103787365_2 DT667367.3
TCCTCCCCCAACTCCTCAACCAACGCCTGCACCAACCCCATCTCCAACACCAGCTCCAGCCCCCGCTCCTGTC
CCTGTACCTCCACCAGAACAAGGATCATGGTCGTACACCAATTCAAGCAATGTTACTTGTGTTCTCATCCAGT
TTGCTGCACAGCTGAAGGTCACTTACACTAAAATGGTGAATTCATCAACATCTCTAGC
AGATGTTCTTATCAATGTACCAAAGAACGCCTCGGTCATCGGCGGTTCCTGCGGTGAGCTCGAACAGTGGATC
AAACTCGCCTGGCCGGTCAGCAACGCGACCAACGCCTCCTACAACAACATGCAGTTCGTGTTCCGAGCCAACG
ACACCACTAAAACTTACGCTGTAAAGAACATTACCGTCTTGATCGAGCCTGAATCGTT
GCCCAATACGACTTCGAAAACACCAGTCTTATTCACTTACGGCGAAGCGTGGAGGACATCACTGTTCCAGTCG
TACAGGTGTGGTGCGCCCACACTCCTCAAGCTTGGATCAGAGACGAGCGTGGCGGCCAATGTCACACTCTCGC
AATTGCAAGAGGAGGCGTTTAGGACCACATCTACTAATAAGTTCAGTGCTGCCCATGA
ATGCGGCGGCGACGACGTCCCCGACGCGGTGCCCATAGCGGTGGGGTGCGCGCTCGGCGGGCTCGTGGTGGTG
GTGCTGGTGGCCTACCTGCTGGGGCGCCGCCGCTCCGCCGCGCGCGGGTA
>103787383_2 DT667080.3
CGGCACGAGGATTTCCAGGCCATAGAACGTTCTATACTTAACACATCACATATCTAATCTACTCATTAGCTAC
TTTTATACTGAGCTAGGACCAAGTTACAAATGACTAGCACATAATAATTACGATAGTATCTCGAAATATTTAA
TATACATTTTTTTTAACATCTCTAGTGATTTTAAACGATGTTTAAATAGAATGTTTCA
ATGACTTCACTTTTATCTGACTTAAATATGAAATTGCGATTTTAACTTATACACAAATTCGTATCGATTAATA
ATCGATTTTTCTTGTCAAAATAAATCGTTAACTAACCAATCGATTCTCCTAGTATGATGATAATATTCAGTAA
CAATTATTATTTTATTTATTGCTATTGCTGTTTACATTATAGATGTTTATTTTTGTAA
CTAAGCTTTAAATAATCGCTTCGTTCGCATCGATTCGGTGCCAGTATCGATAACTGCCAATGAGTAGAACTAA
CGAGTCATATTATAGAAGTAAATAACTAAATAAT
>103787401_2 DT667982.3
GCACGAGGGAAGCACCTCGCGTTTCCCGCGCTCAGTTGCACAGATTAAAAAAAACAGTTCTAAATTCCGCGAA
AAATATAATATTGTGTCACACACAATGCGGTTAGCGTTTTTGGGCGTCCTCACGCTCTTCTCTCTGGTGTATG
GCCAGGAAACCTGCCAGTCCGCTGATGAAGAACCATACCTCCTTTTTGGAACAAAAAC
TGCATACACCTTCGCCAGTGTAGGAATACCATTGAATAAAGTTTATGATGTTCCTGGATGTCAACCCTTAGCC
ATATGGTTGATGAACCGCCACGGCTCACACAACCCAGAAGCGGACGAAATAGCTAACCTACAAAAATTGACGG
ACTTCAGAAATAACATCATAACAAACTATAGAAATGCCAATTTTCGGGAAACAAACAT
GCGTATGTGCACATCTGACGTGAACCTTCTAGAACGCTGGCAATGGAATCCCCGACAGAACATGACCTTCGCT
GGCGACTTAACGAGCGATGGCTATATCTCCACTCAGCAGTTGGCACAAGCTTGGAAGCAGAAGTTCCCTGGAC
TGCTGACTGATAATCCTCATGATTATATGTTTAAATATGTAAATGATCAACGTTCAAG
CACCACGTTCCGAGCGTTCACCGAAGGCCTGTTCCGGGCTCAGGAAGATCCTGCAATTACAACGAAGGANAGC
GATGAGAAATTACTAAGACCTTATAAATTCTGCCCTTCTTGGACACGTGACGTGGATGAGAACAACGATACAC
TGACGCAGCTAAGGATCTTCGACTCCAAACAGAGTATAGAGAAATGATCAGCAACATT
TCAAAACGTCTCGGCTTCAACTA
>103787407_2 DT668677.3

GCACGAGGCTGTTCCTATTCCATTTATAAAATTTATTAGGACTTTTTATTTTTTTATTTACTAAGTGAAACTT
AACCGTTAATTATAAACAAACATAATTTTACACAATGGCACAACCAAAAGCTGACATTGCTCTTATTGGATTG
GCTGTAATGGGCCAAAACTTAATTCTGAATATGGATTCAAATGGATTCATTGTTTGTG
CCTATAATAGAACAGTTGCAAAGGTTGACCAGTTTTTGGCAAATGAAGCAAAAGGTACCAATATAATTGGAGC
AAAATCTTTGGAAGACATGGTTGCTAAATTAAAAGCACCATTAAAAGTTATGTTACTAGTTAAAGCCGGAGCT
GCTGTTGATGAGTACGTAAACAAATTAATACCTTTACTCGAAAAGGGAGACATTATTA
TTGATGGTGGTAATTCTCAATATATTGATACACAACGCTGGTGTAAGCAATTGGCACCTACTGGCATATATTA
CATTGGCATGGGGGTTAGTGGTGGTGAGGATGGTGCTCGCTATGGACCATCTCTTATGCCTGGAGGTCATAAA
GCGGCTTGGCCTTACGTTAAACCAATTTTCCAGGCTATAAGTGCTAAAGTGAATGGCG
AGCCTTGTTGTGATTGGGTGGGCGAGGACGGCGCCGGCCATTTTGTCAAGATGGTACACAATGGTATCGAATA
TGGAGACATGCAGCTCATTACTGAAGCATATCATCTCAT
>103787422_2 DT667939.3
GCACGAGGGAACGACCCTCGAGCGTCGCGATCATTAAAGAAAATCTAGCTTGGCAAAATGGCATCCGAAGTGA
ACGAGTGGTACAGAGGAAGGAAGGTGTTGGTGACCGGCGCGTCAGGTCTGATGGGAAAGGTTCTGATCGAGAA
ACTTCTATACAGCGTGTCGGACATAGGCTGCGTGTACGCGCTGGTGCGCAGCAAACGC
GGTAAATCACCTGAATCGCGAATCGAGGATATGTGGAAACTACCTTTATTCTCAAGAATTAGAGAAGAAAAAC
CACATGTCATGAAGAAACTAATACCAGTAGTCGGTGATATTTTGTATGACAATCTTGGAATTGAAAAGAATCA
ACTGGAAGAAATTTATAATGAGGTGTCTATAGTGTTCCACTTTGCAGCCACTCTCCGC
CTGGAAGCACCTCTTAAAGAAGGCCTAGAAATGAACACCAAAGGCACACTTAGAGTCCTCGCAATTGCAAAAA
GAATGAAGAACCTAGTTTCATTCGTTCATCTCTCTACTGCTTTTTGTTATCCAGATCACGGTAGAATGGCAGA
AAAGGTCCACGATCCTCCAGCAGACCCACACGAGGTGTTGCGCGCCGCAAGTTGGCTC
TCCGAGGAACAGCTGAATCTCCTAGCTCCTTCCATGATGCAGCAACACCCCAACTCGTACACGTACTCTAAGC
GGCTAGCTGAGGCCCTAGTTAAGGAGAGTTACGCGGAAATACGACAGTCGTTGTACGACCGAGTA
>103787455_2 DT667791.3
GCACGAGGTTTTGATTATATATTTTTGTTATTCTTAAAGCAGAGCACGTAGACTCTGGAGTGTGTACTATGGC
CACTAAAATATTATCGCTTAGAAGTGTGATTAATAAGCCGTTAAATTCAATAATAAGCAATGTATCCCTATAT
CAACATGAATGTAACCTAAAAAGTTTTAGTACTACGACTGTTGCTTGCATTACATTTT
TCAATAAATTGCCAGCAGAAAAATTATGGAAATCGGTCACTTCTGTAAGTAATGCTGGAGCTAAAAAAGGACG
TGGAAAAGGTGCTGGCCGAATACGTATAAGAGATTTAAATCGTGGGCAAATGATTGGTATTGGAAAAATTAAT
ATGTTATGGCCAGGTTTGTCAGCGCCTGTTATACGAGGCAGAGAGTTGCTTAAACAAC
AAAGATTACCAGATGACCCAAAAAGGATGGAAAAATTAATTAAATTGAGAGATTCAATGACCAAATTTCGACG
TCTGAGATTGAGTCCAATTGAGAGAGGTTGGTCTGGTTCTCGTATGCCTGGCCGTAGTATTGGACCACCAGAT
GCTGTTGGAGATGAGGAATTCATTGGTTTTGATACAAAAGTTCTACAACTTAGATCTT
TACTTATTATGAAAGGAAGTCTGGGTCGCACAAGGAATTTCCAAGCTATGGTGGTGACAGGTAATGGACAAGG
TCTTGCAGGTTTTGGTTT
>103787501_2 DV501273.2
AATTATATGAAAATGTTCACCTACATTAACAAGCATAAACATGTTTTCAATGCTGACGTCACCTTCGGCACAC
CTTTGGACTATTTCAACGCGATCAAACAACGTCACACTAATATCCCATCTCTAAAAGGTGACTTCTTTGTCTA
CTCGGACTTCTTTAGCGAGGGTAAACCCGCCTACTGGTCTGGTTACTACGCCACCCGG
CCCTACTTTAAAAATTT
>103787507_2 DT668550.3
CGGCACGAGGCCCGTCTTGACAGTGAAATCTCCTTCAAACCAGCAGATTCCAATGGAATTATTCTATACAGCA
GTCAAAATGACGATGGAACTGGAGACTACCTCATCCTTCAACTGATCGATGGTGTGCCAGAATTCACCCTCAA
ATTTGACACATCTGCACCGTTGATTGTAACGGGAGATCGCCCACTACAACTAAACGCT
TGGCATACAATACGATTGAGTAGGAGCGGCTCCAAGATAACAATGGACGTGGACAACACCGGACCATTTGTAG
CAGAGTCGTCTAATGCATTCGCTGTATTAGAACTATTTTTTAATCTTTACGTCGGCGGAGCACCGACACAACG
TCCTCCAGAAATCCACTTCGCTTCAGGATTCGTCGGCTGCGTCAGTATGCTGATCTTA
GGTAAAGAAGAAAAGAAAATTATGTCAGAAAGTATTGAGAAGTACAACGTACACGACTGTGACTCTTGCTCAC
CGAACCTTTGTCAGAATGATGGAATGTGCCAGGAGGCGCGTAACGAGCGGGGCTATATGTGTATTTGTCCCAA
TGGATACGCAGGATTGAACTGCGACCGTCTCGGAG
>103787529_2 DT667875.3
CGAAAGTATACATAACATCGTTCTATAAAATGACCCCAAGAAAGTGACAGTTCCGGAGGGCAGCCTGTCTGAT
TTTGCAACGCGCAAAGTTCACTACACGGCCAATAATCCTCTTAGAAAGGAACTGTTCACGGCTGATACCAGAC
CCGCAAGTTTACGAAAGCTGTACGAAAAGGCAACAACCGATAGAATTTCCGCATCAAT

CCTCGATCAACTAAGAAGAGATTCCCAACATTCCCAGTACCTTCTCTGCACTCCTGTCCTAAGTTCAAAGAGA
AGACGAATATATAATAAGATAGATGTCGACATAGAAACTCATATTCCGTCGGTAGTAGAACATTTAAGGAAAT
GGACGTCAAAAGAAGCTGTGGGAGATGTGACGGCTTTACCGGATGCGTCAATACGCAT
CGCGAGCAGCGTTACTTTAACGCCAGACGAGCTTTCAGAGTGCGGGTCGGGAGACGACGAACCTATAGACCAT
CGGCTGCCGAGCCCCGAGGAACAGCTGCGAGTTATTGTATCTAAATTTCCACCAGAAGTGATAGCGATAGACA
CATCAGGCAAATTCTTCGATAGAATGTGCAGTTCGCGGAAGTCCCTCCACGTAGAGAC
CATTGCCGGTGAAACAGATACGGTGAAGCGTCGCACGCGCACTCGGAAACCACGGGGTAAAAGACGAAACACA
ATTTCTGGCACTGATCAGAAAGAGCTTAGAGAGGCTATAGCTGGTGATACACCAAATGCTGTGGCTTCTGAAG
AGATTGAAGACAGTACAGTTATCCGGTCTCGATCGAGTGATTTGCTTAAGAAAAGTCA
ACAGATCCTAAGAAAGCCA
>103787535_2 DT667478.3
GCACGAGGAAAAGCCTTAAGCTGAATCAACAACTAAAGATGATGAACCTGGTCCGAGTACGGCTGAAAGTTCA
GAAGAAGTACCAGGACCGTCAAATGGTGATGCAAACAATGAGTCTACAGTCAACATGGACACTGAAGACGACG
TTGATAATTTACAACTTGCATGGGAAATGCTAGAACTATCCAGAGGCATATTGCAGAA
GCGTGCAGAGAGTGGTGCGATGGCGGCGCGCGCCCTCTTGGCGGACGTTCACCTCGCGCTGGGAGAGGTGGCT
CTGGAGAGCGAGACGTACGACAAGGCAGTCGTTGATATGGTGAGCTGTTTGGACATACAAAAGGAGTTGTACA
GGAGTGATGATAGAAGGATTGCTGAGACACACTACCAAATTGGTCTCGCCAATTCCCT
AGCATCTAACTTCGAGGACGCCATAACTCACTTCAAAAACGCGGCGAACATTCTAGAATCTCGTATCAAGACC
TTAGAAAATCCAGTTTCGGTGGCGGAGGATGCCACAGTTAAGAAATATTCGACGGCAGACCCGTTTTACTCTG
TGGAAGGCGAGCTGAAAGAGTTGAAGGACTTGTTGCCGGAGATACAAGAGAAAATACA
AGACATGATGGATTACAAGTCCGAGATGATAAAAAGGGTACGTGAAACCCTCTGTCCTTCNTACGGTGAGGGA
TCTCAAGCCTCATCAAGTACCAGTTCAAAGCCAGAAACAAAACCTGCCGCAGATATATCTCATT
>103787542_2 DT668569.3
GCACGAGGGCTACGTAGATGATAGCACGGTGAACGCTGTTTACTCCGGCCGAGCAAGTCTTTCTCGGGAAAAC
GCCGAACAGTGCCGAAATAAACTTGTGTCTTCCATTGAGGCATCCCTCAACAATGTCTCCTCATGGGGTGAAA
GGAACCTAGTTCAGTTTAACCCCCTGAAGACACAAGTTTGCGCGTTAACCACAAAAAA
ATCCCATTTATCGTCTCACCAGTCTTTCAGAACATTCCCCTTACAGCAGCGCCCAGTATCAGTATATTGGGCC
TCAATGTTTCGAGTGACTGCCAGTATCGCAATCTTTTGGAAAGCAAGGCCAAGTTGGCCTCGAAGAGACTGGG
GGTCATCAACAGAGCGAGACGGTACTTCACGCCAAAACACCGTTTGGCCGGCAGGCAG
GCAGGTCCGGCCTCATATGGAGTTCTGTTCTCATCTCTGGGCTGGTTTACCTCAGTATCAGCTTGAACCATTC
GATCGCATACAACGTAGAGCCATTCGAATCATTGGGGACCCAATAATCTGTGAAAGGCTTGATACTTTGGCTT
TGCGTAGAGACGTTTCCTCGCTGTGCGTTCTCTATTGCATTTATCATGGGGAGTGTTC
TGAAGAACTGTTTGACCTATTACCTGCCGCCGAATTTTCTAACCGCACAGTACGATATAAGCTGAAATATCAT
CCACACCACCTGGATACGTGGCAATCCATTACT
>103787568_2 DT667154.3
GCACGAGGATATATCTTTTATTATTTTTCCTGTGTAAATACAATATTAGTAATATAATTTACTTTGATATTTC
ACAGTGCCTTATCCTTGCTTTTTATGCCCACTTAATTACCAATCATGATATTAAATGTTTATAAGTTTTAATT
TTTGTTGTGCGCAAACAGAATATGTTGTTGGGATATCGAACTCAGGTCCCACCCGTCC
TAGTACGATAGTGCCAAGAACAACAGTATTCTTTATTAAACGTTGTTATTATAAAAAATATATAAATTAGGTA
ATTATATAGGTATACTATTTAGGCGAAGTAGTATGGATTTGCATAATGTATTTTCTACAATTTCCAATGAAGG
GGAACTAATGGACATGTTTTTAAAAATATATTAATACAATTTTGTTAATTTAGTGTCC
TAGCAATACAATATAAAACTCATTTG
>103787585_2 DT668721.3
GCACGAGGGCGACCTATGCATCAGGGCGCCGTCGTGCCAGCGCTCCCTCGTGCTTCGCCCCTTCATAACTAAT
GACTTCATGACGGGGATAGCGGCGTTGCCCGGCGAGGACGCTATGCCTCAGGATGTGGTAGACAGAATGCGTA
AAGAACTAATGAATGTGCCAGGCATTTCTCGTGTGCTCTACGACCTCACGCCCAAACC
ACCCGCGACCACCGAGTGGGAATGATTGCGGGACCTCCGTCTTAATAGCAACTGACTACACGGACTCAACTGT
ATAGGATGGAGTTGAAATGCATTGTTTTATGTGTCAAATCCAAGTTTTTGAATTTCGAATTCGAAAGTTTTTA
AATTTCGGATTCGTTCTTTCGATTTTCAAATTGATTCTTTTGATTGAATGAATTCGAA
AATTTTAAATATGAAGTATGAATGAAGTATTACAAATTCAAAAAGAAATGAGTTTGAAGTTCAAAATTATGAA
TTTGATGCGCTAAAAATGAATTTGAACCTATACATACCACATACTTGTATGGACTGGATAAGTTTGACTGTAA
AACTAGTATCTACCATATACAATATATATGGAAAAGCAATGGTTAATAAAAAAAAGGA

TTTTTTGTATAAAAATGTGTAGATAATGAAACTGTTTTATGTTTTTTATTTAATTAAACAATGTAATGTGTCT
TAACTATTTATTTTAAATGATTATTCTTTTTTCTTTCCAATTGTTAGTTTTACAGTCAAATGTAAAAAAAATT
TTAGTACGCATATTTTTCAATGGATGCACCG
>103787587_2 DT667213.3
GCAGAGGAAACAACGCTGCTAATTGTGTAAACTGAGACAAATAATAATGGGATTCTTAACTATTTTGAAGAAA
TTACGACAAAAAGAAAAAGAAATGCGTATTCTTATGTTAGGCTTGGACAACGCTGGTAAAACAACAATTCTGA
AGCGCTTCAATGGTGAACCAATAGATACAATCTCACCAACGCTTGGTTTTAATATAAA
AACATTAGAACATCGCGGCTATAAGCTGAATATATGGGACGTGGGAGGACAAAAATCATTAAGGTCATACTGG
AAGAACTACTTTGAAAGCACAGATGGTGTAGCGTGGGTGGTGGACAGTGCTGATGCAAGACGCCTGGCTGATT
GTGCCAAAGAGTTACACTCCTTGTTAAGAGAGGAGAGGTTAGCGGGAGCCACTCTATT
GGTTCTAGCTAACAAATCAGATTTACCTGGTGCCTTAACATCACAGGAAATTCGAGAGGCTTTAGAATTGGAC
AGTATCAAAACTCATCACTGGCGCATAGTGCGTTGCTCGGCTGTGACCGGAGAGAACTTGCTAGACGGCATGG
ACTGGATGTTAGACGACATTGCTTCCAGAATATTCACATTAGATTGAAATTTTATAGT
GCATGACTATAAAATACACCTCTTATGCTTTTTTATTTATTTTTTAGCTTCACACTATACCCCACACGTAGCA
GGGTAGGCAGTGGGAACATGGTATACTGTTGGTATTTC
>103787599_2 DT667772.2
GCACGAGGCAGCGACACAGTTCACATGTAAACAAAACAGTGTAAACATGTCTTCTAAACTTTGTATTCTTGTG
TTCACTGCTTTAATAGCCGTTGGATATGGTGTTGACCAAACCAATTACGATCTAAGCAGGAACAGGATAAACG
GCGCCCGTAACGGTTTCACTATAAGAGATATATGGGACTATTTATTAGGAGGCAACTA
CGGTTATCCGGGGTACCGACCTGGCCACTATCCCGGAGGTTATCCTGGAGGATCCGGCCTGTACCCAGGGGGA
CAGTATCCCGGGCAGTATGGAGGACATCGCCCCGGGTTGGAAGGACCGCCTGGTGCTCATCCGGGATGCCCGC
TTTGTGATTCTTCAGTCTATAGCTACTGTTCCCATAAGCAAGCTCATGATTCTTGTTG
CTGTGATAATAATAGTTACTACCCATACGGCGGCTGTAGTAAATCAAGTTGCAACTTCCTCTACGCGAATTCC
TGTCAAGAGTACCATCTGATCACTAACTGTTGTTGCGTGGATCTGGGGAAGAACGCCGCTCCCATAGTACCCG
CTGTACCCGCCATAGTGCCAGTTGTGGCCTAAGAACTTATTAATATAAAGCTTTAATG
CCATAATCGTAAATCATTCATAATTTCATTAATTTCTTATAATATCTTAATTTATTACACAAAATTGGAGAAC
CTATCGTTATCATTACAAAATCATTATTTTGAAAAAAAAAAAATACGATGTANGTAAATATATTATAATTTTAA
CTCGTGTATGAAATGATTGAATATTTTAATTTCTAAATTTAAATATAGACTTTTATTC
TACCCTCTATCATTAAG
>103787613_2 DT668485.3
GCACGAGGATTTTACCCTTGAAGGTCGCAAAATATTTCAAATAGCGAATATGAGACTCATTTTATCTCCGTCT
TCAAATGAAAACCAATATCCTATTTATTTGCATGTAACGTAACTGTATTTAATGTCTACAATAACAAAAGTAA
CTTTTTTATCCTGTCTTTGTGCTAAAAACATATTACCTGAGATATCGCAATGTAAGTG
TACAAAAAAATAAGGATTTACTTAAATAGAAATATATATAATACTCTAGTACTAACCTCTGAGCTTGTGCTAC
TGGTCGCCAGATTCTTACGGAAGAGATAAAATAGGAGGTACATACCGATATTCAACACATGGTTACATTAGAG
CAAGGATTCTCAATCATTGGACTGCTGAATTAAGCGGGCAGTGAGCCTTTGGTCGCTT
CGTTGTCAATCTGTTGTTGCCTCAGTCAAACATAAGTTGTGCCGAGATTCAAATCTACCACTAAATATTACAA
TACGTTCATCTATAGTTTTTTATATTTTTTTTAAGAGATCATATTAATTGTATTAATTGAAGAAGATTTTTCT
AAAATATTATTATTGCTTTTTATGTAAGTTCCTATTGAGCTTATTTTTATATAGTTTA
TTTTTATTATACCTATAAATATTATATTAGCTATTACAGTTTATTTTTATTATACCAAGTAGTTCCTGCTAAC
TAGAATGATGTAAAA
>103787627_2 DT667120.3
GCACGAGGGCAGCCTTTAGCGAACCCAAAGCAGTCTCGGGTTGATCGCCTTGTACTCAACCGCTGCACTAACC
AATCGAGAGGACAATAACACACCTACACCTTGACAGCCCCGCTCGCTCTCTGGTACGCCGGCCCAGAAAGCGA
CCCGACTGCCCGGCAAATCGGTCGTGTCATTGCCCTTTCGCTTGGTCTCTGTCACACA
AACGACATCCAAATCTCTATCATCAATGACATCTAATAATTCATTCGTCTTTTCTTCCATCCCTCCTCTTACA
TTCCACGTAGCTATTTGTAGCTCCGTCCTCCACCTCCCGCCGTTCCGGACATGGCGTGTTGGGTGGCGGTCCC
CAGCGCCGCCCATTTAATATACATATATATTTTTATGAAATCGAAATAGTTCTATTGT
ATTTTTTATTATACAATGTGTGAAATAATGGCGCGCCGGTGAAACAATATTTTGTGTATTCAATAGGGCTCGA
TGTGATATATATTTTTTTTATTTATTTTTTAGTTGCTATTGTGAAATTTTAAACATGATCAAACTTCTAACAGA
TTTTGTCATTATTACTAATGGCATTGTTTCTTTATTACTCGAAAGTCATAAGCATTAG
TTTGAATATTATCGTTATCATTTGCGGTAGTTA
>103787639_2 DV501338.2

GGCACGAGGCGACCACCATGAGCCGTGCTGGTCCACCGACCCCTCCGACGAGGAGGAGGAGGACGCTGAACAT
GCGCCGGCGGTGGGGTGCGCGCTGGTGGCGCTGTCGGACTACGCGGCGGTGGGCGCGAGCGAGGTGTCGCTGC
GCGAGGGGCAGCACGCCGACCTGCTCAAGGTGGGCTGTGCCGGCTGGTGGTACGTGCG
CCTCGCAGGTGGGCAGGGCGAGGGCTGGGCGCCGGCCGCATATTTGGACCAACGGAAGACTTCGCGTTCGTCA
AGTCGCTCGCACGACCGACTTCACGACCACTGAGCTCACACTCCTAAATTATACCAATTTATACATACGTATT
ATATCACAAACTTAAAATGAAAATAACCTAAGTTAAAATTTCTGTATTTAAAAATTTT
GCTGATAATGGCAAGTCTATATAAAGAATCTATCATGCTTTCTCGCAATTTTTTTTTTTTAATTTAGGTAATT
TCAATTTTAAGGTTGCGGTATAATTTCCCATACATTTGTTATAACTTTGTCAGGAATGTTATGGAGCTCTGTA
GCCACAACTGAAACTATGAGATATCTAAAGTAAATAACTATTCGACACCACAACCGAA
TCTTATATAAGTTCCGGATAAATTCTGATATAGTTTAATGGTACTAAATGAAACTGGATTTCACGGGTTATTT
TCTAGCTTTCAGTAGAAACTAGCGATACGTAAAAAGAACTTTTGTTTAGCTGTTTTTTATAGTCTTTTTTAGA
CTATAAAAAACAGCT
>103787642_2 DT667070.3
GCACGAGGGAGAGACAGAGAGAGAGAGAGAGAGAGAGAGAGAGACAGATTGACACCGATGTCTTAACAGGCCA
ATTGTACCGGTCGTCGTCCTTTAACTCATCCGGGTGCGGGTCGGGTGGGGAGCCGGCTGACGACATGTACTCT
GATGTATCACTTGAACAGGATGTGCAAGGTCTCAACTACAAGGTCCAACTATTACAGC
AACAAGTGACGTCTCTCGCCGACACTCAAAGTACGGCGGACGAGAGGTACGCCCGCGCGAAGGCAGACAACGC
AGTGCTCCAAGCTCGTGTACATATGCTCGACGAGCAAATACTATAGATCGAGTGCCGATGCGAGGAGCGCATC
GCAGAGGAGCAACGACGATGTAGGGAGGCGATAGCGCGGGTGGAGCGCGACAGGGACG
CCCAGCTCGCCGCGCTCAGCGAGCGCCTGGCCGCTGCGGAGACGGAGGCCTCTCATCTTAAACAGGAGGTGGG
TCGGCTACGTGGTGTAGCAGAAACACTGCGCACGAACGCCGAAACGTGCACGCGCAACGCTCGCGAGGCGCAG
GAGGCGGCGGGCGAACTAACCGCGCAGCTGAGCGCCGCGCGCGACGCCGAACGCTGCG
AACGGCG
>103787654_2 DT668462.3
TCTTCGGTTATTAGAAATCAAGATAGTTTTAAGTTGAGCTAGAATACAGTGTACCAAAATGTTGACCGGGACG
ACGTTAACACTCATACAAATATTAGAAAAAACTATATCTCCAGATCGAAATGAATTGGAAGCAGCAGAAAAAT
ATCTTGATCATGCAGCTGCCACAAATTTTACAACTTTTATCAAGATGCTCTCAGATGT
ACTGGTGCAAGGGGGCAACAGTCAAGTGGCACGAATGGCAGCTGGGCTTCAATTAAAAAATCACCTCACTTCC
AAAGATCCCACATTGAAACATTTATATCAACAGAGATGGCTTGCATTACCTGAAGATATAAGACTTTATATAA
AAAAAAATATTTTAACAACAATAGGAACAGAAAGTGGTAGACCCAGTTCAGCAGCCCA
GTGTGTGGCTTATGTAGCTGTGGCGGAACTTGCAGAAGGACAGAGGAATGATTTAATACCTATTCTTGTTGAA
AATGTTGTTAATGCTCAATCAACTGAATTGAAGAGGGAAGCAAGCTTACAGGCTATTGGTTATATTTGTCAAG
AGATAGATGCTGAAGTACTAACAGAACAAAGTAATCGTATATTGACTGCTATCATCCA
TGGTATGAGATCAACAGAGCCAAGTATCTATGTAAGGCTTGCAGCAACACAAGCTCTACTCAACTCACTTGAG
TTTACTAAAGCTAACTTTGATAAGGAAAATGAGAGAAACTTTATAATGGAGGTAGTATGTGAAGCGAC
>103787674_2 DT667110.3
GCACGAGGCAACATCTTATTAAAATTACTTCATTTATAACTCGTTTCATTTTTATATAAAATGGTGTCAAGAA
GTTTAACAGAAGTGTTTGTGTTGATGCGCAATAATGCTATACACAGCCGTCAACTGTTTTCAGAACAAGGAAA
TGATTCAGAGAGGGTTAGGTTAACTAGATCTAGCACTCATGATCTAGAAGTGGGGTTG
GATCGGTCAGAAGTAGCCCCACCACCATGGAGTGACTGCTTAGAAGAAGCACATTATATTATTACTAGGTTAA
GGACAAAAATATCAGAATTACAATCCCGCCATGAGCGTCATATACGACGACCAGCGTTGGACGACAGTGGAGA
ACAACACCACATAGATAGACTATGTTCAGAAATAGGAAAGCATTTTACTCAAGCCCAC
GCAAGATTAACAGCTATTAAGGCACAAGTTAGACATGGTAACAAAACAGAACAAAAATTAGCTTCCAATGTAG
TTTTAGCTCTTGTATCAATATTACAAGATCTCAGCGTAACTTTTCGTACATCCCAATCAAATTATCTAAAATC
CCTTACGTCTAGAGAAGAAAGATCAAATGCCTATTTCGATTTACCAAATTTTGAGGAG
CTGAGTCTCCAAGACGAACTCATACCTGGTTTAACTAATAACCAAACAGACTTATTATTCTCCTTGCCAAGCA
CAAGTCAGAATTTCGACGATAATTTGGACAATATGTTCGAAAAGCCAGCGTCAAATCAAAAACAGTTACTTCT
AAATGCAGAAGAGAATACCAAAATGGTTGAAGAGAGGGAACAGGAAGTCAATAAATAG
TGAAATCTATAGTCGATCTAATGA
>103787689_2 DT668623.3
GCACGAGGATGCGCTTCACGGTGATCTAAATTATGTGATACTATAATGAATTTATTTGTATATAGCCCTATGT
AATAATTTTTATTGTTTCTGATTCCTTCATAAAGAAAATAATGTAAATGCATTTAAACGACCGTATATTACAT
ATATTCTAAAGCAGCGAGATTTGAATTTCTTTAATATATTTATATATTAATCACACAA

TGCATAATATAATATAATATAAGGTAGACCACTTAATAAATAGTTTACTGCTCAATTTACTTAATAGAATATA
ATAAAACTGCAGATGGTAGATGCCACTTTAGTTTGAAAACAGATGATTTTTACAAATAGACGCTTGTAATTCG
CATCCCAGCGAATTCTACATCACATACAGAGCAAATTTAGATCGGCGCCTCTATTTTT
CTTAGCATTTATGTGCGTCTCGAGGCGACGCAGCGGTGGCGGGCTGTCGGTGTATAGGTGGAGCTCGATACGT
TTTTTAACCGGCATCGGCGGGGCATTGACACCGTTGCCGGGGCACTGCCCGTTCTTGACAGTTCAAATGTTTA
CACTTTACATCTCGAAGGATCACTCACTTCAACCGACTCCGATTCGAATAAGTCTTCC
GAGGATGCGCATTGGCAGTAGATACGTAAAATACTGTTACTGTGACGTCATTTCTGGGTACCAAACGTCACTA
TTTTAGTATTAGAGAACATAAGACATCGGACTTCGGCATTATGTGTTGTTTAGTATTTCGATATGGTTTAGGC
ATAAGCTTAAATATTTGTGCATTATTTTAATAAAGACTAACAATAATAATTTAATTTG
CAAATTTTAGTATTTTATAATGAATGATCAATACACAGTACAT
>103787721_2 DT668948.3
GCACGAGGGCGAAACCTGTTTTGGATTGTCAATTCTGTAAAATGTTTATTATTTTGTAAGCTTGTCGAAGTTT
ATGCGTAAACGACAATGTCTTCGACAGAACCATCTCAAAACAAAACATGGGAGCTATCGCTATATGAACTTCA
TCGGACTCCTCAAGAAGTTATTACAGACGCCACAGAAATAGCTGTATCCCCACGAAGC
TTACACAGTGAATTGATGTGCCCAATATGCTTGGATATGCTAAAAAAGACTATGACCACAAAAGAATGCTTAC
ACAGGTTTTGTTCTGACTGTATTATAACAGCTCTACGTTCTGGAAACAAAGAATGCCCGACTTGTCGTAAAAA
ACTTGTATCTAAAAGATCTCTTAGGCCAGATCCTAACTTTGATTTACTAATATCAAAA
ATATATCCAAGCAGAGATGAATATGAAGCACACCAGGAACGTGTATTGGCTAAAATTAACATGTCCCAATCTC
AAGCTTCCTTGGTTATATCAATAACAGAAGGAATTAAGCTGCAGTCACAAAACAGACCACAGAGAGCTAAGAA
GAACCACGAAGAGAATAGTAACACATCTAATTCAAATGGCGCTACAGAATCTCCTCAA
AATGGAAGTAGTTCATCTGTTCCTAAAGATAGTACATCTTCTGCAAGCAATCCAACTCCACCTGGTCAATCCA
CTTCTAATCCAGCGTCAGTGCGGAGTACACCTTCTCCAGTTCCGTCAAATGTCAGCTCTGTATCAAAAAACAC
AAGCAC
>103787724_2 DT667379.3
GCACGAGGCACAACGTCTAAATTGCTGTGTGGGATACACAATTGCAGTGTTAAGATTAGATATAAATAAAAAA
AATTGCTATATTTAATTTAATAATAATAAAATAATAAATAATTCCTTACTTATATTTTTTTGTAAAATGTTAA
AGCTTGGTATCAATCGATAATATACACTGTACGTATTTTTCTTCCGTATCCGGCATAT
AGTGGGGTAGACAGAGAGAAGCTAGTTTTAAAACACTCCGTTACACATTTTCTCTCCAACTTTCGAGGCATTG
CTTGAGTGGCAGAATTCGAACGCCGTACTTGGACTTGTTATTAGAGCGAGGAAATAAAAATATAAATAAATAA
TAATTAATTTTCCTGAGTGGGAATCGAACGTCTACCCGCTTTTGCCAACGCATCACGA
TGGCCTGTACGTCACGAGTTTTAAAGTTTGCATAAATAAATTCTTGATTGATTATTATTTGCTATTACTTCTT
AATTAGAATATTTGGTTATAATATATTTAGATCAATTAGGCCTGGTTTTTACCATGATAAAAAAAATACGGGT
AATGCTTAGGCGGAACGGACTCCTTTTAAATAAAACAGGTTTACATTTCCTCCAACAT
AGAGTTTTATATTTTGTAATCGGGAACCAGAAACGGGAAACAGGGCTTAAAGACGGATTAGCGCATTATTACG
TCTCTCTTAAGAGAGAAGACGCAATACAACCGTCGAAACACGAAAATCATATAGATTTATATTATATTTTGAT
CTATACTATATCCAAAATACCAAATTACAAT
>103787745_2 DT668723.3
TCACCTCATTCGGTCATTTCCTTTTCAACATACAACGTAGAACTCACCAAATTTAAGAAAATACAAATAAGAT
TAAAAATTTGCCTTAAATAATTATACGGCTGAATTTAAGTTAGATGTATGTAAAACCTTATAAAATGTGTTAG
TGCTTACAGTTCGGTTATTACGGTTCGGTTATTGGTTTTTGAAACTTTTTCTAAGATT
TCGGTTTCGGTCTTATTGACGTCTAGAGAATTTTTACGTTCGAAACAACCATTTTTAAGTTGTGTGATTTTTG
TTTGATTTTGTTACAATGTCAAATAATAATTAGAATAATGTCTAATGTAAAGACAACTGATAAAAACATCTAA
AAAAATAATGAGTTTTTTACGATTTTTCCATTTTCGGTTGTTTCCTGCAACTAATAAC
CGAACTGATGACACTAGATAAATAAATTTCGGACCACTGCAGTATTTTCTTAATGGAATCACATATAGGACCA
CTCTTGTAAAATTTATAGAAAAGCTATCTCTGTAAAATACGCGTTTACATAAATAATGAGAATTAATGTTAAT
ATTGCATTTTTGTATGCGGTGTTAAGAACCGTTACATTTTGGCTTCCATAGTATCGGG
TAGAGATAGGTTCTCGAAAATTTTGTACTTTAAATATATACATATAAAATTAGTGAAATCTGCAAAAAAATCA
T
>103787751_2 DT668185.3
GCACGAGGCAAAACCCTTCAGTAGTTGTCTAAGCGCATTGGTGATATTGCGTCCAGCGGCTATGAGACAAGTG
TCATCTGCGAACTGATACCTCTCACTTCTTTTGATTGCATTAGCAAGGCTATTTACGTAAGTGATATAGTGCA
GTGGGCCAAGTACCGAGCCTTGTGCTGTACCTACTGTTGACTTTATCATGCAGCTATC

ACCATTACCAACCCTTACAGTATGATTTCTATTACAAAGATAGTTTTCACACCATGCAAGTAGCTTTCCTCTG
ATTCCGCTATCGCAGAGCGATTGCAGCAGCCTATCATGTCTGATCGTATCAAATGCCTTCGTGTAGTCAATAA
ATACAACCGATACGTGTTCTTTATTATCGAGATGTTTATATATACTGTCCGTAAACGA
TGATAGCAGTTGGGTTGTGCTCTTATGAGGTTGGAAGCCGCATTGATTTGGCGTTATGATGTTGTTAGCCGCC
TACAATTTATATATTTGTTGACTTATGTATTTTTCTGCTATTTTGTTAATGATAGGTAGTATCGTGATTGGTC
TATAATTAGCGTAATTTTCTCGGCTTCCTTCTTTATGTATCGGTCTAACAATTCCAGT
CTTTAGCATATGAGGATATTTGCCAGTTACGATGCTCGCATTGAT
>103787754_2 DT668053.3
GCACGAGGGTGATTCATGTACTTGTGAACTCTTGGAGAATGAACCTATGCGTAATTGTAAAGCCGATATATGG
GATCGCCCGTGGCTGGTAAATGGAAGGCAAATTAAGGTATCTTGCAATGTAAATAAAGAAAAAGTTGAACAAG
CTAAAAGAGAAAAAAGATCATCAAATTATTTTGTTGGTGGACAAACTACAAAAGATCC
TAACGATCCAGAATATCTTGTATTAGCTAGAGGTTCGCTAAACGATTTTGTGAAAAAAAATGATATTAGTGAA
GGATATGAAGTTATAAATATCGAAAAGGTTACTGTACAAGTTGTAGCCGGTACCCTTACGCGAATGACTTTCA
AAGTTCGTTCTAAGAACAGTAACGCTGTTGAAATCTTATGTCACTCCACGATTTGGCA
AAGACTATGGTTACACAGTATTGAATACAACACAACGTGTGATGATAATTTAAAACGTGTACAAAAACGTGCA
GTTCCAGGTGGTGTTTCTGAGCAGGATCCGAATAAATCAGAATTTAAAGAATTAGCAAATAAATCTTTGCAGA
AATATCTTGAGTCTGCTGGCATAAAACGACATCATAAGTTAATTGAAGTTCAGTCAGC
AACTGTACAAGTTGTAGCTGGTCAGCTTATAGAACTAAAATTCAAAATTTCACCCACAAATTGTAACTTAGAT
GGCACTTTAACAAACTGTGATATAATTGATACTTCGGATGTGTTACTGTGTAATTCAAAAATACATATACAGC
CATGGCTCGAAAAGAGGACATTCAGGTTACGTGTGAAA
>103787764_2 DT668181.3
TATTGGACCCAATGTTGGTATCAAAATCTCAATTTCAAATCTAAGCCTTGAAGCCTATTGCTTTAACTTCGTT
AACAAATTAGAAATATTTATATAAAATAATGTATAATTTACTAACTGAATTAGAGAGCGATTTTTTTATGAAA
AAGGGCAATTGTTATTTTTGGCCTAGTTTTTCTTCAGAAAATGGATAAAAAGTTATCC
AATATATAAAAATCTTCATCTTAATTTTTTTGTTACTCAACGCAAAAGTCTCAATGATTACTTGGTATAGAAA
ATAAGTTTTATGATCGATATTTAGAAGAAGAAGAGTCGTGATAAATATACTAGCGGATTGCAGAATTAATATA
GTATAAAATCTAGATATTCTGGAATATTCTAGAATATGCTTATATATCTTATTAAAAT
ATAAGGTATAAGTATTTGGTAACATGCTGTTTACTATCAAAGTAGTTAATGGCATACCAACTACTGGTACATC
AAACACTGGCATGCCTTTACTTTAATTTAATTGTATTTAGGCATTTTGATATATATTTCTGCCATTTGCAAGA
ATGTATATCGTGATTTAAGAGATTTTATTACAGTACCCACCGTGGTACAAGATTTTAA
CATGCGACCAGCTAAGTTTTATCAAGTGACTTGCAATATATACGGGGCGTGCTATTTATTTGATACTTA
>103787810_2 DT668748.3
ATATAGAGTAAAATATTTCAAACCCGTACCTAAAAAAAATATCAAAATTCCTTAACAGTTTAGACGTAGGATC
GTTATTTTCATTTTATGATTTTTATCTATACTGACTGACGTTAACACCTACAACTACAAAAATACTACTAAAC
ATAAGATGATCTGCCACATTTTATTCTCAAAAAACATACCAGAACGAGAAAGGGATCT
CTCGTTCTATTGTACGCAATTACCAATTAGTATTACTAATTACCAATTTTCTATTTATAATAAACCTTAACAA
CGTTCAACTTCGCTGCCAATATCTGCGACTCGATTTCGTGAAT
>103787816_2 DT667184.3
GCACGAGGGTAAACTTCCCCAATCATATCATCAAAATTTTCATCCTTTCACCAGTCTTTCACATTCACAAAAT
AACACTATTCATTTATTTGCACCCGCCACAAAAAAAGAGGTTGGTGAAATAATTGACAAATTGAACGTAAACA
CAAGTAAAGGCATCGATGACATCGGCGTAAAAGATATAAAGTGTATAAAATACTTTAT
TCTAGATAATCTTACTGACTGTTTTAACAAATGTCTTACACAAGGCAAATTCCCCGAAAGTTTAAAGATTGCT
AAAGTTACGCCAGTTTATAAATCCGGAAGTAAAACAGATCCGGGGAATTATCGGCCGATATCTGTTTTACCAG
TTTTGTCAAAAATTTTAGAAAAATTAATATATGTCCGTCTTTATAATTACCTCAATAA
ACAAAACTTCTTTTTTAGTCAACAATATGGCTTTCGTCCGAAATCTAATACACTTTCAGCAACTGTGGATATT
GTCACCAAAATTAAAAACGCGTTGGATAGTAAGCAAATCGTTCTAGGTATCTTTATCGACTTAAAAAAGGCTT
TCGACACGGTCAGCCATAATTTATTATTACAAAAACTACATAAGGAAGGTATAACAGG
TTCGGCAATCAAAATTTTAGAATCATATTTTTTACATCGTCGCAAGCAAATTGTTAAAATCGGAAAATTTCAA
AGTAGGCCCGCAACGTTAACATATGGAGTACCACAAGGCTCTATCTTGGGACCGCTATTATTTTTAATTTATA
TAAATAGCATTAGTAACATTGATTTATCTGGAGATATAACTCTGTATGCGG
>103787826_2 DV501351.2
TCTTGGGCGAAAGGAGAAAAAGTAGAAAAACAAAAGATCGGAGCGAGAGCTGAATAAGAATGGACGAACCAAA
TTATATATTCAACGCACGACATATAATAGAGTGTGTTCGGTTTAGAATGTATTTATTCAATGTAAAAAATGTT
CACTTGTACAAATGATTGTCCTTTTTTTAAAGTATATTTCATTAATTTTGTATTTCAT

GAAATTATTAAAAATATCCTATTAAA
>103787841_2 DT668355.3
AAAAATGTGAAATTATTAAAAATGTGTATAATTTATTGTATATAAAAGTTCTTAAAACTGGTTGTTTTAGTGT
TATTAATAACAGATGTAAATACTATTGTGCTCCGAGCGTACATATCTCAACATGGGCTACATGGAGAAATTGA
ATTCTCACAAAAAAACGAATCCCTCATTAGTATACGAACCAACTTGAAGCCAACGCTA
CAGTATCCAGATGGAGTATGGCGATGGACTATACACGAGTACGCAGTGGACTATACAGATTTGTCTAACTATT
TTTTTTCTGAAAGAAGTCTTGGGAAGGAGATTATCGATCTTACAGAAGAATTACGCTATTTAATAGTACCTGG
TAAGGATCATGCTGAATTTGAGAGTATAAACAGTTTAACTGGCCCTATGGGACTGTGG
GGCAAGAGTATAGTCCTGGAGACAGCTGAACGTGATAGAGTCATTTGTGCCTCGATTTTTAC
>103787844_2 DT668003.3
GCACGAGGGTTAAACCTTCACCATTGGAAAGAAGTCCAAAACTTTTAGAAATGATCAGAACTGAATATTTGAG
CTTAATCAGAAATATGCAGCTGGAGTACGACATTCCAGATTTTATGAGATTCACCACTAGCCCGGATTGCAGT
AAGCCCGGGGAATGTATACTACAACACGATCAACATATCGATATCGAAGGAACTTTAG
TAATCGACTCGTGCCCGCCAGATGATGTTTTTACTCATACACTTAAAGTCGGACCTAGATTTTTAGATGAAAA
ATTAACAATAATAGTTGAAGTGGACTGTCAATGTGATTGTGAAAAAGCTTGGAAAGGCGAAATGAACTCAAAA
AAATGTAGTTCGAATGGAACTTATCAGTGTGGAGTGTGCAAATGTTACGAAAACAGAC
ACGGCGACACGTGCCAGTGCGCGGGGGAGCGGCTGGACGCGCGTGACTCCGACAAGTGTAAGGCGGCCGACGA
CGCCGTCGCGCTGTGCAGCGCGCGCGGCACCTGTAGCTGCGGCAAGTGCACATGCGATAAAGATTTCTCTGGT
CAGTACTGTGAGTACGATGACAAAGCGTGTGTGACGGCGGACGGCAGGGTGTGTTACG
GGAACGGGAAATGTAAGTTTGGTAAATGTCAGTGCGACGCGCAATGGCTTCCGGAATATTGCTTGTGTCCCGT
CGACGACAGCAGCTGTATAGCACCGCAGTCGCAGGAAGTATGTTCAGGGAACGGTGAGTGCGTGTGTGGGCAG
TGCGTGTGCAACATCAGAAAGAGCGACCACGGCAAGTGCTCTGGAACATTCTGCGACG
>103787850_2 DT667553.3
GCACGAGGAAGTTCATTTTAAAATAGTAAATAATTAAAACATGCAGTTAATTAAATAAACAAACGATGTAACT
CATGCATATTTAAATCATCAGTTTTCCTCGATCCTCAATATTTATTTTAATCCTTCTAGTTTCTATTATATAG
TGTTTAAGTGTATGTATATAAAGATGTTATTCGAAATAATAATATTGATAGTGATTTT
GCCATTACTGTTCTTTGGTGTCCTATGTTTTGGTATATTTGTGATAGTACCATGTGCGTTTATGTTTGCCTCA
TGGTACATGAGAATAAAAGAGAGAAAGTTGCAGAGGAGGAAACGAGATGGTCCTAACATTGCCTTCTTTCACC
CTTACTGTAATGCGGGGGGTGGTGGTGAAAGAGTATTGTGGGTAGCCATTAAAGCTTT
ATTAACTAGATATCCAGATTCCCACATATACATATACACAGTAGAAACAGCAGAACCTATAACAATACTGAAT
AAAGTGCAAAACCAATTTAATGTTAAAGTAGATCATGAGAAGATCAACTTTGTTAGACTTACTATGCAAAAGG
CTATTGAATCTGAGACATACCCATACTTTACTTTGTTGCTACAGAGTTTGGGAACTAT
GGTGCTTGGGATGGAGGCATTTATGACATTTAATCCAGATATCTATATAGACACAAATGGGTTGTGCATTCAC
TTATCCAATATTCCGTTATCTAGCCCAATGTCCGGTAAGTTGTTATACGCATT
>103787878_2 DT668590.3
GCACGAGGGTCATCCTTTAACAATAAATTTCTTATTTATTTTATAAATCTCATTGCATTTAATGCGTCAAAAT
TTTCAAGAAACTCGCTTACACGATATAAAATTGAATATAACAATATAGACTCACGACAGACGATAGATTACAA
TGCTAGGTTCTCACAATAACTGGGAAAGGCTGTCGTAAGGCCTGGACTTCGTCCAGGC
ATCCATATCTGAGGAAAAAATTGCAAGAGTCAACGTGGAATTACATATAAATGTAATATTAGAAGATAATAAG
AAGTAGAAAAGGATATCTGGCTACTGGCGACAATTTTCAGATTTTTGCATTCTCATTTCGGTTGGGTCGTTCT
ACCGTCTCCAAAATTGTGAAGGAAGTATGCCGTGAAATTTGGAGTACCCTGCAACCGA
ATTATTTGCCTAAACCTACGGTGGAGATGTGGAAAAAAGCTGAGGAAGGATTCGCAGAGCTCTGGGGATTCCC
TAATTGCATTGGAAGCATTGATGGAAAACATGTCAAACTGAAATGCCCAGATAACAGTGGGTCGCAGTTTTTC
TGTTACAGAAATTATTTTTCGCTCGTTCTTCTAGCGGTAGTTGACCCATACTACAAGT
TTATGGTAGTTGATATAGGAAGTTATGGGCGTCACAGCGACAGCGGTATTTTTGAAAATTCCCTTTTTTACAG
TGAGTATATTGACAAAAAAAGTATTGTACCGCCGA
>103787893_2 DT668637.3
GACGAGGGTTTGTCTTTTTTTCTAATAATTTTGCGGTTTGATTTTATTAATATTGGATTATATTCACATTATA
TATTAACAGAAGTAGACTAAAATACTTATTGAACTTAAATTTCTACTGTTCTTCTTTTGGTGTTTTTATATCT
TATTGCAGTTCTACCCGGCGTTCGGAGTTGCCAAGTCCAGTGAGACAATCACAGCATT
ATTGCCTATATTCTTTGATTTTAACCTGTTCACAGGGTCTAGTCTTCACCTATAGATGGAACGTATTCTTAAA
GCCAGGTGTAATAAATAATATCGGTATTAACATATTATTATATTTTCTTTTGCGTAGAAAAAAAATATCGATT
AATAATTCGAAATCATATATATTTTTGCTGTCTTTTCGTATCTACGTATTTTTCGTCA

AATTACAGTTGAAAGATCACGCGCAGGTTCCGGTGCTGACCTAAGATGTAAATCTCTGTCCAGTCAGGTATAA
ATTATGTAACATAATTAATTACAAAGTAATAAACATACTAAGTTTTCAGTATATAAAATTTTAGAAGTGGCAT
GTGTTTAAAACAATGCATATGTAACGCATACGCTTAGGTTTATTAGAACTTACTATAA
GCATTTCGTAATATATATATATGTATGTATGTATGTATGNTATATATGGGATGTTTTTGTTATCTTAGTTTGC
AGTTAACCGAGTGACTGACTGGTGATTACGATGGGCACTGTCTGTTTTTAATGTGAT
>103787905_2 DT668495.3
TTCGGCACGAGGTGGTCTTGCTGATTTTGTTATAATTTTTTAAATTAAGTAGTGCAGTGAGTGTATTAGTGCA
AATAATTTACACGTTATACTAGAAAGTTACAGCTGCAATCATGTTTAGCTGGCTCAAGAAAGAAGGTGAAAAA
ACAGAAAGTATCGAGAATGTCGTGGAGGGTCTCAAAAGAATTTATAAGACGAAACTGT
TGCCACTTGAGTTGCACTATCAGTTTCACGATTTCCACTCCCCACAGCTGGAAGACCCTGACTTTGATGCTAA
ACCTATGATACTGCTGGTCGGTCAATATTCAACGGGTAAAACTACGTTCATCAAATATTTGCTAGAAAGGGAT
TTTCCAGGAATTCGTATTGGACCTGAGCCCACCACAGACAGGTTCATCGCGGTCATGT
TCGATGAAAAGGAAGGCATGATCCCCGGAAACGCACTTGTCGTAGACCCAAAGAAACAGTTCCGCCCGCTGAG
CAAATTCGGAAACGCCTTCCTTAACAGATTCCAATGCTCTACAGTGAACTCTCCGGTGCTTCGTGGGATTTCC
ATAGTTGATACGCCCGGTATATTATCGGGTGAAAAACAACGCGTGGACAGAGGATATG
ATTTTACCGGCGTTCTCGAATGGTTCGCGGAGCGTGTCGATCGTATCATCCTGCTGTTTGACGCCCACAAATT
AGATATTTC
>103787908_2 DT667964.3
GCACGAGGCTTAGTCGTAACAGTCAGTCAGTTTTAATTATCAATATGTGGTCTCTGAGTGTGGTTGTGTGTAC
GTTGTTGGCGATCGCTGCAGCCACCGACGTACGGCAGTGCCCCGGTCAGACAGTTGAGGGCCTTAAAGACGTA
ATACAATTGACGCCTTGCAAGAAAGTGCCGTGTCGCCTAAAGAAAGGAACTAACCAAT
ATATCACCATAACTTTTACGCCAGAGGTCGATATGGATGAAGTTAAGGACCATGTGGTAGCTCAAGTTTTTGG
CGTGCCACTACCCTTCGTCGGAGTTGACGGTGCTTCCGTTTGCGATAAACTTGACCACTGAAAGTGGAGAGAA
AACTTCATGTCCTCTTAAGGCTGGCACCAAATATATATATAAAGATAGTTTTCCTGTT
CTTAGTTTCTACCCTTCGATAGACGTAATAGTGCACTGGTCATTGGTGCATAAAGAAAAAGATATCATTTGTT
TTGAGGTTCCCGTAAAAATATCTTAATTATCTTGAACAAATGAATAATTTAAATTACCTATAACAAAAGTAAC
TAAGAAATTTAGAAATGCAAATATATTTTGAACAAAAAATTTTATGATGTGGTTCCTC
AAATATGGCGTTCCGAACGTGCCAGTTGACCATATTATATCAATCCTCGCCAATGTCACGTTTGACAAAAAAA
ATCAAATAGCTATATTTGTCTGTCA
>103787914_2 DT668659.3
GCATGAGGATAGACTTCTTTTTGTATCATTTTATCGTTTCCAATTGTGTGAAACTGACATCATAGCAACTTGC
GATGTAAAAATAGGTCGCCGTTATTATATATTAATATATTAATCCGATCCCAGAGGCCGTTTCGGTCTAAAAG
ACGTTACGCACATTGAGTTCTACGAGAAACTATTGATAAGTGACTAAAATTGCTGTAT
TTTAATCATACAATGTGATTACAAACTTACTTCCCTTATTATACTTCCTTATTTTTAATACTTATTGGACTTG
AGGATTTGAAAAGTAATTTTAGTTGCAGGGGTGATTTGTTTCTTCATATAACTAATGCGCAGATTCCGTGATT
TGTTCGGACTCTTAATTATAGAAATGTTTTCTCTTTTCAGGGCTTTAGAAAATGTCTA
TTAATAGCAAAATTTGATTGTATTGAAAATGTAATTATGGATAGCTTCATTTCTAAGGGTTTTAAAAAATATG
TCTTGGGAGTGAAGTTTAGGTTTTTTTTATTTAAAAAAAAACACTTTTAAGGTATAAGAGTCCAAATTTGGTG
ACAGTGTTATATAAATATCGACGCAGCGTCGTTAGGGCACAAAAATATTATTTTATGC
AATTTGCTCGTAACATGTTAAGCATGAATGTAAAAAATTACAAATTGGATGCATGTTTAACAAAAATCGTAAT
TTATTTGGTGTGTAATTTTTTTTTTGTAATATACGTGATATTTTTATTTGTGTGATGTATTTATCGACAGATC
TAACCG
>103787921_2 DT667732.3
GCACGAGGCGGACCTCCGCCTTTGCAATAGTCTCAAATTGAATGGTTGGCGGGCGGTGACTCGCCTCCCACTA
GACGGGCCCCCACAAAGCGGCGTAGTGCCTTGGGCGCAACATCGGCGTGGTCGGCTAAGGGTGTAGAGAGGTG
TGCACCGGTTTCACCATCACGAGGAGCTCGTGAGCGGGTTTCCCCGCTATTACGCAGA
TTGAGCACTTCCACCCTTTCGAGCATATAACTCTTGCGCTCCACTCTGCCGGCCAATAAAGGCAAGCCAGAGG
CGGGGGATAGATATTATTATTATTATTACTATTTGAGGAAAGTCGAAACGAGGGGCGCAATTGTGCGAGTGTA
AACGCGATAGGTTCGATTCCCACTCGAGGAAATCTAATATTTAATATTTTCATTTTCC
CGCTCTGCTAACAAGGCAAAGTGCGGCAGTGAGTCCCGTCAGCGGCGGCAATTCGGAGGAAAGTGGAAAACGG
AAGTGTCTTAATGGGAACTGAATTATCGAGAATTAACACTACGTTACCAGGTTTTCCTTGCCTACCCTGCTAT
TTGTGGAATAAAAAGAAAAGCAGTATTAATTATATTTTTAGCAAGAAGCTATATGCTT

TTCCAAGTAACTTATATTTATTTCCCACCTTGCCTGGCTCTTGACCTTAACTCTTGTATCATCTTAATCGATA
CTTATGTAACTAGTCGCTATAAAGGGACCGGTCGAGAGGTTGATGACAATGGCTCCGTATTTACTTTTAATAT
GAATTATTCAGTGA
>103787924_2 DT668667.3
CTCTCTTGCTATACATTTATATACGATTAATATCTTCTCTTACGTTTTGTTGGTGAAACTTAATAGGTACCCC
AGTGTCCTTACGATTACAGTGAATGTGCATAGCTAGGAAGTNCAAATACTGTGGTTCTTAGAGGCTTCCTTAT
TCATCCCGCGCTTATTGACGCCGGTCTTTCCAACACAGATTTATATTCCGACCCAGAC
AAGCTGGTTTATATAACAGGCAAATT
>103787927_2 DT668018.3
GCACGAGGCAAAAACCTTTAGGGGTACCATTAAATAATAGGTCACCTGAGGTCGAAGCTAATGGAACATCAGA
TGTTCCTAAGAAAACAGTCCAAAAAGAAGCGCCTCCCACAGATGACCAAGAGCCGGGACCTTCGAAAGCGATC
GATGACGATCATTACAAAGTTAACGATGACCAAGAACCAATAATTGCAGTGGCGACTA
CATTAGAAAACACATACAGCCACTATCCTCAAGTGATCCTTCTATCAATCAAACGATATTTACGCCCCACTCG
TGACTTTTTCCAACGCATGTTGGCGCCGGGCGCCCGGGTTACTTTTTATATATATGCTTATATGTTCCTGTGT
GATTTCTTAAACTTTCTCGTTGTTATATTTGGATTTGCTGCGTTTGGAACACATCAAG
GCGACGGCGGTGTACAAGCGTACCTCGAAGAGAACAAGGTTCCCATACCGTTCCTGGTCATGTTAATACTACA
ATTCGGCCTCATCGTCATAGACAGAGCTCTCTATCTTCGCAAGTTTATGCTCGGCAAGATACTGTTCCAGTAT
GCTCTTATCATTGGTGTACATATATGGAT
>103787951_2 DT668836.3
GCACGAGGCGGATCCTAAAAAAATAAATTGCACTATAGAAACGCTGTTGCATCATGCTTTCTCGTTTGTGCGC
CTCCCGTTCGGCTTTTCCCGATTCTCATACACTTAGAACTCCAGTGCCTCAGAGATTTGTTCCAAAAAACTAT
GTCATCAGGAACTATGCTCGCGAACCTCGTTCTAGAGTAGCAACAAGATCGCAACCTA
CAGTATGGGAAAAATTGATGGCTCCTGCTGGACCAAATGCTTACAGCTTGGGTAAGGGAGCGTTAGCAGGGGC
ATCAGCAGTAGGACTTGTAGCATTATGTTATTATGGTTTTTTATTTAAGCCTGGTACATTACAAGAAGCTCAC
TTATGGCCACAGTATGTCAAGGATCGTATTAAAGCTACATATGGATATATTGCTGGAT
CACTGATGGTGACAGCAGGGAGTGCTATAACTGTCTTTAGAACTCCTGCTCTACTTAACTTAGTGGCAAGGAA
TGGATGGATGTCAATTATTGCAACAATGGCACTGATGATTGGTTCTGGAATGGTTGTTAGAGGCATGGATTAT
ACTCCTGGTATTGGAGCTAAGCAACTAGCTTGGGTTGTGCATACTGGTATAATGGGTG
CGGTCATTGCTCCTATCTGTTTCTTGGGAGGACCTATTCTTATCCGGGCTGCTTGGTACACGGCAGGTGTGGT
CGGCGGCCTTAGTACAATAGCAGTGTGCGCACCGTCTGGGGAGTTTCTGAACATGCGTGCCCCACTTGCTATG
GGACTTGGCGCAGTGTTTGCTGCATCCCTCCTTGGCATGTTCTTACCACCCTACTAC
>103787962_2 DT667514.3
GCACGAGGAAGGACATTTTGATATTTTACTGTATGCTTCGTGGTATAGACAATAGAGAAGAGGTGATGTCCCG
CACGCTGTGCGCGTTCCAGCTGGCGCCGTACTCGCACGCGCGCTGCGGCGCGCTCAGCGGCGGCAACCGGCGC
AAGCTGTGCGCCGCGCTCGCGCTCATGTCGCGCGCGCGCACCGTGCTGCTGGACGAGC
CCACCAGCGGCATGGACCCGCTGTCGCGGCGCTGCGTGGCGCGCGGCGTGCGGCGGTGCGCGCGGGGCGGG
>103787974_2 DT667531.3
GCACGAGGGCCCCCCTCCGGGTACAGCCCGTACCACCAGCCGCCGCAGTAGACCACCACCAGCACCACCACAC
TGACCTCCACTACTAAACTACCTTCACTACTAAATGGATATTGCCATTGCCTAACAGCGCGCGGCGAACAAGT
TCGGCGTAATATATAAGATAAGGTCTTCGATCAACTATCCACTGGATTATAGGTTACA
TACATACATGGCTCAAAAAAATTGCAAGAGACAATAATTTTTTGAGCCGTTTACTAGAAATACACAATATTAC
TACACACAGACTCATGCCAAAAATAATTTTAAAAAATCAACCTTATTACAGCCAGTTAAGGTTTACTCTCGAC
TAACGCGGGATGCTCGTATACTTTTATGTTGTACGCAATGGACACGAAAAATCAACTG
TACTCTTATGAAAATAAAAAAAAATAAAAAACGCTTAAAATGGCAATAGCAATACTTTTTCAATAGTCCAGAC
CAACCAATATTCGATGAGATTGTTGGTAGTGTTGTTGCGAATATTGGCCCAATGACGAAATATTTGTATTTAA
TTGTTTAATTATGATTTTTAAGTACTATAACACTGAATTTGTGCTACTGCAACATATA
TGACAGTGAAAAAAGACTCCTGATACCTTACAGTTTCGGTTGGCGATTCTTGGACGATACT
>103787980_2 DT667460.3
ATTTTAAAAAAGAGGACAATATTTATTTAAAGCACATCATTCACTTCATATGTATCATATGGGCGAGAGACCA
GTCGTGTTTCAGTTCCACTGGCGCGTCGACGACCACTCCTGAAGCTGTCACCACTCCCAGCTGGTACCGAGGG
AAGGAACGGGCATCTCGGTAGAATAATACTTCCATACATTTACGGACTAGAGCCCTGT
AATTATAAAAGAATAAAGATTTGTGTCAAATTATCAATAATTTTGATTTACTCAAAGTTTTTATTTTAATTTT
GTATTCTACTAGTGGTCCGCACATGCTTTTGCCTGCTTGTACTATGCAATTATTTATCTTCTCATCTTAATTA
CTTTCTTGATCTAATGACATTATAATTTTAATGACAATAATCCATACATTGATTGATG

GCCAGTTCAATAAAGCACTTATTTTGTAGCAAATTTTCACATGCAACTCTTTAGATTTATATATTTTTTTTAT
TTGCAAACCGATTTAAATAAAACAAACACTAAATGTTATAAAAAATTACCCAAATAGAATTAAGTAAAGTTAA
AGTTTTTCTATACATAATGTACCATACAAACAAAGTTCATTTCTTGTAATATCATCTA
CTTCTATTAAGTTTTAAGGAAACAAATTCTACTCAAAATCAAGTAAGAAATGTCATACTTAGCAGACTCCTCA
TCAAGAGGACCATTTTGCAAAGCATCTCTCAACAGAGGTGTTGCCATGTATGCT
>103787983_2 DT668840.3
TAAAGAGGAGCAACAAGCAGATACCTTTGATCCAGATGTAATGCTAGGAGACCAGCCTGATGAAGAAAGAATG
AGAAAAAGTAATGAACAAATGACGAAAACTATTGACAAATCTGCAGGAAAATTAGAAATAGGCAAATTTGTAA
TGGATCCAGCATACACAGAATTCATTGTTGTACCAAAACACGAAGAACCAACTATTAT
AGAACCAGATGAAGAATCTTTTTTTTTTCAATGGGGCATTGCTGTTATTGTAATAGCCTTAGCATCTTTATTA
TTCGTCATTATTTTTGGTGTTACAGTGCTTGTAAATCGCCAAAAAAGTACCAAAACTAAGCAGCCCATTCCTC
TTAGTGAAGATATGCTAAGAGAACTTGATAAAAGCCATATGGGTGGATTTGATGACAT
GCACCAACTAAAGGAGCGTGATCTACCCTACTTTACCTTCAGGAAAGGTAAAGCGAATGTCGAGTGCTTTCAT
CGAACAACTAGCATACCCTAATCCTGGAGACTCTTGGCGATCAGAATGGACTCCACCACTGCAGAAATACTTG
CAGCACTACACTCCCGACTCCGGTCGAGGATCACAAATTTATGG
>103787985_2 DT668759.3
GCACGAGGTATTTACATTCAGACAACCTCACGGTTGGATGTTGAAAATTTCTTTAAGGAATTTTTATTAACAA
CTATATAATCTTAAATTATTAGGAATCTGTCTTAGGGTGCTAAAACTATTTACAGACATATTTACATTACAAG
TCTTTCTCTCAATATAATTATTTTGACCTTATTTTGTATATCATACAGAATGAAATCA
CCATATCGTAAAAATGAAACCATATTCTTAGCTATCCAGATAAACCGTAAACAATAATGACTAGACAAACTAT
TAAAACTTAACTATAAATCTAACTTATTGGAAGCTAAGATACGGCTTTATAAAGGCACAGTGTGTTATAAGTC
TTAAAATATTAGTAAATGCTTTTATGGAACTTAATGTTAAACGAACGTTTCAGTCTTG
ATACTGCTATTTCATCAAATCGAGGTACGAAATGTAAGCAGAGTTCGGTCGCTCAGTCGCTCAGTCGCTCGGC
GGAATGCATCGGTGGGATCGTTATAGTCCACCATTATCCCCTCTCATTAGAAATATACTGATTGCGCTTAATT
AAAGGTTCTCCCGGAGTCCCGTGCGCTCCAATGTTCAAGTCCTTCTTGAAGCGAATAC
TTCTTTTTTCTTTCTTCACAATCAAAAAGCCGCTCAATGCCTTGTGATCACCATATGTCACGATACCTCATGG
ATCATTGCTGCTGCCACCAAGAAATTACATCAAGTAACTCTTCA
>103787994_2 DT667355.3
GCACGAGGGTGTCCCTCAATCTACTGCGTCCTCGTGATCATTGACATCGGCCATTGGTCTATCTTTTGTCAAC
CAATCATTGAAAAACCAGTGAGGAATATCCAATTAATTAAAAATGGAACATGCCCAGATCCAAACATCAGACG
GATCTCAAGTGATGACCATCAATGGACAACAACTGCAAGTGCTGCAAATGAACAATAA
CCCCCACATGCTACAAGGGCCTAATGGCCAACATATTGTTATACATTCTATACCATCTTCGGCTCCAGCTATA
CAAGTGGCAGCTCCAAGTGGTCAACAGTTGCAACAGTTACAAGTTCTGCCCTTATCCAGTCTTCAAGGCACAA
GTAACAACATTCAGCCTATGCAATTAGTGCAGACACCAGATGGTCAAACATTTATCTA
TCAACCAACACCGAGCACTTCTCAGCCACAGATTGATCAACATCAGATTATACATCAACCTGGTACCCTCATA
AATCTTAATGGCAATCTGGTGCAAGTGGCCGGAGTGGGCAATACTGTGCAACCACAGCAAATTGTAAATCAAC
CTAATATTGTTATGATGGTAAATGGCAATACCGGTGCAACAGCATCTTCATCAGAAGG
CGCCTCCCAAGCTGGTTCAGACGAGGAGCCTCTGTTATATGTAAATGCAAGACAGTACAAGCGTATACTGAGA
CGTCGAGCTGCACGAGCCAAGCTACATGAACAGGGGA
>103788018_2 DT668265.3
GCACGAGGAACACCCAATGCCTCCAATATTCGGCAGAAAGTGGGGAACAGAGTGTCTTAACATCTGGTTCCCT
CTGCCTACCATGCTGTGTGTGGGATACAGCGTGAAGCTGATTTATTTATTATTGAATAACGTTTTTTCTCATA
CATCTTCCAAGTACATATGTAAGGATATGACAAAATTTAAGTATCTACCCGAGCCCGT
CGTGGCTCTGGAGTACAATACAACCGTGCGAATATAAACGCGGTAGGAAGTGGGGAATGGAATGCCCTAACAC
TAGGTTCCCTCTGCCTAGCCTATAAGCGAGGTACTAGGTGAATCTAAAAAAACAAAGACGATAACAATATAAA
AAACAATAAGAAAAACAACAAAAATTATTTTCATTTCTTTGTGTGGTAGTTCAGACAA
TCAACCAGCAAGCAGTAACCAGCAGTATATTGACATGAGATTATTTTTGTCTGCCATCTCTTGCCTTAGGCTT
TAAAAAGTACTAAAATGTATAAAAAAGTAATAATTATTTGTATATATTTTATTTTTAACATAACATTCTTATA
TTTAATTTGTTGGTAGACAATGATTCATCACTTAATTGTTTTTAACTGCTTTAATTGA
TGCTGAATTCTAAGCATGATCAAGTAAACAATGGCTCAATTGAGTTTTATTTACGCATTAATAAAAGTGATAC
TCTGAACTCTTGAGTTTCCTTTAGATGACAACACTTCTTTTTTAGATATCATTGACTTTT
>103788029_2 DT668875.3

AATGCTTTATTCAATTATTTCGTTATTAATATAAGTGCAAAAAATTATTTATGCAAACATTTTAAGATATAAC
ATAATATAGCTCATGTTACATTATGGCATTTCTGTGTCCTGTAAGAATAAGAAGGGGAAAAAAGAAGAAATCA
AGTAGTGGTGATTCTGACAAAGAACTTTCTAGACCTGGTTCAGGTTTAAGTCCTGGCA
TGGGCCGTATTACTGGCTCAGCCAGTATTGAAACGTTGGTCCGTGTAGGTATTGAAAAGGAACATGGATTGAG
TCCTGATTCAAAAATGGTTTTGTTACACGATTTTGCTCCCTATGTCGATGATGAATTAGAGGTAAAAAGGGGA
CAAATAGTAAATGTACTATATCGTGAAAATGATTGGGTATATGTAATTGTAACCGATT
CTCGAAGAGAGGGATTCATACCACACTCATATTGTGCCCCGTGTGATCACCATGACTTAAAAAAGAAGCTCCC
TAGAAGTAGATCTCCAACAGATTTAGCACATCGAGATGTGTCACAACTATCATTATCTGATGGAGCAACTAAT
GATGGACATTCAGAACTAGGTAGTGAGGGTGAAGCGTGCCCCTTTAGCAAGGACCCTT
CAGGACGATATGTTGTTCTGTATACATTTCCAGCGAGAGACGAGAATGATGT
>103788032_2 DT667406.3
GCACGAGGCGCTGCTGTACTCGGGGCAGTTCAAGGACGCGATGGGCGCGCTGCTGGAGTGGCTGGCGCGGCAG
GCGCGCGCGCTGCGCGACGGCGCGCCCCTGCACGGCGACCTCGACACCGTCATGGCGCTCATCGAGCACCACA
AGGTAACTTGAGGCTGACCCCCTCCCGCGAGCTTACTATTATAATTTTCCCGCTTTGG
TGAGTGAACAATAAAGTGTTAATAATACAGGTTGTATTACAGAATACAGAACACTAGGTTTTCTCTCTCTTTC
CAACGCTAGATAAAGCATGTAAAAAAAAATTAAGTATGGAAAATTGGCCCAATATAAAATATATGTATAACAT
ATCTTGTATATAACATTATGTATTATATTACGTAAACACATAATAACTTAATTAAATA
GAAAATATACGAAATACACTAATACCTAATATATGTATTAGTATATTTCGTATTTTAATAATATTTAAATAAT
ATACCTACTACTCTATATTATTATTAATTCTTTTCTAGCAATTCGAGGAGGATCTTCACAGTCGCGAACAACA
AATGCAATCCGTTCTCAAAACTGGCAGAGATTTAGAAGCTACTGTACCACAAGAAGAC
GCTCTAAACATAAAGCAACAGTGTTCCGAATTGAAACAAGCGTGGGACAACGTGCAGAGCCTCAGCGAGAAGA
AGGCCAGTGGACTTGAAGCTGCTCTC
>103788035_2 DT668154.3
GCACGAGGGCGCCCCTAGAGCCCCGCCATGGTGCAGACCGAGGACCAGTACGAATTCTGCTACCGCGCCGCGT
TGGAGTACCTCGGCTCGTTCGACCACTACGCCAACTGACGATGAGACATGCTCGTCTGAACCGGAGCCTTGCC
TTGCACATACGCCTTATAGTCTATCTATCTATTACTATTTTACTGTTGAACCGCGTGT
TGTTATATCTTTTCTCCTATTTTCTTTTTGTTCATTTGGTATTTTATTCGTATTTAATAAACATGGCAGGTTA
GAAATAACAGCAGCTCTTTCAGTATATTTTAGTCGTAAGTATATACTATCAAACTGTTTTTTACTATCTTTAT
TTATAATACTTTGAAAATGCCAGCTTTGAGAGTAGCGGCTCATTTAATATTTTATAGA
AGAATTTAAATGAACTAATGAATGAATGATTCAGGATCTATGCCACCTGAAATTTTAAATTTTAAAGATTATA
CTCGAAAGCCGGTTTAATGGCGTACTACGTTCGCACCAGACGCCGAGATATTAAAATAATGATCTTTCAATTA
CCATTTTGGTACTTACTCTATACACTTAAAATGGAAAAATATTTAAATATAATCTATA
AATATTTATTTAACGATTGTTTCTATATAACGACACGCGATACAATATTTATAAAAGTAAAACTTTATTGGAG
ATACGTAATCATTATAGTGAACGAAGAAAGCAAGGCTCTACATCAATTCCAAGCAGTGTTAGAAATTGACTGA
TATATTCAAGTTTTACTTGCAAAGAGGC
>103788050_2 DT667920.3
GCACGAGGATTGGATCGTGCAGCTATGAGCTATGAGTTTTAATTCGTGAAAATATTCAGCTTTTCCTATTCTA
CGATGTCTTCGTCAGCAATCTATATACTAGATGTAAAGGGTAAAGTCCTCATATCGAGGAACTACCGTGGAGA
TGTTGACATGGGTGTTATAGACAAATTCATGCCACTCCTAATGGAGAAAGAGGAAGAA
GGCATGTGGACACCCTTATTGCAAACAAGCGAGTGTACATTTGCATATATCAAGACAAACAATCTCTATATTG
TTTCTACTACTAAAAAAAAATGCTAACATTGCCCTTGTTTTTGTATTTCTTTATAAAATAGTGGAAGTCATGAC
TGAGTATTTTAAAGAACTTGAAGAGGAAAGTATTCGTGATAATTTTGTTGTAATATAT
GAACTAATGGATGAACTTTTAGATTTTGGATATCCTCAAACTACTGATAGTAAAATATTACAAGAATATATAA
CACAAGAAGGTCATAAGTTGGAAATGCAACCTCGTATTCCCATGGCAGTCACTAATGCTGTTTCTTGGAGATC
GGAAGGCATTAAGTATAGAAAGAATGAAGTATTCCTTGATGTCATAGAATCTGTTAAT
TTATTGGCTAATTCAAATGGTAATGTTTTGAGAAGTGAAATTGTTGGTGCCATCAAAATGAGAGTTTATTTAT
CTGGTATGCCTGAGCTACGCCTTGGACT
>103788062_2 DT667733.3
GCACGAGGGACAACCCTGACAGTCAGGACAGCCAGGTCAACCCGGACAACCAGACATACAAGGTCAACCTGGA
CAGCCAGGATACCCAGGACAACCAGGACAGCCAGGCCAACCCGGATACCCAGGACAGCCAGGTCAACCCGGAC
AACCAGGCCATCCCGGACAACCAGGACAACCAGGACAGCCAGGATACCCAGGACAGCC
AGGTCAACCCGGACAACCAGGCAAGCCAGGACAGCCAGGATACCCAGGACAGCCAGGACAACAAAGTGGTCCT
GGTGAACAAGGCCAACCAGGATATCCAGGACAGCCAGGTCAACCCGGACACCCAGGACAGCCAGGCCAACCCG
GATACCCAGGACAGCTAGGTCAACCAGGCAAGCCAGGCCAGCCAGGATACCCAGGACA

ACAAGGTGGACAGCCAGGTCAACCAGGCAAACCCGGTCAACAAGGTCAACCTGGACAGCCAGGATACCCAGGA
CAACCAGGACAGCTAGGACAGCCTGGATACCCAGGACAACCAGGCAAGCCAGGACAACAAGGTGGTCCTGGTG
AACAAGGCCAACCAGGCTACCCAGGACAGCCAGGTCAACCCGGACAGCCAGGCAAACC
AGGTCAACAAGGTCAACCCGGACAGCCAGGATACCCAGAACAGCCAGGTCAACCCGGACAACCAGGCAAGCCA
GGACAACCAGGACAGCCAGGACAACAAGGTGGTCCTGGTGAACAAGGCCAACCAGGATACCCAGGACAGCCAG
GCCAACCC
>103788074_2 DT667247.3
GCACGAGGAAAAACCTATATTAGGTGACCCCGATAACGAACGCGTGTGCAAATAGGGATAGGAATTCAAATAA
TTTCAACCGTCGCGTAATTTTTTAATTTTTTTTAAAATTTCAACCGTTTTAAAAAATGCCGAACGACGCGAAC
GAAAATGGCGGCGGAGACCAAGTCTGCAAAATCGGTTTGAAGATTCCTCCGTTTTGGG
CTCAAGAACCTGCGCTGTGGTTTGCACAAATTGAAGGGCAATTTATTATAACGGGCATTACATCGGACTCGAC
GAAATTCTACCACGTTGGTTCGCATTAGACCAACCA
>103788077_2 DT667410.3
GCACGAGGCTATTCTTAAAATACGGCAAGTAACAATTTTTTTCATTTCAGCTTAGATAACGGTTTTATAAAAA
AAAATACGTTGTGGATGTATTAATGTTATTTTAATAATAAAATAAAATTTTCTGCAGCAAACACTAAAGTGTA
ATTTTCTATTGAATAATATTTTTTCTAAACAAAATTTAATATTTTATTCACCAAATAT
CAGTAGTATAGAATTTTTTGAAGTGTTTACTATAATATAATTTATATAATAAATATTAATTAGGCTTATGCGT
AGAATTATGTATGATATAATAATCAATAAATTATTATTCATATATTTAATATATATTAGTGTAAACATTAGAA
ACTAAACTTATCTTCTTTTATTAAAAAGCAATTATTTTCAAATGCCAAATATAAGCAG
ATATGAATAGTCTGAATTCTGTCCTATGTGTAGTTATTTAAGTCTTTAATATAAATACATTTATAATGTAACC
TCTATGTAACGTGACCGGATTTAACAAACAACTTTCGATTAAAAATAATATATAATAGCAAAATTTTATTGAT
TTATTACTCTTCATTTTACGTATAATAACACCATAAAATGCTTAGTCCTTTGAGTGAC
GTTATATGGAGTTTACACTATATCAAAAATATTACAATGTAATTGTGTAGAGAGGTCCCTAAAGGCTGTGTTG
TTTTCCTCCTCACTAATCACGGTAGTATTTTTTTTTATTATTCGTAAATTATATTTTGATTTAAATTACAATC
TTTGT
>103788083_2 DT667928.3
GCACGAGGAAAAACCCTTTAAAATACATGTAACATGTTCTCACTGAATTCTATAAACACACAAAACCAGATTC
TTATTAGTTTTAGCCAGTTATCTTCACGTTATTCGAGTTTATTTAGACAGCGAAAACATAACAATGTTAGGAA
TAAGAATGTGAAAGGTAAAGAGAGCTCAGTTTTTGTGTATGATGCAACAATCTGCCCG
GAGAGTCTCGAGGAAGCTGTACGTCATATAGATAAGAATGCTGTAGATGAGATGTGTAAAATCGATGTGCGGT
CCATGGCCTCCCTGGCCGGGTCCTCCGAAGCTGTTTTAATATATATACCGAATCTGAAATCAGTTAGAAAAAT
ATCACATATATCGTCAAATTCCTTTGAAAAGAACAAAAATGGATGGTCAGCACCTACA
GTCACTGTTGACCTTATAGGCAAACATACAAAGCTTAGTTTCACTGACTATTTTATTGGCTTACTC
>103788102_2 DT668014.3
GCACGAGGGTGAACCTTTTTTTAAGAATTAAGTAAATTTTTTATATATAAGTAAATAAATCTGATTTGAGTAC
TGACATGGATTTATTACAAGATTTTGACAACGCTGATACGATAAGTATTGATACGTTAGCCCTTGAAGAAGCC
TTGCTAGAAGAAAGAACGAAGGAAATATCGGAATTGAATAATCTTAGCTTAAAAGTTA
CCTACCTCCACACAAGTTTATTATACTATCGAAATGCTAAGACCAAAACAGGAACCGTCTGCTATGAAGCATG
GAAAACCCTGGTTAAAAAAGTTGGAGGTTCTCCAAATACATGGAAAGATCTTGGATATGCTTTGGAGATATCT
CATGATGACTTGAATTATATTACAAATTCAGTGAAAGAAGATCCAGCAGATATTGTGT
TAAAAGTTTTTATGCAAAATGAAACTGCCACATTGGATAAAATTGTAGATGCTTTTGTTGAAATGAAAAGATA
TGATATTCTTAAAGCTATTGAAGAACCCTTTTATAACTTAGCCCAGTGTTTTAACAAAGAGGACAGTGGTTAC
CACAGTGCAAGTAAAAGCAGTGGGACAAGAGAAATTGTAACTTTTACTAAGAATTTAA
AAATTGATCTACCACCAGCTTTAAATAAAAAGTTTATTAATAAAAATAGGGATAGAAACCCAAATCCTTCATT
AAGGCCACCAATAAAGACTGACAAAGAAAAACAAGGAAATGATCGTCCAATTCTATTCCTACCATACACAGAA
GATGGTATTGATACTGCCATTAATATTCAACAGTATGT
>103788105_2 DT667832.3
GCACGAGGAATATGTATGTCTAGCGGAAAAATCTTGCCCTGTTGATAAGAGAAGAAGAAATCGATGCCAATTT
TGCCGTTTTCAAAGTGTCTCGCAGTCGGCATGGTAAAAGAAGTAGTACGTACTGATTCATTGAAAGGAAGGCG
AGGTAGATACCTTCCAGCCAAATGTCCCAGATCTCCTCCAGTCCACCTATTTCTTATA
ACAGCCTAGTCAG
>103788120_2 DT668020.3

ATTAAACTTAGCTAGAATTGAAGAAGCTTAGTTATGTATTCTTTTTCAAGGGGGATGAGTTGAGACTAACTTT
TTAAATTGACTAGATCTTTCTCTAGGAGTTATCTAATCTATATAAACTCCAACGTCCAATACTAAAAAGTATT
AAAATTCTATTGTCCATTTATTTTCAAGTTTTATAGAATTAATTATTATAGGGCTACG
AATGCGACCATTAATAAGATTGTTTTCTACTTTTTATATGTTCGACATACAGACAGGCAGGCAATGTTTTTTA
ATAAAAAACATAAGCCACTTTATATTGGCGCTACTTATAATTATTGTTAATCTGTATTTCAAGAAGTTGAGGA
ATAAAAACTAAATTCTTATACAATATCGATTTTCATGCAGAAGAAATAAATACGATGT
ACTTATAACACCTACCACATATATTTTTTTTAATTCTGGACTTACATAAGCCTTATCAATGAATCCTAAGAAA
CGGCAAAA
>103788126_2 DT668524.3
TAAAGACATTGTCGAAATTTAATATTTGCTCGCTCCAAATAATGTCGATTCCTCCACTTGTATGCAGTACTCC
GCCACCACCCGAGCAATGTGAAGACGATATAGACCCAGAAGACTTTGATTTAACGTATAATCTTTCACAAGAG
AATGATGATGAGGAGAACACTGAATACAATTATGGAAATTATTCCTCATATAGTATTT
ATCCAGCAAATTTTTTTTAAACAGACACTGAAGAATGGCAGTCCCATATATGTAAAAATTTGGAAAATACAAA
AGAAGATCAAATTAAATCTGAAGATATTACAAATCCTGATAATTTTATATCACATAATATATTTGATAAAAAT
GTATCCAAATTAGAAGAAAATATTTTGGAAGACTTAAATTTGAAAGTTGAAGCAGAAG
TTGAACATTCCAATGATATTCATGTTAAGGAATATGAGACTGAATCTGATATTTATAACACTGAAGAACTATC
CGACAAAGATGCAGGAATACAAGATCAAGGTGGTACAGACATTAAAATTGATGAAGTAAATACAATGATAGAA
AAATACCACAACTGAAGTGAATCATATAATTAGCCCAGGCAATG
>103788132_2 DT667796.3
GCACGAGGATAGCCAATGCTGTGTGGTGGTACTACTTTTCGAAGCTTCTCGAGTTCTGCGATACATTCTTCTT
CATACTAAGGAAGAAAGAGGAACAATTAACATTTTTACACGTCTACCATCACTCGACGATGTTTGGGTTCTGG
TGGATCGGCATTAAATGGGTGCCAAGTGGATCGACGTTCCTTCCCGCAATGGTGAACA
GTGGCATCCACGTTCTCATGTACACATACTATGGACTTTCCGTATTCGGACCTAGAGTGAGCCAATATCTGTG
GTGGAAGAAATACTTGACTATCCTTCAACTGATACAGTTCACATGTGCCCTGATACTCGGCGTTAACGGCATC
AGAACTGGTTGCGAGTTCCCGCTGTGGATGCACTACGTACTCATCATATACATGATCT
CCTTTATTGTCCTCTTTGGTAATTTCTATATGAAGGCTTATCTTGCTAAGGGTAGCAAATGTATGGAAGTGAG
GTACGGCCAGTGTTTAGACGACGAAGAAACGATTCGTATCAGAAAACTAAAATCTTATTAAATTGTAATGATT
AATTATTATTTATTTCTTTAGAATAATTATTTTATTAAAACACATAATATATTAAGTC
TTAAACAATTATAACAATAAATATTAAAACAATAAATAATAAAATTGTTGTTGGGACATTTTGTTATATTATG
TAATACTACTTTCTTTGTTTACAAGGCTTTCTTTCAGCTTTCTTC
>103788138_2 DT668673.3
GCACGAGGTATTACTTTTAAATATCGAGGCTTATTTTTTATGGGAAAATGATAAATTAATAAAGATTTTTTTG
TGTGGATATGGAATATCTACAGATTGATTTCTAAATAATTAAGGATTTTGATTCCAACCCAATATTTTTTCTA
TGGGTGGCCATCGAGCCAAATCTTGATTTGTTATATCAAATAACACAATTGAAACGAA
CTTGTTAGAAAGAGCTAGTAATAGCTTAAATAAATGTGAAGCATATGAAATTAAACCTTATTGGGACAAGATG
AAATCAATATATTGTTTAAAAATATGATAAAAAGATATTTTTGTAAAGTTTTTTTATATATTAATAATAAACA
AATAATAAATATTTGTAAGTAATCGATGGGACTGGTATAACAAGTATTAATATTAAAA
CCAAAATGGCTGAAGTAACTTATAACGTCATATTACATTATTAACAATATTTTGCTCTAATTTTTTAAGACAT
TTAATACGATATTATTAATATATTTTCATTTTAATATTTTAAATATTAATATTTTTGGTTAAGTGTGACTCCT
TTATGTCTAAATTGAATACAAAATTCAGTCACTTTTAATATTATATCAGTGAAAGTAA
ATAGCATCAGTATGGGGAACGGAGTGCCTTAACACTATGTTCCCTCTGCCTATCCTGCTGCGTGTGAGATACT
GCGTGAAGTAAACAAAATCTAAATGTTATATTTAAATTAATTTTA
>103788147_2 DT667754.3
GCCGAGGGGGCCACGTCTTCAATATATTTAGTTTAAGACCGCCATTCTCAAGTCTTCCAGATGTTAGTTAGGT
TTACGCTCCAGCATCAGTCAGCATGCGAATCGCTCATCAATGAGTGCTATATGATCGGCGTACATTAGTACTC
TGGATGTGAACTGAGACAGTATCGAGCACTTCATTAAATAAGAACGGGGTAAGAGCCG
AGCCCTGGTGAACTCCTACTGTAATCTGGAAGGATCCTCCCCTGGAAACAGATGCCGCAATGATTGCCGACTG
AGCTTGATCCCAAGCATCCTGAGCAGTTTCGGTGTTAGTCGAAAGATTATGATTTCACCTCTGCCAAGAAATC
GGTCTGCATCGGTCCATTAAGCAATTATCACTTGATCCGGGGCCTACGTTTCTCGGCG
AATTTCTTCTTAGGAGTGACATTAGTCCATGACAAAAAGACGATGTTGAGCCGTCAGGTATCGCCTGTAATGA
CTTTACAGTACTCTACTGGTCAATAAGTAGTCAATTTCAGTCGAGTGGGACCCGTTCTTATACGTGGTAAGGT
GCTAAGGATTCTTTTGGAAGAACGTATTCACGATGGCCAGATCAGACGCAATACAGGT

TCTCGGATTATCTTCTCCCTCTGTATTGCAAAGACCGTGTCCAAAACCTCAGTGAACACGTTCATACGACTTC
GCAGCCTTACCGACGTGACCTTTCAAGTCCCCCAACTAAGACCACTTCGGTAGACGATACAGCAAGTAGTTA
GCACCCTTTTATACTGCTCCCCGAATGTCTCCTTTTCGGCCGTGCTGCATCCCGCCTG
CAGGGCATAGGCACTG
>103788156_2 DT668301.3
GCACGAGGATCGTGCATAAAATACTGCTGTTCCGTCGATTAGAAATATTCAAACAAGAAAATACGGAAGAAAA
ACATCAAGACGCAAAGAATATGTTTCAAGTGTAACAACTAAGTCTATTGTGAAAAGTTCAAGAAAATATGGCG
ATACCTTTTCAAAAACTACGGAGCCCTCATCAAATGGCATTATAGGTGAACCCGAAAA
AGTAAAAAACAAGTTTAGTTCTAAATACAGATCATCATATCTTGACAAACCATTTTACAAACCTACTGTACCT
ACAATCACACCATCAACTGTAGAGGGAGAAGAAATACAATTAGGGTCAGACATGAACGCTATATCTATCACTA
AATCACGAAGTCCATTAACTTCAGCTGACCTCAGACTTTCCGAGAGCCTGGCGAAAAC
ATTACAAGTTATGAATGTTGAGGCGTCCCATCATTCACCTTCAGTTACAGTATCAATATTTGATGCTTTGGCT
GAAATACTCACATCTACACCCAAACCTCGTATATCCTCAACAACTGAAGTATTACAATCTCAAAACAGTAATA
ATGACGTAAAACAAACACTTGCAGGCGTGAGTAACAATATTAATGTAAATAGTGTCAA
AGACTTAACAAGTCAAGACAACTTAATGCCAACCGATGTTGTAGCCACTGTTGCTAATTCTGTACAAACCACT
GGTCAGTCTACATCACATGTGTTTAACCGAATGCCGGTTGGGGGATATATTACACCATCACCAATTATTGAAG
CTGGAATAACTTCTACTGTGGATACACTTTCAACATTTCCTTTTCCTACTTCCCA
>103788162_2 DT667501.3
CACGAGGCTCAATCTTAAGGCTGCTGGAGTAAATTCACTATCAGAGCACGGCAAAGTAATAGCGGAAACAGAA
CAAACAAACGTACCCCACATCTACGCAGTGGGTGACGTCCTCGAAGGGAGGCCGGAGCTGACGCCAGTGGCCA
TACACGCCGGTAGACTGTTGGCAAGAAGGATGTTTGCAGGAGGTTCCCAGCACATGGA
CTACGAGAATGTGGCAACCACTGTGTTCACACCGCTGGAATACGGTTGCGTGGGATTGAGCGAGGAGGTTGCT
AGTGAAAGATTCGGTGCTGACAATCTGGAAGTGTACCATGCATTCTACAAACCAACAGAATTCTTCATTCCCC
AAAGAAGTATTCGTAATTGCTATTTAAAGGCTGTAGCCCTAAGGGAAGCCCCGCAACG
TATTCTAGGGCTCCACTTCGTAGGCCCAGTCGCTGGTGAAGTGATTCAAGGGTTCGCTGCCGCTATTAAATGC
GGTATAACAATGGAACAATTAATCAACACGGTGGGTATTCACCCAACCATAGCTGAGGAATTCACCCGTCTTA
ATATAACCAAGCGCTCCGGCAAAGACCCCAACCCTGCTTCGTGCTGCAGCTAAACTAT
AAAAATACCTTTCAAAATCCTAGCCTTTATATAATACGTCTGTCATTGTTACCACCAGTAAAATAGCTTAATA
AAATACGTCTGTTAGTGTTGCCACCATATAGATTAATTCATATTTGTTTATGTGAAATGGCATTGTCAATTTT
GCA
>103788165_2 DT667088.3
GCACGAGGCCAACGATTATCGTTCAAAATAAAATTGACCTCATGGATCAATGCGTGGTCGGGCCGGATGAAGC
AGAGCTAGTGGCGCGGGCTCTCGGCTGCCGGCTCATGCGGGCGTCGGTGAAGGAGGACGTGAATGTGGGCGCC
GTGTTCCGCGCGCTAGCAGCCCGGTGTCTGGCGGACATGCGTCGAGAACATTCCACTG
AGGCCCCACCGTGCCAAGCGCCGGTTATTGGCACATTTACAAATCACAATACAAACGGCACAATCCTGCTGCG
ACCAACCAAGCACCGGTCCGGCGGCAAAAAGAAGAACGTGCTGAGGAGTGCTTGTAGGATATAGTAAAATTAT
AAATAGATTATTAAAAAAATACTGTTAACTAAAATTTTTTAATCAATTTACAATTTGA
ATTTTAAAATATTGACCGAAAATTTAAATGATGTTATAAAAACTGCAGTGTCATTATAGTTTTTTTTTCGATA
TTGATTGGAATGTTGGTATTATGCAGTCAATAAAAGGTGCTTCGTTTCTAAGTGTACATACGTAGTTACGTAT
ATATATATATATATATAGATAGTTTTTATGTAAAATATATTTTATTTTGTACTAATTG
ATACTTAGAATTCTAAATTTGTTTGATACTAAATCATATTTGGACTTTAAAAAGAAGTGAAGTGAACATGAAG
TTTATAAATGATTACATGTTAAAAAAAAATAAGGAGGATGTAAATATTTTTTAAATA
>103788227_2 DT667775.3
GCACGAGGAGAGCACGTTCCGTTTTGATTATTTACTTAAGTTATACGTGATTGTGACAAAGTTATATCATTCC
ATAGTGTAAACTGTTAGTGTATATAAAATAAACAAAAATTGCTTGCATTAGGATTGTATAATAGACATTTAAA
TTTTCTGAAAAGAGTGCTTTTAAGATGCCGGCCCAAGCTCAAACTAAAGTTATTTTCC
GGGAAGTGACACCTGAAATATCACAGCATATGCTATCAGAAGTAACTACGCTGCCTGGCGTGCAGTTAGGTGA
CTTTCTACTGCAGTTTGAGTTAGAGGAACCTAGAGAATCTGTCAGAGAGATCGCTAGACGTGAATTACGTGAA
ACCCCAGAAATTGTGAAGCCAGCTGTGGAGGAACTAAGAAGACTTTTAGAAGCGGACA
AGGAATTATTAGTGCCATTAGATAGCGACGCTTGGATGATTAGATTCCTACGACCATGTAAATTCTATCCTGA
AAGTGCATACGATTTGATAAAGAGATATTATGCTTTTAAAGTCAAACATCACAAGCACTACGAAGGACTCACT
CCAAGCAAAGAACAGAATATCTTTAACCAGAACGTTCTTCGAGTTCTCCCTAATCGCG

ACCAGCATGGCAGAAGAGTGCTAGTTCTTGAATTAGGCAAAAAATGGAACCACCACAAGTGCTCTTTGGATGA
GGTGTTCAAAGGGTGCGTGTTGTTTTTGGAGGCGGCAATGCTTGAGCCCGAAACACAGATATGCGGCGCCGTC
GTCATATTCAACATGGATGGTCTATCCTTGCAACAAGTATG
>103788267_2 DT668017.3
GTTTTACTTAATGAAAATACATTGCTTTTTTAAAATAATGTAAGTCAATGTCTTTAAGATAGATTTCACTGTG
TACTGAAGATGCCAGTTAATTCTCTGTCGGAAATAAAACAAGGTGTCGAGAAAACTAAATTTTCACTTTCACT
TAATAGAAATAATGCAACTAATTTAAACAGAGAAAATAAAGTTGCGGACATAAAAGAG
AATAATACTCTGAAAGAAACTAACAACGATAAGGAGAATAAACTCGGAAAAAGACCCTTGAAACGCACATATT
ACTATACATTGAATGCAGCGAAAAAATTAAAACCTTTGACAGAAGTAGCTAAACCTAAAGAAAAGGTGTCAGA
GTCAAAGTTAATAGTTTTGGAACCTTCAGGAGGAATTTTGTCTCTAAGTAACACTGCC
ATGTTGAATGGCCATCACCCTGACAACCAGTATGGAGGGAGCACTCAATGGAAAACACCCATTGCTACTCTCT
CCAACCTTGGAAACACATGTTCTCTGAATAGTGTTCTATATACTTTGAGCGTATGCCCCACAATTTGTTCATA
ACCTCCATCACCTGGTGTCAGCTCTCAGTCGTGTAGAACAAAAATTGGGTAGCATC
>103788270_2 DT667373.3
GCACGAGGGCGGCCGTAAGAGCGTTCATCAGATATTACATGGAGAAACCCGAGTCTGATCGACCTGACAGATA
CTGGGAGCTGGTGGGCGTCCACGGCAAACTGGATCCTCTGGTGTCGGTGGACTTGCGCCCCGAGAAGCGCGTC
CAGATCACCTTCACTTGCGGTGATAACAGCGAAGTGCGCACCGTCGATGTTTACAGGA
CTGTGTCAGATTTGAAGTCCCGACTGGAACGTCTCGCAGGATTCCCTGCGTCCAAGATGCGTCTCTTCTACGT
GGATCAAGAATTGAGAGACACACAGTTCTTCCAGGGGCCCGAGGAGATGAAGTACCCCACGAAGCAGCTCTAC
TCATACAACATACGCTCCGGCGACGAGATCATCATAGATTCAAAGCTGAAACACTCCA
TTTCGGTCGGATCTACCGCGTAGATTGTTTTACGTGTAATATAAATATACATTATATGTGAAACTACCGTCAT
TTGTTTGGATTTTGTGTTTGTGTTTTTTTTTGTTTCTTTTTTTTAGTATTTTTTGGGGTTGGTGTTAGGGCAA
CTCTCTTATGTTCTTACCGCTGGTTAAAGAAACGTTGTAGAATGAAAATAATTTAAAA
TTATAAATTAAATAATTTGTGGGAGGACTTAATAAAATAGTATTTTTTTTAATTAGAGTTTACATCTTAAAAA
CTTTGCTATATGATTCTCATTAAAAATATACATGACCCTTATATTTCCGATATTTAAAATACTAACCTTTATA
TTTTCAACATTATTA
>103788273_2 DT667560.3
GCACGAGGCGTGACTTTAAATTTTCCTTTATAAACTAAAGGTTTCTTGTTGAAAAAGTTCTTTACCCGCCAGC
AAAATAAAAACTAAACGGGCCGGGCGTCGTGACATGCAAAGGAGAGCACTCTCGCATAATAAAGATAACGCGA
GAGCTGCTCACACAGGTCGAACAAGGGATGACAGCAGTAAGAAAGGCAATGCAAGTGC
AAACAGCGATTCTAGCAAACGAAGCAACATGGAGGGCCTAGCGCAGGGCGAAAATTTCGAGGGCAAGGAGGCC
TCGCAAGAGAAAGGCAATAATGTTGAGAATAGAGATGAGGGAGCCCAACTTAGTAGTGGAGTGGTATCATCAA
CACAAGATGAAGTGGGCACCAGTTCAAAAAGATCATATGTAGAGGATAATCAGGAGGC
AGAAGCCACAGAGGATGATACATCAAAACGAAAAAAGAGAAAAGACACTGAGGAAAAAGAACCCATGGCAAAA
CCTGCCCCTGTGGCAAGAGGAAGCACTGGCCGAATCCTAACTGGTAGCAAACCTCCGGGACCCGCAAGTAAGA
CTGGTGCAACACCATTGGGAAAAGGTTCTCAATCAGGAGGCAAGGGAGGCGGATCCTC
GGGGAAAGGTGGACAAGCGCAGGGAAAGGGTGGCGGTGGAGCAAAGGCTAATGCTCAAAATACGCATCAGGGG
AAGTCTGGAGCCGGTAAAAACTCATCAGGGTCACAATCTGGTAAGCAAGATCGAAAGAGCCCTGTAGCAAGCC
CTAAGCCTACCCAA
>103788285_2 DT667197.3
GCACGAGGATTTTCTGAAGCAGCAAATGCTTTTGAGAATTGTGAAAGGAGATATAGGGCAATATTTGCTCAGC
CTAAAGGATCTAAGAGAACAGAAACTGCTTTTGAAACAAATATAAATAATTTGGCCATATCTACAGGAAACCA
AAGAAATTCCCTTATGGGTATGATGAATGTTAACCCAAAAGGCTATACCAAAGTCTCG
TTTAGATGCAGTCCTTTTTTAACACAAATGCATGTAGAGTCATTATTTAACATAGTCCCTGGTTTGGTGCAAT
GTCGTTACTTTGTTGATTTAATGAGAAATTTTGGTAAAGGAACAGCACATTATTCCAACCCCGTTTCAGCTGC
TTACGCTGTGGAGAGATTAAATGAATTTGAATATCCACCTGGTTGCAAAATTTTTGTA
AGACCCACAAATAATCAGCAAGAAGACAAAGACATGCCTTCTATATCCTCCGCTGTTAGTAATTTAAGAAATG
CTATTTCAGCATCTGACTCTGCCTCTCCAGACTTAGCACAATTGACCCAAGCTATAGCAGAGGCTTCAAAACT
GATTAAAATGGCCACAGCAGGGGTTTCTGATGATAATATACCAAACAGTAATGACTTG
AATTATTGTAGTGTTAGTTTGCCTTCTCCTCAACCGCTAGCAGACATTGACAGTCCGGTTGCTAAGCGATGCT
TCTTAGTTTGCAAACCGCAACCTCCACCACTCACTGTATTAAAAGATATTTTCTGTCGTTTTGGTATCTTATT
AATGTATATACTTTGCCTAGCAAACTGTAGGATATGCTCGCTACTCCGTCAGTGAATC
AGCTGATGAAGCT
>103788288_2 DT667850.3

GCACGAGGATCAGACTTATAGAAAAAATGGCAAAAACTTACGATCATTTATTTAAATTACTTTTAATAGGAGA
TTCTGGAGTTGGAAAAACATCTATATTATTCAGATTCTCTGAAGATGCATTCAATATCTCATTTATATCAACT
ATTGGTATCGACTTTAAAATTCGGACAATAGATCTCGACGGCAAAAAAGTAAAATTAC
AAATATGGGACACGGCGGGGCAGGAAAGGTTCCGTACAATTACAACGGCATATTACCGGGGTTCTATGGGCAT
AATGCTTGTTTATGATGTTACAAATGAGAAAAGTTTTGAATATATAAAAAATTGGATTAGAAATATAGAAGAA
AATGCAAGTGCTGATGTGGAAAAGATGATTCTTGGCAACAAATGTGATTTGGATGAGA
AAAGACAAGTATCAAAAGAAAGAGGTGAACAATTAGCGATAGAATATCAGATAAAATTTGTAGAGACCTCAGC
GAAAGACTCATTAAATGTTGAATATGCATTCTACACATTAGCAAGAGACATTAAGGCGAAAACAGAGAAAAAA
CAGGAGGCGAGTAATCCCCCAGGTGGCCGCAGCGGCGCGCACCAACTGAAGGCGAACG
AGCAACAACGCAAGCCTGCCTCGTGGCTGTCGCGGTGCTCCCTGCTCTGACGCCCCGCGCCCGCGCCCGCCGC
GTAGGGCTCACGGGGACCGACGAGCTTTTGTATTTTTAT
>103788292_2 DT667904.2
GCACGAGGGTCACGCCCGCCTGCAGCACTACCGCGCCGACTGCGTCACGGCGCTCAGCAAGTCGTGCGCGGAG
CTGCGCGCGCAGCCGGTGCGCGACGCCGCGCTGTGGCGCTGGCGCGCGCCGCTCGTGGCCGCCGCCGACGCCG
CGCTCACGGCGCTCGGCTTCCTGCCGCCGCACCGGTGACCGGGGACCACCCCACACCC
CTCCCCCACAACATACTATACACGTATTTCATAGTTTAGTTTTTATTTCTGGAGTTCCTGCTACTCACTGAGG
GGTCACCGACGTCACGCTGTGCCTATGATTTTGGATGACGAAAATTTATCAATTTTAGTGTTTAAGGACGATG
TCGTGGATTACATTACATTTGATAAATATGACTCGTAGCCGCTGGAGCATTTATTTAG
GTATTAATTGTATCTATTGAAATAATATAATATTTCATGTAACTTGTGACATTCAATTGAAGATGCACAAGTA
AGTATTAGGAAATGTTTATTTATATTAAGTTGCGTCGACGGACACTTGTGCAGCTCAACGTTTAGAAATGGAA
ACGTACAAGTGGATAATGTAAATAATAATATTTGTCATAATGTAATGTGACAGGAGTA
GCGTGACGTGACCGCGCGACACGCCTACTGCCTGCTGCTTCAGACAGGGGTCGAACTGAATAAATTTGTAAAA
AAAAAAAAAAAAA
>103788301_2 DT668004.3
GCACGAGGCGCACCCTTTTGATGTATGGCTGGAACACGATGAGGCCGCTGATGCTAATATTGCTCACGGGTAC
AAGGCCGGCGAGGCGCTGCTGGGCACGGTGGAGCTGTCGCGGCTGACCAGCTGCGACGCCGTGCTGCCGCTGG
CCGCGCTGCAGCGCCGCCCGCTGCCCGACCACGTCGACCCCCACCACCTGGAGAGACA
CCTCACCGCTGCTGATTTCCTGGAGGCGTTCGGCATGACCAAAGAAGAGTTCGATGTTCTACCCGCTTGGAAG
CAAACCAACATGAAGAAAGATGTCGGCCTATTTTGACCCACCAGCGGAAGTTATATGGAAAGGAACGTCACCG
CCATTGCGGACACTTCGAGAACCATTTAGAACATTCGAGAACTATCTAAAACATTCCT
CCATAAACAAAATGGAGGTCCTTTTGTACAACAATTTCGTACTTTTTTACTGATTGAATTGAAATCACGCTTT
AAATATAACGATGGAACATTTTTATGTAATAACCGAATGTATTCGTTAACGTGATATTTTTCGTGTATGAAAA
TGTTGAGGATTTGATGTATGGAAATGTATGGAATTAATTTTTACTGCGTTGTATGTCG
CATCCATAGCATGAGGTTTTTTTATTTATTTATTTATTTTTGTTGTTCGTTTATTGTTTTTTTTTTATTTTAT
TTTAAATATACTTATTTATTTGTGTTTTTTTTCCTCTTAATTTTTTATTGTCACTAAACTTTCTGATTTGT
>103788307_2 DT668138.3
GCACGAGGGTTGAGCTTACAAAGAGGTAGAGGTGCCAGGGCCTTGCTATTAGCAGAGGCGCCGGTGTCGTTGT
TAGACAGTGCCATCTGCTGGTGGGTGTTTGTTTCCCTAGCTCATACGATGCGCACGTTACAATTGAGACGAAA
TGCTATAAAGCTTTCTTTATACCGCCATTTCACAAACACGCTGATCTTCGCAGTCATC
GCTTCCGTCATATTCATGTTGTACTCTCTGAAGTCGTACAGACTCTATTTTTGTATTACCGAATGGAAGCATG
TATGGGTGGACTGCGCGTACTGGCACGTCCTGTTCATAGCTGTGCTCGCCGCCATCATGGTGCTGTGGCGGCC
CACCAACAACAACCAGAGGTACGCCTTCACGCCGCTGCTGGACAACGCCGAGGATGAT
GATGATGAAGAAGAAGAACAGTTCGTAAACGACGCCTATGGAGTTAAGATGCGTGCGACAAACGTCTCCGAGT
CAGAGAGGTCCCCAGCAGGCTCCAGCGGGGCTCCCGATGCGGATCTGCGCTGGGTGGAAGAAAACATACCGGC
CAGCGCTCTTCCAGTTGTTGGACTCTGATGAGGAACTGCTCAATACTAAGTTTGAAGT
GTCCAAGATGCAATAGTCCTTCGCCCCCGTTTACTAAAAAAAGATTTGTTGTTCTTTTCTGTTTA
>103788349_2 DV501347.2
TGCAGGATGAACTCATCAAGTCGAAACAATGGCAAGACGCTATAATGTCTGATTCTCAGATCTCTCTGACGTT
AGAGGAAATCGTCCACATCCGGTCGGTGTTGACGAAAGCGGAACTAGAGGTCCTGCCCGTTGAGGGCATGGTC
AAGGAGGACGTTGAGAAGAGAAGGGTATGCTTCCTTTGTTTGAAAACTCGATTCAGCA
TTTTCGGTCCGTGGTGGCATAAGTGTATATTGTGCAAAAAGACTGTCTG
>103788358_2 DT668339.3

GCACGAGGCACAACCTTACATTCTAAAAAAATCGGGGATGATCTCCCTGGATTTGAGAATCCTGTAAACCAAG
ATGTATCAGAAAATAAAACATTATCTCAAAAGAAACAGAAAAAAGGATCAGGGGGTTTTCAATCAATGGGATT
AAGTTTTCCCATTCTGAAAGGAATTGCAAAAAGAGGTTATAAACAACCCACACCAATT
CAACGTAAGACAATTCCTATAGCGTTAGAAAATAAAGATGTTGTGGCCATGGCTCGTACAGGTTCAGGTAAAA
CTGCTTGTTTTGTACTACCCATTCTTGAAAAATTGGTAGCTCCTATGAATAAACCAGTACATGGAAAAAATTT
AAGGGCATTAATTCTATCACCAACGAGAGAGTTAGCTTTACAGACATTAAAATTTGTA
AGGGAATTGGGAAAATTCACTGGACTTAGTTCAGCTGCAATTCTGGGTGGTGAGTCAATAGAACAACAATTTG
GAATAATGTCAGGCTCCAGTCCAGATATAGTAGTTGCAACTCCAGGACGCTTTTTACATATATGCATTGAAAT
GAACCTCAAGTTGGATAACATAAAAATAGTTGTTTTTGATGAAGCTGATAGATTGTTT
GAGCTTGGTTTTGGGGAGCAACTTCAAGAAATAGTGGCAAGATTACCATCTAGTAGGCAAACCCTCTTATTTT
CT
>103788373_2 DT667820.3
GCACGAGGATCAAACCCTACTGACGCCATTCCCGACCCCCGCGCTGTAAACCAGGACAAGAAGAACATTCTCT
TCTCCGGCACCAACGTCGCCGCTGGCAAGGCCCGCGGTATCGTCATGGGTACCGGACTCAACACTGCCATTGG
TAAAATCCGTACTGAAATGTCTGAGACTGAGGAAATCAAGACTCCCCTGCAACAGAAA
CTCGATGAGTTCGGTGAGCAGCTGTCTAAGGTGATCTCCGTCATCTGTGTCGCCGTGTGGGCCATCAACATCG
GACACTTCAACGACCCCGCCCACGGTGGGAGCTGGATCAAGGGAGCCGTATACTACTTCAAGATCGCTGTCGC
TCTGGCCGTCGCTGCCATTCCCGAAGGTCTGCCCGCTGTCATTACCACTTGCCTCGCT
CTCGGCACCAGGAGGATGGCCAAGAAGAACGCTATCGTCAGGTCACTCCCGTCCGTAGAGACCCTCGGTTGCA
CATCTGTTATCTGCTCAGACAAGACCGGAACATTGACCACCAACCAAATGTCCGTGTCTCGTATGTTCATCTT
TGAGAAAATCGAAGGTGGTGACAGCAACTTCTTAGAATTTGAAATTACCGGCTCTACT
TACGAACCCATCGGCGACGTTTACTTGAAGGGACAGAAAATTAAGGCCGCCGAATTCGACGCCCTTCATGAAT
TGGGAACCATTTGCGTTATGTGCAACGACTCCGCTA
>103788389_2 DT667700.3
GNACGAGGTTTAACTTTAAGGACACTCTGCGTGTTTGTGTTCCATCATTTTTATATGTTATACAAAACAACTT
ACTTTATGTATCAGCATCAAATTTAGATGCTGCCACTTATCAGGTCACATATCAGTTAAAGTTATTAACAACA
GCATTTTTTGCTGTAATAGTTTTGAAAAGAAAATTAAAAAAATGGCAATGGGGAGCTT
TGGGGTTGTTAGTGGCTGGAATTGCACTAGTACAACTTTCTTCGACGGATAAAGGAAAATCATCACCAAACTT
AGCTAAACAATCAAAGATTCTTGGTTTTGGAGCTGCCTTTTTAGCATGTTTTATATCTGGTTTTGCTGGAATT
TACTTTGAAAAAGTCCTAAAAGAATCAGATATATCTGTGTGGATGAGAAATGTTCAAC
TGAGTTTATCATCTCTACCCTTTGGTTTGATAACACATGTTATAAATAGTGGCACTTTGAACGACTTACTGAA
GGGATTTGATGGGTTTGTTTGGTATTTAGTAGTGTTACAAGCAGCTGGGGGACTGATAGTTGCAGTTGTTGTT
AAATATGCTGATAATATTTTAAAGGGATTTGCAACATCTGTAGCCATTGTTATATCTT
GTATAGCTTCCATTTATATATTTGACTTTGTTTTAACTGTACAATTTGCTGTAGGTGCCTTATTTGTTATAGG
ATCAATATTTTTATATGGTTATGTACCAAAAAGAGAACTGAAACTCGGAT
>103788398_2 DT667391.2
GCACGAGGTTCCCCACTGAGGTTAACCAAACGTACAATTATTAATTTATAGATCTAAATTAAATTTAAGAAAA
TCAAAATGTTAGACAGTTTATTTGACGATGTTAAGCGCATGAACAAGAGACAGTTTATTTACCAAGTACTAAG
TTTTGGTATGATAGTATCGTCGGCTTTGATGATATGGAAAGGTTTAATGGTAGTGACA
GGAAGCGAAAGTCCTATTGTGGTAGTCCTCTCTGGTAGTATGGAGCCCGCCTTTCACAGAGGCGATCTACTTT
TTTTAACTAACTACCCCGACGAGCCCGTACGTGTCGGTGAAATTGTAGTCTTCAAGGTTGAAGGCCGAGATAT
TCCAATTGTCCACCGAGTTTTAAAGCTCCATGAAAAGAACAATGGAACTGTAAAGTTT
CTTACAAAAGGTGACAATAATAGTGTGGATGATCGAGGTCTATATGCACAAGGTCAACTTTGGCTCACTAAAA
AGGATGTAGTGGGACGCACTAGAGGATTCCTGCCATATGTTGGAATGGTCACAATTTATATGAATGAATATCC
TAAATTTAAGTTTGCAGTTCTAGGGTGTTTGGCAATCTATGTGCTGGTTCACAGAGAA
TGATGTGTAATGATCTGTTAAAATGAGGCATACTGATGTGGGCGATGGACTTGCAGATGTATTTCTAATTAAA
AAGAAGTTCTCTCTACACCCAAATATAATTATTATTTTTTTTTTTTCACTGTCACAA
>103788407_2 DT668584.3
GCACGAGGCCACACCTTAAAAGGTCTGTTCGATTTTTTATTATTTATTAAATTAAATATCCTTTTATCATTTT
ATAATATATTATTTTAACCAAAAGTGTATAGAATATTAAATAATATGTCTGTACCAGAATATTTTGAGTTAGA
AAATGGAGATCGCATGCCACGAATTGGCTTTGGAACCTGGCAGGCACCTCCAGATATT
TTGGAAAAATCTGTGGATGCAGCTTTAGCGGCAGGATATAGACATTTTGACTCGGCTCAAGCTTATGAAAATG
AAGCTGCCTTAGGCCGCGCACTAAACCGCTGGATCGGTGTTTTTCCAAACAAAAGAAAAGAATTATTTGTAGT
CACTAAGCTGGCACCTGGAGGTAATAAGCCTGAGTTAGTAGAACAATACTTTAATGCC

TCTTTTCGTGAGTTGGGTCTAGAATATATTGATCTTTACCTCATTCACGTGCCGTTTGGTTTTGAGCACGTTC
CAGGGGAACTACATCCAAAAAATCCAGATGGTACCATGAAAATTGAATATAATACTGACATCGAAGCTGTATG
GAAAGCTGTCCTTAAATTAAAGGAATCTGGCCGAGTTCGTCACGTAGGAGTTTCTAAT
ATGAACGAGGAGCAGATCAAGCGTCTAAACGCTATAGCGAAACCAGCGTGCCTGCAAGTTGAAATGCATGTTC
TCCTGCAACAGGGCCCCCTTGTAGCAGCTGCGAACAAACTGG
>103788410_2 DT668921.3
GCACGAGGGGTCGTCTTTCTGCAGGGCTCGTTGTAGTTCGAACGAAAGCCCTTAATAGTGCCTTTTCAAATGT
AGTCCCTTTCGTGTGATCCATATTAAAAGCGTTTTGAACAACGAGTGGGTAATCGATGCACAAATCAGTAGCG
GTCTAGATCAATCGACCGTCCGCCACCGTCATCAGCAGTTTCATCCCGATGGGTTGAG
AACGCCGACATTCAGTGTTTGTGGGACACACCACTGACGCTGGTTGAAAGTAGCATAAATAGAGTCAAAATCA
GGTGTAAGCATCCAGCATTAGAGTAAACTCCAATGCGGACTCCGAGGCCAACAGTCGGAGCGTCAAAAGAATC
GTGAACACCGAACGAGAAAACATCTTTGATGGTCGCTTTGCTTTTGGAAAAGATCATC
TATACGCACTGTCACTTCATTATTATTAAATTTCTCAATATATTTTTCATTTTATAATTTCTATTTTCTATAT
CCTTTATAATATAAAGTAAAATGAGCAAAACTTTAAAAGTAGTAGGAGCCTCATGGGTCCAAACAAAAATAAG
TTCTTACATCTTGGGGTTCTTAGGAATCTTGGCCATTTTAGCGCTCTTTGTGGGACAA
ATTGAAAGAATCCAGGAAGACGGAAACTATGCTGCAACCGTGTTCTATTCAGAAAAAACCGGATATCGTATTG
AGTACTGGGGTCAGAGCAACGATCTAAAGGATATCGCGAAAGGAGTGGCTCGTGCACACTTCAGAGCAGATAT
CGATACTACAGGGTGGTCATTGCTTGAAATTG
>103788421_2 DT667822.3
GCACGAGGGATGACAGAGATCTGAGGAAACGGAGGACCAGTCCAGAGGGCAGGCGGAGTATTAGGTTAACAGA
TAGAGTACTAGCGGCCGTACCGCCGATGAGTAAGGAAGAACGACGTCGTGCTTTCGCGGAGGCGGCCGCAGCT
GCGGATGAGAGTAGACGCCAAAGGGACAGTGCGCAGTGGACGCACGCCCATCCACACT
CGCACCCGCACGCGCCCCCGCACCCCCATTACGGTCATTGGCCTCAGGATTTCACTCAGATGTTCCCACAAGG
CTTGATGCCTGGTCCACAAAACATGATGAATGGCCCCCAAAA
>103788445_2 DV501284.2
GCACGAGGATTCCCCTTTCATCAGTATCGACGTGCTCTGCATATTACTTAAGTTGAACTAAACAAGGATACAC
CGGAAACCTCATACCGCTGAAAGGTTCCTGCCTGCTGCTCCTCCAATCTACATGTTATCCTTTGACAAGAAGG
CACAAATGGTGCTGGGCCTACTTTCTCCTACTCCTCCCTTGCCGCCATAATGATTATC
TTACAAAAGTGTATCAATATCATAAGATAGCAGAGAATCTTTTGAGAAAGTATAGAGCATCAATTAATCGAAT
TTTAACATAACACCTTACTACGGCGATTTTCTAAGAGTCTTTACAATTTTTCCAAAATACATTAAAAGAATGA
TAATATGACATTATCACAATTGTACATGCTACTTAACATTACCATGGAATTGTATTTT
AGTATATTTCTATAGCTTTAATATTGTTAAAAATGTGATGTTTTCGGAGAAATCTTACTCTAATATTTTAGTT
ATGTAAACTGTTCGCGTGAGTTGACAGACTTGTAATAATGACTTAGAAATTATCTTCCAATAATACACGCTGG
TTGAAGAATTTTATATAATATCAATATAAGCATGTTTGGATTA
>103788448_2 DV501304.2
CTCCAGTTTTAGGTGAAATATATTTTCCAGGATAATATTTAAAAATGGAATGTTTCTATGGAAGTCCTAGCGA
TCACAATAACAAAAAAAAATGGTTACGGGCTCTACAGTTACCTACTATTCGCCCTATCGGGTTTAATTATTAT
ATCTATGGACTGTACATCATATAGTACCACTATCATAATCCCCACAAGTGCTTG
>103788457_2 DT668857.3
TTGATAATGAATTATATTATATATACGTAAGTTATTTTGAAATGTTGAATATGAGGCGGTTACTGCCTCTTCA
TTTTCGCATACATAGATATATATATATGTATGTATATATGTGTAAATGATGAGTCCTTATCCCGCTTGAAGAA
TTTTTTTTACTGTAATATAGGTATCAGGTATAACAATTCGCCAACGTGGATTTCATAA
CTTATTATATAAAAAAATATTTTTTATAATTCAAAGAAATATAGGTTCTATTTTTTGCACCTTATTTACCTAA
TAACTGCCAAACTTTATATTCCCTAATATTACATACCGGTGTTGGATGTATGTGCACAGGAACATTCAGGCTA
GATAACGTAACGAAGATCCGCCTGCTTTCGGCTCCCCCACTATAAGTAGATTATAAT
>103788460_2 DT667474.3
GCACGAGGTAATCATTTCAAAATTTTTGCGGCCTGTGAAAGTTTGGTTCAAATAATTAGTTTTCCAAATATTG
TATATCATCACATTTTTACTAGTGCTTCAACAAAACTATAAATATAATATTTATTATTTACAAATGTATTAAA
ATAGTATATCAAGATAACACAGATAAACATGTTGCGCCAACACTTTTGGAAGATATTT
GTGTTGGTTTTCCTATGTCTTCATTTATCAACGCAAGTAAAAAAACAGAAGTCGAATATTATATCGGATATCG
ATGACATCAAAGAATTTAAAAAATTGTTAAGAACAAAAACTAATGTCTTGGTGTTATATATAAATAACCAAAA
ATCATCACAATTTGTTCTTGACGTAGTTAAAGAAGCTGCTGATAGTATGAAAGGTCAA

GCTACGTTAGTAGCCATAGATTGTAATGGAAGTGAAGGTAAAAAACTGTGTAAAAAATTAAAAGTATCATTTG
ACAAACCTTATATTATAAAACACTACAAGGATGGTGACTATCATAAGGACTATGACAGGGGCACCACTATAAG
TGCAATGGTTAACTTCCTACGAGACCCCACAGGTGATTTACCATGGGAAGAAGATCCA
AGTGCAACAGATATTGTCCACCTTGCTGATGCTGATGCTTTATCAAAATTTCTCAAGAAAGGCATAGCTACGT
ATAAAAAATCCATAATAATGTTCTACGCCCCATGGTGTGGATATGGCAAGTCCCTGAAACCAGACTATGTAGC
TGCTGCTGCTGATTTGAAAGGTGAAGCATTCTTGGCGGCCAT
>103788478_2 DT668753.3
GCACGAGGGTCGGTCCTTTTGAGGTCTAGGATCAATGCTTCTGTGCTCAATGAGATTTCCCAAGATAGAACGG
TAAAACTCTCCACATTATCTGCGGCACGTGTATCCCGGTTACCAATGGTGGGTCGTCCCAAGGACAACACCTG
GCACGTAAAGGAAGCAGTCACGCTGGTCACGCCGCAGCTTGAGGCGCTCGAAAGCGGT
ACTATACCGTTGTGGGTGATCATACTTGCAGCGGTCGTGGGCGCTTTGTTGCTCCTTCTGTTGATATTTGGAT
TGTATAAGTGTGGATTCTTCAAACGCAACCGTCCATCCGACCACTTCCGAGCGACAACCCCTCAACGGACGCG
ACGAACATCTTTGATATCCGACCACCGACCTCGGCCTCGTCAGTGACGTCACCTCTGA
CGTCGACACCTCTGACGCTGATGCCGCCTCTGACGTCAACGCCACGTCTGACGTCAATGCCACGTCTGATGTC
GATACCTCTTCTAGTTCCACTGACGCCGCCAGTGACGTCACTAGTCAAAACTCGACTAGCTTAGACAGGTATT
GATGAGACAAAAATATATTTTAAATATCGATTTTTGTAGTGATTTATAGTCGAATCTC
GTTTTCAATTCCATTTTATATATTCTTTATCTGTCATAACAATTTATAGAGCGGCTATATTATCTGTTTTTAA
AATACTCTGCCCATTT
>103788481_2 DV501326.2
TTTTCAATATTTTATTAAAAATAGTTATCAAATTTTACATATTTGAACTACAATAAACAATTTCCTTAGGGAA
AAATTATAGAAATATCCAGCTAGTAAAATAAATTTTTGGGGTTAGTAGTAAATTATATTTATTTTTTTAATTT
AGTTTGAATTTGAGAATTCCAAACCGTTATTATTAGCTACAGATTTACATCCACTTCT
TATCTACCAATCTTCTTTTGATCTATGGAGTAGGCAAAATTTATCTACACTGGCCACTTTCCACT
>103788487_2 DT667980.3
GCACGAGGCCAGAACGTACCTGTCAAAATTTCTTTTGAATGAAAGTTTAGTCGGAATCCGCAAAAATGAATTT
GGTCGTACATTTAATTTAGCCTGGAGTAATGTATGTTAATATAAGGCTCTACGAAGATTTTATAACTTGTTCT
CCAATAAGTAGTACTTACGCTGACAACAGACACCATGGTGCAGTGTCATCCGCCGTTG
GAAATAGAGGAATTACCAAAAGAAGACGAAGAAAGATTAGAAAAATTGTTTTCTAAGTTAGACACGGATGGAA
ATGGCAAGATTGACATTCACGATCTATCCGTAGCTCTCAAGGAGTTGGCCCCGCACATGAATCGCAGCTATGC
AGAGAAATTTCTAGCTAAAAGTGGAAAGAATGCTGGAGATGTTACATTGTCAGAGTTC
ATTCACTATGTCCGGGAGCATGAAAAAAATCTTAGGTTGCAGTTTTCCCATCTTGATAAAAATAAAGATGGAA
AAGTTGACCTGGAAGAACTTATTTCAGCCTTTGCCGACTTAGGAATTGCTATTGGTAGAAATGAAGCTACGAA
TTGATTGAAAAGGATGGATCAGGACGGCACCTTAAACATAAGCTACGACGAATGGCGG
GACTACCTCCTACTTGCACCAGCGACTGACATACATGCTTTAATTCATTCCTGGCGTCACTCTACGTATCTGG
ACATTGGTGAGGATATGAACGTGCCAGATGACCTCACTCCA
>103788529_2 DT668564.3
GTAACTAGAGATAGAGAAGAAAAACTAAACTGCTATCTCTAGCGGCGCGTGTTTTATTATATTTTAATATAAA
ATAATAATAAAATTTTGCTCAATTTTAAAGGGAATTCTAAATATTACTATTTAATAAAAATATATGGTAGATA
ATAACTTTTTATTTTACTACTTTAGGTATAGATTTTATAGTAAATATTCGTAAGTTCA
AGTAACAATAAAAACCATTTGTGCAAGTTGTAATAATTCCCATTTCTCGTAAGAATAGTAAAATTTTCCAAAC
GTAACAATGGATTTTTATAAACCAGGCCCATCTCGTATGGACAAAGAAGAAATGGAAGTGGAAAATGATTTGA
CCAATAAGTTCTTGAGCGGCCAAATGTCTTTCGCCGAATACTCCAGTGAGTGGTACGA
AGATGCTGAGGATGAAGAAGATTCCGAAGAAATGAGACAAAACGACGAGGGAACCCCACAAACTACTGTCATT
ATTGGGAAAGGTATTAAAAGGAGACGTAGACGTACTCGTCTGGCACCCGCTCTAGTTGGATTAATGGGTGAAG
CAAACATTAGATTTGCCGAAGGTGATACCGAAATGGCTGAACGCATGTGCCACGAAAT
TATTAAACAGTTGCCAACTGCTGCAGAA
>103788535_2 DT668298.3
AGGACGCCTTCCTGCGCGAGTTCATGCTGGCGCACCAGCAATGGGTAGAAACATCAGAGGAGTTTACCCAACG
ACTTCCAGTATTGCCGGTGCCTTTACTATGTATTGAGGATACGGCGGTACTTTGCGTGGGCCCCGCGCCGTTA
TCGTCCCCGGGCAGGACGCCCGCAACCTCCCTTTCCCTACCACCGCCTGATACAACTG
AAGACGATCCGTATTGGCACAAAATGGAAGAGATACTATTGCAAATAGCCCAAGGCAGCGTACCAATGATTTT
TAAACCGACAACCGACTTTTTTTCTCGGAAGACAAACGCCAATCCAATCGTCGCACGAGGAGAACCACTACAA
ATTTCCATCACATTGTTCAACCCGTTGAAAGTTAAACTACTTTTGAATGATGTAGAGC
TATTATGGCAATTTACCC

>103788551_2 DT667669.3
GCACGAGGAGTTACATCAAATGGTGAAGGTGAGAAAATCAATCAAATATTTTTATATTTTTAACATGTTATTT
CACATAGAAAAATATTTTTAAAAAATGTGTAGCGGGTTTAGGAACCGCGCCTCTTGGTAACCGTCCAAGCGTC
CTGACCACTATGCTATACGTTCTTATTCTTGTTTTAATGCCAATTACCTTAAGTCCAA
ATATTTTCACGATTTCTTTATAATTATATATTTTTTGTATATCTATACTAATATTATAAAGAGGTAAGATTTG
TATTTTTGTATGTATGTTTGTATTGTTTTCACACAAATATTTCCAGACCGATTTCAAAAATTCTTTCACTATT
AGAAAGCTGCATCTTCACTGTCCCACATAGGCTATATTACAATTTAAAAAAAATAGGG
ATCCCTAATAAAAATGCAACAATGTAACCCAAGGTGTGAAGAAATTGATATTAAAAAATTTATCATCGCGTGC
ACTGCGAAAACTATTGATGATATAACAAAATAATGTTCTACAGTATTATAGAACGCATTAATATCTATAAAAA
AGTCCGCGATACAATATGTTCAACTACTATAGTTATAACAATATCACTACTTTTTTAT
ATTTTATAAGTTGATGAGAGTGCCGGCAAAAATCAAACCATTTTATTCATACCTCTGCATTAATCCTTATTCT
ATTAAA
>103788570_2 DT668802.3
GCACGAGGGAGTCGCTCTATTATTCTGTGCCTCATGTTACTTCACCGTCTCGCTTTCCGACAGGCGGAATTTT
TTATTTTTGTTATAGATGCTTTCATAAAATTTTCCAATTATATTGTATAAATCTATTGTTATTTTTTCTAAAT
TCAATTTTAAAAGTTGAAATAAGCATATTAGAATTTCGAAGTATTTCGCTAAGGACTT
ATGGCTCAATTAGCGACGAGCGTTTGGACGGTTTCCCAGAGGCGCGTGTTCAAATACCGCTACGTCCAAATTT
TAATAGGTTTTAATTATAAATTGAAACAATTTCCTTACAGTTACGTTAAAATATTCTTTCTGTATTATCTGTT
GCCCGCATGTCGGGGAGCATGTCGGGGAGCACGGCGCGCGTGCGCTAGCGTGTAAATA
CAACATTAACATAATAAAATTAATGTTTTTATACAAAGTACGTACGTATCGTAGTGGTTTCTCTATAACGCAC
TGTTCCCATCGTGTTCTCACACGGTCACCGGCGCCCGGCGAGTGCTCGCCTCAGATTATCTAATATTGCTACC
TTCACTCGGGTTAAGTAGTTGTAATTTTTTATAGATACGATCGAGCGGACGAGTTAAC
TATAGTTATTGTGAATGTTGATCTGTATAAAGTGATAATTAAACTAAATAATATAAACTTAAATGATTGAAAA
TTTAAGCATTGGA
>103788576_2 DT668783.3
CGGCACGAGGATGAACGTCCTATATCTTCATTGATAGGAGAAAGAGAAACTGAAGCCAAGAGGTGGACTGAGG
AGAGAGGTACATTATTGAGGGAGCGAGATGAAGCTGCATCTCATCTGGCTTCCATGGAGCATGCTTTTAATGA
TGTTCATACAAAGTATGAGAGATGCAAAGTGATCATTCAAGGATACAAAAGCAATGAA
GAGACTTTAAAAAGAGCCGTTGAAGATAATAATGATGCAATTCAAAAATTAGAGGCACGATATGAAACTTTAC
GGCAACATGCCATGCAACAACTGAACAAAGCAAATGCAGAATTAGATTCTGTAAAGAAATCACACCAAGCTGA
AATTTTGAAATTAAATGCTATGCTTAAAAAGAGTGAAGTGCATGCATCTTCTTTGCAG
GAGTCTTTAGCACAGAAAATTAAAGATAATGAGGAGCTTACTGCCATCTGTGATGAGCTAATAAATAAAGTGG
GATAATTATTGGAACACCGCATCCATTGACTAACCCATTAACATTTTCATTGAACTAACAAACATTATTAGTA
TTGTAACAACAACATTAGAGCATCTACTAACCCTTTACAATCAATCTTAAGTATGATT
ATAACATTTACTTAAAGCAAGTAACTACTGTTACCATTTCATAAAAATTACATGGTATTTAATGTATAATTAT
TCTTAGTATGTGCAATTCAAATGTCATTTTTTTTATTTTTGTACAATTATTAAAAATATTATATAGAATTTAA
GTTTTACCTAATGACATTATTATTTTTTTATTCTTTGTGAATTGTGTCATATACCATG
AATTTCAAATA
>103788585_2 DV501280.2
GCACGAGGCAAGCCATAACGGATTACGAATGAATTAATCGAATAATTTATACAATTATAAATTTAATATTTTA
ATCAAAGACACCAATTCGAGTGGAAATATCTCGAGATATTTCTATAATCGAATGATTCTTTTAATAACATTCG
TAATACGATTCTAGTTCATATCAAGATTGTATGGAAAGGTGTTTCGGAAATTAGAAAA
TTTGTAATCGATTATCATTTTCGATTCGACATTAATAAAATATTGTAGAAGAATCGAATAGGAACAAAATATA
TGTAAACAATCGAGTTGTTACGTGACCGGAAATGATAATTTGAATAGATAATGATGATTTTGTTGAAATAGAA
TGATTATAATGATTATTCTCGTTGAAAAAGATGATTAATATAATCATTTTGTTAAAAA
TCGGTCTGTTGAGGTCTGGTAGGGATGGGTTAGGGGAAGTCCACGCGAAGGCTGATTTTTTTTTAATCTTCTG
AGTAACGTCGTATCAACGAATGCCACTAGACTGAAATAAAGAATTTGTGGCCATTTTGTAAACTACTGTAGTA
CGGCGTTGTATACTAAAGTGATAGAACTAGTTTTTTTTTTTTTGGATTCATAATGGAC
TTATCGATTTTTTGATTTTAAAGGTTTAGTGGATGTTATTTATTAAAAACAGTTTTTACCTTTTTATTGGAAT
TTAATTGATTGACTGACATTTTGGCTTTTCATGGCCAATGTTATATATA
>103788610_2 DT667692.3
GCACGAGGCACAATTCTATATTTGATTATGATGCGTATGATGCCCAAGTGTTTCAATTACCGCCGGCACATAG
TACTTGTGTGCAAAGAGTCTCTCGTACGAAGAAAACCTCAATGGCCGGCTTAGAGAGTAGAAGAAGTCCACCC
GCTTCCGCCATACATCATAGAAAAATGTCAGATCCTGACATCCATCATAGCCTGGGCT

CCGATATATTAACAAATTGTACTAAGTACAATGGTAACGAAGCCTCGAGTGATGCCACAGATTCTCCCACGTC
CATCAACCGGCTGTCCCCGCGTAGTTCAGTGTCGAACAAGCCCGTGAAAAGAACCGGGAGGGCTCTTATCAAC
CCCTTCGACCCTTCACATGTTACAGTTAAACTAACTAGTAATAGACGACGTTGGACAC
ATATATTTCCTAAAGGTCCAACGGGAGTGTTAATTCAACAACATCACTACCAGGCGCGTCCCGCCGGAGAGCC
CACTTCGAGAACAGACCGCGAAAACAATTTGAATAAGGAGAATGGCTCTATAAATAACAGTCATTCTATATAT
CAAGTGGATGGTAACATAATGAGCCGCAAGCGCATGATAAGCGCGTCGGGCGCGGCGC
CCGCCACCAACAACGCGTCGCTGGCGCTGCTGTGGGGCGCCACCGGCGAGCAGGAGTGGACGCCCGCGCTCAC
CACCGG
>103788613_2 DT667006.3
GCACGAGGGTATTCCGTTACCTTGAAATATAAACCTCACGTGCATACTTGAATATATATTTTTTAATAATATA
ATAATTTTAGCTCACTATGTTACTCTAGTGATTTTATGTGATATTTTTTTATTATTTATTATCTATATATAAT
ATAATAATAATGGTGGCACACAAACAGTGGTTGTTAATAATTGGAATTTCTGCATTGC
AAGTGTCTGTGCTCCGATCTCAAGAACAGAACTCAGTGAAAGATGAAAGAGAAAAAAAACCTCAGTCTGTGCG
AGATCTTTTTAAAACTGGATCGTGTATTCCACCGCCGTTTCTATGTCCACACCCAAATATGCAATATTTTTTA
TACACAAGATCGACACAAGAGAAAGGAGAGTTGATAAATACACTTGATAATAACTCCC
TTACATTCTCCCGGTTCAATCCTAAGCATCCCACTAAAATAGTGGTCCATGGATTTGGGGGAGGAAGGAACCT
ATCTCCAAGTACTGACATGAGGGAAGCCTACTTTAAAAGGGGAAACTATAATATTATCATCGTGGATTATGGA
ACACTTGTGAAGGAACCTTGCCTTAGTCAGATAGAATGGGCTCCTCGCTTTGCAAGCA
TGTGCATAACGCAACTAGTGGAATACATCCAGTACCACCCAATAAAACCAGTCCCACCAGAGAAAATACACAC
TATTGGATACAGCGTCGGCGCTCATATTCTTGGATTAGTGGCTAATCATTTAGCTGAAGGGAAACTAGGAAGA
ATAACAGGTCTTGATCCAACAATATTTTTCTATATGGGGAACAATAGATCTCG
>103788616_2 DT668505.3
GCACGAGGCGGCACGCGTTGAGTTTTGATCTTTGCTTAAAAAATTGTAATTTCAAAATTTATTTACTAAGTCT
TTTTAGAAGTAAATAACAATGTCTGCTATATCCAAAACTAAAAGTTTAAAAATATTTAGCGTGTTAACCAAAA
AATATTATCCACGAATAATTTCCAGAAGAAATCTAAACCTTCAAGAATATCATAGTAA
GGATTTACTAAAGAAACATGAAGTATCTATTCAAAATTTCCGACTTATTGACTCTTCCTTAGATCCAAAGTCC
TTATCAGATTTTAAAGCTGATGAATATGTTGTAAAAGCCCAAATTTTGGCTGGTGGACGGGGAAAAGGTCACT
TCGACAATGGATTCAGAGGTGGTGTTCATCTAACCAAAAATGCTAATGAAATACTTAA
CTTAGCCAAAAATATGATTGGACACAAATTGATTACTAAACAAACCCCAAAAGAGGGTATCCTTGTTGACAAA
GTAATGGTAGCTGAGAGTGTAAATATTGTCCGTGAAACATATCTGAGCATTGTTATGGAAAGAACCTACAATG
GTGCAGCATTGGTAGCCTCACCAGCTGGTGGCATGGACATTGAAGCTGTTGCAGAAAA
AACACCACATTTACTTAAAACA
>103788631_2 DT668698.3
GCACGAGGTCACCCTTCAATATCAATTTTTCGCCCCTTTGTTGTTTCCACTGGCGCGAATTTCTACTTCGCGA
GTGAGGTAAAGCACGTACAATGAGTTCCAGTGAATTTAAACTCGAGAGAAATGTGGAGTTATCGGAGGAGACA
AAAGCGATTGCTGAGAAAGAACTGAGGGAGACTCCGGAGAGAGTGCGCGAAGCACTTG
AGAGGCTTAGAGAACTCCTTAAGGAAGCCAAAGACATACACTTTGGCGATGATGACGAGTTGCTCACATTATT
CTTGCGTCCTTGCAAGTGGTATCCGGAGAGCGCCTTGGCTCTAATGCGTCGGATCGCAGAGTTTAAACGCGAT
AATGCGAGCCTCTTGGATGGTTTACTTCCAGATCAAGAAAAAGATGCTTTTCTTGACC
ATAAAGTTGTGAACGTCATGAAAGGACGCGATGACAAGGGAAGGAGAGTGCTCATTGTAAATGTTGGAGGGAC
ATGGGATCCTAAAAAGGTGACAGCAGATCAGCTGTTCAGGCTGTTTTACTTAATCCACGAGGCAGCGATGTTA
GAACCTGAATCTCAAGTGCGAGGTACCGTCGTTGTTATGGACTTCCACAACATGGGTT
GGAGTCAGACAATGGGTCTTACACCGGCATTCTCTAAGCGTCTGCTGACTTTCATCCAAGATGCTATGCCTCT
TAGATTAAAGGAAGTACACTTTGTNAAAGAACCAATGATCTTTAATGTTGTGTGGAAAATGTTCAAGCCTTTG
ATCAGAGAAAAACTAAAGAACAGAATATTCTTCCACGGATCAAAAATGAATTCTCTCC
ATAAGCACATAGCCGCT
>103788634_2 DT667693.3
GCACGAGGCAAAACATTAAAGGTCAATATTTAGCTTAATAGTACAAAAATTGTAAAAATTACTGATATTGGCC
GAAATATTCATAACAACTTATTTTACTTTATAAAAGAAATTGTCAAGGCCTCTAACGCGACAGTTATGGGTTG
GATTCCCACTCGAGAAAAGGGATGGCTTTTAATTTCTCGCTCTAGCAAGGAGACAGCG
GCGTTGAGTTCCGCCACTCAATTAAAATGTATAGAAAATTTTGTGGTGTATAGGGAACTGGAAAACTAAGTTC
CCACTGCCTACCCTGCAATATGAGGGATTCAAAAAAGGAATTATGAGTGTTTATAACTACAGTTTGTAACTAT
TTTGAATTAGTTAGTGTAAAAGCGTTTCATTGACAATTAAAATATAATAAGGTTTTGG
TGTTACTTCATATCAGTCACAAATTATTGGCCAGTTTGAAAGTTTGTAAGGAAATTTGTGTTTTTTTA

>103788666_2 DV501341.2
TACTGAGATTTTTTATTGGTTAAAGAGTCTTGATTGATACAAGTTTGAATTATGAAAAGTTGTTGATGATTTG
CGGAAAATTGTTATAACTCTACAAGCCATAGTTGAGTCTCTTGAATCAGCTAATTCTGGGAATAAAGCTGAAG
CCCCTAAGGAACCAGTGCCTCAAGCTCAAGACGATTCAGATGATGGAGTTGATTTATT
CGGATCAGATGATGAAGAAGAAAGTGCAGAGGCAGCTGATATAAGAGAAGAGCGCCTTGCTGCATATAACGCC
AAAAAATCCAAAAAGCCAGCACTCATTGCATAATCCAATATTATATTGGATGTGAAACCTTGGGACGAT
>103788675_2 DT668278.3
GCACGAGGATTTATCATTAAAATAAATTTAAATAAGGAATTTATTTGTGGGNATGGANTTCCCTGCTACACCA
TCCAATGCATCTGATACCAATGATTATGCTGGAGCAACGCCTATGCAATTTGATGCAACGCAAGACAGTAATA
CAAAGAGGCGAAGTTCATCAAGATCTATAAAAAGAAGGCGCTTTGATGATGAACTAGT
AGAGTACAGTTTGGGTTTGCCTGGAGCATCCTTGGGTAAAAGTGAAAAACGAATAAGAACGCAATCCTATACG
AGTCACCTACCAGAATTTCCTATTGTTGCTGCTCCACCAACTACACCTGTTACACCTGCACCAGAAAAACGTA
GAGCTTCCAGCAAAATATCTGGTGGTGGGAGTATAGGGCGAAAACCAAGGCGTAAGGG
GCAACTTAGTCAATTATCAACAAAGGATTTGGGAAGATGGAAACCAACAGATGACTTAGCTCTTATATTGGGA
GTGCAACAGACCAATGACTTACGCATTGTTCATAGAGGAGTGAAGTTTTCTTGCCGATTCACTGTTGGAGAAC
TGCAGTCTCGATGGTATGCTTTGTTATACAATGCTGAGGTGTCAAGAGTAGCTATAGC
TGCAATGCGCAACTTGCATCCGGATTTAGTAGCTGCTGTACAACAACAAGCATTATATTCTACTTCTGAAGAA
GATTTGCTTGGTACATTACCTAGTAATTCACTACCGGCTTCGGAAAAATTTCAAGAGTTATTGGAACAGAACC
CCCATGTGTTTTATGCAACAAGAACTGCTAAATCTC
>103788684_2 DT668885.3
GCACGAGGGTTTGCATCACGTTAACATACAAAATACGCAATTTTTCGTATTTGAAGTGATTTCAAGTGAAATT
TCTCCACGATGGCGTTAACAAATGTATTACTGTTGAGTCAGATAGCTGGCTATGTGATCGGCCTCATTCTATC
GCTTTGTATAATTGTGCCTATGAGTATGCATCAAGACGAATTTAGTGGTCACTGTCTC
CTCTTCTCTAAAGGCACTTGGCAGGAAGAGGATGGTCAGCTGCTAGTGATGTGGGCTTCAAGAGCCTATTGCA
ATTATGTTATTGTAGTAGGCATACTTATGTTTATTGTGTGCTGTGTCCAAATATACAGGCTTTCAATGTTCAT
GTACCGTGGCACAGACAGTTCATTCCTCTCAGCATTTATTGAGGCAGTGGGCTGTGCT
ATACTGTGCGCGCTGGCTGTGTCTGCGGCTGCCATTGTCACTCTGGGCTTTATGACCTGGTGTCAGAATATTG
TGGAGAGGTTTCCCAGCTGTGAAGATGCTACAGGTCAGGATATCGACAAGAAGGACAAGATATCCACTCATGG
CTTCTACATAGAACTGGGGAGTGCTCAATTTGGAGCATGGGGTGCGTTTGCTACTTGG
GTAGGTTTGGCAGTATTTTCTACCTTGAAAGTTTGCCGCTACCACCAGCTGCAAAATATACAAGTGTCGATGT
ACAGGGAGAGACAGAGGCTTGTCAATGAAGGGGGATCTACACCACCATTATTACATTAAATTTA
>103788690_2 DT668129.3
GCACGAGGGCACCTCATTCACTTTAAAATAATAAAAAAAACATTCGCGCCAAAACATTCTTTTGTGTAACTAT
TTTTTGTGTTATTCCATAGTGAAAATGACGTTATACAGTAAAATTGTTCAAATTATTCTAGTTAGTGTGTAAA
ATGTTGTAAGATTATTATTAAATTTTACAAATATTAAAGAATAATGACAGAAGCAGTG
GAAGCAGGTACAGGTCTCGCTTGGCGGCTGTTAGCGACGCTGGAAGGAGGGTCTCTATTAGTTGCCACTTCAG
CTCTCATAGCATATACGGCTAGACTGAAACTTTCTAGGAAAAGGCGGTATAGGATTATAAACAGCGTGCTGGT
TTCAAATACAACAAGTCCGTTAGGTCGCGAGTTAAAATCTAAGTTAGAATCACATGAG
TTTATTGTACGCGTACCAAGCGAGAACACATCTTGTGTGGACAAAGTTGATGCACTTGTGGTTATTGGTGCGG
AAGTGGAATCTGGGCTGGATGGATTATCTCATCAAGTGACACGGGATGTATATGGAAATTTAAAGTTATTAGA
ATCATTGTCACACTCGGTGAAGCGGGGAGGTTGTATCGCGTGGGCGTGCGCGGGCGGG
GCGGGGGACGCCGGTGCCTTTACAGCAGCCTCCAACGCCTGCGACGCTGTAATGAGAGCTAGTTTACAATATA
TTGCTAAGATTTGTCATTGCGATGGTATATGGATCGATCGA
>103788693_2 DT666986.3
GCACGAGGCTAACCCTTTAATAGGTTTCAATAAACATTAAAAATGATTACATTTTTAATCTACAATGGATACG
TTTACAAAGTCAACAGATAAGAAATTTACACCAGTTTAAAATCTCTCAGGAATATTATATTTATAAAATTTTA
AACAACAGACTAATATTAATAGAATTAATTATATTTTTTAAGTAGCGTCATTATCACA
TAAAATTTTAAGTCAAATATTGTAGAATAAGACACACCAATGGACGTTTTAATTTATCTTAAATATATTTTTT
ATAAATATCTCTAATAAATAATAATTAAAATTTATTATGCATGATATGATATTTAGCCAATATGTCCATTATA
AAATGTATAATTTTTACAAAAACAGATTAAATAAATTTTAGTAGAAGGCACTTTTAAA
ATTAAGGCAATAGAATAGATTTATTGTAATCTGAGCAAACAGTAAATTGAGCAAAGATTATTTGAAAATGGCT
ACCAAATGAATAATTTAAACACATTTTACGATTTTTTTTGGCAAGGCTTCTAAATAGTCTTTTTATGAGATCT
TGTGATGTACAATGATATGACCAGACACGTGTTGTAATTCAATCGAGCTAGGAAATAT

AATAAATATAACCCTATAACAAAATGATTGTAACCTACAATAAGAACTAAAATAACAAAATATACATTAATAT
TTTTTTATAAATCACATTTACTTATTAATAGCTATTGCCATATGAATAATCACTAACATTAAAAATATTATAT
TGTAAATAACGTTAATTAAAATATAGATTATTAAATGAAG
>103788705_2 DT668957.3
GTACGCGCCCGTCAGGGACGCACCCCCTGCCCCCGATGATGAAATGCGCAATCTAATGGATACTAAAGAATGC
TTAAGCAGTGCAGAGGAGAGCGACAAAATGCTGGATGGTCGACCGTCGGTCGCGTAAATACAATATGTTTAAT
GTATTTTCTTGCGAACAGACAAAACCAATAATGTCCACCTATATATATATATATATGT
ATGTAATCTTTTAACCTTCTAACGGTACATTAGTACTTAAATATCTGTGTAAGTAAAGAATTTATTTTTATGA
CAATCGACTTTAGAATGGATATCGAGTAACCTGTTTCTTTTTGTAAGTGATAATGGTCTGTAGTAAATGTTGC
CCGAAGAAAAAAATATTGCTGGTATATTTTATCTATTCACGAATTGATTTCGAGGTAC
AAAAATTTTTAAATATATTTTGG
>103788711_2 DT667773.3
GCCGAGGCCGACCCCCTAGTCCCGCGACCGCCCGAGGCCCGAGGCCCGAGACCCGAGTGTAAAGTGTAGACCT
AGACTTGTGTGAAATAAAATGTTGTATTGTGGAGTTGTGGTGCGTGCGACGATGCGACCGCCGCTGTTCGCCG
TCGCTGCTCGCTAAGTCGCTAATCGGCTCGTATCCCTCATCAGTTCGCAGTTGCATTC
GCTGTGTTTAGTGTATTGTCTATCGTGTTGTGATCATTTAATTGTAATCGCTAAGTTTTTATGACTCCATTCA
AATCTGTTTCACTTCAATGAAAATTAATATACCTTTTGGAAACGATTAAGTTAAAACAACGTGTGACAAAATG
GTACATTTGCAACCATTAAATGTAAATACGGCAGTCCAGTGTATAGTTCCCCGATCTT
TACAATATTAGGAGCCATTTACACATGGCGAGAGGTCACGTAGGCATTAGTGATTTTTGAAACGTGAATATTA
AAATTGCTTACGATTTTCTTCTGTTTTTGTTATTAATGTAGTGATCGTAAACATGGATAGCAAAATTATCCAA
AAAAGTGCAATGCAGAAGAGTGCAGAATTTACTTTCACTCCGCCACCCGAGGCACCCG
TATTTGAACCTACGCTTGAAGAATTTACAGATCCTTTAGGATATATTGCCAAAATACGTCCAGTCGCAGAAGA
CACTGGAATATGTAAAATTAAGCCCCCTGCACGTTGGCAGCCACC
>103788729_2 DT668098.3
AATGTGATAGCTGCCGCATAAAATAAAATCACGTAATAATTTTGTGTCACAATACGAACATTTTAACTCTATA
ATAGTTATAATATTAAAAGGTGTAAAATAATTTAAGAAGTGACTTCTCAACACTAACAGGTCATCATGTGCGG
AATTTGGGCGATATTTGGAATGGATCCGAACTGCCATTCAATGTATTTCAAATGTGTA
TTTTGTATCGCCCATCGAGGACCAGATGCATGGCGCATTGAACAAGATAGTAGAGAACCGCTTGCCATATTAG
GCTTCCAAAGATTATCTATTGTTTACGGGTTGTACGGCATGCAACCAATGAGGTTGCATCAATTTCCTCGAAT
AACTCTTATATGTAATGGAGAAATTTATAACTGCAAAAGGCTTCGAGACCAGTATGAT
TTTCCATATGAAACTAACTGCGACGTGGAAGCTATTATTCACTGCTATAAGAAATTTGGGATAGCAGAAACTG
TTAAATTGCTCGATGGTGTATTTGCGTTTTGCTTGGTTGATGGAGAATCACGTAAAGTTTATATCGCCAGGGA
TCCTTACGGTGTAAGACCCTTATATAAATTACATGACGAAGAAAACAATACTATGGGA
GTTTGTTCAGAAGCCAAAGGCTTAATAGAAATTAAACAAAAAATGTAATGAAATCTCAACATTAAGGGCATTT
CCTCCT
>103788735_2 DT667418.3
ATACCGAGATCCAAGGACGTGCATACGTGTGTACGAACAAAAAAAGAAAAATTAAAACTCCTATTATTGAAGA
GAGTGATGTCTCACCGTCTTTAGAAAGTGAAAAGCATGTAAGAATAAGTAAAACTAATGAGAGAGAGGAAAGA
GTAAAGCATTATGGTGATATAACCAAGAGTACTGTCACCACTTCAAAAAATGAAACAG
TAAGATCCACGCAAATCAAACCAGTAGACGGTGACAGGAAACCAAAATTGCGACGAGAAGTATTAATGGAAAT
TTCAGACTCATTTGACGATAAAGTAGATTCAGATATACTTAAAAAATCTAGATTGGAACAAGATACAGTTTCT
A
>103788741_2 DT667430.3
GCACGAGGAAATTCTTTATATTATTATCCGAAGTAGTTGATGTGAGTGAACAAAGTAATATTTAATATTGATG
CCCGAGGTGTGTGAAATTTGGCTATATTTAAGTATTGAGCACTAACTACCTATAATACGTCACAGGTTTCGTA
ATTCTAAAATTTGGCCCCAATATCAATATTCTATAAACTGACTGAATTTTTATAAAAA
AATTTAGTTCACAATTCAGATAACTTTTGATTGAAAATAAAAATAACACTACGTGACTATCTAAAATTATGAA
TGTTTATTACCAATATTTTAAATATTGTAAAGGTATATAGTACTTTACATATTTACTAAAAACAGAGTCGATA
AACGCCTGTGATTAGAAATGTAATTATTTGTTGTATCGTAATTGCTTTCGCTAGTATC
GCTTAGAATTTCGACACTTAGTAGAAATGATTGAGTTTGTTGCCGGCCGGCGTCGTATAGTTTAGGTTCAATA
TTTTTTATTAATTAGGTGTTTGTGAATTCATTAAGCGTGGATGTCCTTTACATTCAGTAGTTAGTAAATTTTA
GAATGCTAGTTAGTATAAATTCACGTTTGCAAACATTCAACGGATCGCCATCGCGATC

CTTGAATTCGGCCTTCTATTCGAACATAAATGTTTGATATAAAATTCTTATAGAGATTCCTTGTAAACGATAA
TAGTGTACTTACCGATACCGGATATTAGTTTTCGTTACCGATTCGTTAATAATGCTTTTTGAGTTTTTATTAA
CTACACATTAGTAAAAGTTTAATAAAATAAGGATGTAGCGTATTAACAATATAAAACA
AATTGAAACCTTTC
>103788779_2 DV501291.2
GCACGAGGCCTCCCCTCGAATCGTTGCGAAGGTTTTGTTAGGACAGATCCGTGTAATTGATCTGCTGGGATCG
ACTACTGCTGTCTCCGAGGGACCCTGACGATGACTTCTATGGATTGCTGGGATGTGATGGTGGATCCACTGGG
TCTCTTGTACTTGCTAAATGTATACTGTTTCCTTTGCTTGGACGTCCTGATCCTAACG
CATGGACATTGGGATGCTGAGGAGTATATTTGCTAAGATGAAGGATCTTATGGACACTTTGGATGCTGCATCT
ACCACGGAGGTCTCTCCCATGTGAACACAAACTGCACTTTCCTTGGGATACTCCCAGTGGCTGGGCCTGTTGG
AACACCTTCGCCTTTCTCTGCATTGATCTACGTCCCTTACTCCGGCCCGGATGTTG
>103788803_2 DT667916.3
GCACGAGGCCTCGTGCCCCTGTTCAAGATTGAAATGTATTAATCTGTTGGCAAATGCAGCTTAATAAAAGTAT
TATGGGTGGTATGCGGGCGAAGCCGCGCGTGGGCCGCTAGTATAATATTGTTAGCATAAGTTACTTATAGTGT
AGTAATAGGTGTCACTGTAATTATGAATACGTCATTTGATATTGTTTCCATTATCGAT
TGTATTAAGTGGCGGTAGTGTGCTGCGTCTTTTCCGGTGGATTTCCAATATAAGCACAAACGACACGGCACTC
GTGTTCATTACGTTCTAAGATATTGTCAAGACAACATGTCATTACAATTCATATTTCAATATTGTGACTACGA
GAGTATATGTGTATATAACAAGTGTAAAAATATTTATTGTAAAAGCTGGATTTTGTGT
CCGATTTGTTGGTTCGTCCGACAGTATAGTTGTTAAAATTTCGTCCTTAATTACCTTAATTTAACTTTTTCTA
GCTTTCAAGTCCTTGCTCCTAGATAAAGCAAGTGGCGACTGCCATACCGAGAAATACACAATTTTTCATTACA
AAAATAAAATTATTATCTACAGTAGCGCAAATAAAGATAAAATTGTAAAACTAACTTT
AAACCAAAGATAAATAAGCTTTGAAGTTTAAGAGTACTTGCTCAAGCTGTAAAAAAACTTGTATATTTATAAT
TAAAAAATCATTTGTACGTTTGTGGAACTATTTTAAATGATAT
>103788821_2 DT668011.3
GCACGAGGCAAAAATATTAAAAATATGTCAAATATTCAAATTATAACACAGGATTTTATAAATGTGCATATAA
CGAAATCGGATAGCGAAAATGCAGCGCCTGTGGAGCGACGATTCAAAAAAGGAATCACGATACAGGAATTTAA
GACAAAATTAGAACTTGTAACTGGCGGCTCGGCTTCCACAATGAAATTAAAAGTGTAT
GACATCAAAAACAACTTTGTATGCGATATAGATAAAGATGATGCTCTACTTGGATCATATCCTATTGACGATG
GAATGACGATACATGTAATTGATAATTTTGCTATGATCAAAGATTTTGCAGATGCAGATAGTGCTGAAAGATT
TCGTCTCTCTGAAGAAGAATATGAGAAGAAAGGAGACACTCTACGCTCTTTTCTCCAA
CGCAACAAACTTGGCAAGTACAATGAAGAAGAGATGAAGAAACTCAAGGAACAACAACAAAAAGAGCTTGAGG
AGGAAGCTAAGCTAGCAGAAGCGGTGTTAGTCGGCGCCCGCTGCGAGGCCCGCATGCCCGGACAGGCGGCGCG
GCACGCCTCGGTGCGCTCCAACGGCGCGCTCGAGGGCGCGCGGCCACTGTGGATCGGA
GTACAATACGATGAGCCCAGGGGGAAGAATGACCGAGAAGTAAACGGCAAACGGTATTTCACATGTCCACCCA
AGTACGGAGGTT
>103788830_2 DT668687.3
GCACGAGGCCATTGCTGCCGACGCCAGTCGTCACTGACAATGAGATAGGGGTCCTTCCTGCCGAATTCCTTCC
TGATCCTATGACATCCACGACTACAACCACAACCACAACGACTGCATCACCAAAGAATACAAGTGAAATTGAA
AATAAAAGAAGAAAAAAAGAAGTTAAATTCATTCTCCGCGGCAACAGGATTGTTCAAG
TGTGACAATTGACTTGACTGACGGTGAGGTATGTTGACGAGACCGACTGACTAACTTCATTTATATATTAATA
TTTTAGATCGACAAAATTTTGACTAACTAATTTTGACTAGAACTAACTTGACTGAACTATAACTTGACTATAA
GCATAAGCAAATTATTATAAATGACACCTCTGATAAAATAATAATATCAAATTTTTGT
TGTGAATTTTCTAATTTTATTTTCTTTTCCATTTTCAGATACTCCTCTATACGGTGCAGTACTCAACGCTGAC
AACCTGCAATACTATCACGTGTAAATAAGTTACTTGTCCACCTTAATATTTAAGGTTTTAGTTAATTTAATTA
CACAATTTACACATTTAATTATACTTTTTTAAATGAAACAATTGATAAATTAAATCTT
AACCAAAAATATTGTAAGAAATAGCTTCGTGTTAATCANAAGTTGATTTTATTTGAAAAAATATTATAAGTAA
CTATACTATTACTTATAGCCATTAGATTTAAGATTTATTTATAACTATTGGTACTCGCTACGGAGTGTTTTAC
ACTTAGCTCCTTCTACCTATAGGTTCCAGTATATTACACTGTGAAATGCGTGAAGCTG
TTATCTTTA
>103788864_2 DT667522.3
GCACGAGGGCAACGTTGAGCTTGCACAGCTCATCCAGCAGAAGCTTGATGCTTACAAGGCAGACGAACCGACA
ATGGGTGAGGGTCCAGAAAAGGCACGTTCCCAGCTGCTGGTCCTGGACCGCGGGTTCGATTGCGTATCGCCGT
TGCTCCACGAGCTCACTCTACAGGCCATGGCCTATGACCTGCTGCCTATTGAAAATGA

CGTTTATAGATATGACGCACAACAAGGCAACATGAAAGAAGTACTGCTTGATGAAAACGACGAGCTTTGGCTG
GAGCTACGTCACCAGCACATTGCCGTAGTTTCCACTTCAGTCACTAAGAACCTGAAGAAGTTCACTGACTCTA
AGCGTATGGGTGGTGGTGACAAGCAATCCATGCGAGATCTATCACAAATGATCAAGAA
AATGCCACAGTATCAAAAGGAACTGTCCAAGTATGCCACCCATCTGCGCCTCGCCGAAGACTGCATGAAGGCG
TACCAGGGATATGTCGACAAGCTGTGCAAAGTTGAACAGGACCTTGCAATGGGCACTGATGCTGAAGGTGAAA
AAATAAAAGATCACATGAGGAGTATTGTACCGGTGCTGCTGGATCAAGCAGTAAAGAA
CTATGACAAGATGCGCATCATCGCACTATACATCATGTCCAAGAATGGCATCTCGGAGGAGAACCTTAACAAG
CTGGTGACACACGCTCAGCTGGGACCCTCCGATAAGCAAGCGCTGCT
>103788896_2 DT668592.3
ATAGACGGTGAGACGTGTCTTTTAGATATCTTGGACACGGCAGGCCAAGAGGAGTATTCGGCTATGAGAGACC
AGTACATGCGGACTGGTGAAGGATTCTTGCTTGTTTTCGCTGTGAACAGTGCTAAGAGTTTCGAGGACATTGG
ATCCTACCGGGAGCAGATTAAACGAGTTAAGGATGCAGAGGAAGTTCCCATGGTGTTA
GTGGGTAACAAGTGTGATTTTTTTTCATGGGCTGTCGATATGACGCAGGCGCGAGAGGTAGCGCGAAGTTACG
GTGTACCGTTCGTGGAGACGTCAGCCAAAACACGAATGGGAGTGGACGATGCATTCTACACACTAGTCCGTGA
GATACGCAAGGACAAGGAAAGTCGCGGGAAGAAGTACAGGAGGGGGAACAAATTAGGC
TCCCGGCGTACGTTTAAGTGTACACTTATTTAAACTATAAGATTTTTGTCATTATAATATCATTTTCTAATGA
CCCTGACGATTTCGGGAATGTCCCGATAGCTCTTTTATCTAATTTTGTTTTTATCTATGCGTGTGGTGTTGTT
TAAATGCATGGGCGGTTTTTAGTTTGTTTGGGTTTTTTTTTAAAAAAGTGC
>103788910_2 DT668192.3
GNACGAGGACCACCCCTGAAGAGTTACCAGCGCTCGTCTCCAATATCGAATTTACGCAAATATTTTTTCTTTT
ACACCAAAAAGAAATAGTTTTATCGTTTCGTTTTTTTTACTATTGTGGACAAGTGTTGTGATATCGTGCTTAG
TGCGTTTTGTGAAATAACTTCTCTTGAACCCAGAAGTGCGAGGAATTGACCGCCACAA
TGCTGCAGCAGATATTGAGAGACATGTGGGTCGACCCGGAGATCCTCGCCGAGCTCGACGAGACACAGAAACA
AACGCTCTTCTGCAAGATGCGTGAAGAACAGGTGCGTCGGTTTTTTGCTTGGGATCAAAAAGTCAGCAAAGAA
GAAAATGGCCGTACAAAGTTTCAAACAAATGACAAGAAGCAGGTGCAGTTTCTTGAAG
GAGAAGATGGAGAACCATGGGTGTGGGTCATGGGAGAGCATCCAGACGACCAGACCATTGAGAACATCCTCGC
TGAAGAAGCTCGTCAACGCGCCTTAGCTCAGGCCCGTGAGGAAGCCCATCAACTTCGCAAATCAGTTGAGAAG
GAATTGACACCTTTGATTGATTACAAACCTCTTGATAGCTTAGAACAAAAGTTCGATT
TATCACCAAAAAATGTGGATTCGTTGGAAGATACCTTTG
>103788953_2 DT667658.3
GCACGAGGGTTCCCTTTAAAAGAAAAACTAATACAATAAAAATATAAAAAAAAATATATTTAAACGTTCATT
TATAAAATAATAAATTATATTATGATTGTATAAATTGTCGGCGTTTTTGATTATACTCTTATAAGTGAAAGGT
TTATAAGGAAAAGCTTTTAAAAGATGCCTACCTCAGTGGTCTTCGGAAATAACGAGAA
ATGGCGAATAAACAGGAACATTCTGGTGCTGGGATTTGCTTTTATGGTTCAATTTACCGCTTTCCACGGCGCC
GCTAATTTACAGAGCTCTGTCAATACTGACGCATCAGCTTTGATCTTCACATTGGCAGCTATATACTTTTCGC
TGATTTTGTCGAATATATTTCTACCTGCTATCGTTATAAAATGGCTAGGCTGTAAATG
GACGGTGGCAGTCTCTTTCATCGCATACATGCCATTCATCGCGGCTCAGTTCTATCCGTACCTCCATACATTG
GTTCCTGCTGGGATGATGGTGGGGTTTGGAGGAGGGCCACTCTGGTGCGCCAAATGCACTTACTTATCTGTGG
TTG
>103788964_2 DT667414.3
GCACGAGGAAATGCTTTAAAAACCTGGAAAAGGACCTGTTTTCGACCCCGGCGTGTGCAGATGCTGTGGAGCT
TTAAAGAAATGTCGCTTATTAAATATAGAATACGAATGGCAACGGCAGAAAGAAATTTACTCTGATATGTTTG
TCGATTGTTTTGGTTTATTGTTATCACACCTAGATGGTGAGGAAATAGAACGTCTTAT
TTGTGCAACATGTGTATCGAGACTTAGAGAAGCTACTGCTTTTCGTCAACAAGTGTTACAGTGTGAAGAAAAA
CTTTTGAGAACAAAAATACATGAAGAAGAAGACTTCAACACAAATATGAAAATGGAGTCCACAGTTAAAGATG
AACTGGAATCAATACATAATGGTTTTCAAGATGATGATATTACATTGGATTACATGGA
CAATGAAGATATAGATGACTTAAAGGACGAGGCGGTTGTTAAACCCAAAAGAAAAACATTACGGTCAAAAATT
AAAACCAAAAAATGTAAAAGACAAAAAAAAGATGTATTAACAAAGGAAATGTTAAATGATGCTCAAAGATTGA
AGAAAAAATTGAAAGAAATGCAAGATATTGAGCTTCCAGAACAATCTATACAATATAA
ACCCAATGGGTACAAAAACAATGAAAATCTTAAATCCTACCACAACATTACAACTTTAGTAGAAAACTCCTAT
GCATGTCCCTTCGATACTTCATTTAGTGATTTCTTCTGCGTTTACTGTAGAAAAGTATTTACAGATCCAAACA
AACTTCGGGGAACATTCCATGACACACGATCCTAGAAATTTCAAAGATGTCCTATCAA
ACACTT
>103788966_2 DT667546.3

GCACGAGGGCAATCCCTACAAACTAGTTCAACGCGTTCGATTTACAAATTTTTGTTTTTTTCATTGTGTTTGG
CGCCAGCCAGCTGTGTATAAACAAGGAAAATGTGTGAAAAGAGGATTAGAAAAATAAAAAAAGGCTCTCAAGT
AACTCGACTTAGGGAGGCAAGGCGCTTGCAAAGAATGCATTGGCTGTCTACACCAGCA
CCTGTGGAGGCCACCACGCCACCACCACCACCGCCTACCACCGTGGACAAGACCGCTCAAAACAGAGTTATAC
TTGAATTAGCATTAAAAACAATAGCACTTATGCAAAAGAACAGGCTAATTCAGCAAAAAATCATTGCTCTACA
ACGAGAAACGTCGGAATTCGTAGCTGCGGTTATGAGTAATCCAGAGAACAGGCGGCGA
TATTTAGAACATGTCCGGCTTTATACCGCTGGACTTATCACCCTTGTTCCTTCAGAAGAAGAAAGAAATAAAC
CACTAAAATTAGAACCTAAATCATAAATATATTTCTTAACTGTGTACGAGAGAAAGATTGAGAGAATTTTATT
CCTTCGTGATTTATACTATTGCATAAGTATGATTATTGTGATTTTTTTAGTTTTATTT
AAGTACTTATTGATGATGGAAAACATCGGCAAGTCTTTTATTTT
>103788969_2 DT668513.3
GCACGAGGCGTGATTATTCAACGCTTGTTTTGTTTGCTGTTTTAATATATTCTGTATAATGTATTACATTATA
TACAGATTTTGAACATTCTGTGTATTGCAAGATGTATCTTGGAATGTACAGTATTCAAGTTGGTCGTAATTCG
TATATTCGATTACAGTTAAGTGTTAAGTAAAATCGATATTTAATCGTCTCTTGTACTT
GTGAGTTTTAATCGATGTCTCGTTTTTATTATTATTATTTATATAGCAAAAAAGATTACGGTGCATTATCACG
TGGCTTACTGTATGTATCGAGTTTGAATGGGATTATTAATTATATATATACTGATGAATTATTTTTATATTAG
ATCCACTTGTCCTTTGTCTTAACTAGATTTTTAATATATATAAAGATCTAAGCTTATT
TAATTTTCCTATTTTTATTAATATATATGCTTGGGTAATCATATTATGCTATATCAATTTCTTCTTAATATCT
TAACTTCTACTAATACTTTATTCCACCTAAGAAAATTGAATCTTATAAAAGCAAATACGGCGATACATTCTTT
TTAAATAAAGTCCAATATTCAATAAACGTTTTTTTTTTATTAAGTGAATATTTTATTT
TCTACTGCTACATATCTTATAATATCTCAGTTACAGCAGAATAATATATGTTCGTGAGTTACGTATTATTTAG
AANACATGTTTTATTTAATTTTCCCAAATATCGATTTATATGGAGATTGCTATTTTTGCTTCTATATGATTCT
ATTATCTCTTATATTATAAAATAATTATTTTCTCTAT
>103788975_2 DT668611.3
GCACGAGGTAGCCCCCCAGTTGGGAGATGCTCGCGTCGACAAGCACGAGGAGGCGCGGAGACGCAAGAAACAG
GAGATAGTGGAGAGTTTCAAGAGAGAGATACCCGGAGTGTACGACCGTATGATCAACATGTGTCAACCAACAC
ACAAACGGTACAATGTGGCCATCACCAAAGTCCTCGGCAAGTACATGGAGGCCATCGT
AGTGGATACGGAGAAGACCGCCAGGAGATGTATCCAGGTGCTGAAGGAGAGAATGCTGGAGCCCGAGACGTTC
CTGCCGTTGGACTATATACAGGCTAAACCATTGAGGGAGAGGTTAAGAGATATAAAAGAACCCAAAAACGTGA
AGCTCCTGTTCGACGTGCTCCGCTTCGAGCCGCCCGCGATCCACCGCGCGGTGCTGTT
CGTCACCAACAACGCGCTGGTCTGCGAGACGCCCGAGGACGCGTCCCGGGTCGCCTACGACCTCGACCGCACC
AAGAATAGTCGATACGATGCGCTGGCACTAGATGGTACATTCTACCAGAAATCAGGTATCATCTCCGGAGGGT
CTCTGGACTTGGCGCGTAAAGCTAAACGTTGGGACGAAAAGCATCTCTCACAGTTGAA
AGCTAAAAAGGAAAATTAACAGAAGAGCTTCGGGAGTCCATGAAAAAGTCCCGCAAGGAATCTGAACTAACGA
CCGTCGACTCGCAGATACGAGGCCTCGAGTCCCGCCTCAAGTACGCGCTCACCGACCGTGACTCCACGCTAAA
ANCAATAAAAGCTCACGATGCTGAGATCGCTGAATTGGAACGCAAAATGGAAATGTTT
GGCCCACAATTGAAGAGATCGAGCATACTATCCGCGCCCGC
>103788982_2 DT667513.3
GGGCTGCAGGAATTCGGCACGAGGATGGACTATTAGTACGGTAATAAGTTTATATCGCTCGAGGCGGCGCGGC
CCCTCGCCGGTGGGTGAGAGCAGGACGACATGCACGGTGGAGTGTTGCGAGATACGAGATACGAGATATGAGA
TACCAATTATACAAGATTGACAGCCGGATGCCCGTTGCCCTTATCAGT
>103789003_2 DT668731.3
GCACGAGGGTATTTTCCTTCAATTTTATTTAACTAACAAAAATTTCATGTAATGTCTTTGTTTGATAACGCGG
TATTTAATTCGACTCTTTATGAGGCACTGTATCTTCAAATAGAAGATACATTGGGCTTCGCGAGAAATTTCTT
CGAATCGTGCGTGTCATCTTTGCCGATCGAAAACTTGCCGAAAACCTTCGTGAATTTG
AGCGATCTGTCGTTTTACGAAGAGATAAAAGCTATATTTTATTTTAATGAGTTCCAATTTTTGTTATTGAAAA
CTTTTTTCCTTATTTTATTGTTCACTCTACTGTTAATCGTTATTTCTTGGAGAGTGTATGGGAAACGTATCTC
TGAGCGGTTTATGAGGCCGGCAACCTCTGCAACAATAGAACAACTGAAAGAAAGTGTA
TCAAAGCTTAAACTACCAAAAGAGCATACACCAAGGATATAAAAAATGGCAGATAAAATTAAAAAGTTCTTTC
AAAAGAAGAAAATAGATGCTAAATTTAAGTTAGCAGGACCTGGCCATAAATTGAGTGAATCTACACTCAGTCA
GTCCTCTAATATATCTAATATATCTAGAAATGTTCCTGTTGTCAAAAGATCAGGGTTG
TCAGAAGAAAGTAAAGTGGCAGCTGAGGCAGCATTATCAAGGTTACAGCAAACTAGGGAAAATCCTGCTTTCA
ATACCTCTTTGGCTGCTATAAAGGCACAAGTAAAGAAAGAATTAAAAAACAAAACACATCTAATTCTAAAAC
>103789006_2 DT668379.3

GCACGAGGCAACATACTAGTGATAACTTCAATAGAAAATATTTCTCTAGCGGAATTTTAGCGGCTACGGCAAC
TGGAGTTATAACCTATTTTAGTTTTAAAGAGAGAGTATCTGCCGCTTCTGCTATCACTTTAAATGGTAGAAGA
GATAAATATAATTTTATAGCCGACGTTGTAGCTGTTTCAGCACCTTCAGTGGTTTATA
TAGAAATAAATGATGGCAAAAGACTTGATTTGTATACTGGAAAGCCAATCACTCTGTCAAATGGTTCAGGTTT
CATAGTAAAGGAAGATGGGCTTATTCTGACAAATGCTCATGTTGTTGTTAATAAGCCCAATGCGATAGTGACT
GTACGGCTTATGGATGGTTCAACTCATACTGGTTTTGTAGAAGATGTAGATCTAAAAT
CAGATTTAGCAACACTTAGGATACCCGTAAAGAATCTACCAACTATGAAATTGGGGTCATCCTCTGACATAAG
GCCTGGCGAGTGGGTAGTAGCAATGGGAAGTCCATTGGCTTTAAGTAATACAGTGACAGCTGGAGTTGTAAGT
TCAGCGCAGAGGGCCAGCGAGGAGTTAGGCTTAAAAGGTAGAGACATGATGTACATAC
AGACAGACGCGCCAATTACGTTTGGTAACAGCGGCGGGCCTCTCGTCAACTTGGACGGCGAGGCGATAGGCAT
CAACAGTATGAAAGTGACGTCTGGCATATCATTTGCAATACCCATAGATTATGGTAAGGAGTTTTTGGCTAAC
GTAAAAGTAAGCCACCACTAGTA
>103789044_2 DT668674.3
AATATTTTAGATTATATTTATTTTTATTGTGTAGAAGTTGGAAATTGGNAGATGCCTCATAGAAATTATTGTA
AAAATACAAACCCAGTTGTTATAATTCTTATTGATACCTAGGTACTTTGATAAAGCTCCACATTTTGTTTGAC
AAATCAATAATCATACATACACTCTTTTGGTTCACTTAGCCTAACTTACATTTAGTTA
TTATTTACAAACACAATATTTTTATTCTCTATGACATTATTTATCTATATTGTTAAAAGAGATCACAGAAAAT
TGTAGCTGGTTATCATTGAGTAAAAGCCCTAGCATATTCTACAAGACTACCACTTATACTTTTATCACAGCGG
ATTTTTAGATTGTTTTCAAGGCATTGTTTAGGTAAGATACATTATTGTCTTTTTAATA
TTTATATAATTTAAAATAATTTTAGAACATTTGTTCCTGTACATAGATTATCTTAATGAATTATGGTTGCCAA
TTGTCTGATACATAATTGCAATAAAATTGTGGTAGCTTTTTTTAAAAGTTATTTCACATGCTACAATTAAAAT
GTATGCCAATAAATCAATGGAAGACGATATTTCGTGTGATATTCTTTGTTAACTCTTA
AACTCTCAACTATGTAGCAAAAATACATATAAACATACATAAGTAATTAAATTAACAAAAATGCAATATTAGC
GCGATCTGCACGATGGTGCAGTACGGTAGTTGATTTTGGCAACTTTTTCACAATGC
>103789056_2 DT668490.3
GCACGAGGAACACCTTCTACAGCTACCGCTATCATCAGCACTACACCCCTGACTACTTTGCCTACAACAACTA
CTATCACTACAACTCCTACAACTACAACTACAGTTCCAACTACTACAACTACAGTTCCAACTACTACAACTCC
AACAACTACTACTACAACTCCTCCTGCGACAACTCCTACAACTATAACTACTACGACC
ACTTCATCTACAATCACCACAAGTCAGGCTGAAAAAACTACATCATCGTTAAATGATGATTTGAGAAAATTAC
AAGAAGACACAAAGTTGTTGCAAGCATTATTGCAGGCTACTGGAAACCAGAATACTGGTAGCTTACCTATTAT
ATCTGGATTAACCTCTAATATAAGAGTAGCATCTAATCCTTTAACTACATCAATTGAA
TCAAATCCTACTACGGCATTTAATCTTCGCCCTATATATACTACTCGTCCATCGCCTGTTTTTAATTCATTTA
CTCCTGGAACTGTTAGTGTATCTACATTACAGCCTCAGCAATCAAGTACAGAAGATATTAGAATATCAACCAC
ATATAGACCGTTCACTGAACGATCAACAACCATACAAAATTTAGATTCTTCAACTGCT
ACTACTCGTCGCTCCAAGGCCGTACGGTTCCAAGCGACAACTGANATACCAAGTACCTCAACCTTCTCAGACG
AAGAAGATTTATTATTTTTGCAAAATCTGAAATCTGTTTTAAGTTCAAAACCAACGGTCAAGATTCTGAAACA
GCACTTGCTAATC
>103789063_2 DT667931.3
GCACGAGGGACCGACCTATCCATTCAGCTTTAGTTAAAAGCTAAATTTTCAAATTATCATATCACACAAAAGC
GAGCTCATACCCTCATCTATTAGTGACATAATATTTAATTATTATTTAAACTAAAATATTTGTTGAAAATTTA
AAGTGTTTCGGTTACCTAAACTTCACGTGAAATATTTTCACTCTGACTGTTTTACGGA
GAATTTTATAAAAAAGAAATTTATTTTTACAACGCACTTATATTTTAAATACCAACTTTCTATATATCGAAAA
TGTTTGATGCTTGTGCTTAAATATTCCGTTAAATCGATATTATCCTAAAGCATATACTATTGTCAAATGAGTA
TGCATAATTCGTGAATGTATTGACTCATAGTCCTAATGGGCTCGACCTGCTGATATAA
ACCGTCTAATTTCGATAACTTTTTATTGCCACAATGGGGAAGTTCTCCGGGAAGAAATGTCGTCTGCTATCTA
TGTTATGGCTTACTGGCACATTTTTCATAGTAGAGCTGGTAGTGGGCTACGTGACCAACTCAATGGCATTAGT
CGCAGATTCATTTCACATGTTAAGTGATGTAGCTGCACTTGTTATAGCTTTCTTATCA
GTAAAGATGTCACCAAAGAAATGGTCGAAGAACACATTTGGTTGGGCGCGAGCTGAGGTGCTAGGAGCTTTAG
TAAACGCCGTGTTCCTGGTTGCCTTATGCTTCAGTATAACTGTAGAGGCAGTACAAAGGTTCATCCCAATTGA
GACCATACACAAGCCCGAGCTACTCGTCGCCGTC
>103789105_2 DT662046.3
GCACGAGGTAGAACCTTTAAGAGAAAAGGAAATGACAAGAGGTAGCGATACTAATGAAGTAATGGCCTTTAAA
TATCACTACCTTGCTTATATTTTAAGAGAAATTAATAATATTAGAGACAAGCAGAAACCAAATACTGAGACTA
AGGAGGAAAAGAAACATGACATTGTTGAAGTCTTTGCTAAGAAACTTTTAAAACCTGG

AAAAGATGGAGTCAATTTAGATCTGATGGATTCATTCCTAAAAGACTGTGTTCGTGAGTTCCCTTACCGGGAC
AGTACAACTTTCCACCAGATGGTGAGTTCTCTGACAAGCAATTTTCCTCCACCTGCATTATCTGTCATTAATT
GTACAATTAATGGACAAAGAGGTTTTATAGATGCCATTCCTTATTGTAGTACTTGCGG
AGAGGAGAAACCAGCAAAGAAGTGCTCCAAATGTAAAACAGTACAATACTGTGACCGTGAATGCCAGCGTTTG
CACTGGTTCGTTCATAAAAAGGCTTGTAACAGGGGATCCTCACCTGTTTACCGTTCAAACCCAAAGCCTAATG
TAGATGCCTCAGAATTAACTGCTGAGCTCCAAAATTTAGTGGCTGGATAATTATATTA
ATTAACTTATATTTCATCTCTAAATTCTGGATGTTAAAATAAATAAATGCAATTATGTAGCCTATCATAATCT
TTGTTATACTTACAAATTTAATTAAACAAATATGCTTAATCACTGCATACAT
>103789112_2 DT662117.2
GCACGAGGCCGTGCCCCTTTGAGCCGCCACCGTGCGCGTGCAGCGCGTGCTGCGCCGCCAGGGCCGCTGGCCC
GCCGAGCGCGACGTGGTGCGCCTGCGCCTGCCGCTGCCCGCCGCCGCGGAGTGCTCGGGCCGCTTCGAGGTGC
CGCTCCAGAACCACAGGAACTATATCGTGTTCGCGGAGAGGAAGGGCCACGCCGCCGT
GGCTCTCGGCCCGCCGCTGAGGCGGACCGGCAAGTTGATCCGGAGGATCCGCGCGGTCTACAGGCCCGGATAC
AGTGAGTGCTTTGTGTATTGTAAGTGCATGGACCCTCGACGTTTTTTTTTTTTTTCCCGTCCGACGTGTTTAT
TTCGGATTTGCATGAA
>103789123_2 DT663044.3
GCACGAGGCCTCGTGCTGGAACTAGTCTCGAGTTTTTTTTTTTTTTTTTTGCTTGAAAACATCGTAACAAATA
CCCCGCTATGGGGGCGATAGAATTGATAGCGGTCGCTGTACTTATAATCACGTTGATAGTAGCCTTAGATCAA
AAATCTACGAACGCTATGTGCAAATCTAACAAACGATTAAATGGCGAGACAGTTATAG
TTACTGGCGGTACCACCGGCGTGGGGTTGCAGATAGCCACGGATTTCGCAGACCGGGGAGCTCGAGTCATAGT
CGCGTGTCCATTTATGAGCGAAGGGGATGACGCCTTGGAAATTATTGAACAAAAAACTGAAAGTGAAAAAGTA
ATTTTCAAACTATTGGACTTATCGTCGATAGATTCTGTACGAAAATTCGCAGCGGATA
TACTGAAAAACGAGGACAGGGTGGATATATTGGCGAACGACGCAGGGGTTGGTGCAGTCACGGAGTTCATCAC
AGACGATGGGATGAGCTTTATCATGCAAGTTAACTATTTCGGGCATGTCCTGCTTACTTTGCTCCTGATGCCA
CTTCTAAAGAAGACCGGTACTGCATCATATCCTGCCAGGGTCATCAACACGTCGTCCT
GGTTCCACTACTTCGGCACAGTTAACTTTGGAGAATTGAACGATATTAAAGATTATTATCCATTACAGTTCTA
CGCGAATAGTAAAGTGTGTTTAGTGTTATTTTCTCACG
>103789162_2 DT662748.3
GAGGGTTTTACCTTTAGTGATAGGAATATTATGTAATAGTTCCTTGCTGTGATTGTGAATTAGTGAAATTTAA
CATACAAAATTAACTAGGAATGCTGTAACGGTGTGTATAATGATTAATATCAATGTTCAATTATTTTTCTAAA
TAAAAAAATGAATTAACTAATTTATAAATTGTATGTAGTGGTTTATTGGTGTCGTGGT
GTGAAGTGGAGTTGTCTTGAAAAGTTTTCTAGATTTTTATTGGCAAATATAGTTAATCTTAGTAAAATGTATT
ATATGTAATGTGTTTAATAATGTGGAAGTGGGTGGTGATTTATTTTGATGTTTAAATTTATTTCTAAGTTCAA
GTTACGTTCGAAAGATATTATAACATAACATTTATGTTAATTTGGAAAATTTCGTAAT
ATACGTTTTTAAATGGCTATCTATTTATTAATTAATTATTCCTGTATGTATTTATTATTTTCAGGCATTTCTT
TAAAATATTATTTATTCTGATGAACAAGTTAAGAAAATGACGCAGGCATAAAGACAGAGCTTTCTAAATTTAT
TGATGCTCTTATCAAAGAAATAAAATGTATATATTTTTAGTAGAATCTGATCTTTGTT
TGTGTTCTTTTCTAATAATTTTGCGGTTTGATTTTATTATTATTGGATTATATTCACATT
>103789193_2 DT661817.3
AAGAGAATTGCGCCATAGTTAAGGATAATTTATCAAGTTATATCATCAATATTCAATAATAAGTCTTTTTATT
TTAAAAAAATAATAAAAATTAAACAAAAATGATTAACGGACGCGCGTTACACTTCGTTTTTAAAGTAGCTGAT
AGGACCCTCACAGCTAAATTCTACAGGGAAATTCTGGGTATGAAGGTCTTACGACACG
AAGAATTTAGTGAAGGATGTGAGGCTGCTTGCAATGGGCCTTATGCTAACCGTTGGAGTAAAACCATGATTGG
TTATGGTCCATATTTATTACACTTTGTTGTGGAATTAACTTATAACTATGGAATAACACATTATGAACAAGGA
AATGACTTTCACGGTCTGACAATTCAATCAAGTGAGAGTTTAAAGAGAGCTGCGGCTG
CAAATTGGCCAGTTAAGGAACAAAATGGATTGAAATATGTAGAAGCACCTGGTGGATACAAATTTTATATTAT
TGACAAACCACAACCTGTGGATAAAGATCCAGTTGTCAAAGTGTCTTTGGCCAGTTCAAATCTCTCCAAATCC
ATTGCTTATTGGAATGGCCTGTTATCTCTCCCAATTTTCGAAAAGAATAATACATCCT
GTAGTCCTT
>103789199_2 DT661893.2
GCACGAGGAAAAGGCCCTTAAAGTGAACGTACCAAAACAGCGCAGGACCTACTGCAAAAAATGCAAAGTCCAC
AAGGTACATAAAGTATCACAGTATAAGAAATCCAAGGAAAGGCACGCTGCCCAAGGTAGAAGACGTTATGACC
GCAAACAGCAGGGTTATGGTGGTCAGTCCAAGCCTATCTTCAAAAAGAAGGCCAAGAC

AACCAAGAAAATTGTGCTCCGTCTTGAATGTGCTGACTGTAAAGTGAGATCCCAAGTTGCACTTAAGCGCTGC
AAGCACTTTGAATTGGGTGGTGACAAGAAGAGGAAGGGACAGATGATCTTTTTCTAAGCTGATTAATAAATAA
GTTTAAAATTTAAAAAAAAAAAAAAAAAAACTCGAGACTAGTTCTCATTTTGGATCTCC
GCAACGAAACAGCGATCCTAAGTTAAGGGACAGTCCTAATGGGAGTTTAAGACGTGTGGACTACTCTGACAAC
GTAATGTCTACTAGCGGCCCATCTAGTTTGGAATCAACCAACCATCGAACACCGAGGAAAGATAGCAAAAATG
GTTCATCAGATTTAAATTCTACCATAAAAAGGAGCTATCAAACAAGTAGCAGCGGTCA
CTCCAGTCTAGAGTCGCACATATCAGATCGCACACTAAAACCTTCACCTAGTCATAGTAATGGAACGGGCTTA
AGCAGAGCACAATCACTTGTCAAAGTTCAAAGCTTACAAAGTTCCCCTAAAAGCTTACAC
>103789202_2 DT662977.3
GCACGAGGCGCAAACCAAAGTTTACTCGAGACCGGTAGAGTCGGTTGCGCCACAGCGTAGAGTTATCAGAGTG
GCGCCTATGGCGGGCAACCCGAGATCAATACTGCTACCAGTTACAATCAAGGATATGAAAGACTTGAAATCCA
TCAAAATTATCAACGCGGCGGACCTGAAGAACGCCTCGAACATCAAGATAACGGCGGC
CAATCTCCTATCTCAAAGTAAATTGCAAGATCTCAAAATCGAAGCCCGAGACGAGTACGAACAAAACAACAAT
TGTGACGACTCCGGCAGCGACCATAGTGATGACGACGACGATCACAATGACACACAAATCAATGATGGCCGTA
ACGGCTATCCCCGTCTCGTTCTGACCGCTGAGGAGAGACGCTTGCTGGCAAAGGAGGG
AATCACCTTACCTACCAGCTATCCCCTGACCAAACACGAGGAGAGGGAACTCAAGAGGATAAGACGTAAAATT
AGGAATAAAATCTCCGCGCAGGACTCTCGTAAACGGAAGAAGGAATATGTTGACGGACTGGAAGACAGAGTTA
AACAATGTACGGCGGAAAATCAAACGCTCATAAGGAGGATAAGGATACTGCAATCACA
GAACCAGTCGTTAACACAACAGCTGAAGAGATTACAGAATGTCCTCACAGGCGTGACGTCATCCGGTGGTAAA
GCTCAACCGGCCACCTGTCTGTTAGTCCTCCTACTGTCAGTGGCGCTGGTCGCTCTGCCCTCGATG
>103789212_2 DT662862.3
CGGCACGAGGATTTTGTCTTGTTGTAAAGAACATTAAATTAGTGATTTTTTTGCGTTAACAATCACTTTTATA
TAGTTCATGATTAGATTTTTTCTTTATTACTTTAATACTCAAAATACGCTAATATCAATAGAAAATGCCTGGC
CAAGAAGTAGTAACCACCAAAGTGGTTGTTCACCCACTAGTGTTACTGAGTGTTGTAG
ATCACTTTAATAGAATGGGCAAAATAGGGAACCAAAAGAGAGTCGTTGGAGTTCTCTTAGGATGTTGGAGAGC
TAAGGGTGTATTAGACGTCTCAAACAGTTTTGCAGTACCCTTTGATGAAGACGATAAAGATAAATCAGTTTGG
TTCTTAGATCATGATTATTTAGAAAACATGTATGGCATGTTTAAAAAAGTGAATGCCA
GAGAGAAAGTAGTGGGCTGGTATCACACAGGGCCTAAACTTCATCAAAATGATGTTGCTATTAATGAATTGAT
TAGACGATATTGTCCAAATTCCGTTCTGGTTATAATAGATGCAAAGCCCAAAGACTTGGGTTTACCTACAGAG
GCTTACCAAGCAGTGGAGGAAGTGCATGATGATGGTAGTCCAACTACTAGAACATTTG
AACATGTACCTAGTGAAATTGGCGCTGAAGAAGCTGAGGAAGTTGGTGTAGAACATCTTCTAAGGGATATTAA
AGACACAACTGTAGGCAGTCTCTCTCAACGAATCACCAATCAGCTGCTCGGCCTTAGAGGTCTCCACTCG
>103789217_2 DT661697.2
TAAATTTGGAGGCGTCGACATTCTTGTTTCAAATGCCGCTGTGAATCCTGCTGTATCACCAATATTAGAGACA
GATGAAAATGTTTGGGATAAGATCTTTGAGGTAAACGTAAAGTGCTCGTGGATGTTGGCAAAAGAGGCGTACC
CGGATTTAATAAAGCGTGGAGGAGGCAGTATAATATTTGTGTCTTCTATTGCCGGTTA
CCAGCCAATGGAGCCTTTGTTTTTTTATAGTGTTAGCAAAACAACTCTTTCTTGGTTTAACTAAAGCCATTGC
AAATGAAGTTTGTGAATGACAATATCAGGGTTAATTGCGTCGCGCCGGGAATAGTCTTCACGAATTTCTTTTC
TGTGATAACGTCTTCTGAAATTGGTA
>103789221_2 DT663189.3
GCACGAGGGCGCGTCTGCACGACTCCACTGAACACTGCATAGTATAAGTTTATAAAACAGGCTTTGGGACAAG
AACGGATCATATAAGGCTGACCTCTAAAGTTTTAGCTAGAAGTTTACAATTTACTTCATACTTATATCACCAT
GACCTCCGTAGATTATGCAAGTGTGGATATTAAGGAAAAGGCTCTATTTACACCGCCT
TTAGAAGAAGTAGTGGATGTGCTCTCTAGAGGCCTATCAGCTACGTTTAAACATGTTGAAGTAGCGGTCGAAG
ACAGTCCAGACTTAACGCAGCCACCGTTCTACCTCACGGCTCCTGGCCTCTTAGGAGATGCTAAACTAATAGA
GTTGGGAGGTCCTCCTTACTTAGTGCCGCTTGTACAAAGGGACAAAGTGTACGATTTG
GAGGCGTTGTTGAAGCACTTGGGCAGACAGCCCGCGTTACTGGCAGGCGCTGGAGCTGGACCATGGCCATATA
TTGGGGTTAATTGTGAGGGAATAATAAACCTGTTCCTGGACAAAGGTGTGATATCACAAGGCACGCGTATAGT
GTCGGTGTCGCCGGTGGGCGCGGCGAAGGGCAGCAGCGGGTACCTGCAGCAGACGCTA
CCCAATAATGAGACGAGGACCGCATTGCTCGGGAACTACCTGCTGTCCGAGGGGAGACCTGGCAAGGTCATCA
AAGTAGTGGCCAAAGAGAGAATCGGCGAGTCTAATTTTATCACCGCCATCAGAGAGACGCTAAAGAACACTAT
GG
>103789225_2 DT661780.3

GCACGAGGAAGCCATGAACGAAAAGTAAAGTTAACTAAAAATGGTAAAGTAGTGAAGAGACGTCTACGAAATC
CAGAAGAATGGCACACCCAAAAACAAAAGCGTTTAAGAATCGCTGGCTTGGAATATATAAATGCAAATGGCAA
AGTAGTCCCTCCAAAGCAACCGGGAGCTGATTGCAATTGCCGCCGAAAATGTTTTCAA
AAAGTCCTTGAAGATGTAAGACTTAAAACGTTTCAAGGTTTTTATTCAATGAAAACCCACGATGAACAAAATG
CTTATCTATTTGGTCTGATGAGGCAAGTGGACGTGATTTGAAAACGAGTTAAGTCAAGTAGTAGGAGAACATG
CACATTTGAGTATTTTATAAGAGTTAAAGGACGTGAAACTCAAGTTTGCCAAACAGCG
TTTAAAAACATACACGCCATAACTGAGAGAAAAGTAAGAGTTCTTTGTAAAAAAATGGATGATGGAGTAATGT
TTCCAAGTGATAATCGTGGTAAAAACAGCCATCGGCGGTCTGGTGAAGTTCGATTGCCCACTGTGATAATAGA
TCAAATAAAAAACCATATATATTCCATCGTTCACACCCACAGACTTAAAGATTTTATC
AGACTTGATAAAATTGCAGGCTTAGAAATAAAATATATCAAAGATGTGGAAAGACTATATTAAGACTTATGAT
CCAGACAACATATCAGCGACTATGCCTAAATCAAAAAGAAATGTTGAATTTAACAATATCAAGTGAATCCTCT
CAACCCACGCAACATGTACCTCCACAGGACACCTTTGC
>103789231_2 DV500990.2
GCGCCGCGCCGACGCGCTCAAGGCGGAGCGGGCGGCGGCGCGCGGCCGCCAGGACCGCGCCGACGAGAGTGTC
ACTGCTGCTCGCAAGGAAGTGGCGAATATACTTTTTTTTTTTCAGAGCATACAGAAGCAGATCTCCAGGGTCG
AAGCTCGCATCGAGAGTCGACGCAGCGACAGACACAATATATTGAGACAATGCAAGAT
TGATGACATTATGATCCCGCTACTAGAGGGCAGTTTAGACGACACAGCGGAGGGTTGAGGCTGAGTCCTCCAG
TCTCTCCAGCGCCCAACAGCATCTCAGGGACTCTAGGCTCCGTGTAGACTACCGGTCATTAAGCGAGGCGCTA
ACAGACTTGGACGAGGCGGATGAAATC
>103789237_2 DT661764.3
GCCGTATTGCACGTGTTCATCCCTCTAAGACGGTTCTACATGTGAGATGGATGGCGAAGTGATAGGCGTGTCA
GTGGGCGCGTGTTTGGCCGCCGCGCTCGTTATAGGTCTCACGCTGGCAGCTCTCATGTCTTGGAGCCGCAAAT
GGTCACGCGAACAAAAAGCAGTGGGCATGGGGTCCCCTGTGTTCAACTACATGGCGTC
CAACACCATAAAGACGCCGCCGGTCGGCCAGCCTCCTTATCAGGTGACGCTTGAAGAGAGGATGCGATGGGCG
CAGCTAGCTGAGGCGATGGCGCAAGCGAGTAACTACGCGTCCACAGATCCTATCTAAACTATGGCGACGCGTC
CATCGTCCGCCATGTTTGGTGGTTATCCTACTCTACCTCCAGTACCTTTACCTAGGTT
GGGCATGCACGGTCCTCACATGGGGACGTTGAACACGGTGACAAGCAGGGCGAATACAAATTCACATCATAAC
TTATATGGTTACTCCAACCACGCAATAGGCACGGAATCAACAAGTTCCGACGCGTCCAGCCACGTCCAGGACC
GCGCGGACCTGCTCGTGCCGCGGCCCAAGTCCAGGGCAAGGAGCATGCATAATCAAAC
CGGTATATATTATGACGTGGACTATGAAAATGCTGAACCTATATATGGCACGAAGGGAATTCCCCTTTCAACG
TACACAGTCAGCCGCGGCCCACCATTTTATAGATAAA
>103789246_2 DT662176.3
GCACGAGGACTGCTTGAAGAGACAAAATAACATTTTGTATTTTTATATATAAACTTTTCAATATGGCGGCTGC
GTTGGACGTGCGATTGCACTGCAATATATGCTTTGTGTCTCGAAGTATATTGTATAGCCCATTAGCCATAGAT
TATTATAGTATACATAAAATCTTTATTTATAGGGATCAGAATCGGTTAAATTTATATG
ATTTAGACGTATTATAATTATGTTATCGTGTGCTTGGACAGACAGTTTTATTGCTCTCTGTTGGATATAAATT
TTACCGAATGACGAATGAATAAAATTTTATAATTATAAACTATTTGTTGTAACTTAATATTTGTTACAAACTG
TCACATAAAATTTAACAGATAGACACTAAGATTAACAACGGGACTTAAAACTCACATG
TACTTACAAAATAATATTTGTACTTTAAAAATGAGTCTAGTCATATTAACTTTATACCAGTTCCAAACGGCCA
TAAAAATATAAATCGAAAATTTTTAAATTCAAACATCGTAATTAAATGGCGATTTAAATAAAATATTAAAGTG
ATAAAAAACATATTTGTAAGAAGACTGAAGTTGTACATATATTTTCACATTTAATTTC
TTTATTTGTTTATTCTTGAAACTTATAAGGAGCGATCCATAAATGACGTCACACG
>103789249_2 DT663006.3
GCACGACGAAGTTCTTTTGAAATTTTAGTTTTATGTTGTATTTGGTGCCCAAATCATTCTATAAGAACTGATA
TATTAAACTTTTCTCCTCCTAAAAAAAGTGAGAAACGTAGTTAATTTAATAGTGAATATTTTTAGTTTTAATA
TAAGTAGCTTTCCAGAAACCACTACCACCATGCCTAAAGGAAAGAAAAAGGGAAAATA
CGAGCACAGAAGAACTGAAGTTCCTTACTCATCAGATGAAGATGCTGGCATAGATATGACTAATGACAACTAT
TCAGAAACTTCCGGACAATCAGATCTTACAAGTAACCACGATGATGCAGAAAACGAAATCCAAGAAAAAATTG
AAGAGAAAGTTCTAGAAACTATTGATGCTTTAAGTGCAAGAGCTAATGCAGCAAGAGC
AGCCGCTTTAGTGTCTCTTAGGAATTTATTACAAAGACGCTATTTAGCACATCTATTGAGCAGCCAACGAGTG
ACCCTCGCTGATCACGTTACTAGGGCTTTGAGAAGAGGCAAAGATGGTGAGAGAAAAGCTGCAGCTGCTGTTG
CACCTATTTTAGCTTTACAGATTGGTGAAGAAGGCACAGAGGAATTTATGGCTGAAGT
CCGACCTGCCCTTGCTGCAGCCGCC
>103789255_2 DT661900.3

AGTTAGCTGTTGACATGAGACCGTTGATATGTACGAGCGTGGTGGCGTGTTTGCGGGTTAAAGAGATAGCGTG
ATAGCGTGCCAGCGTCATCCTACGACGGCGGTCACGTCACTACGTATGCACGATTGGCAAGCATGAGACTGCA
CGCTTGCGGGTTGGAGTGGTAGTGGTTATGTTTACATATACACGACTATTAACCGTGA
AAACAAGAACTGGTCGCCTCCTCCCGCTATCAAAGACGATCTCGTCGATTAATAGCGTGTTAGTACTTGCGTG
TTAGCGCCGCCCTAGGCCAAAAAACCTATCCCGCCCCCGCCCTTGTAGCGTGTTAGCGTATTTAGCGTGTTGC
ATCACAAGTTTAGCGTTTAAGTATTGAGACTAATGCTTGGTCTCGTTCTACCCAAAAT
GTTTATGGTGCAAATTTTATTATTTATATTTTATTCATAGAGTAGTTAGGGTTTTTTTTTGGTTTGTAATTTT
ATTGTAAGATTTATAAATAAAGATTTATTTTTACTGTAAGTTTAGCCTATTTAACCCACTGGCACGCTTTATA
CGCTGTATTGTGATTTTTTTTTAGTTTTGCTCATTTTATTTTGGGAACCATACATAAA
TGATGTCATACGAATTTCATGATTTGCTGAACCTTCCTCGTCACATTTTGGAATTTC
>103789286_2 DT663068.3
GCACGAGGAGCAACCCTTAGACACAGGGACCAGTAAAGGCTCTGCAGACATTGGATTTTCGGTGTTAGGAGCT
GGACCATCCTTAACACAGACTCATGTACGAGAGCTATCTCCGCCGGTATCAATGGATGAATTCCTTGTAAAAA
AAACAGAAACAGGTTTTGTGGATAGTGATGAAGGAATCATAGATTGGTCAAAACTCAC
TTGTTTGATATGCAAACGGAAATTCCCGTCCTTGGAGGTTTTGACGAAACACAAAACATTATCAGACTTACAC
AAACAGAATTTGGCTGAATATAGAAAAAAAAATGGAGTTGTTACCTCAAAGTAATGGTTATAGAGACAGGGCGG
CAGAGAGGCGGTTGAAATTTGGAGAAGATGAACCTCCACCTATTAGGAAGCGATATGA
GCCACAAGAAGTTCATAGTTCTGTTGTATCACATCCTCCACCATCAGTTGTAGATACAATTGGGGGGAAAATG
TTACAAAAAATGGGTTGGTCAGAAGGGAGAGGGCTTGGTAAAGAAGAACAAGGGAGGATAGCTCCCATTGAAG
CTGAGCAAAGGCCAAGTTTAGCTGGACTCGGGCAAAAAAGAGGCATATACACTCCGAC
TCCCGGCTTAACTTATAGAGATACTGTGAAAAAACTCATGATTGCCAGATACAAGGAAGTTGTTGGACAAGAA
GAAGGAAATAAATAGGTGGGTAACTTTTTAATTTTTTATTTTAATCATTGTCTGTTTCAGGTTCTTTGGCTAT
AGGTAAAAAAAAATCTGCTTAATAGACTATGATTACACATTTTTTTGATTTCATTTAA
ATGTCTTTATTTGTTTTTGTAATA
>103789295_2 DT662558.3
GCACGAGGTTTTGACTTATGGTTTTTATCCATAATGGTAAAATTTAAGATTGTCTCAGTCAGTATATGAAACT
AAATCTTATGATAAGACGTGTGTGTGCTTGTTAAATTAAATTTTTTGGATATTTAATATATCAATATATATTT
AATATATAAATGTTTTTTTAATATTTGAAATAGACTTGCATTCAAATTACTTCGAGTC
CTCAAGACACTATGGAGATCGAAATTTTGAGCATATCTGGTTCAAAACCTTTGGGGAAAGTACATGTAAATGC
TGATTCTACTATTAAAGATGTAAAGGACAAAATACATCATAGTATAAAAAAATCTCTGTACCCAGATAGGCAA
TCTATTAAATTAGAAGCAAAAGGAAGATCATTAAAAGACGAAAGTACCCTTAAGTCTC
TTAACCTTGACAATGGATCAAAATTATATTTACAAGATTTAGGTCCTCAAATCTCATGGAGAAATGTATTCCT
CGCTGAATATGCTGGCCCTCTATTTGTATATTTATGGGTATATCAGAGGCCCTGGATTCTTTACGGAAGTGAT
ACAGCTCCTCCTGGGACAGTAGCTACGATAGCTGCTGCTTGCTGGTCAGTTCACTATG
CTAAACGTCTTCTAGAAACATTATTTGTTCACCGTTTTTCTCATGGGACAATGCCACTACGCAATTTATTTAA
AAATTGTGCATATTATTGGCTATTTACCCTCTACATTGCATATCACATCAATCATCCTCTCTTCACTGCTCCT
TGTAATATCGGTGTTTATGTTGGTGTAGCAGGATTCACTATATGTGAACTGGGTAACT
TAAGCATTCA
>103789301_2 DT661873.3
GCACGAGGGAAATATCTTTCAAGAATCATCCTTAACCTCGGGTATAATCCATCCGTTATCCTTAACCTGAAAT
ATAATCCATCCATCACCCTTAACCTCGGGTATAATCCATCCATTACCCTTAACCTCGGGTGTAATCCATCCAT
TACCCTTAACCTCGGGTATAATCCATCCATCACCCTTAACCTCGGGTATAATCCATCC
ATTACCCTTAACCTCGGGTAAGATTAAAATGATTAAAGACGAATGATATGGGACGAATGATGAGGGATGAATG
ACGAGGGACGAATGATGAAGGATGAATGCTTAACTCCTTAACTTTTTGTGGCATCCATCCTCAAACCCTTTCC
TCGGGCATCATCATCCCTAAACCCTATCCTCGGGAATCACTCAATCCCAAACCCCCTA
CCCTCGGGCACCATCCGACCCATAAACCCTATCCTCGGGCATCATCATCCCAAACCCTATCCTCGGGCATCAT
TCATTCTCAAGCACTATCCTCGGGCATCATTCAATCTCAAGCCCTATCCTCGGGCATCATTCAATCTCAAGCC
CTATCCTCGGGCAATATTATTTTCCATGTTCCTGAAACTCAAACAATATAAGCTTAGA
CTTGCACACATCATAATAACTTTTTTATCAACCTAATTACATTAACATAAAATTCCTTCAAATTTATGAATTT
GAAAATCTATTCAGATAACTTTGATATTCGA
>103789310_2 DV501056.2
TATTCGTTTCCCCCCTTTTTGGATTTAACATTCGGATTCTCTGTACGTATTATTTTTGTAATGGCCTGTTTGA
GTGAGGTGTCTATGGACTGACGGTGATTTATCATATGTTAGAGGCGATCTTTTGTATGGTAGTATTTGATTTG
CGTTTAGGAATTATATATTTTTTTAAATCACCCTATTCATCGGTCTAAAATGAATCTC

TTGAAATAACTTACGACAGGGTAACGAATCATTACTTAACGCTTATCTATGAACTGAGCATATTATTTTATAC
AATAATACTCACTTGCCATCACATTTTAATCACTTTTTATATTATTTTTACATTTTTTTACTTTAATCCTATA
TACATACGTACGTCGCTTAGAATAA
>103789316_2 DT662074.3
GCACGAGGATTTAGCGTTAAATGTAACGATAAATTTGTTTTATTTTTGTACAATTAGACTTTTTTTGGGTAAC
TAATATTAGAGCCTTTTAACAATATGTTATGTGAAGTTAAATAATTACTGTTGTCATTTCACACGGGAGCCGT
GGGACTAATTGAGATTAGGCTTGGAAATATTTTGATTAGATCTCACAACGATCTTGGA
TCTAAAATGTCTGGTAGAAGAAAACCTCCTCCTCCAGGCTTTAGTTTCAAGCCGCAGGAAGAACAAGTCACTG
TAACACCACCGCGGAATTTAGACAAACAAACAACAATCTTTTTTTATGATAAAACGTTTACAGTCCATGCTGA
TGATTTGGTTAAAATATGCGACCTCGGCCGTGGGGCTTATGGCATAGTGGAAAAAAATG
CACCACAGACCCAGTAATACAATAATGGCTGTTAAGAGAATCACAGCATCCTTCAATAGTCAATCTCTGGAGC
TTAAACGCTTGTTGATGGATCTCGATGTTTCAATGAGGGCAAGTGCGTGTCCCTATACAGTACATTTCTACGG
TGCCATGTTTAGAGAGGGAGACGTATGGATCTGTATGGAGGTGATGGACATGAGCCTG
GACAAGTTCTACA
>103789327_2 DT662562.3
GCACGAGGTGAACTCTTTAATATGTTGTTTTTTTTTATTTTACTTTCTTTTTTTTAGTCTTTAAAAATATATT
TTTTAAAATTTAAAAAGAATATCTTTGTAATTTGATCTTTTATGGTATTATCTATCTACTTATTACCATTTTT
ATCATAAAAAAATGTAAATTTAATTCCTTCTTACATAACTAGATCTGACATATATCTTC
AAATTATTACGTCTAAATTTCTCATCTACCCACATACATACATATATATCATATTATATACATATATATCATA
TTATATACATATATATCATATTATATACATATATATAATATTTTATAATATCCTTTTCGGTCCGAGTTGCGGT
TGTCATCACTGCCTAAATACCAAGCAATAATAATGTATGACCCGGTTTAATATAAGAC
GCGTAGTATAAGCGGTTTATAGTATAAGAACTACGTACATTGCACATTTATTATAATAAAAAAATTTAATACA
GTTTTTTACAGTCGGCCCTTGGTCTATAATGTTTAATATTTGAAAGTTTTAAGTTATGTAATTAATTATAAAT
AATTAAGTAAAACTACGAACGGCAATATATGTATATATTTTTAGGTTGTTATAGAATT
TCCATAAAATACTGAACCCATTGTCATAATTTTTTTATAAAAAAAATATTCAAAAGCCATTTATAATTGAGAG
TCAGAGCTGTGTTTGTGTTGTTTCGGTTTATACAGGCTGTTTAGTTTATAGGCCAAACTTTGGTCATATCTGA
GAATTGATGTCAAATTTTGGTCACTGCCTTCCTGCCTTAGCTTAGTAATGGCATGACA
TACAT
>103789338_2 DT662700.3
GCACGAGGGTCGTGCCCTGAGACGCGCTGCCTAAGGACTGTTTTGTTATTTATCAAGGGCACCACGGCGACGC
GGGCGCGGCCATGGCGGACGTGGTGCTGCCGGGCGCGGCCTACACGGAGAAGCGCGCCACGTACGTCAACGCG
GAGGGGCGCGCGCAGCAGACGCTGCCCGCCGTCACGCCGCCCGGACAGGCGCGCGACG
ACTGGAAGATCATCAGAGCCCTCTCCGAAGTAGTGGGCGCTCGTCTCCCCTACGACTCGCTGGACGACGTGCG
CGGGCGCCTCGCTGAGGTCAGCCCGGCGCTCGTCGCGTATGGGGAAGTGCAAGATAATAATTATTTTGCGCAA
GCACGTGCTCTTGCTCAGAGTCTTCAAAAACCAGTGTCGGGCAAGTTAGATGTTCAGC
TGAAACAACTAGAGGACTACTTCATGACAGACTCCATCAGCCGCGCCTCCCCCACCATGGCTAAATGCGTACA
AGCAGTACTCAAGCAAAAGCAATCACCATATTAAAACAAACAAACAAACAACAAATCATCACGAATACACGCT
TTTACGCTAAATAAATATAGATGAATAATAGATTTTTCTGATTAAAATATCTTAAATC
ATGTAGAAATCAATATACTATCCATTTTGAGTATTCGTGTTAATTTATAAGTGAAATTGTTCACAGTTACTTT
TGTATCATATTATTATTATTACTTATAATGTAAAAGTAACTGAGGATTTAGCTTAGTTAATAAATTTTTCAAT
TTTATCTTTCTTCAATCATTTTTAACATTGAAATTTATAATT
>103789366_2 DT663310.3
GCACGAGGCCACCCCTTCAAAAGCGAGTTCACCCGCGACCGCGTGGCCGCCATGCGCGCACGTGCTCGCGACT
GTCCCACCTACTCTCATCCTACTTTGGCTTGTCCATTGAATCCAGCGCCGGAGTTGCAGTCCGAGAAAGAATT
CTCTCAAAGATTCGCCCACGTTATCCGTGGCGTGATCGACTTCGCCGGCCTCATACCT
GGATTCCAGTTACTGACTCAGGATGACAAGTTCACATTATTAAAAAGTGGTCTCTTCGACGCACTATTCGTGA
GGCTCATTTGCATGTTTGATGCCCCACTCAACAGTATAATTTTTCTAAACGGGCAGCTTATGAAACGTGACTC
TATCCAAAGTGGAGCTAATGCACGTTTCTTGGTGGACTCTACATTTAAGTTCGCCGAA
CGTATGAATTCCATGAATTTAACAGATGCCGAAATAGGCCTCTTCTGCGCCATAGTCCTAATTACGCCGGATC
GTCCTGGTCTTCGCAATATAGAGCTAGTTGAAAGAATGCATGCAAGACTGAAGGGTTGTCTACAGACCGACGT
TACGCAAAACAGACCAGACAGACCCGGTTTTCTTAGAGAGTTAATGGATACGCTACC
>103789372_2 DT662067.3

CAGCTGATACATCCTCCTCGGATTCAGCACACGCCCCACTTGTACCTCCCGGCACAGCTGCGGCCACTGCACC
AGCTACTAGCAGTAGTGAACTAAAGGAGCCTGACACTAAGGCATCACCGTCACCATACTGCGACTTCTGTCTC
GGCGATGACAGAGAGAACAAAAAAACCGGAACACCCGAGGAGCTGGTCAGCTGTTCCG
ACTGCGGCAGATCAGGACACCCAACCTGCTTACAGTTCACAGTGAATATGATAGTCTCAGTTCGCATTTTTTT
CTGGCAATGTATCGAGTGTAAATGTTGCTCGGGTTGTGGGACTAGTGATAATGATGACCAGCTGCTGTTCTGT
GACGACTGCGACCGCGGCTACCACATGTACTGCCTCGCGCCGCCCCTCGACACGCCCC
CCGAGGGCTCGTGGTCCTGTGCGCTCTGTATCGAGGAGTTCCACTGAATGCGTATGGAGGCGTAATATTAAAA
ATATAGGGGTATGGCTGTAA
>103789378_2 DT663169.3
TGAGGCTAGCATCGACGCACCTTTTAAGTTTTACCAAACGTCATACATGAAACTATGCCAGGATATTTCGTCC
ATCGATTTCTCCACTCAAGCAGGTTCAGTAGCGCCAATAATCATGAGTATTAAGGATAACGTGAATCAAAACT
TCGCGGCTGATGCCAATAAGAACTTCGACAACGAGGATACTGAGCAGCCTCCGGAGAG
ACCTATGAAGCATCAAGTGAAAAAGTACCGTTATTACGGTCCTATCGAGAATTATATCGTGAAACTGTTGATT
GATCACAAATTGTTGACTTTAGTGGACGACCGTACAGGGAAATTGGCATTTTTTTGTATAACATGTGAATATT
ATACACTGCGTTTCCGTTACGATAGTGTTTTGAATCATGTGTTCAGTGAGACGCACAT
ACATAACCTCGCTTGTATCCTGCAATCGGATAAGCATATAAAATTCTCAATGGAGAACTCGACCATTGAGAAA
ATTAAAGATATGAATACGAAGTTCCTCTATAATCACGGTATTATTAGTGACACGAATGGCCTAAACTGTTCTT
TGTGCAAAGTTTTTATTGAAACAAACGAGAATGTGATCATGCATATTCTAGAAGAGAA
TCATTTGGGACGATTGTCTGACAAATTGTATCGCACCATGAAGAACTCGGATGGATCAGTGCCTGATGAATGG
GGACCAAAAAGCCTGGATTGGGCTAAATTCCTAGCCAGTGTTGGGCAACCACTGCACAATCGCGACCACGTGG
TTATAGAAAACTTCATCAAACCT
>103789384_2 DT663046.3
AGTAAAAAAAAATATATTTAATATATAAAAATATCTAATCAACAATAATAATTATATAGTGCAATTTACATAA
TGGAATGATTTGTTTACGAAATCAAAAATGTATAAAAAAAAGTTAAAACTTATTGCACTTTAAAATAACATTG
ACACTTAAAACTGTATTAATTGTGACGTAATATAAATGCCCAGCCCATCCATATCCCA
TAGACAATGTATACATAAATATATATATATATATATATATTTTTATTTAAAATTTATAATTTCGAGCGTATAGAG
TCGATTTTGCCGGCGGCGGATTTCTGTAAGTCTGTCAGTTTCATGGCGAGGCCCATGTTGCCTTGTATTTTAA
TCTTGCCCTGGAAGAAGGCCTTTTGGGGATTTAGTTTTCCAGAGATCAAATCAGCTAC
GTCCTCATCGTTTATCGTGAACGTGACGTCACATTTATCTTTGCCATTGTACTGGACGTTGCCCTTTCCTTCC
TTCGCGTTGATCACCCAATAGCCTTCCTCCCCATTGGGTCCATTCCTGACTTTGAACCCGTATATGCCTCTGA
CCTTCTCAATAAGTTTATCGTCGTCGTTCTTCATCGCCTCCTCGAGTATTTTCATGTA
TTTGTACACTTTGAAGTTGTCCGGGTTCTCCGCGATGGCGGCGACCTGGTTGGGGACGGCATTCGGGAATCCT
TTGCGGTACATGGCTATGACGACTGCTCCACCGAGACCTAAGTTGTGTTGGAGGGCGATCGGGGCGCGAGGTA
CCTGTCTCTCTCCTG
>103789387_2 DV501084.2
GCCCACCTCGAGGAATGTCCCTTATTAATTGTGTCTACTTTTTATTGGCGTCTTTTTGACTATCGGATATTAA
TTAATTTGTTACATCTATATTAGTTTAAATTATTGTCCAGAACTGGGATCCTTCGCATTTAAAGTGAAAAGTA
CAAATGGCCAGGCTATTTTGAATGAATTGACCTCTGAGATGATTGTGGGTGAATTAAA
AGTGTCTCTCGCTAATATCTCAGATGTTGGCTGCGAGAGAGTTTGCGCATAGTGTGGTTATCGCC
>103789420_2 DT662304.3
GCACGAGGTCGAGCTCTAGGCGGCGCGCGCGCACCGTACGACCGCCGCACCAGTGAAGGGACGCATGACTCAC
ATCCACACCGAACATGAACGAGTACGTCGAGTACGACTGGAGGCTGCGCCAGCTGCAGAACGAGCGCCTTTGT
GACGTGTCCAGGAAGAGGCTCTCCGACGGGGACCGGCAGCGCGCGCTGGCCGGCCTCA
AAGACGCCGTCGATTCAAGTCTCAACTTGGCCCTTCGCGACAAAAGTCGATGTCAAGACGACAATTTTCTACG
GCGGTTTTTATACGCGCGCAAACACGATGTACAACAAAGTTTTGAATTGTTGATTCGTTATCATCAACATCGC
CGAGAGCATCCGGAGCTGTGGGCGACTGCGGACGGCGGGGTGTTACGAGCTCTGGCTG
ATGGTCTGCCCGGCGTGTTGGCGCAACGAGACCGGCTCGGCCGCTGCGTGCTTCTCATGTTTGCCTCTAATTG
GACCCCACATGCCTGTCCGCTCCTGTCCGTGTTCAGAGCTTTGCTTCTCACGTTGGAGCGGACCCTCGCCGAG
TCACAGAATCAAGCCAATGGATATGTTATTATTGTTGATTGGACTGAGTTCACATTCA
AACAATCGTGTAGTTTACAAGCAAAAATTTTGAAAATGATGATAGATTGCTTGC
>103789423_2 DT663024.3
GCACGAGGCTTTTTCTTTTTGGATATTTTTGCGTCAATTTTTATTTTTCTAATAAAATGACGACAGCTAGCGT
TCTGCTCACCGTGAGCACGACGTGGTCAGCCAGCAGCGACGTGTGCGATAAAGAGGAGCGAGTGGGTCGCTCC
ATCAGGAACGTAGCCGCTGTAGCTGCGAGGGAACTCAGGGAGACACCAGCCACCAGAG

ACCAGGCCGTACGGATTATGCGTGATTGGATACAGAAGAACAGTGATATCAAGAATGTTAGACAAGATGAAGT
CTTCTTGCTGCGCTTTCTTCGGCATAAAAAGTACAGTATTCCTATGGCACAGCAAACGCTCCTTAAATACTTA
AGCTTGAGAAAGTTTTACCCGAGTATCTTCACTAACTTGGACTGTGAAGATCCAAAAA
TCAAGGAGATCCTCGGTAACGGGTACATAGCAGTGTCGCCGGTCCGCGACAGCAAGGGTCGTCGGGTCATCGT
CTACAACATGGGTAAATTTGATGCAACAAAATTCAGTTGCTGGGACATGTGCCGGGCTCACATTATAATTTAC
GAAGCCCTAATGGAAGACTCCATCGATCAAATATTCGGCTTCAGCCACGTGGGCGACG
GCAGCGGCTCCACCACCGCCCACGTGACCACGTGGAACCCGATCGACTTCGCAAGACTCATGAAGTGGGGAGA
GCAATCAGTGCCAATGCGTCACAAGGAGTTTCAACTGGTCAACGTGCCGGGCGCTCTTAAATATATAATTGAC
TTTGCAAAGAGCAAAGTGTCATCCAAAATTGGCGAAA
>103789436_2 DT662395.3
CGGCACGAGGGGCTGAACCTCCACAAGACACTCCCGCCACTACAGTGCCGCCCTCCACCACCGGCACACCGCT
CACTCCCCTGACTCCTATTGCCTCACACAACATTAGCACGCCATCGCTTCCCGTCTTATAAAGTGGTAAGCTA
TTGTGATTGCAAAGATAGGGTTCATGTTCCTTATCGCATGGCAGAATTTAGAATACCT
TTGCAATGATGCAGTATAGATTTGAATTATTGATTTGTGATTTGTGTTGTGCATCTCCAGTACTTAAGTGTAG
TGGCGTCTAGATAAAGATGTTTGGACTTTTTGAATGAGAAACGTGCTGGGCACTCGATTTTATTAGAATCCAC
GACTGAAGGCTGGCATACCTTCAATTGAAGAAATTCGAGACCAAAGACAAGAATATAC
ACAATGACAGCGACTGCCAATAACACGAATATACTAAAGTTTCTATATGTTATTTTGTACAATGAATAACGAA
GGCTGCCATTTATTTTTATTTACAAAAGCTTGCAGAAATAATTATGCAAGAATTTAATTGCTGGATGCTCATT
TACCGTAGTGAGTTGAGTTGTCCGTCCAGTTTTATACAATGTTGCAACAATAATATTA
TATTGTAGCGAATGAATGANATACGGTGCGACGCCGGAAAATGGTTGTATACTTAAAACGGTATGGTAAAACG
GTACAAATTAAACTCTTAGATTGCTTGTAAATATGATTGAAAAGTCTTCTCTTGCTGATTAATTTGGTAGTAT
AAATACTGTTTTACCTATGCCTTTTGAGCTGAATAAATAGTATCGATTCTATGTATTA
GCTATTGTATATCATAA
>103789451_2 DV501044.2
GCACGAGGGTTTTTCCTTTTATTTTTTTTTTAAAGTATGTTTATTTATTTATTAATTATATATTTAAAATATA
TACATACATCATTCTATTACCATATAGATGCTACTGTCACTTCCATTTGACAGTGACATTGACAGCTGACATT
TGATATTAAATGACTACAGTTTGACATACGATCACTTAAAG
>103789454_2 DT663091.3
GCACGAGGCTACCATAAGTTTGCCCGAAATTGATATAGCATTAAGTTCTCCAGGGGCGGATGTGGTAATTGTA
AACAGCGATTCAGGTTTGAATTCAAATAATGTCACTAACAGTCCTCCTCTAGTCCCTTTCCTAACTAATTTCG
AATTAAATGATTTTAATGACTCAGAAAGTTTGACTTTAGGGCAGATATTACCAATAGC
TCCAATGAGATCTTTACAAGTAAAAGAATTAGAGGAAATTGAAAATATACAGGATCCAAGTCAACTCTCGCGT
TTACAATTATGGATATTATCCAAAAAAGCTCGTGTTTTGAAAATGATTGAAGATATAATAAGAAATCATAACG
AAGAAATTTCAAATGCTCCTTTGACAGAATTAGTCAATAATACTAATCAAAATAACGT
ATTTCTATCTAGTCGCTTAAGTGATATTGTTAACACAATGAATATGACGACTGAAAACATCATGTCTAACCAA
AATACTCTAAATATGCCAACGTCTACTCCTGTGACTATTACTGAAAGTACGACTTCTCCTACAAATCCTTCTA
CTTCCTTTCAAGCAAATATAGATTTAAGTACAAGTAAAAAAACTTTTTCAACTACCCA
AACTGATGTTCCAATGACAACTACATCGATACCATTCGAAACTGCTTCTGATATTTTAACAAGCACTGAATTA
CCAGCTCCAGCAGCATCCCAAATCACTAATGATGGAGCTGACGCTTTCAGTGCTACTACTGTGGGAAGTAC
>103789463_2 DT663389.3
GCACGAGGCGTCCCCTTTAAGAGAGAAGACGCAATACAACCGTCGAAACACGAAAATCATATAAATTTATATT
ATATTTTGATCTGTACTATATCCAAAATACCAAATTACAATAGAGGACTTAATATCTTTTAAATTGTACTTAA
CTCTCTAAACTTAAATATTGGTAAAAATAATATATTGTATTAATCTTTATATGGACTA
AAGTGTTATCGGTATAACCGGACCTCGCCTTGACCTCACCCACCTACCTACAGGTGGACATTGATCTACACCA
CTGCATAGCACTGCTACGCTCTAAGGGCTGTTACGTATTATGTGCCGTAGGTGGTGGGAACATATTTTATTTT
ATTTAAAAGATATTTATTATATACATATTTATGACATTTACACAAAAAATATTTTTTT
ACGATAATATCCGCATTAGTTAATTAATTTGATGTGGAGGGTGACGTTTTACACTGGCTTTCGTGTGAGATTC
CGTTGCGGCAACGGCAAAGCGTCTGACGGTGACCAAACGGTGGTTCGCTTTCTACTAAGGAGAATATTAGTTA
TTTTAATTTACTTCTTTGACTAGTAAGGCAAAGCGCACATTGTCTTCAAAAATAACTG
GTACGTAGAGAACGGAGTATTTTAACACAAGGTATCCTCCTATAAAAGGATACTTTTCCTTACCTTTTCACGG
TATGTT
>103789475_2 DT661865.3

TTTAATCCATAGTCCAAACCATATTAAGCGTATGATATAAATGTGTGAACGTGAATGCTACAAATTCTAAGAG
TCAATAAACATGCTATAATATAATAAATTAGATTCATTTAGTCTTTTTTGCATAATCAAAATACATTTGAATC
CATTTTGTAGGTACATGATATACATTCTCTTCATACATTATACGTATATTAGCCTCTA
ATTCAATCTAGGATGAAGACGAATGGATTGTCAATCGAGCCAATCAATTCATCTTCATTTAATGAAACCGCTC
TATTTGACATTACATGTATTTTTATATAAGTACTCAGTACTGAGTACGTAGCTGCTACTCATACTAGACAAAA
TTGTTTTTTTGAGTTTTTTCTCCTGTTCTTTTCATATCCCGTATACCCGGCGTAACTC
GACCCCGGGCGAAGAAGTGAAATTATTAAATAAATAATAATTCATTCTCCCGAGTAGGGATCGAATCCACAAC
CGTAATGTTATTGTCACATTCTTGTGTCCTTACACCATCACGGCCTTAAAAA
>103789532_2 DT662033.3
ATAATTGGTGTTCCAATGATAACAACAATAAGATTGTATCCAAAAGTGTTACTTATCTATACTTATGCTAAAA
TTAAATTATCACTGTTCTAAAGTAGCTGTGACACGTGATTCGTGCTTTGGCTGAAATCTGAAAAATTTATCTA
ACACATAAATTATGTTTTAAATGAAATGTTATTTAATACAAATTGTACTATCAAGAAT
AAGTAGTCATACAAAAGTCAATTATTTGTTTTGAAATTTATACAAATACGTCACACTTAAAATTATATAATGT
TTTATTATAATATGATTGTATTTTTTTTTTCACAGTAAGTATGTATGTGACCCACGCCTGCAATCAAACTTAA
AACAAAAAAAAATAAAAAAATACTTATAAGAATAGAGCTTTGTATATAATTAATTTAA
AAATATTTCTATGAAGGGCTCAGCAAGCAAACCAATTACGTGGCTCGATTTATGGAAATTTAATATACCTATA
TACAAAGTCGGTCAAAGGTCGGAACCTTGTAAATCCTTAAGTAACTCTAATTAAACAGAGGTGAGAACAAATG
ATGGGTACTTCTAATGTACAATTCTTAGATTACTAAAATTCTCTTAACGGCAACCATC
ATTGTCAGTCTAGACGCAAAGTAATAAATGAATTTAACTAAAATTTAAATTAGAAAAAAGCACAAACAACATG
TACAATACAAATATTTTAAATGTATTTATTTATATTTAGC
>103789541_2 DT662239.3
TTTTTTTTTTACATATATTACTTTAATGTCTGCTTTATGCTATGCTATAATTAAAAAATAATTTAGAATCATT
ATATTATAAAAAAAAATATAACCCAATATTAAAATTAACTTAAGTATATTTATATTTATTAAATAATCTGTGA
TGTTTCAAGTGTAAAAGTGTACAATATATTGAATTTTTCGAGAAATACTCTACAAGTG
TGAGGAAAACTACTTTTTTTTGAACTTAAATTATTTATTAAGTAATAAAAGTATTATAATTCAAACACAGTAC
GATATAGGCTACAATTAAATTGGTGTTTCACAAGGCTAAGTTAAGTTTCTTGGCGCAAGAGACGCGGGATGAC
TCTATTATATTAAATAACTTGTATTGTATGAGACTATTACACTATTATAATAATATGA
CAGATTAAGTACGCTACTGACGACAAACTGACAGCCATCTCGACTTCCCTTACCGATGAATTTTGTAATACTG
TATATATAGCGATTAGCAATGTAAGCGAAATAATCTCAAAATGGTTGTCAGTTTGTTTCATTTCTTCCTATTT
ATTACAAAATGTGAAATGTTTACAATTAAAAAATGTATTCTGACAGTTGTTAGATGTA
ACTGTGGAGTTGGGGAGGGGTAAGGAGGAG
>103789553_2 DT662457.3
GCACGAGGCAAAAACCCTTAAACTTAAAGTAAATGATTTTTATTATTTTTTCATATAAGTTCATCTAAAGGAA
TAATTTATTTTAATTAAGAAAACACGCTTTTTAGAATGATATAAAATGATTATTTAATTTATAATAATACACA
TATTTACTTATTAATCATGATAGAACTCTCAGCTAAACTCAAAACTGGAACTGTATAT
TTAGCTGGTGAAGCCTTAGAATGTTGTATTACATTTTGCCATACACCACAGCCTGAACATAGAAACTCTCAAA
GTCACAGTGATATCTTAGAAAATTTGGCATGGGCTTCTGCACAAATACATTGTTTTTACTCAACATCAAAAAA
TACTCCAGATAAAAACAATGTTGTTGAAAAAACAACGGCTTTGGAAGTTACATCATGT
GATATTGGTGATGTCATCTTTCACACCAAGCCTAAAATTCTATTTTGTGACTTAACCATTCCATTAGGAGAAA
CAAAGACATTTTGGTATAGAGAATCCTTGCCTATTGAAGCTCCACCTTCATACAGAGGTACTGCTGTTAAATA
TTCTTATAAAATTACAATTGCAACACAAAAAGTTGGTTCTCATATAAAAATGGTTAGA
ATACCATTTAGAGTTTTGCCCATAAGCCCAATAATTAATATGCAAGATTTAGCTGTTTTATGTGGCAATGAAA
CTACTGAAGAGTTGCAACCAACAAACCCATTTTCTGAAGAAAGAAAATTGAAACACCTTTAAGTATGGCTTTA
CAAGTACTACAGAACCTTACGGCTAGGAGAAGTCCAACTCATACATGATTACCAAT
>103789559_2 DV501072.2
CCGGACCAAGGCTTAGCCCTTGGAGGTTAGTGACCACGCGAATTGTTGACCTTATATTTGATGATTCTTCTGT
AGGCGACGTCGATACTGATTCTTTAGACCTTGCTCGTTTTGTATAATATATCCTTACGTGAAGTAGTTTATAA
ATCACAATGCGCCGACATGCTCGCGAATCGAGCGGCGACACAGCCGCCCTCCCGCATG
GCCACGACACCGACCACGACATCTGCTCTGACTCTGATCCTCGATCTCCCACGCCTGATTGAACTCTCACTCC
GCTACAGGCGAGAAATGGGACTCAGCACCATATTACGAATCACTTTTATTTGCGGAAGAGATTTGGAAGCGAA
AAAGCAGACGGGAAGCTTATACCTAATATCCCACTTTTATCTGCTGCTATACAAGATC
ATTTACAGTCATATGATCCACGTTCACCTACTCAGGATTTTGAAAGAACTCCCATTATAGTATCGAACATTGA
TGAAGATAAGGACAGTAAAAGAATGTTAAGAATTAACACAGACAACAATAACTGTGCCAGCCCTTGTTTAAAT
AATGATATTGTGGATAATAATTGTGATGATTCTTTTGATATAAATGAAATAAGCCCAG

ATTTCACAGTACCTAAAAATTTGTGTAATGGCTTTTATGACTTAACTTTGAATGGCACTT
>103789605_2 DT662866.3
GCACGAGGGTCACCCCTTTAAACGCCACGACGGCCCCACAACCGACTAAATATAGTAAATATCATTAAAAATG
ACAAACTTTTATCCCTACAGCTTTCATTTGTAAAACAGCATGTGATATGTTGCTGGTTGTTGATTTGCATAAA
TAGAAGGCTTTAAGGCTTAGATTTTAAGATACATCAATATCATTCACATGAAGACATA
TTGGTATCTAAACTTATGACATATTTTAAAGTTAAATATTTTCGTTAAAACTGCTTGACTAACTTTAAAATAG
GCCAAAATATAATTATGAAACTTAAAATTATTTAAAATTAATTAACTGTAACAACATGAATGTGTAAACATAG
ATTTTATTAAAAGCAATATCAATTTTATTTTTTTATAACAGGATAAGAGGAGAAAATT
AAACCAATCCCAGCTTTCGAACTAAATTTAAGATATTCCAAATGGTGGAAGTTAATGCAATATTTAATATTGC
CAGAGTATCAAGATGAATATAATTGATAACTTTGGGTAGGAATCAAACTATAAATATACCATTTATAGTCGGA
CACTGTGCCATAAGGGTCTATCTATAATGAATAATATCAACAATAGGCTGCTGAAGCT
TTCTTAGGTCTCTTTACAAAAATATTTATAAATGAATTATTTTCTTTATTATTTAAGTATAACATATCANAGT
CTAGAAGGAAGAATGTCTTAAATATAACAATAAACATAAATATATAATATATGTTTATAATCTTATTTAAGTT
TTTTAAGAACAAGATCAATCTATATTTCATTCTAACATAAACTTTCTC
>103789611_2 DT663430.3
GCACGAGGATCCAACCCTTAAAAACAGTCGCGATCATTTATATTTATAAATACAATAATTATAGATATTGTCA
AACTATAGCACTTGATACATTTTTATTTCCATACATACATTATTTTTGTTACCATATCCATATTTAAAATGCA
ATATTTTATCGTTCGACGTTTCTATTCCGGGGGGAGTCCCGGAATAAAAACGTATGAC
ACCAGATCACAATATATAATTATTTAAAATAATTTAAATTTGCTCGACCGGGGACGCAAAAGCCGTTTTTGAG
AATAAATCGACTTCACCTAAAGCTAGCAGGATAAAATGGCACCTACTTATATCTCGATGTACTATCACTGCTA
TTTGTTTTCTTTCGACAATACTTTATATTCTAGTTATTTATATTTTTATACAGATTTT
TCGAGAACAATCGCAGTGTGATGTTAGTCATGTCCCACGGGTTTGTTCGTTGTTATTATTTGACTCGGTGAGT
AAGTCGATGGTGTTTCTATCTGTACTCGAGATGAAAATATATACATTTAGTCGCTATATTCTTACGTCAATAG
CTTTAATATATACAGTATCTTCCACTTAATATAATTATTATAATACAGTTATAGCAAT
AGTTTCCAATCTGCCATATTACCTCAGTAATTATACACATATTTATCA
>103789617_2 DT661791.3
GCACGAGGGTGACGTTAAAACAGCTGTTGTTGACACTACTCAAGTGTCAAACGCAATAATTGTGCAATATTTA
AATGGATAATAATAAGTGTCCAATATCGTATGTTTTAGAGTGAGTCATAACAAAGCAGGTCGTGTAATATATA
GGCTAAGATTATATACTTACTAGTTACTACATTAGAGAAATAAAAATATATATAAAAT
AATGTACAGACTGTTGTCAGCACCACTTTAAATGAAACCACAGTTTTAACTACTCTATAAAAGCCTAATACAA
TTAAAAATACTGGTATATTTTTTTTCCAAAAATTAAAAAGTGGTTTTTTAAAAACAAATTTAGTGCAAACTTA
AGACTTATGTGATGTTCTCATGCATACCATTGTCAGAAATGGAACCACACGCATTATC
TTTTAAAAAAAGAATCCTCAAAATCGGTTCACCCAGCCTAAAAGTGCTGAGGTGACCTCAGCACGTAAAAAAT
ACAATCCAATTGAAACCCTCCTCCTTTTGCTTAAGTCGGTTAAAAAAAAGCCTGCATATAGCATGATAATCAA
AAAGAAGCGTAGTGTTAAGGCTCTTTGTTTAATAGGTCGATTGCTAGATAGGAGGTAT
TAAATGTCAACAAATTAGAATATTTCTTAATAATTTCATATAACTTGCTTATTCTCCTCTCTTTATCCGATTC
TATATTTCTATACTATAACTAATATTATAAAGTTAAACAATGTGGTTAAACGCGCTAATCTCAGGAACCAGTA
GGTTTGAATTGAAAAATTAATTTTGTTGC
>103789645_2 DT663419.3
TTGTAAAAAGCCGCAACCATGGGTCGTATGCACGCACCTGGTAAGGGTATCTCGCAGTCGGCGTTTTTTAAAG
GGGGCAGTGTTCCTACTTGGTTAAGATTAACCGCAGACGATGTTAAGGAACAAATATTCAAGCTCGGCAAAAA
GGGTCTTACTCCCTCACAAATTGGTGTCATGCTCAGGGATTCACATGGAGTGGCCCAA
GTGAGATTTGTCACTGGCAAAAAGATCCTGCGTATCATGAAAGCTATGGGCCTTGCCCCTGATCTACCTGAGG
ATTTATACTACCTTATTTTTTTTTTAGTAGCCATGAGGAAGCACTTGGAACGGGATAGAAAAGACAAGGACAG
CATATTCAGATTGATTCTTGTTGAGTCTAGAATTCACAGACTGGCTCGTTACTACAAG
ACTAAGAGTGTGCTCCCTCCCAACTGGAAATATGAGTCGAGCACCGCCTCTGCTTTAGTGGCTTAAGTTTAAA
TAAACTATGTAAAATTCAAAATAAAAAAAAAAAAAAA
>103789648_2 DT661922.3
AGGTTGTCAGTTCAATTCCTGCTCAGGGAGAATTATTATGTACAATTTATTTTTAATCAAACTCTCTGATAGA
TTAAACGCAGCTTTGAGCGCCAACAATCAACACACAATGTCATGAAAATCCGGTGGAAAATTGGGAACGAAGT
GTCTTAACACTAGGTTCCCTCTGCCGACCCTGATATACGGTAATAAACGTAAAGTTGC
TTTTGTGTCCATTTGTTCGCCATTATATAAAATAATATTTAAATAGATTTTTCTTAAGAATATTTGAATCACA
AAGACATTTACCTCCCATTTTTTTTTATCTCTAGGCGAATAAAACATTTACGTGACTTCATCCCAACAATACG
AGTATAAACTGGCGGGAACTCCGTCGCCGCCATGACCTTAACCTATATACAATGGAAA

CGTGTCTTCTTCCCATTTATTCCTTTTTTTTCTTTATCTTTACATATTATCTCTCTATTAGTGGGAGAGATAG
ATAGAGCGATATCATTCTTGTGACTACGTTCTTAGCTTTTAAATTTATTTTTAAATAATTTTCCTTTCTTTGC
TCTGGCAAAAAAAAGCGGCGTTATGTTTTGCTAATCATCACCCAATATTATTATCGAA
AATAATTGTGGATGATTGAGAACGGAGAAGTCT
>103789651_2 DT662119.3
GCACGAGGCGAATACCAATACTACGATTAAATTATTGTTGTTTAAATCTCTATTATATTGTTCTCGTATTATA
AATATTTTTTCATTGCTGGTAAATCGCGGCGGATACCAATAAAATAAATTGCACTATAGAAACGCTGTTGCAT
CATGCTTTCTCGTTTGTGCGCCTCCCGTTCGGCTTTTGCCGTTTCTCATACACTTAGA
ACTCCAGTGCCTCAGAGATTTGTTCCAAAAAACTATGTCATCAGGAACTATGCTCGCGAACCTCGTTCTAGAG
TAGCAACAAGATCGCAACCTACAGTATGGGAAAAATTGATGGCTCCTGCTGGACCAAATGCTTACAGCTTGGG
TAAGGGAGCGTTAGCAGGGGCATCAGCAGTAGGCCTTGTAGCATTGTGTTATTATGGT
TCAGGAGTTAAGCCTGGTACATTACAAGAAGCTCACTTATGGCCACAGTATGTCAAGGATCGTATTAAAGCTA
CATATGGATATATTGCGGGATCACTGATGGTGACAGCAGGGAGTGCTATAACTGTCTTTAGAACTCCTGCTCT
ACTTAACTTAGTGGCAAGGAATGGATGGATGTCAATTATAGCAACAATGGCACTGATG
ATCGGTTCTGGAATGGTTGTTAGAGGCATGGATTATACTCCTGGTATTGGAGCTAAGCAACTAGCTTGGGTTG
TGCATACTGGTATAATGGGTGCAGTCATTGCTCCTATCTGTTTCTTGGGAGGACCTATTCTTATCCGGGCTGC
TTGGTACACCGCAGGTGTGGTCGGTGGCCTT
>103789660_2 DT662742.3
AAAAATATATTTTATAATAAATTAAAATTATTATTTTAAATTATGCGATTATTACGCAGTGATAATTATATAG
TGATATTTTTCGTTATATCTGAAATTTAATAATATTTCATTTTCAATGTTTTTCTACTTTTAAACTAGAACCA
AAATTATTCAAATTATAAATAATAAATAACATTTCAATAAATCAATCATAATCATCCA
CGATGATTTGACTATTTTTTCTTTAAGATTTGCAAAATTTTTTTTTTACAGTAAAATTATATTTAATTAAGTA
CTTTAACTGCTTCAGCATCACAACTAACTTTCTTATGCATTTCTAAGCAAAACATATTTTCAAAGTTTTTCAG
GCTTACATACTAATAAGTAATAACTAATTTCATTTATCCATAGGAGGAGACTGGGTCG
ACATCTTCGTAGAATTATCATGTATCCATTTATGTATTACATTCGTACCCGTTATATCTACAGTACTACACAC
TGCCCTGCATAAACGAACTAAAAATATATATTTTCTTGTTACTAACGAAGTTATTGCTTACTATAAGAAAAAC
ATGTTTTGATAAATGTTTAAGAACAAAATTGGATTATTTATTGTCTATACTTAATTAT
TTTCTAAGTATAGATATGATTAAAAAAAAAATTTATGTATATAACTATTTAAA
>103789663_2 DT662668.3
GCACGAGGGTTGAACCCTACAGACGTGCTTATTTATATATTGTTACCATAGTATAAGCGTCACTCTTCACTGT
CTGCAATTTTCGAAACTTTATATTTTGAGAAGTGGAATACAACGCCGAACTATATCTTTTTACCAGAGCGGGA
AAATTAAATAAATTAATTATTCCGTTTGTGGATCGAACCCACAACCTTTACACCTAAA
CACTGGCACTGTACCACAACGGCCTAAAATAGCCTTTTTAAAATAATTGCTGTCAAGAAACTGAAGGTTAAAA
AATAATTGTTAAACAAACATGTCTAACTGATGTAAATAACATACTCGGCCTTGATAAGTACGTACAATTTTTT
GTATAGTTATATTTTAAATAAATATTCGCAATGTTTTGACTTTCATTTAACACGTTCA
ATAACGCAATGAAACCTTAATCCCGTAAATATACTTTTATGAGAGTAAATTATTTTTTTTGCATTACGCTGTA
TCCCGCATGTAGCACGGTAGACAGAGGCAACCTAGGCCTTAGACATTAGATACTCAGTTCTCCACGCACTGAA
CGATTATCGAGACAAAGCGTGTTAAGTCACTTATTCAACCTACTCACGTATTGTTACC
AAATGATTTTTTTTCCCAAATAGGAATTGAAACCACAGCCTGCAGACCTTTACTGTCAGACGCTGCTACTGCT
GTAGTAAGTATTATGTAAAATATAGAAAGTATTGCCATTTTTATCAACATTTAAAAAAAATTGTAGCTC
>103789669_2 DT662643.3
GCCGCACGCGCACGCACATCATAATGGACAGCAATCAAACTGGGTCTCAACTGGAGTGTCATCGCCACCCACA
GCGACCACCCCCCACGCGCCGTTACCAGTTTTACCAAACGGACATCAACACGTTGTTTTCCCACCAGTTATGG
GCGCGCCGGCCGAGCAGCGCCAGCTCGACGAGGCGATGGTGTTTCTGCGCGAGCACTC
TGATGTTGGGGGTGCTCGTATGGAAGAGCGTTTGGACGACGCCATTAACGTGTTAAGGAATCACGCGGAAGCT
CCTGAGCTTTACCCTTTTTTTTTGCATCATCCTCACCAGCATCAGCCCGCGATGAGTCGAGTGGGCGTGCCCG
GCGCCGTACCGCACCTGCACACGGAGCCGCCGGTCAAGATGGAGAGACATTTGTTGCC
CAATACTAAGAAACGCAAAGAGCCACCAGACTCTGGTCTGGATTCGAAGCCATCATCCTCGGGGTCCGCGGAC
GCGCTCAAGCAGGCCGGCGGGAAACGAAGCCGCAGATACGTACAAAATCAGTTCAGCTGCTCGTCGGCGGACG
AGGACGAGCTGGACCCCGACGCGAAGGCGGCGCGCGAGAGAGAGCGCCGGCAAGCCAA
CAACGTCCGGGGAGAGGATAAGAATAAGGGATATAAACGACGCTCTCAAGGAATTAGGCAGGATGTGTATGAC
G
>103789681_2 DT663166.3

GCACGAGGCATATTCCTTAAAAATTCAAACACAAAATTTCGCATACCATCTTGAAAACAGTTTTATACTTTCA
CATGCCTGCACAGTAACTCTTACAGCAGTAAGCTCCAGACTCCTAAATAAATTTTATCATTTTGGATTTACAC
ATTAATTGAGTAAGACACAAATATTTTTTATATGATATCTATTTGTATAAATTGCATA
CTTCCTATACAATAAATCTACACAATCTTGTTAAACATCCTAATTTATAAGGAACTATTCAATGAACTTAAAA
GGAAATAAATTATGCATAGCCATTTACAGAAAAAAGCTTTGAAAAAACAAAGTTTAATATAAAATAAAAATCA
ATTTATTACAATTTTTATCCTAAGGAGATCTTTATTTAGATTTATGTTTGATCTGCAA
AATTTACAGTCTTTCGTTTATCTGTTGTAAAATGAAAATACACTTATTTAATTTATTAATTATTCCGATTATG
AAAAATGTAGAAAGAAAAAGAAAAGAGAGACAAAGACAAAGTTAATTCTTAATTAGCTATTTTCTTTATTATG
ATATTTAAGAATAGTAAACGAATAGAAATGCAATTTTAATATTTAAAATACACAATTT
ATAAAAAAACAATTAAGTGAATGAAAGAAAGAAAAAAATATTGTAATAAAAACATGTTTTTTTTCGTTAGTAC
AAAGTGCAAGAATTAAAATATCAAAAT
>103789697_2 DT662147.3
GCACGAGGGACAATACATAAATACAAATAGACAGTTGTAATGGCGGGAAAAGTGACATTGAAGTGACGGACAG
CGAAACAAATATATCAAAGGAAAAACTTCAAAAAAATGGTGACTTTAATAATTTTTTACAAGTTACAAACGAA
AAAGATTTATTGGGAATTCCTAGACAAGGACACAGAATGTCTTTTATGGAGGAAGAAA
ACATAAAAGATAAGATACGATTGTCGTTACTAAAACAGTGTTCCGCCATTTTGAAGCAAGGAGACCAGAGATA
TAATAAGGAGAGTTTACATCGAGCTTTCCAGGATGAGGATCTATTGGAAAGGATATTCCGCTTATTTGACGTG
GAGCGGCAAGACCACCTCGTACAAGAAGATTGGATTGAATTTTTGAAGGAAAGATTGA
GAGAAGAAAAAGAGCTAGACTTCGTAGAGCAAGTGGAGAGCGTCGCGTACGTGCTGTGTGGAGACGGCGTCAT
CACTCTAAACACTTTTCAATTGATATTGAAAAATAAAGTGGCCTCACGTGGGCTTCGACAAGTGCAGTATAGA
TCGCCTCGAGCAATTATTCCGTCAGACGGCCGGAGACCAGAAGGAGATCACTAGAGAA
CAGTTTCAGAAGATTGTTGTCTCTAAGAATCCATTTTTCACTGAGCGAGTATTCCAAATATTCGATGGAGATC
ATTCTGGAACGATTTCTCATCAAGAGTTCATAGAAGCAGTGCATAAATTCGGAAGTCAGACGTC
>103789700_2 DT662904.3
GCACGAGGGCAAAACTCTAGCCACACCGTATATTATATACATTTAATAACGATTTGATTTGTCTATAATAATA
GTTAAAAAATAGATCAATGTTACACTTATTTTTAACTCATTTCTTCGGGGCACCCAAATTATTTTAGTATAAT
GTTTATAGGAGAAAGTGTTTTTAACTAAATTATTAATAATATCAAACATCTTTATTTA
TCATATATGCCAGAAACCAGTACAAAACCAATTACAATGGTGTTAGTCTTCATGTTATTTCAGCATCATACTG
GTTGGCTAGATGGTTTAGTTGAATATCTAATTAGGCTGTGCCAACTGCTATAAAGCATGTTCACAATAGGCAT
TTTGGTCATTCGGCAAAATTAACATTTTTTTTTAAAGAAAATACCTTATTGAGATACT
AGTTGTGTAATTATAGTTATCTGATGTTTAAAAGGATTTTATACATAGTCTATTGTGCCTGTGTGTTGCGCTA
GGTATCATGGCCTGACTCAACACCTTAATATAACAAAATAATAAATTCTATAGCAATAAACTATATATGCTTG
TAAGTTAACATAACTATAATTAAAAGTGTTCTTCATACATAATAATAATTATAAAACA
CAAAAAAAGCAAAAAAAACAACAATGCTTTATGATATTGTTTAAATATATTTTGTCTCAACATAGTATGTCTA
AATTGATTTCACTTTTAATTGTGGTATTTATATGAAGTTTATCAGCCGGGCGTGTACGTAGCGACACATACTA
TTTTTAG
>103789711_2 DT662709.3
GCACGAGGAAATGTCCAAAGCAAAAAAAGTTATCGAAGAAGCAAAAGAAATAAACAACCCCGAAATTGACTTA
GTGGATAAAGGAATTTCTAGCCTTGAAGAAATACCTGGCTTGTTTGCTCTCGAAAATATAACACGTTTGACGT
TAAGCCATAATAAATTACAAACTGTTCCTGCTGGCTTGGCAAATCTAATCAATTTGGA
GATACTTAATCTTGCTAATAATCATATTGAGGAGTTGCCAGTTAGTCTATCATCTTTACCTAAGCTTCGCATT
CTGAATGTATCACTCAACAGACTATATACTTTACCAAGAGGTTTTGGAGCATTCCCTGTTTTGGAAATTCTGG
ACCTGACATATAATAACTTGAAAGAGCAAACATTGCCTGGAAATTTCTTTATGATGGA
AAGTCTTCGAGCTTTGTATTTGGGTGACAATGATTTTGAATTTCTTCCTCCTGAAATAGGAAATCTGAAGAAC
TTACAAATTTTGTCAATGCGAGAAAATGATCTGATAGAGGTTCCAAGAGAGTTGGGCCAGTTGTCTAGACTCA
GAGAGCTTCACCTGCAAGGAAACCGTCTTGTTGTGCTACCACCAGAAATCGGTTCTTT
AGATCTTGCCAGTAATAAATCAGTATTGAGGTTAGAAGGCAATTTCTGGGTGCCACCCATTGAAGATCAACTT
AAGCTTGGACCGTCTCATGTTCTGGATTATCTACGATCAGAAACTTATAGAGTGTTGTACAGTCGTCACATGT
CTGCCA
>103789726_2 DV501092.2
GCACGAGGGTTTTTCCTTTTTGAATGTTTACTCGCGAATAAAAAAATATTTAAATGCTATCTATTATAATGTC
TTTTACAATATTTACATTTTTAAAATCATTTTGTAAGTTTGAAAATTTAAATTTGCTTCTGCATTATTCTTCA
AAGACTACGATTAATTTATCCGATCCGGCAATTCCCACTTATTTGAAACATTTAATGC

TAGAACATGAAAATTTACAAACTAAAATACACATAAATATTGAGGATACAAAACGGCTCCGCGAAATAAAACC
AATATTAAATGTACTTGAACAAAGAATCGCCCTTTTTGATAGTATTCAATCTCTTAAGGAACTGAACAATCAA
CAAAGACAATATGAACAAATTAAGGAAATGATTAAGGAAGAATCTGAAATATATTTAA
AACGCCTTAAAGAAGCACATGATGATTTACAATCTATTTTAATACAACCATATTTATCACAAGGTGGAATTAT
GATTGAACTTACCACTGGAGCTGGAGGTCAAGAATCAATGCTTTTCGCGAAAGAACTTTTCGATATGTACAAA
GAGTATGCTAATTATAAAGACTGGGAAGTTGATGTAGCCTCCGTTGAACAGTCTGATC
TTGATGGCATAAAGAAAGGATCACTCATAATACTGGGTGTATGGGCACCTGAA
>103789751_2 DT663158.3
GCACGAGGCCTCGTCCTAAAAAAAAATCAACAGTATAATCAATATAAAGGAAAAATATACACTATTTACACAT
TTTGTACAAATATCGAGATCGCCTTCACAGAAGGCATACATACATACAGGCACTTAACGTTACACTACAAGTC
TTTGAGACGATAACACTTCAGATACACAACAGTCTATTCATTATAGTCTTATACCCTG
GAGACGTGGAGGGGTCTCTTGTAGTAGGGGTGGTGAGGTCTTAGGAGTGGCGGGGCGCAGCACGGCGCCTTGC
GTGGTGGGCAGAGCGCCTTCTTCCAAACGACAGCTGATTTTTTTACCAGGCCCACGCGCCCGCCGCCACTCC
GCACGACAACCACAGACACATAGACACTCCGCGAGATCCGGCGGTGTTATCTATATTT
CCAGCTTCCAAAGCCGCTAACAACGCTAGGCTGGTTTGTGCTTCCCTGTTTCGGGCTTCCATAACCCGCGCTC
CCGTTGCGAAGGCAGCCAGCAGTAGGTAGAGTAGGCCTCCGGCCGCGGCCCGTGCTGCTAAACGTCCAACTCT
TTTAGCTCCTCTAGAATCTAGCGCCCGACGTACTCTCATTATGCCAGGACACGCCCCT
ATCGCCAGTGCTACTGAAATGATGAGCAG
>103789757_2 DT662161.3
GCACGAGGGTCACACGTCAAATATCCAATCAAAATCATAATTTAAATAAATATATATTATTTTAAATAAAAAA
TTACAATGTTCTCTATAGACGCACCCGTAGAAATTCAAAATAGTGAATATTGCAACTATCGGTCGCCGTTGAG
TACAAGATATGCGTCGAAGGAAATGCAATATAATTTCAGTGATCAGAAGAAATTTTCT
ACATGGCGAAAACTGTGGATCTATTTAGCAAAGGCGGAACAGGAGTTGGGTCTTGACATAAGTAACGAACAAA
TTGCTGAACTAGAATCAGCCATCCACGACATAGATTTCGCATCTGCAGCGGAACATGAAAAGCGTGTGCGTCA
CGACGTTATGGCTCACGTCCATACTTTGGCTGAAAGATGCCCTACTGCTGCGCCCATC
ATTCACCTTGGGGCTACATCATGCTATGTTGGTGACAACACCGACCTTATAGTCCTCAAACATGGTCTAGACC
TGCTTTTGCCGAGGCTGGCTGCGGTAATCGCCCGTTTGTCCAAATTTGCTGATGAATATAAATCTTTGCCAAT
TTTGGGCTTCACTCACTTGCAACCAGCTCAGTTGACGACAGTTGGAAAGAGAGCGTCC
CTATGGCTCAGCGACTTATTAATGGACGAGCGTGCTTTATCACGTGCGCGAGATGATTTACGTTTTCGCGGTG
TCAAGGGCACTACTGGGACCCAAGCTTCCTTTTTGCAACTTTTTAAGGGCGATAGTGCCAAAG
>103789766_2 DT661781.3
GCACGAGGCAATGGAATGGGAAAAACGATTAGAGGAAGAAAACAAATTAAAAGCCGAAATTAATGAAAATATG
TCGGATGATGAAATATAAAAAATTGAAGCAAAACTTGAGTCTCAAACAGAAGAAAAATATACAGACAGTTCAG
AGAGTGAAGAGGAACCTATCGAAAATGACGTAATTCTAGAAGATAAGCCAAAAAAGAG
GAATCCAATGGTGGATGATGAAGCTGAAGTTTCAGAAACAGATGAAGTTTATGATAATAATGACAAAATAGAG
GATAATGAAGATAAAATGGATGATGATGAAGATATGGATGATGAATCTAGTGATAGCTCAGATAGCGAAGAAG
AAGCTGAAGAGGAAAACAAAACAAGAAAAGGAAGAATTTTAAAAGCCTTTGAAGATTC
TGATGATGAAGAAAACGATAAAATTACTTCCAACACAAATTGCGAAAATTTACCAACACTAACAGAAGAGTGT
AAATCAACAGAAAACCACTTAAAAGAGTCACAAGACGACGATTTACAATTAGCTCAGATAAACGCATCATCTC
AAGATTTGTTTTCCACACAAGGATCTAATAATAACCCTGATTCAAATGTAGCAATATT
AAATAGGAATAATGAAGGGAACGCCAGCTCCGATTTGGGTACACAGTCGTTTTCTATAGTGAACTCAGCTGGT
GTAAATGGAACTATGGAACTTTATGTCACCGGA
>103789809_2 DT661855.3
GTTGCCATGTCAAACAGAAAATTATGGTTGTGGTAGTTAATTCAGATGATATATTTTCCTTTAGGGGAGTGAC
GTGTTGTTGAAAATTATCTAATACAAATATAACGACCGCATACCCGACTTTGCGTACTTATCG
>103789812_2 DT661913.3
GCACGAGGTGAACCCCTTAAGGGATAAGGTAGCAAAAGAAATATTAGACAAGTACGGAAGTCCCGAAGAGCAA
TCTAAAGCGACGAAGCCGTTCTCCCCACTCGCTGTCCCCGGGACGCCCAAGAGCCAGCCAGCAACCCCAGTGC
CGATTCGACCGAGGCTGGCAATACAGAATTCCACACCGATATCTAATAACAATCTCAA
TATAGTGCCGTTTAACACTCCAACTGCTTTATATAAGGGACCTAGGCTAAGGTCAGATCAACTTCCCCGCCCG
CTAGCTGATAGAAACCGCTCTGCTCTAGACAAAGTGGTCGACTATTTACTGAAGGACGGTCCCAGCAATCGTA
TGGCGTTGATTTGTTCTGAATGTTATTCTCATAATGGTATGGCAATGGAAGAGGAATT

CGAATACGTGTCGTACGCTTGCGCGTACTGCGGCCGCCTCAACGCCGCCAGGAAGCAGCGCCCCGCCGCGCCG
CTGCTGGCCACGGCGGCGCCGCGAGCCCTGCCCGCGCCTTATAGATCTAGTGCTGGTAGCGGTGATGATTCCT
CCGCAGCTAGTTCGGGGAGTGACAGCGAGGAGGAAAAGGGTAATGAGAATAAACTGAA
ACAAAGCGAAGGTGAACCGGATCGTCCCCCCTCCCCGATGGAGGTGGAAACGAAATCCGAACAAGACAAAAAA
GAAGACTGACAGAATTGTTAATTATTTAAATTTAAATTAAATATATTAATAATACACATAATTTACAAACGAA
AATAAATG
>103789878_2 DT662508.3
GCACGAGGTTGAAACCCTTAAAATTCTTCTGTTAATTTGTTAAATATTATTATAAATATTGTATGAACGTAAC
ATTCTTGAAAAAATATCATTCCAGTGTATTATCTAAAGTTTAGGATAGGTGCACCTTATCTATAATTGTTATT
GAACAATGTGCGGAGTGCTTGCATATATTTTTGTAGTATTATTTATTTACATACCCAT
TTTAAAATACCTACTGATTTTGTTCAAATGTGTGCATATTAGTCTTGTTTTAGTATATTATTTGGCGTTCATG
TTCGAATTTCTGAGTGAAGAGCACTATCTAGTAACGGAATCTTTTTGCCATTATTTGCATTTATTTATATTGT
TAATTTTTAATACCGATTTCGACAACGAACGTGGCTCCAGAGCCGGATGTGCCAATGT
TAAAATGTTTACAAAAATTAAAATTTAATGACTTGTTCCATAAGTATTTTACATTTTAAAAATAAATTAAAGT
TCTTTTGTCAATGGTGTGAATGTCTGTTGGTGGTAATATGTGATGGTACTTTAGAGAGTCAATTAATATTATA
AAGTACATTCACATATGGTTTGGTGCTATACATTTGTAAGTATTTATAGTTGTGTTAT
ATGTTTTAGTCACAACTTAAAGGTTGCAGGTCTTGATTAAAAGTGACAGGACATAGCTATACATATTAACCGA
CGATGGGAGATGTATTAATATAACAACTAAAATCAATCAAGTATGTTTTGTTTTTGCTTTACTAAAAATTAAG
TAATATTCCATTATCTAAGTAAATTTAACTAACTATTTTAA
>103789902_2 DV500992.2
GCACGAGGCAACTTCCTTTAGAGGTTTTAAGGGAATTAGATGATCTTTTTATCACTATATTACCACGTAGGTA
AAGGTGCCAGTATGTACAAGATATTAATATTATCCTCTTTTCTACCATATGCGCTGTCCCATCAGTATCACCA
GAAAGTTGCTCCTGGAGTGCCGCCCCAAATCTATGCACAATTACCAACCTCCTCCCCC
TCCACAACAACTGCAAATGCATCAACAGCCACCTCTCCAACAATATCATCGACCGCCGCCCCCATACAGCAAT
ACCGACATCCGCCTCCCCAACAACAGTATCAACAACAAGTTCCTGTTCAACAGGTAACTGTCCAACAAGTACC
TGTTCAAAGTCAACATGGTCACCAGGGGGATCCTCATGTACTTAATGCCGCTAACATC
GCACAGCAAAGAGAACATATCCAAGAACATATGGACGTGCCGATCGATACATCCAAGATGTCGGAACAGGAAT
TACAATTCCACTATTTCAAAATGCACGACGCCGATAATAATAATAAACTCGACGGTTGTGAATTGATCAAGTC
GTTGATACATTGGCACGAACAAGGTCACGAACAAGAAAATCAGCAAGGAACGCCGCCG
GTAGGTGAGAGAATATTTAGCGATGACGAATTGATCATCTTAATAGATCCTATATTAAATATGGATGATC
>103789907_2 DT663396.2
GCACGAGGATCAACTTTTAGAGTTCTGTTTGCTTATCTTTCAGGTAGTGACAGCAGAGCCTCCACCTCCACAA
TGTAGGGGTCCTCCTGCAGGATTGCATAAAAGTCCCAAAGAATGCTGTAAGTTCCCAAAAGTATTCAAAGAGG
AAGATTTCAAAGAATGTGGAATAGAAAAACCGACAGAAGAAGACGGTAGTTTCCACAA
TCGTGGACCACCCGACTGCGATAAACAAAAATGTATACTACAAACGAATAACCTTATGAAAGATGACTCTACC
ATAGACAAAGATGCCGTGGCAGCGTTTATGCAGAAATGGTTTTTACGCTAACGGCGATTTCAAAGATGCTGTG
GATGCTGCCATCGACAGATGTCTTAAAAAAGACCTTCCTGGACCACCAGAACTATGTG
AAGCTACAAAAATGATATTCTGCATTGGTTCTACTGTGTTTATGAATTGTCCGAAATGGGAAGATACAGATGA
CTGCAAAAAAGTGAAAGAGCATATTGAAGAGTGTAAACCTTATTTTAACTAATTCACTGTTGCTATACGTTTA
TTATGTGAATCAGTAACGTTATAATATTATGGCATAAAAAAAATGACAATATGCATTT
GTTTGTAAATAAAATAAAGAAATATAATATATCTATTAAATGCTTAAAAAAAAAAAAAAAAAA
>103789928_2 DT662084.3
AATGACCTGGTGAGCGCGTTACGTGTGGTGGACAAGACACCTCCACAACTCGCTTTCCTTAATCCAGCGCATT
CGAAGGTTTTTGAAGACCTCCGCGTCGAGTATAGAAGTGCAAATCTTATAAATCCAACATCATGCGCCACAGC
TCTCGAACCAAACGGAGAGGACTGGTCGCTGGTGGTGAACTGTGCGGGAGAGACCCGC
GTGGGGCAGACGGAGGCTGTGTACGCGGAAGGCATCTTCACATTAAGTGTGAACGTGGCCAAGCAGTGCGCCA
AGCTCAAAGTGGCACGATTTTTTGAAATATCCAGCGGGCAGATGTATAACACTGATAAGCCTCACAAAGAAGA
TGGTCCAGTAGATCCGTGGACGGTGGAAGGTCGCATGAAGAGCCGCGTGGAACAAGAA
CTAAAGAATATGCAATCTGAACTAAACTACACTATCATTAGACCCGCCATTGTCTACGGAATTGGGGATAGGA
GGAGTTTAACACCACGCCTCCTCTACGGTGGCATATACAAACATTTAGGTGAAACAATGAAACTTTTATGGAC
GGCCGATCTAAAAATGAATACAGTCCACGTAAAAGATGTCAGCCGAGCCATCTGGACT
CTGGGCATGAGGGGTGACGCTAAAGGACAGACGTACAATGTGGTGGACGAGGC
>103789945_2 DT662466.3

CGGCACGAGGCGAGCTGCCCGCGCGCGACGCGCCCGCCTCGCGCGCCGTGCGCGGCCTCGTGCACGCCGCGCG
CCTGCTGCGCAAGAACGCCATGAGGCTAACGGTAATGCGGCAACACGACAAATTAGAAACAGTTCTACGTCAT
TTCCTAGTAGAGGACCGCGGCGTCGACGGCAGCTCCTCCTACGTAGACTACCTGTGCC
ACATTCACAAGGAGATTAGAGCGTTACTCTAGCATTTGTATGAGGATTCATATTAAAAATATAACGTGAAACG
ATTTATAGACTATGGTTTTTAGTACTAGTGATTGTCTATGCCTTGTAGTTTTTTTCTACAGAGTAAATTGT
TAAAGCCTTATTGAATTCTGGTTTAGATATGCCAAACTGAACGAGAAGAAAATATAAA
GTAATAAATATTATAGAGTCAGTCATATTGATTGTTAATCGTCATTTTTCCTTCTGTTTTTAAATAAAATAT
GGTGGTAATAGATTAAAAATAGAAATCGATTTGTAATTCAAAGTAATATTTCATACAATAGTTGACAAAGAAG
GATAATTGATACATTACAAGCCTTATATTACACATTACAAGCCTTATATTAGTTTTAT
ACTTATAATGATGTTTGAGTACTTTTTATGTTAATTATTACATTATTTTCGTTCCTTCATAATATTTGATTGT
ACTGAAATTCAAAAGTTCAACAAAAATTTCAAAATAAATTTTGTTCTATCATATATACATATATAGATTACAG
TATGTGATGTGAGTTGATTAGTTAAATGTTAAGCAGCTGCCACACCACACTATTACTG
ATGCACTAAGTTACTGTACATAGAGCCTAAGTATGTTAAT
>103789951_2 DT663258.3
GCACGAGGCAGAATCCGTGATATACTTATTAAAAATGGATACGGTTTTGTGGAATTTGAAGACTATAGAGATG
CAGACGATGCTGTGTATGAGCTAAATGGAAAAGAACTGCTTGGTGAGAGGGTGGTGGTGGAGCCGGCGAGGGG
CATCGACCGCAGCGCGGACCGCTACCGGCGCGACCGCCACTACGAGCGCGACCGCGGC
CGGTCGCGATACGATGACTACAATTATAGATACGGGCCGCCGACGCGCACAGAATATCGACTTATCGTCGAAA
ATCTGTCTAGCCGCATTAGCTGGCAGGACTTAAAGGATTATATGCGTCAAGCTGGTGAAGTCACTTATGCTGA
TGCTCACAAACAGCACAGAAATGAAGGCGTGGTGGAGTTCGCGACGCACTCGGACATG
CGCGCGGCGATGGAGAAGCTGGACGGCACGGAGCTGAACGGGCGCCGCATCCGCCTCGTGGAGGACCGCCGCT
CCTCGCGCCGCCGCTCGCGCTCCTCCTCCTCGCGCAGCCGCAGCCGCAGCCGCGAGAGGCGCCGCTCCAGGTC
CCGCTCCCGCCGCTCGTCGCGCTCTCGCTCCAAATCCCGCCCGAAGAGCAAGAGCCCC
GCAGCCAAGTCCCGCTCGAGGTCGCGATCCAAGGACCGCAGCCGGTCGAGGTCCGCCAAGTCGGAGCGGCGCT
CGGCGTCGCGGCAGTCGCGGGGCTCCCGTGGCGCCTCGCGGGGCTCCCGCGGCGCCTCCGAGCCGCGCGCCTC
GCCCCGCCCTC
>103789960_2 DT663408.3
GCACGAGGAGTGCCCCTTAAAAAGTATTTAATATGTCTAAATTATGGATGAATACGACATCATCACAAGCTAG
CTCATGTGAGATTTCTCCGTATGGATAATGCATCGAAGCACTTCCTAAATTGTACTAACAATCTCAATAGGTT
CATATATTATATACACCTGAGGATGAAAGTACACATATCAGTAGAAGGTGTAGAAATT
GTTGCTCCATCATTAAAAAATGAGTCTAGAGATGTTGCCCTTCAAATTCTACTAAAAGAAATAGTTAAAGTAT
TAATACATTTTGGTAAAGGGCTTCCGGTAATTTTTTTGTATACATTATTCTTTTGTGGAGTTTACATTACAAA
AGCTTTGGACATGGAGGAAGGATCAGGATTATTCTACGATCCTATGTCAAAAGAAGAC
CCATTCAAAAGGATAACATTATTGCCCGAAATTATAACAGATGACACAAAATCAGCATTAAAAACTCTATTTC
ACAAATCAATAGATGAATTAAATACAAAAGAAGCTAACGAAATTCTAGTGAAAACTTGTCCCAAAGAAAGCAA
TAATGTTTCCAAATTAATGACAAGGTCAATGAGTGCATCCATATCCACAGGAGCCAAA
AGTTCCAACCCTACCGAAATAAGACAAATATTGATATATCCGCCTGGCAAAGGTGGGATCCCCATAAACACCG
AAGACTACATGTGTCTAGCTCAGGATCAATTTTTAAATGATGTTATTATTGA
>103789975_2 DT662247.3
GCACGAGGGAAAAACTGAGTAAAAAGTCACTTGAACGAGTGTCACTACCTAGGGAGCGGTCTAAGGGGCATGC
GTCGAGTACAAGCAGAGAGTCGTCCGTGGTAAATGAAACTAGAGATAAACCCGTTCCACAAAACGATAGTCAG
GTTAAGACAGTGCAATCATCAGACGACGAAGATAGAGATGACTTCATCACAGTACCAG
TATTACGTGAATATTCTAAGAGTCTCTCTCGGACTCCTTCGCCTTTCTTAAGAAAACATGAGATGGAAAGTAA
GAAATCAGAAAGATCGGGTGACGACGCGTCAGGGAAAGAACATAATGAAGAAAACACTAGTAAGGTTCAGGAG
ACTAAGAAAAACAAACGTAAAAGCAAGCAGTCTGAATCAGAAAGTGAGAGTAGTGAGG
AGGTGTCTAAATCTAAGAGCAAATCAAAACTTAAACGCAAAGAGAAACCAAGCTCTAAGAGATCAAAAAGGGC
TAAGAAAGAGAGTTCCTCTAGTGAAAGTGATTCTGAAGATTCTTCTTCAAGCGATTCGGAGGAAGATAGGAAA
AAGAAGAGTAAGAAGAATGCTAAGAAAAAACTAGCAAAGAAAGCGCGTAAGAAGAGGA
CACGTTCATCCAGTTCCGAGTCCAGTTCTGAGGAGGAAGTAAAACATAAGAGTTCCAAAAGCAAACAAAAGAA
CATTCCAGAGGAATCTGATAACGATAAAAAGTCCAAGAAACGAGGAAAAGAAAACAACGTTGACCGCGCTA
>103789984_2 DT661826.2
GCACGAGGAGCCCCTTGACGAGGAGGACTCGGACGACGACGGCGCCGGCGACGCCGACGACATCGAGATCGAG
TACGACGACCGCGCCTAGGCCGAGCCCATCCGCGCCGCCGCCGCGCGCCCCGCGCCCGCCCGCGCCGCGCGCG
ACGACGACGCCGACATCGACGTCGACATCGACGCCATATGAGCTCCGGATGCCGAATT

GTATTGTGTCATTTCATATCTCTTTTTTAATTAAATCATTGATAACCTATCATTATGTTTCCTCATCTTTTGA
ATTTTCAATAACTTTTATTTCATTTATAACGAAATGTTTTTGCGCAGATGGTATACACATAACCTAAAATTCG
TATATGGACCCCATGTGCTGTAAAATGTTTCCTCTATACTAATTAGTAATCGCCGTAC
ACGTAAGTTAGACACTTTTTAAATAGTAGACTCCGAAATTTATAAATATAGGCTTTACATTAGACATTAGAAA
GATGACAGCTTTTAATATAATTAGTGTTATTATGTATAATATTATACTGTAATAGAATGCATTTGAATATCTT
AGAAAATTTTCA
>103789990_2 DT662441.3
GCACGAGGCGCACTCCTTGATTTGGCATCTGCCAAGCCTGAAATGTATAAAGAGAAGGAAGACTTTCAATTTT
CGAGAAGTTCGTCTGACGATGGTTCAAAATCTGGTTACTACGGAGCTCAGAGAGGAAATATGGAGGCAATTA
CGAAAGAGCACATAATATGGATTCCTTAGCACAGCATCAAATGAGTACTCTTGTAAGA
CATGTCGACGGAGAATTAGGTGAAGGTTCAAATACAAGAACCGGAAGTGTTTTCACTTCAGGTAATTCCCGGG
GTATTTATGGCAGCGGAAATTACGATCTAAGTAATTTGAAAGGTTTATATTTTGGAGAAAGCGGTGCATCATA
CAATGATCTGTATTCGTCATCTTCATTATCTCAAAATTCCAGGAACCATGCAAGTTTA
TATTCAGGAACGAATAATGCGAAATATAGTGGCAATCAACTAGAAGCATCTGGGCTTACCAGTGCCGGATACC
AGCATGGAAGTCTAGCATCTCAATATATTCAAGCTCAGGATAAGCAGCAGGCTGAAAATCAATATCATGGATC
CTCTAGCAATTTTCAATACGGTGGTCAAGGCTCAAGTCAATATAAATATTACTCTCAA
GGAAAAAATGACTTGATTGACCAGAGCGCCAATTTGCATAGTAATAATAATTATAATGTCAACACTAAGAGTA
GATTAATCAGTACTGCGCCTCACAGAATTTATCTAAGGCCTGGGACAAGAGTAACTATACCGGTTGCGGCTCA
AACATATGACGCTTCTTCTTTATCTCAACA
>103789996_2 DT662123.3
GCAATATATTTCTTAATTTTATTATGAATAATTAACATATATATAATTAATTACAAGTTACTACTTTTGTTAA
CAGCAATACACTCAGATAAACGAGCAACTAAGCCTTCTATCAAATGGAAGGCATTTAATGCGGAATGTATAAT
TTGTTTCTTATTCTCAACAGGTATTTTGTCCAATACATTTCCACCAACATTAATCGAG
ACTTCTACTTGTTCTCTGGGCGATCTTTTAGCATTTTCTATATCTCTCAACTTTACTTCATAATGTGCTTTTG
ACATATTTAAACCAGTTCTTAAGTTTTTCAAGTAATCTTCTTCTATGATGTCAAAATGACCCCTAATTCCAGT
TTTGTTTAAAGTGTCTTTTAATGCTAGATATCTTAGAAAGTTCAATGCAGTTATGATT
TGGTCTGCTAGTTCAACTAAATCTGATTCAGCTCCATGAGGTAAATAACAGACTTTTTTTACTATACTAATAA
GTTGGACGCCAGTGAAGCATTCTGGTAACTCTGACTCTTGAAAAGCTTTATCTATGGAATTCTTAAGCAAAGT
TATTGCAAATCCTATTATACCTGAATGGTTAGCTGTATCCAAGATAAATTTGATAAGC
ATACATCGACCCTTGTAATCAAATTTGTTAATATGCATTGCAATAAGTTTAACTGCCTCCTTCCGTATGTAAT
CATAATTTGAATAAACAGATATGTTAACTAAATTTTTACATAAGCTATAATGCACCGAATTTGACAAAACTGT
ATGATTAT
>103790000_2 DT661942.3
GCACGAGGGTTTAGACATTTTTATTTATTTATGATCAATTTGGACCCTAATTTGATCACATTTATGAATATTT
TTACTCCAATATAAAGTGTTGTTGTGTGACCTTAATATGTACCCGTAAATCTTTTTATATTTATTATTATGCG
CGCCGTTTCTTGTTAGATTGATTCCAAAATGGCGTCACTACAGGAGTGTGTTTATAGA
TAGACGCGATAGCATGTGTGTTTAAAAAAAATCTAAAAATAGAGAGCTTGCATAAAGCTAGTTGCATAAATCT
TAAAGAAAATGTCTAATACAGTAATAACAAACTATACGGACCTGAATGGACCGTCAACAAAAACTGATAGTTC
TGTCGTTGTTATTGTAAACAAGGATGGGTCCTTGTCAGTAGACCAAAAGTTACTTGGT
ACACTAATGGGAAGCGATGGATCTCAAGCCGCAGGAATAAGTGTAGTACGAGTTGGGCATGAAGGACGCCCTG
ATGATGCCACTGATTCAGAGAATGAAGATGAAAAAGTTCCACATGTTACTCTGTCAGTTGATAGTTATTATAA
TGAGCCGGATAGCATTATAAAATATGATAGTGGTGCAGAAATGTTACAATCACTAAGA
ATAGAAACAAGAGAAAATAGTTTGGTTGGTTTCCAAAATGATCATTGTTATACACCTCTTACATCACCAAGTC
AAGTATTGCTCACAAAAAAATGATACATATGCATCTTATGATGATCCAGTGAT
>103790029_2 DT662439.3
CAAGCATCGCTTACAAAGAAACATTTTAAAAACGCAGAACTACGTGGAAAGAAGGAAACTAAGAAGAAAGAGA
TCAAAGAGAAATCTCTTCCCGCGCTCAACGCTCACAAGCCACCAGTCAGGGAGCCGGAGTTCGTTGACCCCGC
TAAGTTGACTGGTGATCTAAGGAGCTTGGAAGGAAATGCCAATCTTCTCCGCGATCGC
TACGAGTCTCTCCAGCGGCGTGGTGCCCTAGCAGCGTCCAAGATTATGATGAAGAAAAAGAGGAAACTCAAGA
GTTACTTCAAAGCAGGACACAAGGTCACTGATCAGGATGTCCAGAAATTCGTTCAGAGGTCCGCTGGGAAAAA
GTAATGAGGGGTGAAGTAGAGAAGAGGAGAGGGGGGTGTAGTG
>103790031_2 DT662420.3

GCACGAGGGTTTACGCTTATTTTTGAAATGAATTTTAAAAATGATAGTTTTGATAGTCCAATTCGAAATAAAA
AATCCACGCCTCCGACGAAAGGAAAAGTTAAAAATCCGTTTGATAAAGTGTTAATTGAAAAATTACATAAACC
AATATGCAGTCCGGGAATGTGCAAAATCTATAAGAAGAAAAATAATGGCTCATTCAGA
TGGGACATAGATCAGGCGTGTACTTTAGTACCTGCTGATATTGTAGCGTGCAATAGTCAATTTGAACCATCAC
CAGATCCTGCGTTGGAGAAGATCGCTGAAGAAGCGAATGAAAAATTTTTTTCTCAAGACATGGTCATGCCTAG
CCCAATGGAAATTTCTAAAAAAGTTAAACCGCTTCTTGAACCTACTGATACTACTGAC
AATATTATTACAAGTTTTGTTAGTGAGAAAATATCTACCTTTAGAGAAGTTTCAGTGCAAACTGTCCTTACAT
TGCCGCCAAAACTACCTCCCGAAATTGAAAGCATCTTACAACCATTCTGCACTTATACTCAGGATCAACACAT
GTATGGAGAATATGAAATTACTGCAAATGGATCTCTGAGAAGAAAATTATTCTTTGAG
GAGCACAGTGATATGGATCACTATGACTCTGAACAAACAGATGATGAGATTCATATAGAACCCCAAATTCAAG
AAAGACCACCATCTAGCTATGAAGCACACACACCTGTGTTGTTTAGTCCAGACTTGAGTCGAGATTTAGTAGC
AAAAGGAATGAAACGAACATTTGGAACGCATTACA
>103790037_2 DT662393.3
GCACGAGGATCTCACCTTGCTTACTCTAGTATATTATATCTGTACACTCGTTTAATATAAAACAGTAATTATT
AAATTATTATCTGTATGTAATATGTATTGGGAAAGCTGAAGTTCAGATCGTGTCGGCTGGCATTATGCTGTGA
TGTCATAGTCCTGTCAATGTCATTTAATGACATTTGAAATTATGACGTGTCATTAGAA
TGACGTCGTTGTGTTGTTGTGTCATGATGTGAATGTTGTTGCCCACTAATGGGCAAGTCCTTGACATGGACGT
TAATTAGATTAAGTTTGAAAATAAAATTTTTAGCCATTTCGTATTTTATTAAATAAATAAATAGGTATTAGAA
TGTAAATATTATATACGTAATGTCACAATCGATTTGCTCGGTTTTTTCTTTTTTTGTA
TATAAATCATGTTTTTTTTTATATTTACAATCTTAATTTAGGCATTTAAACTATTATTAATTAATCGTTTTAT
TTTGTATAATATTCGTTTCTCGTGTAGTTTCGTTTTCATCAAAATCGATAACCACCGATGTATTTTAATGATA
TAAAATTTCGAATTTATTTAACTTTATTGTGTATCGATTTCTTTTCTTTAAACTGTAT
ATATATATTTAATGCACGCCCAATTATTAATTGTTGTTTTTTTTTCGATTCTATATTTTTTTGTTATAATTAG
AAATTATGAATCCGCTGTTTCGACCTGTTCATTTTAACTACATGACATTCCAGATACTATGGTATTTTTTT
>103790060_2 DT661846.3
CTCCTAAAAGCGCGGGCTGCAGGAATTCGGCACGAGGCATAATTCTTTTAGGGGAAATGGAAAATATTTGTTG
TTGTGATCTATGTATAGAAGATTAAATATAAACACATAAACCCCTATTTACGAATAGAACGGCAATGATTTGA
AAGGATGTTATATTAATTATATTTAACGTGCTCGTTCAATTTTGTTATTGTTTACTTT
TAGTTATTAAAAATAAATAAGTTGCCCTGTGACCTTATTATAATTAATAATGTCTGGAGAAGGAAATGCGGGT
GCTGGTTCCCAAGTGGCTCTCGTAACAGGAGGATATGATCATACCATCAAACTATGGCAAGCCCATAGTGTTT
TTTTTTTACGCACAATGCAGCACCCTGATTCGCGGGTAAATGACTTGGAAATAAGTCC
AAATGGACAAATGGTAGCTGCCTGTGGATATCAACATATAAGAATGTATGACCTTGCCAGTGCTAATCCAGAC
CCTGTATTAAACTATGAAGGTGTTGTAAAGAATGTATCAAGAGTTGGCTTTCAGGAAAATGGTCAATGGATGT
ACACAAGTGGAGAAGACTGTACAGCTAAAATATGGGACA
>103790069_2 DT662186.3
GCACGAGGAGACCATTGAACAGGCAATTGTTGCTCCTGGCAAGGGTATTTTAGCCGCTGATGAGTCCACAGGA
ACCATGGGCAAGCGTCTCCAAGACATTGGTGTTGAGAACAACGAAGAGAATCGTCGCAAGTACCGTCAGCTGC
TATTCAGTACTGACCCAGCAATCTCCGAGAACATCTCTGGTGTAATCCTGTTCCACGA
GACCTTGTACCAGAAGACCGATGATGGCACTCCCCTAGTGTCTCTATTGGAGAAACGTGGAATCATTCCCGGC
ATCAAGGTCGACAAAGGTGTTGTACCACTTTTTGGATCTGAAGATGAATGCACTACTCAGGGCTTGGATGATC
TTGCCCAGCGTTGCGCCCAATACAAAAAGGATGGGTGCCATTTCGCAAAATGGCGTTG
TGTGCTCAAGATCGGTCGCAACACACCCTCTTACCAGTCCATCTTGGAAAACGCTAACGTTTTAGCCCGTTAC
GCTTCCATATGCCAGAGCCAGAGAATCGTACCCATTGTGGAGCCTGAAGTGTTGCCTGATGGTGAACACGACT
TGGACCGTGCCCAGAAGGTGACCGAGACCGTATTGGCTGCTGTCTACAAGGCGTTGAA
TGACCACCACGTCTTCCTTGAGGGTACTCTTCTGAAGCCTAACATGGTGACTGCTGGACAGTCCTGCAAGAAG
ACATACACTCCAATGGACATTGGTCGC
>103790075_2 DT661988.3
GCACGAGGAACGTACCATAAAAGATTTTTTCAAAATAAAAAAACACAAACATCTTAAATTTCTTTATAAATAT
TATATATATAATATATTTATAATTTATTTCAAAATCGTCTTTTCATAATGCATAACTCGATGTTTGTAATTGG
TGTTAGTGCTATAGTGTTGTGTTATTTACAAATATGTAGTTCTGAAAATGAGGCTCTC
GAAATACAAAAAATATTTTCACCCTGCAAGAAGAAATCACCGGACTTCGATGCGTGTATCAAACGAGCTTTCA
ACAAAGTGAGGCCGTATTTCAAAAGAGGTATACCAGAAATGTTTTTTTTGTCCTTCGACCCACACTTCACTAA
GGAGGGGAAACAAAATCACTCAATGTTCGTTATGGGATACACTCTGATGT
>103790118_2 DT661883.3

GCAAAAGGTGACAGTGGTCCTGCAGGTCCAGTAGGCATATCTGGTATGACAGTAGTAGGGGAAAAGGGCTTGC
CTGGGTTACCAGGAAAGCACGGTAGACCAGGTATATATGGTGCTCCTGGAGAAAAGGGAGAGCAAGGGCTACC
TGGACTTCCAGGGCAAATAGGTACACCAGGAATACCGGGTCCTCCAGGACCTAGAGGA
GAAAAAGGTGAACAAGGGCATGAAGGTATCAATGGTCCACCTGGTTTTGACGGTACACCAGGAGCAGCCGGAC
CTCCTGGAATATCTGGGAAATTTTTTCCTAAGGGAGACAAAGGTGCAATTGGGTTCGGATTACCTGGAATTAA
AGGTGAGCAAGGACCTCCAGGAATTCCTGGATTACAAGGTCAAAAAGGCAATACAGGG
GAACCTGGTCGAAATGGTGCATTTGGGGAACCAGGGCAAAAGGGATATGCTGGTGAAAAAGGTGATAGAGGAT
TACAAGGTAGAACCGGTCAAACAGGAATTATGGGCCAAAAAGGTGATAAAGGAGAAGCAGCTGCTATCGTTTA
TGGTGCTAAAGGAGAAACAGGACCGCGTGGTCCACCGGGTATAAATGGACTCCCGGGT
CTTAAAGGAGACGTTGGCGTC
>103790124_2 DT663245.3
GCACGAGGAAAAATCCTAATTAATTATTTTATAATTTATTATTATTACAATTTGGGCATAATATATGTTCTGT
GATATTATTTTTTGTAAATTTTATCTAGGATATAAATACAATACAGATAAAGCTTTAAGGAATTCAAAAATCT
TGTAAAAATAGAATTTCCTATTAGAAATTCTATAGTCCTCAATAAAAAATTAAAAAAA
TTATATCAGATTTGGAAATTAATTTAAATGTCAACATAAATAACTGATCCAATAAATAAATTAGTCTGTTAGT
CATTTCAATAGATATGGAAAATTTATCTTCTTCTTTAGGACAATATGAAGATCAACTATCAATGGTCAGGAAT
TCAATACAAGCAACAACAAATACTAATGAACGTGAACAGCTAGTAGCTTTAGAAACTG
AATTACAAGAGCTTATTAGCCTTACAAAGGAAAGTTTAGATGCTCATAGCAATAAGGAAATACCCAAACCTGA
AGCTGAAAATGACACTGGATCCCAAGAACTTGATGATGAATATGCATTATTTATGAAAGAAATGTCGGAGACG
GGTGCTTATGAAAAGGACAATGATAAAAAAAAATGATGAAACACAAAATAGTGATGGTA
GTGATATTGAGGATGAATTATCTTCTCTTCTTGGTATGAAATGTGCAGTATACCACACTCATACATGGGGGGG
GGCAGCCGAGCCTACATAATGCAATGGTTAGCTCAGTTGTACCACGGCAAGATGATGATCAGTTCAATGACCT
TC
>103790130_2 DT661770.3
GGCTCAAATCCCTGAGCTAATAACAAGAGGTGACTGCGAAGCCCTTTGTTTAGAAGCCGATAGATTACTAGAA
AAATCGCGTCACGCAGAAGATGCTGCAGATTTTGAAACTGCTTTTTTTCTATGCGATGCTGCAGCAGCTAAAG
CGCGAGCTGCGATGGATGCACCTTACAATAATCCTCATACTATGACTTTCGCTCGGAT
GAAACACAATACATGTGTAATGCGAACGCGTAGCCTGCAACGCCGCATGGCAGGAATGAATAGAGTTACTGAA
ATTCCTCAATTAGCTCCTGTAAGAAATACTAAAGGTGGTTTAGAAAATTCACCTACAACGATTGAAATTTATG
CCACTTTACCCAAAAAGAAAAGTTCAAAAAAATCTCCTAAAATGTCAATTGAAGATGA
TATTGATTGCCCACCAAGAGAACGGCCTCCAAGACATAAATCGCGAGAAGAAGAGAAAGTAAGAGAAAAACGT
TCTAGAAGCGAAGATCGGGGCCGTACTCGTAAAGAAATACAATAG
>103790148_2 DT662676.3
ACTTGCTAACGAACTAAATGGGGCATACTGGGATGTGTTCCTGCCGCTGGGTGGTAAACAGAGTGTGGTGCAC
AGAAGTGTTGTGTTGACTAGAAGAGAACCAAAACACGAGCTATGTGCAACCATCTTGCAGTACGAGTTTGGTA
CTGAATATCAAACCCAGATGAGTTCAGCGGAGGTGGTATTCCGGTACCCCTTAGTGGG
CAGGGTGGTGTTCCGGCAGCCGCAGGAGAGGCCTTGGGAAGATACTACTATTATTATTGAGAGTATGGTGCAC
GCGGACGGCGCCAGCATCTTTTTTTCGCGCGAGCACCGCTGGGCGCTGCACGCGCACGCGCCCGGCGCCGACT
ACTACAACTGGACGGCGCGCTGCCTCAGCGCCGGCGCCGTGCTGGCGCGGCACCCCGA
CGCCGTGCTGA
>103790160_2 DT662746.2
GACAAGAAAAGTTAACAAACTTCAGGCAAAAAAACATACCAGCTTCTCTAAGACAAGAAATTGGTGAACCTTT
CCATCACATTTTTGCCGCTATCCCTAGAGATGACTATGTAGATCTACTTGGCAAATATTACAGGGCAAAACAA
ATACAATCTTCATTAAAGAAGAAAAAAATTGACAAGTTGATTAAAACACCTAAGAAGA
CATTTAATACTGTGTTTTCCCCACAAAATGTAAACAAGGTGATAAAAGAGGGTATAAATGAGCTAAATGAAGT
ACCTTCACGAGAGAAAACTAATTTTAAACTTGTTTTCTATCACAAAAGATTGTTATGTACCAAGCCTAATGAT
TCAGTTCTGAAAAGAAAGAAGAATGAACTTTTACAGAAACTCTACTTTAATATAGGCT
TGATGGACACTAGGGCTCATATCTCTTCAAAGACATTGAAGAAAATGAAGAAAGTCCAAGAACAAGCTCAGCT
GTTCTTAACACACGTTTAGGGTACGAATAGAGTTATGTAGGTGTACGAAATACACCTTTTATTGGCGAATAAT
CGCCTTATGCTGGAAAAGCTCCCATATTACATCCCCTAGGTGGATTTTATTTATGTAA
TGGCGGTTTTTTCCATTGGATGTAAGAGCACCTTTGGTGCATGTTGAATAAACTTCTTAGTGTAAAGCAAAAA
AAAAAAAAAAAA
>103790163_2 DT663210.3

GCACGAGGCGGCACCTTGGTTTTCTAATTGTTAATTGTAATCAATTTCAGTTTTATTAAAGCCACTTTTTGTT
TACTTTATATATATTTAATTAGCTAATTACATAATTTCTGTCATGGATACCTTAATATCGTTTAATTATAAAA
TAATGAATAAAATAAGGACTGCAGGCGATGGAATGGAATCAGATGAAACTGTTAGCGA
AAATAGAAACAAATTGGAATCTAAGCCTGTTCTTACAAAGGTGAATACCAAAGAAACATTGCTTCCTGGTCGT
TTAGTGCAAATTTATCCAACCAAAATCATTACTTCCGAAAAAAAAAGTTCTCATATATATTCTAAAAATCCTA
AATTCGTTCCTTATGAGCCTTATCCTGGAGCTGTAAAACCAATGACATCAAATTGTGT
GCCCAAAGGGAATAAAAAATCTAAAAATCATATGGACATAAACACCCTTATTTCTCAAATGTCCAAAATGGAT
ACTAACATAAATGAATATAAACCACGAAATAAATTAACTTCATGCAGTGATAAATCTGATGTGGATCTAGATA
AACAAAAAGAAAATACAGAAATGCAAAGAAAATTAGATGAGCTATCTAAAGAGAATGA
AAATTTAAAGGAACAATTAAAACAACAAGCACAGGTGAACAAAGAACTCANAACAATGTTAGTAGCTTCAATG
GGTGAAGATTTGGAATTCCAAGTGCAGTCTCTCAATGAAGATAAGAAGCACTTGGCCAATGCCTTAATAAACT
CTGCACAACACTTGTCTACTCATC
>103790190_2 DT662714.3
GCACGAGGGGCAGCCTTTTAAAGTAGCTCAATATTATTCAAGTCAAAGTGTTTATATAAATTAGATTCAATTT
GTATATAAATACAGAATCTATATTTTACTTCTTTAAACAATATCCTTGAGAATTGAGGAATGTTCAATACTTT
CCTATAAGTAATTAATTCTTTGTTTGTTAAAAATGGAGGTTTCAAACAACACCGACGC
TGTCAATCTCGGCGTTGGTGAGGTCGAAGCGGAGATTGGAGAAGAACTAAAGCAATTAGAGGAAACAAAAGTT
TATGATACTCGTAGTGAAGTATCAAGCTCATCTTCAAGTGAATTTTGTACACCAAGTATTAGCTCCGTTAGTA
CAAATAAATCTAAAGAAAAACGTGCAAATCGCAAACGTACTAAATCTAATAGCCATTC
ACCATCACAAACTAAACGACGTTGTATTAAATCGACCAGTACAAAAATAAAAACATAGGACTATATGACAAAA
TTGAATTACTTATTCAGAGATACTCGATTTTTTCTTATAAAATCAAATAATGCGGAAAATATCACATTATCCA
AAGCCAAAAGTGTATGGGCAACTTTACCACAAAATGAAGCAGATTTAAACCAGGCATA
TAGAGAGTCAAGACACGTTTTATTGATTTTTTCAGTTAAGGAAAGTGGTAAATTTGCTGGTTTT
>103790197_2 DT662507.3
GCACGAGGCCGTCATCCTATCCCCGTACCATCTGGTGGTAGATATGTGTCACCACCCGGCCGACTTGTTGTAC
CATCGGGTCGACATGTTTCCCCAACTGGCCGGCGTGTGTCTCCATCTAGACAGATGTCTTCTAACAGAGAAAT
GCTAACTGAAAGGAAGATGTCTCCTTCAAGAATAGACTCTCCACAATTAATAAGAGAG
GAATATGTTAGAGGAAGGAGAATGTCACCTCCGTCACGTTCACCGATTGACAGACGTCCATTACGTCAATCAT
CGGACAGAGACGCCAGAGACATACCTTCCCACGTCCGTCACGAGTCACCAAGACGGCAGCCAAGATTTACTTC
GCCACATCGTATTGATCAAAAGCAAGCGTCATATCAAGATGATCATCACGCTGCCATG
AAGAACATAAGACCTGCCTACGAACCACATGTACAGGCGCCAAATGAAGCTATATACTCAGGTGGTTATCGTC
CCAACATACGTGATGAAGCAATTAAATATCAATCGCGGTCGGAACACTGGAAGGAAAGAGAAAATTTTTCCTC
CAAAGGTTTGATCGAAGATAGAAGAGACGGTCCAAGAATACAGCAATTTGAGGTTTCT
AGAGCATCAGAAAGACCTACTGAGCAAGATGCGAATCGTTCCCGGTCGCCGAAGCGTGATCGATCTCCAATAC
GCGACCGCTTCAAGCGCCATTCTCCTTCTCCTCGATCCGCAAGGCGTTCTTGGGCTTTAGAGAAACGTATAAG
TCCAGACATTCGTGAACCGCCGCCGCCTCCGGTGTGGCCGCAAGA
>103790206_2 DT662858.3
CGACCCTTTAAAGGATTTTTAAGATATAATAAACTTGGAAACGATATCACGGTAATCAGTCAAATAGTAACAA
CTACATTGTCCACGCTTAATAAACAAGAAGAAAGTTTGTAATAAGTTGTTTATTGTTTCACTACAACACTGAA
ATTTCCTGCGTAATTACCCGCGCCACAGAGTCTCAAGGGCCAGTCACATTGTAGCAAA
CTATGCAGTTGTATTACTTTGTACTGTCTGTTTATTTTATATAGAAAAATAGAAAATAGTTAGAAATTGGCTT
GGGATGTTCTTTATTTAAGTGAAATAACAAATTTATATTTTAAAAAAATCCCCCGACCCAAAAAATCACTATC
TCGTTTTGA
>103790212_2 DT662321.3
GAAATTTGTAATTATAAATTATAACTATTAAGTACATTAAGCAAGTAAAAATAAATTAAAAATGGCTATGCGA
GACTATACTGCTGATAAAGAAAGTTTTAAGAACTTCTTCATAGATTTTTGCCAAACTGATGATGAAGGCCGCA
AATTTTTTAAATATTCAGAACAACTTACCAAAGTTGCACATAGAGAACAGGTGACATT
TGTGGTAGACTTGGACGATTTACATGAAACAAATGAAGAGTTAGCTGATTTTGTAATGAAAAACACAAGACGT
TATACTAATATGGTGTCAGACGTTGTTTATGAAATGCTTCCAGAATACAAACATAAAGATGTCGTGGCCAAAG
ATGCCTTAGATGTCTACATTGAACACAGAATAATGTTGGAAGCTAGAAACCACAGGAT
ACCTGGTGAAATGAGAGATCCTAGGAATAGATACCCACCTGAACTGATCAGACGATTTGAAGTGTACTTCAAG
GACATGTCTACATCTAAATCAGTGCCCATAATATAAGTTAAAGCTGAACACTTTGGAAAACTGG
>103790221_2 DT662097.3

GCACGAGGCACCATACTATCACTTGTCTACCTGTATACCCTAATGAATTGGACATCGACTCCGAAAGCGAGAC
AGACCCATTATGGCTGCAACAGAAAACGATGATGATGATCGACGAGTTCACGGATGTCAATGAGGGTGAGAAA
GAGCTCATGAAAATGTGGAACTTGCACGTCATGAAATACAACTACGTAGGCGACTGCC
AAATACCTCTCGCCTGTCAAATGTTCCTACAGATGAAGGGCAAGGAGCTGCTCGAGAAAAATTTATATAGGAA
CTTCATACTACATATGTGTTCACTGCACGACTTCGGACTACTGATTCCGGTGGCGCTGTACCAAACAGTGCAA
TTGCTCAACCAAATGCTGGCAGACAATGCCGAAGCCAAAGAGAAGATGAGAGAGTCGT
TGAAGGCGCAACGAGAACATTGGAACGCAGTGGGGAAGTACCAACAACCTGTGATTATAGAACAAAAGCCAAC
GACTAACACTGCCAAATTAAACAATGCTGAAGCGTCACCGTCAGTGCGGAGAAAAACCTCCAACTTACAAAAC
GCCAATAGAATGGCGAGCAATTTCAACAAATCAGCAAGTCCGGGGCCGAGCAATGAAA
ATAAAAGAAAGATGTCCTCCGGCAGTATCCAAAGTCGCAAACGGTCCTCCATATCGGAGAGAAAAAGCTCTGT
ATAGCGAATGTTCAGTATTTAATCGGTTCAAGGACTGGTCTCCATGAGGCTCCACCTGTCTACAGGATCTCTG
TCCGGTATGCGATCGT
>103790233_2 DT662475.3
GCACGAGGCTACCTCAGTCGACTCGCAGCGTTCATCACGCTGCCAGTTACTTATAAAATATAACTTATATATA
TTAATAAAAAATTAAACTTTAAATAAAATTTAATTAATTAATCCAAATTTTAACTATTTATAAGTCAAAAGTG
AAACAAAGTGATCTGTGCAACCGAACTGTAAGTCAAAAATAAACTGACGTTTATTGGC
GTAACCATAAACAATTATCGAAACCAACACAATATAATGTCATCTAGTGATAATTTTAAAAGCGACGCCAGAG
TAAACGGTCTTAGTGTATCCTTAGAAAAGGGCTTACCTTTAAATGCTAGATATCGTTCAAGAAGCCTTTCCAC
TGACAAATTGAATACAAACAACGCCGATCGTGCAGCAATATTGGAAGATATAGAATCT
CACTACGTGACTGATAAATTTGAAACACCAAGCTGGTTAGTTAGGAATAAAATTATGCAGTCGAACGCTTTGG
GCGGATATAAAGATGGCAAAACCACATACCGAACAGACAGTCCAGCAAGGG
>103790236_2 DT662727.3
GCACGAGGTTTTTTCCATTAAGAAAACATTTATTTCAAACACACAACCACAAATAAATAAAACACACACACAT
ACACTATACCTCTTATTAAATAAATAAATTAAAAATGCATTTTAACTTATAAAATAAAAATAAATTAATTTAT
AAATTTTATTTTTATTTACATTGATTGATGCTGAATAAAAATAGCCTAATTCACAATT
ATAGTTGAAAAAATCTTGTTTGACTCAATTTTATTTATTTCTTTTTGTTATTTTAGATGAATAGTCACAAAAA
AGTAGACATATTTTGATAACAACAAAATATATAAGATAAGTTAACGCCATTTTTATTACAACATTTTTATTAA
AACTAGCTTGTATTTTGATTTGACTATAGTAAATAATTAATCGCAGGATAATGTGGTA
TGTGAAAATAATCTTTGTATGTTATCTTGATTTAACTTACAAAGAAATTAAGGAAAAAATTGTATGACTAAAT
GTTATGTATATATATATTGCTTTGGAGACGACTGAACTGATTATAATGAAATTTTCATAAGAAAGTTTAGGAT
TTTTCAAAGAGTAACGAAAATTGGCAATTTATAATGAGTAGGTGACGGTTTATCTGAA
ACCAGTATTATAAATCATAAAAAAATCATATTATGGAAATTTCAATTTTACATAATAAAAACTATATTTTGCT
ACAAATACTGCTAGTATAAATTCACTATACTTAAAAAGTTGTATGAAAGTTTAATCGTATAATTTTTTA
>103790239_2 DT662650.3
GCACGAGGGCCCGTCCCGCGACGCCGCTACCGCCCGACATACGACCGACCGCCTGTGAGTGAGTGCGCGTGCA
GTGAGTGCAATGTGCTCGGCCGCATGAGGATCCTGCTGAGCGAGCTAGGACTGTCTGGCGCGTGCCTCGGTCT
GACCCTGGAGTCGCGCGCCGCTCCTCTGGATCCCGACAAGCCAGCGCCAGGTCAGCGC
CCGCACGACGTTACGCATACGCGTCCTTCCAATTCTGTGTTTATTTTCGCTTTCGTTGTGTAGAACCTCTGGG
TGCGTAGACTTTGTGTGGAACTGTCGTGACGATGGCCATCGGCTAGAATGCGAGGTGCGTTGCTGACGCCCTC
CAGCCAACACTGTGGCCTTAGACAATTCCTAAACACACGGCCGAGCGCCATTGAGCCG
CGCTCGCCAAGCGCTTTTACGGCAAACTCGTCCAGCATGCTACTGCTGCAAACACACAGCAACTACGGTACGT
CCTTCACTGATCTTTTGTCACCACAATATCAAGAAGACTCTTCTGAAATCTTGGAAGAAAATTTAGATCCGTT
CCCAGACGTTGAATTTCATGCGCCAATTCCTCCTGAAGTGAAATCTCAGCGCGCTACT
CCTGTGAGCGAAACACCTTCGCCTATTCTAGGCCCAGCTTTACCTAGCTTTGAAGAAACCTACTCAGTGCGCT
ATCCAAAACAAGAAATGGCAGAATTCGGATTAAAAATGGATGAGGACTGCTATAATGTTAGTGCATACTCTCA
TCCAGGACATGCGTCAACGCAATTGTTAT
>103790242_2 DT662226.3
GCACGAGGAAATGATCTTATATTTTCAACTAATATTAAAGTGTAAAGGGATAAAAATATTTATAAGTAATAAT
ATTATATTCTATTATTTACAGTGTTATCTCTAAAGGAATTAATAATTTGAAGAGGAAAGTATATTAAAGGCAA
AACCATTTCTTCAATAAACAACATGCCTAAACGTGGTGGAAGAATACGAAACTGGAAA
CCTAATGAACATTTTGAACATGACGACCGGACACATAGTAACAATACCAGACGAGTTAGTTTCAAACCAGGAA
CAAACAAAGGAAAAAATAAGTTCCGTACATGGAATGATGCTCAATTATTACTTGATGGCGATATTGACATGGG
AGCGACAGGAGGACCAGGGGTACATCGAAAAGGATTTAGAAATCGAGGGGGTCGTCTA

AGTTCTCCAGCACCAAGACCAGCGTCAAAAAAGAGATTTATACCTGGACCACTGCCATGGTACCAAATCGTAA
TTCCATACGGAGCTAAACATGACAAACAAGTTGTTTTGCAAGCTCTACTAGGATATTTATCACCTGAAGTGTT
TATACCACATTATTATAAAATAAATGGCAATGCGGCAGTTTTCTATGTGGATGATGTG
AAGATTGCAGAACAATTATTTTATGCAGACAGAAAAATTGTAATGTCAGATGGATTTAAATTGGTTGTGATTG
TAAGAAACTCCGTACCAAATGTAGCCATTGATGATAATATGAAGGAGAAAATGAAACTAGCAATGGCTAAAGA
TACAATGCTA
>103790251_2 DV501076.2
GCACGAGGCTACCCCCTTTAAAGGGCCGAGTACTACCGCAGAATCGGCAAGATGAAGGAGGCCGAGGCCATCG
AGGCGCACATTAAAATTAATCAACAACCACAACAACCGGCCACCGTGCCCCAGCCGGCCGCGCAGGCGACGCC
GCCCAACCCCACTCCACCGGGCAACAACGTGGCCCCGCAGTACGGCCAGTACGGCGTG
TACCCGCCCGTGTACCCCGGCTACCCCTACCCGTACCCCGGGCCGCCCGCGCCAGAGCACACCCAACACCAGT
GAATACCCCGGCCGCACCGCCCAAACGACCCAGAAGATACCTCTTTTTATTTATACATATATATAGTTAAATA
ACTATAATTGTATAAGATACTCCACGCCATTGCGACCCACAATAAATAACTAAGCACC
CTATATATGTTTTATAT
>103790257_2 DT662776.3
TTTTTTTTTTGATTTTCATAATATATTTTTGCGAAGACAATAAGTTGTATCTAGAGTAGACTTAAAATTACTA
TTTTACGTTTAATAAATACTTATAACAATCGACATGCATTTATTTTTCGTTTATTATTTTCGCAATTTAAAAT
TTTTAAAATAAAGCAGGAAGGGTCAGACTAAAAATAAATATAAATTTTTCAATTTTAA
AAATTTTACTTTATCAATTTTATATATCTATCACATTTAAATACTTAAGAAGTGAGAAAAAAACATTACAGTG
TAAACGAGCATTTTACTTTACTCCCCAACACATGTCTAATGGCGTAACGTAGACCTCGTCTATTAGACTGGTC
AAACATAAAATATTTTAAATAGATATATTTTTTGTTTAGAATAAATTACCAATTGTAT
AATTTATGAACAACACTTAATAAAAAAATACAAATACACTTTCACTTTATTATTTTACTCAACAATGGTCAAA
TTATATTATATTAAGATGATTTTTAGACTAAGATCCCAGGGCCCCAAGACCTGAGTAATTTTAGCAAAACTGA
AAGTCTCAACTTAAGTTACACGCTCATCTACGTTAAAGTAGAAACGTTTGTCTTTAAA
TATGTTTTTACAAATAATGTTAAAAATGTATATTTATTATTACCAGGTCCTTTCCGTGGGCCTTTACTCCTGC
ATGCGACAAAAATAATTAAAATTGGGGCAAACTACGTTATACTAGCAAGCTTT
>103790278_2 DT663134.3
GCACGAGGCTCCTATCGAAATTGTACTGTAAGAACCGATGGCAAAACACACATGTTTCATTATCCTGTTTTCG
AGAAAGTGAGATGCCACCAATGGCTTATTAATGCACAAAGACTAGATTTTTTAAACCTTAAGGTTTCCCAATT
GAAAAATCGAGTTATTTGTCAGCATCATTTTAGAGAGGAGTGTTTTATGAACTACAAG
AAAGACAAGCTCACATTTGAAGCAATACCAACAGAAAATGGACCTTTTTGTGATGCTAGCAAATTTAAAAATC
AATCTAAAGAAATTATGAAAGCATATTCAAATGTTATACTTTTAGAAGATATAGAAAATGAGATTTTTAATGA
TAAACAAGCAAACTATTCCATAAAATATGGAGATTTCTTAACTAATGGAGAACTTACA
GACTCATCATCAAAATACAATTATGATATAACTCTCTCAAACTCTTTATCTAGTTCTAAACTTGATCAAAATA
ATAAAACAGATATATTTAAACCCCGCTTAACAAGATTATCACTGCCTTCTAAATCCATAAATAAAAGCCCACC
TCAACCTATGCTCGTAGTTCCTCCATATACAGACTTAAGTAATCATTTGAATGCAGGT
AATGAAGTCAATCAAGATTTAGGGGTAACTAATACTGTCCAATCAAACGAGATAAATGTAAAACAAAATAACC
AAGGCAATGATTTCCAAAACAGTGTTGTTAAGAAAGAACCTAAAGTCAAAATTCTATCAGCAAAAAAGATCAA
TTACAATGTCCGTTACCAGTTTCGGGAGATTTAAGAAAGTCTCTCCCATCACACTTTA
ATTTTTACTATAATCTATATAAATATTGAAGATCTAAAATA
>103790284_2 DT662018.3
GCACGAGGGGGAGCCCCCAAAGAGCAGATTGAACCTGAAGTTGACAAACTTTTACGCGAAGGAGATAGACTTG
AGGAGATTTTGGAAAATGTCAAAGATAAAGTCCCGCCTAAACCGTTTACACCAATATCGGCAAGTCCCATAAC
AAATTCGACTTTCAAGCCTCAAGACAAAGGTGATTCTAACCCCGAAGAGGAAAAACTC
TTAAAGAAATAGTATCCCCTACTACCTTCCAATTTAAGCGACTGGCAACACTGCCACTGAACATAGTATGACA
ATTTCGCGGTGAAAGCTTAATTTTTTTATATGTTGTATGTTTATACTGATTATTCATTTATTTTTAATTAAAG
AAAAAATATTAAATTGCCAATTTATTTGGTTAATAAATATCAAGAACTTTTTTATCTG
GAAAATATTTTTCTTTTTCACTAAATTATAATTGTTGAAATGACTCAAATATTTTTTTTTTGTATTAAAATGA
ATAATCAGCTTAATATAGATTTTTTCTTGTATTTATAACAGCATTTGAGTTTAACATTCGTTTTAATGTAGTT
TGTAGGAGACTGTACTTATAGCGTTATTTTTTTAAATGGCAACACACGTTGCCTGGAC
GCGACGTTTTTTTCTATTTACAAATCATACAAAAAAATCATCACAATCTAATTTTTAAAATATTTTTACAATT
TGTATTCTAGTCCTAAACTAGATTTTTTTAAAGTTTTGCAATATTTTATCTGTAATTTTGTTTTTTTAAAATG
GAAGTACGTCCCTCTTTTTGAATAGTTTTTTTTTTATATTATAT
>103790309_2 DT661793.3

GCACGAGGCTACCCTTGAGTTTTTTTTTTTTTTTTTAGCTGTATTTTTTAAATTTTAACTCTAGTTTCGTTTT
TGTTTGTACTATGACTGCTAATGTGCGAGATTAAATAACATAGCCCGTAAATATAAAGAAACAAAGTATGGAG
TGGTCATATAATAAATACAAGCTATACTTTGCATAGAATTAGTGTAATTTTCCCAGCG
TGGAAATTACTCAATAATAATAAAAAAAAATGAAAAAAAAAATATTAAAAAAATAAATGAAAAATAATAAAAA
AAAACGTGTGGACTATAAATTTCCAGTTTAATATTTTTTATTATTTAGGCATGTTTTTGCATTATATTAAAAT
GAATTTATTACCTACTTACCCAAAATATTAGCTATAAATGTATTATTATTATATATTA
TAATGGAATCATGATGTTTTAGTTGAACGTCAAGATTTGATTGTTTGATACGACTTACCTCAGTGTCGACATG
CTGAGCATTAAACGGAAACATTTCTCAGCTGGTGTCTTGTTGACGGACTGCCACCTTTGCCGGCAGCGGTTGT
TCGTATTCGCCAGTGAACGTACAGGTTTTCTTTTTGCAACAAGATGGAAAACTTTGCA
GGGAAGCTGAGAACTTCAGAGGCGTCAGAGTGAATATATCACTGGAATTAAATGGTTGCGGTATTTTGGATCC
CTTGCCTGTGAAAA
>103790366_2 DT663102.3
GCACGAGGAGAACTTTGAGAGAGAAGAGAAGGAACATTCGCAAGAAAAGACTGATGATAATGAAATGACTGCG
TCCATGACTGCGCGGCGTATAACCACGGAGGAGGAGGCGAAGGCGGCGCTGGCGGAGCGCCGGCGACGCGCCA
GGGAGGAGTTCGAGAGGCAAGCGGAGCTTGAGAGACAGAGGTTGCAACGCGAAGAAGA
ATTAGAAAGAGAGAGACAGCGCATAGAAGAAGAGAGACAAAAGAAAGAAGAGGAAGAAGCAAGAGAGCTCGCC
GCGCTACAGCGACGCATTGACGAAGAGAAACTGAAGAAAGCTATTGAGGCACAACAACAACTAGAGAAAGAGG
AAGCGGAACGCCGCGTCAAAGAAGAAGAGGAGCGCAGAGCTAAACTTCGCGAAGACGA
GGAGAGGAGGCGCGCCGCTGAGGACGCAGAGAAGTTGCGGCGGGAAGAGGCCGAGCGCGAGGAGCGGGAGAGG
CTGGCGAGGAAGCAGAGGGTGGAGGCCATCATGGCCAGGACTAGATTGAAGAAGCAAGCAGAGGAAGACAAAA
AACAACAAGAAATGAAAGCGCAACAAAATCCAGAAGTTCCCGCTAACAACAATTCCCA
TATTTCANATAATACAAATAATAGTAATAATGCTAAAGATATACCAACCATAGTGCCACAAGCTAAAGAAAAT
GAACCTGCACCTACCGTTCCGT
>103790369_2 DT662416.3
GCACGAGGGGGTGATGTCACCTATTTTGTTTTGGAGAAGGCTAGTCAGTTGGACATGTTCCCTACTATACCGA
TTGGAGCGCCTTTAGATAACTGCGCAGTATACCTCTTAGACGAAGAGATGAATCCTTCCCGGGAGTCGGAGCC
GGGAGAGGTGTGGGTGTCCGGGAGGAACTTAGCCAAAGGATACGTCGGAGGGCAAGGA
GCGGATAAATTTTGCGACAACCCCCACGCTTCTCATCCTGATTTCGGTCGCCTATACCGTACCGGAGATTTCG
GTATTTTGCACAAAGGCTTGATCCTGTTCGCCGGCAGGACCGACGCGCAAGTGAAGATCAGAGGACATAGGGT
CGATCTGACGGAGGTTGAACGAGCTGTGCAGAATATTAATGGCGTGGAAAAAGCTGTA
GTCCTGTGCTACGGACTAGTAACGGGAAATCCAGAAATACTGGCGTTTGTTACTATAGAAGATAGCGCGCGTC
TGTCCGGACACCATATCGAGGCGGCATTGAAGAATTCTCTCACACAGTATATGATACCGCAGGTAATCGTAAT
AGAGAAGATTCCGCTACTTGTCAACGGCAAAGTAGACAGACAGGGTCTGTTGAAAATG
TATGAAAATACTAACAATAATGATGACGCAGAAATCCCCCTCGAGATGGACTATACAGGCATTAGTCCACAAG
ATCTGGACTCAGCCAGAGTGTTGTTCGAAACCGTGGGAGAGGTGCTGGGGAGGTCCGTGAGAGGAGTCATGTC
AGTCA
>103790381_2 DT662490.3
GTGATGGATATGGGACTCTTGCGGCGCATCACTATTTTTATGCAGCTGTTCAGGAGAAGAATGCAGCGCCCAT
AGTGGAGGCGCTATGTCTACTGGAGTTGGTACTACATCACTCACCTTCAAACTTCCATGTTAAATTACTTCTT
ATTACCCTATATCACGTTCTTGGGAACGCGGTAGCAGCCGAATCGATCTACCAGCGCC
TGGAGGCGAAATTCGTGCAGCTGGTGTCGCTGGGCTGGCTGCACGCGGCGCGCCTGGCGCCCGCGCTGGCGCC
CGCGCGCGCGCTGCGTTTTTTTTTTCGACACGCACGCCTTCCACAAGCACTACGTCAAGGACGGCGCCGAGAA
CCTCACGCACGCCTACAAGTACGGCACCTTCGAGAAGCTGCTGGAGCTGCGCGCGTGG
GGCGGCCGCCTGCAGGCCTGCGCCTGGTGCGCGCTGGCGGGGCGGGAGC
>103790387_2 DT663287.3
GCACGAGGGTTTGGCGTTAAAGAAAATTAAGAATTGAGCGTTCTTTTTATCTTTACCATATAGTATAACGTTT
GCCATTTTTATTGTTTTCTAAAGAGCATTTTTTCCTCGTAAACACCTCTATAATATAGTAAATCATATAAATA
TTTATCTTTAGCACCCTGTGCTAGCTTTCTAACTAGATCAATAGTAAGAAATGGTATC
CGACGAGTCAGATGATGAACCTCTATCTAATTTGGCAGCTAAGAAAAAGACCGGAGAATATGAATATGAGCCG
CCAAGTAGTAGTGATGAAGAACCTAAAAAAAAGAATATTTTTTGGAACCACCGGCAAAAAAATTAGGAGTGAC
AATAAAATTAAATCGAAAATTAAACTCCCTGGTCGGTCCCGCTGTAATAGAAAGACCG
ACCGATGTTTGGCTTTATTTGAAAGATCTGAATCCGAACGGACCTTATAGTTGTTTACTATGTACAGATTGGT
TTATAAATAGATCTAAAATGATTTTACATTATGCATT
>103790423_2 DT663395.3

GCACGAGGCAATAACTTAAAAACTAGATTTGTTATTATTTAAATTGATTAAACCGCATTATTCACTTACTCAT
AAACCTTTTAGTTCACTAATGAGCATTTGTGTTTTATTGGTTGGTGGAATCATACCTAAAAATAGAAGCGATA
TTGTATTGCATCACGCGAAGGTATACTCTATACAGTGCGCGCACTAAGTAGCGTTAAC
GTTGAGTGGTGATTATTTATTTCTTTTTCTAAAAATGGAGAAAAGTGGTAATCCTCCTCCATATGGCTGGGGT
AACGGACATACGATGCAGCCTCCCCCGGCTGCTCCCTTTATCTATTCTCAAGCTGTTGGTGGAGTCGGGCCCT
CGAGTCCCTATACACCACAATACCCACCAACTGCCGGTCCAGCCATAGTGACCACAGT
GGTGCCAGTTGGTCCCCATCCCACCCACATGATATGTCCCAGTTGCCATGCCGAAATGGATTCCTATACGCAA
ACCAAGCCAGGACTTATAGCTTATGTTTCCGGAACAATCATTTGCATGTTAGGAGGTTTCCTCGGATGCTGCC
TAATCCCATGCTGTATAGACAGCTGCATGGACGTCCATCACAAATGCCCGAAC
>103790429_2 DT662153.3
GCACGAGGCGAATTTCTAGATATTGATGATTTAAAGGTACATACGATACAATACCATTTAACAAATACAAATA
AGAATAGATTTTGTAAATTGTGCTCCATCATATTCGCCAGTATAGATGATTTCGCTTTACACGTGAGAAATGA
TCATTTAAGTGACCTAAAAAGCTGTAAATATTGCGCTAAAATGTATATAAACGTAAAT
TCGTTATTAGAACATGAGAAGAGGCATAAAAATGAAGCGCATTTTTTTAAATTGTCTTGTTCCCAGTGTGAAA
CCAGCTTTGATAATAAGAGTGATTTGGAAAATCATGAATATGGTGATCATAAGGACACTGATGATGGTATTAT
GTTACAACACTGCTTTGCTTCTTTATCAGCACTAATTAACATGAATATAAGGAGATTT
TTTGAAGTATCTCATAGAGATTGTCTATATGTTTGTGCCATATGTGATATGTCGACTCCAGAATGTTCCCTCT
ACATACAACATTTAAAGAAGGGTAAATGTCATTCATATGCTTGTAATGAGTGTTTCAATGTATACAATATGAA
AAGATCTCTCCGAGAGCATTGTGGAAGGCATTTACGGGTTACAAACTTTAAATGTCCA
AATTATAAGAAACAATGCCTAAAATGTAGTANATATTATACTACATATGATTTAAAAAGGCATTTGAAAGACT
GTAATATTATAAAGTGTAGACAATGCTCTCTAAGGTTTGATAGTGTTAGTGCTTTAGNCAATCATATCACTAT
ACACTCACAAAAAATACCAGCACAGTTTATTTCATGCAAATACTGTAGAAAACCAGTT
ATTGGAATGAATGCATTGGAAA
>103790432_2 DT661705.2
GCACGAGGCAGAGCCTTTAAAGAGGGAAACATATGTAATAAATATTTATAAGAATCGTGATTTAAACATTTTT
GATTCACGCGTAATAAATTTTTAATTGATTTATTTATATTATAAATCAAATATTTTTCTTCGAAGATTTAGTT
TTTATATCGATACAATTTAAGCGATTTCTTGTAGTTTCCTGTTAGCGGTCGTCGGAAG
ATGATTCATAACGGACGACTTCTTAACTAGTGATATGGAAGCATCATCATCAGACTACAGCGACGAAGAAGTG
GGCGTGTTAGCACGAGGTCAGAAGTTATGGGTGTGATGTTTTTTTGCACATACTACTTGTGCTGATATAGTTC
GAGCTTTACAAGATGCGCCTGAAGCAGGTGGCTTAAGTGATCATGGAGAATGGGTGCT
GGCTGAACGATG
>103790464_2 DT663346.3
GCACGAGGATAAACTTGTATATAAAATTGCGATGTTAACAAATCGAGATCAGCCCGTTAGTCCGTTATTTCGA
TTAAAATGTTGTTTATGGCGAATTACCGTACCGTCACACGTCTCGGTACAGCTCGATACTTATATATATATAA
TATAATTTTAATTGTTAAATTTATTCGATATACAAAAACGGCCTCGTCTCGTGCGCTT
ATTTTATTTTATTATTTTTGTTTGCTCTAAAAATAAGATAGATGATAACTAAGAATGCTGATAAGAGGATTTT
TTAGTAATTTATTATAGATCTATAAGCATTCCTGGTTGTTTTCTATAGTAGATCATTCTCACGCATGCGCAGT
ACCTGGCTCCTTGGCTGTAATATCGCTTATCATCGTATCTAATTTGCACTAATCATAC
ATTAAACGCTTTTTACTTTCTTTACTTTTAACGCTTTATATTTTCTATTGTATGAAGAAATCGGTGTAAAATG
GTTTTACCGAACGATGATTTGGCGCCCCCGGAGGGTCTTAGGTTACACAAGTATAGTGTTGGTTTGTCAAAAA
ATGATCGTGAGATTATAGAAGCGTTTAAGTAAGTTAACCTTGAGCATGTAAATATGGG
TGTAAAGGACGAACATTCGCTCGGAGTTTGTGATTTGTAGCTATTTAGATATGAGAGATCGTATCGTAAAGTT
TGAACAGTAGCATGCTACCCAATGATACCAGCAGTCAAGCAGTTACGTGTCTTACAGGTGAAAATATGCCGAC
GAAAACGATATACCTCAGGATCCA
>103790469_2 DT663250.3
GCACGAGGTTTAGACTATATATATGGCGAGTATCTTCTAAATCGTTTCTTTTGAGGAAATTAGTACAGATAAT
CATTAAACATGATAGTGTTGGACTCCTGTTGTTTCTGCGTACCGTTGAGAACGGGCAGCTTGATTATCGGATA
CCTAAATCTTATCGCAAATATAATAATAACAGTAACAGCAATCCTGTCGCTGAGCGCT
GGTATTGTGATAACGAACTCGGACTTCGACGACGGCGGCGCCGGGGCCGTCGTCATTGCGATCAGTGCTGTAC
TCCTGATATTTGTGCTGATTTTCTTGGCGTTCAGCATTGCTTTGTTTATTGGACTGCATAAGGAGAAACC
>103790497_2 DT662758.3
GCACGAGGACAAATCCTTCCAAAAATTGTTTGTACCGATAAAATTTGTCGTTACAACGGTATTTTTGAGCAAT
ACAATTAACTTTTTTTGCATAGTGTATTTTATATTTTAAGTTAATAATTAAATTTAAGTGTAATTTAATTGTT
TTTTCATTATAAAGTAATTGTAAACGAGACAATGGCAGTGCTAGCGGCAGCACAGCTC

TTAGATGAGTTAATGGGAAGGCATCGTAATACAAATCCAAACGAAAAAATAAAAAAACCGAATTGGGAAGACC
CAGAATATTGTAAATATTACATGGTTAAATTTTGTCCTCATGATTTGTTTGTGAATACAAGAGCCGATCTTGG
GGCATGTCCTAAAGTACACGACGATGAAGTCAAGGAACTCTTTGAGAAAGCTGACTCT
TCATATAAAAAAGCTCAATATGTGGAAGAATTTTTACGATTTTGTCGTCACATGATTAGCGATGTTGAAAGAA
AAATTCAAAAAGGTAAACAGAGGTTGGAATTGATGAATTCAAAACCAGAAGGTCCACCAATGACTCAAGCTCA
GACTGAAAAAAATCAAGAACAGGTAAAATTGCTATCTGAAAAGATAACAGCATTACTA
CGGGAAGCTGAAGAAGCTGGGTCCTGTGGCAATGTGGAACAAGCTCAAGGACTTATGAAGTTATGTGATAGGT
TGAAAGAAGAAAAAGAGCAGTTGTTGAAGC
>103790500_2 DT661754.3
GCACGAGGGTTTATCCCTTAAATGCGTTTGTGTGTCTAAATAAATATTATTTAGTATAATATGTAGACATATT
TTTAAAATGATGACGCACCGTCATTTACACGCTAGCTTAGTAATTAGAATGCTAGAACTCATCACTACAAATA
ATAAAATAATAAGATATATAACTAATAAAGTAGATGTTATCATGTTCAATGTGAAGTG
ACCTTTTATTGTTATTTAAATTTATGAATATAAATAAAGAGTAAAAAGTGCATTTTTAACTTTTGTGTGTATT
TGTATAGGTCACTTTGCCATTGACTGTAATGTGTTATACCAATATTTTGTCTGTTTTTGAAGACGATATGAAA
CGTCCATTCTTCGAAATAACGTGGATGAGACTGATTGTGTATGTCGTGGCACGCCGTG
GTCACTGAATGATGAAAATGGTGAATCTCAATTGGTAAAATGTATAATAATAATTTTATAAAGTAACTACATA
TTAATCTCTAAAAATTTTAAGGTTTTAAGTACAATTTTTTATTTTCAAACTGTTACACATGTTACCACAAGTT
CTTTTATTTTTTTTGTTTTAAAAACTATTTTTTTATTTAAAAGACATTTTTCTTTTTC
TCTACATCACTATTGTTATTGTATTATAATAATATGGCCAATTGAGATTCACTCTTAAAAACCCTATTGTAAA
ACGGGATTTGGCGCGTCACTCATTTCTCATTAATCAAATTTTTTTATGACAAAAATATAAAAAATTAAAGCTT
AAATTTTTATTTTTTACAACAAATT
>103790518_2 DT662602.3
AATACGGCAAGAGGGTTGCCATAAAAATGAAAGGTTGCATTTTTAACATTGATGCTGGGTATTTGGAGGGATT
GTGCCGAGGTTTTAAATGTGGAATACTCAAGCAAGCTGATTACTTGAACTTGGTCCAGTGCGAAACATTGGAA
GATTTGAAACTCCACTTGCAAGGAACTGATTATGGGACATTCCTTGCTAACGAGCCCA
GCCCTCTTGCTGTATCAACTATTGATGATAAGCTGCGTGAGAAGCTTGTCATTGAATTCCAACATATTAGAAA
CCACTCAGTGGAACCAATGTCAACCTTCCTTGACTTCATTACTTACTTTTACATGATTGATAACATCATTCTG
CTGATTACTGGAACTCTGCACCAGAGGCCTATTTCTGAACTGATTCCTAAATGTCATC
CTCTTGGGTCTTTTGAGCAAATGGAAGCTATTCATGTTGCTGCCACCCCTGCTGAACTTTATAATGCTGTGTT
AGTGGACACTCCACTGGCACCATTTTTCGTCGATTGCATCAGTGAACAAGATTTAGATGAGATGAACATTGAA
ATCATTCGTAATACCCTATACAAGGCTTACCTGGAAGCTTTTTATGAATTCTGCAAGC
AGATTGGAGGAACAACAGCTGATGTAATGTGTGAAATTTTGGCTTTTGAAGCAGATCGTCGTGCAATAATTAT
TACAATTAACTCTTTTGGAACTGAACTATCCAAGGATGATCGTGCTAAACTTTACCCACGTTGTGGAAAGTTG
AATCCTGATGGATTAGCAGCATTGGCCCGTGCCGATGATTATGAACAAGTGAAGGCTG
TAGCTGAGTATATGC
>103790528_2 DT663001.3
GCACGAGGGAAAACTCATTCTTATAAAAGTTTAACGACTTCATCTTCTGATATGGGTACGAGCCAATGTAGTT
CTAATCACAAGAAACCAGTTCTTACAAAATCAGTATCAGTGCAAACAGGTATGCCCGACAGCTCATACACAGA
AGAAGAAGACAGTGGCATAAATATGAAACATGGCAGAAAAAAATCTTAGTGTTGATCAA
ATTAACTATTGTGGCTCAATGGAAAGTGACTCAAGTTCTGGTAGAAAGTTAAAATCTCTAGACGAAAATATGC
TTAAAAAATCATCAGCTAGTAAATCTTCATCTAATCTGGAGCGAAGGCAAAGAATACAACCAGATTTAATACC
AGAAAAAGCAATGGTAAAATCAGAATCATACAATCAAATTCAGAGCTGCGAGGATATT
AATATGTTTGGAGCTGGATCTTTATACAATGACAGCGATTTTGAAATCAATTATAAGCAACACTCTTTCATTA
CCGAAAAACTATGTTCAGAATTCCACGTTAAAACGAAAAGAGTGCTTAGCAAATCGACTTCTAATTTACTACA
TCCTAAAAAATCAAACGACCGAAAAGAATCAAGTAATAGTAATAGCATGCCGACAAAA
ACTGAGCGCTCTAACGAAAGTGAAAAGTTAGTANACTTTAGAAAAAAAATTGACAAGCCAAGAGCACCTACAC
CGAAATCAGAGTTGGATGATGGTGTCTCTGTAGACATATCTGAAGAACCACAGATGCAGAAAAAGCTTAGGCG
ATCTATATCCTC
>103790531_2 DT662093.3
AGCATACGTTCCTTTGAAAGCAAAATTTCAGTTTTTCAAGTGAATTTTTGAAAGTTTCTTAAGAAATTGTTGA
CTGTATTAAACAGTGTGTATTTAAATAACTGTTATAAGTTTAAATCTGTAAAGAATTTAGTGTTTTGTGTTAA
AATCTATGTGTTTACGTGAAATGGGATTTCGTGTCGCCGATCTATGAATTACGATTGT

CGACGATGCATCGTAAGGAGTAAAGGGTTCTTCAAAAATGGCGTCACCAAGGACTTCTGATATAATTATACCA
AACAAAGAATGTGATATTTTTTGGTTAACTACATCGAAGCACAATACACAAAAAAACAATAAGAAAGTTCACG
GTAGCTTTCAAATAACACCTATTGACATGAATTGCTACAAACTCAGGCGACCTTATCT
TTTCTATAATAACTTCGGTTATAAAGAAGAACTTTCCAGAAGGATCCATATAAAGAGAGAATATGGATTTTAT
AATTATTGCTTTATAAGCGTATGCCTTAGCTTTATATTAGGACTTGTAATAGGCGTTGCAATAGTCGGATCTC
CGCAAACTAGAGTGATTTCTTCTAATTTTAGCAATAGAAAATTAGTAAGAATGAATTC
AACTAGAAACCTTAACTTTAGAAGTTTAAATTCCATTTAATATACTGACAAATTAGATAATGA
>103790539_2 DT662664.3
GCACGAGGGGAATACATGCGTGCAAACGTGTGTGGGGTTATAAACAAAAATGTCAGTGGTAAGCAGTAAAAGA
AGACTTATATCATTGCTTAACGAAGTTTTACAGCATTCTTCAGATGAAGCCAGCTCTCAACTATAATGATAAA
ATTGATGAGAACACTGGAGATGAGAAAAAGCCGAAAATGATTACGTTTTATAGCTCGA
CAAAAGCTGGAGAAGAGAAACAGTTGTCGACAAACTGAGCGCAACTTATGATGTCTCTCGCAATAGCAAACGC
TGGCCATTGACGATCTTTTAGGGACTTTTAAATATGGCAGCTATCAATGCTAACTTTATATATCAAGAAAGTG
TCAATAAAATGAACAAACGTACAGAATTCCTCCGCAATTTGGGATTGAATTTGGTTTA
TACGTATTTGCAGTTACAAGGAGATCAAAAAAATATTCAATATTAAGAGCACCGATGCGTACCAGTAGCCCAA
AATTAACCCGGACGATATACTCGATGTGCAGATTGCCCACGCAAGCAAAATCGCAAAACAAAATACGCAAGAA
CTGGGTGTGGAAAAGCCACATGCCTACATCATGCAATTTTTTCTTGCAAAAAAAGTAC
TGATTTTGAATGAAAATACTAAAGTGTTCCTAAACTATTTTATAATGTTTTATGCTCTTATTTTGTGTAAGAT
CTCATATTCTTTTAAGTTTTTAGAGTTTTTTTTGTTTAAATAGGACTTGTTATTTTTTTATCATTTTTTAAAT
AGAGGTTCATATTTTTGTTACCAAAAACAAGTGCCTTAGATCTCATTATGTGAAATTA
TAAAATAAATTGAATTTACTGTAAAAAAAAA
>103790579_2 DT663285.3
GCACGAGGGCTGATTTTACAATTAAAGTTTGGGACATCACTACTGGACACTGTCTTCATACTTTATCTGGTCC
CAACAAACACCAATCGTTCGTGACATGCCTGCAGTCGAGCAACCGCTTCGTGATCACGTCGTCAGACGACGGC
ACCGTCAAGCTGTGGGACGTGCGCACCGGCGAGTTCATCCGCAACCTGATGGAGCTCG
GGTCGGGCGGGTCGGGCGGCGTCGTGTGGCGCATCCGGACCTCCGCCACCAAGCTCATATGCACCGTCGGCTC
CCGCAACGGAACCGAGGAGACCAAGCTGCTGGTGCTCTACTTCAACGTGCCCGGCGCCTGTCGCAAGTGCGAC
GAATAGCATCGACTCAACCGGCGCCTGCTGCGTCCATACACCAACCTGTACGCGCCGT
GGAACTGTTAGGCGAACGGTGGACTATATGACACAGTGCACTACCATTCATACATTCTAGTTCTAAATAGTGT
AATTTGTTCAGTGACGCGTTCGGGGGTGCCGGCTCGTCGGTGATGGCGCACGTCACCACCGCTGGAACTGTTG
AGGCGGCGCTTTACCATCGTCCATT
>103790603_2 DT662797.3
GCACGAGGACGTTACTTAACAACACTTAATGGTATTCAATGCAAACGTGAGAAATCCCATTGAAATTAATAGT
ATCGCCTTATAGTACATCAGTGTTGCGGCAAACTTTGAACTAACCAGTCGTTTATAATGTGGAAGGTTATAGC
ATTCGTTGGTTTAGTTACGGCCGCTCCGAAAAATTTACTTAATATAAACTTTAATCCA
ATCAACGAATTTTCTTTAAAGTTACTCCAAAATGCGTATACATTTCAAGAAAATTTCGGTGATAAGAACTTGG
CTCTATCACCGTTATCAGTTTGGAGTATATTCTCGTTGTTAGCTGAAGGCTCGGCGGGAGACACTTTTACGGA
ATTAGTGAGGGAACTTCGACTGGCGGACAATTTGAAGGAAACACAATTATTACATTCG
GCAGTCACTTCCTTGTTGAGGATAAAGGATGAAGATGTCACAATCAACAGCAAATCAGCCATGTTTTCCGACA
ATACACTGAAAATTTATCAGGAATTCTGCGAATCTGCTCTATATTACGACACTGACGTGTTCTCTGTGGACAC
AAACAATACAACAAAACTTGCAAGTGATATTAATTATTATGTATGTGTAGCGACTGAG
GGGAAAATCCGTTCAGCGGTTCGACCGGAGTTACTTGATAATTTAAGACTAATATTGATCGATGCACTTTATT
TCAAGGCCAGCTGGACCAATCCCTTTGACCCAACTCAAAC
>103790621_2 DT661814.3
GCACGAGGCCGTCCTTAGGGAGTTCCATGCGCATGCTGTGTGCTCCTTTACACAGTTGGAGGATTTGTTCCAG
GACATGAAGGGGCGGCTGGAGGCGTGCGCGGCCGCGTTCGGCGAGGAGGCGGCGTCGCCGGAGCAGCTGTTCG
GCGCGCTCGACGCCTTCCTGGCGCAGCTCGGCGACGCGCGCGCCGACTGCCGCCGCCG
CCTCGACGAGCTGCGCCGCGAGCGCCACTACCAGGAGCTCAAAAAACGTACGATGGAGAGAAAACAAGGTTCC
AGCCTATTGAGTTCAGTAGGAAAATCCCTAGGGTTTTTTTTTTAGCACAGATTGCAATGGCCACTCTGATAGA
AGTTCTCGAGACCGCCCTCTCACAAACGGACAAAAAGGGGAATTCGATGATCTAATCT
CCGCCCTCCGTACTGGAGACGTATTTGGTGATGACGTACCTAAGTTTAAAAG
>103790648_2 DT662908.2

TTCCGCACGAGGCGACGCCTTGGTTGTTACCTAACTCTCTGTGTATATCCGGTGGGTTCCAGCACCCGTTGTG
GCAAATTACGACTGCCCAATGACTTCCCCATTCCTTGTTCCTTACAAAAGTCCTATAAAATTTAAGCCTTACA
GATTTCTCCTTAAGGTCAGTTTGGGTGTTGGTTCAGCCACTCGGGATAACCGAATGAG
TGGGTGGATAATACGACCAAGGTTGCTCGATGGTTGGGCAATAAAGCTTCTCTTAAGTGAGACTCTAAGCCTG
GCCACGAGACACGGGATCTGTCGATGGTCTAGAATGCGATCGCCCGCCACAGTGATACATTACTTGACTGTTG
AAGCAATTTCTGTGTTTGATATATGAAGGGTCCTGTAGGAGGCACCCTGAGACAAAGA
ACTGTCTTAATAGCCTCTTAAAAATACTCAAAAAAAAAAAAAAAAAA
>103790663_2 DT662474.3
GCACGAGGTTTTTATCTTACGGAATATCATTTATTTAGTATCTATTACAACATTGCTATACACGGAGTAACAA
GTTTACAGTTAATTTCAAGCCCAATAGTAAAGATTTAGTACATTTAAAACATAGCGACGATCCATAGAACATA
TTTACTGTTCTTAATTTCTAATAAATATTTTGCAGAATATTCTTCCTATTATATGACA
AACAAATTCACATAGATACAAATACGAGACGAAACAAGTACTATTCAGTGTATATTTTGTAACAGACAAGAAG
TACTTTCGTAATAATTGAGTGCATCTACTCCGTTGACAGTTATATATTAAGCGGCTACCACATCAATACTCTA
TATTCGCCATTCATTCCACATACGTAACTATAAATTATATAACGTCATAACAATATTT
ACAAAGCCTAAAAACCTAACTTACCAGTAACCGCAATGTACACCTTCAATAAATTATTAATAGTGTTTACATA
ATATAGTACAATACAAGCGCTACATTTTATATGATATGGCTTTTAATTTATCAAAATCGACTGCTTTAAATCA
ACCCGTGCAGTCGGTTATCACATTAATCATATTTTTTTCTCACGATCCGAATCATTCT
GTATACGCTCCCAGATTCAATTCTAAGCAATTCTTAAATAGGTAAAAATGCAATTCAGTAGGCCGAAATACAT
AGAACATTTATTTATTATATCTTATTTGTTTTAATGACGATGGGTTAATGCGGCAGTANGGCATAAGGAACAT
TACAGAAAATAAATATATTAACAATCTGATTAACTATAAATGTATTATATTTATACAC
TTGAGCAAATAAATTAGCCCATAAATATTTGACTAGTTT
>103790666_2 DV501033.2
GCACGAGGCGATGACCATCTACTCCTCGGGTCGCGCGTGCTGGTGTTCTCGCTGAGCGCGGAGGCGCCGAGCT
TCTCGGCCATGGTGCTGGCGGCGCACTACATGGGCCTGCGCCCGCGCCACTCCGTGCTCATAGTGCTGCCCCT
CGATGCTGCTAAAGCGAAATCCTTATAGCGAGGCGGCGGTCTTAGATTACTACCGAGG
TATACTCTACCTGAGGGACCTCGCGCGGAGGGCGAAAGTTCCCGTGTTCGACTCCGTTGAAGCCGCCTTGGCG
TGTGTAGTGTATCGCCTGCTATCTGCGCTTTACTTAAGTATTTACTCATTTACGAGCACCCACTTACACATAA
TCACATTTACACAATGTATTTGGCCACCTTTATGTATGGATGATGTATAGAATGGTGA
ATTTCCTTGGTGATAACTGCGACTTATAGTATTTGTGAGCTTATTTGTGATTATTGATTTCTCTTTCTAAGTA
CTAAATTTTCATATTAATGATGTTCACCATTATAGAATATTTGTTTTACTAAGTTTAGTTATGTTTTATGATA
TAAATTGCCGTTCTGTTCATTTCATAGTGTATGATCATGGCAATTTCAATTATATATT
TTTGTTCTGTTAAGACATGTATGTTGCGGTCTACTTCCATTATGTAATTTAAGAGGTTATTTGAATTAAATTA
TTACTTT
>103790678_2 DT662642.3
GCACGAGGCACATTTCATTTAAAGATTACGTTGTCAGACAGAGTCTTGTGAACTATAAACCCAACACCGAATT
GGGATCGTTGGTCTCCCTCTCTGAAATACATAAGAGGACTCTAAAGTTACGGTATCCTCCCCCTCTCGTCGGA
CTTCACATACCCCCAAGATGTGCCATCTTATGTTGGTCAGCTCCTCCTCCGTCCGTTA
TACGTAGCCAGAGTCAGTTGGTGTTGGCGGCCTACCATAATCCGGGGATTCTTATTAATCTTTCGCCATAGCC
TCGTTCGTTATTTGGCAGGGTGCACCACGTGCAGGTGAGGTTGATTGAGAGTACTGAGATGGATTTATACTTC
CTTACACCCAATAATATAAATAATATAAATTGACAAAAATATATGAGTGGCAAATTGA
TAAATACTATAAATGCAATTTGCAGGTCCTATCAAAGTCGTGCAAGGTGATCCCGGTGATGCTGATGGGCAAG
CTGGTGTCGCGCGCGTCGTACGAGCGCTACGAGTATGTGACGGCGGCGCTCATCTCCGCGGGCATGGTGCTGT
TCATGCTGGGCAGCGCGCCGCCGCACGCTGCGGGCGCCGCAGCGCTGTCGGGCGGCGC
GGGCGCGAGCCTGCTGGCGCTGTACCTGTGCTGCGACGGGTTCACGGCGTCGTGACAGGGCGCGCTGTTCAAG
CGCTACCCGCTCAAGCCGCTGCAAATGCTGTGCGCCGTCAACCTGT
>103790681_2 DT662691.3
CGGCACGAGGACCATTTCCATGAGGATGTTGTCGGTTTGATTCCCACTCGAGGTAACGAAATATTTTATATTT
TCATTTTATTTATTTATTAGAGCAATCGAACCTACAGTCTTCGTTTTTTAAAGTCAGCGCCGCTCCGCGTTGA
GTAGGCTTCATGATTGGGTGTGGATCTTTGGAGATAGAATATGAATATATATAAAAAT
TATGTAGAATTTTAATGTAATAGATGTTGGTTTGAATACGTCGGATGTGATATTTTTTTTATTTCTATTTTCT
TATTTTAGTTTAGAAATTTAAATAATGATAATAGCAAATATCAAATTTGTTTCGTTTTCAAAAATATTATAAA
TAAGACGAGTTTGTAATTTTTTATTTATTTTACAACCGTTAGTTAATTTAATAAATTA

AATGCCCTATACTATTTTAAAAATGCATTCCTTAAATAGATATAAACAGTTTGACACAAATTCTGAATGATAA
GATTATATTGGTACAGTTTTTTTTACTTCACAACACTCAAACTTTTTTTATTATTATTATTTCGGTCATAGGT
TTTTATTCCTTCCTGCGGGTTCCTTTGGCATTTAGTACTTACACGTAATTGTTTGCAC
ACTGTAATTTTTATTTTGGAAAAGTCTTAAAAGTTATATTTTAAATTAATTTAAAAGATAGGATGTATCTAAA
AGGTGTTTTAAGTCTTGGTTTCCTT
>103790684_2 DT662938.3
GCACGAGGTAATGTATTACAGGTGTTAATATGTAGAGCCATTTCGATGTTTACAAATATAATTTGAATTTTAC
ATGTTAGTAAAATATTGAAATTATTGAATTTATTATCACAACAAAGTAACACCACATATTTTGTTTGTCTATT
TAAAAATTATTTGATTTCAAACACTGTTACATACTATATAGAATTAATTAGATGATAA
TAATTGGCATTATATTATTATTTTATTCTAACGTCAGCCAGTAAAAATATGAAATAGAATAAAAATAAATGAC
ATCACAATTTGTCAATAAATTAATAATTTTGCTTACTATGTATAATAAGATTTCTGTACATCTTAGTAATAAA
GACCAACTTAGCCACAAATACAGTTAGCCACTTAGAAAATATTAAAAAAAGAATATTA
TTGTCCAATTCTTCTCTAATATGTAACAGTACTACATAATATTTGATTCTAATACACCTGTAGTCTAAATCAA
ACCCGTAATATTTATTGGTCTTTTCGATCAAATCAAAGGGTTCGTGTGATATAATGTAAATCTCTCTTTATTC
TCTATTATTGTAATTATTTAGATATTTTATTACATCTTTGTGAGTAAGTTGATTAATC
AAAAGTTTTAATATGTATATTATAAAATATACAATATTTTATATTGATCATTTTAAATAAATAAATCAACGAT
ATATTTACTATACTTACTACTATGCAATAATTAGTTAGTTTACAAATATTGATTTGCACCATTTTGAATCATC
TTATGTGGCACATAAATA
>103790687_2 DT662876.3
GCACGAGGGCGCGGCGTGGGGCGTCGCCGGCGGCGGGGGGGAGTGCAGCACGCTGCAGAGCGACTCCACTCAC
CTGACGGTGCTGGCGGGCTCCGACCTGGCGCACCACACCGACTCGGACGACGACCTCAACCAGATACGCGCCG
CGCATTAGGCCGTTTGGCACAGCGGCGCAAGCGCAGTGTCAACTCTACACCAAACACA
CAACAATTATTGATCACTGTTTGAATTTTGAACTAACACCATCAATATAAGAGGTACTTCCATTGTATCACTG
AACTTACTGTATCTCCAAATGTGTGTCAATGTGAATTTATTCATATATATATATTAACATTTATGTAATAAAC
AAGGTTAGAACGCACAGACGGACCATTATCTTGTACTATAGATGTCCGGGGCTAATTT
TACAAAATTATGATGTCATTTCACATATTCGTGAAAGAGGACAGTGGCCTCACTGTAAAGCTAACACTGCCAT
ATTATTTTATAGACGTAGTATGTGTGGCCTTGGACACCTCTAAGCTCACATTTTGTCCACACTTGTCGCCATA
TTTTATTATTAATAAGAATATTCTAGAGACTTTAAAAGTTTATTTGCAAACTTCTTCG
TGCTTGCTACGGTTGTTATAGATTTATTCCGTGTCATAATAATTAGGGATATTTCATTGTGTTTAATTAGATA
TTCGAGGATGTTCTACGTTGTTCGTATTGTTTATAGTTGTAATGGCGCTTGTGTGGAGACAGTTGTGTAATGC
GGCCGTGTTTTGATGTCCAGTTTCAGTTTCAGTATTGTAGGTCACGGGCAGC
>103790696_2 DT662981.3
CGGCACGAGGTTTTCTACTCACAACCATCGACATTAAAAAATCCATTTAGTATCATTCGTTAATCGATATTAT
TATGGGATTCAGCGCAAGGAAGGTTCTGGAGAACAGGTCGTTGGCCGATGAAGAACGTATGGACGCTCTGGAG
AACCAGCTTAAGGAGGCCAGGTTCCTTGCTGAGGAGGCTGACAAGAAATACGATGAGG
TCCTTTGAGTTGTGTCGTTTTTGCATCTCCCTAAATCATAACCAGGCTATTATTCTGTTGTGAATATTTATTC
TGTTGTTCTATTAACAAAATTTCGCTATCGCAGCATTGATTTCTGTGTGTGTTTATGATGTTTTTCGGTCTAC
ACTAGACCTTTTCAAAAAAAAACAAACAAAATAACTTAAAAAGTACCCACTAACAATT
TAAAATCTTAGGTTGCCTTAAAAAGTAAAACTAATGTAACCATTTAAAGCTGACAATATTTTTTTATGTTTTC
AAATTCATTTCCGTGGCGTGACTTAATTATGCTATAAGAAGCAGGTTCTTAGTGATAAGGTATTTAGGAATCG
AGACGTTATCACTGATAAGATATATTATTCACCTCAAATTTCAACATTTCATATACAG
TATTTTGCCAATCATCAAGAAAGCAACATGTGTTAAAACAGATGGCTTTCATATTACTGTCCTAATTCCCCTG
ACATATGACCCTGTGTCAATTTTAAAAGTCGTGCCCAAAATGGAGACGTATCGTGCATGACCGTACGTTTCTT
TCCCTTAAAAGGGCAAGTCCATGCTTCGTGTACGTAGTT
>103790717_2 DV501097.2
GCACGAGGATGACCCTATTTAAATTTAGAATGTTGTATGTTCCTAATTGTATATACATTTGTGCAACATTTAT
GTATGGAATTCAATTTTTAATTTTTTTATTTTTGTGTGTTTATTTTCTTTTTCTTTGTTAAATTGTTATTTTT
GATACAATATCAATATTGTATGTGTGGGTAATTTATCAGTGCTATACAGTATTGGTTT
GGGATCAAAGCTTTTATTGTAACAGAACTTGGATTTAAAAAGTGCTAACTCGTAAATGCTGGAATGTGAGTCA
TCGACTAAATAAATACGTATAATATAAATGAATGGAATTCATCCATATTGATTAATACTATAGACATTGATTG
CCTCACTTTATGAGTTAGTACGTTTTGCTTGATACTGAGATTTAAACATATTGTGGGA
TGGTAGCTGGCCTACATAGCTTTTGATGTAAAATGACAAAAAATCAATAAAACGCTTATTTTCACAGTAACCA
GTGCCGACATTGATTAATGTAATAAAAGTGTTGGAAGCGCAACGCACTCTATACCATCA
>103790736_2 DT662879.3

GCACGAGGCGAGAACATTCTTTTATGATATTTAGTAATAAAAATTGAAATTAAAGTTCAAGTGTTTATATCGT
CGATGTTCCGAATTTTATTTTGTTACGATAAATATATTATAATATAAGCAAAAAATATATATTGGCACAAAAA
GACATTTTGTAACGGAAAATCTATTTAGAAATATAATAAAAAAGATTTTACTACACTT
ATTAATAATACTTAAAGCATGAATAAAGACAGAGCTTAATATTTATTAAACCTTTAATAAAAGAACCAATGCG
CAGATGCGCGCCAATCGCTCCGCAGTGCGCATATGTTGGTAGCATTGACTGTTTCCTCCAAGCCACTCGACTA
GACGCCATTACCACGCCATCGGATCTCAAATCTGTCACAGTGTAGTTTGTGTGATTCT
TCGTAAAATTCAGCTGAACTCTTAAGTTGTGTCCATAGGAATCAATATTTTTATAATCCTAAAATGGCAGATG
AAAAAAAGGGAGAAAGCGAACACATCAACCTTAAAGTTTTAGGACAAGATAATGCTATTGTCCAATTTAAAAT
TAAGAAACACACACCACTCAGAAAATTGATGAATGCCTACTGTGATAGAGCGGGTCTA
TCAATGCAAGTTGTACGATTCAGATTCGACGGTCAACCTATCAATGAAAATGACACACCAACATCTTTGGAAA
TGGAAGAAGGTGACACAATAGAGGTGTACCAACACAAACTGGAGGGGCTTTAGTGTAAATTTGACTT
>103790751_2 DT661843.3
GCACGAGGGATATTTTTTACAGGTTTCTCAACAGGTATATTCTTCCTACACGAAATGCCTGATGTGAACCTTA
TACACACGCTGAGTATATCCGAGCACAGGATCAGTAGTATAGCTGTGTCGGCACAAGGCGACTGGATAGCATT
CGGTTGCCCCAATATAGGACAGTTGCTTGTGTGGGAGTGGCAAAGTGAACAATATGTA
ATGAAGCAGCAAGGCCATTCCCAGGACATGACCTGCCTCGCCTACTCCCCCGACGGCCTGTACATAGTGACCG
GCAGCTACGACGGCAAGGTCAAAGTGTGGAACACTATCAACGGGTTCTGTTTCGTGACGTTCAACGAACACAA
ATCAACGGTCACCGATATTGTGTTTAGTGCGAACAAGAAGTTCTTTCTATCCGCTTCG
CTCGATGGGAGTGTGCGGTGCTATGACCTCACTAGATACCGCAATTTCCGTACATTAACGTCGCCCAGCCTGG
TGCAGTTCGCGTGCGCGGCGCTGGACAGCAGCATCGAGCTGTGCGCCGCCGGCGGTCAGGACGTGTTCGACAT
ATACTTGTGGTCCGTCAAGTTTGGCAAGCTCTTAGATGTTCTA
>103790754_2 DT662430.3
GCACGAGGCGCCGCTCGAGTCGCACGTGTAGCGGCCGAGCCCCGCAGAGGCTACCTTTGATTCGATTCATTTG
ACTGAATAGATTAAGTCACAAACCTTTATATATTTTTTAATAACGAAAATAATACTCAAAAAACCTCCGCTAC
TTATCGTGTTCGTGTTTCGCGCATTTACGATTCTTACTCAATTTCTTGATTTGATCGA
AACCTTAGATAGTGACCGGTACTCCCGTGTCAGGAAGCAGGAATGTTTTATTGCAACTTTATCTATTGAATTG
TGCAATACACTTATAATTAATTATATGTAATATGGATTAAAACAGAGCATAGATGCTAAACATTAAATGAATT
AGTACGAGCGTTGAAAAACCTAGCTCCTTAAATAATGCCAAAGAGTGATAAGGACAGC
TCTGCTGATGAGAATCGATAGCAGAGCACCGGTGCAGGGAGCGGTAAGAGGAATCATTTATTAGGTGTAGAAC
AACAGACCGCTGTCATCGATATGGACATAATATTTTCTGTAGTAATAAATTGTGCGTTAGTTATACAATAAGT
ATATCGAAACCATTTGAGTCTATATTACTTGTAAGAAGGACTCCCGCAGTAGGTTCTA
AACTACTATTAATGGAGTCGGTGGTATTATAACTAAGTGTTTGAAATAAACAGCATAATATAGGTAAAGTAAG
TGCTAATAGTATGTAATTAGTATGAAATTTTATTGAGTAGGCCCGAGGGCTTCTTTTAGTGTCTTAGGTAAGA
CCGAAACTGTAACACCATAG
>103790773_2 DT663294.3
GCACGAGGAAGAACTTTTACCGTTTTAATTGGAAAATATCTTTAAAATAATTTTGTTATTTTTATATTAAGTT
TTCCGCCTTTTGAATTTATTAAAATGACCGTATCTGAAGCTGGGAAAAATTTACCATGGGTCGAGAAATATCG
TCCATCAAAGTTAGATGATCTTGTATCCCATGATGACATTATTAAAACAATTGCTAAA
TTTATGAAAGAAAATCAAATGCCTCATCTGTTATTTTATGGGCCACCTGGTACAGGGAAAACTTCCACTATTT
TAGCATGTGCCAAACAAATGTACACTCCACAACAATTTATTTCTATGGTACTAGAGTTAAATGCATCTGATGA
CAGAGGAATAGGTATTGTTCGAGGTCAGATTTTAAGTTTTGCATCCACTAGAACTATA
TTTAAAGCTGGACCAAAGCTTATTATACTTGATGAAGCAGATGCCATGACTAACGATGCCCAAAATGCTTTGA
GACGAATAATAGAGAAATACACAGAAAACGTGAGGTTCTGCATAATTTGCAACTACTTGGGTAAGATAATTCC
GGCGCTGCAATCGCGATGCACGCGGTTTCGGTTCGCGCCGCTACAACAGGCGCAGATT
GTGCCGCGGCTGCGGGACATCGTGGAGAAGGAGAATGTCAAAATCTCCGAAGATGGCATAAAAGCCTTATT
>103790779_2 DT663208.3
GCACGAGGCAGAACCGTAAACAGAAGCAATGGATGTGGACGGTGAAGCTGAGGCCAAAGTGAAGGAAGAGCCG
GCCGAGCCTCCCGCTGACGACGTGAAGGACAAGGATGATGCTACGCCGCCGCCTGAAGCACCAGCAGCGGTGG
ACTCCAAGCTCCAATCAGCAGCGGCAGCAGCACTTGCAGCAGCAGCAGTAAAAGCCAA
ACACCTGGCAGGCGTGGAAGAGAGAAAGATCAAATCTCTAGTCGCTCTACTAGTGGAAACACAGATGAAGAAA
CTGGAAATTAAATTACGCCATTTTGAAGAGCTGGAAGCCACTATGGAGCGGGAGAGAGAAGGTTTGGAGTATC
AACGGCAGCAGTTAATTCAGGAACGTCAGCAGTTCCATCTAGAGCAATTGAAGGCGGC

TGAATTTCGCGCGAGAAACCATGCCATTCAAAGGTTACAAGCGGAGTCCGGTGGTGTAGTGCCAGGTGTCCCC
AGCGTGGCGGTAGCGGGCGCCGGCCCAGGAGCCCCAGTACCACCTGAACCACCAACCGAGGCGCCCACACAAC
CGCACCACGCTTAGATTTTATTTAATTTTAAAAAAAATAAATTTAATTCATTTATAGT
ACGGTCGATGAGCTGTGGCCATCCTATGGTTTTTATAACTTTTGCGCATTGTTCGTTTGGAAAGTAATAATCA
AAAGTAAGAAAATCAAATAGGCGCAGTCTTTGCTGTAAACTTCGGGGCGGCATGACTTATCATT
>103790790_2 DT661859.3
TCTTTAAAGAGCCGGGCTGCAGGAATTCGGCACGAGGATTTCAACATTAAAAGTGTCTAATGGTCGAAAACCA
GAAATCTAAACCAGCCTGAGTCGTTAATACAGTACAATCTAGTAACTTTTCAAATATTCGCTTTTAAAGCAGT
TTTTTCTGTCTATTGTTTTTTTTTTTTTTTCAATTAAAAGGTCCTCCACTGACGCCTAC
GATATATGTTCTAAAACTAACGACTGTAAATGCCAGTGTCGCTCGGATCTCTCGTTTATAATATAAATAATAA
TTGAGATTATTCCATTAAAGAATTAAAATCTTATAATCATTTTCTGTTCTCGATACGTATTCGGCGTGGATTT
TTTTAAAACGTGTCCACTGATAGAATTTTAGAAAAGTGTACATAATAAGTTATATTGT
ATTGTTATATTTCATCTTTCAAATGTAAATATTTGCAAAACTCTGTAAATATATGCACGTATTCGATGTTGCT
AGCTATCCGTAAATAGATTTAGATTAGATTTAAGCTAAAATGTTATTTAAGTATTAACGTCTGGAGTTTAATT
TTGTGTGGCCATGTGTAAATCTTAATAATAGGTAATCATTTTGTAGCTTAGGCGTAAG
TGACTTAATGTATCAAACCAAAAACTTTGAAGCGGAATGCTTGAAAATTTATGTGTTTTGAA
>103790810_2 DT663032.3
GCACGAGGGCTGTACGTGAAAGGCTCCCAACACTCCGAGCCCTAGCAGCGCCCGCCATAAGAGACGCCCGATT
AGATCCCGCACCCCTCGCTTTATTACCAAGACTAGAATTACTGCGCCTAAAAAGTCTCACAGGACTATCACTA
ACACGAGATCTACGACCTATATCAGCATTAGACCGACTCCAGCATCTATCATTAACAA
GTATAAAAGAATTGGGCTGGTGTGCTTGTGAAGTGGTTGGTCAGCTGGAACAGCTCGAGTCGTTGGAGCTTGG
CGAGTGCTCGTTCGGAGCCTCCTTCGCTGAGGCCCTCGGCAAGCTGTCCAAGCTGCGTAGATTGAGACTGGAG
CGCGGCATGTCTCACTGTGCCGCCCCGCAGTTGTTGAGAGCGCTGGCGGCGTTGCCCA
AGTTGACTAGGCTGGAGCTTGTCAATTTTGATGTTAAGGTTGGCTTCGATGACGCACTCGCAGAATGTAAAAA
CATACAGAGACTTCTCATCATACCTACATATGTGTCGCAATCTGCTACAACAAATAAACAGGTGCTAAGTGGT
GTACTCCGACTAAAGGAAACCCTAACCCACCTAATGTGGGGCGTGACCATCGAGCTGC
TGCGTGTCACGGAGCTGTTCATAGACCAGTGCGAGGCGGGCGAGGGCGAGGGCAAGAGGCGGGACGTGGGTGA
GTGCATCCCGGTGCTGAAGCCCGTGCCTGGTTGTAGGCTGCCTGACGACCACCGCACTGGTGCTGGGACGCCG
>103790816_2 DT663444.3
GCACGAGGTGCTAACGTTAAAGAGGAATGTAATAACTACAGTAGTGAACTCAATAATTTATTTTCAAAAATGG
ACGAGAGGAGAAGGAAACGGGACGTACCGGATTATTTGTGTGGCAAGATCAGTTTCGAAATTTTGAACGAGCC
AGTGATAACGCCCAGCGGTATCACCTACGAGAAGAAAGATATTGAGGAACATCTTGAG
CGGGTAGGCCACTTCGATCCTGTGACCCGCGTGAAGTTAACAGCTGACCAGCTCATACCCAACTTCACTATGA
AGGAAGTGGTCGACGCGTTCCTGCAAGAGAACGAATGGGCTCTCGATTACTAGTCGATATCGATTATTCGATA
TATTCGGTTTATTCGGTTACGTCACTATCTAGGTGTTTTCTGTAATTCGATTTTTTTT
ACTAAATTGTAATGTTATAACAATTATTAATGATCTCAATATATATTTAACAAAAAGTCAATTGGTTTTGATT
GTTGTAATAATGATTTTTTTACTGCTTATATAGATTTTTTTATTTTTTATTTTTGGGACATTCTGCTTTACGG
AACTCAACGCTGACTTTTGTCTTGTTGCCAGAGCGAGAAAATGAAAATAATTTATTTT
CCTGAGTAGAATTTTATGTATAATCGATTTTGACCACATTTTCTAGTTAGTGACGTAATTAAAATGGCGTCAC
ACGACATCCATATTATTTATACGCCATGGAACAACACTATAGGAATGTTATAATTGTTGTAATGTTAAATTAC
TTATCGTTTATTTATATACAACTGTGTTATTTAACTTATAGATAGTCACATTTTATTT
TCATATCATCATTCGTTA
>103790825_2 DT663377.3
GCACGAGGCGAGTCCTTTAAAATTTTTTTTTTTTTTTGAAGTAAATATGANGTTAATATTTGTTTCATAATGT
TAAGTCGAATCGGGTATTATGAAAAGTATAATAGATAATAGATTTATTTATATTACAGTAATCACCTTAGTTC
CTGAGATGAACGTGTTGTCGTCCCGCTGTAAAGTCTGATTATAAAATAAAAAAAGATG
TTACGAAAGTCCTTCAAGCCATACATTGTACAATGTCACGAGATTCAAAGATGCGCATATTCAAATAAACTGG
ATGCAATTCGAGAAAAGCTTAAAGAAGGGCCAGGCCTATTTTATTTCATATCTGAAGACAGACCAAAGAATTG
GGATGATTATGAAGGAAAGTTAAAAAGGGAGAGAGGAGAGTCAGAAAGGTTAAGGTTA
CCCCCGTGGTTGAAAACTTCTATACCTACTGGATCTAAATTCAGTGCTATAAAAGAGCAATTGAGAAGTCTAA
AACTAAGCACTGTATGTGAGGAAGCCAGATGTCCCAATATAGGGGAATGTTGGAGTGGCGGCAAACATGGCAC
TTCTACTGCCCCTATAATGTTAATGGGAGACACGTGCACCCGGGGTTGCATGTTCTGT
TCAGTTAAAACATCAAGAGCACCGCCG
>103790837_2 DV500995.2

GAACCCCTCATAATTTAAGGAGGTCCCGACTATTAATCCTAATATGCTAATGGACTTGGATATTTTCTTTTTA
GATTTATCCCCTCCCGGTTTTTGTTTTTCCAAAAATTTTTATTTTTTGATATCTTATGTTGATATTGTCAATA
TTGTTGCATATTTCAATTTTTTATTTGCACGTCTCGCTACGCGAGTACAAAGAGGTGAG
CCTTTTTTTATTTGACTCCGCTGCTCTGAATATGTTTGGGGGGTATGTATTTCCATTCCTCGAGTGTGAATCT
AACCGGCGGGCGTTACGTTACGTTCACGAGCTTGGACCCGTGTGCCACGACTTGCCTTTAATATTATGTTTAT
AAAAAGAAGATTAAATTACAGATGTGTATACGCATGTGGCTGGAAGTGGGCCATGCTT
GCGAACCTCGGCTGTCGGTGACGGGGAGCGCGCTGGCGCTCGACTGGATGGTGTGGGTGCG
>103790846_2 DV501088.2
TGATCTGCCGGAATGTTATTCACAAGTTATCACTGAATCTGACATGATAAACTACTACTACATCTCCGGGTGT
TACATCCTACGGCCCTGGTCGTACAGCATCTGGGACGTCTTTACGAAGTTCCTTATCTGATACTTCACGAAAT
TGGGAGTCAAAGATGGATACTTCCCTATATTTGTATCAGAGGCATCGCTAGAACGTGA
AAAGAGCCGCTTACCAGACTTCGCGCCGGAAGTAGCGTGAGTGACACACTCGGGCTCCTCGGAGCTG
>103790876_2 DT662053.3
GCCATTTTGATTTGTGATCGTGTTAATTTTATTGTGGATTTCTTTATTAATTTGATCATTATTACTCACCAAA
ATGAGCTACGGAAGACCACCGCCGCGGATTGACGGCATGGTCTCACTAAAAGTGGACAATTTAACGTACCGTA
CAACGCCAGAAGATTTACGTCGGGTTTTCGAAAGGTGTGGAGATGTTGGTGATATATA
TATCCCAAGAGACAGATATACACGAGAAAGCAGAGGATTCGCTTTTGTCAGGTATATTATTCATCATATTGTA
CCTTAAATATTGCCTTTTTTTTTTAATGCGCTTCCCCATAGGTTATGCCGCCATAATGGCTTTGTCGGTTTTA
GATTTTACGATAGGCGAGATGCTGAAGAGGCTTTAGATTCTCTCGACGGCCGCATGTT
GGATGGAAGGGAGCTTCGTGTACAAATGGCACGATATGGCCGACCATCGTCACCATACAGAAGTCGATATGAT
CGCCGCCGCAGGTAAGCCATGCATTTGTTATAATTATATTTAGGAAATTTATATTATCTTTATTTATATATCA
TTCAATAATTGTAATATTTAATTGAATAAATATTAATTTTAAGTTAACG
>103790882_2 DT662913.3
GCACGAGGCTTTATCACTCTTTACAATACTTTATTATTTGTAATATAATGTAAATATTTTTAAATAGTACTAT
AATTTTAAAAATGCAGCCTCTACTTAATACTAAAGGTGACATACAAATTGATCTGGATCAAGTCAAATTAGAT
ATTTTACATGGTATCAGAGAGTGTAACGATCGTGGACTAATTCAAACTGCAAAATGGT
TAGCAGAATTAAACTTTGCCTTAAAAGATCATAAAATTACTCAAAATGAAACGTCTAAGCCTCCCGATGATGG
AATAATAGCAGAAGAAAAGGAAGCGTACACTTTAGCAAAAAGTTATTTTGATTGTCAGGAGTATGATAGAGCA
GCACATTTTATTGAAAACTGCACAAGTCTAAAATGTATATTTTTACACAGATACTCTC
AATATATGTCTAGTGAAAAAAAGAGATTAGATAATGCTACTGACACAGGAACTGAAAATATGGAATCAACCCA
GGTTCTTTTGGACCTTTTATCGTTTTTTAAGGCAAACCGCAATACCCTTGATGGCTATCTCTTATACTTAGAA
GGAGTAGTTTTAAAAAAATTAGACTTAAGAAGTCAAGCAGTGACCGTGCTCCAAGCTG
CAGTTGCTGCTGCTCCAACATTATGGGCAGCTTGGGTAGAACTTGCAGGGCTAGCTAATGAATATGAGGCATT
AGACTCTCTTCAACTACCAAAACATTGGATGATGTATTTTTTTGCTGCACATGCATTTGTTGAGCTTAAATTG
TCT
>103790900_2 DT662086.3
ACGAGGTGCGCCCTTCAAGTGATGTCTGTCGCGATCGCGCGCCTTCGATAGATCACACACCATCGATTTCCAA
TACGATTTTTTAACTTTTCTCAATGAAAAGTTTGTGTTGAGTGCATTAATATATAAACCAGTGGAAAAGTGCA
GTGCTCTTATAATATCAGGAGGAAATATATCGGTATTTTATAGCTTATTGAAAAAGTG
TCACGTTTCAGGAGCTGAATGTTTTGTAAACAAGCGTGAATCGTCGTATTTAGCGTGAGGTGTCGTTTAACTT
AAAGAGAGCTCCAGTAATGTGCTGACATGCCAAGCACATCTATCTACCAGGACACCAAGGCGGTGACGCCCGA
AGCTCCCACTACATATGAAGAAGCTTTGGCTACTGCTGAACCGGGAGAGACCATAGCC
ACAACCAAAGAAGAGGAATGGGTAACTCGTAAAACGAGTGAAGTGACGACAACACGTCAGATAGCCACCCGCG
TGAAGCGCGAGCTGGTGCTGGAGGACGGGCGCATCCTCAGCGACTCCGGGCCAAAGATATCTACCTCAGTCAA
TGAAGACACGCATACAACTAATTACCAACAAACTGAGCATAGAGTACCAGAGAACCAA
GATGG
>103790905_2 DT661798.3
GCACGAGGGATTCCTCAAAAAAAAAAGTTTGGCGTATATTTATATAGATTACGCAAAAAAAAGTTTGGCGTAT
ATTTATATAGATTACGCAAAAAAAAAAGTTTGGCGTATATTTATATAGATTACGCAAAAAAAAAAGTTTGGCGT
ATATTTATATAGATCGCAAAAAATCGCATTCGATGTTCCAAAGTTCAATCCAACAATC
GTTACATCACTAATGTAAATTACAATTAAATAGATAGAAATATCTAATGTAACGATGTAACAAACACATACAA
AGAGGAAATCACTATGACATAAAAATAGTATCAGCAAATAATATATAGCAAATAATAAGTAAATAAGACATTT
TTTTAATTTTTTTAAATACATAAGAGCATAAATGCCATCTGTCTATATGTCTTTTATA

TTGTATGTAAGTGAAAACATTTTTATTAAGGGAGATTTTGATTAGATATCATCAAACTTAAAAAAATATAAAA
TTTATTGGGAAGGTTCGATACAGGCGTCAAAACCTTTTTTTTAGAACAAACTTTGCACATTTTCCCATAATTT
TTTTTAAGAGAAAATTAAAATCAATTTCAAAGGTATTTAAAATTGTAATCTTTTTTTT
GCGTGGTATTGTAATTACACTTTGTATGTGCTTGTAAATTTAGTTTCAGAGTAGAATGTCTCTACTCGCATGT
ATACAAGTTGCTAGTCACGCGCCCCCGCGCTACCCTCAACGCAGTACATATGATATTTTTACTTGAA
>103790983_2 DT662761.3
GCACGAGGTGTTTACTTATTGTATTTATTAATGAAAATATAAAAAAATATAAAACTTCATTATCATTACTCAT
AATCTAGTATTTGATGGCAGTTCCTATAATTGACCACAATGACTACGTTACGGGAACGCCAACTCAATGCAAT
CAAGCAAATGCTTAACTTAAATCAGCCACTGACAAAAGCAGTAGCAAATGAGCCATCA
TGGAAAGTACTGGTTTACGATAGAATTGGTCAAGACATAATATCACCCCTAATATCAGTTAAAGAATTAAGGG
AATTGGGTGTCACTCTACATGTACAGTTACACTCGGATCGTGATTCTATACCTGAGGTACCAGCAGTATATTT
CTGTGCTCCGTCTGAAGAAAACTTGGGTAGAATATGTCAAGACCTGGATAATGGCATT
TATGACCAATATCACCTTAATTTTATATCACCAATAACTAGACAAAAGTTAGAAGATCTAGCAGCATCAGCAA
TTCAGTCAAATTCTGTTATGAGCATTCACAAAATATTTGACCAGTATTTGAATTTTATATGTTTAGAAGATGA
TTTATTTATTATGAAGCACCAGCAATCTGACTCATTGTCATACTATGCAATAAATAAG
GGAGATACTAAGGATACAGAGATGGAGGCTATAATGGATGAAATAGTTGAAAGTCTCTTTTCAGTTTTTGTTA
CCCTTGGTAATGTCCC
>103790995_2 DT661934.2
TTCTTTCTTATTAAATCTGCGGCCTTCTCACCTATCATGATTTCGGCAACATTTGTTTTTCCACTGATTGGAC
GAGGTATTACACTAGCGTCAGCTACTCTTAAACCGACCACTCCATAAACTCTTAACTCTGGATCCACTACAGC
GTCAGGGTCAGTCTGAGGTCCCATTTTACAAGTAGATGTGAGATCTCGCTGAGAAACG
ACCATAGTTCTTATGGCACATTTTTTTTTTTTTGCGGAATCNGAAGGGGAGTTCGGGGGGGGGGGATAGGGTG
GCCCAAATAACGAAGCTCCATGTCGTCTAGATGCTTCAGATTGGCCTGATTTAGTGACATAATTGACAGCTTC
TTTTAAGGAGGCTAAATCTGTAGGTT
>103791001_2 DT662450.3
GCACGAGGCCAGAAGCTTCCTGTCAAAATTTCTTTTGAATGAAAGTTTAGTCGGAATCCGCAAAAATGAATTT
GGTCGTACATTTAATTTAGGCTGGAGTAATGTATGTTAACATAAGGCTCTACGAAGATTTTATAACTTGTTCT
CCAATAAGTAGTACTTACGCTGACAATAGACACCATGGTGCAGTGTCATCCGCCGTTG
GAAATAGAGGAATTACCAAAAGAAGACGAAGAAAGATTAGAAAAATTGTTTTCTAAGTTAGACACGGATGGAA
ATGGCAAGATTGACATTCACGATCTATCCGTAGCTCTCAAGGAGTTGGCCCCGCACATCAATCGCAGCTATGC
AGAGAAATTTCTAGCTAAAAGTGGAAAGAATGCTGGAGATGTTACATTGTCAGAGTTC
ATTCACTATGTCCGGGAGCATGAAAAAAATCTTAGGTTGCAGTTTTCCCATCTTGATAAAAATAAAGATGGAA
AAGTTGACCTGGAAGAACTTATTTCAGCCTTTGCCGACTTAGGAATTGCTATTGGTAGAAATGAAGCTACAAA
TTTATTGAAAAGGATGGATCAGGACGGCAGCTTAAACATAAGCTACGACGAATGGCGG
GACTACCTCCTACTTGCACCAGCGACTGACATACATGCTTTAATTCATTTCTGGCGTCACTCTACGTATCTGG
ACATCGGGGAGGATATGAACGTGCCAGATGACTTCACTCCAACTGAACTGCAAACGGGGAAATGGTGGCGTCA
TTTATTAGCCGGCGGTATTGCTGGAGCCGTCAGCCGCACGTGCACAGCTCCTCTTGAT
AGGCTTAAAGTATTCTT
>103791014_2 DT662770.3
GCACGAGGCGTTAACCTAACTAGAAATTTAGATAAAGATGTAGAAGATTGTAGAGAACACGTTGTTTATTTGA
AGAATAATACCAGAGACATACAGACGCCTATCAGGTTCCAAGTGACATACAAAATAGTTCAAGTGCAACCGCA
ATATTCAACACAAGGCGATTTACCACGGATAGACGATTATCCGGTGTTGAACGCTACA
GCCAGGACATCTTTTAGTGCAAACTTCTTAAACGATTGTGGTCTTGACGGTGTGTGTGTTAGTGATTTGGTGG
TAAAACCAGTTTTAGAGCTACCAAAAAGTGAAGATAGCGAAGCGTACACATTGACTTTGGGACAAGAAGAAGA
AGTAAAACTTTCAATAACAGTGGATAATTACGGTGAATCCGCCTACGAAGCTCAATTG
TTCGTGCAACATCACAAAAGTTTACACTATATAGCAGCCAATATTACTGGCAAGCATATGATCTGCACCAGCG
TAAACAAAACCACAGTATCTTGTCTACTTGAAAATCCATACAAAAGGCAGCCTGAAGGTACTCCCGCCATCAC
AATGAGATTTGATGCTAGAGCCTTGGAAGATAACGAGGCTTCTGTAGTGTTTAGTGTT
TGGGCTAATTCTACCTCTAAAGAGATACGACCTAAGAAACCTGTTGAAGTAAAGGCATTGGTAATTAAGAATG
CAGAACTCTTGATTAAAGGCGCTGCTCGGCCAGAACAGGTGTTCTATGGAGGTGAAGTGAAGGGAGAGTCCGC
GATGACTCACTTTGACGATATCGGCACAAGAGTAATACATACGTTATCAG
>103791027_2 DT662874.3

GCACGAGGAAAGACCATACAATTACGTACAGAGCAATCGACACTAGAAGATTCTCTAATATCAACACAAAGGA
GTGTCAAAGAAGGAAAAACGACATTACATAAATTAGATATAGAATTGAAGAGTTTGGCCGATCAAGTACCAGC
TTTGAAGATACAAATAAAACAACAAGAAAATAAGGTTTCCTCAAGCAAAGTGGATGCG
AGACAAAAAGCTAAATTAGAAAAAGTTTTGAAGGAAGCCGAGGAAAAATTGGAAAAAGCCAAACACAATGCGG
GTGAAGTAGAAAAAGAGGTACACGGTGTCGATGCACAAATAGCGGCGGTCGCGGGAAATAAAGTCAGAGATTT
ACAAAAAAATGTCGAAGAATTAACGCAAAAGATAAATAAAATATCAACTGAAATGTCG
AAATTGAAGGTCGCCATCAACAGTAGTATAAGAAATGCAAAAAAATCCAAAGACAAAATAAACCAAATGGAAG
GAGATTTTAAGGAAACGGAGAAAAAACTAGAAAACTTAAATGAACAGAAACGAAAACTAACATTAGATAGTAC
ACAGTATCAAAAGGAATGTGACGAATTAGAAAAAGAGATAAGCGAAGGCACAAGTGAA
TTCTCCGGTCTCCGTCAAGAAATAGCAAAATTACAAAGTAAAGAGAACACAGCGAANACGGAACGTCTCGAAG
CAAATAACGCCGTCAACAAAATGGAGAAGTCTATA
>103791036_2 DV501094.2
GCACGAGGCCTGACCTTGAAGACCATTCACTCGATTTCGGAGGCTTCGACAGATTCCCATTTGACAATATACC
AGCCGGAGTTTATATTACCCTTCCGCTCACATTGGAACAGGACGTTCGGGTCACGGGAGGAACGGTTCCCGCC
TGCCCAGTCTCCACTGCCCCACGCCCCGCAGCAGCAGACCGCGGCTACGCTGACCGAG
GAGGACGAGGGGAACGACGAACAGACACCGTTACCACGGTACGGCCTATGGAATACAGTAGACTTAGGGTCTG
ATAGCCCAGCGGACCCCAGTGTGGAGGCTGATGAAAGGTCTCTCCGGTCAATGTCAGCACCTCCCGATCATCT
GCGCTGTGACTCCAAATACTCCCAAAATGAGTGGACCCTGACAAGAGCAGCCTCATCA
CGAGCAACCGTCTAACGTCTTACGTATACCCATTTTCGGCGAATGCTTAGACAAACCGGTGATCACAGGAATG
TTGACCGCGGTACTCACTTCGCAGACCCCATGCCCACCTACGTCCCGCCGCCGCAGCCGCATATCGATAGAGA
GCAGTACTTCGCACATGACGGGCCGGGTTCCTTTTCCTTCGCGAAAGGTTTCGACAGG
CCCTTCTTTATGCAAGGGCCTCAGCCCT
>103791047_2 DT662626.3
GCACGAGGAAATGTTCTATCTAGAATTTCAACATAAAAGAAATATATATTGAACAAAAGAAATGGCGAAGATT
ACCACAAATAAGATAAAAAAAGGACCGAAGAAAAAGTTTACTATGCATAAATGTAATATTTGTAGTAAGAAAT
TCACAGAGAAAGTGGGTCTAGACCAACATATACAAACACATTCATCCGAAAAACCTTT
TGAATGTAATATTTGCACCAGTAGTTTCAAAACTAAGAAATATCTAAGTCGTCATATTAAGAAAGTGCACAGC
CCTGCAACTGATCATGAATGTAGATACTGCAGAAAAAAATTTCACTATGAAAGCCTTTTGAAACAGCATATGT
ATATACACACAGATGAGAGACCATATAAATGTAAAGTTTGTGGCAAAGGCTTTAATTC
AAAATATTCCTTAAAAAGCCACAATTATTTGCATACAGATACAAAACCTTTTAAGTGCGAATTCTGCGACTAC
GCATGTCGAGACTCCTCCACACTGAGAAAACACCAGCAAAGACATATGGGTATTATCAAACGATATCAATGTA
CATTATGTGATAAAAGTTATAGTACAAAAAGTCGACTCACAGTCCACGTGACTGAAGT
ACACTTAGAAATAAATGTGAACAATGGAAATGCGACAAATGCAATAAGGTCTTCAAATCCAGTAAAGTGTTA
AAATCACATGTCAAAAACGTGCACGAACGAAAATACGCATGGTAATGTGAAATTTGCGGCGCTATTATAACTA
ATAAATACAATTTGCCGCTACATATGTCGAGACATGTGAACTTAAGACCCTATATATG
TA
>103791074_2 DT662414.3
CGGCACGAGGGCGCCACGGGCCCGCGCCGCTCCGCCGTGCCGCCGCCCGCCCAGCGCCCCACGCGCACCACCC
ACGCCACCAGCCACAGCTCGCGCGACAGGTCGCTGCTGGACAGCTCCTGACAGGACATCATAGATTCGGTCAG
AAGAACAACACATTCTCAAGTTTCGAAACATATATTAGACATTACGTACTTATAGAGG
AGTTTAACGAATTTTGGCATTGATAGAGCGTTTAACGCCGGGGAAATAATAATAATTTTAATATAACCTTAAG
AAATATAAGAACATGACATTCAATTAATTATTTATGTTATAAGTATGCTGATTACTCCTTTATGTTTAAACAT
AAACAAAAACATTCGTTCGTGTTGGAATTATTTGTTGCAGTAGTTGATATTGATTAAA
CAAACTACCTACTGAGATTTAATGTTTTAACTGTTTTTAATAACTTTGTCCGCATGATTATAAACAAATACAA
ATAGATTTAATGTGGAGGACAAATTATTAATACATCCCGAAAATGACAATTAATTTTGTAACTATTATTGTTT
AAAATAATATTAAATATATATACCTATAACATAATTATGTACCTATGTCTTATACTTG
AGTAAAATTGAAATTATGTTGCATCATTATAGCTCACCGCAAAATGTTGTTAGCATTGGACCACGAAATATCA
CAAACTGTAAACTTAATGTCATCAGACACGTAGACAGTGAGCCAAAGTAAAAATTTACCTATTTTTGTTGGTC
TTCAAGTTACTATCGTATAAACACATTCA
>103791077_2 DT662360.3
GCACGAGGCCCATGCCTTATGGTAAAGGCGCGTTATGTGGTATGATAACATCACATATGATAACCGCTTGGAT
GGCCACTGGGAGGTTGATATATGTGAATGATACGGTCGCTATGCTGCCTTTGTCTGTGGAGCAATGTTCTAAT
GTAACGCACAGAGTATTAACAACCAAAGCTCTCCCAGTCAAAATGAACCAGACTTTAA

CTCAATTTGCACCAATACTCCAAGTTTTGGATAAAATGCATGTTCCTCATCAACCTGAAGAATCTCCTGTATT
TACAGCTCTCATGAAAATGTATTCAATATCGTATATGTGGTACGCTGTGATAGGCACTATCACTTGTATCACA
GTTGGCGTAGTGATAGGCTTTCTTACTAACAGTGATAGTGATAAATTCGACGAATAGC
TCCTACATCCGTTAATTGCCAAATTGTCCAGAAAAATGCCCGGCAAATGTCGCACATTTACAAGTCAAGCAGA
GAAAGAAGCGAAAGCAGAAGAAACTGTAGAGGATAAGATATTTACAACGAGTAGTAGTGGTGTATACGAAAAT
AATAATGTTCCGATAAGAACACATTTGTGATTTAAATGGCTACTTTCACTTTATTCTA
AATAGCGGGATAGGCAAGAGAACCTAGTGTCAAAACACTCTGTTCTTCTTTTTATCTGATACTGGCTTGTACC
CGTCGTTTCTCGCCCGTGGCTAAACGTAAATAATAGCTAAGATAGATAGAAACAATTACTGTCATACCCTTTC
TCGATACATGGAATATTCTA
>103791087_2 DT663043.3
GCACGAGGCGAATGCTTCATATTTATTTACAACTTTGTACCGCCTCTTTACCGGCCACTACCCCTTAAACCCC
CGCCTTATCCTCGTTGTATATCGACGTTGTGCAATATTCTTTTCTTTCTCTCACCAATTAATTTATCCTTCCT
ATACGAGTACAGTAGATCTTGATGAAAATAATATACTGATTAATGTTAGGGAACGTTT
AGTGGCCACTTCGGTACTGACGCTCACTTAGTCAAATACATATAAGTCTCGTGACGGCGTTTCGTATTACATT
ACGTTTTGTATATATTTAGTTTGTTTTTAACGCATAGGTAATACGCAATGGACGCGAGTTGCTAGAAGGCGAT
TATATACCAAATAATGTCAAACGTATGCAGGTTAACTGTCACCGAGGCAGTGTTGCAA
GGTAGGAGGAGTAGATATTTAAAAGAGATCTAAATTAACCAAAGTATAGATGATTAAACTAGTCTAGACTGAA
AACTCATTAAATAATAATGTAATATAATTTCTTAGTAAAGGTTGATAATGTTTAAAGAAAGATTCGAAATAGA
TTGTGGGTTCAATTACCACTTAAAAAAATGTATTTTAATCTTTGGTGCTCTTATGGTG
TGGTTAGGCAAAGTAAGGAATATAGGCTCATGTCCCGCACTAGTTCTATAGTAATTGAGTTAAATGACAATTT
GCCAACATTTTTTGTGTGACTATTTGCACGAAAAAATCATAATAAGCAGTTAACTTATCTAGNAAATATACAT
TTTCATATTAAAATTATCAAACGATTAAATGGTATTCTTTCTTTTTTATAAAAATAAA
TATTTACCATTACT
>103791114_2 DT663334.3
GCACGAGGCCACCCTTTCAACATTCATCCGGCTTTCACGCGAGACCTATATCAGTTACCTTATCGTCAGTGAT
ACCAAGTGGTTGTACTCGCAAATCAGGAATGGGGGTACACCGTGGAAAATGGTCCCGCGACATGGACAGATAA
ATTCCCTGAAGCTAGAGGTGCTCGGCAGAGTCCCGTGGATATTGATACAAATCTTGCC
AGTAGCAGTGGCAGTGCCCCGCCCCTAAAATGGAGATACTCCGTCAACCACCCTCGTTCCGTTGTCAACCCTG
GGTACTGCTGGCGTGTTGACGAAAATGGCTATAACTTTTTTCTCCGCGGTGGTCCTCTCGGCGCTGATGTATA
CAAGTTGCAACAATGGCACTGCCATTGGGGAGCTATCGACGGTGAAGGCTCGGAACAT
ACGGTTGACGGCCGCTCGTTCTCCGGAGAACTACATCTCGTTCACTGGGACACATCTAAATATAACAGTTTTA
CCGAAGCCGCGGGTCAACCCGACGGACTTGCTGTCCTTGGAGTTTTACTAACGGTCGGATCGAATCACA
>103791121_2 DT662141.3
GCACGAGGGGTATACCTTTACTGTTTGTTGACTTGAGATCTCTATACGAAGATCAAACGAAAGCGGACATAAT
AGATAAACTAATCCACCAATACATAGAAAATTTATCAAAATCTGGAACATTCAGTAGTAAGCCGGACGAAGTG
AAACAACCAGCTAGTGCATTACTATGGTCATATTATTACGCGGCACAGCATTACGATT
ATAAGCAGGATACGGACAAGGCGTTGTATTATATAGATTCGGCTATAGAACATACGCCCACGTTGATTGAATT
GTATATTGTTAAAGGAAGGATATATAAGCACGCCGGCGACCCGGTGTCGGCGTACTCGTGGCTGGAGGAGGCG
CAGGCGATGGACACGGCGGACCGCTACGTCAACAGCAAGTGCGCGCGCTACATGCTGC
GCGCCGGACACGTCAAGCGCGCCGAGGAGATGTGCGCCAAGTTCACGAGGGAAGGTGTACCAGCAACAGAGAA
CCTGAACGAGATGCAATGTATGTGGTTCCAGACTGAAGCTGCAGCAGCATATCAGCGGCAGCAACAATGGGGC
GAAGCTCTGAAAAAGGCACATGAAGTTGATCGGCACTTCTCGGAGATAATGGAGGACC
AGTTCGACTTCCACTCGTACTGCATGCGCAAGATGACGCTGCGCTCGTACGTGGGGCTGCTGCGGCTGGAGGA
CG
>103791125_2 DT662861.3
GCACGAGGATTTTACATTAAGAAGCAAGGTTTTTAATTTACGTTGACTTGAGTAGCAAACTCAATGGTTTGGC
TGAAGAAGTGAAGAAGTAAACAGATGTAACTACTAAAAACTAAAATATTAATAATGCCTGAAGAGTCAAAGAA
AAATATACCAAAAGGAAAAAACGATCCTAAAGAACAGCGGCGAAGGCGAGGTCGACAA
AATCCAGCTCCAATTGAAAAGAAAATAGAGAAGGCACCAGAACCACCAAAAGAACCTGAAAAACCGGCGTATG
AACCTCCTGGACCTGAGTTCTATAAAGGTTTAAAGAGGGAAACTGATGAAATTTTGAAAATAACTGAAGAGGC
CAATTCTAAATATAAGAAAAAAGAAATCCAAAGTAATTGGGCCAAATATGAATTGCCA
ATTGAGACATATGAAGAGATTGATGAACAAGAAAATTTAGGAGCTGATTATGAGGCTCTTATCCAAGCTCCGC
TATCAGTGGGTGGACATTTTCAGTTTAAGCATGAAAAATCTTGGGATATTACCACAGGCCCATCTATTTATGA
CAAATATTTTGATATTAATATGGAAAATTTGAATGTAGCTCTGTCCACTATACCATTT

TTTGAACGAAATAATATTGACATGACTGTATTCAATGAAAGTGAAATACAAAGTATGCATCATAGAGCTACAA
AATTTAAGCAAAAGTACTACAATGACAAAAATTTTACAACACCTGAACTAGATGCCCAGGAGAAGATTTTAAA
TTCTTTAAATCTG
>103791139_2 DT661762.3
GCACGAGGGAAATGCCCTTATAGGGACGCTCGGATTTAGAATGTTCTTTGAAAATCTACGTTGAATTAAATGA
AACTTCTTTACGAAGTGATAAGTTTTGTGACTCTTGTGTTCAAAAGTTTTATTTGAGTTCTTGTTTAGTGTGT
ATTAGTGATTGTTTTGGTTGACCAACAAAGTTAACAGAAGTGCGTCGAAAGTTGAAAC
ATGGTGCCGCTAGGACCCGGGCCAGGCCATCCTACCGCACATCAAACTCACGGCGGGCCATCTACAAATTTAT
CCGGTCCAAATTCGTTATCACAGAGCTCGTCACAATCGAACAATTTGTCAGTAGCGAAGCCGAACTACACATT
AAAATTTACTCTCGCTGGACACACAAAAGCCGTTTCGTCCGTTAAATTTAGTCCCAAT
GGCGAATGGCTCGCTAGCTCCTCTGCTGACAAACTTATTAAAGTGTGGGGTGCTTATGATGGCAAATTTGAAA
AGACTATATCGGGACATAAGATGGGAATCAGTGATGTGGCATGGTCATCAGATAGCAGGCTGATTGTTAGTGC
CAGTGATGACAAAACTTTAAAGGTTTGGGAATTGAGTTCTGGAAAATGTCTAAAAACT
TTAAAAGGACACAGCAACTATGTGTTCTGCTGTAATTTTAACCCACAAAGTAATCTTATTGTCTCTGGTAGTT
TCGACGAGAGTGTGCGGATATGGGATGTCAGGACAGGTAAATGCTTAAAGACACTACCAGCACATTCGGATCC
TGTTTC
>103791145_2 DT662884.3
GCACGAGGCTCAGACCTACCACACACCATGAAGGCCTTCGTAGCGTGCATAGCCCTGGCCTTGGTTGCCTGCG
CAAGCGCAGAACCTCCATCTGGATACAACTACAACCGTCCCTCAGGTGGAATTTCGGGTGGATTCAGTGGTCT
TAGCGGCGGCGGTAGCTACCGTGCCGTCTCATCTGGATACCAGACCTCCGAGGGCCAA
AACGTCGACCCCCAACTCCTTGAACAAATCCGTCAAATCCTCCTTAAAGAAGAAGCTTCCTCGTCATCAGGCT
CAATCGGAGTTAGCGGCCCTTCATCATCCTATGGTGTTCCTTCATCCTCATATGGCGTCCCGTCATCGTCCTA
CGGAGTCCCAAGCGGTGGTCGCGTTGTTGGCATCAACCTTGAGGGAGTCCGTCAAGCT
ATCCAAGTAGCCCAATACGAACAGTCCGGTGGAAGCAGCGGCGGATACCCATCAGGCCCCTCATCCTCGTACG
GTGCCCCCGCCCGTCCCCCCTCCAGCAGCTATGGAGCTCCCTACTAAATCCAAACCTGAACAAATGTTCGTTA
TTCGGACTATCTCGTAACATCTCCGGGAGCCCGAATATTTAAACCAATGGGTAGACCG
CCACGTACAATGTAGGTGGCGTCTCCCGGCCCCCCCATCCTGGCCAACGCCTGCGACTGTGCACGCACACGGG
TTACCCAGCTGTCAGCCAGTCGAANACAACTGACGACTGACAAAACTGACACTTGCTTAGCTTTCGATAACGC
TTGTGTCATTTGTGTCAGTCGGTAGCAGTGCACGAAG
>103791157_2 DV501007.2
CCGGACTACTGCTCCATTTCAGAGATAAATGTTCCAGTATTCAGTAACGGCAAGAGGGAGTTGCTACTCACGC
AGCCCACCGTCGAAGATTCACAAGGAACAGTATCCAGTCCACACTAGTCATAGAGACAGCGTAGCTGACGTGG
TCTCTTTACGACTAATACACAATAAAATACCCGACAAAGATATACCGAAATTACCACA
TGTATCTGAGATTATTATCCCGGATGAAGACGATAAACCAACTGAAGATCACAGATCATTCAATCGATCTAAA
AGTTACCTGGCAAATTTTAATATACACGCTCTCCAAGCTAAAATAGATCCGCGTGGCTCTATTCACATGGGAA
ATACAGTGTCTTCTATTCTCTCCGAGATAAACAAAGGTTCCAAGACAGGTGGTAAGCA
TATCAATTTAACAGATCTAGATAACCAATTTAATAATGCACCAGAAGCCGCAGAGCCGCGCAAAGCTGAATAT
TTTGATGACATAGACAAGTTTGATTTGTCT
>103791159_2 DT662566.3
GCACGAGGATAGAATCTTGATAGTTGTGTGCTCTTAATGAGAGTTTTCAGTTTTATTTCTACTTTATACATTC
AACTCTGTTTTAAAATGTCACAAAATTTGTATGAAAACATATATTTTCAATCGTTATAGTTTTATTCCTAGGG
ATAACAGCTGACTCAAGTGTACAAGTGTGGAAAGTGGTGTAGTTATTCAAAATGGTTT
TAGCAGATTTGGGTCGAAAAATAACAACTGCTTTGCAGTCGTTAAGTCGTGCCACCATAATCAATGAAGAGGT
TTTAAATTCTATGTTAAAACAAATTTGTGCAGCTTTATTATAATCAGACGTTAATATTCGTTTAGTCAAAAAT
CTTCGAGAAAATGTACGAGCCGTCATTGATTTCGATGAAATGGCTGGGGGGCTTAACA
AAAGGAGGATGATACAATCAGCAGTTTTTAAGGAGCTTGTGAAGCTAGTTGATCCTGGAGTTAAACCATATCA
ACCAGTGAAAGGAAAACCAAATGTGATCATGTTTGTTGGGCTACAGGGATCTGGTAAAACTACTACGTGTACA
AAACTAGCTTATCATTATCTAAGATAGAACTGGAAATCTTGTCTTGTCTGTGCTGATA
CATTCAAAGCTGGAGCTTATGATCAAGTTAAACAGAATTGTACAAAAGCCAGAATACCATTTTATGGAAGTTA
>103791182_2 DT663429.3
GCACGAGGGAATAAGCGTTTATTTGCTGTTAAAATCCAAAATATGGCTGCTGAAATAAAACCTGCACCGAGAA
CGCCTAACGATAGATTTTCAACAACTAAAAAACCAGCACTTTTAAGTAAACTAGAAGGTTGTACTGATGATGT
AAATGCAGCAGTTGTGATTCCTGGCGAAGACGGAGTTATTAGTGTTTGCGATGATAAG

ACAGTGAGAGTATGGCTAAAAAGAGATTCAGGTCAATACTGGCCAAGTATATGCCAGTACATGCCATCTGGTT
GCACGTCAATGTTCTATACTCCTGAAACACGTCAATTATTCATTGGTCAAGAAAATGGCACTATTTCAGAATT
TACACTCACACCCGATTGCAATAGGATAAACCCAACTCGTGAGTACCTGGCGCATACA
GCGCGGGTTACAGCAGTAGTGTTCTCTTTGAGCTGCGAGTGGGTATTATCAGTTAGCCGAGACAAGCTCTTTT
CTTATCACTGCAGCGAGACTGGAAGAAGGATTGGCGGCTATTCCTTTGAAGCGTGGTGTACTGCGTTGCAATT
CGATTCCCAATCGAAATACGCTTTCATAGGTGATTACAGCGGACAGATAACAATGCTG
AAGTTAGATAATAACGGCGCGGCTTTAGTAACTACATTAAAAGGACATACCGGTTCTGTAAGAGTACTAAATT
GGGCGCCGATACCTCAGTTGTTGTTCAGCGGATCTTTCGACCAAACTATTATTGTATGGGACATTGGTGGA
>103791206_2 DT662756.3
GCACGAGGCGGCACCCTTTAGACAGCGGTGTTACGTCCCCGCACGTCCCCGCCGCCAGCCCGAGTGACCAGAG
TTGCACCAGCACGCGTTCGCAGCTCCCAGCAGCTGACCCTCAACATGGACACCAGGATAGCGCTATGTTTCCT
GCTTTTTGCTTTCCAAGTAATTTACGGTGAAGTGGACATAATAACAGAGCCACGTCCA
GGAGAAGAATATGTATTAGTGAGTTCTGGACAACAAACTCCTTTAAGAAATTTTGAAACCTCACATAAAATTC
CTGAAAAACATCCCAATCATCGATTAACAAAACAAGATGACGGCGAAAAAATATTTAAAAAATTACACAGTGG
AAAAATTAAACACCCTTCTGAGGGTATTATTTCTATAGAGGAGGATAAAAAGGCAATA
TTAAAAAAGGCCCAAAAATCTAAAGTTACTGAATCTATAGAAGTAGATATTCCTGTTTCTAAATTAGAAGATA
TTAAAATAAAAGAGAATGTTAAGGCTGTAGATAACAATCCTAAGCTTTCTGTTGCTCTAGAAACTATGTCGGA
ATCAGGAAAATTAATTGATAACGATCAAACAACTACTCAGAAGCCAGTTTTAACTACG
TCAGCACCATCTAAATCACAAACCTACGATTATATTCCTATTAATTTCGACACTATAGATGATATTAACAGCG
GGTTACTTTCATCGAATATTAAGTTGGAAACAGCTGGTTCTGAACAAAAACATTCAAGAATTCAAGTTAAAAA
AGGTCCTAACGGTCAAGACTATGAATATGAATATATTTACTATTACTACGACGA
>103791212_2 DT662050.3
CGGCNCGAGGGCGCAGTCTTTAAGGGGGGGACGGGTCACCGTTGCGAGGTGTGCGGCAAGCTGCTGTCGACGA
GGCTCACGCTCAAGCGTCACACTGAGCAGCAGCACCTGCAGCCGCTCCATTCGGCCCGGTGCACCCTCTGCCA
CAAGGTGTTCCGCACCCTGAACTCCCTCAACAACCACAAGAGCATCTACCACCGCCGC
CAGCGCGCGACTCAGCAGCAACCGCAGAACCTCTCTGCGGAGGCAAAACTGCCGCCCGCCCAACCTAACATCG
ACTTCTACAAGTTCAAAGATCAGTTTAACGTCTAAATTAATTTATTTTTTTATATTTCAGAAGCTCAGTACCG
CCTTTAGTTGTTCTTTGACAATTATCTAGAGACTTTTTTTTATTTTTTTTAACTTTAAT
ATTTCTCTAATTTAATTAGAGTACCAATTGGCGTTACTTTTAGCCGTTAACCTAAAGCGGGTACTGGGTATTT
AATTTTTCTATATAGTAGATGATTTATATTTTTTATCCCGCGTGGAGATTATTGACGTGTTATTGTCGATTTA
TTTTATTTACATTACTCTTTTAAATGTTTATCTCGGGAC
>103791215_2 DT662280.2
GCACGAGGCTTTGTTGGTGTTGTATATTCCTCCAAGGAAACGTGCGCGTTTTGGGTGAACTATCAATTGTTAC
TTAGTAATAAACGTGTTATTTAGATATAGTTTGGTAAATTACTGTGAAAATGAGTAACGATCCTGAAAGGTTC
GATTCCATGCTTCTTGCAATGGCACAACAACATGAAGGTGGTGTTAAAGATTTACTGG
CCACAATTGTTAGCTTCTTAGCTAGAAAAACTGATTTTTTTACGGGAGGGAATGAAGGAGAATGGGAAAAAGT
TATTAAGGACACATTTTATGCACATGCTAAAAAAGCACGAGAAGAAGCTGATAAAATTAAAAAAAAAAAAAA
AAA
>103791221_2 DV500991.2
GCACGAGGGTGATACCTTTAGTGGACCATCGGCACCGGTGCCTCTCATAATTTTAGATGATGCAACATGTAAT
AATATAGGGGCTAATGTATAAATTTTAGTCACACACCCCACACGGATAGCAGCTTCGGCTCTAACATTGAGAG
TGGCTAAGGAGAGAGGAGAGCCACTCGGCAATTCAGTAGGTTATGCTGTTAGATTATA
GAGAACAAAGAAGCCGTGGAAGTATACCATTTTGCACAACTGGTATATTATTAGCACATTTAAAAGTAAACCA
AGGTTTGACGAAGTATAGTCACATAATATTATATGAGGTCCATGTTTTGGATGTTCATATATATCTGTCTATG
TGTATTTTAAAACAGGTATTATTTAAACGAAAAGACTTATAACTAATATTAATGAGTG
CAACATTACACGCGGAAACTTTATCAAACTATTACAACTACTGCCCTTTATTACATATCGAGGGACTAGCGTA
TCCCGTACAAGACATTTACTTACAAGATATATTACAATTAACTAAATATAGACTACCTCCAGAGGAGCAACAG
AAGCCAAAACCGAAGTGGATGAAGTATAGAAATAGAAATTTTGATATGCAAAATGATA
TGGAGAAAGATATATTGTATAAACAGGACATATGGCCATGGCTTGAATCTTTAAAGGATAAAATGAGCTATGA
CGTTT
>103791236_2 DT662165.3
GCACGAGGCTTTAACAGATTTAGATTGAAAATTGAACCGGTTTAATACATAAAACGGCGATATTGTGTACGAC
GTATTGTACTCAATTGGCGCAATAGTTAATGGTTGTAGCAAAGTTAATATTCAGTCAACTGATGCGATACGTG
TTGAATAATAAAATTGTATTTTAAGGTCGTTTAGAAGAAATATTATTTAGTGACAAAT

TTCATATACAAAACATACATTGTTTATATATAAGACTACGTATTGAGAAAGTTACTGATCCAAATAGACGCTT
ATCAGAATGTCTTCAGCTTACTATGATTATTATGATGGAAGTAAAATATTCCTGTTTTTGTCCAATAGACTTG
GTCTTCCAATAGATTTGGTTAATTTTTTGATTGCACAACTAGCAGCATTGTGTGTGGC
CAGACTTTTTAGAAAACCATTAAAATTAGCTTCACCAGAATTTCGCCATTCATTGTGCCTTATTGTAGGATTG
CTAATGGGATATTTCTGCTTTGGCAAGCAAGCTGTGCATTTATCAGTTCTACCAATGATGAGCTATGTTCTGT
TAAAGACTGTACCTGAGCATTTAGTAGGAAACATAATTTTAGCAGCGTCTATGCTATA
TTTATCATGTTTGCATTTGCATCGACAAATATATCACACGGCAGATTATACCTTAGATATAACAGGACCATTA
ATGGTGATAACGCAAAGGGTTACATCATTAGCGTATACGATACAAGATAATATAACGAANAAGGAAGTAAACT
CGAACTCTGTAGAGACTCAAGATAAGAGGTTGAACGTAGAAAAAATACCGTCGCCACT
CGAATATTTTGCATACACTTTGGCCTTCCAACATTGAT
>103791250_2 DT663309.3
GCACGAGGTTTTTACTTTTAATTTAGATATGAGTGATATTAATTTATGAATTTAGTTTTCAATTTTCGGCTGT
GTCTGGTGACCTGCTTCTCCGGTCCCGGTGTAAGGCAGCCTTTCATCAGATTATCTTTGTTGTTTTATAGATT
TATTGATATTATAGAAAAATTATTTCGTTAATATTGAAGTTGAAGGTTAGCGATCTTA
GAGATGCGGGCATTGGACGTGAGAGTTATGTTTAAATATTTTTGAAGTTGTAGTGGATCGGAAGCACCATTGC
TTTGTTTATATTCTATAACTAATGTGCCTATAAATGTATTTTATATATAGGTCTCGGTATAAGTGACGGAAAT
TGGTTAATTTGAAAATTCTAGTTTTCTGTAAAGTTTATTGGCGAATGTTTTCAAAAAA
CATATCGTCTATTTATCACATGTGTCCTAGTAACGTATTATGTGTTTATTATGTTTTAGAGCTGGCCTAAAAC
ATAATATCAACCTTTGCCGAGTAGGTAATAAGTGTCCAAGTTCAATATAAGGACTCCATTGCAATCACACTTT
GCTCAAGGAGCGAACTTAAACAAGCCCAGTGGATTATTATTTATTTTTGCACACAGTA
TTTAATATTATGGATAAGTAGAAGTTTCAAAATTATCGGCAAATTGGCGTGATCTCCCTACTTGAGGCTTTAA
CTTTCAAAATAATGATAGGTCTC
>103791258_2 DT663266.3
GCACGAGGGACGCTCATATTATTTACATATTTACCTCCTTTGTTAAGGAAATGATAGTTTGATTGTTGTTCTT
CCATTTATTTGTGCAAAATGAAAGTGGATAAGGAGTTAAGCCTCGTTGCCGAGGAATCTAAACCAATGCTATC
TGACAGGAAGGGCAGCTTGAAGGTGACCATCACACCAGCTCAGCCGAAAACTTTAACT
CATCTACCTAAACGGCCGCCAGTGGATTTGGCGTTCACAGATCTTGCCTATAGAGTGCAGGAAGGAAGAAAGA
GCAATGTGAAGACAATCTTGAAGTCAGTGTCGGGAAGGCTTCTTTCTGGCGAGCTGACCGCCATAATGGGGCC
TTCCGGCGCCGGAAAGTCAACACTGCTCAACATTCTTACTGGATACAAGACATCCGGA
ATGGAGGGCAGCATCACAGTAAACGGTATGGAGCGCAATCTGTCTAGCTTTAGAAAACTATCGTGCTATATCA
TGCAAGATAATCAATTGCATGGAAATTTGACTGTCGAAGAAGCCATGGCGGTAGCTACTGCTTTGAAATTACC
GAGTGCTACTACTAGAGCTGATAAGGAAGAAGTGATCCAAGAAATCCTAGAGACCCTT
GGTCTATCGGAACACCATAAAACAATGACATCTAACCTTTCAGGGGGTCAAAAGAAGCGCTTGTCCATAGCCC
TGGAGCTAGTTAACAACCCTCCTATAATGTTCTTTGATGAGCCCACATCAGGTTTGGACAGCTCATCCTGTTT
CCAATGTATATCCTTGTTGAAAACTCT
>103791266_2 DT662942.3
GCACGAGGCAACACCTTTCGTGCATTAGAACGCCGCCGGGAATTTTTGCGGTTAAAATAGATAAAATGAGTGT
ATCTGCTGAATTACAGGCCGAGGCCGTCTCAATCGTTCACAATTTCCTGCTGAGTGTAGATAAATCACTCGCA
GATGAATTTTCAAGAAAAACGAAAGCGAAACCGAGAGCTAAAAATCTACCATCATTGC
TCAATATAATTACAGAATTTAAAAAAATACAAAAGAAGCCTGCACAAAATGCCAAAAAAACAGCAGAGAGTTC
TGACGATTCTAGTGATGAAGAAGAGGAACCAAAAAAAGTGCAAGTAAATAAATCAAATGGATCTATACAAACA
AAGAAAAAAGAATCTTCTGATTCAGATAGCTCTGAAGATGAAAAGCCACCGGTAAAGG
TTGCACTGCCAGTAAATCAAAAGGTTAATAAAAAGGCAGAGTCATCTGACGACAGTGATAGTAGTGAAGATAA
TGCTACATCAAAGCCAAAAAAACAACCAGTCAAACCACAAGTTGTCAATGCATCAAAAGCTAAAGCTGCTAAG
AAAAAGGAATCGTCAGATGATAGCTCAGATAGTAGTGAAGATGATAAGAAGAAGCCTG
TAAACAATGCTGCAAAACCAGTTACTCAAAAAGCAACACCTCAGAAAGTTGCTACTAAGAAACAAGAATCATC
AGACAGCGATGATAGTGATGATGAT
>103791269_2 DT663089.3
GCACGAGGTGATCATTAAGAGAATCTCTTATATTACCTGTTTTGAGATAATAGGCTAAAAAATGGGAAGGTTG
TTAATGACTTATATATTCTTTTTGGTTGTGATTTTTTTCAAATTATATAATTTTTTTAATAATTATTCCACTT
ATTAGTGATTACCTCGAAACCTGTAATATTCACGTTGACGCTCCATAGCAGCAATATA
TAAAACAACATGCTTTACTTTGCTTTGCTTTGAACATAAGTTCAACTTGTAATTTGTGTTCATTGCATTTTTT
GTTCTAATTGGCATATTACAAAAGAAATTAAAACAGATACAGTACGATCGACGAATAGTGACCGCCGCGAGGT
TAGGTTTTCTGATCTTTTTGCATTGAACACTTGGGACGAAAATAATCAAAAGTAAGAA

AAATAGTAAAATCGAATCTGCCCAGCTTTGCTAACTTCATGGTAGCCACATCTCGTCGACCATACTGTACTTT
TTTAATTACTACATTGTGCAAAACTTGTCTTAAATTTACCCTTTACTGGATTCCAAAATAAATAACCATAGGC
TATAATATAATGATTTCTCAGAAATAATGATTTTAATAAAACAATTATCCATTTATTA
ATAATGACAGTGCTTTTTTCATATTTAATTTGAAACTTATAGAAAGAATACTTTCAATAATATAAAGTGAAAT
AAGTACTGCCATTG
>103791275_2 DT663283.3
GCACGAGGGGATGAATTTGCTTTACTTGCTTTTATATGATTCATATTATATATTTAATGTATGTAATCATCCA
TTTTATATGTATGTAAACTTTTTCATGTCCTTGCACCCTTCTGTATTACCATTGTAAAATAATCAAGCATTTA
TTATAACAGTTTGAGCTTGATTTATTTTTAGCTTTTTTATATATATAAATAAAGACTT
TCAGCCTTTAGCCAAATTGAACTGAGTGTGCAATGATGTTTGTAATATATTTTTATCAAGTTACAGCTTCAAA
TCCAAATGTCTTGTGACTGTAAAAGGACTGTAATATGTAAATTTTGTATATCACGTTTCAGTCACAATATGCA
TTGTACATTTTAAACCTGACATACAAAAGATAGTTATTTTTTTTAAGTGACAAACGAA
ATTATTTTACTTTGAAAGATGTGTATTTTATTTTTATATTTTATTATCATAATATGTTGTATATCATTGATCC
TAAAACTTTTGCCAGAGATTTTTTTATATAACAAACATATTTATTGACTGTCTAAATAATATTTATGTGAACT
GGTGGAGGCCTAGAGAATCTTGCACAAGATGTAATTTCTGAGGAATATACTTAAAAAA
TCGAGTGTCGAGTCGAAGTAAATTAATCGATTATATAACCATACATTTAATCTTTAAATAGCTATTTGATAGT
TGATAAATCGACTATATAACGATACATAAAAATCGATTAAGTATAAACCAACATTGTTAGTTACGAAAATTCT
CGAAGGATTATATTGTGATCTAGAGGACTGGTCGGGAGTTAATGGA
>103791285_2 DV501090.2
GCACGAGGGTTTTTTCTTTGCGATTCCTATTTGAGCTCGTATTCTTCGCCTCTGCTCGTAACCTACTACCTTT
TCCTACATTTGTTCCAAGAGGTCAAAGTCACATATATCTTCTATACAGGGCTGATGGTGCTCAAACCATTACT
GCGACTCTCATTAATATGAGTTGATCTTTCGGTACTAATTCTACTAAGCTCTACTCCA
TATTTCCCGATTCCTCTTCAGCTTCTTACTTCTGTATCTTCTTGTATTTTACTTTCTTCCTTATTTTGAGTTG
TTTTTGTGGGACTCTGAT
>103791293_2 DT661882.3
GAGGGTTTGCCCTTTTCATCTTATATCACATCAAAATGTCTATAATATACCAAGGATTTTTGATGCTGTTGGT
GTTCTCTCTTGCTGAGGTTTCTTCCCGGCCTTCAAATAACGATGCTGCAATTCAATACATACCTTTCTATGGC
GGTGCTAAAGGACAATACTTAGAAGTCAGAAAAGATACAAAAGGATCCATCCTTAGTG
AAAAAGTAGTAGCTGAAGAGAGTATAAGCAGTGAAAATATAGTGAAAAATAGTGATGAAAGTCTTTTATCTAA
AGTGCTGGCAGCAAACTTACAAAGCCTAAGATCTCTATCATATTATCTTCTAAAATTACACAATATAGGAAGA
AAAATTGGACGCTTAGGAAATCAAGAGAAAGCTAGATTTAAAAGTCAACTTTTCTCAT
TGGGTGAAGCTGCTTCAAATACAATAAAACTTATAGATGAAATAGGAGAGGATGTCGATGTGCTATTTAAGAA
AAATGCTACACTCGTTAGAAAATATGATGATAACTATGATGAAGATGTTGGTGAAGAAGGTATCAGTATCGAC
TCTCCAGTAGAATCTGGTGAATCTATTATCGACGGCAAAGTCATTGCCGAAGCAAAAC
CTGTCGGATTGGCAGTTATTGGTGACAGTGGACTTGCAGCATCCCGGCCCATGGCGACGGCTGTGGCAGCTTC
TGGTATTGCCATTGCTCGACCCATAGGTACTGCTATTGCTGGTATCGACCCTA
>103791311_2 DT662488.3
GCACGAGGAATCGACCATATGGTTTCGCTGTTGGAAAGGATAAAAAATTTGGTGAACTTCAGCGTGCCATACT
TAAGCTACTTGGCTATTTTAGCTTTGGTAGGCGTTTCAATTGCCTTCTATGTGATACCTGCTAATTATTTATT
TATGGCTTTTGGTATTGTTAAATTCGGGCGTAAATTTTTGAATCCGAAGAGAATACCA
AATAATGATATTTTAGATTTCATTTCTCGAGTTCCTGATAATGAGATATTGAAACAATGGCGTGAATTGAAAG
TTCCAGAACCGATTACGAATCTCCAGCTCGATACACCTAAAAGCAGATAGCTGTACGAAAAGCTCTTTGAACT
TAGTGCCATATAGGAGAAGCGGGGTGAAAAGAGTACACCATATACCGTGTGGGCAATA
ATTTATTTTATTTAAATAAGTAAAAAACATACGAACTAATTAATAATTTAAATAGAATGTCACAAATAAGGAA
TCAATTATTTCATATATTTATTTATCGTGTGATCTATATAGGCATTTTTATATTTAAAAATATAGTAAATATT
TAAAAGTACAAAAAAAAAATTATAAAATTAGTTTAGATCTTAGATATTTGGGTAAATA
ATGCGATGCCAACCTTTGCCCCTAACATATAAAACACGCTCAATCAAATACTTGCCTTTAATAGGTATACAAA
ATGTATATATTTATTTGTATCAATTTAAAATAATTTTATTTTATATTTATTTATTATTTAAATTAAAAATATA
TATATATTTTAGTGTACTTCACTTATAGATCTTGTGATTTTTGATGTATGTTCTTTAT
ATTCTTGGATGGGTTCTGGTACGCCTAGTTCTTTCCATCCTTTCTGTAATAATAAAAAAAATATTCCAAATAT
TTAAAAAC
>103791317_2 DT662463.3

GCACGAGGCGCCCACCTTGCGAGAAACTAAAGATCACGACACAAATAATACTTCGTATAGCAGGTACAACAAT
CGATCATGGGTAGAAACCGCAAATATGCCGGTGCTTCCTATTAGATGTAAAAATACTTCTGCAGAACTTCATA
AACAGAGATTAGGGTCAGGAGGACGTGGGAGATGTATCAAATATGGCATGGAATGGTA
CACCCCTAGTGAATTTGAGGCTCTCTGTGGCAGAGCTTCAAGCAAGGATTGGAAGCGGTCAATAAGATTTGGA
GGCAGAAGTATACAAGCTTTGATAGACGAAGGAATACTCACTCTTTTTGCTACAAGCTGCACTTGTGGAGCTT
GTTGTGACGACCAGACAGCTATGGGTCCTGTCAGACTTTTCACTCCATATAAAAGAAA
AAGGAGAAATCAGGATGGCACCGATGAAAAGAAAGTTAAAGTGAAACTAGAATCTATAAGTGATGCTGAGGTA
GACAACATACACCAGACTAGCAGTAACAGTCATTCCAAAGAGGCTTGGCAATCCTTAGCTGATGGACTAGACA
CGAATACTGATTATCATTTGTTAGACAGTCCCGAAGCCAATCCAGATCCAATTGTGGC
TCAGCCTGACATGAACAAGGTACTCAAGCGGTTAGAAGACATCGGCCAAAGTCTAATACGACTATCAAATGAA
CTAAAGCAATGTGTAGACGACGTANAAACTGTTAGTAGCAGACAATTGGAACGGTTTGAACAGGAAAGAGCAT
CTGCAATGATAGCAGCAAGTGTAGACGCTCAAGC
>103791320_2 DT663218.3
GCACGAGGTTTAATTTTACGTAAAATAAATCGTGGTGTAGTAAATATGGCTGACGAACTCAATGTTGACAGTC
TAATTCATAGGCTTTTATAAGTTCGAGGATGCCGTCCTGGAAAAACAGTGCAAATGTCAGAATCAGAGGTCCG
TGGTCTCTGTACAAAATCTCGGGAAATATTTCTGCAGCAACCAATATTACTGGAATTA
GAAGCGCCACTCAAAATTTGTGGTGATATACACGGACAGTACACAGACTTGCTGCGTCTATTCGAATATGGCG
GCTTCCCGCCCGAAGCCAACTATCTATTTTTGGGTGATTACTTTGATCGCGGCAAGCAGTCTTTAGAAACCAT
ATGCCTCTTACTTGCTTATAAAATAAAGTATCCAGAAAATTTCTTTCTGCTGCGAGGC
AACCACGAATGTGCCAGCATAAATCGTATTTATGGATTCTACGATGAATGCAAACGTCGATATAACATAAAGC
TATGGAAGACTTTCACGGATTGTTTCAATTGCCTTCCCATAGCTGCTATTATCGATGAAAAGATATTCTGTTG
TCATGGCGGTCTGTCGCCGGATTTGCAGGGCATGGAGCAGATTATACGGATAATGAGA
CCCACTGACGTCCCTGATACAGGTTTGCTCTGCGACTTGCTATGGTCAGATCCCGATAAGGACGTTCAAGGGT
GGGGCGAGAACGATCGCGGAGTGTCCTTTACTTTCGGACCGGATGTCATCAGCAAGTTC
>103791344_2 DT662967.3
GCACGAGGCGAGTCTCTTGCAGTAAATAACAAGCAAAAATATTTTTGTAATGTTTTCTGTTATGAATAACAAA
ATGTAGTGAATTATAAATGTGAATATAAATGAACAAATTTTCATAAAAACTATGTGCGTGTGTTTGTCTATCA
AATATGTAAGGATGAAGACGCAAATCGATTATGTTAAATGAGTTCTGCATCTGCACTA
ATGCTTAAAAATGGGAAACTTCTTTTTATTCACGATCACGGCAGTTGTGTTTTTTGTAGGAATATCGTTGCAG
AGTGAAAATAATGAATGCCCTCAAAAGTGTGACTGCTTAGAGCAATATGTAGACTGTTCAAAGAAAAATTTGG
TGTCAGCGCCAAGAATTCCAAAGTGGGTGGAACAATTAGATCTAAGTTACAATAAATT
GAACCAAAGTGTCAGTGATAACTTCGAAGAATTATCATTATTGAAAATTTTAAAGTTAGACAAAAACATATTG
ATTCACATTCCTAACTTAATACATCACAGTAAATTACAAGAAGTCAGTCTTAACAGAAATAACATCAATGCAA
TAAATACAGGACATTTCCCAGTCAACAGCACAATCAAATCACTGAGCCTAAATAGTAA
TCACATTAAAGTAATAGAAGAGGGAGCCCTTGATAACTTAACAAATTTATTGATATTAAAACTTAATAGGAAT
GAATTATCATCTCTACCAAATCAATTATTTATGCACCAGTCCAATTTAAAAGTACTAGAACTAAATCACAACA
ATCTACATAGTTTAAATGGTCTACTTTTTTCAGGTTTATCTAGTTTGACACATTAAAA
TAAAATATAACAATATT
>103791376_2 DT662150.3
GCACGAGGTTTTAATCGTCACAGACAAATATTAGTGGTGCTATCTATGTTTGAAACTTACACAAAAATAAAAA
TCGTAAGAAGGAAACGTCGCCCTGCATTGATTATATCGTTTTGTACGCGAACGTATGAAATGCGTAATCGGAA
CATAGGGTATACTATACACTAAGTATGTATAGGCTTTAAAATAATACCTAATGTTCTA
TTTAGAAAATGCTTATTGTTTACTGTGCTTTCAAATATTTTGATGTTGGGATAAATATTTCATAAATTTAACT
TAAAATCAAGTGTACGGAGTTGTACGAATTGATATTTATTTTCTATTTTGTAGTGTTAAATGTAAACATGAGG
CCTTCGTGCTGCGTGCTGCAGTCGTAGGTTCGATTCCCACTCGGGGGATTTTTTTTTC
ATTTTCTCGCTCTGGTGACAAGACAAAGCGCAACGTACCAAAACTGGGCGGTGCGTAGGGCACAGTGTCTTAA
CAATACGTTTCCTCTGCCGCCTTCCCCGCTATATTATATTCTGCATATTATATATATTCTTTTTTTGCGCATA
CGAAATAATCACTTTAAATTAATCCTATAAATTGATATTAATATTATATGTAAGTACA
AGTCCACTTTGCATTTTCTAAATGGAATAAAATATTTTAGTGTTTTAAGTAACTTGATTTGTCTAATTCGTGC
ATTTACGTTTGTAGTGTGACTTTACATTATCGGCTGAATGACATTTTATTTAATAACGC
>103791382_2 DT662994.3
GACGAGGTCGAGTTCTTTTTTTTTTTTTTATATTATATTAATTTATTAATAACATTCATATATTATAAACATG
TGGATGTAAGGAAATATCATTTGTTTTATTTTTTATAGATACTTGATGCTTTTAAAACTGGGCTGTGTCAAAA
GAAACACAAAGAATTATTGGTAGCAATTGAAAAAGCATGGGATCAAGGATTTTTAACT

TATGATGTCCCATTTAGGGAATATGATTATTCTCTTTACAATAAACAAACAGTTTAATTGAATGTAACATAGT
ATTTAGTAGATATAATAAAATACAATTTAAATATTATATAACAAATTGTGTAAATTTATTTCTTGCATAACAT
AACATTGATTAAAATTTAATGGCACATGGTTTTCTAGTGTTCCATTCACAGATAATTA
TGTGAAATTATCCATTATTTTTATTCTGTTTCTTTGTATTAATAATTCCCTTATTAAAGCAGATTTTTCCCGT
TCCAAAATAGATATTCTTTCATTCTTGCGGTTCACTTCCTGCAAAAAAAACCTATTAAATATTTCTGTTTCAT
TATATCTATTTATGTAAGCATAACATATGAAAATTTTAGTATTGAAGTTGAGATTATT
ATTTTGTTTACAATGGTGTTTTAAAAGCACAAGAATTTATATGAAGTTAAATATTTATGCATATAATAAAAAT
GTAACTAATCAATGTTTGCACATTGACATATATGAGAAAATCTACTATTGGAGACATTTATAAATATAATAGA
ACCCAAAACTCATTGTCCGTGC
>103791404_2 DT662781.3
ATCTTCCTCTTCCAATTTTTGATTGTAGTGATACAACTTGATTTTGTTAAAATACGGCAAGAGGGTTCCAAAA
AAATGAAAGGTTGCATTTTTAACATTGATGCTGGGTATTTGGAGGGATTGTGCCGAGGTTTTAAATGTGGAAT
ACTCAAGCAAGCTGATTACTTGAACTTGGTCCAGTGCGAAACATTGGAAGATTTGAAA
CTCCACTTGCAAGGAACTGATTATGGGACATTCCTTGCTAACGAGCCCAGCCCTCTTGCTGTATCAACTATTG
ATGATAAGCTGCGTGAGATTCTTGTCATTGAATTCCAACATATTAGAAACCACTCAGTGGAACCAATGTCAAC
CTTCCTTGACTTCATTACTTACAGTTACATGATTGATAACATCATTCTGCTGATTACT
GGAACTCTGCACCAGAGGCCTATTTCTGAACTGATTCCTAAATGTCATCCTCTTGGGTCTTTTGAGCAAATGG
AAGCTATTCATGTTGCTGCCACCCCTGCTGAACTTTACAATGCTGTGTTAGTGGACACTCCACTGGCACCATT
TTTCGTCGATTGCATCAGTGAACAAGATTTAGATGAGATGAACATTGAAATCATTCGT
AATACTCTATACAAGGCTTACTTGGAAGCTTTCTATGAATTCTGCAAGCAAATTGGAGGAACAACAGCTGATG
TAATGTGTGAAATTTTG
>103791410_2 DT663078.3
GCACGAGGGCAAATATCACGATGGCTAATTACGACTACCATGAATCAGCTTTCAAAGAAATATCAGATTTGGC
CAAGGATTTTGTTGATCGGCTTTTAGTACTACAACCTGGGAAAAGAATGAAAGCGACGGTTGCTTTAAATCAT
CAATGGATCTTAGAAGGACCTCCGGGAGGAGCCAGAGCCGGTCACATGAAGAGAGCTA
GGGAGAACCTCAAGTCCTATTTAGCCAATCACAGAGCTAGGTGGCAACGTGCTGGCAATGTTATGATAGCGGC
GCATCGTCTTCGCACTCACGCGGCAATACAACGCAATACAAGCACAGCGGACGTCGACGGAGGGTCACCTCGA
GTCACCTAGCGATAGTTTAATTAATATATTAAGTTATTTAACGGCTATCTAATAAGGT
CCTCCTGAGGTACTCCACAAATCGAGCGTTTCCATAAAAAAAGGAAGAATAATATTTATTAGTGCTATTTTTC
TAAATATAAAATATTAAACATAAAACTGTTTAACCTTTGAAACAGGAATCAAATTTCAATTTATTTCACATTG
ACTAAATATTATTTGTGTAAAACCTTATATTCATTTAGTATACCTAGGTTACCTAAGA
GAGACCTAAATTCATTTATGCTCCTTCTTGAGTTCCGTCATTTAACAATGCCTCTATGCCGCAATGCCTCCAG
AATTCAGCGGAAAGTGGGAAACAGAAGTGCCTTAATGGGAACGGAGTGTATTAACACTACTAGGTTCCCAGGT
TTCCTCTGTCTACCCTGATCTGTGCAGGATCGATTGTGAAGCTAACCATTGCCCAAAA
AGATTTG
>103791419_2 DT662916.3
GACGAGGCTGCTTCCGTTCGATGGTGAAGATTTCGTGCTCCAACCGACTCGCGCCGACGGCAGCAGTCACGTG
TTACGCCGGCGCGATGTGTTGCTGTCGGAACAGCCGGCGACGTACAACCTCACTGGCGACACGGTCGTTGACC
TTGACATTGATTTTGCGGATGAGGAGCTGTCGCCCGTGATGCACGTGTACCCGCGACA
CACTGATCACTCAGATGTTGACTACTTCAGAAACATGATCACGCCCGTCACGAGGCCCGTATCAGGCGCCAAA
GGATTATGGCTCGAATTAGCTATCGTTGCGGACCATACGATGTTGAAATTCCACGGTCAGGAAAGGGTGAAAC
ACTATATCTTAGCTATAATGAACATCGTCAGTGCCATTTTCAACGATCAATCTCTCGA
TTCGAACATTACTCTCGTCATCAACAAGCTGTTTCTATATGAAGAAAAGGATCCGGTGATACGGCACGGCAAT
GTAAAAAAGTCACTTGAAGCTGTCAATAAATGGAATTATCGACACTTAATGAAATTACCACCAGACAGTACAG
GGTGGGACGCAACAGTATGGTTAACACGAGCCCAACTCGGCGGCCCATCTGGATTTGC
TCCGGTTGACGGAGTGTGTACGAAGATTCGGTCTGCAGCTATTAACAGAGATGAAGGACTGACCAGCGCTTTC
GTGATCGCACACGAATTAGCTCACCTGCTGGGATTACACATGACGGTGAAGGTCAGTGT
>103791459_2 DT662453.3
CGGCACGAGGGTCGAGCCCTATGGAAATATATGGAACATTTCTTCCAAAAAGACTGGTGATGGAAATTTTGTT
TTCGCACCAAAATTAAAACAAGACCATATATGTCCAAATGCCAAGCAAAAAATGAGAGTGAACCTTGCTGCTC
AAGTGCTAAGTCACTCAGTTGCTGCAGGAATGTTTGCCAAAATATCTCAAGGATTGCT
ATCTGGGGAGGCAGCAGTCACTGCAAATTTAATATCAAATATGGATAAATTATTTGACTGCTTAAATAGTGAT
TCCCCAGATCGAAAGAGAGGAAAACCGCACTCTACCAACATGACTGACAATTCACCCCATTTGGAACATTTGA
AGAAAATGTATGATTTTTTTGAAAACTGTACATTTGTAGGATGTCATAGCTCGCCCCC

ATCTAAGGAAGGTTGGTTATGGACAATTAAAAGTATCCTAAGATTGTATAATAATTTAAAAATAAAACATAAA
ATAAAAGCTTTATGTACTCGCCGCCTTCAGCAAGATTCTCTAGAAAATTTGTTTGGTACTATTAGAGGAAACT
GTGGATCAAATTATAATCCAACCTCTGGGCAATTTGTTGCTGGTCTGAAGACGGCGAT
TCTATCCAATCTTGCTCATTTGGGGTCAGGAAATTGTAAAGTAGATAATTATAGTTCTATGTCAGATTATAAG
GAATTTTTAACCACAACTTCTCCCACAGGCACATGCACAGAATCAGAAAGTCCACTAGTTTCAGACATGCTCT
CATTGGTGGAGTCATCCTTTCAAGAAAGTATTTCTGATGCACAAGAATTACAGCCTGT
GCATATGTCTGTGGATTTT
>103791471_2 DT663330.3
GCACGAGGATATAACTATATATTTTGCATTTGATGATAAATTTTTCAATAAGAAGTTTTTAATCTGTATTCTA
TAACTTTATATTTACTGAATCAATATAATATAATTATTTATTGTTTGATATTTTTTAAACATAACAATTAAAT
AGAGCGAAGTGACTACGGATAATAAACATAGTATTTCGCACTTCTTAAGAAAGAGACT
TTATGTTATATAGTATTATTTTTTTTTACACTGTAGCAAATTTTGTAAATGTCATTTTAAATTAACATAATTT
GTACTGAATCCTATTTTTCGTAAAGTGAGCCCATTGTAAGACTAATATATCAAATATTTTCATTTAAAACCAC
ATTTAATATAGTTATAATTTCAATTCGCACTTAGCTTTAGTTTTTAAGAGTCCAGTAA
CATGGTGCTTTACTTTAAATATTTATCGCTTCGAAATGACTCGTTTTTCAATTATAATACTAACAATATCCGT
TTATTTTTACCTACTATAGATTTTTTGGATGAACGTGCTAAGTTCTTAGTCATAATTTTGTTAAATAAAAAAT
AATATGTAGCCCAATTAGGTGTTATACATTCTTTGTGACTACGGAACTAGCACGGAAT
ATATTCTTATGACGAAACGCTGGTCAACTTAACCTACTCTTTAAATATCTCGACATTTTGGCCATTTTTTTTA
ATTAGTTTATTATGAAACACACAGTTATGATCTTGCAGCCAGTAAAACTTCTTTTATAGCTTTGGATGTATAT
GTGCGATCATAACTCGTTGGTTT
>103791474_2 DT662666.3
GCACGAGGCTCTTATCTTTTGGATAAATGTTTATAAATCGATTTTTTTTAAATGAAGTCTTAACGTGTGAGGA
AAATCGTAGGTTATTGTTTGAAACCTCACTAGGTGATATAATTATATTGTAAGTGTAACAAGAATGTTGACAG
TGCCGTCGGCTGGTTTCAAATAGCACAATTTATTAGCTATAGATAGAAGCTGGACGGA
AAGGAGCGTGTTAATGTTTTGTACACTTTGCGTTACTCGAAACGGGAAGTTAAATATTACTTACATAAGTGAC
ATACTTTCAATGGATTTTTAATTTTCTTCAATTCTCTTAAGTCTTAACGAACTGGAAAATTATAAAGCGTTCA
AAAACATCCATCTTGAAAGATAATTTAGTTTGCTTGTATCGCTCAGTGTACAGAACTA
ATTAAACAGTATGGAAATCGACTATAATGAATACGAAAGCAATAATTTCCACTTTTCCCTCCTCGAGCGCAAA
ATTTCTAATTATTTTCAAGACATATCAAGGACGTTTCAATAGTTAGATTAATTTTATATTCCCAAACGAACTC
TGCATCTTTATGAAATGTTACTTTACTTTTGTTCCATACATTGACGTTTTATTCTGTT
GAATGGCGGACGTTTTGGCGGGAAATTGAGCCTTCCATTGTTGGTCTAGTGACACTTAGAGTTGACTTTAGAC
AGATTAAAAATAAAAATTATATAATATTATAATTGTAGTGTCGTTTTCACCAGTACGAATACACATATTGTTA
ATGATGAATCGATTTAATGTGAAAA
>103791486_2 DT662168.3
GCACGAGGTGTATGACTTAGAGATTTGATAAAATACTGAGCTTATATCTTAAAACCGGAATTATTACAACTGA
GGTTTGGAAAATCGAGGCGTCACTGTACTTAATAAATAAGTGATTGTGAGCTTCATTCCTTGCCTTAAAACAT
AGAATAAACAAAAAAATATATATGTGTAATATAAAATTTTTACATTAATCTATCTATC
TACCTATCTAATTGAACTGTATTAGTATTTCTTTTATATGAGATTGTGATTGAGATTTAATTTTCTATTTCTT
TTAATAAATGTTAAGTTTCTCATTCAGGTTACGAATAAGGTGAATTTTCAAATTGTCCCTTTAAGCCTTGAGA
TAGTTTGCAATTGTGATAAAATAAATGACTAAATTCCTGCGGCGGTTAAGAGGTATAG
GAATTTTAATTTATAACATTAATAATGAAATATAAAAGTAAACATTACAAAGTAATCGTGATCACAAATATCC
AGTAGGCGGCAGCCATTGTTGTATATGGCTTATTATAATATAATTTCTTGGTGCCAAAATTACGCTCACTATA
TAGCACTGTGCGTGTACAAATTATAGCTAAATAAAACTTTTATTAATACGTTATAGCG
TCAAGTGGTTGTGTAGTAAACGGTACGTCTCCGTCAAAAGGAATTGTTTGTGAATTACAATCGAAACCCGCAT
CCTATAGCAGATTTATAAATATTACACAGCCCCGAGTGATGAATGAAGTACAAAGTGCACGTCAAGTACATAC
CGTGACTATACACACAGACCGGACGGATGATGA
>103791502_2 DV500961.2
GCACGAGGAATTACCTTTAAAAGTCAATAAAAATGGTTATTGGAACTTGTAATTTATGATCTTGGGATCGTTT
TCATTTTTAATAAAATTTTTATTTAAACAAGTATTATCTAAAATTAACACTTTAATATGTTTAGTTAATTAGT
GAACGACTTAATTGTGACAAAAATATGTTCTTCTTGATAGCTCAATCAGATAAGAAAT
AAAAAAAACGTATTCAAATAACTTAAATAAAAGAAAAATATGTGGTTAAAACCCAGCACCATACTGTCTATCC
ATGATGGAAAAATATTTAAAAGTTAACAAGTTGCTATTTTTTTTTTTATGGGACTTAAACCTAATATTATTTT
ATTTTATGTAAAATTGTAATAATTTTGTTTAATAAAAAGATATTATATATTATGTTAA
GTCATTAAAAAGTTTTTGTATTG

>103791514_2 DT663252.3
GCACGAGGAATAACACATAAATAAAATTATAAAATTAGTTTCGTATATAAATTTTAAAATTGTGTTCTTTAAT
TCTGTGTTCACTTCTAATTAAAATATTTTTTAAATATTTAATATTGAACTGTGTTAAATATGTGAATCATAAA
TAAATTATAATAACATTTTGTTAATATAATAATCACTTTTGACGAATTTGAATATAAA
AGCATATTTGCTTTGATATTTTTGGAATAAAATCACGTTTCTCTGCTGGTATTGAGTCGCAAGTGTATTTGTA
TGTAAGGATTTGATACGCGCGACGTTATCTCTTACAATTACACTTTATCTTAGTGTTTTATGTTGTTATA
>103791517_2 DT662701.3
ATAGCCGCCGCTATACATGCATTGAGGACGCGCTCTTCAGCTTTGTCGATGGGTTTGGCGTTACTCGTGTTAC
CTTTCCTGCCTGCCTCTAATCTGTTTTTCCCCGTCGGTTTTGTGGTGGCAGAACGAGTTCTTTACATGCCATC
TATGGGTTGGTGCCTTTTAGTGGCACACGGCTGGCGCTTAGTTGCCAAGAAACGGGCC
AAGTTGGCAGCTGGAGCCTTAATTTTCCTTCTCTTGGCATTCAGTGCCAAAACTTATGTGAGGAATTGGGATT
GGAAAACCGAATATTCCATTTTTGCTTCCGGATTAAAGGTGAACCGTCACAACGCGAAGCTCTACAACAACGT
CGGCCACGCGCTCGAAGCTGATGGGAAGTACGCGGACGCATTAGAATTCTTCAAAACC
GCAGTCAACGTGCAACCGGACGACGTGGGCGCCCACATCAACGTGGGAAGAACTTTTAACCACCTAGGAAGAT
ATCAGGACGCCGAAGAAGCCTATGTCAAAGCTAAATCTCTACTACCAAAAGCTAAACCAGGAGAGTCGTACCA
GGCAAGGATTGCTCCAAATCACTTAAACGTTTTCCTGAACTTGGCTAATTTGATATCT
AAAAATGCAACGCGCTTGGAAGAGGCGGACATGTTGTACCGACAAGCGATCAGCATGAGGGCCGACTACACGC
AGGCCTACATCAACCGAGGGGACATCCTCATC
>103791521_2 DT661749.3
GCACGAGGGGGAGAACATAAAAAGAAAGGAATTGGAGGAAGAAGAAAGAGAGAAAATGCAAGTATTAGTATCA
AATTTCACAGAAGAGCAATTGGACAGATATGAAATGTATAGACGAGCAGCATTCCCTAAAGCAGCTGTTAAAA
GATTGATGCAAACTATAACAGGATGTTCAGTTGGCCAGAATGTGGTCATAGCGATGTC
TGGTATAGCTAAAGTTTTTGTTGGAGAAGTTGTTGAAGAAGCTTTGGAAGTATTAGAAAAATCTGGAGAGCCT
GGGCCTTTGCAACCGAAACATCTTCGTGAAGCTTTACGTAGGCTCTTTTTCAGAGGATCATTGCCAACAAGGA
AGGCCTACAGGAGTATGTTTCGTCTTTAGAAAGTATTTTAATGTTTTTTTACATAGAG
AAATAAAGTAATGTAAATAGATGCTATCTAATCATGACTAATATTTATTTACTATAAGTTACTAACACGTAAT
TAACCCTTTACACTCGTATATCGCCTTATCATTAAAGCAGGTTGGCCGGTCTGTGGTTCCCTAAGCTTATTTC
TAATACATCTTAGTTACTTTCATAAGCGTTCATTGAGTACATTTATACCTTAAAAAAA
GATTTAGTTTTAGGTTTCTTTAGCAGCAACACATCGAAACCCACAGAGAAATGTCGTCGTGTAACGTTTGTTA
GTGAG
>103791524_2 DT662949.3
GCACGAGGAAAACATTCTTGCGAGTAAGAAGAAAGAAATTGTATTAGCTAAAGAAGCGCCTATTAAATTGGAG
AAGCTTTCGTTAACCACAACTAATGACATTTCAGATAAGATTGCAGAAAATGATACTAGTAGTTCTAATAAAA
TTGAGGAAACAGAAAATGGAGATAAGACTGCAAAGGCTGGGGAATCTAACGGGGTGGG
AGATAAGATAAGAAAGTTCGAGAAAGCCGCAGAAGATGCGAATGTCAACGCGGGCAGACTGTCGCGGCCGGGC
TCGGTTCGCGGTCGCTCTCGGTCCGAGAGGATTGGCTCTGACAGTAAGGATGATCTGCCACCGCCGGCCACAC
CTTTCAAAGATCATGTTTTTTTCGACATAGGCAACACAGACAAACCGATTGAACCTCC
TGTACAAAACACAGAAACTCAAGAACGACTCGTAACTAAAGGCTTAGTGAGCTCGACCGCTATGAAATGGCAA
CGCAACGAGGAGGAGAAGATACAAAAACCGGAACCACAAAAGATAATCAAGCCAGAATTCAAACCTATGCCGT
CTCCTATCGACGTGACCATACGTTCCCCCAGCCTCGGAACTAAACTGCCAGATCAGTT
TCCATCCCACGGGACCGCGTTCCGCAACGTCGCGTCCCCCGACACCGCCCGTCTCAAAGCCGACGATGTGAAT
ACTCAAAATAGTTTTAAGTGTAAGCTTAAAAATAAGGAGAAGTACTCCGTCAGCAAAGGCTCCAGCTACTTCA
CGCGAGGATCAG
>103791551_2 DT661745.3
GTCTCGACAGTCGCGCTTTGGCCGTCGCGTCGAATGTTTGGCGCATGCGCAATAATAAAACCAACTACGAACT
GACATTTCGAACGTAAATAAACAGTGTTTAAAGTAAATTAATTATAGCGTGATAGTGTATTATTAATATATTA
TTAGGATTTTTATATTAATAATAACATTCTAGTGCCAAAAAGTGCAACGAATTTTCGA
GAACACCAAAAGTAATGAGAAGATAAAACCGAAACAAAACAAAATGCATCTACAAACAATATTAATATTAAGC
ATATTCTTTATCTGTGATGCTTTTATGCCCAGATACAAGCAGCTCACGCAGCCAAATGAACTACCATCGAACA
CCAACCCACAGTCGCTGAGATTAGTGCAAAACGAAAACAAAAACGAGAAAACTAGTCA
AATAGTCAATCAATATAGAACTCGACATAGAAGAGAGTCCTTTGATTCAAAATCCTATGAATCTGGCCTAATC
TGGGCACATACTGAAAAATTAGATGATCACGGGGAAGTAGTGTTACGATGGGTGAACACTGATTCAAGTATAA
CATTTAGATTGGAGGCTAAAACCCGTGGATACGTCGGTCTAGGGTTCAATTCTGTGGG
AAATATGAGAGGGGCTGATTTAGTAGTCGCTTGGGTGGACGACAGACACGGCAACGC

>103791566_2 DT662941.3
GCACGAGGTGGACTCGTAACAGAGATGTTATGCGCGAGATAGCAATATTTTTTGCAAAAATGACTACTTCAGG
TTATACGGTTCTAAACGATGTGCAAGATGCAACACACCCATCTCCGCGTCGGAGCTAGTGATGCGGGCGCGTG
ACCTCGTGTTCCACGTGCACTGCTTCTCCTGTGCTCTGTGCAGTGCGAGGCTGACGAA
GGGCGACACTTTCGGCATTAGAGATTCAGCTGTGTATTGCAGGCTACATTATGAAACCATGCCGGACTATGGT
CCTCATATGGCAGTTCCAGGGCCCCCGCAGATGTGTCCGGGGCCGTACCCCGGGCCTCCGCCTGGCTCTCACT
ACCCTCCATACCCGTCACCCGAGTTCACGAGAGTAGAACACGATGTTCCTAAAGGCCC
TTTCTTTAACGGGGCAACGGCACCTCCTCCCCGACAAAAAGGTCGCCCTAGAAAAAAGAAACCGAAAGATCAA
GACATCATGACAGCCAACCTTGCATGGTCTTCTCCAGATTTGAATCCTGAATACCTGGAGATGGGGTTCCGGG
GTGGCGGCGGGCTGGGCTCGACGTCGCGTACAAAACGAATGCGGACCAGCTTTCAGCA
CCACCAATTGCGCACCATGAAGTC
>103791584_2 DT662686.3
GCACGAGGCAATGCCTTGAAACATCAGAGGAGTTTACAGAACGACTTCCAGTATTGCCGGTGCCTTTACTATG
TATTGAGGATACGGCGGTACTTTGCGTGGGCCCCGCGCCCTTATCGTCCCCGGGCAGGACGCCCGCAACCTCC
CTTTCCCTACCACCGCCTGATACAACTGAAGACGATCCGTATTGGCACAAAATGGAAG
AGATACTATTGCAAATAGCCCAAGGCAGCGTACCAATGATTTTTAAACCGACAACCGACTTGTACACTCGGAA
GACAGACGCCAATCCAATCGTCTCACAAGGAGAACCAATACTATTTTCCATCACATTGTTCAACCCGTTGAAA
GTTAAACTACTTTTGAAAGATGTGGAGCTATTATGGCAATTTACCCCCAGTGAAGGGG
ATGCAGAGACGCCTAGTGATGAAATATTAATCAACGAACCATCATTAGCCTCAGGCACGAGTTTGGAGAATAA
CATAATAATAGGACAAAAGATCAAATCAATTCTCTTGGATGGAGATGGTAGAAAAGCGTTGAATTTTACCATA
ACACCTTTAAAAATTGGTCAATTGATTG
>103791599_2 DT661731.3
GCACGAGGAAATTTCCTTAAATGGAGATTGAAACAAATGGAGATGGAAGCAAGAGAAGAAGAAACAGTTGAAC
CAAATGACGGCCGAGGCAGATGAAAATCGAAGTAGATTAACATCATACTGAAATACTGAATTAATAAATATTT
CTTGACGTGGTAAAAATATTACATTTTTCAAGTTTAAGTATTTATTTAAATTTAAGTC
ATTAATGTTTTCTTTTCGTCAACTATATAATAAATGTTAACTTTTTATATGAGTCCGTCATGGCGCAGGTGCA
CAAACCAAGGTGATGGTTGTGGGTTCGGTCCCCACTCGAGGGATTGATTTATTATTTATTAATATTTTTATAG
TTCTGGCTCTAAGATAAAAAGCCCAGCGTTGAGTTCCGCCACTCAACACGCAATGACT
AAAAAAAGTCAACGGAAAGTGAGGAATGGAGTGTCTTAGCAGTAGGTTCTCTCTGCCTACCCTGCTGTGATAC
AGTGTGAAGCTGATAAAAACAAGATTTTATATACTTTTTGAAAATATACATGTTACTATAAATAGAAGAAAGA
ATTATGTGTATAATAAAATGTTACAAGAAAAAAAGAACTATTGTATAACGAAATAATA
ATTGTTCAAAATTCTATTATTAACTTTCTGCTTAAAATTTTATTTTGTTTCAGGTAAGGCTGGCTGTTATAAC
TTTTATTTTATGAAGAATGTATTAATAAATAATGATTATAAAATAAAAAAAAAAA
>103791623_2 DT663045.3
GCACGAGGCACAAATAATCCTTTTGGCTTTGGTTAGGATACTCTTAGTAATGATATTATTAGGTACAGTCAAT
TTAGAATATTTGTTTACCAGACAAAAGTTACACTGGTCCTTGAGAGAGTATACAACGTATTCGTCAGTTGGCA
CCGCGGTTATGTTTTTTGGTGGATTCTTCGGTGTAATTGTTCTACAGAAGATTTTCAA
GATGGGTGATATCGTATTTAGTATAATATGTTTAGCTTCTGCGGCTGCAGAATGCTTAATAAAGGTGTTTGCC
GTCGAGACGTGGCAAATGTATTTTGGTTCAATAATATCGATGTTCAAAGGACTACCTGGAGCCCTGATCAGGT
CTTTTGTAAGTAAAACGTTTCCAGCGGAAGATATAGCCAAGGTGTTCTTCCTCCTTTG
TGCGGTCGAGAGTGCTGGACCACTCGTGGCGCCTATAATATTTAACTCGTTGTATAGCGTTACTTTGGAAGTC
TTCCCTGGTGCTATATATGTTTTAAGCGCGATCATGTTGATAGCTTCTTTAGTTATGTTGGGTTTCGTGCAAT
ATTATTCTCGAGATAGATCATCACAAAACTATTTAGTCTTTGAGAATACGATCGATAC
ATAATTATATTCTGCGTTTTGATCCTGATATGTTTCTCT
>103791629_2 DT662448.3
GCACGAGGCACACCGCGTAGGATAAAGCAGTTAAAGTTACTAAGAACTCAGCTACTTATTGGGAGGGTAGAGC
AGACGCAGAAAGCCTACAGCGGGTGTACGGAGTTTCGTTCCCGGAACCTAAACAGCTGAAGGAATGGGAGATA
ATACAAGAGGAAGCTGCCAAACGAGATCATAGGA
>103791635_2 DT662467.3
GCACGAGGGCCGAACGTTGGTAAATTCTTTGAAATAATGAAAAAATTGGGAAAGACAGTCCACTTCGTGTCCA
ACAATAGCATGCGTTCAAAACAACAGTATGAAGCGCTGTTCGAAGCTGCGGGCGTTGAAAATGGATATGAAAA
CTTGACGTTACCATCAATTGCAATCGCATCTTATCTAAAATCGGTGGATTTCAATAAA

ACCGCATATTGTGTGACTTGTCCCGAAACTATTGCAGTTTTAAAATCCTACGGTATTAAATGCCAGGATGGGC
CTGACGTATTTACATACGACGAAGGGAATCTCACAGAATACTCGACCGACGTCGGTGAGATAGGTGCTGTTAT
ATTCGACAGCGACTATAAAGTCAACTTGCCGAAGTTGTATAAAGCTATCACTTACTTG
AAGAGGCCAGAAGTACACTTTGTTAACGGCGCCACTGACAGATACATACCTATGAAGAACGGAAAGCTGGGTT
TAGGTACCGGAGTATTCAGCGACATAGTGAGCGAGGAAGCTAAACGAAAACCAACATTATTGGGGAAGCCCGG
AGAATCGTTCGGGGAATTCGCTATGAAGCGGGCGGGTGTTAACGACGCCGGTCGAGTG
TTATTTATTGGTGATATGTTAGATCAAGACGTTGGCCTCGGTAAAGCTATGGGCTTCAAAACACTACTAGTAT
TAACCAACAAAACTAAAGAGGATATGATGAATCATAAACTTAAACCGGATGTGTACGCTGATTCTTTAGGCAG
CTTAGTTCCTGTTTTACTTAAAGAATATCCAGAATTCGCTTAATTTCAT
>103791647_2 DT662936.3
GCACGAGGAAAACATTTATTCATCAGTCATATTGCAGAATTCATCTTAATTACTATTTTAATTAACTAAATGG
CATAGTATAAAATATATTTGCACTTATATATAATTAAAAGAATATCACAAATAAAATTGTGATAACCGATTTG
TACTTAAGGTGAGTAAACAACAAAGAATGCAACATTTGATTTTTAAAAACAATTAAAA
TAAAATATTATAAGGCACATTATATTGGAATATTACATAACAGTGAGGAAATAACTTTTTTTCATGTTTATTT
CAACTGTCCCAAGCTAATTTCTCCTTCACTTTTGTTTATTTTGTTATTCTTTTTTCCATTTATTTGTCCTAAA
CTAGCTTCACCCTCACTTCTTACAGGAAGTATGGCCTCAGAGAGATAGGTGCTGCCTC
TAGATAACGATGATGAGGAACTCAACTCTGAAATTGATGGTATTTTAATTTTCCTAAGCCGTTTCTTATACTT
TTTATGAATCTCACCTAAACTGCTTATGTCAGATGATGAATAATCCCCATCTATAGAGCTGGAACTATATTTT
TGTTGTAAATTGTTCTTTTTGTATATTTTATTAGATTCTTTAGGACCCAGATTGGACA
AAATGTCTCTCGAAGTTTGTGGGAGACGTAAACTGAAGCGTGGTGAAGACAGATTAACAGATATACCAAGATC
ATCATCTTTTTCTATCTCCACTTTTAGATCTTC
>103791654_2 DV501101.2
AGCTTTGCTACTAGCTAATGCTGGATTACAAAGAATGGGTTGGGGAAATAGAATTTCTAAGGTCTTGCCATGT
TCAGAAATGATGTACGCAGTGGGTCAAGGTGCTTTAGCTGTGGAATGTCGATCGGATAATGAAGATATACTCT
AAATGTTAGCGCCATTTAATCATCCCGAAACGTATTGCAGAGTTTTAGCTGATAGAAG
CTTTTTGAA
>103791669_2 DT661783.3
TTTGGAGAAGAATAATCAGCTAGTAATAAAGAACCTTAAGAACGAAGTGTCCAAGAAGATCGCGACGCCCACG
TGCGAGGAGATCATGTACGCCGGCACCGGCATGCTGCTGCTGCGGGAGACCGACGCCGTGCAGCTGCTGGACG
TGCAGCAGAAGCGGACTGTTGCTAGTGTGAAAGTTTCAAAATGCCGCTACGCCATATG
GAATGCGGATATGTCGCTAGTGGCCCTCCTCGGCAAACACACAGTTACCATATGCACTAAGAAATTGGAACAG
TTATGTTTTTTTTTAGAAGGAGCTAGAGTGAAGTCCGGCGCCTTCGACGATTCCACACCACAGCCAGTGTTTA
TTTATACTACGGCAAATCATATCAAATATTGTTGTAAAGATGGTGACTACGGCATCAT
CCGTACGCTGGACGTGCCGGTGTACGTGGTGAAGGTGGTGGCCAGCGAGGCGGGCGCGCGCGTCGTGTGC
>103791678_2 DT662364.3
GCACGAGGCCGTGCTCCTGGTGCTAGTGCTGGCCGCCGCCGCCGCCGCCGCCTGGTTCGTCATACGGAAGAGG
CCATTCGGTAAAGGCGGAGGGCTCGGTAGCCTCGCGTCATCGCAGAGCGTATCTTTCCGTGCGGGCTCCAACG
TGGAGTTCGGTACGGGACTCAGCACGCCCAGCACGGGGCCGGCCGGTGCCGATGTATA
TGCGCTAGATGAACCAGCGAGAAAAGGTCGTGACTTTAGCAACCCTATGTATGAAGCTGTTACGAGAGCTACA
GCAGCTGGGGAACCCGAACCGCCTTTGCCCGGTATCTACGAGGTGCCAGAAGATATAAAGGACAAGGTGGTGT
CGGCGGTGATATCGCCGTCGTCGACGGAGACGCGCGAGGGGCGCGGCAACAAGCGCAC
GCGCGCGCTCGACCCCGCCGCCGACACCGGCGTCGACACGCAGCAGCTCGTGCGCGAGGACCACGACTGCTAG
CGCCGCCCTACCGGACTTACTATAACGTTTTCTACTTATATTGTTTAGTGACGACTTTTAATGATAACAATTA
ATTGTTAAACGCTATTGATGTATCCATCTACCCGCACTACATAGTGCGCGAGGATAGC
GACCGCCCAATATTCGTCACTTGTTAAAATGTTTTATTCTGTTCTGTCACATTCTAGAANATACTATCCTGTT
ATTGTTTAGTGATGACGGTGAAATATTGAATTTATTTTTGAATATATACTGTTTACGGTAGTCTTGTGGGGTG
ACTCGAGTCGATAGAAAGTTAGTGACGGAGCAA
>103791687_2 DT662768.3
GCACGAGGCACCGTGCTTTCCGACCAGACCGTGGCTTTACAGTCATCGTTGAACCCTCCGGGACGTTTGTGTG
TGATCTTTCCGAAGGTAGTTGTTACGCTTGTTTATATTAAGATTATATTAGAATGGTGATTCGTGATGTTGAT
TGATTTGTGAGATCAATATAAGTCGTTGGAACGAACGCACTACCGCTGGGCGGCCGAT
ATGTTAAATAAACGCACGGCACACACCTCACAGTCGTGTCTCGAACCGTAGGCGGACACTACTACATACACCA
GAGTTCCAGGACATAAAATAGCACCACACACCCGTATGAGACAGGCGGACACTACTACATACACCGGAGCTGG
GACATAAAGCACCACACACATACTAGTCGTATACCAAGTCGTAGAAGTGGTATATAAT

ATGTCAATGACAAACGCCATACAAGCATAGCTTACGTATCGCGAGCGTACTGCGAGACGGCGTCAGCCACGAC
ATAGTTAATGTCGCTATAACAGTTCGCATAAGCCCATCACGTCAGCTTCCGCGACGTCAAGCAACATTCAATA
TTATAATATGGATCAAAGATCCACAATATGTGTCGCTCCTACACATATATTGCCTTTA
TAAGAAATTCGATCCAAAAAGATCGTGCCATCATAGACTTCGAATTATCACATTATATTTATTATACATTTGG
TACGAGTCTTTATGTGCATCATATTTATTTGTACCGTAGTACTTAATAGTTCGACTCGACCGCTGCCTTCCGA
GCATCACCACCACCTATATATACGGAACGAC
>103791690_2 DT661977.3
GCACGAGGTGCAAACCTTTTAAGTATACCATCGTATGGTTGTAAAAGTAGTTTTGTATTTTGGCGTTGTTATT
ATTGAGTAACAGATTCTTGTATAAATAAAAAAAAATTTGTTATTATAAGATTTTGAATAAACAACATCTGTAA
CGCAATCCGGCCTATATCCGCGGTCCTTAATCCTATTTCGTAGATATACTTTTGCATG
TGCAATTCCTATGGCGTCATTATCGTTACCAAGAGTGTTTCAAGTGAAAAAGAACAGTATTGACGCTGAACGA
GAACAGTTTGAAAAATTTCAGCCCGGCACACCACTCACTAATGTTTGCTGAGTTCGTGTTGCACTAACAACTT
GCTATGGCCAATCCTGCAGATAAACGGTTCCATCAGCTGACATTAGCAATTCAAAAAG
CTATAAATGCGATAGAAACGCCAGTTAAAGAGAAACATGTACGAAGCACCATCATTGGGACTTTCCGAGAGCA
GAGTGCCATCACGTATTGGATGGTGGCCATCCGTCAGCCGTTACAAGACGACAGGATCGTGGCATGGAAGTTT
TGCCACGTGACGCACAAGCTGCTCCGTGAAGGTCACGCCGCGTGCCTCGATGACTCGC
AGCGGCACATCGGCATGATCAAGGAACTTGGCAAATTATGGGTGCATCTACGCGAAGGCTACGGACGCCTGAT
CCACTTATACTGTAACTTATTGGTAACGAAGTTAACGTTTCACGCGAGAAATCCTAGAATACCCGGCAACATG
CAACTG
>103791716_2 DT663288.3
TTCGGCACGAGGCTTGTGCCTTGAAGCTCTGGCATCTGCTGCTCCCCAGTTGCTCACCTGGCCTGGCGTCGTC
GCCCCTGGGGTATTGGGACCTTCGGTAAGACTAAATATTTGTTACAAAAATGAATAACATAATAGTTTTCTAA
TTGTCTTGTTCTCTTACGGTTCCGTACACTTATCGGAAATCTATTACTAAGATTCCGT
CTCTGTCACCAGGCTCTAATCTCATGATACTTAGACAGTTGATTTAAAAAATGAAGAAGTTCTCATACAAAAA
TTCATCATCATCATAATCACATTTTTATTTATCTTCAGGATGTGTTTGTAGGTACACATAGAATAGTAGGCAC
AGGGAACCTAGTGTTATTACTACGTTCCCCCACTTTCCGCCGATTTTTTGATGCATTG
TGAGTTGTTTGGCGGAACTCAATTCAGTTTGCGTCATCATCTGGCATCAAACCTACAACCGTAGCATGTGCCA
CGACGGCCTCAATCACAATATTGTTTATTTAATATGAAAATTGCATATAAGTTATGTACATCGAATTTGTTTA
ATCCAGACCATCTTAGCCGGACCCACCGTAGTGAACGCAGTGGACCCAGTCGGCCGGG
TCGTTTCTCCCCGGGGTCG
>103791730_2 DT662498.3
GCACGAGGAAAAGATCCTAAAAAAGTTAAATATATGGGATCTAACATTTTTACAAATATTTATTCAAGTAGAA
TATTTTGTAACCACTTATCTAACTCTAAGCATAACCATTAAAAATACTTGTCTAGAGACAACCTGACTAGGGA
CTACCCCGTTAGTCGTGAAGCTACATAAATAACATTTTATTTTAAATACAAAAATATA
TAATTTTGTCTCTCTCTTCTCTTCAACCTCAATATTGTAATTAATTTTAGAATTAAAGTAATTAGTTTTACAT
AAACTTTTTTTCGGATAAGTTGACCAGTGATTTTTCAATACATTAATAACTATGGAAGCCGATAACGGCCGCA
TTGCTGAACAATCAATTCAAAATGAAGAAGTGCCGAAACCTAATAATGAACAAGACCT
CAGTTCCACTGTTAACACTCCTGTTAACGGAAATGATGCTGAAGCTGTTAATAATCCTGTCAGTTCGAAAGAT
TCTCAGAAACCATCACCTAAAAAGCCGAAAAAACGAAAACCCAAAATGCCAAGAGATGTTACTGCTCCACGGC
AACCACTCACTGGCTACGTAAGGTTTCTTAATGAAAGAAGAGATCAGTTACGTGCAGA
GCAACCGGAGTTAGGCTTCGCAGAGTTAACACGACAACTAGCTAGTGAATGGAGTAAATTGCCTGCCGAAGAA
AAACAGCAATACTTGCAAGCAGCTGATCAGGATAAAGAAAGGTATATCAAAGAATGGACTGAATATAAAAAAA
CTGATGCATATAAGGAGTTTA
>103791750_2 DV500968.2
GCTGATTATTCGGCATCGACGTATAACGATGAATACTATAATACGAGTGCATTTAACACTTTCGATTATTTCG
GAAAAGATGGAAAGGAAATTATGGAGAATGAAAGATCGAGTAATGTGGAAATTGTGTACTTACAATGGGGAAG
GGATTGCGGACATGTGGATGAAAAAGCTATAGCTGATACGTATAGAACTAACAGTGGA
GAAAGTGGATGTTCGAGACCCCTAGAAATAATACAATGGATAAAACACAACGTCAACCTGATCACGTCTAAAA
GACAGCTATTGTTTTTTTTTTTGGATGGGCAGATAGGTTCTTATAGTGCGACAACATGCCTGCACCTGAGTAC
AGATATGAACTATGAGAGCGTTGTGTTTCACGCGTACAAGTACCACGGGCATTCACTT
GATTTGTCGGTAGCGGCGACGTTTTTCATGAAACGATGCACTGTTACGCGGAACGGGGAAGTTTTAGATCAA
>103791753_2 DT661884.3

GCACGAGGGAGTCATCGTAGGAGCTGTCGATGAGCGATGAGTTACGGCGCGCTCTGTACTCTTCGGGCGCGTC
TGTTGACATGGAGTTCTCGCCACCCGACCTCATCGGACCTTCCGAGATATTCACCATGGAGCACAGAGAAAAG
CTTTGCACGGTGTTACCAGCGCGCGCGCAGGGCTACATGTGGTCGCTCGCGTTCAGCA
CCAGCCAGCACGGCTTCTCCCTCGCCTCCATGTACCGCAAGATGCAGCGCGTCGACACCCCCGTGCTGCTCGT
CATACAGGATACTGATAATAATGTATTCGGTGCCCTGACCTCATGTGCTCTACGGCCTTCTGAGCATTTCTAC
GGTACAGGCGAGTCCTTACTGTTCTCTTTCCAACGCGTGGATGAACGCCGCGCGTCGC
AGATCTCGCCTGATGACTCCACACACAAAGACGATAAAGAAGAAAAGAAAGAAGATCAAGATGACCAACTAAC
ACCAGTTAAAACGAAATTCAAGTATTGGGGCTGGACCGGCGACAACATGTATTTTATCCGAGGCAGCAATGAT
AATATTTCTATAGGCGCTGGAGATGGCAAGTTCGGTATCTGGCTCGACGGCGACCTGT
ACCTGGGGCGCACGCAGCGCTGCACGACGTACGGCAACGAGCCGCTCACCACCCGCGAGGACTTCGTCGTCNA
GATAATGGAGTGCTGGACCTTCATCTGAACTCCCGAGGCCCCCCCTGAGGCCCACTAGACTATAGCTATG
>103791798_2 DT662231.3
GCACGAGGCGACAGCGTAGGTGGTGTCGTACAAGGGGTCGCCGCAGTCACGACGCCTCGTCCAGACTCAGATG
AAATCAACTAAGTTTAATGTGTTGCTAACTACTTACGAATACGTTATTAAGGATAAAGGCGTGTTGGCTAAAG
TGCAATGGAAATATATGATAATAGATGAAGGTCATCGTATGAAAAACCACCACTGCAA
ACTGACGCAAGTGCTAAACACTCATTACGTGGCCCCACACCGCCTCCTACTAACCGGTACTCCCCTACAAAAC
AAGCTGCCCGAACTTTGGGCACTCCTGAACTTCTTACTGCCGTCCATATTCAAAAGTTGCTCTACTTTTGAGC
AATGGTTCAATGCGCCTTTTGCTACTACCGGTGAAAAGGTGGAACTAAACGAAGAAGA
AACGATCCTTATCATCCGTCGTCTACACAAAGTATTGCGTCCGTTCTTGCTGCGTCGACTTAAGAAGGAAGTG
GAGAGTCAGTTGCCTGACAAGGTGGAATATATTATCAAGTGCGATATGAGCGGTCTCCAGCGCGTGTTGTACA
AGCACATGCAGTCCAAGGGCGTATTACTAACAGACGGATCGGAAAAAGGCAACAAAGG
CAAGGGTGGAGCTAAAGCGCTGATGAACACTATTGTCCAACTTCGGAAACTGTGCAACCACCCATTCATGTTC
CAGCACATCGAGGA
>103791813_2 DT662747.3
GCACGAGGTAACCACCTAAGACAGCAAACACGAAGTGGTAGATATGCCTCAAAATACTCGCTCATCGACCTCA
GCAAACTTAGCCAAGAGACCGTTACCGCCCGAAGTGGAAGTGACCGTAGTGAACCCGAATAAGAAATTACGAT
CTGACGATTCCATAGACTTGGATCATCACAACAATTTTTCTGGAGGACCAGAAGATAG
GTACTGTAATATTTGTGACAAGGAGTTCACCAAGCGAGCCGCATACCAGCGGCACATGGA
>103791819_2 DT663403.3
GCACGAGGTGGATCTTTAAAGTATCCGTGCCAAATATTGGCGCCAAGGAAAAAGTGATTCTGTCAGGAAATAT
AGCAGAATTAGGTGAATGGGATTATAAAAGAGCTATTGTTCTGGAGCATGTAGATGAGACTAACTTGTGGAGC
AAGTCTATTGTTATTCCCAACACTTGTGACATTTACTATCGCTATGCCATATGCTCTT
GTAATGAAGACAGTGAAATTATTATTCGTCATTGGGAAACTAATATAAACCCAAGAATTATCAAGGAAAATAT
GCTACATCCTGTTACTGATACTTTTGGTGAATTTGATGGTAAACATTATATTTCTAGAGGTTGGCTCACTTCC
CAAAGTCTTGTTCAATTTAAATTTATTAATAATCCTTTAAAATTGAAAAGTCGCTTGG
TTGGACGACTTATGAATATAAAAGTGACTCCTGTGAAGCTAACATTTGGAACAGAACCTCATGAAGATGCATC
TCTAAGTACAGATACTATGGATGTTGAAGTTCCAGCAGGTTCATTTGTAGAAGTAGCATCACTTGATAATGAT
CCAGCTATTTGTAATTTGCACCCTCAAGAACAGTTTGGCAGAGAATATAACCAAAATG
ATGTGTTATTAGTAAATGTCTTCACCTCA
>103791822_2 DT662011.3
TCATACTTATTTAAATAAAAACTCTAATTTTACGTTAAGAACTTTTTAAATTACAATACAGTAAGGACTAAAA
TATATTACTCTTTGTGGTGTTCTGGCTTATGGATAGAGAAATCTAATCGTACATAAACAATAACACACAAAAA
TAACATGTATAATAAACCTACAACCAACAAAGGTTCTTGCAACATCAGAAGACGAGGG
AATGTGTAACGAATTTGGAAATCTTTTTTATGATTTTCTACAACATTTCTTTTGGTAAAAGATATTACAGGGC
GGCCTTTGGTATCTAAATAATTGTAATGGAGATTGTCAGGCAGCCTAGTCACTGCAAATGGGAAATTTACTTT
TATGTTAGTTGATCCTTCAGGCAAGATAATTTTTGTGACTAGTTCGTCAACTTGCATA
TCATCAAATACATGATCAACTGCTCTCATTTTAAGTAAATATTCATTGCCTGAGTGGTACAAGTATTCATAAC
TTGAAACATTATATCCTAGGGTGTAATGTGATCTCCATCCTCCAAAACAATGGGTAGCGGGGTCTTAAGTCTA
AATTTCACTAAGTCTTTTCTTCACCCTCATGTT
>103791825_2 DT661683.3
ATAAAAATAATTTTAGAAATTTGTAATCTTTTAATACGTAAAAATGATCGCAAGACCGTGGACGCTCGATTTG
CCGAAATGTCGGAATACTTGTGTAGCATCGTATTTTTCTCCAGCAGAAGGAAATGCTAATACTAATGATGACT
ATTTTTGCTTTTACTGCCATACTGAAGATAACTCTTGCCTGTATGGTGTATTTGAACC

TCACAATGGTTTGGATGCAGCAAGATTCATCATGCAAAGAATGGCAGCTGAAATTTTATTTGCACCACCATCC
AGTACTAATACTGTTTTTTAAATAAGAGAGAAGCTAAGGAATGCATTTATATCAGTTGAAAAAGCATACATAG
AAAATTATGATGAAATGATAGCAGAAAGAACAAGTTTACAATACAGACTGCAAATATT
AAAAGGATATACTCATACTGCACAGGTATCAGACAACATACTGGACCGTCTAAAAGAAATAGACCAACACTTG
TCCGGAGGAGCAACAGTGGTTATAGCATTAGTTCACAATAACAAGCTTTATGTATCAAACGTAGGGGAGAC
>103791866_2 DT662795.3
GCACGAGGACGACCCGTCGACGTGACGTGTCAAATGGACACGTTCTCATTCATACCTATTGTTATTTTTCAAG
TAGCGGCCTACTTGTTAAAACATTTATTACTTTACGCAGTATCATATATCATTACAGTTATAATACGTGAAGT
CATAGTAATAAAGGAAGCGAAATGACTCGGTTTGTCATTATTATAATAATTTCCCTAA
GAAAAGAAAAACTTTAGTTGCAATTATGTAGAGTTCCGAGAAGCGACCGTCATGGGAGCCTCCTCGCTCCGCC
TCTTCGACTCGTGTGTCTCAGTGAAGTGTCGAGACGCAAACCTACTTACCTTTATTAGTAATGGCCAGTACAT
TTTCAATATGATTTGTAATAATGTTATATTTGAATATTTAAAATATTACGAAATTCAT
GCAATTTTGTTGATTTTATAAAGTCAGGGCTCAAAGAGAGCGCACGAGTCGCTAAACAAATGAACAGTATCCA
TTTCCACACGAATCCAATAGCTGTGTATATAATGTTGTATGTCAGAGAAGACTCCCATAATTTTTAATTTGCG
TAGAAATCGTCTGTGATAATTCTGTTTGAGTCAGTGTGCACTCTACCGACGGCAACAG
GTACCCTTAGTTGTAGAGTATAGTGTAAGGCTGGGATCTCGCGGCTGCGCCCGCCGCCGCCGCGCTGGTGGTG
TCGCAGCGCGAGGCGGGTGGTGGCAGGTGCAGCGGACGAGGCCGCGCCGTATGTATATACGTACCGTACGTTA
GGGCTAGCGT
>103791892_2 DT663344.2
TTTTTTTTTTTTTTTTTAAGCTTAAAAAGTTTAATTTCATTTATGAATAATGTATAAAAAAAATATTGCATAT
TTGTTTTTCTTTCTTCCTTATACCTTACATACGTATGCATACCAAATAACAGTCTTTTGCATGTAATCAAATT
GTCAAAGATTTCTGTATCAATATGTCATATATTACAAAATAAATACAAAAATGTAAGT
TTTATTAAATATTTTTAAAATTTTTAAAGTGTTATTCCACACATATAAAAGAAAAATATTTATTTAGAAAACG
TATAAAAATTTGAACGTAGCGGGACTCAAACCCGCGCCTCCTGGAAACCAACCAGGTCGGTTTTCTAAATAAA
TATAAGTCTTATTAAAGCTTTTAAAAAATAATGCTTCTACAAAATCATTCCACTAGTT
AAATTAAAAAAAAAAAACCAATTCGGATTCACATTAATACTTAATTGACAATTAAAAAGATAAAAAAAAACAT
AATAAAAATTAATATCTTAATTGAGAATTTCTATAGTTTTGACGTAGAGCCTGTGCCCTGTTAACGTTCTGTA
TGTCATTATTAAATCTCGAACTAATATCGGATTTTGGTGCACTTTGACTGGCACTGGG
TGAACCTGGGTCAGTGAAGACGCTGGGTCGTTGATTATATGACACGGGATTCTTCCTATTTTCTTCTAGGGCT
TTCCAGTTAATATACCAGAAGGGTTGCTGGTCGACTGGCATCTTGC
>103791895_2 DT662535.3
GCACGAGGATTTTTCCCTAAAGGTCTCACACAATGTATGAAGATGATAACATTATTTCTACTCCTAAGCGATA
TGCATTGAGTGAAGTTACAAATGCTAGTAGCTATGTGTCTCCAACTGCTAACCTTAAGTTACTAACTAATGTA
GCTCTTCAGTATCCCACGCCGCCTCCAAGCACGGTGCACAGAAAAGAAAAGTCCCTCC
AAATACTATGTGATAAATTTCTTAATCTTTACCCACTGCAAGGAAATGGTACAGTGGAAATACAACTGGACAG
CACTGCAGCTCGGTTAGGAGTCGAAAAACGAAGAATGTATGACATTATAAATATTTTAGAAGCCATGCAATGT
GCAGTTCATAAAAGAAAAAATACATACTTATGGCATGGTGGATCTAGGTTAAACTCAT
TTTTAAAAATGTTAAAGAAACAGGGTGAAAATTTAAGATTATCTGAGGCTCTCAAAGGAAAAGCTCCCAAACC
ACCAGCACCAAAACACAAGACTTTAGGAGTTTTAGCACAAAGATTTTTGATGCTCTTTCTAGTTGAACCTCCT
AATACTTTAATAAACTTAGAAATGGCAGTAAGTGTACTTATAGACACATCAAACAAAA
ACAAAACTAATTTATCACCTGAGCAGCTGGATAGACAACACAAATCTAAAGTGAGGAGATTGTATGATATTGC
AAATGTATTTATTTNCATTGGTCTCATAGAAAAAGTTTCTGGTAATTTAATATTAAAAAAGCCAGTATTTAAG
TATGTTGGACCATTTAAGATGAAAAAATCTGAAAAGATTGTGTTGCACACACCATCTC
CGATACACCACTATCAGTCATAGCAACAGCTCACCAAATGACACCATGCCAT
>103791898_2 DT661831.3
GCACGAGGAGACTATTCATGTTGATTAGAGGATGTGTTAGTGGAGAACGGATACTAGCAGATAGTCTAGAGTC
GGTCCCCATGCCTACGGAGTCAGAGGACGGTGTGGGGGACGGCATGGGGGACGGTGCTGGGGATGCGGCAGCG
GAGCGCCTGCGCGAGCTGCTCGTGGTGGGGCTCGGGCACAAGGGCTCCAGGGTGCTGA
TGCTGATGAGATGTGAGGACCAGCTCATCATATACCAGGCATACAAATACCCTCGAGGCAATCTAAAACTGCG
TTTTTCACGCATGTTCGTGTCTTTCCCGTTTGGATATTTAGGAGGGGTGGTCACATGTTCCGATGATAGCTAC
GAGGCCGCTAACCTCAGGGAGGGGGTGCGACAGATGAGGTATTTTGGTAATGTAGGTG
GCTACAACGGTGTCTTCATATGTGGACACACGCCCTATATCCTTTTCTTAGGGGCTCGTGGAGAATTACGAGT
GCATCCTCTATGCCCGGATGAAGGGTCCGTGTCATCCTTCGCGTCATTCAATAATACTAATTGCCCACAAGGT
TTCCTGTATTTTAATGCTAAAGGTTCTCTCCGCATCTCAGTTTTGCCAACCCATCTGT

CGTATGAGGCGGCGTGGGCGGTACGTAAAGTGCCCCTGCGTGAAAGTCCCCACGCGGTCACGTTTCACGCTGA
AT
>103791901_2 DT662975.3
GCACGAGGGTAACACTACTAGTTCCCTCTGCCTACCCTGCTGTGTGCGGAATACAACGTGAAGCTGATTTTTG
ATTTTTTTTTATTAATTTCACTATATCTTTATTATAAAATAATATTTTATATAAAATAATATTTTATATAAAA
TAATAAGTATACATAAAATATTTAAAAAAATAACTTTTCTTCGATATCAGAAATTTTT
GTATAATTTTTGAAATTTTGTAAATTATGCTAACATTTAATTTATTTCTTAAACTCCGCTATCCGCCACTTAA
AATGCAATACTCAATTTATTGAATATTAAGTTGCGGAACTCAACGCCTCATTTGGTCTTCGCGGACGCAAGTA
ATGATAAAGATCTTTCTCCATCAGTAACGATCGAATCAATCATCGTATTTATATTTAA
TGCAGTAGCTGAAAATTTTTAATATATTTCATTTTTCATTTCACCACAATATTAAACAAGATCTATACTTATT
TTAATTTAAGCCTATTTTATTTTTAAATGCAACTTTATATGCGTTTAAATGATTTATTTCTTTCTATGTACAT
ATTTAATGTTATTAACATAAAATTAGTTACCCATTTTATTATCAAAATATTTTATTAA
TGTTTTATTGTCAAATATCATAACAATCTCAAAAAAAATATTTATTTATTCTTTTTTATTATTTATTGAATCA
ATTATTTAAAACACAGTCCCAGTAGTACTTTTAAATATATATAATGTTAAAAATAAACTGTTGTCGGTGTGTT
CATCAGTGTTAAGCTGTTTTAGAATTTTAATATCTAGTGAGTA
>103791906_2 DT662485.3
GCACGAGGTAAAAAGCAAAATATTTATCTCAATATCCTACTTCTTGTGAGAGTCTATCTAAATTTGTCCGTGA
TCTTAACATAAATTGTGATAGACTTTTGATGATGTGGAATGCGTCCCCTACCCATACAGATACATTTGAGCTA
GCACTAGTTTGTTTTTACTCAAAATTAGTAATATAAATATATTAGAACTGGGTATGGG
TAGGCGCTTAAAGTCTAGCTTAAATAGAACATATCAAAAAAAAACTAAGACATTTTACATAAATCAGCATCAA
GAGTGTTGTTTCTGGCCGCTTGAAACAATAACTGTATACTTCAAACCGGTATAAGGGATCAGGTTAAAGCAAT
TTTTTGACAAAAATTTATAATAATCAAGCGGGCACTCTAAATAAATTATATGAGGTAT
CCGTTTTGTAATTATTTGTTTGGACTCGAAGTCAATGATGTTTTAACCTGATTTACTAATGTTTAATTTATTT
TTATAAGCGGTAAAGTCACAGTATTATTAGAAGAAAATATCTTGAATCATCAATGGGTTGTACAGTAACAAGG
AAAGATATATTTTATACTTCGTTTTTATTTGCAAAAGATATTTTTCAAAATAATACTA
TTTGCACCCCTGTCGACCCTTTGTGCTACAAGAGAGATGGTAAAAAAAAATGAAAGTTTTATAACTGTGACTA
TTTAACAAAGTGGAGGAATCAATTCATCATGTTACGGTTTGCATAATGAACATTGCTCTGCTTATTGGGAAAA
GAAAACGAGAGCATTTGTGTTCAATTAATCTATCGTGCTCTCGGGGGGGCTTCAAATT
CGGGGATGTACGTGAGAGTCACTATTTGGTCACATGCTTTAATAAACACTTTT
>103791927_2 DT662667.3
GCACGAGGGCAAGACCTTAAAGATTTATCTAGAGAAGATGTGGAATCCGACTTGACCTTCGTCGGATTTGTTA
TTATATCGTGTCCTTTGACGAGTGATTCCAAGAAGGCGATTGCTGAAATTGTCCACGCGTCACACTCGGTGGT
AATGATTACCGGTGATAACCCTTTGACCGCGTGCCATGTTGCCAAAGAACTGAAATTC
ACACAAAAGAGCGACGTACTGATTCTTACCAAATCTGAAACGGAATGGATGTGGAAATCTATTAATGAGACAG
TGGAACTTCCGGTTCAACCGTTTAAAACGTCGAAGGAGCTTTTTTTTAAATACGATCTGTGCATAACCGGGGA
AGGACTAAAATATTTAAATGACAATTATAATAATTTTTTATTAGAAATTATAGCAAAT
ATAAAAGTGTTCGCTAGATTTGCCCCAAAACAGAAAGAATTTATTATTGTTACACTGAAATCTTTAGGGTACG
TGACACTTATGTGTGGCGATGGAACCAACGATGTGGGAGCCTTGAAACATGCTGACGTGGGTGTAGCAATCCT
CGCAAATGCACCCGAACGTCCGAAGGAGAAGCGTGAAGAGCGCCCAGAACCTGAACCA
CGTAGGCCATTACAGCTCAGGGAACCTCGTGACGCTCGAGCTGAAAGCCGGGCTGAAGCTGCAGCCAGACTAC
GCAGAGCTATAAAGAAGCTCGAGGAGGATGATCA
>103791930_2 DT662542.3
GCACGAGGAACGCGTCGTGTAATCAGCGGATGGCCCCTAAATAACGTACATTATTATATGTATTCTAAAACAT
CTTCCAGTGATCTTGTAATTAATTACGATACGTAGTAGTAAGTTAGTTGTAAGTATCGCTGTACCTTATCGGG
CCTCGATGTATCGACGCTCGAGGACTTCACAAATTAAAACGGATAAGTTAGTGGTAGC
GATGTCGTTTGTGCGTTTGGACTCTTCGACCGCTTCGAACCCTTAGCTATTAGTGAATGTTTCGATATATGTG
TTCAATTAAATAACAATATTCATATTATTATTATTATTATAGCGTTTCTCATTCACCGTGTATCGCGTTTACC
GTATACCGTACAGCCGTTGCGGTCTTGACAGATCTCTATAGGTTGCATATATCTTATT
GCCTTTTCACTAATCGCTTTTCAACCGCAACTGTTCGCCGGCTAGTTCGTAGTTGATATGACCAGTTATATAT
ATATATATATATTTATATTTATCTTATTATACATACATGTCAAATGATATTTTTACTACAAAAGTTTTGGATA
ATAGTTTATTAATTAATTAATTAAAATATATATATAAATATATATATAGAAAGGCACGGT
TTATGCAACGTAGCGTACGGGCAGTAAAGCACGCGAAGGCCCTATGAACCTCGACGCGACACTGTTAGGTGCC
AAAGAATATTTTATCAAAGATATGAGATGTCGTGGTAATCATCGATCGCATCGCCGCTTAGAAGACATATCTT
CCAAAATTATATTTGAAATTAGTTTTTTTTTCTTTTTCGGTACGGGTTTTTGATCTGA

CGATGTTCCTATGCCA
>103791942_2 DV500985.2
CTCTGATAAGGCCGGGCTGTCGATGGGCGGAAAAGCGATTTTGTGTATAAGGAGCTGGCTGAGCAGATCACCT
TTCAAATCAAGCCCTTCGGCAAATGACTGTAAGCCCACCAGCACGCTGCGCTCACCGTTGGCGACGCGTTTGC
GGTGCAGTTCAACTAAACGGTAACGCGGCTGATCGCCCTGAACCAGCAACATCAGACG
TAAATCCGTCACATAGTCGAGAAAGCGCTGCATCGCCCGTCCGCTGGCAAACAGTACCAACATACCGAGATGT
TTTTTGCTCTCCACCTGCTTACGGAAAAAGGCCGCCATTTCGGCAATATGCTGCTCTTCGTTGTCGATGGATT
TTTTTT
>103791951_2 DT662034.3
GCACGAGGGTAAGACATAAACCACTTTGAAACGTTTCGCCGGCAAATTAATTTAGAAAATACACTATTAAAAT
GCCTGCAAAATACAAAATTGTTATGATCCGGCACGGAGAGAGTGAGTGGAACCAGAAAAATCTCTTCTGCGGC
TGGTACGACGCCGACCTTAGCGACAAGGGTCGTGAAGAAGCTATTTCTGCCGGCAAAG
CTCTAAAGGCAGAGGGCTACCAATTTGACATAGCCCATACATCTGTCTTAAAACGTGCCCAAATCACTTTAAA
CAGTGTACTTCAGGAATTAGGTCAAACCGACATACCTATAAATAAAACCTGGCGTCTAAATGAAAGGCACTAT
GGTGGATTAACAGGCCTTAATAAAGCTGAAACAGCAGCTAAATATGGAGAGGCTCAGG
TACAAATATGGCGTCGTAGCTTCGATATTCCACCACCAGCTATGGAGAAGGGCCATGCATACTATGACAATAT
TGTAAACGACCCGCGCTATGCTGGTGACCCGAAACCAGATGAGTTTCCAATGTATGAGAGCTTGAAGCTTACC
ATTGAGCGAACTCTACCATATTGGAATAATGTTATTGTGCCTCAGATTAAGGAGGGCA
AAAAAATCATCATCGCTGCACATGGCAACAGTCTTAGAGGCATTGTCAAGCATTTAGATGAACTTTCCGATGC
CGCCATAATGGAATTAAACCTGCCAACTGGCATTCCGTTCGTATACGAATTAGATGAAAATATGAAGCCCGTG
GATTCAATGGTCTTTTTAGGTGA
>103791954_2 DT661872.3
GTCCCATACTCGACCATCTTAGAGACCATTTGATAGCTAAAAATGTGGCGGCGGAAATCGCTAATAAATTATG
TGACTCCGTGGCAGAGAAACTGGAGGGCAAGGTGATGGGCACCTTCGACACGGTGTCCCGCACCGTGCGCGCC
GCGCTCACCGCCGCGCTGGTGCAGCTGCTGTCCCCGCGGCGCCGCCTCGACATCCTGC
GGGACTGCTTGCACGCCAAGAACCAAGGGCAGCCCTACATGATAGTCTTCTGCGGAGTCAACGGCGTCGGGAA
GTCCACTAATTTGGCCAAAGTATTTTTCTGGTTGATGGAAAATGGCTTAAGAGTGTTGATAGCAGCCGGGGAT
ACGTTCCGGGCCGGCGCCGTGGAACAATTGAGGACACACGTCCGTCGTCTGAGCGCCC
TACATCCCGGTGCCGTGGAGCTGTTCGAGCGGGGGTATGGTGCTGATGCCGCAGCCGTTGCGGCCGCTGCCAA
GAAATACGCCATAACAGCTGGGTACCACGTGGTGCTGGTCGACACTGCAGGGAGGATGCAGGATAACGCCCCG
CTGATGAAGGCACTCGCCAAGCTGGTGACGCTCAACGAACCGGACTTAGTTCTGTTCG
TGGGG
>103791957_2 DT662284.3
GCACGAGGGTTGGATCCACTTGAGGTCCACTTGCTTGACTTCCCTAACATTGTAATAAAGGGTTCAGAACTAC
AGCTGCCATTCCAAGCTTGTCTCAAAGTGGAGAAATTTGGTGACCTCATTCTCAAAGCGACAGAACCACAGAT
GGTGTTGTTCAATTTGTATGATGACTGGTTGAAGTCTATATCGTCATATACCGCATTC
AGCAGACTGATTCTTATACTACGAGCTCTTCACGTAAATACGGAAAGGACAAAAGTGCTCCTAAAACCAGATA
AAACAACTCTAACCGAGCCTCATCACATCTGGCCGACCATGTCTGACGATGACTGGATTAAAGTGGAAGTTCA
ACTCAAAGATCTAATTTTGGCTGATTATGGCAAGAAGAACAACGTAAACGTAGCATCT
CTAACACAATCCGAAATTCGCGACATCATTCTCGGTATGGAGATATCAGCACCTTCAGCCCAAAGACAACAGA
TTGCCGAAATAGAGAAACAGAGTAAAGAGCAGAGTCAACTGACCGCTACCACCACTAGGACTGTCAATAAACA
TGGCGATGAGATTATTACGTCGACGACGAGTAACTATGAGTCGCAGACGTTCAGTTCT
AAAACCGAATGGCGTGTTCGTGCGATATCCGCGACTAATCTACACTTGCGTACGAACCACATTTACGTGAGCT
CTGATGATATCAAGGAGAGTGGCTATACATATATTCTACCNAAGAATTTTACTCAGAAGTTTGTTACTATTTC
TGATTTGAGAGCCCAGATTGCCTGCTACCTCTATGGTACATCGC
>103792005_2 DT661939.3
CGGCACGAGGGTGTTTACCTTGAAAAGCTAGCAACAATAACAATGATGGGCAACAACCTATCAGGAAACCTAA
AGATAACATACCAGCTATTCAATTATTTAGTAAACTATCCACAGCCATGAAAGCTAAAAGTCAAGAAGTTAAG
GGAAAATTAATAGAATATATTGTCAATCCCAATTCAACAACTAATAATGGAGAGTGGC
ATGTGTCTGCAAATGATAGGAAAGAACATAGATATAGAAATGTGCCTCCCGTTTTTAGTATAAATGATGAAGA
AGAAGATTCTCAATCTGATCATCGTAGAGATTTCTCTAATGCTGATAATTTTGATGAAATTGTAGAGGTTCAA
TCGTTCCTTACAAACCAGAATGTTATAAATAATTTTTCTTGCCAAGAGGTTTTATCTA

ATGGCTATATGTTTGACTCTCATTTAGTTGTCACTGAAACACATTTAATTATCTTGAGGGAATTACCGAACAA
AAAAGGACTAGCCAAAGTATTGTCAAGGCGGCCACTTTCGACAATAGTAAAAATTACAGCTAAAAAAAGACAC
CCAGAACTGATCACTTTCAAATATGGCATACCAGATGGTGATAACTTATTAATTAAAG
ATATGGATAGATTTCTTATTCCTAATGCTACAGTGGCAACAAAAATAGTTTCCAATCAAATTGTTTCTCAATT
AGATAACAAATTACAGTGAAAATTAAATAAAAATTAAATTGAATTTTATCATGAAAATGTAGAGATGATAGTA
ATAAATATTAGTTACTAAACTTC
>103792015_2 DT662300.3
GCACGAGGGTTGTACCTCAACCGACCCTGTCCACTCTGTTACTTTATGAAGTGAAGTGCTTTTGTTTTTCGGA
TAGTTGAAACACATCATGGACGAATGACTACAAACTTAAGTGTTGGAAAAAAAATCTAGTGCAAAAACTTTTA
AACGAGTGTGAGTGTTCATTCAGTGCTTTGTTATAATGGAGTTTAAGTCACATTTCTT
GGCGGCTGCGGCTCTCGTAATTATCGCTCTATCCTGGTGTGATGCTGCACGGGATGCATCTTGTCCCAGAATC
TGCGGTCCGACGTTGCAAGGCGAGCCTGTCTGTGCTACAGATGGCTACATATACCCTTCGCTGTGTGAAATGA
GGAAGAAGACATGTGGAAAAGGTGTAAGGCTAGCTCAAGACCAAGGTTCATGTTCGCG
CGCCCAAGGTTCAAAATGCGAACATCGATGCACAGCTGAACGGGATCCAGTCTGCGGTACCAACGGCCGTACT
TATTTGAATAGATGTATGTTGCAGGTGGAGATATGCAGAGTAGGTATCAGTCTCTCCCATCTAGGCGCTTGCA
ACAACATCAGTGCTCACAGAGAGAACTGCCCCGTATATTGTTCCCAGGCTCCTTTAGA
CGGA
>103792021_2 DT662318.3
GGCACGAGGTTTAATCTTAATTAGAATTATTGCGCCTTATATTACTATTAATTATTGTATTTATTAAAAATAA
ACATAACTCGATGCGAGAAAGTTTTAGTAAACCTTTTATTATAAAAAATTGTATTTTGACTTGGCTCGATAGA
AAATATAGCTTAATCGGCTCGAAGGGTTACGAGTTCATATTCAACTAAGGTAATATTC
TTATACAAAATTTTAAATCCGATACAAGAATGCTTGAGATTTTTCGATTAGTTTTTGTAATATTTCGAATGTT
GTTTAGTTATTCATTTTGTTTGTTTGTTTCATATAAATATTTTTTTTTGTTTTATGGATTCTTTCTTTCTTTT
TTATTTTATTTAACCTTCTCAATTTCAAATATTTTAGGTGAAGATGATGATAATAATA
ATTTTCCCTTAAAAGTGTTATTCAATATTCTTTTATAATTCTAACACCTGTGTTAGAAGCTGAATTTGAATAA
CAAATACAAATTTAAGATTGAATACGATATTTGCTAACATTTAAGTGGAGTAATCGCTGGATAGAACAGAACA
TTCTTAAGAGTTTTTATAGAATTAAGAGCGACGTTGAGCTCTACCACTGAACACGCAA
TGCATAAAGAATTAGGAGGAAAGTGAGGAGTGTATTAACATTAGACTTTCTCTGACAAACTTGCTACATGCTG
TATACAACATGAAGCTGAGAGAAATGTAATTAAAAGTTGATTAAAGTATCTTTTTAT
>103792024_2 DT661963.3
GCACGAGGATTTCATCCGTTTTATTAGCAAACGCATATATATAGTTGCACTCATAAATCTTGCATTGTGCGTT
ACAAAGCGGGCCGCGCGTGCGCACCCTTTGTACCGCACCCTCGCGGACACTGTCTCTAACGGTTATATTGATT
TTTTAACTGTCATTCTACCACGTCCGTGTCCATATAGTTTCCTGTAGATCAAGTAATC
ATTTTTGTTTTTTTTTTTTATATATATATATACTCATGTAATTTGGAGACAATGGTTGTGGTTTTGATTCCTG
TTTCGGGGTATTGTTTTTTATATTTTTTAAGTATATTCGTTTTTGTTGTGTGGTTCTACAAAATTAAATTAAG
TTCCTTAAAGTCATAAGGTTGCTTTTCGCACATTTAGAAAGCGGAAAGATAAAAAAAA
AACATATAATAATTTATTTGTAACCTATATTTGGTGATTGAGTGACGATTAATAATTTTTCTATATCGCGCAT
ATCGTATCTTGATACTATGACTGCCGGACCAGCCCAGCGTTGAATAAATTTATTGATAACACATATTCGTCGC
ATAGGTGATAACTAGGTTATTATGGATTTTTTTTTATATGAAGATAACATCCGAAACA
ATAGTGTGGGTGATAGGAAACTTTTTTTTCTCTTGTATTTCAATGCAAACACGTGTGTTGAATTTTGAAATTT
TCTACTGATAGGGGAATGGAAACGGCCGTCGGCATCTGCCAAC
>103792030_2 DT662717.3
GCACGAGGGAGACGCCTATATGTACATATTATATTGAAAAAAAATAGAAAAATGCTGCGTGTCGGAACTTTTG
TGGCGATTTTTGTAGCGCTAGCGGCGGCTGCGCCAAGACCCAAAATCTTCGAACTGGACCTGTCACCGGAAGA
CGCTCAGAAGTATATCAGCAACCCCCCCTACTCTGAACCCAAGCTTGCCGGAAGAACA
GCTGTATTGCCTTTGGTAAAGTACAATGACCCCAGGTTTCGCACGGCTGATGCCGGTCCCACCCTCGGCCACT
ACTGGAAGAATGGACATGAGATCGAAAACCCCGATGACTATGTTGAAGAGGTATACAATGCAAATCAATTCCA
CGGTCAAGACGGTCTAGGCGCATACGCATACGGGTACGAGACACCAGAGTCTGCTAAA
ATTGAGAACAGAGTTCGTTCCGGAGATATTGCCGGATCTTACACTTACAAATCGGGAGACAACGATATTAAGG
TTCGCTACTGGGCAGACAGCCAAGGGTTCCATCAAGAGGACAACATTCCCAAAGTAGAGCTGAAGCCAGTTGA
AGAATCAGAGGATGTAAGACAAGCTCGCCTCGCTCACGAGAAGGCATGGAAGGAGGCA
GCTGAAGCCGCTCGCAAACAACCTGAATTACCTAATGGCTACTGGAGCGGACACCCTGAGCTCGTACCAGCAG
CGTCCGAACAACAAGCTGCATACGTCGCAGCTCCCAGCGAAGTCAGTCAAGTCAGCCAAGGGCAACGTGAAGC
CCAGGCTTATTATGGCGGTCAGGGCTTCAAAGGTCACAGTACCCATCTCCAGCTCCTG

TGAAAATTGAGGAACCGGAAC
>103792033_2 DT662090.3
GCACGAGGCCGATTCATAAAATTCCTCTGCAACTACATTAAATTACTAAAAAATAATTATGGCCCTTAATAAA
CTCAGCATTGATGCCCTCAACTTAAGCGGAAAGCGCGTGTTGATGAGGGTGGATTTTAATGTGCCATTAAAAG
ATGGAGTTATTACCAATAATCAACGCATTGTAGCCGCTTTAGACTCGGTTAAATATGC
TCTGGATAAGGGTGCAAAATCTGTAGTTCTTATGTCACATCTTGGTAGACCAGATGGTCAAGCAAATAAAAAA
TATACCTTGAAACCGGTAGCAGAAGAACTTAAGAAATTATTATTTAAGAAGTACAGTTTTTGTCAGACTGTG
TGGGAGCTGAAGTTGAAGCAGCTTGTGCTGATCCAGCATCTGGTTCAATAATTTTACT
TGAGAACCTTCGTTTCCACATTGAAGAAGAGGGCAAGGGTGTGGATGCTAGTGGTGCTAAAGCCAAGGCTGAT
CCTGAAAAAGTTAAAGCATTCAGAGCTAGCTTAAGGAAACTTGGAGACCTCTACATTAATGATGCATTTGGCA
CTGCTCATAGAGCTCACAGTTCCATGATGGGTGAAGGTTTTGATCAAAGAGCCAGTGG
ATTTTTACTCAAAAAGGAACTCCAATATTTTGCTAAGGCATTACATGAACC
>103792054_2 DT662061.3
CTGGAGAACCCGTACACCGCTCTTACTGCCCATGAGCTGAACCGCAAGTGGTCGGACGTGCCCCAGCTGGTGC
CGCAGCGCGACGCCACGCTGGCGGCCGAGCTGCGCAAGCAGCAGAACAACGAGACGCTGCGGCGCCAGTTCGC
GGAGAAGGCCAACGCCGTCGGGCCCTGGATCGAGCGCCAGATGGACGCCGTCACCGCC
ATCGGCATGGGCCTGCAGGGCTCGCTAGAAGACCAACTGCACAGGCTGAAGGAGTACGAGGCGGGCGTGTACG
CGTACAAGCCTTTTTTTTTTGAACTGGAACGCATCCACCAAGCTGTACAGGAGGGCATGATCTTTGAGAACAG
GTATTCTCAATACACAATGGAGACACTCCGCGTCGGCTGGGAACAGCTGCTAACGTCC
ATCAACCGCACCATCAACGAGGTCGAGAACCAGATCCTGACACGCGACTCTAAAGGCATCACGCAAGAACAGC
TGACCGAGTTCCGCGCTTCGTTCAACCACTTTGATAAGAATCGTACTGGTCGTCTAGCACCAGAAGAGCTAAA
ATCCTGCTTAGTGTCCGTCGGCTACAGCATCGGCAAGGACCG
>103792073_2 DT661719.3
GCACGAGGTTACGGCCATAAAAATCGTTAAAAATCTTCGTCATCGGCAGTTCATTTCTAAAGTGACACTATGA
GTTCTGGAATGCCTGAATTGGGCTCCAAGATAAGCCTAATATCTAAAGCAGATATTCGCTATGAAGGCCGACT
CTTCACCGTCGATCCTCAGGAATGTACCATCGCTTTAGCTAGCGTGCGATCATTTGGT
ACTGAGGATCGTGAGACTCAGTATCCAGTGGCACCTCAGAGCCAAGTGTACGACTACATCTTGTTCAGAGGTT
CTGACATCAAGGATATAAGAGTGTTGAACAATGTGCCTTCATTTTTTTACGATCCGGCGATTGTGCAGATGTC
TGTTCCGCCATCTCTCGGCACTGGTGGTCAGGGGCAGTATACAGGGCAGTACAACCAC
CCAGTTGTGGGACAGACACAGTACCCCCAATATCATCCGATGGGTGGTTTTCCAGGAAGTGTCCACCCTCAAC
TTAATAAGACTAGTGAGTTATCCCCACAAACTTCTGTGGATTTGGCGCAGACACAACCTCAGCCTGTGACAGC
ACCTATAGGATCTGGGGTGCTTCACAACAACCAAGTGAAAGACCAAGGTTCTATGCTA
GATCTTATTGGAGGAGGATCACAACAAAGTGCTTCAAGGTCTGGTACTCCAGCAGCTGTAGGACATAGGAAGA
GCCCCACTGGCTGATCAAGGCACTCAGGCCGGCTCTGCCAGTGGTTCGGCC
>103792076_2 DT662323.3
GCACGAGGTTTTTTTCTTTTTTTTTAATGATACATAAATATTTATTAACAATTAAAAATTATTCGACACCGCG
ATGTTAGACAAGAACCAAATTAAAAATATTCCTCGAGTGTCTGTTGCAATGGAGATTCTTAATAAAAGGAAAA
AAAAACAATAAAAAATGACGGTAATTTGTGTCTGTTGTATAATTAAAAATGTAGTGTA
AAATTAACATTCCCAAGCAGCACCTCGTACGGATAAAAATAAAATCGATATTATAAAAAAATACAATTATATT
TATATAATCTATTCACATAAAGCTTAGACCGAAACGTACGCTACACTGGAATGAGTTAAGCATAACATTTTAA
AAATATAACTACTAAATATACTTCAATATTACCAGAATAAAAAAAATCATGCATCCGA
CAGATAATAAATAATTATAATAATAAAAATAATCATCATTATATTAAAAATAAAAAATTGTAAGCGAATAAAA
CAACGTCGTTGTATCATGCAAAGAATTGAATTAAAAAATAAACTGACACGCCACAGATTCTTCTAAATGCAAA
TAATATTTCAAATATACTTCATGATCATACTATAAAAGAAACGATTACGAATAACAAT
CGAATAACATTATTCAAAATCGATTCAATATCGATATTACCTCATAAAAGCGTAGCAATACGTTAATTATTAA
AAACATACAAGTCTCAAAATTTCCTGTAAAAGTTACAACATTATAACTTATTTGTTACAGTATGAAACTATCG
ATTTCTAAAAATCGATATTAAAT
>103792079_2 DT663186.3
GCCTACGTAACGTAATGTCCTTTATTTATTGCAACTACTTTTTATTGGCGTCTTTTTGACGATCGGTTATTAA
TTAATTTGTTACGTCTATATTAATTTAAATTATTGTCCAGAAATGGCATCGTTAGCGTTTAAAGTGAAGAGTA
AAAATGGCCAGCAGATTTTGAAAGAATTGACCTCTGAGAGTACTGTGGGTGAATTAAA
AATGTTCTCTCTAGTATATCAGATGTGGCTGCAAGAATTGCGTATTGTGTGGTATCCCCCTAAACCTATAGAT
ATTATAATGATAATAAAACTTAAGTGAGATTGGTT
>103792085_2 DT662352.3

GCACGAGGGAGCGCCCTTCACTTGCGTTACCAAATAAAACTATATATTTGCTATATTTTTATAATATATTAGT
TTTAATGGCAAGAAAAATAAATTTATAATTAATTAATAATCTGTTTTATGTCGTACATAGTAATCTCCTTTAT
ACTTTTCGTATAATTCACGTTTTGCTTATCTTCGGTTTTCGTCGGAAGCGTTCGGGGT
CTTTCGGTTTGGCGACTTTCAAAATGGATGCAATTCATTTAATAGTACTGTCATTACATAAATGTTAGTGTTG
CCATTTTCAGTTTTAGAATTTTTGTGTTTTTTATTAAATTATCTCAATTTACTATAAGAAAAAGATTAATTAA
ATATTACTATGACGGCTAACAAAAAAATTTTAGAAGAAATCCATCCCTTGAACATAAT
CAAAGAAGTACAAAAATGGCCTTTACTGTATACGAAAGACAGTCCGGAAAGAACAAATTTGCATTTTAAAACG
AAAATTTGGAAGGAAATTGCAAGAGCATTGTTCAAAGATTGGGAATCTTTGAACGAAACGGAAAAAGATAGCA
AGAGCTTAATCAAAAGATGGCGAAATTTACGCGACACATTCAAAAGACAACTTGTAAT
AGAAAAAAAAAATAAGGGAGGGCTGCAATATAAAAAAGACAACTTATGTTTACTTCAAGCATATGCTCTTCTTA
CTGCCTCATATCGATACGCCCGATACCGTAGAAGAAGAAGATCGCATGCTAGAG
>103792097_2 DT662918.3
GCACGAGGAGCAGACTACCTATGCAAAGAGTTGAACCACTGTATGGAGGGCATGGGCACTGATGAGGAGGTCC
TCGTGGAGATACTGTGTACTCGCACCAAGCCGGAGATAGCAGAGATTGTTGAGAATTATGAGAGATTATACAG
TCGTCCTCTCGCTGAGCACATGTGTTCCGAAACTTCCGGTGACTTCCGCCGGCTTCTG
ACACTCATTGTTACTGGTGCTCGTGACGAAGAGGCGGGCGTGGACCCTCAAAGGGCAGGGGAATGTGCCCAGC
AGCTGTACGACGCGGGGGAAGCCAAGTGGGGGACGGATGAGGAGGTTTTCAATAAGATACTAGCCCACGAGAG
CTTCGAGCAGCTCCGCTTGGTGTTCGACGCGTACAAGCAGCTGTCGGGGAGGACCATC
GAGCAGGCCATCAAGGCGGAGATTGACGGAGAGCTCAAGGATGCGCTCTCTGCCATAGTGGAATGCGTGGAGA
ACGCGCCGGCATGGTTCGCCCAGCGCCTTCGCGACGCAATGCAGGGCGCCGGGACTGCTGATAAGGTGCTGAT
AAGAGTGATCACGACCCGCTCGGAGATAGACCTGGGCGATATAAAGAGGGAGTATGAA
CGATTGTTCGATAAGACCCTGGAAAGTGATATTTCGGGCGAGACTTCTGGCGATTACAAACGGGCCCTCGTCG
CCCTCCTGGGACCTGCTTAGAAGACATAGTTTTAGAAAACGCCTATTTTTTTCTCATATATTTATGATCATTA
ATGGAGTATACTCGAATTTGTATAAGAAAAATGAATGTTAAATTTTTTTTTATAGATA
TT
>103792103_2 DT663103.3
GCACGAGGGATTCTATTTATATCTCGGTGTATTTTTGAATTATTTTCTTTAAACCAAGAAATGTAAGGCGCAC
ATTTAAATAAACTAGTAACTAAACTAAGCCATGTCATCTAATGGTAACGGCGGCAACTATGCACCCCTGAAGC
AAGCACTGACCGACTCCGAGTCCGAGGACGAACATAAGCTAGAAAATGCTGTGCACAT
CAAGGCTTCCGATAGCTGCAACAATTTGGACTATAACAGTGGACTACAATCTTCAAAGAATTACAGCATGAGT
GACATGGACGACTATAATACAAATGAAAAAACTGTAGAAAGCACAATGGATAACGTTAGCTTTTTGCAGTCGG
ATATTTCCAATAAAATGTCGTTTCCACGTCGGTGTGCGCTAATCGCGTCAATTTTTGT
TTGTATTTTTACTGTAAGTATATTTCTATGGGGCATACCTTGTTCAGATGTTGGTAGCTGCGCCAACAACGAG
TGGCAAGATAAAACAACCAGTTGGGAGATTCCATATGATGAAATTGAACTGTCTGGTGCGGTTCAAGTGGTTG
ATGGTGCTATACCCAATACGAAGAATCTTATATTTATATATCGCGGCAATCATATGAA
AGAGGAAAAAAATAATAATGATAATGTGAATGGAGTTTTGTTAATTGTTGGTAACACAGGAAAAGTTGGCTGG
TATACAANGGAAGCCAGAATACCCACAGAGATTAACTGCCCTCTCATTGACGTGAATAGAGATAGGCAAAAAG
ATTGTATAGTGTCTGGTTCTGAAGGTTACTCGCAGCTCTGAACCCCTAT
>103792123_2 DT662798.3
GCACGAGGATTTAGCCTTTGAAAATACATCTGACTTTGACAGCCTAGAAAGGAAAATGTCCAAAAAAAGAAGA
TTTTCTTTAGATTATGGGCCAGAACTTAATCATAAAGATATATTGATACAAAACACTCCAATGTCTCGCGATA
GATTAACACAAAGTTGGCGGGACTTAAAAAATACGTCTCATTTGCCTTCTCAAGTAAT
AAGCTTCAATGTTGGTAATAAGAAAAGTCTCAATAACAACAGTAATATCTTCATTCATTCAATTGAACCTATA
TACAATAACATAGAAAAAGCGGGACCGCTGACTATTGAAGTTCGTGGATCACCTGTAGAAAATAGACGAAATG
AAAATGAGGATTTTGATCCAGACACTCTTGACAGGAAACCAAAAAGATTATTTACAGA
AACGACAGAAAATAAAAAATGTGTTGAGAAAATTCTTCTAAAATCTGGTGGTAGTTTCAAACATAAAATAACC
GTACCTGAAACTGAAAAGCAAAAGAGTCCTCCTGAAGCGGCATTTACACGAAAAATAGGCAGCTTAAGGCAAA
TATATGAAGCTAATAAAAGTAAATTACTTAATGAAAGACCATTATATGAGCGACGAGG
TAGTGTTCCCTATGGCACACAAGAAGTAGCTGCGTTTGCTCGGAATTTCGAANACGAAAATGAAAATGAGAGT
AAACCGCCGTTATCGAAAAATCGAGAATCTAACTCTAAAAGCAATACACCAAGAAATAGTCCGCCAAGTGATA
GATATCGATCATC
>103792132_2 DT662134.3

GCACGAGGGTTTTTTTTTTTATTTTTTTTTTTTTTTTTACACATAAATGAATAGCTTTATTTTGTTAATCTTA
TTTAATTTAATCTTGCAATAAAACTGTACATGATTATACATATTTATTAATCTAAATCAATAAAAATATATGT
TTATTTAAATGGACACGTTTCATTTAAAACAAATGAAAACATTGCCTATATATAATAT
ATGTATATATGAATCTAACTAATATAAACTAATTGTTCAAACAAACAATTGCACTGAAAGCGTGTGAAGATCA
CCAATGGCTGAGGCTTGGATTTTTTTCATGTAGGACCATACTTTATACAACTTCCTTGCTGGGCGTCAAAATG
CCTAAAGCTTCTTCGTATCCTACTTTTGTTCCTTGTTTGTCTTTTTTGGAACTTTTAC
TCTTCTTTGATTCTTTTTCTTTAGATTCTTTTTTTGACTTCTTATGTTTTTCAGTCTTTATTTTCTCTCGAGT
TTTGTCATCGGAGGCTATTTCCATGGTTTTATCCTTAAAGTTATCATCAGTAACAATGAGTAGGTTTTCTTCT
ACACCGTTATCAGTGGGTTCTGCTAACATTAAGTCAAATTCTTTATTATGCTTAAAAC
TCTTTCCCTTCTTCTTATGGCCAGATTTTTCGGAAGATCTTTTTTCTGTTGATTTATTTTTTCTTAAATTCTT
TTTCCTTATTCAATAAGC
>103792138_2 DT662040.3
GCACGAGGGTTACCCCTAGAGCGATTTTAGCGGAAGAGCGAGGAATTTTAGCATAGAACTACTATATCATACA
CAATTGGAAACAGATGGACACGTTGTTATATCACCTTTCGGTATATGGACTCTGATGACTGGCGTCACGCTGG
GAGCTAGCGGTAACAGCAGGAAACAGCTGTCTCGGGCACTACTTCTACCAAGCAATGA
CAATTCCGTCATAAATGGATACACAAACTTGACGAAGACCGTCCTCGACCCGCGGACCGAGGGCGTCAAGTTG
AAAAGTAAAAATTTTGTGTTCCTCGACAAAGGTTTCAATATAAATTCCGATTTCAAAAAATTGCTGACGAATG
ATTTCGATTCTAAAGTGATGTCTTTAGATTTTAAAGATCCCACAGCAGCCAGCGTAGC
AAACGCTTTAATAGAGAACTCTGGCGCCACCGTCTCCAATGTACTAAGGTCAGACGATTTCGCCGTTTCAAGA
ATGATTTTGACAAATGTTATATCGTTCAAGGGTCTCTGGGCGTTACCCTTCAACGCATCAGACACACAGGTTG
AGCCATTTTACAATGAAAATAAAATGGAAATAGGTTCAGTGAAAATGATGTATCAAAG
GGCTCCATTTCCATTTTCAAATGTGCAAAGCCTTCAAGGTCACGTGCTCGAACTGCCCTACGGAAACGACAAC
AAGTATTCTATGTTAATCATATTACCATATCCGAGAATGAAGGTCGGCGACGTCTACAGACGATTATCAAAAG
TATCGCTGT
>103792150_2 DT661806.3
GCACGAGGATTCCCTTTAGGAAACATTTGAATCGTACTTATAAAAATGTAAATAAAGAAAACACAAAAAATGT
TTGCACTCTAAGCCTCCGAAATAGTATTCTAGTTCTTATAAAATAAAATAAATTGATAGTTGGAACCTTTTAA
ACCCAGCGATACAATAGAGTCAGAGAGGAATTCCATATCAAAAGTCTAGATAATGTGT
AAAGACAGCCGCAACCACAGATAGCTTTTATATAATTAGACTGGCAGTTATCTTATCTATGAAGTTACAATTC
TTGATCAACAACTTAAAATCATATACAACTTATGATAATCAATTTAAAGACTCGAGTTAATGGTTGCTAAGTG
TGATTTCGGAAACTTATAAGGGATGAATAAGTTGATAAATTGACTAACATAAATATAT
ATTTTTTAATCTCAACATTCTTTTATTTATACAATTAATAAATTTCTAAACCGTACTAGAAATTCATCTACAT
GTGTTTCCGAATTCATAAGTACTTCTTGGCTAGCATTAGCTAGCTGCATAGTCTATCAAATGTTTCTTTTCCT
ATAAATGAAGTATGCAAAAGAGCAACTGTTGTTTTTGGTCGGCTTTTCTCAGCTAAAC
TGCCGTCCGAGCCGGTGGTTACTTCCTATAGGATTGATGCTACTGACATAATTATGCAATTCGAGTCAAAAGT
GCTTGTATAGTACGGTCGACAATTCGCGGCTGCCCT
>103792159_2 DT662411.3
GCACGAGGATCAGTTCTCACTCAATGGGAACAAAGTAATGTAGAACTGAGCCGTGGTTTGGACTTCATGCCGC
GTGGTAGTGTTAACGCACGTTTTACCCATTTGCAGCACGATGAATTTGAATATAACATTGAAGTAAATAATTC
AAGTGGATCAGGAGTTATGGGCACTTTCCGTATCTTCATAGCGCCGGTTTTGGATGAA
AACGGTAAACAGCTACCGTTTGTGGAACAACGTCGTCTTATGATCGAATTAGATAAATTCTCACAAGCCATCA
AAGCCGGTAGTTCCACCATAAAACGCAGCAGCCTATTATCCTCTGTCACCATCCCCTACGAGAGGACGTTCCG
CAACCAGAACTCAAGACCTGGCGACCCTGGCTCAACAGCAGCTGCCGACTTCGACTTT
TGCGGGTGCGGCTGGCCTCATCACATGTTGATACCTAAGGGAACTCCAGAAGGTTACCCTATGGTGCTGTTCT
GTATGGTGACCAATTGGAATGATGATAGGGTAGTACAAGACCTGGTGGGTACTTGCAACGATGCATCCTCCTA
CTGTGGTATAAGAGACCGGAAGTATCCAGACCGACGCCCCATGGGATTCCCCTTCGAC
AGACCAGCCTCTGCCGCAACTTTAGGGGATTTCCTGACAGCCAACATGGCGGTAACGGAATGTAAAATCAAAT
TTACGGATGCCGTACGACACCCCAACAGTAAGTTTACGATTAGAGTGTAAACGGTTTGCTTTTGTTTTGAATT
CCAAAATAGTTTAAAAAAAAAA
>103792182_2 DV501032.2
GCACGAGGCCACGACCTTGAAGTGCCCCCCTCCGCGTTATAGCCCCATCCCTAGCGATGGGATAGGGTTGCCA
CTTGATGGTTTTATTTTTCTTTTATTTTTTATAACTTTTTAATGTTGTTACCGACCGGTGCGTCCGTATGAAA
TAGTATTTTATGGTCCCCAAATTTGATGTTTTATTTTCTTTATATTTTTATATAATTG

GTTTCCTATTTTTTTTTCTTATATTTGTTATGTGGCAACCCTGGTGTCCCGGTGTCACGTGAGTGACGTCACA
CGCTCGTATCTCGTAGGTATAGTATTTATTGTGATTTACTTTCTACTATAGTTATATTCTAGTAGAACGGGCG
CTCCGGCTCGTTTGTTGTTTTTTTTTTTTTAATTTCATCGCGAATTTAATAATAATATT
TATATCGATATAAAATTGCATGAAATTTCTCATCCGTTGTTCATATTGATCGATAATATGAAACCGACCGAGT
TTATCTTGTATTGAATTCTAAATGGAAAGTTCGATCTAATGCGAATTATTATTAGACTTATTAATTATAGATT
ATTATTATTATTTATTTATTTTGTTGAAAGTAATGTCTACTTTGTTCGGTCACACCCT
AAAGTTAGGTTAACTAGTACTTATTGAAATTATTTCGCGTCGTCGCCTCCTTTATTCGAATTATATTACTTAT
TGCTTTAAGATAAAGACAGATTATTTTAATTACGCCTAAAACCTAGCTTTTTTCCCATATATACATATATATA
TATATAGCTAGAATAATTTTTTGTTGATGAAACTAAAAAAAAGTGAAAAAATCCAATA
AAG
>103792194_2 DT663424.3
GCACGAGGCACCAGCCATAGAGACCCATTATAAGAAATTCAGCGACATACCGGGACCGTTGGCCTTGCCAATT
TTGAGACACCACGCTCATGTTCTTCCGAGGATTGGAAGCTTCCACCACACTGTGGGTCTAGGCCTACTAGAAG
GCTTACAAGACCGCTATGGAGACTTGGTACGGCTGGCCAAAGCAACTAGATCGAGACC
TGTGCTTTACGTGTTTGACCCTGAGCTTATGAGAGAGGTATATCAAAGCAATGCCACCGAGCCTCCCCAATGG
GCCAAGTCGCCATTGTCTACACATAGGAAAACTGCGCACACTCAATGTCCTATGCATAGTGACGAAACAAAAG
CAATATGGGCTGGAATGAGGACATTGTTGCAAGATGGCACGCTTCTTCGAAACTATGA
TAAGGCATTCGACGGTATAGCCGCTGATGTCACAAGGAGGCTCGGTGAACTTCGACACGCCGAAAATGCACTG
AACGAGGAATTTGAAACAGAAATATATCGCTGGGCGATAGAAACCATTGGAGTTATCATCTTTGGTATGCGTT
TGGGATGTCTCGATGGTAACGTCCATGTGCCTTCTGAAGATAATAGAAGGCCTGANAA
AACTTCAATGGACGACCACATAGACGACCTCTGCTCTCTCTCAAAACGTTGTGTGACCGAACTAACCCCAGCC
GAGAGATTCGTGCGATGTTCCTTAGAAATATCCAACGAAAGCTATCTAGTGAGAAGTGAACACACACTGAAGC
CTGAATCAAAGTCTTTTACAGTGCTTTGAAAGCTTTTGATA
>103792197_2 DT662454.2
CGGACGAGGTTACAACTCCCTCTTTACCTGATTCCTGTTGTCAATTAACCTTAATGTTAGGTAATAATAAAGT
TGAAAACCAATCTGTTTGTTTCTATTCTAGTCTTAAAATAACGACTATTATAAATAAACTTTTTGTTAATCAC
CAAGGCATATTAATTTGTATCTGTACAAGTTTTTATTGCACAACTCTATGGTGAGTTT
TTATTTTTTTAATTATATTTTACAGTATTTGTTATATTTAATCTGTGCAAAATTAGTTAACCTTACATATAAT
TTATGTTAATCATTATTTTATCAGGCAGCTTCTTTATTATTGTAACATCCTTGTATACATATCATTTGTAAAT
AAGTATTAGCATATCGAAACGATCAACCATGAGATTGCTGATTTTGAACTGACATGTT
ACTGCAGTTGGTTTCTAGTAAGACGTTGAATATGCTATTTGAACAACAAAAGGTGTGTGTTGTACTCTGTTGG
TTTTGAATATCGTCTAAAGAATAAAATCATGTATCTTGCAACTTTTTTTTAAATAACACAAAAAAATAATCTA
AATAATTAACTGTCATTAGTTGATTAAGTTCATTTGGCCTAATGTAATAATATTAAAT
GGTAAACACGTATGTTAACTGTCAACATTTAAACATCAATCGAATTTTCTAATTGATCTGTGAGATTGCTATG
AATTGGGACCAATATTGTAACGTACGTGACCGTCGTCTGGTTATAGTGAAATGTTTGTTTTAAGGTTAACATG
CTGAAGTTGTGCATTTATTATTTGTACATTTTACAAACTTACAAAATTTGAGTGTCAT
ACAAAATAAATGTTGTTGCGAAAAAAAAAAAAAAAAAACT
>103792212_2 DT662699.3
GCACGAGGGTCTCGACTTTAATTTTTTTTTTTTTTCAATATCAAATGTCAATTTAATCACTTCTTAAACCTAGC
ATTCTTTAATTAAAACTGTATTACAGTTGTTACTTAGTTACATACAATACTACAACTGACTGTAGAGTGACTG
TAGACTCAATATAAACAAACGGTGACAGAAAAACTAAGTATTATAGATTAGTTGGTAA
ATGGGAATGAGGAATTTTAAGTTTATCGCCCTTCAATATATGTAGATAACTCAATTTCTTCAGGTAGTTCTGT
AATGTTGACATCAAACCGGTCTTGGACTTCATTGAGGATCTTTGCATCGTTTTCATCAAACACCATGGTGATG
GCCAGGCCTTTGGTACCAAATCTTCCTGCTCGCGCCACTCTATGTAGATAAGTATCTG
AATCTTCAGGCATATCGTAATTGAACACTATATTGACTCTTTCAATATCCATTCCTCTACCAAATAAGTTGGT
TGCAACTAATATCCTCTTTTGGAAATCCTTAAACTGCTGGTAACGGGAGAGACGCTCATCTTGAGTCATGTTT
CTATGAATGCCAATAGCTGGGAAGTTCTGGTCTGTGAGAAGCTGAGCTAATGCTATAC
AGCGCTGGACAGATTTCACAAATATCACTACTTGGTTAAATTCTAATACATCTAGAAGCTCAAATAGCTTTTT
ATTCTTTTCATTTTCCTTAAGTTTCACATAAGTGTTGTTGTAAACCATGCAATGTCAG
>103792215_2 DT663094.3
GCACGAGGGCGGCCATCCCGCGCTGCGCGACGCGCCCTTCGAGGCCGTGCTGCGCGCCTGCCCCGCGCTGCGC
GAGCTCGACCTGTGCGGCTGCGACGCGGTCTCGGGCGCGCTCGTGTGCGCCGCCAACGACGCGCTGCGCCGCG
CGCCGCGCCCGCTCACCCTAAGGTTGGCCGCCACCGCCGCCGCCGCGCCCGACCGACA

GCCCATAGAATACGACTGCCACAAGTTATTAACTGTGGACCTAGACGAAGACAGAAGCAACCCTCAACTCCGT
CCCGACTTCGTTGATCATATATTTGAAGAGAGCTCCGATGCCTCGCTGGAAGACTTGCTTTACGACCACGAGG
ATTACAATTTCTTTGGTGAAGATGATGACCGGTTTTTGGATGACGATTTAGAAGATTA
TGATGACGTCCTCCAATTGCATGAGGCCATGCACAACGTCATTCTTCTCTAGTGTACATTTATTTGATGACAA
TATCATTCTTTTTTTTTGTTCAATTCCGTTTAGTATTTTTAGTATGAATGTAGTATTGTGTATTAATTATTTT
AAAAGGAGAGGAGAATTTGCTTTTTATACTGTATTTATAACTTTTGAGATTCATTCAA
AAAAGGTTTGTTTATTTCTGTTTTTTTTTTCTCTTTTTATTTTAGTTTTGTAATAATTGTATTGTAGTACTTA
TACTTATAATTTCTTACAAGTCCTGCGATTAATGAATTTTACATATATCTGTGATGAATGTGTTGCCGCTATA
AAATAA
>103792218_2 DT662799.3
TAATTATCCAAAGTTATTCCCTTAAAGGATAAGAGAGGATGAAATATGGAAATTAAGCATGATGTACCAATAA
AATTATTATTTTTTGCTCAATCACCAGAACTAGCTGGCACACGAGAAGCAATTCTACATTTACCTACTAAAAT
AAGTTATGAACAACTTTTAAATACAATCCTCAAAAATTTTAATTTAGAAATAATAAAA
AATAATATCTTGCTGGCAAAGAATGAAGAAGTTTGTTCTGATATAAAAGATATAGAATTAAGAGAAAGTGATC
TTATTGCTGTGATTCCACCATTGAGTGGTGGATAATATTTTATTTATAAATATATAAATATTAAAACATGGAT
CATTTAAAGTTAACTGTAGATAAATTGTCTGTAGAGACTATTAGCGATTTGGTTGTGG
ATGATAGTTGTGGTGCAGTGTCTGTTTTTATTGGAACTACTAGGGATAATTTTGAAGGAAAAAAGGTGCTAAG
ACTGGAATATGAGGCTTATGAGTCAATGGCCTTGAAAGCCATGAAGGGTATTTGTAATGATGTCAGAGACAGG
TGGCCCGCTGTTCACGGTATAGCAATTTATCATAGGTTAGGTAACGTGCCCTGTCGCG
AGGCATCAGTAGTTATAGCGGTGAGCAGTCCTCATAGACGAGACTCCTTAGACGCGGTCTCTTTCTCTATAGA
CCAGCTCAAAGCGAACGTGCCCATATGGAAGAAGGAGGTATA
>103792221_2 DT662794.3
GCACGAGGGGCCAACCTCGTCGGGCCCTGGATTGAGCGCCAGATGGACGCCGTCACCGCCATCGGCATGGGCC
TGCAGGGTTCGCTAGAAGACCAACTGCACAGGCTGAAGGAGTACGAGGCGGGCGTATACGCGTACAAGCCGCA
CATTGAAGAGCTGGAGCGCATCCACCAAGCTGTACAGGAGGGCATGATCTTTGAGAAC
AGGTATTCTCAATACACAATGGAGACACTCCGCGTCGGCTGGGAACAGCTGCTAACGTCCATCAACCGCACCA
TCAACGAGGTCGAGAACCAGATCCTGACACGCGACTCTAAAGGCATCACGCAAGAACAGCTGACCGAGTTCCG
CGCTTCGTTCAACCACTTTGATAAGAATCGTACTGGTCGCCTAGCACCAGAAGAGCTG
AAGTCCTGTCTAGTGTCCGTCGGCTACAGCATCGGCAAGGACCGTCAAGGAGAACTCGACTTCCAACGTATTT
TGGCCGTTGTTGATCCTAATAATACTGGTTACGTCTCATTCGATGCTTTCCTTGACTTCATGACCCGGGAGTC
CACCGACACCGATACCGCTGAACAGGTCATCGACAGCTTCAGGATCTTGGCTGGTGAC
AAGCCGTACATAACGGCGGAGGAGCTGCGGCGCGAGCTGCCGCCGGACCAGGCGGAGTACTGCGCGGCGCGCA
TGCCGGCGTACCGCGGGCCCGGCGCGCCGCCGCACGCGCTCGACTACATGGCCTTCTCCACCGCGCTCTACGG
GGAGACCGACCTCTAGATACAGGTCAATCTGT
>103792239_2 DT662887.3
GCACGAGGTATAATCCATTGCTCGTTCAATGCACTATCCGATTCTCTACCTCTATTATTTGTTTCAAATCAGT
GTATCACTGTAACAGAGTTCGGTGTGATTTAGTTACAACAAATGAGTTCGAAGCAATCGATGGTTATCGATTA
TAATCGATTAATTCGTTTACAGCACTATCCGGTGCTGTTTTACGATCAGTGTGACGTC
AGATGGTGTCAATTAGGTAAAGCAAAATCGACACTCGATGATTATCGATTATTATTCTTTTGGAAATTTCAAT
CGAATTCGACCCAATAAAGCACTATAGCGATTGATAATAGTAATAAAATAAATAGGAAACTTGATGACTTACG
ATTGGTGAACAATGGTTTTTTTTTATAAAATAATTAGAAAGTACTCTATTTTGACCAA
ATGGCTTTTAAAAATGATCAAAATGTAATGTGTTCAACTTGTCTTCATACTGTGACATCTACAAAGCGTTTAC
TATCAATGATCTAATCTTATAATTAATGTAAAATATTTTGTTCGTACTTTAAACGAAAATATTATAAAAATTT
AATTTAATCATAATAAAACTCATTGGATCTATCGTTGCTCACTTCTCGTACGAGAGTA
TTTTATTTATTTGTTACTGTTAAAATATACACACAAAATGTGTAACAAATTTTACGTTCCATGTTTTTGTGAT
GTGTTTATAGTGCTGGATTATGACGTCATAACGCACTATTATCGTTATCAACAATTTATTTGTAGTACCTATT
GTGCTATTATTTATTATATTTTAC
>103792259_2 DT662772.3
TTTTTTTCCATAAAAATATACCTTAATATATCAACTTATTTTTTTTTCACTTCATGTTGTTTCAATATAATAA
AGTGCAGAATTAGTAAGATAGTTCTCTACCATCCCCCCCCCCTCTCATTCTCTTTCTCTCTGCCTACCGTGCT
ATATGCAAGTTACAACAATAAGCCAAAAAATAATTAAAATTTAACATTATCACAATAA
GTTTGAAAGGAAATTTTATGTTCCTCCTTCAGAAATCTCTTCATGCCTTTAAATAAATAGAACAGTCTTTTGT
TTTGTAATAATTCAGTCAACATATTTAAAAATAAGTGATAGTCACCACTTTTCTTATATGACGATATAGATAA
TTGAAATTTCTTATAATTTTCAGCATCTAAAAGTGTTTTCAATTCTTTCACGAAATCT

ATTAGAGAAGTAGGAGCAGCTCTTTCCTCAGACATTGGTGGCAGTTCACCAGATTCTTTTAAATTTTCTTCAA
AGCCAAAATTCTTAATTTTTAACTTTTTTTTCTTTGCCACAGCAATGACATTAGTATCCTCAGCATTTCTTTT
ATGAATTGTTACTAAGGGCTGAGAAGATTGTGTGTTTTCGCTGGAAGAATCTAAAGCA
CTAAACAAATCTTTAGTTTCTTTTGGCTTGAATTCATTCAAATATTTGGAAGCTTGAGCATTTTTATAAAAAT
CCATTGAAAAATCTGCGTAAACTTCATTTGAATTAGGATATTGTTTAACAGATTTTTTATTTTGTACCACCCT
TGTGTTTGTTACAGGAAATGATACTCCAATTAAACTTGTTGCAGTACTTTCTTTATAA
CTTGTATTAACATCAT
>103792269_2 DT662413.3
GCACGAGGCAATATCCTCCTGTCGTTAGATGTGGTTAGACTGTGAAATGTAACAAAAAATAGCAATGATCTAT
CATTGTCCGATGCGGTCCCTCGGTTTGTAAACTTTGGCAACTACCTGACTTTAGAGGGTTGGGGGCGTCGTGC
CACACGTCGAACGACCGATCGGACGACCCGTCGACTTGTTATCTTCTCGTTATGTCTG
ATAACGGAACAACTGCCGTATCGAGGGCGACTACGCCTCGCCCAGTTGCCGACAGTCCTGTGAACGCACCTCC
ACGTTCAAGACATGATCAATTTCTTGCAAATTCCGCTGAAAGAACTCAGCGGCCTGCTTCCAATGGGCGGAAT
CCTAATGGAAATTTTGAATCACCACCTTTACCTTACCATCGTCAAATGACTACAGGAT
CTCTGTTAGCACCCAATCACAGAGAATCATCAGCATTTGTACCTGTAGTTCCAACACGAGCTATGCATCCTGT
TATATATCCTAATGATATGCACCCTCCACTAATACCATCTGAAATTATGGAAAGAGAGCGAATGTTAGAAAGA
GATAGAAGTGAATCAGCCAAAGGGTCACCAGATAGAGCGCCGGGGAATTTTTCGAAAA
GAAACTCTTTTGATTTAATGGCTATGATGGTTGAAAAGAGAAAAGAAGTCGCATTACGGGAAGCTGCAGCAGC
CATGTTGCTTCCTCATCACAGAACACCGTCCATTGAAGGCATGATGTCGGATGGGTCTTCTCAACTCCAATTT
ACGGTCCCACTGGAGCTTTTCTAGGGTGCACGGGTCCTTCACC
>103792294_2 DV500979.2
GCACGAGGCACAGGTCTTTAACAGTTGCTTTTATTCTACGCATTATCGTGTTTGTAAATCTTTTTATATAATT
CTATATTTAAATATGTGCCTGTGAAGTATTTTATTTACTGGAATATCTTCTGTCTTCTATTCTATTTTTTATA
CTACATACGAATGACAAAAATGGCGAGCAAGGAGCTGGAAGCACTGAATAAAACATCG
GGAAACGATGTGCAACTGAAACGAGAATTAGGATTATTTTCTGCAGTTAATTTAATTTTAGGAGTAATGATCG
GATCAGGAAAATTTGTATCTCCAGCATCATTCGTTAGAACATTCTTGTTCTGTTGCTTTGTGCCTAATTGTTT
GGACTGTGTGCGGAATACTATCCTTATTATGATCGCTATCGTTTGCCAAATTACGAAC
AGTCCTCGGAAAGTCAGGAGCTGAATATTCTTACTTACCAGCAGCGTTTGGAAAATTACACAAATTTTGGGGA
CCTTTACCATCTTTTATATGTGCTTGGATATATTGTTGTGATGTTGAGAACAGCTGAAATACCCCTCGATGTG
ATGACTTTCG
>103792297_2 DT662854.3
GCACGAGGCGTTTCCTTACATAAATTCCACTTTGCTTGTTAACTTAGAAATATATTTGATCGTTAGTATTAAG
TTAAATAATGATACAAATATAGGATTAATGCTCAATTGAGATTCAGGTGACAATATTATGTATTAATGTTTAT
GTTACACGTCGTTTTGGGACGTGATCACATTTTTTATATTAGCAATTTGTTTCATATA
GTTTTTTTACACTTACATTTAACTTTTATATATATATTAATATGATGATACTGTCCTTCATCTGTATAAATAT
AGAAGCTTTTTTTTGGGTGATGATGACCTATAATAGAGGATTTCTTATTTAAAATATTGTCGATATTACAGTC
ACCATTTATTTATCAGAAAATCTCTCAGAAAAGCTCGATTTCCAATCCAGCAAATGAA
ATATTTAATATTTATATTACCTCGCTCTGGCAACCAGGCAAAGCACGGCGTTGAGTCTCGTCACTTAACACGC
AATGCCTCCAGAATTCGGCGGAGAGTGGGAAACGAAAGTTGGAATGGAATGTCATAACACAAGGTTCCCAGCT
TCCATCCGCCTACTCTGCTATACGCGGGATACGGCGTGTGAAGCTAAAAGACATTCTG
TGAGGGGCACCATACCAATCTGGAACGGTGGGCACGGCCGACCCGGTCTCCCATTCCTACGCCCATGATGTTA
ATGTTAAAAGAATAATGTCGACTGTAACATAACTTGTAAAGCGATGAGTTGTATTTCATATCAGAGTTATTGA
AGATGATATCTCGACATTCGAATCATTCCATGATGTGTTGTATTTTATTATATTATAT
TATATAGCTTTCGGTGCT
>103792318_2 DT661712.3
GCACGAGGCACGAATCCTTCAGAAAGTGGGGAACGGAGTGTCTTATCACTAGGTTCTCTCTGCCTTCCCCGCT
GTGTGCGGGATACAACGTGAAGGTTAACCGAAAAATATTTTTTTAAAGAAAAAACAAACAAATTGTGTAAAAA
ATCGAGTTAGTTCTCTTAAAACATTCAAATCTTTTATTGATGCAATCGAGAAGAAACT
TCTGTGGCAAAGGTTTTAAATATATTGAAGGCAATTTTTGCACTATTAGTCTCTTGTAAACTAGCACAATAAT
AACTTTGTATAACATAATGGAACATTGTTTTAATACATATTTATTTTTTCAAATCGTGTATATTTTATAATTT
TACTCATTCTTGTTAACTTAGTGATATTGTAAAAGTTTGCCTTGAAACGCCTTGGTTT
TTGCGGTGTTTGTAATTCATTGAATCATTATAAAAAAAAAAGTTTAATTCGACTATAATAGGATCAGTTGTTG
AAATATTAAAAATAATTTATTAATATATTACTTAAGGCTTGGTACGATTGCGGTTAATAAAATAAAAAGCGCA
TATTATAACATTGGATTCAAAGTGTGCTCAATTTTTTAACAGTTCCACTAAAAATATT

TAAAATTGCAATACAATCAAAGAGCTCCCAATAAACAGCACAATTCTATAAAATATTATGAATGTTTGTATCA
TACCTTGGACTATATTTAGTTGATCATTTCTGGAAAAAAAACTGACTTATATATAAATATTTTATATATTAAT
>103792321_2 DT663354.3
GCACGAGGCAAGCCCTATGACTGGCTTATTTTGTATATTTCTACTTATTCATTTAGCAACAGTTCAGTGTTTT
TTTCATCGATATCCTACTATAAATATAGGAGAACCCGATGGAGAAGATGCAGGAAGTCCGCTTTTCCTTACAC
CGTACATTGAAAGTGGCAATATCAGTATCGCCAGGACACTAGCTCGCGTGCCTTTCAC
AGAGAGCTTACGAATCAAAAGCTATGCCGGCTTTTTCACTGTGGACAAGAAATACAATTCAAACCAGTTCTTG
TGGTACTTCCCTGCAATGATTAGTGATAAGAAAAATGCCCCAGTTATTGTGTGGCTTCAAGGTGGACCTGGAG
CATCCTCTCTCTATGGTCTCTTTACAGAAAATGGTCCTCTTAGAGTCCGTGACAAGAA
ATTTGAGAGACGCAAGTACAACTGGGCCTTGAGTCATCACCTTATTTACATAGACAACCCTGTAGGAACAGGC
TTCAGCTTTACTGATGATCCCAAAGGTTATTGCATCAATCAAACTCAAGTTGGAGAACAGTTATACTCCACCC
TTATACAATTCTTCCAATTATTCCCTGAATTGCAGAACAATAAGTTTTTCATTACCGG
AGAGTCTTACGCTGGCAAGTATGTTCCATCATTAGCTTATACAATCTATAAGAAGAAACCTACTGCTGATATA
AAGATTAATTT
>103792341_2 DT663371.3
GCACGAGGCAAAGACTTTAAAATGGAAAGATTTTCATTTGAATATTGATTTGCAAATCGTAATTCGTAGTTCT
CTGGAAAAGTTTTTATCTCTCGAGAAATAAATATCTTTACTAATAAACTTCCATTATTGTATATTGGTACTTT
TTTATACAATCGTAAGAGTATTAAACTTATTTGTCTACACAATGGCAGATGCGATTCA
GGAAGAACAAGTTAAAAAAGTGCAGAGACTTTTAGAAGGTTGGCGTATGGCTCTTCTACCTTTGAAATCCGTA
TTTTTGTGGGAACAACATTGGCATCCTTATGCTATTATTGTTTCCGTATCAGTATTGCACTTCATAATTTGGA
TGATGGATCTTAACACTCTTGCATCACTATCACTTGTGGGCCTGATCTTAAACTTTAT
TGATTTCATAGTACCTATAATATGCAACACTATTTACAAGAGTAACTCATGGACAGGACAGAATGAAAAGATG
TTTGAGGACATCTGTAGGAATATTGTTGTACAATATAATAAATTACTGAATTATGCACATTCCTTTTATGCAA
TGAGAGAAAACAACCCCACACTGTACTATATAAATTCCATCAGCATATTGAGTACTTT
AGTATGGATGGCTTCAAAAATAAACAACATCTTTTTACTGTACATATTTTCGAGTCTAGTACTGCTGT
>103792358_2 DT662064.3
GAGGCGTTCCCCTAAAAGAGGCTGCAATTACATATGTGCAGTGCGATGGATTGGCGGTGATGAAAATAGTAAA
ACACTGCCATGAACAATCTTACAGCAACATGGAAGTTGCCCAGGGTGCGCTCCTTGGGCTAGTTGTGGAGAAC
CGTCTAGAGATAACAAACTGCTTTCCATTCCCTAAGCATGATGACACAATGGATGAAG
AAGAGTACCAGCTGGACATGATGAGGAGACTCCGAAGAGTTAATGTTGATCATTTCCATGTTGGATGGTATCA
AAGTGCGGATGTTGGCAACTTTTTGAGTCAATCTCTTTTTTTTTCACAATATCATTACCAGACGTCAATTGAA
GAGAGTGTTGTAGTTATATACGACACTAAGAAATCTGCAAGGGGATTCCTGACTTTGA
AGGCTTACCGTCTGACTCCTCAGGCAATAGCAATGTACAAAGAGAGAGATTATACACCAGAAGCTCTGCGTAA
CCTTAAGATTGGTTACGAGAGCCTCTTCATAGAAGTGCCAATAGTGATACGTAACTCACCACTCACTAATATC
ATGATGTCTGAGCTGTCCGAGATGATACCTGAGGAGGAGGGTTCGAAGTTTCTGGACC
TTGGAACTGCTT
>103792364_2 DT662220.3
CGGCACGAGGCACAGTCCATATCATGGCCGAATCTCAAGTGAACGACAACACCACTTGCGTAGTGTGTTTTAA
AAATGTCGTGTATTTCTCAATTGGAGAATGTGATCACCCCGTGTGTTTTGAATGTTCAACCCGCATGCGAGTT
TTGTGCCTGCAGAACGAATGCCCTATATGTCGTCAGGATCTTTCCAGGGTCATTTTTA
CTGAAACTGTTCAACCTTACAAGAAATTGCAAAATAGACCTTTTTCTGACCGATTATTTGAAAGGCAGTTTAA
AATTGCATTTTGTAGTGAAGAAATAAAAAATGCATATGAAAACTTATTTGAGAACCGTTGCAAAATTTGTGAC
AAGATACCCTTCCGTACATTCAGCATGTTAAGTGACCATATGAGGAAAGTACATGAAA
GGTACTTTTGTGACCTCTGTGTGACACATTTAAAGATATTTACTAATGAGAGAAAGTGTTATACTCGGCAAGA
ACTTGCCCACCATCGACGGAAAGGAGACCTAGATGACACCTCCCATAGGGCACATCCACTATGTGAGTTCTGC
GAGGAACGTTT
>103792397_2 DT662616.3
TGAAAACGCTAAAGTTTTTACCTTTCTTGGAATAATATAATTAATATGGCTTCTGTTAAAGATGTTGCTACTT
TGTACCAAAACTTGAAAACAGAATGGGCAAAGAAACCTCGTAAACTTGATAAATGCGGCGAGCTATTAAACAA
AATAAAGATAGCCTTAACACAGCTTACTTTTCTACCAAGCAATAACACCTCCGCTAAT
CAAAAAGAATTGATTCTAGCGCGAGATGTGTTAGAAATTGGAGCTCAGTGGGCTGTTGCAGCAAAAGACGTGA
AAGCTTTTGAGCGATTTATGTCTCAGTTAAAATGCTATTACTTTGATTACAAAGATCATCTCCCAGAGTCAGC
ATTTATGTATCAGCTTCTTGGTCTTAACTTACTTTTCTTACTATCTCAAAACCGAGTA

GCAGAGTTTCATACGGAGCTTGAACGTTTATCAGTAGATGTTATTAGAGCTGATCCATATGTGAGACATCCGT
TGGCCTTAGAACAGTATTTGATGGAGGGAAGCTATAATAAAATATTCTTGGCTAAGGACAATGTACCAGCAGA
GAGCTACACATTCTTCATGGACACCCTACTCGATACAGTGAGAGGTGAAATAGCAGCA
TGCATTGAAAAAGCATATTTGTGTATATCTTGTACTGAAGCTGCTAGAAGATTAAACTTACCATCCCAACAAG
CAGTACTTGAATATGGGAAGAAGCGTAACTGGAACTTAGGTGCGGACAACTGCTACCATTTTAATTCCTCTGA
AGAGACTTGTGCATTAGCAGCGGGAGTGTTCCCCTCATCTGAACTTGCTGAACAACCA
TTGAATATGCTAGACATCTTGAAATGATTGT
>103792403_2 DV501085.2
CAAACCACAAAGCCAACGACAGAGCAAAGTAATTCTACGGAAGAAATTCTTTCAACAGAAACGACCCCAAAAA
GATCAATATCAACTGATCAAACTACGACTGAACAAACTAACACATCGACTGAACTGTCAACAACTACAGAAGA
TAATATTTCTCAGATAACTGAAGTTCAAAGTACGACATCTGAAACAACAACTACAGAA
TCGACCACAGAATCCTCAGCACAGCAAGAATGTCCAGAAACTGATTCTGATCAGTCGTTATTTGTGAGCCCTA
CATCATTTAAA
>103792411_2 DT662980.3
GCACGAGGGGTTTTCGTAACTAATGTCATGGTCACTGTTGCCAAGTCCTTCGAAGCGCCTATTAAATTGGTAT
TCCCCCAAGACCTGCTCGTCAACGGTTTCAACGCTAGCAACTTCGCCATGTTGGGACTCGGCGACATCGTGGT
TCCCGGGATTTTCATAGCATTATTGCTACGCTTCGACAAAAGCTTGAAGCGAGGTTCC
GAGTTCTACTTTAGGGCGACCTTCTCGGCCTACATTTTGGGACTTCTGGCCACCATTTTGGTTATGCATATAT
TTAAGCACGCCCAACCTGCGCTCTTGTACCTGGTGCCGGCGTGTCTCGGCACTCCGTTGACTTTGGCGTTGTT
GAGAGGAGATATAAATGCACTGTTTAACTACGAAGACCAACCCGCCATTGTGGAAGGC
ACAGATAATAAAGCAAAGAAATCAGAATAAAACACACAAAATACAAACATGAAGCACAAATCCGAGGTACATC
AAATGGGAATCGAACCTGGGACCATGGCGTGGACACTGGGAAGATTTAGAATTAGTTGTATTGAATTATATGC
ATAACAATTGATAAAGTAGTTTTTATTTATATATTTGATCTGTATGTCACAAATATTG
TGCCAAATAAATGTTAATTTTGAAGAAAAAAAAGATAAAGGATTTATTTAAATAATTATGATTTGGCTTTTGC
AGTACTTCTTTTGTTTGATAATTAAAAGTACATAATATTTTATTACATGAATTGAAAAAAATATTTCTTATAG
TGACAAAGACTATGCATATAATTCAGTAAATATAAAAAAAACTTTCCATATATCTTCC
ATTTTTTTTCATTTTT
>103792423_2 DT663345.3
GCACGAGGGTAATCTTTATATCGTGTATTCATTGTAATATTTCAAAATTATAAATAAATAACATAAAATGCTG
CAGCACTTCTTGTTATTGTAGAAAATAAACATTTTTATACTTCATTGAAATGAGTTGGTATTAATTGTAAGCC
TCTAAATACAGAAATATGTTTCGCACGGTGAAATCTTCGTATGGTATTTTGTGTTTTA
TCGTATTAAATCTATGGACATGCTCGAGAGGAGAGCTGTTCACAGCTATATCGGATGTTGAACCTTTATTAGA
AACACACAAAAAGATTATAGACGACTTAGAAGATTATATTAATAAAGAAGAAAATAGACTGGCAATATTAAAA
AGACACTTGGTCACGTACAAAAATGAACACGAAAAAGCTATGGAAGACATACCCAATT
ATTTAGGAAATCCTATAAATGCCTTTACTTTAATAAAGAGGCTAACAACGGACTTGGATTATATTGAACGAAG
CATAGAAGTAGGGACAGAGTATATAAAGAACATAACTATTCTAAACAATCACACGGACGTCAAATATCCTACT
CTGGAAGACCTCTCGGGCGCCGCCCAAGCTCTGACGCGTTTACAAGAAACGTATCACT
TAGATGTCGGTGATTTAGCTGAGGGAAGACTCAATGGGGTCATTTACAGCACAGCGATGAGTGCGGGCGACTG
CTATGAATTAGGAAAAACTCTATACAATGAGAAAGACTACAAAAATGCACAATCCTGGATGAATGTGGCACTC
CGG
>103792432_2 DT663039.3
TTCGGCACGAGGTCGAGCCCTTTTATGTTTTTTTTTTTTTTTTAGCACCAAAATAATTTATTTGTACCCCGAT
TTCATTAAAATTTATAATACCTGACAAGGATACAGTGCTGATTACTTAAGATTTTTCATTATTTACAATTACA
ACCCAAAATATACCAATTAAATGTATACTTGGTCCATAACTGAACCAAAAACAATAAA
TTGGTAATTTTTTCATCATTCTTATTAACCTAATATTTGAGATTTTTTTTTACACATTAAAATAATTCTTAAT
TAAATGAATGAACTTACAAATTAATGACTTATTTTAACCAGCAACTTTACAAATACAGAATAAAGATAATTAA
TCATCACACTCATAACAAAGAAATATATTGTTTTATGGCAGTAAATTGTTGTATACAC
AAAATAGGAACCTTCAAATATGGTCTTCGTCACAATATTAAAATCGGTAAATTAGGCTCAGTTATTATCAGTT
TATAAAAAAATTAAAAACCACATTTAAAGATTTTAATTAAAGTTTATTTATAAAACTTTTATGGCAATATCAA
TCATACTTACATACATATTCCAATTTAATTTTTTAAGTTATAGATAAAAAAATTTACA
TCTAAAAAAATTTTCACAAACATATTTAACTGAAATAATTGAGTACAAATTTGTATATATTGTATATAGAAAT
ACCATAACAGAGAATTTACAATAATGTCTAAGTCATTTTCATATCAAAATGAAAACTGTCCAAGGCATGTTAT
GAAGCAAACTAAAATAATTAA
>103792439_2 DT662270.3

GCACGAGGTGTAACTGTTTGGCGCGAAAGTTCCCGTTTTATTTACGCGTTTTCTTTTTTTAATTTTTAAAAAT
GAGCCTGGAGCAGAGAAATGAAAAGATCATAGCCACTTTTGAAAAATATAAAATTGGAGAATGGGGATACGGA
ACGAATAGGACGTGCGCCCAAGTAGCTACTAATGTAGTGGAGCAGAAACATCAAGTGT
CCAGAGCTAAGAGGAGCAGGGCTTTAGGCAGCAGAGCGTTCAGAGGCAGCACTGTCTGGTTTACTGGCCTTAG
TGGTGCAGGGAAGACCAGTATTGCATTTGCTCTTGAAGCTTATCTCGTTTCTAAGGGTATCCCAGCCTACGGC
CTCGACGGTGATAATATACGCACGGGTCTAAACAAGAATTTAGGCTTCTCGAAAGAAG
ATAGAGAAGAGAACATTCGTAGAGTGGCAGAAGTGGGAAAACTATTTGCCGATAGTGGAGTCGTGTGTTTATG
CAGCTTTGTTTCTCCTTTTGCGGAGGACAGAGAAGTGGCGCGTAGAATTCACACCGATTCCGATTTACCGTTC
TTCGAAGTATTCATCGATACCCCACTTGAAGTGTGCGAACAAAGGGACACGAAAGGCC
TTTACAAAAAGGCTAGACAAGGGTCAAATTTAAGGGCTTCACTGGTATAACTCAAGAATATGAACGCCCTGAA
GCACCTGAGCTTGTCATACCAACGGGTGGACGATCCTGTGGAAGAATCTACT
>103792448_2 DT662624.3
GCACGAGGCGAGAACCTGCAACGCAAGTGAAGGCAGACTGCGTGCCCGGCAGCGTGTTCAAGCAGGGCTGCAA
CACTTGCCAATGCACGGAAGACGGGAACCACGCGACGTGTACTATAAAACGATGCAAAGAGGACAACCCTGAT
GAGAATGAGGTTAACTTACCAGAATCCGACCCAAGTTTCCGCTGTAACCCCGGAGAAC
AATTCAAGAGAGGTTGCAACGACTGCGCTTGTTCAGCCGACGGGAAAAGCGTGTTCTGCGCATTACGTCTATG
CGACCAGGACATTACACTTACCATTTAAATAAAATACTATTAATAACAAAGTATAAGACGCTTAATATTGGTC
CTTAAAAATCTTTCTTAAAATTCGAAAATATTATAGAATATTGTATCAGGCAGCAGTT
TGTCTTAAGAAATGTTATACCTTCTTTATAACAATGCGTTTTTAAATATTTTTCGTTCACTTAATTTGTTAAT
GTAGGTTTTATTCGTATTTGTTTATTATATTTGTAGGTTAAGCCTAATAATTTTAGAATTTGGAAAAATATGA
ATTATCAGCCAATTAGCTTCCTAATAATAACATTACACTTTATATAATGTAAATGTGT
TTTATCAAACTGTAATTGGATTTCTAAAGTCTCCTTGCTTGTTTGTAATTCATTAAGACGTAATGTTTTCAAA
CAAATTTAAATAATTTATTTCTCCCAGAATCCATTATGGTTTCCAAAGTAGCGTTTTCAGAGAGCTTGAGATA
AAAACTATTCTTGATAGTCAAAAAGAAACACAATTTCCGTATAACTCANAATGTCTTT
ACATTTTTGGTGTATTCCTAAATAAAAAAAAACTCAAATTATATTTAAAATACATCATAATTCAATATTGTTA
TTTCAGT
>103792496_2 DT662816.3
GCACGAGGGAGGCACTCTATTCCTCTAGTCTTGTATCGTGGTGCGTTTAAGATAATACCAAATATAAAACTAA
TGAAGGCTTTCACGTACTGGTAACGGTAGGCGGTAGCGGTAGCGGTAGCGTAGTCTTAGGCCCGAGCAGCTTG
GATATTGTCTAATACAAGGGTATGAATGGTTGGGTAGTATTATTATATTGCATTTATT
TCAATTTTGTCGTAGTCACTCAAGAATATATTTAATTAATTGAGCTAATGCCTAAAATTTGGAATATCAGAGT
TTATTTAGGATGTTTAATATTTTACATTTTAGTGTTTAAATATCTAAAATTTACCTTAATTATAGCGAATGGA
TTTTTGTATTGTCGGTATATATAGCTATGTAAGACTAAAGGTGTTCTCGATTAACACA
ACAACTGATTATTACAAAATAATGTACTGAGTGACTTTACGTGTAAACGGAGGGCCATTGATTAAAATGTTGA
AGTAAGCTAAATCTCTATATGGTAATTGGTAATTTAAATAAATGTGATCGCTGAAGAATATTTTTAACTGCTA
TATTTAATGTTATTATAGTTGAGTAAGTGGACGACGATCAGTGTCGGTAGTCACACAC
ACACACACAGTGCTAGTACAGTGCACTACAGTGCAGTGCAGTGCAGTGCAGTGCTCGTCATCGTCCCTGCACG
CGCGTTGCATCCCATCACTGA
>103792499_2 DT662476.2
GCACGAGGGTGTCCTCCTTACTAGATCCCTCAAGATGCTAGTACTTGATGAAGCCGATGAGATGTTAAACAAG
GGTTTTAAGGAGCAGATTTATGATGTGTATCGTTATTTGCCGCCAGCAACCCAAGTTGTCTTGATATCAGCTA
CTTTACCCCATGAAATACTTGAAATGACCTCAAAGTTTATGACAGATCCCATCAGAAT
TCTTGTAAAACGTGATGAGTTGACACTAGAAGGTATAAAACAGTTCTTTGTGGCTGTGGAACGCGAAGAGTGG
AAATTTGACACATTATGCGACTTATATGACACGCTAACAATAACTCAAGCTGTGATCTTTTGTAACACTAAGA
GGAAGGTGGACTGGTTGACACAGAAAATGCAAGAAGCTAACTTTACCGTCAGTTCAAT
GCACGGTGACATGCCACAAAAGGAAAGAGACAATATTATGAAAGAATTCAGATCTGGTCAAAGCCGAGTTCTT
ATCACAACAGACGTATGGGCCAGAGGTATAGACGTCCAGCAGGTGTCACTAGTTATCAACTACGACTTGCCCA
ACAACCGTGAGTTGTACATACACAGGATTGGTAGATCTGGTCGTTTCGGACGCAAGGG
TGTCGCCATCAACTTCGTGAAATCCGACGACATAAGGATTTTAAGAGACATTGAGCAGTACTACTCGACACAG
ATTGATGAAATGCCTATGAATGTAGCCGACTTAATATAGTTTCGTAATATCCACAATATTGTGTTTAATGTCA
AAAGTCAACATTGGTCCACACATTGCTTAAATTATATTGAATACTTATTGTGTTACAT
TTATATTTAATAAACATAGAACTTATAAAAAAAAAAAAAAAAA
>103792502_2 DV500981.2

CTTGTGGACACATTCCTCCCTCTATGGACCCGTAGGATAATTGCGACTACTTGGCACTCACTAACCGACCCGT
ATTCTCATTACCGTATTGTAATGAAAAGCAGCGGTGCTTAAATTTGAAAATAAATGGTAATTCATCGAATTTT
ATATTTCGCGGTGTCTATAGGACCATGTCAGTCGCAAAGGGTTAATATTT
>103792512_2 DT662472.3
GCACGAGGCACATACCGTATTCAGTAAATAATTTTAAAGAGAGCCTTCGTAATTGATTACTATGTTATTTATT
ACACGTATTTTATCTATATTCTTCACAAAAGAATTATATATATTTTTTTAATATTATCTACAATTATTCTTTG
CATTATATTAGGTCTTTATTACCATTGTGTGTAATGTGGGTTCATTAATAGTGTTTTC
TTTTCTCATGTGTATATAAAATAGTCATAAATATGTTGCTAAAAGAAGGAATTTAGTTTTTTACATATCAAGG
ACATGTATTTCATTTAGACAAAGATTTTAAGAATTCACAATTATTGCAAGTGGTGGTGCACCTTTATTTTTCA
CATCTTATCACTCTTTGAAGGTTTCTCATATTCATAACACCAGCTCCAACAACGGATG
GAGTAATGGACAGCCAGACTTTACTGCTATGTGTTGTACTTTAATATATAGCAGTATAAAAAAGTGTGCCTTG
TATACAGCCTAGTGTTTATACTTTGGCAATAAAATCAATGATAAAATAGAGCAAATAAAACTTAATTAAATGA
TAAGCTTATTTACTTAGTTGCTTTGATCATTAATAACCAATATTCCCTTTATTAAGGA
TCCTTTTCTAAGATGGTAGATCCTAAGAGAGTTCTTAGTTATGAAGATATGCTTAAAATAATTCATCAACCAG
AAAAAGTAATTATTGATGTCCGTAATCCAGAGGAGATAAAGTCATCTGGTAAAATACCATCAAGTATTAATAT
TCCATTGAGTAATGTTCAAAATGTGTTAAAAACCATGTC
>103792544_2 DT662210.2
GCACGAGGGACTCTTTTTTTTTTCCATCACAATTTGGTTTTGCAGTCTGCACTAACTGCAGTTATTATACTGA
GAAATTCTTCGTGAAATATTCCAAAATGTTAGCAATTAAAAGCCTTTTGAGACCAACTCAGGTATTCGCTAAA
AATGCCGTTCAAGCGAGGAACATGACCGGTATCGCTATTCCAGCAAGAAATAAGATTA
CTCGAGCTGAAACCATCATCCTAGCGGGATCAATGGTTGTCGCCTGGTTAACCATCCCCACCTGGGTCTTGGT
CAACATCAAACACTACCGTGACAAGAACTAAATGTCTTTGACAATGTAGTATATTATAGTATTTGTTTTTAAG
TAAATGGTTAATTTAACATTGT
>103792547_2 DT662963.3
GCACGAGGGACGAACGTACCCATCAATTATATTGTAAAGTCGTACACAACGTAAAAGTTTTTTGACCCCAACT
TTTATTTTGATTTTATGTAGTTTTGTATTATATTTTAATAGTGCTTTGTTTTATTACATTAGTTCTGCAGAAT
AGTGTTGTGATATCAAATTTTCATCAACAATGTCTGGCAAGGGCACAGCAATTTCGAA
ACAACAAAACATTTTAGATTCAAATGTTATAGATTCTCAAGAACTGCAATCTGTAGATGATGACAGTGTTAAA
TCTTGCTGGGTATGTTTTGCCACGGAGGCAGATGATAAGCTGGCTGCTTGGGTTCAGCCTTGCAAATGCATTG
GCACTACGAAATGGGTTCACCAAAGCTGCCTCCAGCGGTGGGTAGACGAGAAACAACG
CGGAAATATAAGTCGAAAAGTTCTTTGCCCTCAATGTAAAGCTGAATACATACTAGTTTTTCCCTCAATGGGT
GCGTTTGTGGCCTTATTAGACGCGTTAGAAGAAATCACACATAAAATAAGCCCCTTCATTGCGGGTGGAGTGC
TGCTAGGATCTATATACTGGATTGCGATCACCTATGGAGCAGTTACTGTGATGCAGGT
AGTCGGTCACAGGGAGGGCTTAGAAATGATGGAATCAGCGGATCCACTGGTACTTTTGGTACTTTTGCCAACG
ATACCAGTGACGCTTATCAGTGCAAAGATGTACAACTGGGAGGATTCAGTACTTTTGTTTCTGAGAAAATATT
GCACGAAAATACCTGCTCTCTCTTATATACTG
>103792559_2 DT662847.3
GCACGAGGGTCAATCGTTTCTCCGATAAAAAATTCCTAATTGAATAAATATTTCATATTTAGACTTTTAAATA
AAAATGACTCCTTTAACCGATCTAGAAAAACTCGGCTTGAGCTTTGCCTTTTTTCTTATTGCCGTTTTCATTA
AAATTACAAGTAGTTTGTTGTATACTTTTGTCATCGGACCGGCGATCAATAAAGTGGA
TTTTAAATCGAAGGGAAAGTGGGCTTTGGTCACGGGCTGCACGGATGGGATCGGAAAGGAATACGCCAGGCAG
CTGGCATCACGTGGCTGTGATATGATTCTAGTCAGTCGATCCATGGACAAATTGAAGACAACAGCAGCGGAAA
TTGAAAACGACTTTAAAGTGAACACGAAGATAATCAAAGTGGACTTCAGCGACGACGA
GAGCATTTACGATAAGATATCTAAAGAGATTGCCGATCTCGAGATCGGCACTCTCGTGAATAATGTCGGCATT
TCGTACACCTATCCTGAATATTTTCTGGACATACCCAATTGGGAGAAATCTGTCAGCAATATTTTAAAAGCTA
ACACGGTGTCAGTGACGCGGATGACCGGCATGGTCTTACCCGCTATGGTGAAACGTGG
GAAGGGGGTGGTCATTAACATAGGATCAGGTTCCTCTATAATACCCAGCCCTCTTCTTACTGTGTACGCTGCT
TCTAAGGCGTTCGTGGACAAGTTTACCGAAGGCCTCGAAATGGAATACTCCAAACAGGGTATCACTATACAAT
GCGTGATGCCCGGTTTTGTCTGCTCCAATATGTCCGGCATACGTCG
>103792564_2 DT662610.3
CGGCACGAGGTTTACTTCCTTTGTATTTTATGAATATTATGCTATTATTATTTTTTTATTACATTACCGTGTG
TTTGAGTGTGTTAGCAGAATTATTTTAGTTTGATTTCATTAGTTTTGATTTTTTAGTGAATAATGATTTTAGT
ATTCTTTTTATTTGCTGAATCTATGACAATAATAGATCTGAATCGATCCTCTCCGGGA

TCGGAGATCGTTGTTTAATTATCGATCTGCATAAGTTTATCCTTTAGTTTATGTGTTACAATATGGTGATATT
CATTTTTAATTAAATTATCTCATTTATATATTTACATGAAAATACATTGAAGCCACGTCACATGATGATCGCG
CCTGCGTTTGCCATAGTTCGTTTGCATACCTGCGTAACATGTGCCATAAAACCATGTT
GTGATTTAATAAGTGATATTTAGTGTTGTTTGTAAATATGTACCATAAACTACTGATCTTTTAAATTTAATCT
TAAGCAAACCTACATTCTTTCATGGTTTCACCGCTTCTAGAAGTTAGTGCATCATAGTTGATAACCAAATGAA
TTTTATATTTATAATCTTGCATGACTGATGAATTTACTGTTACTGTTTTATTTTCAAT
TTCAATAGCATCTAAGTTTAAGAATATTGACTACATTTTGCACTAACCATAGAATGTTTAGGATCAAGTTTTT
AATGAATTATAATTTTTATTTCTGCATTTTGCAATCCACGGTGCTCGCAGGTCGCCAGTCATATTGACGTGTC
TTCGATAGCTCCCATCAATTCGTAGTACATTAGATTAGGTGAG
>103792578_2 DT663237.3
CTTCTTTAATAACTAAGAAATCTGATTAAATGATAAATCAGATAAAGAAAATATGAAATAAATTAGACTGTTA
TAAAATAAATACAATGGATCAGAGTAATAGATCAAGAAGAGTTGATTTGGACGTAGATAGTTTCCGTAATGAT
GAACCGGGATATGACAGAGCTCGAATGCGAAGACAATCAGGTGGCTTTGTCGACTTAG
GAAATGAATCACAATATGGAACGGGTGGGCATCAAGCTTCTGGAGCTAATGAGATTTTTGCCGACGTACAGGG
GGATGCACCTTGGTGGAAGTCAAATTTTTTCATATCGCAGCCAGTCCTCTTTGGAACGTGGGATGGTGTTTTC
ACATCATGCCTTATAAACATTTTTGGAGTTATTGTATTTTTGAGATCTGGTTGGATGG
TTGGACAGGCTGGTCTCGTAAATGCTGTGCTCATAGTGATATTTACAGTTTTTGTTGCACTGATCTCAGTATT
GTCTGCTGTAGGAATATGTGAAAGATGTAGGATAGAAAGTGGTGGAGTATATTTTTTATTAGCACATACTTTA
GGTTCAAGACTTGGAGGAGCTTTAGGGATGGTTTATTGCTTTGGCCAGGCTGTGGGAT
GTGCCTTACATGTATTTGGATTTGGTGAATCAATGGCTGATTTAGTTGGTACACAAAATACATGTGCAGCTCG
>103792624_2 DT662639.3
GCACGAGGAATCTACTTATTAAATCTTACGGATTTAATAATATTTTTATGAACTCGCGTAAATTTTATTCGAA
AAAAAGACATTTCGCTTTTTTGCTTTTAAATTATCGGTATATAAATATTAGTATAAATATTTAGTTTAATAGA
AAATCTGTAATGCCTTAGAGGAGAGACGCACACATACACACTGGTTTAAATTTCACTA
TGTATTCAAAATTTGTATATGATGCTCTAATATGTTTATATATTATTTAACATATTAAATAATATATTTCTAG
TTATGCTTGCCTACTATGCAATATTTGTTTAAAAAAAATACTTTAAAACTTCAATTCCTTTGAATTTTGGATC
ACATATTAATCTGTATAATGTAAATAAGATCTAACTATCAATATAAATACCAAAAAAA
AATTAAATAAATATATCACCATTAAAAATAAATGAATAAAGTTGTATAAATTATGTAGAAGCAAATAAAACAA
TCTTCAGTGAGTTCAGCATTTGGAAAATTATATTTTTTTACTTTCACGTTCTATATACAAAGTAAGTAAAATG
AACTTACGACACTCCGTATTTCACTTTTTGGCAATTTTTTTTGCCAAAATTTTGTCTA
TTTACTACAGCAAGAAAATGAAAAATACTACTACAGCAAGAAACGCGCTACGACGATCTCTTAGATAAATAAA
ACATGTTTTCTCTATAATTCCTGTAACTTTGGAACAAATATTATTTGAACTATAACTGAAAGATAAAATAATC
TCTTAATTATATAATAAACAGGAATATATATATATATTTCATGATCTGTTTAATTTGATA
AATAAATATGGATATTGCTGCTTTTGCTTCTATATT
>103792633_2 DT662818.3
GCACGAGGAAAAACCTTTACAAGTACAGGTTATTGAGAATGGAAATTTAATACATGAATCACATACAGATAAA
GAAAATGTAAATAATATGGATGAAAGCGAGACACATTTGACTAATGGCAACAATGATGATGGAGATGAATTCA
CAGAAGCCACAATAAAAGAGGAACCTGTCAGTGAAGAGGAAGACATGGATCCTTGTAT
AACAGATCTATCCCTGGAGTGTATGCTGTGTACTAAAATATTTAATAGTGTTAGTGGACTTAAGGCCCACGTT
ATCGCGCACCATTCTTACAAATCAGTGAAAAGAAAGTCTAGTACTAGTTTATCTCCAGAAAGGAAAAAACGTT
TTTCCACGTCAACTGATCTAATGGTACACGAGACATGTCACAACAAGAGTGTGTGCTA
CGGATGCAATGAGAGTTTCGACACTTTCGACCAACTCACCAAACACAGACGAAAGTGCAAAGCCATTTCAAGT
AAAGTCACCAGGACTAGGACTCTGGATGATGTATTGAGACCTCAATCTAAGGAGCCACCACAAGAATTAAAAT
GTATGCAATGTGAAGAGAAATTCACAGATACATACTATCTAAACATACACCAAGAAAT
ACACCACAGTGCCAATGTGATTGAACATAAGAACCCGTACATGATGTCATTGAAACTTTGTTCTGGAACTGAT
TCATAGGAAATAATGTAAATGTATGTACAGCGATGTAGTACTGCGGATGAGTCGTGGCCGCTCTGAATATAGC
TATAAATGGCTGAACAGATTTTATTTTAGGATTTTCTTACTTTTCTTATTTTTCTC
>103792642_2 DT662216.3
GCACGAGGCCGCCTCTGTGGTGCTAGTGCTGGCCGCCGCCGCCGCCGCCGCCTGGTTCGTCATACGGAAGAGG
CCATTCGGTAAAGGCGGAGGGCTCGGTAGCCTCGCGTCATCGCAGAGCGTATCTTTCCGTGCGGGCTCCAACG
TGGAGTTCGGTACGGGACTCAGCACGCCCAGCACGGGGCCGGCCGGTGCCGATGTATA
TGCGCTAGATGAACCAGCGAGAATAGGTCGTGACTTTAGCAACCCTATGTATGAAGCTGTTACGAGAGCTACA
GCAGCTGGGGAACCCGAACCGCCTTTGCCCGGTATCTACGAAGGTGCAGAAGATATAAAGGACAAGG
>103792672_2 DT662221.3

GCACGAGGGTAGGGCATTGAAAGAGTCAGTATATTATATTTTGTGCTATTTAGGCGTCTAATTCAGTTTGAAA
GAAATGTCTAATGAAAACACTTTAGATCTTCAATATGTCCACGAGAGTTTTAAAAATAGTTTAAAAGAAGACG
ACGATGTTATAATAGACTCGTATATAGACGGTTACAATGAACTAGTAAAGTTCTTAAA
CTTGATTGGTACAGTATTTTCTTTTGTGAGCAGCGATGTTAAAAGTAAAATTAAAATTATGGAAAAACATAGA
GCAGGGGAGGACCAAATTTATTATGAGTCTTTCAAGAAAATGATGAAGTATGAAAAGGAAACAAGCTTACATG
AAAAAAGTGGTTTTGTTTCGGGCTCTAGGACAATGTTGAGATTGCACAGAGGTTTAGA
TTTCATAAGACTGTTTCTTAAGAGATTGAGTGAAGGAGATGAATCTGTGAACACATGCACATTATGCCAGAGC
TCTTACAATGAAACATTAGCTGAGTTTCATCCTTGGTATATTAGAAAAGCAGCTACATTAGCCATGCATGCAT
TGCCTTCAAGACCAGATTTATTAAAGAAGATATTTGGTTCAGAAGAGAAACTAACGGA
GGCGCTGGCGATCTTGCCCCAAACGCTACAGTCGTGTGACGAGGCGTACT
>103792689_2 DT662480.3
GCACGAGGATCGTACCGTACAAATGAAACAGGTGCGAGTATTCCGTTTAATAACAGAAAATACAGTCGAAGAA
AAGATTGTGGAGCGCGCAGAAGTGAAACTTCGCTTGGACAAACTCGTGATACAGTCGGGACGTCTTGTTGATA
TTAAAAACCAACTTAATAAGGATGAAATGTTGAACATGATTCGTCATGGCGCAAACCA
CGTGTTCTCTTCTAAAGATTCTGAGATCACTGATGAGGATATCGATTCTATTCTCGCCAAAGGAGAGACTAAG
ACAGAGGAGCTAAAACAAAAATTGGAAAGTTTAGGCGAGTCGTCGCTGCGTGCTTTCTCAATGGACACTCCAG
GAGCTACCACCGATTCTGTTTACCAATTTGAAGGTGAGGATTACAGGGAGAAACAGAA
GATAATACCAGTCGGTAGCTGGATAGAGCCACCCAAGAGGGAGCGCAAGGCCAACTATGCGGTGGACGCGTAC
TTCCGCGAGGCGCTGCGAGTGTCGGAGCCCAAGGCGCCCAAGATACAGGCTCCAAGGCCTCCGAAGCAACCAA
TCGTTCAAGATTTTCAATTCTTTCCGCCGAGACTCTTCGAACTTTTAGACCAAGAGAT
ATACCATTATCGCAAAACACTTGGATATAAGGTGCCACGTAATCCTGAGTTGGGTCCCGATGCAGCCAAAATA
CAAAGGGAAGAACAACGTAAGATCGATGACGCCGAAGCCCTCACCGAGGAAGAGATACAGGAAAAAGACAACT
GTTGACACAAGGATTCACCAACTGGACTAAACGTGATTTTAATCAGTTCATCAAAG
>103792692_2 DT662328.3
GCACGAGGGTTGGGAATCCGTAGTTACTGCGAGAAGCAAAATATTATTATGATTAAAAAAGATGATGTGTAAA
TGATTTTAAAAGGACAAATTTACTAAATGTTCGTCTATTTTGTTAGATTAATATAATATAAGGATATATTCTG
AATTTAAATATTATATTTAAGTTATATTTTCGTCAGAGTGAGAATTATTATAATAATT
CAGCTGTGAAGTGTTTGTAAACTTAGATAAAATGGCAAATTCCGTAAACATATCAATAGAAGCACCTATCGAA
AATCAAATACAAGAAATTACTTACGATGGACAGGAAATATATCAAGCACCACTATCCATGTCAGAGAATCTCA
CTCGATTAGCACAGAAAATTGACTTTGCTAAGAGTGATGATGAATTCTGTAACATTAA
GAAAGAGGGTGAAAGTAGTTCTGATGTGAAATCAGAGGAAGATAAAGAACCAGGATATCAGTCAAGTTTATGG
CCTTGGGACTCAGTAAGGAATAAACTTAGAAATGCTTTAACTGAAGTATGTGTACTGGCCGATGTGTTAAGTA
TTGCTAAGGAAAAAAGATATATGGTTCTCGACCCCATCCAAGCTGATCAAGTTGAAAG
TCGTCCTATGGTCCAAGTTTACGGCAGAAAGAAAGCTCTATCTGCTGCAGGTGCAGTACTGCTCAATGGTGTG
GAAAGGCTAAGAGCCAGTGAAACTATGAGAATGACTCGTAATATGCCAGACTTTCACATAGATCTGCTAAGAC
TTAGACAGAACTGGAGGCTAAAGAAAGTTTCTAACACAATTGTTGGTGATTTGA
>103792725_2 DT662754.3
GCACGAGGCTTTGGCCCTGTGCTATGGAGTCTGAAGACGATGTGTTCTATGGTTTCCTTAAAGTTTTATGCCC
TTGGGCTATGAGAGATCCTGAAGTAGCTGCGGCTGTTGTTACAGAACCTACCCTAAGTGCATCTAGGGATGCC
ATCTCCAGTATTGCTGGGGACAGTGTCTCGATGTCATGTTCCGTGCAGGCGCCCATCA
GTTACTGTTACTTTAGGGCACGAAACGGCACCGTATTTAATGTTGGACCAGGCAACTCCCACGACCAAGCAAC
TTATCTTGGCTCTGGCTTGGATGCTGGCGAGTGCGGCATCAGGTTCCCCAGTCTCGCAACCTCTGACTCCGGC
CTATGGAGCTGTCACGTTGGCTTCCTAGATCAGAAGGAACCTGAACAACGTTCATCGT
TTAACGTCACTGTCGTTGACCCAATGGTAGCTGAGCAGCGCATGGAGCGCTCGCAACTTATTGTGGAAGCTCA
AGTTTATGACAACAGACAACTTGACTACTGTAGATTTGTTCGCATCGACGGCTTAGGTTTCACAACAGAAAAT
ATGCCAAATAATTACATAAATCTATCTCAAATATCGCAAGGCAAGTGTGGTATTGCCA
TTAATAATCCTACTATTCTTGAATATCATCCGTGGACAGTAGTAGCCAAGATACGAGGACAAGATGAAGAAAT
ATCTAGAGTCACCCCCGTTTTGCTTGAGGGTCCTCGACTACCACCTGTTGTTTATTACCGTATGCCAACACTT
GGATAGTATTTATGATAATGGGTCTT
>103792734_2 DT663131.3
GCACGAGGCTTTCGTAAAGCGGTCATTGAAGAAAAAGACTAAGAAGTCTCAAGGATCATCACGATATCGCAAC
TCGCAAGATGTGGAGCTTCAGGCATTACCTCTGCTAAAGGATGTTGCTAGCAGTGAGCAAGAAGAACTATTTA
TTCGCAAATTAAGGCAATGTTGTGTTGCTTTTGACTTCATGGATCCAGTGGCTGATTT

GAAGGGTAAAGAGATAAAACGAGCCACACTCAATGAGCTTGTAGAATACATCACTCCGGCACGTGGGCCGCTC
CCTGAACCTGTGTATCCAGAGATAATCAAAATGATATCGGCCAACTTGTTCCGCACTCTGCCGCCGAGTGACA
ACCCTGACTTCGACCCGGAGGAGGACGACCCGACTCTGGAGGCCTCGTGGCCGCACCT
CCAGCTGGTTTACGAGTTCTTCCTGCGCTTCCTCGAGTCCAGTGACTTTCAACCTACCATCGGCAAGAAGGTC
ATCGACCAGAAATTCGTCTTGCAGCTGCTTGACTTGTTTGATTCTGAAGATCCTCGCGAGCGCGACTTTCTCA
AGACGGTTCTGCACCGTATTTACGGCAAGTTCTTGGGGCTGCGAGCTTTCATTCGGAA
ACAGATCAACAACATTTTCCTAAGATTTGTTTATGAAACCGAACATTTCAACGGTGTGGGTGAACTGTTAGAA
ATATTGGGAAGCATAATAAACGGATTTGCTCTCCCGCTGAAGAGCGAGCATAAACAGTTCCTGGTGAAGGTTC
TGCTNGCGCTGCACAAGGTGAAGTGTCTGTCGCTGTACACGCGCAGCTCGCCTACTGC
GTCGTCAGTTCC
>103792740_2 DT663314.3
CGGCACGAGGCTCTTACCTTAACAATACATCAACCATCAGCGTTGCTCTTCGAGAAGATAGACTCGATTTATT
GCTTGTCGTCTGGAAACACCTTGTATTCAGGACCTAGACAGCATCTCTTGCCAGCTATGAGTAGTGCGGGACT
GAGATGTCCACCTTACCACAATCCGGCTGATTTTTTAATGGAAGTCGCATCGGGCGAA
TACGATATTGACTTAAAGGAAGCTGCCAAAAGTATGATTCAATACAAACCAGAGTCAACGTCATCAGCTTGGG
AGGATGTGCCAGTCATATCATCATTATCGAATACTTTGAGTATGGATACAGAAATACATGATAAGCAACTTAG
ATGTAGCGGCAAGGGAAAACATAAAACTTATTCTAAACATATATACAGGCCATCAGGA
TATTTGCAACAGACATGGATTCTCCTTTACAGAAATTATCTGATTACTACGAGAAATTATGCTCTCTTCTTAT
ATCGCGTGGCAGCACATGTAGTCATCGCGTTGATATTCGGATACTTATACCATGGAGTTGGTGGAGAGGCCAG
CAGTGTACTTGGAAACTATGTTTATTTATACGGGTCCATGCTTCTCGTTGTTTACACC
GGAAAGATGTCCGTCACGTTGTCATTTCCACTCGAGATGGACATACTAAAAGGAGAATATTTTAATCGATGGT
ACAATTTAGGACCCTACGTCGTTTCGGTTCTAGCAGTAGAGTTACCATTTCAGATGATTTCCTGCGTATCCTA
CGTG
>103792764_2 DT663315.3
GCACGAGGCCTCGCCTATAAAAAGCAGCGCCTCAACGCGCGCACGCAGTACAAGGCGCCGCCTGTGCGCGGGA
GACGATCATCACAAACTCGTGAGAAATCGTCGAACAGCCGGTCGGCGTCCGTAGAGTCGACTCGAGGTATCTC
TCCGCCGAGGGAATTTGGTGACTATAGGCACGGCAGGACGAGAGCCTCAGAATCGACG
TCTAGCAACGATTGTCATGAAGACGTTTCACCGCACATGCCGGTCATAAGAAATTCAAGAGCTTCACAAAACA
ACTCATCCGGCGATGCCAATGTTAAAGCAAGACAAGCAAGACTTGCAAGAGATATTAGCAGTTCTAGACTGGA
CGATAATTATGATCCATTTGCATCGGCAGCTAGACAAATGAAAGAGCTTATGTCGTCA
GACAATAATATTCCTAAAAAATCCCAAAGTTCCAAAGATCTTAGTCGAACCCTTCCCAGCTTACGTCCCTCTA
AAGAAAAAAATATCCACATCTTACCGTACAGATAATACTAAAATGATGAAGGATACTTTAAATAAAACATATCA
AAAGACACCAACTCTACAAAGAATGAAATCGTTTGGTAGCACTAATAACGACAATAAT
AGTTCTTTTGGTGGTAGGTGTAGTTCGT
>103792773_2 DT662687.2
GCACGAGGGTTCAGTCTTGTTGCGAGAATTTAGCCTTGGCCTTATGAGAGTTATTATTAAACTAAATATTATA
TGTAATCATTATATTAATAATTATTAGCGATTAAATGGCGGAGAACGCCATGCTGGATAGTGACAATTATTCT
ATATGCATGCTTCGACGAGCTTTTTGGTAAAGTACTAATTTAAAATTTAGTAGTTACT
TTAATTGTGTAATCTTTAGGTGAATATTACATATATAATCTAGATAAGGGTATTTATTGTAGAGGATCAGCAT
AGTTACACTACAGTTAATTGAAATGTTATTCGTACATACTAATGAAACAGTGTAATGTTAAATTTATTGTGTT
AGTTTAATCAAATGGAAAAAGATTAATATATTTTTTATTTTTAGCTAGTTGTCTGTCG
CAAACAGGACGATACTTAGTTACCCATACGCGAGGGCGCGGTGACCTAAGTGACACCTCGTTGCGATATAGCA
ATAAACCAAAAATGATTCTTAATGTTTATATTATAAGTATCTAGAGTTGTATTCTGCTGATGTAAACATGTAA
AAATTATAGAAAAAAAAACTAAAAAAAAAAAATCCCTGCTTAGGCTATTATATTAAGG
TTTCGGATATTTGTTGGGTTGAAATCTTGACGCGATATCTAGGAAGTTTTCATACGATTGTTGTTGTAAATTA
CCGGAATATCCGCATGTGAATTCTTATGAAAGTCTCTTAGTCAATATTTAATAGAAGTATGTCAATTTTTGAC
>103792836_2 DT661727.3
GCACGAGGTAAAGACCCTTAAAAGTTTCTAAGCAATGTGTACATAGTTTACGTACCTATTAATTTATTTAGCC
AAAATTTTGCTCAATATTGTATACACTCGACACCAAAGTCATAATAGTAAAATAATAAAGTAACATGTTCAGT
ACAAAGAAAGCATAATTTTACAGAAATATAAAATGCCTTTATTATTAAACATATAAGT
TGAAAGTTGATCTGTAATAAATGTTTTTTTTTCAGTAAAGAAAGAAATACAACGATAATATTTTTTTAATCAA
ATACATCACAAACAAGGCATTCAGACATCTCTTTTCTATAGTTCTTTTTTATAGAGCCTGATACGAGCCCTAG
TCCCGTCTAAGTCTATATTTCGTAATATTTTTGCTTTTACTGGATTGCGTGTTATTAG

ATGTTTTTTTATAAAAGAGTGTATTTAATTTATTATTAATATATTTTTAATGTGCTGAAAATGAGCGAGATAT
GTCGTCGCTTGATATTACATCCGTCTACATTTAAAGTTTATTTTATTTAAGTTAGATATAATTAATTATTATT
TAGTTAATCAAAAAATTAACTTTTTATTGTACTTAGCTAAAGAGTACCTTTATATAGA
CAATCGAGTATTTCTATAAGAGAGAGAGGCAAGATAGTTTACCTATCTGATGGTAGAATTATAAACATAGATA
TTTTACTTTTTTTGAAAGC
>103792853_2 DT663081.3
GCACGAGGTATACTCGTTAGCAGCGCGCGCGCATCTCACTAACAAAATGTCCTTAGTCGAAGGTGCCTCTGAC
ACAGACAGACAGGACGGTAACGAGAACAACAAGAAGGAGCGATCATCGATGTCAGACCCTTACATGTGGAAAG
AGCAGATTCATACCTTGATGAAGGGCGGAGACAGCGAGTCCGACATATCGGACTCCGA
ACACTTTCTAACTAAAGGAGCTTTTCTCCTAACGCCATCCCACGGCGTTGAGCATGGAAAAAGCATCGGCTAT
TTGCGAAAAAAATGGAATTCAAAGGCTGTTTCTCCCTCACTAAAACTGCTACTGGCATCCTGTTCAAGTTCTCC
AGCGTAAATGACTACCAGATGGTGTTCAAGAAGGGCTTCCACAAGGTGACCGGCTCCA
GGTTCTATAGAAAGATCGCAATTCCTTGTCGTCCACAAAAGACGTTCACTATTTACGTCTACGACATCCCAGA
TGACGTGCCCGAAGAGGATGTGCGTCACGCGTTATACAAATTCACTTCTGTAGTAGAAGCTGTGCGTTTGCAT
TTTGCTGGGTCACCTCGTGAAGGGAACACAGAAAAGACGCCACCTCGTGCCATCACCA
ACATCGATGGTGAGCTGGTCAGTAGTATGGTACCGAAGGAAGCTTCGAGCGCGATGAGAGTGACGCTAGCTAA
TATCGAAGATGCCAATATTCTACTCCGGAATGGTTTGGACTCTTATGGTGCAACTTACGTTCCTACTGAGTTG
GCCACTCCTGCGC
>103792884_2 DT662348.3
GCACGAGGGTTGATCTTACAACTTGAGATTATTAGATCCGGAGTAAAAGTTCAATTTACTTTTTTTCGTTTAA
AATTCAAACAACTTCGTTTTAATGGTCAATAAAAAAATATATACACAGATAATCCATAATATCGGTAAAGAAA
AGGCACAATTTAATAAAGTTTTTATCTATAAAAGGGAAAATTTTACAAAAAATATTTT
TTTATTTTTAAATACATTAAATTATTTTCACCTTGATCTTCTAAAAACATACTTTATGTGATGTATGGTGCAT
TTATCATATTAATTGTTGCGAATCGAAATTATATATTAAAAAAAGGTTTATAAAAAAACATGACTAAATATAA
ACCTTATAGTGGCTTACAAAGTGAATACAATAAAAGCTAAAAAAATGACGATGACGAG
AACTTTTATACTAAATTTTATATATACTTTGATACTGTTCCAAATTGAAAAAATATATTTGTGGATTATTAAT
TTAACGAGACGGACTATTTTATTTCAATAATAATGAAAAAAAATAAATTAAAAAATTGTTATAACTAAGCAAA
TTAAAAAAGTTTTCTAAGAATTTATCTTTTGTTTTTTTTAACAACGATCTCTTTTATA
TTACCGGAATATCATCTAAAATGGCATCTGAAATAAAAACTAGCTCTTATTGTATTCACTATCATTAAAACAC
TGCCTTAGAAATGTTTATATGTGGAGTTACATTAGAATTAGCCTGTTGATGTTTATTGATGATATATATTGAA
AT
>103792899_2 DT663117.3
GCACGAGGCGCACTCCTGCGCACGCTAAATTTAGATGTCATTGAAATCTAAATTATAGTATTTTTAAATATGT
TAAAGTATAGAATTCAGTGAAAATGTTTGTGTTTAGTAAAAACTCAGTGAGACAAGTTATAAAGTGGAAAGAT
AGAGTGTCTTATCACTCTGGATTAGGAGTGTTCGGACATCGACCGCGGGAGGTGAATG
AAAGTGAAATTTCCTCAGAAATTCTAAACAAAAGAAACGAGAACTGCCGCGCACACCGCGTCGTTGAGGCGTA
TCGCACTTACGGCCACCTGCGAGCATCTATTGATAACGTTGATTATAAACAAGATAACAGAATCATCAAAGAG
CTGGATATATCAAAGTACGGCCTATCACAAACCGACGTGGTGGACGCGGGCTTATTGT
ACGGTTACAATGGGAGACAATCTGCTGGTGACTTGGTCAAACTATTAGAGGAGATTTATTGTGGGCCCATTTC
TTATGAGTTCTCGTATTTGGAGACCGAAGCTGAAAGGGAATGGTTCGCCCGACGTATAGAAAGCGGTACAGAT
ACCATAAACAAAACACGCCAAATAGAAATTCTGACTGAACTGTTGAATTCACAATGTT
GGGACAAGTTTTTATCTGTAAAATATCCTACTGTTAAAAGATATTGTGGAGAAGGCGGAGAGTCTTTGCTGAG
TTTCTTCTCTACGCTCTTCAGGTTGACTGCTTCAGACAACGTCAAGCATGTAGTACTGGCGATGGCTCACCGA
GGCAAATTGAACGCTCTGAGTGGTCTCTTGAATTATCACCGGTGAAAATCTTTCACAA
ATTCA
>103792940_2 DT663080.3
GCACGAGGGCGGCGCCTCGTGGGGAGCTCCAACGGCGTGTACGCGCTCACCGTGTGCATCATGTCCACCGTCA
ACAAGACGAGAGTGTTGGCTTTGCAGCTGCTAACAAAATCGTGTTACCCACCTGCATCCGGCCATACAATGAT
ATCGGAAGCACTCTCCACTCTCCGCCTACGTTTCGGTGAACCAGTTCGCTTCAGGTTC
CTGGTCGGCATGCTTCAAAGTGCTGCCGGTTCCGGGGACCTGCAGGCAGCTGGGCTGGCCTTCATCAACGCGT
TGTTGGCGAGTGCTCCATCACCACAAAGGAAGTTGTATATACAGGCGGAATTGGAACAGGCTGGATTTGATAT
TGTTACTTTGAAAAAAATTGTGGCCAAACTGCCAAATGTGAATGAACATGTCACCAGA

GAGATTGAACACTATGAAAAACAACTCATAGACATCGACACGTTAAACGAAAAAGCGAATGAGGCGCTCAAAG
AGAAGGACGATCTGCGAAGCAAATTGGAATTACTAGAGAAAAGAGTTAAGATATTACAAGAAGAGAAAGGTAT
TTTACTGTCATTAGAAAAATGTTTACAAGAAAAATGCTGCGTGCTTCAGGAGGAGGTG
AGCACGCTGAGGTCCGAACATGGCAACTCCAGTAGGAAGAAAGGGTTTGCCTCTAAGAAAAAAACTCGGCGCA
TAAAAATAGAGACGAATCATCCCCACCAGAAGATGAAGGTATAAGTTCATCCGAGCGATCTCTCAGCCCTGAA
GTCGACCAACGAGACTCGATGGTCTA
>103792949_2 DT662412.3
GCACGAGGTTTCTTACATCTTAAAAGAATATCGTATAAAAAGCTTCAGCCATGGACTCGATGAGAGAAAAAGC
ATTTCAAGACTATCGCAAAAAACTAATGGAACATAAAGAAGTAGAATCAAGATTAAAAGACATGCGAGAACAG
CTAAGAGATCTCACAAAGCAATATGATAAAAGTGAAAATGATTTAAAAGCCCTTCAAA
GTGTTGGTCAAATTGTAGGTGAAGTCCTAAAACAGTTAACAGAGGAGAAATTTATTGTAAAAGCAACAAATGG
ACCGAGATATGTAGTGGGTTGCCGTCGTCAATTAGACAAAAATAAGTTAAAAGGAGGTACCAGAGTTGCCCTT
GACATGACAACACTTACAATAATGAGACATCTTCCAAGAGAGGTGGACCCTCTAGTAT
ACAACATGAGTCATGAAGATCCCGGTGATGTTACTTACTCGGCTATAGGAGGACTGCAGGAACAAATCAGACA
GCTGAGAGAGGTAATCGAACTGCCACTCCTCAATCCTGAACTATTTGTCCGTGTTGGCATCACCCCGCCCAAA
GGGTGTCTTTTGTATGGACCACCGGGCACTGGAAAGACTTTACTAGCTAGAGCAGTTG
CTTCACAATTGGACGCTAATTTCTTGAAGGTGGTGTCATCGGCCATAGTGGACAAATATATAGGTGAATCAGC
TCGTCTCATCCGTGAGATGTTCAACTACGCGAGAGACCACCAACCTTGTATTATATTCATGGACGAGATTGAT
GCTATAGGTGGCAGACGTTTCTCTGAAG
>103792960_2 DT663313.3
GCACGAGGACAGCCTTCAAAATGGACACTTATATGGATTGTTTGCAAAGTGACACTTCTTTATTTATCAATTG
TGCTTTTCTTCTAAGATTCTATTCACAAACTAGGCAAAGAGAGTAACAATTTTTTCTCATCTATAAATAAATT
GTATGAAGCTACCGGAAAGCAGTTTGATAATGATGAAGATATGCCTGCTATAAATGTA
CCATCTGAAAATCCACTTTGTAAAAACGATTCAGCAGAGAACATGCTGTCCTGTAACGAAACAACGTCTGAGT
GTACAGAATCAGAAAGTAGTGAACAATTTTACGAGTGTAACGACAATATACCATTCATGGACAAACTTGACAA
CATTGTTGACAATCTACAACAACCAATAAAATTCGATATGCCTGATAAACAAGTGAGA
AGTGGCTCAGAAGGAAGTTCCAATGTGAAAATCGATAATGACTTAACGAAAGTCGCATTCAAAAGAAGTAGTT
CAGTCCCAAAAACAGACGAACGTATACATTTACCAATGAGACGAATAAGTCTCGAGCACAAAATCGATATTGA
TGATAACGAACCACCGTTAATAATCGATGCACAGTCCCCGACGTGTATGAATACTATT
GCTGAAGTTATCCCTGTGGCTTTACAACCGAGACTCGAGTTGCAAGAATCGAGTAGAAACGAGTCGCAAGATA
GTTCGCCGACTGATCCGCCAACAGAAATA
>103792982_2 DT662892.3
GCACGAGGCGGTGACCTTACTAGTGTTGTGCACAACTTTCATTTTGTTCAACCGCGAGTGCAGTGACAAAGCG
TTGCACGATGCGCTCGGTGCAGTCCATTATTTTCTTCGCTCTTATCTACTTCGGCGTTGCTCAAGACACCACT
ATTAGCGAAGATATTAATCACAAAACAAATACTATAGAAACATCACCTTCGGTGCGTT
TAGATCCATATATACGAAAAGCTTTACTAAAAGCTTTAAGTGATCTAGAAGAGAGTGCGACTTTTACAGATAT
AGAAAGTACAGATAGTACTACGTTATATAAAACGGAAAACGTAAATAATAATAATGAAGTTACACAAACAGGT
AAAGAAAATATACAAATTCATTCTTTTGTTGTAAATGGACAAGCTGCATTTGATAATT
CATCTCAAACGACTCCAATGATAATAGACAATAAAATATCAATTCTAAGCACTACAGTTGGAAATGTTGAAAA
TTATGCTACCGCTAATCTTCCAACTAAAGCGCCTTTTGAAGATATTTTCCAAGCAAAAGAAAGTGGAATAAAT
GTACAACAAATACGAAGCATACCAAGTAACTCTAAAGTAAGCCCTAATTTAGGACATA
GCAGTAAAATAACGACATCTAAACCTCGTGCTGTATCAACAACAACTACTTCAACTACTACTACTAGCACTAC
ACCAAAACCAACACACAACGAAGATGGCGA
>103793009_2 DT662537.3
GCACGAGGATTTGAACGTAAAGTTTATTTCTTTTAGTACTTATTAATTAATAAGGGGAAAATCAAATTTACTG
CCGAGACATATCAGATTAATAATCCCAATTATATTCCTTGAAATATGGACTCTGATGCAGAAAGAGAACAGCT
GCTTGTTAACAAACCTTCAAAACCTTCATTGGTTAATTGTGACATATTAAATGAATGC
CAGATGTTCTTAAAGGCTGAAGATGGAGGTGGTGATGCACATAAAAATATGAGTCAAAGTACTACAAACAATA
GTGGTAAACCTAATTTTATTACTTCAACTCCATATCCCACTAAGGCTACTTTATCAGAGGATGTTATAATAGA
TACTAGTCCAGAAAGTAATGTTGAAGGTGAAAAACAAAATGAAAATATTTCTAAAGAT
CAAGAGTCAGATGATACACTGCCCAGTACTGTAACTATTTTGGATGCCCCCGATGGAGGCAAAGTTTATTTGA
TAGGCACTGCTCATTTTAGCTTGGAATCCCAAGAAGATGTATCTAAGGTGATACAAAAAGTGTCTCCGCACAT
GGTAATGGTAGAATTGTGCGAACAACGAACTAATATATTGTTACTTGATGAAGAAATT

ATATTAAGAGAGGCTAAGAACATAAATATAGCAAAGATAAGGGCAACTATGGCACAGAATGGTGTTTTCAATG
GTCTCATGTACATTCTATTATTAAATATGACAGCTCATATAACAAGAGAACTTGGTATGGCCCCTGGTGGGGA
GTTTAGACGAGCTATGGCTGAAGCCAAGAAGATTCCAAATTGCATAGTAC
>103793018_2 DT662285.3
CGGCACGAGGGACCTTTTCTTATTCCTCGATCCGTTCTTGCGTAACACGGAGTTGGCGACGCCCGTCATGATG
CTGTATAAGGGGACGCTCAAGGTCCTCCTTGTGTTGCTCCACGATTTTCCCGAGTTCCTTTGCGATTACCACT
ATGGTTTTTGCGACGAGATACCACCAAACTGCATACAGATGCGGAACTTGATATTGTC
GGCGTTCCCGAGGAACATGCGTCTGCCGGACCCCTTCACGCCCAATTTGAAGGTGGACTTGCTCGCCGAGATC
ACGCTGCCGCCCCGCGCCGTCATCAACTACGCCACCATCATACCTGCGACGCAGTTCAAGAAGGACTTGGACG
CGTACATCAAGGCGCGGGCGCCTGTCACCTTCCTGTCGGAGCTCCGCAGCAATATGCA
GGTGGTGAATGAGCCCGGTCGCAGATACAACAGTCAGTTGATGAACGCGGTGGTGTTATACGTGGGCACACAG
GCCATAGCCCACATCAGATCTAAGGGGCAAACACCTAACATGTCTACAATAGCACATTCTGCTCATATGGATA
TATTCCAAAACTTCACTGTTGATTTTGACTATGAGGGACGGTACCTGTTCCTCAACGC
GATTGCTAACCAGCTTCGGTATCCGAACAGCCACACGCACTATTTCAGCTGCTGCCTCCTGTACTTGTTCGCT
GAAGCCAACTCTGAAGCTGTACAGGAACAAATTACAAGGATGCTGTTGGAGAGGTTGATCGTAAACCGTCCGC
ATCCGTGGGGTCTGCTCATCACGTTCATTGAACTTTATCAGAATCCCATTTACAAGTT
CTGGACTCATGAATTTGTACATTGCGCTCC
>103793021_2 DT663012.3
GCACGAGGTTAGGACCTAGAGGGCTCGCTGTGCCATTAGCTTTTGCCAATTCAGGATGGGCTGTTGGCATTGT
CGGCACTATTATTATAGCATTTAGTTTGTGGACACTGTGTACACATATTTGTGACCGTGTCGAGGGGATGTTG
TAAACTGTTGAGAAAACCCTTATTGGGTTACGCAGAAACATGTCGAGCTGTTTTTGCT
ACCGGCCCGAAAAGACTTAGACCATTATCTAACGCCGCAGGTGTATTCGCCGAGTTTTCCTTAATATTTACGT
ACGTCGGAGTTTGTTGCATTTTTACTGTGCTCATAGCGGATTTTTTAAAACAGCTACTCGACCAGTATGTGCC
GGATTTTATATTACAAGTAGAATACTACTGCCTCTTACTGCTAATACCACTATGTATT
ATGGTTCAGATTAGATATTTGAAGTGGTTGGCTTCCTTTTCTTTGATAGCGAACGTTTTACTGTGCCTCACTT
TTGTAATATGTCTATATTACATATTCAAGG
>103793027_2 DT663205.3
GCACGAGGCTCCGACATAAAATTCCTAATTGAATAAATATTTAATATTTAGACTTTTGAATAAAAATGACTCC
CTTAACCGATCTAGAAACACTCGGCTTGAGCTTTGCCTTTTTTCTTATTGCCGTTTTCATTAAAATTACAAGT
AGTTTGTTGTATACTTTTGTCATCGGACCGGCGTTGAATAAAGTGGATTTTAAATCGA
AGGGAAAGTGGGCTTTGGTCACGGGCTGCACGGATGGGATCGGAAAGGAATACGCCAGGCAGCTGGCATCACG
TGGCTGTGATATGATTCTAGTCAGTCGATCCATGGACTATTTGAAGACAACGGCAGCGGAAATTGAAAACGAC
TTTAAAGTGAACACGAAGATAATCAAAGTGGACTTCAGCGACGACGAGAGCATTTACG
ATAAGATATCTAAAGAGATTGCCGATCTCGAGATCGGCACTCTCGTGAATAATGTCGGCATTTCGTACAACTA
TCCTGAATATTTTCTGGACATACCGAATTGGGAGGAATCTGTCAGCAATATTTTAAAAGCTAACACCGTGTCA
GTGACGCGGATGACCGGCATGGTCTTACCCGCTATGGTGAAACGTGGGAAGGGGGGTG
GCATTAAC
>103793072_2 DT662910.3
GCACGAGGGTGGAAACAGTCAGTCAGTTTCAATTATCAATATGTGGTCTCTGAGTGTGGTTGTGTGTACGTTG
TTGGCGATCGCTGCAGCCACCGACGTACGGCAGTGCCCCGGTCAGACAGTTGAGGGCCTTAAAGACGTAATAC
AATTGACGCCTTGCAAGAAAGTGCCGTGTCGCCTAAAGAAAGGAACTAACCAATATAT
CACCATAACTTTTACGCCAGAGGTCGATATGGATGAAGTTAAGGACCATGTGGTAGCTCAAGTTTTTGGCGTG
CCACTACCCTTCGTCGGAGTAGACGGTGCTTCCGTTTGCGATAAACTTGAAACTGAAAGTGGAGAGAAAACTT
CATGTCCTCTTAAAGCTGGCACCAAATATATATATAAAGATAGTTTTCCTGTTCTTAG
TTTCTACCCTTCGATAGACGTAATAGTGCACTGGTCATTGGTGCATAAAGAAAAAGATATCATTTGTTTTGAG
GTTCCCGTAAAAATATCTTAATTATCTTGAACAAATGAATAATTTAAATTAAGTATAACAAAATTAAATAAGA
AACTTAGAAATGCAAATATATGTTGAACAAAAAATTTTATGATGTGGTTCTTTAAATA
TGGCGTTCCCAACGTGCCAGTTGGGCATATTATATCAATCTTCACCAATGTCACGTTTGACAAAAAAAATCAA
ATAGTTATATTTGTCTGTCACAATTATTGTAAAATATCATAATTATATTTTATATTGTAGTAGGTTGAGGATA
GAATGAC
>103793126_2 DT663018.3
CAAATTAACAGCTTTATTATCATAAAATATTGATCTTAAAAATAACTTAAAATATGTATGAATTATCACTTTT
TAGTTAAAATTTCACTTAAAACCTTCTTTTTCTTTTGTCCTTTGTTGAATTTGGGTTTTTTCTTCTTCTCTGC
CGTCATACCAACAAACTCGTTCCTCGGTTTCTTCTCTTTGGGGGGTTGATTGACATTT

TGACGTTTGCTAGGAGGTCCTTCATTACTATCTTGATTCTTTCTCTTATTCATAACTCTTCTATAGGCCCCTT
CAGTTTTATCATCATTTCTTTCAAATCTATTTCTTTGACTAGCATCTTGTCCTCCATATTTACTGTGACTTTG
ACCTGGCCTTTGACTTTGACCCGGCTTATGACTTTGACCCGGCCTTTGACTTTGACCT
GGTCTTTGTCTTTGACCTTGACCCGGCCTTTGATTTTGACCCGACCATTGATTTTGTCTATTATTAGGTCTAG
ATATTTTCTTCTGAGTGCCTTGTATACACCTTTTTACTCTCATTATTCTGTTTTTTATTGTAAAATCCTCTTC
TGTTAGTGCTAATGCCAGTTCTACGCCATCTTTGGATTTAAAATTAACGTAGCCAAAA
CCTTTTCCGGCATTAGTCTTCTTATCCCTCACAATCCTGACTGAATCTATTT
>103793132_2 DT662494.3
CTATTAAAACACAATTTCACAGCATATAAACGTTATAAATTATGATATAAACAATTGTTATTAGTTTATTTAG
TGATTTCTGCAAGTTATTGTTTAGTGCAGTGTTATATCTTAAAGTAAAAGGGGATCACTACGAAGAAAAAAAA
CATAGTGCTGTGTCCCGGATAAACAAATTATGTTATTGTAATATATTATATATACATA
TAGTACATAGTGTGACGTGTGTATGAAACGTGAAAGGTGAACGTGATTAGTACAACAATGAGTTCGATGGGCT
CCCACTCGAATTCTTCGCAGGGTTACTCCCGGAAAATTGACTTGGACCCGATATGGGCCGACCTGAGAGAGGG
CATCGAAAAAATGTACTCGCGACGTGCATTGGACAGACAGAGATACATGGTGTTATAT
ACCCACATATATAACTACTGTACATCGGTACACGAACAAAACGCTAACAGTTTGAGATCGGGCTCGAGTAATT
CTCAAACAATGTCGAGGAATTCGTTTGGATATGCAAAGAAACCGGGACAAGTGCACGGTGGCGCGCAACTCGT
TGGTCTTGAGCTGTACAAGAAGTTGCGCGACTTCCTCATATATTATCTTATCAATTTA
CTTGAGCACGGTGTAGATCTAATGGGCGAGGACGTCTTAGGTTTTTACACGACACAATGGGAGGAGTATCAGT
TTTCGTC
>103793141_2 DT663222.3
GCACGAGGGTTGCGCCTATTTCTGTTGGCAAGGAAAAGACGCTTCGTCTAACGAAAAAGGTGCCGCAGCTTTG
CTAACAGTTGAATTAGATAAAGAAAAGGGGCCTCAAGTGCGTGTGGCTCAAGGAAACGAGCCCCCTGCCTTCT
TAAACCTATTCCAAGGCGAACTTGTCATACACCAAGGAAAGAAAGGCACGGATAAGAG
TAGATACAAATTGTTTGTTACTCGTGGAAATATATCGAATGAAGCCTATTTACTTCAAGTGCCGTGTTCAGTG
CGACAGTTGAGGAGTCGGGGCTCATTAGTTCTAGTCGATACGGAAAAAGATTGTTTGTACATTTGGCATGGAT
CTCGTAGTTTAAAACACACTACACAAATAGCCATCGAATTAGCTAACAGATTGATCTC
GAAACAATCGAGCTTACTCAAT
>103793144_2 DT662937.3
GCACGAGGTATAATTTATAATATTTTATTATTATTTATATAGTTTTATATTGTCGCTATGGCGTCAGCCGTGA
TTAAGTTTAGTGCCATATTATTGCTGGTTGGAGTCGCCTTTGCCGATATTGGAGAGAATCCAGTCACAGAGGC
GTGTCTAGGATGTGTTTGCCAAGCAGTGTCAGGCTGTAAAGCGGGTTTAACGTGCGAC
GGAGACGCTTGCGGTCTGTTCCGCATAACTTGGGCGTATTGGGCCGACGCCGGCAAGCCAACTCTGGATGGAA
GGTCATCTGATGCTCCTGATGCGTACCCAAGTTGTACCAACGACCCTTACTGCGCGGCTCGTGCCGTCCAGGG
ATACATGAGGAAATTCGGACAGGACTGCAACAAGGATGGTAAAATCGACTGCCTCGAC
TTCATGGCAATCCACAAACTGGGAGGATACGGGTGCTCCGGGGACTTACCTTTCCAATACGTTAACGTTTTTA
ATCAGTGTATCGATTATGTGTCCTCATACCAGGGCTGATGTCATTACTCTTTGCTCATTTCCTTTGTGATGTA
ATTTTTTTTAAGAATAAAATAGTTTTAACCGATAATGATGTCATTTATTTGATTGGAA
GTAAGAAAAATATGTTACATTTGAATATCGATACATTTTTAGATTTGTAAGTAGATTTGTAGTAAATGAAAGA
AATGATTTACAATATTAAAATAATCATTTTAAT
>103793150_2 DT662409.3
GCACGAGGGCCAGGCTTATTCATAAATATGACACTGAACACGTGCGTACAGTGCAAGAAGGGAACATACCAGA
ACGAAATGGAACAACCATCATGCATTCCATGTCCGATAAACACCAGCACGAGAGACGCCGGAGCTACGTCTCT
AGAAGACTGCATCAACCCGTGCGAGATGAGCGGGCCGCAGCGGCGGCACTGCGACGTG
AACGCGTACTGCCTGCTCATACCGGAGACGAGCGAGTTCAAGTGTCAGTGCAAGCCCGGCTTCAACACCAGCG
GGGACCTTTGTATAGACGCGTGCCTGGAGTACTGCGACAACGGCGGCGAGTGCGTGAAGGACGCGCGCGGCGC
GCCGTCGTGCCGCTGCGCCGGCTCCTTCACGGGCCGCCGCTGCCGCGACAAGAGCGAC
TTCGCCTACATCGCCAGCGGCGTCGCCGGCGGCGTCGTGTTCGTCATCTTCCTCGTGCTGCTCGTGTGGATGA
TCTGCGCCAGATCGACAAAGAAACGTGAACCAAAGAAGACATTGACACCAGCGATAGACCAAAACGGGTCGCA
AGTGAACTTCTACTACGGCGCGCACACCCCCTACGCCGAGTCCATCGCGCCCTCGCAC
CACTCCACCTACGCGCACTACTACGACGACGAGGAAGACGGATGGGAAATGCCCAACTTCTACAACGAGACCT
ACATGAAGGAGAGTTTACACAACGGAATGAACGGAAAGATGAATAGCTTGGCGAGATCTAATGCTAG
>103793166_2 DT662760.3

GCACGAGGCTCAGACGTTCACACGCGCAACCCGTCTGTTCAAACTGTGGAGGTTCAAACGAAGGTGTAATACA
TTCAAAAGGTTATTCAAATGGCGCCTTTAAATTGTGTTTCTTTTGTGTTTTTTGTTCTGGCACTTCACCAATG
CTCGCCACGAGGTCTCCCAGAAGAAGACTCAGTCGTGAGCGTGTATACGACACAGTCC
ACTACAGACGGAGTGACGCACGTCGACGTCTACCACCAGATCGCTAACAATATTGCTGAGAGGATCACTTCGC
CTATATATAGATTCCTAGGCCTCAATAAAAATAAAACGGAAACTACAAAAAAACGTTCATGGGACAAAATCGA
ATTGTTAGACGAACCTGAAGAATCCACCAAAGTCATAGCACCAATCAATAATGATATA
TCTAATAAAGACGTAGAAGAACTATCTGCTGAGGCTCTAAAAAAGATAGATAAGAAACCAGAAAAGATAACAC
TATACTCAAGCTATTTACCCTCAGAAAAGATAGAAGTGAACGAGACTATTAACGAAGTTGATAATGAAACTTT
TGGCTTAGATGATGTTGATGATACTGATGATAACAATGATGATATAGATAATAGTAAA
CCTGAAAGTAAAATAGGTCCTCTAGTAATAGTACTGGAGGCGATCGGTAGTTTCATACAATTAATCTACGGTG
GATTAGTAGGGATTTTCTCATCTTATAAATCTAGTCCAAATTAAGCTTAACGAAAAGAAAAAAAATAAATTTA
TATTATTATATAATAAATGAAATATATATTAAAGATT
>103793172_2 DT662106.3
GCACGAGGCAGCAACCCTATGAGGAGCAGCAGCCTAAGAGAACGGTGACTGGCACTGATGCCGATTTGAGGAG
GCTTAGCTTGAATAATGCTAAGGCTTTGTTAAGGAAGTTTGGTGTTCCAGAAGAAGAGATTAAAAAGTTATCT
CGATGGGAAGTCATTGACGTCGTGCGAACATTGTCAACAGAGAAAGCAAAGGCTGGCG
AAGAAGGCATGACTAAATTTTCTAGAGGAAACAGATTTTCTATAGCTGAACATCAAGAAAGATACAAAGAAGA
GTGTCAAAGAATATTTGAGCTACAGAACCGCGTGTTGACGAGCACTGAAGTACTGAGTACAGACGAAGCTGAA
AGTTCTGTTAGTGAAGAGTCCGATTTGGAAGAAATGGGCAAGAACCTCGAGAACATGT
TGGCTAATAAGAAAACAACGGAACAGTTAACAATGGAAAGAGAGGAAGCTGAAAGAGCTGAATTGAGAAAAAT
AATTTTGGGACAGTCTGAAAAGAAACCACAGATTAATCAAAATGATCAGCAACAATCTTCAAACCAAGGTCGT
GTCCTCCGAATTGTTCGTACATTCCGCAACGCTTCCGGTCAAAGATACACTCGCGTTG
AGTTGGTAAGGAAACCGGCAGTCATAGAAGCGTATACAAAGATAAGGTCTACCAAGGATGATGCTTTCATACG
ACAGTTTGCTACTATGGAT
>103793175_2 DT663358.3
GCACGAGGGTCTACCTTTAAAACGCTAAGTTCTTGCAGTTTCAACTGACTGTTAACATTGTGGCCGTTATCGT
TGCTTTTATCGGTGCTTCCGAAAGACAGGACAGTCCTTTGAAGGCAGTACAAATGTTGTGGGTTAATCTCATA
ATGGACACCCTCGCTTCTCTCGCGCTAGCCACCGAGCTTCCAACCCCCGACCTCCTCC
AACGCAAGCCCTACGGACGAACAAAACCACTAATAAGCCGCACCATGATGAAAAACATACTGGGGCAGGGTGT
CTACCAACTGTTTATTATATTCACTTTGTTGTTTGCTGTTGTTTAAATGTTAAATATCCCTTCTGGAAGAGGT
CAGCAGCTAGGGTCGGAGCCCTCTCATCATTTCACAATCATTTTCAATACATTTGTAA
TGATGACGCTCTTTAACGAAATAAACGCTAGAAAGATCCATGGCCAAAGAAATGTTTTCCAAGGACTGTTCAC
TAACCCTATATTCTATTCTATTTGGATCGGTAC
>103793184_2 DT663070.3
GCACGAGGGTTTCCTCATACGCATATAACACGCTCAGATTAGTTAAACTTTCTCAACTAGAACAGCTTTTTCT
TTAATTCTCAGCTAAAAAATATATAATTTTTAAGTTAAAAAAAAAAAACCTTTTAAAACAAAGGTTTATTATG
TTACTTATCACATTCAAACACTTTGTTACGATACACGCTTTAAGTACAAAAGCTCTAC
GAAATCAAATGTTTCTCACTTATGCAATTCACGAATAAGTAAGTACTTTAAACTGGGCGGAATGACTGGTAAT
ATTTCGTACAAGGTTCCCAGTGACGAATAAGAGCAAAGATATCGTCGACGTCTCCTGCGTAGCTTAAAGACGT
GGAATATTCTTACAAATTTTATTATTTTTGTCTTGTTTCTTCTTTACTAGTGTACTTA
GTTTCTTAATTAAATATTTTAATTGCGTGTAAATTAAAACGAAAGAAAAATAAGGAGAAAATTTTAGATATTC
TGTGATAATATTGATAAGAAAAGCCATTTAATCAAGATTAAATCTGCGTAAATCAAATCAAATACAATAATTT
ATAAAACTCGTGTCATATGTTTGTGCTCTGTTTATAGCGTGTTTATGCGTGCAATGTC
AACGCCTGCGGGCTATCTGTAAATATAAAGATATATCANATAAATATAAAAAAAATATATATAACAATATCAC
CACAAATTATAGAGATATATGTATTGCCTTTGTTTATTAAACCTATAATTTTAAATACACATTAATATCCTCG
>103793205_2 DT662901.3
GCACGAGGCATCAACCCTCATTTTATATATTTATTATACTTGCGTGTAAATATTTTTATCTATTTATTTTCGT
TTTCATGAAGTTTAACCCGAATTAACGTGTGTATTTGCTCGATGTTTTTTTTTTTTTAATATTTATTTCGGGT
CAGAGCCGATTCCATAACACATGTATGCCAAAGTCGATGTCCTATTTGCCATTAAATA
CCGCGACATTCGATCTGTGGATCTCCGACATCACAACAAACTATACTATTTTAATGAACGTACAAATGGGCGC
CATTTTGTTTTTATACACGGCCCGCTCCAAGCTGCTCCGGTGTTATTTTAGCTCAAGTATTCAAATAGTAGTT
TGCTCATATTAAATATTGTTGTTATTATTTTTTATTATTTATAATATCTTTATATTTA

ATAATAAACTATTCGTTGGCGTGCGAGTGAAATTTTTAAAATTAAATAATGTCCGCCATCTTGATTTTGTTAG
CTTTTAATGTACGACAAAATGGAGTATCCTGATGTTGTGTTAATAAGTATTGCATCGTTTTCGTGTTACGCGT
CCAATGTGTATTTGTGTACCGCCTAGAACGCATCTCATAGTAATATTTACTCACCATA
TTTTTTTATGAGTACGTACGTACGACTCCCGCCGCCATCTTCTTTGTGAAAGTTCAAATTCCGCTGAGACTTA
GTCTAATTTAAATTATAATTGGTTATTTGAAGCGAGACGGCGGCGCGGCGTCCACGTCGGCCTCCTCCCCCGT
CGCGAATTTTGATGCGATCTCAAA
>103793213_2 DT662306.3
CTTGCACACGTGTTGGCTTCTAACACGTTGACGTTATCACGTAATATATAAATAGAAAAAAAATAAATAAATA
TATGTATTAAATAAATGAATTATATAAGACTTCATTTATTGTATTTTTGTGTTTTGTGTTGATAACATTGTGA
CTATCTGTGCATAGTGTGTGCTTTTTGTGGATTTACTCCTGGAGGGAAAAAATTTATC
CACCAATCATCAAAATAATGGGCTAGCCCTACCAAGGGAATTCGAAAATGAACGAGAGTCGCGAGCGAATTCG
ACGTGAGAGGGAGAGAGAGAAGAACACATATACTTCTCCTCGCCTAGCTTTGCGTCGCGTGCTGCTTTTGGCT
GAGGGTCGTCAGTTTAGGGAAGCCGCGGCCATCTTGGCGCGCCTCGGCCCTGGCGTAC
TACAGACAGTGGCCACTGAATTACCAGTAGACTTGCTGGTAGAAGCCTTACCTCATTCCGCTCATCTCATTGA
AACTCTTTTGAATAGGCTAATATCATTAGAAATAACGCCCCGTCCTGAGTTACAATGCGAGACAGTGGCGTGG
AGACTCGTCGGTCTTCTGGGTGGAGACCAGGGATCAGGTCTGAGGGCCAGGACAGCTC
GGCTGGCTAATGCTCTGGTCCATTACAGTCCTGATGCTAG
>103793216_2 DT661801.3
GCACGAGGTTATCTTTTGAGTTTTAATTTGTGAAAATAATAATGGGTTTGGCTACGGTTATAGAAAATGAAGC
CCATTTCCAATCTTAAATGGCTAACGCAGGAACAAAATTAGTTGTTGTGGATTTTACCGCTACTTGGTGTCCT
CCATGCCAAAGAATAGCCCCATTCTTTGAGCAACTACCAGCTAAGTTTCCTAGGGCAG
TTTTTCTGAAAGTGGATGTAGACAGATGCGCAGAGACAGCAAGCGCGCAAGGGATAAGTGCAATGCCTACGTT
CATATTCTACAAGAACAGAACTAGGATTGACATATTTCAAGGTGCAGACCCTACATCATTAGAAAATAAAGTG
AGGCATTATTACGGAAGTGATGAATCAAGTGAAGATGACAATGCTGTTGCTGGACATA
TGGATTTAGCAACATTTATTGTAAAAAATGAATGTGAATGCTTAAATGAAGCAGATGATCATCCACTTGCTCA
TGCTCTCACAAGTGGAGGAGGACACCTTGCTAGTGGCTGTGATGAACAGCTTATCATCAATATATCTTTTAAA
TCAAGTTGTAAAATTACATTCTCTAAAAATGAAAGCAC
>103793223_2 DT663284.3
GCACGAGGGTTTTTCTTTAAAATGCGTTTGCAAAAATTTTGGAATATTAAATTGAAACTATAAATATTTTTAG
GTTGCAGCTTCTTTAATTGAAAATGTGTTTATTTTTAATTTCCAACATATAATTAAAATCCAGTAAAGGGTAT
TGTTAACGCTTGTTTCAATATAGAAATTAAATTTGTGTTATATTTTACCATGAACCCT
TGTTCTGTGGCAGCTCCACAACAAGATATTGAAGGAGACGGCAGGTGGAATAGCATTCATAAAAGATTTCTTT
CGGACGCAAAAGGCAAAGACGGAGACGTCATTTTTATTTTATATTCCATTCTTCAAGCATTAGAACATACAGA
AGTGTGGAATCAGTGGTTTGCACCACTTCATTGCCTTAATTTTAGCATCTACAATGAT
CAAACCCAAAATATACTATGGCGTATCAAAAATGGAGAGTTAGATCATGTTGATCCAAGGGTGATAGTTTTAC
ATGTAGGGACTAATAATTTCAATCATACACCAGATCAAGTCTATGAAGGTATTTTAGAAATAGTTCATACAAT
CAGAGAAAAACACCCCAGTGTTTACATTGTACTGCCTAGTCTTCTTCCAAGAGGTCAA
TATCAAAATGTATTGAGAGAGAAAAATGATAAAATTAATCAACTTTTACATGAAAAAGTTGCTCAAATGAATA
AAGTTGAAAT
>103793226_2 DT662820.3
GCACGAGGTAAATACCATATAAGAATAAATAAAAAAACAATTTAAATTGGAGCATTTATTCATTCAAACATTC
ATCAAGTTTTTATCATAACCTTTATTTTATATAAAGAGATTAGCAAAATGTTATTTGTTTTCTTAGAATATAT
TTTAACATATGTCCTAGTATTACCCAAAAGTAATAGGAACAGGTTTATAAAACTAAAA
TTTAAATAAATAACTTACTATATCTTTATCTCTGACTGGTATCTCTTGGTCTGTAGGCTATACCGCGACTCTT
CATTTCTCTGATAACTCCTGCTGGAAGGCGACCCGATACAGATATAGCATAAACTCCTTTACAAAATCTACCT
ATAAAAAAACCAGGTGGTAAATGACAGCAGAAATATAATATATATAAAGGTTAGAGAC
ATCAAATGTGTGGAAACAAGTGTCATTCATTACATCATACAAAGAAGATGATGTAGACTGATTTGGCAGTTTT
TAAATATAGGTTAGGTTAGTTTAGTTATTTTTTAAATTTTAAATACTTAACCTGCATTTAATAAATGACAATT
CTGTAGCTACTGTGGTAATAGTATTGAAAATCTATGACTGTGATTAGTAAGCACTGAA
AAAAGTTAAGTTAGACTTCTCAATTTTACCAACTCTGACTTATATTATAAGTATAATATAAAACAGAAAACAT
TAGTAGTAGGCCATCTCTTTAGGTACACAGATAGGTGTTGATTAATAATAACAAAAAAAATTATATATCATAA
AATGGAAAGAGAGCAAGG
>103793269_2 DT662052.3

AGAGGCATCTAGCAGTCTAAATAAAGAGAACGTGGCACGTCCGTCACACAAGGAAATACCAAGTCCATTTAAG
AGGCACTTCTTTTATCCTGCGTTTGAAGAAAACAAGAAAAAAAGGATAATGAGGGAAAGAATGCCAACGATTG
TGTCCGGAGAATTGTGCCAAAAATATTTCGAAACCAAAAAGAATAAGAAAGAGGATCT
TGAGCAATTGAAGATAGAACGAGCCACTGAAAGGCAACGAAAGAAGGAAGAGAATGAAAGATTTTTTTTTAAT
AGGGTTAAAGTGGTGAAAAATAAACATATTAAAAGTGTATCAAAGATTCTTTCAGATAGCTCTGACTCGAGTG
TCGATGTTTCTTCTGGAGATAGTGTGGTTTCAGAATCCTCGGGAGATGAAAACTTAAG
CAACCTAAACCCTTCTAGAAAACCGTTATTAAAACAAATTAATGAATATGTTATTTTCACATACGATAATAAG
TATTACCCTGGCAAAATAATTAATGTTACTGAATATACTGCAACAATTTCATCCATGATACAGTGTGGTCGCT
TATGGAAATGGCCCGAGAGACCTGATGTTCTTGAATATCAATGGAAAAATGTGACATC
CCATATAAATGAACCCAAAAAAACCAGTAAAACGCGTATTGTTTTACTCTGTTCCTGAGCTAGATTTCATGTG
GG
>103793272_2 DT661775.3
GCACGAGGGCGGCATATATAGATGCAGCTACATCAGCAGCTCTAAACCAAATGCTATGGTTACCATACCTAAG
CCAATTGGAAGCGATGGCCGCGTGCTCTCAAAATATGGACTTTTTAAAAGCTTTAAATTCGTCGGGTTACTCG
GGGCAAAACTATCCTGACCTTTCTCAAATGTTTAATCCTTCGTCACGTTTCATGGGTC
CTGGAGGATTTCCCATGATGCAACCCATGCAGGACTACGATAATCGATTGCAGTTCGCTATGTGGCAGGAAGC
GATGTCGCACGCGAACGCGAACGCGAACGCGTCGTCTTCGCAGCGGTCTAAAACAGTTAGTGATCAGTTGAAA
GAACTTTCAAAGTCAAGTGACGTTCAAAATCCTTATGTGCATTCCACTAAAAATCATC
AAGCAAACAAACCTCATTCTTCGGCTAGAACGAGTCCAATTGCCCATAACTATAACAACCAACGGCCCATGGG
TCACCACAGCCCGTTCCTACAGGGCATGTATCCAGGAATGAATACGAACATGAGGCCTAATATGCCATTACCG
GGTTTACAAATTCCATATTTTAACCCGCAAATGATGGGTAACCAGCGATCCCCGCGAG
CCCCGCAGCAGAAGAGTCCAAACTCTCCATATTATCCGAATAACAACTACTTGCAATCTGTGAAGCAGTCTGA
>103793275_2 DT663054.3
GCACGAGGGAATCGCATATGAAAACACATTAGGTTTCTTTCAATAGGATCGAGTGGTACCCAAATGTCAAGTT
TCTCTCAAGTCAAGTTCTTGTAACCAAATTTTCGTAAATGATGTATAACTGTTTTATGATCTACATCCAGTTC
TTCAGCAATGTCATAACTGCTCAAAGAGGGCGTCTAGAGCGAACCTCATGTTTCACAC
TAAAATTTCCAGACTTGAAACGCTTGAACCACATCTGTGCTACTCTTATTAATACCGCATTATGTCCGTAAAC
GGCACAAATTTTATTCGCGGCGTTCGTCCCATTACCTTATACCTTTTTTGAAATAAAACTTTAAAATGTACCG
AATTTCTTCGTCACATACGTTCATTTTGCTTGGTTGAAAAAGAATTGAAAAATGGCGG
TAACTAGTTTTTTTTATTTTGCATCTTCAGAATGTCAAATTTAATTGAATTAAAACCAGCCAGTTACATTCAG
TACAGTCAAAGAGGTTTGATTACTAATAAAACATTCAAAATACGAAAATACAAAAGTATTTTCGTATTTATAA
AGTATTAAAATATGAATATTGTTGATGAGTTCTTATCTTGGTCATCTAGTAATTATGA
TAGTGTTGACAGTAATACCTCGTCCCTCTCATGAGCTTGCTACTCTCTTCTTTCATTTACTCCTGGATCTCCG
CACTGAGCGGGGCGGTGGTGGCGTTGGTACATACCTCCGTCACATCACTCCTGGCAGTGTCGTCTCTCGCT
>103793278_2 DT662889.3
GCACGAGGGTCGCCCCATGCAACGCGCTCCTCGCGCTGTCCGCCGCGCTGCTCGCCGCCGCCGCCGCCGCCGC
CCCCTCCGCCGCGCACCACCTGCGCCCGCGCCGCCAGCTCGCCGACGACAACACTCTACAGCCGCTCGACGAC
GCGTCGAGCGACGACCAGGCCCGGGAGAAGCGAAAGCTCGAGGGCGTCACGCAGGCTA
AGTTTGGTATCAAGAACGCCGTTCTGGGCTTCGTATTCGGGAAAATCAATTCGTTGATCGACTCAAAGACGAG
GCTCGTGGAGTCGCTGGATCGCCAAAACATCGAGTTGAATAAACTTTATGGCATCGAAGCGCCGAAAGGAGGA
GGTCTGAGCTCCCTGAGCGGCATCGTGGGGCAGGTGATCGGGCCCAAGCTGCAGTTCC
TGGGCCCCAAGATCCAAGCGGTGACGGGGCTGCTGGGTGGCGCGTCTGGCGGCGGTGGTTCAGGTGGGGGCTC
GGGTCTGGGCTCCATCTTATCGCTCGTAACGTCCTTGTCGGGATCCTCGTCCGGAGGAGCTGCGGCGGGCGCT
GCCGTTGAGACCGATGACTCCTCCGAGGAGGAC
>103793290_2 DT662245.3
GCACGAGGGTTTTATGGTGGTTTCCTTTGACGCTCTAAGATTTCTTTAACAATTATTTTATTTCCAGATAATA
TTTCACAAACTTCTTAATATATTTTGTTTGTCATCATAAAAGAAACAAATTTAAGAATGGCCGAAGATTTTGA
TGTGGAAGCAATGCTAGAAGCTCCTTATAACAAATTGAACTTTGTGGTAATTGTCCAG
CATACTTCACCAACCTGAACAAAATGAGAAGTATCCCTGTGTGATGTTAGCGCTGTGTACATCCTTAATCAGT
GGTTTTCCGAATGGATAACACTTCCTCTACAAAACACCGGTCGGACAAGTATTCTGACAAGCATAGAGACAAA
GAAAAGGACAACAATCGAAGACGAAGCAGGTCGAGAGATAGAAGAAAATCAAGAGATA
AGGATTCACTCCGTTCTAGAGAAAAGGATGACAAAAAGGACAAAGATCGTGAACGCGATCGCGACCGAGATCG
GGACCGCGACCGCGGTCGGGAGCGGGAACGGGATAGGGACCGTCGCGATCGCGATAATGACAGGTCTAATAGG
GACAAGGACAGGGATCGTGCGGACAGACCCGACCGGCCCGATCGAGGCGACCGAGGCG

ACCGAGGCGACCGGGGCGACCGTGGCGACCGGGTAGAACGAGCGGAACGGGTAGAGCGAGCGGAGAGAGGGGA
CCGAGGGGACAGGGCAGACCGACCAGAGCGAGGAGACCGCGGTGAACGCGGCGACAAGGACAGAGAACGGG
>103793302_2 DT662484.3
GCACGAGGATTTGATCATTTTCAATATGAATCCTCAAGAACTGCACCTCGACCCGTTGCGCCTCGGCCCGTAC
CATTTGTTCAACGTCCACAACCTACTCCATTCCGACCAACTACAAATGATCCCCGTGATCAAATGTTACGACC
AGGCTCTAAACCTGCCCTTCCAGATAGAATAAAAAGTTCTATAAAACATCATCCGCAA
CCTTTTCCTTCGCACTTACCCCCTCAGCAGCCGTTGCCATTTAATCCATTTTATTACGATCGAAAGCGTAGCG
ATGACTTAATTCCAGAAAATGAATCATCTTTATCAGCTCGATCTGTTGCACCACCAACTTTAAAAGAAAAAAC
GATACCAAAGCCGAAAAGTCCACCTCGCGTTATTGTTACAGCAAGCGCATCCGTAAGT
GACAGTAATGGCAAAAGGTTAAATTACACAGTCGGCAATGTAGTAAGTGCTGTGAAGCCTATAGTGCACATAA
ACTACGACGATTACAAAGAATCTGATCTTATTTTTGATCCATTCTTTCTTGACGTGCCAAAATTACAATCGCG
ACGAAAGACTAGGTCTAGTAAATACAGAAAGGTTGTTACTATACCCGCCCCAGCAACT
ATAATGGCAAAAGTAACGACCGAAGAAACTGCTTCTTCGTTCTCACCAATAATCTTGCAAGCTACAACAACAA
AATTAACATTAACATCAACATCAACACCATCTACAACTACTGCTGCTTGGAACCCCGAAGACTACGTTGACGA
TAATTATGAACCTCATGCATATATTCCACCAGCGCCTCCATTAGCCGTTTTAGTGACT
AATGACCAATTTAATATAAAAAACG
>103793320_2 DT662177.3
GCACGAGGCAAACACTTCGGATTCCAATTAGTAAGTCAGCTCAACAAAACATCGATAATAAAGCGTACTTATT
GAAAACACAAAGCTTAAAGAGGGGACCAAAAACCAGTAATAACAACGGATATCTAACCGATGATCAGCAACTA
CTTCTGCGTAATATGCTTGATATGGACAAAGATTCCCAAAAGATTAAAAGAACTAAAT
CATTCTGGAAATTCGGACGTACGACTTCTGAAGAAATAATGGAAGGAATGTCACTATGGCAACATAGAGATAT
TGTTGACACAGTTCCAGAATATAAGAAGAAAATGTTCGTAAAATTGAAGAAAGAGTTGAGACCAGCTCCAGAT
ATCCCAGAAGCCAAAAAATCTAATAGTCCTGAAAAAACTGCTTTAATACCAACTGACA
ATTCCAATCGTCCTCAAGAAACAATCGATAGGAAAGATATTAAAGTAAATAATGGCATGCGACAGGCCAGAAG
CTTAGGATCAATACAACACGAAGAACACGCTGAGAGTAGGAGAAATAATATGAATTACCACCAAAATCAAATG
AACAAGAAAAGCTTCTCTGAAAAATCAATAGCGGAAGAGAAAAATGATGAGTTTACTC
CTCGTAACGAATCTCGCCATTCAGATCTGACTAACACAAGTACAATCAAAACTAACTTTGAAAATAGTTTTTA
CAACGACGAAGACGGCGATGGCTTTGTAATGAAGACTGTTAAACGTAGAGAAATATTACAAAGGTATGATAAT
GAGAGTAATTCCG
>103793326_2 DT662705.3
GCACGAGGAACCTCCAGTGATTAAACGTACAAATTAATATTACGGACATTGTACAGATTGATAGATAGCTAGT
ATTTATTTAGTTATTACATGAAATGTTTCATACAAAAATGATGAAAGTGAAGTTTGCGCCATTCGGAATCTAA
AATGTATGAATGTTCCCGTTGCCAGTGTAACCTCATGGGCCGTGTAACCACCAATTTA
CGAACGAACAGGCGGTGTTTGTGTCTGCGTATTGTCAATTGTTAAAAATAGAATGTCTATTGTTTGTTAACTT
ATTGATAGTAACAATATTGACTGGTTAATGTACCTCTCAGTATACCGCTTTACTATATGTAATTTAATATTAT
AACTGCATCGTAATATATGTAACTGACTTAAATATAATTTTTGTAACATGTCTGACTA
TAGGTTCAGGTTCATTTTCTATTAAATAACTATAAGGAAATAGCTAATTTACCAATAGTAGACATATTATATG
CTTGCATATATGTACCTATTTTTGTAATATTGACAATTTTAGGTTCATTTAATGTGATTCTGAATTTGTGTTA
AGAATGTATATATGTAGGTGTCACACGTTTTAGCATATAGTTTAACTAGTTAAAACTT
TTTTTGTTTTATTTTTGTTGTTGTTATTTATGTGTGTTTCATTTTAAATGTTCCATTTCAAACTATGTGATTT
TTAGATTATAATTTATGTTTAAAAAAAATCTCTGATACTACTTAGTTTATATTAAATAACTCATGCACACATT
TTTGTAATTCAAGTCAATAGT
>103793338_2 DT662481.3
GCACGAGGGAGATACCGTTGAACCTGGCTTAGATGGCTATACAGGTAATTTAGGAGAAAAAGGTATGAAAGGA
ATAAGAGGTGACGATGGTGAAGAAGGTTTGATAGGATACCCAGGGTTAAAAGGAGTAAAAGGAGATATTGGTG
ACAGCATTTCATTTGCTGATGTTCCTATTGGAGAACGAGGAGATATGGGCCCGGTTGG
ATTTAGTGGCCAAGATGGTCGTCCTGGCGAACCTGGTGATTTTGGACAGAATGGAATACCTGGATTCAAAGGT
GCCATTGGTGATACAGGTGATATAGGATTGGCTGGCTTTCTAGGACCACGTGGGGAAATTGGCTTTCCAGGTG
TAAAAGGCGTAATTGGTTTGGATGGTGCACGGGGTGGACCAGGACTTCCTGGATTGCC
TGCGCCACCGCCTCCAATCCCAAAATCAAGGGGCTTTTATTTCACCGTACATTCACAAACGAGGATGGTTCCT
CAATGTCCGTCAGGAACAACACCACTGTGGGATGGATTTTCACTAATTCATATAGTTGCATATGCAAAAGCAC
ATGGTCAAGACTTAAGAGCGCCTGGAAGTTGCCTTCGAAGATTTTCAACAATGCCATT

TATGTTCTGTAGCATTAACTATGTGTGTGATTTTGCACAGCGAGAGGATTATAGCTTTTGGCTTTCTACACCA
GAACCCATGCCAATGGCTATGACACCCATACAAGCTCGTGATGTAGGCTCATATATTTCAAGATGTCAAGTAT
GCGAAACATCGACGAGAACAATAGCAATTCATAGTCAAAGCAATAGTG
>103793350_2 DT661841.2
GCACGAGGCAGCTTCTGCTACGGGCAGGGCGGCTACGGCGGCGGTTACGACGGCTACGGCTACGGCGGATACG
GATACGACAGCTACGGGGGCTACGGCGGATACGATTACTCGGGCTACCCGAATTACGCAGGCTACCAGGGCGG
CAAGCAGCGCGGCGGCGGCGGCGGCCGGGCCAACCAGAGGCACCAGCCCTACTAGGCG
CGCACCAGTACCTGCCGATAGTCATCTCAGTCCAATAGTAAGGTTAAGCGATCGGTCGCGCGTC
>103793365_2 DT663339.3
GCACGAGGAAAAGACTTTTAGAAAGATTCCCATACATTAGTACAAACCATACTACCAAATCAAGTAGAAAATG
AAAAATCTATAAAATACTCCGAAAAAAAAGGCAAATCTCTAAATCCAAAAGATCTATATAAAACCAATTCACT
ACAACTCGTGTCTGATCAAATATATCAGACATCAGAAAAAATAAAAAATTTAAACGAA
GAGACTAAACAATTGCAACAAAAAATTGTATCGACCAGTCAACAATTGAATAATAATGGTCAAAAATTCGTAC
AAAATGATATACCAGTAAAAGGCAACAAGGCAACATATTCTTTTTTTATTCAATCAGCCAATGACGGGAAAGG
AAAACCTTCGATACCTCACGAACAATTGTTATCTGAAGGTCTGTTACAGAGTATATTA
CAAGCAATCGAGCAGCCTAACTCAAGCAAAAATACAGAAACGTATCAAACTCAAACGAAACAAAGTTTTTCAT
ATCATTCAAATAACCCTCAGGTCAAAATTACTGAAGTCTTTCCATCAAACGTTCATAAT
>103793368_2 DV501103.2
GGAAAACCATGACGTGGAGCCGTTACGGTCAGTTGCTGAGCTTCACTGACTGTTCCGGTTATGTAACCCGCTA
TGACCATGACCGTTTTGGTCAGGTGACGGCGGTGCACCGCGAGGAAGGGCTGAGTCGTACCGCGCATACGACA
GCCGTGGAAGTTAATTGCCGTGAAGACCCAGGGCCATGAACGCGTATGATACACGCCG
CCGGTG
>103793371_2 DT662465.3
GCACGAGGGACTGGCCTCACGTCATGCTGCCGCAACAGGCCGCCAGGCCGCAGTTCAATCATCATCATCAAGG
ATTCAATATGGGGAATATGGGCGGTATGGGTAACGTGAGTGGCGTCAACGTTGGTGGTAGTATGGCGCAGCAG
CAACAGCAACAGCAGCTACAGATGCAGCAAGCGCGGTTACAGCAACAACAACAACAGC
TTATGCAGCATCAACAACAACGGGCGTCGAATCAGCAGCCGTCAATGTCACATCTAATTATGCAACAAAATCA
AATGATGAGCGTGCAAGGACAGATGCCAAACATCCATATGTCGCAGTCGCAAAGTATGAGTATGCAACAAGGC
GGGCTCTCACACGGTCTTGGAATGCAGCCACCCACCTCACACTCTCTACAAAATAATC
TAATGCCACAAAATGGCAACTTCCTTCACTCGTTTGGCCCCACTGCGGACTTTAACTTGGACTTCCTAGACAA
CATGCCCTCTAGTGACGCTGGAAATATGACGGCGCAAGAATTACTAAACTCTTTGGACAATTCGTTCCTCAAT
GATATTCTATAGATGATGTTTACTCTGATCGTCTTCCTATACTAAGGTCTTTAGGCTC
GAGATCTCCTAATTTCTTCCAACCTCATGCCACAAAGTGATGTTGCAATGACGGCGCAGTAACTGATAAACTG
TTTGGACAATTCGTTCCCTAAAGGTATCATGTGAAATGTTAACTTAATTGTCTTCCTACACTAAGGTAAGGCC
TTAGGTAGACTAGGGATCTCATATATTTTAACATTTCTACAAAACAACCTCATGCCTC
AAGATGTCAAACTCCTCCATTCACTTGGCTCCACTGC
>103793374_2 DT663087.3
GCACGAGGGCTGGTCGTGCGCTATGACCCTCGAGGAGAGCGGCGACGAGTGCTACCTGACCGTGTGCGAGTGC
TGCGGCTTCAGCGCCGAGACCTCCCTCTTGTTTCCAGTACGTTTCCTTTCACGATAGTGTACGGAGGACGCGA
CGATGCCCTTCGCGCACGCACCAGCCAGCTCCGAGAGGCGGGAGCACCTCTACAAAAT
CCTGGTGATCGGAGAACTTGGCACCGGCAAGACTTCCATCATCAAACGATATGTCCATCAGTTCTTCAGTCAA
CACTATCGTGCTACGATCGGAGTTGATTTCGCCCTTAAAGTACTCAATTGGGACGCGAATACTGTTATACGCT
TGCAGCTATGGGACATAGCAGGTCAAGAGCGTTTCGGCAACATGACGCGCGTCTACTA
CAAGGAGGCAGTAGGTGCCTTCATAGTGTTCGACGTGTCTCGCGTGGCCACCTTCGACGCAGTGGTCAAATGG
AAAAACGATTTAGATACTAAGGTTCAGCTGGCTGATGGATCACCCATACCCTGTATACTGTTGGCGAATAAGT
GTGACCAACAAAAGGAAGGTATAATAAATTCTCCGACAAAAATGGACGAGTACTGTCG
GGAGAAAGGCTTCGCCGGATGGTTTGAGACGTCGGCAAAGGAGAACATAAATATAGAAGAGGCGGCACGTTCG
TTAGTACATAAGATTCTC
>103793385_2 DT663343.3
GCACGAGGCCATAACTATAAAAAATTATCCATAAACAAAACTTTACGTCCAAACATAATGTACAATAACCGAA
AAAATATTAATTGATATACGATTATTTTGTATGAAAAAAAAAATCCTATTTTTTTTTTATTGTGAGTAAAAAT
AGTCGATACAATATTCGCAAGACGCCTAATCGATATATAAAGTGACTAACTAGTATAC

AAAATATTGTGTTATTACTGAGATTCTAGAATATTCCATATCATATATGATACAGTATTGGGAAATATCGTTA
CAATTACTATTTTACACTCTCAATAGTGATTGTGAACGTATTGATTCTTGAATGGCGAAAAACAATTTAAAAA
CAAAAGATTATTTAATAAAGTTTTTCGTTTAAAATGGTTAACTGACACAGTGTCATAT
TTTTTTTTTACAGTAAACGTCATCATGTACATAACATTTCGAGGTGCGGTTGGTTTTCGTTTGTGTTCCCGTC
CGTAGATTACGTTTCGTGTTTTAAATTTCAATTTTTTCTATTTATTTCGTCGTGAACAATCATACAACTCATT
AAACATTCATTCCAGATCACTCGAGCTAATATCGATATTTATCATTCATTCATTCATT
CTGAACGATTATTTTTGACCGAATGTCATTATGTTGCATTCGATTTTGATACGCTTATATTGTGACCCGTGTG
TTTGTCGAGATAA
>103793423_2 DT663092.3
GCACGAGGAAAAACTACAGCTTAGGTACGTGCATATTAAAAGAATAAATAAAATCCTTACAAATATACAGAAT
CTCGCTGATAAACTACCGGAAGGTAGAAAACTATCAGATAAACTATTACAAAATCTAAAGAAAATTCTTGTAT
TCACTGAAGAAACTTATGCAGCATCTGTTAGAATGGAATACGCAGGCATCGTTAAAAG
GGTAGATAATTTACAAGCTAGTCTAGATACATGGCGAGATTTTCTTACTCGCATACTTGGCCTAATAGGTGAA
TACGAAAAATTAACTAATGACTTACAAAAGTTGTACACTAAAACTCAAAGTGATGTAATGTCATATTCCGACG
AAGACCGATTATCTAGACCTCAAATTAAAAAGGCTATTGATAGATACAGCGACTTGAG
AACAAAAATTGATAGGACTGAAACTCAAATTGAAGCTCTAAGCGTCATACAAGAACAACTCAAAGAGTGTCTC
AGTCCACAAGATATAAGAATAGTTAGTCAAAAAGTATGGCAATTAAGACAACAGCGTGCTGACTTAGAACATC
AACTATCAATAATTATTCATAGGTTACAAGAAAGATTAGAAATCTATACCTTATTTGA
CTCAAGGCTCTCTCGATTCAGCGAATGGATGACAGCACTCGAATCCCGTCTTGATATTGCCATTTCATCCAAT
AATATAGTAGCTTTAGATC
>103793426_2 DT662962.3
TTCGGCACGAGGGTTGTGTTCGCCCAGTGTAAGGACAAATAGCAAGATTAATAAATTGCTCAATTTACATATA
ATGAGATTATAAGCATTTTATTGATTTACAAGTGTTGTTTTCAATGTTAGTGAGTTCCTATAATGATTTTGAA
CAAAGTGCGTTTTTTTATATTTTTAAGTATATTATATTTACCTAGTTTTAAAAATTCG
TTTTACTGTTTTAGTGAGCATATTCCTAAGTTTATGTTGTGTTTATGTCAAGTGTATAGATAGATCTGATATC
GTGCTCGTAGTCTAATTTGATCTCTCTCACTTGGCTAATTTACAATTACATGAATGTGTTAGCATGTTTTATG
TACTTAAGAAACATTTAAAATGTAAATATACATTATTTTTTATAAATATCGTTAATTA
TCGTCTTCCTACGTTAAAAAAAACATATATTTCATATCTAATTTAAATTTCTTAAATAATATATCTATAAAAT
TTTGAAAATAAGACAAAAAAAATACACAGCCTTTTAGTTATTTTTAACTGATATTCTTTATAATTCTTAACAA
TTGTTTTTGTAGTCACGTAACTATTACTACTATATTATATAATTATGATTTTAATTCG
CTCAGCCGCATGCAGCTATATGGCCAGGTTTTAGAAGATTACAGTGCCATTTTATTATTTTGTATATAATGAA
ATTTTATAAAACAGGTTCATTGGTTCATTAATTAATTTTAAGACTTTAATAAAGTGCCTTATGACAATGTGTC
ATTTTCGTATTTACCAAGTGAAAAAAAAACATATTAACATTTATATTATACTATT
>103793450_2 DT662826.3
TTTCCGCTTGGTATGTTTTTCGAACATTTATACATATAAAGTGTGTATAAATATAAAGTAATGTTAAGCCTTT
AATTGATATTAAAAATTAATTTACTTTTATAAACGTGGATGTTAGTTATACTTTTTTTGTACGACTGCGGTGA
AGCCTAACATAATTAAACTATCAATTATACTAGCACTTACATAAATATTTTCTCTTTT
GCTTGAAAAAAAAATGTAAGTATGTCATATGTGTAAGTGTATGTCTGTTTCTTTGTCCCCTTGTAACTGTGAA
ACCAGTAAACTCATTTTGATTTTTGTCAAATATGTCATTGGACTTGTCTTGAAGGCGCGAGTGCAACTTGACA
CGTTCATTATTTTTTATAATTTTATTTTAAGTTTCACGCTGTATCCCTCTATCCTCTA
TCTGTATTACATAGCAGGATACACAGAGGGAACCTGGAAACTTAGCGTTAAGACACTCTTATTCCCCACTTTT
TTTGAATACAACTAACTTGTTTGTTTTTAGTTACACGCTGTATCCCGCATATAGGAGAGTAGGTAGGGAGAAC
ATAGTGTTAAGACACCTTACACCTTTCCGTTATGAGGACATTATAAAGAAATAAACTA
TAAATTTTCGCATGAAAA
>103793472_2 DT662528.3
GCACGAGGCTGAGGCCTTAAAAAGCGAATATTTTTACATTTGTTTAATATATTGTAAAGATTGAGATAATTTC
TGGTTAAAAAAGGTGTCTAATGTGCTGTTTGAAGTGAAATCAATATGAAATTTCTAATTGCACTCATTGCGTT
GTGCATTTTAGAAGTAAAATCACAGCGTTTCAACAATGGTCGACAACAAGTGATCTCC
GTAAGCCCAGTACAAAACTATCAAAATATTGGTGATAATATTTACCTTAGTTCAAGAACCTCAAATGAAGTTA
AAGATTCTTACGATAGATATTTGCAAGACATAGTTAATCAAATATTTACTGCAGCGCAAGCGAATCCCAACAA
AGTTCAAGATTCTTATGATATATATCTGCAAGAAGTAATTAAACGAAAAAAATTCTGAT
GCGCTAGTGAATCCCAATAATGACAATGCTCATCAATTATCATTGACCAATAATCGTGATAATATAAATTCCA
TCAATCGTGTTACAACCAATAGACCAGTCGACTTAGACGAATCTTCGATTGCTTCAAATCCAGTCAGATTTAT
TGGTGTTCCAAAAACTGATGTAGAACAACTTAACAATATAACATACGCAATAACAACA

TTCGGAATAAATTTAATGAAAAGTGTAAATAGTTTACAAAATGGCAACATAATAGTGTCTCCCACTTCAATTG
CAACGGTTTTAGCTCTGTTACAACAAGGTACAGTAGATGAAGCCCAAAAGAAATAACAAGATCTCTGCAAATG
ACTGCTGACCTCACTGCACCTACATATAGACGACTCACTTACGATATGAGAAAAAGAA
ACTCTCGAAATATTTTGACAG
>103793478_2 DT662240.3
GACCAGGATCTCCAGCTAATTTCTTCTATTCAACATTATGTGCCTTATCATGCGACTGATGAACAGAACCTTC
CTTTGCCAATCCACCTTTCCTATCACTCCCTATAACAGTAACCCTACCCAAGATAAATCATCGAATAAGTGTA
CAACGACTACTGACTCCGAACACACTTTGAAGACCGATTTTGATCACGGCGAATTACT
ATCTAGGAATATTGAATATCCCTACTACAACACAAAAGTTATTGTAGAGACTACGTGCCTTCCTGATGGTACT
CGACTTACAAGCACTACAAAAGAGTTTCGTGCTCCTACTGTCCGAACCAGCCAGTCTGAACATTCAGAACACT
CTTACCAATCTAGATCACAAAATAAATTTTCTACTCATTCTTCATCTGAGAAACGTTC
ACATGTAAATGAATCGACGTCAAAGATGGTTCGTAATCGTTATGATGACAATATAGTAGATTCTCAAAAAAAT
ATTGATGATATTGATTTTCAGAGGGTCAAAGAAACTAAATTTGATAATAATGAAAATGATAACTATGTCGACG
ATAATAAATATTCCTCAAAACAAACATATGAAAATAATTTGAAATCGGAAGATACCCA
CCATGTATCATCGCATAATGATAATAATGTTCAACATAAAAGTTATAAAGGGACCAAGTCTGTACATGATATA
AATGACAATAAATACAGTTCTACTCATAATGATAAAGACCAGATACATAATGTTCATAATAACGAAGATCGTT
CGTATCATATTAACAAAAGTGATCTTTCTATGAAGGATACTGAAGATCAAACACACAA
TGTGATTCGTGAAGATGTCGTTA
>103793483_2 DT662654.3
GCACGAGGGTTAAACTTATCGAGATTCTCAGCCGGTGCTAGTGACAAATTAAAATGGTGTCTTCGCGTAAATC
CCAAAGGACTCGACGAACAGAGCAAAGACTATTTATCGTTATATTTATTATTAGTGTCGTGTAATAAATCAGA
AGTTCGGGCAAAGTTCAAGTTTTCAATATTAAACGCGAAAAGAGAAGAAACAAAAGCA
ATGGAATCACAACGCGCTTACAGATTTGTTCAAGGCAAAGATTGGGGATTCAAAAAGTTTATCAGAAGAGATT
TCCTACTGGACGAAGCGAACGGTCTGTTACCGGAAGACAAACTGTCTATATTTTGTGAAGTGTCGGTTGTCGC
CGACAGCATAAATATAAGCGGGCAGAGTAACGTGGTACAGTTCAAAGTGCCTGAGTGC
CGGTTGTCGGACGACCTGGGCGCGTTGTTCGACAACGAGAGGTTCTCGGACGTCACGTTAGCGGTAGGCGGCA
GGGAGTTCCAGGCGCACAAAGCTATATTAGCAGCGCGGAGTCCCGTTTTCGCGGCTATGTTCGAACACGAGAT
GGAGGAGAGGAAAAGAAACCGAGTGGACATCACCGATGTGGATCACGAAGTCTTAAGA
GAAATGTTGAGATTCATATACACGGACCGCGCGCCAAATCTCGACAAAATGGCTGACGACTTATTAGCGGCTG
CGGATAAGTATGCCTTGGACAGATTAAAAGTGATGTGTGAAGAGGCGCTATGCCTCAGCCTGTCG
>103793490_2 DT662473.3
GACGAGGATTTCTGCGTACATTTGCCATTTCACTGGGACTAGGTATCATGCTTTCTCACGTTTGGGGCGCACA
TCTGACATGGAAGATGACTGCGGTTGTCTGCTCAGCATTTCCAATAATAGCCATGGCTATTATAAGCTTATCA
CCGGAATCACCTGGTTGGCTCGTATCGAAAGGAAAATTTGAAGACGCGAAAAAAGCTT
TCAGGTGGGTCAGAGGAGAAGGTCCAGTGCAGCAAGAAGAATTAGAAGGAATGATAAAAGCTCAAGAAAGAGA
AATACATTTAAAAGAGTCTAGAAAGAATCGAGACGTGCAACAGGCGTTTAAATTAAATAGACTGTTTAAATCG
ATATCAGATGCGATCAAAAGTGTTATGAAAATATTTAAGAAACGAGAATTTTATAAGC
CCTTAATAATAGCTATATGTATGTTAATTGTTTTTGAGTTTGGTGGCGCTCATATGGTTCCTGCTTATGGTAA
TTTGATTTTGCAATCAGTACTAGACAAAGACGACCCTATGGATGTGGCATGGCAATTTACTGTTATCGATTTA
TTGAGAACTATATGTGCTTTCCTTGCCATATTCTTATTGAAGAACGTTAAAAGAAGGA
CTATATTATTCACTAGTGGTGTTATGACGGTTCTATCACTAGCGTTGATATCCGCTTTTATATATTTGAGGAA
GGCTGAGGTTTTTACACAAGGATTTATATTAGATACAGTGCCTATGACATTAATGATATTATATTCTTTGTCA
TTTTGCTTAGGTCTTGTACCGCTTAATTGGGTGATATGTGGTGAAGTATTTCAACTGA
CTTACAGGAGCTTAGGATCACACTCTCAACCTCATTTTTACTCCT
>103793502_2 DT662159.3
GCACGAGGACGAGTCGTACATACCGAAGGGAATATTGAAGAAAAACTGGATTCAGAAACGCGTTTCGTTGATA
CCTGCCGTGGTCGTTATATTTTATGATATGGAATGGAATGATCCCCAGTGGAACGAGAAAATTATAGAATGTG
CCTCGAGAGTGCAGTCGATCCGCGCAGCGGTCGAGGGACACGCCACTAAAGTAGCAGT
TGTAGTTGTTCAGAGTGGACTTTCCCCACCCCCTTCGGAGTACATGCTGGGAGCAGAGAGAGCTCAGGCACTT
TGCTCAGCCTGTGAAATACAATCTAAGTCACTGTTTGTCCTGCCCCACAGTGAGCACTTGATGGGATACATAA
TAAGACTTGAAAATGCCTTTTATGATATTGCTCAGAACTACTACCATCATGAGACAAA
GAACATCAAACAGCATAGAGACCATTTAAATAAGACAACCCACCAGTATCTGTTTGTAAGACATCAGTTTAAA
CTGGGCTTTCTTAATGAATTGAAGCAGGACCTAAGCACAGCTCACAAACACTATTTGCATGCATACAATAACT
TATATGAGACCAGGCAAGTTGAAACTAATATGCATGAAATAAGAACTGTGGCTGGATA

CATCAATTATAAACTTTGTAAATTATTGTTTGCATTANATTTAGCAAGAGATGCCATAGCACAACTTANATCT
CATATAGAGCGCTTTAAAAATAGAATTGGATCTACTGAGCTTCTCTTTGAACATTATGCGT
>103793537_2 DT662143.3
ATTTTATATAAGTATTTATTACTAACACTAAATAACAGAATACTGTTTCATTAACTTCAACTGCTTTTTAAAT
CCATCATTTGGTTGTATCGCCGGCCTTTTTGTTTTAACTAAATTATAGGCTTCTTCATAGTTCATGCCCTCAT
ATTGCATTAAATAAGCGATAGTCACCATTGAAGTACGAGAAACGCCGGCATTACAGTG
TACTAGAACATTTTGTTTGTTTTCAACACCATTTCTTATAAATGGTAAACATTCAGTCAAAATGGGCTTTATT
TTCGACTCAGGCAAATCCAAAATAGGAATAAATTTATGATTAATATTTCTAATATCTACATCAACACCTAAAC
TCAGTACATTTTTTATATTATACTTTTCTAAAATTTTTATATCAGCACAATCTTGTGA
GCCAATATATAAATTATTTACAATGAATATTGGTATATCATCTGGCTTTGTATCTACAACAAAGCCATAGCTT
CTATCTACTAAAGTTCCAGAACTTTCCATATATTTTACACCATCAGCACGAGTCACTATTGTTTCAGTTTCTT
TAAGTTTTTCCTTTTGCTTTTTCAATTGATTTAGAAAACTCATTTTTTTACAGTTATC
TTTAATTAATTCAATATAAAAAATATTATATAAGTAGCAATATTAAATTATATTGCACTCATTTTGCTCAATT
GGTTATTTTTTTCAACTA
>103793543_2 DT663399.3
GCACGAGGGTTTACCCTAAAAAAGAAATAGTGACGAATCAAGAGTTGACATTTTTCTATAAAAATCGTGTTGT
GAATATAGAAAACCTGTGCAGCTCATTGGATTTATACTCCTTCCACGTCGCCGAGCAAGACAAGGATTTAATG
CAATAACTGTTACTTTTTATTCCATGATGAGTTGTCTATGATCTATTAACACAAGGCG
TGACAGGCGCCTGCACACAAGATGACTTCTGAACGCGCTATTCCGCACTTGCGCGATATCTTCGGCCTCAATC
AACAGTACGATGGCAAGGCCTACGATGAAAACGAAGACTATTTTAAAAAATCTGGGAAAAAGAAGAAAGAAGA
CTATCGTACGTACGCCAATGGCGACGTCAAAACTCTTGAAAGACATCTCAGTATGAAA
AAAACTATACGCAAGAAAATAATGCGCGACCTTCAACAGGCATTTGTAGAAGACCCTAACGAGTTTAAGGTCG
AAAATACAAGTCCGGAAAAACTAAAAGCGGAACTCAGCGCAGAAGCTGTAAAAATAGAAAAGAATGTTCGAAA
AAATGATCCAACATTCTTAGATATGCTCCGAGGCGAGACAAGGGGAGAGGAGGCTAGG
GAGGAACAGCCACCGGCGGAAAAAACCAGTTTCTG
>103793565_2 DV501071.2
TATGAAAATGGGTAAAATAATGAATATGATGAAGAAAGAGGAGTTGTGAAATAGTCCAAAGAGGCCCAAAGCG
ACTGCAATTCTGATACTGACCATGACCCTGAACTCAGTTATTTAAACATGACACTAAGTGCTGCAAGCCAATA
TGAAATAGCCAAGTGGAAAGCATATGATGATTCAATAGGAAATTTCTGCGAATGTGAT
GATAAAGATGTAGATGCTGAATATGTTGATTTGT
>103793574_2 DT663359.3
GCACGAGGTTTTTTCTTTAAAATTTTTTTAGAAAAAAATGTATTTCACTAATTGCTTTTCAATTATAATTTTA
AAATTCCACCGAAAACTTAATAAAATTAAAGTTTCACAACATACAACTACAAATAACAAATTAAAAGAAGAAA
ACGGAGGCGTCTTAAAGCTTTAATAACAATTTTTTTTGCCGTCACCATACTGAACTCT
AAGACATTGAGTTTTTAACATAACATATTACTACCACTTTATAGACTAAAATAACTATTTCTATCCATGAAGC
ATATAATATACTCATGGGCGTAGAAATAAGGGGAAGGATATTTTTGAGCCCACTATTAAAACAGAAATATAAA
AGAAATTTAAGCCTATTATAAGCCCATCCTAAAGTAAAAAAAAATGGTGTTTTGTTTT
TGCTTACTAAATCATCATATCTATACAAGTAATATTTAAATTTCGTGTCGTATGACTTTACGCCATGTAAATA
AAAAGGCCGGTCCTCTCCCCCTTTCTTCCCCAGGTTAAATATTTAGGG
>103793577_2 DT662946.3
CTTTTCTGGGATGTGCTTATTCTTTTTATTTTATTACTTTTGCATTTGAAATATAAAATGAGTGCTGAAAAAT
TAAGTACTGATGAAGTTCCACAAAAATTTGCCGAATGGCTGCTGGCTATGGGTTGTCCCGTAGAAAAAGTTCC
CTCCAGTGAGAAAATGAAACAAATGTGTAGAGGCCAATACTATATGGTTTGGCGTAGT
CTTATGGAACATGTGCAAGCAAAAGGTGCAATACGTGAAAAAAGATTACAAGTGTTTTATGATGATGTAAAGT
CATGTAAGGAAAAGAATGCTTTTAAGGAGTGTAACTCAAAAATTGTTGTACCTGAACCTGTACAATTATGGAA
GCAACATATGGATCTTAAGGAGAGAGTAACAGATGCTGAAAATAGATTGGAGCTGGCA
CAAAATAATTTAAATGAGTTAATGAATAAAATTTCTTCTAAAGAAAGTCAACGTAACTCCTCACGTCGGCGTG
TCGAAGACTTACAACGACGTGTTTGGCTTTTGAGACAAGTGTCAAATGAATTAACAACTAAAAAAGTTAATTT
GATGGAAACAAAAGCTATTGCAGAGTCATTGTGTAATGTTGATTACGATCAGAATTTA
GAGAGTAAATTAGAAAAATGTCTAATGTCTATGCAGCANAAACAGTCAGCTATACCATTGTCGAATCCTATTG
CAAGTTCTTCTATAGCATCCACATATGAAAATGAAAATGTTAATGATAAAGAAGATAATATAACATCTTTAGT
AAAATGCCGCGGTGATGCTCTATGGCCACTTTTATATGACAAAAGG
>103793589_2 DT663236.3

GCACGAGGCCGCTTCCTCGCGTTAAAAGGATAGAAACATGACATAAAATCTAATTATAATATAAATTCATGAC
AAAAAGTATTTGTTATTTTCAATATTTTGTTTTATTTAGTGTCGTGCAGTGTTTTGTTTGTTGTAAATAAGTG
AAATTTGTTTCTGGGATTACAAAAGTAGAGCGGAAGAATGGCGGGAATGAACCTACGA
CTGGCGTTTGCCATCGTGTCCTCAGCATGCTGGTCAGCTTTCCAACACGGCTATAATACTGGAGTCATAAATG
CACCACAAGCTGTTATATCAGAATGGCTACACAAACAAGCTTTATCCGATACAAACATAACTCTGCCGGCTCT
AGACGATCCCAAAGTAACAACGATATGGTCTGTCGCCGTCTCCATATACTGCGTGGGC
GGGATGATAGGTGGCGTTATAACGGGTGTCGTGGCTGACAGATTTGGTAGAAAAGGTGGCCTTCTACTCAACA
ATATATTGGTTTTCATAGCCGCAGCGTTACAAGGGAGTGCTAAAATAGCAAAGTCAGCTGAAATGTTGATTGT
TGGGAGGCTAATAATCGGTATAAACAATGGTTTAAACGCCGGCTTAGCTCCGATGTAC
TTGGCTGAAATATCACCCATATCGTTACGTGGATCGATCGGTACAGTTTACCAGTTAGTGATAACCATAACCA
TTCTTCTCTCACAAGTGTTGGGCTTGGAGTCGATCTTANGTACAACCAATGGTTGGCAGTGGTTGTTCGTTAT
AACCGTGATACCTGCGGTTATACAATGTTGTACTTTACCCTTATGTCCTGAGTCACCT
>103793598_2 DT663095.3
GCACGAGGTAAACATTAAATAAGAATAAATAAAAAAACAATTTAAATTGGATCATTTATTCATTCAAACATTC
ATCACGTTTTTATCATAACCTTTATTTTATATAAAGATATTATCAAAATGTTATTTGTTTTCTTAGAATATAT
TTTAACATATGTCCTAATATTACCCAAAAGTAATACGAACAGGTTTATAAAACTAAAA
TTTAAATAAATAAATTACTATATCTTTATCTCTGACTGGTATCTCTTGGTCTGTATGCTATACCGCGACTCTT
CATTTCTCTGATAACTCCTGCTGGAAGGCGACCCGATACAGATATAGCATAAACTCCTTTACAAAATCTACCT
ATAAAAAAACCAGGTGGTAAATGACAGCAGAAATATAATATATATAAAGGTTAGAGAC
ATCAAATGTGTGGAAACAAGTGTCATTCATTACATCATACAAAGAAGATGATGTAGACTGATTTGGCAGTTTT
TAAATATAGGTTAGGTTAGTTTAGTTATTTTTTAAATTTTAAATACTTAACCTGCATTTAATAAATGACAATT
CTGTAGCTACTGTGGTAATAGTATTGAAAATCTATGACTGTGATTAGTAAGCACTGAA
AAAAGTTAAGTTAGACTTCTCAATTTTACCAACTCTGACTTATATTATAAGTATAATATAAAACAGAAAACAT
TAGTAGTACGCCATCTCTTTAGGTACACAGATAGGTGTTGATTAATAATAACAATAAAAATATATATCA
>103793606_2 DT663098.3
GCACGAGGAGTCCCTGTAAGTGTATGCAGAATTGATGCTAAATAATACGGAAACAAAACTATACATAAGAAAT
AAATATTTCTGTCAAATGATTAATCAAAATGAAATCTGCCCAAGGATGCAAATTGCATTTTCAAACAGCTTTT
TTTATGTAACTGTAATATATGTTATTTTGATATGTTTACAATAGTATGTTTAATCCGT
GGTACATGCATTTTATTTGATAGCATTATTTAAGAACACGTCTTAGTAACGTAATGTAACATTATTTGACAAA
ATTTGTATATGGATAAGGCCAGTGGTAGGGCGCAGTTGGGGCACAGGAGTGCGACTGTAAATGAGACAGATTC
TGGATTCGTTTCTCAATAATATATTAATATTTTGATTTCCTCCGCAATGTCTCCAGAA
TTCGGTGGAAACTGGGGAACGGAAGTGTCTTAACGGCATCAGAGTATCGTAACAACCTGGAAACCCACTTTCC
AGGTTCCCTTTGCCTACCACACTGGATGCGAGATACAGCATGAAGCTAAAAAGTTATTGTAGATATTGAAGTC
TGAAGGATAAATTAATTTCAACGATTATTATAGACATATAACTACATATATTTTTTTT
ATTTCAGTCACGATCCCGGTCATCCTGTAGCAAGAAAATAACAGTGATATCAGACCCGCCTGCGCACGCGCCG
CCGCCGCCGCCCGCGCCTGCGCTCATGATACCACGCGCTCGTCTGCACCCTTTACACTCCGATAGTCGAGACA
ACTTGTCAAGAAAAAAAGCACAGAAATTTGTAGTGAAACAAAACTTATCAGCTACTT
>103793615_2 DT663374.3
GCACGAGGCGTTTCCTTTAAAAGGCAGGTCTGGTGAAGTACTTAGGAATAATATTAATTATTTCAGCGAAATG
GTTTATTAAAAAATATTCTAAATATTTAGAATAAGATGGAGGAGGACGACCCCGTTGTTCAAGAGATACCTGT
TTACCTATCTCAAGCTCTTTCTGAAAACCTTTATATATATCAGTATCCGGTGCGGCCA
GCCAATAGAGATTGGAAAGAAGTTAAAGTCAATAATGCATCAATAAAACCAAAGAACCAACTTGTGAGGATGG
AAATAGGTTTAGATACTTACAGTGATAAATATTGTTCTTTTTTTTGCGAGCAAATTGCTTTAAATACAGATGG
ACCACAGGAGTCTAGATTTATAAAAGATAAAGAAAAAGAAAAATCACAATATTTTAAA
AATGGAATAATGGATAAAATAGTGTATGAAAGTAGTACTCCATGTGTTGAAACTCAACATTATGCTGTGGCTA
TTTTACAGGACAAGGAACTGCACTGTACACCTATTAAAGGTATTTTGCAACTAAGGCCGTCCTATTCATATTA
TGATAAACAAGATAAAAGGAAAGTGGACAAGAACAAAACTGACAATTCTGATGATGAA
GAAAAAGAACCTGAAGCTCAACAGGTAACAGTTTAAATTTTGCTAGAACAGAAACAGAGGTTGC
>103793635_2 DV501009.2
GCACGAGGGGCCCCTCAACGTGCCGGTCTGGCCTAAAATTTGTTTTTCATTTTATTTTTTAATATATTTCGAT
TTTTAATCTGGCTCACTTATATTGATCAAAATGTACCGGGAGGCTGGCGGTTTGCTTATGGAGGACGCACTCC
TGAACGATCAACTTATGTTTGAGTATTCACCAACTGAGAGCTTAATAAATTTCGAACT

ACCATCAACTGTGAACACGCAATGTGATTATGCAGACTGGCAACTTAGTGATTTGGACTTTAATTGTGATGCT
TTTATTCACGGTTTAGATAGCTCCAACAGGGATCCTACTTTTGACGATAATTCACCGATACTCTTCGATTTCG
ATGATTTTGACAAAAATATTGACTTGGACGATTATCTTCTACAAGCCTTAAATAGTGA
CAAATCCAAAGATGTATCACAACAGAGCACAATACCTTTACGCAATGGCGACGGGAACTCTCTCGGGAGGCTT
TCAGTGCTGGGAATAGATCTAGAAACGACATCTGTCCATAACATTCAGCGCACACACAGACAGACCACCGTAA
ATAAAACTAATCAAAAAGTGCAATGTGCAATACAAGAAACAAAAGCTAGGACGTGGGG
TGAGCCTGCGTTCTGTCCGGAACTAGGAGCTTTCCGGTGTCCGGTGGAAGAGTGCGGCAAGCTTTACGCGAAA
GCGTCGCACGTTCGCGCTCACCTGCGACGTCA
>103793641_2 DV500993.2
TTTGTTCAAAAAATATAATCCGGGACCAATACCTTCCGAGATAGTATTTATTTGAAAGATATATTTTCATTAG
CATAAATATTATTTAAAAGCCACCGACACATTAATTCCTTAAGCATAGAAAATAGATATGATTGTCGAAGAAA
AGTTGCGAATGTAACACATACAGCCTTTATAGTAGGAACGTCACACGAACTACCTCCT
AATAGTAATCAATGTTTCATGAAAGTATTTTTTTTTTTGTAACATTGTGCAAGCGAAGACAGTTGATGTCTCT
AGTCAGAATTGGAACTTAGAATTAACAAATCGTTGTGTTGTTCATTCACTTAAAGTAGTACAAATTGTAACTG
TATTATTCCAGACCATTCAGAAAATCTCTTATCTCAAATAAACTTACACTTTAATTTT
TGTTCCATTTTATCTGACTTTGGCATTTCGCATCTTTTCGGACATTTAAAATCTCATTATTAGTTTTTAATTA
CTTGCGTAATTTCTTAATATAGAAACATTGTGTTCTTAATATTAAATTTCGACTATGACCTTCACTTGTTCAT
GCC
>103793667_2 DT662857.3
TTTCGCGGATTACCATAATCCCACAGGACCCTGTATTGTTCTCGGGCTCGCTGAGAATGAATCTGGACCCCTT
TGGAGTATACACTGATGATGAAATCTGGCGGTCGTTAGAGCTGGCCCACTTGAAAACTTTCGTGCAAGGTCTT
CAAGCAGGCCTCCTCCATGAGATATCGGAGGGTGGTGAGAATTTATCAGTGGGTCAGA
GGCAGCTGGTGTGCCTCGCGCGCGCGCTCCTCCGCAAGACGCCTCTGCTGGTGCTGGACGAGGCCACCGCGGC
CGTCGACCTCGAGACCGATGAACTTTTACAGAAAACGATAAGAACGGAGTTTGCGTCATGCACTGTACTGACT
ATCGCTCACAGACTCAACACTATCATGGATTCTACAAAAGTTATGGTATTGGATAAGG
GACAGTTAGTAGAATACGCGCCTCCAGAGCAACTATTACAAGACAAAAACTCCATTTTTTATAGCATGGCCAA
AGACGCCGGCCTAGTTAACTAACAATAAATACAATTGTCACTTTAATTCAAAAAATTACAGTGGACTCGTCGC
ACTCAACAATTATTTATATTTTTTATTATTATATTAAACATTTTGTTTTGCTGACATT
TATAATCCGTATTTCTTTTGACGTTTTGAAAATAAGGTAATGGGTTCCGCATGTTTTAGTGAAAATATTCCTT
AAATGTATAATTTTGCTTAAAATACTTGACAAGGACACATGCTATAAATTTAAACTTTTTTC
>103793673_2 DT663017.3
GCACGAGGCAAAAATAATCAGACTAAGATTGCGTACAATGAATTTTCTCACACACACATAGAACCTAAAACAT
CAGTTATTGTATCTCAAATACCTTTACAAGTACAAGAAATGCCCAGTATTAATATAAGAAGCCAACTTAAAAC
GCAAAAGGTTTTACAGCTTGACATTTTACCAGATAATAAGGACAACCAAGAAAAAGAA
TCAACTAAGAGTAATGCTTTAGTTATTGAAATGAAATATGAACCGCTGGAAAAAGCAAGAACTCCTAGAGCGA
GTTCATTTGATCATATAAATTTAATTGCTGAAAAATCAACCGACACAACTAAACGAAAAATATCTGTAATGCC
ACCATACGAAACTAGTGAGTCAACAAACAATGTGAGTTCTGAATCCCAACACAATTTG
AGAACGCAGAAACGAAAATCTCTTTCTGATCCTTTATCAAAACAGTCGAGTATTGTGCAGAGTAGTAAAAAAA
GTATTAATAGTAGAAGGAATGAATTGAGTCCATGGAATAAAAAACTAAAAGCGGTTTACGGTAGTACATCTCG
AAATTCACGAACAAAGGCCAAGAAGGTGTCCCCAAAAAAATCAAGTTTAAAAAACAGT
AATGATCAAAACAAAATATATCAAAGCCTAAATAATACTGAATACATACCGTACTCCAAATTAACACTCGGAG
GAGTGTGTGTGAGCGATATTGAAAAGGAAATTTCAAATGTATCTAACAGAAAAGATGTAGTGTTAAGCCCTAT
TTTGGATAAAATTTTG
>103793687_2 DT662838.3
GCACGAGGAATGACCCTTCATGAAATGTTACATCTACGAGCTTTATACACAGCAGCTTTTACCCTCTATATAC
ACATATTCTATTCTAGATATCATATATTTTACATTTATATTATATTTTAACGAATTATACCATTAATAATTAC
TTTGGAGTAAATTGACTTATATAAAAGCAATAAATATTATCCATTAATCGAAGTTAGT
TTTAAAACAAACAGATATATTACACTGCTTCAATTCACAGTTAATATATACCAATATTTGCTTCTTTATGAAA
AGTATACATATTTCTGTATGGATTTAGTCATTTAAATCATATTTTTTTTAAGGAAATACTACTTTAAGTCTTC
CTCTACATAATAGCACATATTCATACGAGTATATGTAATTAAAGTTTAATCGATATAT
AACATATATACAATATACATATCTATTATGTAAATAAGCTTCTACAGGTATGGAAATCAATGTGATATTACAT
AATGCTTTGCTGGTTTATATTTAATAAATGCCAAAGGCATATAATATACCGTAGATTTAGAAGTTATGAGGCC
ATCGTGGCGGTAGCATTGGCTGTAAAAGCAACGGTTGTAAGTTCGATTCCCACTCAGG

GGACTTAATTATTTTCATTTCTTTGCTCTGTTAACATGGTAAAGTGCGGCATTAAGTTCCGCCAATGATTACA
ATGAATCGAAGTCGGGGGGTCTTGAAGAAGCTCCCTCTGCCTACCCTGCTATAAGCGGGATACAGCGTGAAGT
TAATGTATAATTTTAATAGTGCTACAT
>103793705_2 DV501052.2
GCCCAAGGGTCATTCCGTGGAACTTAAACCAACGTTTCCTACTGAGCCCTCGGGACGCATCACGGGATCATTG
TCCTAATTTGGACACCTAATACTTTTGTACTTTCTTCATGGTGATGAGTAAATTATCGCTAACATTTTTTCTA
ATAATGTGTCATTCGTATCCTAAACCCCCATTAATGTACTTTTTTATACTGCATCCGG
AAATTGGACCATTATAGTCATCACCACCTCTAGCCGGACTCGGACATGGATCATGACCCATGATATGGATTCG
ACCAGACCGCCCGTTCGTCGTCCTTTAATAAGTGGCCTTGACCGAATCCAGATCGGGACTGAATCTGGCTGCG
GAATATTGAGACTTCTTCTTGATAATACTTACAGTGAACAGGTCCTTAACTGCAAGCC
AGGATATCTCCACTTTTTGTAAGTTGGTATTAGATGCTTTATAAAATTTATATTGAAGCGGATTTCTTTCTTA
CTTTTTGGGATAATTCTGTTTTTATCTCACAAAATTTTTAGAAAGATTTTTTTCCTTAAATAAATCAGCAATA
TCTCCTGTGTTTCACAACGTTGTTTTATTCCGTGAATATGAGTAATAAATTAGTATAA
AATTTTGTACAGCATCTCTACTCACAAATTATTTTTACAATGTAAGCAGTGTATGTCAGTGTGAATGATTAAT
TATTCAAGTTATAGAACTCTTATTTCATTTCTAGCGTAGGTATACTGATTATCTACATTAATTTTAATA
>103793711_2 DT662260.3
GCACGAGGGTACACATCTTTTAAATTAATATAAAAAAATATACAAAAATACAGCAAAAAAATGTAGGCTGTTA
AGTGTTTTACGGTAAGTAGGTACGTAATAGTTATCGTGACGNTAAGCCTTCGNGGAAGCTATAGTAATACGCC
TCGTTTAATAGTTTATGTTGTTCGACCCTCGCACCGAACGATGTTTGTAGGTGATTGT
ATAATCTATATTAATATTATAAAACTAAAGAAATGGTTTGCTTGAACGCGCTAACCTCAAGAACTTTTGGTTC
AATTTGAACAAATATTTTTGTGTTGAATAGGTTATTTATTGAGGAAGACTATACAATAGGCTATTTCATTAAG
TTAATCGTTTCACTTTATTCCATACGATTCTG
>103793740_2 DT662670.3
GCACGAGGCCCGACCCCTACGGCTTCCGCGAGCGCGCGTTCGGCCGAGACCCCTACCCCCCGCCGCCGCCGCC
GCCCTTCCTGCGCGATCGCATGATGGGAGGATTCGGGGATCCTTACGATGGCTACTATGACAGAGCTCGATTT
GACTCACCCCGGGACTTATTTGAGCGTCGCTACCCAGTGGGCGCGTCCAGGGGCCTGG
ACATGGGGGCTTCGAGGGCCCGAGGGGACTTTGTGTCCCCACCACTCCGCAGGGAACCAATGCCGCCGATGCC
CAACTTGCCACCCATGAGGAGTAGCATGGGCTCAATGAGGTCGTCTTATGATGCTATGTACAGTAGGAGAAGT
CCACCACGAGGGCCACAGATGTCAAGAGGGATGTACGAAGACTTCAGCAGAGACACAT
TTGATGACAGAAGGCCCGGCATGCGCGGTCCGTCGCCGTCTAGAAGATACGCTCCGTATTAAATATATATATT
TATATATAGTATAGTGTTTTAATAAAATAATTTGACAGTCCAAAATAATGGTCAGATTGAAGACAATGTTGTG
TGACTGCGACTGATTTGCAAATAGTTAGGGTTTTGTATGTCTGTCTGTACGTCTGTGT
GACGCGTGACGTGTGACGTGGACGCGTGACGTGACGCGTGCGGGAGTGTATATAGGTTATAATAAATATCATT
TTAAGTATC
>103793752_2 DT662775.3
GCACGAGGCCCACCCTTTGACAGATTGCATACTCCCGCTGCGTCTGTGCCAGGATCTCCTATACACGCGGGCG
CGTCCACTCCTACACTGGATCTTAGTGCAGTAGACCCGGCAAGAGAAAGAGCTGCACAGGACCTATCACTAAA
ATTAGGTATCAATCTAAACAACCTATATTCCCCCCACCTTTCACAAGACTTATTAAAA
TCCATCCATTATGGGAGCTACGGAATTCTTTCAGGCCATAGTTCCCCTTCACCAGGTTCCCCCTTACACCAAA
TAACGGAAGGTCTAAGCTATTTACACACAGGCTCCATAACCAGGGGAACACCCCAACCCCATTCAACCCCTTT
AGACCTCCGAAATAACGTCGAAATGGAAACTAATTACCCCCAATTACAAATACTTCCC
CAAATACATTTGACGCATAGATCATTGAACAATTCACCGATTTCTAGTCCTAGCTTGGATATGATACAAGAGG
AAATAGGCAATGGTAGCCAGAATGAGAAGTTCCTTAAAAGTTATCAGCCCCCCCAAATAAGTTTAACTGATTG
TCTTGGATCGGAAATAACTTTAGTCGCGTCTTCGTCAGAAGACAGCGTGGATAGCTTA
GAAAATTCTAAATATCATATACCTCAATTCGTTATATCGGAACACACAGATTTGGATAATAAACCGTCAATTA
TTAAAGGCATTGGTAGAAAAGTAAGTCAGGAAACGGACAAAGAAAAAATAATCGAAGACGATACGGATATGGA
AAAAGTAGACACTAAGTTAGAGATGCGGAGAAATAGTGACAAATCACTGGGGGTTTAG
TGACGATTCTTTAGTAACGATTCGGCCAACGCAT
>103793761_2 DT662435.3
GCACGAGGAGAATAACTTTGTAATTTCATACAATTTTCGAAGGAGTATCTAATTTTTTCCTGTTAGATTTATT
CAATTATTAATTATGGCTTCAATACTCGTTCCAGAAGTACCAGCTCCAGGTTTTTCTTTCGAAAACTGCCAAA
GAAATGCATTTCTGGCACAAAAAGGATTTCCGGCACCTAAAGCAACTAAGACCGGTAC

CACTATTGTTGGTATAATTTACGCTGACGGAGTAATTTTGGGTGCTGATACCAGAGCAACAGAAAATACTGTT
GTTTCTGATAAGAACTGTGAAAAAATACACTACTTGGCGGGCAATATGTATTGCTGTGGAGCCGGAACGGCTG
CTGACACTGAAATGACAACACAAACAGTAGCTTCTCAGCTAGAGTTGCAGCGCTTGCA
CACTGGTCGCACAGTTGCAGTTGAAACTGCTGCAACATTGCTAAAACGCATGCTGTTCCGTTACCAAGGTCAT
GTTGGAGCTGCTCTTGTTCTTGGGGGTGTTGACAGTACTGGCCCTCATATTTATTGTATTTACCCTCATGGAT
CTGTTGATAAACTTCCTTATGCTACAATGGGTTCTGGTTCCCTAGCAGCTATGGCTGT
TTTTGAATCAAACTGGAAACCAAATATGAGTGAAGAAGAAGGCAAAAAACTAGTGAGAGATGCTATTGCTGCT
GGTATTTTCAATGACCTTGGTTCTGGTTCTAATGTAGATCTGTGTGTTATTCGTAATACTGGACCTGCTCAGT
ACTTAAGAACATATGAAGAAGCTAATGTTAAGGGTAAAAACAAGGTTCATACAGATAT
CCCTTGGGAACACAGCAGTTTTAAAGCAGCGT
>103793787_2 DT663439.3
GCACGAGGCATACACCTTTTTGGTATTTGTGTGTAAAAATTATATTAATAAATGGTCACTTGTTCTGGTTAAT
TTTTTGGTAAACAATAAGTATAAAGAAGTTTAACAAAACAAAATTGGTGCAACATGGGGTCAACCTACAAACC
AACCAGTTCTAATGGTTCACGGTTATATGGACAGTGCTGCAACCTTCATTTATATAGT
GAAACATTTACCTGATACTAATTATTATGTTGCTATTGATATGCCTGGGCATGGCAAGTCAGATTCGTTTCCT
ATGGGTCCTCTGGTTAACCATTCTACATTAGTAGAGGCGATGCGCAGAGTAATTGATCACTTAAAATGGGAAA
GATTTATATTAGTGACACATTCTGTGGGATTTATAACTGGAGTTTTCTACAACAACCT
GTATCCGTTCAGAATAAGTAAAATGGTCAACATAGATCCCGTGCCGCCATTGTCTACATATAATTTCCATCAA
TGGAACCCAATGTTGTGGTATCATTATATTTACGATGATTATTATGACAGTTTTCCTAGATGGAATTTGGAAA
AGACCAAAAAATTTACATATGACCAAGCGATAAATCTTTTAGTGAAAAGTCGGAAATT
TACAAAAGAGCAGTCTGCTATAGTGCTTTCTAGAATTTTAGTGCCTACAGGCGATGGGATGTATAGATTATCA
TGGGAACCCTCAGCG
>103793793_2 DT661789.3
AGAAAATAATCACAACTTATTTAAGAACTACGAGCTATAAGCAACACAGAGCTGAATCAGACCCAGAAAGGGT
TAATGAAATCATAAATAAAAGTATTGAAGATGCTAAGTGGATTGTAAATAAGTATACAAAGGAATGATGGATA
AATTATTCACTTTATATATTAAGAGGACATGTAAGAAATATTTTATTTACTACAAACA
AATATTATAATAATTTATAATCATGATTGGTGAAAGAATAGAAGAATATGAAATACTTGAACATCTTGGGAAA
GGTGGCTTTGCTCATGTATATAGAGCAAAATGTAGGAAAAGTAGCCTTTATGTGGCTATAAAAATGATAGATA
AAGCTTTAATGGCTAATGCAGGTATGATAGAGCGAGTAAGACAAGAGGTCACCATACA
CTCACAATTGAAACATCCTGCAATTTTAGAATTGTATACATTTTTTGAAGATGCACATTATGTTTATTTAGTA
TTAGAGTTAGCACATAATGGTGAATTAGCAAAACATTTTAAGCTAGGAACCCGTGGTCTTAGTGAAAAGGCTG
CAGCAGATATATTTAGACAAGTTGTAAGTGGTGTTTTATACCTACACACTCATAATAT
TATACATAGAGATCTTTCTTTGAACAATTTATTGCTTACAAAAGATCTTTGTGTTAAGATAGCTGACTTTGGG
ATTAGCAACACAA
>103793806_2 DT662290.3
GCACGAGGCAACGGCCCTGATTGCGTCGACACCACAGCGTAGAAATGAAAAATATAAATTTAAATAAATATTT
AATTTCCTCGAGTGGGGTCGAACGATACTTGTGTCCTAGCACCAAGAATGGTCTTTTAAATATATATTATATT
AAATACAAAACTGCCAATCGTAAACAATAATTACACTCGATATTTACAATGTAGTTTC
CACTTGCGACATTATCGATTAATTAAGTAAATTCAGTTTATAAAACTGAAATACTTAACAACCGCTCTTAATA
TTGCTTGATAAGATAATAAAAATACATAATATTTGCTATTTATATATTATCTGTACGTTTTTCTTGAATTTTG
CAAAGTGGGCGGAAACATTAATTATATGTTATAAATATATTGTAATCTGACACATGAC
AACTACATAACCACGACACAATACCATTCAAACGTATTTTATGAACTATAAACATGCCACCATTGCTAATTAT
TAATAGAAAACAGTATAATAGAACTAGAGAATAATCTATCGGCAACATTGTCAGGGCAAAACATACTTATAAT
ATTATAGTTAGCTATAAACACGCGTGACTCGCACGTACCTACTCCTAGCTCATCTCAT
TATCATTTCCAATCGACACTGTTCGTGGAATCCGAAACGGTGTTGGAAAACTTAATGTTTATATCATATATAT
TGGTTAGTTATTGTGGAAAAACTTAAAATTGAGGCTGTGGTGGCTGAGATTGCGCTTGGACACTACTCGATGA
CGAGTTTAATTTTCCAGCTTGACTGTAATTCTGTTTATATCTTTTCTGTCTGTCTTTC
GCTTTTCTCCTCAAAGCTAACGTTCCTTTATTT
>103793812_2 DT663286.3
GCACGAGGGATAGCCTTAAAGGATGCCTTGAACTTTGATGTGAAAGTGGTAACAGTGTGACTGTATCGATATT
TTTTATATTGTTAGACTCTTTATAAAATAACACTATAATGTGTTTACTATATGATACCAGTATTAAATCGGCA
TTTATTTAAATAAATTTTGCATATAGTATGATGACCTATGATGATTTTATTTTTTTTA

AATGATATTACACCAGTGTATGTTATGTGAAATTTTATATTTGTAAAACTTAGCGTCCATTGCAATGGTGATG
GTTTTGTGGGTTCGATTCCCACTCGGAAGATTGAATTATTTTTATTTCCACGGTCTGATCTCAACATGCGAAT
TCTCGAAAATTGGATGGTGCGTGGGGAACAGAGTGTCTTAACCCTAGGTTGCCTCTAT
GCTTACTCTCTTATATGCGGGATACAGCGTGAAAAAATAAAAATGTGACATTTTTGTTTGAGGCTATATTTAG
TTGTGATTTATAATTAAATTGATTGACATTTTTAAATAATTGGAAGGGAAACATTTTATATAGATTTTGT
>103793814_2 DT661947.3
GCACGAGGTGACATCTTACATCACTATCATTATTTACCATTTCAAAAATTGAAATGTACATATCGGTTATTAC
ACAGTATATAATATCTTTTTAATAATAAATAATTACTATAATTACAGTACCAGCTACAATGTGAAGATGAGAT
TTAGTCAATCATTATGGCATATTATATGAATAGGTGGCGATATACAATAATAAATGAC
ACATTATTTCGTCTAGTTGGGGGCGTTATTTTTATTTATATTTATTGTGAATATCTTATTTATTATGTGACAC
AAATTCAATGTGGATGGCCCGCTCTCCAAAATATTCCGGCTAATAATGAACCTGTGTATGCTATGGTGTTAGC
TGATACCCATTTGCTAGGTTCTCACAATGGCCACTGGTTTGACAAATGGAGAAGAGAG
TGGCAGATGCAAAGAGCTTTCCAAACTGCATTAACTTTGCACAGGCCTGAAGTAATTTTTATTTTAGGTGACC
TGTTTGATGAAGGGAAGTGGTGTTCTCAAATGGAATTTGAAGATTATGTAAAGAGGTTCAATACAATTTTTAA
AGTACCACATGATACTTTATTATATGTTGTAGCTGGAAACCATGATATAGGATACCAT
TATAAGATAACTCCAAACTTGGCAAATAGATTTGAGAACAAAATGGATGCGCCTATCGTTAACCTCGTCAGTT
TACGAGGAAACCACTTTATTTTAATAAATTCTATGGCTATGGAGGGTGACGGATGCTCTTTATGTTCTCGTGC
GCTAGCA
>103793817_2 DV501025.2
GCACGAGGCGAAGTCCTTGAAGGTGTCGCGTGAAGTAAAAGTTGGCATTTTTTACGAGAATACTTACGAGAGA
GCTGTGTTTTTACTACGGCGGTAATGAAGATGGCGCTATTCCTAATACGAATAGCTCTAATATTGGGAACCGT
GAATTCTGAAAATTCCACGTTATCTGACGTATCAGAAGACTCCTGCGAACCTCTAGAT
ATAGGACCATGGAGCTTAGCGCTGGAGGACTGGGCCGTCGCCACTACGTCCGGCAGACACGGTCGAATCATGG
CTTTACCGCCATCAAAAGGTTATTACGTAACACAGCGTATACACGCGCCTTACTTGACACCTCCCCCACCTCC
TCCTATACCCCACTTCACCCCCATGCATTACAGCCCAAACCAAAACAACAAAACGTAC
ACTCTAAATCCAGACCAAAAAATATCAAAGCTAGGTCTTGCACCAAATCAAAATCTAAAACCTAATAAGCCAA
TATCCCAAAACGAGAAGCTGTCCTTAAACTCCCAGGAGGTAAATACTGTGAACCAAGGCCGTATCTCGCAGTA
CCCGGGGCAAGTGTACCAAGGATATGGGAACTCACCTCACAGCTACGAGGAGTGGGAG
ACACCACAGTACACTCCACCAGGTGTGAGCAAGATCGTGAATAGACCTCCCAAGCCTCATCAAGACAAGTACA
AGCCTAGCTATCCAGGTCCACCACGCCCAGCATCAGACCGTGTGGACGAGACCTCATCTCCACAAAAGCAAGT
ATC
>103793820_2 DT662228.3
GCACGAGGGAGAGACCTAGAGAGAGAGAGAGAGAGAGAGAGAGAGAGAGAGAGAGAGAGAGAGAGAGAGAGAG
AGAGAGAGAGAGAGAGAGTCAGAGAGAGCCTCGTGCCGCTGAATTTTATGGCGCTACGGAGGGCAGTGCAAAC
ATAGTGAACATCGATAGTAAACCGGGTGCCATAGGATTTATTTCGAGAATTATCCCAG
CAGTTTATCCCATCGCTATTATCAAAGTTGACGAAGATACTGGAGAGCCTATTAGAGATGCTCGAGGACTTTG
TCAGATCGCGCAACCATACGAACCAGGTGTATTTATTGGTATTTTTTTTTCAAACAACCCGTCTCGTGCTTTC
CTCGGTTACGTAGACAAAGAAGCTTCAGAAAAGAAAATAGTCAAAGATGTACTAACTC
ATGGAGATTCTGCTTTTATATCTGGCGATATTTTAGTGGCGGACGAGCTGGGCTACCTATATTTCCGCGACCG
TACAGGAGATACCTTCCGCTGGCGCGGAGAAAATGTTAGTACAACTGAAGTATACGCCGCCATTTCTAGGGTT
GCTGATCAACGAGATGCTGTTGTGTACGGTGTAGAAATTCCTAACGTAGAAGGACGAG
CTGGTGTGTGCGGTATTGTGGATT
>103793827_2 DT663434.3
CGGCACGAGGAACTAACGCTAACATGGATGATAGTGAAGCTAATATGAAATATTTTACAACACCAGCTACACT
CGCAAGTGATAGTGTTAGTGACAAAATAAATGCTATCACTACCATCCAACAACCTGACAATATTGATTTTGAT
GTTGAATGTCCTACCGCAGTAAAAGACTTTGCAGTACCAGCTAGTGTTCATGTTAGTG
TTACAGATGATGATAGTGCGTCGATTGGATGTAATATTACACAAGCTATAGTTGATAGTGATATCAACATCAC
TGATAATGACAGTAATATTGTGAATACCGATTACGTCATCACCGCTGCTGCAACTGACTCGCCTGATAATGAC
AAAAATAATACGCGAGTCATTTATAATGATTATATGATTCGTGATTCTGATGCAGAAG
AGTTGCACAGCGATGACAATGACGTGACTGACTTCGCGATTGACGCTGTGACTGAAGCGTATGACGAAGATAT
CAATATAGAATCAAATAACGTCATCGATATGGATATTGATGATTTAAATGATATTAATGATAATATTAATCCA
AATTTAGGTGATGACAGTAATGATATAGATATAGACAATAATAATGATGAAACTGATG

ATTACTATAGTAATGATGGTGACAATAGTGATGACTCAGTGCAAGATGATAGTGAAGATAATACTGAATATCC
GCTTTGACTATACAATATTGAATAAATAATAGAAAAAATAATTTAAAGGTCTTAAATCAGCAAATAAATTATA
TTATTGTAATCTTTGTAT
>103793863_2 DT662834.3
GCACGAGGGAACCCCTTATAGTTAACACGTTCCATAAAATAGTTCTTAAAATGTTTGTTGCATTTGATGCATT
TGTTTACAGAATTTTGGACTATAACAAAGTTTTCTCATGGCTATAACGATGGATTTAGGAGAGAGTGCGTCTG
ATTCAAAATTTCTCAAAGAAAGCTTAGCGAAAAGAGAATTACGACCGTCAAGCGCAGT
TTCTTTCTCTAAATCGGATCATGGACCAGTTGAAACTTCAACGTCATTGGAGCAGCTCAAACAGTATAAAAGC
TGGGTAAGTAATGAAAGATGGAATGAATTGAAGAAATTAGCGTAAACGGCAATGAAAGATCGAAAAGTGTTTA
TGATAAAAGGAGGGGGATTCCCGGCTGTTCGTCGAGCGCTCCTTGAAAGAGGTTGGGT
GGAAAAGTACGAGTCACACAAGGTTCGTCATCCACCTGCAAACGTTGATCCAAGAAAAGTGTCTGGAAAAGAA
TTGTCCAAGGTAGAAAGGATGATCCTATTTAAGTTTATGGAACATCATTCTGTAGACTTCTTATGGACAACCA
AACGAGATAAATACGATTGGTTGCTGAGTAATAAGGAAGTTATGATAAGCAGATTCAG
TAGATCTATTTTCACTACCAAAGAAGGCTTAACATCCTCTTTAACTCAAATGCATTGGTATACAGAGCCAGGT
GTTGCGATAACACAGTTTCCACGATCGTATAATAT
>103793869_2 DT663410.3
TCAAGTTTTGCCGGAAAGAGCCTGGAACTACCAGCGAACGCGAACGTTTACTATGCAGTAAACACTGCTTATA
ATTTAAACTTTATTCTTCGACCTCTCAAGAGCCAACAAGAAGAGTTAATTATCAACGGCAAAGCCAGAAACAA
GAGAACCTGATTGTACCTCAACATCCCTTCAATATGCTGTTGCCCATTTAAATTATAA
AGTGCTATCCGTGGTTTCTAGAAGGGAAAAATGGACTGAAACACTCATTGCTTGTACAAAATCATTCATTAAT
CTCATTATAT
>103793881_2 DT662544.3
GCACGAGGGTGAATTCTTGAAGGTTGTGTAACAGTGCTTATTTAAAGTATATTCTATCAAAAGCCGAGTGTTT
TTTTGAGTTAACGATGTCGGATGAAATTGCAGAGAACGGAACAGCAAACGGAGATGTCCCGGAGATCGAACTG
ATTATTAAGGCCTCGACCATCGATGGACGGCGGAAGGGTGCGTGTCTGTTCTGCCAGG
AGTACTTCATGGACCTTTACCTCTTGGCGGAACTTAAGACTATCAGCTTAAAAGTTACAACGGTGGACATGCA
GAAGCCACCGCCCGACTTCCGCACCAACTTCGAGGCGACGCATCCTCCGATACTCATCGACAACGGTCTCGCC
ATACTCGAGAACGAGAAGATTGAACGGCACATCATGAAGTCGGTGCCCGGTGGACACA
ATTTATTTGTGCAGGACAAAGAAGTGGCTTCCCTCATAGAGAACCTATACTCCAAGCTGAAGTTGGTACTCGT
GCGCAAAGATGAGCAGAAATCGGCGGCGTTGCGCTCCCACTTACAGCGTATTGACGCGTTGCTCGAACGTCGA
GGCACCAGGTTCCTAACGGGAGATACAATGTGCTGTTTCGACTGCGAGCTGATGCCCC
GCCTGCAGCACATCCGCGTCGCCGGGAAGTACTTTGTTGACTTTGANATACCGGCGAGTTTCCGCGCGCTGTG
GCGCTACATGTACCACATGTACCAGCTGGACGCCTTCGCGCAGAGCTGCCCCGCCGACCAGGACATCATCAAC
CACTACAAGCTGCAGCAGGCCTT
>103793890_2 DT662783.3
GCACGAGGAACATGATTGACGCCTGGAACCTGGCCATCTGCCTGGGCCCCACGCTGCTGGCCGCCTGGGGCGA
GGGCGGCGCGCACGTGGCGGCGCAGAACCTCGTCAACGAGCTCGTCGAGCGCGCCATCCTGCACCACGCCGAG
GTGTTCCCGCAGGACGTGGCGCCGCACGCGCTGTACGTGCGCGTGGAGCCCGGGGACG
CCCCGGACGCGCCCGCCGCGCTGGCGCTACCGCCCGCCACCGCCGCCAACGGGCCGCTCGTCTACTTGGCGTG
TGGAGACAGTACTCTATATGACGATGAAAGGAGCGACCTGTTTCTGTACGAGGACGACGACTATGACCATGAC
ATGGTCAGCGACGGCTACTTGGAGACCGACGTGTGGAGACGCGACCCACCACGCAGGT
CATCCAGCAGCGAGCGCCGCGCCCCGCACGCTGGAATGGAGATTGCATCTGCGGCCTCCGCGACTCCGATCCA
TGATGCATGCACTCGCCTCG
>103793893_2 DT663244.3
GCACGAGGTGATGTCCATCTAAAGGCACCGCCGCGGTGGAGCAATGGCTGGACGTGGGACGGCGACCACTACC
TCTCCAAGGTGTACCTCAATAACGACTCGCGCCTGGACCGCACGGCGCGCTGCTGGCCGCGCATGTCGCACGC
CAGCGGCGACCGCGTGGCGCGCGGCGACTGCGTGCTGCTGCGCGCGTCGCAGGCCCGC
GCGCAGCCCTTCGTGGCGCGCATCGCCAGCCTCTGGGAGAACCCCGACGACGGAGAAATGATGGTATCACTAG
TGTGGTACTACCGGCCGGAGCACACGGAGCTGGGGCGCCAGGCGGCCGACGCGCCCGACGAGGTGTTCGCGTC
GCGCCACCGCGACGCCAACTCCGTCGCCTGCATCGAGGACAAGTGCTACGTGCTCACC
TTCAACGAGTACTGCAGATATAAGAAGCGGCTGAAGGCCGTGGAGGAGGGCATCGCGATGGCGCCGTCGATGG
TGCCGGCGCTGCCGGCCGGCGACGTGCCCGCCGCGCTGCCGCCCGCCGTGTCGCCCGAGCTCGTGCTCATCTG
CCGCAAGATATACGACTTCCGCTCCAAGAAGATACAGGGCCCCAACAAGTGAGCGGCG

ACCACCGCACCTTCGGATTCACATCACGCGGGCGCGTACGGAGCACCAAAGCTTGGATAAGAACTGATATTTC
ATCGTAGTGACCCCCCTATATTAGTACAAAGCCCGATTCCAGTAGATTGTGTGTGGCCATTTCCTTCGAATTC
GCCGATCGGATGTTATTTTGCTTGTGTACCG
>103793896_2 DT662389.3
GCACGAGGGAGAACCCTACGACGCGGTACCGGATGTACCGGCTGCGGTACAAGACCTACTGCTCACCTTATTC
ACTACTGTTTATAATCATCAGGATGAAGAAGGAAGATGCTATTCAGACAGTATGGCAGAACTGCCAGAACATG
ATGAATCGGCAAACGGAGAGAAAGTGCGTGCTATCTCCTTAGACTTAGTGAAAAGGAG
ACTCGATAAAGGATTATACAAGCGTCTAGATCACTTCCAACAAGATATGTTTGCTGTTTTTGAACGCGCACGC
CGGCTGTCGCGCACCGACAGCCAGATCTTCGAGGACTCGGTGGAGCTGCAGGCGTACCTAATCGCGCAGCGCG
ACGCGCTGTGCCGCGGCGTGCTGGCCTCGCCCGCGCTCGCCTACACGCGCGACACCGC
CGCCACCAGCCTCGAGCTCGTCAAGCAGTGCAAGCTGCTGCAGGAGAACGACGACGAGGACGAGACCAGATCG
AGTACGGACGAGTCAATGGCGTCAGGTGGAGCACCACCGCAAACTCAGTATGGCAAAGGAGATTTTGTTTATG
TTCAACCAGAAAAAGCAAATAAAGAACCAGCCATAGTTCANATTGAGCGAGTGTGGAC
GAATTCTGATAACGTGCCTATGATATACACTAATGTGTATTTCAGGCCCCATGAAACATATCACGTGCGTACG
CGCAAGTTCCTCCAGNCAGAGGTATTCAAAACGGAAACCCATCGCACTTTACCATTAGAGCACGTGATCGGAC
GCTGTTATGTTATGAATGTTAAAGAATATTTCAAGTTTAGGCCTGAAGGA
>103793903_2 DT663022.3
GCACGAGGGACAAACCTGACATTACTTATGAGACTGACAGTGAGGATGACGAAAATGCAACAAACCCTAAAGT
AATCGTGGATAATGATGATTTGGACATTGAAGGAAACTCGCCTATCAAGGAAACAATCAGAAGAGAGAGCCTC
AATACAGTATTATCTTCAAATAAAGATGATGAAACATTAAGAAATTTTCTAAGTAAAG
CTGTAATAAGCAAAGGCGATGAAGATAATAGCGATAGTTTATCATTAAATAATAACACGCCAACCGTTCTCAT
GAACAATGATTTTCCATTATGGGCTGGTGATTCTAGTATAAGAAGATCTCCAGAAGGTGGTGAAGATCCAGAC
CAATCAAAAGAAACTGATGTCAAAGCAGAGAAAAAGCACAAAAAGAAAAAATCTAAGG
ACAAAGATAAGGGTGATTATAGCGATAAATCTGATCGAAAAGAGAGGAAACATAAGCACAAGAAATCAAAAGG
TGATAAACAACCTAATCCTCAAAGCAGCTTACTGGAGCCCTCCACTGTCACTACGGAATTTAATTACGAAGAA
TATGACAGCCTTTAGTATTGAATGCAATAATAATAAGGTTAAAATTTGATGAAACAAA
ATAAATCTATAGATGTTGCCAGTCTGCTTGATTGAATGAAAATAAAAGCAGATATCTTCTCTGTAACAAAATA
ATGCATTTTAAAATAGATTATATATATAAATATATATGATGTGTTCTGAAACGCTACATGAATATTTGTTGTT
TAGTATGGGCTAAATTGTTATCTAACAGGCAGATATTTAAATATCTGGCTTTATATGA
AACATTATCAAA
>103793908_2 DT662997.3
GTTTTGAAGAAAAACATTTTTATTTAGATTATAAACTTTGATAAAAATAATTATATTAATAATATTCTTATGA
CAACTTACAAATAATATAAGAGAATAACGTAACATCTATTCAATTTAAACATAGGTAGATTAAAATTTAATAA
AGAAAATGGGTTACCATTTTCGGGATGCATATAATAACTAATGGGACTATATTCACGA
GGCGGCTCGGCCGCGTCCTCCGGCGCCGGCGCGCCCTCTATAAACTCTATCTCCACGGTACGGTAAGCGACCA
CGAATTGTATAACATTTTGTATGTAACATATTTTAAGCCTGGAAATAAGTTTAACTAACAAAAATATAACAAA
TATTGACAAAAACACAACTTCAAAATTATTGACAGAAACACAAAACAAAATATAACAA
AAACGTATGGTGCGTTTAGAAATCTAAGTTACTGAAGTAAAAAGCAAATTATTTATTGATGATGATATTATTC
GTAGACGCGTACCACACTACTAACCCGATTTACATCTCGTCGTCTATTTCAATATACACATACATATTTTATA
GTAATTTCGATAAACTTAAGCATTGCACAACAACGCCGCACGCCGGCTCGCGACCTAC
CGAGACCACGCCTATTTAACTACTAAACTGAATTACGTATTTTATTGCTACTGACGTTTGTGACTTATGACGA
GATTCAGAATCACGATCTAACATCAATGTGAGTAAAATTAAAGCTGTAAATTTATACCATAAGTACATAA
>103793922_2 DT663010.3
GCACGAGGATTTTACTTTAAAGTGTAACTTTTGTAATTAATGCTTGGGTCATCAGAAATGTTGGGCATGAAGT
TATTGGTATAATGAACGCTAGACTATTACTACTTGAGAGTACTATAATTTTTCTGAGTAGGGAACCATTTTAT
CGAGCTTGTATAGGTCAGAAAGATGAGTTTCAGTGGACTCAGATTATAAACCAAATCT
GGCTTTCTGTTCCCATAAGTTGTGTTTTGTCATGTATTTTTGTTTATATTTGGTTACACATTTTACCTCTTGG
TCATCCAGAACATGCTTTCCAATATGAGTTTGGATGTTGTATTGTAGCACTGTCTTGTATTATAGAGCTTTGT
TCGGCAAATATGTGTTTAGTTGCACAACTGTTCTGCTTTGTTAAACTAAAGGTAGTTT
TAGATGCATTACATATATTTGTAAGGACAGTATTATTTTTATCTATTATTCTATATGACAGGTCATTGGCTCT
TATAGCTTTTTCAGTTGCACAAGTTGGAAGTGTTGTTGTTATTGTAGTTGCTTATTATTTATTTTTCTACTGG
TATATCCAATTTAAGCCACTATATGCCAAGAATGCACTTAAGAGTAAATTCCTCCCAA
ATAGTACAACTTCATACGCTTTACAATAACATGGATGACTTTAATTTTACTTCACTCCTAGATTTCTTTCCAA
ATTATTTTGGGAC

>103793930_2 DT663185.3
GCACGAGGCAACTACTTTAAAAAAGCTCACATCGGAGACTACTCAAATAAAAATGCCGTCCAGGGTTTCTTGG
AATTGTGGAAATCTGGTTTTATGCCAAGAAATGTGCCTTTTTCAGTCTTTTACGAAAGACAGAGAAAAGAAGC
TATAGCTTTATTCCAAATTTTATATTACGCCAAGGATTTCGAAGCATTCTACAAAAGT
GCTTCTTGGGCCCGTGTTCACGTAAACGAAGGACAGTTTCTCTATGCTTACTACATCGCTTTGTCCTACCTTG
GAGTAAGGAGCGTCCCAGCTCCTTACGAAGTATATCCTGATTTCTTTGTTAACTCCAATGTCTGGCACAAGAT
CTTTCGTATCAAAATGCAAAATGGACTTTCTTCTGACTTAAGTGCTGAAGACGGTATT
CTCCACGAAAATGACCGTTACATAGTATACGCTAACTATTCAGATTACTCATCTTACCACGGTGACGAACACA
GGATTTCCTACTTTGTCGAAGATGTCGGCCTCAATGCATACTACTATTACTTCAATTTATACTACCCATTCTG
GATAGACAGTGACTTTTTCCCAGGTTATTCCTGGGAACATGTTGGTGAGCGTCGTGAA
GAAATGTACTACTATTTCCACCAGCAACTTTTGGCCCGTTACTACTTGGAGAGACTTTCGAATGGA
>103793991_2 DT661816.3
GCACGAGGGTCCCCTTTTAAATTACGTCGCGTGTACAATGGAAACTAAATTACACTTATTAATTTTATTTCTT
ACTATATTAAAGTGCCTCGGACACGTTCCTATTCTAAGTGATATATTATCTGGAACACATTCCATTATTCATG
GAGTTGCTGGTGGTGTTATAGATCACATCTCACAAGGATTTCGTTTGAACGTAGATGG
GAAATTTGATGTGAATAAAAATGTAGACCAATCACCGCAGTACTCAAACCAACCACACATTGGACCTCAGGAG
ACACCTGAACAAACTATAGTTGTAGTAGTGGAATTTTATCACAATCACCACCACCACCATCGGCCTCATGGCA
ATAACAACGGCCAATACGGACCTGGGTACAATGAATACAGACCTAATCAAAATGGACC
AGGGAGACCTGGCCCTGGACCTGGTGTAAATGGAAATTATGGTAACCAGGACTTTATTCGACCCCGTCCTAAT
GCTTACGATCGGCCATTTGAAAACAATGGCAACTATAACGGCAATCATGGTCAAAATCACTTATCAAAACCCA
ATACAACAAACCAAACAGATATCCGCCAAATAATTATGGAAACCCTGGGTTATGACAA
ACCTTATTACCC
>103793994_2 DT662745.3
GCACGAGGCGACGCCGTGATCGGCGAGTACAAGTACACGGTGACATTCGGGCAAAACTCCATCAAGATAACTG
GACAGAATTTGGATCTCGTCAAGACAGCAAAACTGGTCCTCGACGAGTACTTCGAGAGCGCAGGCGCTCTAGA
AGCAATGGCATTAGGTTCCGGGGATTTCTTCACGCTGTCCCAACGTCCCGCCGCTGCG
CTACTGCTACGCGATGACGCGGCGTTAAACGGCGTGGACGATGACGTGTTCACTGCCAGTGCAACTACTAGTG
ATGCTTCTGCGGCGGAGGGCGAGGCCGTGCCGCGGCGCCCGTTCTTCTCTCGCGCCACCTCCACCGACAAGAG
CGCAGGTGGCGGGTCGCGGCAGACGTACTCGTACGAGACGCTGGTGCAGTACGCGGAC
TCGCCGCTCAGCAAGCTGCCGCCCGCCGGCCTGGCCGCCTTCCCCGCCGAGCTGGCGCGCAAGAGTTGCCTGG
AGTTCGACAGCGTCAGCTACTTGAAGAAGGTGCGCGCGGCCGGCGG
>103794013_2 DT662978.3
TCGAGTTTTTTTTTTTTTTTCTTGTATTTATTCTATTTCACTTATGTAGTTGTTATATATAAAAAAAAATATG
TAAACATGTCATTAAGGTTTTATAAAGTATGAGATTTGTGCAAATGAATTTGTATGGCGGTATATGGCATGTA
TGGCCTAACTACTCAGCATATAAAGTTTTATAATAATAAGTAAATATAATATATATAT
ATATATATATATGTAAACAAATTTTGTACTGCCTAAATCAGTTCTTTTTTTCATAAAATCTATCGGAATATCT
TCGATACCGGCTTGGGTAGTTGCCGAATCATGCTCTCCGCCTGGTATTCTAGGTCACAGGGCTTTATCAATTC
TTGACCACCCTGGTGGATCCACTTGTTGCTCACCCACTTGGAGCCGCGCAGCACGGGG
CAGGCGGCGTGGCGCGTGGCCAGGTCGCCCTCGCCCGACGCGTGCAGGTTGAGCCAGTACGCGGCCGCGCGCC
GCACCGGGAACACGCTCAGGCCCAGCTCCGTGAACACCGTCGCGCCGCCCTGCGCCACCTCCGACATATAGAA
CAAAACTGTAGCTATTCTGTTGCCATTGAACTTTTCAAACGCGTTTTCCTGCTTTCTG
GCAAAATCAAAGTGTGGCTCGTAGTGTCCGCCGATGCCGTAGTTGACGACCTGCAGCTCCTCGGCGGAGCGCA
TGCTGAGGCCGGTGAAGTGCGCCACGCGCGCCGACACGCGCGCCACCACCGCCGACTCCTCGTCCTTCAGCCA
CGCCGACTTGCTGATGCGGTAGTGCGCCG
>103794031_2 DT663200.3
GCACGAGGGCGCCCCTTGAAGCGCCGCGCCGGCGACGACATCGCGGCGGCGCTGCGGCTGGCCCGCGACGCGC
TCGGGGCGCCGCCGTCTGAATTGAAAGAGGGTTCTGATGATGACATGGATACTTCCAGTTCTGATGACTCATT
GGTGGAACCGGACAATAGGCTTGTTGCAGAATTAGAAACTATGGGTTATCCTACAGAG
CAAGCCCGAAACGCGTTAAGTCTCTCACAAAACGATCTCAACATCGCAGTGGAACTCTTGGTGGCTGGTTGTA
CTGAACCTTGTGATACGTCGAATCCTTCCACCAGCGCTGTTTAAAGTTCCAGAAAGAAACAAAGAAAGGAGAA
TAAGGAACTTAGAGAGAAAAAGAGAAAAGATAGAGAGATGGCCCTCCGTCGCCTCCAG
CAAGCAATACGCTCCGACGAGGACGACTATTTAAGTGCATCACTAGCGGAGGAGGAACAGTTCCTAGCTCAGT
ACAAGTCTCTGCTCTAAACCGAGGTGGTTTTTAGGGGGTTACTTAAGATAAGTTATATAAAACGAAATATTTT
TTAATGTTTGAAAAACGATAAAAATATACTCTATTTGGTATTGTATTAGATGTCTATA

TAATCTTATATTTTAGAGGCCAAGCACAAAGTGTGACTGTAAAATCGACTGGTTGTG
>103794062_2 DT663152.3
GCACGAGGCACCGCCCTAAAAAGATAATAGAGATATTGTTGTTGAACAGGAAGTCGACGGCGTAAAAACCTTT
GTACTAAAACCGGGGTCTCTAAAAGCCCAGCTCAGCGAGGAACTGTGGAGATTCTTTACAAACATATATGGTG
GAGGGCCAGAAGTACGGCTTAAATCACCCCCGCACGAGGCCCTACCGCCCAAACGTCT
TTCCAGAAAATACTCCGAATCAGACAAAGAAGACTATTGCACGAAGTCCACCTCAGAAATGAACATCCGCGAC
ACGATACAAAGCGAACTACAGAAGAATCAGTCCCTACTTATTTTTTTCAGACGATATAAACATAGTAAAGCTG
AGTCCGACGAGGAAGTTACATACAGACTCTTCCAATACAAAAAACACGAACCAAACGG
TAACTACAATACGGACGAAGATATGGAAGTCAGTCATACAACCAAAATGGAGAACGGTATCGAC
>103794089_2 DT662261.3
GCACGAGGCAAATAATATTTACAAAATTATTCTGAACTTTCTGGTCAATTTAAATTTATAATTAGTGAAAAAC
CTTTAAGTTATAAAGACAGTGATTAAACCTCAATATGGCTCATTATAAAGGAGCAGCATCAGAAGCTGGCAGG
GCCATGCACTTAATGAAGAAACGTGAAAAAGCTCAACAGGAGATGGAGCTTCAAAAGA
AAAAAATAGAAGGCGATCTCAAGATAGAAAATATAGAAAATAAATTTGCCACCCACTATGACGCTGTGGAACA
GCAACTGAAAAGCTCCACCATTGGTTTAGTAACACTTGATGAAATGAAAGCTAAGCAAGAACATATTCTTAGA
GAAAGAGAAAAAAAATTAGCTCAAAAAAAAGCAGAAAAAGAAAAAGAGCGGCAAAAAG
AAATTGAAGCCAAGCAGGCTCAAAAAAATAAACAAAAAAGACAGATCCAAGCTCTTTCCTTTGATTTAGATGA
AGATGAAGAGGAAGATACAGAAGAAAAACCTGAAATTTCTTGGCAAGATCAGAATGAGACTACATGTAAAAAA
ATTAAGAAGAATCCTGATGTGGACACATCTTTTCTACCAGATAGAGAAAGGGAAGAAG
CAGATTTAAAATTAAGAGAAGAATTAAGGCTTGAGTGGGTTATGACACAAGCAAGCTTAAAAGATGAACCTAT
TACAGTTACATTCAGTTACTGGGGATGGTTCTGGTCATAGACGAAATGTCACTTTAAAAAAAGGTAACTCTAT
ATACCAATTCCTTCAGAGATGTCTAGACACGCTAAGACCTGAGTTTAGTGAATTGAAA
CAGTGTCGGCTGATCAACTTATGTATGTAAAG
>103794099_2 DT662298.3
GNACGAGGGTTGTGCCTTGAAAAGAGAAAAATACAAGAAAATTTTATTTTAATATCTTTTATTTTTGTGTATC
TCTGTTAGTTTGGGCATTATTGCGCTGAACACGCGTCAGGAGAGTCGCTATTTTGGATTTGAAAATGACATGA
AATATTAATATACTGATCTTATCCAGTTTTATTTATTTAAGCAGTGTAATGTAAAATT
ACATTTTTCTTGAGTTTTCATTGCTTTTTTCACAAATTTCAAATGAATAAAAATGTTGATTAAGACCGTCATG
GCAACGGTTGTGAGTTTCAATCCCACTCGGGGTAATAAAATATTTTTCACCCAACATTCAACGTCTCGAAACT
TGGGCGGTGCAGGGGAACAGTATCTTAACACTGGGTTCCTTCTGCCTACTGTGCTATA
TGCGGGGTAGAGCGTGAACTAAGAAATATGACTGGAGGTGTGAAATTACATATTAATATTCTATAATATGAAA
ATTATAAAACAATATTTACATATAAGAAATCAACTTATAAATGATCATTTTTTCAGCTTCACGCATGTAGGGT
AGGCAAATGGAGCCTAGTGTTAAATCAAGTGCGCGTTGAGTGGCGTAACTCAACGCCG
CACCTTGCCTAGACACCAGAGAGATGAAATTAAAATACAAATATATGTATAAATGAAATGAAACAGTCAAGTT
ATGTTTTGGCTTTTGATCAGAGTCAATTCCGTGTCGATGTAGACGAGGGAGGATTCCCTGAGGACTGACAATA
CTTGAGTTTTAACGTTAATTGATGATATCCTGCTAGTTAATTAATATAATTTAAAAGA
ACGTTTCTTTGTTACT
>103794101_2 DT662057.3
GCACGAGGTGCAGCCCTTCAAAGAAAGACAATTATTAGAAATATAAAAAAATGGATATAGATAATGACCTACC
AAGCTGTGTTCAGGCCCGATTTAAGTCAGAAACTGGTGAAGAAGTCGGCAGTCCTTTAGATTTGCCGTTAAAT
GTTACTAAAGATCAACTTACACTAATATGTAATGCTCTTTTAAAAGAAGAAGACAAAC
CTTTCCTTTTTTTTTGTTAAAGATATTGAAATAACATCTACATTACGGGAAGCGTTAGATATTTCCAAACTTAA
CTCTGAAGAAGTGATAGATATAATATACCAACAACAAGCAGTTTTCTTAGTTAGACCCGTTACTCGATGCACC
AGTTCCATACCAGGTCATGCTGAAGCAGTAATATCAACCAATTTTAGCCCATCTGGAA
AAGAATTAGCAAGTGGTTCAGGTGATACTACTGTAAGATTTTGGGACTTACAAACTCAATCACCACTACATGT
TTGTAAAGGTCACACTAATTGGGTGCTATGTATTAGCTGGTCACCAGATGGTAGCAAGCTAGCATCAGCATGT
AAACAGGGTAAAATTATTCTTTGGGACCCAAAAACAGGCAATCAAATTGGTAAAACTA
TGACTGGACATAAACAATGGGTTACAGCAATGGCTTGAGAACCTTATATGAGAAATTCCGAGTGTCGTAGACT
TGCTAGTTCATCTAAAGATGGTGATGTACGAATATGGGATTCAGTAACGGACTCACGAATTTTGAA
>103794141_2 DT663029.3
GCACGAGGATAAGGCGGCAGCACAGAACTGCCGCAAGCGCAAGCTGGACCAGATCACGTCGCTGGCGGACGAG
GTGCGCACGGTGCGCGACCGCAAGGTGCGCACGCAGCGCGACCACCACGCGCTGCTCGCCGAGAGGCAGCGCG
TCAAGGAGAGGTTCGCCGCCCTCTACCGACACGTGTTCCAGAATTTACGCGATCCAGA

AGGCAGGCCGCTCTCGTCCACTCAGTTCTCGTTACAACAAGCGGCCGATGGGAACGTAGTGCTTGTGCCCAAA
ATGCATCACGACCATCCAATGAATCGCACATCCGACGACGAGATGGACAGGAAAACTAAAAACTACGAACAGT
GACGTTTACACACGGACAGACATTATATATATAGAGTTATATAAACAAACACCCAAGT
TTATTGAAGAATATTTAAAAAAGTGCAGATTTTTTTTATTTTCATATTTTAGTACGCTACAAAAAATGTTTGT
CTTATATGCCTTCGTGAGATAAATGTTTTTATTTTTCTACTTATTTACAGTACGGACGACGAGTCGTGACCTC
CCTGAAATTAGAATTTCGGAGCTTTTGCGCATTGTACACTCAGACGAAAAAAATAATC
AAAAATAAGAAAATTCCTCAAATCAAATCTGCACAGCCATATACAGCTAACTTCAGGGCGGCCACGACTCGTC
GACCGCTCTGTACTTATATGTGAATTAATTAGTGAGTTTGGTTATTATTTTTATTGATAACTCGAAAATTGAT
AGTTCTAATTGTTATTTTATTTAGTATTTCCCTTTTGAAGGAGATCTCAAT
>103794155_2 DT662203.3
GCACGAGGGTGACTAGACCGCTAATTACCTTGTCATTTGGTGTATTAACTTAGAAATGGAAGAGAAACACGAC
TTAATGGCACATGTGGCTCATCAAACTATCTGTATGCAATACATGTTGGAGCTCTCTAAACAGCTGGATGTGG
ACCCCAGAGCTTGTATAGGTTCATTTTTCTCAAGGATTCAAGTAGCAGAAAAGACATA
CAAGGATTCATTTGATGATGAGTTAGAACAATTCAAAGCTAGAATCAAAAAACGTGCTGCAGAAAAGATACAA
GAAGCTTTAAGGGAACAGGAGGAGGAGGAAAAGAAAGCAAGACTGGGACCCGGTGGTTTGGATCCTGTAGAAG
TGTATGAGGAACTACCTGATGAGTTAAAGAAATGTTTCGACTCCCAAGATGTTCCAAT
GCTCCAAGCGACAATAGCCAAAATGCCTGAAGAAGAAGCTGTATACTATATGAAGAGATGTGTAGACTCTGGT
CTATGGGTTCCCGGCAAGAACGATGAGCCCACAATGAAAGAAGACACAAAAGACATCCCATCAGCCAGCACTG
ATGACGTAGACTGATTAAGATATTACTTTTATGCATTTTTACTTTTGTATATTTATGG
TAGTATATATGTTATTGTGTATGCAAAAGATTATTTTGTTTTATAATATGTGTTACATATATATGTAGTATAT
GATAATTGTCTAATGTGAAAAATAGTTATTCCCTATGTAAAACCCAATTTGTGATATTGATACACAAAAAGCT
GTATTTAAACATTTCAATTTTTATGTATTCATTAATAGCCTCCATTATGATAGTGGTA
AAGCATTTATG
>103794163_2 DT662427.3
TTTAGAATAGTGTTACAATACCTTTAAAGAAGCTCTCAAAGCATTAGTTCAATACCAATGGAGATACAATATT
ATTGTAACTTAAAAAATCAAATAATATGCAAGTGTTGCACAAAAATAAATGTTAAAGATAAGTAAAGCAAATA
TTTGTGTAAATGTGAACATAATATAATGAAATTATAATTATATTATTATCCTTTGAGG
CTGGACATGCCAGCTCTCATGATTTCATCCACTAATAAAAGGTTGCTAGCAATTACTGAACAGGAATTGATTA
TTTGTTTTTTAACAATATAATTGTCATACACTCCCAGGTCAACTGGTTTGAATGCTTCACCAGAGCTCAAGTC
CAAACCAATCGGATCAGGGCTCAAGCGTGACTCCTCTTGCAATTTCACAATAGTATCC
TGAGCGTCAAATCCAGAGTTAACTGCAAGAGTTTTTGGAATAACCAGTAATGCCTCAGCGTACGCTTGGATTC
CAAGACGGGATTTTCCTTTAACAGTGTCTTTGAACTTTTGAAGTTCCATGTTGGCTTTGATTTCGAAGGCACC
TGCTCCAGGAACATAACATTTGTCTTCGATAGCATTGTTGATGGCTCGCAGACCATCA
CGAACTGCGTCCTTGATCTGGGCGAGTGTGTGCTTGTTGGGACCCTTCATGAGAATAGTCACCGACTGGGGGT
TTTTGCATTCCTCAACAAATGTATACTTTTCTTCCCCAAGAATATGTTCGTACACGACGCCGGCATATCCTAA
GACTTCTTCGTTGAGGTCTTCAACTGAGTTCATGGCTATACCGCCGCAGGCGAGGGCC
AAACGCTCCATGTTA
>103794166_2 DT661711.3
GCACGAGGGCGTGGCGCGCCGCAACCGCGGCGGCAAGTACGACGTGCACGACCGCGAGCGCGCGCACGGCCGC
GCCGACTGCCCCGACGCCGCCTTCCACGAGTACACGCACCCACTGGACAACAAATCACGGCATTCTATGAGCA
GCGGTAAGCCCGGCCCCGAGAGCGACACCGACTCCATGGCCGAGTACGGCGAAGGCGA
AACAGCGGGCATGAACGAGGACGGCTCCTTCATCGGGCAGTACGGCCGCAAGCGCCGCCCGCCCGCCTCCGCC
TCCGCGCACGCCGACTCCGCCTCGCAGGCCTTCGCCACGCTTGTCTGAACACTTCCCCTTCCCTCTCATAGCT
CCCCTCTCGTAGACTCATGCTCTCCTGGTACTGTGGAAAAGCTTTCACAGTCATATGC
CCCTTTGGCACCGCCTACCTCGTTTGACCGACCACCAAGTCCTAACACACCTTGTAATTATTACATTTCCCTG
GGTCCACCTTACACCTTTCATACACCTATCCTCTCCTGGTACCGTAGAATGTATTTCATAACCCGATCACCTC
TTATAATGCTTTGGTCGTCTAACAACAAACTTTTTT
>103794186_2 DT661849.3
GCACGAGGCGCAAGACCTGGCAGAGGACAGGCTGAACAGAATAAGACAACAGGCTGAACAGACAACTGAAGAA
ATCAAAAGAGCCGCAGAGGAAACTATAAATGCTGCTGTAAATAAAAAATCAGAAGTACAAAGTCACATCTCTA
ACAAATTCAATGAAATAAAGGATAGTGGAAGAGATGAAGTACAAAGCATTCAGCAGGC
GGCTAGTGATAAAGGAAATGAGTTACAATCATCAGCGTTAGAAACTTTCGATCAAGTTCAGCATTCAACATCC
AGTGCTTTTGACTCCGTAGAAAGTGCCGCTAAAGATAAAGTGAGCGCCTCAAAGGACACATACGATGATTTAC
AAAGCTCTGCCAGAGACACGTTGGCGGATATTCGAGACGATGTTCAAGGTCTAGAAAG

CTCTGCTCAGGACACGTTGCGCAGCTTCCAGAGTTCCGCTGGAAATACATTCGATAGCTTAGAAGACTCTGCG
CGAGAATTCGTTGGTGGTGTGCAAAGTACTGCATCAAGCGCAGAAGAAAGCTCGAAGGAATTATTCGGAAATG
TTAAAGATAGCTTTGAAGGTTTCCAAGTAGCGGCTGGAAATAAACTGGAGGATATAAA
GGGTTCAGCAAAGGATAGGTTAGAGCAAGTGGATGATAGCTTGGACAAAGCCAAGGCGAGTGCTAAAAAAAGT
GAGGAGGTTGTAGCCGACACTATCGCAAATGAAGCGGACAAGTTCACGAGCAGTCTCAACGACTTGGGCAATT
CCGTTTTCGGAATATCCGGGAAAGG
>103794195_2 DV501002.2
GCACGAGGGTTCCATAAAAGGGGGGAATGAATGAGTTATTTAATATTTTTATTTTCTCGCTCTGATATCAAGA
GAAAGAGCGGAGCTTACAAACGTCACTCATAATACATTGAAATGGCTTATCTCTATGTTACCCTTGCCTACTA
TATATACTTGGCATACAGCATAGAACTAAAAGAAAGAAATATGTATTGATATTTGAAA
GATTTATTTT
>103794203_2 DT662200.3
GCACGAGGCTGCGGTGCGGTCGCGGGCCTTATCCACGCGTTCGTGGTCGGAGGCGGTGCTGGGTAATACACCT
GCGGAGCTGAGGGCGCCACAGGTGTCCTTGTATAGTTAGGGTAGGGTCTCGAAACCCTACCTGCCCTGCCGCC
CTGCCCAGGATGCCTGCGGCGCGGGGCCCTGGGGCACCCGCGGTAATTAGCGGGGTGT
CCCTGGGTCTCGCACAGCACGCAGCTAGGCGGAACGGCGGGGTCTGGGACCTTGCGGGGACAATCCGCAGTCC
CGTGGTCTCCCAGGCATTTGACGCACCTAGGACGTGTTTTGCAGTTTCGGGCCGAGTGCCCGTAAAGCTGGCA
GCGGTGACACTGCCCGATTAGGCCGCGGTTACGAGGAGGCTCCACGCGGAGCCCCGTC
AGGTAACATATCGCCCGGACGTTGTATATTTGTTTTCCCGCGTGGGAATGATCTAAAATTACTAGCACGCTGT
CAAAGGGTTTCTTGTCTCTGAAATGGTACATCCGATGTACCTCGCGGACGGGGAAGCCCTGGGACTGAAGGTC
CTGGAGAACGAGTCCCGGCTCTATCTCCTTGGGGATTCCCTTGATACCTACGCGTAAA
TGGCGCTCTTCTTCGAGAGCGTAAGTGTGGAAGCCAACGTTGGCCTCCCTAAGGTATGCCGTGAGGC
>103794222_2 DT662545.2
GNACGAGGGTCATTTCTTCAAGCAAACTGTAAAAATGTCGTCTCATTTAAACTGGATGATAATTAGAAACAAC
AACGCCTTCCTTGTGAAGAAGCGTAATATCAAAAAGCCTTTCAGTAAGGAGCCCAACAATGTGACTAACCTCA
ACGCTTTCAGATACAATGGCTTGATCCACAAGAAGGCTATCGGCGTGGTCGAGAACCC
CGACAGGAAAGGTTTTTCAGTGGTATACAAGAAAGCTAAGGCTACCAACAAGCCCGCTAAGAACTTAATCCGC
CGTCCATTCAAAGCCGGTGCCAGAAGATCACTCCACAAAGTGAAGAGGTTGATCAGAGCGAACAATTATCGCA
CAGACTTAGCTAAAGCATCCCTTCGTCGTGCATCAGCGATCCTCCGATCCCAAAGGAA
AATTAAGGCGAAAAAACCTGCCGCAGCGGCCGCTAAAGTCGACTAAATTTGTATGTAAAAAATTAATAAAAAA
TATAAAATCTTAAAAAAAAAAAAAAAAAA
>103794244_2 DT663326.3
GCACGAGGCCAGTCCTTGTAAGTCGATCTGGTTTCGTTGGAGACCCTCGTACAGGCTGTTACGATATAGCATG
TCAATCGGACGACCAATGTGCGGATGAAGAAATGTGTATTAACAAACACTGTATCCCCGCCTGCAAAGTAGAA
CCCAACATCTGTGGTCAATTTGCTGAATGTTATGGATTTGAACATCGTGCGTCATGTC
GTTGTGCAATTGGCACATTTGGTAATCCAACTCTAGCCTGCACTCCTATTAGCTGCCGAGCGAATTCGGAATG
TCCACCACTTAAATCATGTATAAATTCAAAATGCGTATATTTTTGTACTGCTAATATTTGTGATGCGCCTGCA
GAATGCCGAGTGCATCTTCACGAATTGTATTGTGTTTGTCCCCCCGGATATCAAAGCA
CTAGCGCAGGATGCAATAAGACAGAGACAATCTGCAATACAGATTTTGATTGTCCACCATCAACTGCTTGCTT
GTCAAATAAATGTGTTAACCCGTGTCTAGAAATAAACCCCTGTGGTATAAATGCCGAATGTACAGTTGTGGA
>103794247_2 DT662869.3
GCACGAGGAAAATACTATACAACTAGATTGTGCCAAAATCATTATACCGAATTATCTTTTCATGCTAAAATAC
CCAAAAGTTACTAAAATGAATCTCAATTTTTATTTTTATTTTTTATCTCATTAAACATTAAGGTGTAGGTATT
ATTATTAATAAAGGTGCGGGTTTTTTACTTTTATTATTTTAATATTCTTATATGGACT
TTTGTAGGGTGTCTTTGCATTACATCAATTTATTAGAATTAAGAAAATGGCACCTTGACTATTTTTAAGACTA
CTTATTTAGGAAACGTAACCTTCCTAATTGTGGATTAACACTTTATAAAAGTTTAAAATATTTTTAATACTTT
TAACAAATGATAAAATAAATGACAGATGTCATATTTTGACAATCACTCTTAGAATATT
TTATTCTTTATTTATGTTGTTTTGTTTGTTATTTAATTTTTGTGTTAAGATTTAAAAAGACTCTGTTTGACTA
TGACTTAAAATGAATTACATGACGGAAAGATGAAAGATGACTGCATAAAGAAAAAGACTGCTAATATTTTTAT
TTTGTTTAGTTTTTTAACTAACTTATTG
>103794279_2 DV501021.2
GCACGAGGGGCCGCCTCTTCGACCCGCGCTCCTAGCGTTCCTTCAAGTACGACCACCTTCGCAAGGAGGCGTC
CGAGTACGAGCCCTATGATGGTGACCGAGTCGCGGAGCCCTGGCGCGCTGCGCTTGACGTGGAGCTATCCGCG
TGCGTTGCGGAGCTCTACAAGCACGGTGCCAGCC

>103794327_2 DT662796.3
GCACGAGGGTGACATCTTACTTAAGATGTTCTTCAATGTTGATTCTAATTCTGAACAAATATTTTATGAATTA
CGACAATTCTTTAAAGCAATGTTGTCTAAATTGAGGTGAATTAACTTGACAAGTTGTTCTTTTTTGTTGAGTT
AGTGAAATTGTTACAGATACTATGTGTTTTGTAAAATATGATGAATGGTTCAAGTAGC
AACTTTTTGCCAAATTCTGCGCTTAACAGTGCTAACAGTGATGTACAAGTTGCGACAAACATGAAGGTCGAAG
GTAAAGCCGCCACTTCGAAAGATAATACACGTGACAGTTCAGAGGATCTCCTTGATCTCAAGGGAAAAGTTGA
ATCACGTTTACTGTCCATGTGGAACAATGTTAAATTTGGATGGACAGTTAAGTTAAAA
ACTAGTTTTTCTAAAGAGTCGCCTGTCTGGCTTTTGGGTCGATGCTATCACCGTAAGTTAAGTCCAAGTGGTT
CTTTAGAATCATCAACAGAGATAGGTACAGAAGCTACAGCTCATGATCCTATGGAACAAATATATGTTGAGGG
TATTGAGGGATTCAAATCAGATTTTATTAGTAAACTTTGGATGACCTATCGAAGAGAA
TTTCCAATCATACCTGGTTCTACATTTACCACAGACTGTGGTTGGGGTTGTATGTTACGCAGTGGCCAAATGA
TGCTGGCACAGGCTTTAGTGTGCCATTTTCTTGGTAGATCATGGCGTTGGGTTCCTGACAAGCCTATACAAAA
TGCTAGAGAATTTCA
>103794330_2 DT662274.3
GCACGAGGATCCGGCGTGCGTGCGCAGTCTGTCGCCATTTTATACTTATTTTACTTTAAAGCCTTCACTTATT
ACGTTGTGTTGTTAAATAAGTTAAGCAGACTTTATAAGTTATGTTAATACAATAGTTTTAACTGTGATGGAAT
TTGAATAGTGATGTGATAGTGCTTTTAGAAATTTTAATTTTCTGGATTACGCTTTACT
TATATATCAAAGAAAAAAATCCAGTGGAATAGAGATTCAATGTATATTTTTTTTAAATATATACTAAGTGTCT
AAAAGTTTAATTTAAATAGTGACATTTATGTGGAAATTTAAATAGTGGCAGTAGTGTCTAGTGACTCGTAGTG
AACAGTGATTGAGTGATGAAGATTCTTATTTTGTGATGTTGTTTGATCGAGGACCAAT
GAAGCGACGAAGGCGTTACATATTCTATACATCTATTCAATGAAATCTTTCACAATGATATTTGCCATTTGGA
TTTTTACAGGATGCATCGCATTAACTACAACACAAATGCCGCCTGGCTTCAGCATACCAACAATTATTCACGT
CGACCGCAACTGGTTCGTGATGTCCACTGACCCAATCGGTGCTGTCGTCACCAGGGTC
TACACTGACAGGAGTGGCACTCAACCAGTCACGTATGGGCTGGAAACGAGTGGACCTCCTACCCCGTTCACTA
TCGATCCTATGTCTGGTACTGTTTCAGTCAATGACACTTTGAAAGATAAGGGAAATAAATCATACTCGCTATG
GGTAACGGCTTCCGACGGAACCTCCAT
>103794333_2 DT662335.3
GCACGAGGGAACGTACTACAGTGTATTTGTAATTAAACTGTAATTGTTAACATTGAAAAATAAAAAAAGATAA
ATTGTCGTATATGAGATATGTGCGTGTGAAAGCTTCTGATAGTATACATATTCGATTTCGCTACCATCATAGA
ACCGGTTCAAATCCGTTGTTCTGAATTGCGACGAACTTATTATTCATTAGACCGGCAA
TCGCGAAGCGTCGTTTAGAGTTAACTTAAAATTCACAATGCAATTATATATTTTTGTGGTTATGGTGACTATA
TTTGGAGTCTCAAATGGAAACGTGTTGTCTGATCGAAATAATACTAATCTGAAGTTGGAAGATTTTGAAGTAC
CTAACAGTGCTTTAACTTCATTGGACAAACTTAATTTGAGACAAATTGCAATAATATC
ACGCTACGCGAGACAAAACCAAGGATCTGCACAAAATTCCACATCTACATCATCAAGACGTTGTGTCTGTAAT
TTAGGAGTTTGTAAGTGTTGCACTGGATATTTAATGGATTTAATCAACCAAAAAGCTTGTATGAAGGTTACCT
ATCACCCCGGAGATTTTGCTTTTGATGTTGCTATGTCTATGAACGATAGAGTTCTTTA
TGAAAATTCCTTGTCGGGAAAAAGTCCAAGACCAATATGTATAAATCCACCACGACTGAATAATTTAAAGGTG
TGCGCAAAGTTTTATAACGTATTCTTTCCGGGAAGGAACTTTCACTTTTGTTTAGCGATGACTGGGAAATGGC
GTATTTTCCATTGTTTGACTTCGTATT
>103794336_2 DV501066.2
AGGGAGATAATAAAGATGTAAAAATTAAAGAAGAATTTATTATGATATTATATTTATAAATTATATTTTGATT
TAAAATTGTATTTTTGGGTTATTGATAATTTTTTGTATGTTGAATATAATTTGAAAATATTATTTGGTGATAA
TATTTTTTTTTGTGAT
>103794341_2 DT663164.3
GTAAATAAATACATAAACATAGGACACTAATTGCGCTAATAAATCAATTTTAGTTTACTCTTCCTCCTTCCAT
GGGGTTATTATTACAATTTAACATTTACGCTTGAACCTTCGTCTCAAACTACCGGCTTTCCGTGATATGTTCT
GTTAAACATCCCACCGACGTTATGGCTTCTAGTTCAAAGAATAATATTATAACTAGGT
TACTTAACGTGAGGCTCGAGGAGAACGGCGGGGAGGAACAGCCACCGCAGAACATGCTGCCGTCTAACCAACA
GTCAAGACAAGGAATTTTTTTCGACGAAGAAAACGGTCACGCAAACGATCCAATGCCTTCTTCTTCCGCAATA
GAAAATCTACAACCGTCGAATAACACTGGCGGTCGTTTACACAACTGGCAAGCTACGA
AGACAACTGTGAAGGAGCGTTTGTCTTTCCTGTTTAATAATGAAATTTTATCTGATGTCCACTTTATTGTGGG
TAAAGATGCAA
>103794344_2 DT663293.3

GCACGAGGGACACCCCTTAATAGGTTTATTAACGAAAGCGAAAAGGTTTATAAAGAGCCTAGAAGAAAGAGAA
AAAAGACACTCAAGTCACTAAGAGCAATTAGCAAGGGAACAACGCTACCTACGACGGAGGTTAGCACAGCTCA
GGGCGCGGAGAGATGTGACACGCCCGTTAGAGGAGAGCGCGATGGCGCACGCGCACGC
AACCAGAGCGGATTCATTGTCGTCGATAGAGTCCTCGTCTGGTGTGTCGACCGCTGAGAGGTCGCCCTCGTCT
GTTTCGGAGAGTGATGAAGTGGACGTGATCGGCTACACGTTTAATCAGAGCGACAGCGAGTCGCTGGTGTCGG
GGCACAGCGGCGACAGCGGCGTGGTGGCGCGCCGGCGTCGCCGCAAGCATCGCGCGTC
GCGGTACACTAGGGTGTAGGTGAGCGATGCGCGGCGCGAGCGCAAGGCGCAGTTACTTCTTATAAAAACAAAC
GTAGGACATTTTGTTGGTACATATATTGAGGCGAATGTTGTTCGTGACTGAGTGGGGAGTGTCTTCTGATACA
GAAATACATTAATATATAAATACATGTTTAAATAAACCACCACCAACCGACCCCCCAC
TGCCCTGACAGTGGTCTGGATATGT
>103794359_2 DT662015.3
GCACGAGGCAGAAATCTTAAAATTACTCGTACAAAAACGTCATCACACGCGGTTCTTCTTGGGTGAAAATAAT
CCACAAAATGTAGACCCAGGAACTGTTGTAGACACAGACATCGTGCATGTTCGTGAATTGGACTTTTATTTGG
TGTCGCACCAGGCCATCAAGGGCACGGCGCGGCCCACGCGCTACCACGTGGTGTGCAA
CGACGGCAACATCCCGTCGGACGAGGTGGAGCAGCTGGCCTACTACCTGTGCCACCTCTACGCGCGCTGCACG
CGCTCCGTGTCCTACCCCGCGCCCACCTACTACGCGCACCTCTTTTGTCTGCGCGCGCGCTCGCTCACCAGTG
AAAAACGCTACAATAATGCTGATCTAGAGAGAAAGCCACAGCGACTTCGAGTATTGGA
CCGCATGTTACAACACAGTCGCATGTTTTTCGTTTAAAATCTTAATATATAAATCAAATATAATATCAACAAC
TTATATAAAAATACAATTGACTATTACAATAGTTACCTATTTAATATTTGTATAATAACATATTCAACTATTA
TTTTATCTCATAATATTAATTCAACAAGGGTAGTTTGATATCCGTGTCTTTAAAAAAA
AGGCACAATTATTTATATTTATTTTTTCAATATAATCTCCTTTCATTTATTAATTCTATTCGATCAATAAAAA
TTGCTTACGTTCTAGCCAAATTTTCATACTCGGGTTTTTTGTCGGAATAATCAT
>103794365_2 DT662800.3
ATATACCTCGACTTCCTAATTTGGTGAATTCTAAACGACAAGTGTTTAACGTCGGAGTCCTCAAAATTAAACT
TCCTCTAAACAATTCCCGTAATAAACGTGTTCTATTCACGACGTCAGCAAAAAAACAGTACCTACCTGTTGTT
TTTTATAGTAGCTGCCCTACAACGTTCTATTGTTAAAGAAATAACATGCCGACGTGGC
ACCACCGGCTCGAAAATAGGGGCGGAACTTTAACGTCGCACAACCCTGCCAACTCCGGACTGCCGCCCGCGCT
CGGCATTGAAAACACTAACAACTACAGCGAATTGCTAACTTTTAATACTTCTTATAAATTAATAATTATGAAA
CTTGAAACGACGTTCATATTGCTATAGGTTTCTTTACTGTCTTCCAATATTTTAAGCA
GTAGACGAAGTAAAACCACGAAATATATTGAGAACGGCACCTACTAAGTCTAAAAGGTGCTACTTTTGAAACT
GATCCAACCCACTTTCATTATTTATTCTAGTTTATATAATATTTAAATGTTAATAATAGTTTCGTATATAGAA
TACGTGCTATTCCTTTAACTATATCTCTAGTACTATTTTATTCTACCTTCTT
>103794401_2 DT663418.3
GCACGAGGACGTGATCTGCAAACCGGAACCGCTCTTCGCGAAACTGGAGCCGGAAATGCTCGAACAACTCCGC
ACCAAATACGCCGGAAGTCAAAAAGAGAGGGAAATTAGAGTGAGTCAATTGTGAATGTCACTTTATACAATTG
TTTGTATTGTCTAACGTGGTAGCGTGTGTTGCGTTTTTTTTATAATTTTTTGTGTTTC
TTATGCTTTTTTGTTTCTTTTAAATATTTCAATAAGCATTCTGCACGCACTACATTTCTATATAAACTATAAC
CGGGGCAGGTTTCATACTCCCCAATTTACGTAGTTTTCATATAAGGAGCACAAAGTTTTCGCTTCACGTATTA
ATATATAGATAAATTTTAAGTGTCTGTCTGTGATCTCAAAATAAAAGTGTTTTTCAAT
ATGGGCTACTTTTTATGCCGTAATTTCTCGTAAAACGCCGCATCGGATTTTTTTGGAGAAAAACGCGTTAACT
GTATTTTTACTTGTTACTGTATTAATATCAAATTTTTGCTAAATACTACGTTTAAATAATATTGAATAAAAAG
ATTAATTAATTAAGGTCTACAGTTGTTATTATTAATATAATAGAGAGACCGTCGTGGC
GCAGTGACGCTGCGACGCAGTTGTAACTAAGCGCAGACAGATGTGGGTTCGATCCCCACATAATGGATAGAAT
CATTGTCAAGACGAATAGCGTTTTGTTCCATCAATCAACAAAAAAAAGACAATGTGTCTCGGAAATAGGGCAT
TGTCTGAGGAACTATGTAAAACAAGGATAACTAAGTACACAATACTCT
>103794416_2 DT661743.3
ACGAGGTTCATCCCGTAAAGGCTCAAATCTGGACAGGTTGTGCCTGGATACGGACACGCTGTACTCAGGAAGA
CTGATCCTAGATACACCTGCCAGCGTGAGTTCGCCCTCAAGCACTTACCAAACGACCCACTGTTTAAGCTAGT
AGCGGCCATATACAAAGTAGTGCCACCAATTCTAACTGAGCTCGGCAAAGTAAAGAAC
CCATGGCCCAATGTGGACTCCCACTCTGGTGTGCTTTTGCAGTATTACGGCCTCAAGGAGATGAACTACTACA
CGGTGATGTTCGGAGTGTCGCGCGCCCTCGGCGTCCTCGCGTTTTTTATCTGGTCCAGAGCACTGGGCTTACC
CATAGAGCGGCCCAAGTCTCTGAGCACTGAGTTACTTATTAAACAGCTCGGCAAGTAA

ATTTTACTTTTGTAAATAAGTTTTGTGATTTGGCTTTTGTTTCGTTGTATTTTTTCTCTACTTGGTTGGCATG
GGCGTATAAATAGGGGGAGCTGGTTGGCCTCGTGCCCACTTCAGAAATTTTACCAACAAAATAATTTTTGTGT
AAAAAAAGCTTTACACGCTCAAAATCTGGGACTTAGACTCCAGAAACTATCTCTATAT
ACGCCCATGTCGGTTGGTATAAATTATTAAGTTGAATTTATGTAGGACATTGGTATATTATTGTACTTATTTG
ATATATGTTTATTATCGGTCCAATCCATTTGAATTAGAA
>103794431_2 DT662864.3
AACAACCACAACAACCGGCCACCGCGCCCCAGCCGGCCGCGCAGGCGACGCCGCCCAACCCCACTCCACCGGG
CAACAACGGGGCCCCGCAGCACGGCCAGAACGGCGCGAACCCGCCCGAGCACCCCGGCCACCCCTACCCGNAC
CCCGGGCCGCCCGCGCCAGAGCACACACAACACCAGCGAATACGCCGGCCGCACCGCC
CACACGACCCGGAAGATACCTCTTTTTTATATACGTATATATAGTTAAATAAATATAATTGTATAAGATACAC
CACGCGATTGCGACCCACAATAAATAACTAAGCACCCTATATATGTTTTATTTAGTACTATGTCTTTTATTAT
TATTATTATAACCGGCCACATATCATTTATTTCTTGTGGGTTCCGCGACGGAGTTTAA
CGACGTTTTGTATGAGATTATTTTCACTTTGACCGCT
>103794433_2 DT662213.3
GCACGAGGCGAGCATATTAAATATAATATTTTCTTTCCTTCGCTTTGGTGTCGAGGCAAAGCGCGGCGTTAGG
TTCCGCCACTAATCACGTGAGAACAATGCCTTTAGAATTCAGAGAAAAATGGAAACGGATTGTCTTATCACTA
TTTTGCCTCTGCTTACCCTGCCGTGTGCGGTATGCAGAGTTAATCTGAAAAAATAATA
ATAAAATAATCTTTCAGGAACTGACGACGAAGTACATGAATATGATTACCTGAGCGCGTGGGGCCCTCGCTTC
GACAAGCTGGCGGACCTGTACGGGCCCGACCTGGACGAGCAGCTGTAAGCTGCAGCGGCCGCACTGTCAATTC
TATCCCGGCGACGCTTCAGTGGGACATGGATCTCTGTATGTTAGGACGGTAACCTGCT
AACTACAATACTTCAATTTTATTGTTTTATATTATTTCTGTAATGAAATCGAATTTCTCTGAAATTTTATTTT
TTTTATTAATAATATTTTATGTTTATTTTGTTTGATATAAGATTGCAGTAGCAAGGTTATCTAGCTATGTATA
ATGTACATCCTACTTGTAAGTCCCACTGGAGCCGCGCCTCCCCCTCGGCTGCTGGATC
CGCGGAGGTCATCACTCGTACGCCGTGACTCGCGTTTCTTATCGATTTTCTACGAGTTTATTTTATACAATAA
AAATCGTTAGCTTAAGTATCTCTATGTTAAGGCATATATGCGTTCGATTGCGTAACTTTTTATAAGGTCTGAG
CCGGTATCTTCACAAATATTTGATATAAGTTTATTCGAGTTAGCATATTACGAGT
>103794439_2 DT661891.3
GCACGAGGCCAAGATCGTTCAAGATTTAACAGTTTAATGAATTAACAGTTATTACTGTTATATTTAACAGAAT
CCGAAATCTGGACGGAACGTATCGATGGTGAAGTACCAATACCTCCTAACTGACATTTTTAACAAATTATCTT
TTTTATTTTAAATGCACACGTTCTACACTATCAAAAATATATGAATATAATATAATTC
ATAAACCATATATTATTATTATTTGAAATAAAATAATAACACTTTTAATTTGTCAAGAAATAGAACGTTTTTT
TTTTTTGGTATGGTTCTTTGATTATGGCGTTTTTTTTTAGTTAGTGTAAAAATTTGACTTAATTCAGTCAGTT
AGATGTTATTGGCGCTGTCGGTATATGACATAGGTAAGCTGGGTAATAGAAAACGGTT
TAATTATTTTAAATATTTTTTATCCCATCCACAGTTATAATATCGGTGTCGTTCATCAATAGACACTCATCTA
TCTGGCAGGGGAGGTTTTGGTCGAACAGGGTGTGGTGCGTCACAGTTGTACTTTTGATATTATTTCAAAGTAT
TTTCACAACGACAATGCTGAGTGCCCGCTATGGCGAGTGTGAAACGTGGTCGCTGTGC
GAATGGCGGCCGCGTTTAACCACGCCCCTCGCCGTTGGATATGCAGTGTCTTTAGTGATGGTGTTGTTGTTGA
ATGAACTTATCAAGTGGCAGGAG
>103794442_2 DT662525.3
GCACGAGGATTTTTACTTTAAACATATGTGTATCATTTGCGAAGGGTCACATATTCTTTGGCAATGATCATCA
TCGCGTCCATCATCAGTTTAGCGAACTTCCACAAATGATTTCTCATTTAATCACTCAAAATCTCAGACCTGCT
CATCCTTATAACCAAGGATTATATCGCCCTGGAATACCAGGGAATGGTAACAATTTCG
GAGCTCCTTCATTCGATTATAGCAGACCATCAAATATTTTTGGTGGAAATTTGTTTGGATCCCCAGATTATAC
AAGTAACGAAGGTGGTTTTAACTCCGAGGAATCAGGTGAACCTTTTCTACAGCCTAATTCAGGTGAAGAAGTA
CAAAATATGCAAATTCCACAAGTTTCTAATGGTGCTAATTTAGATCTTACTTCTAATC
TTAATAGTGAATCTATTCTACCAAATGAACAAAATGTGGATTTAACTGGACAAGGTATTGTGGGCAATATTAA
CGTAGACGTACCCAATATTGAAATCCCACAGATAGAAGGAATAGTTGTAGGTTTAGACAAACCTGTTAATGGA
ATCATTGGTACCGATTCAGGTGCTCAAGCTGAAGATACAAAAGTTGATTTGAATAAAC
CTGCAACCACAATTAATACTGTAGGCCCAATTATTGGACAGTTGAGCCAGGTGGGGATACCAAATAGTGTAGG
TGGCAATGTAAATGTAGAAGGAAATAAACATGAAGTTGGTAGTAATATATCTACATCCGGCCAAGATACATCA
AAGTTAATTTAAATT
>103794475_2 DT663427.3

GCACGAGGGCGGCCGCTGCCACGGCGCCAGTCGGCCTGCGATCAGTTTTGCGAAACCAAATAATGCCATTGAT
CCGTTGTCGATACAGATTTAAGACGCGAGATGCCGCGTAAATAAATAAACGCCGCCCAACACCTCGACGACAA
CAAACGACAATGTGCTCATGGAGGTGGAGGTGCATCGCCGTACTGTTAGTGCTTCTAC
AACTGAATTCGGAAGCGAGAGAGATAACGCAGGACGTTAGCGACAACCATGTATCCACGGGCAATAGAACTGA
ACGATTTTTATTCGACAATATCTTCGGCCTAGAAGTACCACTTCTTGATGAGCAAAGTGTGGAAGATGATGAA
GAGGAACCACAAGTCAAAAATTGCTCCTGTGAATGTGGGGGGCCGAATCAGGAGAACC
GTATAGTGGGTGGTGTACCGGCGGGTCTCAACCGGTACCCCTGGATGGCGAGGCTGGTCTACGATGGGCAGTT
CCACTGCGGAGCGTCGCTCTTGAGCAAAGAGTATGTTCTCACTGCGGCACATTGTGTTAGAAAATTGAAACGC
TCTAAAATTCGAGTCATTCTGGGCGATCATGATCAAACGATAACATCAGAGAGTGCGG
CCATTATGCGGGCAGTCAGTGCCATCGTTAGACATAGAAGCTTCGATCCTGACTCATACAACAACGACATAGC
TTTGTTAAGGCTTATGAAACCCGTTACCTTCTCAAAAATCATCAGACCAGTATGTTTACCTCCTTCAGGCATA
GAGCCATC
>103794478_2 DT662338.3
GCACGAGGGGAACGCGTAAAGCAGAAAGCGATAGAGCGGGAAGAAGAAAAGAAAGCGCGTGAATTAGCCGAAA
TAGAGAAGAAGAGGTTGGAGGAGGAGGAGATGGAGAGGCTAAAGGCAGAGGCGGGCGGACAACCGATGCTCCC
TGCTAAGAGCAAATTTGATTCGGATTCGGAAGAGTCGCAATCGGAGAAGGAGGCGGGC
GACGTGGCGGAGCGCGCGGATCTGTTGCAGGAGGTGATGATGGCCGTGCGCCGCTCGCTGACGGAGATCCTGC
TGGAGGTGACGGACCAGGAGATCCTGACGGTGAGCCAGGAGGAGCTCGCGCGGTATAACACGGCACAAGGTAT
CTCTCTGACGTCGACGCGACCGCATTACATATTATAGCTAATCGAGTTTCGTTTCAGA
TCGACGAGAATTACACCCTTACTAGAAATTAATCGTCCTTTTATTTATATGGTAAAAGCGTCGCGAATCAACG
CGTTGAAGGCGAGCAGGTCCAAGGCGCTCGCTTCCATCGCAACGGGACTCGGTGAGTTATGTTGCCTTCTCTT
TCATTTGTACTCTCTTTGTGTATCTGTCTCACTCTTTCCTTAGGGTATATATGTTCAA
GTATTGTGTAATGCAATTTGGGGTAGGTATTGTAAAATATTANAACCTGCAATACGGAAGGAAGTAAGTGTTC
ATTTCTGTCTTAATTCTTTGTGAGAAAAAAACGCTTGCTTATGCCCACGTGGTCCTAGGCGTATATTAAATCT
AGT
>103794493_2 DT662659.3
GCACGAGGGCTTGGCCTTATGAACACACATAGACGCTTGGTCTGATGAACACACATAGACGCTTGGTCTGATG
AACACACATAGACGCTTGGTCTGATGAACACACATAGACGCTTGGTCTGATGAACACACATAGACGCTTGGTC
TGATGAACACACATAGACGCTTGGTCTGATGAACACACATAGACGCTTGGTCTGATGA
ACACACATAGACGCTTGGTCTGATGAACACACATAGACGCTTGGTCTGATGAACACACATAGACGCTTGGTCT
GATGAACACACATAGACGCTTGGTCTGATAAACACACATAGACGCTTGGTCTGATAAACACACATAGACGCTT
GGTCTGATAAACACACATAGACGCTTGGTCTGATAAACACATATAGACGCTTGGTCTG
AAAAGGCGCTTGGCCTAAGAAAATATCTTGACGGGCGCATGGCTCTGAAAGATTTTGTGAAGGGCGCTTGGCT
CTAAATGACGCATGGTCAATGTGGTGGACGCTTGGTCCTTGCAGACAGAAGGGACGCCTTCTGACTGTTTTGT
TCACAGGTTCCAGAGTTTGGTGCACTGGACTGTAGATACTTGGATAAGAAGATTTTAC
AGGGTTAAAAATGAGAGCTCTCAAAACTTTTTTTATTTTTATTTACTTTTTATATTTCTTTTACAGACATTTT
GATTGCCTGTCTGTTTATGTCTAAACATTAATACAATTTAAATTCTAGATGATGAAGACTTCTGAAGAATTTT
CATCAATACAGCCACGCAACGACGCTAATGAGTTGGAGTCTTATCGAGATGCACCAGC
ATGTGATCAGGCAACTGGATATATATCAGCTAC
>103794499_2 DT661810.3
AATAAAAATGATTGTATAGTTAGTTGATGGTTTAATTAATAATATAATATTATGTAGTGANAATTACATTATA
ATTGATCTTTATGATTTGTGGCCACTATCTCTCCATCTTAGGCGAGAAATATGTTTCTTTTTATTAAGTTTTT
GTTATAAAAAAAATATATAGCTATTTTCTACAACCGTTTACTACAAAATTATTGTACA
TATTTCACATTACGTTTATATAAGTGCTTAATAAAAACCATCGATTTCTTTAAAAATATTCAATCAAAAGATA
ATAATATCATTTGTTGATATGTATGTATGTATGTATGTATGTATGCCTTTTGCCTTTACAACCATTTATAAAT
ATAACCTTTTTACAAAATAAGTATATATTTTTTTAAGTACAATCATAATTAATAAAGA
ATATGGGGTAAAAAGGCAATCATTATTATTTTAGGTTCTAATATGTTTTATGAATTTGTAAATTTATATTGAT
AGATACTTTTAATATTTTTTACACTGTTAATCACATATTAAGGATAGAAAAAAATCATAGAAATATACAAAAT
TTCTAGAAATAATATAATTTTATTCTATTCTATTCTATTCTATTCTAATAATATAATT
TCTAGAAA
>103794519_2 DT663165.3
GCACGAGGGAGCGCCTTAAGAAGCGCCTGTCCTCCGACATCGGCCTCGAGATGGACGAGGCGCAGCGCGAGAT
GGCGGATCGAATGTACTCTATTCTGCCACCAAACAAACGTGCAGCCGCTGCCGGCTGCATCATCCCCCACCAG
CAGCCCTTCGAAGTGCTTTACCGTCTCAACTGTGACAACCTCTGTGCCGACTTCCAAG

AGGACCTGTCGTTTCGCTTCAGTTATGGAATCACGGCCCTCATACAGAGATTCCAAGGGCGGAACACTAACAA
GATTGCGTTGAAAAATCCACCTACTATTACACAGATTCCATTTTCTCTTGGACCAGCAGCGCCTTCTCTGGAG
GTGTCTCGTGGTAGTGGTGGCGTGGGAGTGGGGAGTGTGGGGCTCAGCGCCGAGGAGT
GGGGTATGATATCCAAGTTTGCACTTGGAGCTGTCACGTCACAAGGCACTATGGGTGGTCTGCTGTTGTCCGG
CCTGCTACTGAAAACAGTAGGTTGGCGTGTGGTAATAGCAACTGCTCTA
>103794556_2 DV501091.2
TTTCGATTTCCATACATTTGTCATACTTAATTGATTGTTAATCAATTGTGAAAATCTATTTTTGTTTATTGAT
TCTTTGTTGCCATATGATTGACCATGGATTATCAATTGGTAGTTTTATAATTTTATTTTGGTTGTAATATCCC
TTATAAATGACCTTGCACCTTTTGCTCTTATATTATTAAGATTATCGGGTACTGATGA
TCTTGAATTATCCTCCTGATGATCCTTGACCTTTTG
>103794559_2 DT661951.3
GCACGAGGCAAGGACCCTTTATCATTTTATACAATAAGATTAACTAAGTTATGTTATGAAATAATGACAAATA
AAAATGTTGTAACTTGTTGAATAAACTTAAAATTTTCATTAAATACGATCATTGTGCTTTAAGTGATCAGAAA
TTTAAAAAAATCCTCTTTTTTTTTCAATGGCCTGATTCTATGATGACGACAGGCTTAA
ACTACTTCTCTAGCGCTGTTTTTTGGGCATATGCACTTGACATTAGATAGAGAAATTGTATTATAGATAAGAT
AAAAAATTTTAGGCCTACTTTAAAACTATTTTGTTTCCTCGTTCTTTTAAAACGTATCATGTTCAATCTATTA
AAAATGAACGTGTGGCTTATTTTTGTTTAAATTTATAATATTAATGGATGAATTTAAA
AATTTAAATATATATTCATAATCAAATTATATCGGAAATTGTTTATTGCTTAAGTGGCTTACTAATGTTTAAA
ATAATTAGTTATATTTATTGTAACGCTAGCAAAATTGTTTACGTAACTTTGTTTTAATAGATTATAATCTATT
TGAACTTATAAGTATAACATTGAAAGTTTTGATGTTTATTTAGAGAAAATTAAATTTT
GAAATTTACAAAATTTATCAATATGTTAGGTTCTTTTCGGGAATACATATTATTTTACAGCTTCACCACAGCA
ATAATATTTTTCTTGGCATTTCACGTATGTTATAAGTAAATTTTGTTTTAAATATATTCATTCTCATTTGGCT
AAACTAAAATCTTGCCGAAACTTCCATCCAATTTATTA
>103794562_2 DT661714.3
GCCGGGTCGCGACCCGCCACTCACATTGCACGTACCGCTCGCGCATCACGCCCATGCTATTATAGTGGCGTTT
GCTTTGCAGTGTTCACAGGTACAACCAAAACCAACGACTAATGGCATATCCAGCACGAAAGAAAAGATCACTG
AACGGGAATCGAGGGAAACTCGTGATTCTAGGGAAACTAGAGAATCTCGAGAATCAAG
AGAGTCCAGAGAATCTAGAGATTCCAGAGAGATAAGAGAGCTACGAGAATCGCGAGATGAGTCCGAGATGGAA
ATACGAGATAGAAGAGCCGCTTTTTTTTCCCCAGCGCCGAGAGATAGAGATCGCGAAAGAGATAGAGACCGTG
AACGTGATAGAGATAGGGAGCGTGAACGGGATATGCGGGATGAGAGGAGAGACCAATC
GACACCACAACATGATGGCAGACACCCGCTTCTTCAATTTGCCGTGGATCATTTTAGGCAAAGCCCAGAATTA
GAAATTCTGAAAGCGGACTCCTCACTGAAAAGCAAGGCTAAACGCAACGAATGGACGTGGAAAGCTCAAACCG
ACGTCGTGAAATGGCAGGCCACACCTCTCCGAGCACCACTCCTACGGCTGCCGGCGGC
ACTGGCA
>103794569_2 DT661850.3
GCACGAGGCTTAATTCTTAAAAGAAGGGAGCTCAATAACTTTTATTATTCTCAAGTAAACCTATTTAACTGTT
CTGAAGTAACAAGCCTTACTTGTGGTGATACAGTGTGTGGTACAAACAAATTCGTTTACCAAGACTCGGGACT
CAGAGGAGACCCTCAAATAAAAAACGAGGACTTCGATTGTTTTAGAATAAAGCATAAG
CCCTTCCCTTATTACAATAGATTGTGTATTTTTAACATGTTCGCGTGAACACAAACGCCTTAGCATCCTCTCA
GAACCTGTAAAATATAGTGTACGTATTGTTAATAATATAAAATTTTTTTTACTAATAGTGATAATGAATACGA
TAGGTAATAATATGCGCAATAAGCGCAAGCTTCTTGATATAAACAGTATGTCACTCCA
ATTTCCTATGTCTAACTATTCTACGGACACGCAAAAGCAAGAAATGGAGCAAATTTACGGAGACAACCTAATA
TTGAACAAACTACAACTACTACCAGACCCTTTGAAAAAAAACCTGGCAGATTCTGTTATTACTGGCGAAAAAA
CAATGAACCATAAGGTAGAAAGAGTAGCAACAAAAGAATATCCATCAGATAAGCCATC
TAATAATCAAGAAATTTATAATTCATCTCGAGCTACAATAACTTATATTATGTCTGGAATTTATAATATTGTA
GGTGCATTATTCTCTTCACGCTCACAATACCA
>103794575_2 DT661932.3
TATAACGATGAATACTATAATACAAGTGCATTTAATACAATAAATTATTTCAAAAAAGATGTAAAGGAAATTA
TGGAAAATGAAAAATCGAATAATGTGGAAATTGTGTACTTACAATGGGGAAGGGATTGCAGACATGTGGATGA
AAAAACTATAGCTGATACGTATAGAAATAAAAGTGGAGAAAGTGGAGATACGAGACCC
CAAGAAATAATACAATGGATAAAACACAACGTCAACCTGAACACGTCTAAAATACAGCTATTTTTTTTTATAA
CAGATGGGCAAATAGGTTCTTATAGTGCTACAACATGCCTGCAAATGAATACAGATATGAACTATGAGAACGT
TGTGTTTCACGCGTACAACTACCACGTGCATTCAATTGATTTGTCGGTAGCGGCGTCG

TTTTTCAAAAAACAAATGCACTGTTACACAGAACGGGGAAGTTTTAAATCAAGTAAATATTAGTGAAAATTAC
GACTACGATTCTATTAATATCA
>103794613_2 DT662954.3
CGGCACGAGGGTACATTTTAATACCCTGCACGATACATTACACAATTATTTAAGGAAACACAGATTTTGTGCA
GATTGTAAGACTAAGGTATTAAGAGCTTATCAGTTATTAGTAGAAGAGAAGGAGCCACAGAAAGAGAAAGGTT
ATGTTGGTGCCTTATATGGAGGTATCAAGAGATGTTTGTTAGACAAACATTTACACTT
GCAGGCCAAAACTGATTACATTGCGCATCTCATAGCCAGAGCTGAACCAGAGTTGTTTGGTAATCACAGGGAA
AGGCATGCCGTCACATTAGAAATTGCACAGGAGGAGGTCTTAATATGTCTCGGGATATGTATATACGAACGTC
TACAACGCATCTCATTAAGGCTCCGCGAGGAAGAAGGCACATGTCAAGCCTTAGCGGC
CATTGGAGTTGAAGCTTTATATAGGAAATTTGAAACCGCCGTTGAAATGAAGAGCGGTGTCTCCAAACTTCAA
TTGTTATATGATGAAATTACACAGGAGGAACTCTCGAGACAGCAACGGAAAGAACAGAAAAAACTTAAGAAAC
GCAAGAAGAAGGAGCGGCAGTCAATTGAAAGCAAATGCAAAGAAGCTGATTCAGAAGA
ACCTGAAGACAAGTGCCAATGTAAAGAATGCTTGCTGGAGGATCGTGATATACCTACTAATTTACTATCTACC
CTCAACTATAGCCAGTGTTATGATGTCATGCAATCTAATGGTGAAAGTTGTCAATCAT
>103794625_2 DV500958.2
GCACGAGGACTGTTCCTGCCACCAAACTCCTAGGACCATAACAAAATGTTCGAACAGGATACCCCTCCTCCGC
GCCCTCCTGATTGTAACTGACGGACGCCGTCTCGACTCCCGAAACCTCCTAAGCAAGCCAGCCTTGCCCAAAG
TTGCCCCACGCCACCTCACACGATGCGAACATTCTTCTTAATCCCCACGTCTTTAAGA
AGGCCGTTTGGACGACACAATCGTCATTCACATGAAAATCACGTACCCCTGTCAGATGACCAAAGTGGTCATA
TCACACCAGGCTACGAATCGAGGACGCGGAATGATCTTTACTTGCGTCTTGGACTTCTTTTGGGAGAACGTCG
TGCATCCCGAAGATCCCCGGATAGCTGCACATCACTAGCTTCTTTGGACGCGCATACT
CTGGCTTCGCACAACACTAGTCCAGTATCGACACTCACTGGTTCCTCACAAGTGGACGCACCAACACCGTTAG
GCAGAGATTCACTGGGATCTTTACCTTCTATGTCTTTGTCTGCCGCTAGCAGCTGCTCGTCGTCAAGTCCCGG
AAGTAGGAGGCATTCGGTTACTGCATTAAAATCTGGGCGCGAGGAGCTCACGCGTATG
TCGAGTGGCTTCTG
>103794628_2 DT663176.3
GCACGAGGGTCATCCTTATAGACTTCCCAGCCCGACAAAACAAACTGATAAAGGACAAGTAGAACATATACCA
AATGATGATTTTTTGAGCTATATTAACGATGATAAGAAAAATAATAAATCTGGAGACGACGAGTCATATCCAC
AGTGGCAATTTATTCCAGCAGCTTATGAAAATGAAAAATCTAAGAAAAATGGAGCAAT
AAATAATTTTAACACAGCTGCGCCTCACGCTATATTAACGAATCCTTCAACTCTGCCTTTAGAGGCACTATTT
AAAACAGAAAATCCTTTGCCTAGAAAAGTCAAAGAGATTAAAGACAATAAGCCATTTGTGGTATATTCTGCTC
CTATTCCTGCGCAAGAAGAAGAGGCAAATTTTGTGAAATTAAAAATATTGATACCTCA
AACAGCTAGGAAAATTGTCACTTTTGCCAAAGGGAGAGAGTTTCAAGGACCTTCTGTCGATGAAACTACTATA
AGGCAACCAATTAGTGTATCAATATTGGAACAATCACCTAAATCTGTTAAAGAATCTTCAATACCGACTACAA
AATCGGTTGTACAAATAGCGTCTTCAACAACGGAACTGCCGAGTACAGTTACATCACC
GACAGAAAACACCATGTCCATCAGTCCAATTGTGGAATTACTAACAATGATTCCAGTATCAAACTCCCAACAA
CAATCTGAAATCGTCCAAACAACAGAACTACCTTCCACAACAACAACAACTGAAATATCAACAACTGAAA
>103794631_2 DT662187.3
GCACGAGGAAATCTTTAAGAGACTTACAAAATATTTAGAGTAAGAATATTATTTAAATAAAACTCGATTAAAT
CAAATAAGAATATTTATTAATCAAACTTATACCTGAAAATCTTATTATTTAATTATACTAAAATGAATAAAAA
TAAAAAAAAACTAAATATGCATGTCACCAACTTCTTCCTGAATTTCACACATCTGTTC
ATTAGAACCTGGACCATTTTGTAAAATACTACCATTTAATAAATTCTTAAAAAATGATAATTCTTCAATTTGG
TGCCAATTCTCGCCCCAATGACTTTAGTAGTGTTGATTTGTCTTTTTTTTTTGCATCGGATAATATGCACGAC
GGATTTATTTTAGTTGGGTTTATCATAGAACTTACTTTTCCTCGTTTGCACACGTTTT
TGCGAACTCCATCATCATTTTTATAATGAAGATGTCCAGCAATCAAAACGTTCCCGAATCTTTTAGAACGTTT
CAAAATAAAACGTTTACATAATTTAAATTGAAAATGCCAACTACCCACAGATTTGAGAATTACTTGTGAGGCT
GACTTTCCATCCAAGACATCACATTCATCTCCCAATTTCG
>103794649_2 DT662567.3
CGCACGAGGCACGTGTGCGCGGTTTACGGACTAGCGGTATAATTCTTTTATTATACCTAACTTTTATAACAGC
ATCGCCATTGTTTTCTGTTAATGTGTTAAAGAGAAATTGTGATATAAATAAATGTGTTTACCTTTTGAATGTT
GACGGCGATTTTGATCGGTGGTCGTTGACTCCTGTACCGGGCGTGTGTAATTTAGATA
GTATACAATCTAATTTTATTGGTGAAGATGTAGAAATTGTTGTTGATAGATCGGTAAAGTTGTATTTATGTGG
TGGAACGGGTAAAGAATGGTTCCCGCTGGATTATCATTTGGAAGGTGACGTCATCGCCAGTCAGCGGCAATCA
GAAAATGAAATTGAAGAAACACCACACGATGAGATAATAGACTTGGAAGATGACGTCG

TTAAAAATTACTACAGGGACTTAGCTGTTAATAAATATGGGGACCAAATCGAAGATGTCATGATTGAGACCTT
TGCAAATCAAAATCCTGATCAAAAATCACTTAGAATCAAACGTCAGGCTGAAGACACAACAGTATCGGAAAGT
GCACTAACCACTGAAGATAAAGCTAATGTCACAGCAATACCGCTGCCAGCTGAATCAG
CACAGCCAGTTGACTCAGCACAGGTCAATCCGGTGCAAACAGTTCCTGGACAATTACGAGTCGTGGGCTTAAG
AGTCGAAGAAGCTGACAAAGAACCCAAAGTAGTGGACGGAATGATCCCAAGTGTTCTCAGAAACACCAAGTTC
ACTTTAAGACTTTTCGGTGAAGGTTTTACGAAGGATACTGTGATAGCATTCACTCATG
TTCCAGAGAATTATGGTACTAAATGTCACTTTATGGTCAAGGGG
>103794652_2 DT663223.3
GCACGAGGGTCGTGACCTAAAAGGTTAGTCATTTTTACGTGCAAACTGTAAAAATGTCGTCTCATTTAAACTG
GATGATAATTAGAAACAACAAAGAAGTCCTTGTGAAGAAGCGTAACATCAAAAAGCCTTTCAGTAAGGAGCCC
AACAATGTGACTAACCTCAACGCTTTCAGATACAATGGCTTGATCCACAAGAAGGCTA
TCGGCGTGGTCGAGAACCCCGACAGGAAAGGTTTTTCAGTGGTATACAAGAAAGCTAAGGCTACCAACAGGCC
CGATAGGAACTTGATCCGCCG
>103794682_2 DT662066.3
ACGAGGCGACCCTTTAAGATTTATTAAAATAAAAAAATATATAGTTTTTTTAATATTAGAGGTTTTATATATT
TTTTTAATTACAATATGAATTTATTTAATAGTCTATGGATGTATATGTTGTTTTTGTGTTTTTTTAATTATAA
TGTCATTCAGAGCACCGCACTTTCTTTGCCCGAGCCACACACATGTATCGTAGGCGGT
GGGTACTCAGGGCTCGCAGCCGCAAGGTACCTTAAGGAGTTTGGTATGAACTTCACAGTGTTTGAAGCTGCCA
AAGGATTTGGTGGTCAGTGGCGGTTCGACCCTCTTTTTTGAGTGGATGAAGATGGTGTACCTGTGTTCACAAG
CATGTACAAATACCTTAGAACAAATACTCCCAGGCAGACCATGGAATTTGGAGGCTAT
CATTTTCCAAATGGTACTGCCACTTACCCGTCCGGCCCATGCTTCAATAGATATATCAAGTCGTTCGTGAAGC
ATTTTAACTTGCTACCCCACATTCAAGTCCAAAGCTACGTGACGTCAGTGAAATATGTTAATGGTTATTGGCA
ATTGACGTATTATGACTCAAAGGACAGAAATAGTAATACAGTATCTTGTGATTACGTC
GTCATTGCCAACGGCCAGTATATTGGACCCAATGTGCCTAATTTTCCAGGACTAGATACATTTAATGGTTCCC
AAATTTC
>103794693_2 DT663290.3
GCACGAGGGGGAACCTTTCAAGGTCCCCACGAACGTATCTACTTTTTTCGAAGTTCTCTGTTCAGTTTTTGTT
CTAAAGTTTTACGAGAATGTTTTTGTGCTTCAAGTCTTTTGTCCTGTCGTACTTGAAGGATACGAGACTGCAT
TCGGCGGTGTAGTTAATTGAGTTAATACGTTTAGTGTTTTTTTTTTATAATTTTGTTA
TTGTTAAGTTTTTTGTTTGGTGCATCGTGCCACTGGTTTTATAGAGGCCCCAAGCCTGGACGCGCGATACTTA
CCTTTGGACAAAGCCTTTAGGGCGTGTAGTTGTGGTGGTCGGCGTGAGCGGTCCCCGCTCCAACCGTCAAGCT
GGCAGCGAGCGCGTTGAGCATGAACCCGCACTACCACGGGTACGCGGAGCTCAGCTCG
CCACACCCGCCTTCACCAGAGGCAACATTTGCAGCCAACGGACATGATTACCCACACAACAATAATAACCCGG
CTCTCAAAGAATGTGCAGGATGTGGTGGCAAAATTGTAGAAAGGTTTCTCCTCCACGCTCTTGATAGATACTG
GCATCACGGATGTTTGAAATGCACGTGTTGTGGCCAAGCCTTAGCAGACATGGGTCGC
TCATTTTACTTTCAAGGAGGAATGATATTATGCAAAAGCGACTATACAAGAATGTTCGGCTCAAGCGGCGCGT
GCGCA
>103794696_2 DT662762.3
GCACGAGGATTACCCCTTATTAAGAAATTAACACTTATAAAACTTGAAGTGTAGAACAAAACTCTAATAAGTG
ATATCTCTAGGAAAAGAAGTAAGAGGTCACTCGTAAACTACGTCACACGTTAAAGGGGACGGAGGGGGTCGCT
GAAGTATTAAATTCTGTGACAAGGGTTGGAGGGATGTAGTATGAAGGAAGGGATATAG
GGGTTCTAACTTTCGTTTAGTGTGAACAGAAATCATTTATGATCTGTTCTGGTGTTATTTAAATAATTTCCAT
TTGTTAAATTACAAAATTCACCACAAAATGTGACAAGCTTTGATAAGGGCGGTAGCACAGTCAAAAAAGTTCA
AAACATCATCATTTGTGTGATGTAATTTATGGATGGCTTCAAATTGAAATTAAGAATA
ATTATAATAGTTTACACAATATACTATGACTTGTTTTAAGCATAGAACATAGACAGGTTCTGTTTTCCAAGCT
CCAGTTCTTCTCAGAGACCATTCAAATCAAGTTTGGTCCAAAGTCAATGCGCAGTTTTTTTTATAAACTTCTG
TATCTATATTAAAAATATGTTGCTGACATCTATGTTCTATGATCATGATGAGCCATTA
TATATGTGTTACTTTAGTAAATTTTAGATACAGAAATAATTTAAACTAAACTGTTTTTAGTGATTACATTATG
CCAAGATAGAACACCATACATTTTTCGTTGTTGATTTGCCTTTTCACTTTTTTTTGTGTGATCATAGCAGTTT
TTCACTTTACCATATTATGAGGTTTATAAACACGACAATTTGGGTTATTTCTACGTCA
TTATATTAATTCACACATACATATAACTTCT
>103794699_2 DV501008.2

GCACGAGGATCCCTTCGACCAACGTTTTAACGTTTTTCACCTTTTGCTCATTATTTTAAGACCTTGAAAACTT
ATCAAACCCTTACAAATTTGTTAGAAGTAGGTAGTTATAACCTAGACTATTACTGATGGGATTCCATGAGCAT
TCTACACGTG
>103794710_2 DV500959.2
TTTTTTTTACCTTAAGGAGCGCTTCACATTCCTTGGCACCGATTCCACGCTTCAACTCAATCTTCGCTTCAAA
AGCGACAACAAATTGAAGATACCTATCTGCAACACGATAGGTTATAAGGAATCCTTCACTGTTAAAAGCGTCA
TCTTGTGGAATGACTTGCCGCGATTTATTCGGCATTCAGAATCAATCAGTATCTTTAA
AAAATCTCTTAAGCGTTATTATTTGGCACAACAAAAAACTGTACAGTGTATGTATATATGTATGTATTTATTA
TCTGTATATGTGTAATTTCTATTATGTACCTTTTTTGTATATTTTATAATTT
>103794713_2 DT662968.3
GCACGAGGAATAGGAATCAAAGGGGGCAACGCGTTGGGCGGCATTCCTTTGGGTAACTTGGGTCTTGGAAATT
TAGGAGGTCTTGGACTACCATTAATGGGCGGCGGAGAAAGTAAGTAATAAAGGATAGTATTTATTTTGCTGGG
TATCCAGGGTGAAACTAGTGTTAAGACACTTCATCTTCTGTTTACCGCCCAATT
>103794716_2 DT663333.3
CGAATCTGGCCGGTTACCTCCTAGCTTTTGACGAAAGCTTACGTTCAACTAAAATAATATCATTTAAAAATGA
AAGAACTTATATTTTTGTTAGTGGTCGTTTCAACGGCATGTGGTCTCCAGTACAGTGGGCTCAGAGTGAAATT
CGGGTGGACAGACGCTCTGGCTGACAAGGAATATTTCTTTAAAATTCCTCGGACGATT
GCCGCATCTGAAAGTGAAGGGTGGCGAAGAACGGAACGACCTCCGGGACCTCTCGAAGAACTTAGGCTGTATT
GTCCTCTTGGAAGAGCAGTTTGCCCTCTGTATGATCCTTCCGGATTCTTTGCGGGTCTACAACTAGCTTTTCC
TGTTGATGAATTAATAAGCTCATCGTTCAAGCCTGAGCAAAGATTCGTCAAATGGAGT
GTTCCATCCACGGACGGAGAACCCGGACGAGATTATTGGTCGATCACTCAATATTTTGTATCAGAAGAGTCTC
TAAAAGCGGGGGCTGGTCCACAAGTCGAGAACGGCGCTACTTTACAAGATGGCGGTGTTTGGGTTAACGATCT
TGATGGTCAACTTATGCGCATACCTAGCACAGAAGCTGA
>103794719_2 DT662955.3
GCACGAGGGTTTCTTAGGGAGGGTCGTGAAAATGACCGGGGTGGATGAAATACTGAACTATCAGAGGGACCC
TGAGGATGACTTCTATGGGCTGCTGGGATGTGATGGAAATTCCACTGTGGAGCAAATAAATGCTGAATATAAA
GTATTAGCTTTGCAATACCATCCTGATAAGAACGATGGAGATAAAGATGCTGAAGCAA
AGTTTCAGAAGTTAAAGGAGGCAAAGGAAACTCTGTGTGATCCATCTAAAAGGGCACTATATGACAAGTGGAG
ACAGAGTGGAATTTCCATGGGATACAAACAGTGGCTGGGCATGAAGGAACACATTCAGCAGTCTATGCATTGG
TCTAAACCAAACACTAAGGACCGTATGTTGGAAGAGTCTGGTCGTTCCTCTGGGCCAT
CCAGCTTGGGCCCGACTAATGCCGCAGCTCGGCGAGCTTCAGAGGGTGGAGCGGCCCTATGGGGTCGCTGGGG
CGGTGGCAACCAGGAGCCCCCATCTGAAGTCATCTCAAAATTCCGAAACTATGAAATTTAATTTCATGTCATA
GTTTTCTTATTTTAAAATGAATAAGAGATTGATGTGCCCTAATCTCTTAATTTATTTG
CAATGTTATAGTTTTTAGTTGGAAACTGGGCTGTTTATTTTCTTACCATTTTATTTATAATTAATATTAAGTA
TTATAAGCCTCAAATGGGTACAATATTCATCAAGTCAGCTCTAAAATCTCGAATGTCACTCGTTTT
>103794747_2 DT663228.3
GCACGAGGCAGAACCCGTCTGACCAAAATCAAAGCAGGTGTTCCACAGGGCAGTGTAATGGGCCCTGTCCTTT
ATCTAATTTACACCTTCGACTTACCAACTAAGAAGAAGTTCTCGTGGGGACATTTGTTGATGATACTGCTCTC
ATTGCATCTCATGAAAATCCAGCAACAGCGTCAGTAATGCTACAAAAAAGTTTAAATA
ACATATCAGTGTGGCTTGAAGACTGGAGAATTAAGGTTAATGAAACCAAATCGGTGCATGTAATTTTCGCTTT
TAGACACGATACTTACCCCAGTGTCAAACTTAACGGAGTAATAATCCCTAAATCAAAATATATATATAGATAC
TTGGGCATGTATTTAGACAGACATCTTACATGGAAAAAGGACATTTTTACCAAAAGAA
AAGCTCTAAGTGCACAATTCCGAAAGCTTTATTCACTCTTGTCACGGAAATCAAAACTCTTGTTGTATAACAA
ACTTCTTTTGTACAAAGCTATTCTTAAACCTGTGTGGTCTTGTGGAGTGGAAGTTTGGGGAACTGCAAGCAAT
TCAAACTTAGACATAATCCAACGCTTTCAATCAAAAATTGTTCGCATAATAACGAATG
CCCCTTGGTATGTCACTAACTCTTAGCTACATCGAGATCTAAGGATACCCACAGTGAGAGTAGAAATTACCAA
TAAACATAAGTCATACTCTCATCGCATATAAAACACCCCAACATACTAGCCCGGAAATTAATG
>103794759_2 DV501096.2
CGGCCAGTAATATGTTTATTAAAATAGCTCTATATAAACATTAATTGTAATTTGAAAATAAAAACATTTTAGG
TAAAAAATCACAAATTTAATAAAAACGGGGCATTCAATAAAATATTAGGCGTTCATCATTTCTATGTACCAGG
GACACACAATTCCCATAAACTATGGCATAAAATAAACAAAATGCCATAAAAAAAAACA
ATAAAAGGGCAACAAAACATTCATAAAGTATACGGATAGAACGGATTCTCTAATATTTTAATATCACATTCAA
ATAAGGCACTTTACATAAATAAATTATAAAATTGTCAACCGAGTGTTTATATCATCATAAGTATAAAAAACAA
AGGTCAAAACGATCCACCATTTAAATATTAAAATCACGGGGCCCACAAAACGCAAACA

ATAGTTTGAATCCGTTGGGTACGTCCACAAAAACATCCCTCACCCGTTATCCTGAAAACGGGAACGACACAAA
AACCGTTGCCATGGAAACGGGAACGACACAAAAATTCTAGAGTATATACAAAACGGACACTGTTTTTTTTCTG
CGTGATAGAGGGATAGGAAATCCATTT
>103794800_2 DT663025.3
TTTCCATTGTGTCATCCATTGTAAATGTCAACGCATTATTATAGTCCTCATTATTTTCGAAATTTTGCGCTGT
AATTTGAAATACTTCGCGACCACCATGTCTGCAACAGCATTATTTGGTAATACATCAGGATTGGGAGGTAGTT
CTGGGGGAAACAAACATTTAGTAGAATTTAGAGCTGGGAGGATGACACTAAAAGGACG
CATGGTGCATCCAGATAAAAGGAAGGGTTTATTGTATGTATATCAAGGCGAGGACTCTCTAATGCATTTTTGT
TGGAAAGATCGTACTACGGGAGAAGTTGAAGATGATCTATTAATATTTCCTGATGACTGTGAGTTTGTCCGTG
TTAATGAATGTACAACGGGAAGGGTTTATGTACTTAAATTTAAATCGTTTGCGAAGAA
ATACTTCTTTTGGATGCAGGAACCAAAAACTGATAAGGATGATGATTATTGTCGTCGTATAAATGAAGCTTTG
AACAACCCTCCCACATCAGGAGGTAGAGGTGGAGGTGGGAGTGGGGGCCAAGATGGTGAACTCCAAAACTTGC
TAAATAACATGTCCCAACAACAGTTGATGCAATTATTTGGAGGTGTTGGGCAAATTGG
TGGCTTGTCATCTCTGCTTGGCACTATGG
>103794809_2 DT663390.3
TTTAAATTGGTTATTAATAACTTCATAGAGAGAGATATTTAGATTCTGAAGACTCGATTGGTGATTTTGATGT
ATTTATTAAAATCGATTCATCAGTAATTCAATTTTAAATTTAACCTCAGTATTTTTTCAGTTTCATTTCATGT
ATTTCGTTTATGATACAGACAATGTAATTTCTTTTTGTTAAAGTAACAAACGCTTGTA
ATTAACTTAACCATACATTTGGTTCACTTTGTAGTTAACTGTTCTAAATATAGTACAGTATCTTTTAGTAATT
GTTGATTATTAATAATCTAACGCTTGTATACGTCACAAGATCTCGTAATTTATTAAATTTACGATGTAAAAAA
AAATGATCAAAACTATATAAATGAATACTTTTGTAACGCATTTTAAAACTAACAATTG
TTATAAAAATTGTATTACAATATTATCTACAATAAAAAAGATATTTTGTTTCTTTGTAAAGAAAGTTATTTTT
CAAATTATACAAACAAAAAATATAACCTAAGTTTGATGTACTATTTGATGCATTTATGACCAATTACATTGTT
TCTTTATAGTCTGTCATTGTAATTTCAATACTTTTTCAGAGAAATACAACAAAAAATG
TCAGCATTCGAGATAGCCTCTAACTCATATTTAAAATTTACGTTGATTATAAATTTGAGTTTAATTGTTTGTT
CGCAAAGACTAGCTAGCTAGAGTGTTGGCCCCTCACGTCCTCACGTCATACAGTACGGTC
>103794815_2 DT663409.3
GCACGAGGTAAAGGCTTAACGTTTTGGGAGTGCGCGCTATGCCCGAGCCTCAACTAGAGATCGCGATAACGAC
TGCTGGCAGTCGCGCTCATTATTTATATTCGCATATATTATATTTCGTGTAGGGTTGTTTTTTTTTTCAATAT
TTTGTATGAAATTTTAACGTAATTGACATATTCTTGTTCTGTGGACATCGATCGTCTC
TTATTCTGTGGACGTCGTTCGTCTCTTGTTCTGTGGACATCGATCGTCTCTTGTTCTGTGGACGTCGTTCGTC
TCTTGTTCTGTGGACATCGATCGTCTCTTGTTCTGTGGACTTCGTTCGTCGCGTTGTATAGATTAGACGTGTG
TTCCTATGTCTAAGGTTTCCACACCGGTGGACGTGAGTCGCTCTACAAATTAAAACAT
TGAAAGCAATATATTTAGTATATTTTTTTTATTAATAATTAATTACACAAGGATACAACTAAAAAAACTGTTT
GAATAATTTCAACTCGTTCATATTTATGTAATTCGTGGTGTTGGAAGCGAATAAAATAATTCTAGGTCGTTGG
ATTTTTTTAGAAAATGCAAATTATTTTTTTGTTAATTTTACCTTGTATTGCAGCATAT
ATTATAGTGTTTACATCATATGGAGCTTTTTTTTTTAATTCTGATTTTATAAATAAATT
>103794824_2 EC091207.1
CGTTTAAAGCCCCGGGCGTGGCAACTGGGCGATAAATTGCTCCCTTAAAACCAGGGCGCGTGCTGGTTCGATG
GCAGGCACACCCTGCGGCTTTTACAACCGCAAGGTAAACTGTTGTTCAATGAAATCAACTGGCTCGCTGGTCA
TTCGCCGGATCAAGTTCAGTAATTTCATAGTTACCGGAAAGCAGCGGCTTACAAAGAA
TTTTATAACCGTCGTGATCAAACCC
>103794827_2 EC091208.1
AATAAGGGCGCGTGCGCTATCCACCTTTTTGCCCGTCCATATTCAACGGCTTCTTTGAGGAGTTCATTTTCCA
TCGTTTTCTTGCCGAGCAGGCGCTGGAGTTCTTTAATCTGCTTCATGGCGGCAGCAAGTTCAGAGGCAGGAAC
AACCTGTTCTCCGGCGGCGACAGCAGTAAGACTTCCTTCCTGGTATTGCTTACGCCAG
AGAAATAACTGGCTGGCTGCTACACCATGTTGCCGGGCAACGAGGGAGACCGTCATCCCCGGTTCAAAGCTCT
GCTGAACAATTGCGATCTTTTCCTGTGTGGTACGCCGTCTGCGTTTCTCTTGCCCTAAGACATCAATCATCTG
TTCTCCAATGACTAGTCTAAAAACTAGTATTAAGACTATCACTTATTTAAGTGATATT
GGTTGTCTGGAGATTCAGGGGGCCAGTCTACTCGTTCTTTCTCTTCGCCCATCAGGCGGTAAAACAATCATCG
ACTACTGAAGACAATGCGGCCTTTGCTCAGGTCGTAC
>103794829_2 EC091209.1

GTTAATCAGTGCCTATGCTGTTGGCGTAATGGTTGGCGCGCCGCTGATGACGCTTCTACTTTCTCATCGTGCC
CGCCGCAGTGCGTTGATTTTCCTGATGGCAATTTTCACGCTCGGCAACGTACTTTCCGCCATCGCGCCGGATT
ATATGACCCTGATGCTTTCACGCATTTTGACCAGCCTGAATCACGGAGCATTTTTTGG
TTTGGGTTCAGTCGTGGCCGCAAGCGTGGTGCCAAAACATAAACAGGCCAGCGCAGTTGCCACTATGTTTATG
GGGTTAACCCTGGCAAATATCGGTGGCGTGCCGGCGGCGACCTGGTTGGGTGAAACCATCGGCTGGCGGATGT
CATTTCTGGCAACGGCGGGGCTGGGAGTGATTTCAATGGTAAGTCTGTTCTTCTCATT
ACCTAAAGGTGGTGCAGGGGCACGACCTGAAGTGAAAAAAGAGCTGGCGGTATTAATGCGTCCGCAGGTGCTG
TCTGCATTGCTGACGACGGTACTGGGAGCTGGTGCAATGTTTACTCTCTACACCTATATCTCTCCGGTACTGC
AAAGTATTACCCACGCAACACCGGTGTTCGTCACGGCAATGCTGGTGCTGATTGGTGT
CGGATTCTCTATCGGTAACTATCTCGGC
>103794831_2 EC091210.1
TTAATAACTGCTTAGTGTTGATTTATTGTCATATAAAATGCACGATAAAAAGATGATTTATATGAATGTAATG
AAACAAAATTATAGCGAGGTTAAGCTTCTGAATTACATATTTCTTCAAAATGTATATAAGAACAGTCGTGGC
>103794833_2 EC091211.1
GCACGAGGCAAGTGCTTTCATGTCTCTGCAGGGCTCGTTGTAGTTCGAACGAAAGCCCTTAATAGTGCCTTTT
CAAATGTAGTCTCTTTCGTGTGATCCATATTAAAAGCGTTTTGAACAACGA
>103794839_2 EC091213.1
AATTTGCCCCATTTAGTACCAGAGAACTGAACTAATGCAAGAAAATCAACAAATTACAAAGAAAGAACAATAC
AACCTGAACAAATTACAAAAACGTCTGCGTCGTAACGTGGGCGAAGCCATTGCTGACTTCAATATGATTGAAG
AAGGCGATCGCATCATGGTTTGCCTCTCCGGGGGTAAAGACAGCTATACCATGCTGGA
GATTCTGCGCAATTTGCAGCAAAGCGCGCCAATCAATTTTTCGCTGGTGGCTGTTAACCTCGATCAAAAGCAA
CCGGGCTTCCCGGAACACGTTCTGCCCGAGTATCTTGAAAAGCTGGGCGTTGAGTACAAGATTGTTGAAGAGA
ATACTTACGGTATCGTGAAAGAGAAGATTCCAGAGGGCAAAACCACTTGCTCACTGTG
TTCTCGCCTTCGTCGCGGTATCCTTTATCGTACCGCAACGGAACT
>103794844_2 EC091215.1
GACGTGCTGGTGTCCGCCTCCTACGACTACTCGCTGCGCCTGTGGGGCCCGCGCCCGCTCTATCCCGCCCCTG
CGCTCCTACTGACACCCGTACACTGTTTATTTTTATGGTATATTAACTTCTTGTAAAGTTTGAAATTTTGAAT
TTTATGACTTTTCATATTAATTTTGTCGGATTTTTCTTTGTTTTTTAAATGTGATTTC
TTTGCGTTTTTTTTT
>103794855_2 EC091219.1
GAGATCAGTTTGTAACTATCAAACAAGAAGTATCAGCTCAAGATGCAGCACTAGACTATGACCAAAAACTAAA
AAATGCATTCAATAGTGACATTTACAAATTCGATCTTCTCTTGCTGGGTATGGGACCAGATGGACATACATGT
TCACTATTCCCAAACAATAGCTTACTTGAGGAAGCAAATCTAAATGTGGC
>103794861_2 EC091221.1
AATTTGCTCCTGCCATTTTCCTCATCGCCTGACTCTGTTCCTTCTTCTGCTCACGTCCGGTGTAAAGGCGCAC
GTATCAGGTTGGCATCATGATCCGCGCGACCGCGTTGGCGAGAATCACATCAAAGGCATCGACGTCCTCCTTA
TCGGCAAGCGGCGTGCCTTTCACCTTCTCCCGCCTGTTGATTGGCACGCTTTCCGGCT
GCCTCAGGAGGTTTGCCTGTTGCACCAATACTCCCGCGCGATCTTTTACTGTTTTTTTTAAGCCCACAATGCC
GCCAGAACGGACTTTGATCCCGGCATCGCCCACTTTTTCCAGCGTATCGAGGCGTTCCTGATAAGTGCGTGTG
GTGATGATATTGCCGTATAACTCCGGCGAGGTGTCCAGGTTGTGGTTGTATTATTCCA
GCCCGGCGTTCGCGAGGCGCTGCGCCTGAGATTCACTCAACGTGCCCAGCGTCGTACACGCCTCCTGCCCCAT
CGCTTTTACCCCCTGCACCATTTGTTCCAGGTACGGCATATCGCGTTCGTGGGGATTCTTCCACGCCCCGCCC
CTACAGAATCGCGTCAATCCTGCC
>103794869_2 EC091224.1
CCGCCCGAGGAGTTGCCCTTATGAAGCACATTATGTGATTTCGAATCGTCTTCTTTAATGGATGCAGATCTCA
ATCATATAACTGTCAGAAAATGAAAATTATATCGGATCAAATTGCTAA
>103794872_2 EC091225.1
CACTTTTCCTTCATATTTTTGTTTCCTTTCTTTCATCATCGGTCATTCGTTCGATATGGCCAAACCGTACTGG
GCTCTATCTATCAATCAGATGGAAAATGAAACGCTCAATTACGCTAAAAGTGGAGAACGGAGTGTTTGTTGTA
TTAGCTTTTTTTTTTTTCTCCTGATCTTTATTTCAAACCCTTTGTGGTCCTCTACAGT
TTCCCATG
>103794875_2 EC091226.1

GTTTAAGTATTTTTTTTATATTATTATTAGTCATCGATGAAAAGATGTACTCTTTTTTCTCTTTAGATTTGAA
TCATATACAACAACTATTTTAACCTAGACAAATTTCGACCGTAGCTTATCAAACGTAAACTTATTTTACTATA
AAG
>103794879_2 EC091227.1
TATAATTGTAGACTGTTATTGGCTCCTATCTATTTGTTAATATTAAATTAGTCAAGTTTTAATACGCCTATTT
CACCATTATTGCCGGCTCGGGTTGGATGGTTATTTTTTT
>103794887_2 EC091231.1
ATATAATGGCGGTGCGTTTACCCTAACAGTAATCGACGCAACGATGTGCGCCATTATCGCCTAGTTCATTCGT
GACCTTCTCGACTTCGCCGGACTAAGTAGCAATCTCGCTTATATAACGAGCGTGTTTATCGGCTACATCGGTA
CTGACTCGATTGGTTAGCTTATCAAACGCTTCGCTGCTAAAAAAGCCGGAGTAGAAGA
TGGTAGAAATCAATAATCAACGTAAGGCGTTCCTCGATATGCTGGCGTGGTCGGAGGGAACTGATAACGGACG
TCAGAAAACCAGAAATCATGGTTATGACGTCATTGTAGGCGGAGAGCTATTCACTGATTACTCCGATCACCCT
CGCAAACTTGTCACGCTAAACCCAAAACTCAAATCAACAGGCGCCGGACGCTACCAGC
TTCTTTCCCGTTGGTGGGATGCCTACCGCAAGCAGCTTGGCCTGAAAGACTTCTCTCCGAAAAGTCAGGACGC
TGTGGCATTGCAGCAGATTAAGGAGCGTGGTGCTTTTTAAGATTAACTATAACTGATTTACCATGATGCCCTT
TGCCGCCGGAAAGGGCATCTCTTTCAGAAAATTGCTGATTTTCCCCTTTTCCTTGATT
GCTGTTATTTTTTCCTGGCCCAGCTTTCTTTATGCTGTATCGATACACCAAAGAGAAAATAATGAGGGAGCGA
AGGATGCCAGTTTTGCAGTGGGGAATGTTATGTGTGCTATCACTTCTCCTTTCTATTGGTTTCCTCGCGGTAC
ATCTCCCGGCGGCGCTATTAC
>103794899_2 EC091235.1
GCACGAGGGGTTACCTTTAAGGGACAGAAGTGCGCCCGCGGTATCTCCACCTCCCACTTACAGATCAAACTCA
TCCACTCTAGTCAGAGGATCAACGGGAGCCGCGTGGTGTGGGTGCAAATGAATATGACCTCCTTCA
>103794905_2 EC091237.1
TTTACTTCTTTCTGCCGGAGCCCTGGGGGCTGGTTCAGTGGGAGCTGGGGCCCGTGGAGCCGGTTTTTTAGGG
GCTGGTGTGATGGGCGCTTAGGCTGGGGGTCTTGGCGGT
>103794912_2 EC091240.1
GAAGCCGGTGTAACATCAGAATTTGATGAGCCACCGCTGCTAGAGGAGCTAGAAATATATCCTGACAGAATAT
TAGAAAAAACACTAGCTGTACTTAATCCTTTTCATGGTCAGTCT
>103794924_2 EC091244.1
TTGGCCCCCCCTTATGCTGCACCAATCTACCCTGCTGCTGCGCATTTAATCAAGAAGAGATCTGCTCCAATTC
TGCCCACAATCTACGTTGCCCCTTACTATTCTTCTCCTTTTGTTGACGCCGACGTA
>103794927_2 EC091245.1
CGACATGGGAACGGTGGTATTCCCTGATGCACCAGTGAAAATTTTCCTTGACGCCTCCTCGGAAGAACGTGCG
CATCGCCGCATGCTACAGTTGCAGGAGAAGGGCTTTAGTGTTAACTTTGAGCGCCTTTTGGCCGAGATCAAAG
AACGCGACGACCGCGATCGTAACCGAGCGGTAGCGCCACTGGTT
>103794935_2 EC091248.1
TTCTAAGTTGTTTATTTTGTGGTGGGCGTGATGGTTGGTGATAGATGCCTTTTTTTTATAGGTGGGTCTTGGA
TTGGGATGGGGGGGGGGAAGATTGTGATAGGCAAGTGTTGATGGGGAGTCGGGTGATGATCAATGATGACTTC
GGCATGTTGTAGTATATGTGGGAAGTGCTGGGCTATTAAGGTGTTTGGTATGTTGTAG
AATGGGATATTGATTAGAGGGGCATGCATGAGGGGAGCGTTGAGAAACTCCAATATTTTTAGACAATTTTTTT
TTTTGTGGGTTCCTTTATTTG
>103794943_2 EC091251.1
GCAAGAACCGGAAGCGTCCCTGAAAAGCGGCGCGCAGGCAGACGACAGCGAAGTGGCTGAGATTGAAGCGTTA
ATTCAACAGCGTCTGGATGCCCGTAAAGCGAAAGACTGGGCGGCGGCGGATGCGGCGCGTGATCGTCTTAACG
AGATGGGGATCGTGCTGGAAGATGGCCCGCAAGGGACCACCTGGCGTCGTAAGTAATT
GCGCTATTGCCGGATGCGAGTTTTCGCATCCGGTTATCGTCTGCGCCACCACAACATTCCCATCAGTAGCATC
CCCGGCAACCACACCCACATCAATTCAGAAATAATCACCTGATGCCCGTACGGCGTGGTGTAACGAGACAATG
CAAACGGCGCGACTTTTATCACCTGCCAGGGAGCGAAAAAGCGTTCATCTGACCACGG
CCACAGCCAGCCAACGCCTTTACCGCCAGTGGTTACCGAATCCAGCAAGCTGTGCGATAGCAACGAGACGGTT
AAAAACAGCCAGCAGCGAATCAGCCCAGCCCTGAACCATCGGCGTCCAATAAACACACATAACAGCGGGACAA
CAAACGCAAACACCAGCGAATGGGTAAACCCGCGATCCTCGAATCCTGCGGTCGCCTG
CGTTCCACTCAGCCTAACCTCAGCGTGCGTTACCATGCAGGAATGAGCAACGATTCCCTCGACGCCTGCGTAC
AGGTGATCCGTTGCGGCTTCGGGATGCCGGCGTTCAACAACGACGAAATCGTGATCCCGGAATTTATTAAACT
CGGTATTGAACCG

>103794949_2 EC091253.1
ATTGATGAAAATGATGAGGTGAAATTAAAAAGTAATAATAGGAAAGAAAGGAATTATTTAGATGTTATATTTA
TTGAAGATTTTTTGAATGTGGGTGAGTGTGTGTTTTTATGAGAAAAAAAATATGAAATTAATAATAGTTTT
>103794958_2 EC091256.1
CTATTTATTTGTTTCAAATGTTATGTTTGTATGTTCATTGTTTTTTTTTTTTATTATTATTTACTCAGTTCTA
GGTGTAACAATATTATTGGTTTTGACTTCATTTGAGATATTAAATAATGGTGTTAGTTACATCTGTGTATGTT
GTAAGTAATTTTTTTTAAATATTTGTTGTGTATATATTAAATAATACAGTGAAACGGT
GGGCAGTCTCGGACCACCTTG
>103794964_2 EC091258.1
GCTTCCCCGGATGTGGCGGACATGACGGAGGCTGACGTAATGCTGATGACAGTATCAGAAGGGATCGCAGGAG
GAGTCCGGTATGGCTGAACCGGTAGGCGATCTGG
>103794967_2 EC091259.1
GCACGAGGGCGCTCCCTTTAAAACATCACTCTCCTCTCGGTGGCTGCGGACAATCCCGCGCCCGGACTTCTTG
GTGGATGGGATGGCGGACTATGACTTGGCCA
>103794970_2 EC091260.1
TCTCATTAAAAAAGCAATGGATGGTATTAAGGATATGCCTCTTACACCCTTTAACCCTTTAGATGACTTGGTT
ACACAGAAACCAGTTAAAAATGCTCCTGAAAAAACTGATCCCGTAAAAGTTT
>103794973_2 EC091261.1
GCACGAGGGAGCGTTCGGAGAGTTCGGAGAGTTGGGAGAGTTGGGAGAGTTGGGAGAGTTGGGAGAGTTGGGA
GAGTTCGGAGAGTTGGGAGAGTTGGGAGAGTTGGGAGAGTT
>103794982_2 EC091264.1
GTGCTTTATCGTTCTCACGCTTCCTTTCAGGTTTACCAGAGAACAAAAACTTCAGTAACGGGATGCGCAAATG
AATTTCATACAGAATTATCGCAATCCCTACTACAAATATCAGGCCACAGAGAAAACCAAGCCAGTTGGAGGTG
ATGTGCGGTGTAATGTATGCGCCGAAAAACAGCGTTAACGGGTGGTGAACCAGATAGA
TAAACAGCGATGCGTTAACAAAATAAGTCACCCGCGCTGACTGGAAGTTAAGCAAACGGTGGCCAAAGGAGAA
GACCACATTCACCATCCACAGACCGAGGACCATGGTGATCACCGACTCGGTTTCGTACATCCAGGCATCGCCA
CTGCCATAGCGCTGGTTGAGTAAATAAGCGACAAACGCCAATGCTGCTGCAAGGGTAC
AGCCACGAGACGGCGTGGTAAACAAGGCTTTAAGATGAGGGAAAATGAAAGCCAGTGCGCCGAGGATAAAGAA
CGGCAAATAAAACAGCGTTTGCATGACAATAAAATTGAACATGCCATTACTCAGAATGGGCGGATACACAATA
AAAATCGTTCTTCTTATTACCGCATAACCGATGCCGAGGCATAAAAAAATCACCGATA
GTTTTACCATCGAGAATTTTTTATTCGTTTTATCAGAATTTTCTAAATTATTTCTGATGCGCTTAAATATCCA
TACGCACAGCGTCGTCATGACCACTAGCACCAGTAAAAACCACAGGTGTGATATTAATTCCCAGGC
>103794985_2 EC091265.1
AATTAAAATCCATTTACAAAGTTATGTGTGAATTTGTTTTACAATTTCTCGCTATTCTGTGAATAAATTTTAT
CTTGTAGCTATGAAAGGATGTTAGTGAATTGAGGAAATATGCGAATCTTTTTGAAAATTTATGGAATTACACT
AGTGGAGTGTATCGTCGGTGTGATGGGG
>103794987_2 EC091266.1
AAAAAAAACTAGGTTATTTCAAACCCAAATAGTGATACTACCTCTGAATTTTGAAAACTACTGTTAAGTAAAC
TATTAGTTCCCTAATTATAAGCTCTTTGGCCGAGATTTTTTAAATATAATAAACTTCCTTATTTTTTTGTGAA
ATTTGTCTGCGAAAGGAAAAGATTGACCATCGATTTTT
>103794995_2 EC091269.1
TTTTTGGAATGCTAGAAAAAAATACTGCAGTGGCATTGTTGGGATCTGTTTTAATAGGAGCCTCTACTGAAGAT
AAGCAAAACTCAAGTTCAGAAATACAAACGAAAATCATAGAGGCGGGGGAAAAGAGACCAGATTTACCTACTT
ATAGAGCGGAGGATGTTAGTAAACATGATACTACACAAAGTTTCTGGGTAACTTATCG
TCACGGAGTATACGATGTGACCTCCTTCCTACCGTCCCATCCGGGCGGGGAACAGATATTGAACGCCGGTGGA
CTTCATATAGAACCATTTTGGAATGTCTATGGAATGCATAAGACTGAGGAGATTTATAGCCT
>103795003_2 EC091272.1
ATTCCAGTTCGGTTGTCCGGCGGTCTGCGTGGTGTCCAGGTGAATATGTGGCTCCACAAACGGCGGTATAACT
AAACCTTGTTCGGCATCCAGGCTGTTTTCAGTTATGGGCATCACGCCGGATTGCGCATCAATGGCGCTGATTT
TTCCGTCCTGCAGATGAATCTGCCACAGCCCCTCTTCGCCTGGTAACCGGGCGTTAAT
AATTGTTTGTAAAGCGTTATTCGACACTGTTAGCCTCCACATGCGTCATTGCTGCTGTCGTTTTACGATTCAA
AATCGGGTTAAGGATCAGATAGCTCAGCGCGCCACCTAATACCGCGTTGACCGGAACAATTCCCGGTAACCAG
TGGCCTGCAGCAATCCCCAAGGCGACCGCCAGAATCGCCACCCAATTGACACTCATCA

TACGCGTGGTCGCAAAGTGCTCATAGCGGCGACGGTTCATCAGATAGTCGGCGATGATCACGCCACCCACTGG
AGGAATAGCTGCCGAAAGGAAGGTCAACCAGCCGACAAAATTGTTATACAGCCATAATGCGCAGACCGTACCG
ATAATACCGTTGATTACCGAAAGGGTTTTGCTCGACATCCCGGTAATGTTGGCGAAAC
CTAAACCCGACGCATAGAGTGCGTTATCGTTGGTGGTCCAGATATTCAGCCCCAGCACCACAATCGCAGGCAG
CAGCAGGCCCTGAGCAATCATCACATCAGAGATATCCGCCATGCCCAGTGCCGCAGCGCCCGCTGCACC
>103795006_2 EC091273.1
TTACTCATCGAGTATGGTAGGCCAAGTGTTTAAAAAAATCCTCCCCTTAGATAATATGAAAAAAAAAAATTGT
ATGTGCATTAAGTCAAATGTTGCGCACAATTATGTCGGCGATGTCTCTCTAGGCTCCATAATGTATCACGAGG
AACAACATCCAATATAATATGAAACGGCAAAATATTGAAAGTATCGATATAGATTTTC
TCTAATGTTACATACATACATACATACATACATTATTAATGTAATTTACCTTGTATATACGTATGACCCGGAC
AGTATAGTGGGGCATAACCTATTGTGGCTCAATCCCGGGCGGGCGAGTCTGCGTGACGTCATACGTTTTATTT
TCCTCTTAGTACTCATTGATTGTAAATACATATAT
>103795011_2 EC091275.1
TTTATTTTATTGATAATGGATCCGTTACTAGTCTTATGCTCCCAGTACGAACGACGACTGGTAGCCGAGTTTT
GAGTTCCACAGAGGACGCCCTTGTTGCCGTCGA
>103795014_2 EC091276.1
TCTTCTCCTGCGGCCGTCTCTTCGCAGTCTATCCTACGTCTTGTCCTGCGTCGTGCTGCCCTCGCTGTTGCCC
CCGTTGCCCCCTACTCCGCTGCTCCTGTTGCCCACTATGCTTTTTTTTTTTTTGCTCACTACGCTGCTCCGTC
CCTTTTCTCGTCCTCCG
>103795017_2 EC091277.1
GGTGCACCCAAACTAGTCAATTGTCGTTCGTAGGTGAGTTGCCGCCCCCGCAACGACAACGGTCACGTGTCAA
TTCTATTAAACTGTGCGTTGTTAAGGAAATTTAATAATAATAATAAGAAATAAATAAAATATATAACTTATTT
ATTATATTTGTAGCGATCGATCGATGATGAATAATTCGT
>103795023_2 EC091279.1
GAAAGACCCCTACTAAGGGTGCAAAACACACTTTCCCTAAAAACACCCCTCCCGTGTATATCTACAGGAACAT
GTGTTGTAGCGGCAACCCTCCTTTATAACTTTGTCTCTATATGCT
>103795029_2 EC091281.1
TTTTTTTTATGAAAATAAATATAAACAGTAACTTTTTGATTTGTAAGTTACGATTCTTGCTAAAATAATAACT
ACGATTTTTTTCGACTTAGAAAAATTTACTACAACATGTCACTGTGATTGTTTGTTGTTACATATATTTCTTA
CAGCAAAAAAAATAATAATTACTTAATTTTTTGTAAAC
>103795032_2 EC091282.1
TCAATAAATGCGATACGATTGGCGGCCACTAGAAACTGGCTCAGTTTGCCTTCCTCCGTGTCCTCCATGCTGA
GGCCAATACCCGCGACATAATGTTTGCCGTCTTTGGTCTGCTCAATTTTGACAGCCCACATGGCATTCCACTT
ATCACTGGCATCCTTCCACTCTTTCGAAAACTCCTCCAGTCTGCTGGCGTTATCCTCC
GTCAGCTCGACTTTTTCCAGCAGCTCCTTGCCGAGATGGGATTCGGTTATCTTGCCTTTGAAAAAATCCAGGT
AACCTTCCGCATCATCGCTCGCCCGACCGACGGCCTCCACGAATGCCGATTTGCCAACGGTGTTCACACTGCG
GATATAAAAGTAATAATCATGGCCCGGTTTGATATTGATACTGGCGGCTATCCAGTAC
AGCGCCGTACCAAGATAACGCGTGCTGGTTTCAACCTGTCTGATATCCGCAATCTGCTTTTCCGAGAACCAGA
ACTCAAACTGTACCGTCGGGTCATAAACGGCAAGATGCGGCGTGGCGGTTATCTGAAAATAGCCCGGCGTCAG
CTCAATCCTCGACGGTGCTGCCGGTGCGGCAATCCGGAACGATACCGACGCCGGATCG
CCCTGCTGCCCCCACGCATTTACCGCCCGGACTGTCAGCCTGTAGTTCCCCAGCGCCAGTTGCGTGAAGCGGT
ATGTGGTTTCCGTCGTCCGGGCCGTGCTGACCAGCCGCTCACTGCCGTCGTCCGCTGTTACGGTCAGACGGAG
CANGAAACTCACGCCCTTCACCACCTTCGGTGTGTCCATCGCGCCCGCACCTGATATC
CCCGCTGTCTGCAGTGACTTCTGCGGTCAAGTGCTGCACGCTG
>103795038_2 EC091284.1
CGCGACTGTCGCCAACTAATCAGTCGAGGGGGGGAGAGACAAACGGCGAAGCAGTGTCCCGCCTCGGCGCGTG
GTCCCTCTACGCCGAGAGCTACTCCCGCCGGGACGCCAGCGCGGCCCCGGCCTCCGGCACGG
>103795047_2 EC091287.1
GCACGAGGGCGCCCTCTGCACGCCTCGACCCCCTGGCCTCCGATGTGAGCTTACGATTACGACCTCCCGCCCC
AACTCGCACTTTTGCCACGACGTTGACTTTATC
>103795055_2 EC091290.1
ATGCCACAGATTCTCCCACGTCCATGGGGGGGGTGGGGCCGCGTAGTTCAGTGTCAAACAAGGGCGTGAAAAG
ACCCGGGAGGGCAAAAATCAACCCCTTCGACCCTTCACATGTTACAGTTAAACTAACTAGTAATAGATGACGT
TGGACACATATATTTCCTAAAGGTCCAACGGGAGTGTTAATTC

>103795058_2 EC091291.1
CTTCCAGCGCGTTTATGATTTAACCCACCGTGTCATGCCTGACTGGGATGATGAGCGCGATCTCCCCTCGCAA
ACAGAAGCAGAAATCATCATGCTGGATAACAGTGCGCGTAGCCTGGGAATATTCCGCGAACAGTGGCTGGCAG
ATTACTATCGGCTGAAACGTCCGGCACTGGCGGCGTGGCGCGAAGCGAGGGCTGAACA
GCAGCAAATCATTGCTGTGCATGTTGAAAAATTGGGCAATCTTTGGCTGCATGATGATTTGCTGCCGCTACTC
GAGCGAGCGCTGGCCGGAAAGCTCACTGCAACGCACAGCGCGGTACTTTCGCCTTTTGATCCTGTTGTCTGGG
ATCGCAAACGCGCAGAGCAGCTTTTTGATTTTAGCTACCGGCTGGAGTGCTATACCCC
AGCGCCGAAACGCCAGTATGGCTATTTTGTTCTGCCGTTATTACATCGTGGGCAATTAGTTGGGCGAATGGAT
GCCAAAATGCATCGCCAGACAGGCATCCTTGAAGTTATCTCTCTGTGGTTACAGGAAGGTATTAAACCAACGA
CAACCGCTGCAAAAAGGGCTACCGTCAGGCGATTACTGATTTCGCTAACTGGCAGCAG
GCAACGCGGGTGACATTAGGATGCTGCCCGCAAGGCCTCTTTACTGATTGC
>103795073_2 EC091296.1
AAATCGACTGCTGCTGATTGGAAAGCTGCCTCTACACATTACCTTGCGTGGCGCTACTGGTTTTCAACGTCGC
GGTTTTATTACCGATATCACTCACTAACGAGGCATAAGCGTCGTTAAAGGATTTCGCACCGCCCACCGTTTTA
CTGTTGCTTTGCAGATCCAGCAGGGCCTGACCGTTGCGGTTATCGCTATCACCCGCAT
CTTCTTCGCTCGCCATCGCTATTTTCGCTTCGTCGGTGATTAATACATCCATGTTGACGATGGCGTCACTTAC
TGGTTTCAGCGTGAAGCTGTCGTTAACGGCAGGCGTTCCTGTAAACGTCAACTCCAGACCATCAAATGCCACT
TTACCGTTGGCATCCGGCGTCACCGTAAAAGTGGTATTGCTGGCAAGGCGGGTGACCT
GCCACTGATTATTATCGAACGAGATTTTGTAATCTGTCGCCAGTACCGCGGAGGCATCAGTTACCGTGGCACC
GATCGCAACGTCACCTTTGTTTTTCGTGTTTTGCAGAACCGCGGGCTTACCGATAGCAAAGAAATCTTCACCG
GCATCGCCGTTAGCATCAAATCCGGCTTTGTGTTGGGTGTTGAAAGCCTCGGCAAATG
CCAGCGCCAGTTGTCCAAGCGTATTACGCGTCTGGTCCAGATCCTGAGAACGGAATGTCAGAATGCCGCCCAG
CGACCCGGTATTCAGTAATTTCTCCGGGATCTCAATATTGCCTGCCGTCCCATCAACATAAGCGACAGTCGTA
CGAAAAGGGTCAGCGCTGGAAAGAACTGCCGCCAG
>103795076_2 EC091297.1
CACCTAGATGTGTGACGTAATTTATGGATGGGCCGACGGGCAGTTTAACTATTACGGAACGCAAATTTCTGGA
GGATTTCTAACGTTCTCATGCACCACGCTGTGACCTTTTCTTATACCTCTTTATCTCTTTGT
>103795079_2 EC091298.1
GGCAACGTTGTGTATCGCTGGCGGGAAAAAAGCCAAAATGCGCCCGGGTGATGTATTAGGTGCACTGACAGGA
GATATCGGGCTTGATGGCGCAGATATTGGCAAAATCGCCGTGCATCCGGCGCATGTCTATGTCGCGGTCCGTC
AGGCTGTTGCTCATAAAGCATGGAAACAGTTACAGGGCGGGAAGATTAAAGGAAAAAC
GTGCCGGGTGCGGTTATTAAAATAATGAAATGTTGAATTGCCGGGTGCAAGAGTAAACATCTTATTCGGGATT
GCCGGATGCGACGCTGGCCGCGTCTTATCCGGCCTCCATAAGAGTAGCCCGATACGCTTGCGCATCGGGCGCT
ATCCTGGTTATTTCACTTCAACCACATTCAGCCGTAACTCATCCAACTGATTTTCATC
TTCTTCTGGCTGCCAGCACGCCGGTTGTAGTGGGATCTCTTCGCGATCAAACGCCAGATCACCCCCGTTAACC
ACTTCAGAACCGTGGGTGATGCCTTTGAAATCGAACAGGTTGGTATCGCACAGATGCGACGGCACCACATTCT
GCATCGCGCTGAACATCGTCTCGATACGCCCTGGATAACGTTTATCCCAGTCACGCAA
CATGTCAGCAATCACCTGACGTTGCAGGTTAGGCTGTGAACCGCACAGGTTGCACGGAATAATCGGGAACGCT
TTTGCATCGGCAAATCGCTGAATATCTTTCTCGCGGCAGTAGGCCAGCGGACGAATAACGATATGTTTGCCAT
CATCGCTCATCAGTTTCGGAGGCATACCTTTCATCTTACCGCCGTAGAACATATTTA
>103795082_2 EC091299.1
GCACGAGGGGACCCGTTTCAAGGCGCCACAATACTACTCTTATCCATTTTCAGGATACTTATATGACATGTAA
ATTGATGCCACTAATAAAACTATCATTACATT
>103795087_2 EC091301.1
GCACGAGGTGGCTTCCTTGCTAACATATCTTCATATATTACAACTAGTTTGTAATTTTTCTAAACTTTATGAT
CTGATAACGATCTATAAAATAATGCTTTATTTGTTTAAAATCTTT
>103795090_2 EC091302.1
TAATTTAACTGTGTGAAACTATAAGCTTTGCTTTTCCAAAAAAATTATATATAGTTTTTGGGAATGAAATATG
TGAGAAAATATAAAAATGGTTGTAAAAAAGTACGAAGAAGAGTTAAACTATTTAAAAAAAATAAATCCGCATT
GATGGAGAATTAAAAAGGGATTTCAACCAAATATGAATGTTGAAGGAATATTTTATGT
TAATAATACTCTTGAAAAGATAATGTTGGATGACTTGAGAAATTCTTGCCAGTTTTTTTT
>103795092_2 EC091303.1

CACCGGCACGGCAGTTCCGGCTCTCGCCGGTATTGTTCGGGTTGCTGATGCGCTTCGGGCTGACCATCCGGAA
CTGTGTCCGGAAAAGCCGCGACGAACTGGTATCCCAGGTGGCCTGAACGAACAGTTCACCGTTAAAGGCGTGC
ATGGCCACACCTTCCCGAATCATCATGGTAAACGTGCGTTTTCGCTCAACGTCAATGC
AGCAGCAGTCATCCTCGGCAAACTCTTTCCATGCCGCTTCAACCTCGCGGGAAAAGGCACGGGCTTCTTCCTC
CCCGATGCCCAGATAGCGCCAGCTTGGGCGATGACTGAGCCGGAAAAAAGACCCGACGATATGATCCTGATGC
AGCTGGATGGCGTTGGCGGCATAGCCGTTATTGCGTACCAGATCGTCTGCGCGGGCAT
TGCCACGGGTAAAGTTGGGCAACAGGGCTGCATCCACACTTTCACTCGGTGGGTTCCACGACCGCAACTGCCC
TCCAAATCCGCTGCCACCGCCGTGATAACCGGCATATTCGCGCAGCGATGTCATGCCGTCCGGCCCCAGAAGG
GTGGGAATGGTGGGCGTTTTCATACATAAAATCCTGCAGGTCCCCTGCGTCGCTGTGT
CATGCCGGTCTGCACTTCCAGCTCTGCAATATATTTTTTCAGGTCAGACACGGAAGTGGCCGTAAACTCCACC
CTTCGTCCGTCTTTCTGTACTGTTGCCACCCGTTTACCTGTCATCAGGTCATGCAGTGCCGCACGGGCAGCGG
CAAAGTCTTCCTGTCGCGTCATTCATCCTCTCCCGGATAGGCACGGGCGTAATCTGCC
AGTGTTTTC
>103795095_2 EC091304.1
CCTGTATGCCCGTGGTCAGCAAGAGCAGTATGTGGCGCTCAAACATCCTGCGGTGAAAAAAACCGAAGAAGCC
GCCGCGGCAAAAGCAGAAACTGTAAAACGCAGCAGTAGCAAACTAAGCTATAAATTGCAGCGCGAACTGGAGC
AGCTACCGCAATTGCTCGAAGATCTGGAGGCGAAGCTGGAAGCCCTACAGACGCAAGT
GGCGGATGCTTCCTTCTTCAGTCAGCCGCATGAGCAGACGCAAAAAGTGCTTGCTGATATGGCTGCTGCAGAG
CAGGAGCTGGAGCAAGCCTTTGAACGCTGGGAGTATCTTGAAGCGTTAAAAAATGGTGGCTGATCGCAAAATA
GTCGATTTAAACAGAGACAGCTGACAACGGTAAAATTGCCTGATGAACTGGGTTTATC
AGGCATGAAAACCAACACTTTGTTAGTCATCTGTAAAATAGCGCATCATTAAGGAGTACCAATGTGCGAACAT
CATCATGCCGCGAAGCACATCCTGTGCTCGCAGTGTGACATGCTGGTGGCGTTACCGCGCCTTGAGCATGGTC
AGAAAGCGGCATGTCCCCGGTGTGGCACAACGTTAACCGTGGCGTGGGATGCCCCTCG
GCAGCGTCCCACCGCCTATGCGTTGGCTGCACTGTTCATGCTGTTGCTGTCCAACTTGTTTCCTTTTGTGAAT
ATGAACGTTGCAGGAGTTACCAGTGACATTACATTACTGGAAATTCCCGGCGTGCTTTTTTCTGAGGACTACG
CCAGCCTCGGCACCTTTTTCCTGTTGTTTGTGCAACTGGTTCCCGC
>103795098_2 EC091305.1
TATTATTAAAATTTCTTAACAAACCACACGGTTTTTTTTATTTTGTATGTACTATTTGCTAACTTGCTAATTT
TCTTTTAGAATTAAAGTATTATATTGATTTTCTGATATTGAACATGGCGGTTGGTAAA
>103795101_2 EC091306.1
TTCAATGAAAGCTGGAGGCTGTTTCTATCCAGCAGGCGGAGTACGGCGCGTTCACCATAGATGGACGGCAGTG
TGGATACGCGGACATCTATGTTACGTCGCCCGATACGCAAACTAATTCTTCCATCCTGTGGAATACGTTTTTC
GGCGATATCAAGACGAGCCATGACCTTAATTCGGGAGATAAGCAGTGCCGCCAGTTTT
TTGTTTGGCTGTAAAATTGTCCGCAAAACGCCGTCGATGCGAAAACGGATCGACATTGTTTTTTCATAGGTTT
CAATGTGGATATCAGAGGCGGTTTCTTTGATGGCCTCACTCAAAATCGCATTGATCAAGCGGATAACCGGTGC
CGCTGAATCTTCATTCAGGAGATCTTCATTTGCGGGCATCTCTTCCGAAAGCGAGAGA
AGATCGACGGATTGATCGATATCCTGCGCAATCTGTTGCGACTCACCGGTATTCTGATGGAATACCGCTTCCA
GCCGAGCTTCAAAATCAGCCTGTGACAAGCTGGTCATGGTCTGGCACTGGCCAACCTGACGTTGAATATCGAG
TAACCGCTCGAATGGCGTATCGGCGAGATAAATAATCTCGCCCTCTGAATAGAGATAA
CCAATTCGTTGTGCCAGCGCCCAACTGGCGGGGTACGGTGAGTGAATTCTCATATGAATGCCTCACCGTGACG
ATGGCGCAGGGGCGTGACTGTTGAACGTGTTTTCATCCAGCACCGGCAAATCTTCCGAGCCCACCAGCGCTTT
TGATTTTCCGTCGATCCGCTGTTGTTGCTCCTGACG
>103795106_2 EC091308.1
AGCCGTAGAAACAGCGATCCTCCTCCTTCGCATCGACCGACATAGTCTCCGGTTCCCAAGAAGAAGGATAGTG
AGCCAGTTGGTTCAGCGTAAGCTGTCATGTAGGAATAAATGAACTAATTTTAATATTATATGCTTTATT
>103795109_2 EC091309.1
CGTTAAGCCCCCTCTTCGTGGGCATTAAGCCAGCGGCGTTTCCCTTCATGTTGATCTGAAGTGTCGGTGTCGT
GTTTACTCATTGCTCTCCCTGATTGCTTTAATGAAAAAGTCATATAAGTTGCCATGAACAATGTTTATTCATC
CTGGCGATGATAGTTATCACCATTATTCTTTATGGGTATAAAACTATCGGCGCAGCAT
CCTACCCGTTATCGATAAACGATGCAAAACATCCCCTTACAATCCTGAAGGGGATTAATACAACTGACGAAAA
AATGACAAATCCTTTTGCTGGTTAACCTGTGTACTGTCCTACACTTAATCTTTAAAAGATTGTGAGGGGCATA
TGATTAAGGTGTATGGCGTACCCGGATGGGGCTCTACAATCAGTGAGCTGATGCTAAC

ACTGGCTGATATTCCTTATCAGTTCGTTGATGTCAGTGGTTTTGACCACGAGGGAGCTTCACGCGAGTTATTG
AAAACCCTGAACCCGTTGTGTCAGGTTCCGACACTGGCATTGGAAAATGACGAGATTATGACGGAGACAGCGG
CGATTGCATTGATGGTTCTCGATCGTCGCCCGGACCTTGCGCCACCGGTTGGGCGCGC
CGAACGTCAGCTGTTTCAACGGCTATTGGTCTGGCTGGTTGCTAATGTCTATCCAACATTCACTTTCGCCGAT
TACCCTGAGCGTTGGGCTCCTGACGCACCTGAACAGTTAAAGAAGATTGTTATTGAATATCGAAAATCGCTTT
ATATCTGGTTGAATT
>103795111_2 EC091310.1
TGTTTCTGTGGCGGAAAAGACATTCCAGGCGAACTACCAGTGGATAAAATCCCGCAAATTGTTCTGCTGACAT
TTGACGATTCAGTAAACGATTTAAATAAGGGAC
>103795114_2 EC091311.1
AAAGAAAGAAGTGAAAGCCGCTGAGAAGCAACAGAAAATTTTTGATAACAAGGCAGTCTCATCACAAAACGAA
GCACAAGAGCCTGATATATCATAAGGCAAATATGGTAACTTTTTTTTT
>103795119_2 EC091313.1
GATTAAGTGTTTATTAAAAAAAATGTGATATTTTATTTATTTTATGTTAGATGTAAGTTTATTAGTAAAGTTA
TTAATGTAATTGTATATAAGTTATAGTTTTAATTAAATTATTTTTTTTTT
>103795126_2 EC091316.1
GCACGAGGGAAAACACTTATGATGCTGAAATTACAACTGTACAGCTACTAGCAGCAAGGCGGGACAAGTTGCA
ACATGAGAAATTCAGAATTGGTGCACTTTGCTCCTTCTTTGCTGGAAGTCCAGAGAAGAAAGCTTAAAACCTA
TTTC
>103795129_2 EC091317.1
GCACAAGGGATAACTCTTCTCGAGTTTTTTTTGTTTTTGTTTTAGAATTATTTACTGGTTTATTCTCATAACT
CACTTACATACCGTATATTGCATCTGTTTTCG
>103795138_2 EC091320.1
CCGGGGGAGCGGGGATCAATGTTTCTCCTTCTACTGGCGTCCAGCATAATCGCCCCCTGCCAGACTGCGGAAC
AGTGGGAGGGACGTGCCAGTGAACGGGACTATGTTCATG
>103795142_2 EC091322.1
TTACAAGGCCATAGTGACGCCCATAACAATCTGGCCGATCTTTATGAAGACGGAAAAGGCGTTGCTCAAAACA
AGACACTCGCCGCATTCTGGTATTTGAAAAGCGCACAGCAGGGTAATCGGCACGCCCAGTTTCAAATTGCATG
GGATTATAACGCTGGCGAAGGGGTGGACCAGGACTATAAGCAAGCGATGTACTGGTAT
CTGAAGGCTGCCGCTCAGGGGAGCGTCGGCGCTTACGTCAACATCGGTTATATGTATAAACACGGACAAGGCG
TTGAGAAAGATTATCAGGCTGCCTTTGAATGGTTTACGAAAGCCGCTGAATGCAATGACGCCACTGCCTGGTA
TAACCTGGCCATTATGTATCACTACGGAGAAGGAAGACCTGTCGATCTCCGACAGGCT
CTCGACCTGTATCGTAAAGTTCAGTCATCCGGAACCAGAGATGTCAGTCAGGAAATTCGTGAGACTGAAGATT
TACTGTAGAGATAAAAACAAGGGCCGGGGAATTTCATTCGGCCCATATTCTCTTTATTGCTGTGCGAAATCAG
ACTATTACGGCTCAAACGGGCGTCTCTGGAACTGGTCATGACCACATTTCGGGCATAG
CGTCAGCACTTCCGGTGTGTAGATC
>103795145_2 EC091323.1
GCACGAGGATATCTCCCTAAATGCTCTCTATCATTGACACATCCAACTCGTTACTGAAACATATTCTCATTTG
CATTATCTTCTTGGAATGCATCAAGACCAGCTGGTCCACC
>103795150_2 EC091325.1
CTTTTTTTTTTTTTGTCCGCTCTTACTCTGCAGGTGTTGCTGGGGCTAATTTCCCCCCGGCCCGTTTACGCCG
GTGCTGCTGTTCTGATCACGACGAGGATCGCTGTACTTTT
>103795161_2 EC091329.1
CAACGTTTTTTTTTACAAAAGGAAGTTCCTCATCATTTTTTGGGGTACTCCCCGATACAGAGTTTCCGCCGTT
CCAAGATTTCGACGTCGTTCACAATTACCTGCAAAGTGATTTCCGCAAAGCGCAGGTTAGGTTGCGTAA
>103795164_2 EC091330.1
AATATAGGTCTCGCACATCCAAGTTGAGCCAAGCAGAGATAGAAAACTTGAATACTGAGAACAAGGCATGGAA
TGTTCTTTGCATTCTCAATGTTCTTCATTCACTGGTCGATAAATCTAACATCAAGCGGCAGTTGGAGGTATAT
TGCAA
>103795170_2 EC091332.1
AATATTAGAGAGAGATGAAAATGTTTGGGATAAGATGTTTGAGGTAAATGTAAAGTGGTTGTGGATGTTGGTA
AAAGAGGAGTAATTGGAGTTGATAAAGGGTGGAGGAGGGGGTATAATATTTGTGTCTTCTATT
>103795173_2 EC091333.1

CCTGAACGGAGATATCCAGCGCACATGTTGGCTCATCAAGCACAATCACGTCAGGCTGCGAAGAGAGTGCTCT
GGCAATGGCGATACGTTGCCGCTGCCCGCCGGAGAACGCATGAGGCAGGCGGTCGAGATACTCCGGACGAATA
CCCACCTGCACAGCCAACTCCTCTGCCAACGCTCGCCGCTGTTGTTCACTACTACGCT
TAGCTATCCAGAG
>103795178_2 EC091335.1
GCACGAGGGGTGTCCTTGCAAACAAATTCCACTCCTCTTGTTCCTCTGTGGATAAACTTGATAAATCACCTTG
GGAACAATTTCGTACCGAAATGATAAATGAGAATGGGGTTTCACC
>103795184_2 EC091337.1
CCAACTGGATCAATAGCTGCTTTTCCTTTTGCGAGCGCGTTCTTCTCATCTTCTGTTGCCTTAGTCACTTTCC
CTGCGCTATTTGCAGCAGTTTCCCCAGCCTGCGTCATTTTGACTAATGAGGAGGTCAGATTGTCAGCATTATT
TTTCGCTCCAGTGCTATCAATAATTATTGCGAGACGCGAGGTTTGCTCTGCCATTTAT
TAAAACTCCTGACAACAAAAAACCCACCGCGAAGTGGGTTTCAGGCGACATAATAGTAGATATAGCGATTACG
AGGCCACGCAATGCTTTTCTCCAGGAGCATCATCGATTTAATTAAAGACACCATCACATCTCTGTAACAGAGT
GTACGTAATTAACAACTACACACACTGCTCCTGAAAATACTGGTCATCCAGTGCAAAG
ATCACTGCTTCAAATTCATCGCGCTCAATCAATACCGGATGAGTGGCTAAATATTCATTTATCTCTGTCAGAG
ATAAAGGCAAAGGCACCCCAGCCATTCCAGCATAACGTCGGGCACGGGATATTACCGAATAGGCGTACAACAA
CTCCTTAAGCACCGGGTCTATTTCTGGTTCCGGTATCGGTGGCAACCTGAGTTTTTCT
CGCTTCCATCTTGCCTTTTCCCCCCTTTCTCCCCCGAACTCCGATAACCACCGCTGGGCAGCTATGGCTTTTT
TATCGTATCCTGCTTCTGCTGCTCTTTACCCT
>103795190_2 EC091339.1
AATGGAAGTCGTTAAAGCCACTGCATCCATGCAGCGTGCACAACAGGCTGTAACAACCTCTACCGTTGGCGCA
TCTTCCAGCGTTTCGACAGCGGCAGAATCCTTAAAACGCCTGCAAACGCGTCAGGCCGAACGTCAGGCTCGCC
TGGATGCTGCCGCACAGTTGGAGAAAGTCGCAGACGGTCGCGACCTTGACGAAAAGCT
GGCGGAAG
>103795193_2 EC091340.1
TTAGTTTCTCCGGTGGCAGGACGTCAGCATATTTGCTCTGGCTAATGGAGCAAAAGCGACGGGCAGGTAAAGA
CGTGCATTACGTTTTCATGGATACAGGTTGTGAACATCCAATGACATATCGGTTTGTCAGGGAAGTTGTGAAG
TTCTGGGATATACCGCTCACCGTATTGC
>103795196_2 EC091341.1
ACCGCCGGCACATAGTACTTGTGTGCAAAGAGTGTCTCGTACGAAGAAAACCTCAATGGCCGGCTTAGAGAGT
AGAAGAAGTCCACCCGCTTCCGCCATACATCATAGAAAAATGTCAGACCCCTGACTC
>103795202_2 EC091343.1
CCTTTAAAGCCCGGGCTGCAAAGGGCCTACCACCTATAACGCCCGTTCCCCACACGCTGGAATGATGGCGTTT
CTTTCCCAATCCAGTCTGGTCACGGCTGCCTGGGCTGTGCGGAAAATGGTTTCTGGGATCGCGGTTCGTTCTA
CAGCCGCGTGGTCGATATTCCGCAAATGGGTACTCATTCCACCGCCGATACCGTCGGT
TTAACCGCGCTTGGCGTGGTGGCAGCGGCTGTTGGTGTGCACGCAGTCGCCAGCGCCGTTGACCAGCGCAGAC
GTCATAACCAGCAACCTACAGAAACCGAACATCAGCCAGGCAATGAGGATAAACAGGCATGAGCACTCATTAT
TAAACTCAGGGATACACCATCAATAATGCCGGACGCCGCCTGGTGGTCGACCCGATTA
CGCGCATCGAAGGCCACATGCGCTGCGAAGTGAATATTAACGATCAGAATGTGATCACCAATGCCGTCTCCTG
CGGCACCATGTTTCGCGGGCTGGAGATCATCCTACAAGGGCGCGACCCGCGCGATGCGTGGGCGTTCGTTGAA
CGTATCTGCGGCGTCTGTACTGGCGTACACGCCCTGGCTTCGGTTTACGCCATCGAAG
ATGCTATCGGTATTAAAGTGCCGGACAACGCCAATATCATCCGCAACATTATTGCT
>103795205_2 EC091344.1
GCACGAGGATAAAACCTTTATTTTCTTTTGGAAGATCCTCGCTGTTTCCCGCATATAGCTTATAGAGGGAACT
TACTGTGAAGTCCGTTCATCATATTGATCTGTTGACTGTCGGAATTAAACGCTGCCCTTTCTTTTA
>103795211_2 EC091346.1
GCGTCCCTCTTCCTGCAGATGAACGAGCGCAAGCCCACGTGGCTGTGCCCCGTGTGCGACCGCCCCGCGCCCT
ACGACTCGCTTGTTGTTGATGGGTACTTCCAAGAAGTATTAACATCGTCGCGGTTAGCCAGTGAGTGCAACG
>103795213_2 EC091347.1
TAATCCAGATGCAAACTACTGCTGACGTATCCGATATGCTTTTTGATATCCCAGATGGTTTCGCCGCTGCCGC
GACGTCGTCCGAAAAGCGTCAAATCGTTGCTGTAACCTTGCGGATGATCGCCAGTAACCAGGCTTAATAACGT
CGATTTTCCTGCACCATTTGGCCCGACAATTTGCCAGTGTTCGCCTGGATTCACCTGC

CAGCTAAGGTTATTAAGAATGGGGCGATCGTTATAAGAAACCACGCCATTGTTCAGCACAATGCGCGGTTCGT
TGGCGGGTAAGGCGTGACGTGCTGAAGGTTCATCCGGCTCCGGCAGTTGCACACCTTCAAGCTGTTCACTATG
CGCCAGTTGCGCGACGAGTGCTTGTTGGAGCAGTTCCTCTTTAGCGCCAGTTTCCGCT
AACGTGCAATCCGCCAGCACGCCAGCAAACTGGACAAACTCCGGGATCTCATCGAAGCGATTGAGCACCAGTA
CCAGAGTAATACCGGACTGATGTAACGAGGCGAGTCGCTCAGCCAGCTGCTGACGTGAGGCAACATCCAGGCC
ATCGAACGGCTCATCAAGAATCAACAAGTCAGGCTCCGACATCAGCGCCTGACACAGC
AGGGTTTTTCGCGTCTCGCCAGTGGAAAGGTATTTAAAGCGTCGGTCGAGGAGGGCGGTAATACCGAACTGCT
GCGCCAGTTGCATGCAACGCGGTGCATCCTTTACTTCATCCTGAATGATCTCAGCC
>103795233_2 EC091354.1
TCGGCAAAAGCACCACCCGTTACCATCTGCCGTATGTACTTAACCGCAAGGATAGCTGGCATGTCGCGCATAT
TTTTCGTCGCCATGCGAAGCCGGAAGAACAGGCTCCCATTTATTCCCATATCCATTTCACCAGCGATCTCGAC
GAAGTACTAAACGATCCCGATGTTAAGCTGGTTGTTGTCTGCACCCACGCGGACAGCC
ATTTCGAGTACGCGAAACGCGCGCTGGAAGCCGGGAAAAATGTGCTGGTCGAAAAACCGTTCACTCCGACACT
TGCGCAGGCGAAAGAGCTGTTTGCGTTGGCGAAAAGCAAAGGGCTGACCGTCACGCCGTATCAGAATCGTCGC
TTTGACTCCTGCTTCCTGACAGCGAAAAAAGCGATTGAAAGTGGCAAGTTGGGAGAGA
TTGTTGAAGTGGAAAGCCATTTTGACTATTACCGCCCGGTGGCAGAAACCAAACCTGGGCTGCCGCAGGATGG
CGCGTTTTATGGCCTTGGTGTGCATACGATGGACCAGATTATTTCTCTGTTCGGTCGCCCGGATCACGTCGCT
TATGACATCCGCAGTAACATCACGCTGATCGCGATCGGCCTGCCAGAGGAAATAGCCA
GCCAGGGCTGAACTTAATGCGAATAGAACTAAGATACCGCCTAGAATGCAGGCAAATAACGGAACCAACCTGA
TGTGATGCAAAAAACCTAATCGCTGTGAGGGAGTTGTATTCATTACCGTTGTCTCTCGTCCAGGTTTACTCCT
GATAGAAAGTCATCGGCACTTGACGGTAATTACTTATGACAAAAACCTCTTTGCGTCC
ATTTTTACACAA
>103795239_2 EC091356.1
TGTTGACGATCACCATGTCCCTATCGTCATAGCTCGGTACCACGTCATAACCTTCAGTGCGGAGTTCGGTGAG
AATACGCTCTGAATCAACAAGGTTTTTCGGACAGCCAAGGGAAACAAAGCCGATTTTCGGCT
>103795245_2 EC091358.1
TTTACACCTAGAAGTGACAGTATTTGGGGAAAACCCAAATTAAATTCCACTTTTAGTGTTAACTCGGTGACAA
CAACTAGCCCTAATCCTTTATCAAACTCAGTGTTCATGAAGCCTTCCTTTAATAAGTATCAAAAGTTGGCACA
ACACTCTGATTCTGATTTAGATGAGAGCATAATTTCTTTATGCATAACTAGTCCAAAA
AAGAAAAGTTCTTTTTTTTTTTCCTGTGTTTGCCTTACGCAAATTCGCTGCCTCCCCCAAATTTGTGGCCCCC
ATACCTTTGAA
>103795251_2 EC091360.1
CCGCCAACAGCAGAGCGAGCGGACAAACTGGGAACAGCAAGAATTCCGGAGGAAAACTCCGTCGGTTACAACT
TAAATGGGAGATGTTGAGCAATGCCGAGAGCCCCGTTACACCTTCTGGT
>103795254_2 EC091361.1
CTCCACCACCACCAGCCCGTAGGTTTTCCCGTCGATTTTTAGCGGCAAAATCTGGTACGGTACACCGGGTAAC
GTGTCGGTGCCCGCGCCCGCAGGCAGGCCTTTATCAAAACTCCACTGCGCGATGGCATCGTCCCACGGCGTCA
TTCCTTGCGGATGTGTTAACGGCTGCAATTTACCGTTGTCATCGGGCAACAACACCTG
ACTGCGGGCATGAAACGTGGAGGCAATAAATTGTTCGCTGGTGGCAGCGATATCCTGCGGACTGCGGCCCACC
GCCAGAGCTTTCGACATTTCATATAAGTGCCGTGTGCGTTGCTCGCGGTAACGGGCTACCCGCGCCTGATAAC
GCACGCCAGCAGTAAGGTTCCCGATCACCAGCCCGACGGTTAACATCACCGCGAAGGT
CAGCAGATATTGCACATCAGAGACGGCGAGCGTGCCGCGTGGGGCGATAAAAAAGAGATCGAAACTCACTACA
TTAATGACGGTGGCAACCACTGAAGGCCAGCGTCCATAAAATAGCGCCACCACCACCACGCCAAGCAGATACA
GCATCACCAGGTTGGCGGCATCAAACGCCATCAGCCACTGCATGGCAATTAAGGTGAT
AACGGCGCATAACGCGGCGGCAACCACGCATCCCTGAATTTGTACACGCCACTTGTC
>103795256_2 EC091362.1
AGTATATAAAATTTTAGAAATCGCATGTGTTTAAAACAATGCATATGTAACGCATACGCTTAGGTTTATTAGA
ACTTACTATAAGCATTTCGTAATATATGTATGTATGTATGTATGTATATATATATATATATATATATGGGAT
>103795262_2 EC091364.1
GTTAGTTATATGTAATTATTTATGGATATATATATATGTAAGTATTTATGTATGTAGATATGGGTATATTAAG
AATGGTAATTTATTTATATTTATAAATATGATAGGTATTTAAAAGTATTTAGAATTTAATTTTAGAT
>103795278_2 EC091370.1

TCATCATCACTTCAGTCAGCACGCTCTGATGGCTGAACGCGTTGCCTTTGTGGTACGTAAAATGACGGTGGAA
TATTACGCACCTGCGCGGCTCGACGATATGCTCGAAATACAGACTGAAATAACATCAATGCGTGGCACCTCTT
TGGTTTTCACGCAACGTATTGTCAACGCCGAGAATACTTTGCTGAATGAAGCAGAGGT
TCTGGTTGTTTGCGTTGACCCACTCAAAATGAAGCCTCGTGCGCTTCCCAAGTCTATTGTCGCGGAGTTTAAG
CAGTGACTGACATGAATATCCTTGATTTGTTCCTGAAGGCTAGCCTTCTGGTTAAACTTATCATGTTGATTTT
GATTGGTTTTTCAATCGCATCTTGGGCCATTATTATCCAGCGGACCCGTATTCTTAAC
GCAGCGGCGCGCGAAGCCGAAGCGTTTGAAGATAAATTCTGGTCTGGAATCGAACTCTCTCGCCTCTATCAAG
AGAGCCA
>103795283_2 EC091372.1
TTTATTTAATTAATAATAAGTTAATAGGATGTTTTTAGAGTATAAATAATAAATGATTTAAGGGGAGTGGTTA
GTGTGATTAAAAATAAGGATTGAAATTATGGTAAAAATAATTTTTAAATTAAATTTAAAGG
>103795286_2 EC091373.1
TCGGCCCACCCAGTCATCACAAAAATGACATAAATTCAGGATTTCGTGGACCACCTCCACCGCCAGCTCCTCT
TGCCGATGCCCAAACTTCAGCCAGGGATAAAGTTTTTTAGCATAAGTGGAG
>103795292_2 EC091375.1
AGGGCACCTTAAAATGCGCCCATAAAAGCTGCCCGTTCTCATCATAAATTAGCGTCATGGTGGGGCTTTGCTT
GTCGATATTTTCGGGGAACTCGACATGCAACTTATTGTTTTCCCACTTCGCAAGGGTATAGAACAGATTGCTC
TCGCCACGTAACAGCCGAAACGTAGTTTTATCGAAACTGACGCTATAACCGATCAGCG
CGACCATTCCGTAGGCAAGCGAAAGCACCAGTACTACCGCTGCCGTTGCCAACAGAAAACGTACCCGCAGCGA
>103795295_2 EC091376.1
AACTGGGCGGCGCTAAAGCGATCTTTAGCTCGGGTCAGCCATTGTATCGCCTGAACGGTAGCGCGCTCAACAG
AATGACGGTCAGCCCATTGGTTAATTTCATTTTCGCCCTTGCGCATAAGGGTGCTGATTTTTTTATATTCATT
TGATGAATCCATACAGGTGGCAAAACAAGCAGGACTGCCATTTATACGCGCGCATAAC
AACGCCGCAAGCTGATTATCCGGCTGTGTATTGCCCCGTGTGCTGTTTAACAACTTTTCGAGAAGGGAAAGTG
AAATAGGGTGGAGAGAGAGTATTTCGGCCAGTTGGCAAAGTTCTTTTGTTGAACTTTTTGTGTGCTCTTCAGG
TAGTTTTTCCACCTCATAGCTGCTTCGCCAGCGTAATGATTCATCAGCAAACAGTGAC
GCGTCGGCGTAGTTTTGTATCTCCAGCCCGGGTAACCAGCGTACCGGATGCCCGAGATAAGCCTGAAATGTCG
CAATTGTCCGCGCCTGAAAGCCAATAAAACCAATGATTTGGCTAATGATCACACAGTCCCAGGCAGTAAGACC
GACGTCATTCAGATGCTGGCGCGATCGGTTGTCGATAATAGAAGGCGAGCTAGCCAGT
TGGCGGGCATATTGCGTGATTTGTGCCAACCGATGATTACTTTCCCTGGTGGAATCAGGCCCGGGGAGCG
>103795297_2 EC091377.1
CAGGCGAGTTCCCAGCGGCGTCCCTACAATCGGGCGATTAATCAGAATCGGGTGCTGAAGCATAAAGTCGATT
AACCGATCGTCAGTAAATTTATCTTCCGCAAGGCCCAGCTCCTCATACGGTTCGACGTTTTTACGCAGCAGCG
CGCGTACGGAAATCCCCATATCGGCAATGAGTTTGACCAGTTCATCGCGCGTTGGCGG
AGTTTCCAGATAATGGATAATAGTCGGTTCTGTGCCGCTGTTGCGGATCATCTCCAGCGTATTACGCGACGTG
CCGCAGGCCGGGTTGTGATAAATGGTAATGTTGCTCATATCAGTATCTCTTTACAAAGTGAAAGAGAGACGTA
GCGCCAGCGCAGCCAGCGTCACAAACAGCACAGGCAGGGTCATGATAATCCCTGTACG
GAAGTAATATCCCCAGCTGATAGTCATATTCTTCTGCGAAAGTACGTGCAGCCAGAGTAGCGTAGCCAGGCTA
CCAATTGGGTCATTAGCGAACAGCAACAGATTCAGGAGAAACTGAGTCTTGGTGCGACGCCGAAGCAGGCTTC
AGCGATATTGATTCGCGACAGTATTCGCGCGGCTTTAATTCCGACGGTCGATTCAGCA
AAAACGGTTGGCTTAGTGAGTTTACCAGGAATGATGTCCGGGCTGATATTTGCCGGGATTGATCCGGTGAAG
>103795300_2 EC091378.1
GCACGAGGCCTCACCTTACATGGGTTTGTATTTTAAATTGATATGAATCGGAATTTACAAAAACAATGTAATA
TTATGAAAATATAGTTATTATATTTTATGTTTTA
>103795305_2 EC091380.1
TACCAACGGCGGAAATGGGAGAGATCGACATTCGACGCATCTTCCATCGCCTGCACAAAGTCGTCACAGGTCG
CTGCACTACCATCATGACGCTCGAAATAAAGCTGCATCCCTTTCTGGAAGTTTTCTTCGCCAAGC
>103795308_2 EC091381.1
GCACGAGGGTTTCCCCTTTAGGTTACGTTATTGGTCTGTCACCTTTAACGTGAATGATTTGAGAATCCCCCTC
GTTTCTGATCTTGATCCCTCTCAAGGTTCAATA
>103795311_2 EC091382.1
CCCCCCCCCCAAAAAAAAAAAAAAAATTTTTTTTTTTTTCTCCAAAAAAAAAAAAAAAAAAAAATAAAAAAAA
AAAACCCCCCCCCCCCAAAAAAGGGGGGGGGGGGGGGCCCCCCCCCCCACAAAATTTTTTTTCTTTCCCCCCC
GCAAAAAAAAAAAAAAAAA

>103795314_2 EC091383.1
AAGGTTTATCAACTGTGGCGCGATACTTAATGGAGATACGGTTATCGCCGCATCCGGCAAAAAAGCCCGCACG
TTGTCAGCAACCTGCTTAATATCCCTTCCTCCCTTTCACCCGAAAGGGAGGCACACCAGATTCCTCTCATTTA
AAATCGCCCCTCCTCCAGCATCTATACTTATCTTTTTGCTCTATTTTCTCACTGGAGG
AGTCATGCGGTGGATCCTGTTCATCCTCTTCTGCCTGCTGGGCGCACCTGCCCACGCGGTATCCATACCCGGC
GTTACAACCACAACGACAACGGACTCAACGACTGAACCGGCCCCGGAACCGGATATCGAACAAAAAAAAGCGG
CCTATGGCGCACTGGCGGATGTGCTGGATAATGACAGCACTTTCAGTTGGGTGGTGAT
TGAAAACGCCAGCCAGCGCGGCGAGGCGCGTTCGGCTACTTTGCCGTTGCCTGCGGTGATTCTGGAAAAAGTG
CGTGAAGGTGAGGCGCTTGGTCCCGTGATGTCGCGTTATACCGGTATTGATGAGATTGGTCGTAAGGAAGGGG
CGATCGGCGTGTTTACCGCCGGGAAACTCACTCGCGCCAGCGTCTATCACCAGGCGGT
GATCCTGGCGCTGAGTCCGTTTCATAATGCCGTGTATTAAGCGCCTGATGCCACGCTGCCGCGTCTTATCATG
CCTACCAAACATATTGAAATTACGGGTATTTGT
>103795316_2 EC091384.1
GCGGCAGCAAACGCCAGCCTGGCGCTTCTCGAATCTGGCGACTGGCAGCAACAGGTGGCGGATATTGAAGTAC
AGCTGCGCGAGCAACTTGCCCCCGCCCGTGATGCCGAAATGGTTGCCGATGTGCGCGTACTGGGGGCCATTGG
CGTGGTCGAAACCACTCATCCGGTGAATATGGCGGCGCTGCAAAAATTCTTTGTCGAA
CAGGGTGTCTGGATCCGGCCTTTTGGCAAACTGATTTACCTGATGCCGCCCTATATTATTCTCCCGCAACAGT
TGCAGCGTCTGACCGCAGCGGTTAACCGCGCGGTACAGGATGAAACATTTTTTTGCCAATAACGAGAAGTCCG
CGTGAGGGTTTCTGGCTACACTTTCTGCAAACAAGAAAGGAGGGTTCATGAAACTCAT
CAGTAACGATCTGCGCGATGGCGATAAATTGCCGCATCGTCATGTCTTTAACGGCATGGGTTACGATGGCGAT
AATATTTCACCGCATCTGGCGTGGGATGATGTTCCTGCGGGAACGAAAAGTTTTGTTGTCACCTGCTACGACC
CGGATGCGCCAACCGGCTCCGGCTGGTGGCACTGGGTAGTTGTTAACTTACCCGCTGA
TACCCGCGTATTACCGCAAGGGTTTGGCTCTGGTCTGGTAGCAATGCCAGACGGCGTTTTGCAGACGCGTACC
GACTTTGGTAAAACCGGGTA
>103795321_2 EC091386.1
CGCCGTTCAACGGTCAGCTCAGAACTGGCACGGACAAGGGGAATCCGACTGTCTAATTAAAACAAAGCCTTGC
GATGGCCCTCGCGGGTGTTGACGCAATGTGATTTCTGCCCAGTGCTCTGAATGTCAACGTGAAGAAATT
>103795324_2 EC091387.1
GCACGAGGGTGAACTTGCATTTGTTTACAAAATATTGCTCTAAACAAGATCAAAATAAATCATTTATGGTAGA
AATAATATGGCGCANAAAGATGATAACAAGAAACCCGGTGGAGGGGCGTTACAAAATATGCAATTGCTGAAAA
AAGAAAGTTCCCTTTTTAAGGTTCCTAAAGTGCCTCCAAAAA
>103795327_2 EC091388.1
GCACGAGGAGCCTTTATGGGTTTTCTTTTAAATTAGTTATATTATTCGCATATTTTTTAAACATTGAGTATAA
TATTAGTCTTAACATTTATTTCCATTCTAACTCTGTAACTGGATTACTTTTGCATCTCGCCCGTCTCGGGTAC
TACAAACTGGCGGCGGCGTCGGTAGTGGAAGTGGAGACAGAGAATGGTACGCGCAACG
GAGTACAGCATTCACAGCCGAAGGAAATCTGAACTAAAATAATACCAATGGATCCAGACAGTCTCGTGCTTTG
GCGGAAGCCTTTTACGACTCTAGAATATTTTTTTTGGGAACTTCTAATATTAAGTTCCACAGGACTTAAGAGG
TTATTAGCATACAAGGCACTCGCGTTAGCGCTGCTCTCCGTCATCTCCGCTACGGGTG
TTTTACATTTGATTAATG
>103795332_2 EC091390.1
AAAAAAAAAAAAAAAAATTTTTTTTTTTTCCCCCCCCCCCCCCCAAAAAAAAAGGGGGGGGGGGGGGGGTTTT
TATTTTTTTTGTTGAGAAAAAAAAAAAAAAAAATATTTTTTTTTTTTTTTTTTTTTTGGGGGGGGGGGGG
>103795338_2 EC091392.1
GCACGAGGCAACTCTGTGAAACCTTTGATGAAATAATACATTATTAGGCAAGTCATTTAAATACATTTATAAA
TTTAGCATGCATAATATGGATAATAACACCGATCATGCAGATCCAAACAATTTTAGTCCGCTAACTAT
>103795360_2 EC091400.1
TTTTTTTCGCGCTATAGTACATCGCTGGATATGCGCAACGACTGCACGCGCTCGCCCTGCTCTCACTTGTGCT
TGGCCGTGCCCGCCGGCCGCCGCTGTGCCTGCCACGACCTGCTGCCGCCTCCTAAGCGC
>103795363_2 EC091401.1
CCTTTAATGCCCGGGGCGCGTGGCTGTTGATGGCGTTTACTGCTTTGGCACTGGAACTGACGGCGCTGTGGTT
CCAGCATGTGATGTTACTGAAACCTTGCGTGCTCTGTATTTATGAACGCTGCGCGTTATTCGGCGTTCTGGGT
GCTGCGCTGATTGGCGCGATCGCCCCGAAAACTCCGCTGCGTTATGTAGCGATGGTTA

TCTGGTTGTATAGTGCGTTCCGCGGTGTGCAGTTAACTTACGAGCACACCATGCTTCAGCTCTATCCTTCGCC
GTTTGCCACCTGTGATTTTATGGTTCGTTTCCCGGAATGGCTGCCGCTGGATAAGTGGGTGCCGCATGTGTTT
GTCGCCTCTGGCGATTGCGCCGAGCGTCAGTGGGATTTTTTAGGTCTGGAAATGCCGC
AGTGGCTGCTCGGTATTTTTATCGCTTACCTGATTGTCGCAGTGCTGGTGGTGATTTCCCAGCCGTTTAAAGC
GAAAAAACGTGATCTGTTCGGTCGCTAATCCATTCGGCGCTCCTGCGGGAGCGCTTTTTTCCTGCCGCTATAT
TTATTTGACCCTCAGTAAATCAGAACTTCGCGTTGTATAACGTGGTGAAAGCATGGCT
CGCTTCATCTGCCATTGTTGCTGGATCCCCTGCCCGGCAAAATAGAGTGTTCCTCTGCCCTCTTTAGCATTCA
GTGTATCCATTACCGTCATCAATTGCTCACTCCCGGGGCGCGGTGCGTTGTCATCGAATAAATTG
>103795366_2 EC091402.1
TTTGGTCGTTGGCTGGATCTGACGTTTATGCAGTTGCTACTCGACCACCGCACTGGACCGGACGCGATTGGAT
GAGTCAGTCGCTCGCCCTTGCTATTTTTGATACACTTAGGGCCCAGGGTGTAACGAAAATGTGCTATATGACA
AAGAATTAACGGATAATGAGATGATCATAGCAGAAGATTCTACAGCAGCTCCTGGAGT
GGATTGTTTTTTTTCTTGAGCACCCTATTTCAATCTAAGATATCGCGCAAAAATCTGGCTACAGCAGACACAA
CATCCAGCTTCTGTTCCGAAATTTCATGCGTGTGCCTTTGGGAGAATACATTCTTCAAACGAAAGCTTTGTCG
TGCCGCCATTCTTGTCCGGCTCACCGCGAAATCTATGCTTGATATTGCACTCTCTTTG
CATTTTGATTCACAGCAGTCATTCAGCCGCGAATTTAAGAAGTTATTCTGCTGCTCTCCCCGTGAATACCGCC
ACCGTGATTATTGGGATCTCGCAAATATCTTCCCTTCTTT
>103795371_2 EC091404.1
GCACGAGGCTCGTCCCTTATTCGATGTTTATTGTTTGAGGACGAGCTATTAGTATATTGGTATTAATATGACT
TTTTAACAAAAACCTGCCCAATTGGGACATGTGCTCTTTTCATAAG
>103795374_2 EC091405.1
GCACGAGGGGATAGCGTTATTGGTTTTATTGTTTGTGATAGGAATATTATGTAATAGTTCCTTGCTGTGATTG
TGAATTAGTGAAATTTAACATACAAAATTAAATAGGAATGCTGTAACGGTGTGTATAATGATTAATATC
>103795385_2 EC091409.1
GGCGGGGCACGCCGTTCTTGCCTGCTTAGAGATGGTGCAGTCAGGCCGCCCGTTGAAGCAGGAACCCACCGAA
ATGATTCAGGCGACAGCCTGAACGTATCAGGGATCCACGTCCTTCAGGGCGTGGAGGATGTCAATATCGGTTT
CTGTCAGCGATTAAATTCAACACCACGTAACAAACGCCTCCGGCAATTAATCCCCAAA
ACGCAGAACCAATCCCGACCAGCGTCAATCCACTTGCCGTTACCAGAAATGCCACCACTCCACTGGAGACGAC
GGAAGATCGTTGCCGAAGCGGCGTTCCACCGCTCCCAGCATGACGTCCTGTACTGTTTCACTGTTGAAGCCGC
CGGTAGTGACCGCCCAGTGCAGTGCCTCACGATCACAGCAGTCCAGCGCGAACGTGAC
ACGCAGTCTCTCTCCGTTATCACAGCAGAACTCGAACCCGTCAGAGCACCATCGCTGATTGCTTTCTTTCACG
GCCACTCTGCCTGTATGTGCCCGTTTCGATGGCGGTACAGCAGGTTTTCGCTCAAGCAACAGCGCATTCTGGC
GCATGATCCGGTAAACACGTTTGGCATTGATCGCAGGCATACCATCAAGTTCTGCCTG
TCTGCGAAGCAGCGCCCATACCCGACGATAACCATACGTTGGCAGCTCTCCGATAACATGGTGTATACGGAGA
AGCACATCCGTATCATCAGTGTGACGACTGCGGCGGCCATCCATCCAGTCATCGGTTCGTCTGAGAATGACGT
GCAACTGCGCACGCGACACCCGG
>103795387_2 EC091410.1
GGATCGTGCCAGCGAGCAAGGGCTTCGATGCCGTACAGTTCACCCGTTTCTGCGAAGATTTGCGGCTGGTAAA
CCAGTTTCAGTTGGTTATTGCTAATCGCTTCTTTCAGCGCTGCGCCTAAAACCAAACGCTCTTTTACCATTTC
GTTCATCGCCGGGCTGAAGAACTGCCAGCCGTTACCGCCATTCTTGCGAATATAATCC
ATTGCATTGTGAGCAGTGGAGAGCAAGTAATCGCGGTTTTTACCCAGGTCGTAGCTGATGCCAATACTCAAGG
TAAGCGGGAAGGGTTTATCGTCAATCATTATCGGCTTGCTGACCACATTCCGTAGCTCATCGGCGATTTGGGT
AATGTTACTGACGTCGTTTTCGAGGCTCACGAGGACAAACTGCGTACCTTCGATACGA
CAGAGATACTGATCCGGTTTGAGTTTTTCACGAAAGCGATTGACCACTTCCAGCAATGCCTGATCGGCCCACG
CATAGCCAAGGCTATCAATCACATCCTGAATATGGTCAACACCGATGAGATACACCACGGGAGAGACGGCTTT
GTCGACCAGGTCATCGAGGTAATTGTGCAGGTTATTGCGATTTGGCAGACCGGTCATC
GGATCAAATTGGATGAGTTGTTCAATATGCTGACGGCTTTTTTCCTGTTCCAGCGCCAGCGCGGCCATATGCT
GGCTGATATCTGCCACGCGTTCGATAAAGGCGCTGGTTTCTGCTCCTGACGAGGTTTTAATTTGCAGGATCCC
CGCAGGCGCGCCATCACGCTGACGAATGGTCGCTGACCAGCTTTGCGCATTTTGAATT
TCTGCACC
>103795396_2 EC091413.1
CGGTAACTGGAGACAGGCACTATACGCAACTGGAAGCGTTAGATAATCGCTATCCGTTTGGTCCGTATTCGCA
GCAGGTGCAGCTGGATCTCATCTACGCCTACTATAAAAACGCCGATTTGCCGTTAGCACAGGCTGCCATCGAT
CGTTTTATTCGCCTTAACCCGACCCATCCGAATATCGATTATGTCATGTACATGCGTG

GCCTGACCAATATGGCGCTGGATGACAGTGCGCTGCAAGGGTTCTTTGGCGTCGATCGTAGCGATCGCGATCC
TCAACATGCACGAGCTGCGTTTAGTGACTTTTCCAAACTGGTGCGCGGCTATCCGAACAGTCAGTACACCACC
GATGCCACCAAACGTCTGGTATTCCTGAAAGATCGTCTGGCGAAATATGAATACTCCG
TGGCCGAGTACTATACAGAACGTGGCGCATGGGTTGCCGTCGTTAACCGCGTAGAAGGCATGTTGCGCGACTA
CCCGGATACCCAGGCTACGCGTGATGCGCTGCCGCTGATGGAAAATGCATACCGTCAGATGCAGATGAATGCG
CAAGCTGAAAAAGTAGCGAAAATCATCGCCGCAAACAGCAGCAATACATAACAGAAAC
CTGAAACACAAAACGGCAGCCCTTGAGCTGCCGTTTTTTTATTCTGTCAGTTGTGAAACTGAAGCGATTTAGT
CGCTATCGATCTCATCAAATATGGCTCGCTTTGAGATATTCCTCAAGTAAAAAAACATCTCTTCCTGCGATTT
CTCACAAAAAAGATTCGTTGACAAAAAGTGACAAAATTATGAGATTTTCATCACACAT
TTTGA
>103795399_2 EC091414.1
GCGCTCTGTCTTACGGAACGTCAAATCAAAAATTTGGTTCCAAAATCGAAGGATGAAATTAAAGAAGGAAATA
CAAGCGATAAAGGAGTTAAACGAGCAAGAGAACCAAGCGCAAGCGCCAAAAGCAGCAGCGGCAGCGGCGGCTG
>103795402_2 EC091415.1
CCTTTAAAGCCCGGGCTGCAGTGAATCTGTATACTGCGGCTCCCTCAAACGGTCGTGTAAACGATGGCTAAAC
AGCGAGTTGGCTGCGGCAATGTTGCTGATATAGCTTCCGGCCTCCGGGCGATAAACTGATGGGCCTTCCGGAT
CCACAAC
>103795408_2 EC091417.1
TGCCCGATCACACCATTTCACCCGCCGTTAAAAATGCGATTGCCGCAGCTCGCGCCCGTGGCGTGAATGTCGT
GCTAACGACGGGTCGCCCGTATGCAGGTGTGCACAACTACCTGAAAGAGCTGCATATGGAACAGCCGGGCGAC
TACTGCATTACTTATAACGGCGCGCTGGTACAGAAGGCCGCTGATGGTAGCACCGTGG
CGCAAACTGCTCTCAGCTATGACGACTATCGTTTCCTGGAAAAACTCTCTCGCGAAGTCGGTTCTCATTTCCA
CGCCCTGGACCGCACCACGCTGTACACCGCCAACCGTGATATCAGCTACTACACGGTGCATGAATCCTTCGTT
GCCACCATTCCGCTGGTGTTCTGCGAAGCGGAGAAAATGGACCCCAATACCCAGTTCC
TGAAAGTGATGATGATTGATGAACCCGCCATCCTCGACCAGGCTATCGCGCGTATTCCGCAGGAAGTGAAAGA
GAAATATACCGTGCTGAAAAGTGCGCCGTACTTCCTCGAAATCCTCGATAAACGCGTTAACAAAGGTACGGGG
GTGAAATCACTGCCCCACTGGCTGATTATTATGCCGCGCCCTGAAAACACTACAGTTA
TTCAGGGAAATTATTTCACCATTCATTCGATGATGATTTTTGAGGAATTATGGGCAACACTAAGTTGGCTAAT
CCGGCACCGCTGGGCCTGATGGGCTTCGGCATGACCACCATTCTGCTTAACTGCACAACGTGGGTTATTTCGC
TCTGGACGGTATTATTCTTGGCATGGGCATTTTCTACGGCGGCATCGCGCAAATTTTT
GCTGGTCTGCTGGAGTACAAAA
>103795420_2 EC091421.1
ATAACCCTTTTGCTGAGTATCGCTTTGAACATTTTGGTCACTATTTTTTGTTCCGTCCCGATCATCATTGCCG
GGATTGTAAAACTATTGCTACCTGTCCCAGTCATTTGGCGAAAGGTTTCGCGTTTTTGTGATTTTATGATGTA
TTGCTGGTGTGAAGGTCTGGCGGTATTACTGCATCTGAACCCACACCTTCAATGGGAA
GTTCACGGGCTGGAAGGGTTAAGTAAGAAGAACTGGTATCTGCTTATTTGTAACCACCGTAGTTGGGCAGATA
TTGTCGTACTGTGCGTGCTGTTTCGTAAGCACATTCCGATGAATAATTATTTCCTCAAGCAACAGCTGGCCTG
GGTGCCATTTCTTGGCCTGGCGTGCTGGTCGCTGGATATGCCGTTTATGAAGCGTTAT
TCCCGCGCTTATTTATTACGTCATCCGGAGCGACGTGGTAAAGATGTTGAAACCACTCGGCGTTCTTGTGAAA
AGTTTCGCCTGCATCCCACCACCATTGTCAATTTCGTTGAAGGCTCCCGTTTCACGCAAGAAAAACATCAGCA
AACCCACTCCACTTTTCAAAACTTGTTGCCACCAAAGGCGGCAGGCATTG
>103795422_2 EC091422.1
GCACGAGGGTAGCTTGAAAGAGTCTCTAGTGTTTAGCTTTTACCCTTATTTTATTATTTAAAAGGGATCAACA
CGACGACTAACATGCAACATGGAACCATTTTGGATGTACT
>103795443_2 EC091429.1
GCACAGGCCTTGTACTTTAAAGACTCTCTCGGAAGTTAGTGCTGACGTAATAAGTGGTGAAATTTTAAAAGTG
GATCTTGATTTCCGTAATGTTGGCCCAGTTCCTATGAAAAATTTATATATAGCAGTTTCCCATCCTGATTGTA
TGAGTGTGGTTAGTGCGATTGATTACAAAGATGACTTCAGAGCGCTCTCTGAATATAC
CTATTTTGAACCATGTGAGTAGTCG
>103795446_2 EC091430.1
GCACGAGGGTCTCACTTAAAGAGAGGATGAGGTGAAATACCACAAATACTACCAGTGGGTGTGTTTTGTGCTA
TTCTTCCAGGCTATTCTGTTTTATGTGCCTCGTTATCTGTG
>103795452_2 EC091432.1

CCTTTAAAGCCCGGGCTGCATGCTGGGCATACCAGTCAGCAACGTTGAAGAACCCCGCCATATCTTCAGTCAC
CAGCAACGCTCGCCATTCACCCTCAATTTTCACCGCTTCACCAAAAACGATCTTCGGCACGATGACACCGGCC
CGTTCCAGTTCTTTAATTACCGCAACCTCACGGACAATCGTTGGTCGGCCGAACGGAT
AACGTACGGAATGAAACAGATGATGCGTCATGCGCTTTACATACAGCTTTTTGCCGTTGCGCTCGACGCATTG
CACCCCGCTCATACCATTACGGCGATAGTTAGGCTCTTCAACCCAGTCGCCCTCTGTTGCCCACCAGTGATTA
AATTCGTCGTACTTTGCTGAAACTGCCATACCCTATCGCCTGTCATTTTTATTAACGA
CAATGACTATAGGTGGTTACCTGAGGAAAATCTTAATGAAACGTGTCGTATTAATGAATTTCTTTTGCCACTA
CTAGCTTGACACCGCTTTTACCCTTCATTTGCAGCGCATAGATTCCACCAGCCACTACCACTAATAGTGCGCC
CAGCAGATAAAATGTGAATTTAATCAGGCGGATACAGCGATGAATTTTATTCGACAGG
GATTAGGCATCGCCTTACAACCAGAGTTAACGCTGAAAAGCATTGCAGGTGAATTGTGTTCCGTTCCTCTCGA
ACCAACTTTCTATCGACAGATTTCGTTGCTGGCTAAAGAAAAGCCGGTAGAAGGCAGTCCACTGTTTTTACTA
CAAATGTGCATGG
>103795455_2 EC091433.1
GGAGGAGGCTATGGAAACAACCAGGGTGGGTGGAACAATTCAAATCCTTGGGACAACAACCAAGGAGGTGGTT
GGGGAGGTAATCAGGGGGGTGGTTATGGGGGAGGGAGGCAAAGAGGAAGATGTGGGGGAGACTGGGGAGGCAG
ACATGACTTTGGTAATTATA
>103795464_2 EC091436.1
CTGGCAGGGAGCGTTTGGCCGATCGTCGATTTAACCGGACGTTCTGCATCCTCATAAATTGCTGATGACGTGG
CGGAGTGCCGCGTCTTATCAGGCCTGGAGGTGGCAATTACTGCCCACACACCTCACACCCCGGATTACGCATC
AGTTTCATTTCGCGAAACTGACAGGTCATCGCATCGTACATGACGATTTTCCCGCTGG
CCGGTTTGCCATAACCTGCCAGCATTTTGATCGCTTCCATTGCCTGCAACGAACCAATTACGCCGATCAACGG
TGCCATTACGCCTGCTTCCACGCAGGTTAATGCATTTTCACCAAACAAACGGCTAAGGCAGCGATAGCACGGT
TCACCGTCCTGATAAGTAAAGACGGTGATTTGACCTTCCATACGAATTGCCGCGCCGG
AAACCAGCGGTACCTTCGCGGCAAAACAGCCTGCGTTCAGTTGATTACGTACCGCAACGTTATCCGTACAGTC
GAGCACCAGATCGTGTTCAGCAATCAATGCTGCAAGTTCTGCGTCATCCAGCAGTGCATTGACTGGCGTAATC
GCGATATGTGGGTTGATCCGCGTCAGGGCGTCACGGGCGGATTCCACCTTCGGTTGCC
CGACCGTGGCATCACTGTGCAGTGTCTGGCGTTGCAGATTCGAGAGCGAAACCGTGTCGAAGTCGAGCAGCGT
CAGGTTACCGACACCGGCGCTTGCCAGATACTGCGAGGCTGCACAGCCGAGGCCGCCCAGGCCCACTATCAGC
ACGCGAGAATCTTTCAGCGCCTC
>103795466_2 EC091437.1
AAAATTAAGTTAATCTCCAAATAAAATACAATCTCCAGTATACAAAATGCATGCTTCAAACTCCATTCAAATT
GAAACTCCAAATAAATCTTTAAAAAACCAAATTGAAACTATTTATAATGAAAAT
>103795469_2 EC091438.1
GCACGAGGGTTTTTCTTTTTTTTTTTTTTTATAAAACAAAATATTTATTTAAAACATTATCCACAAGCAGATT
ACCTTAACACCTAAGTAACACCTAGACCAGTCTAAATTAGACACTACATTAAACATATATT
>103795478_2 EC091441.1
ATGCGTGGCAGTCTCCCTTCGGTCCATAATAGCAGAACAAAAGAAAGAGCTCGAAATGACGGTCAGATATTAT
ATCAGTTCTGCTGATTTAACCGCAGAGAAGTTCGCCACAGCAATCCGAAACCACTGGCATGTGGAGAATAAGC
TGCACTGGCGTCTGGACGTGGTAATGAATGAAGACGACTGCAAAATAAGAAGAGGAAA
TGCAGCAGAATTATTTTCAGGGATACGGCACATTGCTATTAATATTTTGACGAATGATAAGGTATTCAAGGCA
GGGTTAAGACGTAAGATGCGAAAAGCAGCCATGGACAGAAACTACCTGTTGTCAGTCCTTACGGGGAGCGGGC
TTTCGTAATCTTGCCCTGGGCATTATTGTCGTATAAACGCTTTACCGGACACATGACA
TTCACAATGTGTGTTAATTTTACTGACATCTTTCAAAAGGAGCGTAATCATGCCGCACATCGACATTAAATGT
TTTCCGCGTGAACTGGACGAACAACAAAAAGCAGCACTTGCTGCAGATATTACCGACGTTATTATTCGTCATC
TGAACAGTAAAGACAGTTCGATAAGCATTGCTCTACAGCAGATTCAACCAGAATCTTG
GCAAGCTATCTGGGATGCCGAAATCGCGCCCCAAATGGAGGCTTTGATAAAGAAACCTGGTTATAGCATGAAT
GCTTAACCCGCCGTAACACGCTTGCCGCTGGTAACAACGTCCCAGCACCTAAATGATGCAACGTATTGAGCTT
ATCATCATCAAACTGCCAGCGGGCTTCGACAAATACCCGTGG
>103795486_2 EC091444.1
ATAATATTTGGTGTGTCGCTATTTTGTATAAAAATCAATAGGTGCAATCATGTTAAAAACTTTTCATTAAAAG
TCAGTGAAGCTTTCCGTTGCTTGTCTTGCTAGATTTTGTATCGTTTTTTTTATTT
>103795489_2 EC091445.1
ATTAAACGGCCACAAAGCGTCACTTCTACTAGTTCCACACGAAACCGCCTTCGAGATCTCGTCGAAAGGAAAA
CTATTTTTTATGATAAACCCGCGGCAAACACCGTCGCTGATATACAGTATTTTGAACATCCT

>103795492_2 EC091446.1
AGGAACTCATATGTACCGTGCTCATTCTGCTGCCCATGCCGCGGACGGGCTTGCTGGAGCTTTCATAGTTCGT
CAATCACCTAGGCTTGACCCACTACGAAGCTTATACGACGTGGATGATACGGAACATACTATATTTATTGCGG
AATGGGGCCTCTCTATGGGTCCTCTAGCTGGA
>103795495_2 EC091447.1
AATTATTCCGTTTCGTTTGTGCCTTTAACCATGGCAGAAGCATTTTACATCCTGATAGGATTTCTGATTATGG
CGGCGATTATCGTCATGGCTGTCCTTTACCTCGAAAACCACTCCTGACGATCATATTGTTCCCCCGGCATCG
CATTCACCAGACCTGAAACCGCCTTCTGCGCCGCCTCTGCGCTTTGTGCACCGGGTAC
CAGAATCACCTTACGGCACTCCTCTTCACGTTTTTCGAAAATCTCATATCCCCGGGCGGCCTCTTCAAACGGC
ATATAGTGGGTAACAATTTCTTCTGGTTTCAGTAATCCTTTCTCAATTAACGGTAATAATTCTCCCAGCCATG
CGTGAACGTGGGTCTGTCCCATTTTAAACGACAACCCTTTATCAAAGGCGTCGCCAAA
CAGGAAACCGTGAATAAATCCAGC
>103795498_2 EC091448.1
CCTTGTTCCCCCTCGGCAATTTTTAGAACTCCATGAAAAACTAAGTGGCTAACCGCGAGAGAGATCAAATAAA
CCATACTAAAAGAATGGTAATAATGTGGCCTCTTTTCATTATCTCCCGTGGTACGGGGAAGGAAAATCATGTC
ACTTGTCACCGATCTACCCGCCATTTTCGATCAGTTCTCTGAAGCTCGCCAGACAGGC
TTTCTCACCGTCATGGATCTCAAGGAGCGCGGCATTCCGCTGGTTGGCACTTACTGCACCTTTATGCCGCAAG
AGATCCCGATGGCAGCCGGTGCGGTTGTGGTTTCGCTCTGTTCCACCTCTGATGAAACCATTGAAGAAGCGGA
GAAAGATCTGCCGCGCAACCTCTGCCCGCTGATTAAAAGCAGCTACGGCTTCGGCAAA
ACCGATAAATGCCCCTACTTCTACTTTTCGGATCTGGTGGTCGGTGAAACCACCTGCGACGGCAAAAAGAAAA
TGTATGAATACATGGCGGAGTTTAAGCCTGTTCATGTGATGCAATTGCCCAACAGCGTTAAGGACGATGCCTC
GCGTGCGTTATGGAAAGCCGAGATGCTGCGCTTGCAAAAAACGGTAGAAGAACGTTTT
GGGCACGAGATTAGCGAAGATGCTCTGCGCGATGCCATTGCGCTGAAAAACCGCGAACGTCGCGCACTGGCTA
ATTTTTATCATCTTGGGCAGTTAAATCCTCCGGCGCTTAGCGGCAGCGACATTCTGAAAGTGGTTTACGGCGC
AACCTTCCGGTTCGATAAAGAGGCGTTG
>103795501_2 EC091449.1
GCCCAAGGCAAGACCCTTGAGTGTAGGATTTTTGATAATTTATTGTTCATTCACCTATATTGAGTATGATTTG
CGGATCCCCAATGTTTCTGATCTTGATACA
>103795504_2 EC091450.1
GCTCCAGCAACACCTGTCGTTTAGTGCCACGAAATGCCTGTCCCTGCCAGTCAATATCTACATTTGCGTCCGG
TTGTTGCGTGATGTTGGCGGTATCATCAGCGGCAGCTGCGCCGTAAATTTTTGCCGGAGCCGTTGCCAGAATT
TCCACCTCATCGCCAACGCGAATCACGCCGCTATTACGGGCAATTAAATTCTGACCAA
AATCGACATCGCCGTTATCCTGGGCAGTGCGGAAAGATTGCAATGTTTTTAATGGTTCGCCTGCCGGATGTTT
TTGCCCTTTTTCTGGGCTGACGGTGGTGAAAATACAGCGGCTATTTTGTTTAACCACATCAAACACCACATCA
CCAATGCGAATCACTTTCCAGCGATCTTCTTCCCAGGCTGACGCGCCGGAAACCACCA
GATTGGGGCGGAATTGCTCCATTTTTACACTGGCCGGACAACGTTGTTGGAGATCACGTAACGAGGCTTCGTT
AGCAAGAAGGTAAGGATAGCCATCAGCAAATGACAGAGGTACAGTGTTGTGGCGTTTCACGCGCCGGGTCATT
TGTGGCCCCACCCAGCGTAATTGCACTTCGCGGGAGAAAAATCCAACTTAGCCATTT
>103795507_2 EC091451.1
CAGCTCGATAGCTTAATTCTTTCTCATCAATCAGCAGAAAATGTTGCCATTACGGAGGCAGTATAATGACCAA
ATTCTCAGTGGTTGTCGCAGGCGGTGGAAGCACCTTTACGCCAGGCATCGTGTTGATGCTCCTGGCGAATCAG
GACCGTTTCCCGCTTCGTGCACTGAAATTTTATGATAACGATGGTGCGCGGCAGGAAG
TGATTGCCGAAGCCTGTAAAGTCATCCTTAAAGAAAAAGCGCCGGACATTGCGTTTAGTTACACCACCGATCC
TGAAGTGGCATTCAGCGACGTTGATTTTGTCATGGCGCACATCCGCGTCGGCAAATACCCGATGCGCGAACTG
GATGAAAAAATCCCGCTGCGCCACGGCGTTGTTGGTCAGGAAACTTGCGGA
>103795510_2 EC091452.1
AAATAAATAAAAATTTTTATTATATAAATTAAATTATTTTTTTAAAAAATTTTAATTATAAAAAAATTTTTGT
TATTGAAATTTAATTATTTTGAAGTGGATGAAAAATGAATAAAAAAATAAATATTTTATA
>103795524_2 EC091457.1
CGGTCTTTTCTCTGCTGGGTGCTAAGGGGCTTAACGAACGTGTTGGCTCGACGCTGGAAATTGAATCCGGCAG
TAATGATCCAATGGCGGTCTTTCTGACGATTACCCTAATTGCGATGATCCAGCATCATGAGAGCAATATTAGC
TGGATGTTCATTGTCGATATTCTGCAACAATTTGGTCTGGGCATTGTAATAGGTCTTG

GCGGCGGTTATTTACTACTGCAAATGATCAACCGCATCGCCCTGCCCGCCGGATTATATCCATTGCTGGCATT
AAGTGGCGGTATTCTGATTTTCTCATTAACTACTGCGCTGGAAGGTAGCGGTATTCTGGCGGTGTATCTGTGC
GGTTTTCTGCTGGGTAATCGCCCTATTCGCAACCGCTACGGCATCCTGCAAAATTTCG
ACGGCCTCGCCTGGCTGGCGCAAATCGCCATGTTCCTGGTGCTGGGGCTGTTGGTTAACCCAAGCGATCTGCT
GCCTATTGCCATTCCGGCGCTCATTTTGTCCGCATGGATGATATTCTTCGCCCGTCCCCTTTCGGTATTTGCT
GGATTGCTCCCCTTCCGCGGTTTCAATCTGCGTGAGCGCGTGTTTATCAGCTGGGTAG
GATTACGCGGCGCGGTGCCAATCATCCTGGCCGTGTTCCCGATGATGGCGGGGCTGGAGAATGCACGTCTGTT
CTTTAATGTCGCCTTCTTTGTGGTTCTGGTTTCACTGCTCTT
>103795532_2 EC091460.1
CGTTAATAGCCCGGACTGGGCGCTGATCCCGCTGCTGATCATCCTCGCACTGGCGACGGTAATTGCCTCGCAG
GCGGTTATCTCTGGCGTCTTCTCATTGACGCGTCAGGCGGTACGTCTGGGATATTTGTCGCCGATGCGCATTA
TTCACACCTCCGAAATGGAGTCAGGGCAAATCTATATTCCGTTTGTGAACTGGATGCT
CTATGTCGCGGTCGTGATTGTGATTGTCAGCTTTGAGCACTCCAGCAACCTGGCGGCGGCGTACGGGATTGCG
GTGACCGGAACCATGGTGCTGACGTCTATTCTCTCGACTACCGTGGCACGTCAGAACTGGCACTGGAATAAGT
ATTTTGTTGCGCTGATCCTGATTGCTTTCCTTTGTGTCGATATTCCATTGTTCACCGC
TAACCTCGATAAACTGCTCTCCGGCGGCTGGTTGCCATTGAGCCTCGGTACTGTGATGTTTATCGTGATGACC
ACCTGGAAGAGCGAGCGTTTCCGCTTGCTGCGGCGGATGCATGAACATGGTAACTCTCTGGAAGCGATGATTG
CTTCGCTGGAGAAATCACCGCCTGTTCGCGTGCCCGGGACCGCGGTGTATATGTCGCG
TGCAATCAACGTCATTCCCTTTGCGCTGATGCATAACCTTAAACATAACAAGGTATTGCATGAGCGGGTGATT
CTGTTAACTCTGCGCACCGAAGACGCTCCATATGTCCATAACGTCCGTCGGGTACAGATTGAACAACTGTCGC
CCACTTTCTGGC
>103795535_2 EC091461.1
AGAGGGAGGGTCACAAGAAATTACACCCATACCGTTCAGATGAAAGACGAAATCTACTTTGTGATAGGGAGCA
AGGCAACCCTATGGAGCCGACGACAAGCCGCAACACTAGTTCAGGACAAGTGAGCCGATG
>103795538_2 EC091462.1
AGGTAAAGACGTGCATTACGTTTTCCCGTATACAGGTTGTGAACATCCAATGACATATCGGTTTGTCAGGGAA
GTTGTGAAGTTCTGGGATATACCGCTCACCGTATTGCAGGTTGATATCAACCCGGAGCTTGGACAGCCAAATG
GTTATACGGTATGGGAACCAAAGGATATTCAGACGCGAATGCCTGTTCTGAAGCCATT
TATCGATATGGTAAAGAAATATGGCACTCCATACGTCGGCGGCGCGTTCTGCACTGACAGATTAAAACTCGTT
CCCTTCACCAAATACTGTGATGACCATTTCGGG
>103795540_2 EC091463.1
AATAACGCTTCAGCTGTTTCCTTAGCTGGCGCAGTTGTGCAATGTCTTCTTCGTAACGGCATTGTTCAGCAAA
TTCGGTCAGCGTAATGGAAGGCTTGCCGTCAAAATCTGCAATCACATCGTAGACATAACCGGTTCCGCAATCC
GTCTCAACGGTACCGTGATAGCGCGGTATTCCACTCCAGTCTTTTAACCGGCGACCAA
GATGCGCGTAGTACTTTAACTCACGGCGGATCTCTTTATCGCCACCATCGCCACGATGGTAGACAATCTTGAT
ACAGCGTTGGGCATCTTCCGGATGCGCATAACATTTCCGATGTCTTCCGGTGCCCAGGGGACTTTGTTCAGAT
AAGCGAATCATGCCATCTCCTGGAAATATTTTCTGACGATCTCCAGTATGGTCGATTA
ACTTAAACAAAACCTAAACAATGCCTCTTAAGGTTTTCTTAAGGTTCTTCTGAAAGTGAAAAGCGCACAAAAT
GTACAAATGACGTTGATTGAGAACGATTTCAGTACGTGATTTTCTTATTGTCG
>103795549_2 EC091466.1
GCACGAGGGTTTTACCATTTATCACTTTTTCTTGTATCTTATCATTTATTTTCTTATCAATTTACAGATCTTG
TTAATTATGTGGTTCTGTTCATATACTTTCA
>103795552_2 EC091467.1
TTGCTGAAATTTTGGAAATGTTGCACATAGCAAAACTTTAATCTACATGTTCAGAAGGGGCACCGCATCAAAA
TTTTTTTTTTTTTACAATTTGAAAAGTGATTTTTTCTGGGGCGGGAAATCAAGGACTAGCCGGTCCTTGATGA
ACTCCACAAGTTACAATCCCTGCGCTCTCACACTTTGCATTTCGAAAAATCCTTGTCG
CTATTCAGCGAGAGACCCAAGAT
>103795555_2 EC091468.1
CCTTAAGGCCCCGTTTTACCCCAAGGCTGGTAATGGCCGGCCCCTTTAGCCGCTGGAAACCATGCTACGCATT
CACTGCATGCAGCATTGGTACAACCTGAGCGATGGCGCGATGGAAGATGCTCTGTACGAAATCGCCTCCATGC
GTCTGTTTGCCCGGTTATCCCTGGATAGCGCCTTGCCGGACCGCACCACCATCATGAA
TTTCCGCCACCTGCTGGAGCAGCATCAACTGGCCCGCCAATTGTTCAAGACCATCAATCGCTGGCTGGCCGAA
GCAGGCGTCATGATGACTCAAGGCACCTTGGTCGATGCCACCATCATTGAGGCACCCAGCTCGACCAAGAACA
AAGAGCAGCAACGCGATCCGGAGATGCATCAGACCAAGAAAGGCAATCAGTGGCACTT

TGGCATGAAGGCCCACATTGGTGTCGATGCCAAGAGTGGCCTGACCCACAGCCTGGTCACCACCGCGGCCAAC
GAGCATGACCTCAATCAGCTGGGTAATCTGCTGCATGGAGAGGAGCAATTTGTCTCAGCCGATGCCGGCTACC
AAGGGGCGCCACAGCGCGAGGAGCTGGCCGAGGTGGATGTGGACTGGCTGATCGCCGA
GCGCCCCGGCAAGGTAAGAACCTTGAAACAGCATCCACGCAAGAACAAAACGGCCATCAACATCGAATACATG
AAAGCCAGCATCCGGGCCAGGGTGGAGCACCCATTTCGCATCATCAAGCGACAGTTCGGCTTCGTGAAAGCCA
GATACAAGGGGTTGCTGAAAAACGATAACCAACTGGCGATGTTATTCACGCTGGCCAA
CCTGTTTCGGGCGGACCAATG
>114324354_3 EE743404.1
GCACGAGGTATCATTTGTATTTTTTGATGAAATTTATGTGTATTGTTCATTGACGTTTTAAAATAATCCTTGA
TTATTTTCTTAAAGGGGGAAGATTGTAATGTTTCATATTTCAATATAATTTTATATTATGTTTGTTAAATAGA
TATAAGTATATTTGTATTCAAAAATAAATTAGAACTCATTATTAATGATTGTTTGCCA
GGATAGTCTAACGACTCTAATGGATTGTTTATATTAGAGGACTTATCTAATGATTTTGTTTTGACCAATTGTT
TACTTTTCCCAAACTGTATCATTCTAGTTTGATTATAAATAGTCCTCAACCCCTTTGTAACATCAGTTCTAGT
TAAACTCTTCCTCGCTGAAGTGTTCTTAGTAATAAATATTATAAATATATCGTG
>114324359_3 EE743409.1
GCACGAGGCTAGTCTCGAGTTTTTTTTATTTCTTTTACACTTGTACCACAGATAAACATATAATGTATGTTGC
ACTTTAAAAAAGTACCTATATTCTATACTTTAAGATTTTTTACTTTTTGAAACCAGTCTTATTGAGATTAATT
AGTTATAACATGTACATATATAAAGATTAAAGTTAAGAATTTTTTTCATATTAAGCAA
GATGAATACTATTTTTTAATATGCAATAGATTTTTTTTATATTTACTAAAATGATTAATTTAAATATTTTTTA
CTTAAAATGATATAAAAAAAATAATATCGGCATGTTAAAAATTTGGATTTATCTAGAGTGTATTTTTTTACTT
TTTTACGATTATAACATAGATATTAACAAATATTTGTATATAGAAACGCGATTATTTA
ATGTATAAACTGCTTGTAAAAATAATTAAAATTTTATATG
>114324360_3 EE743410.1
GCACGAGGCTAGTCTCGAGTTTTTTTTTTTTTTTTTTTTTTTTTTTTTTTTTTTTTTTTTTTTTTTTTTTTTT
TTTTTTTTTTTTTTTTTTTTTTTTTTTTTTTTTT
>114324363_3 EE743413.1
AACAAAAAAAAAAAAAAGGGGGCCCCCCCCCCCCCCCCCAAAAAAAATTTTTTCCCCAAAAAAAAAAAAACCCCC
CAAAAAAAAAAAAAAACCCCCGGTTGGAAAAGAGGGGGAGGGGGGGGGGAAAAGGGGGGGGGGGGCCCCCCC
CCCCAAAACCCCGGGGGGCCCCCCCCCCAAAAAAAAAGGGGAAAAAAAAAAATTTTGG
GGCCGTTTCCCCCCCCCCCCCCGGGGGGGGGGGGGGAAACCCCCCCCCCCCCCGGGGGGGGGCCCCCCCG
GGGGGGGGGGACCCCCCCCCCCCCCCACAAAAAAGAGGGGCCCCCCCCCCCCCCCCCCCCCCAAAAAAAAA
GGGGCCCCCCCCCCCCAAAACCCTC
>114324365_3 EE743415.1
GCACGAGGCATAAGCTCGCGTTGATTACGTCTCAGCCCATTCGCCTACACCCACCCTGTTGCCCGCATCGCAC
ACGTATACTAAATTGACGTCAAAATATCTAAGACGATGTAAGTTGTAAATAAATGTGTTTTATTAT
>114324367_3 EE743417.1
GCACGAGGTCGAGTTTTTTTTTTTTTTGTTTTTTATAATAATGCCAATATTTATTTACACTCTTACAATATAC
AGTAGTAAAATAACTAGATAATTAAGACTAATTAGATACAATATAGATGAGCAGACTATAAATTTCTACATAA
TATCTCATGTTGCAGTGGAAAATGTCAAAAATAAATCTTATATACTGTATAGGTTACG
TTACAACCTTAGAAGGCAAGTGGCCTAACTCAAAATTTCGAATAAAATTAGCAACAAAAATATAAAAAATTGT
TTTAGGGTTTGAACCAGTACATCCACAACCATATATATATTATTTTCTTTAACAGTAATAAGAAAAGAAAATG
ATGATCATATTTGATTTAATAAGACCTCGTGCCGAATTCGGCACGAGGTAAAGAGAGA
AAAATTAGTTATTAATGTTTTAAGAAAATAAATCTACAAAGTTTTACTTACCTATTTTGAAGATTTTGAAAGT
GTTATTACATACTTTAAATACTTTAGTTATTTTTTTTAGTTTTAAAAAATACTTTTTTATCGCCAAAGAGGCG
CGATTTGAAATCCCGTTACGTTTTCTAAAGTAATTGTCTTACCTAGGTATATTTCTTC
TTTATAATTATTTCCATTTATACAATATATTTTTAAAAAATACATCTAAATGTACAAATATGTCAATGGTGAC
CATAATAGGGTGATAGTAAATATAAGCGTC
>114324369_3 EE743419.1
GCACGAGGTAAAAACTTGATCTGACACTCCACCGTGTAGTGTATAAATACTTAGTTATAATAAATATCAGTTT
ATATAATATTTTTGTAATGAAAAATAGATGTTACCACTGCTTTTGTAATTTTCTAGCTTGCCTTAGTTGAAGT
AATATTTATATTTTCCGTGTGATCGATGGATAGTTTTATGATAAATGATTTTGCCATT
ACGATGTATGTACCTAGGGTAAGGATGTACATTATAATTGTGTTAATTGTAAGTTTTTAATAAAAAGAAATAA
ATACATTTTTACTTG
>114324379_3 EE743429.1

AAAAAAAAACAATTTTTTTTCCAAAAAAACCCCCCCCCCCAAAAAAAAAAAAAAACCCCCCCCCTTTTAAA
CAAAAGGGCCCCCCAAAAAAAAAAAAAAAACCCCCCCCGAACCCTTTATTTTTTTTTTT
>114324380_3 EE743430.1
AAAAACCCCCGATTTTTTTCCCCCCTTTTCCATTTTTTTACCAAAACACCAAAGGGCGGACCAAAAAGGGGTT
GGCCCAATTTTAAAAGGGGCCAAAAAAATGGGGCAACCCTTTGTAAAGGGCCGTTTTTTTCCCCAAAAAAGGG
GGCTTTGCCCCC
>114324381_3 EE743431.1
GGAAATTACCAAAGTTTTCGAAATTACCAAATTTTATTCTATAAGTTTTTTAAATTAAAATAAAGCTAGGAG
CTGAGTATCGATCAAATAAACTAGTAAAGAGTACCGTTAGTTTAGTCATGGGAGGAATTTTTAGTATAAGTTA
ATTTATCTTTTAAATTATTTATTAAGAATATGTGAATTATAAATTTCACACAAATTTG
GTTGATTTTAGTTTAGTCTATTTTAAAGTTGATGGTAATTTGGGTGGATGTGAAGAAGATTGGTTTATTTATG
TAAAGTTTTAAGCAATAATAAGTAAAAATTCTTATTAATAACACTTTTTTC
>114324405_3 EE743455.1
GCACGAGGAAGGTATGGTAAGGCTGTTTTCATAAAAGCGCTACTAGCTAAAAATTGTCATGAACATTGAAAAA
AAACACAAATTATATTTTTTCCGAAAAGGGATTACGTTTTTATGAAAACAAACCACTAAACTTTTCTGAATTT
CTTATAAACCAATCCGTCCCATTTGGAAAATTTCTTTCATTTTCATTTTTATAAATCC
CACATTAGTTTAGTGCATAATACGATTTGTGTAACCATTCACTGCGGCATATCAACAGCACCCTAAAATAAAT
GTAACAAATATGTAACAACATTTTTAAAATGTACTTTCATGTTTTGTTTCATGTTAAATAAAGTTCTGAACTA
AGT
>114324409_3 EE743459.1
GCACGAGGGAATTATCTCATTTAGTTTTAAAAGGCGATGTTTGCCATTAGTAGTTACAGCGCTTGCCAAGTAA
CAATTATATTTAATACTTTTATAATAATGTAAAGCAAATTCAATTATAAATACTATTGTATACATTTTCTATA
ATCCAATTTAGTTGAAAAACAAAATGGCAATATATTAGAAATACCAAAATGACGTTAC
CCTTACATATATTTATGTATATATGACATATTGTTATTTACACTGACATATAAAGGTTCTGATACCTTTGGAA
ATAAATTTATTTTGTAGGTAATTTTTGATATGTCATAGTAATATTATGCTGTGTATTAATAGATGTGATATTC
CATTTTTGCCTTATTGCGGTGCTCAAATTCAAATTATTTCGATCAATATTTATTATAT
AGTATTGAAAATTATTTACATTTCTTTAAAAAAATATCTTAAATCAAATGATGTATTGACAATAGTAGATAAT
GCTTANN
>114324411_3 EE743461.1
GCACGAGGATAGAAGGCATGCTTTCCCATCAACTGAAGCAGTTCCGTATTGATGGTGAAAAAAGCATCATTCA
AAACCCATCGGAGGCCCAAAGGAAAGAGCATGAGAAGGCCTCCTTTGAGACTTATGAAGTCTATGCCATGGAT
GTACTTATTTCTACTGGAGAGGGTGTTGGAAGGGAAATGGATACAAGGTGTACAATAT
ACAAAAAAACTGATGAAATTTATCAGCTTAAATTGAAAGCATCTAGAATGTTCTACAGTGAGGTCCGCAACAA
GCATGGATCAATGCCTTTCAACCTGCGTAGTTTCGATAAAGAAACTAGTGCAAGACTTGGTGTTGTTGAATGT
GTAAATCATAAACTCATTGAGCCTTTCCAGGTATTGTACGAAAGGCCAGGTGAAATTG
TAGCACAGTTCAAATTTACAGTACTGTTGTTACCAAGTGGTACTCATCGTATCACAGGACTACCATTTGATAA
GAGTCAGTGTAAAACTGAACGCATTATCAAAGATCCTGAACTTAATGCCCTTCTGAACTCATCTGCAAAGTCA
AATAAGAAGAAGAAAAAGAAAACTGGTGCTGAAGAACCAATGGAAGTTGAAACGGCAG
CCTAAAAACAAATTTCACAAAATATCTGTGGGCACTCAATCAGCTTTCTATTTCCTTTCATGTGGTAAACATA
AAGTGTAATA
>114324416_3 EE743466.1
GCACGAGGACACTAGAGTAAGCAACCAACCATTGATTGAGATGGGTGGAGGCTTGATGGGAGTAGAGGTTATA
GTGCGTCGTGCGACTGCCTTAGCCTATTTACTACGGAGATTCCTTCTTCCACACTATAGGCTAGCTATAACCA
CAGTCACTGGTTTTCCTATGAAGGAAGAACAAAACACAAGTTCCAGTGCTAATTATGA
TGTACAGATCATACATTCAGCGGAAGCGCATGCGGGATTGAGGGCCTCACAGACGGCGCAAGAAGCTACTGAA
TCAGTAGTGCTCCGCTGGTGGACGCCGCGCGGCGCGGATGGTGGTGCGCGCGGCTG
>114324421_3 EE743471.1
GCACGAGGTAAACATGATTCTTAGTTCTCGTTTTTAATGTAAGTCGTTTTATGCCTCATTTTATTTGTAAGAA
AAGATGAGAAGAAGGCCTTTGCTATACAACATTCATTAAAAACTCTTTGTGTTGCTAACGTTTTTATAACATC
ATAAGGTTCTATGAAAATTTTATTGTAATCATTTTCCTTATTTACTAAGGAAGTTGAT
CTTGGCTTAGTTTGTTAAAATGATGTCGTCACAGCTATTTATATACTTTATGAATATAAAATAGAAATAAGTG
CTGAGTGTTGGCTCTTTAGAAAATTAGTTAATAAGTTTTTGTATGTGATTAATTGTGGATTCGATTCTCTCTT
GGGGTTTGTATATTAAAATGTTTTTCTGTTTTTCCGGCGTAGTTGGGTAATTTGCGGC

GTTTATTTCCATCACTCATAGACAAGACAGCCTAAGAGTGATATATAACTTCGCTCTAGGCAGAGGCTAGACT
GATGTGTCATAAGAAGTAGACAAATGTAAAATAAAATATTTTTTTATAGGTAGTACACAAATAAATATAAATT
TCTGCTTTAGTAGTATTTCTTCGAGTTTTTTATATTCTATGGATTTCGTTAAGTTTAT
TATTTTATTACCCGTCTACTCTACGCCCAGAAGGAAGATTTTGTTTTTAGCGTGTATGTATTCTATGTATGTC
CGTTCCTTTGTTGTCAAGTAACTGTTAAACCCGTAAATAAA
>114324428_3 EE743478.1
GCACGAGGTGAAATATGATATGTTACATAATTTGTGGATCCCATTAGCAATGTCGTTTCGGTACTAGTGACTC
CGGCAACAAAACGCTGGGTGCGCCGGCGCAGGCTGGGGCTCGTAACCGTGTACTGATTGGTGTAAATTTTTCT
ATGGGTTCGTTATAAGCGGGTTACATTTCTGGCCGAGAGCGATCTACGCGAATTCCTA
TTTTGCAATGAACTGATAACTGGTTACAGGTGCTCTCCCATTTAATTCATTTGTGAATATTTTATGTCTGTAT
TTGTTTTTTTTTGTATCATTTGAAGTGAATTTGCCTCGAGAATTTCTTTACGGCAGTACCACTACTCCTGGGA
ATCTTGTTTCCTTATTTTAGAGTATGTCTATACTAAATATTTAGTCGATTTAGAATAT
TATTAGAATATTAATTGTATTATTTATCTATGCACTTCATCTGGTGTGTTAAATTGAACGTGTAATTTGAAAG
TACCTAATTAATATACAGATTAGGCCGGTCACAAGAATCTACTGGACTAAAGTGTGTCGCGATTATATATATN
ATGTTTGTAATATCTTCAGAATTTCTTTGGTACGTATTCTCTTATTGTGAATTATAAT
AAATAACTCTTTTTACAGCC
>114324432_3 EE743482.1
GCACGAGGCTTGTCATAACTGGTCATAATATTTGTGAGACCCGTACATAGTGTGACGTCACATATTTAGCAAT
TTTACATCTAGAAATTATTGAATTTAAACAGCAATTCTGAAATTATACAAAACATATTATACGTTTTGAAGTA
CAATATCTTGAATTGAATTGATATTTTGATATTTCACACAATGTCAAGAGATGTATGA
CGTAATTTATGAATGGCCCCCAAATTACTAGATTGCAGCTAATATTTTTCTTTTTTTTTTTACTATTGTCTTC
TTCTCTTCAGTGTACTTGTTTGACAGGTACGTACACACATTGTTCCAATGAGGATACATGTTTTATTTTTATT
TTTACACTGTCTGTGATATGTGTTTTTTTATGCATCACCATTCTCAAATATTATTCAT
CAGTTGCATTTTAAATCATATTATAATAATTATTATGAGAACGGTTATAAATGATTCTAAATTATTATTATTA
TTATAAAAAAATATCATTAAATAATAATATTCATATTGTATAAAACGTATAGTAGTTAACATAGGTTAACTCA
AGTTACGTAACTATTGTAAAAAATGATATTTATAATAAGAATCATTTTTAATCGTATA
AAAATCAAGCGGTAAGTAAGCGAAATGACTGTATTTAGTTATCCTCCTGAGGATAAGCTTTATTTAAATAAAA
AAAACGTGACTTTAAGTAAGTAATCGTAAGTGGTGGGCGAATATGGTACTA
>114324439_3 EE743489.1
GCACGAGGACTATCCTTGTTAGCAGTTTAATACAGCATTCAAAAGTACTTAATTTTTCACTTTTGATACTTGA
TGTGACGATAGACGATAGTTGATGTGTTAAGCAAAAATATTTTTTACTCAACCAATTTTTTAAAATTATATAA
TAAATGCTTATTTGCTTCAAAGCAAGTTTTTATAAGCATAAAAACAAAATTGTGTGTT
AACAGTTAAAGTATTAACTGTCAACTTTATTATATATTTATTTTATTGTTATTCTATGTATACACCATCTATG
GTTATTAATTTATAGTTAAGGAAGTAGGCAATAAACATTAACAATACTT
>114324457_3 EE743507.1
GCACGAGGATTAGATCTTTGCATGCTGCAAAGAGATTAGAACATGTGTACTGCACAGAGACTCGCCCATACAA
CCAAGGAGCTAGGCTCACAGCATATGAACTGGTGCATGAGAAAATACCCGCCACTCTTATAGTAGATAGCATG
GTATCTGCACTAATGCATGCCAGAAATATTAGCGCCGTTATTGTTGGAGCTGATAGAG
TAGCAGCAAATGGAGACACAGCAAATAAAATAGGAACCTATCAAATAGCCATAGTTGCTAAATACCATAATGT
GCCTTTCTATGTGGCCGCTCCTCTTACCTCTATAGATATGTCACTTAAATCTGGAGACAGAATAAAAATAGAA
GAGAGACCTGATAGAGAAATGACTCATATTGGAGAACATAGGATTGCAGCACCTGGCA
TCAATTGTTGGAATCCGTCCTTTGATGTGACACCCGCATCTCTCATCACCGGTATTATAACAGAGAAAGGTGT
ATTCGACCCTGACAAGTTATACAAAGCAATCCCTTAAATATTGTCTTCACACTGGATCACACTTATAGAAGGG
TAGACAGATTGAAACTAGTGTTAAGACACTCCGTTTCCCACGCCGCCGCCGAACTTTT
GAGATATTGCGCGGTGAAATTCAACACCGCGCTTTGTGTTGTTACCAGAGCGATGAAA
>114324467_3 EE743517.1
GCACGAGGAGCAAATACCATTATCGTAAATAATTTATAAAAACATTTCTTAAATATTATTTAACTTATTAAAA
CATATAAAAATGGATGACCAAGGAAGGAAAGTTATTGTTTGCGACAATGGAACTGGCTTCGTAAAATGCGGAT
ACGCCGGTAGTAACTTCCCAGCGTTTATTTTCCCTTCGATGGTCGGCAGGCCAATTAT
AAAGGCTGAAAATAAAATAGGTGATATAGATGTTAAGGATTTAATGGTAGGAGATGAGGCATCACAACTTCGG
TCGATGCTTGAAGTTAGCTATCCTATGGAAAATGGAGTGGTAAGAAACTGGGAAGACATGTGTCACGTATGGG
ACTACACATTCGGTCCTAGCAAAATGAACATTGACCCTAAAGAAACTAAAATACTTCT

AACCGAACCACCAATGAATCCCACCAAGAATAGAGAAAAGATGATTGAGGTAATGTTCGAGAAGTACGGTTTC
GATAGTGCGTACATAGCTATCCAAGCAGTCCTCACTTTATACGCGCAGGGTCTCATATCGGGAGTTGTAGTGG
ATTCAGGTGATGGTGTCACTCACATATGTCCCGTTTACGAAGAATTTGCGCTACCACA
TCTGACTAGACGCTTAGATATAGCGGGTAGAGATATTACACGGTATCTTATTAAGCTCCTTCTGCTCAGAGGT
TACGCTTTCAACCACTCGGCAGATTTTGAAACTGTTAGAATGATGAAAGAGAAACTCTGCTATA
>114324481_3 EE743531.1
GCACGAGGTTTATTTTCACAGAAAAATATGCACAGCATCGTATAAAGACATCACTAGTTAAAAAAATCAACAC
TCCTATATACAATGTAAACGTCAAAATATTACTTACGTGTGTTTATTTTTTTAATGATTCTTTAATCATTTTT
ATGTGCGAATGGGCTTCACTAATTAGTTTCAAAAATAAAATAAAAAATAACAAAAAAT
ATATAATATAGAGAGGGGAACAAATGTCTTAATTTATCTTTTTATAGGTTTAATAATAATTATCATTTAGCAG
TTATTGGTAAGTATGTATACATAAATGAGAAGTGTAGATTTGCCTCCTCCTAGACTGCTGTATGCGGGAAAAG
ACAAAACGCTTATAAAAATAATATATTTATATAATACACCAAACAACGCAATCGGCGA
TCCAAAGGACAGATCAAATGTAAATTATTATATTACAACTTCTGACCTTCATTTCTAACAGATGGTTAATATT
ATAATCAAATAGGGGAATTGTGATCAAGATGGCATACTGTGATAAGATGACACATTACGATATTGATGAAACT
ATTAGCGCTAAGTGCGATCTTGTGACGTTTTAGAGAGTGTCCGTAGATGCAGCTAGTG
CTTAAATTCAGTACGGCTGAAAGTGATTGCAGATAGGCAAGTAATTCATGTTTTCGTTATTTCCTCGTGCCGA
ATTCGGCACGAGGGAAAAATTAAAATA
>126034385_2 EL595872.1
GCACGAGGCACGGGTACAAGGCCGGCGAGGCGCTGCTGGGCACGGTGGAGCTGTCGCGGCTGACCAGCTGCGA
CGCCGTGCTGCCGCTGGCCGCGCTGCAGCGCCGCCCGCTGCCCGACCACGTCGACCCCCACCACCTGGAGAGA
CACCTCACCGCTGCTGACTTCCTGAACGC
>126034387_2 EL595874.1
GCACGAGGAAACAATTTATTCTATTTTATCTTATACTTTGTACATTAATATACATTGTTGTAACGTTGTGATT
AAGTGTATGTTTGTTTTCCTTACGGTTTACAGTTGTAAAATAATAGATATTCTAGACAGGATGACGAGCACTA
GAAAGTTCCCTTGGCTGTGGGCCAGCTTCTTCTTGTTTGTTGGTATTGTGGGTTTAGT
TTCTTATGATGTTTCACAAGCCAATGGTAACTTTGCAAAAAGTTCAACTGGCAAACTATTCAAAGATCTTGGC
ATACTAGAGCAAAGTCAACAAGCATGGAGGAAGGCATTATCAAGTTCAGCCCGAGGCTATTTATGGCTGGAGA
CAAATGCACCCATATATTACACTAACACGGTCGAAACATTGAAGCCCTACGGCCAGCT
ATCATTAGATATTGCTACTATTTCAGTCAAGAAAATATGTGCATTGTATGGAAACATGAAAGAGTATGTCGTA
GAAAAAACACCTGTAGTAGTGGAAACTATAGAGCAGTATGCCCCTGGTGTAGTGGATACGGTCCAAAGTTACG
CAGCAAGTGGCTACAGTTTTGTCAAGAAATATTCTAATGAATATTATCAGATAACTGT
TAATTATCTGTNCACGAAAGTTTTTGTAGGAGAATGGGCACCAGAGATCCTACAGAACAAACACAAGTAGCGT
TAAACGCAACTAAGTTCACATGAGCACCTAC
>126034390_2 EL595877.1
GCACAGGCGAACTCATTCATACCGGCTGTATAACTTTTAACATTAGCCTATAATAATAACGCGCTATTTTTTA
AAACGCACGTTCTATTTTCAAAATCATTACCGCGGTAAAATCGATGCAATTGTCAAATTAAATGTGTCAAATT
TTGTGAATTTAGTTATGTGTAAATCGCGTGTATTTATATATTATTTTATTTGAATGTT
TATATTTATATTTATTTATATAATATAATCGTGTCTTCCGTTAAGTGATAATATAGACTAATTGAGTAATATT
TTGTACACTATGGAAAAGGGTGGAGGCTCTAGACTTCTGCAGATGCAGGCTCGTTTCCCGCGGAAACAGCTAT
CAGAAGCGCGAGCTCA
>126034392_2 EL595879.1
CGGCACGAGGCTTTTTACACAGAGGCTTTGACGGCGGTCTTTGTTTGCTACAAGATCAGAACGAGAGCCGGCC
CGACGCGTGCCTCCTTCTGTGGCGTCTGATATCGATTATTGTTATAGATTCTTGTGTCACCGCGGTGCCTGCC
TCTTGAGAGCGCTGATTGAAATGTCAGACCTTCTTGGATTGGTGTTGTGTTTGGGCAG
TATTTCATTCGTTGTATCGCTTTGTGGTTGAAAATCGATTTTTTTCTATCTGTTTAATATATATTTTTTTATC
TGGATATTACAAAACTAATAACGTAAATTAATACGATTTAAATAAATATAAAACTCGACTTTTATGCTAATAG
CTACGTTACAATTTATAGGATTATTTGTCCAAAGTGATCACAACTCTAGCAACAATTC
TCGTTGACAATGCAATTTTAGTTAATTCTATAGCGCGGCGCGTGGCGCGGGCGCGTCCCGAGCTCTCTGTCGG
ACGAATATTAAACTAATTATGAACAAACCGCCGGCCGCGCAAATTGACCGTTGAACTCCATTAGCCCTGTTGT
CCAACTAGGTATAGGTACTCCGCCGTGGGGATGTCAGGTGAGCCACGTCAAACTGTGG
CTTACAGTGTTCCAAGTTATTGTCATCCCGCGTA
>126034394_2 EL595881.1

GCACGAGGCGACAATGTCAACAACGACGCTTGTTTTCAAGTTTTATTAAATAATCTAAATATTCAATTCAAAA
TATTCTATAAAATTTAGTGTTTCTTATCATTCGTGTTTCTCTAATAAATACGTGACACTGGCAGATAATTTTG
CGTCATTCATTTTTCCTGTCATCGCAAAATCAGGCCATTTAAAAAAATATATCTAAAT
ATTATATTTGTTGTTTTGTTCTATATTTGAAATTAATTTATTTATATACCTGTGTTGTGAAATTAGTACTTAA
TTTTGTGTATATAGAGAATAGACAATTATACTTAGTGAAAATGACTGGGAGAGGAAAAAAGGCGGCGGCGCCA
AAGAAAAAAGCAAATGGATATAAAATGCCGGCTCCTATTCCCTTAGGAGAAATAATAA
CTGATTCTATAGCTAAGAAGAAATGGCGTATCGGGCCTTCCATAGGAGTTGGTGGTTTCGGTGTAATCTATTC
AGCGTGTGAACACAATAGCTCTCCTAAAAAAACTTCTAATTATCCATATGTTGTAAAAATTGAACCACATGAG
AATGGACCATTGTTTGTGGAGAAACATTTTTATATCAGAAATGCAAATAAAAACGATA
TTGCGGATTTCATGAAATCAAAAAAACTAACAACATTTGGTATGCCATTATACTTTGGTAATGGAACACATGA
GTATAAAGGTG
>126034395_2 EL595882.1
GCACGAGGGTCGCGTTGCCCCTCACAGCATATGTTTCAGACAATTTTGGTCGTCGCATAGCATTTATAGCCAC
CGCAGTGGCACCTGCCTTCGTTGGCATCGGCCGCTCCTTCACACAAAACTACATCACATATGTGATATTAGAG
TTTATAGAAGCTGTCGTCGGCGCCGGGGTGTATAGCTCAGGATTTATTCTCGCTTTGG
AAATGATGGGTCTTAATCGTAGAGTGCTTGGTGGTAATATAATTTCTTGTACATTCGCTGTCGGACAGGCTTT
GGTAGCGCTGATCGCTTGGGCCATACCCGAATGGAGGACACTAACTAGAGTGATATACGCACCGTCACTCATC
TTTATAGTCTATTACTTTTTCATAGAAGAGAGCGTACGATGGCTCCTCAGTAAAGGAG
ATAAGAAAGAAGCCGCCAGAATTATTTTTAAAGCAGCAGCTGTCAATAAAAAGAAATTATCCCCAGAAACAAT
TAGGCAGTTGACGGAAGAGGAAAATACGGCGGAAAATTCTAAAGCTTCTCAACCTGTAGATGAGAAGGAGCAA
TCCCTTGCACTCCAAGTGCTTAAGTCCAGGGTGATAATGTCCAGATTGGTGATTTGTT
CTTTCTGGTGGATCACAGTGACATTTATATATTATGGACTGTCGATTAATTCGGTGTCTCTCGCTGGTAATAG
TTATGTGAACTATATATTGACAGCGTT
>126034396_2 EL595883.1
GCACGAGGGCTGAATCATGGAATACATGTGTGTTTAAAACAGGTATTTTAGCTCAATTTGTTGTCTTTAGGTT
CATCGGATTCTACATTATCATATATATTATTGTTATAATCGTTGTGCGTTGTTCTATTATTACACTTTTTAAA
ATATTCTTGCAATTTTAATCCGTCTTCTAAATTATGACGACTGATGTATCCTTTTATT
TCAGAAAAATCTTTTAATATTTCAAACATATTAAAATTGTATTATTTAGTTTTAGTTCTAGTATATTAGCAGA
CTTAAGGACGCTACGCAAATTTTGTGTACTGTTAAAACTTAATCAACGAAAACGCATTCTTCTTCGTATTTAA
CTTTCTATGTACTCGCTATTCTGTGGTAATATAAATTATTATTAATAATACCTATGTT
ATAGACCCGTAGTTCTAATAATTGACGCGTAGTTTGAATGTCATTCTGTATATTTAAATAAAATTTACAGGAA
TGGGTTCTCGTTGATCGAAAAATATTTCTCAGTTCTACCAATTTGTAATAGAC
>126034403_2 EL595890.1
GCACGAGGACGAGGCCGCGCGCGCCGTGTGCCAGCTGCGCCACTTCACGCAGTGGCTCAAGCGCACCGTCGAC
AAGCAGTGACGAAATCTCAGTCAATGTCAACTCACAATACCTAGCGTTGGCGGAATTAGTATCTTGTACTTGA
ATAATATTTAATATATAAAATAATATCCAAAGAGTAACTATGTGAAAT
>126034410_2 EL595897.1
GCACGAGGTGGACACATTTCTCATGATGATGAGATCGAGAATGATGATCAAGATTTTTTTGATCACATTAAAG
TAACAACTTATAATCCTACTAAGCTTGCAAGTAGACAGAAAGTCAACATAAAGCCAAATAACTCAAGATTTTC
TCCATCAGCAGATATAAAGAAAGCTGTACAGAATGTGCCAGTAAATAAATATAATATA
GAAAGTAATTTTAATCCATCAAAAAATAATCCAAATATATATCAAGCTACTTCGACTGAATATAAATCACTCT
TAGATATAGAACCTCTTCTTGAAGACGACATCAGTGATATAGGTTCATTCAGAGAATTAATGAAAAATACTCA
AGATTCTTTTGATGTACAAGTCATAAAATCGAATAAAACTGAAGAAACAGATGATAGT
AAGCCAGATGCAACTTCCATGGATACTGACAATGTACCTAATGATACTGCCGTAATGGCTATGTCCGTTGAGT
CCTCACCGCAAAGGCATAATATTACACCTACTTCTACTTCTACAACAGAAAATATTAGTCATAATAATGAAAC
GCATGAAGACGATACTCATGAAGGTACAGATGGAGAAGAAGGAGAAGAAGTTGAAGAG
TATGTTGAATACTATGAGGATGATGAAGAAGCTGAAGTAAAACATACTTCTACTTCTAAACCCACCAATACCA
>126034411_2 EL595898.1
GCACGAGGTTTCGTCGTAGTTTGTATTTTAGGCAAAATTTCTGGGTTAGACAATGGGTTAGCTTTAACACCAC
CAATGGGATGGCTTGCTTGGGAGAGGTTCAGGTGTAACACCGATTGCAAGAATGATCCCGACAACTGTATAAG
CGACCGGCTATTCCGTACGATGGCCGACATCCTCGTCTCAGAAGGCTATGCGGCGGCG
GGTTACGAATACATCAACGTGGATGACTGCTGGCCAGAAAGAGAACGCGATCCCAGAGGGAAGCTGGTGCCAG
ACCGAGAACGGTTTCCGTACGGCATGAAGAGCTTGTCTGATTATGTACACAGTAAAGGATTGAAGTTTGGCAT
ATACGAGGACTACGGCAACTTTACCTGCGCTGGATACCCCGGCGTGGTCGGCCATTTG

GGCGGAGATGCTGCGACATTCGCGTCTTGGGGTGTGGACTACGTGAAGCTTGACGGGTGCTACGCTCTTCCCG
GGGACATGGATCATGGGTACCCGGAATTCGGTCGCCAGTTGAATTTGACTGGACGTCAGATGGTCTACTCTTG
CAGTTGGCCCGTATACCAGATTTATGCTGGTCTCCAGCCTAACTTCTCTTCGATCATC
GAACACTGCAACTTGTGGCGTAATTTCGACGACATACAGGACTCGTGGGCATCCGTCGAGTCTATCATAGACT
ACTACGGAAACCATCAGGACGTCATCGTGCCCCACGCTGGGCCTGGGCACTGGAATGACCCGG
>126034413_2 EL595900.1
ATAATATAATGATTCTAAATTATTTTTAATTATAGCATAGCATAAAGCAGACATTAAAGTAATATATGTATT
TTTGTTTCTTTTATTTATTTTTATGTAGAGTTATATTTGTTTTTGGATTTTAAATAATAAAAAAATATTTATA
GATTAATAACGAAAAATAAATATGCCCTATGTATATTTTAATATCAATATCATGG
>126034419_2 EL595906.1
GCACGAGGCGCGTGCGTGCTACGCCGCCGGACCATGATAACCGGGTGAAGCCACCATGCTCGTCCCACCTGAG
ATGATGGCGGCTCAATCCAAGCTAGTCTACCAGATGAACAAGTACTACAACGAGCGGGTCGCGAACCGCAAGG
CTCAAATCGCGAAAACCATCCACGAAGTATGCAGGATAGTACAGGACGTGCTCAAGGA
GGTGGAGCTGCAGGAGCCGCGGTTTATATCCTCATTGACCGACTACAATGGCCGCTTCGACGGCCTCGAGGTT
GTCTCGCCTCATGAGTTCGAAATAATCATATACCTTAATCAAATGGGAGTTTTGAATTTCGTCGACGACGGAT
CTTTACCTGGCTGCGCTGTCTTGAAGCTCAGCGATGGAAGGAAGCGTTCCATGTCCCT
GTGGGTCGAGTTTATCACTGCCTCAGGCTATTTATCTGCAAGGAAAATCAGATCGCGATTCCAGACTTTGGTG
GCTCAGGCCTGCGACAAGTGCTCCTATAGAGATTGTGTTAAGATGATTGCAGAAACTACTGAAGTGAAGTTGC
GTATACGCGAAAGATATATAGTGCAAATAACGCCAGCTTTTAAATGTGCCGGCTTGTG
GCCCAGGTCTGCGGCACACTGGCCCTTACCGACCATACCGTGGCCGCATCCTAACATT
>126034421_2 EL595908.1
GCACGAGGTTAATTTCTAATAATTGAAACCACTAAATCATGTCATACTTTTTAAACATGTCACATTTCATTTG
ATATTGATAAATAAAATCATATATAAAAATCCTACAAAATGTGATAAATAAAAAATAGTCAGTATTTGTTACT
TTATATTAAAGATCTAAACTCTCATTCAGTACAAAATGAAATTATATTAAAATAATAA
CTTTTTGATGTCAAACAAATATCTGAGGAAGAAAAAGTAATAAATTCGAATTCAAAATTTTTAAAGCATCTTC
TTTAATAAATAGAATTAAATTGTAAATTAGCATTTTTTTTTATTTTTGTGACATTTTTCATATTATCCTAACC
TAACAAAATTTTCAGCTCGAGAGCTAACGAAAAATTATACTTAATT
>126034428_2 EL595915.1
GCACGAGGCACCAACTATATTCAGTTTGTTTCTTTCAAAAATGGCAGATTCTTGGTCAATGGGTCCTGGTACT
TTAGATGTGCCTTTGGCGCTATATGCGAAAAATAGAGAAAGGCTTGCGGAAAATCTACAAAGCGGTCAAATCG
TAGTTCTTCAAGGTGGAGAAAGTATCAATCATTATGATACTGATGTGGAATATGTTTT
TAGACAGGAAGCATACTTTACTTGGATATGTGGTGTCCGAGAGCCAGGTTGCTATTTTGCTTTAGATATAAAA
ACAAAAGAATCCCATCTGTTTGTGCCGAGACTCCCACAAGAATATGAAGTTTGGATGGGAAAACTGCTCTCAT
GTGGAGAATTTCAGAAAATATATGGAGTACATAATGTACATTATGTTGATGAGATTGC
TGATGTTTTGAAATCTTTAAATCCTGAGAAATTGTTGACTCTTAGTGGACCAAACACTGATAGTGGTTTAACT
GCTAAGGAAGCTGTATTTTCTGGCATTGATGAATTCGAAGTGGATAATGAAACATTGTTTCCAATCATTGCAG
ATCTTCGAGTGTTTAAAACTTCTGAAGAAATAGAAGTGATGCGGTATGTTTGCAAAGT
ATCCTCCGATGCTCACAAACAGGTAATGCTGTACA
>126034431_2 EL595918.1
ATATATATATTTATTTTAATAAAAAAATACAATAGCATAATAATACTTTATTCCATTACATAATATTGTGCGC
ATCTACGTTAATTATTGTCGTCATTAAGACGAACACTATTTACTAATTAAAAATATGAAATAATTTTACATAT
ATATTTTTTGTGATTTAATGTCAAATCAACTACCTATGCCTTTTTTAACTATAATTTT
TTCACCACCGAAAAATCAACTTCAAGTATAGCCTCAATTAGTGTTGTACCTAGATTATAAAGTCAAACAAAAA
AGGATAAATAATTGTAATAAATTAAAAAGAATTGAAAGCTGAAATTTGAAGCTCATAAATGAGCTTCTCTATG
AGAAAGAATTTGAAGTAATTTGTTACTGAGTTTCTGAAAACTTTTCTCCTTCCAAATA
AAATATCTCTTCAAGTTATATAATGTACGATACATTTGATCAAAATGATGGACGTTAAGTCGATATCATTTAT
TTAGTTATTAATCGGTATTAATTTGACAGTGTAATAGCAATATATATTTAATATACATATGAGTTAGGGTGCT
TGATTTTTGTTTTTTAAGTAATTCTAAAGATTTCAGTAAAAACTCTATCATATAAATA
TATGGATAATAAAAGCAAAAAATTAGGCCATTCACATTTTTTAT
>126034434_2 EL595921.1
ATTTTTGGTTTCTTGCTTTATCTCGCATATAGCAAAGAAGACAGATGGAACCAAGTGTTAAGACCATCCGTTT
TCACCCAATTTTCCAGACATTGCGTGTTAAGTGGCGGAATTCAACGCCGCGTATTTTCATGTTACCAGAACGA
GGAAAAAAAATTATATTACCGTTGCGCCACCGCTGTGTGTTAATCGAACCCACAACCA

CCGGGAGGGAGCAAAGAATGTATAAACTACCACGATGACGTCACTAATAACTTTAAAAAAATCGTGTATTTTA
ACAAGTTATGATTTACAAAATTATAGCATAAACGTGACCCAATTAAACATATTATAATAAATAAATATTTAAA
TGACGTTATCAGCTCATGTAAATTGGATATATTAGTAGTAGAAAACTAAATACTTTTT
TATATTATAACATTAAACACCACAATATGGCAGTATGAAACAGCCTTAGCAATATACGCACAGTAATATATCT
TTGAAAG
>126034435_2 EL595922.1
GCACGAGGGAGATAGCAGCCAAGCCAGTAGAGAGCTGTCGAGCGGCGTGTTGCGTAACAATTGTTGTTGCTCA
GTGAGAGTGAAAGTGGCGAGACTGTCTCCATATTAATGAACGCGAATTGGGTGCGTCGCGCTGGCGTCTCCGG
AATCCGTTCTGGGTTAAAGTGTCAAGATACCACTTGCTTTCTTACTTCCATTACGTAA
TTTTTAAATAGCAGTTTTGAGTTCAAACTTATTTTGCATAATAACTTTGCTATAACGACAACAACTCCTTGTT
GTACTTACTTATTAAATGTCGATACAGTTTATTGTTTCGTTTTTTGTGGGCATTATTAGAAATAATTATAAGA
CAGTCATTCTTAAATCAGCTTACTATATAAATCCAAATTATAATGAAATCAAATTTCA
TAAATACAATATTGTAAAAACGCTGTTGCATTATAACGTTTTTTGTTATTTTTCTTAGTGAATGCGCTCTAGT
AACATTTTATTTATTTCATTATATGTATGCAGAATTATTGTCAATACATTTTTTTTATGTATATTGTTTACAT
AAATAAGAATAAGGCCAGTCGTGGCGCTGTGGCACAAGCATGTGACTGTAAACGGGAC
AGTTTATTGGATTCGATTCCCACTCGAAGAAATGAAATATTGAATATTTTCATTTTTTTAGTCGAGATCGAG
>126034437_2 EL595924.1
GCACGAGGGGATGTTGGTGATGATTTTTTTTTAATTTCGCGGCCAGTCCAAAAATTTGTTTCGAACGACTGCC
GCCATTATTTTAATTAGTATTATTATTATTATATAAATGTTATTATTTAATACTGTGACTTATCGCTAAGGTC
AAATAATATTTAAAATG
>126034443_2 EL595930.1
GCACGAGGCGAACTAAATGTTGTTCAGGTGGAGACCATGTGCCTCCAAGAGTCTATCAAAATCCCTGTGGCCA
TCCTCAAAGTTGGTGAAACGAGACAAGCACGTCTTGACATTGAGTTTCCTGATGCACCAGTCACTTTTACACT
CATACAGGGCTCAGGGCCAGTGCATTTAATTGGGCAACATTTACTCGGTGCACTGGTT
GAGGAGTTTGAGGACATGGAGGAGATGGAGGAAGAAATGCTGGATGAGGAGGAAGGAGAAGATTGCCAACTGA
AGGATGAGGATGATGAGGAGGGCGAGCCGAAGGGCAAGAAGGCGAAAATTTCAAACAATGCTAAAGGCAAGGC
GCCGTCGCCCAAGAAGAACGCTAAAAAGTGAACCTTTAAAACAGCCGGCATCGCACCC
TGCCACGCGCCTGCCCTCGCTCGCCTCAACACACGCACCAGAAAGACTGATCCGCTCTGGTCTAACAGCCGCG
CAGATGGCGGGGGCGGGGTCAGGGCGCCGTAGGCGGGGGGCGGAGCCCTAGTTTACGCATAGTGTGGTAATTC
TCTAGGTTAGCGTTGTATCTCGTGCGTGCGAGCGTGGGTGTGTGCGTGTACCGCCAGG
TACACTCGTTGTATATCGATCACGATTGAGTGTGATTTATAAACGTTTTTTCTTGAACTTATCATTTTTTTTC
ATTCCT
>126034444_2 EL595931.1
GCACGAGGCTTGATGTCCCACTTCCTCCTCCACTTGCAACTGAAGACATTCCTGAACCATTAGAGGAACCAGC
AAATGCTAATAGTAAAGAAAGTGAATTGGAGAATATTGTCCTAAGTGACAATTCTTCAAACTTTTTAAAATCT
TCTTCAATACAAACAACTGCAGGAACATGGCCTGAAAGCTTAGAGAGCAGCTGCAAAT
AAAGAT
>126034448_2 EL595935.1
CGGACGAGGATTTATTTATAATTATTATAATAATTATAGATTTTTTATTACTTAGTGTTATATTGATATATAC
GTGGTCGGTGTTAGATATTTTATTAATAGCATGTGAAGTGCGCGCTAAACTAACAGTATCCTCTTATTTATAC
GAGTGACGCTGACGTTACAAAAATATGTTTTCATTAAAATTACCATTTTCTTTTTCGC
ATCGATAACCCTCTGACCGCTTGTTTACATAAATTTTGAAATTCACGCACTCATTGAATTTGTGCCAGTGTGA
AAAAAAATAGCAATGTCGTTATGTGATTAATATGAAGTGGACAGTGTTTTTTATTTTGCTCTCATGTTATTTA
GTAAAAGGGGATTATGATGTTAAGTGTAGTTATAATTCGAAAACTGGTTTGACTTTCG
AGCCTTCAAACGATGGAGTTGAGGGACGTTATAAAGTGGAATGGTGCACTACAGCGGATAGAAATGTTACATC
TTGGGAGCTAGTATTACGATACGATGATCGCGCCAAAGAGAGCTGCCTTCGATACCAATTAGGTCCACTAAGC
TCGGTGCACTCGCGCTTCCATCAACCAGTCAATCTTAATTTATCCTGTACTAATGTAT
GCTTCACAACTAACTTAGATCTCATATTTAATG
>126034454_2 EL595941.1
AAATAATGACAATATGCTTAGCAATTGCTGCAATCATTGTACTTATAGTGGTTCATTAAAATAGTTTTATTAT
TTAAAAACTATTATTGTATATTATTAATTTATTATTATTATATTGGATAGTTTTTCATTATTATTTTTTATTT
CTTAAAAACATCGATTTTGTGATTATTTTGTGGATTTCTTTTTTATTTTTTCACAAAA

AATGTCCACAAAGTAATCATAAAAAAATCATATTAAATTTTAAGCAGCTATATATATATAAAAAATTACAGTG
ATGATATTCTATACAGTGATGCATGTCCATTGATTATTAATTTAAAGTTATTAAGTTAATTATTATTATTTTT
AGTATTTAATTATTATTTTGTACGTTTTTTTGTCTATCGTGAATTATTTATTTGTAAG
TGTAATTAAATGTTGACAATATATGCTT
>126034458_2 EL595945.1
GCACGAGGCTGAAACCGGCTGTCTTTTATTTGAGTGCATGTTTCGTTAAAATTTAAGATTATACTCAAGTGAA
TATATAAATGCTATAAATTTGTGTTTGAATTGTAATTTGATAAGACCTAAACAAGCAGTATGGGTATTATAGG
TTTATCAAAGTTAATAGCTGATATAGCACCGCAAGCAGTAAAAGAAATGGAAATCAAA
AACTATTTCGGAAGAAAGGTGGCAATTGATGCATCCATGAGCTTGTATCAGTTTCTAATAGCCGTAAGAAGTG
AAGGTGCTCAGTTAACATCAGTTGATGGTGAAACTACATCACATTTAATGGGAACTTTCTATAGAACAATTCG
TTTGGTAGACAATGGTATCAAACCAGTTTACGTATTCGATGGTAAACCACCAGACATG
AAATCTCATCAATTAAACAAAAGGGCTGAAAGGAGAGAGGAAGCTGAAAAGGAACTTCAAAAGGCTACAGAAG
CTGGTGATAAAGCTTCAATAGAGAAGTTTAACCGACGTCTAGTTAAAGTGACAAAGCAACATGGTGAGGAAGC
AAGACAATTGCTAAAATTAATGGGAGTTCCTGTAGTTGAAGCTCCATGTGAAGCAGAG
GCACAGTGTGCAGCTTTAGTGAAAGCTGGTAAAGTTTATGCAGTGGCCACAGAAGACATGGACGCCTTGACAT
T
>126034459_2 EL595946.1
GCACGAGGCATCGTTATTAGTTTGTATGATAAATTAGAAATTAATAGTTCCTATTCCTTTGAAATAGATTTTT
TTTATGAAATAACTAATACAATTTACTAATATTGATTTAATTCTTGTGTGTATGTACCCCTATTGCTTTAATA
TAAACAGTATACAATTATAGAAGAATATTTTGGTTAATACTATAGGCGTCAGCATGCC
TAAAGAGAAATCTGTAATTGTGGCCTATATACTGTGGTTATTCGGTGGTTTATTCGGCGTGCATCATTTTTAC
TTGCGCCGTGATAGACATGCCTTCGTCTGGTGGACAACCCTTGGGGGTTTCGGTATCGGGTGGCTGGGTGAAG
TGTTGCGGATACCTCGGTACGTTAGAGATGCCAACGAAGATCCTCAATACATACAAGA
ATTGGTAAAAAGGATGCTCCAAAACAAGAAGCCTCCATTCTCCATCAATCGTTTCTCTGGCATGATGGTGGTT
GCCTACTCATGGGGGCAGATGATGATGCTGGCCATCCCTCCTGATGACTTCTGGGGCATTAATTTTAGATACT
TGGATCTCCTCGTGCCTTTTACTGTCGCCCTCGGTGTGTGGACAGTGGGTAACATAGG
GCGAGAGCGTGGCGGGTTACGCTGGGCGCTAGCTGCCGCATACGTGGCCTACCCCGCTCGCTACTACCTCTAC
GATGAAAGCCTATGGCTCAACGCTGTCGTACTTGCCTCAGCCCTCGCGTTCGATTCCTTCTCTAAGGA
>126034463_2 EL595950.1
GCACGAGGGGAAAATGGGCACCAGATGATTTTACGTCAATTTAACGCAAAAACCGATGTATCGGTTCAATAAA
ATAACATAAAAGTGTAAAAGAAATTTTATATAAAGTGACTGTGTGAAAAATATAACTTAACTAATTATATATA
ACAATGCATTCATGTCGGCAATTATTATACAGCCCAGCACGTTGGAAATGCGCTTTGC
CAATAAATTTAAAGACTTCAGTTATTACTATTAAAACAGAACAGTGTCCAGTCCAAGAATGTCGTTTTTATTC
AGCGAGAAAGGGTTTCTTTTCATCCATAATAGAAAACATAAAAGAAGACATTGCAAAAAATAAAGAAATGAAA
GATAATATTAAAAAGTTTAGAGAAGAGGCCCAAAAACTAGAAAACTCTGATGCTTTAC
AAGCAGCTAGGAAAAAGTTTCATGCGGTAGAATCTGAAGCATCTAAAGGATCAGAAGTATTAAAAGAAACTCT
GGAAGGTATCAAAGGCAAAGTGGAGCATGTGTTTGAAGAAGCAAGTAAAACAGAAATAGCTAAAAAAGCAGGC
AAAATAACAGAGGATCTATCANAAACAGCTAAAGGTGCAGCAGAGTCATTGGCGGGTA
AAAGTCAAGCACTGGGACAAACATCAGCTTTTAAGACTATTTCACAGGCTACAGAAGTAGTTA
>126034467_2 EL595954.1
ATTTCAAAAGCTTTTGTACAAACATACATATAGTTTAATATTGTATGATCATCTTAAGTACCTTTGCCGATGC
CATATTAATTGTTTATACAAATATAGTAAAGCAACATTGAGTACAGAAACTATTAGATGGGTGTCCGTTTTTT
GTTTCTGAGATTTTCAAAATACACATACATACATACATATATAAATATAATAGGTTAG
TCTGTACATAAACCTGTGTATGTAAAAGTAGTATTAGTGAATGCCATTATAGCGTCGTTTCAGTTTAGCTTAG
AAAATTATGTTACGTTCAAATTGTCAATTTTCCGTTTGAATATTCTTTTTCACTTCTTAATGTATATATTGAT
TATAATGTTGTTAATGCTGTGTAGAGGATGGCTCTTTCTTTATATTATTTTTTTTTTA
TATTTCGAAAGATATATTAAATAAAAAGCACACATAATTTTGTTAATGACCATTTATAAAATAAAAACTAAAG
>126034468_2 EL595955.1
GCACGAGGTTACATTTTTACGTTTTAACAGGCTGAGCGCGGGTACATTACATTATTAATTGAATAATATTTCA
CTTTAGCCCAGCATATTCCCATTTAAGTGTAAATATACTGTACAGAAATGGGCGATGAAATGGACCCGTGTGA
ATGCCTGTGGAACCACGAGCTGGCGATGCGGAGGCTCATCTCTCTTTTACGTCAAGGC
CAATCATATTGTACGGACAGTGAGTGTCTCGATGAGCTGCCGGGACTTCCCCCGGCAAATTCGGCGGGTAACA
GCTTCCTTATGATGTTCTTGATGATGGCGCTCGCTGTGGCGATGTACATGATGCGTCCGAGACGCAATCAAAT
ACAGGATTCAAAGCCAGTGCCTAACTCTCAGGACCGTGATGGCGCCCCTCCAACGCCG

CCAAGAATTTAATGAAGAAGAAAGTTCTGGAAGGTTCAACAAAGAAAACTGATTAAATATGTAAAAAACGATA
AATAACATCCTTAAGTATAGCGTTATAAGATATATATAAGGAATTATATGAATATGTATAATTGAACTTCAGT
GACTTAGTGTTGCTTGAACGCTGGTATATGTACCTAGAAAAACAATCATTCCAATACT
TCCACAACTTATGTATTATGTTAAAAAAGAAGGTAAAATTTTATTGAATTTATATTTTGGTGCTATAATTTAT
ATAAAAA
>126034469_2 EL595956.1
CTGAATCTTCCAAAAAAGAAACCTAAGTAGGTTAAGTTTCAATGAATAAGTTAATAGGCAGATAAAAAGTATT
GTACAATAAGCTATTTTTACTTTTTTATGTTAATTTTAAGATTTTATTTATTCTTTATATACTCTGTGGCGGT
ACCTGCATGGAAGTGACCAATGATTCTTTTTGTTGGGCTGAGATGCCCTTAGCTTTAG
TTGTTGATTTACATTTATCATTCCAATTTTGTGATTTCGTTTTACCTTTTTTGTCTTGATTTTTTTTCACAAG
CACCCTTTTTTTTTGTTTCAGTTTTAATTTCTTCTATATAAGCTTATATTTAAGATTTTTGATACATTTACAAG
AAGACAGAATTTAATTTTAAAATCTTTATATTTAATAGTAAATACTAAAAGTAATCTC
ATTTATCAAAATTTTTACAATTTTTATTAGAAAGTGGGGAAAACAGAATTATTTAAGGTTTATATCATTTTGA
AAAAAAATATATATCTAGTACTTAACTCAAATATATATTTGAAG
>126034470_2 EL595957.1
GCACGAGGGGAAAGATGTGTTAGCGCTGAAAGATCTAAGGACTTACAATCCTTTGGCGAAACCGTTGTTAAGT
GGATGGGAGAGACAAATAGTAAGATGGAAATGCAACAAAGAGGTGATGTACAAAGCACCCTGCGGCAGACGGT
TGAGGAACATGAAGGAGCTCCACAAGTATTTGAGAGATATACAATCGGATATGGCGGT
GGACCTATTCGATTTTTCCCCCGTCACCCATTGTTTAGCCGAATTCGTATTGAATAACTGTTTTGTAGGGAAA
AAGGTAAATATCGATTTTAATTTTGGTTGGAAAAAGGTAATATCGGTTTTAGTTTGCACAAAGAAGAAGATAT
ATGACGATGCATTTGTTTGATTTTCCCCCTATGATATGTTGTTTGGCTGAATTTGTAT
TTAATTACTGTTGTGTAGGGAAAAGGTAAATATCGATTTTTGCACAGTTGTTTTTTGTCGAAAAATAGTGAAT
ATAGTATTTTTTGTTCAATCGATTTTAATTTTGATTCTACAATTTTGGTTCAAAAAAATTTTTTTTTGGTGTA
AGAACATTCTGGTTTTAAAATAATTTTACAATATTGCGTCCGTCTTGCGATTTTACTT
AAGAC
>126034471_2 EL595958.1
TTAGTTTTATACATGTTTGGGAATCCGACGTTTCGAACATTTTACAGTAGTCCGTGGTCACATGTGGTGGTAG
ACTGAGGTGGCATTCGTCTGTCAGTTGACATAATTATTATATCAAACTACCTCCAACGATTTCTTAATTGCCT
CGCGTACTGCCCTGGATTCCTGCAGAATGACCTGATAATATCTTGCCCTCTGGTGTGG
TGGGTTTGAGACTTCAATGATTTTATGAGAAGGTCCCAGGTGGTAGCAAGCTTCGAGCCGTACTTTCTATTGA
AATTAGGATGTTTTGTATTTTAATAGCCTCGGGATCATTCTAGGAATATATCTGTTTTCCTTAACGAGAATTC
CGGGTTTATCAAACCTGATGTATGCTGGGTTTATGTCCACGTTTG
>126034474_2 EL595961.1
GCACGAGGGTTTGATCGTGATGTTATCACTAAAATAACGCGTCACCGGCCTGTAATTAATCATAATAAATATG
TTAACTAATAGTAATATGTGACAAATATTTTCTGTGAAAATAAGTGTAATTTGTGTGAGAAATGGCAGGAAAA
ACTACGGAATGGCGACCGGGGCCTACTGTTTGCCGATGTTGTTTAAGCGAAGGTTGCT
ACAAAGACATTTCTACTGAATATTTTTGGATGGGGAAGAGAGAAGTTTATGCAGATATGTTGTCCGAAACGTT
TGATTTAAGTATATCGTATTCCCAATCTGGAGGGCCTAACAGCCATAGTCGTCTTATATGTGAGCCTTGTATA
TCAAGGTTGCGAGATGCGGCAGATTTTAAGAGACAAGTGCAAGAGTGTGAACGCACAT
TCACAACTTACTTGGATCCTGGGAACTCAGTTGCTGATATGGAAGTGAGCTTAGAAGTGGACAGAACTGACAG
TGATATCAAAGTGGAATGTGTGAAGGAAGAGAAGGGAGTGTCTGATGATGATGATTTTGATGATCGGGCCGAT
TTTGGTGATGATGATGATGATTATGATGATTTGGATGACCAACCCTTAACAAAGTTAG
CAAGTAAAATACCCAAAAAGGAATCTGTAGAAATATTGGAACTTTTAAATAACGACAAAACAAC
>126034475_2 EL595962.1
GCACGAGGGCGGTGCGGCGCGCCTGGAGGTGCGGCTGGGCTCTAGGATATTGCCGTGGCGTAGCGCCGCGCCA
CTACTGCTTTCTCTGCTTGCGTACCGTCGTCCGTTGCCCAGTCGTATTCTCGAAGACTTAGAGAAAGGTGATA
AAGAAGAGAGAGCGAGTGGGACGGACGAGGCAAAACCGCGCTCGTACTCGAGATATGC
ATTCTGGTCGTGGCGACGATCCAATCAGGACAAGGGATCTGAATCACAAACAAAGGATGAAGAATTAAAAGAC
TCAAACGTGTTACATGTTGATAATAAACCATTAAAGTCCGAATCCACTGACGAAGACCAAGTGGACCATTGGA
TTGAAGAGAAAGTTGAGACGCCCAAAGAAGTAAAGATTGTAGAAGAAGCCATCAAGCC
AGAGGAATTGGAGCCAAGACAACCTACGCCACCAATGCCAACTCCTGATGTGTCAAGCCGGCATGATCAAAGT
TCATCGGAATCGGACCAAGAAGGTCAAGAGCAATCCTCTCGTCGACAGTCGTCTTTTCGTAAGACGTTGCGAC
TTTCCTCCGAACAAATTAAGCAGCTGCACCTGCGCGAGGGCATGAACGAGATGGTGTT

CAGCGTGACCACGGCCTACCAGGGCACCACGCGCTGCAAGTGGCACGTGTTCCGCTGGAGGCACGACCCACAG
GTCGTCATC
>126034478_2 EL595965.1
GCACGAGGGAATCAAGAATGGGACAGAATGGTGTCATATTCTAGTAAACCAGGCAATCGAAAAAGCGTTGTCA
ATTTCAATATTTCGTTAGACGATAACCATATTTCTATCGACGTAGTAAATGCTTTGGATAAAAGTACTAAGCT
AATGGAGAAAATTACTGATTTTTCCGAATTTCTAAAGAAGGAAGTCTTGATTCCTATG
ATTAAAAACGACTGCGTAGTTTATGCGGATTCTGAAGAGTCTGCTTCGGTCACGTTAACACACAAAGCTAACT
ACATGCCTTCCTACAGCAATACTTTAGCAAATTTGAGGCTATTGTTCCACTATCTGAACAACAGGTTCAATGT
CCCTTTTCATAACGAGCGCAGCCTAATGAAAATGTTTGGACATATAGTAGGCGAAGAG
CTTTGTGAAATGTTAATAAAAGATTGTTTGATCGATACTATACCTTATAATATAAATGATCTTCAAACCTACG
GTCGAGTGACGGCAGAAATAGAAGATTTCCAACGTTTTCTTATAGTGACCAATATATTCCCCGACAAAGGATT
ATCTTTACTAAAATATATGAAGAACATCGACGTCTTATTCGCGGAACGGTCTAGTCAA
CATTTCCTAGAGACGGCGCGCTCTATTATGCTCAAAGATTTAAGTTTAACCAT
>126034481_2 EL595968.1
GCACGAGGCTCAACTAACCGGTCAATAGTAGAATCAGAATCAGCGAAGGACAATATGCTATTAGATTTAAAGT
AACGTATTCATATTAAATATTTTTTCCACCGGTCACTACGAGGCGATAGCGGTAAACCGACTGGACATTATTA
TTAAAGTAGATCAAGAATATCAATATTCACCAAAACTTGTTTTTTTATCTAAGAATTT
TAGATTAATGGCGTCATTTAAGCGCCTGTTCATGTATATGTATTGATATATTATATTTTGTTCTGTGCTAGTT
TAAAACTGAATTAAGTTTATGAATAGTATGTAGCCGTAGTGGTGCAGAGGTATAGTGACTGACCGTAAACACG
ATTTGGGATTCGATTCCCACACGTTGGAGAAATTTTAGAAAGAAGACTCTAGCCTGGT
TCTCCGTGAGCGAAGCGGCCACGTCGAGGACTACGTTGTAGTTGCAAGAGTAGCCTCATATTTGTATTTCACC
AATAAATAGCTGAAAAAGACCGAGCGACTAGAAAGAGTTGAAATCAACTCGTCATTTCTAAGGAACGGAGCGA
AGTAATTACTAAATATAAAATGTTTTGTTTGTAGAAATGGAAACCAAATCTATTTAAA
AATGTCCGTTCCGCATAAGCATTACCCGTTAATTTCTATTTCGTAGATTCTCATCATCCAATGTCTCCGAAAT
TGGAGGGAGAGTGAGTTTCTTAACA
>126034483_2 EL595970.1
ATGAGTACGTACGTACGACTCCCGCCGCCATCTTCTTTGTGAAAGTTCAAATTCCGCTGAGACTTAGTCTAAT
TTAAATTATAATTGGTTATTTGAAGCGAGACGGCGGCGCGGCGTCCACGTCGGCCTCCCCCCCCCGTTCGCGC
ATTTTGATGCGATCTCAAACTTAAATA
>126034489_2 EL595976.1
GCACGAGGGCGGTGTTATATCTGAGGAAAATTGTATTTTTAAAAAGAAAACAACATAATGAGTTCCTTTCGTC
GGGATAAAAAAGAAGAAGAAGATGGAGGAGCAAACATTTTTCAAAATCTTGATAAAACAACTTTGCTGCAAGA
AGCTCGCTATTTTAATTCCACTCCGGTAAACCCAAGAAAATGCATTCATATATTGACT
AAAGTTTTATATTTACTCAATCAAGGAGAAAAACTTACAACTCAAGAAGCTACTGATATGTTCTTTGCAACTA
CCAAATTATTTCAATCTAAAGATGTTGTACTGCGTCGGTTGGTGTATTTATGTATTAAGGAACTAAGTTCTAT
GGCGCAAGATGTAATTATCGTTACATCTTCACTGACCAAGGATATGACAGGAAAGGAA
GATTTGTATAGAGCAGCAGCAATTCGTGCCTTATGCAGCATAACTGATAGTAGCATGCTACAAGCTATAGAAC
GCTATATGAAACAAGCTATTGTGGATAAAAACCCCGCTGTTAGTTCAGCTGCCCTTGTTTCCGCTCTTCACCT
GTCCGCTTCTGTTCCTGACTTGGTGAGGAGATGGGTTAATGATGCACAGGAAGCTATC
ATGTCTGACAATGTGATGGTGTCTTATCATGCTTTGGCTGTAGTTGCCGGAGCCCGACGTAATGATCGCCTGT
CGACAGTGAAGCTTGTTACAAAATTGGCCCGTTTCGCATTGCGATCTCCATATGCACTCTGTCTTTTAGTACG
TCATGCTGCAAAACTCGCGGAGGA
>126034493_2 EL595980.1
GCACGAGGCTCTTGGGGTAGTTTCACTAGTTAATATTAAAAAATGGCTTGTGTTTATACGCCACCCAAAATGG
TACCTATTTTACTACCAATAATATTCGAAACGTTTACCAAAATGTATAAATGGCCACCAAATTAAGTTTCCTA
TCATGATAATGAATAAATGAAATTTTTCTTATTATTGACAGCCATAAATGCATTGGAC
GCATTTAAAATTCATTT
>126034495_2 EL595982.1
CGGCACGAGGTGTCGAATGAACACGTTATAGTATTAACTTGTACATACCACTGTGATTCAATAAAATTTAAAT
TTTTCCTAAAATAATATAATATATAGTGATATTTTTACAGTAGGAAATAAGGTGAATTTATTATGATATAGAG
TTAATGTGCTTTGCAATTAGTTGTACGTGTTATTTGTTTGTGACATTAATTAGAGAAG
ATGAAGAGCACAAAGCTGGCGACATGCGCGAGCCGCTGCCTGAGCAGCCGGAGCAACAGGCTGTTCGCCGTCC
GCAGGTCAGACACATACGTATTGTTTTGAAATCATAGTCCAACGTTACTCCGTTGTTCTGTTTCTGTCATTGG
GAACCTTGGACTTTGTACTGTTGCCCAAACACGTATGAGCACTTTAATAAAAACATTA

TAACTATGAAGAATACTTCATAATTAATCTAACCTTGACAATTTTAACTATTTTACAATATATTGCAAGATGA
AAAGAATGATGTCTATGATAAAGATAATCTATAATACATTAAATGTTTATAATAAATTATTAGTTCTTTGTTG
TATAAAAAAAAATTCTTATAAAAATAGTAGGGACAGTTATGTTAGCTTTAATAAGAAAT
CTTGTATATTGTTTCCATTAATAAATACCTGAATAAGATTTAAGAAAGCCAAACAATAATTTATAAAAAAAAG
TAGTTAAATGAACTTGT
>126034500_2 EL595987.1
GCACGAGGATTTTATGAGGTAGAACTGGAATACCTGATTGCTATCCCGGAATGAATCGTATTACACCACAATG
GCAAAACATACATAAATGGACTCCACTTAATTTCAAGGCTATGTTAGTCAATTTTTTTTCACATTCCACAATA
CAATAGTTTCCAATTACATTTGATTTAATTTGAAGACTGAGTTGGGAGATTTTTGTAA
CAAAACAAACAAATACGTATTACATTTTTGCTCACATTCGCGGAGTTTATCTGCAGTTGTGAAAAGTTCCCTC
CTATGTTATAAATAATTTCGAGTGCCATTGTTTATAGGACTTAGGATTTTTTGAAATCTGACAATATTTTTTA
AAATATGTTATGTACTCAACGCATCAGTTCAAATCGTACAATTTGTAACGAAAAACAA
TAGCAATCAACGGAAGCCTTATTTTATCGACGTTCATACTGTACAACGAATAACCTTAAAATATTATGAAAAT
TTCAATATTCGAGAAATATTAGAATGTATAAGATTTAATATTATAATGCTATATGAGAGTATCACAAGATTTA
TTAGAGCTACGTAATTATTTGAGATAGGCATGTAGGGTTAACGCATACGAATATATTA
TAGTTGACGTGTCTAAACTTAAAATTCTTATTAAATGTACTTAAAGTTA
>126034506_2 EL595993.1
GCACGAGGCAAAATAGTTATTAAATTCTTACTCTATTTACCTTAATGGAACGACTTACAAACTTATGTAGACT
ATTTTGTCTGCTTTTATTTTATAGACAAAACTCCAAATAACGTATTGTTTAGACAATGATTAAGTTTTGGAAA
AGTGCAGCCAAAGGAAAAACTGGCTGCCCAGTACGACCACCTGAAACTCTTTTGGGTG
AAAACTCCCATTTTTCTCTCTTGGATACGGATCTGCACGAAAATAGCAATGATAATTTAAATGTTTTTGACAA
AAAATCAAAATTATCAATAAGTCTATATGACATACTCATTGATAAAACTGCTATTAATTATTTTATGATGTAC
ATGGAAAATATTGGTAAACAAAATCTTTTAAAGTGCTGGCTTGAGTTAGAAAAATTCA
AATTGGAAACATCATGCGGTAATGTAAATGAAGGTAGACATCCTAAAATAAGTCATACAAAAAGCTTAGATAA
CCATGCAAATGATAATGAGCTTGAAAAAAATTGTGATAATTTTGTGTTTCATAAATTTGGTTGGAAAACAAAC
ACAACTAATTTAAGAAAAACTAAAAGTGAAAGCTCTACAAGTTCTTGCATAGATTTAA
CAGAAAATTTAGAAACTAATTATCAATTTACAAATGAAAAT
>126034511_2 EL595998.1
GCACGAGGCCTCGTGCCGTTAGATTTAAGTGATTTTGTAGCATCAATATTAATGAGCGACTTAGTAAGATAAA
TCAATTCAAGGTAATATTAGGTAATCCTTAATTTTATTTATCGATATTTTATTAATTTTGTATATCATTAGAT
ATAGTCACATCGTCATATACATACATGCAGTTATGATAATAATTCATAGAAAATAAAA
TATGGATATTAGCGGTAATCTTTGGGGCAATATTTTTATATGTTTTATTACTAATCATGACTCCATGTGAAGG
GCGTGTCGTCCTCCTTACCTCACTCGCTCTTGCTTTTTATAATAGACTTACCTCTATGTCTCATAGATGAAAT
GGCGACGTCGACTTCGTTCTATATAATTAATGCTATTTAGTGGTTTGTATATACAGAT
CTGTTGTTCTATTGCTACCATAAAATTTGCTTTACCGCCGTTCAGTCACTTATCTAACAACTGTTTAGAAACT
TTTGCAGATAATCAGACAGTTCACATTTGTGTCGTACAGTATTACAATAACTTGACTGTTTTTTTTGTAAAGC
ACCATTGCTGCGATTTCTATTTTTTTTAGAATTGTTTTGTTAATCTCATTTTATTTTN
AAAATAAAAAATCATAGAGAAAAAAAATCATACAAGAATCAAATTGATTCTAGATTTTCATTTTACATATTAT
GAAA
>126034514_2 EL596001.1
ATATTATAAAAAATTTATTTATCTTAAAAATGTCAGTGACTACAATAAATACCTCAAAATATTAATATTATGG
AAAAAATGCAGCGAACAATATTTTCTATTATTACAATATATAAATTATAAAATATCCAATATATGTAACTGTT
AACTAAACTATTCATACACAATGGATTTATAATAAAATTTAAAAAAATATAACAAATG
GATGATTCTGACTGACAAATTATTACTAAAAAAATTTAAAAAAAGCTATTTTTTATTTTAAAACCAGTCTTTA
GTAAACAAAATAACTTTTGAACATATAACTAATTGTTGATGATTTTTATAAATATAAGTTATCTTAATGTATG
GCAAAATAAATATATATATACACTTATTTTAAATGCAAAAACATTAATAAGTGTCTAT
TTGTTGTTAAATAAGTACATCTATATTTATTATTGTAAGTTTAATGGCGATTAAACGGTAATTAACATTCTTG
AGTATGACTGTAAAGTATAAGGAAAACTATTGATAAGTTATGTGATAGTTTTGGATATGCACAGTTGAGATAT
TGACTAAAAATATATATAGGCATCACAAAAAATATCCAAATACATTAAAAAAGATTTT
ATATCTACCAATTAC
>126034516_2 EL596003.1
GCACGAGGTAGGAACATAGTATTTTGTGATTTTGTAAAGAAATTATTCAAAATGATAAGTGTTTTAAACACAA
TAGATACAGGCCACGAGGACATGATACACGATGCGGAGTTAGACTACTACGGGCTAAGATTAGCAACTTGTTC
TTCTGATAACTCCGTTAAAATTTACGACATCAAGAGCGGTACACAAACTCTAGCTGCT

GATTTAAAAGGACACTTTGGACCTGTGTGGCAAGTTGCCTGGGCACATCCAAAATTCGGTAATCTATTAGCTT
CCTGTTCTTATGACAGAAAAGTCATCATTTGGAAGGAATCTGGAGAGTGGACTAAACTGTATGAGTACTCAGG
ACATGAAAGTTCTGTGAACTCTGTTGCGTGGGCGCCCGCGGAATACGGTTTGATATTA
GCGTGCTGTAGTTCAGATGGTTCTATATCAACTATAACTTATAACCAAGACGGTGGAAATTGGGATGTAAAAA
AAATTCCCGGCGCCCATGCTATTGGAGTGAATTCAATTAGCTGGTGTCCTGCTCTCTCTGCCGACCTAAACTT
GGATCCTTTAACAAATAAGGATGCACCAAAAAGGATGGTCTCTGGTGGATGTGATAAT
T
>126034517_2 EL596004.1
GCACGAGGCCGGAACGGAGTATTTTAACACTAGGTTTCTAGGTTCCCTCCACCTACAATGATATTTGCGGGAT
ACAGCGTGATGTTAAAAAAATGTATTTAAAATAACGATAATCGTTCAATATCCAAGCAGTAACATATATTGGG
TATAGTTTTTACGATTTCATCCGACTGCGCAACGTAAAGGAGGTTAATGTGTTTATCA
GTCTATGTATGTATGTTTATTCCTGCGACATTGTAGAGAACAACCGATTGTAACGACTACGTCAATCGATTCG
TCTGAACCTTACGAATATCACTAAACTACTATACATGGTAATATTCAAAAGTGACTATAGCGACCATATGTAT
ATTGTCAATATGATATTATTTAAAAAGTCTTAATCTAAAATGATTTTAAGGTTTCTTC
ATTAATTTTGCATTTTAAGTAAGAGTTACAAATATTAGGGTTAGGTAACTTCTATTGTGTAAAAATATATGGC
GTGTCACTAATATAAATAGATTTCAATTATGTACCTATTAGTTCAGTATTACTCTTAACAAAATTAATAACTG
TTTGGCTATATCCAATTCAATACGATAGTGTAACCAAATAGGACCTACTAATAAGATT
GANNAGGCCAGTCGTGGCGCAGGGGTACAAAGTATGGCTATAAACCGACTGGTTGTGGGTCGAACCCACCACT
CGAGGAAATGAAATATTTATTCATATTTA
>126034518_2 EL596005.1
CGTGTCTCTTTTTCACAAATAGGCTTAAGCTGTATAATATAAAGCGTGTTTGTGTAAATATTGGACGATTTTT
GGTGTATTTTCGTGTGTTTGATATCTTTTTCTGTCTTATTATGTGAGAGTGATGTAGATATATTAATAAAAAT
TGATTGTCAAATGTAATCGATTTATAACATATGAATAATATGAAGTTATTTTTAAT
>126034519_2 EL596006.1
GCACGAGGGTTTATCGTGACTTCGAGCGTATCATGTAAATTGTGGCGTTTATTAGCAGTAAATCGTTGCTGTC
AGCTGTTGTGTGACTTTAAGGACTTTTGAATTTAGTTCATTTTAGTGTTACGTGTATTTGTTGAAAAAGTTAC
AACAATGGGAGTATCAGATACCGGTTCACCCACTACTCCAGATCTCTCACCTCACGCC
CCTGATCGCCCAGGGTCGGGAAGTGGGATGGATGATGAAGACATCCCCAGAAGGAAGCAGAGACGTTACAGAA
CTACCTTTACCAGTTACCAGCTCGATGAATTGGAAAAAGCATTCGGAAGAACGCATTATCCTGATGTTTTTAC
AAGGGAAGAGCTGGCTTTGAAAATCGGTCTTACAGAAGCAAGAATACAAGTATGGTTC
CAAAATCGTCGGGCCAAGTGGCGTAAGCAAGAGAAGGTGGGACCACACGCGCACCCGTACAGTGGTTACTTGA
GCAGCGGGCAGCCGCTACCGACGCCCACGCTGCCTGTGCCGCCTCACTCTTTAACGCAACTTGGATTTGGATT
GAGAAAACCTTTCGATAACGCATTGGCTTCATTTAGGTACGCAAGTTCACCGTTATTC
GGCGCACAGTACCTACCACCTTTAACCCGACCTCCTCTATTCGGGACACCATTGTACGCAACCTCCCCGCATT
TCCATT
>126034520_2 EL596007.1
CACGAGGGGGCACAGCAACCATGATTCCTCCTCCTCGGAGAGCGGCGACGTGCGGCCGTGCGCCGACGCGCCG
TCGTGGAGCGCCTCGCGCAGCGCCAACACCGACCTGCTGTTCTAGTGCTCCGACTGCTGTTAAAGACGCTCAT
CTAAACAAGCCTATTAGAAGAAGGAAACTAAAATATACGCGAAAGACGTCTATATATT
TGGAAATGTCTAAATAAAACTGTACAATTCAGTTCATATAAAAATTTAGAGTCACTCAATCAAAATATGTAGT
TCATTCATTTGTGTCGTATTGTTTATTGTTATTTATATTTTGATTAATAATTCAATTCGTACATTTCATTCCA
TTCATTCGACAATG
>126034521_2 EL596008.1
GCACGAGGGCCATCTGCAAAGCCTTCTCCGACGGCCGCACCATCGACGCCACGCAGGAGATGATCGCACTCGG
CGTCGCCAACGTCGCCAACTCCTTCATGCAGGCCTACCCCGGCGGGGGCTCCCTGGCGCGCTCCGTCGTGTCC
AACGGCTCCGGAGTCAAGACCACTTTTAACGGAATCTATACAGGTGTGATGGTGATCT
TGGCGCTCCAGTTCTTCACGCAGTACTTCGAGTACATCCCGAAGGCGGCGCTCGCCGCCGTCATCATGTCTGC
GATACTGTTCATGGTCGAATATGACGTCATAAAGCCAATGTGGCGAGCAAAGAAGTTGGACTTAATTCCGGGC
ATCGGTACGTTTGTGTTGTGCTTGACGCTTCCCATCGAGCTGGGAATCCTGACGGGAG
TGGTTGTCAACATAATCTTCATCCTCTACCACGCCGCGCGGCCCAAGTTCTCGGTGGAGATGCTGAAGACGGA
GCAGGGCGTGGAGTACCTGATGATCACGCCGGACCGCTGCCTCATGTTCCCGTCCGTGGACTACGTGCGGCGC
CTGGTCACCAAGTGCGCGGCCAGCAGCTCCGTGCCCGTGGTCATCGAGTGCACGCACA
TCTACAGCGCCGACTACACCGCGGCCAAGAGCATCGAGCAGCTCACGGCCGACTTCCACGCGCGCCTGCAGCC
GCTGTACTTTC

>126034530_2 EL596017.1
GCACGAGGATAAAGGTGAAATTTTGATAGAAATTGAACCAGTGAGTTATAAGCTAGAAAAAGTCGAATTGGAT
TTAACAACTCAACATGAACGCCAGACAGATCCACAAATATTTGAGGAAAGAGTTTTGAGAAATGATGACGACG
TTGCCGCTACTGTCACAACCGAAATAGAATATAAATATAATTATACCTTGTCGTGGGG
TCACGGTCACGGTATTGCCATTGGCTTAAACACAACTATAGAAATGAGCGACGGAACTGTATTTCCACAAATA
GAATGGGCCAATCAGTTTTCGGAAGAAAGGAAGGAATTGTACAAATTAGAAAAGTACCTAGAACCAGGAACCG
CCTGCAATGTAACATTTAGAGGTAATAATACGGATCGTGATGTGCAATACACGGGAAA
GTTGGTTACCTTTTACAAAGAAAACAATGCGCGATCGAGACAAATGCGAGGGGAAAGAATAGAAAACACCGTT
GAAGTTGAAGCTGTTTATGGTGAAATATACTATACCGTTAATAATACCTTAGTACCGACGACGACGACCACTA
CGTCTACAACTACCACTACTACTACTACCACAACACAAGCGCCAATGCCTCAACCGCC
TTCAGTAGAANAGAACGATGTTGGCATGATAATGGATAGTAATGATATCCTGAGCGATAGCGGTGAAGATATG
GTGAAATCGCCTCCTGTCAAAGAGGACATAAATCG
>126034537_2 EL596024.1
GCACGAGGGTGGATTATAAACAAAAAATTAGACTGCATAAATAAATTTAATGTATCAAATAAAACTCGCATCA
TCGAGAAGAATTCAAGGACGTATGATTTTTAAAAATCGTTTCACTGGTTTAATATTTACAAGAGAACCAAAGA
ACAGACACTCATACATAAATACGCACCCGCTAAAAACATAACCCGGTTTTTAGCATAT
AACATATTTAGCGACCTGATCAAATAATGTTCAATAATGAGTTCCGCCACGAAATATCTGAAGCTATCATAGC
TATAAATATTACATTTAGTATCAATTTGTCACATACGTAATAAATTTTTAATTACTTTAACATTCTTTGTAAT
TTATTATTTTGTACTAAATAAACAATATGTATTTATAAACAAATCACAAATACATATT
GATTATTAAATACAAAGATTGTCTAATATAGATCGACTTTCAAAAATCTATCGAATAACTGATGTTAGATGGT
AATAAATAATTCAATTATAACGTCATACCTTCTCCAATAAGCATATGTAAGCAATTTAGAATTATTAAGGTCA
TAATCCCATTAAATTGCACTCGGGCAATATCTATCACATATTTATCGTGTTCAATAGT
ACCCCAAAAGTCTCAGATAAAATGGTTGTTCTTCTCATTTCGGTTTTTATATAATGAAAATTGCAAACT
>126034538_2 EL596025.1
GCACGAGGGTTCACGTGGGAAGGAAATTATCGTTACAATACATTGGACGGTTACCGGAACCATACGAAATTGA
ACAACGTTTACACTCGCATTATTCTATAACATGCGTCACACTCTGTGCGCAAGCTCCACATACCTACATATTA
AATTAAGCAATCCTTTCTATTACGAATGTACAATGCAAAATAAAAATAAAATATATTT
ACAGCAAAAATTAATATGTTAAAATAGTACCAAACAAGAAATTTACTCCATATGCATTTCAATTTATCATCTC
ATTTATTCGAAT
>126034541_2 EL596028.1
GCACGAGGCTATACCCTCCCCCGCGCCCGACGCCACCTACGTCACGTGGCCCTGCGGCCTGTTCCCGCAGGCC
ATCGGACGCAACGCTCGCGCCTCACATCTATCTAGAGTCAAGGCTAAATTCCGATTCCTTGGAAAGTTTATGG
CTAAAGCTGTAATGGACTCCAGGATGGTGGACATCCCGCTGTCGGTGAGCATGTACCG
CTGGGTGGTGAGCGAGCATGCGTGGCTGGGGCTGAGCGACGTGCGGCACGTGGCGCCCGAGCTGTGGCGCTCG
CTGTGCCGCCTGCGCCGCGTGGTCGACCGCGCGCAGGCGCTCGCGGCCGACGCGCGCCACTCCTCCGACCAGC
GCACGCAGATGATTAACGCACTCGAGCTCGACGGGTGTCCGATCGAGGAGTTGGGTCT
GGACTTCATTTTGCCGGGCGACGGCTGCGCGGAGCTGCGCAGGGGCGGCCGCTACCTGCCCATCACTGCGCAC
AACCTCGCCGAGTATATAGCTCTTGTCACACATTGGTTATTATACGAAGGCGTATCTAAGCAATTGGATGCAT
TTAAAGAAGGTTTTGAATCTGTATTCCCTTTGAATAATC
>126034543_2 EL596030.1
GCACGAGGCGACAATAGGTAAAAAAGAGCTATAGACCAGTAGTTTTGTCTTTATTAGAAAGAGATAGGGCAAA
CAACTTCACAGAGGCCCCTAAATGACCTGCTAACTGCTGCTATGTAATCTGGACTATCAGTTCAGTCTATATT
ATAAGAGCACACTGGCTCCCGAACGGCCTGTTTACAACTGCTAAATAATCAATCATAA
TTTAATACACTTTAAATGGGCCCGAGGTTTGCGTTTCTACAAGGGCCCCAACAAGTTCTAAGATAGCAGTGGA
AACTAGGTATGGTTGTAGTTAAGTCATTCATTTAGTAGTGGCTTAAGAATGTGCTATAATGGCTATCGACTAA
TGTATAGAGGCGTCTTCCCTGCAGACAAATGATAAACGACGACAGTAAGGTAAGCCAC
AGTACAGCTAGTTGTCATATAAGTTATAACTACAGAGGAAACTCAATTATCCGGACTAATTGTTCTAGTCGAT
TATCTGGATATTAGAATATAGAAAGCTACTACAGCTTCACTAATGGATTTTCATATAATGTAGGTTTTTAAAT
TATTATTTTTTCTATTGTTTTATTTTTTGGAATTTTAATTGTATAATCATGAAATAGA
TGTTAATGAATTTTCGTTCCTTATTTTATGAAGCTTGTTTCAAAGTCGGCCTTCCAATATTCTCTGGGTATCT
GAATTATCGTAAATAC
>126034547_2 EL596034.1

GCACGAGGCTATCTTGTTATTTTTAATATTACCTGATGTTCGTCTAATGTAATTAATAAATGAAATTCACATT
CAGTAAATTAGCGTATCAATGCGTTAGTGATACTCACAAATAAATTGTAATTGCCTACTATATTAACAATTTA
CGTCATTTGTAAAGGTTGTAACTTCAAAAAATATTTGTAAACATGGAAGAAGAATTCC
CCATAAACCCTCAAAAAATATTGCGAAGTTCACAATTAAATAAAATCAATAATAAAACATCCGTTACATGTAC
CAAAGTTCCAGAAGGATTATTTGATTCTAAAATTGTAGATAAACATTTTAGTAAGTTTGGCAAGGTTGTCGGG
ATTAAATTGTTGCCTAAGAAACAAATGTGTATAGTAGAATATGACCAACAAAGCTCAG
CTGAGCGGGCTGTATTGAATGCAGGAGCTTTTGATGGTTTTATGTTTGATGTAACACGTACAAAGCCAAGGGT
AAGAAGAAAAAGTAAAAAAGATGATGATCCCGAGTGGTTACCAGATCCAGAGGTTGAGGAAGAATTAAATGCA
ATGAATGGTGCACCAACTTATCGACTTTCTAGACAAAAAACAATGGATGTTGATACTA
CTAATGTTCAGTTAAAAATTAATAAATCACCAATTCGAAAGAAAACT
>126034555_2 EL596042.1
GCACGAGGCTCTGATGTATGCCTCATTTCATTTAGTTCAGTAACACAAAGCCGCCTGGAAATTGTACTTTCTA
GTTTATTGGGCGATAGAGTTATAAAAACGAAAAAAAAATCAAAAGCTATAATCAAAAAATATAATCTCACCTA
CCAAGGTGTCGCTCCCTAGATGTCTATGGAGATCGCCAGAACTGACATTTTCTCTGTC
AAGTTTGGTCGTAGATGGTCATCGAGATCATTAAAAGCATCTTCCTAGATCATCTTACGACGATGGAAAACGA
AGTTTTTGACAAATTACAAATTGTTAAAACAACATATTTTTCCAAGTATTTGCGTTTATGTAGACAAGTAATG
TAATATATTATACTTATTTTATAAATAAGTATAATATATAAAACAAGTTTCAAGCACG
AAGAGCTTCCAAGACGGTGTACTGTGGAACAGCATCTGTCTG
>126034565_2 EL596052.1
GCACGAGGGTTTCTGTAGCTGTAATTGTTTGGTAAACGTTATTTTTCGTAAACAGTATTTTAAGAATGGAAAA
TGCCGAAGAAATATTTCAATTCCTTGAAGAGTTTTCGAAACGTAAACCTAAAACTATACCACAAGAGTTAAAC
GATTATTTAGCATATGTGGCGCGTACCGGCGACCCCGTCTATCAGTGGGCTCTCGTTA
AGAGTTTGTTTAAAGAGAAACTTCTGAATGTTTTAACAGACTTCTACGAAACTAGTCCAGGTATAGATATCCC
ACCATATCCAAATGTTGATCCGTTTAACTACGACATTATGAAAAATAGTTTAATGGAACGACTAGATTCTTTT
ACCTCTGCTCCCTTTACTGTACAAAGAATTTGTGAACTATTGACATACCCACGTAAAC
AATACAACCGTGTGGATAAATTTATGAGAGCAATCGAAAAAAATATTTTAGTCGTAAGCACACGAGAACCTGG
TTATCAACGTCATCCAGAACCAGAAAATGGTGAACCTATAGAGCCAGTTGTAAATGGTTCAGACAATAATTCT
GAATATAATGTTGATGTAGAAATGGAAGATATGACCTGGAAAGAAAGTAGTGGTTCTC
GGCAGAACTCTGAACCTCAACCAGGCTCTTCAGATGCTCATATTTC
>126034570_2 EL596057.1
ACTTTGACAACTTGACTTGTTTATTTATATTTGAGATACAAATAATTATTGTGAATACCAAAATTAGTTTTTA
CATTTACACGTATCGTCATATCAGTGTTGTGTTACGTTAATAGTTACGATTTCTTTAAATCAAATACAAAAAA
GAATATTTTGGTTATTTGATAATAAAATTGAGATCAGCAGCAGAGCGTCTGGCTGTAA
ACGCGATGGTCTTGGGTTCGCTTCCCATTCGTGAAAATGACTTATTTTCACTTCCTCGCGCGCAATATCGCGA
AAAATCGGATACAGAGTATCTTAACACTAAGTTTCGTCTGTTTTCCCTGCTATACGCGGGATATAGCGT
>126034571_2 EL596058.1
CGCACCTCTACATCTTCGGCGGCGAGGCGCGCGGGCACCCCACCAACGAGCTCTGGAGGTTCCACTTTGGAAC
AGAAACCTGGGAACAAATAATGCAATGTATAAAATGGCCGTCACCGCGCGTAGACAGCTGCGCCTTACTCATA
AGCGGGGGCGACTCACGTACTCGCAGCGCTCCACCGGTCAGCCCCCGCGTCGTAGCAC
AACCCCCGACCACCGGGTTCCTTCGAGAGATCTCCAAGTTATCCGGTTTTCACATCCGACGAGCTGCGCGATG
CTCGTACAGTGTACTAGCTGCGGATCAGGACTCCACCGAGAGCCTCCTACGTACAGATCGCTCCGACCTTACT
AAGTCGCGGTCGGCCTACATAATCGACGAACGACAACCGGCCGACGGCGACGAGGGGC
CGCTGTCCCGCGACCCTCTGTCGGTGCCCGACTTCGAGGCGCTGTCTCCGCGCACGACGCTGGTGTACGTGGA
CTCCGACGACTCCACGAAACTGACGAAGGACGAAACGACGACGTCGGACTACGCGAGCGCGGACGCCGTGGAC
GCGGCGGCGGGGTTCAGCAACCCGCACTACCTGGGCCCCCGACGTGCGCCGCCTCGCC
GCCGCGCGCACGCCCGACTCGGGCGCCCGCGACATGGAGCTGCGGGAGCTGCGCCCGCGCG
>126034573_2 EL596060.1
GCACGAGGGTTAGATCAGGCGTTTGTTAATCTTAAATATTTCACATATTGTATTAAAAATGGCAAATGTTGAA
GAATTACAAAAGAAAGTGATTGAATTGGAAGCTAAGTTAGCAGCGTTGCAAGGCAATGCTGGGCCAGTGCGGC
AGAAAATCGATGTTATGTCCTCCGAAGTGGTAGACTCTAATCCGTACAGTCGCCTCAT
GGCCCTCAAAAGGATGGGCATAGTTGATAATTATGAAAAAATCAGAGAACTGTCTGTAGCAGTTGTAGGTGTT
GGTGGAGTTGGTAGTGTTACTGCTGAAATGCTCACAAGATGTGGTATTGGAAAGTTGATCCTATTTGACTATG
ACAAAGTGGAACTGGCAAATATGAACAGGCTTTTCTTTCAACCTCATCAAGCGGGGTT

AAGTAAAGTTGATGCGGCGGCGGCAACACTACAAGCAATCAATCCAGATGTGACTATAGATACTTATAATTAT
AATATTACTACAGTGGATAATTTTCAGCATTTCTGTGACACTATAAGTAAAGGGAGTCTATCCAAAGGCCCAG
TGGACCTTGTGCTAAGCTGTGTGGATAACTTCGAAGCTCGTATGGCCATTAATACAGC
ATGCAATGAATTAAACCAGCGCTGGTTTGAATCCGGTGTGAGTG
>126034575_2 EL596062.1
GCACGAGGGCGCTTGTCGCCGTTTTTGCTGTATTTATATATTTTAAGTTTTATAAAATGTCACAACAAGGGCC
GGCTTTTGATGTAAATGCTATGACCTTAACCCGTTGGGTTTTGGCTCAGCAAAGAACGGCACCAGCGGCTACG
GGCGACTTAACACAGTTATTAAATTCCATACAAACAGCGGTAAAGGCGATACAAACAG
CTGTGAGGAAAGCCGGTATAGCTAAACTTCATGGAATCTCAGGAGATACTAACGTCCAAGGCGAAGAAGTCAA
GAAACTGGATGTTCTCTCCAACGATCTCTTTATTAATATGCTCAAATCGTCTTTTACCACCTGCCTACTGGTG
TCTGAAGAAAATCCGAATGTCTTACAGGTTGAAACGGAAAGGCGAGGAAAATACGTAG
TATGTTTCGATCCACTCGACGGCTCCTCCAACATTGACTGTCTCGTGTCTGTTGGCTCCATCTTTGCCATCTA
CAAGAAAACCTCAAACGGCGAGCCATCAGAATCTGACGCCTTACGTCCCGGCAATGAGCTGGTTGCGGCTGGA
TACGCATTGTATGGATCAGCGACCATGCTCGTTATATCCTTGGGAAAAGGAAAAGGCG
TCAACGGCTTTATGTACGATCCGTCCATCGGAGAATTTATTTTGACTGATCCTAATATG
>126034579_2 EL596066.1
GCACGAGGGAGATTCTAGCTGGATAGCAAATCATTTTCTGCAGTAAAGATTTATTTAAAGAAAAGTCTGAAAG
ATATGTCTATGATTAATTTTGAAAAAAAAAAAACAAAAAATATATGGACCTCATCGATTCAGGGTTTTAGGAT
ACGATGGGCCACCATGGATGGATGGATGGATAGATACCAACAAGCTTCGCAATGTAAC
CAAACATAATATAAATAATTAACTAAAGTAAATATGTATCTATATCATTCTAGATTTAATATGATACACTCAA
AAAGTTTTTTAAAGAAAAGGCTATTTTTAAAGATTTGCTAGGTTGATATGATACAATGCACTAAGTCGATAAC
ATATCTTAATTTTGTCATTGCGGCTATTACATTACAAATGGATTTTTAATGATCATAA
ATATAACGAGTGCGGCGTTGGTTTCATCACCCAATACGCAATATAGTATACATTTACTGTGCGGTACCCTCTA
ACAAGCCCTCATTAGACAAGCATTAACTTATTATAGTAAATAATAAAGAAAGATAAATGAACCTTTAAACAGC
TTAGTCTATTGCAAGAAGTTGTCATTGCAATATTACATGAAATTCGGCACCACATTTT
>126034583_2 EL596070.1
CATTTTTAAAACTAAATTTATTTCAAAAAATCGTAGTGTACAAATATAGTGCCTACTAGCCGATGTACTTCTG
TAATACAACTGTCAACTTTGATTTTAGCCCTCCAGCTTTATAAACGGGTTAAAACCATTACATTTATAACTAG
TACAGTCTTAAAGACAGTTAAACTTACCCGCTTGAACAGCCATTATCATCACATAATT
CTTGCAAATGAACAAAAAAGGAAAAATGGGGAAAAAAATATATATTTAAAACAAAATTAAGGCGGACTGCTTG
TTTTGTCAAAGGAATGTCAATTTCATTTCCTAGGGGTACTTCTAAGTTTCACGGAAACTAATTCAACAACAGA
GAAATCAACATCGGGACGGACAAAACAACAGTGCTGTTACATATATTGCAAAATAATA
CGCATTAATGTTATACCGATTGAATTTTATAGCACCAAGATGCGATTTTATATAATTATAATAACTAGCTAGT
CGAATCCTCGAGCGCCGTACGTTTCTAGTTTTTATAGAACAAAGTATGAATTTTGTAAAAAGCATTATTTTTT
TTTCATAGCTTCAGCTGTATACGAATATAGCATTGTAATTTACGAGACAGTTGAATAA
CTCAACGCAGTGCTTTGTCTTGTTATCA
>126034587_2 EL596074.1
GCACGAGGTTATAACAATATAGTCCGTGTATTGAATATCTTTAACGACTTTAGTGTTTGAGGTCTTGTTCTTT
TATATATTGCATTAGTAAATAGTAATTAGCTCAATTTATAAATTAGTTAGTTGTCAGTCATTACTTTGAATAC
CGCATTTGTACCCTGCGAAGATGCCGAATATCAGAATACAATCATCCGACAATGAAAT
ATTTGATGTCGATGTAGAAATAGCTAAATGTTCAGTTACAATCAAAACAATGTTAGAAGACTTGGGCATGGAC
GATGACGAAGAAGAAGTAGTCCCACTGCCCAATGTCAATTCAGCTATTCTTAAAAAGGTTATACAGTGGGCCA
CTTATCATAAGGATGATCCTCCAATCCCAGAAGATGATGAAAACAAAGAGAAACGCAC
TGATGATATCTCATCTTGGGATGCAGATTTCTTAAAAGTTGATCAGGGAACTCTCTTTGAATTAATTTTAGCA
GCGAATTATTTAGATATTAAAGGTCTTTTAGATGTTACATGCAAGACAGTCGCAAATATGATAAAGGGTAAAA
CACCAGATGAAATTCGTAAAACTTTTAACATTAAAAATGATTTTACAGCATCAGAGGA
AGAACAAGTCCGTAAAGAAAATGAATGGTGTGAAGAGAAATAAAGTATTTATTAATCCTTACA
>126034592_2 EL596079.1
GCACGAGGGTTCATGTCATCATTAAAACATTGTTTTCTTGTTCCTTCATATTTAAAATAAAGTATAGCTTTTG
TTAAGGTTTAATTTCATATTAATTACAACATTTTACAAACGATATATTCGCTTACGAAAGCAATTTTTCGTAA
GAAGAACTCAGGTTGTTAATTATGGATCGTCTTTTACGTCTTGGTGGTGGCATGGCGG
GTTTGTCACAAGCGCCTCCACCTACTGATGCTCCAGTCGTTGATACCGCTGAACAAGTGTATATATCATCATT
GGCGTTACTTAAAATGTTGAAACATGGTCGTGCTGGTGTACCCATGGAAGTCATGGGACTTATGTTGGGTGAA
TTTGTTGATGACTATACAGTACGTGTTATTGATGTATTTGCTATGCCCCAGACTGGTA

CTGGAGTATCAGTAGAAGCTGTCGATCCTGTATTCCAGGCAAAAATGTTAGATATGCTGAAACAAACTGGTCG
ACCTGAAATGGTTGTGGGCTGGTACCATTCACATCCTGGATTTGGTTGTTGGTTGTCTGGTGTTGATATTAAT
ACACAACAGTCATTTGAAGCATTATCTGAGAGAGCTGTAGCAGTTGTTGTTGATCCTA
TTCAGTCAGTCAAAGGCAAAGTTGTTATAGACGCATTCCGCCTTATTAATCCTAACATGATGGTCCTAGGACA
AGAGCCACGTCAAACAACTTCTAAC
>126034594_2 EL596081.1
GCACGAGGCTTACGACAGAAACTTAAAATCATCGAAAGATGATATTCATTTACCATTCAATTCAAGCCCAGCG
AACTTAAAGCTATATAAAGATTTAATTAACAACAATGGTGAACGCAGCTTAGAAAGCCAATTAGTATGTAAAC
TACAGCATTTTGGTCTGATTCCTTCTAAGATGACATGCCCTGAGAACTCGCCCGATTG
TAAAGTTTTGTGCAAATCAGCCCGAGTTATTGATAGAGTCCAATGGATATGTGAAGGTTGTGGTAAGAAACAA
CCAATAAGAAGTGGTTCATTTTTCTTCAAGCTACAGTGTTCTATTTTACAGGCCTTGCAAGTGATCCTTGCTT
GGTGTGAAGATGCTGATATTACTGTTTCGGCTCAACATTTTGTTTGATATAGGAATAT
ATTTTTAAACACATTTGTAAATTGTTTCCTGCTGTCCTGTTTGATAGTCTGATATTAAACCCAGAGTGGTCAA
CTCTATCTATAATAAATTGGATGACTTAGCCATCAGGGAGTTATATAAAACAAAATTGGGAGGTGAAGATGCT
GTAGTTTTAAGTGAAATGTATCCAGATTGCTTGAACCGATTGTCTCCCGATACAACTG
ATCAACCTCATGTGCATAGAATTCTTATGATTGCTGATACTAAGCACATTCCCACACATTATAGACTCCATGT
TATTAAAAATGAACAGAAAAACATTACAGATATAAATC
>126034596_2 EL596083.1
GCACGAGGGTCGCGTCCGTTCACCGCGTATACGATACACAAAACAAAATGAGGGCGCTGTTGATTTTAGCCGT
ACTAGCGTTTGTGTGCAGCGCGTTGGCGAAAAACAAAAAAGAAGAAAAAGTAAAAATCGCAGTATACTACGAA
TCCCTATGCCCAGACAGTAAGAAATTTATAACAGAACAGCTTGCCCCCGTGTGGAGGG
ACTTTAGAGGACTTGTCAAAGTTAAACTGGTGCCTTATGGAAAGAGTACGCATGACCAGGTGAACGGTAAGTG
GGTGTTCAACTGTCACCATGGACCCGATGAATGCTATGGAAACAAGGTTCAATCTTGCATCTTGAAGGATCGT
AGCCTCCAAGACACAGATAAAATGGAAATGATCATTTGCTTGATGAAACAAGCCAGCC
CCGACAAATCTCTGGATACGTGCCTCCAAAGTGTAAAATCCCAGGGTAAATCAGATAAGCTGTCGTCGTGCGC
CAGCGGGGAGCAAGGCGACAGTCTCCTCGCGTCGTACGGCGACAAGACCGACGCGGTGCAGCGGCCGCTGTCG
TTTGTGCCCACTGTTGTTATCAATGAGAAATTCGACCAAGCTGTCCAAGACGAGGCCA
TTTCTAACCTTAAAGCTGTTGTATGTCGCGTCTTACCTAATAAACCTTCGATTTTGTAATATTATTAATAAAT
ACTATTTATATAATACATATTAAATAAGTACATTTTTTATACATTTTATGT
>126034598_2 EL596085.1
GCACGAGGTGAATCTCTCCTTTTTATTTTAATGTTATGTTTTTACAATACAATAACCAGTAACAATTTATATA
ATAAATATATTTTTAGTTCTCAAAACGTTGCTACTTAAATAAATGAATACAACTGTCTAATAAAAATGAAAAG
AAACTGATAATTTTGAGAACTGAAAGGAATTATTAAGAACGCTGAATTTTCAATAATA
ACTGACACCACTAAATTATAATAAACTTGTTGTACACTATTTAAATTTTATAAAAGATATAAAGCGATAACTC
AAGAAGAAACATCTAGAATTTTTTAACACTTTCATAATGTCAGACGATATTGATATAGAGGAACATGATGTTT
TAGACAATCCGCGTATTAAGAATATATTTCCGGACATTGCAAGTATTAAGACTGAGGT
TATTGATTATGAAGAAGAAATGTCACAACCAGACTATCAAGAAGAAGCAATTGAATATGAAAATTATAGAGAT
CTAAACGAATTTGAGCCCATTCCCATAGAATTTGAAACAGAAACATCAAATTCACACCAAAATAAGATCATCT
TGGGCTCAAAACCATGTAACTCCCCTATACCATCAGAAGATGAGTCTTTACCCACCAT
CCAAGAATGCT
>126034602_2 EL596089.1
GCACGAGGCAATCGTGTATGTGGAATGTTTTGTGTTGTGAGTGAATGCTAACTTATAGGAATTGTCGCTATAT
TATTTGTCATGTGGTTATGTGCGCGTATCTTTAGATAAGTAGTGCTGTGATAGAATTCTGAAGCTAACAGAGT
GGAATGGTTCCGGTGTGTCGGCGGTGTCTTGTCGATTTGCGCGGTTGAGGCGAGCTCA
GCAACATGGGGAAGCTGCTGTCGAAGATCTTCGGCAACAAGGAGATGAGGATATTGATGCTGGGTCTTGATGC
TGCTGGAAAGACTACTATTTTATATAAATTAAAATTAGGTCAGTCAGTCACAACGATACCTACAGTCGGTTTC
AACGTGGAGACCGTGACGTATAAAAATGTCAAATTTAACGTTTGGGATGTCGGCGGGC
AGGACAAAATACGACCACTATGGAGGCATTATTATACAGGCACTCAAGGTCTAATCTTCGTGGTGGACTGCGC
AGACCGCGACCGTATCGACGAGGCGCGGCAGGAGCTCCATCGGATCATCAACGATAGGGAGATGCGTGATGCT
ATCATACTCATATTTGCTAATAAACAGGATTTGCCAGACGTCATGAAACCCCACGAAA
TCCAAGAGAAGCTTGGGCTGACGCGCATCCGCGACCGCAACTGGTACGTGCAGTCGTCGTGCGCGACGTCCGG
CGACGGCCTGTA
>126034604_2 EL596091.1

GCACGAGGTGCATTTGATGCATTTGTTTACAGAATTTTGGACTATAACAAAGTTTTCTCATGGCAATAACGAT
GTATTTAGGAGAGAGTGCGTCTGATTCAAAATTTCTCAAAGAAAGCTTAGCGAAAAGAGAATTACGACCGTCA
AGCGCAGTTTCTTTCTCTAAATCGGATCATGGACCAGTTGAAACTTCAACATCATTGG
AGCAGCTCAAACAGTATAAAAGCTGGGTAAGTAATGAAAGATGGAATGAATTGAAGAAATTAGCGGAAACGGC
AATGAAAGATCGAAAAGTGTTTATGATAAAAGGAGGGGGATTCCCGGCTGTTCGTCGAGCGCTCCTTGAAAGA
GGTTGGGTGGAAAAGTACGAGTCACACAAGGTTCGTCATCCACCTGCAAACGTTGATC
CAAGAAAAGTGTCTGGAAAAGAATTGTCCAAGGTGGAAAGGATGATCCTATTTAAATTTATGGAACATCATTC
TGTAGACTTCTTATGGACAACCAAACGAGATAAATACGATTGGTTGCTGAGTAATAAGGAAGTTATGATAAGC
AGATTCAGTAGATCTATTTTTACTACCAAAGAAGGCTTAACATCCTCTTTAACTCAAA
TGCATTGGTATACAGAGCCAGGTGTTGCGATAACACAGTTTCCACGATCGTATAATATCCACAATTCCGACAG
TTT
>126034612_2 EL596099.1
GCACGAGGGTTTTCTAAATAAGTATCCACAAGCCACAAGTGTTATTACCACAAGCTGTATTTTGTCATTTTTT
AAATATACATTTTATAACCTAAGCGATTGTTGAGACATATAACGACTGTACTACGTACAATATCACTATTAAA
TAAAATTGTGGTTTTAGATAAAATCTTTTTAACGCTTCATCCTCATCTTAACCTATAA
GCAGAGAGTGATACGTTTTTACTTAAATTATATTTACGTTTCTTATTTTCATTGTCTATTTCAGAAATAAATC
TGACAGGTATATATAGATATTTATTGCTATATAAAACAAAGCTAAAATCATTTGGCTTTACTTTTACTTTGAT
TTTAACTTTAACACATATATCTGGAACAAAGTGTTTATTGTTGTTGTTTCTTATGTTA
AGGCTTTTAGGCGTGCCAATTGAAGTATTTATTGTGATATAAACTTAAAATCCGACGTCTTTTACAGTCAATC
AATAGTGAATGGCAATCAATAAGGACATATAATACATCAGAGACGTTCCGGCATATTTTGCCATTCATTTATA
GTAGTCGTCCATGCTTTTACCAAATATGGACAAAAGCGGTTCTTGACAAAACAGCCCT
TATTTAGCCTAGAAACGTTGAGTCAGTCGCGTTCGTTCCAAACAGTCACTTCACGTGAACTATTGGTTG
>126034627_2 EL596114.1
GCACGACGTATTACTCTAACAGTAATATAACAACTGCTGACTTTGTTGTCGGCTTTACAGTTGTGCCTTTTAA
GCCGGTAGGCGGTAGGGGTCGCAAATTGGAATCCCGCAATTTAAAAATACCGAGCCTTAAAAATATCGATTTT
AATTTTACAGCACACAAGATTTTAAAATTTTGATTTTTACTAAGATAAAAGTTACAAT
GCTAATTAAAACATACCTGTAATTTTAGCTTCTTTAAGATATACGATGGTTGTAAATATAGGCACGTTACCGC
GAGCAACTTATAGTTAATTCCAAGAAATTCCTTTGTTTATGTTTAAAAACTAGAGACATGATAAAGTTGTTTG
TGACCTTAACACCTTTTTGTAACAAAAATATAAGAAACAAGCGTACTGGTTGACTCAA
CTTTAAATTATTTATAATATAATAATTCATTACATTAATTAAATTTTTAAGTATTCTAAACATTATATTTATC
TTTAATTTTACTTTATATAATAATTTTCGTGGTTCACTTACGTATAAGCCATTGTGAACAGTGTCTTACTACA
AGCACACATTATGTTACAAACAAACATAAATTCCATACCATGAAACGGAATTTAAAGC
ACTTCAGCCTAATCATTTTCTTGCAAAATATTGCAAAAAT
>126034629_2 EL596116.1
CGGCACGAGGCATATGAGACTACACAGCTGTCAAATTGCAAATGTCAGATTGAACAAAAAATAAATCCAAATA
GGTTATCAAAATTGTTGCAACAATACCAAATAATGTATATGTAATATAAAAAAATATTGTCCTACACAGTAAT
AATATGTTTTGTAGAAATGTTAAGTCGGCAGCGGAATTGACCATTTGCAACCCATCAT
TATTCCAGCCAAAATTGATTAATTTAAAGTGTGTTCGAGTTCAAGTTCGTAATGCAATTCATAACACACGCCG
CGGCGGACGACGACCCAATCCAGAACCTCCTAATCCCTTAGAAATTCATGTGGACTCAGGGCCTTTACATGCA
CGAAATTTAGTAAAACCTTTACTGTTCACAGTTGGGGTGTCATCATTAAGCCTGGCTG
GCTGTGTGATTTGGGAATATGAAAATTTACGTTCTCATGCCACTTCATTTCTAAAAAGACCTGGCACTTGGTT
AAATTCACATCAGAAGAGGATAACTTCACCATTTCAATCAGAACCAGGCTCGATAAGAAGATGGTGGAATTCT
CTTAGAGAAAGTGAAAAAGTTTTTTATCCTATCTTAGCACTGAATGTATTGGTATTTG
GTGCTTGAAAATTCAGGCCTCTTCAGCCATTTATGGTTAAATATTTCTGC
>126034630_2 EL596117.1
GCACGAGGCCCTCGAGGAGGTTTCCGTGGGCTTCCAATGCGTGGAGGTGGCGGCGACAGAGGAGGACGATTTC
CACCAGGGCCACAAGGGCCCGGCCCTCATCCTGTCCCTACTATTGAATCTCGAGGAGGAGGGTTTAATAACAA
TAGAGGTGGTCCTGGTGGAATGAGAGGAAGAGGTGGTAGAGGAAGAGGAGACTTTGGA
CCACGAAGTGACCGAGGTGGACGCGGAATGCCTATGAGAGGCAGGGGAGGAAGACGTGGACATGGACCGCCAA
GTGGACCACCTGGTGGTCCTGGACCCCATCCAGGACCACAGAATGCGTGGTCCACCTGTGCAAGCTCCTGGAC
ATAATATTCCAACTGGAGCAACGTCTGTACCTGC
>126034633_2 EL596120.1

GCACGAGGACATTGCGTATAAAATAAAAATATTAATAAATAACAATTCATTCCCTCGAATGGGGATCCACAGC
TATGTATATGTGTGTCCATTTTATTGGTAGTCTATATTAATTGTTAATTGTAATTTAACTTTAATTGGAGTTT
TATTCTAGAAATAAAGATTTTATATTATTATTATGAATGCCTTAATTGACCTTCTTTA
CTTTTAATAAATAGTACGCGGATAATAGACGATCGACCTTTTTTAATTATCTTTTGAAATTTATATTTAGATA
TTAATTAAAATATATATATATATAT
>126034636_2 EL596123.1
GCACGAGGCATCAGTTCCTAGCTCACGTGGACCAGCTCCACATATCGGAGAGGGAGCACGCTCATCTACGTGC
CCTGTGCTTGTTCTCGCCCGATGGCGCACCAGAATTCCTAACCCGCAAGCTCCAAGATTACCAATCTCGAGTA
CTCCGCTCCCTGGGATCCCCCGCGGGAGAACGAGCGGCCGCACTCCTGCTACGACTGG
CACCACTGAAGGGATTCCCCGCCGCCTTCATAAAAGACGTGTTCTTTGTGGGCTTTGTGGGCGATGTTAGTAT
TGATGATGTTATACCTTATCTGTTAAATGCTGAACGTTGATTGAGGATTTTTTAAGAATTGCTACTGTAATAC
TTAGAATGTTCTTACAGTTCGCTCGAAGAGTCGTGGCTGTCTTGAAGTTATGTATATA
CAATTTTCTTAGTTTAGTTTGATTATTGTCTTCCTTAGTGAACAATTCACAAAAGTTCAAAAAACCTAACTCA
GGGCGACCACTACTAATCGACCATACTGTATGAAATTGCAAAAATGAGTGGAAGTTCTAAACATGGTGCTTTT
TTGAGGATTCCTCGCCATTTTCATATATGACATGTTCTTTGTGGGCTTTGTGGGTGAT
TGTCAGCATTGATGATGATACCTTATTTGTTGAGTTTATAGTTGGGTATTTCGGTGGATATCCCCA
>126034645_2 EL596132.1
GCACGAGGGACGTGTTCTTAAAACAATATATATTTGTTTGCATTAAATTACATATATTATTAATTTTTATTCT
TCATGTAGCCAATAGCAATAGAATATGCTTTTAATAATATTATCTATAGTTGCGGGGGCGCTCTCTGTACCGC
TGCAAAGCAAATGTGGATACGAGAGTTGTACCCAAGCTGAGCCTGGACTTCTCAACGT
GCATATAGTACCCCACACCCACGACGACGTGGGGTGGCTGAAGACGGTCGATCAATATTATTATGGAAGTAGA
AACAACATACAAAAAGCAGGTGTTCAGTATATTTTAGATTCTGTTATAAAAGAATTATGGCAGGACCCTAAAA
GAAGATTTATATACGTAGAAACAGCGTTTTTCTGGAAATGGTGGGTGCGTCAAACTGA
CGACACACGTCACAAAGTGCATGCTTTAGTACGCGAGGGGAGACTACAGTTCGTGGGTGGCGCGTGGAGTATG
AACGATGAGGCAGCGAGCCATTATCAGAGCACTATTGATCAGTTTACCTTTGGATTGAGAAAGCTAAACGAAA
CATTCGGCGAATGTGGAATTCCTAAAGTGGGATGGCAAATAGATCCGTTTGGACATTC
TCGAGAATTTGCT
>126034646_2 EL596133.1
GCACGAGGCGATGACGCCGCCTTGACTAATAATAATTTTACTGAAGTCAAGACTTTTGTTGTAAACGGCTCCC
TGAACGAGTTTTAGTTTAGTTTAGTTCACTATACTTGACCAAGACTAACTTGAACTTTAAAATCAACAAATTA
CATATTGATGAAAGAGATTTAGATCAGTTGACCAAAACTAACGTCACTTTAAAGTCTA
AGAACTGTGGATTGGTGAATGAGTCTTAGTTCAGTCTAGTTAAACTTTTTAATTACTGTTATAGTATGGCTAT
TCCTGTCAGATTCTTTTTATTTTATTTTTAATATAATTTTATACAAATATTTTCTTTATTTTCTATTTTCAAT
GAAATTATTTCTTGTATTAAGCTTAAGGTGGAGTGTGGAGGTTCCGTTTTTAGATTTA
CTTTTAAATATGCCTTTTATATGTAATAAATAATAAATATTGTTGAAAATATTATTTAGATATTGATATATTA
AACGAAGGCTATAAAATATACGATTTTATATTTACCATTTTCAAGAAATTCCATAATATATTTTGTCATCAAT
TATTGACATCGTTTTTTTGTGATTATCGTTTTTTTTTTTAATAAAATGACGATAAGAA
AATTGAGAGATCGTGATTTAA
>126034650_2 EL596137.1
GCACGAGGGTAAAAACAGCACTTAGGTTGTCGAATATTTTGTTTAGAGTTTGTTATCCATATATAAGATTGTA
ATGAATAATTGCTAAATCATATCAACCTTTAAACATTAAATAATCATTCTAAATTATTGTACTAGTGTTTGTC
CGTCAACTTTTAGATTTAGATATGCTTTTCTTTCAACTAAATTTAACATCAAACACTG
TATTTCTTGATTAATGAACTTTCCTTCGAAAACAATGTCTTATCTGGTGAAGTAAATTGGTAAAAAAATACAA
AAAAAATATACAGCTGGTTCCTTAAAATGTAGTAGAGTAAGTAGTTGTGTTCGGTGCCATAAAGTTTATAATC
TTTCTCATGTAAATATTTATATTACTATTTTATATATTTTTTTCACTATTTAAATACC
TATTTGTCACAGTTAAAGTGGTTGTTAGGCGTCGTTTTTTGTTGCAGTATTAAGCTCTAGCACCATCTGTTAA
GTGATTAATTAATTAGGACTCGACTTAGTTGTAAGAG
>126034652_2 EL596139.1
CATTAGAATTGATTTTATTATCCTTATAAAACTCATTTTTACATTATATACACTTCATCTCTATCTAATCTAT
CAAGTATAAAAGTTTTGAAAAATAAATATAATTTTTCGTTCTATTATAGAATATTCATATGAAATGGTAACAA
TTTTTTTATACATATTTAGTAAGTAAAAAAACTAAAAAACCCATACTTCAATCGTGAT
TTTGATATTAGTATAGTGGTAGAAATAGGGATTGCTTTTAAAATTATGGCTTCCTCAGTATAGAGGTTTTATA
AAACTACATATTAAATTATTAACAGTACAGAATTAGTAGAATATTTCCAATTTACGTAAAGATAGAATCTACT
TTAAGTGCTTTGTCCAAATTACCTTTTTAATTAAAATGTATATACAGCAACAGTAAAT

TAATATCGTTTCTATATCCATTTCAAATAAATATAATTAAAAATTTTTTCGACCAATCAGTTATCTTCGATTT
GTATGGGACATGCTTCTGGATGACTGTCACTGTGAGTTCGCTTGTATTTATAAGGATCTATAACTTCATAATC
GTATTCTCTATAATGGAGCCAATCATCCAAACGGTTTTGTGTAGTAAAGTCCCTTATT
TCTGCTA
>126034656_2 EL596143.1
GCACGAGGGTGATCAAAAGTGTAGTTAAACTAGTAAGATATTAAGAAACTCGCGAAAGACACTATAAATTACG
ATAGAACAGGTGTAATCACAGTTAAAACTTAGTGTTTTGAGCTAAAAACTCAACATAATACAGTGTTTAAATA
TCCAAAGTTATAACCTCAAATTAATAATTGTCAACGTCAAAGTTAACAACATACGTTC
CGTTTGCCGAATCTAAAACGTTTTGTTTTTGCATTGATAAAAATAAATACGTAATATGTCGGAGGAAATGTCA
ATGTCAACATTATATAGCATGATGAAGCAAGAAATGGAGAAACAAGCTAATCTTATTACTGCTAATACAGAAA
AAATTATGTTATCTATTGATGAGAAAATAAAACCATTAATAGAAGAAAATAAAAATCT
TAAAATAGAAATTGAAGTACTCAATAAAAAAATTACTACATTGGGAGATATGAACAAAAAGAATAATATTATA
ATACATGGGATAAAGGAATCCGAAACGAATTATTTACAATTATACAATAATATTAAGCAGTTTTTTCAAAAAG
TAGATGTAAATATTGAAAATTACGATATTAATAAAATGTATAGAATTGGAAAAAAATAA
AATAGAAAAAGTGAGACCAGTGCTGATCTCTTTTACTAGTTACAACAAGAAAGCTGAAGTAATGAGAAACAAG
AAAAAAGTACTGAGGACATTTATATAAC
>126034661_2 EL596148.1
CAATTCATTTGTATCGATCATATAATGTATTGCAAGGGAGATGAAATTTCTTCTTTTCATTTTATGTAAATAA
TGTGTTTTTTCTTTTCAAAGCGATACATGTTTTTTGTATTAAATTTGACAAAAAAAATATGAAATATGACATA
AATATAATGCATTGTTTATTCAAATTAAAGGCTTAAACAGTTTTTTAACATAAAGTTA
TATAAAGGCTCCCTAGATAAGAGGCGCCTTTGCAATTTAAAAAGTAGAATATTCTGTCTATTAATAATTATTT
GCTAAATTTGTTATGGAATAAGGATGCGGATTCAAATTTTTATATGAGTCTTTAATATATACATATTGAAATG
AAATACATTTTTTTTATTATTGTTTCATACTAACTTAAAATATTATTTTTTATTGAAT
TAAATA
>126034664_2 EL596151.1
CACGAGGGATGGCGAGAACTTCGGCAAGGACCAAAGATTTTGGGATATCCCTTGCTTCCCCAGGAAAAAGAAA
GAAAACAGCACAGATATACCTACCCAGCCACAAGAGGATCATTTCATCAGATCGCTCCCAGCAACAGGGTCCA
GAGCGTCCGTGCGGGCCTCAATAAGACTGTCGACGAGATCCACAAAGAGAAATCAAGA
CGAAAACAAAGCGTAAACTAAAAATATTAATTATTAATAGTAATTTTATTTATTATTTTGTAATTTTTATTAA
TTAAAAGAGATCTCTGTATAGTAATTTATGATTGGTTGTAAATTTCGTGAAAGAAAATGATTAAAATTGGAAA
ATTAAGTTATTTTAAAATATTAGGCTAAATTTCGTTGATTTCGTCATCTGATGACTCT
TGAGTGGAAAAGGAAATAACACGCAATAGTGTATCTCTAGGCAGGGATGTTACGGTGTTCCGTAATTATCGTA
ATATTAATACGTAAATTATATCGTAATAATATACCGTAATCGAAACCCATTATATGAGTGATGAGTGAAACGT
TTTTGATTTGGCCGGCCTGAGTAGG
>126034667_2 EL596154.1
GCACGAGGCCAAGAATAATGTCTTCTTTAGTAAAATTGTTTAAAGTGGATGGTGAAAACTGGGAAGACAGTCC
AAAACAAGATATGGTATCAATATGGTCAAAAATCCACACATTGATAGCCCAAATTGTAGCTGTTATGCCTTTG
ACTAGTGATCTCTTAATACATACTGTAATTGAACAGTTTCCTTACTACAAGGCAGGGT
GTTTTCCAAACAGAGCTTATATTCACAATTTAATCTGGATGAGTAAATATATACCGTCTCTAAGGGAGCAAAT
TATGATTACTATTATTAATAAAATGGTAGACATGGATACAAATATGGTAGATCGTAAAAAAAATAAACATGAA
ACAATATTTGACATGGAGATCGATGAAAAGGATGAAATAAGTGAAACTATCGACTATT
GTATGTTAGAAATGCTAAAATGGCTTGAAGATGAAAGAGATCCCGTTTTAAATATAATGTGCAATGTTTTTGA
GAGAGTTATATTACCTACACATGGGACAAAGCACATCCAATTTCTCTTATTATACACAATATCAATCAACCAG
CAGTGTGCGGACCGTGTGTTCACAAACCTGTGGATAGTTGCCGCAGGCTTACACGGCC
TAGGTCCTGGGGCATTGGCAACTAGACGGACAGCTGCTGG
>126034672_2 EL596159.1
GCACGAGGATTTTTAACTACAAGCATTCATTATTAGAGAGAAAGTTTACATTTCAATGAAATTTTATTAAATA
AAAATCCAGACATTACACACAAATTTATAATTTAGTAAGATATTTAATCACTATTTATATAAAAAATATTTTT
CCTGAATAAAGGTTAAAAGAAACTCATTTGTCAATAGAAATAAATGCCCAAAGTTTAT
AAAATAAAAAAACAATCGTTGCCTAACAATAACAATTATTATCGGTATAGATGAAGGCTAAGCCATTCTAAAA
TAATTTTTACATTTTTTAATCGTTATGCGATAATTTCTTTTTAGGGTTCCGTAGCCAAATGGTAAAACCCTTA
TAACTTTGTTCCCCTGTCTGTTCGTCCTTCCGTATGTCACAGCCACTTTAGTTGGAAA

ACATGGGTACCGTATGGTTCAAATTCGGAAGGCAAATGTATTTTACGAATCTCTACAAGATTATGCAGTATGA
TTTTGAAAATTAAAATTATTAAGGATCTCCATGGTAACTGAGAGATGAAAAAACCGTGTGGGAGTTTAATATA
GGTCTTTTCTAGACTTTTTTGATTACGCTATTACTTGCTATTTGATTTCGTGGCTT
>126034673_2 EL596160.1
CGGCACGAGGTTCAAATTGTATTATAAAAATGCGACCATTAATAGTGAATAACAATGACGCTCCGTCGATGTG
ACAGCTTACCCGCAAAACTTAGGGAGGTGCCGATCTCAAACTCAGAAATCCCTCCAACAAATAAAATATCCGA
TTCCAGCACTGACCCAAATTTCACCGACACGGCTATACGGGACGAAGTTATCGCCACT
TGGAAATCCCTTCACACCGACATCAAGAATATAAAAAATGGCTTCCCTACGAAGAATAGCCCCTCGGACTACG
GCGTGCACAAAAGGAAACCCATTACGCACAATTTCAGTCCAACATCAGTGCGCTTCAATAGAGAAAGGCAAAA
GAAAATCATTTATAATAATAAGCACATCTCGGTCGGCTCCCAAGACGTCGATCGTAGC
TCGTCATTCCCACTGGACACGGGCCAGAGGTGTTCCGCTGATAAAAATAATTCAGCAAGACGAAAAACGCTAT
TGGAACGACTCCTCTCCTGGAGAACTCCAGAATGTGATTGCCACATTAAATATCAGCCGCCCCAGAAATTGGC
ACCGACAGATTTCTGCACTTGTGGTGTATTGCAATCGATTAAAACGAACACT
>126034678_2 EL596165.1
CGGCACGAGGATTTCCTCAAATATTTATTTTATAATCTTGACTGTTGTTAAAAGGTCTTTTAGAACCAAACAA
GCAAGCAAGGTTAGTATGTAAATCTATTATATATCGATTTATAACAATATTAGTTATCAAGCTGATCGACGAT
TATAATGGATATTTCATACTAACTATATTACTACTACTTATAATTATATTTTTTGTAT
TTAGATCAGTTGCTCAAACGCGGTGTTTTTCATCTTGATGGATTGTTGGTATTTTATCTAGCTACTATATGTT
TTAGTTTTAAGTTTAGTAGGTTTTTTAGGTCGTGACCAAGTAACTGTACATTGCAAATATTTGTGTTTTCTTA
TCACTTATCGTTTTAAGAAAATGTACAATGAAATGCTTTGTTTATCTATTAAAAATTG
AATCCGGTGTACTATGTATTGTATGTATGTATTCACGTATTAATGTCGCAAAAATTTTGTAACAACAACATTA
ACATTATGTTTCTTAAATTAAATTCGAATTTACTTATGAAACATAAATTATGATAGTTACAGGGACATAAATT
CTAACAAATATTAACTCTTTAATCTTTTCTTTTGAACTACAGTCAATCTGGGTATCTT
TGATTCTTTGGGTAACATTGACAATGGGAACCTCGAACTAGTTTCGATAATTTTTCCGTATGACACAATACTA
TTGAATATGTTAATTTTAAAACGAAAAGAAACTTAAGAATG
>126034681_2 EL596168.1
CACGGCAACTTCATTGATCCAGTTTTTCATATTTTAAGTAAATTAAAAATAGTTTTAGTTTCTAAATCGTAAA
ATAAAACATAACATACAGTTTAAGTGTAGATTTACTTGCCCCTACATTAAAAAAAACATAAATCACCATGAGC
TCAGGAGGAACTCGCGTGTACGTGGGCGGGCTCGTCGAGGGCATTAAGAAGGAGGATT
TGGAGCGCGAGTTCGATAAATACGGCAAGCTCAACTCCGTGTGGGTCGCACTTAACCCCCCAGGTTTTGCGTT
CATTGAATTTGAAAACATGCAGGAGGCGGAGGATGCTTGTATGGCATTGAATGGATTCGAGATGTTAGGAGCT
ACCTTAAAAGTAGAGTTATCGAGGAAACGAGACGGCCCTCGTCGGGGTGGTGGTAATT
TCAGAGGCGGGCGCGGGGGTAACTTCAGAGGGCGTTCCTTCGGAGGGGGCCCTCAGGGCGGCGGCCGGTCCTA
CGGCGGCGGCAGGCCCTTCAACCGCAACAACCAAGGCAACGGGTACGGCTCAAGCTATGGTGGAAACTCCAGA
TCTAGGTCACCGATCACTCGATGTTAATTTAATTCTAGCATTTAGTTAGGTTTAGTGT
TATCGTAATCTGAAGCCTTCCAAGTG
>126034683_2 EL596170.1
CGGCACGAGGGTGGAAGAGCGGTCCTAGCGCTAAGGGCAAAAAGACTGTGCTACTGTGCCATGGGCTACTTGG
TAGTAGCGGTGATTTTCTCATTATGGGACCAGACAAGAGTTTAGCTTATTTATTAGCGGACGCTGGATATGAT
GTGTGGCTTGCCAATTTGCGTGGGAACATTTATACGAGTCACACAAAATATAAGAGAG
ACGATCCGAATTTTTGGGAATATAGTTTCCATGAACACGGCATATATGATCTACCAGCATCCATCGATAAAGT
GCTCCAAGTCACTGGACTTCATAAATTACTATACATAGGATATTCAATGGGGACCACGTCAGCTTTTGTCATG
CTGTCTGAACGGCCTGAATACAACAACAAGCTGATATCTTTTGTGGCTCTCGCACCAG
CTGTGTATATGACAAATATAAAATCATTCGCCGAATCTATGCTGAAGGATTATTCTATTACTGAACAAGCGCG
GTCGGGAGGATGGCTGGCAGTAAGCATACGGCGTGAGGCACTAGACCTATTGGTCCAGACTATGTGCTCAACT
AGAAGACCAGAAGATAATATATGTTTGAGATTCGTTTACCAAGTTATAGGGCACGATG
AAGACCAATATGACCCGGATATTCTACCGATAGTATTTTCACGCATACAGCCAGCCTCTTTTAAGC
>126034690_2 EL596177.1
GCACGAGGCACAAGCCAAATTATAGATGGAAAATTATTGGAAATTTTAGAGAAACTAAGTGATAAATCTAATG
TAAACAAAGAAAAACATAATAATTTAAGCAAATTAAATGAAAAATTTGTTTTAAAAAGGTTTGATAACAAGAA
CACTAATGCCAAACAATGGTTGCAAAATTTTGAAAGTGAATGTACTAGATTTGATTTA
AATGATGATACAGAAAAAATTAGTGCTTTGAGATTATTTTTGAATGACTCAGAAAATGACTGGTATGAGGCTA
TGTTAATTAAACACAGTTTAAATTCGTTGTGGAAAATATGGCAAGAGAGTTTTCTACACACGTTTGCAGATAA
AAGCTGGTCCTCTGTAGTGTATGCACTCAGTTTTAAACATTTAAATGGCTCTCTGCTG

GAGTATGCATTGAAAAAGCAGAGATTGTTACTAGAATATAACAGTGATATAGATACTAGAACTCTTACTGACT
TAATTGTCGCAGGATTGCCAACTTATATTACAAATAAACTTGATAGACAAGAGGTTACAAACCCAACTCTTTT
ATTTAGTGCCCTACGGATGCATGAAAATCAGAATAAAAATTTACAAAAACATTTTACT
GAAA
>126034691_2 EL596178.1
GCACGAGGGTTAAATCAAAATAAGCCTCTTATTTATACGACCTATAAGTCCTGCCCAGACAAACGCAACTATA
ATGATGTGATTAGACTTCTGTCAATGACCCTTCCTGTTACTGTATATCAGGGTAGGCAGAGGGAACCCTTTGC
TAAAAGACCCTGATCACTACTTTCCGATGAATTTTGGAGGCATGCGTATTGAATGGCG
GAACTCAACGCCACACTTTAACTCGACACCAGAGTGAAGAAATGAAAGTGTTTAATATTTCATTTCCTCGAGT
GGAGATCGAACCTGTTGCTTTTTCAGTCACACTTTATGCCCCGGCGCTACGACTGGTGTCATTCTCTATTAAA
ATCAAAAAATAATTTTAACAAAAATACGTAAATGACAATTTTTTATGAAAATTTCAAA
AATATTTTCGTTTATCTAAAAAAATCACAAACATGCAAGGTCAGTTTACTTCATAGACAAAGTATATATTTTT
TAATTAGTTTGAATATTTTTTATCACTCTGTATCCCGCACATAGCTGTGAGAGTAGAGTTAAAATACTCCGTT
TACCAATTTTTAAGAGCGAAGAAATGTAAATAATTTATTTCTATAAGTGGAAATCAGG
AAATGAAAAAATAGTTCTTGCCTCTAGGT
>126034694_2 EL596181.1
GCACGAGGGAGAGCACTTTTGGTGTCTCAGCGGAGTATTGGTTGCAAGTGTGGAAGCTGTGTGACGCGTTGTG
GGCTGCGGATTTGGACAATGACGGCATTCCGGGCACAGACGAGCTATCAATAGTGAATAGACACAAGCGTTTG
CTCGAGTGGTTCAAGTATGCGGTAAATGATATAACTGATAAGAAGTTGGCTGAACCGA
GACAAGGGGAAAAGGAAGATGAAAACGACTTCCACAGCGCTAGAGTACTTACTTTACTTCTCGGTGGGAGAAT
ATTAGAAGCGTGCAAGTTGTGTAGAGACAACGGTGACTTGAACATGGCGGCGCTTATTGCGCAGTGTGCAGGT
GACCCAGCCTTCAAATCCCTCATAACACGTCAGCTAAACACGTGGCGTGAATGCGGCG
CTGTAACCCTCTTGGCCGAAAACCGCATCTCGGCATTGAACCTATTATCCTCCGGTGGCAGGACACCCGATGC
GCATTGGCTGCATGCTCTACACGCCGTCGCCAGATACATAACTCCACAAATACCTACGCTAGAACAAATAATA
AGAACGTACGAAAGCAACTTCAGTTCAGAAGATGAAGTGGATTTGACGACCCTGCCCG
ATGATGAGATGCTACAGTTTCCTGTGCCACCGTACCTCGAGGGGTATTGTGTCAGTGTGAATGGTAAGACCCG
CCGTATTCTGGACCTACGCTATGAGTTACTAAGAGCTAGGGCGT
>126034696_2 EL596183.1
GCACGAGGAAACGTTTCAACGCATGTTGGATAGGGAGAAGGCACTTACTGGTTTATTGAAAAACGAACCGAGC
AAACACGAAGAGCTAGTCACAAAACTACAAAAAAACATAAAAATATCAAACAAAAATCTACAGAATGTTCTAG
CAGATTTAGCCCAGTACGAAATAGAAAAGATTAAAAATACACAACCTAAGCCCAAATT
CATATTTATGTTTAAAAAGGAAGCGACGCCCGAGTTCAATAGAAATATATGCAAGGGGTTAGAAAATGAGAAT
TTGTTTATTTTCATGGCGTCTGAGGAACCAGACAAGGTTAAAGAAGGTCAAATTATTATACAGGGTCCGGAAG
AGCATTGTTTAGCGTTGGGACAACAAATAACTGATATATTGAAAGGCAAAGGTGCATT
CAAAAATGGCAAATTCCAAGGGAAGGCGGGAGACTTGAGCGGTATTAGCAAATGCAAAAAATTGATTGAAGAT
TACTTTATTAATATACCATAGTACAATAAATGCTATTTTATAAATATAAATTAACAGACTTTTTTTGTGATTT
ATAAATAAATTGTTTTTTTTTTATTAATATTTTGTTAAGAAAAACGTAACAGAATAAA
CGAAAGATTGATGACCCAGAGGCGTAAATTCAAATTCCACTACATTTAATTTTTGTTTTTAAAACAAGTATAA
ATGTATATTTTTAGTATCTTTCTTTTT
>126034697_2 EL596184.1
GCACGAGGCGAGAGTGAACGTTCGAACGGTTATAACAGTGCACTCAATATTATTAGTTTCCATTGTCGTGAAC
TTGAACTGCGAGACTAATAATTGAAATTCATCACAAGGAATTTAATTTTAATAGATATCAAGCCAAAAATGTT
CTTCCAAAACTATCCACAGCTCAGAAATCTGGAGGTTACATTGAACCGCAGATAGACA
CATAACTGTCATGATCTAGTACAAATAGATCATGGTTTAATCATTAATCTTATGAGCATACCTTTTGCAACGT
TACTTCCTTTTTTCAATAAAAACATTAAGTGCCTCTATTTAAATACCAAATATAGAAAACAAAAGTGAATTGA
AATCTATCATATCAACATACGAGCATAGCACACTGCCTTAAAGTACCTAATAAATAAG
TGCCAAGTAATAATAGTTTTGCATCAAGATGTCGCATGGTGCGGTGAAGAGTGCGAAGAATGGGACCTCGCAG
GCAGTCGAGACGTCGAGAGAGGGCTCCGAGAGCCTGCCACCCCCGCCAGACGGCGGATGGGGTTGGATGGTGG
TTTTCGCATCTTTTATGATTCACATAGTCACTGATGGTATGACCTATTCGTTCGGCGT
GTTCTACGCAGAGTTCCTGACCTACTTCAACG
>126034705_2 EL596192.1
GCACGAGGCATAGTCGTTTTTACTTTTAATTGTCTAAGTTAAAACTTGAGAGGAATTTAACTTGTTTTTTATT
TTGTGATAAGAATAAGGAGCATAATTAAAGTGTTAAACGCTTACAAATATTTTAAAAGCAAATTTTGTTGGTG
TCTATATTTCGTTAAACAATTTGATAAACCAATACTAAAGTATTAATCCTGTTATAAT

AATCCAAGGACATAAACATTTAATCAATTATGTTGAATAAACATAAAGGCAAACTCTTTGTTGTCAAGAGGGA
TGAGAGAATGGAGGAAGTTCATATAGACAAAATAACTTCTAGAATAAAAAAATTATGCTATGGATTAAATAAC
GACTTTGTTGATCCTGTAAGCATAACATTAAAAGTAATAAGTGGTATATATTCTGGTG
TAACAACTGCTGAGCTTGATAATCTAGCTGCTGAAATTGCAGCTACTAAGACCACAGATCATCCTGATTATGC
CATTTTGGCTGCCAGAATAGCAATATCCAATTTACATAAAGACACCAAGAAGAGTTTTTCAGCTGTTATAACA
GATTTGTATAATATCATAAATCCACATACAAAGAAGAGGACACCAATGATATCAGAAT
ACCATCACAACATTATCATGAAAAACAAGGACAAATTAGACTCT
>126034715_2 EL596202.1
GCACGAGGATTAATTTCATTGTGCCTTATCTTAACTTTTATTTATTTCAATATTATTATAACTTACATACATG
ATAGCAGTTATTGCATTAATGGAATTTCAAATTTATTTATATTGTATTAGTTTGAGGAAATGTTATTTTCTAA
TAAAGTGTTTGGCATGTTTTTTGCTCCCTAATTGTTTTGTGTTTATATATTTCCTATT
TCTGAATTTCAGTTCTCTAGCCAGAATATGGATTGGAAATGTTGTTTCCTTTATGTGGAAGTAAGATTCATTA
TAATTGATGATTCTTCACATCTGCTTCTTTAACATTCAATAGTTTTATTTTCAGTTTGAGCGTATTTGATATT
TATTTTACACCTTATTTCATGCCTTTTTCGTTGTTCTTATATTATTTACATTGTCCAG
ATTTGATTGTTTAATTATGTATTTTTGTGTGCTTGCTTTCAGAAATATTGTCATTATTTCTAATTCTAATTTT
GTAATTTTCATTTTGTAATTAACTCTTTTATTCAAATGTGCTATATAGCAATTTAATGACAAATATATTTCTT
TTCATTAAAACAATTAGGTTTTATTTTTTTTAATTTTAATTATTTTGCAGTTGGACCG
GAGGCTGGTTCAGCTGGTCTCGTCAGTCAGATGTGATGCTACGCAATATTGAGCAAACTATTCTGTCTTGTGT
AAAGACAGCCTATAAGCGTTTCTACGT
>126034719_2 EL596206.1
GCACGAGGGTGACGTGGACCACAATGTTCAGCTTCAAGTCACGTAGGTTACTGCTGGTAAGCAGCGTGTTGGC
GGCGCTGGTTGTGGGCGCGTTGGTGGCGGTGCTGGTCCTGCAGCCTTGGGCCGGCCGCGACCAGATACGCTAC
CGCCGCGCCGTCGTCGCCGCCAATGGATACGAATGCGCGTCTGTTGGACGTGCGATAT
TGGAAAAGAACGGATCGGCAGTAGACGCAGCAATAGCAACTTTGTTTTGTGAAGGAATCGGTTGTGCACAATG
CATGGGGCTAGGAGGGGGATTTCTGGCAACGGTGTACGACGCGAGGACGGGCCGAGTACGAGTTCTCAACGCT
CGGGAACGAGCTCCAGCAGCGACGACTCGGGACATGTTCGCGAACGCTTCCTCAACTA
TAGGCGGGCTAGCCATCGCCGTGCCAGGAGAACTCAGAGGTTATGGCGCCCTCTATCGGGAGTATGGACACCT
GCCGTGGGCAGATCTCGTGCGACCAACAACAGAACTTTGCCGCAAAGGACACCGCGTCAGCGAATATATGGGG
CGCGTCCTCAAATCGTACAGCAGCAGAATCCAAGACGAACCATCTATGAGGGAAGTTT
ACGTGAACCCAGAAACAGGCGAAGTGTGGAACGAAGGCGATTTGATTCGCGAGCCCACATTGGCGCGCACGCT
>126034722_2 EL596209.1
GCACGAGGGTCATACGCCGGAGGTAGCTCGCGGCCGTTTGATACAGTGCGGTGCGTTTGTTTTGTTTTAGTGT
AAGGATTTACTATATTACTGTATAAAAATGCTGCATCAGCGACGAGCGTCGACTTTGACGGCGGCACTATGGG
TGTTTTTTACGGTTGTATTACCAATTGAAACACAAATGGCGAATCCTATTTCATCAGT
AATGCAATTTTTACAAGAAGGTACAAATCAACTAGATAATGAGCCACAAGATCAACAGAATCTCCTTTCCGAA
TATGATTTCATAGTAGTGGGCGCAGGCACGGCTGGATGTGTACTAGCAAATCGATTAACAGAAATACCCGAAT
GGAAAGTGCTTTTAATAGAAGCGGGTTTCAATGAAAACTTTGTTATGGATATACCAAT
TTTAGCAAATTATTTACAATTTACAAATGCTAATTGGAAATATAAAACAAAACCTTCAGATAAGTATTGTGCA
GGATTCGAAAATAAGCAGTGTAACTGGCCACGTGGTAAAGTCATCGGTGGATCTAGTGTCTTANACTACATGA
TTTATACTAGAGGGTCACAATATGATTACGACAATTGGGAAGCAATGGGCAACCAAGG
CTGGGGATGGGATGACGTCCTTCCATATTTCAAAAAAATTGAAA
>126034724_2 EL596211.1
GCACGAGGATTTCAATGAAATTAAATTTTAAATTATGTTATTATTATTTATTATATTTCCTGTTGTTTCCTGT
ATCATAATGATTTTCTTGCTTATACTCCTTTTCTCTACATGCACAATGACCTAAGCCATATGTAATGGCCACA
TTTATTTTTCAAAATATCATGTTTGATTATGGTTATCATTTAAAATATATATTATTCA
TTTAAAATATTATTCTTATGCAGGTAAAATTCGCCATAATAAAATACGTTATTTATTTTGTCAGGTCATCCAA
TAATAAGCAGCAGCTATTATTATTATCACTTTTATCATAATTTCAATTATATTTGTTAAATTAATAACACTCC
AAACACATGCATTTAGTACACGTGGAATTTCTATTAAACAGGACTGATATTGAAATCT
GTTTATTGTGAAATTTGACAAATTATATATTATATAGGAATTAAGCAATAAGCATAACAGTACAATAATCTGC
ATTTAAATTATCCAATAACCAATTTCCACGACATTATGATTAGTAAATAAAAACAAAACGACTACCACAGATC
ACACTTGGTGATGTTAATCGATTGCTTAATATTTGGATCGATTTTAAATTATGTCGAT
TTATT
>126034726_2 EL596213.1

GCACGAGGCGCCGCTTTGATTTGGTACTCAGCGGACACGTGCTTGGACTTCGAGTGCGCACGCGCCGGTCGGC
TTGGGATCATTTTTCGCGGAAATATCTAATTGCAATGGGAATACGGATATTGGTACTGTTGACGCAACTTGCG
TTAGCGATGGCGTCTCTGGAGGTCATCAGCCAATGGAGTTTGCTGCAATTCGACTTTC
CTCCAGACCCAGTGTTGCTAGAAAAGTTTCAGCCCGAGAATACGGTGCCGACTGGACTAGAAATTGGATGGGA
TCGCATATACTTAGGGATTCCCCGGCTGCGTGCTGGTGTACCTGCTACTCTAGCGTGGATCCCACGGTCTCTA
CCACCCGGCGTCAGTCCTGTTCTACAGGCGTATCCTGATTGGTCATGGCACACCGCCG
GGCGCAGTGACATCAACTGCACAGGCCTGATCTCTGTGTACCGAGTGCGCGCTGACCGTTGCAACCGCCTATG
GGTGCTCGATGCTGGCGTGATCACCAGCCTCGATGACTTCCCGCGCGTTTGT
>126034729_2 EL596216.1
GCACGAGGCTTTCTTTTTGGCGTGGCGTCACTCGGTGGCGGACGTGTAGTGATCGCTTAATCGTTCACATCTC
AATCACACTTGTTTAACGCTAAAAAGGCTTTATAGTTTTTTTCATTTAATTAACAGTGCTTGTAACTTTTCGG
ACATAATGAGGCCGCAACAGAATGGTGATGACGGTTTTGATGAGCCAGAAGTTCATCG
TAAAATTTTCATTGGAGGTTTGAACTATCGTACTTCTGATGAATCTTTAAAAGCCCATTTTGAAAAATGGGGA
GAAATTGTGGATGTTGTTGTCATGAAAGATCCCAAAACGAAAAGATCTCGAGGATTTGGGTTCATCACTTATT
CCAAGGCGCACATGGTAGATGATGCCCAAAATAATAGACCACATCGCATAGATGGCCG
AATTGTAGAAACCAAAAGGGCTGTAGCTAGACAAGACATTAAGAATCCTGAAGCAGGTGCTACTGTTAAGAAA
TTATTTATTGGTGGCATTAAAGATGACCATGATGAAGATCAACTGAGAGAATACTTTTCAAATTATGGAAATG
TACAAAATGTTAGCATAGTTACTGATAAGGGAACAGGAAAGAAACGTGGCTTTGGTTT
CGTAGAAATTGATGATTACGACCCAGTTGACAAAATATGT
>126034731_2 EL596218.1
GCACGAGGCTCCAACAAGCGGCCAAGCTGCGTTGCCGCGCGTTCGTGACGCCGGCTGATGTAGTCGGAGGAGT
CTACAAGCTGAACTTGGCCTTCGTTGCCAATCTGTTCAACCAGCACCCAGGGCTGCAGAGGACCGATGTGGAC
AATGAGGCCTATCACCAGCTGGATGAGACTAGGGAGGAGAAAACATATCGTAACTGGA
TGAACTCCATGGGTGTGGCGCCACACGTCAACTGGCTGTACTCGGATCTCACCGACGGCCTCGTTATTTTCCA
GCTCTACGACATTATCAAACCCGGCATTGTTAATTGGAAAAAGGTTCATCGCCAGTTCTCCAAGTTACGTAAA
TTCATGGAGAGATTAGAGAACTGCAACTATGCAGTGGATCTCGGAAAACAGCTTGGTT
TCTCTTTAGTGGGCATCGCCGGAGCTGATATCAATGAAGGAAACGCCACACTTACTTTAGCTCTAATTTGGGC
ACTGATGCGAGCTTATACACTGTCTGTACTGACGCGGCTCGCCAACAACGGGGAATCCCATTATTTGAAA
>126034735_2 EL596222.1
GCACGAGGATTTTTCAAATATAATGTAATTTGTGAAACGTATATATCTTATAATTTACTTCATGTAACTATTT
CTAATAGTATTTTATTAATTTATATTAATCATTAAACGATTTTAATTGGATATTTCGGGTTTAAAGTCCATCG
CATCTTAAATTATCGACAGTGCGCTTCTGATAATTGTTTTTATTATTGATATTTTCTA
TTTTGGATTCACAAACGAAAATGGTTGCCTAAATTTTCCATAATAATTTGAAAAATCGTACTTAAACGTAATA
TTAGCTTTATTAAGTGAATAACCATTGATATAGTGAGATTTTTAATTAATAAATTTTGTATACAACACATATT
AATATCTAATAAAAGATAGCAAGATGTCTGACGGTCATATAGAAGTAAACCAGCAGAG
GAAAGAGAGTGTAGCAGCGGCTAATGTACCCAGTGATAGTCCAGTGACAGACCCTGGCCAGGTCCGTGGTGGA
AATCCTGATGGTTCAGCAGGAAAATTCGAAGGGCAACGAGGAAAATCTGGTGCATTGAAGAAGTCTGCTAGAT
ACAGAAGTAGATCCCTTTCAGCATCCTCAGCTGATTCATACAGCTCTACATCATATAC
TGGGTCATCCTCCGACGAAGATGAGGGTGTACAGCCCCACGAAAAGGCAATAGCAGCAGGACAACCAAACCCA
CCACA
>126034739_2 EL596226.1
GCACGAGGCAAGGTCTTATATAAAGTTATTAGACAAAAAACCTATAATAGGCAATAATTTGCAGACAGGCGGT
GCGGACAGCGCGAGCGGTGGCTCCGGAGGCTCGGGCATGGAGTGTATGCAACTACGATCGCCTCCTGGACCCT
TCCATTACCTCATTTCGGAGCAGGCTAAGTCTCTCGTCGCGTTACAGGTAATTTCTAT
TATAATAAAATATATATCTGTGTTAACATTTTAAAATTATTATTACTTTTTTTATTACTATTTACTTGGTAGT
TTATAAATCCTGTACAACTGATTTCAAAACATTTAGTTATATATCTGTTATTCTGGTACTTACTACATAAACG
GAAAATTGACATTTAATGACACATTTATTTAACCGTTTTTTTTAAATATCATTTTCAA
TCATCTTCAGGAACTCCAGAATGAGGTCGGGGCATTACTCGAATTTCGCGATCTCGTCATCGAGACCTTCCCC
AATTTACGTTCCAAAATGGCGCCGTCGACGCCGGCGAATGAGACGCGTCGCGACTGGGAACCCGGAGTGCGAG
TACGGAGAANACTCGCAGGAGGCAGCGGAGACACTGCCAGGACGAAACCACAACCCTC
TGTTNCAGACTCTGGCTTTAGCACAGAGACATCTTGTGGAAAAGACGCTCATTCCTCTGCCTCCGCATCCGCC
TCCCGCACACGCCCTCGCCCTCTCGAAGATGAGCTGTGGGCATTACTCGACGTTATAC
>126034741_2 EL596228.1

GCACGAGGCAAACTTCCTAGGATTTTTGTTACATAAAGAATAGACAGTATACTTTTCATAACCTGAAAATGGC
TGTTTTCAGTAAAAATGTGTACGCAGTTTTTAAAAAATATCCCTTACTAAGGGGTATGATTTCGTATGGTATA
ATGTGGCCTACATCATGTTTCATTCAACAAACTTTTGAAGGAAAACGCTTTGATTCAG
AAAATAAATATGATTGGCTTAGATGTGCCCGTTTTGGTTTATATGGATCATGCTATGTAGCACCAACATTATA
TAGTTGGTTAACTGTAGCTAATATTATGTGGCCTGGAAGTTCTATTAAAGCTGCATTTATCAAGACCTTTCTT
GAAAGTGTTACTTATACACCATTTGCAATGCTCAGCTTCTATTTCTGCATGAGCTTGC
TGGAATTGAAGCCATTTAATGAAGCTGTGGCTGAAGTGAGAACAAAATTCTTGCCTACCTATAAGATTGGAGT
ATCTACTTGGCCTATCGTAGCAATGATTAATTTCTGCTTTATACCACCAAAAAACAGGGTACCATTTATAAGT
GCATGTAGCCTTATATGGACATGTTTTCTTGCTTACATGCAACAATTGGATAAAGATA
AATTACCAGTAAAAAATCCTAATAAGTTGTTAAAAATATAAATTTATTATGTCAATACAATACAATAGCAG
>126034742_2 EL596229.1
GCACGAGGGTTGGGTGTATGTTACTCTCGACTCGAGTTCACGTAGTATAGTCATTTAATATTGTATTTATTAA
TTAAACTTGTGATTTTTAATTTCTTGAGTATTCTATAAGCTTTGAGTGGCCTACCTATTAATTGTTAAGTGTA
TATTTTTAAAGTTTAGTGTTATAATAAAAGTCGACGACCTTGTTTGAAACATGGCCAA
AACTGAGAAGTCTTTTCCAAGAAACTTTACAGGATGTCTGACATGCGTCCAAGTCCTGAACATCATGGTGCTC
TTCGGCTTCATGCTGAACTACGCGCTCCGTGTGAACCTCACTATAGCTATAGTGGAGATGATCTATGATGAGA
AGGACAACCACACACTTATCACCAATGTCACGGATTCTCTCAATGTTACCCAGCACCC
CGGGCACATAGAACAAACCCGCTACCATTGGGATACAAAGCAAAAGAATCACCTATTGGGTAGCTTCTTCTGG
GGATACATACTGACAGAACTGCCGGGTGGTAGATTAGCTGAAATCATTGGAGCTCGGCGAGTTTTCGGCTACA
GCACACTTCTGGCTAGTATCTGCACACTGCTGTCGCCAGCTGCCGCGGCTGCTGGTTA
TTGGTGGATTGTCGTTCTTAGAGTATTCCTTGGCTTCTTCCTTGGAGCCACATGGCCAGCTATCCTGCCTCTA
GCCTCAAAATGGGTCCCGCCCATGGAC
>126034743_2 EL596230.1
GCACGAGGCCAAATCGAAAACAAAAAACGATCAAGTGAACCAAAGAAACGACATTCCGACTTCGATTTACCAA
GAAACAGTCACATTAAATTCCGTACAGCAACTTACGAATCTCCAAAAGGGACGATCGTTACGAGCACGGACTT
AGAGAACAGACGACTTTCACAATTAGATCAATTGCACAAATCGAGTGAACCAACACAA
AAACCGATAATATCCGCGAAACCGAGTAACATACCGGTTAAATCGGTAGATAAAAAGCCGTTTTCAGGCTTAG
TTTCGTCTAAAATACCGGTTTTCGCCACACAAAAATCGTTAAGTCAGGAAAATTTGTCGGAGAAAACTTTCTC
GTTACCCCGAACTGAGTTTGGCGGTAGTGCTGGAAATATATCGATTACGTCAATAAAA
AGCAGCTCGAGAAGTCCAAGTGGTGGTCCATTATAGGCTTAGTTTGTTGTATTTAGAAATATTCGATTGTAAG
TTATTAGTTATTCTAGGAAGATATTCGATAGGATAATTTCGAACCTTTTGATATGTTTTCTTTATACATGCAT
ATCTTGTATAATTTCTTATATTATGAATTTTCATCACTTCAGTTAAATAGATCCAAAG
CATTGTCTATATAAATGGAATCTACGTTTCATGTTTATTACAAATATCATATTTTCCCTGAGGATTTTTTTTA
CAATATCGGAGTTCTTCCTAAAATAATGAAAGTATATTATAAAACTAGTTATACTTTG
>126034744_2 EL596231.1
GCACGAGGCATAATCAGGTGTAGTAATAATGAAATGATCCAATCTTTACATAACTGTTTGGTAAAGCGATATA
AAATAATATAAATCACACATTTAAGTGTTTGGTGGCTGAAGTAGACATAGCACGATTTTACTTCAACTTTGAA
TTAATTACGCAGGCCGGTTCGATTCTCGGGCGAGGAAAGGAAATATTTATTATTCCAC
GCCCTGGTAAGGTGGCACAACGCGGCGTTTTGCCACTCAACACGCAATGCCTCCAGAATTTGGCGGAAAGTGG
AAAACGGAAGTGTTCCAACTATATATTTACATTCAATATTGTTCATACTATGCAATTAATAACAATAAACATT
GTTATCTCCATGTTACATGATTATGACTTCATAACATGGATTTAAACATTTTTAATAC
AACCTACAAATGACCATTTATATGCAATCATTCCGAACTGTTACTATAGAATATTTTAATTACATATACATAA
AGTTGCCTAAATAATAGTTTCTTTTTATATCTTGAATATGTTTTAGATTTAGAATAATTATTTCTCATATTAT
ATTTACCTTTATGCACGTATACTTTTAAATAAATTTTATGGAATAT
>126034749_2 EL596236.1
GCAGAGGGTCCGGCGGGGCCGCCTCTGCGTCATCTTACCATCCCTATAGGCGTTAATGACTAATTTTACATTG
CACTGTTTACCATTCAAAACTTAAATTTACCACTCCCTGGAACATTCTGAGTATGTTTAAAACTGTGACTTGA
CAGTTCCAACGTATTAAGCAATAGGTATTTTGACACAAAGTCGATTAATATGACTAGA
TAGTCTGGTTTGCTTTAGTTCTGTGTTATAACTGTTAGATAAGTTCCTGTTAAATTTGGTGATTTGTAATGCT
ATCTCTTTCTTTTTACTCTTAAAAGACGGCGACGTTGATATTGTGTCGGATAAATTAGCAGGAGCATATCAAA
CAGATAAACCTAGCTCTTTAGACTACGATCTAAGCAGATTCCATATTTCGTCTTCTTA
TATGAATATATATGAGTTTTAATTAACACATTTGTAAAATATGTACTTTTTCTATAAAAACAATCATTGAGTG
TATAGTTGGTATAGCATGGCGGTAACGACACTTTAACCAACTTTTAATTCTTATAACTAAGTTGGTAGTATAG
ATACTTTCATGTTTGTATGTATTTCGTGTACCTTCTTATTAATTTTGTGGACGGCCGG

TTTCATTAGAAACGTTGAAATTTAATAGTTATAGTTACTACTGTATAAGTTCACAT
>126034751_2 EL596238.1
GCACGAGGATTGTGAGGTTGCACTTAACAGCGTTTTACGGTGTTGTTGAGTGAAGTTAAAGACATGGCGCTGA
TAACAGAAAATATGGATTTAGAGAGGGAGTATTCTCCAAGTATGTGGTCAAGAAGATTTTCGTCGGCGCAAGA
GGTCCTACAGTATCACGTGAATTTTGTAACCACAGCAAGCGAAATAGCAGTTAACAAC
GTGCCACATAAATTAGAAATTGAATACGGATCAACCCCAGGGCAGAAACTCGATATTTTGGGAACGGATCTTC
CAGATGATTCACCAATTCTAGTCTACGTACACGGAGGCTACTGGCAGGAGCTATCCCGGGAAGTATCCCGTTA
CCCAGCGCAACCTCTGCACAGGTTTAGAATTAAAACTATAGTTGTCGGCTACGACCTG
TGCCCCGCCGCTACACTACCAGAAATTATAAACCAAATCCAAAACGCTGCGCGATTCGTATTTGAATATGCTG
AGAAAATGGGATCAAGAGGCGTATACTTCGCTGGTCACTCCGCCGGAGCCCATCTGGTAGCAAAATTATTGTC
CAATTCCGATTTCCTAGAAAACACGCCAGGATCTTACAGACTTCAGGGTGCATTTTTA
ATATCTGGCGTATTTGATTTGC
>126034755_2 EL596242.1
GCACGAGGGGAACCTAGGAACATAGTGTAAGAAATTGTGTCCCTCATTTGGATACTACCATTTCCCATGTTCC
AGTGAATTCATCAGGCATTGTTGATTGGCTGAACTCAAGGCAATGGTGACTTTTTACCAGAGCAAGATAATAA
AAATATTTAACATTTCTTTTCTTCAAATTGAAATCGAACCCACAAGCATTGTATTTAC
AGTTGCATATTTTTGCCCTCTGCTACAACTGCCTATTTTTATCTTCCTTATATGTTTTCGAAATATTCTATGT
TAAGTAGTGAAATCCAAGAGCAAAAAGTCTCAAGTTTTAGCCCCACTATATTATTTTTTTTATATATTTTCTA
AAATATTTGCTTTCCTTTGTGTAGAATAACTGCAAATGAAAAGTTTAAATATTTCGTA
AAAATAGCTTTATTGTAATTGTCAGTATATTATTTTTGTTATGATTAGATAACAAATGCAAATTGTAAACAAA
ATAAAGTACAACTGATAGTGACTTCATTTTGCTGCTGTTTGTTATCACTTGGCTTANAGGTGATTTTTAAATT
TAATAATAAAACAAATTAATTTTTAAATTTAATACTTACTGTCGTGGCACAGTGGGAG
TGTCTTATGGTAAACATAATGGTCTGACTCCTACTCAGAGAATTGAAATTGTCAGAAAGATCTAATTTTTAGA
GGGTCTTAATAATAAATTTTCTCTACAAACATATGTATACCTAACAC
>126034756_2 EL596243.1
GCACGAGGCTCCCACTCCTAATTCTGCCTCAACTGGCTCCACGCCTGCTTACACGCCGCCTTCTGTTGGTTGG
ACGGCGCTGGTGTACGCGTGCAGCGGCGGCGGCACGGGCAGCGCGGAGGTGGCGCCGCCGCGCCGCCGCTCGT
GCAGATCAACGATATACGATACCACATCTTTGAGCAAGTAATGAAATACCTCTACTCC
GGAGGGTGTTCTTCACTAGAATTACCAGCAGGAGACGTGCTCGAGGTCCTCGCTGCTGCCTCCTTCTTCCAGT
TACTGCCGCTTCAGCGACACTGTGAAGCGAGGGCCGCTAAGACTGTGGACTTGCATAATTTGGTGTCTGTTTA
TATTCATGCTAAGGTATATGGCGCTACACAACTATTAGAATACTGCCAAGGATTTCTT
CTACAGAACATGGTCGCTTTACTCACATACGACGACTCTGTCAAAAGACTTCTGTTTGGCAAACGGCTGCCGG
GCCATAATGTACTCGCTGGCTTATTAACTACACTGCAAAAAAGAATTGAATCCAGGAAAAACCAGGCCAAGAA
TAGGTAGCAAGATAAAAACTAATATTATTATCCTGTTTAATTTATTATTTTTCTAAAT
TATCTGTGCGTTTTTCTTTGTTTGTATTAAAAAAGTTGTTGAGATGTACAAAAATTATAAATGACTGATACTT
CTTTATTTAACTTGA
>126034762_2 EL596249.1
GCACGAGGGTCAACGTTGCTATTGGCATTAAGCAGCTGAAGAAGGGATACGAACTGAAACCTGGCATTAAGTC
GTCTTCAAGGAGGATCACGGTTAACGGATGTGATCTCAACGATATTCCATGCCAGTATTCTGTCGTTGGTCTT
GAGAGATATGAACTGCCCGTAGTTGGAACCTACGTGGATCCTCGTATCATCCCTGGAT
TTTATTACCGAGTAAGGCCAGCGACAAGCCGTCGTCATTTGTTTGAAGGCCGCAGCTTACTATTGCAATCTGT
TGGCATGGGCTACGGAAAACGTATCACTTTTAAACCAGACACTCTAAATGCCCCAGAAAACTACTTCTGGTCT
GACTCTCACCCTGAAGGATTAGGAATGGAACCACGCGCTGTTCATCCCAATATGAAGT
TTACAATTACAGGGAACGGGGGTTTGCTTGGAGAGGCGAGTGTCTTCAGAGCGGACCTGCCCCAAATTGAAGA
AAAGACTGAATACGTAGATGTGCCGGGGCTAAAAGGAAAAATTGTTGAGAAATATATTCAAGTGGATGTCACT
TGCCACGTGAAACTGGCGACGACCGGTGGCGGCTCAGTCGACTCTGAGGTGCATCTAA
TGAAGGTGTCCGGAACAGCTCTCGTCCGTAAAGAGCCGGGGCCAGCTGCTGCCAAACTTATGAAAGTATACAA
CGTCGGCTTGGACTCACAGCTCAACCTGCTTTTCGCTCACTCCCAAACTGAGCTCACCTTTCA
>126034763_2 EL596250.1
GCACGAGGGGGGCTTCGGAGCGAGAGATTTCCGTACTCAACCGAGACAGGCGCCGCGCTCTGCTGGACCCTCG
GCTGTTGGCTCTGGATTCGGTTACGGCGGCGGCTCGTACGGCGGCAACTACGGCGGCTCGTACGGCGGCGGCG
GCGGCGGCAACAGCGGCGGCCCCGACTGGTGGGACTCGTAAGCGCGCGCACCGCCCCC

GCGCGGCACACGGCCAGGCCCACATACCGCGTCACAAGTTTTCCAAATTGTCGAATTACCGTTTATAAAATAA
TACTACTACTAACTACTACTACTCTGGCCCAACGCCGTCCCCTTGTGCCACAGCGCGGTGCGACCGCTGAATC
TCTCTTAAATCATTAATTATTATATATTACAATTACGATATGCCGACGGGAGGCCCCC
CGCACCACGCGTGGCTCTCGATCGTTATCATTTTTCGATATGATGTGAGTAGTTGATTAATATAGAAATATTT
TTGATGTTCAATCTGTATCGTGAATTTGTTCGAATGTATTACGATTTTTTCTATTTTCCCGACTGTCTGTCTG
TCTCGTCGCTGATTCACCACTACCGATATAAAATTTTGAAGCATTTACGCACCCTAAT
GTCGATGTAGCTATTTAACGCAGTAGATATGGTCGAGATTACCGATACAGTATCATAGTTATGTACTAAAATT
TGTCGTAGATTACCGTAATTGTAACATCTGTCAACATCGTGGTCTATTAT
>126034766_2 EL596253.1
GCACGAGGGAAATCTATGATGTCGAAATATCAAATAAAAGCTCCATACAAAATGTGGAACCGCAACAACTAGA
TGTATTATGCAAAAATGTTGGTACAATAATAAAAACTTCTTCAGAAGAAATGTCGGACGAGATTTTGAATATA
AAAAGTATCTCTCCTATTGAAAGTGAAGCTATAAGTACTCAAATTGTTGAAAAACTGC
AAGTTACTGAAGATATTTCATCAAAAATTGCGTTAGATGATATGATTTTATGTACAGAAGACATTGTAGTACC
AAAAACTGAACTTGTATTGACAAAACAAAAAAAGAAAAGGAAAAATAAGAAATATGAAGGCGTTTCATGTGAA
GATAAAAATAAACCAGAAATATATGTAAATAAAAGTGATGTTTGCTTCATTGAAAATA
TAGATAATCATGAAACATCTAAGATGTCTGCGTTAAAATCAGAAATTGTCAAAAAAGATATTGACATTATAAA
AGAAAACGATAAACCTACTAATATTATTACTTGTGCTAACGAAAAAGTAAATGAACCTAAAAAGAAGACAAAA
TCTAAAAAAACTTCACTTAAAGACAAACAATTATTGGTAATTGAAAAATATTTAAAAG
ATAATAAAGATACAGATGAATATGGTACAGATACTAAATTCGAAAAGCTATCAAAGACTTCAAATGACGACAG
TAATACATTGCTTAAAAAAATTGATCAAGTGAA
>126034771_2 EL596258.1
CGGCACGAGGGAAAATTTATTCTACCAGAGTGGTCAATTTGACCCTTGTAGATCTACCAGGTATAACAAAGGT
ACCAATAGGTGATCAACCTGAGGACATAGAAAATCAAATTAGAAATCTTATTATCAAATATATTGCAAATCCG
AATTCCATCATTTTGGCTGTAACAGCTGCTAATACTGACATGGCGACAAGTGAAGCTA
TTAAAATGGCAAAAGATGTTGATCCAGATGGTAGAAGAACTTTAGCTGTAGTCACTAAGCTTGATCTTATGGA
TGCAGGAACTGATGCTATCGACATTTTGTGTGGACGAGTTATTCCGGTGAAATTGGGAATCATCGGTGTCGTT
AACAGATCGCAGCAAGATATTATTGATAATAAATCTATTGCGGATGCATTAAAAGACG
AAGCCACATACTTACAAAGAAAATATCCAACGATAGCGACTCGCAACGGAACTCCATATTTGGCTAAGACTCT
AAATCGACTTCTTATGCACCATATACGGGATTGTTTACCGGAGCTTAAGGTGCGTGTAAACGTGATGATATCA
CAATTCCAATCGTTGCTTAACTCTTATGGAGAAGATGTATCCGACAAATCGCAAACGC
TGTTACAAATAATAACTAAGTTTGCAAGCGCGTATTGTTCCA
>126034780_2 EL596267.1
GCACGAGGAAGATACCAAGATCTTCAAGCAAAAATTATAACAGGAAAATAGATTTTACAAAGGCTTATGAGGA
ATACAATTCTGAATTTGAAAAAGCAGAAAAAATTAAAAGAAAAGTAGAAGAGAAGAAGGCCATCTCCAATATA
ATGTGTTTTTATTTATTATTATTAAAATAATTAGTTTTGCTATAAAATACATTAACTG
TATGCACTATAGTAAATGATTAAAAAAATCTAATCTCACAGTATCTGATATGTTACCATGTGGGTTGTAGTTT
TGACTCCCTTGAGGGAATAATTTATTTTTATTTCCTCACTCTGGTAATATGACAAAGAGCAGTATTGTTCTCC
CACTCAACACATTTCTCAGAAAATTGGACTGAATGTGCCCTGTCATGGAATGCTTCAT
ATAGCAGGGTAGGCCCCTTGTATATGAAGCATGAAGTTAAAAATAATATAATTTTAACAGACATATCTATAAA
ATTTTATGCTTTAAAATTTAAAACTTTATGCTTTTAAATTTCAGCGAATGGATAACGGAAAATAGTAATGATG
GAAAGAAAAAATATGAATTAACAATGCAACAAGGCATCAAGTAAACTCTCTCATAGA
AGAACTCTGCAGAACTTATGGTTAAGTATGATAGTGGTTGGAACATAAGTCAT
>126034783_2 EL596270.1
AAATTACAACTAACTATACGATTAATATGTACGTAAAACTAAAATTTATTCAATTTAGTATCAGTAATAGAAT
TTAGAAAACTTGAAAAACTTCTTCAGAAATGTAGAACTAGTAAATTTGAAGAATTTCGTACAAGTGACGCGAT
GCTTGAATCGATTTTGAAGCCAAATCGATTCGTCACGAAATTTAATATATTTGAATCT
AAATTGTATATACATTTTTACTGCTTCTCACACAGGGTTACTATATGTATATATTTAATATGCTGTACGTTTG
GCATGCATTCATTCGCTTTAAATGAATGATATTGACTGATACAATTGACTTACAATATTTTGAGATGATAAAT
TTTAAATTTGATATTTGGCTATAATTATGCACATAAACGAAGATGCAATTTTTTTCAT
AGTATTTGTTTAAAGCAGCAGTAAACTCATCTCTAAATAGATAAAATATAAATATCGAAGCTCACTGAACTAT
GTATCACTCGCCTGCGATAGGTCATCGTCACGTAAAAATATACTACTGTATGAGAATACTCGTATGTAAATAA
TAAATCTATTTAGATTCATTTTATTTTAGTCTTTCCCTTTTCGTACAGAGTGCATCTT
AAGTAAAATGGTAGTCGATTACGTTCCCGGTGTATAATGATTTTGTTCCCTTAGCATATAAGTAGATGGGCAG
AGGGAAACCTTATAAGATACTACGTT

>126034786_2 EL596273.1
GCACGAGGAACTATATTTTTGCTTGTTGTGTTCGCATTTTAAACTTATTATTATTTATTTTTTTTCCCGACTG
ATCTTAAACGTGAACAGGAGGTACTTTGTTACACGTAATAATTGTGAAAATTTAATACCTATAAGGCACAAAG
GGTCCCCTGTTTTGTATCAAAAAAAATTTGCGTGAAACATCGTAACAAATGTTCACAT
TTTTATAAAAGTAGTATTTTGAACTTAAATTAATTTAGATTTATTTTAAATATCATATATATATTTAGCAAAA
AGTAGCTTAATTTCGTTTCGTTATAAACTAACGTATACGTGTATGATGAGTTTCATATTTGCTTACGATATGT
TTTATGTTGTAACGCGGATTCCCTTTTTTGTAGTAGTTTGGTATGTTTTTTAAATTGT
CAATATATTGTATAGTATTTTATACAATTTTGTCCCACGTCTGGGACTCGCGGTATTGCCGCCGCGTGAAATT
CAAAATTTACCATAGTCCATTTTATATCGATTGGCTAAGACGTTTCAAGTGACCTAAAATAGTCGTTAGACTA
CAGATTTATTAGACAGATTGTAGATGACAACCGTCGATTCACTATTTTTGATATAATC
TTGTCGAAATTTGCCGTTTCATGGATGTTTAATTAGTAAGGAGTTGCGGAAATACACGTGACGTTCGTTGGAT
TAGCTAGAGTAACTTTATTTACAACATTTTTATATTA
>126034788_2 EL596275.1
GCACGAGGTGTAAATTAAAATAACGAAGGAAATAACGACAAAAAATTCTGTATCAACATTTATATGAAAGTAA
CTAATTTATATCTTTAAAAGTTTAAATTGAAAAATATTAATAACTTATATACACCATATTTACTTACAAAGCA
TATTTGTATTGTCTCCCTCTGGCAGAGAAAGTGCGGCGTTATATTTTGCCATTTCATA
CACAACATCTCGAAAATTCGACGTTGGGCATCGTTGCATGGGACAAGAAGTGTTTTTTCACTCAATTCTTTCT
CTTTGCCTACCCTGCTACATGGGTGGGTACACGTAAGAAAAATGTATATCTCATCATTGTTTCTTATATTATT
AGTTTATCAAATAGAAACTATTATTAATCTGCGATAAAATTGTTTATTAAAATTAAAA
CAATTTTACTTTTAGTAGTATAGAAATAAATAATATTCCGTTGAATTTATAAATAATATGTTATAAATGTTGA
GCGTAAAAATTAAATTATTTAAATTGTCGACAATAAATTAGTCATATAGCANAGAATGTTTTGTATTAATAAA
TACGTGTCAAATTCCATCCATTTTTGTTGTTACGAGTTCATTTTTAAAAAAATATTGT
TGCTGTGAAATATGAA
>126034790_2 EL596277.1
GCACGAGGGAAAATATCAATGTGGATGAATTTTTACCTGCCAAGAATCCTATAAAATTAAAAAATGTTCAAAA
TAAAGTTGTTTCTGAATCGGGGGAAGGTATGGACGTAGAACAACAAAATGATGCGGGAAACAAACGAAAACGT
CGATCTAAATTGAAGAAAATAAAAAAAAGTGGAGAGCCAAATGAAAGTAAAGAAAATA
TGAGGAAAGTTGCAGTACCAGCTCACAGATACACACCATTAAGAGAAAATTGGTTAAAGATATTCACACCAAT
TGTAGAACATCTTTTGCTCCAAGTGCGTTTTAACACAAAAAGTCGCAATGTTGAAATCAGAGTTGGACCAGAA
ACTAAAGATATAGCTAATTTACAAAAAGCTGCTGATTTTGTTAAGGCATTTGTCTATG
GATTTGATGTTGAAGACGCACTAGCTTTACTAAGATTAGATGACTTATTTGTCGAGTCTTTTGAGATAAAGGA
TGTGAAGACTCTACAGGGAGATCATTTAGGCAGAGCCATAGGCAGATTGGCCGGTAAAGCAGGCCGCACTAAG
TTTACTATAGAAAATGTAACGAANACTAGGATAGTTTTGGCTGATTCAAAAATACATA
TATTGGGTAGTTATCAAAATATTGCCTTAGNCAGAAGAGCTATTTGCAATTTGATCATGGGTTCACCGCCTTC
CAAAGTGTATGGGAATTTAAGGAATGTCGCCAAACGAGTTGCTG
>126034792_2 EL596279.1
TTTTTTTTAAAATGTCACATGTTGTCTTCATTTATTTTGTTGTATATATTAACAATCTCGAATGGTGTTGCCA
TTATTATATTAAATTGTGAAAGCAAATGTTATTCTAATAATAAACTGATAAATCCCAAACCCAAAAAAAAAAC
TAAACTAAATGACATATCACAAGCTTGCCATGAAACAAAAAATAAAATGTTTCAATAT
TAGAATACTTTATTTAGTATAATTTGTAAATGTAACAGAAAAATATGAAATATAAATAGAATGGAAGAGATAC
GTGAAAAATATAATAATATAAGAGTCAGATCTGAGTATTGCCATATTCAAATAATAATCAGTTTTATAATCAA
TTCATAATAATGAAAGTTTGAAGCTGAAGAAAACATTCTTGATTTAGAATTTTAAATC
CTTTGGCGTTTTTATATAAATTATAGCACCAAAATATAAATTCAATAAAATTTTACCTTCTTTTTTAACATAA
TACATAAGTTGTGGAAGTATTGGAATGATTGTTCTTCTAGGTACATATACCAGCGTTCAAGCAACATTAAGTC
ACTGAAGTTCAACTATACATATTCATATAATTCCTTATATATATCTTATAACGCTATA
CTTAAGGATGTTATTTATCGTTTTTTACATATTTAATCAGTTTTCTTTGTTGAACCTTCCAGAACTTTCTTTC
TCATTTAATTCTTGGCGGCGTTGGAGGGGCGCATCACGGTCCTGAGAGTTAGGCACTGGCTT
>126034801_2 EL596288.1
GCACGAGGAACCATCGCAAATTGTATCGTGATTTGTGAGGCGGTTTGCGTTTGATCAATTTTTTCACGTGGTT
GAGCGTTTTATGATAGTTAATTTACACGCTTTAAAAGATGTCTGGTGAAGTTCCTGTACCGCCTGCAGTTTTA
TGGGCGCAGCGCAAGGAAGAGGTTTTGCTCACATTTAGCGTGGAATCCAAAGATCCGT
CTATTAAAATAGAAAAGAATTCCGTATATTTCAAAGGCGTCAGTGCACCAGAGAATAAAGTCCATGAGGTAAC
TATTCAATTATATGACGCTGTAGTTCCCGAAAACAGCGGTTTTGTTAATAAGGGTCGTTGCATAGAAATGGTA
CTCCGGAAAGAAAAAAAGGACGCGCCGTATTGGCCTTCATTAACGAAAGACAAAAAGA

AACCACATTACCTTAAAATAGATTTTAATAAATGGAAAGATGAAGATGATGAACAGGATGATGAAGAAGGTGG
TGGTGGTGGTATGGGTGGTAATCAATATAATATTGAAGAATTGCTTCAGTCGATGGGTGGCGGCGCCTCTGAT
AAGGGTGATAAGCCATCATTTGATGACCTGGAGAGTGACTCTGACGACGAAAACATAC
CTGATTTAGAATGAGGCCCACTGT
>126034813_2 EL596300.1
GCACGAGGGTTAAATTCAAGAGCCATTTAATTATTAAAATGGAAGTTACTACGGAACAAATTCCAAAAGAAAA
CTCAGATAACCCCCCACTGGCAAATAATATTGGTGAGAAAGCACCCACACCTCAACCACAAGAACAGCCAAAG
AAATCAAAGAAGGATGATAGATCTAAAAAGGATGACAAAAATGTTGAACAATTCATGA
AATCTTTGAACTCCGTACCAACACTTGATGAAAAACTGTCTTTAGTATGTAAAAAATATGTACAAACAGCTGA
TGATAATAAAAAAATGCAGTTTTATATAAAACAATCTGATAAGAGGTATGCTTTACTAGTGAAGGAAAAGGAA
CAGCTCCAACATGAATTTAATAAGACAGTATTAGTTAAGTCTAAACTAGAGAATCTGT
GTAGGGAATTACAGAAACAAAATAAGGCTATTAAGGAAGAATCTCTACTCAAAATACGTGAAGAGGAGGAAAG
GCGTAAGGAAACCCAAGCCAAGTTCCAAAACACATTGACAGAAATAACTTCTTTATTGCAACAGAATAATGAG
AAGAATGCTAAACTAAGAGATGACAATATTAGTATGACAGAAAAGTTTAAAAGTGTAG
TTCAACAATATCAACTGAGGGAGCAACAGGTAGACAAAATGG
>126034815_2 EL596302.1
GCACGAGGCTGGAAAGTTTCCCAAGCCCACTAAAATACCTGGCAAAAACTATTCTAAAGATGAGGTTGTTATA
CGCAATGCTGATTCGGGGAAAGTGATGGGTATCAAGGGCAGGCGGGTGCATATGATCGAGGAGCTAAGCGATA
CGATCATATCGTTTCAGAGAGTGAGTCCTGGAGCGAAAGAGCGTCTAGTGCAGATCAC
TGGTCCAAACGAGGAAAATGTAAACCACGCGAAGCACCTGATCGGCGACACGATCCGCCGCAACGCGTCGCCG
GTGCGCCTGGAGGGCGCGCTGGAGGGCGCGCTGGCCACCTCGCGCGCCTCGCTCGACTCGGCCGGCTCCGACG
AGCGCCCCCGCGAGAAATCTCCTCACAATAATGCTAGGGCTTTGCTGCACAGCTTCTC
CACTAACGACGCCGCGCTCGGCGAGTACAAGTACACGGTAACGTTCGGGCAAAACTCCATCAAGATTACTGGA
CAGAATTTGGATCTCGTCAAGACAGCAAAACTGGTCCTCGACGAGTACTTCGAGAGCGCAGGCGCTCTAGAAG
CAATGGCATTAGGTTCCGGGGATTTCTTCACGCTGTCCCAACGTCCCGCCGCTGCGCT
ACTGCTACGCGACGATGCGGCGTTAAACGGCGTGGACGATGACGTGTTCACTGCCAGTGCAACTACTAATGAT
GCTTCTGCGGCGGA
>126034818_2 EL596305.1
GCACGAGGTGAGGTTTTGTTTCTGAAATTTCTTTCAGTACAGCTTCTAAAGTTTATATATTTGTAGTGTGTTT
TGTGTACAGTCAGTTTATTAAATTAAAATGGGAAAAGATAAAAAAACGGAGACCAGTGAAGGTTCAAGTGAAG
AAGAATATGTTGTAGAAAAAGTGCTTAATAAAAGAACCGTCAAAGGAAAGGTGCAATA
CCTTTTAAAATGGAAAGGCTACAAAGAAGAAGACAGTACATGGGAGCCAGAAGAAAATTTAGATTGTGAAGAA
TTAATTAAAGCATTTGAAGATAAAAGGAAAGAAAAGGAGGCAAAGGCAAAAAAATCAGAGCCTGAGAGGCCAA
AGAAACGAAGCCGAGACTCTGGTGATGATGCAAGCACTACTAGTCGTAAAGGTAGAGG
GACCAGCGTATCATCTGTAGAAGAAAGCAAAGAGTCAAAGAAAGAAAAGAAAGAAGATAAATTGAAATCTGGT
TTTGATAAAGGGCTTAAAGCAGAGAAAATTATAGGGGCATCAGATGCAACAGGAGAATTGATGTTCCTGGTGA
AGTGGGCGGACTCCGATGAGGCGGAGTTGGTTCCCGCCAAAGTAGCTAATGTGAAATG
CCCACAACAGGTAATAGCGTTCTATGAGGAGAGGCTGACCT
>126034820_2 EL596307.1
AAAATGCACAGTATAATTAAAATTCCTCGTTTCTTTTTCTTCTTATGTGTGAATTTTTTGTTGCAAGCTTTCA
GAACTTGTCTTCTTTAATCGAATGTACACCGGCGCGATGCATACAGTGCAAATTGCGGTGATGCTCCTCGAGC
GCGCGCGAACGACACATGCATGCCGCCCACGCATCCGGTGTGGTTGTGAAGTCATGGT
GGTGAGCAGTGACTCTTCTAACGCTGGACGATCACGACTCGCCC
>126034823_2 EL596310.1
GCACGAGGGCTTTATGACCGGCAGACGCGCAACGGACGCCGTGCGGAAAACGCGACTCGAGCCGTGAGTGACT
TTCGACCTTTATCGGTGATTGAAAAATATAGTGCACATATGTCATCTTTATTATGACTCGGGTAAAAGATTTT
TGTACCGTGCTGGGATTCTTATTACTAAGTATCCACACAGTATATCCAAATCCTAATC
ATGACTTGGGGACAACAAATAAGCAGATCTCAGATATGACATATGCATCTGACAAGTCTGATTTAAGTGGAAT
AGCCTCAATTTTTTCTCCACGTATGAATTTCGATCAGTGGAAGCCTTTGACTGGTCGCGGAGATCCTTTAAGT
AATGATCCGACCTATGATTATGAACCTCCGGTACTTGAAAAAGTTCATTACTGGGTTG
ACGATACCAGACCTGAACGCGAAAAATTTCCGGAAAGAAAATCTGAAGTTTTAGTTTTAGGAGTGTCATCACG
TAAGCCAAGTGTTGCTTCTAGACCACCTGCTCCACTAAGAAGACATCCACGACCACCTTCATTTGCAATGAAA
TATGAGGATTTTTCTTACAAATTTAGTGATCACTATCCGATGACCATACTTGTACCGC

CACCACCTCCACCACCTCCAGGTCATATATCTAACATGTTTATACACTCAGAAGATACATTGTTATTACCTAC
TATACCTTCT
>126034824_2 EL596311.1
GCACGAGGATTGAACAGTTTCCTTACTACAAGGCAGGGTGTTTTCCAAACAGAGCTTATATTCACAATTTAAT
CTGGATGAGTAAATATATACCGTCTCTAAGGGAGCAAATTATGATTACCATTATTAATAAAATGGTAGACATG
GATACGAATATGGTAGATCGTAAAAAAATAAACATGAAACAATATTTGACATGGAGAT
TGATGAAAAGGATGAAATAAGTGAAACTATCGACTACTGTATGTTAGAAATGCTAAAATGGCTTGAAGATGAA
CGAGATCCCGTTTTAAATATAATGTGCAATGTTTTTGAGAGAGTTATATTACCTACACATGGGACAAAGCACA
TCCAATTTCTCTTGTTATACACAATATCAATCAATCAGCAGTGTGCGGACCGTGTGTT
CACAAACCTGTGGATAGTTGCTGCAGGCTTACACGGCCTAGGTCCTGGGGCATTGGCAACTAGACGGACAGCT
GCTGGACATTTGGCGGGTCTTTTAGCGAGATGCGTGAGAGTGCCGAATTCAAGAGTGATACATTACTTAAGAA
CAATGGCGGAGTGGTGCCACGCCTATATAACAGCGACACAAGAGTCTACTGTAGCCGA
TAATATGAAAGTGCATGGAGCGTTTCATGCTATATGCCACGCTATATTTTAT
>126034827_2 EL596314.1
GCACGAGGCTCCAGTCTAGTGACTTTGAACTTCATATAACAATACAAAAATGGCTCAAGACAAACCTATTTTT
GAATACCACGAGGAAAGCCAGGGCGAAAGATTAGCGCGCAAAACTAAAGAATCTCCTTTCATGGTAGTTGGAT
TGGCTGGCCTAGCAACCGCTGTAGGCTATGGAGCCTACGCATACAAGAACAGAGGAAA
GATGAGCACCAGTGTCTACCTCATGCAGTTCCGTGTCATCTCACAGGGCCTCGTAGTGGGCGCGCTCACCGCC
GGCATGGCTTACACTCTCTACAACAACCATTTCAACAAACCTAAAGCAATTAGCAGTAGTGTCATATCCAGTA
ACGAGCACTAAGTCATACGTTTTAGACTTACTTATAGAGTAAAGCTGTATTATTAGAT
TATTTCCAAAGTATAATTAATTGGAATGATTTGCGTAAATTGAACTATACAAGATAATATCATGTAAAGCACT
ATGGCACACCGTCCTGAGCACAGAGGCAGAGGACCTAAACGCTTTACATATCGCACGTGATGTAGAAGACAGT
GCTAGACACTGAAAAACCTTTTAGAAATGAACACTTAAACTTTAAATAATCCAAAAAG
AGAAGAATGAGATTCTTGATCTCCTAATTTAAACTAAATAAATAAAAACAAAGACATATGCAAATTACGGAAT
ACCTAGTTAATTT
>126034829_2 EL596316.1
GCACGAGGGTCACACTAAAAGAAATTTTAAAATAAAACATGAACAAAATCAACGTATTGCGTTGTATAATTTT
AAATATTTTAATTTTATTAGGTTTTTAGCGCAGTTAATGTGATAAAAGATGCTTTTTGTAAGGGACTTTTTTA
TACCTATATAAAATGTATGTAATTTAGACGATCTATAAATGTTTGTAAAATTAATGAA
ATATTCTAAGCTATTATTGAATTAATAAAAAATGTATAAACCGAGTAACTATAGCGCAGTTGGGGGAAAATCT
AATTGTAGATTCGACGATAATTCAAAATGTATTTTTTATTAGTATTTTTTTATTTAAACACCGAAGTCCTAGA
ATATTATATAGATGAATGTTTCTGATGATTTAGATGAAACTTTACAGCAATATATCTT
ATACATTCAAATAGTATATTTATGTAAGCATTTTGTAGAATTTGGTTATAATATAAAAATAACACTAAGCTGG
CGAAGCCGCATTTGGTCCGTTCCTATGTGATAATTGATAATAGCTTAGAATATTTAACGATGTATATATATAA
TATTTATTATGTGTTCATTATGTTTGTTTCCTTATATTCGTGTTATATTTTTACATTG
TCACATAAAGTATTTTTTATATAAATGACAATTTTATAATACAGATTATATATAAAATTAGTACAGAATAAAA
ATGTTGGAAGAATTATAAATATTATTTCCGCTTAAATTATTATATATACATAAT
>126034831_2 EL596318.1
GCACGAGGCTCAGACGTACTTCCCAGACAGACAAATAAATATCTAGATTTTATAAACAACCCAGAGACTAACT
TAGTCAATTTTCATAAAAGTCAAGTGAAAATGAAATTACTACTGGCGGCTTTGCTGACCAATTTGCTGGTACT
TTCTATTTGTGAGGAATCAAAGGTCCGCAGGCTGGGTCGTTCGAAGAATTATTTTATA
GGTTACATTAAGGATATTCAGGATAAATTAAGAAAAAAGTTTGATTGTCCGATGCTAGTCGACCAACTGTTGC
CGGACACAGAGGATGTTATATTCCGAAATCGCATTCAGACTTTACTCCGAATGAAAACGGAAAACGAGAGCTG
TACCCAATCAGAATTTGAACCTGAACCCGTTCCAACGATGATGATAAAAAACAAGTTC
AAGACCAAAAGGCTGTTATACGACAAGGAGTCGTTGCAGAAATGTAACGTGACTATCATTTTCATTGATCCTG
CCGAATGTGGGGACTTATGTAATGATCCTTACGCTGAAATGGTTTTTGATCCTGAAGAGTTAGATGTATATAA
TATGTAAATGTTTGTCTGTCCATCTTTTACTTTCTTCTTTGGTGGGAGAGGGATTTGG
TGTCTAAACTCTCCACTTTCCTGTATTTGATGT
>126034838_2 EL596325.1
CGGCAGAGGGTCATTAACTGGATTGTTAGTGTATACTTCCTTCGTGTTATGAAAAATTATCACCAGAGAATGT
TAGAAGACAAGCCTTTCACCATACAAAATGTTTAAGCGTTTTAAATTCATGTTATAAATATAAATTTAAGTTA
ATTTAAAATTAATTACATTTAAGAAATGCATTTAAATTTTGTCTATTTTTGTACTTTG

TAATAAAACAATCTGTAAGATTTTAATGGTATTCTAGATTTATTTATTTATGAGAGGATAGTGAAAGATTTTG
TTGATCACATTTATCTATAGATTATCGTAATAGAACATATACATACATGATAAGATAACCTACCTATAATATT
TTAATAGATATCTAGATGATTTTAATTGACTTAAGACAGTCAGGCATGCGTGCGGCAC
TGCGGGTTGAGTGGCGGAACTCAACGCCGTGTTTTGTCATAAATATTAATTAATATTTGCCGCCACTATTACA
TACATTATTTTTATTTACTTTGCCTCGACACGAAAGAGCACACATAGAGCGAAAATATGAAAATATATAATAT
TCATTTAAGACTATTTCATATTAATTATATGTTACAAGAGGTGTAAGACACAAACTGT
GATTTATGAGTTCTTTGGAAACTGTTTCCAAGCTTTAGGTTATATATAAAATCTTTGTGTTTTTGTTTACGAT
GAATTATTTCATAGTAGATATGCATATTTAATCTACGTTTACTAAA
>126034839_2 EL596326.1
GCACGAGGCCGACGCGGCGGACGGCCCCGCCGCCCCCGCCACGCCCCTCTCGCGCCGCACCACCCCCGCCTCG
CGCACGGACAAACGCGAACCCTTCCGACTTTAAACGAATACAAATCGACACCATTCCCGTCTGAAATCCGCCA
CCTTGATTAATCCTTTTAATGTCATATAATGGCGGATTTTTGACATTTTTTTGTAAGA
CGCAAGCCGCTCCGATCCGTCACTAATATAACCTAATGCCGATGCAAATTGTATTTTTTATTTTTTAAGCTAG
TGTTTGCAAAGATGAGACGGAGCGTCTAGCGTTTCTGATAAATTGTAACTACTAACAATAAATTAACTCGTGA
TTTGAGCCAAGCTTGTATAAAAATGGGCGAAATTGGCTTATGTTTAGTAACAAACAAC
TCTAGATGTAGTAAACCATAAAGTCTGCACTTGTTGCCTTGTATACTTATTTTCTATGATCCCAGCCAACTTT
ACACTTTGAATAATTTTTGTGCTGTCCTTCATTACGTTTGAAAATTCTATCAATTACCTTTTTTCAATGTGAA
TATTTCTTATTTATTGTCAAAGTTGCCATAATTTTAGTGTATGTTATATACGTATTAG
TCTCGACTGGAGAC
>126034841_2 EL596328.1
GCACGAGGGCCGCGAGCGCCGCGACGCGCGCGACACCCGGGACGGCAGGGAGCGGGACAGGCGCGATAGGGAT
CGAAGGGAGCGTCGCGAGGGAGATAGGGAAAGAGACGACAGACGCGACCGGGACAGAGACCGTGGCCGGGACC
GGCGGGACAGGGACCGGGATCGGGACCGGGACAGGGATAGGTTGAACAATAGGGATAG
CAATAATCGTGAAAAGAGCCGCGAAAAATCACCGCGCAACGATCCAGCACCGGAAAAAAGTCTCCAAGAGCGA
CTGTGGGAGATGGCCAACGGGAAGGCGGGCGACGGCGACGGAGACGCGCCCGCGCCCGCCGCGCCGCCGCCCA
GCGCACCGCCAGATAATGAAGGCCCCACAGAAGATGACGGTTGGACGCAACTACCTCC
GCCTCGAGCACTACAGCCCGCTGCATTTATAGCTGGTCCGTGGATGGAGGGAGGCCCCGGACCGGGACCGTTT
GGACCACGGTTCA
>126034842_2 EL596329.1
GCACGAGGTTTACTATCTGTGTCAATATAATGAGTAACTATAATGTTAACTTTGGCTTCGATTGTTAATAAAA
TTCAGAGCAAATTCATGTTGTCGAGTTTTGTTAAAACGTTTTTTTTATTATTTTAATTTAAATTTATCTTTGT
GTAAGTGGGAAAAATATAAAACTTTCTATTTGAATTACACCTCACTCCTGTATATAGA
GTGTGGGTTTAAGCGACGTATTATTTATGTATAAAAATATATATTTTTAGCCAAATCCATTTCTAATTATTTT
ACTTTTTTATAGATTTACTTCCTTTCATTTTTTGTTTATAAAAATAAATTTATATATTTTTATTTTATAATAT
ACATTTGCATTTTATTGTAAGTTAGCAACCTTTAATAATTATATATTGTAACAACGGC
GGCTCGGAGGTCAAGTAAATGGGGCTCAGTTGTTACACAATTATTTATTTATATTTAGGGTTGCCATCATTTA
TAATAAATAATATATATATTTTTTATATGTGTTATTTTTATATGTATATTC
>126034847_2 EL596334.1
GAGCCTTCAACGCGCACGCCCTGTCCGGCATAGCTGATATTTTCAGGGTCATCAAAAACACCACGTATCACCG
CACCTGACTGCTCACCGGATGTAATGGTGGCTGACGTTCCCATGTACCCGCGTATCGTTTCATCGGCGCGGGC
AATGGCAGCATCGAACAGGTTATCGAAATCAGCCACAGCGCCTCCCGTTATTGCATTC
TGGCCAGGCCGCGCTCTGTCATTTCGGCTGCCACACCGGCAGAGACACGAAACGCCGTTCCCGGCAGCACAAA
TGCCACAGGTTCATCCCGCGTGGCGTGAAGTGCATCAGTATGCAGCTTCACCAGTGCCACGACCGTGACCAGT
TCAGACGTATCCAGAATCACGGTATCCGGCTGCGCTGATCCCACCTCATTTTCATGTC
CGGTCAGCACATTTTCCCGGCTGAGAGGGGTGTCCTGACCGGCAGTTTCATCCGTGTCATCAAGCTCCTCTTT
CAGCTCTGCCACACGGAGCGCCAGTTCTTCTTTCGTCCCCGTCAGGCTGACATCACGGTTCAGTTGTTCACCC
AGCGAGCGGAGACGGGCAATCAGTTCATCTTTCGTCATGGACTCCTCCACAGAGAAAC
AATGGCCCCGAAGGGCCATGATTACGCCAGTTGTACGGACACGAACTCATCAGGGTCAGCCAGCAGCATCAGC
GGTGCTGACTGAATCATGGTGAACTCACGCGCCGG
>126034848_2 EL596335.1
ATTAGGATACTCTGCTACCGCCATTTTAATATTGTTTCGCGGCTTTTGTCATACATAATACACATACAAAATA
TACATACATTTTTGTATGTAATTAATGTTTTGTGATTAGATTAAATCTTATCACGTTTTATTTATTTTATTTC
ATTGTTTCTATGTTTTATGTATA
>126034850_2 EL596337.1

GCACGAGGGAATCATTATTGTCAAAACATCGAATTATAAATAAATTTCACGATTATTATTTAAAATGTCTTCA
ATGCGAATGAAAGCAGCAAAATGTCCCTCGGACGAATTGGCTATCACCAACTGCGCCCTTATCAACCCGGATG
ATTTTCCCAGCGATGTGAAGCATATTGAAGTTTCCACTGGACCTTCACAACACTTTGT
ATTCAGCATCAGGTTCTATAGTGGTGTGGACAGAGGAACTGTTGGTTTCTCTGCACCTCAAAGAAAATGGGCT
ACATTATCTATTGGACAGACAATAGATGTGAGGCCATTCAAGCCTCAGAGTGCAGAGTGCTTATGCAGCGTTA
CACTGGAAGCTGACTTCATGCTAAAAAAAACGACATCTATGGAACCCTACGATTCTGA
ACAAATGGCGCGGGACTTTCTGATTCAGTTTTCAAACCAAGTGTTTACCGTTGGACAACAACTTGCTTTCTCG
TTCCAAGAGAAGAAAGTTTTGTCATTATTTGTTAAAAATTTAGAAGCGGTTGACGTACAAGCCTTAGCGGCGG
GTTCGAACGCAGTACCTCGACGCGTAAGAATGGGAAGACTATTACCGGACGCCTGCAT
ACAATTTGATAAAGCTGAAAACTCTTCACTTAACCTCGTGGGCAAAGCAAAAGGAAAGCAGCCGCGTCAGTCA
TCATCAACCCCGA
>126034852_2 EL596339.1
GCACGAGGTCCGTCCGTCATTAAAATCAAATTAATAGTCAGTTGATGGATTTAATTGTAAAATAAAATTCTAG
TATTTTAATACTAGAATTAGTGCTAGTGTATTTATTAAAATAAATAAAACAATGCTTAGGCCGCTTGTAAATG
CGTTTTGGAATGAGGATGTGTGGTTACCTCCTAATACTACATGGTCGGACCTCGCGCC
CGGGCCCGATAAGGCAGTGGTGTATACGGACCATCGTCATGTCTTCTTCCCTATACCGTTAGCCCTGGTTTTC
ATAACAATACGTTATGTACTGGAGAGATTTGTGTTTGCACCATTCGGTCGATATCTAGGCATTAAGGGATCAA
AACCTAAGAAACCACCACCAAATTCTAAACTAGAGGCTGTATACAAATCACCTATAAC
CAAACAGCAATTATGCGCCCTAGCGAAGCAACTGGATATGTCGGAGCGTCAAGTGGAGCGTTGGTTGCGGAAA
CGGAAGTGTCAAGACAAACCGTCTACTCTCGTAAAGTTCTGCGAGAACGCCTTCAAATGTACGTTCTATATTG
TCAACTTCTCCTTTGGACTCTATATACTGTGGGACAAGGACTGGCTGTGGGACATCGA
TCAGTGTTACATTGGATATCCTCATCAGGGTCTAACAACAGACGTATGGTGGTACTACATGATCAGCGCGGCG
TTCTACTGGTCGCTGACGCTGTCGCAGTTCTGGGACGTGCGCCGCAAGGACTTC
>126034853_2 EL596340.1
GCACGAGGGCCGCCCGACCTGGCGCCGCCGCTCGCGCTCGCGCGCACCGAGCCGCTCGAGCTGCAGCTGGACT
ACTGGCTGGTGGGTGCAAGATGCGCGGAGGGTGGTGGGGGCGGAGCGGAGGGCGGAGGGAAAGTCACTCTGAA
GGGCTCGTTTAGAGCGTTGTTGGTGACGCGACACAACCACCATCTGTGCATCACTTAT
CTCACTAAAGAGAAGAAGCAGAAGATAATGAGACTGGGTAAGAAAAAGGAGAAGAGTGGGGAAACAGAGACAG
GACGCGCTCAGACGGTGGAAGGAGTCGCCAGGCTCATCTGCTCAGCTAAGGGCTCGCATAATGCACCTCTAAA
AGTATACATCGACGGCACTGAATGGAACGGAGTTAAATTCTTCCAACTGTCCACGCAA
TGGCAGACTCACGTGAAGACCTTCCCTGTTGCAACTTGCGGGGCTCCCCTCGCACCTCCTGAGACCTAAGCCC
TTTGAAACCCCTTTCGACGACCACGCAAAGACCTTTCGAGTGACCCCTGGGACCCCTCGAATGACCTGGGAAC
TCTTGGACCGCTTTGGTAAAGGTCGAGTGTAGTCAAGGTACACTTGGCTGATATCTTA
ATAGAGATCTCTTTGTAACGTCTTATAAGGGACTCGTATGAAACTAATACCGACTTCTTCTTAAT
>126034855_2 EL596342.1
GCACGAGGTGAAAGTGATATAGAAGTAGACATAGATAGTGACTCCGAAATCACAAATGCTGAAGAAACTGTAA
ACAAAGTCGAAAATACAACCAAGGAAGCTCCTATTGCAGTACCTTTGAGAAAATTTGAACCAATGCCTAAAAG
GAATCGTAAAATACATTTAGATGGTGGTGGTGGTTATACAATAATGCACACGGAGTCA
GGTGATTTATATGAGATAGGTCAAGAGCCTAGAAAAGAGAGACAGAATAAAAAAACTGCAGTACAATTAATAC
CATGTCGTTTCTATAATTCAGAGAAACCTGCTCCCTTCACAATTCATTTATCAACATCAGCCCTAATAACAAT
GGACGTCCATGCCCATACATCGCGTGGTGAGGTCATGGGGTTGTTAGGTGGAGTGTGG
ACTCCTCCATCTCTACGCTTGAGGATATATCGTCGGGTTTCCGCAGCCGCCGAAGTAACACATTGCGATATGG
ATCCAGTGTCACAATCGCAGGCCTCCGAGTGGCTGGCGGGGCGCGGCACGCCGGTGTGCGGCTGGCACCACTC
GCACCCGCGCTTCCCGCCCGCGCCCTCCGCGCAGGACCTGCGCTCGCAGCGCGCCGTG
CAGGCGCTGCGCGCGCGCCGCCCGCTCGTCGCGCTCATCTCCTC
>126034856_2 EL596343.1
GCACGAGGATCATAATACATTCACAAACAGTCGCGGCAACTAACAGCAATGGCACGTGTGCGCGGTTTACGGA
CTAGCGGTATAATTCTTTTATTATACCTAACTTTTACAACAGCATCGCCATTGTTTTCTATTAATGTGTTAAA
GAGAAATTGTGATATAAATAAATGTGTTTACCTTTTGAATGTTGACGGCGATTTTGAT
CGGTGGTCGTTGACTCCTGTACCGGGCGTGTGTAATTTAGATAATATACAATCTAATTTTATTGGTGAAGATG
TAGAAATTGTTGTTGATAGATCGGTAAAGTTGTATTTATGTGGTGGAACGGGTAAAGAATGGTTCCCGCTGGA
TTATCATTTGGAAGGTGACGTCATCGCCAGTCATCGGCAATCAGAAAATGAAATTGAT

GAAACACCACACGATGAGATAATAGACTTGGAAGATGACGTCGGTAAAAATTACTACAGGGACTTAGCTGTTA
ATAAATTTGGGGACCAAATCGAAGATGTCATGATTGAGACCTTTGCAAATCAAAATCCTGATCAAAAATCACT
TAGAATCAAACGTCAGGCTGGAGACACAACAGTATCGGAAAGTGAAGATAAAGCTAAT
GTCACAGCAATACCGCCGCCAGCTGAATCAGCACAGCCAGTTGACTCAGCACAGGTCAATCCGGTGCAAACAG
CTCCT
>126034862_2 EL596349.1
AATTCTAATTTTTTATTTAATCTACAAAACAAATATTATTTCCAAACTAAAAACACTAAGCTTTATATACATC
AGTAGGGTTATAATTGATCTTCTTATCAATACCTAGGAGTGGGGGGATTAGGAACTCAGGAAGACGTTTAGGT
CCCCATCGAGGGGGCAAGGGAGTAGGATGGGCTTTGGCAATTTTAGTTTTTACATATT
CTTCGGGGGAAAATTCATTTGCCGGGTTAACCATGGGTAATAATTCATTTGGGAATATTTTCTCCTTCTTTAG
GGGAGGGTTCTTCTTTACTCTAGCCCAACGAGGGCTCATCTTCTTCCACAACTTCCTTCTCGGAGGCTTCTTC
ATTTTCTTCCTTCTAATAACAATCAATCTAACAGCTGGGTTTTCTTCTTGCTCAATAG
CGGGGAAACCAATCTCATCTTTCCTCAAATTCGAATCAATTAAGGGCAAAATAGATCTAGGATTTACTATGGG
TATATAAGTAAGAGGATCTTTCTTTCTATTATCTTGTTGTTTGTTTATATCAAAAATGATACTACGGGGGCTG
AAATTCATTGTTTTCGCCTTCG
>126034863_2 EL596350.1
GTTGAAACAACGCTGTTTTTACATTATTATATATTTTAATGGACGTTGCTGTTACGATCCCTCTAGTTTTATA
TGCGTCGTTAAGTTAAGCTATCATAGTCTGATACTAAAGTTTTTGGTAAAGTTATTTATTTATTACAGTGTTA
GTCTACCTGTTGTAGGTATATGTGTACTAAAATAATCTACTATAATTTTTTTTCTACT
CGGCTTCTAATAAGTTTAGATTAGGATCATATAACGCCTTAAAATTGTTGCATACTATCTATTCAAAGTAACG
TGTAATGGAACTAAATAAAAATAAGAACAAAATATCCAATGTATAAACATTATTCCTTATAAAAAAAACAGTG
CATTTTTATATTGGTACATAATTTAAGTAGTTAATTTGTATTTAAGTCTATACTATAT
AC
>126034869_2 EL596356.1
GCACGAGGGTCGAGGGCACCGTGGAGGGAGAGAGCGAGGGTGTCATGTACGTCACCAGAGAGGATGGACAGAC
ATTACTAGTTGCCACGCAAGGTGTCGACGGCACAGTTCAGGGCGTGGAAGGCGCGGTCGAAGGCGAGGCTGAG
GGAGTCACGTACGTCACGAGAGAAGACGGACAGGACGTGCTAACAATAGACCCGGCGC
AGCTCGCGCAGCTGATGCCGGGCGGCGAGGCGGGCGGCCTGCAGCAGGTGGCCGTGCAGGTGGAGGGCGAGCC
CGGAGAGGACGCCACGCAGGTCATCGCCCAGCTGGTGCAGGCTGACTTACCTTCGCCAGGTGGTACTCGGCGC
GTAGTGCTTCTATTACCAGATGGCAGCTTCACAATGACGGAACTTGACAAAGAACAAT
ATGAATCAATCACAGAGGCCAGTAAAGCACAAGAATGATCCAGTGAATGTGATTGTGCCCTAAAAGAACTGAA
TTTTTTGCAAGTGCTCTAAAGTGGTTATATATTTAATTTATATTATAAACCGACAAACACTACATGGAAGAAA
GTAATATTCATTTTTTAATTTAATTAACAATATTTTTTGTCTTGGTCCATTCTAGTAT
GCATTCAGATGAGATCAGTTTTATTGATATTCCA
>126034874_2 EL596361.1
GCACGAGGGAAATTAAAAGTGCTTTTCTTACCTAAGAAGTGGATTAATAGTTTAGAAATATTGAAAATATTTA
ATAAATAACATGCTCTTTTGTCAAATGATAATTCTAAGCAAGGGCAAGGCTATATGCTAACAACTTAAGCAAC
ATTGTAATAACAACATACATTCATATACATATATGTATATTTGATTTGCCTATATTTA
TTAATATTAAATAAGTGTATATGAAAACAAGTATTATTTATTTTTATTTTCAAAATTTCTAAATGGCTGATAT
TTTTATATATAACCCACAAATTTTTAACTATGTCTTGTTTTATCATAAAAATCTATTTAAGAAAAGCCTAATT
TTATTAATGATATTTTTGAAATTAGTCCACGTTTAAATATTTTTGTATGAGAAAAAAT
AATAATAATGTTGTTTCGTAATTATAATCTTTTTTTTGTATTTTCCTTTTCGTTATATCCTTATTTTTTATTA
AATTGCTTTAGATATATGGCTCCCATGCATGAATTATTGTTTAGTGCACTGTATGCTTAATAATAAATCACAC
TTTTGAAATAACTTAAATTTTATTGTATTTATTTTATTGTAAGCTTTTTAATCTTATC
TAATACTAGATTTTAAGTCCTAGTTTAATACATTTTAGCATGTTTATGTTAATTTGCTTATGTAAATAGTAAT
AGAGAAATGGAATTTCTATTTGAACTTCTA
>126034876_2 EL596363.1
GCCGAGGGTACTAGATCACCTGATAACAAATTGGTGGCAACTGGAGATGACTTCGGCAAAGTAAAATTATATG
CATATCCTGTCACACAACCTAAGTCGCTGTGCGCGGCGCACGGCGGGCACTCGTCGCACGTGACGTGCGTGCG
CTTCCTGCCCGAGCGCCGCCTGCTGTCGGCCGGCGGCCGCGACGCCGCGCTGCTGCAG
TGGCGCCTCGACTGAGCGAGCCTCCTCAACCAACACCTAGTACCCGCTATTCAATCAGTCAGTATATGTAATG
GAAACGCAATAGTTATTTTGGATCAAAATTTAAATTTACTAATATATCATTGTGCGTTTCCATTTTTTTGTTA
TTTTGCCTCAACTTAAAGTATGTGAAAATAATTGTTTTAATGTATAATGCTTAAAATT

TGGTCTAAATCACTATACAATGTTTAAAATCAATAATAAATCTAACTCTAAATATGTTTCACTAAACATTTTA
TAATAATTCGCTCTTGTATTGTAAAAATGCTTTATGTGCATAATAAATAATGTTAACGTACGGACAGATTGAC
AAACAATAAAGTTATAAAGGTTCTGTTTTGTCCTACGGAAAAGTACAGAACCCTAATT
TTAATATAAAGCAAGTTATTGTTAAAGATATTTTGTCATTATTTTAATATTTTATAAATATTGATAATTAATA
GGAAAATTG
>126034878_2 EL596365.1
GCACGAGGAAAATGGATACTATTTCCTTTTTTTTGGGAATGTTACTGCTGCCTGGCCCTTCCTACACGAACTC
CGATAATCTAGCTCTCCTTGCTGGGACGCTCAGAACAACGAAGCGTGCTGCCTGCGATGATCAAATGGTGTCC
TTATCATGTCCATCAACTACGATAATAAGCGTACAGTTCGCGCAGTATGGGAAGGCTA
TACCTGGTGGTCACTCTTGTGTTGCTGATCCGCATTTGAGTATTGAAACCGAGGAATGCTTGTGGCCCAATCC
TATGCAGTATTCTCTATTACAAACTGTGGTGGAAGCGTGTCAGAAGAAGCCCCAGTGCAAGTTTAGTACTAAG
TTGAAGCCGGGGCAGAAAGACCCCTGCCCACGATCAAGGAAGTTCGTTGAAGTGGCTT
ACAAATGTCGACCTCAAGAATTCCGGGGTCGCACCGGATGCGAGGAGGATGTTGTCAAACTGAGTTGCAATGC
ACACTCACGTCTGGCCATATACGACGCTCAGTACGGACGGACGGCGCTCGAGGCCAACATATGTTCTCAACCA
CCAGGGGCCTCGGATGAAACGTGTTTTGCCCCACATGCAGTTGAAAAAGTAATCAAAA
TATGTCACGGGAGACGATCATGTCAAATTTTAGCAAGAAACGACACGTTTGGGTCTAACTGTAGACCTCGATC
GAGGACACCC
>126034880_2 EL596367.1
GCACGAGGATTTAAAGATAATTTAAAAGTTAACCCTGAAGAATCCTGTAGGACAGTATCACCCGCGCCATCAG
CTCAAAAACCTGACAAAGAAAAACCTGGTGCAAATAGACCTGACAGGTTTCCTGTCAACTGGTCGGTAAATGT
CAATTGTCATATGGACCTCAGTCGTTATCCTGATGGTGATTACGACAGCCCCAGAGCT
ATAACATCTGTTAATCTGACAGTTTGTACGCCCACTTCAAACATGGCCAGTCCAGTAAGGGATATAAGAGAGG
AGGATAATCCTGGTTTCGAAGGTCATGTCACAGTGACAGTGAGTCCAAGTACGACACGACCTCCAAGACGTAG
GGCTCCACCGCCACCTCAGTCTCGTATAGAGTCACCAAGACCCACCAGAGTAGCGCCA
GATCCACCTAGTCATGGATATGATCGTTCTTTAATAGAACGTCAAAAAGAGCGCCTATCGCGTTTAGCAAGTG
CCTTAGCCCAAGAGAAGGGTCGGTTAGCTCAGCTGAACAGAGAAACGCAAATTCTCTCCGCCCCATCACCGAC
GCCGAGTGTCGCCCAAAACCTAAAGGAATATGTGGAGAGGTTGCGCGATGAATGCGAT
ACTATGGCGAAACGGTTGGAAATGGGCAGTAATGCCCGTGTAATGCCCGTTGCAGAGGCAGAAGATACAGGGA
ACTTCTATAGTAACATTTACACCGGACAGCGGCAGCCGACGTCGTGGCAGTGCCATATGTG
>126034885_2 EL596372.1
GCACGAGGCTACTTCAAGTTCACGGACCCCGCGCTGCAAGCCAGCGCCGTCACTATTAAAGAGCGCTTGCTGC
ACTGGTGTCGCGACAAGACCCGGGATTACGAGGTACACCCCCGAGCCCTGCTGCGTACCCCGGGACACACCGC
TTGTAATGACCCCCTAACACACTGAAAAGTTGCATCCCAACAGGACTGTTGTATCTAC
ACTAGAAATATTAATAATAATTAAAATTCCAAATTCCTTTAAGATTTAACTTACATATATTACAAAAAGGTAC
GTATCAGGGTTGTCACAATTACAAAAAGGGCCGACGCACATTAACTTTCTTACGAGCACATCGAAAATGGCGA
ATTTTAATGATTACTTAGTAATTTGATTGGAAAGTTGAGCAAAAAGCGGGACAATGCG
CGTCGGCTTATAGGGAGCGCGCACGTACCGCCGACGTCGACACCACACACCACTCAGACGTACAATGTCGGTA
CTTGTTTCTGTTCAATAACACCTTTCTACTTTTTATCTGATTTTTTAATTTTCACAACATATTTTTGTCTCTA
ATAGATTGTTTAGATCAATAAGCCTAACTTATAAAAGATAAAGAAATAGTCCAAATAT
ATAACATGTATAAAAAAATATTAAACAATACACAACTTATGTTTAAATGATATACTATATTGATTTATAATGA
ATTAATGACTATT
>126034888_2 EL596375.1
GCACGAGGAAGAGATATAGAAATGCTCAGAGGGTGTTAGACGTTGAAGCAAGGCAAGTGGCTTCTGCTGTAAG
TGATATAGACTCGGCTACCAAGAAAGGTGCTACTGCTACTGAAATTGATAATTTCCTAGGTGGAGTGGTAGAA
AAGCTGACTACAATGAAGCGTAAAGCATCAGAAGCAATAACTGAGGAAGTTCAAGCTG
CTTTTGTTTGTAAAAAACGTTTGGAACATCTCAAGGAACAAGCTGGTGCCCTCGCTGATGCAAATGCACCACA
AGCAAAGACGCAAATGAACCAGTGGCGTAAGGTCAGGTTGGATAGGATGCTCGTGGATTACTTCCTACGCAAC
GGTTACTATGATTCTGCCAATAAGTTGGCAGATGCTCGTAATCTAAGAGATTTGACTA
ATGTCGAGTGTACATCTTTGTTCTTCTAGAATATTTTGATAATATTATTGTGGGTTCCGAACCAAATGTTACC
AAAATATTGTACAGTACGGTCGACAAGTCGTGGTAGCCCTGACTTTAATGAAAAGTAAGAAATATCCGAAAAT
CAAATCTGCGCAGCCTATAGCCAACTTCGGGGCGGTCACAACTCATCGACCATACTGT
AATTTTTGGTATTTTCATTCATTGTTTAT
>126034912_2 EL596399.1

GCACGAGGCATATTTAATTTATACACAATATAGTGATATACAGTAGTTTTTTAGACAAACCAAACGTTTTAG
TGATAGTGAAAGGTTATGAAACGAACTGTCTGTAGTGTCAAATGGACTGTAACCATAGTTTAGTGATTTTTAT
ACGCAAACATTTGGATTTTGTTGGAAGGAATCGTGCGACGTAACCGAGGAACAGGCTG
TGATTTTTGGAATACAAATCGTGAAAAGTGTTACGTTTGACGTTTGAACGGTGTAAAGGTAGCCTCAGGTGAC
ACTGACGCGGGGCTTTCACGGAGGCGTTTCATCGCCCGAGTCTTAGTACAGTGCGATAGATTTTTAAATCGGC
CGTGTAACCTCGCAACGGCGCCCTCTGCGAGCCGCACTTGCGACAACTTCAGTACGGT
CCATTGCAGGTTTGAGAATGCTCGCGAAATACATTACGTGTCGTGTTTACTTTCCCTAAATTGGATATAGTTT
TGAAATTAATACCAATTGCTCGATCGCGACGACGTGCGGGCTAGATATTCGGTGAGGCTACATGGTTTTGAAG
GGTTGAACCATGGCGACTGCAGAGATGAGTCTGCCTGACAAGAAGCCTGAACACGAGT
CGGGATACTACGAAGGCAGCGATCAGGAGGGCAGCATGGAGTGCGGATGCCCCCTCACAGTTAGCTCGAGGAA
CTCCTCGCATTCGACCAAC
>126034913_2 EL596400.1
GCACGAGGAGAATTTTTACTTTCGTAAAAACATGGCAAACACGGGCTTGTTACCATTTGTTCGTGGTGTTGAC
TTTACATGCAATGATTTTAGTGGGGAAAAATTCCCAGATGCTATACGGCACATGACTGGTTTGCAATGGCTTA
GACTCGATAAAACAAACTTGGAAGAAATTCCTGAAGAATTAGGAAAACTTATGAAATT
GGAGAATTTATCTTTAAAGAAGAATAATTTAGAAAAGCTATTTGGAGAATTGACAGAATTGAAGTGTTTGCGT
TCATTGAATGTGAGGCACAATAATGTAAAGAGTTCTGGCATACCAGCAGAATTATTTAAATTGGAAGAATTGA
CGACATTAGACTTATCTCATAACAGATTGAAAGAAGTACCTGAAGGATTAGAAAAAGC
TAAATCATTGCTTGTACTAAATTTAAGTCATAATAAAATAGAGAGCATTCCACCAACACTTTTTGTCCAGTTG
ACAGATCTATTGTTCTTAGATCTTTCTAACAATGCACTGGAGACATTACCTCCTCAGACTAGAAGACTGGCGA
ACTTGCAGACTCTAATACTTAATGATAACCCACTTGGACTATTTCAGTTGAGGCAGTT
ACCTTCACTGCAAAGCCTGGAGACTCTACATATGCGTAACACTCAACGAACTCTTGCAAATCTTTCCACTTCT
CTTGACACACTCTGTAATCTGTCAGATGTCGATCTATCTAAAAATGCTCTCACTAAAGTTCCTGATGCCCTGT
AC
>126034915_2 EL596402.1
GCATAAATATTTTTATTATCATTATTGGATGTAAAAACTTTTTATTTAAATAAAATAATTTAAAATATAACTT
AAAATTATTACTACCGCACCAATTAAAATTATACATAATAAAAACATTGCAGTAATTTTTTTAAAAGCTTTTT
TATAAATTAAATAACTTTTTTGTTAAAAAGATACCTTAAAAGCTATGTTAAGAGTTGT
TATTGCTAAGAGCCATAAGGTAACGTAATATAATTAAGAAGATACAATTAAGAAACTAATACAATTAATTAAA
GCTTTTTGAAAGCATAAAATGAGTGGGT
>126034918_2 EL596405.1
AGTATAAAATACCTAAAGACTGTATAAAAAACTTTATTTTTAATTTTTAAAACTTTAATTTTTTTAAATTATA
ACAACAGTGAAATTATTGCTACCATCGTTGCTTCAGCGCTGTGAATAAAAATATATTTTCGTACCTCTGGTCT
GAACCTTATTGTTGAGCGGCAGAACTAAACGCCGTGCTTTGTCTTTGTTACTAGAGCG
AGAAAATGAAAGTGTATTCTCCTGAATGGGAATCAAACCTACAATCGTCTCAATATACAATCAGAAGCTGTGC
CACTGCACCGCGACCGCC
>126034920_2 EL596407.1
GCACGAGGTACACACTCAACACTTTAATTAATTTTCAGTTTACTTTGTTGCGGAATTATAAATGGGGATTTTA
TTAAATATGTAGAGTGTTTAGTGAATTTAATATCCTGTGCAATTAAATAATCCCCGCAAATTTGTTATGTAAT
AGTAGAATTGGGTAAGTTTTAATTGCAATTTACAATTAAATATTAATTATTGATCATT
TGTTTTTGTTAAAATAACAATAATTGATAATATACAAATTATCATTTAATTTTTGTCATTGAAATAGACAAAA
AATAAAGTACATTTTTGTAGAATTAATTAACTGTTAATTCAAATTTCCGCACTAACAAATGATTAGTAGTAAA
TAATTAATTGTTAATTGCAATTGAAATTTGCCCAATTTTGCATAATAGTAATTGTACT
TTAATTTGAATATTTCCGCTCATTCTAAAAATTTGGTGCTTTAAAATGCGGCGCTAGTAGTATACGCGTGTAA
TGCTATTGGCAAAATTTTTCTTTTGTATAAGTATAGTATATGTTCCTTGGCTACTATGTTGATGATGTCAGTA
GGTCTTGTAAACAAATGCATTGACATCAGTGACCGCGGCTATACATATTATTATTTAA
AAATGAGTTACTTTTATTATCATTGGTGTGCTGTTCCTAAATGCGAAAATACTAGTAAAAAAACACCGAACAA
ATTATGAATCCATGTGCCAAATGAAATCAATATG
>126034922_2 EL596409.1
GCACGAGGACCTGACGAGTTGCGAGGAAAATTTGATCTCGTTTTTTCTAATTACACCCTACATCGGACAACAC
AGCAAGAGATAGCATTCACAAATATATACAATTTAATGACGAGAGGTGGTACTTGTCTACTTTCATTCTTAGG
ATACTTTCCGATGTATATTATTTATAGAACTTTAGCTCGCCAAAAAAAATGGAATAAT

TGGGTGAGGGACGTTGAACATTCCGTGTCTCCATACCATGATTGTCAGGACCCTGAAAAAGAAATTAAGAAAA
TGATGACTTCTATCGGATTCAGCAATATTGAAGTACGTTGTACTGAAAAATCGTATACATACATCGGCTTGGA
GGCTATTAAAAATATTATAGAAGCAATCGATCCGTTTAAAATACCGAAGGAACTGATG
GAAGATTTCCTAGAAGACTATATGCAGACTGTTAAAGATTTGAAACTCGTAGACTTTGTATACTATAATGAAG
AAGATTCAGTCAGAACAAGAATAGACTATCGTCTTATCACAGTATTTGCCCAAAAATAAATAATAGAAATTGT
TACATTATATGATATATTACTTATTATAAAATATTTTACTGTATATTGTATGTATATA
TGTAGAATTTAATATCTATATATGTATATAATACTAGCGGCCCGCCTGCGGCTTCGCTCGCTTGAACTACATG
ATTATTGATTTAA
>126034928_2 EL596415.1
GCACGAGGGTTTGATGTGGTAGTCTCGGGACCTGGCTCCAGAGCATTACCTGTCAGATGTTATCAGACCAAAT
CTGGGTTATTGCAAGCTGAATTCTCTATTAATGAAGTCGGTCAAAGTACTATAGAAGTTCTACACCTTTCAAA
ACCGGTCACGGGTAGCCCGTTCACCTGTGAGTCTTTCGATCCAAATAAAGTTATCATA
TCCGGATTGCCAACTGGGAACATCATAGTGCAGACACCTATCAACTTTACAGTGGACACAAAAGATGCAGGCG
TTGGTGAATTAGAAGTGGTATGCGAAGCCATGGGTGATAGAAGAGTGAGGTTACCCATCGACATAAGGGAAGA
CGGACACACGCACAAAGTTCGACTGAGACCCACCGTTCCTGCACATTATAGAATATAT
ATTACTTATGGAGGTTCACCAATAGTAGGAAGTCCAGTTTCCTTAGGGGTCCTCAGTGGCAAAGCGTAACGCA
GCTAAATGTTCCGGTACGGGTCTCGCTCACGCACACGTTGGAAAAGAAGCCGCTTTCACTATACACTGTGTCG
AAGATACTGCACCCACTGTTNCAGTGGAAAAACTAACTGATAACAAGGAAGAAACAAC
AGACTCTGGTTTACTGGAATGTAGAGTATTAGCGGGAGCGCCTCGAGAATGGCTCTGTGCATACAT
>126034929_2 EL596416.1
GCACGAGGAGTGAATGGTCAAGTATATCTTACAACACCAGCGGTAGAATTAAGGCAGAACTACAAAATCAGCC
AACAATTCTCGAAATTACCGATTTGAGGGGTGACGATGAAGCGCTATACCATTGCCGTGTGGACTTTCTTCTC
GCTCCCACAAGGAATATTGGCATTAATTTAACAATTATAATTCTACCAAGCCAACCAT
TTTTCCTGGATGAAGTTGGTAACAAAGTTGAGAGCAAAATAGGCCCATACCACGAAGGGGACACACTGGTACT
CAGCTGCCTTGTTATTGGAGGTAGACCACCTCCTCGTATAAGCTGGTATTCTGGCAATGAATTGGTAGATGCC
TCAGACGGAGCTAGCGATATACCAGGGGTCAGAGAAAACGAGTTATACTTGCCGTTAA
CAAGAGATAATGCAGAATCGTTATCTTGCAGAGCAAGCAACACTCATCTCTCTCCACCAGTTGTATCTTCTCT
ACAAATTGAACTTTACTTACCTGCACACAACGTGACTATAAACTGGATGCGGGGATTATTCGAAGATGCGCTA
AGAGCTGGTAGGACGGCCCTTGTACAGTGTGTGGCGCTCGGCTCATATCCGCCACCTG
AACTGTCCTGGTGGTTAGACCACCGGCATCTAACGCAACGCAGCAACCAGAGCTGGGAACATGTCAGTCGTAG
GGCGG
>126034931_2 EL596418.1
GCACGAGGACTTGACGAAATCGAAACTTACCACAATCGGTATTAGATCCCGGTACCACATGCATGTTGAGTAA
AGACAAAATTGCTTAACAAGATTAACAATACAATAAATATATAAATATTTTTTTTTGGTACATTTTTGGATTG
TATATTTTG
>126034933_2 EL596420.1
GCACGAGGAGGATACGCACCTGATCCAATTGCTGATTGGAAGAAAAGGGCGAGTAAAATGCAAGGATTGACTG
CTTTGGAGGACGATGATCCTCAAGAATCTGAAGATGAACCTGAACCAGAAGAAGTTAAAGGGAAACCTGTGGA
TCCTGACAAAGCATTCCAGCAATTAAAAGAGGTCAAGAAGTTTAACTACCTGCTCGGC
ATGTCCATGTGGATGCTCACCAAGGAGAAGAAAGATGAATTGCTCAAGCAAAGAGATCAGAAGCTCTCTGAGC
TCACTATTTTGAAGGCAAAAACACCATCGTTACTTTGGCGTGAAGATTTAGACACATTCTTGATTCGTTTGGA
AGAAGTAGAAGAGAAAGAGAGGCAAGAGGAGGCGAATGTCAACAAGAAGACCTCTAAA
GCTATGGGTGCAAACAAAAAGAATCGCAAATCCCTAATGGACATCGCTCCATCTGAGAACGGCAGACGTGTCG
AGCCGAAGATATCGGAGGACCTTATTAAGAGGATACAAGCAGCTGAAAAGGCTAAGATTAAGAAGGAGGTTAA
GAAGGAATATGATCCTGATGACCCCACTGGTATAAGTCCGGCAAGTGGTGAAAAGAAA
CCAAAAGCTCGCGTGAAGAAAGAGAAACCCGAAAAGGCCGA
>126034934_2 EL596421.1
GCACAGGATTTATGTAAATGTAATATTAAGTTTAAGCGGGCGAAGCCGCGTTTCAGTTGTCATTTATAAGTAA
ATATTATACTTACTCAGGACAAAATAGCAGGGACAGTATGGCGGGTAAGGCGCGTATGTTGGGCATTAACGTG
GTGTGCCGCAACATTAACTTGTTACTCATTTCATTTTGACCTACTTCTGCTGCCACTA
GCAACCTGGAAAAAATATTTATTATTATAAAATATAAAGCAGCTCATTCTAATAAAAGGGCTTTTCGAATAAA
ATATGACGACTAATCATCTTGCTGCATTTTCCAAATAATAGGGCAGTCTGAGGGAACCCGTGAACCATGTAAT
GACACTCCTTTTCCCGTTAGGCCTCACCATTCTCAAGTTTTCTCTGAATTTTTGAGCC

ATTGTGAGTAGAGTAGCAGAACTAACTGCGCTTTTGCTGCGTTACCAGAGCGAGGAAATAATACACTTTAATA
AATAAAATTTTGAATAGGGGTGAATAAATCAGTAATTGCAATGTTCTGCTATCTAGAACTTTTTAAAATTCTT
AATTTTTTTATGGTGCAATAAAGTTTTTTATTCATTTAGAATTTGATTTGTTTTTCTT
ATTTTAGTTTGTAATAAATACATAAAATTATTAAGTATAATTTTATTCCTTCATTACATAGAACTAGCAAAAA
TAAATTGTAATTTCATGCAAGTAAAACAAGTAGTTTTAAACAAAAAACTTTACCTCTCGTATACTTC
>126034935_2 EL596422.1
GCACGAGGGGTCAATGCAACTTGCGAGTTATTGCTGAAATTTCGTTTTATTTTACTGAATCGTAGGAAAAAGT
TAAGAGCACGTTTAATTTACTACTGTAGTAGTGTTTTTCTTTTGTAAACCTATTATATAGAGGTGAGGCATGA
TGGTGGAAGGTTCGGCATTCACGAGAAACGATGTATTCCAAATGGCAATTCGAGTTGC
TTTTGTATCCGCAGTGACTTATTTCTCAATAAAATGGCTAGTAAATCAAATAGACCCTACATCCAAGAGTAGG
AAAAAGGCCGAAGAGAGAGCGCGGGAACAGTTACGCAAGATATCTTGCAGAGCGGGCCTTAGCGGGAAGCATG
CACTGGCATTGGACAAATTGACAGATCATGAAATGATTATAGCATCACAGCTGGTAGT
GCCTGAAGAGATAAATGTAAACTGGAAAGACATCGCGGGTCTAGATCATCTTATCAAGGAGTTGAGAGAGACA
GTCATCCTGCCGATACAGAAACGAGAGTTGTTCGCGGATAGTCGTCTCACACAGCCGCCCAAAGGTGTACTGC
TGCATGGGCCACCAGGCTGTGGCAAAACGCTAATAGCCAAAGCGACGGCAAAGGAGGC
GAACATGAGTTTTATAAATCTCGATGTTTCCCTCCTCACTGACAAGTGGTATGGCGAAACGC
>126034937_2 EL596424.1
GCACGAGGCGATTACAACCTAATTAGGTATCGATTTAATTGGATCTATTCAATGAGCTGTATATTTTACGTAT
TAAGGTTCCGTCAATATTTCTTTTTAATTTAGATTGTTTTTTAAAGCGATTCATTCCAACCGTAGTAGTGCCG
TCACACGGTAAGTTCTGTATATACGAAGCTTGTTTTTGTAGCTGCTACGTACTTCTAT
GAACATTATAAATATATCGTTATTTTGATATGAATATATATAATTTTAATTTATTTAAAGTTCTATATTGAGA
ATTAATTTTATGTGTTTTTAATCTACTGCTTGAAACTCGCGCTCTATTATTTTTTTTACAATTCTTGTTATGA
TCGAAACATACCAACACCAACTCCTTTGATTTTTTTTAGATGATAACAATAAACAAAT
ACATCATGTAGACTAGTCAAATGGTCTTATAATATCCTGAAAAGCGGATTCAAGTAAGATTTACCTCTCATTT
TCAAACTTCATATTAAAATTTAATGTTATTACAAGAAAAGAATTTATAGTTGTTGTAAAAATACTGAAATTTC
TCACTGATTTCTCGACTGGCACTAAGAAGTATTTTGAAG
>126034939_2 EL596426.1
GCACGAGGATTCCTCGGTCCATAGCAGCTTTGGCAAGTTGTACCGTACACATAACTCGCGTAGTGCATAAATA
ATCAAAAGAGTTTCGTAAAAAAAATAAAATGGGTATGGCTATTAATCATTTGCCGCAGATTCGCGAGGAATGC
GAAATCCTCCAGTTATCAGATAAACAACTAAGGGAAATTATGAGAAGACTCCACGGCG
AACTAGTTAAAGGCCTCGGAAAAGAAACACATGCCAATTCTATTGTCAAATGTTGGATAACATACATACAAGA
TTTACCTAATGGAAAAGAGCGCGGTAAATTTTTAGCGTTGGATTTAGGTGGAACTAACTTTAGAGTGTTGATA
ATAAACTTAGGGGAGAACCACTTCGACATGCAGTCCAAAATTTATGCTATTCCAAACC
ACATAATGACGGGAACGGGTATCGCCCTTTTTGACCACATTGCAGAGTGTTTAGCCAATTTTATGAAGGAACA
TGACGTTTATGAGGAACGTTTAGCTCTTGGTTTCACGTTTAGTTTCCCGCTGCAGCAGCTCGGGTTAACTAAA
GGGATATTACAACGTTGGACGAAGGGCTTCTCTTGTTCGGGCGTCGTAGGGGAAGACG
TGGTACAGAGGCTCAAGGACGCTATAGCACGAAGAGGGGACGTACAGATAGATATTTGCGCTATACTGAATGA
CACAACGGGTACATTGATGTCTTGCGC
>126034941_2 EL596428.1
GCACGAGGCAAACGTGAAATAGTGGCTGATATCAACACTCTAGTAGACCTCGCTTTATTATGTGAGAGTATGA
ATTGGCTAAGCACAAACATAAAGACGATTCCCTCCACAATAAGTTCTCCGTCGCCTTCCGGTGGAGGATTGAA
GTTACCTGAGAAGTTTAACAACGATATATCAGCTGCCGCCGCCATATTTGATGAAATT
GCACATAAATGCCTTTTATTTTTACATTTGGAGATGCGCATAGAGTGCTTCCACTTCCTGGGCGGCGAGGAGC
GCGGCGAGGCGGCGGAGGCGGGCGGCGCGGCGCGCCTGGCGCAGCGCCTGCTCGCGCTGCACGAGCGCGCGCA
CGGCCTGCTGGCGCCGCACGCGCGCAACTACATAATGTGCGGTATCGGCGAGATGATG
TCCTCTGCTGTCGTCTGGCGCTGGCAGAGTGCTGGCGGCGGGGCCTCACAAGTGGGCGTGACTTCTGTATCTG
GCAACAGCACTGCGCCAATAGGAGGGCTTGCATCGGCAGGGGGCGGGGCTTCGGCCGGGTCTGCAGCTTTAAG
GGGTGGAGCTGGAGGGGATGGAAGGAGCTGC
>126034942_2 EL596429.1
GCACGAGGCAAAGCAGTCGTGTTATTATAACATTACTCATCACTTGTTATATCGAAAACAAAGGAAATCATGA
AACTAAACACGACGTAAAAACCAAAAAGACGCAGTTTATTGATAAGATTATAATAATTCGTAGTAAAAAATAA
AAAAATCGTATTCGGGGTGATGTTTCAGTAATTTCTTAGTTTTTAATTAGAAATTACG

TGTTTAAGCCATGGCCGCGGCCCTCGCGAATGATTCTCCCAGATATAAGAACAGTTTAATCTGCTGCAGCCCT
AGAAGTGCTGACCTTGTTTCTAGATCTTCCTCATCCACCTCAGGATGTGTCTCCGCTGACGAGAGCCTCGACT
CGAACTACATCCCAAAATCGATAGCCAATAAAAAACTAAAAATTCACAGCTCAGCCAC
ACGAGAAGAAATAGAAAAAGCAATTAATCAATGCAAGGAGCTAGTTTTAAGCAGCCCCCAGTGTTCAGACGAG
CGTAAGTGGCTTGTACGTTATTTAGTGGAACTACGACTTAGGTTAGAAGATCTAAAGGAGAACGATGGACAAA
TAAAATCCAAAGTTGCTATAAAAGGACACCATTTCGAACAACAAGTTAATACAGGTAA
TAGAAAACAGTATTGTGACCATTGCAGTGGAGTGATATGGAGTATTGTTCAAAGTTCTTACATTTGCGCGGAC
TGTAGCTATGTCTGTCATTAT
>126034943_2 EL596430.1
GTTATTTCTGAGCGCTTATTGTAAAGTTTTTAAAAGAAAGAAAATTTGTGATAAGTATGTTTATTTCACATAT
ATTTCAAATTTTGGTAATGTCTTATTAAACTACATGCTTGGCTGAAAATAAAGTATCTTATCATGCGGCATTG
GATAATATCTCTTTTAAAACGAATAACACATTTTGAAATAAAAAGACATATCAGCACA
ATGTCTAAAAGAATAGCAGTATGTCAAATGACATCGGTAGCTAATAAGGAAGAAAATATGAGTGTAGTCAGTA
AAATAATAAGTGATGCTTCAAAAGATGATGTACAGATGGTATTTTTTCCTGAAAGTTGTGATTTTATATG
>126034949_2 EL596436.1
GCACGAGGCCTCGTGCCGTCTGCCCTTTCAATTTAGGGCAGTATGTACAAAAGACCTACGTATGATAACTAAT
ACCTAATCTGCTATATAAAGCGAAACTCAAGCTTAAGTATAAGGGTCAAGGGCTGTTTGGACCCGTTCTCACC
CGCGTGGTATTCCCATCGACCAACCCAGTGGCGGTACTCAACTCAAATCATTTACTGT
CTTTGACTATGAAAAGTTCCTAACTTCATATTCAATATATGTAACATGTAAGTACATTTTAAAATGTTATACC
TACATAGTTGTTACTCCTAGCTGTCTGTCTGTGTGTAATTGCAATGTTATTTTAACTAGTCGAATTTGAAGTT
CGCAACTTGTTCCTGGACGATGATAGTTTAGATCTGCAAGATCTTAAAT
>126034961_2 EL596448.1
GCACGAGGGCTGAACGTCGTAAGGACTTCTATGAAAAGATTAAAGCTGACCCGACCCAGTTTCTGCAGCTTCA
CGGTAGAGTGTGTAAAATACACCTGGATCCAGCTGTAGCAGCTGCCGGCGAAGGTCCAGCTATAATGATGCCG
TGGCAGGGCGACACCAACAACATGATAGACAGGTTCGATGTTCGTGCCCATCTGGATA
TAATCCCTGATATAAAAACACCCGAAATACCACCAGAAGAATTATCTCAGGAAGAGAGACAATGCAATTATGA
AAGATACAGAATTTTGGCTCAAAATGCTTTTCTAGGAATAAGTGAAGACAAATTTCTTCAACAACTTGCTATC
GAAGAACAATTTGGAGTGACTGTTGAAGAAAAAGAAACGCAAAGGGAGAAATTGAGTG
AAAAAAGAGGGACAGGGGCTGCCATAGGATATAATTATAATGAACCTAATCAACCGTCTACTTCTAATGCTGC
TCCAGAAGTAAAGAAACCTGAAGCTAAGGATTCTGATGATGACTCTGATGTTGACATGATTGATGTGGATTTA
AGCATTGACGTTAATAAGATGGAGGCTTCACAGGCACATGAGTTAAATGCAGTTGGCC
CTCAATTTGGAATGGCTGGTTGTGATTTATTTTCATTTTTGACGGGAGATGCAGATGATGCCGAACATCAAAA
ACAATTGTTAAGGGAGGAGAAGGAAAAAGCTATGTTCTCTGGAAGAAAGAGTA
>126034964_2 EL596451.1
GCACGAGGCCCCATCCACAGGATAAGAACTCAGATATGCCACTTAAGAGTTTCTACCGCTACGTGCTGGAGCC
GGAGCTGCAGTTCAGCGCGCAGGGCGCGCAGGGCGCGGGCGCGGCGGCGCGCTTCTCGCGGCTGCCGCACGCG
CCGCTGCTGTCGCTGGAGCTGCGCACGCCCGCCAACTGGCTCGTGGAGTGCGTCAAGT
CCGTCTACGACCTCGACAACATACGCCTCGCCGACGTGGACTCCGTCGTGCATAGTGAATTCGAACTCGAATA
CCTGCTACTCGAGGGTCACGCGTGGGACACGTCACTGGGCACGCCGCCGCGCGGCCTCCAGCTGGTGCTGGGC
ACGCGCGCGGCTCCCGAAACCGTAGACACCATAGTGATGGCCAATCTCGGCTACTTCC
AGCTGAAAGCCAACCCCGGCGCCTGGACGCTGCGCCTCAGGCCAAGACGCTCGGACGAGAATCTATGAGATTG
TTGGACATGAAAA
>126034965_2 EL596452.1
GCACGAGGTGCCGCTTCTGTCCATCTGCACATGCTTTCTCCGGCTTCACAGGGCTTATTCCACGGTGCAATAG
CGCAGAGCGGAGTAGCTATCTCGCCGTGGGCGCTCGCGCACGGCCCGAAGAAACATGCCTTTGAACTGGGAAG
GGAACTGGGCATCGATACTAACAGTACAGCGGAACTGCTTGGTTATCTCCGCGCGACA
CCAAGCGAACTCCTAGTAAAGGCAGGTGCAAGGATTGCTGGAGCGCCGGGCAAGTCAGCGGACTTACAAAGCA
CTGTGGCGTTACCCTTCTTACCCGTACTGGAACCAAATATATCAGGTGCTTTCCTCACACGCTCACCACGGGA
ACTGCTTCCCGGCGCCGACGTACCGCTGATGACCGGGTATAACGCTCAAGAAGGAATC
ATATTGTTTAGACGTCTCCAGCGCTATCCTAAATTACTAAGCGAATTAGAACATGAATTTAGACGAGTCGTAC
CTCCTGAGCTGATATCGTCAGATGAAGAGATATCAAACAAGGTCACGGATTTCATACGCGCTTTCTACTTCCA
GCAACGACCAGTTGACATGAGAAACATTGACAGTCTCATTGATTTGTTCACAGACGTA
ATGTTCTTGAGGCCAGCATTAGAAACGTTAAGATTACAGG
>126034967_2 EL596454.1

GCACGAGGCCTCGTGCCGAATCGCACGAGGCATATCGATTTCGGTTTCATGTTCGAATCATCGCCCGGTGGTA
ATCTCGGATTCGAACCGGACATAAAGCTGACCGACGAGATGGTGATGGTGATGGGCGGTAAGATGGAGGCGCC
GCCCTTCAGGTGGTTCTGTGAGCTGTGCGTGCAGGCCTTCCTGGCTGTTAGGCCGTAC
CAAGAAGCGATAATATCGATGGTCTCACTGATGCTGGACACCGGCCTGCCGTGTTTCCGTGGACAGACGATCA
GACAGCTGCGTTCCCGTTTCGCTCCAAATTCAACGGAAAAAGAGGCGGCCGCCTATATGCTGTCGGTGATAAG
AAACTCCTTCCTTAACTTTAGGACCCGTACTTACGACATGATACAGTATTATCAGAAC
CAAATACCGTATTAGTCATGTATGTATCATTTCGAGGTGTATGTTGTCGGTTTGATTCCCACTTGAGGTAACG
AAATATTTAATATTCTCATTTTATTTATTTATTAGAGCAATCGAACCTACAGCCTTCGTTTTTACAGTCAGAC
GCTGCGCCGCCGCGTTGAGTAGGCTTTATGATTGGGTCTTTGGAGATAGAATATGAAT
ATGTATAAAAATTATGTAGAATTTTAATGTAATAGAATGTTGGTTTGAATACTTTAGAT
>126034969_2 EL596456.1
CGGCACGAGGGCCATATTGATCTCTTATAGTATACGGTGCACGTCCGTTGCATCGGGTTTACTTGTTAAGAAT
ATTTTAGAAGATCAACTGCAAGTATACAGACCCCCCCAGGCCAGTGTAACGTTGGCAATTTTAGGTACTACGC
GTACTATGTAGTTCTTTAAGTGACTGGTTTAATTTTTAGTTTAGGATTTTTATTCCTA
TTGTAAAAACGTGTGACTGTGTGATGTATATATCTATGATACGCATTTCCTTTTGGATATATGATCTGTGAAT
GTCACTAGAGATGTTAACTATTGTACGTCTCGCCCGAGACGACCCTGAATCTAAACGGCATTTCTAATCATTC
TCTCCTTCTTAACCTC
>126034975_2 EL596462.1
GCACGAGGGTAGTTATTATTTTACTTTATTAGTCGTTGTTTATCTTTCGTAAGGAAACGTTATTGAATTATGG
AAGTTAATTACTTTCATTTTTAAATTAATTACCAAATCTAGGTAAAGCTATGCTGTATTAAAAAGTGATTGTA
TCAAAATACAACTTTAATTATAGTGTTTATATTTCCTTGTTGTTCATAATTTATTCAA
CAGTGCAAAGTCTACTGTGTTAAAGTTATTTGTTGCGTTGATGTAACCTTGAGATAATAGTTTTTAAACATTT
AAACTGGTTTTGATTTCTTATTTGAAGTAAAATTAACATAAGCAATGGCTAGTGTAGTCCGTCGAACAGTGTG
CCAAGTGCTAAACAAAAGAAATACAGCACTACCTTCATGTAGATTAAAAAGTACTGAA
GCTAATATACTTAAATCTATTCATAAAGATGTCAAGGTAACCAATCAAACTGTATGGGACTTTGCTTGGCAGA
ACCTGGATCGGTGGCCAGATAAAACATTTACTGTATGTGCGGTAACTGGCCATGGCTACACATACGCTCAAAC
CTACAGAATGTCGGTCTCGTTCGCCGCGTCACTACGTAATAAACTGAAATTGCAGAAT
GATGATGTAGTGGCTATCATCTTACCCAATGTGCCGGAGTATCCGTGCGT
>126034982_2 EL596469.1
GCACGAGGCTCTTGCTGTCCGCAGGAGGAGAAGAACTGTTGATTTGCGACTTTGGCATCAGTCGTGCGATACA
GCCTGGAGCACATGTTAGAGAAATTTTAGGCACAAGAGATTATGTTGCGCCAGAAATCCTATCCTATGAGCCA
CTTTCCCTAGCGGCTGACATTTGGTCAGTGGGGGTGCTGGCCTACGTGCTGCTGAGCG
GCTACTCCCCCTTCGCGGGGGACACCAAGCAGGAGACGTACCTGAACATAGCGCAGTGCCAGCTGTCCTTCCC
CCGCGAGCTGTTCCGCGGCGTGTCGCAGCGTGCCATACAGTTTATACAGGAGACACTGGTTGTTGATCCGAAG
GGTCGTCTTACAGTGGAAGAGTGCCTGGAGCACCCCTGGTTGAAAGACGAGGCGGACA
TCCCGCACGCGATCGTCGGCGTGGGCTTCGGAGCCACCGACCTCGCCAGCGAAGACGACAGCGACGAAACGAA
TGTTAACGGATTTGATGAAGTTAATGGAGTTAACGGGGTTAACGGAATTAATGGAGTTAACGGTGTTAATGGA
ATTAACGGCGTTAATGAGACTAACGGAGTTGGTTCCGTTAATGGAGTCAACGGAGTGA
ATGTTAACGGAGTTAATGGTATCAACGGAATCAACAGACACAATGGTGTTAACGAATGCAATGGTACAAATGG
TACACATGA
>126034986_2 EL596473.1
GCACGAGGTAGAAGCTTTGTATATAACCTACGGTCTAATTGCTGGCTTAGGTATGGGGCTATTGTATGTTACT
GCCGTCGTTTCAATCGCTTATTGGTTTGAAAAACGTCGTAACTTAGCCGTAGGCTTAGGATCATGTGGAGTGG
GCTTTGGTACTTTTGTTTATTCACCTTTGACTACCTATTTATTAGATGAATATGGCTG
GAGAAGTGCTCTATTGCTGTTAGCCGGCACCGTACTTAATGTGTGTGTATGTGGTGCTGTAATGAGAGACCCA
GAGTGGCTTATACTGGAACAGAAAAAACAAAGAAAATTAAGTAAAGCAAAGAGGGCATCCAGCTCTACATCGA
TATCAGCGAAATCAGGAGGTGGAGAATCTATGTATCCAGACGCTGAAGAATTAAAAAC
TATTATTAATAGTGGCGAAACTCCTGAACATATACTATCTGCTATTGTAGCTGCAATAGAAGAAGCAGAAAAT
ATAGAAGCAGTGACACAAATGAACGCCGATTTATCAAGTCAGCATAAAGTTAATTCTGTTATAAACCTTCCAA
CATTTTTAAGACAACATGAAAAGGTGCCACCAGAAGTTCTAGAGCAGCTGACATCAAA
TGAGCGCCTATACAATATCATTTTGC
>126034988_2 EL596475.1

GCACGAGGCTCGCGTCGACGAGCACATGCACTTGTAGTTGCGCTAAACGCTAATTTTGAAGACGATTTCGGAA
TATTATTCGAAAAAAAAATTGTGCGGTGTGAATTTCGATATTATATATTTATTTCATTTAAATATAAAATATG
GCGACTTTGTGGTTTGCAGTGGTAGTTGCAGTTTTTACACAACAAGTTATCGCAGTTG
AACGAGATTGTTCGAATTTCGATAATTTAAAAAGTTGCAACGAATGCATCAGATGCGGCGGAAATTGGTGCAA
AGATAAAAATGAGAACCGCCGATGTTCTCTATTGGCCTACGACAACTGGTGTACTCACGACAAGGAAGAACTA
TTGTACATCAATAGCAACCAAGAGGAAGGTACAGAGTTCAATCCACGGTTTCAAACAC
AAACGCTGAAAGTTGGTAATGATGATAAATTCAGCGTCGTGTATTCTTCTAAGATGGAAAATCCAAAGACGCA
ATTAATAGTGAACTCAACACAAAGCTCCGACTTCGTAGTATCCAACACGACGAGCTGTTACGGCGGCAGTTGC
ACTACTACCATCTACGCTAAGCCAAAAGTAAATTTTTGTGGCTTCGATGCTAGCCCAT
CTGAGTACTTTAATGTAAAACTGTCAGTGGAAGGAATCAAGGAAGAAGCTGAGCTGAAAATCCACGTGCCCTG
CGCCTGTTCTTTTGATGATAGAATAGATGAAAGCATTTTTTCCTGTA
>126034996_2 EL596481.1
GCACGAGGCCCAAAATGAGAGTGTACCCGGCAACTAATTATAATGATCACTATCAGTATATGAATCACCATAC
TAAGACACTGCCCAATGGTTTGCTAATGGGCGGTCAGTTCCACTTCGGCGGTATATGGGTGAACGCTGAACCA
TTCGGCGAGGGTGCCTCAGCGGAGTCCTGCAGCACCTACCGCGGGTACCGCCGCCTCA
GCAAGGAGCCCTCCTTCCGCATCCGCAGCCTCGAGGTGTGGGGCGTGGGAGACAAACCTATGCTGGACAAGGA
CCTGGATGAACGTGACACAAGTATCTTAGATACGAATCCGGAAGCTAAAGCCATATTGGACATGGCGGGACGC
ACCAGACACAGCGAGGGTATTAGAGAACCACCGCCTTTATAATGCATCTTCAAATCGA
CGTCTATAATAAAAATATCTAAGCAACGTACAAGTGTTCTTACTACCGCCCGCCTTATGTCACATATTATTAT
AATTATATGCATTTTTATATATATTTTTTCTATATAATTAAATCACTAGTAATTTTGTAATAATTGAAATAGT
CAGAATTTAATTTGAAATGTATAATTTTATATGGTCATGATATTTTTTTATCTATGGA
TCTTTTTTGGTTAAATTGCTGTTTAAAAATTACTATAAGATTTTTTAAAAGAAATATGGT
>126034998_2 EL596483.1
GCACGAGGGGTTAACACACGTGTTAGTGAGGGATTCTTGTTTTTGTGTTATTTTTCACAAATTTTTAGTGCAA
ATTGCGGTTTTTAAATAAAAGATCTGTATTTCTTAGTTATTAGGTTGTTGTGAGTGTTTGTGGTGTTTTTGTC
AGTAGATTAGTTATATTATAAGACCACAACATATACATGAATATAAGTTAACAGCCTA
CAGTTTATACGCAGCAGTGTCGGTGGGTTTGCAAACTAACGACATTGTTGAGTATCTGCAAAGACTAAGCAAG
TGTGCTGTACCAGCTGGCATCATAGAATTCATACAACTTTGTACTTTGTCATATGGAAAAGTGAAACTTGTTC
TTAAACACAATAGGTATTTAGTTGAAAGCAAGCATGTAGATGTACTACAAAAGCTATT
GAAAGATCCAGTGATCCAACAATGTAGATTGCGGCGAGATGGCGACGAGGAACTGCTTGCCTCCGCTTTACCG
AACAAGCCAGCAACCACTTCTGCTAAACCTGGTGAAGACAAACCTGCTGCAAATGGGGCGGTTCCGGAAGATA
TAAGTCAGTTTTACCAACAATTGGATAAAGATGATGATGACGACGAGACTACTGATAT
TACTGCTAAGAATTCGGCTGTTGCCTTTGAA
>126035006_2 EL596489.1
CGGCACGAGGCTGAAGATGATTCAACAGATAAGGAAAATACCTTAACAGACAGCTCACCTAATGTTATCAAAC
CAGTATCTTTTGTGAGTGCTGGCCAGCTGTCTAGCACATCAACTGTTCTAGCCCCACCCAAATTTACTGGCAT
AAATGTAATATCAAATAGTAATGGGTCGCCAGCTCCTGAATCTGTATTTGGACAAACG
GAAGCAAAATCTATATTTGGATCAACAGAGACTAAGTCCATTTTTGGCCAAAGCAACCCAGTTTTTGGTGAAA
GTAAATCTATATTTGGAGAATCAAAACCTTTGTTTGGTTTGTCCTCAGTCAAACCTGCCGTGGGCTTTAATCT
CAATGCTAGTAAAATCGATACGCAATCAAACGATGTTAAATCTATATTTTCGGCTGAT
GGGAAACCTTCTGTTAACTTATTCAGTGGAAACTCTCAAGGAAGTTTATTCGGTCCTGCAGCCTTAAAGAGTT
CTCAACCTGGAGGTATTTTTGGTAATACTGCAACATTTACCTTTGGAACTGCACCAGCAAAATCAAATGAAAA
TAATCCAGAATTAAGTGTTAAACCAGAATTTTCTTCTGGTAAACCTGATGATCATTCA
TTAGAAGTAAATAAAAACTTGTTTAGCAATTTA
>126035008_2 EL596491.1
GCACGAGGCAAAAATAAAAATGCTATAAAACTGATGAATTTGCTTGGCAAGCCGCTAATCTCAGAAACTCTTG
GTTCAAATTTAAAAATATATTTATTTTTTGAATTCGAACCTGTAGTTCTTCGTTCGTTCTCGTTTTATTGGTT
ACTGACATTAAACTACGAGTACCTTCATGGTTTCGACGTAACCTACCTACGGTCAGGG
TTACAGCTAGTAAAAGCTATTATATAATGCATTTACAAAGTACATATTACAAAGTATTAATGCAAAATTGTTA
TTTAAAGTAAAATTAACTTGAAAACCTTGATTTATAAATGTTTCTACTAACCTTTAACTTTTAAATGGTGATC
TATTTCTCTACTAGAATTATAAAGCTAATGAGTTTGTTTGGTTGAACGCGTTAATCTC
AGGAACTATTTGTTCCAATTGAAAACTTCTTTTGAATGTTTGGAAAGATCAGTTCTCGAGAAAGTTTTCCTTT
TTTTTATACTATATTGATCGAGGGTCGTTCTTGTATGGACGGCCCGAGTGTCGTAATCGGTGTGTGGTATGTA
TGGTTGAGTTGCCGATCACACACAATCGTCCTGGAGGGTTCATTAAAGACTATTGCGT

ACTTTGGGTGTTCTTCTTTTTCTTATAACTAACGAATGTAGACAGTTGTAAT
>126035014_2 EL596495.1
GCACGAGGGTGGCTTAGAGCTATAACCTCTGTATTTGTAAAGTATTAGATAAATAAATTAAAATGGAGTTGAA
TGAAAGGATTTTAAAGTATTTAGAAGGTAGCGACAAAGTCGATACCCTAACATTAGCTAGTGAATTCAACGAA
GATCACCAAAAGATAGTCGGGGCTGTTAAAAGTTTAGAAGCACTTGAAATGGTCGTCT
CAGAGCCAGTAAAAAGCACAAAATGGGAATTAACGGAGGAAGGAAATCTAGTAGCTGAAAAGGGCAGTCATGA
AGCTGTTCTATATAGAAGTATTCCCGAAAATGGAATGCCGCAAGCTGAAGTTATGAAGACTGTACCAAACGCA
AAAGTAGGCTTTAGCAAAGCTATGTCCCTTGGATGGATATACATGGATAAAACTGGGG
GCCCTCCATTAGTTAAGAAGAAAATTGAATCCATCACTGACACAGTACAAGATTATCTTAATGAAATCAAGAA
GGGTATCGACAACCTACCTGATAATGTAAGAAATGATTATAAAAAAAGAAAGCTTCTTCAGGAAATCACTTTA
AAAAGTTTCTTATTATCTAAAGGGCCACAATTTTCTACTTCAATAAAAAAATTAGAGA
CTGATTTAACTAGTGAAATGTTATTAACAGGATCTTGGAAAGACTTGCAGTTTCAACCATATAATTTTGCTGC
TCT
>126035021_2 EL596500.1
AATGTATATTTTTATATTTTTACGTATCGCATATTTGTTAATTTTGTTCTATCTGTTCTCTTGAACAATTAGA
ACAAAACTAAACTGTTTGTTAAGTTTGACCGACTGTATGTTGTGCTGCTAGACACTTTGTTTACTCAGACGTT
CAGAAACTTTGTTAATTTGTACGTAGATAGCAAGAAATTGTATATAAGTCAATTATTT
TCCTATTTCTATTCACCCATGCATGATTAATATAAAAAAATTATATGCGGTAAAATAAATCTATTAATGATTT
TTCCTTGAAAAGTAACTCGCAAAATCTTACATAAATTATAATAATTGTTATTTGGCACAGACATTTTTATTCC
ATTTTTTTGCTTTAGCCTTTAGTGTTTACTAGAATTGTTGTTCTGTTTTATTCCTACA
AATATAATATGCTCCATAATATTTTTATGTTTTAAACATCATTTAGTCTTGCTTACTTATATGACTATATAAA
GAAAAT
>126035025_2 EL596502.1
GCACGAGGGGGCGGTTTCCAAGAGGCGCAGGTTCAAATTCCGCTACGTTCAAATTTTTACACGTTTCTAAAAT
AACTCGGAGGTTACTTAAAAATTATGTTCCAATAATAAATGTATCGTTTCAATTGTTATTGTAATTGAGAGTT
TTAACTAAACATTTTAGTGGATTTAACAGTTTCGGATGGTTGGTTTTATATCCAAGAT
AAATAACTGAAGGAACCTTCCAGTGTAGTTACTTAACTGTTGACATAACTCGTCCTGATGACCAAACAGGTAA
CCGATAATCGATACGGTGATTTAGTAATGCATAGTTAAACGATTACAACACCCCACCTAGGTTGTGTTGGACA
TGGCCAGTTTAAATTGGGTTTCCAGTAAAGGTCGTCGTGTTACGGATTAAAAAGAAGG
GTCGTGCTGAATCAAAGATAAGATAAGATAAGATCGGAAAAAAATGAAGGTGATAAAGATTTTTTATATCTGGA
CAATAATATCGTTTGTTGTGTTGCAAAGTGTTGTGGTTGGATCAGAGCAAACAGGGTTTGAGCTTCCCATACA
GTTGGTCGGGTTCCCATTCATAATAATTGCTGTGAGGCTCACGAATTTCATCAAGAAA
CTGTCGTATGCGCTAAGTCCAGCAACATACCGTTCCCGTAATCGGCGAGAGCTAACTTTCGCAAGTGAAGTTC
CCGAAATCGTCGACGCGCA
>126035029_2 EL596504.1
GCACGAGGTCAAAACCTAAACATAACCAATAAAACTTTATTGAAATAATGATTTTATAGCACTTTAATATAAA
TAATTTTAAGTTATCAGTGTTCATTGTGGTACTCTGTTTGTTATTCTAGGTAACTTTATTTTGTCACGTGATG
GATTCAGACTTGAAAGAGAAAATAAATCAACTTCAATCGAAAATAAAAAGTTTTCCCG
ATTTTCCAAAAGAAGGAATATTATTTTGGGATATATTTTCAGCTTTACAAGATGGTGCAACTTGTAAATTATT
ACAAGATATTCTTGTAAATGTTATTAGAACGAAATATCCAGATGTTGAAGCAATAGTTGGTATAGAATCCAGA
GGTTTTCTATTTGCATTCTCTATAGCTGCCGAATTAGGTATAGGATGTCTAGTTATTA
GAAAGAAAGGAAAACTGCCAGGGGATGTATTCTCCTATAAGTATGATTTGGAATATGGATCTGACATTTTAGA
AGTTCAAAAGAGTGCAGTGAAACCAAATTTAAAATGTTTAGTCATTGATGATTTAATAGCTACTGGAGGTACA
ATTACTGCTGCAAGTAAA
>126035037_2 EL596510.1
GCACGAGGCTCATTCATACCGGCTGTATAACTTTTAACATTAGCCTATAATAATAACGCGGTATTTTTTAAAA
CGCACGTTCTATTTTCAAAATCATTACCGCGGTAAAATCGATGCAATTGTCAAATTCATGTGTCAAATTTTGT
GAATTTAGTTATGTGTAAATCGCGTGTATTTATATATTATTTTATTTGAATGTTTATA
TTTATATTTATTTATATAATATAATCGTGTCTTCCGTTAAGTGATAATATAGACTAATTGAGTAATATTTTGT
ACACAATGGAAAAGGGTGGTGGCTCTAGACTTCTTCAAATGCAGGCTCGATTCCAGCAAAAACAGCTACAGGA
GCGCGAGCTCAAAATAGCGTCTCTCTACGAAGCGCAACAGGCGCGCGCGCTCGACCGT
GTCAGGCACTCGCCCAGCAATGGCGTGTCGGGCACTCCGCCACAGCCGGCCGTCACGCACCAGCCGGGAAAGG
TACGACAAATGTTCGAAGAGCGAAGAACTAAAGCTGGCATAGACAAGAGTTACCCATTACAGCCCATACAGAA
TACAGAACGTATACCACCAAAGAAAATTCTGCCAAATGGATATACGAAGGTGTCAACA

AATCGGACTCTGGAAAAGAAAGCTTCTACCCGTCAATCTCACACGAGTATCAGCTCGCATAGTATTAAA
>126035185_2 EL596512.1
GCACGAGGCCGACGCGTGTGTGCACCAACGGGTTTTTTGTAACGGTACACTTCGTGACGTCAAAAATGTCGCG
GCAAAAAAGTGTTAGTGTAAAGTGGACTTTAGTTTTAGTGTTAGTTTTATTCGGTCAGTTTGTATATTCGCGA
CCTCAAATTGAATCAACTACTTTAGAAGTTAATGTTGACATCGATAAATCAACCGATG
TCACACCAAGAATCAACGAGGGTCATGAAAAGAAAAATGGTATGCTATCAAACGAAAATACTGCTAGAGATGT
CAAGAACAAATTACCTATAATTGGAGAATCTATAAAAAAAGTTGCAACACAATTACTAGACAAAAGTAATAAC
CAATCCAATACCATTGCAGCGGTGCCAGATACAAGAATACCACCAAAATTAAAAACGC
AGATTGCACAGAAACAAAAGATCAAAACTAGAAAGGACGACAGCAATGAATCAGTTGAAAATTCGCATGCAGG
TGTCGCAGTACCAGTCACGATGGAAGAATTGGAATTAGAAAACAGAGAAGCGTCAAACGAGTCTTCAACAACT
AAAGAAGGAATATCTACTTGGATATTACTAAGCAATCCTGTCAATAAAGATAACGATT
CTGTTTCAACAGAACCAACAAAGGTAGAAGAAACTCAAAAGAAACAAAAACCAGTTGTAAGCAAAAATAAAAC
GAAAAAGCCACAAAA
>126035186_2 EL596513.1
GCACGAGGATGTTAGGTAACCTTACGCCAATATGAGATAAACATTGTTTTAAAAGAGACTTATTCTTTCTAA
ATTTATTGTGCTTTCAATTTTGTGATCTAAAATGGATTTCTTATCGGAAATTATTGGTGAAACAGTTATACTT
GGCATAGATTCCTTGATTCTAGGATTTTGTGTAAAGCAGCTGAGCAAATGTAAACATA
TATTGAATGCTTTACAGTCTGCTCCAGTACTCGACATTGACTCTACACTCTCTAAGGAAATCAATAAGTATCC
AAACAATGTTATCCCATATGTAGTCATAAGAGGATTAGTGAAGCCTCTTGGTACTCCTATCAAAAGTAATTAC
AATCAGGCTGTTACAGGAGTGATACAACGATTAACAATTAAAGAGCATGTAATAGCAA
GAACATCCGCGGGGTTTTGGTCGGATCAAACAAGGACTATACATGAGGTTTACAATTCTACTCCATTTGTTTT
GAACAATGGGAAGTACAGTATTGAAGTGGTTGATGCCTTAGCTGCTGAGTTATTAGACTTAGATGTTATATCA
GATAAATTTGAACCAATGTCACCTAGTGTAATAGATCATGTATGGGGTTTCTTTTCTG
GAGTTCGACAAAGGGGTTTGCAAACTATGGAAGAAATGCTACGAGATGGATCCTATATCACAGCTAT
>126035193_2 EL596519.1
GCACGAGGAAGACGCGTCCGTCCACCCACCGTACAACGCGCCTATTGCCAGCAGAATCTGCTGACCAGCGGAC
GCCACTGGCAACTACGAAAAAAAAAAACAAAAAAGAACAAAGAAAATAGTGAATTTTAGTGCTGTATTTATCA
ATTATTTAACAATCGAGTTGAGTTGTTAATAATAAATCGAGATGAATCAATGTATTTT
CAAAATTCTCTTCGTTATCTTTTTCACGATCGTTTGTCATAATGGGGAGGCTGCGGAGAACGATCCGGCTATA
CCGGAATACTTGCTGCCGTGCAGTCGAAGCGACCCAGAGCTGGACAACTGCATAAAGAGATCCTTCAATCACC
TACGGCCGTACCTGGCGAGGGGTCTCCCTGATCTGGACGTTCCCCCTGTGGAACCTTT
GTTCATAGATAAGCTGGTTATGGCAAACGACGCTGGGCCTGTGCGAGTGAGAGCTACCTTCAGTAATATCACC
GTAGTGGGACCTAGTAACTATACTGTGACTAAAATAAGATCGGATTTGAAGAAACTACGAATTGATATGGGTC
TGGTGCTTCCTCGTATCGAAGTGACCGGTCACTACGAGGTTTCCGGACAAGTGCTGCT
GTTCCCCGTCCGCTCGCAGGGAGACTTTTGGGCCTCATTTAACGACGTCGTGGCTATCGTAAAGATATTCGGC
AAGGAAATCGAACGCGAAGCCGTCAAATACATGTCAGCTGATCGCATGCAGGT
>126035194_2 EL596520.1
AAAACTACTTTATTTAAAAGCTTTACATTTATATTAATTTTTAAATATAACACATTTAGATAGTTCTATTAAT
AATTAGTTAAAAAATTAGTTTTTTAATATTTATACTACAGTCTATATAATTAATTCAGTTTAAAATGCTAAAG
AGAACACATTCTGAAATATAATAAAAGAATAATTGAATCTAAATGAACAAAAATAGC
AAATACGGGCCGAATTCATTTAAAAACAAGCACACAATATACTATGACTAGGAAATACAATTTTCATTACATT
CAGTAATATAGCTTGAATTTGCATATAAGTACATAATATTCGAGAGGTAGTTACTTAGTTAAGGCGTAACAAA
AACACGACTAAAGTATGATAAAACGGAGTCAACAAACAAAAAGGAAATACAATTTAAT
TACATTTAAAATGTATATTTAACAC
>126035367_2 EL596521.1
GCACGAGGCGGTCGTTCAGTTTGTTGTGCAGTGTTAAGTGATTTTATTTAAAAAAAAATGGCTGTAGCTGTTA
AAAAAGGAATATACGTAGTGGCAGCAAAAAGAACGCCATTCGGCAAAATGGGCGGAATGTTAAAAGATTTTCA
TCCGGCTGACTTATTGGCTGTGACCGCGAAAGATGCAATGAAATCTGGCAATGTCGCA
CCGACTGCGATAGACACCGTCAACGTAGGAATCGTTAATGCTCTAAGTGTAAGTGCTGATGGGGGCTTATGCC
CTCGACATGCTGCCTTGAAGTCAGGTATTCCTCATGAGAAACCAGCTCTTGGGGTCAATAGACTGTGTGGATC
TGGTTTCCAAGCTGTCATTAATAGTGCTCAAGATATATTGACGGGAGCTGCCCAAATA
TCCTTAGCAGGTGGTACAGAAAACATGTCTGCTGTACCTTTTGTAGTACGAAACGTAAGATTCGGAGTTCCTT
TTGGAGTAACTACGGAGTTTGAAGATTTGTTATCTAAAAGTTCTCTGGACACGTATTGTAACTTCACAATGCC
ACAAACGGCGGAGAATTTAGCTGACCAATACAACTTGAAAAGGGGAGAAGTCGATGAA

TTTGCGTATAATTCTCAGCAGAAGTGGAAAGCTGCTTATGACAGCGGTGTCTTCAAAGCGGAATTAACACCAG
TTTCTATT
>126035376_2 EL596530.1
GCACGAGGGATACTCCTTGAACAAAGTCGAATCATTCAGGATGTCGGAGACTCCCGCTAGACCCATGAAATAT
CCTTACACCTTCAGTGCCAAGATAGCACAGTTCCCCTTCAAATACTACGTCCAAAACCAATGGATCTGGCGCT
ACTGGATGATCGGAATTGCGGTTGCTACTCCCGTGTTCTACAAGATTCATAAATTGGC
TAACTCCCCTGAGAATGTGAGCAAATGGGCTGAAATGAGGAAGAAGGAAGCGGCGGGACACCATTAACATTCT
AGTTTATTTTGCTCTGCAGTTAATAAATGTTATATTTATAATAAGAATTAAATCATGTCTTAGTT
>126035378_2 EL596532.1
GCACGAGGCCAAGATGTTGATAGCCAGCTGCAACCACAACTGTTCCAATGAGATACTGTCCATCACTGCCATG
TTGAGTGTGCCACAATGCTTCGTGCGTCCCAATGAAGCAAGAAAGGCTGCGGATGAAGCTAAAATGAGATTTG
CACACATCGACGGCGACCATCTTACTTTGTTGAACGTTTATCACGCCTTTAAACAGAA
CATGGAGGACCCGCACTGGTGCTACGACAACTTTATTAACTACAGATCGCTAAAATCCGGAGATAATGTTCGC
CAGCAACTCAGCAGGATTATGGACAGATTTAATTTAAAAAGGACAAGTACAGAGTTTACAAGTAAAGACTATT
ATATAAATATAAGGAAAGCACTCGTAAATGGATTCTTTATGCAGGTGGCACACTTAGA
GAGAACGGGTCACTACCTAACAGTCAAGGACAACCAGGTGGTGCAGCTACATCCCTCCACCTGTCTTGACCAC
AAACCAGACTGGGTCATTTACAATGAATTTGTATTGACCACCAAGAATTATATAAGGACAGTCACTGATATTA
AACCGGAATGGCTCCTCAAAATAGCGCCTCAATACTACGAACTGAACAACTTCCCACA
GTGTGAGGCCCGAAGACAATTGGAACTGCTACAGGCGCGTCTAGACTCAAAAGCTTATCAAAAAGGCTTTAAA
ATGTCTGGA
>126035379_2 EL596533.1
CGGCACGAGGGCTAGCGATAACGATATTATTGGCGGTAGCCATCGCCGCTTCTTTCGCCATACCTCTGAAAGA
TGAAGCACAAAACGTAGAAGTGTCCAGAGAAAAACGCTCACCCACTCCTGGCTGGCATTCAGAACCTGAGGGG
GTGTGGGAAAAGAAATTAATTTGGAAAGAAGATTGGAAACAAATTTGGAAAACTGAGA
AAAAACTAATTTGGAAAACAGAATGGAAAAAAGAAAAAGTTCCTGCGTGGAAAACCGAAAAAGTTCAACAGTG
GAAGGAAGAGCAAGTACCTGCTTGGAAAGTTGTCAAAAAACCCATATGGAAAGAAGTTCAAGTACCAATATGG
AAAGAAGTTCAAGTTCCAGATTGGAAAAAAGTATGGAAACCAGTGTGGAAAGAAATTC
AAGTACCAGTGACAAAAGCAATCCAGGTGCCAGAATGGAAACAAAACTTTGTTCCTGAATGGGTAAAAGAAGG
CGTTCCCGGTGAAAAATATTTAGGAAAGGATGATCATGGATGGGAATACACTAGTCACGATCTATGGAGAAAG
AAATTGATATGGAAACCAGTTTGGAAGAAAATTTGGAAAACGGAGCACAAACAAGAAT
GGGTAACTGACAAGAAATTAGAGTGGAAAGAAGAATGGAAACAAATCTGGAGAACGGAAAAGAAACAAGCGTG
GGTCACTG
>126035380_2 EL596534.1
GCACGAGGGCGCCCAGCGAGCAGGGCGTGCCCGACGGCGCGCTGGTGCTGGAGGCGGCGGCGCGCGCGCACGC
GGGGCTGTACCGCTGCGCCGCGCCCGGGGCCGCCCCCGCCGACACCGTGCTGCGCGTCAAGGACATGTACGCG
GCGCTGTGGCCCTTCCTGGGCATCTGCGCCGAGGTGTTCGTGCTGTGCGCCATCATCC
TGCTCTACGAGAAGCGCCGCACCAAGCCCGAGCTGGACGACTCCGACACCGACAACCACGACCAGAAGAAGTC
CTAGGTCACTTGTTATTTAGTTAAGATTATCTTACGAAATTATAGAGAATTTTTATTTTATCCGAACTCTTCT
ATGTTCTATACAAACTTAATATAATGATCACTTAGCTTAACCTGAATTTATTTGTATT
GTTTACTTGTGTTGTTTTCGCACATATCGTTATATGCTTTTATTATATTTCGCGCAGTCGCCATTTTCATCTC
GTTTTAGCGTACTTAAGAGTAATTGTTAACAAAGTAGTGTAATGATCACCCCGACTGGAGGCCCCCGGTCTCC
GCCCCCGGCCTCCTTCCCCGTACTGCCACTGATACCACAACTATCACTACTACTACTT
CACTTATCAA
>126035383_2 EL596537.1
GCACGAGGAAAACATCCAATTTATATATATGCCCGTCGATCCCTGCAAGAAATTCGCTTGTCAGATCCAGAAA
TGTCTAACTGAAAATAACTTTCAAGAGATTCGATGTGAGCGAGTCTTCGAAGCGATGCGCCAGTGCTGCCTAA
AGCACAAGTCAGTGTCTTTGGTCTGTGAGGGATACCGGCTGGAGCCACGGGTGTTCGC
GCCGGCCACCGATAGGCCGATCAAGGGGTCCAATTAATTATGGAACATCGTCAACGTAAAAAAACACTTAAGC
AAAGAAGTTGGATATTTCGATACCCGAGGACATTTCAAGTGACATTCACCGTTTTGGGTCTTGGGATATTTTT
CTCGAAGCCCATCTATGACATTTGCATCCGAGAACGCGAAGAGCCGGACCTATCGGAG
CCCCCGACCACCTTTTTGTACGTAGAACCAAAGGAAGCTACGTAGATTATGGAATCTGTGAAACGCGCTAAGG
AGAGATTTGCTAAATACCCGCTTATATACGCAAAGTGCACTAAACAAGGATCGCTATACGCCCGATGCGTTTT
ATTACGAGAGGACTCAGTGAAAAAGGACGAATGTGCTAAAGAATTTCAGGAATTCAAA

TCTTGCTTAGTATCTGCTGCCAAAGAACTAAAAACAAGGATTTAAAATGTGGGCGTTTATAAGAAAATTTGTT
GATCAGGACTTTAGGAGGCATTTAAACATCG
>126035384_2 EL596538.1
GCACGAGGAAGTTGCCGTAAAAGCAACCACTATGCCGCAGAACGAATATATTGAACGCCATCAAAAGCTTTAC
GGTAGAAGGCTCGACTATGAGGAGAGGAAACGGAAGCGAGAGGCTCGCGAACCTCATAAGAGGGCCGCAAAGG
CGCGTAAACTCCGGGGTATCAAAGCTAAAATCTTCAATAAGGAGAGGCGCAATGAAAA
GATTCAAATGAAAAAGAAAATCAAAGCGCATGAAGAGAAAAATGTTCGTCAAAACACAGAAAAAGTCGCTGAA
GGCGCTGTGCCAGTGTATCTTCTTGACAGAGATGTACAATCGAGAGCTAAGGTTCTGTCTAACATGATAAAAC
AGAAACGTAAAGAAAAAGCAGGCAAATGGGACGTGCCTATACCTAAAGTCAGAGCTCA
AGCTGATGCTGAAGTCTTCAAAGTTTTAAAATCTGGAAAATCCAAGAGGAAAGCTTGGAAACGAATGGTCACT
AAAGTTACTTTTGTAGGTGAAAATTTTACAAGGAAACCCCCAAAGTTTGAGAGATTCATCAGACCTATGGCAC
TGAGATTCAAAAAGGCACATGGTACACATCCTGAATTAAAGGCAACCTTCTGTCTACC
CATCATAGGAGTTT
>126035385_2 EL596539.1
GCACGAGGTTTGTATCGAAAGGAGCATTTATTATATAATATAATGTAATGTTATTCAATTGTAGTGATTCTTT
TGTTACCACTTTGTTAGGTGTAAAAGAAATTGTTAAGTGGATTCCAATTTTTCATATTATTCTATTATATCTG
TATTTCCATAGCTAAATGTCGATGGAGCGAAATTAATTGTTTTAAAAGCTAACATTTT
TATTTGTCGTAAATTCAATTTGTTTGTGACGAATTTTGATACTTTCGTCTTTATTCTATTGGATTTTTCCGTA
TTATAGTTATGTTGTTTGATCGTTTTAAATATATTTACTTTTTTTTGCATGTCGTCTCTCTTTTTGCAGGATA
AGGATTGTATTTTTACCAAAATTGTGACGAGTGACGAAACTTAAAATCACACTTTGTT
TATTCAAAACATCAAATTAATTAAATTCCCATACATTTCGCTCTGTCATCATATATTTTGGCTTCATCTACTG
CACCCTCAATATCTATCTATAGTTCAACATTATAATGTAATGGACATATTTTCTTATTGTTCGAATGTTGTCG
GAATTTTAATGTAATTTATGTAAATATAATGGTTGGTAAGAGTTTTTTTATAATCGCC
GTAGATTATACTACTTTTTTTTTTCGTTTTTTAAAGTTAGTGCCGTACGATTGGTCGAACGATTTGCTGTAAT
ATTTGTAATAAA
>126035386_2 EL596540.1
GCACGAGGAGTTAAATTTTGGCGTGTTGAACTAAAGGACAGCATCAATGCCGCCGCTACAGCAATGTTAACTA
TCGATTCAGTATACACCAAAGCGTTGCTGCCTTATCCAACAGCTATTACCCAGCAAGAAGATCAACTAGTCAA
ATACATTGGTAACTTATACTTCTTTTCTCCATACGAGGTTTTGTCACAAAGAACAAAT
GTTCTTCTTAACACAAAATCCATTGAATCGTACACGAAAGTGAAACCATTTTCACAGCCAGATGTATCTATTT
TATATGGACCATATCCAAACATTGCTCCATTCACAGAGAAAGAATTAAGTATTCACTATAAGAATAACTCTCC
TTTCTTAACTGTAACACGCTTAGAACGTCTTATTGAAGTTTCCCACTGGGGTAATATC
GCCATTGAGGAAGTAATTGAAGTTGAGCATACTGGTGCCAAGTTAAAGGGACCGTTTTCAAGATATGATTATC
AACAAGATCATCACAGTGGACCTGCTAGTGTACGCAACTACAAGACACTACTCCCAGCATCTGCCTCTGACGT
GTACTACCGTGACAGCAATGGCAATATCTCAACATCAAACATGAGAGTGAAGAAAGAT
TTAGTTGAATTAGACTTAAGACCCCGCTACCCATT
>126035390_2 EL596544.1
GCACGAGGCTCTTTTCGATTTCTTTTATAATTTTTAAAACAAATCTGCTGATGTGACTTTATAATGTTAAGAT
ACTATGGACTTTATAAGTCACATTAGCGGTTAGCCCGCACTCTATTTATGTGTATATAAGGCGTGCTTCTAGA
ATGATAATGTGTTATTATCTATAACGTTCGCCAAATTGCTTAATAATGGGTAACCAAA
ATAGTTGCTGTTGTTACCGAAGTCCTTCGCCAATCAGGAAGGATATTGTTAAGCTTGAAGACTACCTTCCTGA
AGGAGAAGTAAGCTCCAATAATATTCAACATATTAGTGAACGAGAACCGGACGATGGTGATATTGATCCATCT
CAAGATCCAATTGCAGGAACAATTTTTATGGAAAGGTCCAAAGCGTCAATTGAAAATG
GAATCACAAGAAAACGTAGTCAACATCAAATTGCAGATAATAAATTAAAGAAAAGCAGTTCCTGTTCTACAAT
ATACTTAGATGATAGTACTGTGTCTCAGCCAAATTTAAAGAATACAGTGAAATGTGTTGCACTTGCAATATAT
TATCATATAAAAAATAGAACATCAGAGAGACGGCTAGATATATTTGATGAAAAACTTC
ATCCTTTAAGTAAGGAAGGAGTAAGTGACGATTATGATAAATATAATCCTGAACATAAACAAATATATAAATT
TGTTAGAACATTGTTTAATGCAGCCCAGCTAACAGC
>126035392_2 EL596546.1
GCACGAGGCTCCTGTTTCCATTGCGGACACAAGTATGCAACGGAAATGAGGCTGAGAGAACACTTAAGGGACG
CTCACGGAGTACACTGCGTTAGTTGTATGGAGGAGGGTTGTAATAAAGTGTTTTCAACACCTAAAGATCTAAT
CAGCCACCAGCGTGTCCATCGCTCCCAACAAAACTGGTGTAGACAGTGCGGCCTTCTC

TTCACTTCCCTCAAAGCTTGCGAACGACATCTAGACGTACACAAAAAAAGGCTGTATGTCTGTCCCGTCTGCA
ACCGAAACTATAGCGAAAAATATCTCATATTGAAACACGTGCCGCAACATTTTGAAACTGTTATCCATCTTTG
CAAAGTCTGCGGGAAAGTGTACAACGCTAAAAATAGATTGATAGAACACATAAAAACA
CATTCGGAAACCAAAACACATAATTGTTCATACTGCGGCAAGGGGTTCGTCAAGCTTGGACAATTACAGCAGC
ATTTGAACATACACACTGGATCGAAACCCTATAAATGTCCCGTTTGCGAGAAATCTTTCGCTAGCTATCCCAA
TTGGCATAAACATTTGCGTCGGATGCACAAAACTGATGGTAAAAAATATAAAAAATCT
GAGAATGTTCCAGATATTAATGAATCTGATGATGAAGAAAGCGATGATTTTATTAAAGAAATTGAAACTGAAA
TAAGTAATAAAACAAATGATTTCAACGCGCATTTACAAGTTGACATATCTCAATTAAC
>126035398_2 EL596552.1
GCACGAGGCGCCTGCGAGGCCTACGTGCTCGTCTACAGAAAAGTTAACCCGCAAATGACAATACTGCGGCAAA
AGGCAGCCGAAATATTAGAAAATGCCAGCTCCGAGCCCAACGACATTAAATTCTACATCTCAAAACAATGGAT
CAATAAGTTCAACACGTGGGCGGAGCCGGGCCCCATCGACAACAGCGACTTCGTGTGC
GTGCACGGCGGCGTGCGGCCCGAGCGCGCGCCGCACCTGCCCGCGCTCGCCGCGCGCCTGCCGCAGCAGCTCT
GGGACTTCCTGCACGCGCAGTTCGGCGGCGGGCCGGCGGTGTCGCACGCGCACGAGTGCGGCGTGTGCGCGCG
CGCGGCGCAGCGGCTGCGGGCGCGGCGCGCGCGCGAGCTCGCCATGTTCGCCGACTTG
CACACCATGTTTCAGGATCAAGAAAATCCTCTCTCAGTGTACGCGATCAGCATGTCATGGTTCCGACAGTGGC
AGGCCTTCGTGCGCAGCAAACGTAACCCACCGCCGCCCCTTGATAATAGGGATATTGTTGTTGAACAGGAAGT
CGACGGCGTAAAAACCTTTGTAC
>126035399_2 EL596553.1
GCACGAGGGCCGCGTCTGCGCCACCTCTACCAGCACCGCTACTGGGACTGCAAGAAGCGCCAGGACTGGAGCG
ACTGCCCCGACATCTTCTAACTATTGGGATATTTGTACAGTTGTCCTTTGTTGTTATGGATCGACTCTGTTCG
CGCGCTACTGGCTTGTGAGGCCTAAATTTTATGTTGCCGACCGTTCAGTTTCCACGTT
AACATTTTGAAAATTACAACGAAATTTAAATTTATTAATATTATCAACAAGCTGATGTTTTTATATAAATTCT
TTTTTTAGAATAATGTTGTAAATAATTTTGAAGACTGGAGACGAAATAGCGTGCGTACGATCTTAGCCTTGTG
ACGTTGGTTCCATACTTTTTAGTGATTTTCCATACGACATACGTTCATTATATAATCT
ATTATTGCTACAGTCTGTAATAACATTATTGTAAAATATTTAATAACAATGAAAAATTTATAGAGAATTATAA
TATAATTCATTATGATTATTTTTTTTATTGAAATTGATCGTATGTCGTGTCGCACGACGTGTGAGGCCATATG
GAAGATCACTTATTTCTATTTAGAATTAATAAAACAAAGTATTTAACGTCTTAAGCTT
ACATTTGAACTTAATAACAGT
>126035404_2 EL596558.1
GTGGACGAGACGTACCCCGCCTGTATCTACACGGATTTCCATTGGCTTATGAAGGTATCGTGTTCCTGGGACG
AAGTGAAGGCGCTAAGATCAGATATGGAGAAACATACTTCGTCGTCAATACATTTTAGATTGAAACTTTTGAC
AGCCGCCGCTCAAATGCAATCCGCTTTGTGTATACAGGACTTAGGCCAGCTGTACCAC
AAGCCGCTCCGGGACGCGCACGGTACGGTGGTGTTGTCCTGCATCAGCTCGGTCAAGTCCGCCAAGTGCGTGT
CGGCGCTGAACTCGCGCTGGGCGCCCGTCGCCAAGTTGCGGCGGCGCGTGCTCAGCGAGGACAACACTATGGG
GGAGCTGCTTATGGCGTCTGTACACGAGCAAATTGCTTATCATCAGGTATCCCGAGAC
CAATTACCACGTGGCTTGTATCTGGGCTATCTGAAACTGCAGTCTTCAGTAGAAGTAGTAAGGATCGTTGCGC
CGGCGCGGACTCCAAACGTACCTCCACATACACGGATTAGAGATAATCCACATGTTTCTGCGGAAGAATGGGA
ATATCTCANATCTCAATCAGGCAAATTAACAGAGGAAACTCAATCAATAAATAATTTA
GTATCGATCAACACTAACGACAAACCTTCAGAATCTCAAGAGATGTTTCTAGAACAAATCGCCTCAGCATCAC
GCAGACTGTTCAAAGATATGGA
>126035597_2 EL596564.1
GCACGAGGCTAACGACCACGTCAACAAGTCTCAGAGTTCAAACGACACGTACCCCACAGCGATGCACATCGCC
GTGGCTATGGAACTGAGAGACCGCCTGATGCCCGGTTTAGTCGCCTTGAGGGACACCTTAGAGTGCAAATCGA
AGGATTTCGATAAGATTATTAAGATTGGCAGGACGCACCTGATGGACGCGGTGCCGCT
CACCCTCGGCCAGGAGTTCAGCGGCTACGCGACGCAGCTCTCGTACGCGGTGGAGCGCGTGTGCGCGACGCTG
CCGCGCCTGCACTACCTGGCGCTGGGCGGCACCGCCGTCGGCACGGGGCTGAACACGAGGAAGGGCTTCGCGG
AGAAGTGCGCTAAGGAGATTGGGAATCTTACTGGCATTCCCTTCGAAACGGCCCCCAA
CAAGTTCGAAGCGCTCGCGTGTCACGACGCTATGGTGGAAGTTTCTGGAGCTTTGAACACTATTGCCGTGTCA
TTGATGAAGATTGCCAATGATATCAGATTTTTGGCGTCAGGTCCACGATGCGGTTTAGCCGAAATTATGTTGC
CAGAAAATGAACCTGGATCCTCCATTATGCCTGGCAAGGTGAACCCGACGCAGTGCGA
GGCGCTGACGATGGTGGCGGCGCAGGTGATGGGGCACCACGTGGCGTGCTCG
>126035598_2 EL596565.1

ACAAAAAAGTTAACCGCATAAATTAACATCTTAGTCTAATACATATGTACAGTCTTAAAATTATATTTTTATA
GTGGCCACATTGGCGGTATGCTGATTCTAAATTAAGATTTTTTGTTTAGTACATGAAAATGGACGAAATTTTA
GTCCTTTTCCCAAAAAAAAT
>126035608_2 EL596571.1
GCACGAGGCAACTGTGACATAGTACATGTTCTTGAATAAACGTTTGTTCATTTTATTCGCAGAGGACCTTTTG
CTGCGAGCGGCGATCACTGAACCTGTGCTAGTGATGTGCACTCTCCCGGAATATAACTCCTCGCCATCCAGTC
CGAGCAAGCAAATGGATCCAAGATACCATGCAAAGGAAACTCTACAGCTTCTTCCATT
GAACAGCAATATAAAAATTCGAAGAACTCAACTCGGATTTGAAAGTGAAAAAAGGATGTTCCTCTCTGCGCGC
CTTCAAAAAGCACTAACCTTCTGTCAATTGAATGTTGACAACTGGATCAGGCAGATCTCTTATGCAAACTCGA
CTGTCGTTAGCAAAGCGAAGACAGTTGAAGACCTCATCAAAGAAGACCACGAACCAGT
GAAATTTGAAAAAGAAAAGAAATTTACTCTGCAAGGACTAAAAATAGACACGTCTAGGCTTTTCGGGAAAAGT
GATAAAGTATTAAAAGATATACCAGATGAAACAGAAGGTTCAATCATATTTTTAAGTAAAAATGAACTCGAAC
ATATGAATTATGATGTATACAGCGATGACGAAGGTAAAGACAATAAGAGTGAGGAAAA
AATTGAATCAGAACAATTTGCCAATGATAAAATGCATGTATT
>126035612_2 EL596573.1
GCACGAGGGTTTATTAAAAAAAACTGAATAATTATGGCCTAGTTTTTTCTAAGACGTTTTGCCCCAATTTTTT
TTACTAAATATCATTTTCAGTTTAATTTCTAGAGTTATTGGATACATTAATTTAATATAATATATTAAAATAT
TAAAGTATCAAATGTGTTTAGAATTATATGGGCAATATATTTAATGTTTGTTTATATA
GTGTTAATAATATTATTTAAATAGTAACTGTACTAATAACCTATTTAATTATCATATTAAAAACAAATAACTA
TGCTTAATTAGATAGAGAGAGATATCTAAAAACCAACATTTTCATTAAATAATTGATTTCTCACATTGAAAGT
TTATGTATATATTGCCTACAATTTAAATAAATTTGATATGTAAATACCCATATTGAAT
GTCTAATTTACGTTATTCTTTCGGCATTAGTATAGGATTGCGGGTGCTAAACCAGAATATTAAATCTTACTAT
ATAAAATCGAATGTATATAATTAAGTGAAATTAGTGATAATAATAAATTTTAATAACTACAGTCGACCCGACT
ATACAATTTATCATTTCAGTCACATTTAACACGGCTTTGGTTTATTTGCACTAAATTT
GTTCATTCCAAGCACTTTAGCTTTGTATCGATAAATATAAATAAATATTGAGTAGTTAGTCAATGTGACTGC
>126035613_2 EL596574.1
CGGCACGAGGCTGAGCCTGAATATGAGCCACCTAGCTTAGAATCTAACCCTGGAAAACTTATAGCATCACCTG
ATGAGTACAAAGAAAAAAAATATAAAGCAAAAAAACATTTTACCTCTAGTCATGATCTTTTGCAAGGTGGCCA
AAAGTATAAAGTCAAAGAAGATTATTTTGGAAAAAGAAAGGGTCATTATGCCTCTAAT
CCAGAAATAGCGCAAGAACGAGAAGAAGAATACGCCAACCTTGGATATAATAATAAGCATGAATCGTATCACA
ATTCTTTACGTCGTGCTAAAAGTAAGGACAACGACTTCAAAAGGCACGCCAGTATTCGACGCCACGATTCTGG
CGACAGCAGAGACTACTATCGTGAGCAAATGTCTCCGCGTCGATTTGATTTTGATAGT
CGCTTAGTTGATTCTGGGATTGAAAATGATTTTAGACACAACTCAAGTAGCGAGATTCACAGGTCTAAGCCCA
GACGACACGAAAGTGACGAAGACGTCCGAAATACTTCACTATTATTAGCAAGTGAGAGACAACACACAGAAGA
TAACTATCCTAGCGAACAAATTTATGCAAATGGAGAATATTTGGCCAATCGGAAAAAG
TATCAAGCAGAAAGGAAGGATTATATTCCAAGGGACAGGAGCGTTGATGAGGGCTCTCTTATAAATCGTAAAA
ATAATCGTCATG
>126035616_2 EL596575.1
GCACGAGGGTTTGACTTGTTGAAGGGTTTTTTTGCAATAATTTCCTAGTTAATGTGCTTTTTAGCAGTGTAAT
CTTTGATTTCAGTTAATTATATCATTATAACAAAATAGTTAATAATGCGCGAATACAAAATAGTCGTGCTAGG
TAGCGGAGGCGTGGGAAAATCCGCCCTGACAGTACAATTTGTTCAAGGCATCTTTGTG
GAGAAATACGACCCCACCATCGAAGATAGCTATCGGAAACAAGTGGAAGTTGACGGTCAACAATGCATGCTCG
AAATTCTTGACACAGCTGGCACAGAACAGTTTACCGCTATGAGGGATTTATACATGAAGAACGGACAAGGGTT
CGTGTTAGTGTACTCGATCACAGCGCAGTCGACGTTCAATGACTTGCAGGACTTGCGG
GAACAGATCCTCCGGGTCAAGGACAAGGACGACGTGCCAATGGTACTGGTGGGCAACAAGTGCGACCTGGAGG
CGGAGCGCGTGGTCGGCAAACAACAGGGCGCGAACCTTGCCAATCATTTCAACTGCGTTTTTATGGAGACCTC
CGCGAAGGCTAAAATCAGTGTGAACGAAGTGTTCTACG
>126035621_2 EL596578.1
GCACGAGGCTCAAGTAGAGTGGTACTGAATTCTACAGCTCATATAGGTACACAAGAAAACAAACAATGGGTGA
GTTGTTTACACATAACTACTTATTTTATATTATTTTCTTAGAATGAATTTGGTGATTAATGCTTCAAGATTGG
ATAAATTATATTTAGTAACTTATTTTTTCAAGTTAATTTAACACTTGTGACAACGAGA
AATAACATCGTATTGGTTTTGATAAATTGGGAAGGTAAGAAGATTTTCAATCAAGATTACTGACAAAATATAT
GGAACATACTCGTATATAATTTTATGTACCTTAAAATATAGCATTTCAAAGAAAAAAATATTTTATTTGTTAA
TTAATTTGGTACAAATCGATATATTAGTAGCCTTTTGTCGTTGTAAGCCAAAGAAAGA

TATATCCTATAAATGTTTTGGTCCAATAAATTCGGATGTTCTTTCAGCGTTCTTTTCGTTTAGCAAAACTTAT
TTTCCTGTGTCGAGTCTATTAGTAAACGGCAAAGGTAATATTTCAAATTTATTTCTGCCAATTCAAAAGGGAT
CAATGGATATGAATTGAATTTCTACTCCGGTAAATAAATATTTACAAATTATTATTAT
TATTTTTTTAATTTTTTTT
>126035645_2 EL596592.1
GCACGAGGTGGGCAGTGCCTCTCTCTCCGCAATTTGACTGCTTCACTCACCTTCAAGCGAGGAGAGTCCGTGT
CTACATTTTAGCTGGACAAAACGACAGTCCCACATTCAAAAAACAATCTAGAGAGTTCTACGAGCTGCTGTAC
AACACCTGCTTAATGCAGAACATGTATTTGGAGATAAAGGATGATATGGACCATTTTG
ATATTGTCGAGTGTTTCGCCAACGACGACAACTATCTTAAGAATCTTCTTGTATACGATGTTAGGAAACATTT
GTGAATGTTTTAATAATATATATTATATTTTTTGTGGTTGTTATTACTGATAGTTAATCTTAAACTCGAATGA
TAAATTAGATTTTAATGTTTATTTTACTTTGCTGCCGCATTCATAGATGACATGTCAC
ATCATGCCACGTCACGTCACTGCCAATGGATAAATAGTAATGCGTATATGTAGATTAATATCAGTTAAACACT
AATTTACATCTCTGAGTGCGGCTGCTTGGTGTATCAACTGACCGTAATTCAAATTATTTATTTAAATGCCAAC
GTTATTATAAACTATAGTGAGTAACATCCATATTGCACTCTATTTTCTTGTTGAATGT
CCGATAAAAATTATATTACCTACTCATGTTTGCTATCACAAAACACACATACCACTTAACCCACTTGCCCTG
>126035646_2 EL596593.1
GCACGAGGCACAGTCCAACATGCAAAGGATGCAAATGATTCAACCAGGTCCCTCCAACGCAGGGGTAAATGTC
GCCCCCAATCAGGTCACAAATGTTGGGGGAGTCGGGAATGTTGGGGCAGTAGGGAATGTGGGAACAGTGGGGA
CAGTCGGAAACATGGGAGGAGTGGGAAATGTGGGCGTGGGTTCAGTGTCAGCGCCGTT
ACAGCGGACATACATATGGAGTGGAGCTCTGGAATGGATGGAAAAAGGCAAAGCACCTGGAGATCAACAGAAA
GTTATCAAACATTTGCCTTGTCAAGTTTCAGCAAACTCAAAAGATATAGAGCCTGAACTCAAAGTGGACACAT
GGCCTAGTAAACTACTGATGCAATTGATGCCAAAACAACTGATAAGCAACATCGGCGG
ACAGTACCTAAAAGACAGCAAGTCGGTGCTGTTCCACCTGCAGCAGAACGAGGCGCTCGACGCGCTCACCAAG
GTCATGGTGGGGGGCTTCGCCGGCTGCGTGCACTTCTCACCGATGCCCTCGCCGCCGCAGTGCGACATCAAGG
TGCTAATCCTGCTGTACACGCCTGATAAGAAGGCGTATCTGGGCTTCATTCCCAACAA
CCAGGCTACGTTCGTAGACCGCCTGCGCAAAGTGATCCAGCAGCAGAAGCTCAGCAAGCAACTGCCGCTGGGG
CGCACACACAGCAGCAGCAGCA
>126035651_2 EL596596.1
GCACGAGGTGGTAGACTATTTTTTTTCTTTATACTGGGATGAGCACCGCAAGCCCATTCCTGATTTAGAAGAC
AAACATTATAATTTGATGTTAAGTGCTGTGTCTTTAGCAGAGAAAATCAGAAAAAAGGAACTAAAGTCAGAGG
ATCTAATTACAGCTTGTATTGAAAGAATTAAACAGGTCAATCCAATTTTAAATGCAGT
AACTGATGAAAGATATGAAGCGGCACTTAAAGAGGCTAAAGAAGTAGATGAAATGATAAAAAATGGATTATCT
GAAGAAGAGTTCAAACAGAAACCATTCTTGGGTGTCCCATTCACTGCAAAAGAAAGTCACGCGGTAAAAGGCA
TGTTACACACTCTCGGCGTGAAATCGCGGAGAAACGAACGTGCTGAATACGACGCGGA
GTGTGTGCGCCTCCTGCGCGCCGCCGGAGCCATACCACTAGCTGTCACTAATGTACCTGAGATAAATAAATGG
CAAGAGACCCGCAACATGGTGTTCGGTCAGACCAACAACCCGTACCACGCGGGGCACATGGTAGGCGGCTCCA
GCGGGGGCGAGGCGGCGTTGTGCGCCGCGCTAGCCACGCCTATATCGCTTTGTTCCGA
CATAGGCGGATCGACGCGAATGCCTGCATTCTTTCTGCGTCTATTTGGGT
>126035657_2 EL596598.1
AATATTTTCTTTGTATTGTAGTTATTATATATTTGTATATAGTACAGTTAACATAAGTGAACTAATTAGTTTA
TATAGAAAATACCTACTATTAGTATCTAATTAATAATTATTTAATAATAAACGTTCTCAAATATATTAATATT
TAAGAATTAATAACAATGCACTAAATAGGATAAATTGTACATATATATATATATATAT
ATTTTCTGGCTGTGTGTTACAGCGTTTTGTGAAATTATTTATTTTATATTGAATCGTTTATTATTATTTTTTA
TTAACAATTGTGTTTTAGGAATTTGCTTTATTAAAATTGTTAACTGAAACACATTTATTTGTAT
>126035658_2 EL596599.1
GCACGAGGTTCGATTCTATCAATAATCCCTAAATAAAGTGAGACGTATTTTACCAAATTTTGTTATATTCTAA
ACCTATACAAATATTCAACTAAAATGTTATATAAAATAACATTGGCTGTGTTTATTGTTTCGTGTGCTTCGTT
AGTATCCGCATCATATGAGGACAAAAGATGTAAATGTGTGTGCCCCAGTCCTGCCGCA
GTCCTCAATAACACAGTGGGCTCAGACCGGAAACTCTATATTGGGAATGTACCACCCAACAAATGCAATTGCG
ATGGGGTAATTCTTCCTCGTGTTGGTGAACTGATAAAAGGCCGTGAGCAAGAGTTCTGTCCGCGATGCGAATG
CAAGTATGAAAATCGAAATACTACTATTATAAAGGTAGTAGTGATTATAGTGATATGG
GTGATAATGCTGCTAGTAGCCTACATGGGATTCCTTATATGTCTCGATCCACTTATCAACAAGAGAGCTAAAG
CCTCTTATCAAGAACACACGAACGAAGATGATGAAAGCACCATCAGCGGACCGTCTCAAGCTATGGGTGTTCG
AGGAAATGTTCTCAACCGCGTCACCCACCAACAGGACAAGTGGAAGCGTCAAGTGAGG

GAACAGAGGAGGAACATATACGATAGACACACCATGCTCAACTAGCATGGTGACAATGGTAACCGTAGCTCAT
ATAACCATGTACCCTTTAAACCCTAA
>126035661_2 EL596600.1
CAATATTTTTGTCAGAATGTTATCTATAATAACTATTGAATATATTAATAATATTCCATTAAAAAAACCCACT
TAGCACGTAAGGTATTGTTTAATAATATATTTTTTAAATGATAGATGATGATATGATATAGAATATTATTTTA
TTATTATTTTGAAATATATTTTTTATTATTTCGGTTCCTATACTGTTAAAGAATAGTA
CGCATTCAGCCTTAATTGCATTATACCAAAGTATACATTAGATCTCTACTTTTTTTGGTAGCTCAAAAATAAA
CATTTAAGAACGTAAGACAATCTTTTGGTAGCTAATAGTTATTAAAAAATATATTATTTCTTATTATGAAAAT
AATTCTATTATTAATATTAACACAATTTGTGATATGTTTGTTATTTTCTATTTATTCT
TGTAAAAAGAGATGATTTTTAAGATTTTTTTTTTTTGATAATTTGACATTTCGAAAACGTCAAACATTGTCTA
TGAATATTAAGTTTTTTCGCCTATTATATTATTATTAT
>126035665_2 EL596602.1
GCACGAGGGGCGTATTAGTTTTTAAATTGCGTTATACAGCGCCATTTGTTTGGTAGACGTTGGTGTAGCGTCC
GCCATTTTGAATTATATCACAATTTTTTCATCGACATTTTGTACAATTATGATGTAAATGGATAGTGCTATAT
ATATCGTGTACATTTTTACGTTTTGTTAGAAAGTAACATGATTTCTAGTCAAAATATT
GAGCATATTAAATTGTAGGTAACGCGAATTGACAATAAACAGGCCTGGTGACCTACTTTTCATAAATAAATGC
AGTATTTTATTTACTTTCGGACAGTATGTATATGTGTTTGCGGTTGTGTTTGAGCTACTTTTGTTGACCACTG
GATAGTTTTAAAGTTGGCACGACTGTC
>126035669_2 EL596604.1
GCACGAGGCTTATTTGTATTATACTGTATCAAATTCAAACTTATTGTTTACAGTTTGCTTTACCCATCTTCCC
ACATTTGCCAAACTATAATTAAAATAAAGTCATGTCTAGTGCGCGGACTTACCGTTTGTTACGTGAAATTAAA
GGCTTAGAGACTCAACCTCCTTGGGGTATTACATGCAAGCCAGACACAGAAGCAGGAT
TCGATATATTACACGTTAAACTATGTGGACCAAAAAGCTCGCCATATGAAGGAGGTGTATTTGAACTCGTTAT
AAATGTACCAGAGAGATATCCTTTCGAGCCCCCGGTAGTAAAATTTGTTACTCCTGTCTACCATCCCAACATT
GATAAAGCTGGCCGTATATGTATGAACATGTTAAAAATGCCACCAAAAGGTTCATGGC
TGCCGACTATCACAATTGAAACACTGCTCATATCTGTACAAGCTTTGTTGTCATCACCTAACCCTGACGATCC
TCTCATGATGGACTTGGCTTATGAATATAAGTATGATATAACAAAATTTTTAGAAAATGCCAAAAAACACACA
GAAAAGTATGCTACTCTGTCTTTAANGTGAATATAATATGAAAATTGGTTCTTAATTG
ATCTCCTTAAATTCTAAATTATCTAAAGTTTTATAAATTAATTTTTATTTTTATATATTTTTTAAATAACTAT
TTTCTCTTAATATTACACCTACATCATCAGTTTGATG
>126035681_2 EL596612.1
GACGAGGCTTTTACAACAATTAGATAAGCTTTTTATCACTATATTACCACGTAGGTAAAGGTGCCAGTATGTA
TAAGTTATTAATATTAGCCTCTTTTCTACAATATGCGCTGTCGCAACAGTATCAGCAGAAAGTTGCTCCTGGA
GTGCCACCACAAAGCTATCAGAATTACCAACCTCCTCCCCCTCCACAACAACAACAAA
TGCATCAACAGCCACCTCAACAACAATATCAACAACCGCCGCCCCCACAACAACAATACCAACAGCCGCCTCC
CCAACAACAGTATCAACAGCAAGTTCCTGTTCAACAGGTACCTGTACAACAAGTACCTGTTCAAAGTCAACAT
GGTCACCACGGGGATCCTCAACTACTTAATGCCGCTAACATCGCACAAGAAAGAGAAC
ATATCCAAGAACATATGGACGTGCCGATCGATACATCCAAAATGTCTGAACAAGAATTACAATTCCACTATTT
CAAAATGCACGACGCCGATAATAATAATAAACTCGACGGTTGTGAATTGATCAAGTCGTTGATACATTGGCAC
GAACAAGGTCACAAACAAGAAAATCAGCAAGGAACGCCGCCGGTAGGTGAGAAAATAT
TTAAAGATGACGAATTGATCAACTTAATAGATCCTATATTAAATATGGATGAT
>126035687_2 EL596616.1
GCACGAGGCAATTATGTTATTTCTTTTGACGAGCTTTATTTACCCAAAGTAATTATAAGAATCAATAATATTT
TATAAAATAAACCCAAATATGTTAAATATACAAAACTACGGTAGCAGCTCTGAAGATGATACAGATAATGAAG
GAGAAACGAGTAAACAGCATAGCAACGAAACGTTGTTATCACATTTAAAACCTATAGA
TCCCGATTTATCAATTGCAAAGAAAATGCAAGTTTGTGCAGCTCCAGCAGTTGTACCTACGAATAATGAAGAT
ACCAGGATGTTACTAAATAATACACAGGAATTAATGCATAACCCCAAGTATGAAGAACTATATGCACCCACAT
ATGGTCCCGAAAATCCATTTCAAACTCAGCAAATGAAGGCACAAAGAAATATGTTGTC
CGGGTATGTTGAAAAGGCACATATAAGTGAATTTCAGTTTGAAAATCAAAGACGGACATTTACAAGTTATGGT
TATGCTATGGATCCCTCTACAGAACCTGTAGAAGATGAAAATGGGGATGCATTAGTTGTGTCGGCAATGGTGG
CAGCACCAGTTGAAGCAGAAGGTAAATCTGTATTCGAAACTATAAAGAAAAGGCCATT
AGATAAAAAGAAAAGAAATAAAAATGACAATCCGGATGATATCCCAGGATTTTTAGGTCCTTGGGGAGGGTAT
GAAGGTGAAA
>126035688_2 EL596617.1

GCACGAGGATTTTAAATAGTTAATAATTTACATTTACGTAAACATTGCTCATTAAATATTGTAAGTTTGTATA
TGAATAAATTTAGACATTATGAATATTAAATCTTCCAAATATTTCTAAATTAAATGCATATTAGTTAGTATAT
AATGAAAGCAACAGATGGTTTATCAGAACAATGGAACAGAAAAAGTAAATTTTCTAAT
ATGCAAACTCACAAAGATTGTCTAATATGAAAAACATCAAAAGGTGCGTGAAAATAAAAATCTTATTAAAAAA
AATGATACGCAAACAAGAGTTATTTTTTATATGTATTATCGACTTTTTTAAGTGCTAAAAATATTGCAACGCT
TGATTGCAACACCGCTTTGTCTAGTATAGGTGGTTTGTGGTAGACGTTGGGTAAAATA
TTTTGTCAATTGTAGTACATTTTATACATTCATATCCGTTACTTAATATTATATAGAAATTGAAACAATATGA
ACGCCCCCAAGTAAAGTCGATATTGATTTTTATGTATATGTGTGGTGTTATACCTTGTACAAAGTGTTTATAT
GTTATTACTGAACCTACAACTATATAGACTAATTATTGGAGGAAATAAATGTAGCAAG
TGTTATTAGCGAACAATATATTTTTTTTCAATTGAGTAGAATCTTTCATATTTATATAAAAAACGTCGACATC
TTCTC
>126036491_2 EL596623.1
GCACGAGGGTTCAACACAGGGTGCTCGTGACGAAGAGGCGGGCGTGGACCCTCAAAGGGCAGGGGAATGTGCC
CAGCAGCTGTACGACGCGGGTGAAGCCAAGTGGGGGACGGACGAGGAGGTTTTCAATAAGATACTAGCCCACG
AGAGCTTCGAGCAGCTCCGCTTGGTGTTCGAAGCGTACAAGCAGCTGTCGGGGAGGAC
TATCGAGCAGGCCATCAAGGCGGAGATTGACGGAGAGCTCAAGGACGCGCTCTCTGCCATAGTGGAATGCGTG
GAGAACGCGCCGGCATGGTTCGCCCAGCGCCTTCGCGATGCTATGCAGGGCGCCGGGACTGCTGATAAGGTGC
TGATAAGAGTGATTACGACCCGCTCGGAGATAGACCTGGGTAATATAAAGAGGGAGTA
TGAACGATTGTTCGATAAGACCCTGGAAAGTGATATTTCGGGCGAGACTTCAGGCGACTACAAACGGGCCCTC
GTCGCCCTCCTGGGACCTGCTTAGAAGACATAGTTTTAGAAAACGCCTATTTGTTTCTCATATATTTTTGATC
ATTAATGGAGTATACTCGAATTTGTATAAGAAAAATGAATGTTTTAATTTTTTTTATA
GATATTTAATTATATTTTTTTTAGACTACTTGTAGCCTTGACTGTGCCTTTGATAAGTTATTTTGCGAACTGG
TTGTTGACACTGATTAAATTTTAA
>126036840_2 EL596634.1
CGGCACGAGGTGTTTAAAAACATTAAAACATTTACTTTAACCATATAAAATCTTATTTCCACATGATCCATAT
AATAAATTAGAAATAAAAGTGCTTAAAGTTGTTGTTCTGAAATACACTCAAATATGTTTATGTGATATAAAGA
TTTCTTAATGAAATGCTTATTTTATTTAAAATGGATCAATCGATTGAGAGACGATTAG
CGATAAGTTGGTTCATAAGGTCGGGGCAAAAATATTTTCGTAGTGTTCATACAAAGAAAAATATTTTTTTAGG
TATAATTTTTTATTTATTTATAATATATATGCGCCGCTTTGATCGATGACTTGTTGCCATCTTATGGGTAGTG
CCATGATTCTATTCCTGTAGAAATTTTGAGTTTTGATTAAAAACAAAAAAATCTGACA
GTGTTCACAGTGTTCTTTTGAAGTTAGCTTTATGCTTCCTAAAGAGTTCTGCATTGACCGAAACAGGTGGAAA
TCTGATGGAGCAAGGTCAGGGCTATACGGAGGATGCATCAACATATCCCAGCCAAGCTCTCTTAATTTTTGCT
GCGTGGCTATAGATGAGTGCGGCCTGGCGTTGTTATGGTGGAACACCACGCCCTTCCT
ATTCACCAGCTCCTCATCAGTTGCTGACAATAGAGGTCAGAATCGATAGTTTTACCCGGTGGCAAAAGCTCAT
AGTGGAGGATTCCTTTTCCATCGCACCATACACATAGCATCACTTTGTTCCGAGTCAGGCCGGGGC
>126036841_2 EL596635.1
GCACGAGGGTCGGCTGTATTTTTAATTATTTTTTCAAAACAACTTCTCTAAAAATATACATTTTTTGTTTATT
AGATAAATCCAAGTATACAATTAGTGTTGAATCATGGAACATCTAGCGTCTGAAAAAGGAGACACCGCAGAAA
ATTTAGACCCTAATTCAATAGCCAATGGCAGCTCAAAAGTAGTTGCGGAAAACAAAGC
ATGTGTTGATAATGGCAAACCGCATATCGAAGCCTCCACTGAAACCGCTGCGTGTAGTTCAGAATCAACAGAA
GATTCAATATGTGAGATCCCGGAACAAATCGAATTTACCGTAGTTTTTAATAAAAGTAGACATGACATCACAT
TTGCTTATGATGCTACTGTATTAGAACTCAAAGCTCACCTAGAAAGGATATGCGGCGT
ACCACAATCAGCTCAAAAATTAATAATTAAAGGCATGGCCCGGGATAATGTTACATTAAGAAATGCTGGAATA
GTAAAAGGTGGCAAAGTAATGCTTGTTGGATCTAAAATGGATGATATATTGGCTGTAAAAAGTGCACCTAAGG
AAATTTTGGAAGAAAAAAATAGTAGTCAGTCAAGTAAGGAACCACTGTGCGTACAAAA
AATCCACAGAAAAGTTCTTGATAAAGGAATCCCTCCAGATGTTATG
>126036845_2 EL596639.1
GCACGAGGCGGACCAACAAGCTTTGCACACGCCAACACTTAATATTTCTACTGAAGGCTTCGTGCCCACGGAG
CCGCAAACGTGGCGAGTTGACTTGCCATGTACGCGCACGGTGGCCGCCGAAGTCGACGTTACCATATCTCTTA
ATGTGTCTACTGGTTCATCATCTACTGCTTTACACTTCCGTCGGCGCAAAATATGTTT
GAAAGAGGCACCTCGACCGGCGGTGAGGGTGGACAGCGCGCCGCATGCACCAACATCTGCAGATATATTTTAC
GCGGGTGCAGGTTGCGCCGGCGCAGCACTCCTAATAGCTGCAATCGCTGCAATTGTATGCCGAGCACGAGCCA
GGAAATTACGAAGAGCTCGAAGTGATGAACAAGACGCTCATGCTTTCTTGCCAGATTC

GTCATGTAAATCGGCCGCTAGTTACACTAGTTACAGGCGCCCCACGTCCCTACCAGCCGCAGCTGCTGAGGAG
AGAGCTAGAGATTTGCAGCAAAGAATTGCGGAATTGACGATACAAAGATGTAGAGTGAGATTGCGTTCAGTTG
CCATGGAGGGCACTTTTGGTAGAGTATATC
>126036846_2 EL596640.1
GCACGAGGGCCGCCTGCAGGCCGCGCTCGCGCCCGCCTCGCCCGCGCACCGCGCCATCGCCAAGCGTATGGAG
CAAACTGAGAATGAAGTAAAAACTTTACAAGAATCTTGCCGCGCTTTAGTCGAGCAGAGCATTCCCGACTTGA
AAATTGAGGAAGTATCACGCGACCACGCGATCGCCATATCAAACAACAAAACAGGCTC
CGGCGATCCCGACTACACTCCCCCGAGTGGTTGGGTCTGGCGCGTGCTTCGATCTTCAATTCCTATACAATTA
TGTCTCGTAGCAATCCTCCTAGCAGCTTGGTTGGTGGAACGACCGAGGTGCTGTGATGCGCTCAACTCTTTGG
CGCAGACATTAACGCCGCAGTTACGTTACGTGCGCGGACCGCCGCCGGTGTGAATGCC
CACATTGAAGTGGCTGTCAAATGTCAATGTCTTGGCGGAAGTGACAAACGTCAAAGTGACAAGTGTCAAAGTG
ACATCTGTCAAATCTAATTTGATACAGTTTTTACGTTAGATACGTAGTATTTCACATGTGCAGACTTTGATAA
TTGCATAATTAAACATCTTATTTGTACTTTTTCAATTAAATGTGGCAATAAATCAAAA
ATAATTACGATAGCACATTTTTTAGCTATACTCAAGCTATTATCGAGATGATTACTGAAAGTAACAGTATATC
GATGGAAAAATTGGCGTTTACTAACAGTA
>126036848_2 EL596642.1
GCACGAGGATCAAGATGTTATTACCTTCATTTTTAGTTGCATTCGGTGTTATAGCAGTTGCGTCGTTAAGAGT
ACCTGTCGAGAACACAGAAGATATTCATGAAAATGTCGAAGAAGAAGAAATACCAAATAATAGAGAACAAATG
CACATGCAACCTGTCTACTATGCTACTGAAGAAGCAAATGTTGATAGTTACTTAGTTC
CACCAAATCCTCATAAACCCCAGGAGGTTGCCCATGATATGCTCGTGACGCCTGCAACTTTTTTGCTACCACC
AGCTTTAGATACGCAAAATGAATATTACTATAAGCCTACAGAGCCCGGAGATCAGAGTGATTGGTATCCTATA
GCGGCGTCGCCTCAGAAACAAATAGAAGTCATACCAATCATGTCAAATGATCGTTTAG
AACAACATATAATTAAAGAAAATTTAAGGACGGGTAAAGCACTAGAAAGTCAAGTAATTCCGGTACCCTCAAG
AAGTTTACTACCACCACGTGAAGACGCACCCAACGATTTTATAATTTTATCCCCGTCTCCAGATTTAGAGTTA
CCGTTGGAGGAAATTGATCATACTCTGAAAAATGATGCAAATGCAGAAAAAGCTTTCA
TCGTCCCTGTAGCACAGCTTGAGAAAAAATATCCATTGCATTTGCAAACAAAAACGAGTGTACCACTTCCATT
TATCATTCCGCC
>126036851_2 EL596645.1
GCACGAGGATTGAGGTGGTTATTTACTCGATTTCGAGTAAAAAAGTAATATTAAAACAAAATAATGTCCACAA
GCATGTTCAAATTATCGTCTGCGCTAATAAATTCTTCTAAGAATTACAGATGCGTAGTGGGACTCATTAATAA
AAACAATTTTTCAACAACAAATGTTACGAAGAGAAAAGAACGTGACACATTCGGCGAG
CTCGACGTGCCCGATGACAAGTTGTACGGCGCACAGACCGTACGGTCTGTCATGAACTTCCCCATCGGAGGGA
TTGAAGAGAGGATGCCGTACCCAGTTATTGTTGCTTTCGGTATATTGAAGAAGGCGGCCGCTAAAGTCAACAT
TGAATTTGGTCTGGATAAAAAACTAGCCGATGCGATAATGCAAGCCTGTGACGACGTG
ATCTCCGGCAAGCTGTACAGGGAGGGGCACTTCCCTCTGGTCATCTGGCAGACGGGCTCCGGCACACAGTCCA
ATATGAATACCAATGAGGTAATTTCAAATCGTGCCATCCAAATACTGGGTGGCAAACTAGGCTCCAAGGACCC
CGTGCACCCCAACGACCACGTCAACAAGTCTCAGAGTTCCAACGACACGTACCCCACA
GCGATGCACATCGCCGTGGCGATGGAACTGAAAGACCGCCTGATGCCCGGT
>126036859_2 EL596653.1
GCACGAGGGAAATAAGAAATTGGTTGTAGGTTTGTGAATGTGTTAGGTACGAGGTGCATGTCAAATGTAGCGA
CACGTATTTCATACAATTATTAATAAAATGTGGATAGAAATTATGTAAACGAAGCATGGATTATACATAGATA
GTGTGAAAAACCTAGACATAAAAATGTGTTTTGAATTAAGACAGATTATACCTATAAA
TGTTTTGTAAAGACTGACTGATGTTATATT
>126036860_2 EL596654.1
GCACGAGGCCAAGATGCTTATAGAGTACCTCTTACTGACTGCAGAGACATTGAAACTGGTCGTATCGGCAAGT
GCTGTCGTGATCCATTCTACACTGACCCCTGGCCAACGAATCAGCTTGGGAAGTGGGTTCCCGGGGTTTTCGG
TGGAAACGATGGAAAATACGTACCTGATAGCCGCGTCAGTCCCAATAACATCCAACCA
ACCATTACAGCAAGACCTCCAGTTACAGGATCTCTACTATTGAATAATTTCATGACGCCCAAACCACCTGTTT
CTCCATTCAATCCTAACCTAGTTTCCGGATTCCCTTCATCCACTCCCTTCCCTATCAAGGGTCAGTTTGGACA
AGGAGGAGTCGGTATTTATAGCCAAGGAGGCCAAGGACAATATGGTATTGCTGGACAA
GGATCACAAGTCAACACCGCTATCCAAAAGGGACAAGGACAGATTAGGAAGCAAGGTCAAGGTCAATTTGTGA
GTCAAGGCCAAGGAAGCGCTATCAGCCAAGGTTTTGGTACTGGGATCCGTCAGGGACAAGGTGTTGGTGTTGT
CCAGGGACAAGGAGTACGCCAACAAGGTGGATTCGTATCACAGGGACAGGGTGCCGTT
GTATCCCAGGGCGTCGGGCAGTATGGTGTTGGAGGACAAGGAGCCATAGGAGTGCAAGGCCAGGGTGTGCAAT

>126036866_2 EL596660.1
GCACGAGGTAATTGTGAACCTCTGTAGAAGTAAGGATCCACCACCACCCGTCAAGACTATTAAGGAAATTTTA
CCAGCGCTTAATGTGCTCATTACACACACAGACACTAATATATTAGTGGACACAGTTTGGGCGATAAGCTACT
TGACAGATGGTGGCAACGAACAAATACAAATGGTCATAGACTCTGGTATTGTGCCTAA
ATTGATACCACTACTGTCACACAAAGAGGTGAAAACGCAAACGGCGGCGTTGCGCGCCGTGGGCAACATAGTG
ACGGGCACGGACGAGCAGACGCAGGTGGTGCTCAACTGCGACGCGCTCTCACACTTCCCCTCTCTGCTGTCGC
ACCCCAAAGAAAAAATCTGCAAAGAGGCCGTGTGGTTCTTATCAAACATAACAGCAGG
CAACAAGCAACAAGTCCAAGCGGTCATCGACGCTGGACTTTTACCCAAAATAGTGGAGAACCTCAGCAAAGGG
GAGTTCCAGACGCAGAAGGAGGCGGCCTGGGCGGTCTCCAACCTGAGCATCAGCGGCACTCGCGAACAGGGTG
GCGACGTTGATACAGTGCGGTGTTATACCGCCGTTCTGTAATCTGCTCGCTTGGCAGG
ACACGCAGGTTATTAATGTGGTGCTCGATGGGCTGAGCAACATGCTGAAGATGGCGGGCG
>126036870_2 EL596664.1
GCACGAGGCTAGAAATGCACATGGAACAACTGTTCCTGGACACGTTGGACGCGTACGTGTGGATATACGACCC
CATGCCGTGGTACTACTGGCTGTGCGGCGCGCTCGTCGTGTTCGGCACCATCGGCGTGTGCATGTTCCCGCTG
TGGCCGGCTACCGTGCGCAAAGGTGTATACTACCTTAGTGTGGCGGCCGCAGCCTTCC
TAGTACTGATTATAGCATTGGCCGTACTAAGAGTGGTCGTATTCTGCCTCGTATGGATATTGACATTGTCCAG
ACATCATTTGTGGTTGTTACCCAATTTGACAGAGGATGTGGGCTTCTTTGCTTCGTTCTGGCCCCTTTATAAA
TACGAATACCGCGGCCCGGGCTCAGAGAGCGATAAGTCGTCTAAAAGCAAGAAAAAAC
GCAAGAAAGACAAACATTCTGATGACGAAGAAGAGAAACAATTATTACAGGAACCAGTGAAGGAAACTGGTGC
TAAAGTGGTAGAGAATGAGGATAAAGAGGCAAAGGAAGACACACAAGAGCCTCCAGAAGCATCAGAATCACCA
GAAGCACCCGACCCGACCGATAAGCATTCAGGATCAGAATCTGATTCTCAACGCTCAT
CCACTGACAGGGACTTTGAAATAGTTGAACCGGGAGACGTAGAACCCGATGTAAAGATAATACTGCTTAGATT
ATAGTGTTTAGTTTCATAATATCTAACTAACTATTCTGGACAGGTGGATTATGGGTGGTTATAGATGATT
>126036882_2 EL596676.1
GCACGAGGCGCTAGTCGACACGCAGTCTCGAAAATTAGCCGGCACATGGGGAACGTATTGACTAAACACTAGG
TTCCCTTTGCCCACCGTGCTATATAAAAGCTTGAATCGAAAAAAAAAATATATTGACATGAGTTTAAAGATGC
ACAGTTCGGTAGTTGCGAAACGCGGCCCCGTATTACTTTAATGTGATATCACCTGCAC
AAACATTGTTTTTATACAAGAATTTTAATACATACATTTTTGAATGTGATTTTCGACTATTTTATCATCAATT
TACACGATTTTAAAAGGTGTTTTTTATTAGATAATTTAACAAGTCACAAGCGCTCATTACGAATACCTAATTA
GAACTAGAATAACGTTATAACTGATTTAGTATTATGAACAGTGACACCAATATGAATA
AGATATGCCACTAGTTCCGTCCCGATACGGACTTTTTTTTGTTTAATTTAAATACATTTTTTATTGTGTGTGA
TTTAGACAACGGCTATAATAACAAATAATAATGCTCAAATCAAAGCAATAGCTATAAACTGGCAAACGAATCT
TGTCGATATGTTATTATATGTATTGATATTTAAATGTTTGTGTAGAGTCTATTTACGT
CTTTCGACGGTACAGCTCAGATAGTGATTTGCCAAAAATTATAATAAATGTGATCTTTTATAGTTGGAGTGAT
ATATATATTTGAACGACAAACTATTGG
>126036884_2 EL596678.1
GCACGAGGATAAAATACACCGTTTTTTCTCTATAAAATTTGTTCAAATGTCAGTATTTTATTATAATTTTACG
TTGAGTTCCAGTACTCGACTGGCAATGCCTTCAAAAATCGGTCCTAGGTTCCCTACATCATGAAGCTAATATA
CATAAGCTAAGTGAAGCTAATATATGCTTTATATCAAATATCCGACATTTCTAGGCCT
ATAAATTATTTATTTGGAACCGCAAAAAAAATCATAAATAATTAAATAAAATGATATATTCTGTATATGTATA
TTAATAATGTAAAACAATCTTTGATACAACGTCGGATAGCCCATACAAATAATTAAACACTCAGTTTCAGTGA
TTTAGGTCAAGCACCGCCTTGAAGCCTAGACTACAGGGAATTGTACTGTATGTAGCCT
ATAGGTTTACCTATTGTTGAATTCTGGTTATAATTAATAATGTTATTTATGTTTTGAAAGTCATGGATGGTGT
ATTTGGTTTATTTAAATTAGAATTAATAGTATCTTGTTATTTGTTGTTCGTCTGCTGTCGTTTTGAGATTATA
GTTTTATGATTTTTTAATAACTTTGTTCGAATCGAATGTATATTTGATATAAATAATA
AGTATTAATATAATTAAAAATAACTAACAATTTTCAAAGGTATTGATTATTCAAAATATTATTATATTATTTC
AAAACACTAAAATGAAAATCTTTTCCGAACTATTTTCAATTGCCATCAATCGTACATATT
>126036893_2 EL596687.1
CGGCACGAGGTAGAAATACAACAGCAAAGCACGCAGCCCTAACTCCCAACGGCCGCCCCCTTGTCGTCGTGCT
AACAAAATGGCGACGCTTCAATGCCTTCTCTATGTGACAGTGTAATCCGCTTTGCTATACAATATATCGTACG
ATATATTTGACGCATGGGAGCTTAATGCGGTTGGAGACGTGTATGATGTGTTGTACGA
CATATCGTACGATATGTCATAAGATTCGTTTTATGATATTATGTCGCATGATGTGTCGTACGATATGTCGGAC
GATATGTTGTACAATTTTCTTATGATAGATTGTTTCGTATGCATATATCGTAAGATGTCTCAGGATACGATAT
TTTCTACGATTCATTGATTCATTTATCACATGACATGCCTTAAGATAAATCGTACGAT

ATATCGTCGGATAGGCTTTCGATGTTTCCGAGACATGTACATGAAACAATATATAGTTGTAAGTTACATTCAC
TGCGAATATTAATTAAGTTAATAATTATTATCGATTAATTAAATATTAATCGATTTTTTTATATATCGATGT
TGTGTATAAACGCACTCTCGATTCTTTTTTAAGTATGCAATTTAAATTGTATGTAAAA
AATGAATTGTATGTAAAGCATTTT
>126036900_2 EL596694.1
GCACGAGGTGACGGTTGTGTAACAGTGCTTATTTAAAGTATATTCTATCAAAAGCCGAGTGTTTTTTTGAGTT
AACGATGTCGGATGAAATTGCAGAGAACGGAACAGCAAACGGAGATGTCCCGGAGATCGAGCTGATTATTAAG
GCCTCGACCATCGATGGACGGCGGAAGGGTGCGTGTCTGTTTTGTCAGGAGTACTTCA
TGGACCTTTACCTCTTGGCGGAACTTAAAACTATCAGCCTAAAAGTTACAACGGTGGACATGCAGAAGCCACC
GCCTGACTTCCGCACCAACTTCGAGGCGACGCATCCACCTATACTCATCGACAACGGTCTCGCCATACTCGAG
AACGAGAAGATTGAACGGCACATCATGAAGTCGGTGCCCGGTGGACACAATTTATTTG
TGCAGGACAAAGAAGTGGCTTCCCTCATAGAGAACCTATACTCCAAGCTGAAGTTGGTTCTCGTGCGCAAAGA
TGAGCAGAAATCGGCGGCGTTGCGCTCCCATTTACAGCGTATTGACGCGTTGCTTGAACGCCGAGGCACCAGG
TTCCTAACGGGAGACACAATGTGCTGTTTCGACTGCGAGCTGATGCCCCGTCTGCAGC
ACATCCGCGTCGCCGGGAAGTACTTTGTTGACTTTGAAATACCGGCGAGTTTCCGCGCGCTGTGGCGCTACAT
GTACCACATGTACCAGCTGGACGCCTTCGCGCAGAGCTGCCCCGCCGACC
>126036902_2 EL596696.1
GCACGAGGCACTCGCTCTGCGCGCGCATATTTAAATCTAAATTATGTCCTCTCTAGCAGTTTTTGATTTTGAT
CGCACGATAGTAAATGATGATTCAGATGCGACTATCATTAATAGATTACGTGAGAAGAAACCACCACCTGAAT
GGGAGGCTGGGAATCACGACTGGACCCCTTATATGAGCAATGTATTTGAACATGCGTA
CTCAGCTGGTTTCCATCCATCGGACATTTTGGACAGCATCGCCTCGATGCGCCCGAACCCTGGCATCATCGAG
CTTATCACCACATTAGCGCAGGAGGGCTGGGAGGTCCTGATCCTGACTGATGCTAACAGCGTGTTCGTTAACC
ATTGGTTTAAAGTGCATGGATTGCAGGAATCTATAACAGCAGTAATAACCAATCGCGC
GTTTTGGAAGAACGACCGCCTCTACATAGAGCCGTGTATGAATCAAACTTCGTGTCCAAGGTGCCCATCAAAC
CTGTGCAAGTCGCTCGCCCTGGCTCAGTGGAGCGACGGTCGCAGGTTCGACAGAGTGGTGTACAGTGGGGACG
GCAGGAACGACTATTGTCCTGCCACAAATTTGCCACCTAGTTCTACAGTATTCCCA
>126036903_2 EL596697.1
GCACGAGGCCTAACTCAAGCAAAAATACAGAAACGTATCAAACTCAAACGAAACAAAGTATTTCATATCAATC
AAATAACCCTCAGGTCAAAATTACCGAAGTCTTTCCATCAAACGTTCAAAATCCAAGCAATGGCCAAATTTAT
CAATCAAACGTTCAAACCCAGTTTAAGACAAATGAAAACCAAAATCTGGACTATCAAT
CGTTTGATTTAGACGGGTTGGTTCTTCCCGCTGGTTATGAACCTATTGATAGAACGAAATCCGATCAAATAGT
TCAAAGTTCGAATCAAGATATTAAGCATATATTGAAAAAAGAAATAAATCATCAACACCATTTGCATAATTTG
CATGGAGATTGTGACTTAAGAGCTGATAACTCTGTCACACACACAATAGTGGTACCAC
CACCAAATGTAGACAAAGTTGAACCTGTAGATGAAGTCGAAGATAATGACGTAGCCATATACTTTGGCCAAAA
CGAAGAAAAAACTGAGACTGTCACAGAAATTTCAGTAGCAACTAGTATAAGTGAGGACGTTGATCGTAAAACA
CCAGAATTCGGTGAGAAAATTAAGAAAACGTGACCGTTCTAGTCTGAGAACCAGTAGG
CTGAACTGATTTTAATTATATTCGGTGTTTTAATACAAATTAAATTAACAAATCGGTTTAAACAAAGCCAC
>126036918_2 EL596712.1
GCACGAGGAAGGTATTTTAGGACCAATTGGAATTCCTGGAAAACCTGGACTTGTTGGTGACGTTGGCGATTAC
GGTGAATCTGGTAAAATGGGTACGCCAGGTTTGTCTGGTGATTCTGGAATACCTGGTTTACAAGGCATTAGAG
GTGATAAAGGTTCCAAGGGAAAACAAGGTGATATTGGCCCTAGTTCTTATTTACCTGC
AACTAAAGGTGACAGTGGTGATATAGGTATGGAGGGCTTACCCGGCCTACCAGGTGACTACGGTGATATTGGA
ATTAATGGATTGAAAGGTGCCAAAGGAGAATTGGGTGATATTGGTTTCCAAGGAGAATATGGAGACGATGGGC
TTGAAGGACTAAAAGGTACGCGTGGGAACGTAGGTCCTCCTGGTCCACCAGGTCTTGA
TGGAATGTTTGCAAAACAAGGTGAACCGGGAAATCCTGGCATTGACGGTCTCTCAGGTTGGCCGGGGCCCATG
GGTCAAAAAGGTGCACCTGGTCATTATGGAAACGATGGTCCTGAAGGAGCTCCAGGACAACCAGGTCTAACAT
TCCAAGGTGCAAAAGGTTTTAAAGGAGACGTTGGCTACCCGGGTCGGCGCGGTAATAT
GGGAATTCCAGGTGCTCGTGGAATGGAAGGTGCTCCTGGTTTTCCGGGACCTAGAGGTGAAACTGGTGAACCT
GGTATGGCG
>126036921_2 EL596715.1
GCACGAGGGTCATCATTACCAGTTCAAAGGTCAGAATCCAGTAGTTCTAATGATAGTCCTAAAATACATGGTA
GTAGAGTTCACTGTATGCAAGCATCAGCGCCCAGTTCCGCATCTAGTTCTCCTGGTGGAGTACCACAAGATAA
TACAGTATCACGAGCAGCACCACCGTCACCAAGCCAACTCTGCGCTGTTTGCGGGGAT

ACTGCCGCTTGTCAGCATTATGGTGTCCGCACATGTGAAGGCTGCAAAGGTTTCTTTAAAAGAACCGTTCAAA
AGGGTTCAAAATATGTATGTCTAGCCGAAAAATCTTGCCCTGTTGATAAAAGAAGAAGAAATCGATGCCAATT
TTGCCGTTTTCAAAAGTGTCTCGCAGTCGGCATGGTAAAAGAAGTAGTACGTACTGAT
TCATTGAAAGGAAGGCGAGGTAGATTACCTTCCAAGCCAAAATGTCCACAAGAATCTCCTCCAAGTCCACCTA
TTTCTTTAATAACAGCACTAGTCAGAGCCCATGTCGATACGTCGCCTGATTTTGCTAACCTAGATTATTCACA
ATATAGGGAGCCTAATCCTATGGAACCACCTATATCTGACCTGGAAGTCATTCAGCAA
TTTTATTCATTATTAACAACATCTATAGATATGATAAAGATTTTTGCTGAAAAAGTACCAGGATATGGTGAGT
TATGTCCAGAGGA
>126036930_2 EL596724.1
GCACGAGGATTTTATAAAAAAGTGTATTTTGCAATGAAATGGAGGGATCGAACCGTAAAGAAATACTTAGTTG
TGCCTGTTGTCTAAACGATATTGGGGATGAGGAGTACGTATCGGCACTCGGCCAGGAATGGCACAAAGATTGC
TTTAGGTGCTCAGAATGTGACGTTCAGCTGACCACGTGGTACTTCGAGAAGGGCGGCA
TGCTGTTCTGTCAGGCGGACTACTGGAGGCGGTACGGAGATAACTGCCAGCAATGTGGACAGGTAATAACGGG
CCCAGTAATGGCGGCCGGGGAGCACCATTTCCACCCGGAATGTTTTGCGTGCGCGTCTTGTGGCGCTCATCTA
GAGGACACGGAGCCCTACGCGTTGCTCGACCGATCTGAATTATACTGTGGGTCGTGCT
ACGCGGAGTGGGGGCGCCGCGACGCGCACTGCATCCGCGTGGTGCGCACGGCGCCGCGCGCGCTGCGCCTCGC
GCCCGCCGCCTGCGGGCACTTCATCGTGGCGCAGATCGACTCAAGCTGCAATGTCTTAACGCTGCAAATAGGG
GACAAAGTGTTGGAAGTGAACGGGACCCCGGTGAGGAACAGGCCGCT
>126036942_2 EL596736.1
GCACGAGGCAGTATTGTTTCTATGGACGATGTCTTGGATTCCAGTTCTTACCTTCAATGCGTGGTATTCTCAA
AGGGATATCTATTTGTATGGCGGGATTCTCGGAAGCGTACTACAGTCATGGGAACCTCATAAGCTCTGTTTGG
ACGGGAGGCCAGTATTTAATAGACCCCGAAATGCGTGCAAGGAGAATTGTTAATATAT
CACAGTCGGCGAGCGTAGAATTTTGTAAGGCGTTCTGGTTTTTAGCTGAAAGTGAAATAATGAAACGTGTGCC
AAGTCTTATGTCGTCAAATGTAGCTGTAAATAAAATAATAACTTTGAACCCTGAACCCCTAGTGGTTACTACT
AAAGAGGGTAAGGAGCTGACGGTGCTACCGCCGTCCGTACATATAGGATTGCAAGAGC
TGAATCTTAGGTTAATTAGCGTTAATAAGAGGGCTGGGATGTCAGGAGAAGATTCATCCTTACCGCCAGCAGA
AGGTATAGTGTTCCACTGCCACGGTGGTGGTTTTGTAGCACAAAGTTCTAAATCCCACGAAACTTATCTACGA
GAATGGGCAGCCAAGCTGAACATGCCGATATTGTCGGTGGACTACAGTTTGGCCCCCC
CAGCGCCGTTTCCTAGAGCATTAGAAGAAGTTTTCTATGCATATTGTTGGATGATATCGAATTTCAAAGAAAT
TGGTACTACGGGTAAACGAATAG
>126036947_2 EL596741.1
GCACGAGGTAGCGAGCGGTCGTGTAACGTGCCTACGTATTGCATCGCCCGCTCTAATAACGTAGCTACTTAGC
TCAGCTGCGTCTGTCAAGTCGTCGCGCAAAGAGTTCACTGGCAGTCTCTGGCCGCACGACGGGGTCATTGCCG
TACGACTGAGGCTTGCGACATGAAGCATACACCATGGAGGAGTCTCATCTGTTCAATT
CAACTTTACTTCATTTTACTACATGGGTCTCAAGCCATTAGCAGCCTTGATTTATCACCAGAAGTAATCCAAC
TGAAAGATAGTGTAAGCCAAAATTATCAACTATCAGAGAGGGAGCCCCAAATTGATCGCAATATTAGCATAAT
AGAAAATGATTTTGTTTTGGATGGCAAAACTATTCGGATAATGTCTGGGTCCCTGCAC
TACTTCCGCTTACCGTCAGAATATTGGCGAGATAGATTAAGAAAGCTGAGAGCAGCTGGTCTCAATACAGTAT
CTACATATGTAGAGTGGAGTAGTCACGAACCAGAAGAAGGTTTGAATTTATTTGATGGTGACAATGACATCAA
ACGCTTTATTCAAATCGCTGCAGAAGAAAATCTCCATGTACTGTTAAGACCGGGACCG
TATATATGCGCTG
>126036954_2 EL596748.1
GCACGAGGATTTGGGCACACGCGATGATGTTAAAATGGAAGATAGCATCGGATTACGCCAACTATCTAGTTAA
TCACAGTAAATGGTCAAAAACCATTTATGTATATACGGAAGCTGCGTGTTTGATGGAACTCGGACATTTAAGC
AGTCTCGAGGAACGCAAGCGCAGCGCTGATCTTGTAATGAACGCGTCATGTTATAGGC
AAAAAATACTTGGAAGATCTCTGCCGATGGAAAAGTTTGTTGTCCGACGATGTGAACGTTGGGCCAAGCGAGG
GTACCTAGTGCTCCCTGGGGTCGAGCTAGTCTGTCTATGGAACATATTTCCTTCACTGGCCATGGAGCCACAT
TATGCTGATCGCATGCTAAAGCTTATAGAACAAACCCACGAGGAAGTAGTTAACGTGA
TGAATTCAAAGTATGAAATAGATTCGGAGAGACGACACACTCCACCTTATGATGCTGACGACTTGGCGCTAGT
GTGCTTCCTCCGTGGAAGCTTATTGGCGGCTATGTGCTTACCGCGCTTAGGCATTAGGCACTTGGAAGCTGCT
ATGAAAATGAAAGATGACTTAAAAGATAATACACACCTTCTCCCGTACGCTACAGTAG
AGATCGCCATGTGCCATTACGCATTAAGGGATCGGTATCGAGCTGCTGAGTTGCTTCACGACGCGAGAAAAAA
GTACTCCGG
>126036959_2 EL596753.1

GCACGAGGGATGTTTTGGTTTTTTTATACGAAATAACTCTTTATAACATTCAATGTGTTAAATTACAAAGTAA
ATTATTATAAGATTAAATCATTAAAATAAATAAATTATGATTTTACAAGGTTAATGAGTGCAAAAATGACTTC
TGAAATTAAAAGTGGTAGAATATAACATCTGATGATAGAACGAGAAAATAATTGCTTA
AAAATGCCGTGCGAGAGTTGCGCGGTTCAGTTTAGTGTGTTTAAAAGGAAACGGGCTTGTTCTGAATGCGAGA
GGTACTATTGCAGCGGGTGCCTTCGTCGAGGAGGAGGCACTATGTGTGTTCCTTGCAGGGGCCTCTCGACTAG
ACCTTTGGGCAGACAAACAATAAGCCACTTGAAAGTCAGTGACCT
>126036960_2 EL596754.1
GCACGAGGATTATATATATATATATAATATTTCTTTAATTAAGCTTGTTAAAAAATCTATATTATTTACAGTAAT
ACAAAAGTGCAAAATGTTTGTCGCAGTTTTAATTTGCTTGTTTTGTTGTTTATATTATCTACATTTTCGTCGT
AAGAGAAAACAATTATATGAATTTAGTAAAAGTATACCAAGTGTGGGAAATCTACCAG
TTGTTGGACATAGTCACTGGTTTATTGGAGGACCTGAGAAAATATTAAAAAACATTCAGACACTTGCACAGAT
GATTGAAGATTCCGGTGGTATAAGTAGCATATGGCTTGGCCCTACCTTGTATGTTGTTACAATGAATCCAGAA
GATGTGCAAAAAATTTTAGAGAATAGTCTTCAAAAAGATTTTTCGTACAAATTTCTTC
AAGAATGGCTCGGAAATGGTCTTTTTGTAGCACCAGTTGACTTATGGAAAGTACATCGTCGACTTCTCCTACC
CGTATTCCATAATCGCATCATAGAAGACTATATTGACGTGTTTGGAGAGCAAGGTTCCGTATTGGTAAAACGT
ATGGGTGAAAAAGTTGGCAAATCTGAGTTCGATGTGTTCCCATATATCACTTCGTGCA
TGTTGGATATTGTATTTGAAACGGCAATGGGTGAAAAAATGGATGTCCAGCATAACCCTGACACACCGTACTT
GCGCGCGCGCAACACTGTCATATCAATCAT
>126036962_2 EL596756.1
GCACGAGGGTACGTAGTACGTTATATGTAAATATGGTACACGTAAGTAGCGGTCGTCGGACCTCCCTAACTCC
CAACCCCCCTCCCTCCCCTAGAAGTCCACTCGTTCGCTCAATTTCTAGTCCTTACCCCTATTCCTTCATGTTT
TATATTTTGTAATTACATTTCCCATCTCGTCTCATTATGTCCTTTTTTTGTTTTATTT
TTAAAGTATATAACAAAAGTTATATGATTTTATATACTGTGATTGTTTATATCGTTTATAATACGCAACAAAA
ATGTTGACTGGTACAGATTTTTTCTAACTGCACGCTGTATCCCGCATTTAACGGGGCAGGCGGAGTGCACCCA
GTGTTAAGACACACCGTCAAGTTTTCGAGACGTTACACCACCAGAGTGGCGAAACTCA
ACGCCGCGTTTTATTTGTCAAACCAGAGAAAGAAATAAAAATAATGAATCCTGCTTAAAGTAGGACGTCGCGC
CGTGCCGTCCTCCAAACGAAGCTTACATCGTAGCGGTAGAATGTTTCTGTTAAGTTGCCATCTGTTCGTTCAC
GACATCACTCTTAGATCATTCCTATTTTAATTTATTATGATTTTATATTTTATTTTAA
GTTGATCTGTATCCTATTGTGTTATGTATGCTTTATATTTTATATTGTCCCGTAATATGAGTATTGTTT
>126036963_2 EL596757.1
GCACGAGGGGAAGGCGAGAGTCCGCGTTGTCCGCGCTCAGCGGAACCTGCCTCGCCGGCGCGAGCTGACGCAC
CGTGCCCGCCGCCTTTACCTTTCTTAGTTCCCAGTGATTCTGTTGTTGGCATGGCGTCAGTACTCGGAGCGCC
GTTAAGCCCCGAGCAAACTTTCGGATCCATAAAATCGCTGGCGATATCTCGAGAAGTC
GCTAGCTTTCCGCTATCTAGGTCACCTACACCGCCCCCGTTAAGTTATCCGTCTTTCAAAGACGAAACAAGAA
TAATCGAGAATATACCGAACTCCGTTCCACATTCGGACTTTGACTGTGGCACGATTAACTATCGAGTTGTCGA
ATGTAAGCTCACGACCACAGATGAAATAAAAACACACAGTGCGCCCCACCCTAGTATA
CACACAGACAATAAATATATAGATAACAAATGCACAGATACAACAGAAAAACAGAGACCCTCAACAGTGTGCA
TCGTTGTACCCGACGGCAAGGGCTAATATTTGTATGAAAGTGTTCGTGTTGTGGCAAATGATTGGTGTTTTAT
AAATGTTTGTAGTGCTAGACGACTAGTTTATTTGAGGTCGTCCAAAGAGACCATTCCA
TGGATGTGTGGTTTATTTTTTTTTATTAATGGCTATTTCTAATCTGCTCAATCACGTTGGTCTTCCGCTATCG
CTTTCTGAATTCAAAAATATTGTATCATTTTGATGTTGCACTACAGGTTTGCTATGATTTTTTGA
>126036964_2 EL596758.1
GCACGAGGGTTTAAGGCTTTAAAAACACGATATGATCAACATCAGGACAAGTTAAAGGAACGGGATAGAGTAT
CGGGCAGCGTAACAGTGGCGGAGGCGGTGGTCCCGTCGCTCCTGCGCACGCGCTACCGCCGCGAGGCGCGCCA
GCCCGACGACGAGGAGGAGATGTGGTTCAACGACGCGCCGCTCGACGCCGCGCACGCG
CCCCACGCGCACGCGCACCCGCACGCGCACGCGCACCCGCACGGACATGCGCACGCGCACCACGCGTTGGACG
CCATTGGTAAAATAGTAGAGAAGAAAGTATGTTCGAGCACTGAAACAGTGAACGGTCCGTCGCGGGTGTTGTT
AAACGCCGCCAAGCCGCCGCACACCGACGTTCAGGGTCTCGTAGACTACGACGGTGAT
TCGGACGAGGAAGAGGAAGCTAGTACCACTACTACCGCCACTACCACCACAACCACAACCACTACCACGAGTA
CGGACGAGAGGGACGCCAAGAGACCGCGCCTTGCGTAGTCGCCCATACCTTGTATGTAGATACAAGTGTTTCA
AGGAATATATGTAAAAGTCTCGCTCTACTGTGTCATGGAATGCCGGATGGTGAACGGT
CGCAGGTTCAATTTTTTTT
>126036969_2 EL596763.1

GCACGAGGAAGAAAGCAAAATCAGTGTAACAATTCACCACAGTCGTCCAATTACTTGGGTTCCGGCAGACACA
ATAAGGATGATAAGGATAAAGGTGATCAGCAGAGTCAAAGTACACAGGCCTCACCCACTGGTACTCTCAGGCC
GCGTCGCGCTCGAGCTCGTTCCGCCGACGAGTCATGGAAACACGCACTGTCACCGCGA
GAGAGCTACGACGACTGGGTCATCCATGCAGATGAAATACTTATTGGAAATAGGATAGGTTCAGGCAGTTTCG
GAACCGTGTATAAAGCACACTGGCACGGACCCGTCGCAGTTAAAACACTCAACGTAAAGACACCCACAGCGCT
ACAGTTGCAAGCATTCAAAAATGAGGTAGCAGTCCTCCGTAAGACTCGACACTGTAAC
ATCCTGCTGTTCATGGGCTGCGTGTCCAAACCCTCGTTGGCGATAGTGACGCAATGGTGCGAGGGCTCGTCCC
TGTACCAGCACCTGCACGTGCTGGAGACGCCGCTGCCCATGCTGCACCTCATCGACGTGGCCCGACAGACCGC
GCAGGGCATGGACTACTTGCATGCCCAGAATATTATACATAGGGACCTGAAGTCAAAC
ACATATTTTTAAGAGACGACTGGTCGGTCAAAATAGGCGATTT
>126036970_2 EL596764.1
GCACGAGGCGCAGCTGTTTCGATTAATATAATCATTTTTTGTGGATGTGGAGTTCATAAAATAGGGGAATATA
TTTTGTACCTAAGTTAAAATAGATTAAAAATGTTGCTTATAACATTATCTTGTGTCGCATTATGTATATATTT
GTGGATATTAAGAAAACAAAATAAAGACGATCCACCGACGCTTTCCGGTAGTTTACCT
TTTATTGGACATGCCCGCTTGCTCTTAGGGGATAGTGTGAAATTATGGAACACAATTAAAGAGTATTCAATCA
AAACAACAGAAGTTGGTGGCGTAGCTGCAGTGTGGATTGGACCAACAAAAATTTATCTTGTGAGCGATCCAGA
GGATTTTGCAACTGTAGCCAATACGTGTTTGAAGAAAAGTAGATATTATGAATTTGGA
AGGGCTTGGCTAGGGCAAGGCCTAGTCACAGGAACTTTTGAAAAATGGAAAATCCACCGTCGTTTGATAAACC
CAGCATTTAACCAAATAGTGCTCGATGGTTTTATTGATATCTTCAACAGTCAATCTCGTCGTTTAGTTAAAGA
TTTAGAGCAAAACATTGGAATGGGCCCATTTGATCATTTTCCTTATATCCGTCATAAT
GCTTTGGAAACAATTTGCTTAACAGCAATAGGAGTAGACTTCACTGATG
>126036973_2 EL596767.1
GCACGAGGGTTCTTACAAAGTCTTATAAGCTTCGCGTTTCATATATGTTATGATTTCTCATTAAATTCGAATA
ATGACGCACTATAATTAAATTCGGAAATGTAATGTGTCAGTGTAAACGCTAAGTGGCTTAATTTTTAGTAACT
TGTAGAAAAAATAAAAAATATAAATTTGTCGGCGTTATAGGTTACACATTCGTCATCA
AAATAACGTATGTGTTATGAAAAAAACAATTTCTGGACCAAGATTGGCTAAAATTATTCTATAAAAGTGTTTA
TTAAAGTGGACATTGTAATTTTATATTAATCTAAAAGCCGCCTGGAAAGAAAATGGGTCTGATCATATCGAGA
TTTCGCCGCAAGAAAACAACCATAGAAATCCTAGAGAAACTAGAAACCAAAATCAAGA
ACATAGAACAAGATGGCCAATACAAGGAGCAGACGCACAAGCGTGTCATCGGCTACGTGATGGCGTACTCTGT
CGGTCTGTACGTGCTGTTCGCCGTGCTCTACTACTACATGTACGTCGGACGGAGCCAGCACTGGCTGCACTCG
CTCCTGTACGCCTCCCCGTTGCTGTTCATACCT
>126036975_2 EL596769.1
GCAGAGGAGACGGACAGACAACGCACGCAGCTTCACGAGCTTTAGACGCTTTATCGAATGCAAAGCTGATCTG
TGATGGTATGACATAGTTAGGTACATTTAGGCTGTGTGGGTTTACTTGTCCGTACATTAGGTACAGGCACAGA
TCGACAGATATTGTGACCAATTTTATACTAACTTCAAATTGTCCATGTCCGCTTTAAA
TTTGGCTTTTCCTACGCTGAAAGTTAATTAATAATGTTATTAGTAATCTGCGTGTGGTTCTGTTTGTGTGTAG
CGACCGTATTATATGTATATTGTATTATATATATATATATATATATATACATTTATATTTACA
>126036981_2 EL596775.1
GCACGAGGGTAGAGTAGAAGATAGAGGCAGTTCGTCGGAATTACGAAACAGCAAACAAGAGTTGAGGGAAATG
AAGTCCCGTCGTCGTACACCAGTAGCCACCTCCGCAGAGAGCAGTACTGAAGGCGATTCGAGTCAGCAGTCTC
AACGAAGCGTTGTTTATTTACATGCCACTACAGTTGGTGATATCCCCGACCCCGGCCG
TCTAACAAGAAACCGTTCCCGAGATGACGTTTCCTCCGTAATGTCTTCCAATATCCAAGTCCGAAGCAGTACT
AAGAGCTTCTCCCTCTTCGCTCCTTGGACTCCACGCCACTACAACGATCAATACGACGTACACTACGAACAAA
AACCGAGAAAACCCAAAAAAGAAAATGAGCGATTTAAAAAATCATCATCGACACCGCG
GAATGAATCGATTTTGCAAAAAAATAAATCGAATAGTCAGTCAACGTTAAGCCGCAGACCAAATAGTCAAAAT
CGATTAACAAGCAGCAGCACAACACTATACAGACGAGATAGGGAACGAGGGAAGGAAAATGATGCATCGCACA
GCACTATGTCAAGAAAAAGAGACAATAAGAAGAGTCAATCGAGTGAGATTTTGAATCG
AGATGATCG
>126036984_2 EL596778.1
GCACGAGGAATTAATATAAAATATTATCTTGCCTCCAATTTTCATATTTATTTATGCATGGTTCATACAAGTT
TTCATTTGTGATGCTTTTCATTTCAAAGAGTGAATAAATATATGACATGATACTTAAAATTATTATTAATTAA
TTAAAAAAATATATATTATTTCTCAATATGCTTATTGAGTGTAATATTTATGACATTC

AATTGTAGTTTTGTATGGTGTGATTTTTTTTAATATTAAGTTTTACCCCTTGAACTGTTCTTACACATTTGGT
TAATTTACGTCCATGTTTTATTTTTGCATATTTTATGTTATGTATTGTTTTGTCAAAATAATTTAATTATTGT
TTTCGAATGCATAATAGAAATTGTTTTATATTTTGTTTAAAAGAGTCATTATATTTTT
TATGATATTAATATTTCGGAATCTGCAATATATTTGGTGTCGATATGATTTCGACTTTTGATGTTTAATAACT
AGGTCCAAATGAATTCAGTATAGTTAGTATTTAGATCTATTCAACGTAGCATTTATGTAGTTGATAATATCTT
CAAATAACATTAAATCCTCTGGTGCTTGTAGCGGTAAGTGTTTTGACTCCATTCCATG
TGTGTATGTATACCT
>126036988_2 EL596782.1
TTCGGCAGAGGGTCGTTTGCACGTCGTTATCGCGCGCTTTGCTCCTCGTAAACAAGATCGATCTGAGTTCAAG
GAACGTTTTAACATGGGCAAGGTTTTGGATCCCACTGGCATCTATTGAAGCATTGCGTATTTTTGGGACAGAT
TTTGTTTTATAATTTTTTTAACAAAATGCTCGGCTTGGACCCAACTGGTGTTTATTGA
AGATACAAATTCATATCAAGATATTGCAATCAAGATGTTTAGCCTTGACCCTACCGGCGTCTACTGAAAAGTA
AAGAAGCTGTGAACTGAACTCTACATTTGTTTTAAATTGAAAATTCTGCATTTCAAAGCCATCATCATGTTTG
TGCTCGACCCGACCGGTGTTTACTGAAAAAAGAAGAAAAGTGACAAAGTGATAATAGT
TAAATATCAAAATGTTCGTCCTAGACCCCACGGGAGTGTACTGAGAAAAGACTCTTAATTTTGATAAGTGATT
TATATTTTCAAAATGGGCCTCGACCCGACTAATGTTTACTAGGTTAATTCCATCTCCATATTTTTTTTCTATT
ATTTAATATTTAGTTTAATTCTCACCATGGCTATCTATTTAGATCCCACTGAGGTGTA
TTACAACAGTTAAGTTTCCCTTTTAAATGTGTTTTCGCTGTTAACTCTACTTTCGTATTTCAAATGTATTATA
TTATGCCGGAGTGCACGCATCGTCGCCACAAAT
>126036997_2 EL596791.1
GCACGAGGAATCGCTAGAGAGAAGGCCCCAGCCGCTGCAACTGAAGACATGTTTCATGGAAATTATGATAAAG
CAACGAAAGGTCCTTTAGCAGTGGGCGTCCCTGGTGAGCTTCGGGGGATGTGGGCCGCGCATCAAAGATGGGG
AAAACTCCCCTGGAGTATGCTAGTCAAGCCTACTCTGGAGTTCTGCAAATATGGATAT
CCGATATCGAAAGCTCTTTACGATGGACTCGAAAGTGCACCGTTCATCAAGAATGATCCACACTTGCGGAAAA
AATATTTCAACAGTGCTAAGAATAAATTTCACAAACCAGGCACGATAGTCAAGCCTAGTGAGGCGTTTTGCAA
CACCTTGACAATTATAGCGGAGAAAGGTGGCGATGAACTGTACAATGGCACCCTCGCC
GTCGAGTTCCTGGAAGATCTCAACAGAGTTGGCAGTATTATAACAGCGGAAGATTTGAAAAATTATGAAGCAA
AGATCACAGAACCTATCTCAGTGCCGTTAAGCAACGGGGACGTTCTGTACACCCCACCACCGCCCAGTAGCGG
AGCTGTTCTGGTCAACATTCTGAACATTCTTAGTGGTTACAACTTTACCTCCGATAGT
ATTAATGGGACAGACAACAAGATACTTACTTACCACAGGAATATTGAAGCTT
>126037000_2 EL596794.1
GCACGAGGGTTCAACACTACTGTTGTGTTTTTGTATGTTTTTTAATAATTATATTAAAAGTAAATAACAAATA
TGTCTAATGCAATCTCCAGTATGGAATGTACAGATGATATAAATATAATAGGAACTTGTCTCACCATGTGTCC
ACTAGAAGAATATATCTTGCGGAAAAAGGAAAAAATGATACATATTCTAGAAGTAACA
CAATCAAAATTTGTATTGGTGAAAAGTTATAGTCGCTCAGCTGCAGATAAAAATATGACAGTTTCTTCTTTGT
TAAGGCCTTTCGACGTACTTCTGGAAACAGTGCACTATTTATTATTAGAAGTTTCAAAAAGAGAAGATGTTAA
GATGTCAGTAATGTACAACTTTATACATAACAGACTAATGGCGGTTTTACAAGATATG
ACTATTCAGAGGATGCCTACCCAACAATGTGACAAATGTACCTTGTTATTAGAGCCTATGATAAGATTTTATG
TTTATTTTGGTTACAGAATGTGTGACAGTCCACCTAGTGAGTTTGATCCAGTATTAAACAAGAAATATTTATT
GCAATGCTTAAAATTATTC
>126037006_2 EL596800.1
GCACGAGGAGAAAACGTAAAATAATGATTAATATATTAGATTTATCGTTTCTCATTTTGACTACTTCGGTTAC
ATTAACTGGACTTTTAATTGATCGTTTTGAGAAACATTTGCCGCCAGTAATTTTGAAAGGTTTTAAATATGGA
TGTTTCGCCTACAAAGGCGAAGGGGCTAGTTTCCTTCAAACAATCGAAATGCCGAAGT
CCGCATATTCTCATTTCTATTTATTTTCAATGGTTTTTGGCACAATTACCTTAGTGTATGGCAATTTTGTATA
TATCTTTGGTTTTGATGTGCATAGATATGTTGTGAATATGTTATATTTCTTCTTAGAGCAGGATGAACCTTCG
GTTCCTGCACCCGCTGCTCTTTTAGTTCTTTTTCTTTTAACCATACAATGTACAAGAA
GAATGTATGAGACAACCAGTGTACAAGTGTTTTCCAAGCGAGCCCAAATGAACTTAAGTCACTATGCCGCAGG
AATGTTGCATTACTTAGGAGTCATTATAGCCGCTGTTGGACAGGCACCTTTATTTTGTGGACCTCACGACAGA
TCTTTAGTTGTATGGAAAGACATTCGAACTTTAATATTGGCGATTCCCTGCACAGTAG
TCTTCCTTTGGGCTTGGATTGAGCAGTACAAATCTAACATGATTCT
>126037012_2 EL596806.1

GCACGAGGTGACGATTTACATTACAGAATCAATTCTTAAAGTTTTTCTTTTCGTAATTTAACAAAGTTAGAAT
TACGTGTGTTGGAAATTTGTTTATTTAATATTTTCGTTAAATCTATAAAGCTCATATTCAAAAGTTCACATTT
TAATATTCCTCACCTGTGCCCTTATACATTTACCGCAGCCTGTAACTTTCCCGTTACC
TGCATTAAAATCAATATTTTATTTGTGCCGTGCCACGTTCTTGCGGCAGTTTATTTTTATATTCTTAAAGGGA
TGTAATTTATTTTTCGAGACATTTTTCATTTAAAGAAGCAGTCATGTCTAGCATGTCAGATCGTAAAGCTGAA
CTGGAGCGTAAGAAGGCCAAGCTCCAGGCTCTAAGAGAAGAAAAGGATCGCCGTCGAA
GGGAAAAAGAACAGAAAGATGCAGAGGAAGCATTGCAAAGAGCAAGCACAGCCTCAAGTCTTGACAGCCGCAG
GGACTTAGATGAGATGCTGTCATCTCTAGGAGTGGCTCCAGTTAAAGATGTTCTATCGTCTTTATCCTCAATG
ACATCTTTGACACCACCGCAAACAGCATCACCGGATGCCAGCCTGCCACATACTGATA
AATCTAGCTTACCTCCACATGGAGGTGTAAAGAAGCCCCCACAGTTGCAAGTC
>126037014_2 EL596808.1
GCACGAGGCTCTAGTACAATTAAATATACTAAACATGTTTATTTTGCTTAGAGTAATTACTATTAGCTTCACT
CTTTATCCGTAGCGGGGTAGTCGGAGAGAACGGAGCGCCAAACGCTACGGTCTCTACATACCTCTTTCGCCTC
GTCCACTGTCATCAACCTCTTCATACATGCCCTCCGTGCTTTTTACGTGACCTTCCTC
CAGTATCTTTGATACCGTGTTTTCGAAGGTCAACCGAGATCTCCCCCTACCTCTCTTAGAGTAATTAAATGTA
TATAATAATATGATTAATTAATTTGTACATTATTGTGATATACGAGTATGTATACATACAAAAATTGTTCATT
ATTATCTTGTCAATGTCATTAACACTTTAATCATACTAGAGATAAGATATATTTTTTT
AAGTATATTAATGCAAATATTCCTCTGAATATTCTTAGGAATTTTTCTCTACAAATATTACTAGGAAATCATA
TTTGTTTGCAGGTTGCATTGTCAACAGTCCTTGAAAAAAAATCTCAAGAGGTTGTTTTAGAAGATGTTGCTGA
GGATTCGTGGGTGAAATTGAATCCGGGCACGGTGGGCTACTACCGCACGCGGTACCCG
GCGGGCATGCTGGAGCAGCTGGTGCGCGC
>126037015_2 EL596809.1
GCACGAGGCTTAGATATTTTTTATTTTCGTAAATTCGATGACATTAAGTGACAATAAATAATATTTTATAATA
AATAAAAAATCTAAGATGAAGTGTGTCACTGTAATAATACAGATTTTATTCATGTCAAGCATTTGTCTCGGGA
CTTTGTCACCACCGGTTGATAAAAAGTCACAAAAAGGGGTGAAACCTCAAGAGGGATC
TCGAAAAAACAATGTTCTCGATCGAAAGTTGGTTGTAGAGACACCATATGTAAAAGATATTCTTAAATATTAT
GCTACTTATCATCAGGATACATCTCTCAGAAATTTTAAGAACACAGTACTGGGTTATGTTACTCCATGGAATA
ACAGAGGCTATGATGTAGCTAAAACATGGGCTCCAAAATTTAATTATATATCTCCAGT
ATGGCTGCAGGTAAAGCGGCAAAGCCCTAATATTTACATTATTTCGGGATTACATGATGTGGACAATGGATGG
ATGAAATCAGTTAAACAAAAAGGAACTGAAAATAATTTAAGAATTGTGCCAAGGCTTCTATTTGAAAACTGGC
AGCCATCAGATTTAAAAGCTTTCTTTATGGAGCCATCATCAATGACTGAACAAAAGGC
ATTAATAGCAGAAGTTAAAAAAGCATGCAAGTTATGAAAATTTAATGGCATTGTTTTGGAAATTTTATCACAA
ATTGGGAAAATATATTGATAAATCTGTCAAGTTTGTT
>126037016_2 EL596810.1
GCACGAGGGTTGTTTCCACTGGCGCGAATTTCTACTTCGCGAGTGAGGTAAAGCACGTATAATGAGTTCCAGT
GAATTTAAACTCGAGAGAAATGTGGAGTTATCGGAGGAGACGAAAGCGATTGCTGAGAAAGAATTGAGGGAGA
CTCCGGAGAGAGTGCGCGAAGCACTTGAGAGGCTTAGAGAACTTCTTAAGGAAGCCAA
AGACATACACTTTGGCGATGATGACGAGTTGCTCACATTATTGTTGCGTCCTTGCAAGTGGTATCCGGAGAGC
GCCTTGGCACTAATGCGTCGGATCGCAGAGTTTAAACGCGATAATGCGAGCCTCTTGGATGGTTTACTTCCAG
ATCAGGAAAAGGATGCTTTTCTTGACCATAAAGTTGTGAACGTCATGAAGGGACGTGA
TGACAAGGGAAGGAGAGTGCTCATTGTAAATGTCGGAGGGACATGGGATCCAAAAAAGGTGACAGCAGATCAG
CTGTTCAGGCTGTTTTACTTAATCCATGAGGCAGCGATGCTAGAACCTGAATCTCAAGTGCGAGGAACCGTCG
TTGTTATGGACTTCCACAACATGGGTTGGAGTCAGACAATGGGTCTTACACCGGCATT
CTCTAAGCGTCTGCTGACTTTCATCCAAGATGCTATGCCTCTTAGATTAAAGGAAGTACACTTTGTAAAAGAA
CCAATGATC
>126037017_2 EL596811.1
GCACGAGGGAGACCTAGTCATAACGTATACAACCTCAAAGATAAAATAAGTAAAGTCTATTCTAATAGCAAAA
GCAAAATCGACGACAATAAAAATAAAATACAAGATGGGTATTCAAGTCCTATTGGCTTCCACTGCACTTAAAA
CAGTAAGTGTAACTGGGCTCTGGCTTATACCACTGCTTTTGGGGGCTTTTACATATCA
TAATTACAATTCATACGATCCCGAAGCAAAAGTTCTCGACAAAGAACCTAGAAAGGAGTACGACTTTATAGTT
GTTGGAGGCGGTTCTGCTGGAGCTGTTGTAGCAAATAGATTAACAGAAATCAAAGATTGGAATACCCTTTTAT
TGGAGAGTGGTCCTGATGAAAATGAGATTACGGATGTCCCTTCGTTAGCCGGTTACTT

ACAACTAACGAAGTTGGACTGGCAATACAAGACTGAACCCACAGAATATGCATGTCTAGGTTTTAAGAAGCAT
AGATGTAGTTGGCCCCGAGGAAAGGTATTAGGTGGATCTAGTGTTTTAAACTATATGATATATGTAAGAGGAA
ATAGATACGACTTCGATCAATGGGAATCATTTGGAAACCCCGGTTGGGCGTATAGAGA
CGTTCTTAAATATTTT
>126037028_2 EL596822.1
GCACGAGGAAATACCCTAGCTATACAAGTTTTAATCAGGATGGAAGATTAAAAGAATCTGCTTTAACAGCTGA
AGATGTTGGATATGATACAAAAAATGATAGCTACAAGGATGTTGCAGCATTGAGATTAGAACTGCAAAAACTC
GCACAAGGAAGGAGCTTCGGGATTACCGAAAGACCACAAGAACCAAAGAAATCAGTAG
TAGCAATACCTGAAGTCAGTCAAAAAAGAATTACGGGAACGGATCTTTTTTCAAAACCAACAAGTGATAACAG
TCAAACTAAATTGTTTCAATTTCAACCGGCCATACAAGTTGCGCCAGTCGAGGTTATTGATAATTCACCAGAA
AAAGTTTTGGAAGAAAGTAGAAAAAATGTTTTTACATTCTCTAACCCACAAAAAACAG
AATCAAATATATTTTCAGCTAATCTTAGGGGAAATTTGTTTTCGACGACCACAGACGCAACATTTGCTTTAGA
AACAAATAAAAAACAAAATATTTTTGGTAAAGAAATTGAAACTGATAGAAATTCTAAACCACCAACTAATTTA
TTTAAAATGGAGGATAAAAATGATGTATTTAAAAAGTCTACTACAGCTGTAAATCATT
CTGTGTTTGGCCAGTCTTCCGAAAATCGACAAGACGATAAAAAATTGTTTGTCAGTAGCAACACAAATATATT
TGCCAAAAGTGATGGTGT
>126037033_2 EL596827.1
GCACGAGGCTTGATTTGATAGGAACCTATAAAAACCCGATGTCTTATTTCTGCGATTTTTGAAAAGTACAGCC
AACATTTAAGTGTTTCCAATCAATTGCTTTGACAAATATTGTGTCTGTTATTATATTTTTATTTACATAAGGA
GAGAAACTAATGATATAACCAATAGTAACTAATGCACTCCAATCTTAAAAATGTCAGT
AAAAAGGCACGCAACCACAGAACTGAACCACGACAATTGGGATCAAGATGACCCTACAGACCAAGAAGAAGAA
GGTAGGTGGTTTTAAAATGGCATCAAAGGAAATTCTTGAAAAACGTGTTTTTACAACCGCTAAAAGACGTTCA
CAAGCTACTGATGATGAGCCAAATAGAAGTGTATTCAGTGGATTTAGTGGATTTAATA
GAACACATAAAACATCTTTTGATTTTCTAGCATAGTTGACTAATGGTAACAAGCCAAACAATAGCACCTCTAC
TACTAACAATGATGTATCCGAGACCTCTACTACTGATACCAATAAACCCACAGCTAGTCCCAGTATAGGATTG
TTCAGTTTATCAGGTACATCAACTCCTCTACTCAAATCTACATTTGGAATACCATATA
CAGAAGCTCCTTTGTTTGTAAACATACCTACAACTTTTTCACA
>126037035_2 EL596829.1
GCACGAGGGTAGAACTGGAGTACCAGGACCAGTGCGACAAACGAGCGACAGCTTCGTGGGAGGAGTTGTTAGG
TAGCACCTCGGGACTGTCAACAAAGCTGGAAAGCGATAAGGCCTTCGGTATATTTGTACGTAAGCAGAAGACG
GATGTAGCCAGCGTGTCCACGGGCCACGCGTTGGGTGCTAACGACGATACATTGCGTC
GCCGCGTCAAATTAATTCTACAACCTGGTGATGCTTTACTGGATACGCAAAATTGGATACGGCTAGTAACGTT
CGCAGAAACAGCATCACATCGCCTACGTTGGGCGAAAGATTTTGATTGTGGATCAAATACTACTTGTACATTA
AGAGAGCTGCATAATAGCATGGCTCGAGGTCAAGATGAAGATGCACTCCGCCATATGA
AACAGTCGTGGGAGAATCACCTGCCGGATTTGCACGAATATCTGGAGACCATACTGCCTTTGTTAAGGAATGC
CACTAAAGAAAATCACCACACATTAGTAGAAGACTATTGGGATGAACTAGCCGAACAAGAAGGAGCTTTATTA
CTAGCTAAAGAACTATGGCAGCGCGTTAAGCCCTTATATTTAAAACTGCACAAATATA
CGGCGCTACGACTACGAGGCGCGGCCGAAGTCGGTAAACCACTTCCGGTACATTTTGTGAGATCACTTAAAGG
>126037037_2 EL596831.1
GCACGAGGATTTTCACACTTGCTATTTTGTATTGTAATTTGTTGTCTTAACTCTTACGTCCACTTTATTTAAT
AAGATTTCACTATTAAAGTATTGTAGCAGTATTCAAACAAAACCTAGACAAAATGACTATGGTGGACGATTTA
CGAAAATACCTAAACCTTTTACTAGAAAAGGTAAATGGACTTCACTGTATATTAATTA
CTGATCGTGACGGTGTTCCTGTAGTAAGATCTGTGACAGAAAAGGCCCCACAGCTGGCCTTAAGACCTAATTT
TATATCAACTTTTGGAATGGCTACAGACCAAGCAAGTAAACTCGGGCTTGGGCGTAATAAAACTATTATTTCT
ATGTATTCTAGCTATCAGGTTATACAAATGAACAAGCTTCCATTAGTGATTACTTTTA
TTGGTAGTGACAATTGCAACACTGGCCATATATTGTCACTTGAAAGTTTAATAGAACCTTTTCTTAAAGATTT
AGAAACAATTGTAGCTGAAGCTCCATGAGCATAATTTCCTTTATGATTTTCTTAGCATTTTTGTATAATATTA
CTAATTTGGGGGAGTTCAAGTATTACATATCAAAATTT
>126037045_2 EL596839.1
GCACGAGGCGCACAAGCAGGAGCCCGAGGAACCCGAGACGGAGGTTCCGCCGCCGCCCAGGATGTTCCCGCCG
CACCAGGACATGTTCCCCCCAGTGCCCCCGCCTGCCGTGTCCGCGATGTCGTTAACGCCACCTCACATGTTCG
GAATGGAGTCCCCCCTGGCGCTGTTCCCGCCAGGTATGGAGCACAAGATGTACCCACT

GATGGACGTCGACCACCGCGCGCCGCTCGACGCGCAACTCTTCACCAACGTCAAAAAGAAATGGCGGCCGAAG
GGGCAACACAGCGCGCCGCGCGGCGGCCCGCCCCGCTCCTGGACCAACGCAGAACTCACGGAAGCGCTACAAC
ACGTCTGGAACAAGAAAATGACAACGAGCCAAGCATCAAGAATATTCGGCATCCCGTA
CAACTCACTTCTGATGTATGTGCGGGGTAAATACGGCAAGAGCTTAAAACTGGAACAACTCCGAAAAGATTGC
ATCGGCGGTCCGCTGGAGGTGTTAAATTTAAATTCGGGCAACAACAACAACAACCACCCGCCGGGCAAGCACC
ACGACAAGGACGACCAGCACCAGAATGCGAGCCAGCGGCCGGCGAGCTCGGAGCCGGA
CATCTCCTCTCACCCGATGTTCANCCCGTTCCAGCAGGGCTTCTACCCGGAGTTCCCGCCCGGGTTCCCCATG
CCGCTCAACATGCTGCACCTGC
>126037047_2 EL596841.1
GCACGAGGGGAAAGTGAGAGCGTGAGAGTTGAGAGTTCGAGGTCTAGCAGCAACTCGATCGCTCCCGTTGTTC
CCCAGGGCAGCATTATATCACCTGCTCTCTACGTCCTATACACTGATGACATCACAACGCCCTAGCGAGTATC
ACTCAACGAGAAATCCTGCAGGTGGTCCAGAACCTCATCCGTCGAGTGACGCAGGCGT
CATAATAAGTGAGGAACACCACGCATCATCGGAAGATAAAAATAGAAAGCCTGGAGGTACACATTGGTCGTCT
CGCGTCCAACGCATTCCGCTGCGCCAATGTATCACCTGCACCTACGGAGCAGCACTCCGCGGCATACTCGGCC
GCCCGAGGTCAGACTACTTCCGAGGGTCTTTCTATTCTCCTCCAAATCCGACTCATGA
GCCTTTGCTTTCATACTGTGTCAACGCCCAGATGTCGTTCGGAGCTACTACGGAGATGGACGAGAGAATGCTT
CAATTCTGCGCCTGGCTCTATAGCAAAGTAAATTTAGAAGCGATTGCACGTATTTTGGAGTTATCCAATAATT
ATAATGTATTTCGTCGCGGCATTGTTGGTTACATC
>126037054_2 EL596848.1
GACGAGGGTTTTACCTGATGTTGACACACAAGCAATACGACATATTAGTGATAGTTATAGTGTTCATAGTGAC
GCCCCCCCTTCACACAGCCCCTCCCCCTCCCTCTCCTCTACCCGAAACTCCACTCGATCCACGTCTATATGTC
GCCGTATTTGATTATCTTCATACGGTAATTGTGTATTAACGCCACCGCAGCTACTGGT
GTAGCGGTTTTGAAGTGCAAGTTCAGTTAGGAACTTTCACATATAAAAATAATCTACACTTAAGAACCGCCCT
ATCTGTAGTTGAGGCGGCGCTCATACATAAACACCTCTCGTACTATAGTTTAGGGTAATGGTACATTTATTAA
AGATGTTTTTGTATTTGTAACGTATTAGTGTTCATTTAAGGTGAGTTTTAGCCGAACA
TTGACATAGTGTTGGTTGTTATTACACGTTGTTCTGTTTAGGTTTATTAGTGAGAGTAGACGGCGTCGCTGCT
GCTGGTCAGTGCTCGTGTCCCGAGGTGGAGCTTTGTCATATATATACGTTTAATATATTATAAATATTCAAGT
TATATTAAAATTCTGTCATATTTCGATGGCATGTCTCGTATTATCTGGTCGTCTGAAT
AGATGCCGTTAGTCTTGTCAGTAATAAAAAACATTGAAGAGTGCCAGTTGCGTCAATACTTCGTCAGATCTTT
CTAATTTGTTTTA
>126037055_2 EL596849.1
GCACGAGGTAAAGAAATATTATTCATATTAATTTTATTATTAATATTAAATAAAAAATAATTATTTATATATT
AATTTACTTTTATTTTCAAGATGCTTAAAGTGGACAATGTCAAAAGTAAGATAGACTATAATTATAATAATTG
ATAGAAAAATAAACAATACAACGAAAACATAATATGTCTACTACTAACTACTAGATAA
GCCTATTTGAAATGTATTGAGGTGAATTATTATTTTATTTATACCATTGCTCAAACAAAAGAAAAATACTTTA
AACTCCTATAAAACTTATAAATTACGAAATTTGCCAGTTTTCATGTGTCTTCTTCCAGGTGTCTGATCAAATT
TCTAATTATTACAATTTTTGTATTAGTTATAGGTTAGATACTCTATAGAAGAAGTCAC
GGGTTCAAATTCTGCAGCACAAATTAAAAAATATATATCTGTTAAAAAGTTTTAAGTCGATAATTTATTATGT
AATATCGAAATATTTGTTTCAACAATGTTCTGAAATAAAATAATATCCTAATATTTATCTAATTCATACGAGT
CTCACATATTATTATTAATAAAAAAACACTGATTCGGATTAATGTATCTAGTGGACTT
TTCGCATAAATGTTTCATCATATATCATATGATTATGATATATTGATATAACACGCCTTTATAGTTTGTCATT
AAGCTTAACACATATTTTTAAACAAATACTACTAAATTAACAGCAGATTTTATGTTTTG
>126037056_2 EL596850.1
GCACGAGGGTAATAAAAAGATAAACAACACATAAAAAAACTGCCAGTATTAGAAAAATAAGTTTCTTAAAATC
TAACATCTGAAATAATCCTATATCCCCATCGAACTGACATAATATTCCTCATATACGTATCCGTAAGATACTG
GCTAAAGGCCTGATGAGAGCTACAAAAATCAGAATCTCAAAAGGTTAAGTGTGATGCT
TCCTTAGTTTTTATTTTAAAAAATTAAGTACAGTGATTAAATTTACTTTTGTCGAGGTTAAATTTCAATGATT
TAGATAACATTTTTAAGGCGTTTGTGACAGCACAATGACTGACCGTAAGGATGTGGATTCGATTCAAACTCGA
CATTAAGTCCTGCCATTCAACTCGCAATGGCTCGAAAAATTAGCGGACCTGGTGTTAA
AACACTCCGTTCTCTTTCCTACTCTGCTATATTATGATTGATATTTCAAATGTTCTTGTATTTTTAGAGTTTT
CTCCTAAAGGAAATCTGACAAATTAGTAAATAATATTATCCTATTAAATACGTAAGCCATATCCTTGATCCTG
AAGTTGATATGTGTGCCTTAAATTAAAGGTTCCTATTTTGTAAAAATATTGAATGTAT
AAATTTTTATAAAATATGAAATAATATATTACTTA
>126037058_2 EL596852.1

GCACGAGGAAGAAAAATCACATTTAAAATCAATAATATTTGATTACCGATTCACATTTAATTAATTTATAAAT
CGACGTTATATAATTATTAATTTGTGACAAATCTCTTATCTGTCATGGCTAGAATAATTTCCACAGAAGAAGT
AAAAAAACACAGGCGCAAGAGTAGTGTTTGGATTATTATACATGGTGATGTTTACGAT
GTTACAAGCTTCTTGGAAGAGCACCCTGGAGGTGAAGAATCCCTGCTAGATGTAGCCGGCCAAGACGGATCAC
AGGGCTTTGATGATGTAGGTCACAGTGATGATGCAAGAGAATTATTAAAAAAGTATAAAATTGGTACATTATC
TCCTGAAGAGAAGACAAAAGCAAATACATCTTGCTCAAATTGTCGTCTAAAATGGGCG
GTACTAGCGCTGGCTGGGGCGTTGGTGATCGGCTTCGCTTTAAAGAAATATCTCGCTCATTAAAAATGAAAAA
TGTACTGGCTTAAACTGTTTATAGTCACTGTTTGCTAACAAAAGTAGATATACATTTAAATTATTATATATGG
AAATCCTAACTTAACTTTAAAAAAAATATTTACCAATAAAAAGACTATTAAAACCCTA
TACAATATATAATCGTGATTTCTTACAAATATAATAAATGTCATATACAGCCAAGTGGCCCTAACTAGAGAGA
AGCTGTGTGTGACGGATTTTGTATTATATCGTCAGCTT
>126037065_2 EL596859.1
GCACGAGGAAACTGTTCACTTTACAAAATTATATCATAATTAAAAATAAGAATAATTAAATACACTTAGTAAT
TTCTCTTATAAAATTCCTTTGGCAATAATATTCCTTTCTACGCATGTGAGACCTTTCGTAGAATAAGATTGTG
CAAGACAATATTACAATGAATTAAAAGTGAACGTCATACACGACTCCATATTAGTTTG
GTAGGTTTATAAGGTTACTGAACCTTAAAAATTACGTCACACATGTTGTAATTTTTTGACTGATCCCTTATTC
TGGCCACAGCTACTCATATTTGTGACTTAAATCTAGTGTGACATCTCATATTTAGAAATTTTGCACCTGAAAT
TATTTAATTAAAACAGCAGTTTCGATATAATGTTAAATATTTTTTATATGTGAAAAAA
AATCACGAAATTTAGTTCCCTTCTCCACCTCTTGACACACTCCGTTGACCACTTACCTCCCCTTAATGTGTGA
CCTTATTAGTAGAGGTCCTCTAACAATAAAATCTCGATAGTTCGATTAAAAGTCCCTTATAGTTATCGTCAGT
TTTTATATTTCTAGAATGTGTTGCAAATGCGGCACTAGTAAAATTATTTCGAATTTAG
TTTCGGTAGAGAGAGGAGCTCGAATAGTGGAAGCGGGCGTTAATGTCCGTTTGGAGAGCTTTTTAAGCCTAGC
CAAAGATGATAATTGCCTTTCTACACATAT
>126037067_2 EL596861.1
GCACGAGGCTCGCGTACCAGCCAATCCCCTTCAGCAAAACTGGTAACCCCGTGATGAGCACAGTAGCCTTCCT
CGCGTCCGTAGTGGACCCTCGCATCGCGTCTAAAGCCACTCGCGCCGCTATGGACGAGTTTGCTGCTATCAAG
GACGAGGTGCCGGCGGCCATGATGGAGGCGCACGTGAAGGCGGCGGGCACGCACGGGC
CGGCGGCCGCGCTCGCCGCCACCGGCATCGCGGGCACCGCGCCGCCCGCGCCCGCCGCGCCGCCAGGCGAAAA
GAAAGAAGGTGCACCAGAAGTGAAAACAGAAGCAATGGATGTGGACGGTGAAGCTGAGGCCAAAGTGAAGGAA
GAGCCGGCCGAGCCTCCCGCTGATGACGTGAAGGACAAGGATGATGCAACGCCGCCGC
CTGAAGCACCAGCAGCTGTAGACTCCAAGCTCCAATCAGCAGCGGCAGCAGCACTTGCAGCAGCTGCAGTAAA
AGCCAAAACACCTGGCAGGCGTGGAAAAGAGGAAGATCAAATCTCTAGTCGCTCTACTAGTGGAAACACAAAT
GAAGAAACTGGAAATTAAATTACGCCATTTTT
>126037068_2 EL596862.1
GCACGAGGGGCAAATATTTACTCAGGAACAACCTTACTAAGATGAATGGAATACGTGCTTTCGAAATATTTTC
TTTTACAAAGAGTACTTTAACGAACATCACAAATATTTTTACCAGAAATATTCTTTACTTTTACTTTGTTGAG
TAACTCATATAACAACGTATCAATACAGATGAGTTTTCAGTTTATTTGTTAATTCCGG
CATTCTTTAAGCCCGTAAAGTATGTCTATATACCTTATTAAGTGTAATCAAAATAAATAAATTTATTTTGGCT
ATGATTCATAGTTTTTTTAGCTTAACGATATGTCCCGCACATAGCACTGTAGGCAGAGGGCACGTTGTTAAGA
CCGCTCCCATTAAGACACTTCCGATCCCACTTTCCACCAAATTCTCGAGGCATTGCTT
ATTGAGTGGCGAAACTCAACGGCATACGCACGCATACTTTGCCTTACCATCAGAGCGAGGAATTGAAAATATT
AAACAATTCATTCCCCCGAGTGAGAATCGAACCGACAACCGTCACGTTTACAGTCACACGCTTGTGCTCCTGC
GCCACGACGGCCTCAATACGGATTCATAGATCATAGCATAGAGTTTCTATACGTACGT
ATATACTTATTTTGGTAGAGATAAACAGATAGAAATAAGCTTATATTTCTATAAAAAAGCAAGTATATAAATG
TAAAGTATTAGCGTTCAAAGAGCTT
>126037069_2 EL596863.1
GCACGAGGGTCAGGTGATAGATGCTGGTATTAAACTGCAACAGGAAAACGCTAAACTAATATTGAACAATTGT
AGCTTCAGGCAATCTGTTCAAAACTGCTCAATTAATTCACTTCCAACTTACAAACCTAGCAATGTTGTGGTTA
GTGACGCTTTGGAGAGAGCATCAGTAGTCATACATGGGATTAAACAATTTGTTTCTAT
AAATAATATCAACAATAATACTTCTTTAGAGGCCATCACACAAGAGGATTTACAGTTACAAGAAGATATAGAT
ATTCTCAATAAACAGTTCAATGAAACTTTTGATAGTTTGAAACAAACTCAAAAGAAAATCATTTCATCCATAA
CTCCAACTAATTTAAATACTGAAAAAATTATTCAATCAACACAAAAAACTGATGTGTT

TCCAGAAATTCCAATAATAAAAGAAGCACAGTTTAAAATCATAGCACCAAATATGGAACCAAGTCCAAGTAAA
TCAGATTCAAGACCAGTTGCAAAAAAGAAATTGAATGTTTCTCTTAGTGAAAGTTCAAAAGCTCGTGTAGTGC
CATCATCACGTATTGGTAGGATGATATCTTTTGGGTCATTAGCTGCTGGCTTAGGAGT
GGGCACTATTGCGCAATATACACGTAATACAC
>126037073_2 EL596867.1
CATAAAATGATTAGTATTTTACGAATAATCTCTTGATTTTAGTACCAATAAATATAGTAATAATTTTAAATGT
TTGTTATTATTAATAATAACGTAATTATAACATTATGTTNCATTGTTTAGGTGTTCTTCTTCTTGTGCTGTGT
TGCTCTGGCATCTGCTGCTCCCCAGTTGCTCACC
>126037074_2 EL596868.1
GCACGAGGTGTCAATATATATATTTATAAAATTCGTCATACATCTGAAATAAATATCTTTGCACGACTGGTGC
CGGTGTGTGTAAGCAGCGCCGAAAGTTGCCCAGCAATTCTGAACAATAACGAGTGAGGTCGAAAAAGGCCGAC
AAAGCCCGAGTGGCGCTTAGTATGACCGAACGAGGCCGAATAGTGCCGAGTAACATCG
GGCGGGGCCGAACATTGCCGATCGGGGCCGAGCGGCGCCGACTACGGAGCACACCTTTGTTGTTTATTGTGCT
TGATTGAGTTTGTTGTAATCACGATAATATGATAGATTCTCTCTAACTACTTACCTATTTTTTATTTGTTATG
TAATTTTAAGTTTTACTATTTTACATTGTAAATAGGATAATATAAGTCGTAACGTAAC
TGGCCATGAGTGAAAAAAAAGGGTCTTAATATCGTTATTCTTGTAATGCACTAATTATGTCGGCTGAGTCTTA
AAAATGCCTTTGAAAATATAATATTTATATATGAATGATTTATTTTAAGTTAATCAACTGTGCACTAATATGA
TAAATATTTATTTTATATTTCTCTTAAATTGGCTCGCTAATTTGATTCACTTCGTTCT
TTGTAGTGACTGAACATTCTTTATTTG
>126037079_2 EL596873.1
GCACGAGGCATATTTCATCTATAAGATAATATTTTATTAACTAAATAAACAGAAAAGACAAGTGTAAATAGTT
TCGCTGTTTTTCATTTATACTTTTTAAAATTAAATAAAACATGGATATACTACCAGATTTTCATAAGTTTACG
TGGTGCACAAAAGATTGGAATTTATCTGAATTAAATGAAAAAGAAAAAGATCTACTAT
ACCAATGTAAAGATATAGAACCAATAACATTAGTGGATGATATCGAAGAAATAATTCAAAACAGTAAAAAGTT
TCCTATAAAGTTCCCCATTGATACTATAAGATTAGAACAATTAAAAGAACGGAAACCCTTAGAGAGACTAAAA
AGAAATATTATATCAACATACCCTTTAATACATGAAAGAGTTTTACTCTTAATGGCAC
ACTTTTTAATTTATAAGAGGGAACATGGCTCTGATATTGAAAAAGAATTTTATAAAGACATAACGATACTACA
ATTGATAGATAGAATATTGAAAGAACAGAGCTATATCATTTGTTGGACCTCGCGAATAATATAGATTAATAAC
AGGGGAAAA
>126037081_2 EL596875.1
GCAGAGGAAAAAAGAAAGCGGCGTACTAGCGGATCTAAAGATGCTCAAAGTGATGGATCTCCTCCACAGACTC
CTACTAGCGCATCTAGTCCCAACAGTGAAAGTAAATCAGTGCCCCCTAGATTTTATCAAGATACCTTAGATGA
AGAAGATAATAAAGATAAATCCAATGATAAGCAATCTGATATCAATGAAAACCCTGAA
AAAACGATATCTAATGAAGTCAAGCCTGAAGAAGATATGGACACTTCTGAACCACACACTTTGACAGTAAATG
GTTTGAAAGGAGTGCTATGTTATCATAAACGAAAAGGCCCCAAGAAAAATATCAGATGGAAACCAGATTCTGA
ATTAGAAGAAATTCAATACTTCGAACTTGATGAGACTGAAAGAGTAAATGTTACCAAA
ACTTTCACAGATATGAAACATCTTGAAAGAATACACGAGAGAGAAGCATTCCAAAAAGCAAGAAATCTCAGTA
ATGATGATATTATGGAAGAACGCACAAGCTGGCGACCTTTAGTACTTGTTGATGGTGGAGGACAAGTGAAAAT
AGAATATGGTAAATCTAGCACAGAAAAAGATACCCAAGCAATTCGTCAGAAAGGCACT
TTACAGCCACTATATTTCCATAAATCTATGATCCCCGATTCTCCTCATG
>126037088_2 EL596882.1
GCACGAGGTACATATTCACCCTCATGTCCCCGTTAACGAGACTGGTGTTTCACCCTCATGATGATCCTTTGCT
TGTGCACGAATTTGAAGACAATCAAAAGATTGAGCCCGTCCACTACATTCCCATATTGCCTATGGTTCTAGTG
AATGGAGCTGAGGGCATCGGCACCGGCTGGTCCACTAAAATACCCAATTACAATCCTA
GGGATATTGTTGCTAATATTAGGCGAATGTTAGACGGCGAAGAGCCAGTAACAATGCATCCCTGGTACAAAAA
CTTCCGGGGAACAATCGAAGGTTTTAGCGATAAATACGTAATTTCCGGTGAGGCCGCCATCTTGCCTAATGAG
AAAATAGAAATAACCGAACTTCCGGTTGGCACGTGGACGCAGAATTATAAGGAGAACG
TTCTAGAACCTATGCTTGGCACGGATAAAGTGAAGCCTTTGATATCTGAATATAGAGAATACAATACAGACAC
AACAGTGCGTTTTGTAGTTTCCCTATTACCAGGTAAACTGGCGGAGGTCGAAGCCGAGGGCATTCACAAAGTG
TTCAAATTGCAAACAACCATATCTATGACTTGTATGAACGCATTTGATCATAATAATT
GTTTAAAAAAATTCGACAAAGTAGAAGAGATACTCCGCGATTTTTACGATGTGAGGCTAAAGTACTACGCAAA
ACGTAAGGA
>126037094_2 EL596888.1

GCACGAGGCTATTACGAGGAGAACTGGACGAGAGGCACTCTGAGACCGCCGAAGAGTCCTTTGCTCGAGCATT
ATCAACTGGATGTGGTGATTGTAGAGACTTGGTGCGATTACGAAATGAAAGAGATACCCTGCTCGCCCAAAAG
GCTCTCCTGGAAGCCACCGCGGCAGCAAGTCCACCTCCGGCGCCGACCACGCCATTGG
GCCGATTGGACACTGCTCTCGGAACTCCTTCTCGGCCGCCGCAAGTCGCAACACCCGATTCAAAGAAGTTCGC
TGCGATTCTTCTCGAGAGCAATCCCGTTGAACTTCAAAGACAACTACTCATCTCTACCGTTCAGAATCAGGTG
ATAAGTTCACAGCTAAGACGAGCTACAACTCAACGTACTCAACTATTAGAGAAATTGG
AAAGGGCGAGAGACCAAAATGAAGATCTTAAGTTTAGACTGGAAGAGAAAAGCATCGAGCTCGAGGGGGCCCT
AGCGCGCGTGCGGCAGCTGGAGGGCAGGCGCAGCGGCAGCAGCGAGAGCCTGGAGCG
>126037096_2 EL596890.1
CAATTTATGTATCTGTATCCACAGCTGCACTAACTTATTTCTTGAGTATAGACGTGCCATTATTCCTGACTAA
CTTTTAGTTGTAAATGTATATAATTTTTTTCAAAAAAATCACACAAAATATTTAAAAAAAAGAAATACTATAT
CAGTTCTAAACTGGCCCATCACACTGCAATCTGCTAAGATAAATCGGTTATGTATGTA
AGCTAATATCCATTTTAACACCAAGAAAAAAAGCTTTAATATATTTTTCCACCAATCGAATAATGTGATGTTG
TTATTACTAATTTGTACTATCTACCTCCCGAGGTCAATAATATAGATAACCTATTTGCTCTTTTATTAATACG
TCACCTTGACATGTCACAGGTAAAATAAAATTTATTTGAGGCCTTGTATATCCGCAAC
TAAATATATAAATAAAATACGAAAATATTAGCGTGTCTAATTTCGTTTATTTTCTTAATACACTGACCATTTA
ACAAACAAATATATACATAATGTTCAGGCTTTTCTGTTAGCATTCATTGTTTTGCATTTAAATAATATAGAGT
TCACAGTCACCAAACCATTTATAGTCTACGTTTTTTAAGTTTTAGAAAATATTATTAT
TTTTTTGTTTCGTCTATATCGATTCAATAATATGCAAT
>126037101_2 EL596895.1
GCACGAGGCACTTGACCTGTGAAACGTGTGTGTATAATAAAAAATAGATTTTTACACCAAAATGTGCATAAAC
AACTATTAATTTAACCGAATTTAAAAAGTTTTAACCGAATGAATAATGGCGATACAAATGGATATCGTAAATG
TGATTATTATAAGTGATATTAAATGATTAAAACTAAAATGAAATTATAAAAAATTGAG
GCTTATAAACAAATATGGTGATAAAAGTACCAAATTTTGAAGGATCGGACCTTATGCAGTGTGATTATGTCGA
AGAATAAAAGAAGTCCAACAAACAAACTAAGAGATTTTTTTGGCTCCCAGCATTTTACATTTGTAAAAAGTAA
ATTGAAAATTTACTTGTCAATAATATTAAATTATAGTTTATAT
>126037103_2 EL596897.1
GCAGAGGGAAGGCGCGAAAGTGTTTGGCACCATAGAAGACAGTTTTGATGAATTCAACATGACCAACTCTACA
ATTTATGATGAGAGTTCTGGGTTTATGTCGTCGTCAATAGAAGAGCTTCAAGTTCGCAACTCTCGCACCGAAT
CGACCGAAGACGACAAGCGTAGCGACGACAGCCGCCCGCTTAGCCACGCGTCCGACTT
CAGCAGGGATAATATACTGGGCAACGCACCCCGCCGCAGTCGTCGTGCCATCGCCAACGAGTGGCAGAGACCC
CTCACGAGGTACTTGCCTGTCGACCGCGACGACTTCGACCTGCGGCGACATGTTGAATCTGCAGGTCACCAAA
TCGAGCTTTGTCCCTACGTGACGATCAACTCGACGTCGTGCCGCGGATACCTGCACAA
GTTGGGCGCCAAGTTCCACACCTGGTCCAAAAGGTGGTTCGTTTTCGATAGAGAAACGAAAACGTTCGTCTAC
TACTGGGACAAGAGCGAGAAGAAACCGCGCGGTGGCGCGTATTTCCAGGTAATCGAAGAAGTGTACCTCGATC
ATGGCAACACATCTAAATCACCGAACCCGCAAACAACGTTCATAGTAAAAACGCGACA
AAGAAGATACTACTTAATGGCGCCTTCTGGAGAAGCTGCTAGAATATGGATAGATGTTATATTTACAGGGGCA
CAGGGGTACACCGAGTATTTGGAATGAATA
>126037107_2 EL596901.1
GCACGAGGACTGAGTGAATTGCAGCAATATAACTAACATATTTTATTAAATTTTCCATTAGTTTTTTTTGTTA
GAAAATCTTTCGAGCACAATGGATGAAAGAGCTGAAAAATTGGCAAAAGCTCGTAAAAAGTTAAGAGATCACC
AAGAAAAGAGATCTACAATAAGTCACAAAGAAGAAGTAACTGCTCAATCAAGTCATAC
ATCCATGGTACAAGAATCTGTAAACGATAATATGAAAAAAAATACTGATAACGTTAATGAAGATCACAGTTCT
TCTGCCAATGGTGAAACTGAACATTTGTACCATGACAAAAATTTAAACGACTTCAGTGCAAAATCTGACATTA
ATATTGCTGAAATTTTAATTTCAAATAGAACTAACCTTGAAACACAAATTAGTGATTT
AAATGGAAAACTAGCACACATTGAATACTTATACGGCTTAGAAGTAGCAAATCATAACATCTGTAAGCAAAAA
TTGACATCTTTACAATCTGATGTCACAGATATTTCAAATAAATATTTTACTGCACTTGAAGAAATTAATGATA
AAAATGAATCTTCAAAACAAATAACCATTTTATATCAGTCTTTAAAGGACGAAAATAA
TAATTTATCTGAACAGCTTGAATTCACAAAATCAATGCTAACATCTAAAGAATTTGAAAATGCACAGCTTCAG
AATCAAATATTCACTTATC
>126037108_2 EL596902.1
GCACGAGGAACGCGTCAACACCTTCAGCAAAAAAGTTAGATGAGAAACCGAGCACGCCCATCTCAAAGCCCGT
GACTCCAACATCTGGAGCCAGCGGTGTGGGCGCAGCGGGTGCACCTTTAAAAGCTGCGGTCAAACCTCCAGCA
CTTCAGTATCCGTATTTGGGAAATGGGGCTCATGACGCGTACGGCCTCGCCGGCTACT

CCGCTAGGGCGGCGCTGGCGTATGAACCCCTACGTCCCCCTATCGGACCGGCGGCGTTAGCGCCTATACCTGG
TGGCAAACCAGCGTATTCCTTTCACGTATCAGCGGAAGGACAGATGCAGCCCGTGCCCTTTCCCCCCGATGCC
CTAATGGGCCCCGGGATTCCCCGACATGCAAGACAAGTGTCTGCATTGGCACACGGCG
AAGTGGTGTGCGCTGTAACTGTGTCTTCCCCCACTAAGTACGTGTACACGGGAGGCAAGGGTTGTGTGAAAGT
GTGGGACATCAGTCAGCCCAGCAAGGCGCCAGTCAGTCAGCTGGATTGCTT
>126037109_2 EL596903.1
GCACGAGGCGGCAAGATAAGGCAATCCCTACAACCAGTTGTCTCCGATGTGATCTATGATAAGAAGGCGATGA
GCGATTTGGCCATCATGAGGCTCAGCCAATGTTCGGACTATAGCACTGGTGGGGCTGAGATTATATTACTTTG
TGAGAAGGTAACACGCGAAGACATAGCCATAGTGTTTTTCGAACAGGAGGGCGAGCGC
GTTATATGGGAGGAGTATGCTAACATAGTGCTAGTACACAAATTAACTGCTATAGTGTTCCACGTGCCGCCCT
ACCGCGACCGGCATGTGCCGCATCATGTACAGGTGTACCTCCAGTTGAAAAGGACGTCCGACAACGCCAGAAG
CAACGCGATGCTTTTCGAGTACATCCCGAAGAGCCAAGATCCGAGCTATTTAAAACAT
AAACGACAGAAGACATTGTCCCCCACTCTGCAGACGTACGAGGACAGATTTGCAAACGACAACATTAAGGCAG
AACCAAGAGATAAAACACCGCCTCACCAAATGAGCAGTCCGCGGCAAATCTTCTCGCCGGCGTACGACGAGCC
AATGCCGGAGACCTCCTACCAGTGGGATATGCAACCGGCTATCACTGGACCTAGCCAA
ATGTCATACGGACAAGACATGTTACAATGGAACCAAAATAATTATATGCAAGTGCCCCCAAACATA
>126037112_2 EL596906.1
GCACGAGGTATGTCTAAAACGATTGGATACATAGTAGATTTTATGTACTTATAAACTAAGGTAAATTAATTTT
ACGCTTATTTTACAATTTCCTTTAAAAATAATTAGATACAATATACGTAAAGATTACAATACAGGTATTTTTT
TCATATTCAAGCTGTATTCCATATGTAACAGTGTAAACAGAGGGAGTCTACTGTTAAG
ACACTCCATTCATCACTTTCTGGAGGCATTGCGTGTTGAGTGGCGGAACTCAGCGCTTTGCCTGAGCAAAGAA
ATACACCAGAGCAAAGAAATGAAAATATTAAATATTGCATTTCCTTGATTGGGAATCGAACGCACAACCTGTC
GCGTTTACAGTCTCACGCTTGTACTTATATAAGACCAATGATTTATAAACTAGACCTA
CTCTAAAATTACACTCCAAATTTTATTTTGTGTAATATAAAATAAACAAACAGTAAACAGTATATATTAATTA
CATAAGCATATAAGTACCTAATTCAGATTTTTTACCGATTTCTTTACATTCTTTTTCTAAGCGTACTGATTTT
TTTTATATTGAATATGATTTTTTATGTCGCTCGTTGTTTATTTGAACCTCACTGGTGA
TAGTTATGTCAGGTTGACTGGTACTTCCAAAATGATGATATCCACGTTTGTTTCCAGAT
>126037134_2 EL596928.1
GCACGAGGGCGACCACGCGCGCCAGCAGCAGCTGGAGGAGCAGCGCCGCCAGGACATCATATCGGCCCGCCAA
GCAATGGCAGCCAAGTTGCCGTCGGAGCCGGCGCCGGGCGCGGCGGCCGTGGCGCTGCTCATCCGGCTGCCGG
GCGGCGAGCGACTCACGCGGCGGTTCCTGCTCGATCACACCACACAGGATCTGTACGA
CTTTGTGTTCAGTCACCCTCAAGCTCCTGAAGAATTCGAAATCACTACTAACTTCCCCAAACGTATAATAGAG
CGTGGCAACTCAAATTTAACAGCTGTCGGTCTTAAAGACCGAGACGTACTCTTCGTCAATGACGTCAATGCGT
AGATGTATG
>126037141_2 EL596935.1
GCACGAGGGAACTAGTCTCGAGTTTATTTTCATTCATTTATTTATTTTCTATTGCTATTCAAACATTTAAGTT
TGTATATTGTTGTCTTATAAAGTATATATTTATATTTAACAAAGCCAATTGTAATTCATTATAGGCGATAATA
TTTTAAATACTAACTTTTTCGTAAAGCAAATATTACTCGTTACGGTGTACAATCACCT
AAATATGTTAATTTATAATAATTAGTTAGAAATGTATAAATATATATGTATAGGCATATTTATTCTACGTTGT
AAGATTTTATTTTTATATATTATTTTAAATATCTTGTCATTTTATGTAATAA
>126037146_2 EL596940.1
GCACGAGGGATTCATATTAACTCCTACATATCTTTATTATTTATTTTAATGAGTACTGTTACTTTAAAAAGTA
AATATTCCCATTGTATATAAATTTATTATCTTTTTCCATCTTTACTACAATAAATAATTACATCTAATTACAT
ACAGTATGTAAGGGTCATAGATAAAAGAGACCTTAATATTTATTTATTTGTGTGTATG
AAAATTTGAGAGTTTGAGATTTAGTATTGAATAGCGTATGACTGTAGGTGTTGGTGGTGGGTTCTTTCATGTA
CACTCAACGAAAATGAATATTTTTACTTCTTCGGTCTCATAAGATATAGCGAGGCATTGAGCTCCGCCACTCA
ACACGCAGTATCTTGATTTCTGGCGGTGAAAAAGTGTCTTGACACTAATTTGCCCCTG
CTTAAACAGGGTGGAGT
>126037148_2 EL596942.1
CTTTTAAAACATTGTTTGGGCATAAATTCGATACACACATATTTATTGTATATTTCGGTAGAAAATGGTGACA
TAACTTTTATGCGAGACTGCAAAAGCTTAAATTTAATGTTTGAAAAAACTAAATTCGAATAAATTCTTCTATT
TAAATTATTAAGAAAAGATATTTTTGGATTTTCTAAATTTGACGAATCATTTGTCATT
>126037151_2 EL596945.1

CGGCACGAGGAAGCAGTGTACTAAATTTTTTGATATACCAACGTGGCCATCCCGACGATTACAACGATTGGGT
TAAAATGGGTAATGAAGGTTGGAGTTATGATGAAATTCTGCCATATTTCAAAAAATCTGAAAATATGAAAATA
AATGAATTACGCAATTCCACTTTTCATGGAATTGGTGGATATTTAGATATAGATCATG
CAACGTTTAACTCACCTTTAAATGAAGCTTTCAAAAGTGCTGGCGAAGAACTTGGCTATGAATGGAATGATCC
AAATGGAAGAAATGTGATTGGATTTTCAAAACCTCAAGCGACTATTAGAAATGGTAGACGATGTAGCACGTCT
AAGGCATTTTTAGAGCCAGTAAGGAAGAGAAAAAATTTAAAAGTATCAAAATATTCTA
CGGTAACAAAAATTCTCATTGATCCTCTCTCTAAAATAGCTAATGGCGTAGAATTTATTAAAAACAATCGAAG
GAGAAAAATATTTGCTCGTCGCGAAATCATTTTAGCAGCAGGGACCGTTGGGTCTGCGCAACTTCTGATGCTC
TCTGGGGTAGGGCCAAAGGAACATTTACAAAACCTTGGCATCAAAACTCTAGCAGATC
TGCCTGTAGGATACAATCTACAGGATCACATAACTTTCTCCGGAAACGCATTTATTGTGAATACCACTGGACT
TTGTATAAACGATATTACAGCAGCGTCACCAGCAGCATC
>126037152_2 EL596946.1
GGTACAATAAGAAAAAATTTAAGACATGTTATTATGCCTTATTCTGAAAGTAGTATGCTAATTTAATAATTTTA
ATGAATATTTACCAAAACGTAAAAACATTATTCAATAGATTATAAACACCCAAAAAAGAGATCCATCCATCTG
TAATGGTTGACATAAAACATATCAATGTGCAAAAAATGATAAGATTTATTATTCTTTA
T
>126037161_2 EL596955.1
GCACGAGGGTATTTCTGTGAAATGAGAATATATTATTTTTAAGATATAATTATGTGAATACAAATATCTAGGA
TCAATACGTATTTTTTTTATAATCTAAATATGAAGTCTTTTAAATTAATTGTTCATCAGTCTAGTTTTAGCAC
GGAAGACCTCATTATAAATCTTAAAGATTACACAGGGCTGAAAGAAAAAGATATAGTA
GAAATCTATCACCCAGAAAATGATTATCCACGACTACTCTTACAAGTTACAAAAGTCGATGCTCCGGGGCGAG
GAAGAGATACAATCAGTGTAGAACAAAGCATAGCTACTACATTCCAGCTACGGACATTTGCTGATGTTTATGT
CAATATTGTGAATGTGGCAGATGTAGCTCTAGATTCTGTGGAACTTACATTTAAAGAT
CAATATTTAGGCAGATCTGAAATGTGGAGACTGAAGAACCATTTAGTGAGCACTTGTGTATATCTTAACAAGA
AGATTGAATATTGTGGTGGTGCAGTAAGATGTCAGGTATATGAAATGTGGTCACAAGGTGATAGAGTCGCTTG
TGGTGTAATTACAGAGGACACAAAAATTGTATTCAGGTCTTCCACTTCCATGGTATAT
CTTTTCATTCAAATGTCTTCAGAGATGTGGGATTTTGATATACATGGTGACTTGTATTTTGAAAAG
>126037165_2 EL596959.1
GCACGAGGGTTTTCCATTAATTTTATTTGCAGTTCATGGTTGAGGTAACGGCGTTGTTCTAGAATGACAATGG
AAAGGAGGATAAAGCTAAAGACACTAAACAGCCACATAACTTGCAAGATTTGCCGCGGATACTTCATAGACGC
GACCACCGTCACCGAGTGCCTTCATACGTTTTGTAAAAGCTGTTTT
>126037168_2 EL596962.1
GCACGAGGCGAGTGCCCGTGCTTTGAACGTCTTTACAAAGTTTGTACTTCAACGTTCGTATTTAAAAGTTGTG
CTATATAGGTAATTAGAGTTAAACCTTTCGTTAAATATTTTTGAATAAGTTCTCAAAGTTTGGCCTCTATACC
AAGGTCTTCACAAATTGCACACCACAACCCCAAATTGGAATATAAAAATAAACAAAAT
AAGACAAGATAAAATATGTTTTACACAAGTCCATTACCGTGACGCTACCATTCAGTTCAAGCTATCAGTGTCA
GTGACAGTTATAAGCAAAAGTGAACATGCGTGTGTGGTGGTTATGTGCCGTTGTAGTCTTTGTGTACGGACTG
ACCGCGAGTGATGCACTGCCTCCTGCGGCGTTAGGTGACGTCGTGGCGAATGCAGCGA
GCAGCTTGAACTCTATGAAGGTAACGGGAGCATGGCGGAGACTATCAAGTACTGTGAGCGAGTCGGTCGGCTT
GAAGCATGCGTTCCGACGACTCCAGGCGGTGCCAGCCCGAGCCGCTCGTTTGGTCCACGCCTTAGTAGACCGT
ATGCGAGTAGGTGGTGGACCCATTATTGCAACCAAGTTTGGATCTTTACGTGGTCGCA
GGGTCGTGGCGAGGACCAGTGCTCAAACACCGTATTATAGCTTTAAAGGCATAAGGTACGCTCAACCACCCCC
GAGTCCATTACGTTTT
>126037174_2 EL596968.1
GCACGAGGCGCATTCGTCGCGTGCGTTGTCGGGATTCTTTGTGCCATTCTTCGCATACCAATAAGAGTATATT
CTTTAAATTCACTCTTAAATTGACATTTTATCAAAATTCGCCCCTTCACTACACCTTCGAAGCTCGAAATAGG
TGTAAGACTCCAAATGCGGGGGACGGCGTGACAGCATCTTCACGATGCACCAATGCTT
ATTTACGCTGACACTTCTCTGTATAACTGTCAAAGATATATCAGGTAGCTGGGAGGAATGGTGGACGTACGAT
GGCATATCAGGTCCGGGATTCTGGGGTCTCATCAACCCGCAATGGAGTATGTGCAACAAGGGACGACGACAGA
GTCCAGTCAACATAGAACCAGACAAACTGTTATTCGACCCGTGGTTACGAGAGATACA
GTTTGATAAACATAAGGTCAGTGGTGTCCTTCAAAATACTGGGCAATCACTAGTATTTCGTGTGGAGAAGGAC
AGTAAGCACCAAGTGAATATAAGCGGGGGCCCGCTATCCTACAGATATCAATTCGAAGAAATATACTTCCACT
ATGGACTTGACGACAATAGAGGATCTGAACATCAGATAGACCATCATACGTTTCCTGG

TGAAATTCAACTATATGGGTTCAATAAAGAGCTGTACCACAATATGTCAGAAGCCCAGCATAAGTCACAAGGC
GTCGTAAGCATCTCGCTAATGGTCCAAATT
>126037176_2 EL596970.1
ATGAAAAATAATTATAATTACTTTATTTATTAAAATTAGCATAATTTGGAGATTATAATTCATTTTTATAATT
TAAATATTATAGAAATTATTTACGTTATGTATACTTTAGAACCGTAACACTTAATCACCCGCATGCATCAGCC
TCTGCCATTTTATTTTTGGATTAATATTATTGCTAATATTCTCCACCTAGTTCTTGAT
TAAATGATGAAGAAGAATAATGTGGGTGGGTTCGTTGACTGTATAAGAAGAACTTGAACTATGCATTTGGTGT
TTACGGTATGTTATATACATACATTCTCATTATACATATTCTCTCTGATATAAACTTAACAAAATAGTTAATT
TCCCAATAATTTTTAACCTTATCGTTACAACAATTAGTAAATTCCTTGATAACCAACA
TCCTTGTCTAATCTCACAACGCATTCGTGGCGGTCTGCGGCCTGCGGTCTGCGGCCTGCGGTCCTCACGCTCA
CTAGTTAGTGCCTTTATATTTAGCTACAAACAAATACAATAAACAAGTAATATTTATACTACTCGCGAGCACT
CACCTACGCTACAGCGACTCGGGGTGTGTTTGAGGACACTGACACTGACACTGACAGA
ACTGCTTATACTTGGTAAATATATATTACATAAAAA
>126037181_2 EL596975.1
GCACGAGGTATTACATTCATATTTATACTTTTAATTTAACCAAACCTTAGTATTATTTTAATCTCATTTCAAT
AACAATATAAAGCTAAAAATGTCTGATAGTGCAGGATCAGCTCCGCCCTCTGCAGCACCTTCGGCCGCTAGCG
TGGCAGAAGAACAACGTTCAGGAACAGCCACTGGTTCAGGGTCAGAAGAAAAAGTGTT
TGGAGGATGTGAAGGTCCAGATGCCATGTACGTAAAACTGGTATCTTCAGATGGTCACGAGTTTATTGTGAAG
CGCGAACATGCACTCACCTCTGGCACAATCAAGGCTATGTTGAGCGGACCCGGACAGTTTGCTGAGAATGAGG
CTAATGAAGTTAACTTTAGGGAGATACCATCCCACGTTTTACAGAAAGTATGCATGTA
TTTCACATACAAAGTGCGCTACACAAACTCCTCCACTGAGATCCCTGAGTTCCCTATCGCACCAGAAATCGCT
TTGGAGCTCCTGATGGCGGCAAATTTCCTTGATTGTTAAATATTTTCAATAAAAAAATAGATACTATATTTGT
TTTTTATAATTTTTCTTTAGAAACTTGAAATGTAAAACGAAATAATACTTACAAATAC
ATAATAAGTTGAGACTTTATAC
>126037182_2 EL596976.1
GCACGAGGCTTTCAACAGCAGCTGCCTAGTGGCGATCCGACGCGCGTTATTTCCGAGATGTGTTCAGCCGCCG
GCCTCTCGTGCAAGGTGGACCCCACGCTCGCCTCCGCTCTCCGACAACATAAGGCTGAGCTGGAAGAGGAGGA
GCATCTAATTGTCTGCTTACTTATGGTATTCGTAGCAGTTTCTCTTCCAAGACTCGCT
CGTAGCGAAGGCTCATTTTACAGGCCGTCTTTGGAAGGGCACGCGAATAATATTCATTGCATGGCACCTGCAA
TTAATCATATTTTCGGAGCTTTGTTCACGATTTGTGGACAGGGCGACATCGAGGATAGAATGAAGGAATTTTT
AGCACTGGCTTCTTCGTCTCTATTGCGACTTGGACAAGAGACGGAGAAAGAAGCTATA
AAGAACCGCGAATCTGTTTATTTACTACTCGATCTTATAGTACAAGAGTCACCTTTCCTTACCATGGACTTGT
TGGAATCGTGCTTCCCTTACGTGCTGATTCGAAATGCTTATCATGAAGTATACAAACAAGAACAAATGCTGCT
GCACTCTTAACTCTGTTCAAAAGACATTAATTAAGTAGTCATAGTAATAATAACGAAG
TCATTATTATTAATGACAAATTCCATTGTATTAATATTGTAACTAATTATTATTATTATT
>126037197_2 EL596991.1
GCACGAGGTACAATTCAACTTAGCCAATACTGTGGCTATAAAGAACACTGTACCTCTTGAACGGTATACTTTG
ACTTGCTTGGAATCCGTTCCAAATAATTTAAATTAAATAATAAGACAATAGAGGCCATCTCTGGCAAAATTTT
ATACAGTAGTAAAAATAAAAGTAACAATGATGTAAAAATAAATACTATTTGTCATGAT
AATGATACAAACTTGGTGCATCAAAACATACAAGGGATGTTAAATAAAGACTTAGAAATTGAGCTATTCTTAA
ATACTAATAATATTCATGTACTTTGTATTACAGAGCACTGGCTTCGAAGCTCTGAAGCTATATTTAATTTTAA
TAATCACCAAGTAGCTAGTATGTTCAACAGAGAAAAAGCAATTCGCGGTGGCTCCCTA
ATAATTTTGAATAAATCTTTCAAGTTTAAAGAACGTAAAGACATTGTGAGCCTCTCTGTTGAGCAGACTATTG
AAATGGCCTGTGCTGAGCTAGAGCATTTCATTGTTGTAAGTGTATACAGACCTCCCACTGGGTTATATGAACA
CTTTGAAATAATTTTAGAACAGGTTCTTGTAAAATTATCTGGAT
>126037199_2 EL596993.1
GCACGAGGGTCGAAAAGACACCCACTCTGTGTTCAAGTGTTAAATATTCAGTGATAAATTTTTAATTGAAAAT
GGCACCTATAGATGGGTCTTTTGACTATGATTTGGCCGTCATCGGTGGTGGGTCTGGAGGCCTGGCCTGTGCT
AAGGAGGCAATTAATCAAGGGGCAAAAGTTGCTGTATTAGATTATGTTCAGCCATCAC
CACAAGGATCAAAATGGGGTCTTGGAGGCACTTGTGTTAATGTTGGATGTATTCCTAAGAAGCTGATGCATCA
AGCTGCATTACTAGGGGAAGGTATTCATGAAGCCGTCGCGTACGGGTGGGAGGTACCTTCACTGCAGCAAGTC
AAAATAAACTGGGCAGAACTAACACAAGCCGTACAAAATCACATCAAATCAGTCAACT

GGGTTACTCGGGTCGACTTGAGGGAAAAGCAGATTGAGTACATCAATGGACTTGGTGAATTCAAAGATCCTCA
CACAATTATTGCAACTATGAAGAATGGGAGCAAGAAGGAACTTACGGCTAGGAATATAGTCATAGCTGTCGGT
GGAAGACCTCATTATCCAGATATTCCTGGAGCCAAGGAGTACTGTATCAGCAGTGACG
ATATTTTCAGCTTGAGCAATCCTCCAGGCAAGACCCTCGTCGTTGGTGCTGGATATATCGGCCTGGAATGCGC
TGGTTTCCTCAACTCTCTAGGCTTCTCAGCCACAGTGCTCGTCAGGTCTGTTCCTC
>126037202_2 EL596996.1
GCACGAGGATGGCCCAGCGCCTGGCGTCACCGTCCCGTCAACCCCGCTCAGTGTCCCGCCTGCACCCCGTGGC
TCGTCCGCTCAGCGTTAAACGCAGCCAGCGGTGCCGCGCCTGCGACCACAACCTCACCAAGCCGGAGTACAAC
CCTGGATCTGTCAAGTTCAAGATTGAGTTGCTTGCATACTACCATGTACCAGAAGTAA
AAATAATATCATACGAGACACTAAAACCCGGCGTTACTTCAGCCCTTCTGTTGAAGCTAACCAATCCTACAGT
ACACGAAATACAAGTGAAGCTATTAAAGCCCGAGGATTTACCAGAACAGCCTCAAACTGAAGAAGAGGCTCAG
ACCATCGAGAAGTCTTTGGAGAAGTCTCTTAATTTGGAGAAGGACATGTCCTGGACCA
AAGCGCTGCAGCGCCAGCCTCTGTTGCCGTCCAGCACGGCGCTGTGCGCGCCCGACCTCGCGCTCACGCTGGC
GCAGCGCGACGACGCGGCCGAGTACGACGACGACCCGCAGCAGGATAGTAGCGACGTAATCCTCTGGAGGAAA
TCCAACAAGTTAGCACTCCGACTGAAAGTGCAGACGGATCAAAACGCCGTAGTAAGGG
AGGCCGCGCGGGTCGCCCTCTCGCTGCAGTACTCGTTCAGGAACACGGTGGCGCCCTCCGCCACCGCGCCGCA
ACGCCGCGACTACACGCTCTACACTACCGTGTTGCTGGAACTGG
>126037212_2 EL597006.1
CGGCACGAGGTTAAATTTTAAGTTACATGTGATATGACGAATTTCATTATCTCAAAAAATCATATTTAATAAA
TAAAAGTGTCAATTCAACTGAAAATCTCGAAGTACATTTGCAATTTATAAACATGGAAGGGATTTTGTGGAAA
TGGACAAATTACTGGAATGGTTGGCAAACCAGATGGTTCGTTTTAGACAATGGCATAT
TATCGTACTATAAATCGCAAGAAGAAGCAAATCAGGGATGCAAAGGATCTGTAAAAGTATCTGTTTGCCAAAT
AAATGTCAATAACATTGACAACACTCGACTAGATTTAGTTATACCTGGACAACAACATATGTATCTTAGAGCT
CCATCTCCTCAAGATAGACAAAAATGGCTCGTAGCTTTAGGAAGTGCTAAAGCTTGTG
TTGAT
>126037219_2 EL597013.1
GCACGAGGGACTCCGACTCGAGTTCAGATGATTCTAGTGATAGTGATAGTGATGTAGATTCTGTTAAAGACAT
CGAGGAATTTTCTAGCGGTGATGAACTGGAAAGTAATAATCGTTCTGGAAAACTAGAGCCTCCTAAAGTACAT
GGTGAATTGGGTCTTGATGACTTACCGCCTATAGAAGATCTTGCCATAAGCTTACCGT
CTCAAGAAACTACTAAAATTGGAACCATTACCAGTATAGTAGATAGATTAGTTATAATTCGAGCATATCCGGA
AACACCATTAGTAGATCTGGACAGTATTCTGTTTTTGGATAATGGAGCTAAAGCATTGGGAAAAGTGTTTGAT
GTTTTTGGACCTGTAACAGAACCAAACTATTGTGTCCGTTTTAATTCTGAGGATCATG
TCAAAGAGCGAGGTGTTCAGCCTGGTATGGAAGTGTTCATTGCGCCTCGGACCCAACACACTAAATTTGTTTT
TCTCACTGAACTTATGAAAGTCAAAGGATCAGATGCATCATGGCTAAACGATATAGAACCTCCGCCAAGCCAC
GTCGACTTCTCTGACGATGAGGAAGAGAGGCGCGCTAACAAAGCTAAAAAGGAACAAA
AACAAAACAAGCAAGATACATCAGAAAATGGTGAGAATTCCAAAACCCCTAGAAAAATGCTTGAAAGCAAACG
TAATCAAAGACCATCGG
>126037222_2 EL597016.1
GCACGAGGCGCGCACCTCGCGTCTCGTTGAACTAAGAGAAACTATTCAACATGATGTTCAGCTACTTCATTAC
GGTACTCTCCGTGGTGGCCTACACCCAGGCGTACTCTTCAGGGGCCCCTGAAAGCGCTTGCCAAGATATGATC
CCGCGTCACCCTGTTGAGCCCCAAAAGCACCCAGCTCCGTACATAATTACTACTAGCA
CTAAGGAAGTCAAGGCGGGCAGCCCCATGGAAGTCACTATATCTGGTAAAAGCTCTTCCGACACAATCCGCGG
TCTGATGCTGCAGGCGCGTGCAGGAGAAAAGGGAGACAAAATTGTTGGTGTCTTCAGCAAAGTGTCACCCAAC
GATCCTCTTGTCAGGACGATGAAGTGTAACGTTGATGGGGACACAATCACCCACAAGA
AACACGAGCACAAGGACGACAAGCAAACCGTCTCTTTCGTTTGGACCCCTCCTGCTGACCTCAATGACATTAT
CAAATTCAGAGCCACTATTGCCCTTAACGGTGCAGTCTTCTGGGTCGGCGTGGAGTCTGCTCCAGTGAAAGTA
TACCAATAATAAATTATAATAAT
>126037224_2 EL597018.1
ATTTGAAAGCTAGTGCTTTACGTGTGCTCCCATTTATTTTTTGTCAATTTCTGATAATGGCATCTATGAGAAA
ATCACACGTCTTAAATTTGAATTAGGTTTGTGCGCGACAAATAGGCGAATAACTCAATATCACGCCAACCGAT
TCTGATGATTCTTCTTTTATTTGAAAGGGTACACTTTAAGGATAGTTTAGTTAAACTT
TAGTTAGGTTTTGATTATGGGATCCGGTTGAAATAGATTTTTTTTTTTTGTAAAAAATATAATTATACTGTAA
TAGTTACAAGGAAAAACATTGTTAAAATTGAAATTTCTCATCGTTGCGTTCG
>126037225_2 EL597019.1

CGGCACGAGGCTTTTTACTTGGAACCATCTGTCATTGTAGTCATTACTGATGGTGGAAAACTGACAAGCAATG
CAGGAATTCAGGAAGAATTTAATCTACCTATGAACTGTCCTATACCTGGATCTGAGTTAACAAGGGAACCATT
CAGATGGGATCAGAGGTTATTCTCCTTGGTGCTTAGGCTGGCTGGCACTCCTGCTGTT
GAAAGAGATACTGGACTTGTACCGTCTGATTCATCACCTATAGATGCAATGTGTGAAGTTACCGGAGGTCGTT
CCTATTGCATAACATCACATCGTATGCTAATGCAGTGTATCGACAGTCTAGTACAGAAGGTGCAGAGTGGTGT
AGTTATTAACTTCGAGAAAATTGGCCCCGACCCTCCTCCAATAGATGGTGGAAATAAC
AGAGAGGGCGAGGAGGATGTACCAGAAGGCGATAAGGAAATGGATGTCGATGGAGATAGAAATGGACGCTGGA
ATGGAGGACCCTATCAAACAGGAATACCCAATCAAGCGACTGCACCTCAAGCGTGGCATTCATGTCGGCGTCT
CATCTATGTACCGCGTACAGTGTCGCAGAAGGGCTTCGCTGTTGGCTTCTGGCCGATA
CCTGAATCATTCCGACCTGACCCCAATGCACCATCTTTGCCTCCACGATCAGCACATCCTGTAGTAAAATTCA
CATGTTCATCTCAAGAGCCGATGGTGATCGACAATTTACCTTT
>126037233_2 EL597027.1
GCACGAGGTGAGCTTCCACACACGCAATATAATTTTCATAACAGAAAACTGTCAAACTATCATATCGTATACA
AGTGTTACAAGTAATCTCTTCATTGACAACATCTAATGGACTTTAAAGTTGTTATGGACACTCACGTCAAGAA
ATGCTAGCAAGTTTTTCTTCAGTGTTAAATTGTGCTACGTACTTTGTTTCGGAAATGG
TGATATCGACCCACGGGGAGTGTTTATGAGTGGAATCGTTTTATAGTGGAATGAAATATCTGTGATAATGTAT
GCTGTAACGGCTGTGTTGTGTGCGCTCCTGCATGTAGGATGGACTGTGTTGCCCTACGGCTTGGACAAGTGCG
TTCGAGTAAATGACCGGGACTCGGAAAGCAGCGTTATATGTAAAGTTAGAACTTTGGA
TGGCGATGGCAGCAGTATATCTTCAGTACGCCCAGAAACAAGTAGATTAACTATAGAATGTAATCATCTGTTG
TTATTCGAAAGCTCATTAAGAGCACATTATTTTAGTCCAGTGATGGGATTGACAGACTTGAATATAGAGAACT
GCAAACTGTTAACACTTACGGATTATGTTTTCCAAGATTTGAATAAATTAAGACGACT
T
>126037235_2 EL597029.1
GCAGAGGGTCAAAGTTAGTTTTTTTTATTTTCTCTGTAATCTGTGCTGTGCTATACGTTATTGTGCCGAGAAG
AAGGATAAGTCCTAGTTAAATTCCTATTGGATTAACAATTCCAGTGTCGTGAAAAAAAACAACATGGCGTTTT
TAAAACTAGTATTCTCTTTAGGCTTTATACACATGGTAGCTGCTAAAGGTTCAGAAGC
TCAAGTAGAATGTACGCCAGAGTACATGCGAGTAATGGTGCCGATGGATGGTGATAGAAGTATATCTTATCTG
GACCAATTGAAAGACTATTCACCTTGCCAGCCAACTATAGAAGGACCCATGCACACGTACGTGCTCGACCTTG
TCGACTATCACAAGTGCGCTGTTACCCGCGTTCTTAATACTTACACCGGCAAACGGAC
ATACTATCACAAAATCGTGATAGAGGACTCATCAGGAGGCAGAGAGACTGTTAGAGCGCGGTGTGAGGTGACC
GGCAAGACGCGCGCCATCGCAAAACGCGCTGTCGAAGCTTTCCCACTGGATTTTAATGAACCTGATGTATTGA
ACATCACAAGGTACATGGAGGGTCACGCTGGTGATCCTATTTTAGGAGCAGCTGTAAA
GCAAAAT
>126037240_2 EL597034.1
GCACGAGGAATAAAGATTGTTTTCATATTTAAAGCCGAGAATATTTTATCTTAAAATTTAAAAATGTTGGTAT
GCACTCAGTTTTTTTTATTTTCAATAATATTTTTTAAATATGTCATGTACATGTAAAAACACCTAAAAGTCAA
CTTCTTCTCAAACTTCAGACACATTTTAGTACTATAAAAATAAACTATATCTCGTAAT
TAATATTTATATTAGAACATTATTGTTCTAATAAAAAATACAACAGAAGAAGCTTGGTAAAGCTGTAGTCACC
ATGTGATAAAAGAAGAGCAAAAGATGAAAAATAAAATTAATTTAATAGATAATGTGGTTAACCGTTTCGTCAT
TAATGTTTGTGACAGTTCCGCAGAATCCTCCGAATTCTGATAAATAATTGAATAATAG
ATATATTAAGATTTATTATCCTAATAGTTAAGTAACTTTTAGTAAATTATAAAAGACAGATAGCAAGTATGCT
ATCATATTATTTACAATTCAACTTAGCCAATACTGTGGCTATAAAGAACACTGTACCTCTTGAACGGTATACT
TTGACTTGCTTGGAATCCGTTCCAAATAATTTAAATTAAATAATAAGACAATAGAGGC
CATCTCTGGCAAAATTTTATACAGTAGTAAAAAT
>126037241_2 EL597035.1
GCACGAGGCGCGTCCGCCCGTCGGCTGGGCCTCGCTGCAGGTGCCAGATAAAAACCAGTTGGTGAGCGACGCG
GGAAAACTTACAGTATTAGATTCGTTGTTGAGAAGACTGAAAGAGCGCGGTCACCGTGTTCTTATATATAGCC
AGATGACGAAGATGATTGATTTGTTAGAGGAATATATGTGGCATCGTAAACACAAGTA
CATGCGGTTGGATGGCTCCAGTAAAATATCGGCGCGACGAGACATGGTCGCCGATTTTCAGGCTCGCACGGAT
ATCTTCGTGTTCCTACTATCAACGCGCGCCGGCGGTCTCGGTATTAATCTAACTGCTGCTGATACTGTGATAT
TTTACGATTCCGACTGGAACCCAACAGTGGACCAGCAAGCCATGGACCGCGCGCATCG
CCTCGGTCAAACCAAACAAGTGACGGTCTACAGGCTTATTTGCAAGGGCACTATTGAAGAACGGATTATGCAA
AGGGCCAGGGAAAAGAGTGAAATACAAAGGATGGTGATAAGTGGTGGTAATTTCAAGCCTGACACACTTAAAC
CTAAAGAAGTTGTATCACTACTTTTGGATGATGAAGAATTAGAGTTGAAATATCGTCA

AAAG
>126037246_2 EL597040.1
CATTATTGTATTACATAAGTATGTCTATTTAATTTATTTAGTTGTAATGTATTTTATATATTTTTTTGTAAAT
TATCTGTGTATTTTTTTATTTTTTGTTAATCTTTAGTACTTCAAATTTCTTATCAAATGAATCTATATTAAGC
TTAACATAAAACAATATATTTCGTTTTACATATTATATATATCTTCATTTTTTAAATA
ATATTAATTGTTTTACCATTAAAGTATTAATTGAGACATTCCTTTATATTTATCTGCATTTAGTTTTAGC
>126037248_2 EL597042.1
GCACGAGGACAAGACATTCTAATAGTTTGAGTATTGAAACAGAGAAAATATTCAAGTTTAAGTGACTGGATGT
GAATTTATAAGGGGAAATGGCTCTATTTATCAAAATGTATAGTGTAACGTGCTTGGCATTATTGTCAATACTT
ATAAGCCAAGAAGTAAATGCAGCAGCAGTTATATCCATTGATTTCGGATCAGAATGGA
TGAAAGTTGGTATCGTGTCACCTGGTGTCCCTATGGAAATTGTCCTCAATAAGGAATCTAAGAGGAAAACACC
AGCTGTCGTAGCATTTAGAGATGATGTTAGGACATTTGGAGAAGACGCTGTTACAGTAGGCGTGCGATTCCCA
AAGAACTCATACAAATATCTTCTGGACCTCTTAGGAAAACCATATGATCATCCTTTGG
TGCAAGCATACAAAGAAAGATTCCCATACTATGAAATACTACCAACAGACAGGGGGACACCAGAGTTTGTTCA
TGATGACAATACTAAATATACACCTGAAGAGCTTGTTGCACAGTTGTTGGCTAAGGCCAAAGATTTTGCTGAA
ATCAGCCATGGTCAACCAATATCTGAGTGTGTCATAACAATCCCTGGTTACTTCAACC
CAGTTGAGCGTAGAGCTATGAAGGAGGCAGCGGCACTTGCAGGTCT
>126037249_2 EL597043.1
GCACGAGGGACCCCCTTCCCCTGTTGTATTTCCAAACCAAATCTACGAAGGACCTCCCCCTCCGATCGGCAAA
CCAAAATTAATCTACGGACCCCCAAAACTTCATGTCCCGTCCAAGCCCAAACCGATATACGGTCCACCGAAAC
CTGTGTATGGCCCGCCAAAGAAAACATATGGCCCTCCCAAGCAATCGCTTCCAAAGCC
AACCTACGGGGTTCCATTCAAAATTTCCAAAATTCCTCCAAAACCAATATACGGTGTTCCAAAACCAATTTAT
GGGGCCCCAAAACCTGTGTATGGACCACCTAAAGTCACTTACGGGCCACCACTTATAAATGTTCAAAACATAA
AAGTGCCAGAAGTGCCCATTTCTTTCCCTCAAAACTATGCTTCTATAGGTAATATTGG
AACCGGCTTTGGTAGCATCAGTACTATAGGTTCCAATTTTGGTACATTAGAAACCAGTGTTGGGACAAGCATT
GGGACCAATTTAGATTTGGGTTTAAATCTACCAGCTCCTATATACGGAACTCCAATATCTAGTTTACCAGTTA
ATTTGAAACCAAATTTCCCTATACCATCTGATACTTACGGGCCACCCGGCCTCTCTCT
AGGACCTATTGGACCAAATGACCAGATTGTATTACAAGATTTCAGCAATATCGGGCATTATGGACCTCCTCAA
CCTGACCCGAACCCACGTCCACCACACCCAGGTATTCCAGCACCACCA
>126037256_2 EL597050.1
GCACGAGGGTTCAACACTAATTGACAGCATCATGAACACTAAACGCACTACTAAAAGCATAGTTTCCTTTGAC
TGTGAAGGAATTAACTTGGGTTTAAAAGGAGTTTTAACTTTATGTCAAATTGCTACAATGAACGGTGAAGTAT
ATATTCTTGACATTATGGCCTGCCCGGGAATGGTCATGGAGGGTAAACTTAAGGATCT
ATTAGAAAGTGAAAGTGTTATTAAAATTATTCACGACTGTCGGAATGACTCGGTGAATTTACACAACCAATTT
GAAATTACATTAAAAAATGTTTTTGATACTCAAGCAGCACATGCAGTGCTGCAGCTGCAGCAGCAGTGTGTTC
CAGTATACAAAGTTAAGAATCTTAGTCTTAATGCACTCTGCGAGCTGTATAACGCTCC
TATGAATCCAATGAAGGAACAATTGAAAAATGTTTATAGAAGGGACCAGCGGTACTGGGCTCGGAGACCATTG
ACTAAAGACATGATTATTTATGCTGCATCAGATGTGCTTTCTCTTGTAAACTCCGCAATATATGCTTATATGT
CAAGTAACATAAAGCCCGAAAATCAACAGTTATTTGAGGAATTGTCAAATGAACAGGT
CTTTATGCACATTCAGCCAACTGAAGTGAAACTAAAGAAGCGTCAA
>126037261_2 EL597055.1
GCACGAGGATTACTAGGCACTACAGTGTTGGACAGGAAATAGAAATAGAAATAGAATTAACGGCGAATCACCT
CGGTACCTTTGTGCTGAAGCTCTGTCCGAACAATAACCCCAAGCAAGAAGCCACTCAAGAGTGCTTTGACAGG
TATCCTCTATACATTTCTGGCACTCGCGAAGACAGGTTTTGGATTCCTCTAGATACGG
CGAAGAAGGACACTTTCAGATATAGAGTTAGACTACCTCCGTACGTCACTTGCACGCAATGCGTTCTGCAATG
GACCTATTATACTGGTAACATGTGGGGCATATGTCCAAACGGCACAGAAGCAGTTGGTTGTGGTCGTTCGGAG
ACATTCAGGAACTGCGCTGATGTGAGTGTGATTACCAGTACTGGTGGGCTTCCTCCTG
CCTTCGCTGGAGACCTTCGACGTGACAATCCCTTCTTACTCTACTATAGAGACTACAGTATGCCGCAAAATGT
ATATCCTCTTGTCGTCAGAGATCAAGTTTGTGTACCGTCTGAAGGATTCCGCCTCATCCCCGGCATGTTGAGC
TGGTGCGAGACTAATTGCTTACGGTACCCTCCCAACTGCCCCGAANATCTCTGCCACT
GCCCGCAAGTTTGTGAAGCAATTGGAGAGTTGGAAGGTAGAGAAGGGGCCGACGTGTATTGTATGGACCAGTG
CATCGTGTACCCACCACGCTGCCCTAAGCACAGATGTCGCTGTTACT
>126037262_2 EL597056.1

GCAGAGGACGGTATACACTGTGGTTCCTTGTTTATCACGCTGACATGTGAACCAGGTAGGTAGCTTAGTCGAA
TCCTATAGATTTTTTAGATTTACTTTAAAAATCATGTCAACAATATAACAATTCGAACGTTTCTAGCGGCTGA
GCCCAAATAATAATATACATTTAATTTCATAATAAATTTATTAAGTTACCCCTTTTTT
TTCTTATTTTTAATAGGCAATTATACAATTGTAGTCAAACCATCTTATCTTATTATTGAATAATAATTTTAAG
AAAATCTTATGAAATGGTGCATAGATTTTATTTGCCATTTTTTTTAGTAACATGCGACATCACATTCCATTTA
TGTACAAAATCTGTTTGATTAGCTTCAGTTTTGTGCTGTCCGTACATTTTGGGCTAAC
TTAATATGACCAATGCGTACTCAGGTTGGCCCAACTTACTAGTAATTGTCGCATACTGTAGATTTTTAACTGG
TTTATTATAATTCTATATTGTTGTAAACCACAGTAAAACGGTAGTTAAACCACTGTTTGTCTTTTCGGTATGC
GGCGTTTTTTTTTAAATAGCTCATTCCTACCTTTTGTCCATAGTCAGCATTTATGATT
TGGAATTGAAAATAAATTTATAAATATTTGTTTTCATA
>126037263_2 EL597057.1
GCACGAGGCACACTGTCATGCGTGACATAAGACTTATATGTAATATAAATTGTGAAACAAAAGTAAAAGGCCT
AATTCAATATAGACATTAGATATTATAAAAATACAAGGACAAAACATCGTTGTCATTGCTTGAAATAATTTGT
ATTCAGGATGTGTGGAGGGTTTACTTGTTCAAAAAATGCTTTAATAGCATTAAATATT
CTTTATGTTGTGGTAGCTTCTATCCTTATATCAGTTGCGGTGTACGGGCAGGCATCGTCGTTAGTGACCAATC
TCCCCATAGTCGGAGGAATTTTAGCATGTGGTGTATTTCTAATATTAATATCGCTTTTGGGACTAGCTGGAGC
TGTAAAACATCATCAAGTGATGTTGTTTTTTTATATGGTCATACTTTTCCTACTATTT
TTAGTGCAATTTTCTGTTGCATGTGCTTGTTTAGCTGTAAATTCTGAACAACAAGAGATGCTGGCTCAACAGG
GCTGGAATAAAGTAAATATGGATGTAAAAGAGCAAGTACAAGAGAGATTTCAATGTTGTGGATTCCAGAGTAG
AATAATGCCAAATAATGCTACAATTGATTATCCATCTTGCAACTTGGTTGATAGAGTA
TGCTGTAAAAATATGCCCATATCTGTAGAATGGTCATGTCAGCCATGTATGGAAAATCTTAAACAAACAATTG
ACTATGCATTTAAAC
>126037270_2 EL597064.1
GCACGAGGTTTCATTCTACTAGTGCATTGCGTTCATATAGCGTCGGCGCAAATTCAGCGTGACGCCATTTTGA
TTTGTGATCGTGTTAATTTTATTGTGGATTTCTTTATTAATTTGATCATAATTCCTCACCAAAATGAGTTACG
GAAGACCACCGCCGCGGATTGACGGCATGGTCTCACTAAAAGTGGACAATTTAACGTA
CCGTACAACGCCAGAAGATTTACGTCGGGTTTTCGAAAGGTGTGGAGATGTTGGTGATATATATATCCCAAGA
GACAGATATACACGAGAAAGCAGAGGATTCGCTTTTGTCAGATTTTACGATAGGCGAGATGCTGAAGAGGCTT
TAGATTCTCTCGACGGCCGCATGTTGGATGGAAGGGAGCTTCGTGTACAAATGGCACG
ATATGGCCGACCATCGTCACCATACAGAAGTCGATATGATCGCCGCCGCAGCCACTCGCGTTCACGGTCTCGT
TCACGTCGTCGATCCAGGTCTCGTTCGCGGAAGCGTTCGTACTCCAGAAGCCGTTCCCGTTCTCGCTCCAGGT
CCAGGTCTCGCTCGGACTCGAAGAGCTCTCGCGGCCGCTCCCGTAGCCGCAGCCGCAG
CCGTTCACGCTCAAGACATTTGAATTCTGGGCAAAAGTACCACTGAGAGCT
>126037271_2 EL597065.1
CACGAGGGGAAGGCACGCGTCCGCGTCTCCTCCGTTCATACGTCGTCCGTACGCCGCAATGTAAACATTGATC
AGTGAAGTGTATAACAAACCAGTGAATTCGAACAAAATTTGATACAGTGTAGAAGTGAATGAGCACAACTTTG
AAATTGATTAAAGACAAATGTATATAGAAAATGTTTCAATTGGTGACTCTTTTGTTAG
TGACTTTGGGTGTGATTGCCGCGTATATTCCGAAAGGCTTAGATAATTGTAGAAAAGATTTAAATGGTGATAA
AGGGACTACTTTGTGTCACGTGCGAACTCTTGAATCCAATGCATCCAGTCTCGCATCTGCACCTTCGGAGAAT
ACAAAACGTTTGACCGTTGAGTGCAACCAGCTGCTACTGTTTGAAAGTTTTCTTGAGG
CAAACTATTTTCAAAGATTTTCAACGTTAGAAGAGCTCACCCTTCTCAACTGCAAACTACTTCGAGTACCAGG
CAACGCTTTCGAAGGATTATCCAAGCTTAAGGAATTATCAATAAGATCTAAAAACTTTGACTGGAGTCAACAT
AAAAACTTAGAACTTTCACTCGGCACTTTTAACGGCTTGCGTGAATTGCAGACACTTG
ACTTAGGAGAAAATAATCTCAAAGTTATACCATCTGATGTGTTTTGTCCTTT
>126037274_2 EL597068.1
GCACGAGGATTTTAATTTTTTAATTTTAAGCATTTGTTTATGTTTTACTATCCTAAATTAACCAAAACAACAA
TAGAATTTTAATCATTGTATAAATTCTATATGGTGTGTTAAATAATACAAGAAACAGTTTATAATATTAGACT
CCAAAAATGGGTAAGGATGATGTTTCTAACAAGAAAAGACGCAAGATAAATACAAGCT
CAACGGTCGATAATACAAAAAATTCACCAATTGAAATATATAAGAAAGTAGTAGAATTTGAGGAATCTGAAGC
TCAATTACGTTCTGCAGAAAAAGATGCAGTTTTATTTAAAAAGATATGTCAAGATCTAAGGCAGTTATTTGCT
GATATTGCTGAATTAAAAGTTAAAGCAACAGAAGAGGCAAAAGAAAAAATAAAAGCAA
AGAGAATAGAGGCATCATTGCATCTTGTAGCATTGAAAAAACTTAATCGTTTAGAAAAAGTTCGAACACGCGG
GGGACGTGATGCATTACATAAGGAGAAACAAAGAGTAGACTCTACACACTTGTTGTTGCAAAATCTCCTATAT
GAAGCTGATCATTTGAATAAAGAAGTAACCAAGTGTTTGCAATTTAAATCAAAAGATG

AGGAAATTGAATTAGTACCATTAGAAGAATTTTATAAAAATGCTCCAAGTGATATTTCACGTTC
>126037279_2 EL597073.1
GCACGAGGAATTTATTATGCAGCGAGAATACAATATAAAAAAAAATACAATTGCAGTATAGAATACTTCTTTC
CAAAATAAACACTTATAAATAGATATTAAAAAAGAGAAAATTATTATGTAAAAATACTGTCACCACAATGTTA
TACGTACATACGTCATGTAGCACCGTACAAATTTTATTTTTATACTTTCGTATTTATT
ATGCTCAGTAATAAGGCACATTTTACATATAAAATGCTACCATTGTACTTATTAAGTTAGGGCTCATCTTTAT
TGCTCGTTAGCTATCGTTATACAGCATTCGGGGAGTCCCCTAATACGGGCTATTGACTAATGAGAGTAGACTA
TCTACATACGGCATGCGGGTCTTCCCCGCGTTTTCACCACAATGTTTTACGCACATAC
GGTGCGTAAACGGAAATCTCCTTCAGCCTGTATACGTGATGTAACGACCAATGGAAATGTGCTCTTATTTATA
AAGGAATGCTGCTTTATCATCATATATAGAGGGATTCTTAAATGTGGCGTTAACATTAATTCAATAAAATTAA
AAGGAGCATCCTTATTATTATTGTATGTTAATGGAGTGACAATATATTTTATTTTCAT
TAGCCTTATTT
>126037281_2 EL597075.1
GCACGAGGCAAATATAAACGTAAACTACTTGTCGCCGACTTTGTAGTTTGTTTATTAAATCATTTATTTAGGC
TTTATAATATTATAAGTTTGGAAACTGTGATTTACAATGAGCATTGACGTGAACAGAAAACGTAAAATTAATG
AAGAAAATGGCAATGGATCTTCAAGATCCGCAGCCAAAGTTCTCATTCAGTGTCCGTA
CTTGGATACCATAAATCGTCATGTTTTAGATTTTGATTTTGAAAAACTATGTTCAATTTCTCTGACAAGAATC
AATGTTTATGCATGTTTAGTATGTGGCAAATACTTTCAGGGTCGGGGTACTAATACACATGCTTACACACATT
CAGTGGCCGAAGGACATCATGTCTTTCTGAATTTACACACATTGAAATTTTATTGTTT
ACCAGATAACTACGAAGTTATTGATTCCTCTTTAAATGACATAAAATATGTTTTGAATCCCATTTTCACACCA
GAACAAATAAAACAATTAGATGAGAATACTAAAATGTCAAGAGCTATTGATGGTACTATGTACATGCCAGGTA
TTGTGGGTCTTAACAACATTAAAGCTAATGACTACTGCAATGTTATATTACAGTGTTT
ATCTCAAGTGAGACCTCTTCGTAATTACTTCTTGA
>126037284_2 EL597078.1
GCACGAGGCAGAAACTCAGTTGGCTTATTTTAGTCAGAAAAGGGAAAGCAAAGATGTTAAACTACAAAAACTA
GAACCAACTGGCTTACAAGGTTCAGTTCCATCTTTCATAACTCGAGCAGAGCCAACGTTGGCCGGTATGCTTA
AAACTATGGGAGGTGATTTTCATAAAACTCAGGATTCCTTGTACCCAATGCTATGCCA
AATTGTTGGGGGAACTACATTAGATACTATACTTGCTGTTGCCACAGTAGAGGGAAAACTAAAATTATTTGCA
TCAAGATTAATTAAATTTAATGAATTCGCTCTCCTGACAGCCAGTGAAAAAGGAGCTTCACAATCAAAAGTCT
ACATATTCGATATATCATTTTTAATATTGTGCTCTATTGTACAAGAATATGGAGCTGA
AGCTGTTCTTGATGAAAATGGAGATTCATTTTTTGAACTGTGGGTTAAAGACTGTATGGCACACAAAGGTGTA
TATAAATCACCTGATCATATTTTACAGAAATGTGATTCTCAATTAGTAGACATTATTATTCATCACCTCAGTG
CTTCAGATTTTGACTTTAAAAATACTAATTTAAAACCACATGATTTGTGCACAAATGT
GGCAGGTGTTGTGAAAGAGATTTTATTTGCATGGGAACAAGGTTCAATTTCCTCTA
>126037286_2 EL597080.1
GCACGAGGAATTAAAAAAACACAAGTTACGGTTTTTAGAAAAATGTTTTAAATTATTTAGCTTTAATTGGTAC
TGATTGGCCTAGCAAATTTACATATTATATAAATTTTATTAAGTAATTATTAAAGTATCAATGAATTCTAATT
TTACTATCTAAATATTATTAAACATTGTTTTTTAGAACTAAAATAAAAATGGCAGCTC
GAGTAAAAAAAATACTTTTGATTTGACATATTGGCAACCAATTAAGGTTTACTGACCTTCATTATTAATTAAC
CTGTTACTACAGTATTAAGGGAGAATAGTCAGTTTTGTCAGTACAACAAGAACTTACTATTAAAAAGTATCAA
TAAATACTTTAAAGCTAATATTCAAATTATTACTTTTATTGTAATGACTGATGTCTTA
ATTAGGGCTTATTATGTATACTGCATAGTAATATGGAGATGAGTATAAAAATAATTTCTTATTGGAATTTTAT
ATTTCTTTCAAATTGTTATTGTATTTTATTAAAAAATCTAGTTTCAAATCCTGCTGATATACAGGGTGGAAGA
GGCAGCGCTCCTTTTACGGAAACAGCGTATAGGGTAAAATATTTTAAACCTATACCTA
AAGAAATTA
>126037287_2 EL597081.1
GCACGAGGCAGTTATTCGTGTTTTCGTGGGAACAGTGTAAATTTCGTATAAAAATAACATTTCATTACAATTA
TTGTTAGATACACTATCTGAATTATTCATTTTTACGTTGCACATTTATAAAATGTCCCCCCACCGTGAGCCCA
CTGTATATGGTGTCACCAACTTCAACGCAGCAGAAGATGCAGCTACCCTTAGAGCAGC
AATGAAAGGCTTTGGAACTGATGAGCAGGCTATTATTGATATATTGACTACACGGTCTAATGCCCAGCGTCAA
GCTATTTCTCAGGCATTCACTCATGAGTATGGCAGGAACATAATAGAAGATCTAAAATCTGAGCTTGGTGGTC
ATTTTGAAGATGTGATAGTAGCACTGATGCTACCACCAGCAGAGTATCTATGCAAAGA

GTTGAACCACTGTATGGAGGGCATGGGCACCGATGAGGAGGTCCTCGTGGAGATACTGTGCACTCGCACCAAG
CCAGAGATAGCAGAGATTGTTGAGAGTTATGAGAGATTATACAGTCGTCCTCTTGCTGAGCACATGTGTTCCG
AAACTTCCGGTGACTTCCGCCGGCTTCTGACACTCATTGTTACTCGTTCAACACAGGG
TGCTCGTGACGAAGAGGCGGGTGTGGACCCCTCAAGGGC
>126037291_2 EL597085.1
GCACGAGGCTCATCCAAACAACAATGGCCTTTAAGATCGTCATCTTCTCCTGCCTCGTGGCTATGGCCCGCGC
CAATCCATCTATCACAGTAGCCGCGCCACTTGCAGCGGCTCCTGTTGTCGCAGCACCAGCAGTCGCAGCCAGA
CTCGAAGAATTCGATCCTCTTCCACAATACAGCTTTGGTTACAACGTTGCTGATGCAC
TCACTGGCGATTATAAGAGTCAACAAGAACAACGTAATGGTGACTTAGTACAAGGATCGTATTCGCTCGTGGA
CCCTGACGGCACACGCCGCGTTGTTGATTATTCCGCTGACTCTGTTAACGGATTCAATGCGGTCGTGCGCAAA
GAACCTTTAGTAGCCGCTGCTCCAGCCGTCGTCGCCGAGCCCGCGGTGGTACCAGCTC
GTATTGCTGCTGCTCCAGTTGCTGCAGCCCCTCTTGCCGCCGCCCCCGTATTTGCCGCCCCAGTAGCCAAATA
CACAGCTGCATACACAGCCCCATTGGCTTACTCAGCGTACAGTGCCCCAGTAGCAAAAGTAGCGGCATACAGT
GCGCCATTCGCTTACTCGGCTCCCGTTGCCAGATATGCTGCATACTCTTCACCTTTGA
TAGCTGGCAGATACGCAACGGCTCCAGTGGTAGCTGTCTAAATATTTGCTATATACAATTATGACTGATTTCT
CTCTATATTTGGGCCATAAATTTTTTATTTGTGAC
>126037292_2 EL597086.1
GCACGAGGGTCCAAGAAAGTCGGGCGCACAGTTGAACGAGAATGATAAGCGAATTGCTTTGCAAGTGGATAAT
GTAGGTCGGATATAGTAGTTTGTTTTGGTTTATCATTGGTTATATGCTGTGACTGGATGCCCGTTGTCTGGAT
TGTACTTAGTGATCTATGATATTTCGAATTTTGGACTCCGAGATACTGTGACAGGAAC
ATACTGTAAACATTTGCTCTGTGGTTAAGTGATTTACTATTTTTTGTGCATCACAATGGCCGAAGCTGCAGCA
GCCACTTGTACCAATAATCCTAATGCCGAAATTGTACGAGGCCAGGTGTTTGAAGTTGGCCCTAGGTACAAGA
GCCTCCAGTATATTGGCGAAGGAGCCTATGGCATGGTTGTATCCGCTTACGACAGCAT
GAAGTTAACGAAAGTAGCTATCAAGAAAATATCCCCATTTGAGCACCAGACATACTGTCAGAGGACGCTGAGA
GAAATCAAAATCCTCACCCGATTTAAACATGAGAATATTATTGATATAAGAGATATTTTAAGAGCAGAAACCA
TTGACCAAATGAAAGATGTGTACATTGTACAATGTTTAATGGAGACGGACCTTTATAA
ATTATTAAAAACACAGAAATTAAGTAATGACCACATCTGTTATTTCCTGTACCAAATCCTGCGAGGGCTCAAG
TACATCCACTCGGCGAATGT
>126037294_2 EL597088.1
GCACGAGGCAAGTGTTCTTCTTAAACAATCCGTCCATTGACTTCAATTTGGTTGGTGCAGCCGATATACTGGA
TATGCCTGGATTTAGTGACATATTACGTAGGTGCATTGTAGAGCAAGTGGCTAAAATGATGGTGCTACCAAAC
AAACTACCTATTAAATTAAGCGATGAAATTCCTACTGTTGATCTGCGCATGCCTGAAC
CAGAAGGTGTGTTAAGAATTCACTTGGTACAAGCTCAGAATCTTATGAAGAAAGATGTCTCCATGTTAGGAAA
GGGTAAATCAGACCCCTACGCCATCATAACGGTCGGCGCGCAGCAATGGAAGACAAAACATATTGACAACAAC
ATCAACCCGAGATGGGAGTTTTGGTGCGAGGCGCGGATTATGCAATCGATCGGGCAAT
CATTGGATATTGAAGTATTCGACAAGGACGAAGGCAACGATGACGACAAACTGGGCAGATGTTCTTTGGATAT
CTCACAAGTTGTTCGTGCCGGTCGTTTAGATACGTGGCAAACGCTCCAGCAAGCCAAAACCGGAAAAGTACAC
CTAAGATTGTCTTGGCACCGTTTGTCTACTGACCTCATGGATCTCAGCCATGCTATTA
CTGAAACTCAACTGGTGAAAAATGCTGAGCTAAGTTCGGCGGTGGTATCCGTGTATATTGATTCTTTGCAACA
CCTACCGAACGCGCGCGCACAGTCCCGCCCCGACCCGTACCTGACGG
>126037298_2 EL597092.1
GCACGAGGGATTATTCAGGGTTCGAAGAATCATTTAGGTTTAATTGGACCATTAGCAAGTGTCAACAATGGTT
CTGTTAGATTCCAACAAACCCAAAGCACTAAGACCAAGTTTGGACCTTTGGCAGATAGCGATAGAATCTTTAG
TAATTTGTATGGACGTCATGATTGGCGATTGAAAGGTGCACTGAGCCGTGGGGACTGG
TACATGACCAAGGAGATCCTACTCAAAGGAACAGATTGGATTGTTGGTGAACTTAAAACATCTGGACTTCGTG
GACGTGGTGGTGCTGGTTTCCCAACTGGTATGAAATGGTCCTTCATGAATAAGCCATCAGACGGGCGTCCCAA
ATACTTAGTGGTGAATGCTGATGAAGGAGAGCCAGGGACATGCAAGGATCGAGAGATC
ATGCGTCATGATCCACACAAGCTTGTTGAAGGTTGTCTCATAGCAGGTAAAGCTATGGGAGCTCAAGCTGCAT
ATATTTATATAAGAGGAGAATTTTATAATGAAGCTAGCAACTTGCAAGTGGCTATTGCTGAGGCATACCAAGC
TGGTTTAATTGGTAAAAATGCTTGTGGCTCANGCTATGATTTTGATGTGTTTGTACAC
CGTGGTGCTGGAGCCTATATATGTNGGGAGGAAACTGCTCTAATAGAGTCTATTGAGGGGAAGCAGGGCAAGC
CCCGTTTGAAGCCACCTTTCCCAGCTGATGTAGGGCTCTTTGGGATGTCCACTACT
>126037300_2 EL597094.1

AATATTTTACAAACGTCGCGCTCATGTCGTGACGACGTCACACTCACGTCACTGCGACGTCACTGCGACGTCA
CGGTGACGTCACGCGCACGTCACGCGGCGCCGACGGCAGCTCACTCATACATATCTAACGAGTAAAGCAATCA
TTACATGGCATTGTATGACTTAGTAGTTTTAAACATACAGTGGCTTTTTGATATTTGC
TGATTGATATATGAAATAAATAAGTATATCTCCTTTCATACTCTCGTATTGTGACGGTATTTCGATAAAATTT
TGACGTTAACCGTTCGTAAAGTCTTATCTGTATTCCTTAAAGTTATTTCTTTAATAGAATAAAAGTATTTTAA
AGTAAACAAAATACAGTCAATTATACAGTTATTTAAATTTTCTTCATCATTTTCATAT
GTA
>126037308_2 EL597102.1
CGGCACGAGGAATTGGATTCTCCAGCAATTTTTATTAAAGGGACGGCGCACTGCGCGAATATGTATCCCGCCG
CCGATTCAGACAACCCCAACTTGGTGGCGGCGCGCTTGGACATCAAACAGTACCTAGACGCCTGGATCAATCT
CCCCTGACCCACTGCCAATATAACATTCTGA
>126037316_2 EL597110.1
GCACGAGGCGGTGAGGCACGGGCCTACCGTGGCCCCCGCTCCCGCCGCCGCAGCCCCAGCCTCCGCGCCCGCA
ACCCCCGCGCCGCCCAACAGCACGCCGGTTAGACGAAAAACGAGGAGTGCGGTAGGCGAAGGCCCTCCAGGCG
CAGGCGCAGGCACGGGCGCGGGCGCGGTGCGTCGACGGCGCGCCTCCCGTGACGGCAA
GTGACGGCGGCGACGCGCGGCTTGCACCGCGCCTCCAATCTCCTTGCCCCCACTCACACCCATGACGCAACTG
GCGCCCCTGCCCACCATGCTCTACCCCCTCGAGTGACAGTGATCAGTGACTAGTGACTCTGCGTGATGTCAGG
AAACACCGTGGTTTGCGACATTTTGTGGAACCTTAAAATTTTATGAATCTCAAGTGTT
TGTAAAATCATTGAATACATTGAATTGTGCAATATCGATCCTTTGAGGATGCTTACAGAGTTGGTATATGTGT
GCTTTTTTTATTATAACAAACGTGTTCAACGGTTCCTGACTGCGCTGCACTGCGGGACTCGCGCTTTGCCAGA
ACTATATATTTTTTAATTAGTGAACAAAAATCATGTTACCATTCACTATTGTAGACAC
TCGACGTTTTGTTTAATTGTAATTTTCCTTTTTTTCATTTCGTGTACGAAGTATTTTTTAAGATGGTCTTTTT
ACCAGGCTGGACTCGGTATCAGGAGTCGC
>126037317_2 EL597111.1
GCACGAGGTTAATGTTAAAAAATGTATCTTCTGTTACATTTGTTATTATTTGTTGAAGAAGAAAAAAACATTG
AATAATACAAGCTACCTTCGCGCGACTTCGCCCGCTTCAACTATACATATATTTAAAAAATACTTTTTAAGTA
TATTTAAGGAAAGTTGGTTAATTATATTAGTATGGTTATCTATACTGTTGTACACTTT
AGAAAGCTTGCAAAAAATCTTTAGTTATTTCATATCTACCTAGGAAAGTACGTGCACACGTTAAACGCGTCCT
GTTCTAGAAAATAAAAATAAAAAGTAGCCAATATACTGTTCCATAACCCCCTCTTTCCATGAAAGTCCCATAA
AATTCTAGTTGTTCCGGAGATAAGGCCGGACAATCAGAAATAGAGATAAAAAATTCTA
ATTGTTTGTTTGTTATGTATCGTGTAAAAAACTACATATATATTATATTCTCTTCAACAAACACTTTTTGTAT
TTTGATGATTATTTAGAATTCACAGATAGTTACTTCAATTTTTATTTTTAAGTATTTTTATATTAATATCTAA
TAAATAACTAAATGTAAGATTAAATCACTATTTTAATTAATAACAACATTGTCGACCG
TCCTTTTGCTGCATGAGGCACGAAGTAAACGAAACCACTAAGCGAAAATATCTCTCGACCTC
>126037319_2 EL597113.1
GCACGAGGCTTACGTACACCCTTGTACGCCTATATTTATTAATTTATTATATTTACGTTTTGTGTATAAAAAA
TATAAAAAAATCAGCAATGGACGAAAGAACATTGGAAATACGGACATCAAATGCTGAAAAAGAAGCAAAAATA
ATAGAATTGGAGCTGGATAAAATAATAAACGATGATCTTAGTTCAGTTGGTGATCCGA
GGCTGGATAAATTGCTATCCGATAATGAGAAACTCAAACATCGTCTAACTATTCTTGAAAATGCTATAAAATC
AGAACAGGGTAGTTCACCGGATTCGGTGAAAAGAAAGAAACCTAGTTTAATAGTCGACATAAAGTCTGTCGAA
GGTACTATTTGCATAATGGATGAATTAAAAAATGTTTTCGAAGTCGCTATAACTTCGG
CATATCCTGATTTGGTGGATCCACCAATTGTTATATCATTATCTGGGAATAATCCAAAGTTTGGTGATTATCA
GTGTAATTCTGCTATGCCTATATCGCAACTCCTTAAGGCCAAAAATATAAAAACGAATCCTAGAGAAGTAGCA
AATAATATTTTAAATAAGACTCCTTCTTCACCACTTATACAAAAAATGGAAGTTGCTG
GTGCTGGATTTTTGAATATCTACTTAAATAAAGCTATGGCTGAACATGTCTTAAAAAGTATTCTTAATG
>126037322_2 EL597116.1
GCACGACGCTTCGTGCCGAATTCGGCACGAGGGGACGGTGTTGATGACGCTCACTACTAATGCGGCTGATAAA
CCGCTGCAGTACTACGTGTCGGAGCGCACCTTCCTGCAGCAGTTCGCCATCAACGGCGCGGGCGACGTGCACC
TGCGGCGCTCACTGCCGCGCGACAACGTTACTGAGTACAAGTACCAGGTCATGGCCAC
CGATGGGAACACGAACGATACAGCGTCCATACAAATAAGTGTGCAACACGTGAACGAGTGGGAGCCGCGTTTT
AAGCACGCGCACTACGAGTTCATGCTGGACGCCTCCTCCGCTAACACGCACGAACACGCTAACGGCACGCTCG
TGGGCACGCTGCAGGTGCACGACGGCGACGACGAGGACAACGTCACGTTGTACCTCAC

CGGACCTGATGCCAGGCTGCTGTCGGTGAACGCGCTGGGCGAGGTGCGCGCGGGCGCGGGCGCGGCTCGGCGC
GGTGCGGCGCTGCACGTGGTGGCCACGGCCGTCGATTCCGGCACGCCGCCGCGACAGACATCGGTCCCCGTCG
TCGTCCGCATCACCTCCAAGGGCGGTGCGCCGGGCGCGGCGTCGGGCGCCGCGGCGGG
CGCGCCCGTACTGCTCGCGGCGTTCAGTTGCGCGCTGGCGGTGTTGGCGGTGCTGGTGGCGGCGCTGCTGT
>126037323_2 EL597117.1
GCACGAGGCGTTCTCTTCGTGGTCGGCGACCGCTTTGCCTTACTTAACTTACGTCTCCCGCGTACTTTTTTCG
ATATTTTCTCGCGCTTCCAGAGAACTTTTTAACGGATTGTCTCCAAGTTTTTTTTGTGTGTTTGAACATAGTT
TTTTTTACACGTGTGCCGGTGTGTTTGCACAGTGTTTTGTGATCACAGGTGTCGCATT
CCAGTAGGAACGCACACAGCCTCCGTTACCCGTACCCTACACTAGTAAGCTAAACAGGTGGACAAACAATTTA
CAACACATTGGAAGCAGTTATGAAGAGCAAGCTAAATATAGATGGGAACGGCGCGTGAAATGAGGAATGCCAA
CAAAAGGGATTTATAATTGTTGCTGAGATGGCGCGATGGGTAGCGCCTATCCGGCACA
CGGCGATTTGGCTTCTGATATGTCTTCTGCCGATAACACAAGCCACACCAGGCCCTTGCGAAGTGGAAACTGG
ACAGTCGAGCATTATAGTGGACATAGAAGAGAGTAGAGGCGATCAAGTCAACCAAACGACAGTACCACCCGAG
CTACCAATAGTCGGTGATCCAGACGTCGACGTGGTACTATCGACGGTGTTTCCCAAAG
GCCCCACGTTGTTTCAACTCGATGGCAAACGGTTGCAATTGCTTCAGCCCTTGGATAGAGATGCTGATAACTT
ATCGCATATG
>126037329_2 EL597123.1
GCACGAGGTTTTCATGTGACCGACATTATTTATTATCAATATCAAAATGACAATTAAAAAAATCAAGGTTTCT
GTTGCTTATTGTACTCAATCTGCGCCGCGGCGACGGACTTCATGGAGTCCTGCGCGTGGTGCACGTTGGCGCG
CCACGCCAGCAGCACGTCGCGCAGCTGCTGCCACTGCGCGCGCCCGAACGTGCGGTGC
ATGGTGCTGGACACGCGCACCGCCCGCGACGCCTGCTCCATGCGCGCGCGCACCAGCCGCGTCTTCAGTACTT
CTATGATGAATGCTTCTACATTCTTTTCTTCAATTTGCAGTTCAGAAACAATATCATCAAAGGATATTTCTGG
ATTAGACTCTGCCATCTGCATGAACGAGAGTATCCTCATCTTTTTTACATTTTGTTCA
TGGTTTAGACCTTGAGAATGAACAAATTCCTTGTGATTTTTGTAAAATGCCTGATAACTTGACAATTTCTCTG
ATACAAATATAGTTAATAAATCATGAATAAGTTCTCCTTCTAAGAAACGGACAGGTTTGAGAGCTAAAAGTGG
ATCCAGTAAGAATGAATTAGGATCTGCTAATGCAGTTACTATGCATTTTATAGCATCT
TCTCTTGCATATGAAGCATTTTCATCTGTGTATGTACCAAGCAATTCAATCATAACCTTAGATGCCAATTCAC
TGTTTTGGTCTTTCAGTACAGAGTGAAGCAGTCTGTACAATTTCTGCATTT
>126037331_2 EL597125.1
GCACGAGGTCACAATCTGCGTCTTGTTAAAACAAGGGTGCAGGAGAAAATAAATAGATATAATATTATAGTTA
ATATTGTATTGAGGCGAGACGCGGGCCGCGCATGCCATGCACTGCGGTGTTGTGCATGTATAGTTCCTCGCAC
TGCCGCACCTACAGTGGCGCTCCTAAGCCCCCTATATTCTAAACTGTAGTTTAAAATG
TAACACAAATTAATGATGTATTAATTCAACTTCTTATTCGATTTGATAATATTATTATCAATTAAAGTGTTTT
ATTTGTGTCCATATACTGTGAATAGTTCACCACTTGTATATAAAAGAATATATTTTATTCATATTCGTTATTT
CGGTAACATACAGGACGTCGCACCGCCTGTACATATAAAGTAAAGCATCGCGACGGTC
GCGGGAGGCAGCATGAATCTTATAGTTCCTATTTAATGCGAGCGAACTATAATGTTCAATTGCCGTTTAATGT
AAGATATTCATTGACGTTTTCGCATATTCTGCATTTTTAAATAGTAAGGTATAGGATACGTGAGTATAGCGCT
ACTCGCTTTTTGGGTGATATATTCTAAATGAATTGTTTCAAATCAACAAATTGTAACG
AATTAGTTGTTACAGATCTATATTGTAAATGTGAGTGCAAAATTGAACTCTGTTAAATTATTTTGCGTTATAT
>126037332_2 EL597126.1
GCACGAGGGTCGACACCACGCTAGTGTGTTACAAGTAAATATATAGATAAATAAAAATGTTCGCGTATATCTT
CTTAACAGTGCTGTTTGCGTGTGCGCAGGCCAAGTTTTATACAGATTGTGGTTCAAAACTAGCGAGCATTGAC
AACGTGGGCGTAAGCGGGTGCGCCGAGAACGCCAAGGAGTGCGTGCTCAGGAGGAACA
GTAATGTATCCATATCCGTCGATTTCACACCAACGGTGGACGCGACCGGCTTGGAGACGGTGGTGCACGGCGT
CATCATGAACCTGCCCGTGCCGTTCCCGCTGCCGCAGCCCGACGCCTGCAAGGACAGTGGACTTGCATGTCCT
TTAAAAGCCGGAGACAAAGAATCGTATAGAACTACAATGCCAATCCTCAAGTCATATC
CCAAAGTGAGAGTGGACGTGAAATGGGAACTGAAGAACCAGGACGGAGATGACCTCGTCTGCATCATGATACC
CGCGAGAATCAATTAGAAACTATATTTGAAAAATCATGATAATAATCTTCTCTTANAAAAATGAGTAAATGTT
TGGAAAAAGATAATTTGTTAATGTGTTTCGAATTACCAGAATTGATAAATTCTTTTTC
AATAATATTAAATTTTATATAATA
>126037335_2 EL597129.1
GCACGAGGCAACTACCAAGTGAAAGACCAAGGTTCTATGCTAGATCTTATTGGAGGAGGATCACAACAAAGTG
CTTCAAGGTCTGGTACTCCAGCAGCTGTAGGACATAGGAAGAGCCCCACTGCTGATCAAGGCACTCAGGCCGG
CTCTGCCAGTGGTTCGACCCACCGTGACACTAGGCGTACAAGCACAGGCGGTCCTTCA

AAAGTGGGTGCAGGCAGTACAAGCACAGGTGCGGGTTCAGGGCCTCCTCGTGCGAGGCCAACATCGCGCACTC
GCCAGCGACAGCCAAGTGGGTCCCAGGGTCAACAGCCTGGTGAGCATGAATCTCTACAACAGCATCCAATGCA
AAATTACAACCGTGGAGGTTGGAGGGGTAGACCACGTGGTCGCGGCCGCGGCTTCGTA
CCACGCAACAAGAACACCCTCAAATTTGACAATGACTACGACTTTGAACAGGCCAACACCGAATTTGAGGAAC
TGAGGAGTCAACTAGCCAAGACTAAGATATCTGATGGGGAGGTTAAAGTTGAGGCCAACACGGCGTTTGAGGA
GCTAAGGAGTCAGCTGGCCAAGACTAAGATAACTGATGGGGAGGTCAAAGTTGAAGGT
GAAGTTGACAAGAAAGATGATTCTGGTAATGAGACAGGCGCT
>126037336_2 EL597130.1
GCACGAGGGTAATTTGTTGTCGTAACTCTTACGTCCACTTTATTTAATAAGATTTCACTATTAAAATATTGTA
GCAGTATTCAAACAAAAGCTAGACAAAATGACTATGGTGGACGATTTACGAAAATACCTAAACCTTTTACTAG
AAAAGGTAAATGGACTTCACTGTATATTAATTACTGATCGTGACGGTGTTCCTGTAGT
AAGATCTGTGACAGAAAAGGCCCCACAGCTGGCCTTAAGACCTAATTTTATATCAACTTTTGGAATGGCTACA
GACCAAGCCAGTAAACTCGGGCTTGGACGTAATAAAACTATTATTTCTATGTATTCCAGCTATCAGGTTATAC
AAATGAACAAGCTTCCATTAGTGATTACTTTTATTGGTAGTGATAATTGCAACACCGG
CCATATATTGTCACTTGAAAGTTTAATAGAACCTTTTCTTAAAGATTTAGAAACAATTGTAGCTGAAGCTCCA
TGAGCATAATTTCCTTTATGATTTTCTTAGCATTTTTCTATAATGTTTGTAATTTGGTGGAGTTCAAGTATTA
CATATCACAATTTAGGTGTTTTGTGAAACATTCCAGAATGGTGAAAAGTCCTGGCCGA
CCTGAAGTTAATTTTTCTGAACTTTTTCACATTTTTCACT
>126037341_2 EL597135.1
GCACGAGGGTTTATTTCTTTTAGTACTTATTAATTAATAAGGGAAAAATCAAATTTACTGCCGAGACATATCA
GATTAATAATCCCAATTATATTCCTTGAAATATGGACTCTGATGCAGAAAGAGAACAGCTGCTTGTTAACAAA
CCTTCAAAACCTTCATTGGTTAATTGTGACATATTAAATGAATGCCAGATGTTCTTAA
AGGCTGAAGATGGAGGTGGTGATGCACATAAAAATATGAGTCAAAGTACTACAAACAATAGTGGTAAACCTAA
TTTTATTACTTCAACTCCATATCCCACTAAGGCTACTTTATCAGAGGATGTTATAATAGATACTAGTCCAGAA
AGTAATGTTGAAGGTGAAAAGCAAAATGAAAATATTCCTAAAGATCAAGAGTCAGATG
ATACACTGCCCAGCACTGTAACTATTTTGGATGCCCCCGATGGAGGCAAAGTTTATTTGATAGGCACTGCTCA
TTTTAGCTTGGAATCCCAAGAAGATGTATCTAAGGTGATACAAAAAGTGTCGCCGCACATGGTAATGGTTGAA
TTGTGCGAACAACGAACTAATATATTGTTACTTGATGAAGAAATTATATTAAGAGAGG
CTAAAAATATAAATATAGC
>126037344_2 EL597138.1
AGATAAGATATATAATAAAATATCTCGTCATTCTCGATCGTGTCATATGAAGTATTTTATAATTATTTTCTTA
TAAATACAAGTATTCTACTTCAAACATACACTAATTTATTAACATGTTATTTCTTGAGCTTTTAAATTGATTA
CGATCAATGATTTCAAAAAAATATATTTTCATATTAAGCAAGTCCCTTCAAATAATCG
TCAGATTATGAGATAGCACGTTTAGGGCATGAAGATGCACCATATAAATCTTAATCTGACGCGCTTGAGAATT
TCAAAGTGGCGAAATTATTTTTGCACATTCTAACAACGACTGCAAGTTTGCGTCACATTCAAATCGTCAGATT
ATTATATAAGAACTTTTTCAGGCTCACTTAACATCCCCTTAGAAAATCATTTACGCAA
ACGTAGGCGTATTTAGAGATATTCTTTTTGGTGCTGGACTTGTGCTTTGAACAAGAATGTTCGTGT
>126037350_2 EL597144.1
GCACGAGGAGTTGTTATTTGCATTTTACTTTATTTTTTATTGTAGTATTTCAAGTTTTTTGGTAGAAAACAAA
AAATACACACGCAAATTCACTACAGCTTCGATATTGTGTTTCCTATTTTGTTTCAAAACGTTTCTGTATTAAT
TATAATTTGGCTGTTTTCCCATTTTCGCATACGTAATTTAGTGTTTTCTCTAAACAAG
TGTTATTGACTAATGCATCATTTATAGAAATAAATATTTTTTACAAAAATGACTATGATGTTAGCACAATCAC
ATATGCCAACCGAGTTAAGCCTAAATCTTAGAGAAATAGAGCTGACTATTTTGAAATTAGTCGACTTATTCGA
CATAAAAATAGTATGGGACCAAAATGCAGCAATATTAACTGGCACATTTGCTCTTGTC
GGAGGATTTGTGGGAGGATATGCTGGTGGTAGAATGGGAGCAGCACTTGGAGCTGGGATTGGAGGAGCAGCTG
GATATGGAGTATCTACTGTGGTATCACTTCGTGAAATTTGGTCCTTCATCAAAGAGCAATTGACCGAGCTGTA
CTACATAGTCTTCAATTATTTAAGACGATTGGATCCCGTAGATTGCATTCGAGCTATA
GAACTGTTACTGCGTTGCTCATCTAGTAAAACAGAACTTGTACTTACTATTTTAGATTTTGTTGCGCATAAG
>126037356_2 EL597150.1
GCACGAGGGTCAGCCATAAAGATTGTGGACTTTGGTAGTTCGTGTCAGCTGGGTCAAAGGATATACCAATACA
TACAATCAAGATTCTACAGATCGCCGGAGGTGTTACTGGGTATTCCGTACGACCTGGCGATAGACATGTGGTC
TCTCGGCTGTATCCTGGTGGAGATGCACACGGGTGAGCCTCTGTTCAGCGGCGCCAAC

GAGTTGGACCAAATGAACAAGATAGTTGAGGTTCTGGGTATGCCGCCCGATCATTTGTTGGACCAGGCACACA
AAACAAGAAAGTTCTTTGACAAATTACCAGCATCTGAGGGTGGTGGTTATGTTCTAAAGAAAGTCGCCGGTAA
AGATGGCGGCTACAGGAAGTACCGCGGCGCGGGCTCGCGGCGCCTGCACGACATCCTG
GGCGTGGAGGGCGGCGGGCCGGCGGCGCGGCGGCGCGGGGAGCCCGGACACTCTGTATCTGATTATCTTAAGT
TTAAGGACCTAATCCTACGCATGCTCGAGTACGAGCCGAAGCAGCGCGTGACGCCATACTACGCGCTACAGCA
CAACTTCTTCAAGCGCACCGCCGACGAGGCCACCAACACGCAGCAGGCACAGGCGCAC
CATCAGCACGTGGGCGGCGCTCGGCTGTGGGGCGGCGCTGAGCGCATGGACGTGGAGGGGGTCCGCCGCGACG
ACGACATGCTGCCCGT
>126037373_2 EL597167.1
ATTTGTACTAAAAAANNNNNNNNNNNNNNNNNNNNNNNNNNNNNNNNNNNNNNNNNNNNNNNNNNNNNNNNNNN
NNNNNNNNNNNNNNNNNNNNNNNNNNNNNNNNNNNNNNNNNNNNNNNNNNNNNNNNNNNNNNNNNNNNNNNNNN
NNNNNNNNNNNNNNNNNNNNNNNNNNNNNNNNNNNNNNNNNNNNNNNNNNNNNNNNNN
NNNNNNNNNNNNNNNNNNNC
>126037375_2 EL597169.1
GCACGAGGCTTAGCTTATGCTTAGTGGTCAGAGCGCCTGATCGGTTTCCAAGAGGCGCGGGTTCGAGTCCCAC
TACGTTCAAATTTTTGTACGTTTTTCTTTCTTAAGTGTAGAATAATATTTACTCTAAAAATTTTAAAAAATAT
TTTATATGTTGTTGGCTTGTTTAAAGTTCGGGTTGTTACACACTATAATATATGATAT
TAAGTTGGCAAGTCTTCAAGGAAACAAACATATAATTGGCACGTGTTAAGTTATTTCTCTGTACAGAGTATTC
GACTTATATAACTATGCAATTTGGTTGTTTATATATAATTATTAGTCATTTTATGTTAGTACTCTATATAATA
TTTACCATTCCTATATGCTCCATAATGTAAGAGTGCTTGTGCATATACATTATATCAT
CGATCTCAATACAAATACTGCTTTTTGTTTCTGATTGTCAAAAATAAAAAGAGCTTTTCTGCCTTATTGGTCA
ATAAAGTAATAAAAAAGCAAAAAATAAAATAGTTATATAGTATAGTGAGACTCAAAAATTTTATTTGAATAAT
TATGTTTGGATACGGGGGAACCGCGCTTCTAAACTTACTAATATTATTATTGAACTAA
AGAGCTTATTCGGTTGAACGCACTAATCTCAAAAACTACGGTTCGATTTAAAAAAATATTTTTGTATT
>126037376_2 EL597170.1
GCACGAGGAAAACATTTGAAGACAATGTTTACGCTAAATTGGAGAGCAAGCACCATTATAAGTCAACGTTTTG
ACATGTGCCTGATAATGTCAAAGGAACATATAAAGAATTTATTATTTTTACTTCATTGCGTGGCTAGCCACTG
TTTGCACTGTATAGGTAAAGACATATAGTAGGTAATTGTTATATTTCATTAACGTCTA
AAAAGGTGATAGGGTCAGGGTGGTCTCTAAATGTACCAGTGGGTTATCTAAAACATGCTTAAGGCATATGCAC
ACTTAAGTGGTCAATAAGCATATGTTTTCTCTATCTGACCCGAGACTCCACGAGGTCACAACTCTTATCGTTA
TTACTGTCTTACGCACATAAGATAGTGAACATAATAAATGGGAGTTGTTTGCGGATGC
CACCTACAGTATTGATTGTTAAGTTAGACATTTGATATTGTCCAAAGAGTATTGTTGAGTTCTTGTAAGGTTA
TATCCGTAAATCTTTTAAATAAGAAATATGTGTAAACAATAACATTAATTATAATTTTTTTTTCTTTCGCAGG
GTGGGACCGACCAAGGCCGTCGTGGCCAACAAGACGAGGAACGCTTATACCGTTCTCG
>126037382_2 EL597176.1
GCACGAGGACAAAACAAAATACAGATAAAAGAAAAATTAATACTGTACACAGATAATAATCACAATCATATTA
TTTGGCTAGAAAATAATATTAAAATCATTAACTAACATTATGGTCCATACAAGAGTACTACCAGCATAAATAG
CTAAAAATAAATTCTAGAATTCGAGATCTAAGACTAAGTTCTATTTTTAATGCTGGAG
TGCTGCCAACTTACAAAAAAAATATTTGGACAGATTAATAATATTACGTTGTTTTCAAATAAAATATAAATAA
CGAAAAATTATAACAAGCAAACTTTAAGAAAATGAAATATTATTATACGAGTTTTAGAAAGAAATTGTTGTTG
TAATTTATAACTATGACAATAAATACTAAGCAAAAAAAATCTTTAATAATATTCTTT
ATTTTAATAGATTTTCGGAACGTTCGGAAAGTAATACATTTTATTATTTGCGCTATCTAGCTTAAAAATTCCA
TTAAATTTCATAAAGCAAATTGTTTAATAGTCTGTTTGTAGGCGTTAAGTTAGAAATATGATAATACCAGTGG
CAGTGGGTTAGTTACGAAAAAAAATATAATCAAAAAATTCAGTATTTACACAAAAATG
TATGCATATTCTGTACCTTCGGAACCAAAATGATGTTATTTTCTCTACGTTTGACTATATTTAATACTGACTA
ATAATTATATGATTCCATCGAGAGTTTC
>126037389_2 EL597183.1
GCACGAGGCAGTGTACCGTTAATCTTGGATAATATACAATAAAGCTTTACGGCGAAGTAAATTTTCGTAAATA
TTTAATCATGTTCCAATATATTTTAACGTTAGTATTATTTAATACGTATTATCTTAGTTTCGGATTACCTATG
CCAGCGAAGGTAGCCCATATACCCATAGGAAATAAATTACAAGCTCTTGCGCAAACAT
GTGAAAATGTAGTTATTTCTAGTTTGATTAAAAGCCAAGTTGAACGTTGCAATGCCAGTACTCTATTGGATAT
ATATTTGGAGCCCAAATGCTTAATGTTCTATGATATTAATAAGCAACTTTGTGAAACATTCGATAAATCCAAG
TTTGATATACAAAGTGATTATTCATCTAAAATGAAAGAAACACAAAATGTCCAAAGCT

TATGTGAGAAAGCTGTATCATGGAAACCCAAATATCTACAGGAATACCCAGAGTTAGAGCTGGAAAAATTGTT
TCAAACTGAAGTTTTATGTGTGAGAATGTGTGCCACTAAAGAAAATCTCCTAAGTGTAGATGTCAATTTCTTT
TGTAAATATTACAAGTGGGGAACTGAAGCGATTAAAAATGTTACAGCACCACTAGCAC
AAATAAGCAATGAAATGGATACTGGGACTAAAACAGTCGAAGCAACAGTAAATCAAAATAATAAGGCTAGTGC
TGACATTTTG
>126037394_2 EL597188.1
GCACGAGGCACACACTTAAAGATATGATATCGAGCAGATTCAAGAATAAAGATGGTAATGAAGAAAAAGCAGA
AGAAGCAAGACTTAATAATATAGAAGAGAGAAAAAGGCCGGAGCCCGAACAGGTGACTCCTAGAGAAACTCCA
CGGAAAGTGGAACAAGGGATATATGGACGACCAATGCCACAAAACCGACCGGATATGC
AGTACGGGCATGCTATGGCTAACAACATGGCATATCCTAGCCCTTCGCCACATAGACAACTAATGCAACAACA
ACAACAAATAGTGCAACAACAAATGATGCTAAATCAACAAGCCAGGTCCCAAGAAATGCTGGCGACGAGGCCC
CAAGCGTTAGGAGCTGATGCCCTGTATCAACAATATGGACCTCCCGGAAGGAGGAGTG
CAGTATACCAACGAGACCTGGATGTTAGGTCTATGGCTAACTTTACCTCCTTGAAGACTGGACCTCAGCCACA
GTTCGACAACAACTCCCACTCAAGGCTAGATCTTCGAAGCCCTCAGCAATTAGAAAGAGAGATAATACGACAG
CGAGGCTTAGTGGAGGGGAGGAGAGCAGCATCACATCCACATCTATTAGATGAACCAC
AACATAGACAAGAAGTCACAACTCCTCAAATACACGACCGAT
>126037398_2 EL597192.1
GCACGAGGAAAAATTAAAGAAAAAAGCTAAACGCCAATCAATGCTTCTTAAGAAACAAATGGAACAAATTGAA
GAGATGGAAGAACAAAAAAAGACTGGCAACAACAGTGAATCTGCACCTATGTCCCAAGATAATGATGATTCCC
CGCCAACACCTGAAGAAGTTAGTGTTATAGAAAACACTCGTTCTGAAAATGATTCAAA
TATAAATGGTATTAATGACATGCAGAGAGCAGATGATGATGTTTTTGATACAGATGCAGAATCAGTCCAAGTA
AGAAGTAAGCAATATGGCTGTGGAGATGATGCAGGCACACCTATGTCTGAGGGAGAGATGACCGACACCCCAC
CTTCCTTTAACTCACAAAACAAAAGTAAAATGGATAAGAAACCAGACCCTGCATTTGA
TGAATGTGAAGTAGAAGTGAAAATAGCTGATCTAGGTAATGCTTGTTGGGTACATCGTCATTTTACTGAAGAT
ATTCAAACAAGGCAATATAGGTCATTAGAAGTGTTACTCAGTGCTGGCTATGGCACTTCCGCTGATATTTGGA
GTACGGCATGCATGGCTTTTGAATTGGCCACTGGTGATTATCTCTTCGAACCACACNN
CTGGGGATGGTTATAGCCGAGATGAAGATCACTTAGCTCATATTATTGAGTTGCTTGGTGATATTCCTAAACG
TATTGCTGCATCTGGAAAGTACTCT
>126037400_2 EL597194.1
GCACGAGGCAACAACATAATAATATATTCTTATCGAATATAAAATGCCATATAAATAAAAATAAAGGCTATAC
ACATTTCGTAATAATATTAGTTAAATCAAGGTTACGCAAAATTAAAATAAAAATATTGTGATCTAAACAACTA
TATCAGAACCGAGTCTGTCGAACTCATTTGCGAAATAAAGCATATCCTTGTGGTCGAG
TGCGGAGTTTTGAAAGACAATACGGAAAAAGTTAACCAGGGTACCTTGCGGCTGATACGTGACCATCATGCTC
CCTTCTTTTATCATACGTTCCTTTAGTTTGGGCGCAACTTTGTGTAATCTCTTTTTGTAGTCTGGCCCATTTT
CACATCCTCGAAGACACTTTGGTATATACCAGAACATTATATTGGTACACTCTGGTTT
CTGCAGCACCAACTGAAAGCCTTCCCGTTGCCTAATATGCTCAAGGAAAAAGTTTGCATTATCGAATAACTTG
TCGATGTGCTTTTCAAATCCTTCGCTGCCTTTCGCTTTCCACATAAACCAAAATTTCAGTACATCGGCCCTTC
GTCCGCACTGTATGTGTTTGTCGCCGGTGTCGTATGAAGTATCGTAAAATTTGTCTTT
TTGGAACAGGTATTTTGCATTGGACGAATGAGCTTCTTCGAGTACACGCTTATGCCTGGTCAGGAATGTAAAG
CACTGTTGTGGGGCGGCTAATAACTT
>126037403_2 EL597197.1
GCACGAGGCTCAAGATAGTGTCTCCACAGAAGAAACCACATCGAGTGTGATAGCAAAAGTGTAAACAAAAACT
TTTATTTTTTTTCTTAAGCTTTATTTAAAAAGCCTGATCGTTTTTAGATATACATTTTGAATTTGTTTAGAAA
TGTCGAAATATGTGAAATTAGTAAGATACTGTTGCTGTTTAGTATAAAATCTTGTAAA
TGCATGTAAAAAACTTATTATATGATAATAATGTGTGTTTAAGTTATACATTTGCAGAAATAATTTAAATTAA
TAAAATTAAATATTTTACATTTACTAGGTTTTCCTATATAGCGACAGTATTATTTATAATAAAATTTTAGGCC
GTTTTCTATAAAAAAATATATAGCATGCTTAAGAATAATAATTTATAATAATTTACAA
ATTAGATATTTTTTGTTAGATTTTCACTCAACAG
>126037407_2 EL597201.1
GCACGAGGATTAAATATTATACTTCTCCTGTTCAATAAGACAAAGTGCGGCGTTGAATTTCAATTTTCAACGT
AAAAATATGTTATTTGAAGGTATATGAAAAAAAAGAACCATTCCTTTATAAAATTCGTTGAGAAAAATATATT
AAGTTAAACGTACGTATTAAATATATTATAGTTTTAAAATAATTATAATGAACATTTA

ATATAAACATTACTTCAATAATCAGATTATATCATTTTCAACGCTTTAACAGCCTTGACAACACGAATAGGCT
TTATATTTTAATAGTAGCGTACAAAAACTGGAATGACGTAAATCGTTCATGAATGTTTTTGCTATTGTAATGC
ATTTACTTGTTACTATAATTAAAATGGCAATGGTTTTTTTTACATGATTTTATTTAAA
TTTATAAATGGTAATAATAACAGAGACGCACAGACAGTAATTGTTTTTGTATATATTTAAAATGTTTTTTTTT
CATTATTGTATTACATAAGTATGTCTATTTAATTTATTTAGTTGTTATGTATTTTATTTTTTTGTAAATTATG
TATTATCTGTATATTTTATTTC
>126037410_2 EL597204.1
GCACGAGGATCTATTCAGGCTCGAGGCAAATAAGTATATAAATTGGAGCAATTTAAACTACATAGGCGTTCTA
CTCTGATAAATTAAACTGCGCCATCGCATGAGCTGATAATACAATGGAGCTGAAATCATACTTAGACACTTGT
AACACTTAGTTGAATATATGTAATTTAATCGAAACTTAGCTGTAAGGATAATTCGGTA
AGTTATCGCTGTCGATTTTTTCTCATGCACGATTTTAAAAAACAAAGAATTTAAAAAAATAATAGATTTATAT
GTGCATTTTTACAGCTTTCCCCGGGTTCTGTGGGTGCCAAGACGAACGGTTATTTAGCTTTCATTCCAAAGCA
AACTTCAAGCAGTCTTAAATTTGTATACGTATCGAAATTACCTAGTAACAGTGAGCTG
AACCTAAATTAAGAATTGCAATTGATAAATCATTTATTTAAAATATAAAAGTAATGGATATACTATTGTAAAA
CAAATAATTACCTATAACAGACATACCGACTCAATTTCGGGTAAGTGGTCATATAAAAAGAATTTTTGATTTT
GTGATCACAATAAGATTTATCANATACTACTGAATTGATCAGATGAACTAACTTATGC
CTAAGTTATTTTTTTTACACAGTTTTTTTTACACATATCCTGTGCAATTACTACTTACTACTACAAATTACTT
TTT
>126037411_2 EL597205.1
GCACGAGGACAATTCGAAAAGATATGAGATGCGCTGAATTACGAAAGTACGACGGGACTCAATCTGATGGTCG
TGTGTTGGTAGCGGTAAATGGGTGGATATTCGATGTGACACGAGGACGCCGCTTCTACGGGCCCGGTGGTCCA
TATGCAGCATTTGGAGGAAGTGATGCATCTAGAGGTCTTGCCACATTTTCAGTGACCA
AATCAGACAAGGAGTATGATGACCTCAGTGACCTCAATTCCATGCAGATGGAATCTGTTAAAGAGTGGGAAGC
ACAATTTAGAGAGAAATACGATCTCGTGGGCAAACTGCTGACGCCAGGCGAAGAACCGACGAACTACTCCGAC
GAAGAGCCCGAAGAGACCGACAACTCCACCAGTCCTGG
>126037414_2 EL597208.1
CTAGGCATGAAAATACTTTATAGTTTAAAAATGTCAAAATAATAAGTGGAATTTTTTTTAGCAAAATTTTAAT
TCTTTTTACTCATAACGTTACACAAGGCTTATTGTCTTAATTAATTTGTTTTCTGTATGTTTGTTTCAAAAAT
TTTCAAAAGTTTCAAAAGTTTTTTTACATCG
>126037423_2 EL597217.1
GCACGAGGGTCTCATCAACTGTTCATGGTCTGAACTATAACCGCTCTTATTGCATATTCCATTCTCATTTCCT
CTCTTTGCAACATTACTAAGAGCGTGATTGCGTTCCTCCTTTCTTTACTCAATGTCTCTTAATTATCTCTATA
TTTCTATATGTGGGATACTGGCATTCATGATTACTGATACTCTTTATTTTATATTCGT
GTATTTGCTGTTGTATGATTCATATTCTTACTTTTAT
>126037424_2 EL597218.1
GCACGAGGTAGGTAGGAAAATAATACATAAAGATGGTTTTAATCAAACTGGTCTATTAAGATATATGGAACAA
AAAGATCTTGACGTTATTTGGACACCAAAACTCGAAACAAATGTTTCTATTAGAACTTCACTTAGAGAGAGTA
AGCAATATGATGAAGAACATAATTTAAAAAGCTTAAATCTCATAGATAACAATTACAA
ACCTAGGGACTTTGTAGATATATTTGAACAAAAGTCTGCGTCTGAAACGAATTCAACTAAAAGTAATTTAATA
TTTAATAATGATAATGTAGTACAAGAAGAACCAAGGAATAAAGAAATGGAATGCATCCATTCAAATGATAACC
AAGATAGCTCTTCAAATTATATTGTTTACAGTACAGAAGAAGAAACAGCCAAAAAGAC
AAACTTAGTAGATACCACAAAAGTAATGGACACATATGAAAAAGTTATTAAAAATAATGTCCTCTACTTACAA
GCACCAAAAGAAATAACGAATTTTTCTAATGTCAAAAAGTTGCTGAACACTAATCCTGTAGCTAATCCAAAAA
AGCTGACAAAAAAGGCAGTTTTAGAACTCACAACAAACAGTAACAAAAATGATGAAAC
AAATGTCAACTATATAACTGCTGATAGCTCCAGTGTCTTTGATAAATGCTCGCGTTTACCAATCATTACATCT
ACAGTATCTTTAGCACTGTCTGG
>126037426_2 EL597220.1
GCACGAGGATTCTAGTCAAGTTAAAATAGATTATCTCAAAAAAAACGCACGCCCAGCCGCGCTGAGCCCAGCA
GAAATGGCTAATGATAATAAAGAGATAGGTGACATCGTGAAGAAGTGCAAAATTTTACTTCTGGACGTCGAGG
GGACAACAACATCGATTAGCTTTGTTAAGGACAAGTTGTTTCCCTATGCCGAAGAAAA
TGTGAAGGAGTTTCTTGAAAACCAATGGGAAACCAGCGAGGTGAAAGAGGCGGTCGCTGCATTGAGAAAGTTG
GCTGTTGAAGATGAGAAAAACAGTGTCGAAGGGCTCGTCGCTATTCCTGGAGAGGACGCGTCAAAAGAAGATC
AAATACGGGGTCTAGTGGACAATGTGAAGTGGCAGATGTCCTGTGATCGCAAAATGGG

GGCCCTCAAGCATTTACAGGGACTTATTTGGAAGCAAGGTTTTGATAAAGGGGACATTAAGGGACACGTATAC
GACGACGTATCTTCGGCCCTAGAACAATGGCACGGAGTTGAAGGCCAGAAGATATACATATATTCATCTGGCT
CAGTCCAAGCCCAGAAGCTGCTCTTTGAGATATCCCTCGCTGGTGACCTTTTAAAGTT
TATCGATGGACATTTTGACACAGCCGTTGGTCCCAAACAAG
>126037427_2 EL597221.1
GCACGAGGAAATAAAATGTGTTTTTATACTAAAAGCATTTAATAAAGTTAAGAAAAAAATGTTTTTTGTATCA
TTATCTATAAAAAAAACTATTTGGTCATATAAAATCATAAGATATTTATATGTTTGTTGTACTTGTTGTAACG
ATCTTATTAATTTTATATTTCATAAATGAATTTTATTGTTCTTGTATGTTACTTTTAA
AGAGCCGTAGATATATTGGTAAATAAAATTTTTAGGGCTCCGTAACTTTTAAGGAAAAAAGTAACCCTTATAA
CATTACTTTGTTGTCCATCCGTCTATTATTTTCAAATTTAAATATAAGAATACTGCCTTAAATTAAAAATTGA
GGTTTATAATTTTATTTGTGGGATGTGGGACTAATAATTGTTAATAGATTCACTGCAA
ATAGTATTATTAAATATTTATGACCTGCTTAACGTTTTTACATTACTATGTATCCAGTGATCAGACAAAGATT
TTTATTAAGAGAATGTGCTATTATTATATTATATTATTACTAGTAAATTAAGAAAATATTTTATATAAACTAC
ATATTTAAATTGTTTATATTTTCCTCGTGCCGGTTCTAGTGAGATACCACCCAACACC
TATAACGCCATCAATATGAAATTCTTCGTGTTCTTCTTCTTGTGCTGTGTTGCTCTGGCATCTGCTGCTCCCC
AGTTGCTCACCTG
>126037428_2 EL597222.1
GCACGAGGCAAATGAATGTATAAAGCTGATTATGAAAAGAGATAAAGGATACAAGTCGATAGAAAATAATACT
GGTAAAATAGTTACTCAATCTGGTTCTAGTAATACTTATCCTTTTATAAGTAAATCAACGAAAACTAGGAAAG
AATTACTCTTAAGAAAGAATTTATCAGAAAATCGTAAGGAATTGTTAGACCCTAATAA
TAGTCGTGTCGTTCGTATTGGATCTAATTTTAGTTTTGACATAGAATTTTTTAAGGATGCAAAAATGTTAAGA
AATAATAATGATATTTATGAACGTACCTTGATGTCACCATACCCACCTTATAAGAAAACAGGAAAAAATAAAA
ATAATTTCTCAAAACCATTCAAAAGTTTGGGAACAAAAATGAACGAACAGATATATCG
TAATATGCAATCACAGTCAACACTAACTAATGGGGCACATAAGGGGTCTGCTACTTTGCCTATATTAAATAGA
TGTTTTTGCACATTAAAACTACATAAAAAAGGAAAGATGTACAAAAAACTTGCTTCAGAAGATGCGAAGCCTA
CTCTTTTTTATGACCAAAACGCTTTTAAAATAAAAACTGCTAGTANAAGTCAAAATAC
AAAAAAAATCAAAGTTCCATATCAGTTACAACCTTACGAATGCGAACCAAATATATGTGATCCAGATAACTGC
GATCCATA
>126037429_2 EL597223.1
GCACGAGGCCTGTCAGAAGTTAACTAAACAAACATGAATCGTTCAATTATTATATTAATAATTTCCTCCATAT
TACTCATCGAGGTTTCTACTTATTTAATAAAAGCTAGGAGCTTTGATAGTGTCAAGGATATCCCGGAATCCTT
GTCTAAAGATGGTGTTGACAATAAGTTGGTGGAACCGGACGTGGTCTCGGAGGTAACT
TTGAAAGTGGACTGCCTGAAGCCGGGGCAATTTTGCATGAATCATAAGGACTGCTGCTCCAACGCCTGTCTGT
TCTACTTGAAGAAGTGCGTAGGACAATAACAATGAAACAACAAACTATACAAACAAACATTATTATATTACAT
AAAAAATAAATAAAATCAGCTTCACGCTGTATCCCGCACACAGCAGGGTAGGCAGAGG
GAACCTAGTGTTAAGACACTCTGTTCCCCACATTATATTTTATGTATTCACTTATTTATTTTGCGCGCCTTGA
ACAAGGGGTGGTTGAGGTGCGCCAGGTCCGGGGGGTTGTCCACCAACGCGCGAATAATGTTCATAGTGTCTTC
TGATATCTTCCATAGATCGGGATGGTTTTGTTTGAAATTGCTGTACCATTTTTGGATC
TGTAATTTTTTTTTGTATAAAACAAATTATTTTTATTAATTATTTATAGTAAGCACTAATATTATGTTATGTT
TTCGTAAATGTGATT
>126037432_2 EL597226.1
GCACGAGGTCTAGACCGGCTACTAAGCATGACTATTATGACAAATACCCCCATGATGAAGTGCAAACGAAAAC
GGCCTGCAGTCGCCAGCAACACTGATAATATAGTAGCTTTGAAGCTACGATAACGACTGGTGCATCAAATCAC
CGAGTGTGTTGCGGCTATCAACAACGAGGAGTCGACGCGGCAACTCAACATGCGTACA
CTAAAGACTGACGAGACATTAGCAGAGTTGGAAAAAGCATCAGTAGGAAACACTGGGATATCATTGTATTATC
TGAAGTCCGACTAAGAGCGACTAACAGTCATAAGCAGTGTCAGATACTTGTGTTTGTAGTGTCTCGATCAGGA
TAGCGTACTTGACACTCAGAATGCCACATGCCATATGCCACATGCCATATGCCACATG
CCACACATCCCGTGAAACAATTCAATTATTGCATGAAACATTTCCAGATTGTGTACTTGCTCGTTTCGTCCTG
TGATTTAACACCTTTATACGAGAAGAGCGTTCGAGCTGCATCAATGGAGTTCTGCCACATTTGTACCAACGAG
TGTATAATCATGTTGCCAACTTGTAAAGTTATGGAGGCTATTTGTCTGATGTGTTATT
TCAAAAATAGCCCTATCCTACATGTATATATGTACTTAATTTTATTT
>126037433_2 EL597227.1

GCACGAGGGAGAGAGAGAGAGAGAGAGAGAGAGAGAGAGAGAGAGAGAGAGAGAGAGAGAGAGAGAGAGAGAGAG
AGAGAGAGACAACAATCAACGAGATATAGTATTCTTTAAAAACAAAGAAACAATCGTTATTGAACTATAATTG
TATTATTGCTTATTAAACGGTCGCAATTTTTAGATTCATCTGTGTAATATATTTACTA
ATTTTGTGTAATTTTAAAGATTTTTTTTCGTATCATGAACTTGTATGAGCTACAATAACGTTTAAAAGAATTA
TCTAACCAGTGATAATCAAGACGAATAAAAAAATATTATGGCTCACATCCAATTAGTTGATGAATTGGTTCGT
GAGTATTTACTGTTTAGAGGATTCAGTACTACTGTGAAAGCCTTTGACAATGATTTAA
AGTCCGATAAGGATAAGGGTTTTAGAGTTGATAAAATCGTTGAACAAATTGTACACTACATAAATGTTTCTGA
TTTGAGTGGATTAAAAGAATACTGGTCCCATTTAGATAGTCTTATTTTTTCTAAATTGGAAATTCATGTACAA
CCTGCCGTAAGGAAAATAGAGTACAGCTTATACAAATTATATTTTGGTACAGCCTC
>126037438_2 EL597232.1
GCACGAGGCTTATTTAGAAAATAAGATAATGTAATTGAAGCAGACCGACATGGACGACACTGAAAGTACAACA
ACATCCGCTGATGAAAACACTGGTAATTTGTGTGACAATCCCACAAGAGACACCTTAGCTGAAGGTCTTTTAG
GTCTACTTAAACCTACTGTTGACCAACTTGATGATAGAGTTAGGGCCACTAGGTAATA
TTTCGTATATATATATATTGTTCATTCAATCAAAATGACATCTATACAGGGTTATTAGTAATACGACCAGAAT
ATCATTTTATCGTTGTTTTTGTATGAATAATGACCGAAGAAAAAAAACGATCTAAAGCTTTATAATGTTCCAA
AAAAAGTAATTATACATTTCCCAGAGAAATTTTATATTTTAATAGAGGTATCAGACTT
ATCTTTATAAAGTATAACAGGTAACTCTTACCTAATAGTTAGTAAATTATATCTGGCATATCCTCTAAATAAT
AACAAAAAAACAGTAACATTCTTGAAAACATTTTTTTTAAATTAATTTTTAAACTTAATAATAGGTTGTTTCT
TAAAAAGAGACCTAGAATTTATGATATCTCAGATCATCCTGCATTCATATGCAGCTCA
NATATTTTATAGGACCAAATCCGTTTGTAATCCCATCTGCTTACTCTTTATAGGAATTACCAATAACCTTGTA
TCTAATTTGATTT
>126037439_2 EL597233.1
GCACGAGGCTCAATAATTTAGTAGAAGCTGGTCTTTTACTCGAAAATGAAAAGATAATTTTAGATAATTTAAA
TGATAAGTTTCCGAAGCCATCAAAACATTGGTTACCAATAGTATGGGCCACTAGTATAGTGACGAGAGCCCGA
AAAGAGGGCAGGATTCGTGACGATTTCGCTGTGAAAACAATTATTGATGAATTAAATA
AATTCAGAGCTCAAGCAGGACTTTTGCTTAGCTACGACACTATAAGTGTCCCACTAGTCTACACACAGGTAGT
AACCATTGCTGTTTATTCATATTTTATAACTTCTGCATTGGGTAGCCAATGGATCGATAATAAGCTGCAATCA
CATTCTTCGTCAGTCTATATAAGTAATATAGACCTTTATTTTCCCATATTTACAACGC
TAGAATTCTTCTTCTACATGGGATGGCTGAAAGTAGCAGAGTCTCTTATAAACCCGTTTGGTGAAGATGACGA
TGACTTCGAAGTCAATTGGATCATAGACAGGGATTTACAGGTTTCATACATGATTGTCGACGAAATGCATCAC
GAACATCCAGAGCTGATCAGGGATCAGTATTGGGACGAAGTGTTCCCATCGGAGTTGC
CATACACCATAGCGACGGAGAACCAGCGCGAGGAACATCCGGAGCCCTCCACGGCGCGGGTGGCGGTTCCCCT
TCGACAGCGCAGCATGGTCACCGTGGCCCCC
>126037447_2 EL597241.1
CGGCCGAGGGAAATTTGCAGCACTGTCGATGACGGCGCATGGCCAGGAAAGTACTAGACATGCTAATAACTGA
AATGGTTCAGAATGTTTATCACGTGCCTCAGGGTGCTTTATGGGTGACGCTCATTCCTGGTTAAATGGGGGTT
ATAAATGACCGTACATGATCGAGTTTTTGCCATTATTCCCACTAAAGTTAGATTGTGC
TAACATTTTGTTTGACGTGATGCAAGCTAATTCTGGCAAATTTTGTTGTTATGCTGAGTACACTAGTAGTATT
ATATTGCGACTTAAAATAGTGAGAGGATTAAGTTATGGATTAAAGAGTACTAATAATTATATGTGGGATCATA
GACCCACAGGTTAGAGCAGCGGCTGCAAACGCCAATCTGGCTTGTGATGGTTTCATTC
AATCTGGTTTGATTTTTATCAATATACTAGCTGCCCACTCGTGGCTTAGCCCGCATACCACCTTTAATACTTT
TATTATGCTGTATTTGCCAACAGATTAATACATTCAATCATAACCATTACATGGCTGCTCCCAGTATAGAGAT
ATTCAAGTCTAGGCTGGTGACCGTAAAGAGAGCATCGAATTATTTTAATTTTACATTT
TCTCG
>126037449_2 EL597243.1
GCACGAGGGCTGTAGATGCTACCGTCGCGACGATCGTAAAAAAAATGTGTAAATTTAATATTTCGATATCAAT
TATTGTTTTATTGGGTGTTCTTTTGGATAACACACAAAGTGCGTGGTTGAGATGTTACAAATCCTCTCTCAGC
AACTATACGGTTGTATCTCTATACAACCCAACACCTCTACTAACTGACAGTTGCTTTG
ACCCAAATCTTCAAACGATGATATATATACTTTTGGATTCAGAGGAAAATGTAATGGACCAGCGACTACTGCCGT
TTTACAAACGTATATAAATATGAACAAAAGAAATGTGTTATTATTGGATTGGGAAGACGAATCTAGAAGTGAT
ATTTTGAGTATATCTGTTGGATACGCCTTGTTAGTTGTGCCTAATGCAAAAAAAGTCG
GTCAAATATTTGGCGAAGCATTAATGACCCTGGCTAAAAACGGCCTCAAAATGAACGAAGTGCATCTCATTGG
ACATTCTTTGGGCGCCCACGTCCTGGCTTACGCGGGCCGGTGGACCCGTCAGAGAGGCCAGCCTGTTGCTCGA
ATAACCGGTCTAGATCCTGCGCGAGCTCTCTTCGAAGGCACGTTTGCCATATATAACG

GCTTAGATCGGACTTGTGCAACGTTTGTGGACATCATCCACACTAATCCAGGG
>126037451_2 EL597245.1
GCACGAGGGAGACGACCGCTCGCCTGTTACGCGTGACACGTTTCTCGTACGGCGTATTTTGACGTCAACTCCA
CTAGTCTAGTTTTGGAGATCGTAAAAATGGTAACAGAAAGTGAATCAGTCGTGTAATGAATACTGTGGGGAAC
TAACAAATCCGTGTCTTAGTGTAACGTGGATTATATAGTACCGATCACCGTGCACTCT
ATTTATCAGTTTCGTGATTGATTTTTGCGGCATGGGTGAAGATACTCAAGCCGGTGGTGACTCAAAAGTTACG
CTAAAACGTAAAATAACGTTGTTTAATGGCGTTGGAATTATTATAGGAACTATAATTGGTTCCGGAATCTTTA
TATCTCCAACTGGGGTTTTTCTTCACACTGGATCGGTGGCAGCATCGTTAATAATATG
GCTTGTTTGTGGACTTTTGTCTACACTAGGGGCTCTATGTTACGCCGAATTANGGACTTCAATATCGAGGTCT
GGCGGTGACTATGCCTATATTTATACTGCCTTCGGACCATTGCCAGCCTTTTTGAGGATGTGGATAGCCCTGT
TGATTTATAGGCCCACTACACAAGCGATCGTGGCGCTGACATTTGAACATTACGTTGT
AAAACCATTCTTTCCCGATT
>126037452_2 EL597246.1
GCACGAGGGCGATCGCTTAATCTAGATTTTCTAAATCTGTAGTTGCGCCTACGTTGCCGTTTACACCCAAGGA
TTTTTTAGCTTCTCCCTCTGTCCTGGATATGGTCTATGGTACTGACATAACTATTATTAGATGTGTTTCCCTA
CTTAGGAACCACACATACGTATCCTGCTCTATGTCCATATTTTAATACTTTGTCTAAT
TTGTCAACTAAGCTAACTGTCTAGAGGATCGTATCATCGTTTTCACAACTTTTCCATTACAGTGCGTTTTGTC
TTATTACCATACGCGACCAGTTCAGCGTATTTTCATTCCCACTATCGCTATTCTGAGCCGCCGACACCGACTC
AACAACATAAGTGACGTATATATCATTACTTGTTTTCTTCG
>126037455_2 EL597249.1
GCACGAGGGTCCGTGGTACACCGACGAATGGAAATTAGAAATATTTATATGTGATATTTTTGTGGTATAAAAA
TTAAAACTAGATTAACTATAAAAATGTGTCAAGTCCTCACATATATACGGCTCTTCATGGACAATGTGATCGA
TTTCATCTTCGGGCTTTTCTGGGGTTGCAGTAAAGTCGCCATTCCAGATGTTGAGAAA
AAACATTCCTTCCTAGCCGAAAGCGCGTCAAGTATGGCGAGAAAAATTAAACAAAAGGAATTGAAATCTGAAG
AGCTAGTACAAGCCATGATTGATAGAATTAAGCAGGTCAATCCGTATTTAAATGCGATTACTGCAGATCGGTT
TGAAGAGGCTTTAAAGGAAGCGAAGGAAATCGACAAACAAATAGCTTTAGGAGCCTCC
GATGAACTTTTGAAGAAGCCATTTTTGGGAGTGCCTTTTACAACCAAAGAGTGTCAGGCAATGAAAGGAATGC
CGTTGACGATGGGTATCTGGTGTCGCCGCAACGAGAGGGCAACAGAAGACAGTGATGGAGTAGTGCTGCTGAG
GAACGCTGGAGCCATTGCCATCGCCTGCAGCAACCTTCCTGAGTTACTTATTTGGCAA
GAGACCCGCAACCCCGTGTACGGCATGACCAATAACCCGCACCACACGGGCAGGTCGCCGGGCGGGTCCAG
>126037463_2 EL597257.1
GCACGAGGAATTGCTGTAATACAAGTATTTACACTAAGAAGTGATTGCTGATTTCTAAATCAGTTTGAAATAT
TACGTTTTGATAGTATATATAACGTTATTAAAATAATTGTTATTATATTGTGTCACACTTGAAGTAAACAATT
CCAAATCATTGATAAGATTATCTTATAGTTGGATTCTTAAAAAACAGCATACTTATAA
ATAGTAATGGATAAAAAATTATCATTCACGGAGTTGTATGCTATTTATAAGAAATGGGTAATTAATAATCCAG
GAATAGTTACTGATGTAGAAACTGTTGCAACATGGTCATCTTACTTTGTAGCAGGTAAAATAAACAAATCACC
AATCATTTCTGAACTTGTTTATTCCTTATCGAAATTATTGTCACTATTTAATGACAGA
CTCATAAGAGAAGCTTATGGAAATGAATTAAATGACTATGGTTTACAAGAGCAAATAAAATTATGGCTTACTG
TAATCCATTATTGTGAAGTATTTGTAGAACTTGTTGTAAAAAACAGGTGGGGTACAAAAGGAAAATGGACCGC
AACATCTTTACTTCAGATGTTCAAATGTGCTTCGGCCATTGTGTTATTGTATAGATAC
AAGGAGATACCTATTCAACATCCACCTATACCAGCTTTACAAAGAAAAAAGTTTACCGAAAGTAAAGATGTAG
ATGAAAGCTCTAATGCCTTTTTTACGTTACGAAGGTCAG
>126037465_2 EL597259.1
GCACGAGGGTTTATAGTGTAAGCTTGTTTTAACATATATTTTTATATATTGTGATAAGTCGTCGTGCCGCCAC
GCAGCACGACGGGCATTGGTCTCCGAGCGTGTGTCCTGGGCGACTTGCTGCAACGAGCAAGTTCCAAGCCTCT
AGGTACCGTCTGCGCCAGATGGATTCGCGAAATTTTAGTGGGTGGTTTTTATATTCTA
GAGCGGAATATGAAAATCTCTATCAACTAATACAAACATTGCCTGGTTACAGCAACTAACAATAAACCAATGC
TTAATCTTGTTTTCCAATACAATATTCGTAATATTTGATGGTTTCTTCACTACAATACAGTGTAACGGCGCGG
GGCTTCGTCTCCCGCCGCGTTCTCTTTGGTCCATCGCAACTGCGTCGTGTTCACGTTG
ATTTTGAGCTGTGATGTTTTAGTGGAATTCGATCGCGCTTGCACGGTCGTGTTATCGTATCGTAGTCACTTAT
TTATGTATCGAGTCGAAGGCTCGTATCCGATTAGCACACGCGCTAACTGAAGCTTTGTAACTGTTATTGACAG
ACCCCTAGGCTTATCATTTTTCATTTCGGTAGTATTTGACTTAGATGCGCCTTCGATT
TGGTTAAAGTTCTCTATTTGCTTATAAGATGCATTAGCTTCATAGT
>126037466_2 EL597260.1

GCACGAGGCGATGGCGGTCGTTAATGCTAAGAAATTGAAGTTCTCCACTCTTAGAGGATCTTTCTCTGAGAGC
GGAATTAATGAGTTTTTGAGAGACCTTTCCTTCGGTCGCGGACAAACAGCACCCGTACGAGGAGCTGAAATGC
CCAAGATTCAATCCACTGAGCCCTGGGATGGCAAAGATGGTGAACTGCCCCCAGAAGA
GGATATTGACCTCTCTGACATAGACTTGGAGAAAGATGAACTGTAAACTAGTGATGTGAAAGTGATATGAAAC
TTTTTTTCTTTACAAAAAGCTGTCTAAGATTGCAATGCTATGTATGATGTGGATTTTTAATGTGTTTTGTAAT
TGGTAGATATAAAATCTTTTTTAATGTATTTTGATATTTTTTGTGATGCCTATATATA
TTTTTAGTCAATATCTCAACTGTGCATATCCAAAACTATCACATAACTTATCAATAGTTTTCCTTATACTTTA
CAGTCATAATCAAGAAAATTAATTACCGTTTAATCGTCATTAAACTTACAATAATAAATATAGATTTGTACTT
ATTTAACAACAAATGGACACTTATTAATGTCTGCATTTAAAATAAGTGAATATATATA
TTTTTTTTGCCATACATTAAGATAACTTATATTTATAAAAATCATCAACAATTATTTATATGTTCAATAG
>126037467_2 EL597261.1
GCACGAGGGTACAGCACCACTGGCCGCAGCAAGCGCAATAAACATCACAGAACCATTGAACAGTATGAACGTG
AAATAGAGAGGCTAAAGTGTTCAGTGGAGATGTTGCGCGGACGCCTGGGACCCACGGACTCTGGTCAGGATCA
TACTGATGCCAAAATGAAGGCTATTATATCCAGATTAATCTGCGTGGAAGAAGAGCTA
AGGCGGGAACAACGCAAAATGGCCGCCGCGCTCTCCCACAAACAACGCGTCATAGAAGCCCAGGAACATCGTA
TCGCGGCCCTGGACGAGGCCAACACGAGGCTGCTCTCTGCTCTGGTACACTTGCAGCAGCGAGCCCCACACCC
CCCCGCCCCCCACAGTAACTCACACTCTCCCCATTCACCGCATTCTCATCAGGAACTG
CAAATATAATTGTGAAGTAGGTTTTCTCATATATCATATTATATCATCTTTTATGGATTAAAAAGAAACAATT
GACGAATCACTCTTTCTTTGTCGTTCATTTACAATTTAGTACAATATGGTCGACGAATTGTGGCCGCTCTAAA
ATAGATTTTCT
>126037468_2 EL597262.1
GCACGAGGGTTAATAATTCAGTTGTAACAATTAATTTTACTTGGATATGCATATAATTGAACGTTGAGTATAT
TTTATACTTCATATTTTCTTCTTTTTTTTTTTTAAACTGTAAAAAAATAATGCATTAAACTGTCATTTTTGTA
TTTTGTAATTGTCTGCAATTTATATATGTATTTGGAATATG
>126037477_2 EL597271.1
GCACGAGGGTAATCAAGTATCAACTAACAACGTCTTTTGAAAGTTTATCGAGTTTCCATAATTCCATTTCCTT
CCCAATTTGCTATAGAGTCTTTGTGGTTTTGTGATTCTGCTTATTGTTGTAAAATTTGAATTAAACCTATTTT
CTACACTTTAAATCAAGTTGGAGTGTGTAAAACATACGTTCTAATTAATAAAAGATTT
AACGCGTAACTGAATATTTTCTAAACCTTGTGCAATAGTAAGGCGCTATCAAAATGGCTCTATATCCATCATT
AGAAGATATGAAAGTGGATAATATGATTCGAGCACAAATGGCTCAACCAGACATGGCACAGTCTTCTTATCAT
CAATCCTACCAGACACCACCAGTATCAGCTCCTCCAAGCGCACCTCGACAAGTATATC
CTGCCCTTGGTGAATATATGGGATTAGAACTGTCACCAGAAGTTATTGCTATCAATATGCCCGAGTATCAAAT
CCAAACAGTACAACCTATTGGATCGGCATCTACTATGATAGCTCCACTTTCTTCACAATCTCCCAGCATATTG
AAGGCAACTGTCACTCAAGGAATAAGACAAGTCATTCTTTGCAAGGATAAAGATGGTA
AATGTGGCTTGAGATTACAC
>126037486_2 EL597280.1
ATATATATATATGCTTAGTAATAAAAAATATGAAACGGGAACAAATCAGTTATCAATGTCGCTGAAGCTCCCT
TATAATTAATATATACCTACATTTTCAAATTGATGTGATTTTTTTTTTAAATATGATCATTATTTATCCCCTA
TTAGCGTATGATGCTCATTTTTGAAATATTTACCATTTATTTGTTTAGTTTAGTGGTA
GTTTTAAGTTCGTT
>126037487_2 EL597281.1
GCACGAGGGTCGATCTGACCGAACGCCGATTTTAAGACGAGGTAAAAATCTAAATATTGAAGCTGAAGCGATC
AATGCTTTATCAAATACACCAACAACAACAGAAAGTCTACAAACAGAGGAGACAACGCAATATGAAGTAACTA
CTACAGTACGAACTACTCCGAGCCTTAGAGGAGGTAGGAAGTACGTTGCTAAAACCCAC
TGAAACAGAGCAATTTAGGACAACGGTACCAGATCGACCTATTGCAGAAGCAAGAATCGCTCCACAAGGAGAA
CTAAAAGTCCAATCTGATACTCGCGCGTTAGAATTATTAAGATCTCTGTACACAATCGCAGCGCGATGGGGGT
GATTAGAATGATATAATATTTATTCATAATTAGGGTTCGTTACTTCTCAAATAAGACT
ATTAAAAGTTTAAGACTCGTTTTGTTATAATTAGGTTATTCGTGAAAGTATTTTTTTAATGTTAAGTTATTTA
GAAGTAGCTCTTTCAAATTATGTCCGAAAATTTGTATTTATTTAATCGAGCCCGCAAGCAAGAAAATATTATT
TAAAAAAAATATCCAAGTGCACTCGACGGACCAAATAGTTAAATAACTGTTAAACAAA
TCGTATTTTCTTACAAAATTTTACTTAAAAAAATGCTCAATATCGAACGAACCCTAGATATAAACTTCATTTG
TAGATAATATCACGACTGCGCTTATTTT
>126037488_2 EL597282.1

CATTGTAAAAATTGAAATTTTTTAGTCTTCCTCGTGTCCGCATCGAGACTACTGGACAATACTCGAAACAACT
TGCCCAATCACGATTTTATTAAATTTGTAGATAATGTTCGTTTTGGGTAGGAATTTCATATTAATTTTCGATA
TAAAATAAGTAGAGTTCTGAATAATGTCCATTCACCTCCATTGTAAATATTAGATATT
AATTAATTATAGTAAAAACAAATCCTATATATTGTAAAAAAGTAAAACTCCAAAAAAAGCTAGATTGTTACGA
TTCCAGGAGCATAGCCGCAACAATCGTAAGGGGCCGTCCGTAAATTACGACACGAATTTCATTTATTTTTTGA
CCTCTT
>126037489_2 EL597283.1
GCACGAGGCCACATTGGAAGGTCTGGTAGGACTCTACTCTGACGGAGTGAACGAGAGGGGCTACTTCATGGCA
GTTCTAGAAGCTTCCAGGGAGTGTCTTATGAGGAATCACGACAAGTTCTCAAGAACTGTGCCTATGGACAACG
GTCGCAACTGCGACATATCGTTCGACATCTTCGAGTGCATCTCCGACCGCATCGGCGA
GTATTGCGGAAATGCTGGACTTTGAACTTAAATACTCATTTTTATATCTATTTTAGTAGTCCTATTCACATTC
TTTATTTCATATTTCATTATATCACTTACTTTATGTATATATAATATATATTACATGTATTATATCACTTATA
TGTATGTATAACATCGTTAAAACGTGTTTATTATTTTCTTATGAATCATATATGGTGA
TATATTTATTTCTATACTTGCTTTAATTATTTATTTGTGTATTTTTTATTTGTTATAATATTTTCTATATATT
TCTTTTTGTTGCTGTTATTATATAATATTAGCTTAATAGAGTATTTATTTTGTTCTAATATAAAAGGTTTTTG
TTTGATTTTTTATTTTTATTTATGCTTTTTATTTGTTTATTTGAGTTTGTATACTTAA
ATATATATGTTTGGACACATACATATAAGTACAATGTCTCGTTACCACAATACAACATAATATCATATTTACT
T
>126037495_2 EL597289.1
GCACGAGGTATAAGCGACCTTTCAACTCATGAAAATTATTTTAATGTGAAAAGAAATATTTTATATATGTTTT
ATGAAATTACAAAGTGAAAATAATATTTATGTTGACTTTTGTCAATTTTTATATACTTTTATAATAAGGTATC
AAATATTTGACTCAATGTGCAAACAACAACAATAATCTATGCATTTGCTAGTGATGCA
ATTAAAGGACATACACTACAGCTACGTAGTACATTCACATATGT
>126037497_2 EL597291.1
GCACGAGGGAAGGCTCGAGGGGAGGCGGAAAAGGGCGAGGAGGCGCGTCTACACTGCCCTACGTAATACCTTG
CACGAGCACTGACGCTGGACAAACATCTGATAGCTCCGATGGCAATATTGAAGACAAACGATCCGGAGAGGGC
AAGGCAGGACAAAGAGTACTTCGCTCCCACAGAACCAATGACGGGGATAAAGATAAGG
GAAGGAGTTCCCCGACAGGTTCGGAGACCCGGTCGGGACACGGTCAGGCTTTGAACTCCATCAAAAGTCCACA
AGCCTCTGGTCAAGATGGCGATCATGCTACGTCCACGTCTGGTTCTGGTGGAAGATCTGAGTCAGGTCCGGGC
CCGTCCGTAGAGTTGCACCCGAGGAAGCGCAAGATCAAAGCATCCAAGGACAGCCACT
CGCGGGAGTCCAAAAGTGACCAGGTGCCCGACAGCACCACTCTAACCCACAACATCACCCATTCCAACCCTTA
CCAGATGTACATACATATTCGCAAACAGATCGAGCGACGTCAGAAGGGCCTGTTTCCTGTAAAACCTAAACCT
CCAAAAGATTTTAATAAATACCTGATGAACCGATGCACTTACACACTCCAAAATAATG
TGAATCCTGAGCCCCAAGTCGAGATACCGATAAATTTGCCTTCCCAAATGGTCAATGAATTTTTATCG
>126037503_2 EL597297.1
GCACGAGGGCGACCCGTACAGACTTATTTTATTTTTGTGTCGTGATTAATTTAAGGATTTTGTGAAATGAAGT
GGATAGTGATAGCCAGCTTTATGGTGTACCTGGCGGCGTTTAGTGAAGGCAGCGCGTTAAGGTGCTCTCGTGG
AACGGACAATGATTGCCACACAGATAACACAGAAAAAGAAGCTGAAAGTAGTGTTGAT
AGTAAAAAGCCAGAGTGCCTCGCCGGCAGCGAGTGGGAGAGCTTATGCCATAGCTGTCGGTGTTCTGAGGACG
GCCAGCCAGAGTGTGCGAGATTAGGCAGTTGTAGAGATGAGACAAAGAGTGAGCCAATGGTTTGTAAGCCACT
CACAGAGTTCCGTAGAGACTGCAATATATGCATTTGCTTCGAGAGCGGTCTGGGGAGC
AGCTGTACATCAATGGAATGCGCACCTAAAACTGATATTTCAAATGGCAAAGAATGTAAAGTGGGTACAAAAT
GGCAGAGCCAATGCAATAACTGTAGTTGCTCGGACGACGGGATGCCTATGTGCACTGACATGGCGTGTCCTGG
ACAAGAGGAAGAGCCCGAGATGTTGTGCGCTCTAGGCACGTCGTGGAAACTTGACTGT
AATACTTGCTATTGCCCCCCGACTGGACGTGCGGCCTGTACCAGGATAGGCTGTG
>126037506_2 EL597300.1
GCACGAGGCAGAATCTTGCAGTGATCTAGGCAGACCGGTTCAGCAAGTTTTAGAACGAACTGCAGCTAACGTG
CAGTGGACGGAGAGGAACTACCAAACTATAGTCAATTGGCTTTTAGATACAGAGAAAACTATGAAAAACGTTA
AAGCGTGATTTAATTACAAAATATTTTTATTGTTGTATGTATAAAAAATGATTTTTTT
AATTATCACTTTCAAAACCTCTGATAATATAGATAATATAGTATATCGATAACATGTTGAGTACTTATAAAGT
TTTGACCTTTGAATACTAATTTAATGAAAACGATATAAAAAATTGTAACATGAGTCATAAATGTACAACTTAA
ATGTTTTAAATTTATGTTGAAAAAAAATGTATAATCAAGTCATATTTTAACAAGTCTA

AGTTAAAAATCTATTTCATAAACTCATAAAAAAACGTACTATTTTATTCGAAATAAGGTATAATAGATTTAAA
TACATCAAAAGCTACTAAATACTTGTAAATATTTGTAATCCCTTGCAGTAATATTGCTTAAAGATGAAGACCA
TCAGTAAATGTAGCAATGTCGAACGTATTCAGTTATGTATCGACTAACATTTGTATAA
NATAAGTATTAAATGTAATAAAGTATTAAATGTAATATGAATTAGCATAATTTAGTAAATTTTTTT
>126037507_2 EL597301.1
GCACGAGGGGTCGCCGAGTTCATATTGAGTCAGCAGCAGCTGCTGAGCGCGGGTGCGGGCGCTGCCCTCGGCC
CGTCGCCGACACCATCCCGCGTGCCTCCACCACCACGCGCGAGACTGTCTGTCTCACCACACTTCCCGATGGA
TCAACCACCCAGCTGCCCCTCTAATGGCAGCCCTGGAGACCCTCATGATTATGGCAAC
TTCGATTTTTCTAAGAGGAAGGAGTTCTTTGGACAACGTAAGCAAAGGGAATTTATTCCCGATAACAAAAAGG
ATGATGGATACTGGGACCGTAGGCGGCGCAACAACGAAGCTGCTAAACGCTCCCGTGAAAAACGTCGTTTCAA
CGATATGGTTTTGGAACAACGTGTTGTTGAACTCTCAAAGGAAAATCATGTACTGAAG
GCGCAATTGGATGCAATTAAAGAGAAATACGGCATTTGTGGAGAAACTCTTATTAGCATCGATCAAGTACTGG
CAACCCTACCAACATGCGATCAAGTTTTGTGTGTAACTAAGAGAAGTAAACTATCAGGCGGTGCGCTCTTTCC
ACCTGCACCACCACCACCACCGCCGGTGCCCCCAGCGTCACCGCCATCTCCTCCCCCA
CAACGTGCACCAGAGCCATATCAA
>126037508_2 EL597302.1
GCACGAGGGTTGAAGCTGTTACAAATAATATAAATGGTGATGGTATTTCAGAAGAAGAAGTATCTGCATTAGA
CAATGAAAATGATGATGTATCTCCGCCAAATGGCAATTCAGATGATGTTGAAATACCAGTAAAAGGTCGTAAA
AAGGCTCCAAAAAAAGATGAAAAGCCAAAAGAAAGCAAACCAAGAGGTAAAAAAAATG
TAAAAGCAGAAGTAGATACAAATAACAAAGATGTTAATGAAGAAAAGCCCAAAGGCAGAGGTAGAGGTCGGAA
GGCTCAACCAAAAGCTGAACCTGAAGATGATGAGAATGCTGCCGAAGAAGAAGAAAAGCCAGTTGCAAAAGGT
AGGAAAAAAGCACAACCTAAGAGTCAAGAAAAATCAACCGATCAGAAAGTGGAAGACA
TAGAAATGGAAGTTGAAAATGGAGATGAAAAGGAAGTGCAGGAGAGCATTGACAATGAAGAAAGATTGGTAGA
GCCAAAAAAGAAAGAACCTAAAGGACGGAAAAATGCCAAAAAGGCTCCACTTAAGGAGGAACCACAAGAAAAT
GATGAAGATTTTCTAGAAGAAGCTCCTACCAGCAAAAAACGGAAAGTTACTAAAAAAG
AAGATAAAGGAAGAGGTGATGCTAAAAACGAAGGTGAA
>126037509_2 EL597303.1
GCACGAGGGTGAAGTTCTTCAAAAATGGACTTGTATTGAAATTTTAGATACCCACGAACTAAATGTCACTTAG
CCACAGTGAAGTGATAGTCAAATGTTTACAAAATAATGCACGAATCTAGATGTATTTTACTTTTAATTTTTTT
TCAATCCCTTTCTGCCCTATTGCTTAAGACGATTGACTGCTGAAAATGGGAGAACCAA
TTTTATATTGTCCTCTTTGTTGCAACGATACATTTGAATCAAAACAGGCTCTTTATGATCATTTATTTAATAT
ATCATCTAATTTAGTTTGCTCCTTGTGTACTAAAAGATTTTCGACATTGGAACAGCTTGTAGATCACTTAAAG
TCTGATAATTGCAAATTAATAAATGCTACTGAAACCAATGCTACATTTGAGGAAAGCT
CTCAAAATGAAACTAATGAAAGCTCTTTAAATAACACAAGTGTGAAGTTAGAGTTACATAATGGTCATATAAC
AAATAATGATTTAGAAGACCAAATCACCTCTCACACAATAAAATCAAATTTAAACCAGGGCTCTGAAGTAGGA
AAAATGTATGTAGAATTACTTAGTAAGCAACTAATGAAACCATGCTTGCAAACTCAAG
AGTTAAAATTAGTAAAAGAAGATGGACAAAGTCGCTATGTGATTTTAACAAATGAAGAATCTGTTTTAAATTC
AGGTGTTGCCATTGTAACAA
>126037510_2 EL597304.1
GCACGAGGATGTCAAGTACTAAACTTTTTATAATCTGTGCTATATTGGCTGCCAGTCCTAATTTACAAATGAA
AAAAAAAATTAAACATTCGGTATAAAGTATTTATTAAAAATATAATAAAAGATATATTGTGAGATTAGATCAG
GAACATAACATAGTTATGTTATGTTACTGAGAAAACAGTAAAACTGAAAACACAATAT
ATAATATATAGAAAATACGAATTGTTATATTGTTCAATACAATTAGATTGTTGCTTAAAGATTTGTCTTTAGA
CTCGTATAAATGTTTAGCTTTTTGCATTATAAGTAAGATGTTTACAAATTATAATGACAGTATAGGATTAATA
ATAAATGAAAATAATATAATATAAAATAATACATATATTTATCTGCTTTTCTTATGCA
GTTTTTCTTTTTTAACTTAATGTGAATCTCTTATATACTTGAGGACATGGAGCAAGCATATAAGCACCAATAC
ACTAAATGTTGGATTTTATGTACTTCAAATATATGTCACCAATATAATAAAGTACAATTTTTAAACCATTTTC
GATGTTACTTGTTACTCACAATTTTTAGCACTAAATATATTATACAAACTAAGTATTA
AACATACTGAATGAAAATGTAGCAAAAAATCTTATAGAAGGACATTTTTGAACTCTATACATTCAAG
>126037511_2 EL597305.1
GCACGAGGCGATCGACGTGCCTTAGACGAAACGTATCGAGAAGAGTTAAATGGTATAATGGGGACCCCCAGCA
CTATGAATTCAGATTCGGAAAACTGTGTTGCTAGCGCAACAGTGGCTAACGATATGCAACCGTTGGTAGAGAG
GATTAGGGAGCTGGAGGCGTTGCTGAAGCGACGCGATCAGGAAATGCTGGAGTTGCGC

TCTCAACTCGATAAGTTACAGAGCGTGTTCCCGTATCAGCAGTATGCGAGGTCCAGAGGTCCCAGGAAACAGA
GGGCCCAAGGTATCTCAGCAGAACCACAGACGAGCTCGTCTCTCCAAGACTTGGCTCATCAGACATTCCCGAT
ATACGACAAAAGTGAAGGATCCCGGGAACTTATAAAGAGCGCTATCCTAGACAACGAC
TTCATGAAGAACTTGGAAATGACACAGATACGTGAGATCGTGGATTGCATGTATCCAGTGGAGTATGCCGCCG
GCAGCCTCATCATCAAGGAGGGGGATGTCGGCAGCATAGTGTATGTTATGGAAGAGGGAAGAGTGGAAGTGTC
ACGCGAGAACAAGTACCTAAGCACAATGGCTCCCGGCAAGGTGTTCGGTGAGCTGGCC
ATCCTGTACAACTGCAAGAGAACCGCCACCATCAAAGCAGCCACCGACTGCCGGCTGTGGGCCATCGAACGCC
AATGCTTC
>126037512_2 EL597306.1
GCACAGGGGAACTGGAAGTACTTATCAGGGAGTTCTCTGAAACCCTCATAGCCGAACTGGCTTTGAGAGACGA
ATTGGAATATGAGAAAGAACTGAAGAATACCTTCATATCATTGCTACTGGCGGTGCAGAACAAACGCAGACAA
TACCACATTGAGAAGAAGAAGCGGCCTGGTGCTCCTAGATCTCCCAATTTAAATACTA
GCCACTCTGAATCAAAGTACTTGACAACAGTTATCCCTTTCCACTTGGACAATGGTCCTCCCGACAACCAGTC
CCTTCAAGTTCTTATCAAGATCTTGAAAGCAATAAATGAAGACAGCCCTACCGTGCCCACACTGCTCACAGAT
TATATTCTCAAGGTCCTATGTCCAACATAAACATTCAAATTATACTTAGCTAATTTTC
ATTATAAATTTACAAATGAGTACATTTTCACTAATAATTTTCAACATAATATAGAAATTACATTAAATATAAC
AAAAGAATCTGTTTTCATATCTGTTCGTGCCTAAATCCCTAAATTGATAATTTAACGTTTTCTTATGATCTAT
CTGTGTTATAAAAAAAGGTGAAATATTTTATTTAAAGTGGAAATGTACTTATCGCCAT
TAAAACGATTCAATTTTCAAATGATTCGATGTCAGCACATTATATTTAATATTAAATAATTATTTCATATCAA
ATGAAATGTGAAATATTTTC
>126037518_2 EL597312.1
CAAAATTTACTAAAGCAAATTTATGATTCAAATTAAATATTTTCATTGTGCTAAGTTATTATTGAAGTTAATA
TAATATTAATTTTATATAATATTGTAGTAAACTACTTTACTACAATATTATATAAAATTTAATTTAGATATAG
ATAATCTGCTCATGTAACTATAAAAACATCAAAGAAACTAATTGATTTGTTAATATAT
TTTAGCATTTTTATATTATTTAAGGATTTTTGTACTGCTTTGATAACAAAAATAACATATTTTAAAATAACGT
ATAAAGATTTTCATGCAGCAGATTGGATTTCAAACCGCGCCTCCAAACTTTGAAAAGGTACAACCTACGTCGA
AATTCCTGTTTTCTTATAAATTAAGTTATACTTAATTTAATTAATGAGGTGTAGGTTT
GTGGCCATNCACATAAAAATTGCGAGTTGCTTTCGGTAAGGTAAGCTCCATTCCTTCCATGT
>126037526_2 EL597320.1
GCACGAGGCTCCAATGTGATGAGAGTGTAAATTAACAAATTTATATGAACTCGATTTAATTTTTCTAACTAAA
TATTGATTTGTTTAAATCAAGCAGCAATTTATTTGTTAATTATTTTTTTTATTTCGTAATTCATACTAGTAAT
GTGGAGATTTTGGAATACATGCTAAAAAATATATACTTAGTTAGCAAATTTAAAAAAG
ACGTGAATAGTCGAAAATATTT
>126037527_2 EL597321.1
GCACGAGGCTTTATTGTGGGTAAAGATGCAAATCAACAAGTGATACCTGCTCATAAGTTTGTATTGTCAGTTG
GAAGTGCGGTGTTCGATGCTATGTTCAATGGAGTTTTAGCAACTAAATCTGAAGAGGTGGAGCTACCTGATGT
AGAACCGGCCGCATTTTTACATTTATTAAAGTTTTTATATTCAGATGAAGTCAGAATC
GGTCCAGAAAGTGTCATGACAACTTTGTACACAGCTAAAAAATATGCAGTGGCTGCTTTAGAAGAGCATTGTG
TGGATTTTCTTAAAAGCAATCTTGGTACAGATAATGCATTTTTACTTTTAACCCAAGCGAGGCTGTTTGACGA
GCCACAGCTTGCTGCATTGTGTTTGGAAATGATTGATAAAAATACTACAGATGCTTTA
AATGCCGAAGGTTTTACTGATATAGATCAAGACACATTAAATGTTGTTTTAGAAAGGGATACATTAAGAATTC
GTGAAGCGAAAATATTCGCGGCAGTTCTAAGATGGTCAGAAGCGGAATGTATACGGCGACAGCTGCCCGTAAC
GCCAAACAATCAGAGAATGGGTATTAGGGAGGGCCTTTCACGCGATACGTTTTCTCCT
CATGTCGGTAGAGGAATTCGCAATGGGTCCCGCCCAAAGTGGACTACTCGATGATAGAGAAATAGTACAACTG
TTCCTATATTTCAC
>126037530_2 EL597324.1
GCACGAGGAAAACGGTGAATTCTGGACTTTCTCCGTTTTAGGGATGAAGCTATATCCCAAAAGTGTGAATATA
ATTTTTAAAACATCATTTTATATGAAATTCTATGGATATTTACGCATATGGATATAGTTCCTAGTGATTTATT
GTACAATTGTCGATTTGCACCACATCTCAAAAACTGTTAATGTAATGTACATACAAAT
ATATACCTATATAGATATATAAAAGGTGAATGCTCTATGAAAATTACGAACTACTGCATATTTTAACATATTG
TAAAAGCGTATGCCATAATGTCATTTGTTTTGTAATATTTTTTTAAATTTTTTTATAACTTAAGTTTTTAATT
TTCTGCCGCATATTGGAAGGTTTTTAAGAATATTTGTGTATTTATGTTTCTATAATAA

ATTAATTTAGAAGTACAAAGAATATATATGCAATATTCATTTTTTACTAGTAAAAATTATTTGTGGCTATAGA
GGCAATTAAAATGTGTAAATATGAGTCTTCTGTTTTGTGTGCTCGATTTCCATTTGCGAAAATGAAATATTTT
CATTTCATCGCTTTGGTAACGAGAAAATCGTGGCATTTAGTTCCGCCACTCAATATAA
AATGTCTCGAAAA
>126037531_2 EL597325.1
GCACGAGGTCAACCTCGAATTACTAATAACAATAATATGTTTAAGTTCATAATAAATACATATTTATCTTGTT
GAATATAATATGTTATATGAGTAATATGTGTAAGATTAGATTGTATTTAGTTGTTAAAAGTGGTATAACTGAA
AAATTGGTGCAAGTAAAAATTATTACTTTTTATCAGAAAAAAAATTTTTTTTGATGCA
TTTTAACAAACCTAAAAATATAGTTTTAACCCTTTTATCACAGTTGTCAACATCAAATATTTTTATTCAAAAT
AAGAAGTTAAAAACTTATTTTTTC
>126037535_2 EL597329.1
GCACGAGGATAGGACAGAAAATGGCTAGTAAAACAGTCATCACATTCTTCATAACCTATTTGCTAGCACTAGC
GCTATGTGCGCCAGCGCAGGACCAAAAAATTGAGAACGAAAAGGAAGACCTTCAAACTGCTGCATCCCATTGG
GGAGGATTTGGTTACGGTGGGTACGGACACTATGGACACTATTATCCCCATTACGGAT
ATGGATACGGATACGGATTGGGGTGGCCGGGATATGGATATTATTACGGAGGCTATCCCTATGGGTACTATGG
CCATGGGTACTGGTGGTAATCGCGAACAATATTACTGAGTAATGTAATCCAAAGATGATCAAATGTACATTAT
TTAGGAATACTTCGATAAATGTGTGATACGTTTTAGTTTTATAAAAATTATCATGTTT
TTGTAAATTATACTTAGTGTTGAGGTTTTGTAATACTTTTTTTATAATTTTCTATTAAAAAGTATTTTTATAT
AGATTAATAAAAAAATTAAAGAAAAAGTGTGACGTCACTTTCGCAATTGTTATGTGCGATACGTCCGCATGGA
TATCATTTTAAGTTAGCTGTTTGAGCACGCAGGATTTTTTATAAAGCTGTCTCTACTG
TATTTGGTCAAGTTATGATCTTTACATATGAGGACGTAGTGATTTACTTCTATGTAATAGTACTAGAGCGCTG
ACTTCACAAAATTATCGGATTTAATCCTTCAATATATTGTTGCTTAAATAAA
>126037536_2 EL597330.1
CGGCACGAGGGACACCAAGGCCTCCTTCGTGCTCTGTGCGCGACTGTACGCGCTGCAGGGTACAATAGGATCA
ATGTCATGGCGCGCCGCCCCGCTTGCTGCTGAAGTGTTGAAACGTTTAGATGATGCCAACTTCTTAGAGCATC
CCTACCAGAATGTGCGCGAGAGCGTCGGCAGCTTGCTAATGACAATATTCGACATGGA
AATCACTTTCCCCGGAGGTGGTCCCAGCACTGTACCACAACTGAAACAGTTCTTAGCGTCGGTGAAGCCTCGG
CTCGCGGCATTGTACGATGAAAACGGCGATATAGTAATAAAAACTGCGCCGTCAACATTACCACTAGCATCAA
CGTCAGCGTCTTCAGCTTCGGTGTCAGCGTCGACGTCATCATCTGTGACGTCATCGCC
GTCAGTGTCTGCGTCGGCGTCGGCGTCGGCGAGCGAGTCCGTTCCCATGGCAACGTTGACACCAGTTGATGAT
TTGGATCAACGGCCGGGGCATTGTCATC
>126037538_2 EL597332.1
GCACGAGGCTTGCTATATATTAAACTCAAGAATTGATTAAATCATGTTACATTTTTATTGTTTATTCTTTATA
TTGATTTATTCAAATTTTTATTGCATTGATTTTTAATGTTGAATTCTCGAATTATGATTGTCTACACAAATAT
TTTTTCATGGCCTAAATAAAACTGTTAAAAGCAATAATTGTCACAAATTCGTATAAAA
ATATTATGGACATACATATATATTTTGGTACCTCTAAAATTGTAAGAATAATGTTTAGTTTGTAAAATACTTT
AAAAGACATGTAAAAATACTTATTATTACCTTAATAATAATG
>126037542_2 EL597336.1
GCACGAGGCGAACCCACAACCAGTCGCATATACAGTCACACAATTGTGCCCATGCGCCATGACTGGCGCACAT
GTATGTTTAATTAAGATAACAGTCTTAATTTGTGCTTAATTTACATATAATCCATAAATCTGTTTATATAGTG
TATAATTTACTGTTTTATATGTTATTTGTTATTAATCGACCCCGGACTTTTATTCTGC
GATGAGCCAACAACTGGGCTGGATTCCTCGTCAGCTCAAAAATTGATGACACTCCTGCGGGCGAGTGCGGTGC
AAGGGAAAACTGTGATATGCACCATACACCAGCCGTCGTCGGAGTTGATGGCGCTGTTTGATAAACTTGTACT
TTTAGCTGAGGGTAGAGTCGCTTTCGCCGGAAACGCTTCTGCAGCTCTAAGTTTTTTT
GAAAGCTTAGGCTACCAATGTCCCATCACATACAATCCAACAGACTATTTTATAAAAATCCTCGCCCTCACTC
CTGGGTCCGAGGGTGCATCTCGACAAGCTATCAAAAGTGTTTGCGACAGGTTCGCCGTCAGTGATGCTGCTAA
AGAACTGGATATGGAGATACAATTAGAATTCCATCTTATGGATCACGAGGACGAGGAA
ACAAAGCGATCAAAATTCAATAACAATTTCAAATCTCCTTTACTACATACGAAGATCGTTTGGCTCGTGTACA
GATATCTTCTAATCATACTAAGGGATCCAAGAGTGCAATTTTGTTCGAATATTACAAAAATTGGC
>126037545_2 EL597339.1
GCACGAGGCCACGGCGGACACGCGTGTTTATAGTTTTATTAATAAATGTTCGAAATACAATATAAGATTTAGC
TATGTATCTGTTTTTAGCATTTCTCCTCGCATGTATACCATACCATTTGGATTGTCTTGAATACGTGCCACCG
GAAATATCACGTTTGAACTTCTTCGATACCGACCTGCTTCGATATGCTGCTGAAGATA

AAGTGGGCAATGTGATAGTGTCACCGGCCAGCATCAAGTCTATCTTGGCCATGATTGTAGAAGGATCTCATGG
AGATACTGAAGCTGAAATCAAAAGTGCTCTAAGATTATCACCTTATAAAGACGAACTTAGAGATCAGCTGAAC
TTATATTTGTCGGCGCTTTACGCAAACACATCGGGGGTGTCCCTTCAAAATGCAAACG
CAATATTTGTTTCTAAAACACTAAAACTTAAAAAAAATTACGAGAATATCGTTCGCAATGTGTATTTTTCTGA
TGTCGGAGTGGTTGACTTCAGTGATACTTATAACGCATCCGCTTTTATTAATAGTTGGATTAGTCAGCACACT
AAAGGTCTCATATCCAATCTTGTTCAGCCGTCTGATATCAATCCAACTTCTGACTCTC
TGTTAGCGAATGCTCTATACTTCAAAGGAGCTTGGC
>126037547_2 EL597341.1
GCACGAGGAATCCTTACTAAACTTTGTTTTTTATATAGACTAGATTTTCAAACTTTCTTACAAGAACAAATAT
TAACTTTACAATTTGTCATGGCCTTAACGGGAGTAAAAGTTATAGAAATGTTAGGATCAGCTCCTGGCCCTTT
TTGTGGAACAATATTGGCAGATTTTGGAGCAACCGTGACGGTGGTTCAAAAGGTAATA
TTTATCTGCACATTCGCTCATTGGAGATAAAAGATAATGAAGTCGCCATTTAATTAATACGAGTGCAAACATT
ATTACAATTTTCTGAGTAAGAGTGTTTAATACTTACTTTTTTTTAATAAATAAAGTTCACATTTAAGATAAAC
ATACTTCGGCGAAGTTCATTATATGACAATTCGCAAAACATATTTAGAACATTGTACT
AGAGTAATATAAAGTAATATATATATATATATTTTTTAAAAATGTCAGATTAACTGTCACTCGGCAGGGTATGCA
GAGAGAATCTGGGAGCACTGGCATGGTCGGAATGTCATAATAAAAGAACAGAACAGAGAACCTGGGAACCTGG
TGTTGAGACTGTTCCTATTAACATACTTCTACCAAATTCTGCAGGGCATTGAGTGTTG
AGTGCCAGAACTCCAAGCCACTGTTGTCTGTGCCACATTTGTTTTTTTAATATA
>126037558_2 EL597352.1
GCACGAGGTTCGCTAGGGACACGTGTACTTGTTGTATTTAGGTTTGTTTGAATCACGGTTGCCGAATGAAGAT
CAAGTGTTGAAACTCGAGTCTGTGTCATCTACGAATAAACATTACTATACAATAAAGTCACAAACATGACTGA
ATTTAAGGACTGGTACAATAGTGTACCATTTTTTACCCGTTATTGGCTGACATTCACT
ATAGGTTTAAGCATTATTGGAAGATTTGGTATAATTAGTTATTATTATTTCATTTTGGACTTCTATCCATTCA
TATATCAATTTCAGATATGGCGACCCATCACAGCTTTATTCTATTACCCAATAGGCCCCTCCACGGGATTCCA
CTTCTTGATAAACTGCTACTTCCTATACAATTATTCACAAAGATTGTAATCAGGCATG
TTTGCAGGAAAGCCTGCAGATTACTTTTATATGTTGTTATTTAACTGGGTCTGCTGCATTGCTATTGGCCTTT
TGGTGAATCTACCTATACTTATGGACCCTATGGTGTTATCAGTTTTGTATGTTTGGTGTCAACTGAACAAAGA
TGTGATTGTGTCATTTTGGTTTGGCACTCGCTTCAAAGCCATGTATTTGCCGTGGGTG
TTGCTTGCATTTAACTTAGTTATTAGTGGAGGAGGTGTAATGGAGCTCCTTGGCATCCTC
>126037561_2 EL597355.1
ATTCTCATTTAGTCGCCAATTTTATAAATAAACAAAAATATATTAACTTAATTTACATGGAAATGTATAAACA
AATGCCTGATTAAACTCATTGAAAATGCAACAGACCTTATAATATCTATTTTAGCACCTCAGGGTCTTTTTGA
GATTATTGAAATCCATAGTCTAACTT
>126037563_2 EL597357.1
GACGAGGGCAAGTTCTTGTAATTGTTTCAATATTTTATTATTTTATTTATAACCATTTTTAAGAGTCCTATAT
TATAATGGACCACAACGAGGCTGTGTTAAGGGCTCATCTTTCGAACTACAAGCACCGAGATGTTACATTAAGA
GAAGTGATTAGTGTGATTCAAGTATATCGCAGTCTTACTTATCGTTTAGAAGGCTTTG
TTTTCAACAACGGAATTAGAAAAGACTTGTTAAATCTTGAAGGTACTATACCGGTTAATTTCAAAGGTGCATA
CTACAATATCCCAGTGTGTATTTGGTTGATGGATAAACATCCACAAAATGCTCCGTTGTGTTTTGTTAAACCG
ACGGCAGATATGTCGATTAAAGTGTCTAAATACGTCGACCATAATGGAAAAGTATACT
TGCCTTATTTACATGAATGGAATCCAAAAACCTCTACATTACAAATACTTATACAATGCATGATATCAGCATT
TGGAGAATTACCTCCAGTGTTTTCTAAGCCTCGCAATGCAACTCCACCATATCCTATTAGTCCTTTTATGCCT
CAACCATCAGGGTATCCGTACCCACAGGTTAGCTCCTCTCAGACAGGATTCCCAAATT
TTACTTCATA
>126037567_2 EL597361.1
GCACGAGGCTGCTAATCAGTCGCTCGGCACACGTGATAAAGACCATACGCAATTTTAACATTCAAAATGAAGT
CAGTTCTGGTTTTCTGTGTAATTTTTGCTTTTTACCTCTCCAATAGTCACTGCAAAGATCTAATTATTGGTAC
CAGTTTCAACAATAGATTAATATGGCAAGAAAAAGCTGAATACAACTCCATTCCACTC
AAAAAGAGGGTCAAGGAGGTTTTCTTTTCTGATCCTGGCCAACAAATTATAAAGGGCATTGTTGCGAGGGATC
TAGATCATACGGACGCCGTAGCAACGGTCACTGCGGGAGGAGTGGGCCAATCCTATGCCAATATTAGATTGAA
GAGTGAGCGGGGATCCGGCCTCAATTATCAACTTGAAATATATGTTTGATTATATAAT
GCCAAAACAATTTATTTTCTATACGTCAATATTGCCAAGAATTCTACCTATGAAATAATAAATATGCCTATTT
AT
>126037569_2 EL597363.1

GCACGAGGGCTAATTATTGTATATACATATTTTTATTAACATGGGTACCGAGATAGCTATATCATTTAGTAAC
TTACGAGCTCAACTAGAGAATGAGAGAAGCAGTCTGTTCAAAATAGACGAAAATATTAAGAAAATTGTTCAAT
CGTCCGGACGGTTTAGTGATAGATTTAACGCAACCGGAGACTATACGAGAGGCGGAGC
TCGTCCTGGCGGTCGGAATTCCTATCCTGATGTAACGAATAACTATAATAAAAATGAGGATCAATTAAAACGC
AAACATGAAACTAAAACTGTGTTTAGTAGATTATCAGCCAGAGTTCCCGATAGTGATGGAGAAGAGGATGGAC
CAAAGAGGACTAGAATGTCTTCAGCAGTCTCTAGGGAGCTTCCCACCAGGGCTGCAGT
CCTCCGAGCCCAGGGTGATGATGAACAGGCTAGGACTAGGAATAGAAGAATATTCGGTTCTCTACTTGGAACA
TTACAAAAGTTTAAGCAAGAAGAAATTGTGTTACAAACTAAGGAAGAAAAAAAAGCTCAAGTTGAGAGAAAGA
TTGAAGAGCAAGCAAGACTGGAAAAAGAAAGGGAACAAAAAGAAAGAAAAACATTATT
TGCGGAAAGAGAACTTAAAAAAGCTACCATAAAGGCTTTAGAGGCCAAAATGGGACGAGTCCAGGAGTTTGAA
AAGTGGGAAGCCTCGCAGCAGTGCCTGAGTAACTTCATACTGACAAAAGCTA
>126037572_2 EL597366.1
GCACGAGGCTCGTAAATATATTTCTTTGAATGTCTTAATACACTTCGTTTGTAATTCATATTTTTCCCGTGTA
TATATATAATTTTTTACACGATATAATAAAATAAATTTTCAACCAATGTTTTATATCGGACTATGTAGGTAAT
ACTTTTGTACCAAGATGTCGGTAATAATCTTCTACGTTTTTGACAAGAAAATGTTTGT
ATAACAATACAATGCTTTTGTGAAATTTATATGAGCCTGCATTTGTTTATATGTGTACATAAAATAGATAGAG
TCACAGCAACATGAATCAAGTATCTGATGAAAATGTTCAAAATATGATGTGCAGCCAGAAAAATGCAAGCGCG
AAAAATAAAAGTAATATAATATCCAAAGATCCTCCTGCATCACCAGGAGACATATGTA
AATGGAGTGAAAAAATTACACGAATCTTTAGCAAATACTCTTCATATTAACAAATTTAATAGTAACCTAACAAA
TGGTACCAGTGACCATTCTAAAGATAGTGTCAACGACTTANATAAAGCACAAGACGCAACACCTAGTGAAGTC
GAAAAAAATGTAAATAGTGTACAGTGTAGTGATGCCGTAGAAGATAAAAATGTC
>126037575_2 EL597369.1
ATTCTCATTTAGTCGCCAATTTTATAAATAAACAAAAAAATATATCAACTTAATTTACATGGAAATGTATTAA
CAAATGCCTGATTAAACTCATTGAAAATGCAACAGACCTTATAATATCTATTTTAGCACCTCAGGGTCTTTTT
GAGATTATTGAAATCCATAGTCTAACTTATATTTAGGAGAAAAAAAATCTTATACAAA
AGATTAGTTTGGCATGTTATATGTATATAAGGATAAATAATATTTTTATGACACATTGATCAAATTAGGCAGG
TTGAAAATTAAGTACTTAAACAATAAAATTCATACATAAAAAAAAGAAGCTTAGTATTGACCATCTAACATTA
TAAAGCATAGCAAAATACCAATAATAATTTAATAAAATATATATATTTTGTTATAAAA
TATAATACAGGGATGTTATGAATTTCAGAGAAGAACAATATATTAGTTTTGGTTACAGCTTTGAAAGTATCAG
AGTATTTGCTTGTTTTGGATATTAATTACAATAAGATGTTTGAGTAAATTATGTTTTAAAGAATGTGTTTATT
GACTTGTTTGTGATTTGTTATTTTTTTTCTAGTAGTAAGTTCTTCGTACTTACGCTCA
TTCATACCAAACACTAGTGCAGGGGTTCC
>126037582_2 EL597376.1
GCACGAGGCAAGAAACATGCATTACACCAACAGGTAAAGACGGTGTCTGTATATTGATAAAGGACTGTAAGGC
TCTCAGAGATAAAATAAAGAAAACAGCAGAAGAAAGGGAGACAATAAGGCAATGGCAATGTGGTTTTGAAGGA
GAGGTGTCTAAGGTATGCTGTCAATCTGCCACACCTCTCATAAGCATTCTTTCGACCA
CCACAAGTACCACCACAATTACCACTACTACTGCTACAACCACCATAAAACCTCCAAAACCTGCGGAGGAGAC
ACGTTGTGTTACCAGATTTGGAACAATTGGTACTTGCATTCGAATTGAAGAATGCCCAAAGCTTCTCATGATG
ATAGCAAACAAGACTAGGCAAAATTTAGATTTTATACAGCGATCAAGGTGTCCTGGTC
GGGGTCTTAGTGTGTGCTGCGATGAAGTTATTCCACTGAACAGAATTCAGAATTCCCAAAACTGTCCTAATTC
GGTAACGCCTCCTGCACCTGACAAAAACTGCTGTGGCATAGAGAGTCCTTCAGGGAACAAAATTATAGGTGGT
ACACAAACATCTATAGACCAATATCCGTGGTTAGTTCTAATAGATTACAATAGGCCCT
ACAGTTGCGGAGGAACACTTATAAGCAGTCTGTACGTCCTGACCGCCGCACACTGTCTAGTCACACCAGATAA
AAAC
>126037583_2 EL597377.1
AAAAAGTAATTAATTTGTGCTTTTTGGTATTCCATATGAAATTAAAGCAAACTATTGGTCAAAATGTATTATA
GAAATATTTGTTTAAATAATAAAGAAGATATACTAGAATTTTCCTTTAAGCTTTTTCTTGAGCTTTTTAAGGC
TATTCAGAAGTGCTTACAAAAATTACATTGAATTATTCAGTTTACATAAGAGTTATAT
AATCATTTTATTATTTTTATATGGTATGTTTTTTTTATATGTGTATTAATAACTATTACAGTAAATGAAATTA
TGAACCCTTTCTTTCCAAAGCTGTCTTGCCCAATGTAAGTCAGTCATTTCAGAGAAAGGAGATCAAACTGTGA
CATTACCAAACGGCGAGTGTCATCAATACCTCACTTGTTTGAAGTGTACACTACATAT
GAATAAATATTAACAGTTTCAGTCTTTATTACATAGATTGGGTAGGCAAGCTTTAAATATAGTTGGTCTGCAC
CACTATTTT
>126037589_2 EL597383.1

GCACGAGGAAAACATAATAGTAAATAATACAGTGATATCAAAACAAAAGTGAAGTGAGAAACTAATTAAAATT
CATAATGTCATTCAATCGAAAAAATCCGAACCAACATGACTCTCGTTAATGAAGATATGAGTAATATCCTGGA
TTATTTATCCAGAAATGAAATAGAATTTGACGAAATAAATTCTAAAAGTGACAAAATG
GAAAGCTTCCATACGGGAGCTCTATTGTCTGATTCCTGGCAGGCATCAGATGATATCCTGGAGAACATATTCT
CGTTGGAACAGGGGTCGTTGAACCTTTTAGATGAGACCTTGCCTGATTTCAACTTGACAACTGATAATGCCTC
GCCTCCGGGACTTAGTAGCTCTTGTTCGGACAGTGGATTGTCCAGTGACCCAGCCGAA
TTCGATTTCGAGCAACAATTGTCGCCGTTTCTCATACAAAATTCGTGTGATGAAGATCTACCCACACCGGTCT
TAGAACCAATGAGCCCGTGCAACACCCAAGATGTCATCATACAGGATACGAACGACGGCTCGAACAGCTCCAC
CGACCAGGATACCTTCGATATGGATGATTTCGAACAAAACGTTGTGCCCGGATTCCTT
AATAATAATACATTCCAAAGCCCTGGTAAAGGTAGGAAACGCCGTCTCTC
>126037591_2 EL597385.1
GCACGAGGGTCTGTTCTAAGTACTTTTAAAGTAAATTGATTACAAACCGAAATTGGCGACTTCGCACGTATAG
TATATATATAATATATTTTTCTTCCATATGTCTATGTTTTATTATGATTTTGTCATGTACAGTTGTGGTGTAC
TTATTGAAATATTTTTTTAAAACATATGAAGCCCGTGTATATAAGGGTTATTTAATTC
TCATAATGCCTGGTGCATATGAGATAATCTATTTGTATATATAGCGTCGGTGTCGAAGTCGCCCCCCGCTAAA
TGGCCGCTTTACAAGTACAAAAGGCGTCCCCGTGTGTCGGCTCTACATTAGTTTACGTGGCAATGATTTGATC
TCTCTTTTAGTCAAATATTTTTAGGGTCCTCCCGTCTACGCCCGCGCTCCTTGGTTGA
ACATCGGTCAAACCTCCGTAGATGATAGCATTTCAATACATAATTACAACAATTCATTAATTAATCATTAATA
CATACATATTTTCGATAAATAAGAAATGATAATTTCAATACACGAACGATTTCTAGTTCGACATACGCATCGT
CTATGGAGGAACTTATGTGCATTACATAGCGTTTAATTTGAAGACATGCGGTANGAAA
AATATTATCATAGTTAATTTATAGAGATTTTTTTGTATTTATTTCATAGATTTATATAATTATT
>126037597_2 EL597391.1
CAACGCCGAAGGTAATGTCATGGTCGAGAATATTGGCGATGTACTGCGCCTGGTTTGGCGTGCGCGGCTTAAT
TACGCCGCGTTTGGTTTTGATATTGACCGCTTTGCCGTACTCCGGCACGCTCTCCGCGCTTTGCTCCAGTACC
CGCGCTTCTTTAATCGCAAGGTGGATCTGTTCCGGTTCGATATCCTGAATCTGACCGC
GCATCGGGGCAGTATCGACATACAGGCTACGCAGAATGTCTGCCGCAGCGGTGACGCAAATCGGACGGCCGGT
CAGTTTAAAGTGGTTATCGCGGCGATTGATCTCGATGCCGAGACGGCGTTCGAGCTGCTTGATGTTGTCATCA
AACGGGCCGCACAGGCTCAACAGACGCGCATTGTCTGCTGGCTCCAGGGTGATTTCGC
GAGTGTCTATGTTCAAACCGTTCCTCTTATCTGTATGCCGCCGGAAGCTGAACATTCACCGGCCTATAAGGAA
ATTATTCACGCCACAGGAAAAAGGCGCAAGCATTGCAATAAAGAT
>126037600_2 EL597394.1
GCACGAGGAATATTTGAAGCCAAGTAGAAAATCTTTTTAATACATTATCTAAATTTGGTAAGACAATAAAGTA
TCAGCGAAGTTGATAAAATTTATATGTAGTGACGTTTTGCTCTCTCTATCTCTTATAAGCTTAAAAAAACAGT
TAGGGCTAGTAGTGTTAGTAG
>126037601_2 EL597395.1
GCACGAGGAATAAAAAATTGTGACGCCAGTATAAAAAAAATGGACGAACCGGAAACCATTCATACAGTTATAA
AAAAATATATAAATTGTAAAGTATCATAATCAGTAAACAAATCGCTAGTCTAATTGTGTTATAGATTTATTTA
TTTAATATATAAAGTAAAACAGAAAAATAATTGTTAATTAATTTATATATAATATAAA
TAGTTTCACTAAAATAATTCCACATGTATAAAATACAATGTTAGTATAAGCCAAAGACGTTATAAATAATAGA
TGCTATTAAACGTATGGAATACGAGGAAAGAGAAAGAGGTCGACAGAAAAGAGGCCAATAGGAAAGAGGATAA
AATGGCGGCCACAATGGTGGAGACGCCTCCACCTCAGCTCATAACTAACATTGAATGG
GGTATGATGGATAAAAGCAAGTTTTATCCGTTGTATACGTTGAGCAGTTTTACAGTCCGCTGCGCTCTTTATC
CTCTAACGTTAATCAAAACGCAAATACAAGTTCAAAGGAAGAAAGAAGCGTATAACGGAGTATCTGACGCTAT
TTCCAAAATCTACAGAAGTGAGGGTGTGTCCGGATTATACAGGGGATTCTGGCTTTCT
AGTTTTCAGATAATCTCAGGTGTATTCTACATTTCGACGTATGAAGGTGTTCGACATGAATTGGGCAAATACG
ATATAAATCCAAGAATGAAGTCATTCATCGGCGGCGGCTGTGCGTCCATAGTGGGGC
>126037603_2 EL597397.1
GCGACAATTAATATTGTGATACGTTTTGAAAATATTGTGAAAGAAGAACTATAATGATAGCAGAATGAAGTGC
CACTACGAAGTTTTATCCTTGGAGAGGGAAGCTAGCGCATCGGACATAAAAAAAGCCTACCGGAGACTTGCAC
TGCAGTGGCATCCGGACAAAAACTTAGACAACTCGCAAGAAGCTAAAGAACAATTTCA
ATTAGTCCAAAATGCGTATGAAGTACTCTCAGATCCCCAAGAAAGAGCATGGTATGACAATCATCGTGAACAG
TTGTTACGTGGTGCTGGCAGTTCCTATAACGATGATAGCCTTGATGTTTACCCTTATTTTAGTCCGTCCTGTT
ATAAAGGTTTTGGTGATGATCCCCAAAGTTTCTATGGAGTTTACTCTGAAGTATTCTC

TAAATTAGCCTCAGAGGAAACTGATTTCCTTGATGATCCTGAAGAAGTTGGTAAATTTCTAAGTTTGGGAATT
CACAACTCCCTATGAAGATGTTAATGAGTTTTATGCATTTTGGAT
>126037605_2 EL597399.1
GCACGAGGGCCAGTTCCAATCCTGTGTCGAATTTATATAATTTAATTGTTATTTTTATAATGAAGATCCTTAT
AGTATTGGTGTGTATTGGTATTGTGTCAGCGAAAATTTTACCGGACGATTTTCCCCAGTGCAAGCGGAATGAC
CCGGAACTTGACAAATGTGTTATCAAGGCAGTGGACGTTGTTAAACCAAGACTTCTTA
ATGGAATACCAGAAGTGAATGTACCAGCTTTAGATCCATTTTTAGTACCAACATTGAAATTAGACAGAACAGC
TAACAACTTAAGATTGAAGGCTACTATAAAGAATATGAAGGCGTATGGTGGTTCAAACTTCAAAATAGAGAAG
ATCAAGTTCAACCTCAACAACAAGTACGTGGGAGAAATCAGACTGATCATCCCCAAAT
TATTTGTAACTGCTGACTACGATGTCCGTGGTTCCAAGATCCTTACTCTGGACATCAGTGGAAAGGGAAAGCT
CAGCAGTAATTTTACTAATATTGCGGTAGTGGCTAAGGGAAGTGCCAAGCCAATCATGAAGGACGGTGTCGAA
TACTTGCAAGCTGAAAAAATTATTACAAAATTGAAAATTGGCCACGGACAAATCGCCA
TCGATGATAGCGAAAGACCAGTGGCTGCGACTTCAGCCGCCACCTTCTT
>126037606_2 EL597400.1
GCACGAGGGTGCGGTGTAACGCGTGCCGACGGCAGCATGACATACTGACGCAAACACCCGATTAAATTAAATA
TTTACAAAAAATTGCGTAAAAAAAATTGTGAATAGTATTGAGTGCGGTAATTTATTTTGTTTTTATTATTTTT
AAAAAATCACATCAGTGAAAATGGGGTTGAAGTCTGAAGATGATAAAAGACCCAGCAA
GCTGGCGATTATGGATGACATTTCATACACGCCAAGTGGTCTCGGTCACCGTCACTGGGTGACGTTCATGCTG
TTCCTGGGCATGGCGAACGCGTACATCATGAGGACGAACATGTCGGTGGCGATCGTAGCTATGGTCAACCACA
CGGCGTTGCCTGTCGACAAGGAAGCTGTAGACACTGAATGTGGGAATGTTAATCCAAA
TGCA
>126037610_2 EL597404.1
GCACGAGGGCGGCGCGCGCGCACCGTACGACCGCCGCACCAGTGAAGGGACGCATGACTCACATCCACACCGA
ACATGAACGAGTACGTCGAGTACGACTGGAGGCTGCGCCAGCTGCAGAACGAGCGCCTTTGTGACGTGTCCAG
GAAGAGGCTATCCGACGGAGACCGGCAGCGCGCGCTGGCCGGCCTCAAAGACGCCGTC
GACTCAAGTCTCAACTTGGCCCTTCGCGACAAAAGTCGATGTCAAGACGACAATTTTCTACGGCGGTTTTTAT
ACGCGCGCAAACACGATGTACAACAAAGTTTTGAATTGTTGATTCGTTATCATCAACATCGTCGAGAGCATCC
GGAGCTGTGGGCGACTGCGGACGGCGGGGTGTTACGAGCTCTGGCCGATGGTCTGCCG
GGCGTGTTGGCGCAACGAGACCGACTCGGCCGCTGCGTGCTTCTCATGTTTGCCTCTAATTGGACCCCACATG
CCTGCCCGCTCCTGTCCGTGTTCAGAGCTTTGCTTCTCACACTGGAGCGGACCCTCGCCGAGTCACAGAATCA
AGCCAATGGATATGTTATTATTGTTGATTGGACAGAGTTCACATTCAAACAATCGTGT
AGTTTANCAGCAAAAATTTTGAAAATGATGATAGATTGCTTGCAGGACTGTATGCCAGTGAGATTCAAAAGTA
TACATTTTATAGGACAGCCGTGGTACGTAGAAACAGCGCTCGC
>126037614_2 EL597408.1
GCACGAGGCTTTAATACATTTAGAATACGTTGTGCGTCATGTGAATTTCATTTAAACCGGTTAGTCTACTGAG
GAAATATATTATACAAGTTGTTTTAACTTTAGAAAATAAGTTTTAAGTGTAATAAATGTATATAAAGCGAATC
TTTGAAAATTATGGGTATCGCGTTATATTGCTGCATCATCTATCTACTAGTGGTAATG
GTCTTGTTGGTAGAGGCATGTCAAGCCTGTAGACCAATCATCGACGTAGATGATAATAAAGTTTGTCCTATTT
CTGAGAAGTTGAAGTCCTTGAATTATGTTTTAAGCCAAGGAGTAGTTTCTGACAGTAACGAAACTTTAGGGCT
CACTTTACGAAAATGTGAAATTGCAACCGTCGAATTGCAAGCCTTTCAAAATTTATCC
TCACTTAAGTACGTTGATCTATCACAGAATTCCATTTCTTATCTAAAAGTAGGTGTTCTTGATAGTGCCAGGC
ATATAACATACTTAAACCTATCTAATAACGCGCTGACTGACGTTGCGCTTGGATTGTTTGATCAGACAACGAT
CTTGGAAGTATTAGATTTGAAAAAAAACAAAATACAGCAACTAAAGTTAGGATTATTT
GATCCCTCTAA
>126037615_2 EL597409.1
GCACGAGGATTTGACTCTAATAATACATTAATTAGCCGTTTTACGGGTACAGTGAGTGCAATAAACATACATA
ACAGGTCGTGACAGCCATGCCGATGTGAGTAATATCGAAGGTGTGGCGCGTCGGTCTGGGCGTTGTTTCTTAG
TGCAGTGACGTTTAGTGTTACGAAAAATACTACTCATATCTCGTCGCAAGATGACCGA
GTACAAGCTAGTGGTGGTAGGGGCTGGCGGCGTCGGTAAATCCGCATTGACTATACAGCTTATACAAAATCAT
TTCGTGGACGAGTACGACCCGACTATCGAGGATTCGTATAGGAAGCAAGTTGTGATAGACGGTGAGACGTGTC
TTTTAGATATCTTGGACACGGCAGGCCAAGAGGAGTATTCGGCTATGAGAGACCAGTA
CATGCGGACTGGTGAAGGATTCTTGCTTGTTTTCGCTGTGAACAGTGCTAAGAGTTTCGAGGACATTGGATCC
TACCGGGAGCAGATTAAACGAGTTAAGGATGCAGAGGAAGTTCCCATGGTGTTAGTGGGTAACAAGTGTGATT
TACAAGCATGGGCTGTCGATATGACGCANGCGCGAGAGGTAGCGCGAAGTTACGGTGT

ACCGTTCGTGGAGACGTCAGCCAAAACACGAATGGGAGTGGACGATGCATTCTACACACTAGTCCGTGAGATA
CGCAAGGACAAGGAAAGCCGC
>126037620_2 EL597414.1
GCACGAGGCATATACATGGCCATCGGTGGTTACGTCGGTGCGGCGACAGTTGGCGCCGCCTCCTGGTGGTTCA
TGTACTCGCCCTTCGGTCCTCATATGACATACTGGCAGTTCACACACCACTTGCAATGCCTCAACAATGCTGA
TGACTTCAAGGGTATCGACTGCAAGATCTTCACCGACCCCCATCCCATGACCATGGCC
CTCTCCGTACTTGTGACCATTGAGATGTTGAACGCCATGAACAGCTTGTCATAGAACCAGTCGCTGGTGACGA
TGCCCCCGTGGTCAAACATGTGGCTCGTCGGCTCCATGGCCCTCTCCTTCACCCTTCACTTTGTCATTCTTTA
TGTTGAGGTCCTCTCGGCCGTGTTCCAAGTGACGCCGCTGTCTGTGGACGAGTGGTTG
ACGGTGATGAAGTTCTCGGTGCCGGTGGTGCTGCTGGACGAGGTGCTCAAGTTCGTCGCGCGCAAGATCTCCG
ACGGTGAGCGCCACTCCCTGCTGGACTGGCTCCACGGCATGCAGTGGATTGTGCTCATGTGGGCCGTCTTCTT
TGGCATCATCATCTACAGACCCCTATAAGACGCCCGCCCCTGTTTGATTCACACCTAA
TACCCCCCTCATACCGCCAC
>126037624_2 EL597418.1
GCACGAGGCTCAGATGATCTCGTGTTTCCGAGTATCTTCGAACTTCTTTTCCGATTTGTTTGTTCTTATCGCG
TTGGTGGTCGAAGTCCTATATCCTATAACATGGCAGTGCCAAAGCAATGGATGGCACGGAGGCTCATTCGTGA
GATTGTACCTATGTGGCACTTTGGCTTTGCAAGCCTGCCTACTGCTGCTGCTGGCTGC
ACTGGCCCAACAATCGGCCAGAGGAACCATCACGGAGACAGATGCAAGAAAACTTGTATCACCTTTATTATTA
CTTAAGGTATTTCTAGTGTTACCAGAGGTGGCTTTGAATGTAATGGGGACCATGTGGATGTTCTGTGAAGTCA
TAGAGTGCACTGTATCGGATAAGTTTTCTAGTATTGTTATACAAAGTATAGTTCTATT
TAATTGGGTGCAACTGGGGCTAACGGTGTTCGGACTTTTTATGGTATTCGACCCCTTGGGATCAGTTAATTAT
GGAGATATGCAGGACACGCCCAACCAAGTCCGACATCATAGAAAAGTAACTGGGTTATGGTCACGGCGGTTCA
GGTGGGCCTTTTGTTGGCTGAAAAGGGATGAACA
>126037629_2 EL597423.1
GCACGAGGATTTAATTCATATTAGGTAAATGTTTTTAGTGTCTGATTCATTATATAATGCAAAACACATATGC
TAAGGCTGATCGTCCAATAGATGTAAAAGCGGTATTTATCATTAAAAATTTTTTATCTATTCTTTTTTAGGAA
ATATTTTATATTTTAATGACAAATATCGCTTGACCGGTTTTTGAAGGAAAAATTTATA
AAAAATTAGAACTTAGCCAAATTTAGAATACGAGACTTATCTACATACGATTGTTAAATCTGAGTAATAAACA
AAAGAAATAATTTGTAAATACATAAGAACATATATAGACTGGTTAAAATCTACTATATTATTTTCAAAATTAT
TCTGATTTTAAATTACAATTCACTCTTGTATTATATTAATTATCTGATGGACGATCAG
CACAGGCCTTATTAATTGAATGTTTTGAAGTAGCTCAAAATTGATATTGATGGGAATGTTCCGGCATTTTCAT
ATCCTCTCGTGTTATCGTCGTTATTTATTATTACAGTGTTGATGTACGTTTAATACTACCTTCAACCATTAAG
TGTAATGAAAAAATTTAATATTTTTTAACCAAAATGTTATATTAGTTATACCTGCTTT
TTTATTTTTTATTAATTTACTGCTA
>126037633_2 EL597427.1
GTTTTTGTGTTTTAAATTTCCTAAATATTTAGTAATTATCCATTTCAAAGGTAATTTGCTAGTATTTGTTCAG
GTCATGATAGTTTTTATTAGACGTAGAATTGTATTTAAAAATGAAACAAAGGACGCGTCTAATGCACAATGGG
GATGATCTATCCACAAATATGAACGGTTTGAAGGACGCTAAAGAGCTACACAGTGAAA
TTCAGCTATATAGAATAAAGCCTCAAGACCACTTTATCGAAGCTCAAGTTCAAGAAGGCGATACGTTACAGGC
GATCGCGCTACGGTTTCATTGTTCTATTTCAGAATTAAAAAGGATCAACCACATACATAAAGATAATGAAATA
TTTGCAAGAAGGACAATCAAAGTGCCTGTCACACCATACTCAGTTCTTACAGAACTAG
TCCCGCTGCAGCCAATAGAACCAGTATTTTCAACATCCAGTCAAACTTCAACAAGTTTGACTATGAACAATTT
GCTACTAAATCCTGTAGAAAATAAATCATCCCAACAGTCCAATATTATTAATAGTACTGAAAAGAAAGAAGAC
GATTTTGCAATTGATTGCAATGCAGTAGTGTTAAATAGTACATTGACTCCATCTGTAA
TTCCCTATACAGATACAGATAACAATGAATTTGCATCAGAAGATACCCAGCTGTTGTCCAACAAGCCCAAGGT
GCCAGTAGAGGCTG
>126037634_2 EL597428.1
GCACGAGGTTTAAGAATCCCATATTTTATTTCTTTATTTAAATTATGAAATAAAGATGGCTAATATTTCATTT
ATCATTTTATCTACGGCAATAACAATATCAATATGTTCAGTATCGTTAGTATCAGCAGAATGCTCAATTCCAA
TAGTTTTAAGAAACACTTGGTTTTCTTTCGAAAAAGGAAAACAAACAATAACCGACAT
AAATGCCAGTGAAATGACAGACAGAGGTGTTTGTGTGGATATCAAGGCAGACTACCATGTGAATTACACAATG
GTTTTCAAGCATGCAGATTGCTATTATTGTGTTAAATTAATTGTGAGAACTGTAAATGTGTTAGAGAAAATTG
AAACCCCATGCATAAATCTCAAACCAGGAGAAGAACCAACAGTGCAGACAGTGTGCAG

AGGCCTTAAAAATGACCAATATTTGATTACATTGTTTTCTGAAAATTCTGTTCCTGTAAACTGTAGATCATCC
CTTGAAGGTGTGTGGCAGTTTGCATACCAAAATCGTTTTAGATTCACAGGTGAATGTAATCATCCCGATGCCC
AAATAAGATCGTGTCAAACAGCTGGCACACAATTTCTCATAACAAACCAAAAGTTTAA
TATAACATATAGAAAATGTTCTGGAATGTCTGGCACGTTTGATGGAGTTGTTGAATACAGTTGT
>126037635_2 EL597429.1
CGGCACGAGGCAATTTTATGTTATAAATTATAATGGCAGATAAAAGGAAAACTAAAAAGCGCAAAGAGGAGTT
TGGTGAACCTCCAGTACCTGAAAAACCGACAAGTGAAGAGTATTCGGTCGCGAAATGGCTGAAGGCGAACGTT
CCCACTAAGAAGACGAAATTCCTTAATCATCACGTTGAGTACTTTACAGGCACAAAGG
CCGTAGACGCATTACTAACATCAAAATGGGCGACGGGCAAGAACCCAATATTCACCACGCGCCACGAAGTAAC
GGATTATTTACACCAGATGTTAGTGCACAAATTATTTCACAGAGCTAAGAAGGTTCCAGTCAGTGAACAAGAG
TTAAAAGGAAAGTCCAAGAAGAAAGATGTAGATAAGTCCAGCAAGAGTGGAGACGAGA
AGGATGGGGACAAGAGTGCTGCTGAAAATAAGGATACAAAAGACACCAAAGACAAAGAAAACAAAGAGAAAGA
AAAGAAAAAACGTANAATCCGCCTAGAAATGCACATGGAACAACTGTTCCTGGACACGCTGGACGCGTACGTG
TGGATATACGACCCCATGCCGTGGTACTACTGGCTGTGCGGCGCGCTCGTCGTGTTCG
GCACCATCGGCGTGTGCATGTTCCCGCTGTGGCCGGCTACTGTACGCAAAGGCGTATACTACCTGA
>126037641_2 EL597435.1
GCACGAGGGCAGCTTGGTAATAAATTTCGAGTAAATTAAGCTTAAAGTGAAATATTTTTTATATGTATAATTA
ATAAACAATTAAATTTTAATGTACCTATTTCAGTTTTGATTTAATGTCTTACGTTTGATGTCTTTATGTCTAA
AATCTTAATGTTGAATGTATTTCCTAAATCAAGGACAGACGTGGACACTTTTTTTATA
TATATAAATGGATTCAATAGTGTTTTTTGTCTCACTTCTAGATACTATTATGAAATTGTAACAAAAAAATAAA
AATTCTTTTTGGTGCATTGCATATAGTAATTAATAAAACCAATATTTACTCTAAAATTATCAATATTTCTATC
AAATCTTAAAGAAAAAAATAAATACGTTTAAAATCACCAGAAATTTCCATATAAACAA
TAAAAACATATCTCTTATATAAATATTGCTGTCCTTTTCATGTTCATACAAATAGAAAGAGATAGATAGACTC
GAGAAAATCCCATACATAAATAATCTTTTGTATTATATTTAGTTAGCAAACAATAATCTTTATACTTATTTAG
TCTTTGTAATAGTTTTTTTTTTTTATTTTTTATTTTATCTATTATCAAAATTGCTTGT
AACTAATAATTCAAAATTGTAGTATAAGAATCGGCGTGTTTTTTTT
>126037651_2 EL597445.1
GCACGAGGATCAAATGAGAAAAATGTTATCCCAAAAGGAATCCAATTATATAAGATTGAAACGAGCAAAGATG
GACAAGTCTATGTTTACGAAAATCAAGCCTATTGGTATTGGCGCGTTCGGGGAAGTGACGCTTGTGAGAAAGA
TCGATACTAGTCATCTCTATGCCATGAAAACCCTTAGAAAAGCTGATGTCCTAAAGAG
AAACCAAGTGGCTCACGTCAAGGCTGAAAGAGACATCCTGGCTGAGGCCGACAACGAGTGGGTCGTGAAACTA
TACTATAGCTTTCAAGACAAAGATAATTTGTACTTCGTAATGGACTACATACCGGGGGGAGATTTGATGTCGC
TTTTAATAAAATTGGGCATTTTCGAAGAGAACTTGGCGAGGTTTTATATAGCGGAGCT
TACGTGCGCGGTTGAGAGTGTCCACAAAATGGGATTTATTCATAGGGATATAAAACCTGATAATATTCTTATA
GACCGCGATGGTCACATAAAGTTGACGGATTTCGGTCTGTGCACGGGCTTCCGGTGGACGCACAACTCTAAAT
ATTATCAGAGGAATGACCACGGGCGACAAGACTCAATGGATCCCGTTGACGGTGAATG
GGGCGCTATGGGCGAATGTCGGTGTCACCAGCTAAAACCCTTAGAAAGGCGGCGTAAAAGGGAACATC
>126037656_2 EL597450.1
GCACGAGGCGCTCCCGCCTCTGGAGCGACGCGCACCACGCCCACGCGCACTCCGCCGACAACTTCAGCTTGAA
TTCAAAAAATCCAGTGCTACACAAGATTACGGAACATCTCATAGAAGAGGCATCAACTGAAGAGGAGGAGTTG
TTAGGGCTGGAGGCGTCATCAGAGTCTGTAAACCAAGATCAACCGGACCCGGAGTTAG
TGCGTGTTCTCGACCGCCTTGTGCTGTACTTGCGTATAGTTCACTCGGTAGATTACTACAATCACTGTGAATA
TCCCTATGAGGATGAAATGCCTAACCGCTGTGGAATTATGCATGCACGAGCTGGAGCACCTGCTAATAAGCCC
ACTCAACAAGAAATCCAAGATTATATCAAGACATTCCAAGGCAAAATGTCTGCTTTTC
TCCAAGATGTTAAGCCATTGACTGACGAAGAGCTGCAGAAACTTGGTATTAAGGATCCAGATGCAGAAGTGGA
AAAATTCATACAGGCCAATACACAAGAATTAGCTAAAGACAAATGGTTGTGTCCACTCAGTGGGA
>126037658_2 EL597452.1
GCACGAGGGTTACTTGTGATAAATAATGTAAATTAGTAAAGTGCAAGAAGCGTAATGTAATAATTTAATTTAT
GTATTTACCAATTAAAAGTAACAACGTGTTTAAAAATAATTAAAATAAAATTTAGAATCATAATAAATAAACT
GTGGTGTAAAGTGATATTGCAATTTGTTTGCACTTAAGACTTATTTCCGGCAAAATGT
CTACGAGTGAAGCAGAAACGAGTGATTCCAATATGGAGTCTCCGTCTCCATCATCGTTTAATTCTGAATCTCC
ATATAGTTCACCTTCTCAGCAAGTGCCTCAGCTCACTGCTAACATATCACAACTATCATGGGTGGGAAATTCT
GTTGGAAAAAATGGTAAAAAACCAGAGCTGGTAATAGTGGAACAACCACAAAATCATT

TCAGATTTAGATATAAAAGTGAAATGATTGGCACACATGGTTGCCTTCTTGGCAAATCTTCTTCTAATAGTAA
AACAAAAACTCATCCAACTGTTGAGTTAAAGAACTATGTTGGAAACGCCCTTTTAAGATGTACGTTAGCCAGG
CATAATGGTTCAGATGAACATCCACACAAATTACTCGAAGATGATCAAGATCGTGATG
TCAGCAGTATGGTGCCACAAAATGGATCGTACAGAATTGCA
>126037661_2 EL597455.1
GCACGAGGGTTCGGTATCGTTCAGAAAATAATACAATAAAAATATAAAAAAATAATAATAAAATTATATTTTA
AATATTCGTTAATTTATAAAATAAATTATATTATGATTGTGTAAATTGTCGGCGTTTTTGATTATACTTTTAT
AAGTGAAAGGTTTATAAGGAAAAGCTTTTAAAAGATGCCTACCTCAGTGGTCTTCGGT
AATAACGAAAAATGGAGAATAAACAGGAACATTTTGGTGCTTGGATTTGCTTTTATGGTTCAATTTACTGCTT
TCCACGGCGCCGCGAATTTACAGAGCTCTGTCAACACTGACGCGTCAGCTGGAATCTTCACATTGGCAGCTAT
ATACTTTTCGCTGATTTTGTCGAATATATTTCTACCTGCTATCGTTATAAAATGGCTA
GGCTGTAAATGGACAGTCGCAGTCTCTTTCATCGCATACATGCCATTCATCGCGGCTCAGTTCTATCCGTACC
TCCATACATTGGTTCCTGCTGGGATGATGGTGGGGTTCGGAGGAGGGCCACTCTGGTGCGCTAAATGCACTTA
CTTGTCTGTGGTTGCAGAACCGTATGCAAAACTTTCGGGAGTGTCGGCGGAGGTACTG
GTGGTACGATTTTTTGGCCTCTTCTTCATGTTCTATCAGATGGCACAGATTTGGGGCAACCTGGTCTCTTCTG
CAATTTTATCGTCATCTTTAGGTGAAATATCCACTTTTGTCAATGAAACTT
>126037669_2 EL597459.1
GCACGAGGTGGCAGTCCTCCTAAACCCAAACCCCCACCCAAAGAAAAGAAACAAAAGAAAGAAAAAGAAAAGA
AAACCAAAGACAAAGAAGGAAAAGATGGAAAAGAAGGCAAAGACGGGAAGAAAGATGGCAAAAAAGATGGAAA
AAACAAGGAGAAGGACAAGAAAGAAGATTCTGGAAAGGATAAGGCAGCCAAAGAAACC
AAGAAGCAAAGCCAGTTTCAGCAACAAGATGTAGTGGAGCTAGAAGCGGGACCGGTGGCGCGACCAATGCGAC
GTCGTAAAACATCTCAGAACGAACACAGTCATGGCAGTTTCTTCCTTCGAATTGGTGCTATTGCATTCGGCCT
CGGAACAATGATTTACAATGGCCTGGAGTTTGGAACGTTCTTTGAATTGCCATTAGAG
TCCCCCTGCTATTTGATATTGAAAGGTGTAAATCCTGTTCTACAAATGGTTTTTACATTCATGCAAATGTACT
TTATATTTATGAATTCTCGTCTGAATATTCATCGATTCAAAGTAATTGCTCGTTTTGGTCTAATGCACGTGGT
TGCTACAAATATTTGTGTATGGATACGTACATTAGTTTTGGAATCTCTGAAGGAAATT
ACTGATTACCACGTGAAAAATCCTCAAGGTGTGCCCGGCGAGGGTGTACTTGGAAAAGTCATCCGGAAGCATA
CTTTAAGACACTCTGGAAAAGTATTCGGCGCTATTACTACAGCTGCTACG
>126037676_2 EL597464.1
GAGTTAATATTAACGTATATTTATATTGACTTAAAAATAAGATTATTGTTATTTAAATAGGTACATATTATTA
AATTTTCAACAATTTTTAAGTGTTACTCCACACTGAGGAAAGAAAAATATTTATTAAGAAAACATAAAAAAAA
TGAATGTAGCGGTATTTGAACCCACAACCCTATGGGAACCGTCCAAGCGCTCAGACCA
ACTAAGCCGTACATTCCGAGTCATTTGGCTTAATCGGTAAGAGCGTTCGGACGGTTTCCAAAGAGGCGCGGGT
TCATATCCCGTTCAATTTTTTTAATAAATACATATTATACTGCTCATCCAATCACAAAAATCCAATT
>126037679_2 EL597467.1
GCACGAGGATTTGAGTAGCAATAAGTAAGTGCAACAAAAACTGTGAAATTATTATGCATTCCCAATACATATT
CCATTTTACTATTTGAATGTGAAAGGATAATAGATCAATTATTTTTTATCAACAAGGTCTATAGCTCTATATG
AGGCTATTGTGGTGGAGTGAGATATTGTCTGAATGTAAATGCGATGTCTATGGGTTCA
ATCTCTTTTTTAAAGAAATTATTATATTTTCATTGCCCAGCTCTGGTAGACTAGACTCGAAATTTGGAAACGG
TATCTTAACACTAGGGCCCTTCACCCTAAAAATGTACAGCCCAGCTAAATGATCTCTTACCCTATATTATATA
AAAATATACTTGAAGAATATGTTTTTAAGTAGTACTTTGATCTGTTA
>126037690_2 EL597472.1
GCACGAGGAGATATCTAGTTTTAGCGAAAATAGGATAGTTTCTTTATTCTTATTTCTTGGTAGTTATTTTTCA
TTTCTGAATCTTGCTTTATAAATTATTTTCTAGTTTCTAAAACATACTCCTAAGTGTAATTAGAAAGCAACTT
TTTAATAAAAAGAAATAATTAAATTGGTGTGAACGTCATCCGATGGAATTTCTTTAAG
AATTGTGAACTTACGAGTATACATCACTGAAATTAAAATGATTAATAGTACTATATAATAACAAAGATTTTAT
ACAATGGGGAATGATACTTCTCAATTGAATGGTTTGGAAATTGAAGAAAAACCAATTGAGCTAACTGATTTTT
GGTCACACTACCAGGCTACAATAAATGACAGTTGCAGGTATTTCAGCCTCAAAAGTGA
TGGTTCCGTCTCTGTTTTTAAGGGGGAGTCAGCTTTAGGTCCTTTGTGGACTGTATCCACACCCTTGGAGAAG
TTTTCTAATAATCTTTTGAAATATCGACATCCATATATTGTCAGATATATTTCTGCTTGGCAACAAAAATCAA
CATTTCATTTAGCAACAGAATATGTTCAACCATTGGCACACGTCTTAAGTACACAAAC
ACCTCTACAAATATGTATAGGCCT
>126037694_2 EL597476.1

GCACGAGGGCGACACGGCCCTCTGTACGGGCGGCTGGTCGCCGAAGAGCAGCTCGCGTCTATGAGCGCGCCTT
CGGACATTCAGGCAATGCAAGCCCGAATACCACACCACTTTCGGGACTCCTCGACGGCGCCGTTAAGAAAACT
TAGCGTCGACCTCATCAAAACCTACAAACACATAAACGAGGTATACTACGCAAAGAAG
AAACGAAGAGCGCAGTACCTAGGCGACGATGGTTCACACAAGAAGGAAAGGAAGCTATACAACGAGGGCTTCG
ATGATGACAACCACGACTACATCATCAAGCACGGGGAGAAGTTTCTCGATAGATACGAGATCTCATCACCGAT
CGGCAAAGGCTCTTTTGGACAGGTTGTCAAAGCGTACGACCACGAGGAGCAATGTCAA
GTAGCGATAAAAATAATTAAGAATAAGAAACCTTTCCTAAACCAGGCACAAATAGAAGTCAAGTTATTAGAAA
TGATGAACAGAGCAGATGCAGAAAATAAGTATTATATAGTAAAATTGAAGCGTCACTTCATGTGGCGGAACCA
CCTGTGCCTAGTGTTCGAGCTGCTCTCGTACAACCTGTACGATCTGCTCCGCAACACC
AATTTCCGTGGTGTCTCCTTAAATCTCACACGCAAGTTCGCGCAGCAGCTCTGCACCGCGTTACTGTTC
>126037697_2 EL597479.1
GCACGAGGGCGCGATGTGATCATATTCATTTTTATAATTTTCGTAATCACGCCGAGTCAGCCATTAACAAAAG
AAAAGTATCGCACTTATTAGACTTTTGTTGCAGTGGATTAAGTCAATATACTATTATATTAAAAATTGTATTG
AACAATCGAAAATGCCAGCAAGCAGTATAAATTTGTACCATATGCAAGATTCAAAAAA
TAACTTTCAAGCTCTGAGACCTGCTCCTGTCTGTCCTGATTCTAACATGTATATGGGCCAATATCAGAATATC
CACATATCCACAAATTGTTATTATGGTTACCATGAACCGAAAACTGCTCTAAACAATACTTCCGAAGCCGTTG
ATTGCGAAATGATAGAAGTGCCGCATGGAGGAGACATAGTCACGTCGTCATCTCACAC
CAATACGAGAAAAAGAAGTGCAGAAGATTGTTCATACCCGAAAAATAAGCGCCCACGCGAAGGTAAGAATTTT
TATATATCACTTTTTGAAATCTCATTGAAGTATACATACTTAGTAAAAATTTAAATTTAAATATAAGACAAAA
TCTATTCGGATAACACATTAAAATATAGACTTNCATGAAATTTTATATTTTTTTAATG
ATATACATATATAATGTTAACCTTACATATTGCGAA
>126037700_2 EL597482.1
GCACGAGGAACAACACGGCGCACGCGCACCACAACGCCGCGACTGCTGAGGAGGTCGCTTTCAGCAGACTGAT
GGCCACTCTGTCGGTGTTGTTCATGATTTGCTGGTTGCCGCAGATGGTGACATCAGCGCTGTACCTGACGCTG
GGCGAGGGCCTGTGGGTGCGACTCGGCACGCTCGCCAAGCTGTCGGACGTGCTGATGC
TGCTCAACTACGTGCTGGACCCCATACTGTACGTCCTCATGAGGCAGCGTCGGCAGCTGTCCCCCAGCGCCCT
ATGCCACTCCATCGCGCATTGCTTCAAGCGGAACCACAAAACATCGACTGAGTCGATGAAGACCTCGTGCTGT
CCGCAGGAGACCGTCATCATCAGCGAGAGCTCCGGCGCAGAGTTACGCCCCCTGCGCC
CTACCGCGCAGTCGCATCCCGCCACTATCACACCCCTCGCGCATTCACGCAATCACTACTGAAGTACTGATAC
TGAGTGACGCAGCTCTCCGAGATAATAGATACTAATACTCAAGTTCTATTTAAAATGCGCTTTCACCATGACT
TGATGAAAGGTTATATTGAGCAAATAAACGACTTTTAATTTTAATAATGACAGCTATC
AATTAATATAAATAATCGGACGACGCGACGCCCACAGCATAGCATACACGTGAAATTTGACCATTCAACAAAA
CC
>126037701_2 EL597483.1
GCACGAGGTTTAAATTTGTATTAAAAGAAAGTACAAATATATTTTAACACCTACCTTTTTATAATAAAAATTA
ACACGAAATGTGTAATTCTGGCTGTGCTATTATAAGATTGTTAATTTATAGTTTATTCACTTATTGTTTTGCT
TTAGCGAATACAATAGAGGGCATAACAAATGGTTATTGCGATACCAAAAACAGTTGTT
CAGAGGAAAATAACGAAAAATATCTGCTATTCGATGTAAATCCACCAGAAGGTTTTAATTTAAGACGCGACGT
GTACATGAGATTCGCTATAATGGTAGCTGAAGCTCGAAAAAAGGAAAATAAAAGAAATTGGAACTTAGTACTA
CCTCCTTGGTACAATCTATACCATTGGAAAAACGATGGTTATATGAGTAAACCTATGC
CATGGGGCAAATTCTTCGATTTAGATTCCATGAGACGTTTTCTCCCTGTTCTTGATTTATATGAAATGTTTCA
TAAAACTGATACTAAAAAGTTTCATATAGACAGAGTATATGTTTTACAGAACTATGAAGACCCTTTTGGACCT
AATGGCGAATTTGTTGACAAATGGGAGGTAAATAAAAACTGTAAATACAATGGATATT
ATTGGGATTATAATAATATAACAGTAGGTGAAGTAGTATGCATTAAATTTCAGGAAAGAATATCTAAATTATG
G
>126037702_2 EL597484.1
GCACGAGGACATGTCGAAGCTCTCCGCGGATGATATCATCTTCGCATCATTCAACAACCGTGTCTTTGAATTG
CCGTTTTGCGTGATAGCTGATCACGATAGAGAAAGTATTGTGGTGGCGGTTAGGGGGTCCATATCTCTGAGGG
ACATCTTTACCGATTTTACGGCTGGCTCCGAGAAATTTGAAGCCGACGGCTTGCCAGA
AGATACAGCAGCACACAAGGGTATGTCAATGGGGGCAGCCAAAATGTTAAGGCGTCTTACGCCCATACTGGAG
AGGTCGTTTGCACAGTTTCCACATTACGATTTAATTCTTACAGGACATAGCCTTGGGGCTGGGGTAGCAGTAT
TAGTAGCATTAAAGTTAAGACCGAAATATCCTCATCTTAAAGTATATGCATTTTCAAC

ACCAGCCGGACTTATCAGCAGGGAGGCGGCGCGGTTCACGGAGACCTTCGTGATGACCATCGGAGTAGGAGAC
GATCTCGTCATGAGGCTCAGTGTCCACAGTATAGAGAATCTACGGACAAAAGTCATACAAACCATTCACGCCA
CTAAATTGCCTAAGTACCGTATAATGCTGAACGGCTTCGGCTACGCCCTATTCGGTGT
TCCGGCGCGCGACCT
>126037704_2 EL597486.1
GCACGAGGAGAGCGAGTCGCAAGACTGGCTGCCATTGTAAGTCGCAGTTATGATTTTCCTCGTATCAGTATTG
CGTTGTCTCATTTAGAATAGTGTAGAAACTAGAAAGGATAATCGTCCGACGTCACTCGACTGCAGACCGGCCG
AGGCTGAGGCGGCGTTGCCAGCTCTCCGGCGAATTTCCCGTTCCGGCGGTGTTGCCGT
CCCGCTGCGGCTCGCGTCACGAGCGTTCGCCCGCGACGTAACCTTTGCAAATTCATATTTTTTGCAGTTTCAT
TGCAACATAAATGTGGAAAACATCGTTATATATGATAATAAGGGACACGCGCCCGCGCCGAGCTGGCAACGAC
GCGCGACGTCGCACAACACAAGTCACGAGCGGATGTTCTGTTAATTAAAATTCCGCCT
GCGCCGCCCTCGCGGAATGGCGGCCATGTGTTCGACACTCGGCTGTCGGACGTTCCGAATCACGTTTTGTTGC
ACAAGGTTAACCTTTTTTCATTATAATAATGGCGCCTCGAAGCCTGACGGACCCGTCACGCCCGACGGCAGAT
GCTGTGCTTGCGCGAGAGACTGCCAGAGGCCAGAGTGAAAGACGGGAGTGTTGCGAGA
TGAATGTGTCTCAGTAAGTCGGTACTGACCCGGTGCTCTTTAAAGGTCTTGTAACGTGAGG
>126037705_2 EL597487.1
GCACGAGGGTGTTATCGCGTTCGATTCCTATCAAACTTTTACGTCACAAAACCGGTTTTAAAGTAAAAGGTGG
TTAAAAAAACTTAAGTATCGATGATTTTTTTTATATAAACTGTGCCCTTTTAAGTGCTAGTGGTTTAATTTTT
TATCAAGAGTGAAAAGGACAGTCATACATCCAGAATAACTGCGCGAGAGAAACGGAAG
AGTCCAGTGTAAACCAGTTAAGGTT
>126037713_2 EL597495.1
GCACGAGGGTCGAGTCAGATTATTTAAAATATGTTTAAGCCATCTTTCACCTGTACCTTGATCGGGTAAGATC
CCCTGAGATTTTTGTTTTACTTTTCCAAAGCAATAATATATATTATAGATAAAATATAGACGTAGATAAGCAT
AATACAATTTAGTAGAACTGCCAAAAGTGAAACTTCTTTAATGAATTGTGACGTTGGC
AGTATTGTAAAACTGCAGTTTAAAAATATTTATTATATAAGCATGAGTTAATGAGATTAAATTTCTTTTCAGC
ACATAGTTTTGTATCGACTCACCGGACTTTATAGAAATGTTAAATGTACTTTGACAATATTTTAGATTTTAAT
GTCATTCCAATAATAAGATCCTTACTGTTTTGGAAACATCGGAGTTTTGGTTTGGTGA
CCACATATTATTGGAGGCTGACTTTTCAGATTTACAAATAAAAATATTTAAAATCCAAAAAATATAAGACTTA
CGAATTAAAATACAAAATTACTTTTATTTAAAAAAAATGTGAAGAATCAGCCAAAATGTAATGTTGGGTCCAG
ACACAGATAATCAATGTGGTATGCCGCTAGGTATGGTGCGGAGGGCGAGTCGGCTGAC
GCAGGTGAGGGAGCACCCTTACCAGCGCACTGCCTTTACAGCGCCTTTATTTCCGCAAAACGCTCT
>126037714_2 EL597496.1
GCACGAGGAAATCATTTACATAATAGAATAAGTCATATATTTTATAAAAAACAGTACATACCTATGTATATAA
AATACCTTATGCTGAAGGAATATATTAAACACACTTACCACTAATAAACACGTAGACTCATAAAATTCTTATT
AATTCTTTGTTTAATTTACCAAATATTTTGCAAATTAACTTTGTAACGCTTTTTTTTG
TATATAATCTTTGTAATAAATAAACTTTTGTAATACTTCATTTAATTTTTCTTTGACGCAATAGCTTTCAATT
TTCAATTTCCCGCCATTGCGGTCGACCACAGATAAAATATGTTCTAATAACGTATAGCGTCAGACATTCCGAC
ACCTATTTGTATTATTTACTTTTAAATTTATATAATAATAAAAACAATCGTTTTTAAC
TTAACATGTTAATACAAAATAATGATTTTGATTATAATAACTTGCCTTACAAACGTTCTCGATATTTACGTTT
GTATTTTTTTTAGTTTTATTTTATTTTGTAAATGTCTTGTAAATAAAATTCATTATAAAAAAAACAAGACGGA
CAGACAAATGTTATAATACACCGCACTTAAAAATAAATTAATATCAGTTAAATGCTAA
CTTACATCTCTGAGAGCGGCAATCTGTTAACAGTTACAGTCTACTATATGTTGTATTCCTATTCGCTGTAAAT
TGCATCCT
>126037720_2 EL597502.1
CACGAGGAAAAAATGTTGCTATACACTCAAATGAGCACCATATCAAATGTAATCACTCGAGTGATCATAGCGG
CATTGATGTCTCTCATCACTGGAACTATATTTTGGGACCTGCCGACCACTGACGCAAAGTTAACACAAAACGA
CCGCATCGGCTACCACTATTCCGTGATGTGTGTGAGCGCGTGGCCGCTGCTGCTGCTG
AGCGCGCGCGAGGCGAGCGCCACGCGGCGCCATGTCGAACGAGATATAGCTGTCGGCTTGTATTCTAGAACTA
TGTTTATTTTGTTTGATCAATTTCTGGAAATATGGCTGGCCATATTGACGTGGCTCGCGTACTTAGTGCCGAG
CTACGCCATGAGCGGTCTGTATGCGCAGGCACCGGGATCATTCGACGGATTTTATGTT
TATTTAGGTTACATGCTGCTATACCTAATAAGCACACAAATGTTATGTCGGGCGTCTATATTCATCATACCAA
TGGAGAAGGTAGCTTCAATCGTATCAGGGTTGTGTTTCCTACTAACCACACTCGTGAACGGCGTGTTGTTGCA
TCAACAAGACTTGCCGTATTACGTGAAATGGTTAGAATACGTATCGCCATCGAAATGG
ACCGTTCCAGAAATATTAAGGCGGGAATTGAGTGAGATTGCCTTGAGAAGCAGTA

>126037723_2 EL597505.1
GCACGAGGCTATGTACTCATCCTAAAGGAAATGATGAGAAAGTACAACTTCTTCATATCGCGACGACCTTAGA
CAAGTTAGAGAAAAAGTTAGAAAATATGGAAAGAATTGTCGACCCACATGGACAATCAAGGGGGCGTAAATTG
TCATTAGGATTAAGAGGTAGTACTAATGGAGATGCCTTGGGACCCGTGGCTGAAGGTG
AAGAGGAAGATCATGATTCTGATTCGGAAACAGACACAATGTCTTCAACACCGAGGGAAAAAAGAGATGACCT
TATTAATCCATATTGGATAGAAGACCCTGAATTGAAAAAAGGAGAAGTAGATTTCCTAAGTCAATCTGAACTG
TCATTCTGGAAAGACCTTATTGATAAATATCTATATCCTATAGATGAAAATAAGGAAG
AGAAGGCTCGAATTGCACGTGATTTACTTGAGCTTCGCAATAAATCAGTTTTTGCGTTTGTCATGTTCAATGC
TTTATTTATATTGATAGTATTTTTATTACAACTCAACAAAGATCAACTCCACGTGATTTGGCCTTTGGGTGTA
AAGACAAACATTACGTACGTCGAGGATACAGGCGAGGTGCTAATATCCAAAGAATACT
TGGAACTGGAACCTATCGGTCTAGTCTTTGTGTTTTTC
>126037724_2 EL597506.1
GCACGAGGGCGTGTACAAAATAAAAGTGATAGCCGATAAAAATTGTCAAGAGAATTTAGAAGGTGAAGCGGAA
CTGATATATAAAAACCAGGAGCAAAAAGTTCTGGTTAAGAACGAGGACAGTGATGCTGCGCCAGTGAGCCGCG
AATTTACAACAGTAATTAGTGACGAATGTGTTAGTGATGGTGTGTTGTGTTTGAACGA
GGTGCGTTCAGCTGATAAATTACCCGTGGAGCTGGTCGCCGCTATCGATGAAACTATTTATGTGCCATTCAAT
TACTCTACGCGACGTATTTCAGCAAAACGACTTGAGAGCTGGGGTCAAACAGATGGGCATCGCTTCACGTATC
CGGCGTCACCACTGCTAACTCAAACTGCTGATGTGGCTCCAGAGGCAATGTGCCCTGA
TGGTCCTGGGGATGGAGAGGAATGCGTTCACGTCAAGCATGTCCCACTGTATTCTACTGTCGAATTAATAATG
TTCGATCAAGGAGGTGAATCAGATCATATATTCCATCTCCACGGCTACAGCTTCTATGTGACTGGTGTCCGAC
AC
>126037725_2 EL597507.1
AATTAAAAAATCACTGTCTTAGTATAAAGTTGACTTTCTAAAATTCCTAACCGATTCTTGCATATCCTCTTAC
ATATCTGCTTCTATGAACTGATCAAACACTCAATATGTCTATAATCCTAGTATTTAAATTAATGTTTATTGAA
AATTTACCCAGAAATTTATTTTCTATAAAAATGTAGCATCAATTGTTTTATCTATGTT
TTTCCTTTT
>126037735_2 EL597517.1
GCACGAGGATAACCAACAGGGTTATAGCGGTGGCCCCACTCGTAACCAGCAATATGGAAATAATAGATCAGCA
CCATACAATGTCAACCAAGGCAGCGGCGGCGGCGGTGGTGGCTATGGCGGCGGCAACTATGGTGGCAACCAAG
GGCGTGGCGGACGTTTTTAACGTTTCGTGCTAAGTACACTAGTAGTGCAGAATATGTC
TAGTGTAGGCAGCTTCGGAGCACTAGTTACTCGACCTTGGTCCACATGCGAGACCCCATAGAAGCCGTCAGGA
ATACCATAGACATAGTTTGGACCTCGTATATTTTAGAAATATTCATAGATTTTAAATATGTACTGATATGTAA
GTTGATCTATTTATTGTAGTACTTTAGGTACTTACGTGACATACTTGTAAGGAATATA
TAGTTTGTGAAGTCGATAGTTACTTTTTTACGGATATAAGTACAATGTAGGATTATCTTGTAAGAGAAAAATG
AATTAGAAGCTAGGAAATACACATTTTAAGATGACATTAGATTATAAATGATCATATTTTTTTGCTGACTTAC
TCAGTATTAAGTTTTCAAATAGATGTTATATCAAAACATAGGTGTATAGTGATTTATA
GGTTAATTTAATTAATAAAAGTACTACAAAACTA
>126037736_2 EL597518.1
GCACGAGGTTCATTGCCTCGCTCTTGTTGATAAAGCGCGGCGTTGTTTTTCGTCAACGGAGTGTCTTGACGTT
GGGTTATCTCTGTCTACCCCACAGAAACCCTAAAGCGTAGTGTAGCTGAAAGAAAAATGAAATAAATCCAGTT
GATATTAGTTAATTTTTTGTGCCTTTGTAAAAGTCAATTTGACATTAAAACAGATTCT
TTATTCATATTTATCTTAAAAATAATATTAAAATTAATGAAAAAATATGTAAAATTGTTATTAAAACTAATGA
TTTTTTTTAATTATATTTGAAGATATAAATTCTTTTAGTGAAGAACGGAGTGTCGTAACACTAGGTTCCCTCT
GCCTACCCTGCTGTGTGCGGGATACAGTGTGAAGCTGAAATTCTTTTAACAATATTAA
TAAAGTTTTTATAATAACTTTTAGTTTTCAATTTGGTTATTAACAAATTTTAAACTGGTTAAATGTCATATGG
TCTTTAAATTTATACTTAAAGTGACACCCCCCTTATATTATTAATATTGTAAATATAACGTACATAATTTAGT
TGTATTTATTATTATTTAGTAGAGGCCCTATTTCAAAGTTGATGTAAATAAAAGTATA
TTAGCCATTATGCAATTTATACTCTATGTAATCTGTATACTGTATATTTGCTATCTGTATTCCGCGCATTTGT
GTGATTTTCGTAAATTCTGTGTAATAGAGTTTTTAATAAACGGTCTTGAATAAAAA
>126037743_2 EL597525.1
GCACGAGGGAGACACTTAACTAATATTTTATAAATTCTTTACTATACTTAATTGACTCAGGACCATTGATACA
AATATTAACAGTATTGTACAATAAATTAATAATTAAAATGTGATATCAATCTGACTGGACATTGAATATTAAA
AATCCGCCACTGTTCAGTGTCACACGCCGCGATTTTTCATTATTTTTAAGGCTGTTTC
TTATTTTGTTTAGTTATGAAC

>126037752_2 EL597532.1
GCACGAGGCCCAGGCGCTTTTGCGTTTCGGAGCGGTATTTTAAAAGATAAATAATTAAAAATTCAATATTTTT
TTGGTTGACTTTTGTGATTTTAAGCAGTGATCGCTTAAACTGAGGAGTGCAGTGTGATCAAATGCAGTGATGA
GGCCCCCTACGGACTACGACGGTGCGAGGGTACCAGCGGAGGCGGAAGAAGGGGACCC
GAGGGCTCCAGCGATGATGATATGTGCGAGATTAGCAGGAAGAGCTATCATTAGAGTGGTCGGCAGGTGTGAG
GATGCACAAGAAAATAGTCAATTAGATCTATCTGAATGTCAACTTATGCAAGTACCTGATGCAGTGTACCACC
TGATGCGACACACAGAGCTCAAAAGTTGTGATCTCAGTGGGAATGTTATCACAAAAAT
TCCACCTAAATTTACTGTTAAATTTAGTTTAATTACAGATTTAAACCTCTCAAACAATCAAATGGCCAAGTTG
CCAGATGAGCTCTGCACACTGGCATGCCTCCAACGTCTGGACATCTCCCACAACACATTTGTGGCCCTTCCAA
GTATTGCTTTCCAGTGTCCCAGTTTGCACACATTATTGGCACACCACAATCAAATCAT
TGAAGTGGATGTGGACAGGCTAGCAAGATCTCGGGCACTCGAATATGTAGACTTAAGTGACAATCCTATTCCA
CCAAGAATGCATGAAGAATTAAAACAGTTGACTAGGCCTTCTATATCTGTATCGGA
>126037754_2 EL597534.1
CGGCACGAGGGAGAAACCGCTTTGTTGTATGATAAGAAGGAGTCATCGTTTTTGAATAAGGAGAGGGTTGCTG
AGGTCGTAGCTCACGAACTAGCTCACCAATGGTTCGGCAACCTGGTGACCATGAAATGGTGGTCGGATCTATG
GTTGAACGAGGGTTTCGCAACCTTCATGGCGTCCGTAGGTATGGCTGCCGTGGAGCCT
GAATGGAATGTCGAAATTAATAATGCAGTGGATCATACATTAGCTGTTTTGGCATTAGATGCCCTGGAGTCTT
CGCATCCGGTTTCAGTGCCTTTAGACGATCCCAAGCGTATATCAGAGATATTCGACACGATCTCCTACCGTAA
AGGCGCGACGCTCATACGTATGATGACCATGTTCCTCGGAGAGGATGTCTTTAGGAAA
GCCTTGCATAACTACCTAACAAAATATTCTTACTCCAACGCGGAACAAGATGATCTGTGGTCGGAACTGACAG
CTGTCAATCGCCAACATGGTGGCCTCAGCAGGGACGTCACTGTCAAACAAGTGATGGACACGTGGACCAAACA
GACCGGATATCCGTTAATTACTGTCACCAGGGATTATTCTGAGAAATCGTTGACGATA
TCGCAGAAAAGATATCTCTCTCTCGGCTCATCGACTCGCTCATCCTCCTCCTGGTGTGTACCGCTCAGTGTTC
TCTGTC
>126037756_2 EL597536.1
GCACGAGGGCCAATTTTTATTTCAAAGGAGCTTAATATAATAGGCGATTGAGTCACTATTCATGTTCTTCATA
TACATATTTACATTAACACATAAATTGCTTCTATTTTTATCTGGCATGAATAAATTAAAGTAAAACATCACAT
TTTATTATTGTTATATTTCTAAAAATCTAGTTTGATCTGTGTAAAGAAATAAATTATA
TATTATTCTTTCAAGGATTTGAATTTAATATTTAAAAAGTACATGTATAAAATATTGAAAAACGAATATGCTT
TATTTGTTTCATATATTCAACTAGGAACTGTGTGTTAATTATTTAATATTTAAAAATTAAATTGTCGTCAATG
TCACCCTTGATACAACTAATAATATCTAAATTTCTTATACTTTAAGTTACTCCTAATT
TACATGATGAATTTGTGACATACATAAAGATCAATATCATAGGAATTACAAGTACATTTTATTGCCAACATAT
GTAAAATATTAGTAATAGTTTCATTTAAATATATATATTTCATAAATTCAATAAAAATATGTAGATGTACAAT
AATGTGTTAACTTTAATTTTTAAACTTATAATTTAAAGTAAAAACAAAAGTGTTTATA
GCTTGTCACATTATTGTATGTCCATTCATTTTATAAAATTAAATATTATAATTATTTTTAAAAAACTTCTCAT
ATCATTATTTTT
>126037758_2 EL597538.1
GCACGAGGCATCAACTTGACAATCAATTCAGAGATGAAGTAGACATCGACCCCCGCGTCCATAGGAGGCTCAT
AGGCCTGAAAGGAAAGAATATTCGTCGTATAATGGATGAGTATAAAGTGGACATTAGATTCCCCAAGCAGGGA
GATGACAGCATTGTCGTCATTACTGGGGACGAAGACAATGTGCTAGATGCAAAAGATC
ACCTGCTGAATTTGGCTGAGGAATATCTGCAAGATGTGGTAGACCGTTACCAGAGACCAGCGGCGCCGTCTTT
GGGTGATTTTGGGGATGTGCTGAGTGGGGAGGTCGCGAGTGGTGCTCCGGCGGCTCCGTCCACCGGCTTCGTG
GTTAAGGGTGGGCCCTGGGAGCAGCGCGCGCCCGACACGGCCTCCACGCACGAGTTCC
CCACCATGCCAGGGGCCCCACGCGCCGCTGCGCCCGCCGCCGCCCCCGCTCCTTCCTCCGCCTGGGGGCCCCG
CCGCTAACCATCTACAAGCTCATATCTCTTACAACTAAGCTTGACTTTTAGATCCAACTTATACATTCTCAGA
TTAAGCCATTAACTGTTGTAGATGCAAGTTTTAGTTTCATGCATGATGAGAATACAAT
TATAATCATAAGTAACTATTGGTATTTCATTAAAACTTTCGGTTTTATTGGAATTTTTATATCATATATT
>126037760_2 EL597540.1
GCACGAGGGAAACGCGGAAAAACGCGTAAATTAGAATACATTTATGATGTAGTATGATATTGTTTTTGTAATG
CCGCAAGCACTGGGGCCGTATTCAAATGGATGGGCGATAGACACTGCTGCGCCCGTTGACCTCAACAATCCTG
ATAAAGACTACCACCCAGTCTTCAAGGCGTTCCTGGCTGGATCATTTTCCGGCACCTT
CAGCACAATCCTCTTTCAACCTTTGGATTTAGTGAAAACAAGACTCCAGAATCCTGGTCAGCATGTTGTTGCA
GCGACTGTCAACGGCCGAATCCAACCCGGCATGATATCGATCTTCATGAATATAGTACGCCAGGAGCATATCG
TCGGATTATGGCGGGGAATGGTGCCTTCCGTTGCCAGATGTGTACCGGGTGTTGGTTT

ATATTTCTCATCTCTACATTGGTTGAAGTCCAAGATGGGCAAGACCAGGCAGGATCTGGGGGCAATGGAGGCA
GTCACTTTGGGCGTAGTCGCCAGGACCATGAGTGGTGTTGCCCTGATACCGATTACGGTTATTAAGACTAGAT
ACGAATCAGGTGTATACAAATATACCAGCCTTAGTGGTGCTCTAAAAGCTATATACAA
AGCGGAAGGAATTAGAGGATTATCTTGTGGCTTGGGGACGACGCTGGCAAGGGATGCGCCATTTTCTGGCTTA
TATCTTATGTTCTACACTCAAACGAAACAAGCTATACCTAAAGAATATC
>126037762_2 EL597542.1
GCACGAGGCAGAGACGCCCTCGCCAAGGCGGTTTACAGCCGCCTCTTCGACTACATAGTACGGCGTATCAACG
AGAGCATTCCGTCTTCAGCTTCAGCTTATTACATTGGTGTTCTGGATATTGCTGGGTTTGAATATTTCCAACA
GAACAGTTTCGAACAATTCTGCATAAATTACTGCAATGAGAAACTGCAACAGTTTTTC
AACGAACGCATTCTCAAAAACGAGCAGGAACTTTACAAACGCGAAGGATTGAACGTTCCCGAAATACGATTTG
TGGATAATCAGGATTGTATAGATCTAATTGAGAGTAAAAATCATGGTATCTTCCACATTTTGGATGAAGAATC
AAAACTACCAAAACCAGAGTTCGGACATTTCACTAGCGCCGTGCATAAAGATCTCGGC
AATAATAATGGAAGTTCACGGCTACAAGTCCCACGCGCGTCGCGTCTGAAGGCGCACCGCACTCTACGAGAAC
ACGAGGGCTTCTTGCTCAGACATTTTGCTGGCTCCGTCTGTTATAACACTACTCAATTCTTGGAGAAGAACAA
CGACGCACTACACGCCTCCCTAGAGTTCCTAGTACAGGAATCTCAAAACTCGCTTGTA
CAGCAGTTGTTTGAAACTATAGACAATAATAACGCAAAAGGAAAAGTGAATTTTATATCCGTAGGCGCTAACT
TCCAGCGGCAATTGGCTCTATTGATGGACAAATTGAA
>126037771_2 EL597548.1
GCACGAGGGTCTAAGGATAACGGCGAAAGAAGCCCAACACTATCCGAATCGGCAACAACAGCACGAGCGTCAA
GTGTCAACGACAACAACTCAACCAAAATACACAACCACAACACAAACAACACAACCATCGCACGAAACACACA
AACTTTGAACCGTAGAGTTCAAGGTGTGTGTTCAAATCACGCAATGAACAACGCACTG
GGTACTTTGCCACGTGTCAATAATAATAATATAGGTTCCGGTTTGGTTACAACGCTGGGGAGAAAAATTGGCA
ATAATAATAATAAGAAGAAATCTCAGCCGATTATGGCGTATGATGAAATACCAGGATTGCAAAGATCATCAGA
TAACGATAATGTTACGTTTGGCAAACGTAACTTCGGTGGATTCACCTACGAGCCTTCC
CACGTAGAAACTACTACAGAACTCTAGTTATTAACAATATAGTGCAAAAGTGAATAAATATTATTAATATATA
ATATACTTGTGTATAAGTGTTATAAACAAAGTGTTTAAAGTGTAGAACGAAAGTGTAGCTATTTGTGTTCAAC
GAAGTTTTTAATATATTGTTAATATATCTACAATGACTAGTGTTGCCAGAATACGGGA
TATAAAATTCAAGGAGAATTTTTTTAAATTAAATATTAACCAAAAAAATACATACGATATATTTTTG
>126037772_2 EL597549.1
GCACGAGGGCCGGCCCGAGCTCGCGACGTCAGCCACAGCGGTTATTTACAAAAATATCATTGTTTTACAATCG
CGTTGTGTTAAATATTCTGTGCAAGTGCTATACCCCCTGTGTGTGTTTCTGTGCGTTTTTCTCATAAACAAAC
TGAGCTGTATACCTATGTGCATGCGCTGAACTTGCAAGATCATACGATATGATCCACG
TTGATGAAACAGATCCTGTCGGAGAGATAGAGCCGCCATTTCCGTACAGAGATGTCACAATCAAAAGGAATAC
AGATGTTAACGACTTCTTCGATATGCTATCGGAGATCGGCCGCGGTAAATTCGGCACTGTATATCTATGCCGA
GAGAAAAGCACCGGATTGGAGCTAGCAGCTAAGCTGGTTTCCGTCAACCGGAGAGACG
AGAGGAGGAACGTGGAACGTGAAGTCGAAGTGATGAGAAGACTAAGACACCCGAGGCTGATCCAACTCTACGA
TTCCTACGATTGGGGGAAATATATGTGCGTCGTTTTAGAATTGATTACAGGCGGCGAGCTATTTGAACGTGTC
ATCGATGAGGACTTCGTGCTAACTGAGCGAGCCTGCACTGTGTTTATGCGGCAAATAT
GTGAAGGGATTGAGTTCGTGCATAGGCAGAATATTCTCCATCTTGATAT
>126037782_2 EL597559.1
GCACGAGGCTTTTTATTTAATTCAAACGTAGATATCGGCGGTAGCAGCGCTGTGTCTTCTATAAAACAACGTG
TAGTTTCCTTTGCACGGACAGGGTCACCCGCACCCGTTAACATGACGGAGTGGAAGGCAGTAATGAAACCATT
GATGAACGTGCTTTACCTCAGTGGTGGAGAAGTAGCTCCCAATGTCACAATTTACAAA
GAAGAAGCTTTGGGTAATAGGCATGTGGAAAGGACTGACTTCTGGGATAATATCCTTGAAAAAAGATACTTGC
CACCAAGAACCGTCTCGTCTGCTGGGAGCTTGGTCAGCTTTGGACTAGTGGTTTTATTTACTCAAATGTTAGT
TAAGGCTCTTTAGATTACTAAATAAAAACCTAATACGTAATCAATAAGTTTTTATTTA
AATGTAAGATTTTTAATTTTAATTTTGTTCTACTTAACCGTCCTCTTGTCAAAAATTAAGTATTATTTTGTAC
GTAATACTATATATGAAAAACAAAATTTAACATTACAATGTTACAAAAAATCATTTTCAAGCAAATTTTCTAA
TGGATTGTATTGCATTTATTANATTTTAGGTTTTAAGAAGTAAGGTTTTCGAACAGTA
TTTTAAACAATTTATTTTTTTTATACCTACTTGTGTATATGATATACGTTATAACAAACTG
>126037819_2 EL597567.1
GCACGAGGCCGGCGTGGTGCGCACGCCGCCTCCATCCCACTCACACCTCTACTTCTAATATACATCGTGAATT
ACACCATATCGTACTCCATCCTGCTTATTTTGAACCGCAGTCATGTTGAACGGCTTCATGAGATGGTTCCTGC
GGTTCTTGGTGTCACTACTGCAGATTGTAGTTCGCCCATTGACATATTTAATAAATAT

CAAAAGAAATAGAAAATGTCCGCCTCCCACCAATCCTATATTATTCAAGTCCGCTACTACACTAGCTATGATG
ATCAGAAACAAACAGATAACATCAGAGGAAGTAGTAAAATCTTATATTGATAGATGTAAGGAGGTTAATCCTT
ATTTGAACGCCATTGTTGAACCGCGATACGATTTGGCGATAAAGGAAGCGAAATGTAT
AGACAAAATGATAGCATCCAATGAGCGATCTCCTGAGGACTTGGCTAAGGAATATCCCTTACTTGGTATCCCA
ATGACTGTGAAGGAGAGTATTGCGGTTGAAGGTATGAGCAACGACAGCGGCACCGTTTTCCCATACAGAAACC
CGGCTAAGAAGGACGCTGATATCGTTCGTAGGGCGAGAGCAGCTGGAGCAATTCCTAT
AGCAGTGACTAACACACCGCAATTGTGCATGATTTGGGAAACATATAACAATGTTACTGGCTTAACAATGAAC
CCTTATGATC
>126037821_2 EL597569.1
GCACGAGGAAACTTCTTCAGAAGAAATGACGGACGAGATTTTGAATATAAAAAGTATCTCTCCTATTGAAAGT
GAAGCTATAAATACTCAAATTGTTGAAAAACTGCAAGTTACTGAAGATATTTCATCAAAAATTGCGTTAGATG
ATATGATTTTATGTACAGAAGACAATGTAGTACCAAAAACTGAACTTGTATTGACAAA
ACAAAAAAAGAAAAGGAAAAATAAGAAACATGAAGGCGTTTCATGTGAAGATAAAAATAAACCAGAAATATAT
GTGAATAAAAGTGATGTTTGCTTCATTGAAAATATAGATAATCGTGACACATCTAAGATGTCTGGGTTAAAAT
CAGAAATTGTCAAAAAAGATATTGACATTATAAAAGAAAACGATAAACCTACTAATAT
TATTATTTGTGCTAACGAAAAAGTAAATGAACCTAAAAAGAAGACAAAATCTAAAAAAACTTCACTTAAAGAC
AAACAATTATTGGTAATTGAAAAATATTTAAAAGATAATAAAGATACAGATGAATATGGTACAGATACTAAAT
TCGAAAAGCTATCANAGACTTCAAATGACGACAGTAATACATTGCTTAAAAAAATTGA
ATCAAGTGAAAGTTTGAATATTGAAGATGAAGACAAACCCATTCTAGTACAAGAAACTAAAACAGTACTTATA
AATCCTACAAATAAATCGAGTATTGTA
>126037831_2 EL597575.1
GCACGAGGGCAACACGAGCTTTATTCTTTCTACTGGCAACACTGCCCCATTTAAATACAGAGTATATACCGCC
AGGCCCCACTTATCCATGCCCAAAAGACAATCTTCTCTTACACCCTTGCATCTGCCAGAAAGGTACCGATGAA
GGCTTATATGTAGTATGTGAGAACATTGGTTTAGCCATGATGAGTGTCGGCCTGGGCA
ATATAGCTGGTATGGGTCTGCCTATAGAGAGATTGGAAATACGGGAAGCAAAAATTGCACATCTCTTCGGCCC
ACTCCTCCATCGTTCAACAGTGAGAACTTTACACATAATGGACACGCCTATCAAGTCCATAGATCAATACGCA
TTTGCCGGGGTCAATAGAACTCTGCAAGAGATTTATATGGTCAATACGAAGATAAATC
AATTCCCTAAAGACGCTTTTCAGATCCTAGGCAACCTATCAATCCTATCAATAGATGGTCATCTGATGACGTC
ACTTAACAAAGACATCTTCTCAGGCAGTCTGGCTGTTGGTAAATTGGAAAAATTACATCTTATCAATGGGCTT
TTATCGGATATACCCATTGAAGCTTTTCAGAATCTCAAAAAACTTAAAACGCTAGATC
TTCATAGCAATAAATTGGCAAATTTAAAAAGAAATCAGTTCAAAGGATTACGGGACACGGAGGTTTTAGATCT
GAGTTACAACAAAATAACCAAACT
>126037833_2 EL597577.1
GCACGAGGATCAAATCTTAACGAAGAGATGGTCACGCCTGACGCTCTATTTACCCTTTTCGGCGTGTATGGTG
ACGTGCAGCGAGTGAAGATACTTTACAACAAAAAGGATTCAGCACTAATTCAAATGGCAGAGCCGCATCAGGC
TCATCTAGCCATGACCCACATGGACAAACTGCGCGTGTTCGGGAAGGCGATGCGCGTG
ATGCTCAGCAAGCACCAGACTGTGCAGCTGCCGAAGGAGGGACAGCCGGACGCTGGACTCACTCGGGACTATT
CTCAGAGCCCTCTCCATAGATTCAAGAAGCCGGGCAGCAAAAACTATCAGAACATATACCCGCTGAGCGCTAC
ATTACATCTGTCCAACATTCCAGCCACCGTTTCTGAAGATGACATCAAGGAAGCCTTC
ACCAAACGGGGATTCACGATCAAGGCCTTCAAATTTTTCCCGAAGGATCGTAAGATGGCGTTGGTACAGCTGC
CGTCGATCGATGATGCAGTAGCGGCACTTATCAAGATGCATAATTACCAGCTCTCTGAGTCCAACCACCTGAG
AGTATCTTTCTCANAATCAAGTATTTAAACTAAATGACCAGCGCGTCCAAGGAATATC
ACACAACAGTCGATGTAAACATTATTAATGAGATTGGCCACTANGTATGGGCAATTATAATCGATTTACTGAT
AGATTCTCATAAACATAAGTTCTAATTAGCTG
>126037851_2 EL597591.1
CACGAGGACCGAATGTATATCCGACGCAATACAATACAGTCGGGAGAAGCAAAATACGCCGTCGAAAAACGTC
ATTTGTACGGAGCTTTGCATTTCCCTAACAACTTCAGTGACGCGCTCTCCATCAGAGTCACCGAAGGGAGGGT
CGACAACGAGACCGTCGACGAGAGTACTGTTAGCGTTTGGGTGGATACGACTGATTAT
CAACTATCGTATTTCGTCAAAATGACAATGCACGAAGCGTATGAAAAGTTCGCGAGACGCCTGATGTTGGCGT
GCGGGAAACATGAGGAACTTGTACAGATACAACTACGATTCCAAAGAGACGATATATGGTAAAA
>126037853_2 EL597593.1
GGCACGAGGTTTATATAAAAGAAGAATATTTTCGTTTTTGTATATATTCCAGTAGTGGTTGTGATAAAGACCA
TGGGTCTAACAATTTCTAGTGTTTTTACGAGACTTTTTGGCAAAAAACAAATGCGTATCCTAATGGTTGGTCT
CGATGCCGCAGGCAAAACCACTATACTTTACAAACTAAAACTTGGTGAAATAGTGACA

ACTATTCCGACTATCGGTTTCAATGTGGAGACAGTTGAGTATAAAAACATTAGCTTCACTGTCTGGGATGTCG
GTGGCCAAGACAAAATCAGGCCTCTTTGGCGTCATTATTATCAAAATACGCAAGGATTGATATTTGTGGTCGA
TTCCAGTGATACAAAGAGGATAGCAGAAGCAGAAAATGAACTAGCTAATATGTTAAAA
GAAGATGAGCTGAATGATGCAGTAATTCTGGTATTTGCCAATAAACAAGATATGCCTAATGCCATGACAGCTG
CAGAATTGACAAATGCTTTAAATTTGAACAATTTGAGAAATAGACGTTGGTACATCCAAGCAACTTGTGCTAC
ACAAGGACAAGGTCTCTATGAAGGTCTGGACTGGCTTTCAAATGAATTGGCGAAGAAG
TGAGACATTTAATATTTACATGAGTGGGAGATAACATCAAAAATTCCCCATCTTG
>126037859_2 EL597597.1
GCACGAGGCCTCGTGCCGAATCGGCACGAGGCAACTGTTGCAGTAAAAAAAATAACAAAGTTTGGAAACACTA
CCTTTAAAAAGATCTTTAAAAGAATCATATGATATTTATTTATAACTAAGGAACATATTTAATGTCACAATGT
TTTATATCTATATATATAGCATTTTTACACTAAATGCTTGAAATAAAAATTACTTTTA
AATTATTTTTTCTAAAGTCTTATGAAAAGTCCAAGGAATTACATACAATTTTTTAAATGTGATAACATGTATA
AATATGTGGACTCCGACTACACGTACGAAAAGCATGTCATCGTCACGGAATAAGCGATAGAGCGAGCGAAGCG
TTCGCCTTGTGACGCATCTATCCACTCATTTTTACCTGTAGTTATGTTAACGAGTGCA
AAATAAATGTTTCTC
>126037865_2 EL597601.1
GCACGAGGGTTTCCAATGGTAACTGTGTTTCGGTAGGGTGAGGACCGCGCTTGCTGTGGGGTAGTGGACCACG
GTCCACTGTCTGCGATCTCTGTAGGTAACGGTGAAGCGCTGCTCGCGCGGTCTCGGTCCGCCGCGCGACCGCG
CACTGGCACGATACTAAACACTGCACTGTCACTAGATATATACAAACCACACTATACT
ACTGTCTCTTCCAGTCATTCCATTTCCGAATCTGTCATGCCTCGTTCACACTAATCGTTCAAACGCTCCTTCC
ATTTTTTTATTTCTCATGAAATGGAAGAATGGTGTAGGATCGTTTGCGTGTTCCTTTTTTACTCCTAAATTAA
AATATATATTCTGTGGTGATTTATTTTCTTGTGACGTGTCGGTGGTCAGTATCGCATC
TTTAATGTTTCCCGCTGTTCCCTTCTGTTGGCTTGTATAAACCAAATAATGCATTCTGTTATTACTATTACTT
GACCAATTATAATTATTGTCATTGTTCCTATGATTATGATTGTTTTTAAAGCTAAAAAAAATGTAATTTAAAG
CTAAAAAAAATTCTTGTTGCGGTTTTTCCT
>126037866_2 EL597602.1
ATTATATTAATTAATTAATTAAACCAGGCTGAAGCCAGTCGTCCCACAAACTGTCGCCAAACGCGTCAGTTTG
CGGGTTCAATACCCATTTTCTTGCTCCCTCTTCTATTTTCTTTTTTATAGTATCGAGGCAAATGGCGGCGTTC
AGTTCTGACACTCAACGCGCAATGTCTCCAATAAAATTCGGCGAAATGTGGAGAGTGA
GTGTCTTAACACTATACTCCTTCTGCCTGCTCTAGCTCTACATGTGAGGATACAGCATGAAGCTTTGATATTC
ATTTGTTTTAAACTTAGCTACAAATTAATAAAATTAAATTCTATACATAATATTATGCATAGAATGTTATTTC
ATACTGTCTTATACCTAATCTTGGTTAGCTGTGGCTGTTTCTAATGTGCAATCTTACG
TATGCAACTTACGTCAGTGTGCGTTGGGAACATAGCGCAGCGTAGGCGGTCGGCATTGCGGTTTAAGATTGGC
CTCGGTCACTATTTATTATCTTTGTTTAATTTGATGCATTTATTAGTAAATCGA
>126037869_2 EL597603.1
GCACGAGGCACGAAATCATCCGGAGATACCTCTCACGACGAAGACTCGTGTCCTCAGTTACGAACAATCCTTT
AAGTTTGCGACCACGTTCAGGCCGCGCGGCATGTCCCCGACGCGTTCACAGGTCGGTGCGCCGTATGACTGAT
TCCTACTAGTATGTCCCAACTGTCTTAAATTATTGCTCTAATTTAAGTTTATTTAATT
GTGGATATCGGCGGTAATATATATGTAAATATTTGTCGTAATTTATTATCGAAATCATTAGGTGTAATGTGAC
TTGTGTATATTTGGGCCGTCAACTTAAACGCCGGCCTCGTAAGAATGGACTTGTTATTCGCCACCCGCGTGTT
CACGAAGCCCATTCCGGTTGGTAACTAGGCAATTTGTAGCTAAATTAATAATTATTTA
TTTTATTTTAATTATTTGCCAACGGAATGCGACTATTGTACAGATTATAAAAAACAGTAGACAAATATAGAAA
GAATCGTTTCCACCCTTGCTACATGTGCCGATGAGTGAGCGAGTTGAGATAAATGTAAAATTGTTAGTTTGCG
AGTCAGTGTAGTTAGAGTGGAAACATTCTTTGTATAAGAAGGATAGCTTCTTAATTTA
GGAAGGGATGTACGCGGACGAATGCGAGGTCCGCTCGTCCTGCTCCACCTCATCGACTGCGAAGTCGTTGAGT
TAGTGCTTTACCATACACGTGCGTTACATGCACGCTGCACTA
>126037878_2 EL597610.1
GCACGAGGCACCCGTTCCCGGGTCCGGGATTGGCGGTGCGTGTGCTTTGCCAGGAGGAGCCCTACGCGGACCG
AGACTTTGCTGAGACCCAGGTGATAGTAAAAATAATGGTGGAATACGCGTCGATGTGTGTGAAGTCCCACGCG
CTGCTCGGCCGCGTGTCCAACGCGACCACGCCGCAGGAGCAGGCCGACCTCAAGCGTA
TCTCCTCCGCCTCGCCCGTGGCCGCCACGCTGCTGCCCGTGCGCAGTGTGGGCGTGCAAGGCGACGGTCGCTC
GTACAGTTACGCGGTGGCGCTGTCGACGGAGCGCTACCCGCCCGACTGGCAGGACATGCACTACCTCGCCAAG
ATCATACCACGGGTTTGCCACAACGTGAACAGGGTGTGCTACGCATTCGGTGGTCTGA

TCAAGGAACAAGTGACAGACATCACACCCACTTTCTTATCTCAGCAAGTGATATCCACCATACGCCAAGCGGA
CGATATTGCTACACAGATACTAATGTCGAGCGGCGGCGGCCCCCGCATAGCTCAGATGCCAGTCGTCCTCATC
CCGGTGCACTTCGACCGCGACGCCGGCATCAGGGCGCCGTCGTGCCAGCGCTCCCTTG
TGCTTCGCCCCTTCATAAACCATGACTTCATGACGGGGATAGCGGCGTTGCCCGGCGAGGACGCTATGCCT
>126037881_2 EL597611.1
GCACGAGGTTTAGACACGCGATGTTGTGACCGACATCGATTTTAGTAAATTGAGTTCCTATGAGCATTTTGAT
ATTTCCCCGGCGTTATTATATGAATTAAGAACTATGGCCTCTTAACATCCACTTAATTATATTTATATTTAAG
TGCATTTTTGCCCCCCCCAGATTGCTCAGGACTTGGCGCGGTTGCCAGCTTGTGATAG
TTTCTTGCCACCGGAACTAGATATCGTGAATTTATTTTTCTACGTCACTAAACTAGCGTTTTCGCCCTAATAA
TTTTCGAAATAAATTTGAAATCGTCCAATGCTACATCTTCGTCCTTTTAAAGACTTGTACTAAGTACACCGAC
AGCCATACGACGGACTAAATATGGCCAAAAACAGTGAGATCCTGTTGCGCGCCCGCGC
CCTTATATCGACTAGGTTGTGTAGGTATTTTGAAAATGTAATGTAAAATAAGTGTTTGTAGTCGTTTTTGGTG
AGATGTATATTAAAATCATAATCAAATTTTTGTCCTCAAATTATATCTCCATCCACATTCGAACAATAAGCGG
TGATGTGTTGTACATTTTCCGTGACGACGCGTCCAGGCGTAGTGTAGCTCACCTAATG
TATATTCCATCCTCTTAGAGTAATCGTAAGTACATATATAGATAGCACTGTGAGAACACTCAGCGTCGT
>126037899_2 EL597625.1
GCACGAGGGTAAAAATGGAAAATCCCATTAGTTTCTGTTTTCGTAGAAATTTTTAGAAATACTTCTCTTACCA
ACTAACTATACGAATTTTATATCTGTACTATCTGTTCTAATATTGCAAGGTTGAAGACCTTGTTTGGATAAAC
TCGCTAATCTCAGGAACTTAGAAGTTTCTGAACTAGAGTTCAAATTGG
>126037901_2 EL597626.1
GCACGAGGCTATTGCGATATGTGCAATTATGGGCTGTTGACCCGCCATTCTAATATTTAATACGATCCATATC
TTTCACGATATTTTTGCGTAATGCATTTGTAAGAAATTTTCATTTTTCACATATAAGTTTGCAGTGTTTTTAT
TTTTTTTATTGGATTAACAATGCTTCTGGCCTTGTATCCTTGTAAAATGGCGCCTCGC
CCGACGTTATTATTATTACATTCGAAGCGAACTTCGTGACGATCTCCATTCTCCAGCCCCCCGCTCCAACGCT
CCCCGCGGATGCGTATCCGATTGTCGTGAGTCGCGACTGGCGTTTACGACTTGTCAAACAAGTTAGGTTATTC
GAATTGTTGAAGCGAATATTCATAAATATAATTAAAAATGGACTTCCGTATACGTAAA
TAGCTAATGTCAGCTGGGTCACAAATTCAGATGGCACCACAAACTACTGTCAACACGGGGCAGCCACTACATA
ATCAGGACTCCAATATGAGCACAGGGTCTTCACATAGTGATAAAGAAGTAGATGCTTTGACACCTGAAAAGTC
AGCTGCTATGAGAGGTTCAAATGTAGGCCCAGGCAGTGCCCTTGTACCACCCATCGGT
GCCACACCAACTATAGCAATGCCAGAAAGAAAAAGGGAACGGAAAGGTGATG
>126037905_2 EL597630.1
GCACGAGGGCGGTAGCAGGCGGAGTACATGCCGGTGTCATATCGTACGCGACGTTAAACGCCGCAGTCGTTGG
ATGGGACTTGAGGGCTCCGGGCAACGCGTGGAAACTCCAAGGCGATCTAAAACAGGGCGTGTACACGTGCCTG
TACGCCAGCAGCAGCGGGTACCTGGCGGTGGGCACCTCCAGCGGCGCCATCTGCGTGT
GGGATCTAAGATTTGGATTGCCCATCACGTCTGTGAAACATCCTAATTCGGAGCGCGTGCGGCGCGTGGCGGG
CGCGGGGCGCGGCTCGCGCGTGTGGGCGGCGGGCGCGCGGGACGCGCACGCGTGGTGCCTGGAGTCCACGCAG
CGCACGCACGCGCTGTGGCCCGCCGGCGCCGGCGCCACGCCGCTGCACTACGCGCCGC
CGGCGTCTCACTACATAAGCGCAGTGTACTGCGGCACCCGCGATGGTAACAAGTTCCTCATAACGGGAGGATC
GGATCAAAGGCTCAGGTTTTGGGATCTACAGCATCCATAGGATTCTCATGTTTTATTGAATGCTCCACA
>126037907_2 EL597632.1
GCACGAGGGAAATGTTGAACATGATTCGCCATGGCGCAAACCACGTGTTCTCTTCTAAAGACTCTGAGATCAC
TGATGAGGATTACAGGGAGAAACAGAAGATAATACCAGTCGGTAGCTGGATAGAGCCACCCAAGAGGGAGCGC
AAGGCCAACTACGCGGTTGACGCGTACTTCCGCGAGGCGCTGCGAGTGTCGGAGCCCA
AGGCGCCCAAGATACAGGCTCCAAGGCCTCCGAAGCAACCAATCGTTCAAGATTTTCAATTCTTTCCACCGAG
ACTCTTCGAACTTTTGGACCAAGAGATATACCACTATCGCAAAACACTTGGATATAAGGTGCCACGTAATCCT
GAGTTGGGTCCCGATGCAGCCAAAATACAAAGGGAAGAACAACGTAAGATCGATGATG
CCGAAGCCCTCACAGAGGAAGAGATACAGGAAAAAGAACAACTGTTGACACAAGGATTCACCAACTGGACTAA
ACGTGACTTTAATCAGTTCATCAAAGCAAATGAAAAATATGGCAGAGATGACATAGAGAACATAGCANAAGAT
GTTGAAGGGAAAACACCTGAAGAGGTAATGGAGTATTCAGCTGTGTTTTGGGAAAGAT
GTCATGAATTACAAGATATAGATAGGATAATGGGGGCAATAGAAAGGGGTGAAGCTAAGATACAGCGACGGGC
CTCCATTAAAAAAGCGCTGGAC
>126037914_2 EL597637.1

GCACGAGGCTGAGTATCGCACAACATTATATAATTATTTATTTATATATAAAAAAACTATTTAAAAGACACAT
AAGACGTAACACTGCGGTCGTAATATTCTAAATTAGATAAAAAGAAACGTTATTTAAAAGATTTTTTAAAATA
TCCCTATACATTAGAATATTTCTATTTGAAATACATATATTAATCGATATATACATAG
AAGTTCATACTATACAATTAAATAGAATGTTAGATATTGTTAGTTTTGATCGCAGCGACGTCGGAATCTCCTG
CAATGACAAATAAATATTATATGCAGCAACACTCAGAAACGGCAATTATCGTTTAATTGACATTAAAAGTATT
GAAAAAATTGTCGTTTCTAAGTGCAGCTGATAGACTAATATTATTAGGAAATTTATTA
TCTATGCATTGATCATAGACAGCCAATTGGAAGCAATATCTTAAGTAAGTTTTTTTAGCCAATATGACTATAG
TACAACAAAATTTTTCACCAATTTCTTCAAAGTTCATATAATTCATTTCTAGTAAAACTGAAGACTTATCTTG
ATCTGATTTTTTAAAAATATTTAGTGTTTGTTTTCCGAGTATCTTTAAAAATAGTGAA
CGTTCTAACAAAGGAGTCTAGTAATTGTCGCAGTTAATATTTATCGGTGCGGCCAAAAAGCGTGTCAATGTAA
CTTTTTATACTCTTCTAGGTCTTTTCTGTCATTTC
>126037917_2 EL597640.1
CACGAGGGTCGTGTACCGTGATGCCATATCAAGAATGACAGACGAGAAGTCATCCACGGAGACCGTCACCGTC
GTGACGGCCTTTCCGACTGTGTCAGAAAAATCTGAGGATTCCACAGAACAATTGATATGTAAAACACCGCAGA
AGAATGAAAAGAAAAGTGTTAAAAGTGTCAAAAGTGCGAAAAGTGTTAGAAGTGAGGG
TAGTTCTAAAAGGATTGTAGTTTTCCTGGGTCTGGGACAAGTGTTTTTGGGGGTGTTGCTGGTGGGCTCGGGC
GCCTTGGCTGTGGTCAAGGGGGCCTCGTTGTCCCGAGTAGGAGCAGGCCTGTGGTCGGGATGCGTGGCGGTGG
TGGCTGGAGTGGTAGGAGTGCTTGCCGGCATCAATGATTGTTACGGATTGAATAATTC
CTCTAGTAACGGGCTGTTAACAGCGTTCTTGGCGCTGTCCTTGCTGTGCCTGGCGTGTGGCAACAGCGCCGCG
GTGCTCGCCGCTACTGGCCTGCAGCGGGACTCGCTTCGACAGCCGGAGACGATTAGTAGTTTTGAGGATGAAA
TGCAGGCATGGACTCCAGTCTTAGCTAACATAGCGCTCCTTATCATAGCATCAGTCCA
CTGCCTCGTCTGTGTATTCTCCATTTACCACCTATCCCAGAGGGTGTGCCCTTGCTTCAGACCGAAGCAGGCC
TTCGACCACAATCAGTTCGACCCCAACTTCAAGCCGGTACAGCCTTGCGTGTTTCATGTCGATGAAATAAAGA
GAGCTCAGGAGG
>126037922_2 EL597644.1
GCACGAGGGGCAGGTCGGTCAACACAGCTAATTTATGAGATTTTCGTAGTCCTAATTGTATATGTTTGTACTC
AAGATTATATAAATTATGCCGAAAAATAAAGGAAAAGGTGGTAAAAACAGAAGGAGGGGAAAGAATGAAAATG
AAACAGAAAAACGTGAGTTAGTGTTCAAGGAAGACGGACAGGAGTACGCCCAAGTCAC
AAAGATGCTCGGTAATGGCCGCTTAGAGGCCATGTGCTTTGATGGCATAAAACGTCTTTGCCACATCAGAGGA
AAATTAAGAAAAAAAGTCTGGATAAATCAAGGAGATATTATACTCATTGGTTTAAGAGATTATCAAGATGCCA
AAGCTGACGTCATCTTAAAATATACTCCTGACGAAGCAAGGAATCTGAAGACCTATGG
AGAATTCCCTGAAACCGTACGCATTAATGAGACTGTTGTATATTCGGTTGATGGTTTGGATGAAGACATAGAA
TTTGGTGACGAAGTTAGTTCAGAAGATGAAGCAGATGCTGTTGATGCTATATAAATATTTTTACATATTTTTA
CCATCTACAGTAAGTTAAATGTAATTTTATTTTCCGTTATAAATAAATTACCTTGTGT
GTTTGTAATTTGTTCTTTAAATTACAAGTAGCTGTATACAATAGTGTCTAGATTTTTTAGACCAGTATTGTGT
TAAATTCAACCATGTTTAAAG
>126037924_2 EL597645.1
GCACGAGGAGGAGGAATTAAAAATTAAAGTTCCTAAAGAACTTCGTGCTTAGGTTATGAATCTAAGATTTACG
TTAATTTAAATATATTTATATGAAAGTATTTCAAAATTAGTATCAAAATTCTTTCATCTTATCGACTTCATTA
AGAATCATTCTGGAGAAAAGAAAGATGCAGTTGGACGTATCGTTTTTTTCGGGGCTGG
GATGCGGCATATGCATCGGAATATCTGTGTATGCATTAATTAAACATTTTGGATCAGTTTCCAAAACTGTTAA
AGCAGCTAAAAAGATAGCTACAGATTCCGAATATAAATTAATTTTAGTTGTAAGAACAGACCTAGGCATGGGT
AAAGGAAAAATAGCAGCTCAATGCAGTCACGCTGCAGTCGGTGCTTACGAAAAAGCTT
TACGCAGGGACCCAGAAAGTTTAAAAATTTGGCAAATGACAGGTCAAGCGAAAGTAGCAGTTAAAACTGACTC
TGTAGAGGAAATTAAACAATTAGCAGATAATGCTAAGAAATTAGGACTGGTTACTTCACTAATAAGAGATGCA
GGAAGAACTCAGATAGCAGCTAATTCCATAACAGTACTAGGTATAGGACCAGCGCCCT
AAAGTGTTATAGACAAAGTGACGGGACAC
>126037937_2 EL597656.1
ACTACGTCCGATGAGGACTATAATTGTGGCTACCACATCTTTCCGATTACATTCCGAAACCCGCAAGATGTAT
TCAGACAATTTTTTGGCGGTTCTCCGTTTGGCGAATTATTTGCTGACATAAACGGACACGGCTGCCACCGCCT
CACCCACCACCCGGTCACGTCCCTCAC
>126037938_2 EL597657.1

GCACGAGGTGTAAATTTATTTAAAAAAATATAAATTAGTATAAATATGGCATCCTTAAAAGTGTATTCAGTGG
GCAGACTAGCTAAATATGTTACGGATTCATCTTTAAGGACTTTACGGACAAATATCTATAGAACATCAGCTTA
TAAATATAGTACAGAAGAAAAAGCAGCAGTTTCAGATGATAAACAACCACCGTCTTCT
ATAGAAGAATGTCACAAACAAATAGAAAATTTGACAAATGAACTTGGTTCAGTTAAAGAGCAAATTAAAGACT
ATGATGATAAATATAAAAGAGCATTAGCAGATGGAGAAAATGTTAGAAGACGAATGTTAAAACAAGTTGAAGA
TGCTAAATCATTTGCCATACAAAGTTTTTGCAAGGATTTATTAGATGTAGCAGATACG
TTATCAGCGGCGGCGGAGAGCGTACCGGAAGGTGTGGAGGGTGAGAATGCGCCCGCGGCCCTCCGCTCTCTGC
GAGATGGACTGAATCTTACCAGAGCTCAGTTGCAACAGGTATTCAGTAGACACGGTCTGGTCGCAGTGTCGCC
GTTGAGGGAAAAATTCGACCCCAACCTCCATGAAGCATTATTCCAACAAGAAGTGGAA
GGAGCTGAACCCGGCACAGTGGTAACAGTTAGCAAAGTCGGCTATAAATTACACGAGCGTTGCGTG
>126037950_2 EL597666.1
CACGAGGAGACAACTTGATTACTGTAGATTTGTTCGCATCGACGGCTTAGGTTTCACAACTGAAAATATGCCA
AATAATTACATAAATCTATCTCAAATATCGCAAGGCAAGTGTGGTATTGCCATCAATAATCCTACTATTCTTG
AATATCATCCGTGGACAGTAGTAGCCAAGATACGAGGACAAGATGAAGAAATATCTAG
AGTCACCCCCGTTTTGCTTGAGGGTCCTCGACTACCACCTGTTGTTTATTACCGTATGCCAACCACTTGGATA
GTATTTATGATAATGGGTCTTACTCTACTTTCAATATCAGCTCTTCTAAGCACTAAAAATAACAGGCAATGGA
CCTACGACAGAGCGAACGCCATTAGACACAGCTTCATGAGAAAGAATATTCATCAAAA
CGCTGAGAAGAATACCGCCATGGCTGCTTAAAAATTTAATATAGCATTTAATATAATCAAAGCTAAATTATAC
ATCTTTTATTTTTTTAAATAATAATATTTTGTTTCGCTCAAGACCATGCACGTAGGAAAAGGGGGGACCAAGC
CGGCCATGCCCACCCCAGAAATTTTACTAATATTTTTTTTTGCCAAAGAANATGAACC
AAACATTTCTTTGCCCTAGAAAATATTTCTAATTACGCCCAAGCTCTCAAGACGGCTTTGGCTAAAATGGTGA
TATGGTTTCAATTTTAAGCCCAGGTTTTGGTTGCAACTTCTGG
>126037952_2 EL597668.1
GCACGAGGAACGCGATCGCTTCTACGACTCGACAATAACATAACGCAGGGTTTTTGGAAACGGCAGTCAGAGA
TTAAGTAGTCGGTATCGAACGCCGCCGCATGCCGCCACTCCGTGCTTCAAATAACAAAGTGAACACTTTTACC
AACTCTCACTTTTACCGCAACATGAGTGCTAGTGCTAGCACCGTCAACGTCAGTGCCA
ACGATCTTACCAAATCAAACATACGTCTCACGTACGACGACGAGAAGAAAACGTGGCGCGAAAAATATAAAAG
TGCTTTGAAACAAATCAAATATTGTTTAAGTGAAACGACAGTTGAGCCATGTCTTTTTGTTTATATGTTCTGT
ACGGCGTTGTCGTCGTTGGCCGTGCAAAATATGCATTTAGAAAAATCTTGCCGCGTTC
ATTTTCAATTCGGTGATGAGATATGTGATCGTATTAGAGATAGAAACACCACTGGACTTCAGAACGAAGTGAA
CCAGGTGCAGTCCTTGGTGGCTAGTGTTATGGCCTGGAAGTTTCCTTTGCAGACCAGTATACCTGCTGTGGCG
GTGCTTTTTATAGGCGCTTGGAGTGATCGTACAAAGAAAAGAAAAATATTTATTCTAA
TACCATTTATTGGAGAAATAATCGCGAATATCGGTTTAATATTTGCAACTTATTATTTTTATGAATTTATCGT
GGTGGATACGGCACTAATAGAAGCTTTACCAGCCGCATTC
>126037954_2 EL597669.1
GCACGAGGATTTGAAAATCCGCACAATGGCGTCGAGCATAATGGTAATTTCGATAAGAAACCTCGCCCTTCTA
ACGAAATTAAAATGGTACAAAATAATAATCTTAATCCTAAAGATGTTATAAGAGCGAACGATCCTCCAGAGGA
CGATTTACCAAAAAATATAGGTGTTAATAAGTTTGTCAACTTTTTCGAATCTTTGGGC
GGTAAAAAGTAATTTCTTTGCGTATTTTTCCAAGTCATTTATAAAATATTGTAGATTTTTTCTTCATTATATA
TTTTTATGCTTATATATTTGTGTACAGGTTGGTTCAATAATGCAAACTATAAAAAATATCGTTTTTGTATGAT
TGGATGATTTAAATTGATTTGTTAATTAGTTTCAATTATAATAATTTTTTAACTTAAA
AGTATTCTACGGCTTGCTCTTTTTTTCCGTTACAAGTAAAAATATATATGTTATTT
>126037960_2 EL597674.1
GCACGAGGGAAACAATAATAATTTATAATCTTAATAATTGTATTCAATATAAATTTAATTGTTAATAAGTAAT
TCCCAAAAAAAATAAAAACTTTTTCTTTGTGTCACATTGAAATTTGTAAAAATCATAAAGTTATTCCATATTT
TTATGCAGTAACTGAACAGTTTTAATTGATAACAAATTTCAGCTGCTGGCATTGTTAA
TTATTGTTCAGTGAAATAAAATATATAATACTAATCACCTATCAACATGTCGGCCCTATTAACTGCAGATTGG
TTCCAACTCGACTCTTATTATCGTAAATTTGATCTGTATAATATGATTTGGTCCATGGATGAAGGTTTAGGCA
ATATGATAGTAAGCGGAGCACCTTATGGTGGACCAGTAGCAGTTGTTAGGGATAGGAA
ACAATTTGTAAGAGTAATGGCAACATCTAAACCAGTGATCACAATATACAACTGTGCAGGAAATGTTATATCT
AAAATATTGTGGAACAGTGGTGTGCTTATTCATATGGGATGGTCTGATGGGGAACAATTGTTGTGTGTCCAAG
AAAGTGGTGATGTACTCATATATGACATGTTCGGAGCTTATCAAAAAACTTTTAGTAT
GGGCCAAGAAGCAAGAGACACTA
>126037970_2 EL597682.1

GCACGAGGCTTAAAGGAAGCTGTAATTCTTCCCATTAAATTCCCACATTTGTTTACAGGAAAGCGAATTCCCT
GGAAGGGTATACTTTTATTTGGCCCTCCTGGTACTGGTAAATCATATTTAGCTAAAGCAGTAGCAACAGAAGC
TAACAATTCAACATTTTTCTCTGTATCATCATCAGATCTTGTATCTAAATGGCTTGGT
GAATCTGAGAAACTTGTGA
>126037973_2 EL597684.1
GCACGAGGCAACGGTCCAAATCCGCCTTTCATGGGACCTAATTCGATGTCGGATTGCAAATCCTTAGATATAC
CACCTCCTGAGCCGATGAAGCTACAGAACATACCACCTCCCATGCCCATGCCATTGAATCACATCCCGAAACC
CAAGGACATGGAGCCATGCAACATTCCGGCACCACCAATACCAAATCAAGTTCTCTCT
CCAGATGTGGCGAGTGCAGTGCGCCGTGCGGCTGTCGAGATAACGGCTAACGGCGACCAATGGGAACAAGCAC
TGAAAATACAAGACAATATGTGGTTCCTTCGTAATACCAACTCGAATGAATATAAGGCGTATCGGGAGCTGGT
GAATCAAATGCGGAGGGAGAAACAACCTGAGATCAAACCAGAAGATAAATATGAACCC
GAGTTCTCTTTGGAAGACGATGATAGTAATGATAATAGTAAACCTATAAAGGAGGAAATTAATGACAGTTACA
ATATTTATACAAATCAAAGTAACATAAAAAGAGAGCATACAAGTTCTCAAAGTGACGAGGACGAGAAAAGGGA
ACGTAGAAGGAGGAAGAAGTCCCGTTGGGGAGACACACCAGATGAGAAACCTGGTACT
GTAGCTACATTACCTAATTTTATGCCAGGTGCAAGTTTAGCTA
>126037974_2 EL597685.1
GCACGAGGGCGCGGCGAGGACGGCGACATGGAGGTGTTCCCGCCTTTCCATGCCGCGCCATTTTCCAAAATGC
TTACTTTCTACAGAAAAGAACCATTCACTGTTCGCGCTTATTACTCTGATCAATCAACCTATCCCGATAGTTT
CATTGGACAATGGAACATAAGAGACGTTCAGCCAACCGCCGAAGGAGAATCGCAAAAG
GTTAAACTGAAAGTCCGCGTAAACATCCACGGTATCGTGACCGTCGCGTCCGCGTCCCTGGTGGAGAAGAAGC
AGGACGCGGCGCAGAGCGAGAACGTGGAGATGGAGAACTCGAACGAGGCGAGCCAGGACGCGCCCGCGGACGG
CGCGCCGGAGGCGAACGGGCCCGACTGCCAAGAGGAACAAAATGATGACAAAAAGGAT
AAGTCCAAGGACAAATCTAAGAAAGTATTGGTGAAAACCGTCGAGCTACCGATTGACGCTCAGACTCACGGCT
TTTCCCAGCACGAGCTTAATTCATACATGGAACAAGAGGGTAAAATGCAAGCCCAAGATCGTCAAGAAAAAGA
ACGTGCAGATGCACGTAATGCTCTCGAGGAATATGTATACGAATTGCGAGGAAAGTTA
TCTGAAGGAGAAGTTCTTCATGACTTTGTGGC
>126037976_2 EL597686.1
TGAAAGTGACGGCCCACGACGGTGAATACTTTAGTACGAGCGAAGCTAAACTGGTTGATACTAATGTTGACCC
GCGCGTCCGCCCGCGCGAGGAGGCGACGGCCACGGCGGGCTGGTTCATCGGCGTGATGGTGGCGCTGGCCTTC
CTGCTGCTGGTGCTGGTGCTGGTGTGCGTGGCGCGCCGCAACCGCGGCGGCAAGTACG
ACGTGCACGACCGCGAGCGCGCGCACGGCCGCGCCGACTGCCCCGACGCCGCCTTCCACGAGTACACGCACCC
ACTGGACAACAAATCACGGCATTCTATGAGCAGCGGTAAGCCCGGCCCCGAGAGCGACACCGACTCCATGGCC
GAGTACGGCGAAGGCGAAACAGCGGGCATGAACGAGGACGGCTCCTTCATCGGGCAGT
ACGGCCGCAAGCGCCGCCCGCCCGCCTCCGCCTCCGCGCACGCCGACTCCGCCTCGCAGGCCTTCGCCACGCT
CGTCTGAACACTTCCCCTTCCCTCTCATAGCTCCCCTCTCGTAGACTCATGCTCTCCTGGTACTGTGGAAAAA
CTGTCACAGTCATATGCCCCTTTGGCATCGCCTACCTCGTTTGACCACTAAGTCCTAA
CACACCTTGTAATTATTACATTTCCCCTGGATCGGCGTTACACCTTACATACACCTATCTTCTCCTGGTACCG
TAGAATGTATTTC
>126037978_2 EL597688.1
GCACGAGGATAATGTGGGAATATATTTAAATTCTAATCATTTACAAAATTCTGGATAAAATATCACTGGCAGT
ACTCAGATAATACTTAAATTTTATATTAAGATAAAGATAAGCTCCTTAAAATTTAATATTAACATACATCTCA
AGCCTTTTAAATAGAGCTAAACAGTATGGAATGCTCAAAAAATTTACATCGTGAATTA
TTTTGCGTGTTGTTTCCTGGTTACGTTAAAAATGAAGAGAAAGCTATACAACACATGGGAGGAATTAAGGGAA
TTTCAAATGTGTATTCACAGGCAAATAAAAAGAGATTAGGGATGTCGTTTCAACCTGAGAATCCTTTTATAAA
AAAGATATACGGAGATGTTAAGCCAGCTGCAGGAGTTCTCTTAAAGATAAAAATTAAA
AAAACTAAATCAGGTAATGAAGTGAAACGAGAAGTTATCTCTACATCAGTTGTTGGATCGGTATCACAAATCA
CAAAATTCGAATCTTTGTCTGATTTTCAATATATGCCTGTGAATACTTCTACAGCTGAGAAACCACAATGTAT
GTTAGAACAAATACTACCAAGTGGTGTTGATCATTTTAATTCATTATTGGAACCAGCA
TCAGCTTATATAACACCATTTATCTTTACAAGATCAGATAAGGCTACTAGCTATTCTTATTCAGAACAAAGAT
ATACTGTCAAAGATCTAGTTAAAGAGGATTCTGCAAACAGTGATGAAGTACATAAA
>126037999_2 EL597704.1
GCACGAGGGTTGAAGGTGTATTTAAACATTTAATGTTTTATATATATTAACATAAAAATATAATTCCATTAGC
CTAGTCTTTATAGAAACTTTTTTAAAATGGAAAATTTAGAAGTTGATTCTGATGTTATCAAGGAAAAAATTCA
CACGGAGCCAGTATTAAACGATCCTAAATATATAACCTTATTGAGTTTGATTGTGTTA

TTATTCACTTTCGTATTTTGGTGGTGTTTCTCACGACGTCGTCATTTACGCCGCTCCATTTTACTTGCGGGAC
TTTCTGACGCTGGCAAAACATTGCTTTTTGTAAGATTGGTATACTCGCAATTCAGACAGACATTCACATCCAT
GAAAGAAAACCTTGAAGAGTATATAACATCCCATCAACCTCTGAGAATCGTGGATTTG
CCTGGTCAAGAACGCCTAAGAAATAAATATTTTGATCATTACAAGAATAATGCCAAAGCTATTGTTTATGTTG
TTGATTCTGTAACTATACAGAAAGAGATTAGAGATGTTGCTGAATATCTATATACTATACTCTTGGATCCTGT
GGTGCAGAGCTATAATCCTCCATTGGTAATATTATGTAACAAACAAGATCAACCTATG
GCAAAAGGCAGTCAAGTTATCAAAAGTTTACTTG
>126038010_2 EL597712.1
GAGTTTAAAAATTTAATTTTGAGTCAAAAATGTCTCAAGATAATGATAACAGTTCATCAGATAGGAGCTTGGA
AAGGCTGAATGAGGTGAAGTCGTTTATAGAAGCAAGCAAGCGGAGTGCTGAGAAAGTTGGTGAAATGCAGGTG
GACGCGGAAAGGAGCTTGGGAAAGCTCAACGAGGTTGTTTTCGAAGATGCATTTGAAC
TAACAGGTCACGGCCGCTTCAACTACATAGTGTTATTAACCTGCGGTCTGATAATGCTCAACGTGTCAGTGGA
GTCAGTCGGTATGAGTTACGTGATACCGGCAGCGGAGTGCGAGCTAGGATTGACAAGTGAACATAAGGGGCTT
ATCAACGCTGCTGCTTTCTTAGGTATCATAAGCACATCATTCCTGTGGGGTTACTTGG
GGGATCGATATGGCCGACGAGCTGTCATGTTACCTGCGATGGTAACGTCAGCAGTGTTCTCTATAGCTTCTTC
ATTTTCCACTAACGTGTGGATACTACTCGTTTTA
>126038015_2 EL597716.1
GCACGAGGATTATTGTCAATATGCTTTATTTTAATCAGATATACATATATGTATGTCGCGATGTATATATTTA
ATAATCGTTGAAAAAAAAAATGAAATAATAATAATTACAATATATTTTTATCTTTACGAATAGGCATTATAAAT
CGAACGAAATGTATTCATTATTTTGACACACCAGTGGTGAT
>126038019_2 EL597718.1
ACAAAATTCTTATTATTTTCGAAAAAAAATTTTTTTAAATATCTTGTTATTATCGAAATATAATTTAAATATT
TATATTTTCAAATTTTATAAAATTACACTCTGTATATATAGTAAATAAAAAGAAGTTAAGTTAAAATAAGTAA
AAGTAAAAGTAGTAAGTAAAAGTAGTAAGTAAAACAACAATAATAAAAATAAAGATTG
TTTTCATATTTAAAGCCGAGAATATTTTATCTTAAAATTTAAAAATGTTGGTATGCACTCAGTTTTTTTTATT
TTCAATAATATTTTTTAAATATGTCATGTACATGTAAA
>126038023_2 EL597721.1
GCACGAGGAAATAACACGTTATTTATATTGTATTTGCTAATAAAATACTTAATAAATACAAAATAATCTCTTA
TCAAATGGGAATACTATTGGAAAAATCGGTGACTTAAAGTTATAAACTTTAGAGTTCTTGATCAGTGAAAACA
TCTTCATCACTTACCGCTTCATTTATTTACTTGTCTACTTTACATTTAAATAATATTA
AATTATTACTATATTAATTATATTATAATCAATAACTATAGAAAATCAATTGTTGTTTAATTTACGGGTTTTT
TGAAGCAAGGCTTAAACTAATCATAAAAACATGTCGATGCCACATAGCAGTTCAAGTACAACAAGTTCGAGCA
CAAAACGCAAAGAAATCTACAAATATCAAGCTCCATGGCCTTTGTACTCAATGAACTG
GTCAGTGAGACCAGACAAGAGATTTCGACTTGCTCTCGGCAGTTTTGTTGAAGAGTATAATAATAAAGTACAA
ATCATCTCGCTAGATGAAGAGACAAGTGAATTCAGTGCAAAAAGTACTTTTGATCATCCATATCCTACTACTA
AGATTATGTGGATTCCAGATTCTAAAGGCGTTTACCCAGATTTACTGGCCACTAGTGG
TGATTATCTCCGCATATGGAGAGCTGGAGAACCATACACTCTATTTGAATGTGTGCTAAATAACAACAAAAAT
TCTGATTTTTTGTGCCCACTCACTTCCT
>126038031_2 EL597727.1
GCACGAGGGTTCTAGCGCAGTCCAATTCGCCGTTGGAATTCAACGCTATTTTCGTTCAAGGCAGTGATAAGCC
AGTAATCAAACCTCGTGTTTTGTAAGTGCACGTACGTACGTTGCTCTAGCGATAACATTTAATTCGCGTTCTT
TAATCTATCGCCTATTTTGAATCTGAATATTGACGGATGACTGTCCTGTTGCCTTTTT
TCTGGGACCGAGAAAGAGGCGCAATGGAGAGTTGTTACAAGTTGTACACATGGATCAACCACACGCTGCTGAT
GCTGTTGGTGCAGATGTTCTGGAAAGTCAGTCAGATATCGGACTATTTCAAGCACAGGTCTGTAGAAGAAAAG
AAAACACAGAGCCCTTTCATGTTTTCTCCGCTCACAAAAGAATCAAAATATAGAAGGC
ACATCCGATCTATGTCAGATGTACAGAGTAGTACTCCATACCGCCTACCATTTAGATCAAGAAAAGGATTCCA
TCCCACTCTCACATATGATTCTGACACAAGGGCGAGAAGGATAGCGTCCTACAACAGAAAAAGGCTGCAAGAT
TCTTCAGACTCTAGTGACTACTTTGAAGCCCCAAAACATTTAAGCATAGGACGCAACA
TCATTAAGCATTCTGACTCTATGAGGATACTCTGAGAAGAAATGATCTGAAAAAAAATGACTTACT
>126038034_2 EL597729.1
GCACGAGGATTACACTGTAGGTAGGCAGAGTATCTTTTTTAATTATATTCAAATATACGAATTATAGTAAAAG
TGCATATATGGGATATACGTGAACGTATTAAGTGTCGTTATTCACATATAAATGTTGTTTAAAGGTACTGTGG
TGTAATGTCATATGTAATAGAATCATTTATCGCATTCAATGTTCGCGTTCGTGCCGCC

GATGTAATACTTTGTCATGGACATAAGCTTATTTTTACGCACGAATATTATCTTAAATACGTGATTAATGCTA
ATAATATTCTGTGATTCTCTCATTCTGTTCTTTCTATATGATATTTATACGTATATTTTAACGTTATCAAGAC
ATTCCTACTTTTCTATTTCAAATTGAATAAAAATGTTATTTGGCTGAGATGACAGTCT
TCGTGCCGTCCCACTGCGGAGTGATGGCCGCGAGCACAGCTTGCGACGTTATTGCGACTATCTGGTTAATTTT
CGAAAGTTTTATAATCCTAATGTGCAATTGTCAATTTATTTATTTATGGCTTAATATTTATTAGAAAATATAT
AAATGTTCTATAGTGTCTTGTANAGGTGGGATCTGATGTTCCTAGATATGTAAACGTT
TTGTATGATACACCGATCGCGAGCTGAAGTGTATTACAGATACAGTCATGCATTCCAACTTTGTATTCCACTT
TTGTAATAGGCCTAATGAATTCTACGAC
>126038040_2 EL597733.1
GCACGAGGGTGCAAGTGTTTAAACGTTTGTTATCGTTATTCGTTCCCAAAGTTTACTCTATTTCTACATGAAA
CCTTCAACAAACAATTTTATACACATAATCTTATCCGACATCGTCATTGAATGATAAGTTATCGTGAATAATG
ACGTTGTGATGTTAACATTTAAAGTTCTCGCGAGTTATGTGGCTCGGCTGTGATTAAT
TTTGCAGCAGGCGAGTGTAATGAGTGCGCGTTAACTTTTACATTATAGAAAACTTATTATTTACCGTTATATA
GTGTTATTCGTGATGTCTTTGTTC
>126038044_2 EL597736.1
TTTTAGTCCATATAATTTTTGATAGTACATCTTTGCTTAGCCTATGTTGGTAACATTTGTTTGTATGAATGTT
ATTCCAGTGCCTCCAGGCTTTCCATATTTTATATGTCAAATAATGAATTCCTTAAATGAGAATTTAACTTGCA
ACCATCAAATAGCATTAGACAACATCCTACTGCACCACAATAGCCTGAATAAGGGGTA
ATCGTTTAAGCTTTGTTTAATTGTAAGTTCAATAATTTATTTACCTCTATATCTATAATTATCATTGTACCAA
ATTTATTAAGAAATTTAAAATTATTGCGTAATTTGTACATTTTGTTGCCTGAGCAGGGCACTTAAAGGATACC
CCAATTATAGGATAGATTGAACCCAATACTAACTACATTTACAGTAACACTAAATGCG
CCACAATGACCTGTGTTTATTAAGAGGACCATAACATAAGAAATATTTATATTGGTTGAAATAAAACAAACAA
ATGAACTAACAATTAATTCTTTAATTCTGACCAAGCGACTACACCTCATATTAACTATTTAATTTAAATGAAA
GCATTACCTGATGTGTTGGAAATACAAATCAACCACTGTATCATATTTTAGAAGTCTT
AAAACAAATAAATCGTTTTTTTTTATTAAGTCATCTGTGACTGACTTGTAACTTTCATGTAAAAAAAGCATGC
ATTTAAGAACGCAGCTTAATGTAAGCCCTCTTGAACCACTCAAA
>126038051_2 EL597742.1
GCACGAGGTATTTCATCATTGTTTTAAATATTGTTTTCTATTAATATTTCGTTAGAATAAATAAAAACACATA
TAAAGATGCCGTTGTCGTGTCGGTTTTATCAAGAGAAATATCCTGAGGTGGAAGATGTAGTTATGGTAAATGT
ACGGTCCATCGCTGAGATGGGTGCGTACGTCCATTTACTAGAATATAATAACATAGAA
GGCATGATATTGCTTTCTGAGTTGTCGAGAAGACGTATTAGGTCTATCAACAAATTGATTAGAGTGGGCAAAA
CTGAACCAGTAGTCGTAATAAGAGTTGACAAGGAAAAAGGTTACATAGATCTTTCAAAACGTCGTGTCTCAGC
AGAAGATATAGATAAATGTACAGAAAGATATGCTAAGGCTAAAGCAGTTAACTCCATC
CTTAGGCATGTAGCTGAACTTCTGAATTATCAAAGCTCGGAACAGTTAGAAGAATTGTATCAACAGACAGCTT
GGCATTTTGAAGAGAAATATAAGAAAAAGGCATCAGCTTATGATTTCTTTAAACAAGCAGCTGTAGACCCCTC
AATTCTCAATGAATGTGGCTTAGACGAAAACACTAAAGAAGTACTCCTTGCCAATATC
AAGAGAAAGCTAACTTCACAAGCAGTGAAGATCCGTGCCGACATTGAATGCGCATGTTAT
>126038055_2 EL597745.1
GCACGAGGATGTGATGAAACTATTGATGATTCGATATTCACATCAGGGTTAATAGTAGGCGCGTGCTGTGTGT
TAGGCAACGCTGCCTGCGCACTGGTTTCCTCCAGAGGAGGTTACGGTGTCAGAAGAGCAGCTGCACTCTGTGC
ATTAGCCTGTGCACTGGCATGTGCCTGTTTAGCTGCATGTGTGTGTGCTTGCACAGGC
ACTTCTCATTTAGCAGTAGCTGCGGCTGCGGCTTTAAATGCTGCGTCGTTAAATGGCAATATTCTGTTGATTA
GAGTGCTGTTACATGCTTTGCCTGCTAAGTTGGGTGCTTTAGGTGTATGCTGGGGAGCGTGGTGGGGTCGCGC
TGGAGGAGTAGCATCGAATTTGGCAGTGGGTGTACTCCTGGACTACTCATGTCCAGCT
CCCTTTGTGTCGGTTGCCGCTCTTTTAGCATTATCCATCGGAGGCATAATGATGATCAAATTGGAAGACGTTA
AGTTAGAAGAAACCAAAATTGAGGAGAGTCCCGACAAGAACTTAGGTTTAGATAGATACATATCAACTCATAT
GTAAATAAATTATTTATTTATAGGTTTATTGTAATTACAAAAGAGTTATATTACTGTA
AACAAAATATGACATTTGAAGAAGAATTTTGTAAATCAGTTGTTATATGAAATGTATAGCTAAAGATCAA
>126038062_2 EL597750.1
GCACGAGGCAAACATACATAACCACGAGGAATAAGCGTGATAAAATTTGAATCTTTTTGTAAGAAATTCAATT
AATACTTTTTAAATATATAAGTATTTAAGTAAAGAGTTGTGTCCTGTGAACAAATATAATTTATTCAAACATG
AAAGACAAGAAAATAAGTAAACCGCCATCTGAGAGCGGTTCAGACTCCGAAGAAGAGC

TATCACCAGAAACCCATAAAAAGTCACTAGAAAAATTAAAAAAGATAGATCCAGACTTTTATAATTTTCTTGA
AGAAAACGATGAAAATTTATTAAATTTTGAAACCGATTCCGACGATGATGCTTCTAATAAAGATGATAAGGAT
GACGACGATCAGCTGCATGTACCCGGACCAATCACTGGAGACAGTGATGAAAGTGATT
TTGAGGATGAAACTGCTAAAGTTGTTCATGGAAAAATTACACTTAAAATGCTAGCTCAATGGCAATCAGAGCT
ACAAAGTGAAGGTAAAATAAAATTAACAACATTAACTACTGTTATAAAAGTGTTTAATGCTGCCATGATAAGA
GCCACAAGTGAAGATGGTGCATCTGTGGGAGAGTTTAAAGTTGAAGGTTCATCAATTT
TTAATGCAGTCATTCAAATGTGTGTCTTATATTTGCCACCTGCCATAAAAAAATACTTAAGCATGGAACAATC
TGGCAAAGACCCACAAAAATGTAAACATTTTATAAAAATCAAAGGTCCTCTT
>126038067_2 EL597754.1
GCACGAGGTGGAATTGCTACGTTTTTGCAAATGCAGAGCACCACTAAGAGTTACAAAGAAAAAGATGCCGGCA
AAGTCGTTCTATATACAACTACGATGGGCATTGTTCGCAACACTTATCAAAGATGTGTCCTTGTTAAGAAGAT
ACTACGGAACCTGCTGATTAAGTATGAGGAACGTGACGTCTTCATGAGCACTGAGTAC
CAGGATGAGATTAGAGATAGGATGAAGAGTGATCAAATTCTTGTCCCGCAACTGTTCATAGATGGACAACATA
TTGGGGATGCGGAAACCGTTGAAAAACTGAACGAGAGCGGCGAGCTGAGGAAAATGTTGAAACCGTATAAGAG
TCCAGACGCATGTAACACTTGTCAAATGTGTGGCGGCTTCCGGCTCTTGCCTTGTCGA
ATCTGCAATGGATCTAAGAAGAGCTTGCACAGAAACCATTTCACTGCTGAATTCGTCGCCTTGAAGTGCATGA
ATTGTGATGAAGTCGGCCTCGTCAGATGCGAGGCTTGCTCTTGATTGTTGTTTAATTTTATTAAATATAGCTT
CGCTATCGCAGCTTTATTGTGCACCTGGCCTTATATATATTATCGAATATAATACACG
GCATAGTGTATTTTGGTTCATAAAAGTGTCAAGTTATATGGAAGTGTTTTAGTGTAATAAATTGAACAGAAAT
GATTAATTTGTACAAAAAT
>126038069_2 EL597755.1
GCACGAGGGTGAGCCGGCTCTTTCGATCTTATTTTTAATAATTTGATTATAAAAACACTAAAACATGGACGAA
GTAGAATATAAATTAAAAACAAATAACAGCGTATTAATTGTTAATGCAATAGATAAGTTAATATCGACTATTA
AATCAAAATATAAGCCAGCTGAAAGACAAAAATTTGTGCTAGAAAATGAAGAACTAAA
ATTTCTAAGAGAAAAATGTTCATCAACAGACTGTATGGTCAGTTTGACCGCGTGTCAAGGATTACTAGCGTTA
GTAGAACTGGGAGTTTTAGAAATCGCTCACACTATGTCCACTGTTGTAACCTTACTTCCTACTGCACATAACT
ATTCGGCAATAATATCAACAATGGCTGGGCTGCTCATCCTAGATCTAAAGTCCCGTCT
AACACCTGGCCAGCCCTACAAATGTCAATTCTCTCTAAGATCACCACCCCATCCCTTTATAACTGTTTTAGAC
AAAAATAAAGATGTTGAAGACAATGTACTGGTACAAATGCAAGCATTGTGCACTCATCCTGAATATACGGTGG
CCTCGAATAGTCTAGAACTATTAAGACCTGTGTTCCTCTGGCTAACATGTAGTCCGCA
CAGCCACGGAGTTAGACCGTGGCAGCTGTTGCTGAGTTTACCTCAGAGTAACGCTCAGACAAACTTGTTGTTG
GCTTGTATTAGTTGTCA
>126038075_2 EL597760.1
GCACGAGGTATTTGTGTTTTTTAAATAAAATAAAATTATTTTAAAATGAATTCACGAAATGTATTTCGATTGA
GCAAACCTCTGTTAATAAATAACATATATAGAAGACACAAAGGATACAAAGTCGTAAAGCGACCAGATCCAAA
GTTATTAAAGATGCCTAACCTAGCCGATGCACCGAGTATAATGGACATAATTAAAGAA
AAAATACGATTGAATGGTCCTATCACTGTGGCTGAATACATGAATATAGTAATAACAAATCCTACCGAAGGAT
ATTATATGAAAAAAGATGTTATAGGTGAGACTGGAGATTTTATAACGTCACCTGAAATAAGTCAACTATTTGG
GGAAATTTTGGCTATTTGGTTCTATGCTGAGACACAGAAGATGGGTCCTGGAAAACCT
TTACAAATTGTTGAATTAGGACCAGGAAAAGCCACACTGTTGATAGATATTCTTAGAGTATTAAATAAATATG
CCTATAGAGCTGATGATCTCAGCCTTCATCTTGTAGAAGTTTCAACAACTATGCAGCTAGTACAGGCAAATAG
ATTGTGTGTTTCTCACCGGGCTACTGATATAGATCTACCATATAATTATGAGGGTGAA
ACAGTGAGTGGCATAAAAGTATACTGGTACAATGATCTAAAGAAAGTGCCAAAAAACTTTTCGTGGTACATAG
CCCATGAATTTTTTG
>126038089_2 EL597771.1
CGGCACGAGGAAGGTGGAAGCGTAAAAACTTTATTGTACGACGAAAAGCTTAGACATTTTATTTAATTCTGCA
ATAAAACAGAAAATAATGGCAGAGAACTCTGAAGGTCCACTAATACCCTTACAATCCAAGTTGGCTGCTTTGG
CATTGGCAAATTTATTTTTATATAGTGTGGGAATGTTTACATTACCTTTTGTTGCATT
TTTTGGTGTAAGACATTTACTTACAGACTACTATCCAGTTGAACCATTTATAAGAACCTGCTGGTCAGTAGTT
GCTGCTGTTGTAGTAGTTAACATTATTATATGCATGTATGCATATAAAGCCTATCATGAGAAGGAATATGATG
AGGATGGCAATGAAATTGACCAACATTCATACAAACCATCTACTGATACAGCAAAAAG
TTCATTAAATGTTAAACAAGACTAAGTGTGACATTCCCCTCTGTGTGCAGTATTACATATAAATATGATATTT
TATGAACAATTAGCCTAAAAATAAAAGTGCATTTACAGTACAAGTACTCTTTTATTTATTAAAATATCTTTTT
AATCAGAATAATAATATAATATTGGAAAACATTTATACTTCAATATAACAATATTTTT

ATAAAAAGGCCATTGTTGCATTGTGGGATATGGTCTGGCTGTGTCAGTCCC
>126038090_2 EL597772.1
CGGCACGAGGTGCATAGATAAAATATAATACTAGATGAAATAAATACTTTCAGTGTTAGTTTGAATAGATACA
CTATAAAAAGCAGTTGTGACTGTGTATTGCCTTCTTTGGCTTCGATCCCCAATCGTGGGAGTGATTTTTAGTT
TAGTTTAATTATTCTAATATATCCCGCCATGGAAGACAAAACACAGCGAGTATGGCCA
CTCCAGTACGAAGTGTCTTGATACTAGGATTCCTTAGGATTTACTATATGCGAACAGACACGTGAAGCGGTTT
AAAATTATTAAATTTTGTATATTTAAGAGGATAGTTATTATAAAGATATGTAGATTAGAGATATCACAAAACT
AAATTTTACAGTATTCTATTTTATATTGTTATAAATGATTAAAATAACGTTTCCGCCT
TATAATATTAATTGACTTGTTGAAATGTCAAGTGATTTTTTTATTATTTTAATTTTTACATTATTGTGGCTAG
AAGAACATTCACGTTCACGTTTAGAAAATAGCTAACTGTAAAAGTCTCAAGTCTAAAAAAGTTTTTTTAAGAA
ACAAATTAAAATATTATTTTCCTAAGTGTGGTAACTTTAAAAATGTTAAAATGTCCCA
AGTGTTCTTCAAAATAGTTGACTTTTTTTTTT
>126038111_2 EL597788.1
GCACGAGGGGCGGCAAAGTTCCTCGGCGAGCCGACGAAAGTAATAACTGGAAGTCACGACCGAACGCTCAAAA
TTTGGGATCTAAGAAGTAGAATGTGTACAGAAACTAAGTTCGCAGGATCATCTTGTAACGACCTCGTAACGTC
AGACGGTGCAGGTTCGACTATCATCTCTGGACACTTCGATAAACGCATTCGTTTTTGG
GATATACGAACGGAAATGTCGGCCAACCACATCATGCTACAGGGCAAGGTCACATCGCTTGACCTCAGCAGAG
ATAGCAATTATCTCCTCGCGTGTGTCCGAGACGATACGATACAGCTCATAGATTTGAGAATGAATACAATAGT
ACGATCTTTCAGTCACGAAGCGTTTAAGGTGGGCTGCGACTGGTCGCGAGCGGCGTTC
GGGCCGGGCGGGCGGCTGTTGGCTGTGGGTGCGGCGGACGGCGCTGCGTATGTATGGAACACACATTCTGGAA
GACTTGAAGCGGTGCTGAAAGATCATGGGTCGGCAGTAACGGCGGTATCGTGGCATCCACAATCTGGATTATT
GGCGTCGGTAGATCGAGGAAAGCGGGCTGTCATTTGGGGTGACTTGTGACACGCCTGG
GACCACGTCCCGCTCCCGTCCGCCT
>126038112_2 EL597789.1
GCACGAGGTCTAGCTGTGTCGGTTACATTCATTCAGTGATATCTTGCGTTCCAAGGTGATAACTCCAGTCTAG
TGACTTTGAACTTCATATAACAATACAAAAATGGCTCAAGACAAACCTATTTTTGAATACCACGAGGAAAGCC
AGGGCGAAAGATTAGCGCGCAAAACTAAAGAATCTCCTTTCATGGTAGTTGGATTGGC
TGGCCTGGCAACCGCTGTCGGCTATGGAGCCTACGCATACAAGAACAGAGGAAAGATGAGCACCAGTGTCTAC
CTCATGCAGTTCCGTGTCATCTCACAGGGCCTCGTAGTGGGCGCGCTCACCGCCGGCATGGCTTACACTCTCT
ACAACAACCATTTCAACAAACCTAAAGCAATTAGCAGTAGTGTCATATCCAGTAACGA
GCATTAAGTCATACGTTTTAGAATTACTTGTAGAGTAAAGCTGTATTATTAGATTATTTCCAAAGCATAATTA
ATTGGAATTATTTGCGTAAATTGAAATATAGAAGATAATATCATGTAAAGCACTACGGCACCGTCCTGAGCAC
AGAGGCAGAAGACCTAAACGCTTTACATGTCGCACGTGATGTAGAAGACAGTTCTAGA
TACTGAAGAAACATTTTAGAAATTAACACTTAAACTTTAAATAATCCAAAA
>126038113_2 EL597790.1
CGGCAGAGGGCAGCGTATAGCTTACAAAGACTAGAACAAATAATCCGCAAATTGAACAACATCGAATCGTCAT
TTATTATAATTAATAATTATGTAACTTTTAAAATTTAAATCATAAAGCAAATCAGCACGCTTAAAGATTTCAA
TAAGACTGCAACATAAATAGAATACATTAAGAAATATATATTAATATGTATTATGTAT
GTATATATAGTTATAGGAACTTATTGTTGAAAGTAAATTAGTAACAACATATCTTGTAATACCTTGTGTAGGT
TATGTAATTGATAAACTGCTGTGTCACTTGTATGAAATCGTAGATAGGTAGTAGTGTCAAAAAAAAGGTTTAC
GTCACAATGTAGGTTTCATGTCTGAATTCAATAAATACGTACGGGGGTGGCTGTTGCA
TATCAGATGAGTAAATAAAAATTATAATATATAAGTTTGAGTGTGTTTGTTAATAAAAATATACAATTATAAC
AAATTACAAATAAATATATATACAAAATTTTGTAATTAAGATGCATTGTTAATTACGGATATTTTTATAATTT
CGTATAGAAAGTGCTATAAGTTAAATTCTTCAGTGCTTCATAAACATGAAGATTACAG
ATTGGAAAATATACTAGATACCAATAACACGACGTGACACTTCCGTATGAGTTTAGTAGTTGGTTAAAAA
>126038116_2 EL597792.1
CATAACTTGAGAGTCATTCTCGAATGTTGTACCTGCATTTAATTTTTTTTGTTAATCTTGACATAAATAATAA
AGATAATAATGATTTAGTTATTTGAAAGTAACATTGGTTTCATACAGTGTTTAATGGAAAATAGCGCTTGCAT
AAGAAAAAGTCAGTAAGTTAATCGTTAATAGTAAGAAAATGACAGCTTTATATTCGGT
AGACAGATCGACATAATTAAAGCGTGAGTAATGCACATAATCTGTGACAAGGCACATTTCCACCACTGCCAAA
TG
>126038121_2 EL597796.1

GCACGAGGTTTTAAAGTCACTAAAGGCAAAGATTTTGAAGGTACGCCTTATTCACCTGGAAAATTGATGGAAG
ACGGACGCAGAGTTTGCGACTTTTGCAACAAGACATTTACTAAGCCATCTCAGTTAAGATTGCATTTAAACAT
TCATTATATGGAACGGCCGTTTAGATGTAGTGTATGCGCCGTCAGCTTCCGTACCAGG
GGACATCTTCAAAAGCACGAACGTTCTGGATCTCACCATAATAAAGTTTCAATGACATCTACATTTGGTGCAG
CGACATCGTTTAATCCACGTCCATTCCGTTGTTCTGATTGCAACATTGCTTTCAGAATACACGGGCACTTGGC
AAAGCATCTACGGAGCAAAATGCATGTTATGAGGCTCGAGTGTTTATTTAAACTGCCA
TTTGGTACTTTTACGGAGATCGAGCGAGCTGGCCTAAGTCTAACAGATATCGATACTACAGATTGCGCCAGCT
CGTTGGCAAGTTTACAGTCTCTAGCCAGAAAACTCCATGAAAAAGATCCTACGAAGTTAGAATACCGGGAGCC
TAGCGCAGTTCCGGCGCTGCCCCTGGCGGGCAGAGACTCCTCCGAGGACGAAGATGCT
GTGATCTCGGATAAAACCTGTGACAGTGCTAAAGATACTGAGATAAAAACTATTGAAAATTGTGAGAAATCAG
AAACAAACAGACAAGTTAACTATAGTGC
>126038124_2 EL597798.1
GAGTAATAAACTCAATGTATAATTAATCTACTATTTAAAATATGACTTACAAAATATTATTTAATATTAACTT
TCAGGGACGTTTATTGCTATTAATTATATAATATACCATATTTTTTAATAATATAAATAGTATATATATGCT
ATTAATATTATAAAAAATACTTTTTTCGTTTGTATACATATTTCTGTATAAATTACAT
ATTTATT
>126038134_2 EL597808.1
GCACGAGGCATTAAAATTATAATAAAATCACTTTATTGATATTTGAATAATAAGGTAATATAAAACCGTCACA
ATTGCGAAAATATCATCTTCAGATACAGCCATGTCTTCAGATTTCTATATACGATATTATGTAGGCCATAAAG
GTAAATTTGGTCATGAGTTTCTCGAATTTGAGTTTCGGCCGGATGGTAAATTAAGATA
CGCTAATAATTCAAACTACAAAAATGATACAATGATTCGTAAAGAAGCATATGTTCATCCATGTGTGATGGAT
GAACTGAAAAGAGTTATTGTAGATTCTGAAATTATGCATGAAGACGACCGGCTTTGGCCACAACCAGATCGTG
TTGGTAGACAGGAACTGGAAATTGTAATTGGTGATGAACATATTTCATTTACAACTTC
TAAGACTGGATCATTAGTGGATGTTAATCAGTCTAGGGACCCAGAAGGATTGCGGTGTTTTTACTACTTGGTA
CAGGACCTAAAATGCCTTGTGTTTTCCCTTATTGGTTTACATTTTAAGATTAAACCAATATAATATATGATTA
CAATTACATTAAATAAATCTCTTTTCACTAT
>126038160_2 EL597826.1
CGGCACGAGGCGAACCCATACACACACAAGTGTTGTACCGTGACGGCTTTGTTGTAAGCCTATATTTTATATC
ATGTTTATTTCTGCCTTTTTCTTTCTGTTTCTATAGTTTATTTGTAAACATTTTATATATGGAATTGATTTAA
TTATTAATAACATTGTGCCCCAATTTTTTCTTCATTTTAATATTGTTACATAGTTTAA
TAAAAATGTACAGTCGTGTTCAAGTTACAATTGGCAATTAGTATAGTTAGTGTCTTGCCAAAATTATTAAGAA
AATATGTGTAATTGTAATGAAGATATGAACAGATTAAAGTTTTATGTTAATATTTTAACAGAAGGTATCAATA
AATAGTCTAATGATTTTACAATTAGAGAAGAATAAAAAAAATTGAAAACTCAATTATA
AATATTAAAAAAGACAATGAACTTGATCTCTTTCTTCATAAAGAAAATTTTGTATACTATACTTTGCTTAAGT
TTGTAACATTTTAATATCTTAAAACCCTTTTTGCACGAAAATAAGTAAAAAAGAAAATCTAAAAAACTAAATA
CACTACTACAGTGGCCAATCTGATACTCAATACAATGCCTCCAGAATTAGTTGGAAAG
TGGGGGACCGAGTCTCTTAACANCAGGTTCCCTCTGCCTACCCTGTTGTGAGCGGGACAGCGTGAAGCTTATA
AAATAAATATAAAAACATCACTACATCCTTAACTTAATCTATTCATATCTTTCATTATATTGTTTATTCCTAA
CTT
>126038164_2 EL597829.1
GCACGAGGCAAAATGAGTTTGGTCTTTCCAATTATAATCGGAGTTGGATCTGTAGTCATCATAGCAACTGTTT
TTGCTCATTTTGTTTTTGGCAAGAAATCAAAATCCTCCAAAAGTAAGAAACTTGTTGCTTTAGTGGACCCAAA
CATCAAGTATGCATTGCCTCTCATTGAAAGAGAAGAAATAAATCATGACACAAGAAGA
TTTAGATTTGGCTTACCATCACCTGATCATGTACTTGGATTGCCAATTGGTCAACACATACATTTATCTGTAA
AAATTGATGATGAACTTATAATAAGAGCTTACACTCCGGTGTCAAGTGATGAAGAAAAGGGTTATGTTGACTT
GGTCATTAAGGTATACTTCAAAAATGTTCATCCAAAATTCCCTGGAGGTGGTAAAATG
TCTCAATATCTCAATGATATGAAAATTGGTGACACAATAGATTTTAGAGGGCCTTCTGGTAGATTGCAATATG
CTGAAAATGGCACATTTTTAATCAAGAAATTAAGGAAGGACCCTCCTGTGAAGATAAATGTGAAGAAAGTCAA
CATGATTGCAGGTGGTACGGGCATCACGCCAATGCTACAGCTCGTTCGCCACATTTGT
AAGGCCGCAGGTGATCAAACTGAGCTCAGACTGCTTTTTGCAAATCAAACTGA
>126038169_2 EL597833.1
GCACGAGGATAGCTTTTAGTTTATAGATGTGTTTGTATTTTATAAAAATGCAACTGATTCTAACTATACATGC
AGCTGCTTTAGTTATCATTAAATATTTTCTACAACAATTCTATTATGTCTTCTTTAATTAAAAACATAAAAAC
ATATTTTTCTTAAGTCGTTATGTTTTGTCCTTTAACAAATATATTTAAAGCAACCAAA

ACATATAACTTGCAATTTTTTTTCCTCACAGTGCATATAATTGTTAACTAATGACTATTACTATGGCTTTAGC
ATAATTATATAATTTATTAGTATTCAAAATTATAGCAAAATTAAGGAAATGTTTATAGGTAAAAATATGACAC
CTCTGAACCTGTTTGTAGAATATATTGAGATGTAATGTGGAATTATATTTTCCTAATA
CCTTAATTCTAAAGATGGTTAAATCATGTCACCAATTTTAATAAGTCACATATTGGTAAAAAAATTATGCCTT
TGATTTGTACTAATGTTATCTTATGTGATAGTGTAATAATGGCTATTAAGGATTTATAATATATAAAAACGAG
GCAACATANAATGTTGAATATTCTTTATATGTATATTTATATTAAACTGTTTTTTTTT
TCATACATATATTGA
>126038170_2 EL597834.1
GTTTCGATATAAGAATATTATGTAATTGAGGCCTTTCGTGGCGCAGAGGCACGAGCGTGCCTATCTGACTATA
AATGTGATAGGTTGTAGGTTCTACTCCAACTCGAGAAATATTTAAAACTCTCATTTCTTCGTTTAGGTAACGA
GGCAAAGTGCGGTGTTGAGTTTCGCCACTTAACACGAAATGCCTGCAGAATTCGGCGG
AAAGTGGAAGCGCGGGTGTTTTAATGGGAACGGAGCCAACCCTATATTCCTAGGTTTCCTCTGCCTACCCTGC
TATATGCCGGATAAATCGTGGAGCCAAAATAC
>126038172_2 EL597835.1
GCACGAGGCTAATATTCATTACGGTAGCGTTTGTTAGTTATTTTCTTGAGACTTAAGAGAATATCGTATAACA
AGCTTCAGCCATGGACTCGATGAGAGAAAAAGCATTGCAAGACTATCGCACAGAACTAATGGAACATAAAGAA
GTAGAATCAAGATTAATAGACATGCGAGAACAGCTAAGAGATCTCACAAAGCAGTATG
ATAAAAGTGAAAATGATTTAAAAGCCCTTGCAAGTGTTGGTCATATTGTATGTGAAGTCCTAAAGCAGTTAAC
AGAGGAGAAATTTATTGTATTAGCAACAAATGGACCAAGATATGT
>126038174_2 EL597837.1
GTAATTTTAATATAAAAGTTGATTAAATTAAAAAAGAGTAAATATTTTTTTCTTTTTATTAAGAATCTCTACT
GTTGATATTTACTTCAAATAATAAATATTTTAAAGACCACCGTGGCTCAGTGGCGCAAACGTGTGACTGTAAC
CGCGATGGTTGTGGGTTCGATTCCCACTCCTGAGGGAATGAAATACTTAATAACCGGA
CAAAGCGCGGCGTTGATTTCCACCACTCAACATCTCTAGAATTCCTCTAGAATTTGGTAAACAATGGAGAATG
GAATAACAAAAGACTGTGTACCCCTTTCTCACCCTGTCATATGCCGGACACAGCATAGGGCAAAGAANAAGAC
TTTCTACTCAAATATAAACATAATCAATACTTAACTCATGAATTTATAAAAGATATTT
TCCGCCATTTCTCATGTCAAACAAATAT
>126038177_2 EL597839.1
GCACGAGGATTCAATCAGCACCTGTACCTATGCTCGTTCCGAACTCTAACGCTCAATATAGAGCACTTATTGG
CGATAGAGATGCTGACGATACCACAGACCTTTCCGACAATGCTAGTGATAACGAAGAACCAGACGTTGAATAT
AAAAGTGACATTAAAAAAGTTCTGGATTCAAAACACGTGATTGGTGGCAAACCCAAAA
TAAATGAAACATTAGATTTTGAACCAGATAGTCTGATGATGATTGAAGATAAAGATGTTACACAAATTACAAA
TGAGCTTACAGAATCTGAAGATACAGTAGATTCTGGGTTAATTCAAAAGAATATCGGTAATGGTAATATTATA
TATAACGACAAAGATGAAAGTCACAATTCAATATTAGTACTTGGAGTGAACAGTGGGC
CACTGCCTAAAACGGAGTTTAATCTCAAGGAAGAAATACACAGAACAGCGTTAAATGGACACGCTGGGAATGA
TGTAGATGAAGAAATGAGAGAAGACAGCATTGAAGCTAGTTTTTCTCTTAGTAAAGGTAAATCAGAATTGAAT
TAAATTATTAGGAAACATAACTTAGTTTCATGCTGTATCCCTAGCGGGGTAGTCAGAG
AGAACAGAGCGCCAAACGCTACGGTCTCTACATATCTCGTTCGCCTTGTCCACTGTCATCACCCTCTTCATG
>126038178_2 EL597840.1
CGGCACGAGGAGATGTTGTAATGTTATTGTGATATTTAGGTTCATTTAATTTTATTCAGGCCGTGGGTGGTTA
TGGGTTCGAGGTCCATTTAGGGGAATGGATTATTTGATATTCTCGATTTCTCGCTCTCTTAACGCGATGTGGC
GTTGACGTCCGCCGCTCAACAGGCAATGCCTCGAGAATTCGGCAGAAAGTAGATAAGG
GAGTGTCTTAACACTTCCTTTTGCCTCCACGGTCATATGCGAGATACAGCGTGACTCTAAAAATAAATATCTA
ATTTTATATATAAATGGCCTTCAAATAACTTCGCTCTATTTCACTTAGGTTATACCTACCGCCTACCTTTCAT
AGTAAACAGGTAGGTTAGGCTATTTCCTACTAAAGGGGAACTATTTTCCCTTATATTA
GGTCATGGCTA
>126038180_2 EL597841.1
GCACGAGGGCGTGCGCACGCGCAGTGTTGTTACGTGCTCGGCGGTTCAGGATGTCTCCGGCAGCGTCGTTTGG
TGAACGATGGGCCCGCGATAACCTAACCAGATCCACATCGGACGAAGAAGTAGCAATTTTAAATATTTTTCAT
GCAACCAGTGATGGCAGCGAGATAGATGCAGCTAAAAGGGCAAGAAAGTACTACGTGG
CTCGAGGCCCTGGAGGTTTGACTGAACTATGTCACAAGAGACACCCTGATGATGAAGAAATTTTATCTAGCTG
TCAAGATGTATATATAGTACCTCTCAGAGCACGCTCAATGAGTGGGAAGCGAATGACAATGGTGAGACTCCCC
GCACCCACAGCGCAGGAGCGGCCGCTCTCTTCAAAGGCCTTGCTGGCTCGTTGGCTGA

TGATCTTGGATATAAGACTTCGCGAAGACCCTACACCCGGCGATGAAATTGTATTTATAGATGTCAGTGACTT
ACAACCGTCGCATCTCAAACATCATTTCCGAGGCTCCTATTGGAAGCACTTCGTATGGTGCATGAAGTCAGCC
TACCCTATCAAGTTTTCCGAAGTGCACATAATAAATACATATCGTTTAAAGACAATGT
CTCTGGTGCTGTTACACCTTGGACTGTACCCATGGCGACGCAAGATGGGTCATATGCATTCTGATGCT
>126038189_2 EL597849.1
TCCCGGTTCCGCGATGGACACGCATTTTAATGGAGTCTTCGCAATCCTGACTTGCACCGAAGTAGTGCTCCGC
CAGCGCCAGACTACGGATATCGTGACCAACGAAACAGGTCACTTTAAAACGTTCTTCCAGAGCTTCTACCAGC
CCCCAGTTTTCTACCTGAATATGCGGCATGTAATGAATTTTGCCGCTGCCCGGGTCAA
CAAGCCCTGGCAGGATCACCGAAATCGCGATCAGCTCGCGCAGTTTGCGCTGGTAGCTATCAATAAACTGAGC
AATGGCATTCAACAGGGCATGTTCCAGCGTTTGCTGGGTACGTTCCGGCAGCGGGTAATGTTCTTCTGCCAGC
ACTTTGCTGCTGAGATCAAACAGAGTGATGGTGGCGTCATGACGACCAATCCGTACGC
CGATTGCGTGGAAATTGCGGGTTTCGGTGACGATGGAGATAGCGCGGCGGCCCCCGGAGGAGGCCTGCTGATC
AACTTCTTTGATCAGCCCGCATTCGATAAGCTGA
>126038201_2 EL597859.1
CGGACCAAGGAAAAAACGTTTCCACTAGTTATCATCACTCGCTCGATGCTGTTTGTAGATGGTGTGAAGTGCG
TTTTTCGTACTAGAAAGACTCGTATATATCTCGTCCTTGATCACTCATCATGAAAGTAACGAATAATTATGTT
TATATTACTGATATCACGTTTTTATTTTATTACCATATATTTGAATCCTGTCTTCAAT
ATTACCTTATTGATGCGTTTATAATAATCCTTATTTTGGTCGACATATTTTTTATGTTATAATCTCATAGCAT
AGTTTACGTACTGTATAATTATAGTGCTGACATTTTATGCTCTGTTCTTCTCGCACACTGGCAAATATTGTAT
ATTAGAAAGAGATGACGTTATTTTTAACTTTTATAATAAAAGTATGTGCTGAAAGAGA
CGGACTATGGGCTCAGTTGTATCATTTCACATGATTAGGTTAAGGGTTGAAATATATTTTTTGTAAACGTTTT
TCTAATATTTATGTTTTATATTCATTATTGTGATAATATTTTTATGTTAACGATTTTGTTTTTAACCTACTAA
TTTTAATTTTATTTTTCTGTTTTTAGGTTCATTTTTGTATAT
>126038202_2 EL597860.1
GCACGAGGTCCGGTACACAGACGTATAAGTTTCTTTTATTTATATAAATAAACCGTTTTAATCTTTCCGCTGT
TTGATACTATTGTGAGATTTTTGAGTGCAAAATAATGGCGTCCAATCCAGAACACATGAAATGTTTCGAGGAG
CTCAAGGAATGGATAAATCGTCAACCACATTTACCGAAAGACTTAGACGATAATCTCA
TACTACGCTTCGCGCACAGCTGCTATTATGATTTGGAAAAGGCGAAGACGACAGTGGAAATGTTCATTTCCAT
CAGAGGAACTTGCACGGATCTTTTAACGAATAGAGACCCTCAATCAGCTCAAATGCAAAAGATTTTAAAAATT
ATAAATATTGGACAGATCTCGATGGCAGGCAATCGAGGTTTGTGGTTGTATCAAATAA
ATGACCCGGGTTTGGACAACTACGACTATATCTCAGATGCTAAACTGTTCTTCCTTACAACTGATTGTAGATA
TATTGAAGCGGAAGATTTACCCGAGGAAGACATCGTTTTAATAGATGCCAAAGATTTAAATTTAAAGTTTTTA
ACAAAATTGAATCTCTCAACTGCACGAAGACTGGCGAAGTATCAGGAAAATGCAATGC
CGATCCGCCTGAAACAAGTGCACGTTGTGAACGCGCCATCTATTATT
>126038207_2 EL597864.1
GCACGAGGCACCTGTAAACGAAGCAATAATACAAACAACGCCTATTGATGACAAAGAAGCTCATACACAAACA
ATTGAAGATATCACAATTTGTGAAAAAATTGAAGTATTAGATTCACAAATTCAGACGAGTAGAACAGAGACAC
CTGTACCACTTGTAACCTCTGAAGTTACTACGCAAGTCATTGCAAGAGATATTGAAAT
TCTTGATGATAAATTCTCACAAACATCATCGCCTGAGATAGATGATGATAAAGAAATAGTTGAGAAAAAATAC
CGTGACATACAAACATCCCCCGTACCAAAAACAGAAATAGAAGAAAAAGGTACAGAAGTTATTACGGAGACAT
CGGAATGTGTGATACAAACCGATAAACAAGAGGTAAAAGATCAAGAAACCCTTACTTC
ACCTATTAAATCTGTGAAAATACAAGAAATTAGCATTCAGACTCCTGAAGTGCCACTAGTTACTACATATACT
CAATCTGATTCACCAACTAACGAATTAGATATCATTTTACAGAGCAGTCCACATTTACCCGAAATTAATCTTA
CACCTAAAATTGAAACTATTGATAATACTCAACAAACGTCTCCGAGAGATTCTGAAGA
GGCATGTCCTGTTATGAAAAAAGAAGTTGTTATGATTGATAATGTACAACAAACATC
>126038213_2 EL597869.1
GCACGAGGAGGATGTACTTAGAGAGCGAGTCGCGAGACTGGCTGCCATTGTAAGTCGCAGTTATGATTTTCCT
CGTATCAGTATTGCGTTGTCTCATTTAGAATAGTGTAGAAACTAGAAAGGATAATCGTCCGACGTCACTCGAC
TGCAGACCGGCCGAGGCTGAGGCGGCGTTGCCAGCTCTCCGGCGAATTTCCCGTTCCG
GCGGTGTTGCCGTCCCGCTGCGGCTCGCGCCACGAGCGTTCGCCCGCGACCCGCCGTGGACGTAACCTTTGCA
AATTAATATTTTTTGCAGTTTCATTGCAACATAAATATGGAAAACATAGTTATATATGATAATAAGGGACACG
CGCCCGCGCCCGCGCCGAGCTGGCAACGACGCGCGACGTCGCACAACACAAGTCACGA

GCGGATGTTCTGTTAATTAAAATTCCGCCTGCGCCGCCCTCGCGGAATGGCGGCCATGTGTTCGACACTCGGC
TGTCGGACGTTCCGAATCACGTTTTGTTGCACAAGGTTAACCTTTTTTCATTATAATAATGGCGCCTCGACGC
CTGACGGACCCGTCACGCTCGACGGCAGATGCTGTGCTTGCGCGAGAGACTGCCAGAG
GCCAGAATGAAAGACGGGAGTGTTGCGAGATGAATGTATCAGTAAGTCGGTACTGACCCGGTGCTCTTTTAA
>126038214_2 EL597870.1
GCACGAGGGTGCCCTTCTTGATGAAGCGCGAAGTGCGAGCTGCCGTCATGATTATATTCTTTGCGTGGCTGTG
CAGCTCTGTTGCTGTGGCTCCACACATAGACATAGGTCTCGACCAAGAGTTATCGATGCCTCAAGATAGTTTC
CAACTGAAATACTTCCAGCATCTCAATAAGTATCTCAACATCGGTCCCCCGGTCTACT
TTGTGGTGACAGATGAATTGGATTATTCGGACAAGGATGTACAGAACATGGTCTGCGGTACAAGGTTTTGTAA
TCCTGACAGTGTTGCTATGCAGTTGTATACAGCTTACCGCATGTCGAACGTGACGTACATAGCTCAAGCACCC
AACTCCTGGCTGGACGATTTCTTCGACTGGACCTCTTTACCGTCCTGTTGCAAATATT
TCCCCACCAATTCTAGCTTCTGTCCTAATGATTATACATCAACCTGTAAAGAATGTAACATACCACTAATTGG
GCCAGAGGAGCGTCCAAGTCCCTCCGACTTCCAGCATTACGTGCCCTTCTATCTGCAAGACAATCCCAGCCTC
AACTGCGTTAAGGGAGGCCACGCGGCGTACGGACACGCGGTCAACTATAATATGTTTA
ACAGAAGTCAGGCTAAGATTGGAGCTACTTATTATCAAGGATACCACACAGTGC
>126038219_2 EL597874.1
ATACACAGAACATTTATTTAATATTTAAATCACTTCAGTATAGTATAGTAACTCACTTCAGAATCAAGGTATG
TGATAAATAATAAGATAACCAAAGTAACCATTTAAAAAATATATTATTTTACAAAAACATATAACTACACAAT
TTCCATAAAATATTTTTATAAGTCTAGAAACTTTAAAAAAACTATGTATTTAGGGTCC
CATTTAAAAAAAATTAAACAATACATCTTAATTACTTATTTAATATTGCCTAAATAACATTTCCGTAAATATA
TACAATAAGAAAAAATTGGTATTTTATTTTATATTTTCTTAACGAAATACGAAAAAAGGCTGAAAACGACTTC
ATTTATTCTATAACTTGAAAAATCCCAACATATGGCATATTTTAAATAGTATTCATAT
TATTTTAGGCCTTTTTATATTCAATCCTCTCAACTTTAACTTCATCACCAAGTAATTTATAAACGTAGGTGAC
AACTGTTGAGCTTTGTATATCCATTAGAACAAATGATGGAGTTGCGTTTCTATACAAAGGGCTATACGCTCCT
GTAGCAGACCCCGGGTTGATATAAAACTTATTCTCATGTTCATAAGCCTCAAATCTAT
GAGTGTGACCAGATATTAAGATATCCACATCCAGCTGCCTCTGGACTAGAGCCAGGGACTCTTCA
>126038222_2 EL597876.1
ATAAATCGTCTGGATGCGGGTTCTGTCGTTCTGTATTGTGGTCAGCCGGTCGCCGTCCCAGCCGTACCAGGTC
ACTTGCGGTTTCCGTGACAGCGACATCCAGCCCGTCAGGTCCCGTTCACGCCGCCACACCCGTTTTGCCACCC
TGCGGCCCAGCGGGTCGTAAAGATAGCGACTTTCGACCAGCGGCTCTTCATATTGTGT
CCGCGTGTAGTGCACCAGCCGGTGCTGACTGTCGTAATGGTACCGGTGAGTCCGCTCATCATCCGTGCGGATA
ACCCCTTCCGGGATGAGGTCGGTTTTCTCTGTCAGCCTGCCGTGACGGTCATACCGGTAAAGATAGTGCGCGT
CACGGGCGATACGGTTATCCGGCCACATGCTGAGGGTGCTGTCCGGGTGCAGCTCCGG
GTCGGGCAGGCGGTTACCTGCCGGGTCTGTGGCATACGGGATGCGGATATCCAGATTCGCTGCGGTGGTGTGA
ACGCCGGTCAGCCTGCCGGTGGTGCTGTAGCTGTAACTCCGGGTCTGGCGCGGGCTGCTGATGCGGATGAGTT
CGCCGTTGTCGTTCCAGGTGTAATCGCGGTCAGACAGCAGGCTGTTCAGGTGCTGGCT
CTGTAACTGCCCGGCAGGGGTATAAGCGGTGGTGAGTTCATAACGGCCGAAGCTGCGCA
>126038223_2 EL597877.1
GCACGAGGCTCCGATCAGTGACAAGTAGTGTGACTTGTTGACCGTATACACGGATCTTTCTTCTCAAAATGGC
CTCGTTACGCTTTTTGATGATCTTGCGTGTGGCATATAATTTAACGATCTCGGATCTTGATTGTTGATCTTCA
GACTCCCAACTCCTCGATAATGAAATAT
>126038226_2 EL597879.1
GCACGAGGTGAACGTGTATCAAGTAAATAACATCTCGGACGCGCGCTCGAACTCGGTATACAATAATAAATAT
TAAATAAATTTAATTACTACGTAAAAATGTATTGACTTGCTATGAACTTCAAGTGATTTATTGTTTATTTATA
AAACTGTGACTTTATTATATTTTTGAACTGATGTTATAACAGCAAAAAGTTGTTAAAT
CTAGATTTTTCATAAATAAACTTTTAAAAAATATTTACAATGATGATGGCTAGTGCAAAAATGATACGAGACT
GCAGCTCGGAGCTTAGCTCTGATGTGCGATCTCTTTCCGTGGCCTCTTCTACCGAAGAGCAGGACTCTACCAC
CACCGATTCTGAGTACTCTGAAGAGGAAAAAGAATACGAATTGGAAGACCTTCAGATT
CTTAAAACTATTGGTACCGGTACGTTTGGCCGCGTGTGCCTTTGCAGAGACAAGGCGGCGGATGAATACCTAG
CTATGAAGATTTTATCAATGGCGGATGTCATCCGCTTAAAACAAGTCGATCATGTCATGAACGAGAAGAGTAT
ATTGGCAGAGATAAATCATCCTTTTATAGTTAATTTACGCTGGTGGACCCACGACGAC
TCCTGTATTTACATGCTTTTTGACTACGTGTGTGGCGGGAAATTATTTTCTTACTTA
>126038227_2 EL597880.1

GCACGAGGTAAGAAGAAAGACTGAAGACAGGCTTGCGTTTATAATATGATTTAAATAATACTGTAGTAATTTT
ACAATTTTATAAAATAATTAATTTTGCAATGTATAATTTCTTATAAGTTATAATTCAGTGAAATTGTGTACTA
TATACTATTTTTATTGTCTTAAGCTGAACAAATAAATCATCAAAATGCCAAGAAAGTT
GCTTAAATTTTTGATTGTTTTTGACAACACATCGCTATTATACTTCCCTGGCCAGTTTTTATCAGGGAAAGTT
TTAATGGAACTACAAGATGATACTCCTGTACTAGGTCTCCATTTCCATGTAGTAGGTGAAGGTGTTGTGAGAG
TAGGTTCAGGTCGTCATGAAAGGCTTTTTGACAAGGAAAACTACATTGATTTTAGAAT
GCGACTACTTGGAGAACCAGGTCACAGCACATCAGTCCTTTCACCAGGCATACACAGTTTTCCTTTTAAATTG
GGTCTACCTATGGGCTTACCTTCCACATTCCTCGGTACTCATGGGTGGGTACAATATTATTGTAAGGCGGCAC
TAAGAGAACCCAACGGCTTGACACACAAGAACCAACAAGTGTTCATTGTTATGAATCC
GATTGATTTGAACCTGGAACCTCCTGTTTTGGCTCAAGAATTCGAGCTAAGCGTAGAACACAAACTGGGGTGT
AGGTTGC
>126038234_2 EL597886.1
GCACGAGGCAGGCGCAGTCGTCGCTTCGCGGCTGTCCGAGATAGGCAACTGGACAGTATTGCTTCTCGAAGCC
GGACAAGATGAGAACGAGATCTCCGACATCCCAGCCTTAGCTGGATATACCCAACTCTCCGAAATGGATTGGA
AATTCCAAACAACGCCTTCCAAAAATCGTTCCTACTGCCTCGCTATGAATGGTGATCG
CTGCAACTGGCCCAGAGGCAAGGTACTCGGCGGTTGCAGCGTCCTCAATGCTATGGTATATGTCAGAGGCAAT
CGGAACGACTATGATCTGTGGGAGGCACTGGGCAACCCTGGTTGGTCGTATGATCAAATATTACCCTACTTTT
TAAAATCTGAAGATAATCGAAATCCATATTTAGCAAACACACCTTATCATTCAACAGG
TGGATATTTGACCGTACAAGAAGCTCCGTGGCGAACACCGTTATCAGTAACCTTTTTGAAAGGTGGCATGGAA
CTAGGTTACGAATTTCGCGATATAAACGGTGCAAAACAAACTGGTTTTATGTTAACACAAGCTACTATGCGTC
GAGGAAGCAGATGTAGTACCGCAAAAGCGTTTCTTAGACCAATACGCAATAGAGATAA
TTTACATATAGCGGTACAATCGNCAGTAACTAGAATATTAATAAATCCAGTCAAAAAACAAGCATACGGGTGT
AGAATTCTATCGCAACGGTGAAAAACATAAAGTACGT
>126038237_2 EL597888.1
AATATAATATTCTATATATTAACTGAAACCATAATATATATAATACAAAATTTACTTGGAATCCAGTACAAAC
TAACTTATAATAGGACAAAATGCTAGTTAAGGTAGGCGTACACATTGTAATTATAAAGTAATTAAATTTATAG
CCGGCATATATTGGGTAAAAATTTAAAATCATGTCATAATGCCATACGGAGTTAAAAA
TCGCAAGTTGAGAAATAAAATATCTGCGTAACTGAATCAATTATAATTTAATAAACAATAAAGACTAGCAGAA
CAAGTTATCTTACATTAGTTTGAACACTACACAGTGTACAACTGCGGTGTACGCCGACCTCACTCGGCGAAGA
GTTACGTCACTACGAGGACAGTGGACAGCAGACGGTGCACGGCGGACTGTGGGCAGTC
CGCTCTATTAGCTACGTCTTAACTACCGCTTTCATCAAAAGTAAGAGCAAGACGATTGTCGACATATTCCCAT
ACATACAATAGATTCTCCCATACAAATATATATTATCATACAGTAATAATGAGATATACAATGAACAATCAAT
AATACACGTTTAATATACATAGGTCCCTTAATATATATATATATTTGAAATTACATAAAA
AAGTATGTC
>126038240_2 EL597890.1
GCACGAGGAGTGTGCAATATTACATTCATAAAACCATAAAATTAATCAATACTTATAACAAGGTGTGTTTTTA
AACTCTTTAAGTCCAAAAATGGTCAAAATACTGTTTTTGCATCCAGATCTCGGCATCGGTGGAGCTGAGCGGT
TGGTGTTAGATGCTGCATTAGCCTTCAAAAGCAAAGGCCATGATGTAGCTTTTTACAC
AAATCATCACGATCCTTCTCATTGTTTTGCGGAGACCAAAGATGGAACTTTTCCAGTGACAGTTATTGGTGAT
TGGATCCCGCGTGCTATTTTCGGGCGGTTCAAGGCGGCATGTGCGTACGCTCGTATGGTATTCGCAGCCGCTT
ATTTGGCATGGCACGTCATTCCAGCTGAAGAGCCTACTTTGATATTTTGTGATCTTAT
TTCTTTATGCATTCCATTTTTAAAGATGGCACGTGGGCCTTTCCGAGTAATATTTTATTGCCATCATCCAGAC
AAATTATTGACAACTGAAGGTGGTTTCCTAAAGAAAATATATCGCGCGCCTTTAAATTGGTTGGAAGAGCTAA
CTACTGCTAAAGCTGACAAAGTTCTTGTGAACAGCAAGTATACTGCTAGAGTTTATCA
AGATGCATTTCAGAAAATAAAAGATATTCCTGATATTTGCTATCCTTCAATAAATACTGATTTCTTTAATAAA
A
>126038241_2 EL597891.1
GCACGAGGATATTGTTTTACAAATGAAAAAGAGAAGATGTTTAAGTTTTTGTGTTTTTTGTGTGGTCTATATT
TAAATATAATAGGCACAGATGGCCAGAATACCCAAAAAATAATTTTGGAAGTAAATGGTAAAAGAATACTGGA
TGAAATTATAAAAGTTGATGATGAAACTTATGATATACGTC
>126038242_2 EL597892.1
GACGAGGCTTTATCATCTCTAAAGGACAGGTGAAAGTAACGCAAAAGCAGCCTAATAGCAACGATGAGAAGTT
TATCAGAACTCTTACCAAAGGCGACTTCTTTGGTGAGAAGGCATTACAAGGAGACGATCTACGAACGGCGAAC
ATTGTCTGCGACTCACCAGATGGCACCACGTGTCTGGTCATCGATCGGGAGACCTTCA

ATCAACTGATTTCTACCCTCGACGAAATTCGTACCAAATACAAGGATGAAGGCGACGATAGGCAGAGACTCAA
CGAAGAATTCGCCAACTTACGGCTATCAGATCTGCGCATCATCGCCACACTGGGTATAGGCGGTTTTGGTCGC
GTAGAGTTAGTGCAAATACAGGGTGATTCGAGCAGGTCGTTCGCCTTGAAACAGATGA
AGAAAGCCCAGATCGTGGAGACCAGACAACAGCAACACATCATGTCAGAAAAAGAGATAATGTCCGAAATGAA
TTGTGAATTTATTGTGAAATTGTATAAGACGTTCAAGGATCGTAAATACTTGTATATGCTGATGGAGACTTGC
CTGGGTGGAGAGTTGTGGACTATTCTAAGAGATAGAGGACAATTTGACGACTCCACGA
CGAGATTCTACACTGCATGCGTTGTAGAAGCCTTCCATTATTTACATTCTAGAAATATTATTTACAGGGATCT
TAAACCG
>126038243_2 EL597893.1
ATTAATTTATGGTTACTGAATTAACTTTCAACTATAAACATCATATTGTAGTCTCCAAATGATAGGCTTCCAT
TTTAATCATGTGTTGTTTGTACACATTCATGTTAAGAGTAAATTACAAGATATTTTTCAGCATAGCTAATTAT
AAATTAGAGTTTAGAGTTAATTTGTGCACAGTAGGAGTAGGAGCGAAAGGTGTAACAG
TAAAGTCTCTATAGTTTGCTAACAGATCACGGCCTGTACACAATTATAATCAATCATTACTAGAATACTTCTA
AGGTGGTACTAGTAGATTCATATTGAATTTGGTGTTGGTAGTGAGAGAAGCTCATATAGTGAAAGCCTACACT
TGGGTTGTATACCCCAGGCATTGTCTGAACTCCTCACCCTGCAATGCCACTCTCATAC
AA
>126038245_2 EL597895.1
CGGCACGAGGAGCGAACTAAAAACAAGGTTATGTTTAAAATATTTCTAAAAGATTGAATTCAGAAGGTGATCC
GATATGAGTGACATTTATCGGAGAACCGATAACCCATACTCAGGTGCGCTTTGGGTATATGTGTCCTGTGCTC
AGGTGCACCCAAGGGACTGTGAGACTATACTGCCGATAACGTTGGAGTGGAGAAAACT
GGCTGCACGCTGAAGAGTGGAGGCACGTCTTGAGATATGTAGTATAAGACATTCTCTAGCTGCGTGAAGTGAC
ATGAAGATTGCGAGTTTTTGTAGATATGCTTAGTTTATATTTTACAACTGTAGGTAATAATTATTTACATTTC
AGCGTAACCATTATTTGAACAAAAATTATGAGTAGATAGTAAGTAATACAATTAAAGA
TTGACAGATTTAAATAAAAAAGGTTGTTTTAAAGATTATAATTACAAAAAATTTTAATTGCCTGTTTACAGTG
GTTTCGGTTTGTTACGTAACTTTATATTAGTAAAAACATTTTGTGAACGTGTAATTTTTTTTATCATATTAAA
ATAAAACAACTTATATATTCCGTATATTTTATTATTTATGAACGTGTTGATAAATAAT
TAACCATTGTCTTATGATGAGAATTAAATTTAGATACTTCTTTATATTATAATTAATGGGTAATAACAATGTT
TTATTTGAAATAATATTTGAAACACATTTGCTACGACCCAGCTACCGAAAGTTATCATGTTTTTTAAACG
>126038248_2 EL597898.1
CTAATATATGGCCTTCTTTACCATATAAAGACGAAATATTTATTACATCAATTTATGCCAATTTAATGATGCA
GTTTAATGGAGAAATTGAATTTCTAACCTAGTGTTAAGACACTTCGCTCTCACTTCCCGCCGAATTTCGGAGG
AATTGCGTGTTGAGCGGCGGAACTCAACGACGTGCTTTGTCTTGTCACCAGAGCGATA
GAATTAAAATATTAAGTATTTCATTTCCCCGCAAGGGAATCAGCGTCAACTTGTCACCAACCTGTGGCGTTAA
CAGTTGTACCACAACTAGTTGACCCCTATGCAGCTATTGGAAACAAATAAACACATTTCTTT
>126038249_2 EL597899.1
GCACGAGGTGCTACTTGGCTGTTCATATTGCGAGTTCTACAAGGCATGGGAGAGGGTCCTACTATGCCAGCCT
TGATGATCATGTTGGCAAGATGGGTTCCACCACATGAGCGATCGTTCCAAGGTGCCTTAGTATTCGGAGGGGC
ACAAATTGGAAACATTTTTGGTTCCTTTATGTCTGGAGTTCTATTATCTGAAGGTAGA
GACTGGGCTTATGTATTTTATTTCTTCGGTGGATTCGGCCTTTTGTGGTTTACCTTGTGGAGTCTTCTTTGCT
ACAGTACACCAAATACACATCCGTATATTTCAAAAAAAGAACTCAACTACCTCAATAAAAATGTGACAACGGC
AGAAAATAGCAGCTCTAAAGATCCCGTGCCCTGGAAAGGAATATTGCGATCAGCACCA
GTATGGGCTCTTGTATGGGCAGCAGTGGGCCACGACTGGGGATACTACACCATGGTGACAGATCTCCCCAAAT
ACTCAACAGATGTTCTAAAATTCAACATAGCCACTACTGGCACACTCACTGCCCTGCCATATATAGCGATGTG
GCTTTCCTCGTTTTTATTCGGGTTCGTTTGTGATTTGTGTATCA
>126038251_2 EL597901.1
GCACGAGGGAGAACTAGAATCAACGGTAGATGATTTTACAACGGCGAGTGGGTTCACGCAACCGGCGGCCGAG
AAAGAAGACGATTTATCTGAGGAAGAAGAAATTGAAGAAATCGAGGAAATTGAAGAAATAGAAGAGATTGAAG
AGGAGGAAGAATTAGAAGAAGAACCAATTATTAAGAAGAAATGAACTGATAGCGTTAG
GAAGGATGGGAGGCTAGTTGTGTATGTGTTGATTAGATTTTCGTACAAACATACAAACATACGGACAGATTAA
CAGGCAAACCGAG
>126038254_2 EL597904.1
CGGCACGAGGCACCTTTATAGTAAGTAAACACAAACACGAGACCAACATTCACCAACCATCAATCATCATATT
AAAATAACAGCACAAAGAACGCTCCCTCACAATTTGCCCTTACATAAAAAAAGAGCCTATGCCAAATTACACC
ATAACTACACGAACGAACAACGAGAATTATAAAAAGAGAAGAAGAACACAGAACTTTA

GCGTTCCAATCTCTGCTAATCGCGGCCTTCTGTGCCAGTTTTTACAAATCAAAATATCTTGAGTATTCACAAT
GACGTCGCTCGCCGCCGGGGGTGTATTCTCGCAGTTTACGCCGGAGATCACGAAAAGCGCCGTCTGGGCCGTT
TTAATTGTTTAGGGCTAATCCGTACCCGGCGTTTTTCGAGCGTCTAATTCGTTTCAAG
TAGCACATTGTGTTCAGGAGGTATCTACTGTCTGTGGGTACAGCTGTTGCAACTGGTGCTTGTGTGTTAAATG
GTTGTGGCAGGGCACAAGCGTGAGTGGTTTGGGTTCGATTCCCAGTCGAGGAAATCAAATGTGTAATAATTTC
ATTCAATCGCTGTGGTAACTTGGAGTTTCGCCACT
>126038257_2 EL597907.1
CTCGCGGCATATCCCGCTATGCCATTTTATTTTTGAACGCAGGCCAATCATGACTAACAGCAATCGCATCAAG
CTCACATGGATTAGCTTTCTCTCCTACGCACTGACTGGTGCGTTGGTTATTGTCACCGGGATGGTGATGGGAA
ATATCGCCGATTATTTCAATCTGCCTGTTTCCAGTATGAGTAATACCTTCACCTTCCT
CAACGCCGGCATTTTAATCTCTATCTTCCTCAACGCCTGGCTGATGGAAATCGTCCCGTTGAAAACGCAGTTA
CGTTTTGGCTTTCTCCTGATGGTGCTGGCGGTTGCCGGTTTGATGTTCAGCCACAGCCTGGCGCTGTTCTCGG
CGGCGATGTTCATTCTCGGGGTGGTCAGCGGCATCACCATGTCGATTGGTACATTCCT
GGTAACACAAATGTATGAAGGGCGTCAGCGCGGTTCCCGCCTGTTATTTACCGACTCCTTCTTCAGTATGGCT
GGGATGATTTTCCCAATGATCGCCGCGTTTCTACTGGCGCGCAGCATTGAGTGGTACTGGGTTTATGCCTGCA
TCGGGCTGGTGTATGTCGCTATTTTTATTCTGACCTTCGGCTGTGAGTTCCCGGCGCT
GGGCAAACATGCGCCAAAAACGGATGCTCCGGTAGAGAAAGAAAAGTGGGGGATCGGCGTACTGTTTCTCTCT
GTTGCGGCGTTGTGCTACATCCTCGG
>126038270_2 EL597916.1
GCACGAGGCGAGGACCTCCTCGCCCTATCCCCCGAAGCGATCCTCCTCCGCTGGGTCAACCATCAGCTCCAGA
GTGCGGGCATCACGCGTCGCTGCACCAACTTCCAGCAAGATGTCGCCGATTCAGAGATATATTCTTATCTATT
GAAGCAAATTGCACCGGACGATGCAGGCGTCACTATGGATGCGTTGAGGGAAACAGAC
CTCCTCCGTCGTGCCGAGGTCATGCTCCAACAAGCGGCCAAGCTGCGTTGCCGCGCGTTCGTGACGCCGGCTG
ATGTAGTCGGAGGAGTCTACAAGCTGAACTTGGCCTTCGTTGCCAATCTGTTCAACCAGCACCCAGGGCTGCA
GAGGACTGATGTGGACAATGAGGCCTATCACCAGCTGGATGAGACTAGGGAGGAGAAA
ACATACCGTAACTGGATGAACTCCATGGGTGTAGCGCCACACGTCAACTGGCTGTACTCGGATCTCACCGACG
GCCTCGTTATCTTCCAGCTCTACGACATTATCAAACCTGGCATTGTTAATTGGAAAAAGGTTCATCGCCAGTT
CTCCAAGTTACGTAAATTCATGGAGAGATTAGAGAACTGTAACTATGCAGTGGATCTC
GGAAAACAGCTTGGTTTCTCTTTAGTGGGCATCGCCGGAGCTG
>126038273_2 EL597918.1
GCACGAGGCGATGGCGAGGTCAACCACGGCTCGCCCGTGGTGGTCGCACGGCGAGCCTGGACGCAATATGTTA
AGTTTTAAAAAAATAATTAAGTAAACAACACTGACGTTTCTATCGAAATATGTAATTTATTTGATACTAAATA
GCATGGGATTATACTACAAACGCGCCTCTCTAAGATTTACCCTAAGAATATTGAAGAA
GGAAGAATAATATTTGATGTACCCTGCCACAAAAAATTCTCCACTGTTTTTTATAAAAAATCTTGCATTAAAT
TCTCTATTACAAGAGAACTAAAACTGCATTTAATTCTAAAATGGAGGTTCACTGTTAAAGTTATATAACAAAC
AACATTTGTAATAGATTAGTTCAAATGGAATGTTCTAGATATGTAAAAAAATAATATT
AAGACTGAATATTGGAAAATAATTGACTTTATAATGCATTTCATATATAACATAAAATTATATATATTCTTTT
ATGCATTTTAAGGTCAATAACATTTATTTAATGATACACTCAATGGCTGTGGATAAAAAACTCTTTTTAAAGA
CCGTCGTAACGCAACAGTATATTGTCTGTAAACAACGGTTGTGGGTTGATTCTTATTT
TTTCTGCTTTGGTACAAGACACAGCGGCGTTAAGTTCTATCATTAAATAAGTAATGTTTCGAAATATGGCCT
>126038276_2 EL597921.1
GCACGAGGTTCATAATGGTGTATTTAATAATAATACTAGCTATTATTTTATTATACTTATATGGTGTCAGGAA
TTTTAATTATTGGAAAAAAAGAGGTGTTAAACATGTTTCTCCTTTGCCATACGTTGGGAATAATTTATCTAAC
TTTTCGCAAAAATGTAGCATTGCCATGATGGCCACAGAAACTTACTATAAATATCCTA
ATGAAAAAGTGGTAGGATTTTTCAGAGGTACTGCACCCGAATTAGTGATTCGGGATCCTAAGCTTATAAAAAG
AGTGTTGATAACAGATTTTCAAAGTTTCTATGCAAGAGGATTTAACACTCACGATACTGTAATTGAACCTTTA
ATGAAAAATTTATTTTTTGCTGATGGAGACTTATGGCGACTAATAAGACAACGATTCG
GACAAGCTTTCAGTACTGGAAGATTAAAAGCTATGTTCCATATAATAACTGAAAGGGCACAGAAACTGCAAAC
AATAGCCGATGAGGTAGCGAACATGGAATATTATGACATGAGAGAATTAATGGCACGGTACACTACAGATTTC
ATTGGGGTGTGTGGATTCGGGTTAGATATGGACTCATTAAACAATGAAATGTCAGAAT
TTCGA
>126038280_2 EL597923.1

GTATAAAATAGGGATACTTTTATTTCCTCATTGTATTCACATGAATAAACTGCACATTATGCATAGTATATAT
ATCAATATATATAAAAAGTTACAAAGTACAAAATTATACTTAGACTAACTAAACAAAACAAATTTGTATAACA
ATAATTTTGAAGAGCATTCTATACTATTGCTTATAAAATTTTGTCAAATCGAGAAAAA
TAATAACATATACCATAAAATGGAGGCAAAACATTTTCAGTTCCCATAAAACATGTACAAAAATAATATGTGG
AATGTTGATATTCGTTAAGAGGAATTATTTGTTATTGTTTGTTAGCCAAATTTTAAATGATAAGTTAATTTTA
AATTTCATTATATAAGATAAGAAATAAATCATATACCAACATAATAGACAGACGGTCA
AGACTTCATTTATCCTTTCATCTGTACTGTTTTTACAATACATTTCAAACTCCAATATATAATGCCATAATAT
TACAAGCTGTAATATTTTTGCATTGTCAATATTACTAACAAATATTGGACACTACCATTACTATACCTAATAA
TAGTCTTAATATTACAAATTTCAAGAAAAATTAGTAATAATTTTCTGTTTTATTACAT
TGGACTGAATGGATGGTGAGTTCATTTTTTATAAAGGCACACT
>126038285_2 EL597926.1
GGATTTCCTCCTAATAAGCGGGCGAAGCCGCGCGCGCCCTCTAGTATACAATATATTTCAACCATGATCTTTA
TTGTATAGCTTTGATTACATATATATATGTATATATAAATATATATATTTGGGGCCATCTATCAGAATCACGT
GATGTTTCCTAGCATGTTTTAACTCCCCCAGGTATAAGACTACAACCCCCTAATTCAC
AATCACGTATATTTTTTTTAAATGTTAATAAACTACAGATATATGTTATCTAATTTTATTCTAGAATATTAAT
AACCATAGTAAAAATGTTTAGACAGAGAGGTTTCACATTGTTTTTGACTGGCATAGATATAATAACAGCAGTA
ATCTTGCGAAAATATTAATACTATTAGTACCTGAACATTAATTTAATGTACGTTGTCC
TCGGACTACGGATCAGTAGGATTTTTTTCAACACCAACAACTGGACATGCATCTTGCAAGTCTGTTGC
>126038298_2 EL597933.1
GCACGAGGGTTTTTACTTCGGGGAATATTACATGTGAAGCTCAGAATAAAATAGATACAACTAGCGAAGCAAG
AAAATTTTTAGTTTGCGAAGTTCCTGGTTGTTTTGGGGTTAAAGATGCCGATGATATGTGGTTTTCGGAAGGG
CAACCTGTTACTGTTGACTGCTATGCATCTCTGTATTACTTCACTGATATCAAATGGT
ATAGGAATGGCTCTGAGTTAAACACTACTATTACCGTAACACCATATTCGATAATAGCCCGTCTACAAATACC
AAAGATAGCTTTAGAAGACGCGGGCAACTATGAGT
>126038302_2 EL597935.1
GCACGAGGGTTTTGTGTTGTTGTACTTGTATTATTGCGCTGAATTTTGCGATTTTTATTTCTTATTATACAAA
TATTACTCGCGGCAAACTGACAATTAGAAATATTTGAGAGTCTATTTATGAAAAATGAACATATACGGATACC
CGCCTTACCAATACAGCCAGTCAGATGAACTGGCAAAACAAATGTTCGCACAACAAGC
ACTGGCTTCATCAGTGCCTTACAACCATGGGACTCAGGAACCTCATACTATGCCAGTACCAACAGGCAGTCCA
TGGAACGTCCAAAGTATACCCTGGAGTATGCAATCCCCACCAAATTTAGTACAATTTACGGCACAAGCACCAG
AAGCTAAGGTTACACAAGTAGTCCATTGTAAAAGGAAGAGTTTAGATATTGAACCTCC
AGTTCCAGCAAAACAATTCATAACTGCGGAAAAAAATGGCAGCACATCTTAGTGGACTTCACATATCTTCCAAC
TATACTAAACATTCACTGGCCTGTGAAGATGTAATGGAAATGAGTGTTGACACAAGTTCATTGAGTGAGAAAT
TGAAAGGACACACTATTGTACTATCAGACGACGTAAAAAAAGTCAAGACGAACCCCTT
TTACCTGCATCTTTAATTGAGAGATTACAAAAGCCACAAATGTCACTAG
>126038303_2 EL597936.1
GCACGAGGGTTTAATTCATTTAAACAGTTTTTTAGCAAAGACTTGTGTATTTTTTTTCCAAAATAGTATTTC
TCATATGAGTATATGTAGGTAACATATTTTTCATAATTGTTATTTAACTATTAATTTTTCGGCTTATCTGTCA
TATTATAACTAAATAATACTAGTATACTAGAAGTATTCAATACACCTATTATTATATA
ATCAATCTTGTGTTGAGTTATATTACAATATCTGCTTCATATAAAATATTACGATATCTCTTACTGTTCTTTA
ATTACGTCTTCCAAACTTACGGTAATTTTGTAATATTTTATAAATAAATATTTTTCTTCAGGCAACATTTGAC
AGCTGTCACATTTAATCGACACCCTTAGTGTTGTCATCTATCTATAATATAATAATAC
AGTAGAAATTCACTAATCTAGCACTATTAAAGACCAAAGCATGCCGATTTGTTGGAATGTATGCTAAATAAAG
CAACACATAAAGTATCTTACTGTAAATTTATTACAACTGTTTTAAAATTATCCTTTAATGATGTATGTTTTTA
AGCAAATTGTTTTAAAAAACAATTTTATATATATTTTAAGTAAACACTTTTTAACCGT
TTAAAAATATAAATGTTATTTGTAATAATTTAATACCTCAGAGGCCACATAATTGAATCGTCTAAATAAATTA
CTAAATATTAT
>126038306_2 EL597937.1
CACGAGGTGCATTGTCAAAAAATATTTGACAAAAATTTATATAAAAAATGTAATCATTAAAACAATCCGTATT
TCATTAATGTAAAACTAAGTGCTCCTTACTACAATGTATCATAAAATCTATTAAGAAATTAACTCAACAATTC
TTAAATATAAGAAACCATTTTTTGAGGTTACAATGTACACTCCGAGGCAGAAAATATT
GTGGTTTGCATTTAGTACCAGAATCTTCATATTATTTCTACAAGCTGTTTCCAATATTATTATACCTGATCAT
GATGCAAATGTTTTTCTGAGCCCTGAAGATCCACAATTGCGTAAAAACAAAGGTGACGTTATTATTGGTACCA
TCTTAGGCGGTCTAACTCGATGGGATTCACAGTATTTTATGCATATATCACAATATGG

ATACACATACGAAAATTGTTTGGCATTTTTTCCATTGTTCCCGTTAATTGTAAGATATGTCGCATATATATTA
AATAGTATATTGGGTACCTTTTTGAATTTCCATAGTACTTTATTGATTTCTGCTACTATTGTAAATTTAATTG
TATTTATCANATCTGCAGATGTACTCCACAAGTTAAGCATAAGAATTCTTAGAAGTGA
ATGCAAAGCATATAAATCAGTGATTTTGTACTGTGTTAATCCAGCGAGTATATTTTTTTCTGCTCCATATTCA
GAATCACTTTTTGCATTAATGTCATTTTTATACAATGTAAAATGTAC
>126038315_2 EL597941.1
GCACGAGGGCGCGATACACACGTATTATTATTTATATAAAATACTTCTTCAAATTGTTTACAAATAAAACACG
CTGTGAACTCCTCGGATAACCGTGACAGGTTGTTTGTTGAACAAAAACGACAATTTCCAATTTACAAACATGG
AGAGCTGGTTAGATGATGTGAAACGAGTCAGAATACCTCGGCCCAAATTCGAGCGCAA
ATGCCCTGTGGAACCCCCAAAAGATTACCTTGAAGATTGTAGTGACCTCAGTGGGAAACGACATCATCGCGAC
AATAAAGATCTAGTAAAGGCTACGGAAGAGCTCAGACGGACACTACAGGAAAAACTATAACCCGTTTACCATA
ATTTAAGTTGAAAAGACTAATTTATATTACCAGAAAACTAAATTACTTTAAAATATTA
AATTAGGTGGCTTACGTTACAGTAAGCCTATAGGTTAGTTCGTTATAAGCACAGCATACACCATGGCCGAATG
TGGTCATGTCATAATAATTTCTATTTGTGAATAATTTTAATATTCCTGATTTTTTAAGAAGAATTTTATG
>126038316_2 EL597942.1
GCACGAGGCGTATTTCGGAGCTTATTTTTAAATTACTTAATTTGTATTATAATTTATTTAATTAATATTAAAA
AATGTCGGAAAATAAAGATGTTGACAATAAGAAAAACGACGCAGACCCTGAACTCGACGATTTATTGGATAGT
GCCTTACAAGACATGACGAAAAAGGACCAAGATTCAAAACCAGAAGACAGTGGACAAC
AAAATATGTGGAGTGAAGAGTTTATTAAGGAGGCTGCTGCACAGTTCGAAAGTAATATGGCAGCCATATTGGG
TGGATTTAGTGGTGTTCCAGAGGATCAAATCAGTCAAGAGCAAATAGCACAAACATTTTCCAAAATGGCAGAG
GCAGCAGCCCAAGTGTTGAAGCCTTCAGATAATGTCAGTGAACCTGCACCTACCGATC
CTGCTTCACAGGAAAAAATAGACGAGGTTTCCGCAGCCATATCACAAACGCTACAAAATTTGAACACAAATTC
GGAAAATCTGCAGACGCCATTCTCTGAAGCGGACTTAGCGAACATGTTCAACAATTTCAATCTCGGTGAAGGG
AATCAGGAAGGCAACATGTTTTTGCCATTCATGCAAGGTATGATGCAATCGCTTCTAT
CAAAGGAAGTTTTATATCCATCTTTGAAAGAATTAGTAGATAAATATCCAAAATGGCTGGA
>126038331_2 EL597951.1
GTTTTATCTGTGACTTTTTTTAATGTGTTTTGCAAATTTCGACTTTAATTTTGATTTATATTAAATTATTTTT
AATTTGTAAACTTAAAATACTGTTAATATTATTTTTTTCAATTTTGTAAAGTTTTAATATATTTGCCCTACAA
TAATTAACTTCTTAAAATCTTCTCAAATAGTTTATCCTGAACAACGAATAATATTTCA
ATTATATAACACTTAAAATGAAGTAAAATAATATTACTTGACTTTAATTTTGATTTATATTAAATTATTTTTA
ATTTGTAAACTTAAAATACTGTTAATATTATTTTTTTCAATTTTGTAAAGTTTTAATATATTTGCCCTACAAT
AATTAACTTCTTAAAATCTTCTCAAATAGTTTATCCTGAACAACGAATAATATTTCAA
TTATATAACACTTAAAATGAAGTAAAATAATATC
>126038332_2 EL597952.1
GACGAGGCCGCCTTGATTTTTTTGTGAACAAAGTAGAAGGGTCCATGATGACGGCCCAAGTACAAGCACTATA
TGACTTCACTGGCGAGCAGGGTACCACAGAAATGTCAATAACCTGTGGAGAAATATTAACATTAATAAGTACA
GATATTGGTGAAGGATGGTGGGAAGGAAGGAATGCACAAGGCCAAACTGGACTATTTC
CAGCAGCATATGTGAGAAAGTTAACACCTGAAGAATTAGCACCAACAAAAATGGCACCAGCTGCACCTAGATA
TGACCAAGCTGCTGATGACTGGGGTGAGCAACAATATAATGCAGGTGATAATAATATACAAAAATCTTCACAT
GACGATGGATGGGACGATGACTGGGAAGATGACACTTATTCAGAAATAGGCCCAGGAG
CCCCCCAACAAAGACAAGCAATGAACCAACAACCATTAACTCCTTTGCCAGGAATGCCTATTGGTGATTTTAA
TCATCAAATGGATGACACTTCCTCAAATTTTGGTTCTTCAGTGGGTACAGTTAGAAAAAACAAATTTGCACCT
TCGTCTAAAGTTAGTGGTGAAAGTTACCTATTGGCAACATTAAATGTTGAAGTCCCAG
ACTCTGACAAAGTGTATATAGTCCAAGAAGGAGACAGTTATATATGGGCACCCATTACCCAGCCATATCATGT
GACTGTAGCTT
>126038344_2 EL597960.1
GCACGACGTCTCGTGCCGCTTAGGTGTAGTTATTTTAATTTTTTTCCTTTGGCTTGTATTAAGTTTCTATAAA
TTTATATCTTCTTGACACTCGTGTTTATATGACGTGATACCATTTTATGGGAATTTTCGTAATTAGCTATTTG
TACTAGAGTATGATAGTCACATCCGATATGTCTGCAAAAGAATTTATTAACGATTTTA
AAGAAAATTATGGTAAGGTACGAAAGTTATTAGATGAAGACAGCAAGAATGATCCTGAGAATGAGCCATACGT
GTCTAAATACAAAGCTAGAGAAATATTAAACAATATGCGCGATGCACTGACTAAAGTGACTGAAACTGAGATG
AATTTGGACCAATTGAAATTGGATGCTATGCTGGGGGTTGTTTTATTAAATATAGGAG

TTATCGACATGGAAACAGAGGAACTTACAGCTAGTGAGAAAGTGTTAAGAGAAGCTGAAGATCTTCTCACTCC
AAATGCATCTAATCCAGAAATTGTTACCACTCTCATAAATATTGAAAACAACCTCGGTATATTGTGGTCCAAT
CGAGATGAGCCTGCTAAAGCTAAATTATTTTTGCTCAAAGCAAAAACATTTTATGAAG
ATTTCAAATGTACTTTGCAAATGCCT
>126038349_2 EL597963.1
GCACAGGAAAAAATATAATTTAATTATAAATGTTATAAATAACTATAATATTTTGATAATATGTTTAAAATGT
AGTTCAAAATTACTTAAGTTTTTAGCTTCACGCTGTATTCCGTATAAAGCAGAGGACACCTGGGAACCTAGTG
TTAAAACACTCCGTTTCCCACGCACCGCCCAATTTTCGAGCCATTGCGTGTAGAGTGG
AGGAACACGGCGTCGTGCTATGTCTTTTTAACAGCAAAAGAAAATGAATATAATTTATTCCTACGATGGGGAG
TTGAATCCAGAACCATCGCGTTTACAGTCAAATAAATTATAAAAACGTAAATT
>126038359_2 EL597968.1
GCACGAGGGTAATGTGTATCTATCTCAATCCTTGTGTATTCATTCGTTTTATTAAATGTAGTGTTAAATAGTT
TATGAATAAATTGCTATTCGTGTGAAAATTCTTGTTTTATAAAACCAACTGAAGCTAAATTACAATGTTGTCT
TTCATAACAAAAAATTTAAATGATATCATATATTTATCATTACTTTTGTTTAGTGTTT
TTATTGGTCCATACTATAGAAAATTAAAGACAGTTGAGGCAAAAAAATGGGGCGGTTCAATTTTGGGCTTAAC
ATTAATAGTAATAGTCTCAGGATACAGTTCTGTTCATCCTATATTATCAGCTTTACTTGGAATTATTTCCATA
AAAGTATTCACAATCAAATACTGTCATGTTGTGACATTCTTTCTTATGTTTGGTTATT
TATTCTTCTTCCGTCTTGCTGATAAATTTGGCTTCTCTCCTTCCTCGGGTCAAACTAATCTGATTGAAATGAT
ATTGGTGTTAAGAGTTGTTGGAGTAGCATTTGAAATGAATGGCTCTTGGCTAGCTGTCGCTGAGAGAAAGAAA
AAAGAAGGCAAGGAAGATAGAGAAGCAAAGCCAGTGAAAGATAGAGATGATGATTTCT
TAGAATTGATTAACCCTGATTTTATTGATCTCTTTCATTACTCATTTAATTATATTGGATTATTGACAGGTCC
ATATTACAGATACAGAACATTTGATGACTACTTTCATTTGCCCTATAGTAAATATGTGGAACTGTTTTGGTT
>126038363_2 EL597970.1
GCACGAGGACTATAAGAAAAATGTTTTAGTATTTATGCATACGAGTACATATATTCTATATAAATAATATTGT
ATTGGGTTCACTTTTATTTTATCAGATATGTATGCTCAAAATTAATGGATATCTCATTAATATTTTTTACATT
TTTAAAGTGTTATCCCACACTTAGGAAAGAAAAATACTTTTTAGAAATCATATAAAAA
TTTGTACGTAGCGGGATTTGACTTTTTTTTAAGTTCATGAAACGTATGGCTTAGTCAGAAAGCTTAGACGGTT
TCCCAAGAGGCGCGGGTTAAAATCTCGCTACGTCCATTTTTTTATGTTTTTTAAAAAGTATTTATCTCATTAG
TATTTACATCACATTGTCTGCTTATATCATTTTTGACAACCAAACCGCCTACAATAAT
TCTCACTTTAAAATGTAGTAATTAAGTTATCAAATGTTGGCGCCCGTTTAAGTAAACGATTTTACGGGCATAC
ACTTATGGGGAAAACTGTTCAATTCTTCAAACAAATATTTTCAATTATAAATTTGTCTTTCACATGATTTTAT
AAATATTTTTAATTATAATTTATGCTTAATTATAGTACGTATATAAGTTATTAAATAA
TAATTATAAAAAAAATGTTCTTATACCATGCTATATAAAATAATGACATGATCTTATATGCTGTACGATTTTT
ATATTATTTTATGTTCACAAAATTAATGTACTATCCATTGT
>126038364_2 EL597971.1
GCACGAGGAGCAGTTGAAGTATGAACATGTGTTATTGTTTTTGTATTTCTTGATACTATTGTGATTTAAAATA
ATTACTTTTTGTTTGAGTCGCATGTGAATCGCTGGGGACGTTTGTTTTGGTTAACACACGTGTTAGTGAGGGT
TTAGTTTTTATTGTTTTTTGTCGTACATTTTTAGTGGAAATTGCGGTTGTTT
>126038369_2 EL597974.1
GCACGAGGGTTGATTAAAATTTGTAATAAACAGTATAAAAGAATATTGTAATTCCAGCTCATGAAGATTCACT
TTGTTATGTTATCAATACGTCTATGTGTCCATAAATATAAATAATTTGTGTCATCATAATTTGTGCATTAAAC
GAAGGATGGTTTAAGTTGTGAAAATGAAGTGCGATTTCTATGCTAAATACAAAAAAGA
AAAATCTATTAAAAGAT
>126038372_2 EL597977.1
CACGAGGAGATAGACCCCGAGGTCAAGGAGGCCGACGAGGAGACGGTCATTGAACAGGAAATATCAGATCAAG
AGTTAGAAGTAGAAGAGGTGGAAGGTACTGGCATGGTTGGAGGAACATATCGCTTGTTGGCTACTGGTGCATC
AACACTCACGTCTTATATGAACGAGGAGTCGGGGGACGATATCAGTGATGTTGAGGTA
TTGGATCAGTCGTACGAGGAGATCGACATAGATGTAACGAAACCCGTCAAATTCGATTTGTCCCTCGCAGAAA
ATTACAGAATCCATCCGGCAAAAAGAGCGAAAACCAGTTTCATATATCAACCAGTGACCACACAAACACACAC
AAGCAGGCGGAAAACTCCACTCGTCAACCGAATAGCACTAGTTTAATATAAAATACGT
TTTCAATCACTTAAAATAAAATTGATTTAAATTTAAATCGTATTTTATTTTATGTGATTTAATATCAAATGTA
TGGGCTAATAATTAATTAATAATCATATCTTTTCTTTTTAACTTTGTTGCTAACTTCAAATTTGTTTGTGATA
AACAATATATGTATGTGTGGTATGTAGAGATGTTATGGAGCTTCTTAACTGTAAACAT

CAGATATCTAAAGTAAAAATGTTTCTGTATCGGTTATTGATGCTACGGATGATTTCTCATAACAAGTCTCTCG
TAACTTATGATCGGGCTTTTTAAATTTTGC
>126038375_2 EL597978.1
ACACGTTTCTAAATATTTTCCTTTCTTTAGAATATTTAATTGATAATAATAAAATATACTGTTATTATTAATT
GCAATGTAACTAATTAGAAAATAGATTTTTTTAGAACAGATAAAGAANAACAATACAAAGTTTAAGTAAGTGA
CTAAACTAAATCTAGAATTGACTATGGTATATTCTACAGATTTTTCTCATTTCATTCC
TTTATTTGATAAAAAAATCATAATTATCTAAAATTAGCCATATTAT
>126038377_2 EL597980.1
GCACGAGGATTAGTCTCATAGTTTGACCATATTTATTTACTTTATGTAATTCTATGAATTCTTTGAAATTTTA
AATTATGAAATATAATATAATAATGGGTTACCATTCAACATTCATTTATATAACGTTTACGTAGAGTGGCATC
ATATTAAGATAAAAAATCATGGAATATCTATGGTAGTTTCATAACTTTTATAATTATA
CCATGTCAGTTTAAATATTTCTTTATATTCCAATCTTAGTAGCACTGTTCTTATCGGTCCCAGATGGAACAAG
GCAAATATTTGTTGTCGGTTTGTATGAATGACGACAATACGTAACTTTAAATAGAACTACAAAGGTTCTTATT
TATTTCGAAATTTTAAAAATTATATTTCTTTAACGAAACCGAGGCAATTGGATTCAAT
TTAATTACAAAGTGAGGAAAGATGTAAGTAAATACATTAATTTTAAATTTTAAGATTTATAAACAGAGAAGCT
TCATATCGTATACTCATTGGGATTATATTTGAATTTGTTTCTTTTCTTCATATATTACACTGTCGCATTTTGA
AG
>126038380_2 EL597982.1
GCACGAGGAGGACATCCAGCCAGATCTCTGCACCCACATCATCTTCGCCTTCGGATGGATGAAGAAGGGGAAG
CTCAGCTCTTTCGAATCAAACGACGAAACTAAAGATGGCAAGACTGGATTATACGACAGAATCAATGTGTTGA
AGAAAGCAAATCCTAAATTGAAGACGCTTCTTGCTATTGGTGGCTGGTCTTTCGGTAC
CCAAAAGTTCAAAGAAATGTCCGCAACAAGATACTCCCGACAGACTTTCATTTACTCCGCCATACCTTATATG
AGGGAACGCAACTTCGACGGCTTGGACGTGGACTGGGAGTACCCTAAAGGAAGCGATGATAAGAAAAACTACG
TGTTGCTACTGAAAGAACTTCGCGAAGCATTCGAAGCTGAAGCTCAAGAAGTTAAGAA
GCCACGTCTACTTCTTACGGCCGCTGTGCCAGTGGGACCTGATAACATTAAGAGTGGATACGACGTACCTGCT
GTTGCTAGCTACCTGGACTTCATCAACGTCATGGCATACGACTTCCACGGCAAGTGGGAAAGAGAGACCGGTC
ACAACGCGCCTCTGTACGCGCCATCATCAGACTCGGAATGGAGGAAACAACTCAGCGT
GGACCACGCTGCTCATTTGTGGGTGAAACTTGGAGCACCTA
>126038382_2 EL597984.1
GCACGAGGCGAATAAGTGTTTATTTGCTGTTAAAATCCAAAATATGGCTGCTGAAATAAAACCTGCACCGAGA
ACGCCTAACGATAGATTTTCCACAACTAAAAAACCAGCACTTTTAAGTAAACTAGAAGGTTGTACTGATGATG
TAAATGCAGCAGTTGTGATTCCTGGCGAAGACGGAGTTATTAGTGTTTGCGATGATAA
GACAGTGAGAGTATGGCTAAAAAGAGATTCAGGGCAATACTGGCCAAGTATTTGCCAGTACATGCCATCTGGT
TGCACATCAATGTTCTATACTCCTGAAACACGTCAATTATTCATTGGTCAAGAAAATGGCACTATTTCAGAAT
TTACACTCACTCCTGATTGCAACAGGATAAACCCAACCCGTGAGTACCTGGCGCATAC
AGCGCGGGTTACAGCAGTAGTGTTCTCTTTGAGCTGCGAGTGGGTATTATCAGTTAGCCGAGACAAGCTCTTT
TCTTATCACTGCAGCGAGACTGCAAGAAGGATTGGCGGCTATTCCTTTGAAG
>126038391_2 EL597991.1
GCACGAGGGACAGCACCATAGTTAACTAGATATTTGACAGATACTGAAAGTCGTTTTAGTTTTCCCAAAGCGA
ATGATGTATACTAACTTTGATCGATGAGATGTAGGTGAGCTCGTCCGTACGTACTGCGCGGCCGCGGTGAGTC
CCGGCGCAGAGCGATATTTTTATCGCGCGCCTCTCATTGTAATTCGGCATCGGCGGTC
GCTTGTTCGTGTCGACTGTCGTGTTCAGTTCGGTGCTTATTGTCATCTGAGAAATGTAAACGGGGTTCACGAC
TATTTCCCATGTTTCGGTTAAATGTGGATTCCGAGCACTAATAGCAGTGCCTATTTAAATATGTCTATATTTA
TTTTACGTAGACGCTAACGTCGCAGTCGCTGCAAACTGCTCCTTGTATTTTGTTTTGT
TTTTATTGATTATTTTATATATTTTTTTATCATTATGACCAAATGTTAAAGATATAGATAGATAATTAAAAAT
GTTTTCCTTTCTAAATAATAAAAATCAACGTTATAAAAACAAAAAGAAAACAAAAAATAGTCCAAATTTACCA
AATAATCGGTACGGTTCATTCTCAATGAACATAAGAATTACAGGGAAATAGTGACGTT
AGTTTAGATTTAAGTAAAAAAGATTGAACTAAAAGACTAGTGGAACGTTACTCGCTCACTTTCTATACATTAC
TCTATTACATATTTACACTA
>126038392_2 EL597992.1
GCACGAGGAAACCGTCCGCACGTGGCACTCGCACCGCGCCTCGTCCGCCGCGCAGCTCGCCGACCTGTCTGCA
GCACTTACTGCAGCCAAGACAACACTGTCTCAGCGCGCTGCCGCTGCCGACGAGGCGGCACAGCGAGCGCTGC
ACACGGTCACACAGCTTACTGTTGATAGGGAAATGCTGCAGAAGAAGTTGGATGCTTT

AGAAAAAGATAACAATATGTTAATTGGACAATACATTAAGAAGGCGGCCGAGATGCAAAATGAAGTCATCAAT
CTACCCGACGATGTTGCGGAGCTGCAGGAGCAGGCGCTGCAGCTGCGCGAGCAGCTGCTGGTGTGCGCGACGG
GGCGCGAGCGCGCGCTGGCGGCGGAGGCGGAGCTGCGCGCGCAGCTGCTGCACCACGC
CACGCTGCTGCTGCGCAAGGACGACGACCTCGCCGGCCTCGCCGCCGACCTGCGCGCGCAGAGTGAGGAACTG
GACAGGTTACAAACAGAGCGTGAACAGATGAAGGAATTGGCGGACAAGTTGCGGCACTCCAACGAGATGATAG
AGTCATTATTAGAGGACAAGAAGCGACTACAAAACGAAGTGAGTGAAGTGCGTGCAAA
AGTGAGCGTGCTACAGCAGGAACTTGACAACGGAGAGAAAGGTGCACAGGACTTTGTTAGACTGTCGCAGTCG
TTGCAGGTGCAGCTGCAGCGTATCCGC
>126038394_2 EL597993.1
CGGCACGAGGGTAGTTATAACAAATCCCTAATAGCAAGCCCCCTAAAAATTACAAGGAAAATTCTACTTTAAA
ATCTAGTCCACTGCTCAACCGATCCCTTACTAAAAAATATCTAAATGGTAACGACCATGATTTCAAAAATGTT
GAAATTAAAATATGTACTGCTACAGATATTATTGACAGTGATAACGATTCAGCTATGA
GTTCCACGCAGTCAAGCTATCGTTCATCAGCATCCTCACCCATGCATAATATGGATAACGGAATAGAGTCAGA
TAGTTCCCGTTTATCACCACGAATAGTACATTATAACTCTTATTCACTAATGAAAATCGAGCCTCAAAATAAA
TCACTTTCTACTGAAAAAAATAATGAAGTATATATCAAAAGAAATGTTTGTCGCTCGG
TGTCTTCTGATACAAACGTTTCCCTTACCTCTTCAGCAGGATCAGCAGCCACGTCTGGTTCGCAAGCAAGTTG
CAGTTCCCTCGAGAGTTCTGTAGCTGACTCAGATAAGAGAAAAGTTTCAAAAATATACAACGTTGATACCATA
AATAGACGAAATATTGTCGCTTCCGCAAAATGCAGAAGTGGAAGGGATGTTAAACTCG
AGTCACCCGTTGTTGAAACAAAGTTCTCACATGAGTCTTATAAC
>126038396_2 EL597995.1
GCACGAGGGGCACATCCTCAGGAGCGACGACAGCCGGCTACCAGGGTGGTTACTTATCGTTCCAGCAGCAGCA
ACAGTACCACGCGGGCTGGGCGCCATCTTGCAGCCTCGCCAAGTTGCAGCAAATGGCGGACGCGCCTCAGCAC
CCGCCGCACACGCCGCCCGCTCCCGCGCCTCAGTATGGACAGCAAGCCGGCACCCCAC
CGGCTGGGCACTACCACGCGCCTAAATATTATGCTCCAGCACACAACCAACTCGAGTCTCCACGGAATACAAG
GAATACTACTAGCAATCTCAGTCCCATGCAACATGTGCAAATGGGCCCCGGCTCCCGCATGTCACCTAACCTG
AACACCCACATAATAAGCCAGTACGGCCTGAACGGTTACCGAGTGCCACCGCAGCAGC
AGTTCAACAACCTGCCAGTCCAGATGATGAATGTCCAACCTGGAGTCCAGTACCCTGGGCCTGATCCCAGGGC
TCAGCAGCCCAACGTTTACGCCTACGCAGGTTATATAAACCCACCTCTGACCATGCAGACCCTCAACTCGACG
ATGCGTCGGTAGCGACCCAAGTCCCAGTGCCGGAAACAGTAAATGACAGTGACATTGA
CAGCTGAGTGACAGTGACATTAGTGTGAAGTGATTATTTAATCTGTGACCAAAAAGTGACTGTATCATATAGT
TAAGTGGAAGTACA
>126038403_2 EL597998.1
GCACGAGGACTGTTTTGTTAAATCCAAATTTAAATGAAAAAATCTTGGAATGTTTCAATAGATATTAGCGTTT
TTATTTAACTTCAATTATTAAATAATATGTCTACACCAAATATCCTATAAATTAAATTAATGATATTGTATAC
AATATTACAATATTATATTTTTATAAAAAATATGGTATGTAAATGTTTCCAGAAGTAT
GTCAAAACAAACAAACAAAACAAAGGAAAAGTAAAAACTTCTTAATGTAGAGTTTTAGTGTAAAATGAATATT
ATATAATAATAAAAAGCAAAGCGCATGAGTGTAACGCGCTGCACTCGAAGAAGT
>126038405_2 EL598000.1
TTAATAGAACAAATCATTTTTTTCCATGATCATACATTAATTATTTTAATTATAATTACAATTTTAGTCGCAT
ATTTAATAATAAATTTATTTTTTAATAATTATATTAACCGATTTTTATTAGAAGGACAAATAATTGAATTAAT
TTGAACTATTTTACCGGCTATTACTTTAATTTTCATTGCTCTTCCATCTTTACGATTA
TTATATTTATTAGATGAACTTAATAACCCTTTAATTACTCTTAAATCAATTGGTCATCAATGATACTGAAGAT
ATGAATATTCCGATTTTAATAATATTGAATTTGATTCTTATATAATTCAATCAAATGAAAATCTCTCTAATTT
TCGACTCCTAGATGTCGATAATCGAATTATTTTACCAATAAATAATCAAATTCGAATT
TTAGTGACAGCAACAGATGTGATTCATTCTTGAACAATTCCCTCATTGGGGGTAAAAATTGATGCCAACCCTG
GTCGTTTAAATCAAACAAGATTTTTTATTAACCGCCCTGGAATTTATTATGGACAATGTTCTGAAATTTGTGG
AGCAAATCATAGTTTTATACCTATTGTTGTTGAAAGAATTTCAATAAAAAATTTTATT
AATTGAATTAATAATTATTAAG
>126038409_2 EL598002.1
GCACGAGGAGACCGGATACAGTGTTACTATTGGCTCTATAATGACTGCTAATTTAAAGGTACCACACACCCAA
TGGCAGGAAGCGTCGACGGGTGTATCAAGAAGAGAAATATTACTACTATCAGCGCGATCACTCGCCGGATCTT
ATAAAAAGTTGTTGGCCGTCTCCGCTGATGAACCACAATAAACGTTAATTTTAGAAAT

TATGGTATCTATATCTTAGGTGAGTATTATTCTAAGTAAAAATAGATAGTTTAATATAATTAAAAGAAATTTC
CGCTACTGTAACTAGTTAATAAAGTTATAAATGGTTCTGATAGCTTTGTTGCAGAGTTTAAATAAGAGTTTTT
ATGATAGATTTTTGATTTCGTTGAGCCTATATACTCGATTAATACGAATGGAGATAGA
ACGAAGAACTTTTGTTAATAGAAAAAGATAAATAACAAATATTTATTTAGAAAATAGTTATTTTTATCGACAA
AATAACGAATCCGTAACCAAAATTATCCGAATCTAACAGACAATTCGATATCATTTTATATCTGTAACATAAT
AATTTTATTATGTTACAAATATTGATATAAAATTTTACATTTGTAACATTGCTGGCTT
ATAGAAATCTCAAATTTATATTATGCATATGTTATGTGACTATAATAAAAAAAATCAATTTCCATATT
>126038413_2 EL598004.1
TGAGGCCATCGTGGCACAGCGTATGACTGTAAATGGAACGGTTGTGGGTTCGATTCCAGCTGGGGAAATGAGT
TATTTTCATTACCTAGGTAATAAGACAAAGCGCAGAGTTGCGTCATTCAACACGCAATATCTCAAAAATAGGG
AAGAAGGCCAAACCAACAACAGATAGTCCAACTATCTCAGTGACGCCACCGCACAGTT
TAGAGTACGACTGTCTGAAGGGAGGTTGCACCCCTAACGGTAGTGTAACACCTACAGGGATGGTTTCTAATAG
ATCTGGAGCGACTACAGTATTATTAAGCCCAGGGTACTTGCAGAGCCCTTGCCAATGCAATCAGTCTATAAGG
AGTGTAAGTGAAGTAGCACTCGCTATGCCGGCGAACACGTGGAACGAAGACCTCGCGT
CGCCGTCCTCACCTGATGGTCTTTCGTGGAGAAGACTGCATATGTCTCGTGCAAAGCTTAAAGCTACCGCGAC
CACTTCTGAATTGTTATCGGGTTTCGCGATGGTAGCAATGGTGGAACTTCAGATCAACGAACCGACGAACGTG
CCAGAATGGCTCTTCGTAATGTTCGCAGTGTGCACTACAGTGTTAGTGGCTGTACATA
TATTTGCTTTGATGATATCCACATACTTATTGCCTAATATTGATGCAGTCAGCAAAATGGATATAC
>126038415_2 EL598006.1
GCACGAGGACCTGGAGTTCCACAACACGTACTGGCAAACCCTCCGCGTCAAGACTGATGTGTTTCACTTTTAC
GGCGCATACTTAGATGCACGGAACACGTCACGCATCGGGCCGGCGGTGCGCGTGCTCGGCGTCCACGACCGCA
TCAAGCCCACACTCACGACGCACTGCCAGCTGTGGTTCGAGGAGCGCGAGGCGCCCGT
GGTGGTGCGCAATCTGGAATACAAGTACGTGTGGAACAACAAATGGGGCAACTACCGCGACGGGGTGCTGCAG
CCCTACCTACTGGCGTGCGTGCTGCCCGCGGAGGTGCGCCACCTGGTGCCCGCCTCCGTGTCCATCGTGGTAG
ACCCCTGCGACCGCGCCACCAACAACTTGCGCGTGCACAACGACCGCCCCAAGCCGCC
TGCGACCCAGAAGGAGTTCGCAGTATGCGTGAAGGGTCTAGACTTTCTCCACGAGGACCTCTCCGTGCGTCTA
GTCGAGTGGATTGAGCTTATTCGGTTGTTAGGTGCAGATAAAATATTTTTCTACGAACTCCAAGTACATCCGA
ATATTACAAAAGTGTTAAACTACTACAATGAGCTCGGTGTGACCGAAGTGACGCCTAT
AACCCTGCCCGGNGGTCAACCGAACCTGCCCGGCCTCCAGCACATGTACCTAACAAAGAAGACGACTCACAAG
CGACAGATGGAACTGATTCCGTACAATGACTGCCTCTACCGCCACATGTACCAGTACCG
>126038417_2 EL598008.1
GCACGAGGCGACATTAACATTTCGACTAAGAACAATATATCGACACCATTATGTACTACAAAATAAGTTTTAC
AATTAGTTTAATGAACCTGCTACCCACCTCAGTTCTGAGTTCCGTTCTGTTGAAATAAAATACACATAATTAA
AGGTAGAGACCGTTATATGGGACCTGATGAAGAATTAGTGCAAGTAATTTAATTCGAA
AAAAATGATTATTTTCTTTATAAATATTGTTTTGATTGAAATAAAATATTTCGTATTGCTTATTAAAGAAATT
CAGATATTTTTTAACGAATACTTCATCATTTGTTCAGTCTTTCTCCCTAAATACATGATATAGTATAAAAAAT
ACTTGCACTGACAACCTGACATGCTATATTTTTATTATTATACATTTGTAATATTCTT
TATAGTGTTATTTAAACACAAACTATATAAACTTATAAGACCTCGACTTTTATTCAAATTAAAATTTAATG
>126038420_2 EL598011.1
ATACTCAGGATTCATGTCCTGGGTATTTACTACTTACTACTACAAATTACTTTTAATAATTTTCATAAATTAT
TAAAGGTAACGTCAAAATTATAACTTTGGAAATAACAATATTAAGAATCAATATTATAAGCCATTTTTTATCA
CATACTTAGCTATATGTTTTTACTATGTCATGATTTATATATTTATTCATATATATGG
AATCTTGTAGGGCAAATATTTTTATACGTGAATATTGGCGAAACTATTCGCCGTTATTTTCTTTGCTTTTGAG
TAATGTACAGGAACGGAATTGAACTTCGCATAAAATTGAAAATGCTCGAGGAAACACCGCATGCTGATCTGAT
GCTGGCTTAGCTGTGCCTCGTAAGCGGTTTCTCTCGACGTAAAATGATTTATATTGTA
AGGAAATTCTGAAGCTAGTAGAAACTACATTTTCATACACTACTACTAAAACTTTGCCGTGTATTTGGGATAA
TGGATGGATATTATAAGTACTCTTGAAA
>126038423_2 EL598014.1
GCACGAGGGTTAAACTAAAACTATTTTGATAAACGTTATTACCCTAATTTATAAATAAAACATTATGGATAAA
CAAAATAAATTAGAAGAATTGGCCCTTAAATTATACGAAATAGATGCAGTCAAGTTCGGCGAGTTTATGACTA
AAAGCAAAATAAAAACACCTGTATATTTTGACTTGAGGGTTATTGTTAGCTATCCCGA
TGTTATGGAACTAATTTCGAATTTGCTATACGATTTTGCGCTTAAAGAAAATAACTGCGATCACGTGTGTGGC
GTACCATATACTGCGTTACCCATTGCTACTTTACTTAGTGTTAAAGTGAAGAAATCCATGTTGATGAGACGGA
AAGAGGCAAAGTCCTATGGTACAAAGAAAATGATAGAAGGCCATTATAAACAAGGAGA

TTCATGCCTTATTATTGAGGATGTAGTTACATCAGGTTCCAGTGTATTAGAAACCATAACTGATTTAAGAAAA
GAAGGTCTTAAGGCAAATGAAGCAATAATTATTTTAGATAGAGAACAGGGAGGGAGACAAAATTTGGAATCTA
ATGATGTACAAATGAAATCTTTATTTACAATGACTGAGTTGATTAGTATTCTTGTTAA
AAACAAAAAATAACTGAAGAAGTTGGCAAAAATGTTATTAATTATCTGCAAAATATCCAAGCACCTGTTTCTG
CT
>126038439_2 EL598016.1
GCACGAGGGTTTTATAAAATAACGGCGTCAATTTTTAGTGGATTATCTATAAAAATATTATTATCTGTGCAAC
GTTTTAATTATCTGCGCATCGAGGATTATTTCGCGCCAAAAATTCTATCACAAATAATAGTGACCGTGACATT
ACTATTGTAAATTGACTGAAAAATGTGATTTTGTAAAAAAAGTTTTTGTTTTTTTATT
TATTATTATTCTGTAGCGTGACGGATTTGAAATATTTGTATTTAAATTACAAAGTGAGGTCGGAGGTGACATT
ATGCCCCAGATTTTCTTCAAAAAGGTAATGGTGGTGCGGGATCGTGGTGATTTCGAGAATGCCAGTTATGTAA
TCAACAGACGCTATACCAAGAGTAAAATGTCACCGGTATGCCACAACTTCGTACAGAA
TGCGTGGAAGAAGGATTTCTGCTCAAATTGTTTTAAATCACGCGAGGAACATTCAAAGCAGGACAAAACATCA
GAAGGCAGTAAATATTTAACCACTCCATTTCAAAACAGGAGTACATTCTTTAAGAAAAACCCTCCGAGTATTC
TTAAGATACACCCTAAGAAAAAAGGAAAACGTAACGTACGATTTCCTGAAGAAGTATG
CAGCGTTATAGGATATGGAGGTG
>126038440_2 EL598017.1
GCACGAGGCTGAAACGACTAATTTTTATGGAATTGTAGTTAAAATATAACTTTTTTTATTTATTTTCCACTAT
ATTTGCGAATGGTGTAAAGCATCAAAATGGCTTCATCTAATACCTTACAAAAGGCCATTGATCTTGTCACAAA
AGCTACCGAAGAAGACAAAAATAAGAATTATGAAGAAGCATTAAGACTATATGAACAT
GGAGTGGAATATTTTTTGCATGCAGTGAAATATGAAGCACAAGGTGAAAGAGCAAAAGAGAGTATCAGAGCAA
AATGCTTACAATATTTAGACAGGGCAGAGAAACTCAAGGAATATCTTAAGAAGGATAAGAAGAAAAAACCTGT
GAAAGATGGGGAATCTAATTCCAAAAGTGAAGATAAGAAAAGTGATAGCGATAGTGAT
TCTGATGATCCAGAAAAGAAAAAATTACAGGGCAAGCTCGAAGGAGCAATTGTTGTTGAAAAACCACATGTTA
AATGGAGTGATGTAGCTGGACTTGAAGCTGCTAAAGAAGCTTTAAAGGAAGCTGTAATTCTTCCCATTAAATT
CCCACATTTGTTTACAGGAAAGCGAATTCCCTGGAAGGGTATACTTTTATTTGGCCCT
CCTGGTACTGGTAAATCATATTTAGCTAAAGCAGTAGCAACAGAAGCTAATAATTCAACATTTTTCTCTGTAT
CATCATC
>126038442_2 EL598019.1
GCACGAGGGCAAGTTTCTTTCAATTCAAATTCAAATTATGTATAGACATTCAATTATTTCTTGATACTTGTCG
CTCCGCTACGCTTCCTCTCTGAAGTTGTTGTTTTTATAAAGTAACTAACAGAAACAGTACCCGGTTATATTTG
TGCACAATCTAATCTTCCTATCTAAGAAAGCATGGAGGGTCCCGCAGGACCATGTTGC
GTAGGAGATACAACGGAGCCCATGAACAGACTTAGTATGAACAGCAATTGCGAATGCGACAAAACACAAAATG
AAGTCAAAGTGAAGAGTAAGCGATATGGAAGGGGTGCAGTTTCATCAGGATTGACACACGAATGTGGGGTTTT
CGGTGCAATAGGAACAGGTGATTGGCCAACGCAAGTGGACGTAGGTCAAGTAATATGC
CTTGGACTGGTCGCATTACAACACAGAGGACAAGAATCAGCGGGTATTGTGACATCAGAGGGCAAGAGCGCGC
GCACCTTCAACACTCACAAGGGCATGGGGCTTATCAACAATATATTTAATGACGAAGCCATGAAAAAATTGAA
AGGCAACCTTGGGATTGGTCATACAAGATATTCAACATCAGCGGCATCAGAGGAAGTG
AATTGTCAGCCGTTTGTGGTGCACACGGCGCACGGGGAGCTCGCGGTGGCGCACAACGGGGAACTTGTTAATT
GTAGCAGTTTGCG
>126038457_2 EL598020.1
GCCGAGGTGGAAGAGGCGGCCGCGTCCACGACGCGCGACGCGCTGGGCCGCCCCGAGCGCGCGGCGCTGTTCG
CGGAGTGGCACGAGTGCGCGCGCCTCGGGGACCTCGTGGCGCTGCCCTACGTGGTGATCGACTGACGCGCGCC
CGCGCGACCCGCGACCCCAAGTGTCCTTATGCTAGGTTAAGTGAGCGAGTGCGCGGAG
AGGCGCCCGCCTGACTCCGAGCTAACCTCTAGACTGTCGTACTGAGTTATCTTGTTACCGATTGTGATTTATA
AAGAATTAGGTCATTAATAATAATAGGAGTTCCAATTGTATATTATACATATATATTGCTATATCGAGTCAAA
TATCAGATACGAATTAGATGGAGTTATCGAAAGAAATAGTTATTTTCAATACATTGCA
AGTAATTTTTATTAAAAAAAATGTTAAGCGCCCGGCTTAGTGGGTCTGAACTGTTCCGATGTGCCAGATGATT
TGTGCCAGAACTCAGTGTTAACTCAAATTTAAAAAAATTTGAAATACCACAAAAAAAATGTTTATCAGTTTTC
GAAAAAAAACTTTTAGTCCAAATTTTGTTACAAATATACATACATGTTGAAGTGATAT
TTTATGATTGTGCTTTGTAGTTTAGTAATTAGTATCACTTAGCTTATGTTAATTTATTGTCATGTTTCTAACG
>126038473_2 EL598022.1

GCACGAGGGGTGATCAAACCTTCTGGAAAGTTTCCCAAGCCCACTAAAATACCTGGCAAAAACTATTCTAAAG
ATGAGGTTGTTATACGCAATGCTGATTCGGGGAAAGTGATGGGTATCAAGGGCAGACGGGTGCACATGATTGA
GGAGCTAAGCGATACGATCATATCGTTTCAGAGAGTGAGTCCTGGAGCGAAAGAGCGT
CTGGTGCAGATCACTGGTCCAAACGAGGAAAATGTAAACCACGCGAAGCACCTGATCGGCGACACGATCCGCC
GCAACGCGTCGCCGGTGCGCCTCGAGGGCGCGCTGGAGGGCGCGCTGGCCACCTCGCGCGCCTCGCTCGACTC
GGCCGGCTCCGACGAGCGCCCCCGCGAGAAATCTCCTCACAATAATGCCAGAGCTTTG
CTGCACAGCTTCTCCACTAACGACGCCGCGCTCGGCGAGTACAAGTACACGGTGACGTTCGGGCAAAACTCCA
TCAAGATTACTGGACAGAATTTGGATCTCGTCAAGACAGCAAAACTGGTCCTCGACGAGTACTTCGAGAGCGC
AGGCGCTCTAGAAGCAATGGCATTAGGTTCCGGGGATTTCTTCACATTGTCCCAACGT
CCCGCCGCTGCGTTACTGCTACGCGACGACGCGGCGTTAAACGGCGTGGACGATGACGTGGTCACTGCCAGTG
CAACTACTAGTGATGCTTCTGGTGGCGGGTCGCGGCAGACGTACTCGTA
>126038490_2 EL598025.1
CGGCACGAGGCACACAATACTTGGCGTCGAGTAATTCCACATTTCAACTAAGTAATTTCCTAGATAAGAGCGG
AGTGCAAGGCGCGGCTGGGGCCAGAATCGGTTATGACATGTCACCATTCATCCGGCGGTACGCCAAGTATCTG
AATGAGAAGGCGCTCTCCTACAGAACTGTTGCCTTCGATTTCTGCAAAGTAAAGAGGG
GCAAAGAAGAAGGTTCACTTCGTATGATGAATGCGGAGAAACTCCTCAAGACCCTGCCCGTGCTTCAAGCTCA
GCTCGACGGGCTCCTCGAGTTCGACTGTACCGCCAACGACTTGACCAACGGAGTTATCAATATGTGCTTTATG
CTTTTGTTCAGGGATCTTATAAGGCTGTTCGCGTGCTACAACGACGGTATAATCAATC
TCCTTGAGAAATACTTCGATATGAACAAAAAGAACTGCCGCGAAGCTCTCGACCTCTATAAGAAGTTCCTCAT
TAGGATGGATAGGGTTGGAGAGTTTTTGAAGGTAGCAGAAAATGTGGGCATAGATAAAGGTGATATCCCAGAT
CTAACTAAAGCTCCGAGCAGCCTCCTCGACGCCCTCGAAGGACACCTCGCCACTTTGG
AAAGCAAAAAGGGATCAGCAGCCAATACTCCTACGCAGACTGC
>126038509_2 EL598030.1
ATTTTCCATTCTATATTTAGAAAAAGCACCTTCTGTAAAACATACAAAATTTCGATAAAAAACTTTTATTGGC
ATTGTTAGCTTATTATAAAATTGAAAAAATTACTGTATGGATATAATGCAATACGACAGTATAAAAATTTATT
GTTATTGTTCACTTTCTTATAAGAATAAAACAGAATTGCTTCTAAAATTATTAGAAAA
TCTTAACTTTATACGCAGTTGAGCATTTAGTATCCAATAATTAATCCAATAATTT
>126038545_2 EL598038.1
GCACGAGGCTTATAAGGCTAAAACTGTTTTAGACAAATATTTTAAATTAGCAGCTATATTTTAAGTAGATATT
CGATGGGTGACTCTGATAAAAGACCACAATTTGGAAACAGATTGTTAGAAAATGTGGACGAGGTCTTCAAACA
TAATGCTTGGGACAATGTTCAGTGGGATGAGGAACAAGAAAAAGCAGCTGCGGAAAAA
GTAGAGCACAACTCTCTTGTAACATTTACTGAAGAGCGCTTAAAAGATTTAGAATCAAATGCTAATAAACATT
GGGATGCTTTCTATGATATTCATCAAAATAGATTCTTTAAAGACAGACATTGGCTGTTCACTGAGTTCCCAGA
GCTGGCGCCCGACAACACTTCAGCACCAGTCAGAGTATTTCCTAATAGTGATAGTAGT
CAAGAAACTAATAGTATAGAAAGAAATGAAATTGTGACTTCTGATATTAAACGCTATATTTTTGAAATAGGAT
GTGGTGTTGGAAACACAATTTTTCCTATTCTACAATATAGCAGAGATCCAAATTTATTCATTTATGGCTGTGA
TTTTTCATCAAAAGCTATCGATATTATGAAAGAGAGTGAACTCTATGATACTAAAAGG
TGTAAAGTTTTTGTTTTGGATGCAACATCAACTGTTTGGAATGTGCCTTTTAAGGAAAATTCTTTGGATATTA
TTGTTTTGATATTTGTATTATCTGCAATTGATCCTGCAAAGATGCCCACTATCGTGAACAACATTTACAA
>126038547_2 EL598040.1
AATTTCAAATTCATATTTATAATAATAATATAAAATGTAAGCTAACGAATTGTTAAGCAAAATAAATAATTAA
GTCATTGAAGCACACATTTTTCTAATTATTATTGATTAATTTTGCAAGTCTTCTGCACTTCTGAGAGCTATTA
TAGTACAAACACACAAATTCTCATCAAACACACAACTAACAGATAAATCTTCTAAAAC
ACAATTGTATTCATGGTCTGGATATCTAGTCCTTACATAGACCGATCTAGGATCCTCTGAGAGAAGGCTTTCT
ATATTACTCTTGAAGGACGGCGCCCTGTCACCTATCAAATTATTAAGTCTATTAAGAGCATCTTCTGTAAAAA
TCACTTGATATTTAAGGGCTGGTGGGTTATTAACCCACGAAGCCACGCGAATGCCATC
TTGATTGACATTTAAATTATTCTGGGGTGGCGTAAATCTCTCTTGGCCATCCGGAGCTCCTCTTTCGGATTCC
ACTCTCCGCATCGGTGAAGAAACTGGTCCGTCGAGCAAATC
>126038584_2 EL598049.1
CGGCACGAGGCGTCGATGCCGAACGTTTCTCCTTTGTGGCTGAACAATATTGCCGTGTCCGCTGGAGGTATAG
CGACCATGTTGAGCGGCCTTTACTACCACGACGCTTATCAGTTCACATATTGCGGCATATTTGGATTGGCTAT
TGCATGTTTCGCGTCTCTTCGTTCCATCCTCGTAGTGGAATACATTGGATTAGAGCAG

CTCACGAATTGCTTTGGATTGTTCTTGTTGTTCCAAGGCTTAGGAGCGCTATTGGGGGCACCTATTGCTGGTG
CCCTTATGGATATAACGAAGAGTTTTGAAATTTCCTTTTGTGTATCCGGCGGTTTCCTTCTCTTCTCTGCTCT
GATGTGCTATCCTGTAAATTACATCAGCAGGTGGGAAAAATCAAGGAACAAACTAGCG
TCATCGCCCAGCGCGTAAAATAAATGTATATTTAGTATTGACCAACCTCTGCGAAATTACTCTTGACACTAAA
CTGACTACGACTTACAATCTGACATTAACGTTATTTATGTCATTAGCAGTAACATTATATTTTATTATCTATG
GTCCGTTTTAGGACGTTTCCATTGGTTTATTTAGTGTGTAAACTTTGTTTTTTCACTT
GACACTGCCGAGTACTTTCCAGTTTTAAAATTATACAGTAAATACTTTACGTTGAGATTCTTGTATACTATAA
TTATTTGGA
>126038586_2 EL598051.1
GCACGAGGATCTCACTTTGAGTTGCCTGCTAGGCACACTGATGTTGTGCGCCCTTCATTTGATTTGCCGCAAA
ATTACTTTAAAGAGTTTGATGCTTTTAAAGCTGGTTTTAACGACAACAAAAAAGCTATTGAAGATAGTTATGA
TATTCCCTATGTTCTGCATAAACGACATATAATGAATGGGACGGCTCCTTCTCCTCTT
GATGATAAAAGACCACCGCCAGATATTATAACTTTGCCTCCGCAAAATGGGCGTCATATAGTAAATGCCCATA
CTCGATGTAAATATCATGATCATCCTAAGTCACCACGCTCACCCAAGTCACCAAAATCACCAAAATCTCCCAA
ATCACCTAAGTCACCAAAATCACCTAAATCTCCAAAATCACCGAAGTCACCAAAATCG
CCAAGGAACAGTGTTCAATGTGATCACGTCTCTAAATTTCTTGAAGTTGAAAATCGTGATTTCTTATTTACTC
AAAATATAGATTTATCAAAAGTAGATCCTATTACTTTAGAAGTCGATAAAAGTGGGCAATTGCAGCACCTCCC
AAGTCCAAAGAGAGATATTACCAAGTCTTTAGATTCTGCTTTGCTAGAAACGCTGAAT
TCCAAACTAAGTCAAATCGAAAGTGATTCCGCTACGAGTTC
>126038588_2 EL598053.1
AAACAGCTGATATATCACATAACCTTTAACAATATTCCGTTTTATGTAAATAATTGATATTTTACTTTTAATT
AAAAGAGATACAATGGAGAAAATTATAAAAATTGGTAAAACACTACGTAATAATTGGAAAAAGTCGGTGTTTG
GAGCAATCGTCGTTTATTACGGTGCGGCAAAACTGAAAGAAAAATATGAAATCAGCAT
TTTAATGCAAGCAGCATGTAAGGAAGCCTCAAAATATGGTGACTCATTAATACCCATGGATAGGAACCCAACA
CTTGTTACTGTTATCCTAAATCCTGTTGCAAATAAGCGTAAAGCTAAAAAGGAATATGAGAAATACTGTGAAC
CAATATTGCACTTGGCAGGATTACAAGTGGATGTGATACAGACTACGTCAGAGGGACA
TGCCAAAGAAATTGTTGAAACATTAAAGGGTACAGAAGCTATAATAGTTGCAGGTGGAGATGGTACTGTGTCT
GAAACAGTTACAGGCTTACTAAGGAGAAATGACAAGGCCAACAGATTTCCATTAGGTGTCCTACCTCTCGGTC
GTACCAACAGTCTCGGCAACGATCTATTTCCTGGAGGCGATGGTGTAGAAAAAGTAAA
ACAATTAATTCAAGCTTGCATGGCTATCATAGAAAATAATACAACTTGAAAAGAT
>126038589_2 EL598054.1
GCACGAGGCGGCTTACCAGTTTCAAATTAAATTTTAATATTAACGTTTTTTTAATATATATATTTTATACTA
TTATTGTGTATTTAAAATTGTAAAGAAAGTAAAGCATAATGACTGACAAGAAGCATCTGTTACTGTTCTTCGA
CAGACCGACAGAACCATGCTTCATGCCAAAAGACGGCTCCGTCTTCCAATTACCTGAC
AATTATTTCCCGGAAAAATACAAGGAATCCAGCAATGTCCTCTCAAGTCGTTTCGGGGAGGGTGTTAGCCCCA
TACCAGTGAGAAACATTGCGTTGCCAAATCTGGATCTGCCCATGGAAATGCCCTATAATGACCAGTTCTCATT
ATTTATACCTAAACATAGGAGAATGGCTGGGAAGCTTATTGATGTGTTTCTTGGTATG
CGTGATGTCACCGACCTCGTATCCATCTGTTCGTTCTGTCAACTGAGAATCAATCCTTACATGTTTAACTACT
GCCTTTCTGTGGCTTTACTGCACAGAGACGACACGAAAGGTCTAGACATCCCGACCTTCGCTGAGACCTTCCC
CGATAAGTTCTTAGACCCAAAGGTGTTCAGGAAGGCGCGTGAAATATCTAGCGTTGAA
GAACCGAAAGAAAGGATACCAATTCCAA
>126038591_2 EL598056.1
GCACGAGGGTTAATGTCTTTAGATATAAACCAATGTCCGGACAAGTATTATGTGCCTAACGCATTCAAAGGCA
CTGATAAGTGTGATAGAAGTTCCTCTTATTGTGTACCAATACAAGGTCGCGGGTTCGACACCGGCGGTTACAA
ATGCGAATGCCTCCAAGGATTCGAGTACCCATTCGAAGATCCCATCACATACTACGAC
GGCCAAATAGTTGAAGCGGAATTCCAAAACATAATTGAGGATAAAGTAACGAGGATCGACATGTTCAAATGTA
GGTTGGCCGGTGCGAGTAATATACAAGTTAGTTTTGTCACGTTGGCAGTATTGTTGACATTTTTGTGGAACAT
TAGGTAAAAATCATTCATATTTAAGTTTTTGTCTCGAGATTTTCAAGTTTTTGTCAGG
TTTTGTGATGGTATTTTAATGTAAAAGATTAAAATAAACTAAGAAAGGCGTGATTGAAATATTTCTAAATATC
TGAGCTAAAATTATAAGAACGAGAATTATTACTATAATTCTCGTTCTTATATTACAACATGAAAAGTGGACAT
GGTGTGTAGTGTGGAAAAACAAATATGTTTACTATATAGGTAGGTATAGGTGTTAATG
CTTTTTTTATTTCTTCAAACTATTAAGAAAAAACATATTCAAAAGTTTTCTATAATATAATATTTTTATACTA
CTAATTC
>126038611_2 EL598062.1
[truncated: 442,157 more chars]
